# Supplementary material for: Protein degradation by human 20S proteasomes elucidates the interplay between peptide hydrolysis and splicing
Source: Nat Commun. 2024 Feb 7;15:1147. doi: 10.1038/s41467-024-45339-3 (PMC10850103; doi:10.1038/s41467-024-45339-3)
Supplement: Supplementary file 1 — Supplementary Information [file 41467_2024_45339_MOESM1_ESM.pdf]

Protein degradation by human 20S proteasomes elucidates the interplay between peptide hydrolysis and splicing

Correspondence to: Michele Mishto (michele.mishto@kcl.ac.uk) & Juliane Liepe (jliepe@mpinat.mpg.de).

Supplementary Table 1	Proteins investigated in the study
Supplementary Figure 1	Proteolysis of proteins by 20S proteasomes
Supplementary Figure 2	Data processing workflow of the inSPIRE 1.5 - aSPIRE pipeline
Supplementary Figure 3	Weights of the features considered by Percolator for peptide and PSM assignment
Supplementary Figure 4	Comparison of inSPIRE 1.5 and invitroSPI methods for the identification of non-spliced and spliced peptides produced in the processing of proteins by 20S proteasomes
Supplementary Figure 5	Benchmarking of inSPIRE 1.5 against DN1 and DN5 methods in a (pseudo) ground-truth dataset containing both non-spliced and spliced peptides
Supplementary Figure 6	Benchmarking of inSPIRE 1.5 in a spliced peptide-free dataset
Supplementary Figure 7	Representative generation kinetics of non-spliced and *cis*-spliced peptides
Supplementary Figure 8	Correlation between protein length and peptide products
Supplementary Figure 9	Average and relative quantity of non-spliced and spliced peptides produced by proteasomes from each degraded protein during the digestion kinetics
Supplementary Figure 10	Quality check of PSMs assigned to spliced and non-spliced peptide sequences
Supplementary Figure 11	Comparison of PSMs assigned as spliced peptides to the best possible isobaric non-spliced peptide competitor
Supplementary Figure 12	Ffh degradation and peptide production by 20S proteasomes varying the proteasome to target molar ratio
Supplementary Figure 13	Length of non-sliced and spliced peptides, splice-reactants and intervening sequences produced during the *in vitro* degradation of synthetic polypeptides
Supplementary Figure 14	Preference for protein regions by peptide hydrolysis and peptide splicing during 20S proteasome-mediated degradation of proteins and correlation to protein features
Supplementary Figure 15	Schematic illustrating the meaning of SCS-P_1 and PSP-P_1
Supplementary Figure 16	Correlation between protein's SCS-P_1 and PSP-P_1 upon 20S proteasomal digestion
Supplementary Figure 17	20S proteasome purity

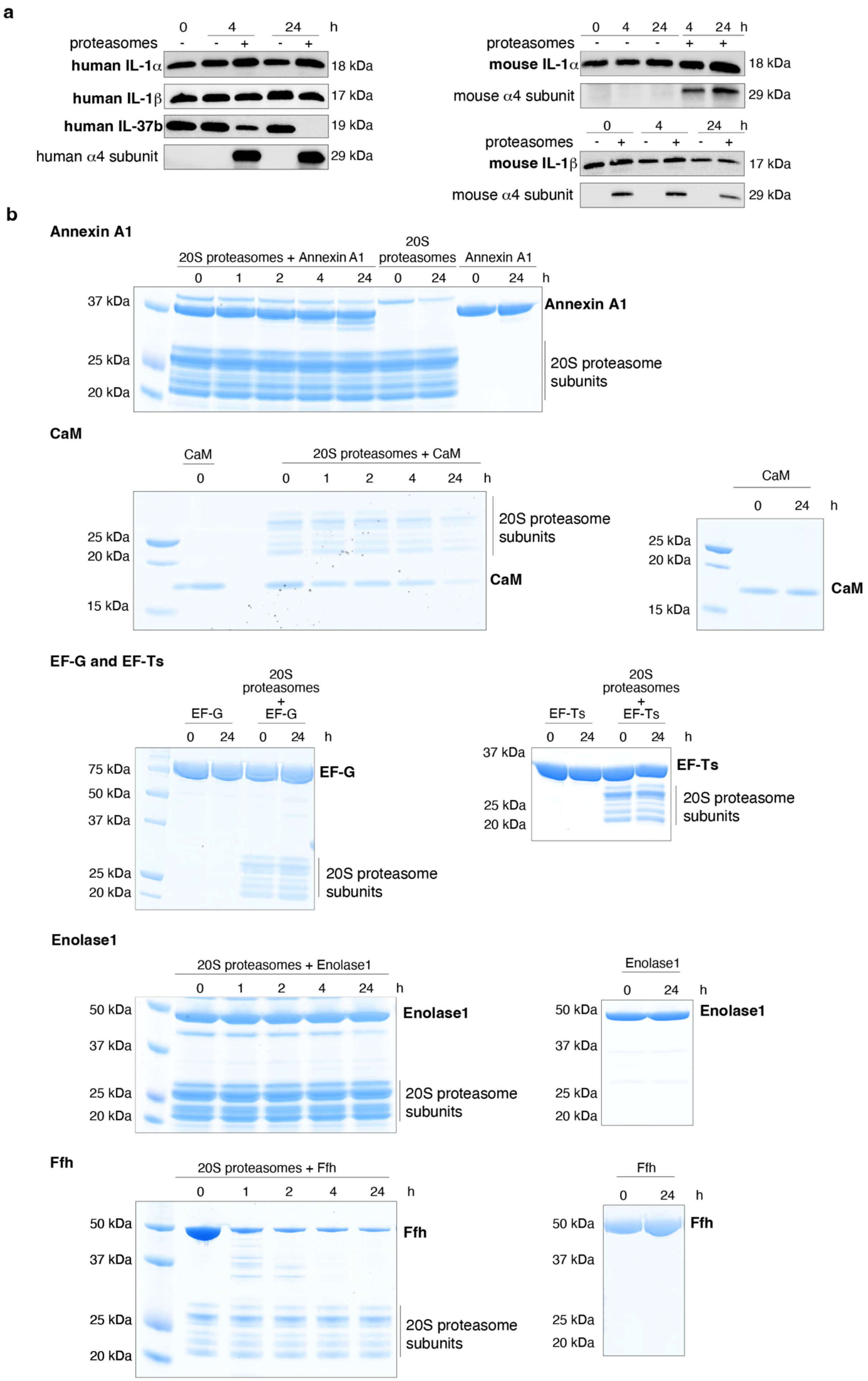
a
0
4
24
h
proteasomes
- - + - +
human IL-1α
18 kDa
human IL-1β
17 kDa
human IL-37b
19 kDa
human α4 subunit
29 kDa
0 4 24 4 24 h
proteasomes
- - - + +
mouse IL-1α
18 kDa
mouse α4 subunit
29 kDa
0 4 24 h
proteasomes
- + - + - +
mouse IL-1β
17 kDa
mouse α4 subunit
29 kDa
b
Annexin A1
20S proteasomes + Annexin A1
20S proteasomes
Annexin A1
0 1 2 4 24 0 24 0 24 h
37 kDa
25 kDa
20 kDa
Annexin A1
20S proteasome subunits
CaM
CaM
0
20S proteasomes + CaM
0 1 2 4 24 h
25 kDa
20 kDa
15 kDa
20S proteasome subunits
CaM
CaM
0 24 h
25 kDa
20 kDa
15 kDa
CaM
EF-G and EF-Ts
EF-G
20S proteasomes + EF-G
0 24 0 24 h
75 kDa
50 kDa
37 kDa
25 kDa
20 kDa
EF-G
20S proteasome subunits
EF-Ts
20S proteasomes + EF-Ts
0 24 0 24 h
37 kDa
25 kDa
20 kDa
EF-Ts
20S proteasome subunits
Enolase1
20S proteasomes + Enolase1
0 1 2 4 24 h
50 kDa
37 kDa
25 kDa
20 kDa
Enolase1
20S proteasome subunits
Enolase1
0 24 h
50 kDa
37 kDa
25 kDa
20 kDa
Enolase1
Ffh
20S proteasomes + Ffh
0 1 2 4 24 h
50 kDa
37 kDa
25 kDa
20 kDa
Ffh
20S proteasome subunits
Ffh
0 24 h
50 kDa
37 kDa
25 kDa
20 kDa
Ffh

b (continued)

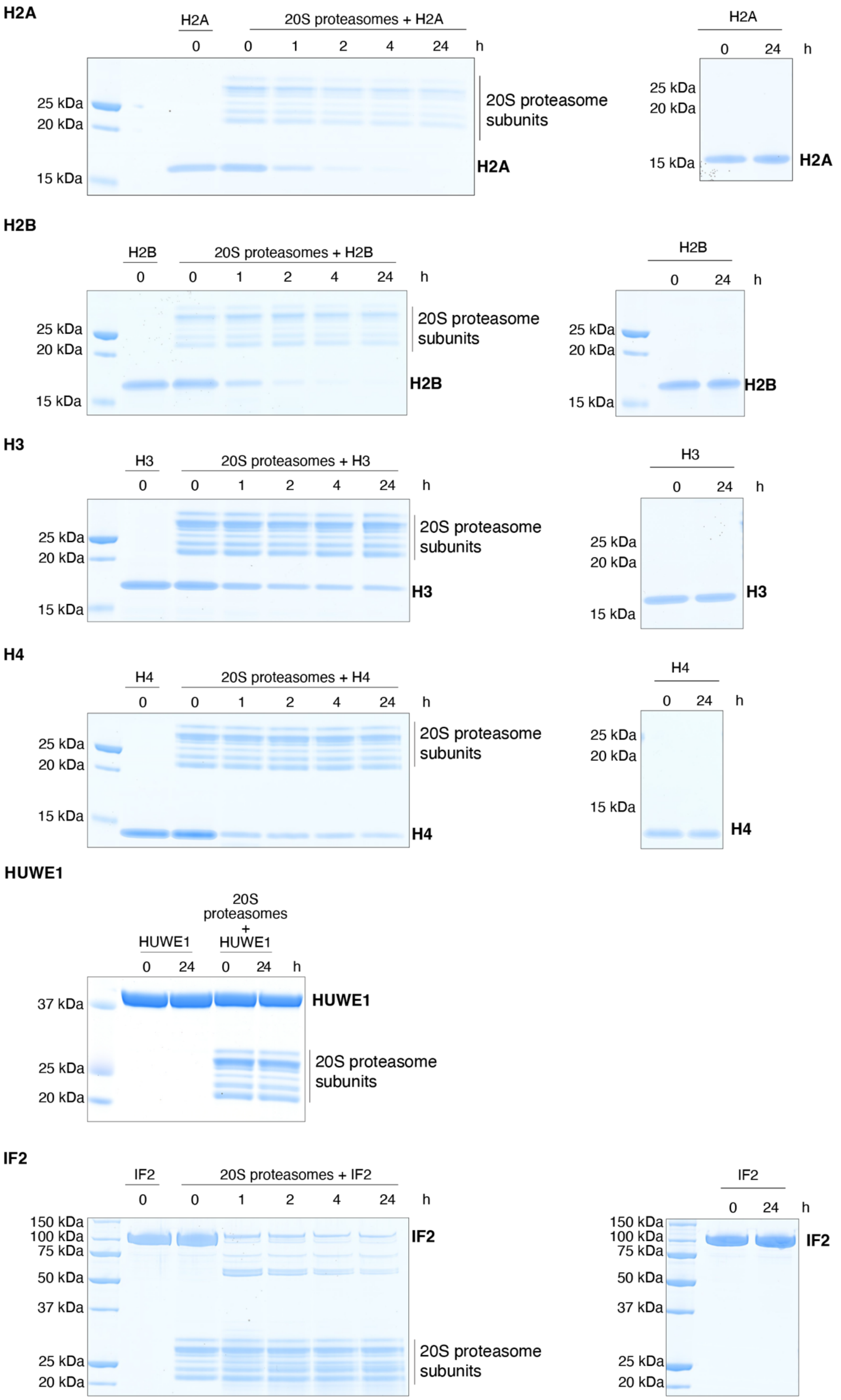
H2A
H2A
20S proteasomes + H2A
0 0 1 2 4 24 h
25 kDa
20 kDa
15 kDa
20S proteasome subunits
H2A
H2A
0 24 h
25 kDa
20 kDa
15 kDa
H2A
H2B
H2B
20S proteasomes + H2B
0 0 1 2 4 24 h
25 kDa
20 kDa
15 kDa
20S proteasome subunits
H2B
H2B
0 24 h
25 kDa
20 kDa
15 kDa
H2B
H3
H3
20S proteasomes + H3
0 0 1 2 4 24 h
25 kDa
20 kDa
15 kDa
20S proteasome subunits
H3
H3
0 24 h
25 kDa
20 kDa
15 kDa
H3
H4
H4
20S proteasomes + H4
0 0 1 2 4 24 h
25 kDa
20 kDa
15 kDa
20S proteasome subunits
H4
H4
0 24 h
25 kDa
20 kDa
15 kDa
H4
HUWE1
HUWE1
20S proteasomes + HUWE1
0 24 0 24 h
37 kDa
25 kDa
20 kDa
HUWE1
20S proteasome subunits
IF2
IF2
20S proteasomes + IF2
0 0 1 2 4 24 h
150 kDa
100 kDa
75 kDa
50 kDa
37 kDa
25 kDa
20 kDa
IF2
20S proteasome subunits
IF2
0 24 h
150 kDa
100 kDa
75 kDa
50 kDa
37 kDa
25 kDa
20 kDa
IF2

b (continued)

IL-37b

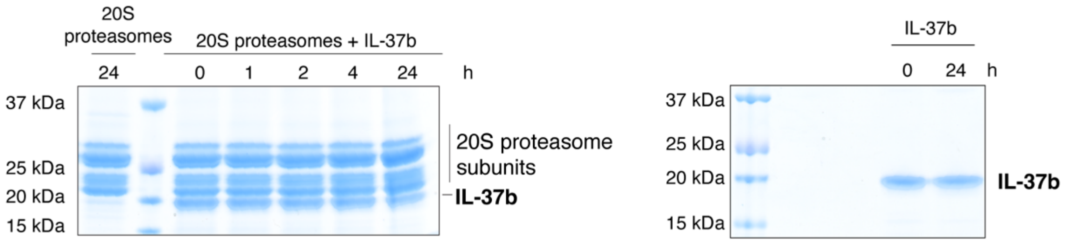

LEDGF

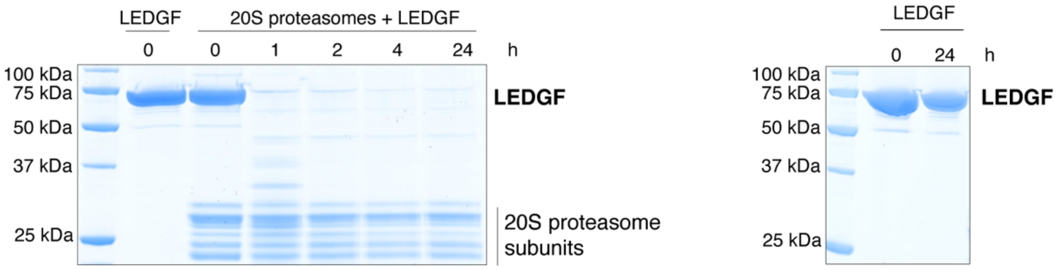

LRP130

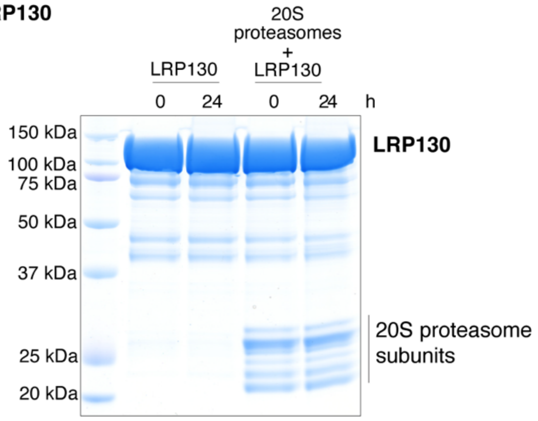

Ovalbumin

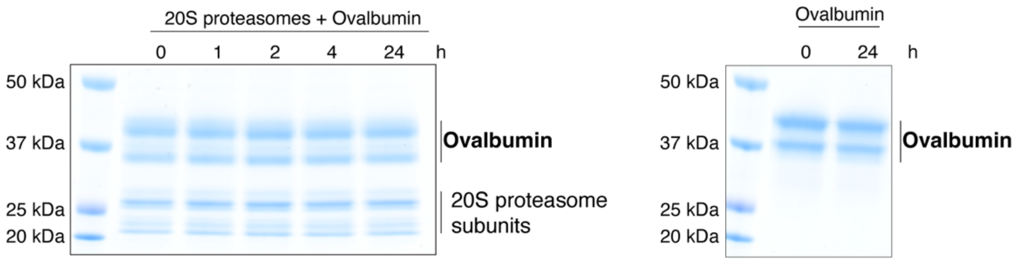

PDF

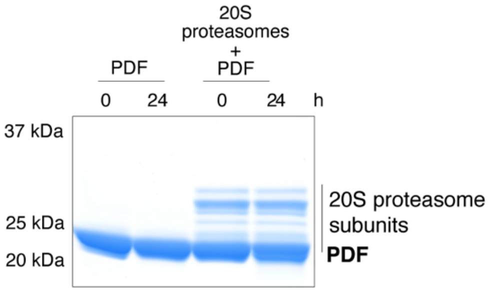

b (continued)

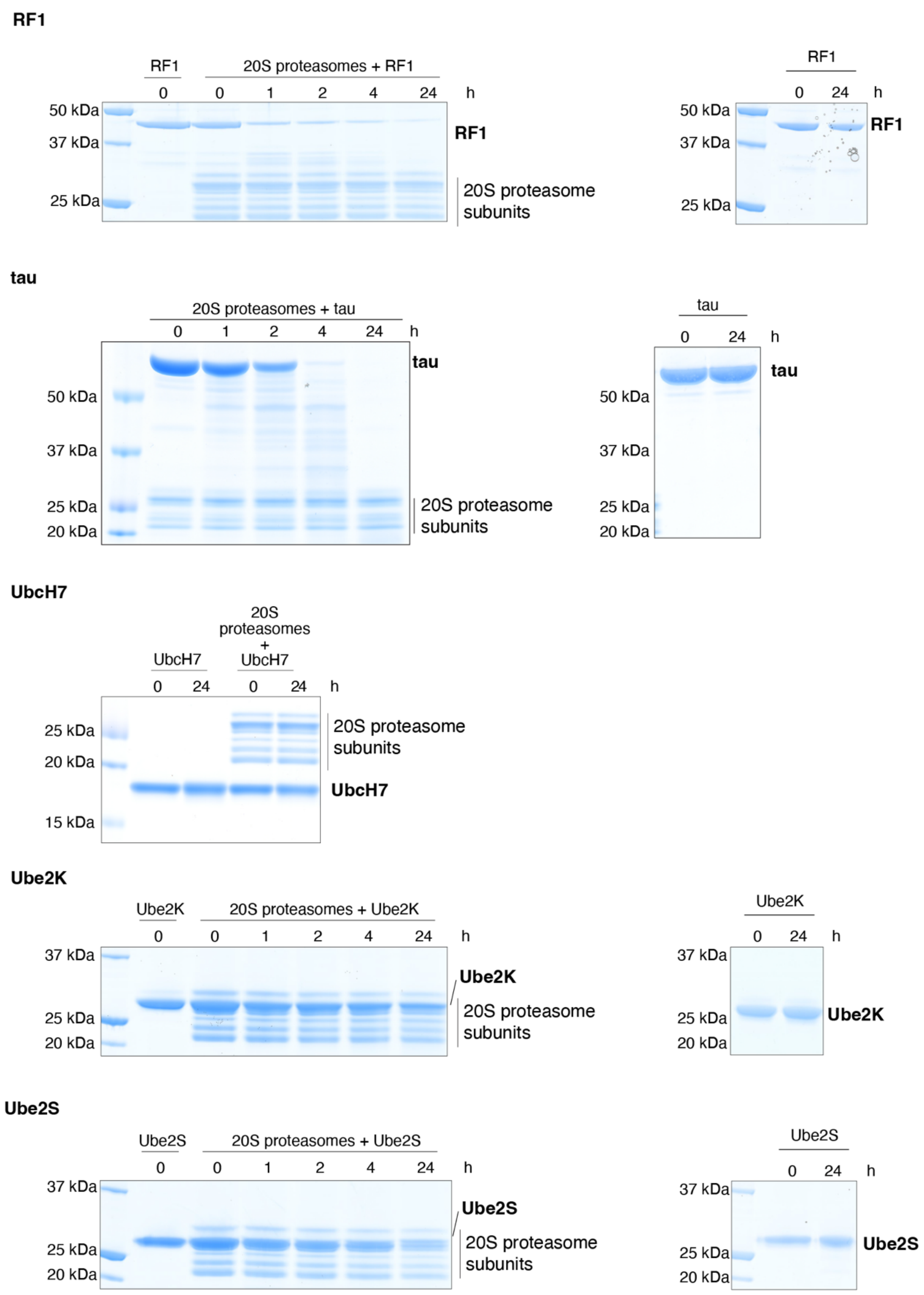

Supplementary Figure 1. Proteolysis of proteins by 20S proteasomes. (**a**) Degradation of human IL-1α, IL-1β, IL-37b and mouse IL-1α, IL-1β by purified 20S proteasomes, measured by western blot upon protein separation on SDS-PAGE gels. Either human or mouse proteasome α4 subunits were used as proteasome controls. (**b**) Coomassie blue stained SDS-PAGE gel of Annexin A1, CaM, EF-G, EF-Ts, Enolase1, Ffh, H2A, H2B, H3, H4, HUWE1, IF2, IL-37b, LEDGF, LRP130, Ovalbumin, PDF, RF1, tau, UbcH7, Ube2K, and Ube2S processed by human 20S standard proteasomes. In (**a**,**b**) both substrates and 20S standard proteasome subunits are visible. Source data are provided as a Source Data file.

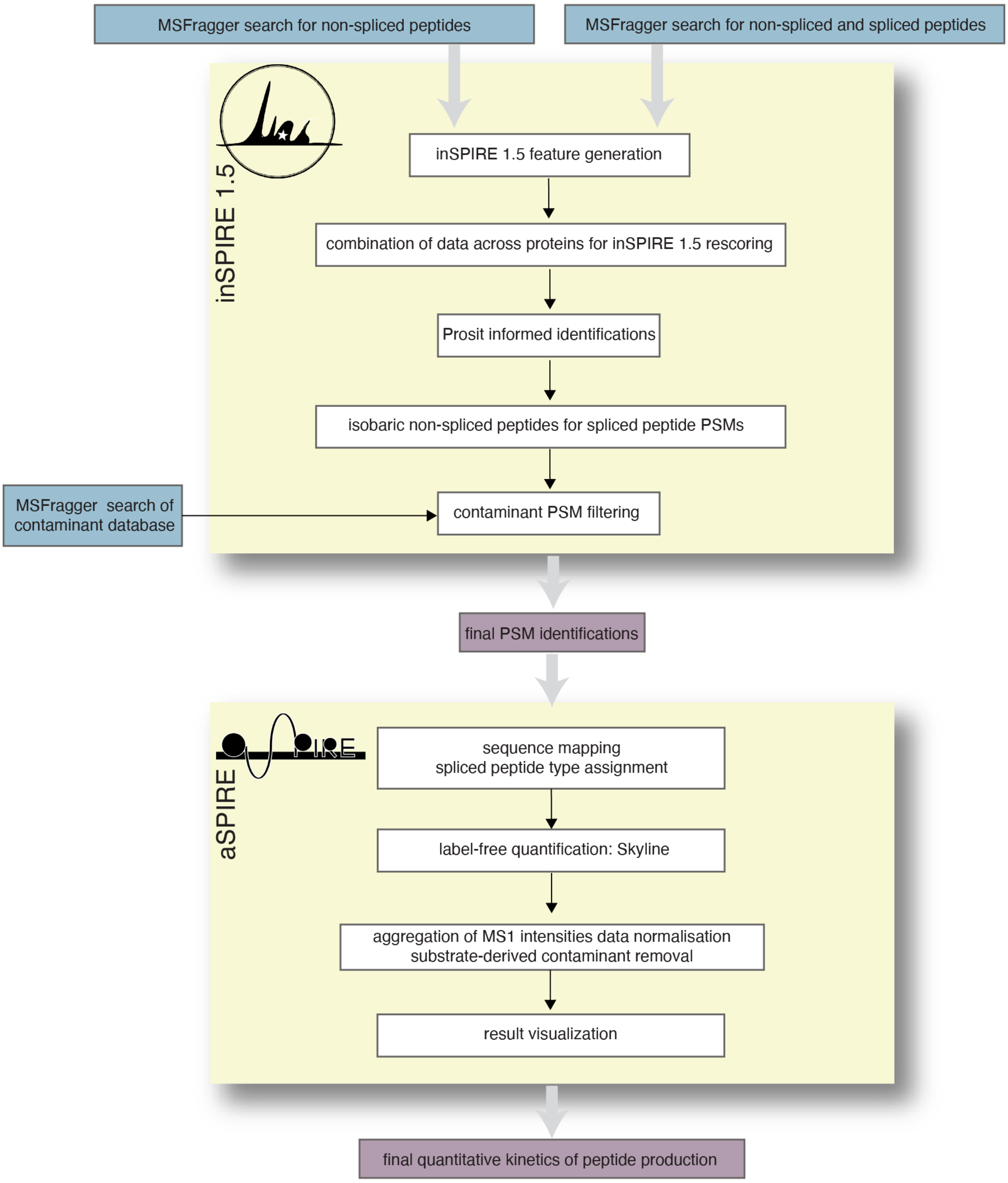

Supplementary Figure 2. Data processing workflow of the inSPIRE 1.5 - aSPIRE pipeline.

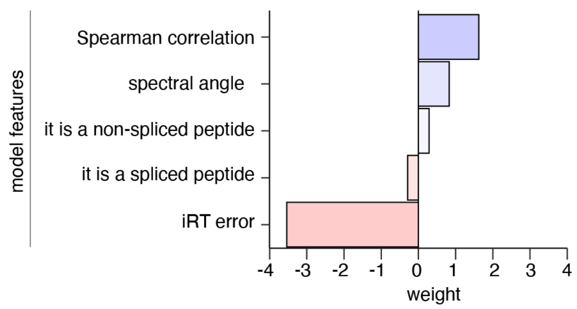

Supplementary Figure 3. Weights of the features considered by Percolator for peptide and PSM assignment. The weights learned by the Percolator model(s) to separate target and decoy PSMs in the analysis of the MS measurements. Percolator trains 3 models to avoid overfitting, the weights of which are shown, along with average weight across the data. PSMs with higher values in the positively weighted features (Spearman correlation, spectral angle, and a peptide being non-spliced) are more likely to be assigned by the model. PSMs with higher values in the negatively weighted features (retention time prediction error and a peptide being spliced) are less likely to be assigned by the model. Source data are provided as a Source Data file.

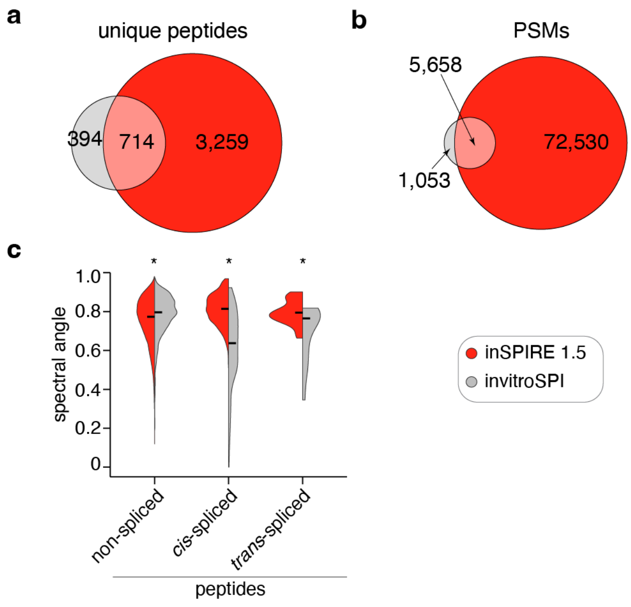

Supplementary Figure 4. Comparison of inSPIRE 1.5 and invitroSPI methods for the identification of non-spliced and spliced peptides produced in the processing of proteins by 20S proteasomes. (**a**,**b**) Number of unique non-spliced and spliced peptide products (**a**) and cognate PSMs (**b**) identified by applying inSPIRE and invitroSPI to the MS measurements of the digestion kinetics of 3 out of 15 degraded proteins (*i.e.* H4, IF2 and RF1) by human 20S proteasomes *in vitro*. The results are the sum of unique non-spliced and spliced peptides identified in at least one of the two biological replicates and the two technical replicates. (**c**) Spectral angle distribution computed between measured and Prosit-predicted MS2 spectra of peptide products identified by applying either inSPIRE 1.5 (n = 35,242, 1,411 and 20 non-spliced, *cis*-spliced and homologous *trans*-spliced PSMs, respectively) or invitroSPI (n = 3,671, 338 and 31 non-spliced, *cis*-spliced and homologous *trans*-spliced PSMs, respectively) to the same samples as in (**b**). In the violin plots, horizontal black lines represent the median. Statistically significant difference between pairs is labelled with * (Wilcoxon rank sum test with continuity correction; p-value < 0.05). In (**c**) only PSMs of peptides shorter than 13 amino acids are included, because of the dependency of Prosit performance on peptide length. Source data are provided as a Source Data file.

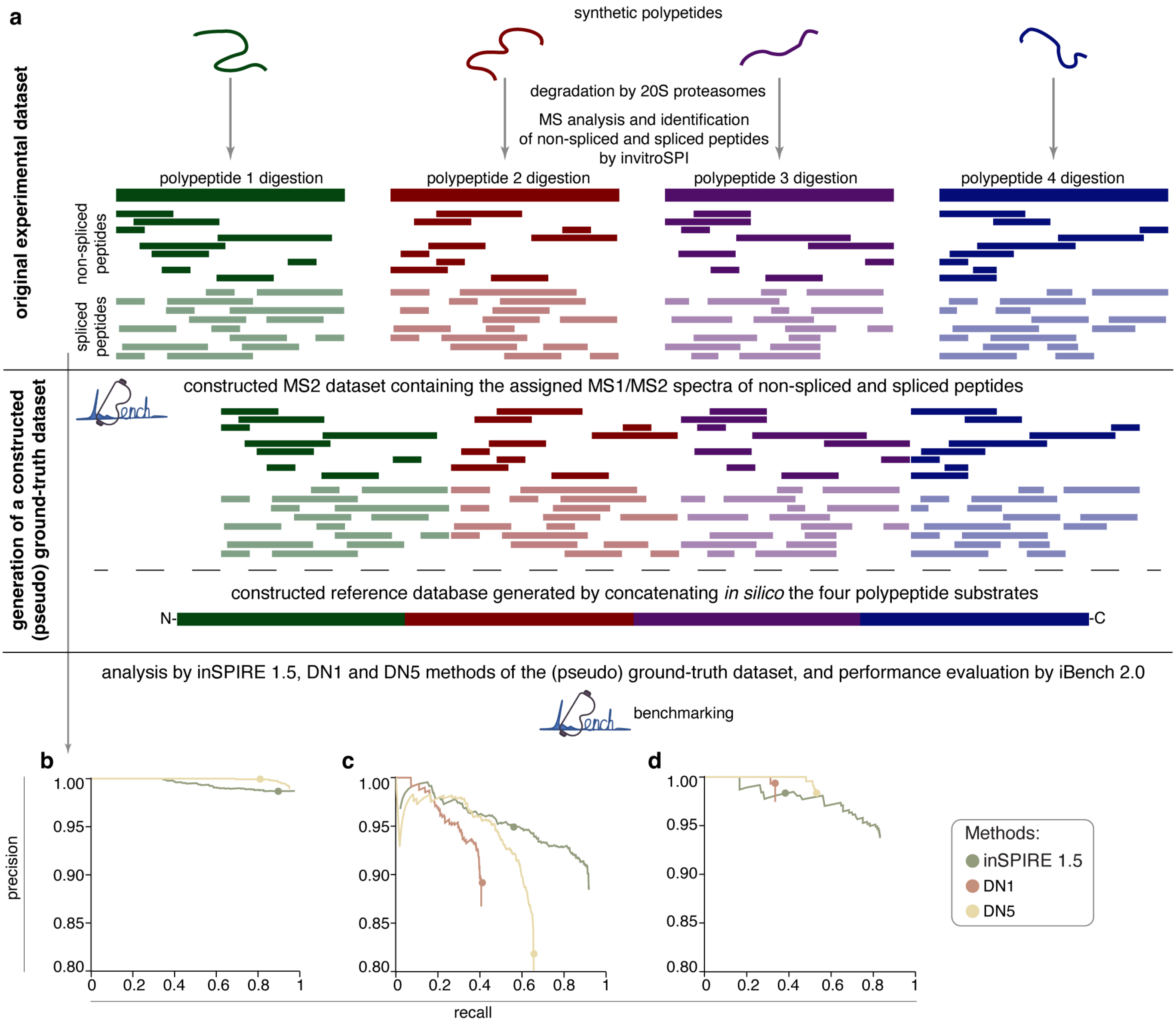

Supplementary Figure 5. Benchmarking of inSPIRE 1.5 against DN1 and DN5 methods in a (pseudo) ground-truth dataset containing both non-spliced and spliced peptides. Performance of inSPIRE 1.5 against two *de novo*-based strategies, DN1 and DN5, for the identification of non-spliced and spliced peptides in the (pseudo) ground-truth dataset generated by iBench 2.0 containing both non-spliced and spliced peptides, as represented schematically in (**a**). Performance is measured via precision-recall (PR) curves for non-spliced (**b**), *cis*-spliced (**c**), and homologous t*rans*-spliced (**d**) peptide identifications. The PR curves report precision – *i.e.*, number identified peptides labelled correct over number identified peptides – on the Y axis, and recall – *i.e.*, number identified peptides labelled correct over the total pool of peptides – on the X axis. The dots in each line correspond to the estimated 1% FDR. Source data are provided as a Source Data file.

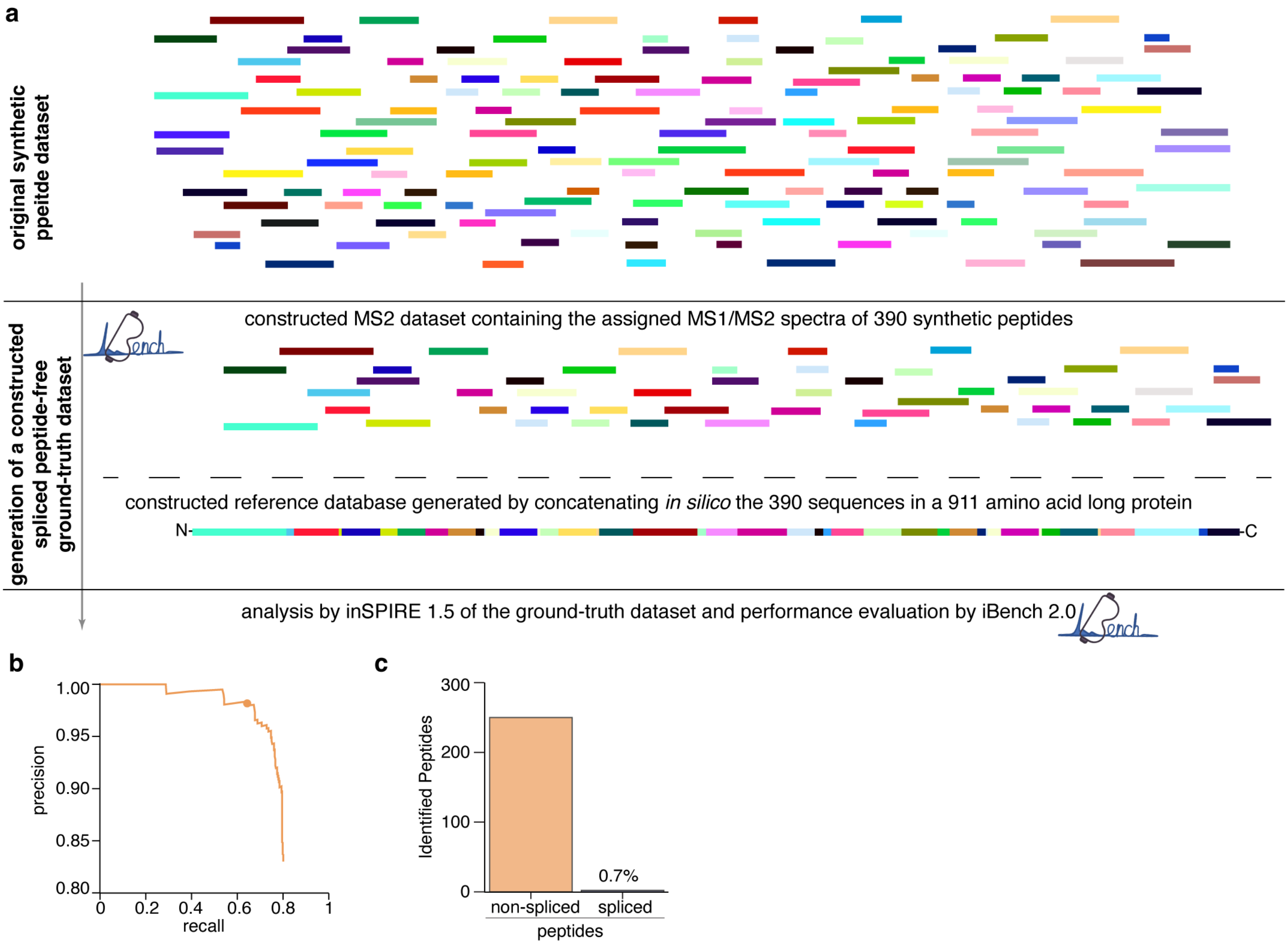

Supplementary Figure 6. Benchmarking of inSPIRE 1.5 in a spliced peptide-free dataset. (**a**) Schematic representation of the pipeline for the estimation of the performance of inSPIRE 1.5 in a spliced peptide-free dataset generated by iBench 2.0. (**b**) The PR curve for all peptides identified by inSPIRE 1.5. The dots in each line correspond to the estimated 1% FDR. Since spliced peptides were not present in the ground-truth dataset, the PR curve for the spliced peptide identification could not be generated. (**c**) The number of non-spliced and spliced peptides identified by inSPIRE 1.5 at the estimated 1% FDR threshold used throughout this study. Relative frequency of the wrongly assigned spliced peptides is reported. Source data are provided as a Source Data file.

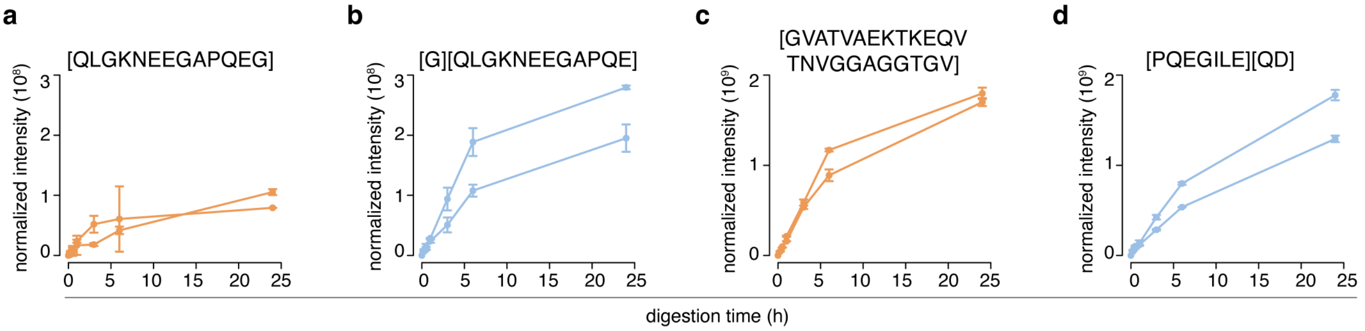

Supplementary Figure 7. Representative generation kinetics of non-spliced and *cis*-spliced peptides. (**a-d**) Generation kinetics of four representative peptides produced by 20S proteasomes whilst processing human α-Synuclein. The peptides were identified by inSPIRE 1.5 and quantified by aSPIRE. Because of the semi-quantitative nature of the label-free quantification of aSPIRE, the quantification of the area under the peak can be compared within the same peptide but individual peptides cannot be compared among each other. In the charts, each line depicts a biological replicate and the bars the SD of 2 technical replicates. The peptides in (**a**, **c**) are non-spliced peptides. The peptides in (**b**, **d**) are *cis*-spliced peptides. The assigned peptide sequence is reported. For the peptides in (**a,b**) the MS2 spectra and comparison with synthetic peptides as well as the peak in the MS extracted ion chromatogram is reported in **Fig. 3f**. Kinetics of all peptides identified and quantified in the 15 protein digestions are available in the online repository (see Source Data Section). Source data are provided as a Source Data file.

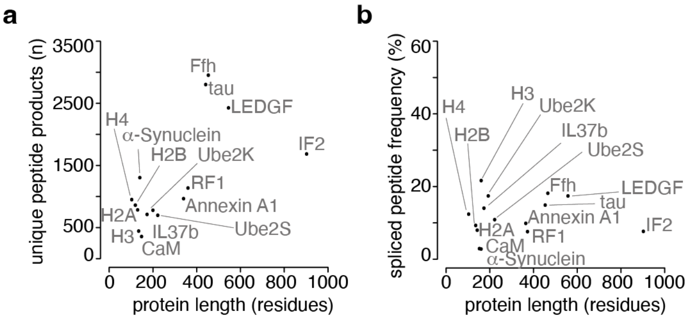

Supplementary Figure 8. Correlation between protein length and peptide products. (**a,b**) Correlation between the length (as number of residues) of the 15 degraded proteins and either the number of peptide products (**a**, PCC = 0.62, p-value = 0.01) or the relative frequency of spliced peptides (**b**, PCC = 0.12, p-value = 0.68) identified in the protein degradation. P-values were obtained via algorithm AS 89 on Fisher-transformed PCC. Source data are provided as a Source Data file.

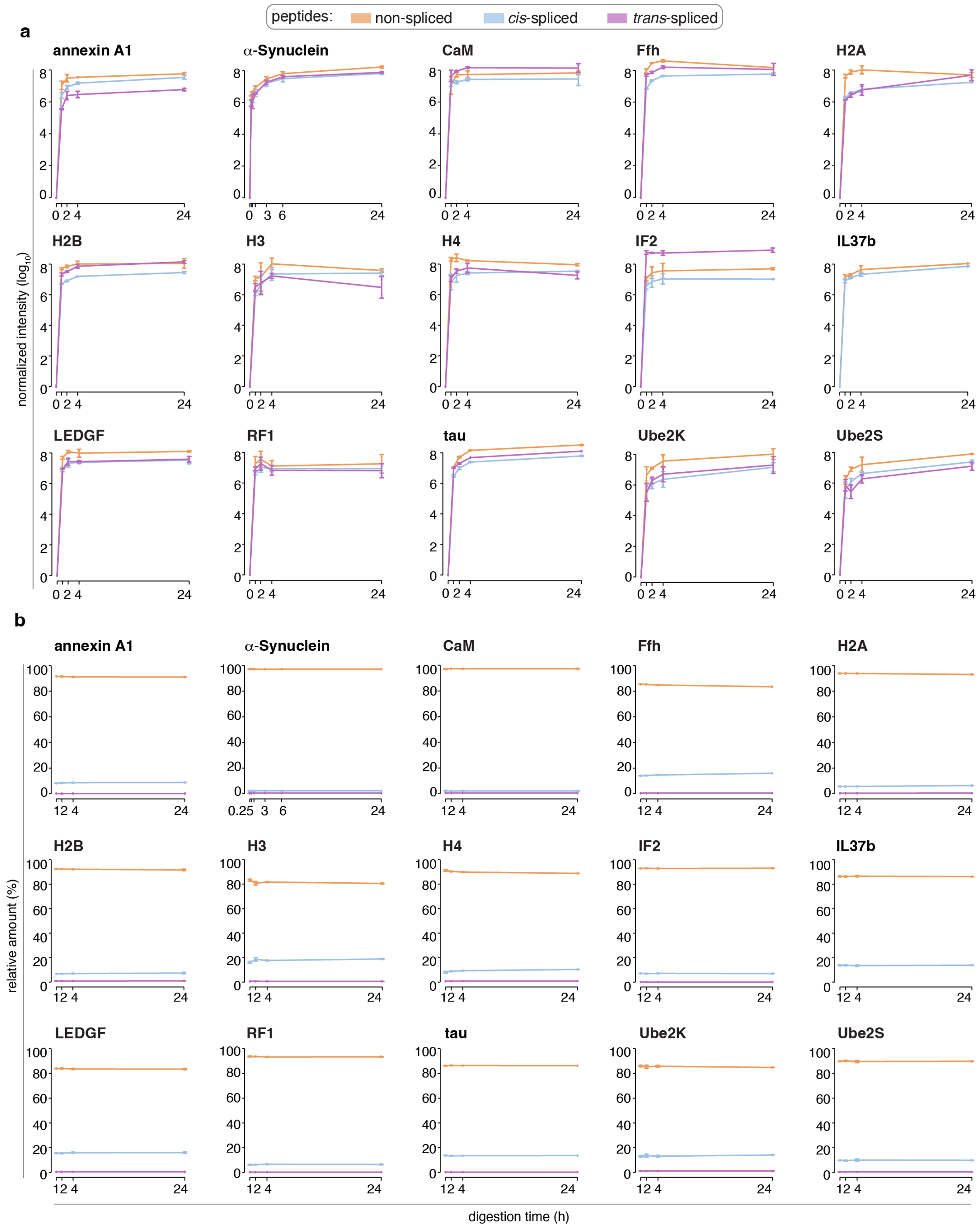

Supplementary Figure 9. Average and relative quantity of non-spliced and spliced peptides produced by proteasomes from each protein during the digestion kinetics. The average quantity of a non-spliced, *cis*-spliced and homologous *trans*-spliced peptide produced over time by 20S proteasomes during the *in vitro* digestions of the 15 proteins (**a**) and the relative quantity of all peptides of a given peptide type over time (**b**) are reported for each digested protein. Source data are provided as a Source Data file.

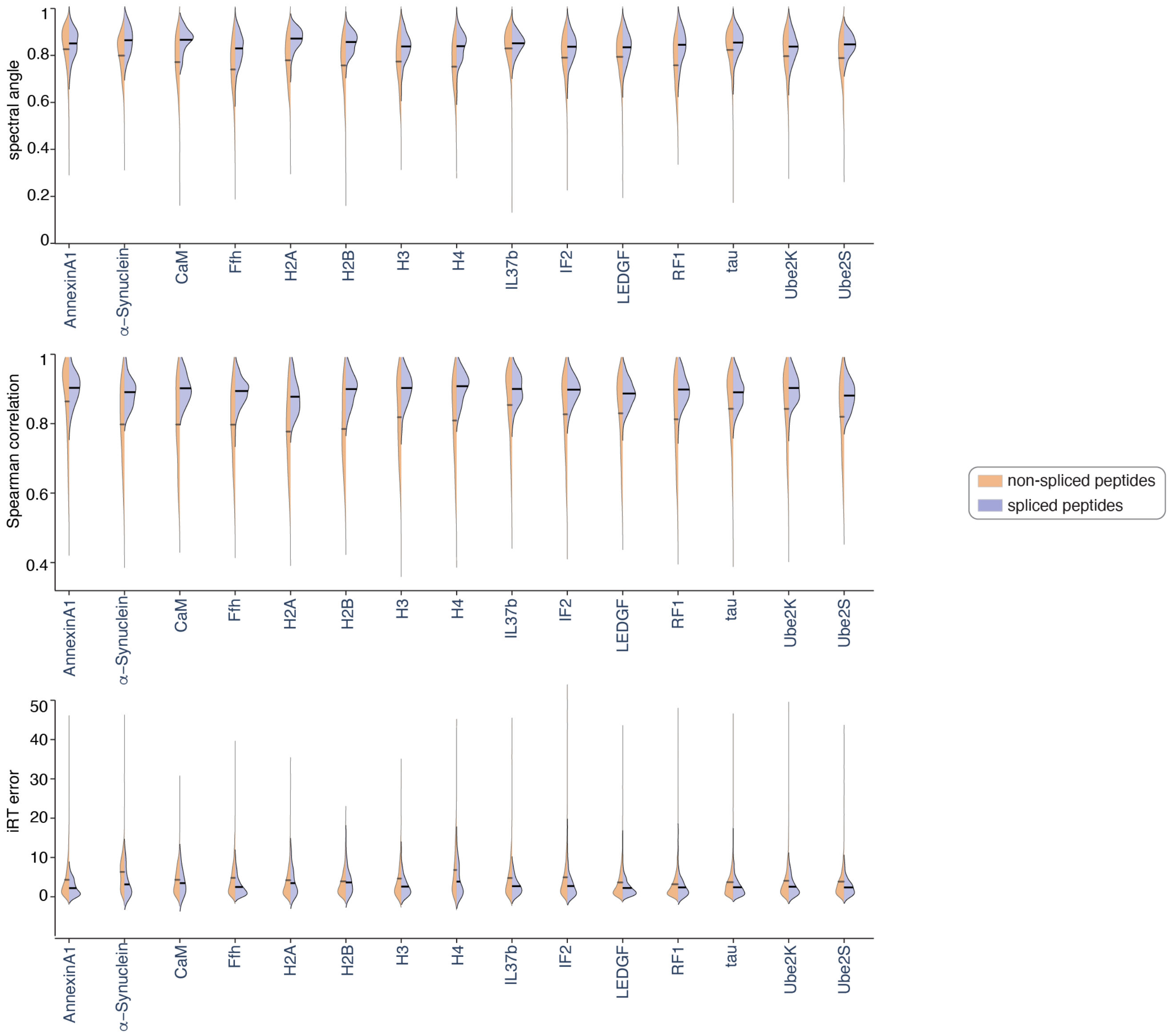

Supplementary Figure 10. Quality check of PSMs assigned to spliced and non-spliced peptide sequences. Distribution of the spectral angle, Spearman correlation and iRT error between the Prosit predicted spectrum of the non-spliced and spliced peptides and the cognate experimental MS2 spectra and the observed iRTs from which the peptides were assigned, for each protein digestion kinetic. Spliced and non-spliced peptide sequences were assigned at 1% FDR by inSPIRE 1.5. Details are reported in **Suppl. Data 3, 4**. In the violin plots, horizontal black lines represent the median. Briefly, high spectral angle, high Spearman correlation and low iRT error are markers of high precision in the PSM assignment. Source data are provided as a Source Data file.

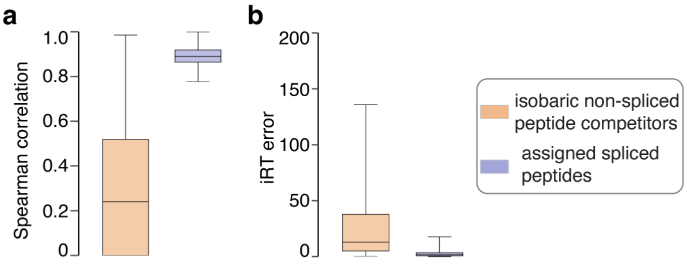

Supplementary Figure 11. Comparison of PSMs assigned as spliced peptides to the best possible isobaric non-spliced peptide competitor. Comparison of the PSMs which were assigned to spliced peptide sequences (n = 699) by inSPIRE 1.5 although they had an alternative isobaric non-spliced peptide sequence as competitor for the same MS2 spectrum. The box plots report the comparison of the assigned spliced peptides to the best possible non-spliced peptide competitors based on Spearman correlation (**a**) and iRT error distribution (**b**) of the relevant putative PSMs. In the box plots, the horizontal black lines represent the median of the values and bars the SD. Details are reported in **Suppl. Data 7-8**. Source data are provided as a Source Data file.

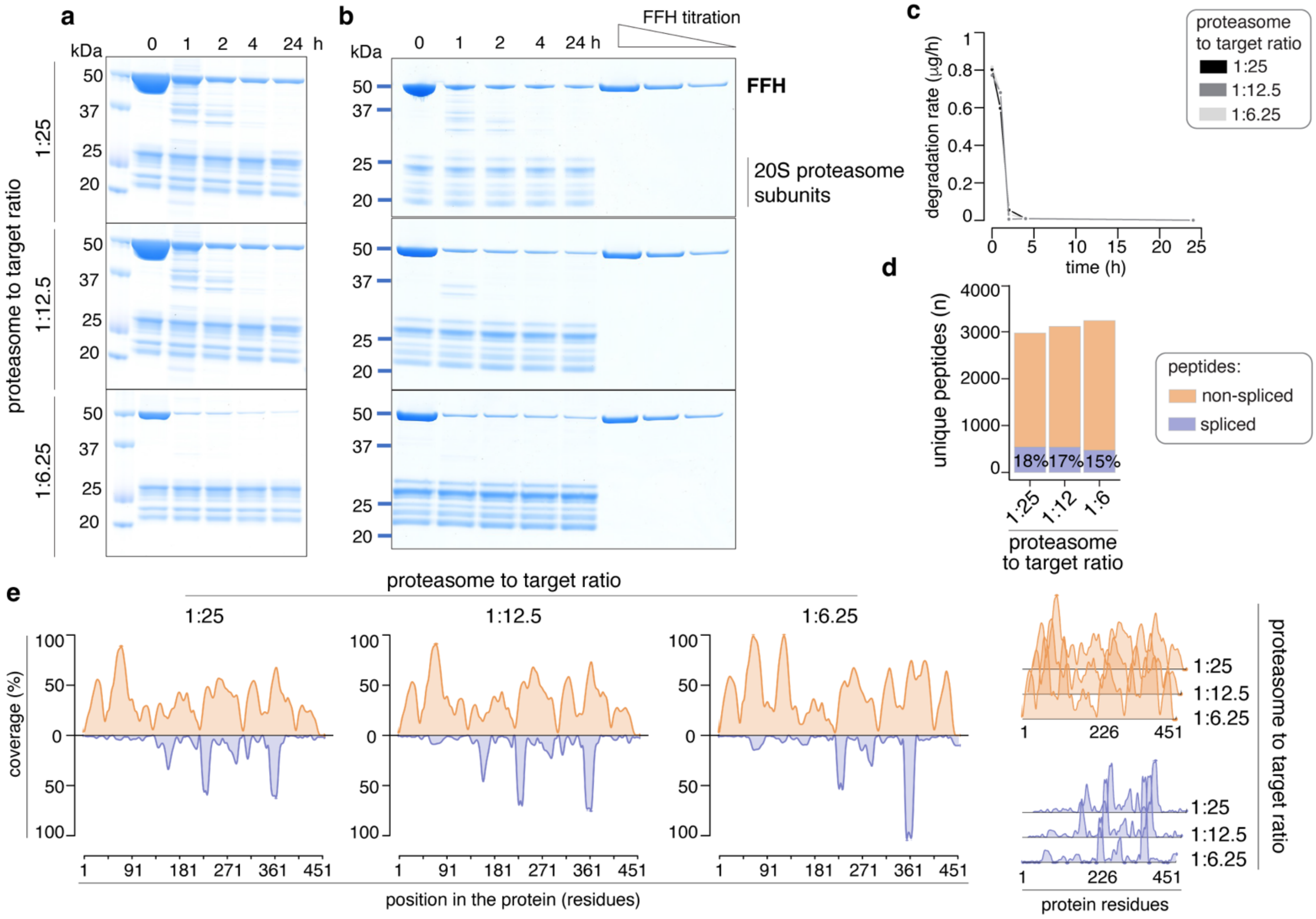

Supplementary Figure 12. Ffh degradation and peptide production by 20S proteasomes varying the proteasome to target molar ratio. (**a,b**) Coomassie blue stained SDS-PAGE gel of Ffh processed by human 20S standard proteasomes using three proteasome to target molar ratios, *i.e.*, 0.2 µM proteasomes and either 5 µM (1:25), 2.5 µM (1:12.5) or 1.25 µM (1:6.25) Ffh substrate. In the gels, both substrates and 20S standard proteasomes subunits are visible. In (**a**) same sample volume was loaded into the gel, thereby corresponding to different initial substrate amount and same proteasome amount. In (**b**) equal initial amount of the substrate has been loaded in the gel. Ffh titration (*i.e.*, 467 ng, 233 ng and 118 ng were loaded) has been used for the Ffh band quantification in the digestions. (**c**) Ffh degradation rate upon quantification of the Ffh substrate band at 50 kDa in the gels shown in (**b**). (**d**) Number and relative frequency of unique non-spliced and spliced peptides identified in the degradation of Ffh. (**e**) Ffh substrate coverage profiles by the amount of non-spliced and spliced peptide products identified in the three conditions. In (**d,e**) the same amount of initial substrate present in the reaction was loaded in the MS, similarly to what is shown in (**b**). Source data are provided as a Source Data file.

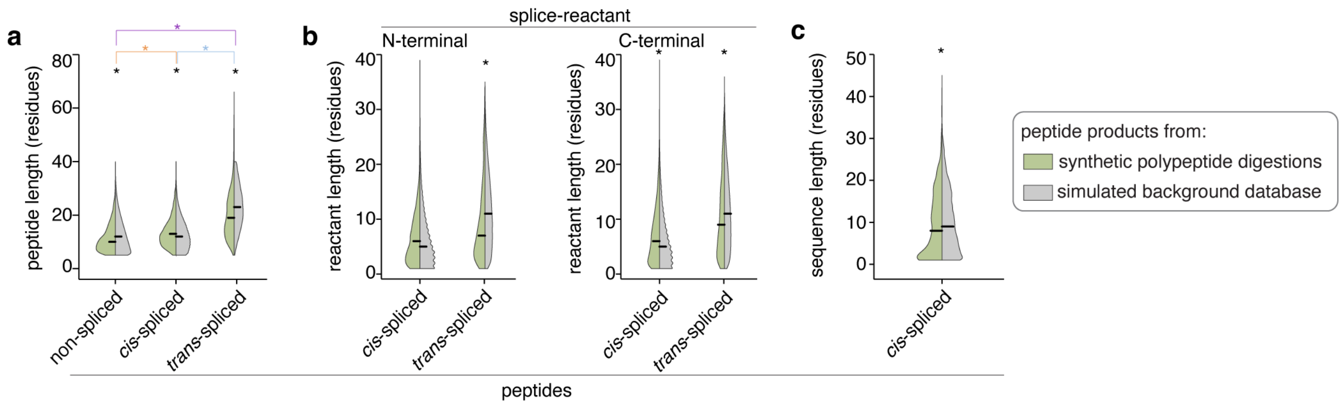

Supplementary Figure 13. Length of non-sliced and spliced peptides, splice-reactants and intervening sequences produced during the *in vitro* degradation of synthetic polypeptides. In (**a-c**) the observations in the experimentally identified peptide products were compared to those derived from a random database computed from the same substrate sequences. Experimentally identified peptides were detected by applying invitroSPI to the dataset of 80 synthetic polypeptides degraded by human 20S proteasomes. In the violin plots, the horizontal black lines represent the median of the values. (**a**) Peptide length distribution of non-spliced and *cis*-spliced peptides. (**b**) Splice-reactant lengths among *cis*- and homologous *trans*-spliced peptides. (**c**) Intervening sequence lengths among *cis*-spliced peptides. In (**a-c**), qualitative analysis, *i.e.,* counting the number of unique peptides, was used. Statistically significant difference between pairs is labelled with * (Wilcoxon rank sum test with continuity correction; p-value < 0.05). Source data are provided as a Source Data file.

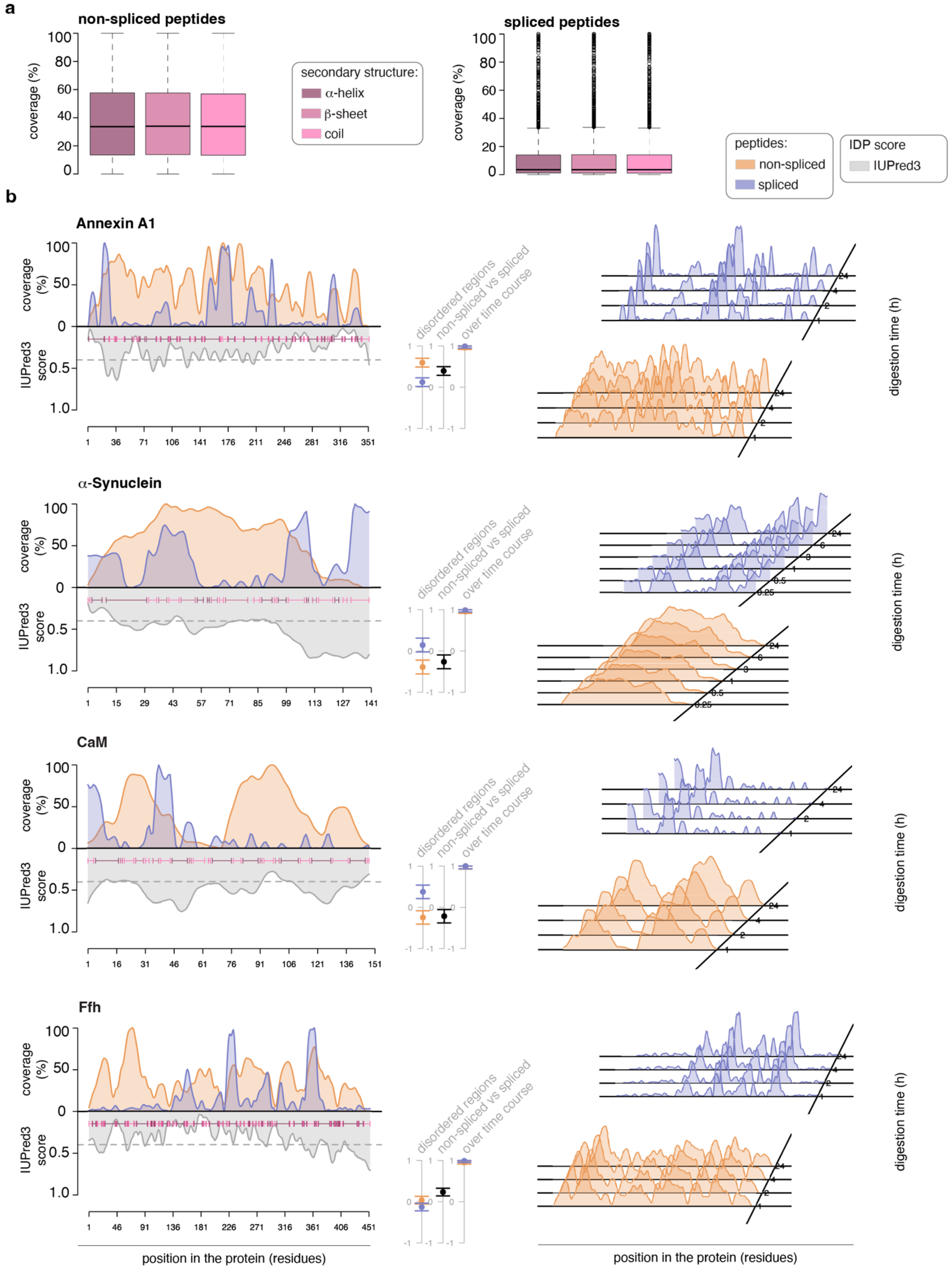
a
non-spliced peptides
spliced peptides
coverage (%)
secondary structure:
α-helix
β-sheet
coil
peptides:
non-spliced
spliced
IDP score
IUPred3
b
Annexin A1
α-Synuclein
CaM
Ffh
coverage (%)
IUPred3 score
disordered regions non-spliced vs spliced over time course
digestion time (h)
position in the protein (residues)

b (continued)

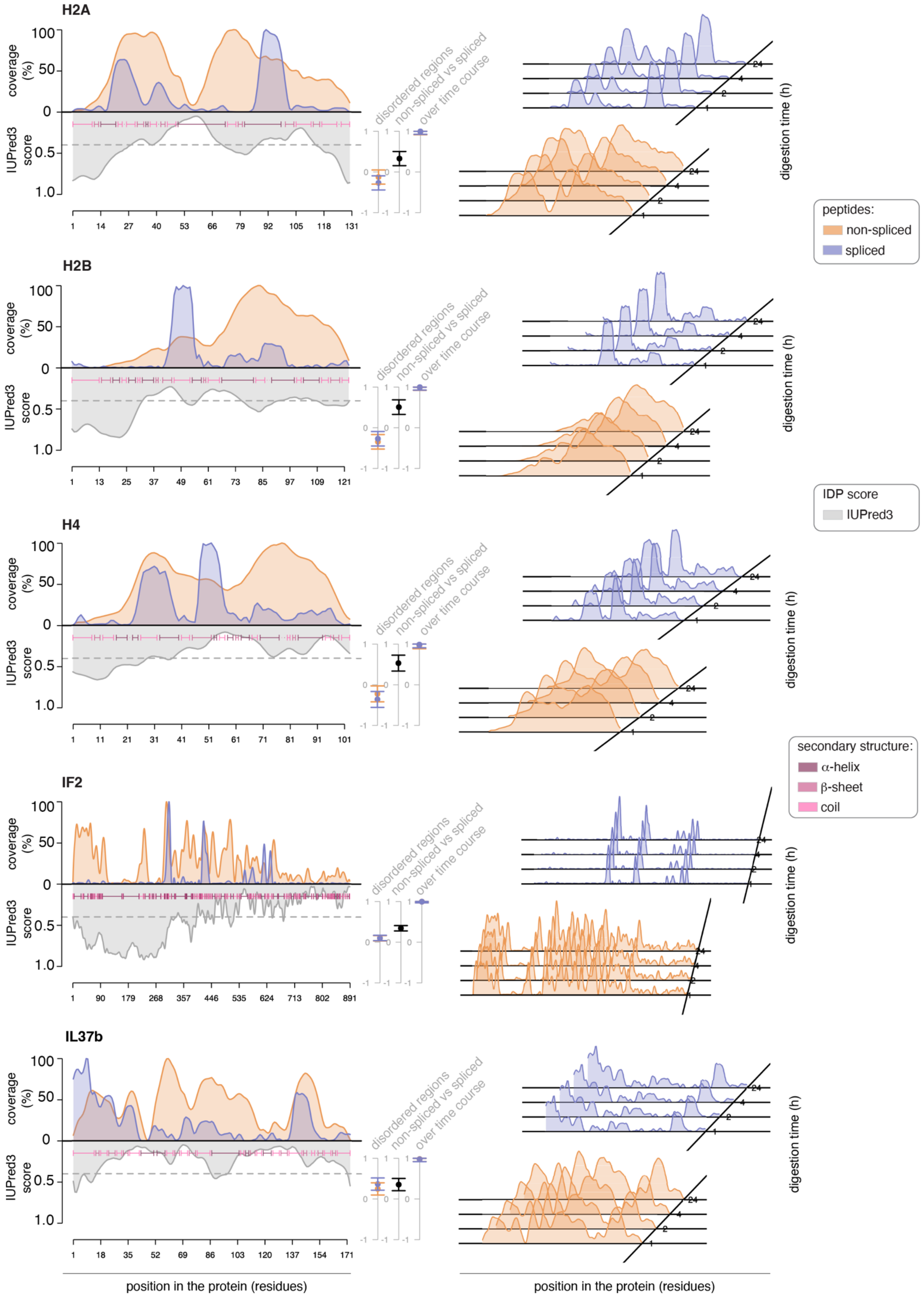
H2A
H2B
H4
IF2
IL37b
coverage (%)
IUPred3 score
disordered regions non-spliced vs spliced over time course
digestion time (h)
peptides:
non-spliced
spliced
IDP score
IUPred3
secondary structure:
α-helix
β-sheet
coil
position in the protein (residues)
position in the protein (residues)

b (continued)

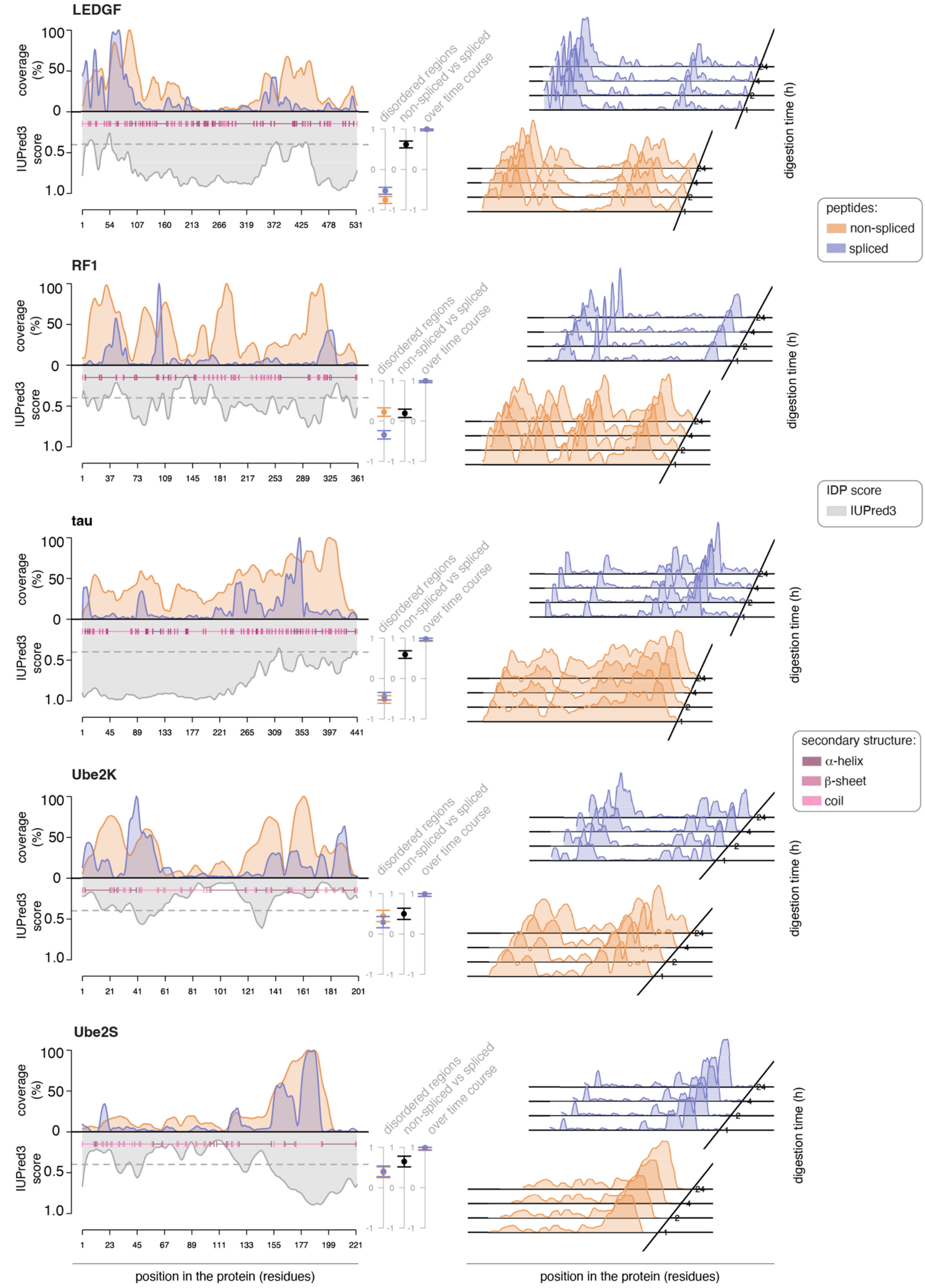
LEDGF
RF1
tau
Ube2K
Ube2S
coverage (%)
IUPred3 score
disordered regions
non-spliced vs spliced
over time course
digestion time (h)
position in the protein (residues)
peptides:
non-spliced
spliced
IDP score
IUPred3
secondary structure:
α-helix
β-sheet
coil

Supplementary Figure 14. Preference for protein regions by peptide hydrolysis and peptide splicing during 20S proteasome-mediated degradation of proteins and correlation to protein features. (**a**) Distribution of normalized coverage values across different secondary structure elements. (**b**) Protein coverage profiles by non-spliced and spliced peptide products of *in vitro* 20S proteasomal digestions of 14 out of 15 degraded proteins. The results of H3 processing are shown in **Fig. 6a-b**. The coverage is compared to the IUPred3 score predicting the presence of disordered protein segments. The grey dashed line represents the score = 0.4, which is used as threshold for a disordered segment. Predicted secondary structure elements are depicted as pink bars. The Spearman correlation coefficients and confidence intervals in the inlets denote: (i) correlation between spliced (blue)/non-spliced (orange) sequence coverage and IUPred3 score, (ii) correlation between spliced and non-spliced sequence coverage and (iii) correlation between spliced coverage profiles at all time points (blue) and non-spliced coverage profiles at all time points. Dots represent estimated Spearman correlation coefficients and error bars denote the confidence interval. Coverage profiles were normalized for spliced/non-spliced peptides separately. In the right panel, the protein coverage profiles at different digestion time points are shown. Here, coverage profiles were normalized for spliced/non-spliced peptides across all time points. Source data are provided as a Source Data file.

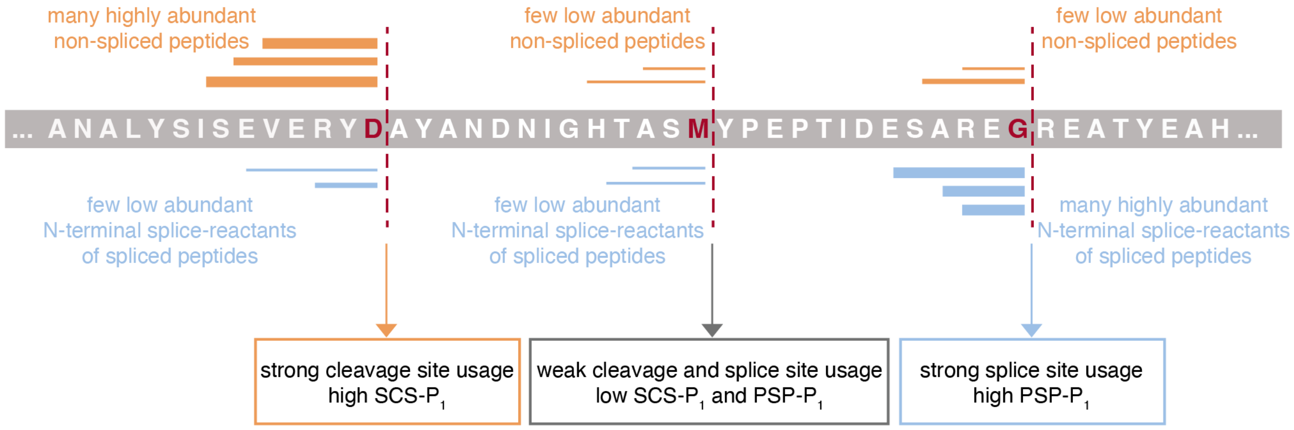

Supplementary Figure 15. Schematic illustrating the meaning of SCS-P_1 and PSP-P_1.

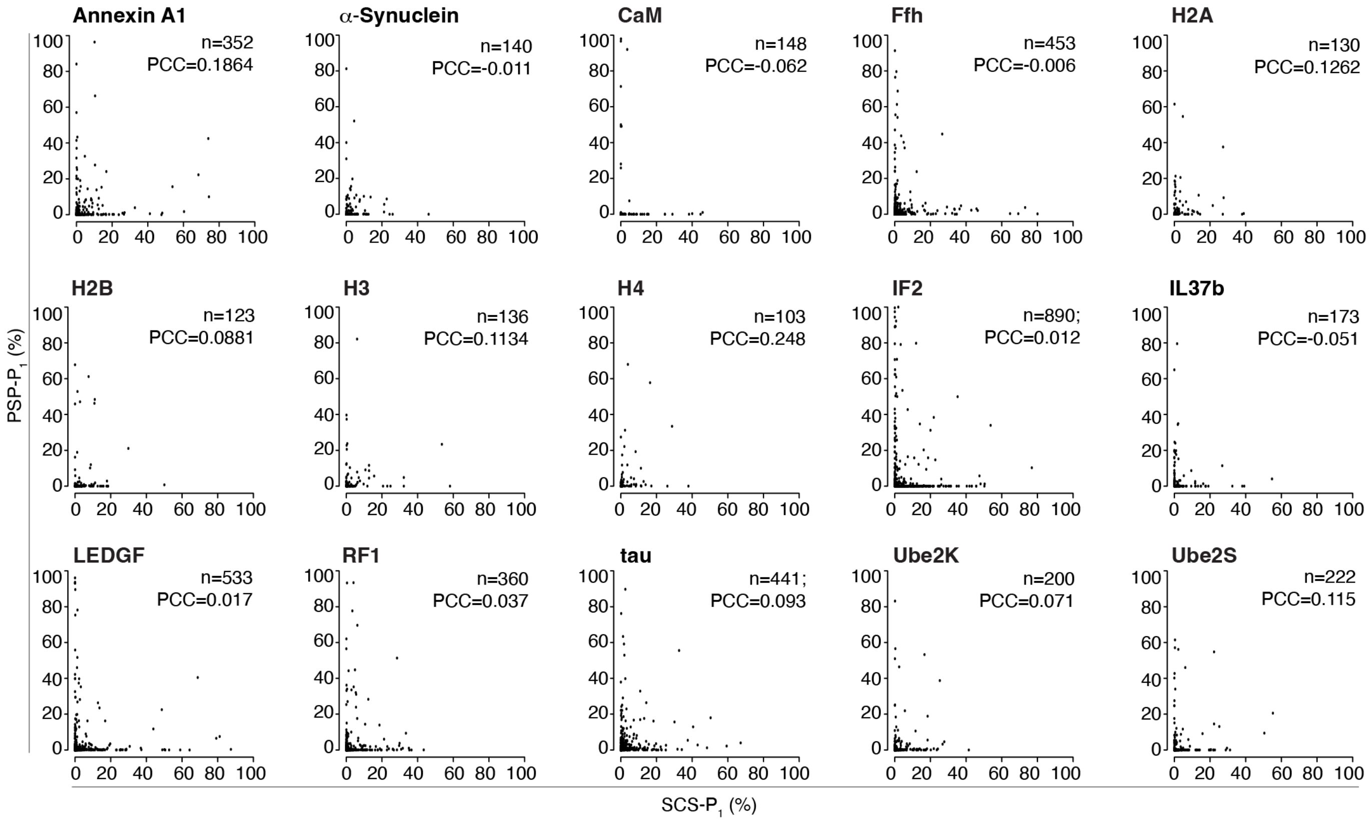

Supplementary Figure 16. Correlation between proteins' SCS-P_1 and PSP-P_1 upon 20S proteasomal digestion. Correlation between SCS-P_1 and PSP-P_1 of the 15 efficiently degraded proteins. Analysis has been done using the estimated quantity of each peptide by aSPIRE. Each dot represents a substrate residue. The number of residues and the Pearson's product moment correlation coefficients (PCC) are reported for each protein. Source data are provided as a Source Data file.

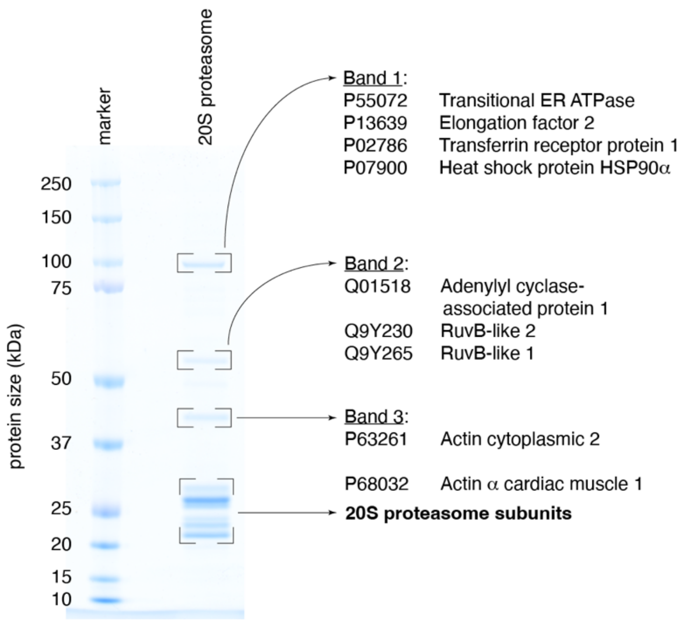

Supplementary Figure 17. 20S proteasome purity. Coomassie blue stained SDS-PAGE gel of human 20S standard proteasome purified from HeLa cell lines. The blot shows the standard 20S proteasome subunits pattern between 20 and 30kDa. 4 μg 20S proteasomes were loaded on a 12% SDS-PAGE gel. The identities of the various protein bands migrating between 37-100 kDa were determined by excision of the individual band for in-gel tryptic digestion followed by LC-MS/MS analysis. The protein identities shown are based on the top intensities, molecular weight matches and a high number of PSM. The PEAKS DB search results can be found in **Suppl. Data 9**.

Protein	UniProt ID	Protein name	Organism	Length (amino acids)	Notes
Annexin A1	P04083	Annexin A1	Human	352	6-His tag at C-terminus
α-Synuclein	P37840	Alpha-synuclein	Human	140	
CaM	P0DP23	Calmodulin-1	Human	148	
EF-G	P0A6M8	Elongation factor G	*Escherichia coli*	704	
EF-Ts	P43897	Elongation factor Ts, mitochondrial	*Escherichia coli*	325	
Enolase1	P00924	Enolase 1	*Saccharomyces cerevisiae*	437	
Ffh	P0AGD7	Signal recognition particle protein	*Escherichia coli*	453	
H2A	Q6DKE3	Histone H2A	*Xenopus laevis*	130	
H2B	A0A1L8FQ56	Histone H2B	*Xenopus laevis*	123	
H3	A0A1L8EMY5	Histone H3	*Xenopus laevis*	136	
H4	A0A1L8G0S8	Histone H4	*Xenopus laevis*	103	
hIL-1α	P01583	Interleukin-1 alpha	Human	159	Mature form
hIL-1β	P01584	Interleukin-1 beta	Human	153	Mature form
HUWE1	Q7Z6Z7	E3 ubiquitin-protein ligase HUWE1	Human	384	HECT domain, D_{3993}-A_{4374}
IF2	P0A705	Translation initiation factor IF-2	*Escherichia coli*	890	
IL-37b	Q9NZH6	Interleukin-37	Human	173	Mature form
LEDGF	O75475	PC4 and SFRS1-interacting protein	Human	533	
LRP130	P42704	Leucine-rich PPR motif-containing protein, mitochondrial	Human	1338	
mIL-1α	P01582	Interleukin-1 alpha	Mouse	156	Mature form
mIL-1β	P10749	Interleukin-1 beta	Mouse	152	Mature form
Ovalbumin	P01012	Ovalbumin	Chicken	386	
PDF	P0A6K3	Peptide deformylase	*Escherichia coli*	169	
RF1	P0A7I0	Peptide chain release factor RF1	*Escherichia coli*	360	
tau	P10636-8	Microtubule-associated protein tau	Human	441	
UbcH7	P68036	Ubiquitin-conjugating enzyme E2 L3	Human	156	
Ube2K	P61086	Ubiquitin-conjugating enzyme E2 K	Human	200	
Ube2S	Q16763	Ubiquitin-conjugating enzyme E2 S	Human	222	

Supplementary Table 1. Proteins investigated in the study.
