# Supplementary material for: Protein degradation by human 20S proteasomes elucidates the interplay between peptide hydrolysis and splicing
Source: Nat Commun. 2024 Feb 7;15:1147. doi: 10.1038/s41467-024-45339-3 (PMC10850103; doi:10.1038/s41467-024-45339-3)
Supplement: Supplementary file 3 — Supplementary Data 1 [file 41467_2024_45339_MOESM3_ESM.pdf]

Supplementary Data 1. Target protein sequences. Sequences are provided in single letter amino acid code.

Protein	Sequence
Annexin A1	MAMVSEFLKQAWFIENEEQEYVQTVKSSKGGPGSAVSPYPTFNPSSDVAALHKAIMVKGV<br>DEATIIDILTKRNNAQRQQIKAAYLQETGKPLDETLKKALTGHLEEVVLALLKTPAQFDADEL<br>RAAMKGLGTDEDTLIEILASRTNKEIRDINRVYREELKRDLAKDITSDTSGDFRNALLSLAKG<br>DRSEDFGVNEDLADSDARALYEAGERRKGTDVNVFNTILTTRSYPQLRRVFQKYTKYSKH<br>DMNKVLDLELKGDIEKCLTAIVKCATSKPAFFAEKLHQAMKGVGTRHKALIRIMVSRSEIDM<br>NDIKAFYQKMYGISLCQAILDETKGDYEKILVALCGGNHHHHHH
α-Synuclein	MDVFMKGLSKAKEGVVAAAEKTKQGVAEAAGKTKEGVLYVGSKTKEGVVHGVATVAEKT<br>KEQVTNVGGAVVTGVTAVAQKTVEGAGSIAAATGFVKKDQLGKNEEGAPQEGILEDMPVD<br>PDNEAYEMPSEEGYQDYEPEA
CaM	ADQLTEEQIAEFKEAFSLFDKDGDGTITTKELGTVMRSLGQNPTEAELQDMINEVDADGNG<br>TIDFPEFLTMMARKMKDTDSEEEIREAFRVFDKDGNGYISAAELRHVMTNLGEKLTDEEVD<br>EMIREADIDGDGQVNYEEFVQMMTAK
EF-G	MARTTPIARYRNIGISAHIDAGKTTTTERILFYTGVNHKIGEVHDGAATMDWMEQEQERGITI<br>TSAATTAFWSGMAKQYEPHRINIIDTPGHVDFTIEVERSMRVLDGAVMVYCAVGGVQPQS<br>ETVWRQANKYKVPRIAFVNKMDRMGANFLKVVNQIKTRLGANPVPLQLAIGAEEHFTGVV<br>DLVKMKAINWNDADQGVTFEYEDIPADMVELANEWHQNLIESAAEASEELMEKYLGGEEL<br>TEAEIKGALRQRVLNNEIILVTCGSAFKNKGVQAMLDAVIDYLPSPVDVPAINGILDDGKDTP<br>AERHASDDEPFSALAFKIATDPFVGNLTFFRVYSGVVNSGDTVLNSVKAARERFGRIVQMH<br>ANKREEIKEVRAGDIAAAIGLKDVTTGDTLCDPDAPIILERMEFPEPVISIAVEPKTKADQEKM<br>GLALGRLAKEDPSFRVWTDEESNQTIIAGMGELHLDIIVDRMKREFNVEANVGKPQVAYRE<br>TIRQKVTDVEGKHAKQSGGRGQYGHVVIDMYPLEPGSNPKGYEFINDIKGGVIPGEYIPAV<br>DKGIQEQLKAGPLAGYPVVDMGIRLHFGSYHDVDSSELAFKLAASIAFKEGFKKAKPVLLEP<br>IMKVEVETPEENTGDVIGDLSRRRGMLKGQESEVTGVKIHAEVPLSEMFGYATQLRSLTKG<br>RASYTMEFLKYDEAPSNVAQAVIEARGK
EF-Ts	MSLLRSLRVFLVARTGSYPAGSLLRQSPQPRHTFYAGPRLSASASSKELLMKLRRKTGYSF<br>VNCKKALETCGGDLKQAEIWLHKEAQKEGWSKAAKLQGRKTKEGLIGLLQEGNTTVLVEV<br>NCETDFVSRNLKFQLLVQQVALGTMMHCQTLKDQPSAYSKGFLNSSELSGLPAGPDREG<br>SLKDQLALAIGKLGENMILKRAAWVKVPSGFYVGSYVHGAMQSPSLHKLVLGKYGALVICE<br>TSEQKTNLEDVGRRLGQHVVGMAPLSVGSLDDEPGGEAETKMLSQPYLLDPSITLGQYVQ<br>PQGVSVVDFVRFECGEGEEAAETE
Enolase1	MAVSKVYARSVYDSRGNPTVEVELTTEKGVFRSIVPSGASTGVHEALEMRDGDKSKWMG<br>KGVLHAVKNVNDVIAPAFVKANIDVKDQKAVDDFLISLDGTANKSKLGANAILGVSLAASRA<br>AAAEKNVPLYKHLADLSKSKTSPYVLPVPFLNVLNGGSHAGGALALQEFMIAPTGAKTFAE<br>ALRIGSEVYHNLKSLTKKRYGASAGNVGDEGGVAPNIQTAEEALDLIVDAIKAAGHDGKIKIG<br>LDCASSEFFKDGKYDLDFKNPNSDKSKWLTGPQLADLYHSLMKRYPIVSIEDPFAEDDWE<br>AWSHFFKTAGIQIVADDLTVTNPKRIATAIEKKAADALLLKVNQIGTLSESIKAAQDSFAAGW<br>GVMVSHRSGETEDTFIADLVVGLRTGQIKTGAPARSERLAKLNQLLRIEEELGDNAVFAGE<br>NFHHGDKL
Ffh	MFDNLTDRLSRTLRNISGRGRLTEDNVKDTLREVRMALLEADVALPVVREFINRVKEKAVG<br>HEVNKSLTPGQEFVKIVRNELVAAMGEENQTLNLAAQPPAVVLMAGLQGAGKTTSVGKLG<br>KFLREKHKKKVLVVSADVYRPAAIKQLETLAEQVGVDFFPSDVGQKPVDIVNAALKEAKLKF<br>YDVLLVDTAGRLHVDEAMMDEIKQVHASINPVETLFVVDAMTGQDAANTAKAFNEALPLTG<br>VVLTKVDGDARGGAALSIRHITGKPIKFLGVGEKTEALEPFHPDRIASRILGMGDVLSLIEDIE<br>SKVDRAQAEKLASKLKKGDGFDLNDFLEQLRQMKNMGGMASLMGKLPGMGQIPDNVKS<br>QMDDKVLVRMEAIINSMTMKERAKPEIIKGSRKRRIAAGCGMQVQDVNRLLKQFDDMQRM<br>MKKMKKGGMAKMMRSMKGMMPPGFPGR
H2A	MSGRGKQGGKTRAKAKTRSSRAGLQFPVGRVHRLLRKGNYAERVGAGAPVYLAAVLEYLT<br>AEILELAGNAARDNKKTRIIPRHLQLAVRNDEELNKLLGRVTIAQGGVLPNIQSVLLPKKTESS<br>KSAKSK
H2B	MAKSAPAPKKGSKKAVTKTQKKDGKKRRKTRKESYAIYVYKVLKQVHPDTGISSKAMSIMNS<br>FVNDVFERIAGEASRLAHYNKRSTITSREIQTAVRLLLPGELAKHAVSEGTKAVTKYTSAK

H3	MARTKQTARKSTGGKAPRKQLATKAARKSAPATGGVKKPHRYRPGTVALREIRRYQKSTELL<br>IRKLPFQRLVREIAQDFKTDLRFQSSAVMALQEASEAYLVALFEDTNLCAIHAKRVTIMPKDI<br>QLARRIRGERA
H4	MSGRGKGGKGLGKGGAKRHRKVLRDNIQGITKPAIRRLARRGGVKRISGLIYEETRGVLKVFL<br>ENVIRDAVTYTEHAKRKTVTAMDVVYALKRQGRTLYGFGG
hIL-1α	SAPFSFLSNVKYNFMRIIKYEFILNDALNQSIIRANDQYLTAAALHNLDEAVKFDMGAYKSSK<br>DDAKITVILRISKTQLYVTAQDEDQPVLLKEMPEIPKTITGSETNLLFFWETHGTKNYFTSVA<br>HPNLFIATKQDYWVCLAGGPPSITDFQILENQA
hIL-1β	APVRSLNCTLRDSQQKSLVMSGPYELKALHLQGQDMEQQVVFSMSFVQGEESNDKIPVA<br>LGLKEKNLYLSCVLKDDKPTLQLESVDPKNYPKKKMEKRFVFNKIEINNKLEFESAQFPNW<br>YISTSQAENMPVFLGGTKGGQDITDFTMQFVSS
HUWE1	GPDFDVKRKYFRQELERLDEGLRKEDMAVHVRRDHVFEDSYRELHRKSPEEMKNRLYIVFE<br>GEEGQDAGGLLREWYMIISREMFNPMYALFRTSPGDRVTYTINPSSHCNPNHLSYFKFVGRI<br>VAKAVYDNRLLECYFTRSFYKHILGKSVRYTDMESEDYHFYQGLVYLLENDVSTLGYDLTFSTE<br>VQEFGVCEVRDLKPNGANILVTEENKKEYVHLVCQMRMTGAIRKQLAAFLEGFYEIIPKRLISI<br>FTEQELELLISGLPTIDIDDLKSNTEYHKYQSNSIQIQWFWRALRSFDQADRAKFLQFVTGTSK<br>VPLQGFAALEGMNGIQKFQIHRDDRSTDRLPSAHTCFNQLDLPAYESFEKLRHMLLLAIQEC<br>SEGFGLA
IF2	MTDVTIKTLAAERQTSVERLVQQFADAGIRKSADDSVSAQEKQTLIDHLNQKNSGPDKLTL<br>QRKTRSTLNIPGTGGKSKSVQIEVRKKRTFVKRDPQEAERLAAEEQAQREAEEQARREAEES<br>AKREAQQKAEREAAEQAKREAAEQAKREAAEKDKVSNQQDDMTKNAQAEKARREQEAA<br>ELKRKAEEEARRKLEEEARRVAEEARRMAEENKWTDNAEPTEDSSDYHVTTSQHARQAED<br>ESDREVEGGRGRGRNAKAARPKKGNKHAESKADREEARAAVRGGKGGKRKGSSLQQGFQ<br>KPAQAVNRDVVIGETITVGELANKMAVKGSQVIKAMMKLGAMATINQVIDQETAQLVAEE<br>MGHKVILRRENELEEAVMSDRDTGAAAEPRAPVVTIMGHVDHGKTSLLDYIRSTKVASGEA<br>GGITQHIGAYHVETENGMITFLDTPGHAAFTSMRARGAQATDIVVLVVAADDGVMPQTIE<br>AIQHAKAAQVPVVVAVNKIDKPEADPDRVKNELSQYGILPEEWGGESQFVHVSAKAGTGID<br>ELLDAILLQAEVLELKAVRKGMASGAVIESFLDKGRGPVATVLVREGTLHKGDIVLCGFEYGR<br>VRAMRNELGQEVLEAGPSIPVEILGLSGVPAAGDEVTVVRDEKKAREVALYRQGKFREVKLA<br>RQQKSKLENMFANMTEGEVHEVNIVLKADVQGSVEAISDSLLKLSTDEVKVKIIGSGVGGITE<br>TDATLAAASNAILVGFNVRADASARKVIEAESLDLRYYSVIYNLIDEVKAAMSGMLSPELKQQ<br>IIGLAEVRDVFKSPKFGAIAGCMVTEGVVKRHNPIRVLRDNVVIYEGELESLRRFKDDVNEVR<br>NGMECGIGVKNYNDVRTGDVIEVFEIIEIQRTIA
IL-37b	VHTSPKVKNLNPKKFSIHDQDHKVLVLDSGNLIAVPDKNYIRPEIFFALASSLSSASAEKGSP<br>ILLGVSKGEFCLYCDKDKGQSHPSLQLKKEKLMKLAAQKESARRPFIFYRAQVGSWNMLE<br>SAAHPGWFICTSCNCNEPVGVTDKFENRKHIEFSFQPVCKAEMSPSEVSD
LEDGF	GPGMTRDFKPGDLIFAKMKGYPHWPARVDEVPDGAVKPPTNKLPIFFFGTHETAFLGPKDI<br>FPYSENKEKYGKPNKRKGFNEGLWEIDNNPKVKFSSQQAATKQSNASSDVEVEEKETSVSKE<br>DTDHEEKASNEDVTKAVDITTPKAARRGRKRKAEKQVETEEAGVVTTATASVNLKVSPKRG<br>RPAATEVKIPKPRGRPKMVKQPCPSESDIITEEDKSKKKGQEEKQPKKQPKKDEEGQKEEDK<br>PRKEPDKKEGKKEVESKRKNLAKTGVTSTSDSEEEGDDQEGEKKRKGGRNFQTAHRRNMLK<br>GQHEKEAADRKRKQEEQMETEQQNKDEGKKPEVKKVEKKRETSMDSRLQRIHAEIKNSLKI<br>DNLDVNRCIEALDELASLQVTMQQAQKHTEMITTLKKIRRFKVSQVIMEKSTMLYNKFKNM<br>FLVGEGDSVITQVLNKSLAEQRQHEEANKTKDQGKKGPNKKLEKEQTGSKTLNGGSDAQD<br>GNQPQHNGESNEDSKDNHEASTKKKPSSEERETEISLKDSTLDN
LRP130	SNAAIAAKEKDIQEESTFSSRKISNQFDWALMRLDLSVRRTGRIPKKLLQKVFNDTCRSGGLG<br>GSHALLLLRSCGSLLPELKLEERTEFAHRIWDTLQKLGAVYDVSHYNALLKVYLQNEYKFSPTD<br>FLAKMEEANIQPNRVTYQRLIASYCNVGDIEGASKILGFMKTKDLPVTEAVFSALVTGHARA<br>GDMENAENILTVMRDAGIEPGPDTYLALLNAYAEKGDIDHVKQTLEKVEKSELHLMDRDLL<br>QIIFSFSKAGYPQYVSEILEKVTCERRYIPDAMNLILLLVTEKLEDVALQILLACPVSKEDGPSVF<br>GSFFLQHCVTMNTPVEKLTDYCKKLKEVQMHSFPLQFTLHCALLANKTDLAKALMKAVKEE<br>GFPIRPHYFWPLLVGRRKEKNVQGIIEILKGMQELGVHPDQETYTDYVIPCFDSVNSARAILQ<br>ENGCLSDSDMFSQAGLRSEAANGNLDFVLSFLKSNTLPISLQSIRSSLLLGFRRSMNINLWSEI

	TELLYKDGRYCQEPRGPTEAVGYFLYNLIDSMSDSEVQAKEEHLRQYFHQLEKMNVKIPENI<br>YRGIRNLLESYHVPELIKDAHLLVESKNLDFQKTVQLTSSELESTLETLKAENQPIRDVLKQLILV<br>LCSEENMQKALELKAKYESDMVTGGYAALINLCCRHDKVEDALNLKEEFDRLDSSAVLDTGK<br>YVGLVRVLAKHGKLQDAINILKEMKEKDVLIKDTTALSFFHMLNGAALRGEIETVKQLHEAIV<br>TLGLAEPSTNISFPLVTVHLEKGDLSTALEVAIDCYEKYKVLPRIHDVLCKLVEKGETDLIQKAM<br>DFVSQEQGEMVMLYDLFFAFLQTGNYKEAKKIIETPGIRARSARLQWFCDRCVANNQVETL<br>EKLVELTQKLFECDRDQMYYNLLKLYKINGDWQRADAVWNKIQEENVIPREKTLRLLAEILR<br>EGNQEVPFDVPELWYEDEKHSLNSSSASTTEPDFQKDILIACRLNQKKGAYDIFLNAKEQNIV<br>FNAETYSNLIKLLMSEDYFTQAMEVKAFAETHIKGFTLNDAANSRLIITQVRRDYLKEAVTTLK<br>TVLDQQQTPSRLAVTRVIQALAMKGDVENIEVVQKMLNGLEDSIGLSKMVFINNIALAQIK<br>NNNIDAAIENIENMLTSENKVIEPQYFGLAYLFRKVIEEQLEPAVEKISIMAERLANQFAIYKPV<br>TDFFLQLVDAGKVDDARALLQRCGAIAEQTPILLLFLLRNSRKQGKASTVKSVLELIPELNEKE<br>EAYNSLMKSYVSEKDVTSAKALYEHLTAKNTKLDDLFLKRYASLLKYAGEPVPFIEPPESFEFYA<br>QQLRKLRENSS
mIL-1α	SAPYTYQSDLRYKLMKLVRQKFVMNDSLNQTIYQDVDKHYLSTTWLNDLQQEVKFDMYAY<br>SSGGDDSKYPVTLKISDSQLFVSAQGEDQPVLLKELPETPKLITGSETDLIFFWKSINSKNYF<br>TSAAYPELFIATKEQSRVHLARGLPSMTDFQIS
mIL-1β	VPIRQLHYRLRDEQQKSLVLSDPYELKALHLNGQNINQQVIFSMSFVQGEPSNDKIPVALGL<br>KGKNLYLSCVMKDGTPTLQLESVDPKQYPKKKMEKRFVFNKIEVKSKVEFESAEFPNWYIS<br>TSQAEHKPVFLGNNSGQDIIDFTMESVSS
Ovalbumin	MGSIGAASMEFCFDVFKELKVHHANENIFYCPIAIMSALAMVYLGAKDSTRTQINKVVRFDK<br>LPGFGDSIEAQCGTSVNVHSSLRDILNQITKPNDVYSFSLASRLYAEERYPILPEYLQCVKEL<br>YRGGLEPINFQTAADQARELINSWVESQTNGIIRNVLQPSSVDSQTAMVLVNAIVFKGLWE<br>KAFKDEDTQAMPFRVTEQESKPVQMMYQIGLFRVASMASEKMKILELPFASGTMSMLVLL<br>PDEVSGLEQLESIINFEKLTEWTSSNVMEERKIKVYLPRMKMEEKYNLTSVLMAMGITDVFS<br>SSANLSGISSAESLKISQAVHAAHAEINEAGREVVGSAEAGVDAASVSEEFRADHPFLFCIK<br>HIATNAVLFFGRCVSP
PDF	MSVLQVLHIPDERLRKVAKPVEEVNAEIQRIVDDMFETMYAEEGIGLAATQVDIHQRIIVIDV<br>SENRDERLVLINPELLEKSGETGIEEGCLSIPEQRALVPRAEKVKIRALDRDGKPFELEADGLLAI<br>CIQHEMDHLVGKLFMDYLSPLKQQRIRQKVEKLDRLKARA
RF1	MKPSIVAKLEALHERHEEVQALLGDAQTIADQERFRALSREYAQLSDVSRCFTDWQQVQED<br>IETAQMMLDDPEMREMAQDELREAKEKSEQLEQQLQVLLLPKDPDDERNAFLEVRAGTG<br>GDEAALFAGDLFRMYSRYAEARRWRVEIMSASEGEHGGYKEIIAKISGDGVYGRLKFESGGH<br>RVQRVPATESQGRIHTSACTVAVMPELPDAELPDINPADLRIDTFRSSGAGGQHVNTTDSAI<br>RITHLPTGIVVECQDERSQHKNKAKALSVLGARIHAAEMAKRQQAEASTRRNLLGSGDRSD<br>RNRTYNFPQGRVTDHRINLTLYRLDEVMEGKLDMLIEPIIQEHQADQLAALSEQE
tau	MAEPRQEFEVMEDHAGTYGLGDRKDQGGYTMHQDQEGDTDAGLKESPLQTPTEDGSEE<br>PGSETSDAKSTPTAEDVTAPLVDEGAPGKQAAAQPHTEIPEGTTAEEAGIGDTPSLEDEAA<br>GHVTQARMVSKSKDGTGSDDKKAKGADGKTKIATPRGAAPPGQKGQANATRIPAKTPPA<br>PKTPPSSGEPPKSGDRSGYSSPGSPGTPGSRSRTPSLPTPPTREPKKVAVVRTPPKSPSS<br>AKSRLQTAPVPMPDLKNVKSKIGSTENLKHQPGGGKVQIINKKLDLSNVQSKCGSKDNIKH<br>VPGGGSVQIVYKPVDLSKVTSKCGSLGNIHHKPGGGQVEVKSEKLDFKDRVQSKIGSLDNI<br>THVPGGGNKKIETHKLTFRENAKAKTDHGAEIVYKSPVVSGDTSPRHLSNVSSTGSIDMVD<br>SPQLATLADEVSASLAKQGL
UbcH7	GPMAASRRLMKELEEIRKCGMKNFRNIQVDEANLLTWQGLIVPDNPPYDKGAFRIEINFPA<br>EYPFKPPKITFKTKIYHPNIDEKGQVCLPVISAENWKPATKTDQVIQSLIALVNDPQPEHPLRA<br>DLAEEYSKDRKKFCKNAEEFTKKYGEKRPVD
Ube2K	MANIAVQRIKREFKEVLKSEETSKNQIKVDLVDENFTELRGEIAGPPDTPYEGGRYQLEIKIPE<br>TYPFNPPKVRFITKIWHPNISSVTGAICLDILKDQWAAAMTLRTVLLSLQALLAAAEPDDPQD<br>AVVANQYKQNPEMFKQTARLWAHVYAGAPVSSPEYTKKIENLCAMGFDRNAVIVALSSKS<br>WDVETATELLLSN
Ube2S	MNSNVENLPPHIIRLVYKEVTTLTADPPDGIKVFPNEEDLTDLQVTIEGPEGTPYAGGLFRMK<br>LLLGKDFPASPPKGYFLTKIFHPNVGANGEICVNVLKRDWTAELGIRHVLLTIKCLLIHPNPES

	ALNEEAGRLLLENYEEYAARARLLTEIHGGAGGPSGRAEAGRALASGTEASSTDPGAPGGPG GAEGPMAKKHAGERDKKLAAKKKTDKKRALRRL
