# Supplementary material for: Protein degradation by human 20S proteasomes elucidates the interplay between peptide hydrolysis and splicing
Source: Nat Commun. 2024 Feb 7;15:1147. doi: 10.1038/s41467-024-45339-3 (PMC10850103; doi:10.1038/s41467-024-45339-3)
Supplement: Supplementary file 5 — Supplementary Data 3-annotation [file 41467_2024_45339_MOESM5_ESM.pdf]

inSPIRE Spectral Plotting for experimental vs. prosit comparisons of non-spliced assignments

Experimental Spectrum Colour Code:

- Experimental peak matched to a Prosit predicted peak.
- Possible ion unknown to Prosit.
- Precursor matched peak.
- Experimental peak not matched to any potential ion.

Prosit Spectrum Colour Code:

- Prosit predicted peak matched to experimental spectrum.
- Prosit predicted peak not matched to experimental spectrum.

Additional Notes:

° indicates an ion with loss of H_2O.

* indicates an ion with loss of NH_3.

Loading [MathJax]/extensions/MathMenu.js
