# Supplementary material for: Protein degradation by human 20S proteasomes elucidates the interplay between peptide hydrolysis and splicing
Source: Nat Commun. 2024 Feb 7;15:1147. doi: 10.1038/s41467-024-45339-3 (PMC10850103; doi:10.1038/s41467-024-45339-3)
Supplement: Supplementary file 7 — Supplementary Data 4 [file 41467_2024_45339_MOESM7_ESM.pdf]

Supplementray Data 4. Experimental vs. Prosit-predicted MS2 spectra for all identified spliced peptides. Plots comparing the experimental spectrum for an identified peptide on the positive y-axis against the Prosit predicted spectrum for that peptide on the negative y-axis. Comparisons are shown for the best scoring spectrum of all spliced peptides identified at 1% FDR (both *cis*- and homolgous *trans*- identifications are shown).

inSPIRE Spectral Plotting for experimental vs. prosit comparisons of non-spliced assignments

Experimental Spectrum Colour Code:

- Experimental peak matched to a Prosit predicted peak.
- Possible ion unknown to Prosit.
- Precursor matched peak.
- Experimental peak not matched to any potential ion.

Prosit Spectrum Colour Code:

- Prosit predicted peak matched to experimental spectrum.
- Prosit predicted peak not matched to experimental spectrum.

Additional Notes:

° indicates an ion with loss of H_2O.

* indicates an ion with loss of NH_3.

Loading [MathJax]/extensions/MathMenu.js

Source WSoh_301121_021221_HFGoe_M2_AnnexinA1_2h_R2 **Scan** 7974
Peptide GEELRAA **Charge** 2 **Spectral Angle** 0.96

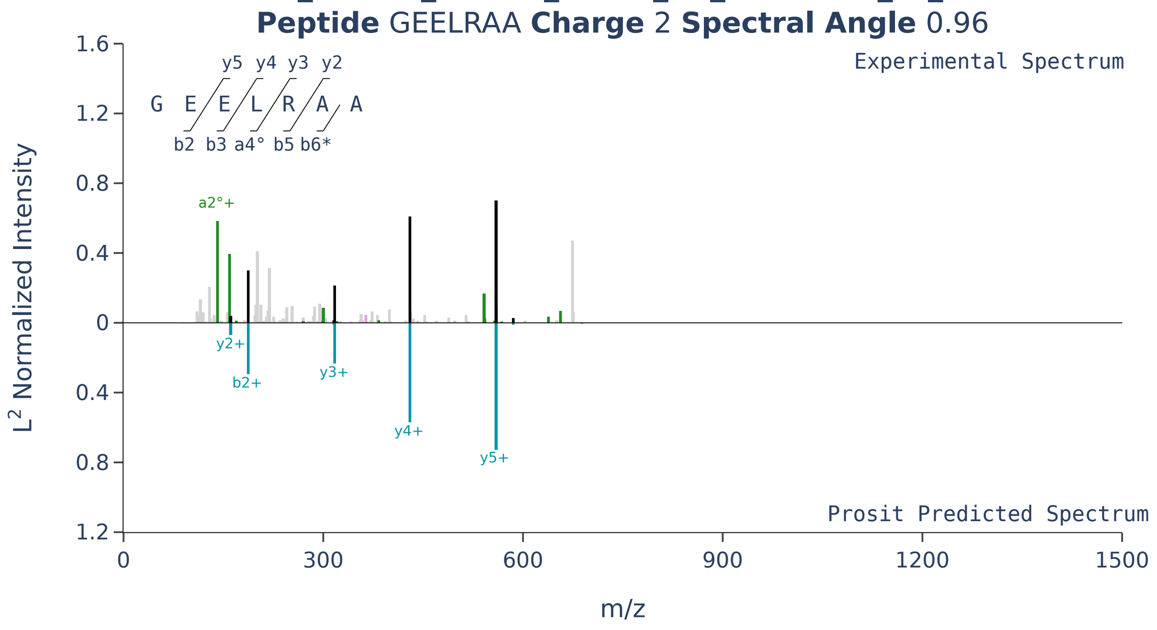

Source WSoh_301121_021221_HFGoe_M1_AnnexinA1_24h_R1 **Scan** 8408
Peptide ILDESRT **Charge** 2 **Spectral Angle** 0.94

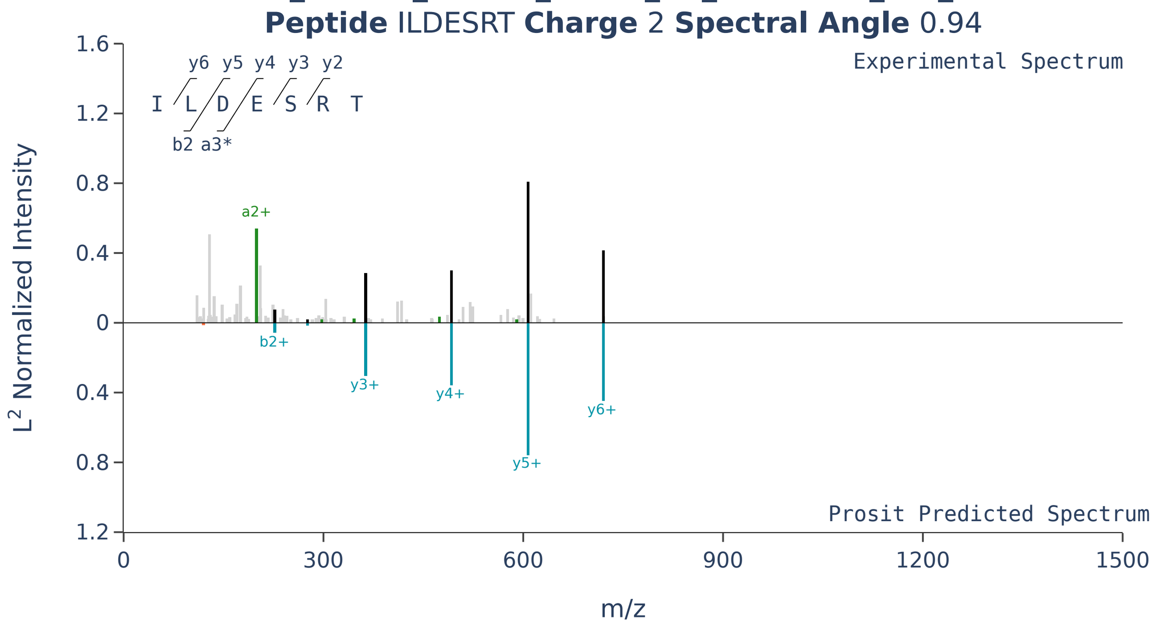

Source WSoh_301121_021221_HFGoe_M1_AnnexinA1_24h_R2 **Scan** 15565
Peptide DEFGVNE **Charge** 2 **Spectral Angle** 0.91

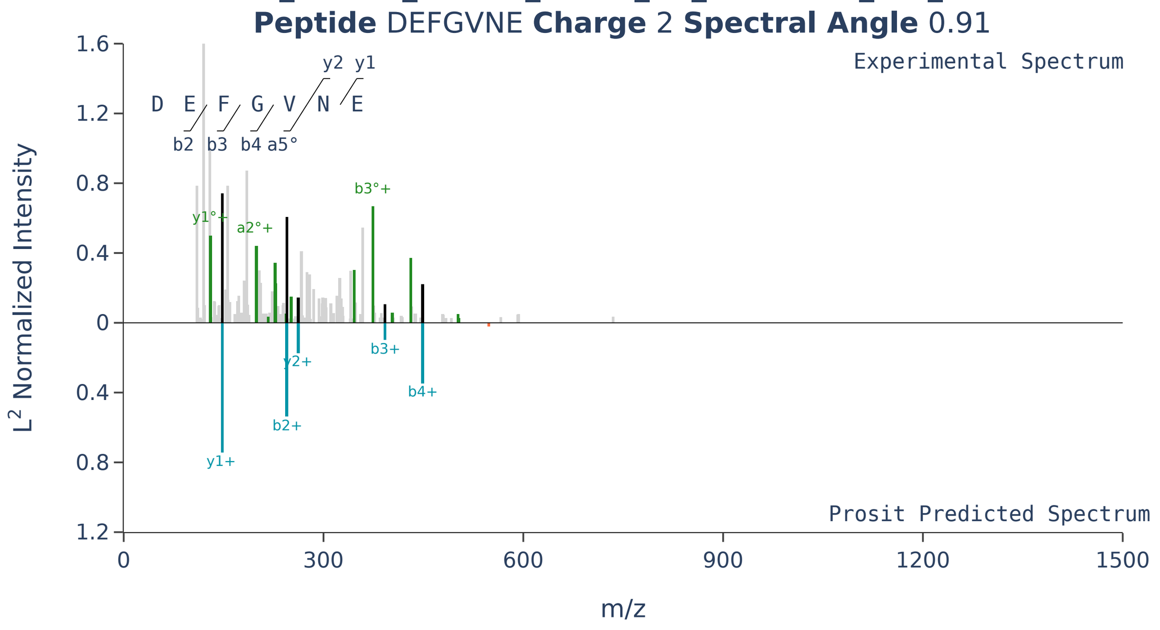

Source WSoh_301121_021221_HFGoe_M2_AnnexinA1_24h_R1 **Scan** 17786
Peptide SVSEFLKQ **Charge** 2 **Spectral Angle** 0.91

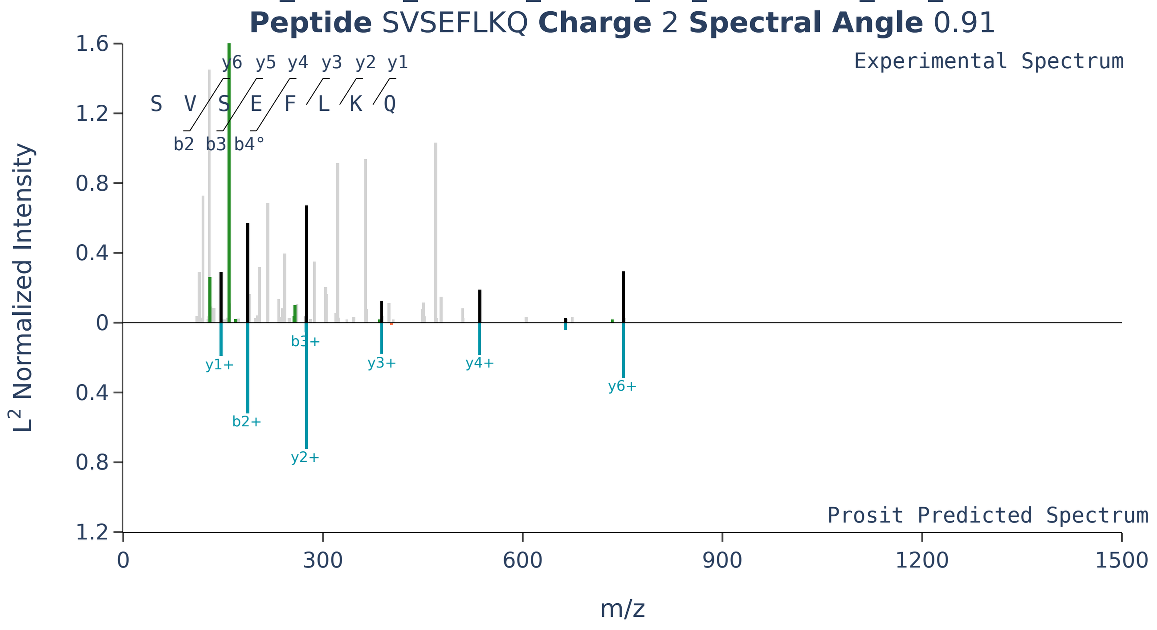

Source WSoh_301121_021221_HFGoe_M1_AnnexinA1_24h_R2 **Scan** 11504
Peptide SSDARALYE **Charge** 2 **Spectral Angle** 0.89

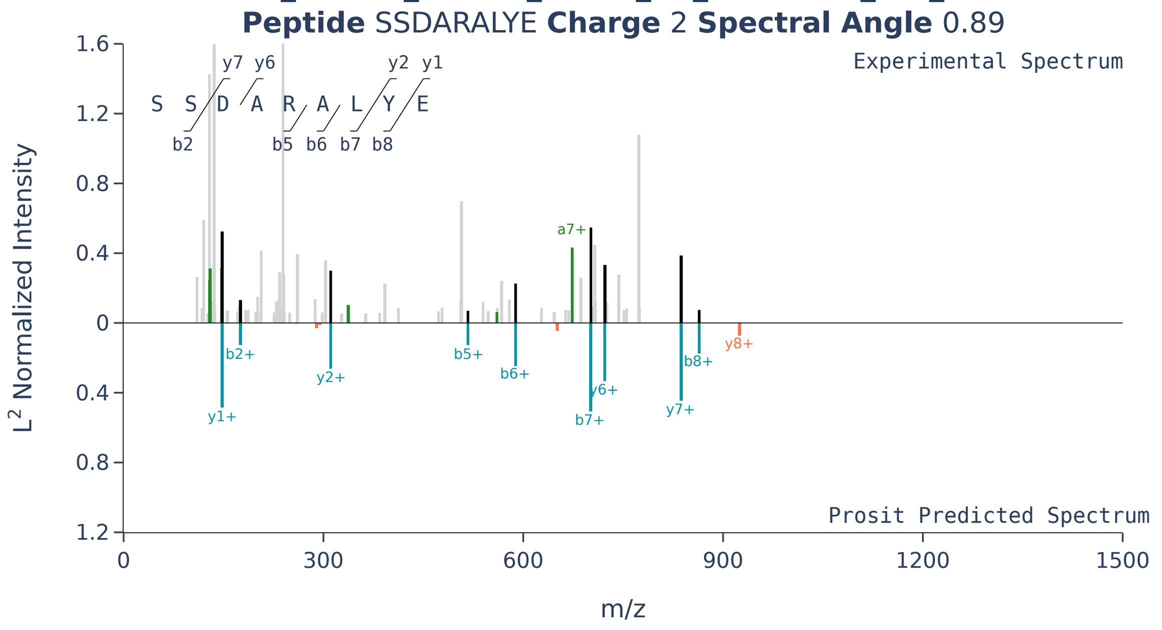

Source WSoh_301121_021221_HFGoe_M1_AnnexinA1_4h_R2 **Scan** 18851
Peptide RASEIDMNDI **Charge** 2 **Spectral Angle** 0.91

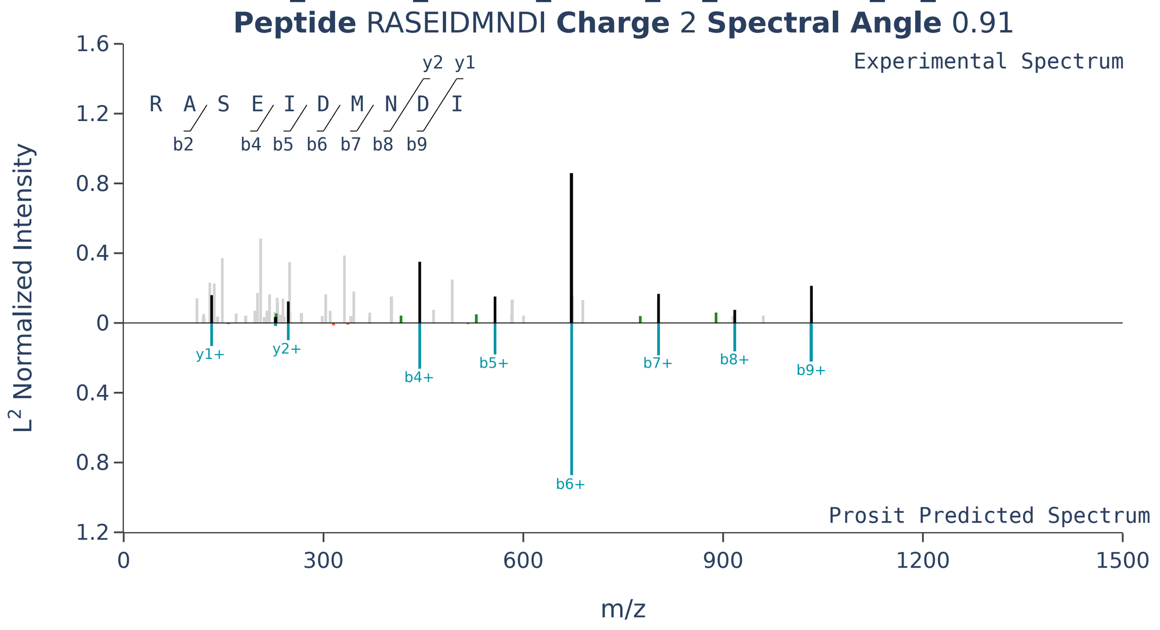

Source WSoh_301121_021221_HFGoe_M1_AnnexinA1_1h_R2 **Scan** 13459
Peptide RDLAQQI **Charge** 2 **Spectral Angle** 0.93

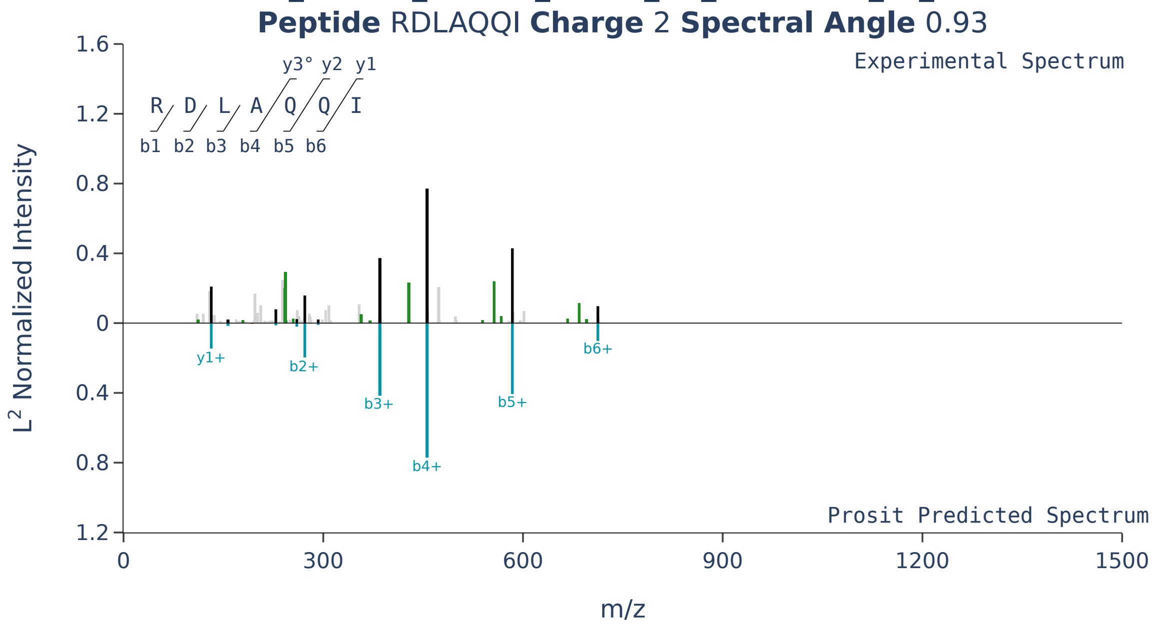

Source WSoh_301121_021221_HFGoe_M2_AnnexinA1_24h_R1 **Scan** 13674
Peptide EKILVGD **Charge** 1 **Spectral Angle** 0.87

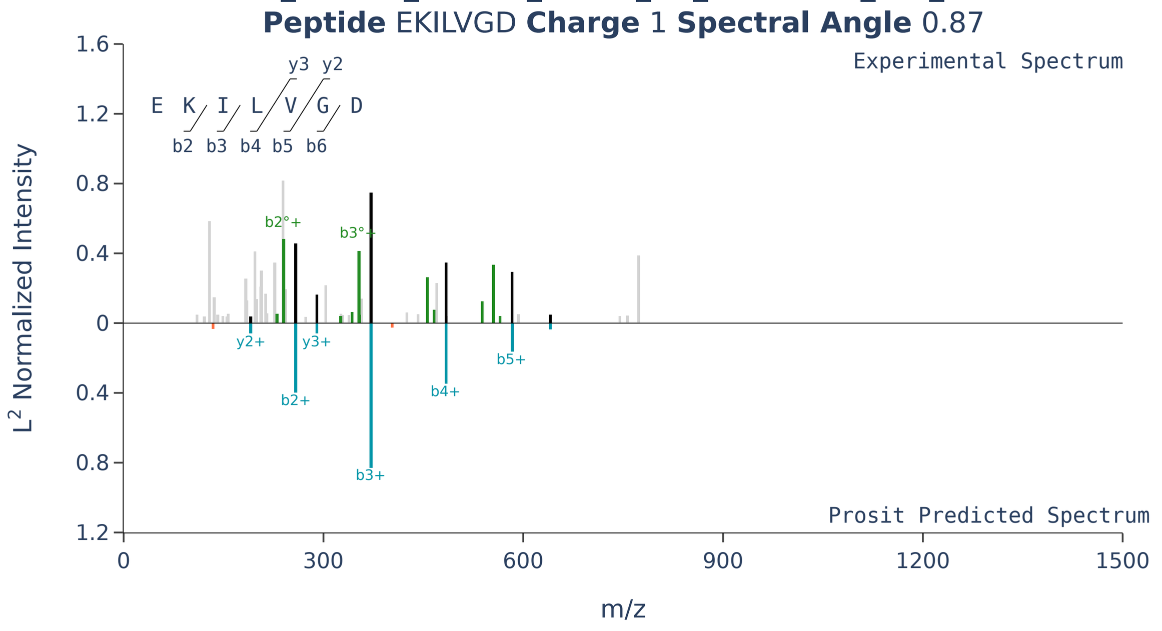

Source WSoh_301121_021221_HFGoe_M2_AnnexinA1_24h_R1 **Scan** 14307
Peptide KSVSEFLKQ **Charge** 2 **Spectral Angle** 0.86

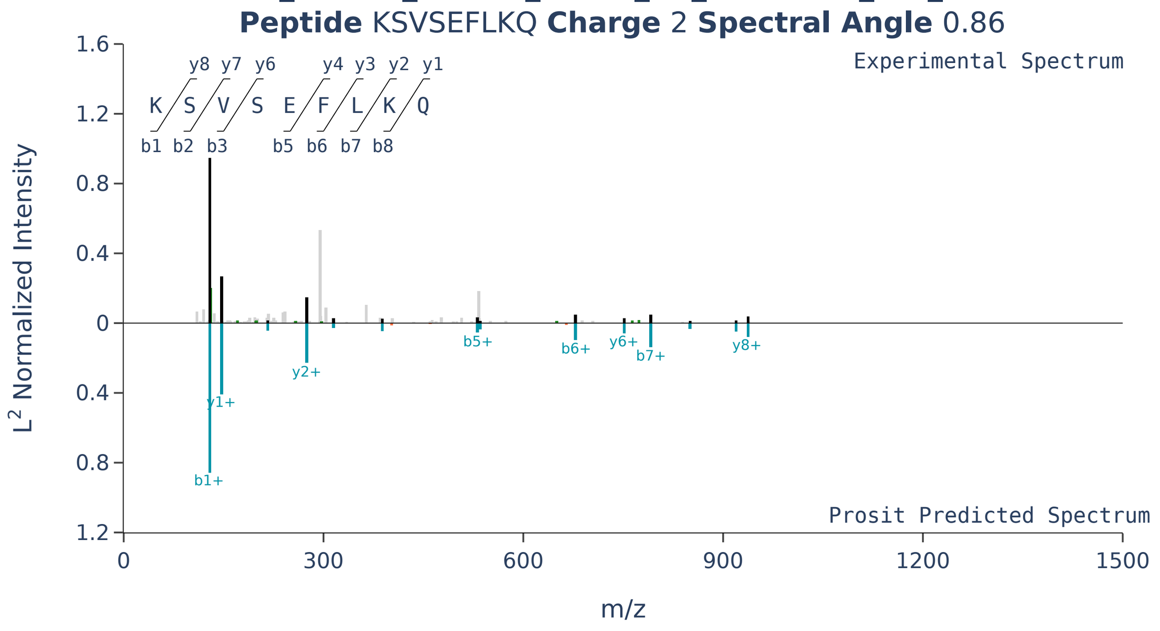

Source WSoh_301121_021221_HFGoe_M2_AnnexinA1_24h_R1 **Scan** 6565
Peptide KDINRVYRE **Charge** 3 **Spectral Angle** 0.9

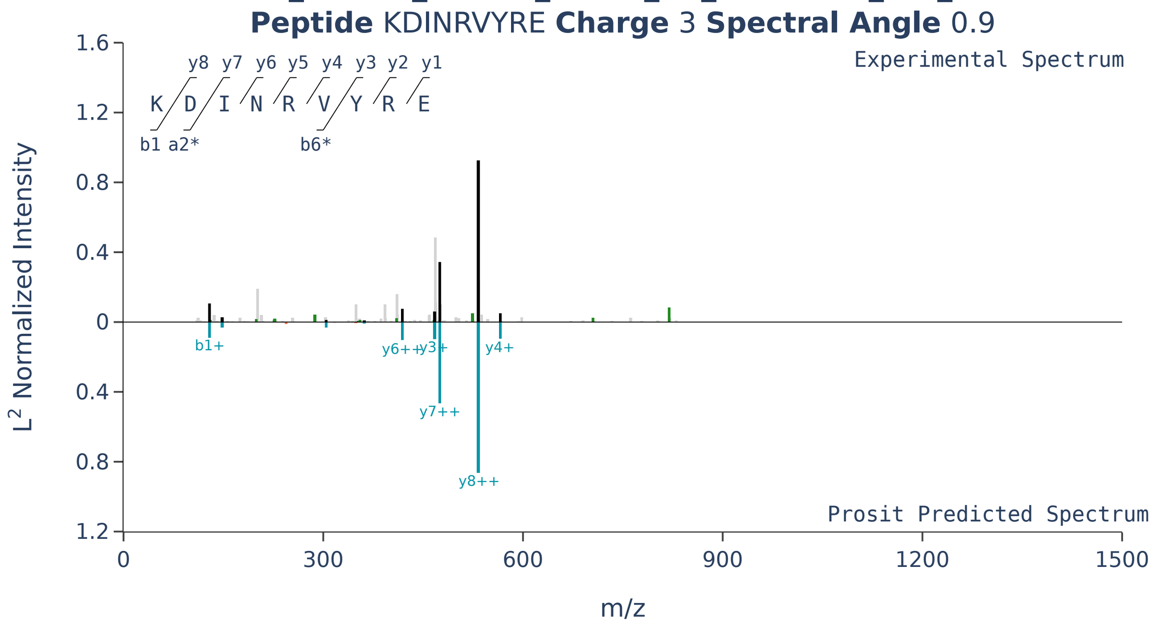

Source WSoh_301121_021221_HFGoe_M2_AnnexinA1_1h_R2 **Scan** 18716
Peptide ARSEIDMNDI **Charge** 2 **Spectral Angle** 0.91

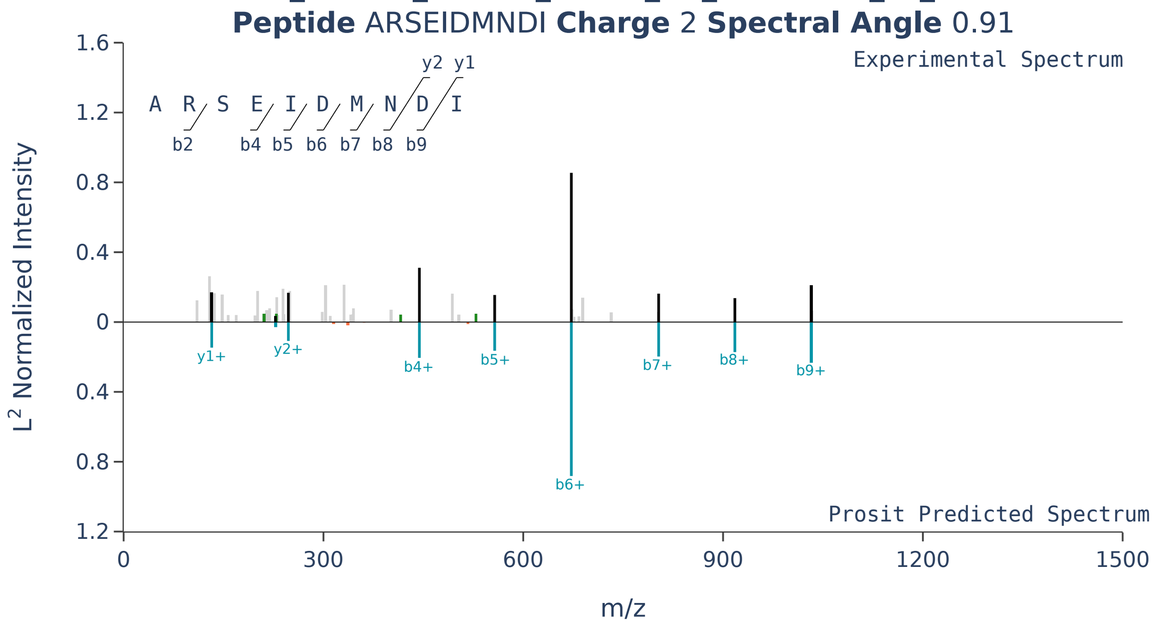

Source WSoh_301121_021221_HFGoe_M1_AnnexinA1_24h_R2 **Scan** 14670
Peptide ILDETRSEID **Charge** 2 **Spectral Angle** 0.92

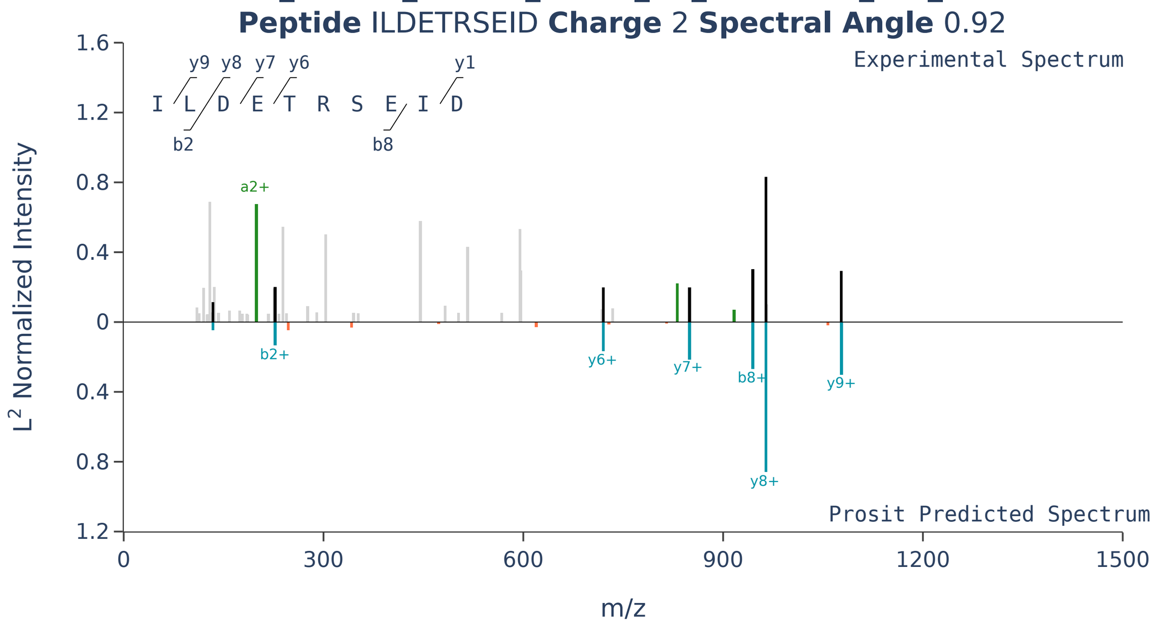

Source WSoh_301121_021221_HFGoe_M2_AnnexinA1_24h_R2 **Scan** 14215
Peptide KGDYEKILVKA **Charge** 3 **Spectral Angle** 0.96

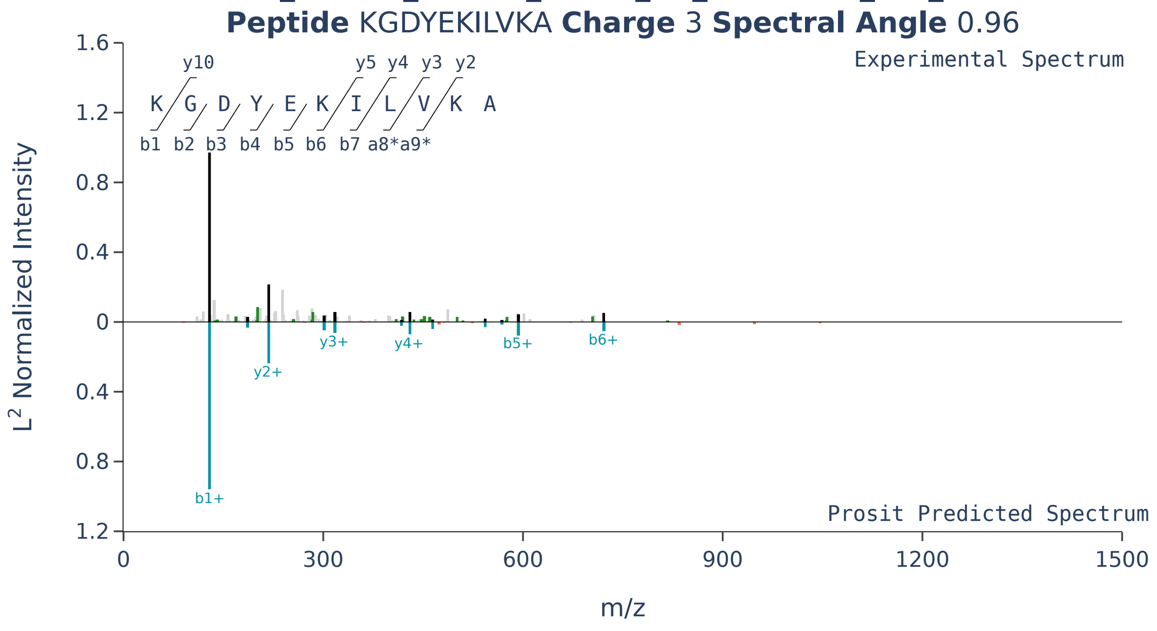

Source WSoh_301121_021221_HFGoe_M2_AnnexinA1_4h_R1 **Scan** 14436
Peptide ATSKPAFFAERS **Charge** 3 **Spectral Angle** 0.91

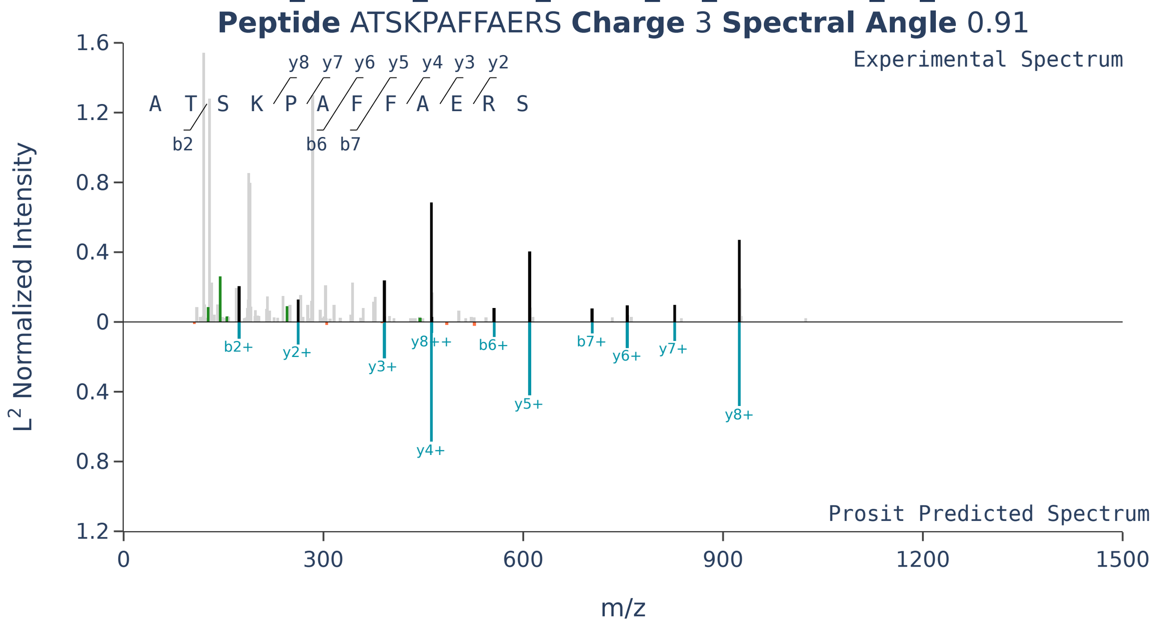

Source WSoh_301121_021221_HFGoe_M1_AnnexinA1_24h_R1 **Scan** 14676
Peptide SKGGPGSEQEYVQTV **Charge** 2 **Spectral Angle** 0.84

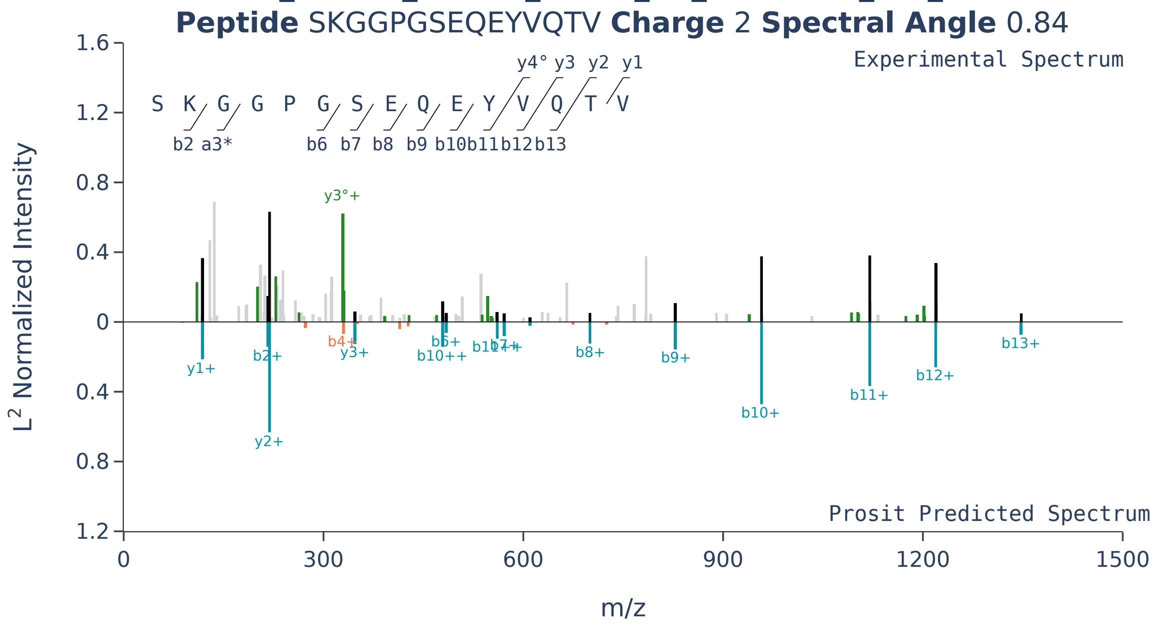

Source WSoh_301121_021221_HFGoe_M2_AnnexinA1_24h_R1 **Scan** 10702
Peptide TKGDALYE **Charge** 2 **Spectral Angle** 0.89

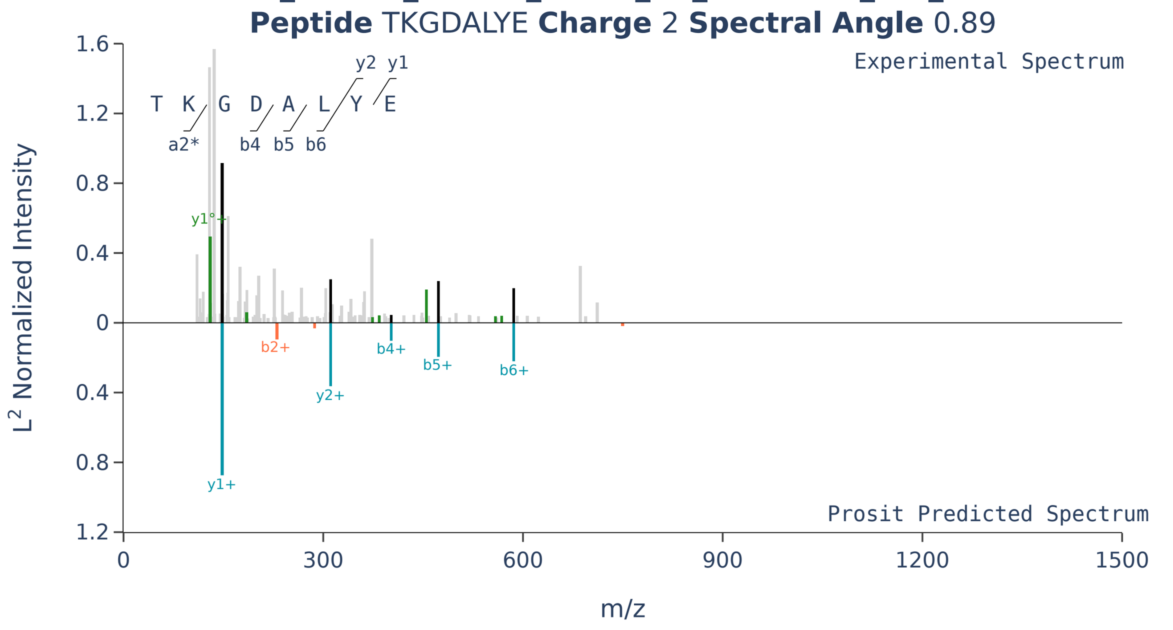

Source WSoh_301121_021221_HFGoe_M1_AnnexinA1_1h_R2 **Scan** 14184
Peptide KQEATII **Charge** 2 **Spectral Angle** 0.82

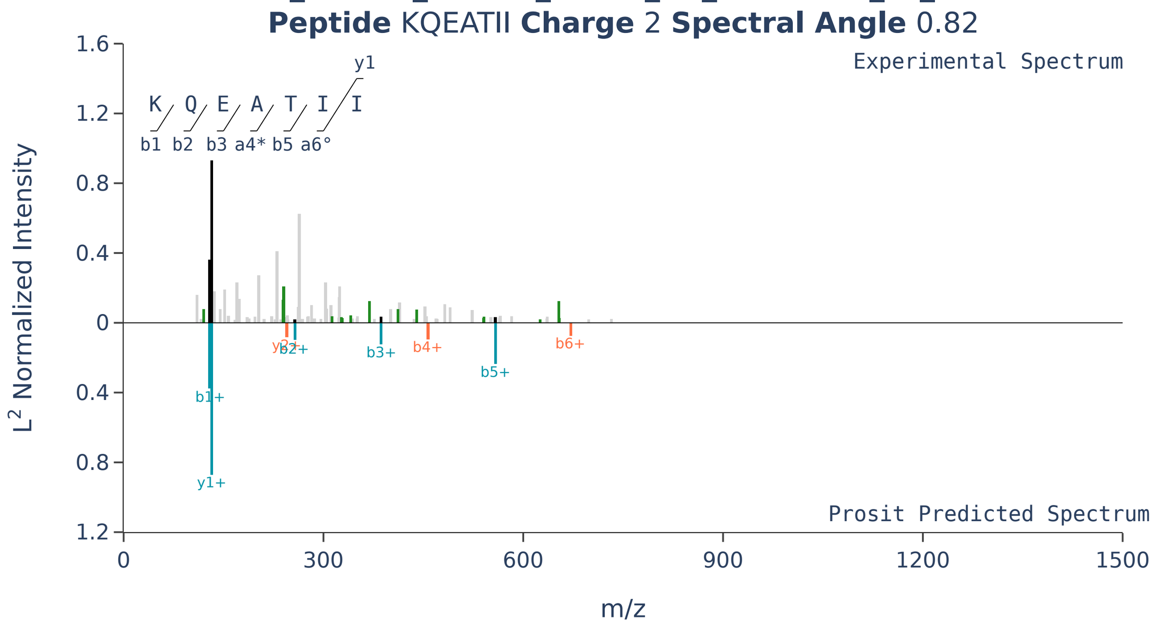

Source WSoh_301121_021221_HFGoe_M2_AnnexinA1_24h_R2 **Scan** 18710
Peptide GTRSEIDMNDI **Charge** 2 **Spectral Angle** 0.9

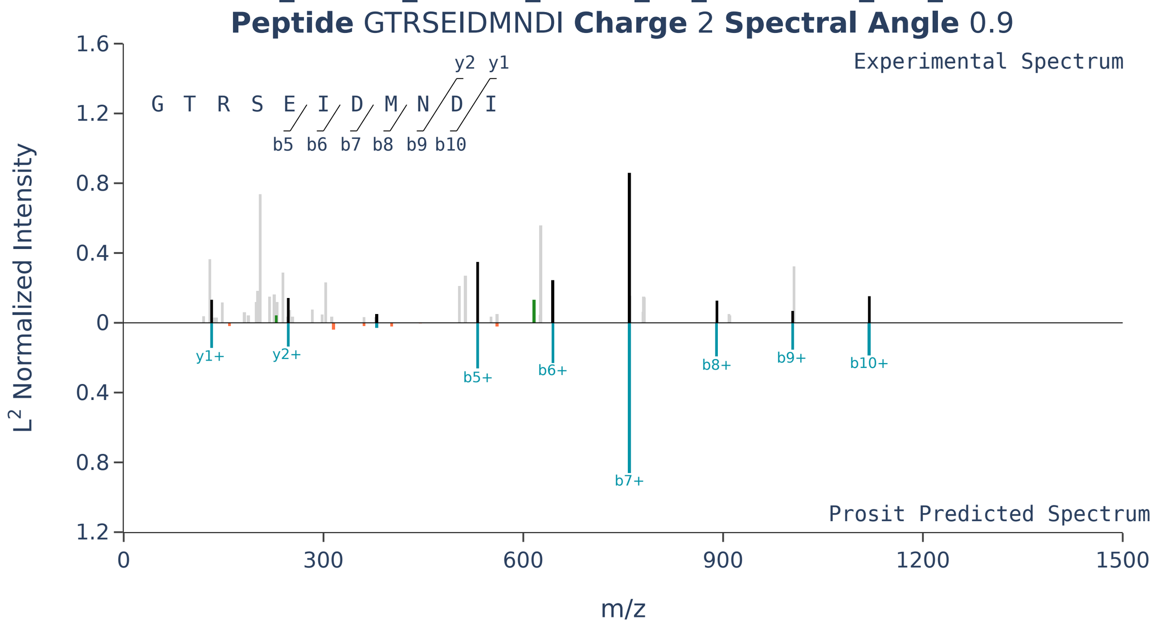

Source WSoh_301121_021221_HFGoe_M2_AnnexinA1_24h_R1 **Scan** 15702
Peptide EQEYVQTVKSV **Charge** 2 **Spectral Angle** 0.9

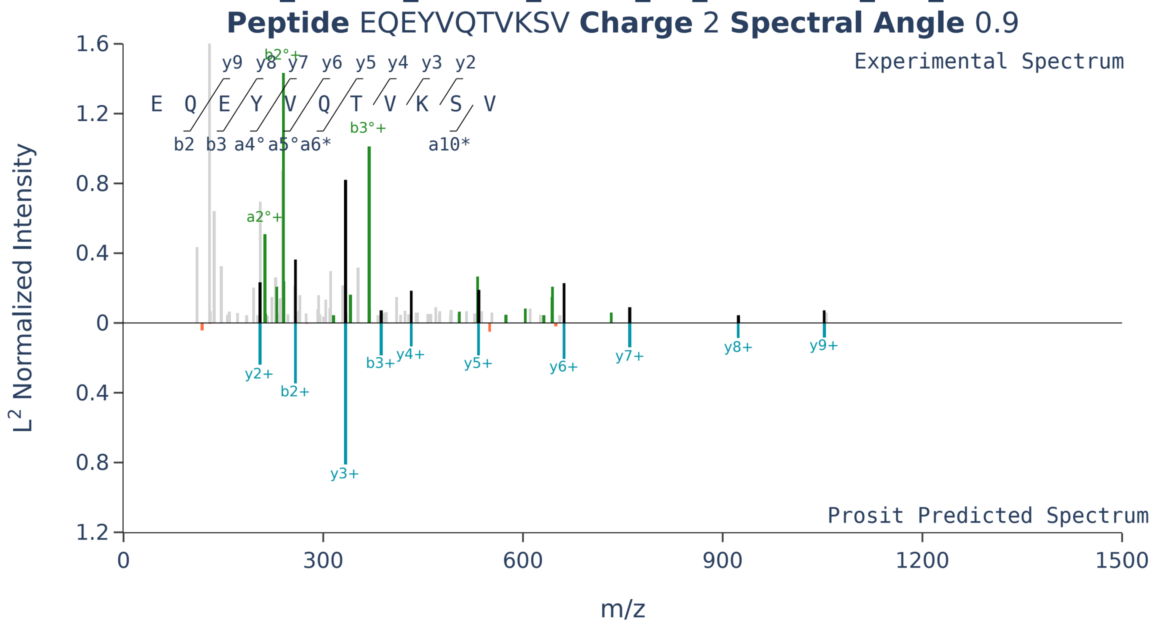

Source WSoh_301121_021221_HFGoe_M2_AnnexinA1_24h_R2 **Scan** 11980
Peptide EQEYVQTVKSA **Charge** 2 **Spectral Angle** 0.9

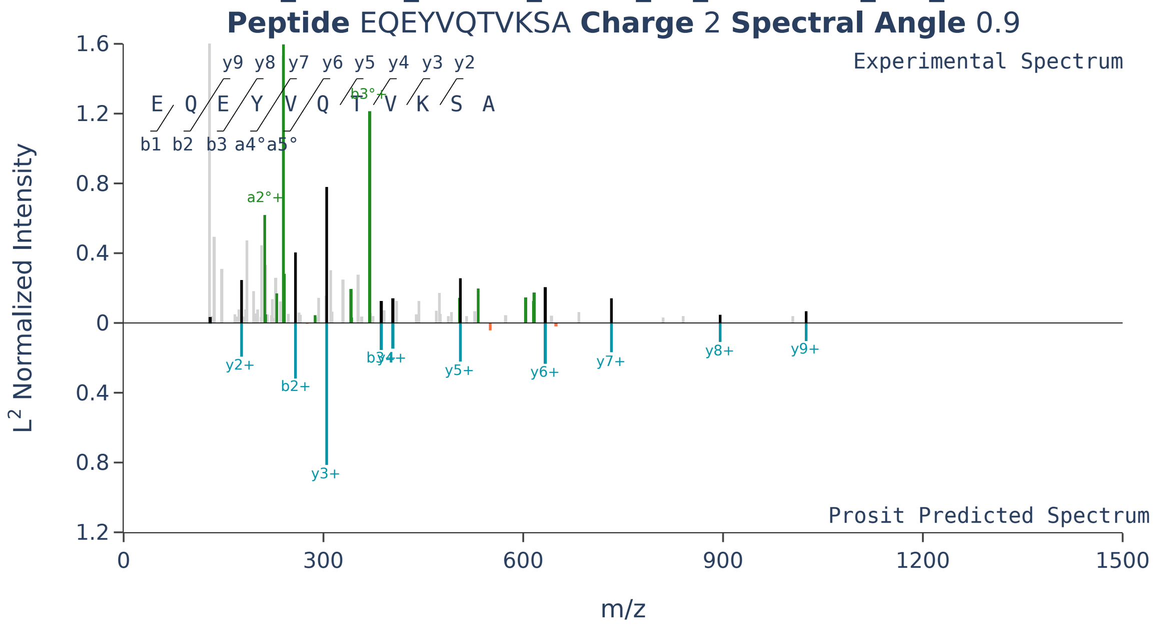

Source WSoh_301121_021221_HFGoe_M2_AnnexinA1_4h_R1 **Scan** 16220
Peptide LQDITSDTSGDFRNA **Charge** 2 **Spectral Angle** 0.83

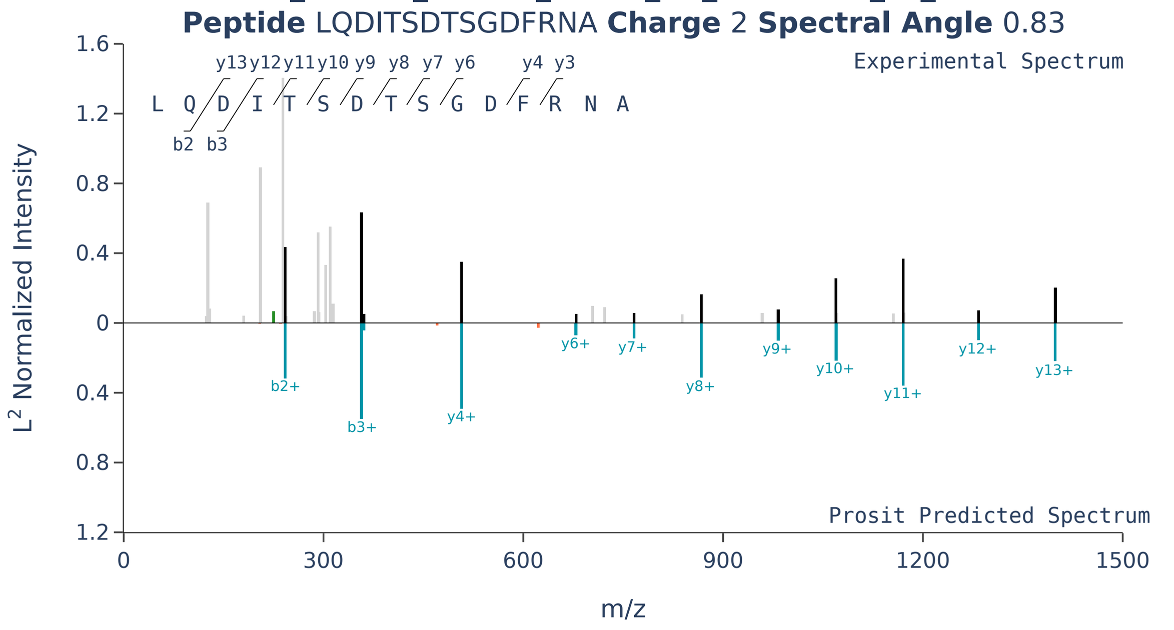

Source WSoh_301121_021221_HFGoe_M2_AnnexinA1_4h_R1 **Scan** 12047
Peptide AAKDITSDTSGDFRNA **Charge** 3 **Spectral Angle** 0.87

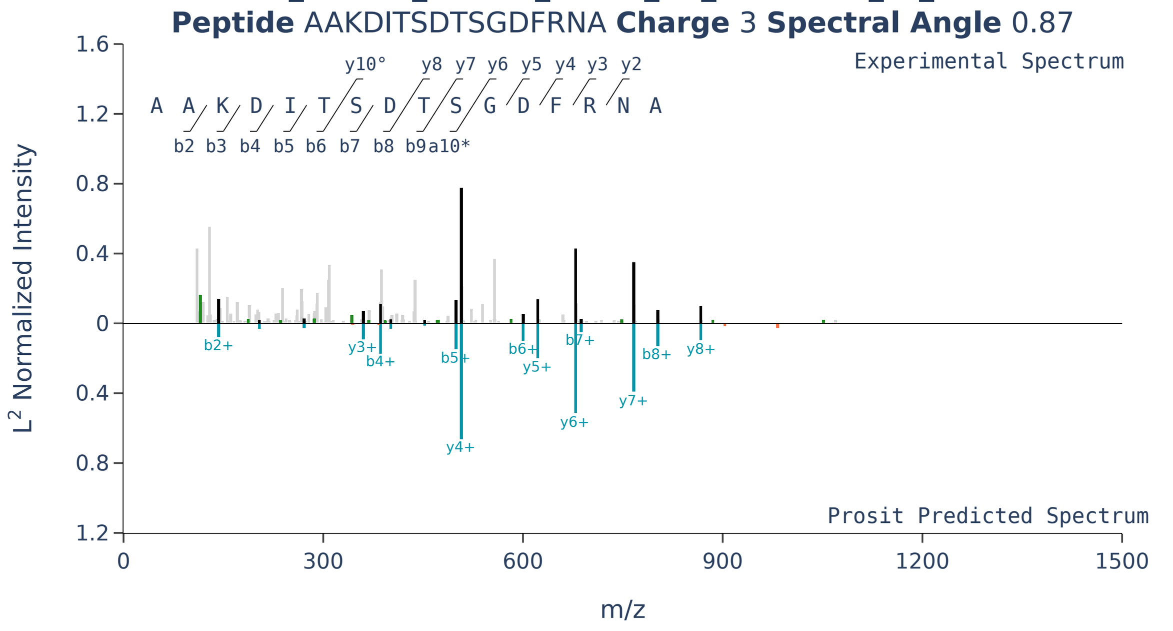

Source WSoh_301121_021221_HFGoe_M1_AnnexinA1_24h_R2 **Scan** 9128
Peptide RSYPQLRVK **Charge** 3 **Spectral Angle** 0.89

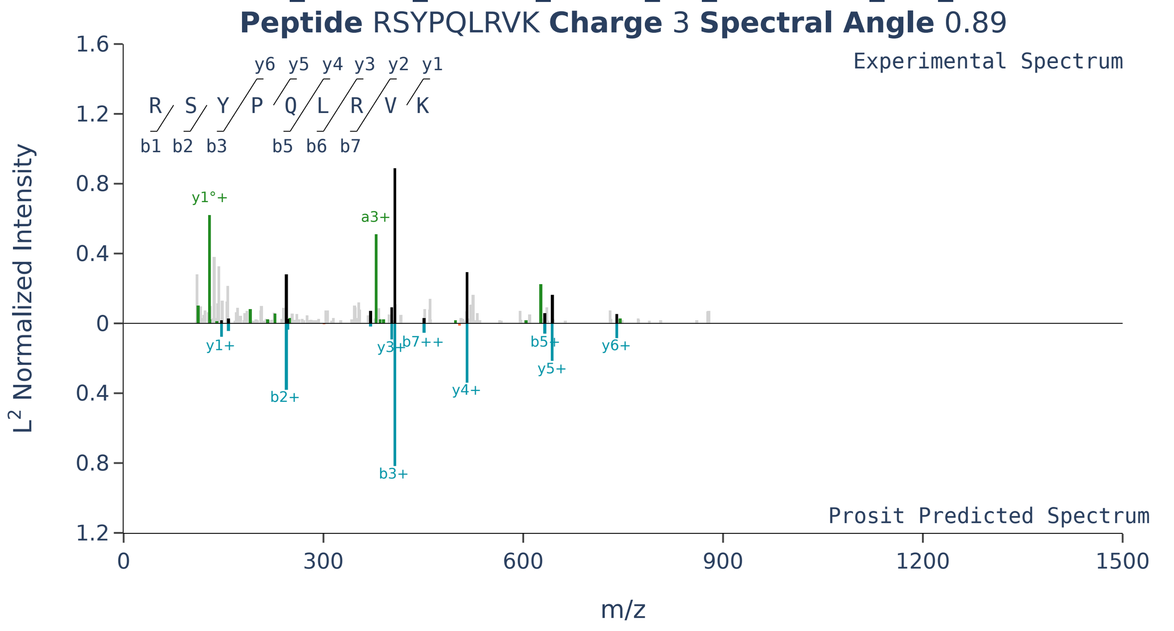

Source WSoh_301121_021221_HFGoe_M2_AnnexinA1_24h_R2 **Scan** 9007
Peptide RSYPQLRKV **Charge** 3 **Spectral Angle** 0.86

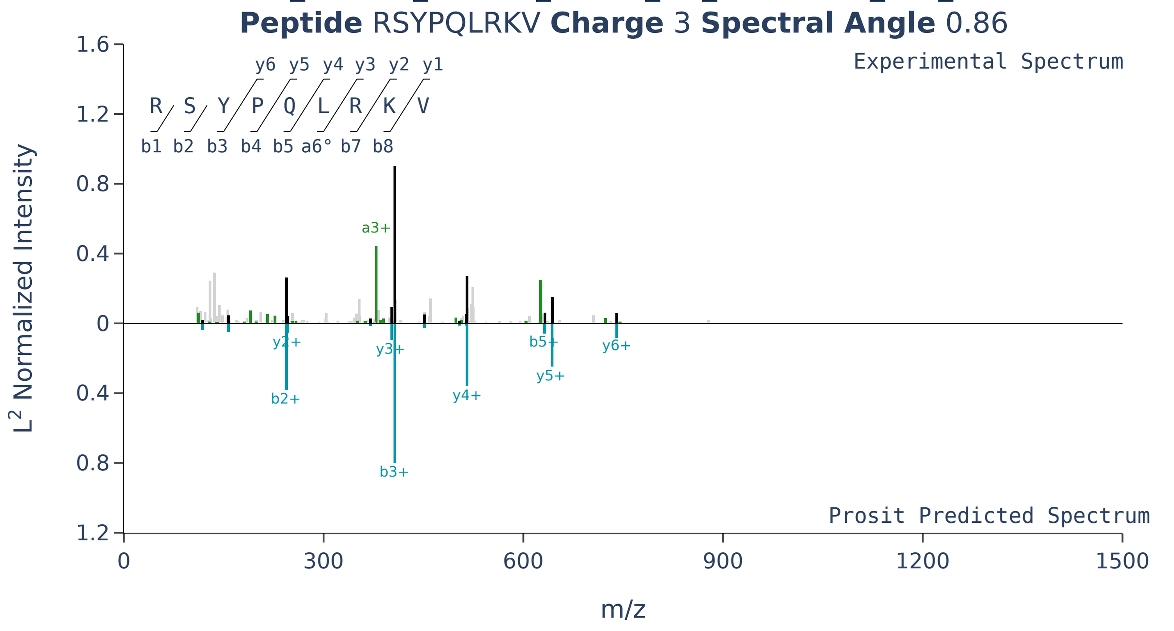

Source WSoh_301121_021221_HFGoe_M1_AnnexinA1_24h_R1 **Scan** 8144
Peptide KDINRVY **Charge** 2 **Spectral Angle** 0.85

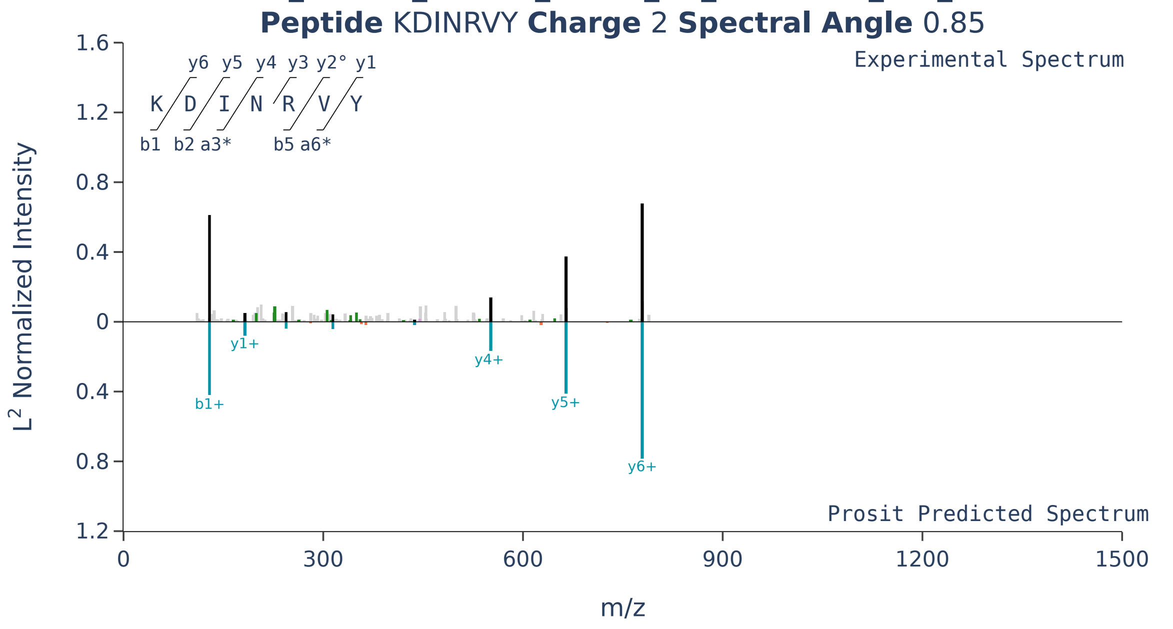

Source WSoh_301121_021221_HFGoe_M1_AnnexinA1_24h_R1 **Scan** 12129
Peptide EQEYVQTVKTG **Charge** 2 **Spectral Angle** 0.89

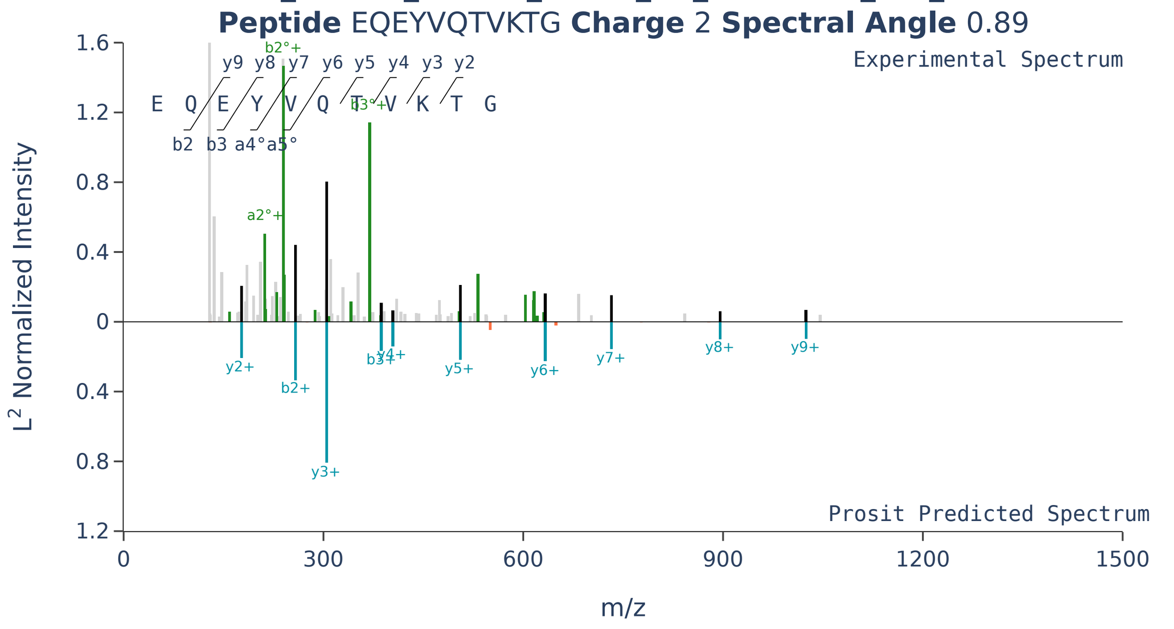

Source WSoh_301121_021221_HFGoe_M1_AnnexinA1_24h_R2 **Scan** 13653
Peptide SKGGPGSQEYVQTV **Charge** 2 **Spectral Angle** 0.82

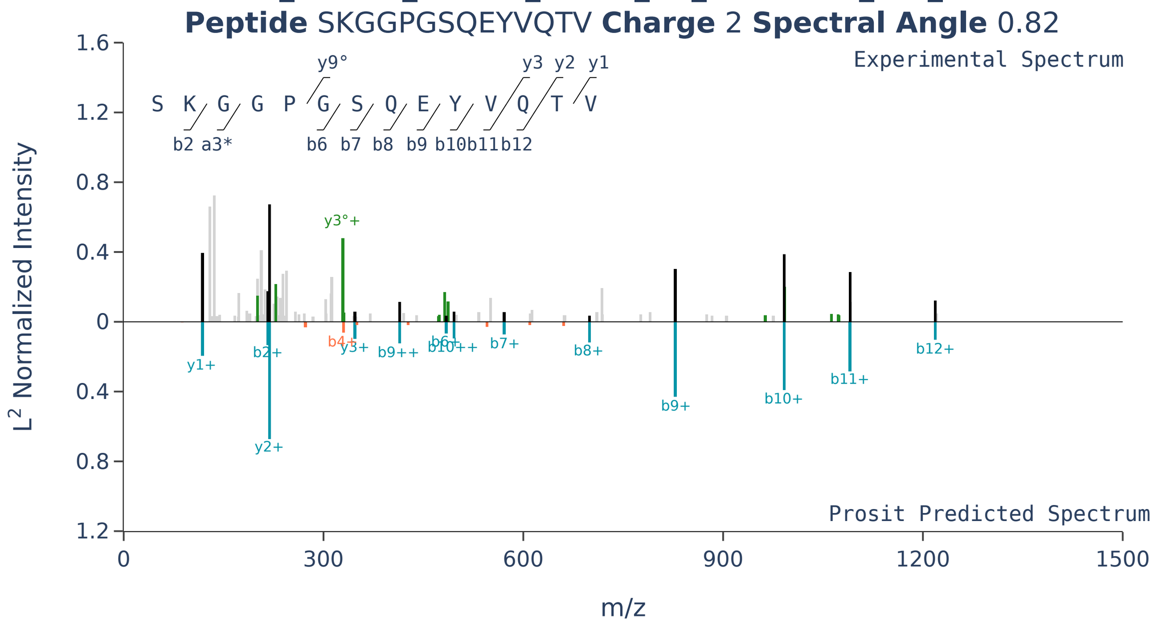

Source WSoh_301121_021221_HFGoe_M1_AnnexinA1_24h_R1 **Scan** 16500
Peptide KSVSEFLKQA **Charge** 3 **Spectral Angle** 0.89

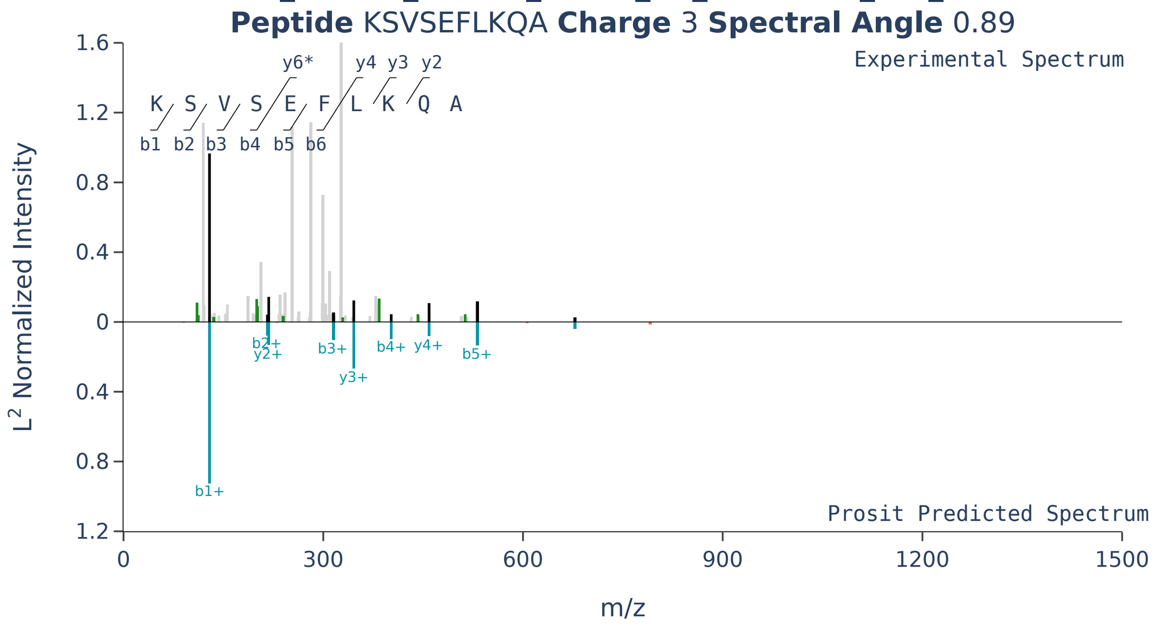

Source WSoh_301121_021221_HFGoe_M2_AnnexinA1_4h_R1 **Scan** 6537
Peptide RDINRVYKE **Charge** 3 **Spectral Angle** 0.82

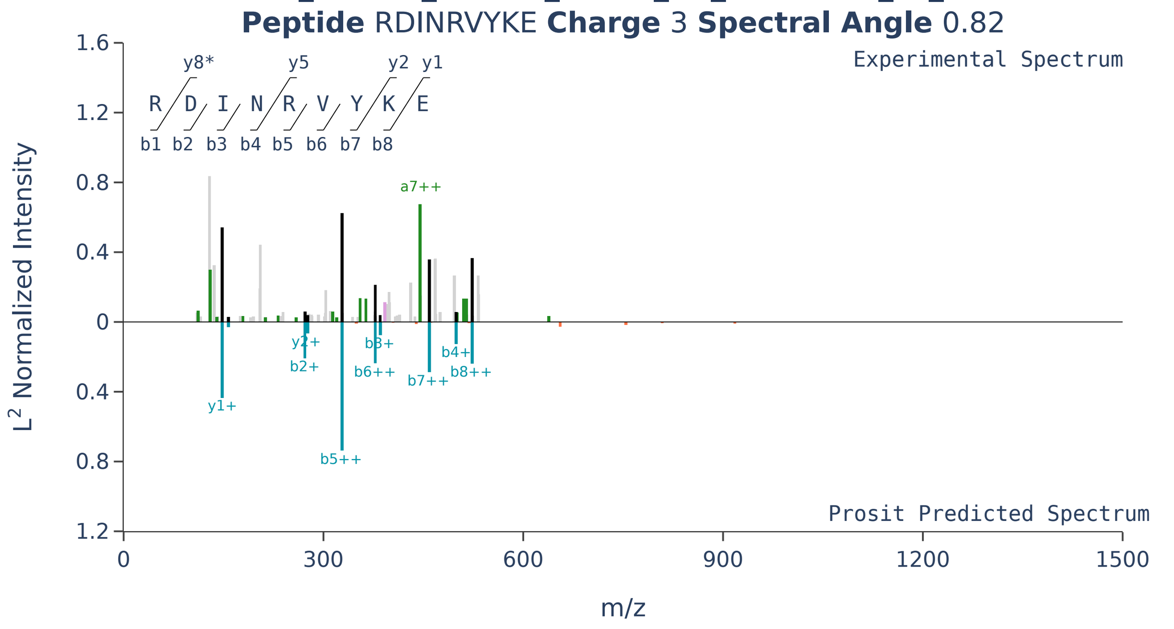

Source WSoh_301121_021221_HFGoe_M2_AnnexinA1_2h_R2 **Scan** 11583
Peptide ASDARALYE **Charge** 2 **Spectral Angle** 0.83

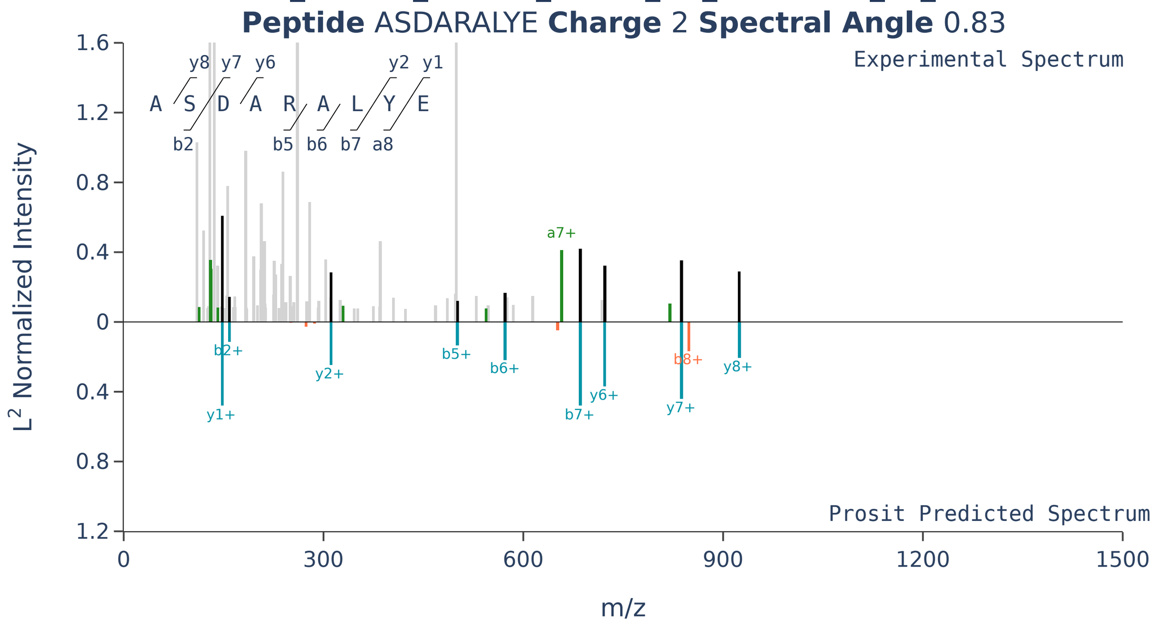

Source WSoh_301121_021221_HFGoe_M1_AnnexinA1_24h_R1 **Scan** 12636
Peptide ILDETKRDL **Charge** 2 **Spectral Angle** 0.9

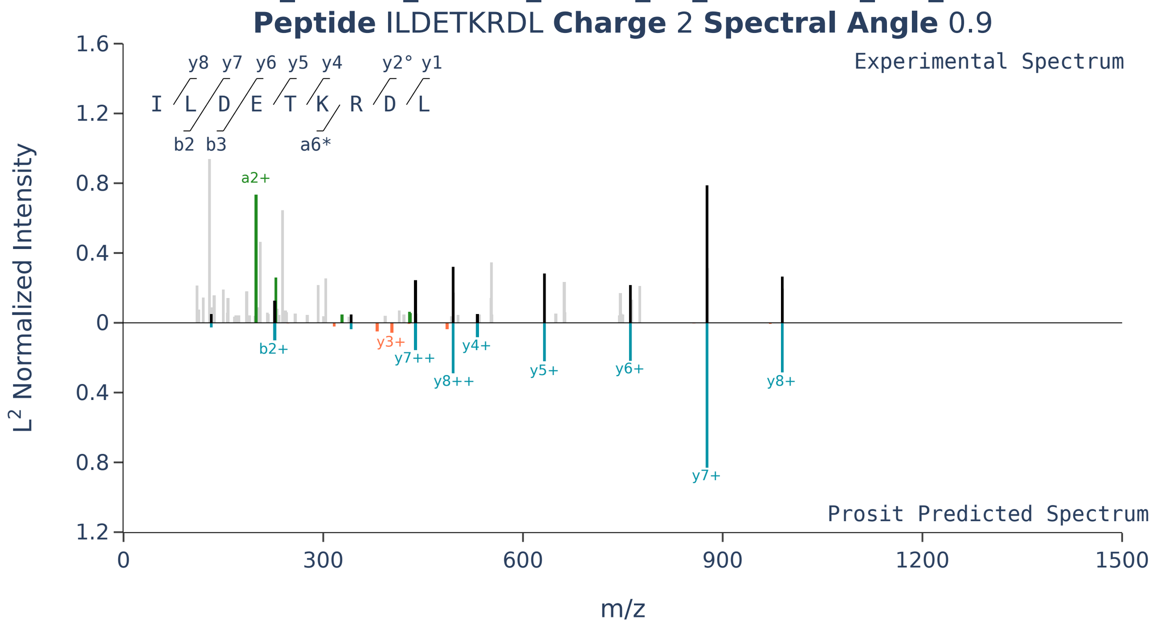

Source WSoh_301121_021221_HFGoe_M2_AnnexinA1_24h_R2 **Scan** 10908
Peptide QEYVQTVKTG **Charge** 2 **Spectral Angle** 0.87

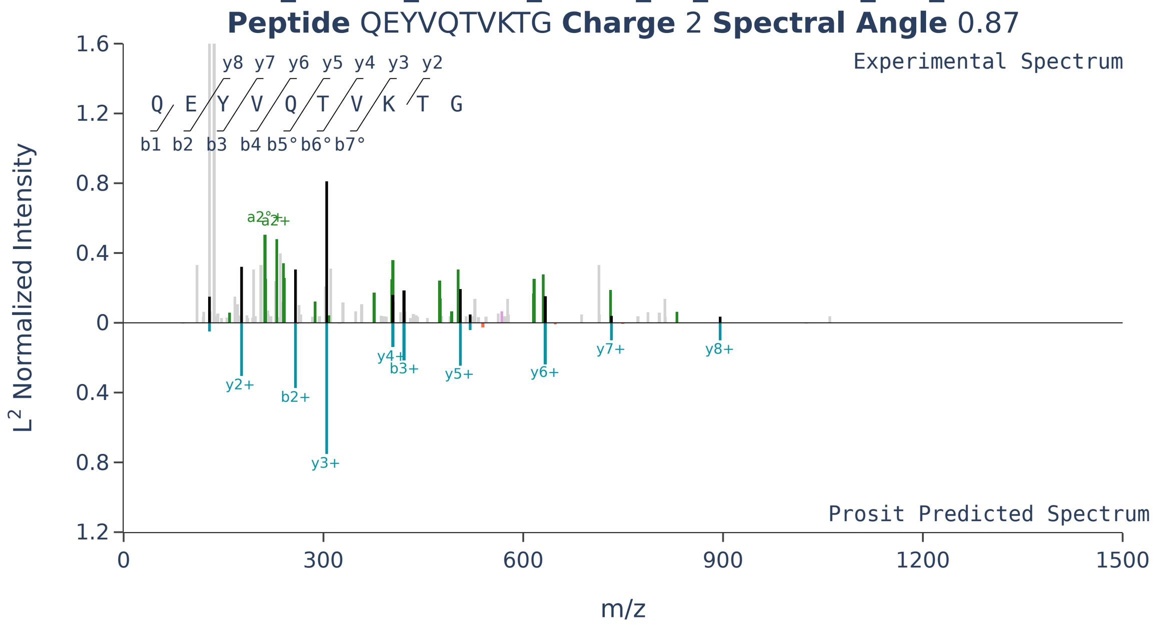

Source WSoh_301121_021221_HFGoe_M2_AnnexinA1_24h_R2 **Scan** 18672
Peptide SRSSEIDMNDI **Charge** 2 **Spectral Angle** 0.87

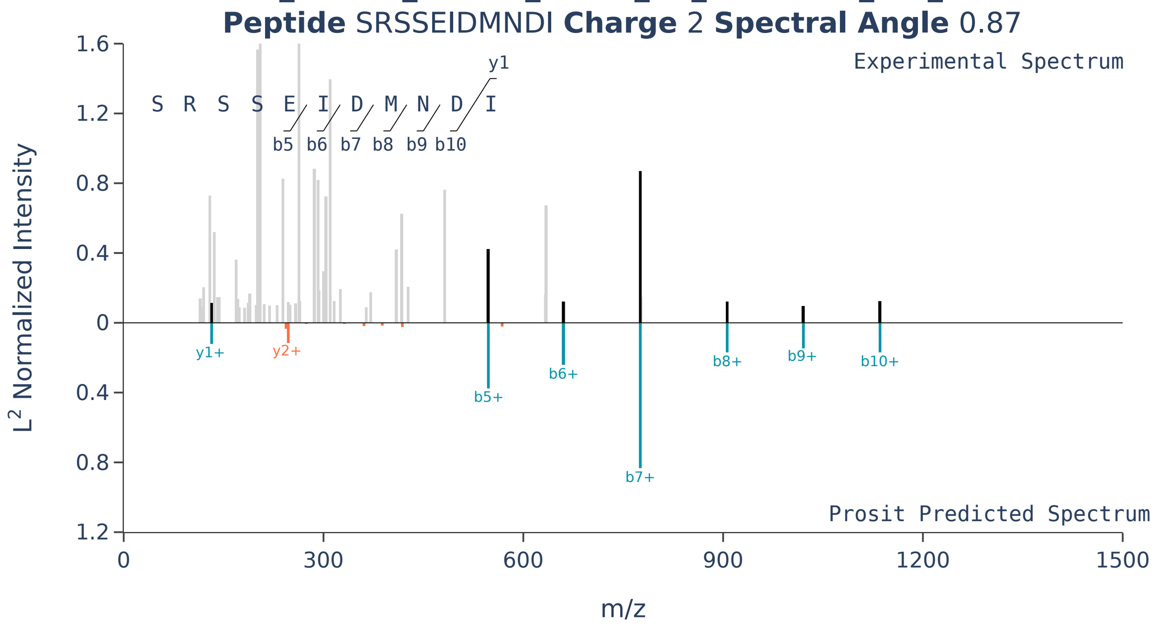

Source WSoh_301121_021221_HFGoe_M2_AnnexinA1_24h_R1 **Scan** 16313
Peptide SDINRVYREEL **Charge** 3 **Spectral Angle** 0.87

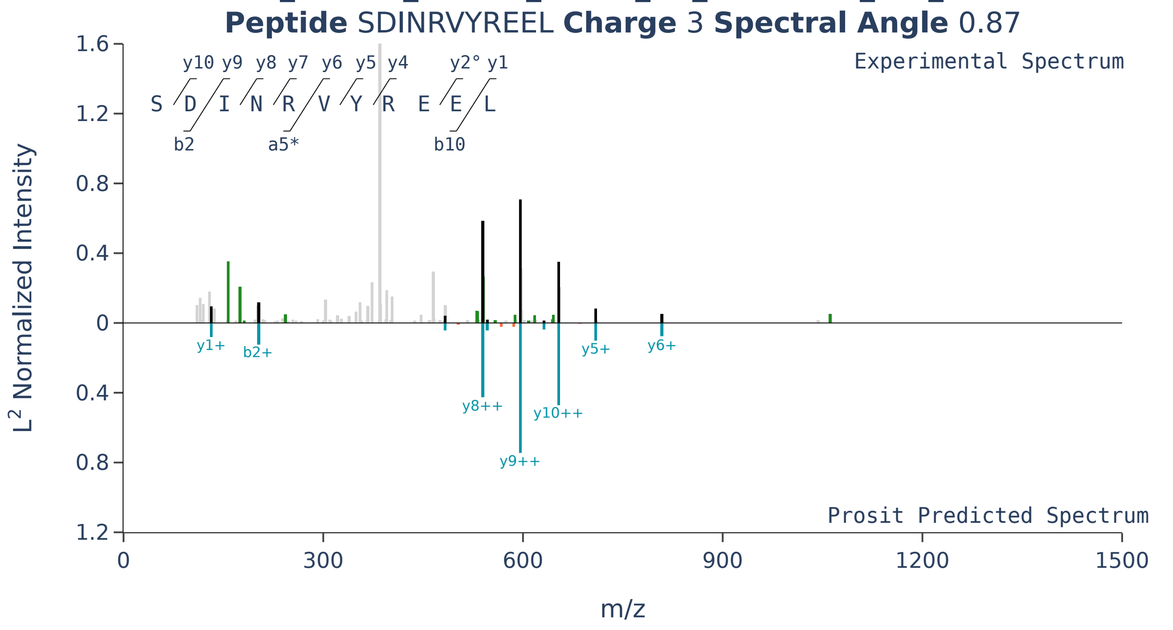

Source WSoh_301121_021221_HFGoe_M1_AnnexinA1_24h_R2 **Scan** 10880
Peptide SKGGPGSEQEYVQTVKS **Charge** 3 **Spectral Angle** 0.86

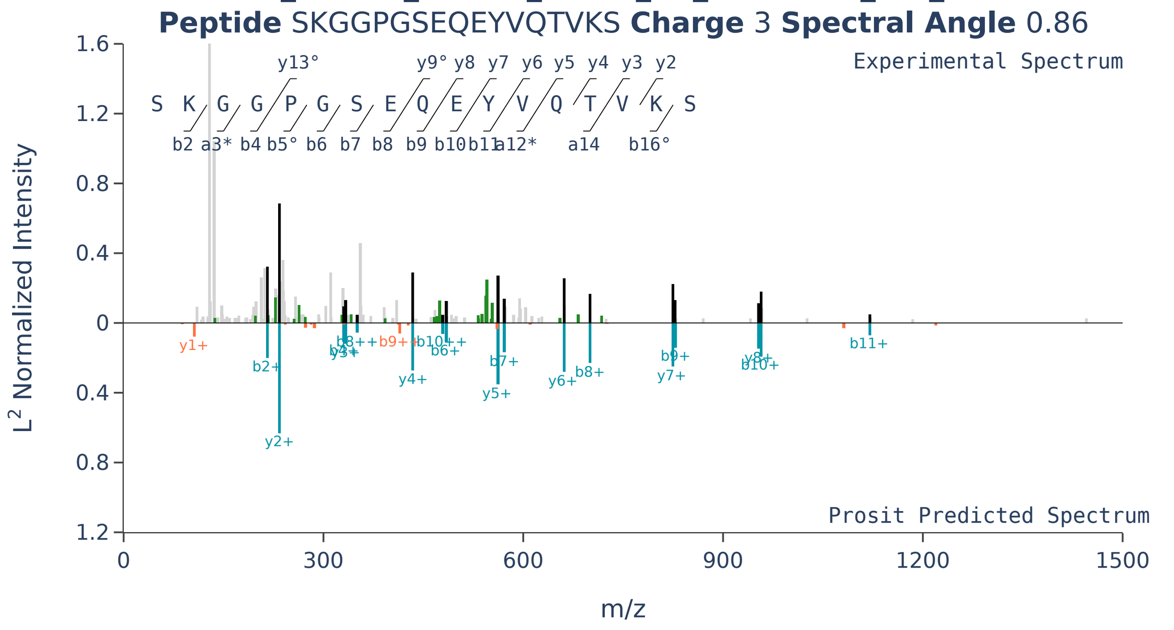

Source WSoh_301121_021221_HFGoe_M2_AnnexinA1_24h_R2 **Scan** 14577
Peptide KGGPGSEQEYVQTV **Charge** 2 **Spectral Angle** 0.79

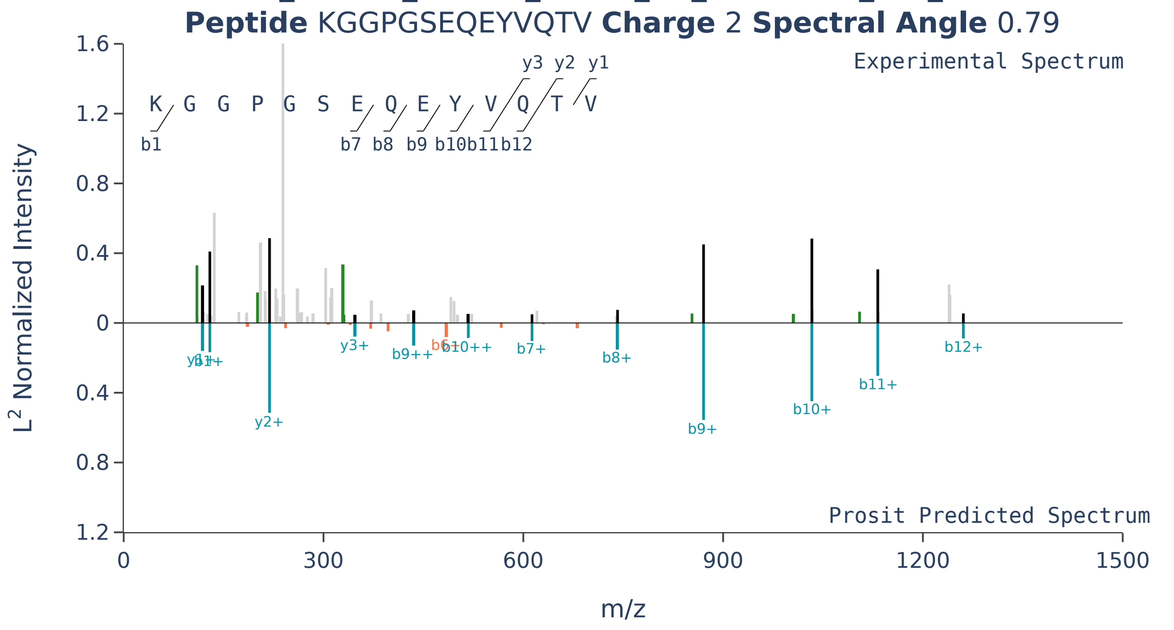

Source WSoh_301121_021221_HFGoe_M1_AnnexinA1_4h_R1 **Scan** 18702
Peptide SARSEIDMNDI **Charge** 2 **Spectral Angle** 0.87

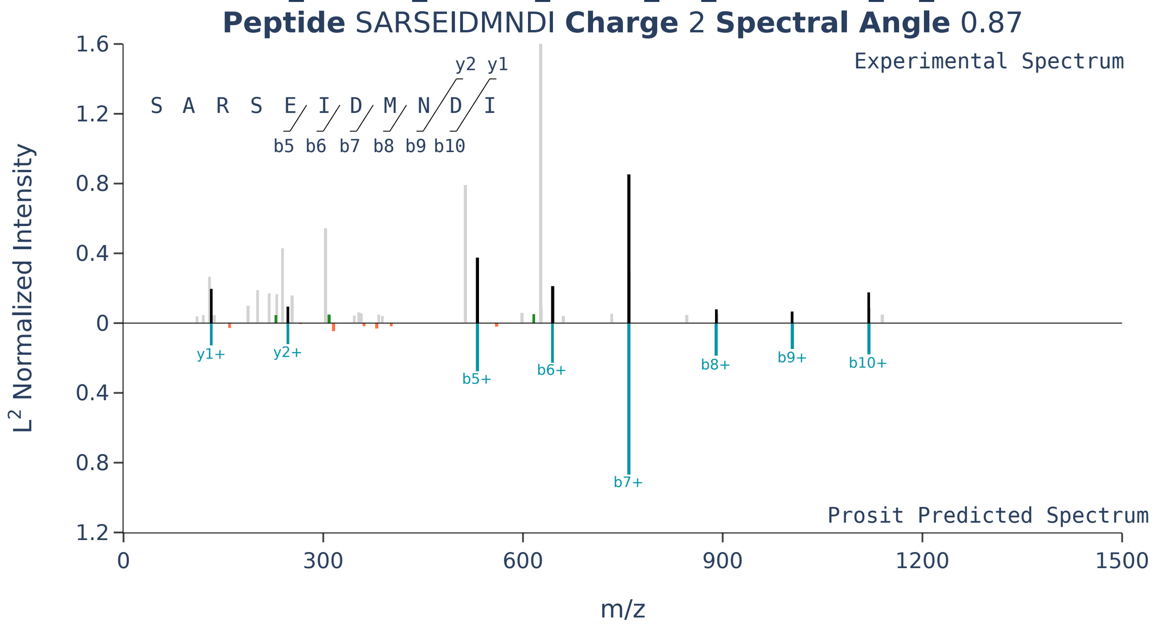

Source WSoh_301121_021221_HFGoe_M1_AnnexinA1_24h_R1 **Scan** 18722
Peptide ASRSEIDMNDI **Charge** 2 **Spectral Angle** 0.89

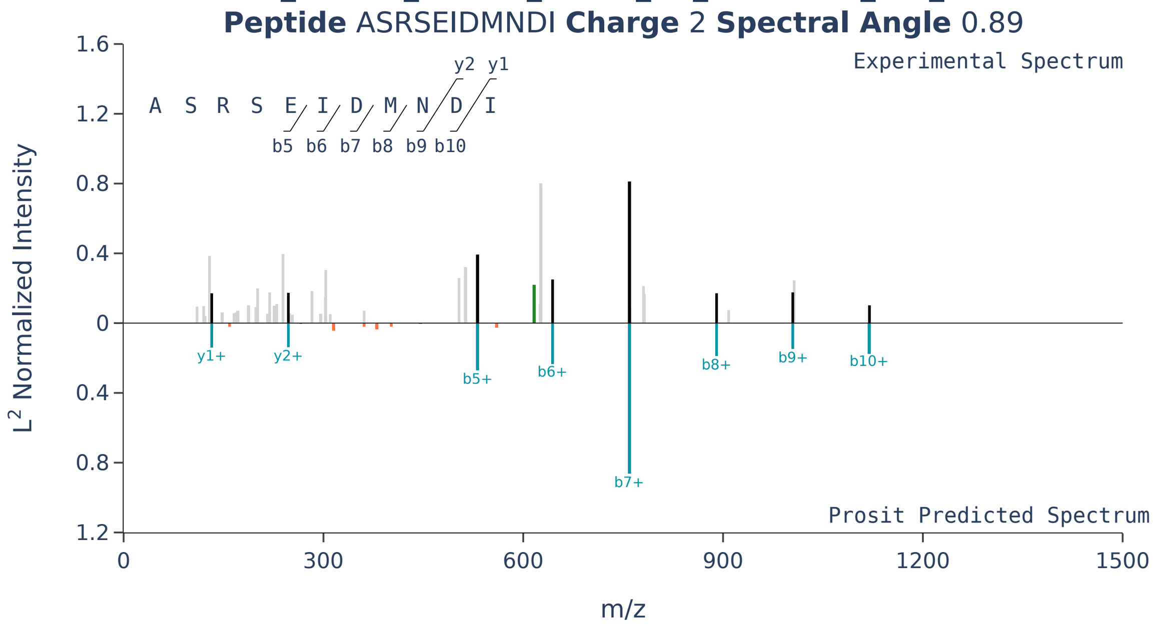

Source WSoh_301121_021221_HFGoe_M2_AnnexinA1_24h_R1 **Scan** 15979
Peptide KGDYEKILVS **Charge** 2 **Spectral Angle** 0.8

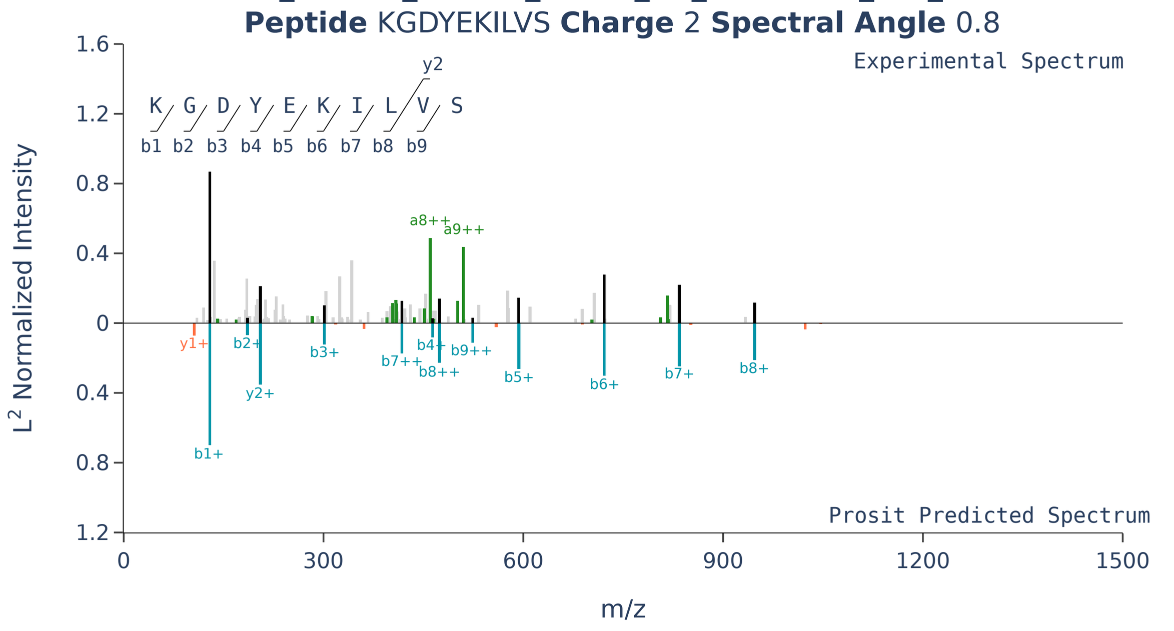

Source WSoh_301121_021221_HFGoe_M2_AnnexinA1_24h_R1 **Scan** 20000
Peptide SVSEFLKQA **Charge** 2 **Spectral Angle** 0.83

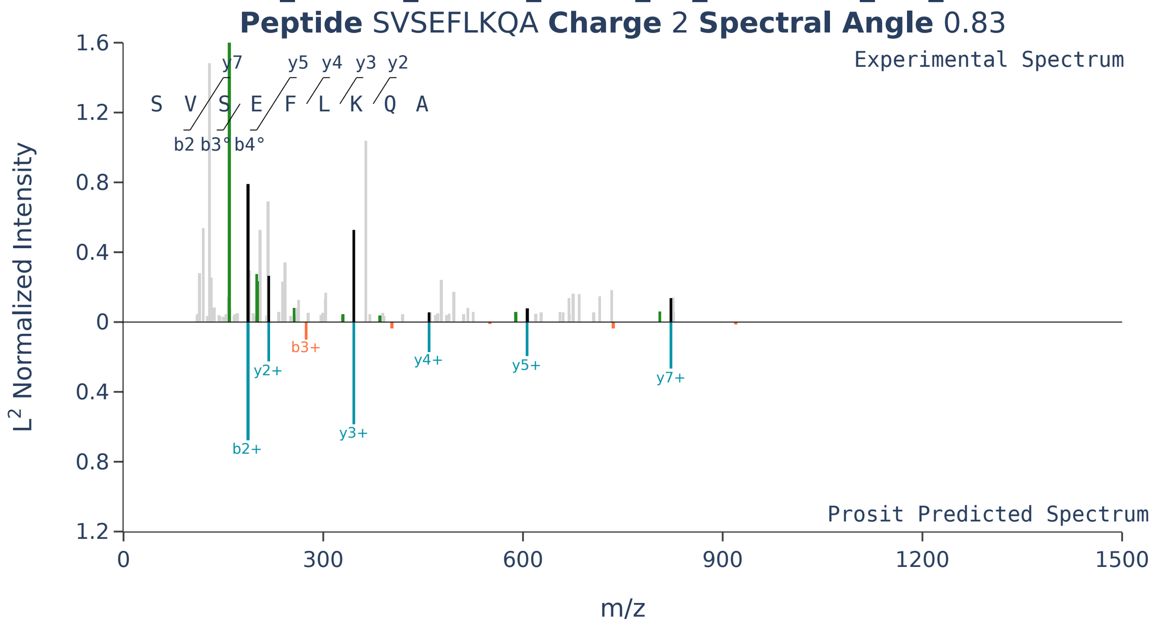

Source WSoh_301121_021221_HFGoe_M2_AnnexinA1_24h_R2 **Scan** 10911
Peptide AKDITSDTSGDFRNKRDL **Charge** 4 **Spectral Angle** 0.93

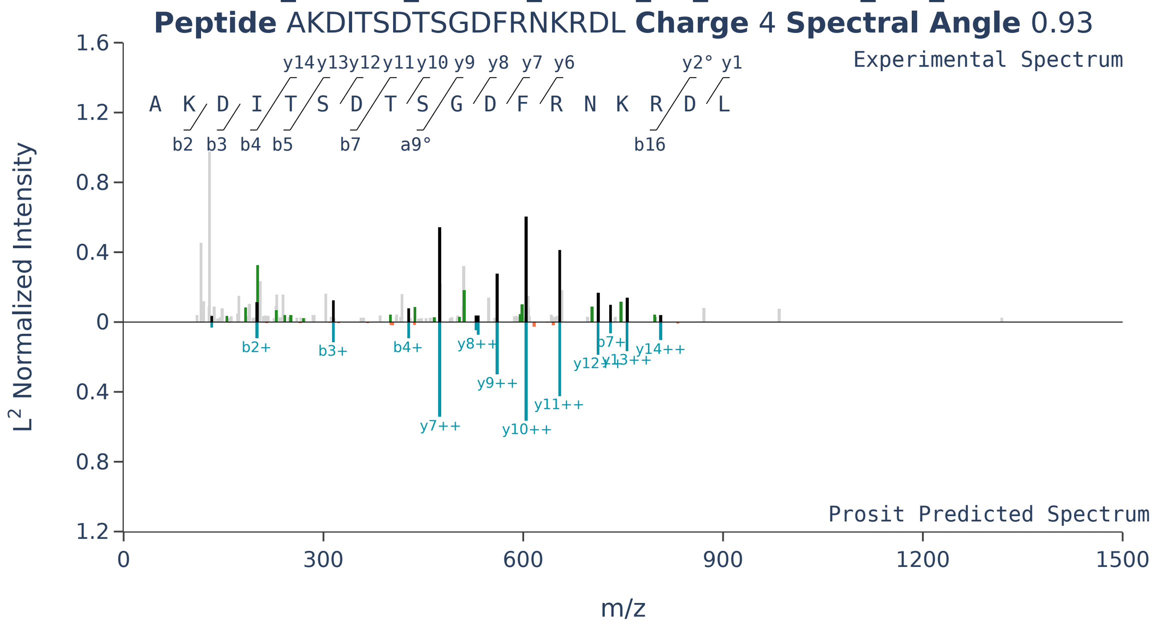

Source WSoh_301121_021221_HFGoe_M1_AnnexinA1_4h_R1 **Scan** 11429
Peptide ILTTRGDY **Charge** 2 **Spectral Angle** 0.94

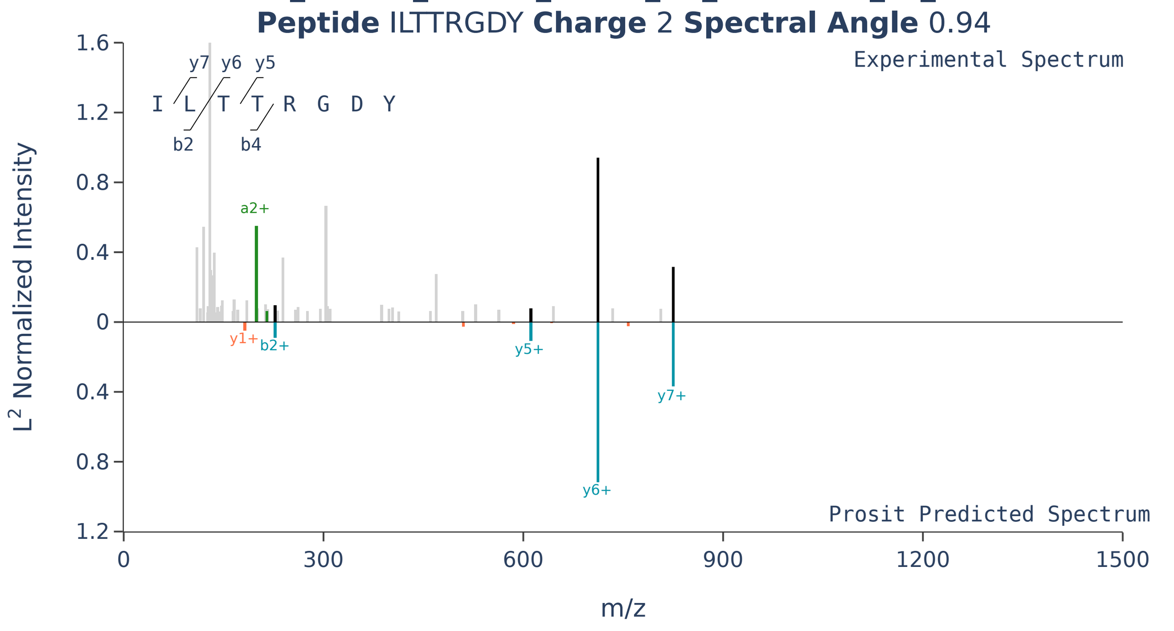

Source WSoh_301121_021221_HFGoe_M2_AnnexinA1_4h_R2 **Scan** 9907
Peptide GEELKRDLA **Charge** 2 **Spectral Angle** 0.84

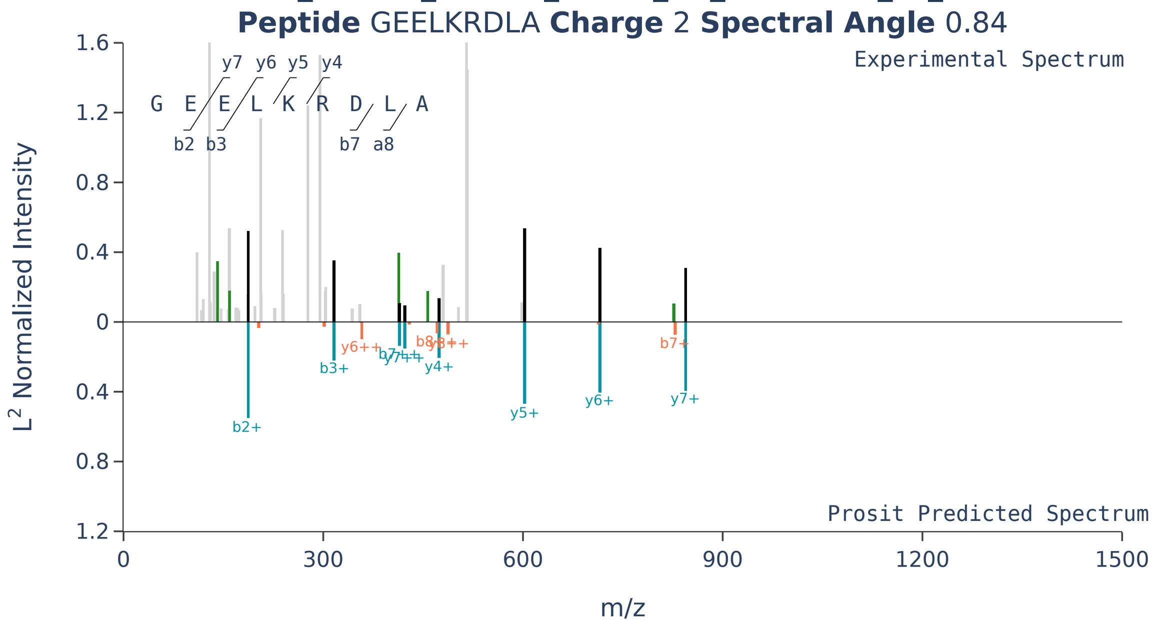

Source WSoh_301121_021221_HFGoe_M1_AnnexinA1_24h_R2 **Scan** 6899
Peptide KGVDEAKRDL **Charge** 2 **Spectral Angle** 0.76

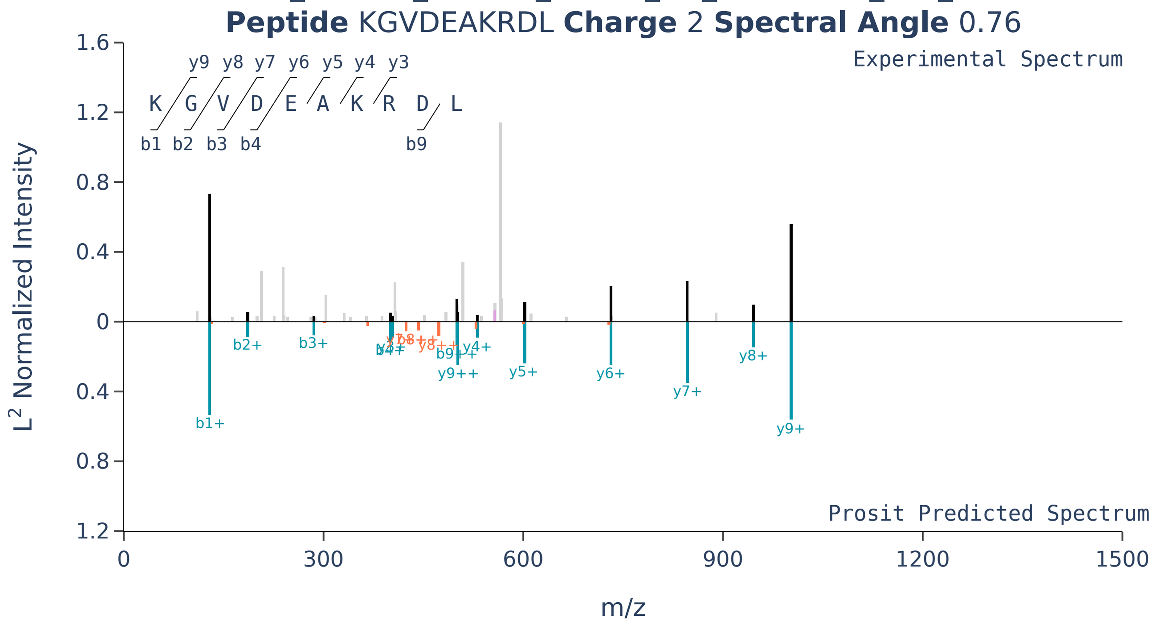

Source WSoh_301121_021221_HFGoe_M1_AnnexinA1_1h_R1 **Scan** 13465
Peptide KEIGDYEKI **Charge** 3 **Spectral Angle** 0.87

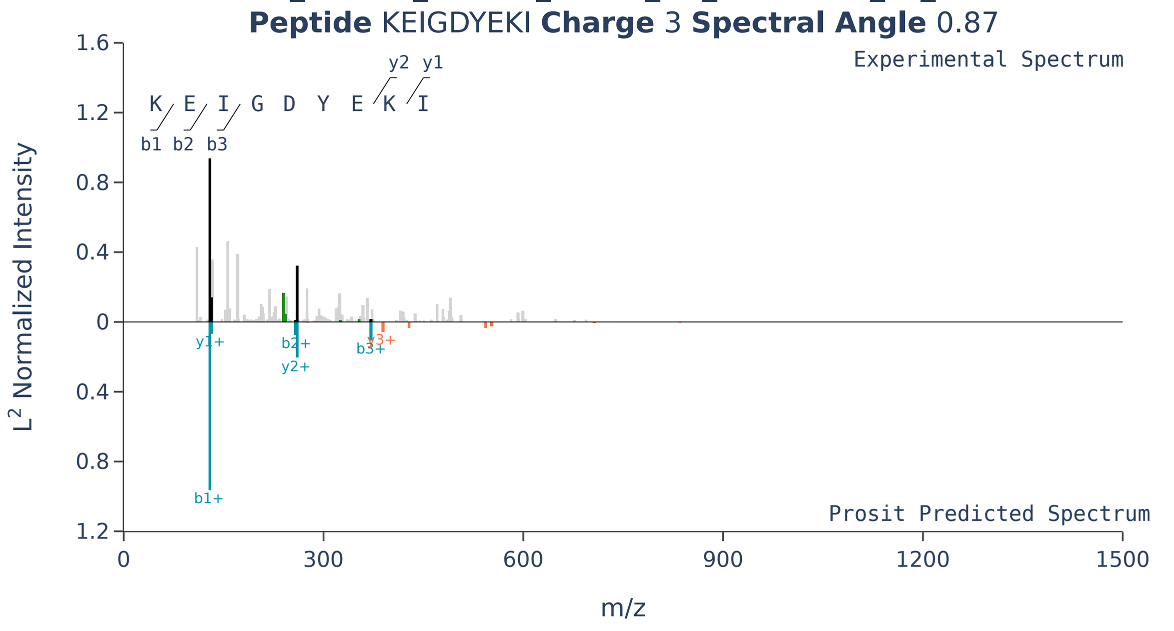

Source WSoh_301121_021221_HFGoe_M2_AnnexinA1_24h_R1 **Scan** 18761
Peptide KEDYEKILV **Charge** 2 **Spectral Angle** 0.79

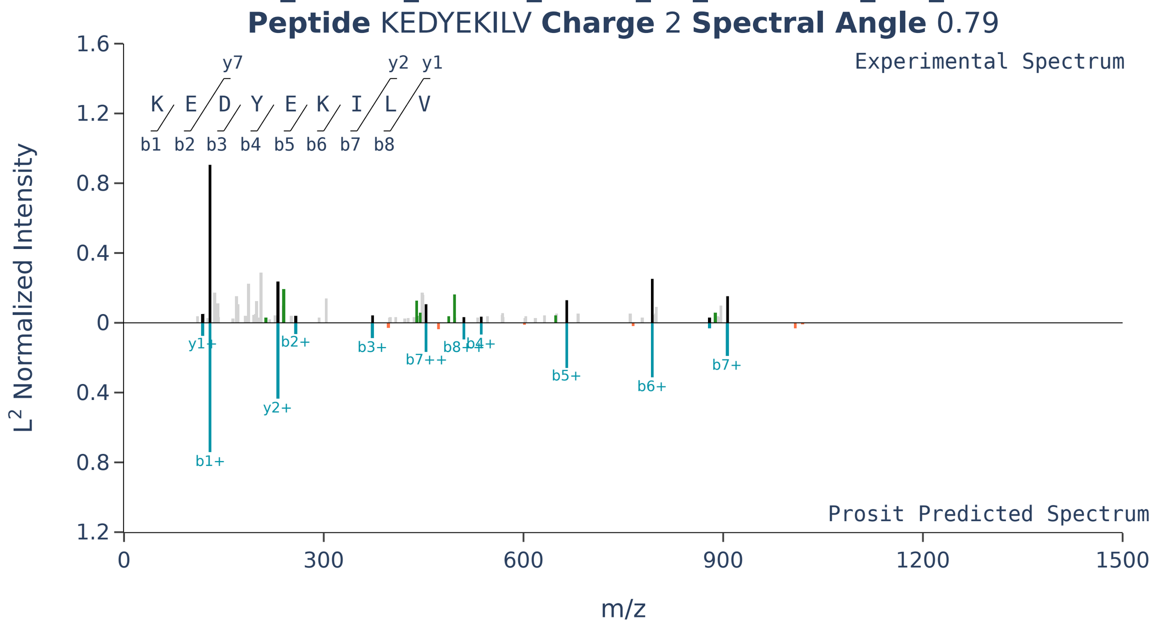

Source WSoh_301121_021221_HFGoe_M1_AnnexinA1_24h_R2 **Scan** 11180
Peptide DQEYVQTVKS **Charge** 2 **Spectral Angle** 0.85

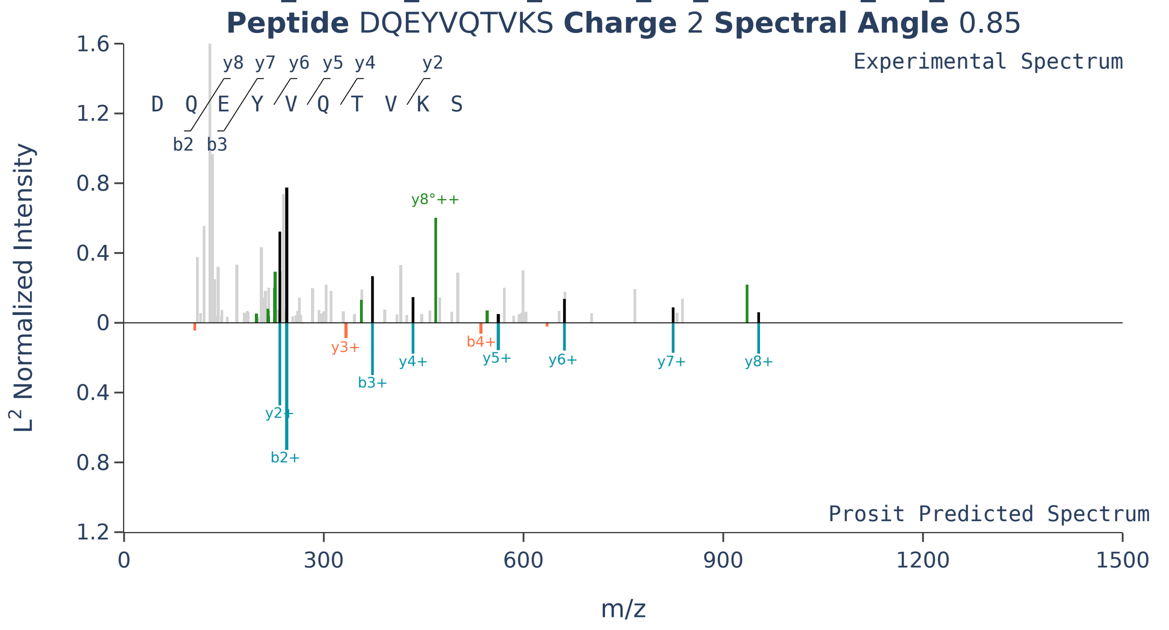

Source WSoh_301121_021221_HFGoe_M2_AnnexinA1_24h_R1 **Scan** 9665
Peptide SKGGPGSQEYVQTVKS **Charge** 3 **Spectral Angle** 0.86

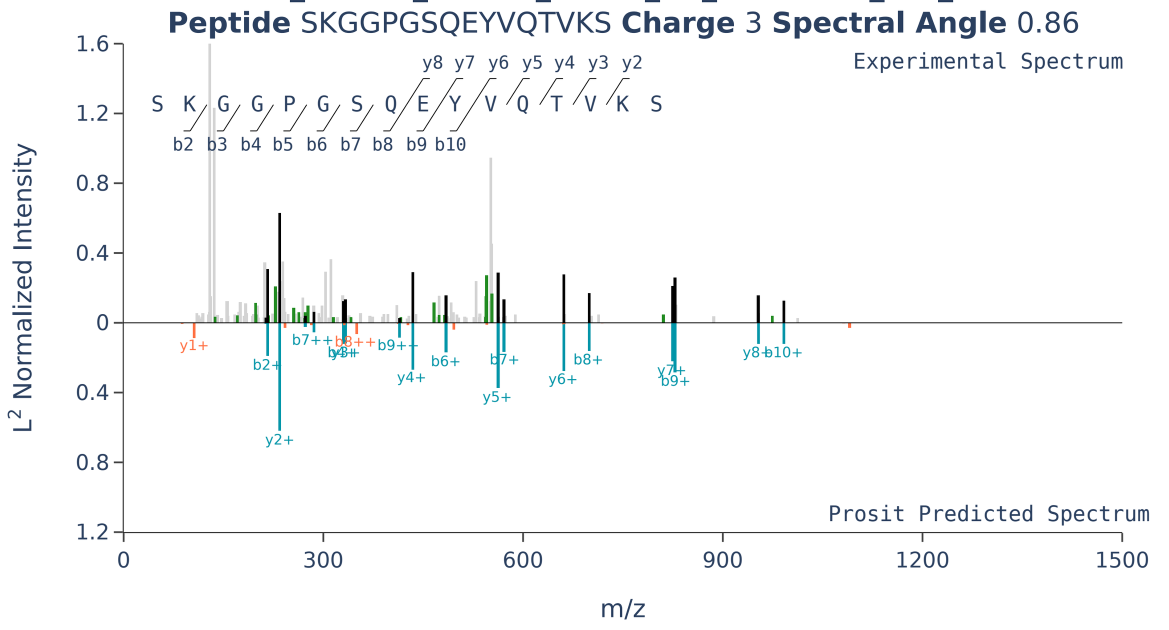

Source WSoh_301121_021221_HFGoe_M2_AnnexinA1_24h_R2 **Scan** 14519
Peptide QEYVQTVKSV **Charge** 2 **Spectral Angle** 0.89

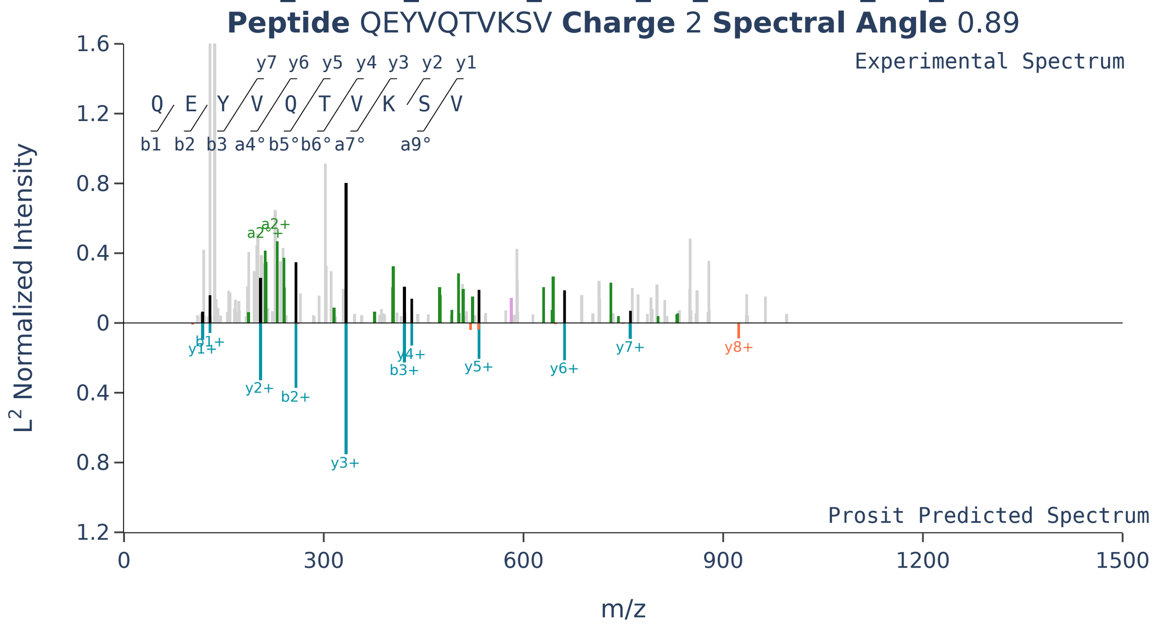

Source WSoh_301121_021221_HFGoe_M1_AnnexinA1_4h_R1 **Scan** 16305
Peptide SYLQETGKPLD **Charge** 2 **Spectral Angle** 0.85

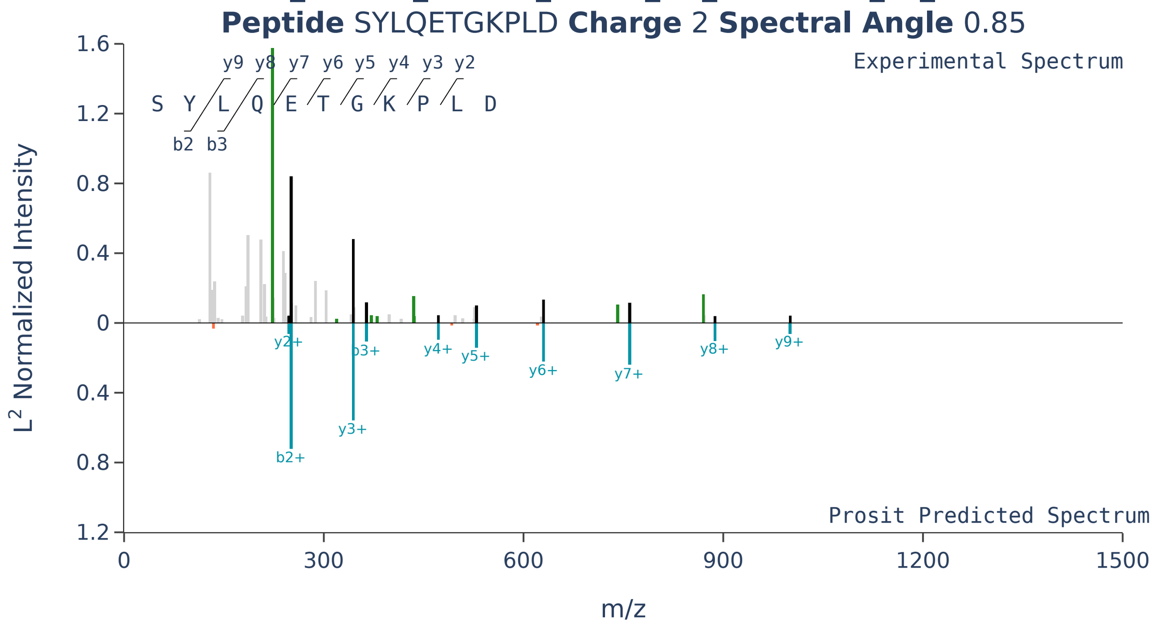

Source WSoh_301121_021221_HFGoe_M1_AnnexinA1_24h_R1 **Scan** 12286
Peptide KDITSDTSGDFRNAA **Charge** 3 **Spectral Angle** 0.75

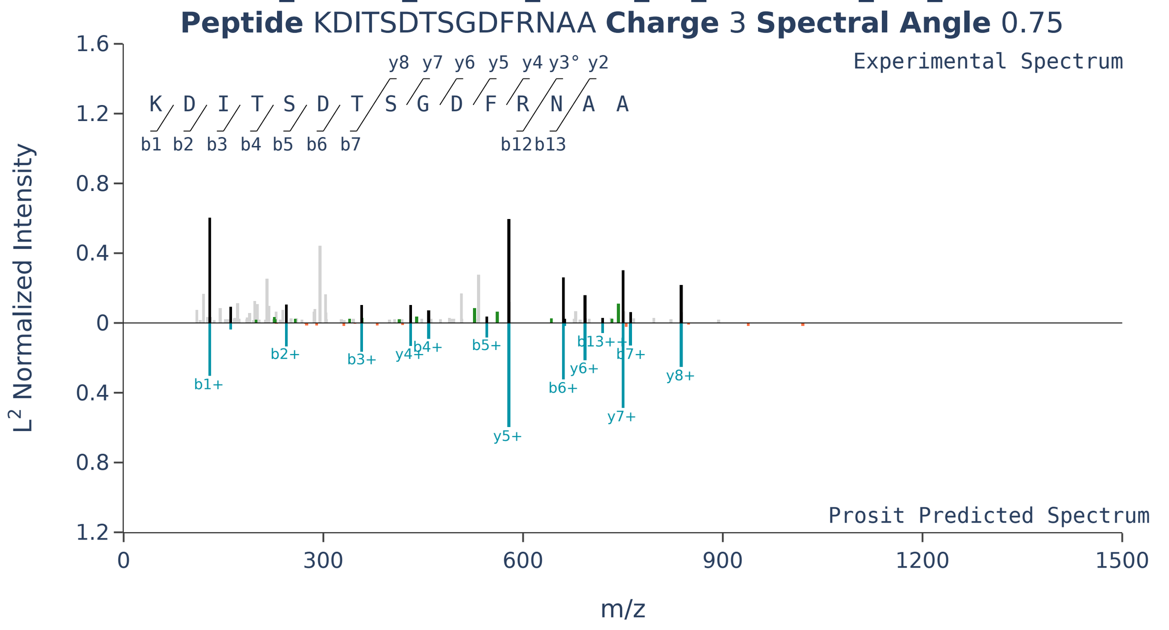

Source WSoh_301121_021221_HFGoe_M2_AnnexinA1_2h_R1 **Scan** 18929
Peptide KSAMVSEFLKQ **Charge** 3 **Spectral Angle** 0.82

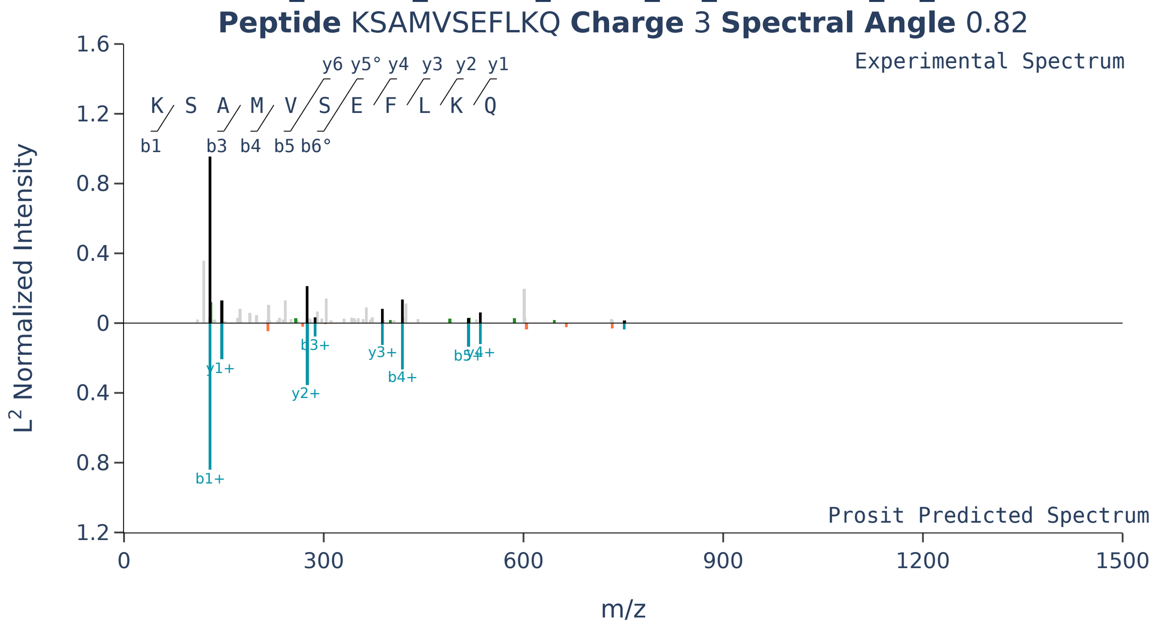

Source WSoh_301121_021221_HFGoe_M2_AnnexinA1_24h_R1 **Scan** 8240
Peptide AKRDLAKDITSD **Charge** 2 **Spectral Angle** 0.85

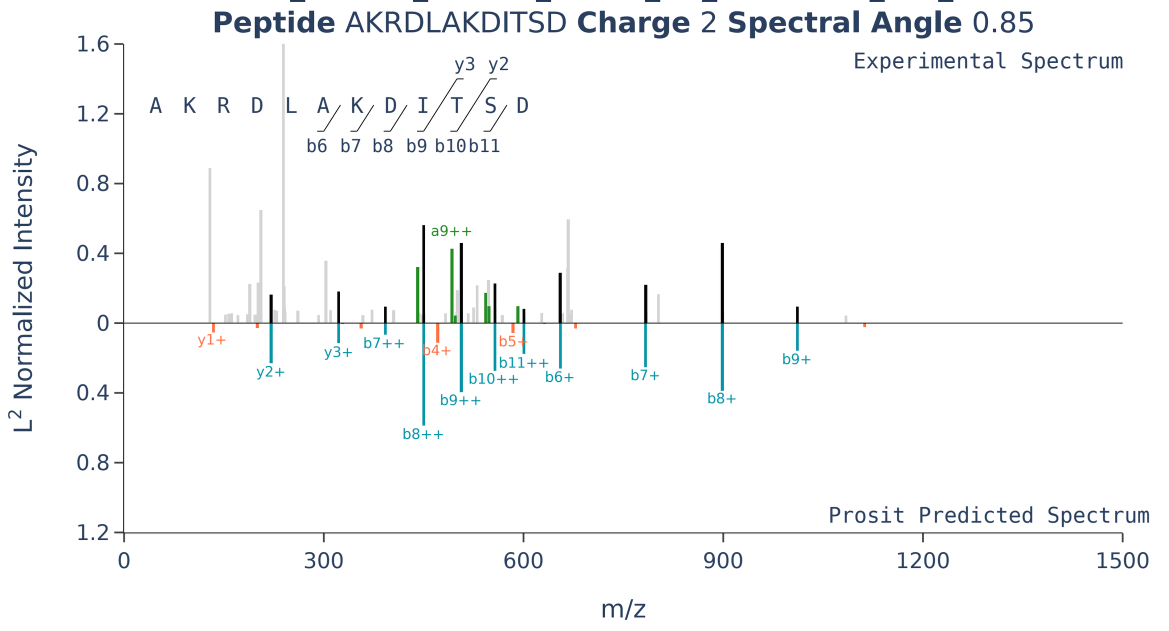

Source WSoh_301121_021221_HFGoe_M2_AnnexinA1_24h_R2 **Scan** 10534
Peptide SKGGPGSEQEYVQT **Charge** 2 **Spectral Angle** 0.86

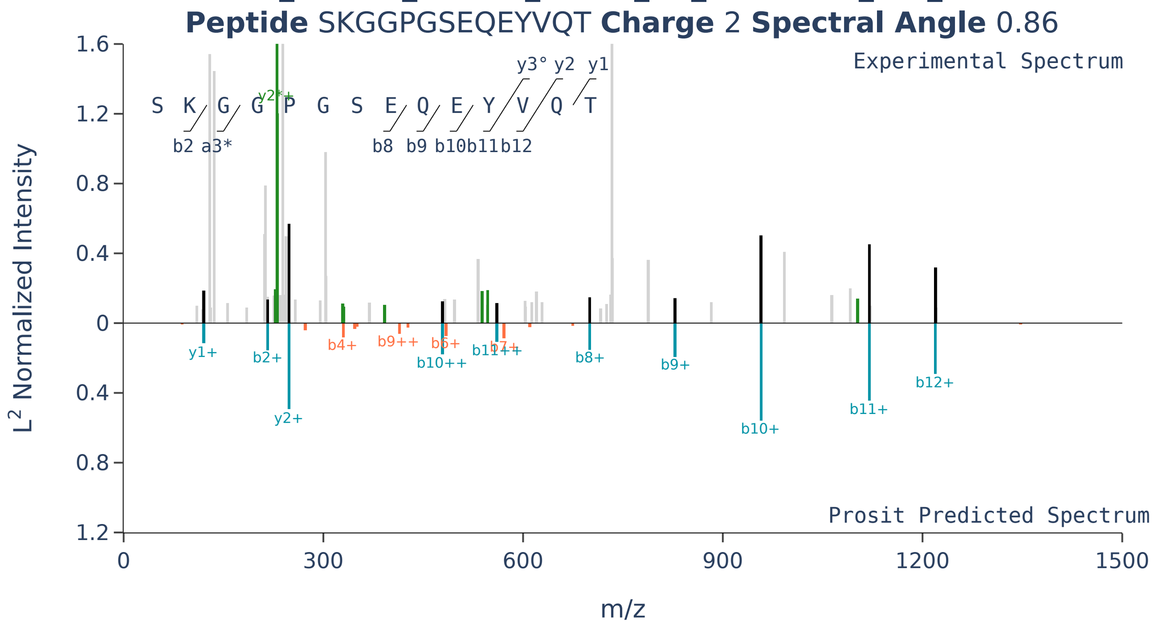

Source WSoh_301121_021221_HFGoe_M1_AnnexinA1_4h_R1 **Scan** 9042
Peptide NDLKKALTGH **Charge** 3 **Spectral Angle** 0.92

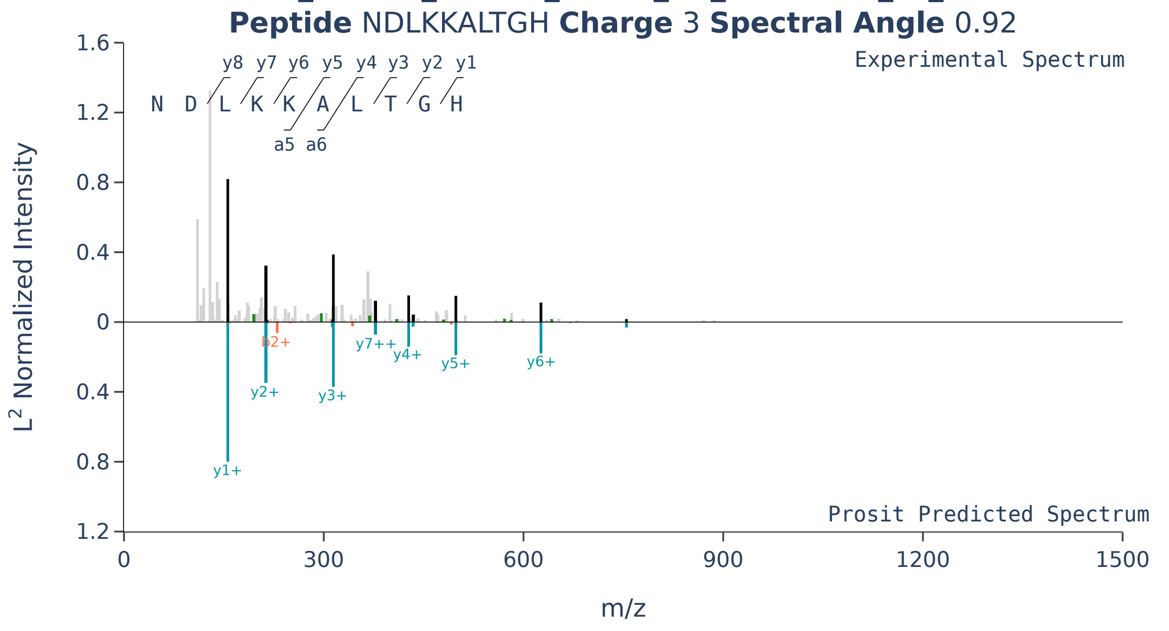

Source WSoh_301121_021221_HFGoe_M2_AnnexinA1_24h_R1 **Scan** 12246
Peptide AYLQETGKRDL **Charge** 3 **Spectral Angle** 0.85

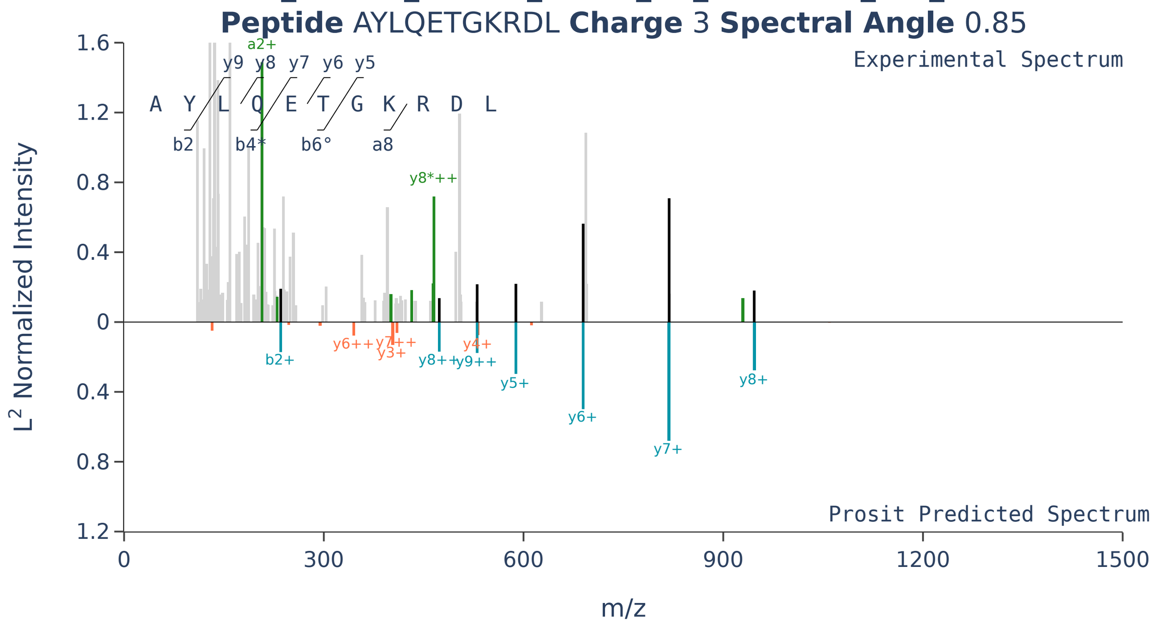

Source WSoh_301121_021221_HFGoe_M1_AnnexinA1_4h_R1 **Scan** 14528
Peptide ARSYPQL **Charge** 2 **Spectral Angle** 0.91

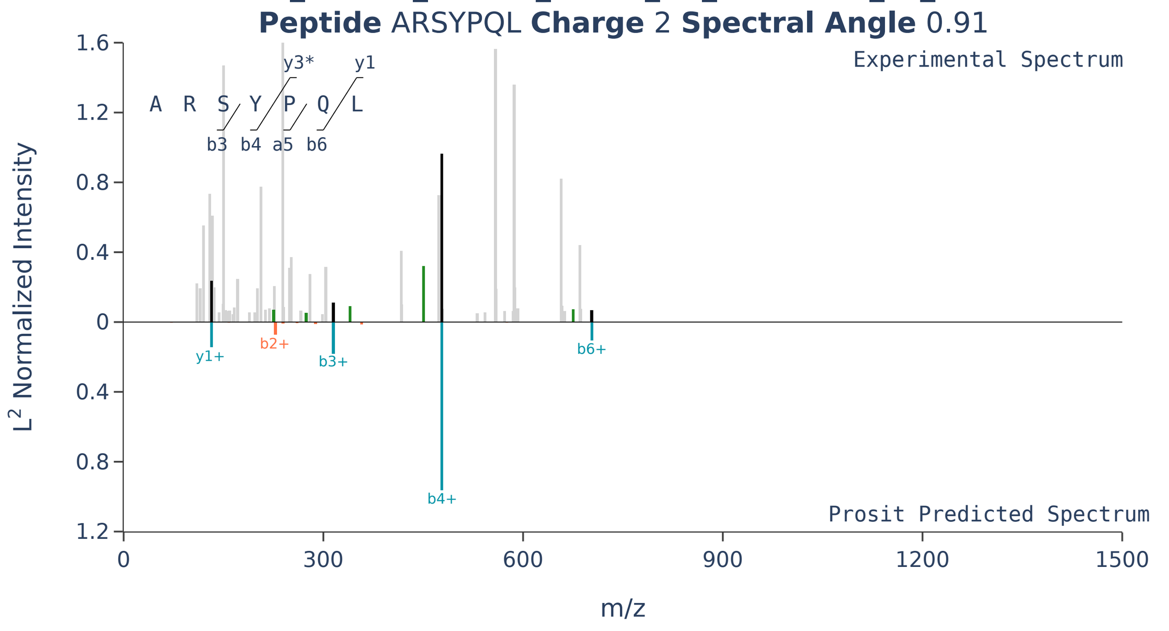

Source WSoh_301121_021221_HFGoe_M2_AnnexinA1_4h_R2 **Scan** 20269
Peptide KSPAFFAEKL **Charge** 3 **Spectral Angle** 0.84

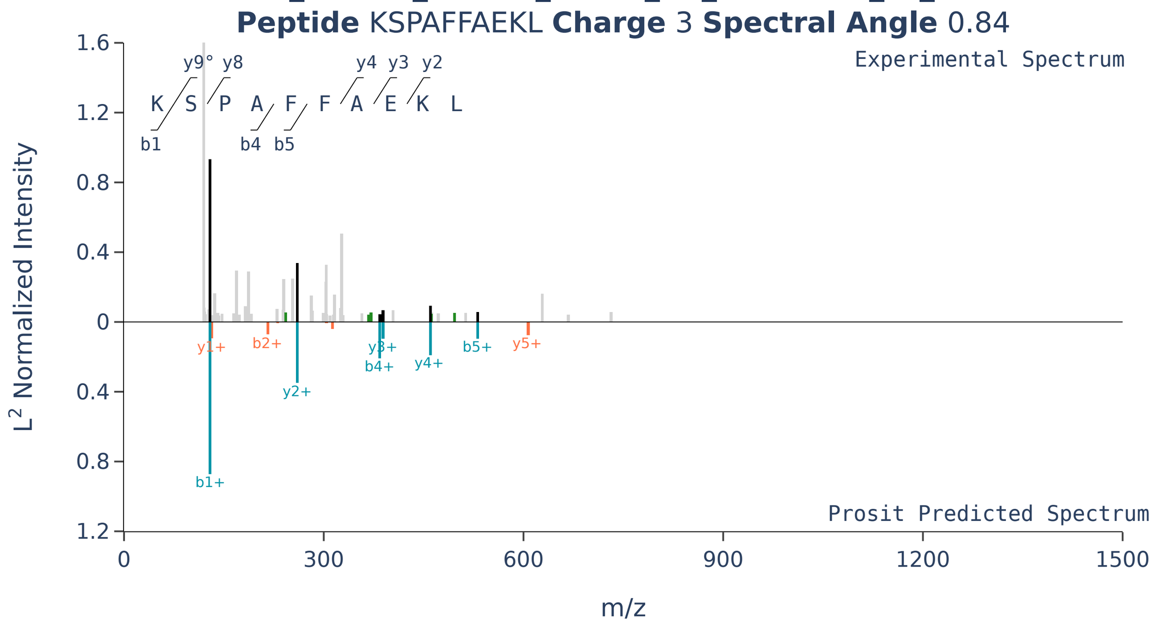

Source WSoh_301121_021221_HFGoe_M2_AnnexinA1_24h_R2 **Scan** 12750
Peptide AGERRKGTDDVNVF **Charge** 3 **Spectral Angle** 0.89

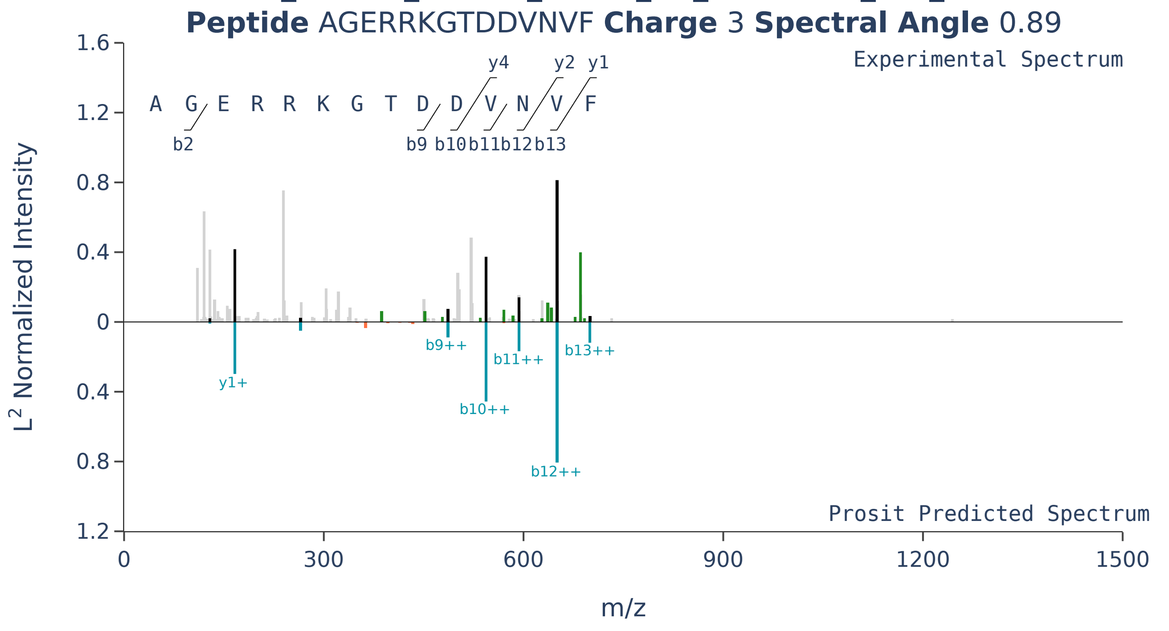

Source WSoh_301121_021221_HFGoe_M1_AnnexinA1_4h_R1 **Scan** 13663
Peptide SKPEEQEYVQTV **Charge** 2 **Spectral Angle** 0.78

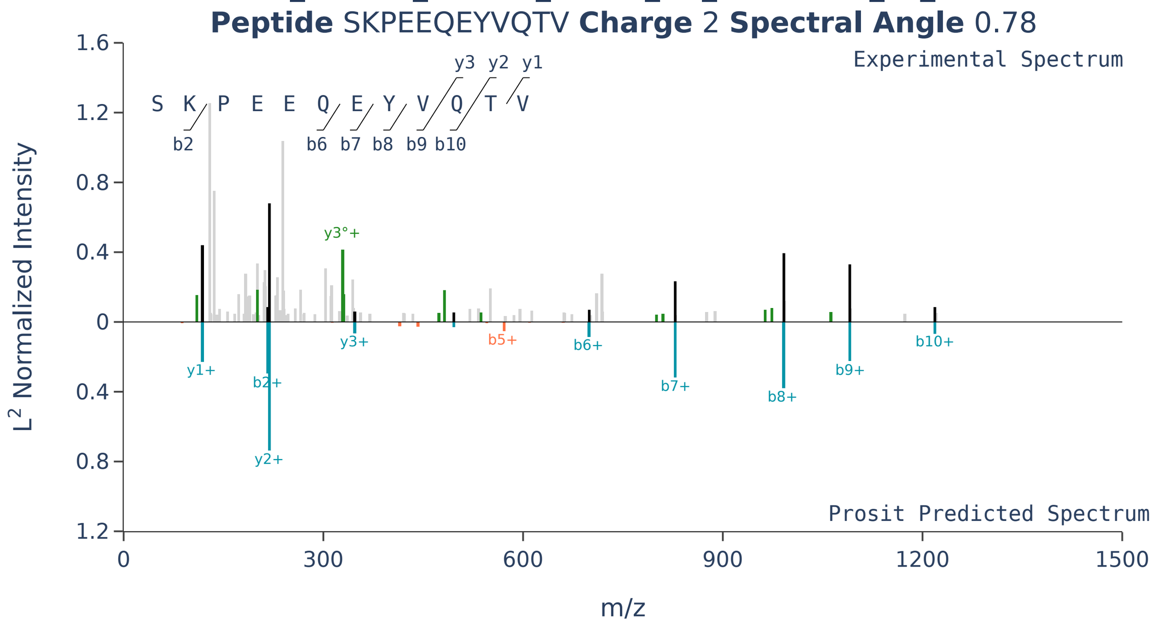

Source WSoh_301121_021221_HFGoe_M2_AnnexinA1_1h_R1 **Scan** 10913
Peptide AGEEYVQT **Charge** 1 **Spectral Angle** 0.82

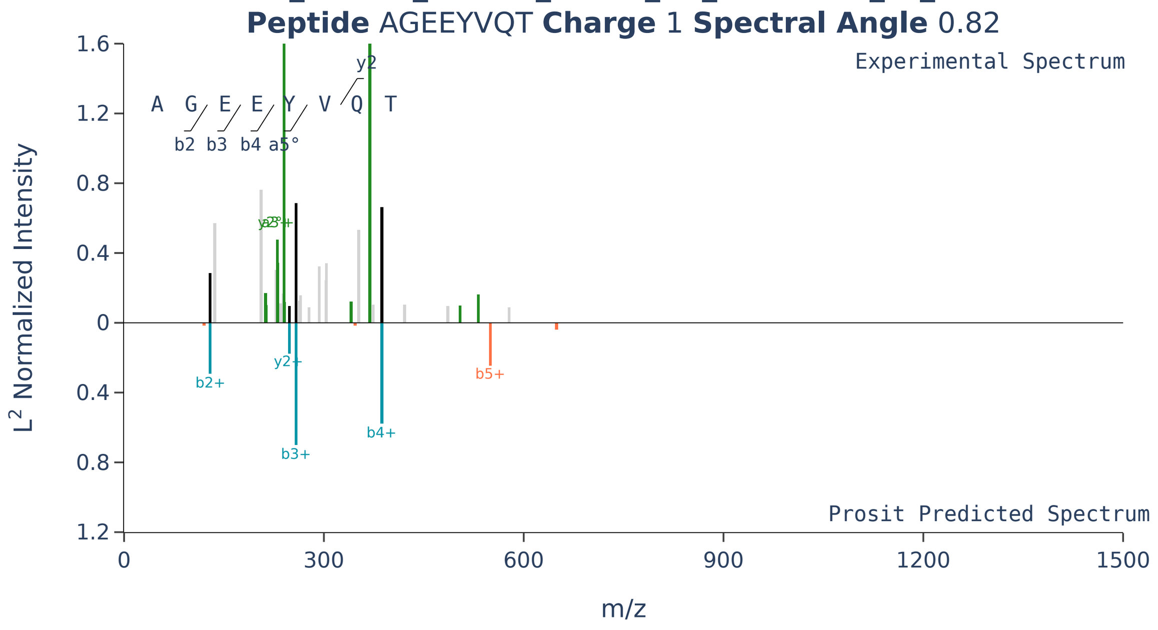

Source WSoh_301121_021221_HFGoe_M1_AnnexinA1_24h_R2 **Scan** 10305
Peptide ADELKRDL **Charge** 2 **Spectral Angle** 0.82

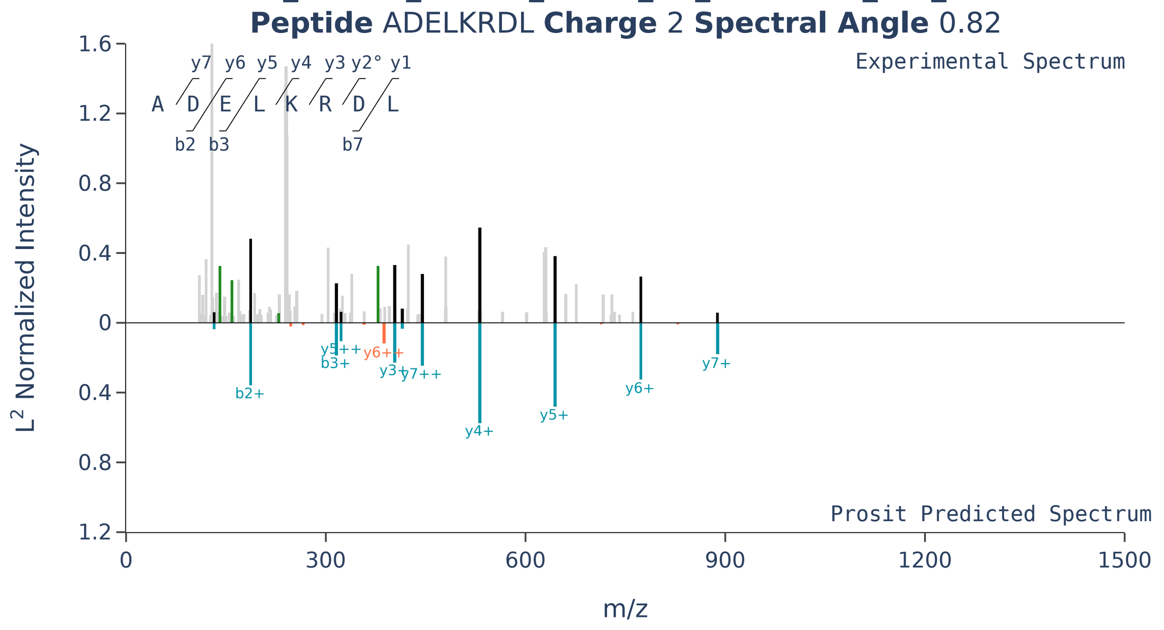

Source WSoh_301121_021221_HFGoe_M2_AnnexinA1_24h_R2 **Scan** 12534
Peptide DEQEYVQTVKS **Charge** 2 **Spectral Angle** 0.8

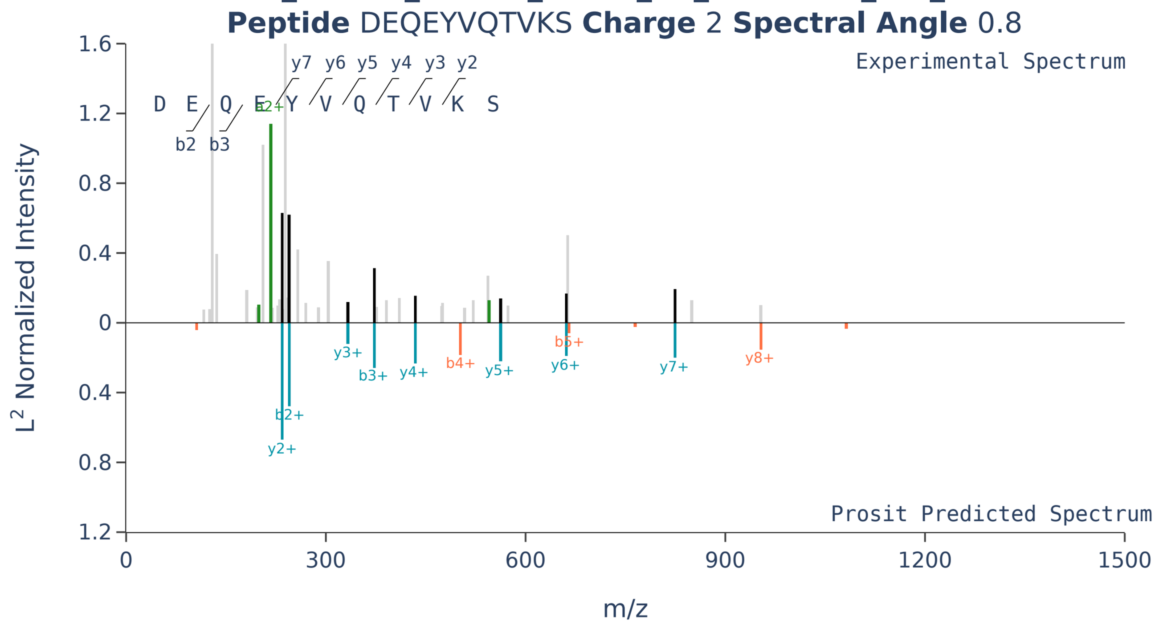

Source WSoh_301121_021221_HFGoe_M2_AnnexinA1_24h_R2 **Scan** 16243
Peptide DRSYPQL **Charge** 2 **Spectral Angle** 0.89

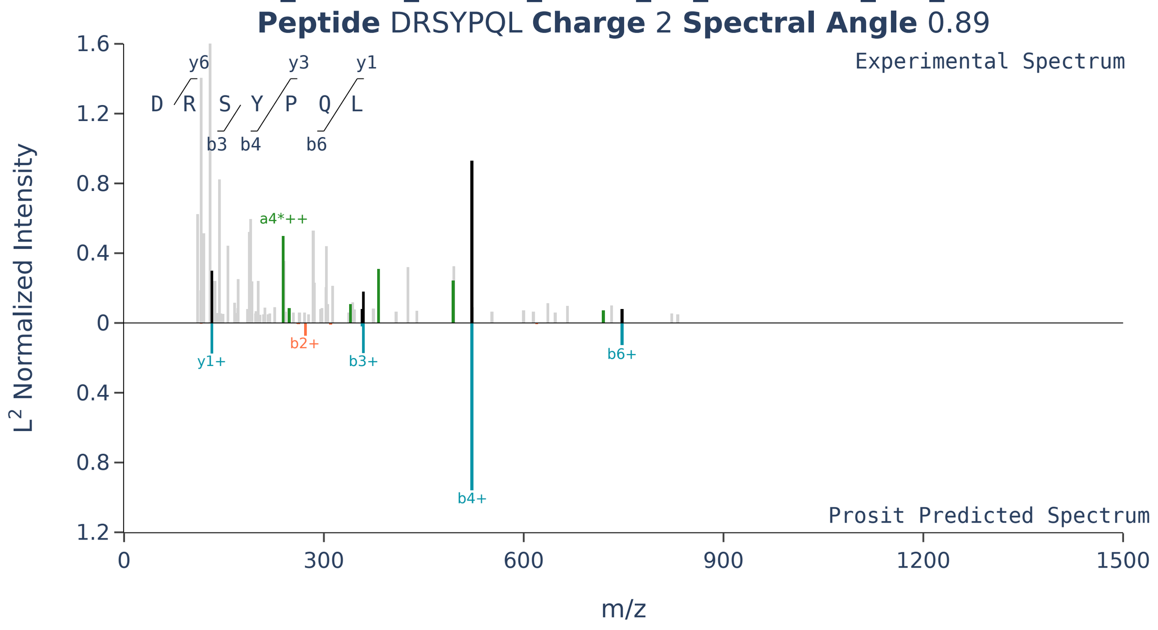

Source WSoh_301121_021221_HFGoe_M2_AnnexinA1_1h_R2 **Scan** 9454
Peptide DIKEKCL **Charge** 2 **Spectral Angle** 0.82

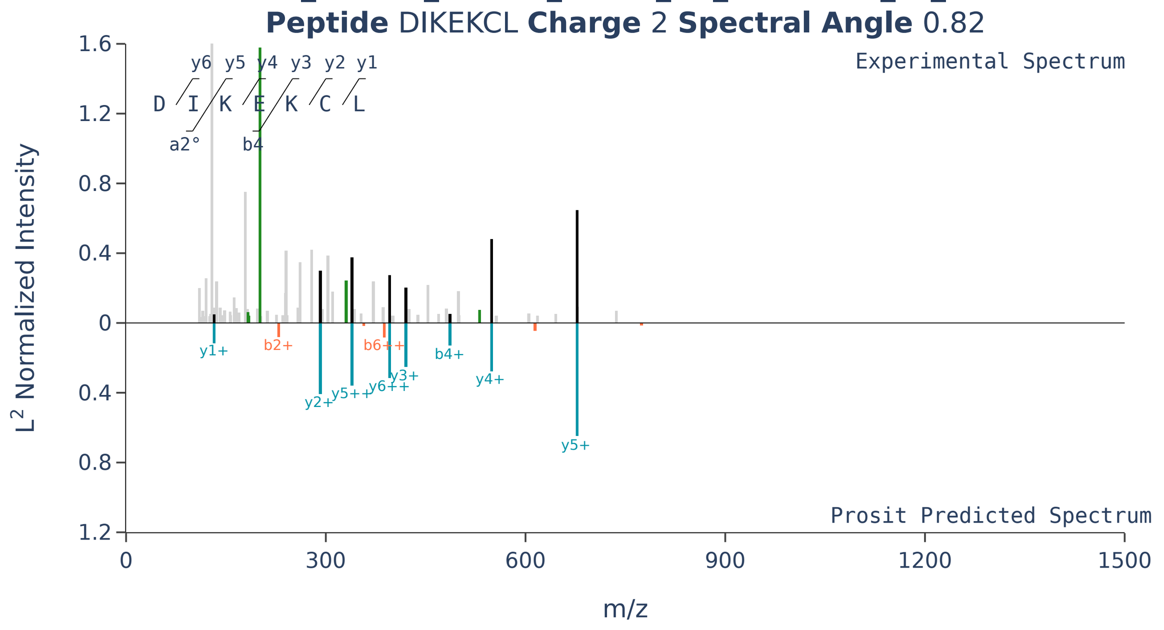

Source WSoh_301121_021221_HFGoe_M1_AnnexinA1_24h_R2 **Scan** 7989
Peptide TSGDFRQQ **Charge** 2 **Spectral Angle** 0.91

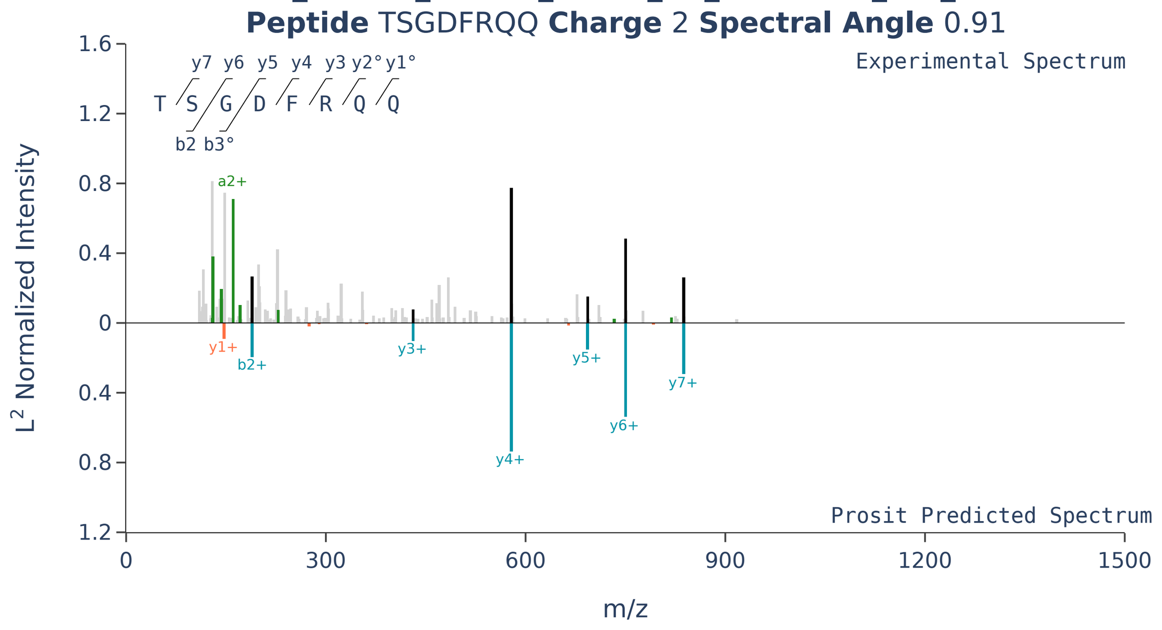

Source WSoh_301121_021221_HFGoe_M1_AnnexinA1_4h_R2 **Scan** 8949
Peptide KGSDARALYE **Charge** 2 **Spectral Angle** 0.7

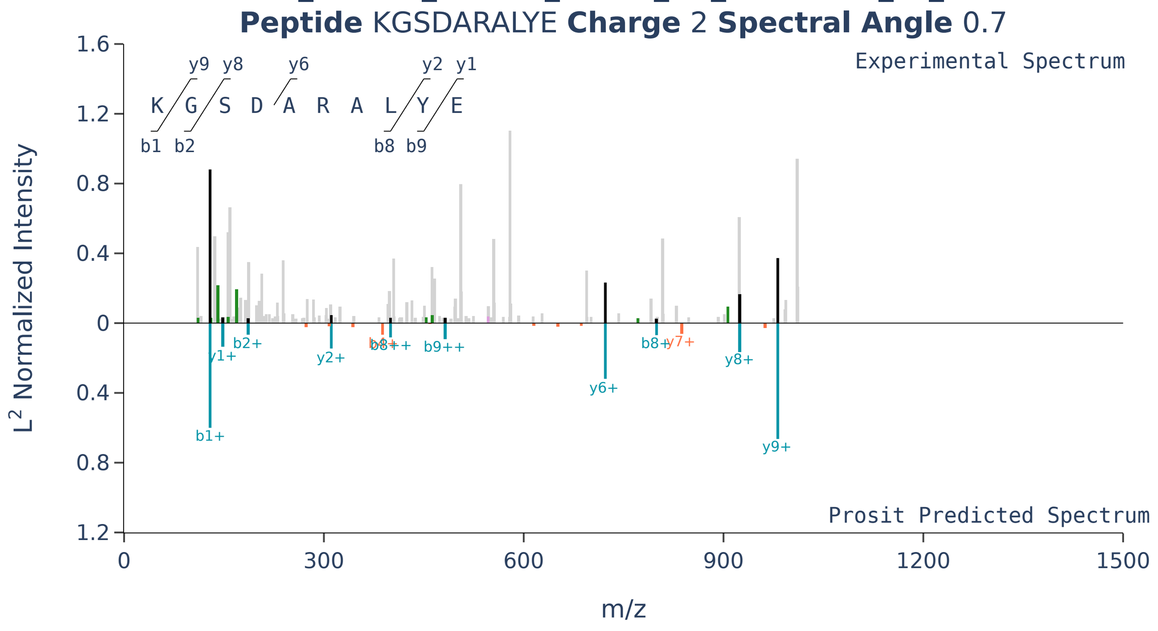

Source WSoh_301121_021221_HFGoe_M1_AnnexinA1_24h_R1 **Scan** 13991
Peptide VSEFLKQS **Charge** 2 **Spectral Angle** 0.79

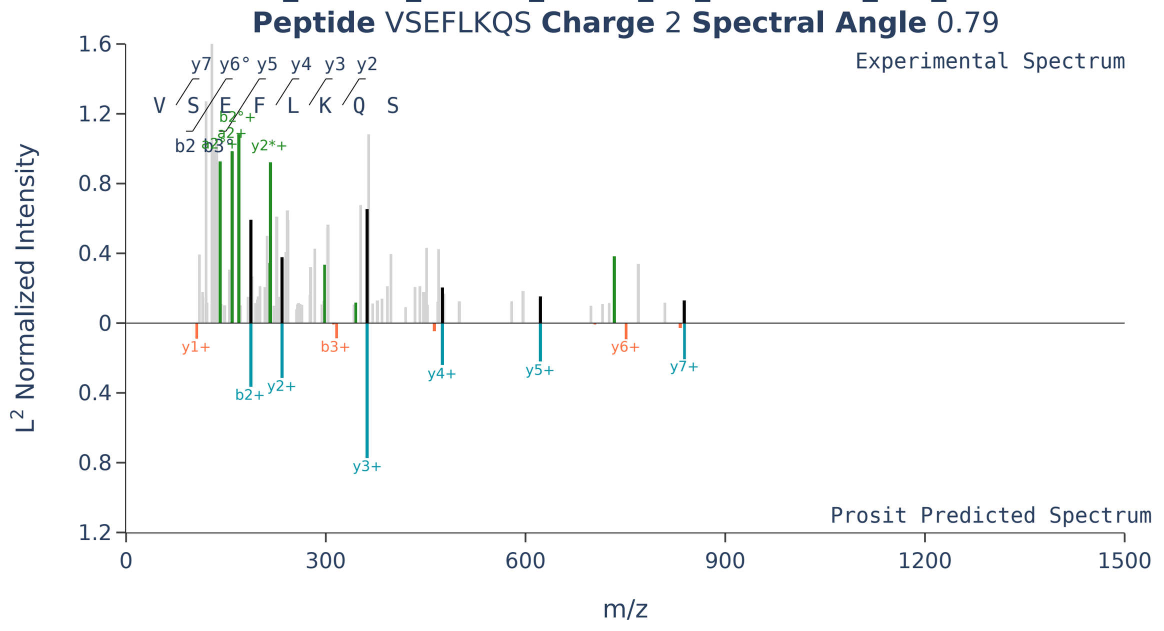

Source WSoh_301121_021221_HFGoe_M2_AnnexinA1_24h_R2 **Scan** 15479
Peptide KGRSEIDMNDI **Charge** 2 **Spectral Angle** 0.75

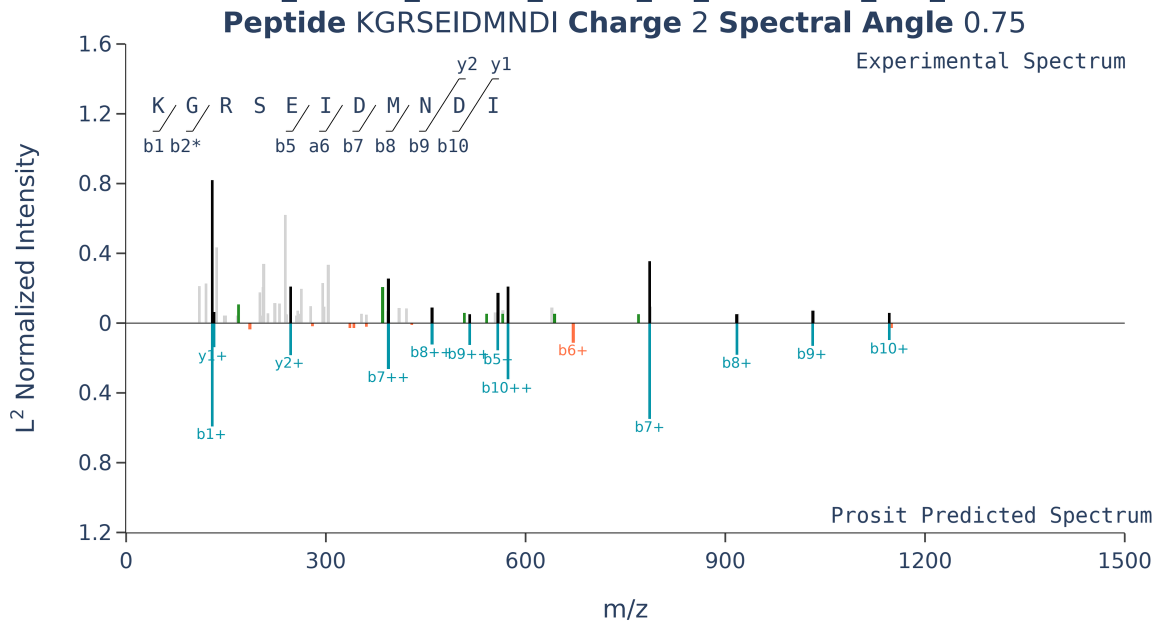

Source WSoh_301121_021221_HFGoe_M2_AnnexinA1_2h_R2 **Scan** 11090
Peptide ETLKKALTGA **Charge** 2 **Spectral Angle** 0.79

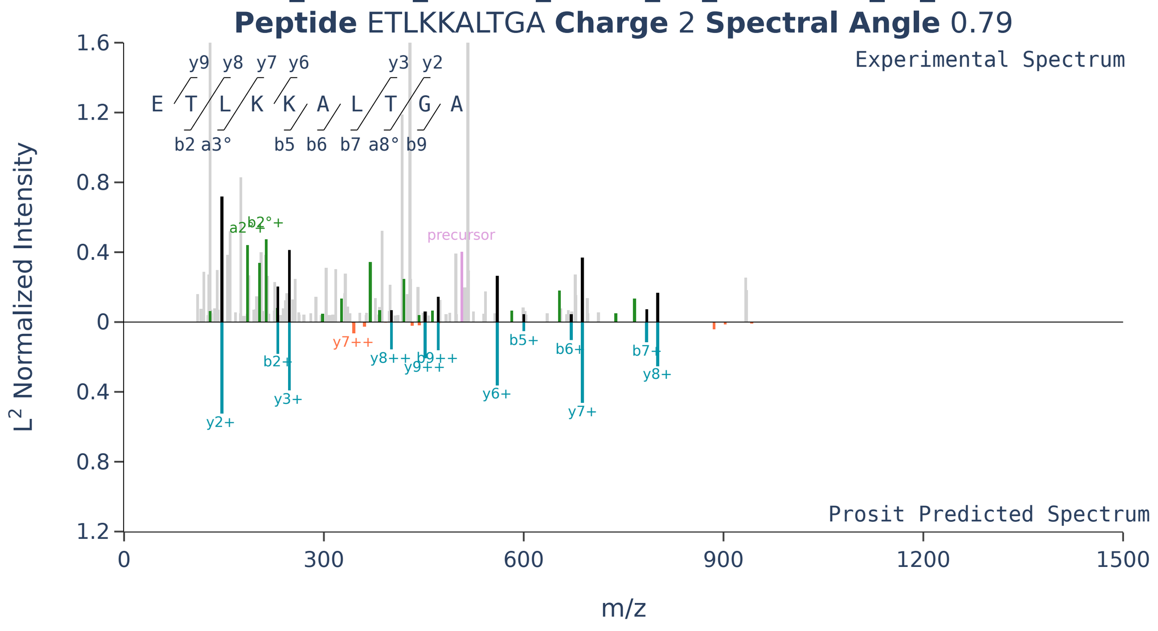

Source WSoh_301121_021221_HFGoe_M1_AnnexinA1_24h_R2 **Scan** 22836
Peptide TGMVSEFLKQ **Charge** 2 **Spectral Angle** 0.81

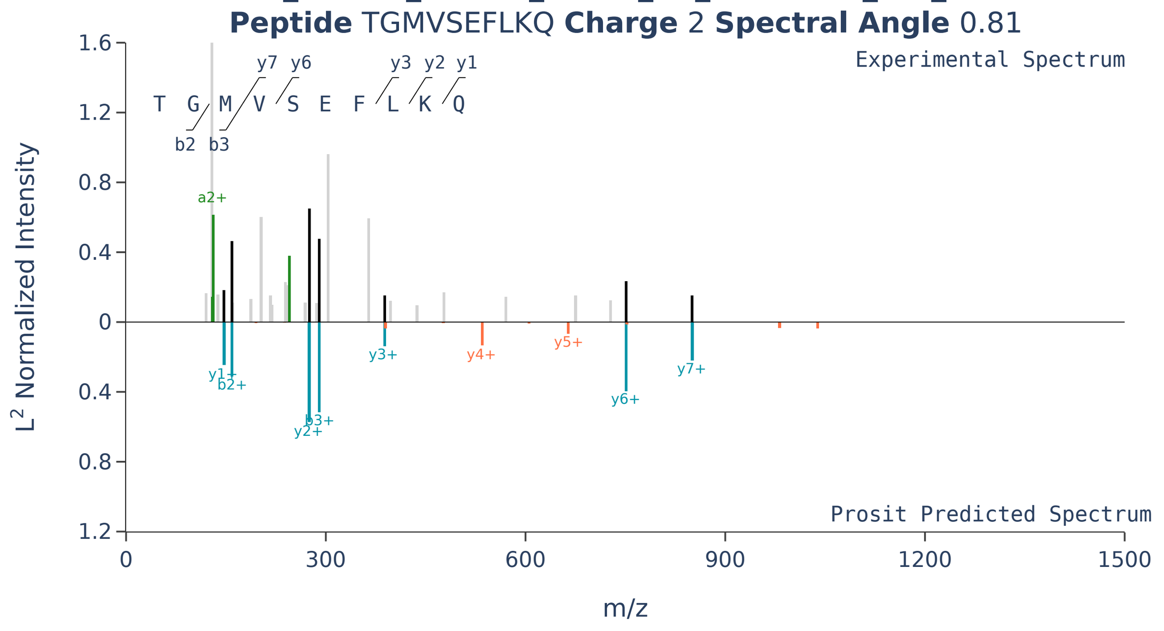

Source WSoh_301121_021221_HFGoe_M2_AnnexinA1_2h_R2 **Scan** 18848
Peptide KGTMVSEFLKQ **Charge** 3 **Spectral Angle** 0.83

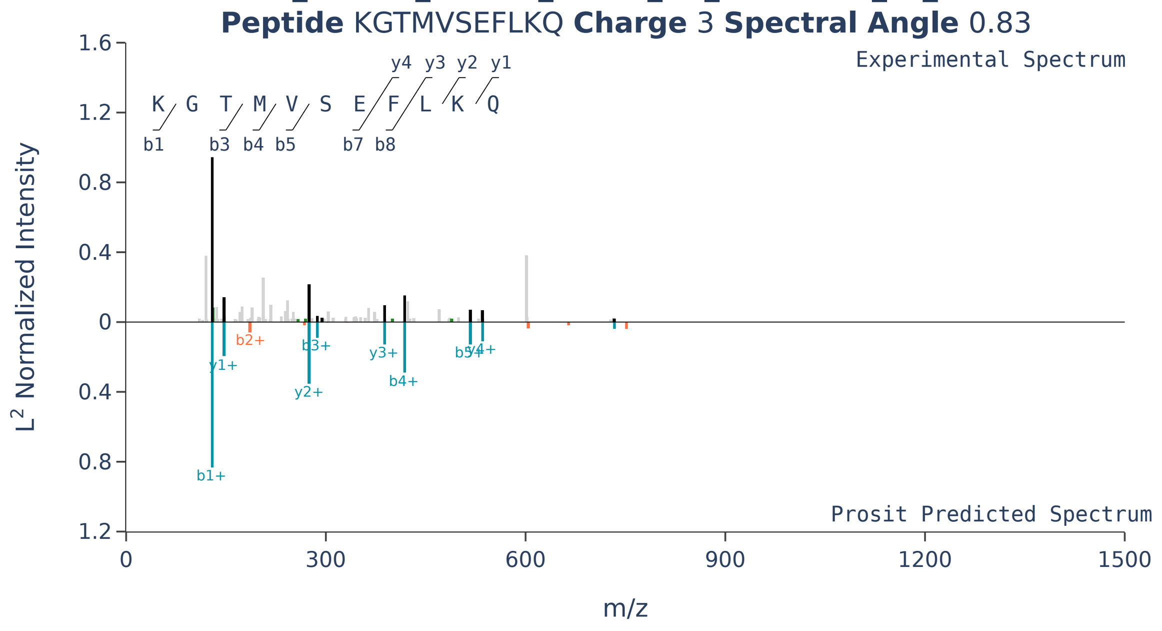

Source WSoh_301121_021221_HFGoe_M2_AnnexinA1_24h_R1 **Scan** 9068
Peptide KGGPGSETL **Charge** 2 **Spectral Angle** 0.71

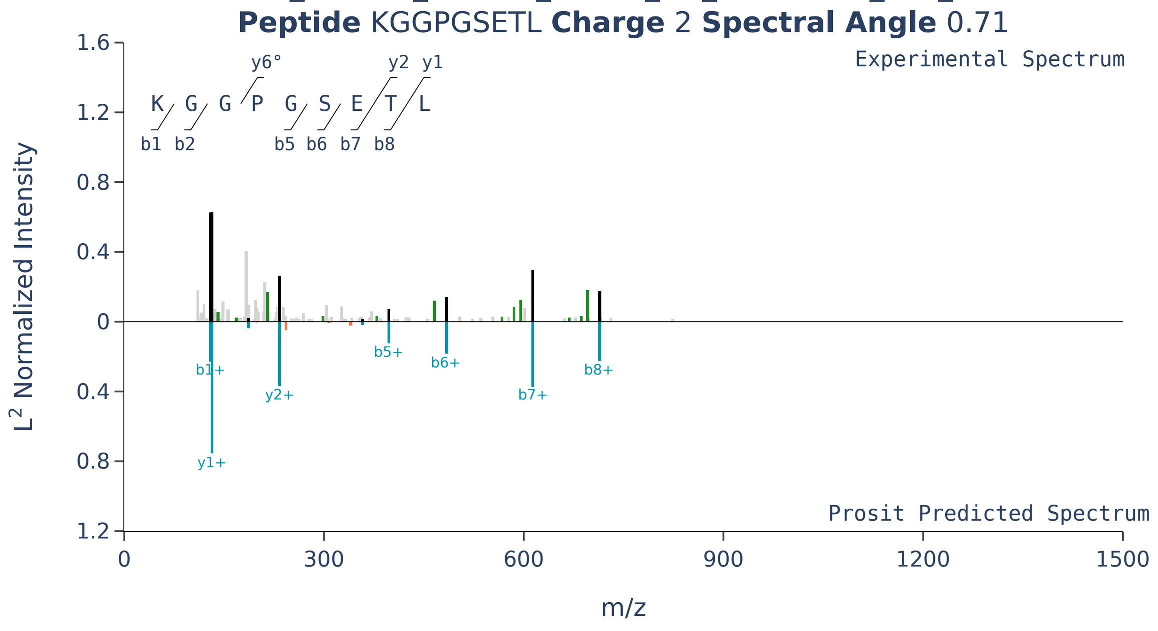

Source WSoh_301121_021221_HFGoe_M1_AnnexinA1_4h_R1 **Scan** 8841
Peptide RSYPQLRS **Charge** 2 **Spectral Angle** 0.84

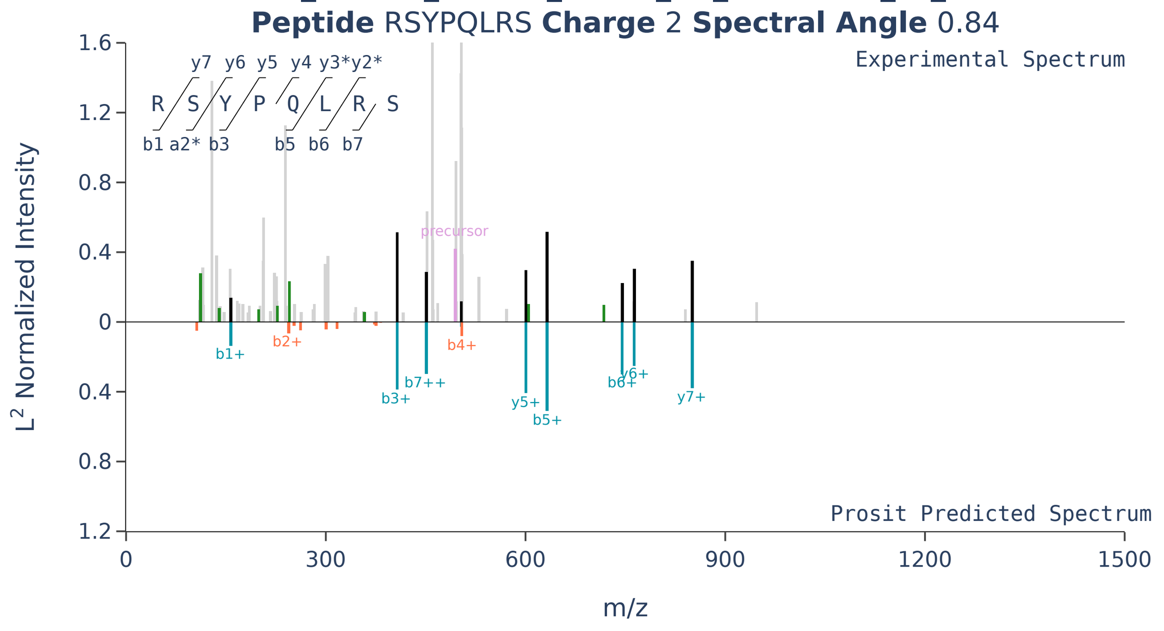

Source WSoh_301121_021221_HFGoe_M2_AnnexinA1_24h_R2 **Scan** 9818
Peptide LASRDLAD **Charge** 2 **Spectral Angle** 0.73

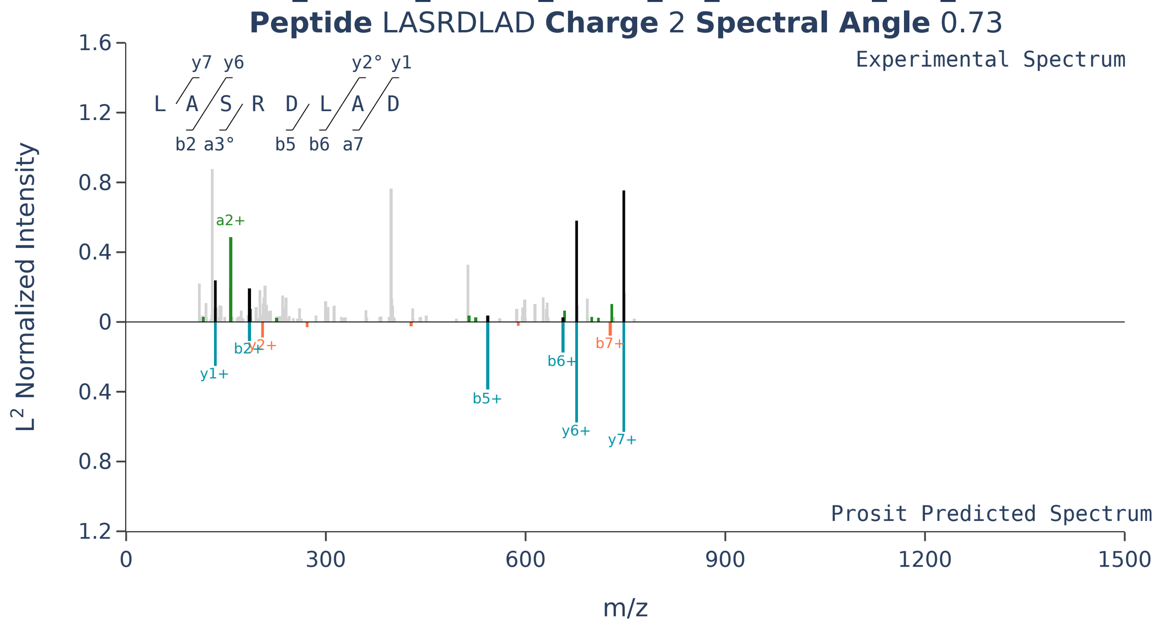

Source WSoh_301121_021221_HFGoe_M2_AnnexinA1_24h_R1 **Scan** 17744
Peptide LELKGDLAD **Charge** 2 **Spectral Angle** 0.76

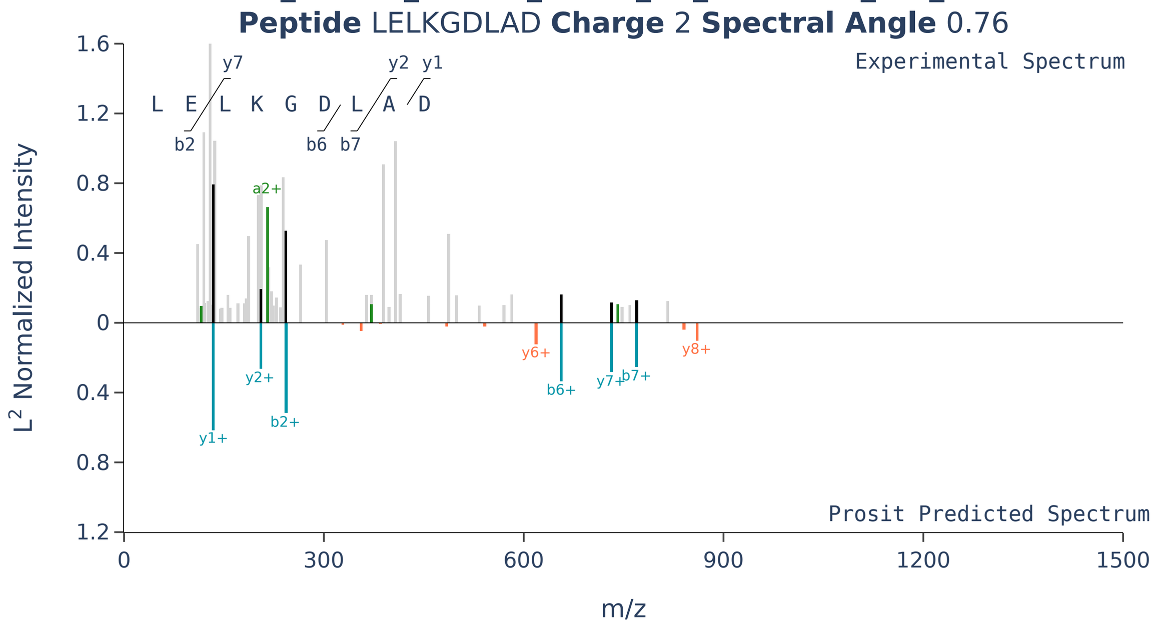

Source WSoh_301121_021221_HFGoe_M1_AnnexinA1_24h_R1 **Scan** 18706
Peptide LLAKDITSDTSGDFRNA **Charge** 3 **Spectral Angle** 0.9

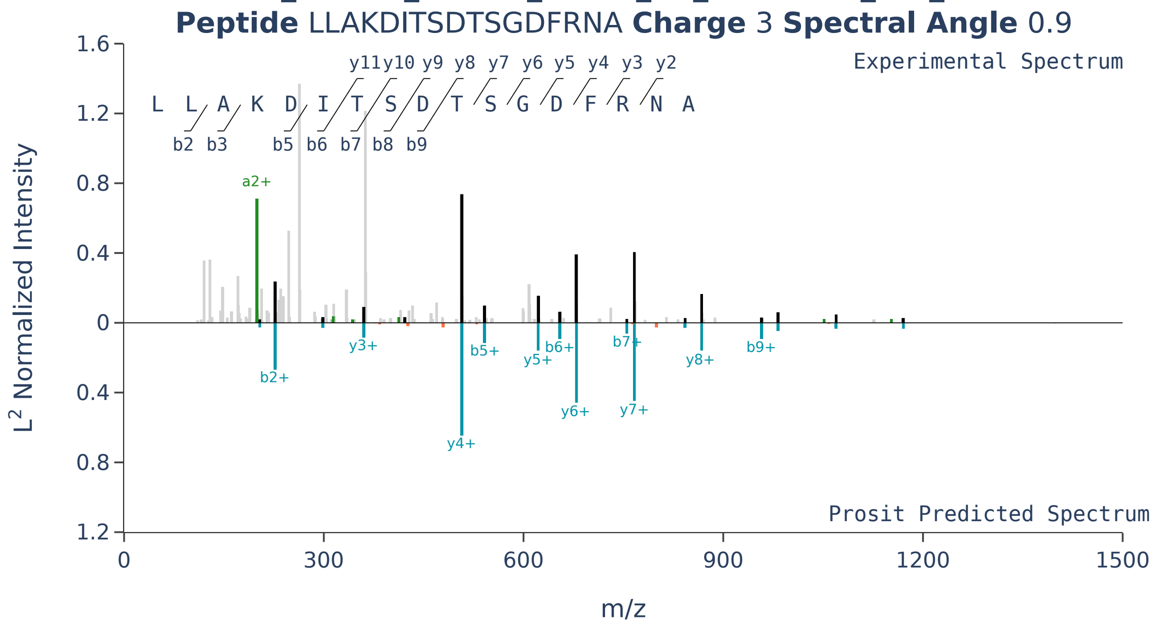

Source WSoh_301121_021221_HFGoe_M1_AnnexinA1_2h_R2 **Scan** 12601
Peptide RSYPQLRVS **Charge** 2 **Spectral Angle** 0.82

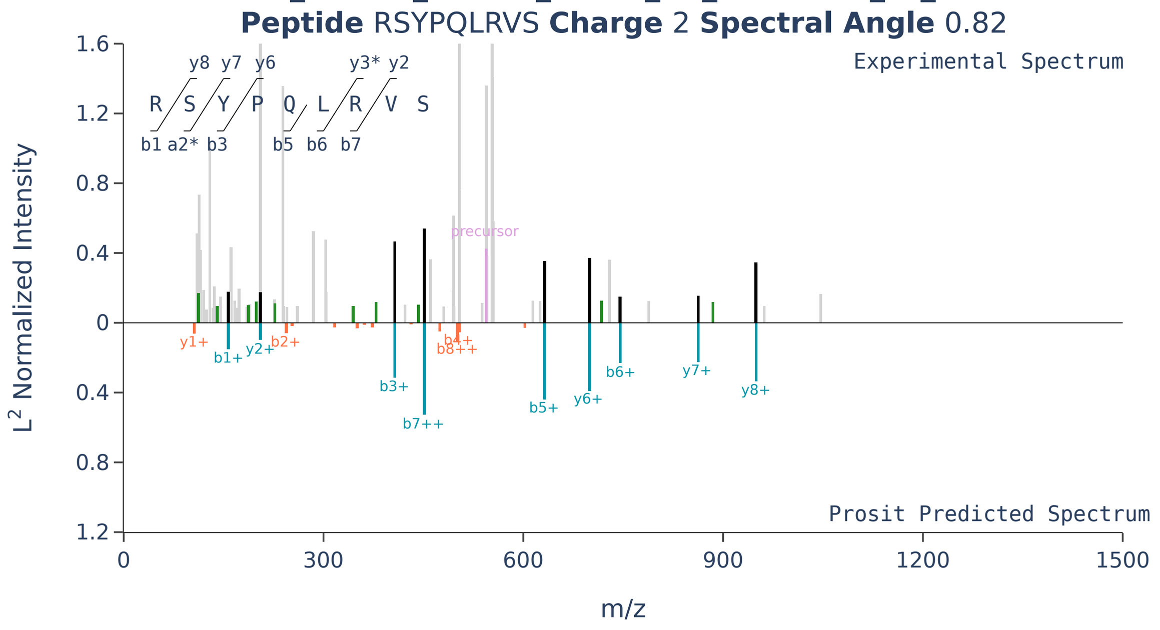

Source WSoh_301121_021221_HFGoe_M1_AnnexinA1_4h_R1 **Scan** 9226
Peptide LAQETGK **Charge** 2 **Spectral Angle** 0.8

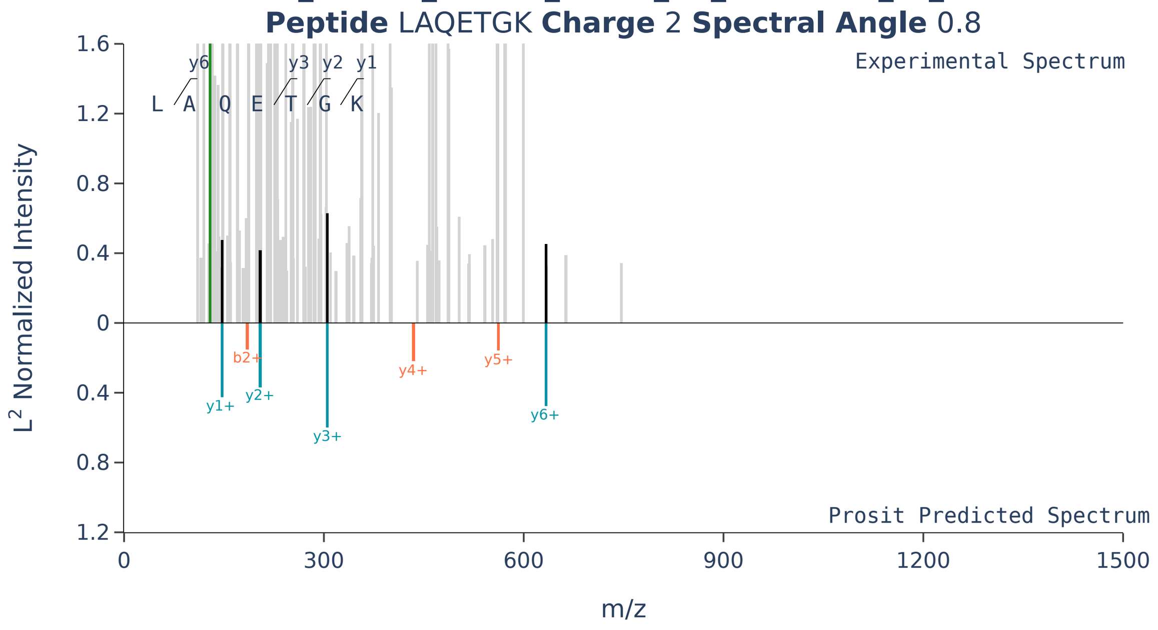

Source WSoh_301121_021221_HFGoe_M2_AnnexinA1_24h_R2 **Scan** 9748
Peptide SKGGPGSQEYVQT **Charge** 2 **Spectral Angle** 0.79

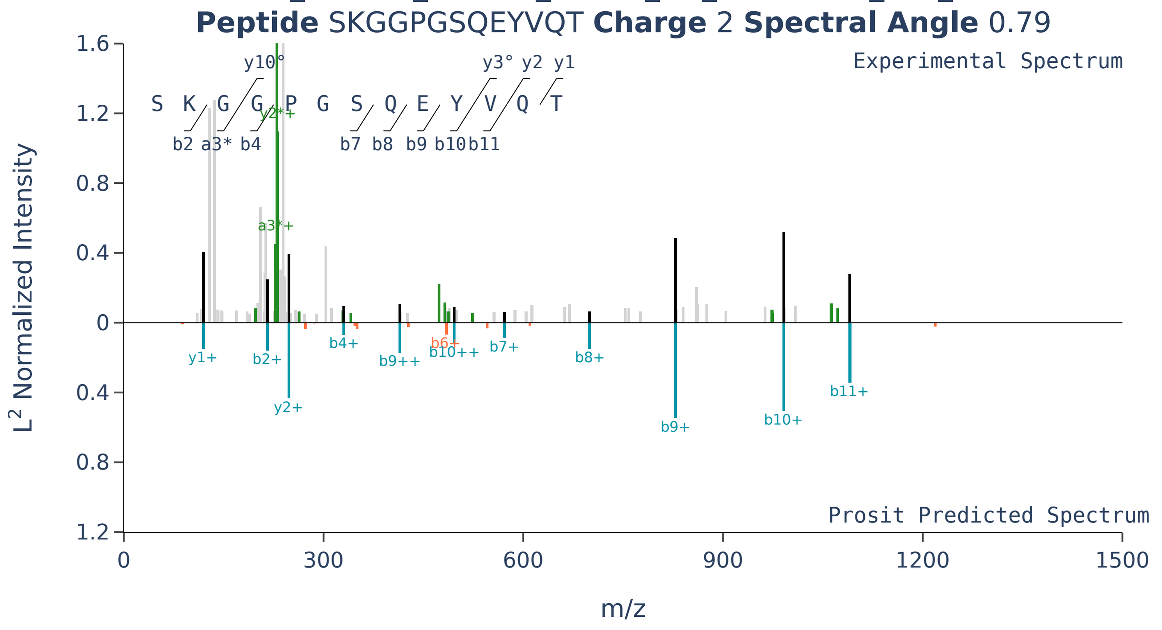

Source WSoh_301121_021221_HFGoe_M2_AnnexinA1_2h_R1 **Scan** 9597
Peptide TRSYPQLRKV **Charge** 3 **Spectral Angle** 0.84

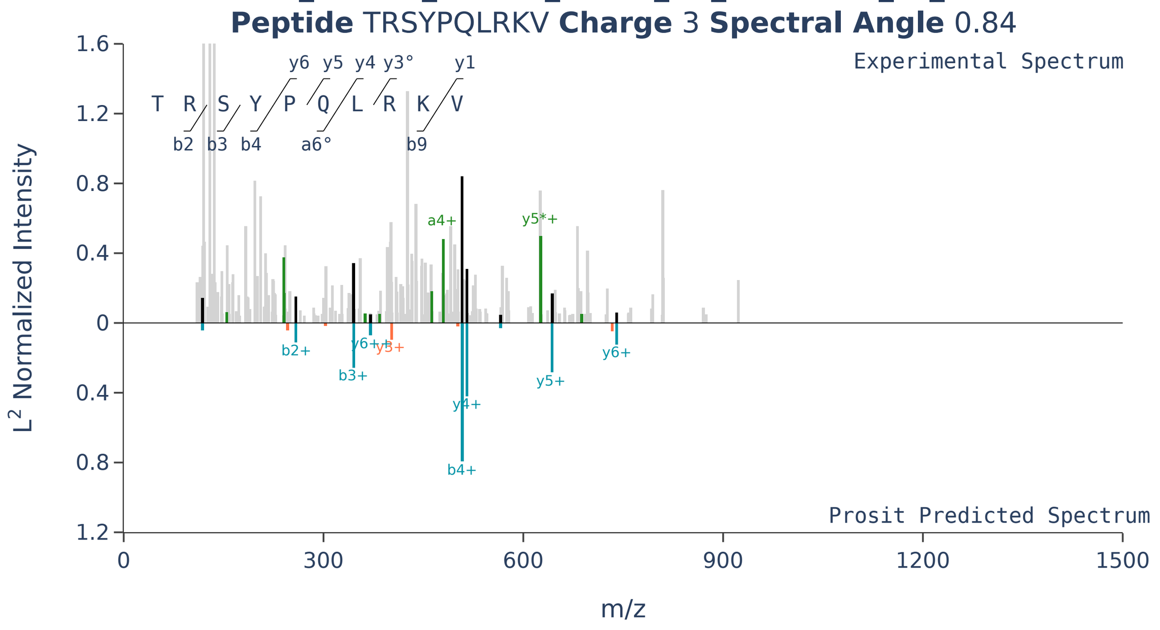

Source WSoh_301121_021221_HFGoe_M1_AnnexinA1_24h_R1 **Scan** 12906
Peptide DAGERRKGTDVNVF **Charge** 3 **Spectral Angle** 0.91

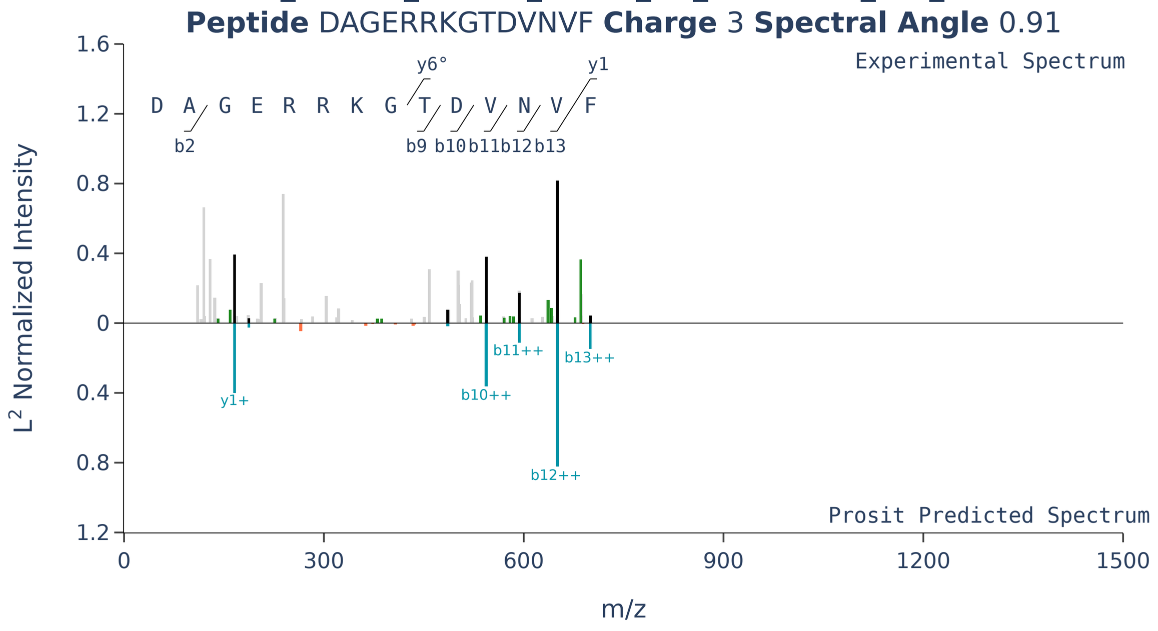

Source WSoh_301121_021221_HFGoe_M2_AnnexinA1_24h_R2 **Scan** 13658
Peptide SKGGPGQEYVQTV **Charge** 2 **Spectral Angle** 0.81

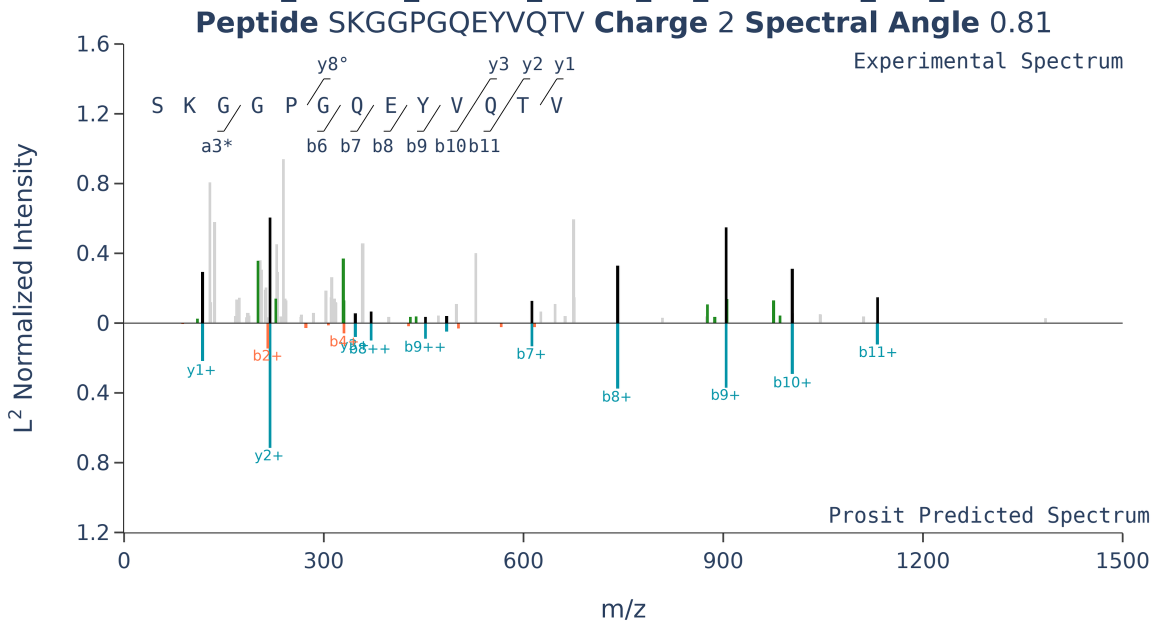

Source WSoh_301121_021221_HFGoe_M1_AnnexinA1_4h_R1 **Scan** 12969
Peptide AKDITSDTSGDFRNAKRDL **Charge** 3 **Spectral Angle** 0.89

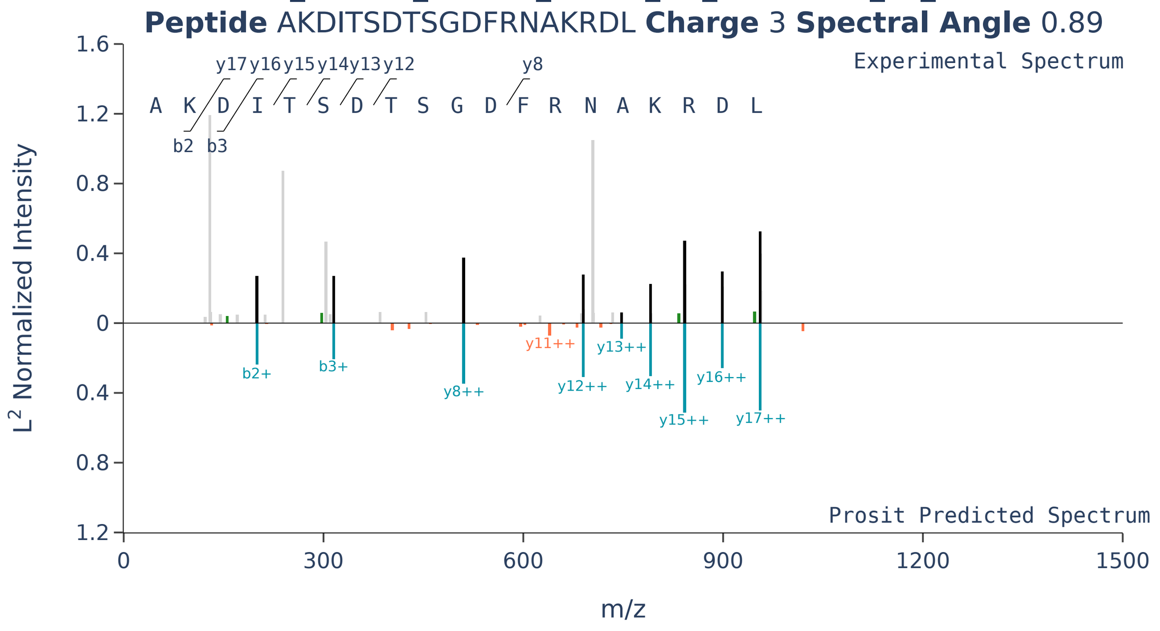

Source WSoh_301121_021221_HFGoe_M1_AnnexinA1_2h_R2 **Scan** 18821
Peptide TKGMVSEFLKQ **Charge** 3 **Spectral Angle** 0.77

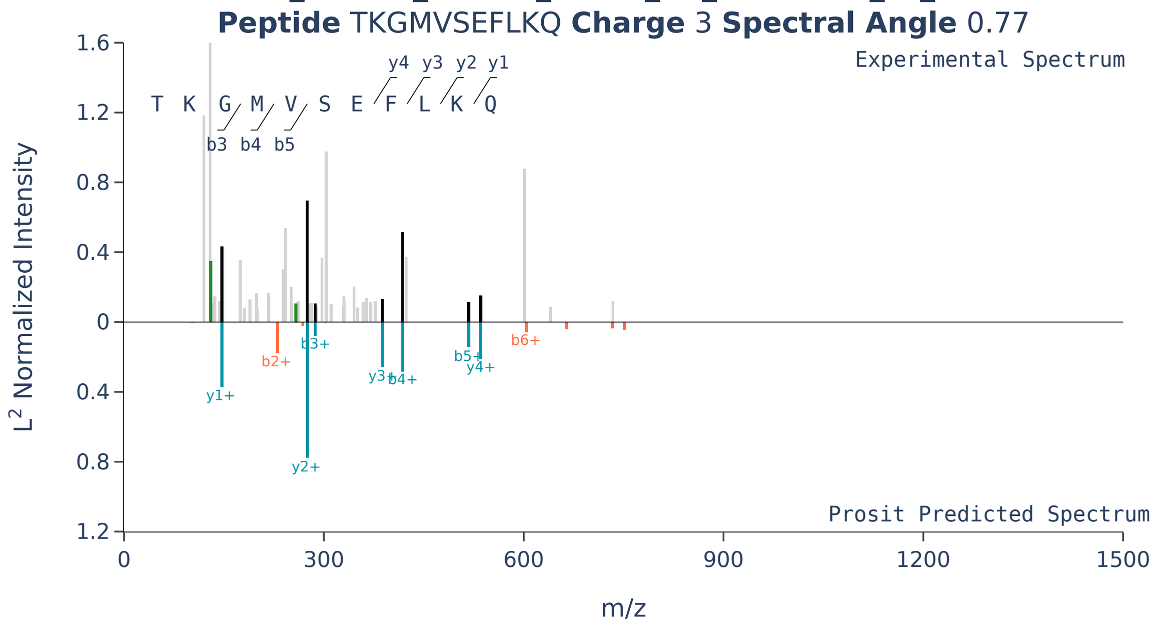

Source WSoh_301121_021221_HFGoe_M2_AnnexinA1_1h_R1 **Scan** 13941
Peptide VDLKRDL **Charge** 2 **Spectral Angle** 0.82

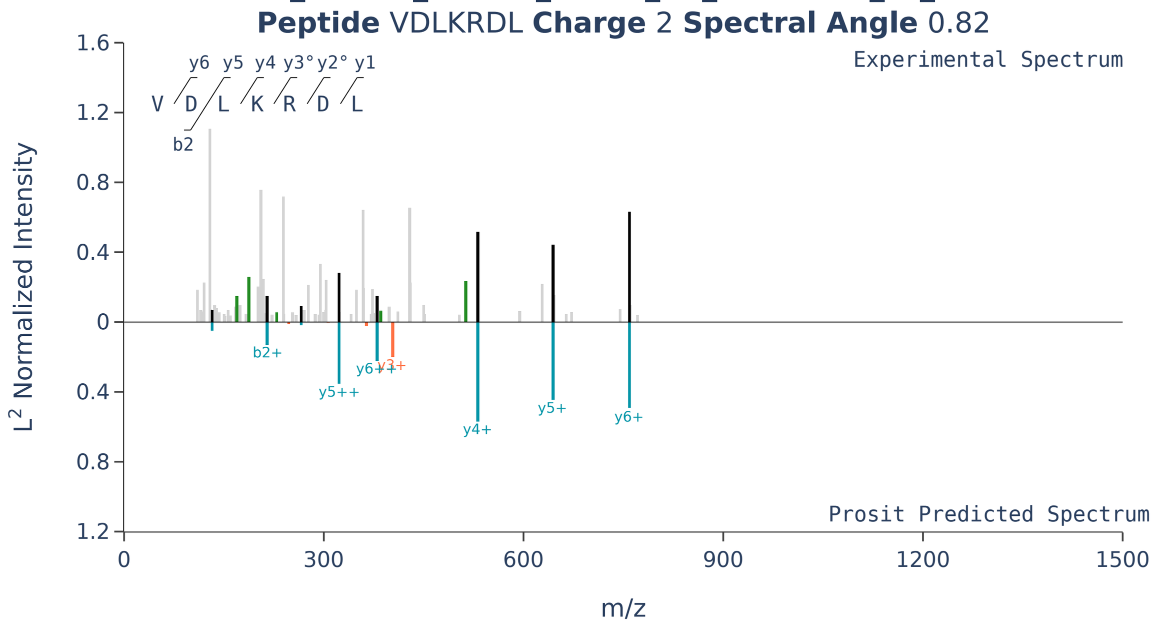

Source WSoh_301121_021221_HFGoe_M1_AnnexinA1_24h_R1 **Scan** 14488
Peptide GRSYPQL **Charge** 2 **Spectral Angle** 0.87

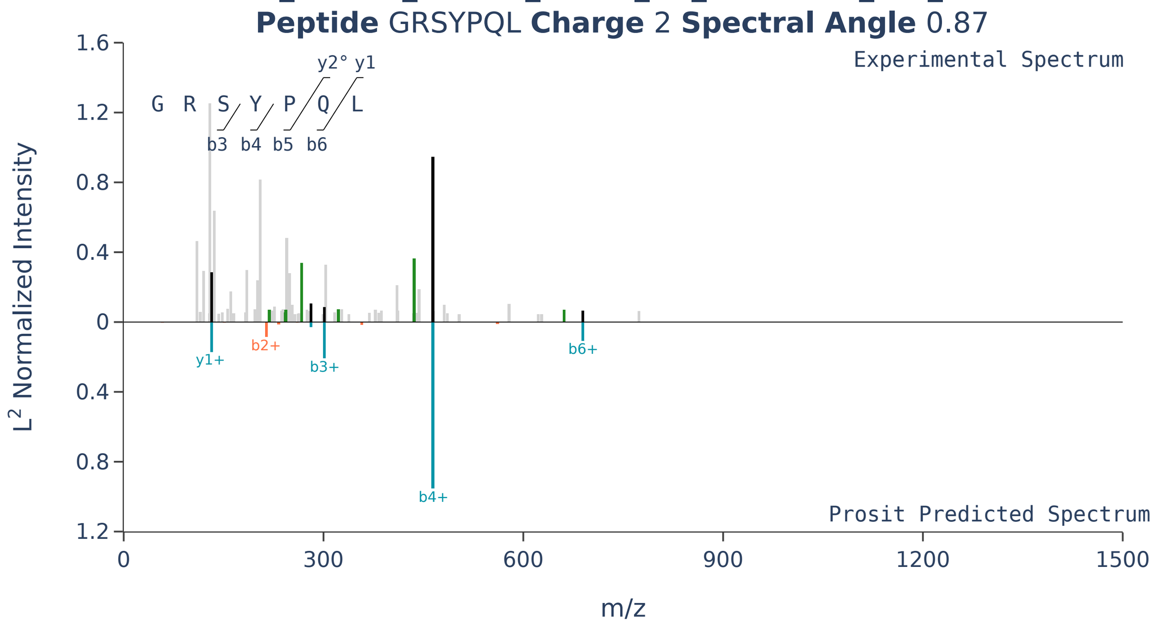

Source WSoh_301121_021221_HFGoe_M2_AnnexinA1_24h_R1 **Scan** 18378
Peptide EQEYVQTVKSETL **Charge** 2 **Spectral Angle** 0.81

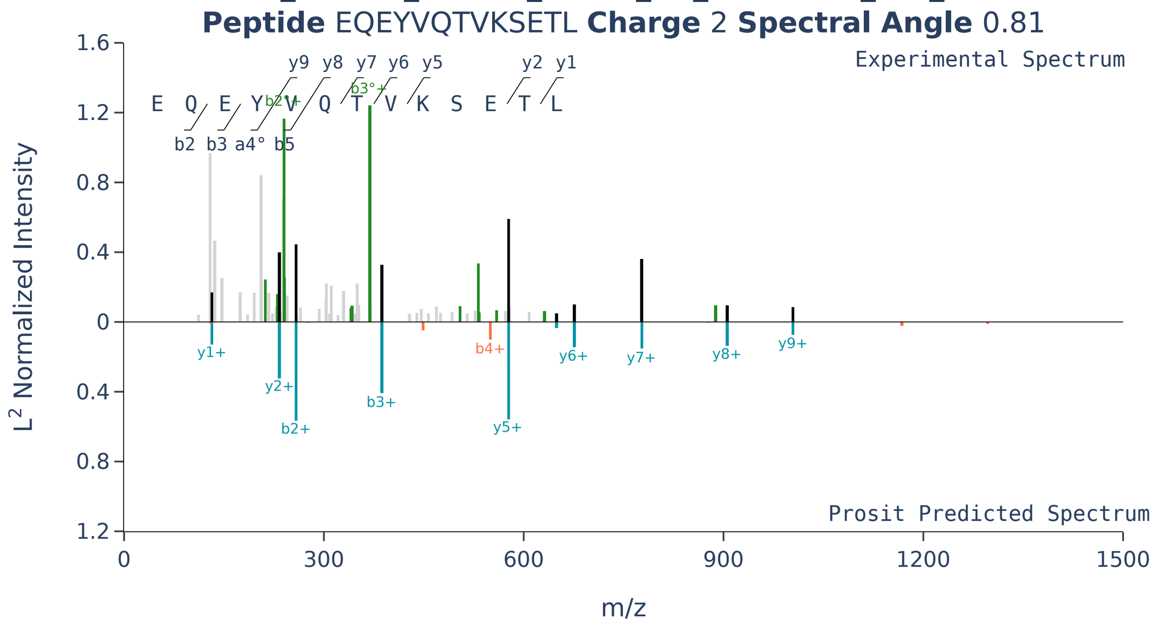

Source WSoh_301121_021221_HFGoe_M2_AnnexinA1_24h_R2 **Scan** 13875
Peptide VSEFLKNT **Charge** 2 **Spectral Angle** 0.75

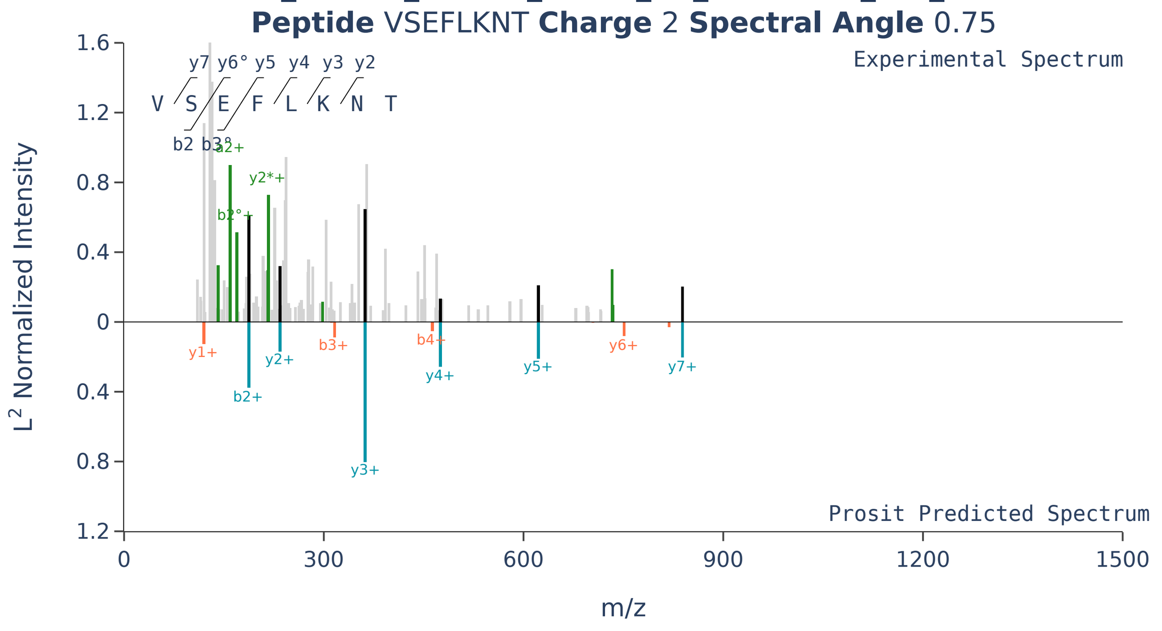

Source WSoh_301121_021221_HFGoe_M1_AnnexinA1_24h_R2 **Scan** 9887
Peptide SKGGPGQEYVQT **Charge** 2 **Spectral Angle** 0.81

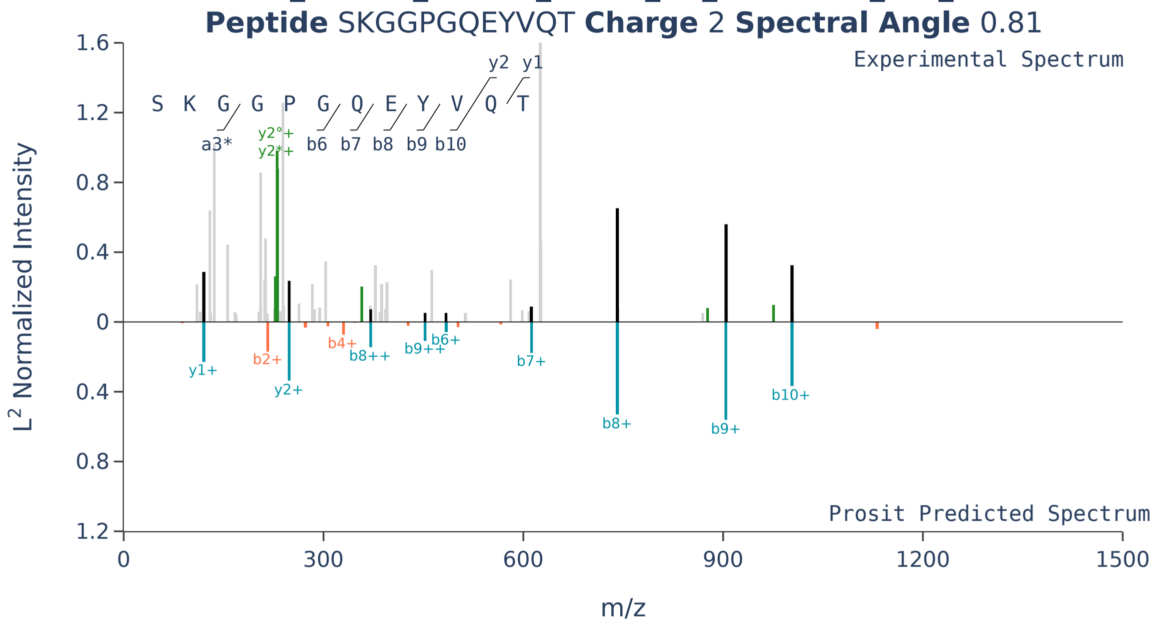

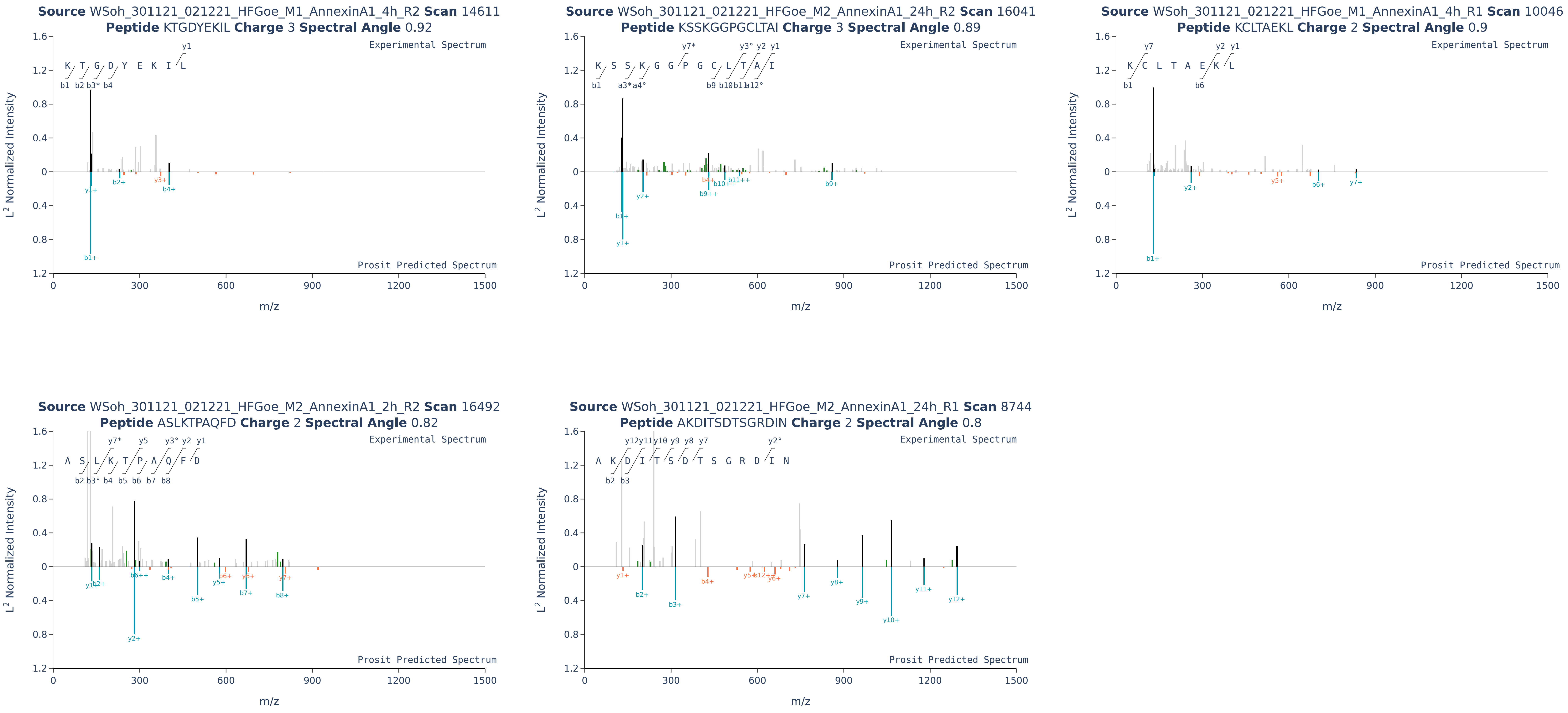
Source WSoh_301121_021221_HFGoe_M1_AnnexinA1_4h_R2 Scan 14611
Peptide KTGDYEKIL Charge 3 Spectral Angle 0.92
Source WSoh_301121_021221_HFGoe_M2_AnnexinA1_24h_R2 Scan 16041
Peptide KSSKGGPGCLTAI Charge 3 Spectral Angle 0.89
Source WSoh_301121_021221_HFGoe_M1_AnnexinA1_4h_R1 Scan 10046
Peptide KCLTAEKL Charge 2 Spectral Angle 0.9
Source WSoh_301121_021221_HFGoe_M2_AnnexinA1_2h_R2 Scan 16492
Peptide ASLKTPAQFD Charge 2 Spectral Angle 0.82
Source WSoh_301121_021221_HFGoe_M2_AnnexinA1_24h_R1 Scan 8744
Peptide AKDITSDTSGRDIN Charge 2 Spectral Angle 0.8
Experimental Spectrum
Prosit Predicted Spectrum
L² Normalized Intensity
m/z

Source W_Soh_160821_170921_Goe_aSyn_A1_6h_R2 **Scan** 13749
Peptide NEYQDYEPEA **Charge** 2 **Spectral Angle** 0.87

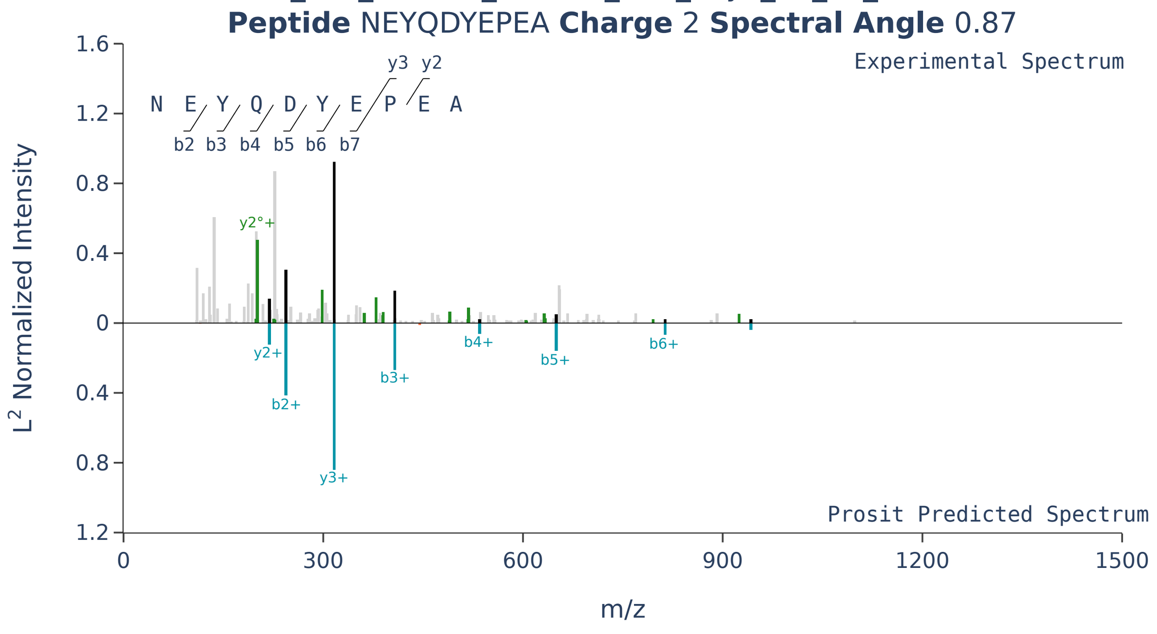

Source W_Soh_160821_170921_Goe_aSyn_A1_24h_R1 **Scan** 16985
Peptide PQEGILEEGA **Charge** 1 **Spectral Angle** 0.96

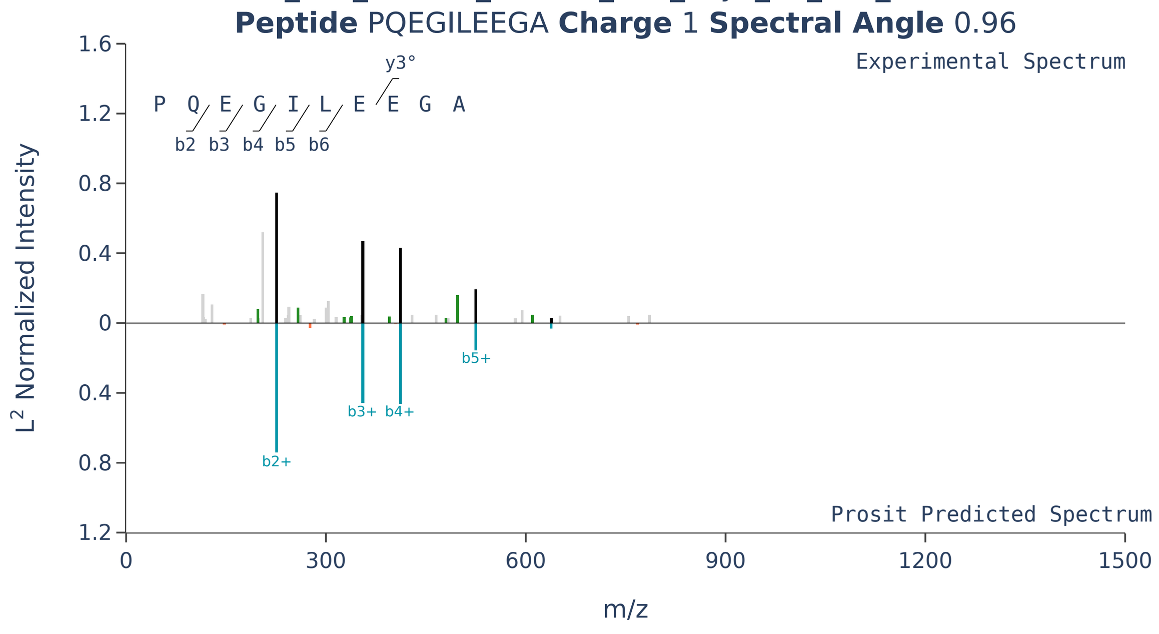

Source W_Soh_160821_170921_Goe_aSyn_A2_6h_R2 **Scan** 13263
Peptide GEGYQDYEPEA **Charge** 2 **Spectral Angle** 0.88

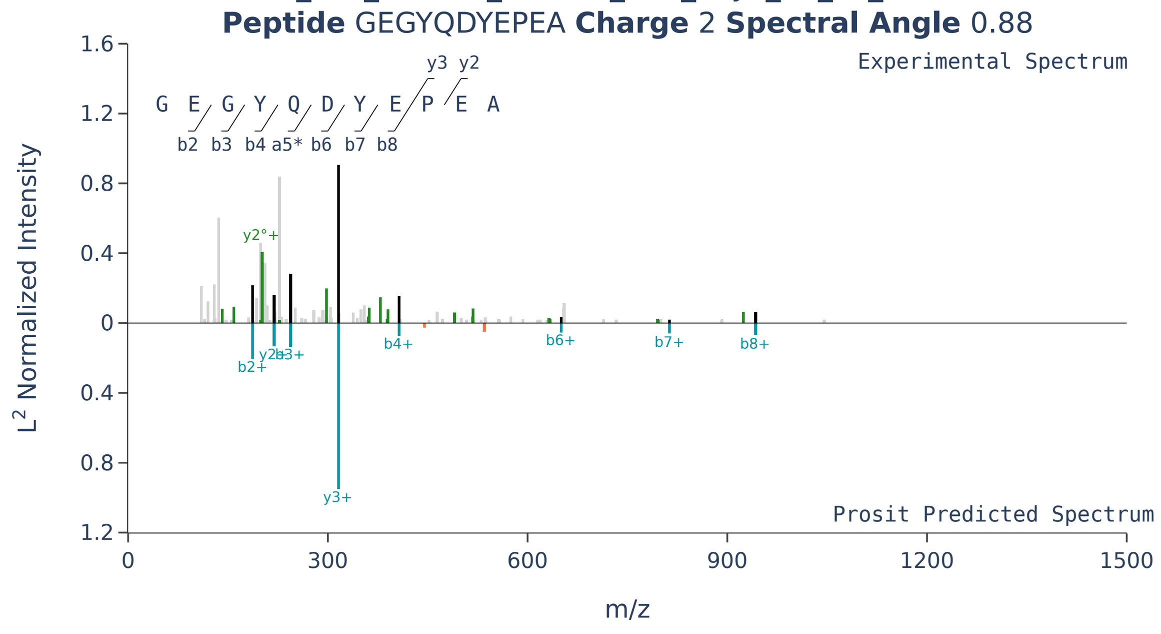

Source W_Soh_160821_170921_Goe_aSyn_A2_24h_R2 **Scan** 16725
Peptide PQEGILEDEGA **Charge** 1 **Spectral Angle** 0.91

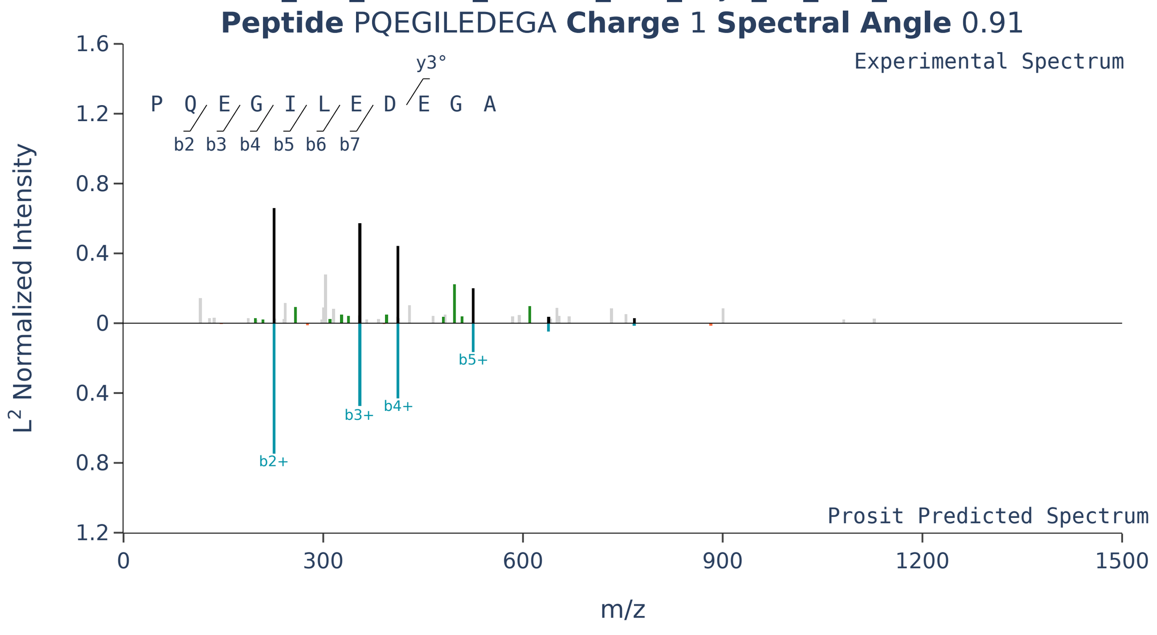

Source W_Soh_160821_170921_Goe_aSyn_A1_3h_R2 **Scan** 6430
Peptide GQLGKNEEGAPQE **Charge** 2 **Spectral Angle** 0.95

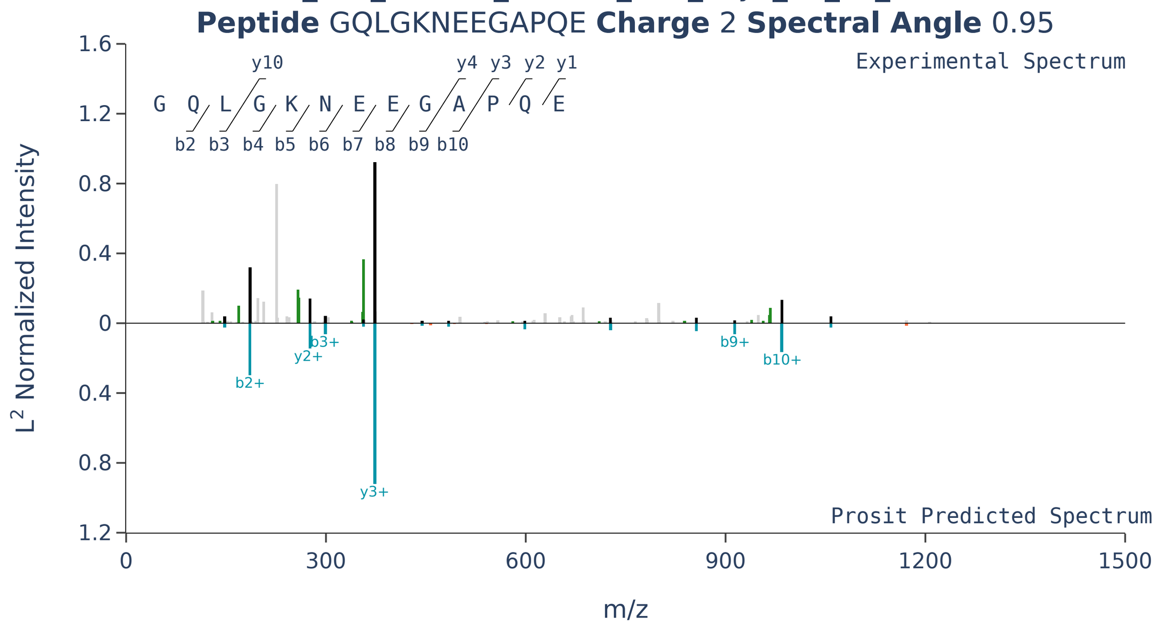

Source W_Soh_160821_170921_Goe_aSyn_A2_30'_R1 **Scan** 13589
Peptide PQEGILEQD **Charge** 1 **Spectral Angle** 0.9

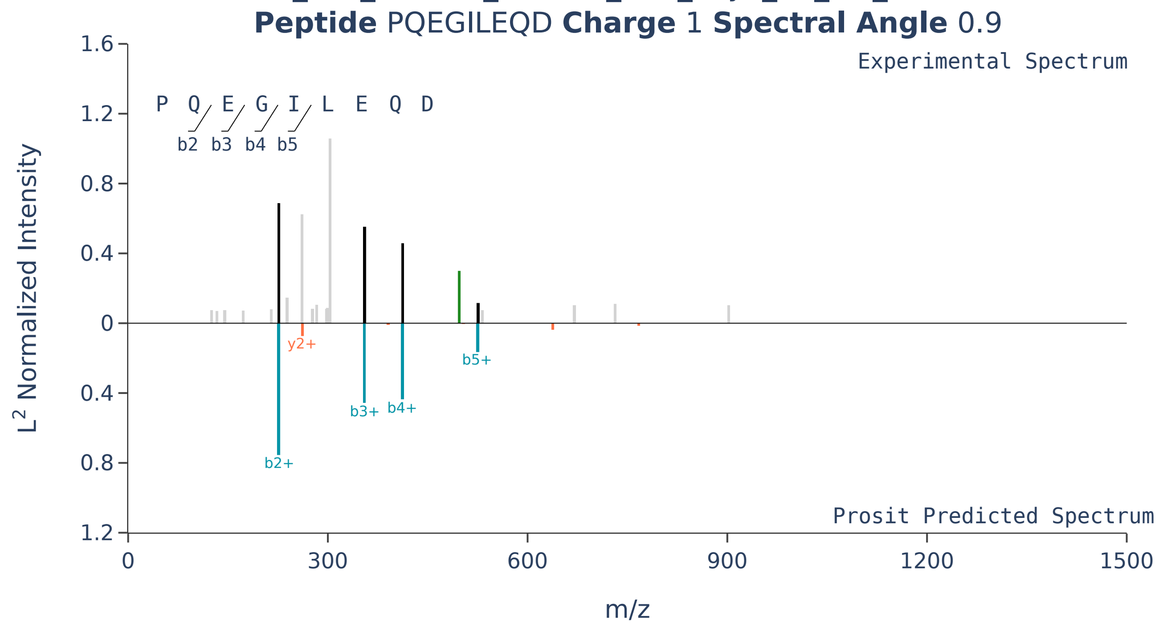

Source W_Soh_160821_170921_Goe_aSyn_A1_6h_R1 **Scan** 16528
Peptide PQEGILENEE **Charge** 1 **Spectral Angle** 0.94

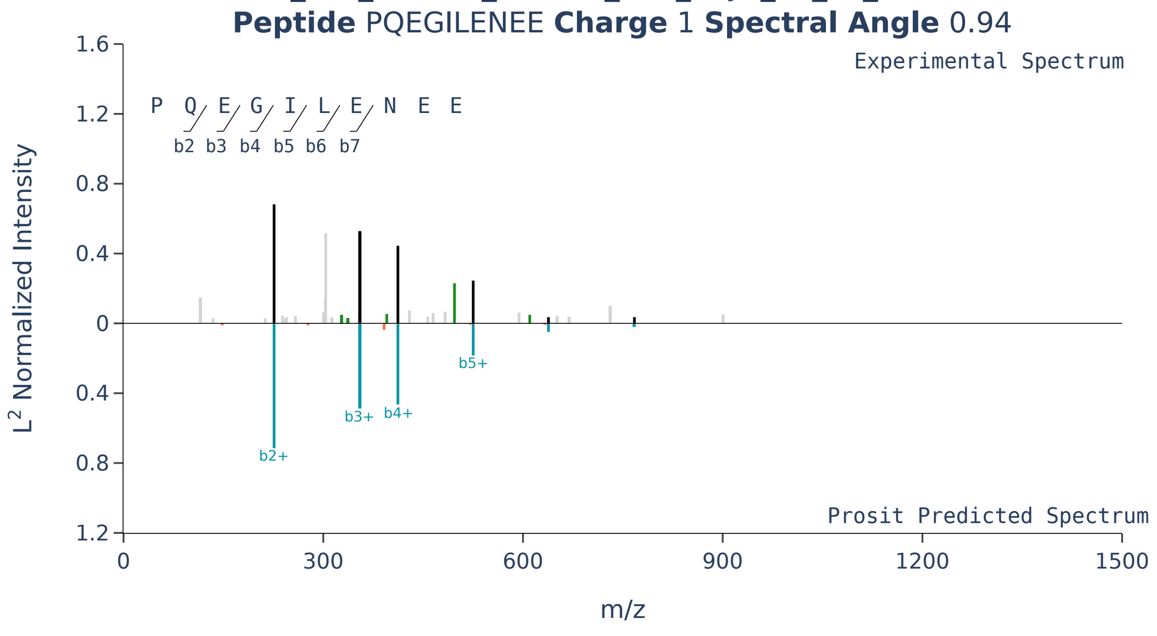

Source W_Soh_160821_170921_Goe_aSyn_A2_24h_R2 **Scan** 9511
Peptide KEGVVAMD **Charge** 2 **Spectral Angle** 0.92

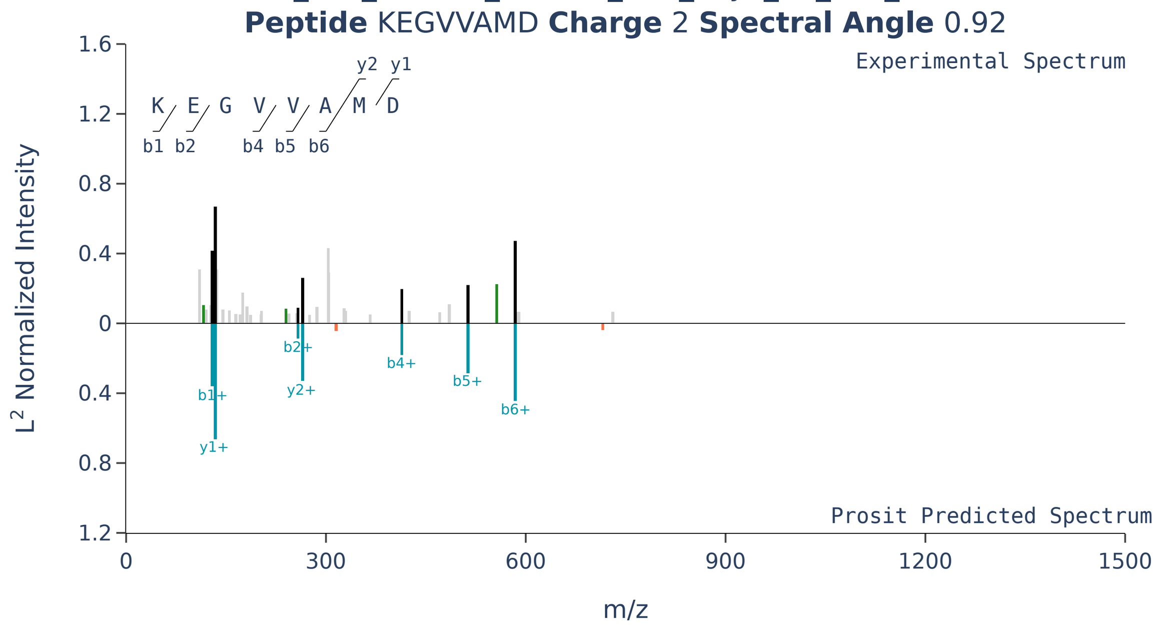

Source W_Soh_160821_170921_Goe_aSyn_A1_24h_R2 **Scan** 16566
Peptide PQEGILEDGA **Charge** 1 **Spectral Angle** 0.92

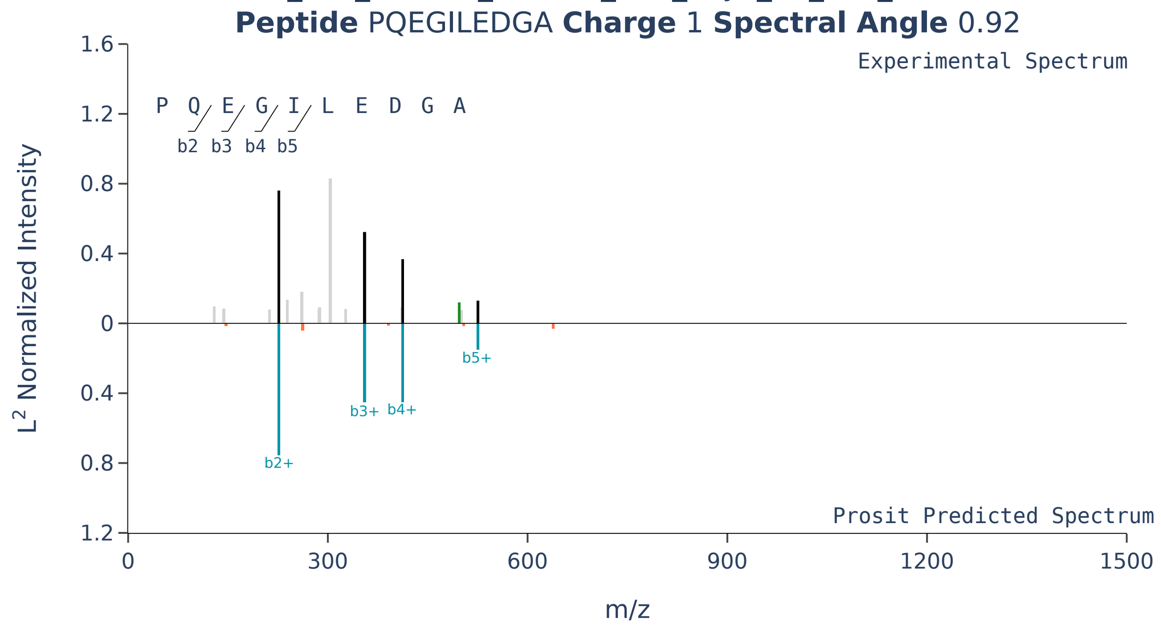

Source W_Soh_160821_170921_Goe_aSyn_A1_24h_R2 **Scan** 17951
Peptide PDNEAYEMVDPD **Charge** 1 **Spectral Angle** 0.85

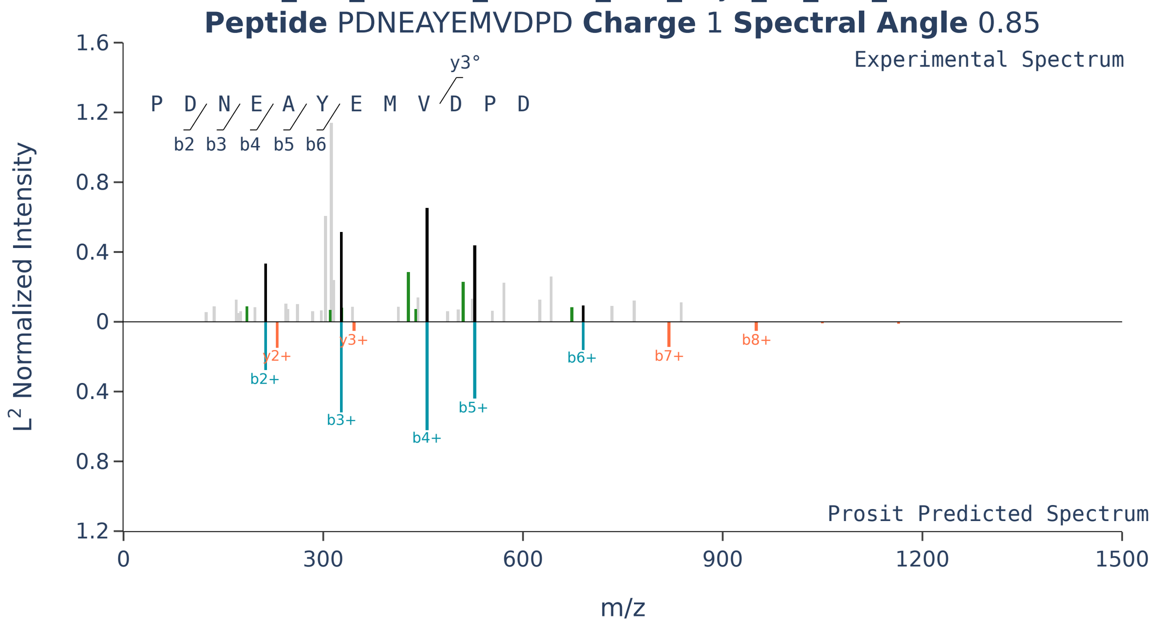

Source W_Soh_160821_170921_Goe_aSyn_A1_24h_R2 **Scan** 16174
Peptide NEAYEGYQDYEPEA **Charge** 2 **Spectral Angle** 0.9

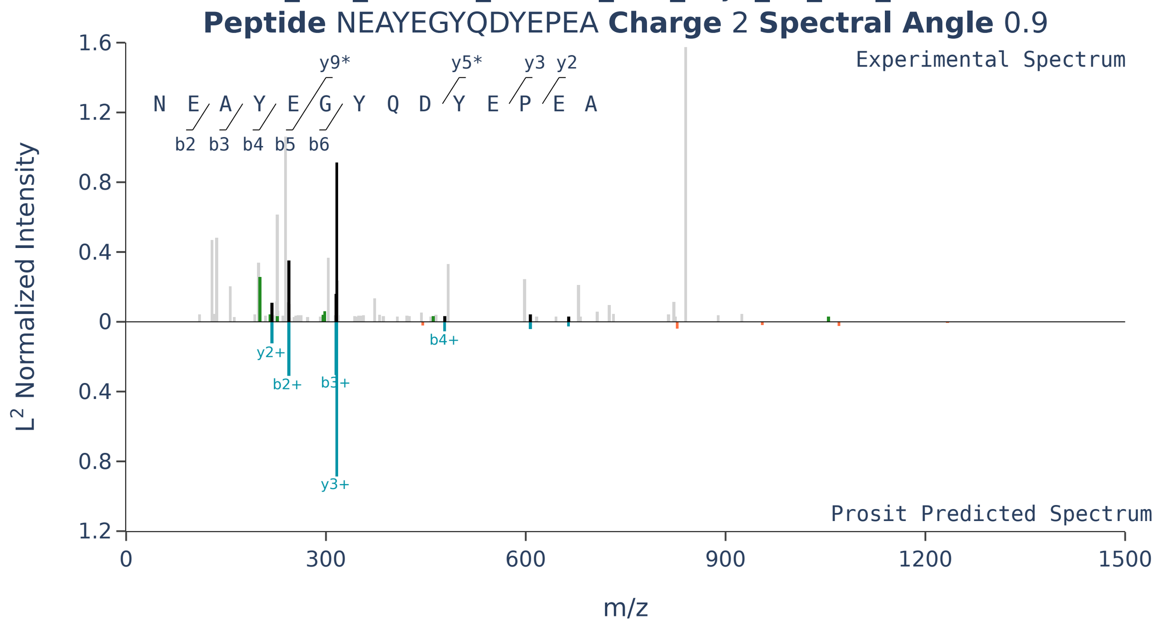

Source W_Soh_160821_170921_Goe_aSyn_A2_1h_R2 **Scan** 12282
Peptide ATGNEAYEMPSE **Charge** 2 **Spectral Angle** 0.9

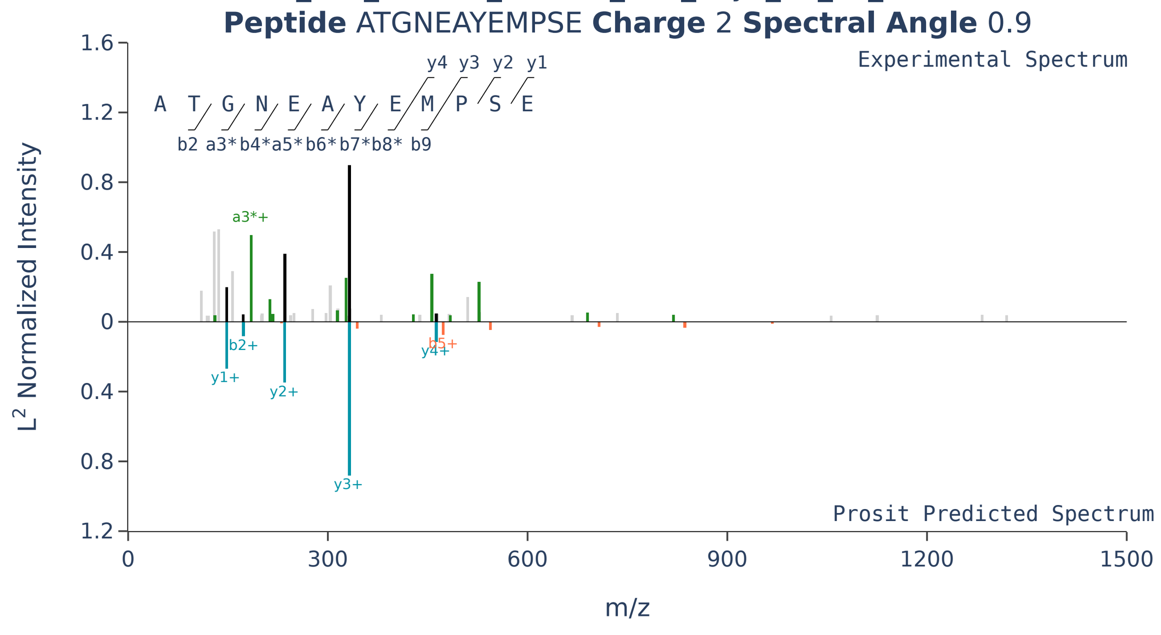

Source W_Soh_160821_170921_Goe_aSyn_A1_24h_R2 **Scan** 14198
Peptide AEGYQDYEPEA **Charge** 2 **Spectral Angle** 0.75

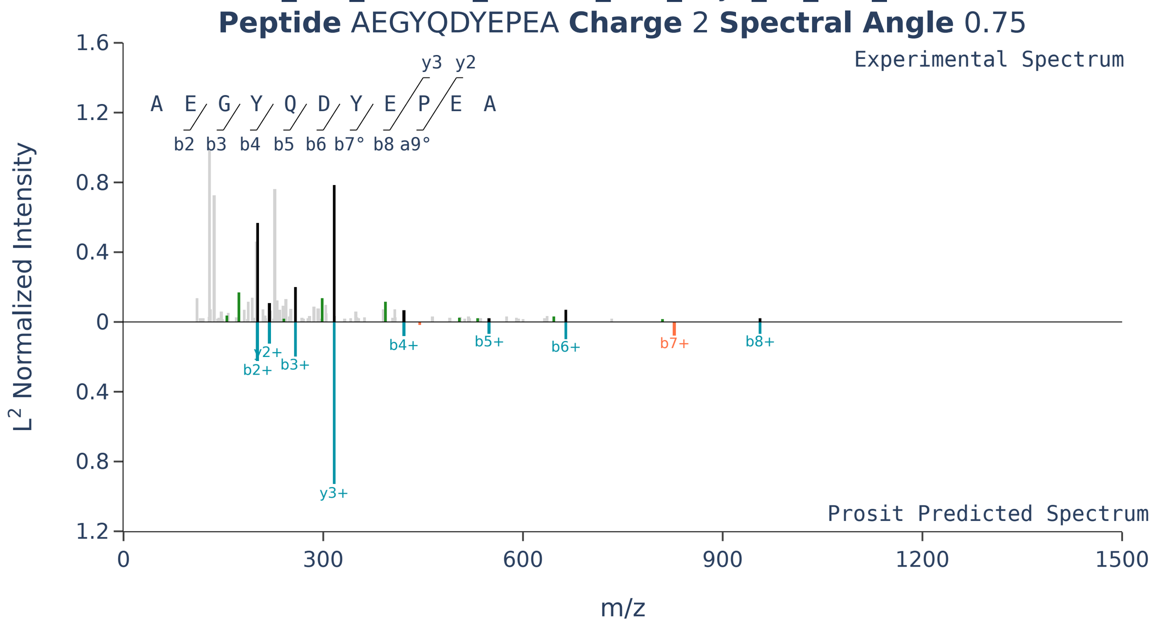

Source W_Soh_160821_170921_Goe_aSyn_A1_1h_R2 **Scan** 14829
Peptide PQEGILEDQE **Charge** 1 **Spectral Angle** 0.89

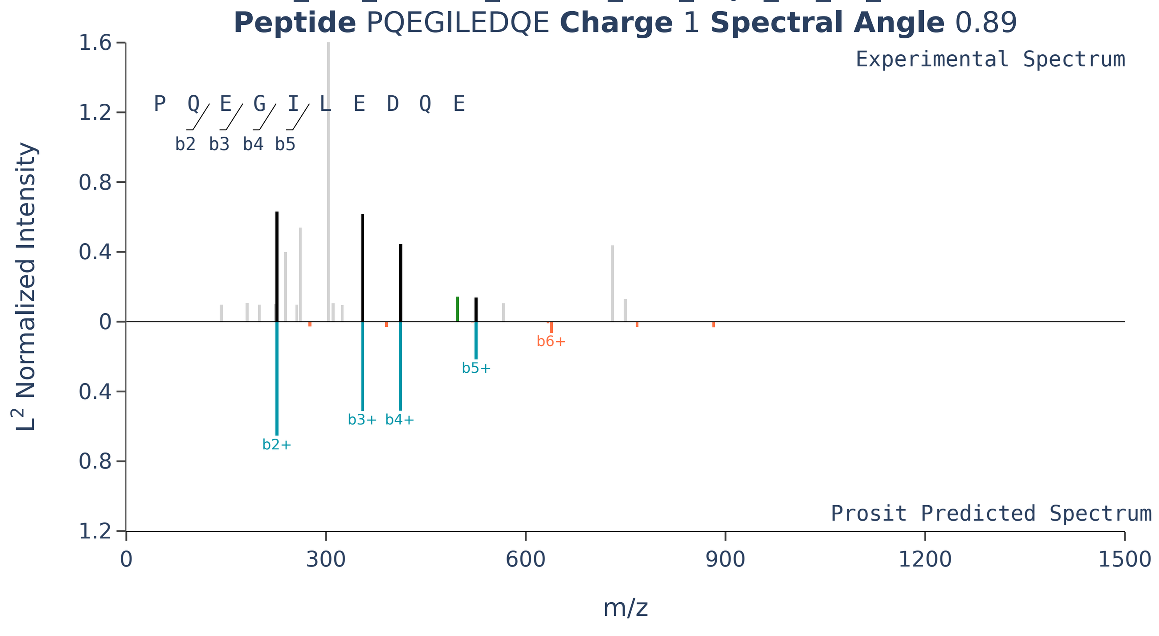

Source W_Soh_160821_170921_Goe_aSyn_A2_6h_R1 **Scan** 13204
Peptide EGGYQDYEPEA **Charge** 1 **Spectral Angle** 0.81

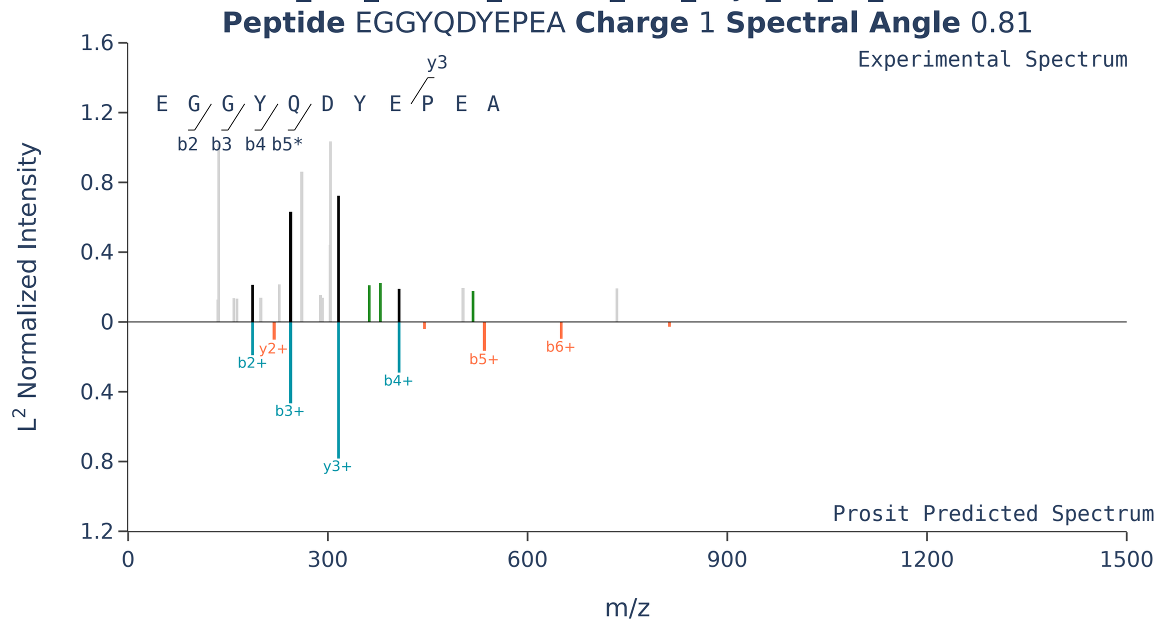

Source W_Soh_160821_170921_Goe_aSyn_A1_24h_R1 **Scan** 6105
Peptide VAEAAQKTVE **Charge** 2 **Spectral Angle** 0.89

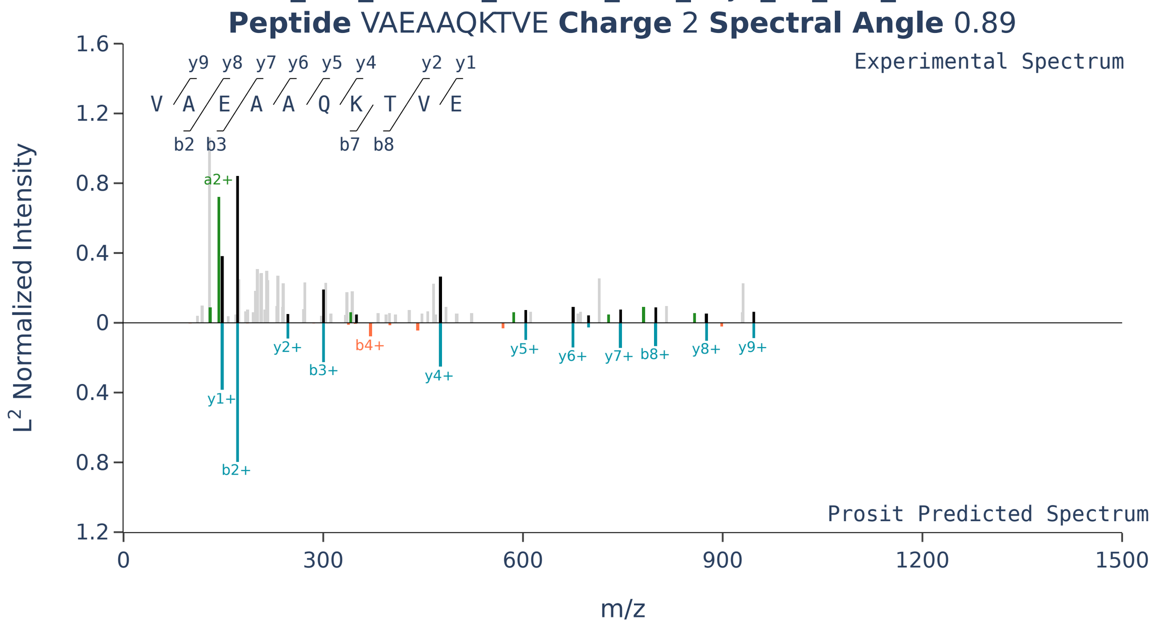

Source W_Soh_160821_170921_Goe_aSyn_A1_24h_R1 **Scan** 21152
Peptide PDNEAYEMFM **Charge** 1 **Spectral Angle** 0.84

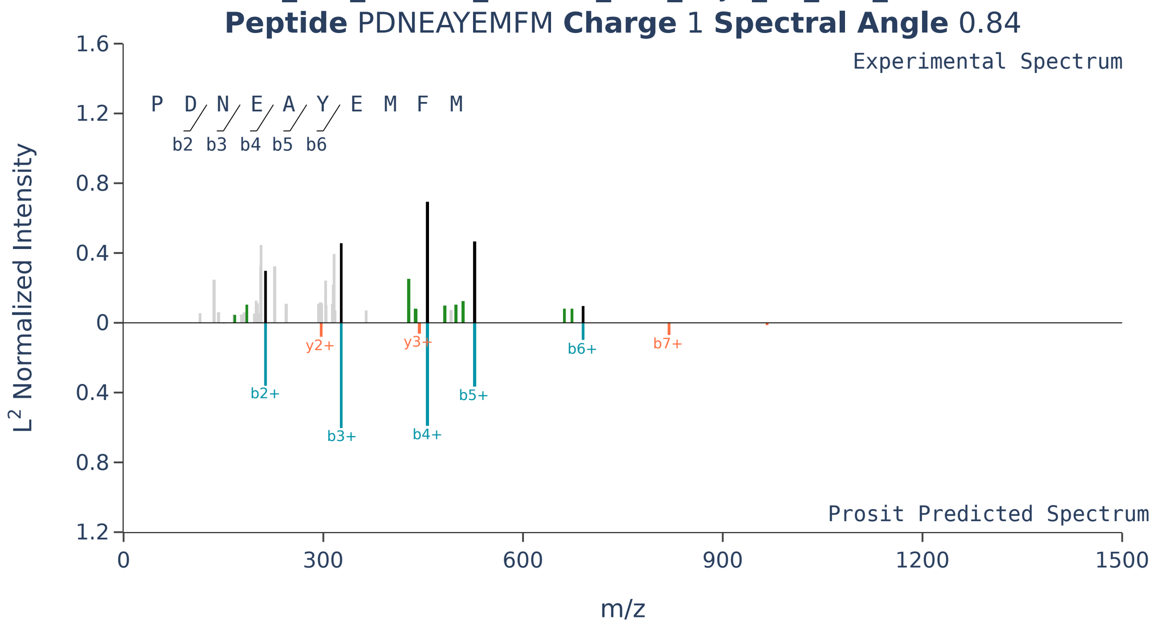

Source W_Soh_160821_170921_Goe_aSyn_A1_24h_R2 **Scan** 6537
Peptide ALGKNEEGAPQE **Charge** 2 **Spectral Angle** 0.93

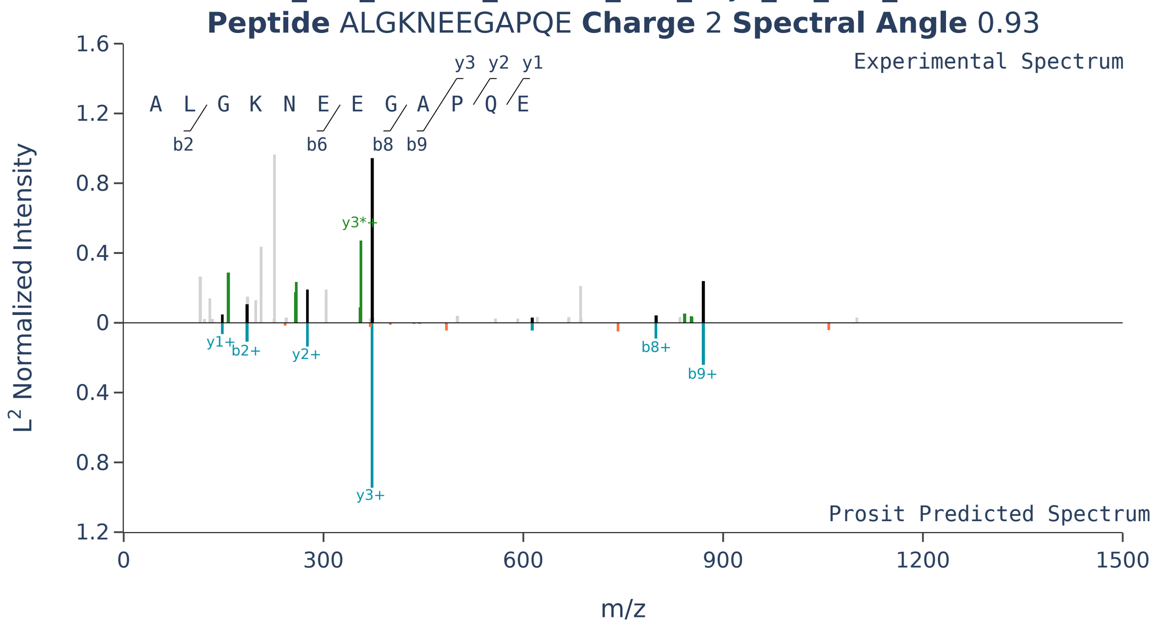

Source W_Soh_160821_170921_Goe_aSyn_A1_24h_R2 **Scan** 10619
Peptide DPNEAYEMPSE **Charge** 1 **Spectral Angle** 0.76

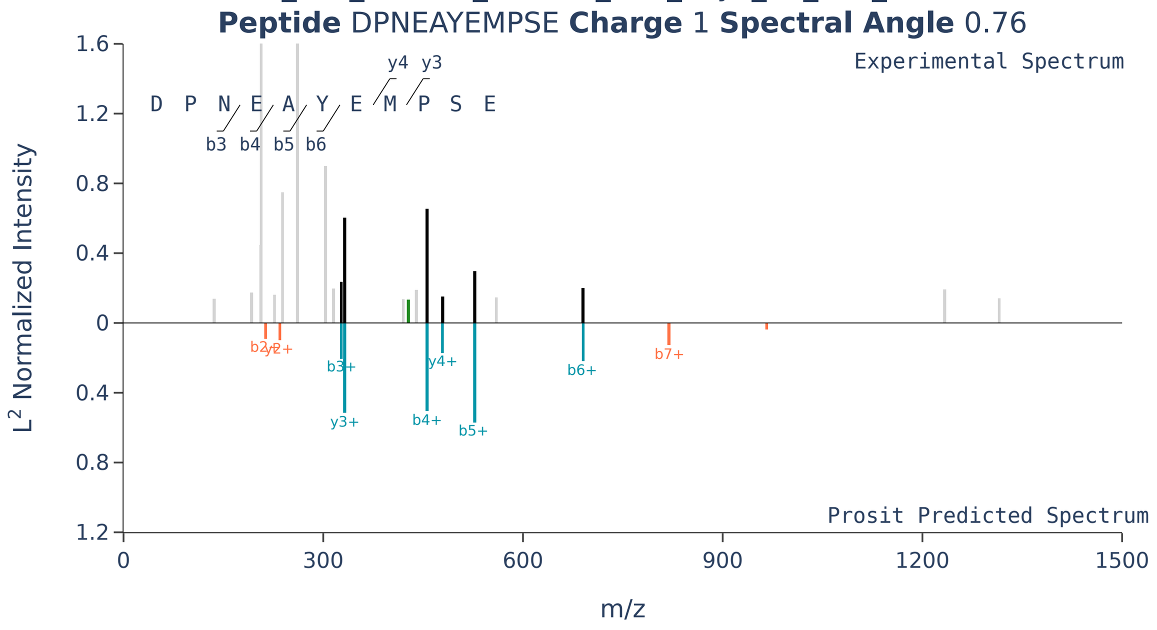

Source W_Soh_160821_170921_Goe_aSyn_A2_6h_R2 **Scan** 18817
Peptide PSEEGYQDYEPEGYQDYEPEA **Charge** 2 **Spectral Angle** 0.87

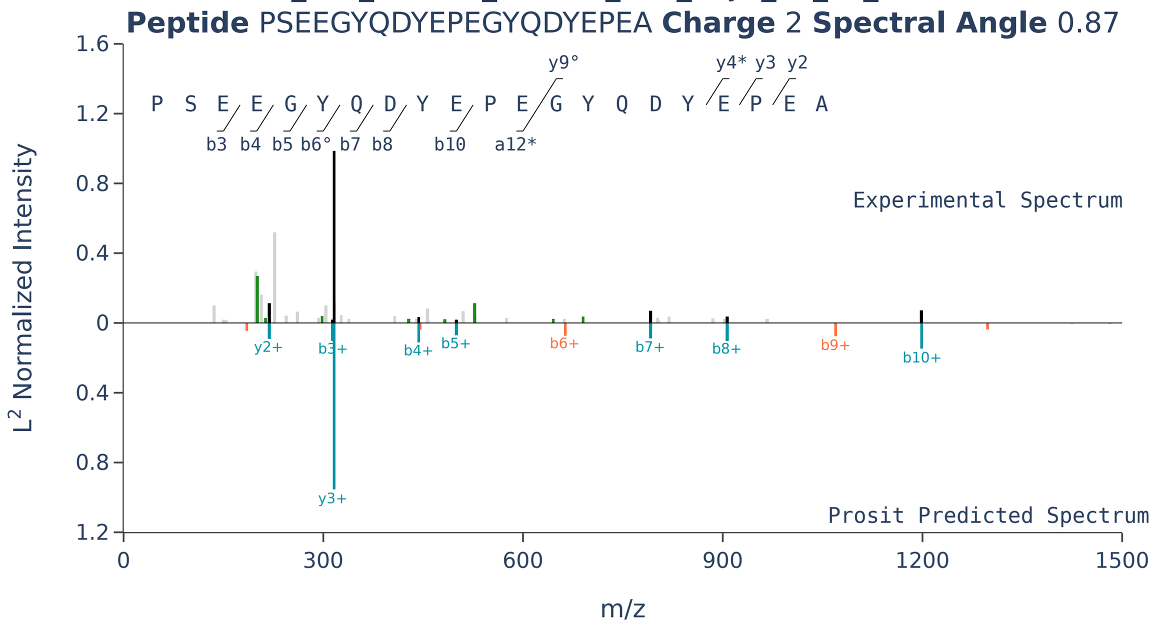

Source W_Soh_160821_170921_Goe_aSyn_A1_1h_R1 **Scan** 17060
Peptide GYQDYEPEPSEEGYQDYEPEA **Charge** 2 **Spectral Angle** 0.95

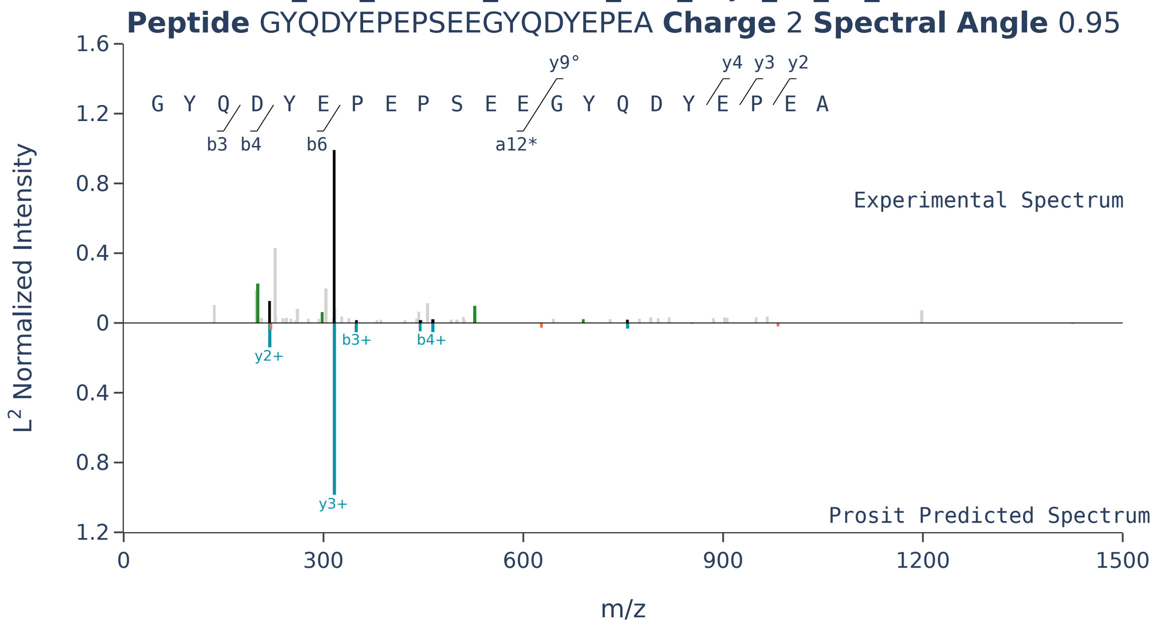

Source W_Soh_160821_170921_Goe_aSyn_A1_24h_R1 **Scan** 10212
Peptide AVLYVGSKTKEGVVH **Charge** 3 **Spectral Angle** 0.84

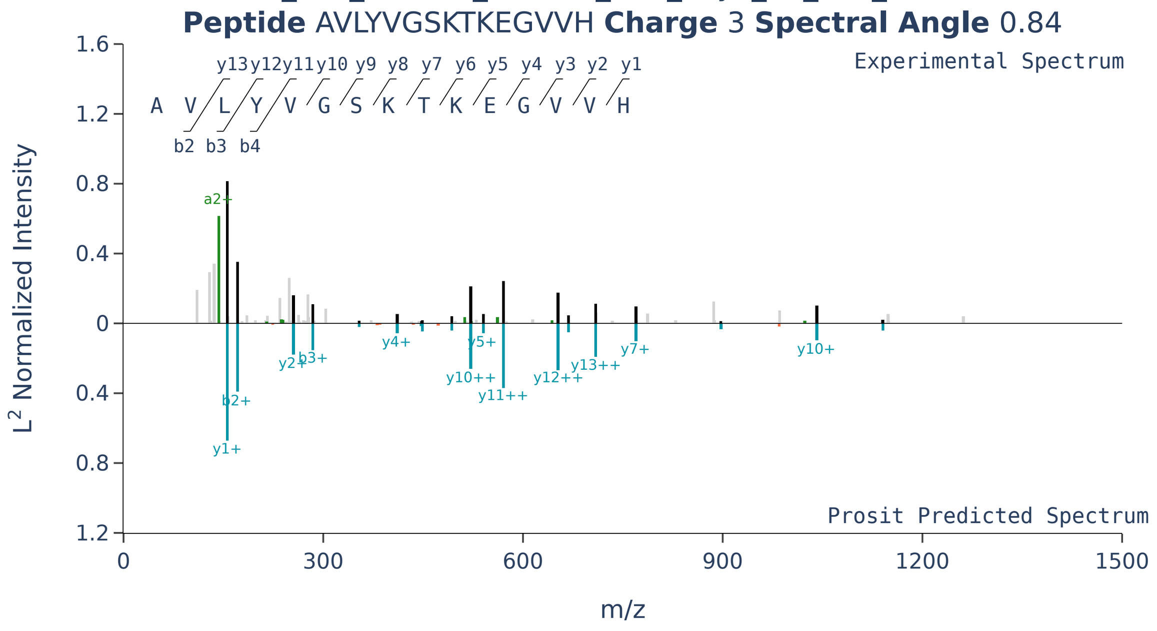

Source W_Soh_160821_170921_Goe_aSyn_A1_3h_R2 **Scan** 15357
Peptide DMVFMKGLSKAKEGVVA **Charge** 3 **Spectral Angle** 0.87

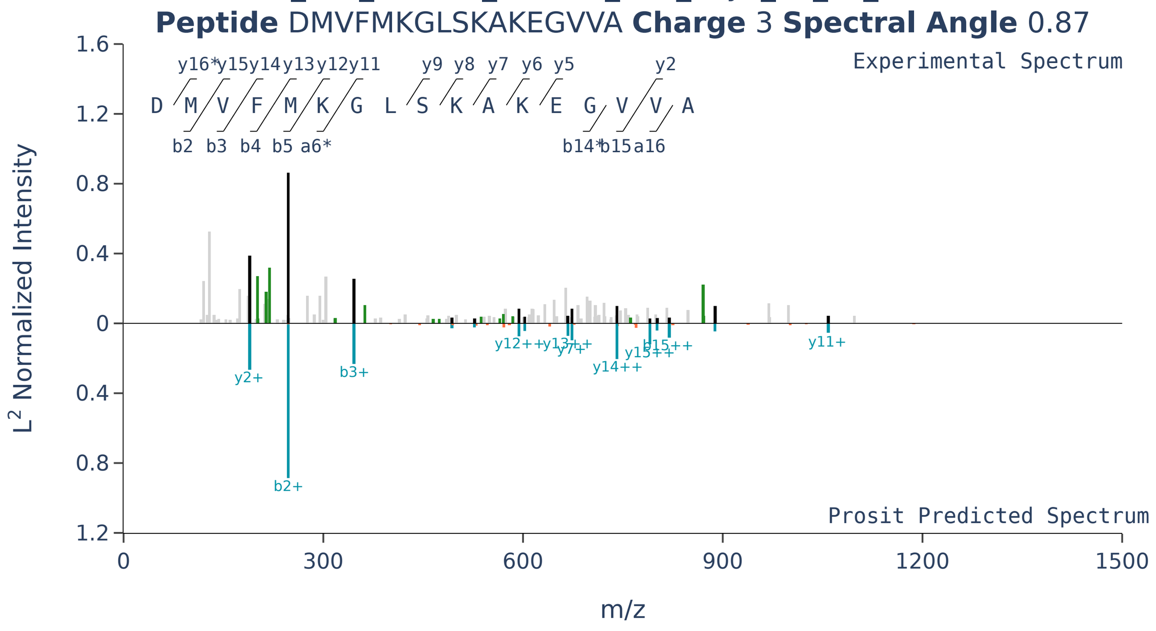

Source W_Soh_160821_170921_Goe_aSyn_A1_24h_R1 **Scan** 7161
Peptide EGLGKNEEGAPQE **Charge** 2 **Spectral Angle** 0.94

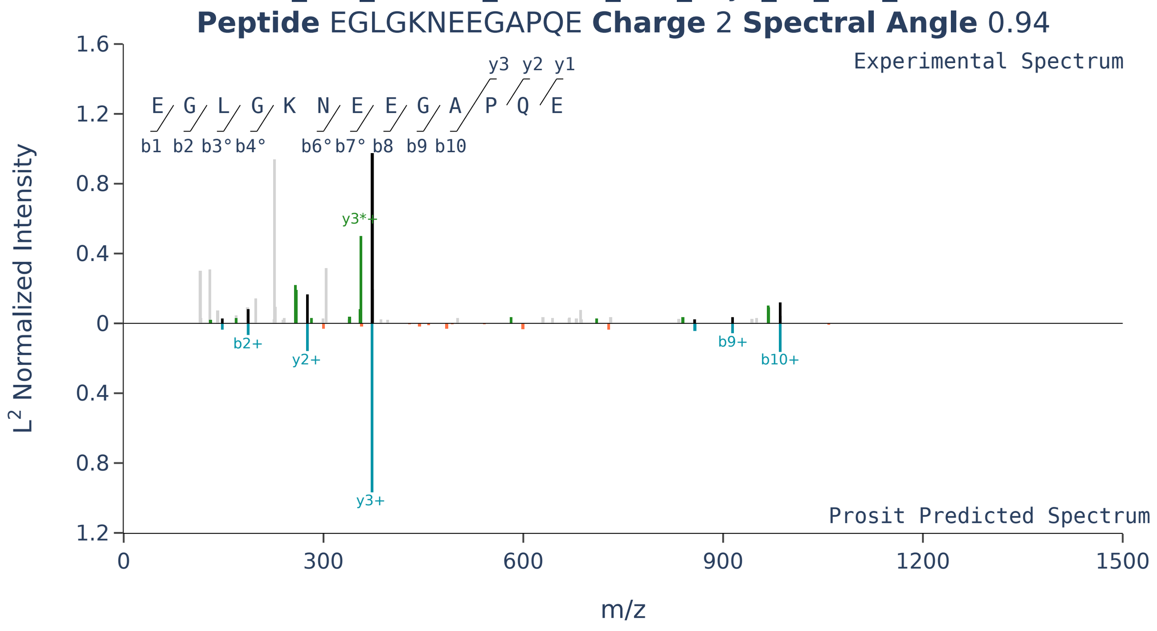

Source W_Soh_160821_170921_Goe_aSyn_A1_24h_R1 **Scan** 7762
Peptide SKAKEGVVAMD **Charge** 2 **Spectral Angle** 0.85

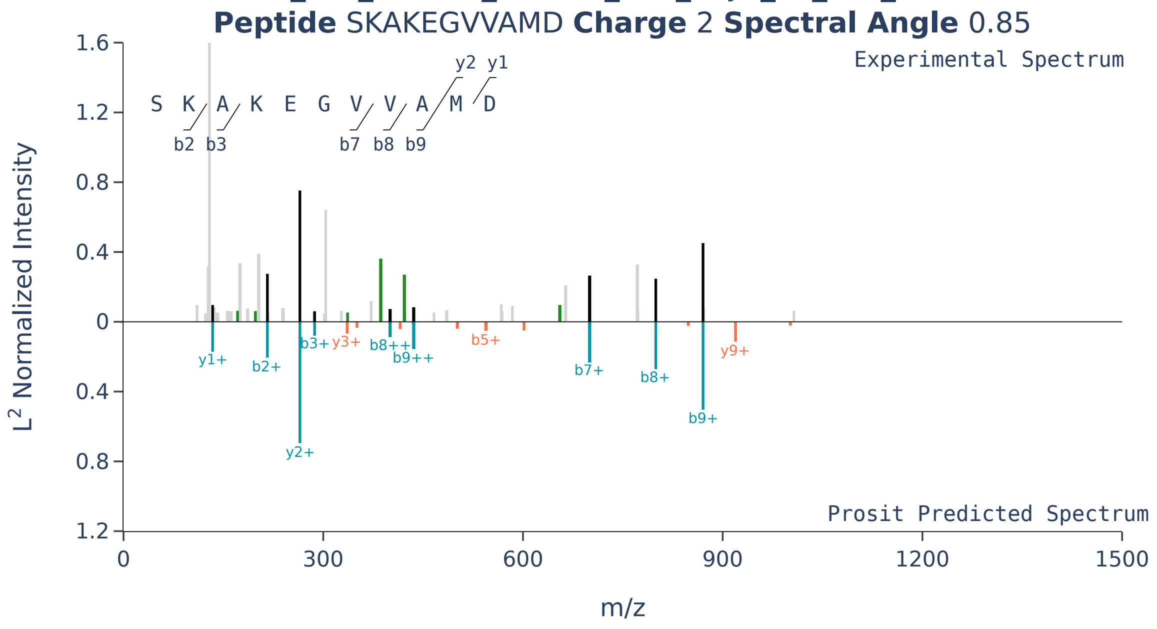

Source W_Soh_160821_170921_Goe_aSyn_A1_3h_R1 **Scan** 13508
Peptide PDNEAYEMDPD **Charge** 1 **Spectral Angle** 0.81

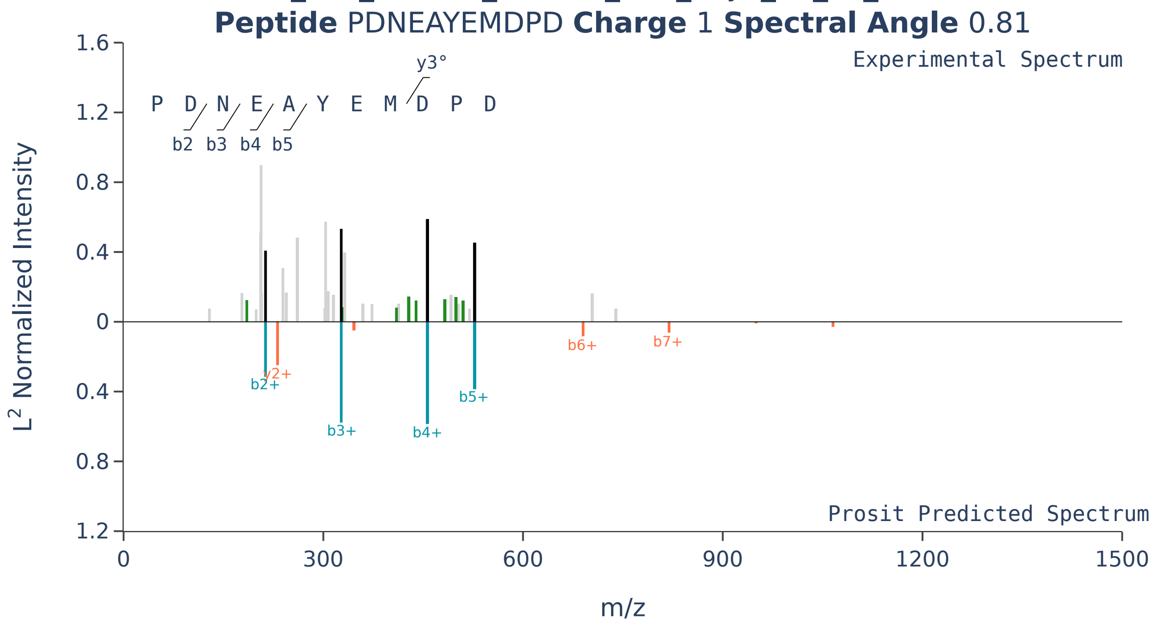

Source W_Soh_160821_170921_Goe_aSyn_A2_15'_R1 **Scan** 17157
Peptide PDNEAYEMPSEEGYQDYEAAAT **Charge** 2 **Spectral Angle** 0.87

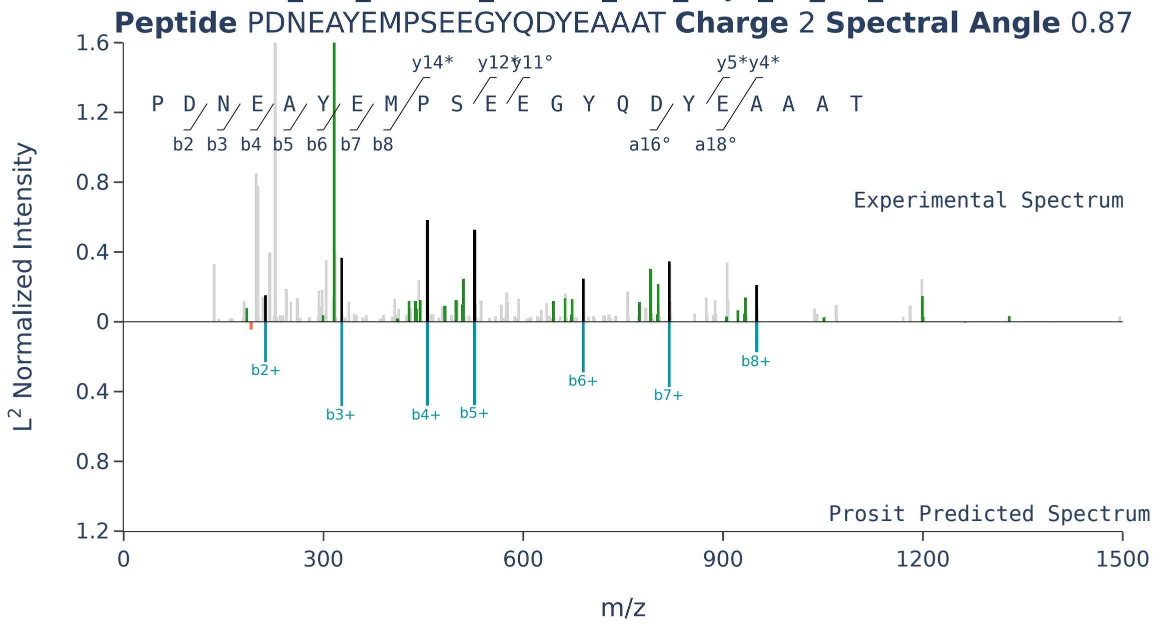

Source W_Soh_160821_170921_Goe_aSyn_A2_15'_R1 **Scan** 24928
Peptide MDVFMFV **Charge** 1 **Spectral Angle** 0.91

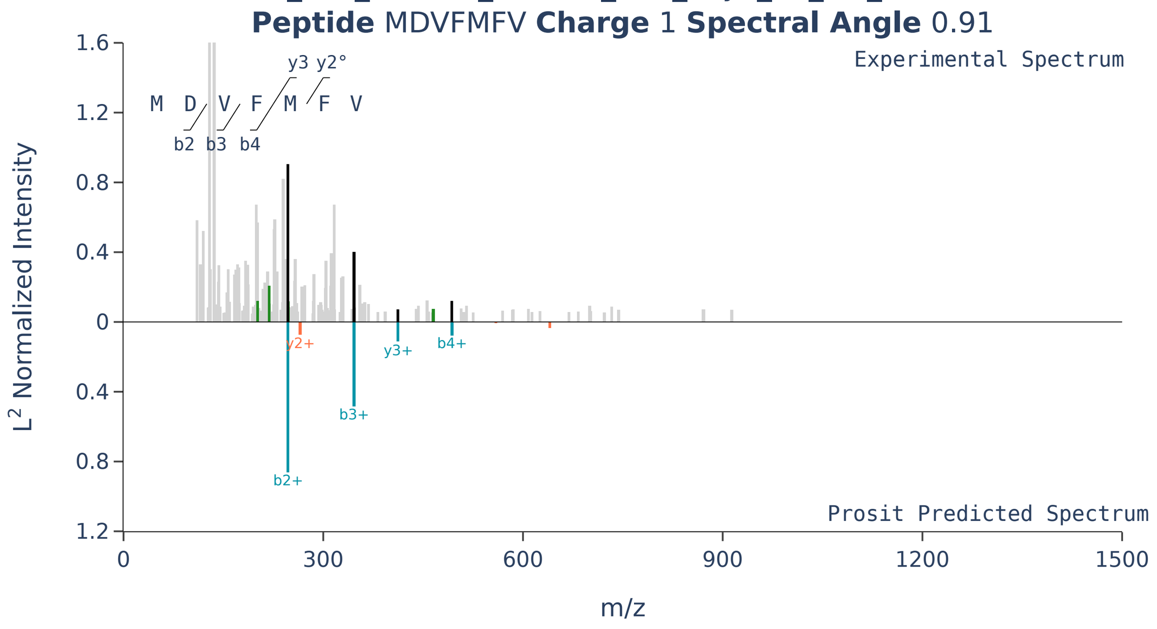

Source W_Soh_160821_170921_Goe_aSyn_A1_3h_R2 **Scan** 19625
Peptide AMDVFMKGLSKAKEGVVA **Charge** 3 **Spectral Angle** 0.83

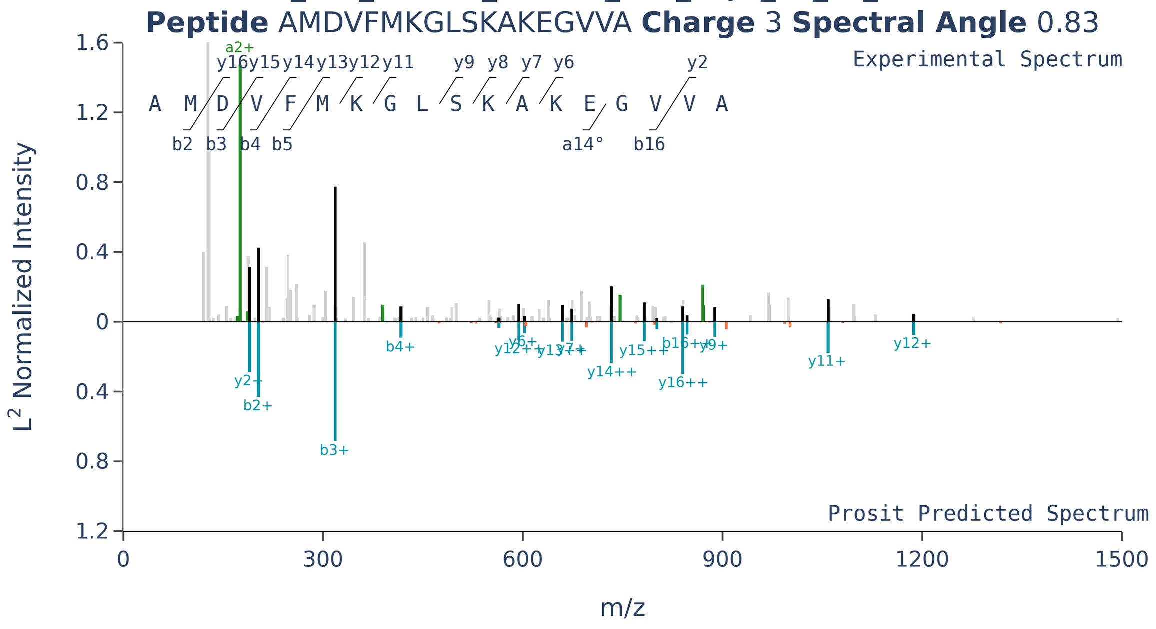

Source W_Soh_160821_170921_Goe_aSyn_A1_6h_R2 **Scan** 17655
Peptide PDNEAYEDMPVD **Charge** 1 **Spectral Angle** 0.78

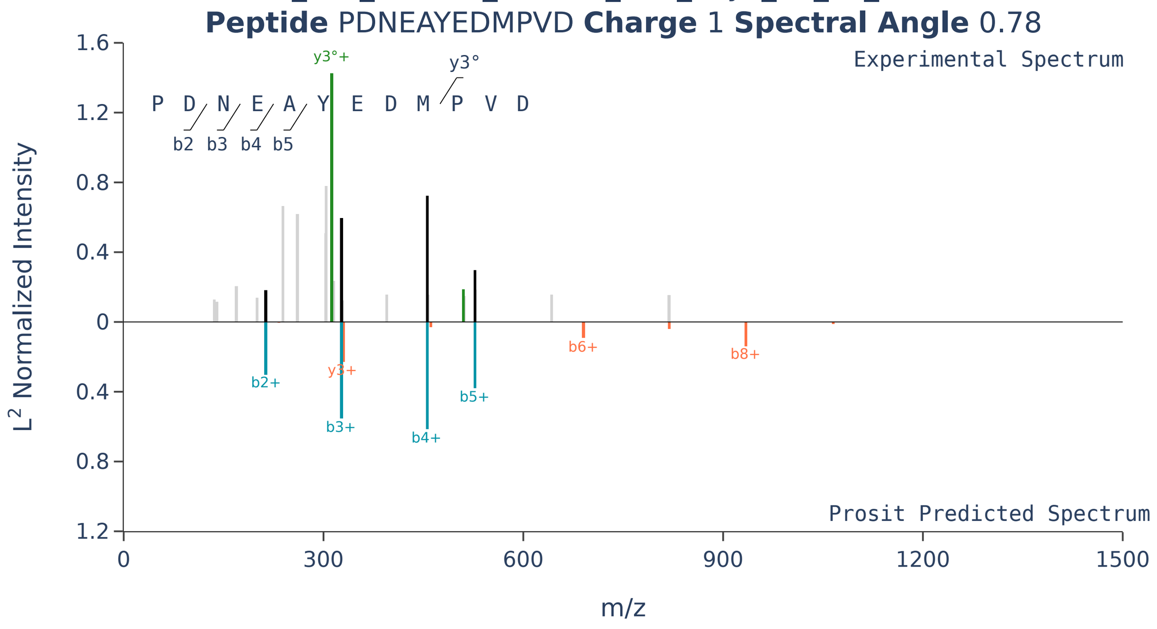

Source W_Soh_160821_170921_Goe_aSyn_A1_24h_R1 **Scan** 13983
Peptide MDVFMKKGLS **Charge** 2 **Spectral Angle** 0.8

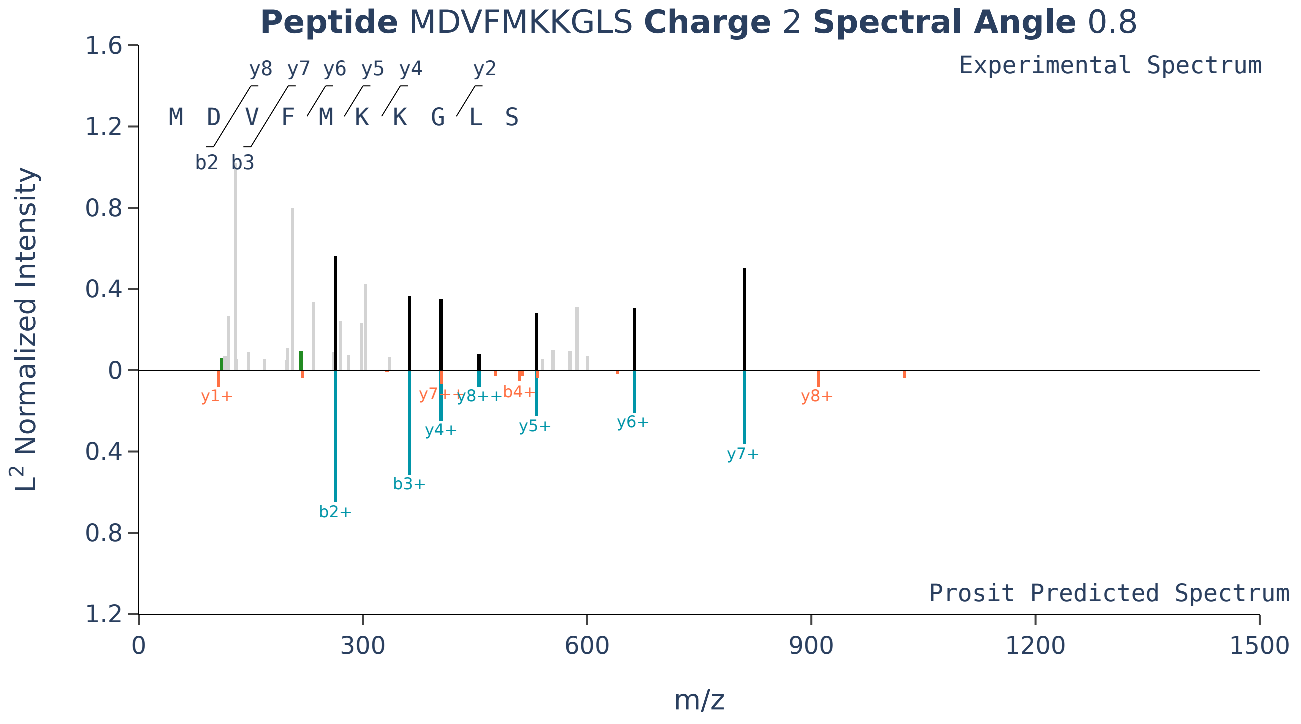

Source W_Soh_160821_170921_Goe_aSyn_A1_1h_R1 **Scan** 18229
Peptide PDNEAYEMPSEEGYQDYEQLG **Charge** 2 **Spectral Angle** 0.81

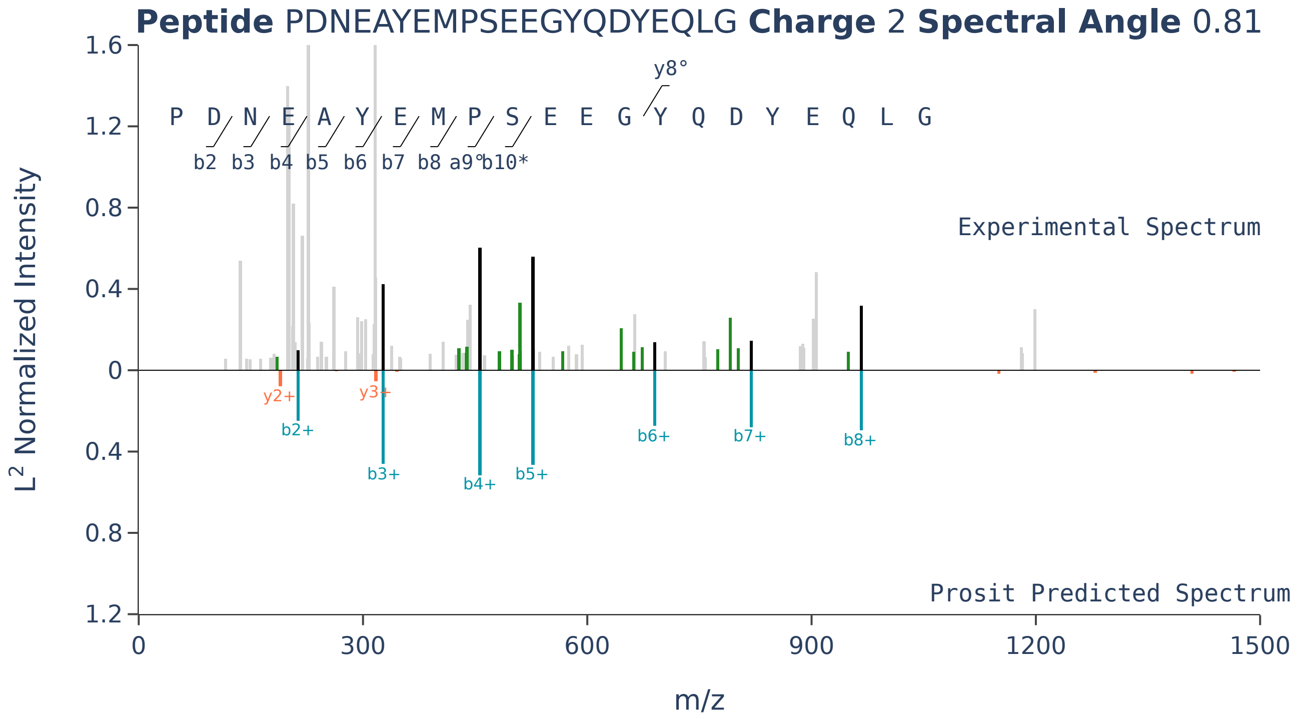

Source W_Soh_160821_170921_Goe_aSyn_A2_24h_R1 **Scan** 14349
Peptide ATGNEAYEMPSEE **Charge** 2 **Spectral Angle** 0.8

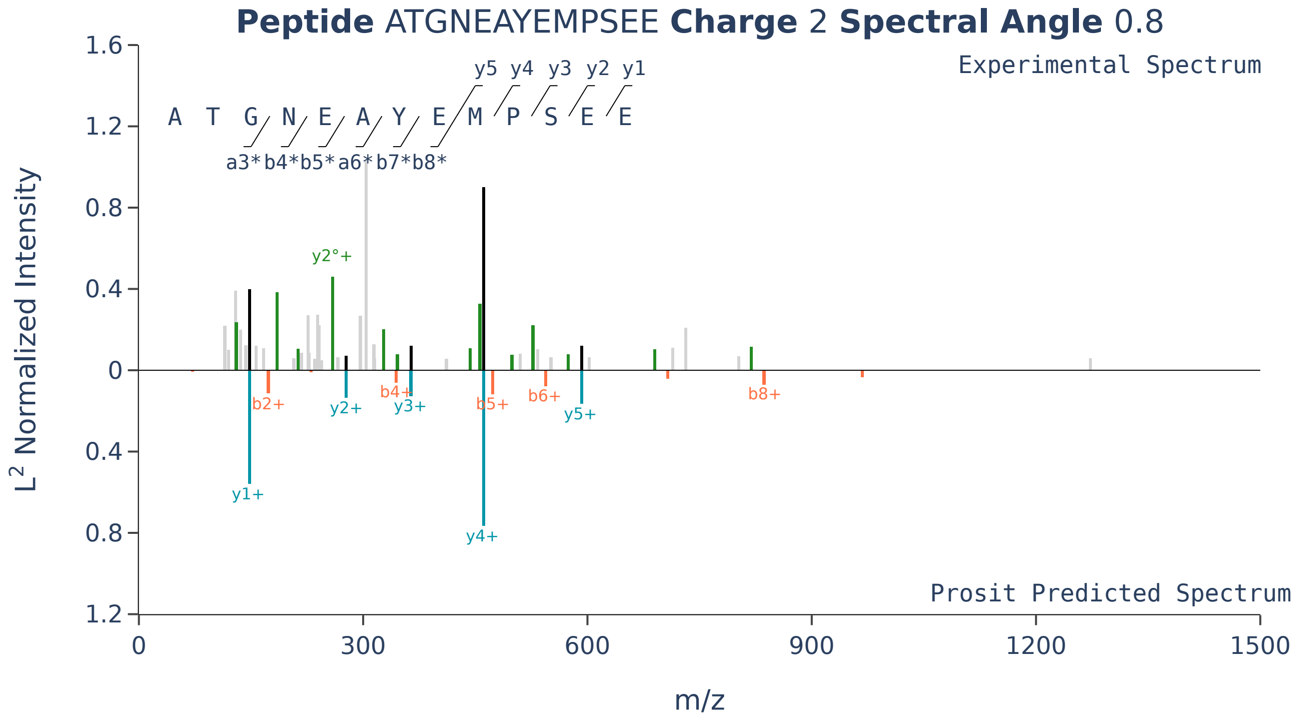

Source W_Soh_160821_170921_Goe_aSyn_A1_24h_R1 **Scan** 6922
Peptide EGATGFVKKD **Charge** 2 **Spectral Angle** 0.83

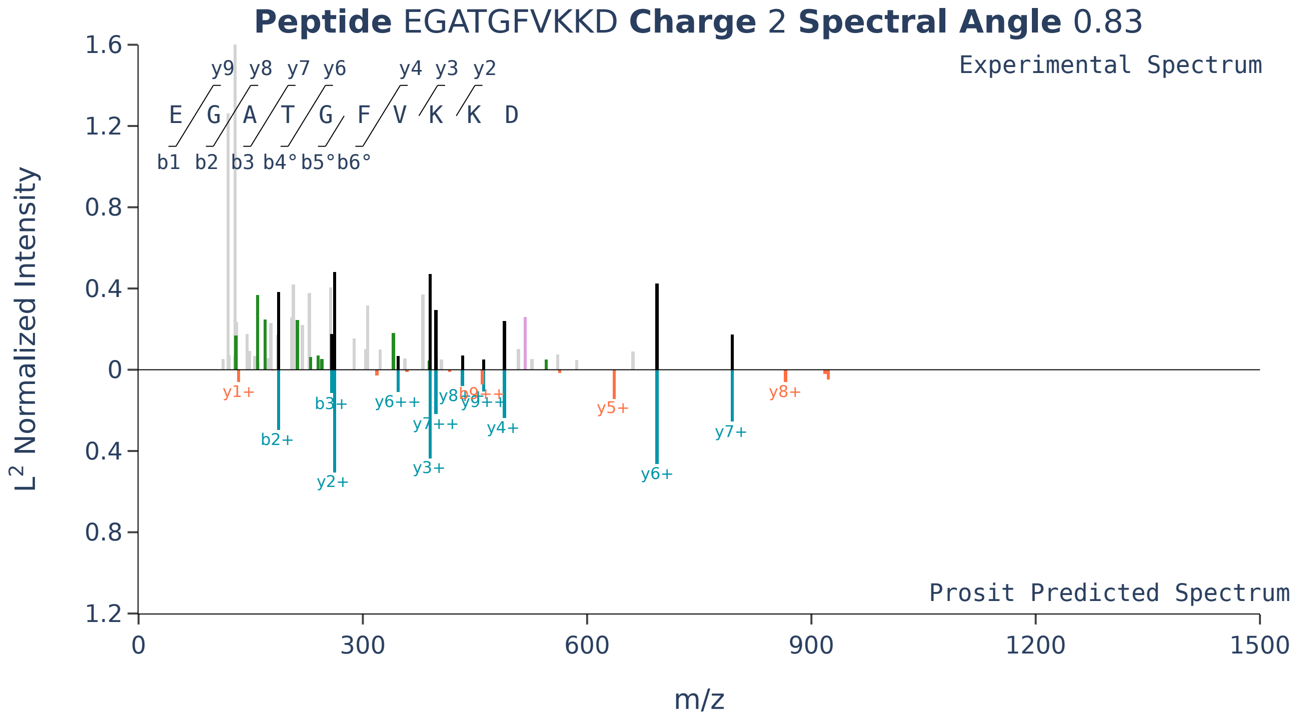

Source W_Soh_160821_170921_Goe_aSyn_A1_24h_R1 **Scan** 10269
Peptide KEGVVAM **Charge** 2 **Spectral Angle** 0.9

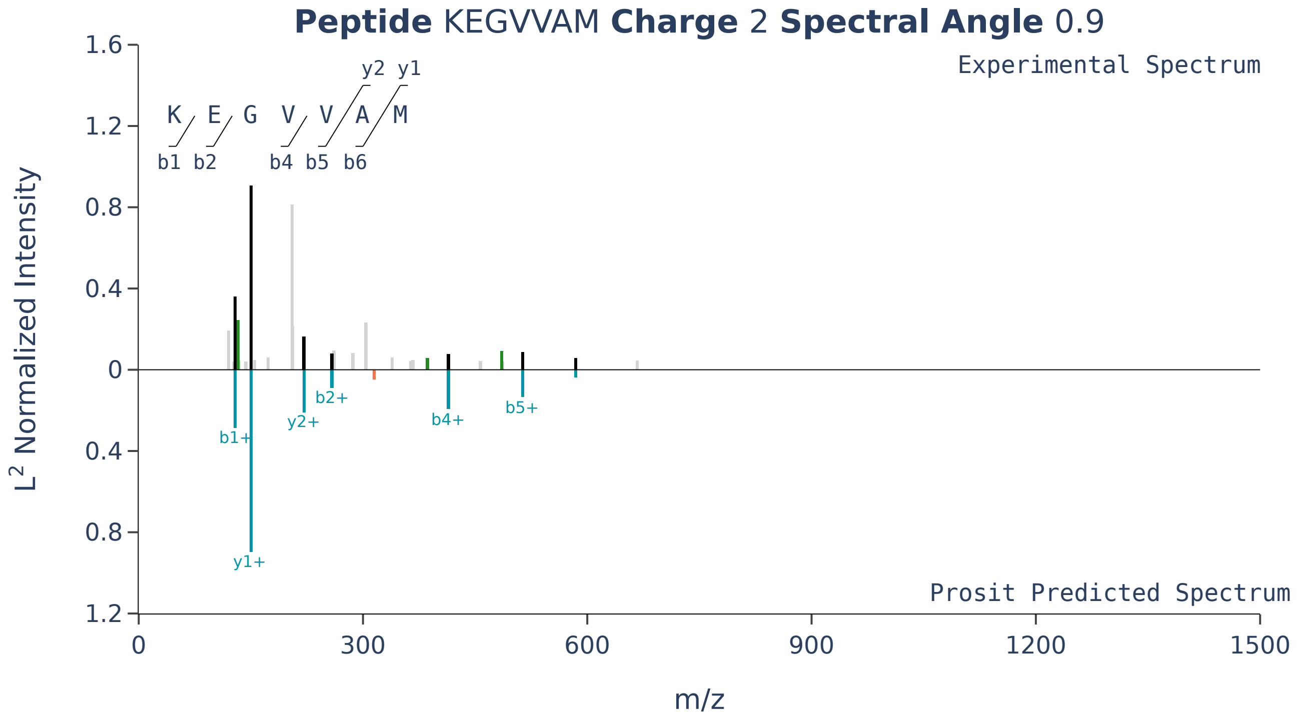

Source W_Soh_160821_170921_Goe_aSyn_A2_30'_R2 **Scan** 10640
Peptide AGKTKEGVLYVGA **Charge** 3 **Spectral Angle** 0.85

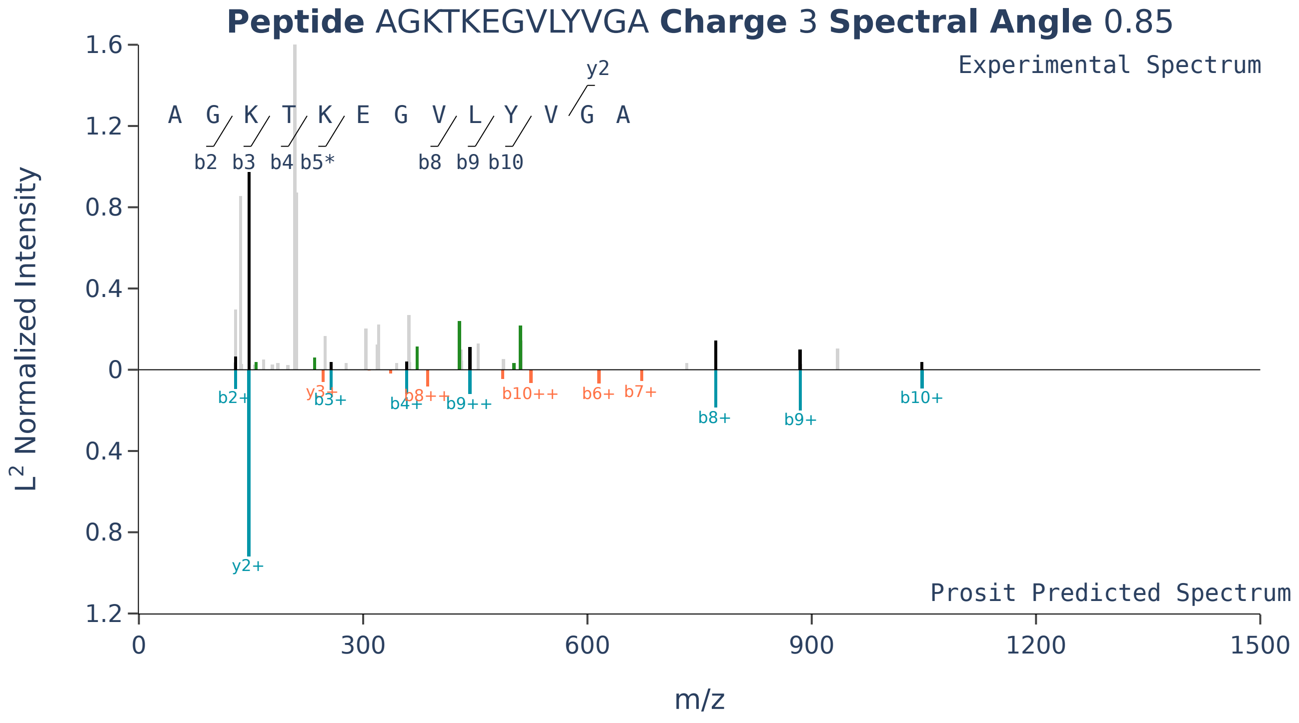

Source W_Soh_160821_170921_Goe_aSyn_A1_6h_R1 **Scan** 6143
Peptide SKTKEGVVHGVATA **Charge** 3 **Spectral Angle** 0.84

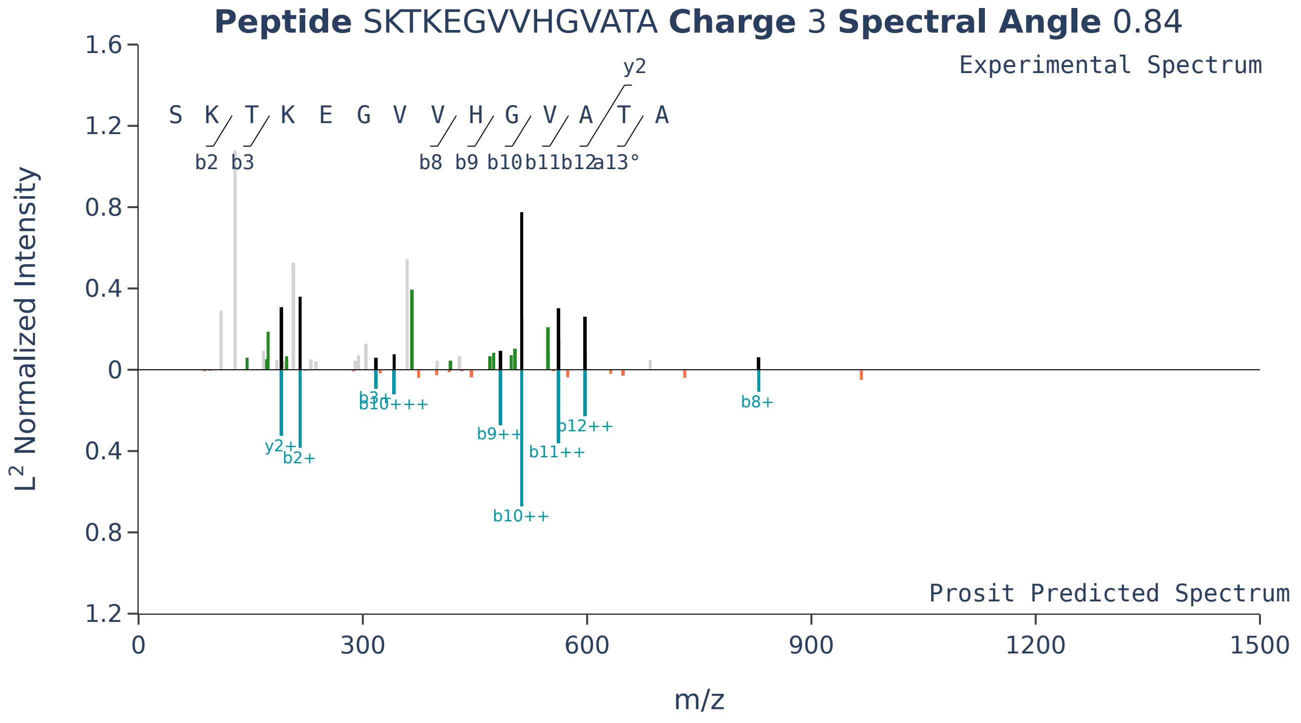

Source W_Soh_160821_170921_Goe_aSyn_A1_6h_R1 **Scan** 21197
Peptide NEAYEMPMPSEEGYQDYEPEA **Charge** 2 **Spectral Angle** 0.81

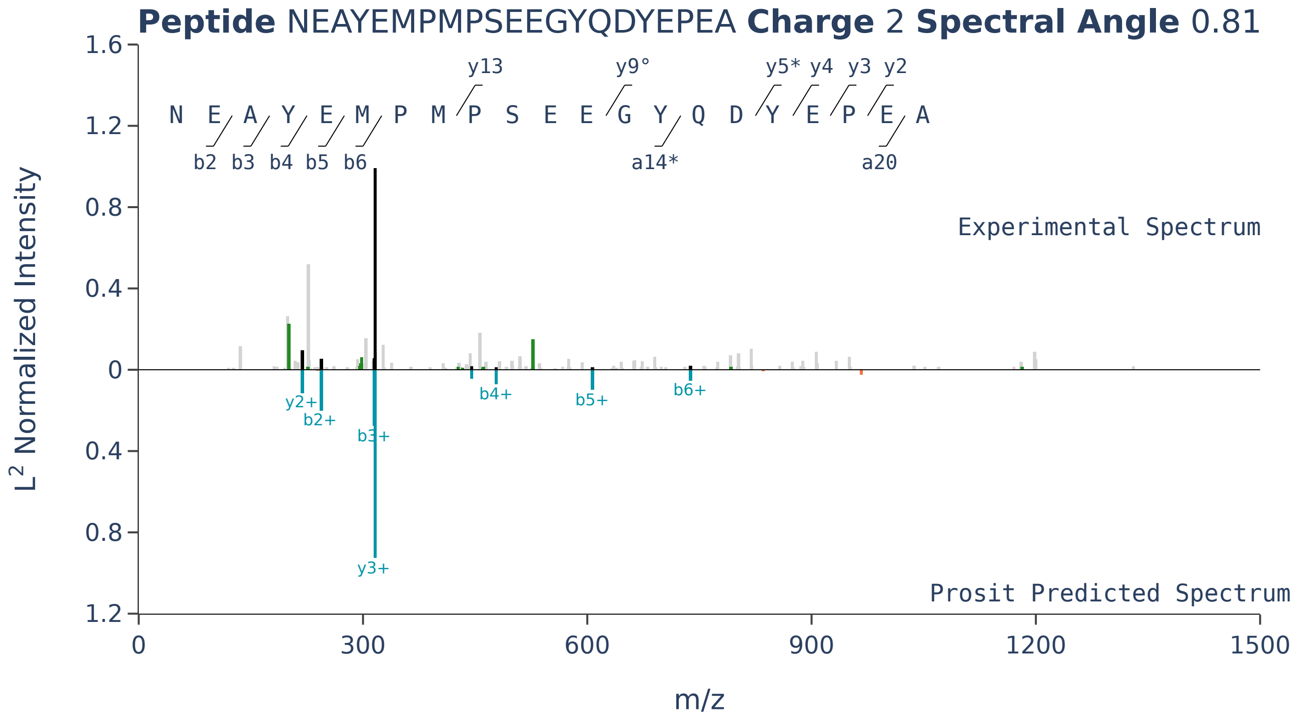

Source W_Soh_160821_170921_Goe_aSyn_A2_1h_R1 **Scan** 18091
Peptide PDNEAYEMPSEEGYQDYETNV **Charge** 2 **Spectral Angle** 0.8

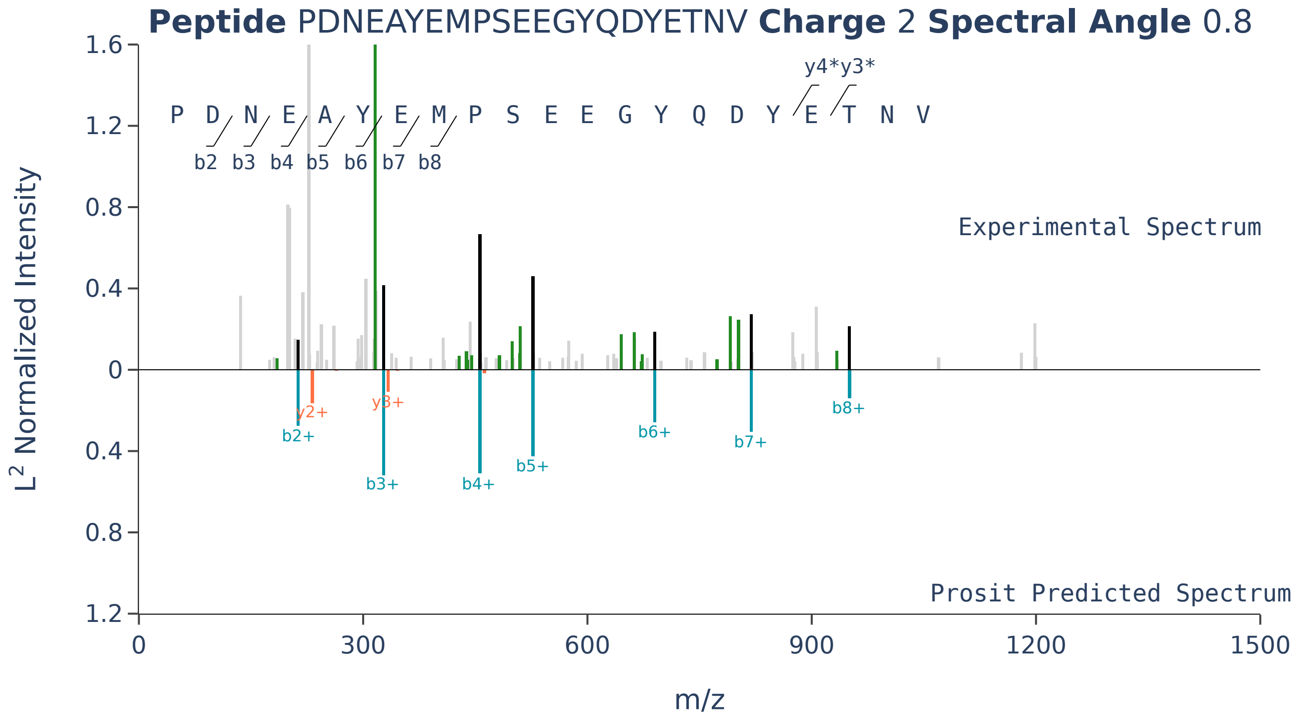

Source HRoetschke_291222_040123_Fu_CaM_b1R2_24h **Scan** 5816
Peptide ADQLTEEQIRE **Charge** 2 **Spectral Angle** 0.94

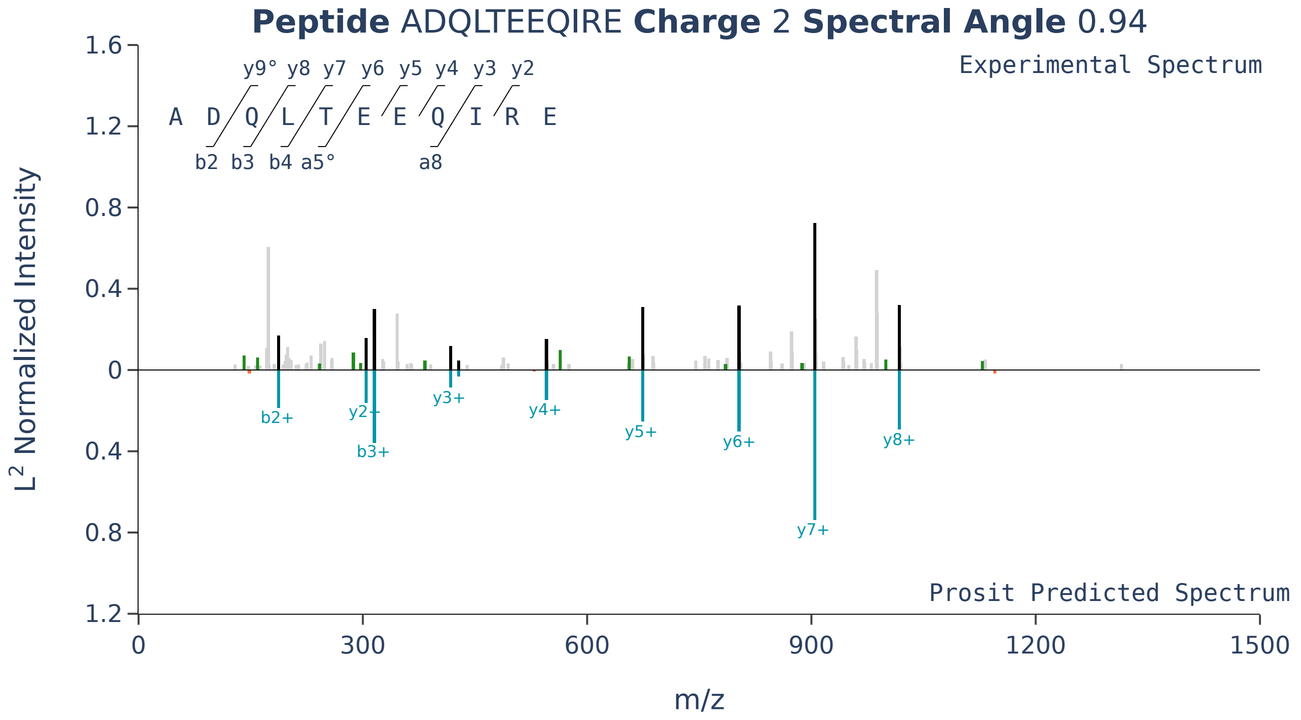

Source HRoetschke_291222_040123_Fu_CaM_b1R2_4h **Scan** 4910
Peptide DKMINEVD **Charge** 2 **Spectral Angle** 0.92

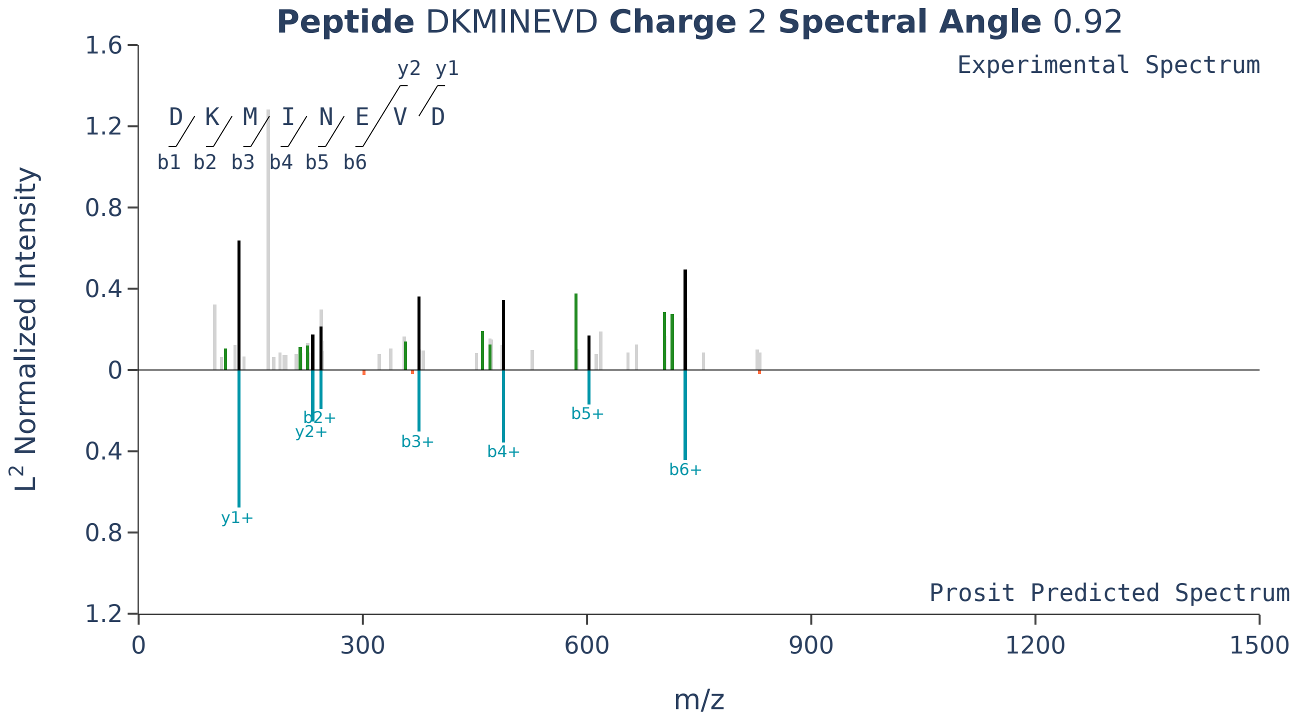

Source HRoetschke_291222_040123_Fu_CaM_b2R2_2h **Scan** 11217
Peptide PEFFLTM **Charge** 2 **Spectral Angle** 0.89

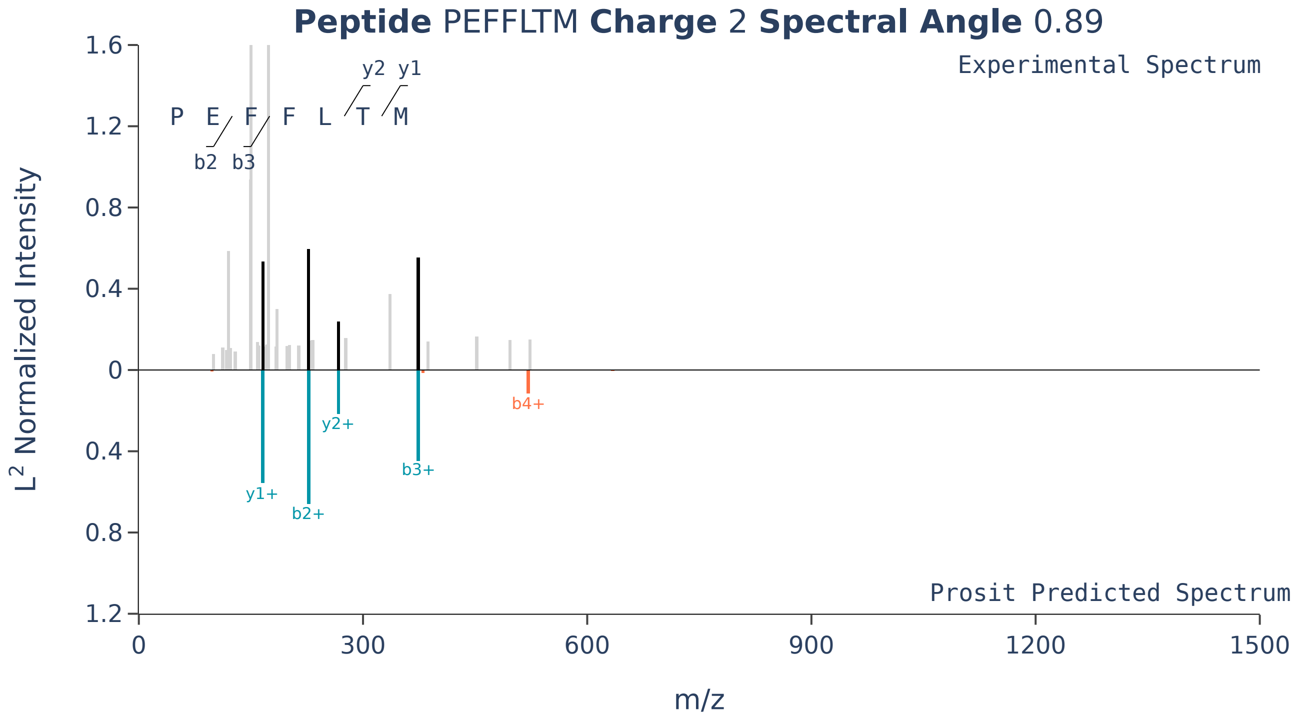

Source HRoetschke_291222_040123_Fu_CaM_b1R2_2h **Scan** 7533
Peptide ADQLLTEEQ **Charge** 1 **Spectral Angle** 0.87

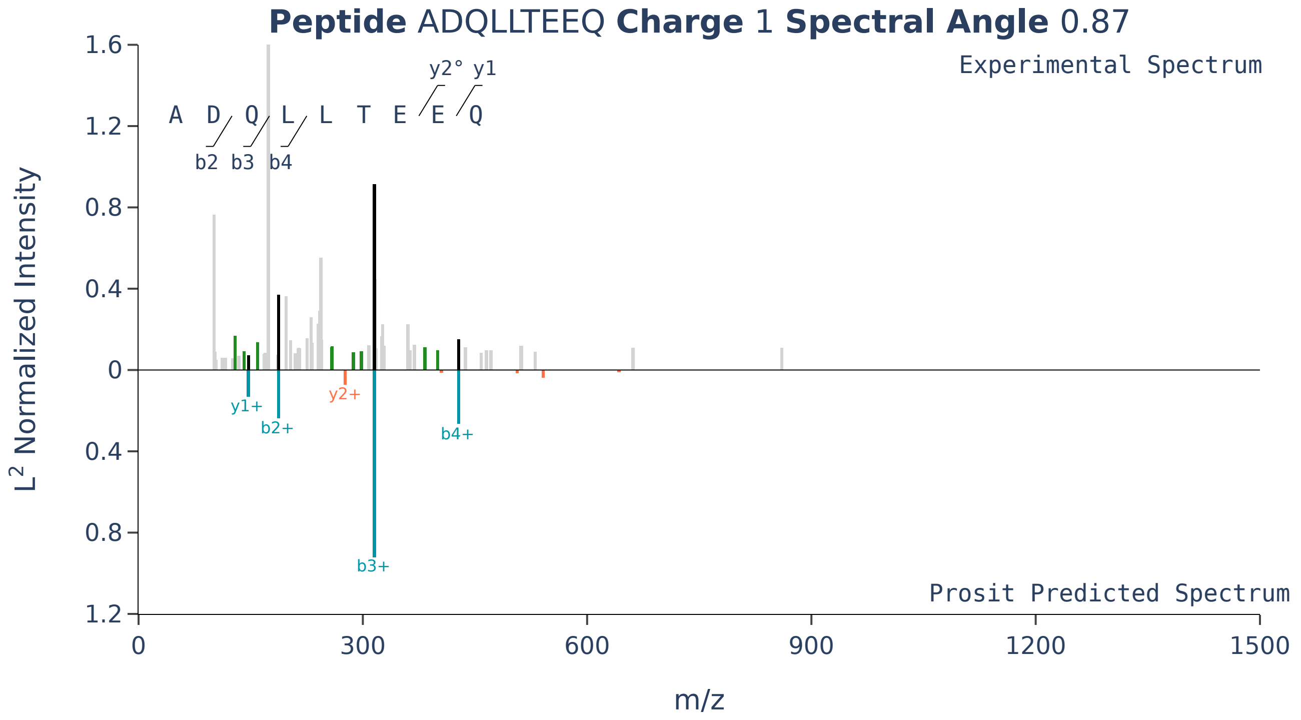

Source HRoetschke_291222_040123_Fu_CaM_b1R1_1h **Scan** 5492
Peptide RSAEFKEAF **Charge** 2 **Spectral Angle** 0.88

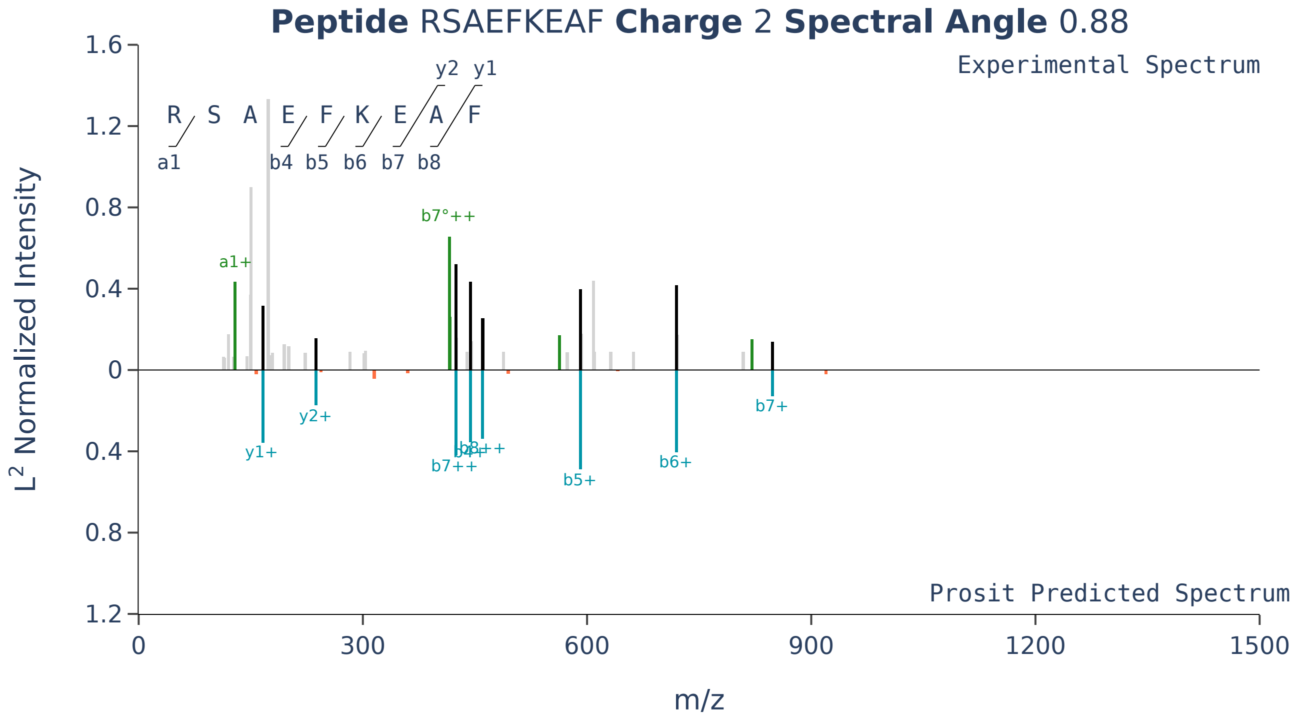

Source HRoetschke_291222_040123_Fu_CaM_b1R1_24h **Scan** 2719
Peptide ARSLGQNPTE **Charge** 2 **Spectral Angle** 0.89

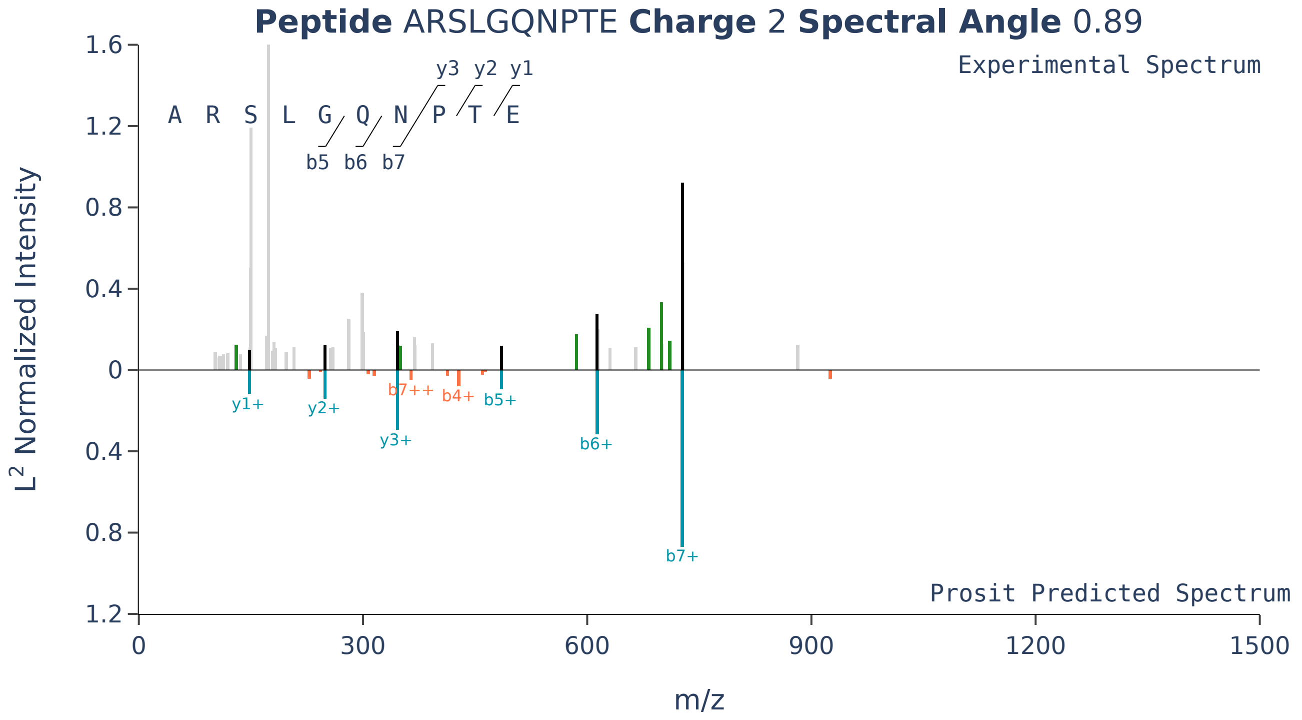

Source HRoetschke_291222_040123_Fu_CaM_b2R1_1h **Scan** 2364
Peptide EKLGTVMR **Charge** 2 **Spectral Angle** 0.76

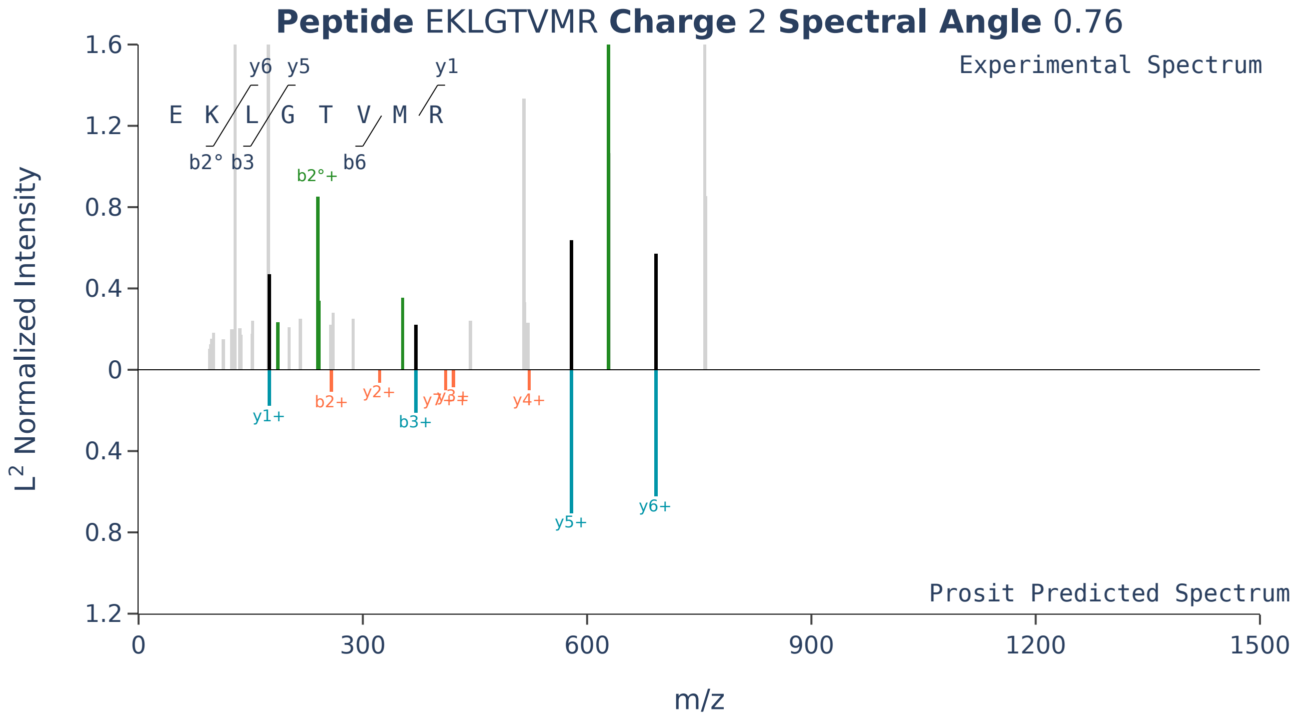

Source HRoetschke_291222_040123_Fu_CaM_b1R1_24h **Scan** 5555
Peptide KELGTADQLTE **Charge** 2 **Spectral Angle** 0.88

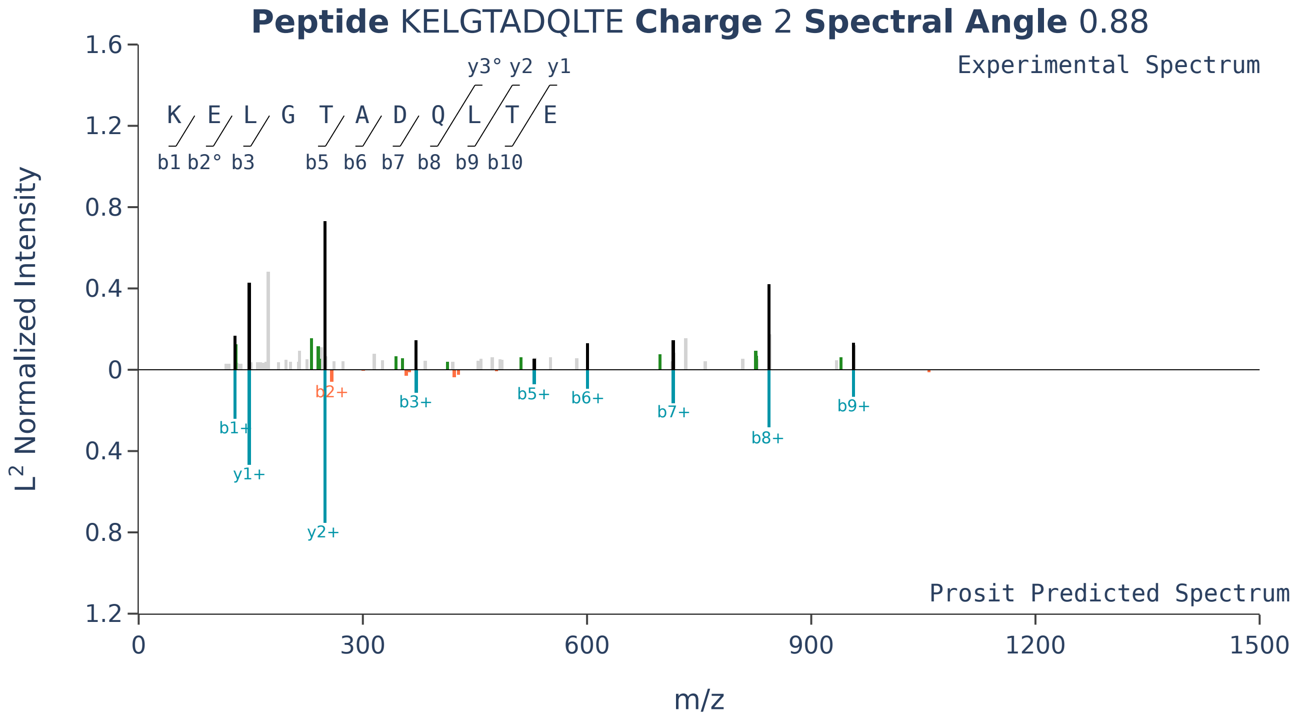

Source HRoetschke_291222_040123_Fu_CaM_b2R1_1h **Scan** 5750
Peptide RSADQLTEEQI **Charge** 2 **Spectral Angle** 0.86

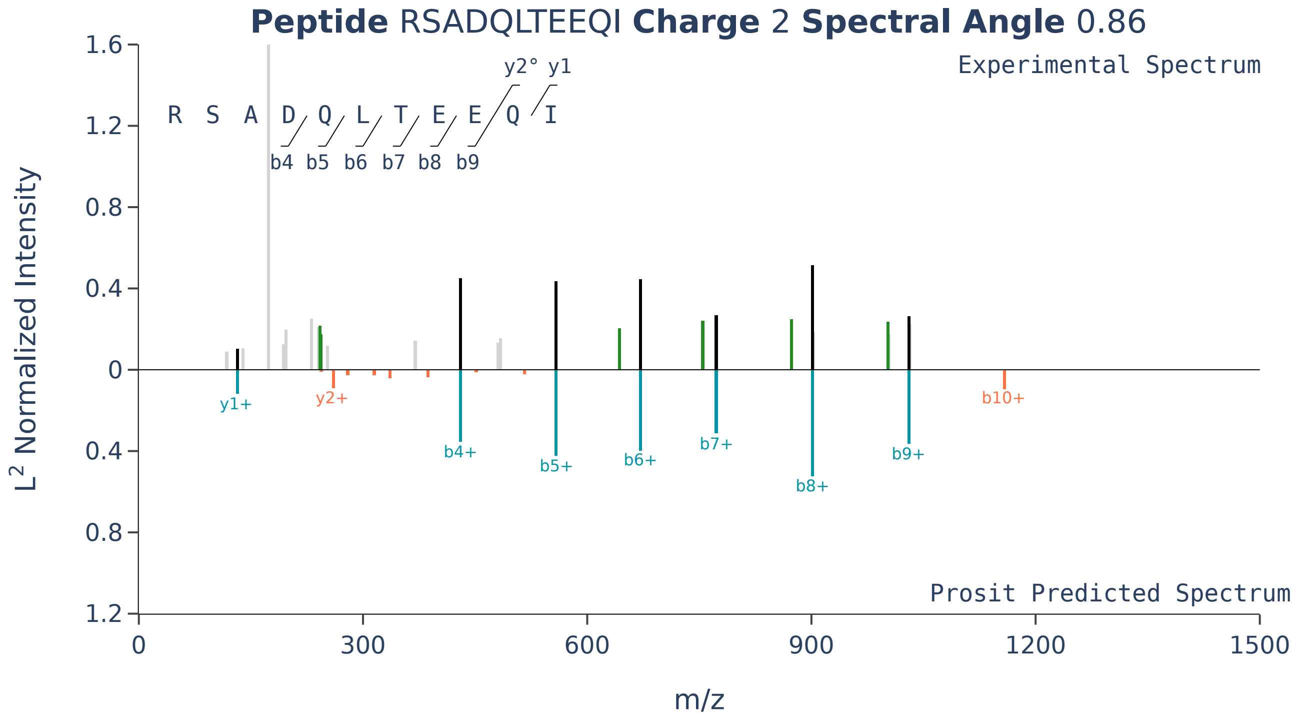

Source HRoetschke_291222_040123_Fu_CaM_b1R2_24h **Scan** 5225
Peptide MKDINEVD **Charge** 2 **Spectral Angle** 0.81

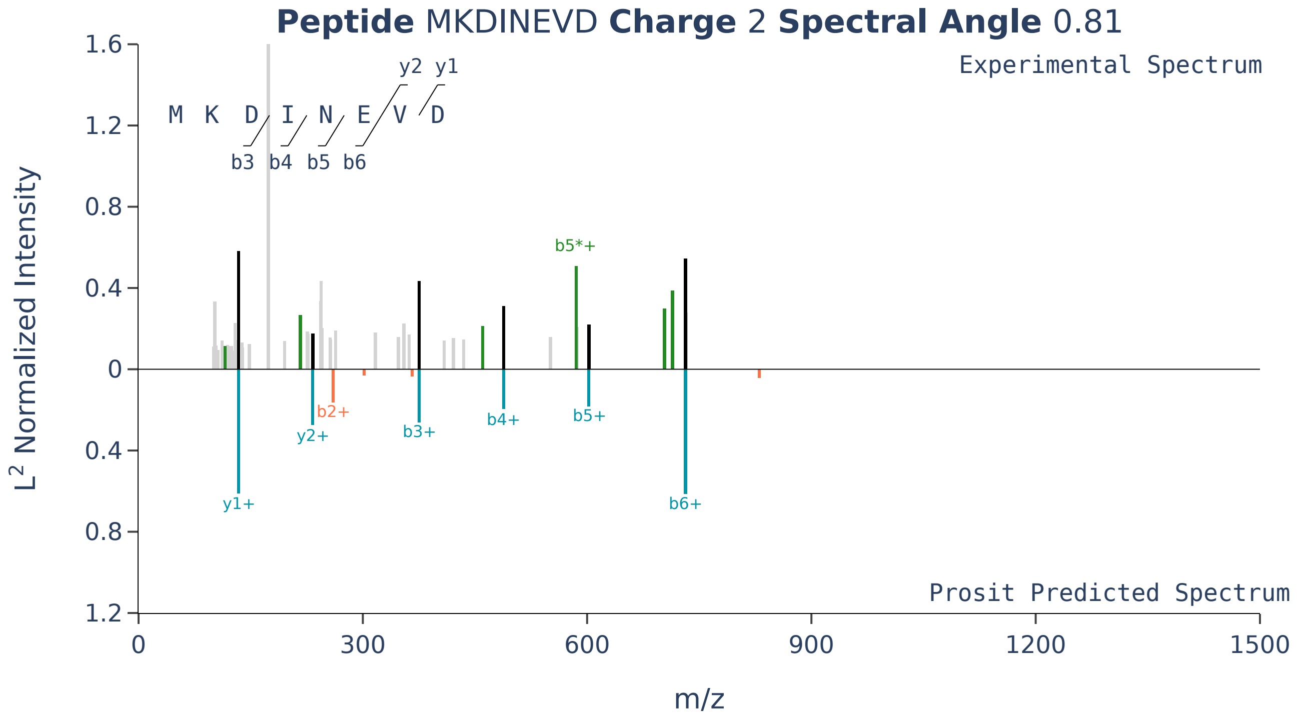

Source HRoetschke_291222_040123_Fu_CaM_b1R1_24h **Scan** 4362
Peptide KELGTVMRGT **Charge** 3 **Spectral Angle** 0.84

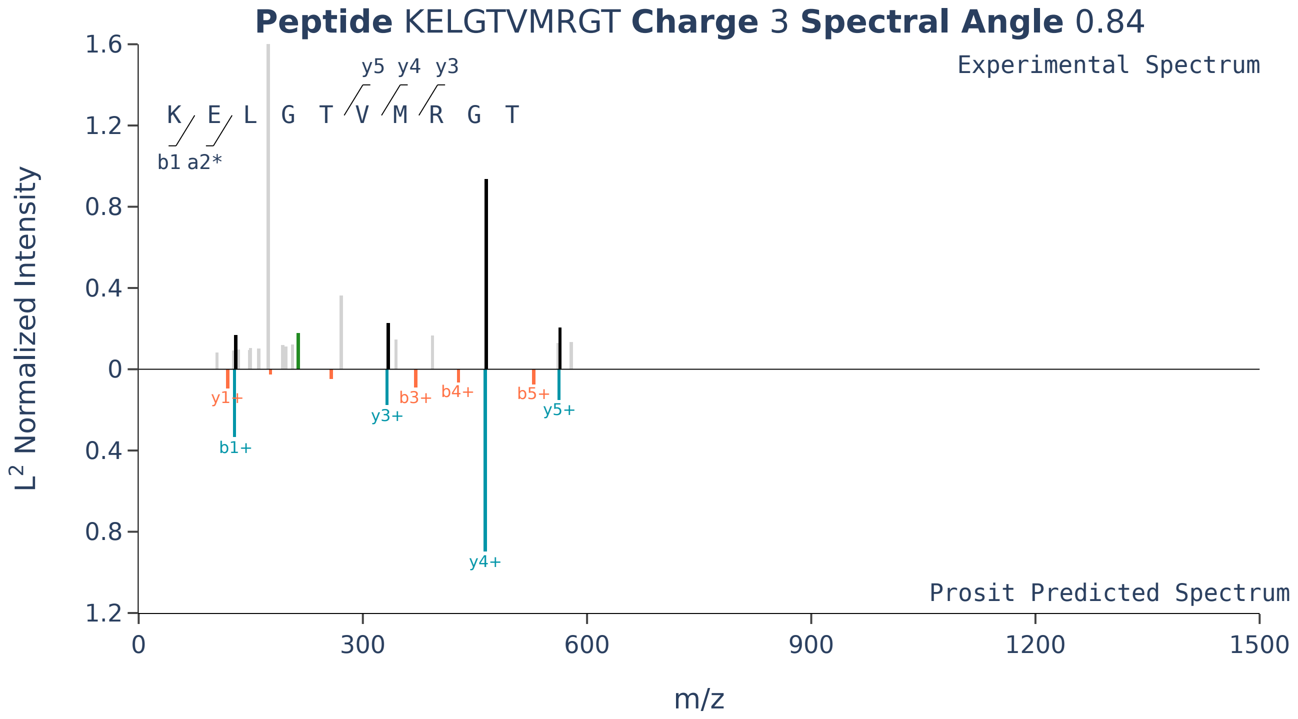

Source Ncheng_210623_230623_HFGoe_FFH_20S_25_1_A1_4h_R1 **Scan** 28811
Peptide DVDFFPSD **Charge** 1 **Spectral Angle** 0.93

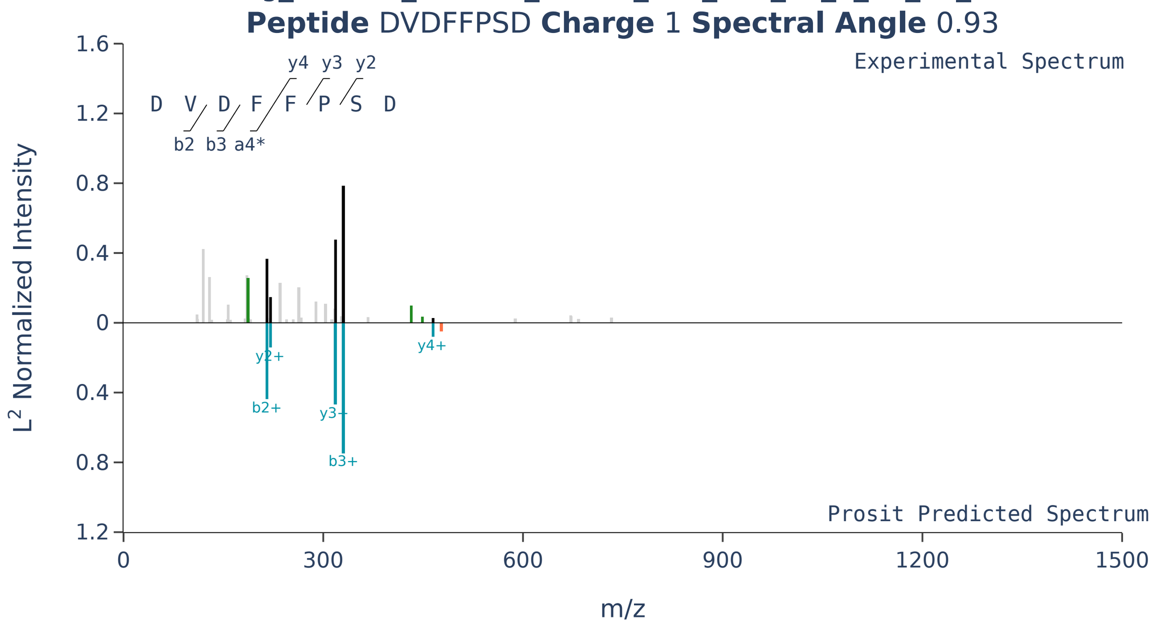

Source Ncheng_210623_230623_HFGoe_FFH_20S_25_1_A2_2h_R1 **Scan** 29151
Peptide DGVDFFPSD **Charge** 1 **Spectral Angle** 0.91

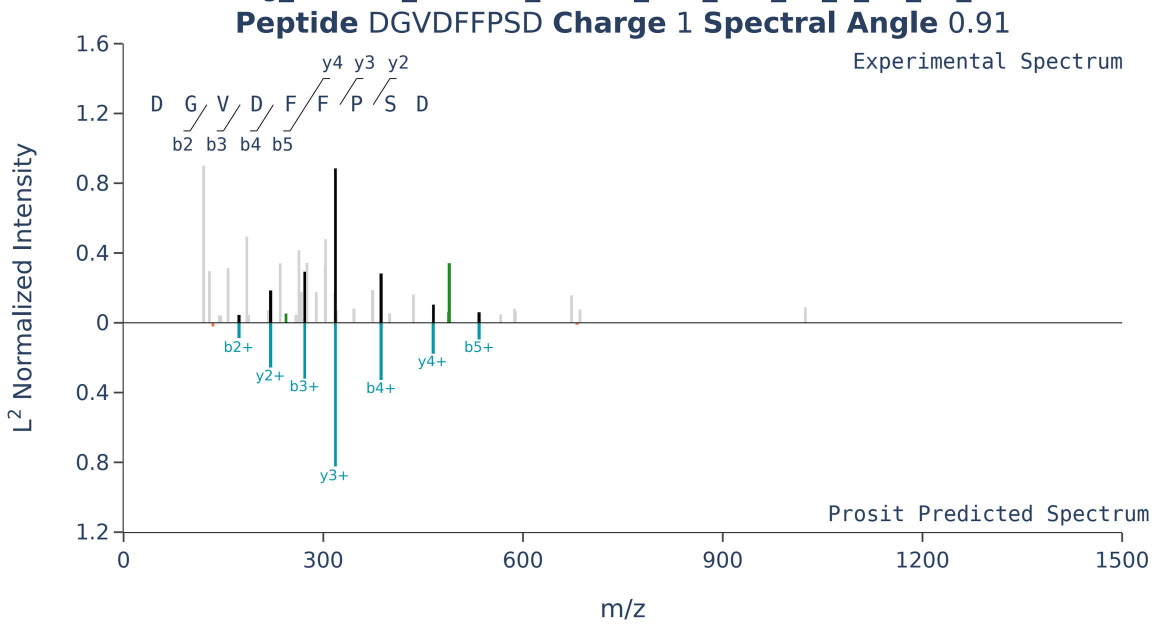

Source Ncheng_210623_230623_HFGoe_FFH_20S_25_1_A2_24h_R2 **Scan** 26799
Peptide SVDFFPSD **Charge** 1 **Spectral Angle** 0.91

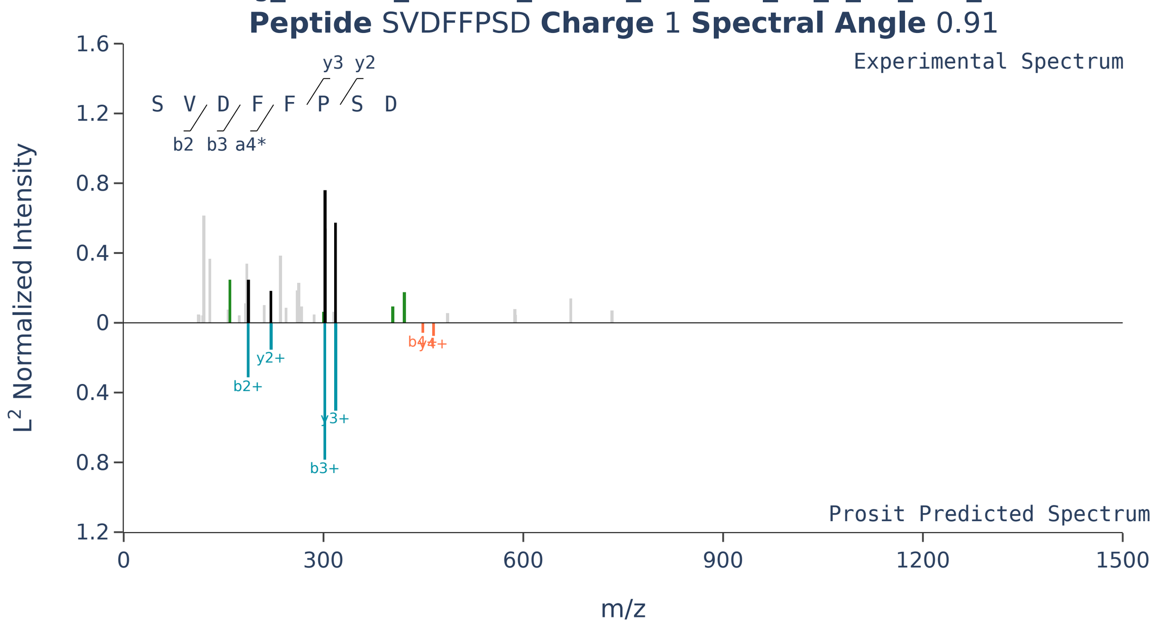

Source Ncheng_210623_230623_HFGoe_FFH_20S_25_1_A2_24h_R2 **Scan** 26793
Peptide VDFFPDT **Charge** 1 **Spectral Angle** 0.89

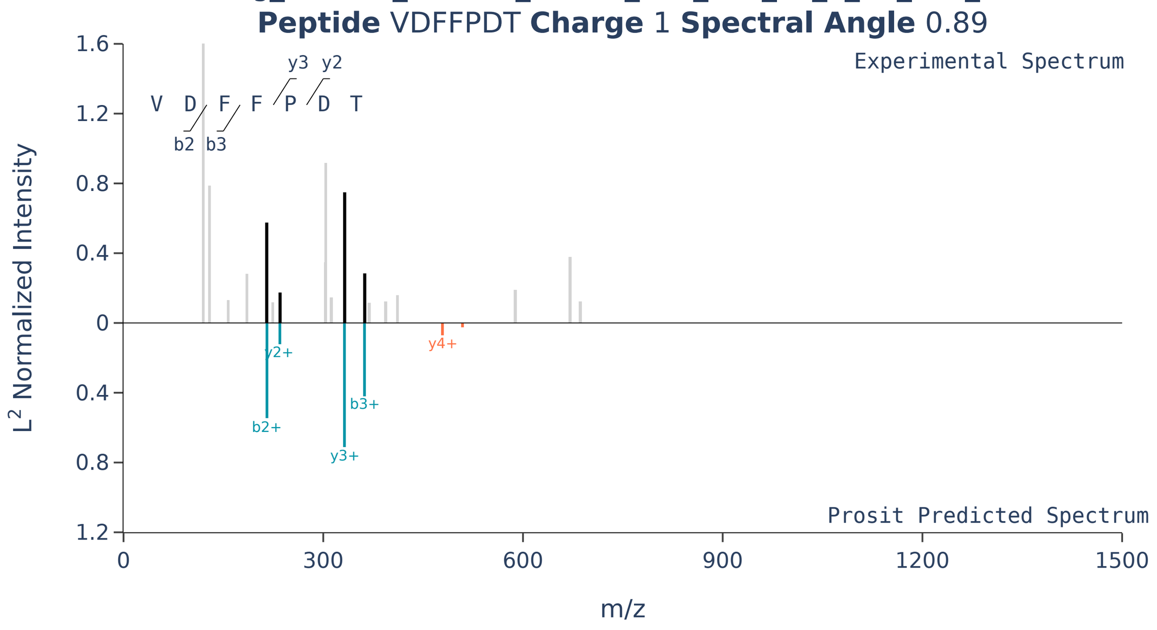

Source Ncheng_210623_230623_HFGoe_FFH_20S_25_1_A1_24h_R1 **Scan** 15273
Peptide GEKTEVVLTKV **Charge** 3 **Spectral Angle** 0.93

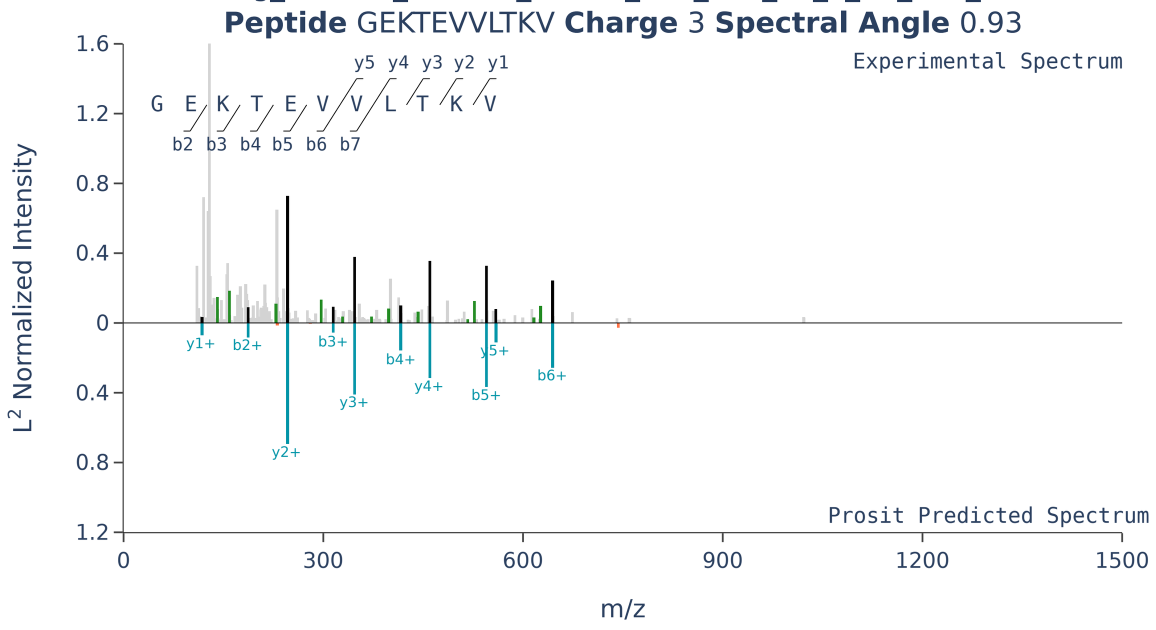

Source Ncheng_210623_230623_HFGoe_FFH_20S_25_1_A1_4h_R1 **Scan** 26636
Peptide VADFFPSD **Charge** 1 **Spectral Angle** 0.91

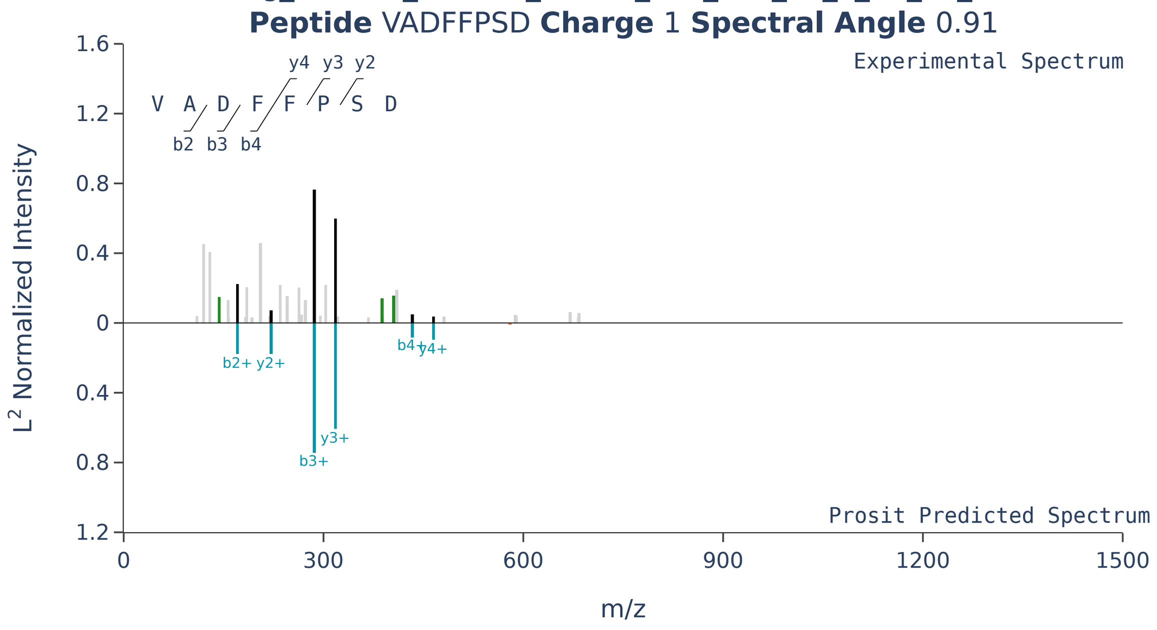

Source Ncheng_210623_230623_HFGoe_FFH_20S_25_1_A2_4h_R2 **Scan** 11649
Peptide TLAEQVQD **Charge** 1 **Spectral Angle** 0.93

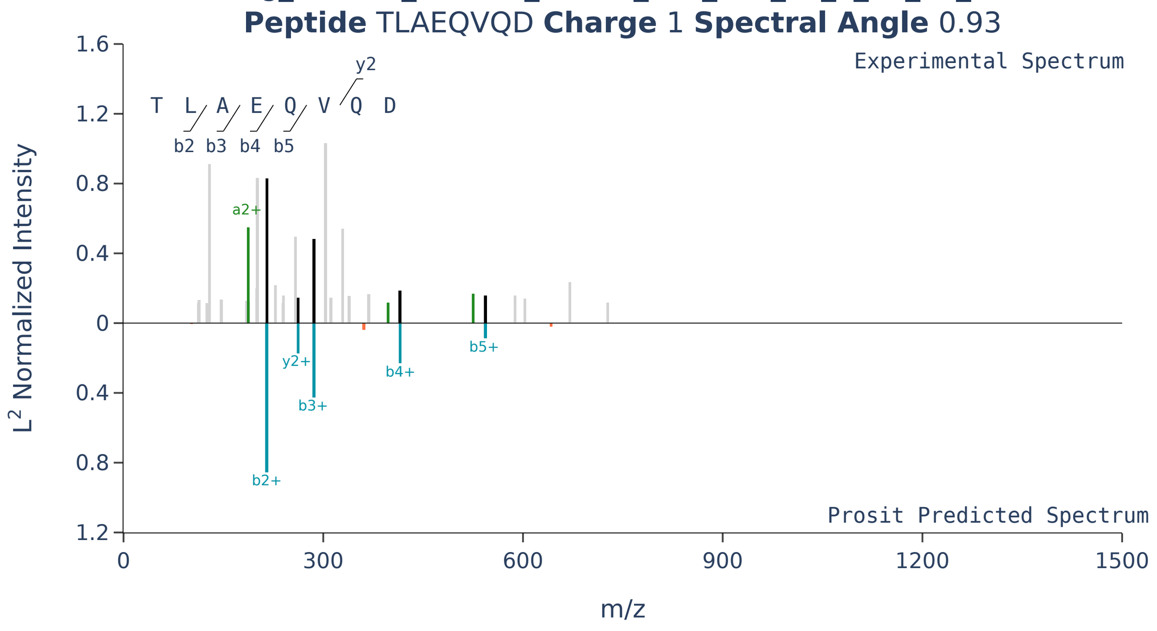

Source Ncheng_210623_230623_HFGoe_FFH_20S_25_1_A2_24h_R2 **Scan** 9245
Peptide EQVGMTGQD **Charge** 1 **Spectral Angle** 0.97

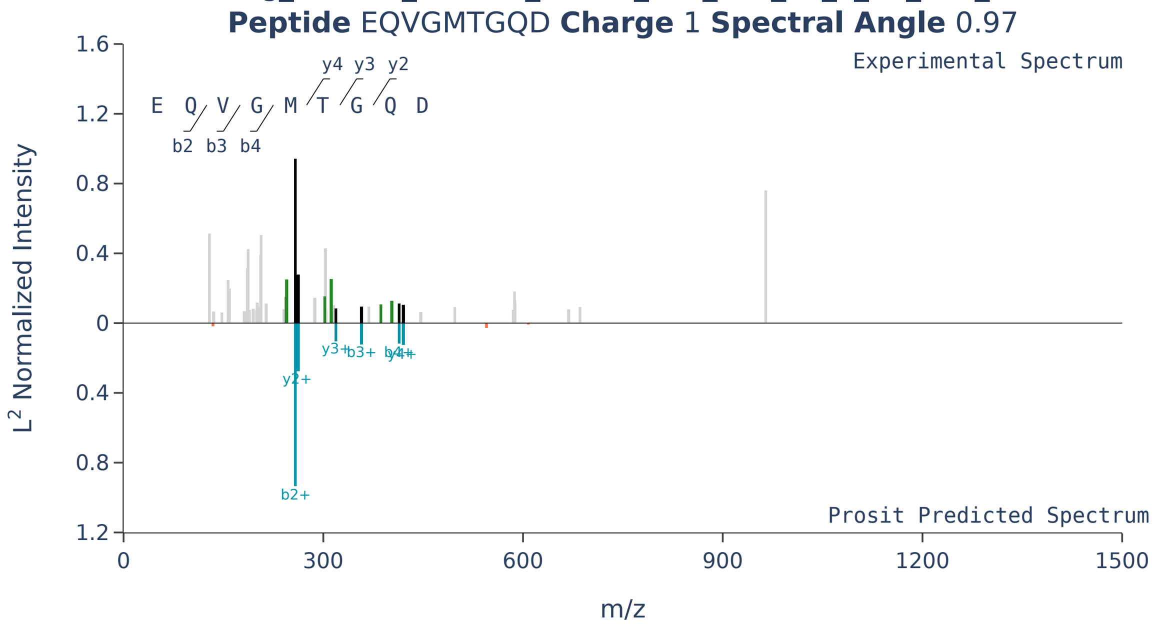

Source Ncheng_210623_230623_HFGoe_FFH_20S_25_1_A1_24h_R1 **Scan** 14006
Peptide SAAMTGQDAANTAKAFN **Charge** 2 **Spectral Angle** 0.92

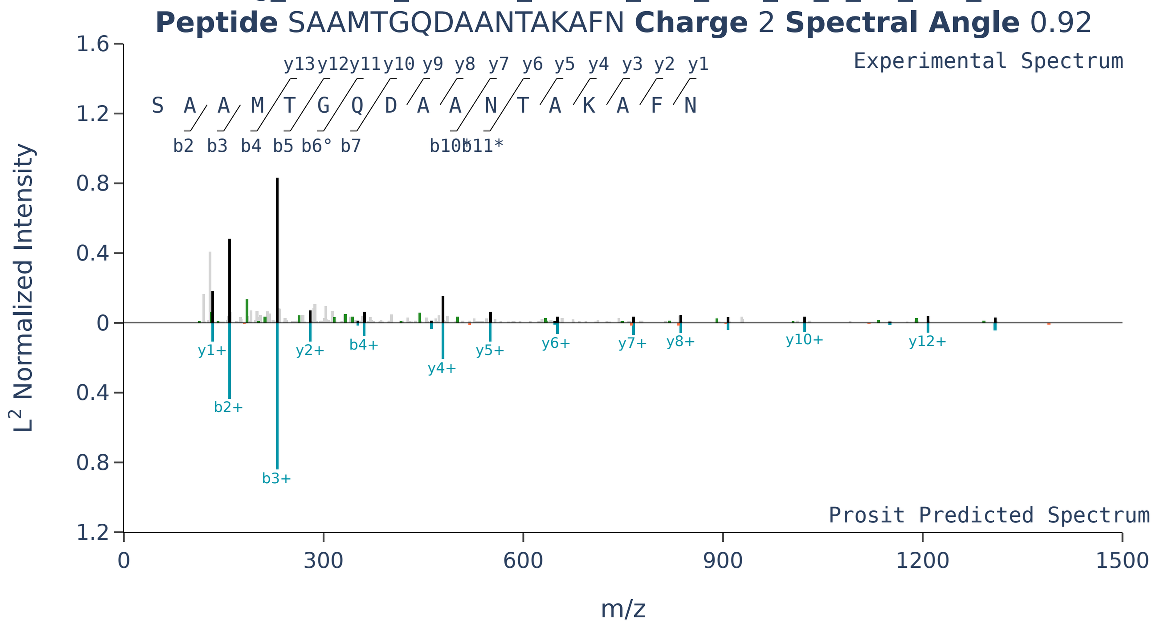

Source Ncheng_210623_230623_HFGoe_FFH_20S_25_1_A2_4h_R2 **Scan** 19420
Peptide GEENQTLNLVGQKPVD **Charge** 2 **Spectral Angle** 0.87

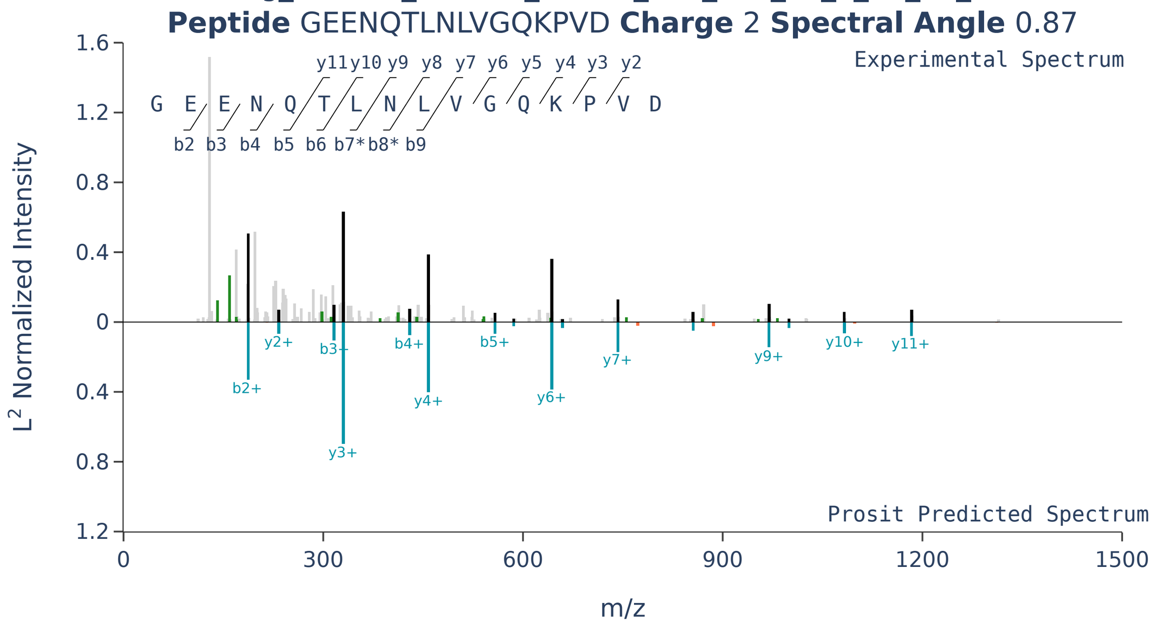

Source Ncheng_210623_230623_HFGoe_FFH_20S_25_1_A2_4h_R1 **Scan** 14290
Peptide QTAMTGQDAANTAKAFN **Charge** 2 **Spectral Angle** 0.89

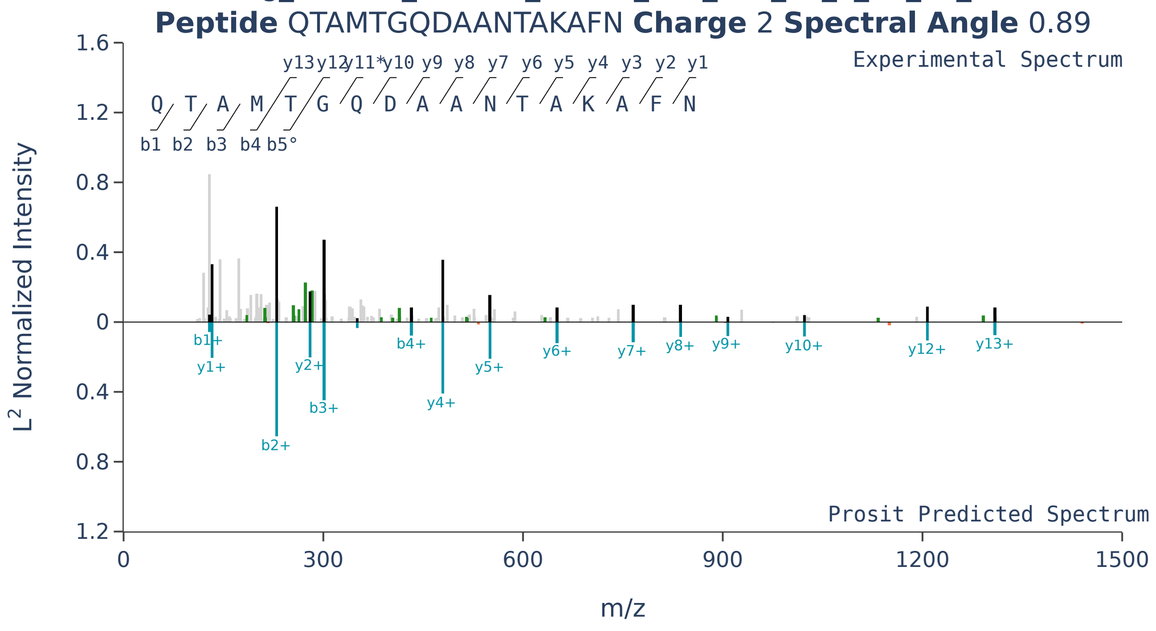

Source Ncheng_210623_230623_HFGoe_FFH_20S_25_1_A2_24h_R2 **Scan** 25259
Peptide VEFFPSD **Charge** 1 **Spectral Angle** 0.94

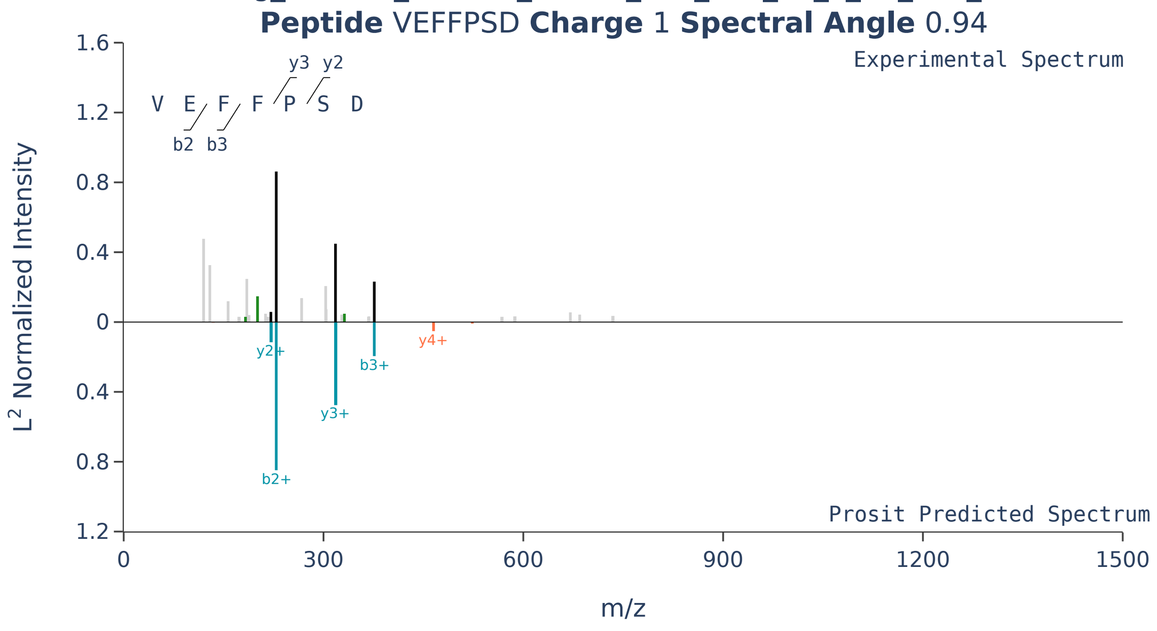

Source Ncheng_210623_230623_HFGoe_FFH_20S_25_1_A2_24h_R2 **Scan** 27329
Peptide AVDFFPSD **Charge** 1 **Spectral Angle** 0.94

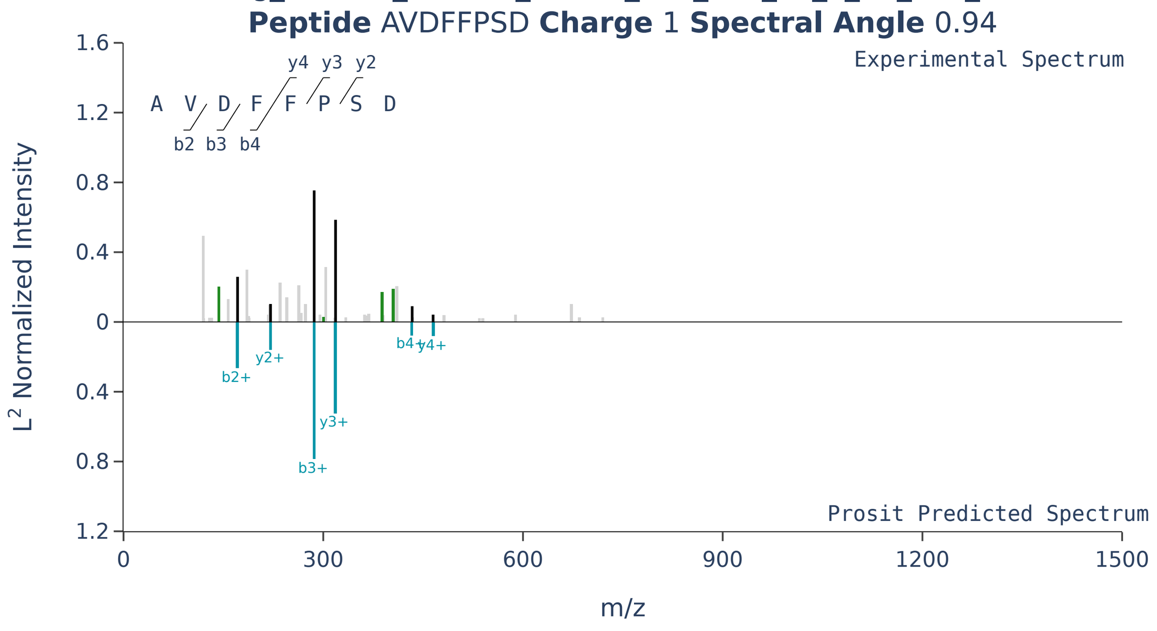

Source Ncheng_210623_230623_HFGoe_FFH_20S_25_1_A2_24h_R2 **Scan** 16308
Peptide VNQKPVDIVNA **Charge** 2 **Spectral Angle** 0.9

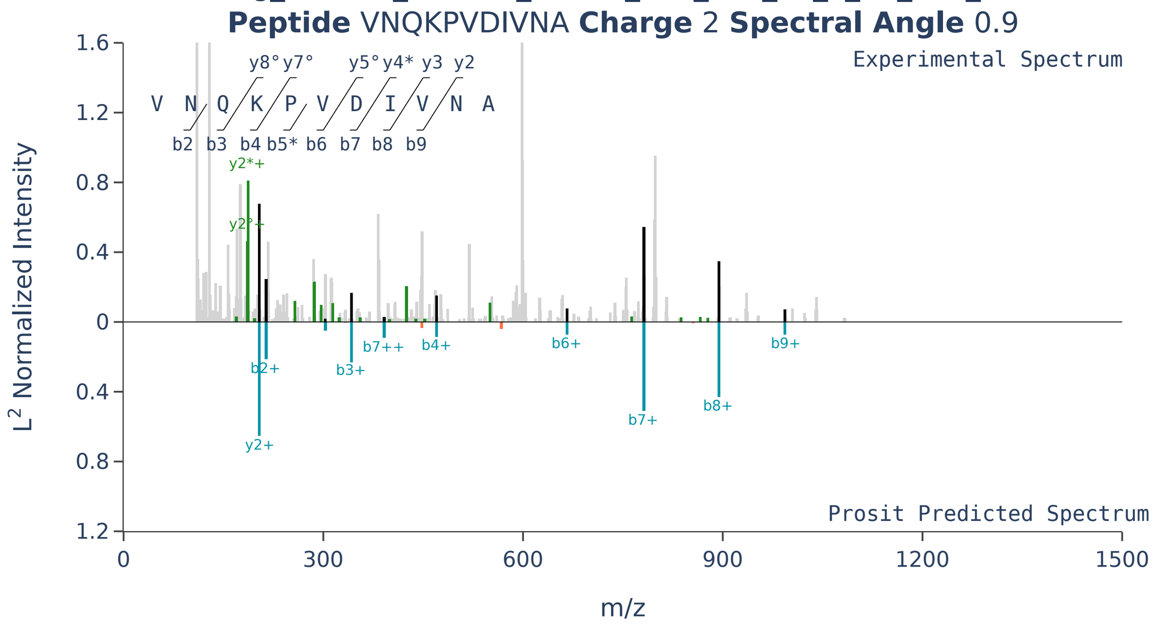

Source Ncheng_210623_230623_HFGoe_FFH_20S_25_1_A1_1h_R2 **Scan** 21612
Peptide GHVDFFPSD **Charge** 2 **Spectral Angle** 0.9

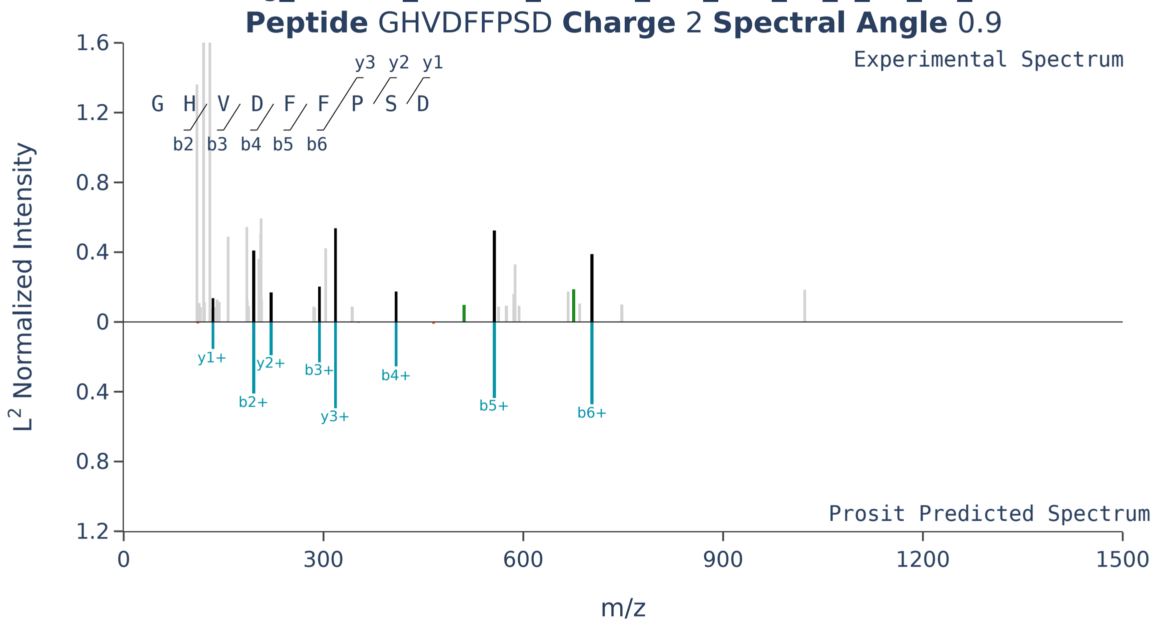

Source Ncheng_210623_230623_HFGoe_FFH_20S_25_1_A1_4h_R1 **Scan** 11778
Peptide ASSINPVE **Charge** 1 **Spectral Angle** 0.9

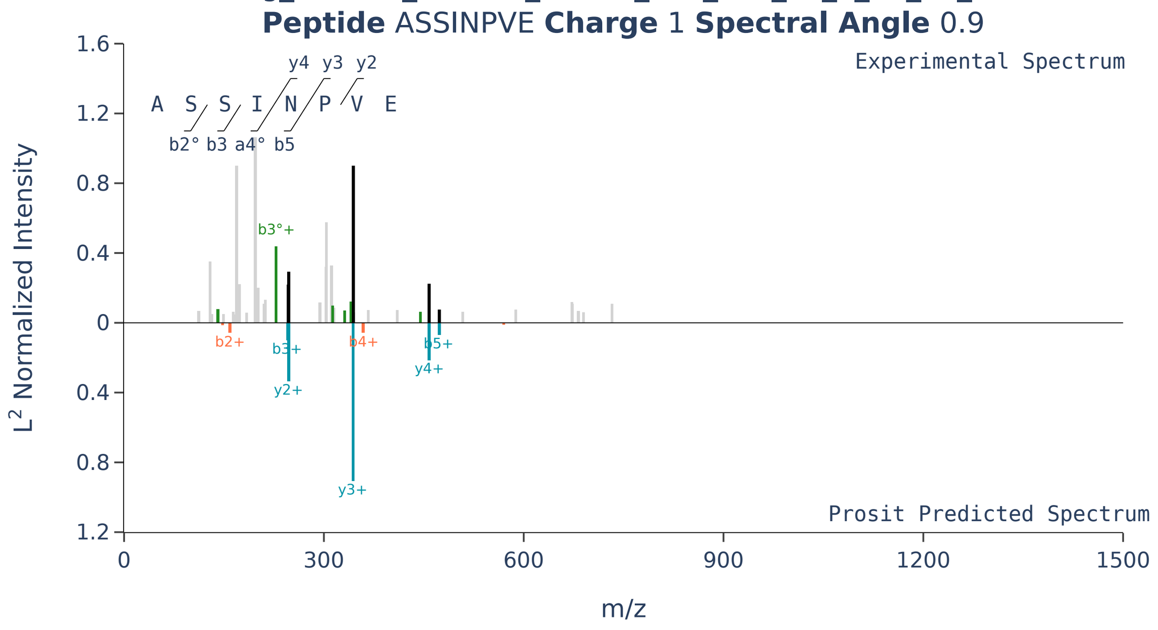

Source Ncheng_210623_230623_HFGoe_FFH_20S_25_1_A2_2h_R2 **Scan** 33068
Peptide PPAVVLMMM **Charge** 1 **Spectral Angle** 0.94

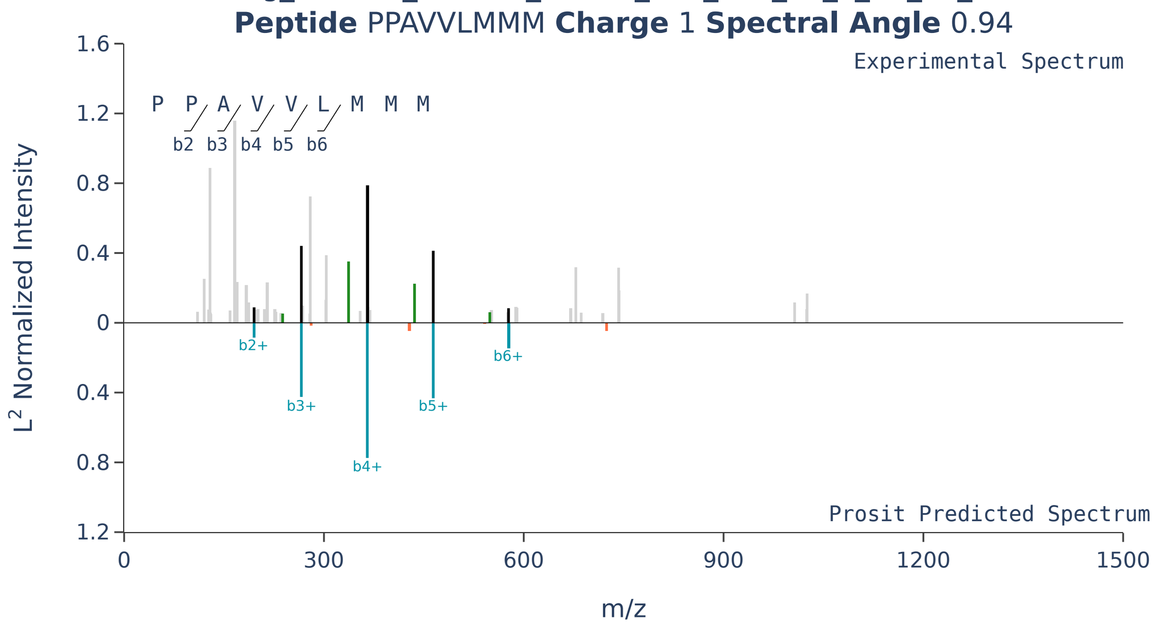

Source Ncheng_210623_230623_HFGoe_FFH_20S_25_1_A1_4h_R1 **Scan** 15255
Peptide TLAEQVGVGQKPVD **Charge** 2 **Spectral Angle** 0.86

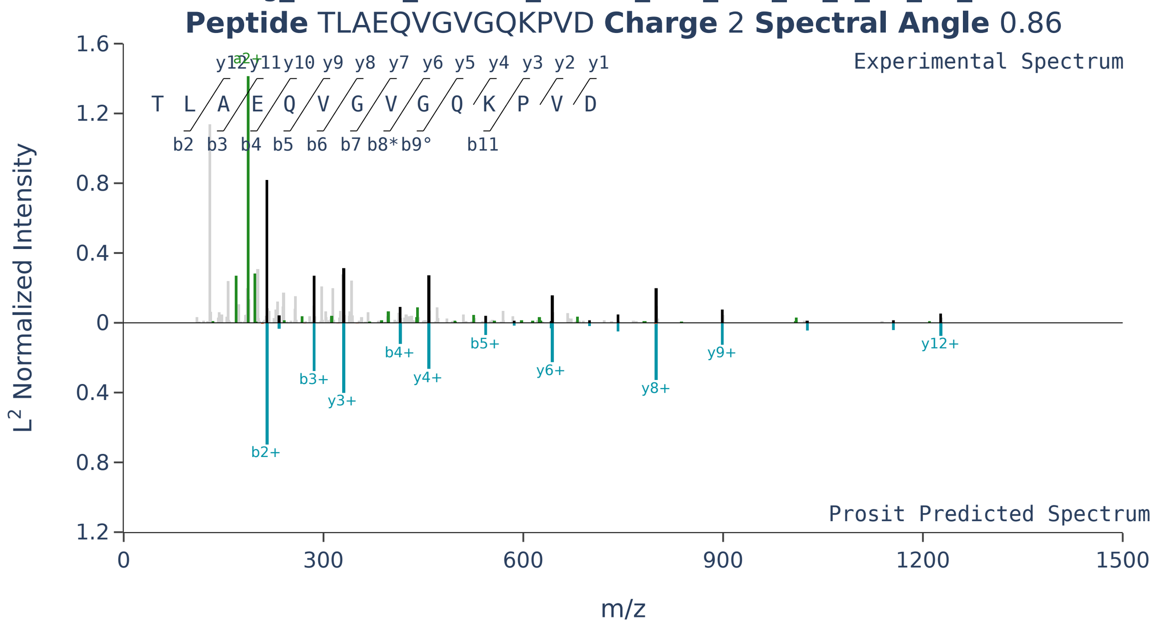

Source Ncheng_210623_230623_HFGoe_FFH_20S_25_1_A2_4h_R1 **Scan** 22412
Peptide ELVAAMTGQDAANTAKAFN **Charge** 2 **Spectral Angle** 0.91

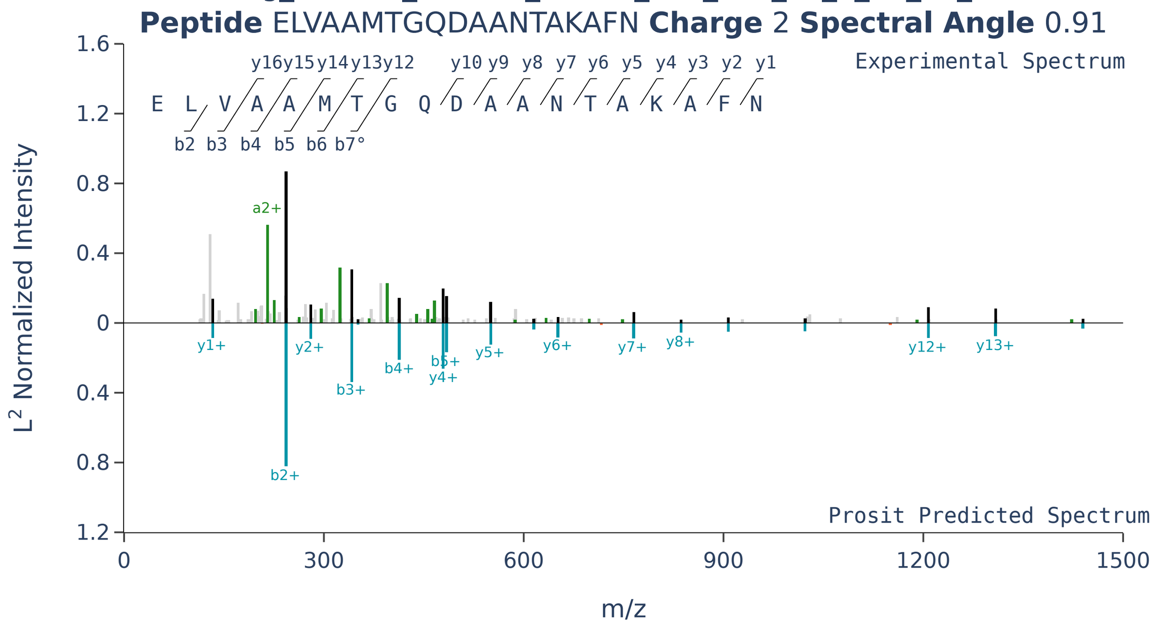

Source Ncheng_210623_230623_HFGoe_FFH_20S_25_1_A1_4h_R2 **Scan** 11184
Peptide TLAEQVGE **Charge** 1 **Spectral Angle** 0.91

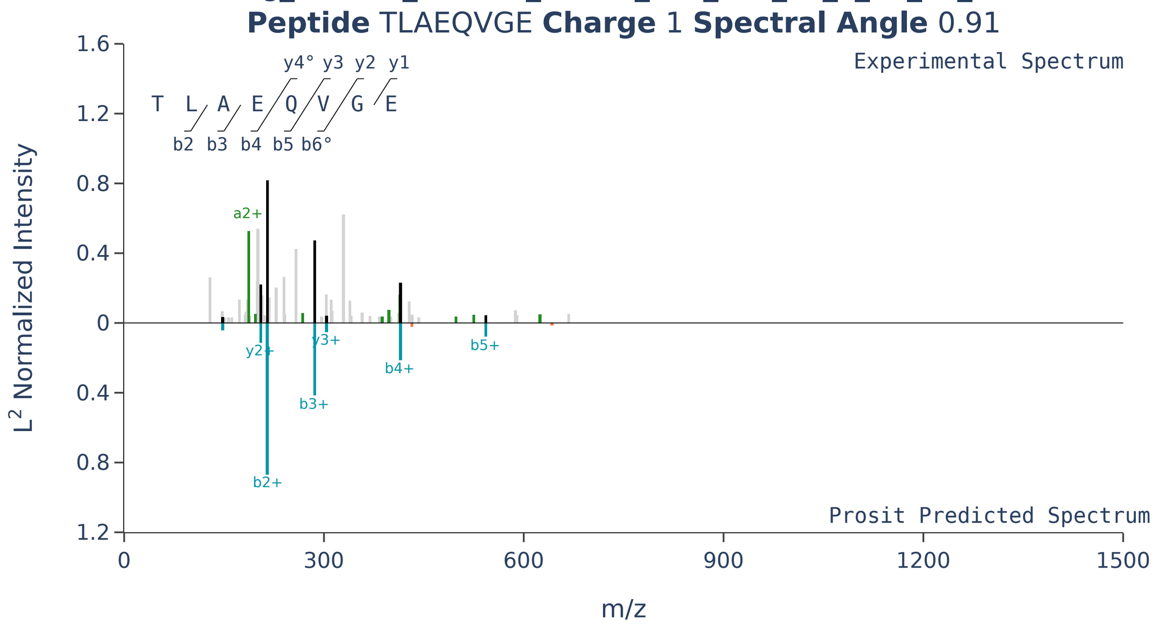

Source Ncheng_210623_230623_HFGoe_FFH_20S_25_1_A2_4h_R2 **Scan** 21175
Peptide LETLAEQVGVGQKPVD **Charge** 2 **Spectral Angle** 0.93

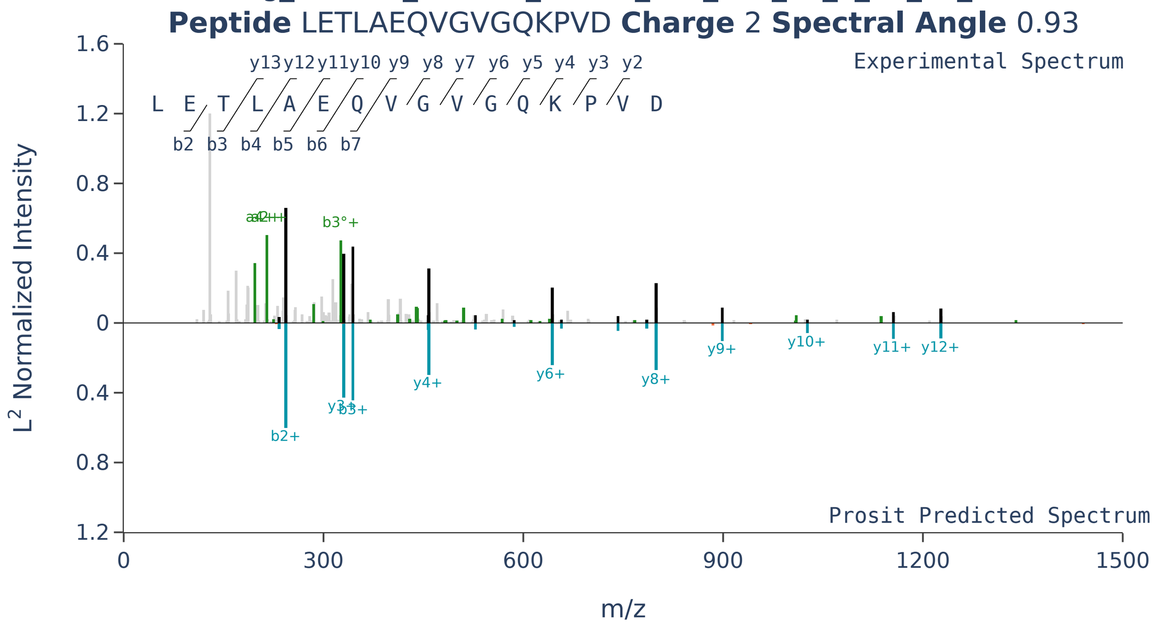

Source Ncheng_210623_230623_HFGoe_FFH_20S_25_1_A1_4h_R1 **Scan** 29229
Peptide TGQVGVDFFPSD **Charge** 2 **Spectral Angle** 0.91

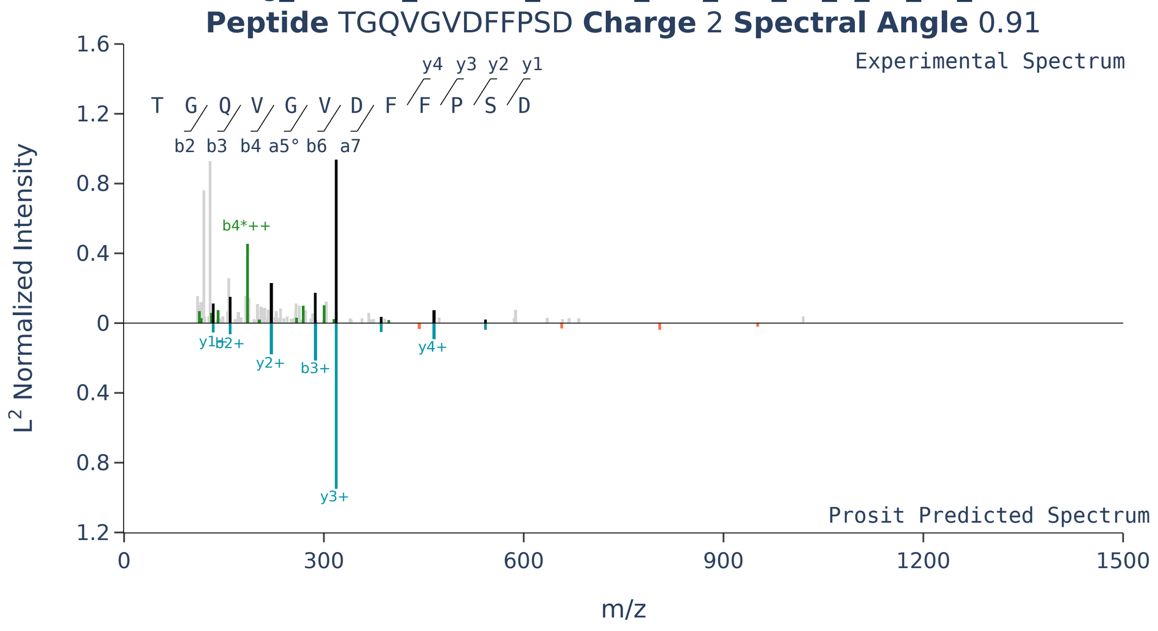

Source Ncheng_210623_230623_HFGoe_FFH_20S_25_1_A2_24h_R2 **Scan** 14669
Peptide ADAMTGQDAANTAKAFN **Charge** 2 **Spectral Angle** 0.92

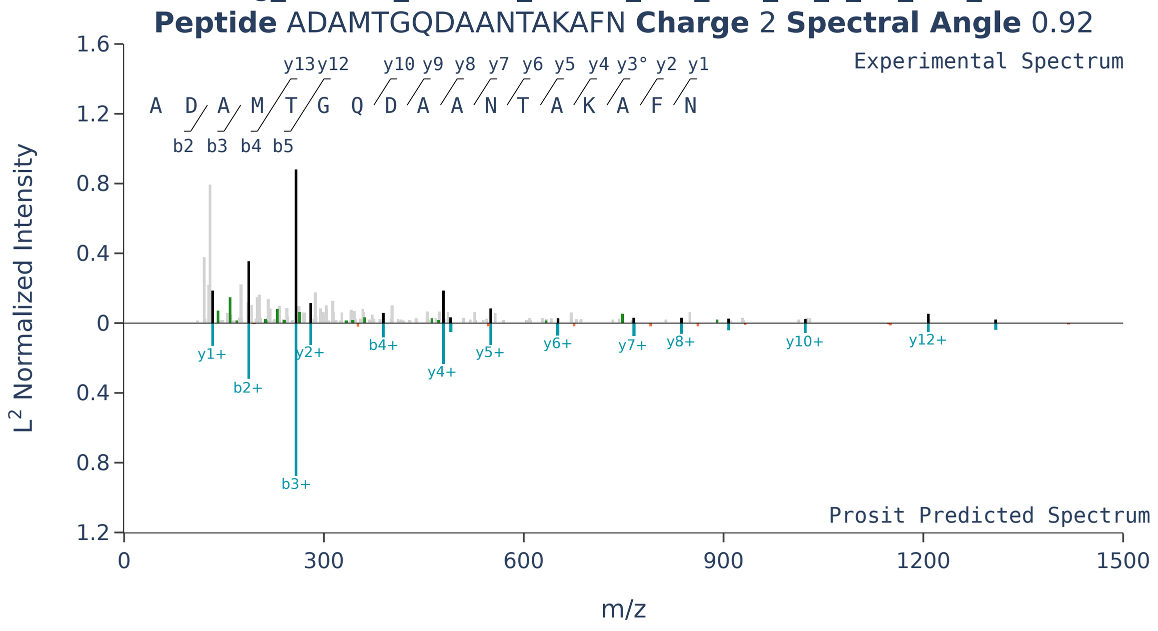

Source Ncheng_210623_230623_HFGoe_FFH_20S_25_1_A1_4h_R1 **Scan** 27225
Peptide SGVDFFPSD **Charge** 1 **Spectral Angle** 0.86

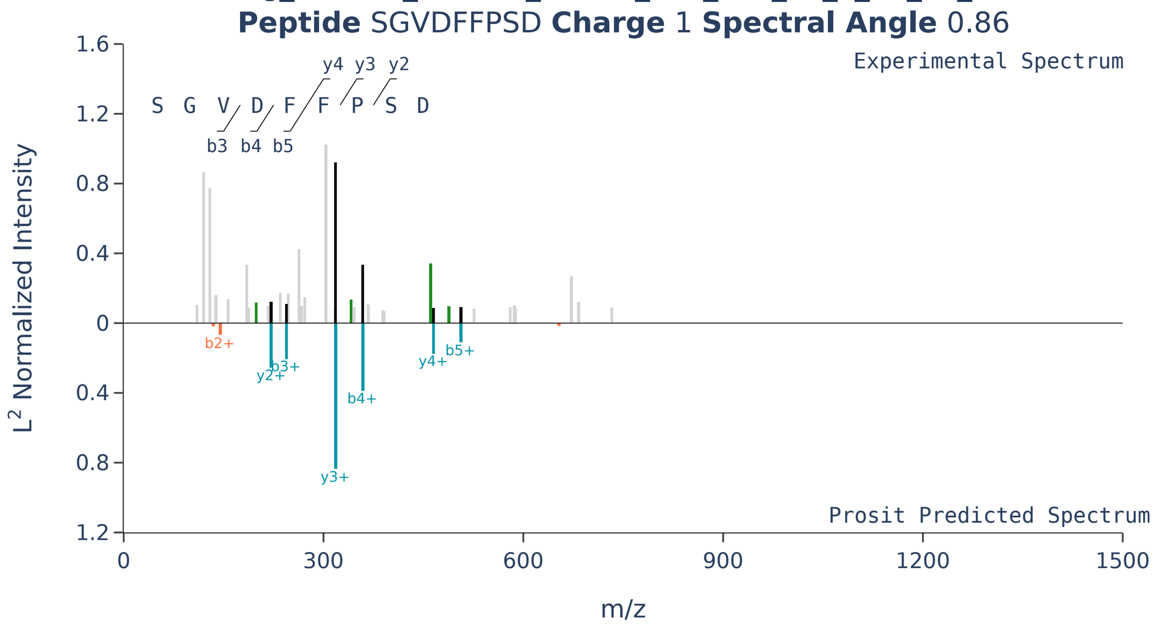

Source Ncheng_210623_230623_HFGoe_FFH_20S_25_1_A1_4h_R1 **Scan** 27104
Peptide EVDFFPSD **Charge** 1 **Spectral Angle** 0.85

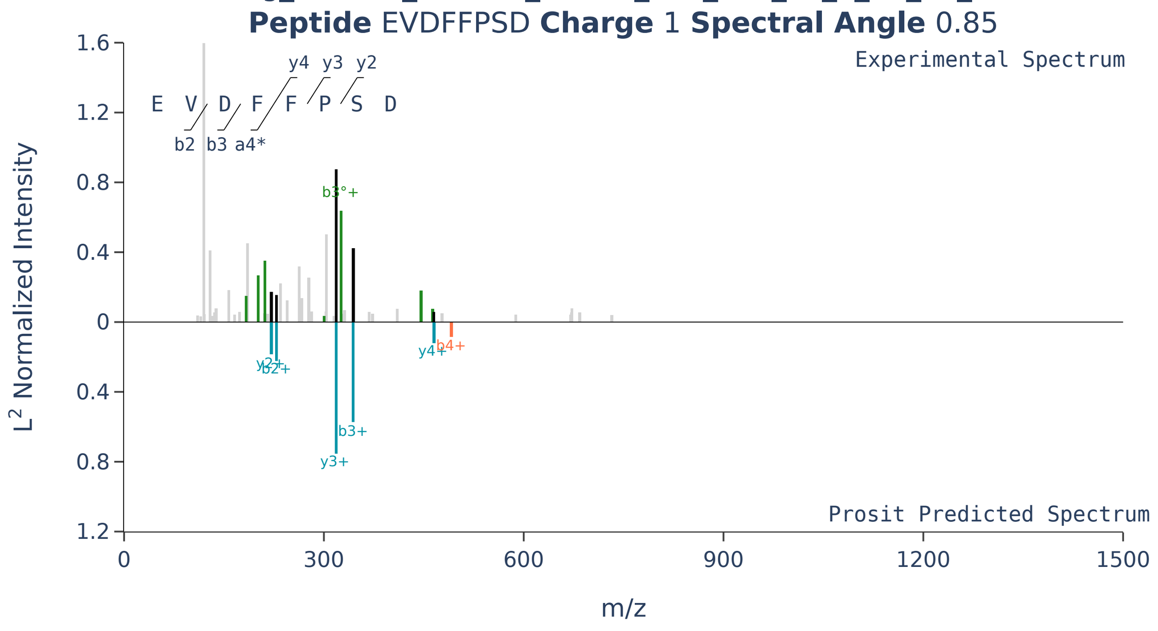

Source Ncheng_210623_230623_HFGoe_FFH_20S_25_1_A2_4h_R2 **Scan** 23408
Peptide TKETLFVVD **Charge** 2 **Spectral Angle** 0.85

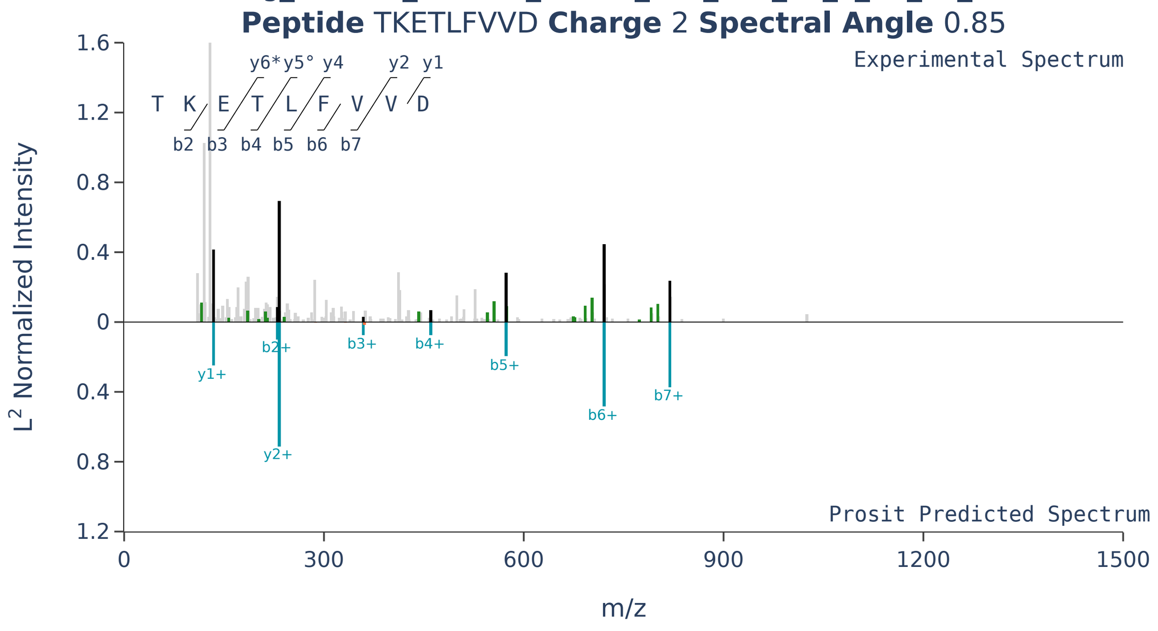

Source Ncheng_210623_230623_HFGoe_FFH_20S_25_1_A2_24h_R2 **Scan** 12087
Peptide TGSINPVE **Charge** 1 **Spectral Angle** 0.81

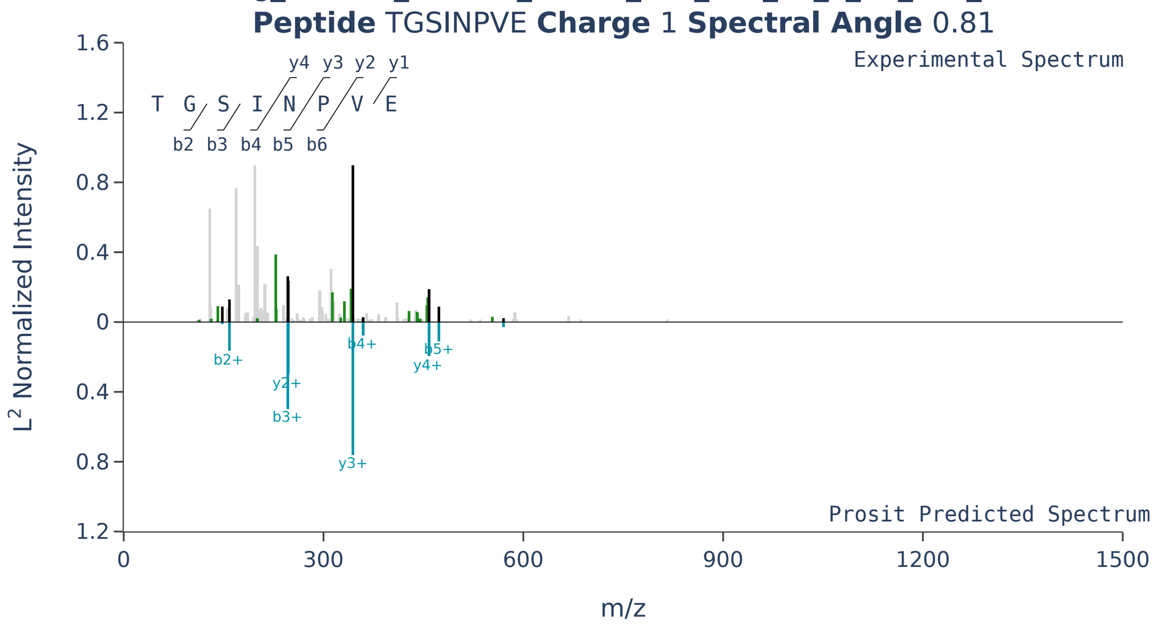

Source Ncheng_210623_230623_HFGoe_FFH_20S_25_1_A1_4h_R2 **Scan** 27743
Peptide AGVDFFPSD **Charge** 1 **Spectral Angle** 0.86

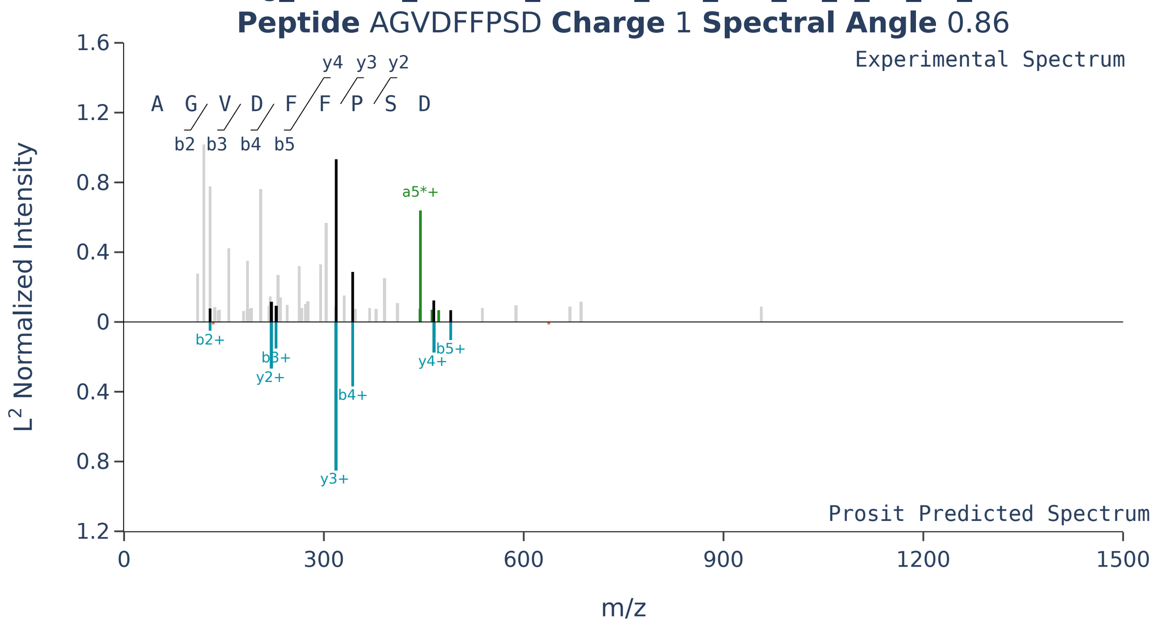

Source Ncheng_210623_230623_HFGoe_FFH_20S_25_1_A1_4h_R2 **Scan** 22455
Peptide NVKSLTPGQEFVKIVRNE **Charge** 3 **Spectral Angle** 0.86

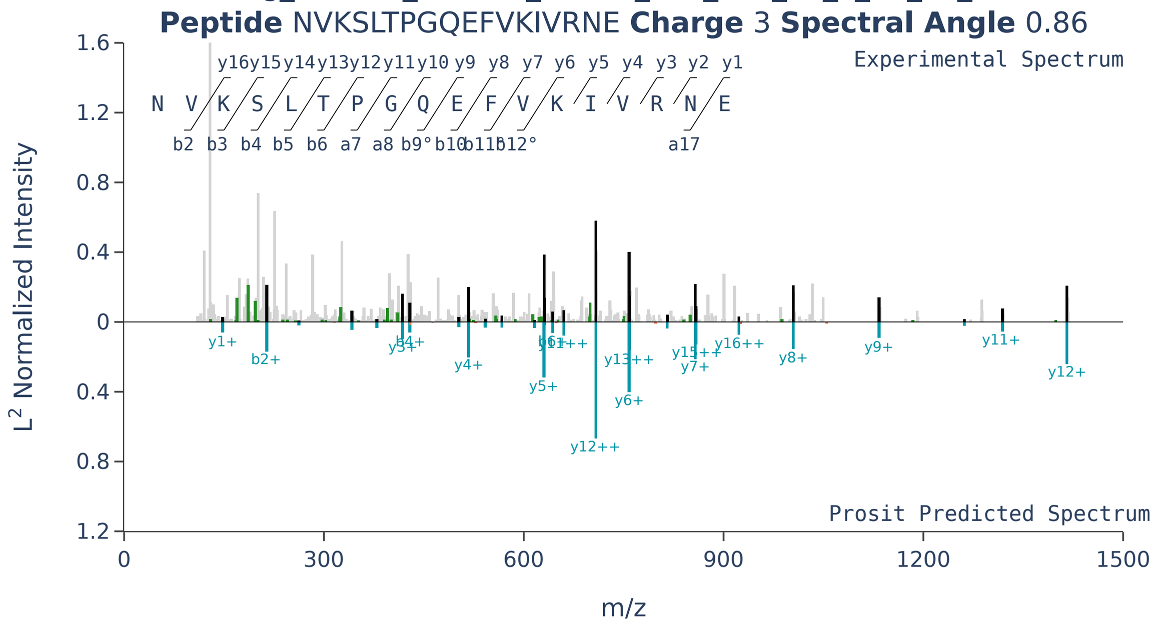

Source Ncheng_210623_230623_HFGoe_FFH_20S_25_1_A1_2h_R1 **Scan** 30213
Peptide GKTLAEQVGVGVDFFPSD **Charge** 2 **Spectral Angle** 0.96

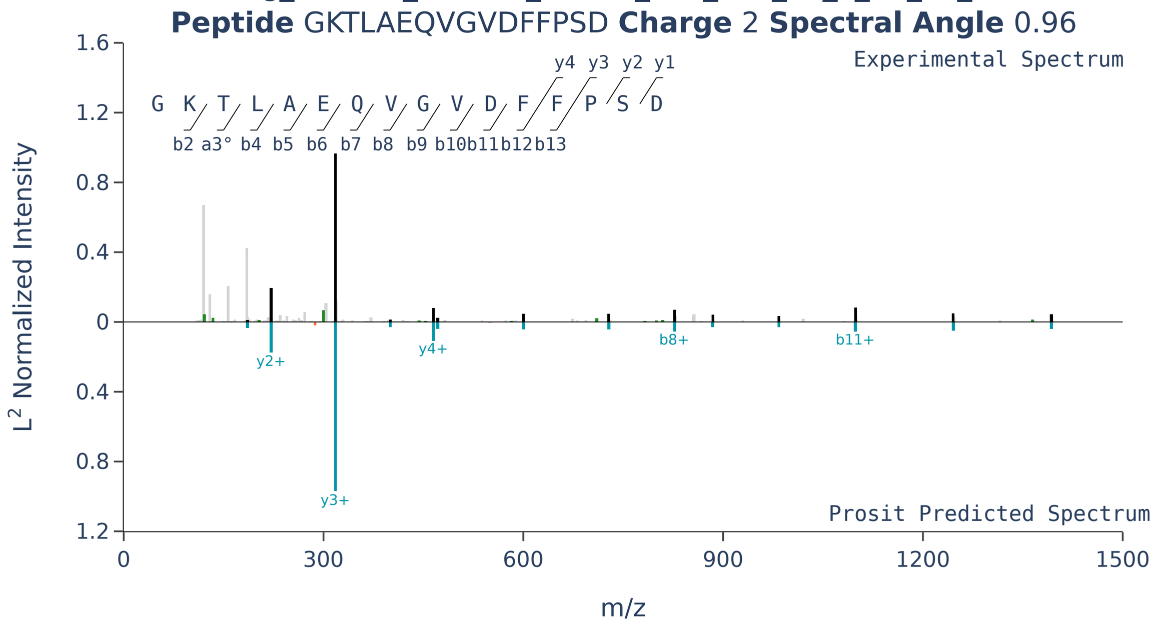

Source Ncheng_210623_230623_HFGoe_FFH_20S_25_1_A2_24h_R2 **Scan** 16652
Peptide MGDAMTGQDAANTAKAFN **Charge** 2 **Spectral Angle** 0.88

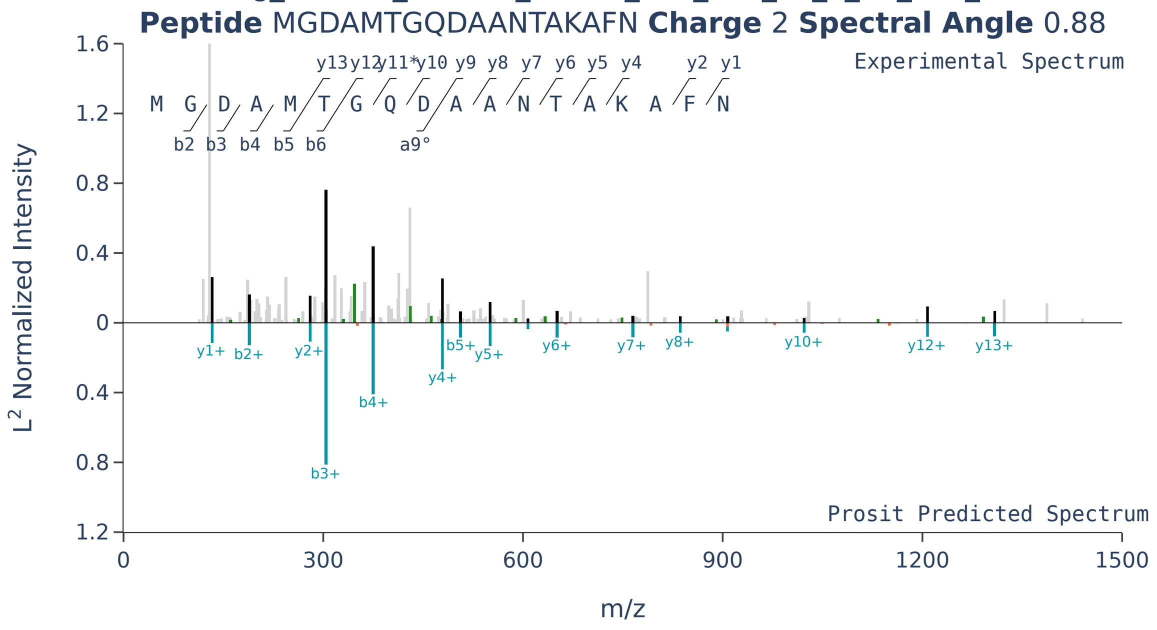

Source Ncheng_210623_230623_HFGoe_FFH_20S_25_1_A2_24h_R2 **Scan** 22284
Peptide NKGVDFFPSD **Charge** 2 **Spectral Angle** 0.91

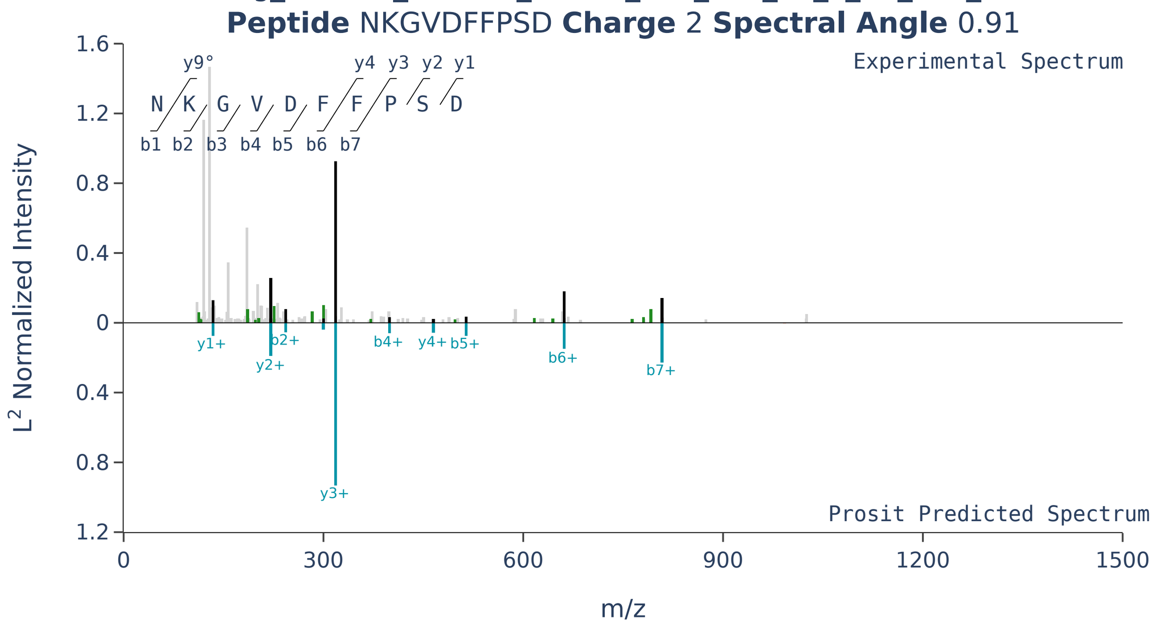

Source Ncheng_210623_230623_HFGoe_FFH_20S_25_1_A2_4h_R1 **Scan** 28248
Peptide AALKEQVGVDFFPSD **Charge** 2 **Spectral Angle** 0.93

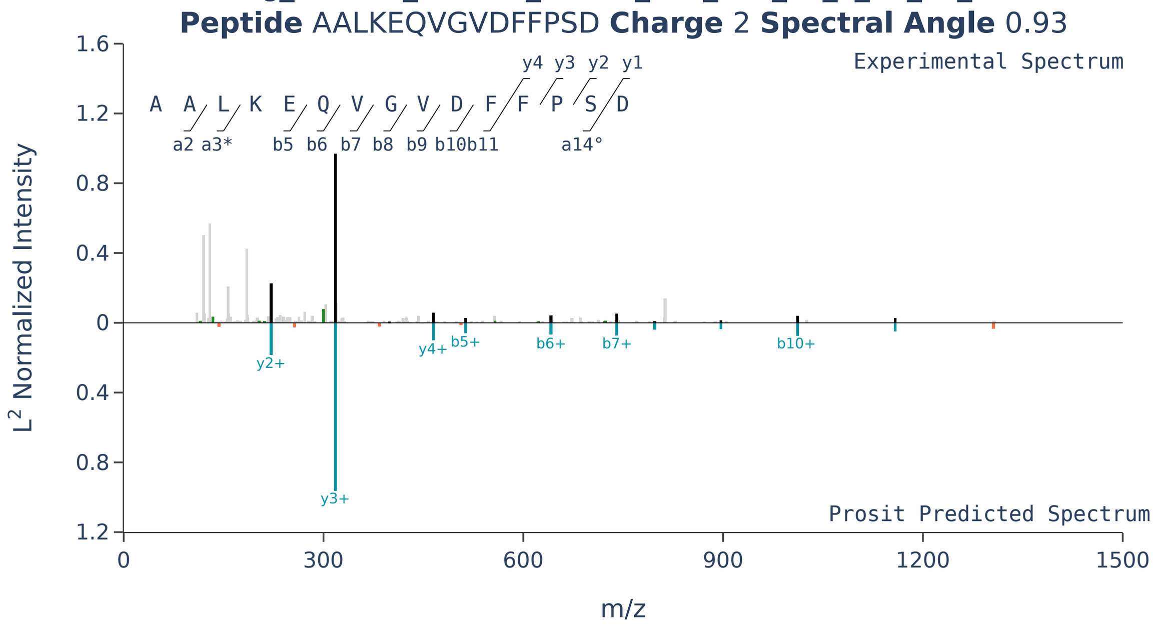

Source Ncheng_210623_230623_HFGoe_FFH_20S_25_1_A1_4h_R1 **Scan** 12044
Peptide PDNVKSQMDADVA **Charge** 1 **Spectral Angle** 0.89

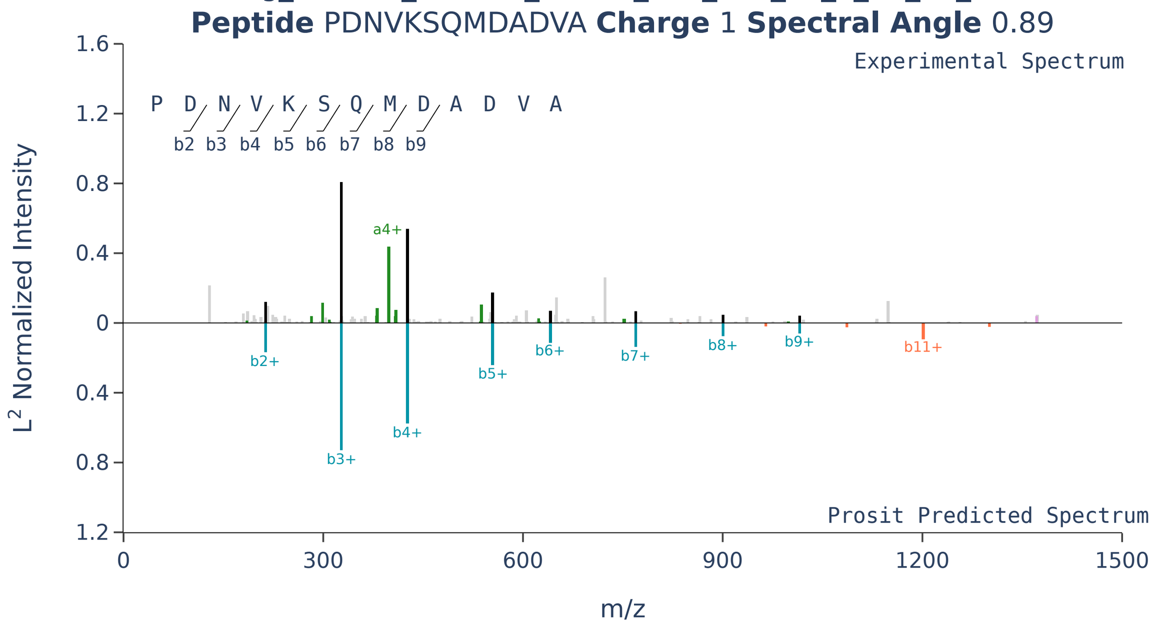

Source Ncheng_210623_230623_HFGoe_FFH_20S_25_1_A1_24h_R2 **Scan** 16252
Peptide MGAMTGQDAANTAKAFN **Charge** 2 **Spectral Angle** 0.9

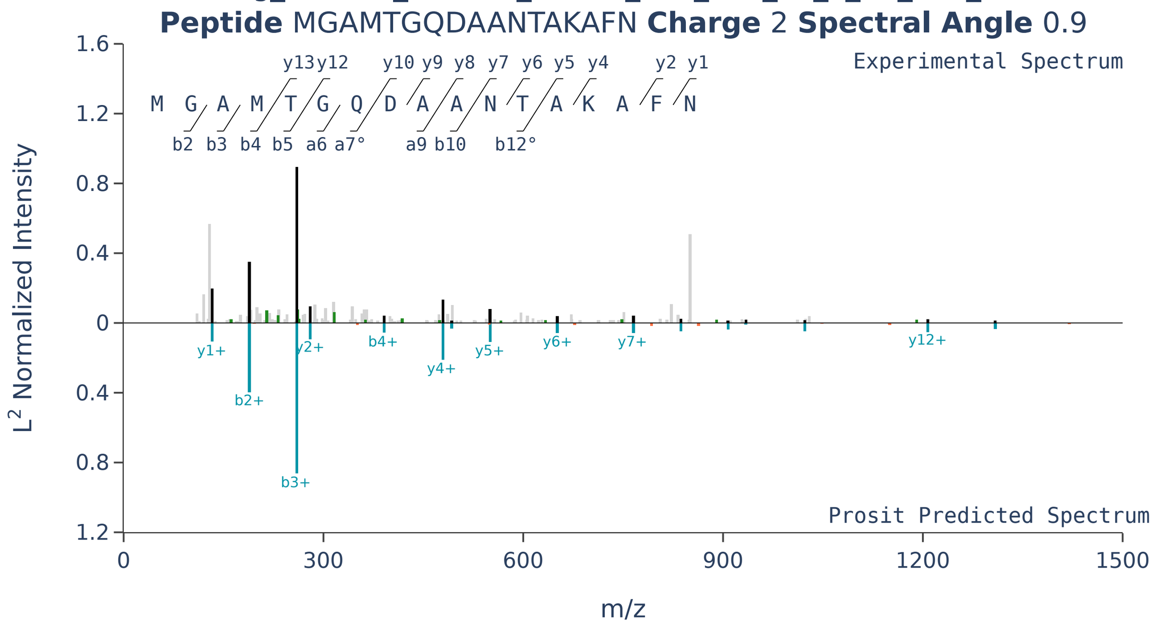

Source Ncheng_210623_230623_HFGoe_FFH_20S_25_1_A2_24h_R2 **Scan** 14132
Peptide KNMGAMTGQDAANTAKAFN **Charge** 3 **Spectral Angle** 0.9

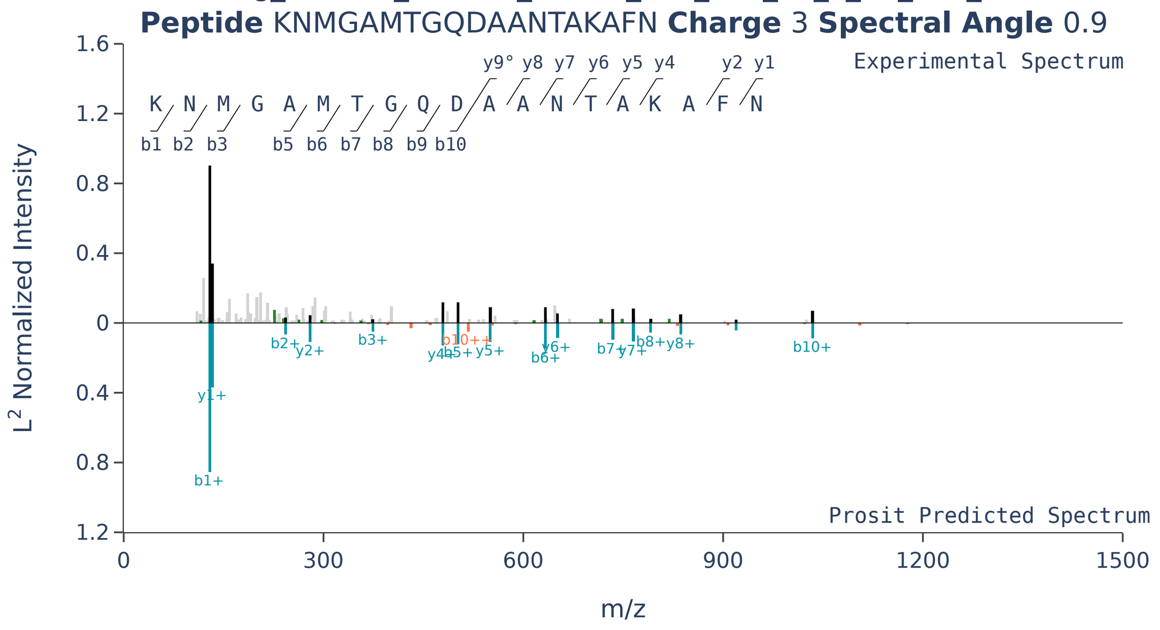

Source Ncheng_210623_230623_HFGoe_FFH_20S_25_1_A2_24h_R2 **Scan** 28381
Peptide PPAVVLAAMG **Charge** 1 **Spectral Angle** 0.86

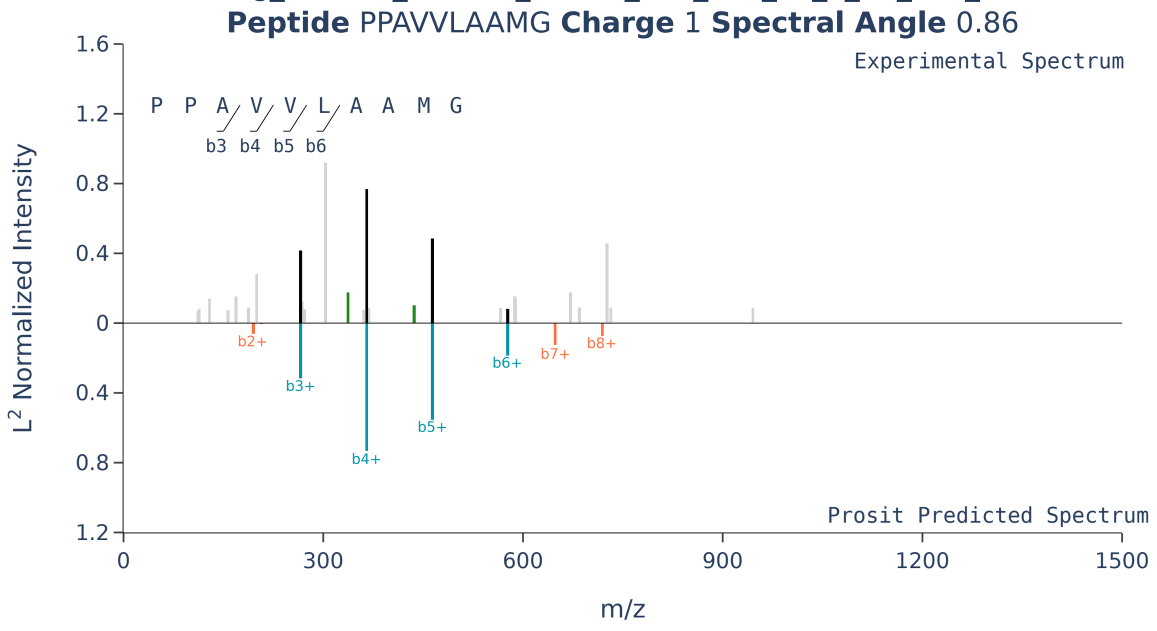

Source Ncheng_210623_230623_HFGoe_FFH_20S_25_1_A1_24h_R1 **Scan** 22398
Peptide SKGVDFFPSD **Charge** 2 **Spectral Angle** 0.88

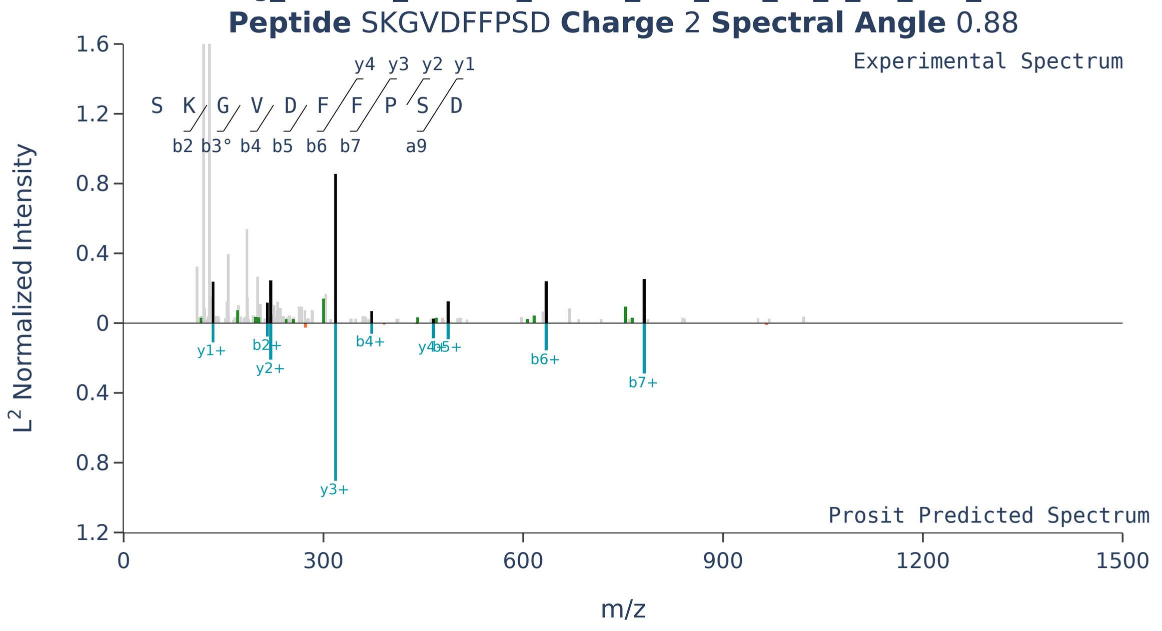

Source Ncheng_210623_230623_HFGoe_FFH_20S_25_1_A2_4h_R1 **Scan** 17094
Peptide AMTGAMTGQDAANTAKAFN **Charge** 2 **Spectral Angle** 0.8

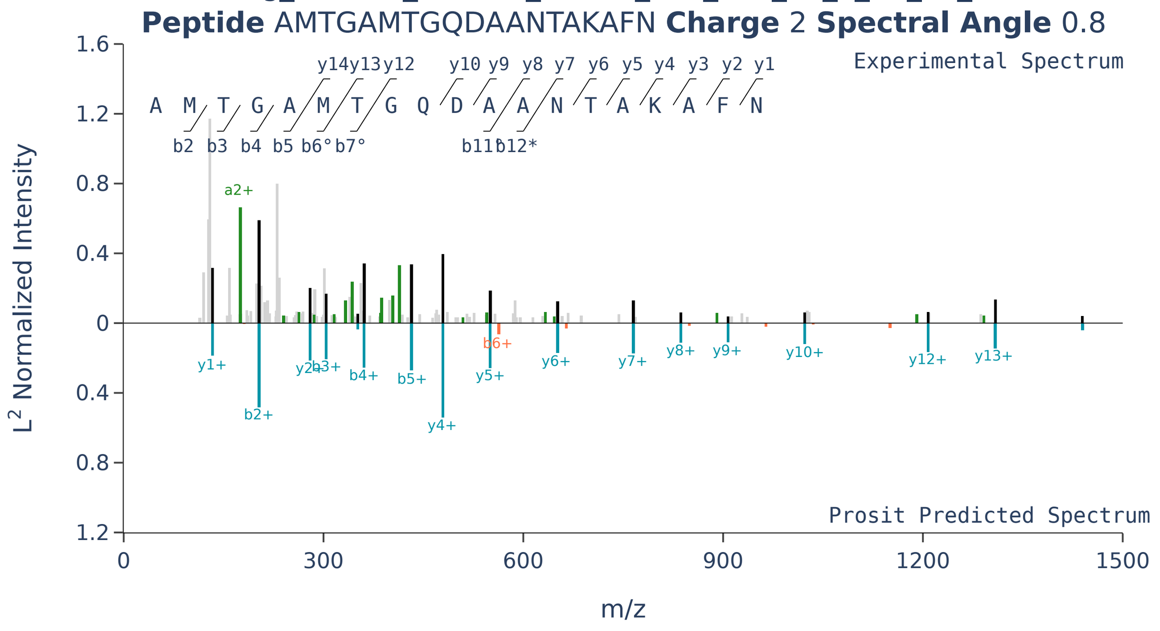

Source Ncheng_210623_230623_HFGoe_FFH_20S_25_1_A1_2h_R1 **Scan** 26279
Peptide EKTAEQVGVDFFPSD **Charge** 2 **Spectral Angle** 0.95

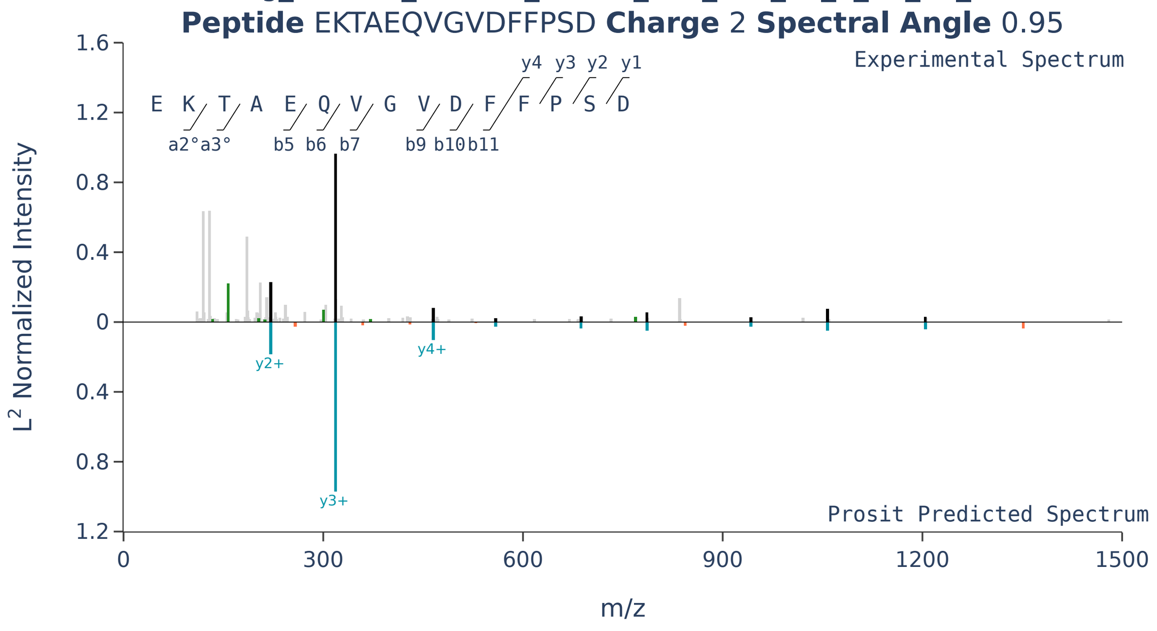

Source Ncheng_210623_230623_HFGoe_FFH_20S_25_1_A1_4h_R2 **Scan** 7173
Peptide KTEDIESKVD **Charge** 3 **Spectral Angle** 0.81

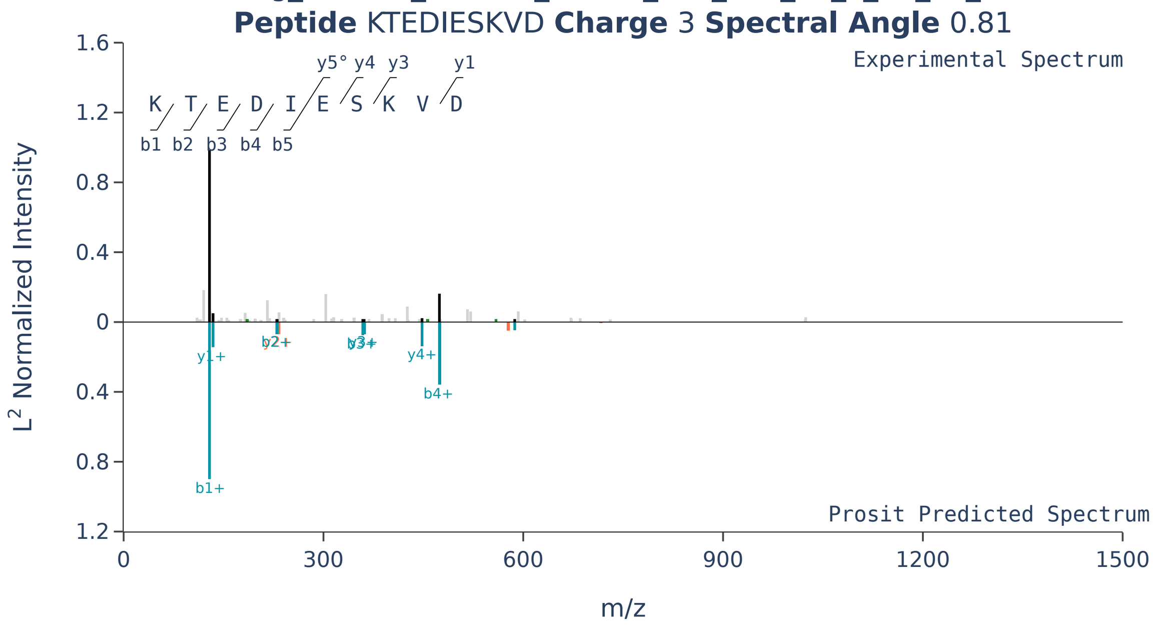

Source Ncheng_210623_230623_HFGoe_FFH_20S_25_1_A1_24h_R2 **Scan** 11979
Peptide RNISGKLKF **Charge** 3 **Spectral Angle** 0.88

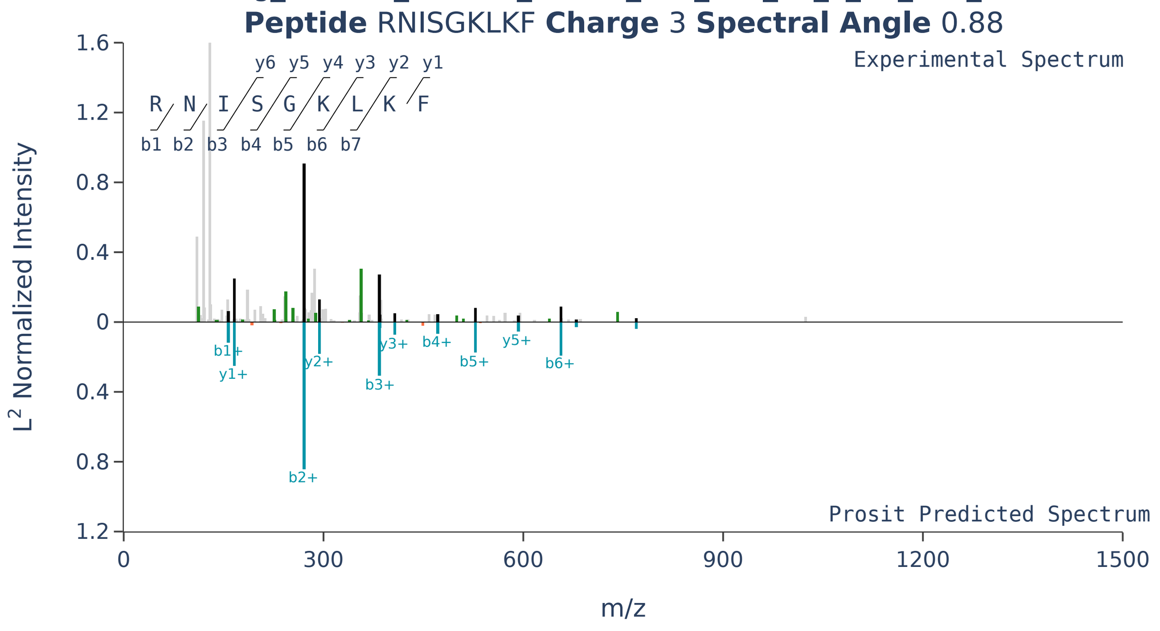

Source Ncheng_210623_230623_HFGoe_FFH_20S_25_1_A1_24h_R2 **Scan** 16240
Peptide QVGAMTGQDAANTAKAFN **Charge** 2 **Spectral Angle** 0.81

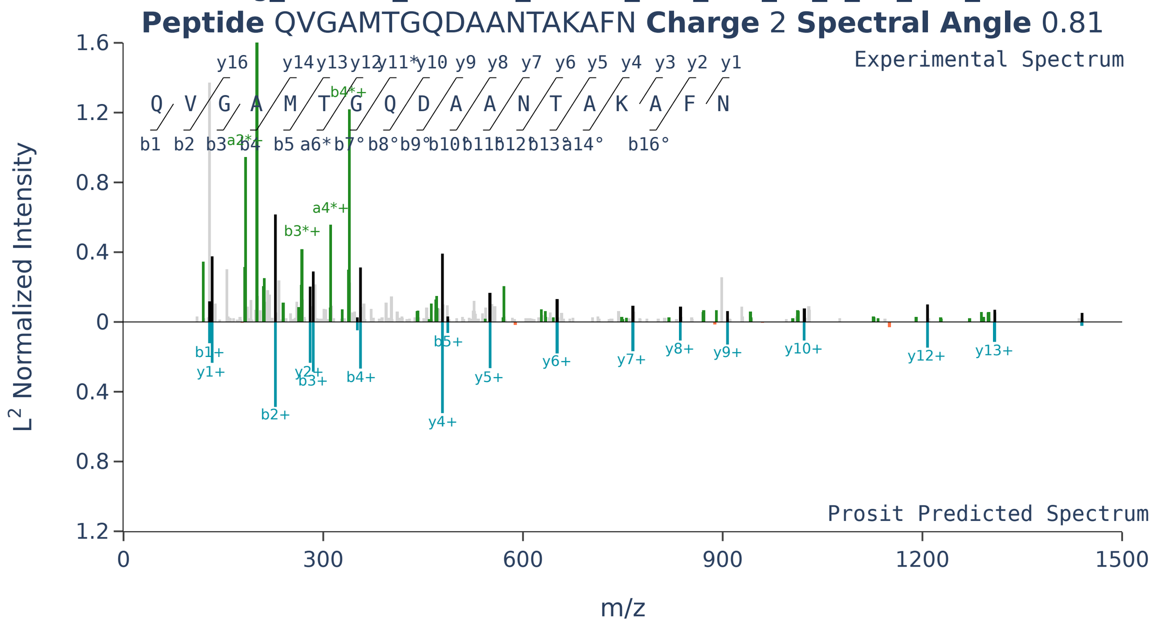

Source Ncheng_210623_230623_HFGoe_FFH_20S_25_1_A1_24h_R1 **Scan** 8915
Peptide GRLTEDNP **Charge** 1 **Spectral Angle** 0.91

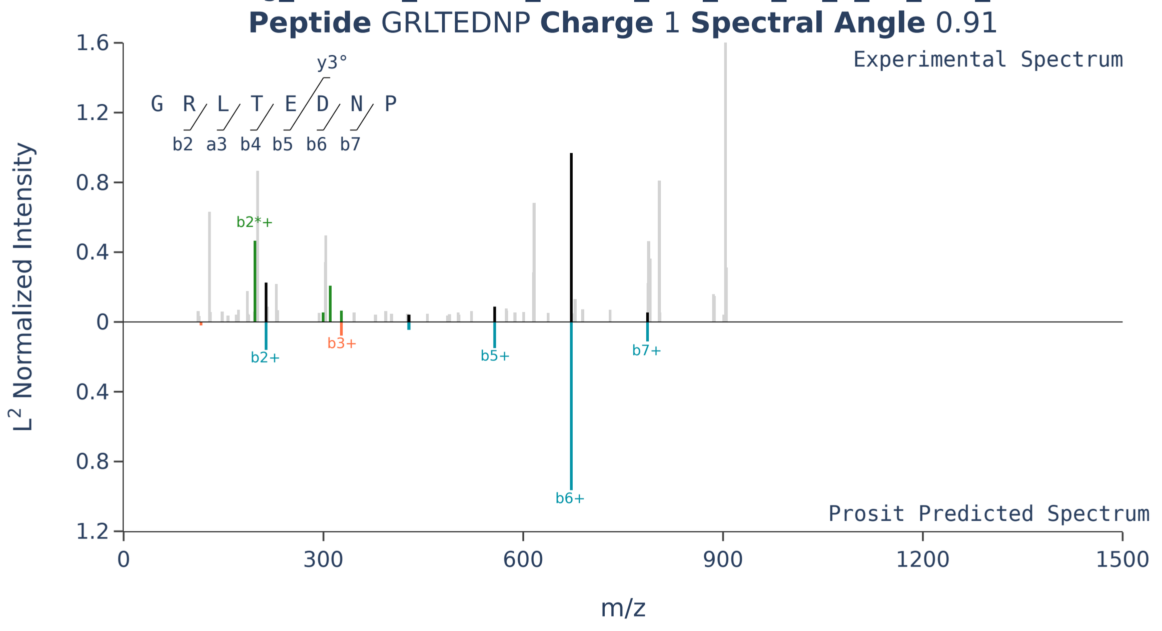

Source Ncheng_210623_230623_HFGoe_FFH_20S_25_1_A1_4h_R2 **Scan** 9252
Peptide ASKLAEQVG **Charge** 2 **Spectral Angle** 0.88

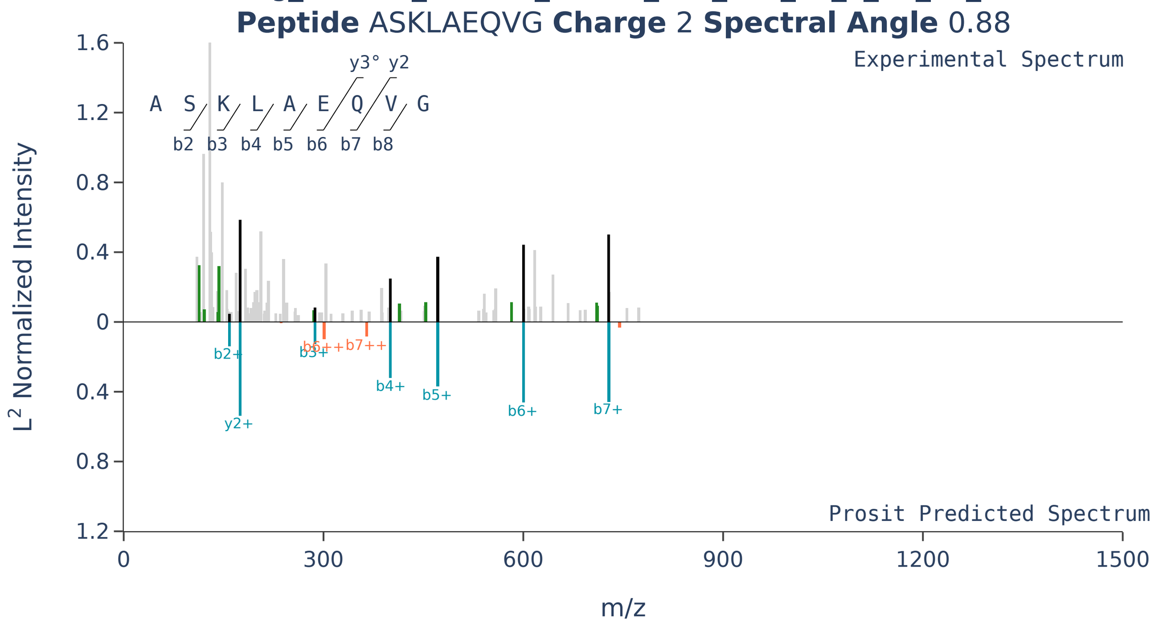

Source Ncheng_210623_230623_HFGoe_FFH_20S_25_1_A2_24h_R1 **Scan** 7516
Peptide SAAMTGQDAANTAKA **Charge** 2 **Spectral Angle** 0.83

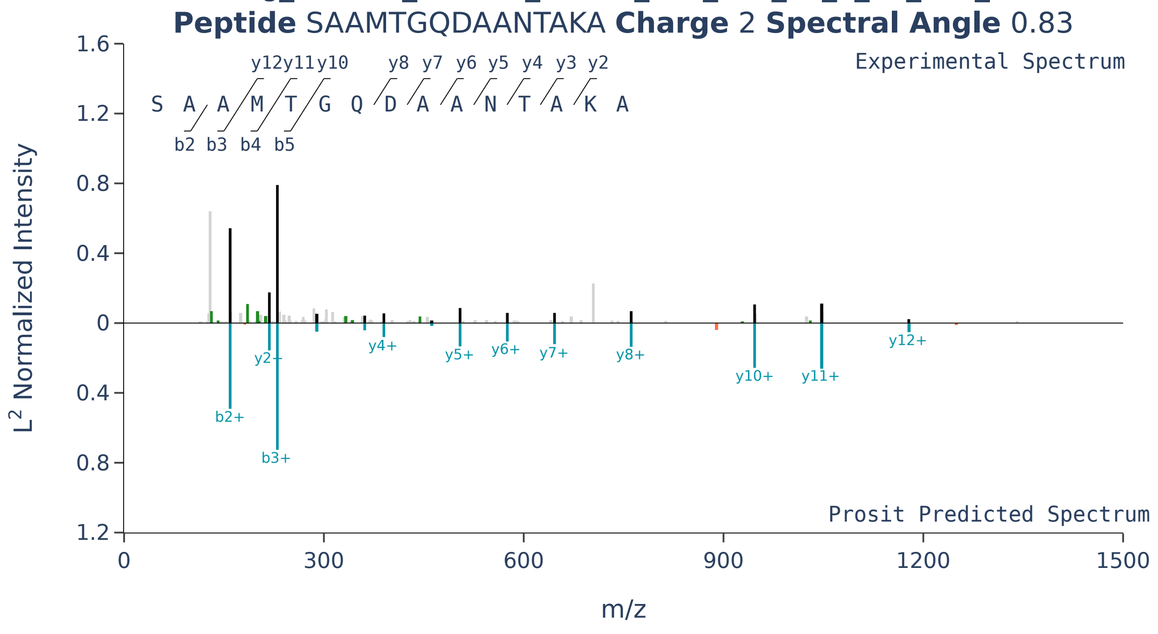

Source Ncheng_210623_230623_HFGoe_FFH_20S_25_1_A2_4h_R1 **Scan** 9233
Peptide DDIESKVDRA **Charge** 3 **Spectral Angle** 0.84

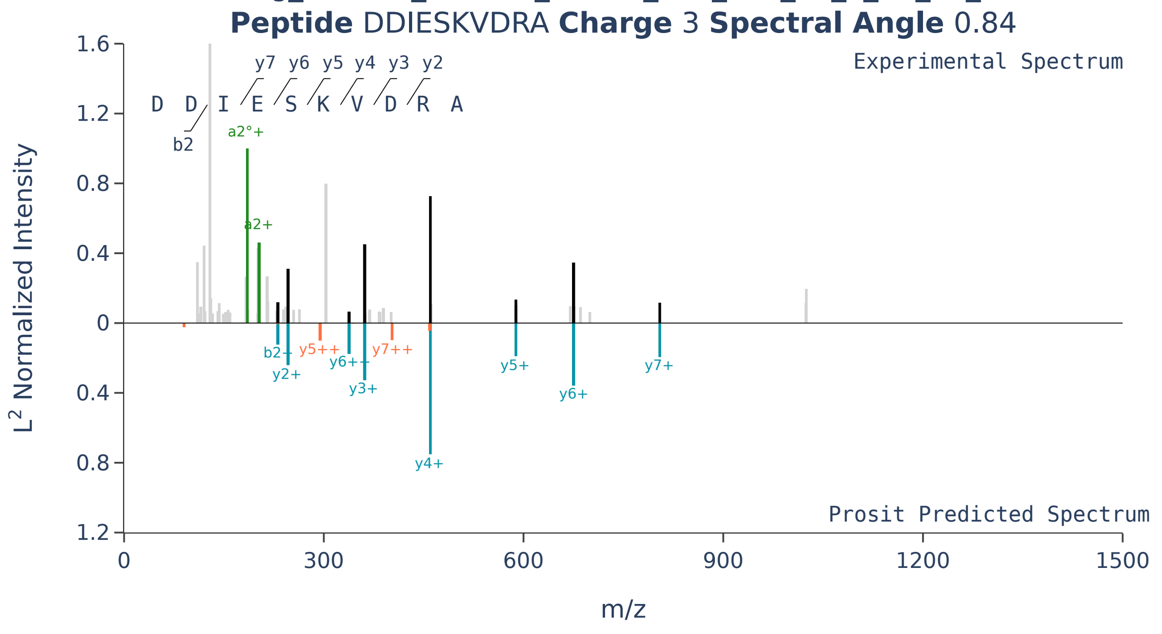

Source Ncheng_210623_230623_HFGoe_FFH_20S_25_1_A1_4h_R2 **Scan** 21555
Peptide TDKPIKFLGVGE **Charge** 3 **Spectral Angle** 0.91

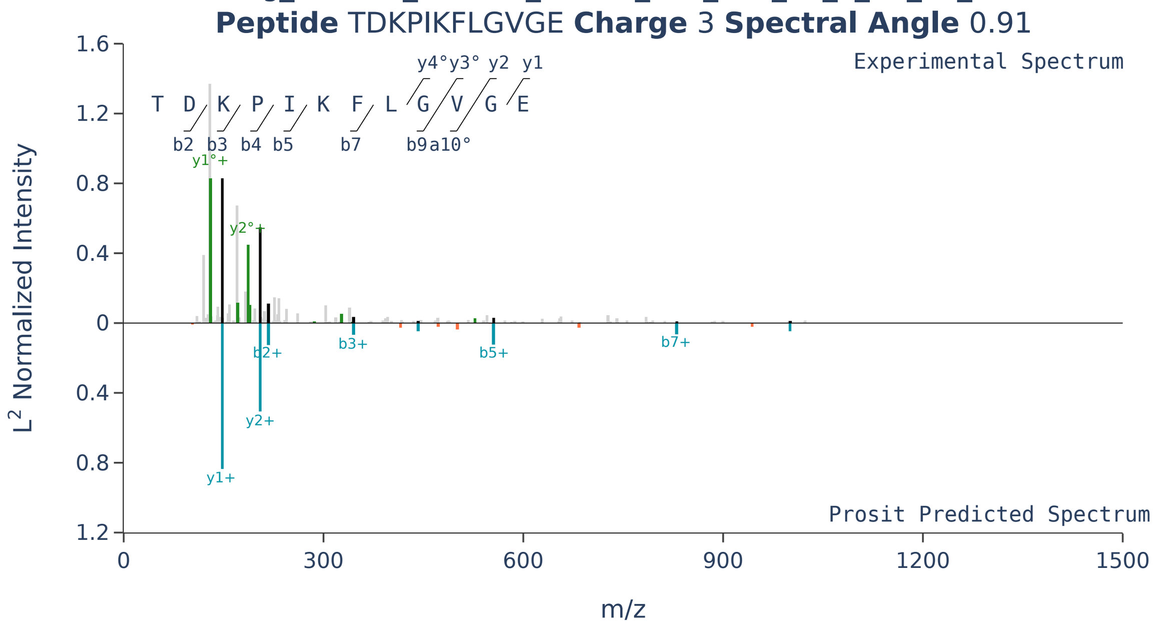

Source Ncheng_210623_230623_HFGoe_FFH_20S_25_1_A2_24h_R1 **Scan** 15586
Peptide AVMTGQDAANTAKAFN **Charge** 2 **Spectral Angle** 0.85

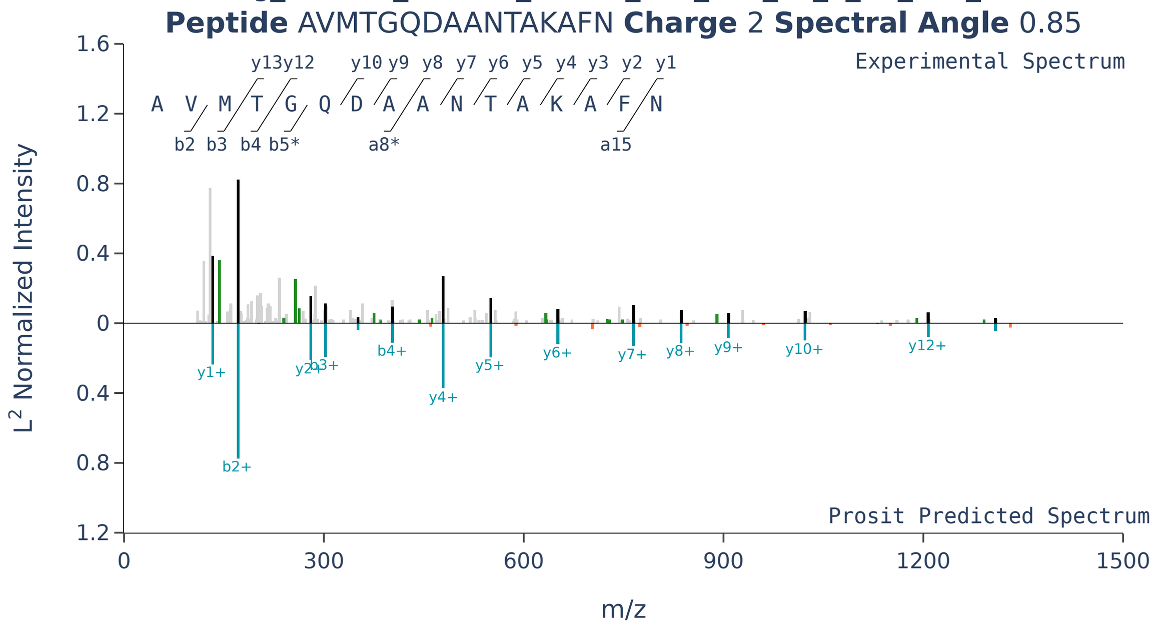

Source Ncheng_210623_230623_HFGoe_FFH_20S_25_1_A1_24h_R2 **Scan** 23407
Peptide GDARGGVDFFPSD **Charge** 2 **Spectral Angle** 0.86

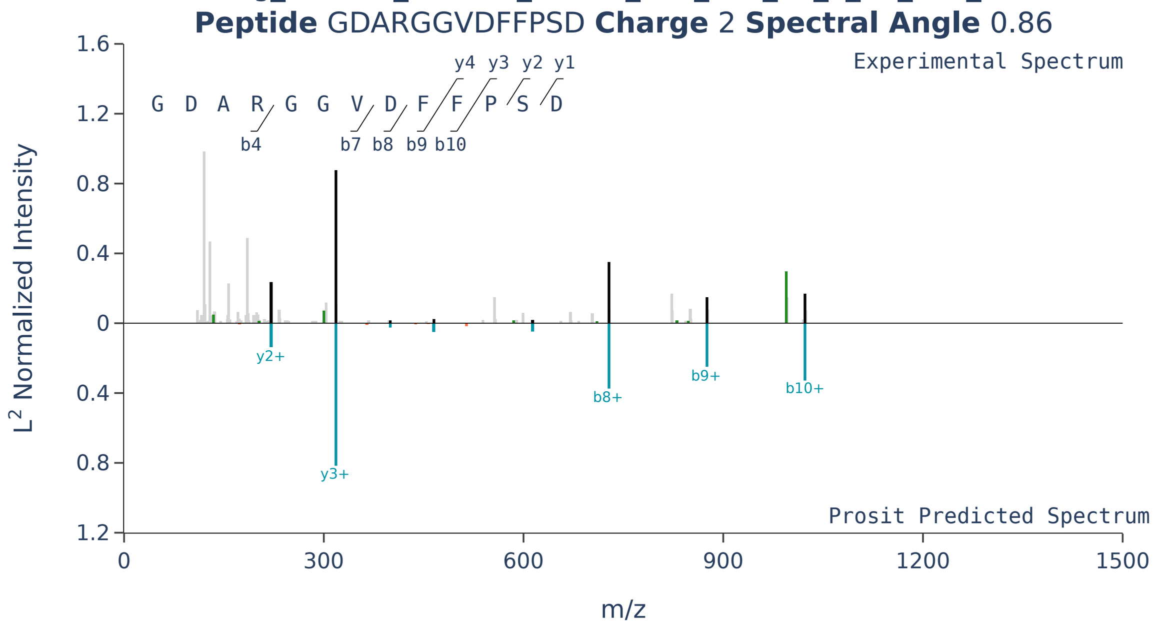

Source Ncheng_210623_230623_HFGoe_FFH_20S_25_1_A2_4h_R2 **Scan** 13470
Peptide SQMTGQDAANTAKAFN **Charge** 2 **Spectral Angle** 0.9

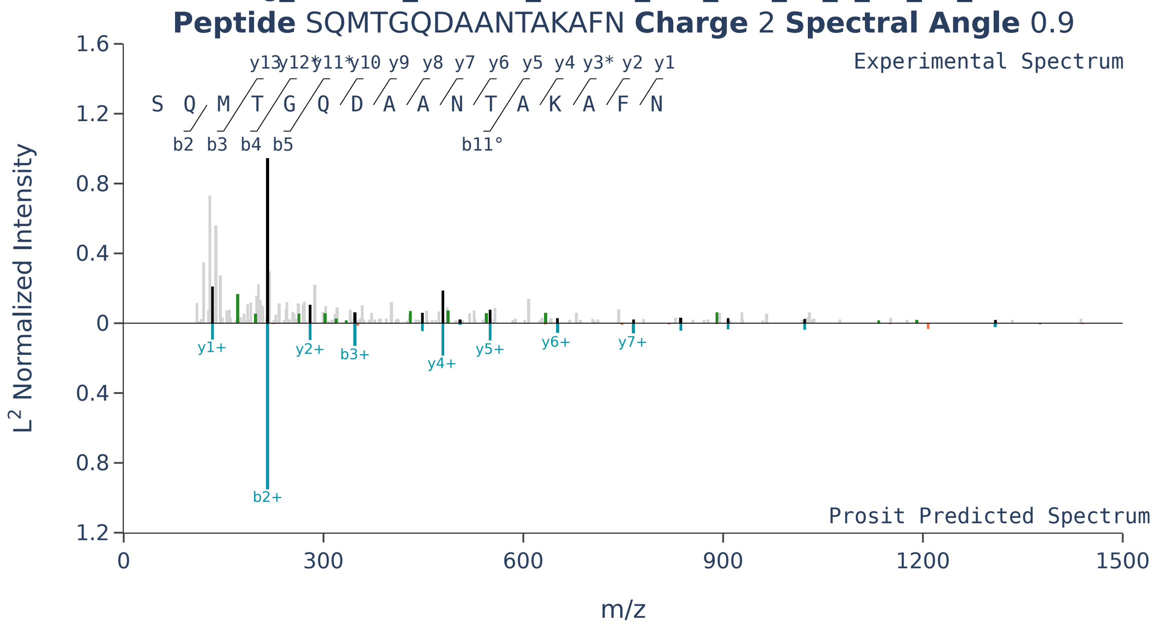

Source Ncheng_210623_230623_HFGoe_FFH_20S_25_1_A1_24h_R1 **Scan** 22200
Peptide GKTLFVVD **Charge** 2 **Spectral Angle** 0.77

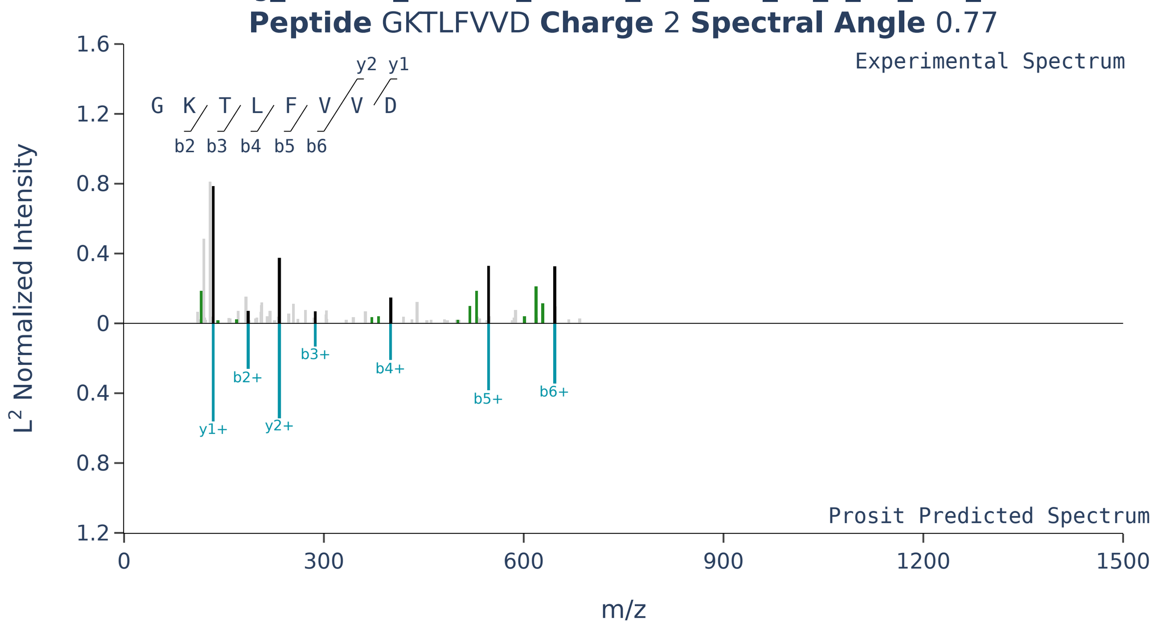

Source Ncheng_210623_230623_HFGoe_FFH_20S_25_1_A1_24h_R1 **Scan** 20972
Peptide NVKDFFPSD **Charge** 2 **Spectral Angle** 0.87

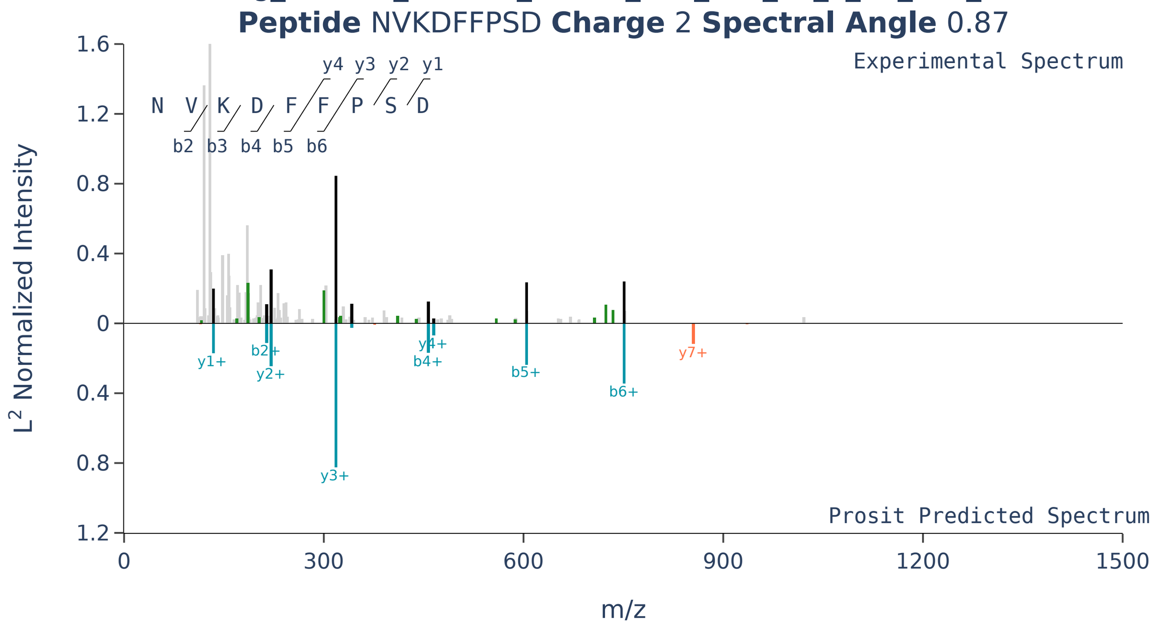

Source Ncheng_210623_230623_HFGoe_FFH_20S_25_1_A2_24h_R2 **Scan** 17691
Peptide ADVAMTGQDAANTAKAFN **Charge** 2 **Spectral Angle** 0.9

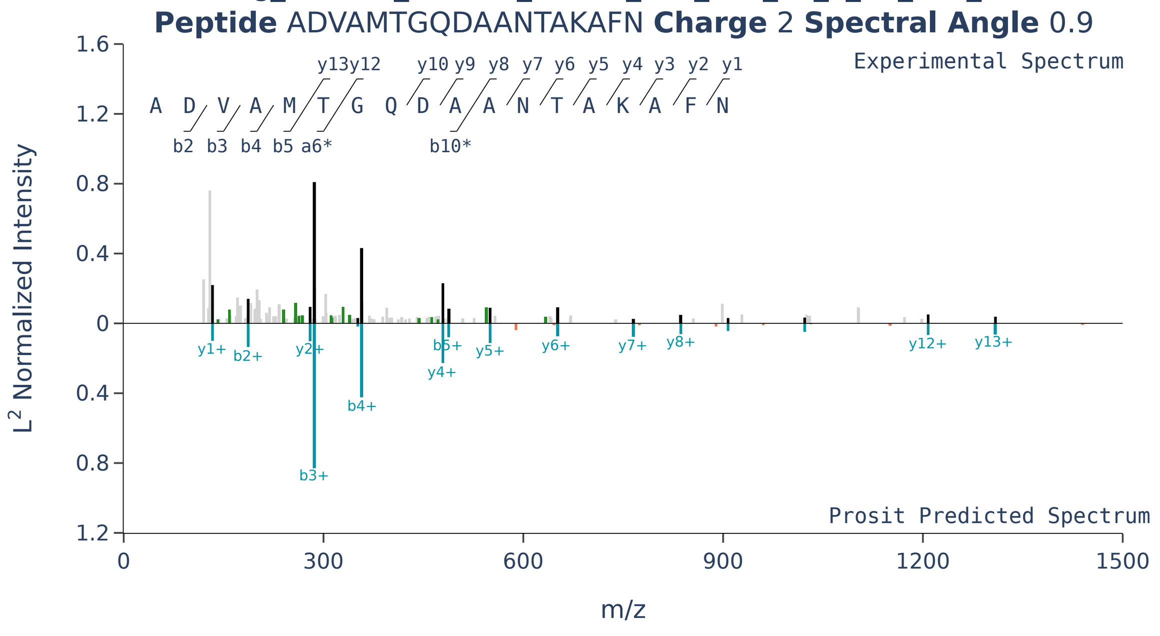

Source Ncheng_210623_230623_HFGoe_FFH_20S_25_1_A2_24h_R2 **Scan** 17277
Peptide LEPFHPDRIASD **Charge** 3 **Spectral Angle** 0.87

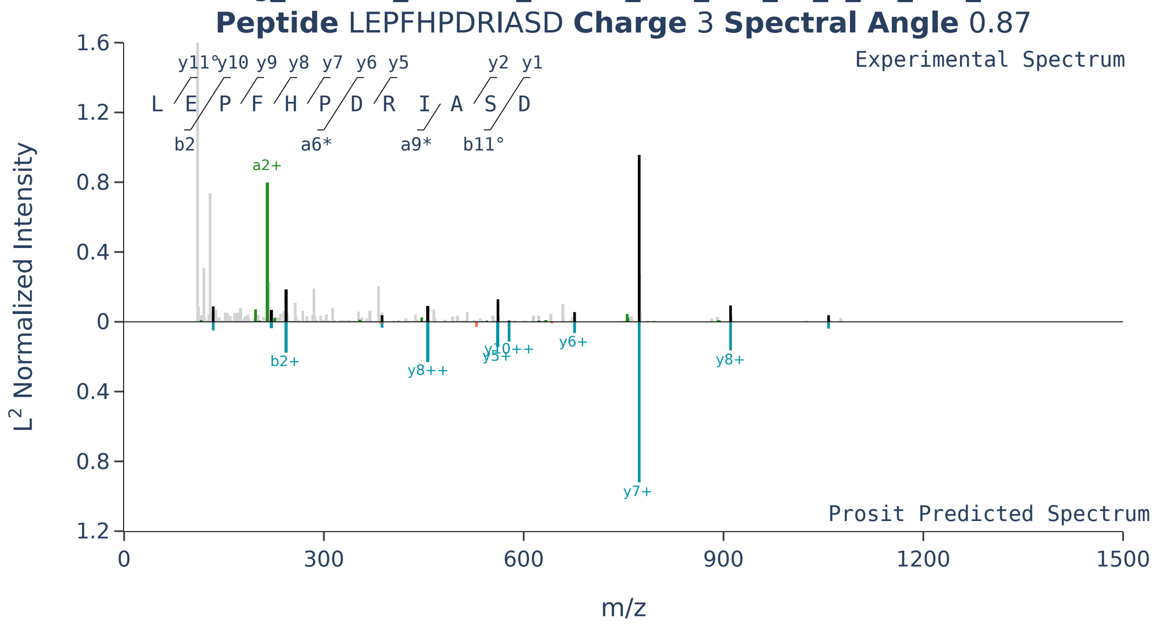

Source Ncheng_210623_230623_HFGoe_FFH_20S_25_1_A1_4h_R2 **Scan** 15031
Peptide SAAMTGQDAANTAKAFNE **Charge** 2 **Spectral Angle** 0.93

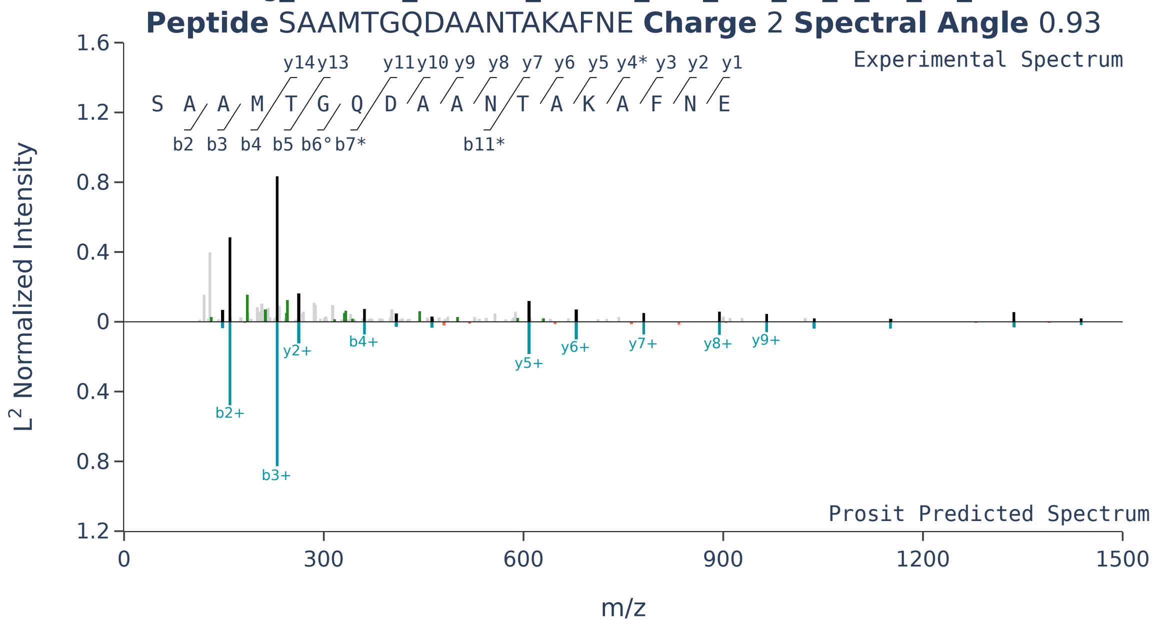

Source Ncheng_210623_230623_HFGoe_FFH_20S_25_1_A2_24h_R2 **Scan** 16069
Peptide PGMGQIPDNVKTG **Charge** 2 **Spectral Angle** 0.76

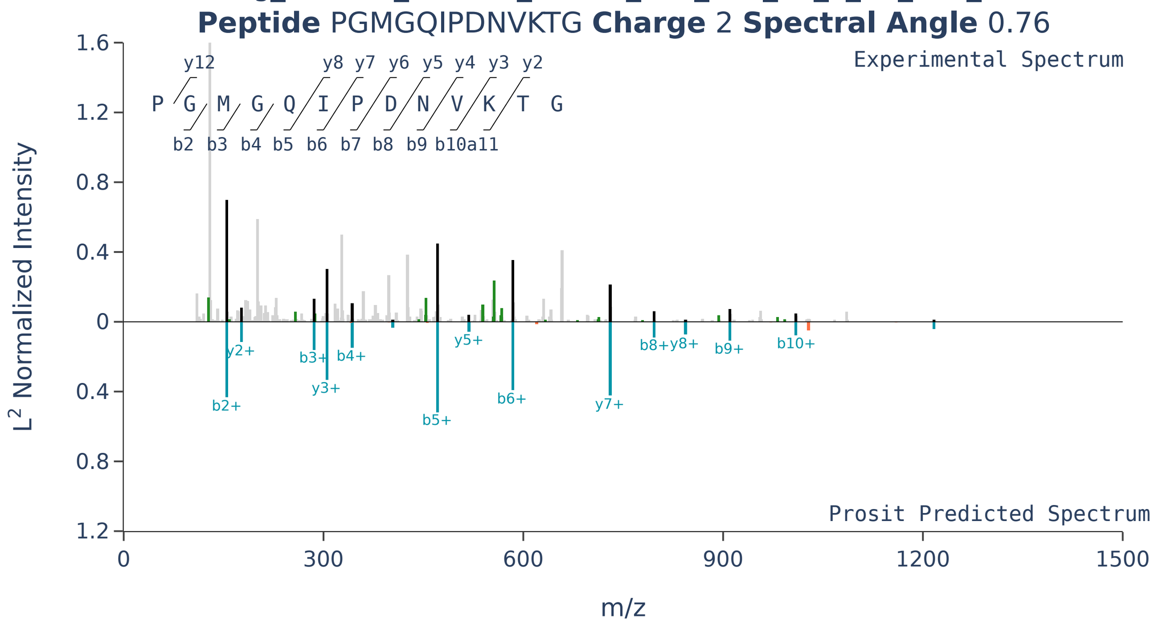

Source Ncheng_210623_230623_HFGoe_FFH_20S_25_1_A2_2h_R1 **Scan** 24820
Peptide AMTGQDAANTAKAFNEALPLTGKTE **Charge** 3 **Spectral Angle** 0.87

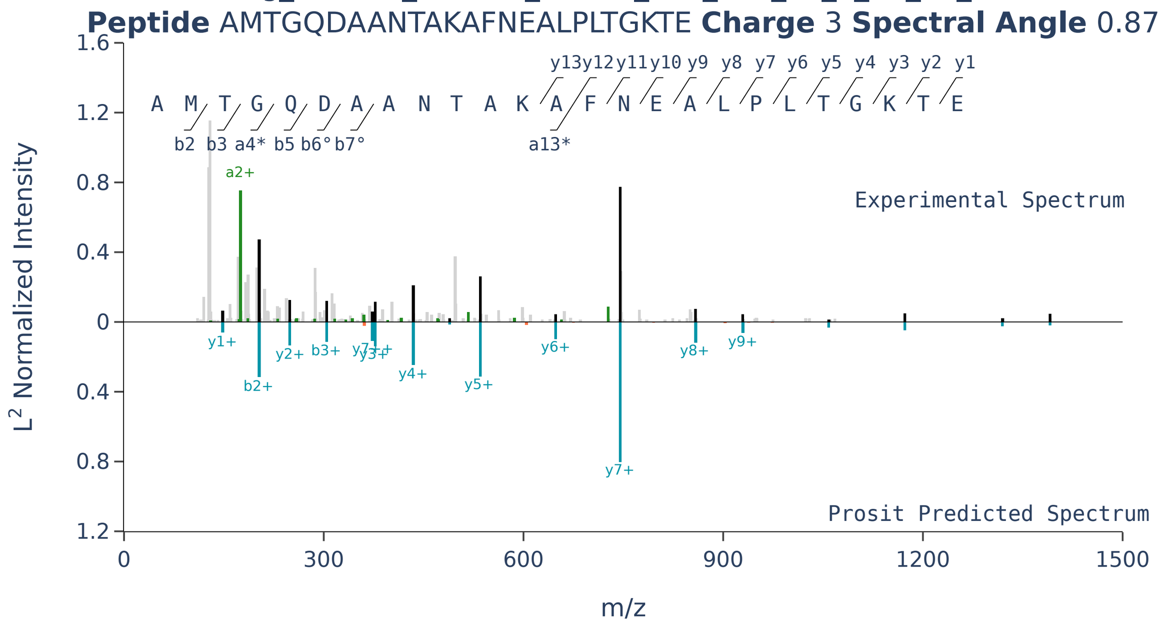

Source Ncheng_210623_230623_HFGoe_FFH_20S_25_1_A2_2h_R2 **Scan** 26217
Peptide EKTEAQVGVDFFPSD **Charge** 2 **Spectral Angle** 0.95

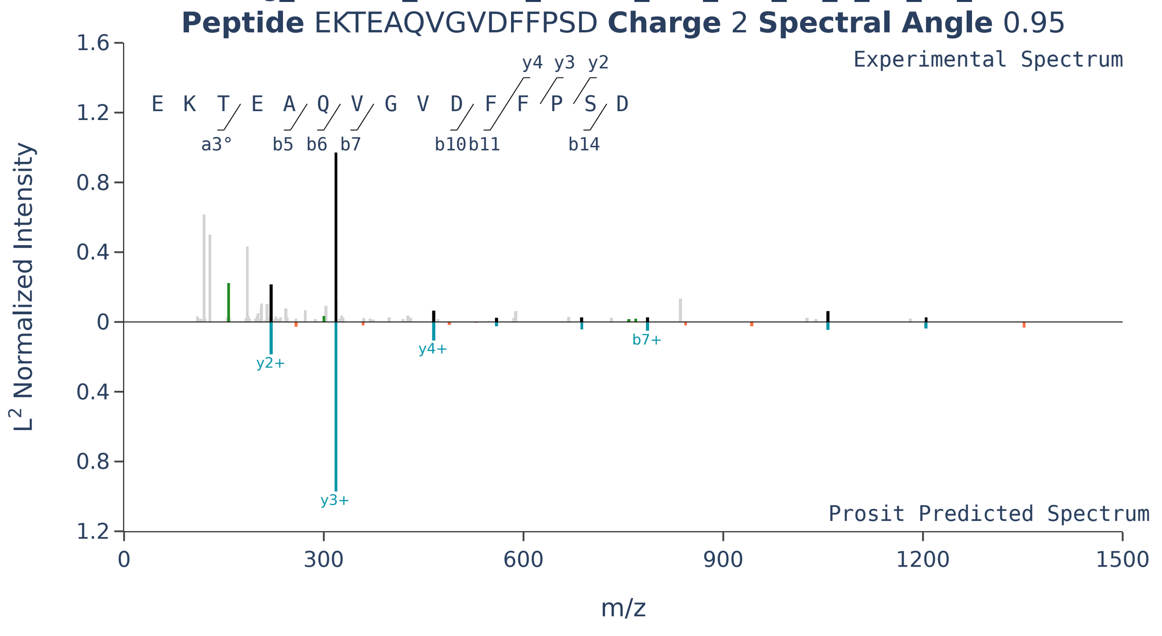

Source Ncheng_210623_230623_HFGoe_FFH_20S_25_1_A2_24h_R1 **Scan** 17350
Peptide LEPFHPDRISAD **Charge** 3 **Spectral Angle** 0.89

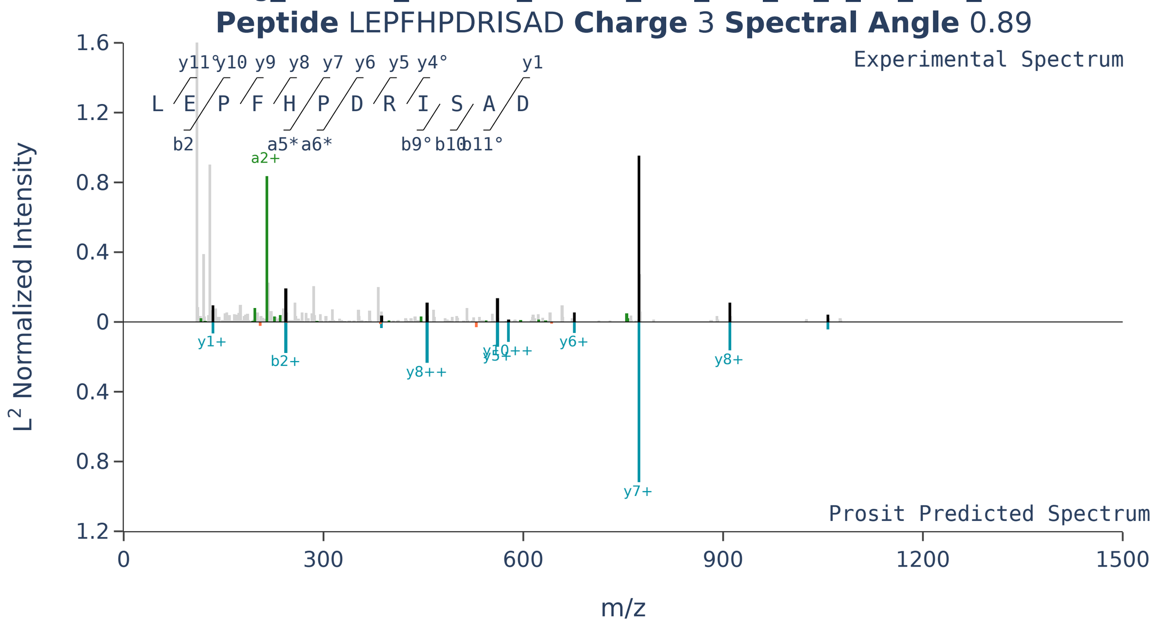

Source Ncheng_210623_230623_HFGoe_FFH_20S_25_1_A1_24h_R1 **Scan** 28310
Peptide PPAVVLMAAG **Charge** 1 **Spectral Angle** 0.96

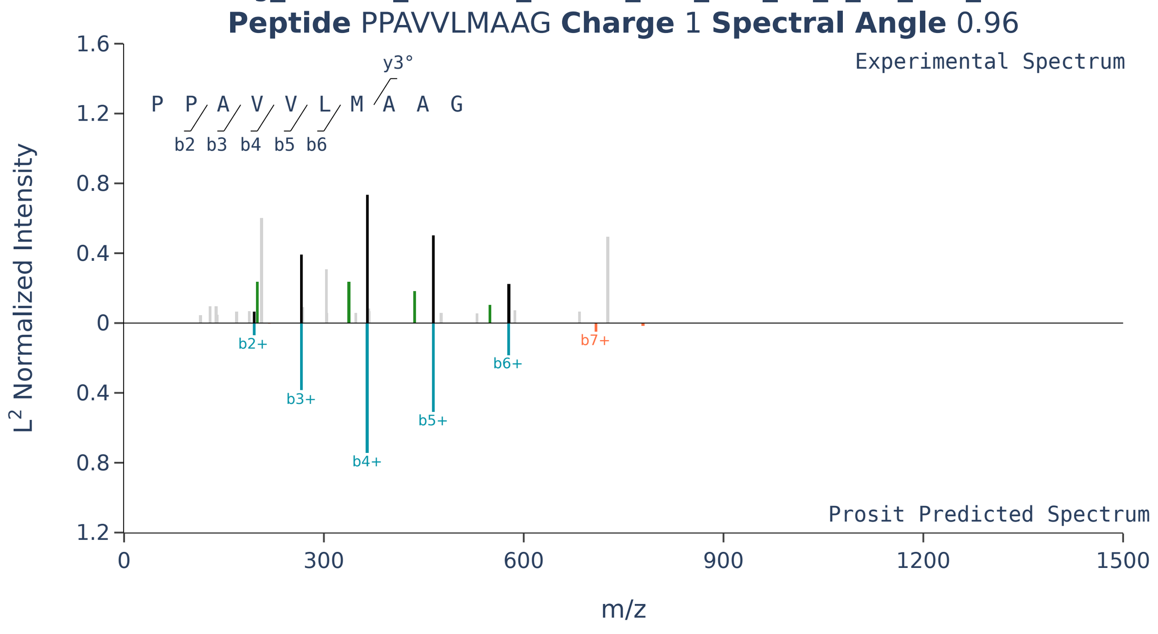

Source Ncheng_210623_230623_HFGoe_FFH_20S_25_1_A1_2h_R1 **Scan** 11036
Peptide TLAEQVDA **Charge** 1 **Spectral Angle** 0.89

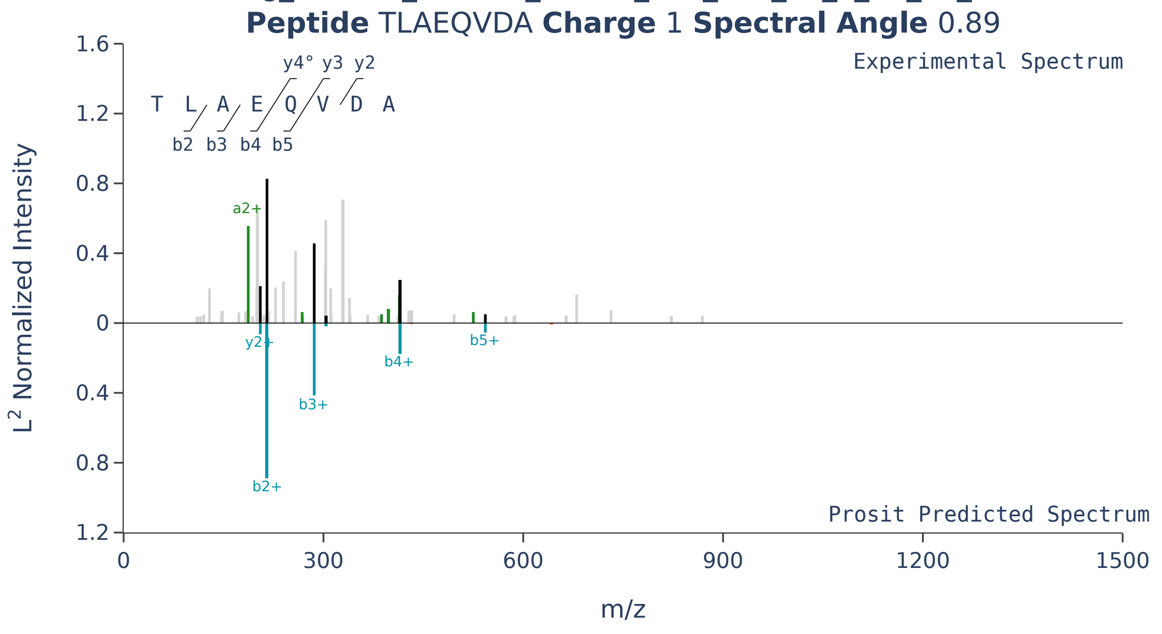

Source Ncheng_210623_230623_HFGoe_FFH_20S_25_1_A2_24h_R2 **Scan** 9099
Peptide GVGEVGQKPVD **Charge** 1 **Spectral Angle** 0.82

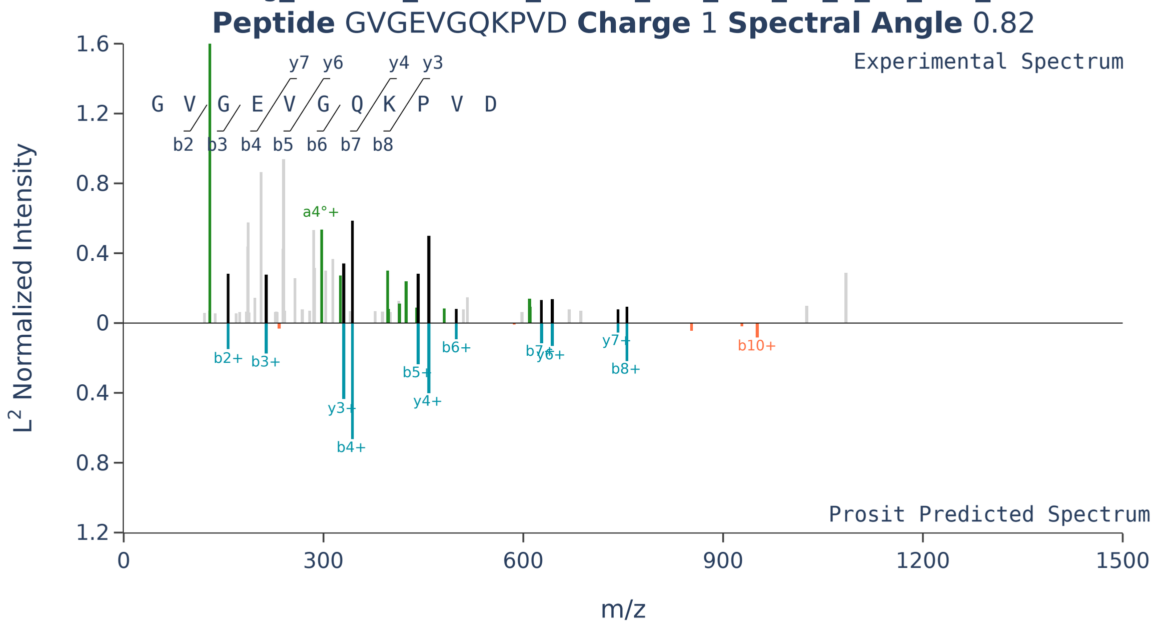

Source Ncheng_210623_230623_HFGoe_FFH_20S_25_1_A1_24h_R1 **Scan** 7513
Peptide TGAMTGQDAANTAKA **Charge** 2 **Spectral Angle** 0.84

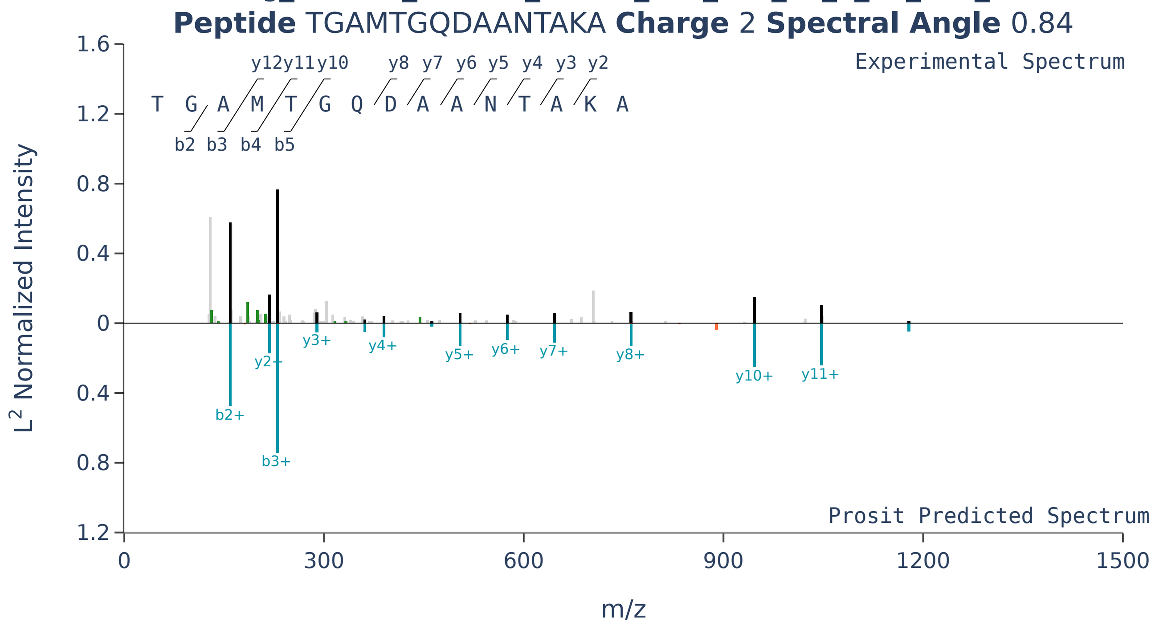

Source Ncheng_210623_230623_HFGoe_FFH_20S_25_1_A2_24h_R1 **Scan** 15017
Peptide DKVLVRMEAA **Charge** 2 **Spectral Angle** 0.9

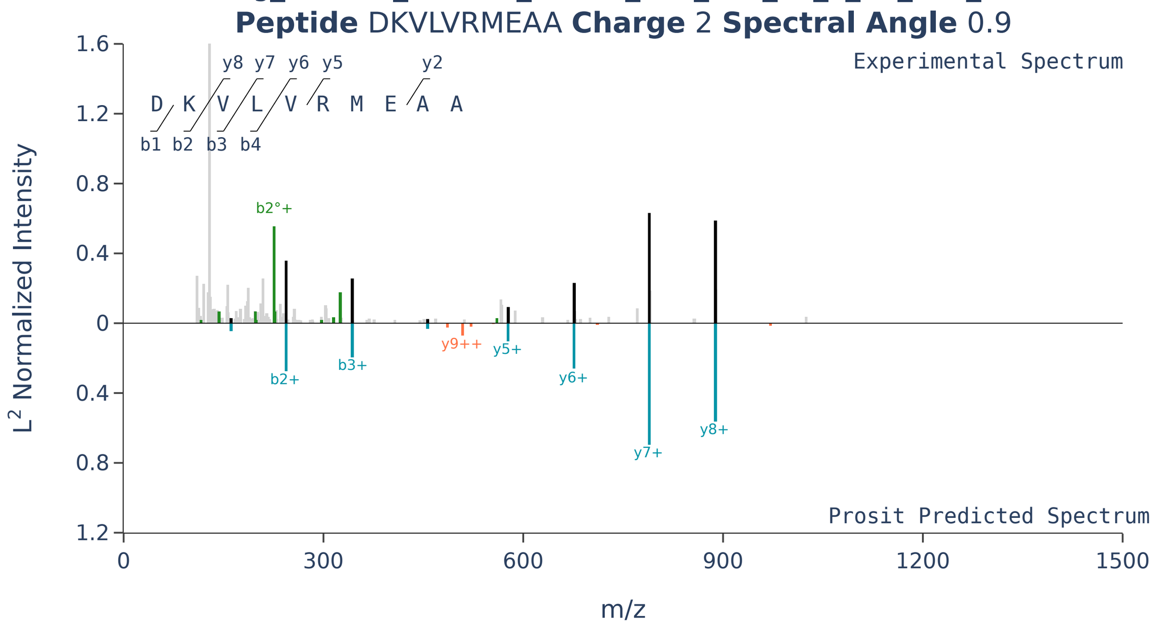

Source Ncheng_210623_230623_HFGoe_FFH_20S_25_1_A2_4h_R1 **Scan** 28666
Peptide RIAAGQVGVDFFPSD **Charge** 2 **Spectral Angle** 0.88

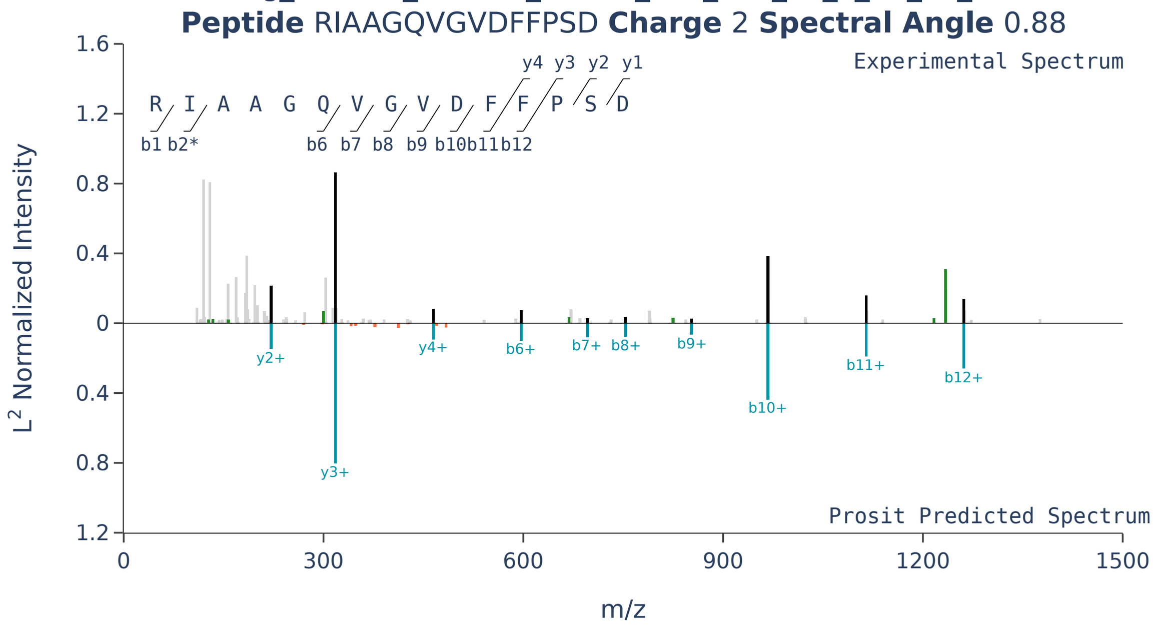

Source Ncheng_210623_230623_HFGoe_FFH_20S_25_1_A2_24h_R1 **Scan** 15438
Peptide TGAMTGQDAANTAKAFNE **Charge** 2 **Spectral Angle** 0.93

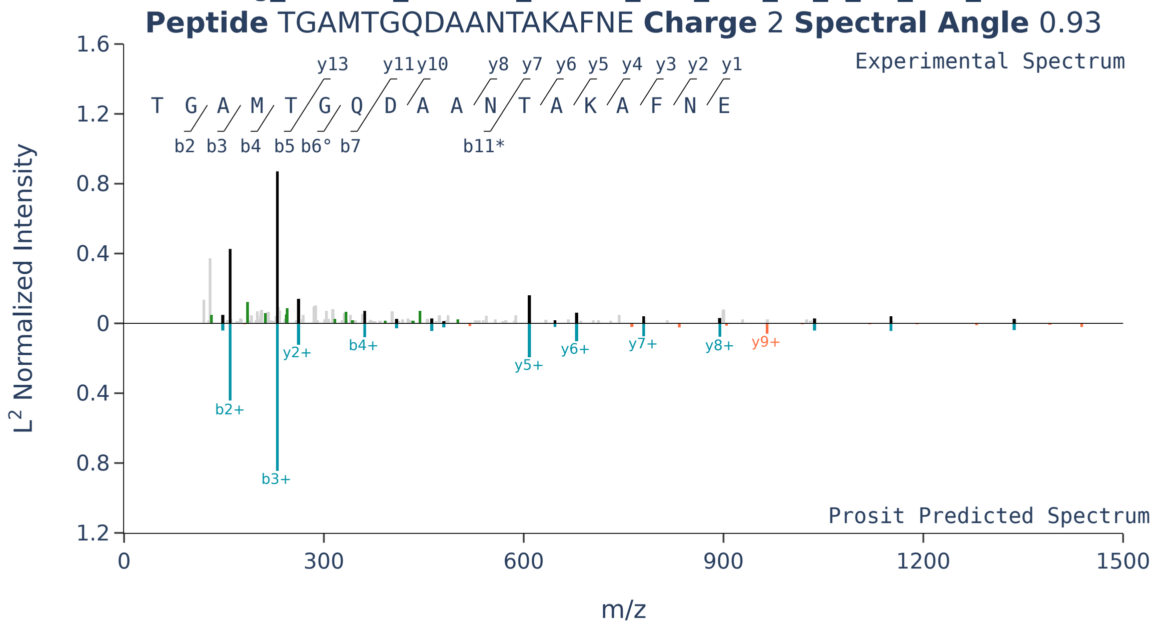

Source Ncheng_210623_230623_HFGoe_FFH_20S_25_1_A1_4h_R1 **Scan** 28201
Peptide PPAVVLMAAAG **Charge** 1 **Spectral Angle** 0.87

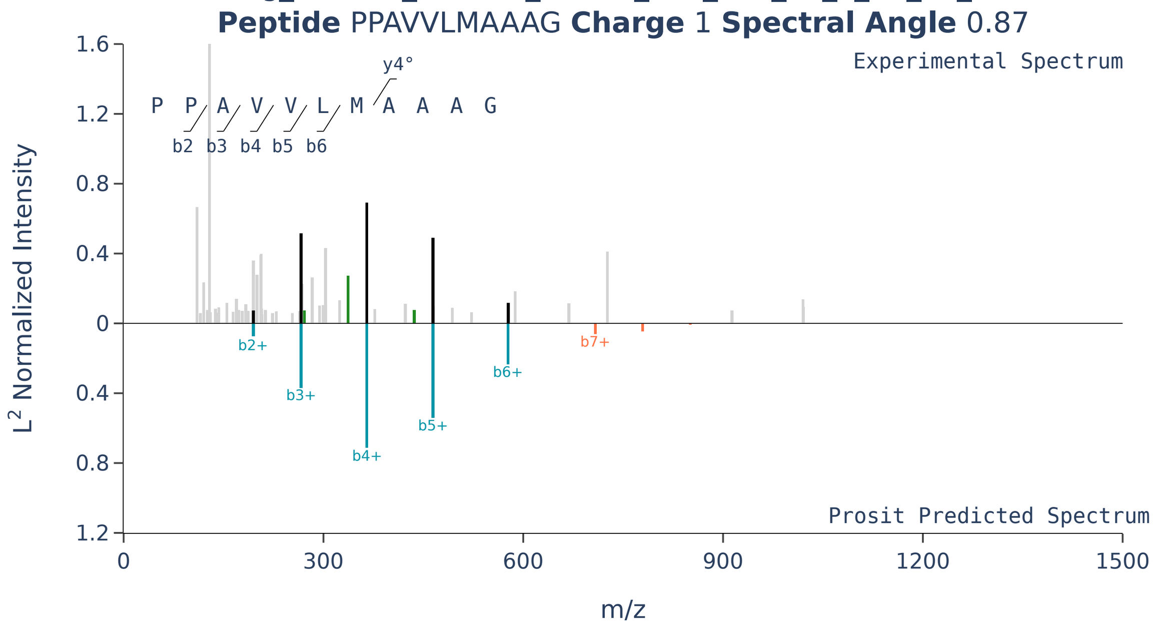

Source Ncheng_210623_230623_HFGoe_FFH_20S_25_1_A1_24h_R2 **Scan** 9751
Peptide RQMQDVNRL **Charge** 3 **Spectral Angle** 0.8

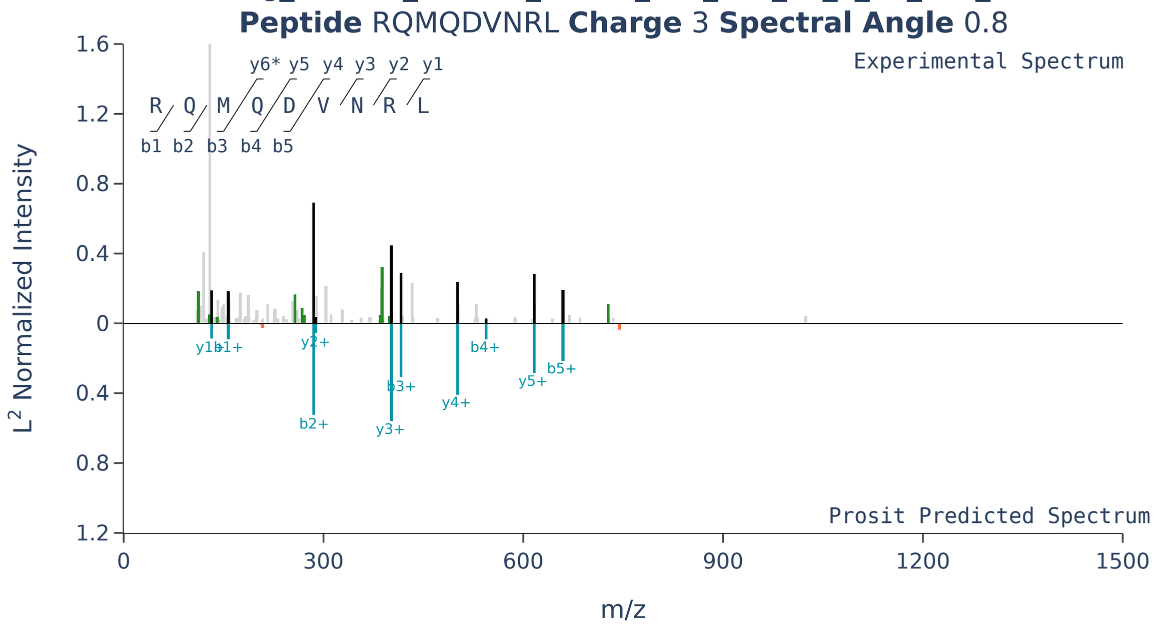

Source Ncheng_210623_230623_HFGoe_FFH_20S_25_1_A2_24h_R2 **Scan** 12714
Peptide PDNVKSQMDDGLQ **Charge** 1 **Spectral Angle** 0.86

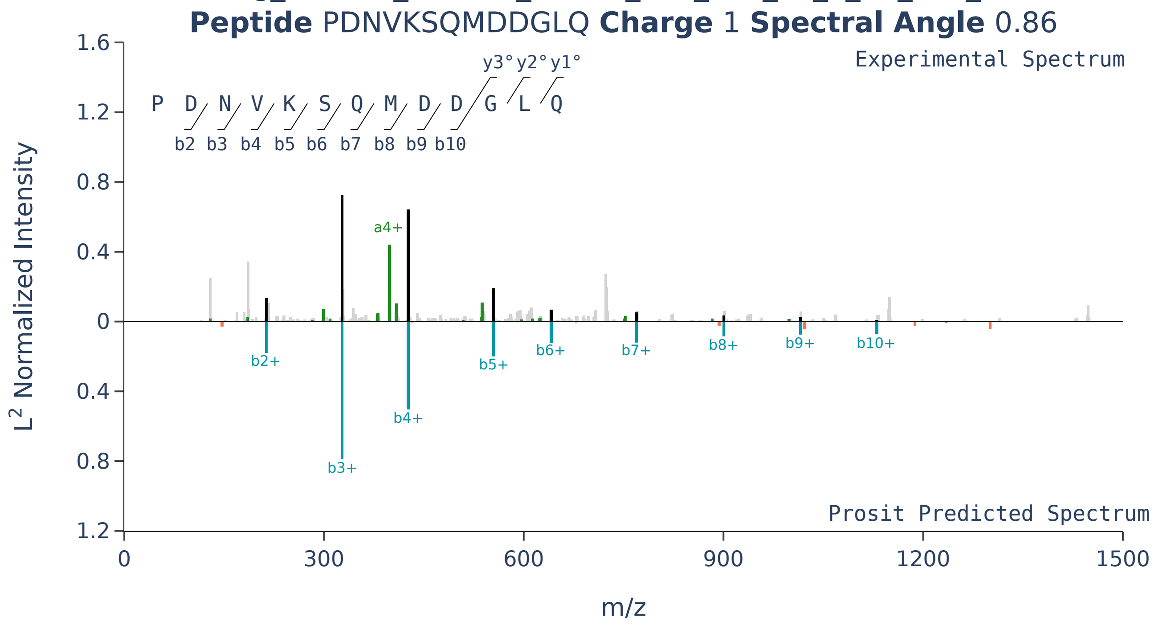

Source Ncheng_210623_230623_HFGoe_FFH_20S_25_1_A2_1h_R1 **Scan** 22012
Peptide KTEGVDFFPSD **Charge** 2 **Spectral Angle** 0.86

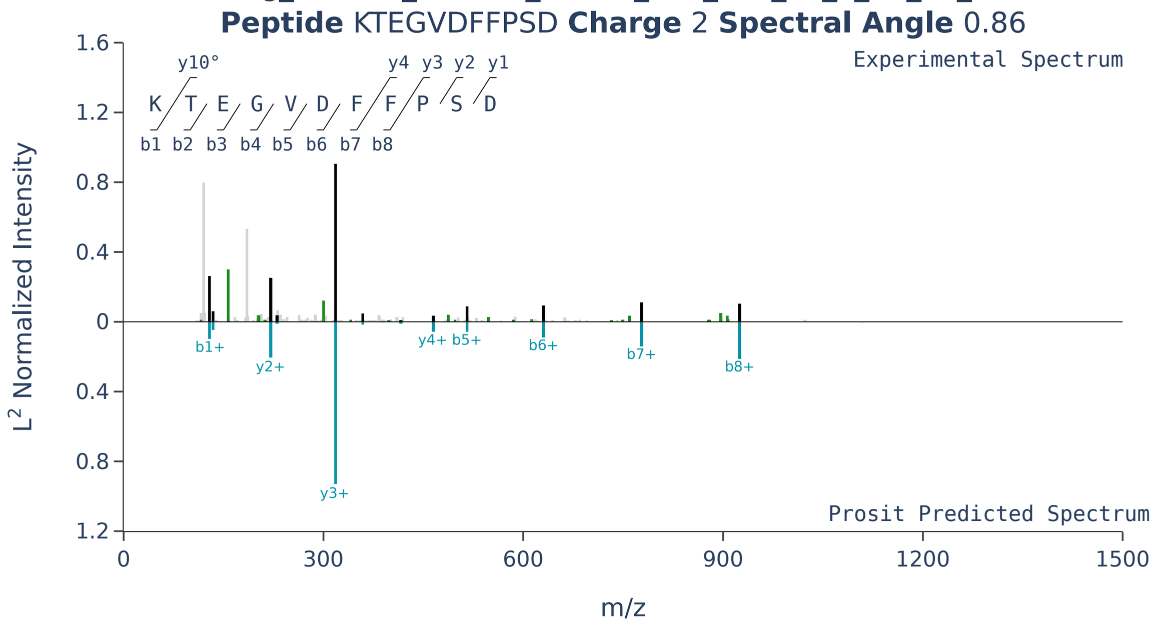

Source Ncheng_210623_230623_HFGoe_FFH_20S_25_1_A2_4h_R2 **Scan** 13968
Peptide GEAMTGQDAANTAKAFN **Charge** 2 **Spectral Angle** 0.9

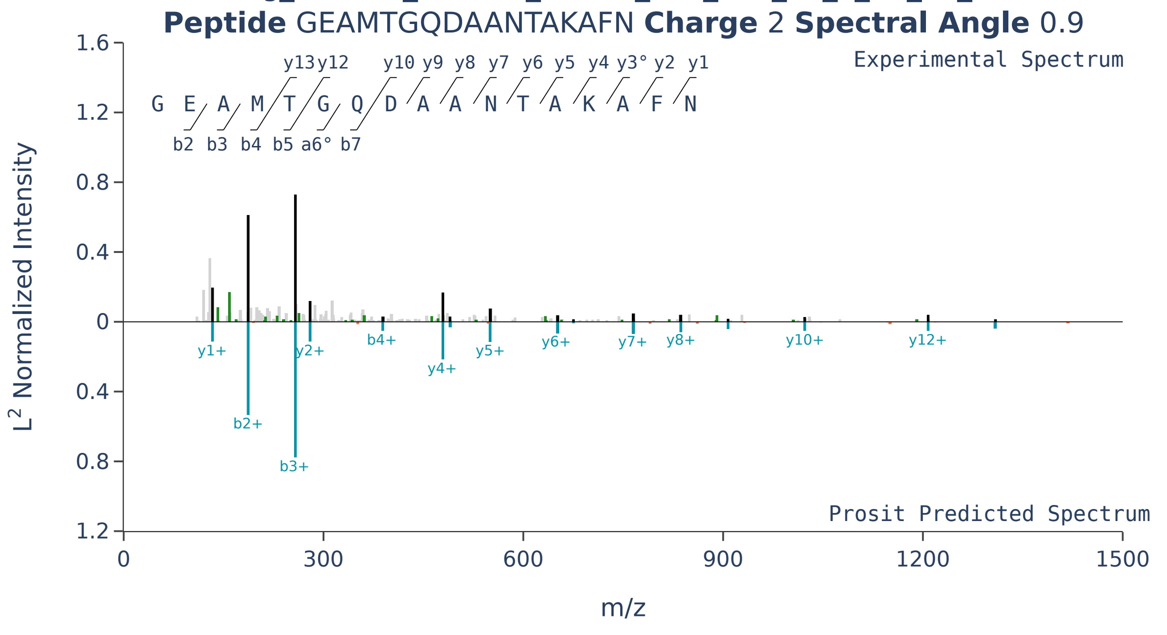

Source Ncheng_210623_230623_HFGoe_FFH_20S_25_1_A1_24h_R2 **Scan** 21858
Peptide DGDARGGAALVL **Charge** 2 **Spectral Angle** 0.88

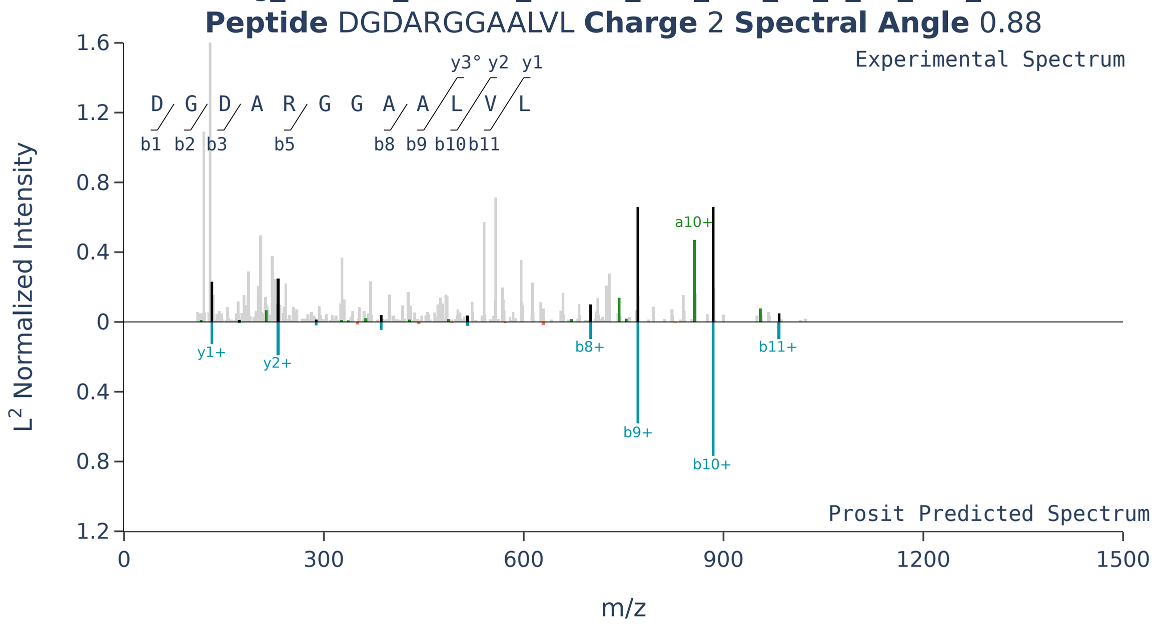

Source Ncheng_210623_230623_HFGoe_FFH_20S_25_1_A2_24h_R1 **Scan** 12409
Peptide GEENQTLVGQKPVD **Charge** 2 **Spectral Angle** 0.83

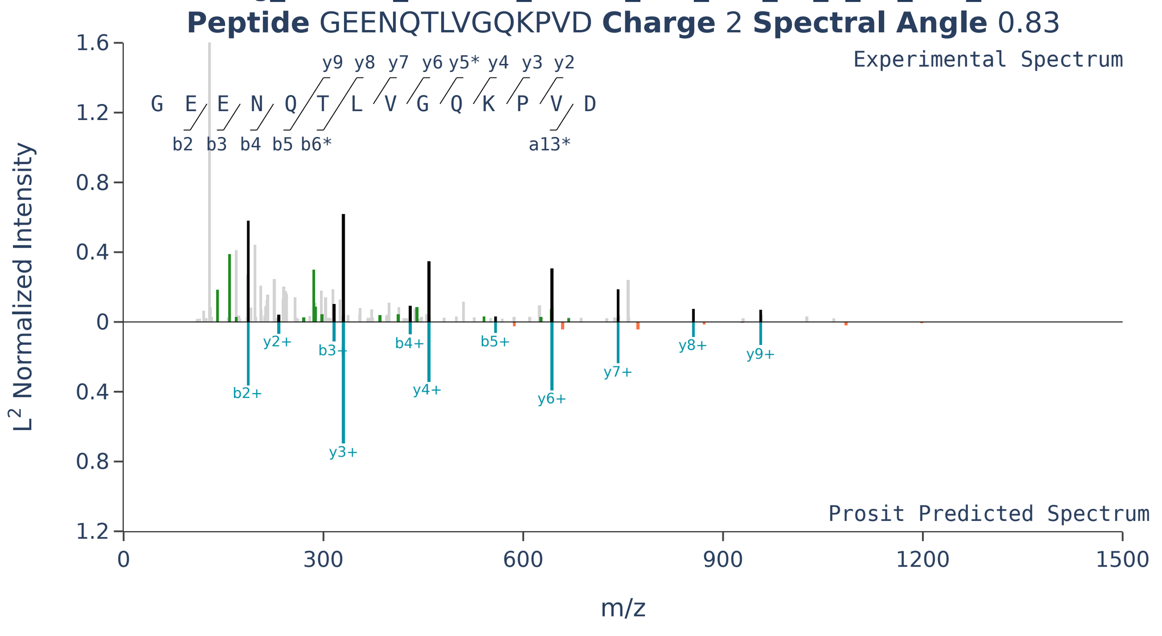

Source Ncheng_210623_230623_HFGoe_FFH_20S_25_1_A1_2h_R2 **Scan** 17481
Peptide KNMGGMASLVGQKPVD **Charge** 2 **Spectral Angle** 0.85

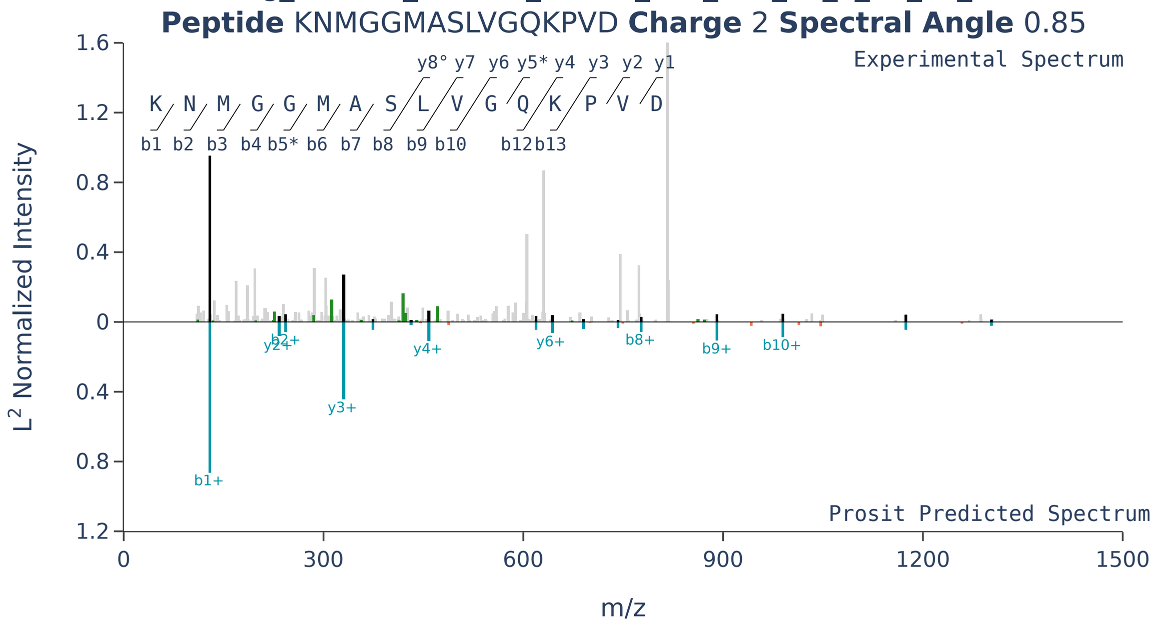

Source Ncheng_210623_230623_HFGoe_FFH_20S_25_1_A1_24h_R1 **Scan** 9285
Peptide GKSINPVE **Charge** 2 **Spectral Angle** 0.88

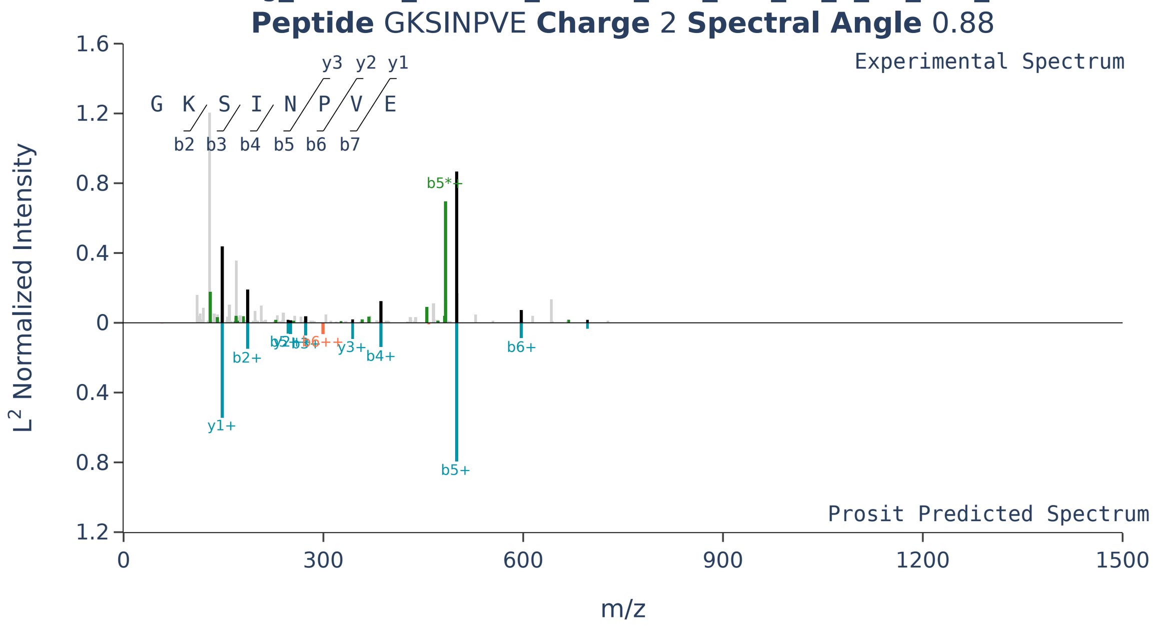

Source Ncheng_210623_230623_HFGoe_FFH_20S_25_1_A2_2h_R2 **Scan** 10165
Peptide DKVLVAD **Charge** 2 **Spectral Angle** 0.86

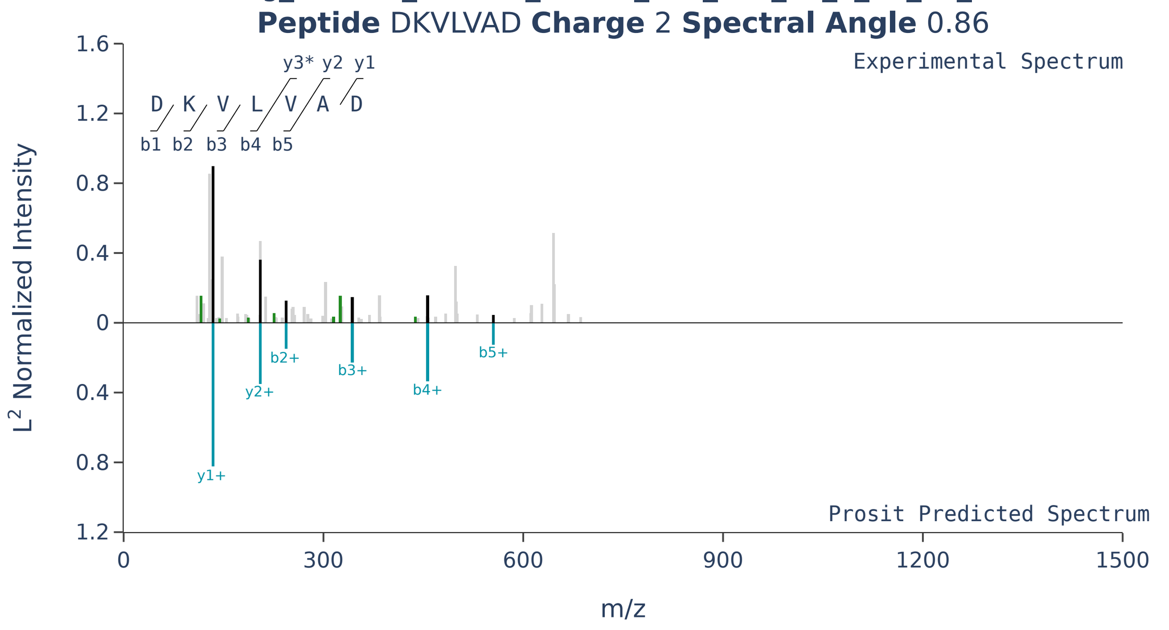

Source Ncheng_210623_230623_HFGoe_FFH_20S_25_1_A2_2h_R1 **Scan** 15523
Peptide MMPPGFPGRRS **Charge** 3 **Spectral Angle** 0.92

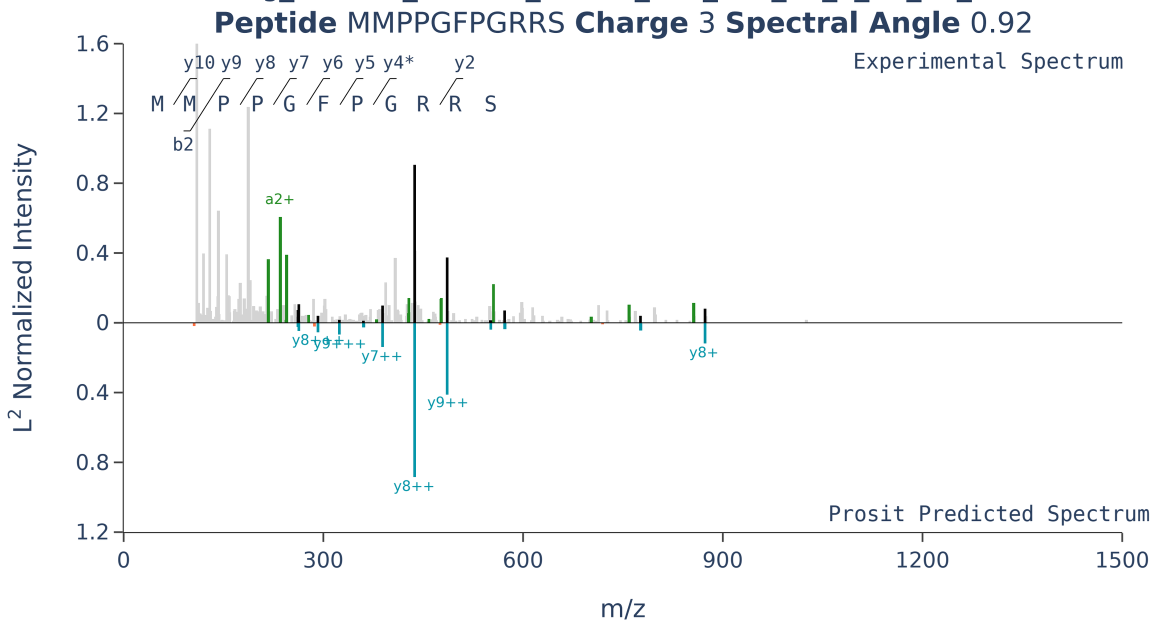

Source Ncheng_210623_230623_HFGoe_FFH_20S_25_1_A1_4h_R1 **Scan** 22422
Peptide GKVDFFPSD **Charge** 2 **Spectral Angle** 0.77

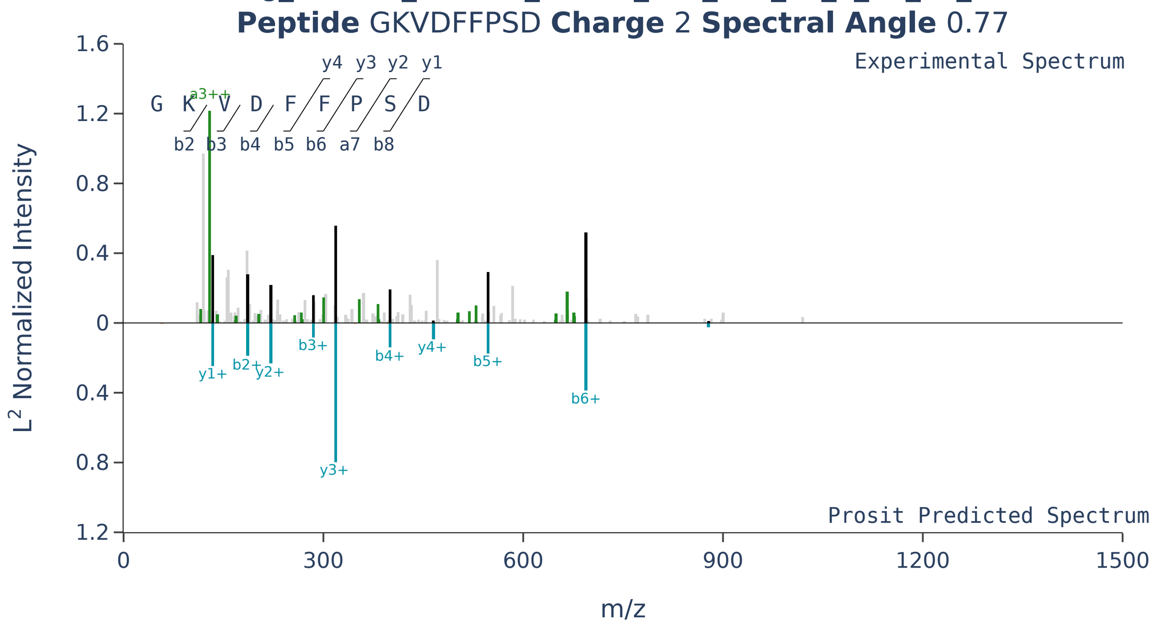

Source Ncheng_210623_230623_HFGoe_FFH_20S_25_1_A2_24h_R1 **Scan** 15309
Peptide PGMGQIPDNVKSE **Charge** 2 **Spectral Angle** 0.74

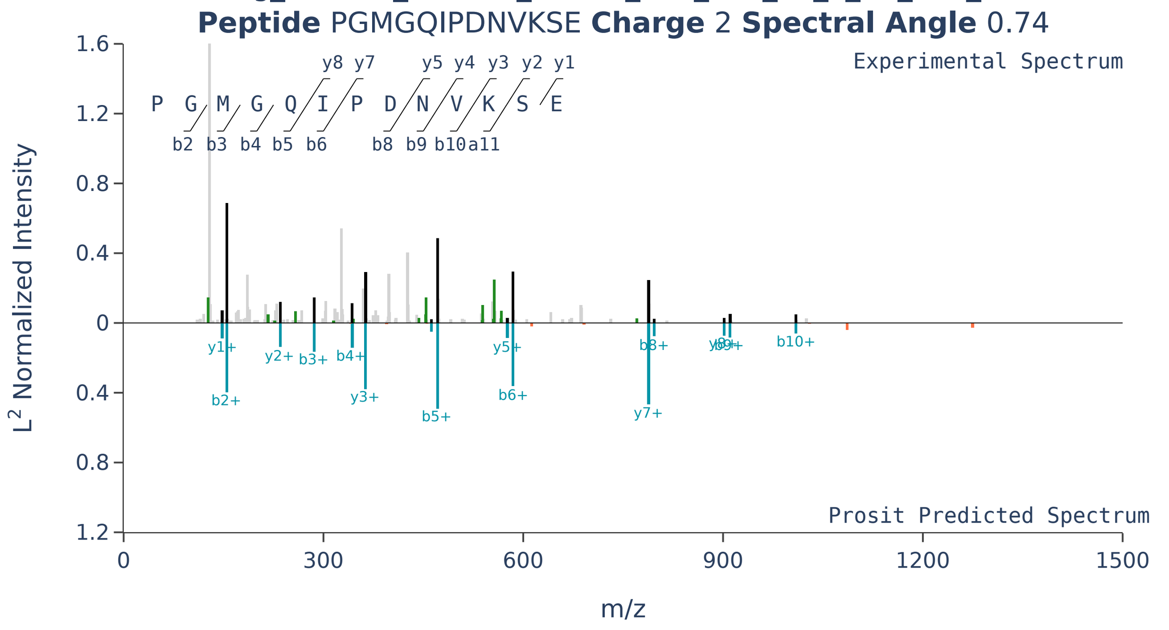

Source Ncheng_210623_230623_HFGoe_FFH_20S_25_1_A1_24h_R1 **Scan** 11250
Peptide LGVGEKTEVGQKPVD **Charge** 2 **Spectral Angle** 0.86

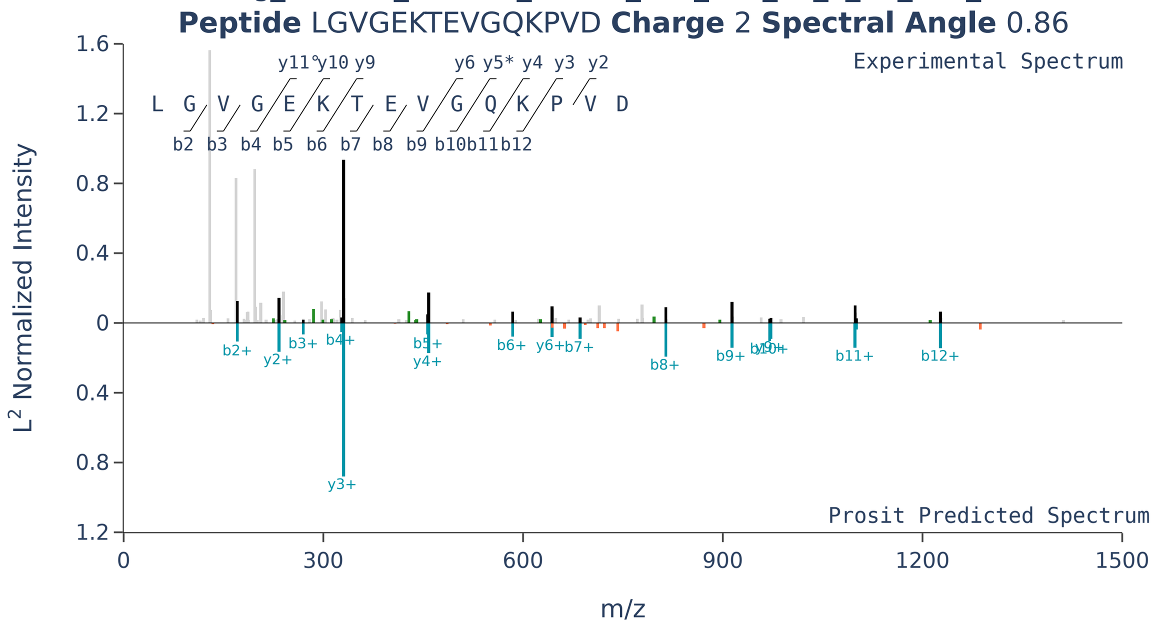

Source Ncheng_210623_230623_HFGoe_FFH_20S_25_1_A2_24h_R2 **Scan** 6926
Peptide KNMGVGQKPVD **Charge** 3 **Spectral Angle** 0.86

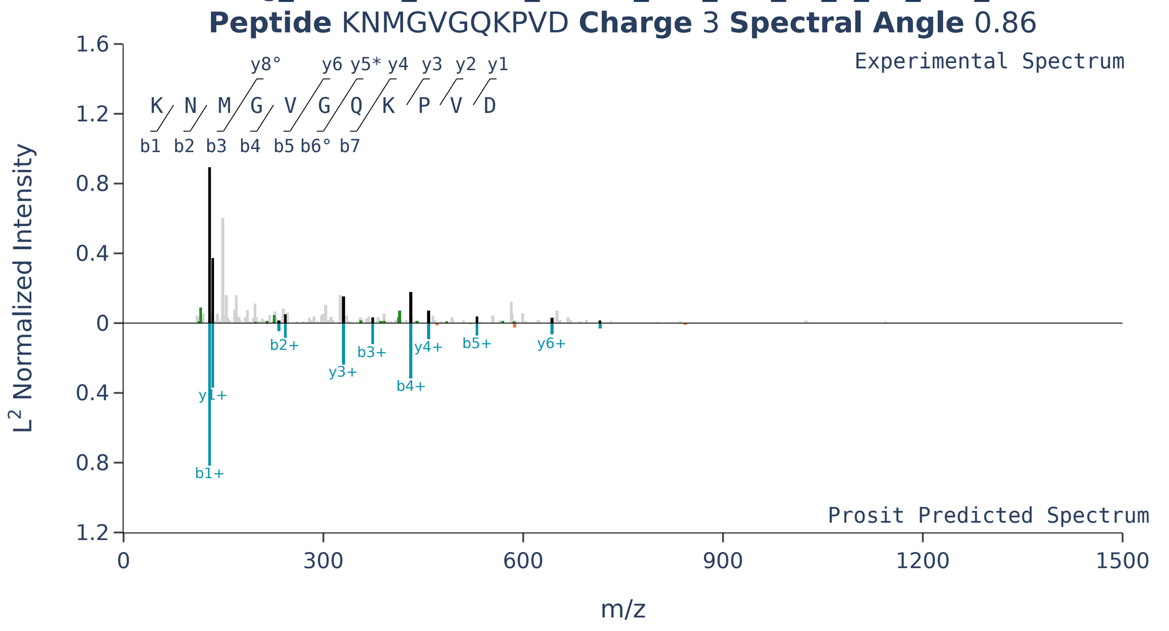

Source Ncheng_210623_230623_HFGoe_FFH_20S_25_1_A2_24h_R1 **Scan** 27913
Peptide GVDFFPES **Charge** 1 **Spectral Angle** 0.89

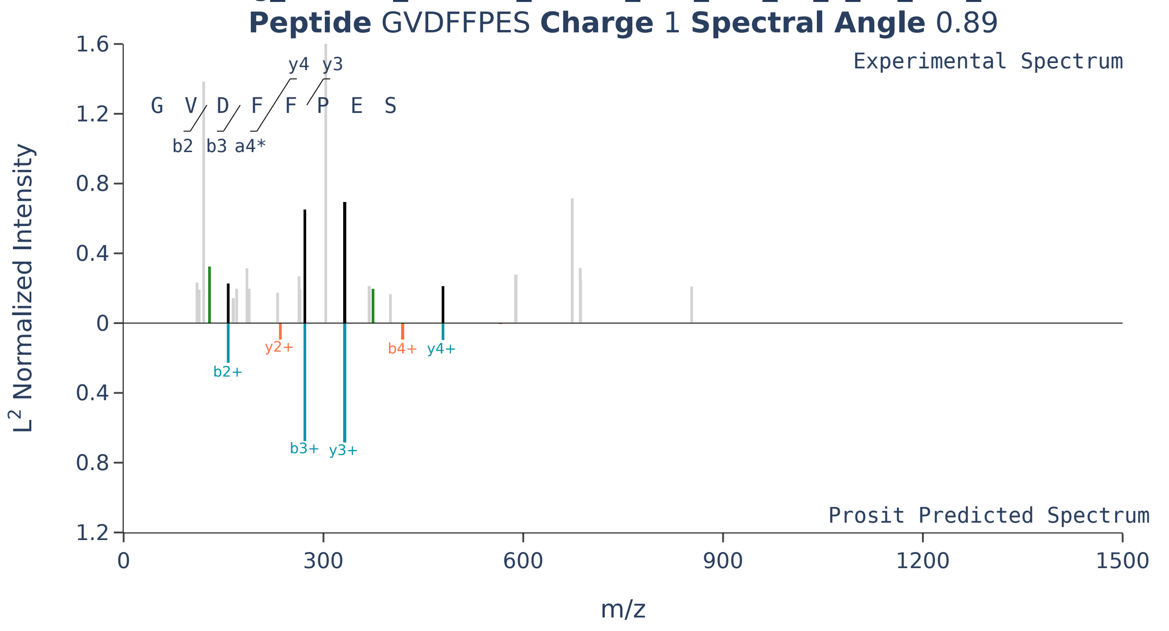

Source Ncheng_210623_230623_HFGoe_FFH_20S_25_1_A1_24h_R1 **Scan** 21336
Peptide TKLFVVD **Charge** 2 **Spectral Angle** 0.74

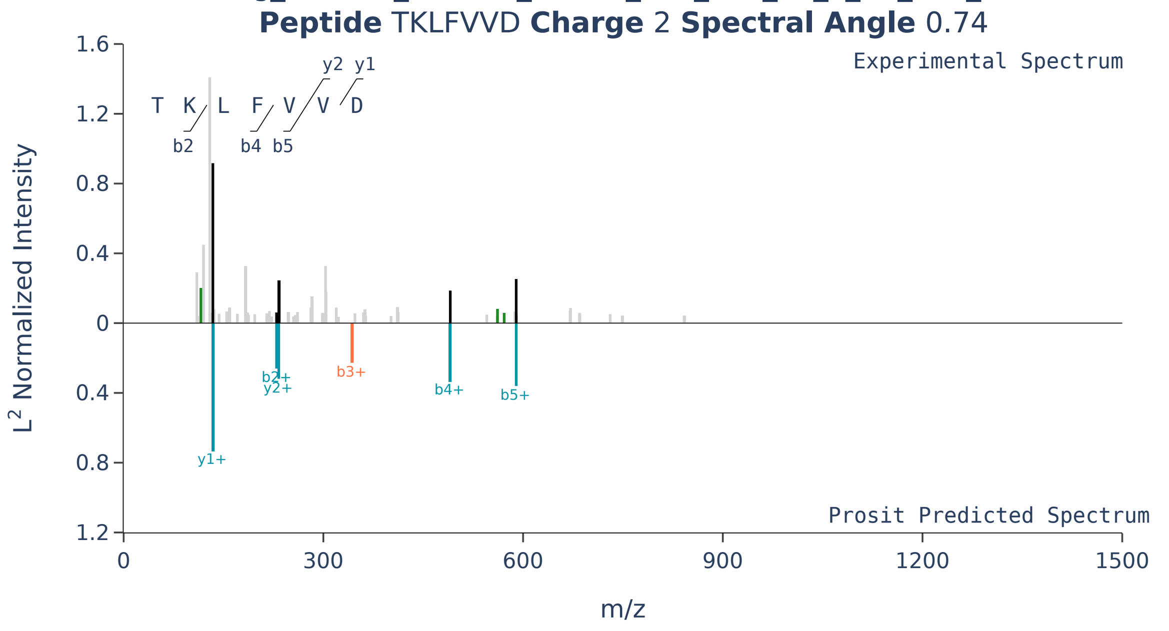

Source Ncheng_210623_230623_HFGoe_FFH_20S_25_1_A2_24h_R1 **Scan** 29929
Peptide ANTVGVDFFPSD **Charge** 2 **Spectral Angle** 0.89

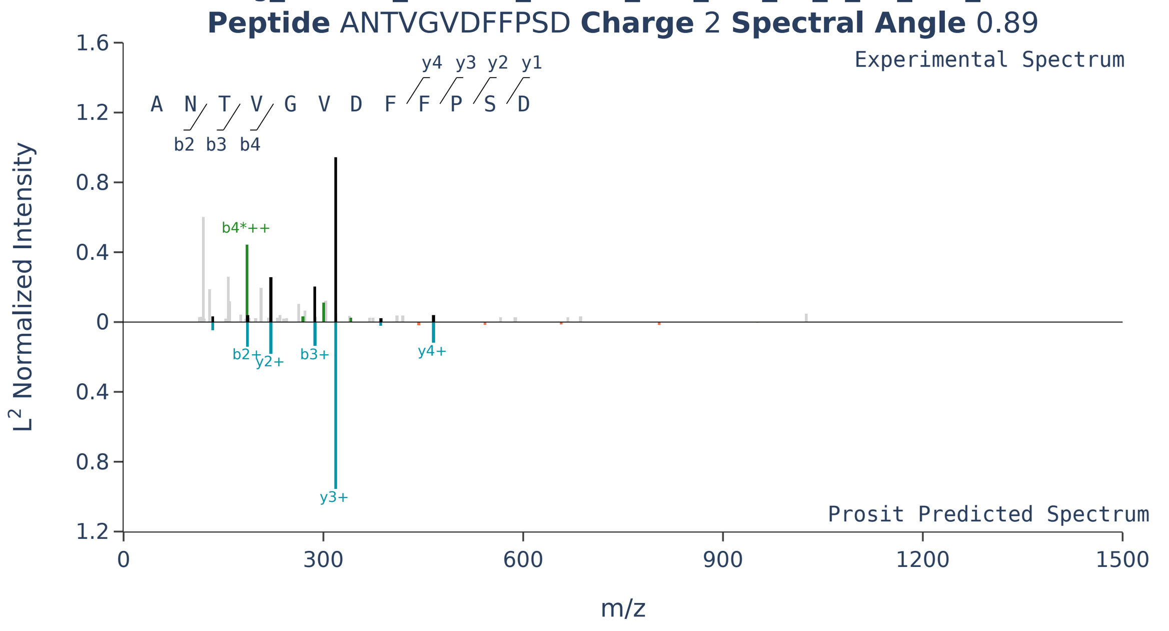

Source Ncheng_210623_230623_HFGoe_FFH_20S_25_1_A1_24h_R2 **Scan** 27457
Peptide GGVDFFPSD **Charge** 1 **Spectral Angle** 0.92

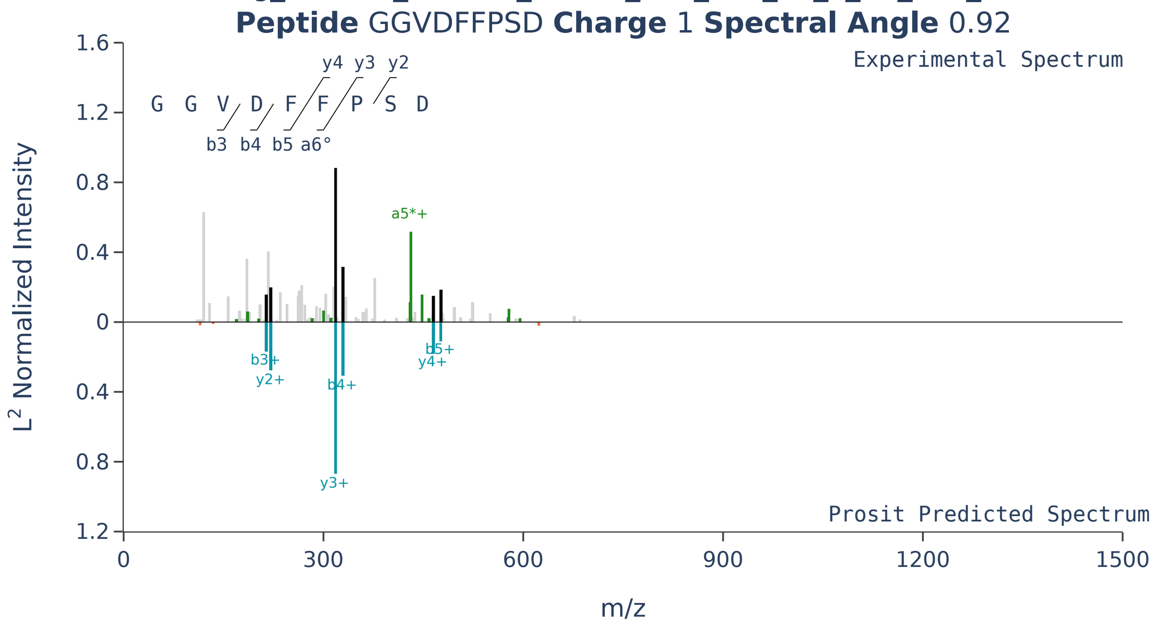

Source Ncheng_210623_230623_HFGoe_FFH_20S_25_1_A1_24h_R2 **Scan** 10659
Peptide KNMGGMAKL **Charge** 2 **Spectral Angle** 0.91

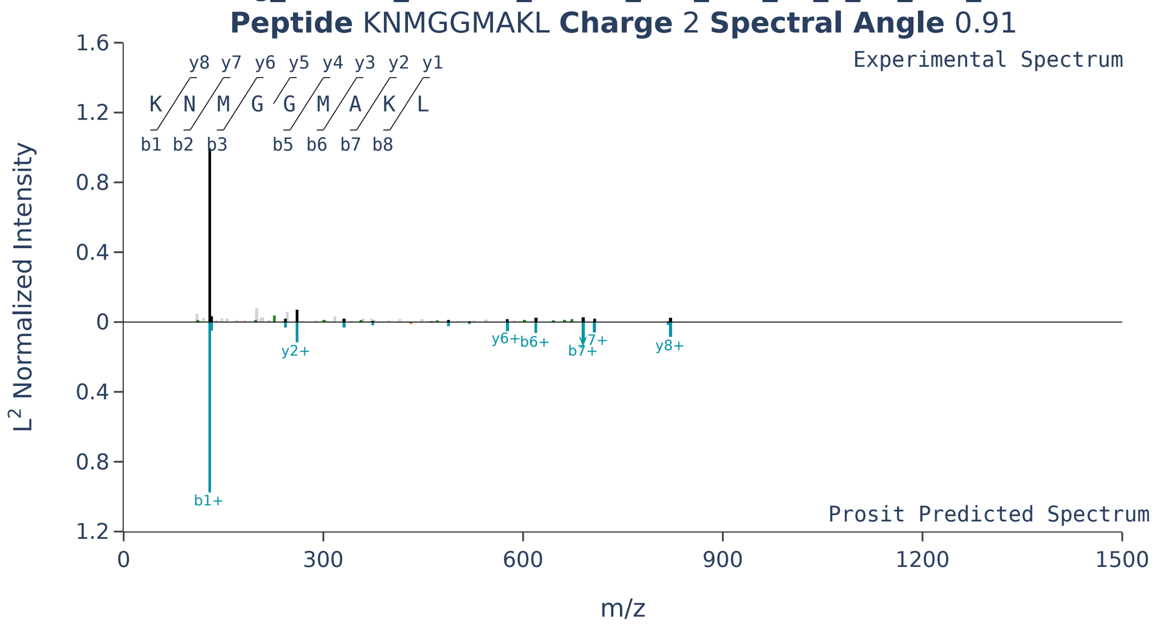

Source Ncheng_210623_230623_HFGoe_FFH_20S_25_1_A1_4h_R1 **Scan** 20842
Peptide SAALEPFHPDRI **Charge** 3 **Spectral Angle** 0.82

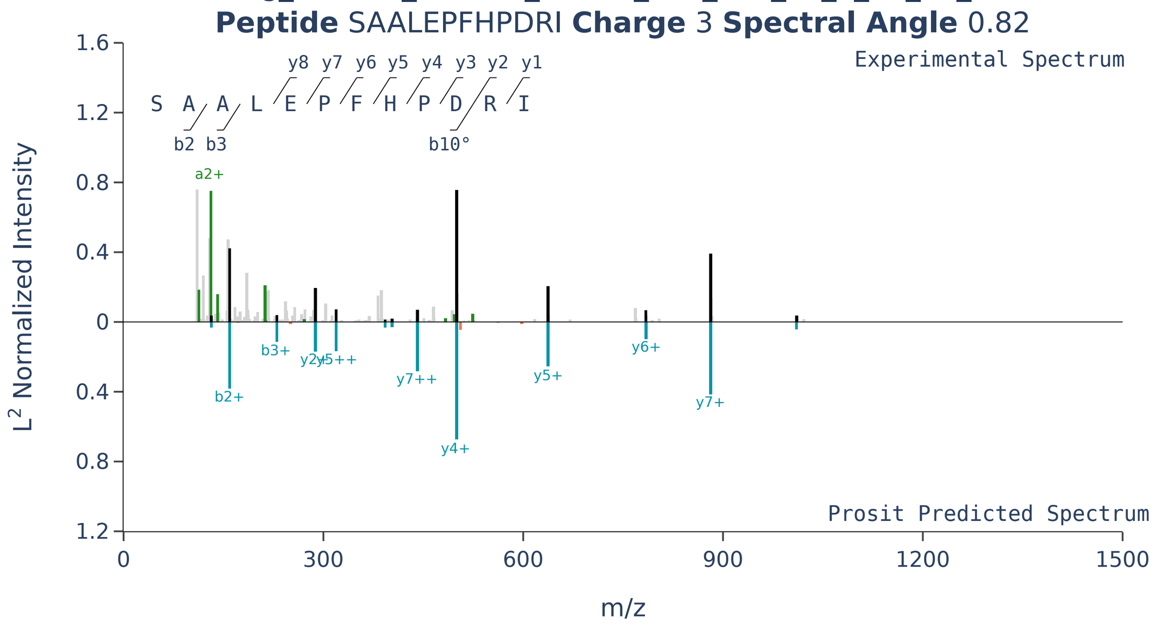

Source Ncheng_210623_230623_HFGoe_FFH_20S_25_1_A1_24h_R2 **Scan** 13868
Peptide MMPPGFPGRSR **Charge** 3 **Spectral Angle** 0.88

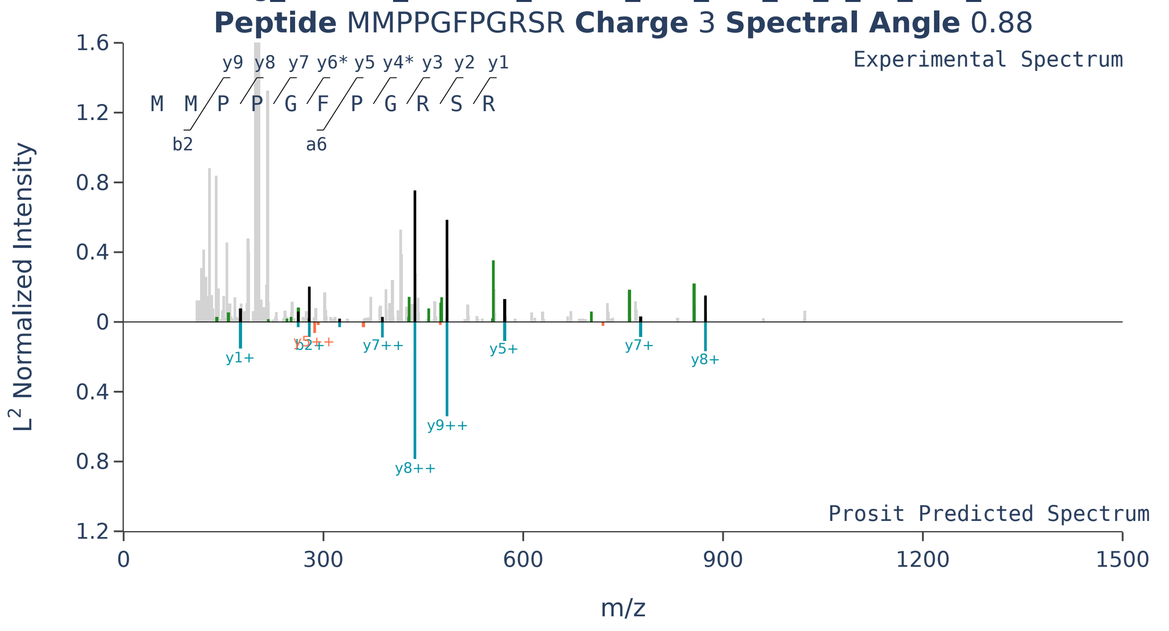

Source Ncheng_210623_230623_HFGoe_FFH_20S_25_1_A2_24h_R2 **Scan** 23222
Peptide HGVDFFPSD **Charge** 2 **Spectral Angle** 0.82

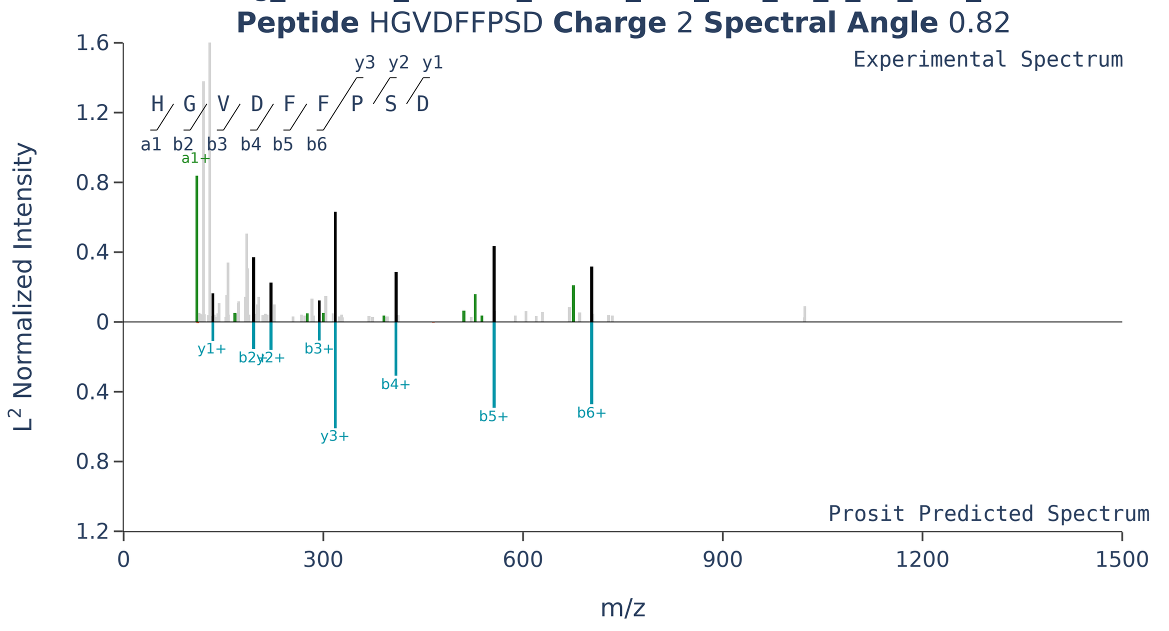

Source Ncheng_210623_230623_HFGoe_FFH_20S_25_1_A1_24h_R1 **Scan** 5629
Peptide KNMGGMAR **Charge** 2 **Spectral Angle** 0.8

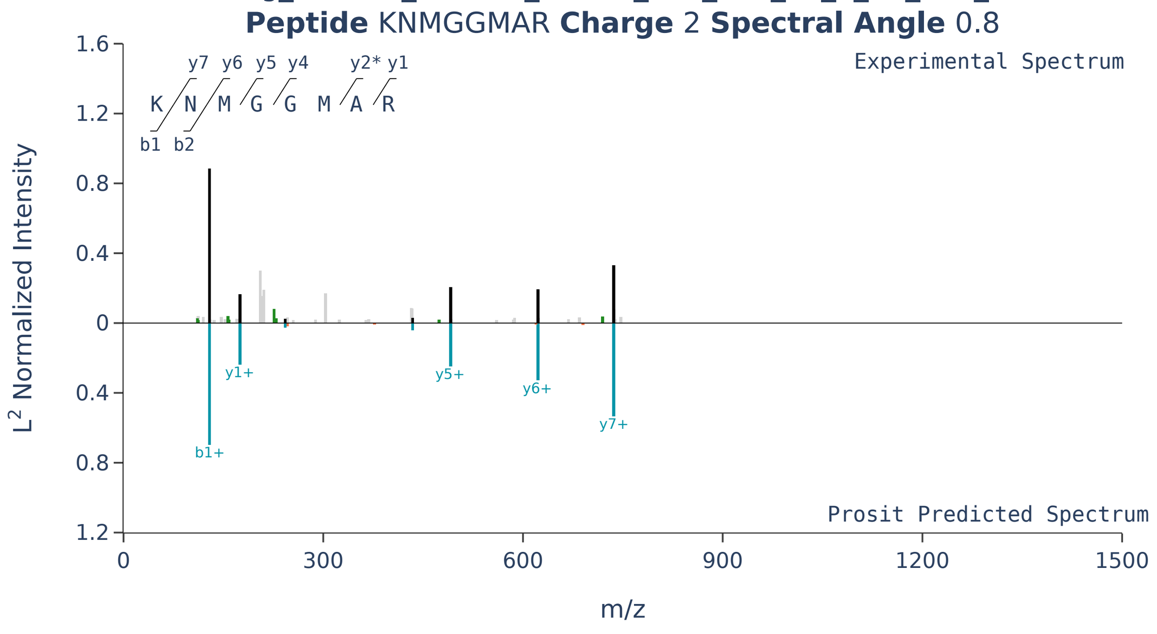

Source Ncheng_210623_230623_HFGoe_FFH_20S_25_1_A1_4h_R1 **Scan** 11948
Peptide PGMGQIPDNVKSK **Charge** 3 **Spectral Angle** 0.78

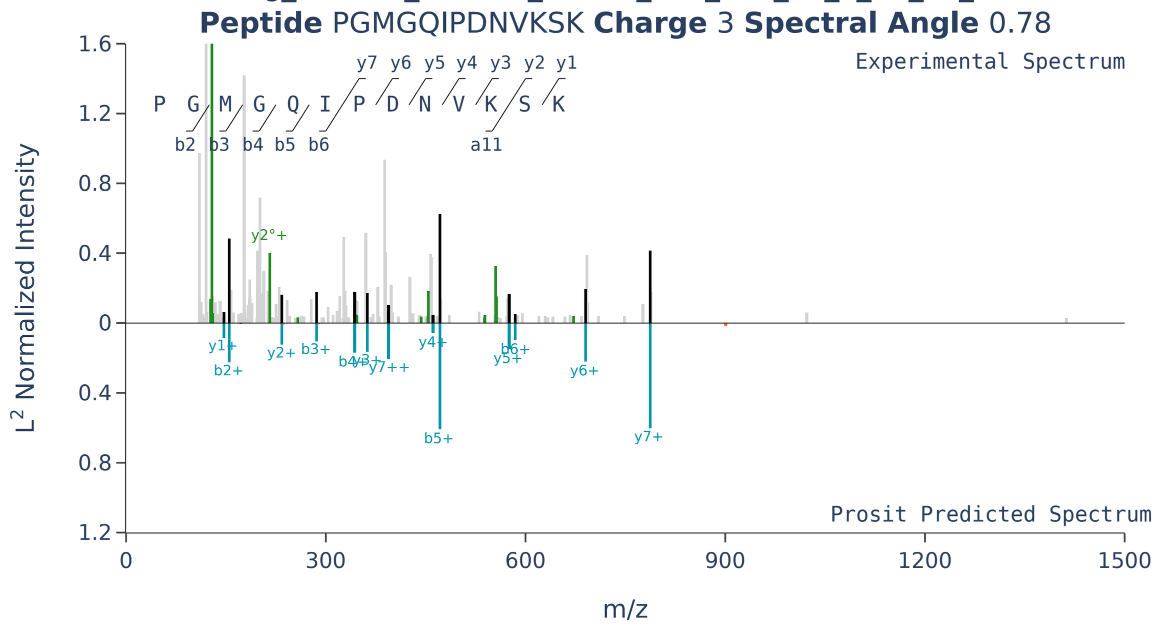

Source Ncheng_210623_230623_HFGoe_FFH_20S_25_1_A2_24h_R2 **Scan** 9932
Peptide GQIPDNVKTG **Charge** 1 **Spectral Angle** 0.85

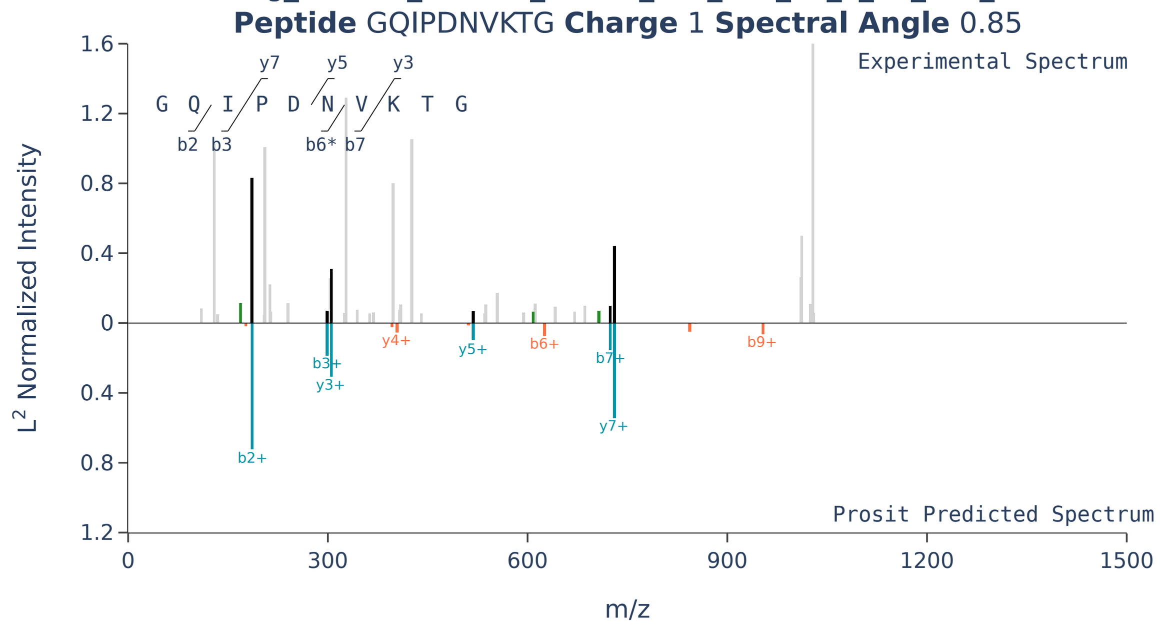

Source Ncheng_210623_230623_HFGoe_FFH_20S_25_1_A2_24h_R1 **Scan** 17491
Peptide GVGEKTEVVLTKV **Charge** 2 **Spectral Angle** 0.8

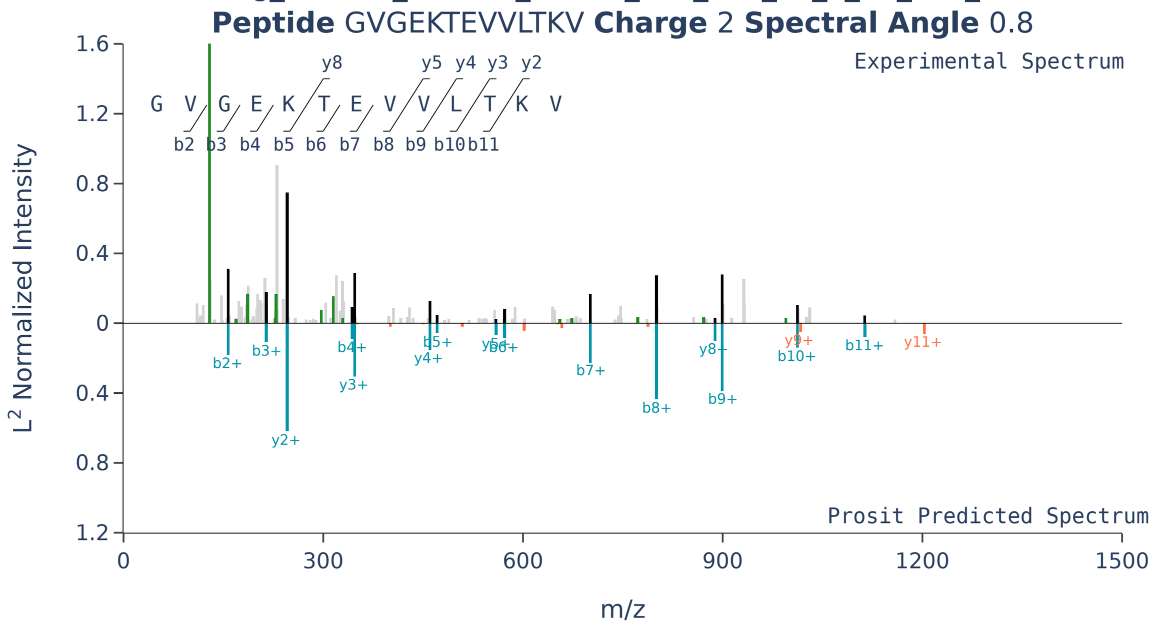

Source Ncheng_210623_230623_HFGoe_FFH_20S_25_1_A1_2h_R2 **Scan** 9432
Peptide RSTLAEQVG **Charge** 2 **Spectral Angle** 0.89

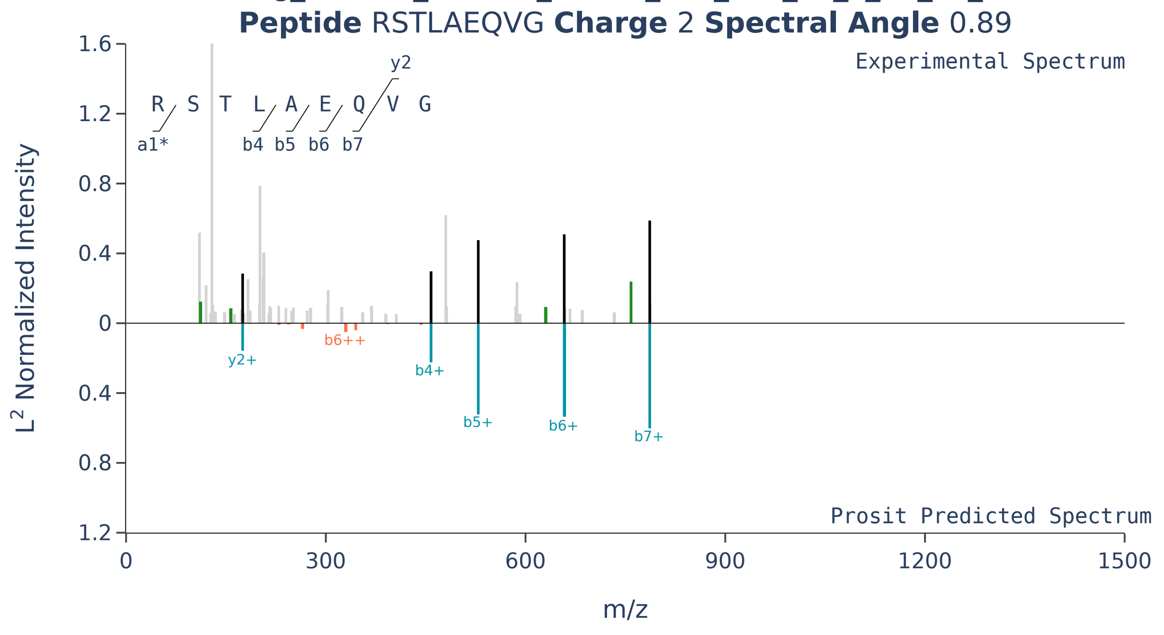

Source Ncheng_210623_230623_HFGoe_FFH_20S_25_1_A1_24h_R2 **Scan** 20869
Peptide EALPLTGA **Charge** 1 **Spectral Angle** 0.86

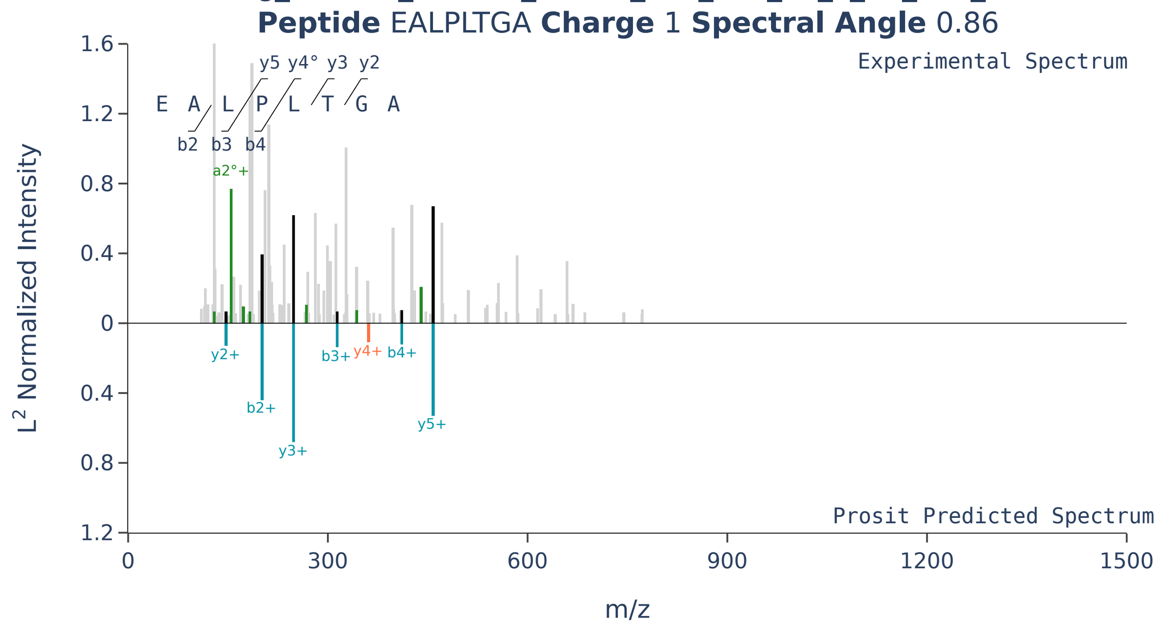

Source Ncheng_210623_230623_HFGoe_FFH_20S_25_1_A1_24h_R2 **Scan** 16277
Peptide MAMTGQDAANTAKAFN **Charge** 2 **Spectral Angle** 0.87

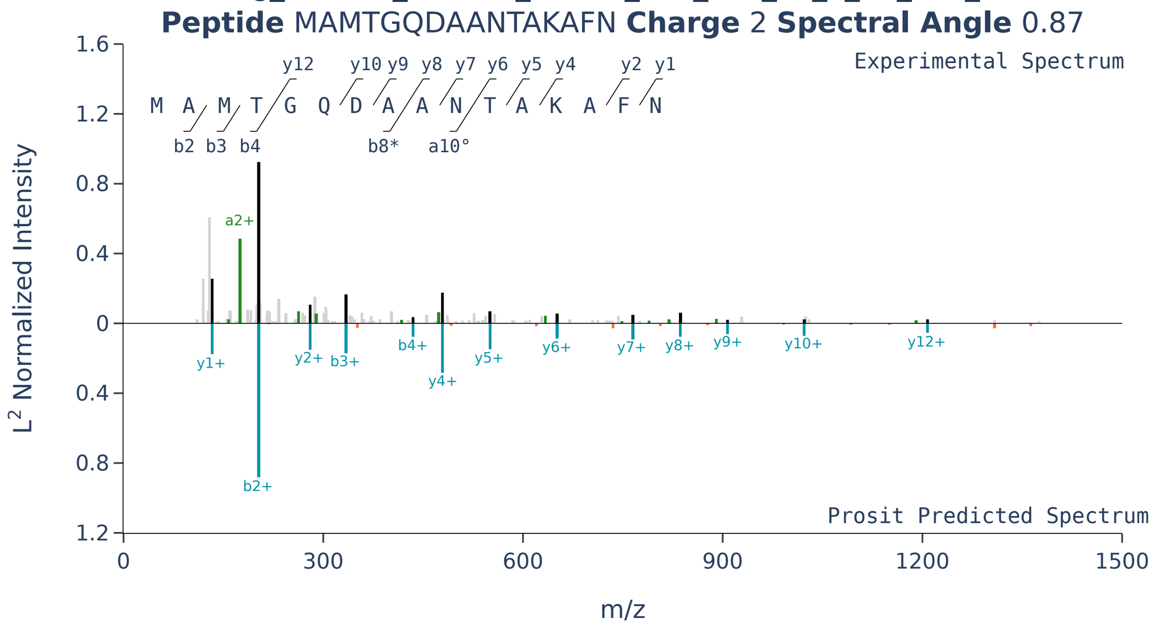

Source Ncheng_210623_230623_HFGoe_FFH_20S_25_1_A2_4h_R2 **Scan** 12086
Peptide RIAAGDIESKVD **Charge** 3 **Spectral Angle** 0.91

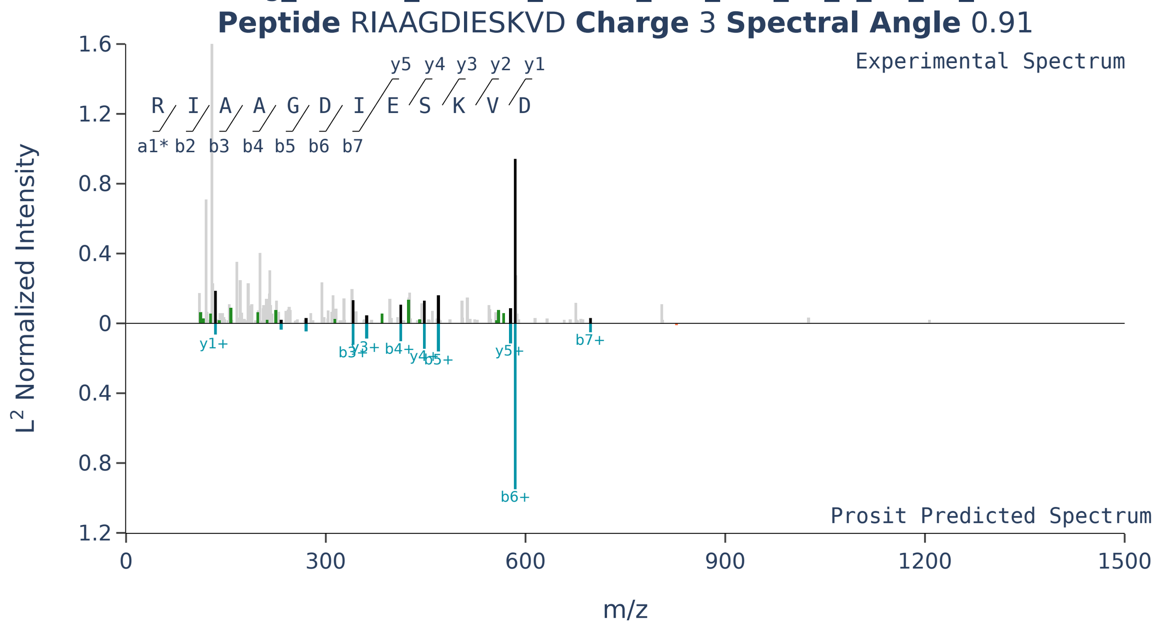

Source Ncheng_210623_230623_HFGoe_FFH_20S_25_1_A1_24h_R1 **Scan** 24046
Peptide EKATLFVVD **Charge** 2 **Spectral Angle** 0.83

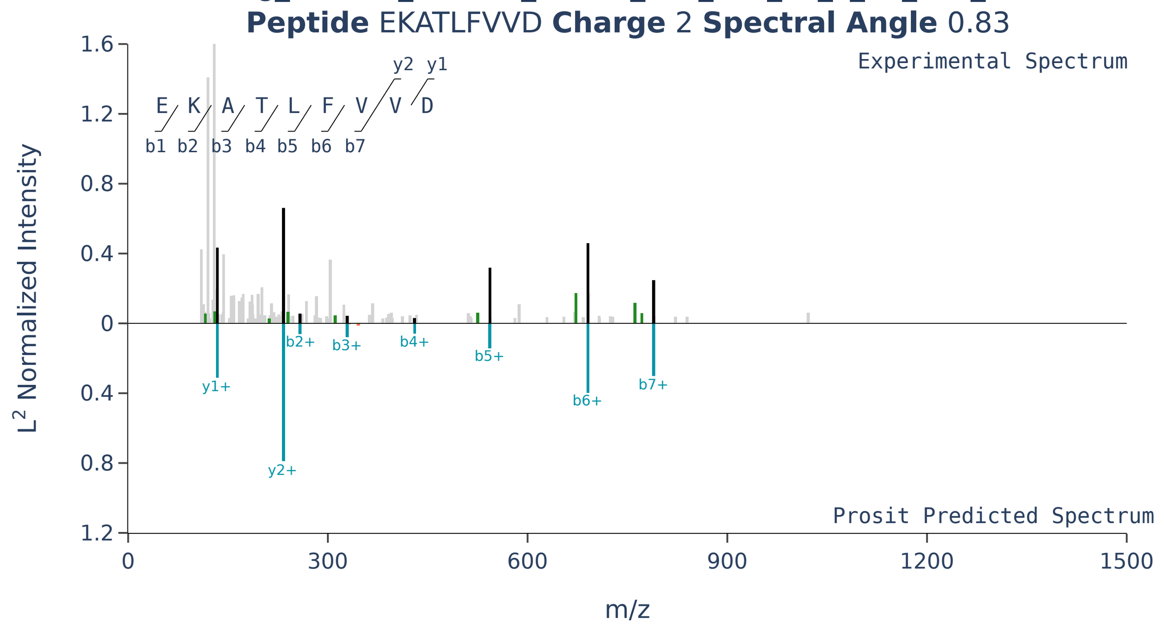

Source Ncheng_210623_230623_HFGoe_FFH_20S_25_1_A2_24h_R2 **Scan** 13952
Peptide TAMTGQDAANTAKAFN **Charge** 2 **Spectral Angle** 0.87

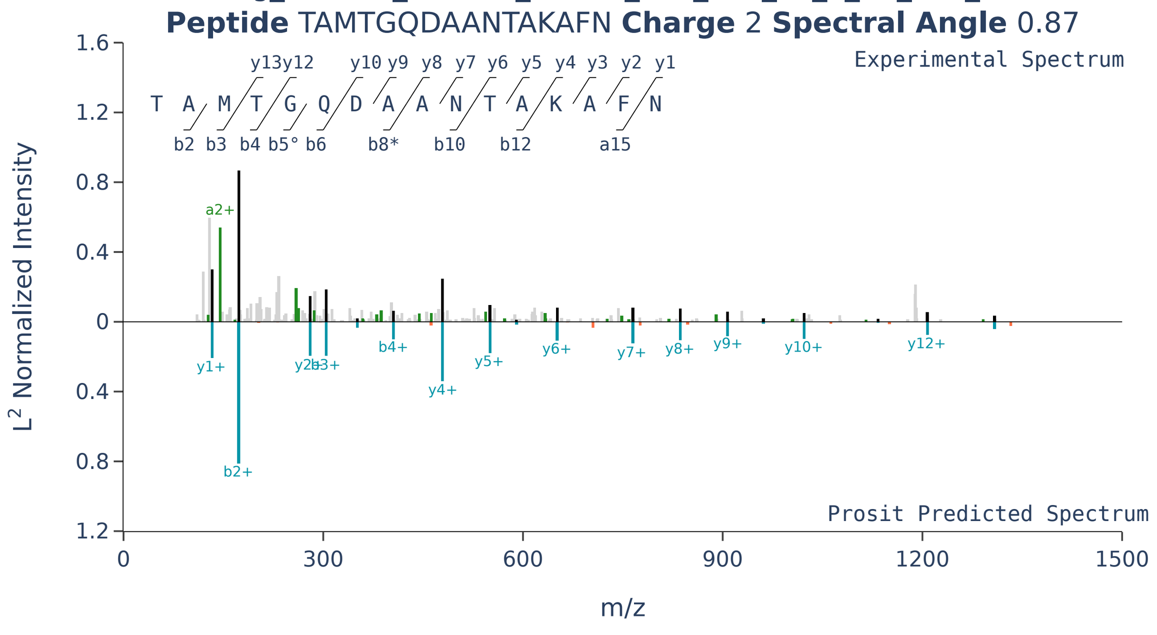

Source Ncheng_210623_230623_HFGoe_FFH_20S_25_1_A1_4h_R2 **Scan** 10141
Peptide ASRILGE **Charge** 2 **Spectral Angle** 0.91

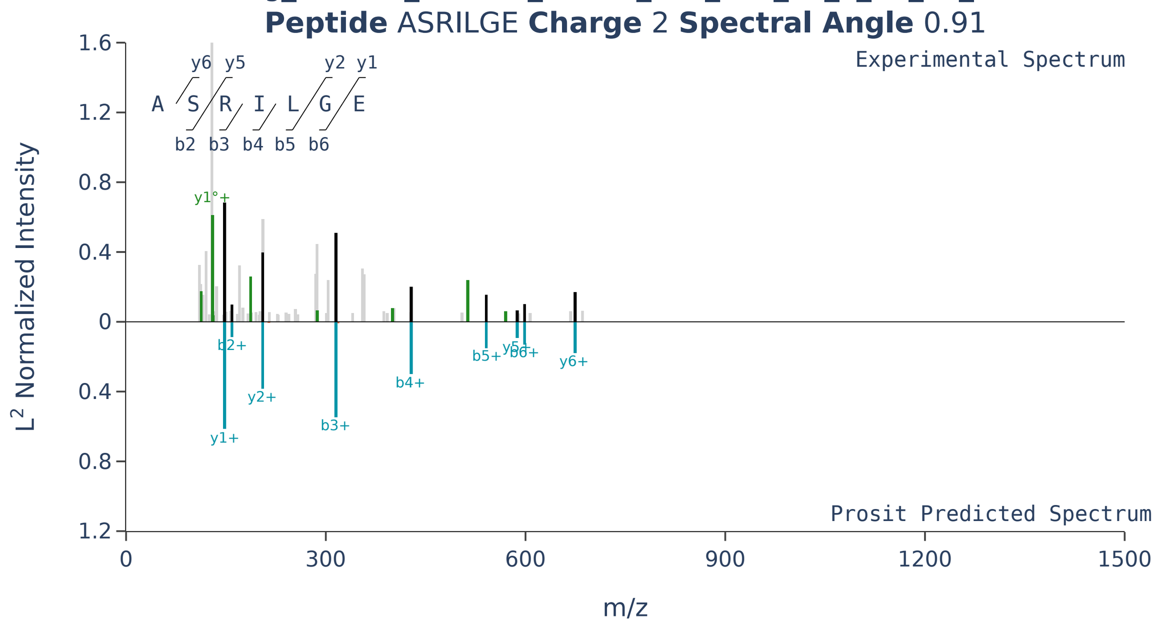

Source Ncheng_210623_230623_HFGoe_FFH_20S_25_1_A1_4h_R2 **Scan** 14111
Peptide GDAMTGQDAANTAKAFN **Charge** 2 **Spectral Angle** 0.88

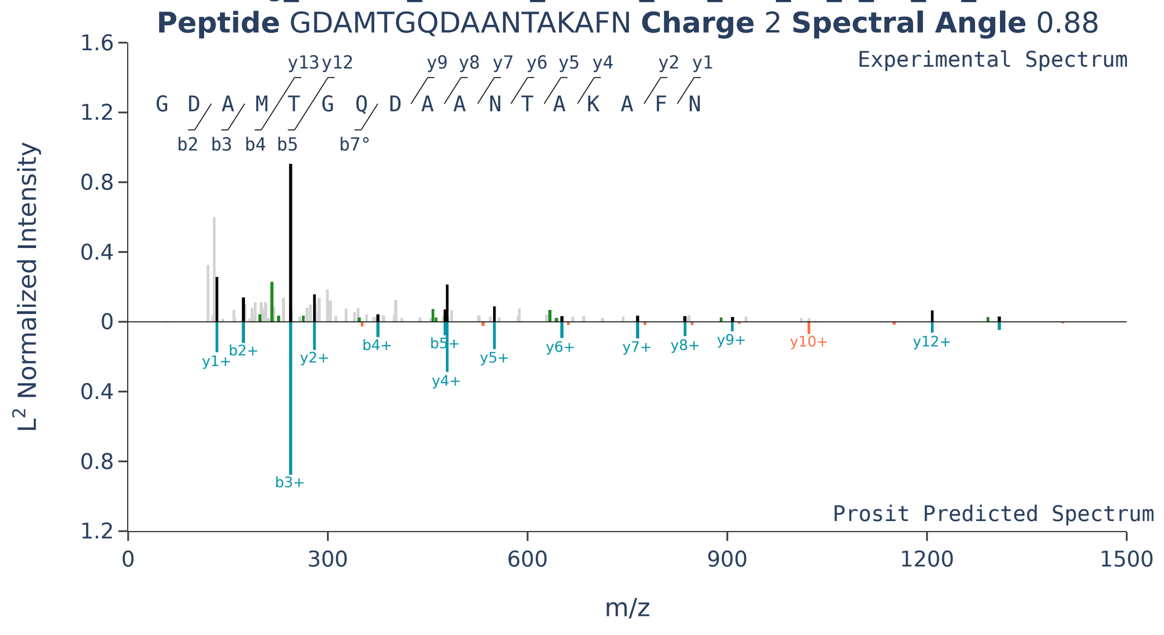

Source Ncheng_210623_230623_HFGoe_FFH_20S_25_1_A1_4h_R1 **Scan** 15645
Peptide MGKLPGMGQIPDNVKNT **Charge** 2 **Spectral Angle** 0.88

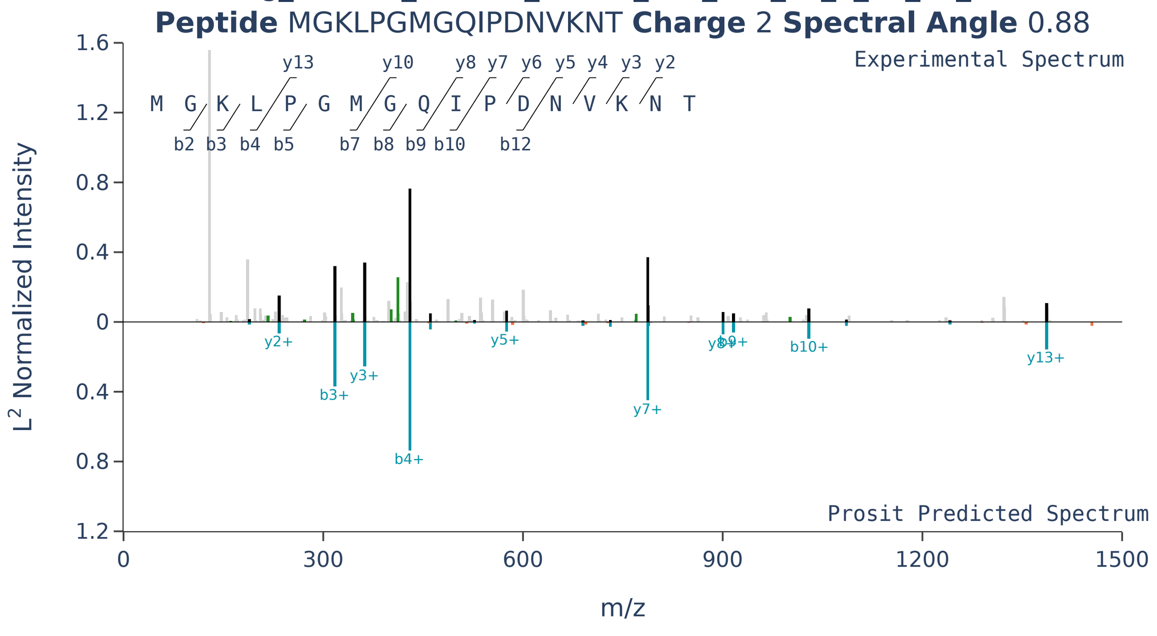

Source Ncheng_210623_230623_HFGoe_FFH_20S_25_1_A2_4h_R2 **Scan** 27662
Peptide TKVAEQVGVDFFPSD **Charge** 2 **Spectral Angle** 0.9

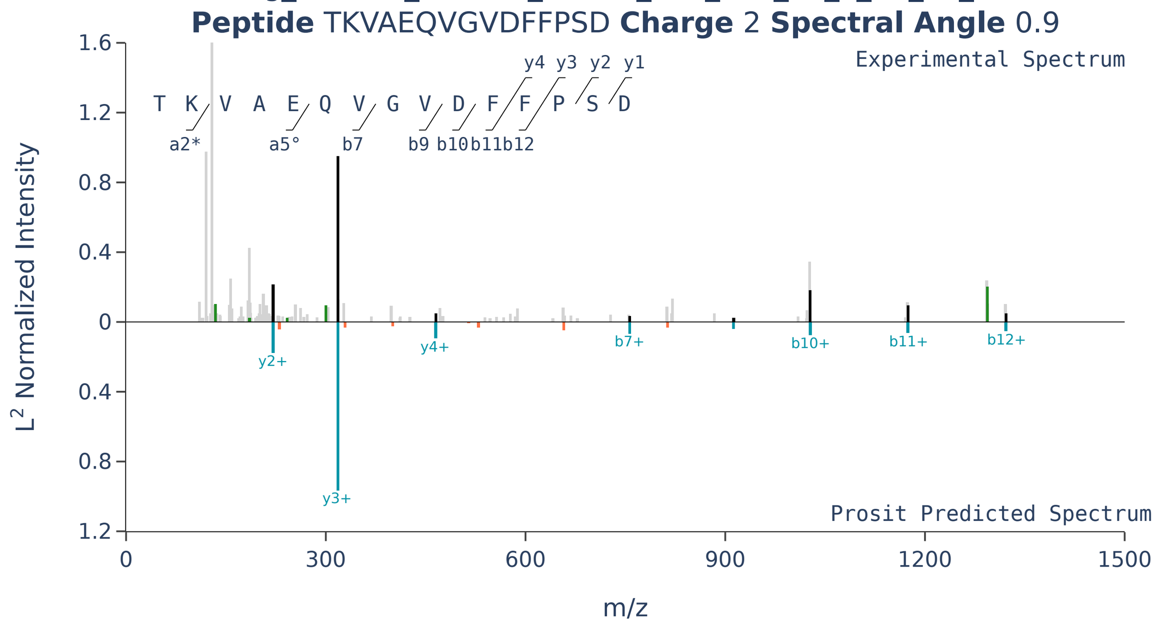

Source Ncheng_210623_230623_HFGoe_FFH_20S_25_1_A2_24h_R1 **Scan** 19701
Peptide AEQVGVGQKPVDIVNA **Charge** 2 **Spectral Angle** 0.88

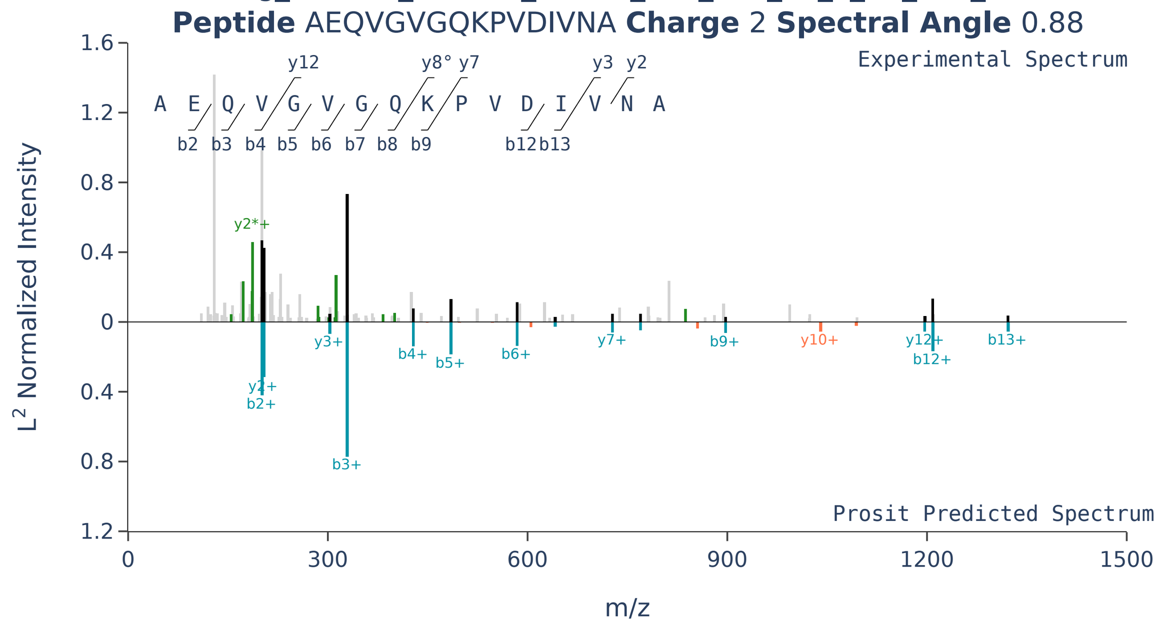

Source Ncheng_210623_230623_HFGoe_FFH_20S_25_1_A1_4h_R2 **Scan** 24925
Peptide EVFFPSD **Charge** 1 **Spectral Angle** 0.89

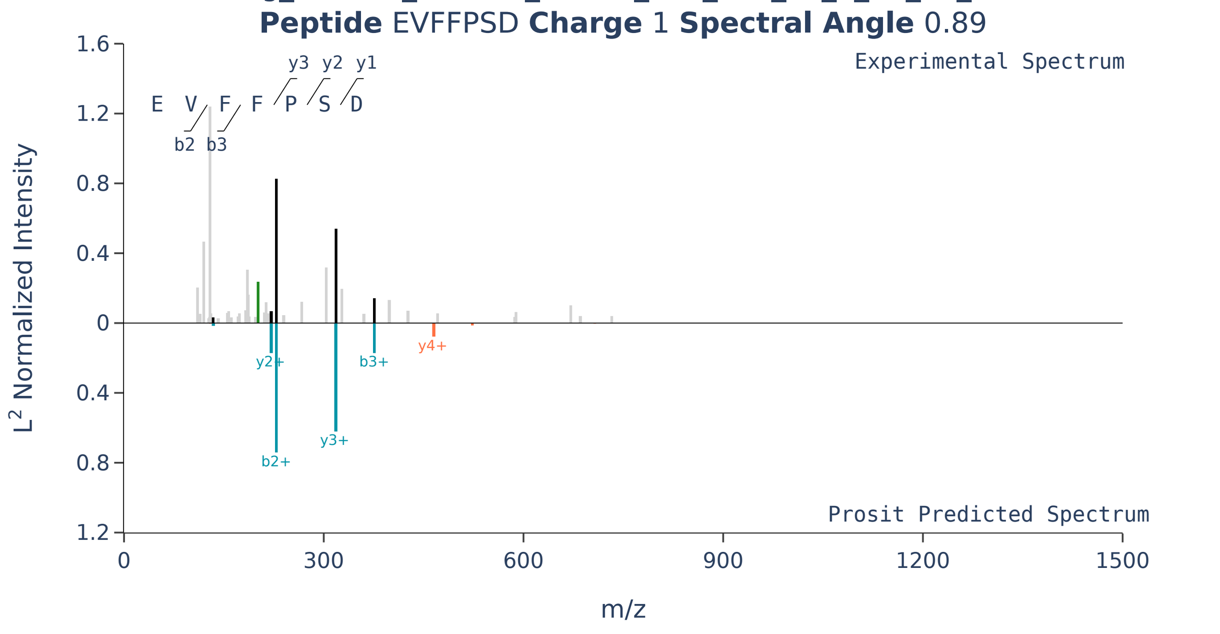

Source Ncheng_210623_230623_HFGoe_FFH_20S_25_1_A2_4h_R1 **Scan** 39025
Peptide PVETLFVVDEAL **Charge** 1 **Spectral Angle** 0.8

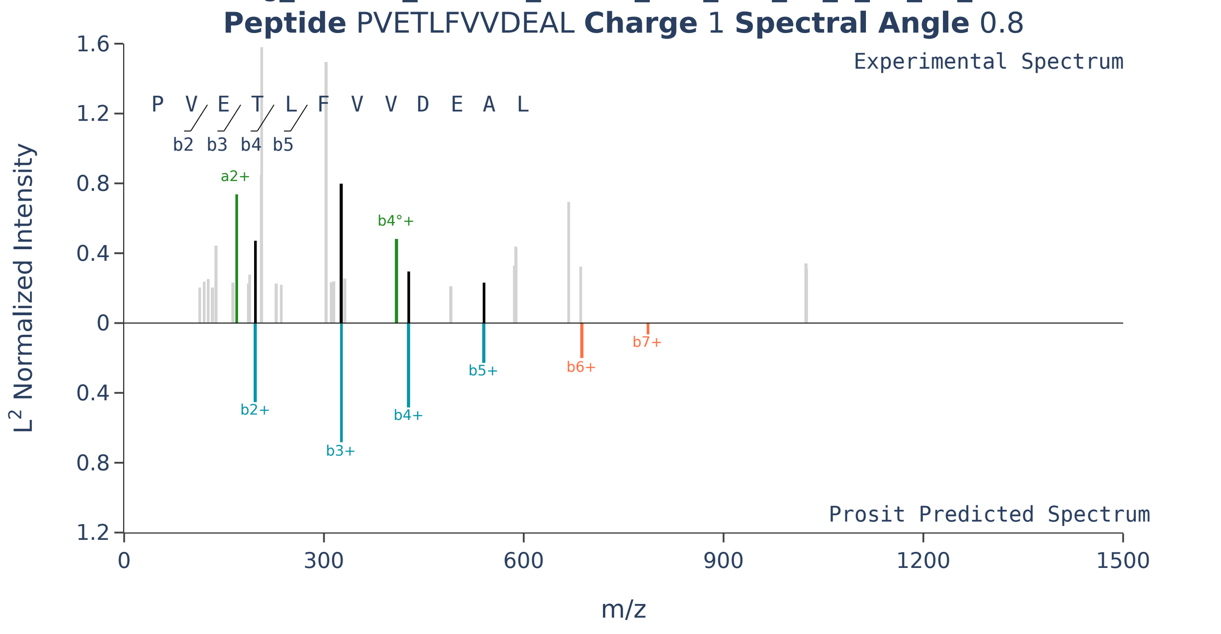

Source Ncheng_210623_230623_HFGoe_FFH_20S_25_1_A2_24h_R2 **Scan** 9509
Peptide GKTLAEQVG **Charge** 2 **Spectral Angle** 0.84

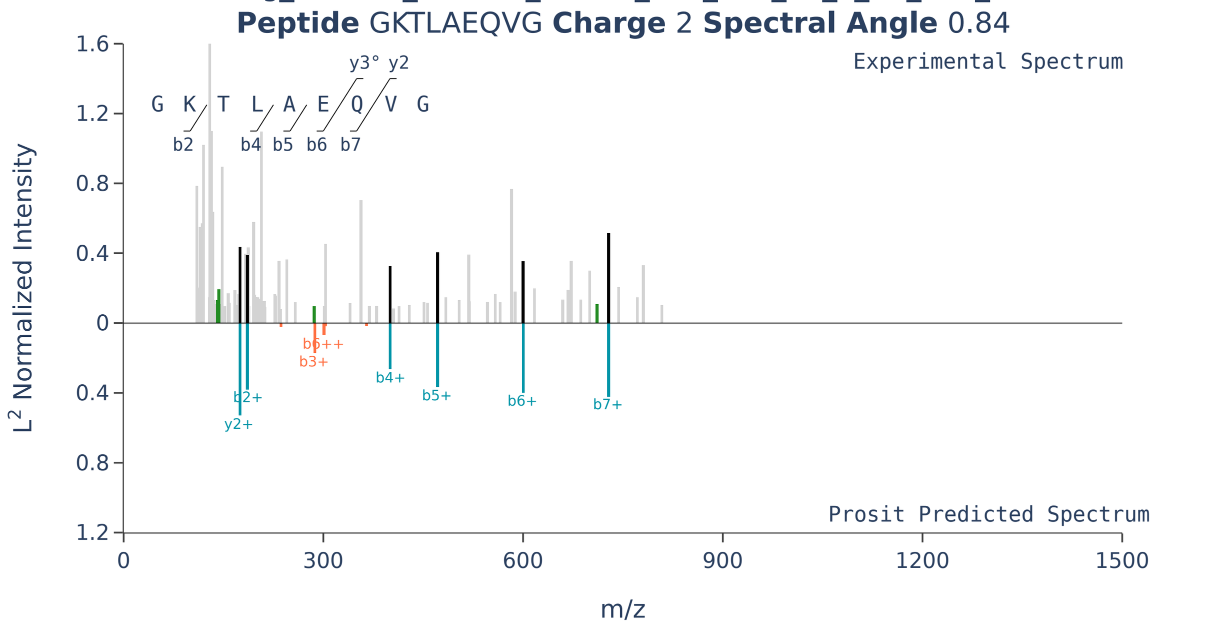

Source Ncheng_210623_230623_HFGoe_FFH_20S_25_1_A2_24h_R1 **Scan** 16828
Peptide DIESAANTAKAFN **Charge** 2 **Spectral Angle** 0.85

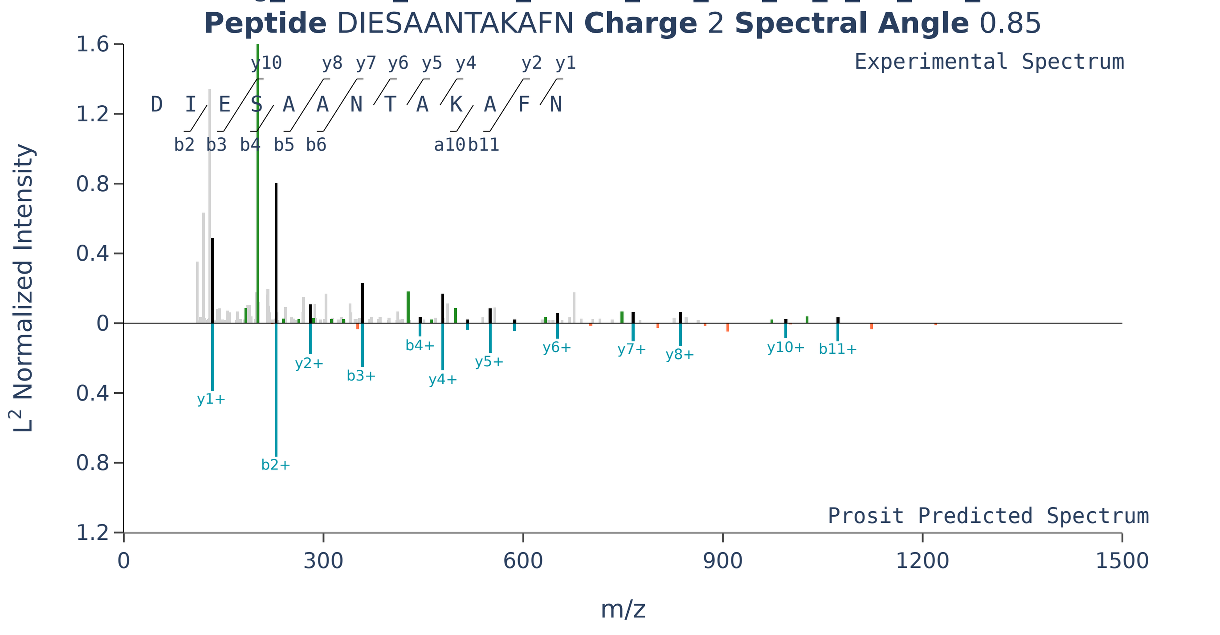

Source Ncheng_210623_230623_HFGoe_FFH_20S_25_1_A1_24h_R1 **Scan** 8466
Peptide KNMGGMAKV **Charge** 2 **Spectral Angle** 0.9

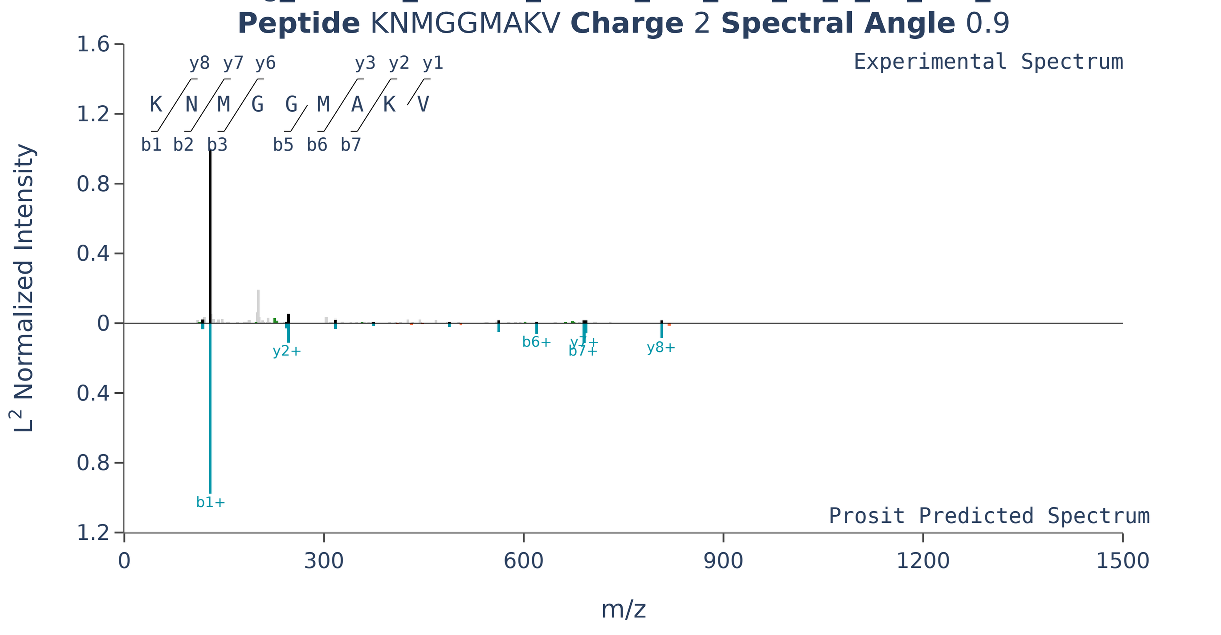

Source Ncheng_210623_230623_HFGoe_FFH_20S_25_1_A2_2h_R1 **Scan** 19960
Peptide LQEPFHPDRI **Charge** 3 **Spectral Angle** 0.9

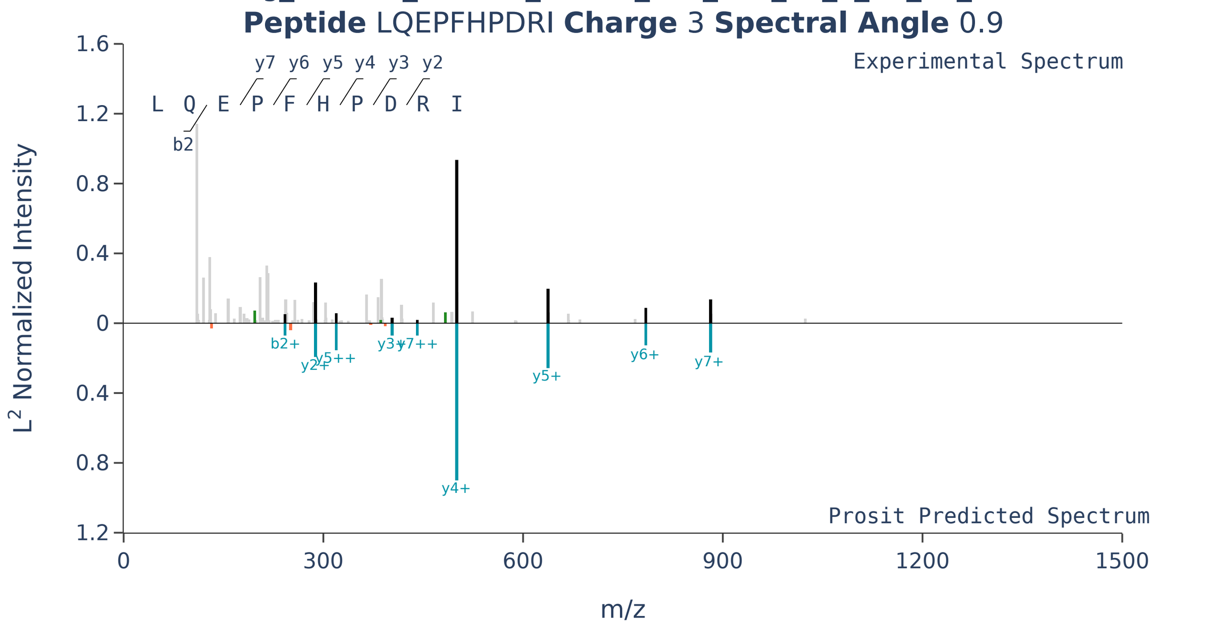

Source Ncheng_210623_230623_HFGoe_FFH_20S_25_1_A1_24h_R1 **Scan** 15387
Peptide KNMGGMADIESKVD **Charge** 2 **Spectral Angle** 0.92

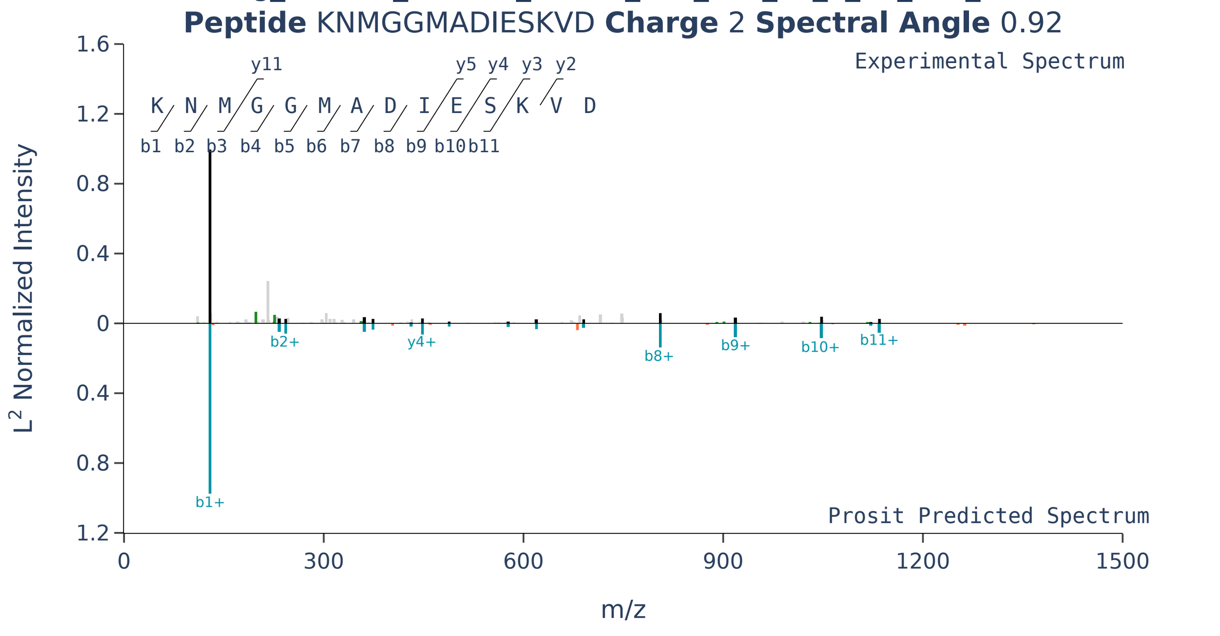

Source Ncheng_210623_230623_HFGoe_FFH_20S_25_1_A1_4h_R1 **Scan** 9545
Peptide TLAEQVGKTE **Charge** 2 **Spectral Angle** 0.83

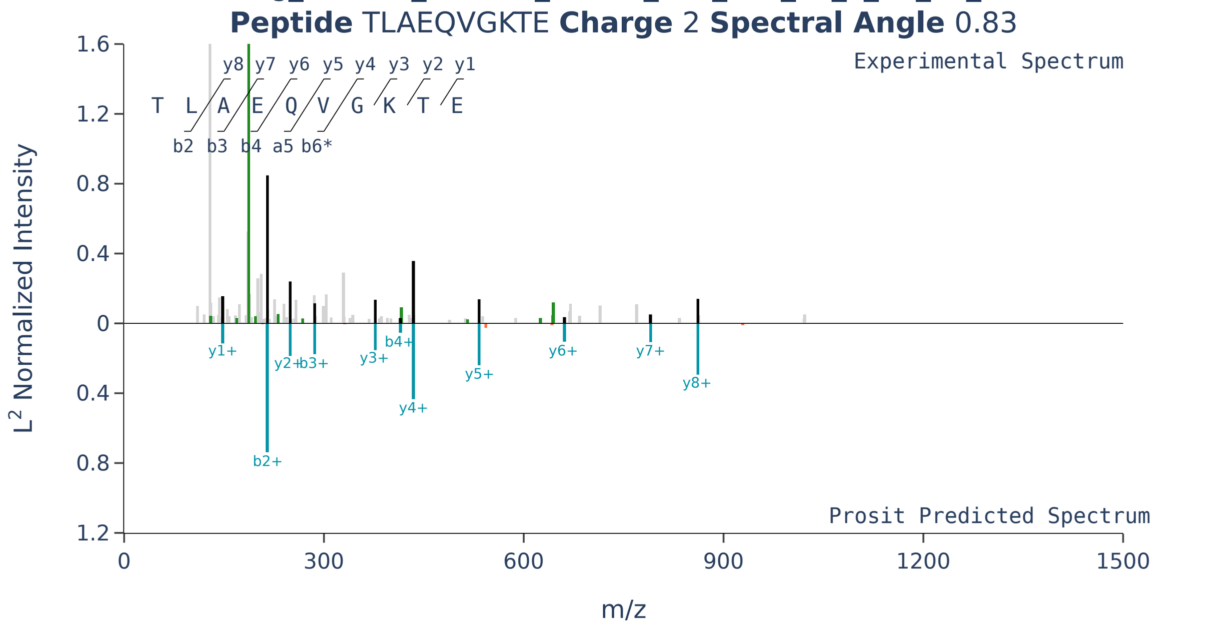

Source Ncheng_210623_230623_HFGoe_FFH_20S_25_1_A1_4h_R2 **Scan** 22309
Peptide GKGVDFFPSD **Charge** 2 **Spectral Angle** 0.81

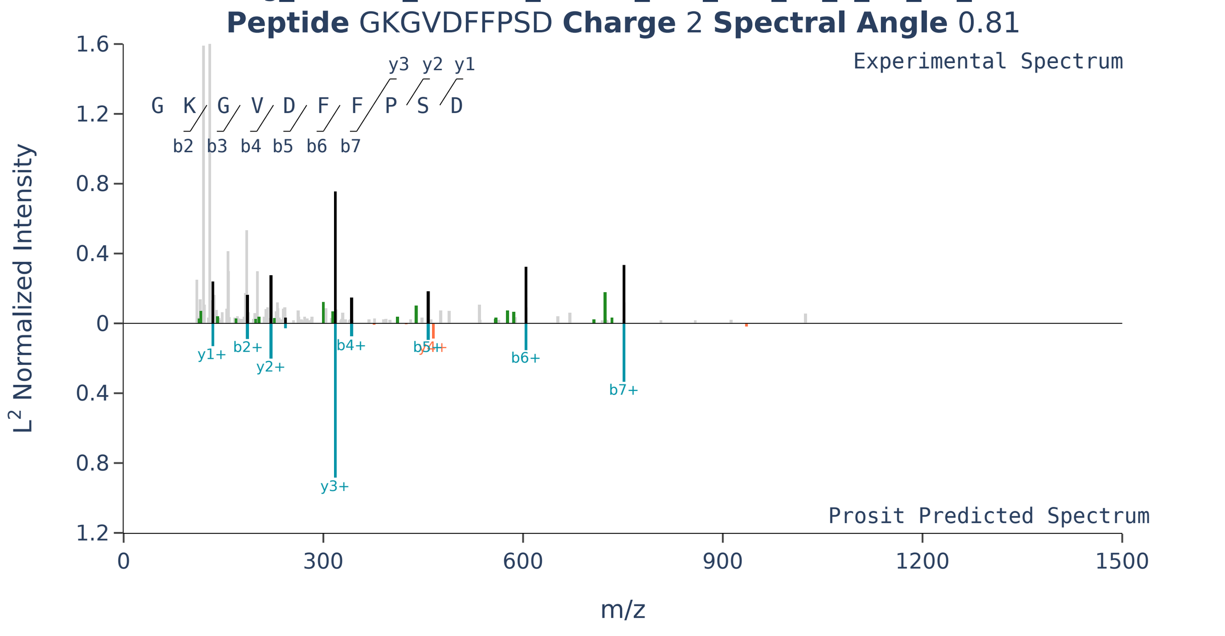

Source Ncheng_210623_230623_HFGoe_FFH_20S_25_1_A2_24h_R2 **Scan** 9486
Peptide RQMKNMGGMARM **Charge** 3 **Spectral Angle** 0.85

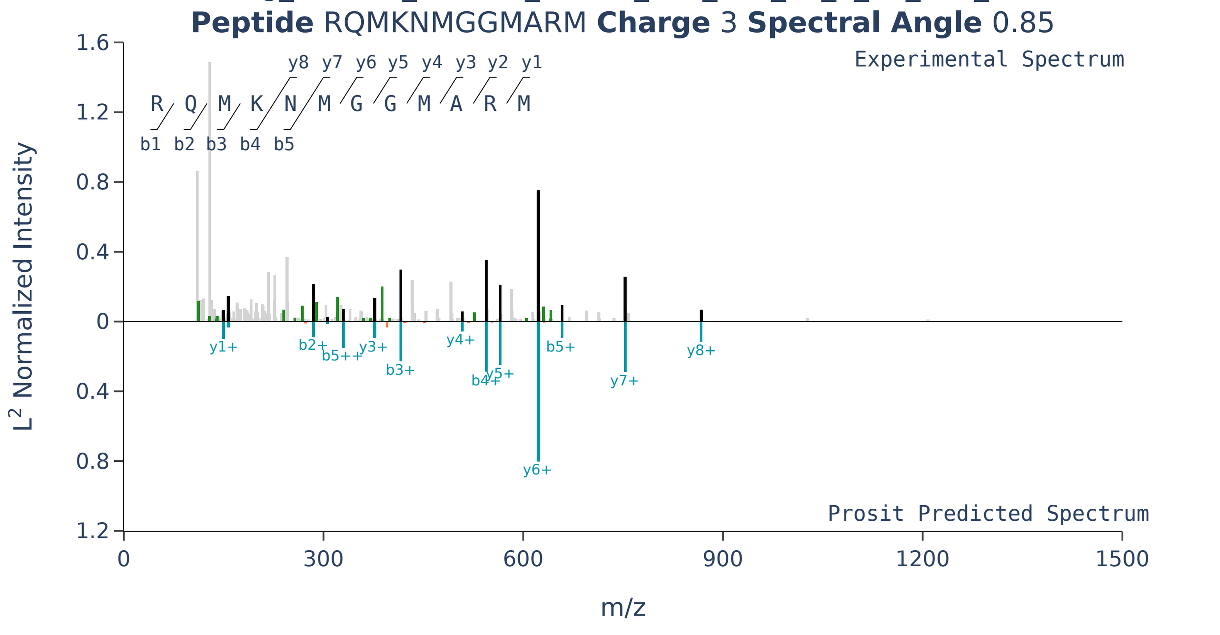

Source Ncheng_210623_230623_HFGoe_FFH_20S_25_1_A2_24h_R2 **Scan** 23499
Peptide PGQEFVSLT **Charge** 1 **Spectral Angle** 0.88

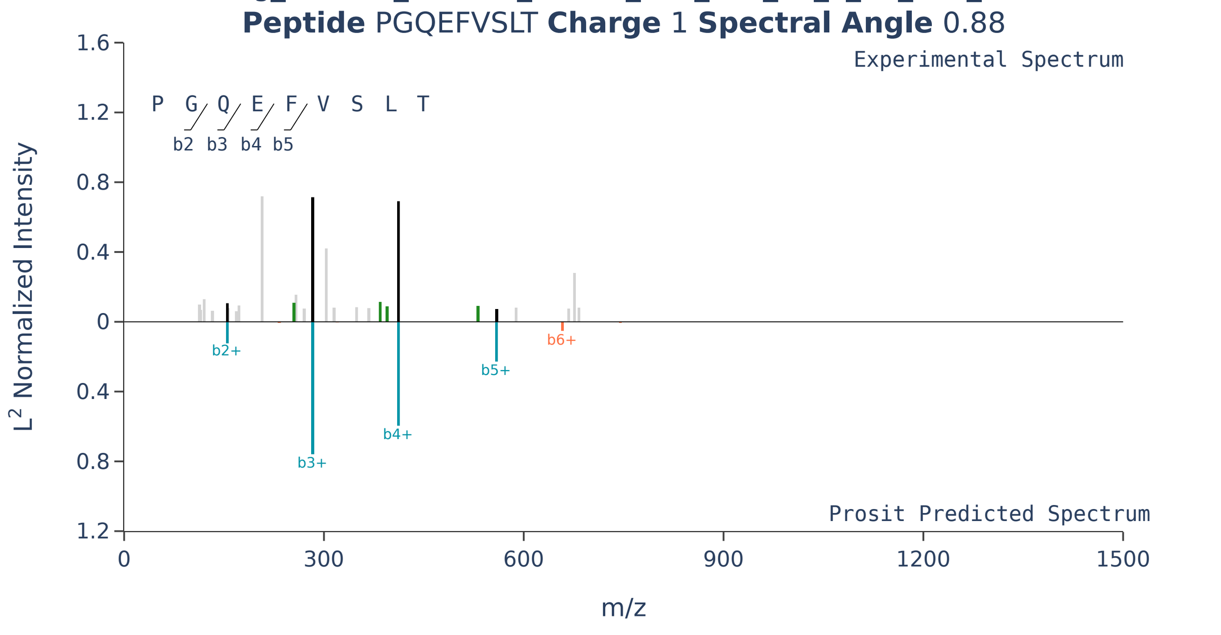

Source Ncheng_210623_230623_HFGoe_FFH_20S_25_1_A1_4h_R2 **Scan** 32197
Peptide DVGLSLIE **Charge** 2 **Spectral Angle** 0.88

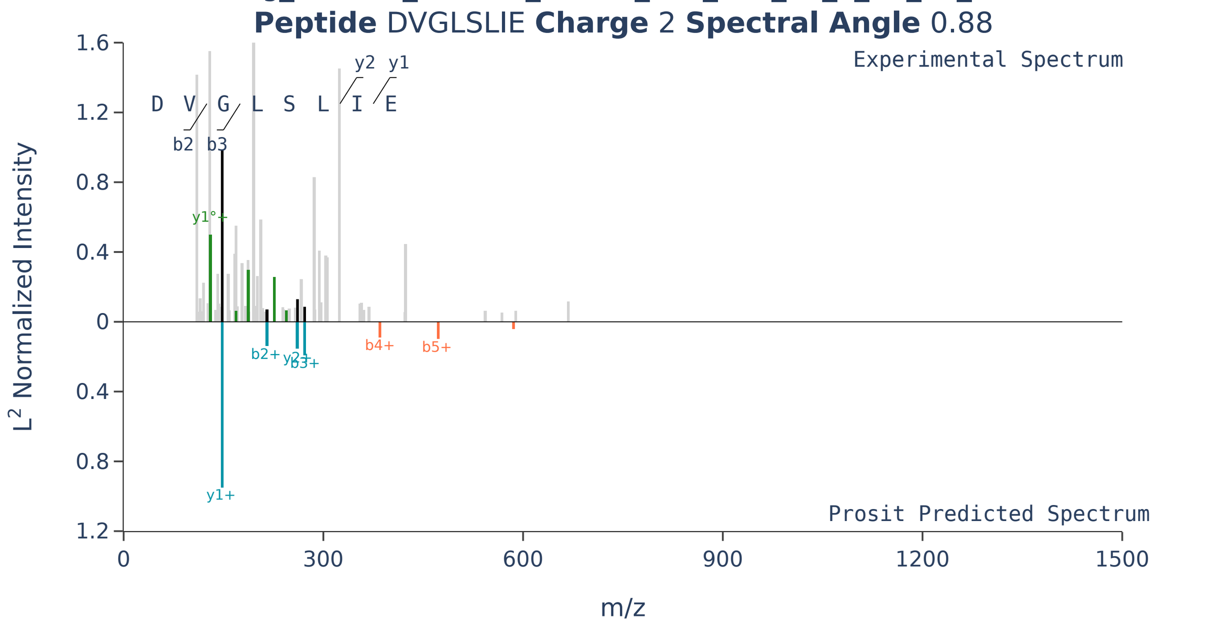

Source Ncheng_210623_230623_HFGoe_FFH_20S_25_1_A1_24h_R1 **Scan** 7039
Peptide KNSLTPGQE **Charge** 1 **Spectral Angle** 0.84

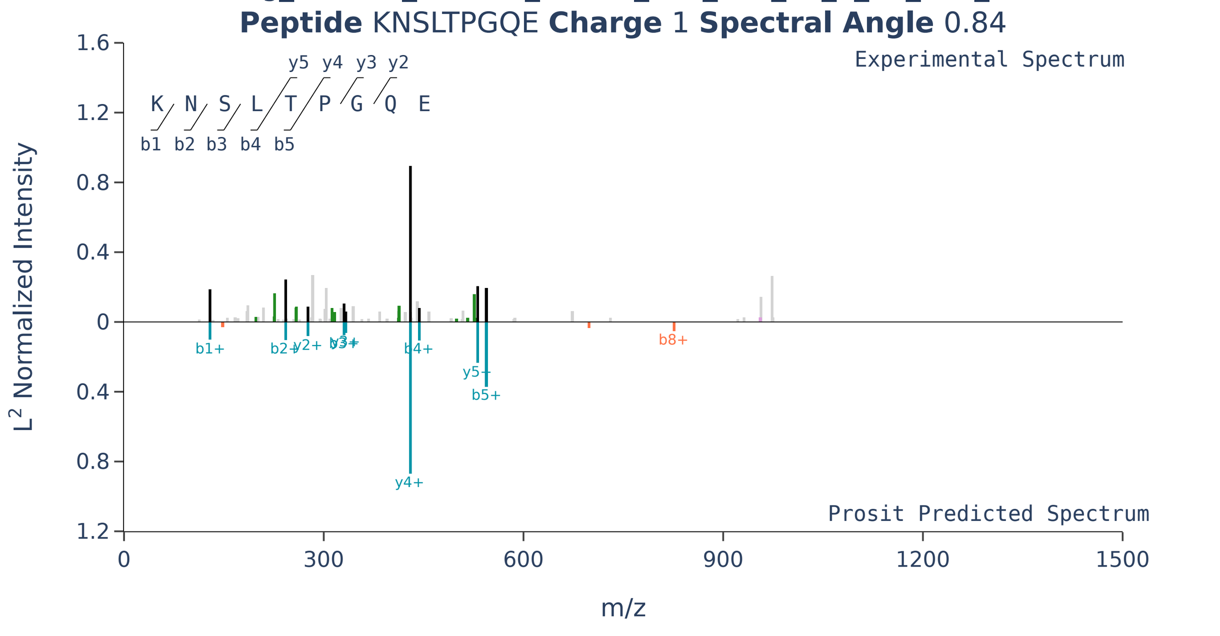

Source Ncheng_210623_230623_HFGoe_FFH_20S_25_1_A1_24h_R2 **Scan** 27602
Peptide GVDFFPDT **Charge** 1 **Spectral Angle** 0.91

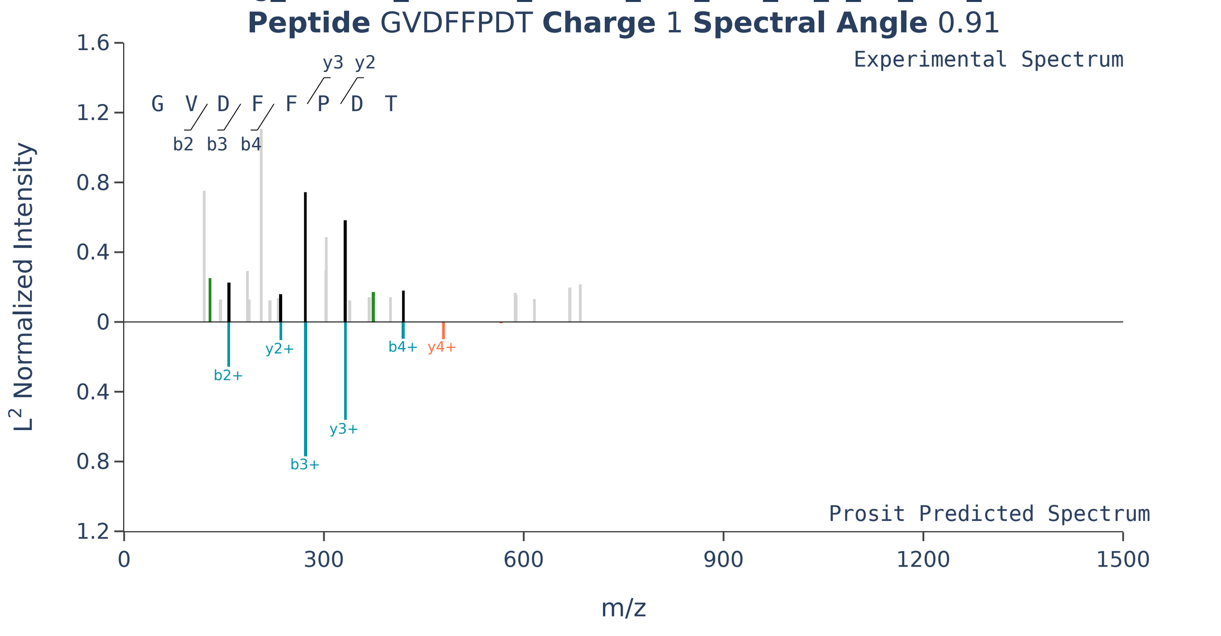

Source Ncheng_210623_230623_HFGoe_FFH_20S_25_1_A2_1h_R1 **Scan** 11614
Peptide PDNVKSQMEQVG **Charge** 1 **Spectral Angle** 0.85

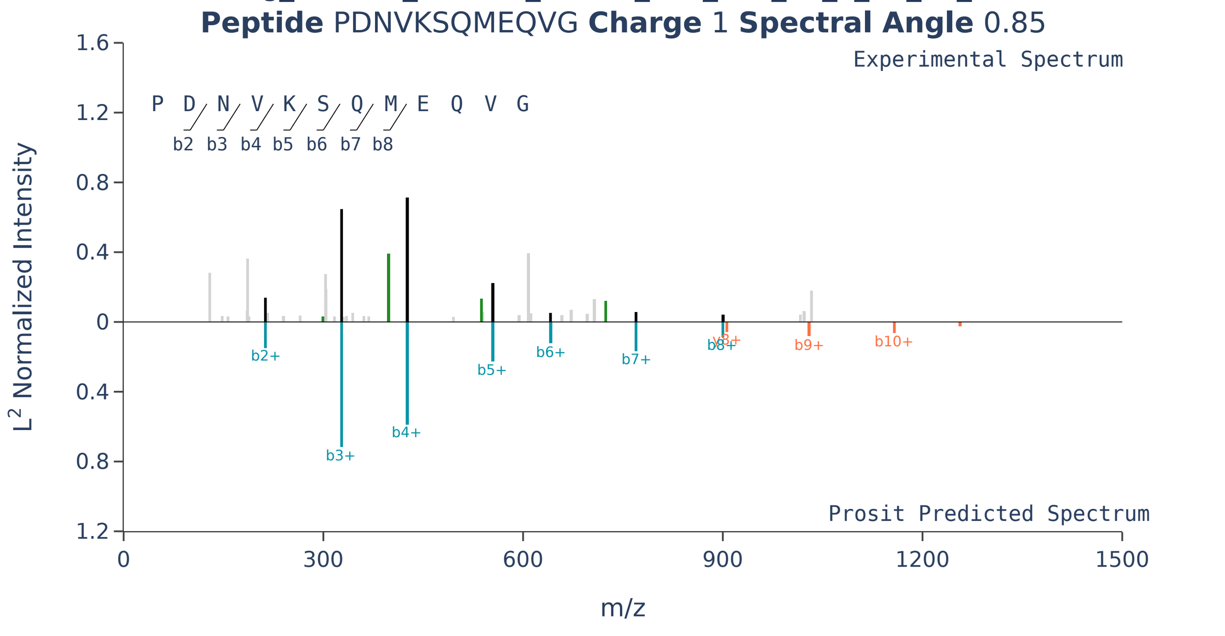

Source Ncheng_210623_230623_HFGoe_FFH_20S_25_1_A2_4h_R1 **Scan** 15871
Peptide ALKEAAMTGQDAANTAKAFN **Charge** 3 **Spectral Angle** 0.8

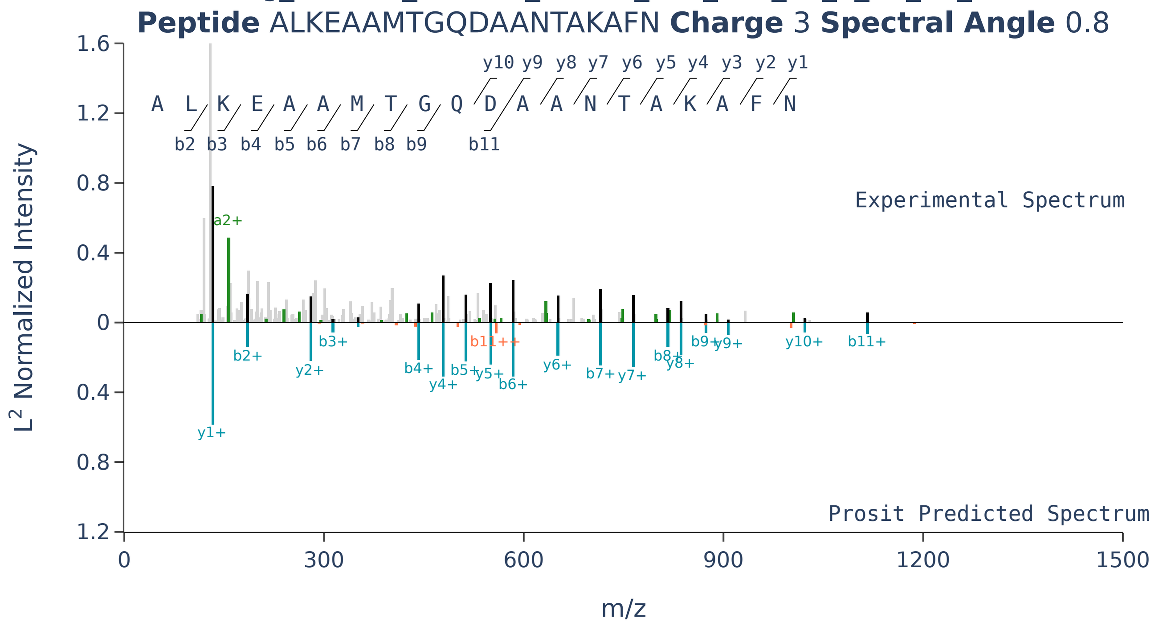

Source Ncheng_210623_230623_HFGoe_FFH_20S_25_1_A1_4h_R1 **Scan** 10314
Peptide EDNGMQVQDVNR **Charge** 2 **Spectral Angle** 0.79

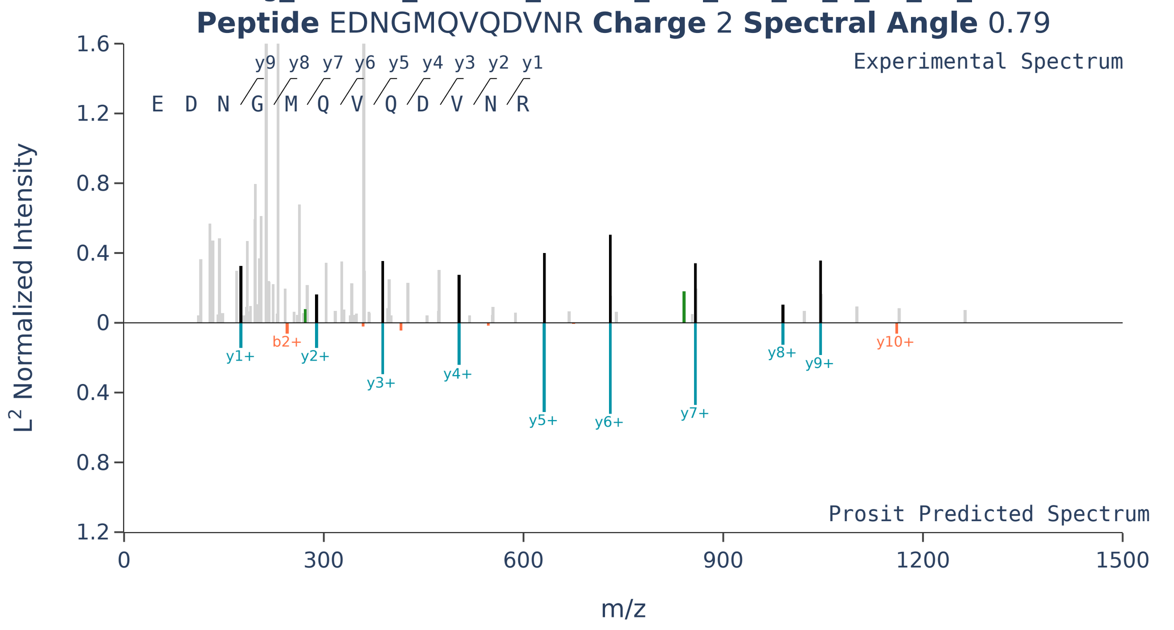

Source Ncheng_210623_230623_HFGoe_FFH_20S_25_1_A1_24h_R1 **Scan** 8925
Peptide KNMGGMARM **Charge** 2 **Spectral Angle** 0.74

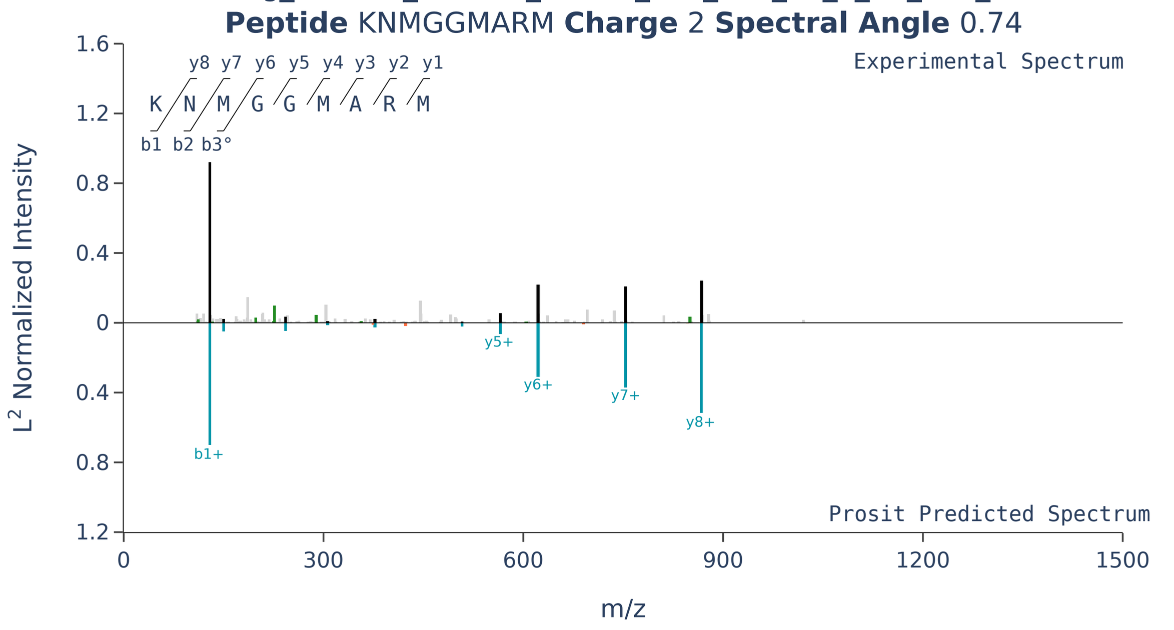

Source Ncheng_210623_230623_HFGoe_FFH_20S_25_1_A2_24h_R2 **Scan** 18129
Peptide SRTALEPFHPDRI **Charge** 4 **Spectral Angle** 0.85

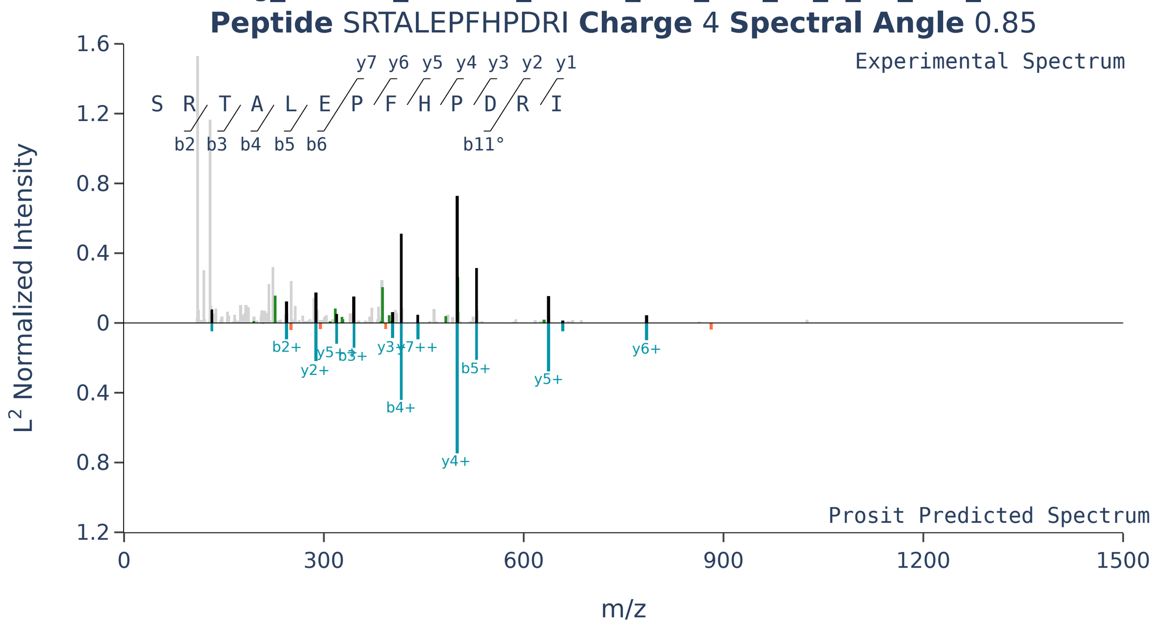

Source Ncheng_210623_230623_HFGoe_FFH_20S_25_1_A1_4h_R1 **Scan** 9500
Peptide GVGEKTETLAE **Charge** 2 **Spectral Angle** 0.85

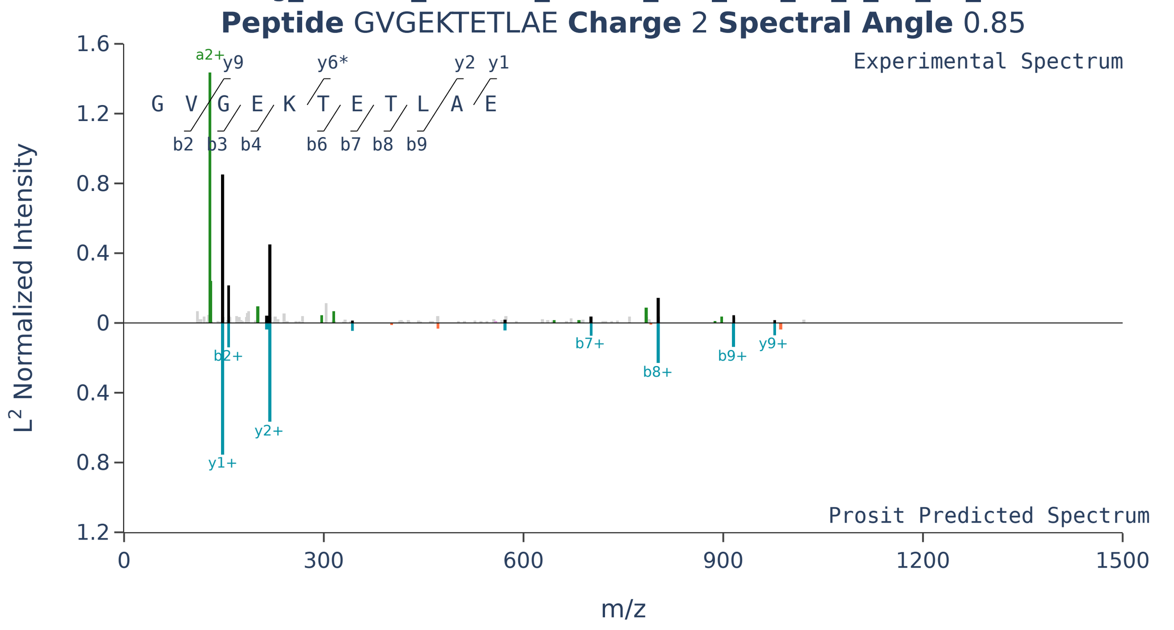

Source Ncheng_210623_230623_HFGoe_FFH_20S_25_1_A2_24h_R2 **Scan** 18269
Peptide GMAAMTGQDAANTAKAFN **Charge** 2 **Spectral Angle** 0.84

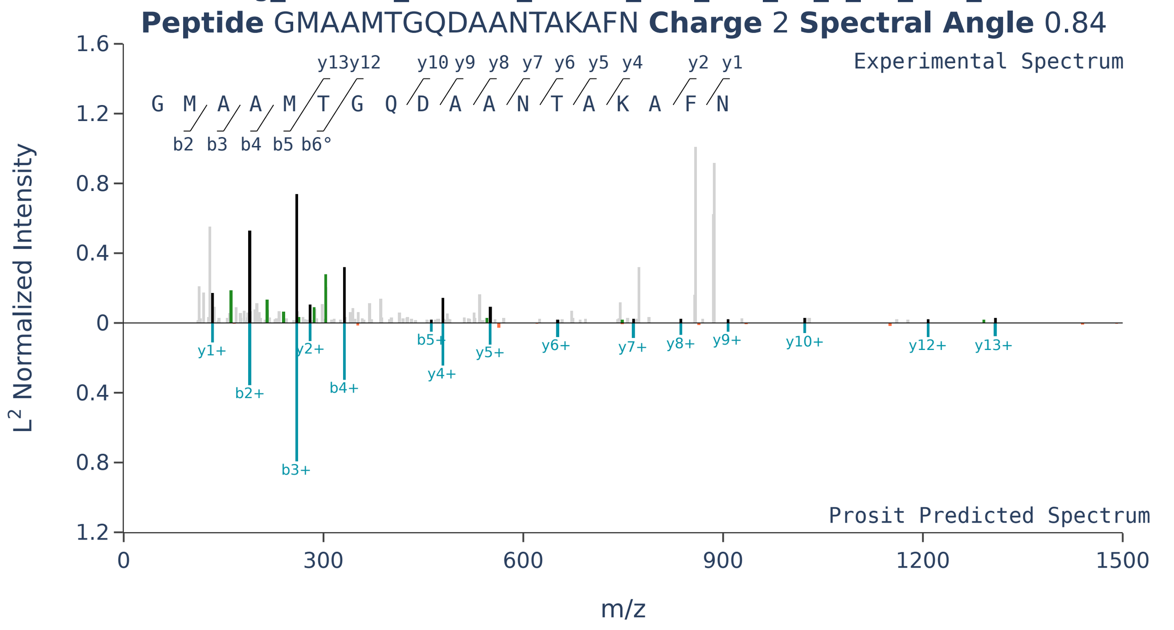

Source Ncheng_210623_230623_HFGoe_FFH_20S_25_1_A1_24h_R1 **Scan** 23875
Peptide EKTTLFVVD **Charge** 2 **Spectral Angle** 0.86

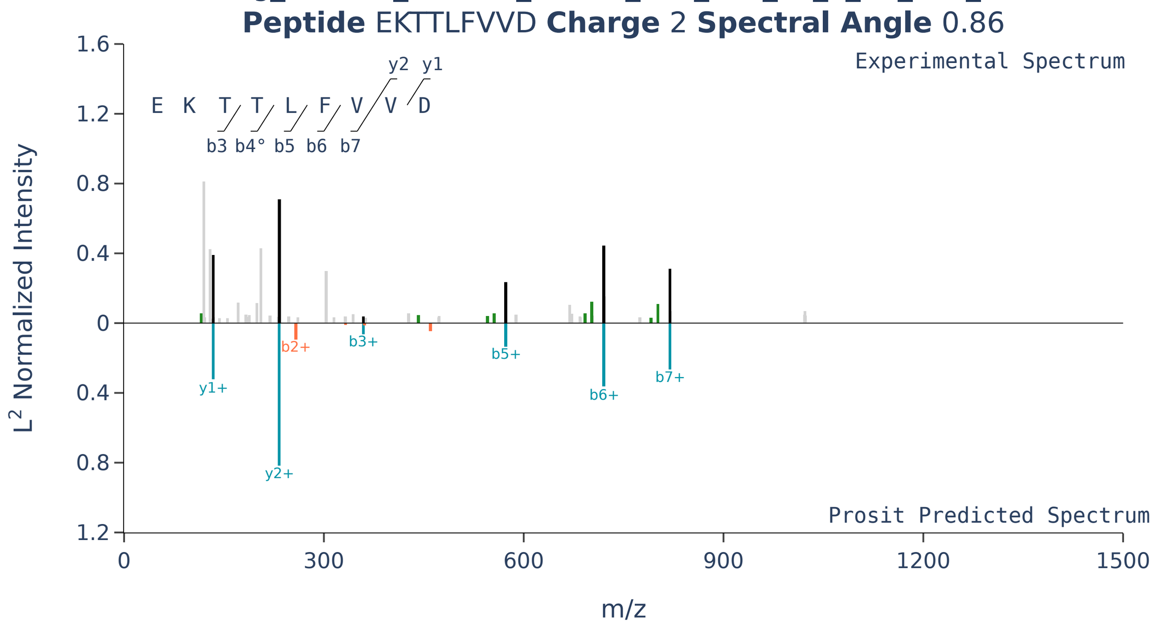

Source Ncheng_210623_230623_HFGoe_FFH_20S_25_1_A2_24h_R1 **Scan** 10510
Peptide GHASINPVE **Charge** 2 **Spectral Angle** 0.89

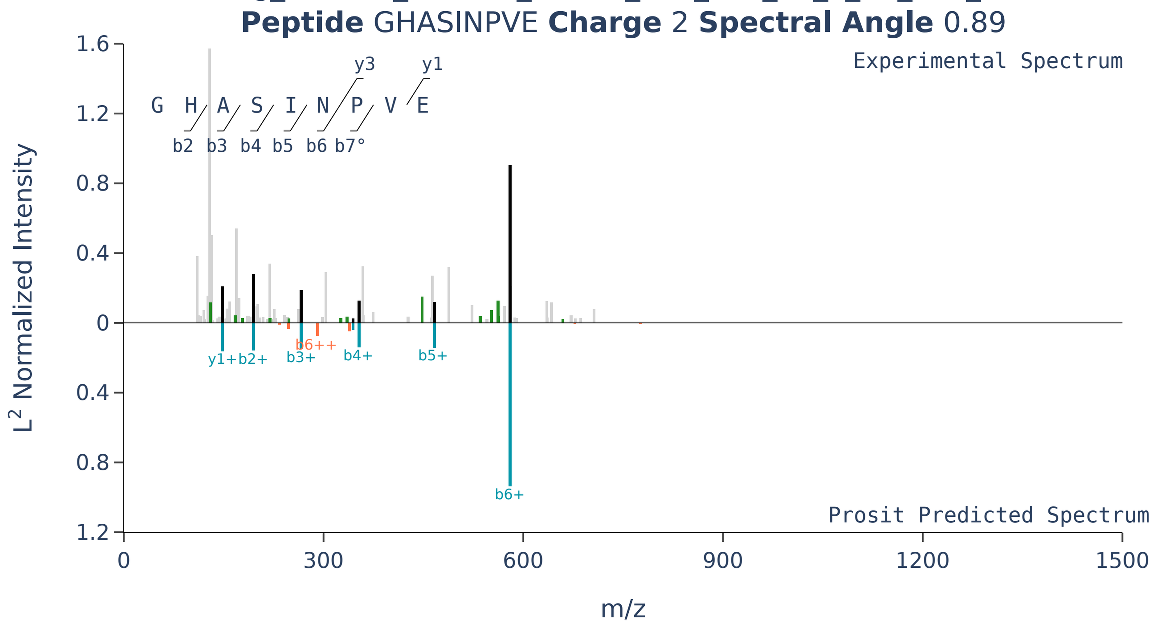

Source Ncheng_210623_230623_HFGoe_FFH_20S_25_1_A1_1h_R2 **Scan** 30908
Peptide EKTTLAEQVGVDFFPSD **Charge** 2 **Spectral Angle** 0.95

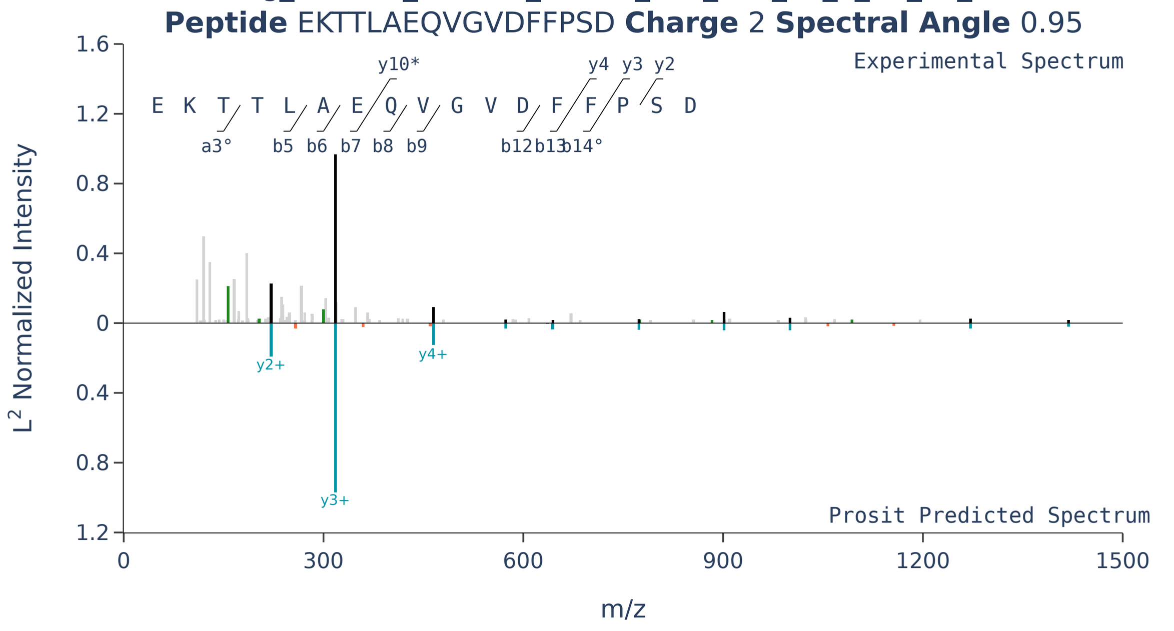

Source Ncheng_210623_230623_HFGoe_FFH_20S_25_1_A1_4h_R2 **Scan** 29293
Peptide AEQVGGVDFFPSD **Charge** 2 **Spectral Angle** 0.92

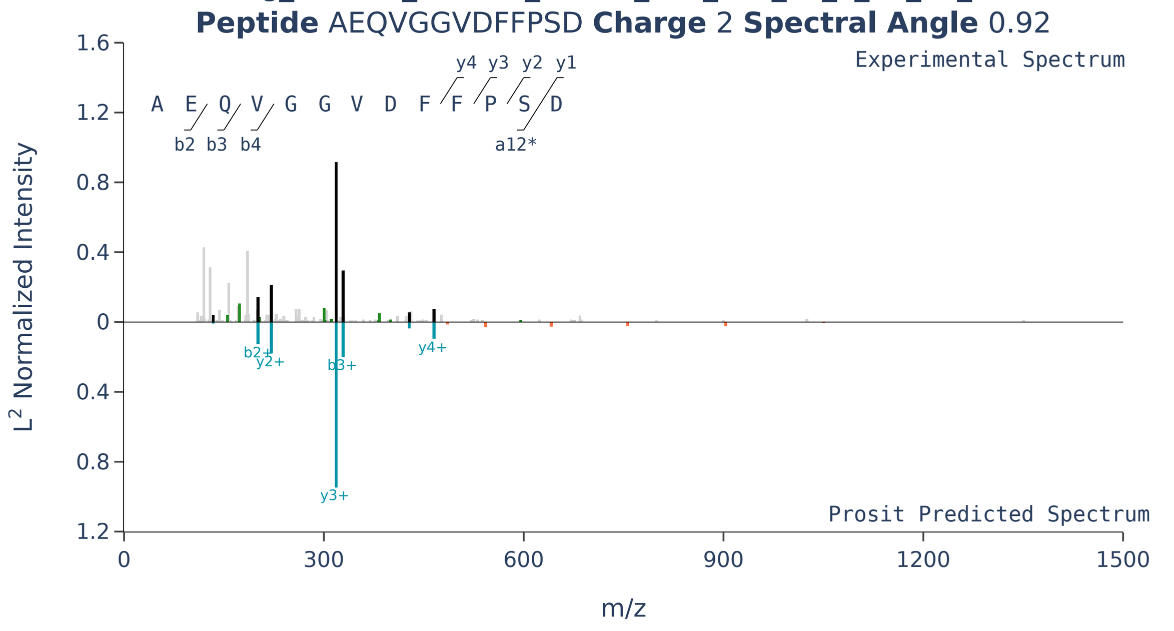

Source Ncheng_210623_230623_HFGoe_FFH_20S_25_1_A2_2h_R2 **Scan** 26870
Peptide LREEQVGVDFFPSD **Charge** 2 **Spectral Angle** 0.86

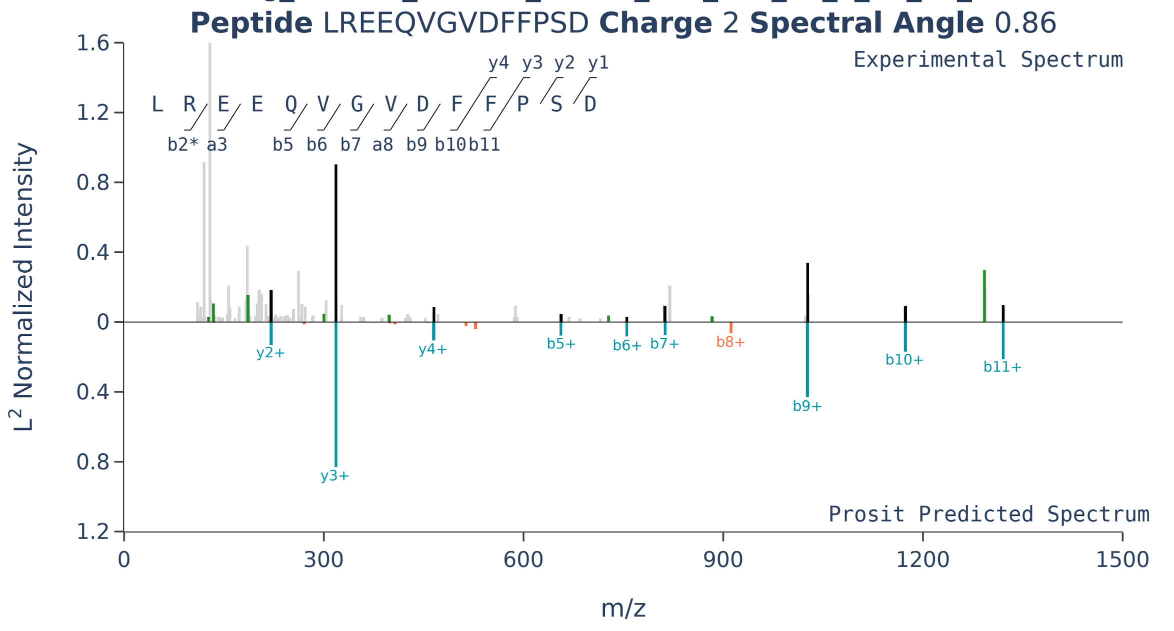

Source Ncheng_210623_230623_HFGoe_FFH_20S_25_1_A1_24h_R1 **Scan** 12217
Peptide TDKPIKF **Charge** 2 **Spectral Angle** 0.75

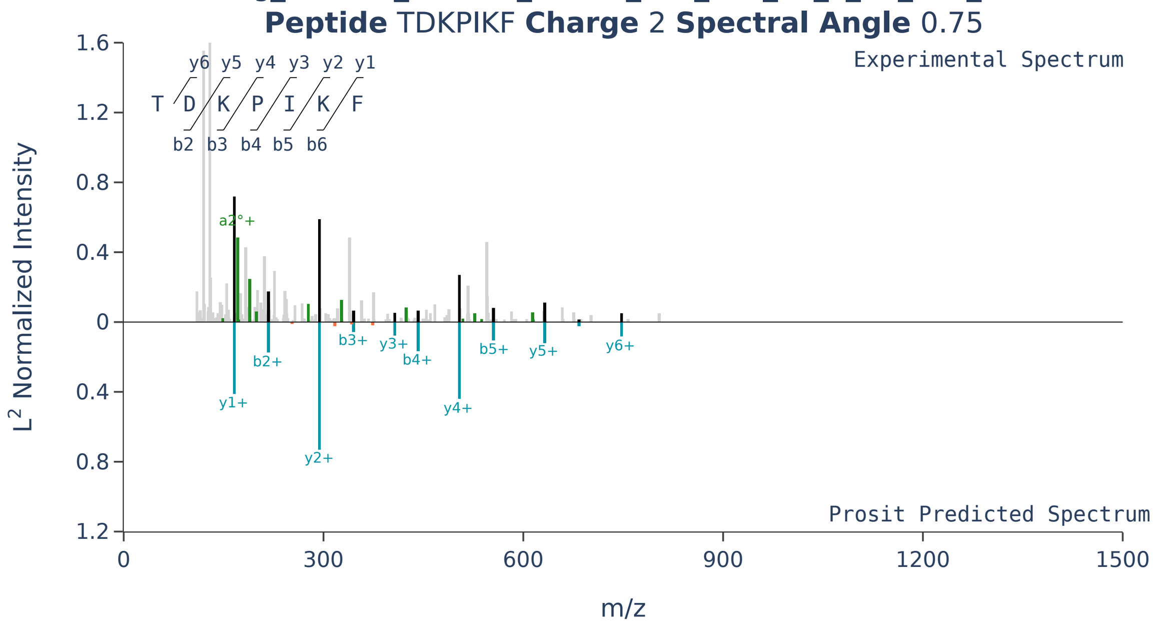

Source Ncheng_210623_230623_HFGoe_FFH_20S_25_1_A2_24h_R1 **Scan** 16396
Peptide AANTAAMTGQDAANTAKAFN **Charge** 2 **Spectral Angle** 0.85

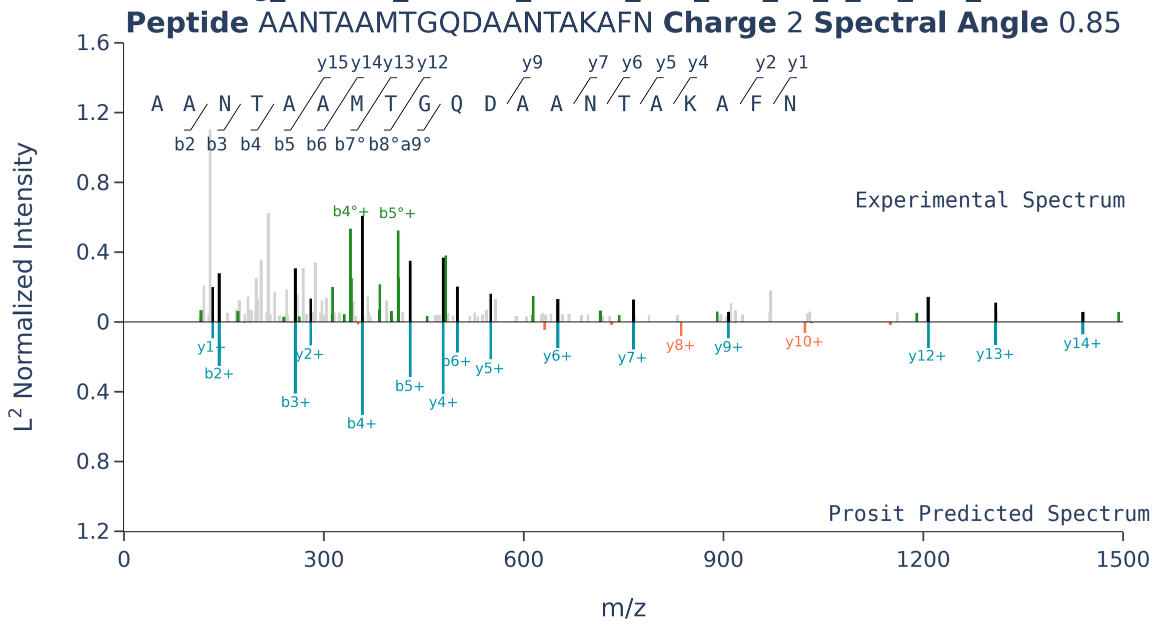

Source Ncheng_210623_230623_HFGoe_FFH_20S_25_1_A2_24h_R1 **Scan** 21012
Peptide NKVDFFPSD **Charge** 2 **Spectral Angle** 0.87

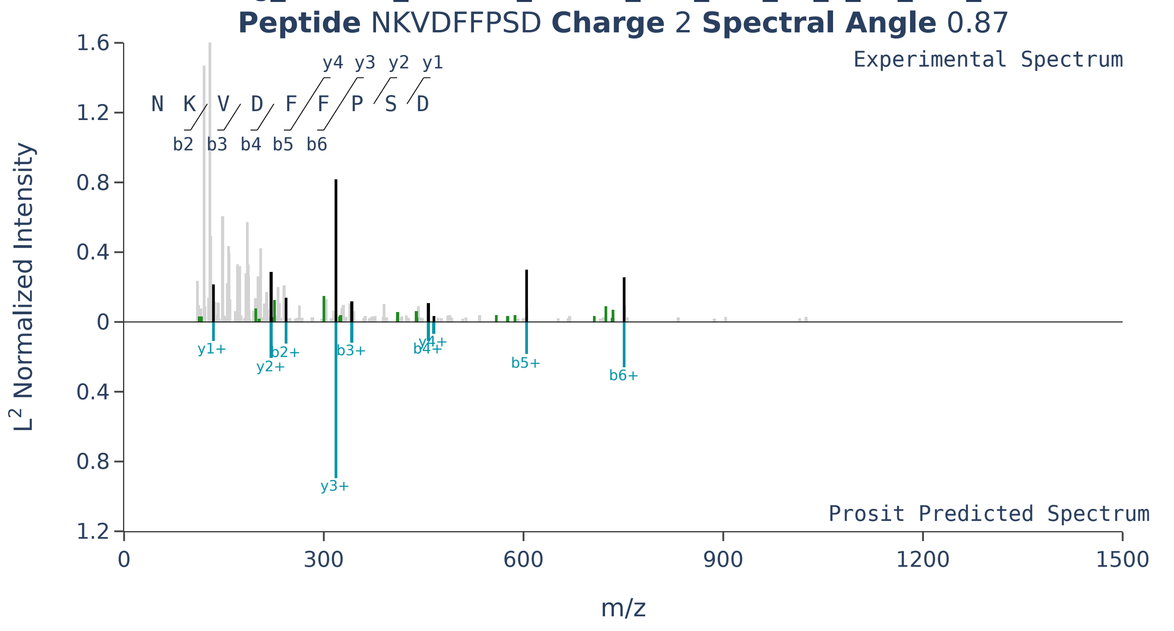

Source Ncheng_210623_230623_HFGoe_FFH_20S_25_1_A2_4h_R2 **Scan** 17219
Peptide MREAIINSMTMKE **Charge** 3 **Spectral Angle** 0.81

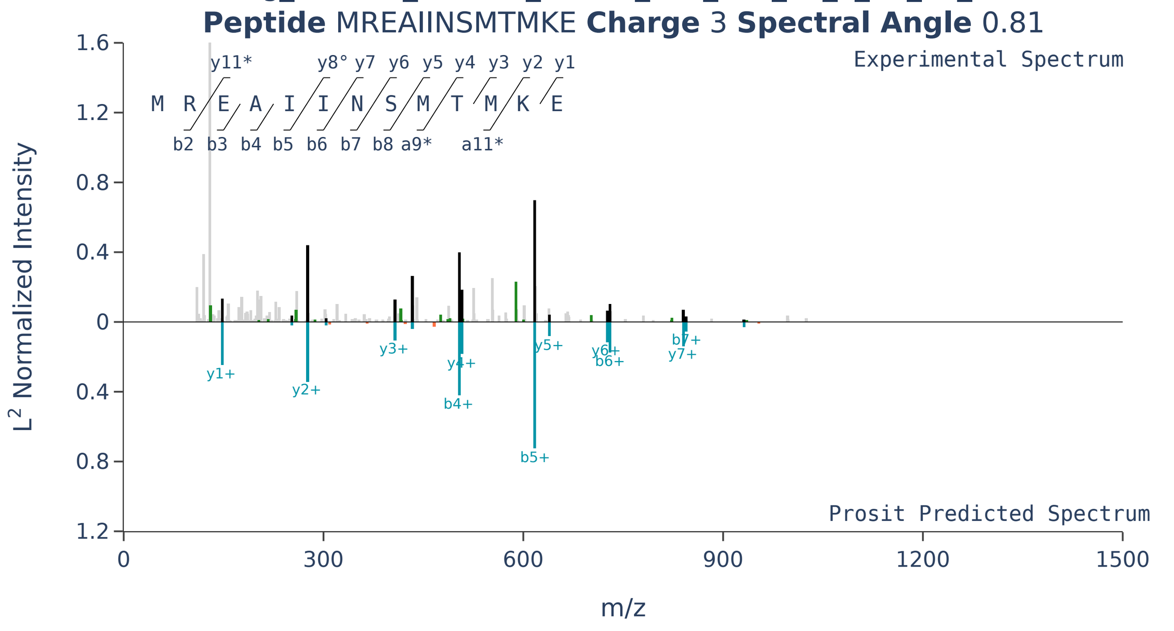

Source Ncheng_210623_230623_HFGoe_FFH_20S_25_1_A1_24h_R1 **Scan** 14870
Peptide GVGEKTEAMTGQDAANTAKAFN **Charge** 3 **Spectral Angle** 0.8

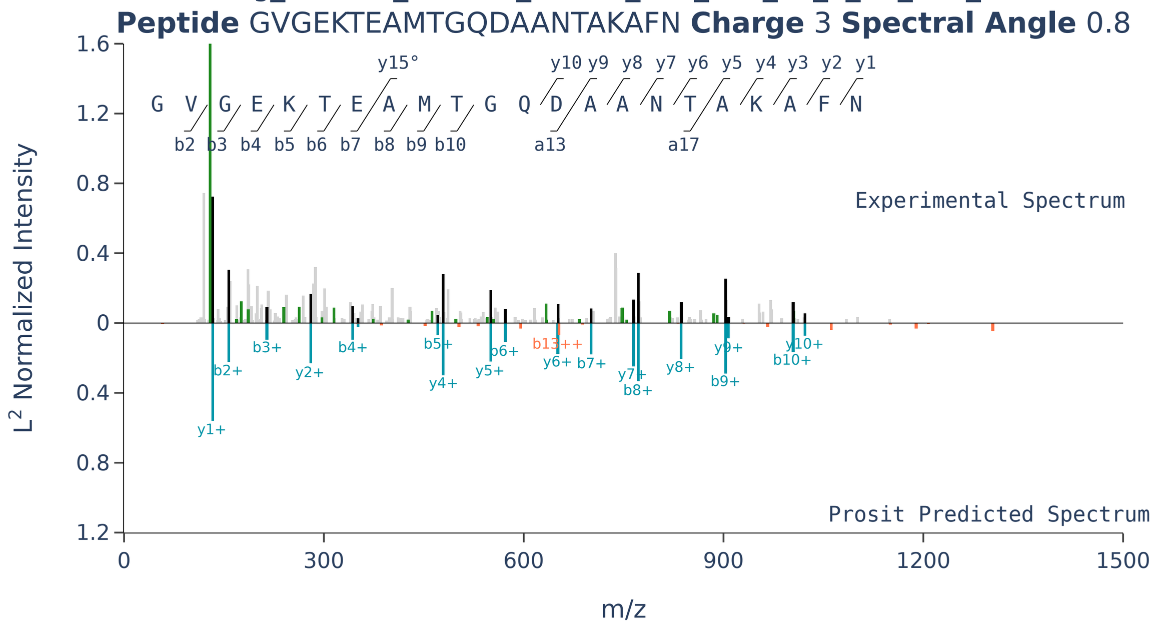

Source Ncheng_210623_230623_HFGoe_FFH_20S_25_1_A1_4h_R2 **Scan** 9528
Peptide SRTLAEQVG **Charge** 2 **Spectral Angle** 0.85

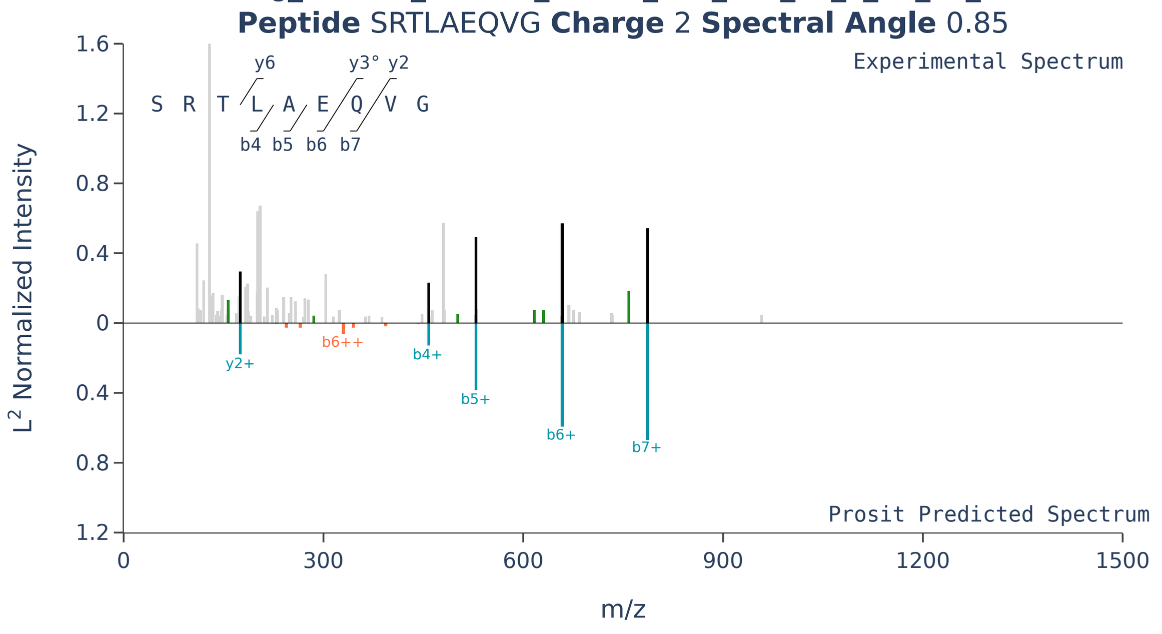

Source Ncheng_210623_230623_HFGoe_FFH_20S_25_1_A2_4h_R2 **Scan** 29429
Peptide PGMGQIEF **Charge** 1 **Spectral Angle** 0.8

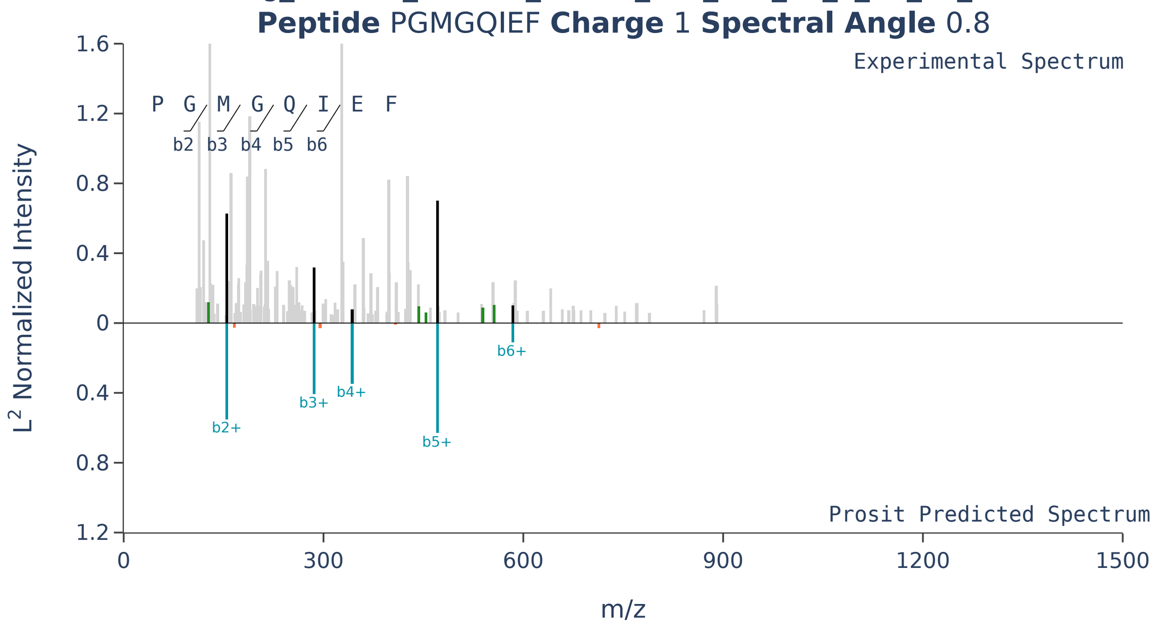

Source Ncheng_210623_230623_HFGoe_FFH_20S_25_1_A1_4h_R1 **Scan** 25141
Peptide RNISGGVDFFPSD **Charge** 2 **Spectral Angle** 0.86

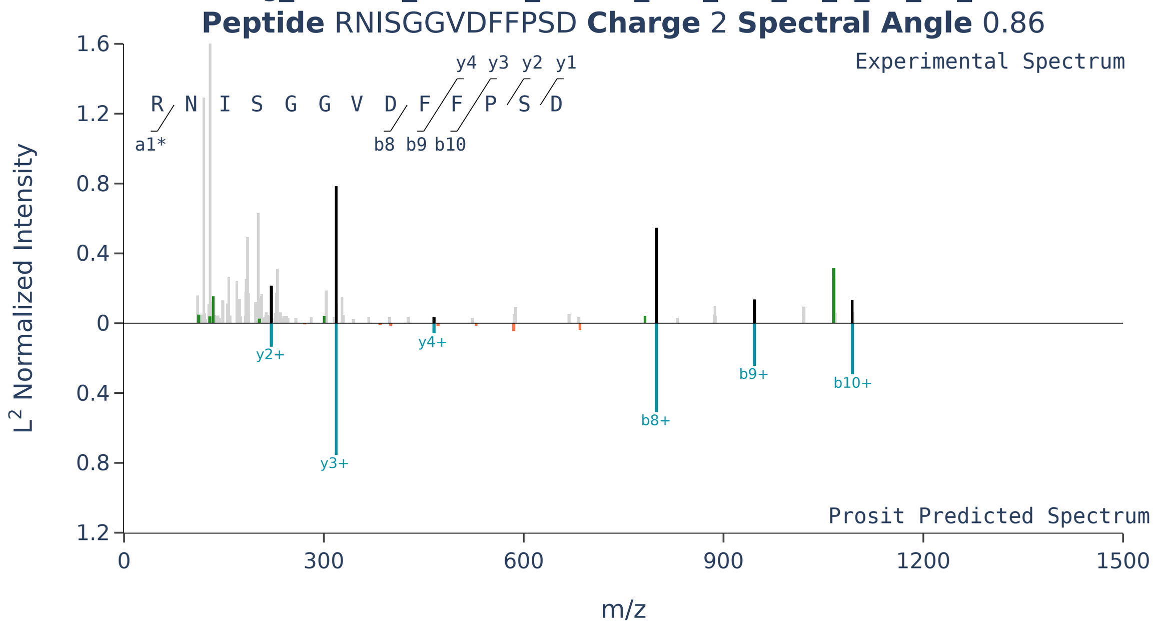

Source Ncheng_210623_230623_HFGoe_FFH_20S_25_1_A1_2h_R1 **Scan** 22721
Peptide SMGKLPGMGQIPDNVKSQMDD **Charge** 3 **Spectral Angle** 0.83

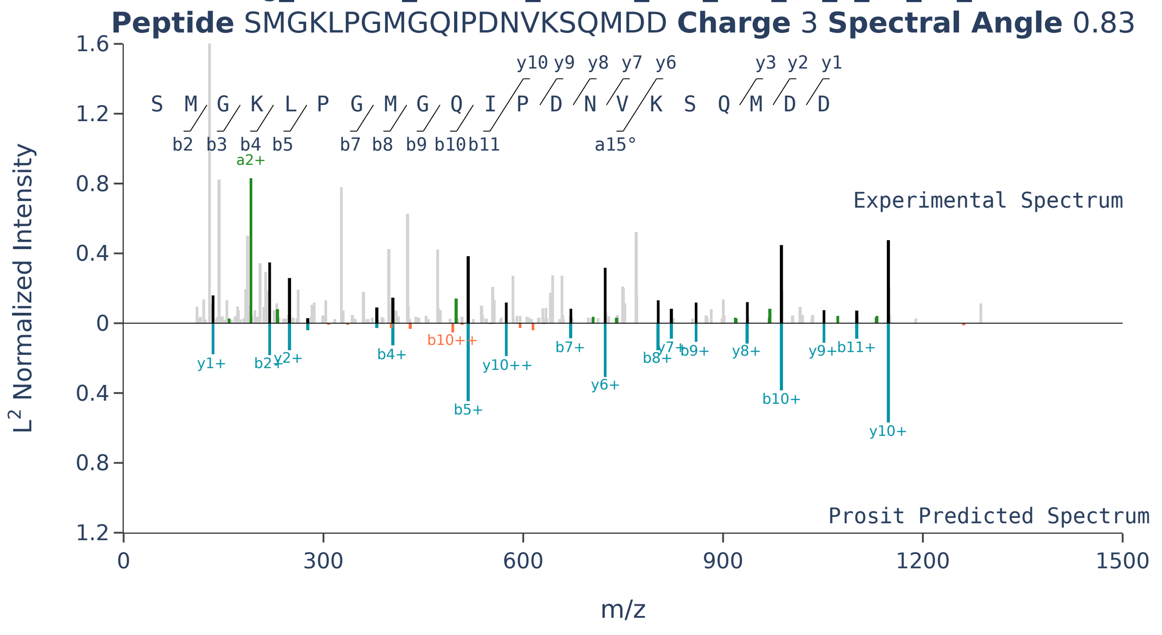

Source Ncheng_210623_230623_HFGoe_FFH_20S_25_1_A1_24h_R1 **Scan** 16243
Peptide KNMGGMADIESKVDRAQAE **Charge** 3 **Spectral Angle** 0.89

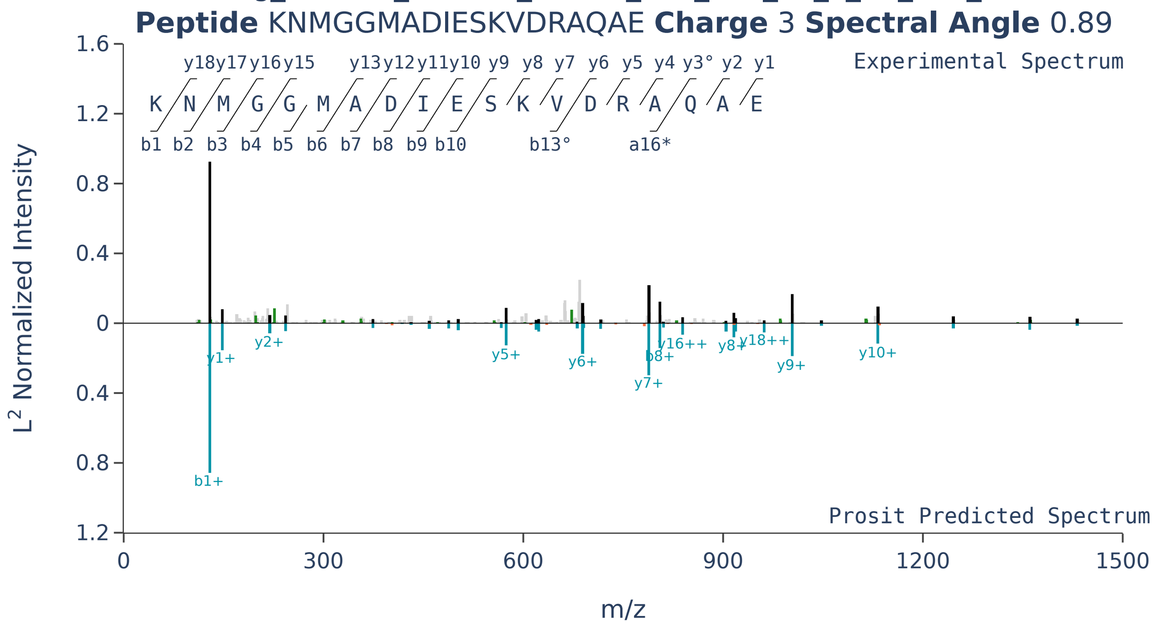

Source Ncheng_210623_230623_HFGoe_FFH_20S_25_1_A2_24h_R1 **Scan** 19363
Peptide KLPGMGQIPDNVKTG **Charge** 2 **Spectral Angle** 0.9

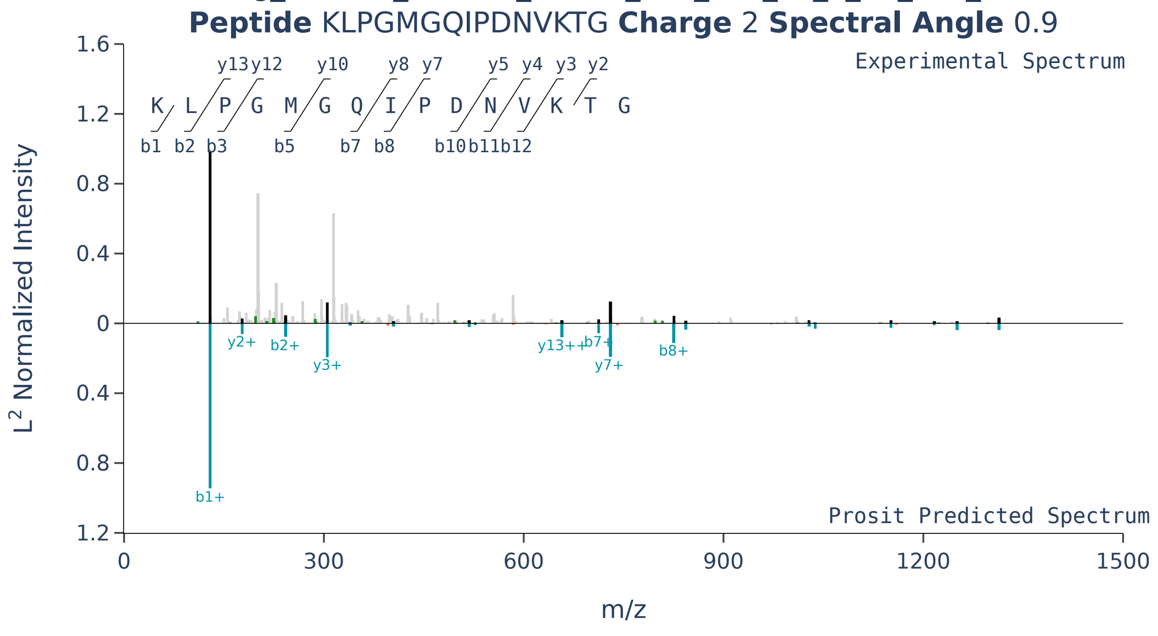

Source Ncheng_210623_230623_HFGoe_FFH_20S_25_1_A1_4h_R2 **Scan** 15332
Peptide ALEPFHPDRASKL **Charge** 3 **Spectral Angle** 0.86

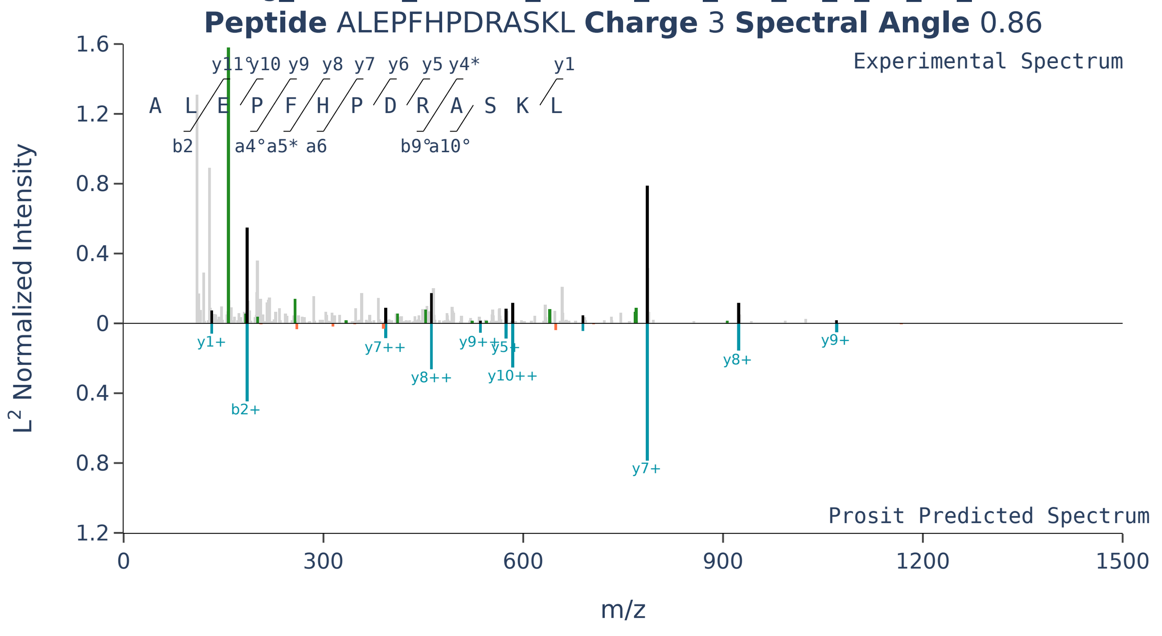

Source Ncheng_210623_230623_HFGoe_FFH_20S_25_1_A1_4h_R1 **Scan** 32660
Peptide TLAEQVGGVDFFPSD **Charge** 2 **Spectral Angle** 0.94

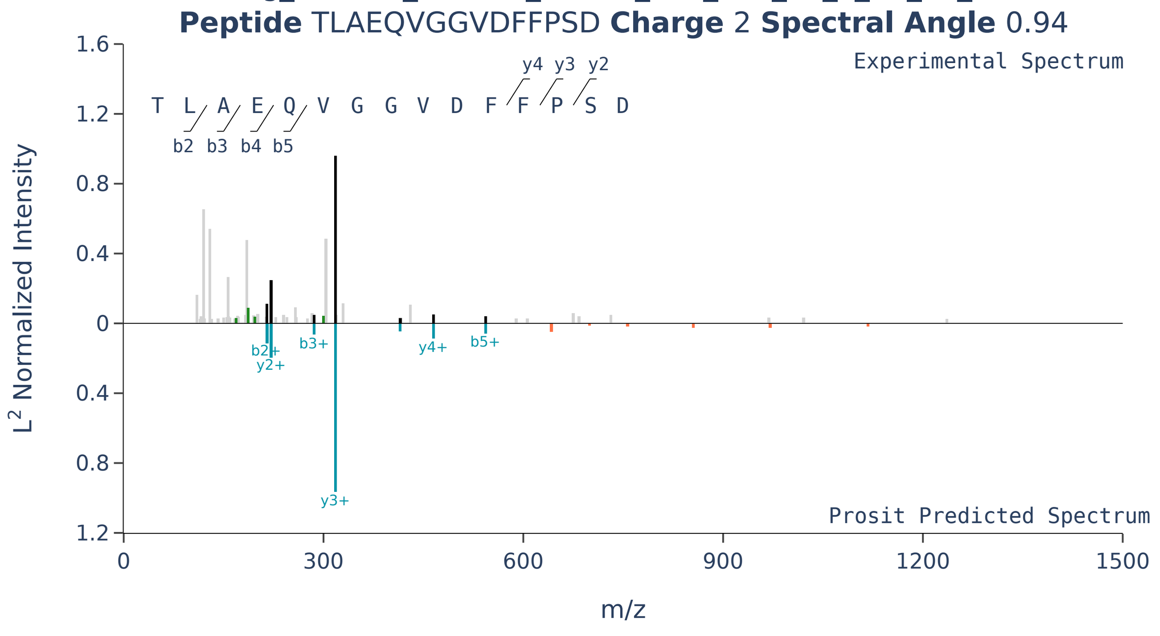

Source Ncheng_210623_230623_HFGoe_FFH_20S_25_1_A2_24h_R2 **Scan** 29805
Peptide ASINGVDFFPSD **Charge** 2 **Spectral Angle** 0.93

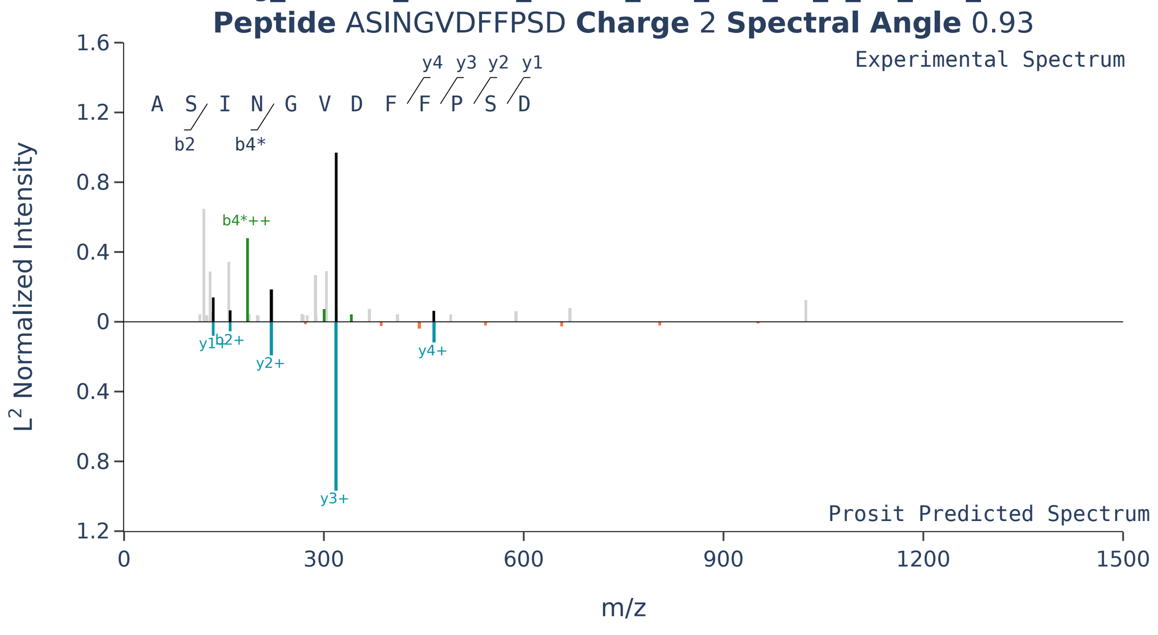

Source Ncheng_210623_230623_HFGoe_FFH_20S_25_1_A1_2h_R2 **Scan** 28475
Peptide AANTAKAFNGVDFFPSD **Charge** 2 **Spectral Angle** 0.91

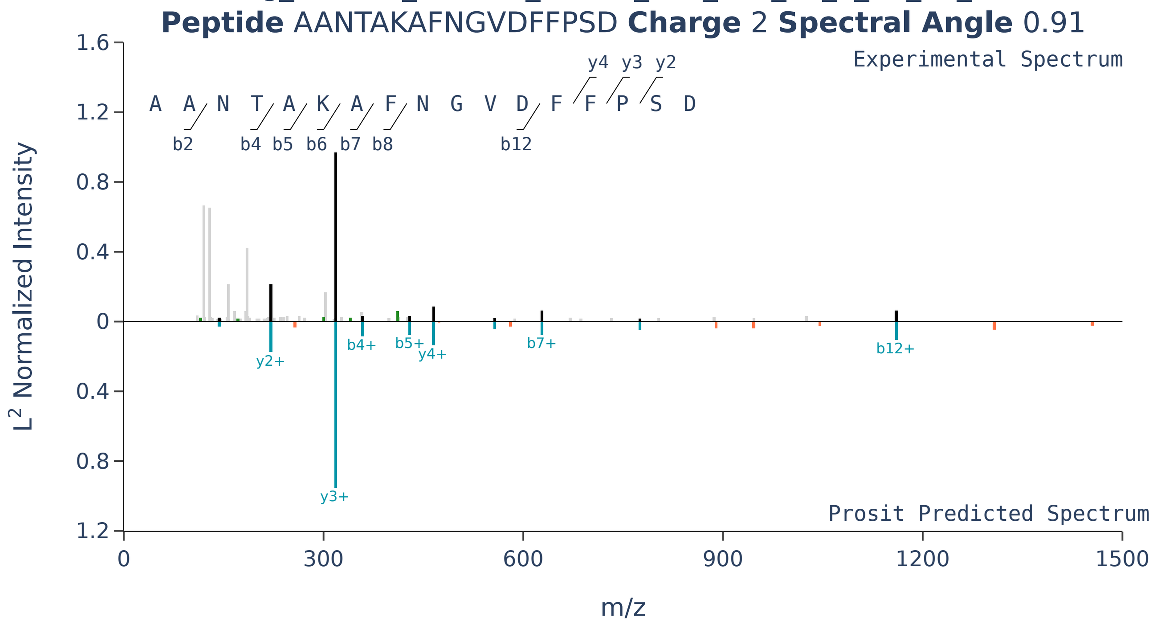

Source Ncheng_210623_230623_HFGoe_FFH_20S_25_1_A1_2h_R2 **Scan** 27413
Peptide AMTGQDAANTAKAFNEALPLTDG **Charge** 2 **Spectral Angle** 0.88

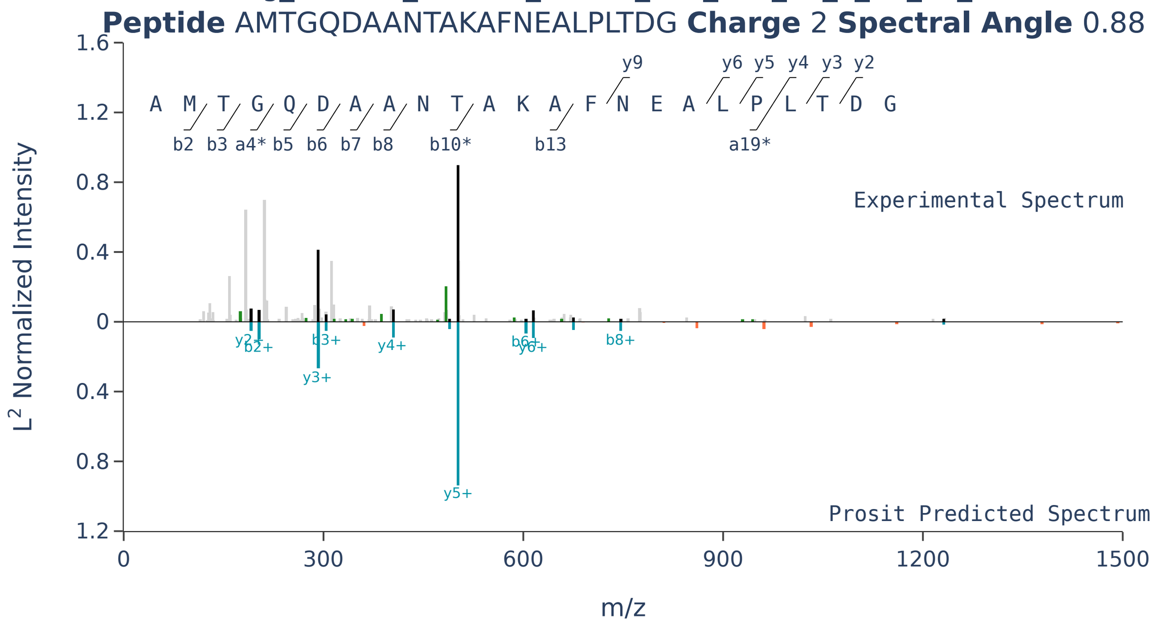

Source Ncheng_210623_230623_HFGoe_FFH_20S_25_1_A1_24h_R1 **Scan** 8559
Peptide KGGMAKMMD **Charge** 2 **Spectral Angle** 0.89

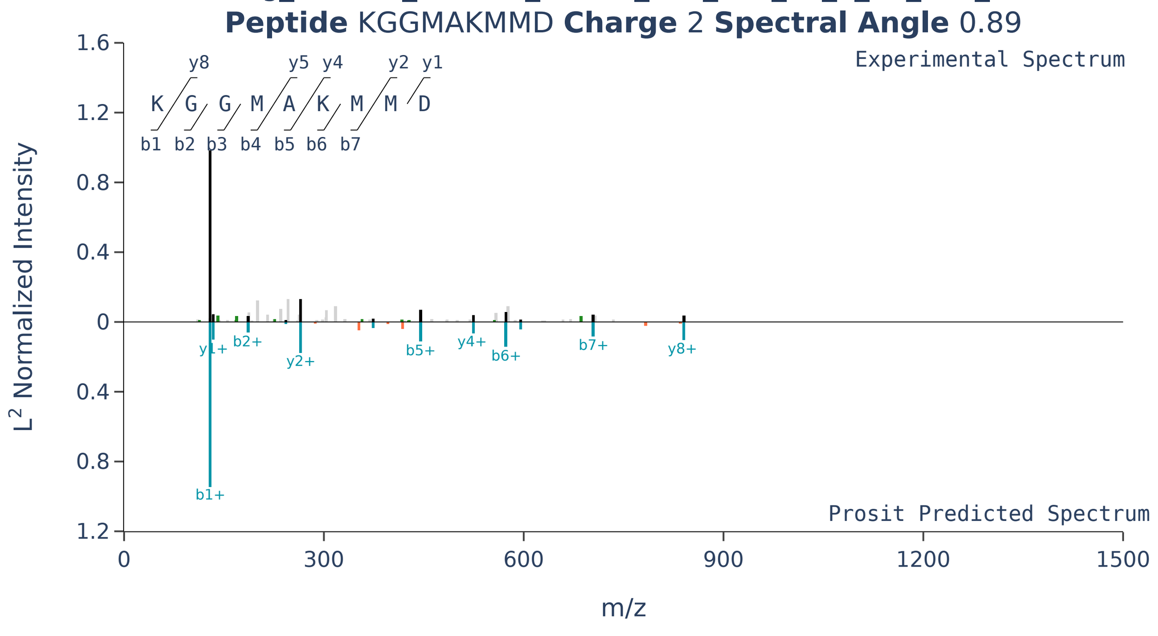

Source Ncheng_210623_230623_HFGoe_FFH_20S_25_1_A2_4h_R1 **Scan** 22489
Peptide DMGKLPGMGQIPDNVKS **Charge** 3 **Spectral Angle** 0.85

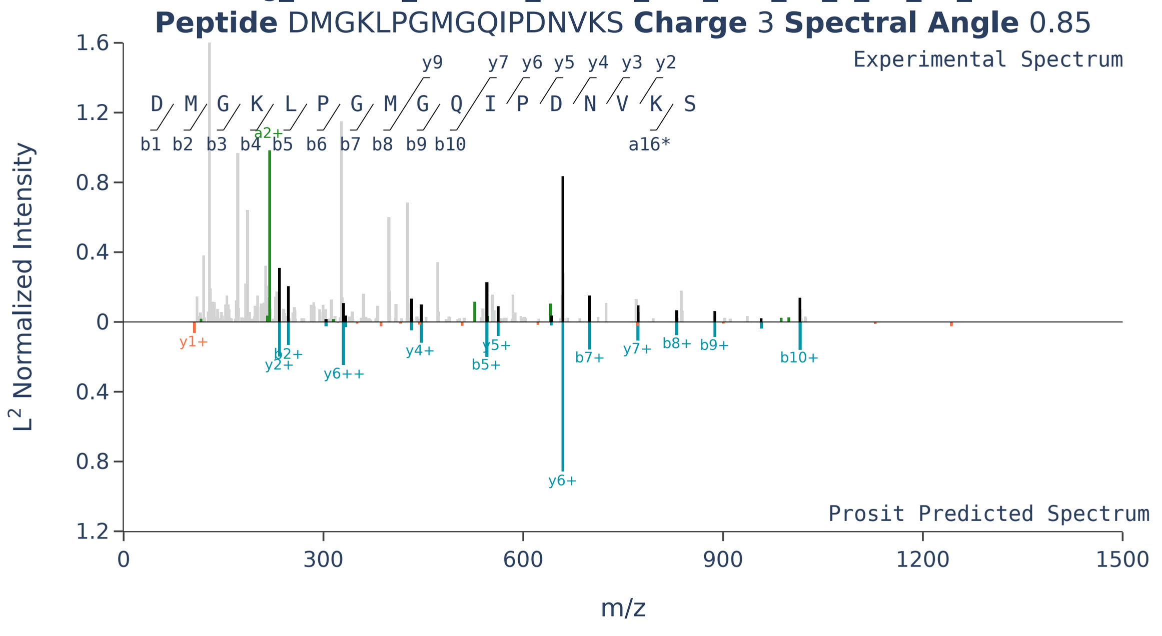

Source Ncheng_210623_230623_HFGoe_FFH_20S_25_1_A2_2h_R2 **Scan** 18966
Peptide EALPLTDG **Charge** 1 **Spectral Angle** 0.83

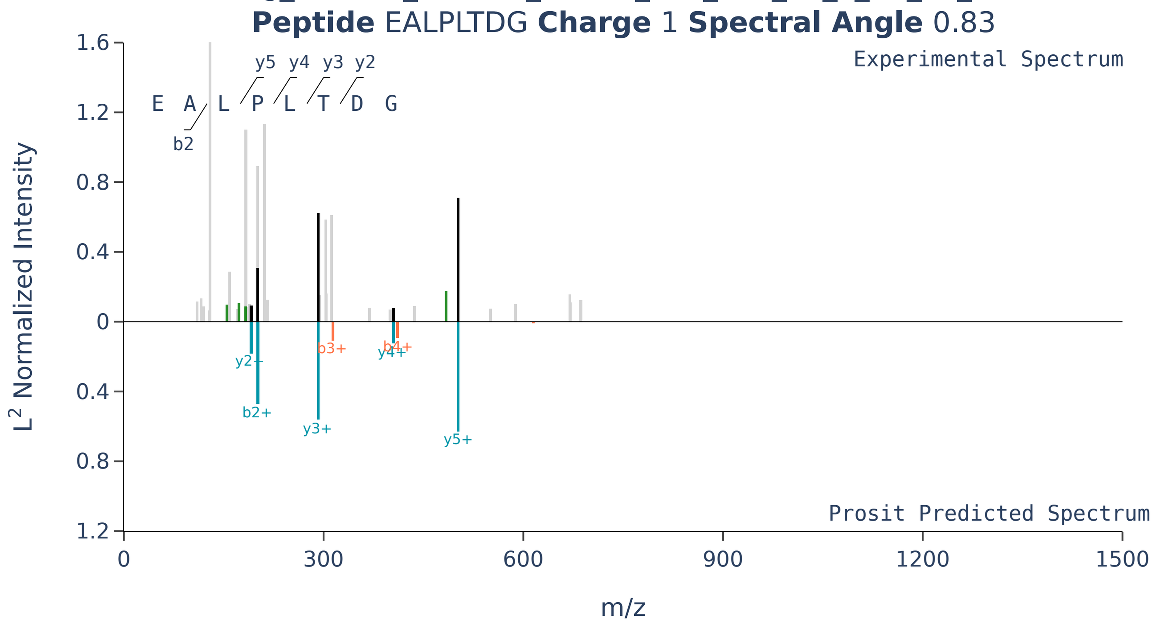

Source Ncheng_210623_230623_HFGoe_FFH_20S_25_1_A2_24h_R2 **Scan** 24170
Peptide PPAVVGAAL **Charge** 1 **Spectral Angle** 0.87

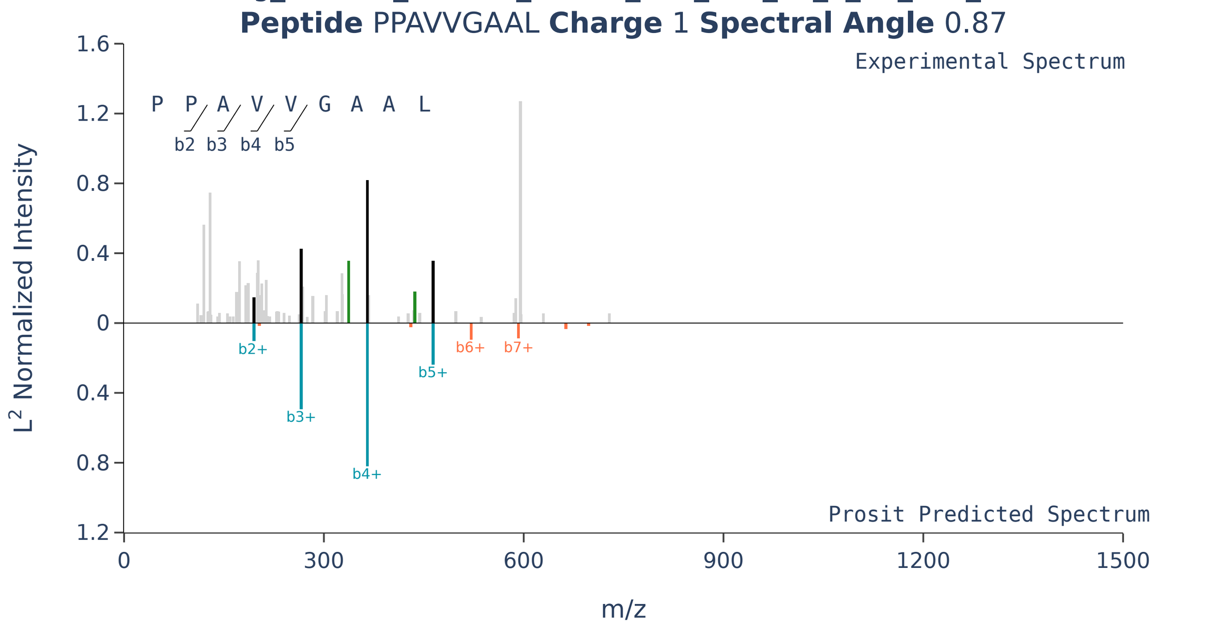

Source Ncheng_210623_230623_HFGoe_FFH_20S_25_1_A1_4h_R1 **Scan** 13640
Peptide AQMTGQDAANTAKAFN **Charge** 2 **Spectral Angle** 0.8

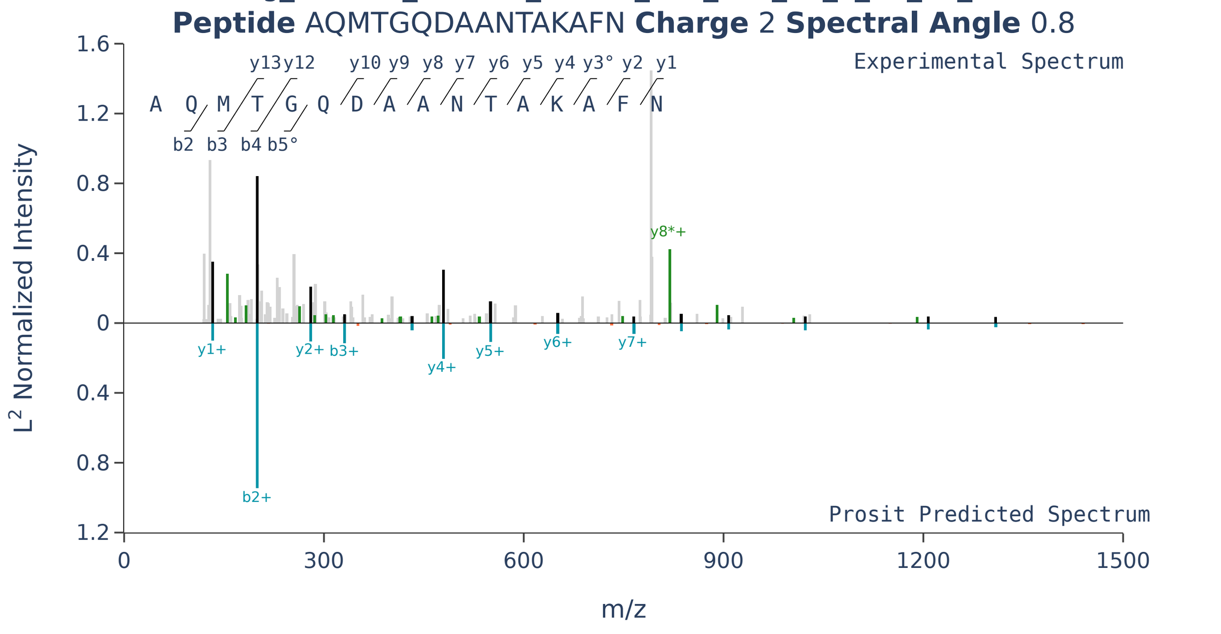

Source Ncheng_210623_230623_HFGoe_FFH_20S_25_1_A2_24h_R1 **Scan** 29461
Peptide SGQVGVDFFPSD **Charge** 2 **Spectral Angle** 0.95

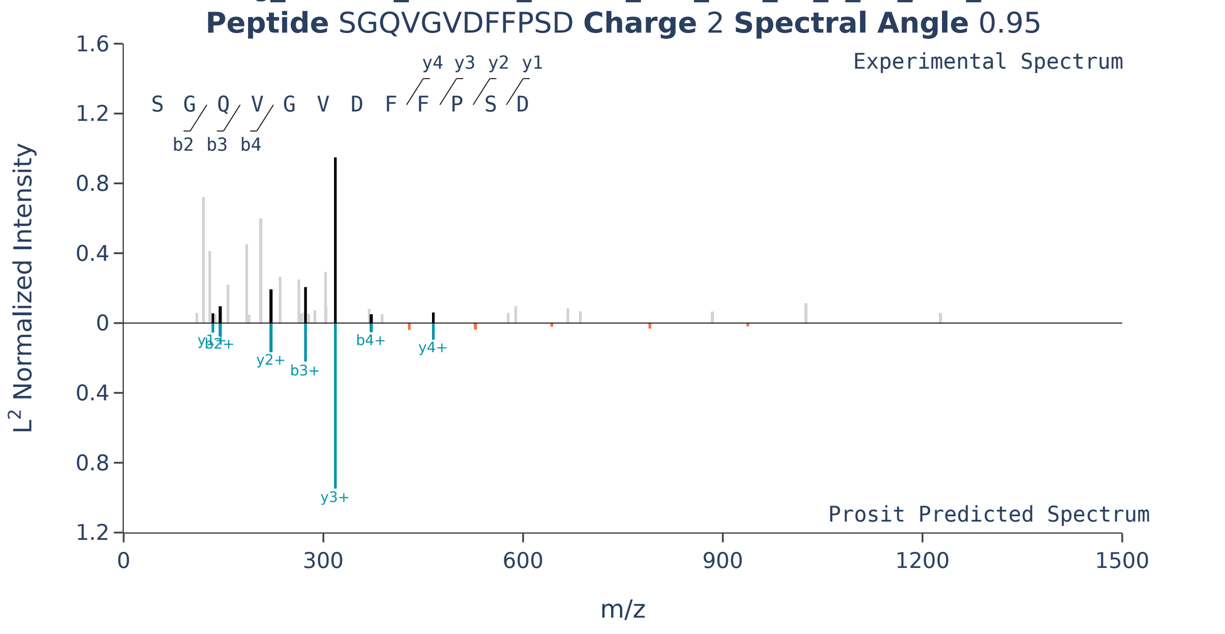

Source Ncheng_210623_230623_HFGoe_FFH_20S_25_1_A2_24h_R1 **Scan** 7351
Peptide GDIESKVD **Charge** 1 **Spectral Angle** 0.89

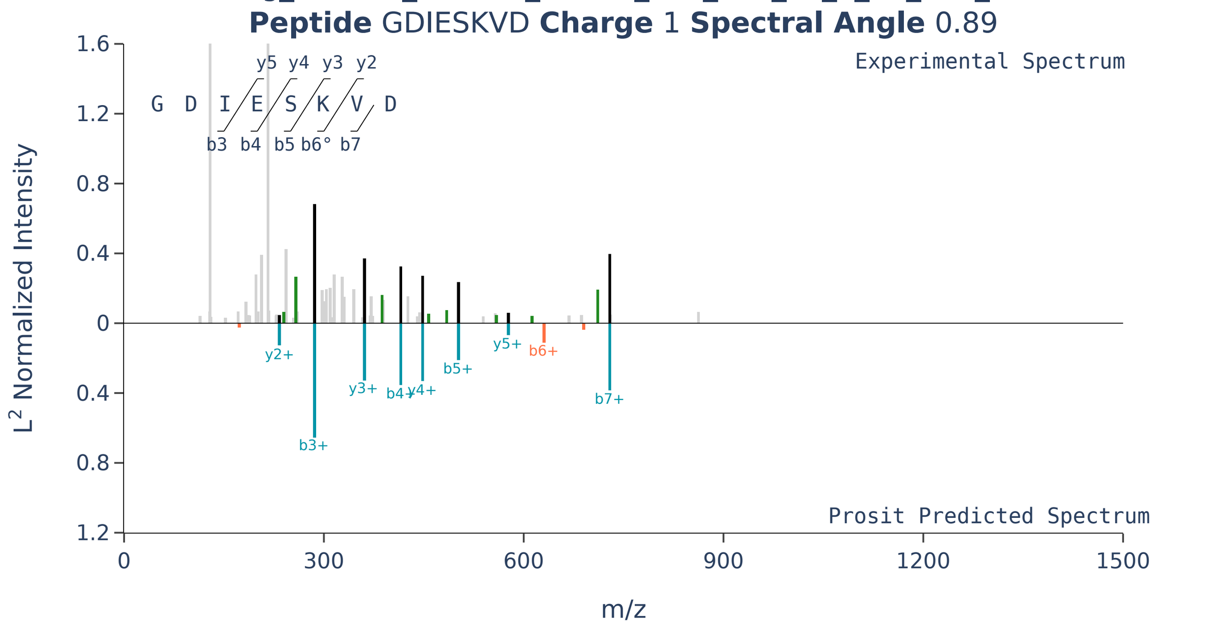

Source Ncheng_210623_230623_HFGoe_FFH_20S_25_1_A2_2h_R2 **Scan** 32174
Peptide NMMASLMGKLPGMGQIPDNVKSQMDD **Charge** 3 **Spectral Angle** 0.75

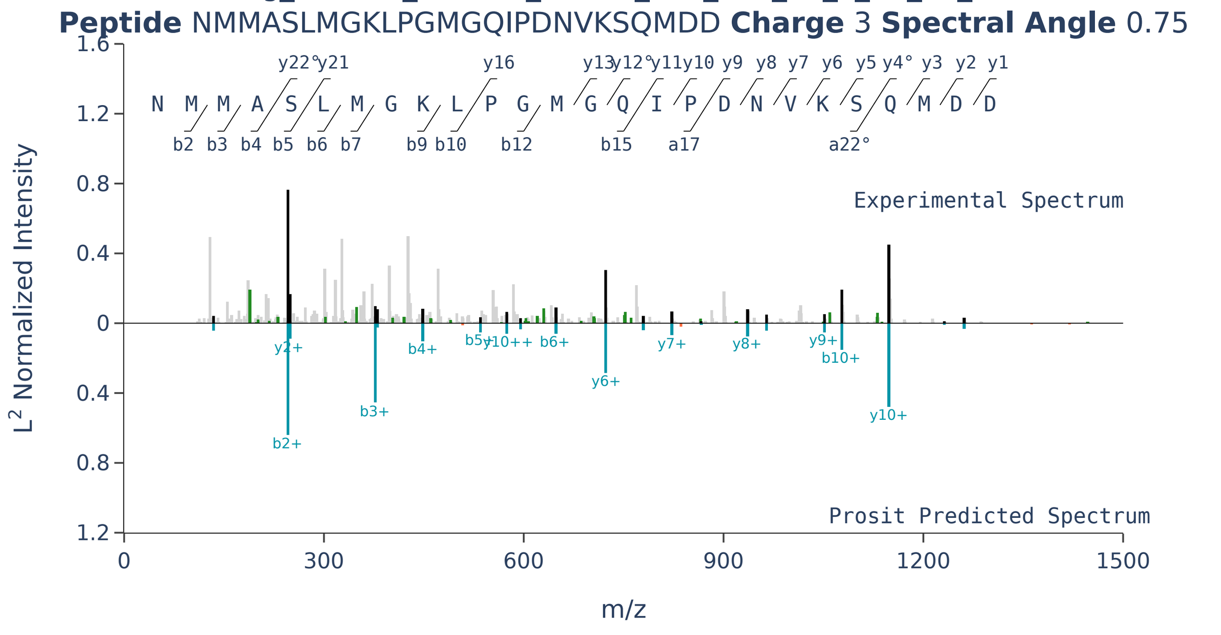

Source Ncheng_210623_230623_HFGoe_FFH_20S_25_1_A2_4h_R2 **Scan** 20687
Peptide TLAEAMTGQDAANTAKAFN **Charge** 2 **Spectral Angle** 0.86

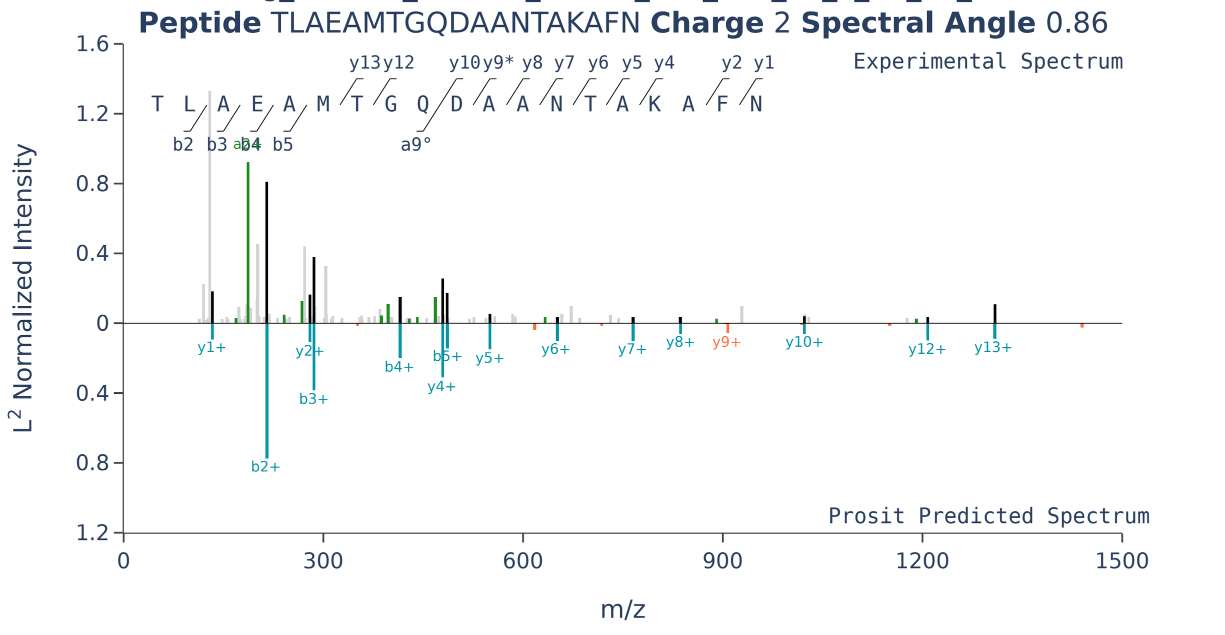

Source Ncheng_210623_230623_HFGoe_FFH_20S_25_1_A2_4h_R1 **Scan** 20502
Peptide GKMGKLPGMGQIPDNVKSQMD **Charge** 3 **Spectral Angle** 0.84

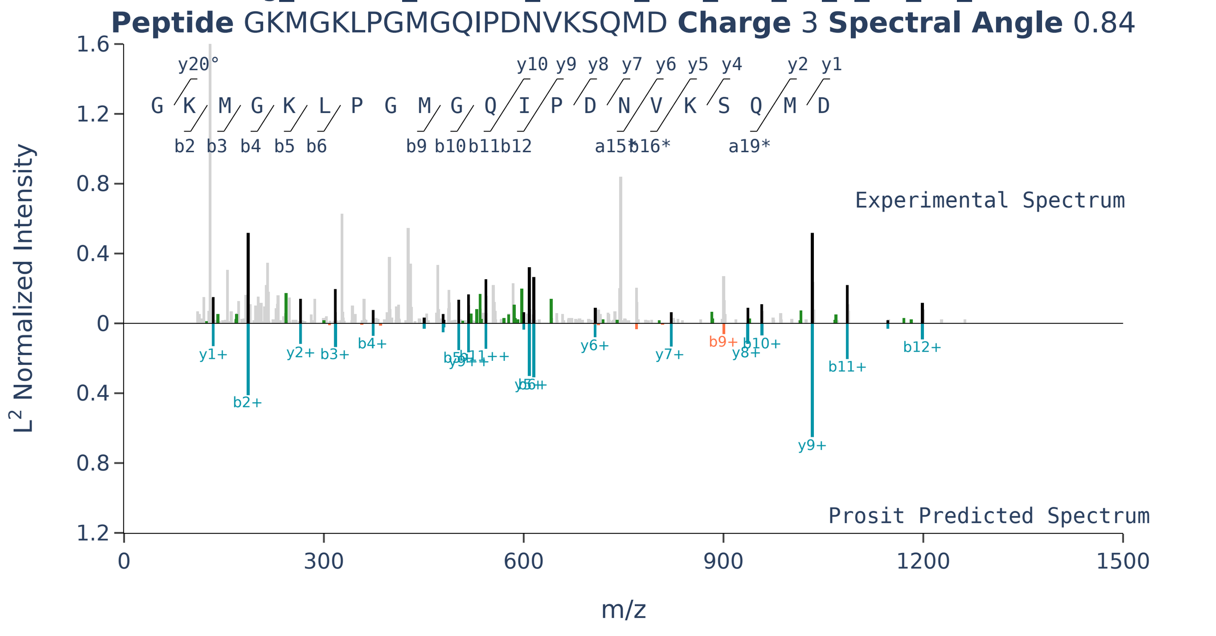

Source Ncheng_210623_230623_HFGoe_FFH_20S_25_1_A2_24h_R2 **Scan** 10004
Peptide GKSINPVET **Charge** 2 **Spectral Angle** 0.84

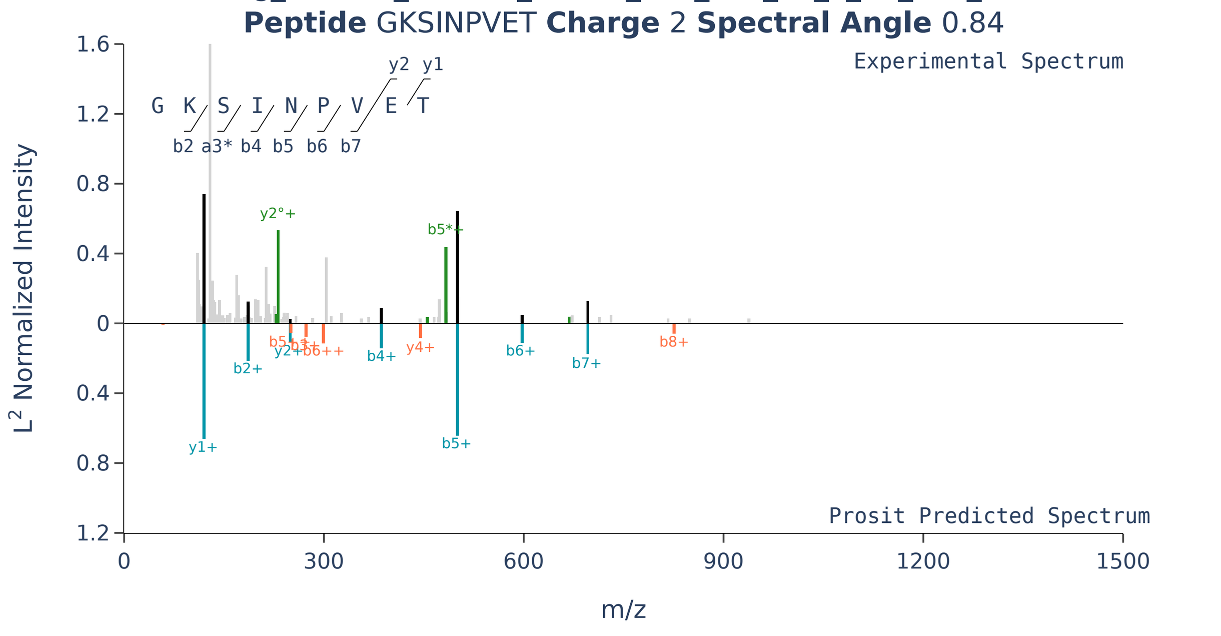

Source Ncheng_210623_230623_HFGoe_FFH_20S_25_1_A1_24h_R2 **Scan** 22266
Peptide SRTLFVVD **Charge** 2 **Spectral Angle** 0.76

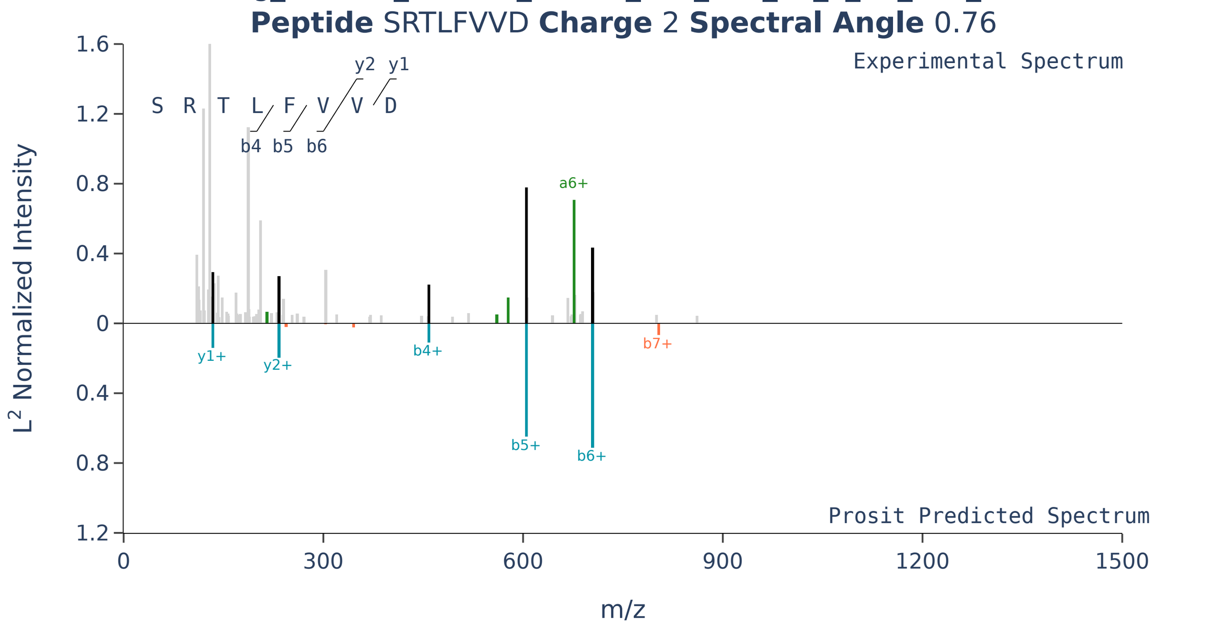

Source Ncheng_210623_230623_HFGoe_FFH_20S_25_1_A2_4h_R2 **Scan** 13694
Peptide AGAMTGQDAANTAKAFN **Charge** 2 **Spectral Angle** 0.85

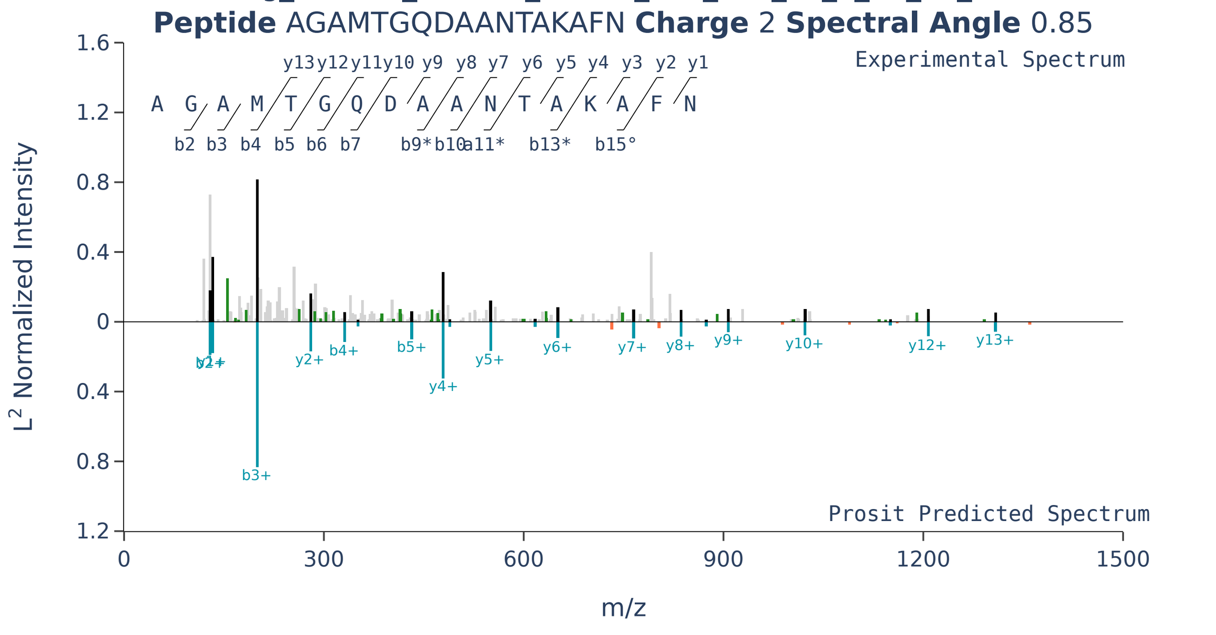

Source Ncheng_210623_230623_HFGoe_FFH_20S_25_1_A2_4h_R1 **Scan** 13525
Peptide KNMGGIIN **Charge** 1 **Spectral Angle** 0.78

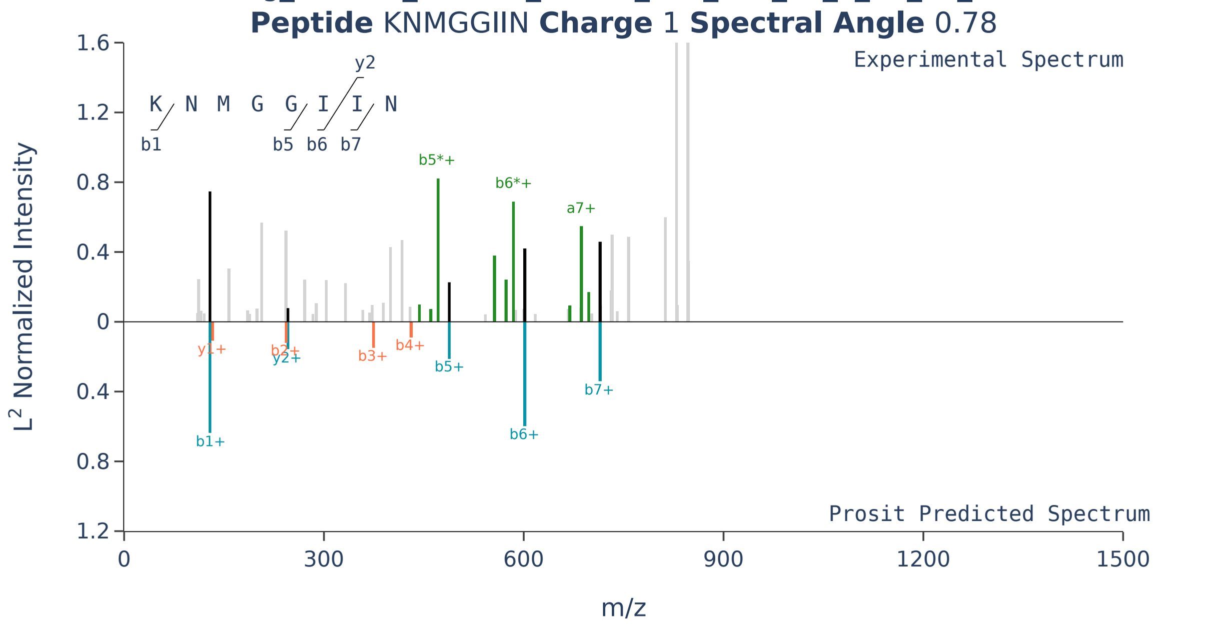

Source Ncheng_210623_230623_HFGoe_FFH_20S_25_1_A1_2h_R2 **Scan** 6546
Peptide KVLVKTE **Charge** 2 **Spectral Angle** 0.87

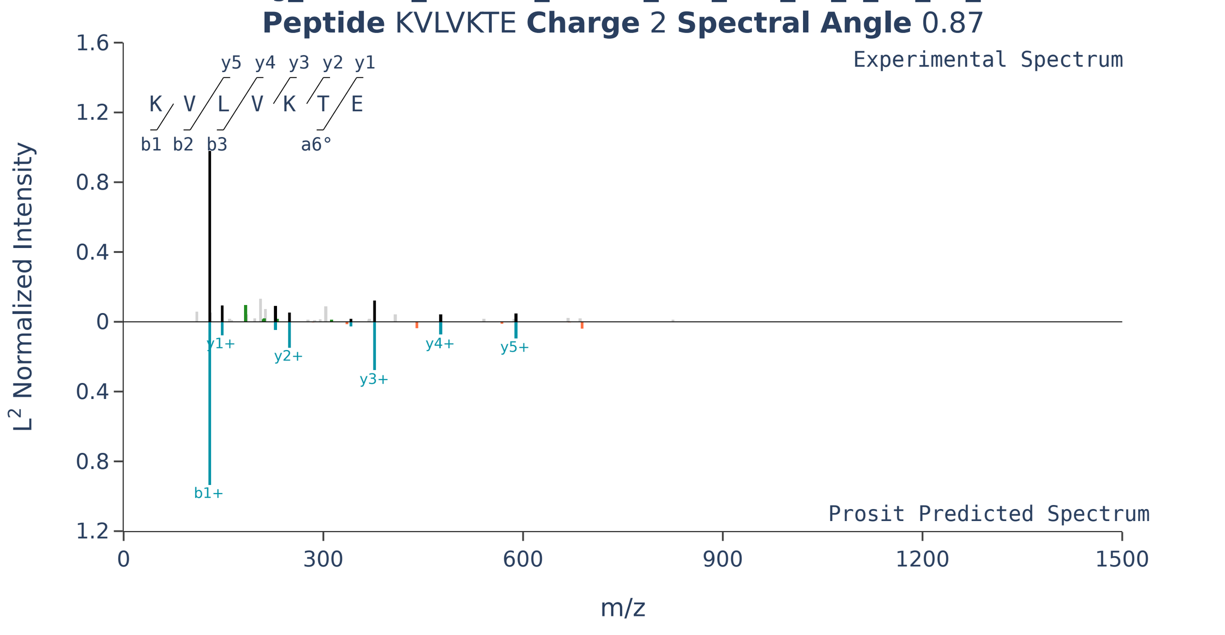

Source Ncheng_210623_230623_HFGoe_FFH_20S_25_1_A2_4h_R2 **Scan** 8646
Peptide ASQDVNRL **Charge** 2 **Spectral Angle** 0.77

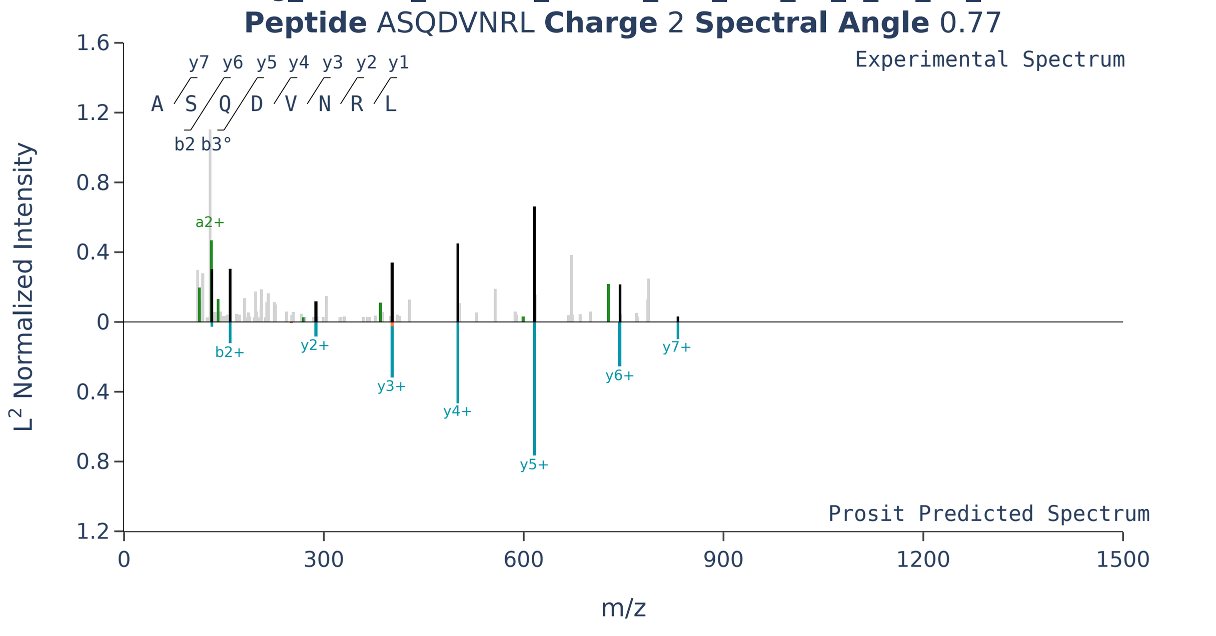

Source Ncheng_210623_230623_HFGoe_FFH_20S_25_1_A2_24h_R1 **Scan** 12066
Peptide ALKEAQDVNRL **Charge** 2 **Spectral Angle** 0.82

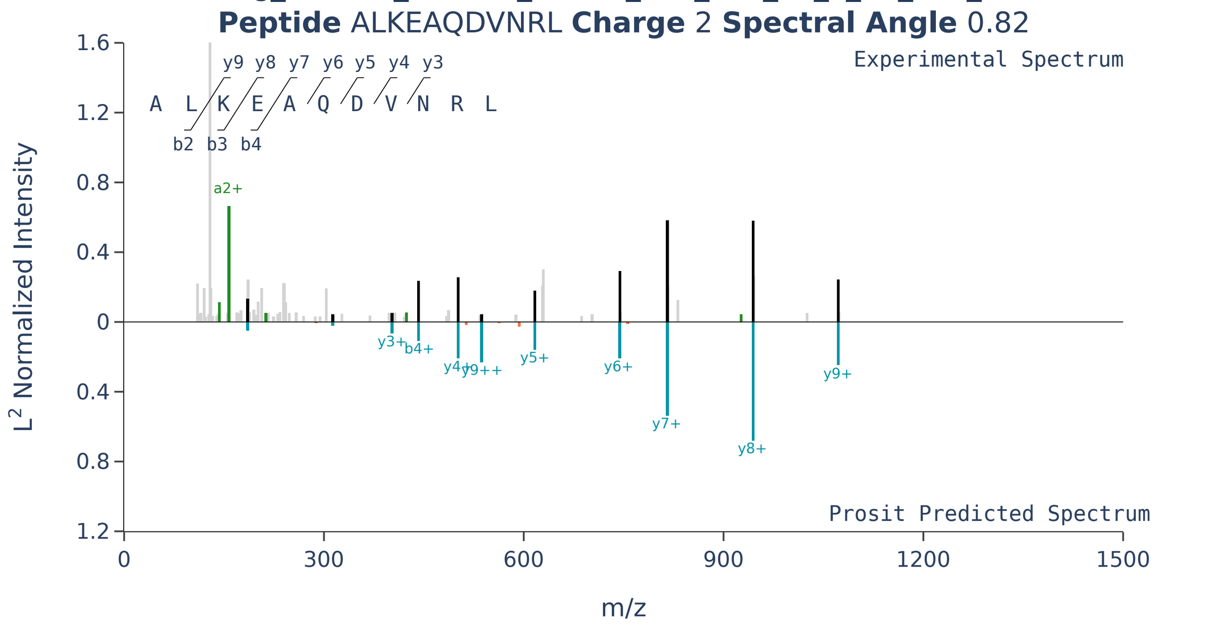

Source Ncheng_210623_230623_HFGoe_FFH_20S_25_1_A2_24h_R2 **Scan** 22031
Peptide RSGVDFFPSD **Charge** 2 **Spectral Angle** 0.85

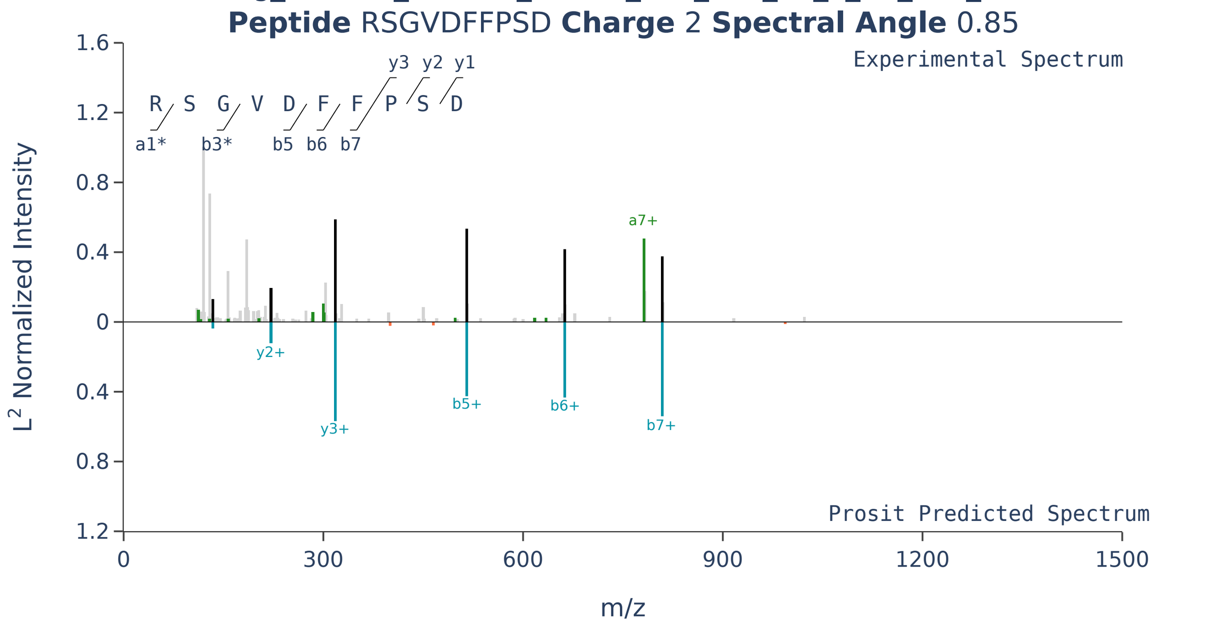

Source Ncheng_210623_230623_HFGoe_FFH_20S_25_1_A2_1h_R2 **Scan** 25800
Peptide RSMTLFVVD **Charge** 2 **Spectral Angle** 0.85

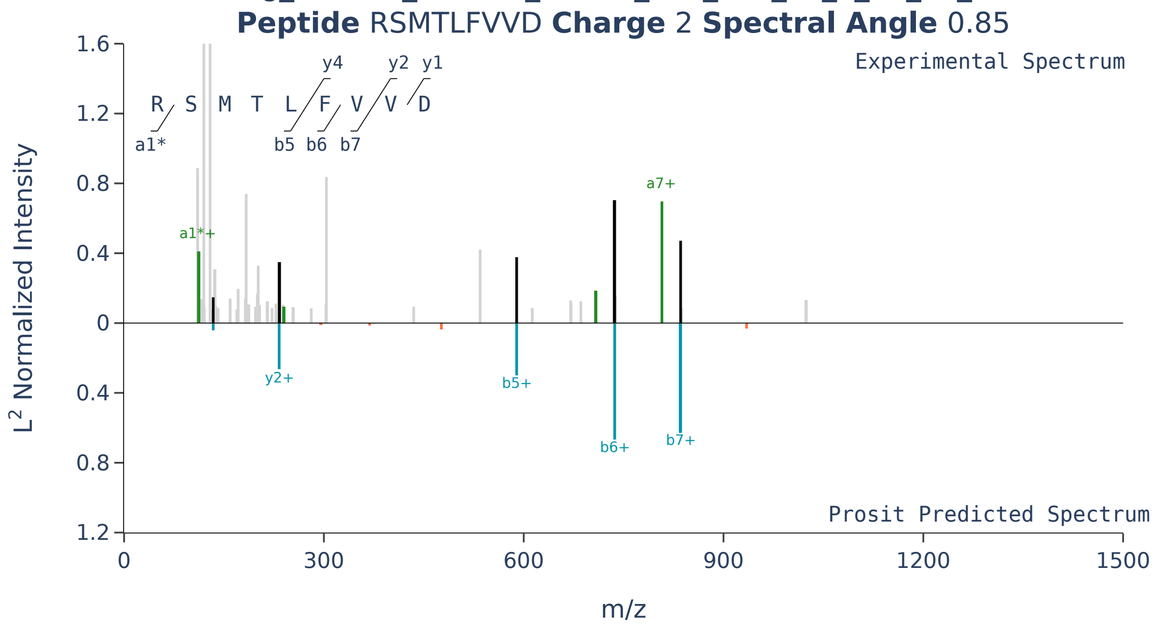

Source Ncheng_210623_230623_HFGoe_FFH_20S_25_1_A2_24h_R1 **Scan** 6695
Peptide ALKEAMRS **Charge** 2 **Spectral Angle** 0.88

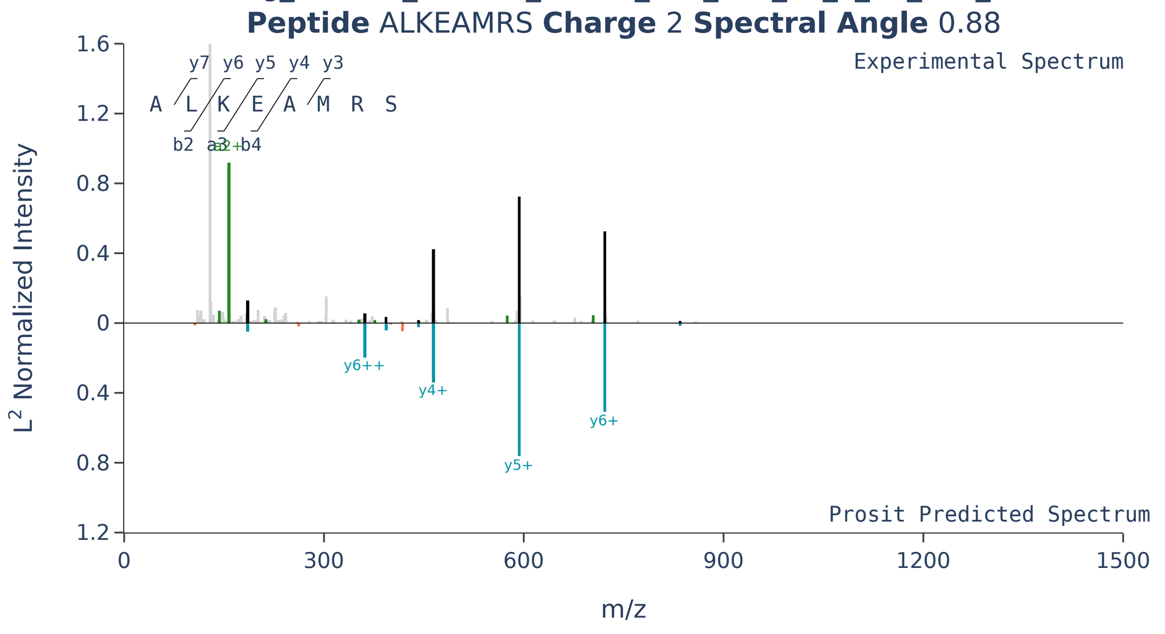

Source Ncheng_210623_230623_HFGoe_FFH_20S_25_1_A1_2h_R1 **Scan** 28025
Peptide AANTAKAFNEALPLTGAMTGQD **Charge** 2 **Spectral Angle** 0.81

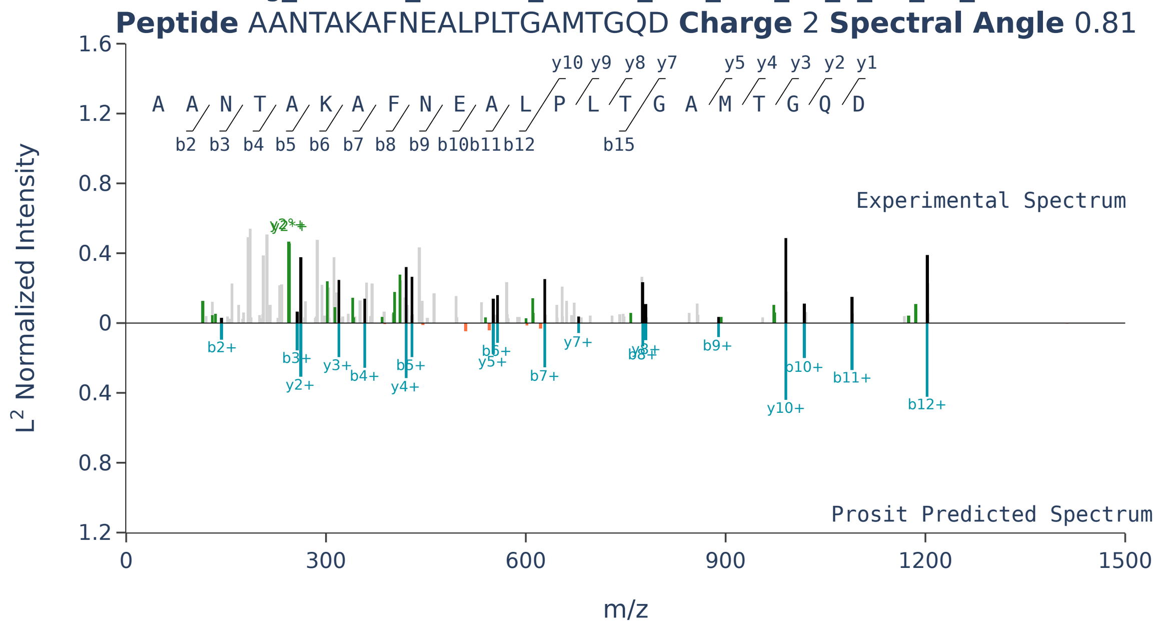

Source Ncheng_210623_230623_HFGoe_FFH_20S_25_1_A1_4h_R1 **Scan** 23018
Peptide GMAKLPGMGQIPDNVKSQMD **Charge** 3 **Spectral Angle** 0.82

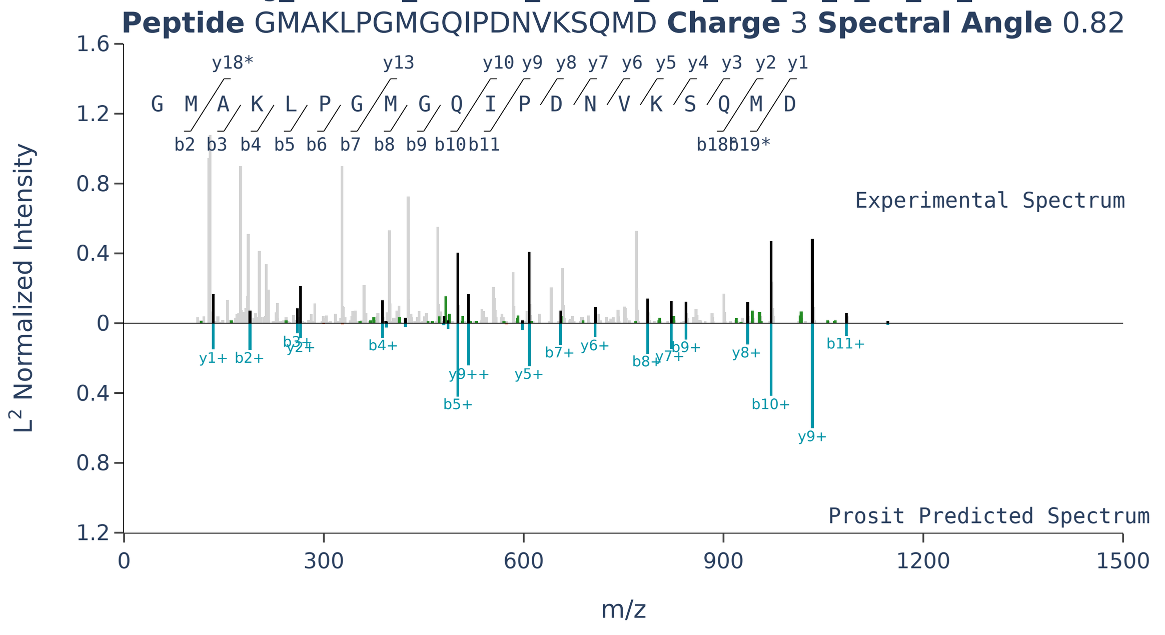

Source Ncheng_210623_230623_HFGoe_FFH_20S_25_1_A2_24h_R1 **Scan** 14074
Peptide GAAMTGQDAANTAKAFN **Charge** 2 **Spectral Angle** 0.82

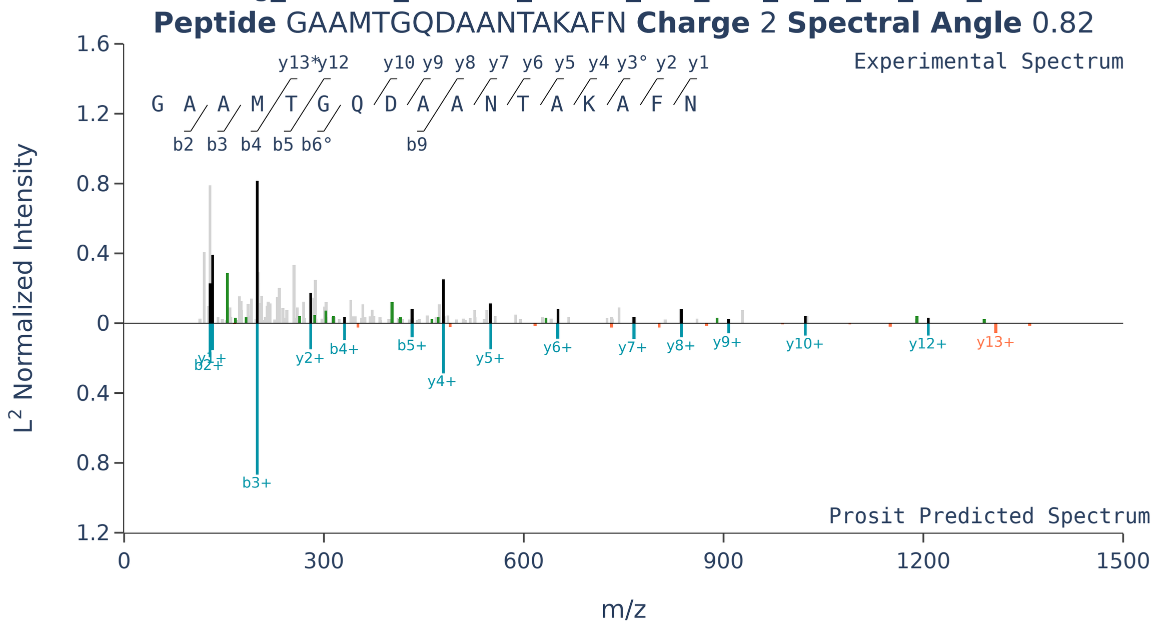

Source Ncheng_210623_230623_HFGoe_FFH_20S_25_1_A2_24h_R1 **Scan** 9772
Peptide GMGDVGQKPVD **Charge** 2 **Spectral Angle** 0.76

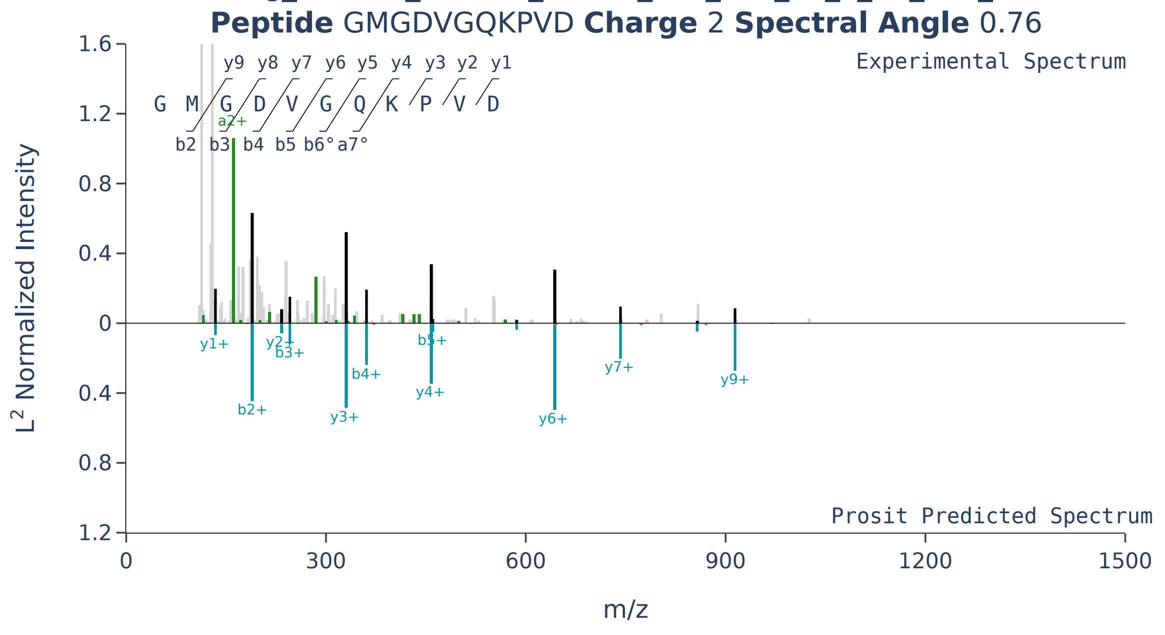

Source Ncheng_210623_230623_HFGoe_FFH_20S_25_1_A1_4h_R1 **Scan** 19497
Peptide TGALEPFHPDRIAS **Charge** 2 **Spectral Angle** 0.86

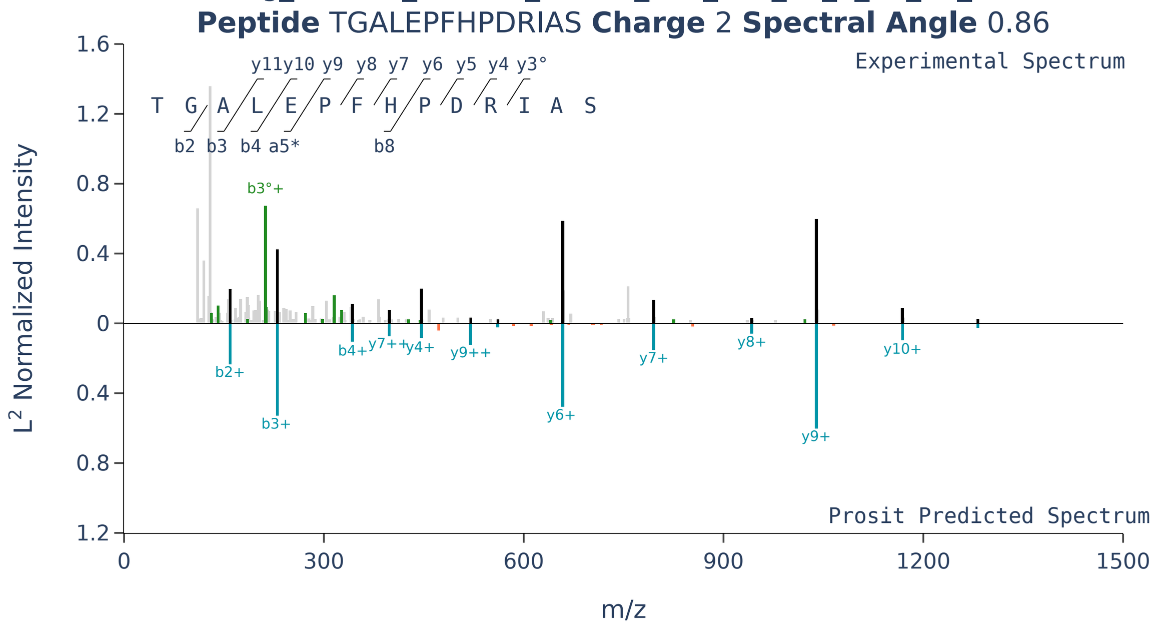

Source Ncheng_210623_230623_HFGoe_FFH_20S_25_1_A1_24h_R1 **Scan** 10711
Peptide PFHPDRIASD **Charge** 2 **Spectral Angle** 0.83

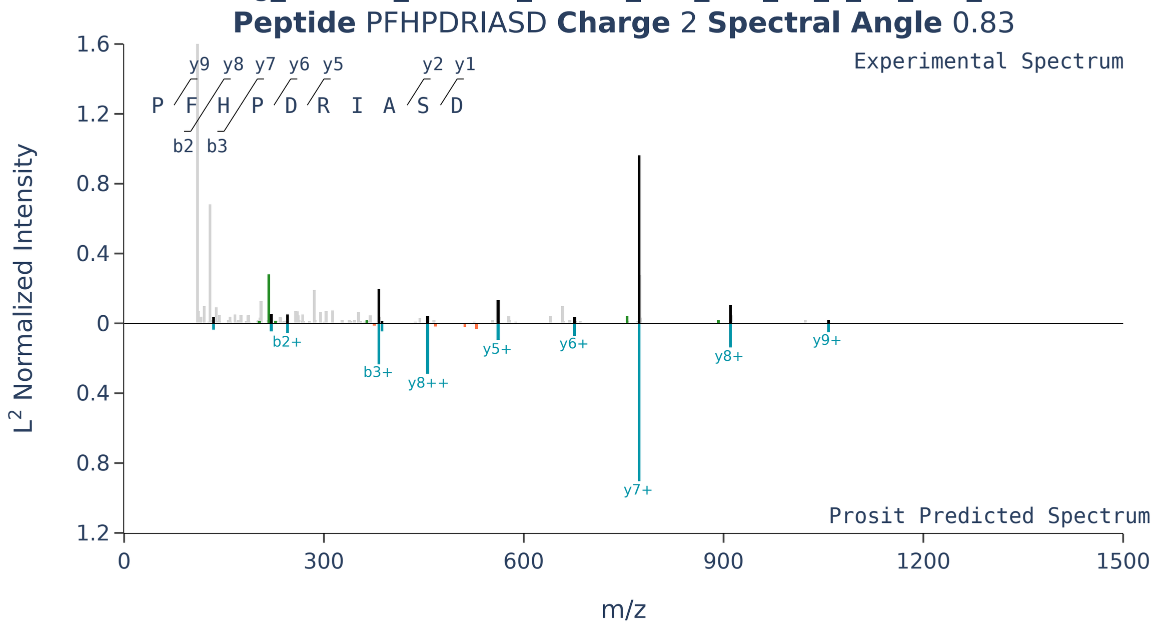

Source Ncheng_210623_230623_HFGoe_FFH_20S_25_1_A2_4h_R1 **Scan** 15334
Peptide ALEPFHPDRLASK **Charge** 3 **Spectral Angle** 0.77

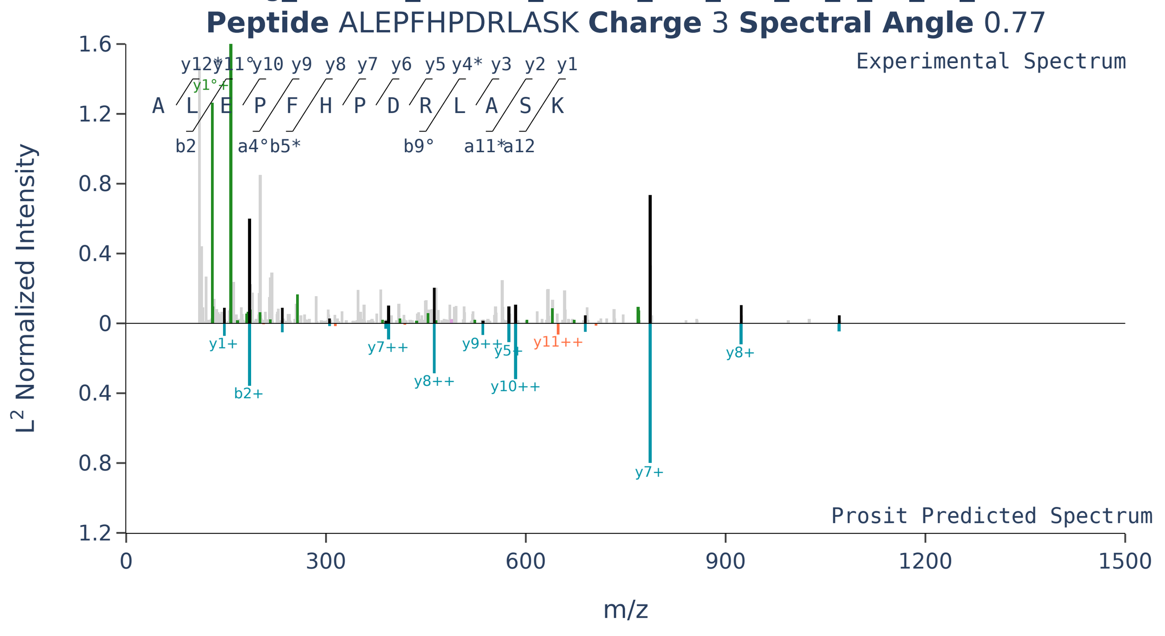

Source Ncheng_210623_230623_HFGoe_FFH_20S_25_1_A2_4h_R1 **Scan** 23571
Peptide AKETLFVVD **Charge** 2 **Spectral Angle** 0.79

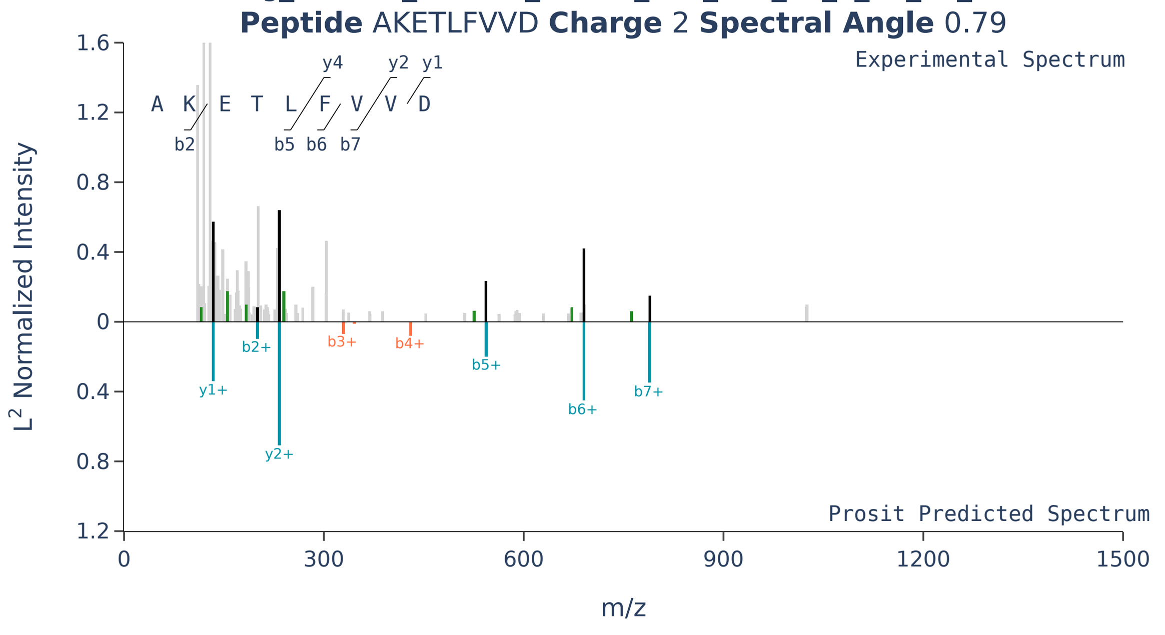

Source Ncheng_210623_230623_HFGoe_FFH_20S_25_1_A1_24h_R1 **Scan** 12835
Peptide GKLKFYD **Charge** 2 **Spectral Angle** 0.73

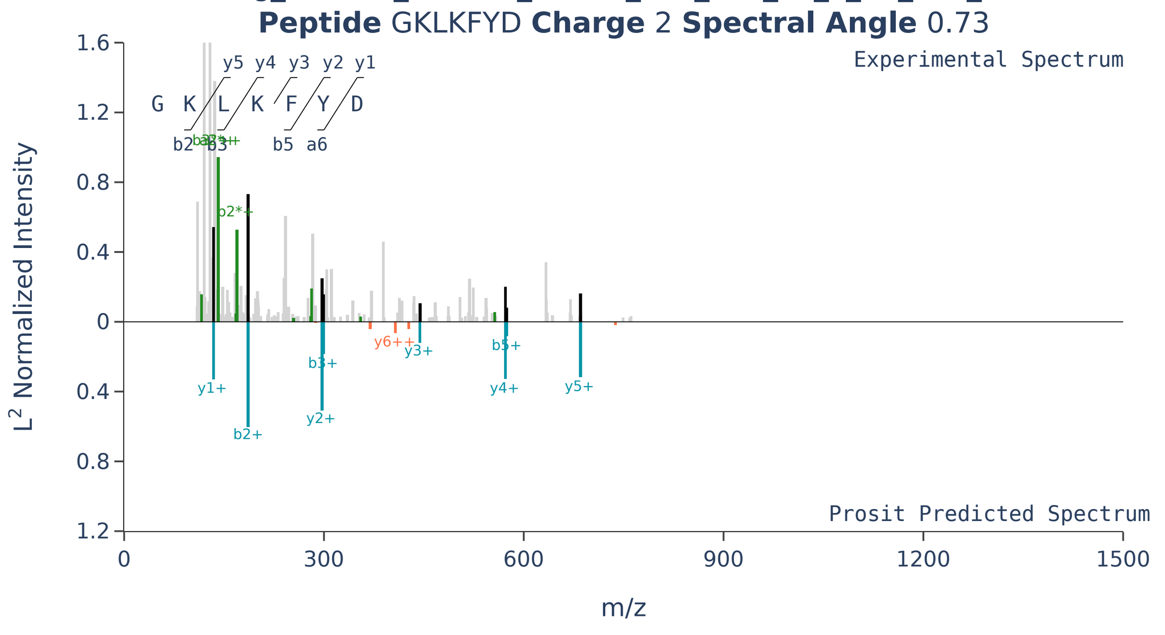

Source Ncheng_210623_230623_HFGoe_FFH_20S_25_1_A1_2h_R1 **Scan** 26910
Peptide RNISGQVGVDFFPSD **Charge** 2 **Spectral Angle** 0.82

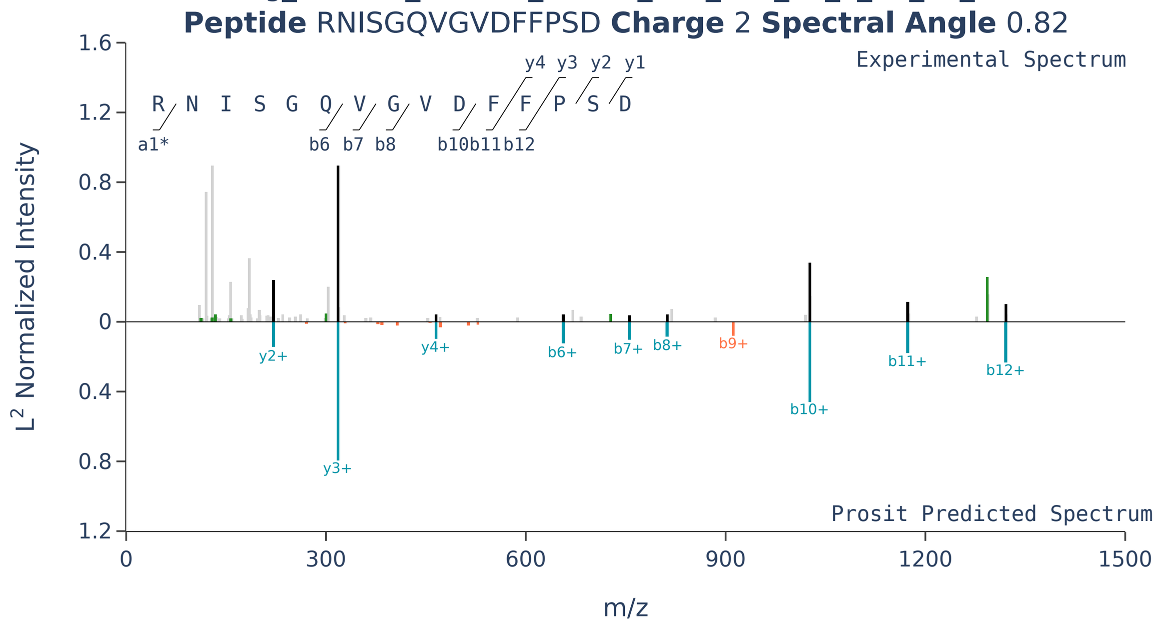

Source Ncheng_210623_230623_HFGoe_FFH_20S_25_1_A2_4h_R2 **Scan** 21666
Peptide MGKLPGMGQIPDNVKGMAS **Charge** 2 **Spectral Angle** 0.88

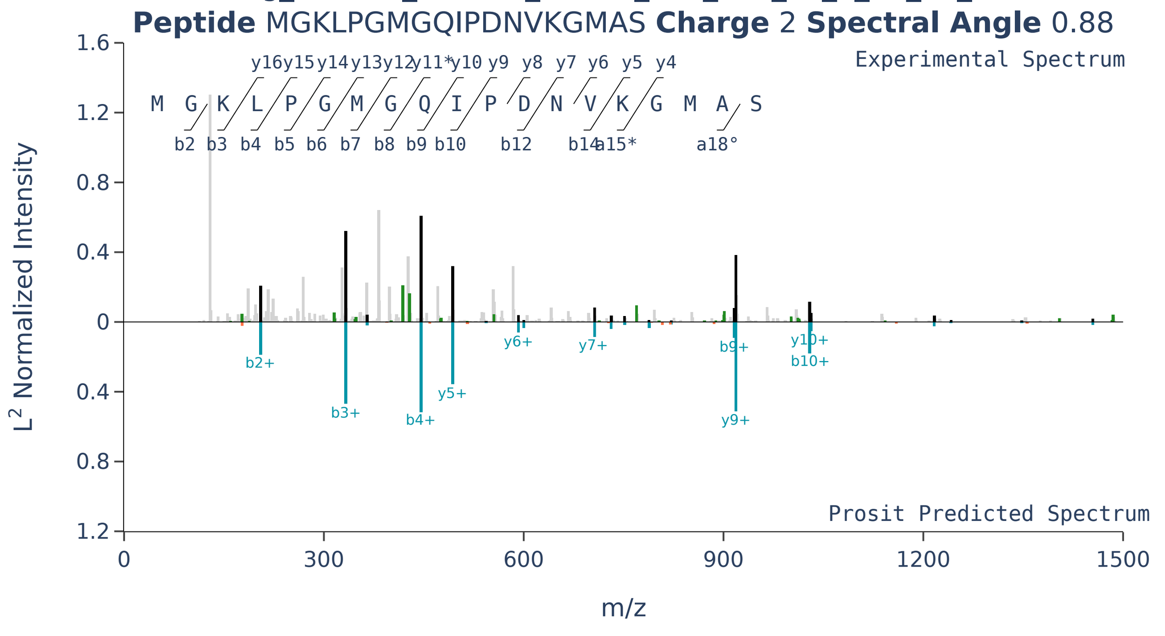

Source Ncheng_210623_230623_HFGoe_FFH_20S_25_1_A1_24h_R2 **Scan** 25968
Peptide ADVALPVVD **Charge** 1 **Spectral Angle** 0.91

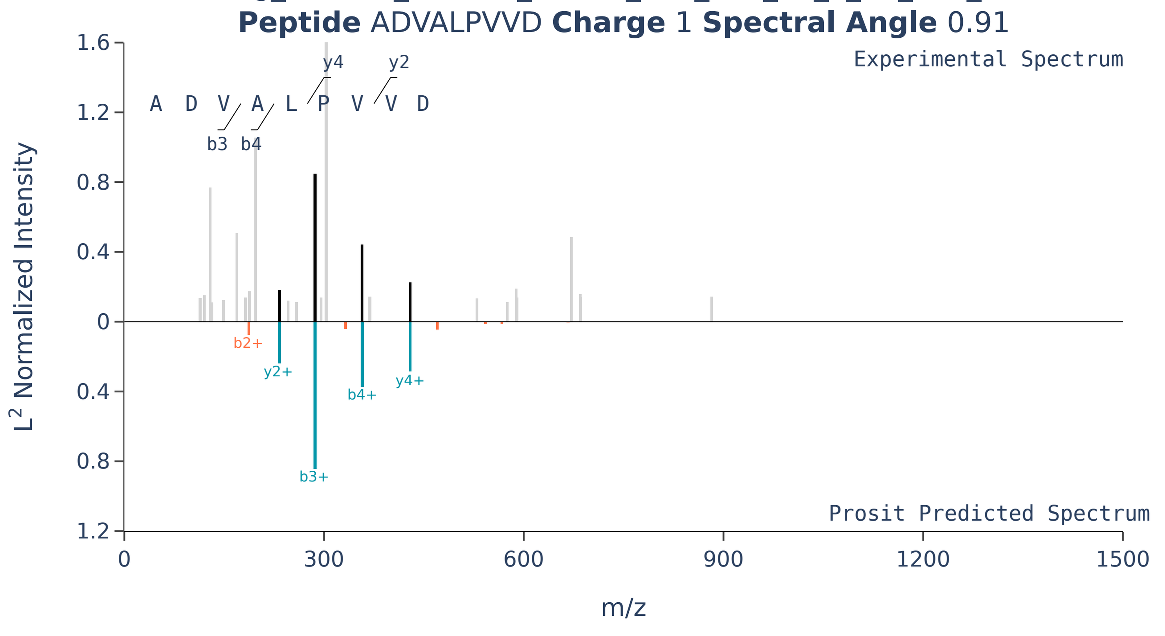

Source Ncheng_210623_230623_HFGoe_FFH_20S_25_1_A1_4h_R1 **Scan** 16055
Peptide QVQMTGQDAANTAKAFN **Charge** 3 **Spectral Angle** 0.82

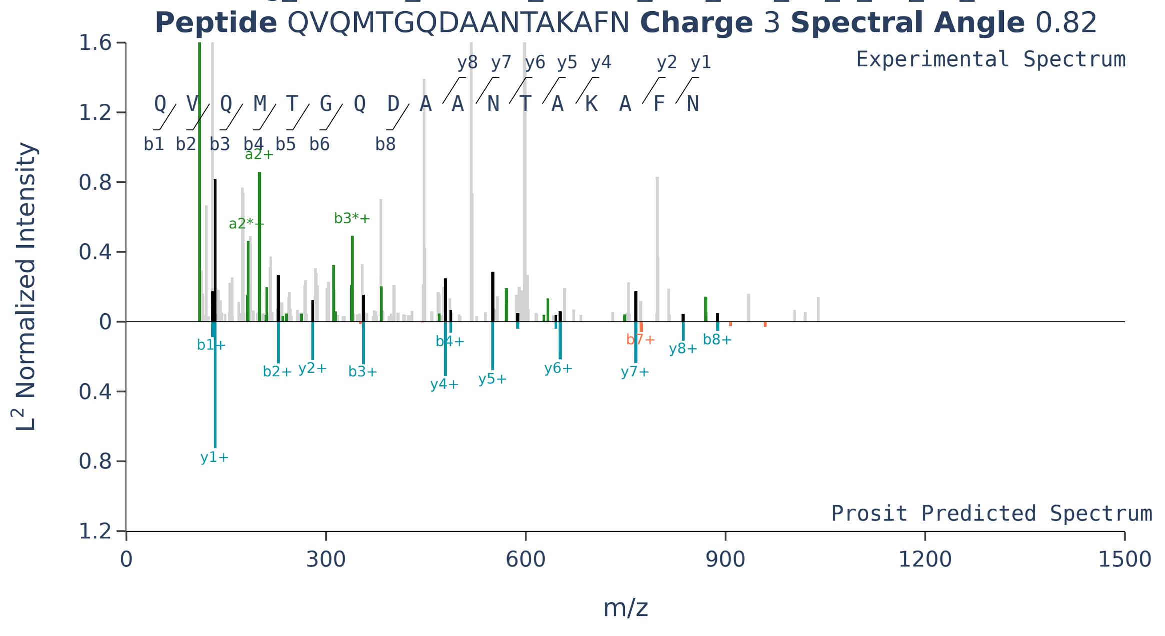

Source Ncheng_210623_230623_HFGoe_FFH_20S_25_1_A1_24h_R2 **Scan** 8661
Peptide KNMGGMARME **Charge** 2 **Spectral Angle** 0.78

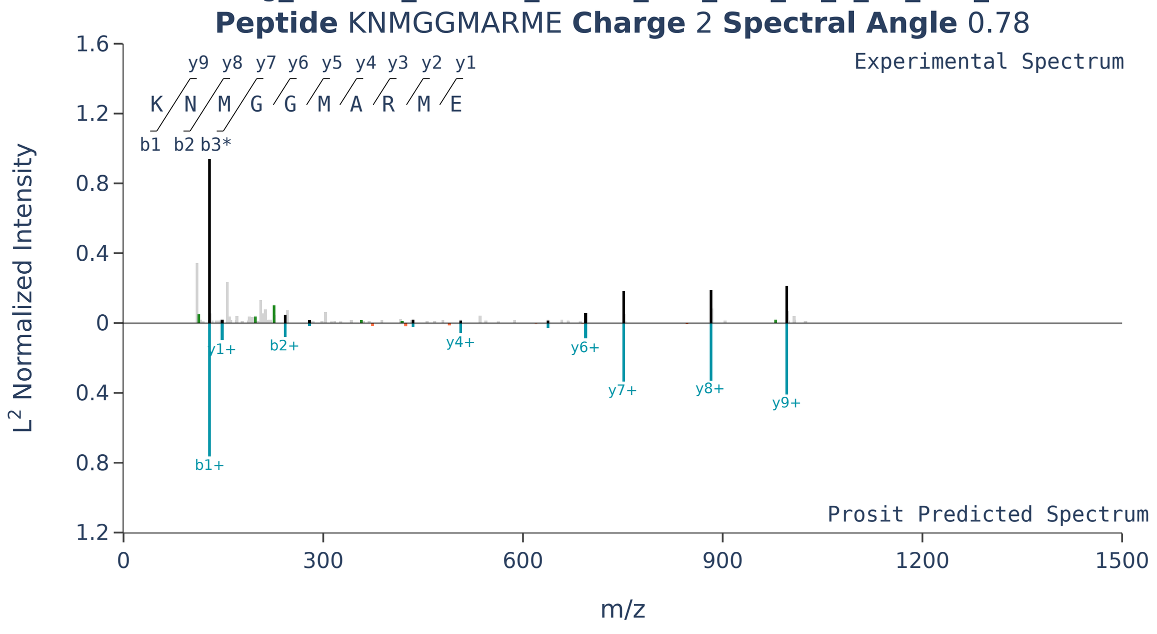

Source Ncheng_210623_230623_HFGoe_FFH_20S_25_1_A2_4h_R2 **Scan** 14342
Peptide AQAMTGQDAANTAKAFN **Charge** 2 **Spectral Angle** 0.82

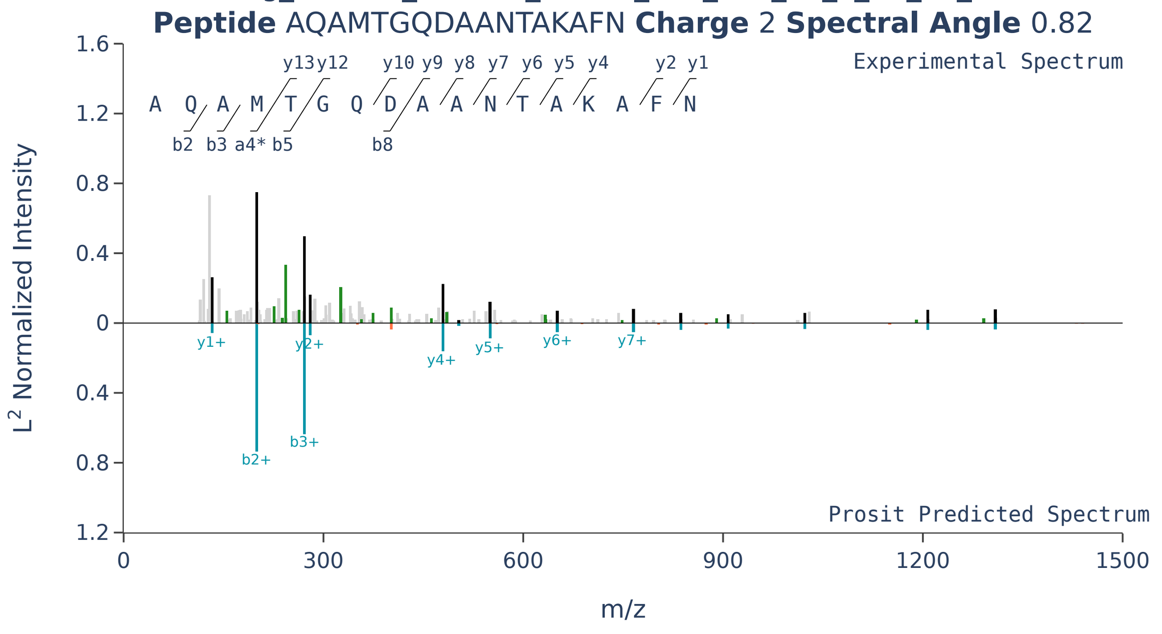

Source Ncheng_210623_230623_HFGoe_FFH_20S_25_1_A2_2h_R2 **Scan** 15329
Peptide TGKPIKFLGVGEKTED **Charge** 2 **Spectral Angle** 0.85

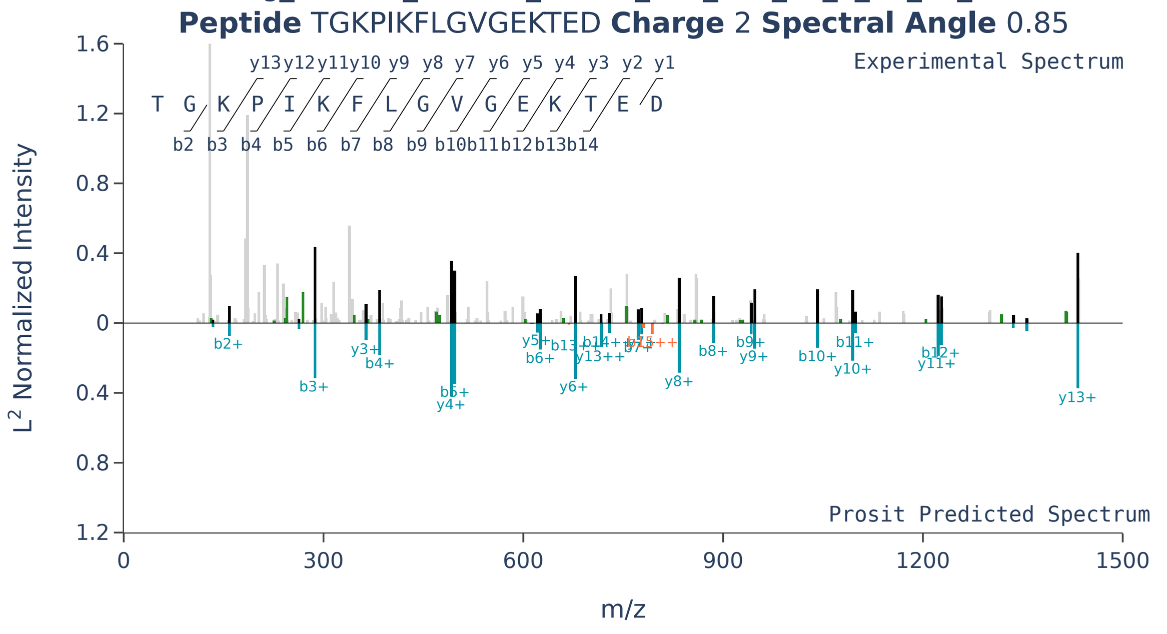

Source Ncheng_210623_230623_HFGoe_FFH_20S_25_1_A2_2h_R1 **Scan** 26158
Peptide EALPLTGAMTGQDAANTAKAFN **Charge** 3 **Spectral Angle** 0.91

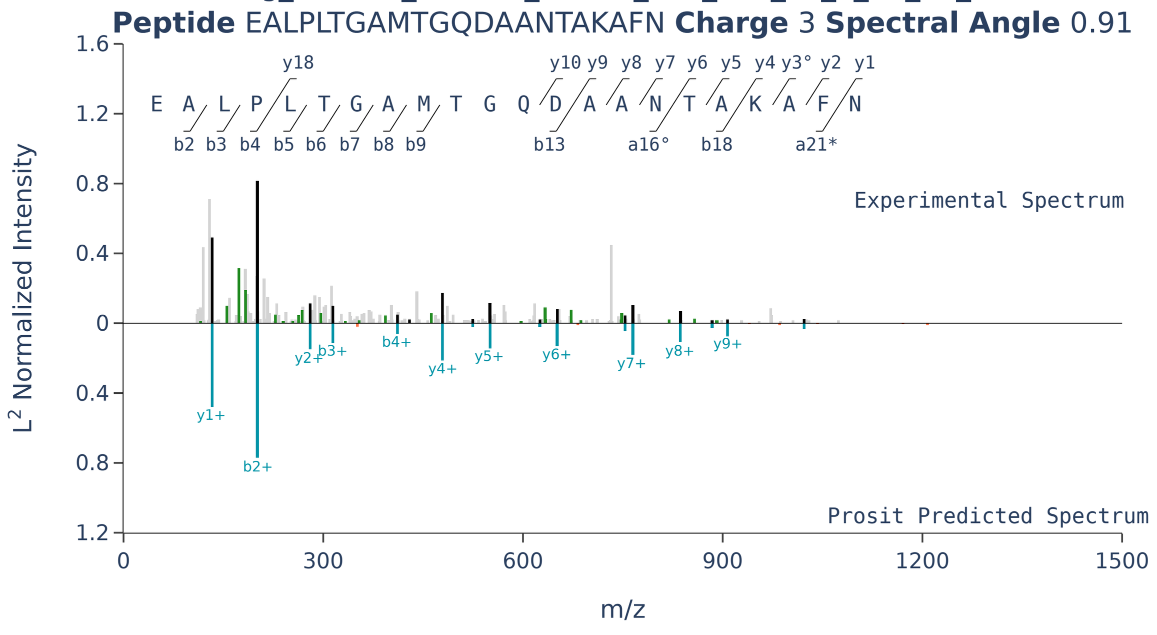

Source Ncheng_210623_230623_HFGoe_FFH_20S_25_1_A2_24h_R2 **Scan** 17333
Peptide GVGEAMTGQDAANTAKAFN **Charge** 2 **Spectral Angle** 0.75

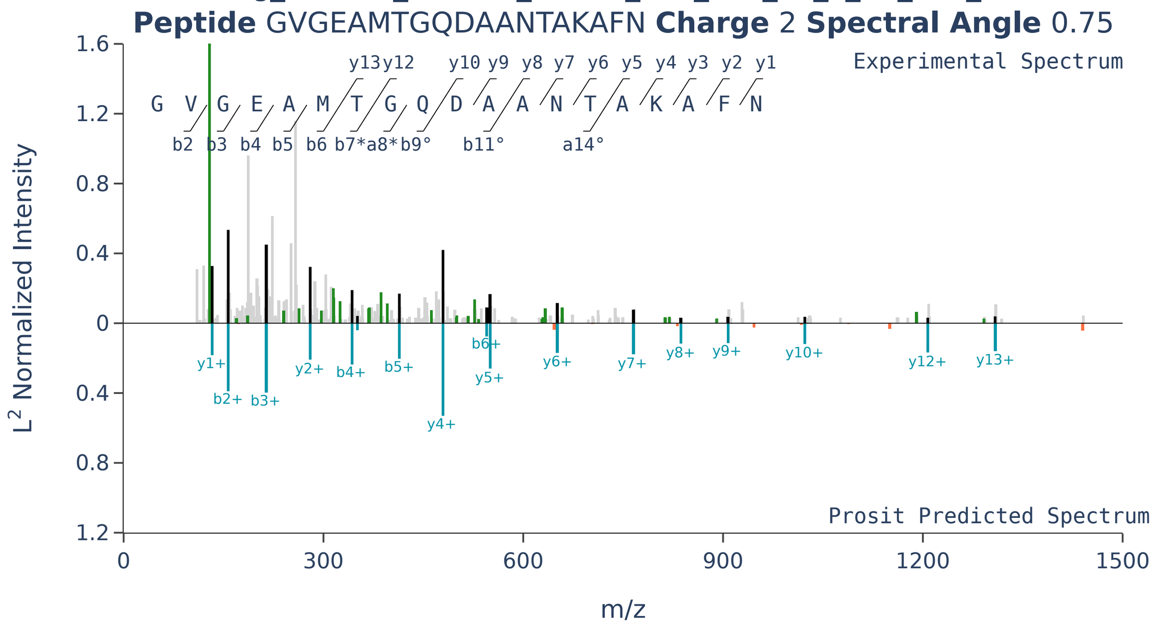

Source Ncheng_210623_230623_HFGoe_FFH_20S_25_1_A2_24h_R1 **Scan** 15066
Peptide GAMTGQDAANTAKAFNE **Charge** 2 **Spectral Angle** 0.82

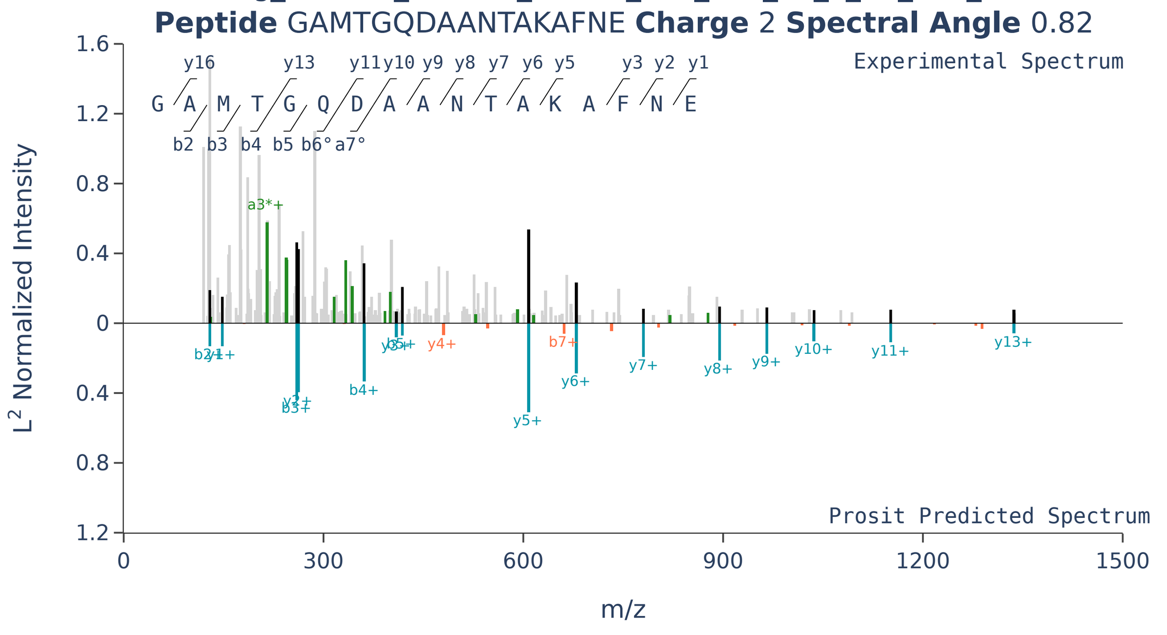

Source Ncheng_210623_230623_HFGoe_FFH_20S_25_1_A2_2h_R2 **Scan** 10421
Peptide AAGCMQVQD **Charge** 2 **Spectral Angle** 0.79

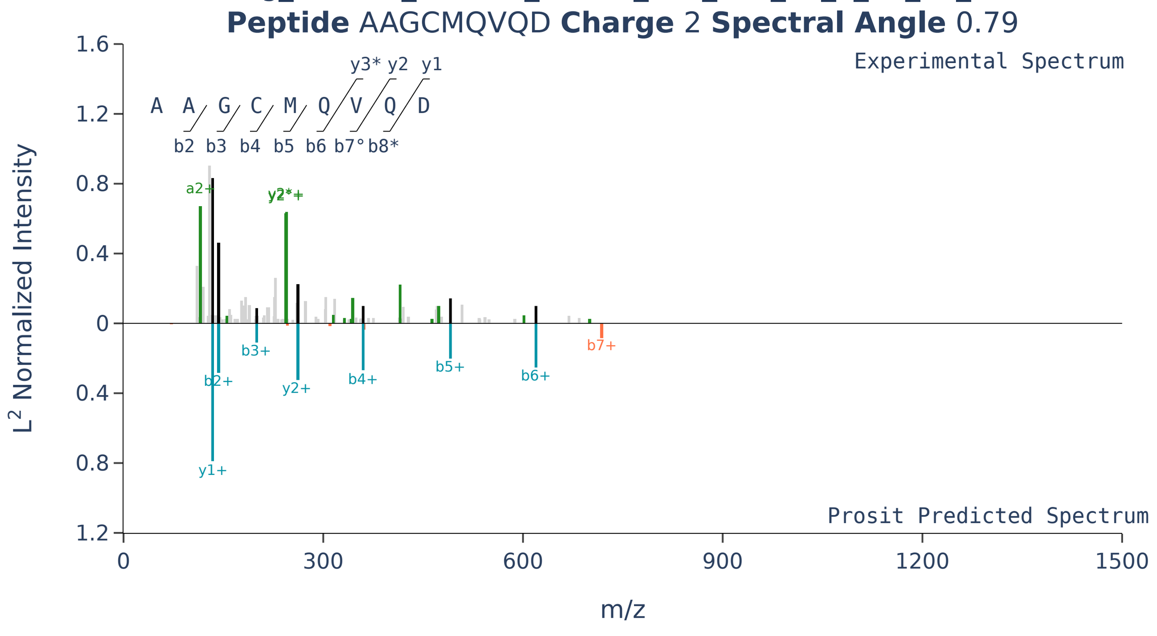

Source Ncheng_210623_230623_HFGoe_FFH_20S_25_1_A1_24h_R1 **Scan** 9986
Peptide AEQVGAMTGQD **Charge** 2 **Spectral Angle** 0.91

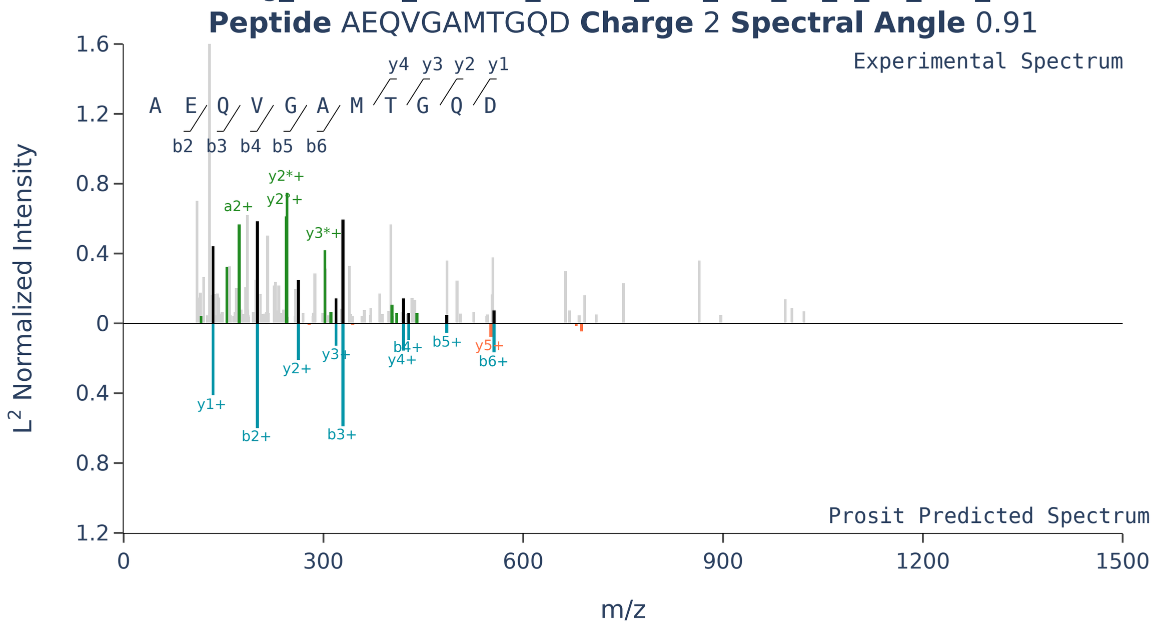

Source Ncheng_210623_230623_HFGoe_FFH_20S_25_1_A2_24h_R1 **Scan** 6554
Peptide GAGLQGAGKTT **Charge** 2 **Spectral Angle** 0.76

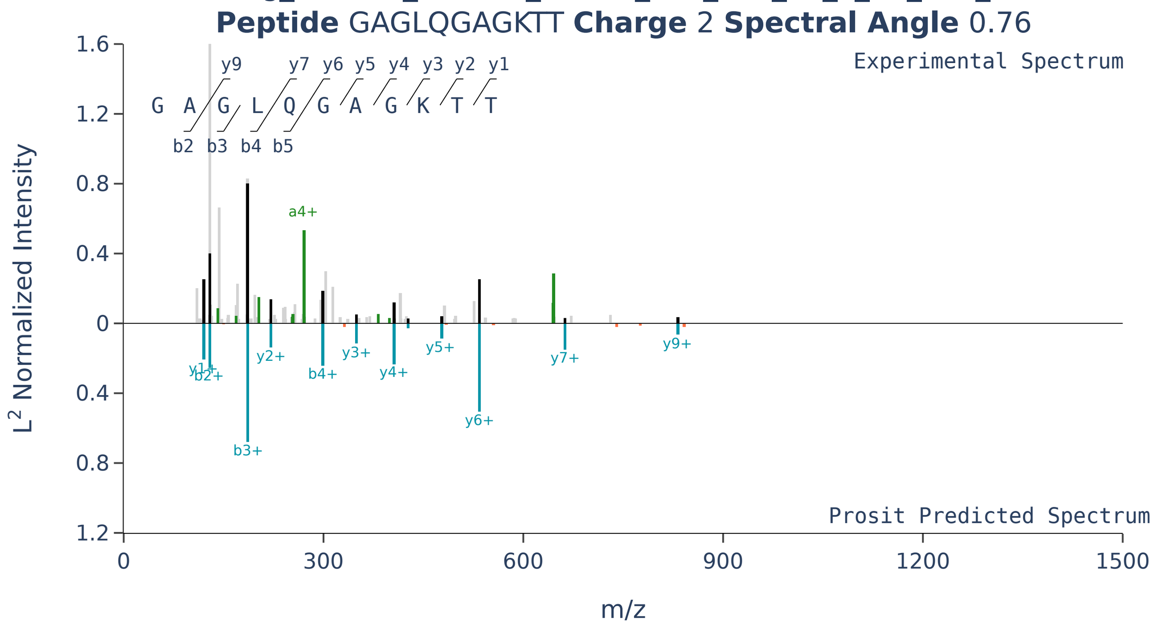

Source Ncheng_210623_230623_HFGoe_FFH_20S_25_1_A2_24h_R2 **Scan** 9799
Peptide GMQVGQKPVD **Charge** 2 **Spectral Angle** 0.78

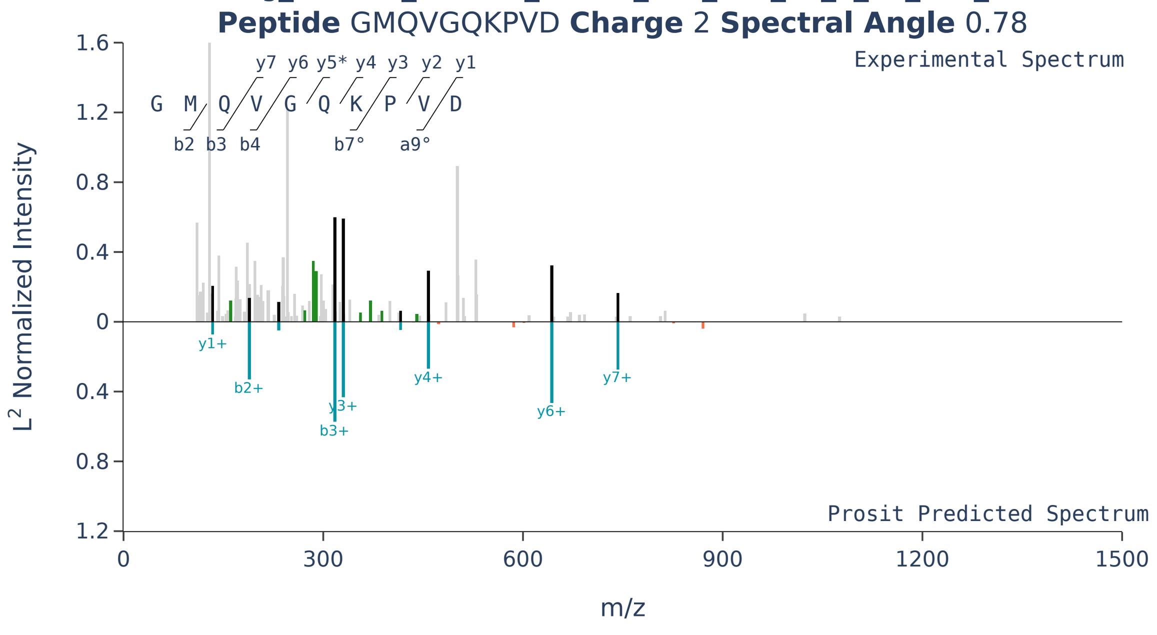

Source Ncheng_210623_230623_HFGoe_FFH_20S_25_1_A2_4h_R2 **Scan** 12047
Peptide TLAEVGQKPVD **Charge** 2 **Spectral Angle** 0.81

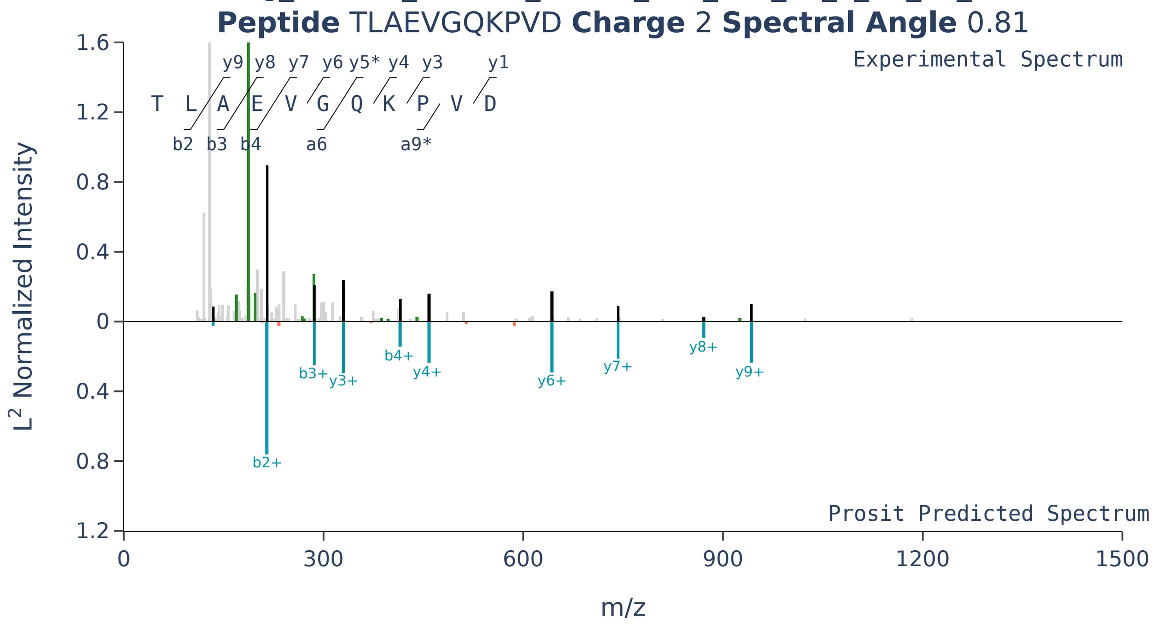

Source Ncheng_210623_230623_HFGoe_FFH_20S_25_1_A2_4h_R1 **Scan** 11483
Peptide DIESVGQKPVD **Charge** 2 **Spectral Angle** 0.8

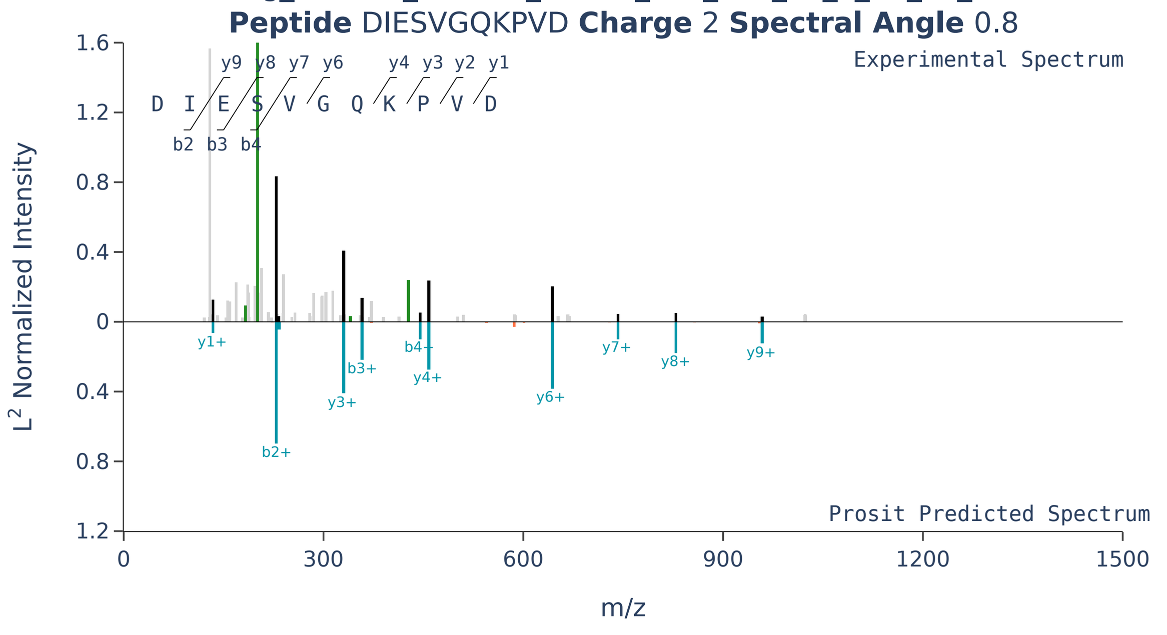

Source Ncheng_210623_230623_HFGoe_FFH_20S_25_1_A2_4h_R2 **Scan** 14245
Peptide RSMAMTGQDAANTAKAFN **Charge** 3 **Spectral Angle** 0.83

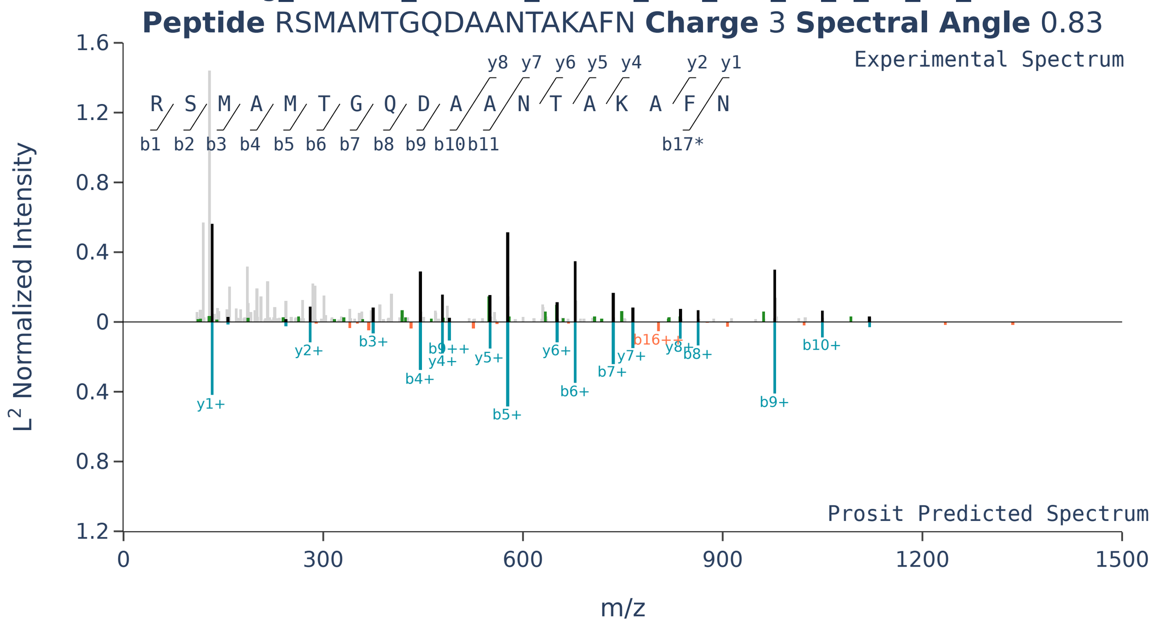

Source Ncheng_210623_230623_HFGoe_FFH_20S_25_1_A1_24h_R2 **Scan** 9645
Peptide KLKFYDKTE **Charge** 2 **Spectral Angle** 0.86

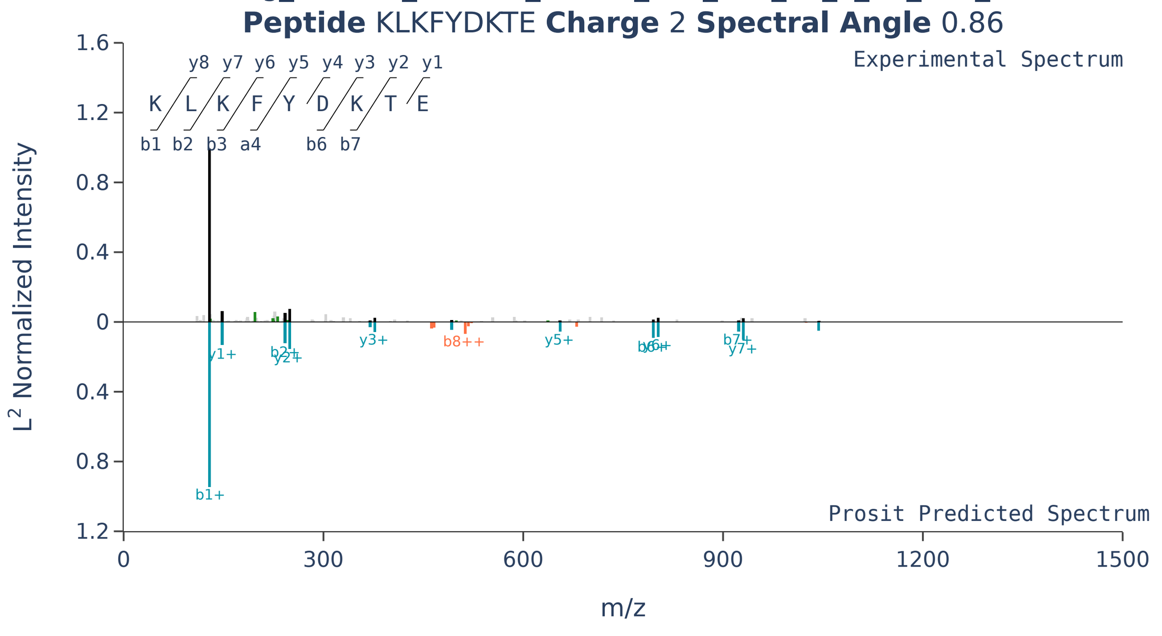

Source Ncheng_210623_230623_HFGoe_FFH_20S_25_1_A1_24h_R1 **Scan** 7533
Peptide GVGEKTEVGQKPVD **Charge** 2 **Spectral Angle** 0.83

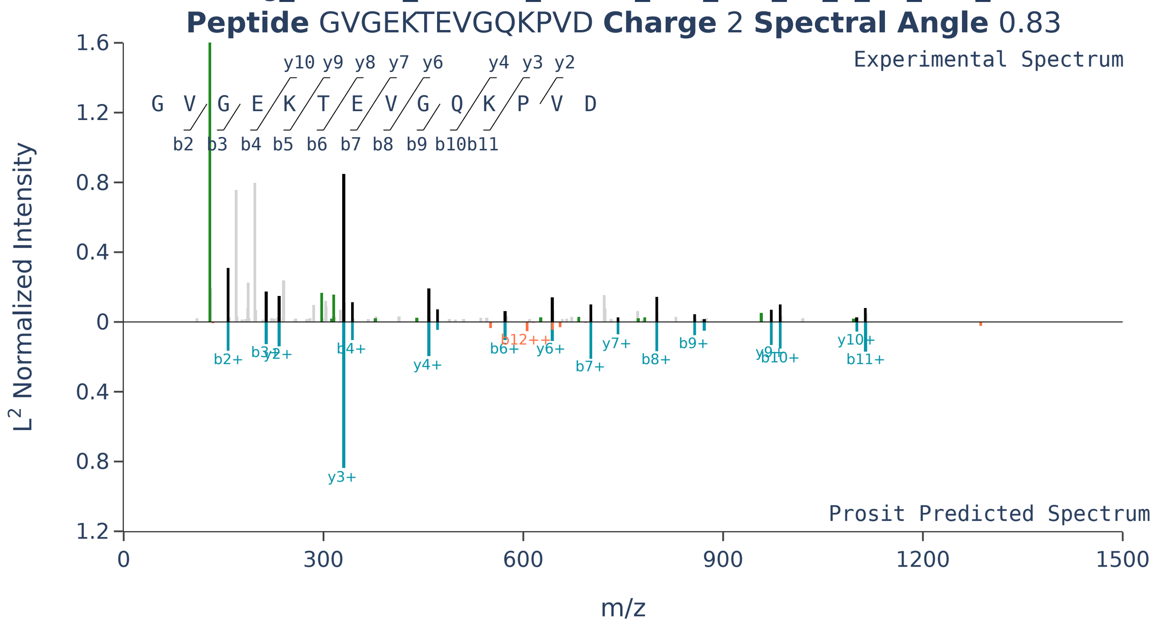

Source Ncheng_210623_230623_HFGoe_FFH_20S_25_1_A1_2h_R1 **Scan** 25416
Peptide TKEQVGVDFFPSD **Charge** 2 **Spectral Angle** 0.92

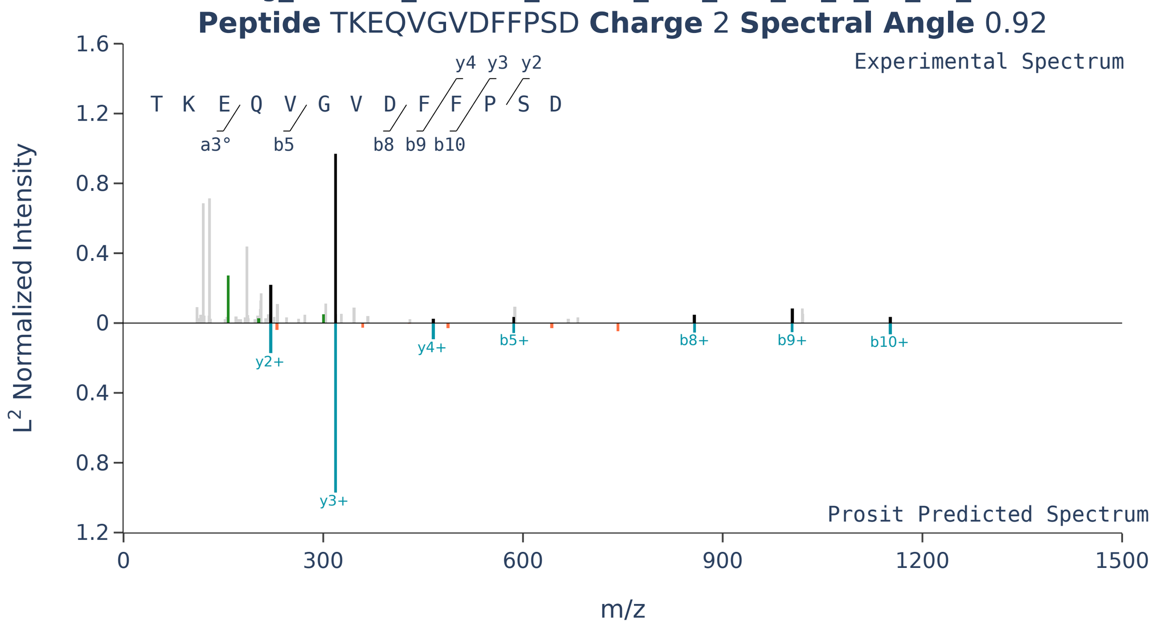

Source Ncheng_210623_230623_HFGoe_FFH_20S_25_1_A1_4h_R1 **Scan** 22260
Peptide DMGKLPGMGQIPDNVKSQ **Charge** 3 **Spectral Angle** 0.76

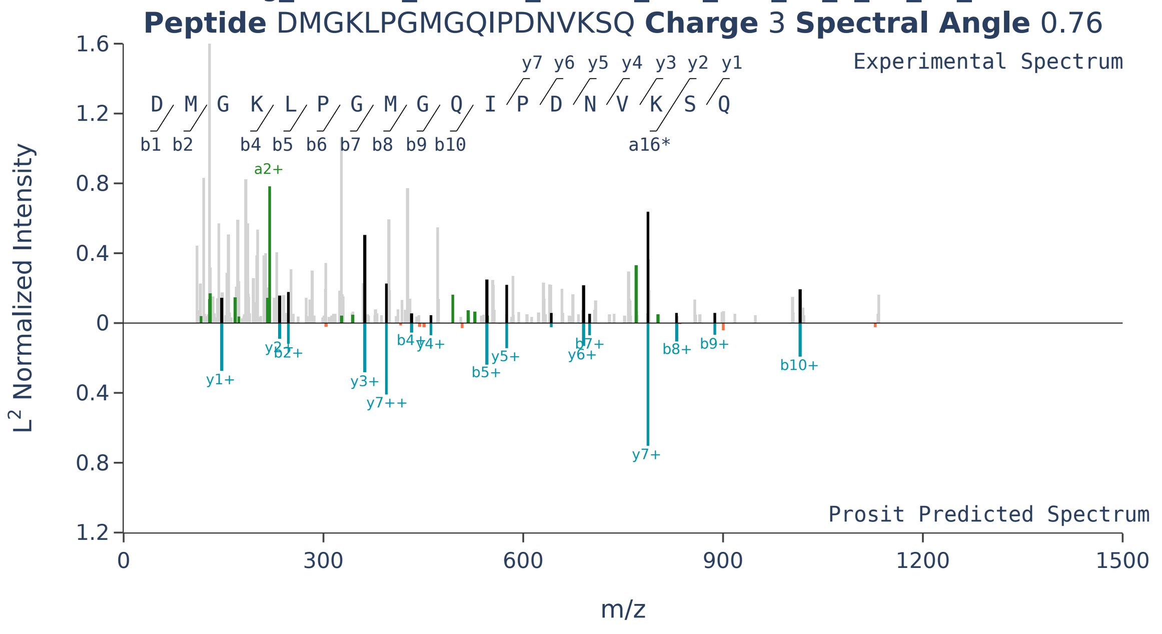

Source Ncheng_210623_230623_HFGoe_FFH_20S_25_1_A2_4h_R1 **Scan** 23534
Peptide SAKLPGMGQIPDNV **Charge** 2 **Spectral Angle** 0.86

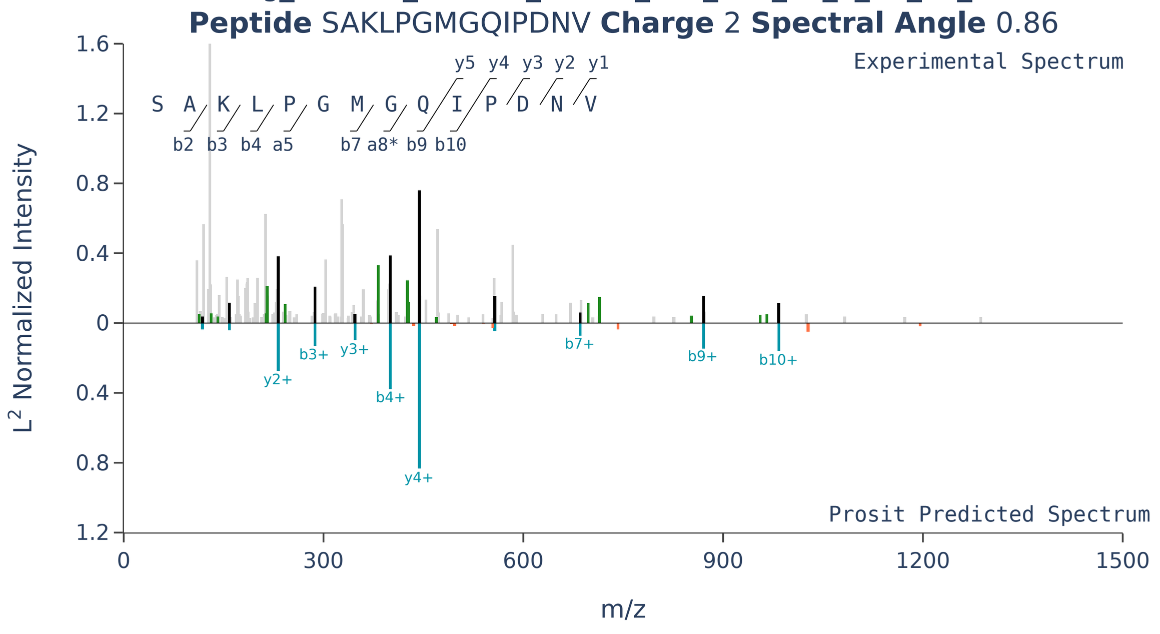

Source Ncheng_210623_230623_HFGoe_FFH_20S_25_1_A2_24h_R1 **Scan** 9286
Peptide RNISGAGLQG **Charge** 2 **Spectral Angle** 0.88

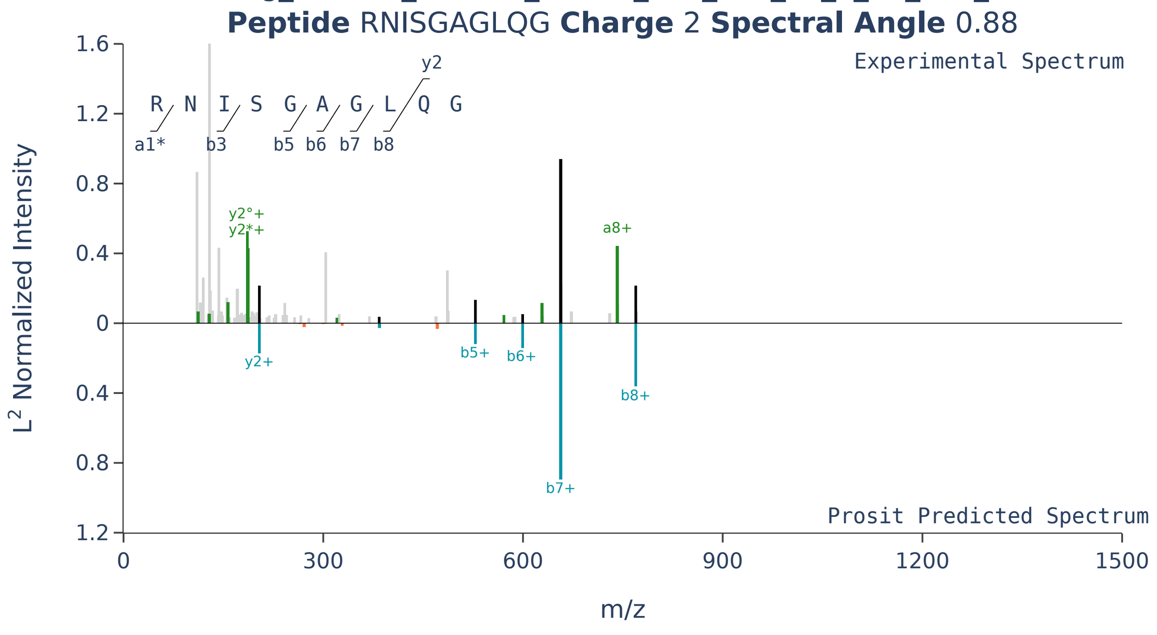

Source Ncheng_210623_230623_HFGoe_FFH_20S_25_1_A1_2h_R2 **Scan** 17782
Peptide DEAMMDLTE **Charge** 2 **Spectral Angle** 0.87

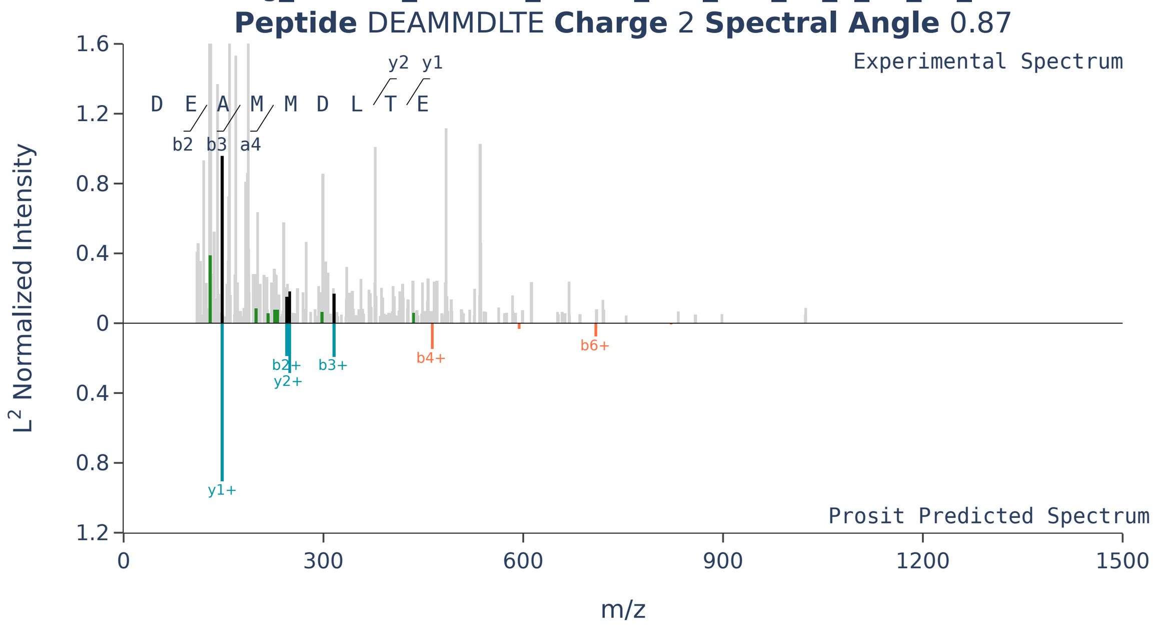

Source Ncheng_210623_230623_HFGoe_FFH_20S_25_1_A2_24h_R2 **Scan** 12142
Peptide DVYRPAAIKQA **Charge** 2 **Spectral Angle** 0.78

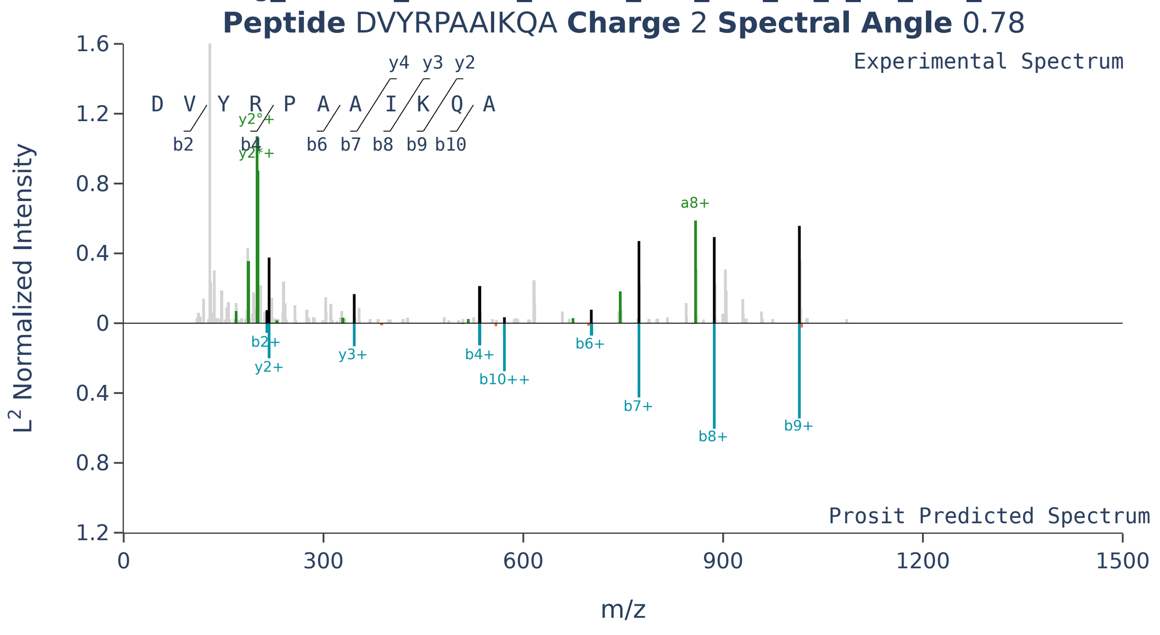

Source Ncheng_210623_230623_HFGoe_FFH_20S_25_1_A1_4h_R1 **Scan** 21470
Peptide DIESAMTGQDAANTAKAFN **Charge** 2 **Spectral Angle** 0.8

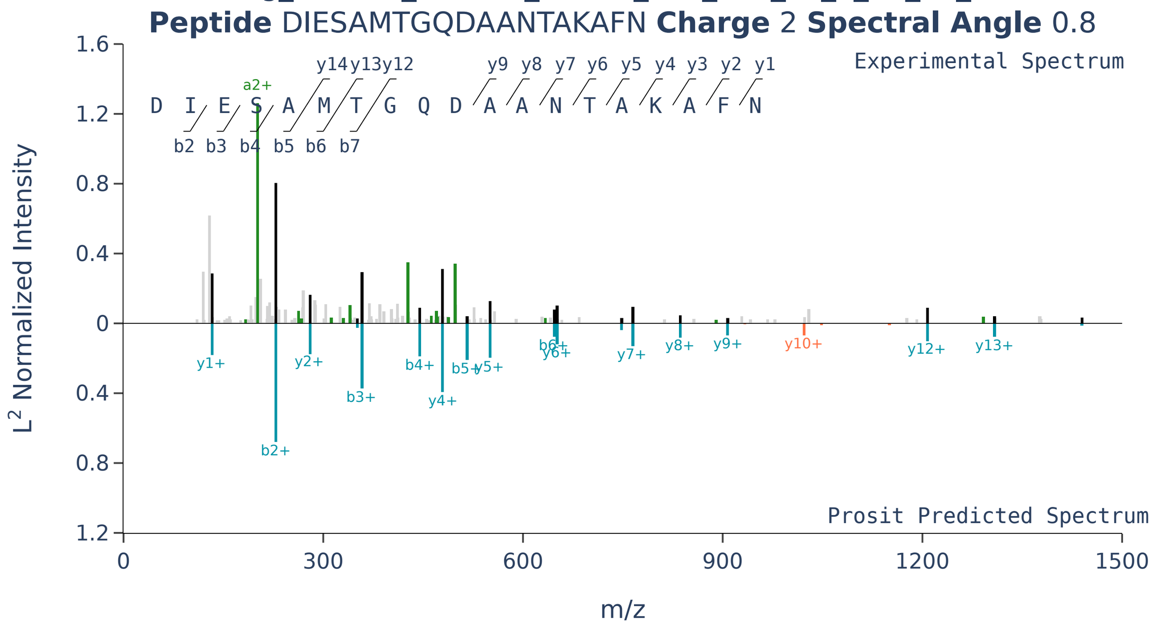

Source Ncheng_210623_230623_HFGoe_FFH_20S_25_1_A1_2h_R2 **Scan** 10534
Peptide KVLVRMED **Charge** 2 **Spectral Angle** 0.69

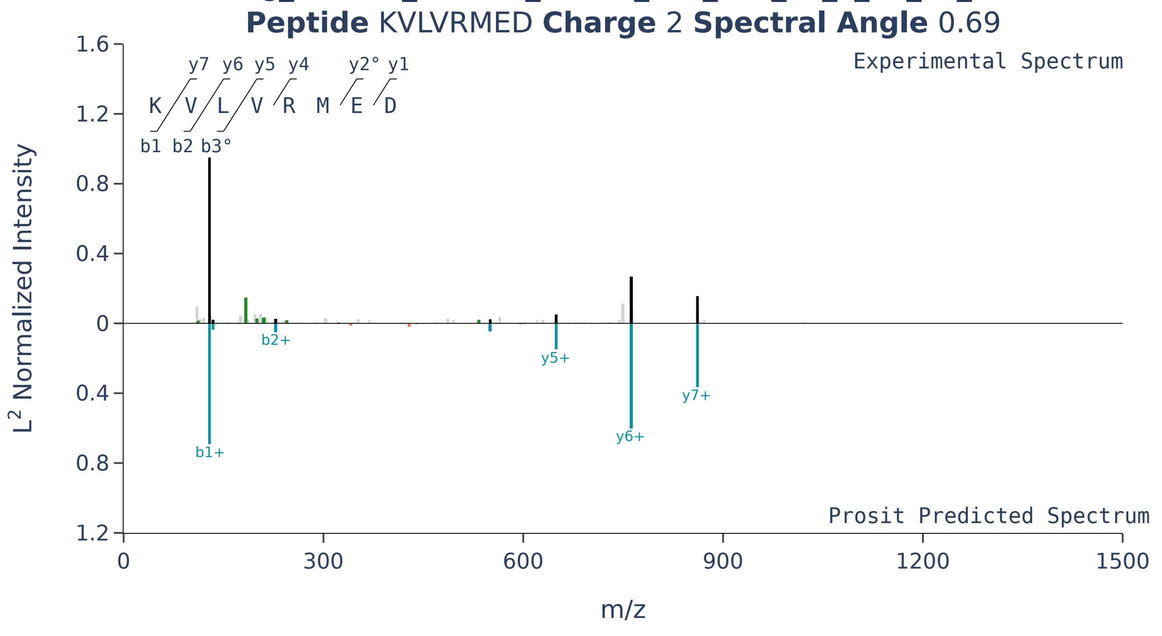

Source Ncheng_210623_230623_HFGoe_FFH_20S_25_1_A2_24h_R2 **Scan** 9431
Peptide RNISGSIRHI **Charge** 3 **Spectral Angle** 0.81

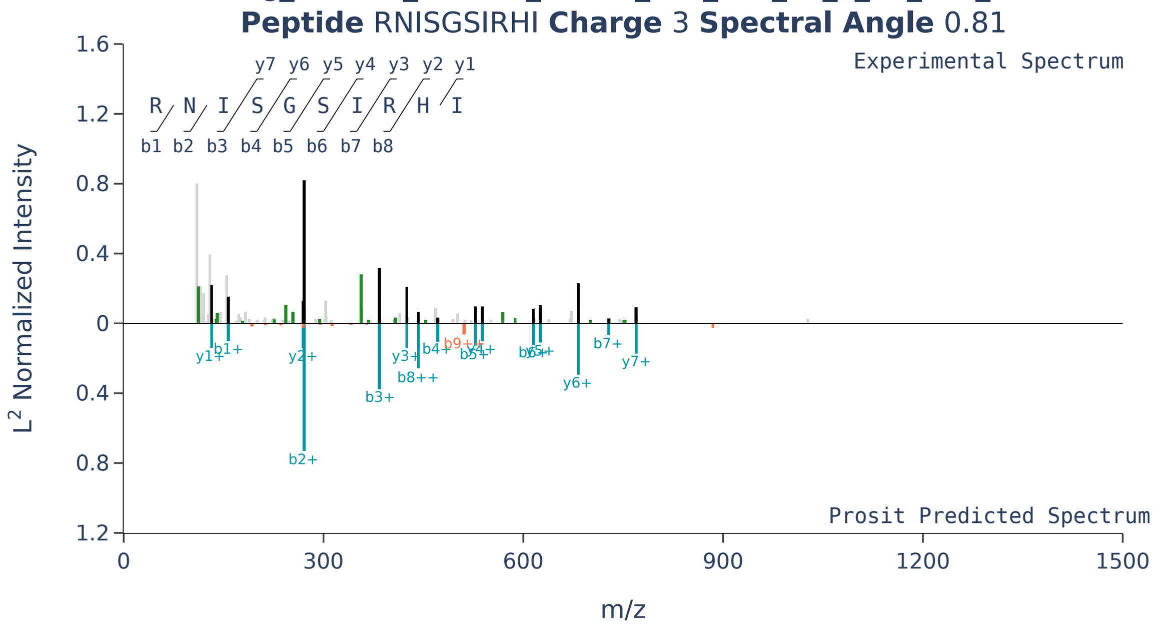

Source Ncheng_210623_230623_HFGoe_FFH_20S_25_1_A1_4h_R2 **Scan** 23812
Peptide DGMGKLPGMGQIPDNVKSQMD **Charge** 3 **Spectral Angle** 0.77

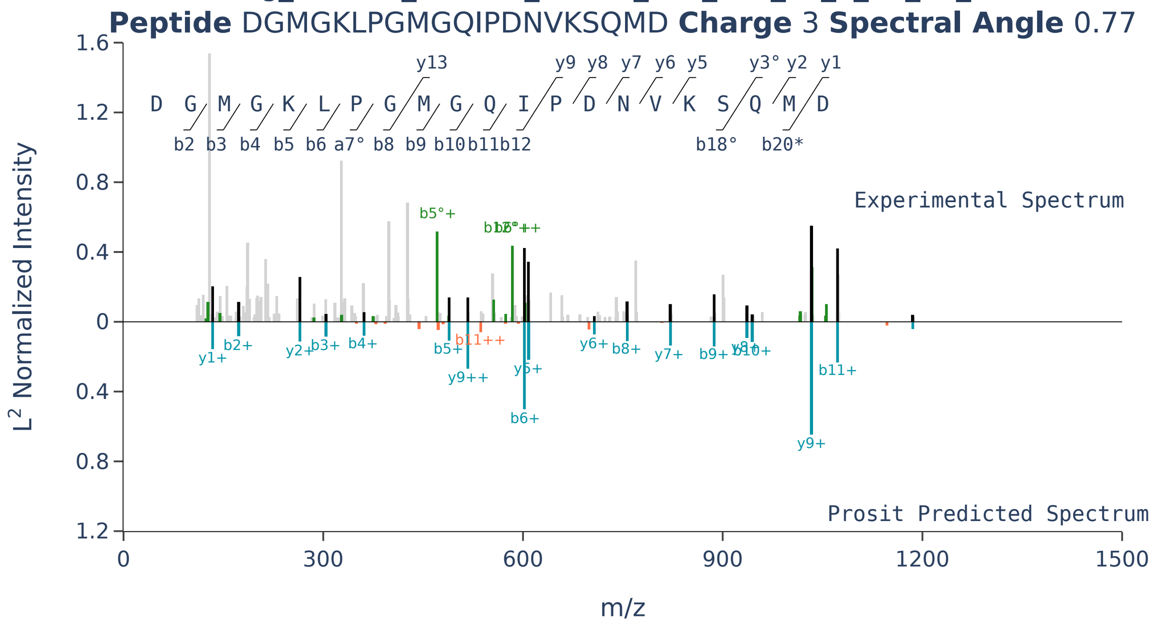

Source Ncheng_210623_230623_HFGoe_FFH_20S_25_1_A2_24h_R2 **Scan** 7595
Peptide LGVGEKTED **Charge** 1 **Spectral Angle** 0.83

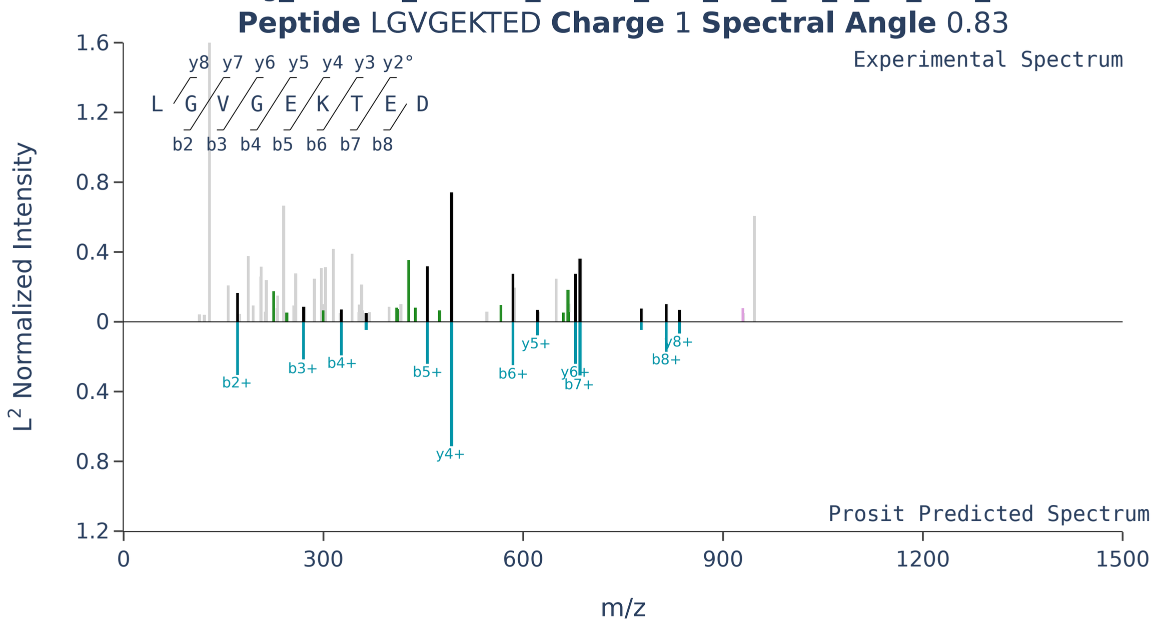

Source Ncheng_210623_230623_HFGoe_FFH_20S_25_1_A1_4h_R2 **Scan** 10367
Peptide QHASINPVE **Charge** 2 **Spectral Angle** 0.86

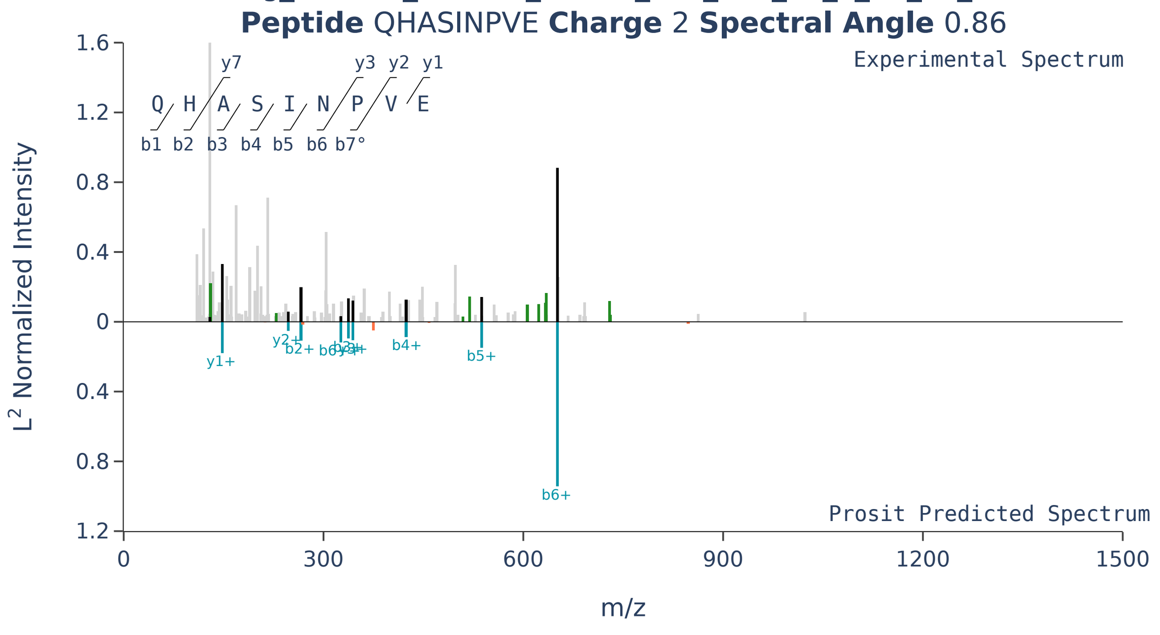

Source Ncheng_210623_230623_HFGoe_FFH_20S_25_1_A2_24h_R2 **Scan** 13171
Peptide AMTGQDAANTARL **Charge** 2 **Spectral Angle** 0.78

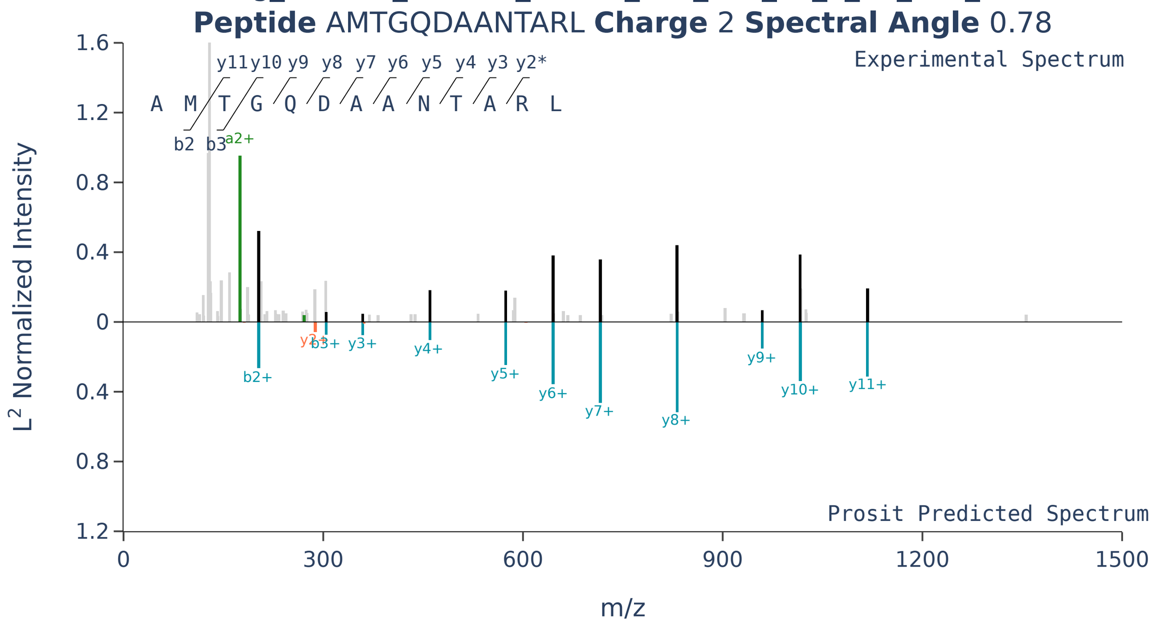

Source Ncheng_210623_230623_HFGoe_FFH_20S_25_1_A1_2h_R2 **Scan** 27541
Peptide AANTAKAFNEGVDFFPSD **Charge** 2 **Spectral Angle** 0.93

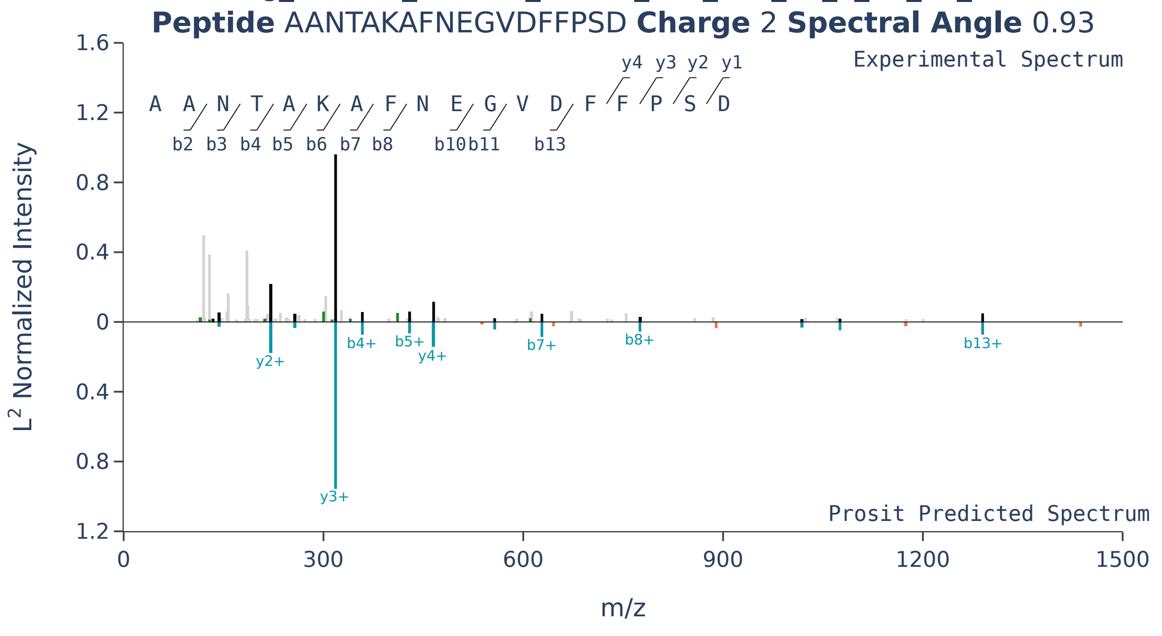

Source Ncheng_210623_230623_HFGoe_FFH_20S_25_1_A1_4h_R2 **Scan** 12085
Peptide KDTPIKF **Charge** 2 **Spectral Angle** 0.9

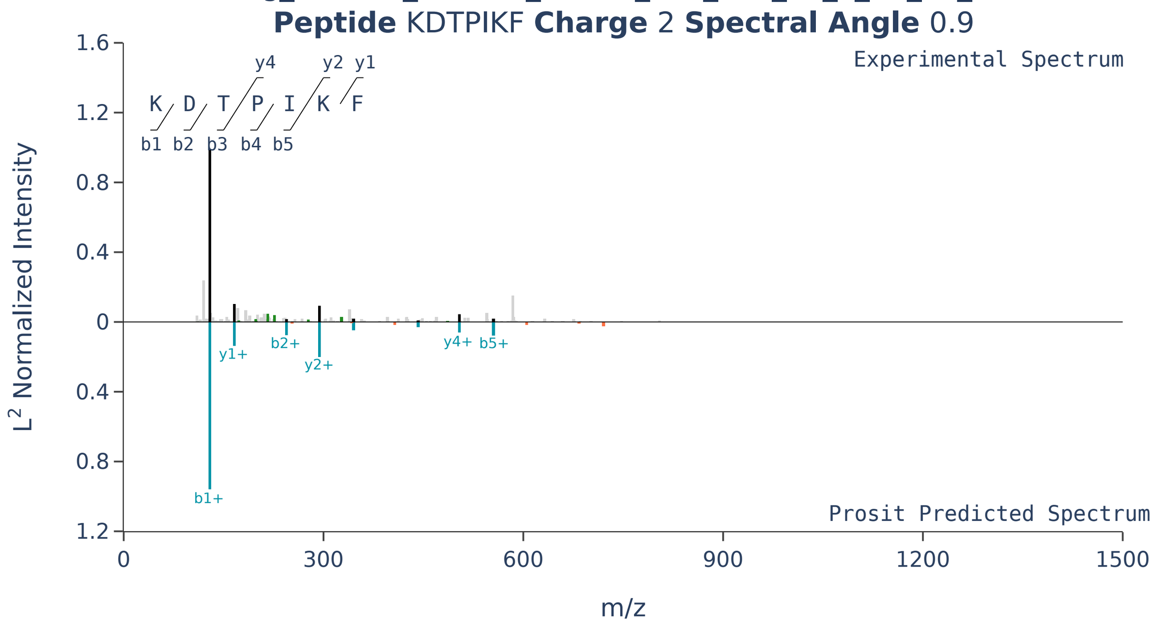

Source Ncheng_210623_230623_HFGoe_FFH_20S_25_1_A2_2h_R1 **Scan** 17849
Peptide GEENQTLENQTL **Charge** 1 **Spectral Angle** 0.78

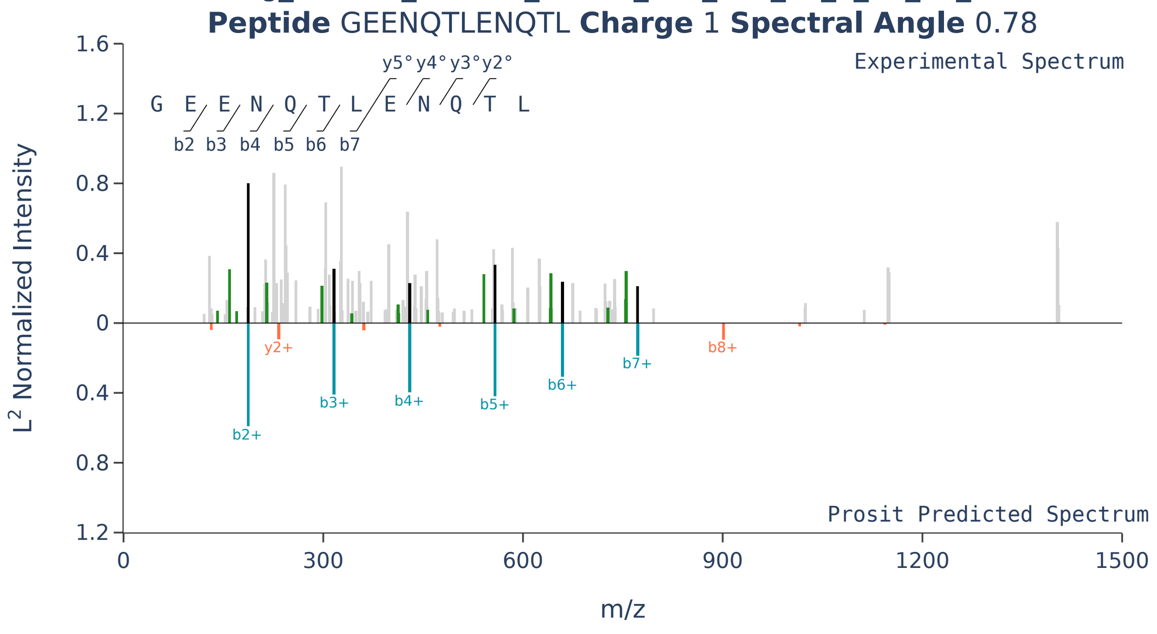

Source Ncheng_210623_230623_HFGoe_FFH_20S_25_1_A2_24h_R1 **Scan** 13153
Peptide KVDGDARGGAALKL **Charge** 2 **Spectral Angle** 0.84

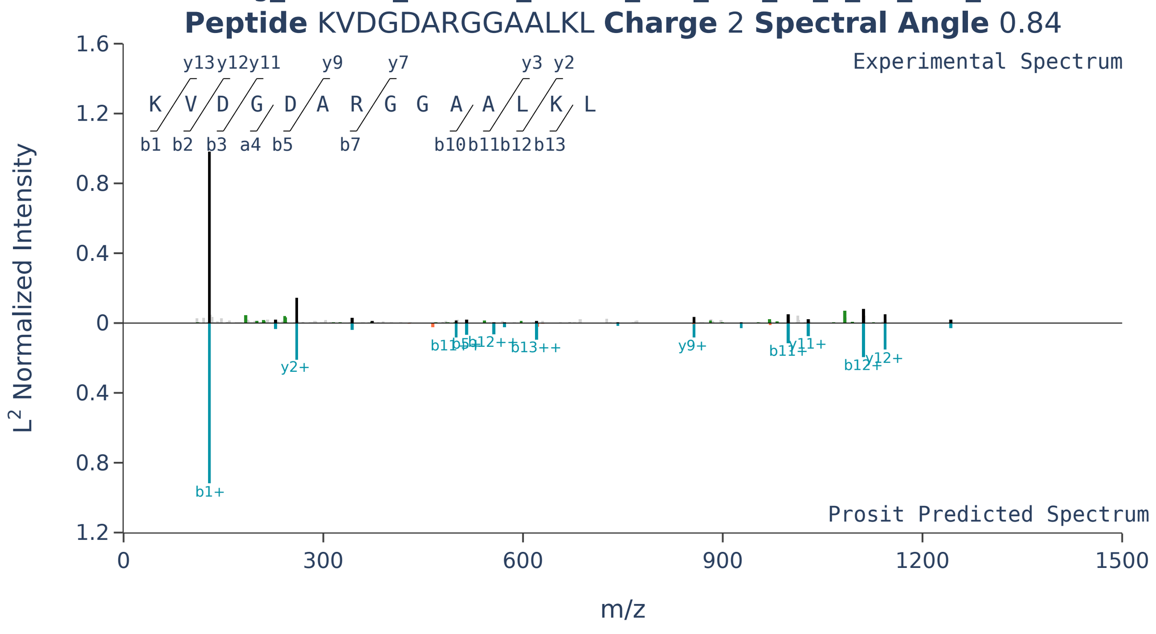

Source Ncheng_210623_230623_HFGoe_FFH_20S_25_1_A2_24h_R1 **Scan** 14319
Peptide EAMTGQDAANTAKAFN **Charge** 2 **Spectral Angle** 0.8

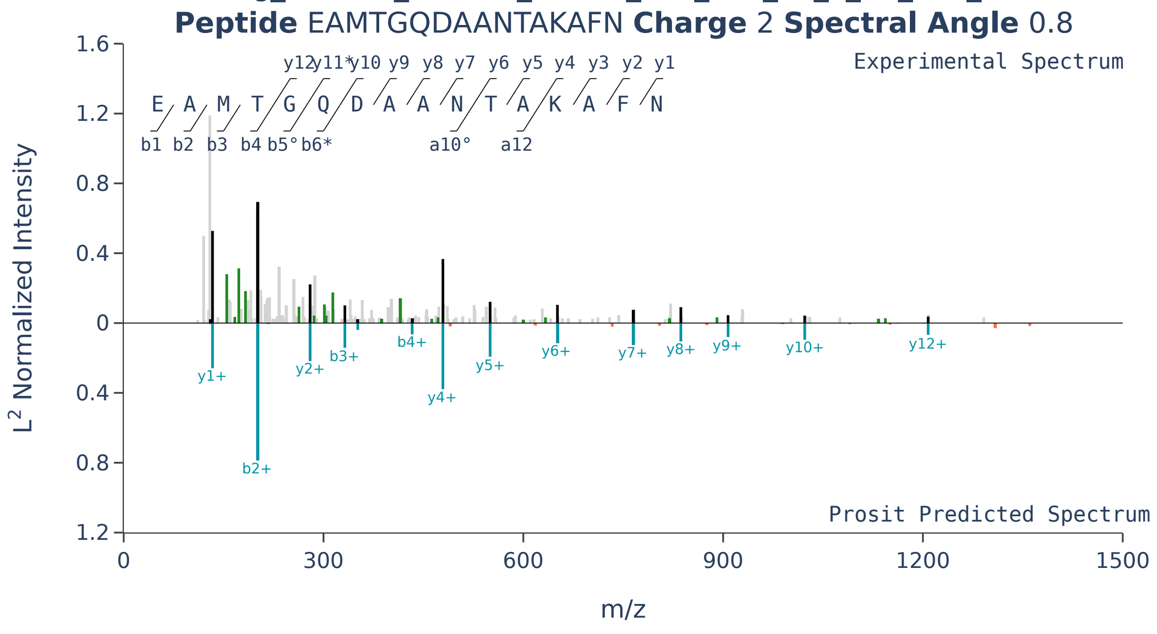

Source Ncheng_210623_230623_HFGoe_FFH_20S_25_1_A2_4h_R1 **Scan** 20775
Peptide AMGKLPGMGQIPDNVKSQ **Charge** 3 **Spectral Angle** 0.75

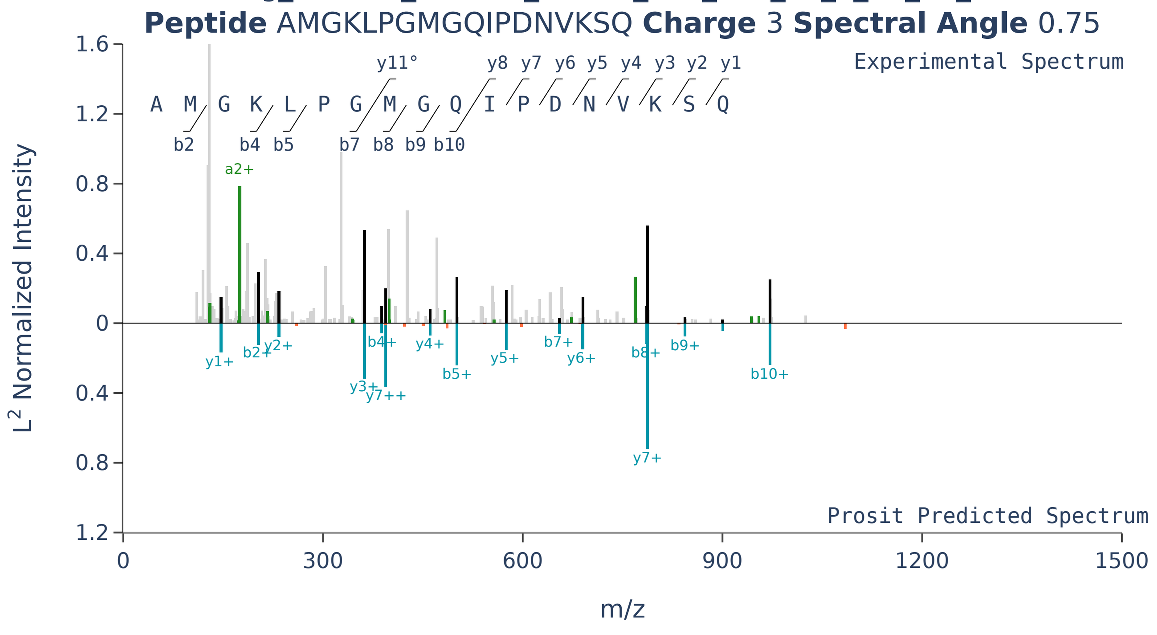

Source Ncheng_210623_230623_HFGoe_FFH_20S_25_1_A1_2h_R2 **Scan** 17593
Peptide KLPGMGQIPDNVKSQDM **Charge** 2 **Spectral Angle** 0.84

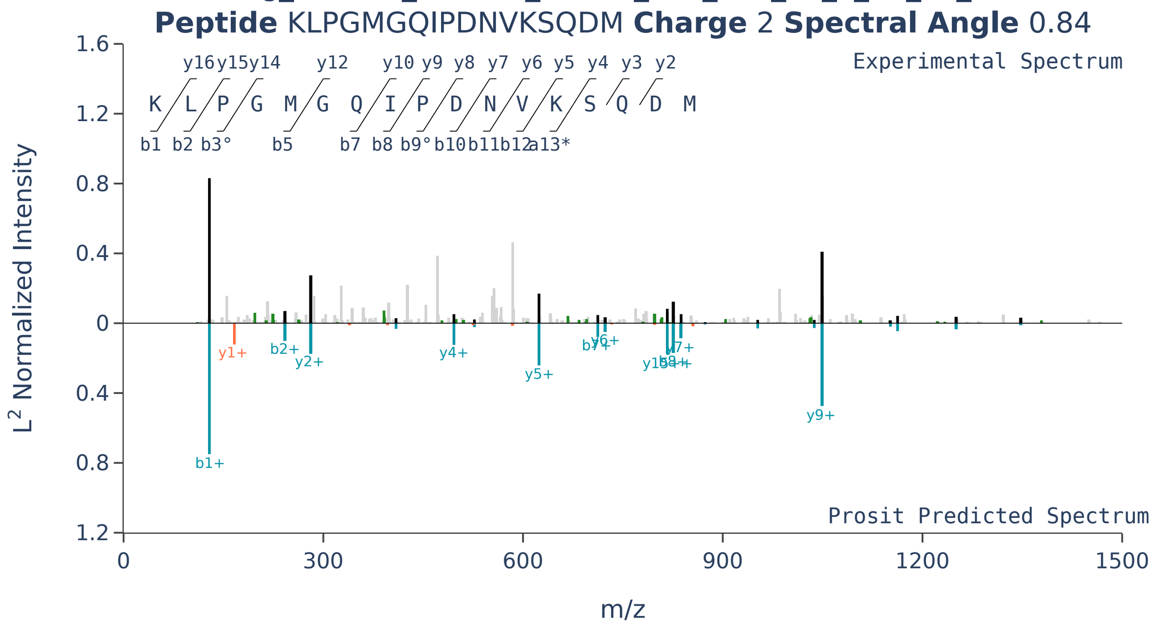

Source Ncheng_210623_230623_HFGoe_FFH_20S_25_1_A2_2h_R2 **Scan** 18045
Peptide KLPGMGMGQIPDNVKSQMDD **Charge** 3 **Spectral Angle** 0.84

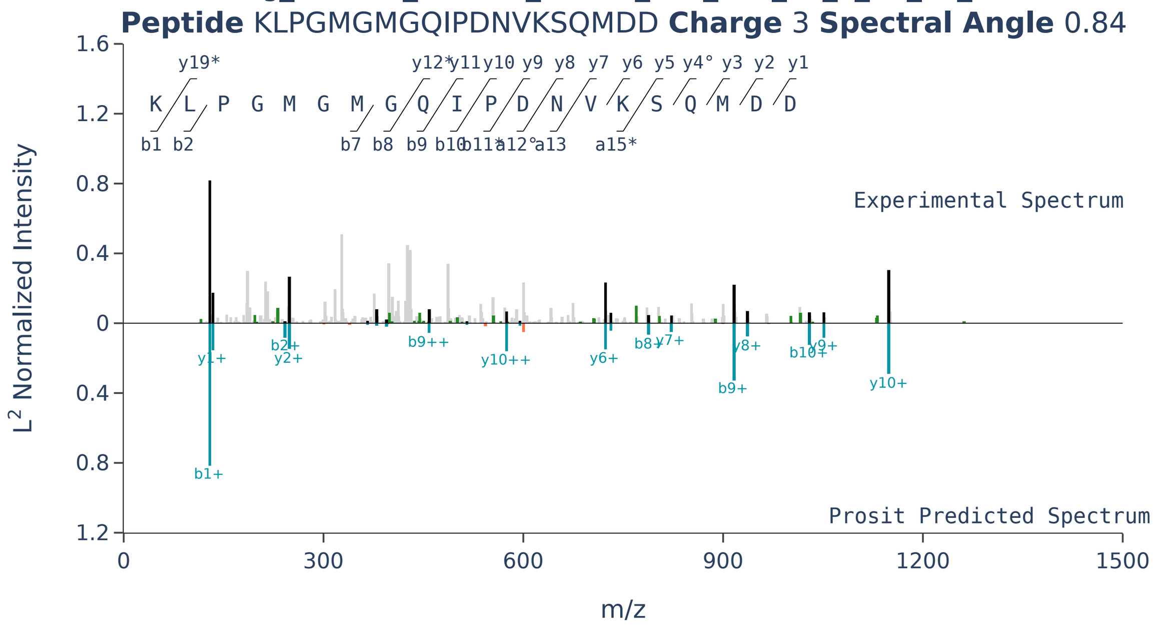

Source Ncheng_210623_230623_HFGoe_FFH_20S_25_1_A2_2h_R1 **Scan** 20172
Peptide GKMGKLPGMGQIPDNVKSQMDD **Charge** 3 **Spectral Angle** 0.84

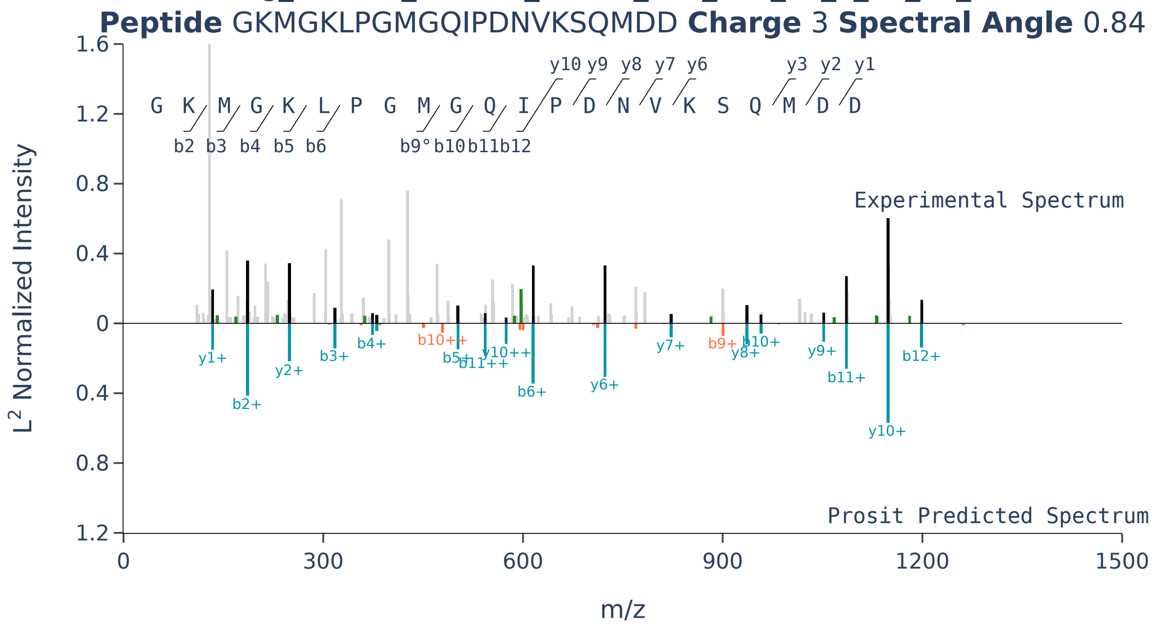

Source Ncheng_210623_230623_HFGoe_FFH_20S_25_1_A1_4h_R1 **Scan** 12404
Peptide EPFHPDRIASD **Charge** 2 **Spectral Angle** 0.91

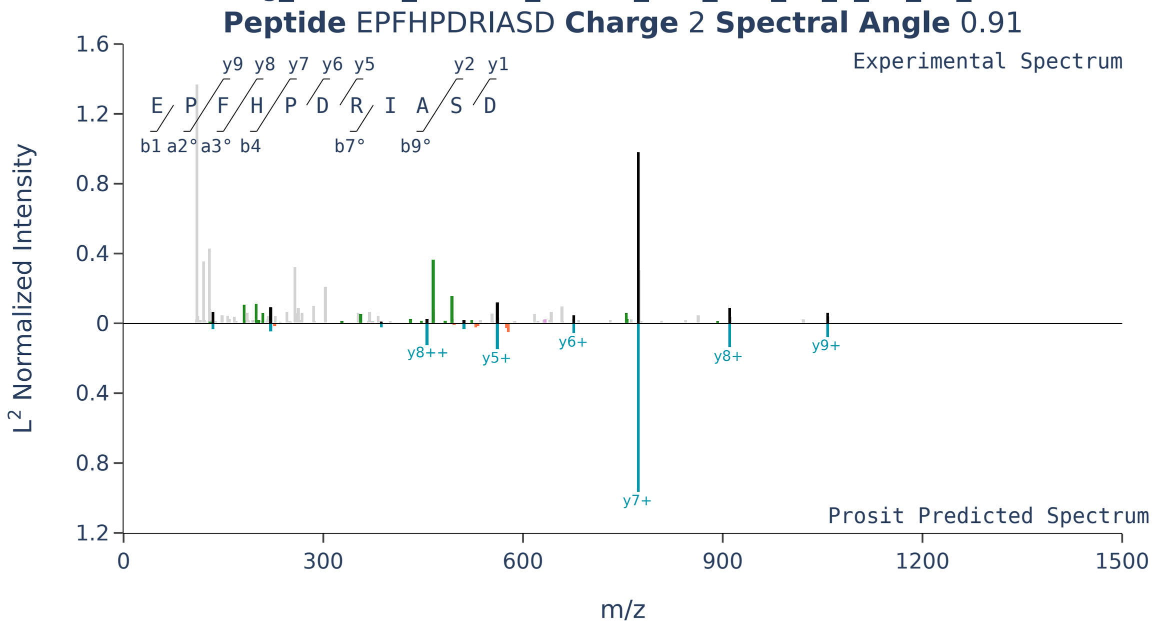

Source Ncheng_210623_230623_HFGoe_FFH_20S_25_1_A2_4h_R1 **Scan** 21048
Peptide TMGKLPGMGQIPDNVKS **Charge** 3 **Spectral Angle** 0.87

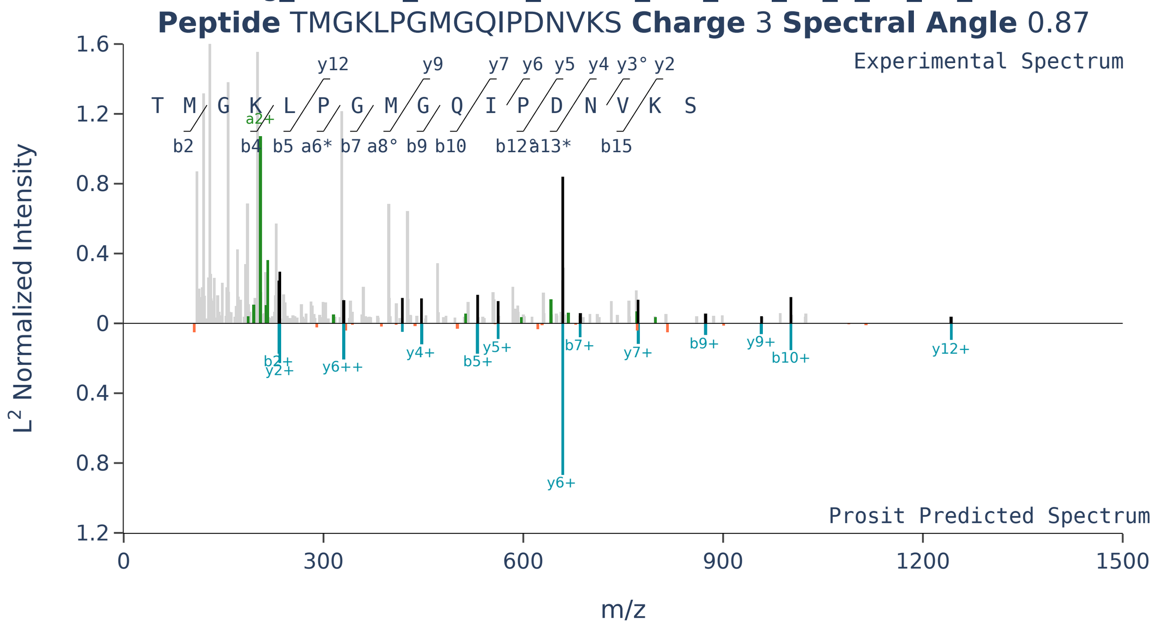

Source Ncheng_210623_230623_HFGoe_FFH_20S_25_1_A1_4h_R1 **Scan** 28619
Peptide KNMGGMAGVDFFPSD **Charge** 2 **Spectral Angle** 0.78

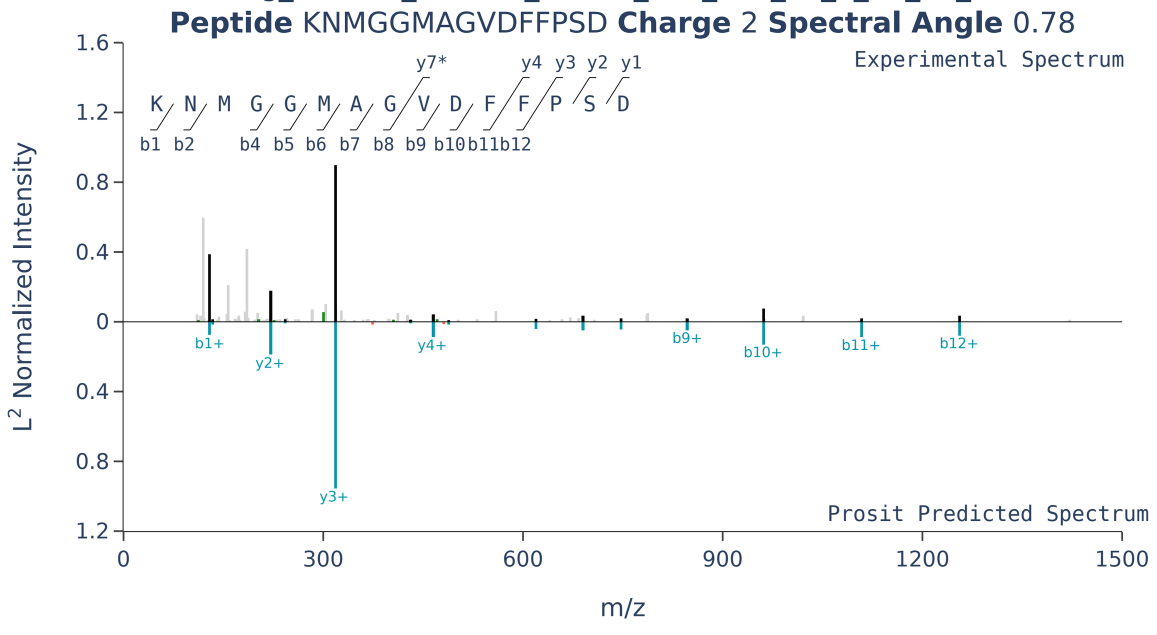

Source Ncheng_210623_230623_HFGoe_FFH_20S_25_1_A1_2h_R1 **Scan** 10863
Peptide TKETLAEQVG **Charge** 2 **Spectral Angle** 0.77

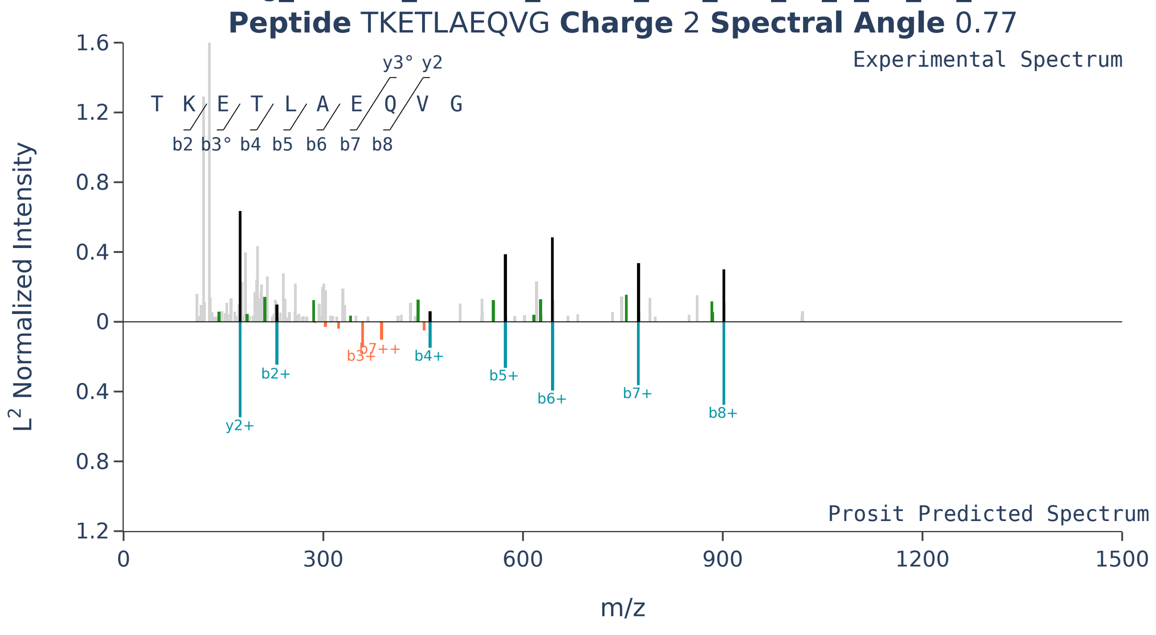

Source Ncheng_210623_230623_HFGoe_FFH_20S_25_1_A1_4h_R2 **Scan** 18824
Peptide ALEPFHPDRIASE **Charge** 3 **Spectral Angle** 0.84

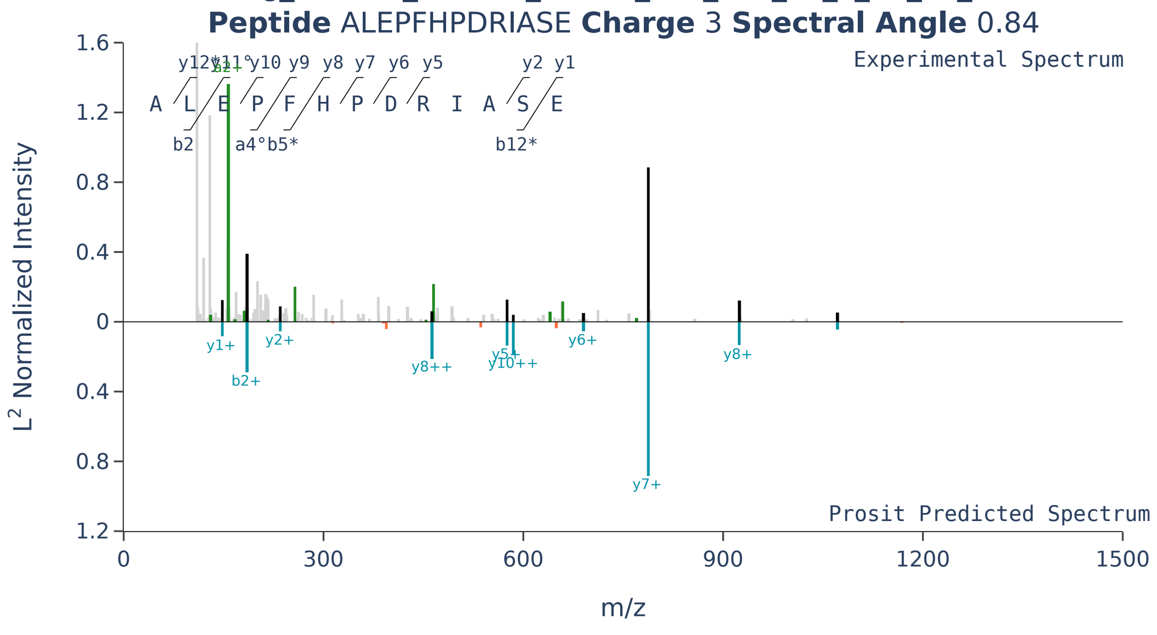

Source Ncheng_210623_230623_HFGoe_FFH_20S_25_1_A2_24h_R1 **Scan** 8068
Peptide SAAANTAKAFN **Charge** 2 **Spectral Angle** 0.83

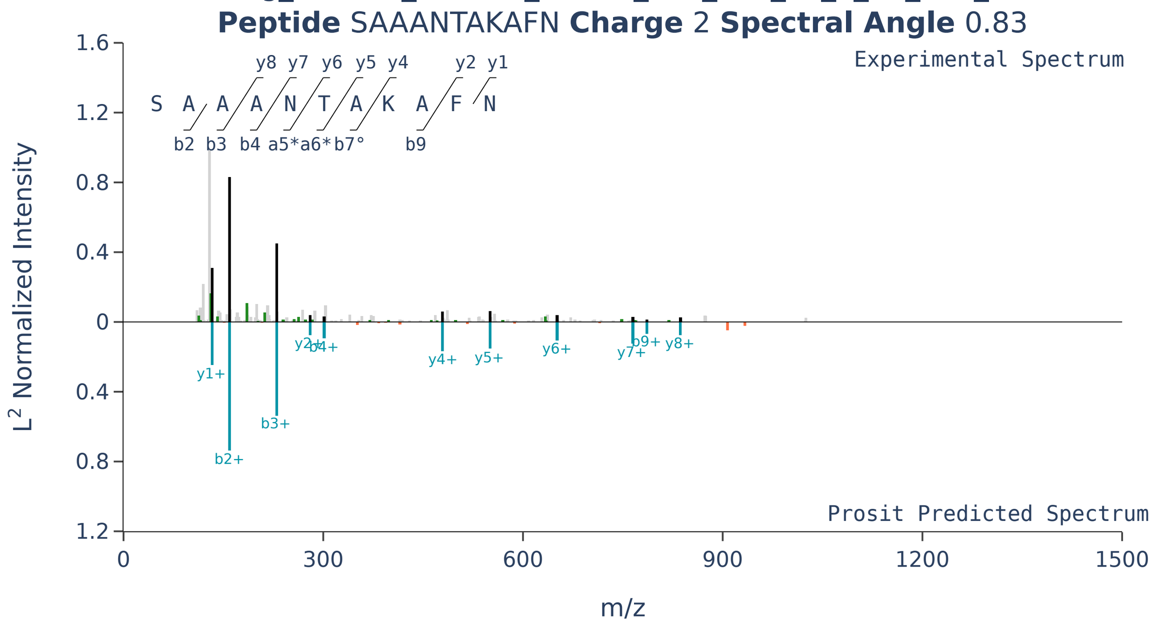

Source Ncheng_210623_230623_HFGoe_FFH_20S_25_1_A1_24h_R1 **Scan** 25900
Peptide EALPLTGAMTGQD **Charge** 2 **Spectral Angle** 0.91

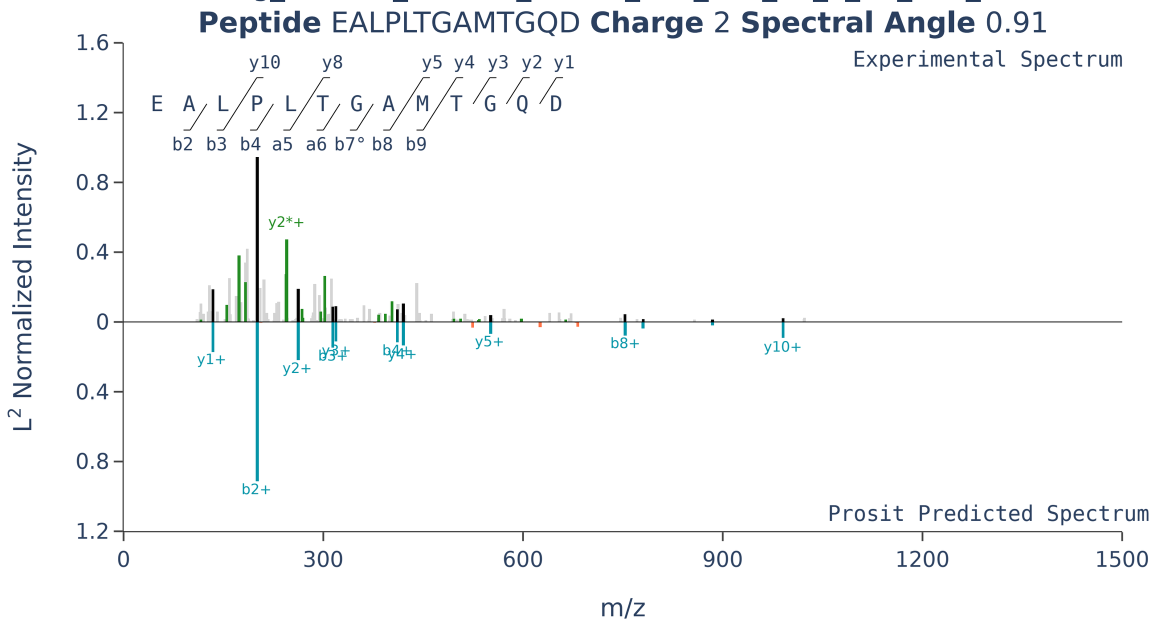

Source Ncheng_210623_230623_HFGoe_FFH_20S_25_1_A2_24h_R1 **Scan** 10015
Peptide GGMAVGQKPVD **Charge** 2 **Spectral Angle** 0.76

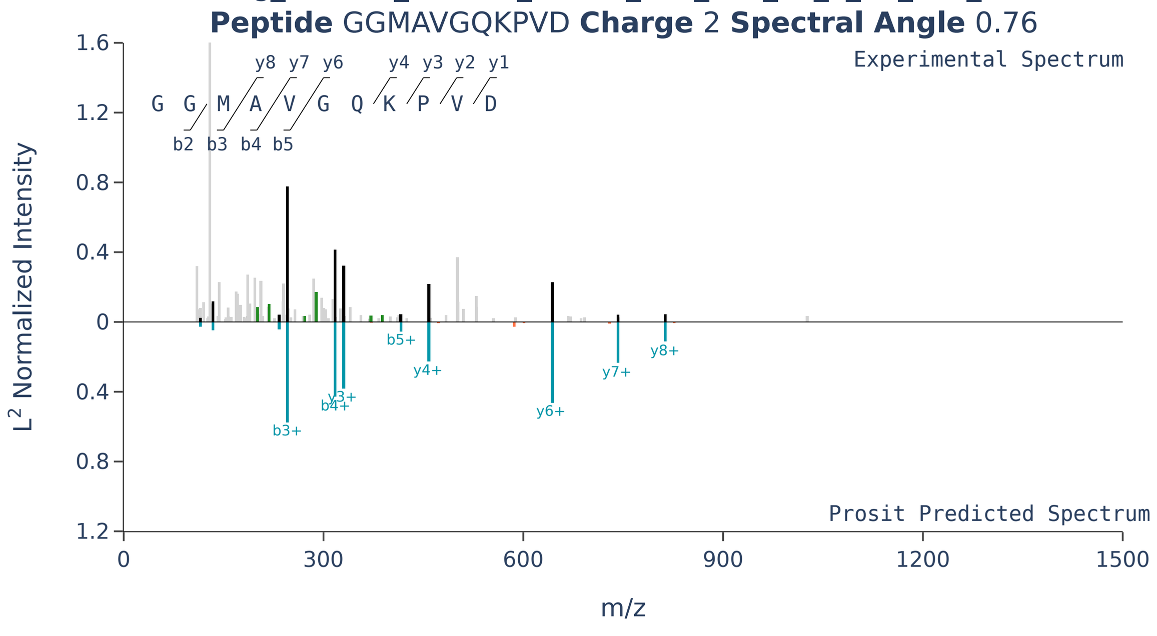

Source Ncheng_210623_230623_HFGoe_FFH_20S_25_1_A2_24h_R1 **Scan** 13667
Peptide SAMTGQDAANTAKAFN **Charge** 2 **Spectral Angle** 0.78

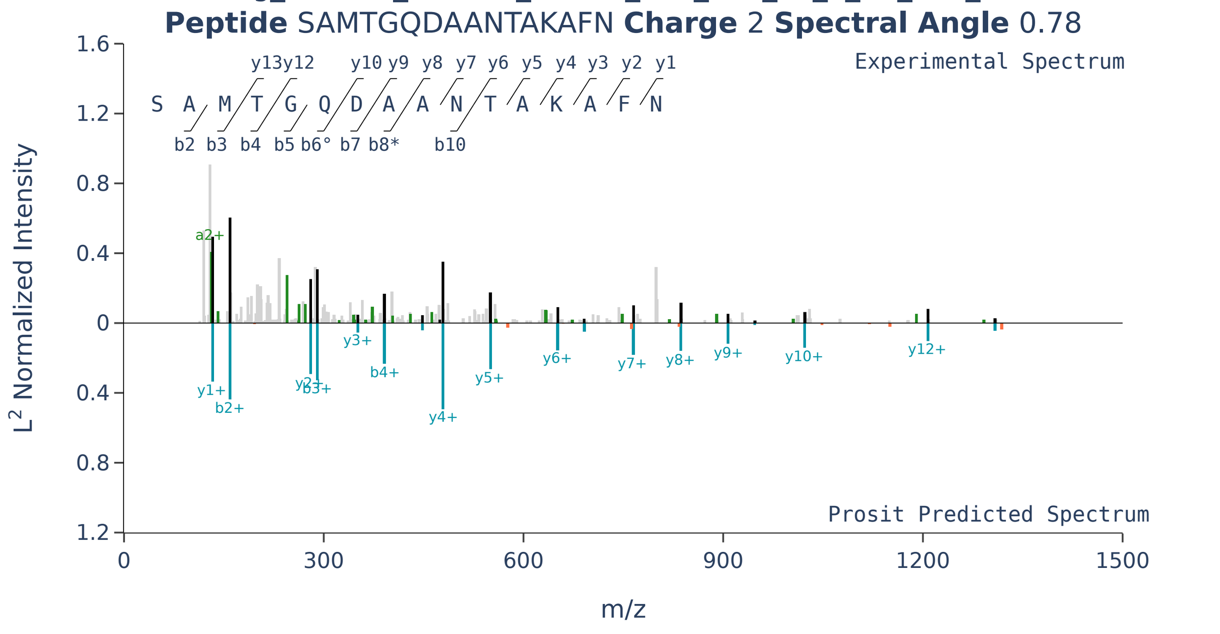

Source Ncheng_210623_230623_HFGoe_FFH_20S_25_1_A2_24h_R2 **Scan** 17732
Peptide AEQVGAMTGQDAANTAKAFN **Charge** 2 **Spectral Angle** 0.9

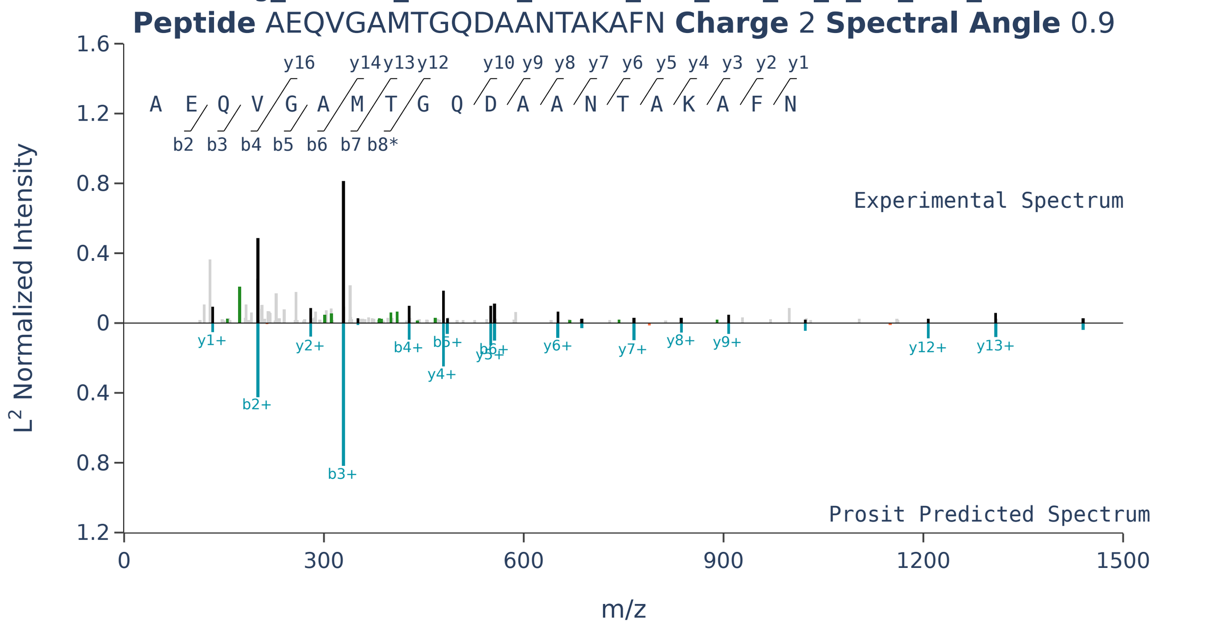

Source Ncheng_210623_230623_HFGoe_FFH_20S_25_1_A1_4h_R1 **Scan** 9400
Peptide AEQVGVGQKPVD **Charge** 2 **Spectral Angle** 0.79

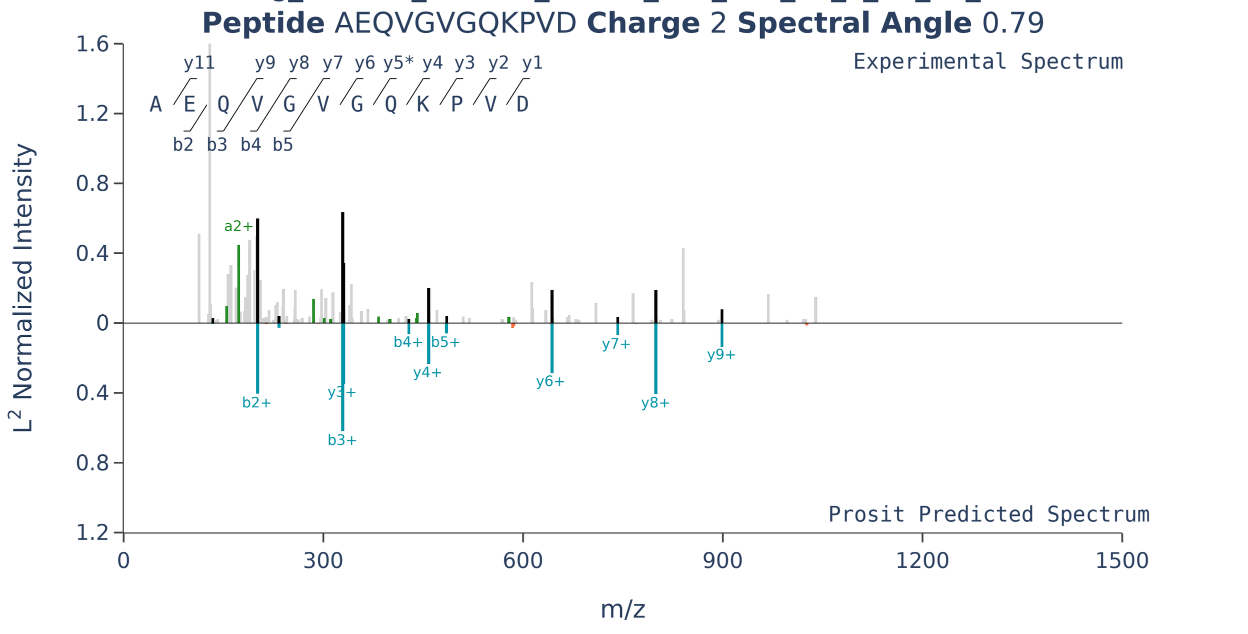

Source Ncheng_210623_230623_HFGoe_FFH_20S_25_1_A2_4h_R2 **Scan** 29152
Peptide AANTAKAFNEVDFFPSD **Charge** 2 **Spectral Angle** 0.91

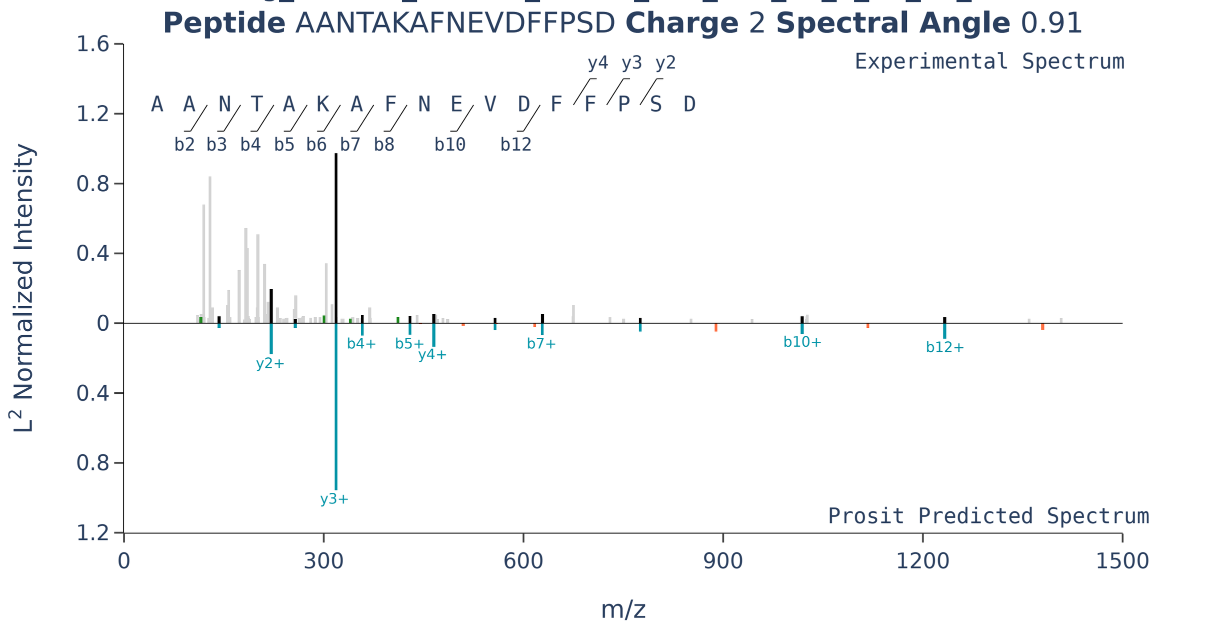

Source Ncheng_210623_230623_HFGoe_FFH_20S_25_1_A1_2h_R2 **Scan** 17535
Peptide ETLAEQVGVGVGQKPVD **Charge** 2 **Spectral Angle** 0.88

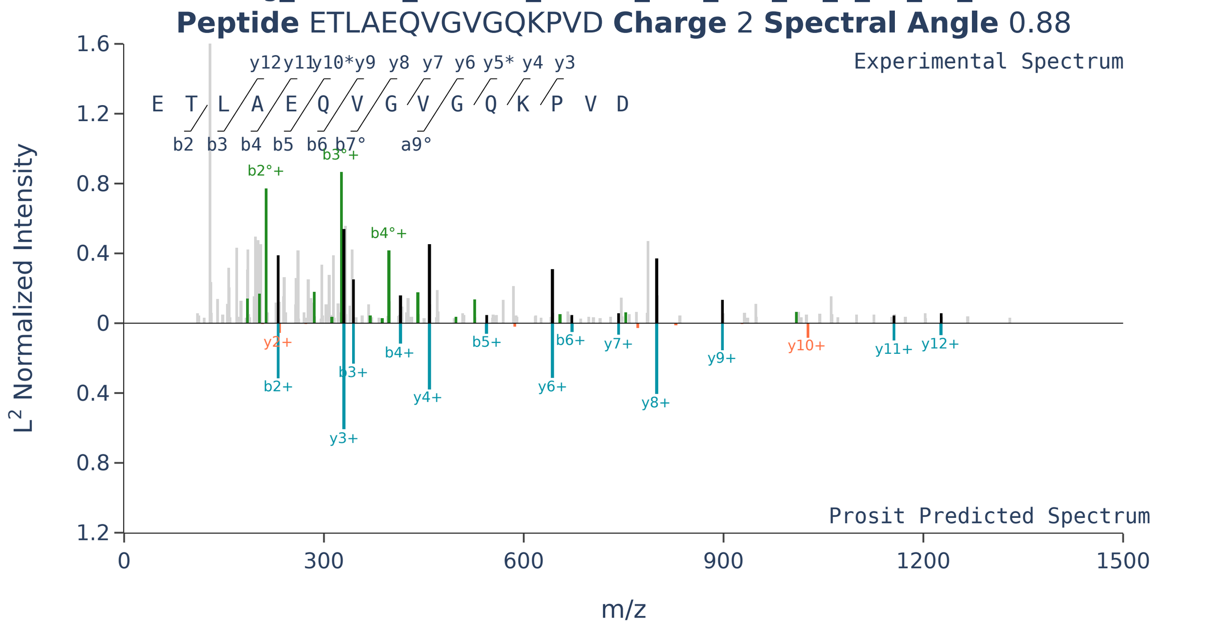

Source Ncheng_210623_230623_HFGoe_FFH_20S_25_1_A2_4h_R2 **Scan** 21511
Peptide SAMGKLPGMGQIPDNVKS **Charge** 2 **Spectral Angle** 0.88

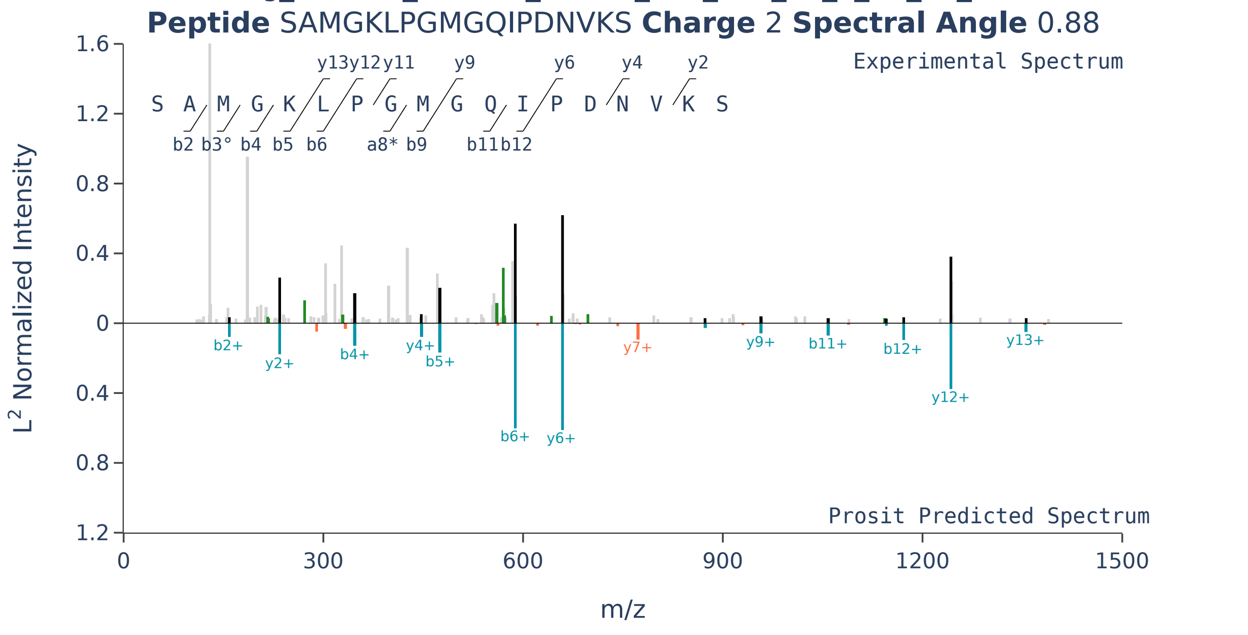

Source Ncheng_210623_230623_HFGoe_FFH_20S_25_1_A1_4h_R1 **Scan** 17130
Peptide LEAMTGQDAANTAKAFN **Charge** 2 **Spectral Angle** 0.9

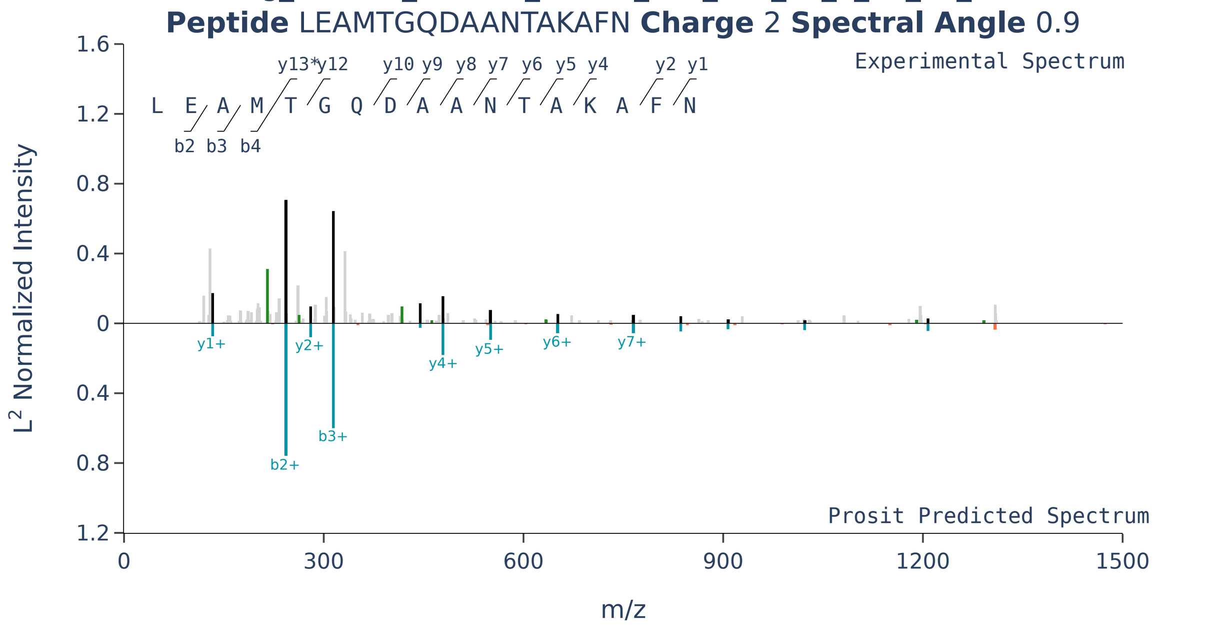

Source Ncheng_210623_230623_HFGoe_FFH_20S_25_1_A1_4h_R1 **Scan** 6412
Peptide RILGKTE **Charge** 2 **Spectral Angle** 0.79

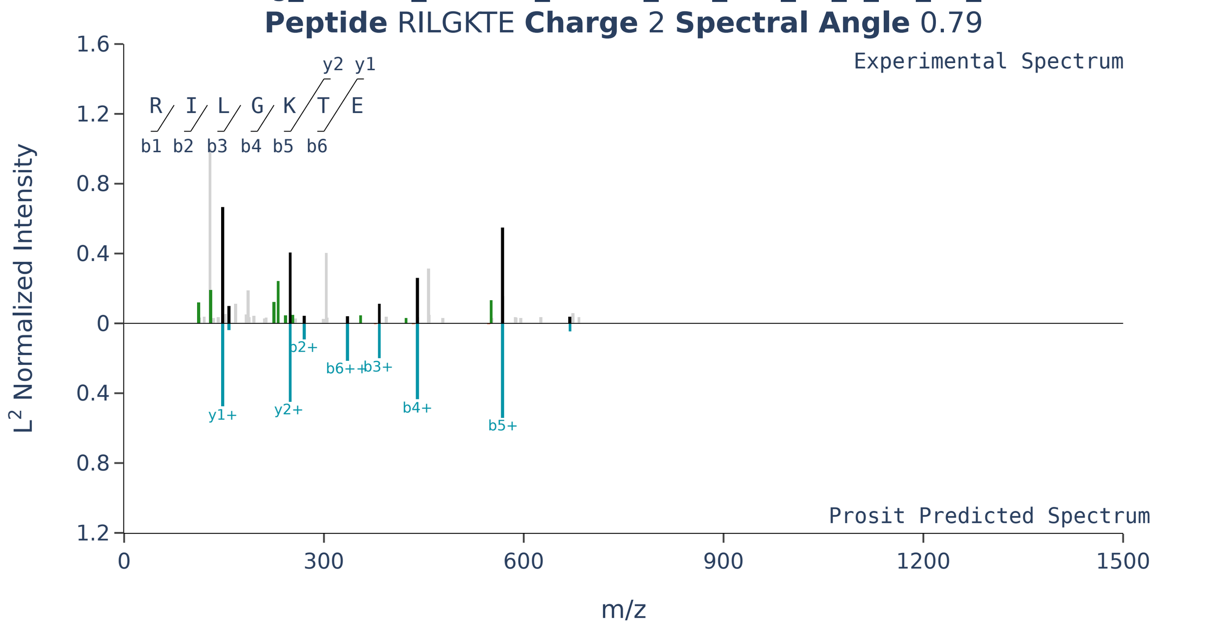

Source Ncheng_210623_230623_HFGoe_FFH_20S_25_1_A2_2h_R2 **Scan** 10850
Peptide DHASINPVE **Charge** 2 **Spectral Angle** 0.91

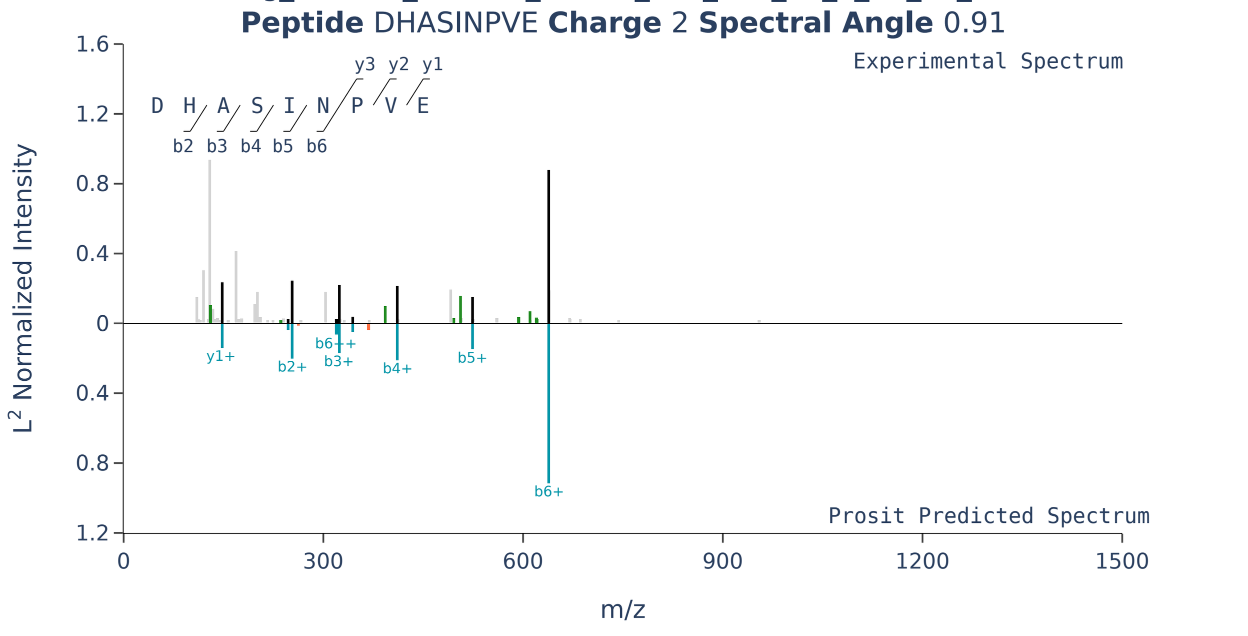

Source Ncheng_210623_230623_HFGoe_FFH_20S_25_1_A2_2h_R2 **Scan** 18096
Peptide GMKLPGMGQIPDNVKSQ **Charge** 2 **Spectral Angle** 0.8

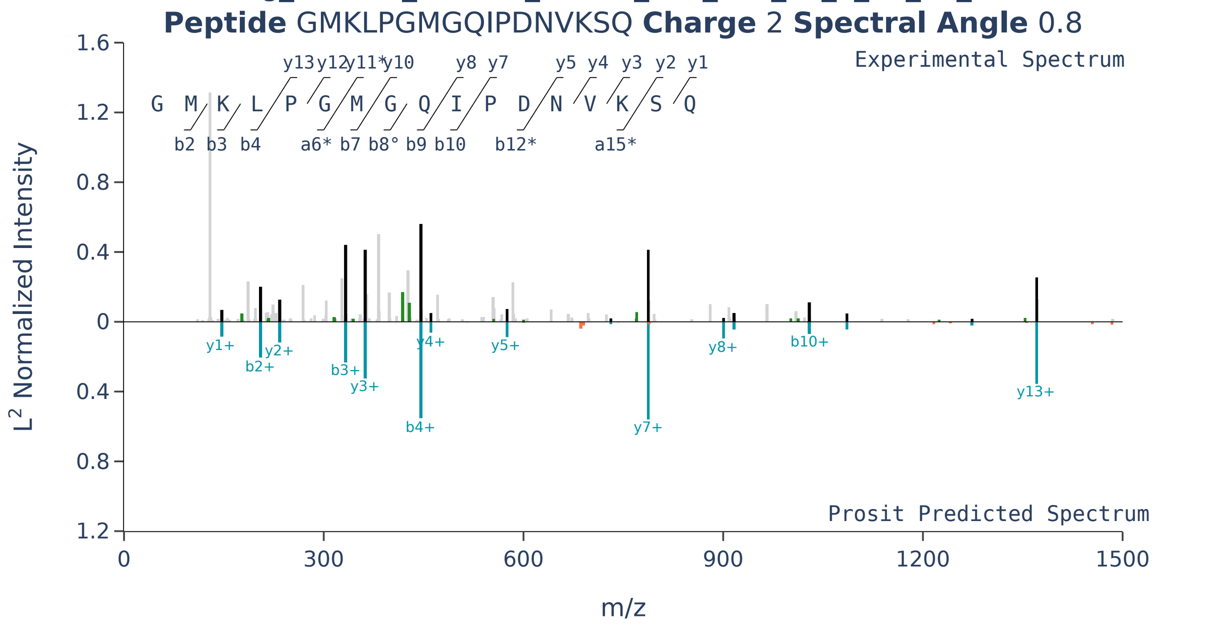

Source Ncheng_210623_230623_HFGoe_FFH_20S_25_1_A1_24h_R1 **Scan** 26131
Peptide ADVALPVDV **Charge** 1 **Spectral Angle** 0.86

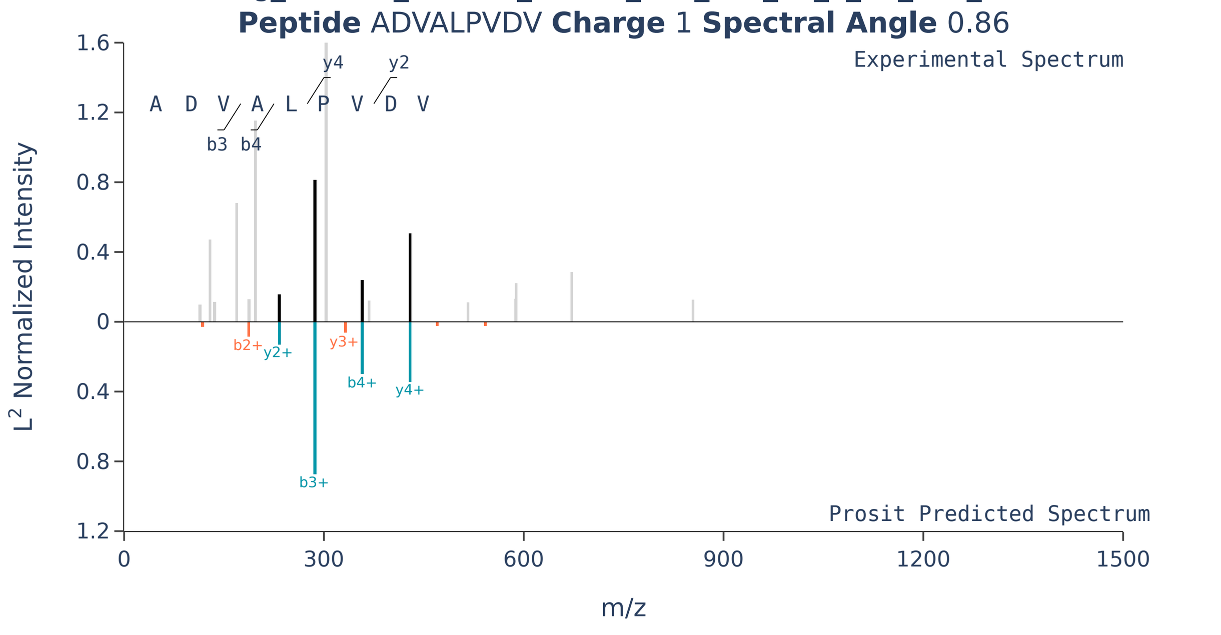

Source Ncheng_210623_230623_HFGoe_FFH_20S_25_1_A2_1h_R2 **Scan** 8809
Peptide TGKLAEQVG **Charge** 2 **Spectral Angle** 0.81

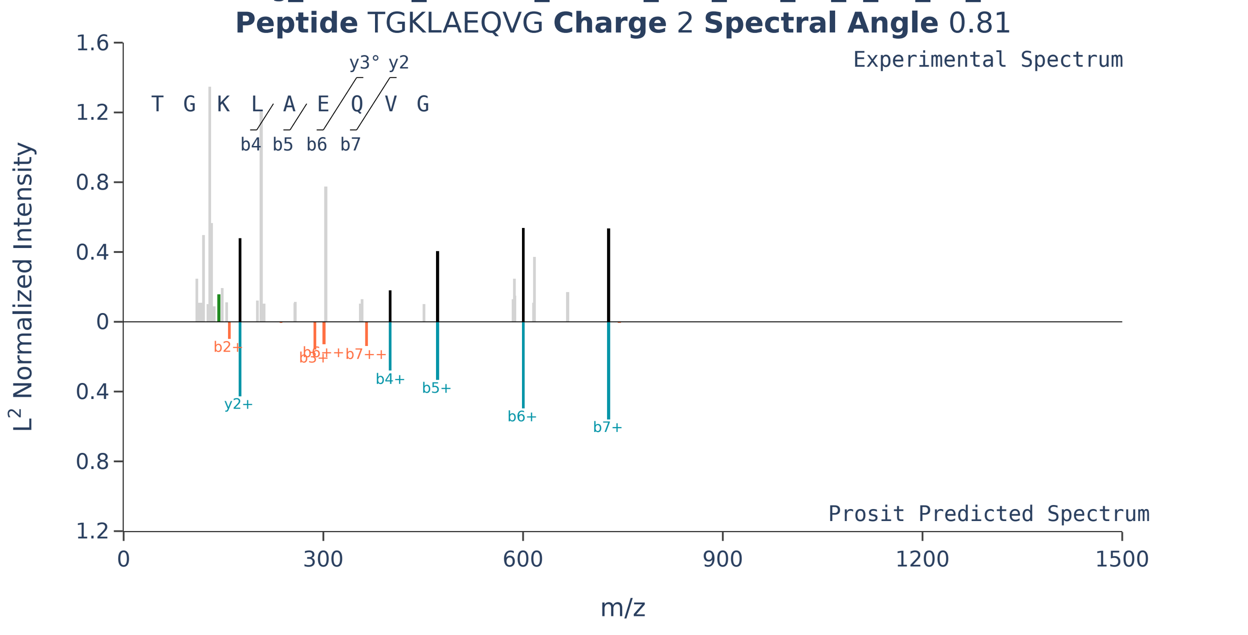

Source Ncheng_210623_230623_HFGoe_FFH_20S_25_1_A2_1h_R2 **Scan** 8358
Peptide RNISGNLTD **Charge** 2 **Spectral Angle** 0.81

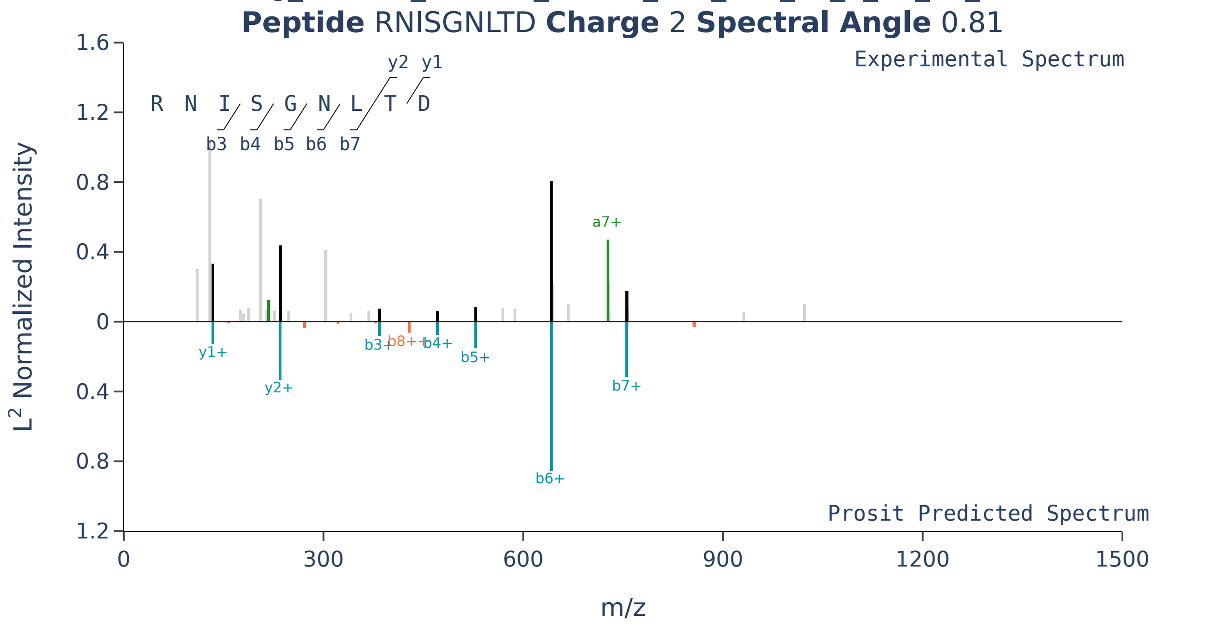

Source Ncheng_210623_230623_HFGoe_FFH_20S_25_1_A1_24h_R2 **Scan** 13418
Peptide KTEAMTGQDAANTAKAFNE **Charge** 3 **Spectral Angle** 0.83

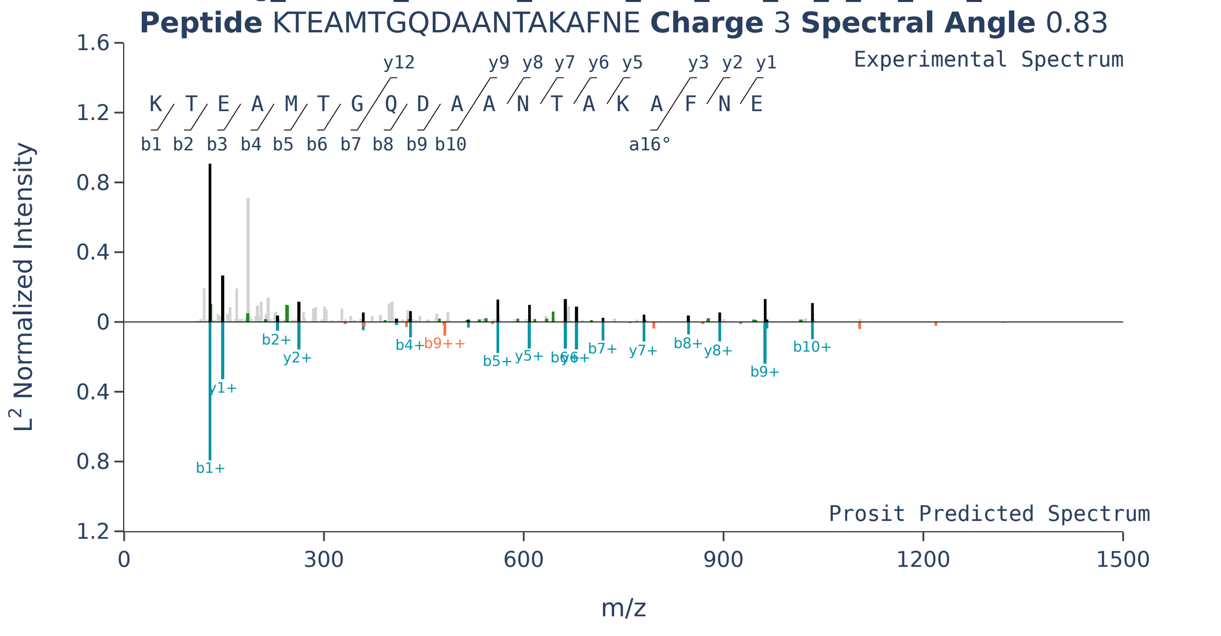

Source Ncheng_210623_230623_HFGoe_FFH_20S_25_1_A1_24h_R2 **Scan** 22068
Peptide AGKGVDFFPSD **Charge** 2 **Spectral Angle** 0.86

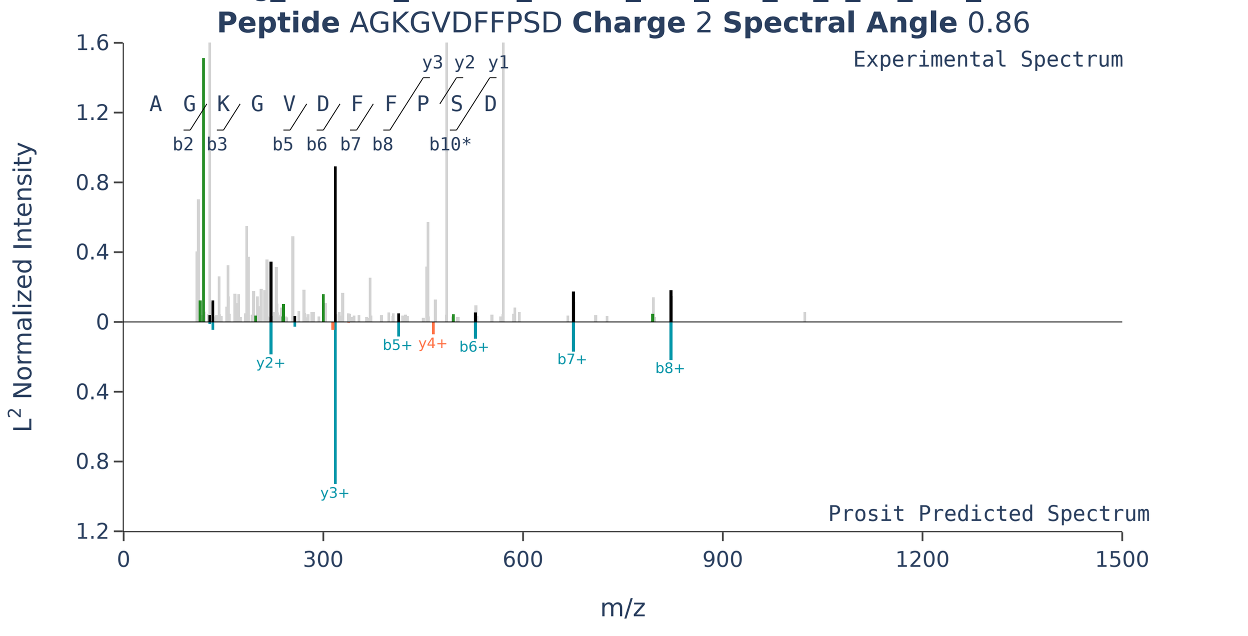

Source Ncheng_210623_230623_HFGoe_FFH_20S_25_1_A2_24h_R2 **Scan** 15927
Peptide TGKPIKFLGVGEKTEE **Charge** 2 **Spectral Angle** 0.82

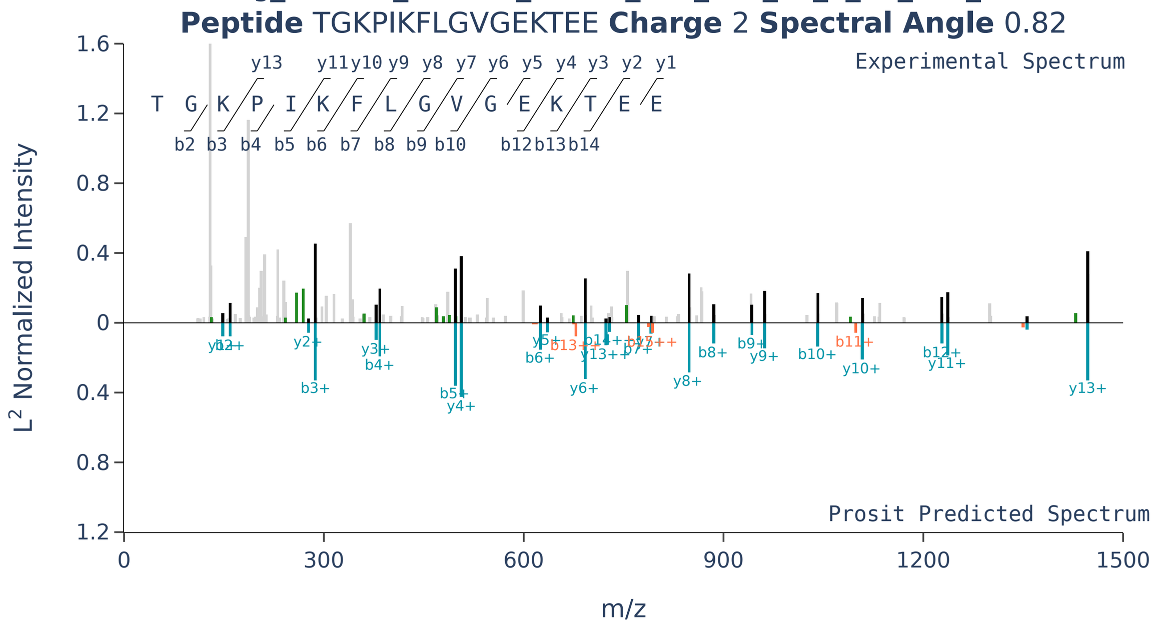

Source Ncheng_210623_230623_HFGoe_FFH_20S_25_1_A1_2h_R1 **Scan** 25773
Peptide SVDFFPSDVGQKPVD **Charge** 2 **Spectral Angle** 0.89

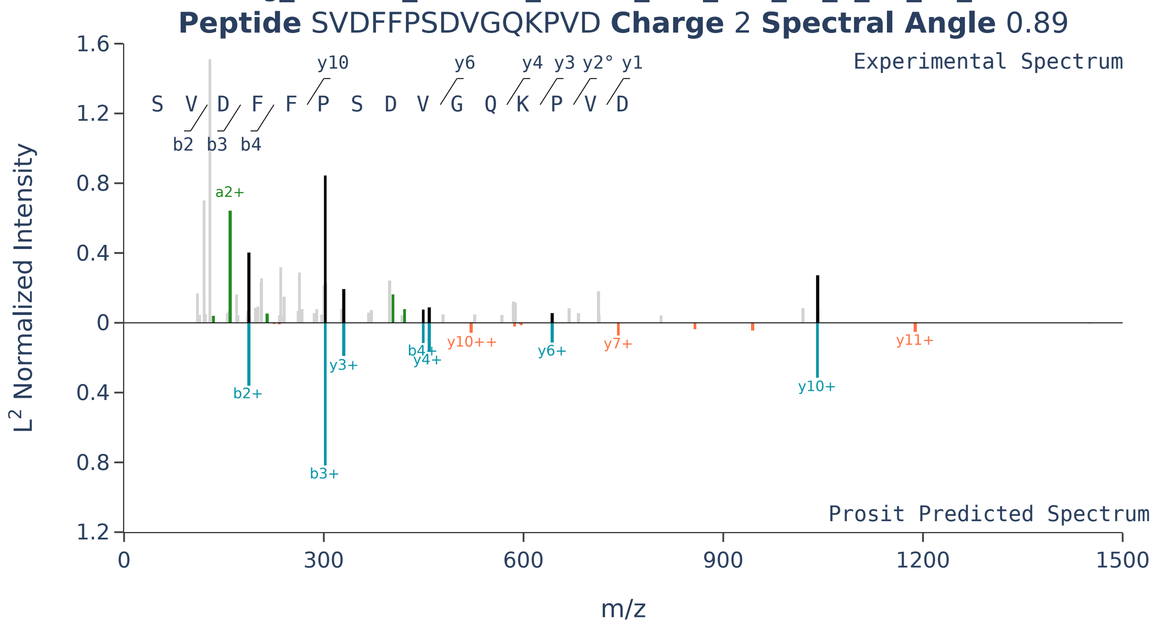

Source Ncheng_210623_230623_HFGoe_FFH_20S_25_1_A1_4h_R1 **Scan** 20564
Peptide SMGKLPGMGQIPDNVKSQ **Charge** 3 **Spectral Angle** 0.8

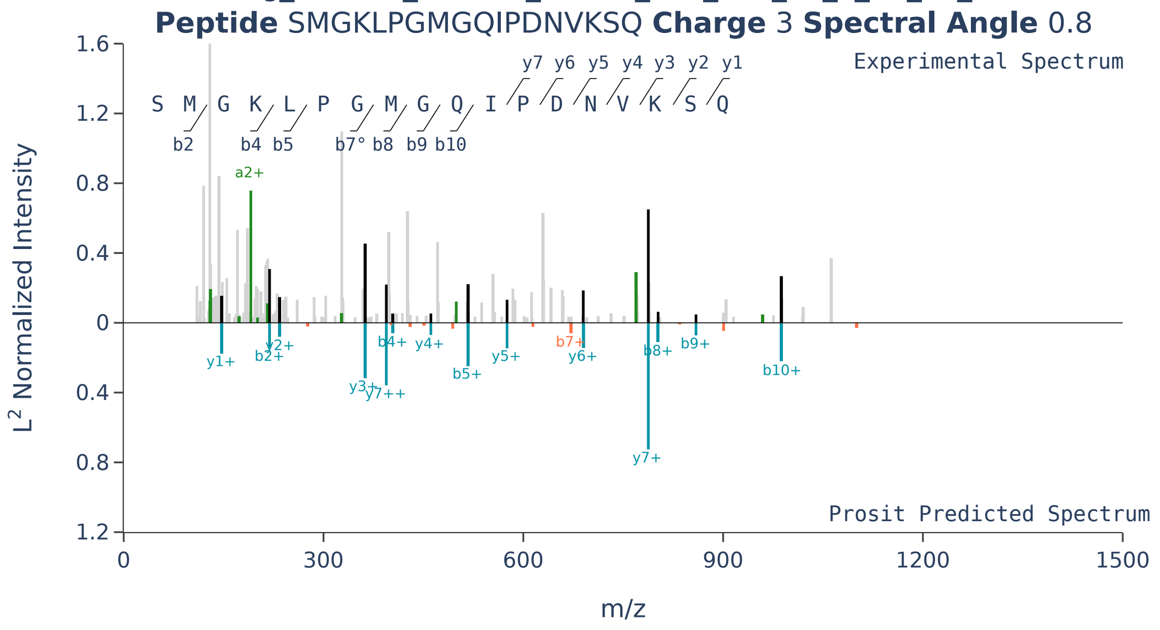

Source Ncheng_210623_230623_HFGoe_FFH_20S_25_1_A1_24h_R1 **Scan** 20044
Peptide TGVNRLLKQFD **Charge** 2 **Spectral Angle** 0.79

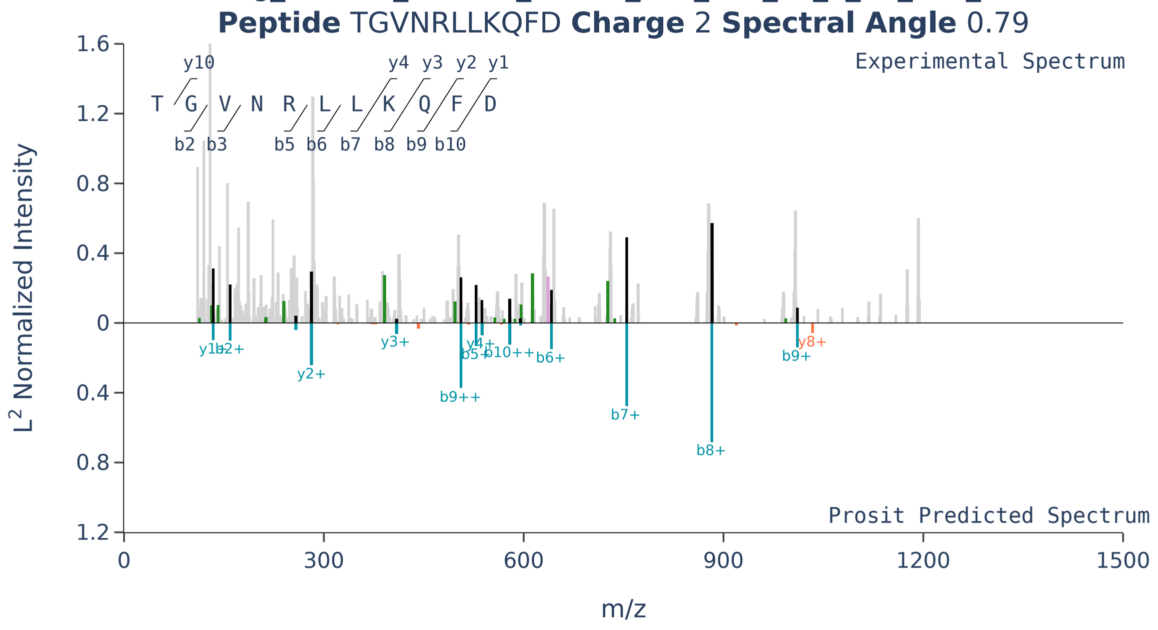

Source Ncheng_210623_230623_HFGoe_FFH_20S_25_1_A1_4h_R1 **Scan** 10235
Peptide KNMGGMASLR **Charge** 2 **Spectral Angle** 0.71

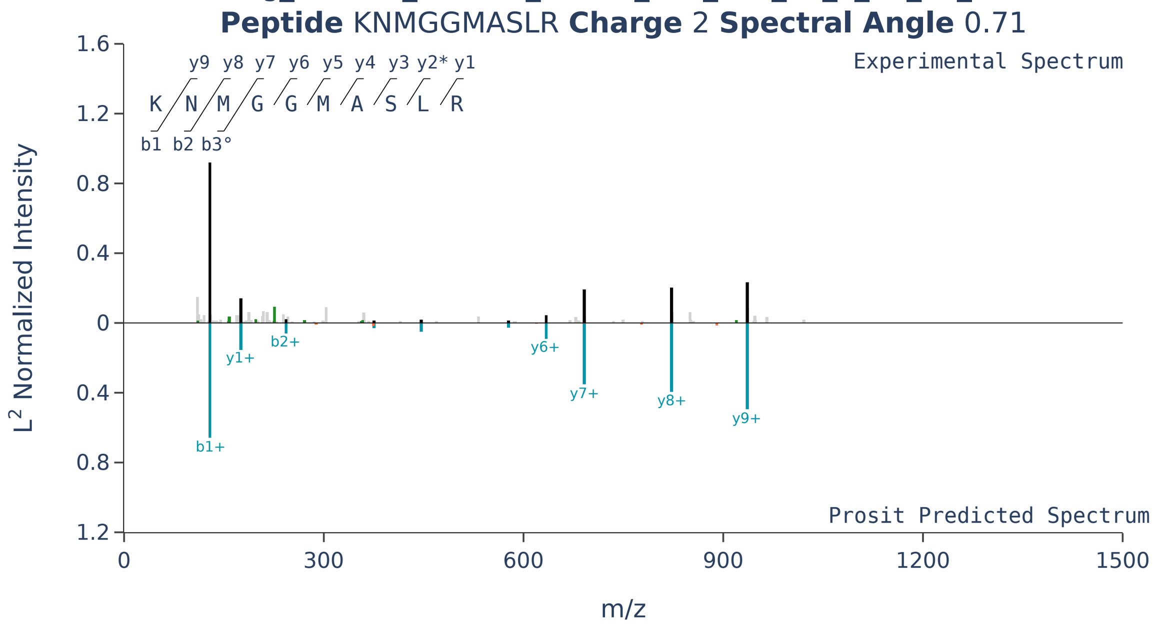

Source Ncheng_210623_230623_HFGoe_FFH_20S_25_1_A2_4h_R2 **Scan** 20370
Peptide MGKLPGMGQIPDNVKSMAG **Charge** 2 **Spectral Angle** 0.84

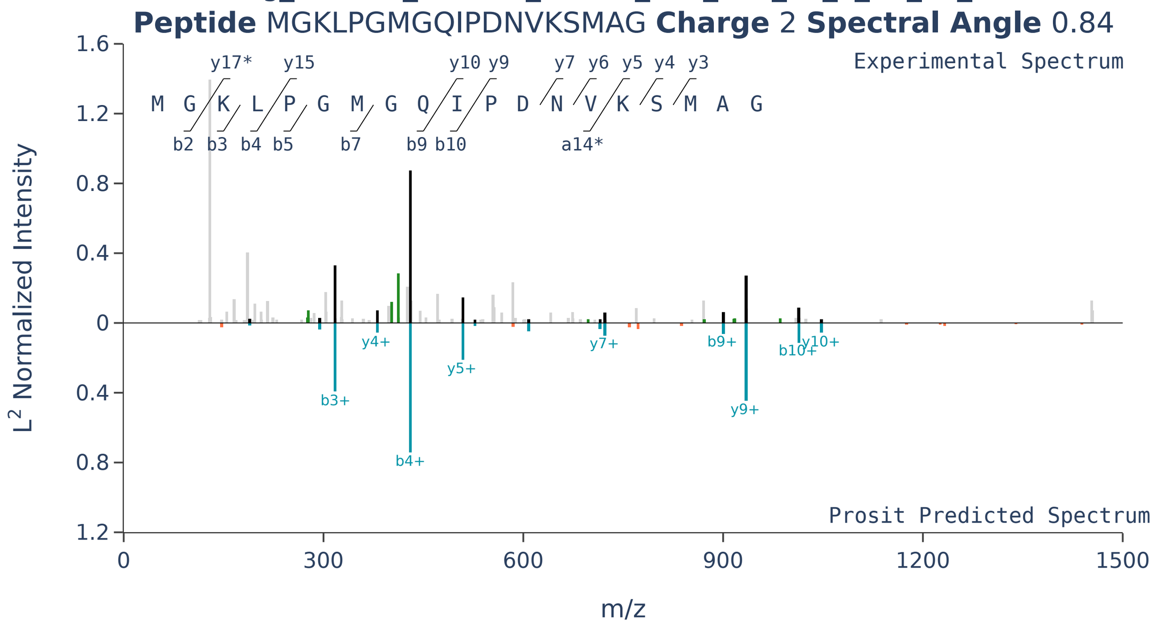

Source Ncheng_210623_230623_HFGoe_FFH_20S_25_1_A1_24h_R1 **Scan** 11136
Peptide AMTGQDAANTARQM **Charge** 2 **Spectral Angle** 0.78

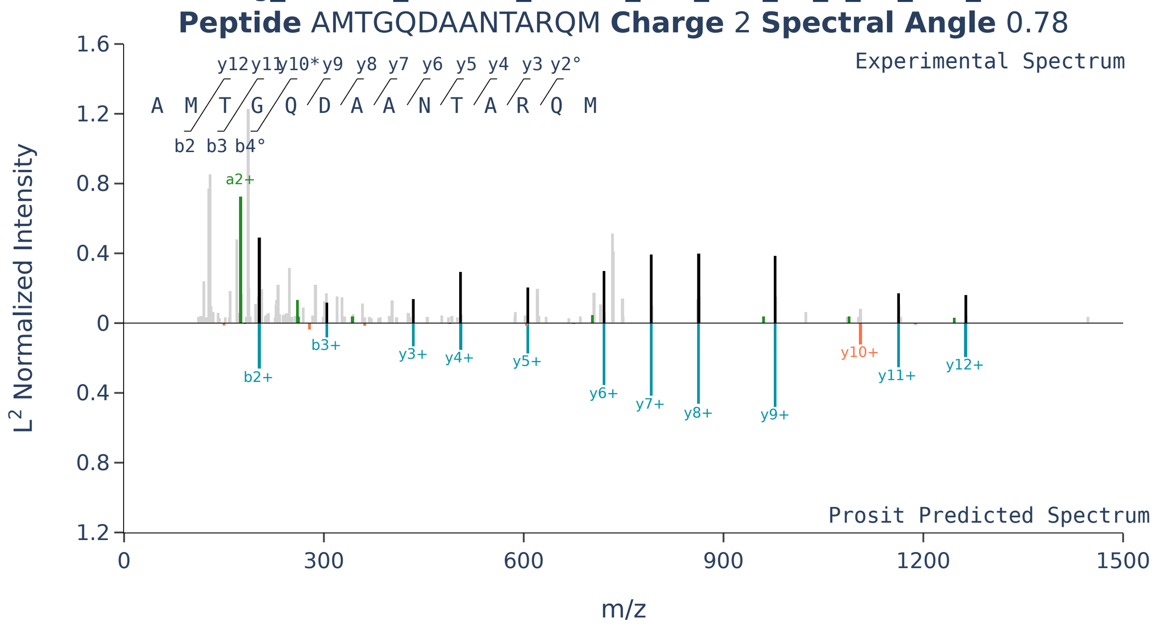

Source Ncheng_210623_230623_HFGoe_FFH_20S_25_1_A2_4h_R1 **Scan** 6987
Peptide DNVKDVGQKPVD **Charge** 2 **Spectral Angle** 0.91

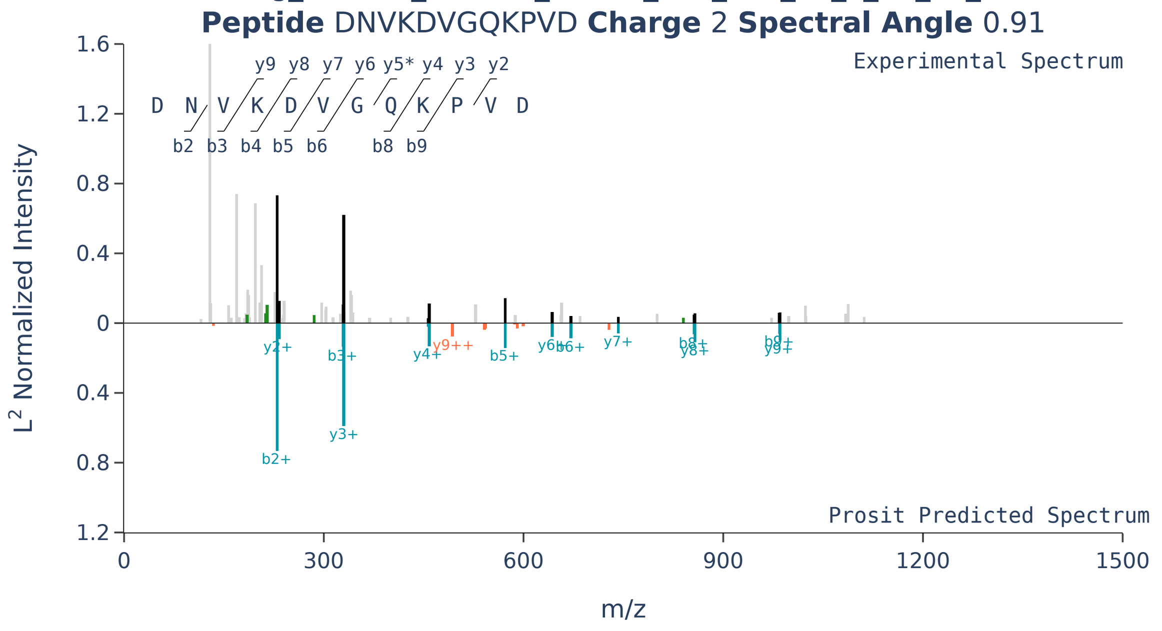

Source Ncheng_210623_230623_HFGoe_FFH_20S_25_1_A2_24h_R2 **Scan** 7640
Peptide DAIESKVD **Charge** 2 **Spectral Angle** 0.85

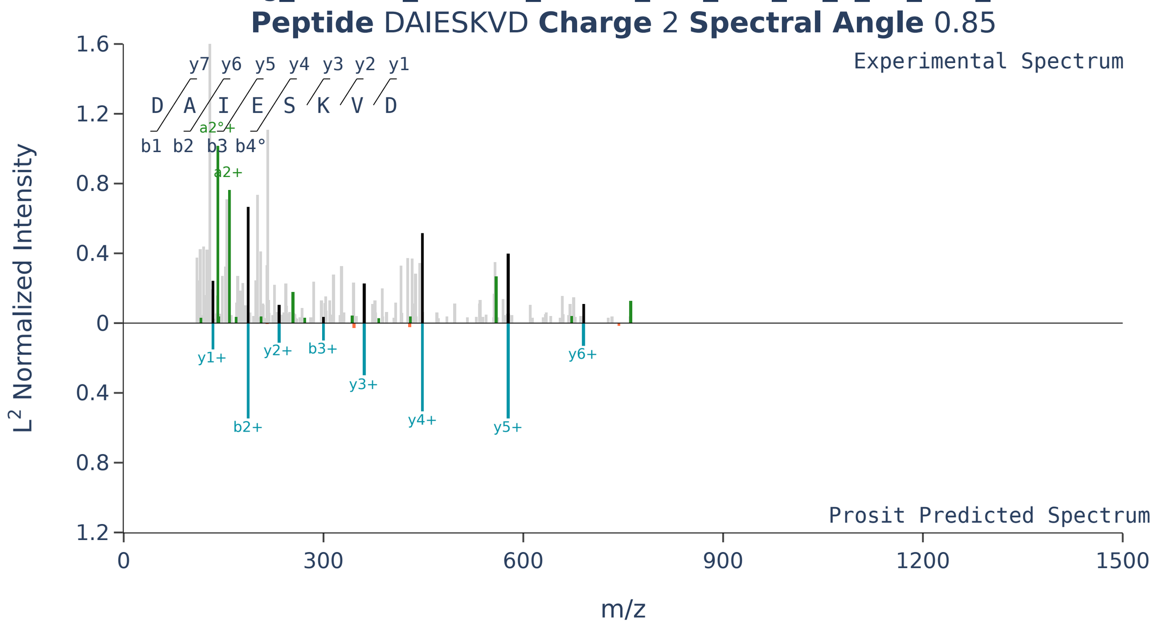

Source Ncheng_210623_230623_HFGoe_FFH_20S_25_1_A1_4h_R2 **Scan** 10253
Peptide HASINPVED **Charge** 2 **Spectral Angle** 0.86

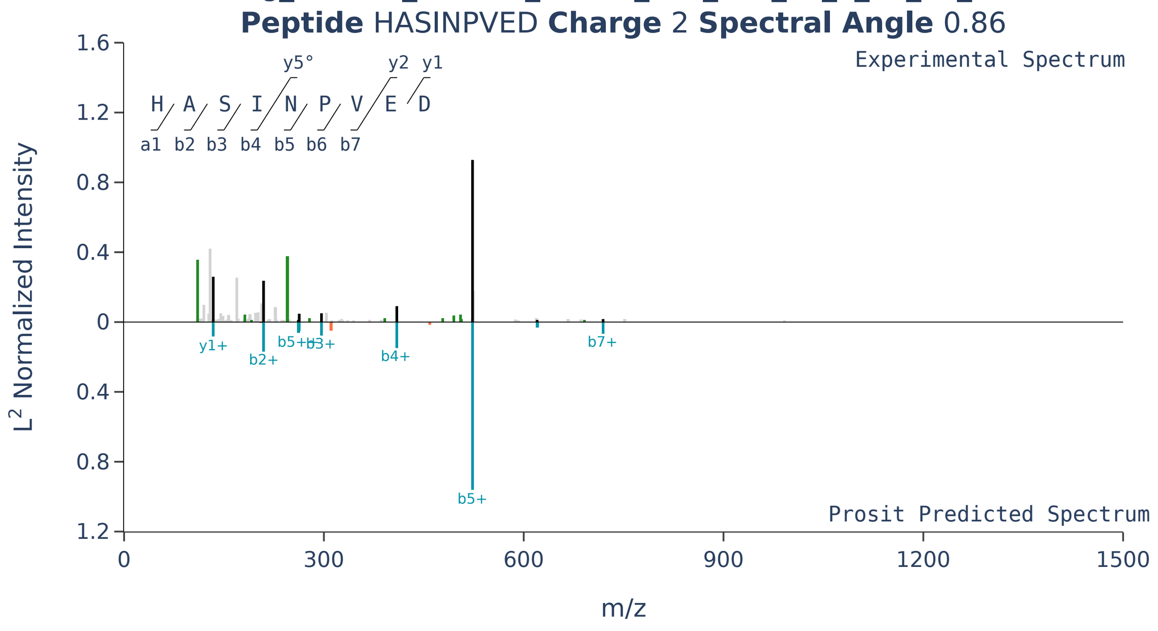

Source Ncheng_210623_230623_HFGoe_FFH_20S_25_1_A2_4h_R1 **Scan** 36572
Peptide PLTGVVEAII **Charge** 1 **Spectral Angle** 0.84

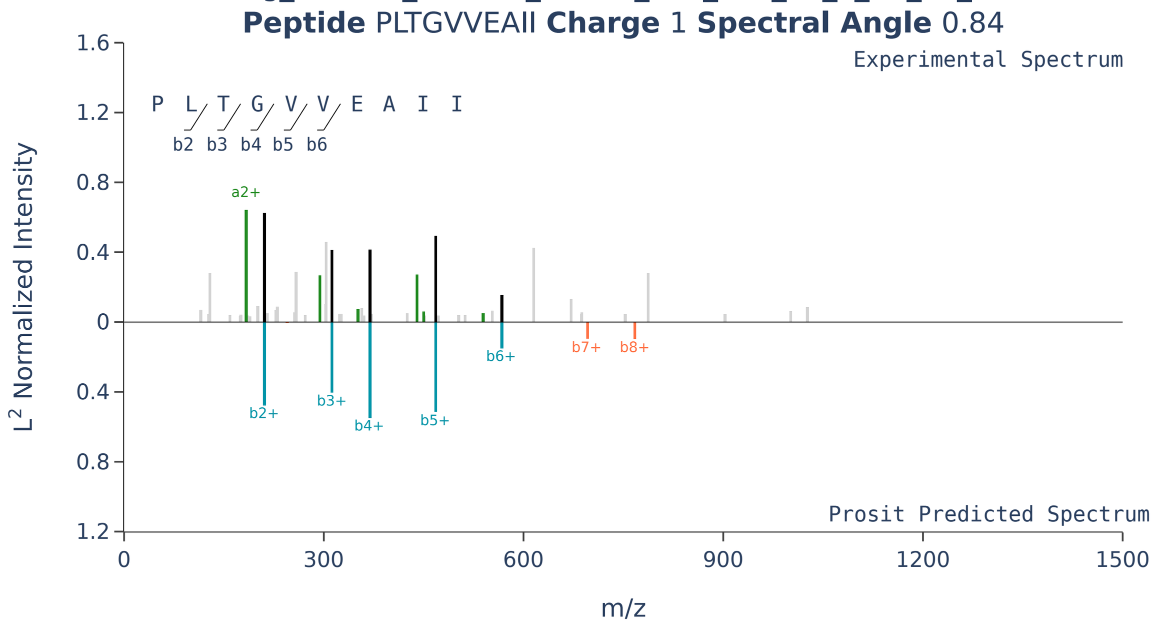

Source Ncheng_210623_230623_HFGoe_FFH_20S_25_1_A1_2h_R1 **Scan** 26760
Peptide LGVGEKTEGVDFFPSD **Charge** 2 **Spectral Angle** 0.92

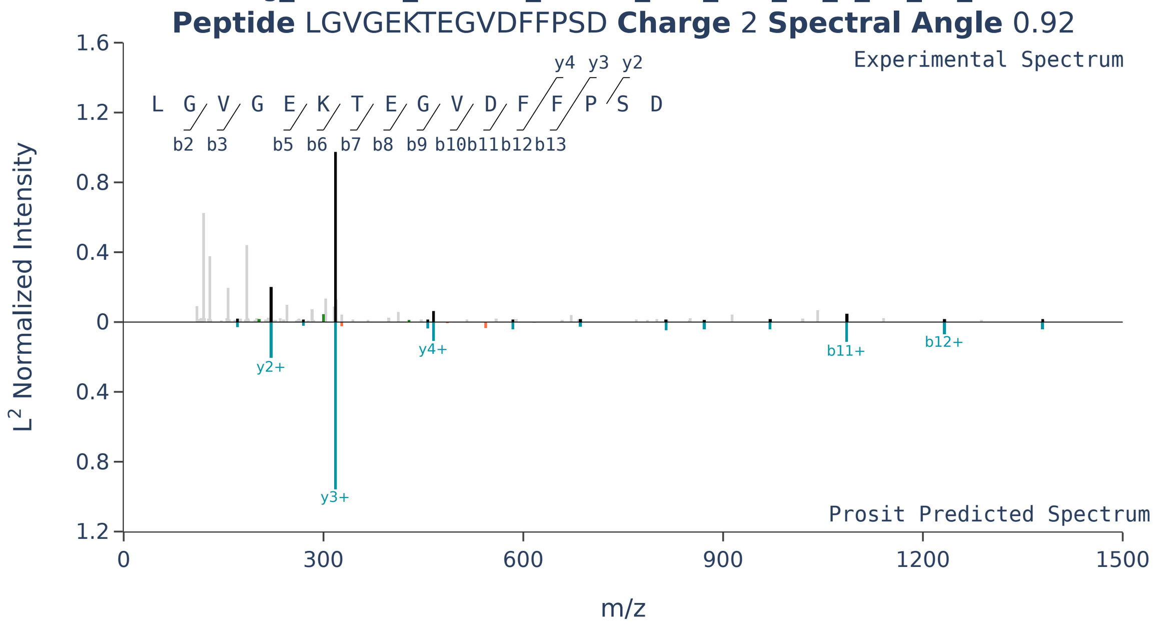

Source Ncheng_210623_230623_HFGoe_FFH_20S_25_1_A2_4h_R1 **Scan** 8017
Peptide DDIESKVD **Charge** 1 **Spectral Angle** 0.81

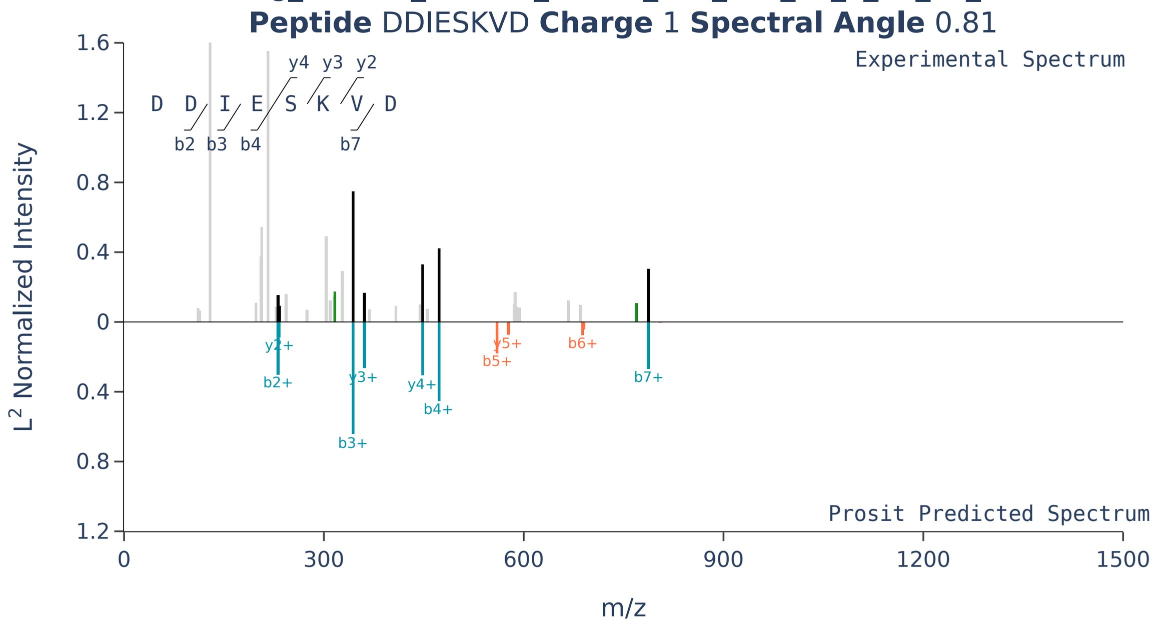

Source Ncheng_210623_230623_HFGoe_FFH_20S_25_1_A1_24h_R1 **Scan** 7082
Peptide RSMVGQKPVD **Charge** 2 **Spectral Angle** 0.85

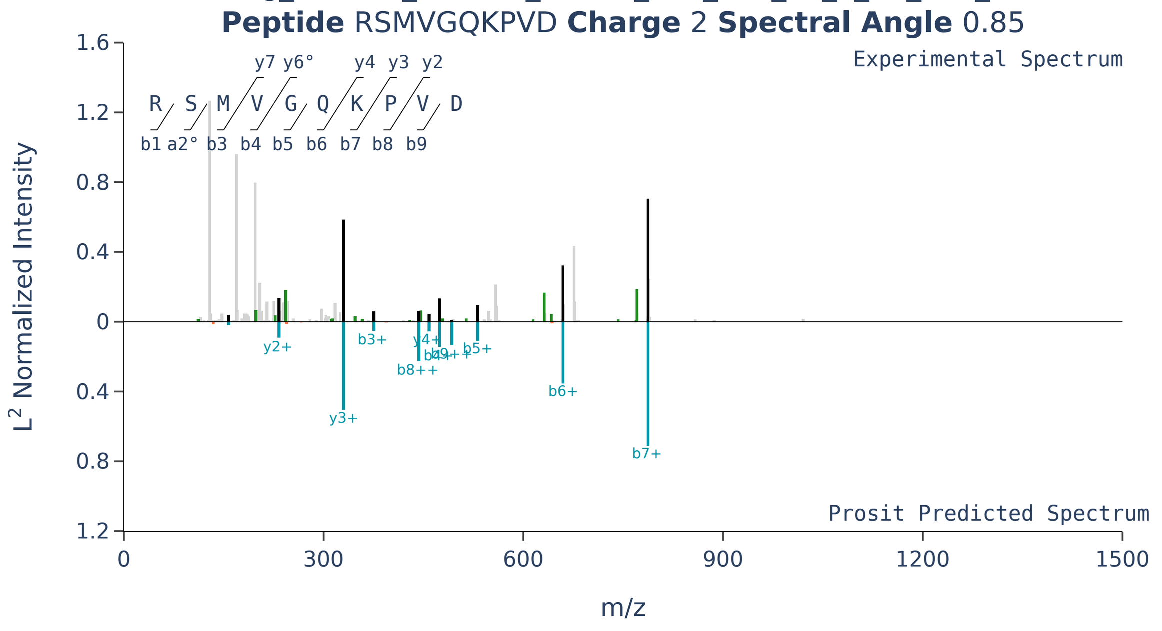

Source Ncheng_210623_230623_HFGoe_FFH_20S_25_1_A1_4h_R1 **Scan** 10368
Peptide VDIESKVDRA **Charge** 3 **Spectral Angle** 0.85

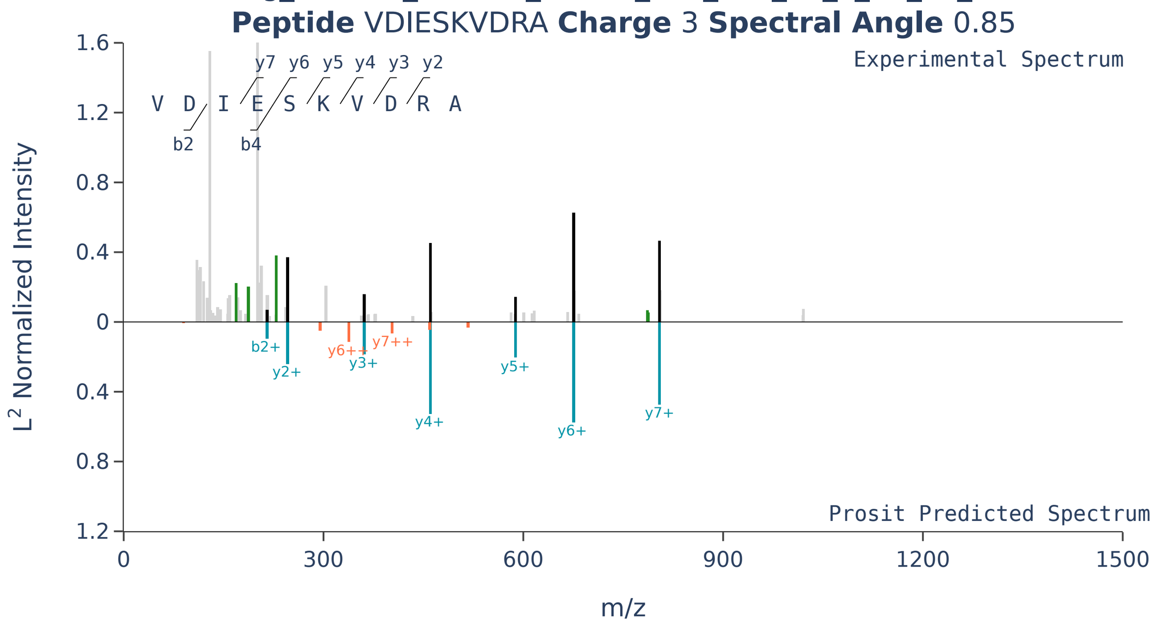

Source Ncheng_210623_230623_HFGoe_FFH_20S_25_1_A1_2h_R2 **Scan** 12931
Peptide PDNVKSQMNLA **Charge** 1 **Spectral Angle** 0.87

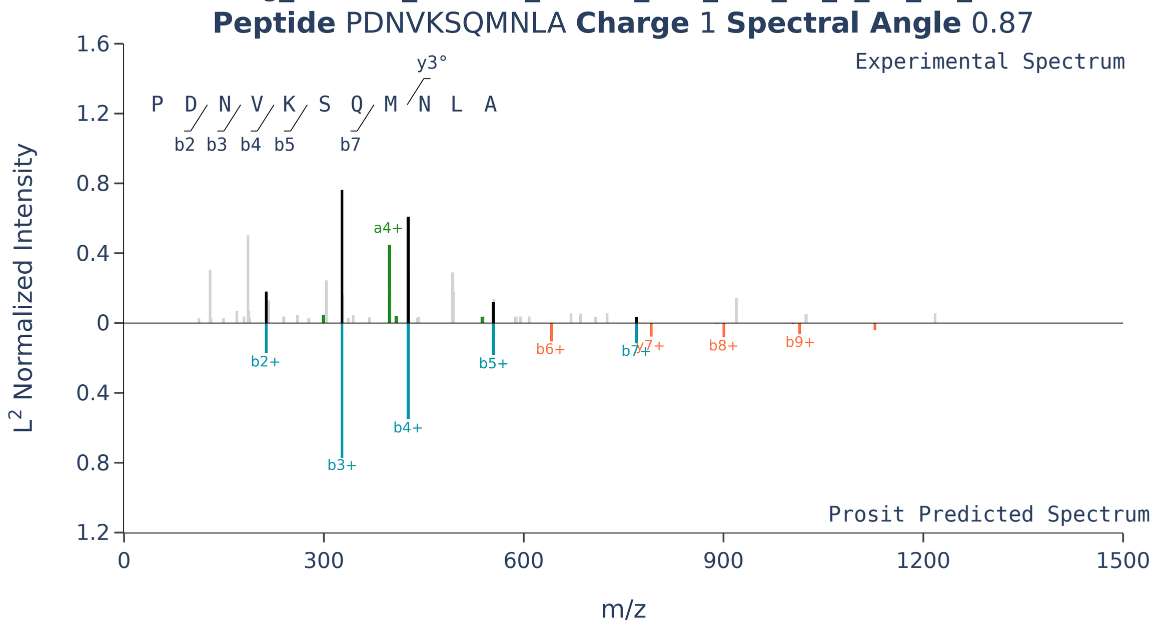

Source Ncheng_210623_230623_HFGoe_FFH_20S_25_1_A2_2h_R1 **Scan** 17397
Peptide TLAEQVGAANTAKAFN **Charge** 2 **Spectral Angle** 0.9

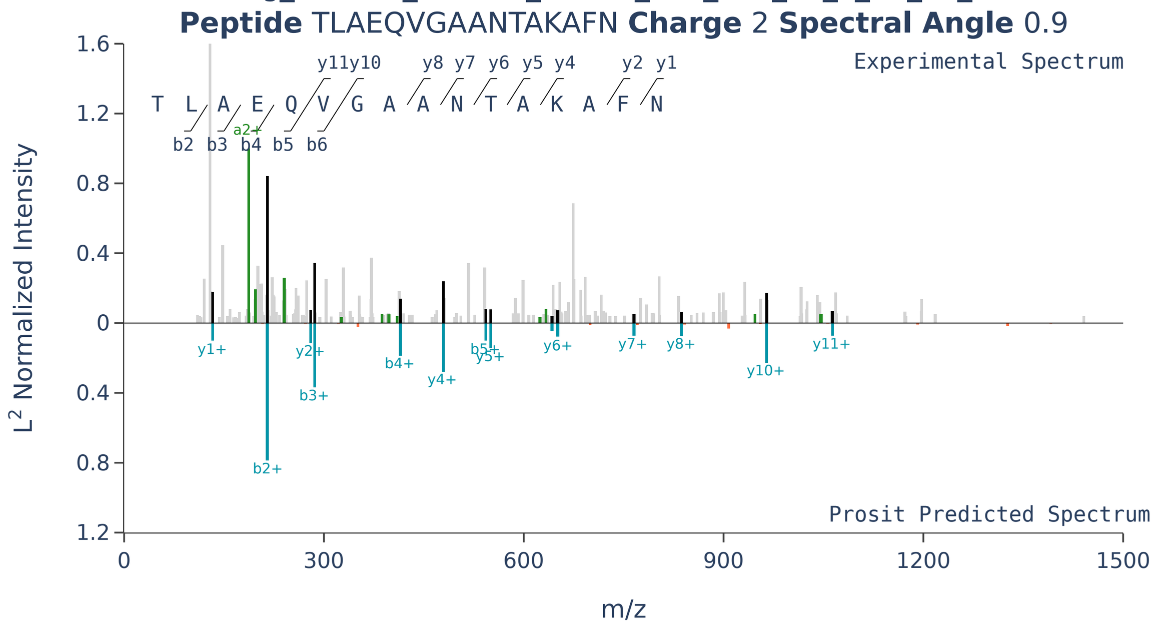

Source Ncheng_210623_230623_HFGoe_FFH_20S_25_1_A2_4h_R1 **Scan** 24771
Peptide DMGKLPGMGQIPDNVKSQMDD **Charge** 3 **Spectral Angle** 0.77

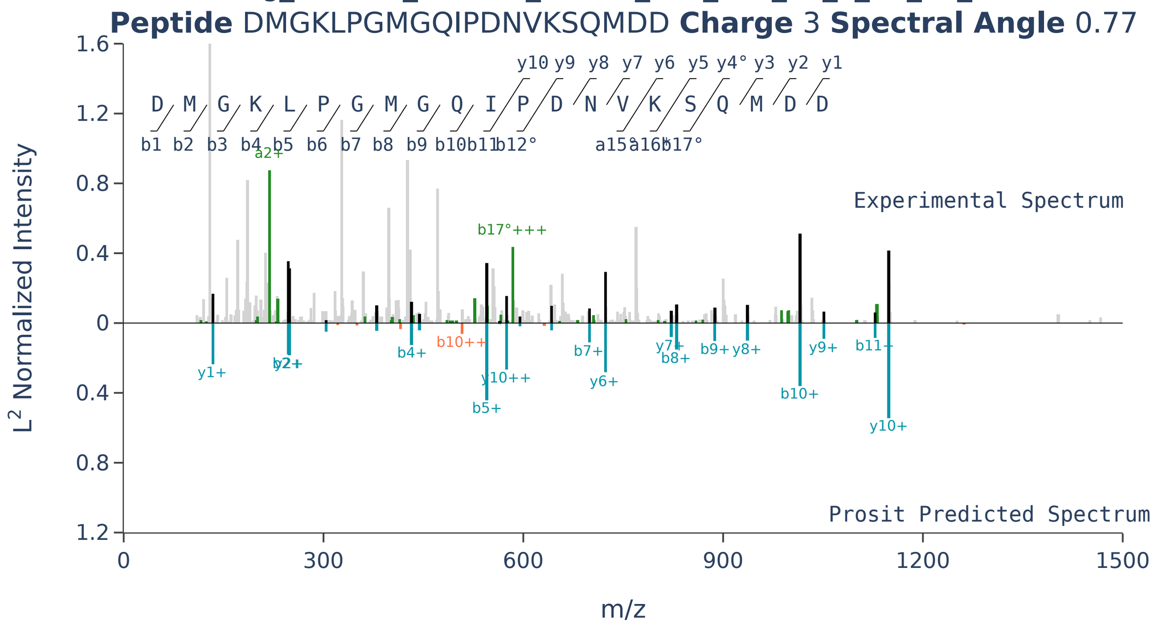

Source Ncheng_210623_230623_HFGoe_FFH_20S_25_1_A2_2h_R2 **Scan** 10347
Peptide TLAEQVDG **Charge** 1 **Spectral Angle** 0.85

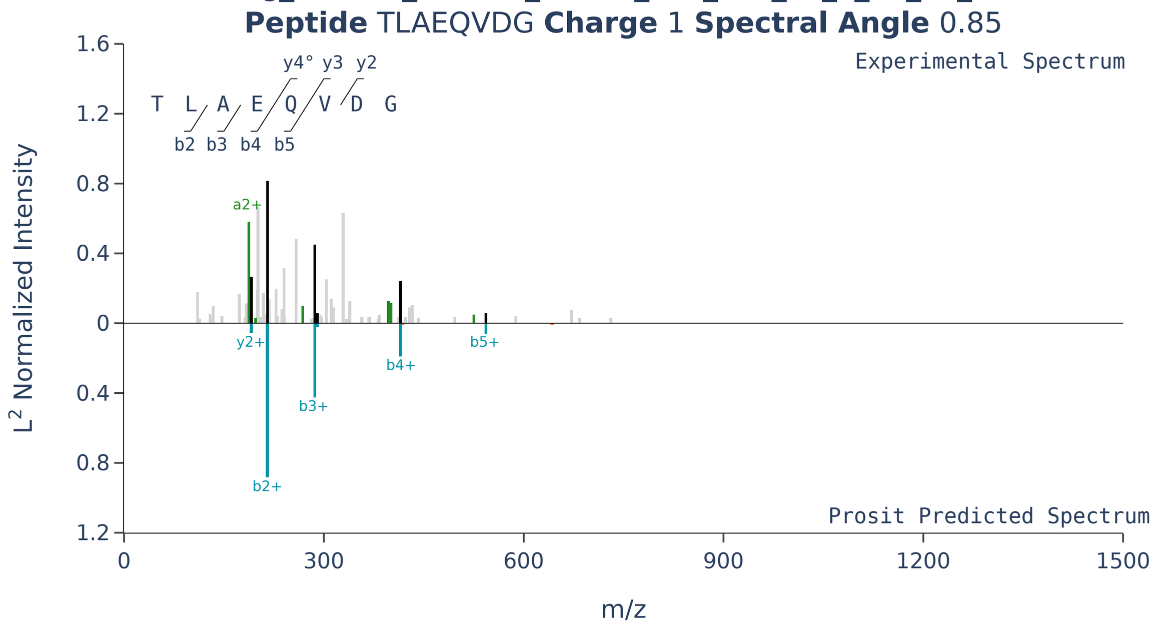

Source Ncheng_210623_230623_HFGoe_FFH_20S_25_1_A2_2h_R1 **Scan** 17874
Peptide GEENQTLLAA **Charge** 1 **Spectral Angle** 0.81

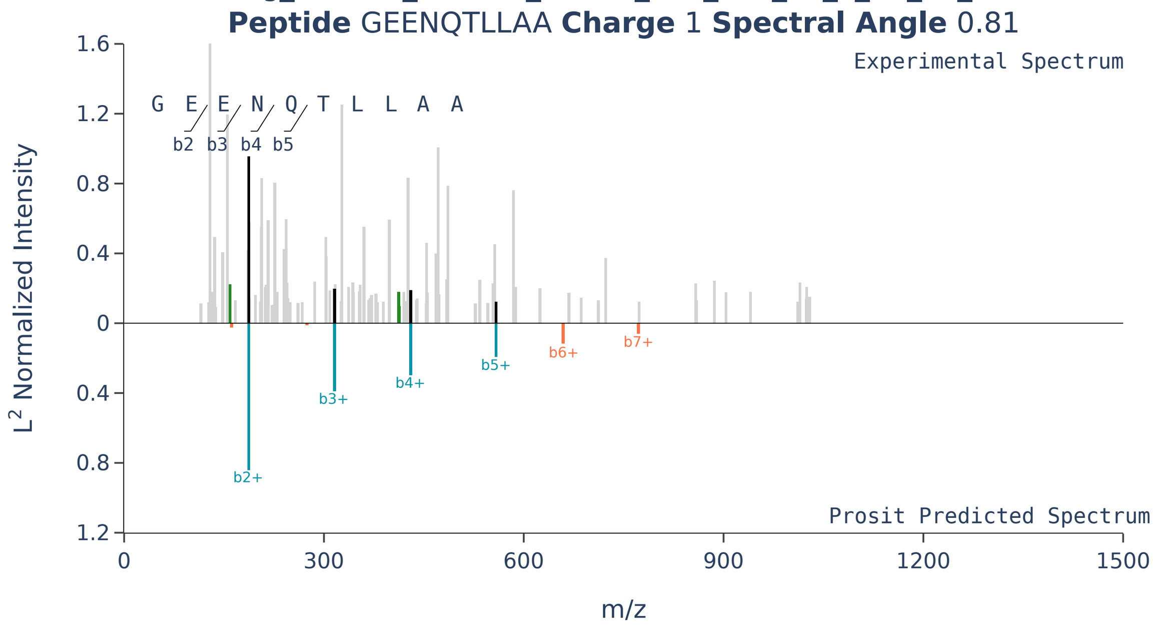

Source Ncheng_210623_230623_HFGoe_FFH_20S_25_1_A2_4h_R1 **Scan** 10293
Peptide TLAEQVGD **Charge** 2 **Spectral Angle** 0.78

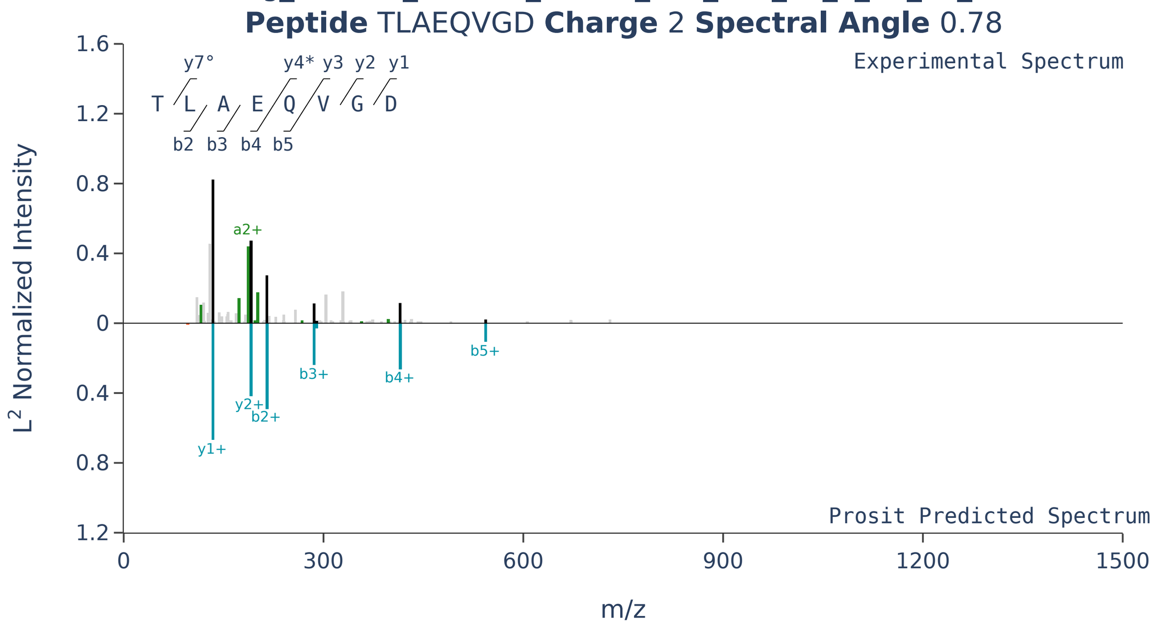

Source Ncheng_210623_230623_HFGoe_FFH_20S_25_1_A1_24h_R1 **Scan** 7951
Peptide LGVGEKTEE **Charge** 1 **Spectral Angle** 0.85

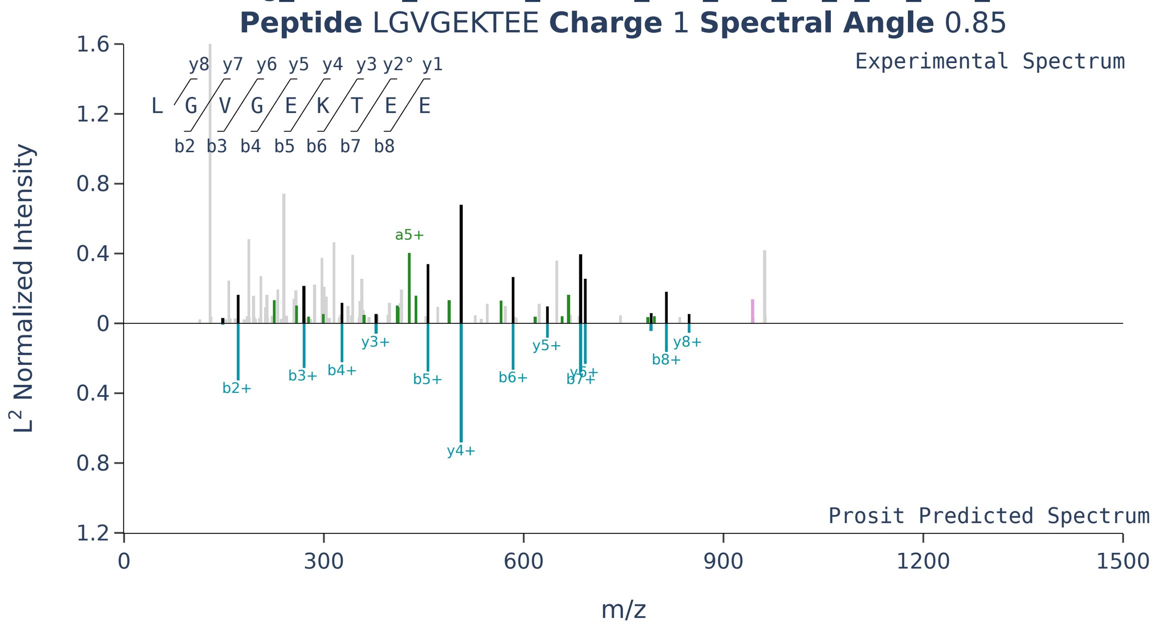

Source Ncheng_210623_230623_HFGoe_FFH_20S_25_1_A1_4h_R1 **Scan** 18179
Peptide ALEPFHPDRIASD **Charge** 2 **Spectral Angle** 0.76

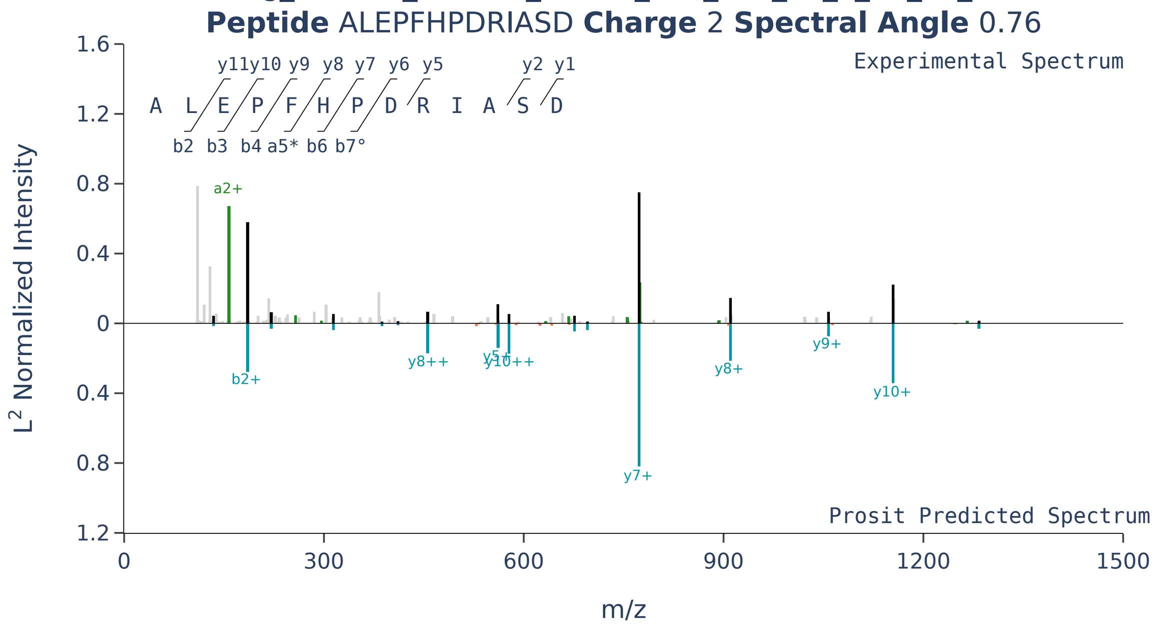

Source Ncheng_210623_230623_HFGoe_FFH_20S_25_1_A2_4h_R1 **Scan** 23826
Peptide AEKLAMGKLPGMGQIPDNVKSQMD **Charge** 3 **Spectral Angle** 0.82

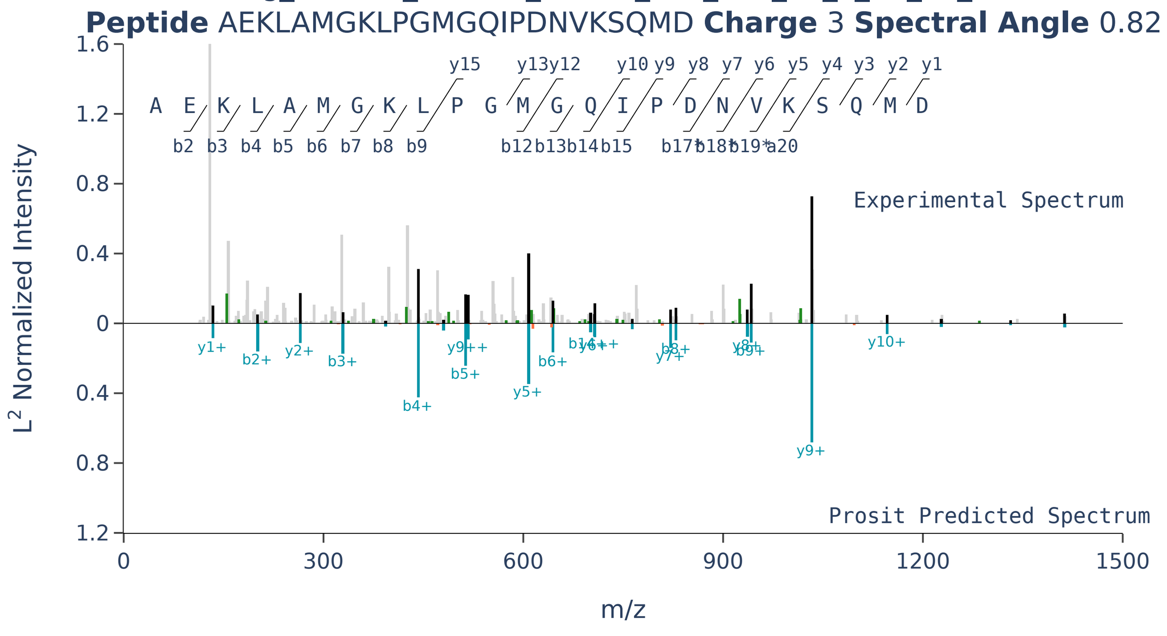

Source Ncheng_210623_230623_HFGoe_FFH_20S_25_1_A2_24h_R1 **Scan** 15804
Peptide DGDARGGAALRL **Charge** 2 **Spectral Angle** 0.85

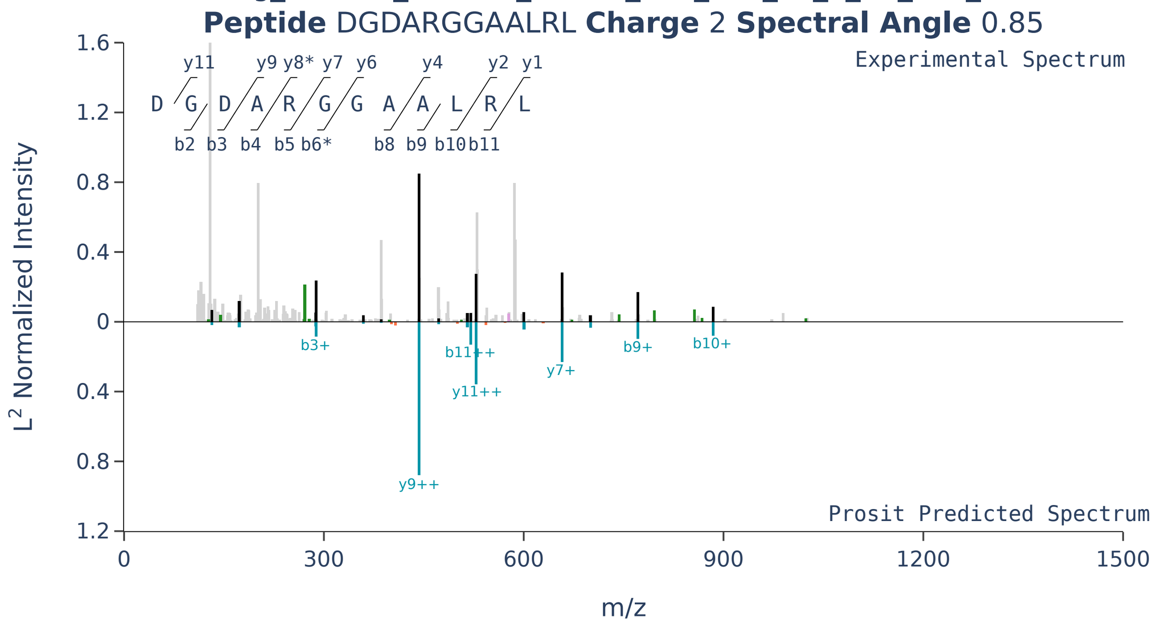

Source Ncheng_210623_230623_HFGoe_FFH_20S_25_1_A2_24h_R2 **Scan** 12384
Peptide FNAMTGQDAANTAKA **Charge** 2 **Spectral Angle** 0.87

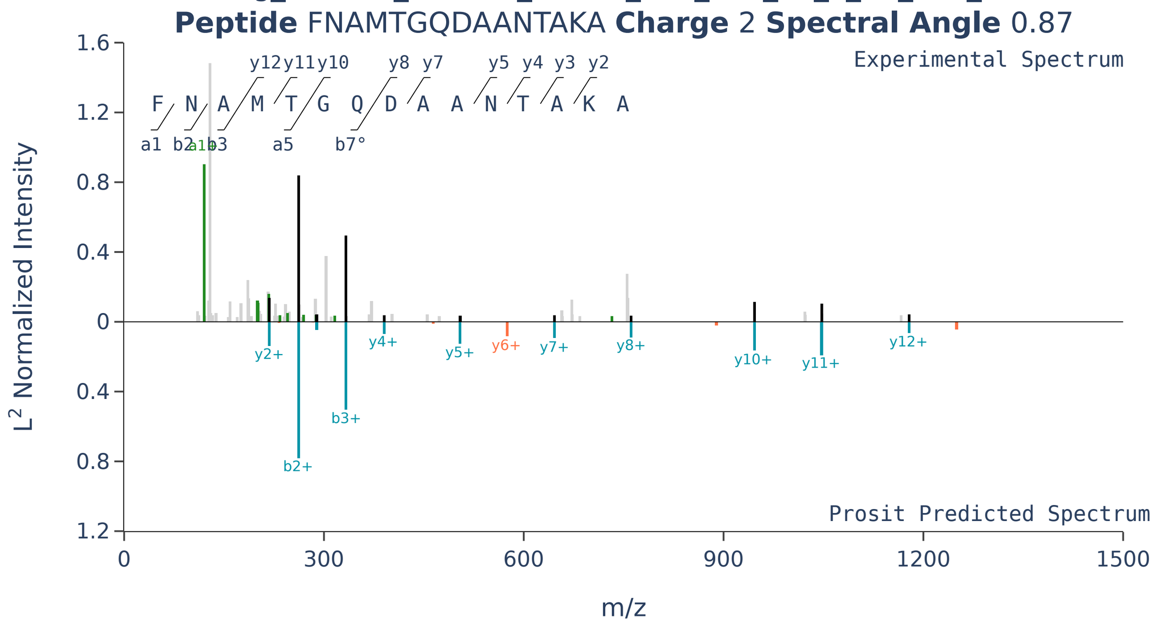

Source Ncheng_210623_230623_HFGoe_FFH_20S_25_1_A1_24h_R1 **Scan** 18753
Peptide GEENQTLNLADTA **Charge** 1 **Spectral Angle** 0.78

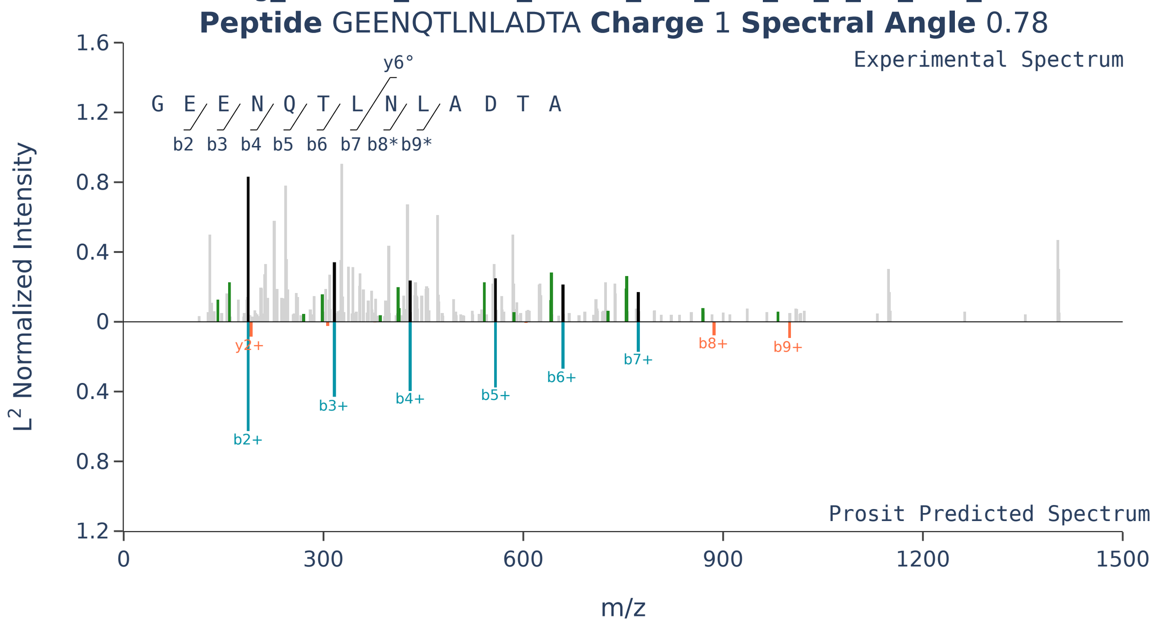

Source Ncheng_210623_230623_HFGoe_FFH_20S_25_1_A1_24h_R1 **Scan** 19320
Peptide KLPGMGQIPDNVKSA **Charge** 2 **Spectral Angle** 0.87

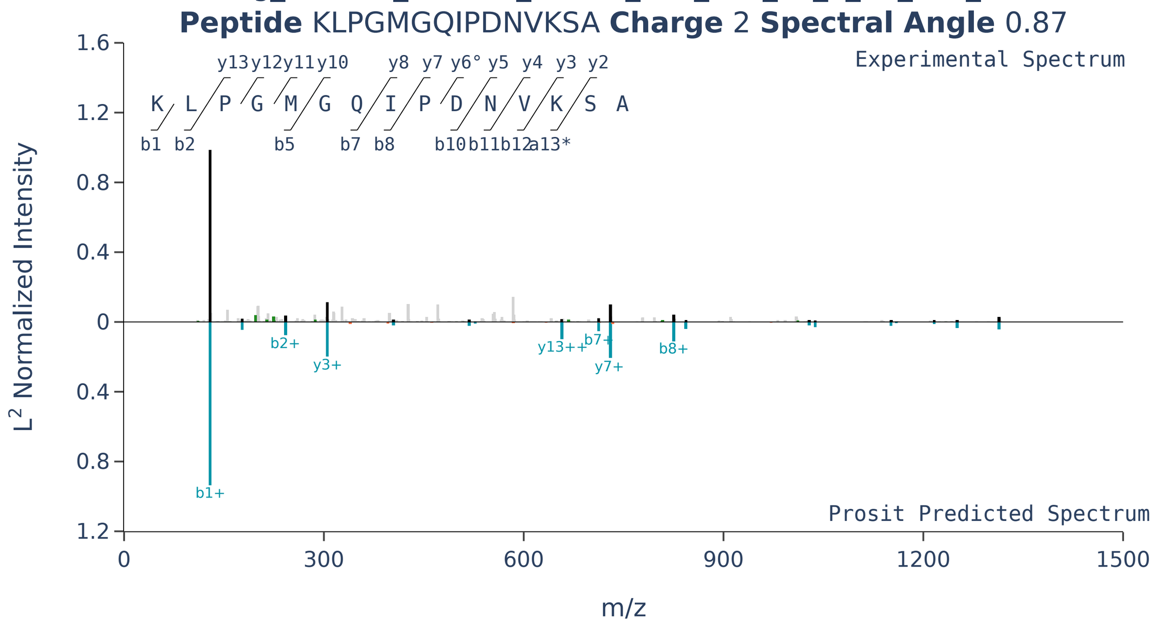

Source Ncheng_210623_230623_HFGoe_FFH_20S_25_1_A2_24h_R1 **Scan** 8041
Peptide RNISGVGQKPVD **Charge** 2 **Spectral Angle** 0.86

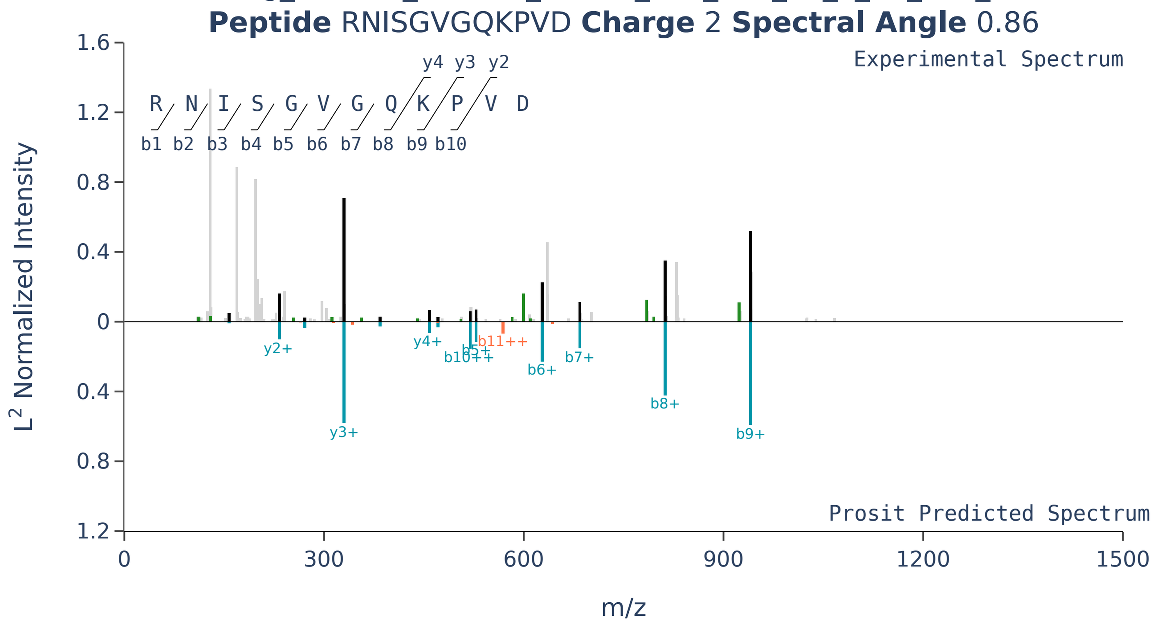

Source Ncheng_210623_230623_HFGoe_FFH_20S_25_1_A1_24h_R2 **Scan** 26438
Peptide DAALPLTGV **Charge** 1 **Spectral Angle** 0.91

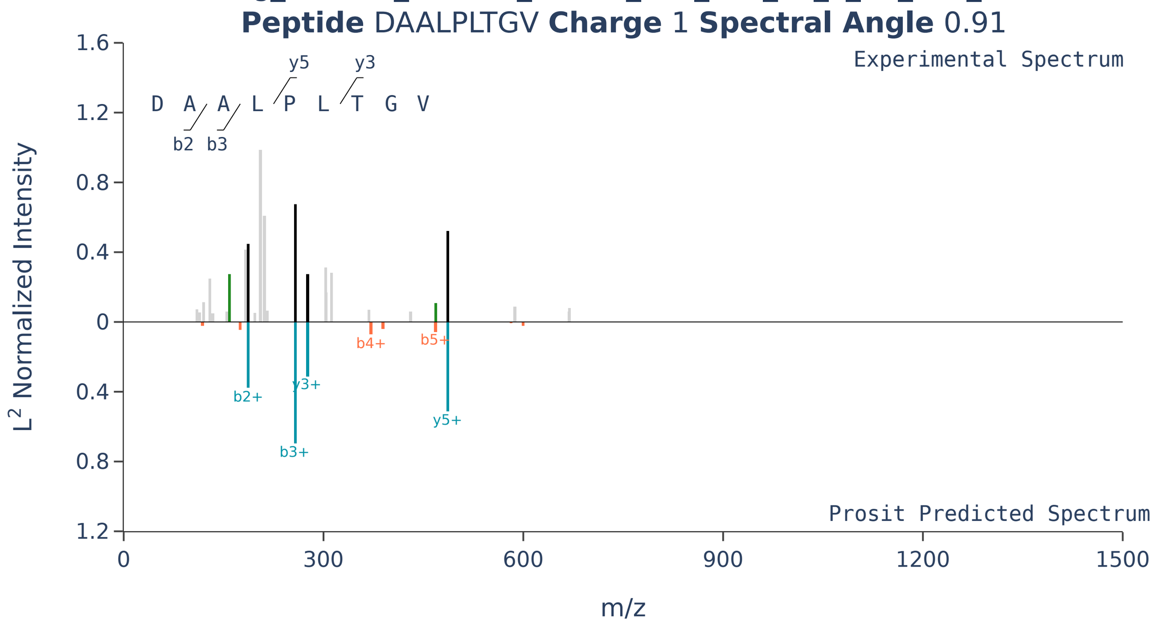

Source Ncheng_210623_230623_HFGoe_FFH_20S_25_1_A1_4h_R2 **Scan** 23197
Peptide GGMKLPGMGQIPDNVKSQMD **Charge** 3 **Spectral Angle** 0.76

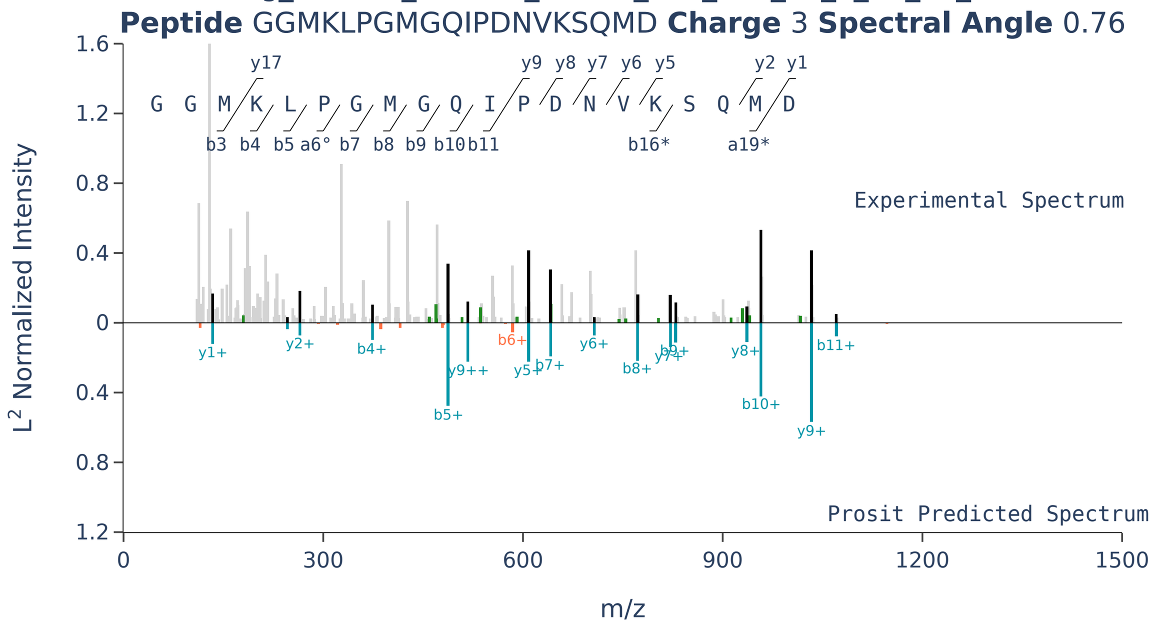

Source Ncheng_210623_230623_HFGoe_FFH_20S_25_1_A1_24h_R2 **Scan** 12148
Peptide DGDARGGAALVGQKPVD **Charge** 3 **Spectral Angle** 0.85

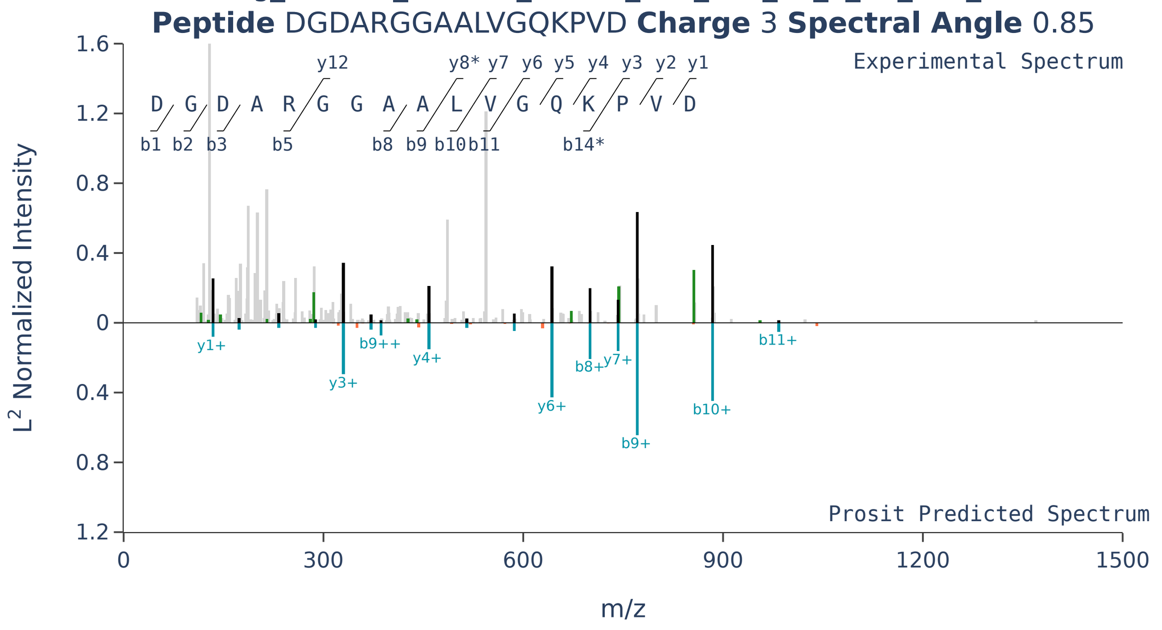

Source Ncheng_210623_230623_HFGoe_FFH_20S_25_1_A1_24h_R1 **Scan** 20689
Peptide DGDARGGAALSINPVE **Charge** 2 **Spectral Angle** 0.83

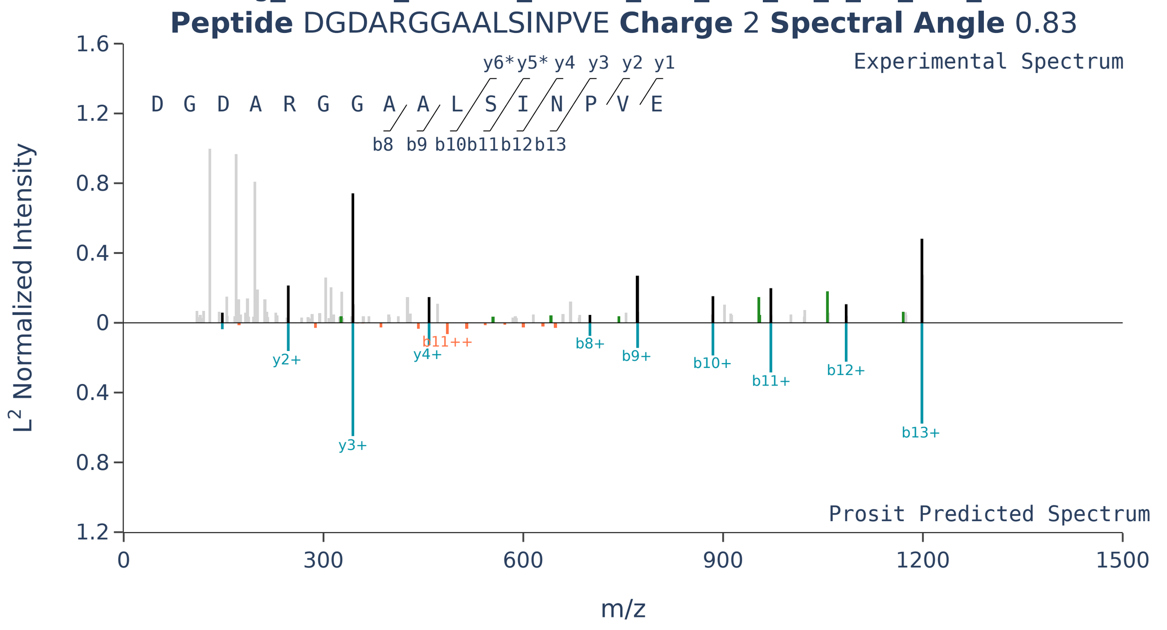

Source Ncheng_210623_230623_HFGoe_FFH_20S_25_1_A1_24h_R2 **Scan** 11957
Peptide QVGDIESKVDRAQAE **Charge** 2 **Spectral Angle** 0.89

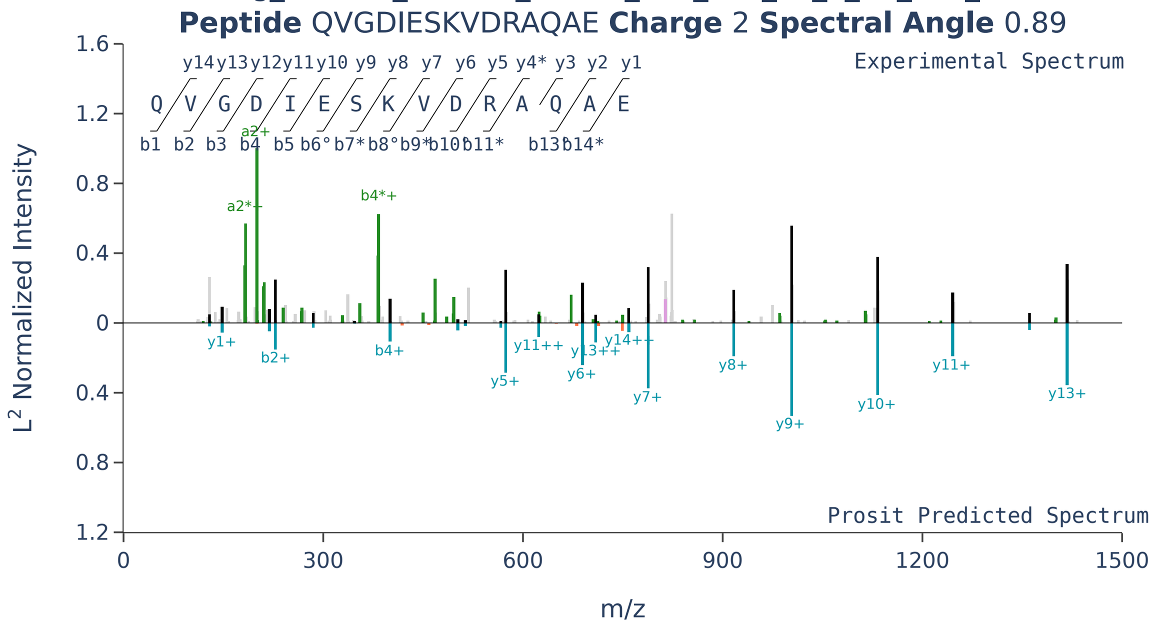

Source Ncheng_210623_230623_HFGoe_FFH_20S_25_1_A2_4h_R1 **Scan** 14560
Peptide HASINPVEVGQKPVD **Charge** 3 **Spectral Angle** 0.85

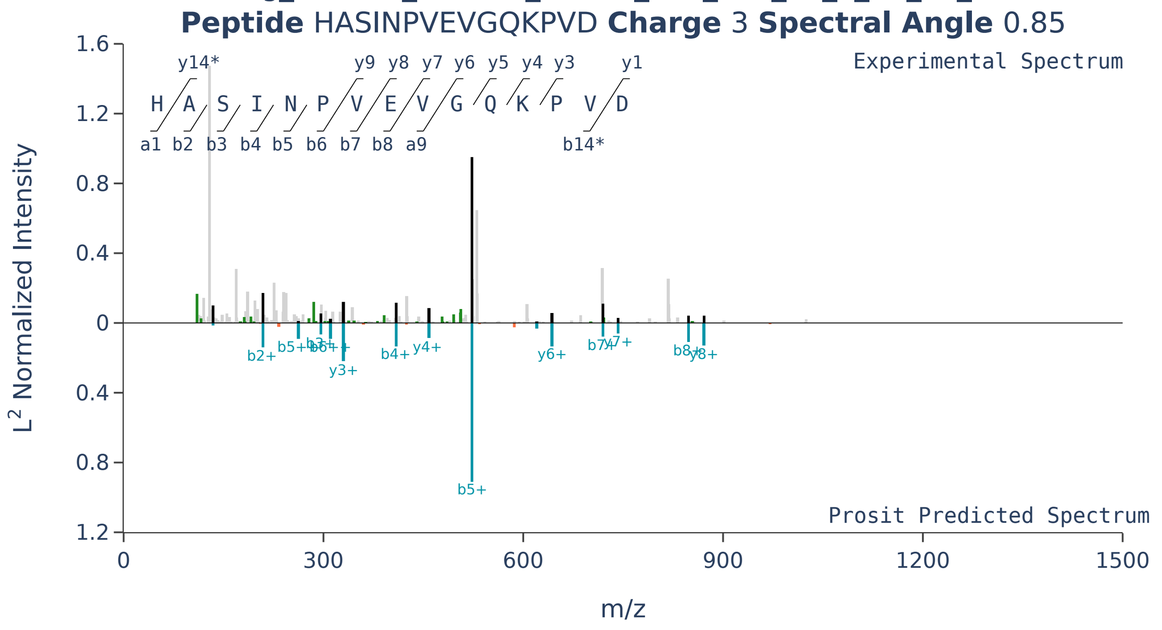

Source Ncheng_210623_230623_HFGoe_FFH_20S_25_1_A2_4h_R2 **Scan** 28206
Peptide RIAAGTLFVVD **Charge** 2 **Spectral Angle** 0.86

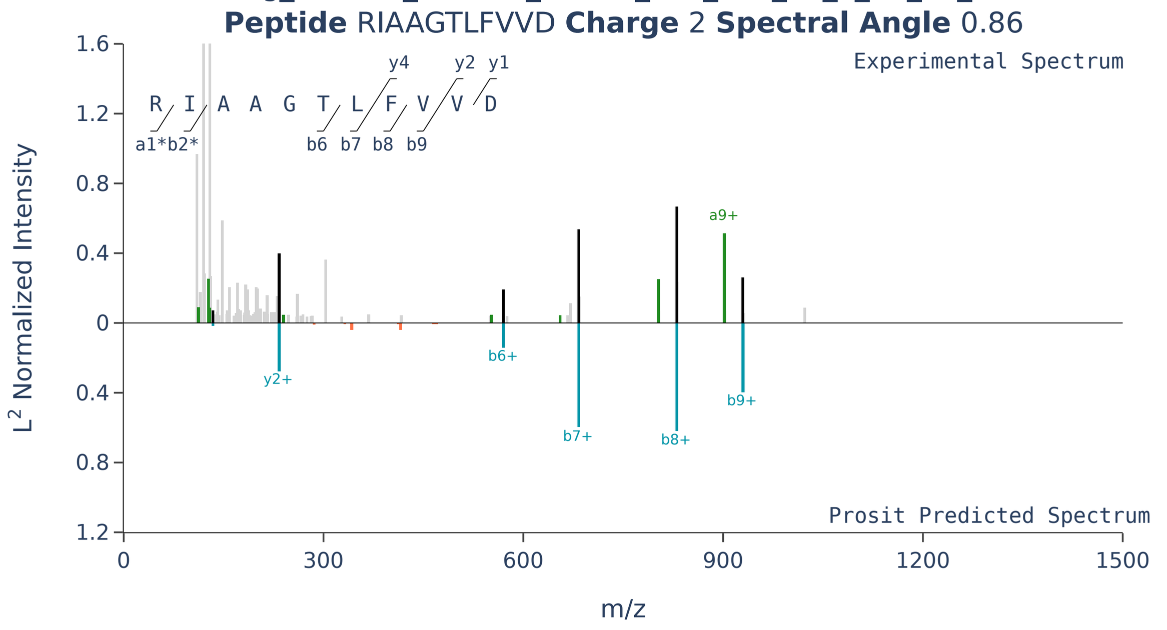

Source Ncheng_210623_230623_HFGoe_FFH_20S_25_1_A1_24h_R1 **Scan** 26538
Peptide GEALPLTGV **Charge** 1 **Spectral Angle** 0.9

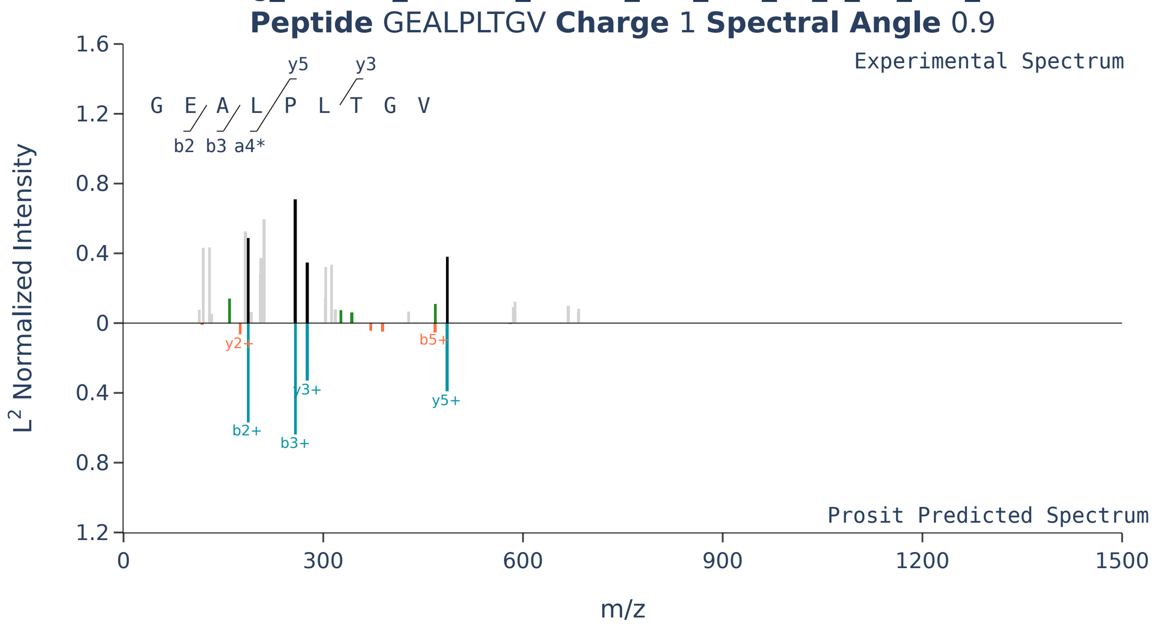

Source Ncheng_210623_230623_HFGoe_FFH_20S_25_1_A2_24h_R1 **Scan** 12230
Peptide PGMGQIPDNVKKS **Charge** 3 **Spectral Angle** 0.82

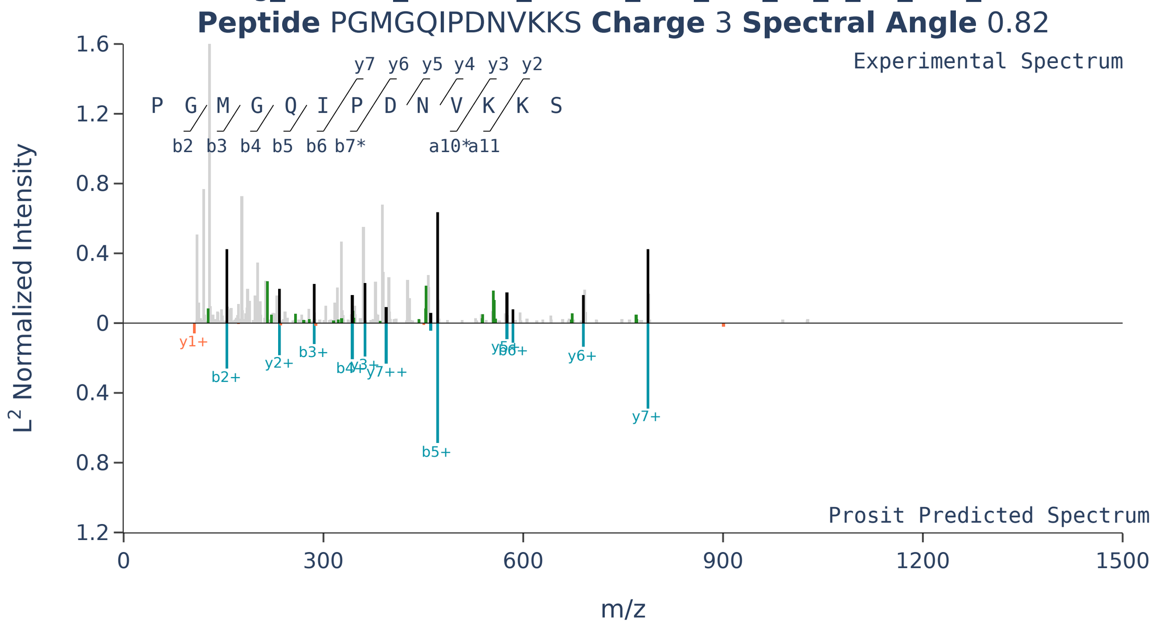

Source Ncheng_210623_230623_HFGoe_FFH_20S_25_1_A2_24h_R2 **Scan** 10282
Peptide KNMGGMALSR **Charge** 2 **Spectral Angle** 0.69

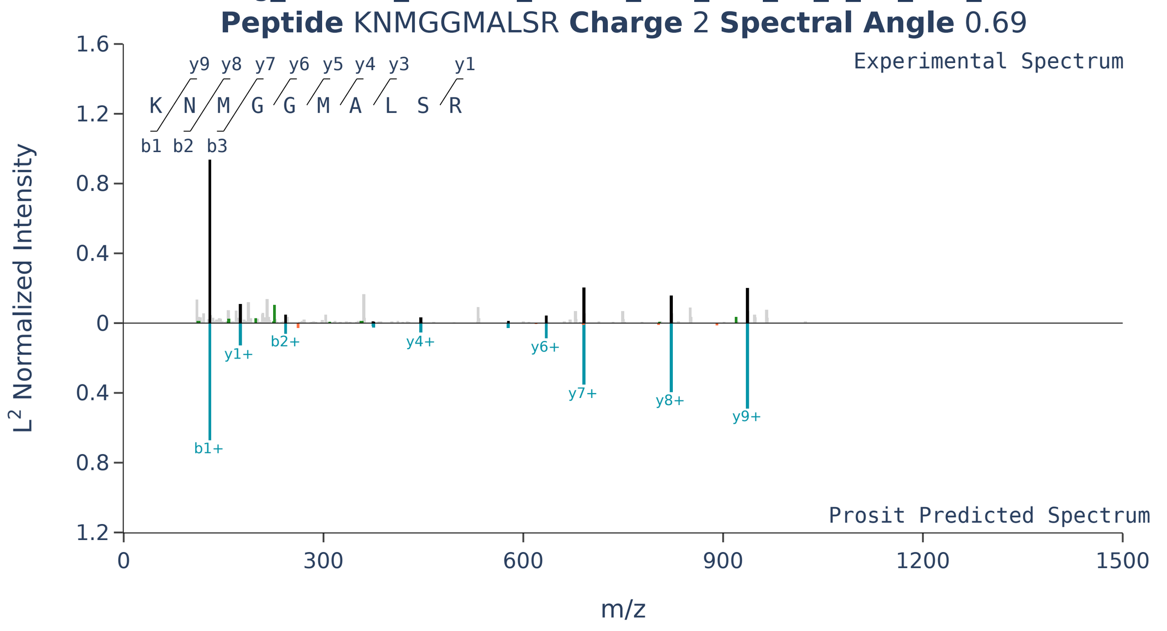

Source Ncheng_210623_230623_HFGoe_FFH_20S_25_1_A2_24h_R2 **Scan** 12030
Peptide EPFHPDRASIN **Charge** 2 **Spectral Angle** 0.92

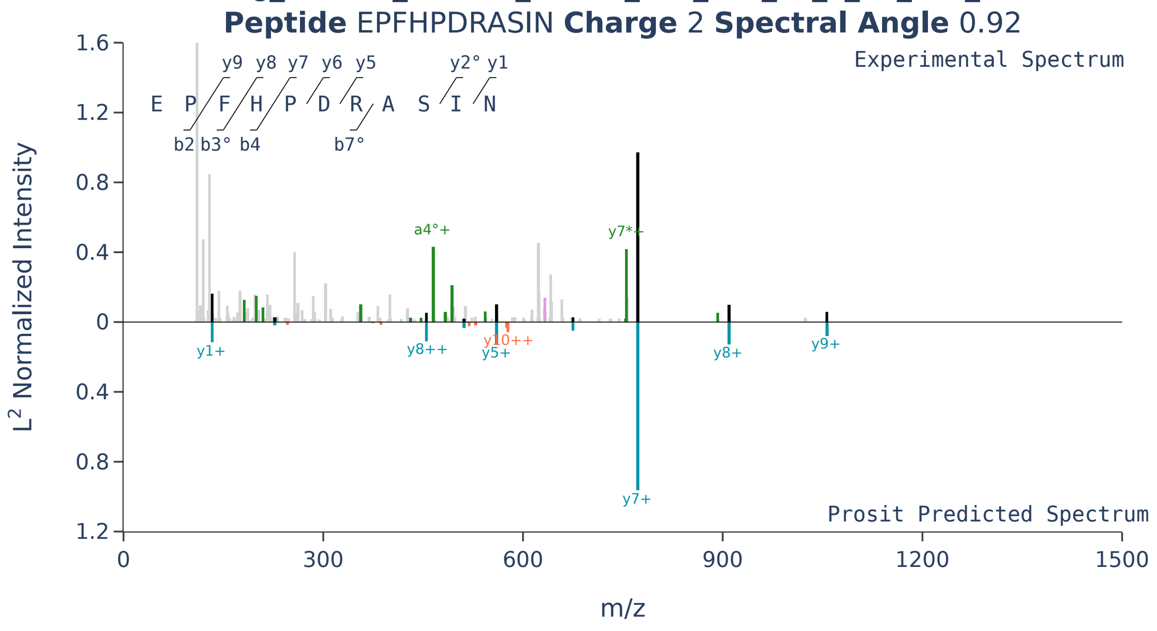

Source Ncheng_210623_230623_HFGoe_FFH_20S_25_1_A2_1h_R2 **Scan** 9739
Peptide DKVLVGE **Charge** 2 **Spectral Angle** 0.84

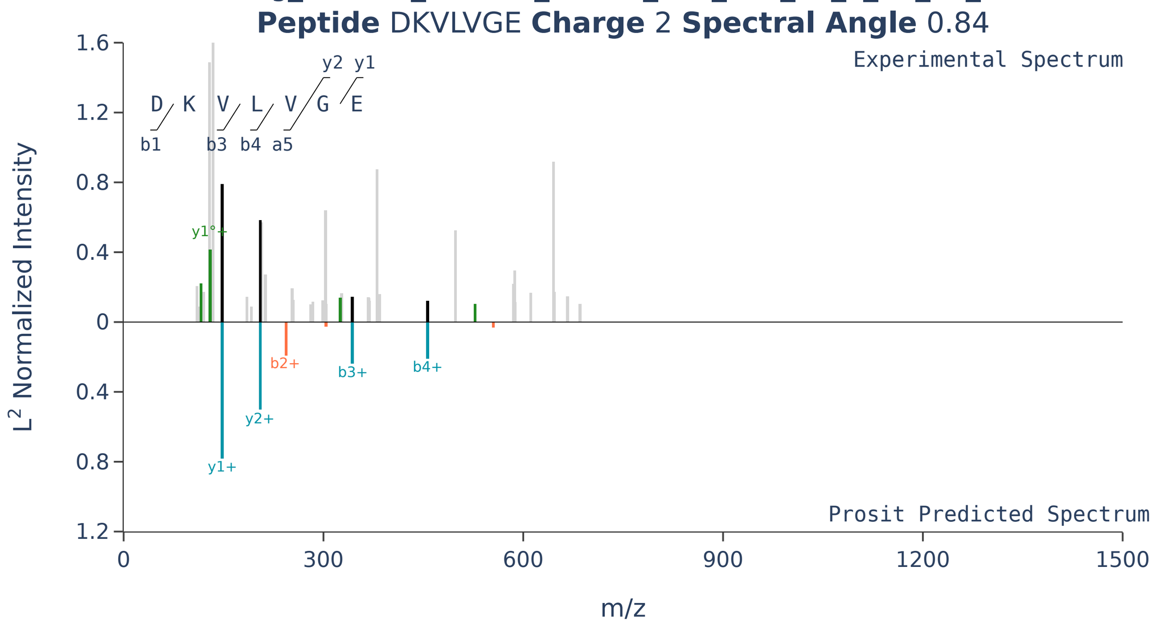

Source Ncheng_210623_230623_HFGoe_FFH_20S_25_1_A1_2h_R2 **Scan** 19329
Peptide GMKLPGMGQIPDNVKSQMD **Charge** 3 **Spectral Angle** 0.75

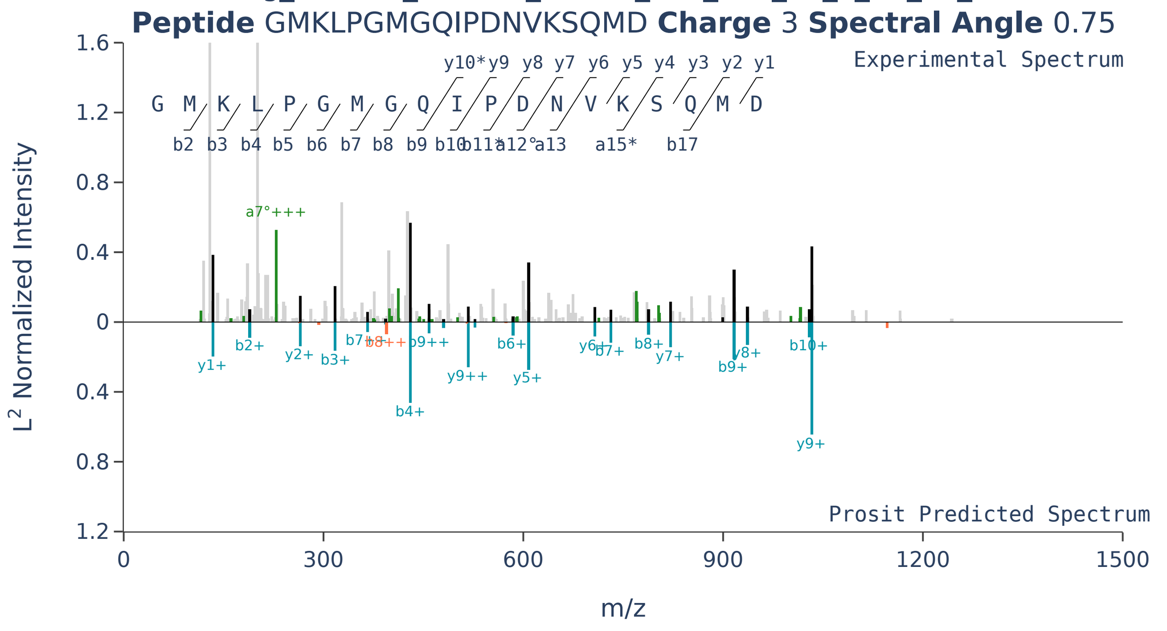

Source Ncheng_210623_230623_HFGoe_FFH_20S_25_1_A1_2h_R2 **Scan** 11881
Peptide KTEAMTGQDAANTAKAFN **Charge** 3 **Spectral Angle** 0.72

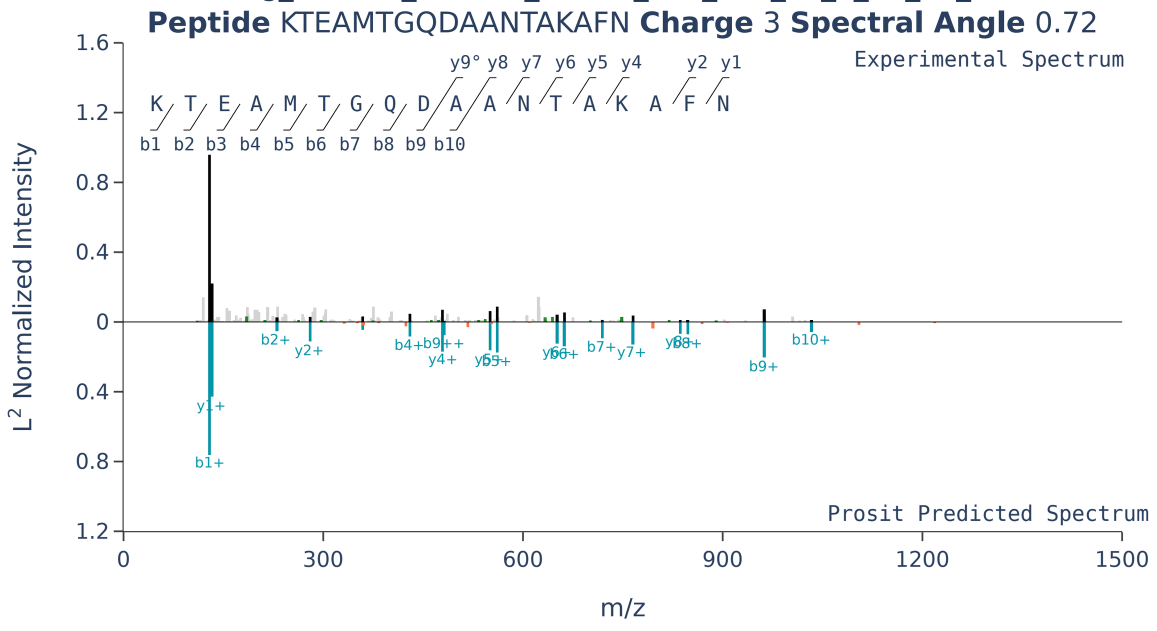

Source Ncheng_210623_230623_HFGoe_FFH_20S_25_1_A1_24h_R1 **Scan** 16577
Peptide SAVGQKPVDIVNA **Charge** 2 **Spectral Angle** 0.78

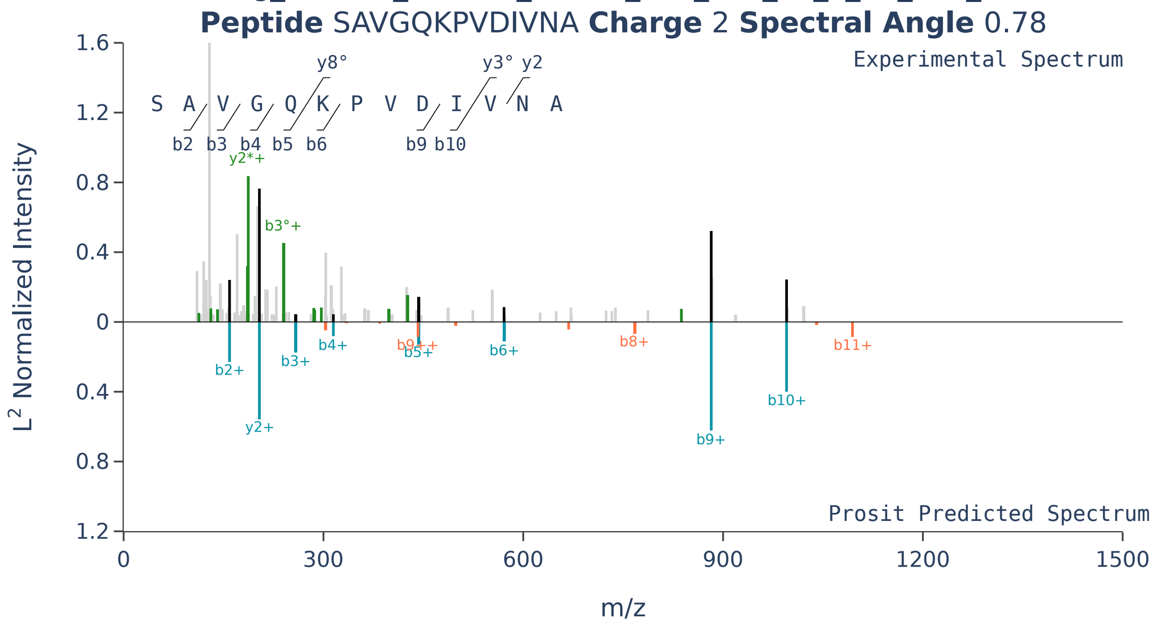

Source Ncheng_210623_230623_HFGoe_FFH_20S_25_1_A1_2h_R1 **Scan** 15407
Peptide EIKQVHASINPEV **Charge** 2 **Spectral Angle** 0.85

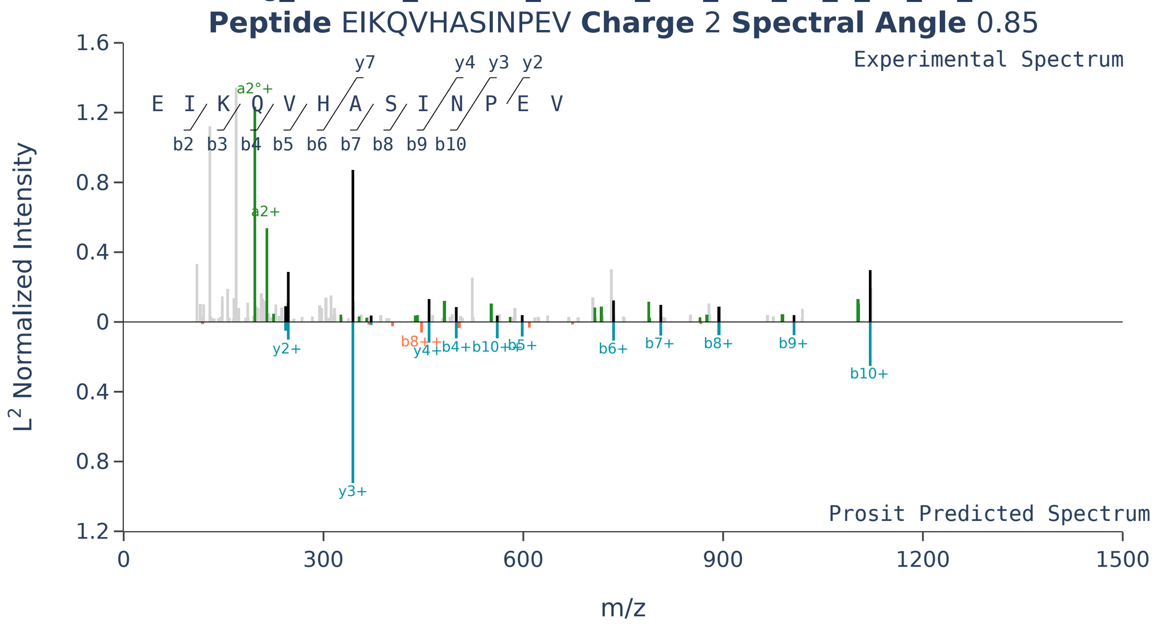

Source Ncheng_210623_230623_HFGoe_FFH_20S_25_1_A1_4h_R1 **Scan** 15069
Peptide RNIAMTGQDAANTAKAFN **Charge** 3 **Spectral Angle** 0.81

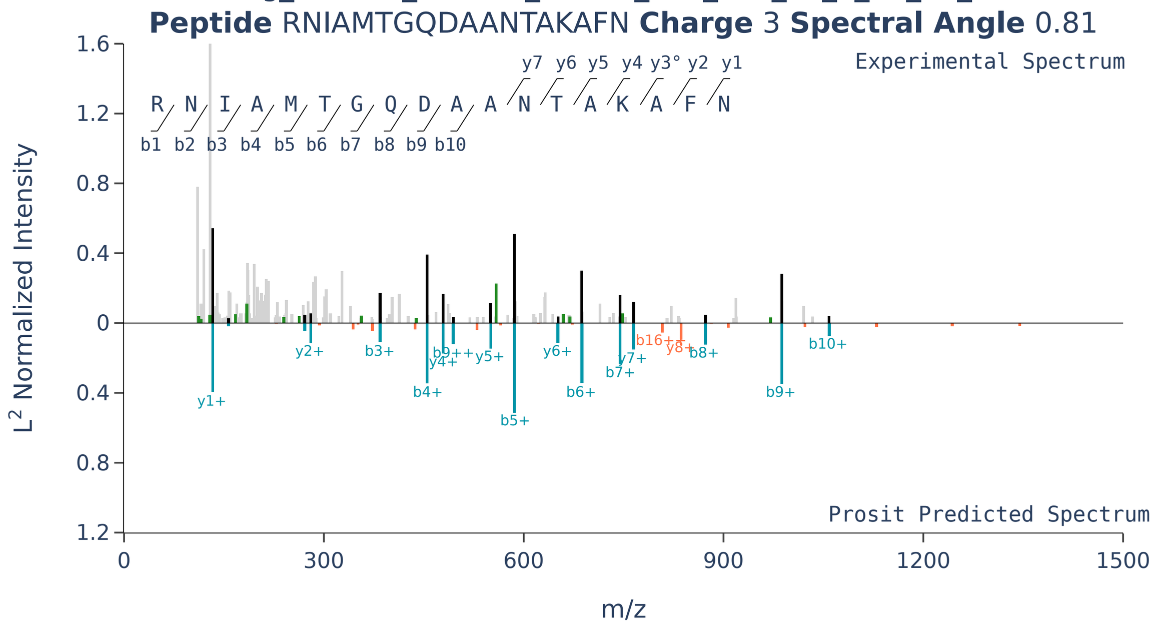

Source Ncheng_210623_230623_HFGoe_FFH_20S_25_1_A2_24h_R2 **Scan** 9758
Peptide KEATGKPIKF **Charge** 3 **Spectral Angle** 0.85

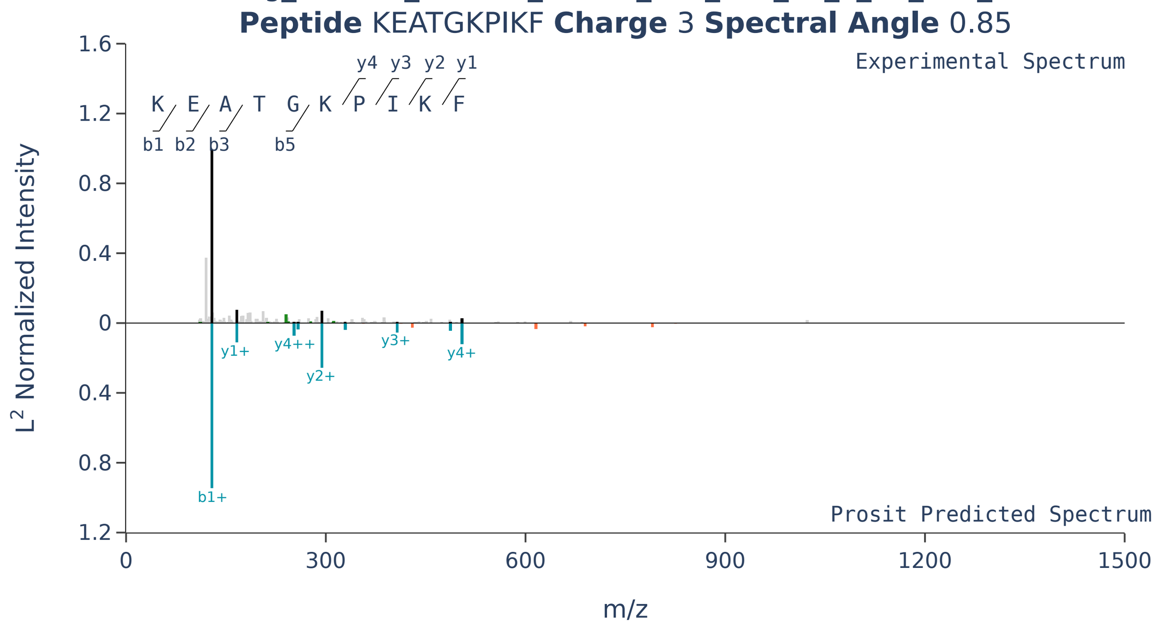

Source Ncheng_210623_230623_HFGoe_FFH_20S_25_1_A2_2h_R2 **Scan** 9431
Peptide VHDEAMMD **Charge** 1 **Spectral Angle** 0.75

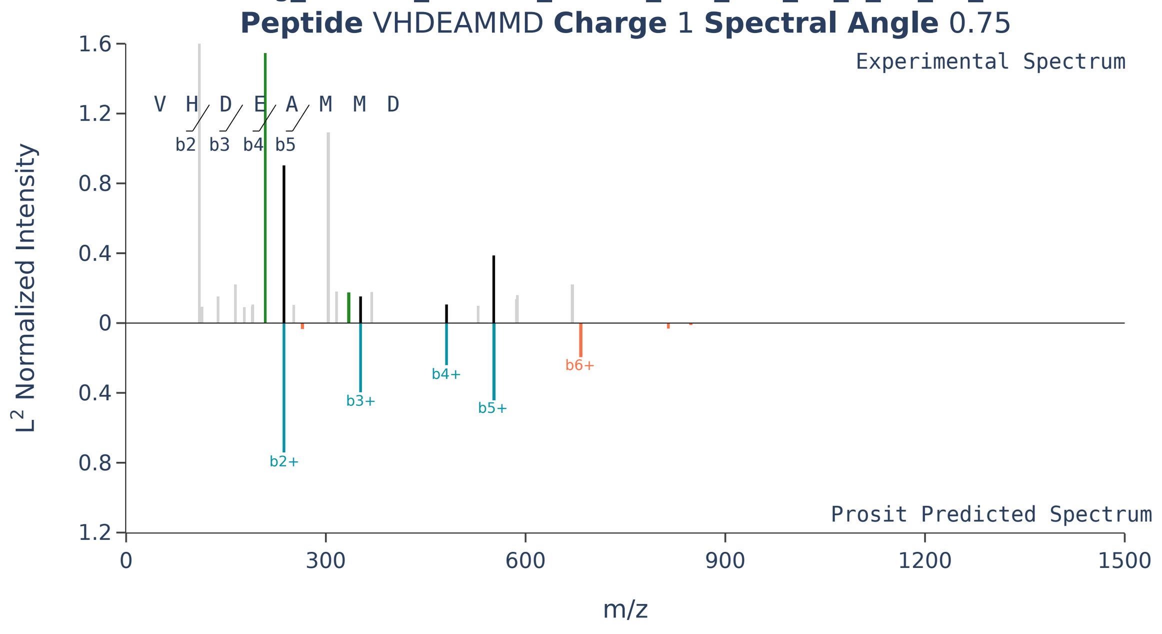

Source Ncheng_210623_230623_HFGoe_FFH_20S_25_1_A2_2h_R2 **Scan** 15013
Peptide RIAAMTGQDAANTAKAFN **Charge** 3 **Spectral Angle** 0.81

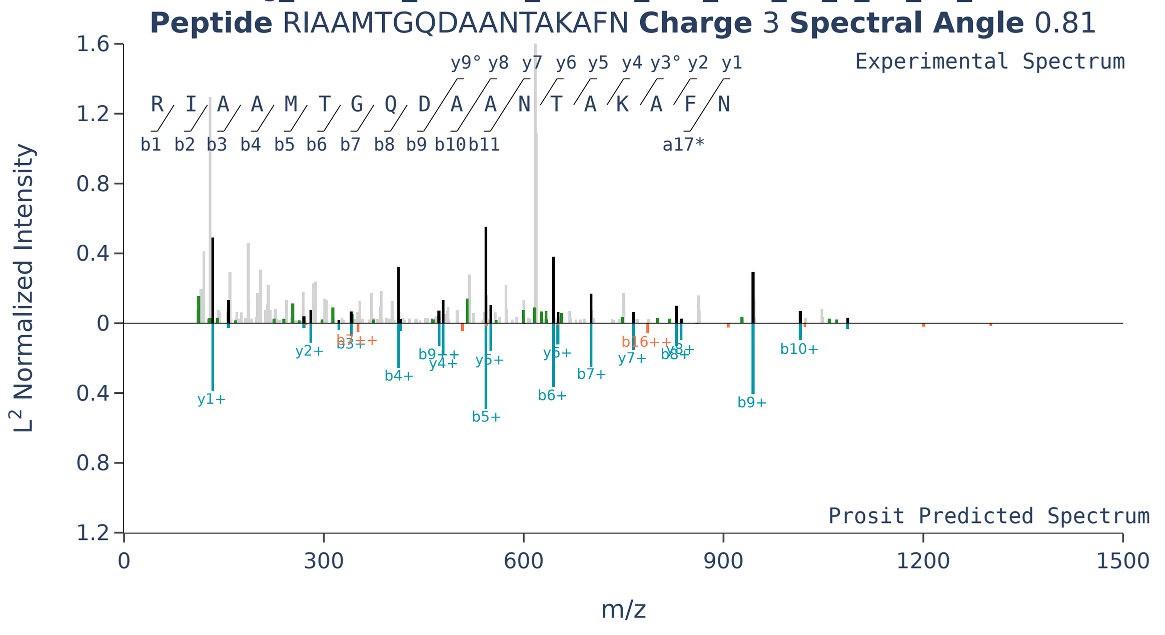

Source Ncheng_210623_230623_HFGoe_FFH_20S_25_1_A2_2h_R1 **Scan** 14832
Peptide MRSTLAEQV **Charge** 2 **Spectral Angle** 0.83

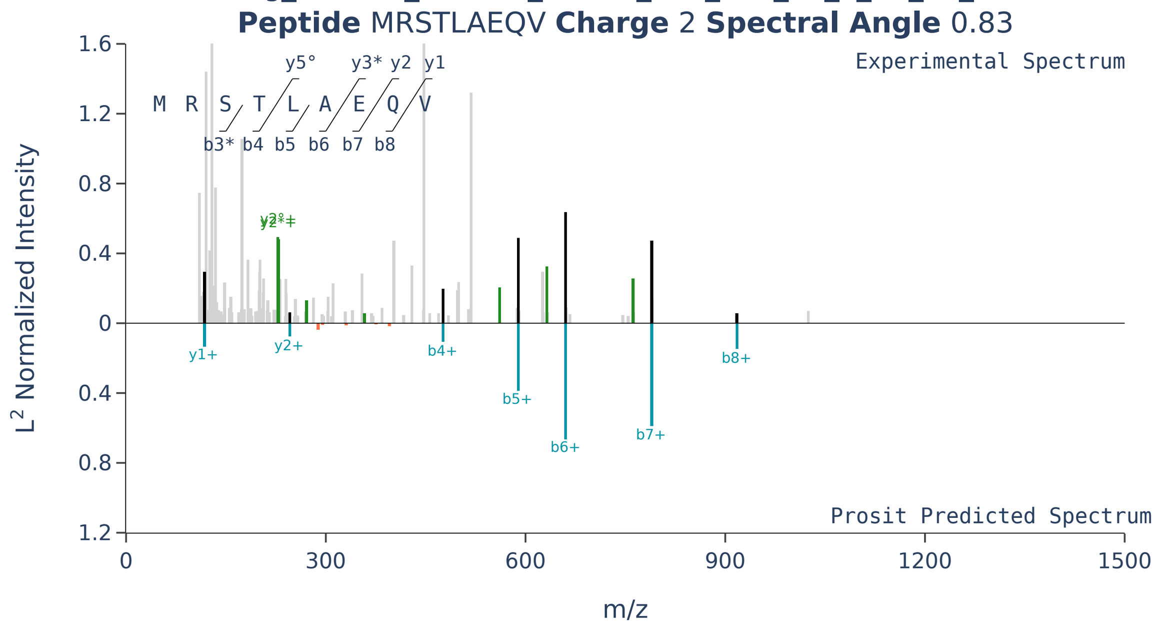

Source Ncheng_210623_230623_HFGoe_FFH_20S_25_1_A1_2h_R1 **Scan** 6805
Peptide GGKSLTPGQE **Charge** 2 **Spectral Angle** 0.75

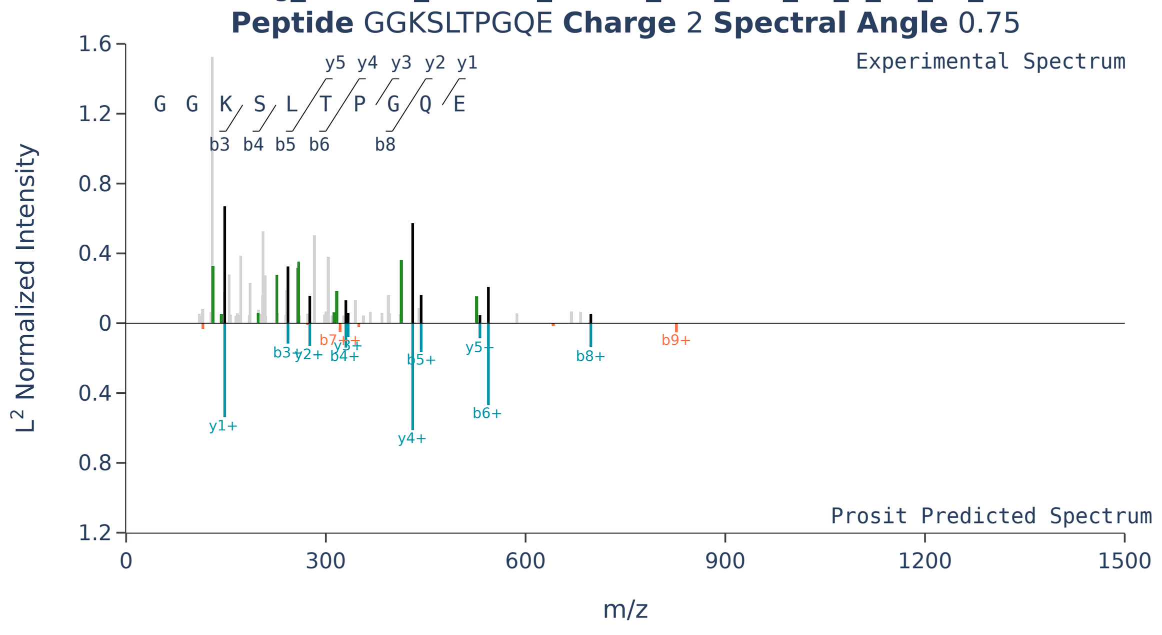

Source Ncheng_210623_230623_HFGoe_FFH_20S_25_1_A1_24h_R2 **Scan** 25388
Peptide ARGVGVDFFPSD **Charge** 2 **Spectral Angle** 0.88

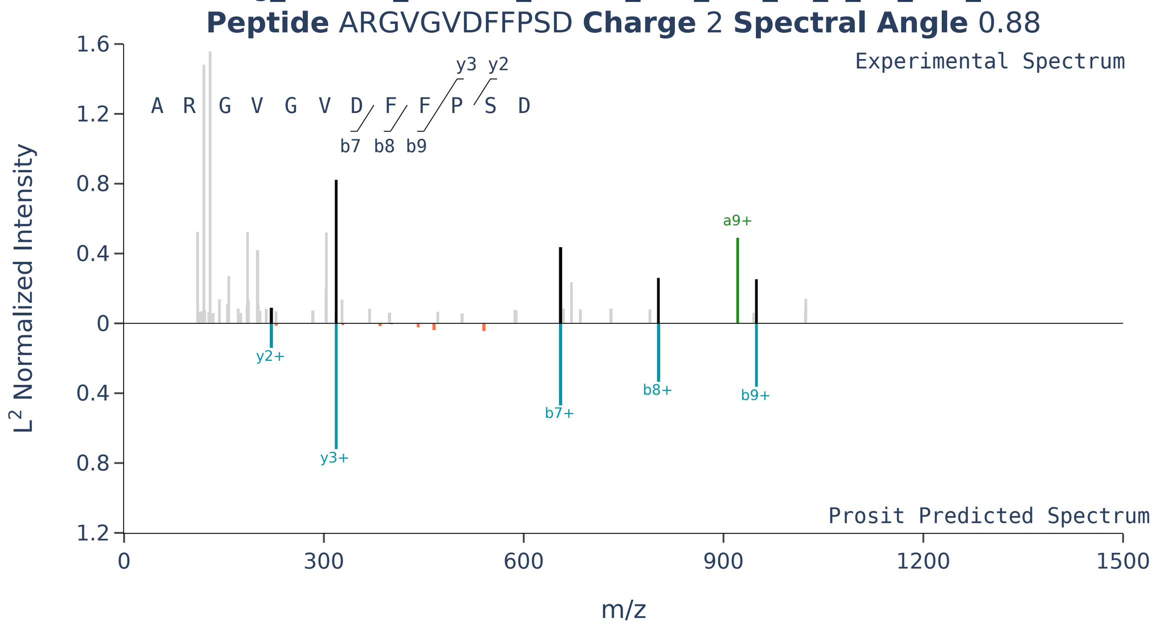

Source Ncheng_210623_230623_HFGoe_FFH_20S_25_1_A2_2h_R1 **Scan** 31532
Peptide TKETLAEQVGVDFFPSD **Charge** 2 **Spectral Angle** 0.95

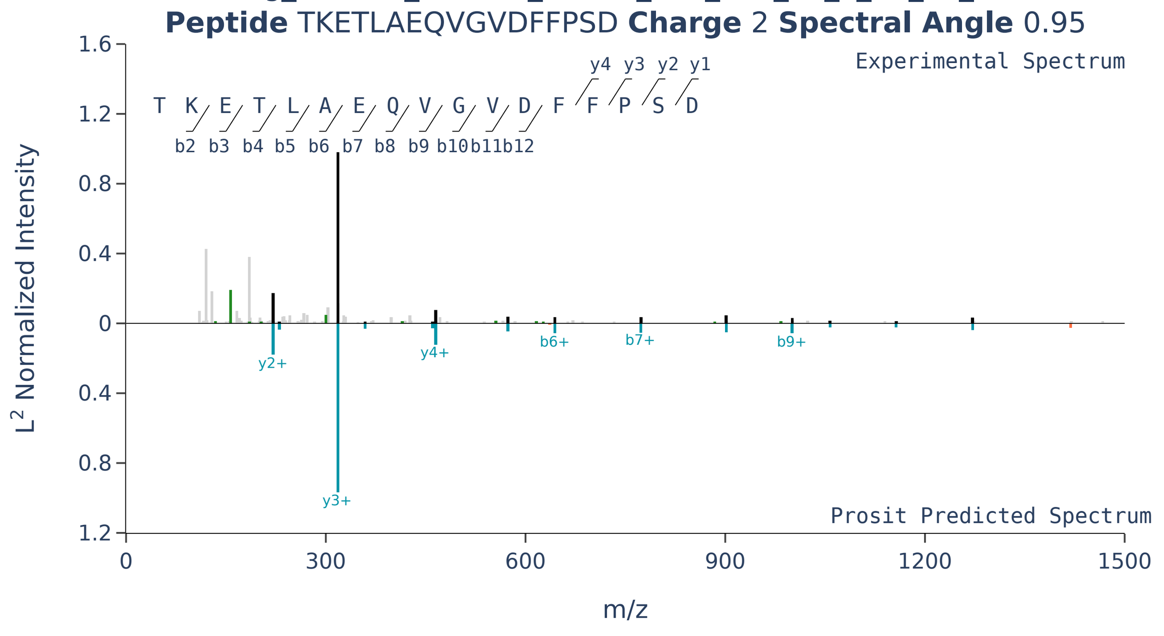

Source Ncheng_210623_230623_HFGoe_FFH_20S_25_1_A2_24h_R2 **Scan** 18651
Peptide ALEPFHPDRISAD **Charge** 3 **Spectral Angle** 0.84

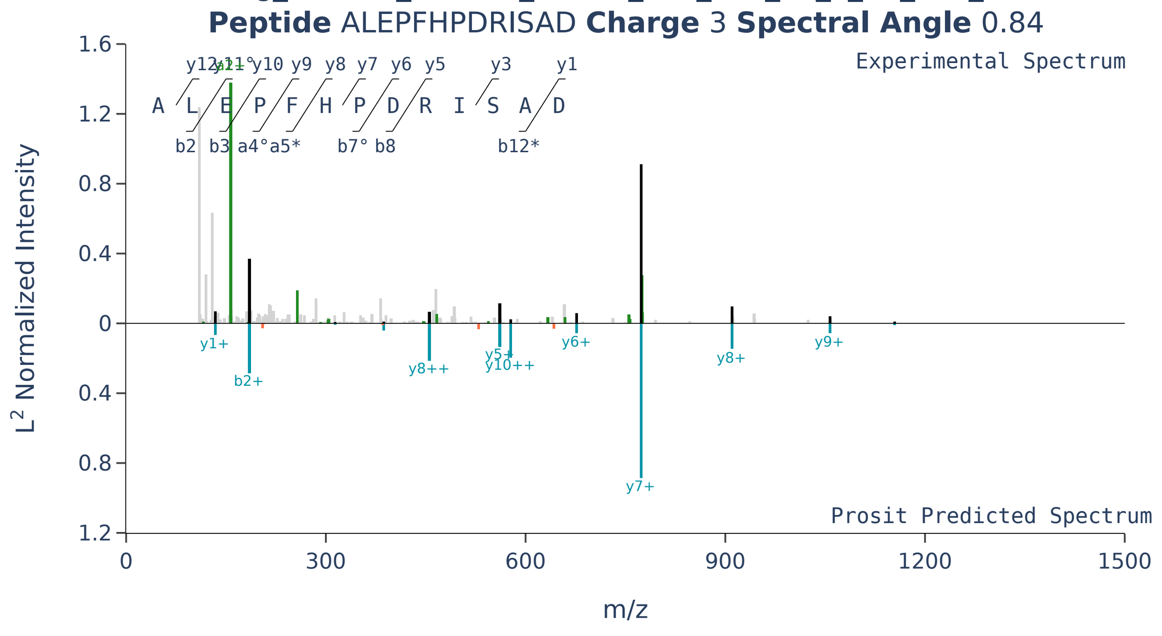

Source Ncheng_210623_230623_HFGoe_FFH_20S_25_1_A1_24h_R2 **Scan** 28766
Peptide TDLFVVD **Charge** 1 **Spectral Angle** 0.88

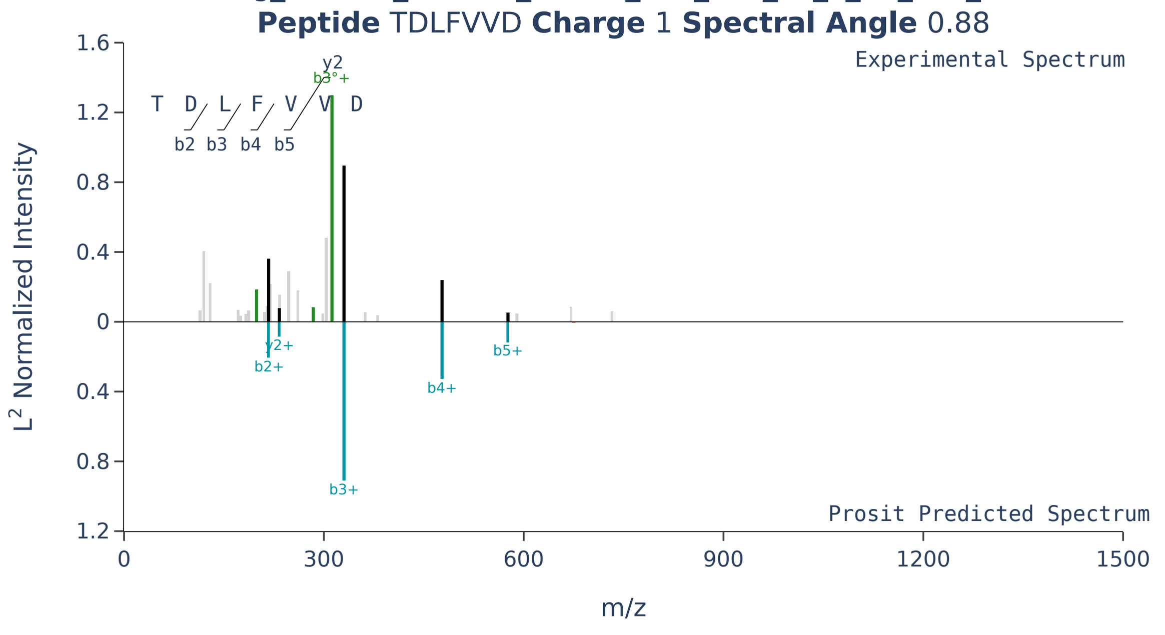

Source Ncheng_210623_230623_HFGoe_FFH_20S_25_1_A2_24h_R2 **Scan** 24520
Peptide DIESQDVNRLL **Charge** 2 **Spectral Angle** 0.74

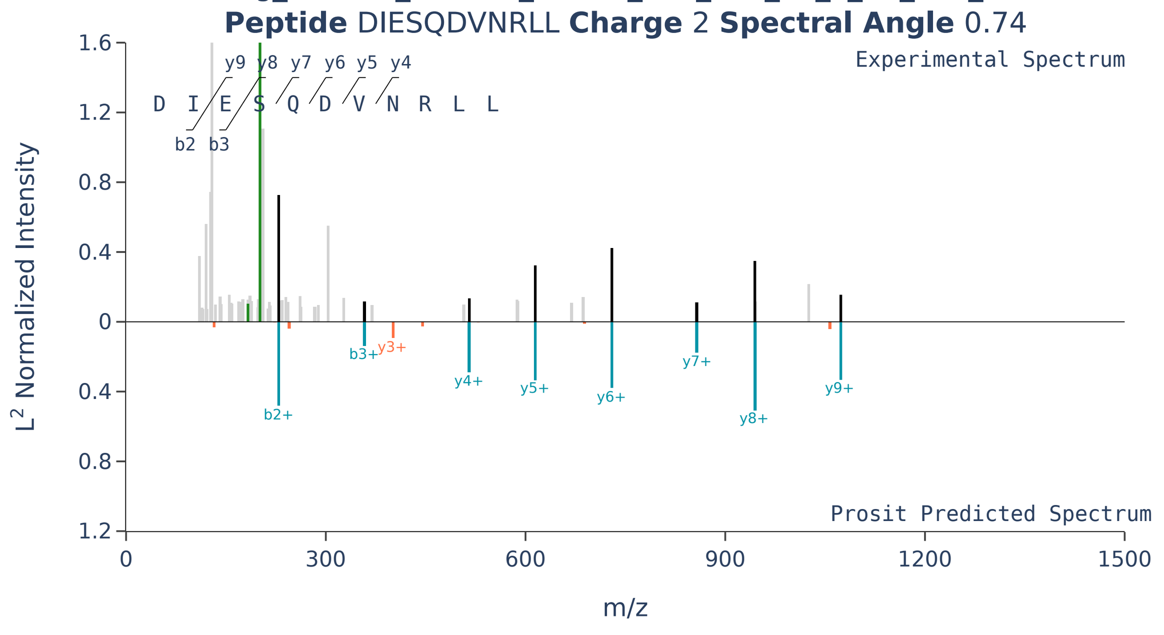

Source Ncheng_210623_230623_HFGoe_FFH_20S_25_1_A2_4h_R2 **Scan** 21017
Peptide LGVGEAMTGQDAANTAKAFN **Charge** 2 **Spectral Angle** 0.78

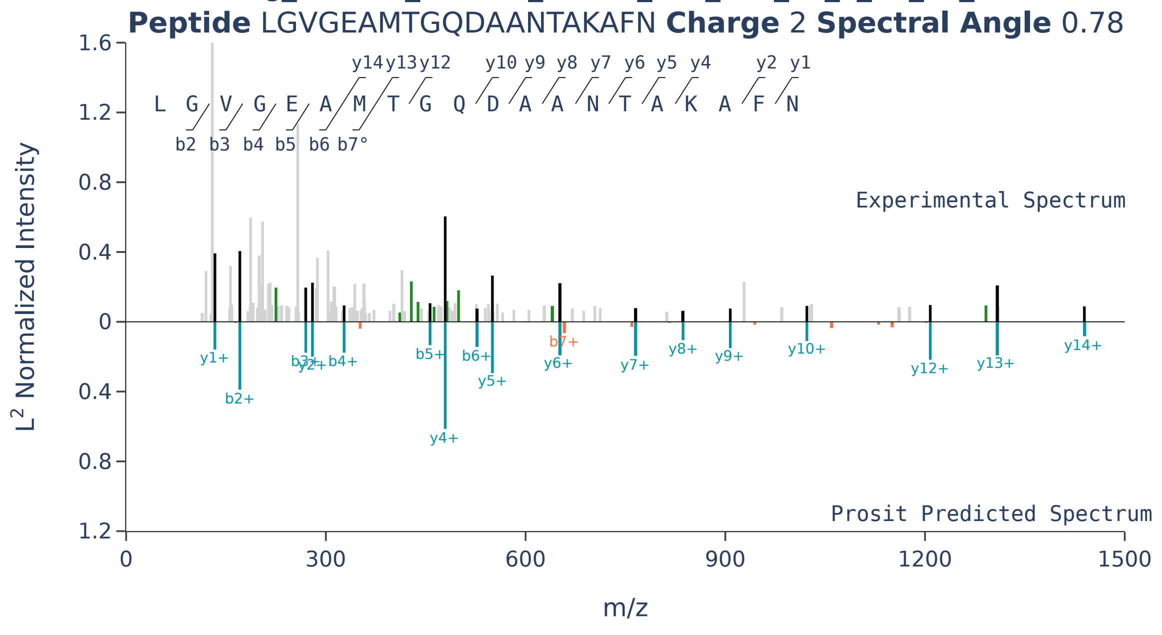

Source Ncheng_210623_230623_HFGoe_FFH_20S_25_1_A1_2h_R1 **Scan** 17367
Peptide ANIPDNVKSQMDDKVLVRME **Charge** 3 **Spectral Angle** 0.81

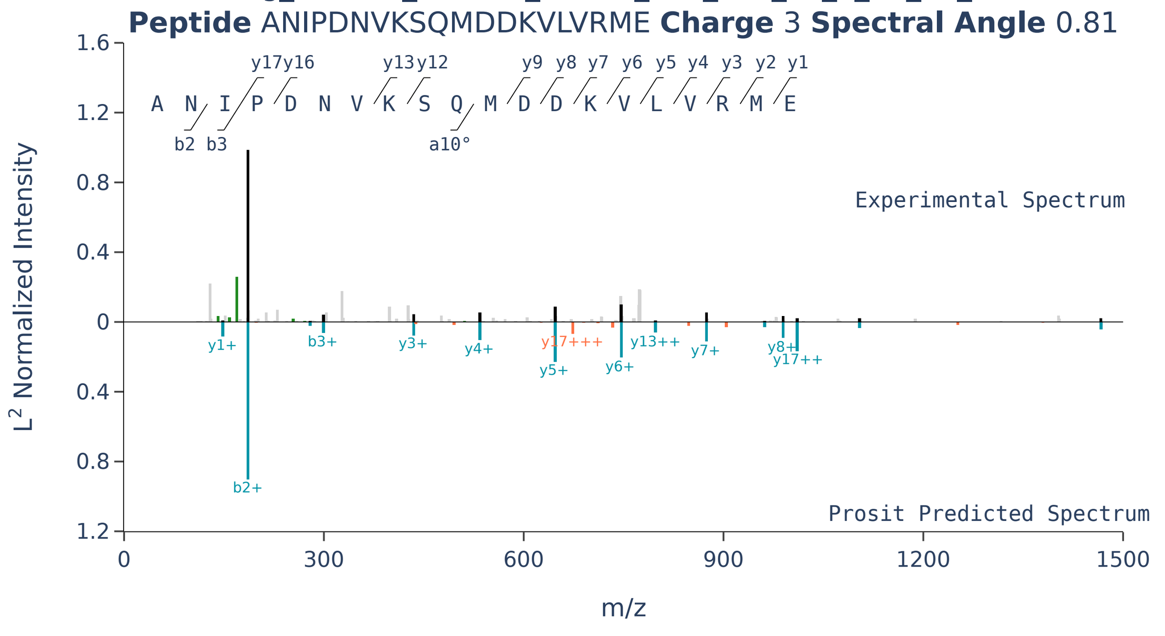

Source Ncheng_210623_230623_HFGoe_FFH_20S_25_1_A2_24h_R1 **Scan** 7264
Peptide DTAGVGQKPVD **Charge** 2 **Spectral Angle** 0.75

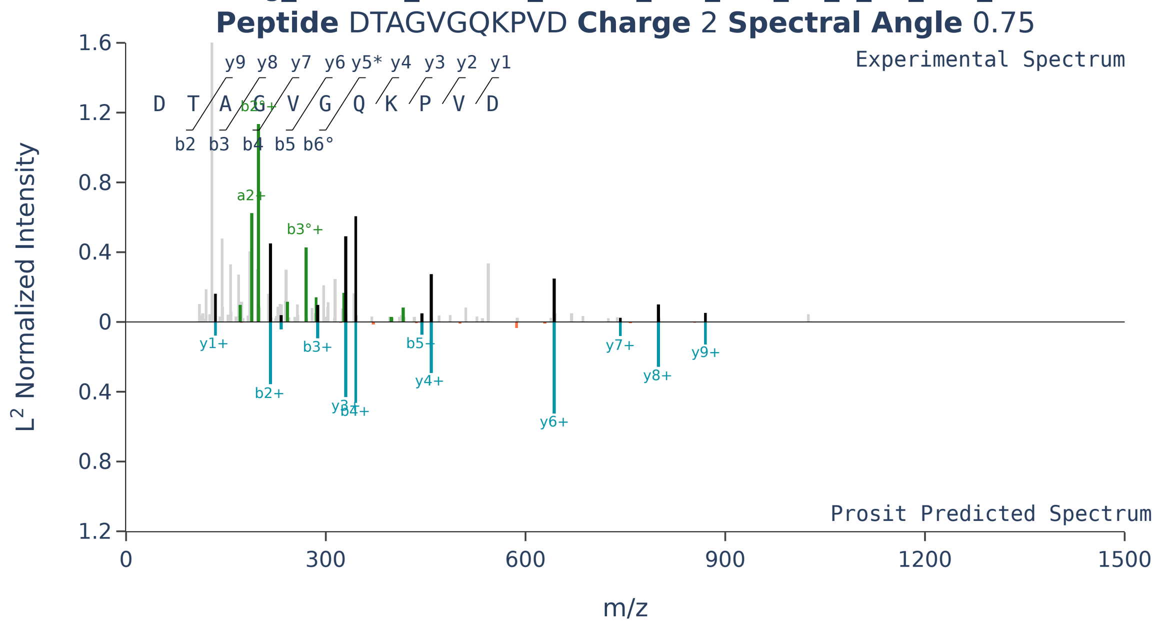

Source Ncheng_210623_230623_HFGoe_FFH_20S_25_1_A1_4h_R2 **Scan** 13175
Peptide EAMMVGQKPVD **Charge** 2 **Spectral Angle** 0.8

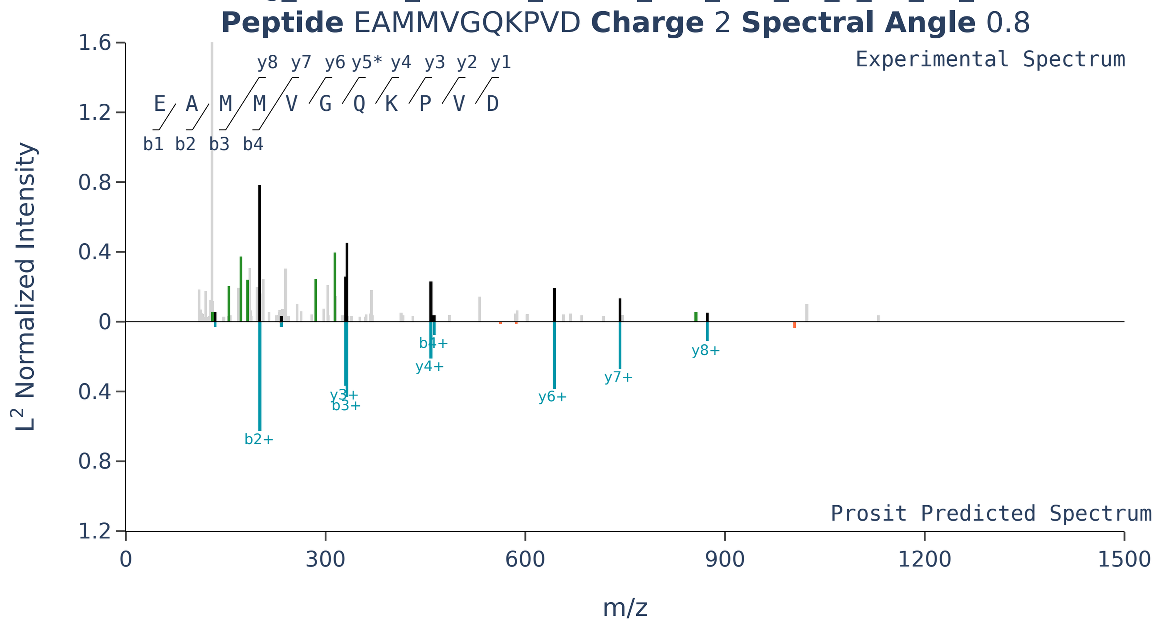

Source Ncheng_210623_230623_HFGoe_FFH_20S_25_1_A2_24h_R1 **Scan** 13892
Peptide AMTGQDAANTAKAFND **Charge** 2 **Spectral Angle** 0.79

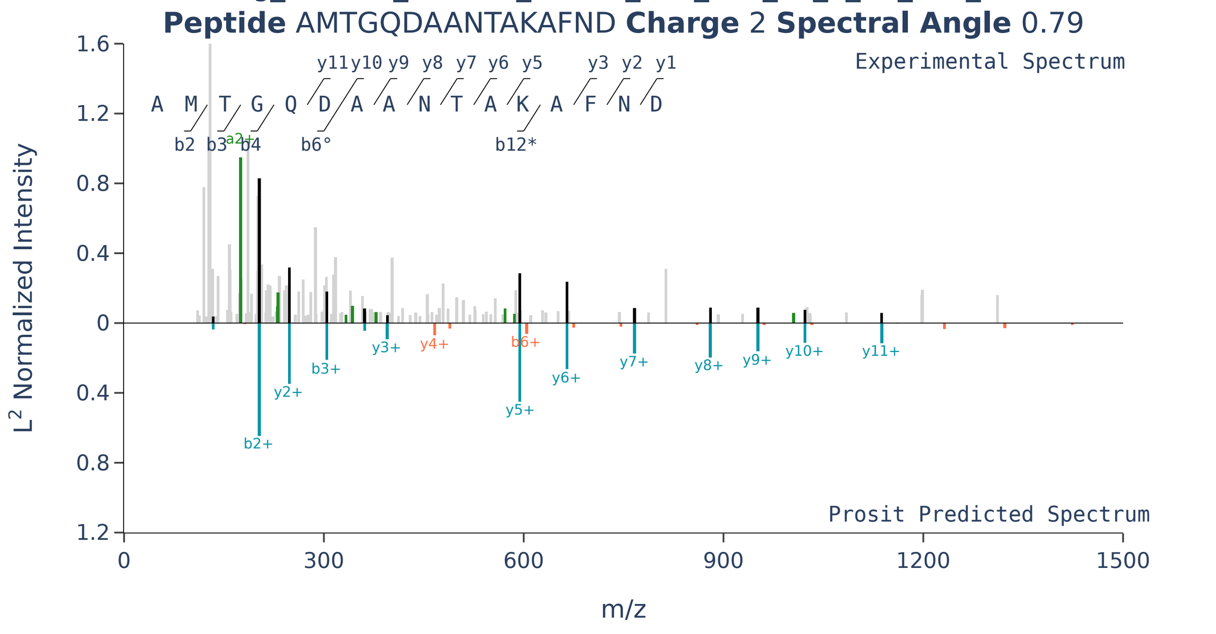

Source Ncheng_210623_230623_HFGoe_FFH_20S_25_1_A2_4h_R1 **Scan** 11750
Peptide RQMKNMGGMARL **Charge** 3 **Spectral Angle** 0.82

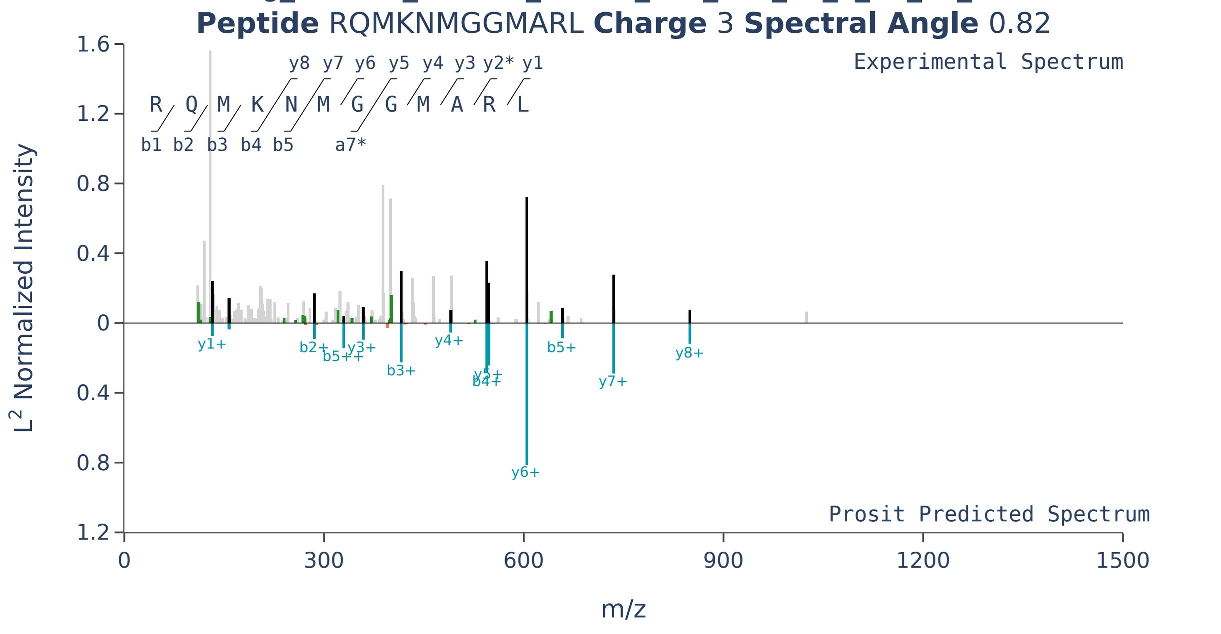

Source Ncheng_210623_230623_HFGoe_FFH_20S_25_1_A2_24h_R2 **Scan** 8658
Peptide DEIKQVQA **Charge** 2 **Spectral Angle** 0.81

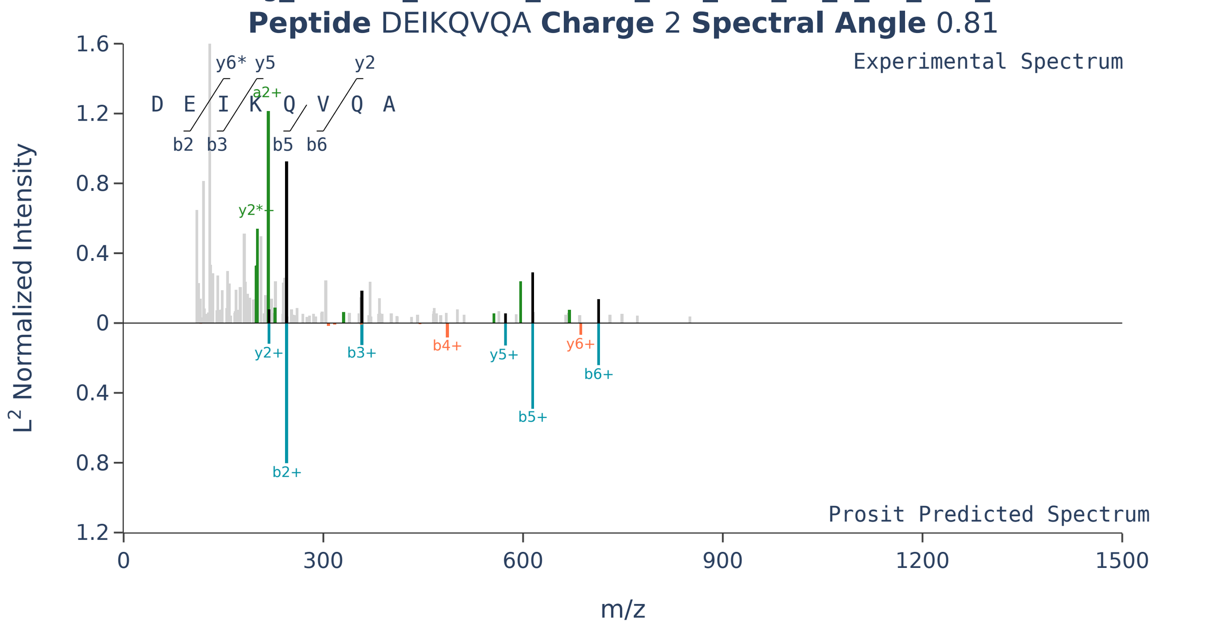

Source Ncheng_210623_230623_HFGoe_FFH_20S_25_1_A2_1h_R2 **Scan** 12553
Peptide DKVLVRMEE **Charge** 2 **Spectral Angle** 0.88

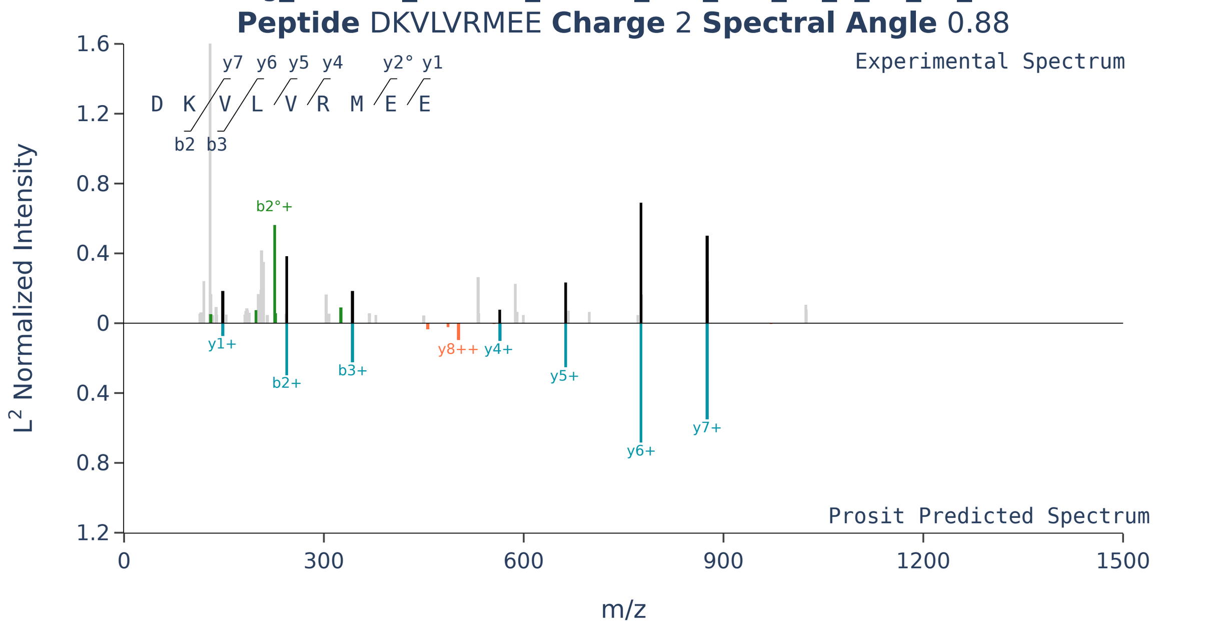

Source Ncheng_210623_230623_HFGoe_FFH_20S_25_1_A2_4h_R1 **Scan** 20918
Peptide GHEVNKSLTPGQEFVKIVRQD **Charge** 2 **Spectral Angle** 0.8

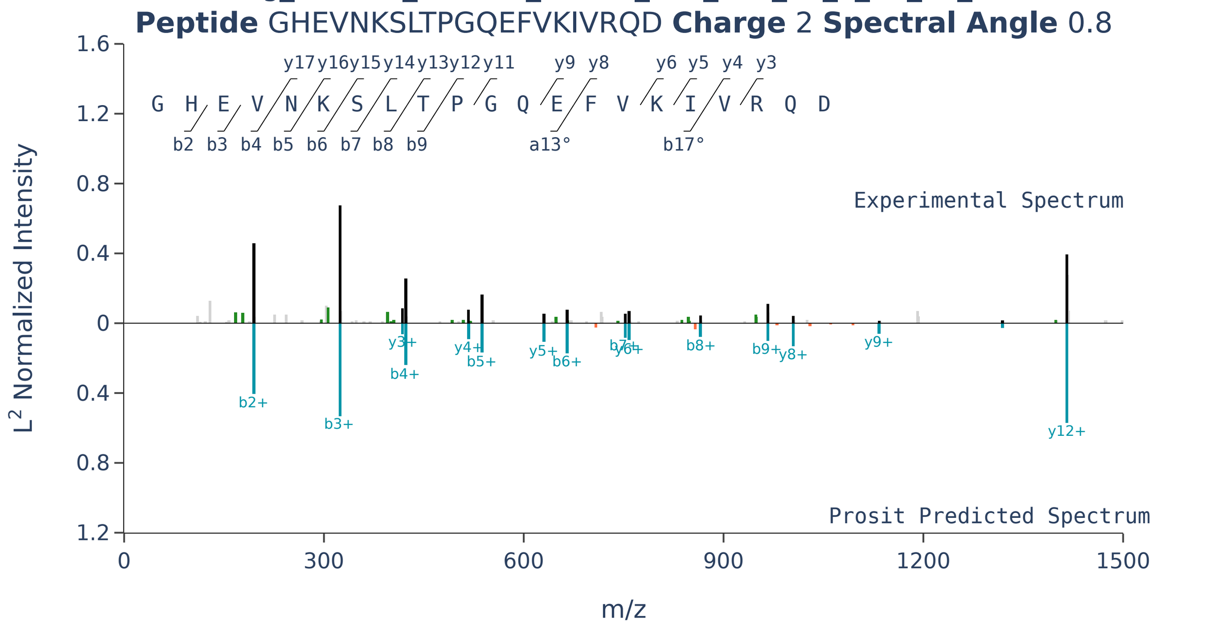

Source Ncheng_210623_230623_HFGoe_FFH_20S_25_1_A1_2h_R1 **Scan** 22821
Peptide GMAKLPGMGQIPDNVKSQMDD **Charge** 3 **Spectral Angle** 0.79

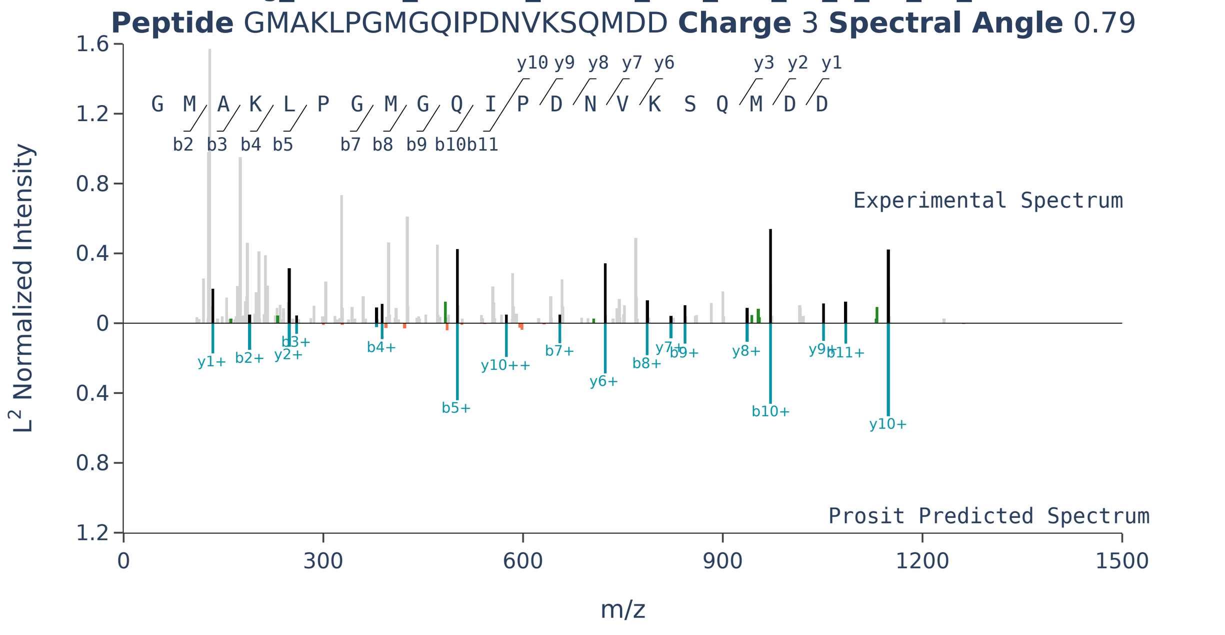

Source Ncheng_210623_230623_HFGoe_FFH_20S_25_1_A2_4h_R1 **Scan** 18978
Peptide QVQLEPFHPDRIASR **Charge** 4 **Spectral Angle** 0.82

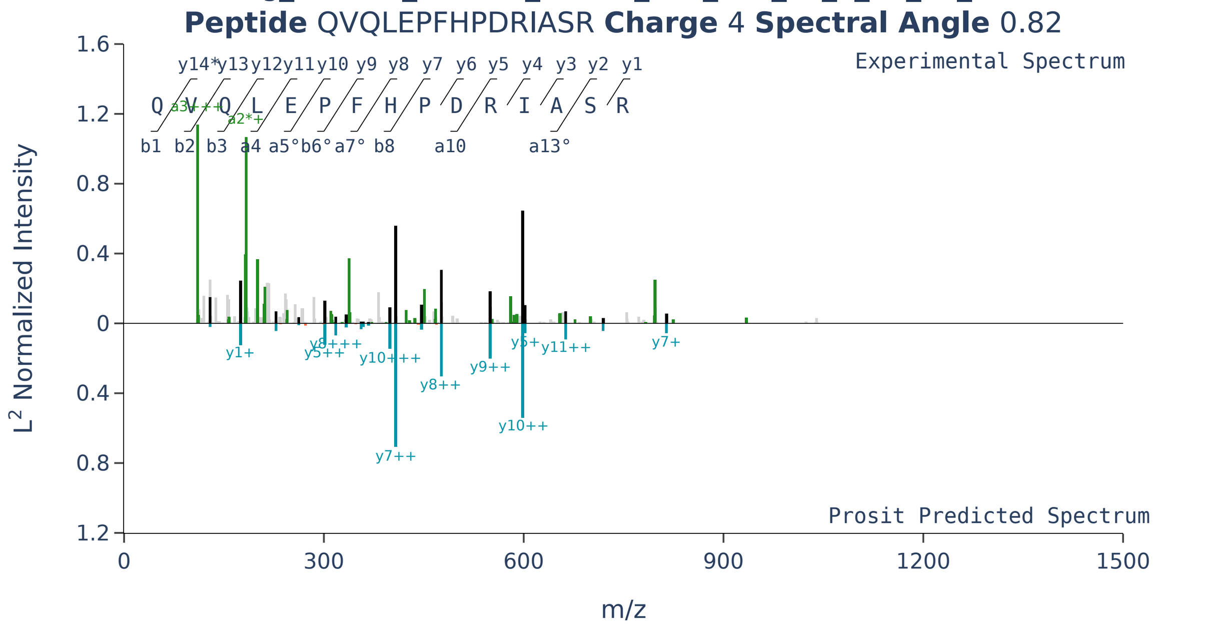

Source Ncheng_210623_230623_HFGoe_FFH_20S_25_1_A2_2h_R1 **Scan** 18566
Peptide MKGLPGMGQIPDNVKSQ **Charge** 3 **Spectral Angle** 0.78

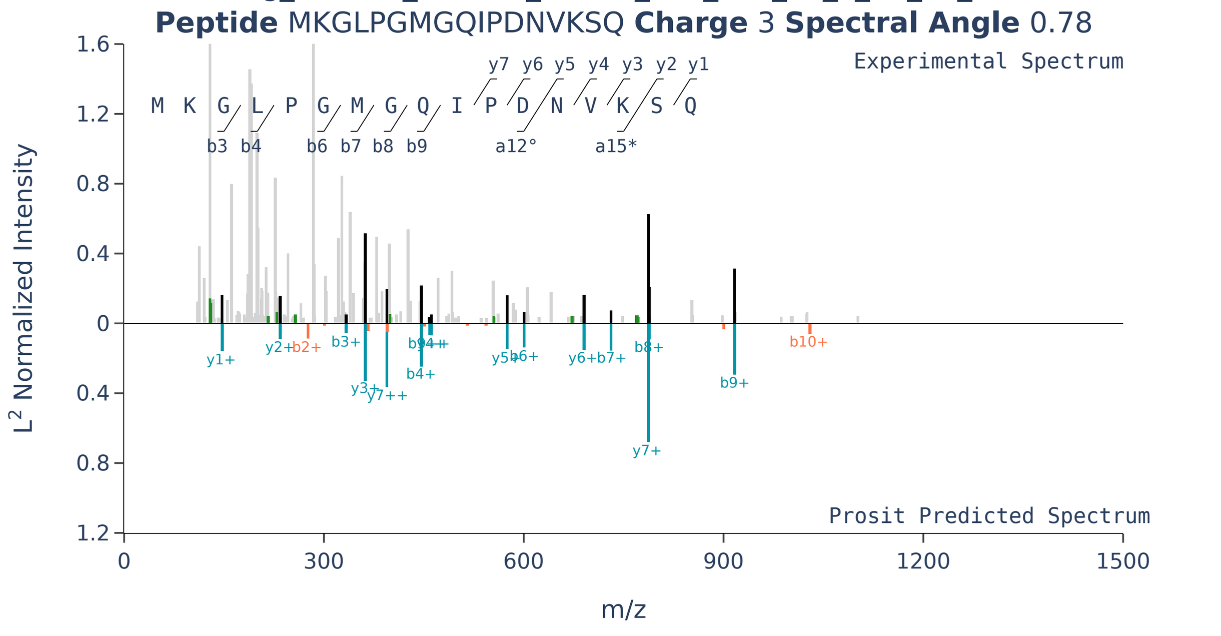

Source Ncheng_210623_230623_HFGoe_FFH_20S_25_1_A2_24h_R2 **Scan** 8078
Peptide GEAANTAKAFN **Charge** 2 **Spectral Angle** 0.82

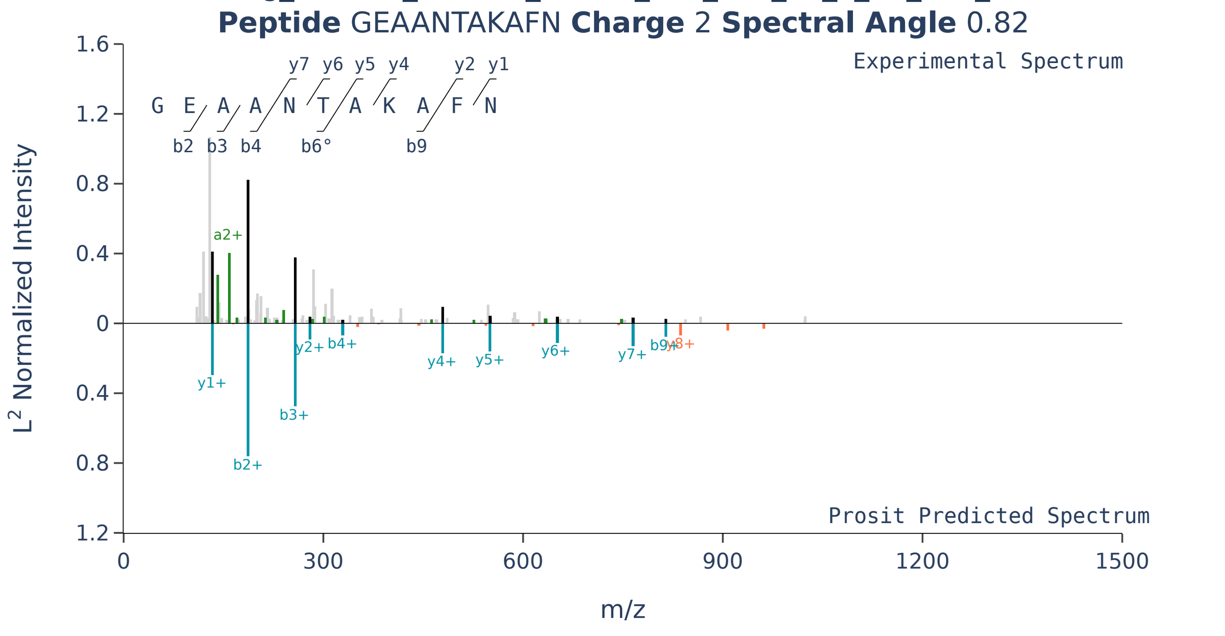

Source Ncheng_210623_230623_HFGoe_FFH_20S_25_1_A2_4h_R2 **Scan** 23936
Peptide GDMGKLPGMGQIPDNVKSQMDD **Charge** 3 **Spectral Angle** 0.77

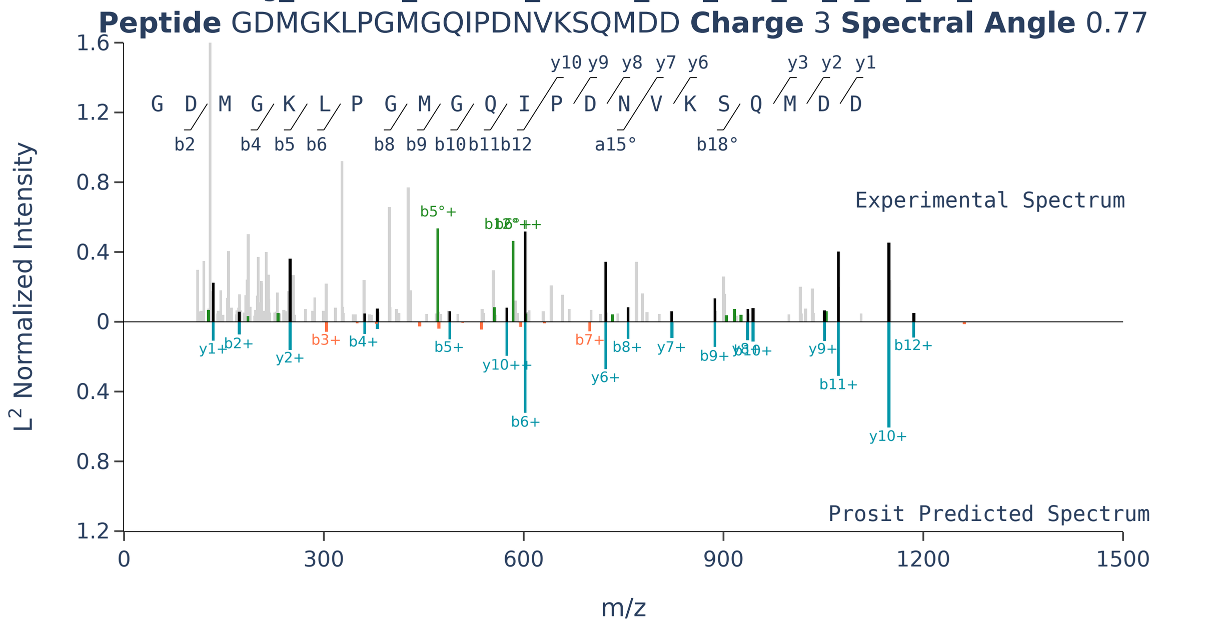

Source Ncheng_210623_230623_HFGoe_FFH_20S_25_1_A2_24h_R1 **Scan** 12586
Peptide MPPGFPGRSR **Charge** 2 **Spectral Angle** 0.79

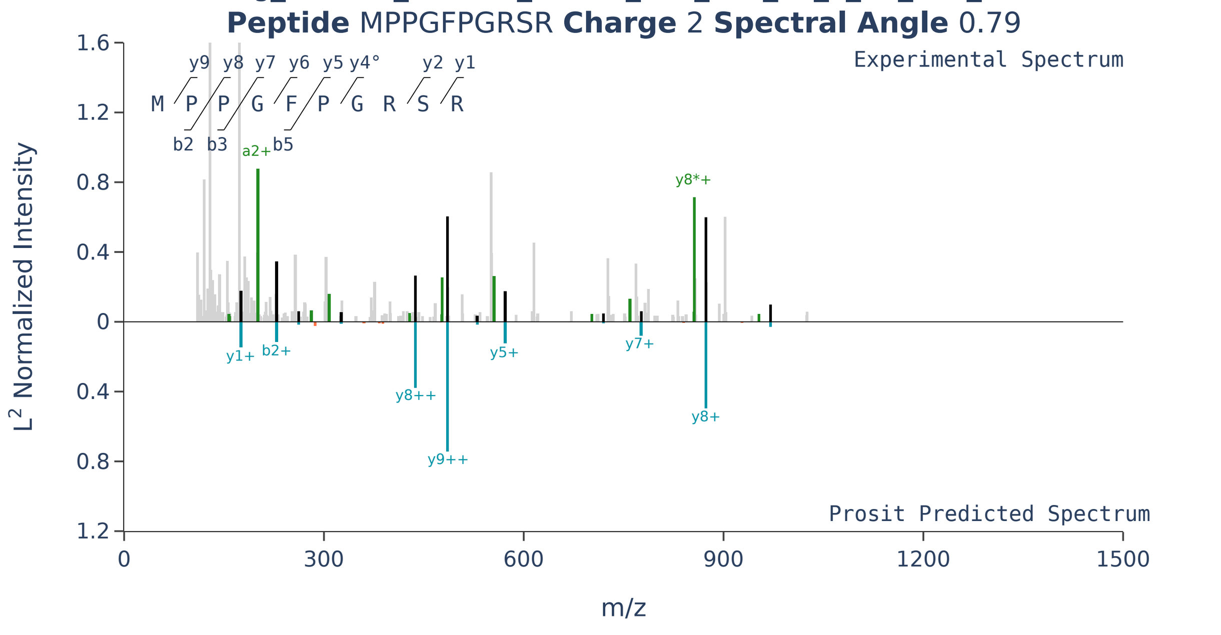

Source Ncheng_210623_230623_HFGoe_FFH_20S_25_1_A2_24h_R1 **Scan** 13188
Peptide EPFHPDRIASE **Charge** 2 **Spectral Angle** 0.91

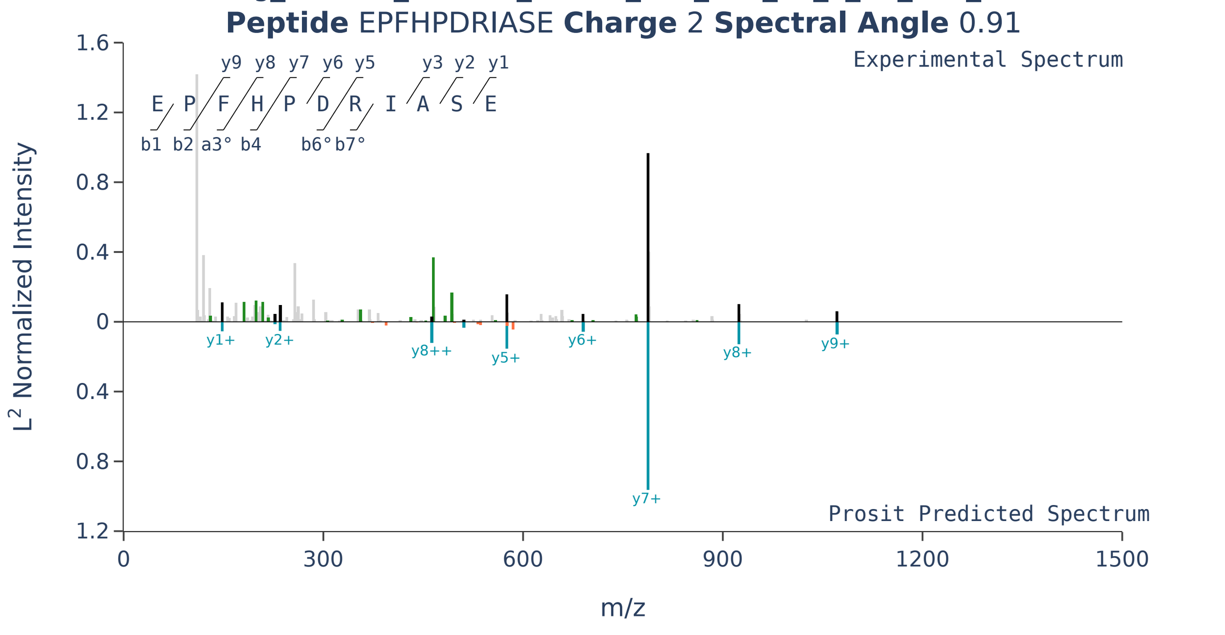

Source Ncheng_210623_230623_HFGoe_FFH_20S_25_1_A1_2h_R1 **Scan** 8256
Peptide GTKVDGDARGGAAL **Charge** 2 **Spectral Angle** 0.85

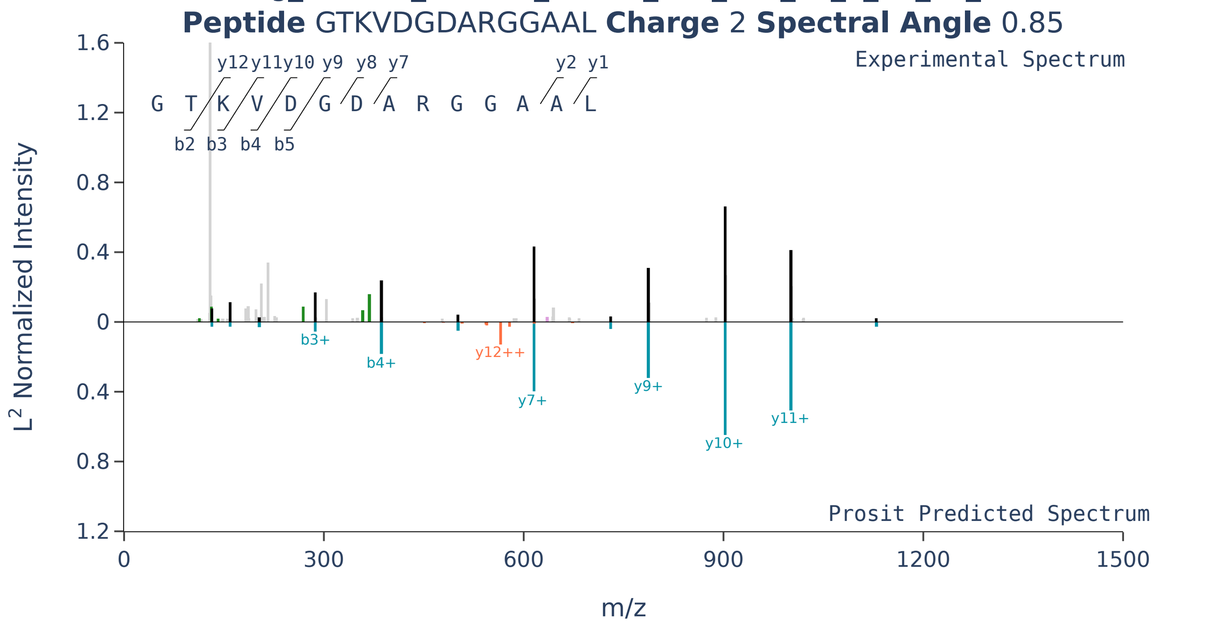

Source Ncheng_210623_230623_HFGoe_FFH_20S_25_1_A1_4h_R1 **Scan** 29016
Peptide QVGGVDFFPSD **Charge** 2 **Spectral Angle** 0.92

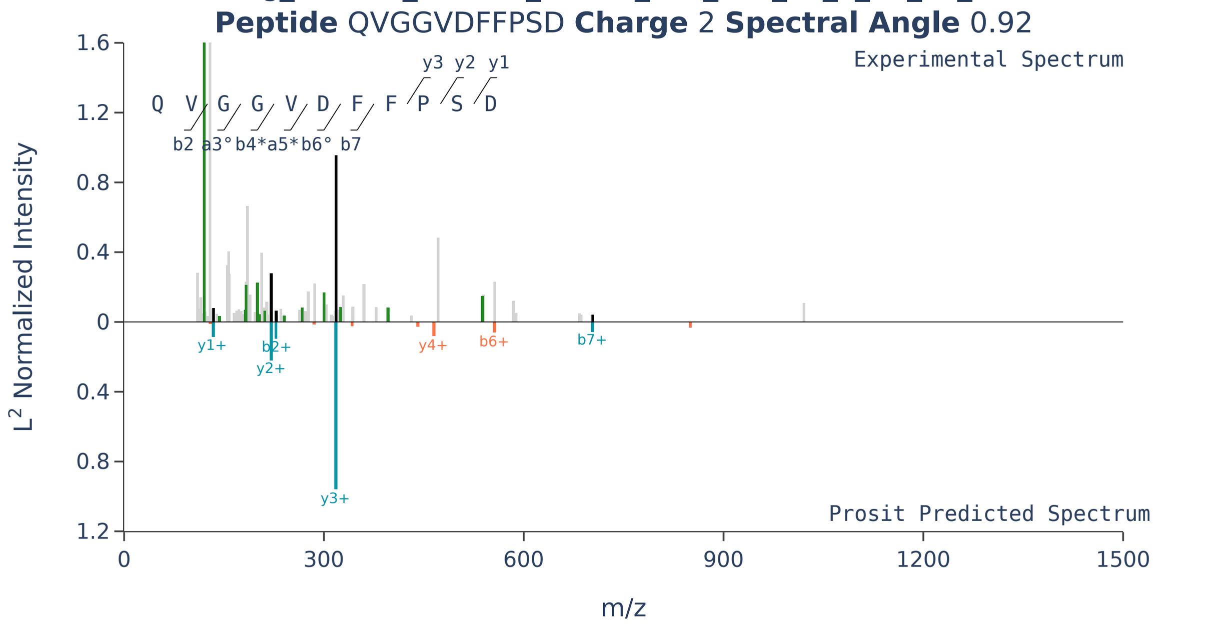

Source Ncheng_210623_230623_HFGoe_FFH_20S_25_1_A1_24h_R1 **Scan** 12625
Peptide EPFHPDGRLT **Charge** 1 **Spectral Angle** 0.89

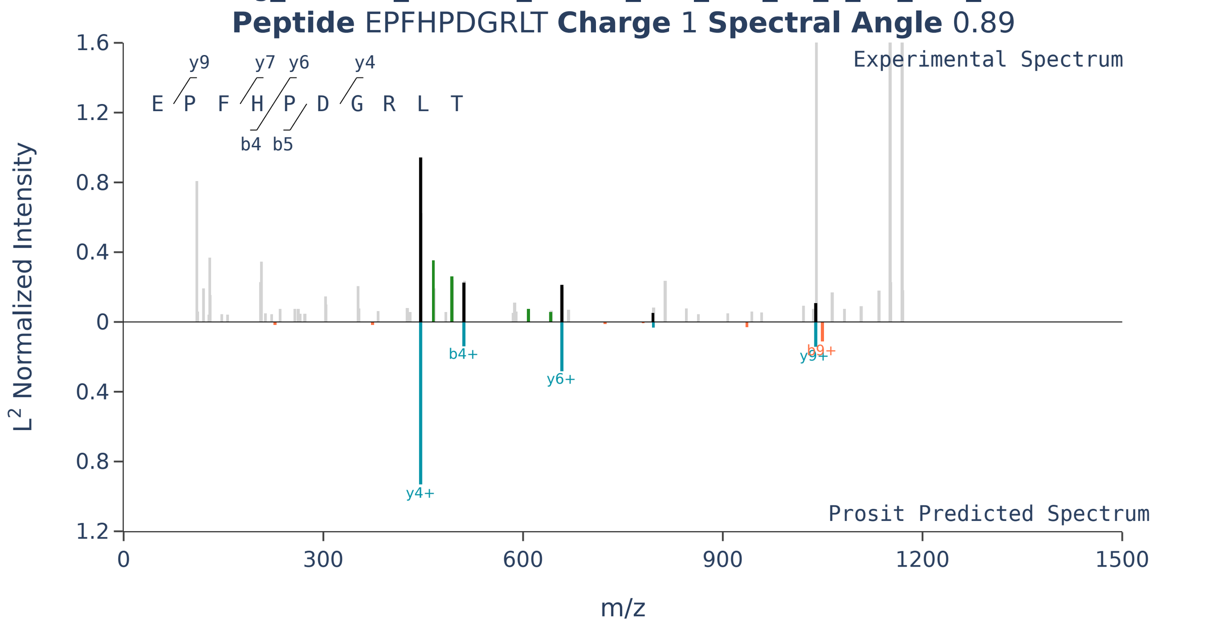

Source Ncheng_210623_230623_HFGoe_FFH_20S_25_1_A2_2h_R1 **Scan** 26788
Peptide DVDFFPSDVGQKPVD **Charge** 2 **Spectral Angle** 0.86

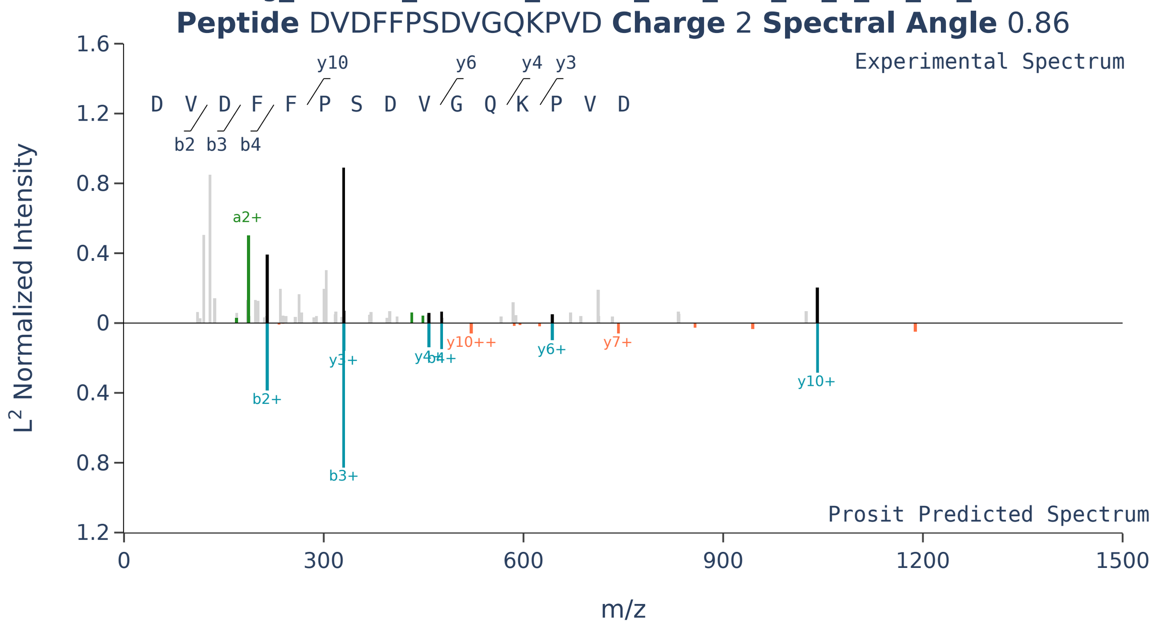

Source Ncheng_210623_230623_HFGoe_FFH_20S_25_1_A2_24h_R2 **Scan** 12264
Peptide KEAAMTGQDAANTAKAFN **Charge** 3 **Spectral Angle** 0.86

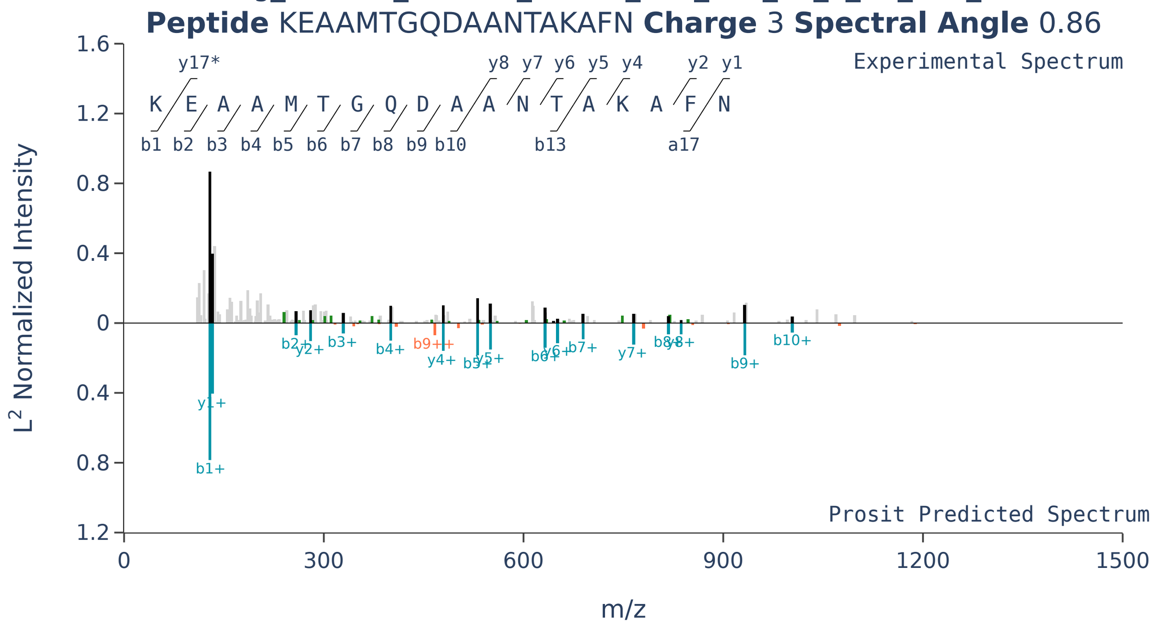

Source Ncheng_210623_230623_HFGoe_FFH_20S_25_1_A1_2h_R1 **Scan** 26182
Peptide AANTAKAFNEALPLTGDIESKVDRAQAE **Charge** 4 **Spectral Angle** 0.77

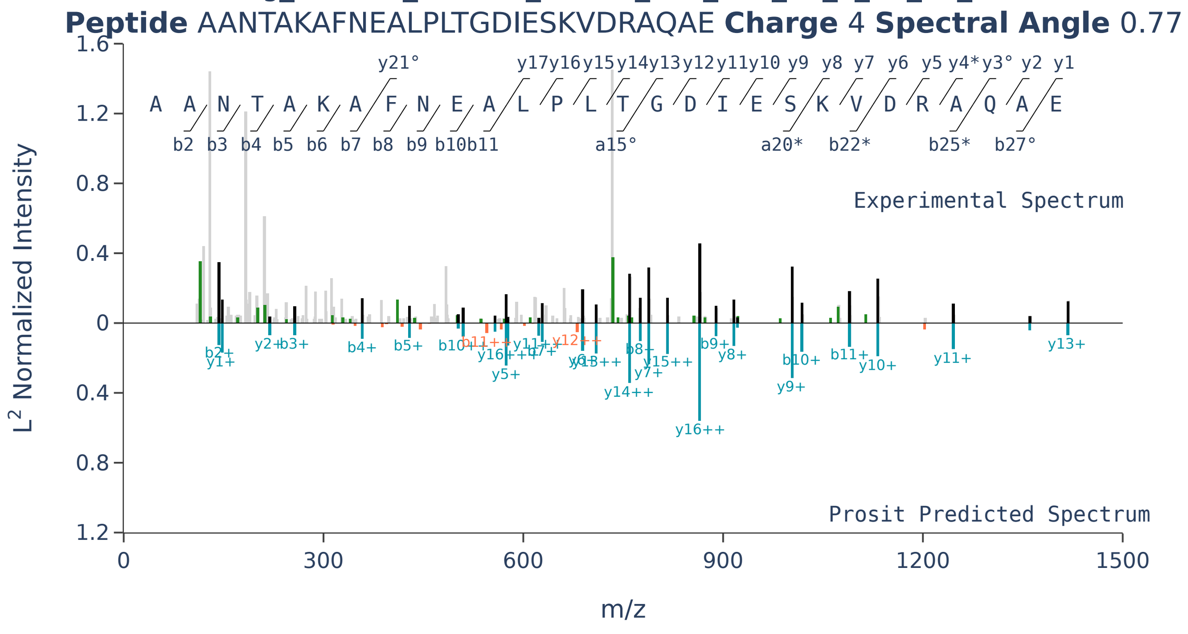

Source Ncheng_210623_230623_HFGoe_FFH_20S_25_1_A2_24h_R2 **Scan** 27389
Peptide SAGVDFFPSD **Charge** 1 **Spectral Angle** 0.77

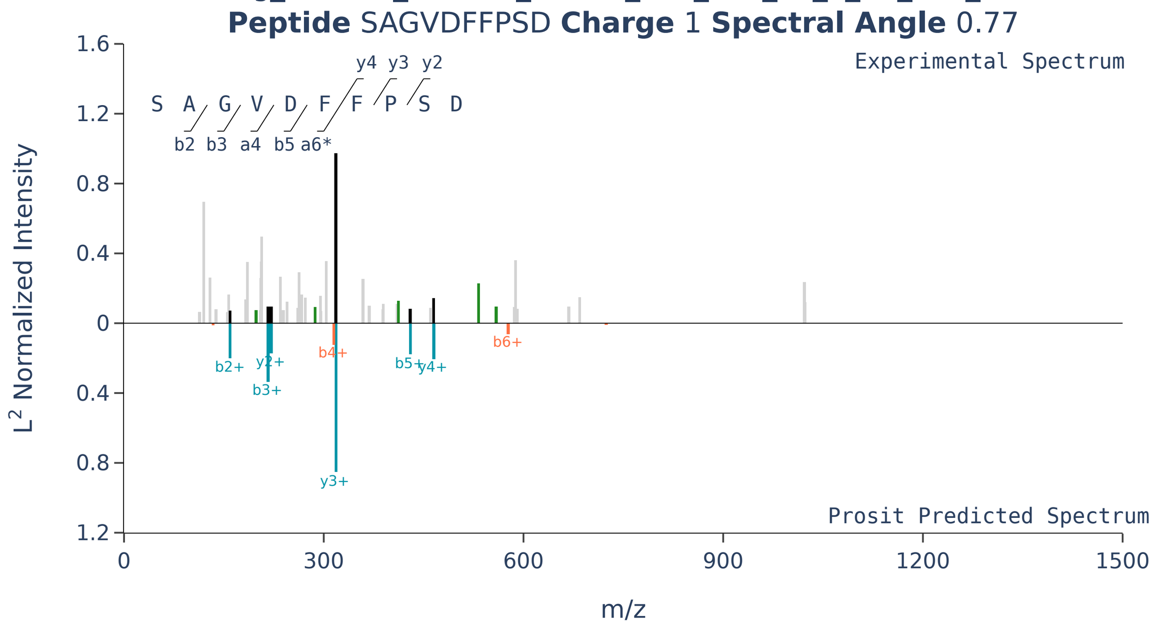

Source Ncheng_210623_230623_HFGoe_FFH_20S_25_1_A1_24h_R2 **Scan** 19150
Peptide ALEPFHPGRLTED **Charge** 2 **Spectral Angle** 0.82

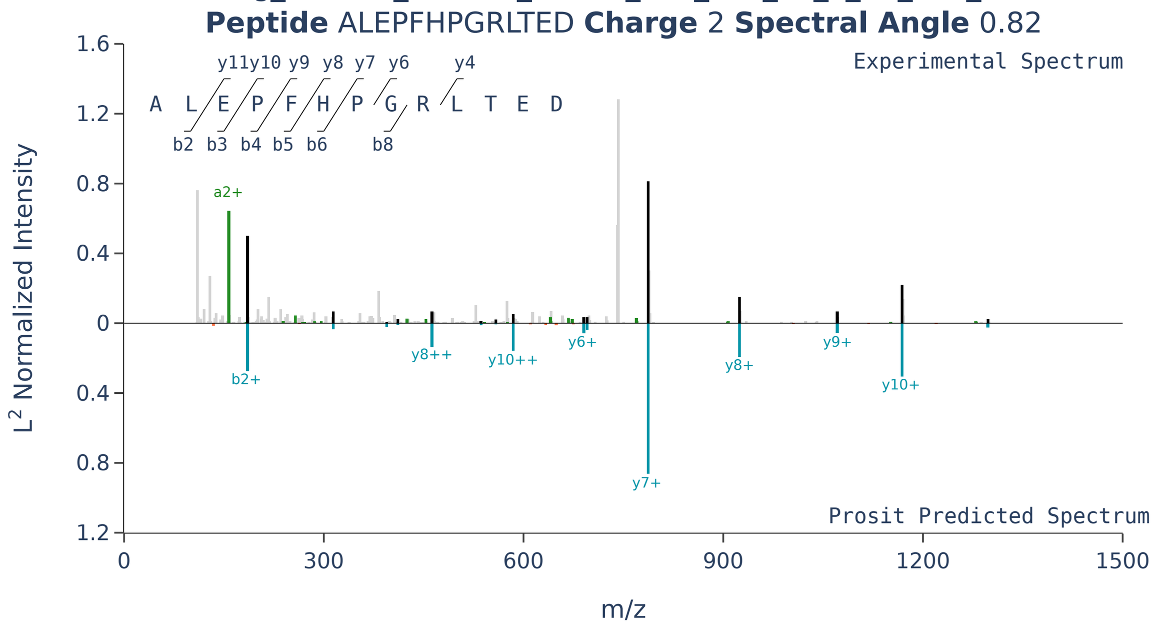

Source Ncheng_210623_230623_HFGoe_FFH_20S_25_1_A2_4h_R2 **Scan** 21749
Peptide GMQKLPGMGQIPDNVKS **Charge** 3 **Spectral Angle** 0.87

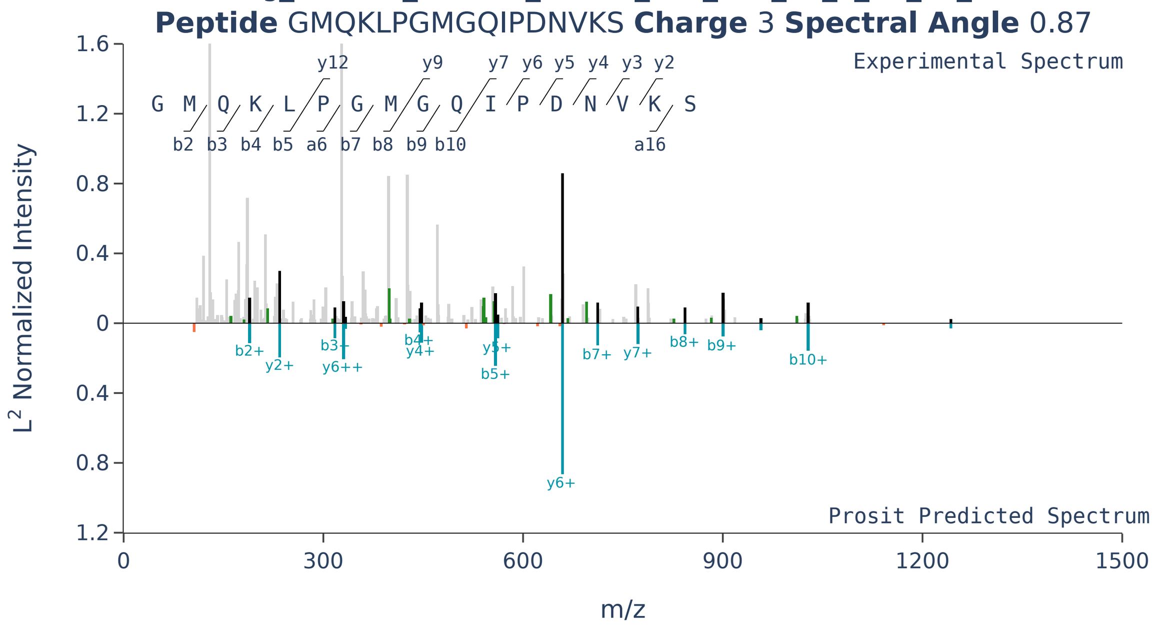

Source Ncheng_210623_230623_HFGoe_FFH_20S_25_1_A1_24h_R2 **Scan** 9269
Peptide GFPGRRS **Charge** 2 **Spectral Angle** 0.73

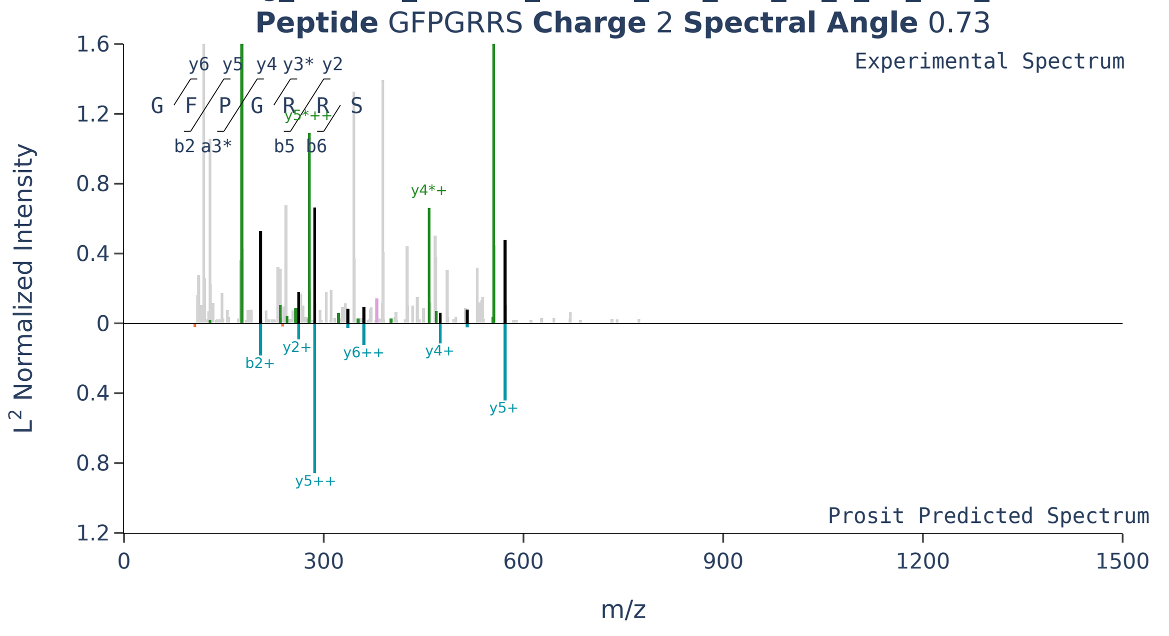

Source Ncheng_210623_230623_HFGoe_FFH_20S_25_1_A2_4h_R2 **Scan** 34440
Peptide PLTGVVLTLEA **Charge** 1 **Spectral Angle** 0.79

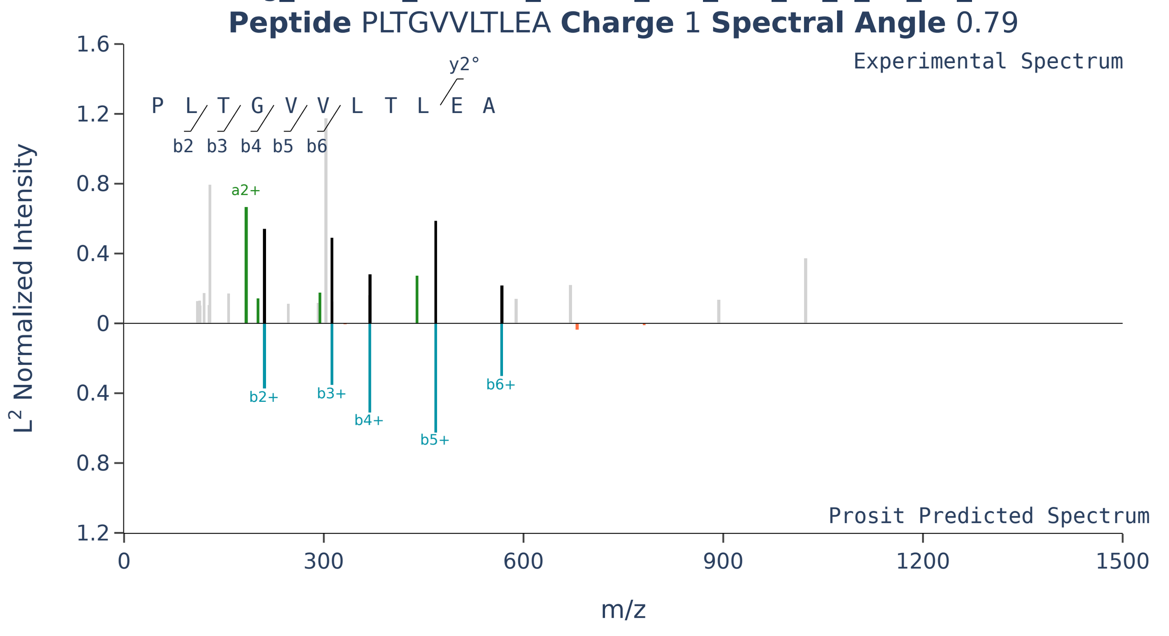

Source Ncheng_210623_230623_HFGoe_FFH_20S_25_1_A1_2h_R1 **Scan** 6134
Peptide EKLQVHA **Charge** 1 **Spectral Angle** 0.76

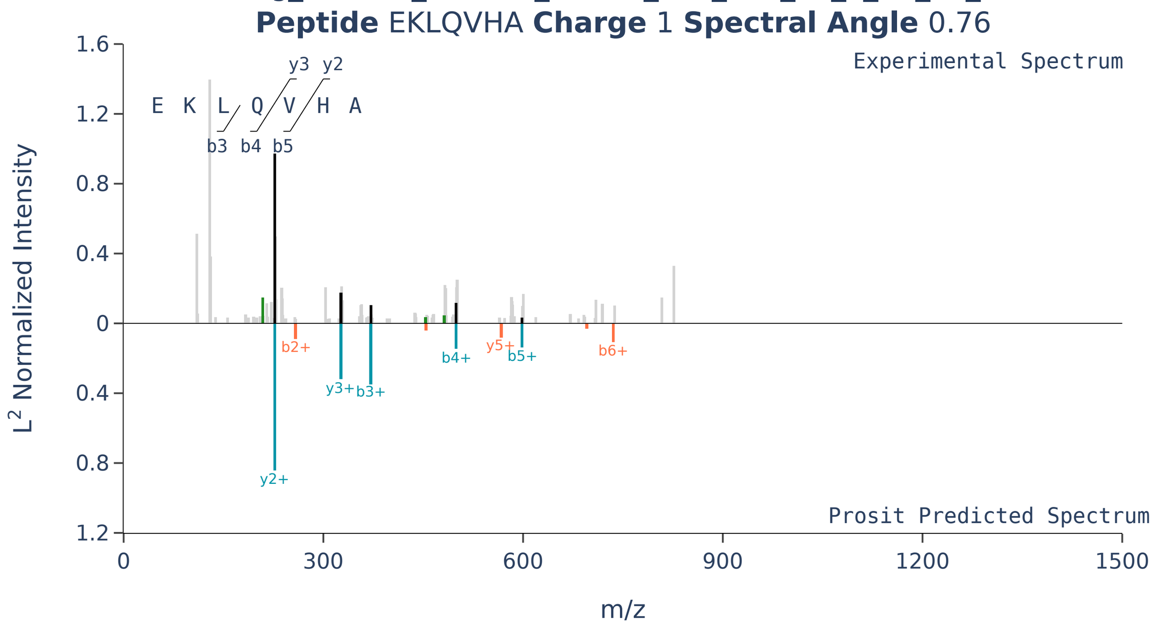

Source Ncheng_210623_230623_HFGoe_FFH_20S_25_1_A1_24h_R2 **Scan** 13966
Peptide TGAMTGQDAANTAKAFN **Charge** 3 **Spectral Angle** 0.84

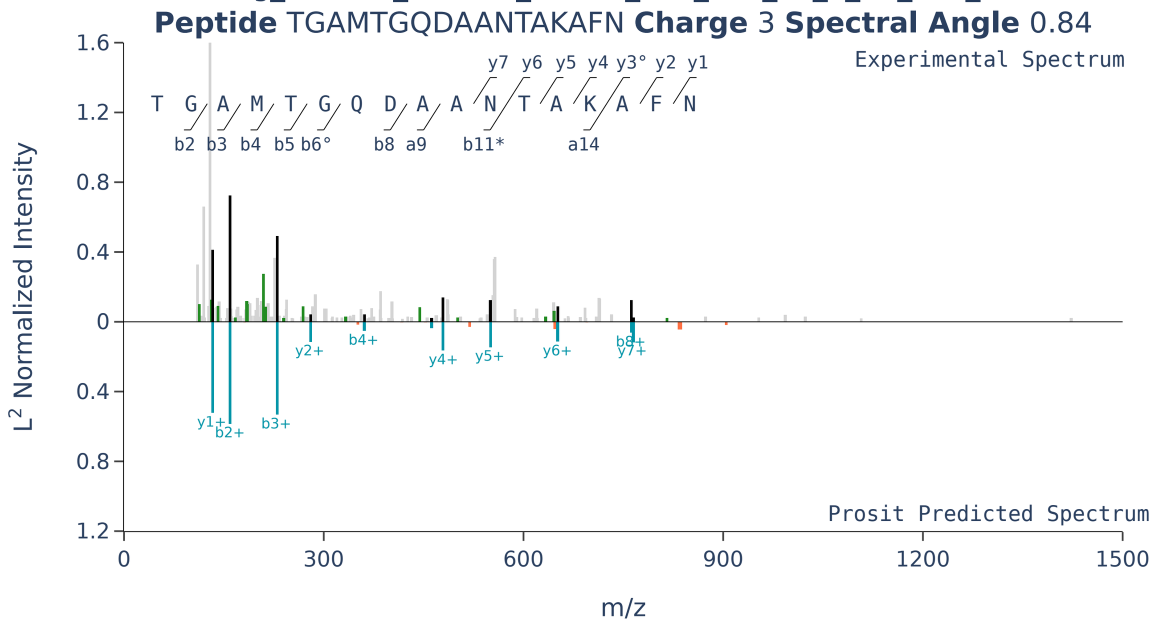

Source Ncheng_210623_230623_HFGoe_FFH_20S_25_1_A2_4h_R1 **Scan** 23669
Peptide GDMGKLPGMGQIPDNVKSQMD **Charge** 3 **Spectral Angle** 0.76

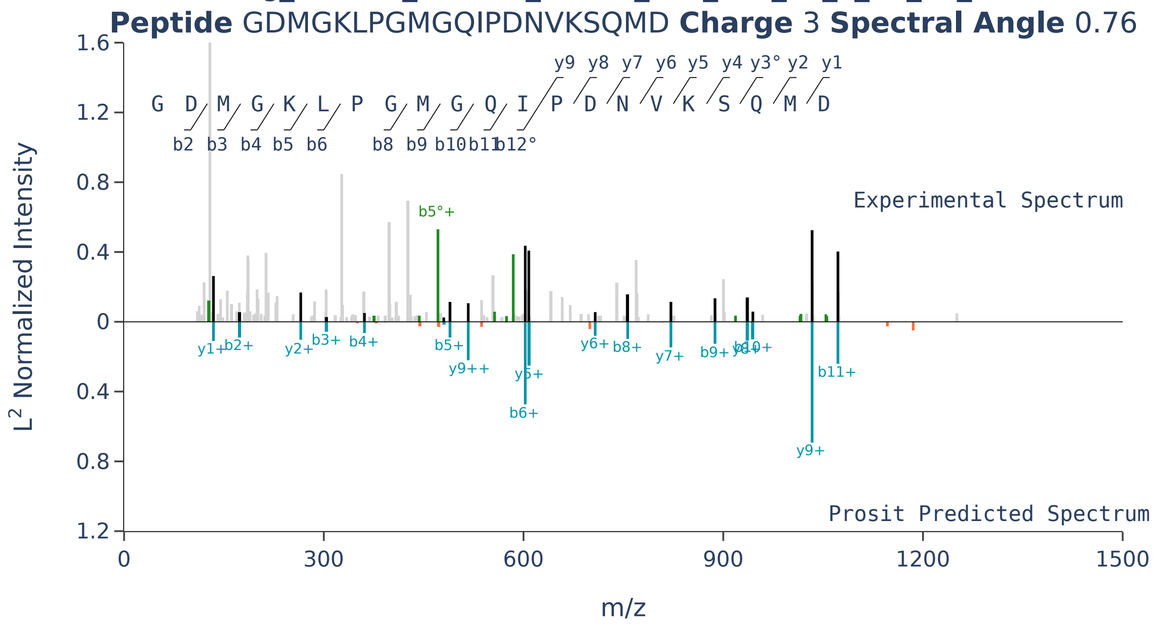

Source Ncheng_210623_230623_HFGoe_FFH_20S_25_1_A1_4h_R2 **Scan** 13976
Peptide QMDIESKVD **Charge** 2 **Spectral Angle** 0.73

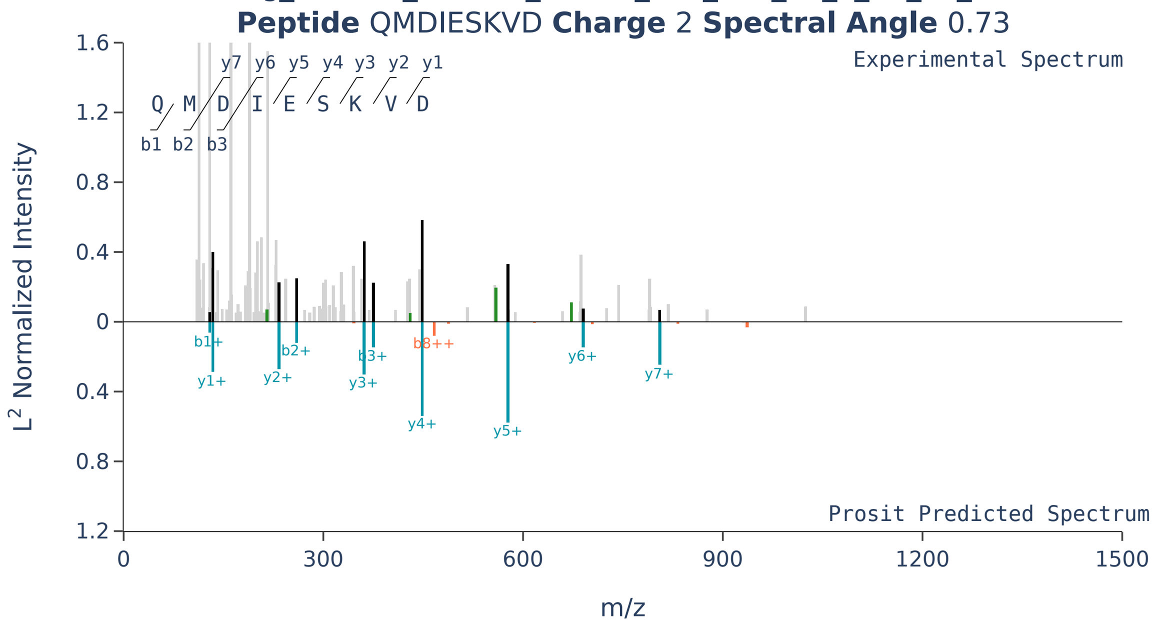

Source Ncheng_210623_230623_HFGoe_FFH_20S_25_1_A1_4h_R2 **Scan** 15446
Peptide RIAAGAMTGQDAANTAKAFN **Charge** 3 **Spectral Angle** 0.82

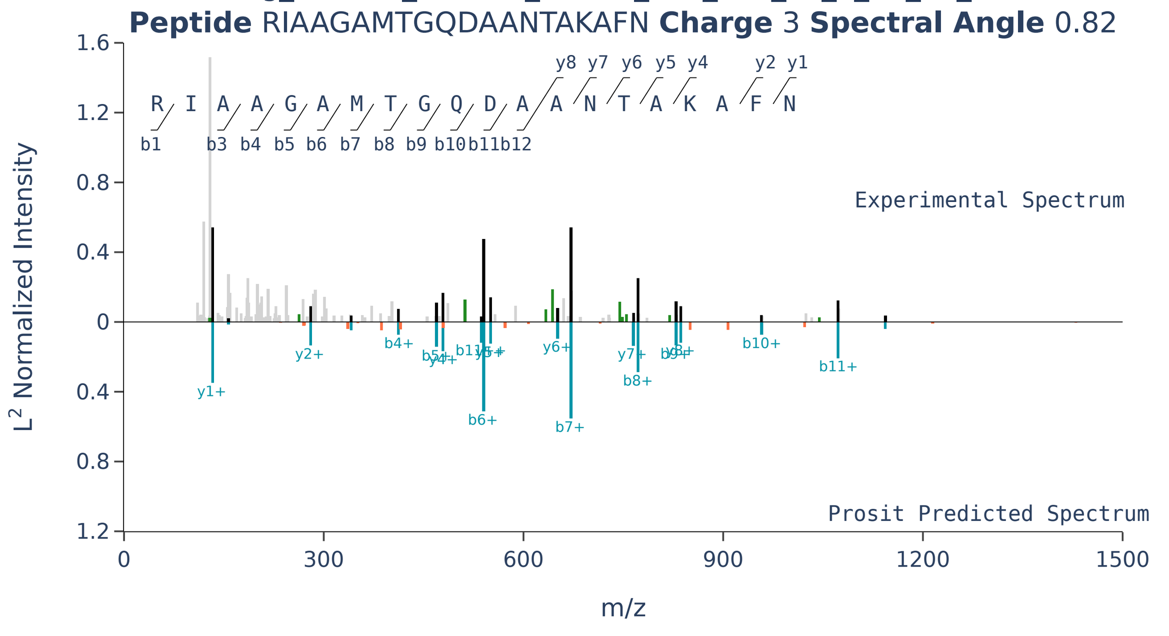

Source Ncheng_210623_230623_HFGoe_FFH_20S_25_1_A1_24h_R1 **Scan** 14587
Peptide KMDAMTGQDAANTAKAFN **Charge** 3 **Spectral Angle** 0.81

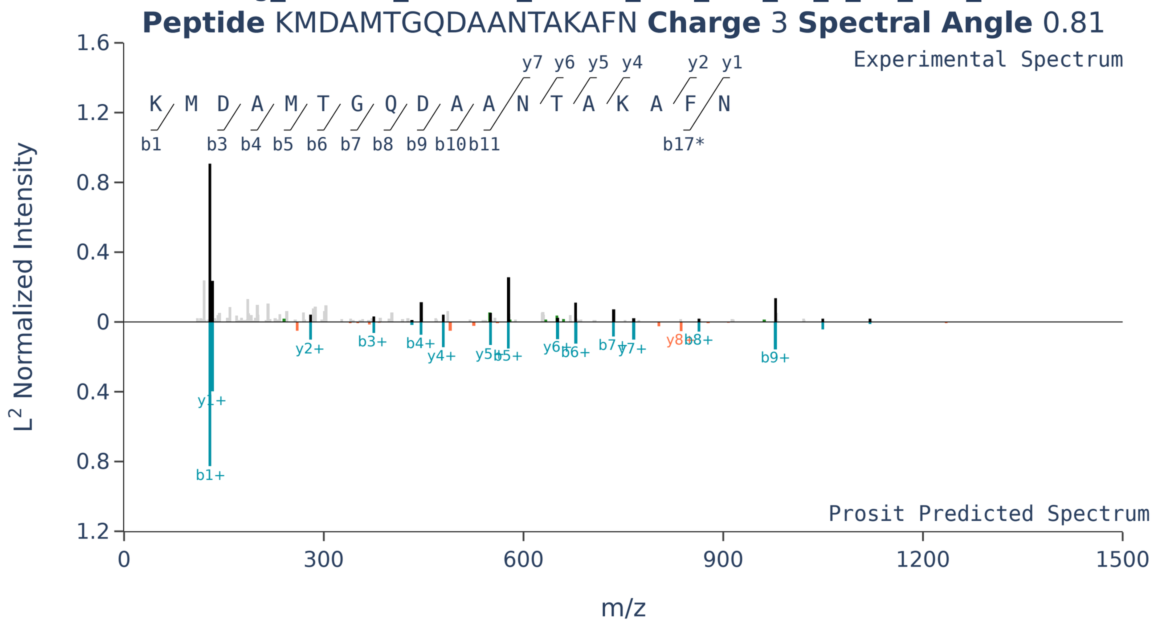

Source Ncheng_210623_230623_HFGoe_FFH_20S_25_1_A2_24h_R2 **Scan** 8439
Peptide DVVGQKPVD **Charge** 1 **Spectral Angle** 0.79

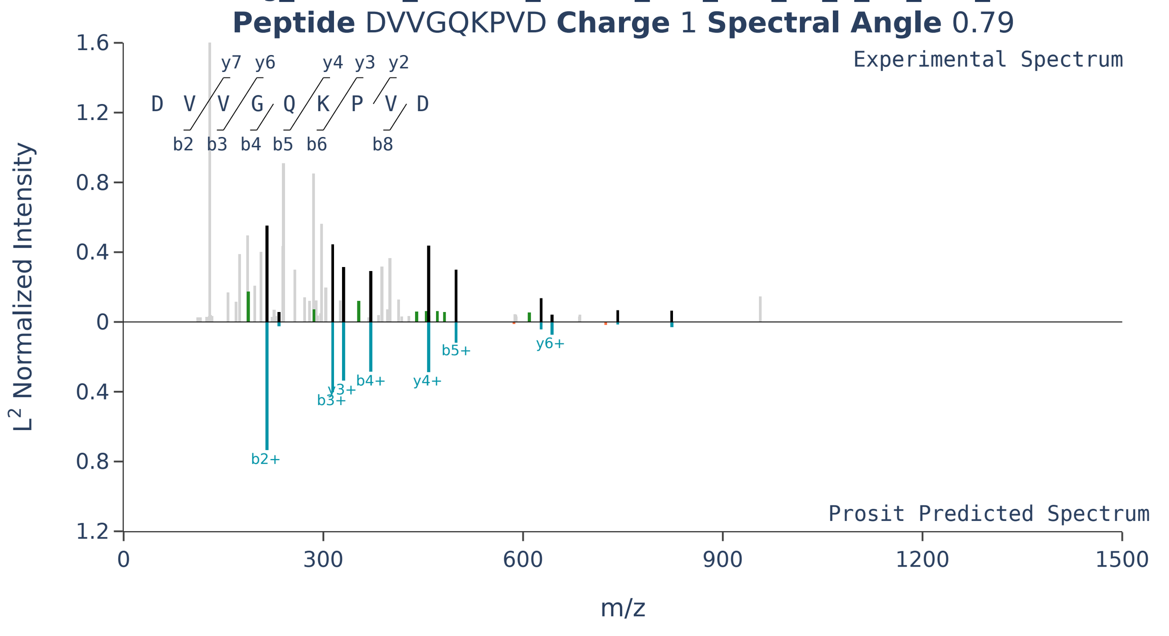

Source Ncheng_210623_230623_HFGoe_FFH_20S_25_1_A2_24h_R1 **Scan** 20487
Peptide PGMGQIGDV **Charge** 1 **Spectral Angle** 0.76

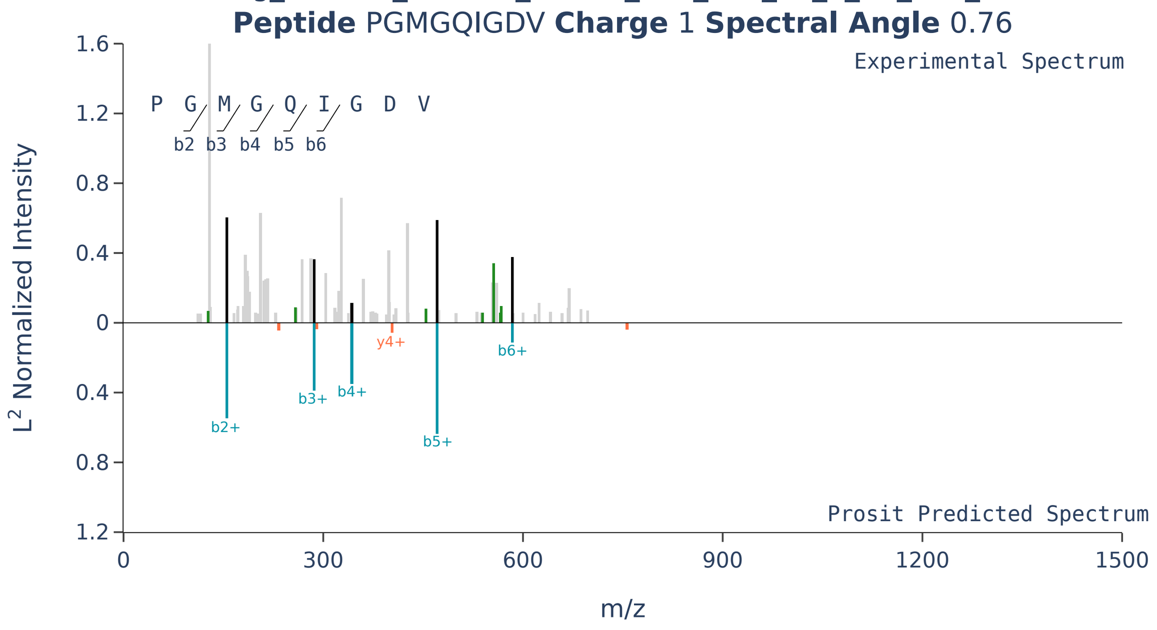

Source Ncheng_210623_230623_HFGoe_FFH_20S_25_1_A2_4h_R2 **Scan** 14898
Peptide ASNRLLKQFD **Charge** 2 **Spectral Angle** 0.77

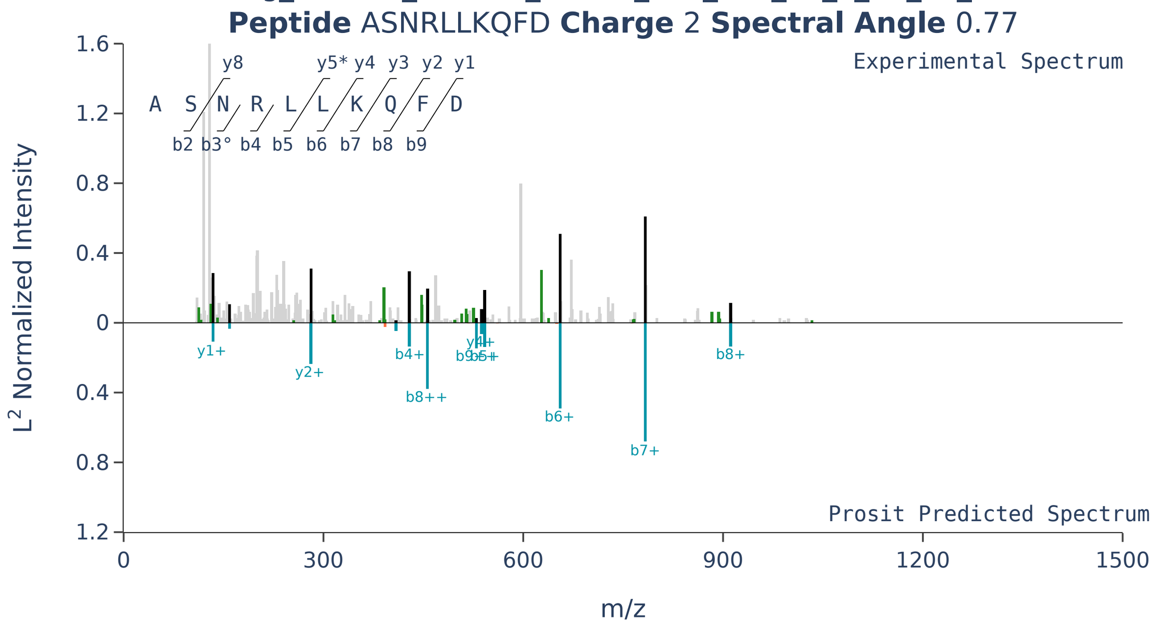

Source Ncheng_210623_230623_HFGoe_FFH_20S_25_1_A2_24h_R1 **Scan** 27137
Peptide VGEGVDFFPSD **Charge** 2 **Spectral Angle** 0.87

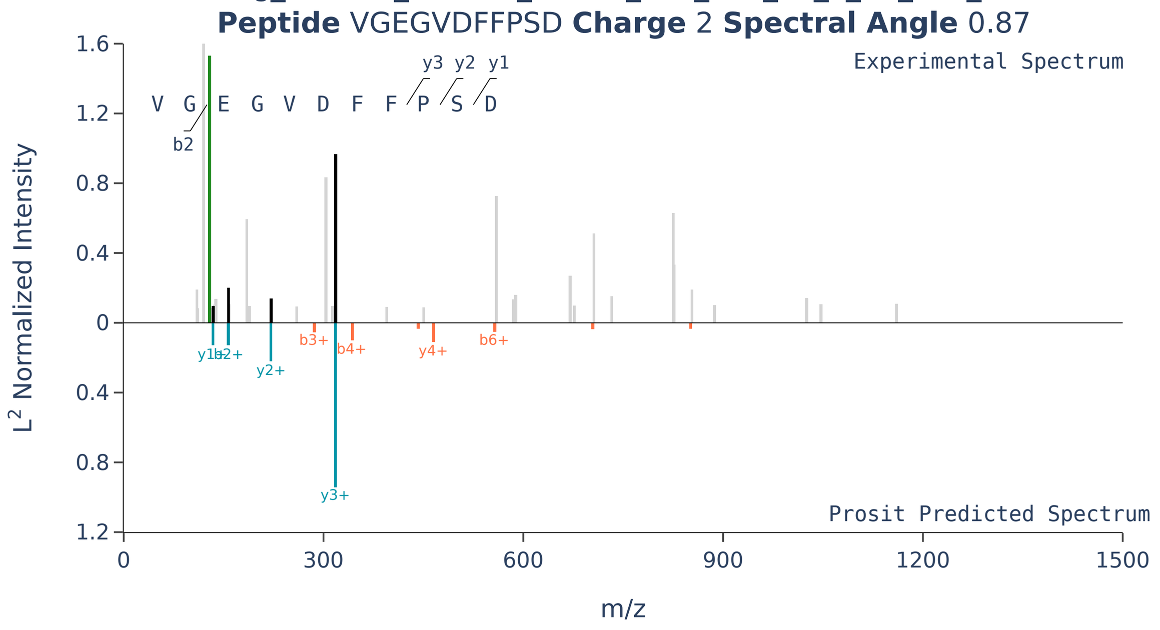

Source Ncheng_210623_230623_HFGoe_FFH_20S_25_1_A1_24h_R1 **Scan** 17734
Peptide TDKPIKFLGVGEKTE **Charge** 2 **Spectral Angle** 0.81

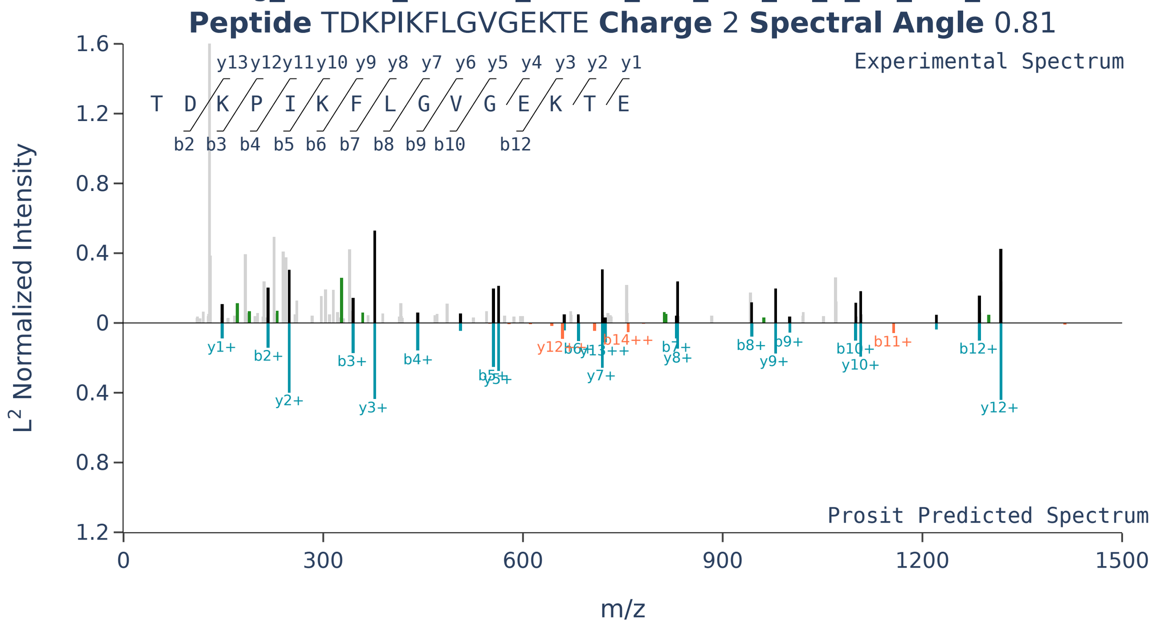

Source Ncheng_210623_230623_HFGoe_FFH_20S_25_1_A2_24h_R2 **Scan** 17114
Peptide LAMTGQDAANTAKAFN **Charge** 2 **Spectral Angle** 0.81

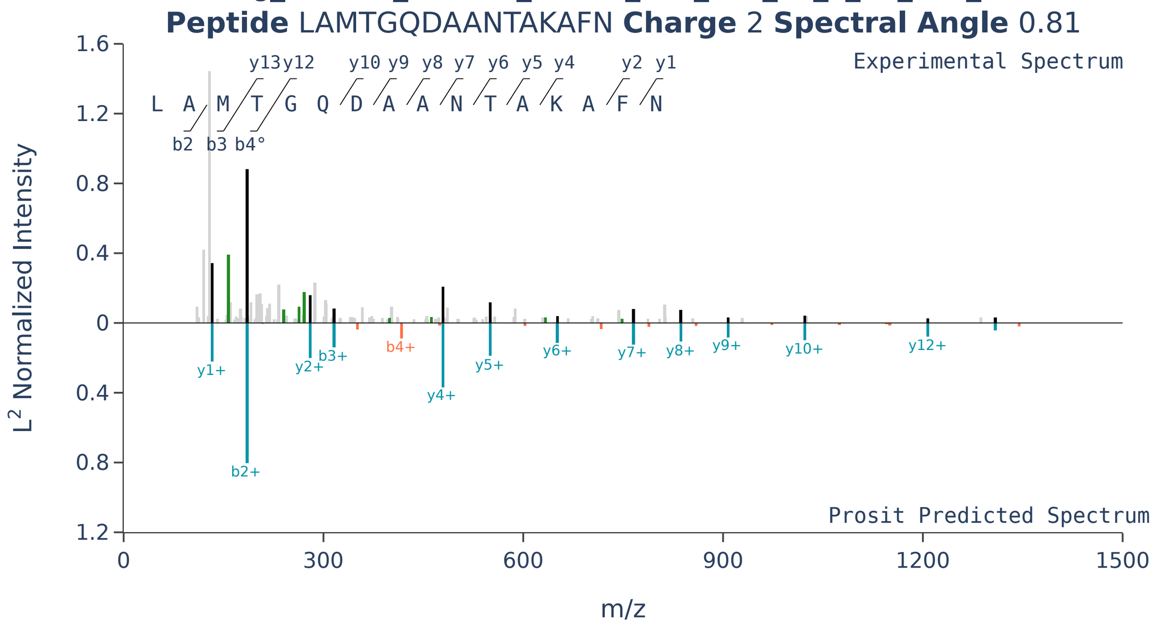

Source Ncheng_210623_230623_HFGoe_FFH_20S_25_1_A2_2h_R2 **Scan** 32804
Peptide PVDIVNAALNPV **Charge** 1 **Spectral Angle** 0.92

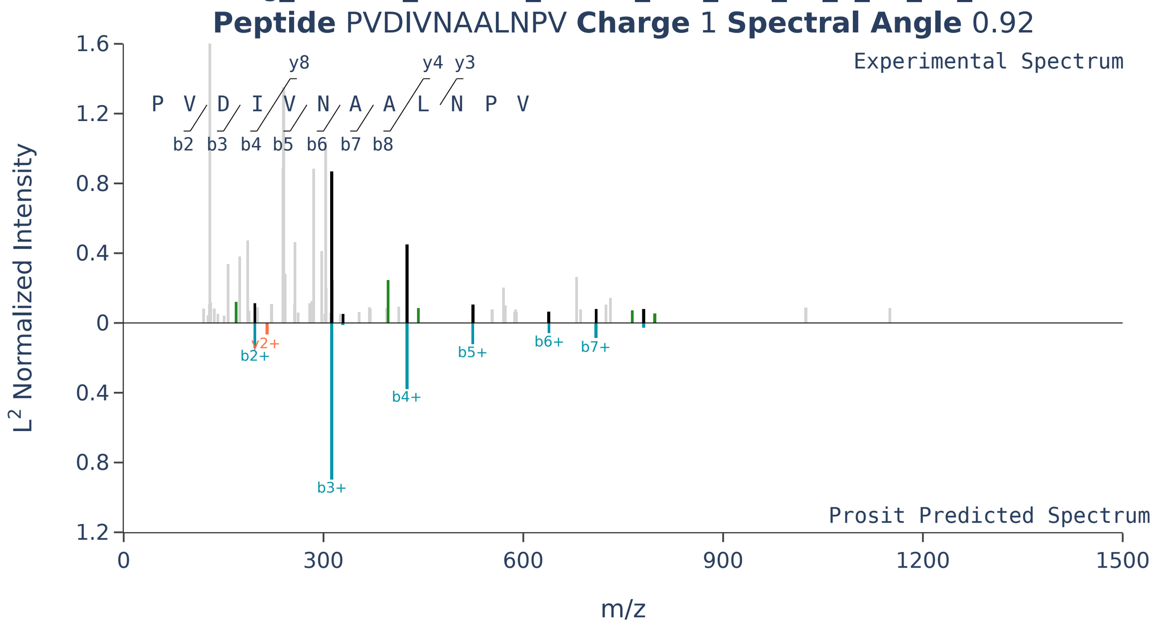

Source Ncheng_210623_230623_HFGoe_FFH_20S_25_1_A2_4h_R2 **Scan** 13009
Peptide RIAAGCMQVQ **Charge** 2 **Spectral Angle** 0.69

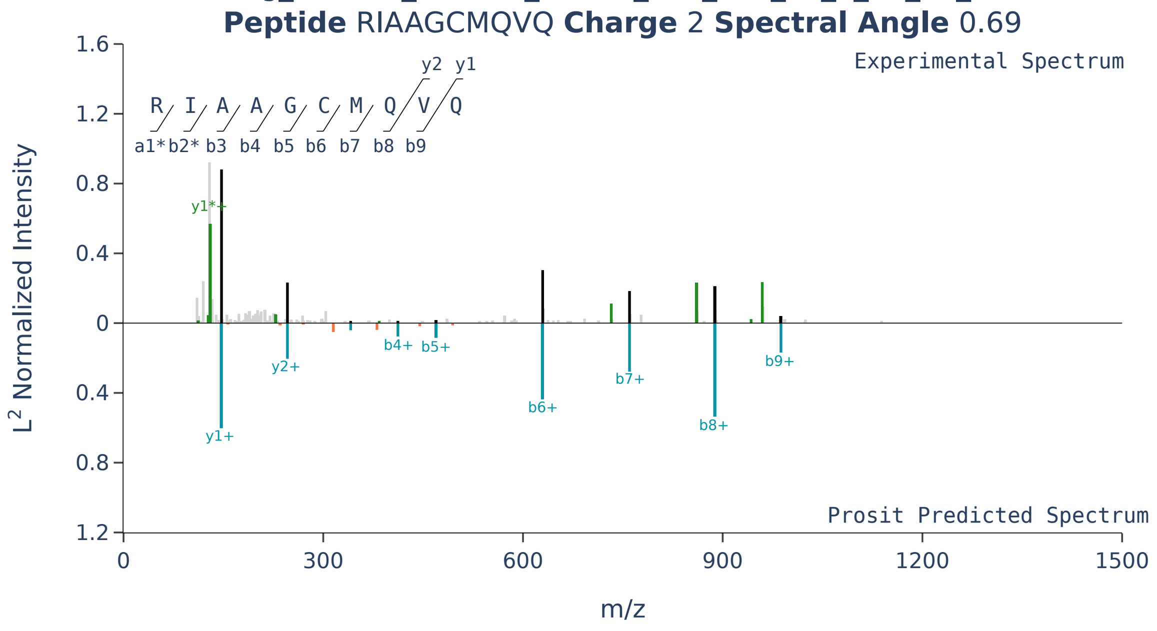

Source Ncheng_210623_230623_HFGoe_FFH_20S_25_1_A1_4h_R1 **Scan** 8845
Peptide RIAAGVGQKPVD **Charge** 2 **Spectral Angle** 0.85

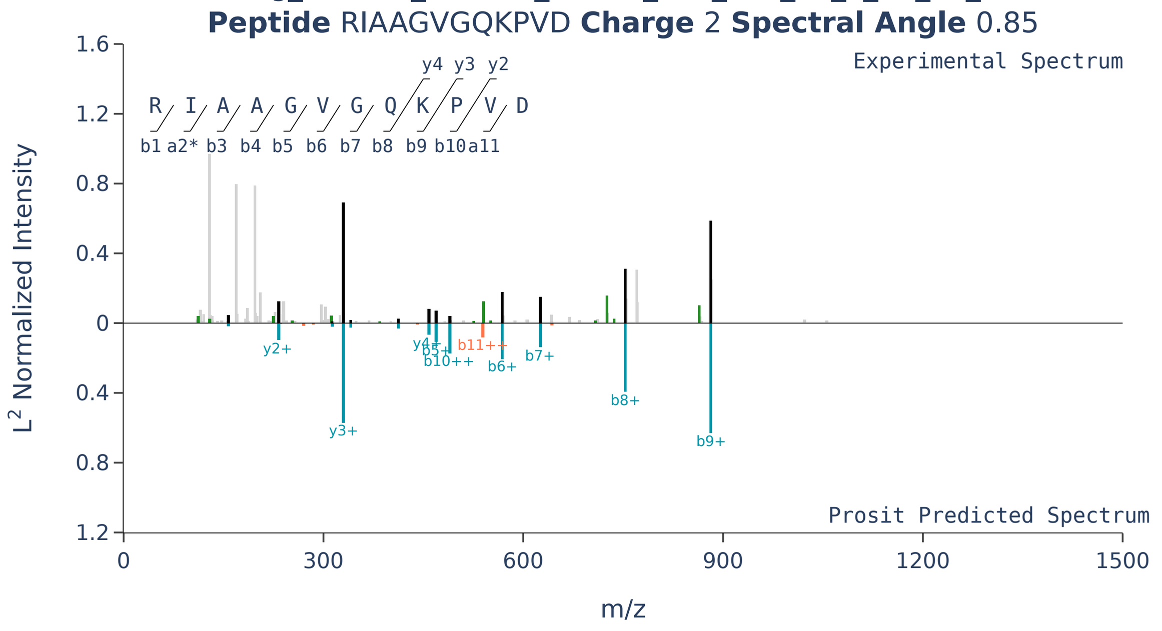

Source Ncheng_210623_230623_HFGoe_FFH_20S_25_1_A1_1h_R1 **Scan** 11824
Peptide FHPDASRI **Charge** 2 **Spectral Angle** 0.85

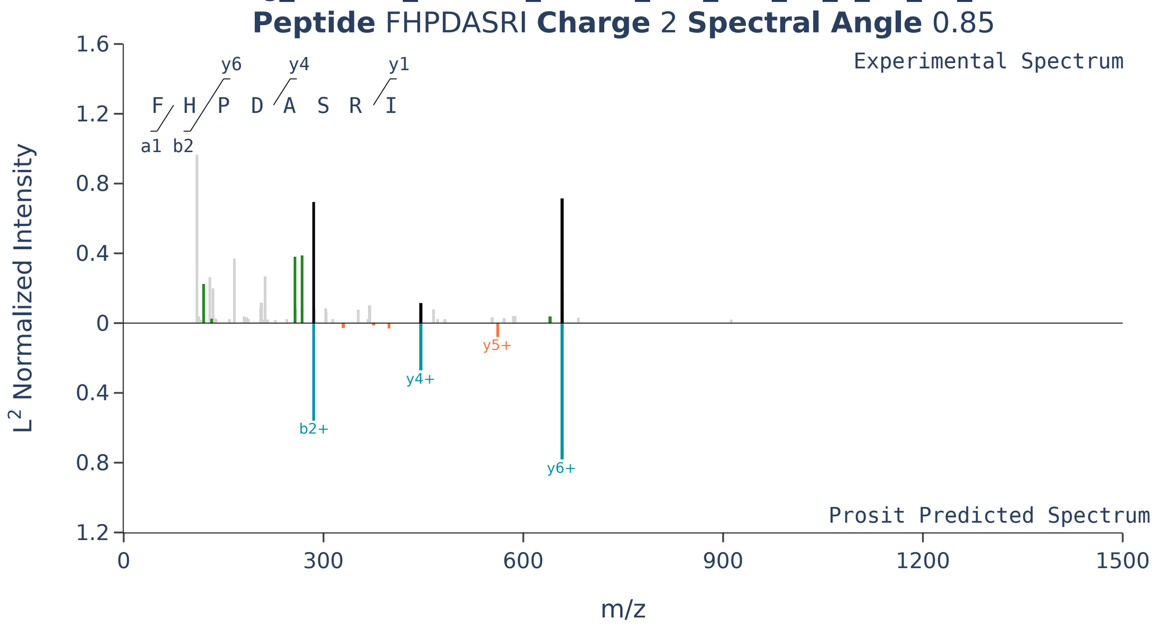

Source Ncheng_210623_230623_HFGoe_FFH_20S_25_1_A2_24h_R1 **Scan** 7971
Peptide KEAKFYD **Charge** 2 **Spectral Angle** 0.81

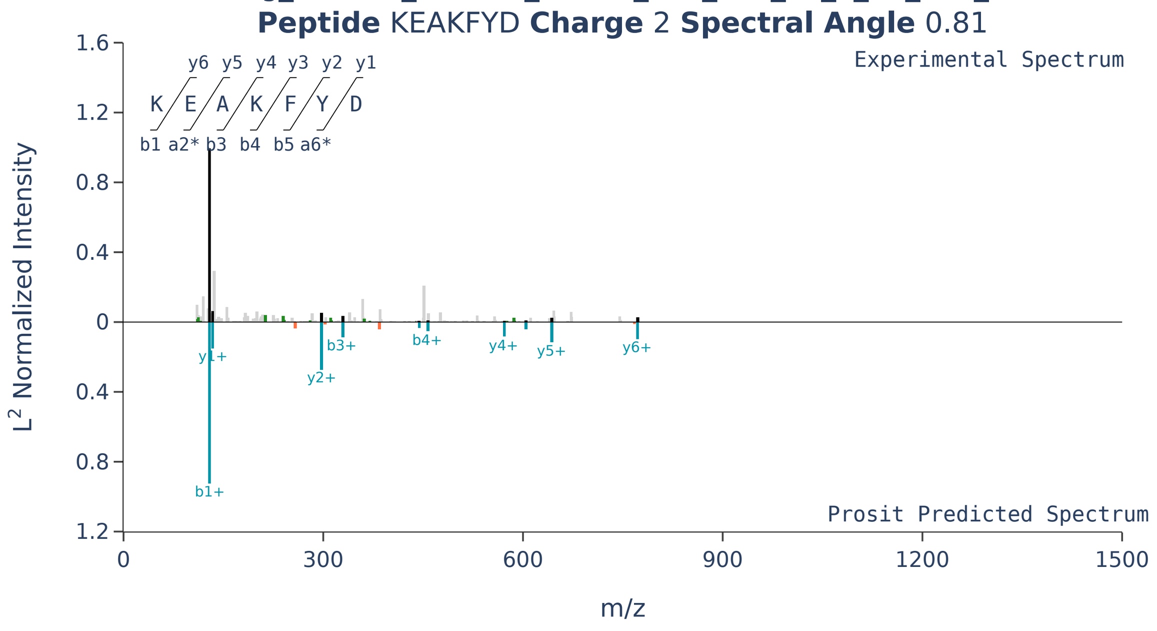

Source Ncheng_210623_230623_HFGoe_FFH_20S_25_1_A1_1h_R1 **Scan** 11245
Peptide PDNVKSQMADVA **Charge** 1 **Spectral Angle** 0.84

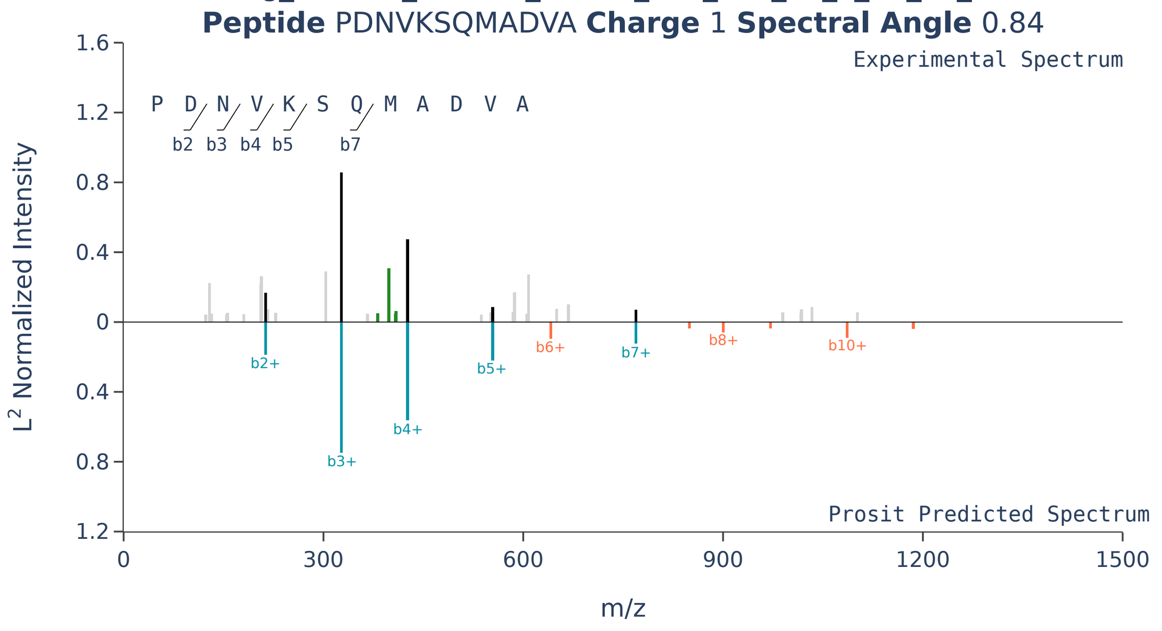

Source Ncheng_210623_230623_HFGoe_FFH_20S_25_1_A1_24h_R2 **Scan** 17836
Peptide LGKGHEVNKSLTPGQEFVKIVRN **Charge** 2 **Spectral Angle** 0.8

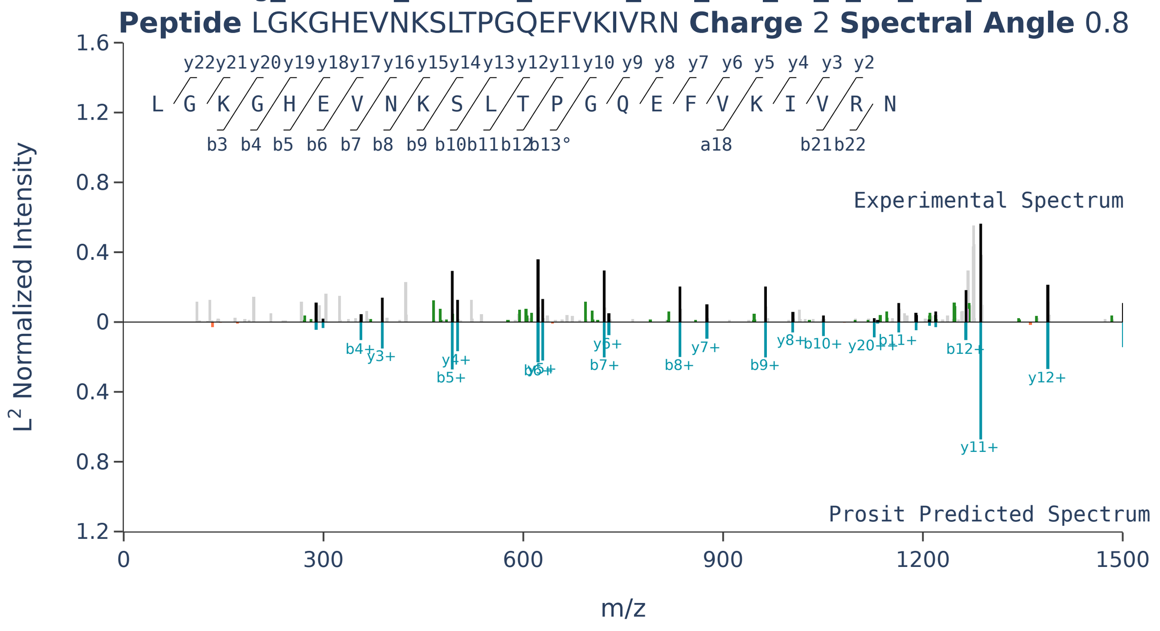

Source Ncheng_210623_230623_HFGoe_FFH_20S_25_1_A1_4h_R1 **Scan** 23678
Peptide GAAMGKLPGMGQIPDNVKSQMD **Charge** 3 **Spectral Angle** 0.82

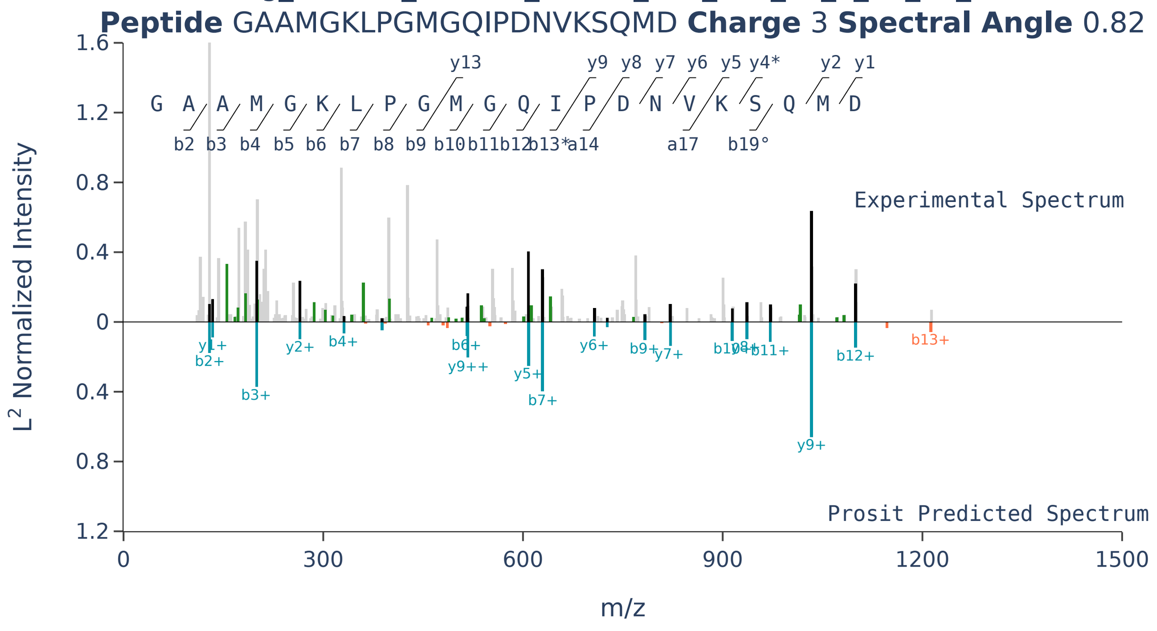

Source Ncheng_210623_230623_HFGoe_FFH_20S_25_1_A1_24h_R2 **Scan** 14765
Peptide PGMGQIPDNVKSD **Charge** 2 **Spectral Angle** 0.75

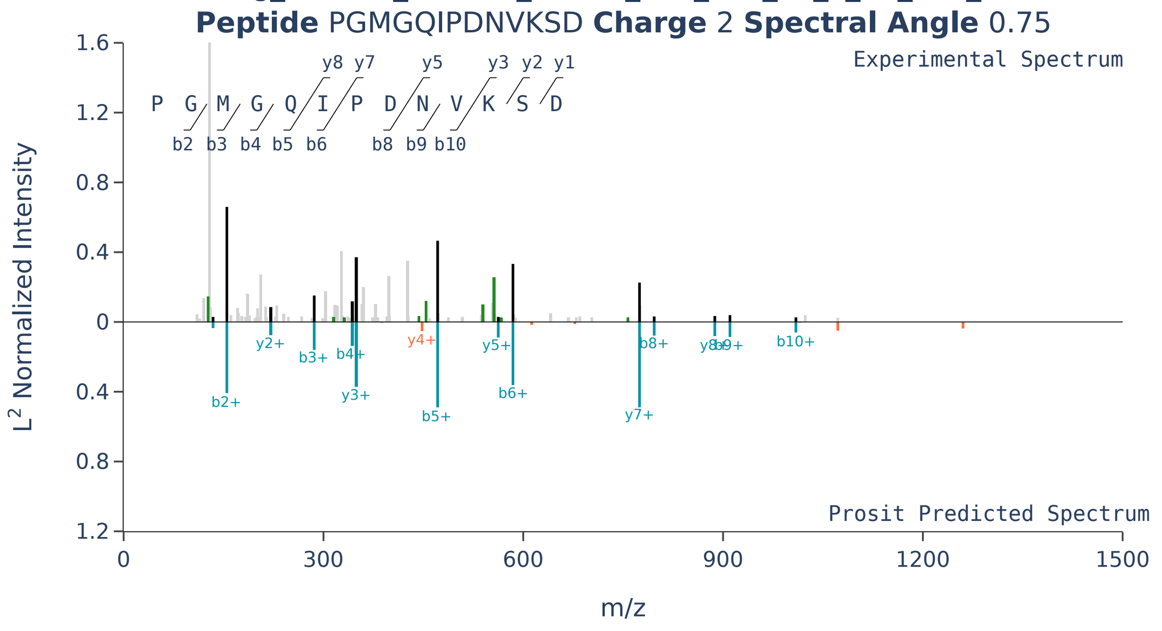

Source Ncheng_210623_230623_HFGoe_FFH_20S_25_1_A2_4h_R1 **Scan** 20304
Peptide MGKLPGMGQIPDNVKSG **Charge** 3 **Spectral Angle** 0.82

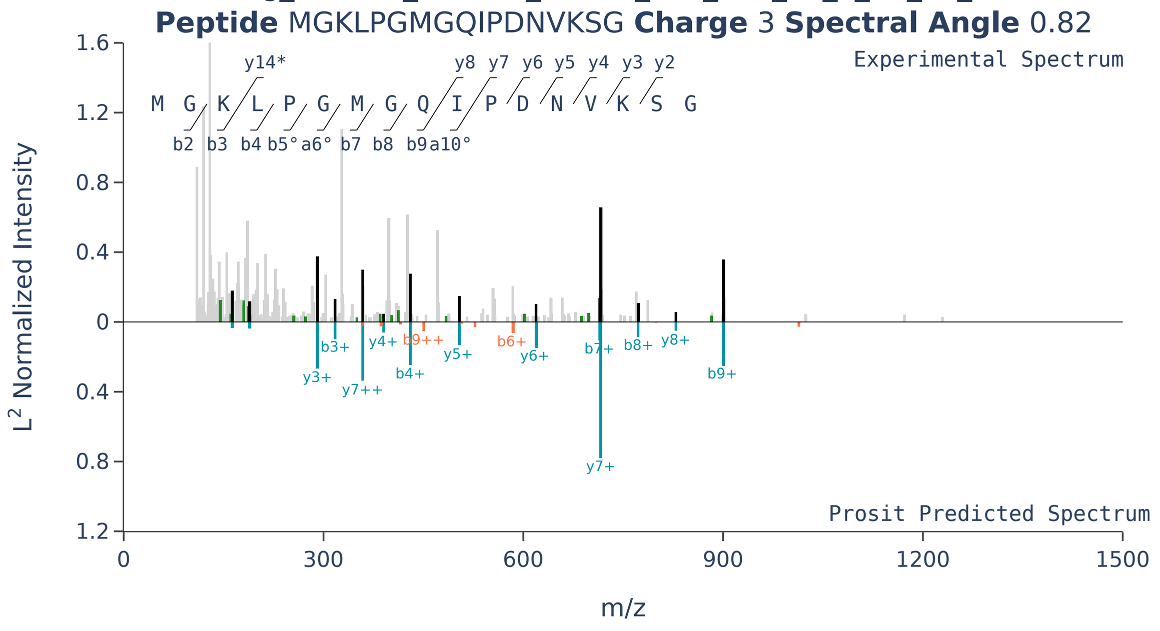

Source Ncheng_210623_230623_HFGoe_FFH_20S_25_1_A2_24h_R1 **Scan** 25916
Peptide GAGKVGVDFFPSD **Charge** 2 **Spectral Angle** 0.93

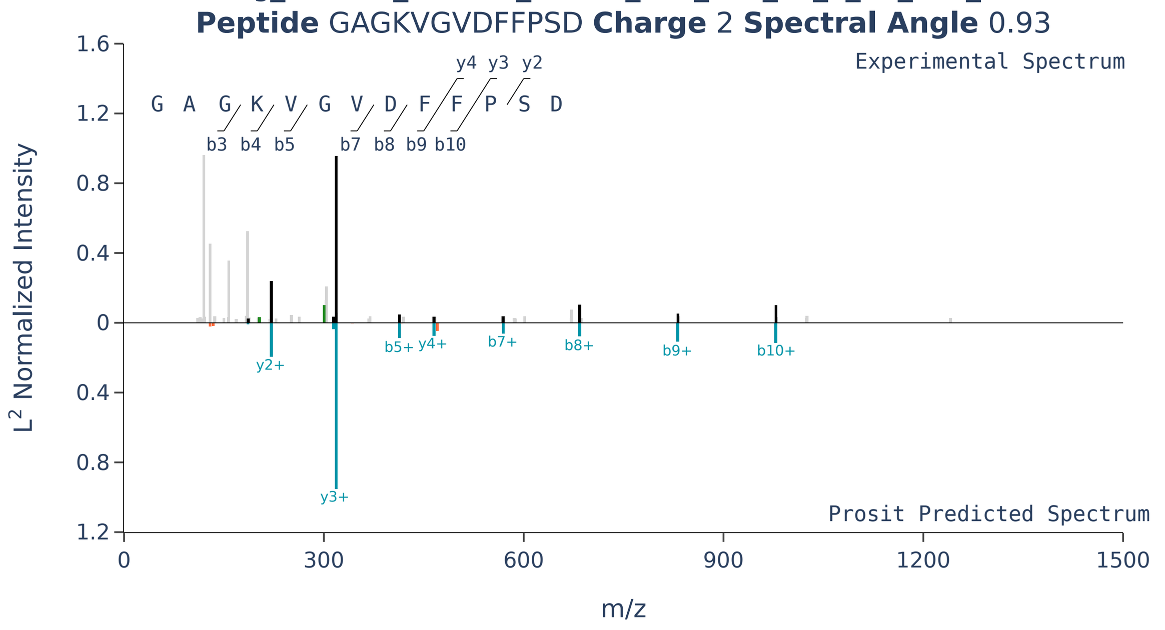

Source Ncheng_210623_230623_HFGoe_FFH_20S_25_1_A1_4h_R2 **Scan** 26654
Peptide SAMGKLPGMGQIPDNV **Charge** 2 **Spectral Angle** 0.89

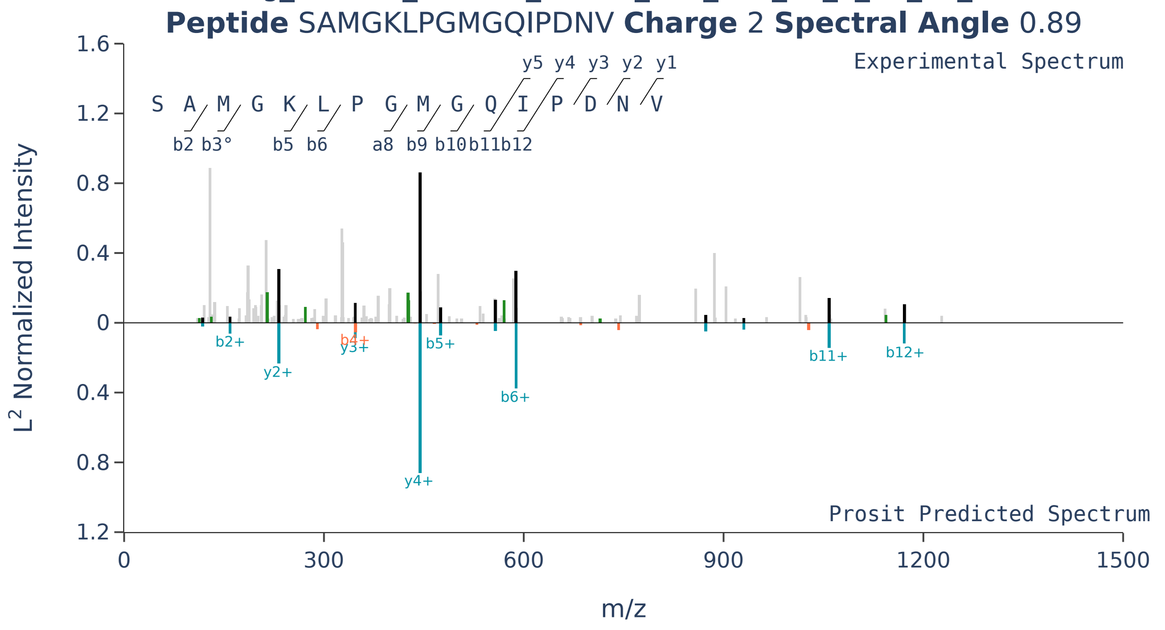

Source Ncheng_210623_230623_HFGoe_FFH_20S_25_1_A1_24h_R2 **Scan** 10647
Peptide CGMQAEQVG **Charge** 1 **Spectral Angle** 0.83

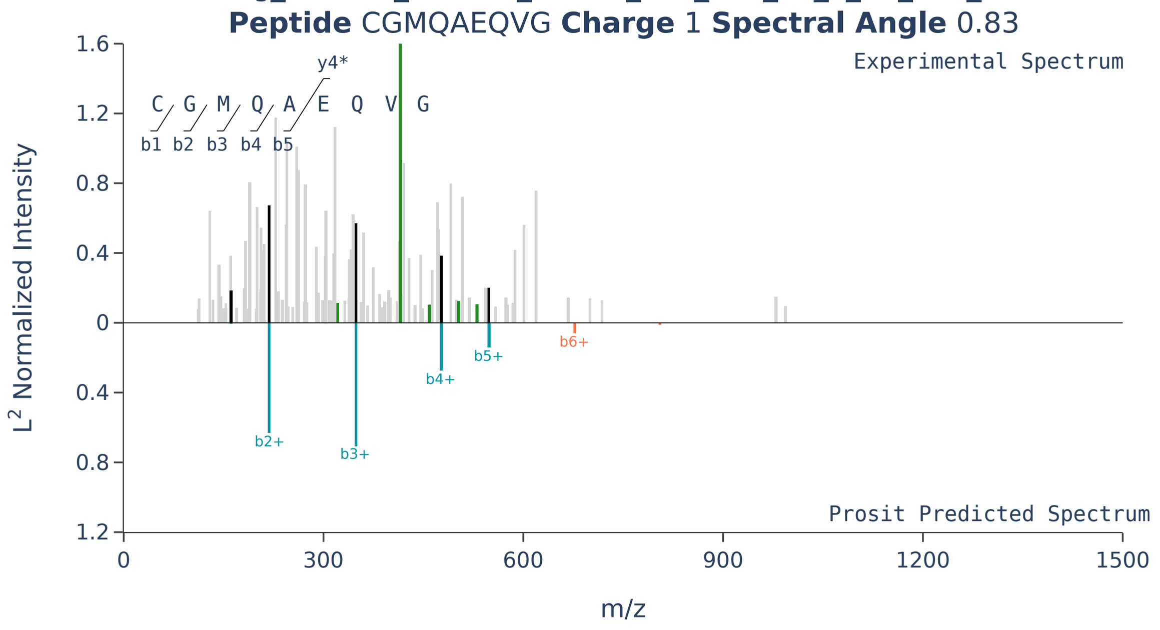

Source Ncheng_210623_230623_HFGoe_FFH_20S_25_1_A2_1h_R1 **Scan** 30536
Peptide NLHEVNKSLTPGQEFVKIVRNELVAAM **Charge** 5 **Spectral Angle** 0.89

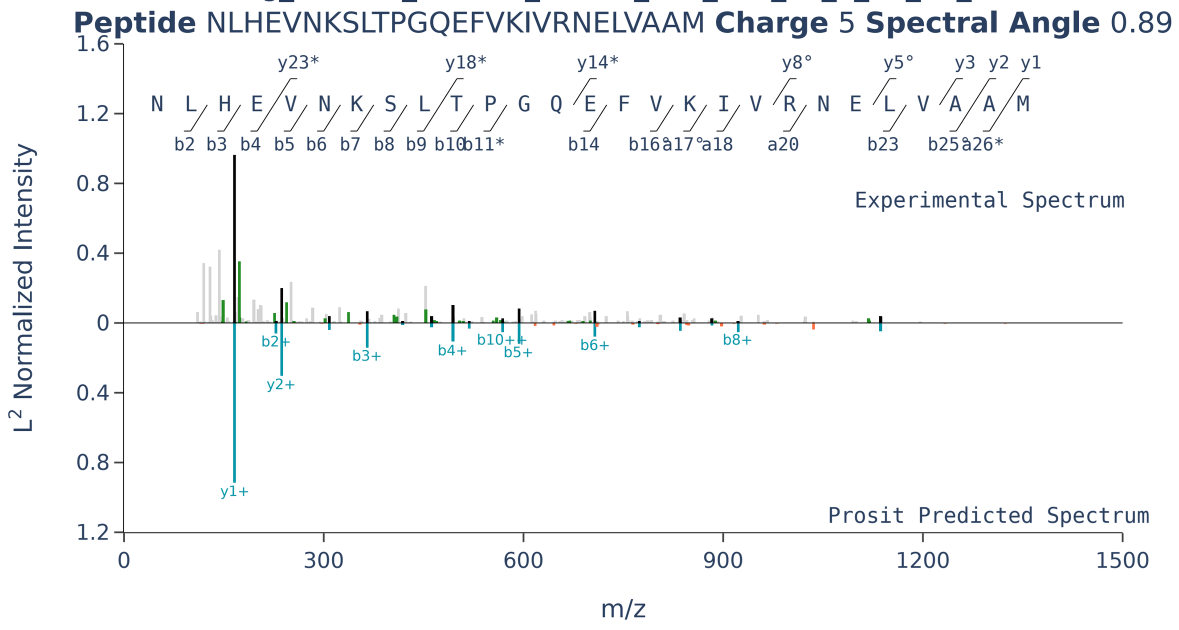

Source Ncheng_210623_230623_HFGoe_FFH_20S_25_1_A1_24h_R1 **Scan** 20280
Peptide DIESKLKF **Charge** 2 **Spectral Angle** 0.71

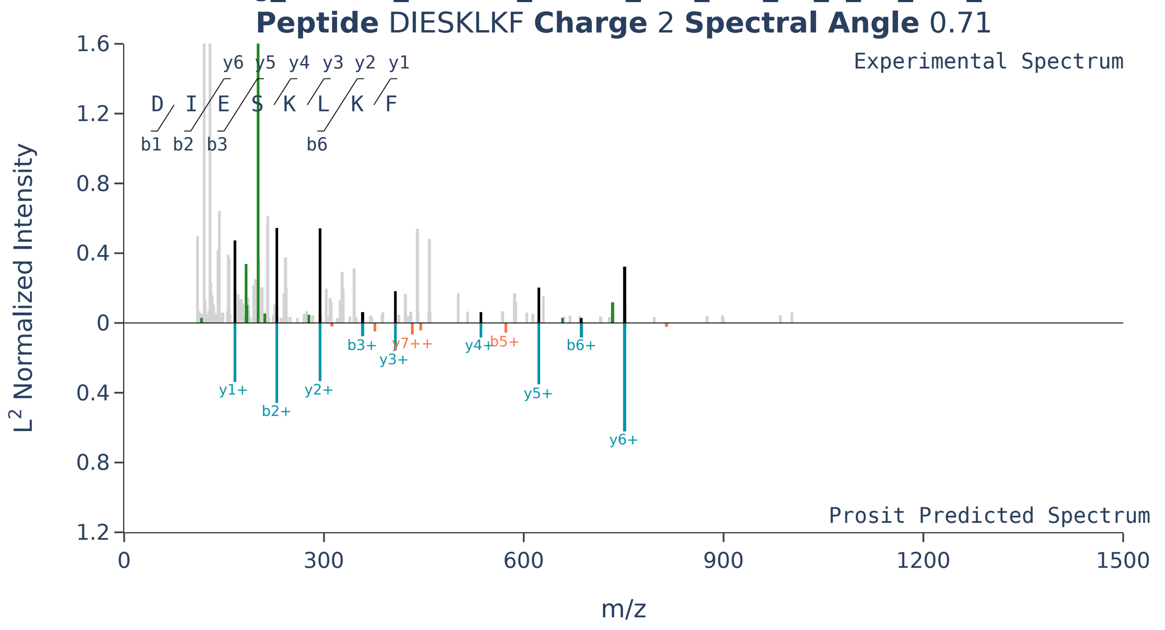

Source Ncheng_210623_230623_HFGoe_FFH_20S_25_1_A2_2h_R2 **Scan** 9209
Peptide ESKVDNL **Charge** 2 **Spectral Angle** 0.91

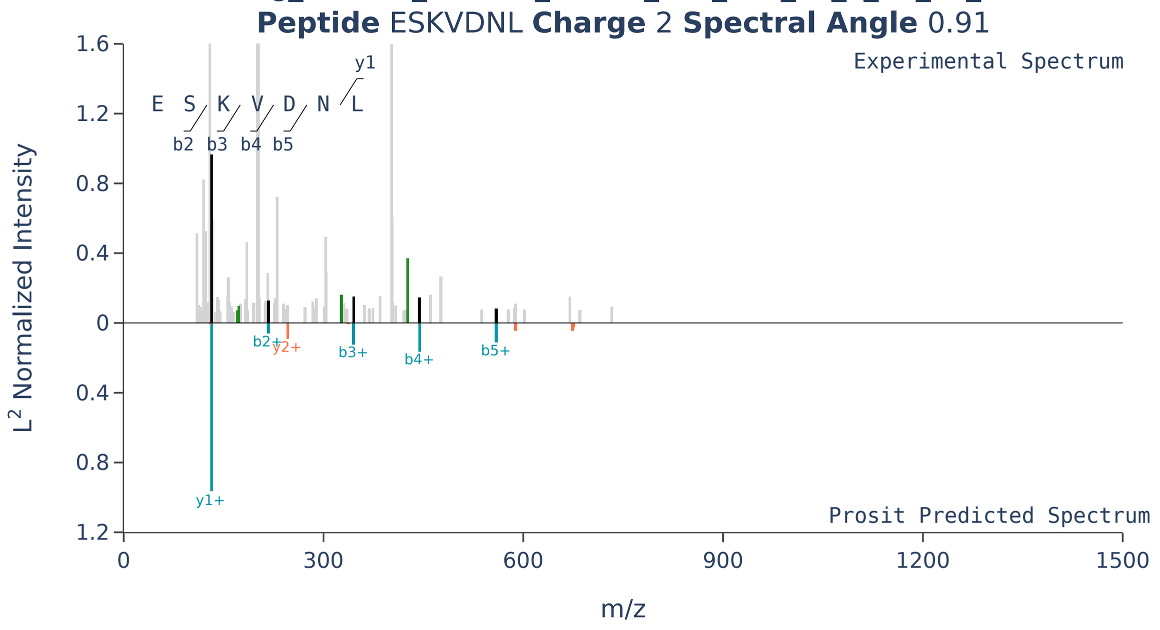

Source Ncheng_210623_230623_HFGoe_FFH_20S_25_1_A1_2h_R2 **Scan** 12726
Peptide GEENQTLNLKTE **Charge** 2 **Spectral Angle** 0.8

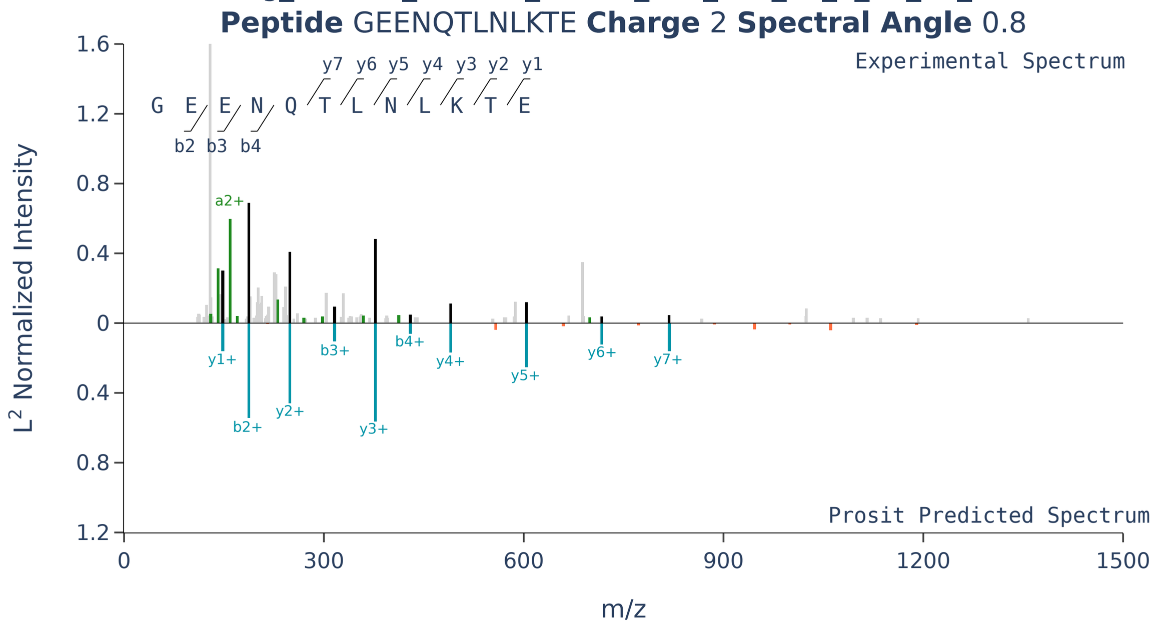

Source Ncheng_210623_230623_HFGoe_FFH_20S_25_1_A2_4h_R1 **Scan** 8342
Peptide ALKEARIA **Charge** 2 **Spectral Angle** 0.87

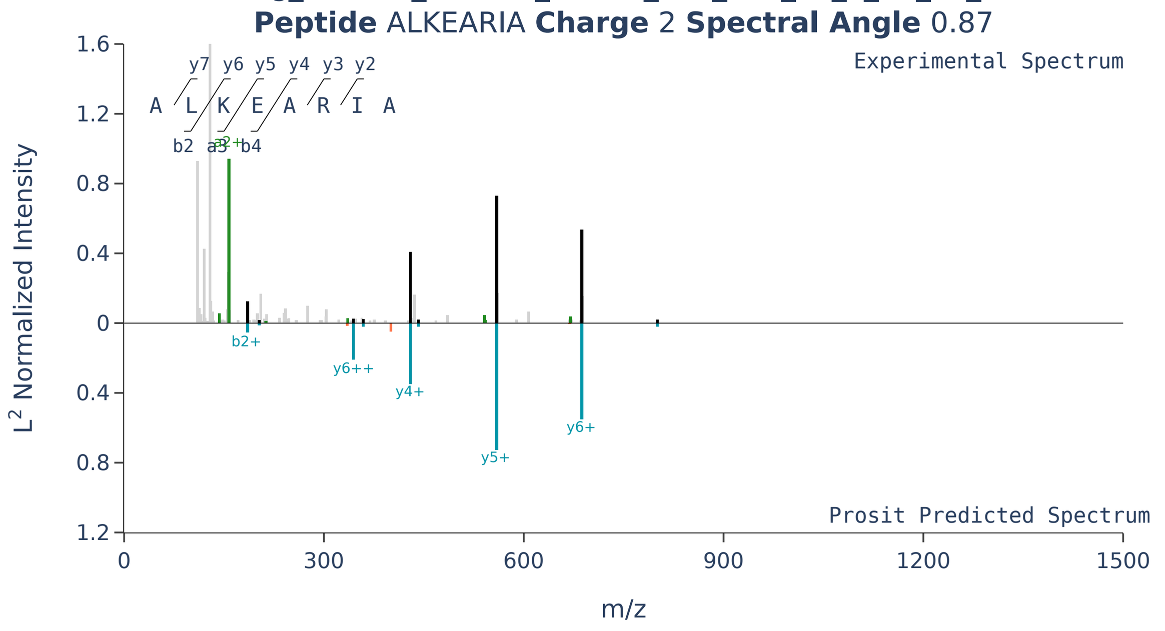

Source Ncheng_210623_230623_HFGoe_FFH_20S_25_1_A1_4h_R2 **Scan** 10101
Peptide AAGCGMVQD **Charge** 2 **Spectral Angle** 0.8

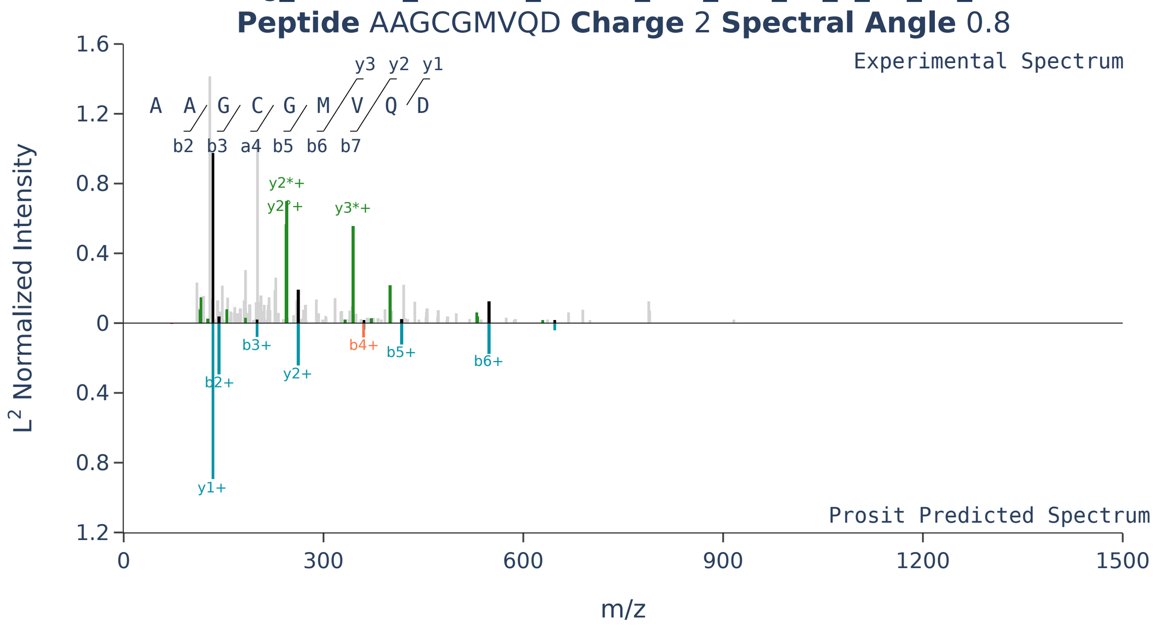

Source Ncheng_210623_230623_HFGoe_FFH_20S_25_1_A1_4h_R1 **Scan** 16728
Peptide MTGMTGQDAANTAKAFN **Charge** 2 **Spectral Angle** 0.69

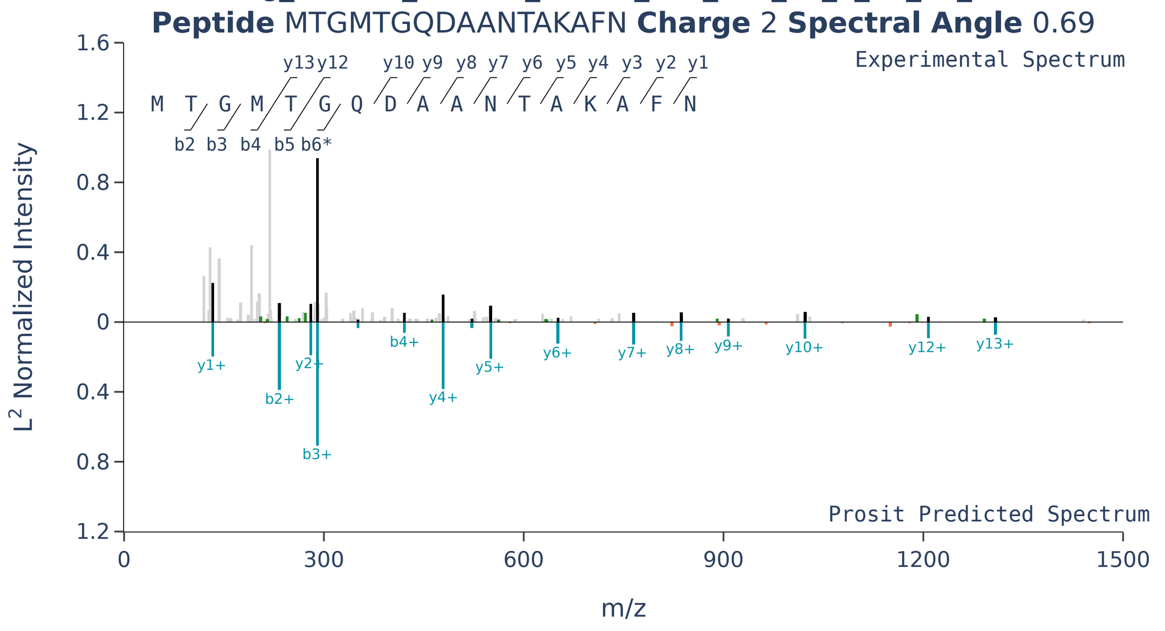

Source Ncheng_210623_230623_HFGoe_FFH_20S_25_1_A2_24h_R1 **Scan** 12769
Peptide AMTGQDAANTAKL **Charge** 2 **Spectral Angle** 0.67

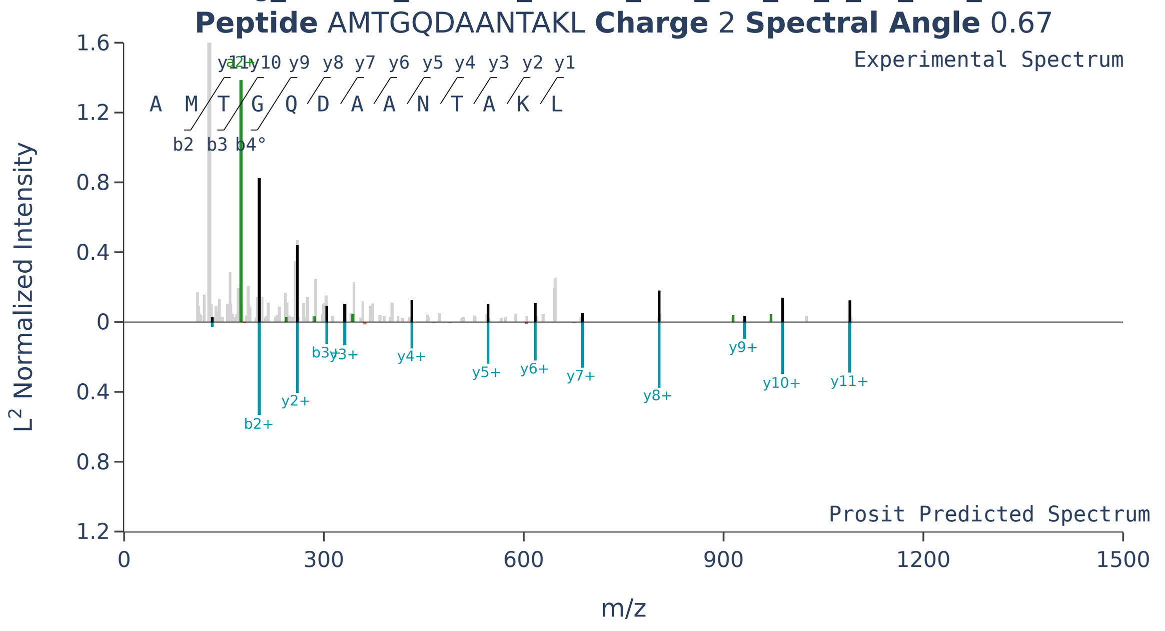

Source Ncheng_210623_230623_HFGoe_FFH_20S_25_1_A2_24h_R2 **Scan** 8926
Peptide ALKEALR **Charge** 2 **Spectral Angle** 0.82

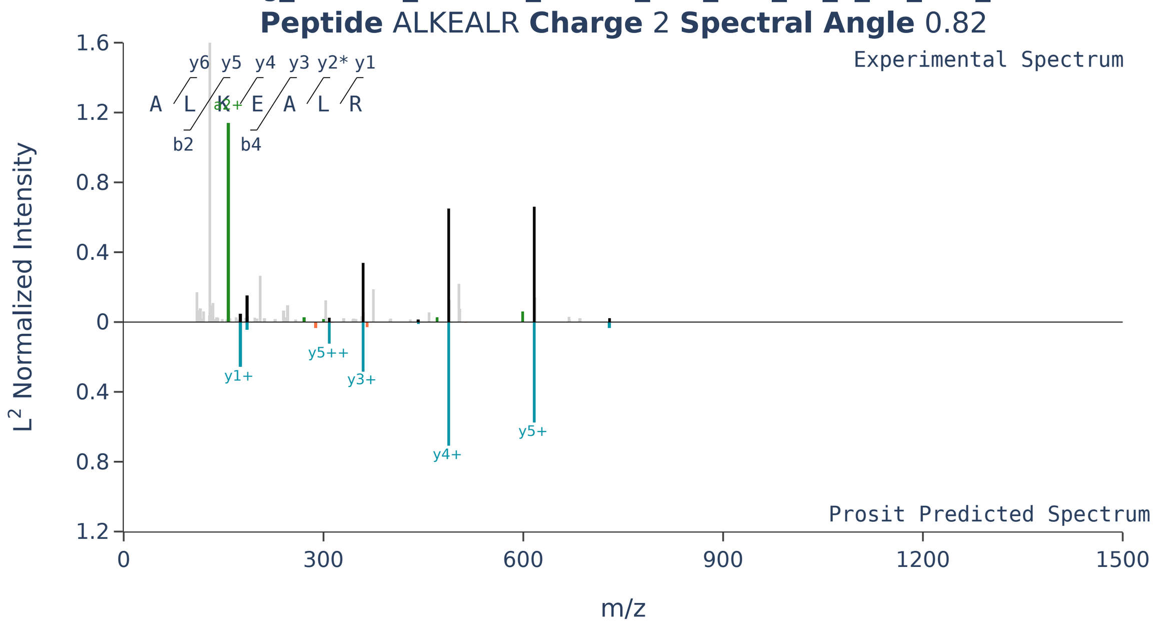

Source Ncheng_210623_230623_HFGoe_FFH_20S_25_1_A1_2h_R2 **Scan** 22888
Peptide KNMLPGMGQIPDNVKSQMDD **Charge** 3 **Spectral Angle** 0.74

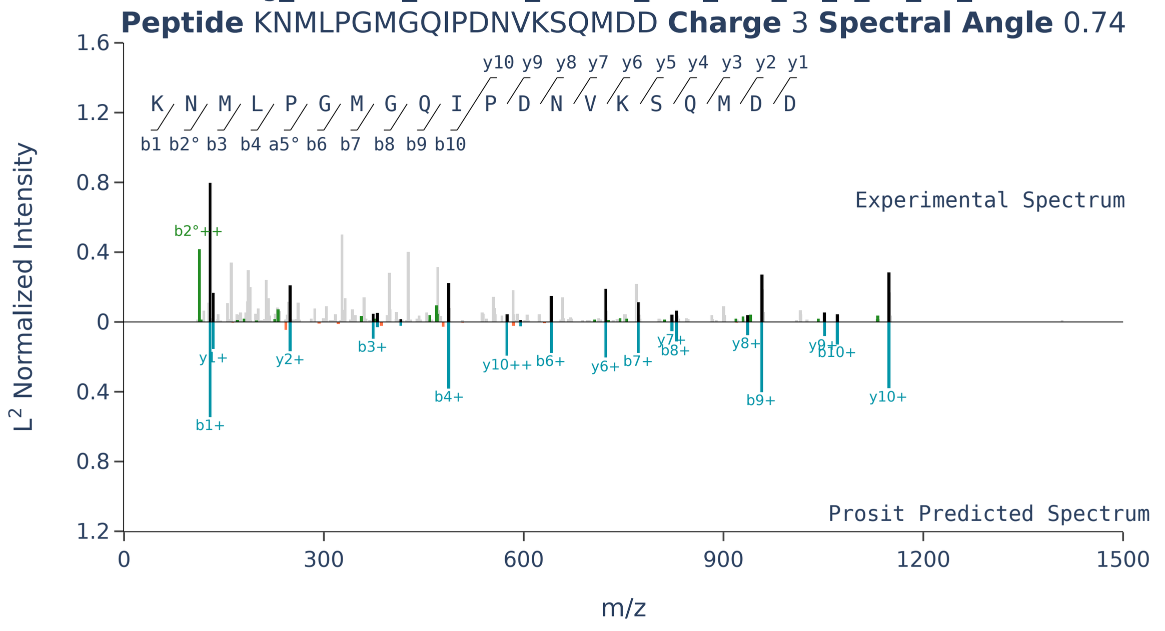

Source Ncheng_210623_230623_HFGoe_FFH_20S_25_1_A1_24h_R2 **Scan** 13704
Peptide KTEVVLTKV **Charge** 2 **Spectral Angle** 0.87

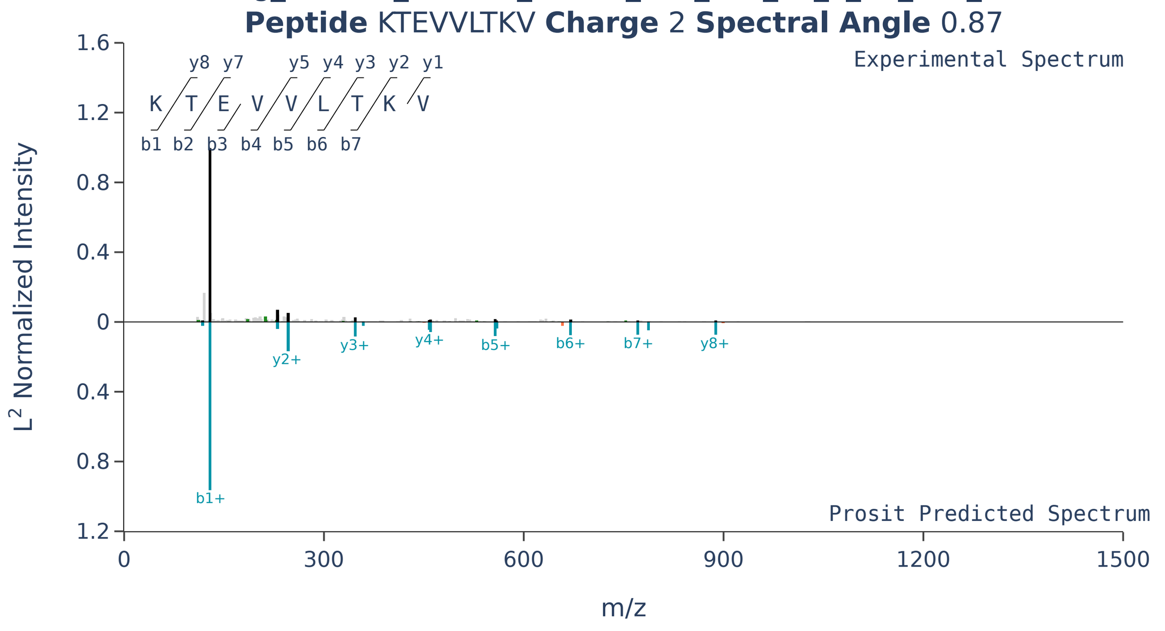

Source Ncheng_210623_230623_HFGoe_FFH_20S_25_1_A1_24h_R1 **Scan** 14727
Peptide LGVGEVGQKPVD **Charge** 2 **Spectral Angle** 0.72

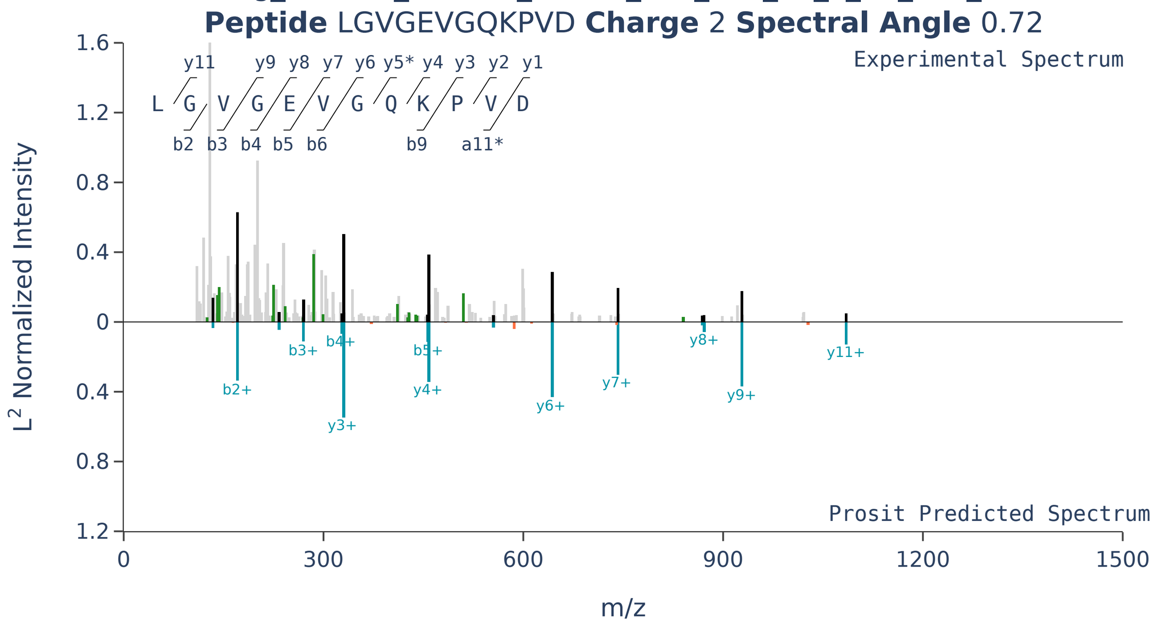

Source Ncheng_210623_230623_HFGoe_FFH_20S_25_1_A1_24h_R1 **Scan** 21928
Peptide DTKPIKFLGVGE **Charge** 3 **Spectral Angle** 0.91

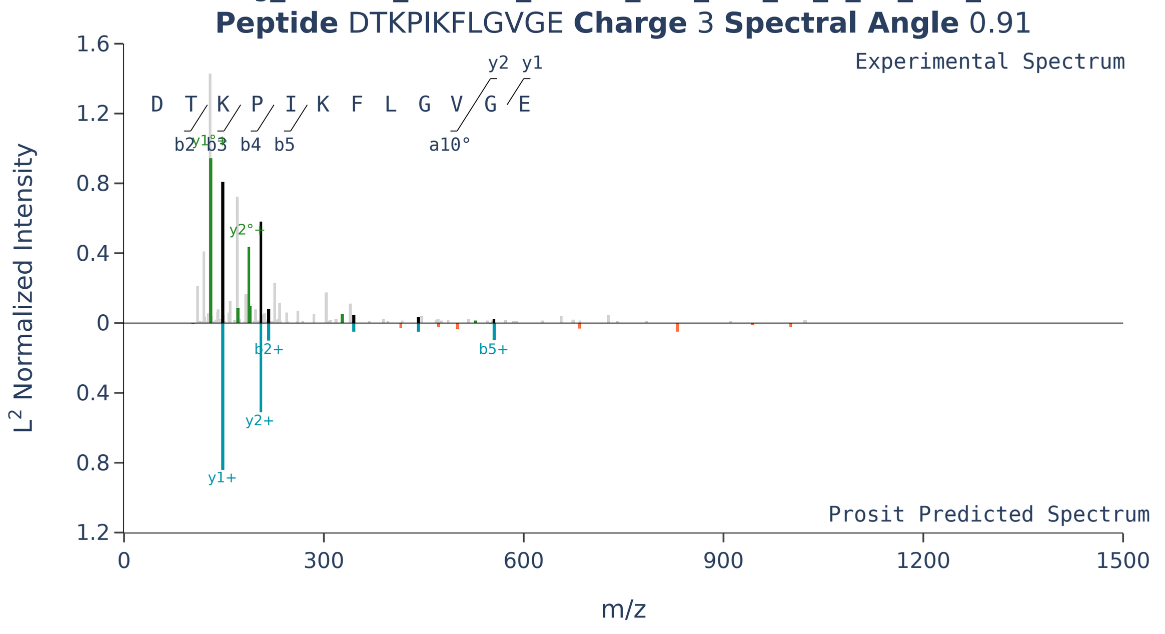

Source Ncheng_210623_230623_HFGoe_FFH_20S_25_1_A2_24h_R2 **Scan** 17549
Peptide ALKEAVGQKPVDIVNA **Charge** 2 **Spectral Angle** 0.8

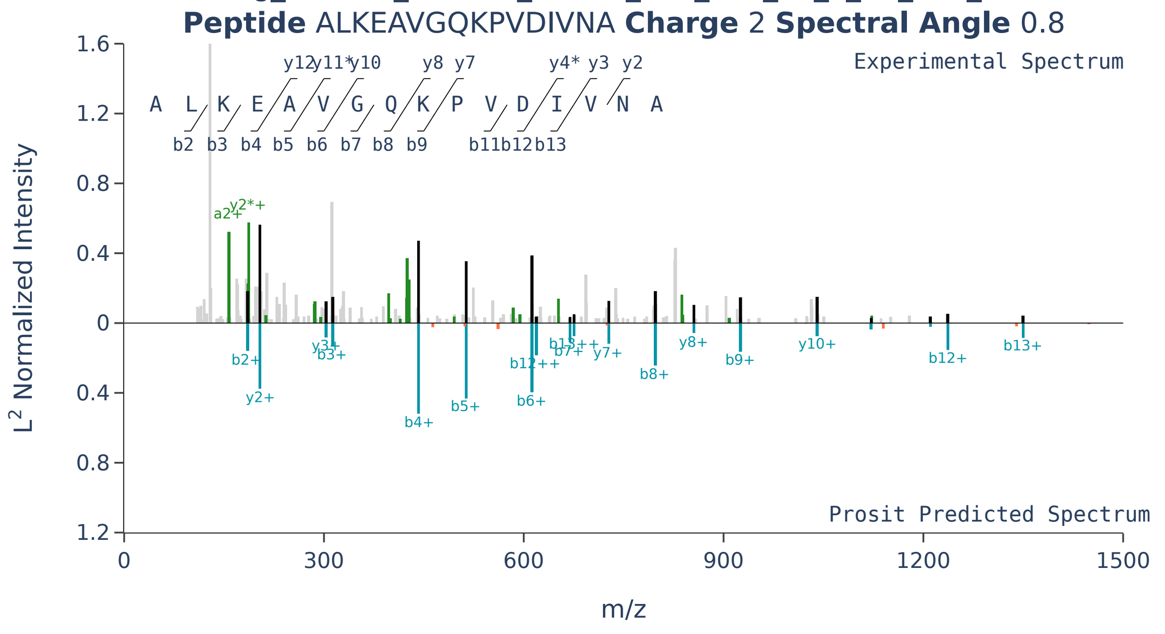

Source Ncheng_210623_230623_HFGoe_FFH_20S_25_1_A2_24h_R2 **Scan** 22123
Peptide TGMGKLPGMGQIPDNVKS **Charge** 3 **Spectral Angle** 0.85

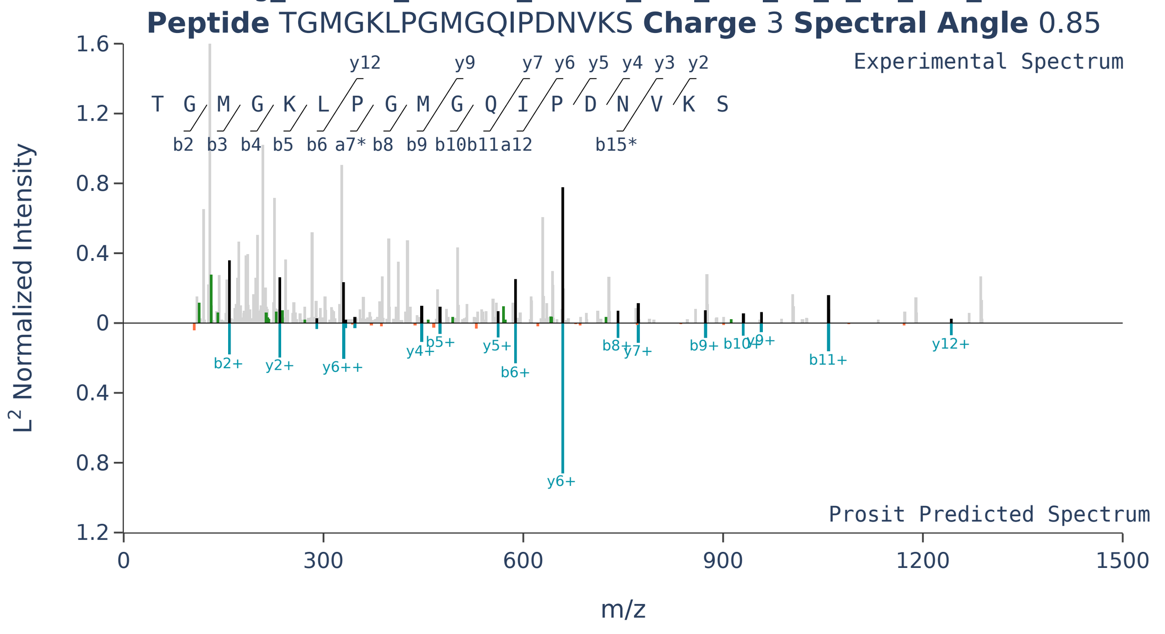

Source Ncheng_210623_230623_HFGoe_FFH_20S_25_1_A2_4h_R1 **Scan** 23629
Peptide SAMGKLPGMGQIPDNVKSQMDD **Charge** 3 **Spectral Angle** 0.77

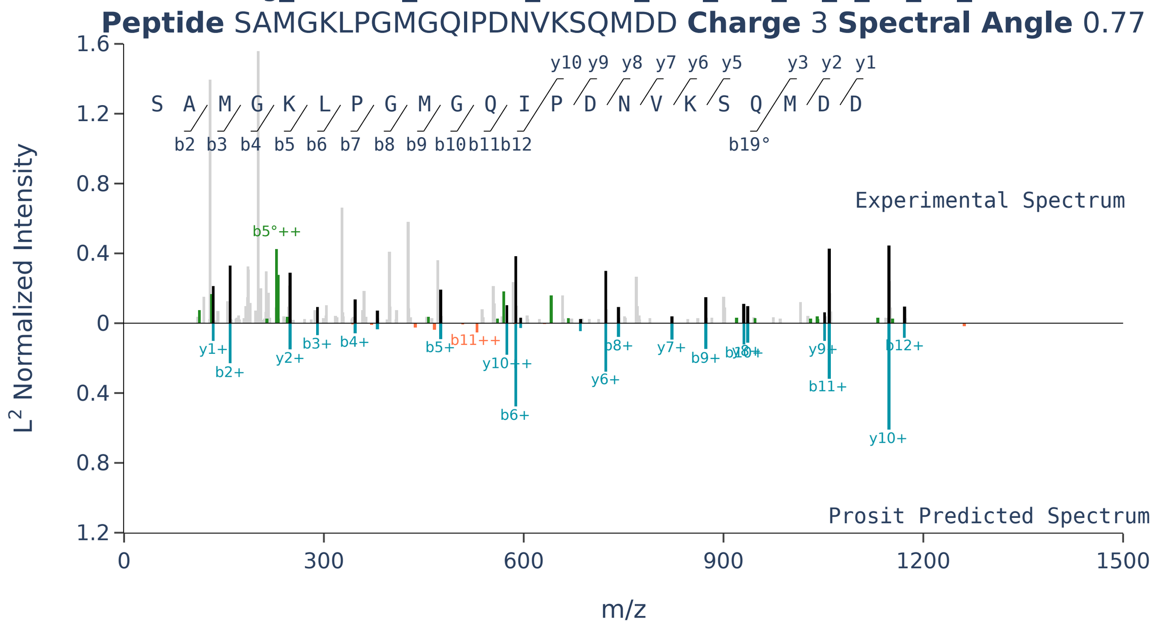

Source Ncheng_210623_230623_HFGoe_FFH_20S_25_1_A2_24h_R1 **Scan** 12960
Peptide GEKTEAMTGQDAANTAKAFN **Charge** 2 **Spectral Angle** 0.79

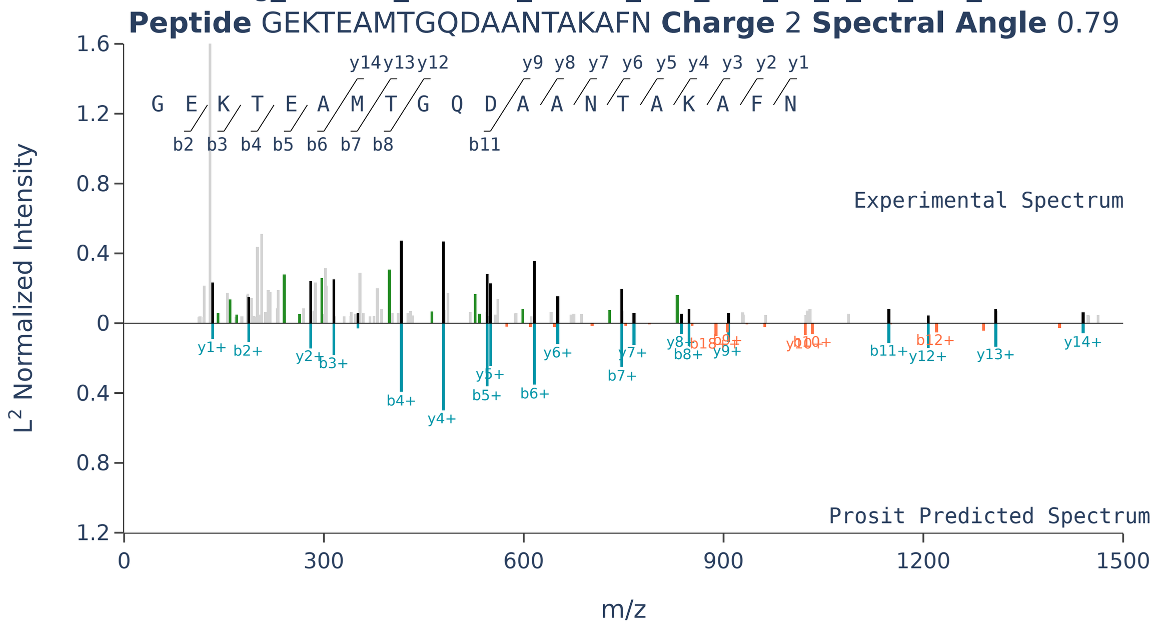

Source Ncheng_210623_230623_HFGoe_FFH_20S_25_1_A2_24h_R1 **Scan** 9125
Peptide AQDAANTAKAFN **Charge** 2 **Spectral Angle** 0.8

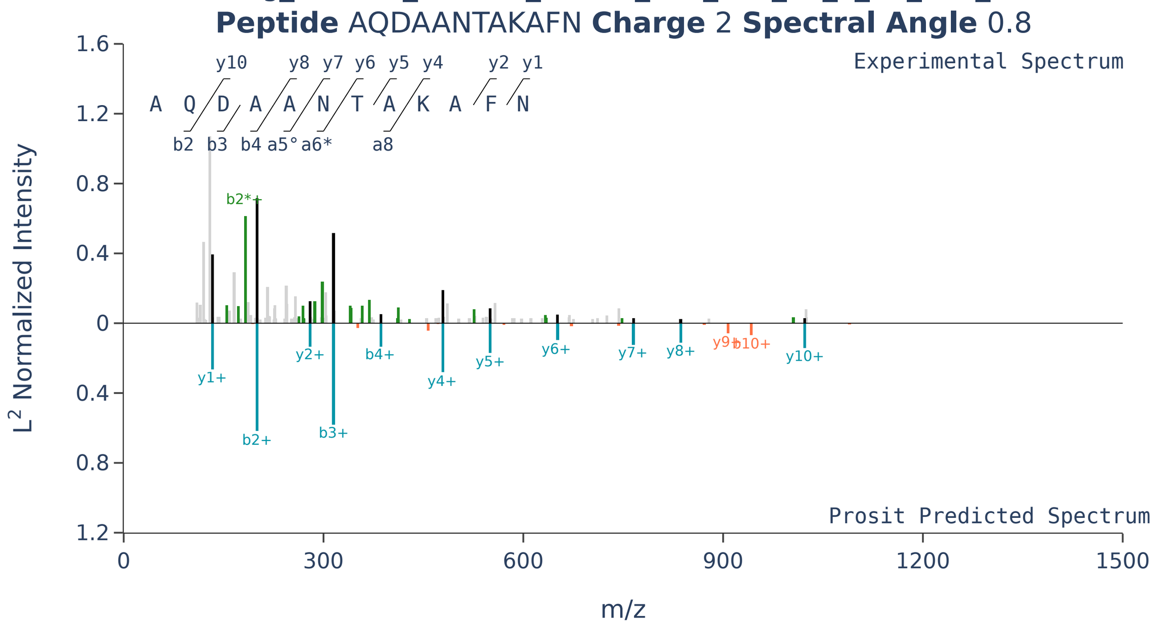

Source Ncheng_210623_230623_HFGoe_FFH_20S_25_1_A2_24h_R2 **Scan** 14077
Peptide AEQVGDIESKVDRA **Charge** 2 **Spectral Angle** 0.76

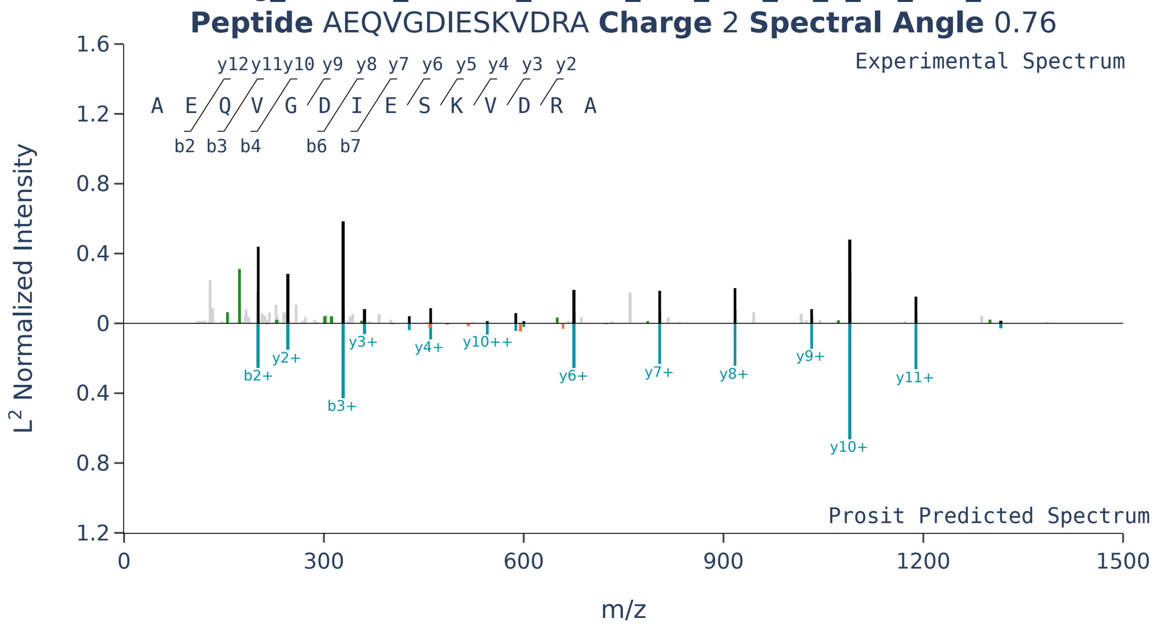

Source Ncheng_210623_230623_HFGoe_FFH_20S_25_1_A2_24h_R2 **Scan** 18421
Peptide SADVYRPAAIKQLED **Charge** 2 **Spectral Angle** 0.81

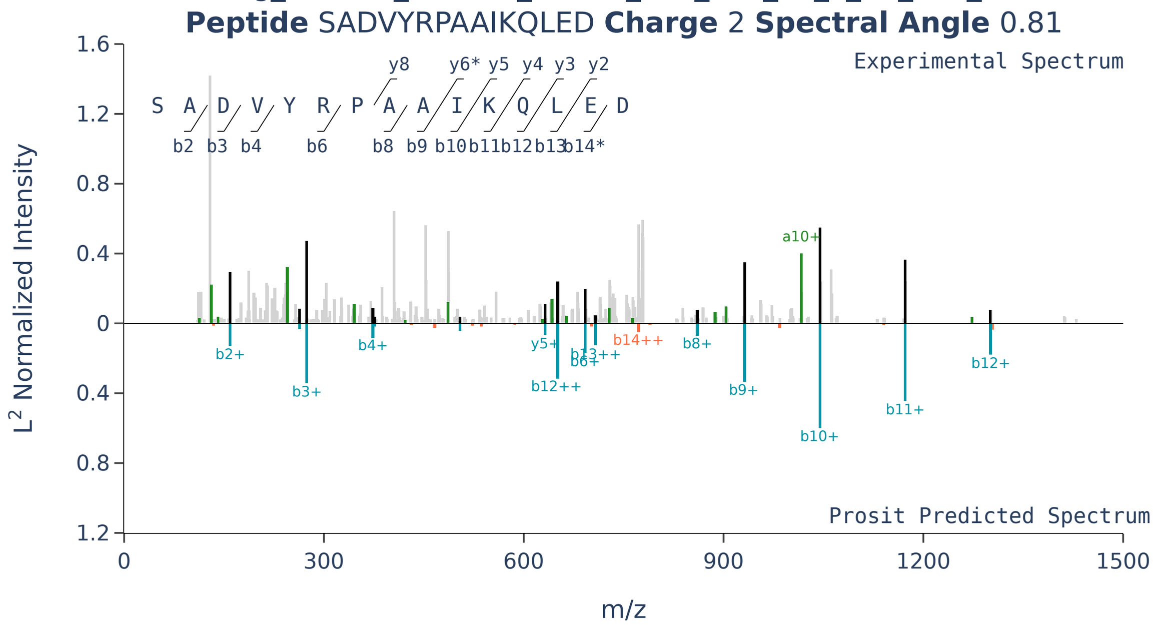

Source Ncheng_210623_230623_HFGoe_FFH_20S_25_1_A1_2h_R1 **Scan** 10496
Peptide KVLVRMDE **Charge** 2 **Spectral Angle** 0.69

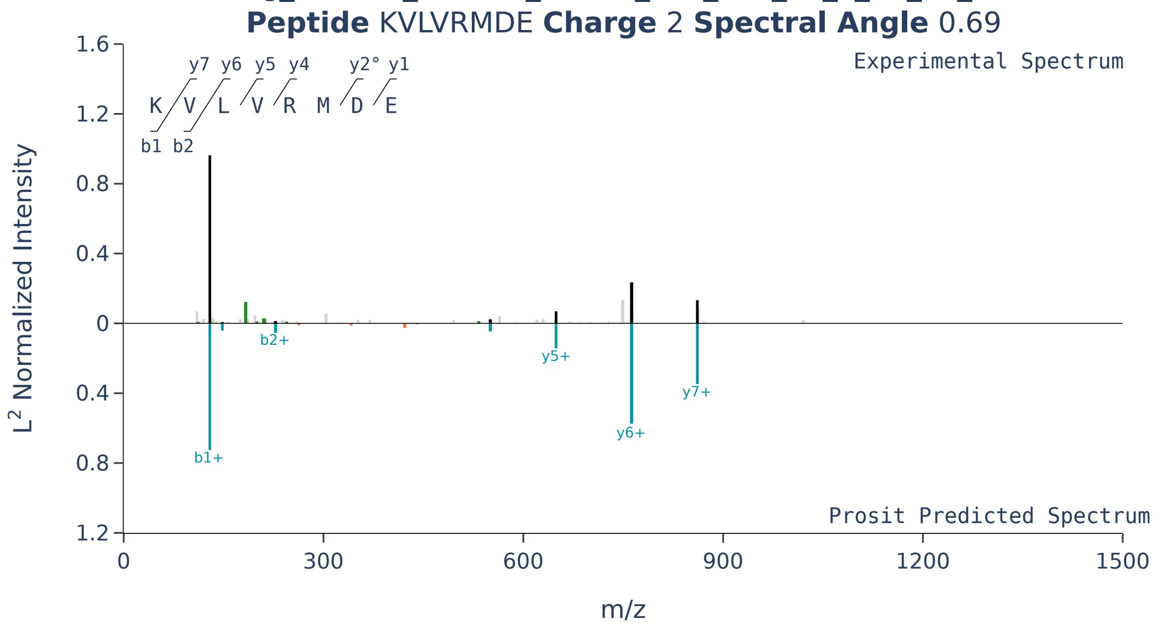

Source Ncheng_210623_230623_HFGoe_FFH_20S_25_1_A2_2h_R1 **Scan** 22465
Peptide AMGKLPGMGQIPDNVKSQMD **Charge** 3 **Spectral Angle** 0.74

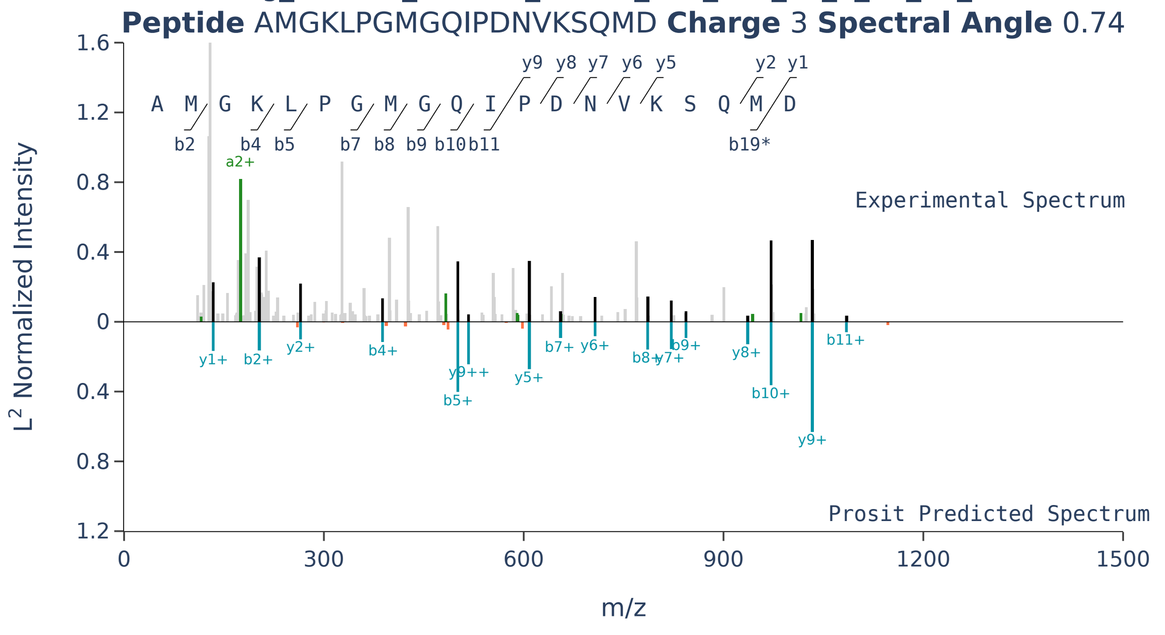

Source Ncheng_210623_230623_HFGoe_FFH_20S_25_1_A1_24h_R2 **Scan** 13575
Peptide SGAMTGQDAANTAKAFN **Charge** 2 **Spectral Angle** 0.87

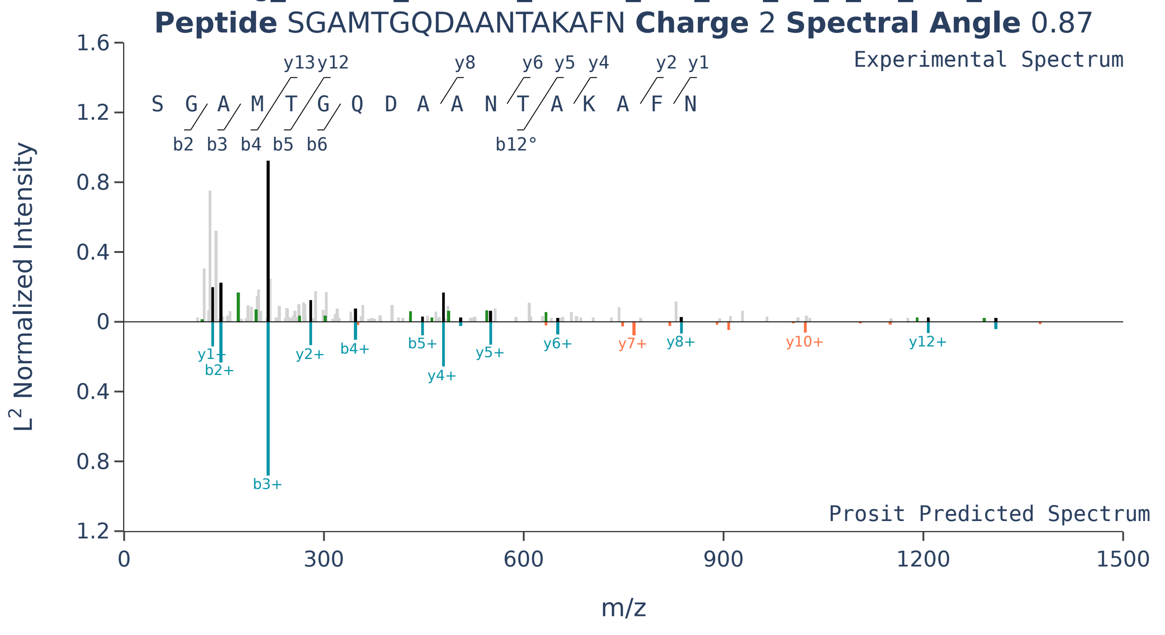

Source Ncheng_210623_230623_HFGoe_FFH_20S_25_1_A1_4h_R2 **Scan** 9628
Peptide MAANTAKAFN **Charge** 2 **Spectral Angle** 0.81

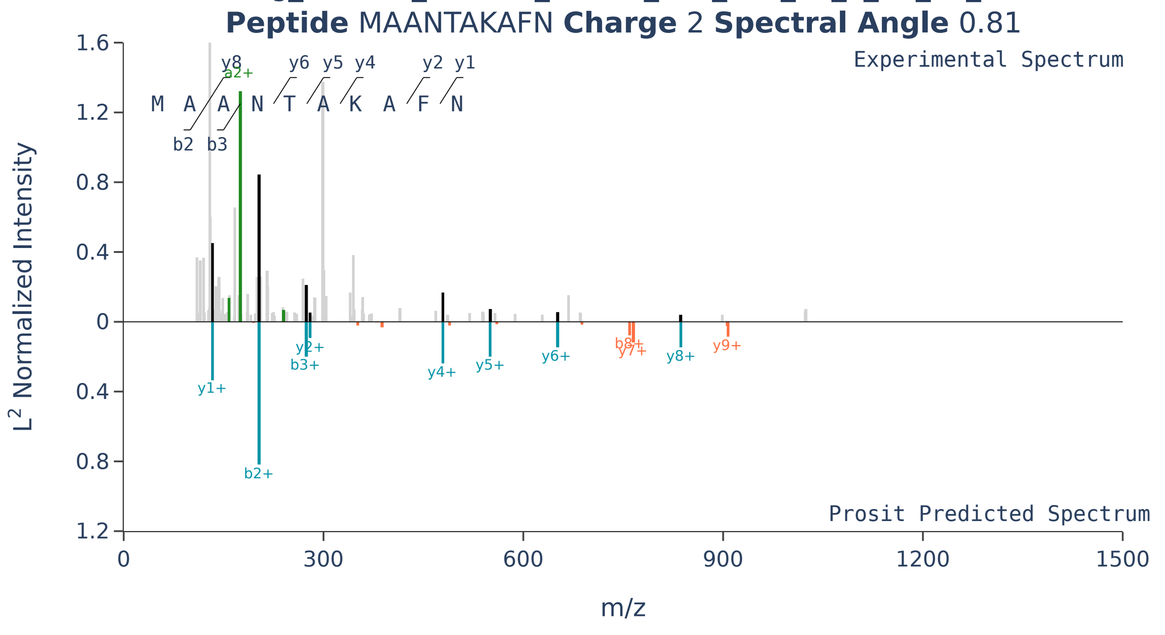

Source Ncheng_210623_230623_HFGoe_FFH_20S_25_1_A2_24h_R1 **Scan** 9776
Peptide DIESKQLE **Charge** 2 **Spectral Angle** 0.86

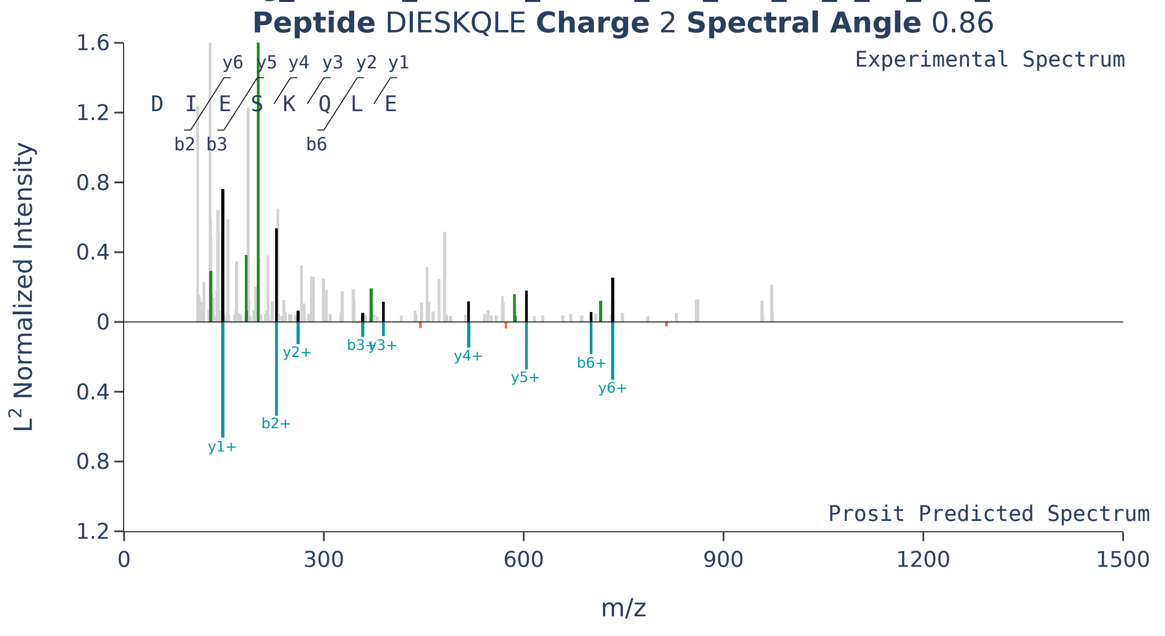

Source Ncheng_210623_230623_HFGoe_FFH_20S_25_1_A1_24h_R1 **Scan** 18817
Peptide GMAKLPGMGQIPDNVKSQ **Charge** 3 **Spectral Angle** 0.79

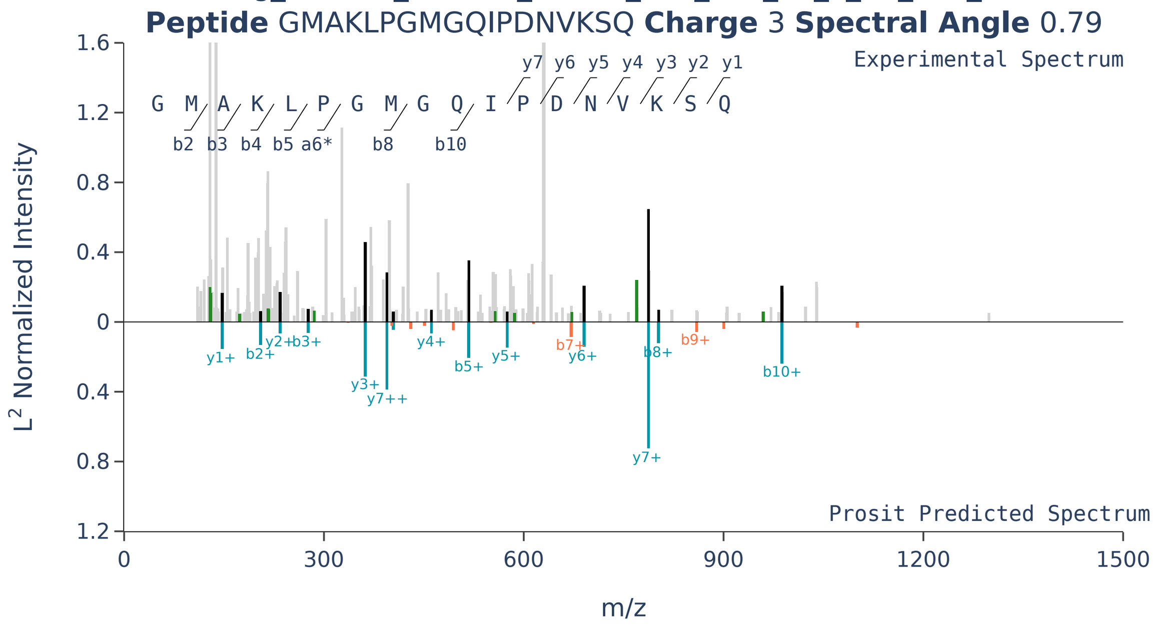

Source Ncheng_210623_230623_HFGoe_FFH_20S_25_1_A1_24h_R2 **Scan** 12401
Peptide TKETLAEQV **Charge** 2 **Spectral Angle** 0.68

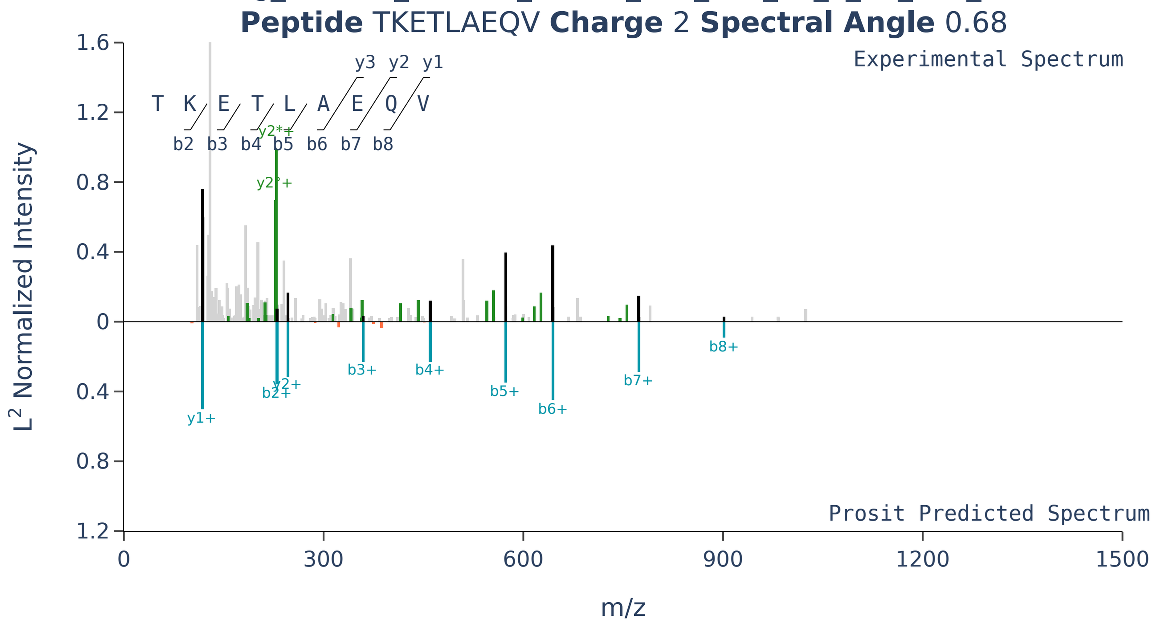

Source Ncheng_210623_230623_HFGoe_FFH_20S_25_1_A1_4h_R1 **Scan** 9498
Peptide QAEKLASSRTL **Charge** 2 **Spectral Angle** 0.85

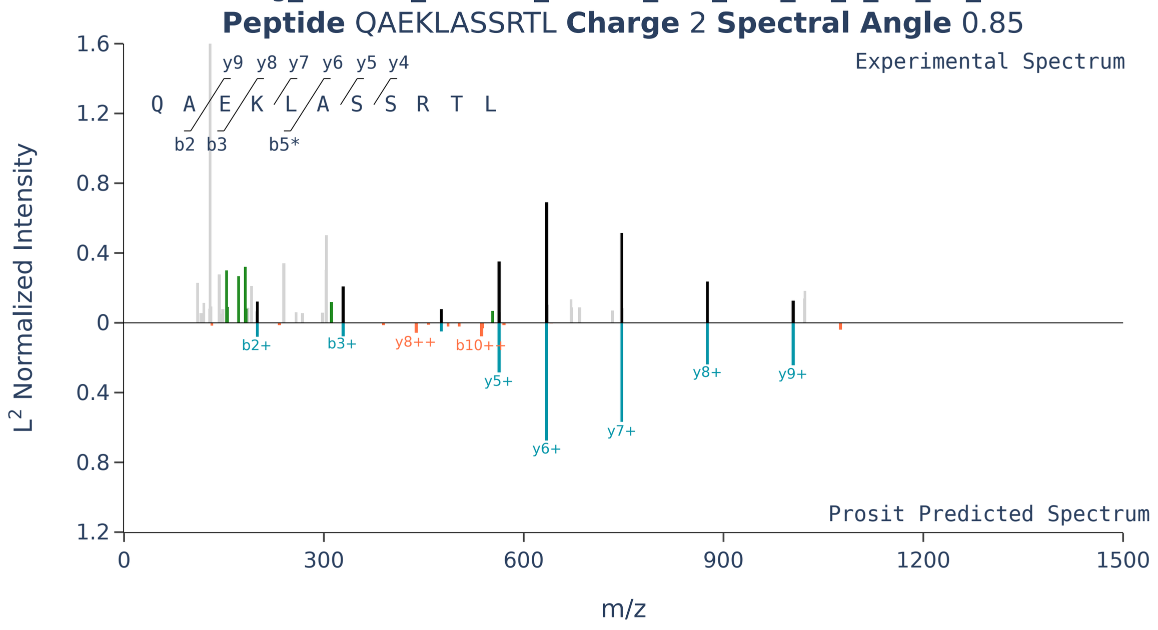

Source Ncheng_210623_230623_HFGoe_FFH_20S_25_1_A1_24h_R1 **Scan** 23470
Peptide GVGEKTVALPVVREF **Charge** 3 **Spectral Angle** 0.81

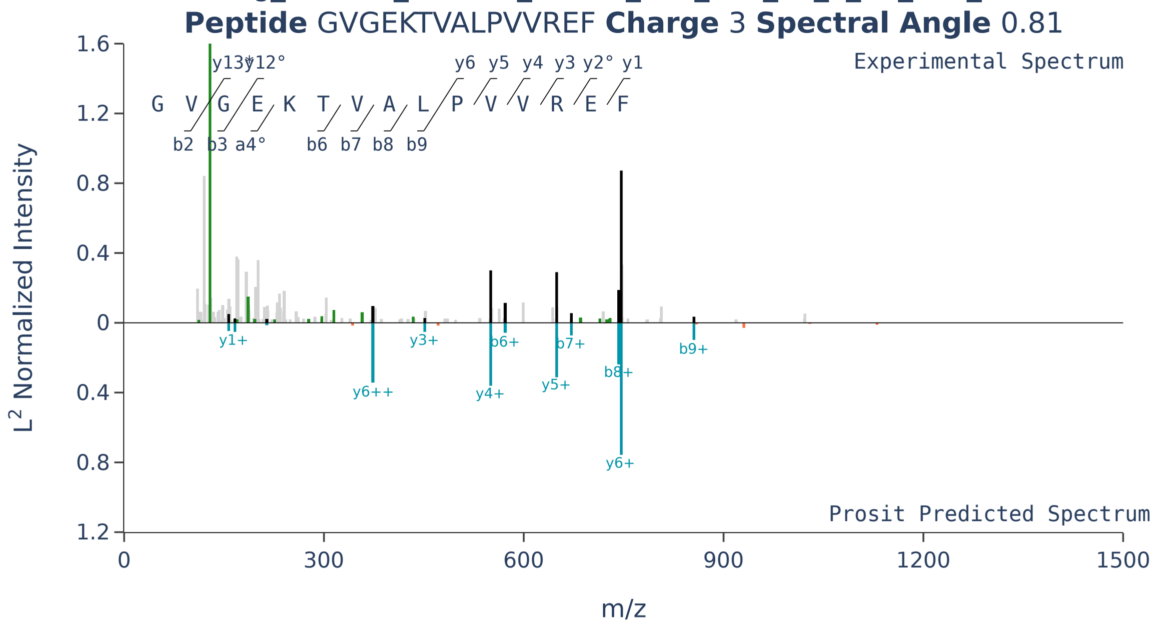

Source Ncheng_210623_230623_HFGoe_FFH_20S_25_1_A2_24h_R2 **Scan** 9140
Peptide AVGHEVNKSLTPGQD **Charge** 2 **Spectral Angle** 0.86

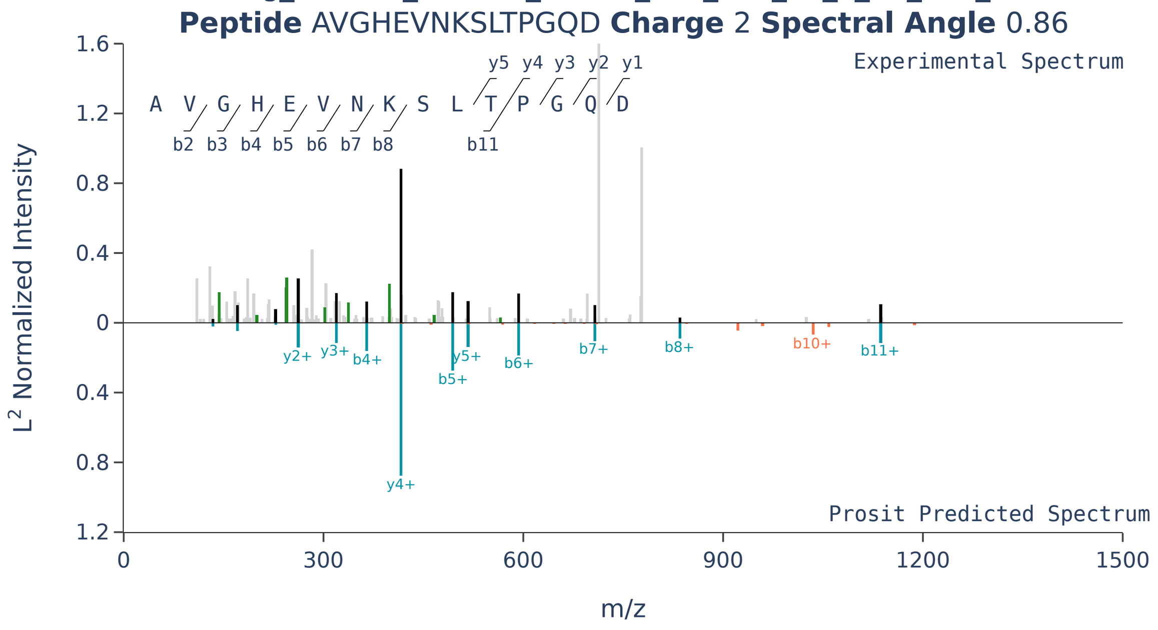

Source Ncheng_210623_230623_HFGoe_FFH_20S_25_1_A1_4h_R1 **Scan** 10874
Peptide ASKKGDGFDLND **Charge** 1 **Spectral Angle** 0.77

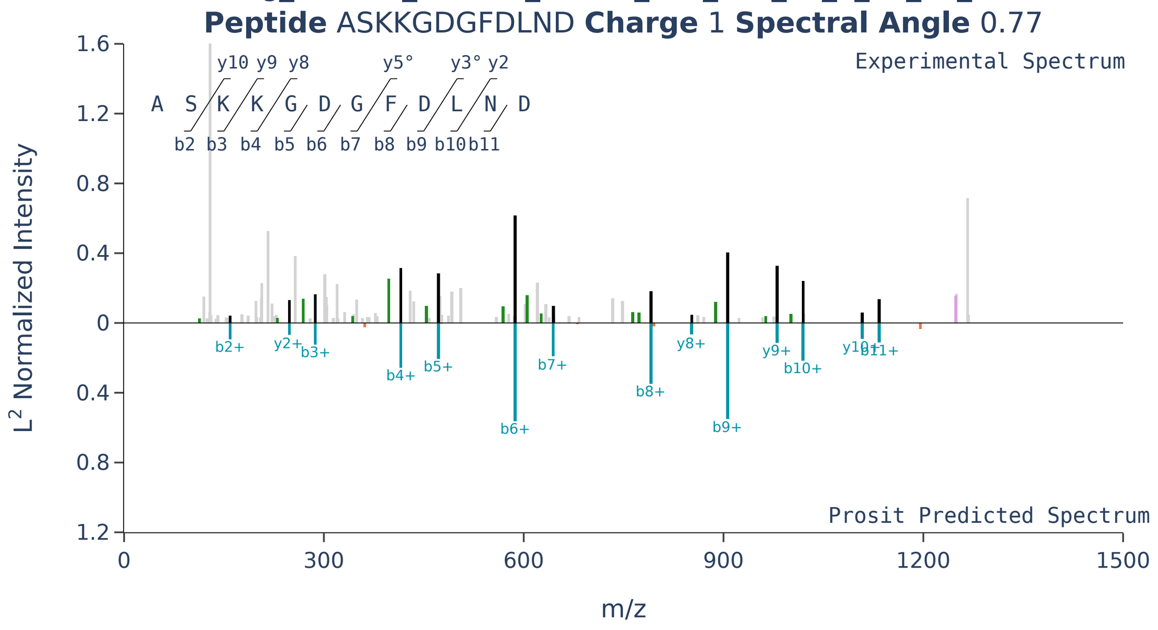

Source Ncheng_210623_230623_HFGoe_FFH_20S_25_1_A1_4h_R1 **Scan** 15330
Peptide KLKFYDVGQKPVD **Charge** 2 **Spectral Angle** 0.82

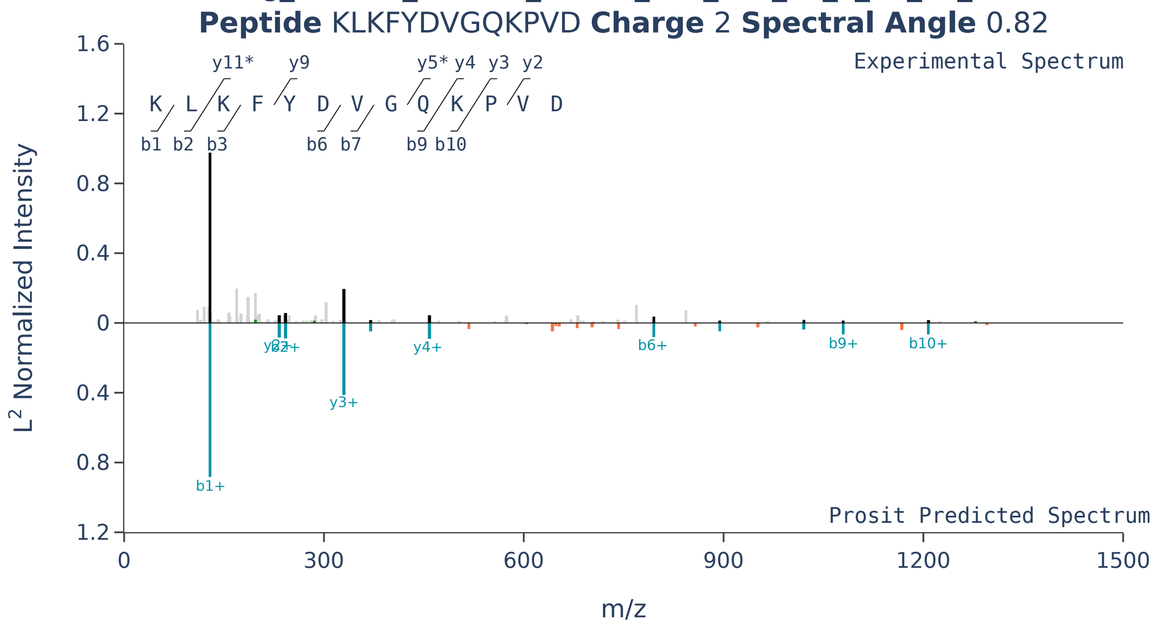

Source Ncheng_210623_230623_HFGoe_FFH_20S_25_1_A2_4h_R1 **Scan** 8709
Peptide AEQVVGQKPVD **Charge** 2 **Spectral Angle** 0.8

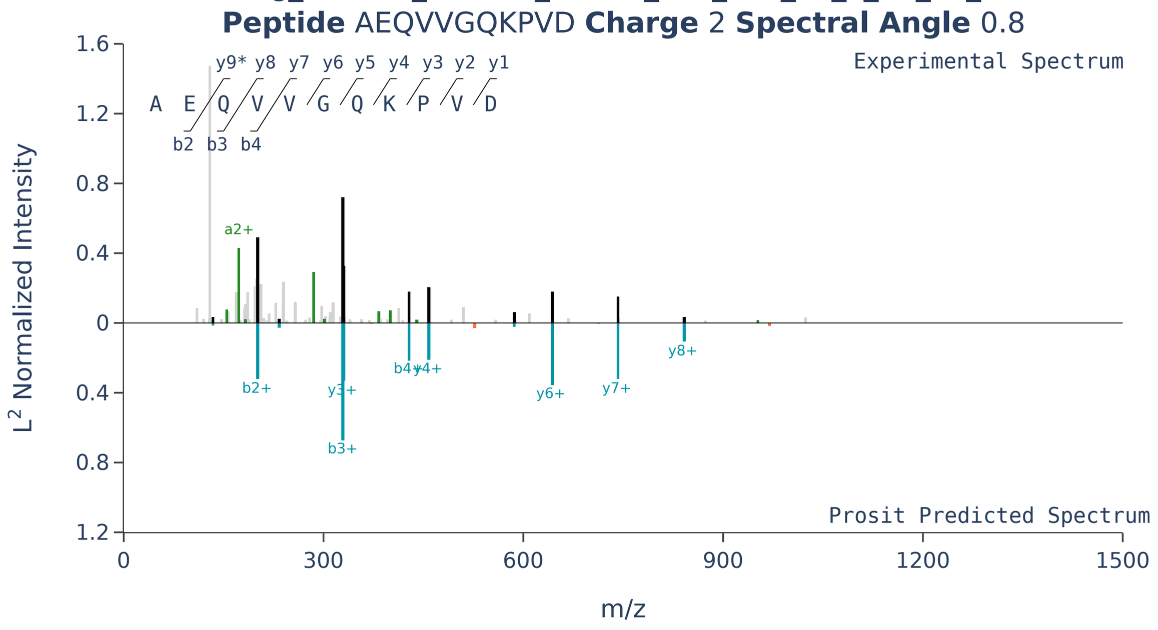

Source Ncheng_210623_230623_HFGoe_FFH_20S_25_1_A2_24h_R2 **Scan** 12252
Peptide GEENQTLRM **Charge** 2 **Spectral Angle** 0.77

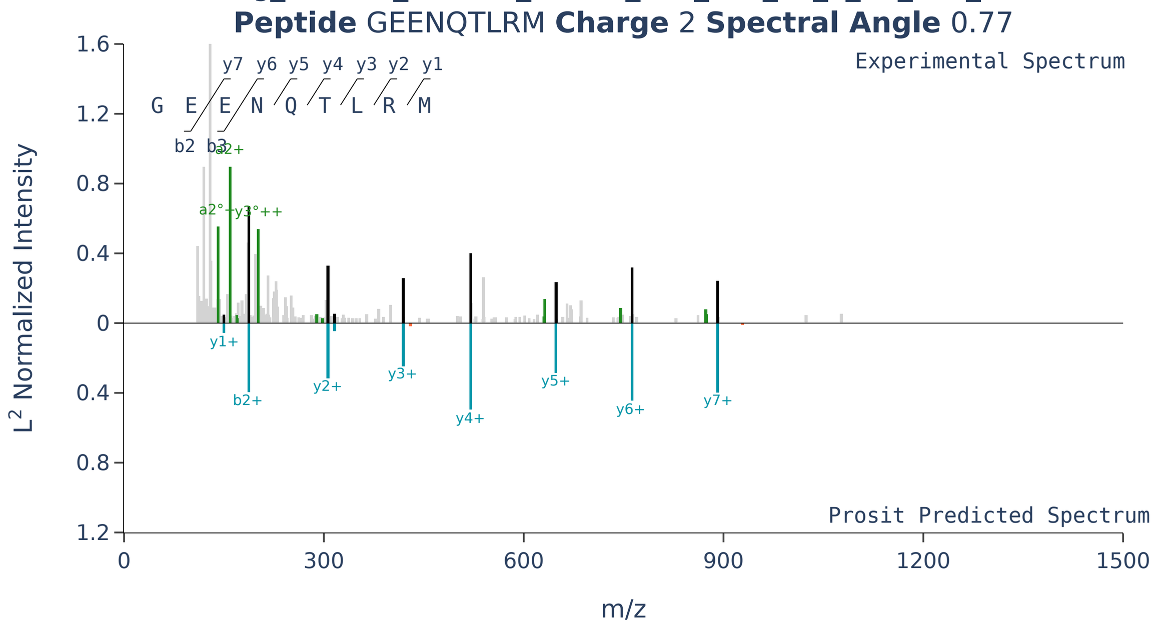

Source Ncheng_210623_230623_HFGoe_FFH_20S_25_1_A2_24h_R2 **Scan** 15240
Peptide TAMTGQDAANTAKAFNE **Charge** 2 **Spectral Angle** 0.84

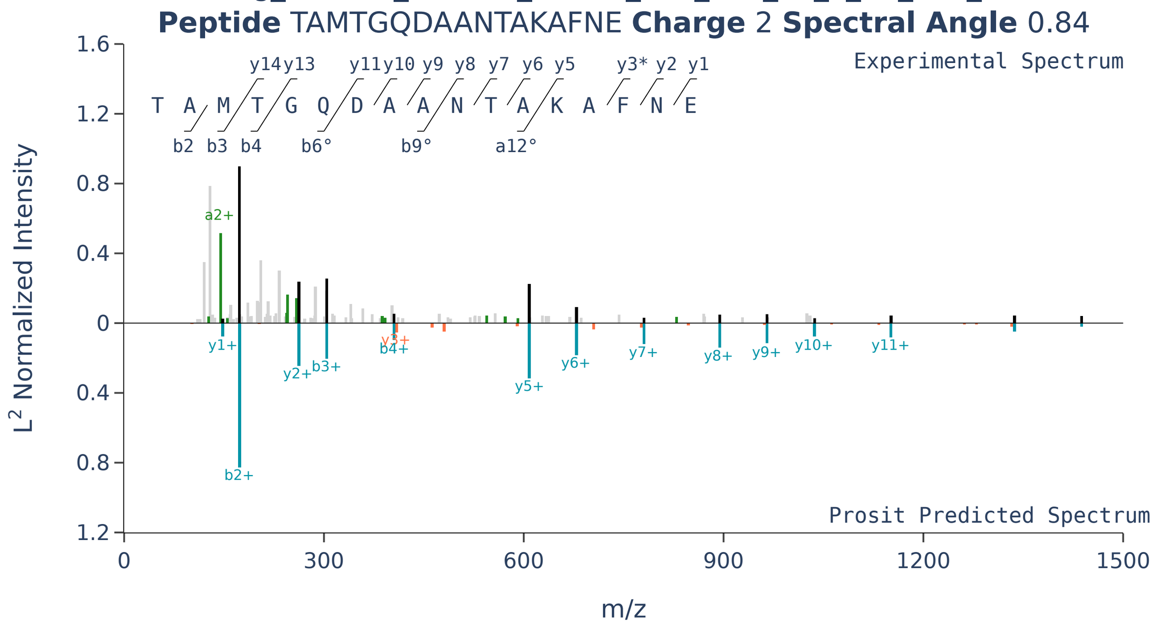

Source Ncheng_210623_230623_HFGoe_FFH_20S_25_1_A2_24h_R2 **Scan** 13441
Peptide KFYDLKE **Charge** 2 **Spectral Angle** 0.86

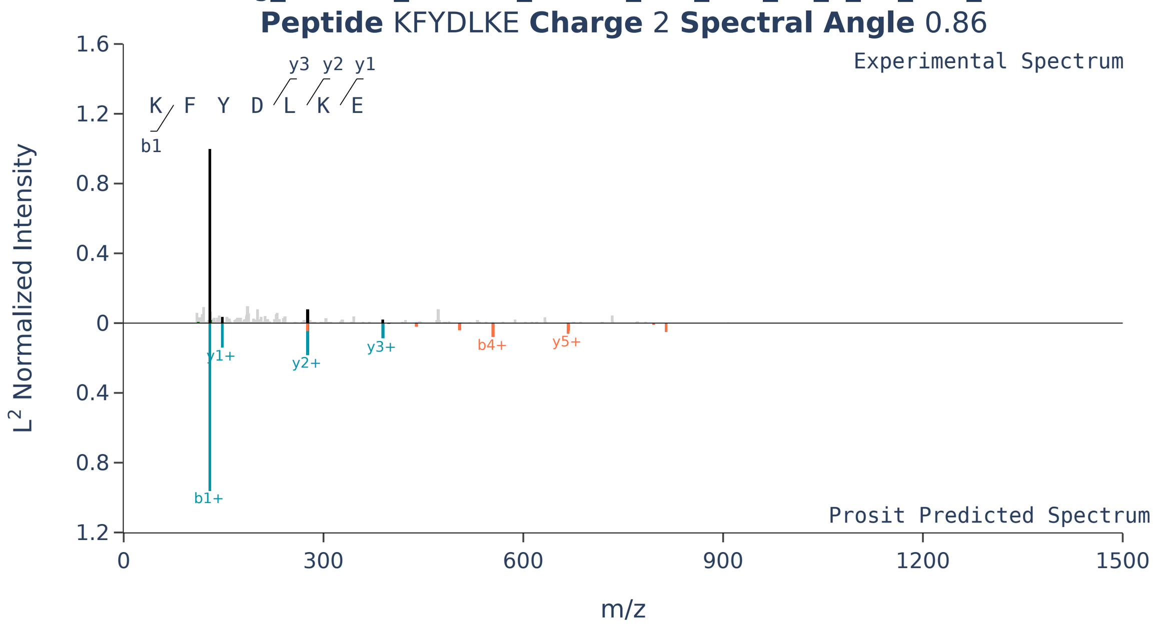

Source Ncheng_210623_230623_HFGoe_FFH_20S_25_1_A2_24h_R2 **Scan** 17719
Peptide KGSLKFYDV **Charge** 3 **Spectral Angle** 0.84

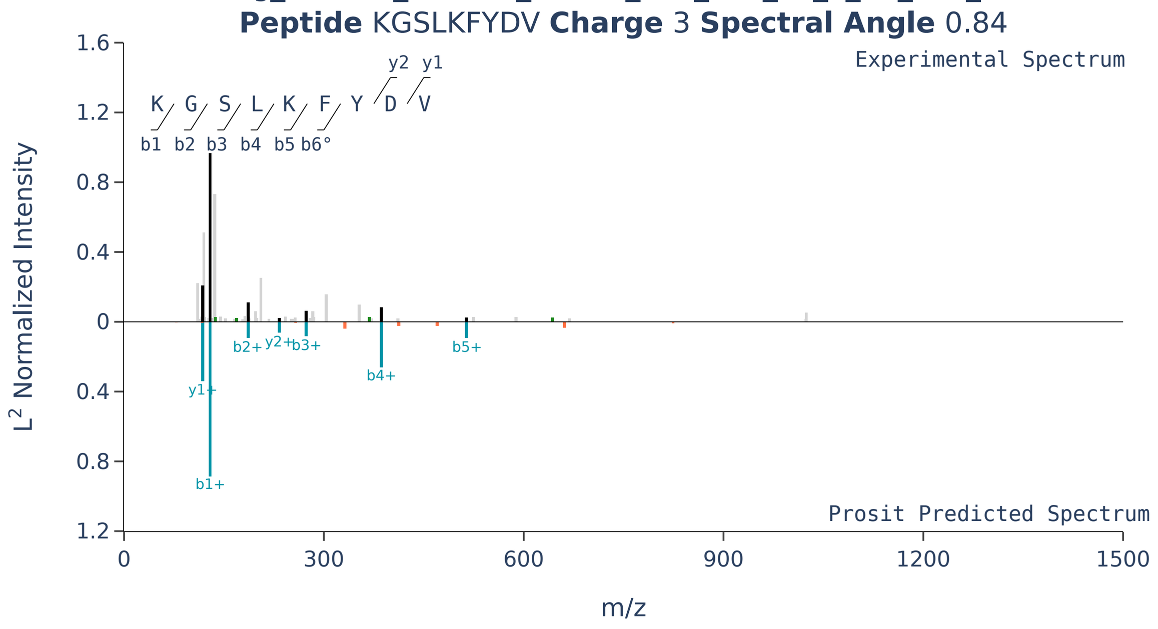

Source Ncheng_210623_230623_HFGoe_FFH_20S_25_1_A2_2h_R1 **Scan** 19140
Peptide GMKLPGMGQIPDNVKSQMDD **Charge** 3 **Spectral Angle** 0.76

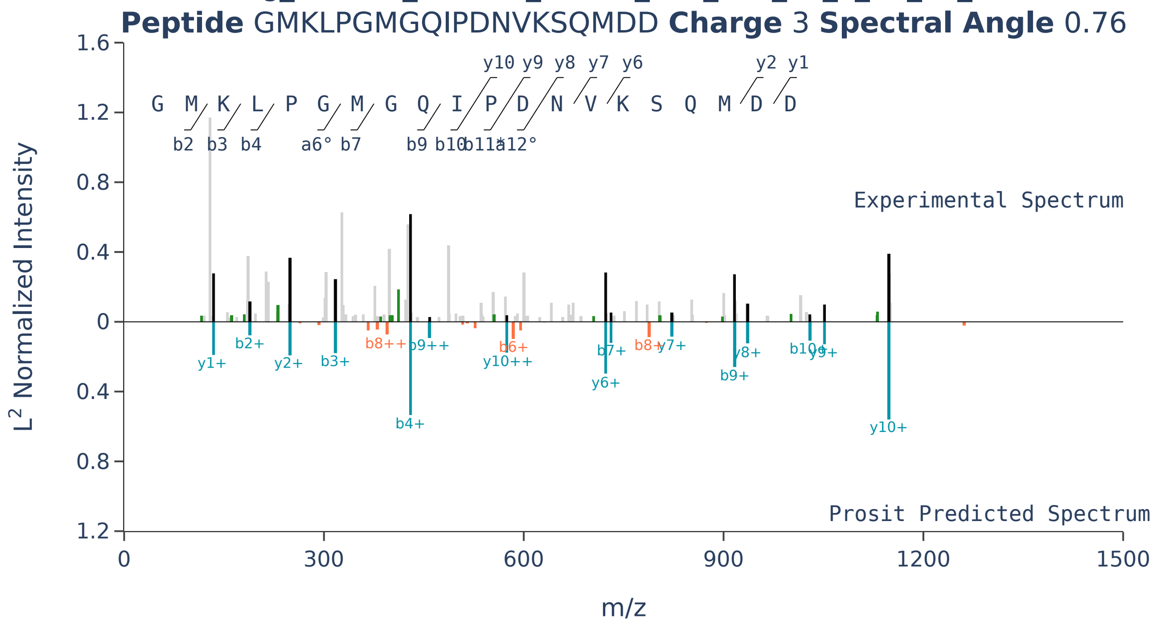

Source Ncheng_210623_230623_HFGoe_FFH_20S_25_1_A2_4h_R2 **Scan** 8206
Peptide SMVGQKPVD **Charge** 2 **Spectral Angle** 0.78

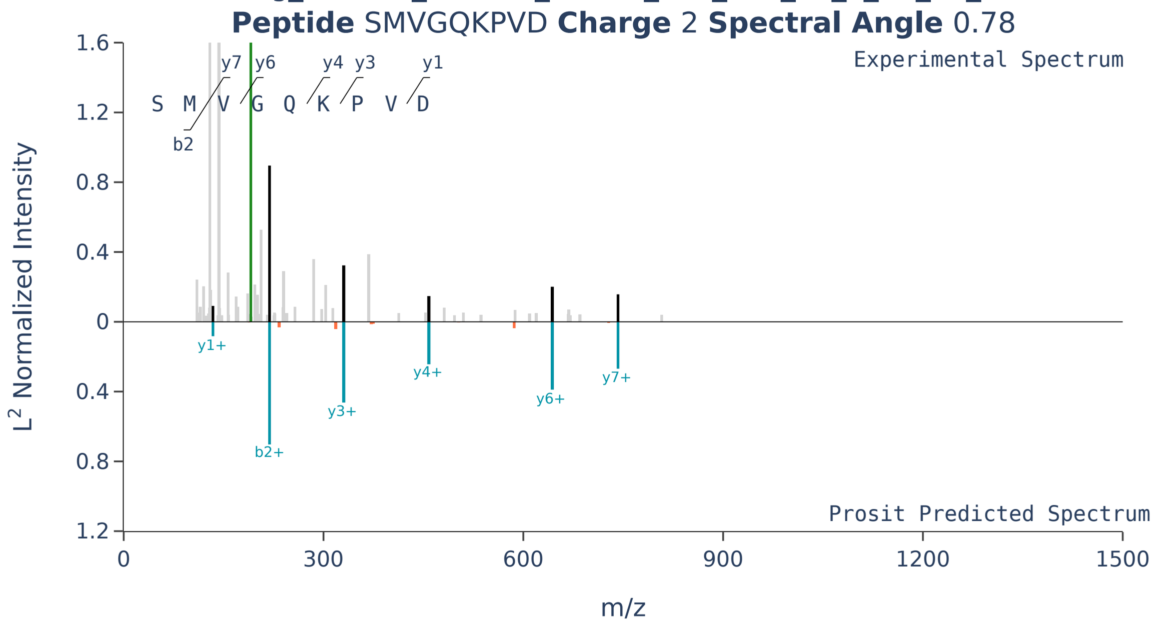

Source Ncheng_210623_230623_HFGoe_FFH_20S_25_1_A2_24h_R1 **Scan** 11845
Peptide HAAMTGQDAANTAKAFN **Charge** 3 **Spectral Angle** 0.73

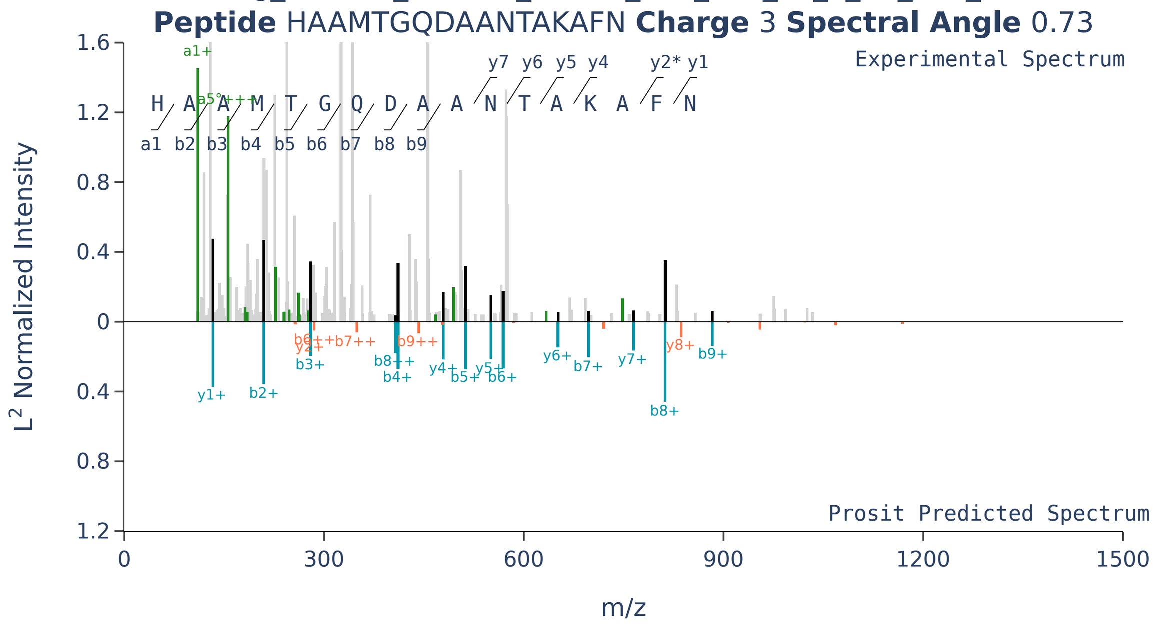

Source Ncheng_210623_230623_HFGoe_FFH_20S_25_1_A1_24h_R2 **Scan** 6468
Peptide VKIVRGDA **Charge** 2 **Spectral Angle** 0.89

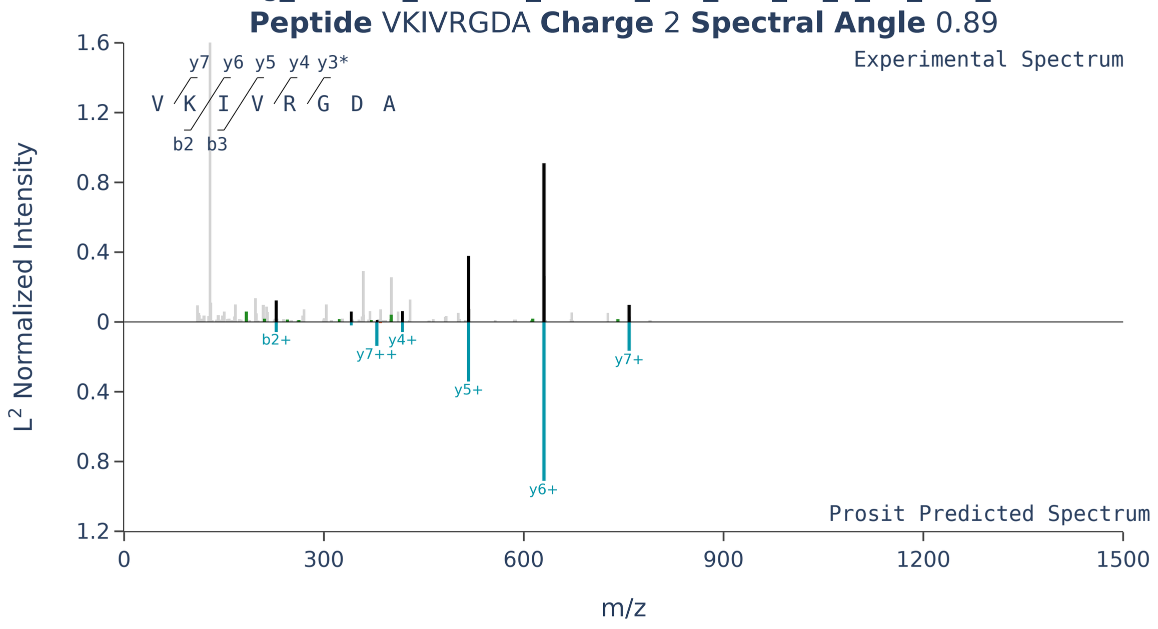

Source Ncheng_210623_230623_HFGoe_FFH_20S_25_1_A1_24h_R1 **Scan** 15572
Peptide VGQKPVDIVNANA **Charge** 1 **Spectral Angle** 0.79

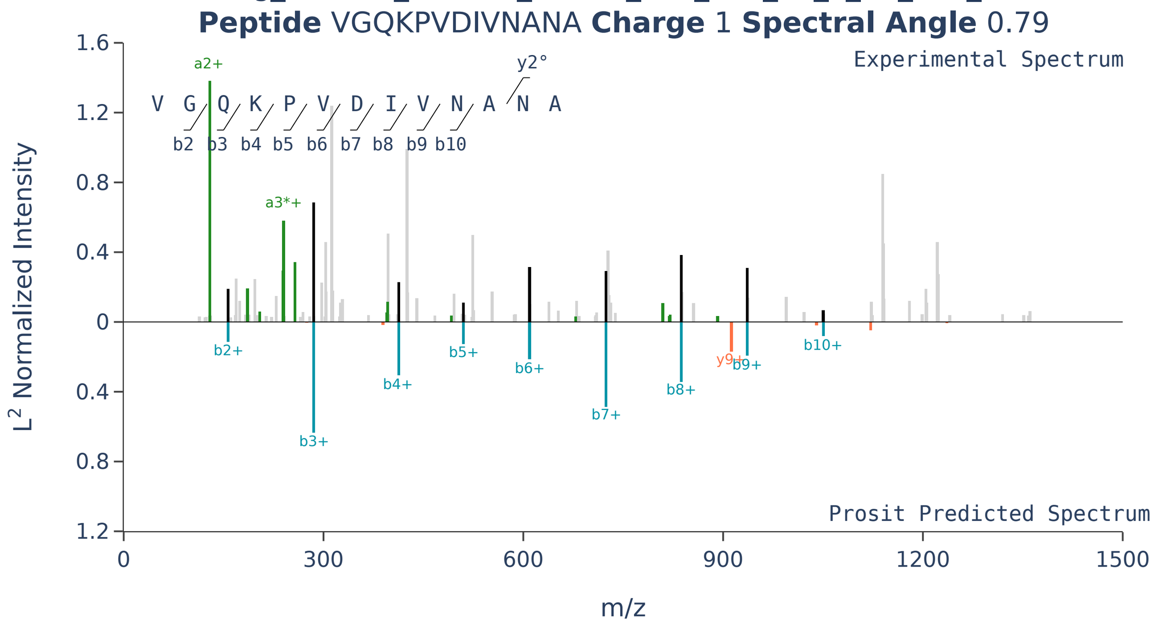

Source Ncheng_210623_230623_HFGoe_FFH_20S_25_1_A2_24h_R2 **Scan** 12287
Peptide KVLVVGQKPVD **Charge** 2 **Spectral Angle** 0.73

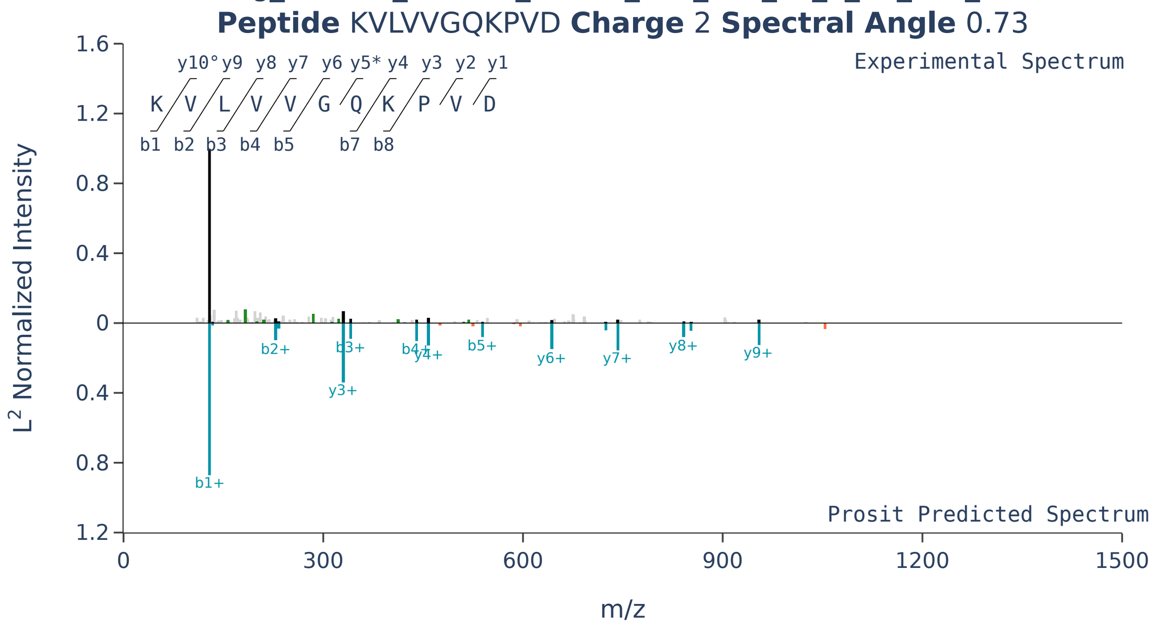

Source Ncheng_210623_230623_HFGoe_FFH_20S_25_1_A2_24h_R1 **Scan** 12184
Peptide ADVYRPAAIKQA **Charge** 2 **Spectral Angle** 0.77

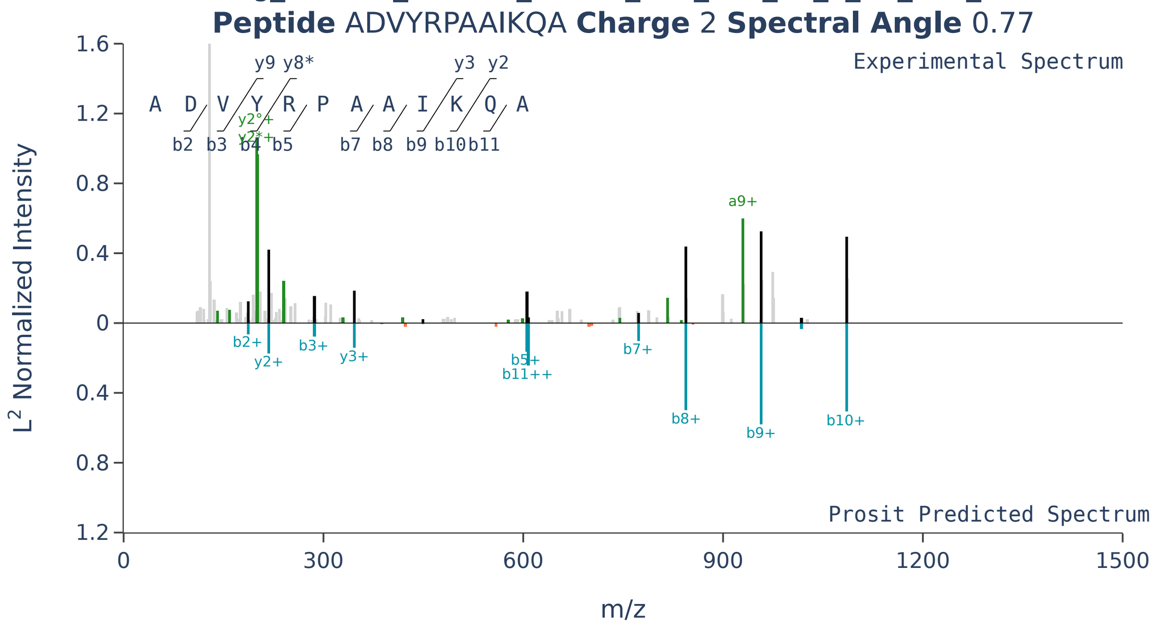

Source Ncheng_210623_230623_HFGoe_FFH_20S_25_1_A1_24h_R2 **Scan** 10595
Peptide PFHPDRVDTA **Charge** 2 **Spectral Angle** 0.85

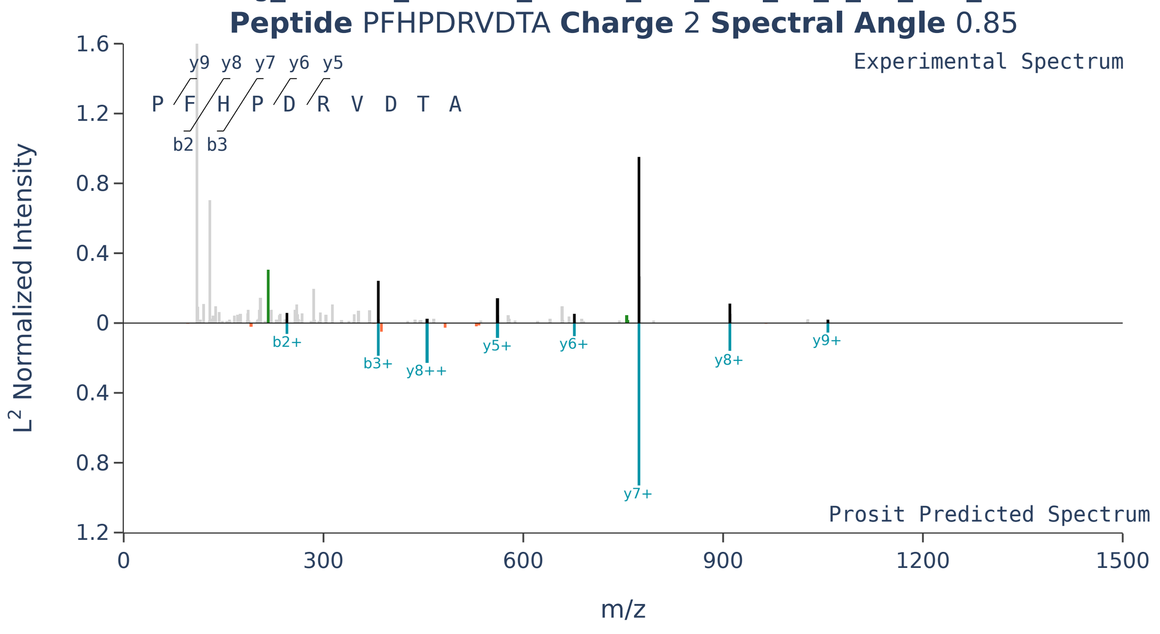

Source Ncheng_210623_230623_HFGoe_FFH_20S_25_1_A1_24h_R1 **Scan** 27461
Peptide RLVDFFPSD **Charge** 2 **Spectral Angle** 0.88

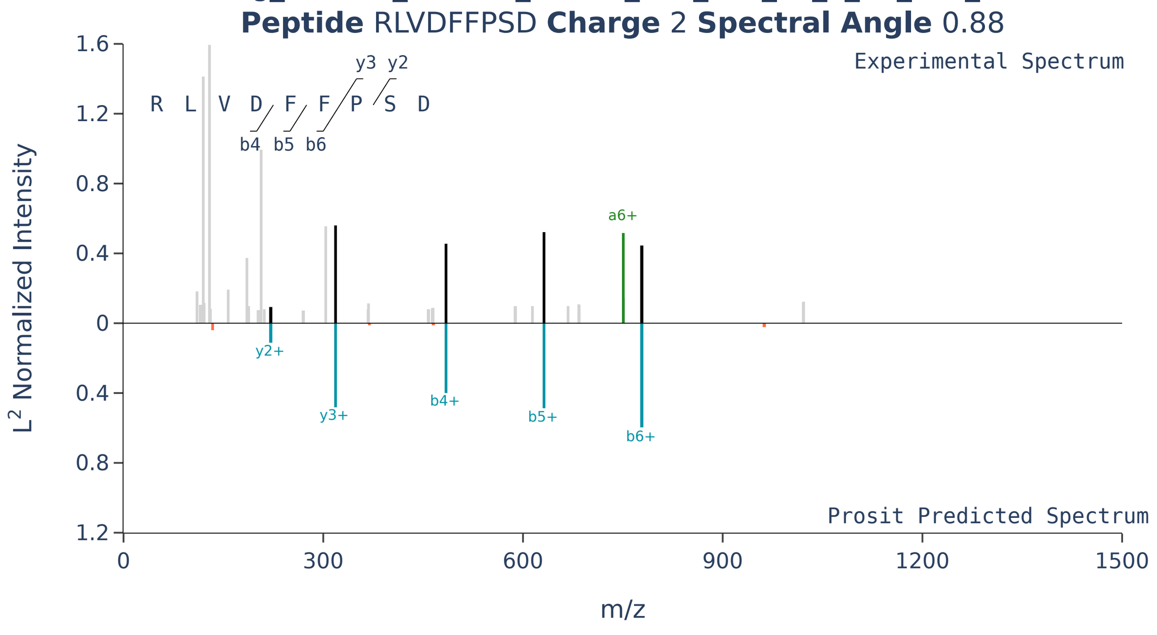

Source Ncheng_210623_230623_HFGoe_FFH_20S_25_1_A1_2h_R2 **Scan** 22962
Peptide TLAEQVGDIESKVDRA **Charge** 2 **Spectral Angle** 0.77

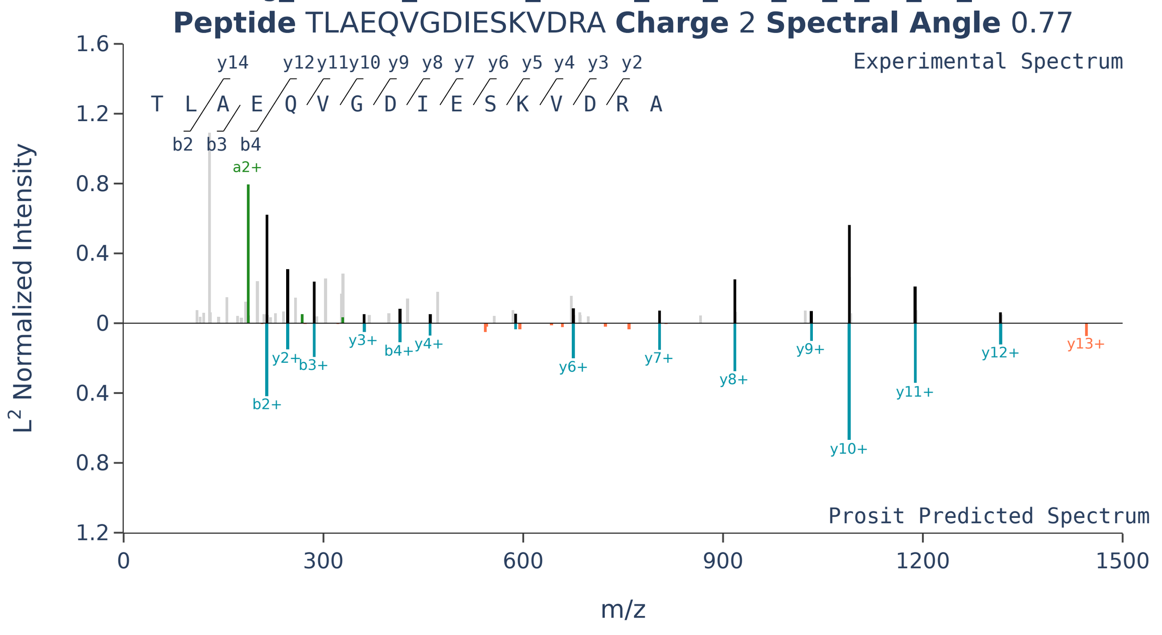

Source Ncheng_210623_230623_HFGoe_FFH_20S_25_1_A1_2h_R2 **Scan** 11160
Peptide MDIESKVDRA **Charge** 3 **Spectral Angle** 0.79

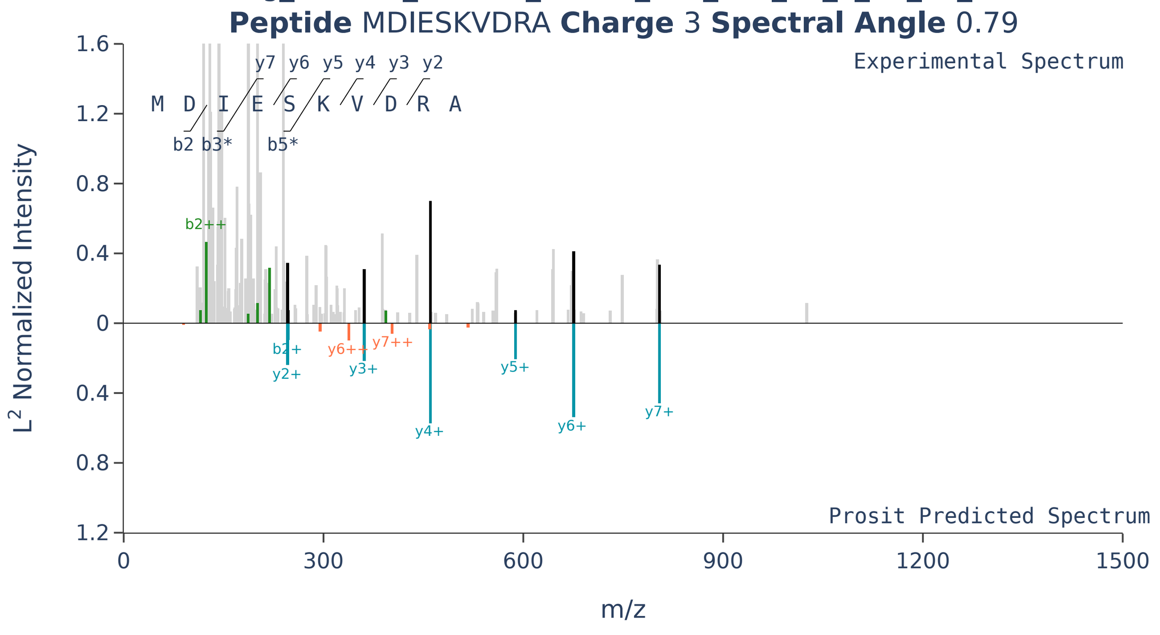

Source Ncheng_210623_230623_HFGoe_FFH_20S_25_1_A1_4h_R2 **Scan** 8796
Peptide ALKEARL **Charge** 2 **Spectral Angle** 0.87

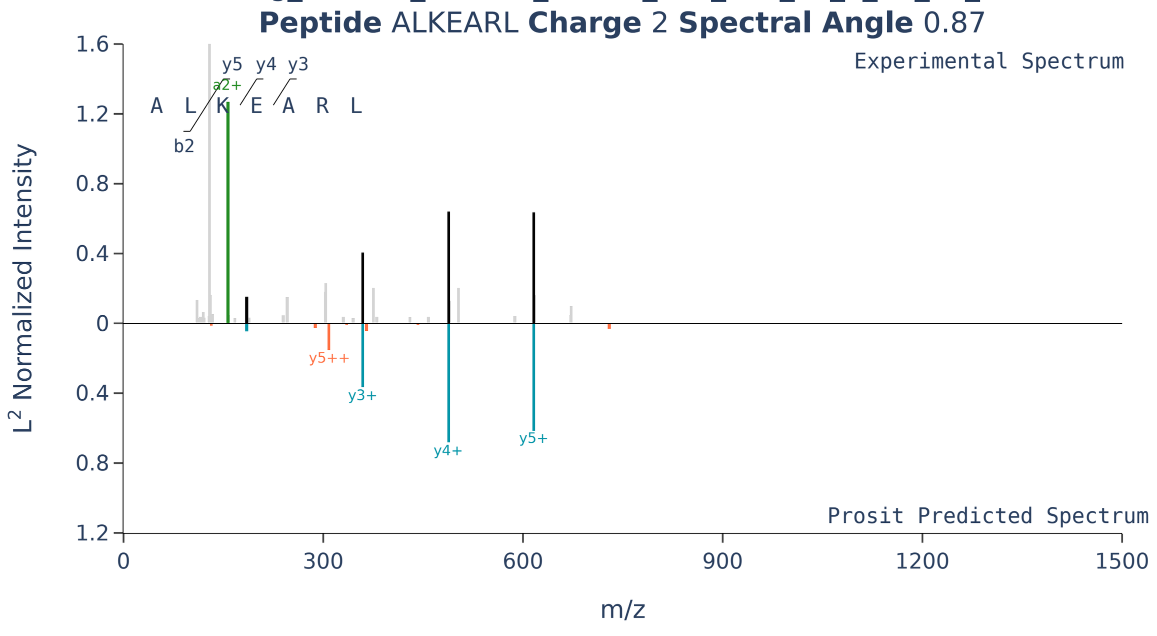

Source Ncheng_210623_230623_HFGoe_FFH_20S_25_1_A1_24h_R1 **Scan** 19240
Peptide ALEPFHPDRIDTA **Charge** 2 **Spectral Angle** 0.79

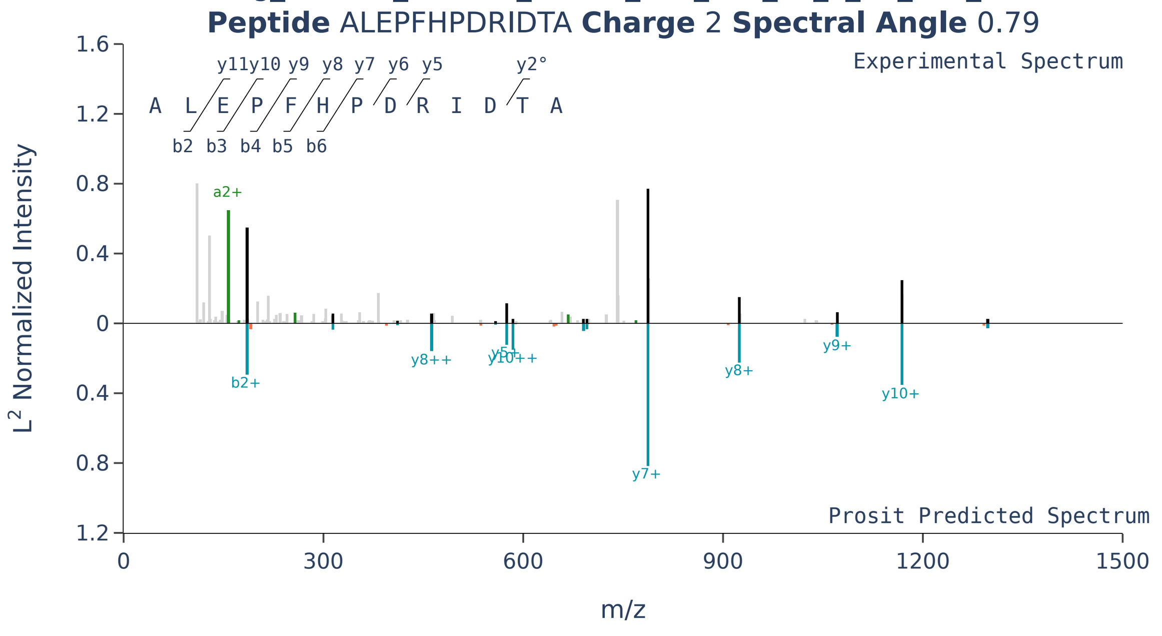

Source Ncheng_210623_230623_HFGoe_FFH_20S_25_1_A1_24h_R1 **Scan** 9747
Peptide KAFNEVGQKPVD **Charge** 2 **Spectral Angle** 0.74

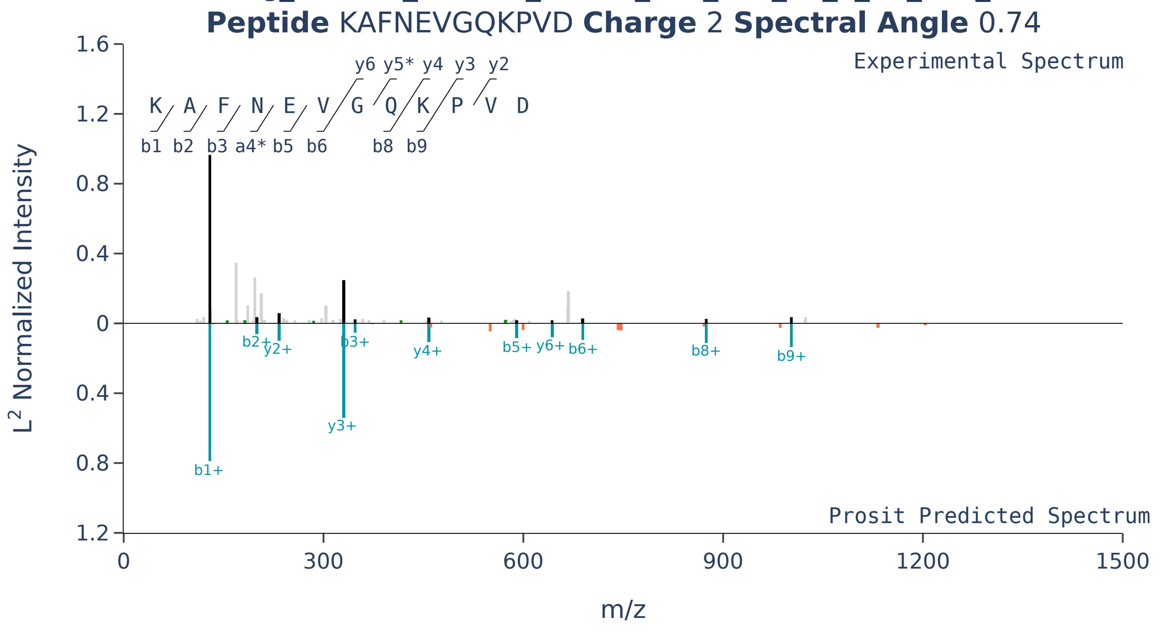

Source Ncheng_210623_230623_HFGoe_FFH_20S_25_1_A2_4h_R1 **Scan** 21465
Peptide ESKPIKFLGVGE **Charge** 2 **Spectral Angle** 0.76

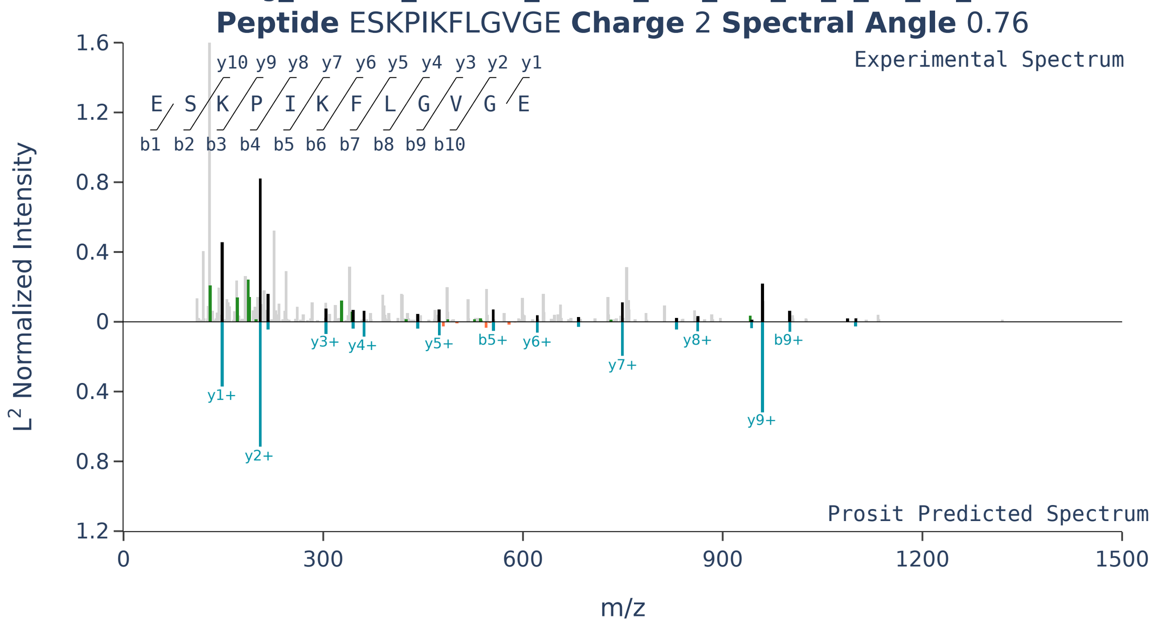

Source Ncheng_210623_230623_HFGoe_FFH_20S_25_1_A2_24h_R2 **Scan** 15267
Peptide PGMGQIPDNVKST **Charge** 2 **Spectral Angle** 0.76

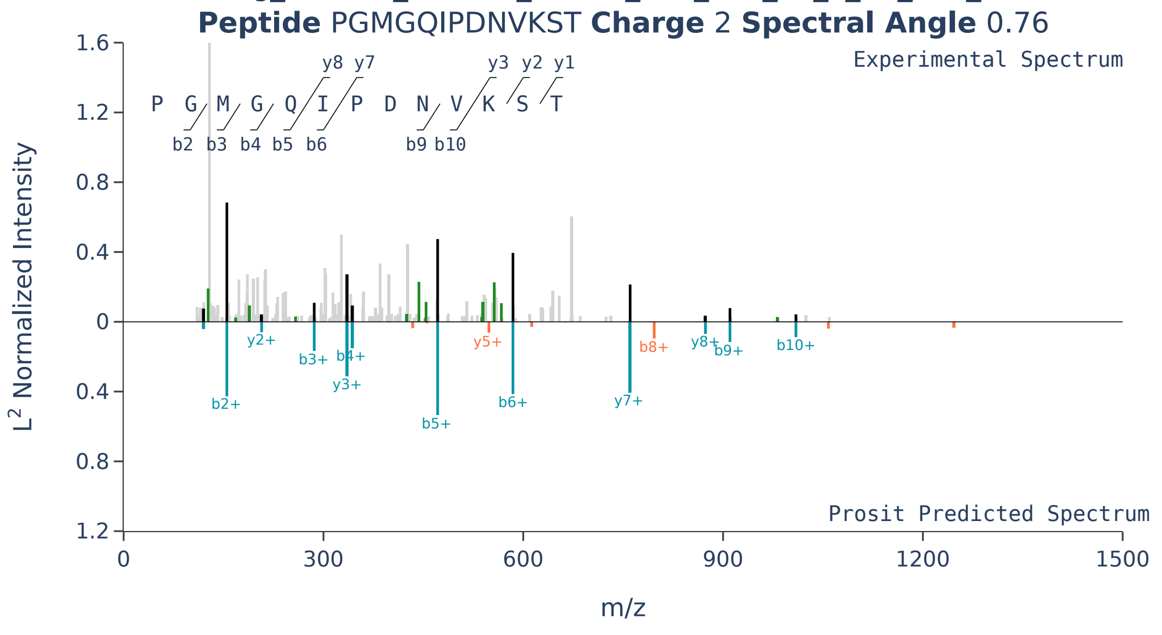

Source Ncheng_210623_230623_HFGoe_FFH_20S_25_1_A1_2h_R2 **Scan** 9957
Peptide AGCMQVQD **Charge** 1 **Spectral Angle** 0.62

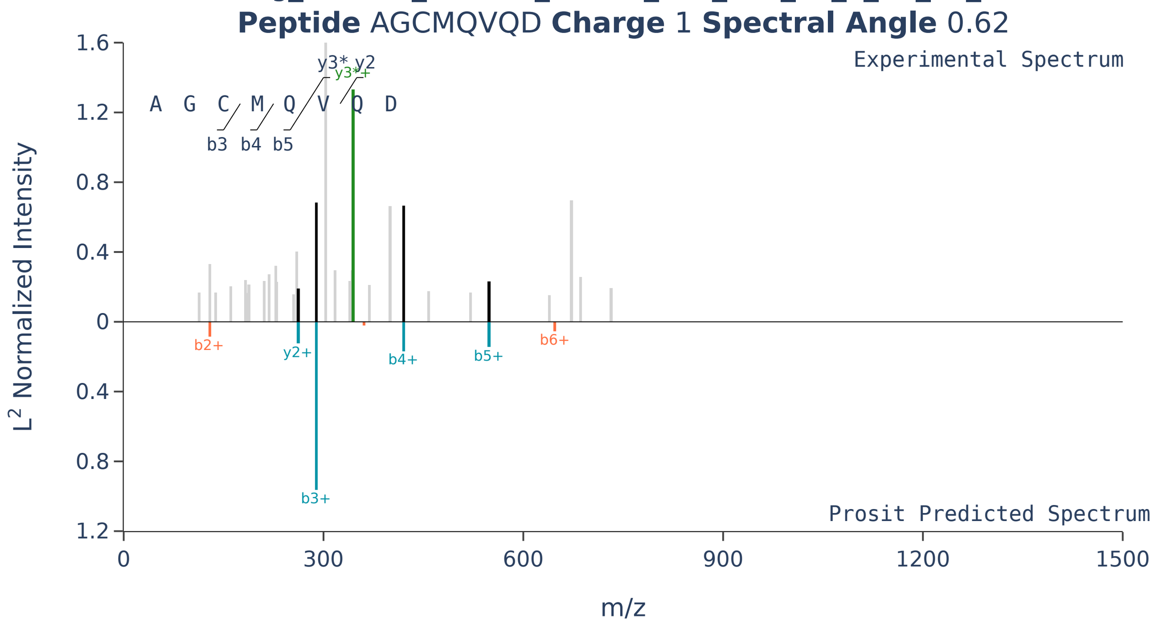

Source Ncheng_210623_230623_HFGoe_FFH_20S_25_1_A2_24h_R2 **Scan** 6831
Peptide ALKEARME **Charge** 2 **Spectral Angle** 0.84

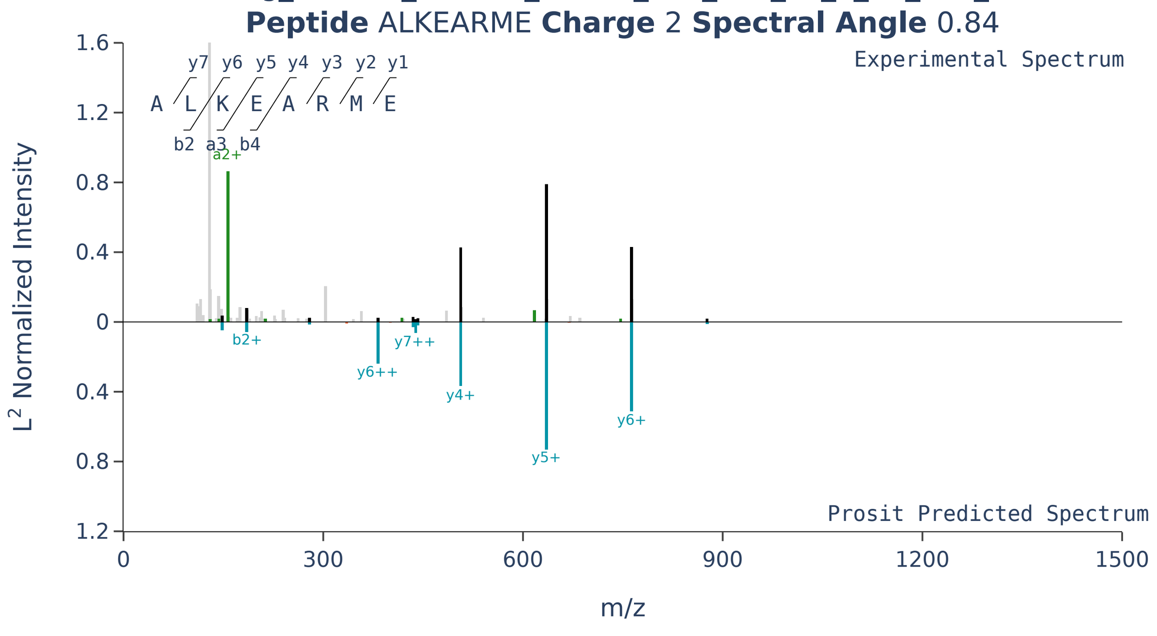

Source Ncheng_210623_230623_HFGoe_FFH_20S_25_1_A1_4h_R1 **Scan** 11014
Peptide TAGRLHVDEVGQKPVD **Charge** 2 **Spectral Angle** 0.76

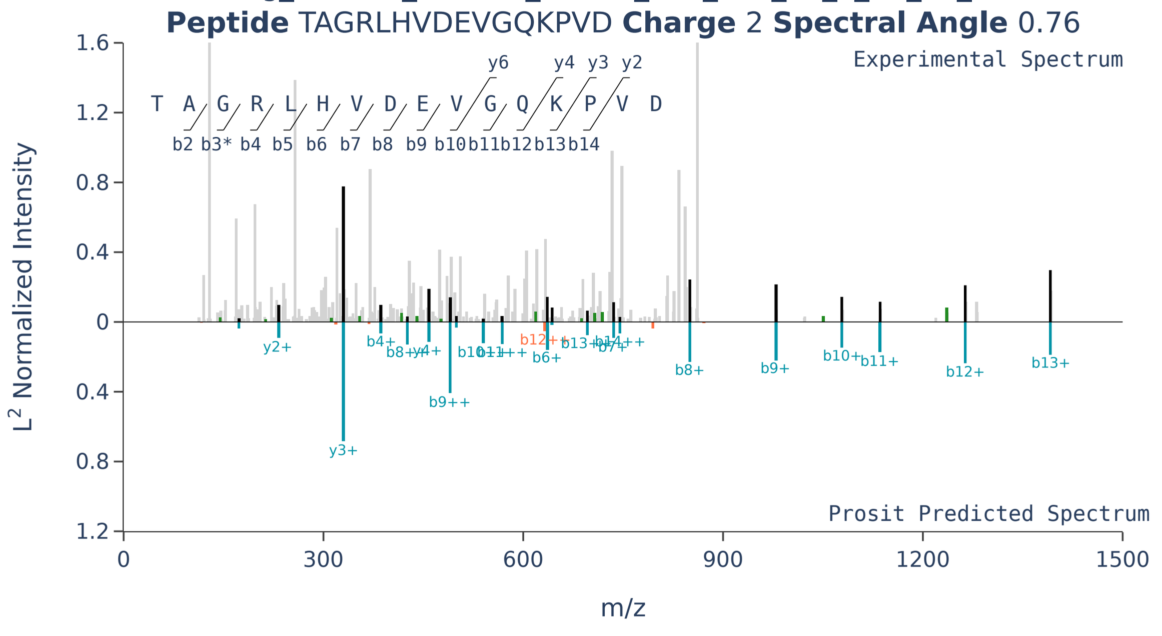

Source Ncheng_210623_230623_HFGoe_FFH_20S_25_1_A1_4h_R1 **Scan** 25670
Peptide DIESKLKFYDV **Charge** 2 **Spectral Angle** 0.8

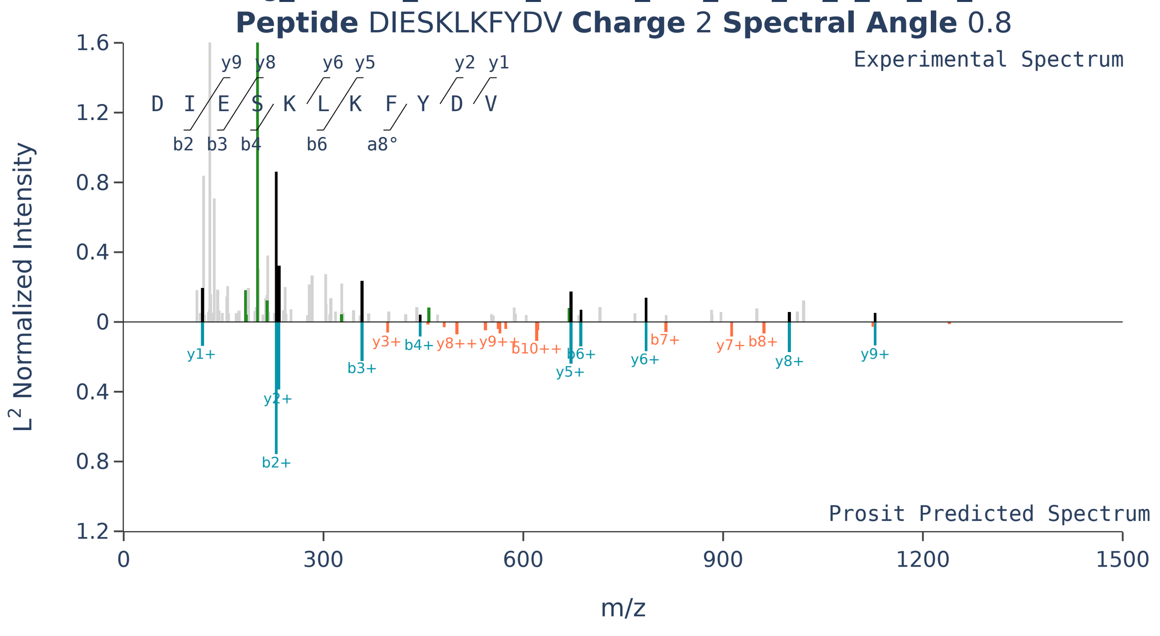

Source Ncheng_210623_230623_HFGoe_FFH_20S_25_1_A1_24h_R1 **Scan** 13632
Peptide GQIPDNVKVS **Charge** 2 **Spectral Angle** 0.88

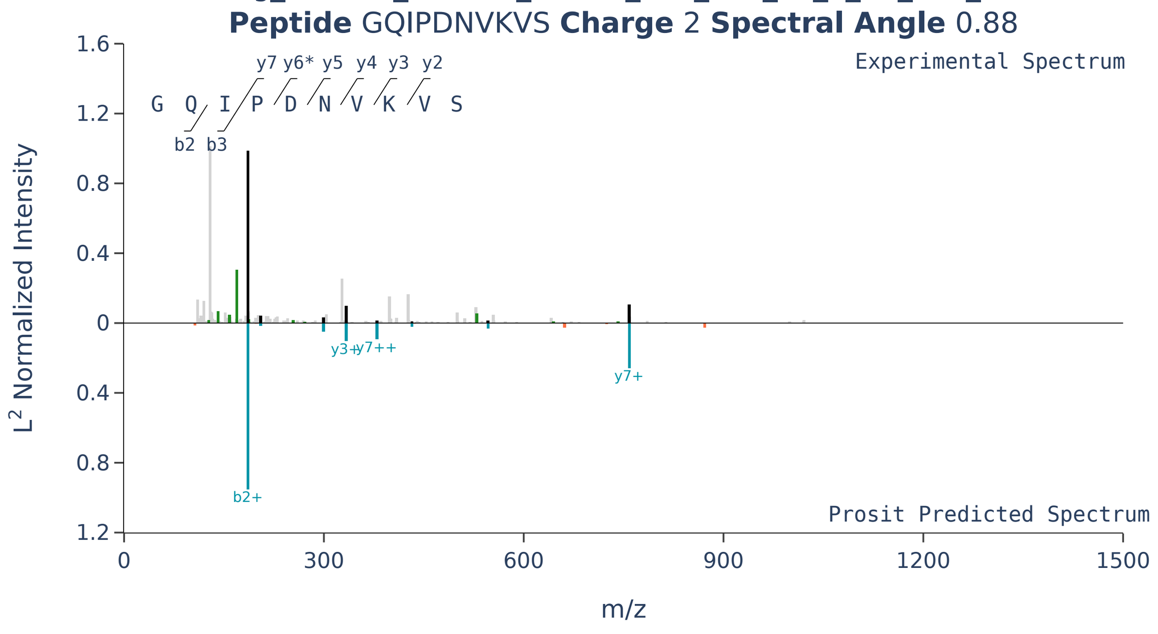

Source Ncheng_210623_230623_HFGoe_FFH_20S_25_1_A2_24h_R2 **Scan** 10292
Peptide NKMGGMASL **Charge** 2 **Spectral Angle** 0.74

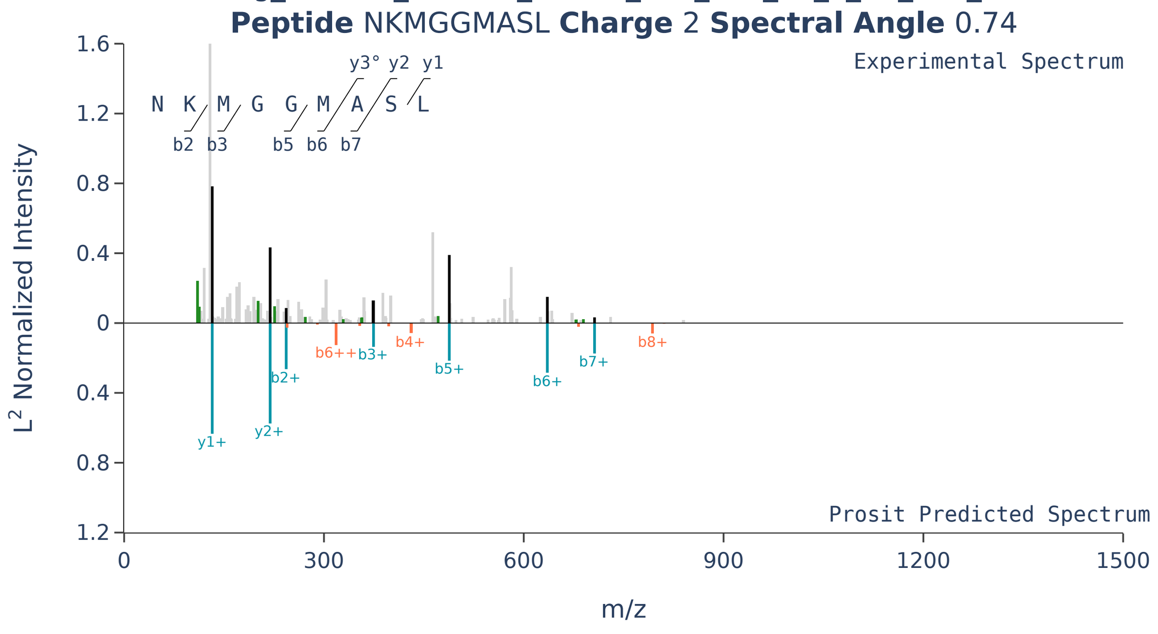

Source Ncheng_210623_230623_HFGoe_FFH_20S_25_1_A1_24h_R2 **Scan** 8031
Peptide ADIESKVDRA **Charge** 2 **Spectral Angle** 0.76

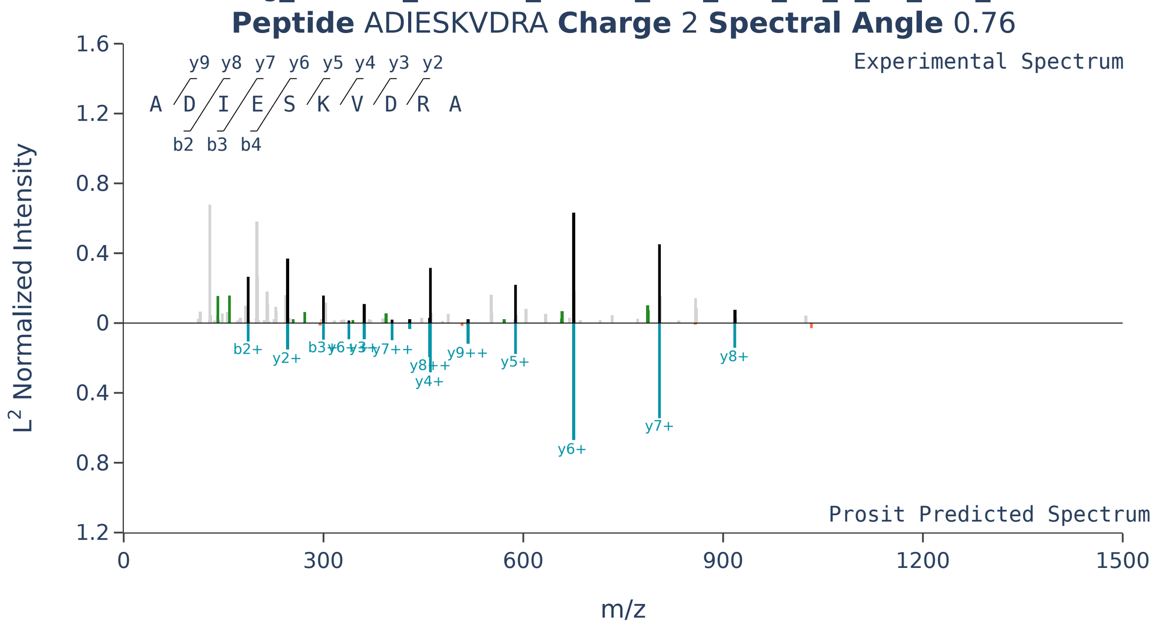

Source Ncheng_210623_230623_HFGoe_FFH_20S_25_1_A1_24h_R2 **Scan** 14090
Peptide KVDAMTGQDAANTAKAFN **Charge** 3 **Spectral Angle** 0.75

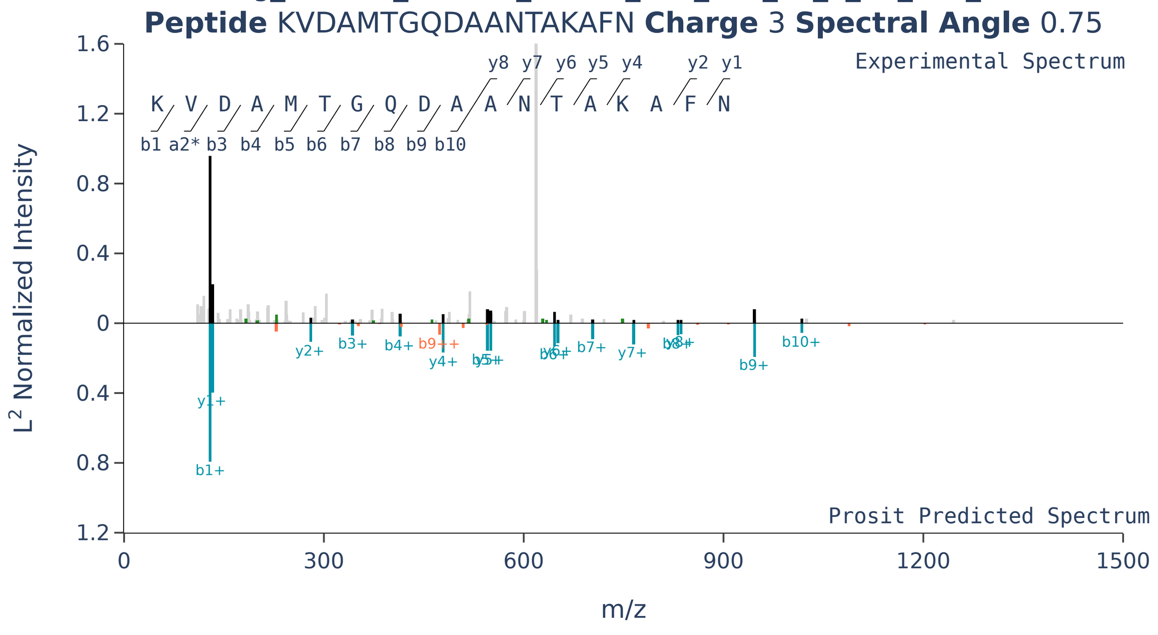

Source Ncheng_210623_230623_HFGoe_FFH_20S_25_1_A2_24h_R2 **Scan** 21605
Peptide GGMKLPGMGQIPDNVKS **Charge** 2 **Spectral Angle** 0.87

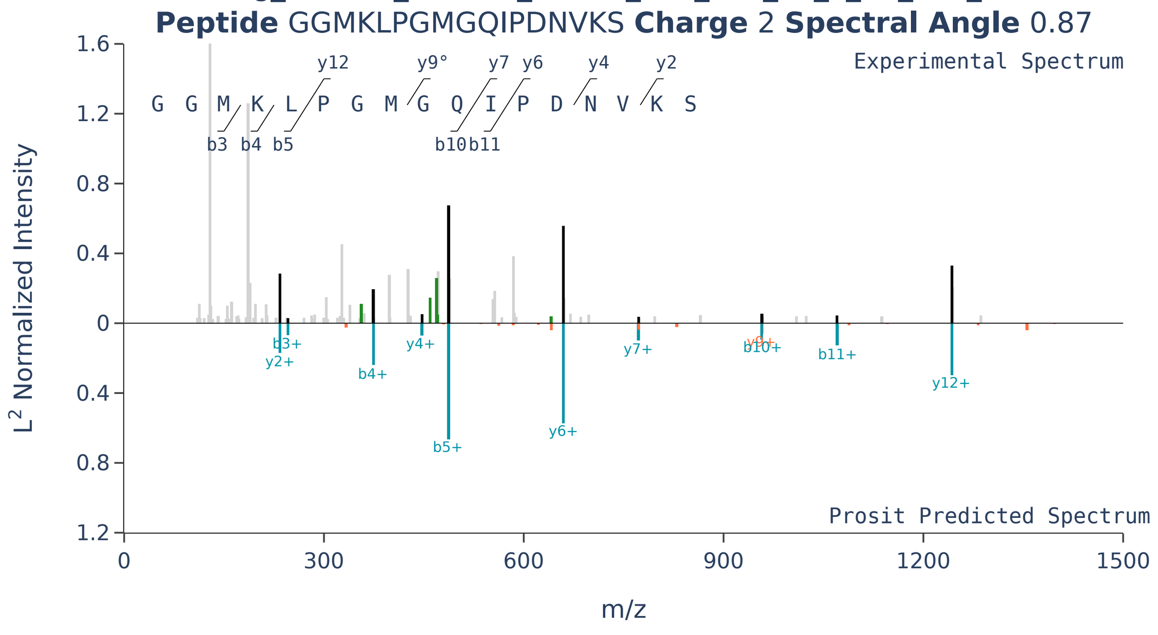

Source Ncheng_210623_230623_HFGoe_FFH_20S_25_1_A1_24h_R1 **Scan** 15683
Peptide GEKTEENQTLNL **Charge** 2 **Spectral Angle** 0.83

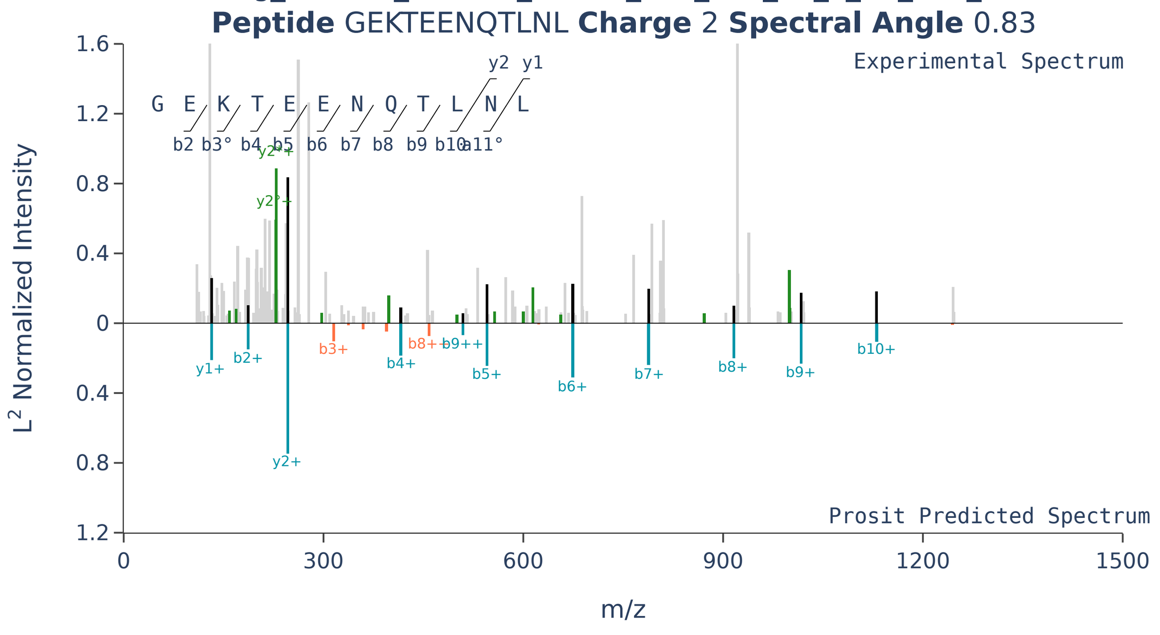

Source Ncheng_210623_230623_HFGoe_FFH_20S_25_1_A1_24h_R2 **Scan** 8418
Peptide VDVGQKPVD **Charge** 2 **Spectral Angle** 0.75

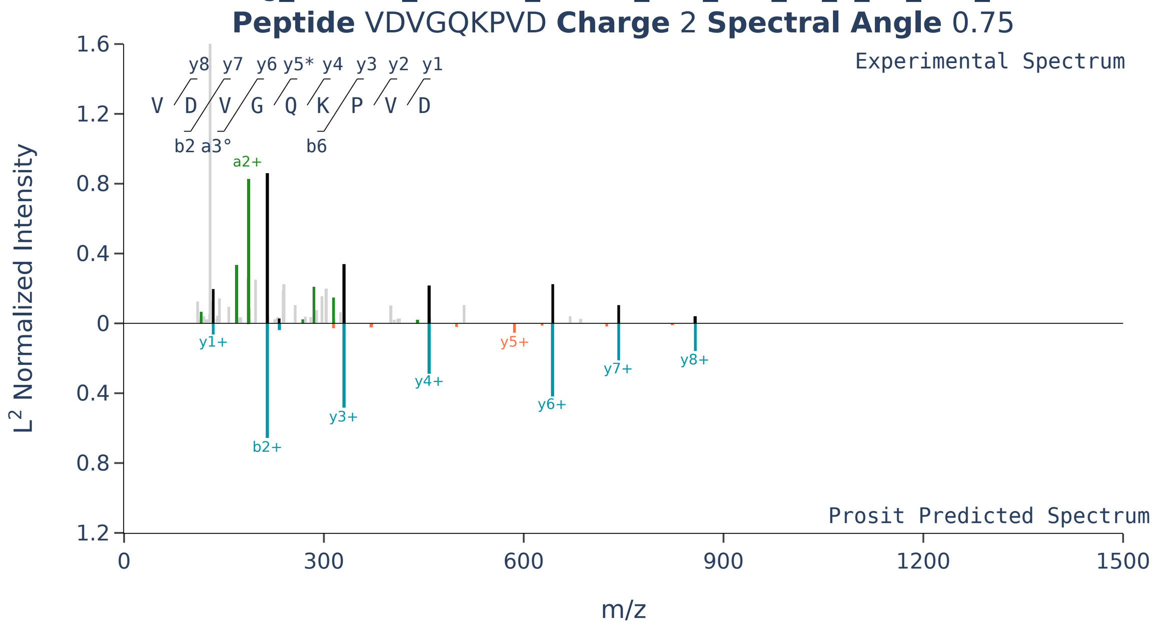

Source Ncheng_210623_230623_HFGoe_FFH_20S_25_1_A2_24h_R2 **Scan** 9057
Peptide GLQGAPGQ **Charge** 1 **Spectral Angle** 0.82

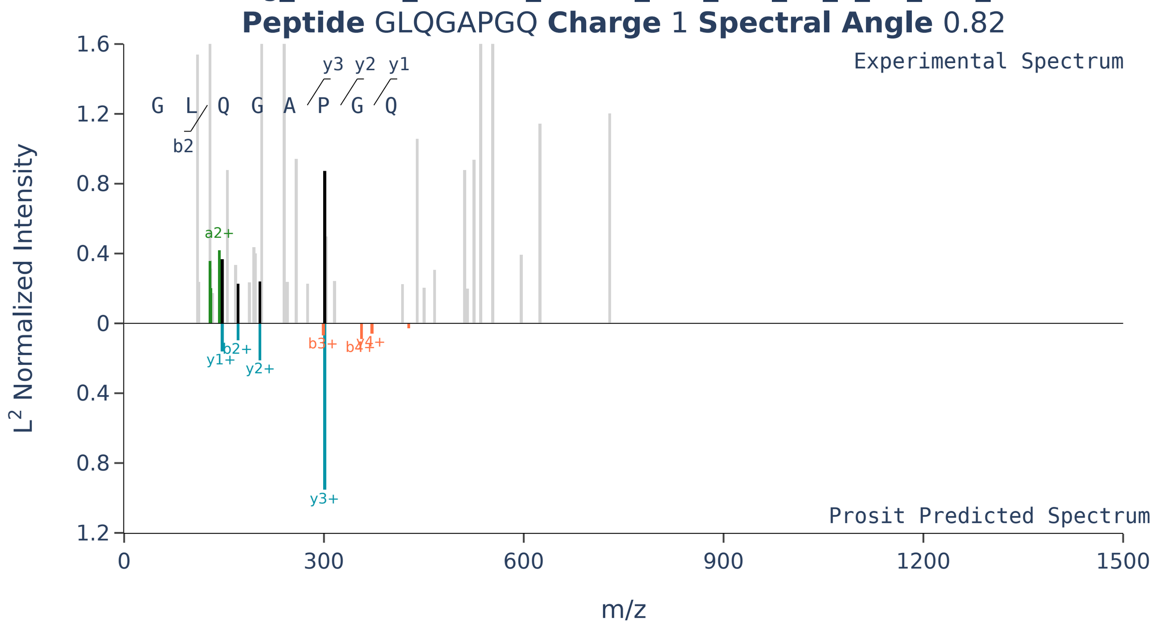

Source Ncheng_210623_230623_HFGoe_FFH_20S_25_1_A2_2h_R2 **Scan** 17909
Peptide KLPGMGMGQIPDNVKSQMD **Charge** 3 **Spectral Angle** 0.78

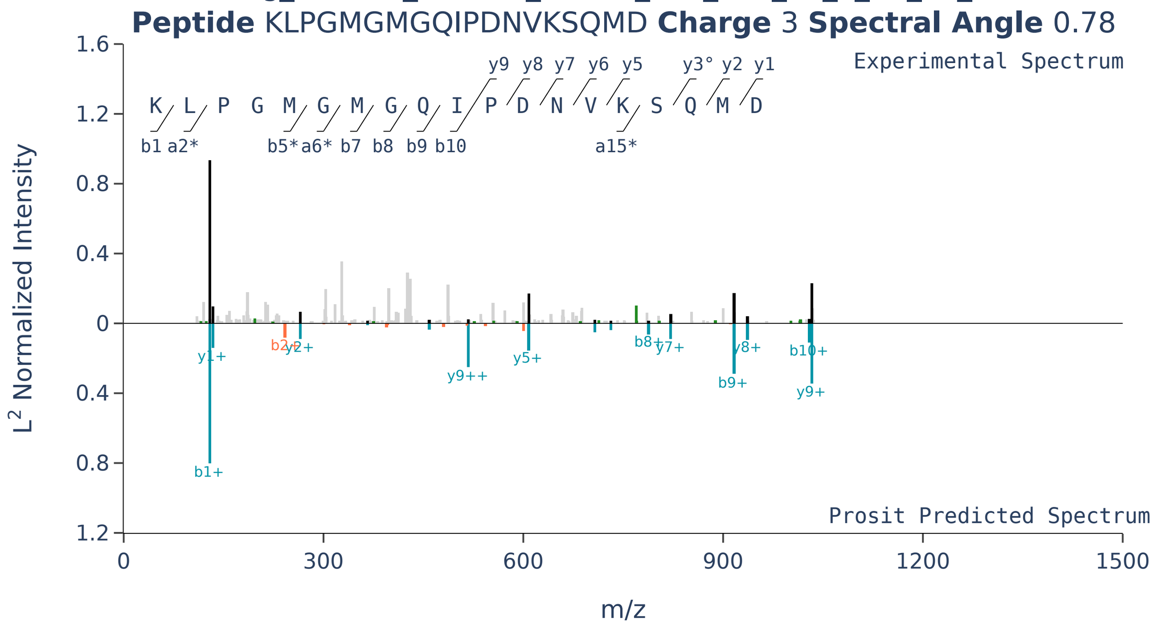

Source Ncheng_210623_230623_HFGoe_FFH_20S_25_1_A2_2h_R1 **Scan** 20592
Peptide GMGKLPGMGQIPDNVKS **Charge** 3 **Spectral Angle** 0.8

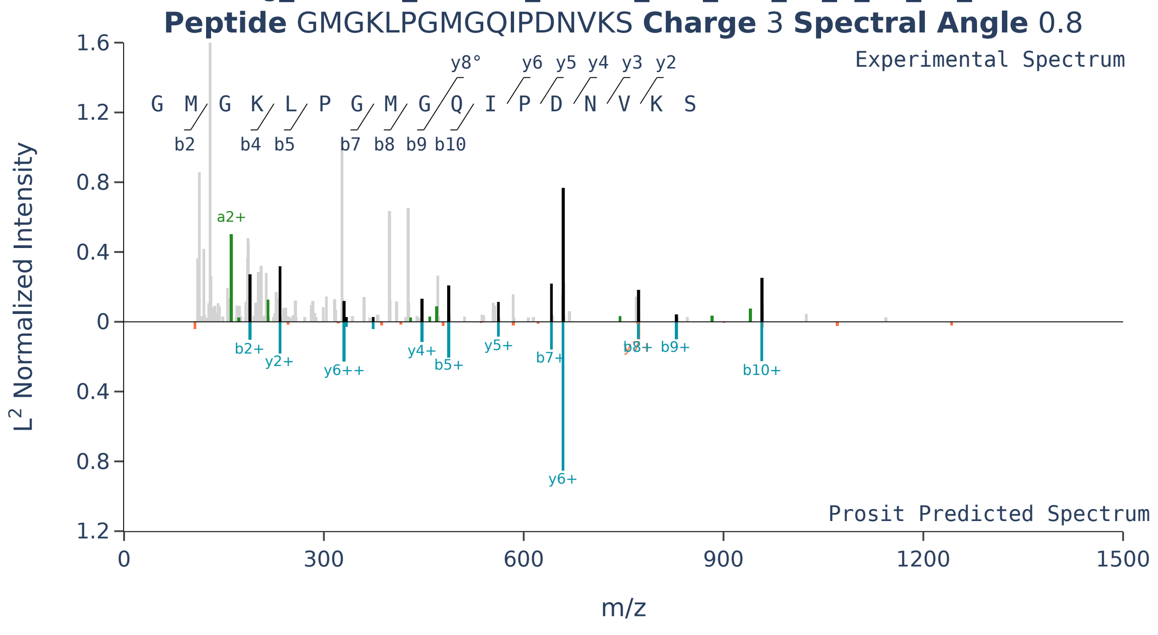

Source Ncheng_210623_230623_HFGoe_FFH_20S_25_1_A1_24h_R1 **Scan** 6791
Peptide TAGVGQKPVD **Charge** 2 **Spectral Angle** 0.77

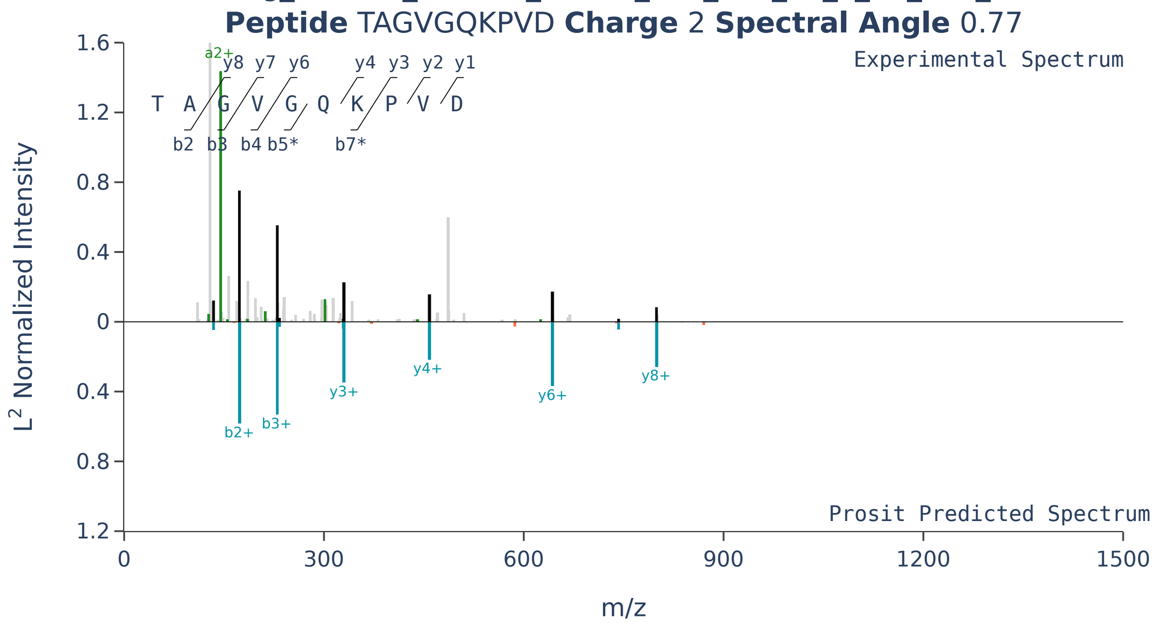

Source Ncheng_210623_230623_HFGoe_FFH_20S_25_1_A2_4h_R1 **Scan** 23853
Peptide AEKLAMGKLPGMGQIPDNVKSQMDD **Charge** 3 **Spectral Angle** 0.82

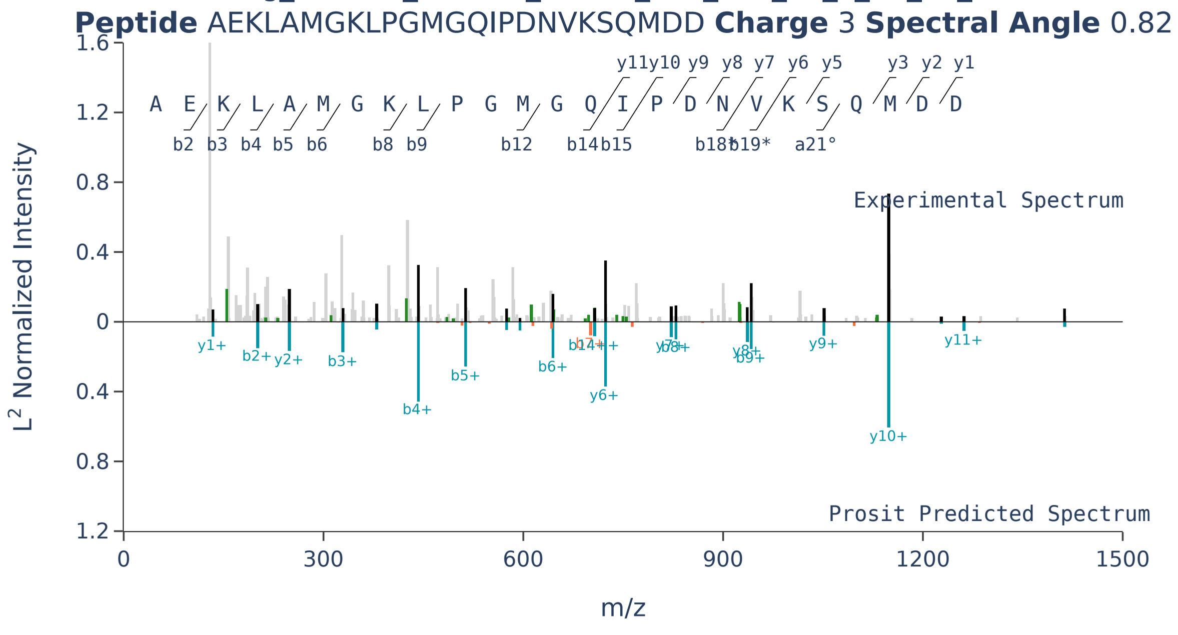

Source Ncheng_210623_230623_HFGoe_FFH_20S_25_1_A2_4h_R1 **Scan** 9136
Peptide DIESRME **Charge** 2 **Spectral Angle** 0.91

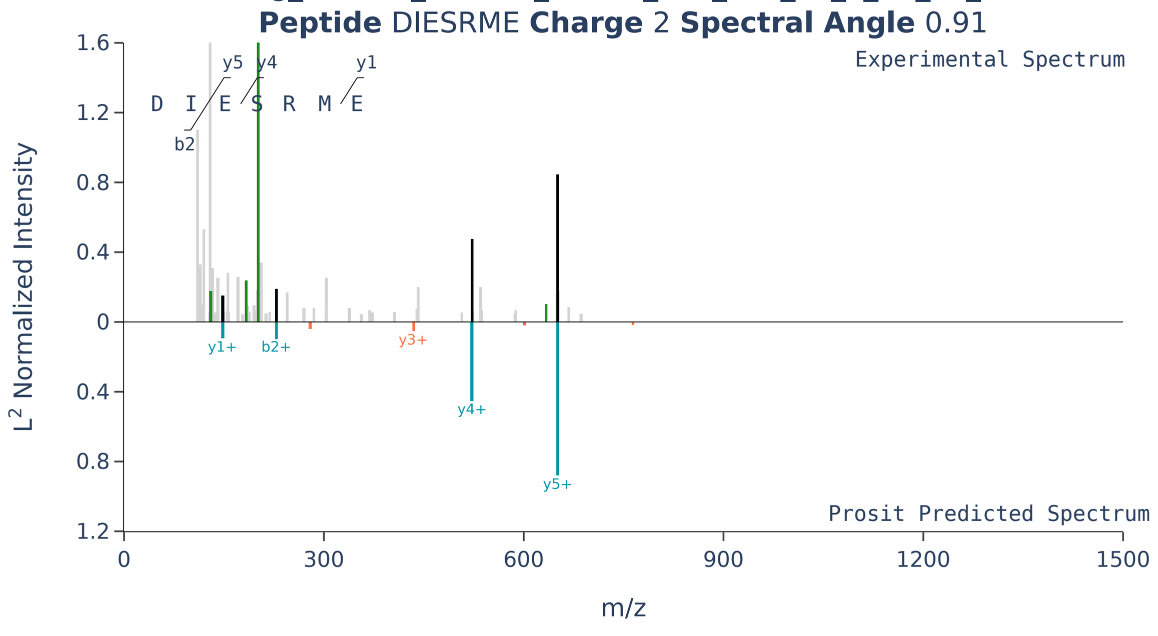

Source Ncheng_210623_230623_HFGoe_FFH_20S_25_1_A1_4h_R1 **Scan** 30183
Peptide AVGHEVNKSLTPGQEFVKIVRNELAV **Charge** 2 **Spectral Angle** 0.78

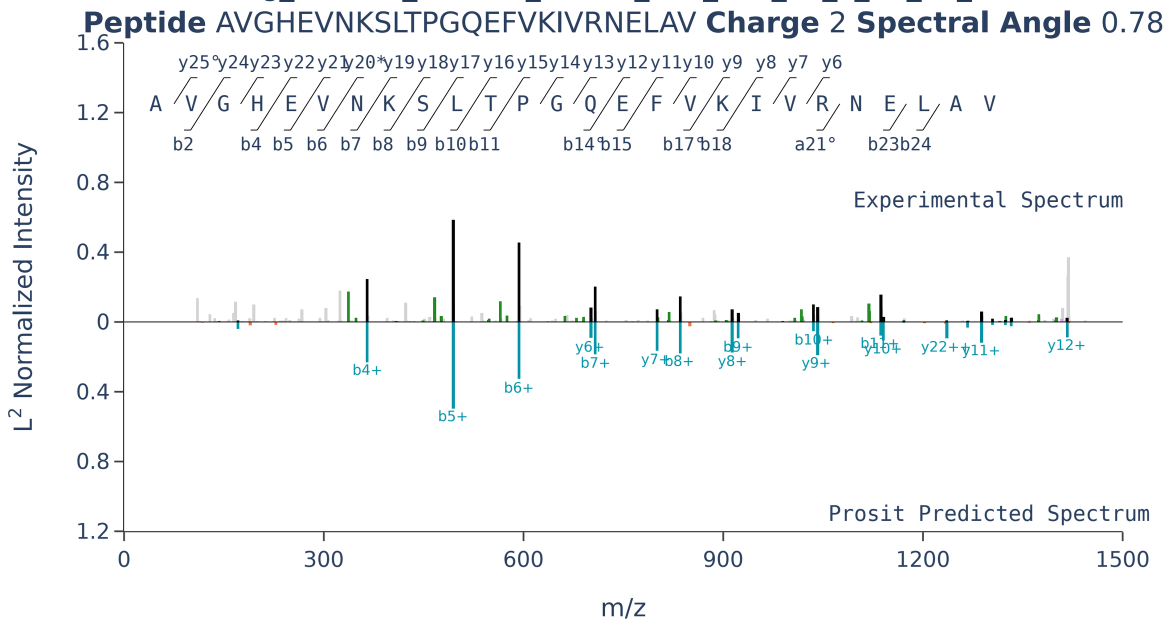

Source Ncheng_210623_230623_HFGoe_FFH_20S_25_1_A1_24h_R2 **Scan** 9873
Peptide GQIPDNVKAS **Charge** 2 **Spectral Angle** 0.85

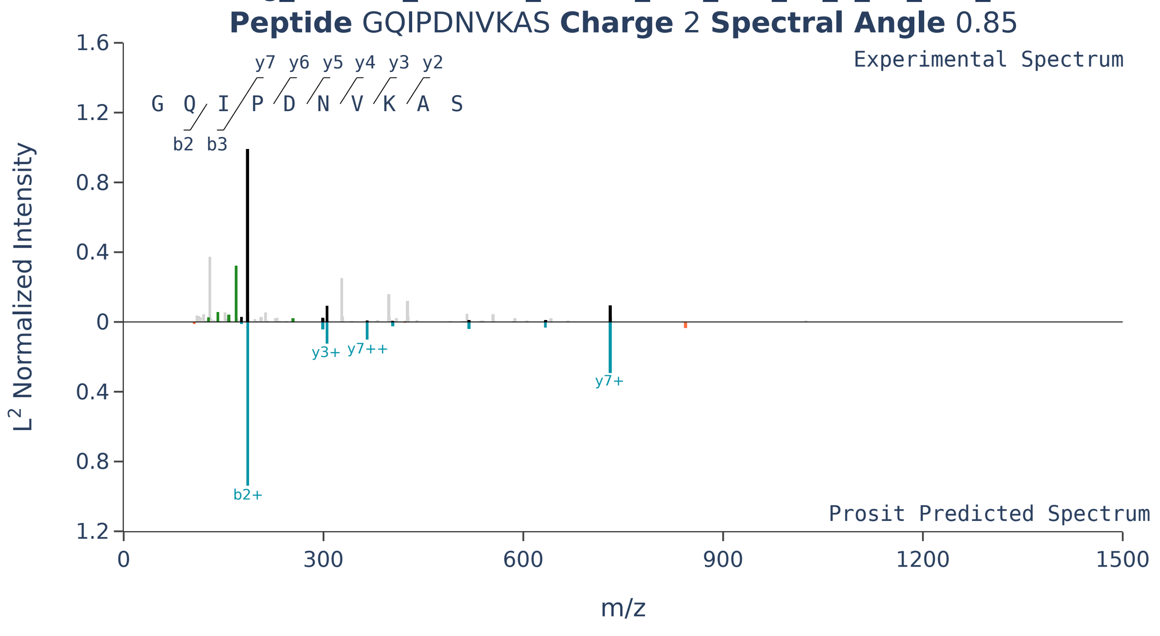

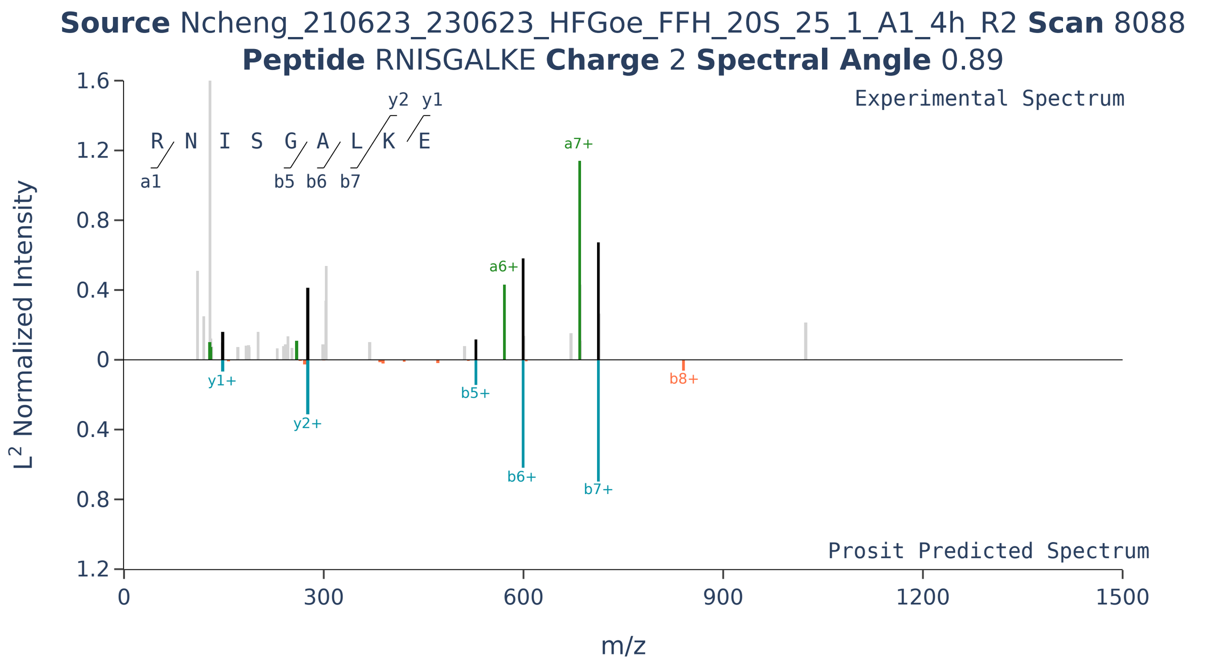
Source Ncheng_210623_230623_HFGoe_FFH_20S_25_1_A1_4h_R2 **Scan** 8088
Peptide RNISGALKE **Charge** 2 **Spectral Angle** 0.89
Experimental Spectrum
Prosit Predicted Spectrum
L² Normalized Intensity
m/z

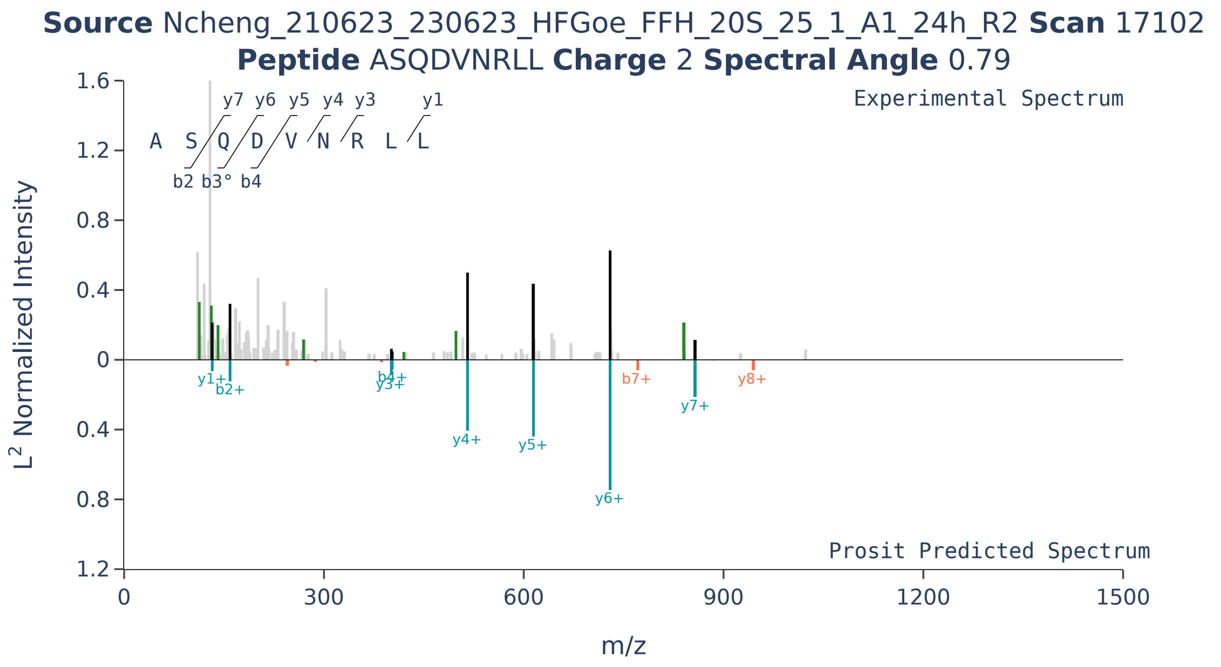
Source Ncheng_210623_230623_HFGoe_FFH_20S_25_1_A1_24h_R2 **Scan** 17102
Peptide ASQDVNRLL **Charge** 2 **Spectral Angle** 0.79
Experimental Spectrum
Prosit Predicted Spectrum
L² Normalized Intensity
m/z

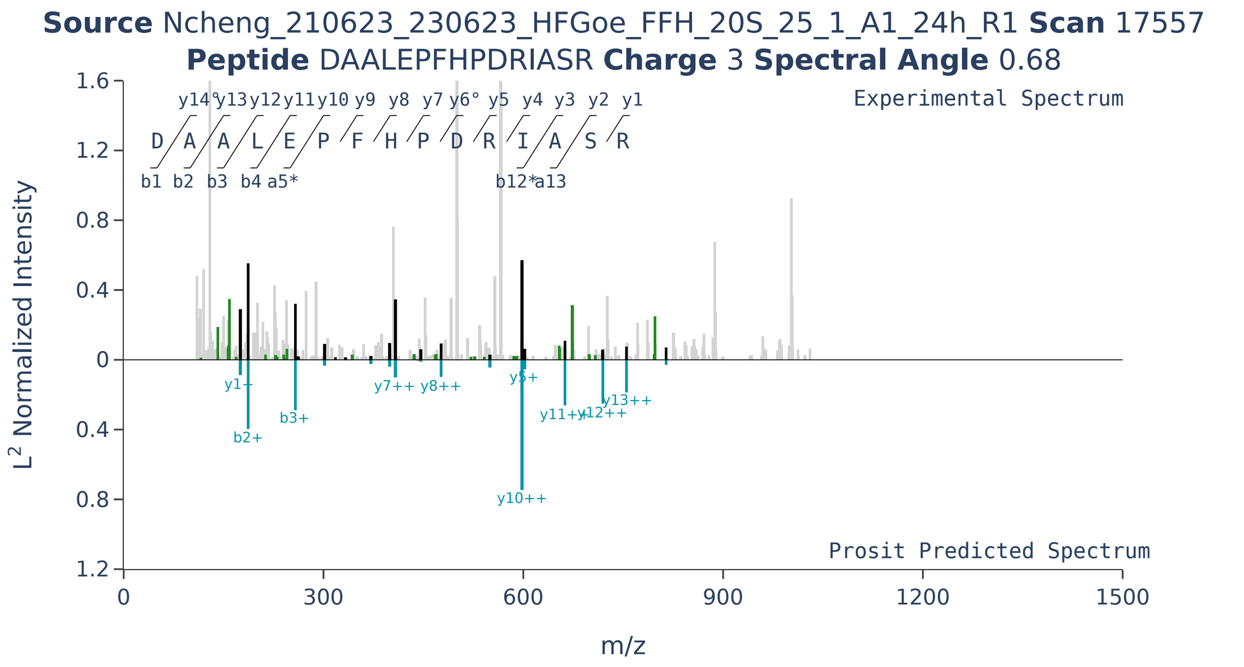
Source Ncheng_210623_230623_HFGoe_FFH_20S_25_1_A1_24h_R1 **Scan** 17557
Peptide DAALEPFHPDRIASR **Charge** 3 **Spectral Angle** 0.68
Experimental Spectrum
Prosit Predicted Spectrum
L² Normalized Intensity
m/z

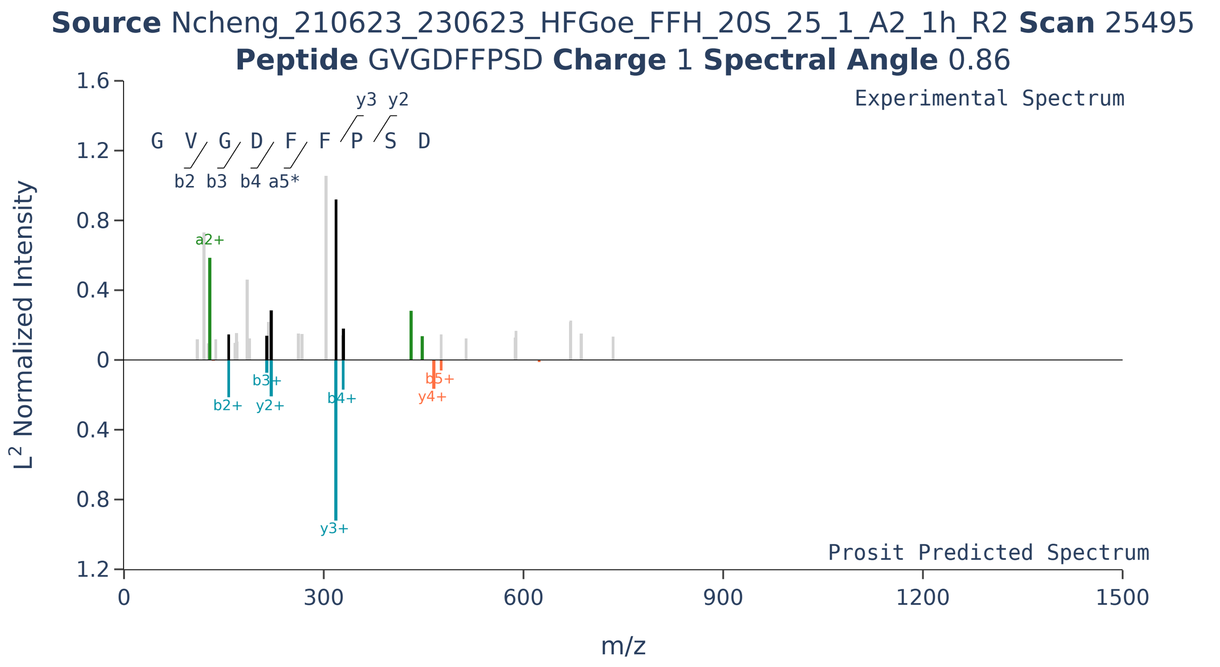
Source Ncheng_210623_230623_HFGoe_FFH_20S_25_1_A2_1h_R2 **Scan** 25495
Peptide GVGDFFPSD **Charge** 1 **Spectral Angle** 0.86
Experimental Spectrum
Prosit Predicted Spectrum
L² Normalized Intensity
m/z

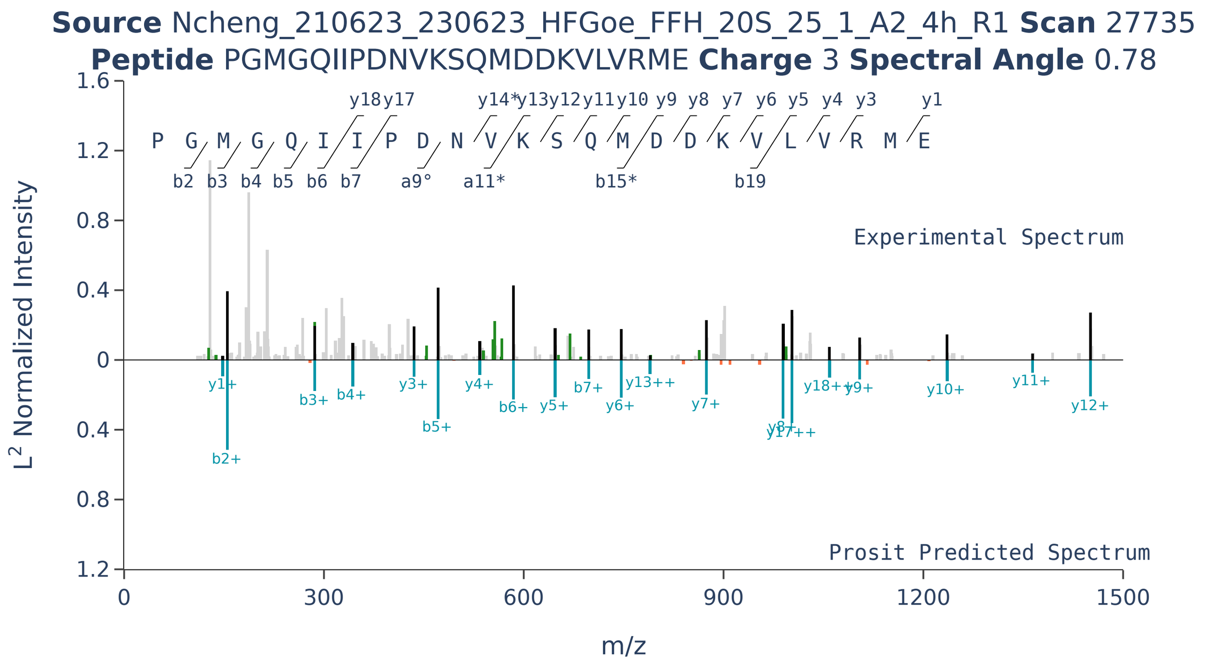
Source Ncheng_210623_230623_HFGoe_FFH_20S_25_1_A2_4h_R1 **Scan** 27735
Peptide PGMGQIIPDNVKSQMDDKVLVRME **Charge** 3 **Spectral Angle** 0.78
Experimental Spectrum
Prosit Predicted Spectrum
L² Normalized Intensity
m/z

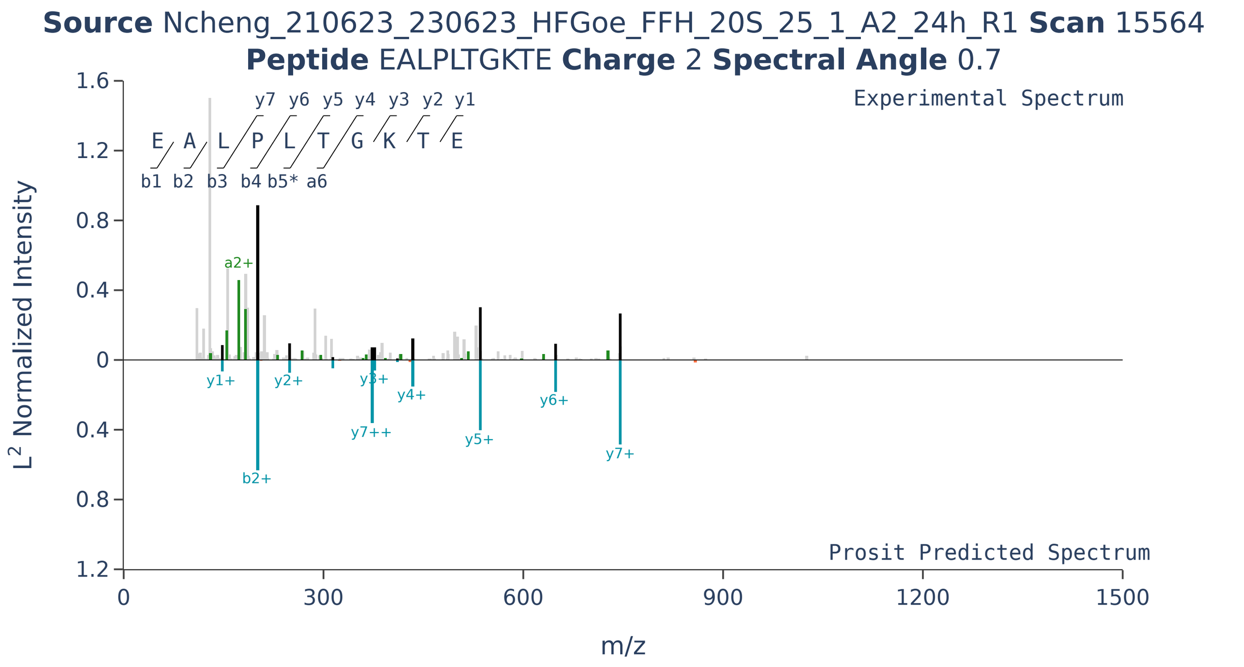
Source Ncheng_210623_230623_HFGoe_FFH_20S_25_1_A2_24h_R1 **Scan** 15564
Peptide EALPLTGKTE **Charge** 2 **Spectral Angle** 0.7
Experimental Spectrum
Prosit Predicted Spectrum
L² Normalized Intensity
m/z

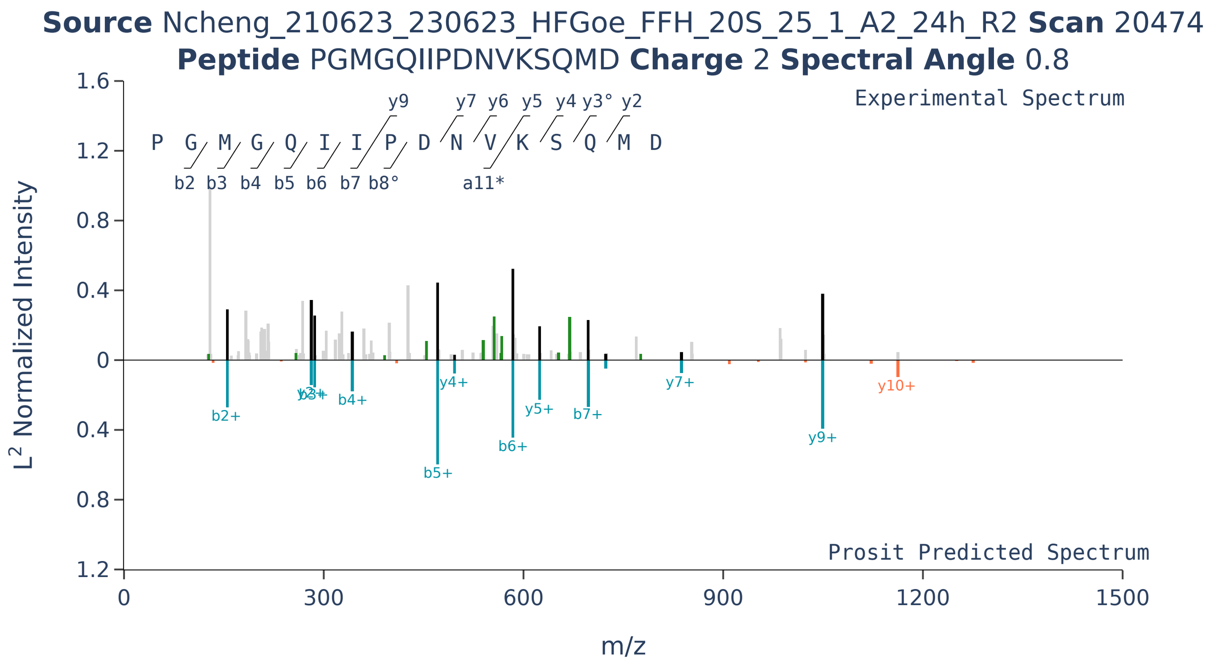
Source Ncheng_210623_230623_HFGoe_FFH_20S_25_1_A2_24h_R2 **Scan** 20474
Peptide PGMGQIIPDNVKSQMD **Charge** 2 **Spectral Angle** 0.8
Experimental Spectrum
Prosit Predicted Spectrum
L² Normalized Intensity
m/z

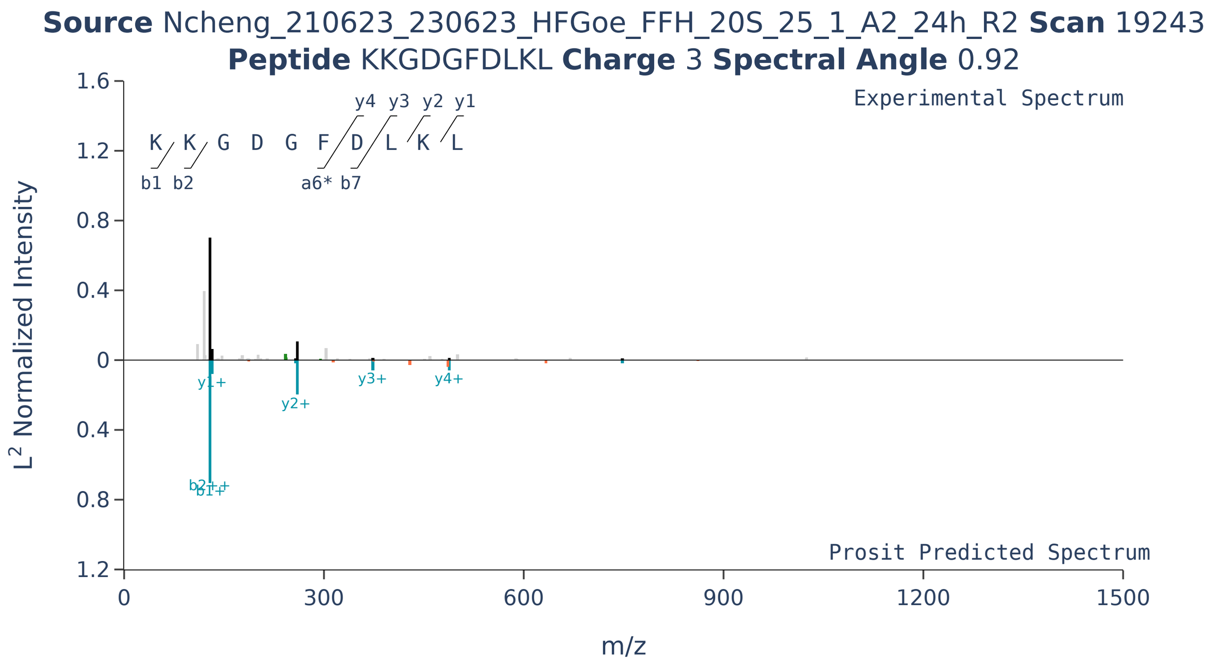
Source Ncheng_210623_230623_HFGoe_FFH_20S_25_1_A2_24h_R2 **Scan** 19243
Peptide KKGDGFDLKL **Charge** 3 **Spectral Angle** 0.92
Experimental Spectrum
Prosit Predicted Spectrum
L² Normalized Intensity
m/z

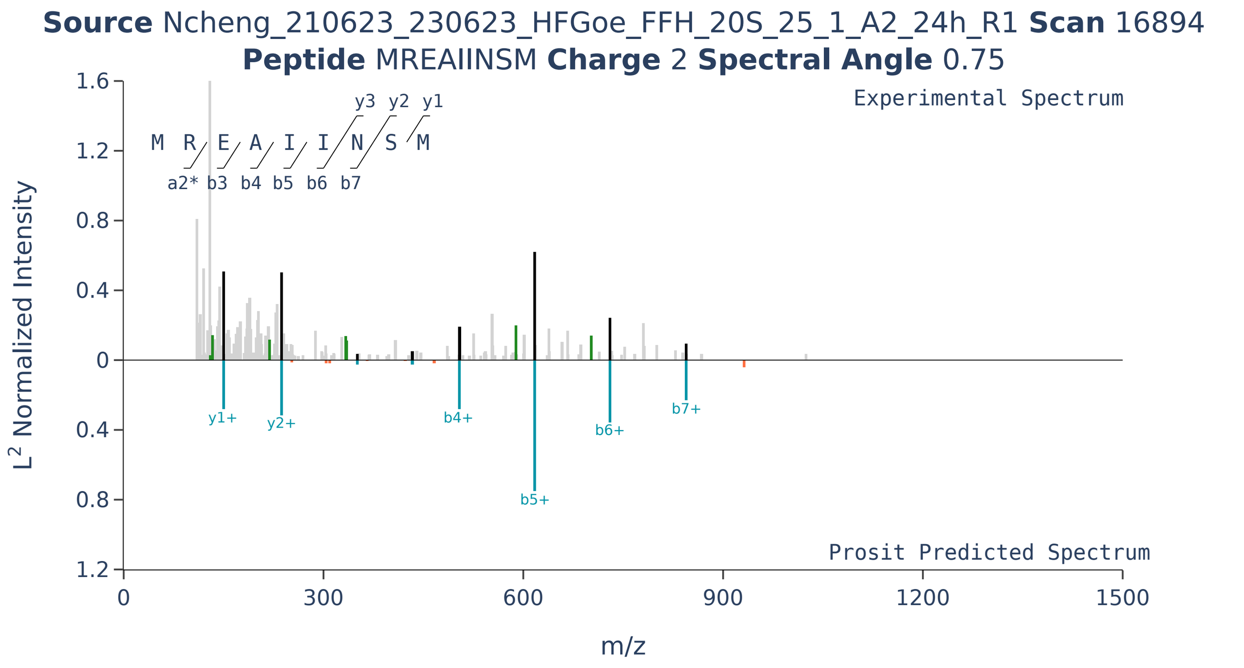
Source Ncheng_210623_230623_HFGoe_FFH_20S_25_1_A2_24h_R1 **Scan** 16894
Peptide MREAIINSM **Charge** 2 **Spectral Angle** 0.75
Experimental Spectrum
Prosit Predicted Spectrum
L² Normalized Intensity
m/z

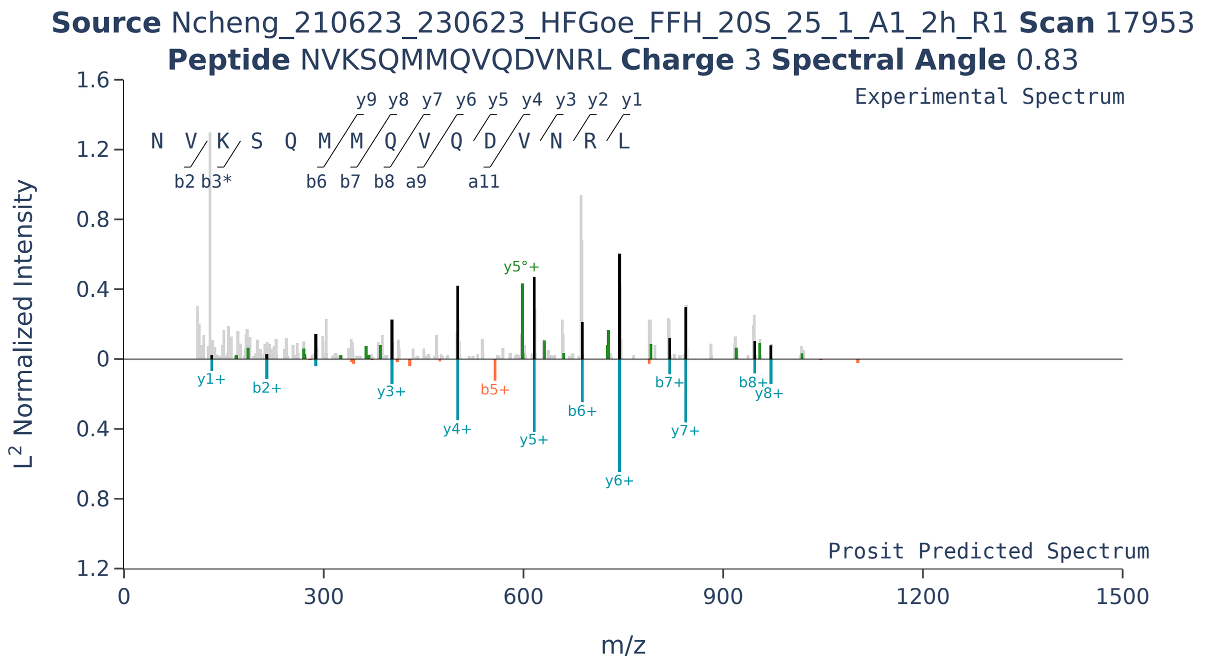
Source Ncheng_210623_230623_HFGoe_FFH_20S_25_1_A1_2h_R1 **Scan** 17953
Peptide NVKSQMMQVQDVNRL **Charge** 3 **Spectral Angle** 0.83
Experimental Spectrum
Prosit Predicted Spectrum
L² Normalized Intensity
m/z

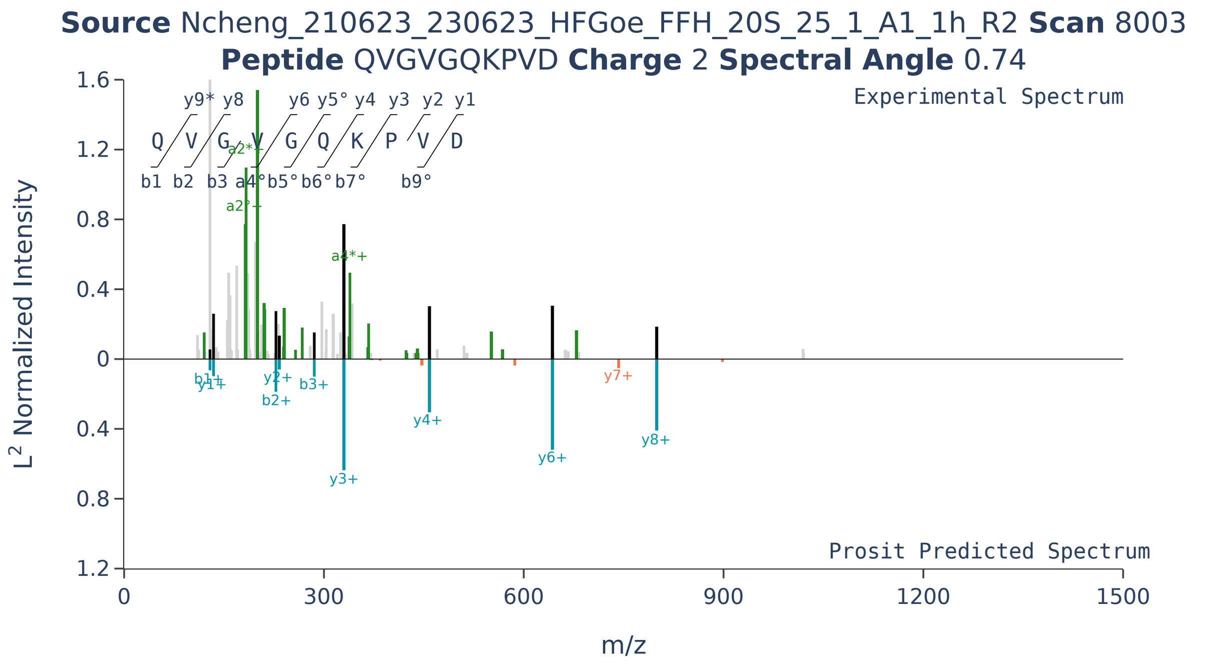
Source Ncheng_210623_230623_HFGoe_FFH_20S_25_1_A1_1h_R2 **Scan** 8003
Peptide QVGVGQKPVD **Charge** 2 **Spectral Angle** 0.74
Experimental Spectrum
Prosit Predicted Spectrum
L² Normalized Intensity
m/z

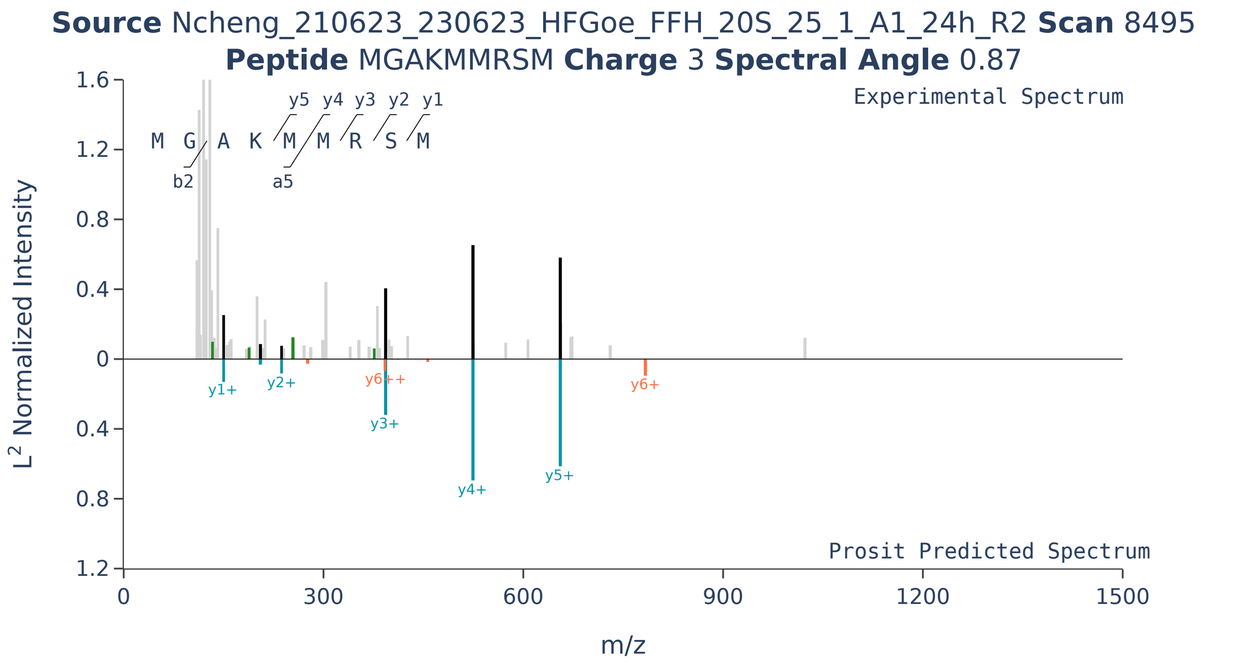
Source Ncheng_210623_230623_HFGoe_FFH_20S_25_1_A1_24h_R2 **Scan** 8495
Peptide MGAKMMRSM **Charge** 3 **Spectral Angle** 0.87
Experimental Spectrum
Prosit Predicted Spectrum
L² Normalized Intensity
m/z

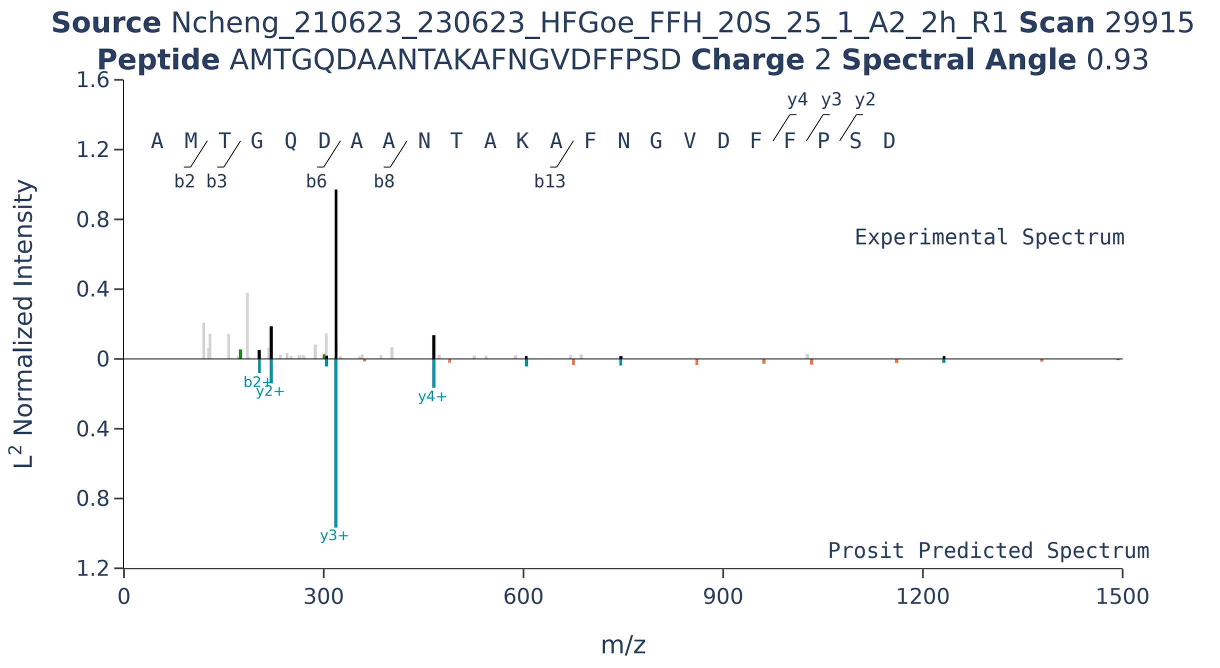
Source Ncheng_210623_230623_HFGoe_FFH_20S_25_1_A2_2h_R1 **Scan** 29915
Peptide AMTGQDAANTAKAFNGVDFFPSD **Charge** 2 **Spectral Angle** 0.93
Experimental Spectrum
Prosit Predicted Spectrum
L² Normalized Intensity
m/z

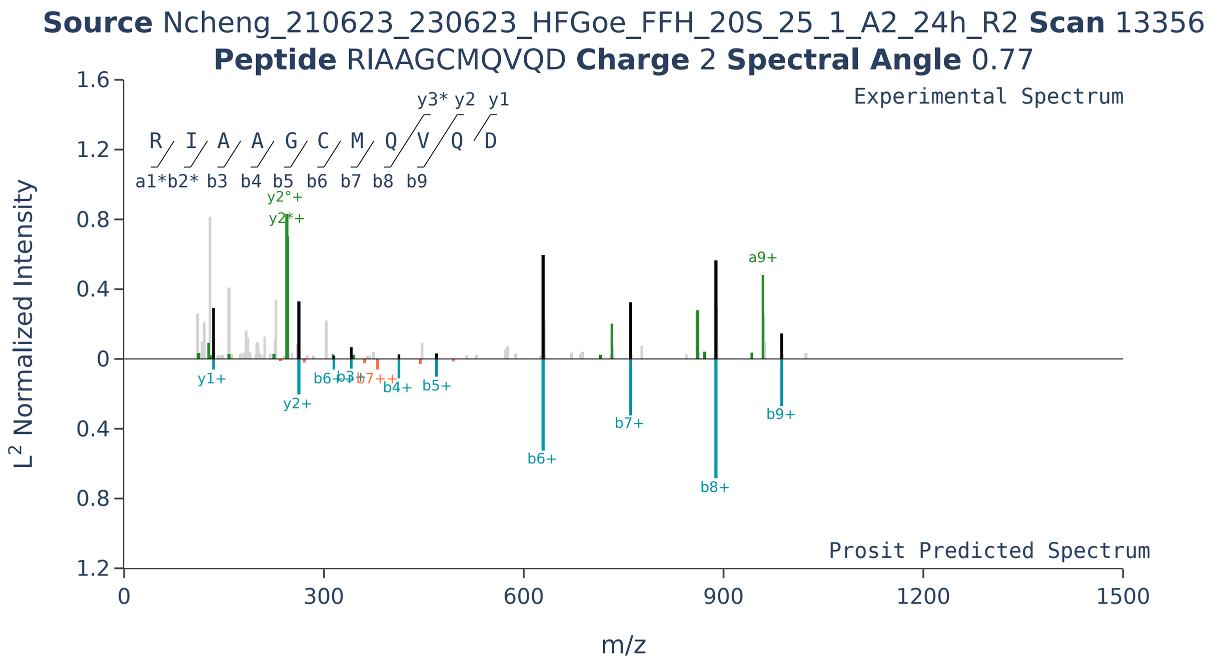
Source Ncheng_210623_230623_HFGoe_FFH_20S_25_1_A2_24h_R2 **Scan** 13356
Peptide RIAAGCMQVQD **Charge** 2 **Spectral Angle** 0.77
Experimental Spectrum
Prosit Predicted Spectrum
L² Normalized Intensity
m/z

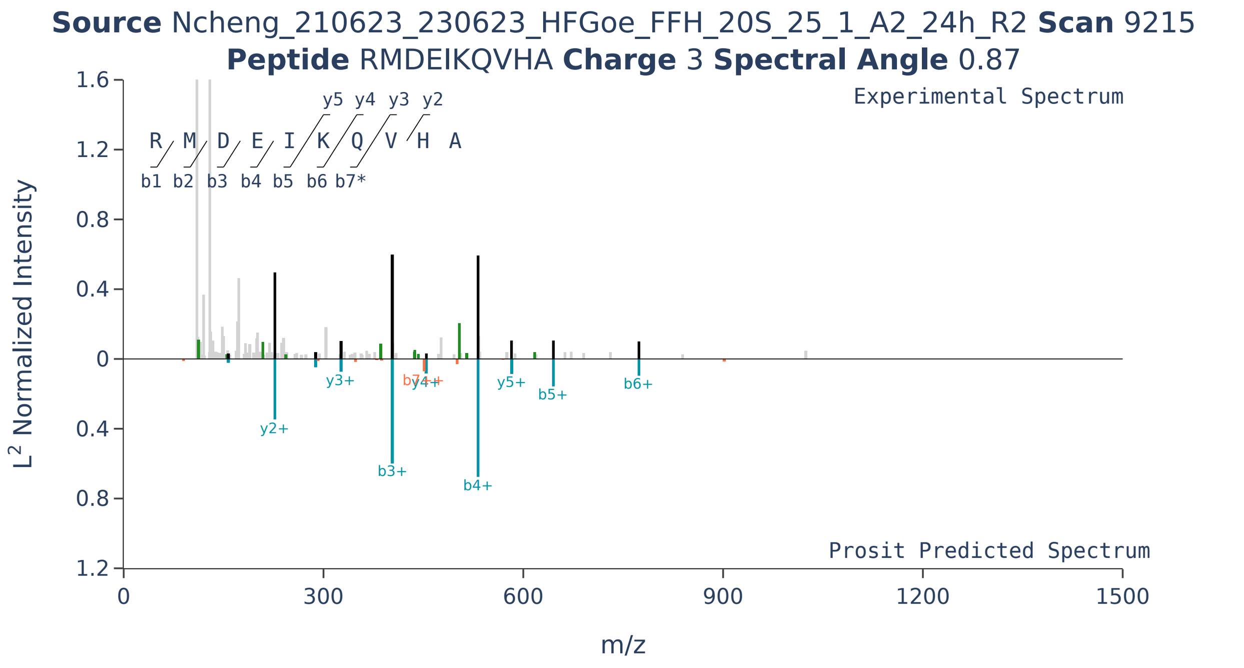
Source Ncheng_210623_230623_HFGoe_FFH_20S_25_1_A2_24h_R2 **Scan** 9215
Peptide RMDEIKQVHA **Charge** 3 **Spectral Angle** 0.87
Experimental Spectrum
Prosit Predicted Spectrum
L² Normalized Intensity
m/z

Source Ncheng_210623_230623_HFGoe_FFH_20S_25_1_A2_24h_R1 **Scan** 29919
Peptide GAEQVGVDFFPSD **Charge** 2 **Spectral Angle** 0.9

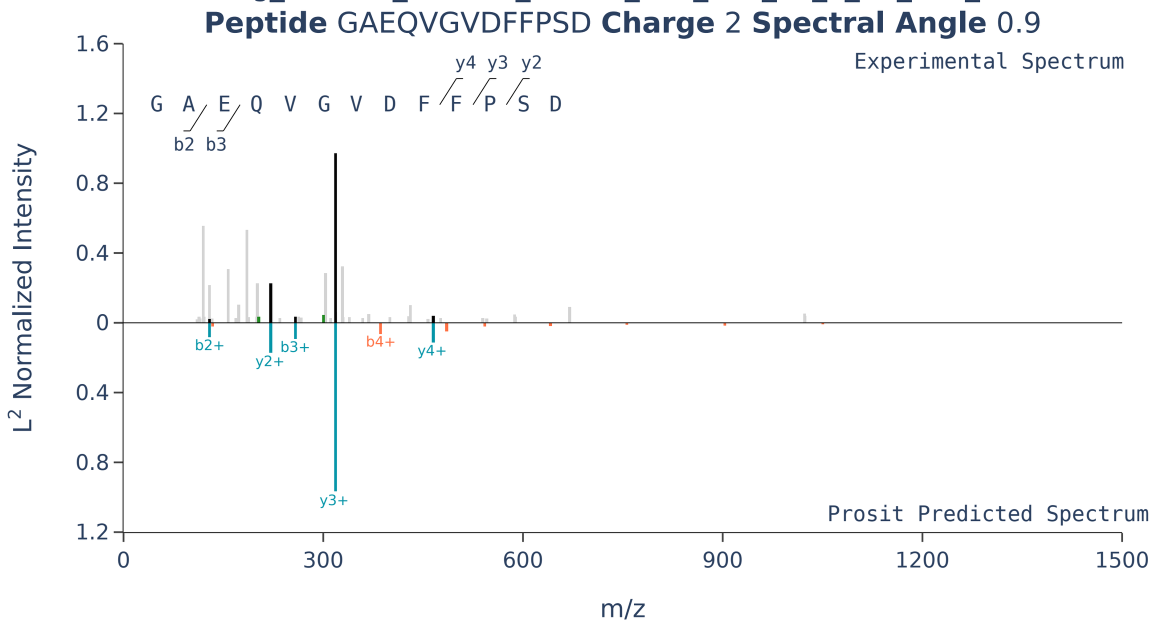

Source Ncheng_210623_230623_HFGoe_FFH_20S_25_1_A2_24h_R2 **Scan** 8747
Peptide KNMGGMRMA **Charge** 2 **Spectral Angle** 0.73

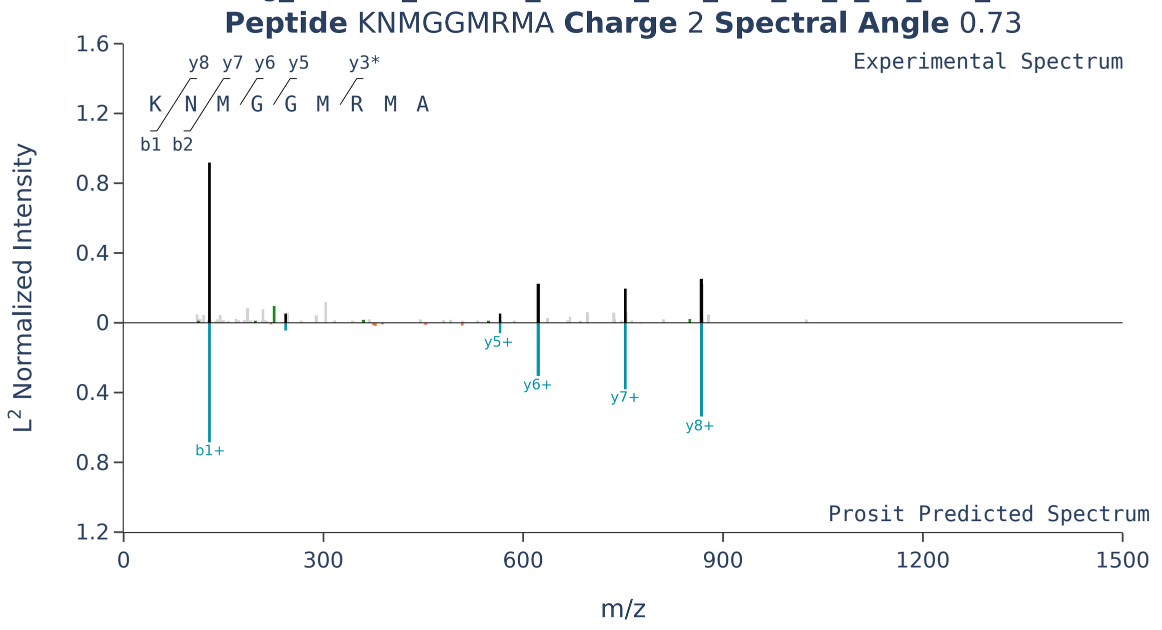

Source Ncheng_210623_230623_HFGoe_FFH_20S_25_1_A2_24h_R1 **Scan** 17165
Peptide GEKTEALPLTG **Charge** 2 **Spectral Angle** 0.87

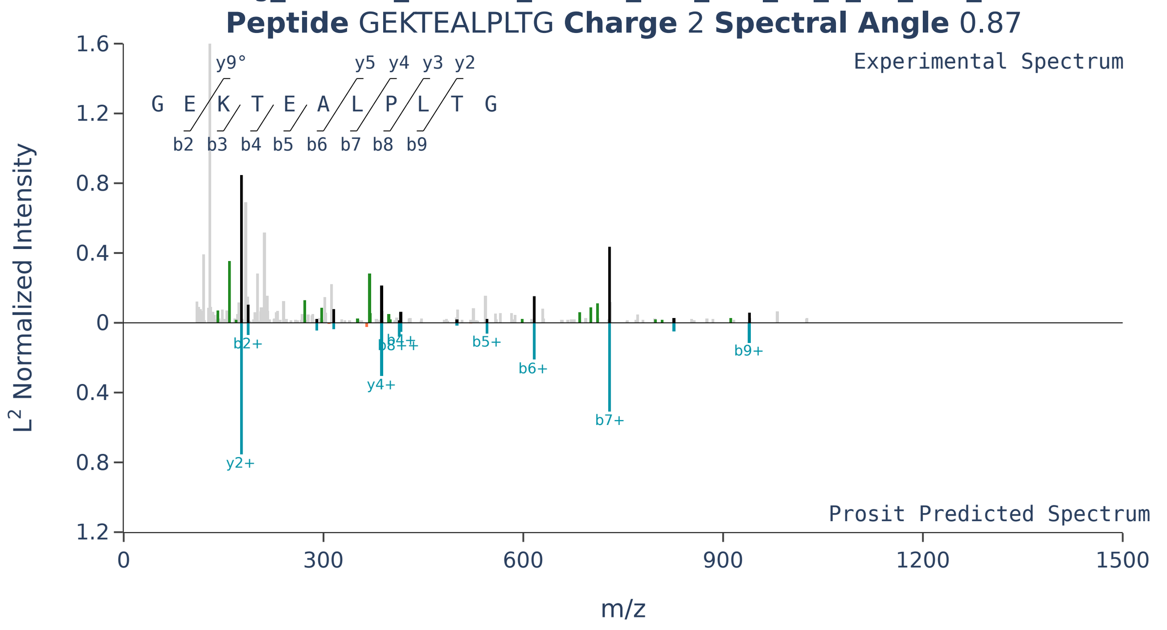

Source Ncheng_210623_230623_HFGoe_FFH_20S_25_1_A1_24h_R2 **Scan** 24608
Peptide DGDARGVDFFPSD **Charge** 2 **Spectral Angle** 0.76

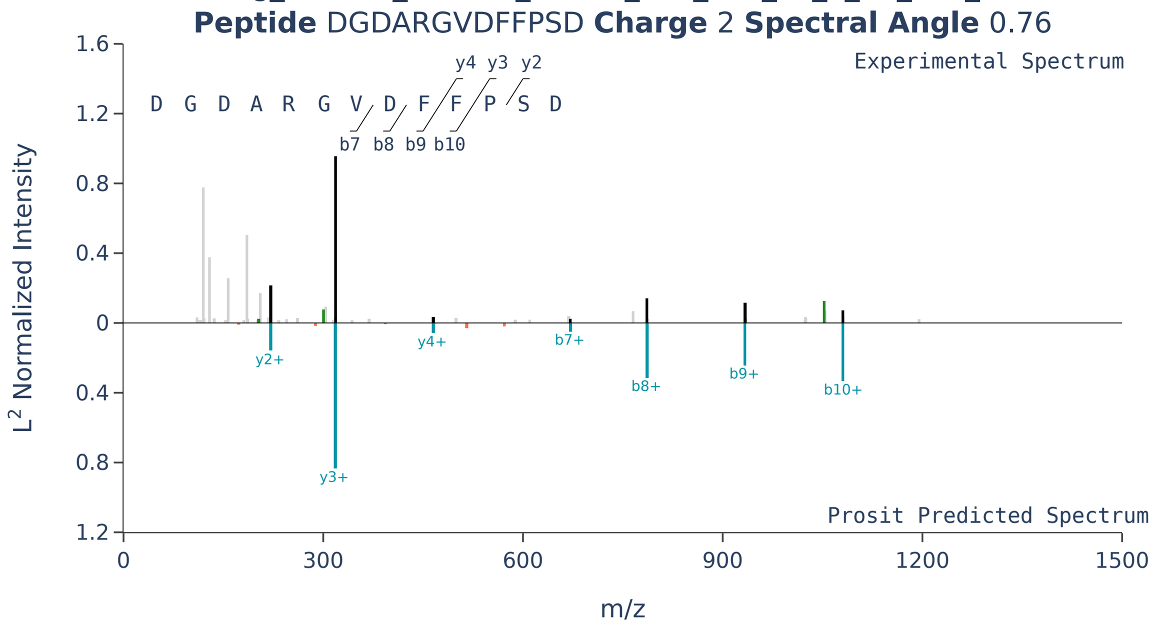

Source Ncheng_210623_230623_HFGoe_FFH_20S_25_1_A2_24h_R2 **Scan** 20718
Peptide TGLEPFHPDRI **Charge** 3 **Spectral Angle** 0.75

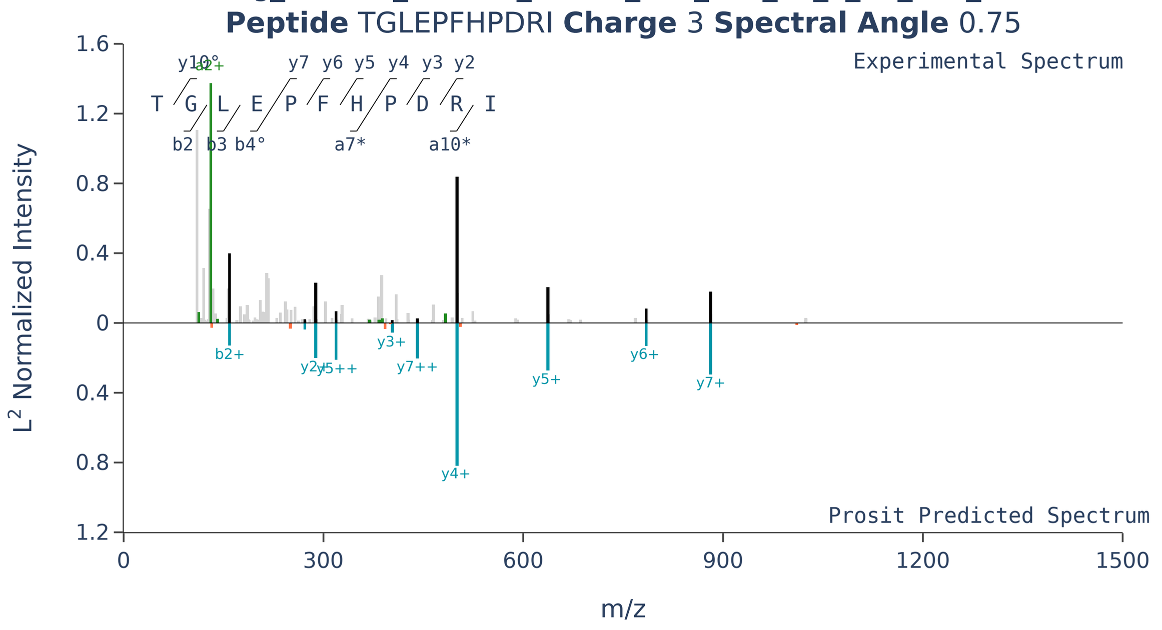

Source Ncheng_210623_230623_HFGoe_FFH_20S_25_1_A2_24h_R2 **Scan** 6127
Peptide LKEQVHA **Charge** 1 **Spectral Angle** 0.8

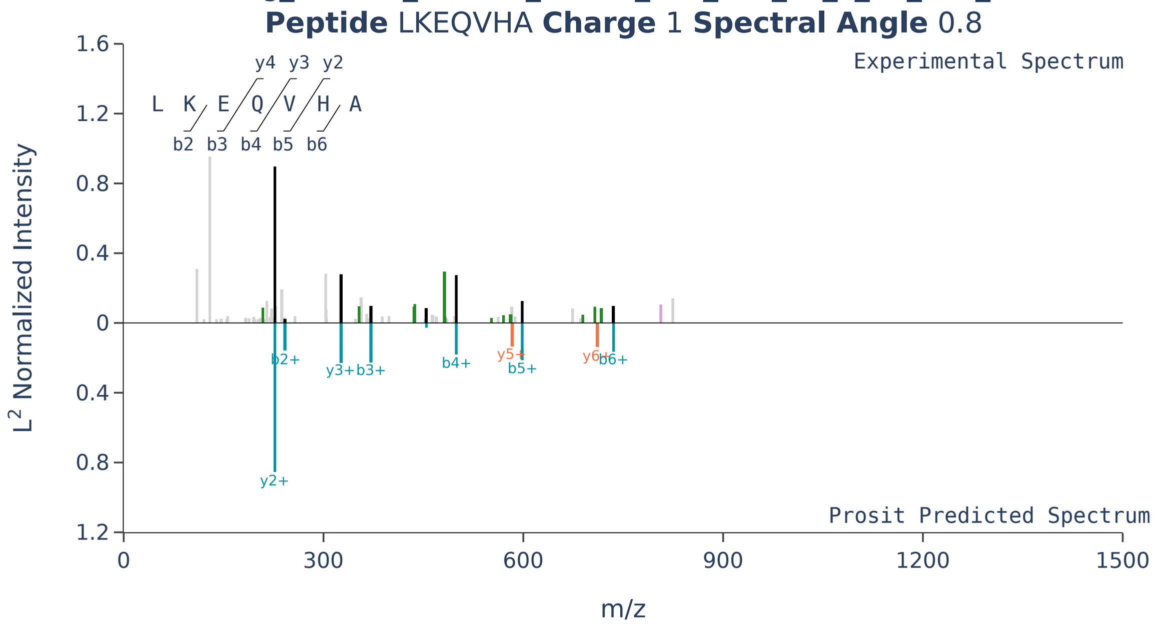

Source Ncheng_210623_230623_HFGoe_FFH_20S_25_1_A1_2h_R2 **Scan** 20322
Peptide MGKLPGMGQIPDNVKST **Charge** 3 **Spectral Angle** 0.8

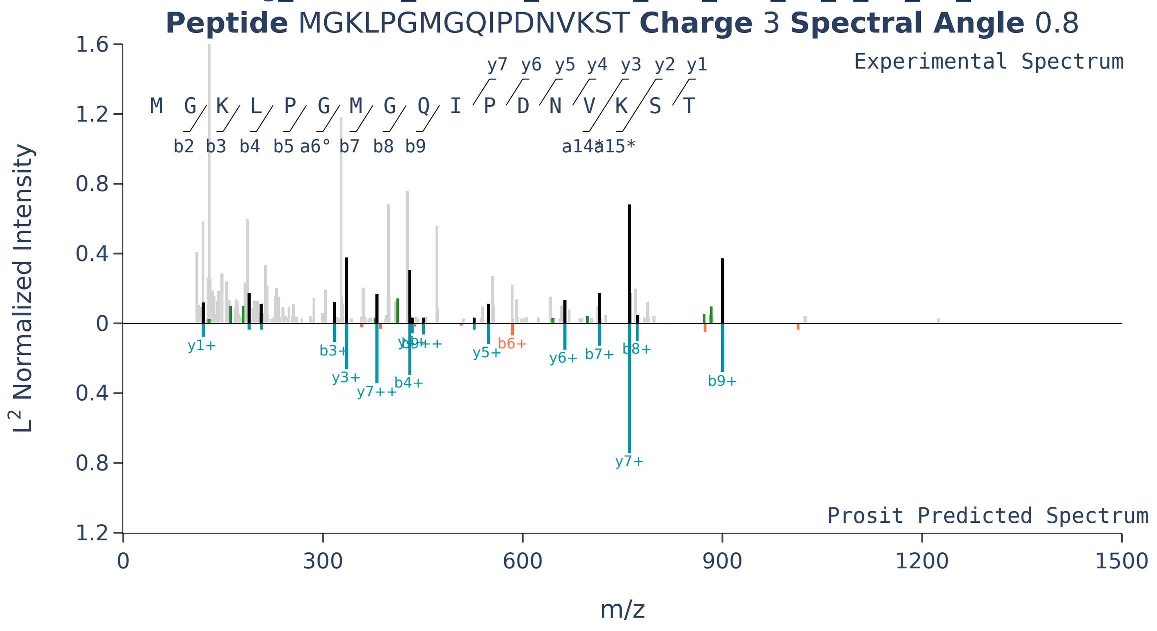

Source Ncheng_210623_230623_HFGoe_FFH_20S_25_1_A2_4h_R2 **Scan** 17017
Peptide MKGMMPPGFPGRSR **Charge** 3 **Spectral Angle** 0.88

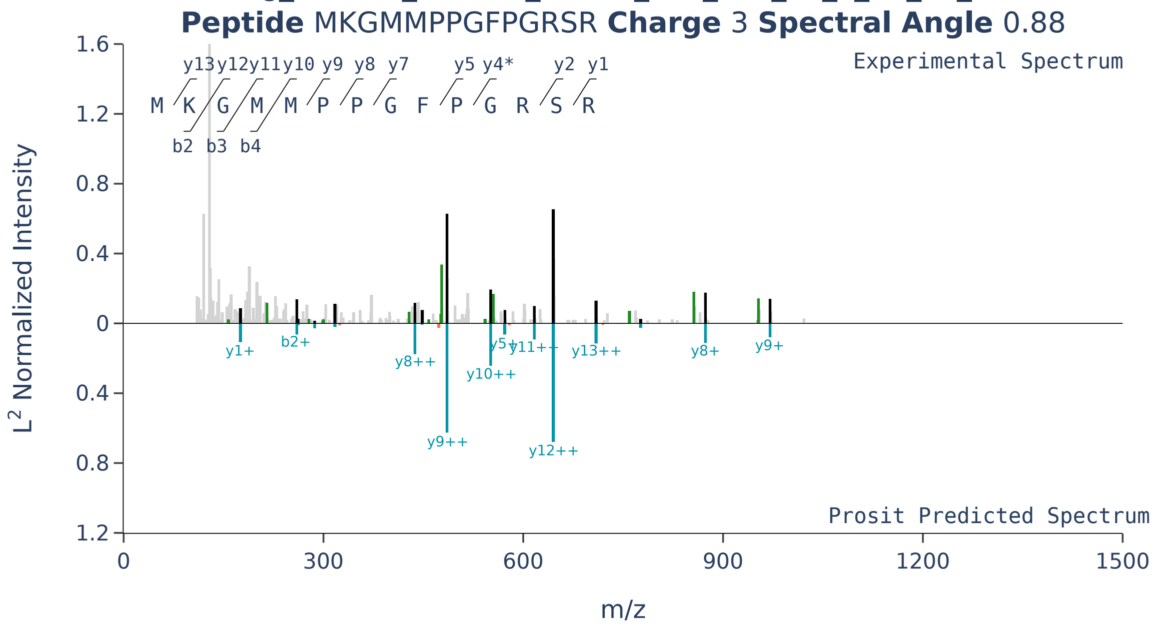

Source Ncheng_210623_230623_HFGoe_FFH_20S_25_1_A1_4h_R1 **Scan** 12244
Peptide RMEAIINSMTMKERKA **Charge** 3 **Spectral Angle** 0.85

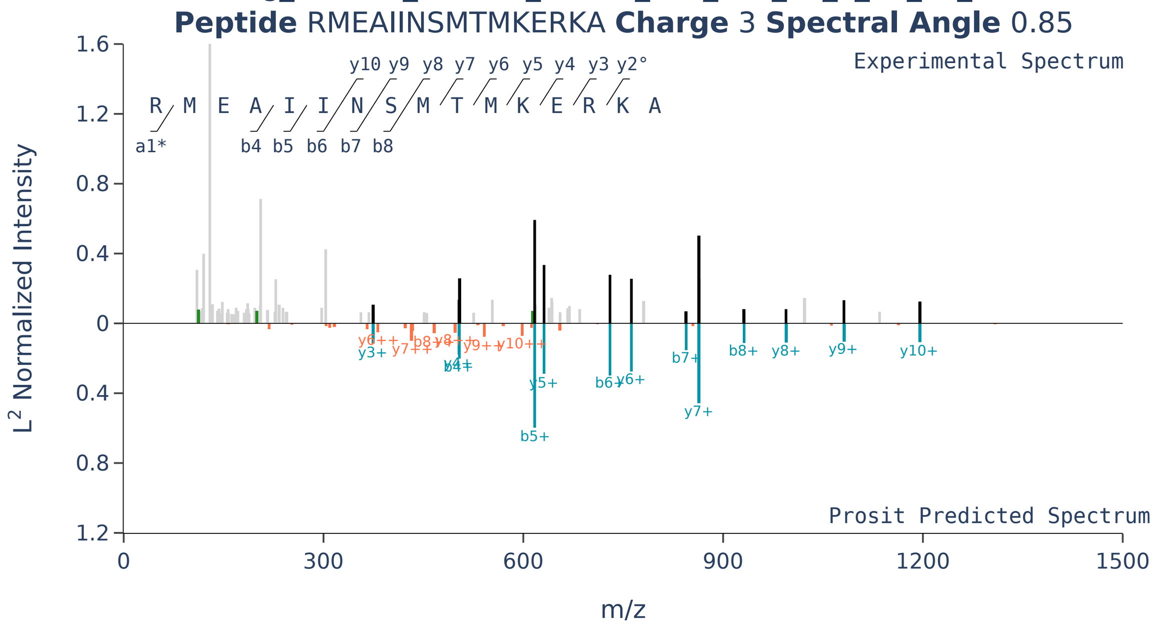

Source Ncheng_210623_230623_HFGoe_FFH_20S_25_1_A2_4h_R2 **Scan** 19091
Peptide KLPGMGMGQIPDNVKSQM **Charge** 3 **Spectral Angle** 0.79

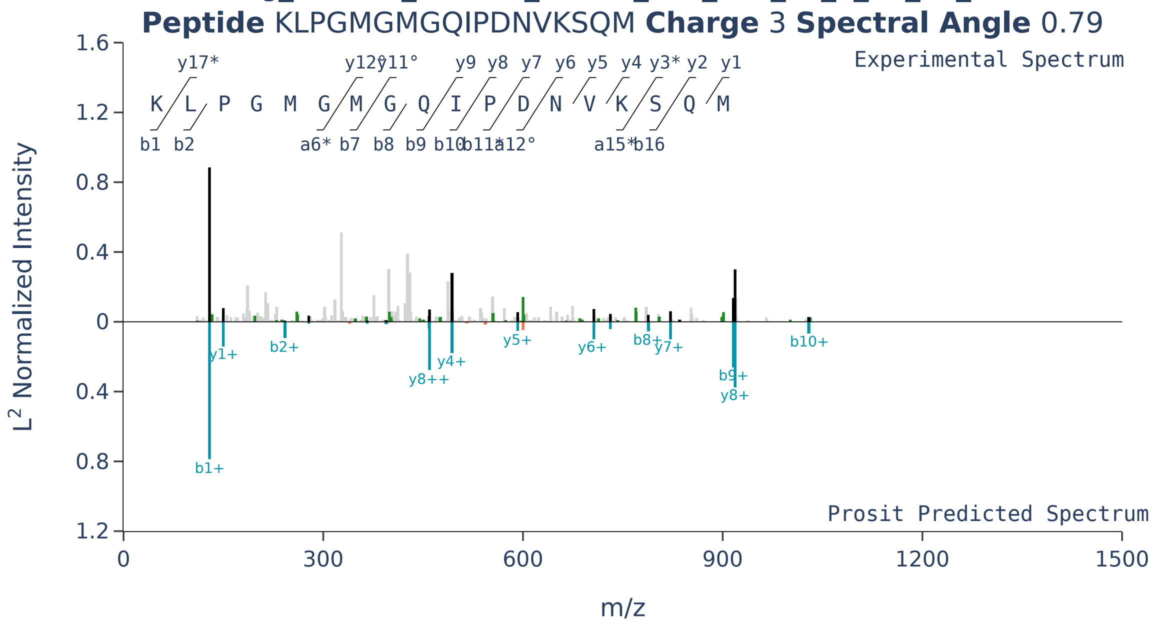

Source Ncheng_210623_230623_HFGoe_FFH_20S_25_1_A1_24h_R1 **Scan** 17003
Peptide KNMGGMADIESKV **Charge** 3 **Spectral Angle** 0.74

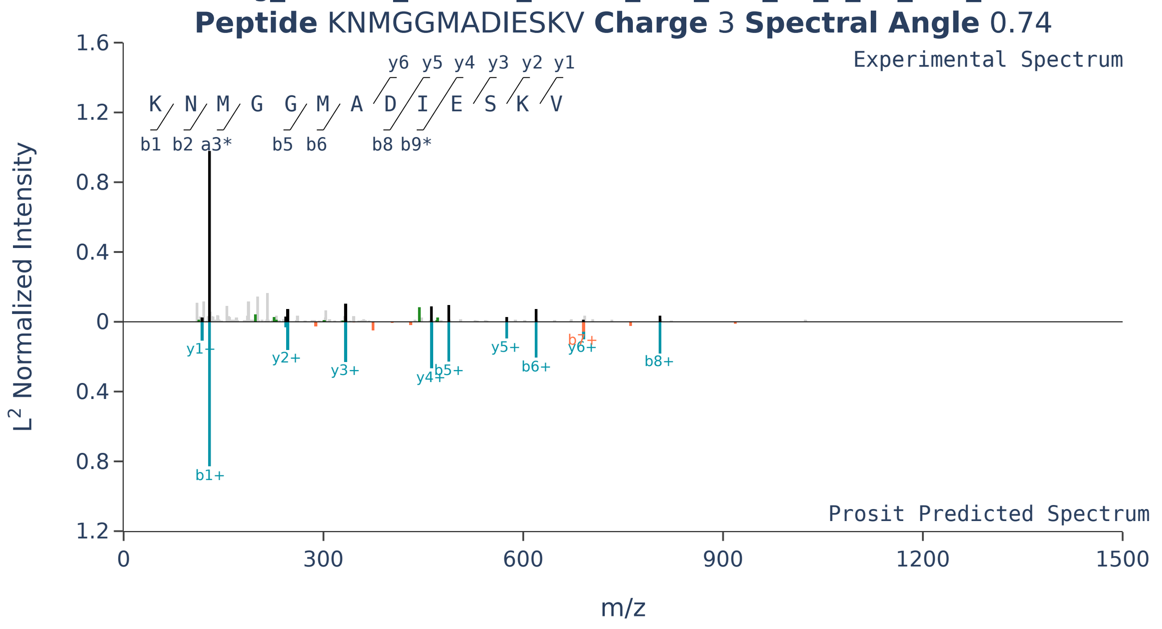

Source Ncheng_210623_230623_HFGoe_FFH_20S_25_1_A2_4h_R2 **Scan** 13481
Peptide AAMTGQDAANTAKAFN **Charge** 2 **Spectral Angle** 0.73

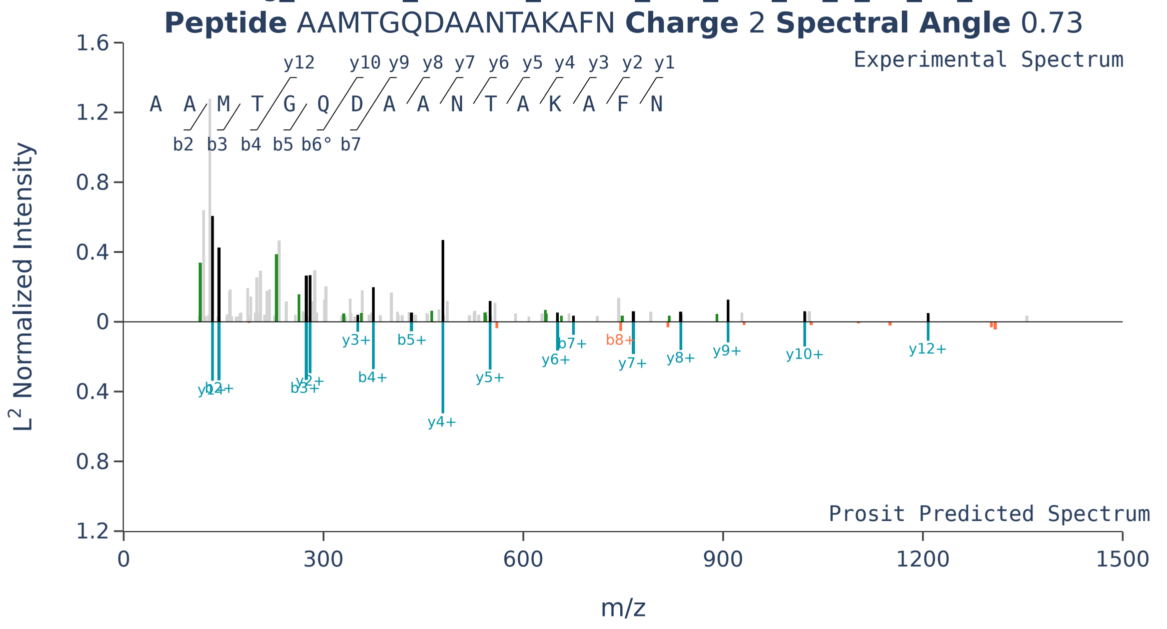

Source Ncheng_210623_230623_HFGoe_FFH_20S_25_1_A2_4h_R2 **Scan** 10538
Peptide KNMGGMASVGQKPVD **Charge** 2 **Spectral Angle** 0.88

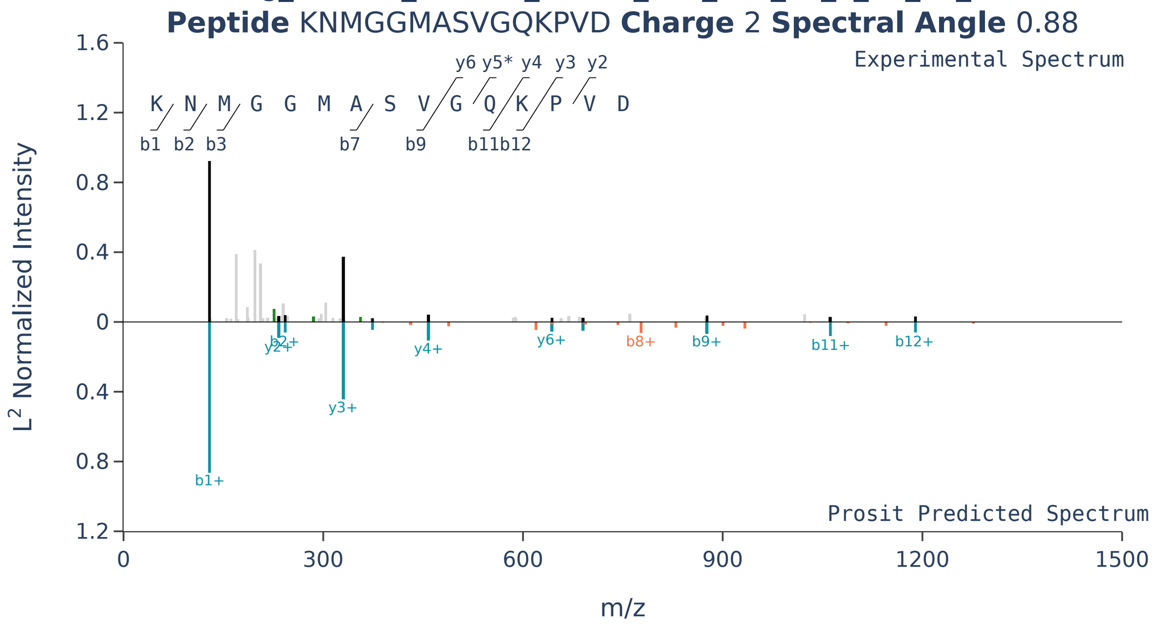

Source Ncheng_210623_230623_HFGoe_FFH_20S_25_1_A1_24h_R2 **Scan** 17166
Peptide TAGVGQKPVDIVNA **Charge** 2 **Spectral Angle** 0.77

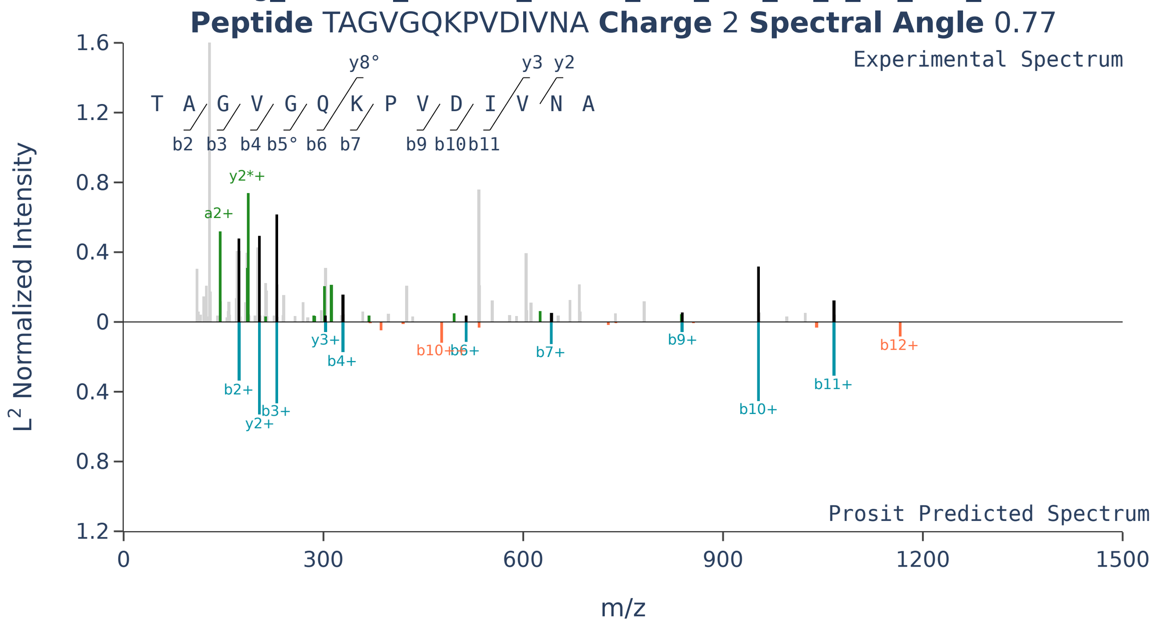

Source Ncheng_210623_230623_HFGoe_FFH_20S_25_1_A2_24h_R1 **Scan** 10725
Peptide VVLTKVDGDARDN **Charge** 3 **Spectral Angle** 0.8

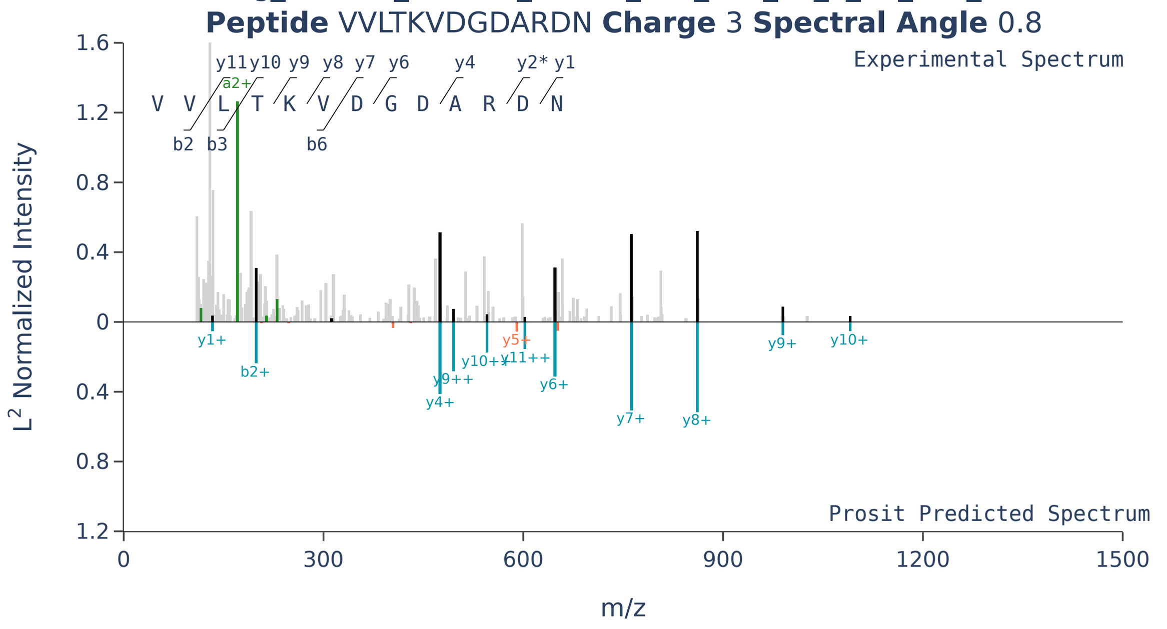

Source Ncheng_210623_230623_HFGoe_FFH_20S_25_1_A2_1h_R1 **Scan** 23336
Peptide DMGKLPGMGQIPDNVKSQMD **Charge** 3 **Spectral Angle** 0.73

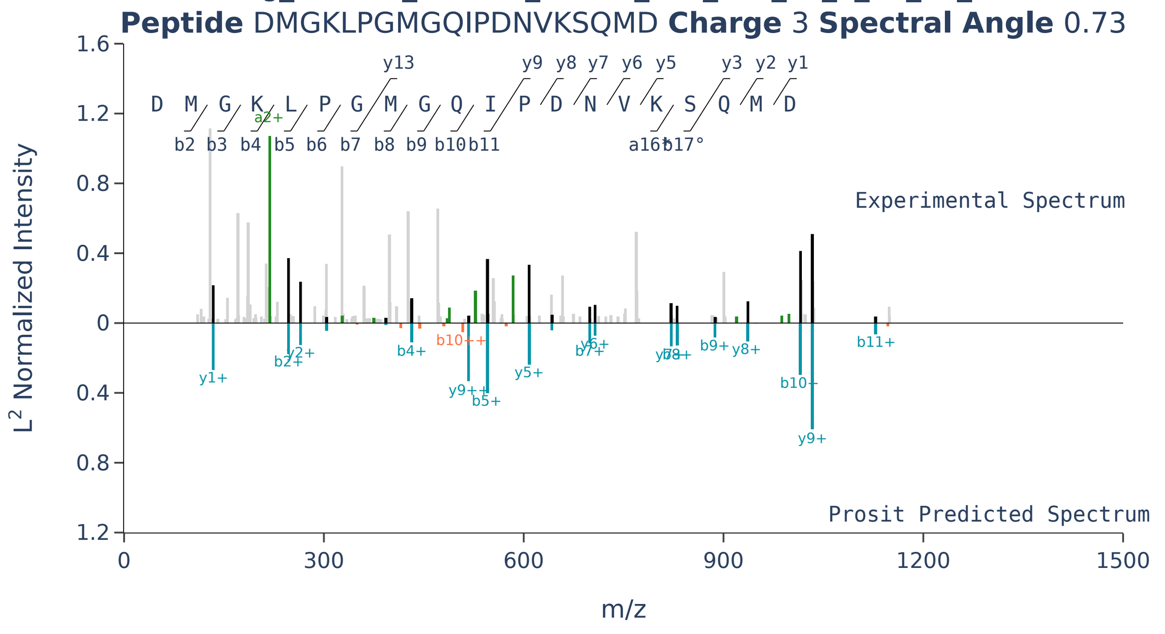

Source Ncheng_210623_230623_HFGoe_FFH_20S_25_1_A2_24h_R2 **Scan** 17668
Peptide GKLKFYDV **Charge** 2 **Spectral Angle** 0.74

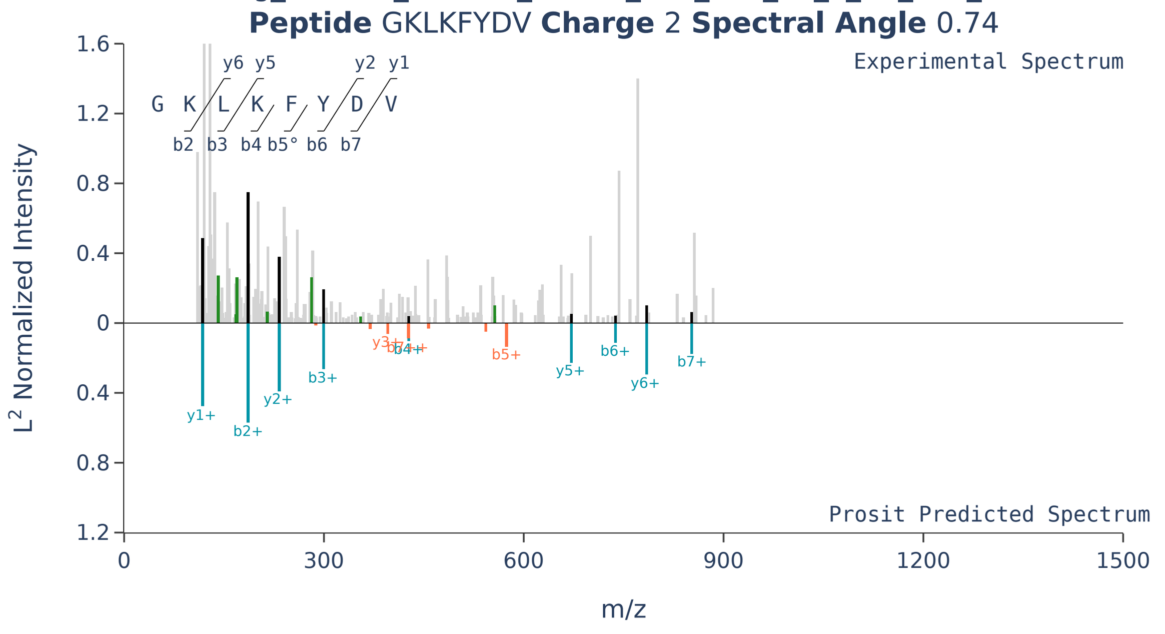

Source Ncheng_210623_230623_HFGoe_FFH_20S_25_1_A2_24h_R1 **Scan** 12697
Peptide EPFHPDDRIAS **Charge** 2 **Spectral Angle** 0.86

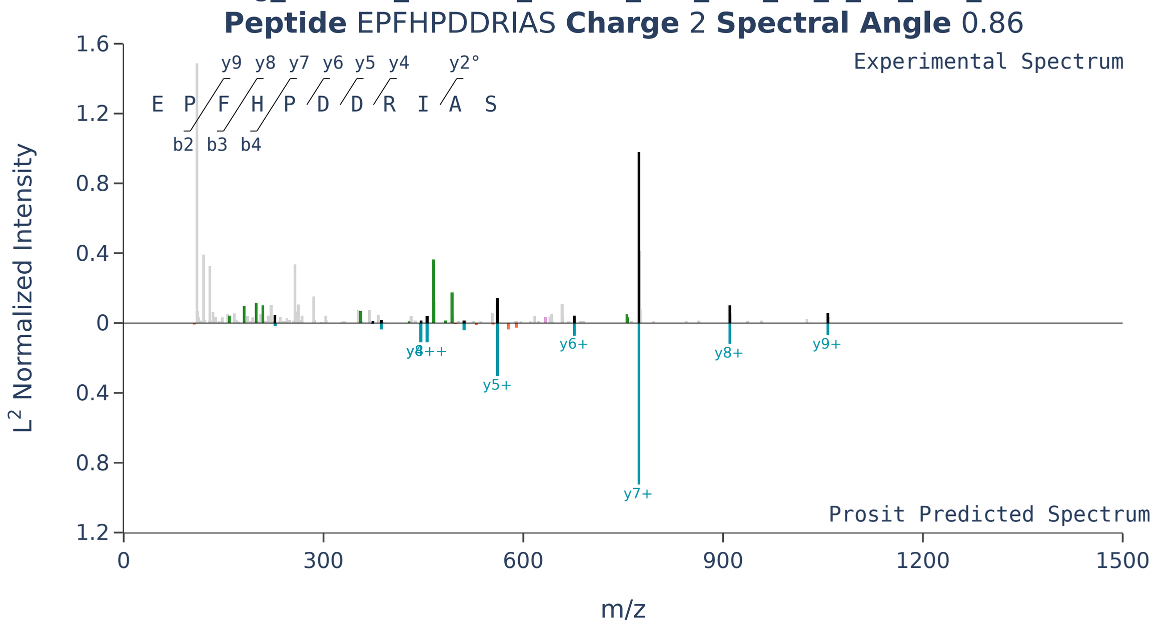

Source Ncheng_210623_230623_HFGoe_FFH_20S_25_1_A1_4h_R2 **Scan** 10504
Peptide GEKTINPVE **Charge** 2 **Spectral Angle** 0.88

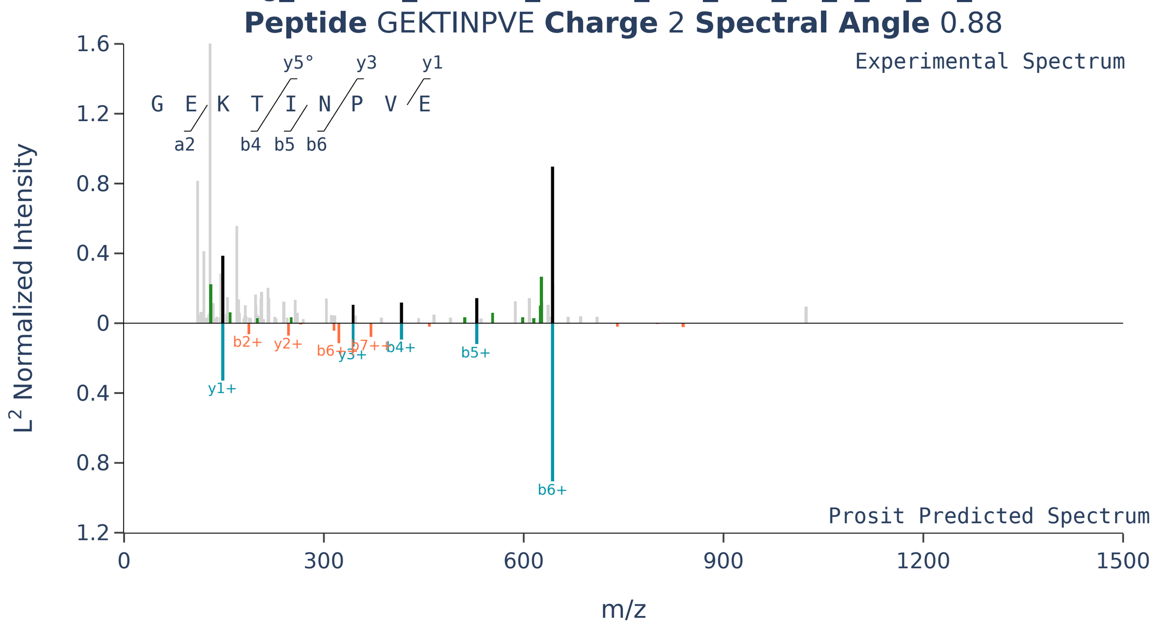

Source Ncheng_210623_230623_HFGoe_FFH_20S_25_1_A1_4h_R1 **Scan** 12481
Peptide KNMGGMAAMTGQD **Charge** 2 **Spectral Angle** 0.84

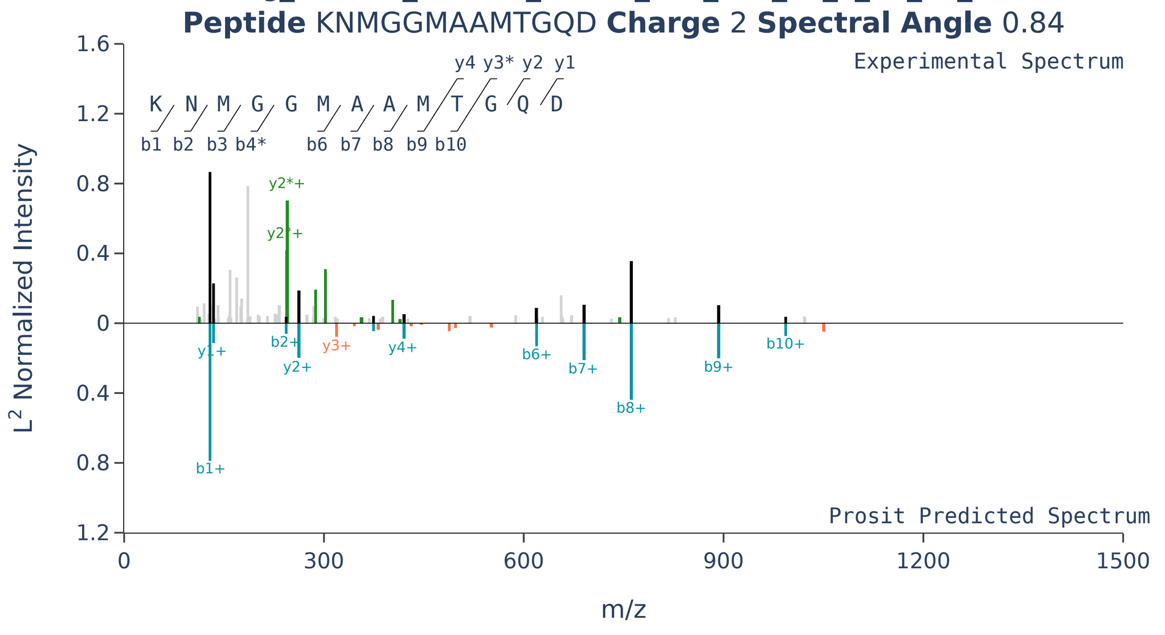

Source Ncheng_210623_230623_HFGoe_FFH_20S_25_1_A1_4h_R1 **Scan** 20545
Peptide AALEPFHPDRI **Charge** 3 **Spectral Angle** 0.76

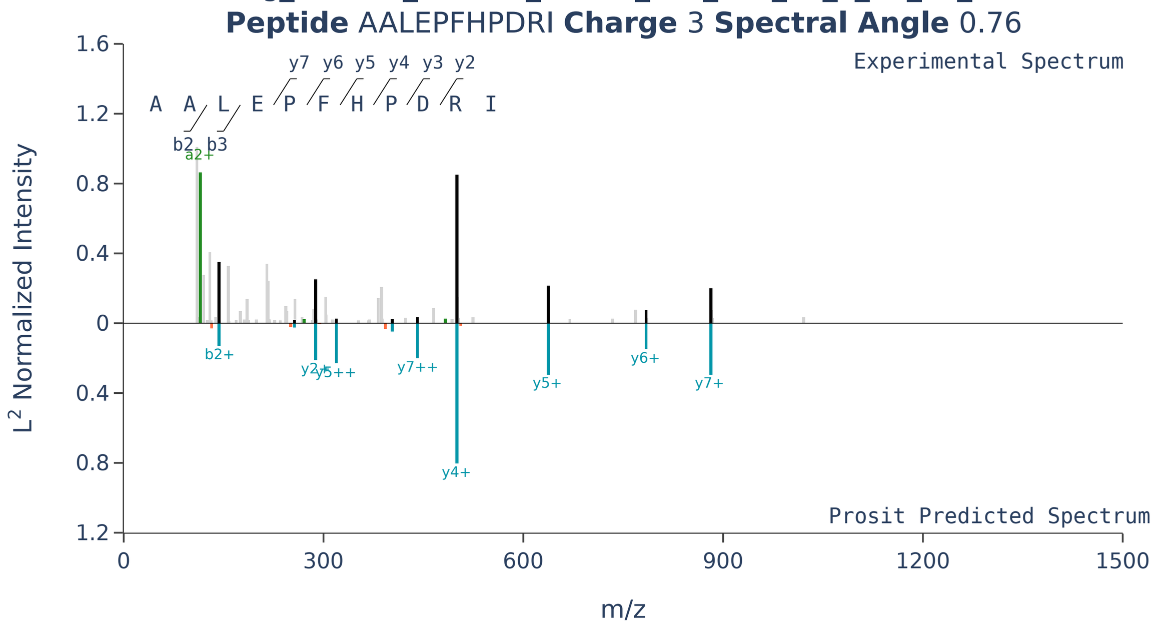

Source Ncheng_210623_230623_HFGoe_FFH_20S_25_1_A1_4h_R2 **Scan** 19281
Peptide EALPLTGD **Charge** 1 **Spectral Angle** 0.87

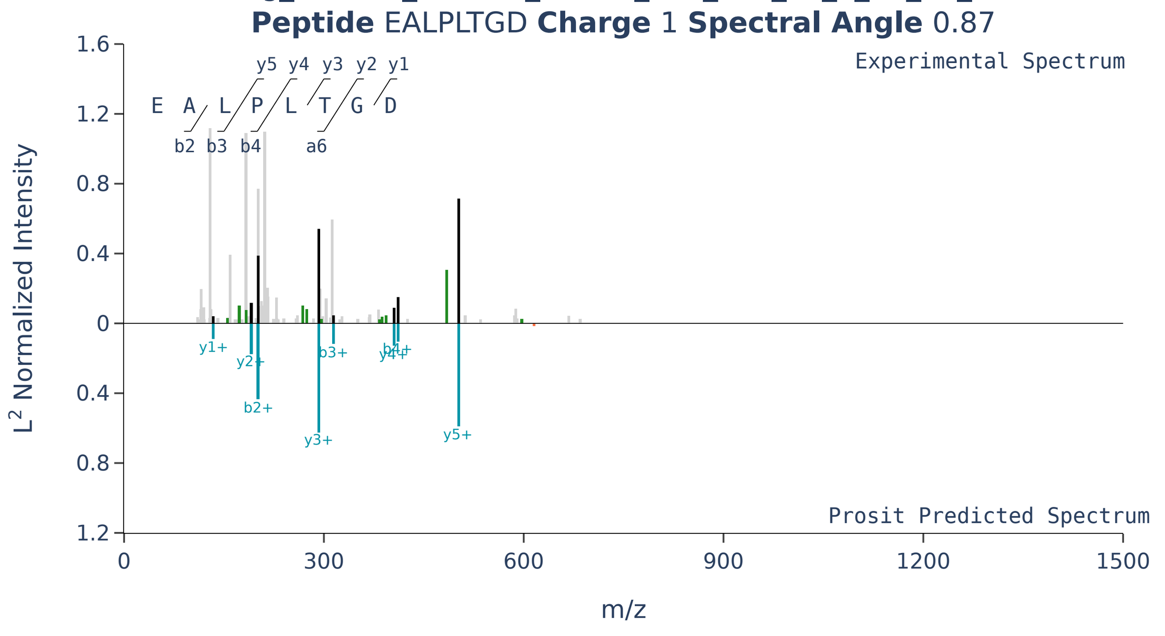

Source Ncheng_210623_230623_HFGoe_FFH_20S_25_1_A2_24h_R2 **Scan** 11464
Peptide DVYRPAAIKQT **Charge** 2 **Spectral Angle** 0.73

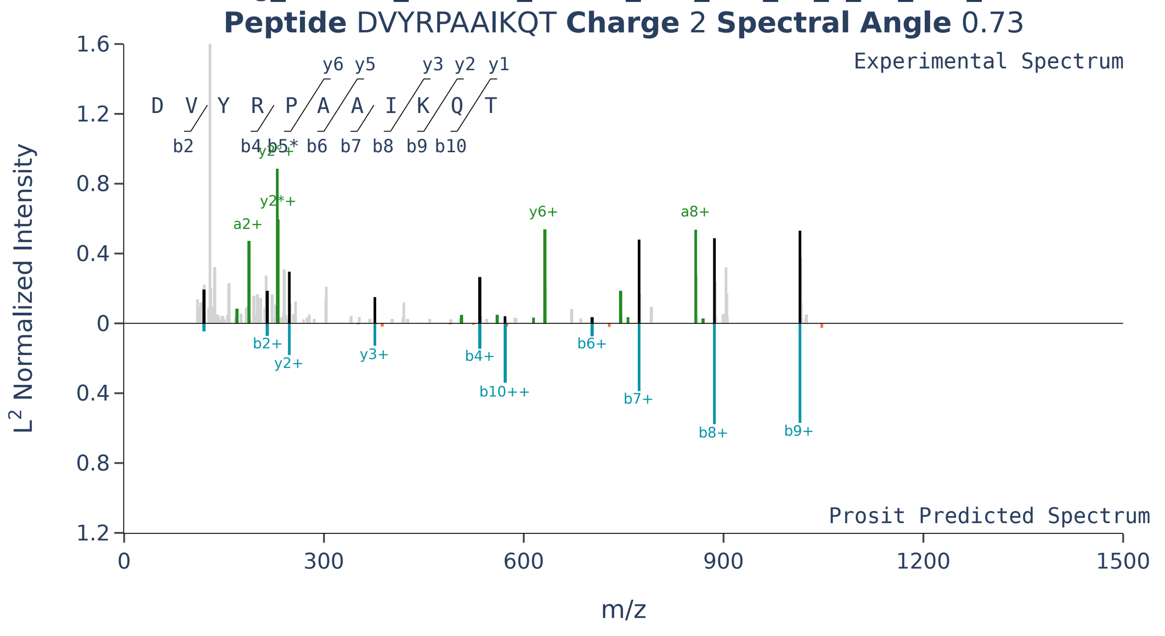

Source Ncheng_210623_230623_HFGoe_FFH_20S_25_1_A1_4h_R1 **Scan** 10910
Peptide RQMKNMGGMASRTL **Charge** 3 **Spectral Angle** 0.84

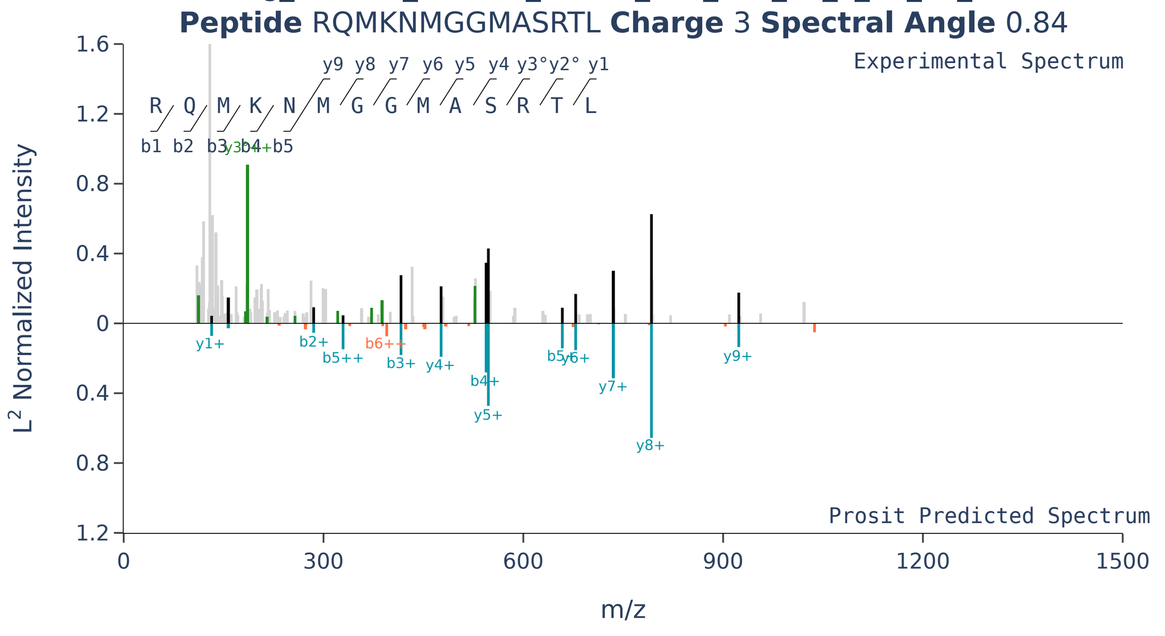

Source Ncheng_210623_230623_HFGoe_FFH_20S_25_1_A2_1h_R2 **Scan** 6795
Peptide SVKAVGH **Charge** 2 **Spectral Angle** 0.81

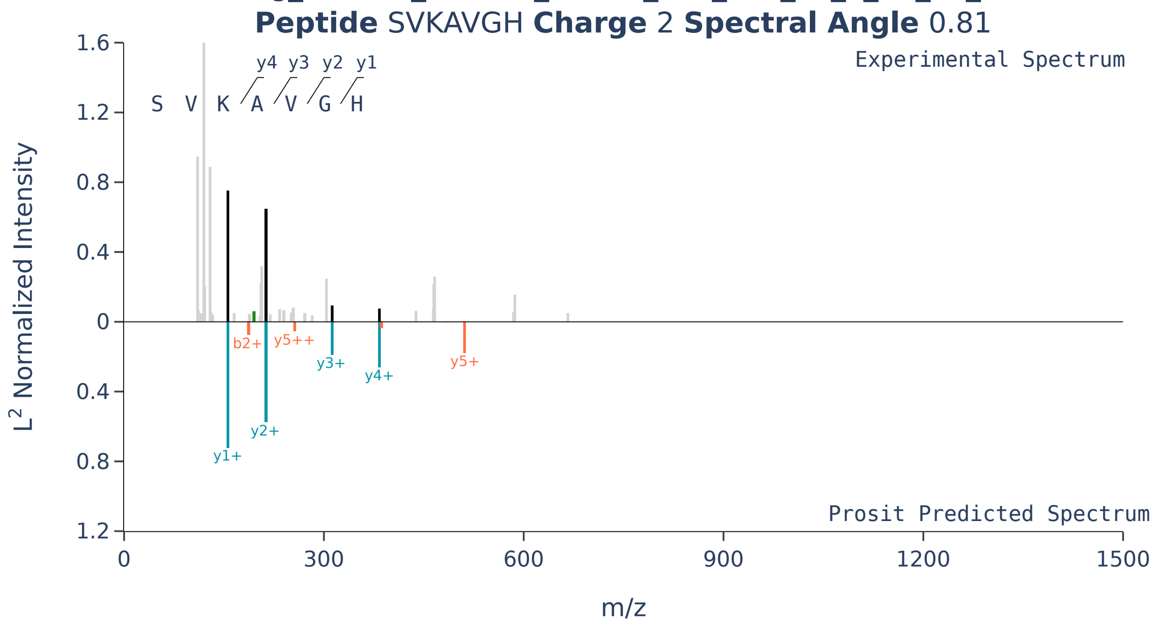

Source Ncheng_210623_230623_HFGoe_FFH_20S_25_1_A2_24h_R2 **Scan** 10795
Peptide AKKGDGFDLND **Charge** 1 **Spectral Angle** 0.79

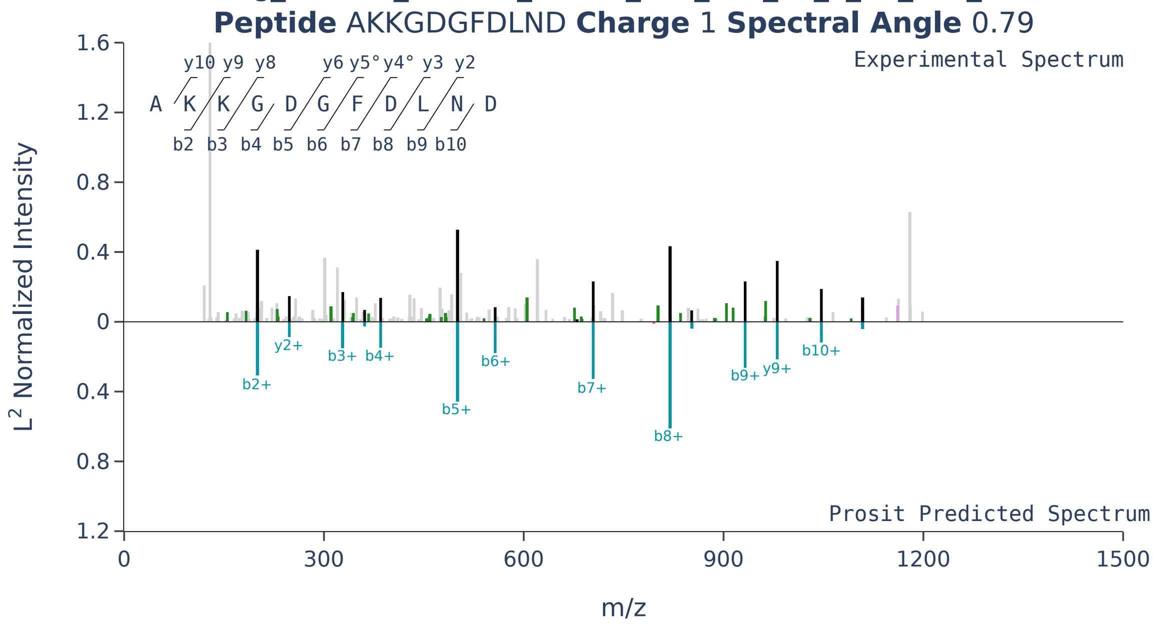

Source Ncheng_210623_230623_HFGoe_FFH_20S_25_1_A2_4h_R2 **Scan** 23800
Peptide EMGKLPGMGQIPDNVKSQMDD **Charge** 2 **Spectral Angle** 0.81

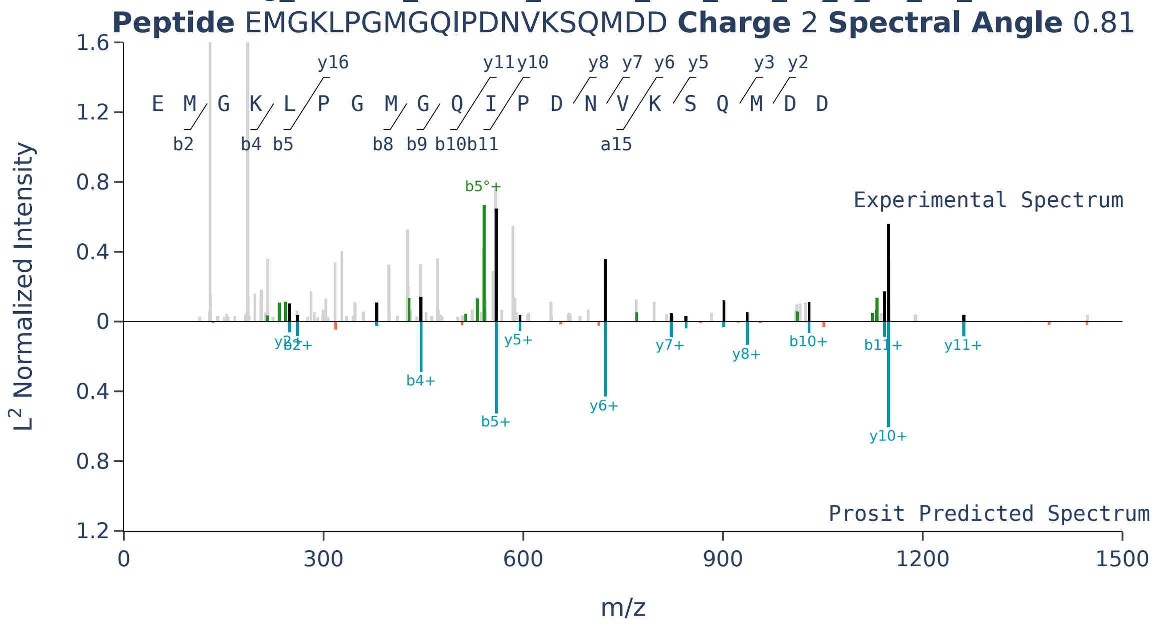

Source Ncheng_210623_230623_HFGoe_FFH_20S_25_1_A1_2h_R2 **Scan** 10156
Peptide HASINPVDE **Charge** 2 **Spectral Angle** 0.82

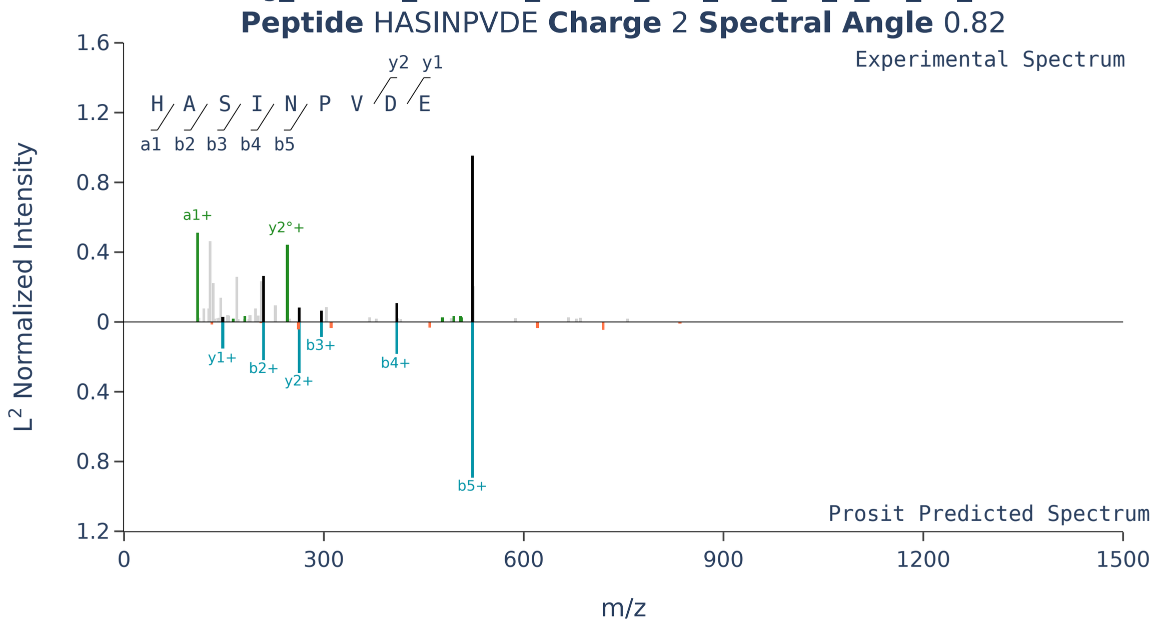

Source Ncheng_210623_230623_HFGoe_FFH_20S_25_1_A2_4h_R1 **Scan** 15998
Peptide KLPGMGMGQIPDNVKSQ **Charge** 2 **Spectral Angle** 0.84

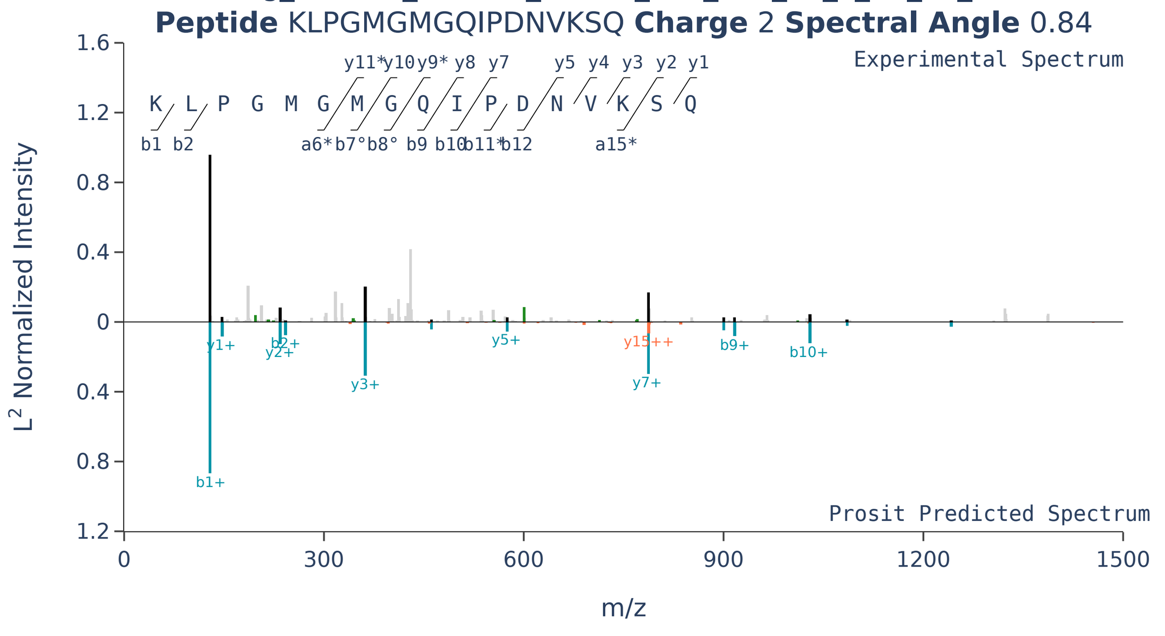

Source Ncheng_210623_230623_HFGoe_FFH_20S_25_1_A2_24h_R1 **Scan** 7085
Peptide GEENQTVGQKPVD **Charge** 2 **Spectral Angle** 0.8

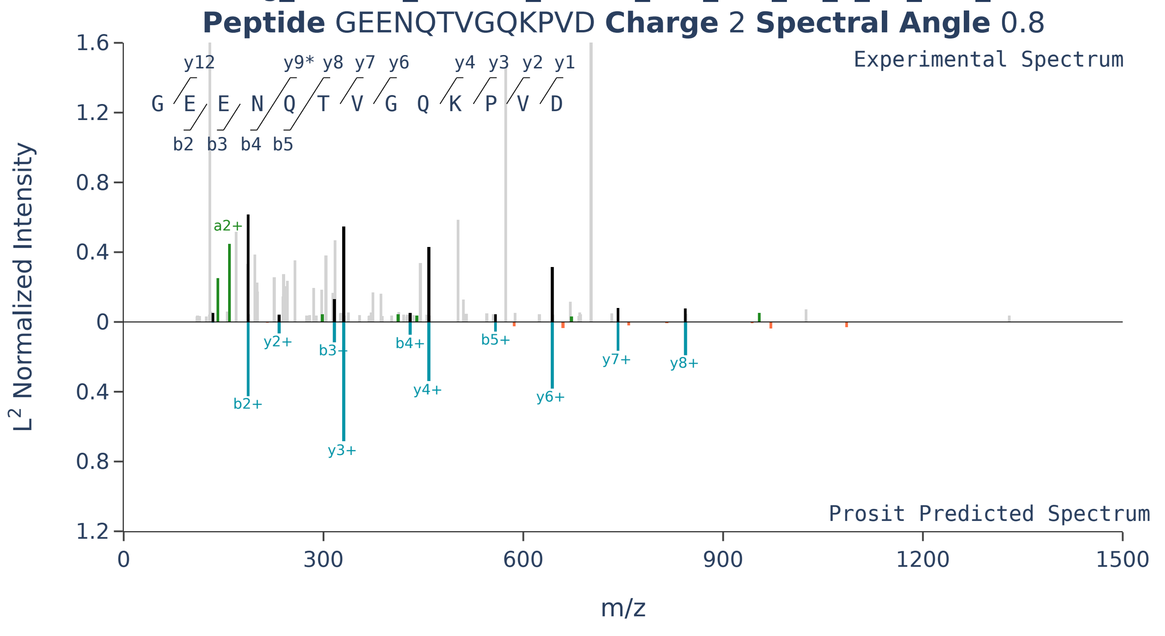

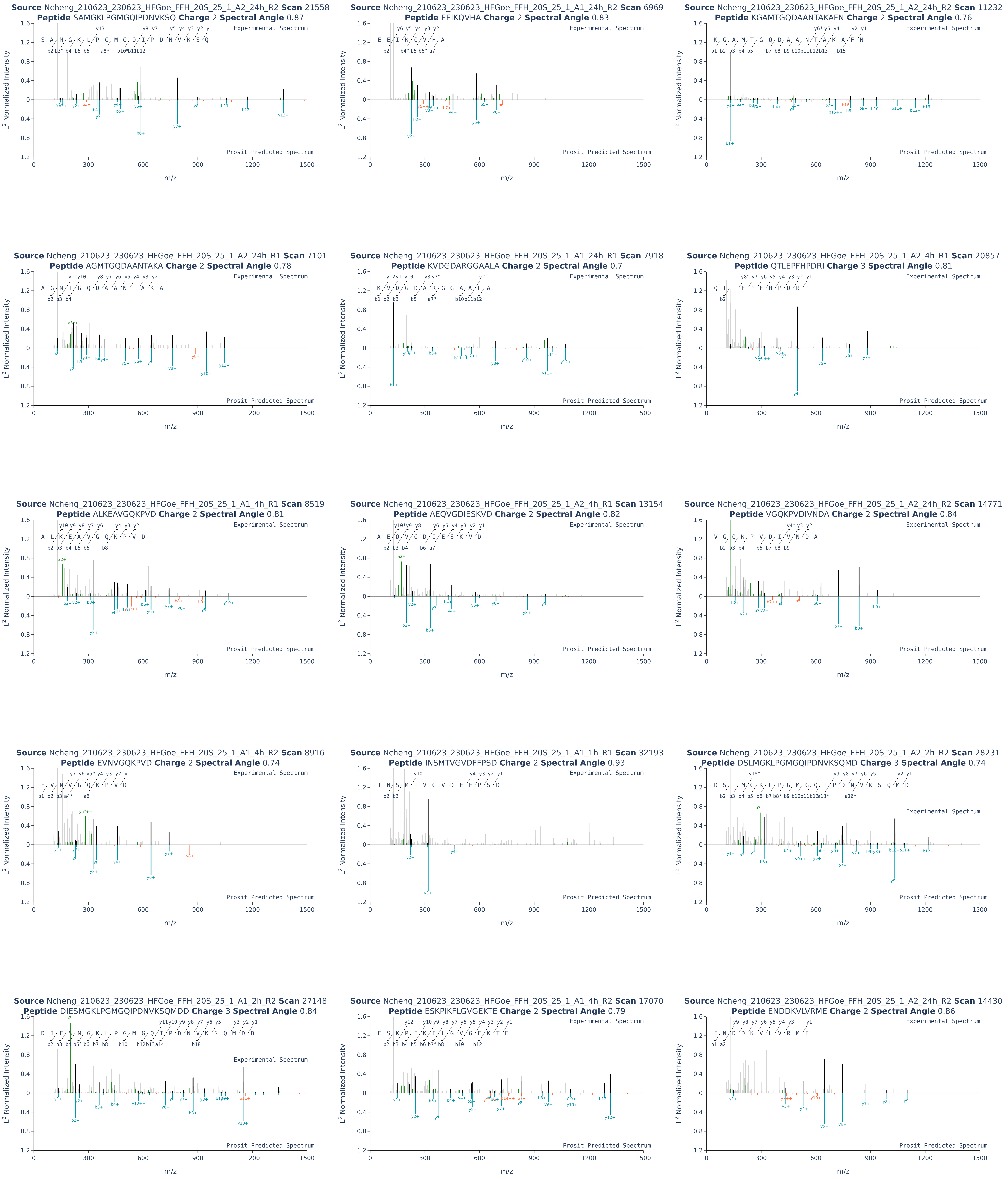
Source Ncheng_210623_230623_HFGoe_FFH_20S_25_1_A2_24h_R2 Scan 21558
Peptide SAMGKLPGMGQIPDNVKSQ Charge 2 Spectral Angle 0.87
Source Ncheng_210623_230623_HFGoe_FFH_20S_25_1_A1_24h_R2 Scan 6969
Peptide EEIKQVHA Charge 2 Spectral Angle 0.83
Source Ncheng_210623_230623_HFGoe_FFH_20S_25_1_A2_24h_R2 Scan 11232
Peptide KGAMTGQDAANTAKAFN Charge 2 Spectral Angle 0.76
Source Ncheng_210623_230623_HFGoe_FFH_20S_25_1_A2_24h_R1 Scan 7101
Peptide AGMTGQDAANTAKA Charge 2 Spectral Angle 0.78
Source Ncheng_210623_230623_HFGoe_FFH_20S_25_1_A1_24h_R1 Scan 7918
Peptide KVDGDARGGAALA Charge 2 Spectral Angle 0.7
Source Ncheng_210623_230623_HFGoe_FFH_20S_25_1_A2_4h_R1 Scan 20857
Peptide QTLEPFHPDRI Charge 3 Spectral Angle 0.81
Source Ncheng_210623_230623_HFGoe_FFH_20S_25_1_A1_4h_R1 Scan 8519
Peptide ALKEAVGQKPVD Charge 2 Spectral Angle 0.81
Source Ncheng_210623_230623_HFGoe_FFH_20S_25_1_A2_4h_R1 Scan 13154
Peptide AEQVGDIESKVD Charge 2 Spectral Angle 0.82
Source Ncheng_210623_230623_HFGoe_FFH_20S_25_1_A2_24h_R2 Scan 14771
Peptide VGQKPVDIVNDA Charge 2 Spectral Angle 0.84
Source Ncheng_210623_230623_HFGoe_FFH_20S_25_1_A1_4h_R2 Scan 8916
Peptide EVNVGQKPVD Charge 2 Spectral Angle 0.74
Source Ncheng_210623_230623_HFGoe_FFH_20S_25_1_A1_1h_R1 Scan 32193
Peptide INSMTVGVDFFPSD Charge 2 Spectral Angle 0.93
Source Ncheng_210623_230623_HFGoe_FFH_20S_25_1_A2_2h_R2 Scan 28231
Peptide DSLMGKLPGMGQIPDNVKSQMD Charge 3 Spectral Angle 0.74
Source Ncheng_210623_230623_HFGoe_FFH_20S_25_1_A1_2h_R2 Scan 27148
Peptide DIESMGKLPGMGQIPDNVKSQMDD Charge 3 Spectral Angle 0.84
Source Ncheng_210623_230623_HFGoe_FFH_20S_25_1_A1_4h_R2 Scan 17070
Peptide ESKPIKFLGVGEKTE Charge 2 Spectral Angle 0.79
Source Ncheng_210623_230623_HFGoe_FFH_20S_25_1_A2_24h_R2 Scan 14430
Peptide ENDDKVLVRME Charge 2 Spectral Angle 0.86
Experimental Spectrum
Prosit Predicted Spectrum
L² Normalized Intensity
m/z

Source Ncheng_210623_230623_HFGoe_FFH_20S_25_1_A2_24h_R2 **Scan** 18058
Peptide KLKFYSADV **Charge** 3 **Spectral Angle** 0.82

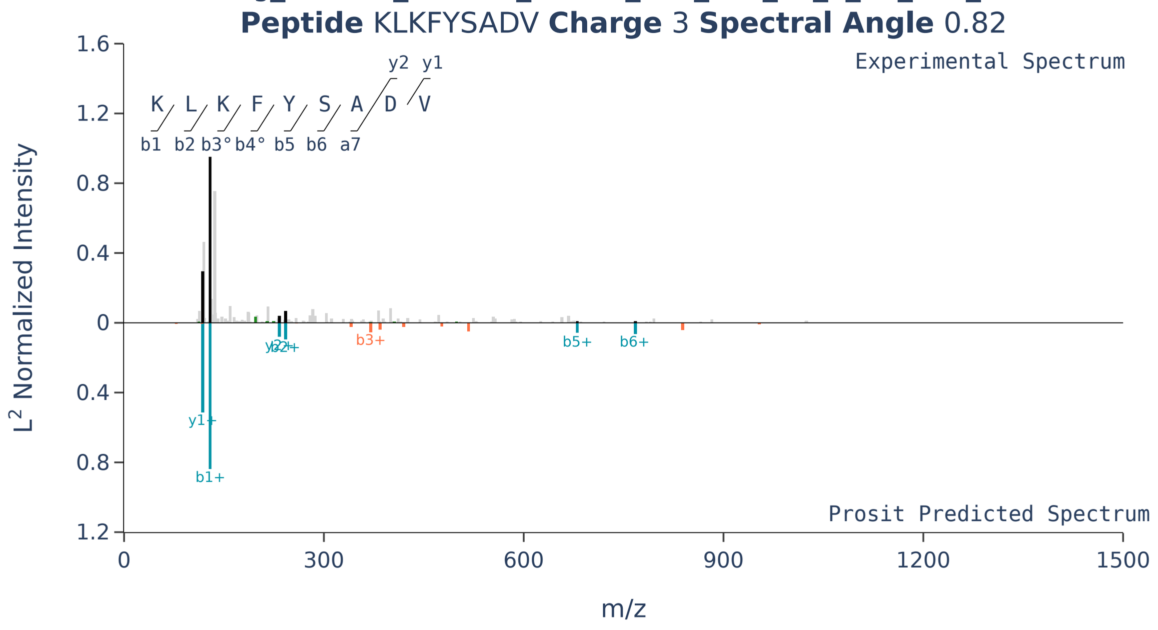

Source Ncheng_210623_230623_HFGoe_FFH_20S_25_1_A2_4h_R2 **Scan** 24055
Peptide ALKEAMGKLPGMGQIPDNVKSQMDD **Charge** 3 **Spectral Angle** 0.87

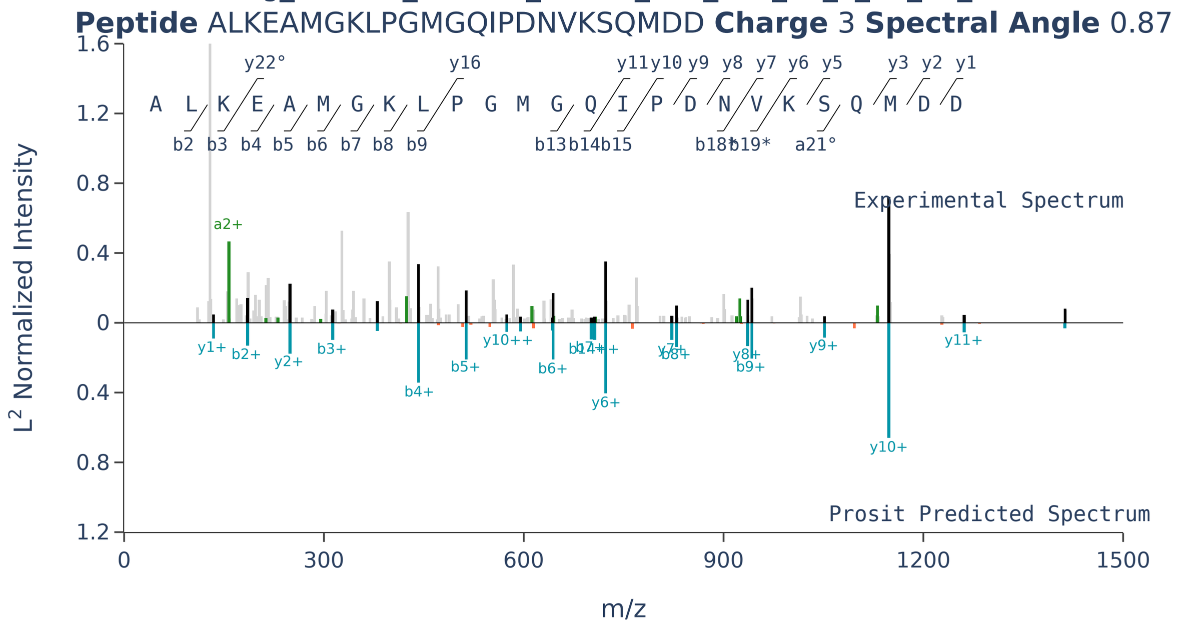

Source Ncheng_210623_230623_HFGoe_FFH_20S_25_1_A1_24h_R2 **Scan** 14247
Peptide PGMGQIPDNVKSN **Charge** 2 **Spectral Angle** 0.72

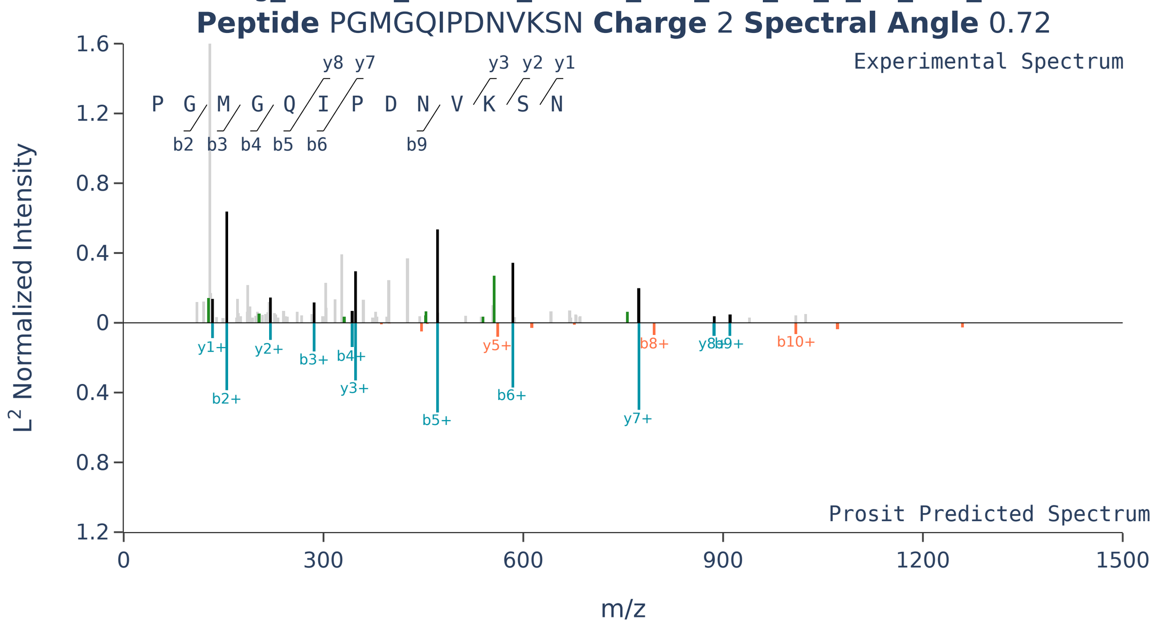

Source Ncheng_210623_230623_HFGoe_FFH_20S_25_1_A2_4h_R1 **Scan** 6886
Peptide NKSLTPGDAA **Charge** 1 **Spectral Angle** 0.8

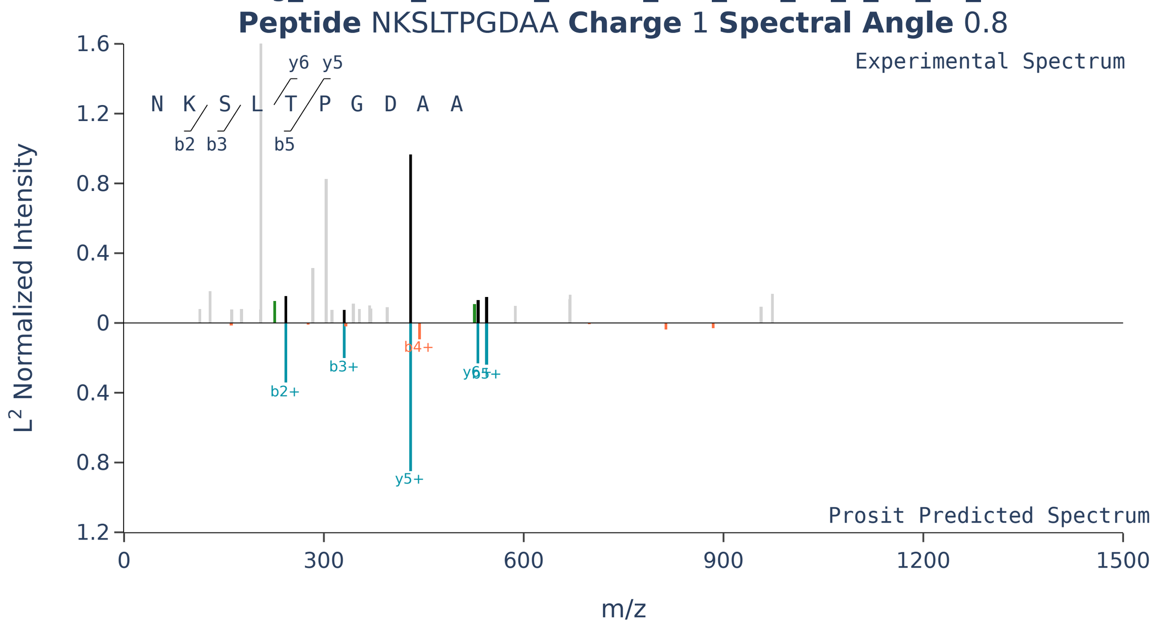

Source Ncheng_210623_230623_HFGoe_FFH_20S_25_1_A2_24h_R2 **Scan** 15707
Peptide RQMVGQKPVDIVNA **Charge** 3 **Spectral Angle** 0.68

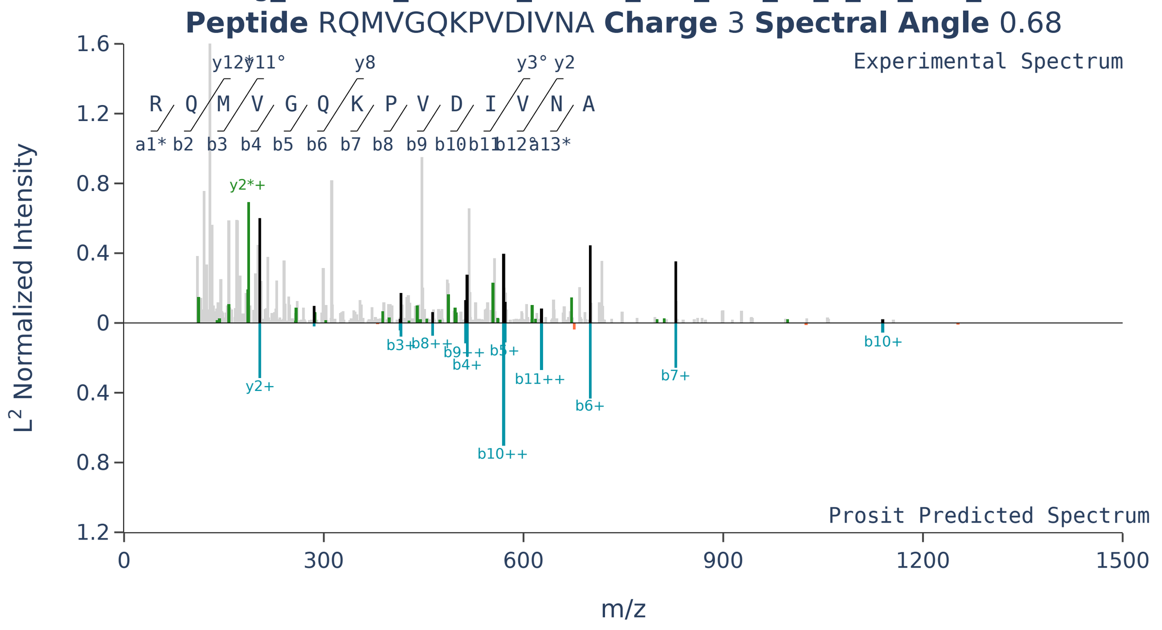

Source Ncheng_210623_230623_HFGoe_FFH_20S_25_1_A2_24h_R1 **Scan** 8682
Peptide DDEIKQVHA **Charge** 2 **Spectral Angle** 0.82

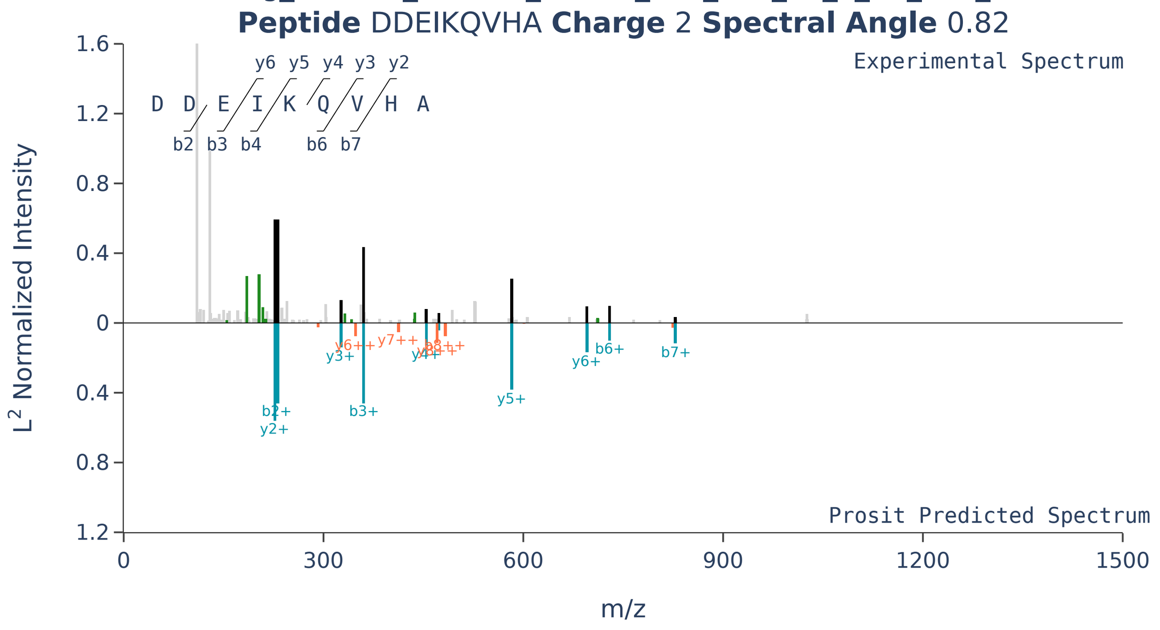

Source Ncheng_210623_230623_HFGoe_FFH_20S_25_1_A1_4h_R2 **Scan** 18893
Peptide GKLPGMMGQIPDNVKSQM **Charge** 3 **Spectral Angle** 0.74

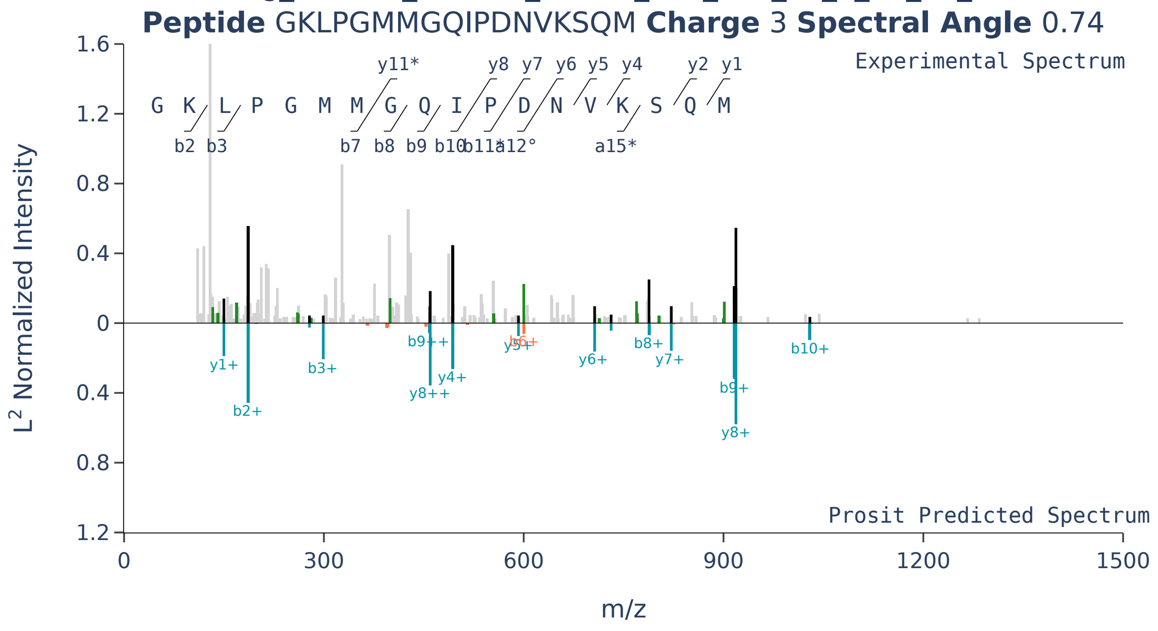

Source Ncheng_210623_230623_HFGoe_FFH_20S_25_1_A2_24h_R2 **Scan** 16993
Peptide HALEPFHPDRI **Charge** 3 **Spectral Angle** 0.77

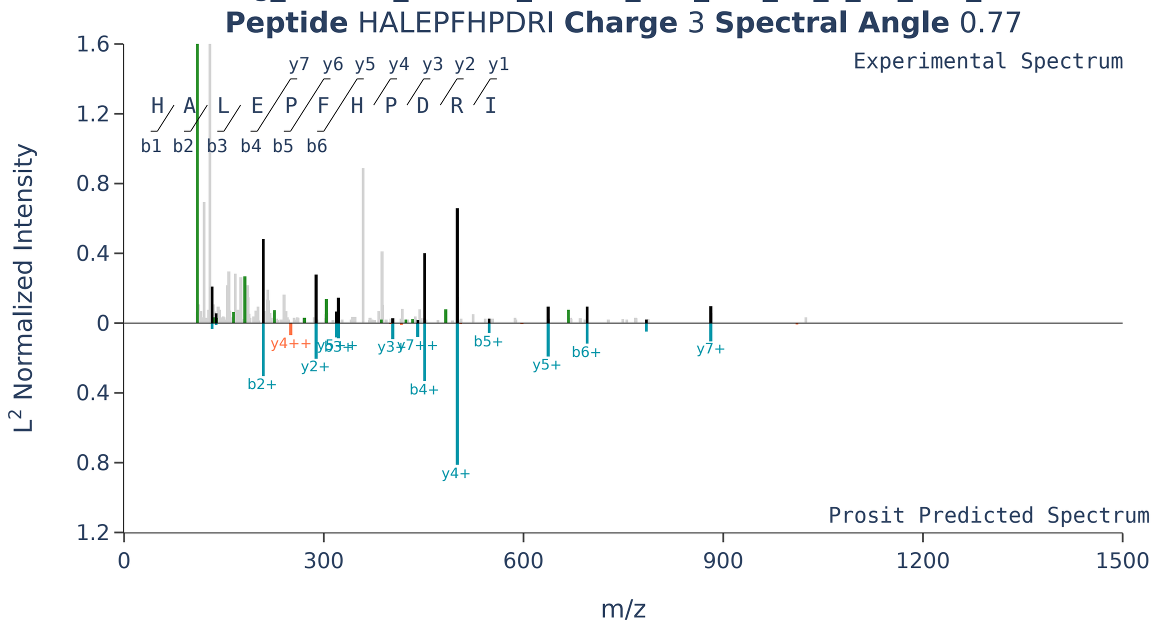

Source Ncheng_210623_230623_HFGoe_FFH_20S_25_1_A2_24h_R1 **Scan** 7361
Peptide KNMGGMAA **Charge** 2 **Spectral Angle** 0.72

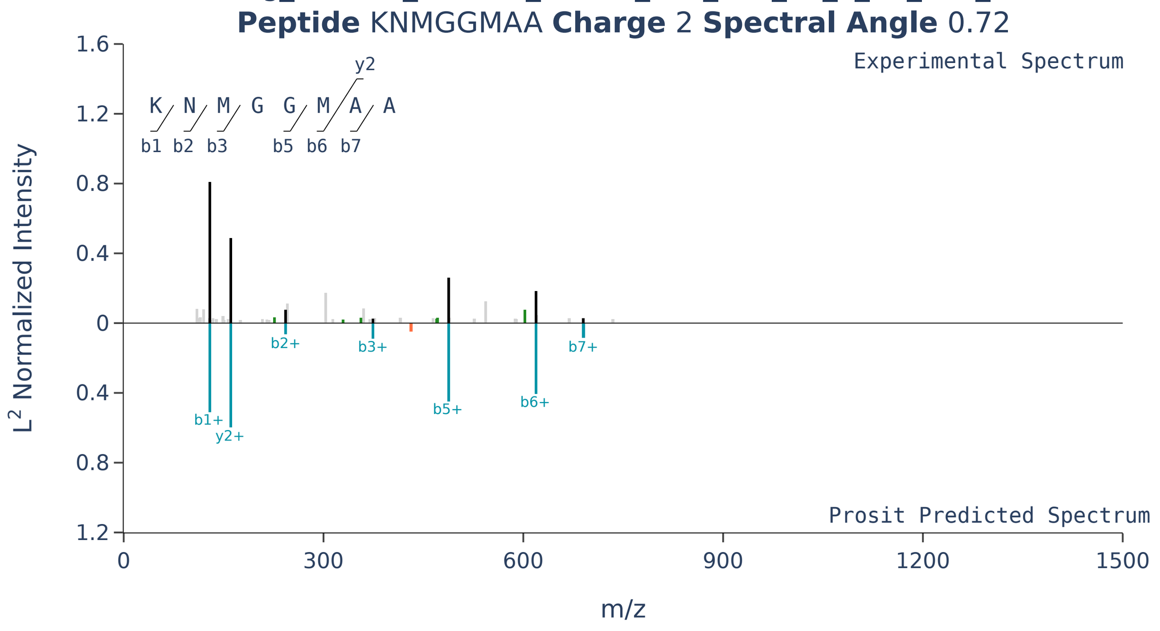

Source Ncheng_210623_230623_HFGoe_FFH_20S_25_1_A2_24h_R1 **Scan** 11373
Peptide ASKAMTGQDAANTAKAFN **Charge** 2 **Spectral Angle** 0.78

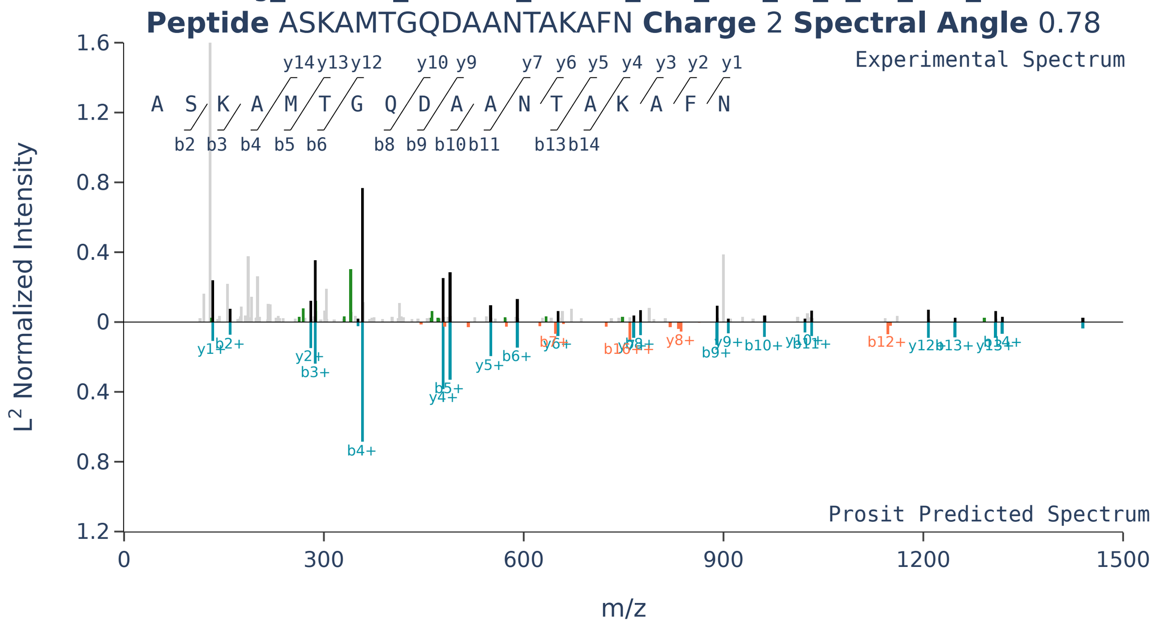

Source Ncheng_210623_230623_HFGoe_FFH_20S_25_1_A1_4h_R2 **Scan** 18019
Peptide SADVYRPAAIKQLDE **Charge** 2 **Spectral Angle** 0.81

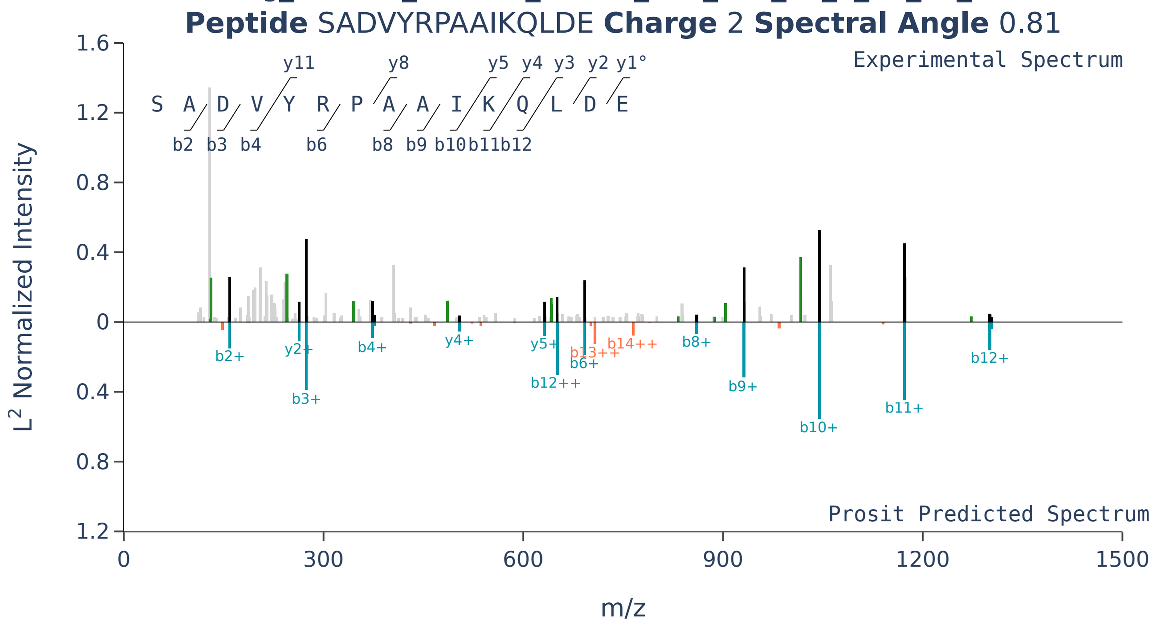

Source Ncheng_210623_230623_HFGoe_FFH_20S_25_1_A1_24h_R1 **Scan** 23749
Peptide HAGVDFFPSD **Charge** 2 **Spectral Angle** 0.77

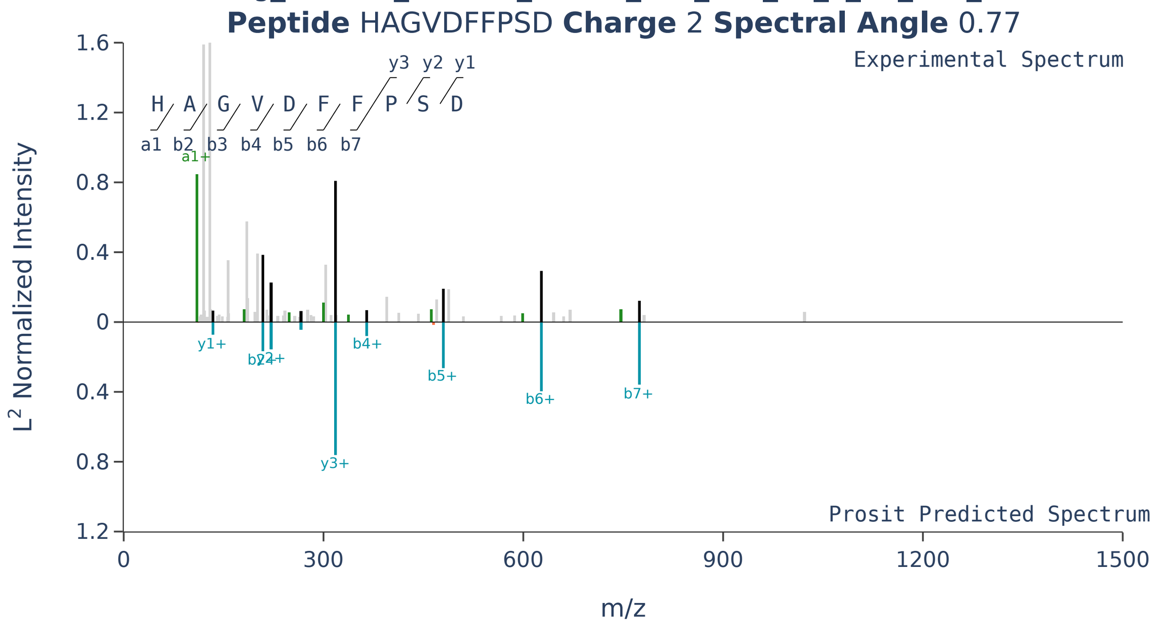

Source Ncheng_210623_230623_HFGoe_FFH_20S_25_1_A2_4h_R1 **Scan** 10615
Peptide PFHPDRISAD **Charge** 2 **Spectral Angle** 0.81

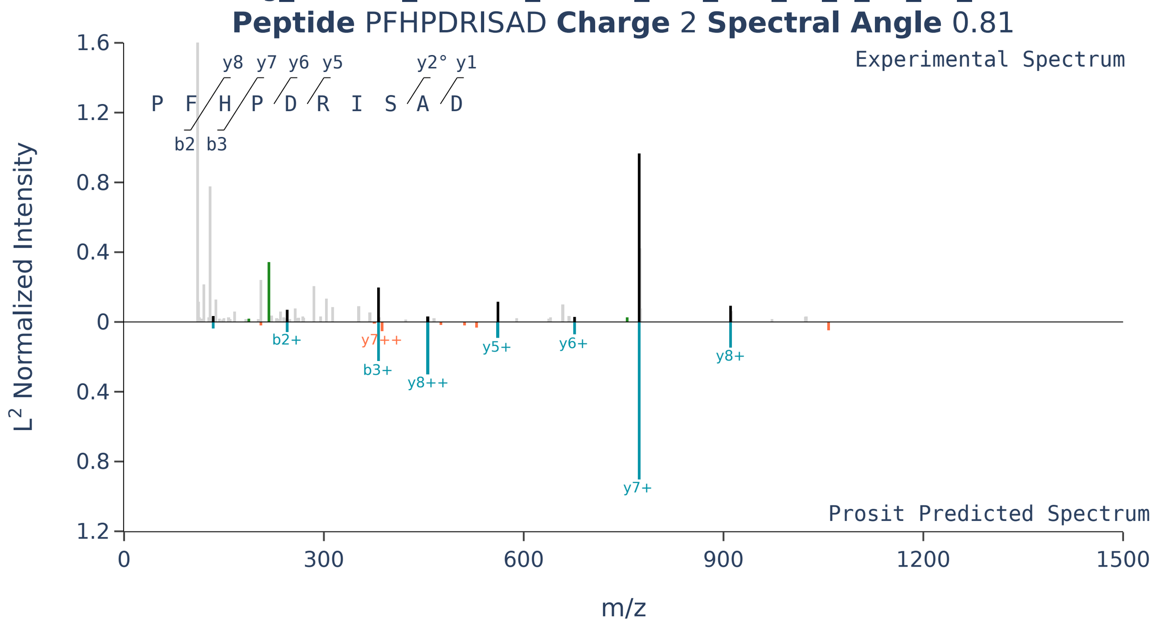

Source Ncheng_210623_230623_HFGoe_FFH_20S_25_1_A2_2h_R2 **Scan** 7108
Peptide RMALQVHA **Charge** 1 **Spectral Angle** 0.8

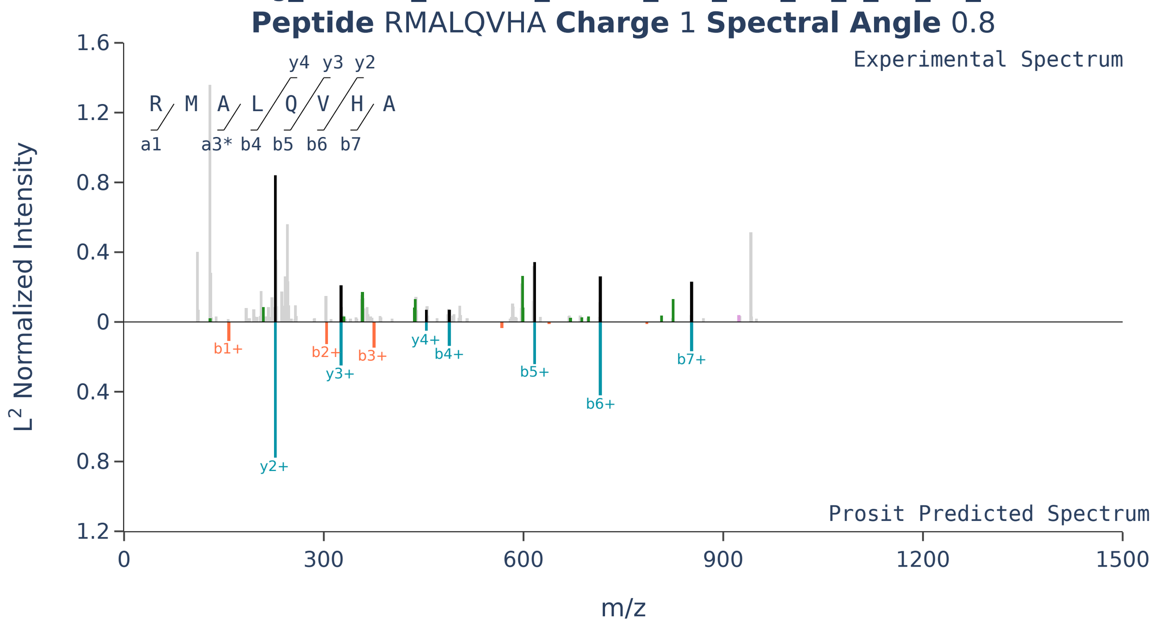

Source Ncheng_210623_230623_HFGoe_FFH_20S_25_1_A2_24h_R1 **Scan** 18071
Peptide VVLTKKGDGFDLND **Charge** 2 **Spectral Angle** 0.87

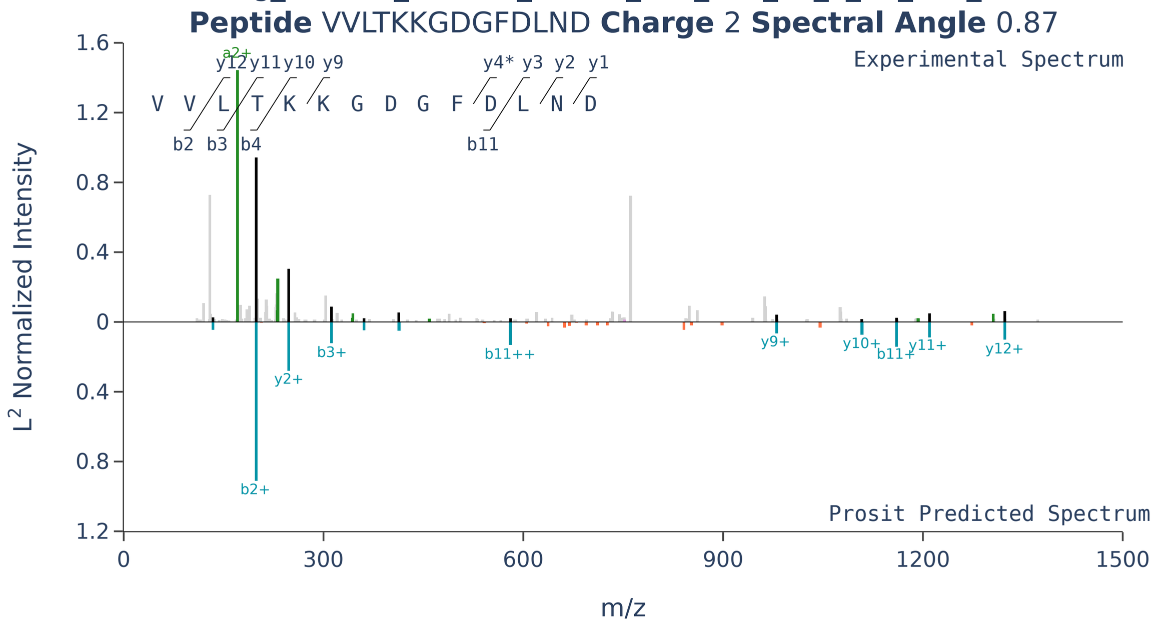

Source Ncheng_210623_230623_HFGoe_FFH_20S_25_1_A1_24h_R2 **Scan** 16060
Peptide MGKLPGMGQIPDVNKS **Charge** 2 **Spectral Angle** 0.75

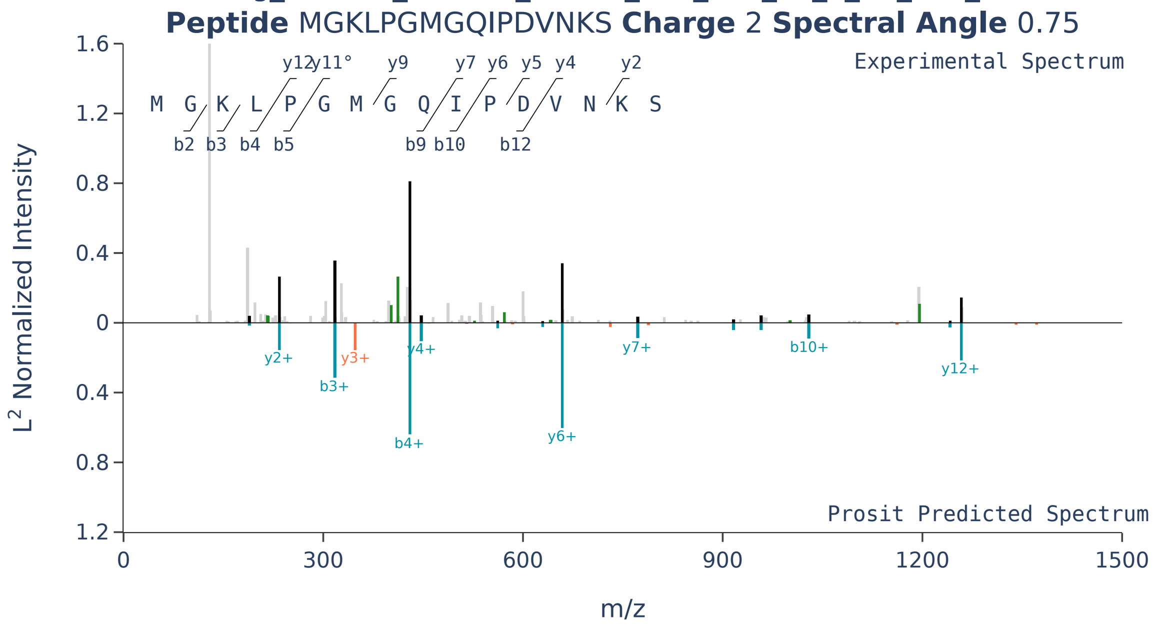

Source Ncheng_210623_230623_HFGoe_FFH_20S_25_1_A2_24h_R1 **Scan** 7030
Peptide GQIPDNVKKS **Charge** 2 **Spectral Angle** 0.82

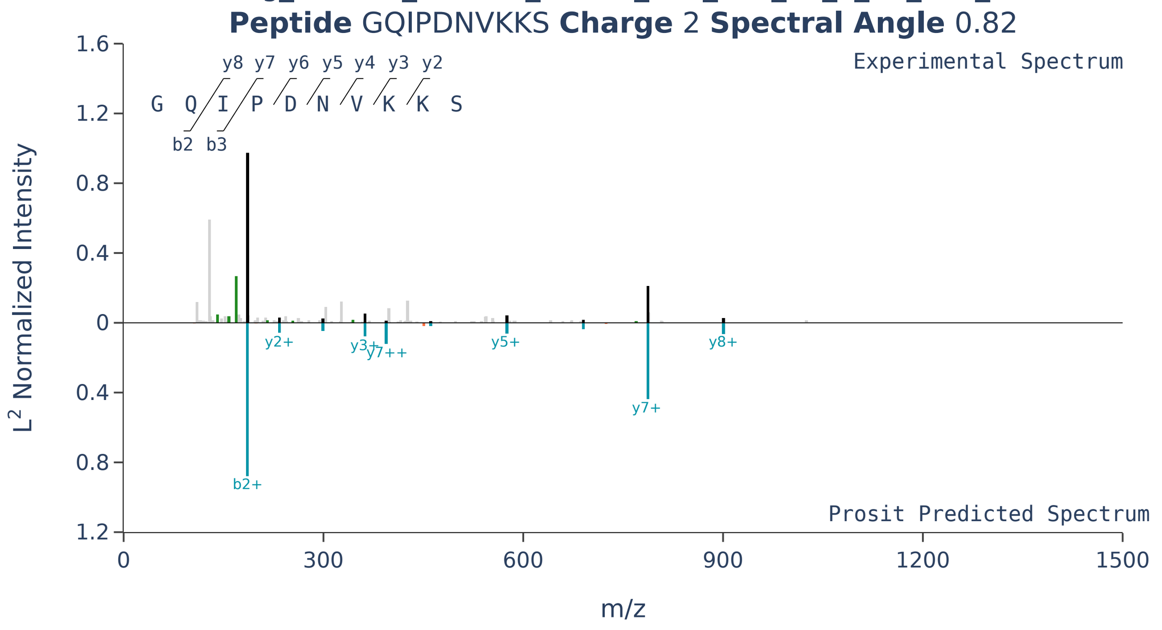

Source Ncheng_210623_230623_HFGoe_FFH_20S_25_1_A1_4h_R1 **Scan** 15574
Peptide ALEPFHPDRIASKTE **Charge** 3 **Spectral Angle** 0.71

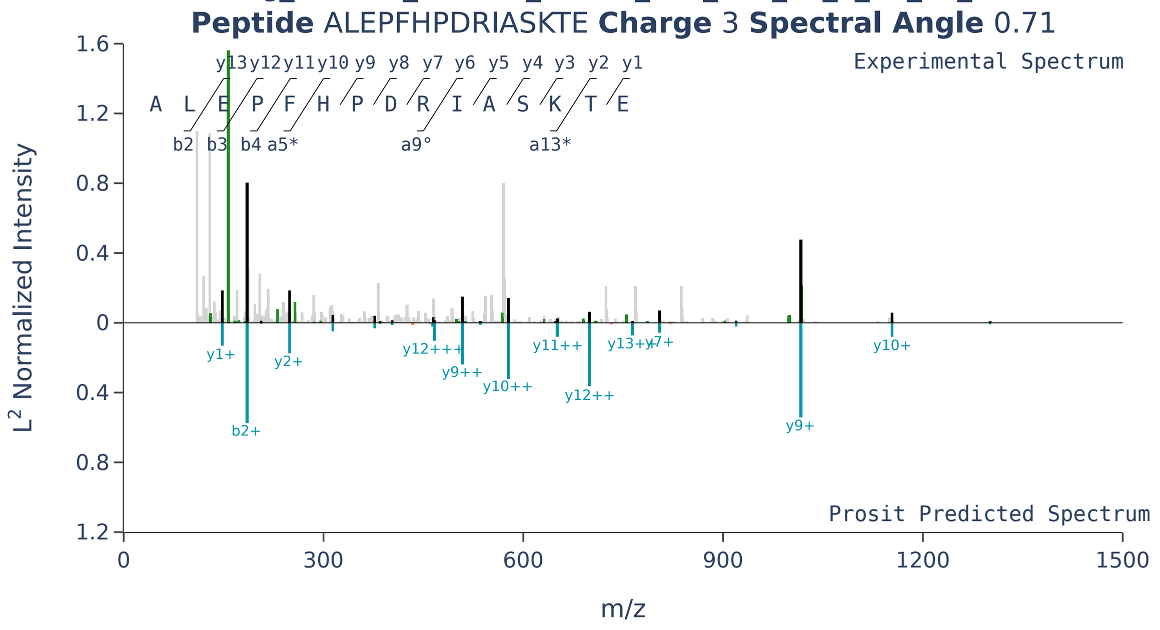

Source Ncheng_210623_230623_HFGoe_FFH_20S_25_1_A2_4h_R1 **Scan** 10333
Peptide MGDIESKVDRA **Charge** 3 **Spectral Angle** 0.8

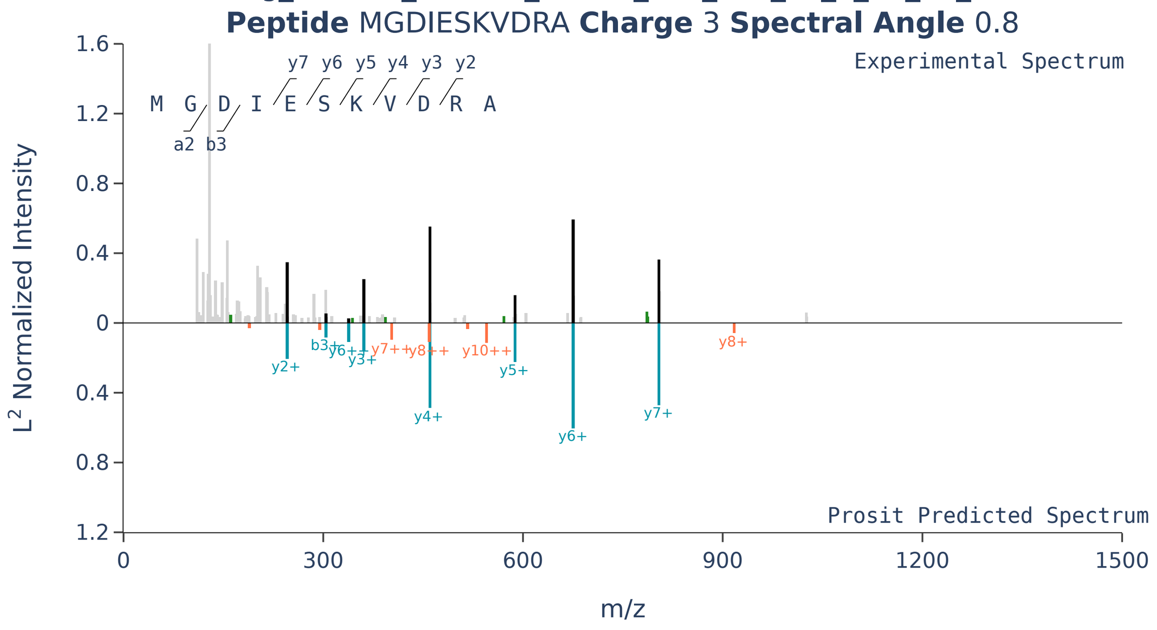

Source Ncheng_210623_230623_HFGoe_FFH_20S_25_1_A1_24h_R1 **Scan** 14672
Peptide AAGAMTGQDAANTAKAFN **Charge** 2 **Spectral Angle** 0.87

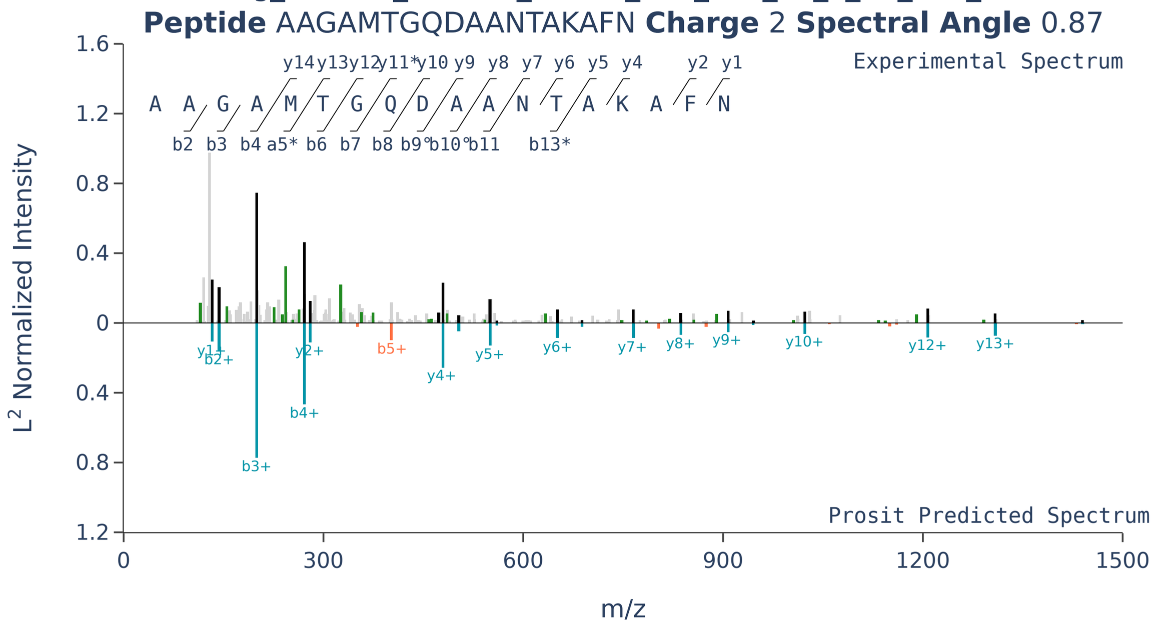

Source Ncheng_210623_230623_HFGoe_FFH_20S_25_1_A1_4h_R1 **Scan** 17737
Peptide KTEDIESKVDRAQAEKLASKL **Charge** 3 **Spectral Angle** 0.95

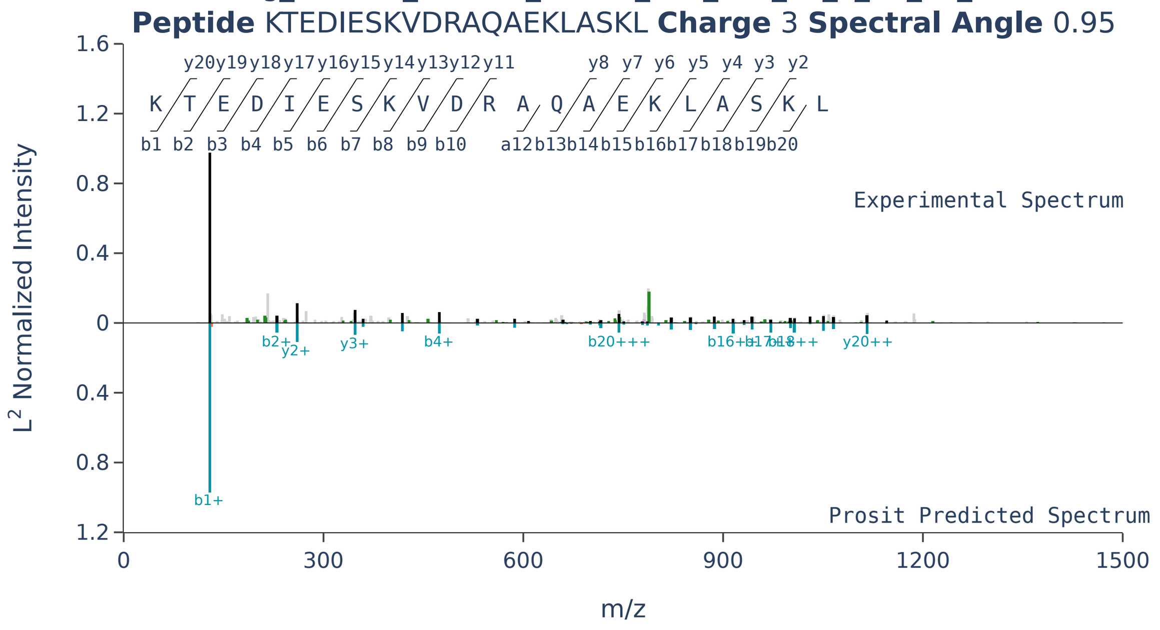

Source Ncheng_210623_230623_HFGoe_FFH_20S_25_1_A1_4h_R2 **Scan** 17749
Peptide TSDVYRPAAIKQLE **Charge** 2 **Spectral Angle** 0.71

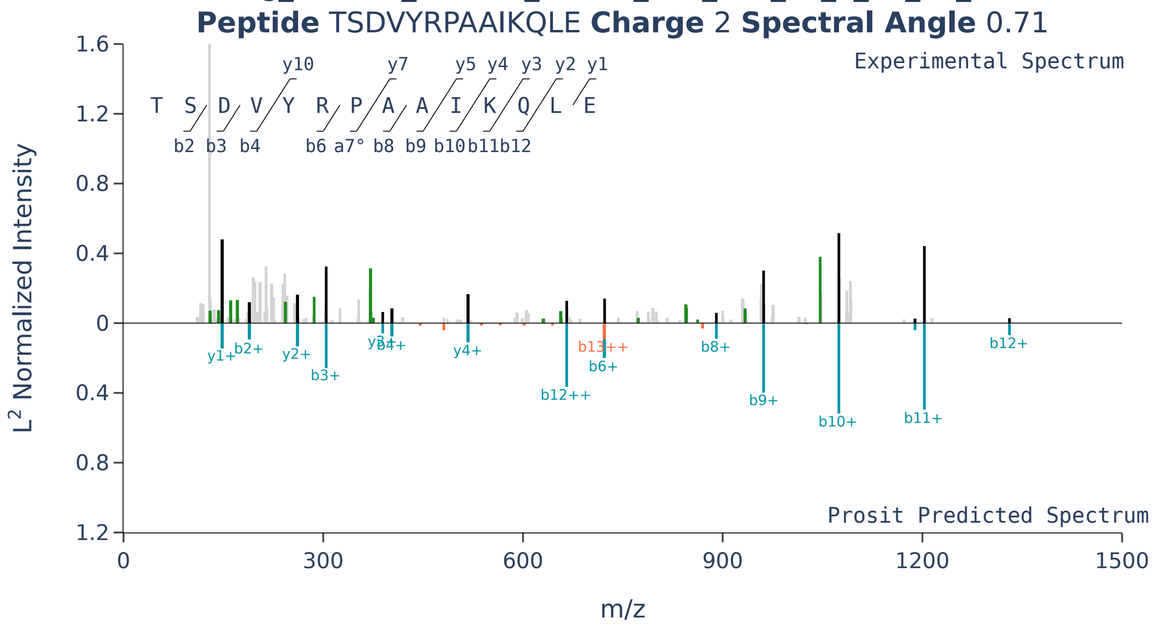

Source Ncheng_210623_230623_HFGoe_FFH_20S_25_1_A1_24h_R1 **Scan** 25716
Peptide ARGGAALSDFFPSD **Charge** 2 **Spectral Angle** 0.83

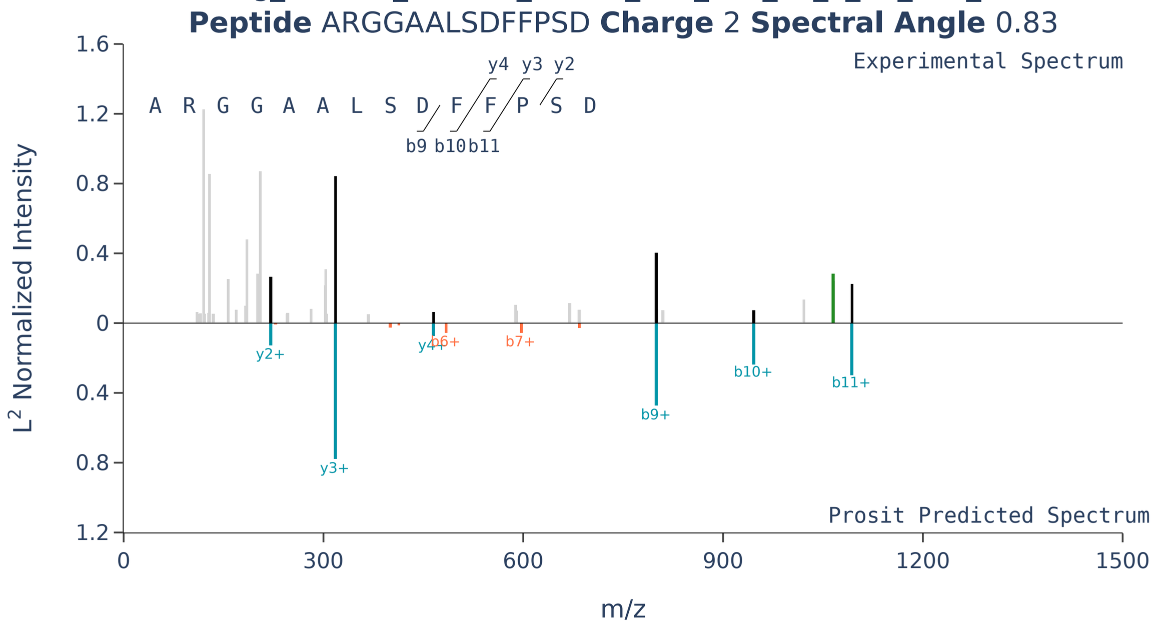

Source Ncheng_210623_230623_HFGoe_FFH_20S_25_1_A2_2h_R2 **Scan** 19686
Peptide PGMGQIVDG **Charge** 1 **Spectral Angle** 0.67

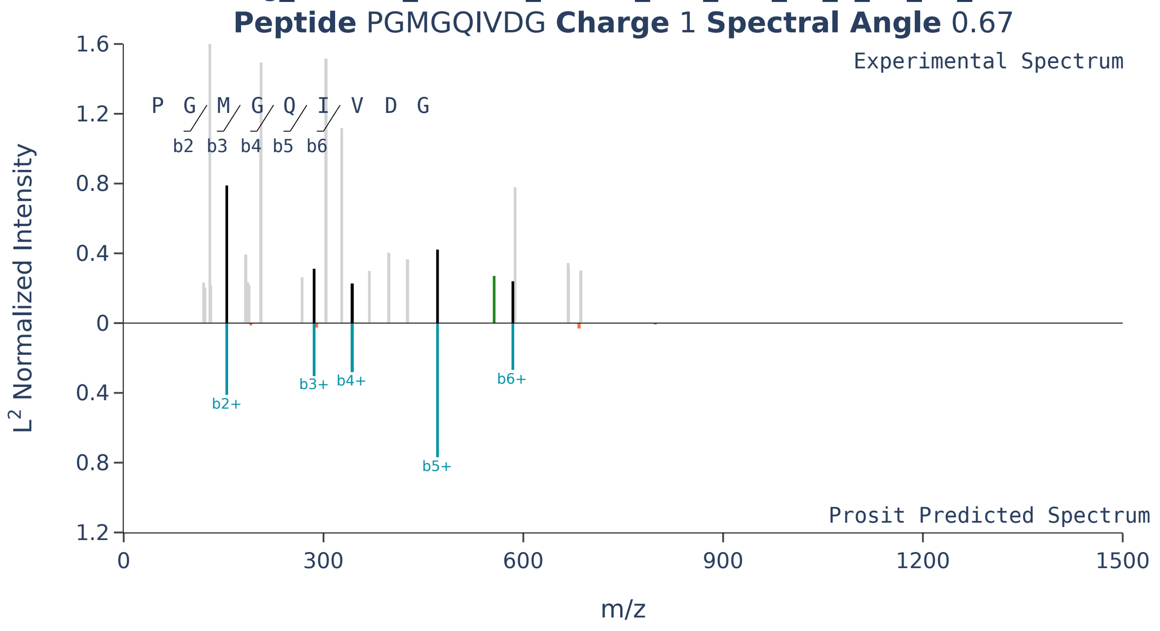

Source Ncheng_210623_230623_HFGoe_FFH_20S_25_1_A1_24h_R2 **Scan** 26404
Peptide QRMVDFFPSD **Charge** 2 **Spectral Angle** 0.82

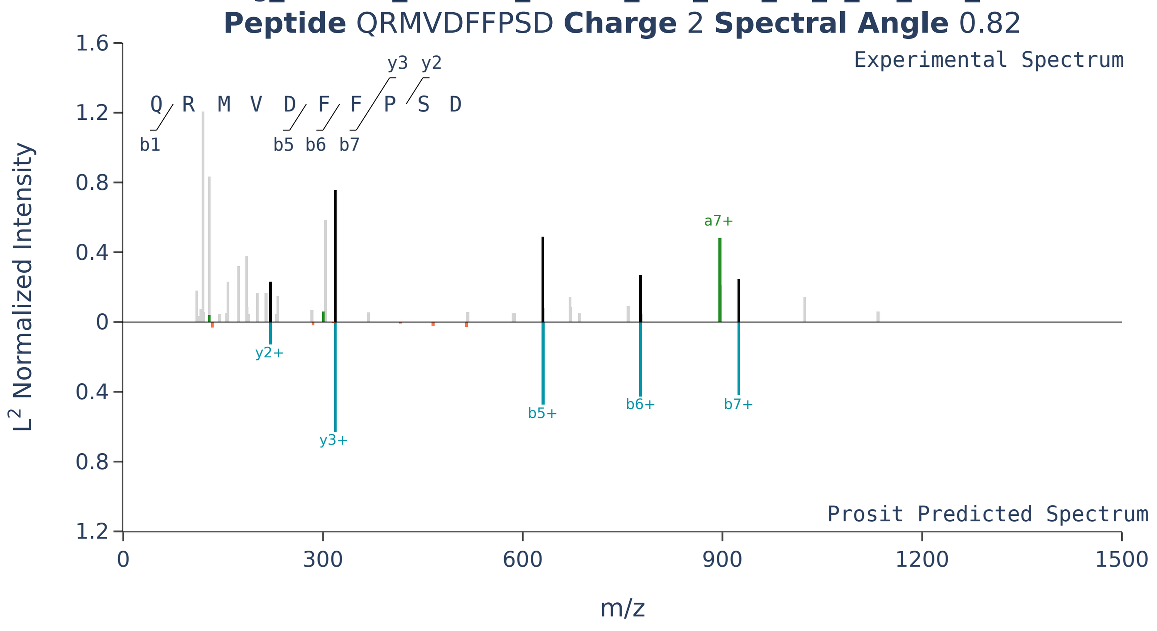

Source Ncheng_210623_230623_HFGoe_FFH_20S_25_1_A2_4h_R1 **Scan** 16095
Peptide SINQKPVDIVNA **Charge** 2 **Spectral Angle** 0.77

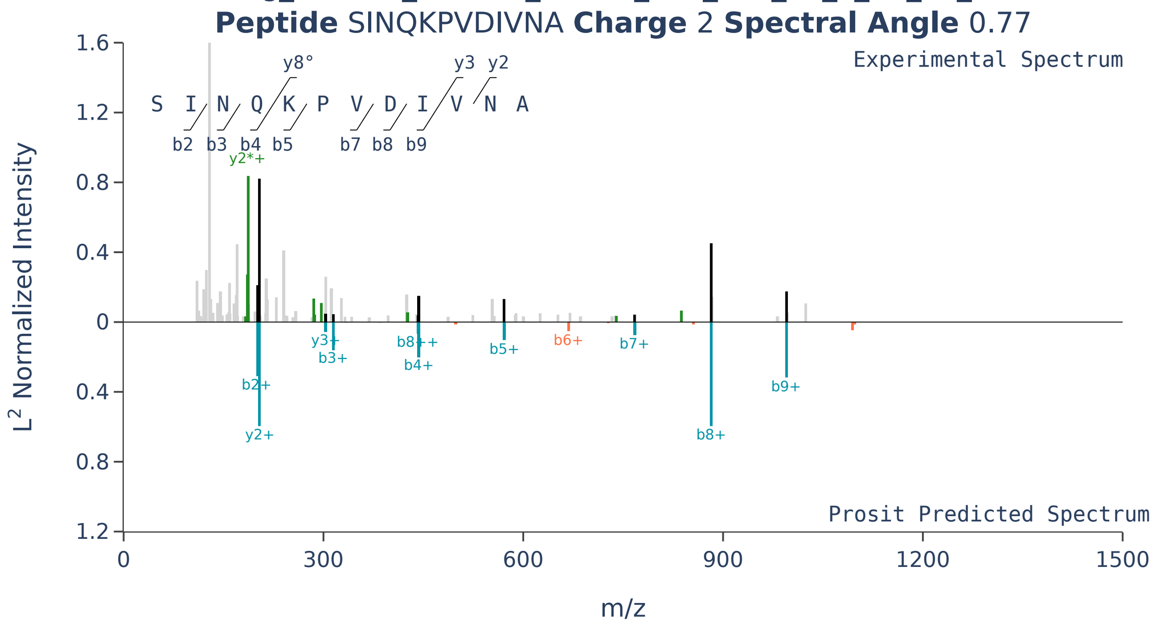

Source Ncheng_210623_230623_HFGoe_FFH_20S_25_1_A1_24h_R1 **Scan** 16972
Peptide LNGQKPVDIVNA **Charge** 2 **Spectral Angle** 0.81

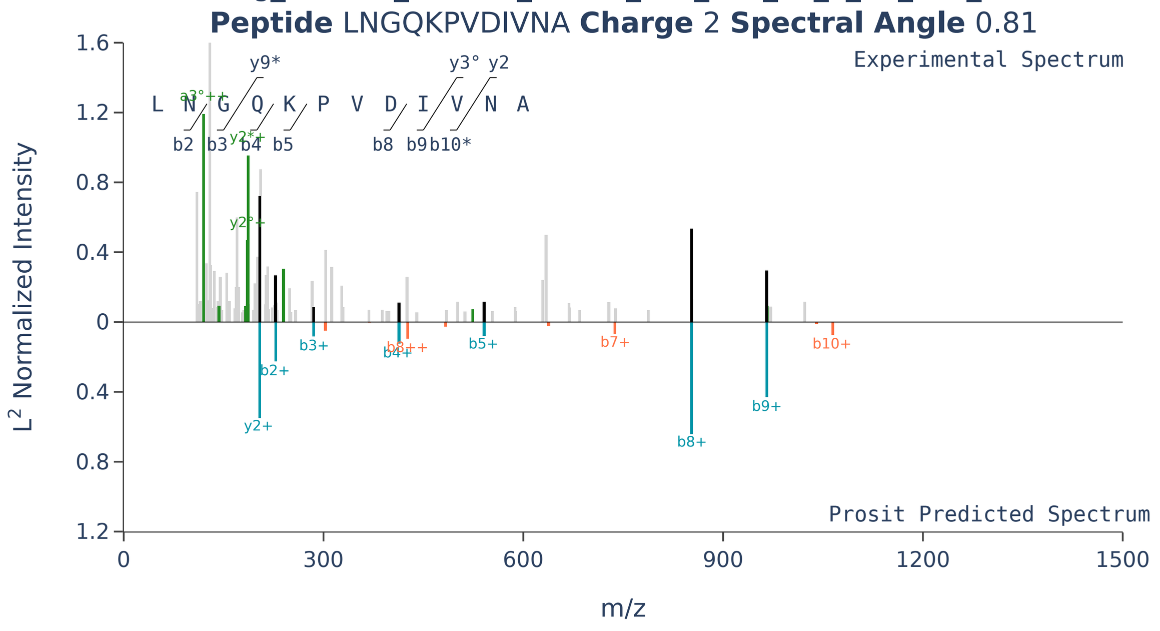

Source Ncheng_210623_230623_HFGoe_FFH_20S_25_1_A2_4h_R2 **Scan** 23478
Peptide SAMGKLPGMGQIPDNVKSQMD **Charge** 3 **Spectral Angle** 0.73

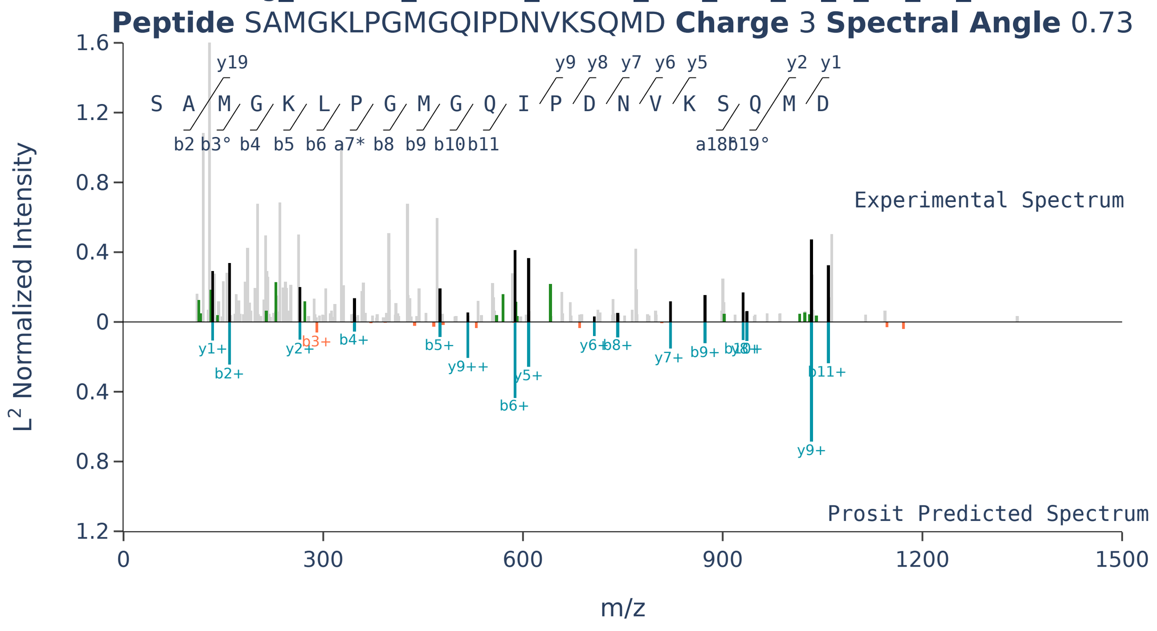

Source Ncheng_210623_230623_HFGoe_FFH_20S_25_1_A1_24h_R1 **Scan** 19244
Peptide GMKLPGMGQIPDNVKSQM **Charge** 2 **Spectral Angle** 0.83

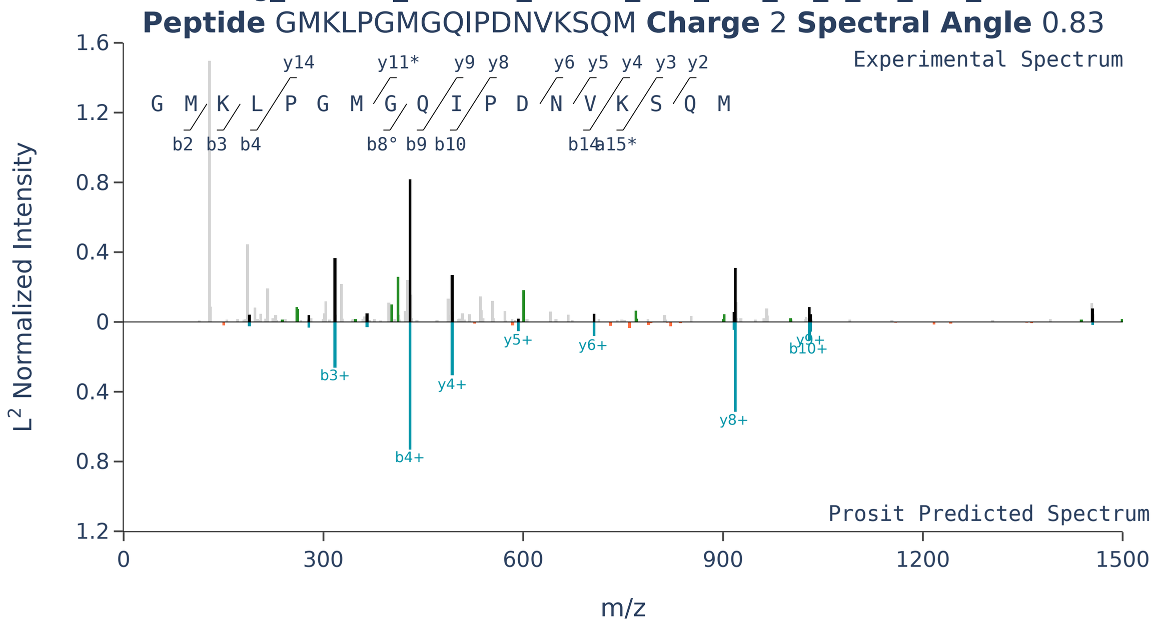

Source Ncheng_210623_230623_HFGoe_FFH_20S_25_1_A2_24h_R2 **Scan** 7174
Peptide SIRHITGA **Charge** 2 **Spectral Angle** 0.87

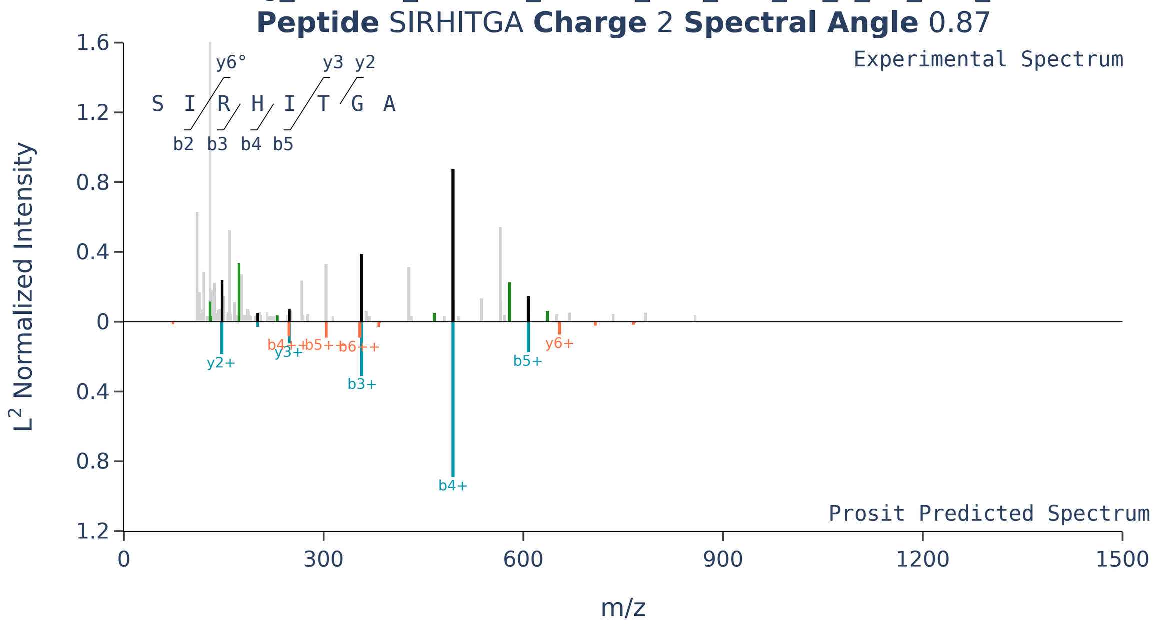

Source Ncheng_210623_230623_HFGoe_FFH_20S_25_1_A2_4h_R2 **Scan** 30387
Peptide LGGHEVNKSLTPGQEFVKIVRNELVA **Charge** 2 **Spectral Angle** 0.72

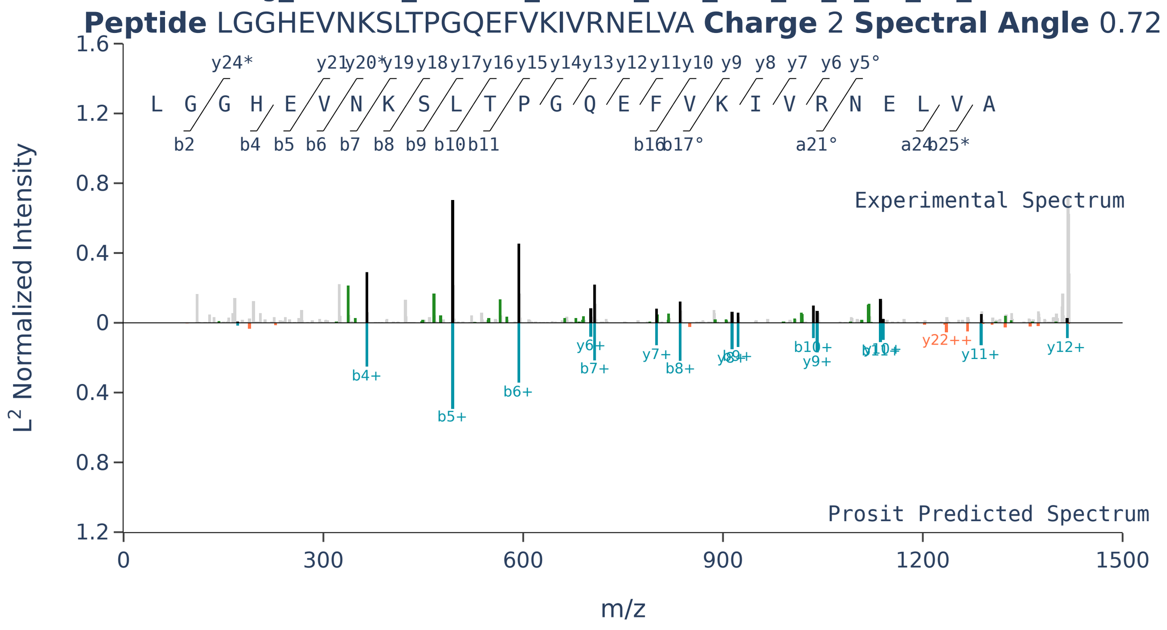

Source Ncheng_210623_230623_HFGoe_FFH_20S_25_1_A2_4h_R2 **Scan** 10233
Peptide ADVIESKVDRA **Charge** 3 **Spectral Angle** 0.8

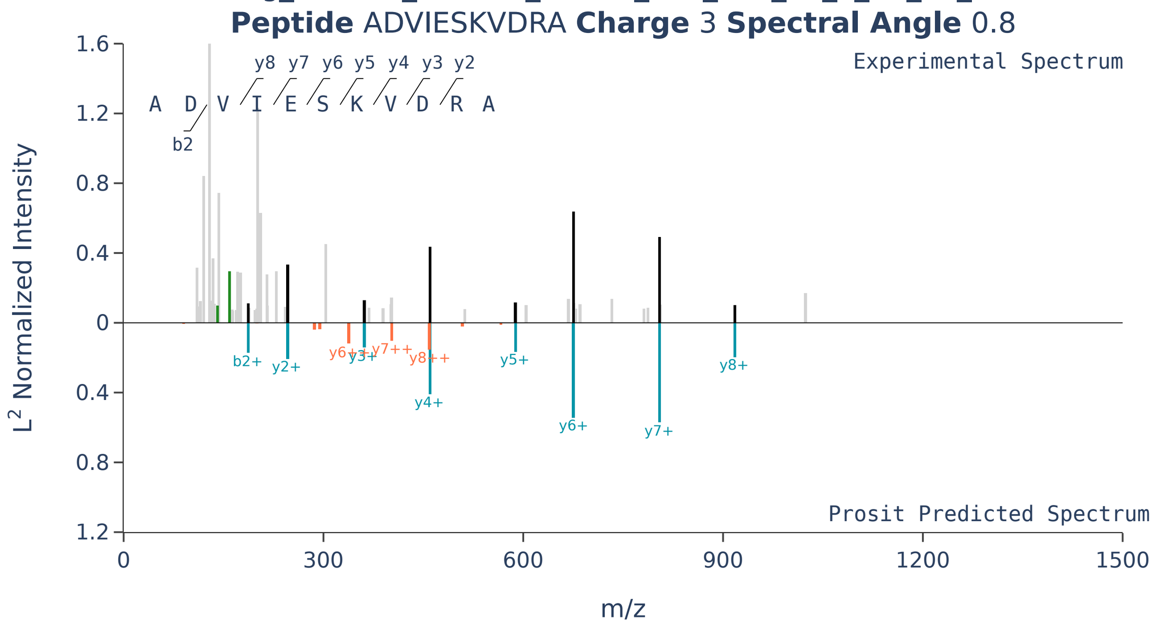

Source Ncheng_210623_230623_HFGoe_FFH_20S_25_1_A2_24h_R1 **Scan** 6501
Peptide AEVGQKPVD **Charge** 2 **Spectral Angle** 0.82

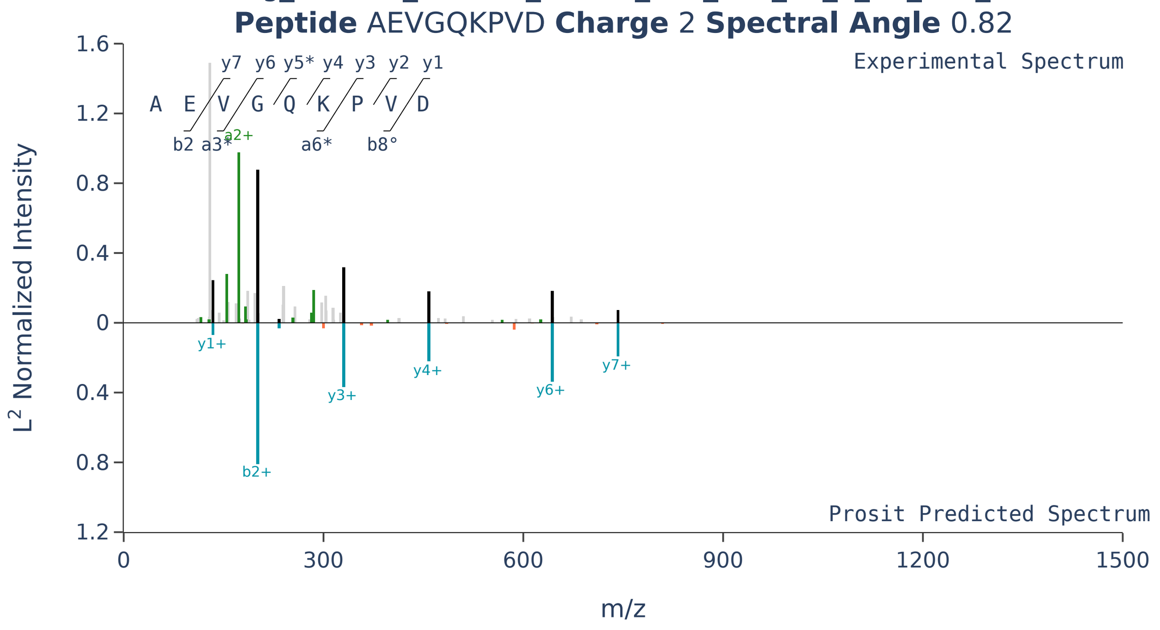

Source Ncheng_210623_230623_HFGoe_FFH_20S_25_1_A2_24h_R2 **Scan** 7444
Peptide SAAMTGQDAANTA **Charge** 2 **Spectral Angle** 0.84

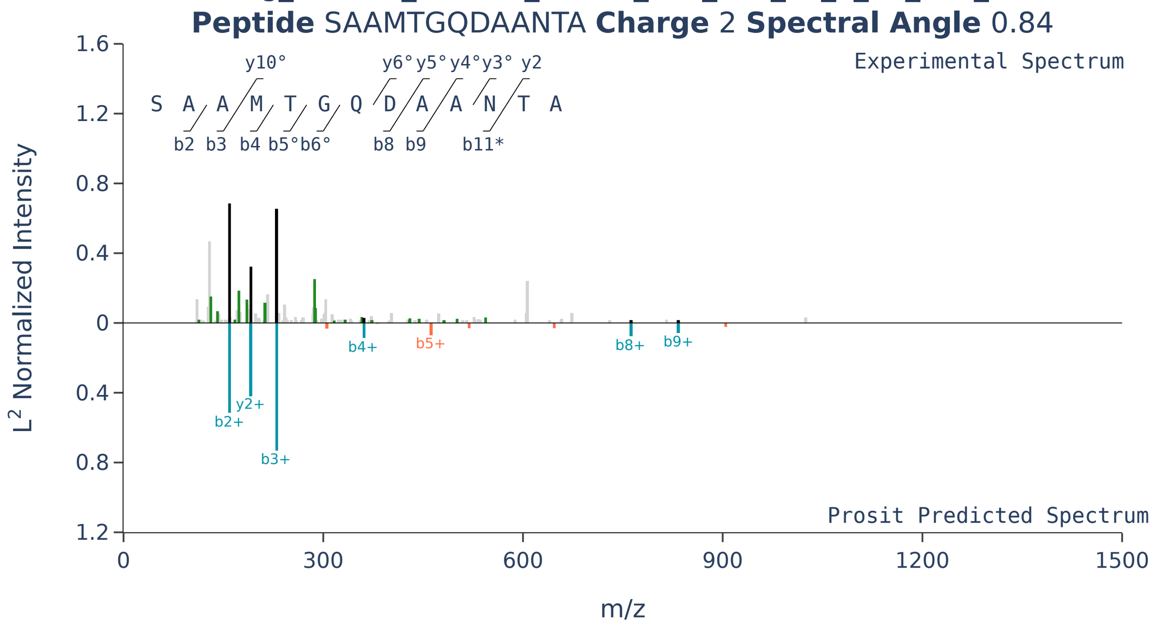

Source Ncheng_210623_230623_HFGoe_FFH_20S_25_1_A1_24h_R1 **Scan** 7259
Peptide MVGQKPVD **Charge** 2 **Spectral Angle** 0.76

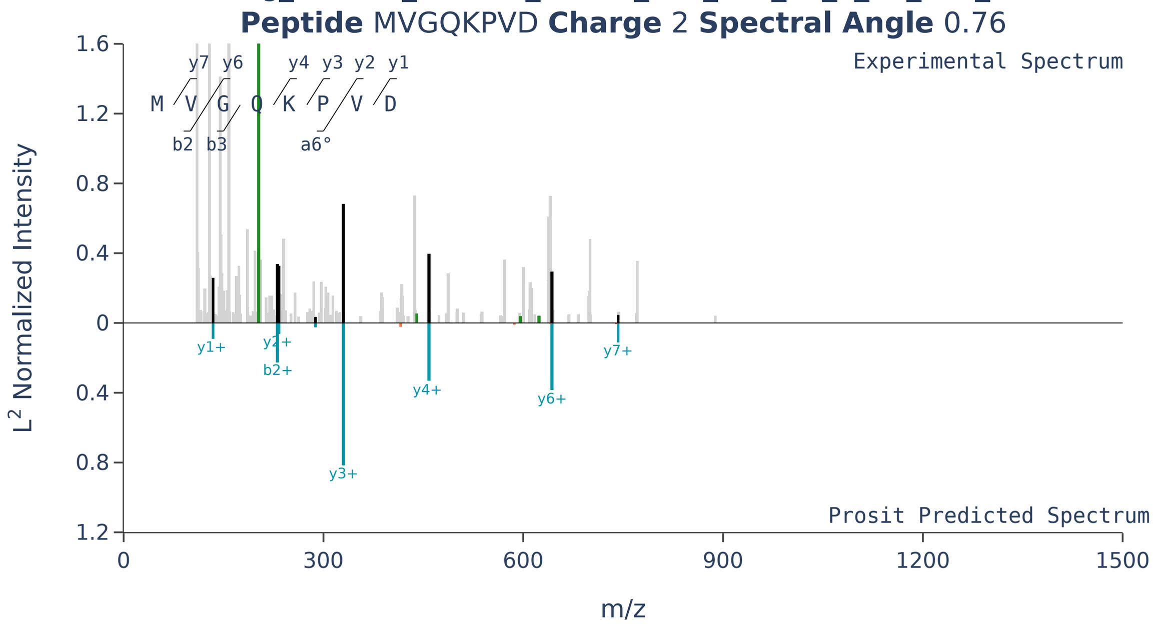

Source Ncheng_210623_230623_HFGoe_FFH_20S_25_1_A1_24h_R1 **Scan** 13153
Peptide DEIKQVHLA **Charge** 2 **Spectral Angle** 0.77

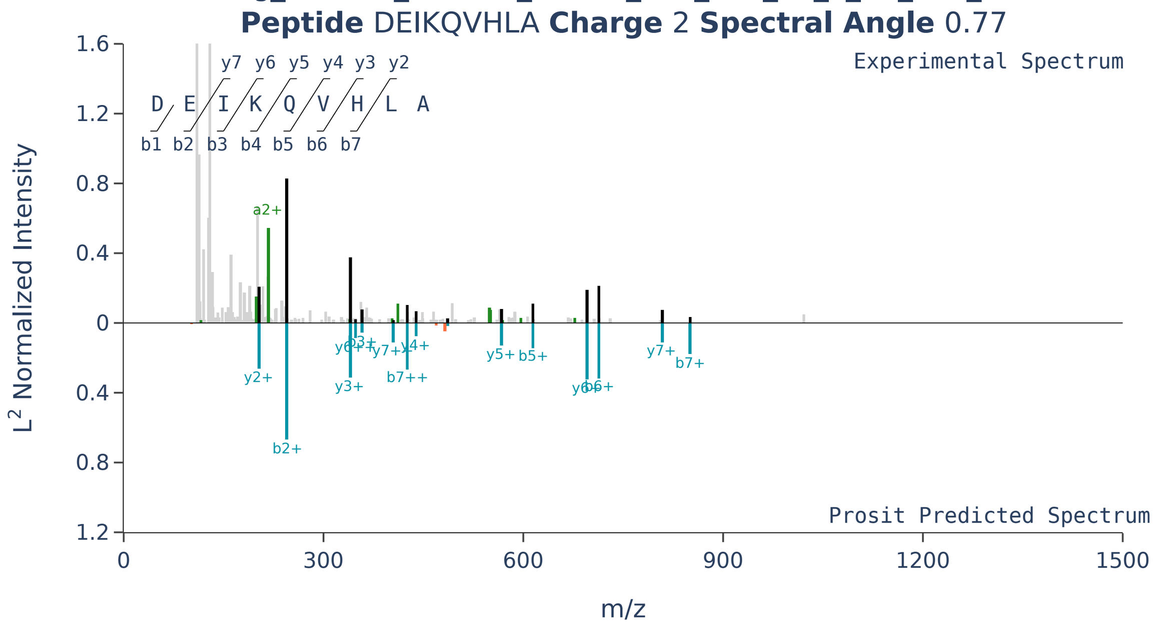

Source Ncheng_210623_230623_HFGoe_FFH_20S_25_1_A2_2h_R2 **Scan** 18366
Peptide GKLPGMMGQIPDNVKSQMDD **Charge** 3 **Spectral Angle** 0.74

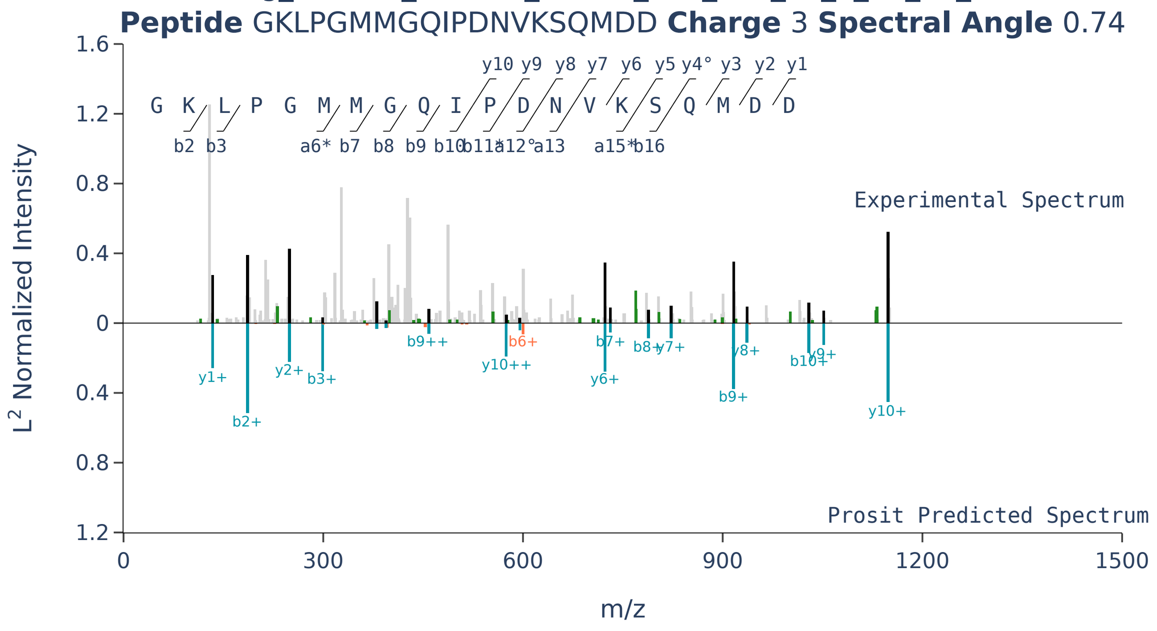

Source Ncheng_210623_230623_HFGoe_FFH_20S_25_1_A1_4h_R1 **Scan** 18506
Peptide ASKLPGMGQIPDNVKS **Charge** 3 **Spectral Angle** 0.81

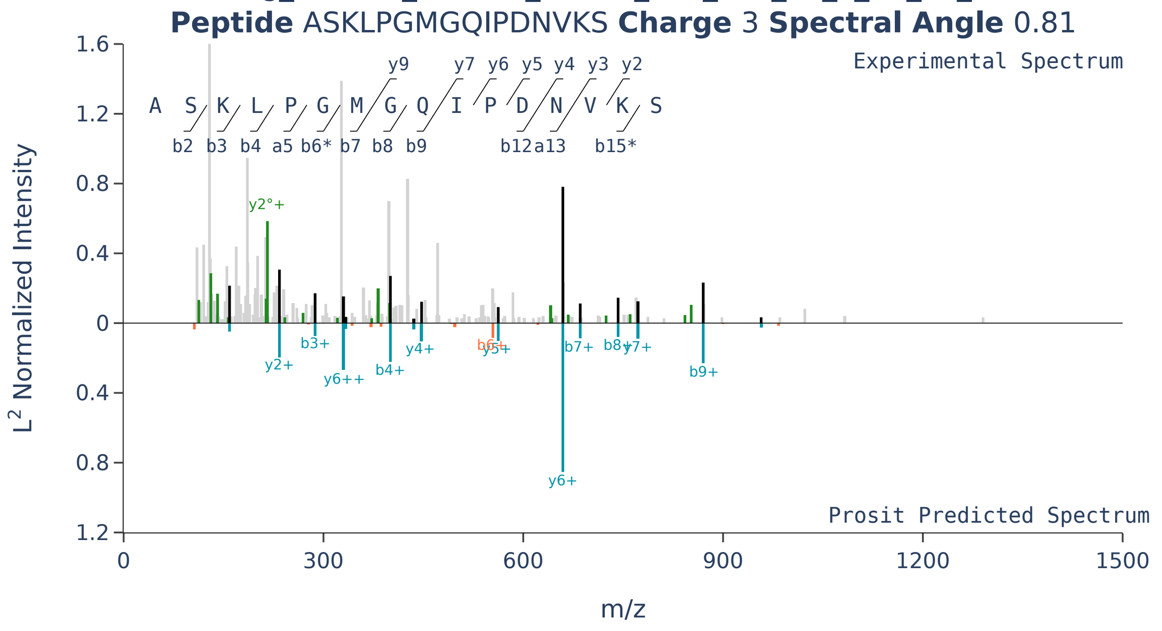

Source Ncheng_210623_230623_HFGoe_FFH_20S_25_1_A1_24h_R2 **Scan** 23561
Peptide GEKTEGVDFFPSD **Charge** 2 **Spectral Angle** 0.95

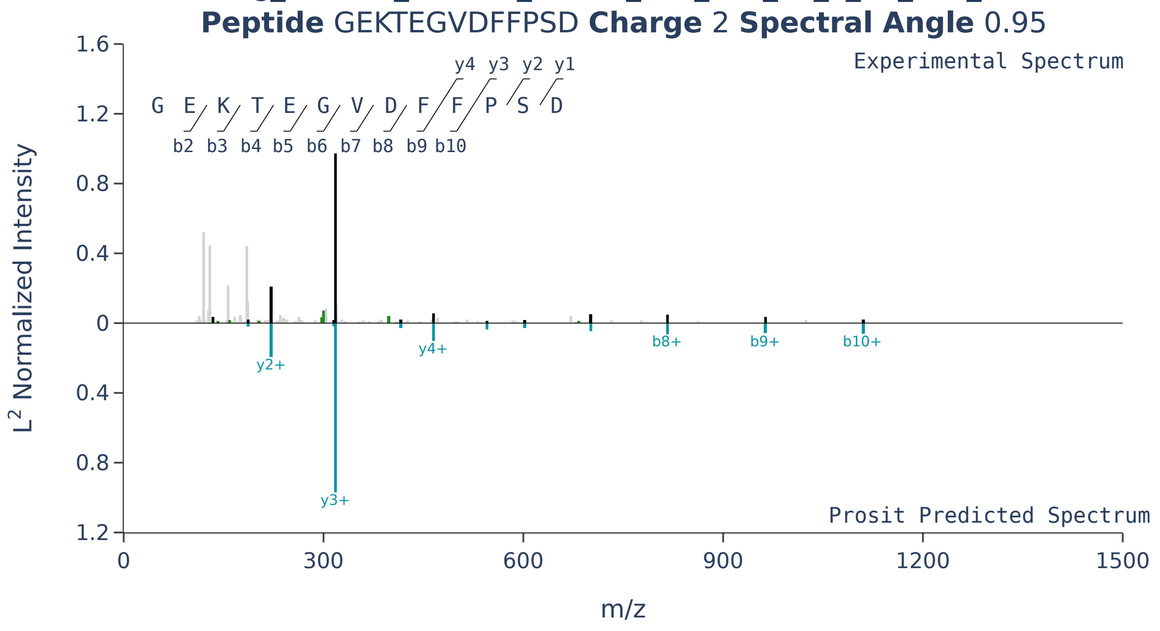

Source Ncheng_210623_230623_HFGoe_FFH_20S_25_1_A2_24h_R1 **Scan** 11550
Peptide RQMTGKPIKF **Charge** 3 **Spectral Angle** 0.81

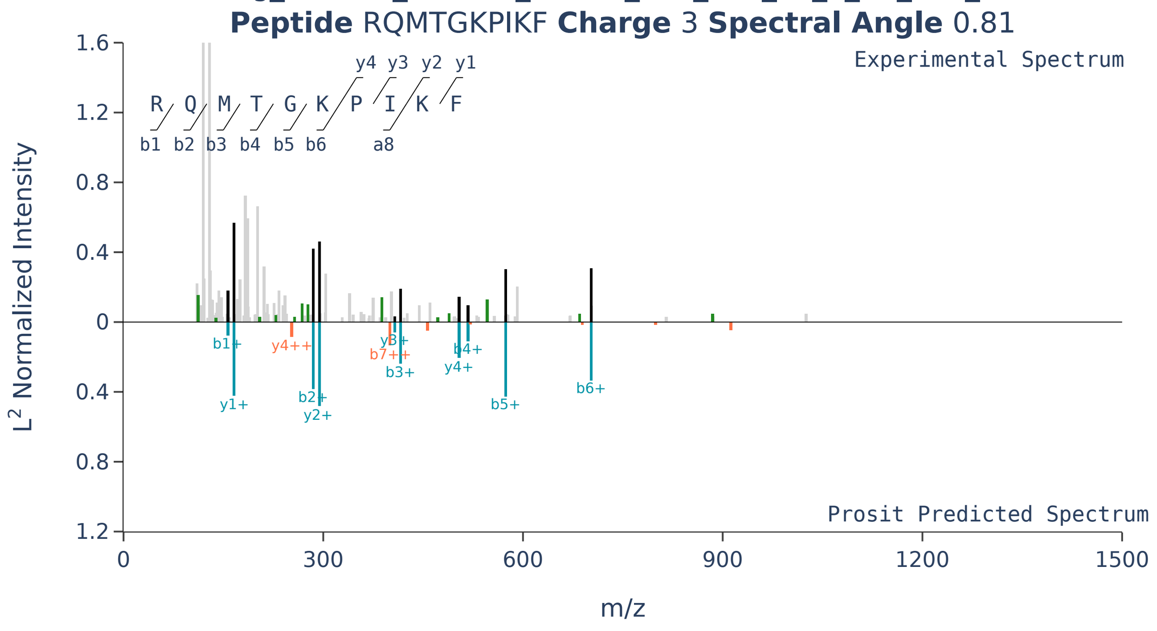

Source Ncheng_210623_230623_HFGoe_FFH_20S_25_1_A2_24h_R1 **Scan** 29670
Peptide NAAVGVDFFPSD **Charge** 2 **Spectral Angle** 0.87

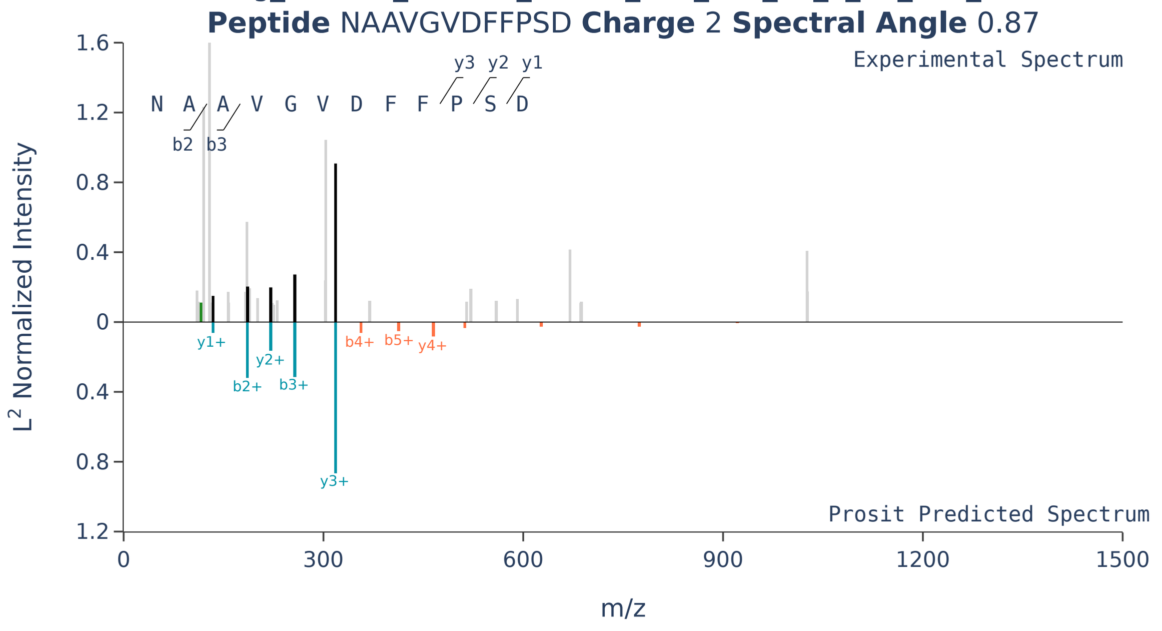

Source Ncheng_210623_230623_HFGoe_FFH_20S_25_1_A2_24h_R1 **Scan** 20786
Peptide SALEPFHPDRI **Charge** 3 **Spectral Angle** 0.76

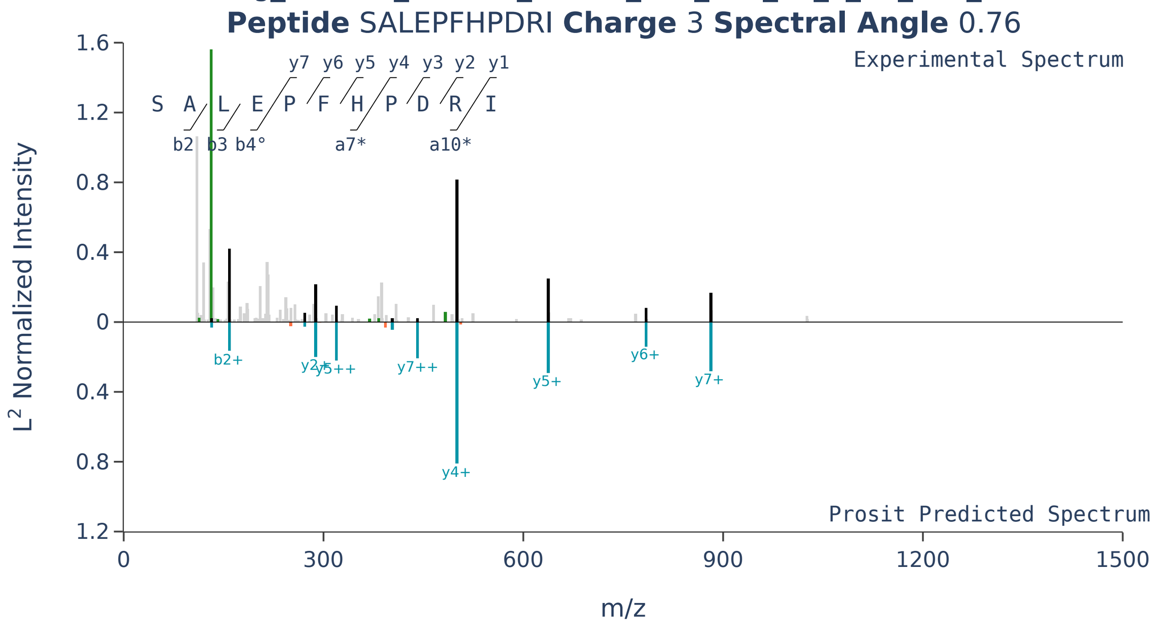

Source Ncheng_210623_230623_HFGoe_FFH_20S_25_1_A1_2h_R2 **Scan** 38104
Peptide QLETLAQVGVDFFPSD **Charge** 2 **Spectral Angle** 0.92

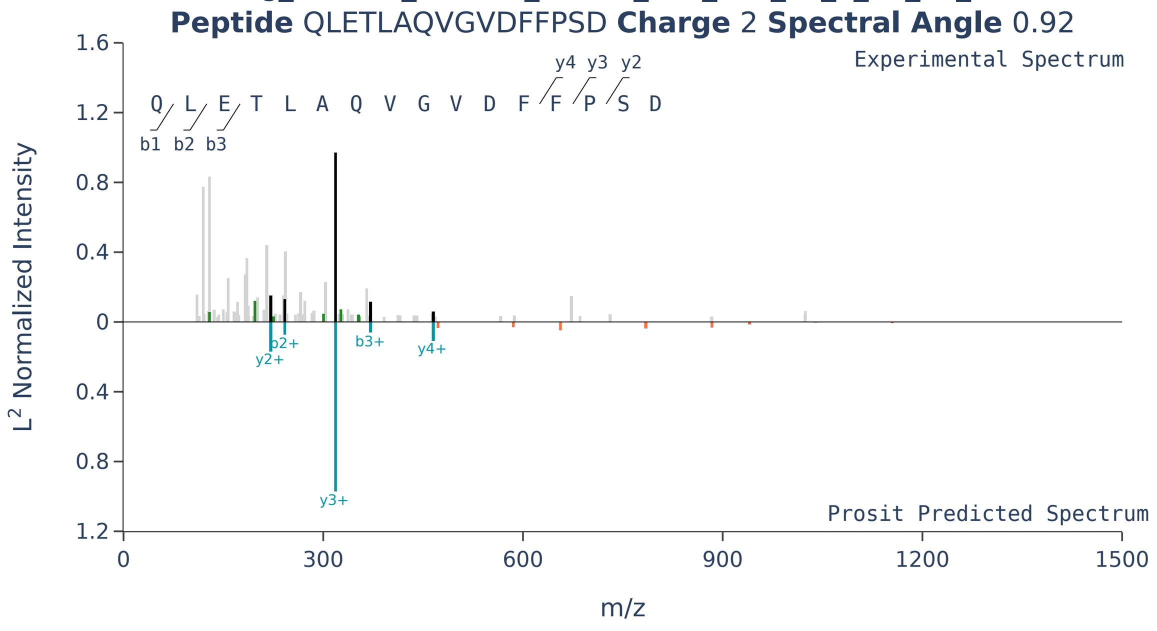

Source Ncheng_210623_230623_HFGoe_FFH_20S_25_1_A2_4h_R1 **Scan** 9971
Peptide RMDIESKVD **Charge** 2 **Spectral Angle** 0.75

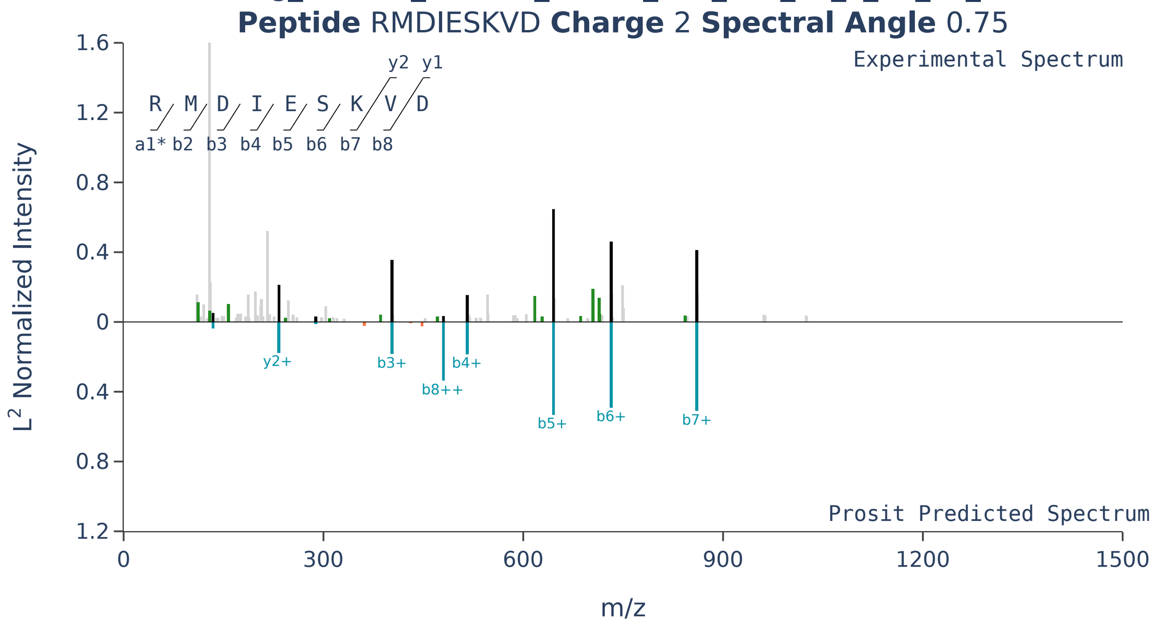

Source Ncheng_210623_230623_HFGoe_FFH_20S_25_1_A2_4h_R1 **Scan** 10426
Peptide MAGLGAG **Charge** 1 **Spectral Angle** 0.86

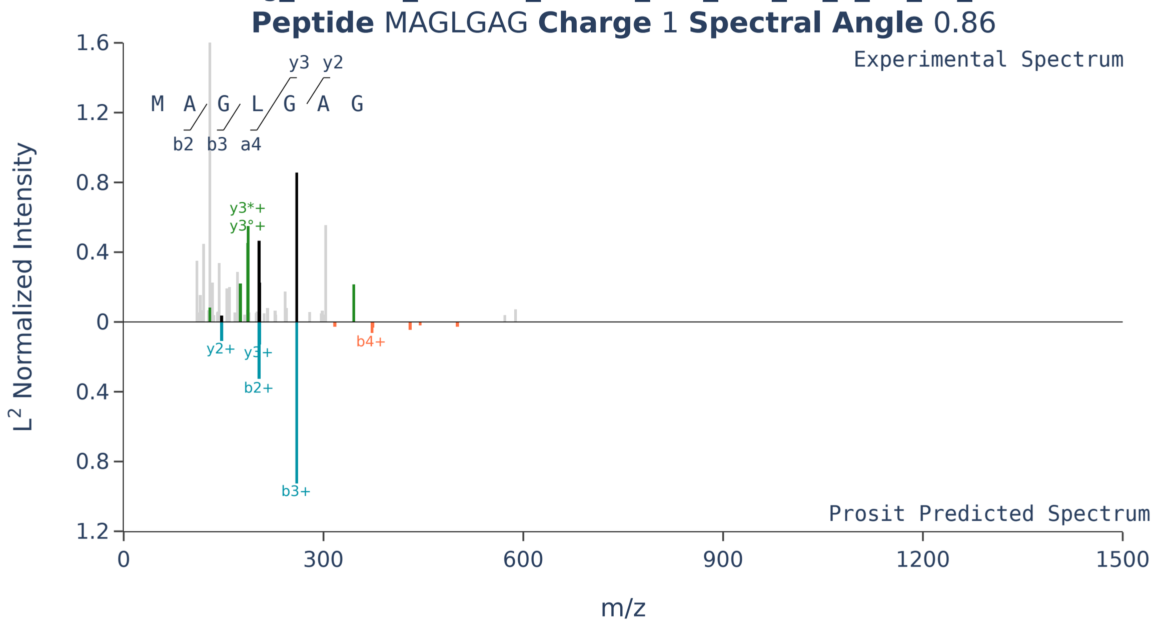

Source Ncheng_210623_230623_HFGoe_FFH_20S_25_1_A1_24h_R1 **Scan** 10843
Peptide NISTLRE **Charge** 2 **Spectral Angle** 0.78

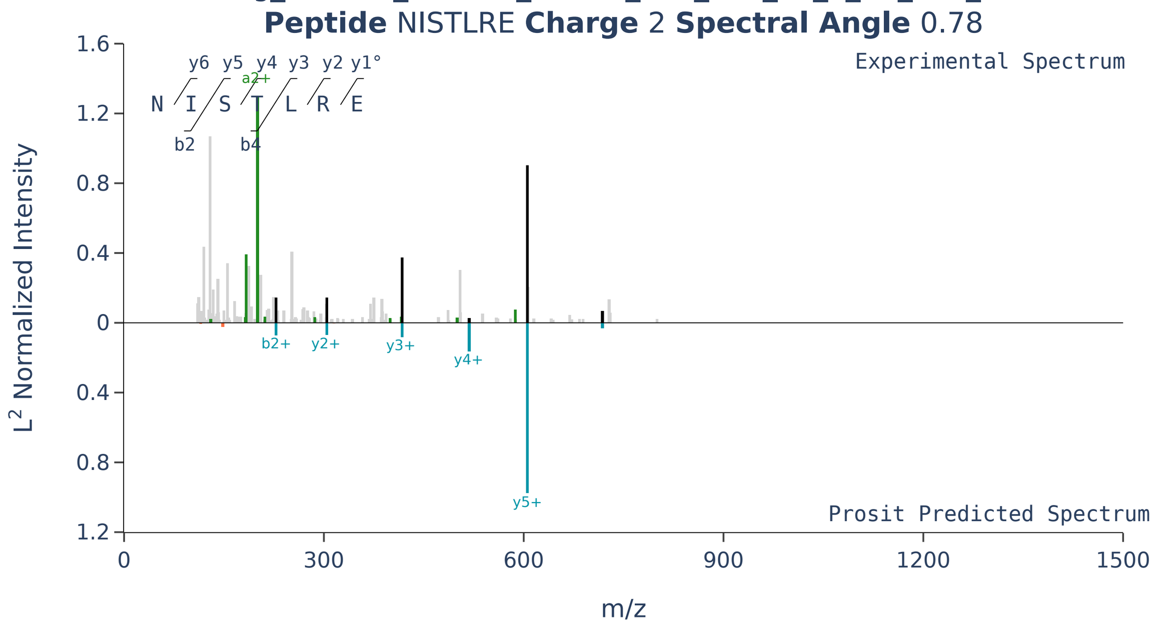

Source Ncheng_210623_230623_HFGoe_FFH_20S_25_1_A2_1h_R2 **Scan** 23837
Peptide GVGEKTETLFVVD **Charge** 2 **Spectral Angle** 0.8

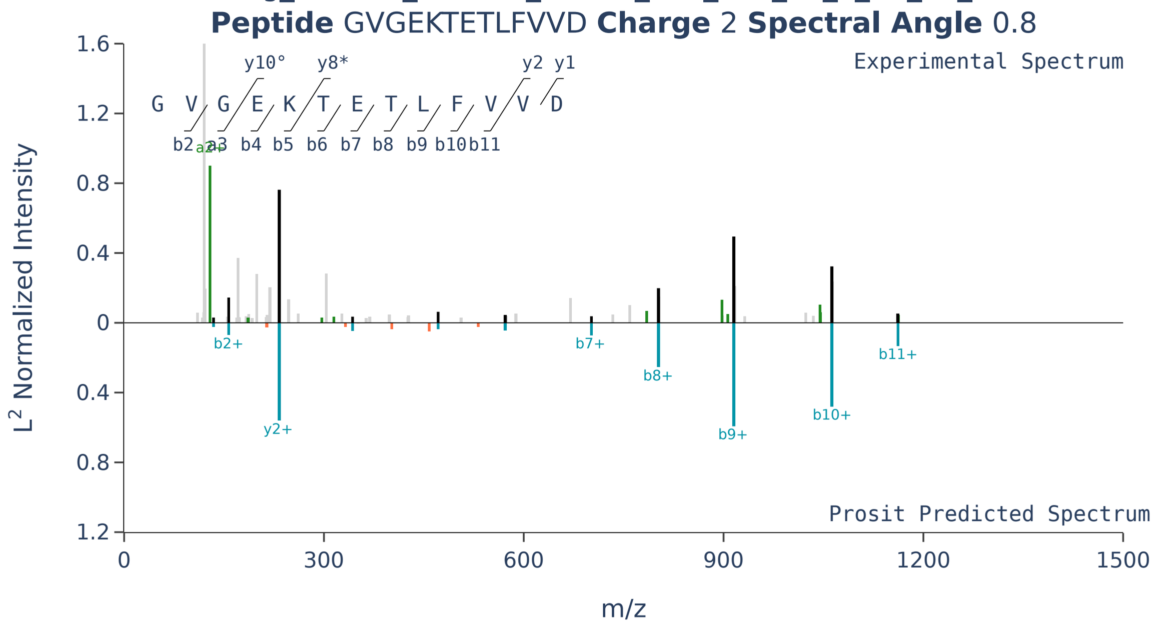

Source Ncheng_210623_230623_HFGoe_FFH_20S_25_1_A1_4h_R2 **Scan** 13996
Peptide SMKGMMPPGFPGRSR **Charge** 4 **Spectral Angle** 0.87

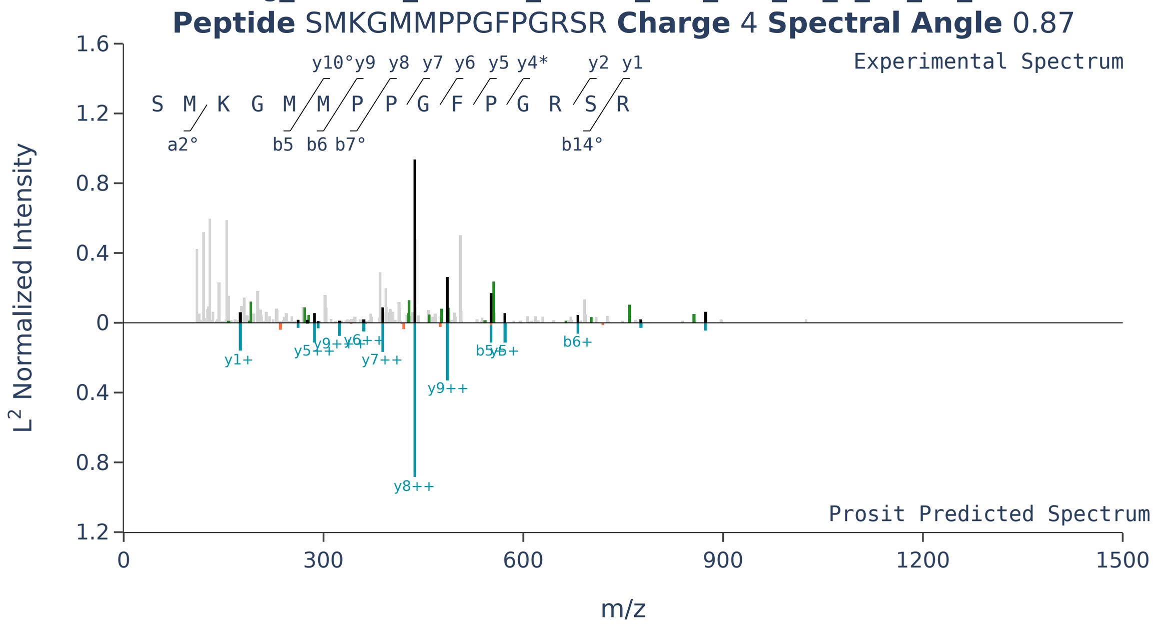

Source Ncheng_210623_230623_HFGoe_FFH_20S_25_1_A1_2h_R2 **Scan** 33468
Peptide RIAAGTLAEQVGVDFFPSD **Charge** 2 **Spectral Angle** 0.87

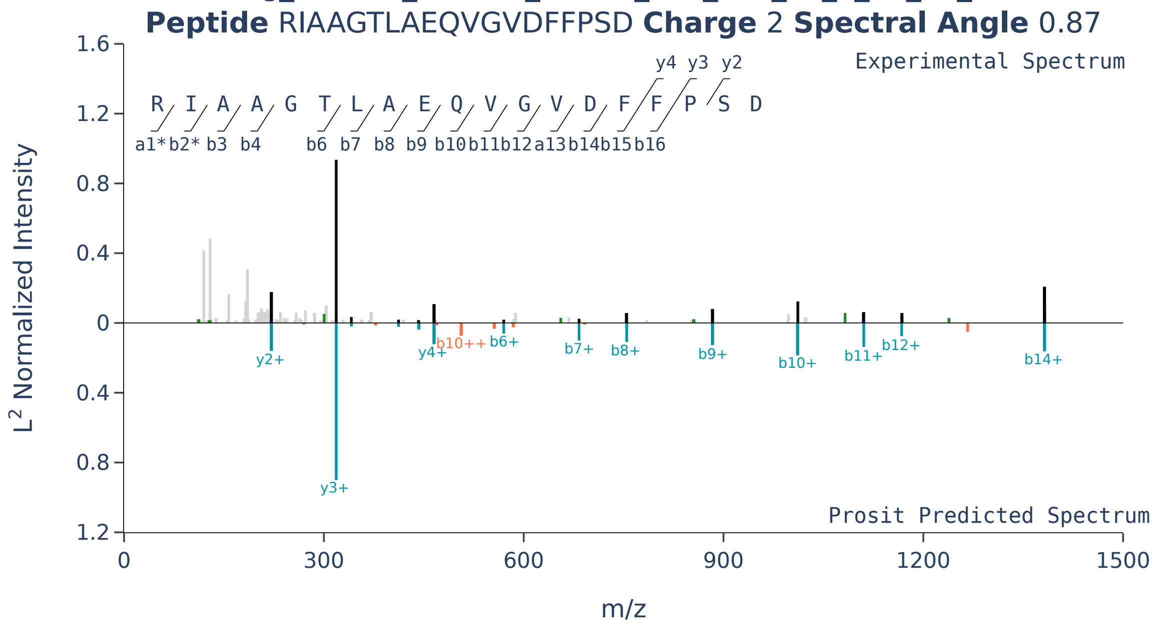

Source Ncheng_210623_230623_HFGoe_FFH_20S_25_1_A2_24h_R2 **Scan** 9209
Peptide MKNGGMASL **Charge** 2 **Spectral Angle** 0.76

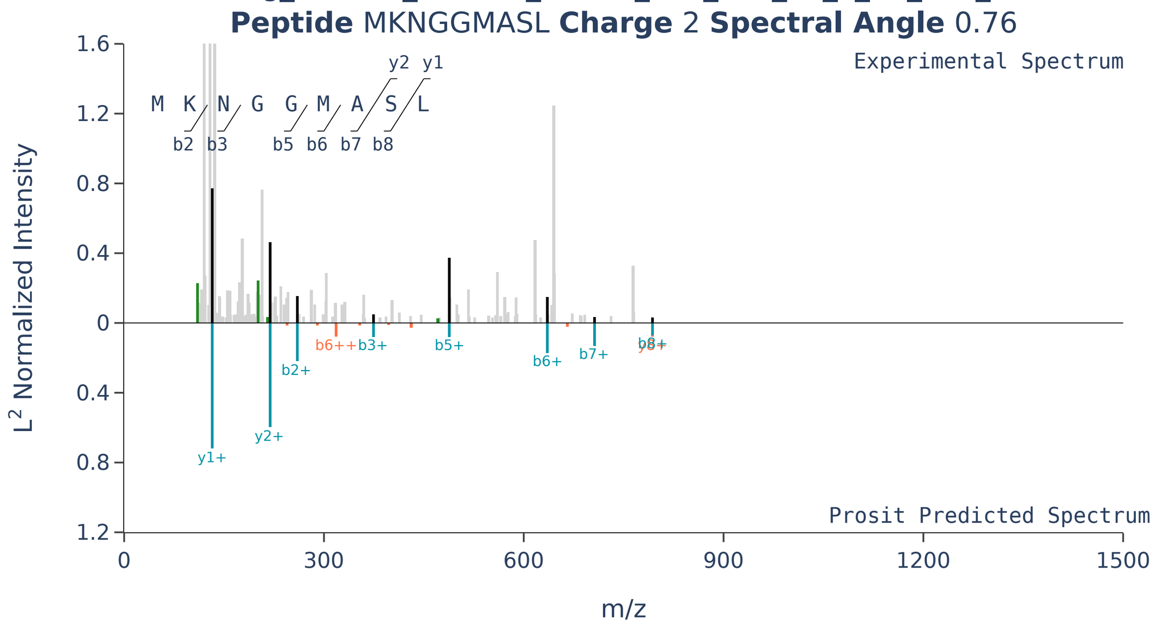

Source Ncheng_210623_230623_HFGoe_FFH_20S_25_1_A2_4h_R2 **Scan** 36873
Peptide PLTGVVLEAL **Charge** 1 **Spectral Angle** 0.82

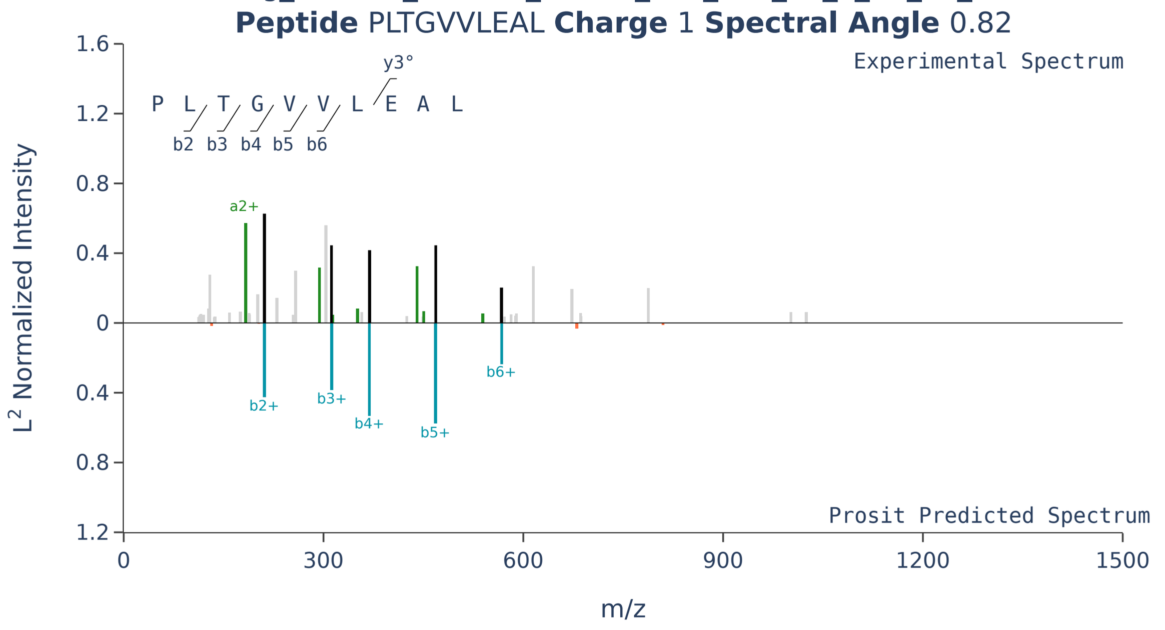

Source Ncheng_210623_230623_HFGoe_FFH_20S_25_1_A2_24h_R1 **Scan** 13662
Peptide DGDARGGAMTGQDAANTAKAFN **Charge** 3 **Spectral Angle** 0.73

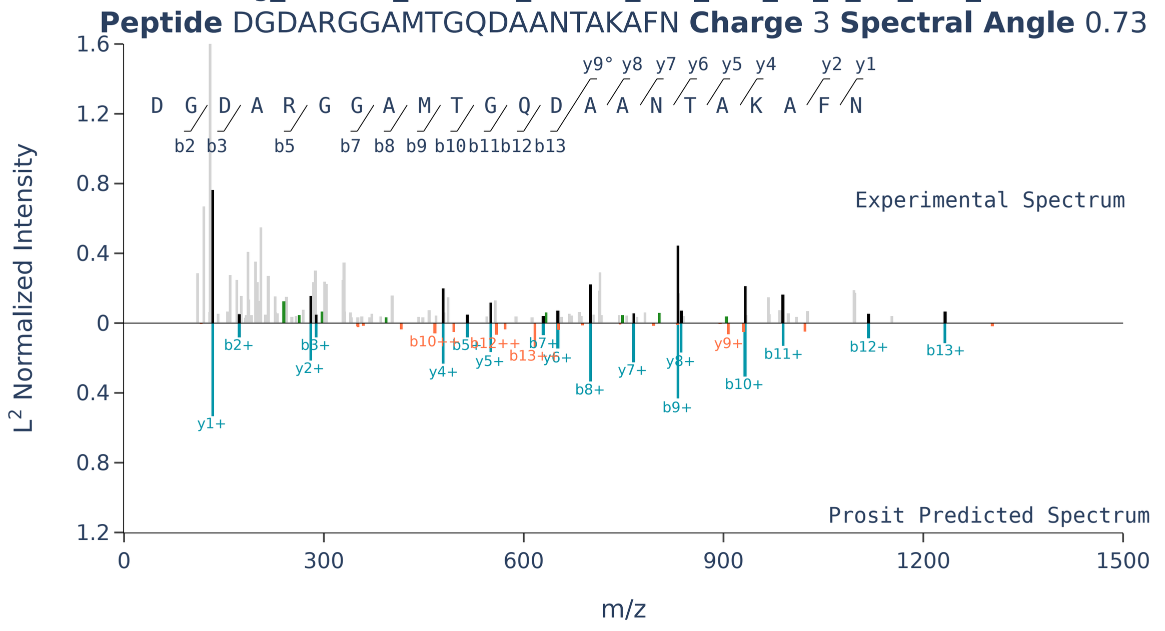

Source Ncheng_210623_230623_HFGoe_FFH_20S_25_1_A2_24h_R2 **Scan** 17256
Peptide MAGLQGKKGDGFDLND **Charge** 2 **Spectral Angle** 0.8

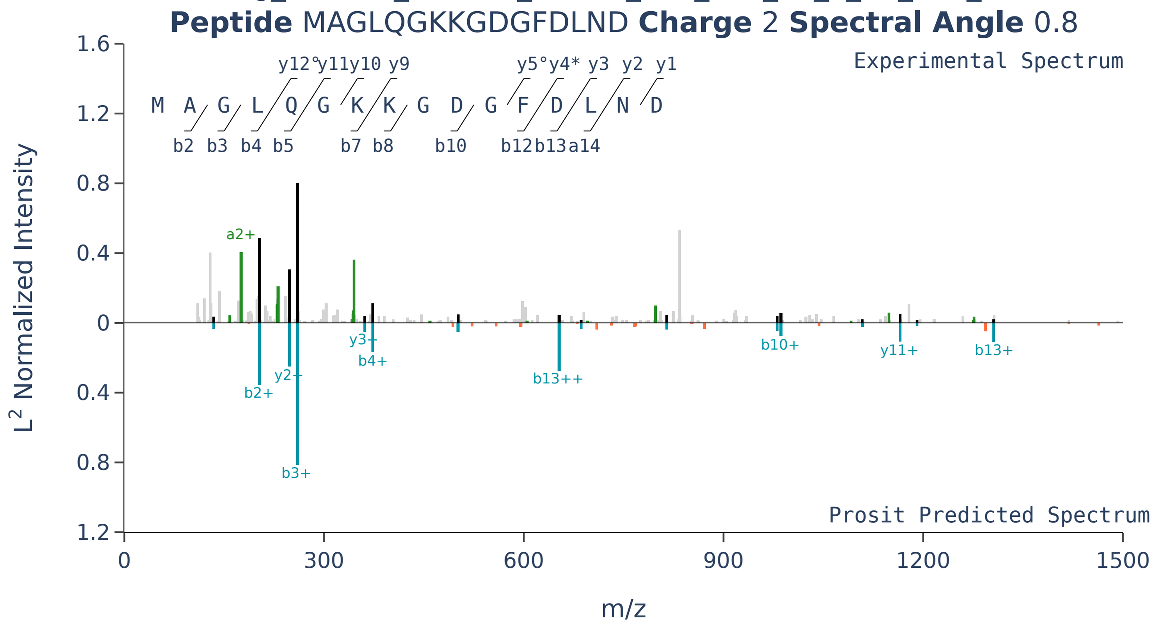

Source Ncheng_210623_230623_HFGoe_FFH_20S_25_1_A1_24h_R1 **Scan** 27424
Peptide GSVDFFPSD **Charge** 1 **Spectral Angle** 0.86

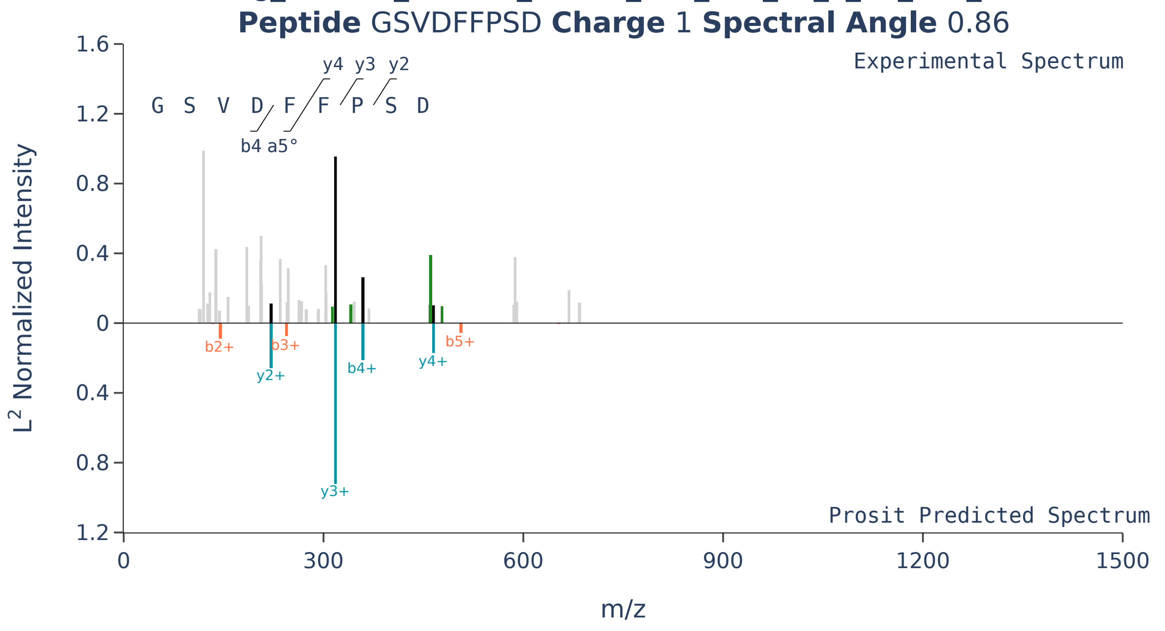

Source Ncheng_210623_230623_HFGoe_FFH_20S_25_1_A2_24h_R2 **Scan** 14599
Peptide QAAMTGQDAANTAKAFN **Charge** 2 **Spectral Angle** 0.77

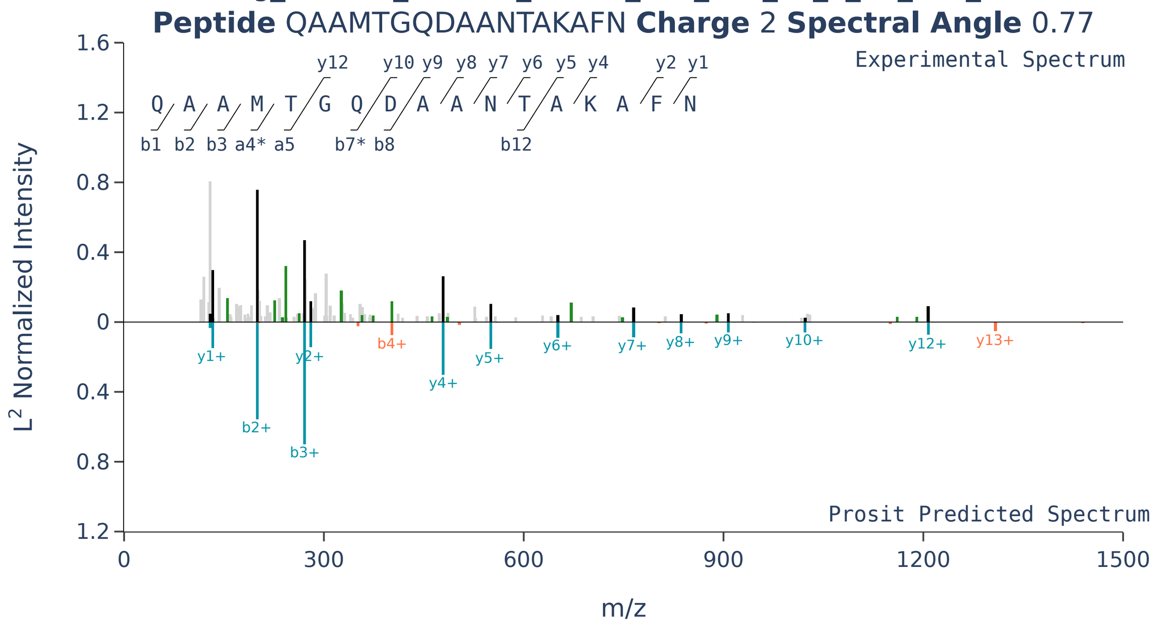

Source Ncheng_210623_230623_HFGoe_FFH_20S_25_1_A1_24h_R2 **Scan** 26817
Peptide RMGVDFFPSD **Charge** 2 **Spectral Angle** 0.77

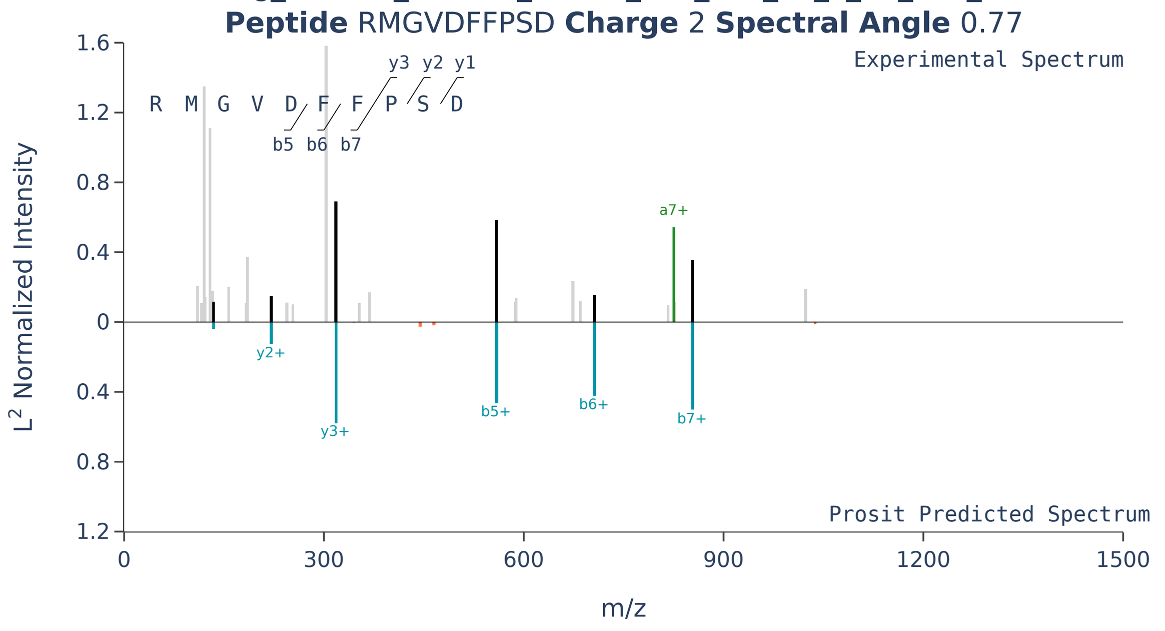

Source Ncheng_210623_230623_HFGoe_FFH_20S_25_1_A2_4h_R2 **Scan** 15115
Peptide EAMTGQDAANTAKAFNE **Charge** 2 **Spectral Angle** 0.87

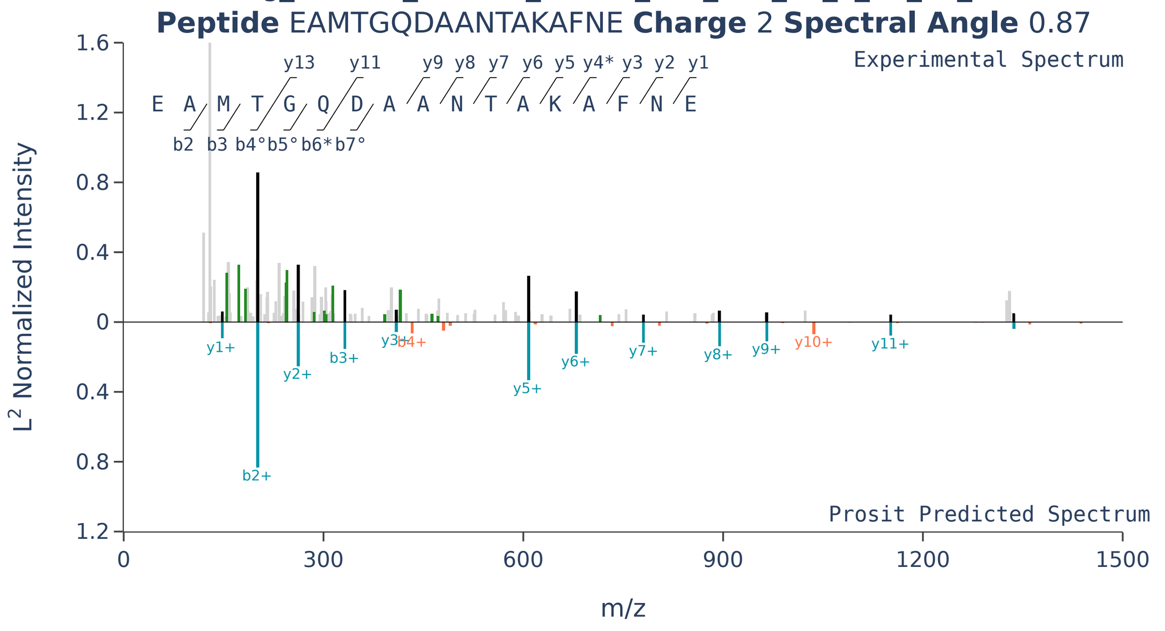

Source Ncheng_210623_230623_HFGoe_FFH_20S_25_1_A1_2h_R2 **Scan** 22938
Peptide GEENQTLNLAAQPPAVD **Charge** 2 **Spectral Angle** 0.94

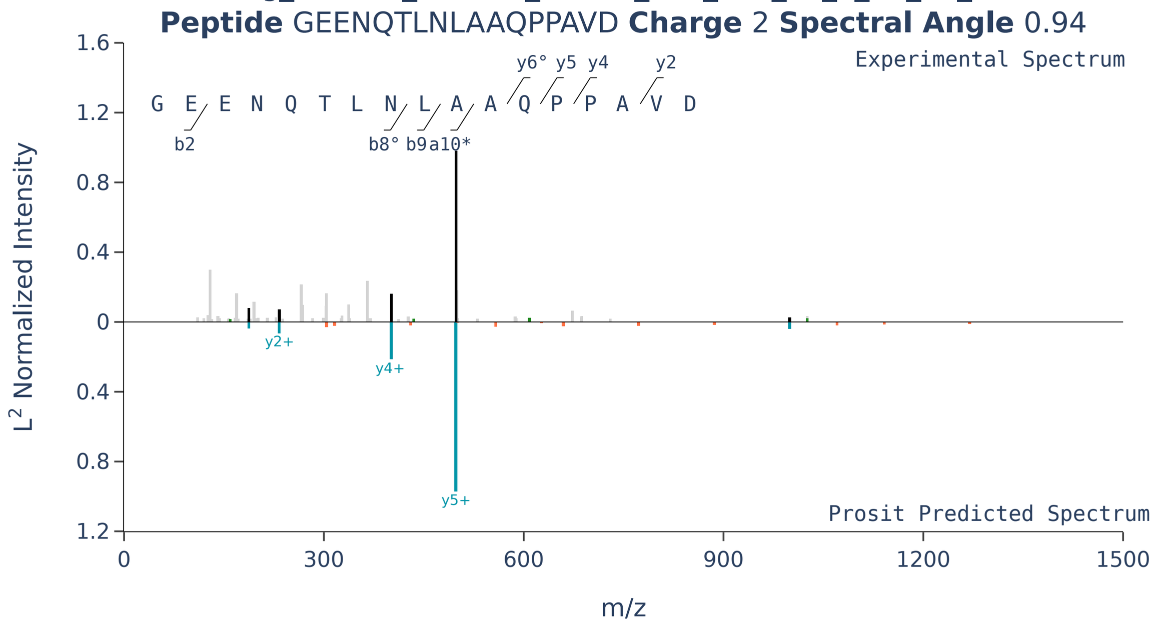

Source Ncheng_210623_230623_HFGoe_FFH_20S_25_1_A2_4h_R1 **Scan** 28493
Peptide DLMGKLPGMGQIPDNVKSQMD **Charge** 3 **Spectral Angle** 0.71

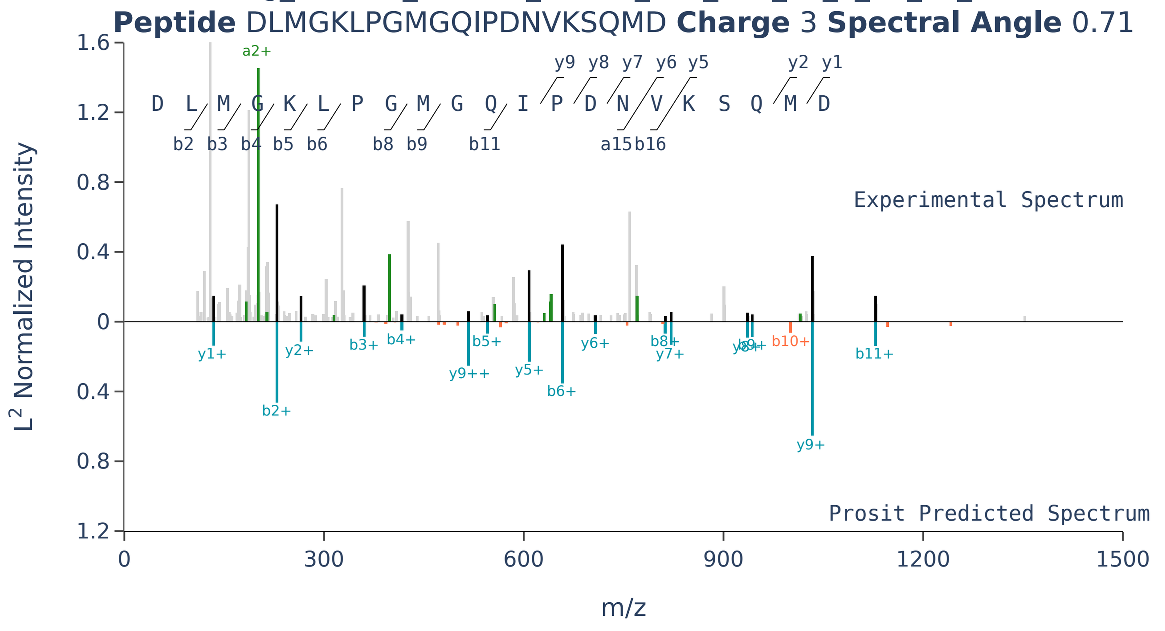

Source Ncheng_210623_230623_HFGoe_FFH_20S_25_1_A1_24h_R2 **Scan** 21739
Peptide NEALPLTGVGQKPVD **Charge** 2 **Spectral Angle** 0.93

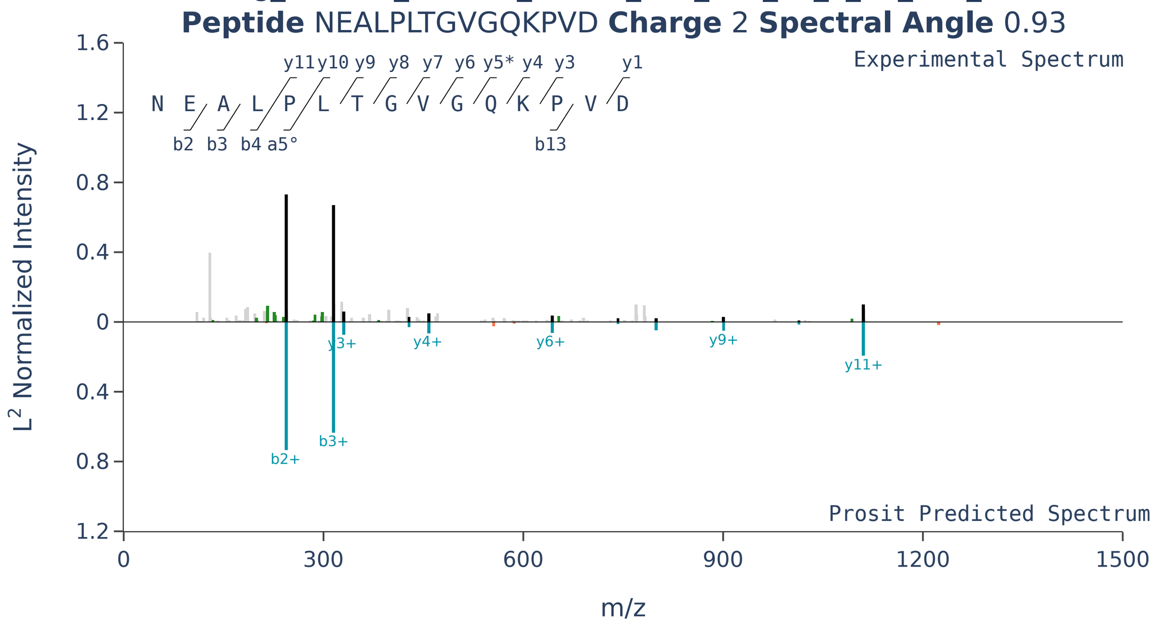

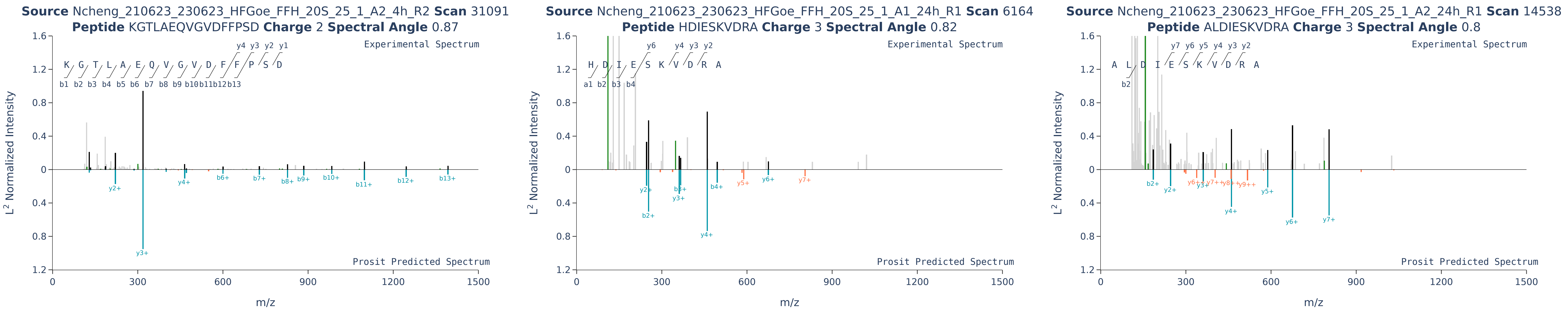
Source Ncheng_210623_230623_HFGoe_FFH_20S_25_1_A2_4h_R2 Scan 31091
Peptide KGTLAEQVGVDFFPSD Charge 2 Spectral Angle 0.87
Experimental Spectrum
Prosit Predicted Spectrum
L2 Normalized Intensity
m/z
Source Ncheng_210623_230623_HFGoe_FFH_20S_25_1_A1_24h_R1 Scan 6164
Peptide HDIESKVDRA Charge 3 Spectral Angle 0.82
Experimental Spectrum
Prosit Predicted Spectrum
L2 Normalized Intensity
m/z
Source Ncheng_210623_230623_HFGoe_FFH_20S_25_1_A2_24h_R1 Scan 14538
Peptide ALDIESKVDRA Charge 3 Spectral Angle 0.8
Experimental Spectrum
Prosit Predicted Spectrum
L2 Normalized Intensity
m/z

Source HRoetschke_291222_060123_Fu_H2A_b1R1_2h **Scan** 13671
Peptide SSSSRAGLQFPVG **Charge** 2 **Spectral Angle** 0.91

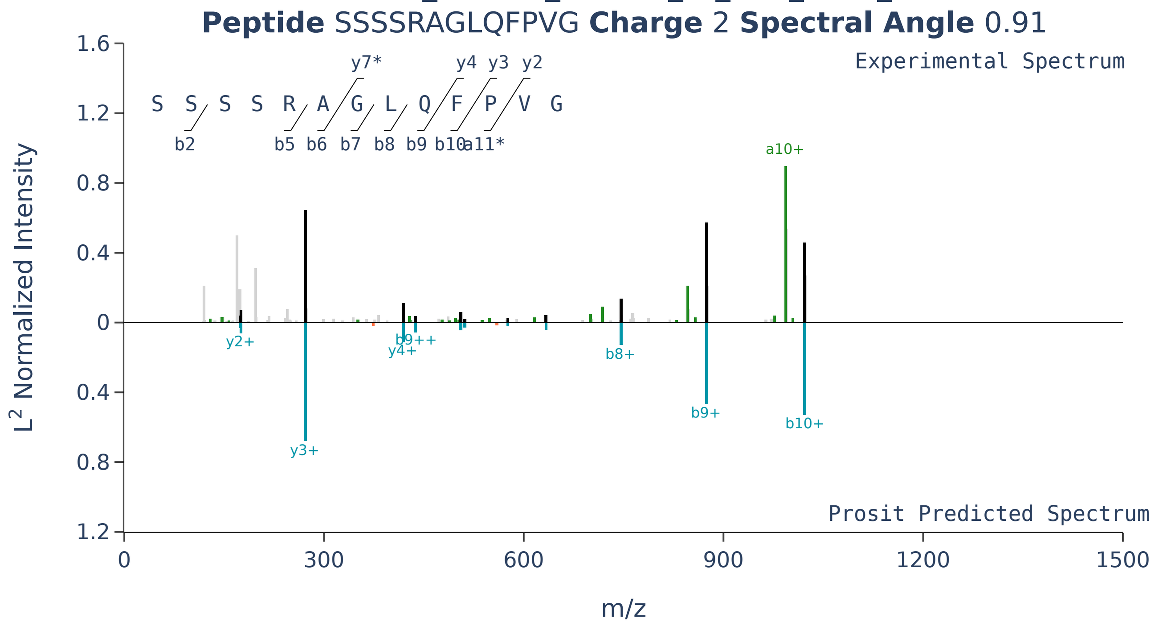

Source HRoetschke_291222_060123_Fu_H2A_b2R2_1h **Scan** 9385
Peptide QGGVLPNIQSRA **Charge** 2 **Spectral Angle** 0.91

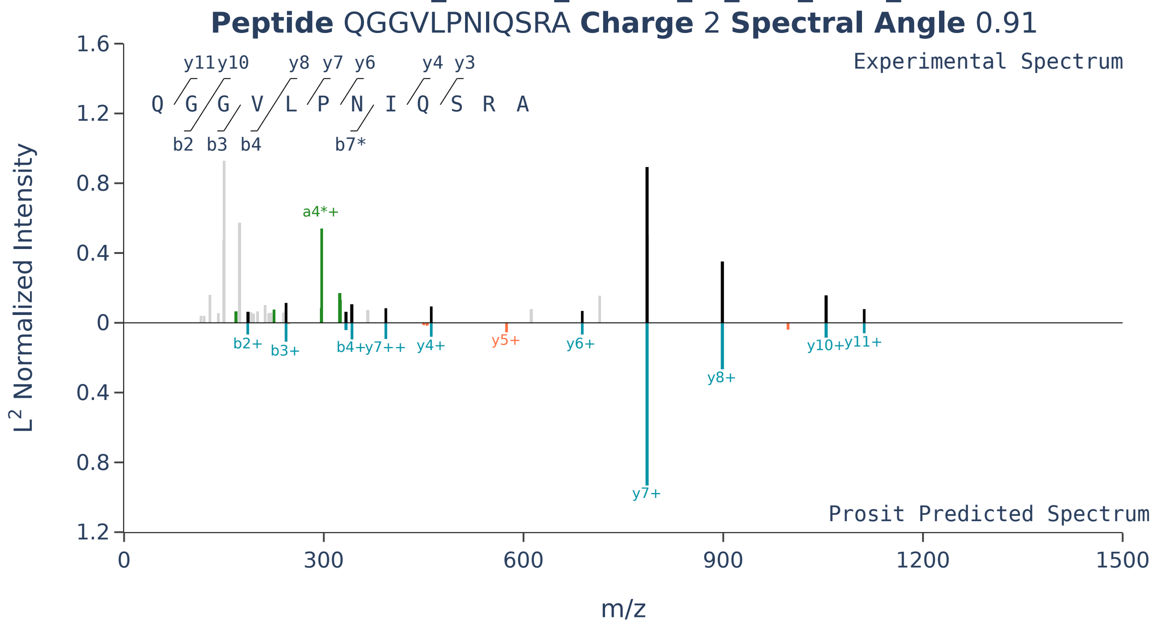

Source HRoetschke_291222_060123_Fu_H2A_b1R2_24h **Scan** 10629
Peptide AGLQFPVGSGRGK **Charge** 3 **Spectral Angle** 0.92

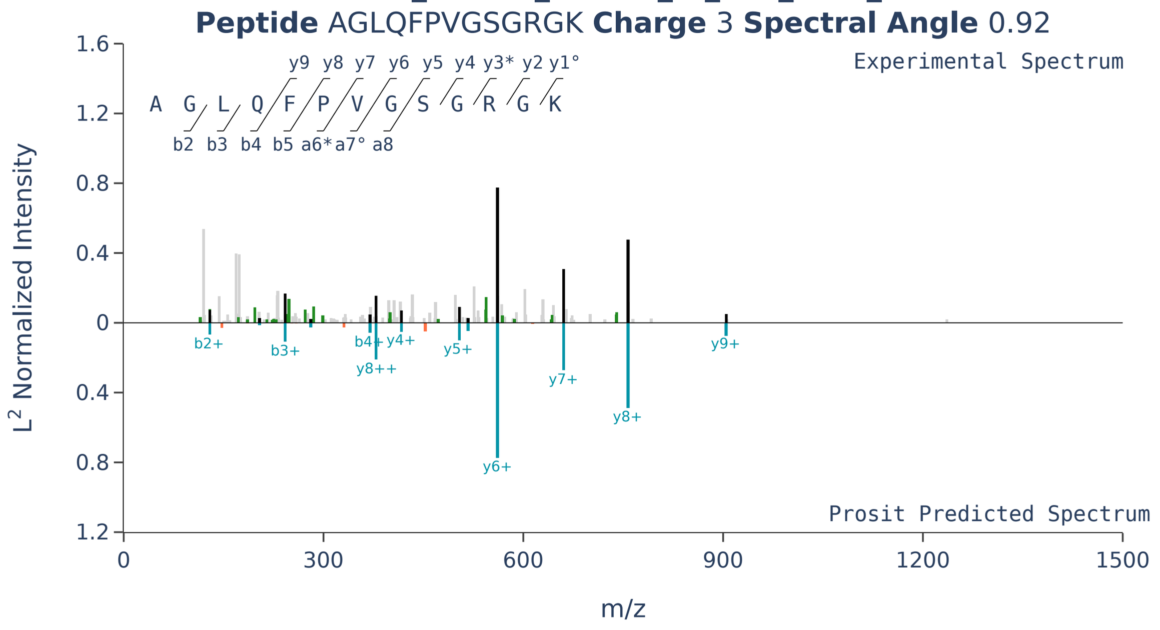

Source HRoetschke_291222_060123_Fu_H2A_b1R1_2h **Scan** 9331
Peptide HLQLALR **Charge** 2 **Spectral Angle** 0.92

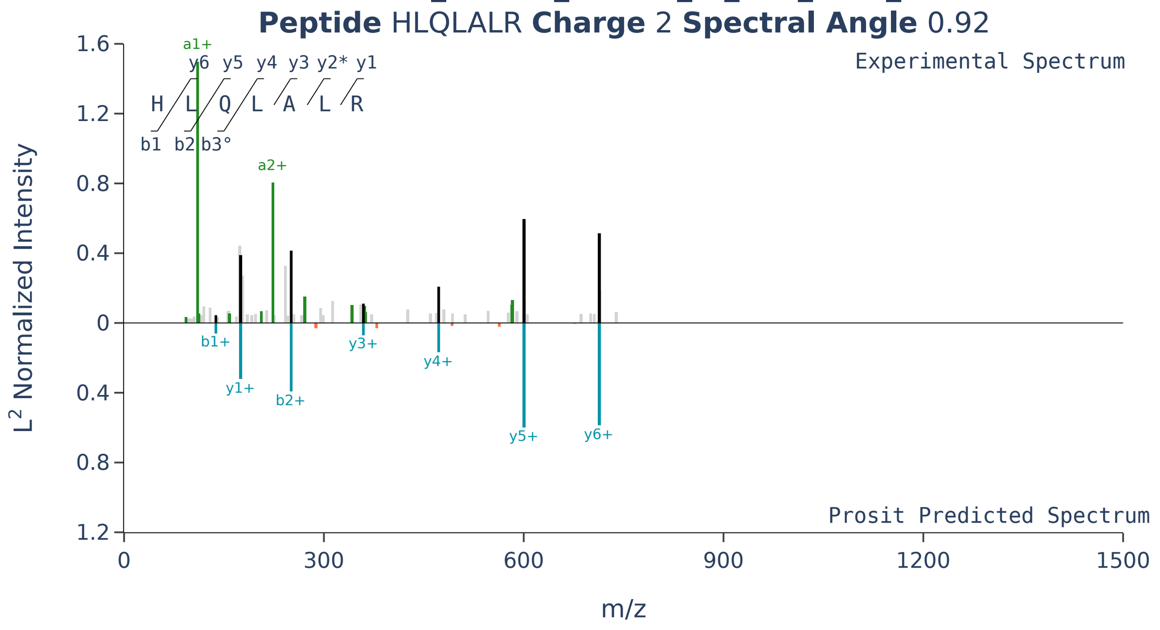

Source HRoetschke_291222_060123_Fu_H2A_b2R1_24h **Scan** 10700
Peptide RAGLQFPVGSSR **Charge** 3 **Spectral Angle** 0.87

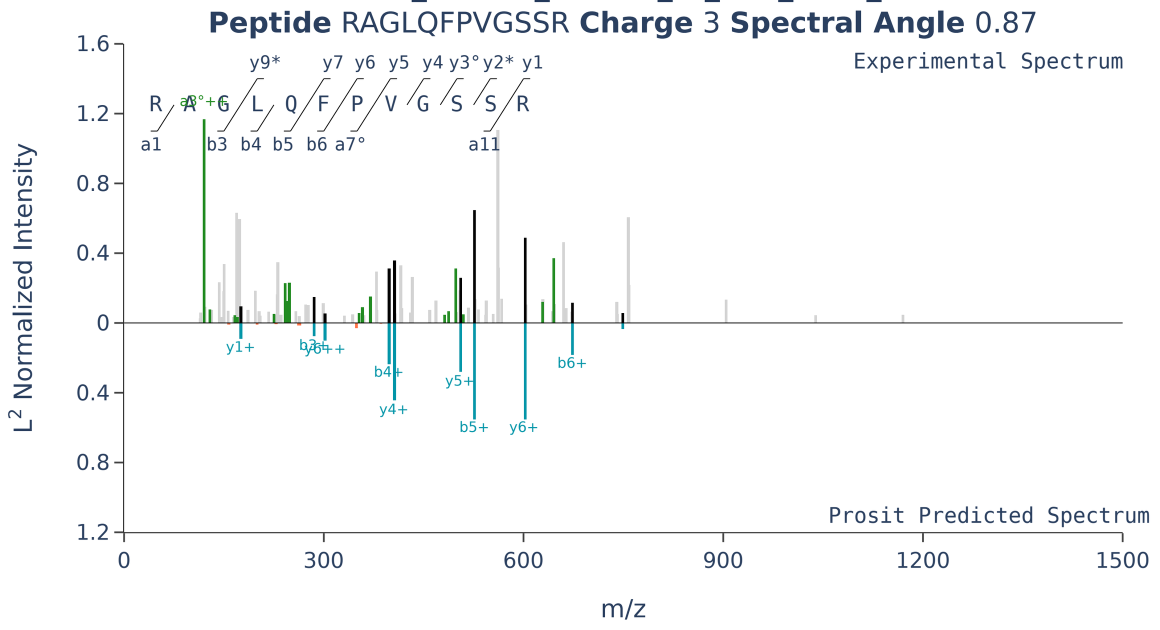

Source HRoetschke_291222_060123_Fu_H2A_b1R1_2h **Scan** 5513
Peptide VGQLAVRND **Charge** 2 **Spectral Angle** 0.9

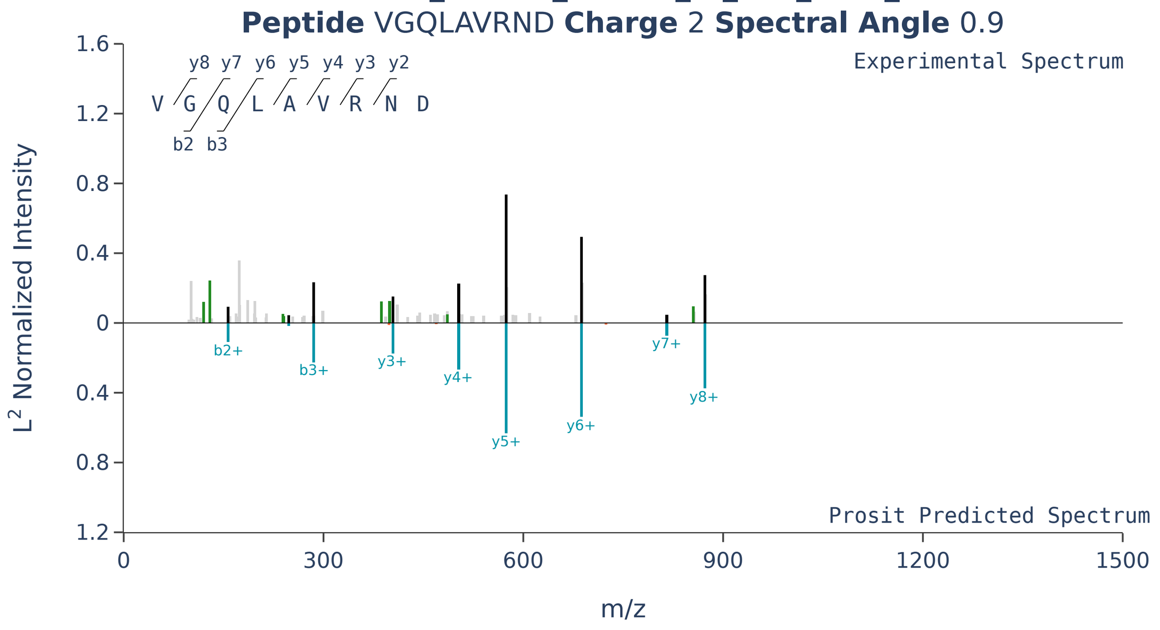

Source HRoetschke_291222_060123_Fu_H2A_b2R1_24h **Scan** 10573
Peptide SSRAGLQFPVGSSR **Charge** 3 **Spectral Angle** 0.92

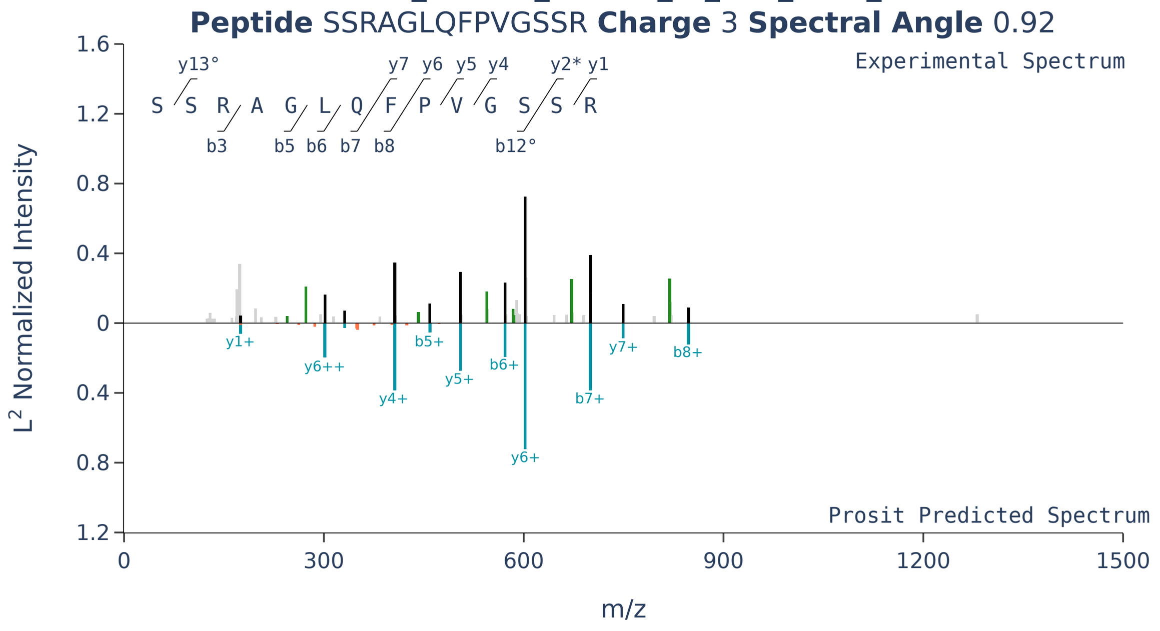

Source HRoetschke_291222_060123_Fu_H2A_b2R1_24h **Scan** 10597
Peptide QGGVLPNIQRS **Charge** 2 **Spectral Angle** 0.93

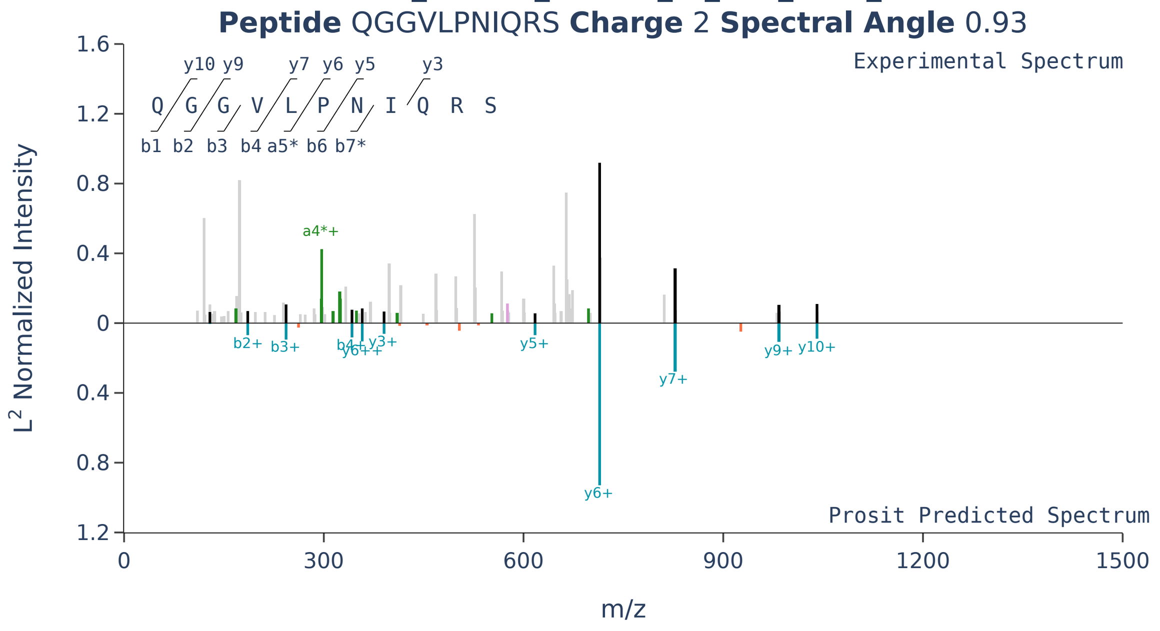

Source HRoetschke_291222_060123_Fu_H2A_b2R1_2h **Scan** 4291
Peptide GNYAERVGE **Charge** 2 **Spectral Angle** 0.94

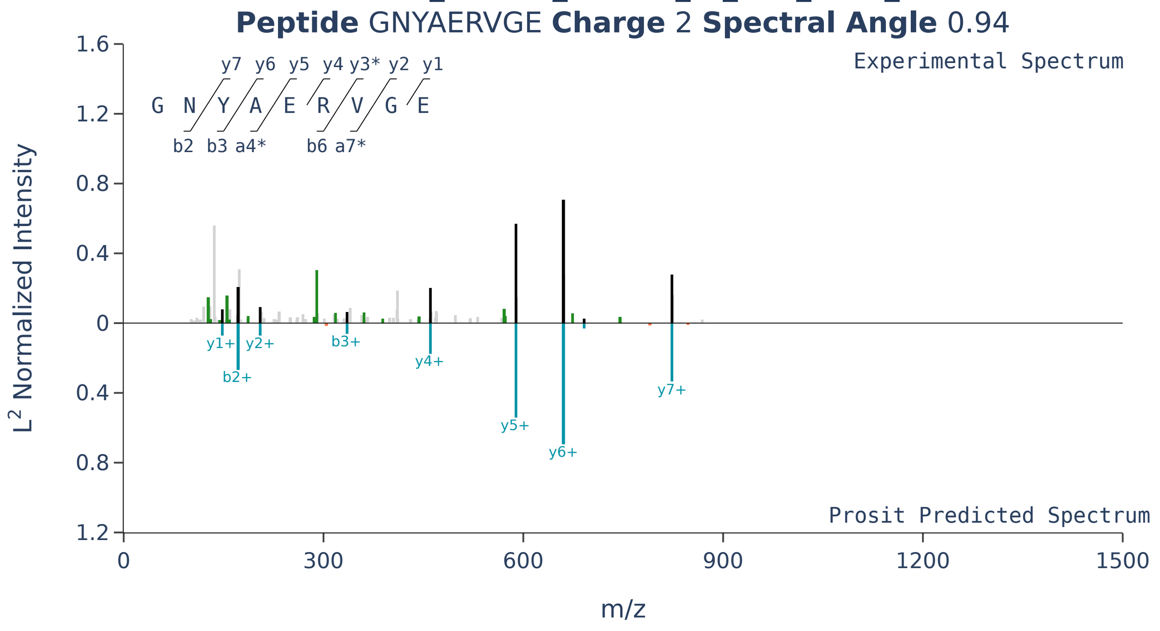

Source HRoetschke_291222_060123_Fu_H2A_b1R2_24h **Scan** 10854
Peptide RAAGLQFPVGRVH **Charge** 3 **Spectral Angle** 0.9

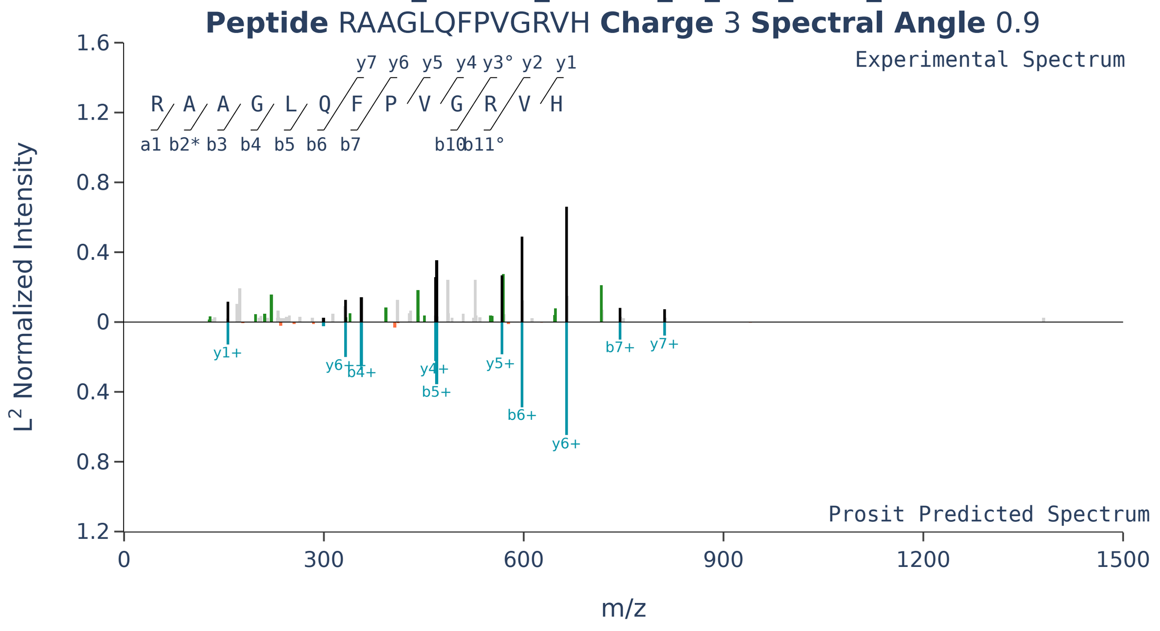

Source HRoetschke_291222_060123_Fu_H2A_b1R2_4h **Scan** 11084
Peptide ARAGLQFPVGRVH **Charge** 3 **Spectral Angle** 0.89

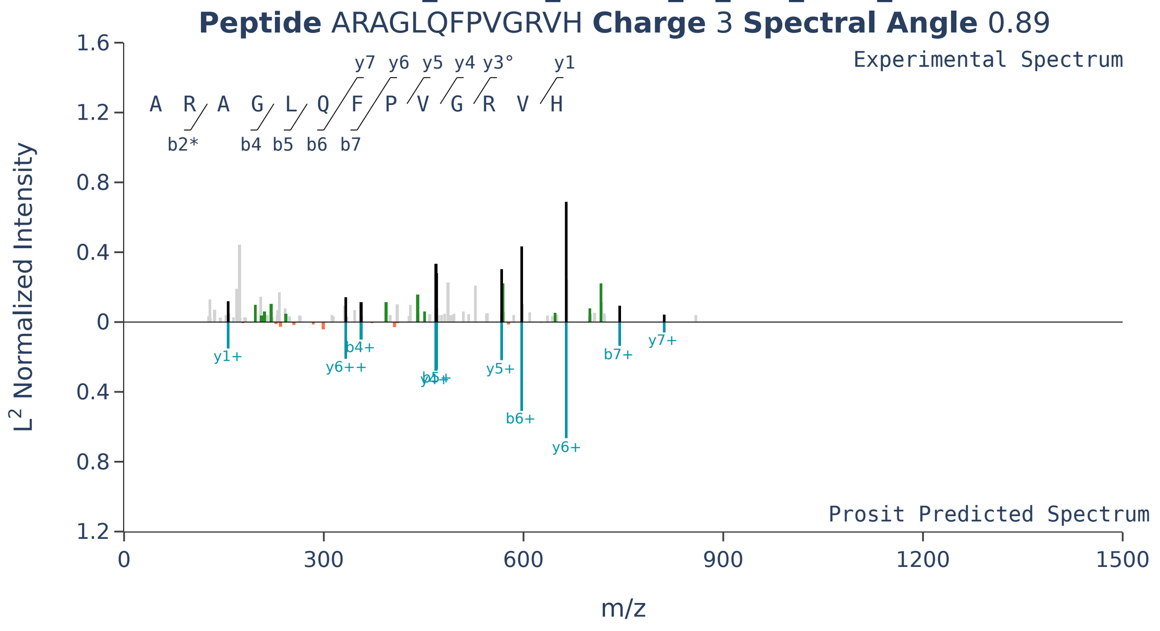

Source HRoetschke_291222_060123_Fu_H2A_b1R1_2h **Scan** 3662
Peptide QLLRKGNYAER **Charge** 3 **Spectral Angle** 0.89

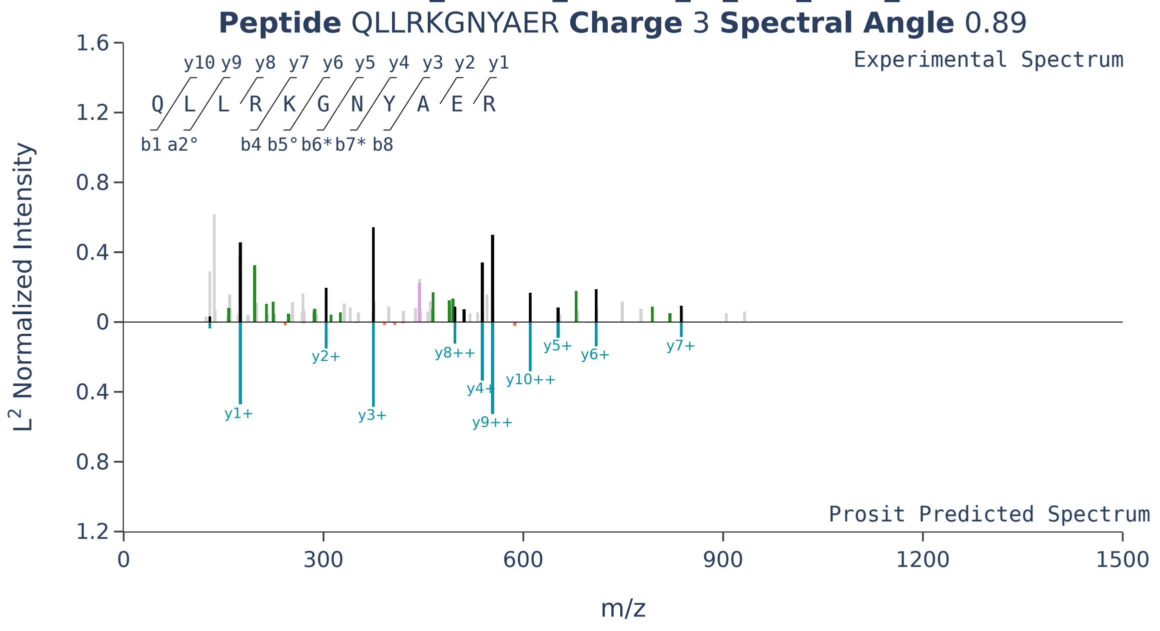

Source HRoetschke_291222_060123_Fu_H2A_b1R1_24h **Scan** 10841
Peptide GGVLPNSRAGL **Charge** 2 **Spectral Angle** 0.91

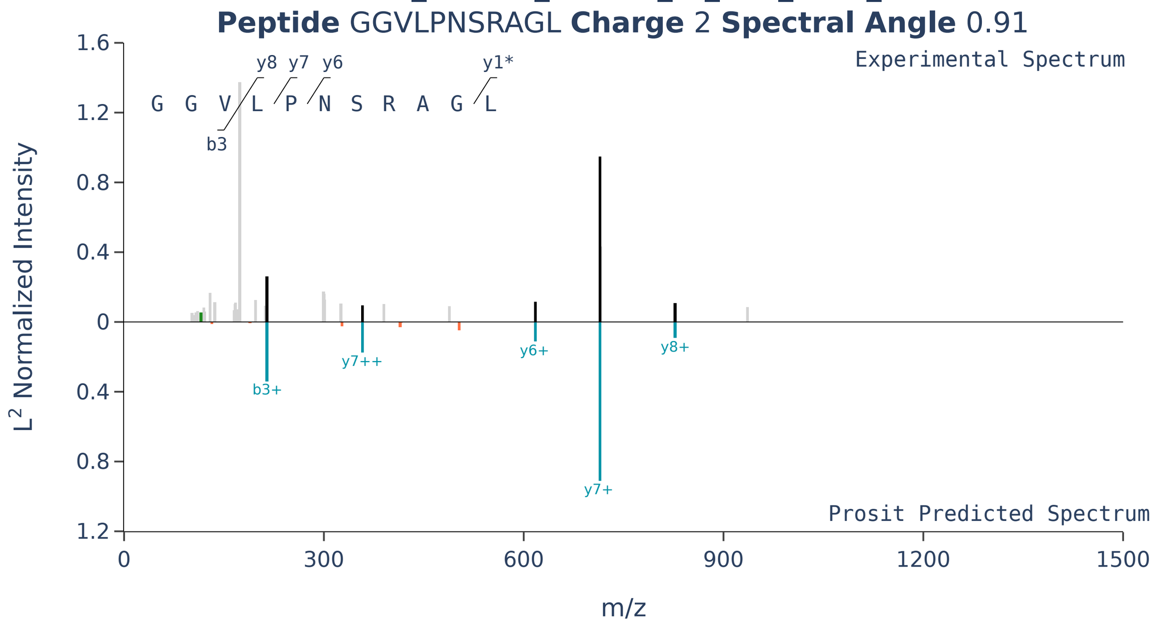

Source HRoetschke_291222_060123_Fu_H2A_b2R1_24h **Scan** 6712
Peptide RANDEELNKL **Charge** 3 **Spectral Angle** 0.91

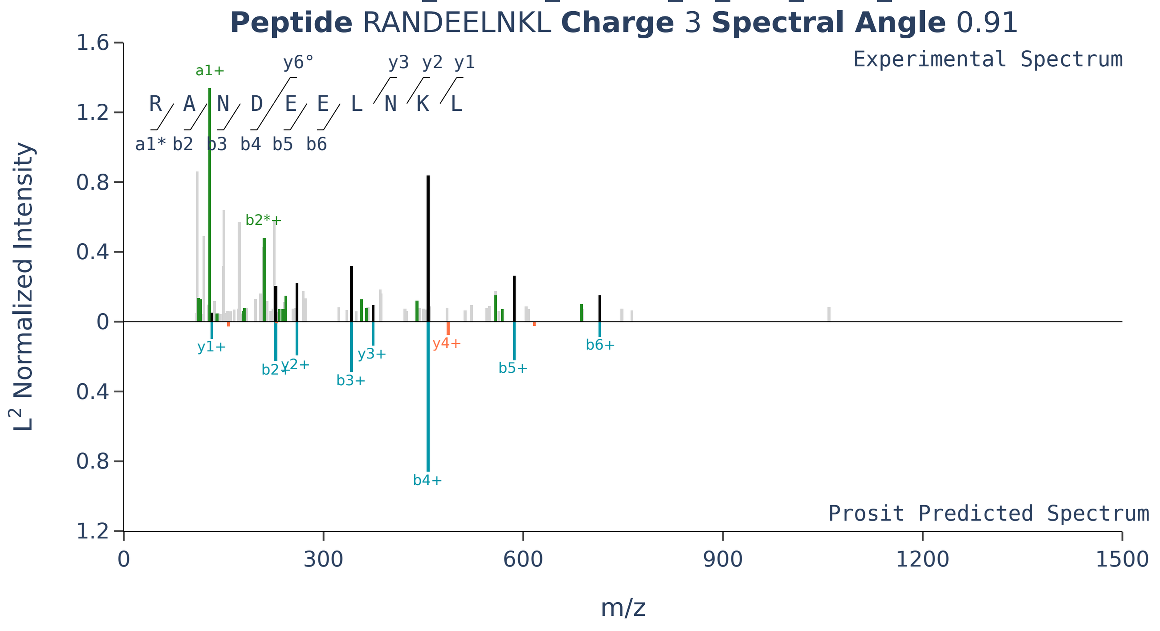

Source HRoetschke_291222_060123_Fu_H2A_b2R1_24h **Scan** 4481
Peptide GNYAEERVG **Charge** 2 **Spectral Angle** 0.93

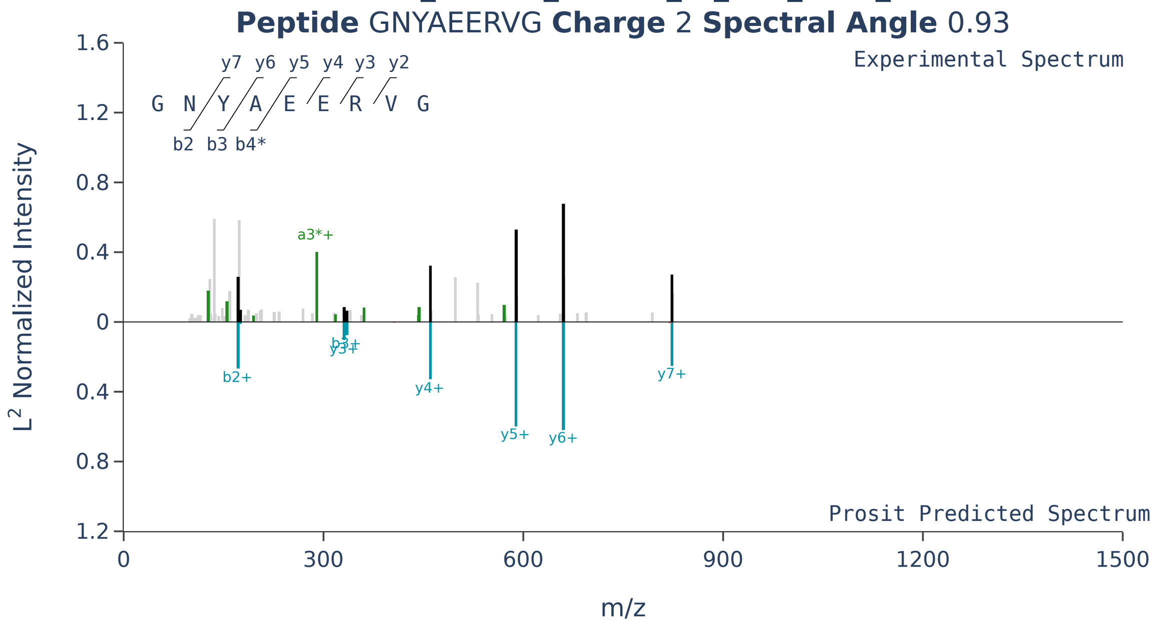

Source HRoetschke_291222_060123_Fu_H2A_b2R1_24h **Scan** 8686
Peptide SSRAGLQFPVGSGRGK **Charge** 3 **Spectral Angle** 0.89

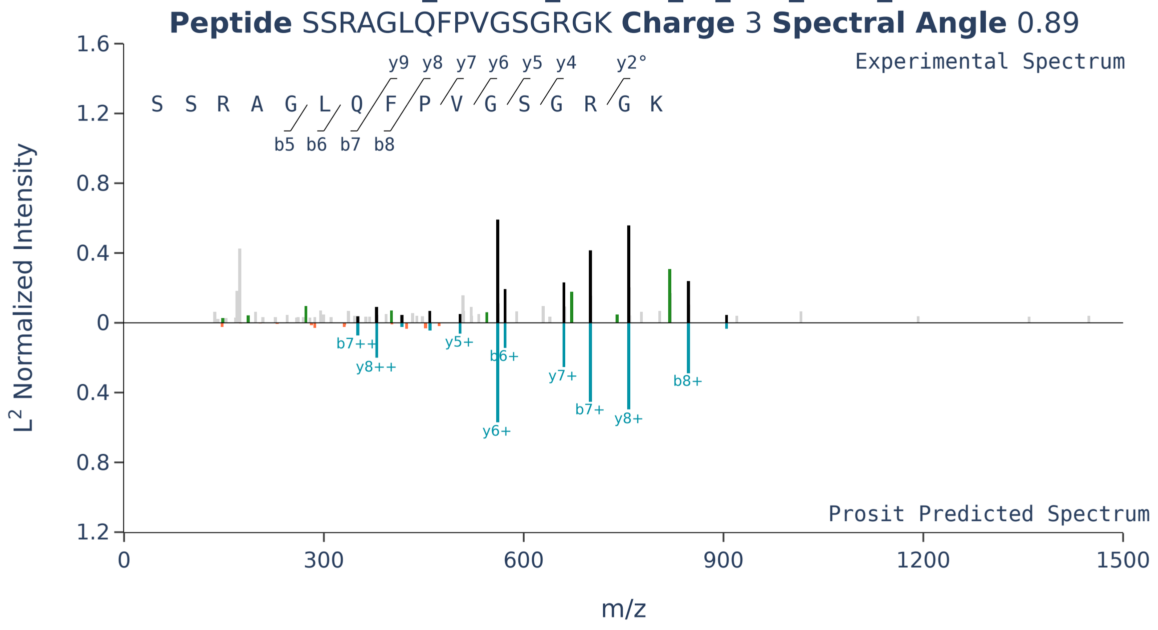

Source HRoetschke_291222_060123_Fu_H2A_b2R1_24h **Scan** 13511
Peptide RNDEELNKLLGRGL **Charge** 3 **Spectral Angle** 0.9

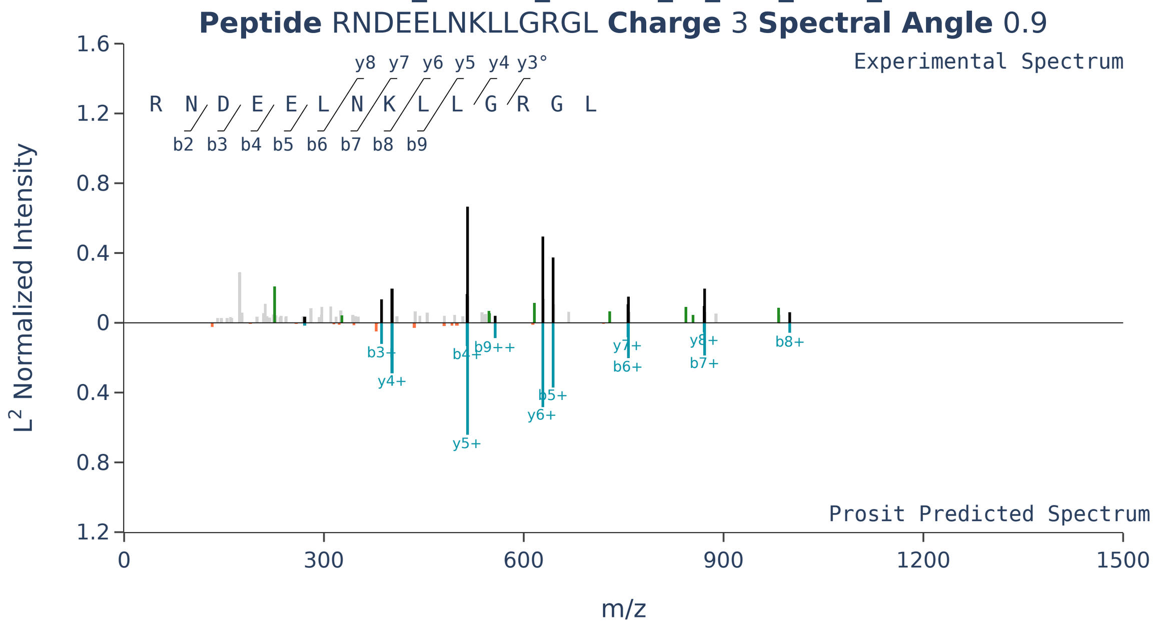

Source HRoetschke_291222_060123_Fu_H2A_b1R1_24h **Scan** 4295
Peptide GNYAERVE **Charge** 2 **Spectral Angle** 0.92

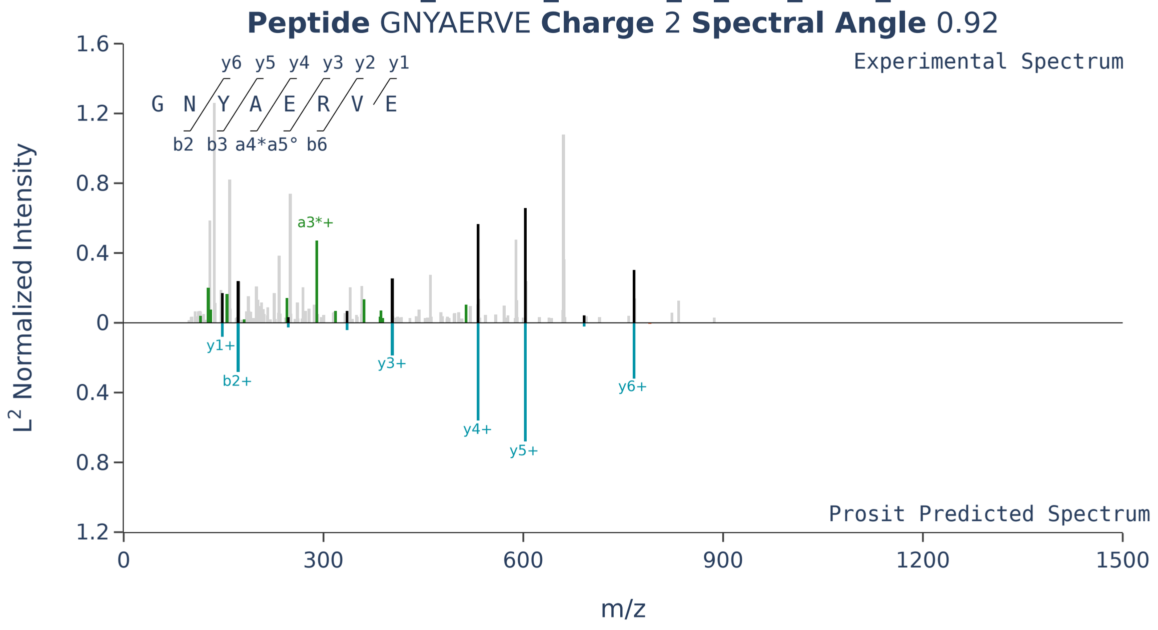

Source HRoetschke_291222_060123_Fu_H2A_b1R1_4h **Scan** 4429
Peptide VRNNYAERVG **Charge** 3 **Spectral Angle** 0.85

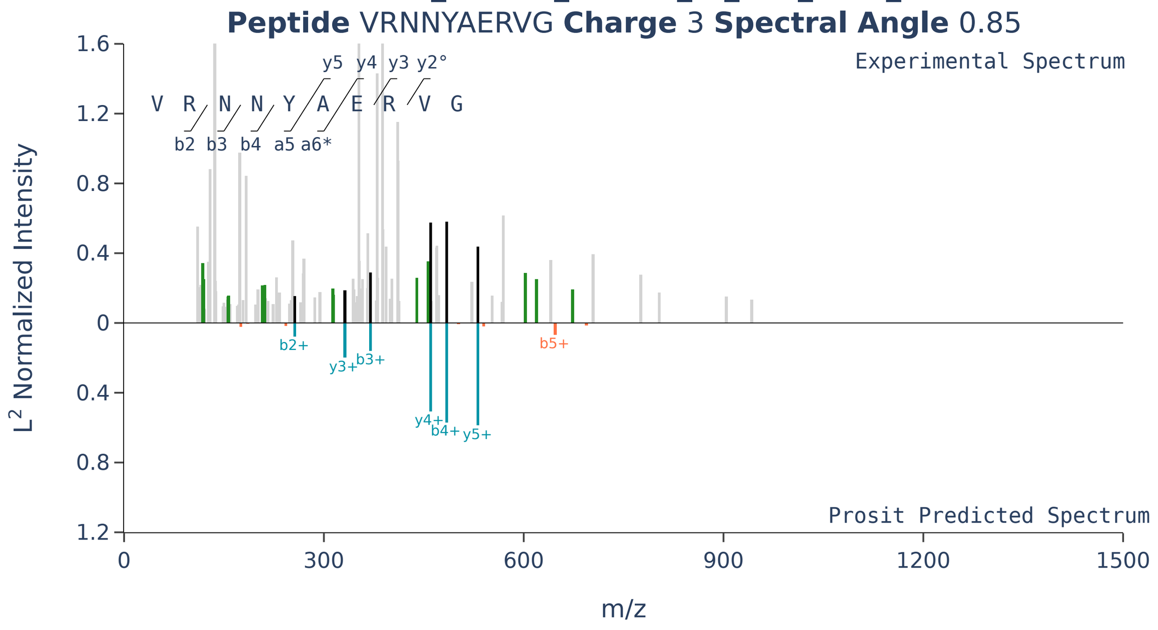

Source HRoetschke_291222_060123_Fu_H2A_b1R2_1h **Scan** 4227
Peptide AQGQLAVRNDE **Charge** 2 **Spectral Angle** 0.9

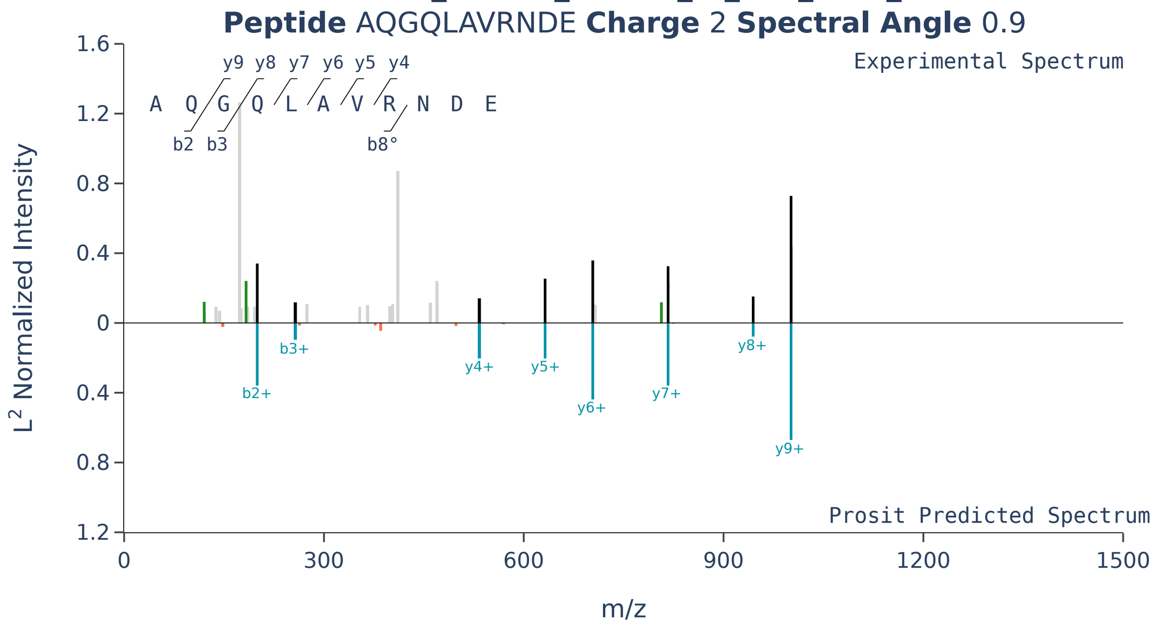

Source HRoetschke_291222_060123_Fu_H2A_b1R1_24h **Scan** 8414
Peptide QFPVGRVHLR **Charge** 3 **Spectral Angle** 0.88

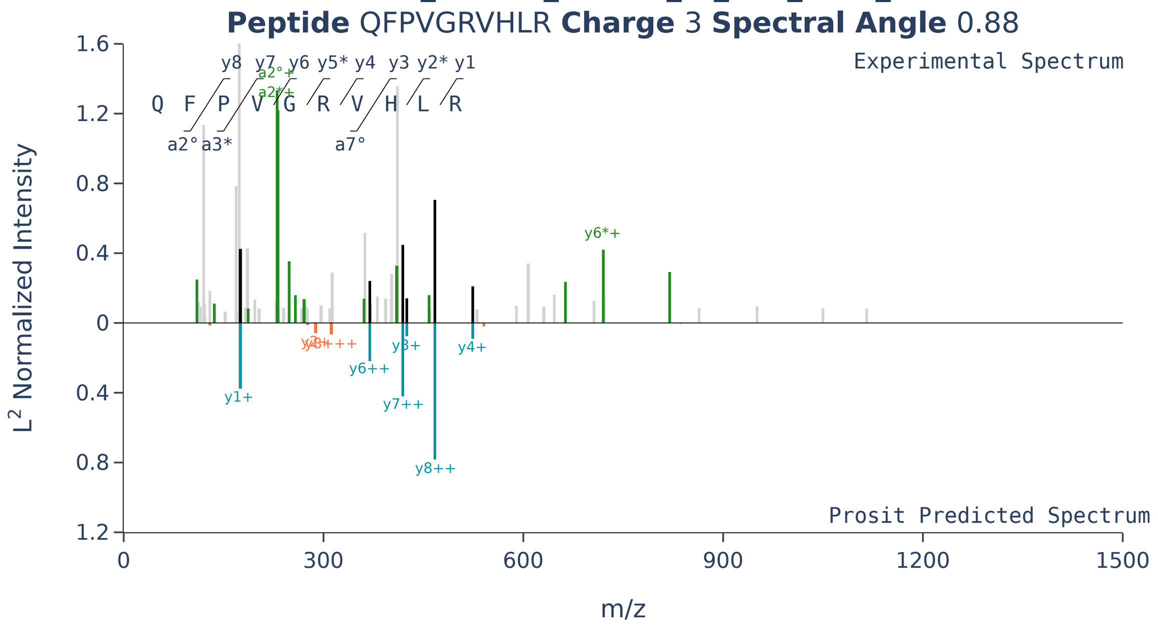

Source HRoetschke_291222_060123_Fu_H2A_b1R1_24h **Scan** 7089
Peptide SSRAGLQY **Charge** 2 **Spectral Angle** 0.8

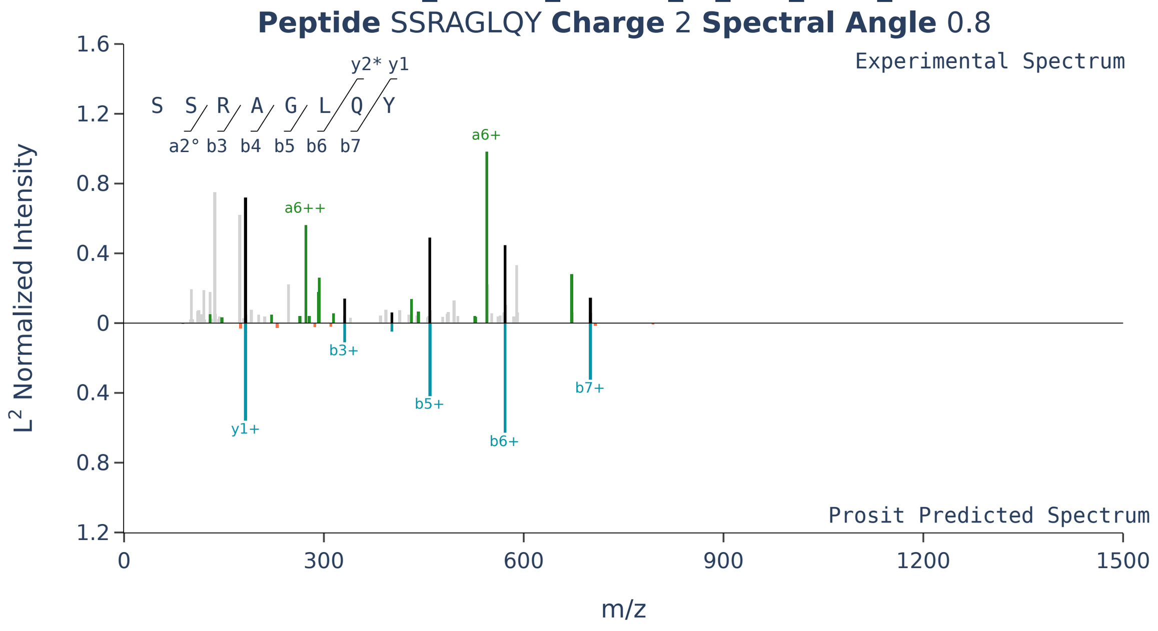

Source HRoetschke_291222_060123_Fu_H2A_b2R1_24h **Scan** 3083
Peptide GNYAERVGSSR **Charge** 3 **Spectral Angle** 0.82

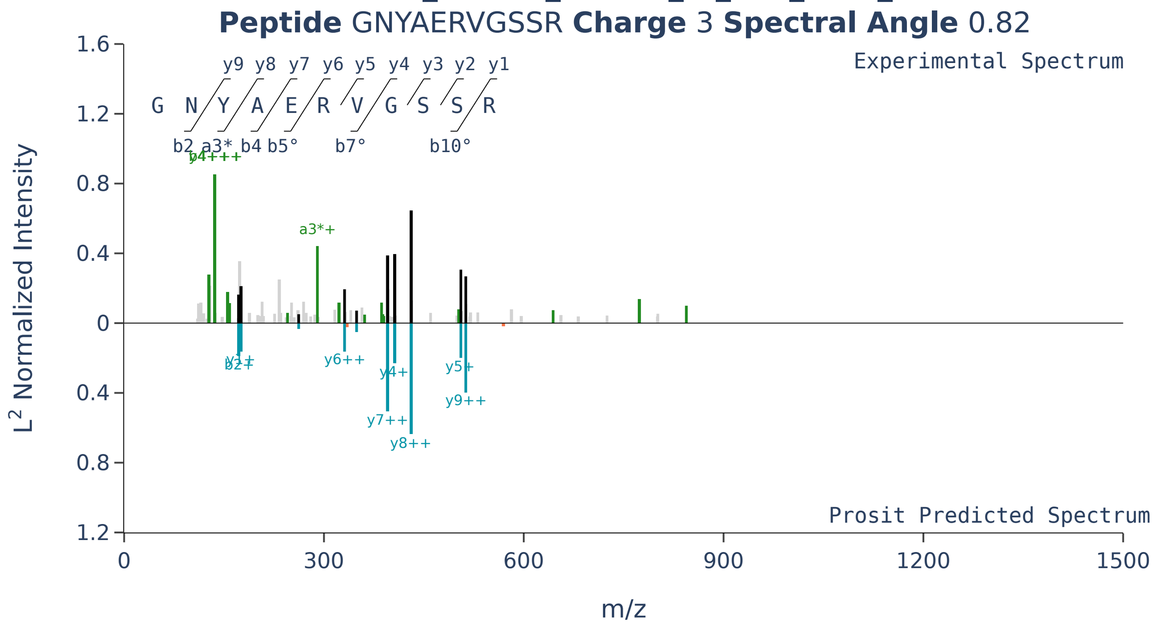

Source HRoetschke_291222_060123_Fu_H2A_b1R2_24h **Scan** 7607
Peptide GNYAERVGALGR **Charge** 3 **Spectral Angle** 0.85

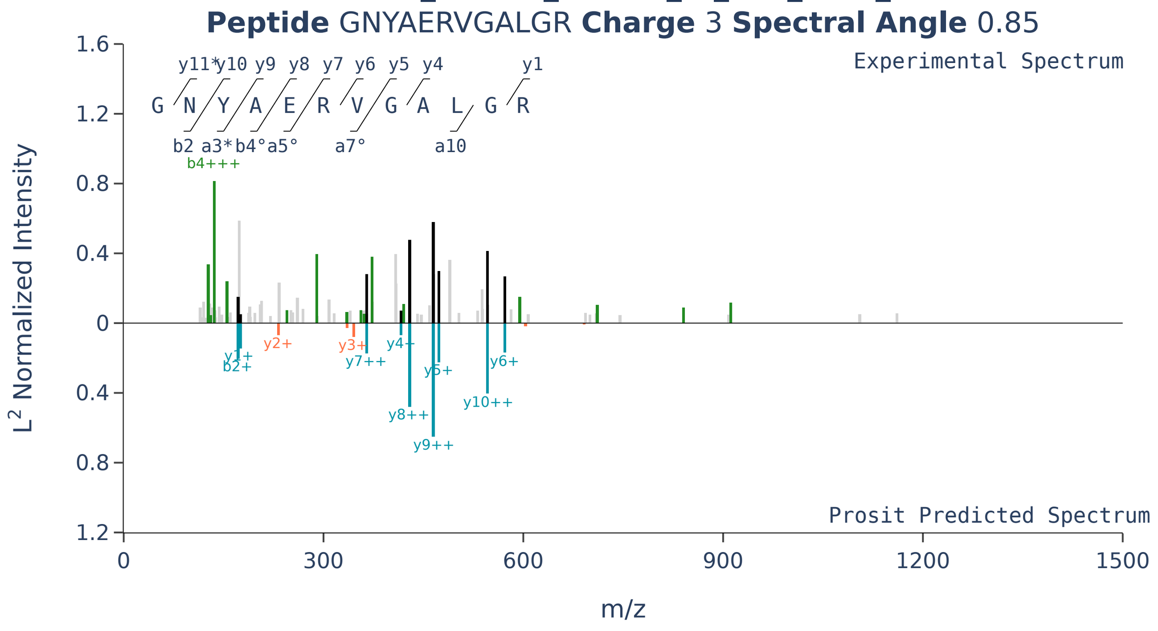

Source HRoetschke_291222_060123_Fu_H2A_b1R1_24h **Scan** 6682
Peptide ARNDEELNKL **Charge** 3 **Spectral Angle** 0.86

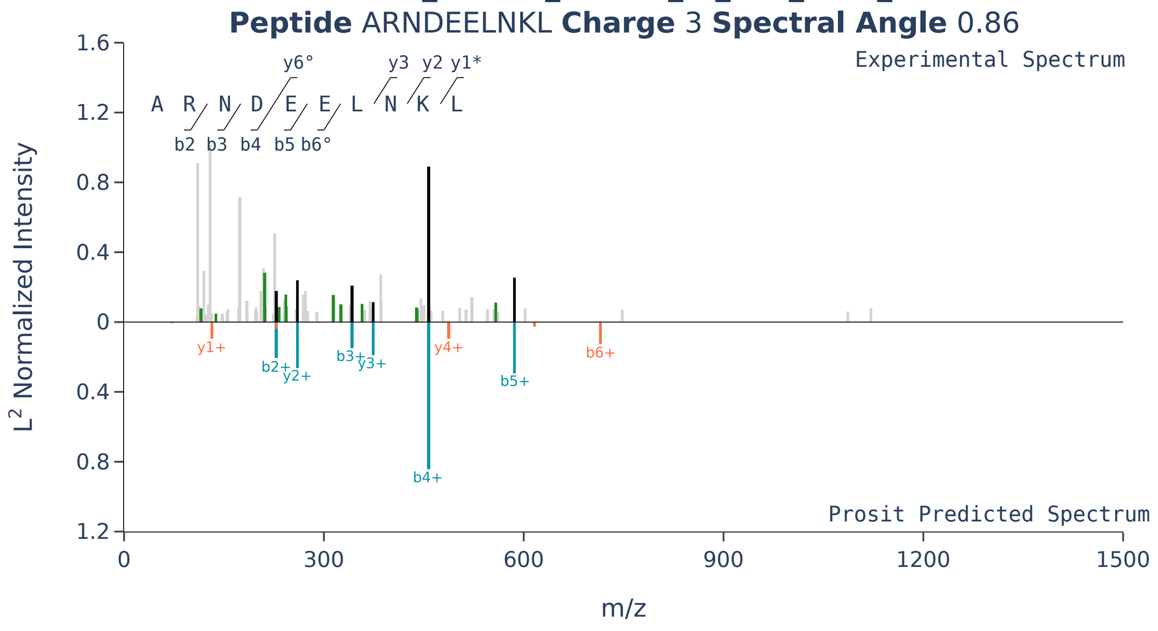

Source HRoetschke_291222_060123_Fu_H2A_b2R1_24h **Scan** 12952
Peptide GNYAERVGEYL **Charge** 2 **Spectral Angle** 0.8

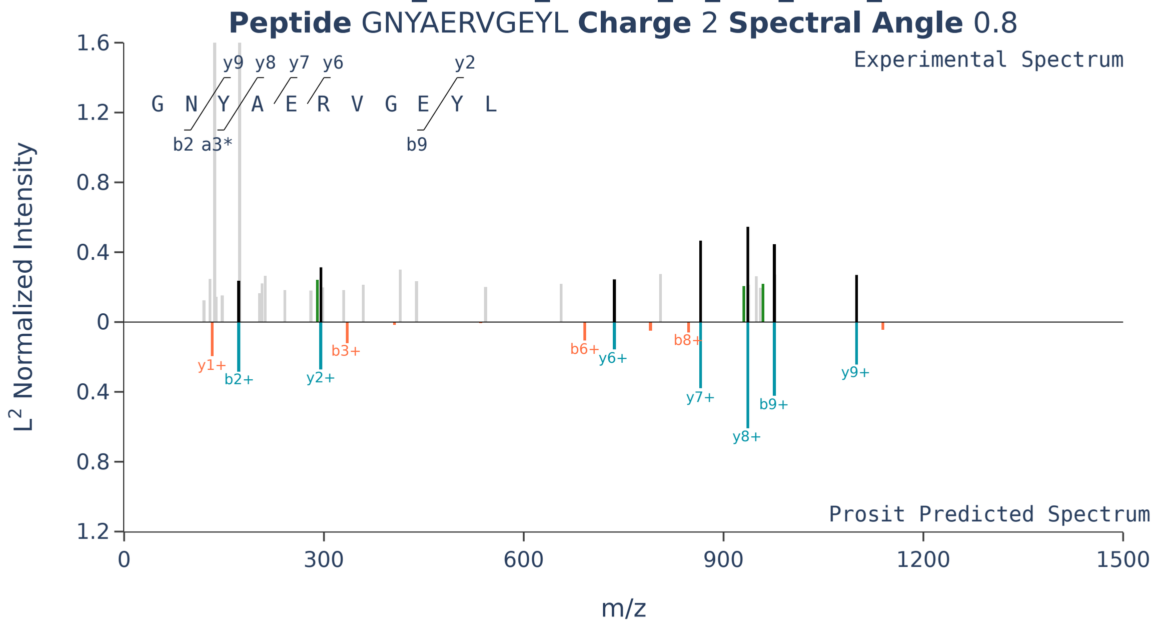

Source HRoetschke_291222_060123_Fu_H2A_b2R2_24h **Scan** 7600
Peptide GNYAERLAVRN **Charge** 3 **Spectral Angle** 0.85

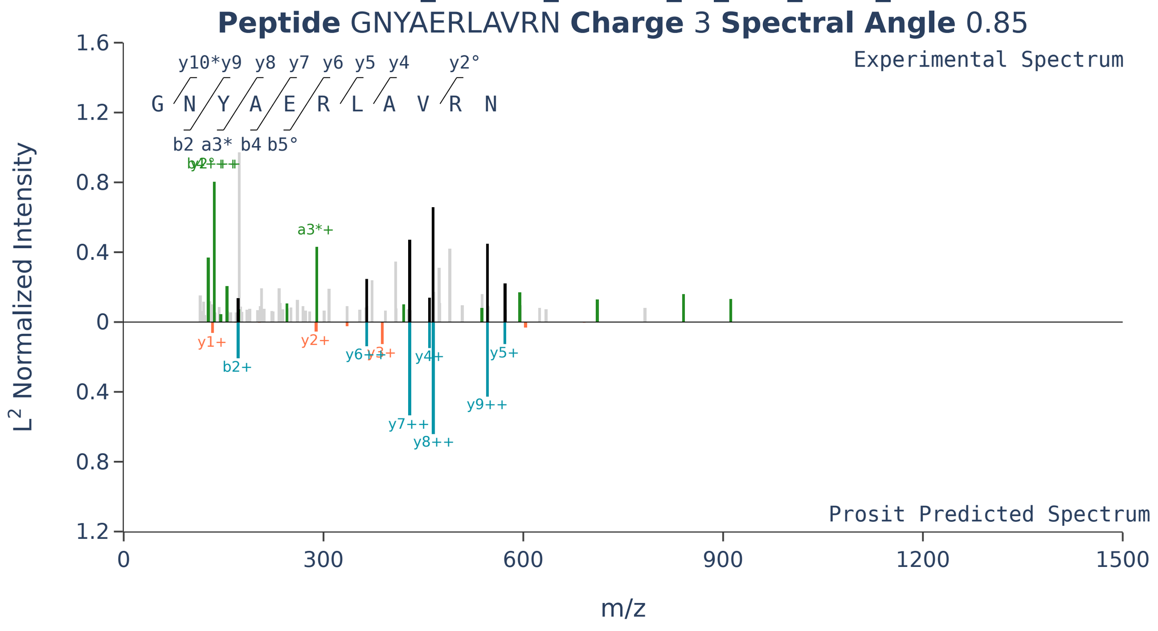

Source HRoetschke_291222_060123_Fu_H2A_b1R2_24h **Scan** 12333
Peptide SSAGLQFPVGRVH **Charge** 3 **Spectral Angle** 0.91

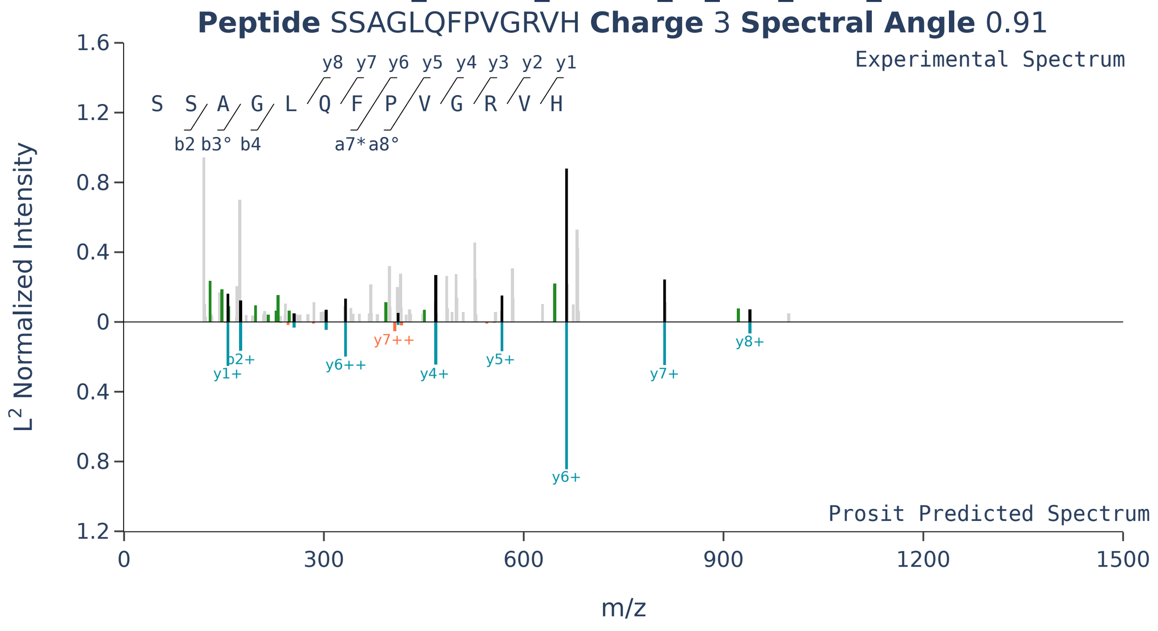

Source HRoetschke_291222_060123_Fu_H2A_b2R1_24h **Scan** 11830
Peptide RNDEELNKLLGRA **Charge** 3 **Spectral Angle** 0.88

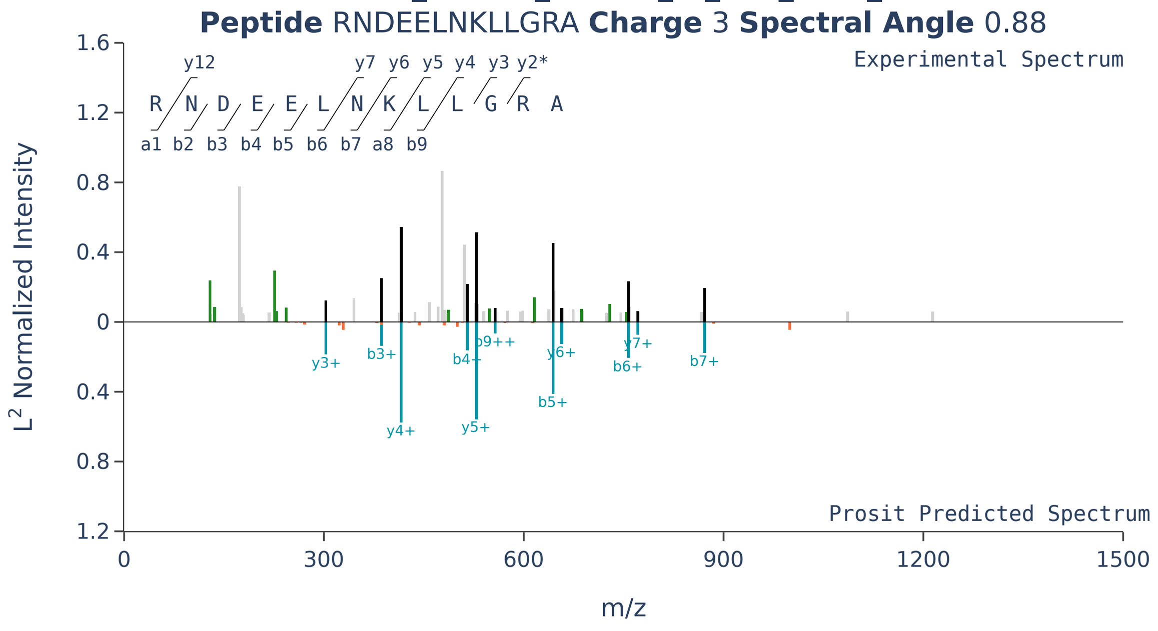

Source HRoetschke_291222_060123_Fu_H2A_b1R2_1h **Scan** 3872
Peptide AQGQLAVRND **Charge** 2 **Spectral Angle** 0.91

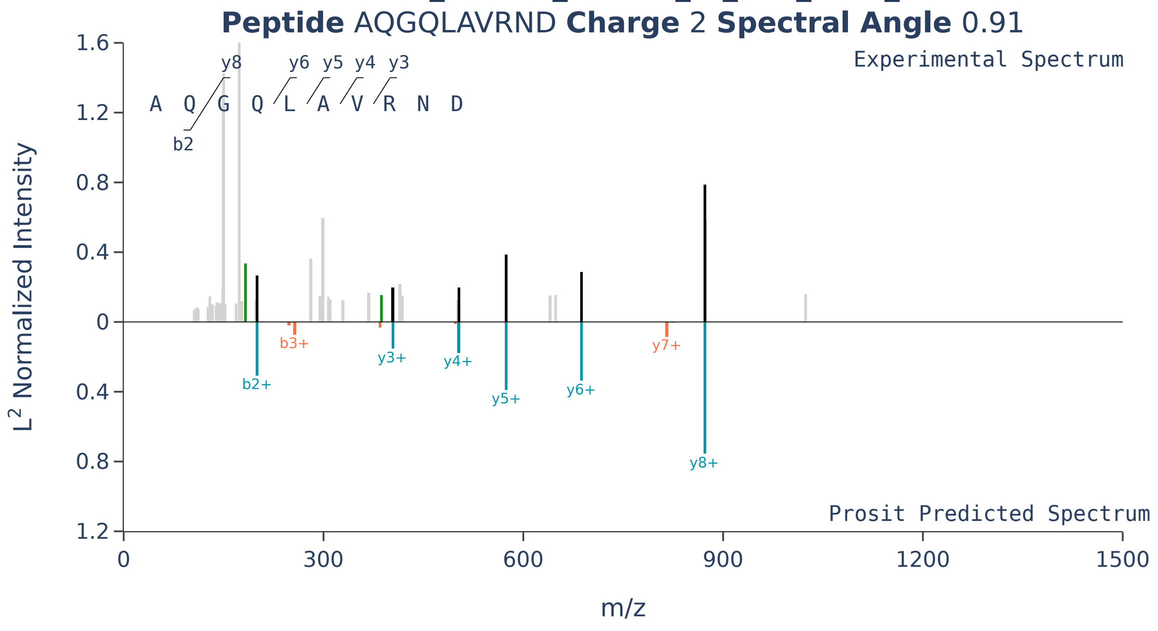

Source HRoetschke_291222_060123_Fu_H2A_b1R2_24h **Scan** 4243
Peptide NYAERVGE **Charge** 2 **Spectral Angle** 0.9

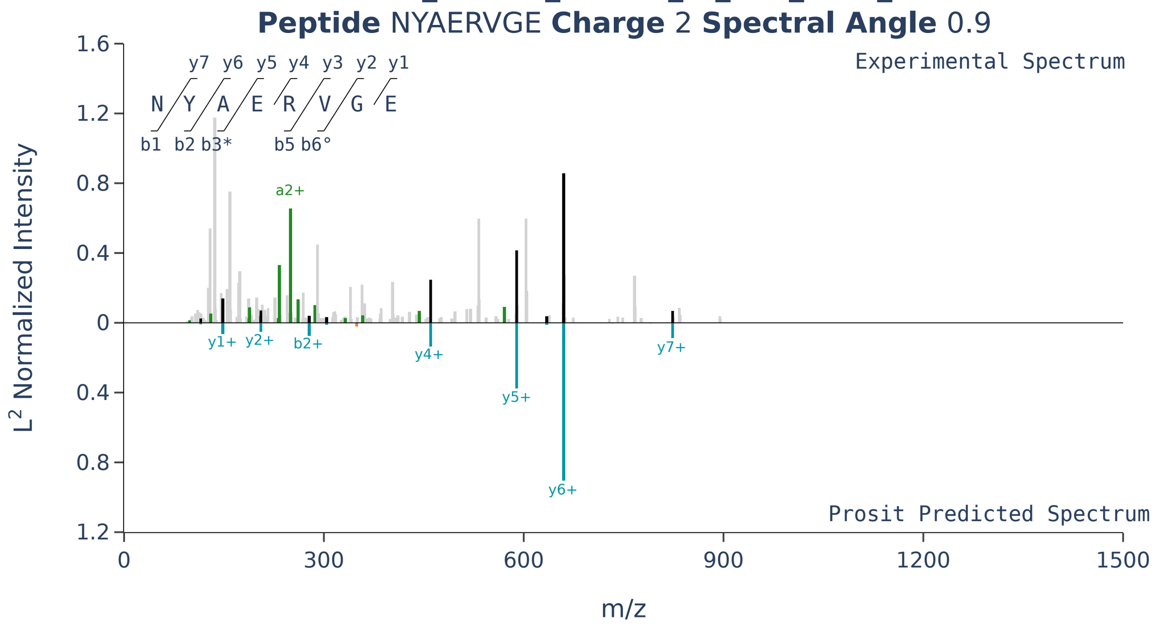

Source HRoetschke_291222_060123_Fu_H2A_b2R1_24h **Scan** 13403
Peptide SSSRAGLQFPVG **Charge** 2 **Spectral Angle** 0.89

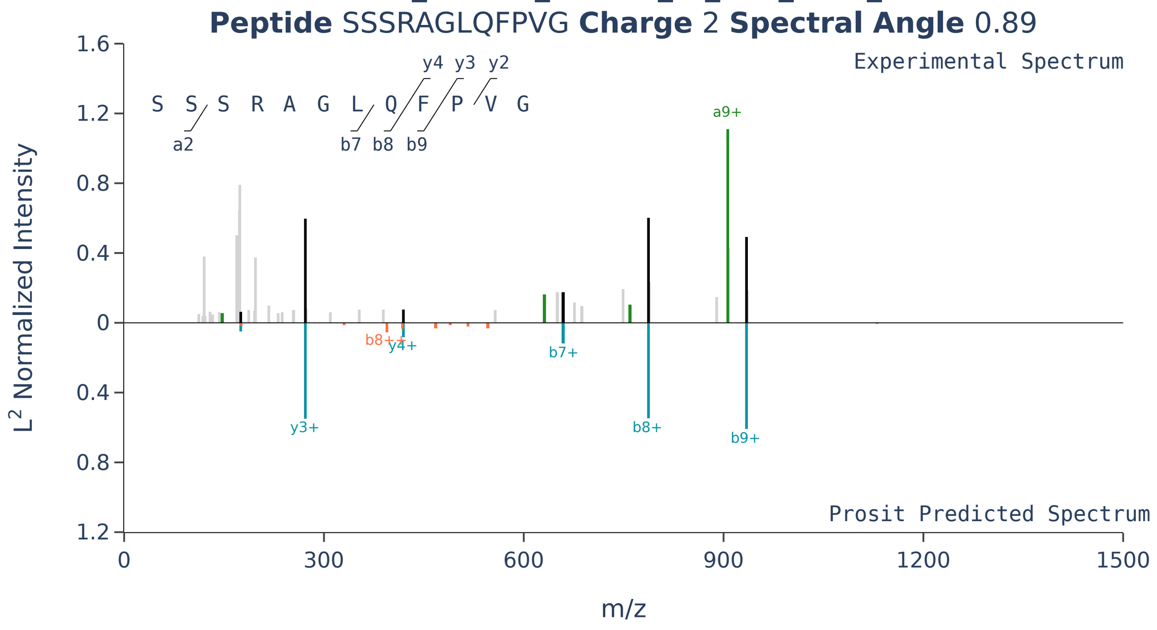

Source HRoetschke_291222_060123_Fu_H2A_b2R1_1h **Scan** 9928
Peptide KAKTRSSVLEYL **Charge** 3 **Spectral Angle** 0.78

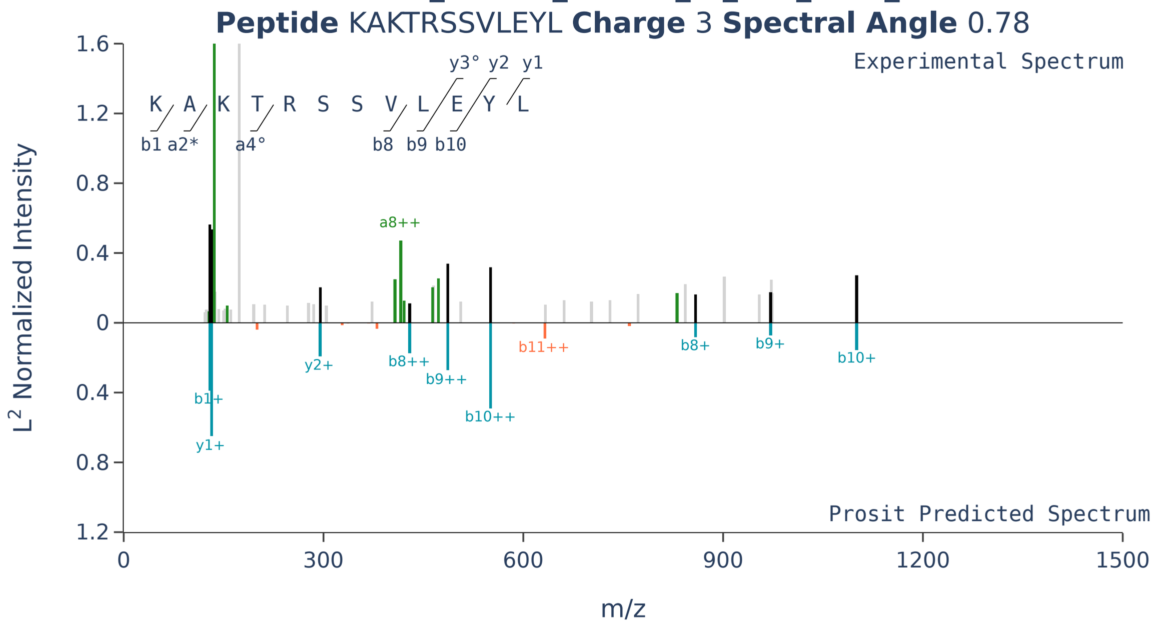

Source HRoetschke_291222_060123_Fu_H2A_b1R1_1h **Scan** 9889
Peptide AVRNDLQF **Charge** 2 **Spectral Angle** 0.92

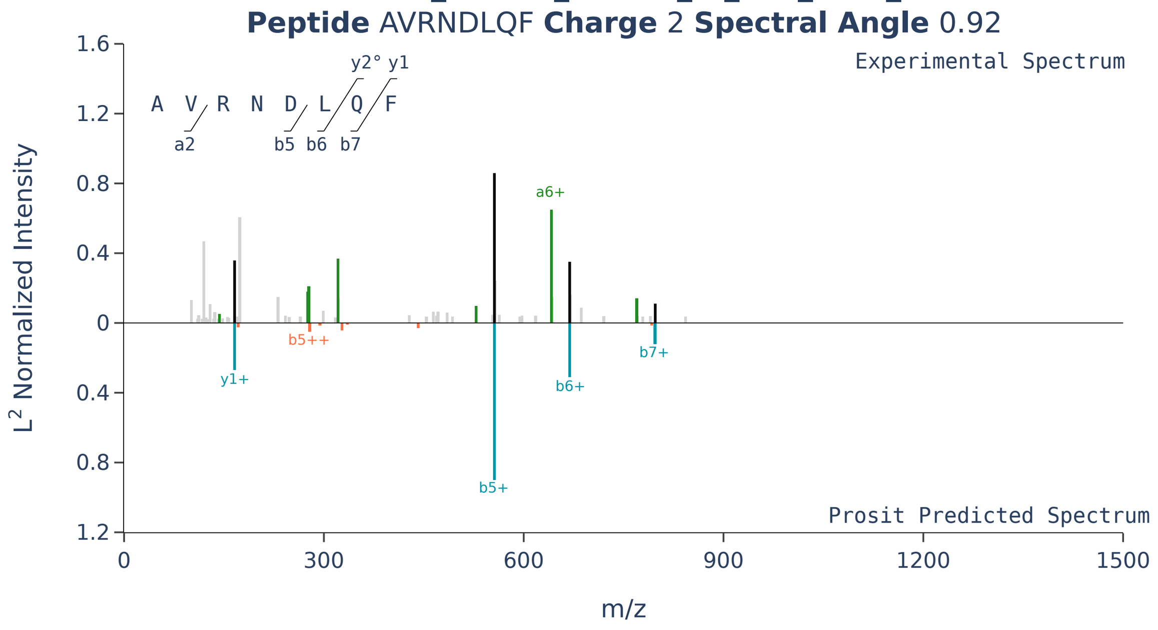

Source HRoetschke_291222_060123_Fu_H2A_b1R1_1h **Scan** 7025
Peptide AAVRNDEELNKL **Charge** 3 **Spectral Angle** 0.85

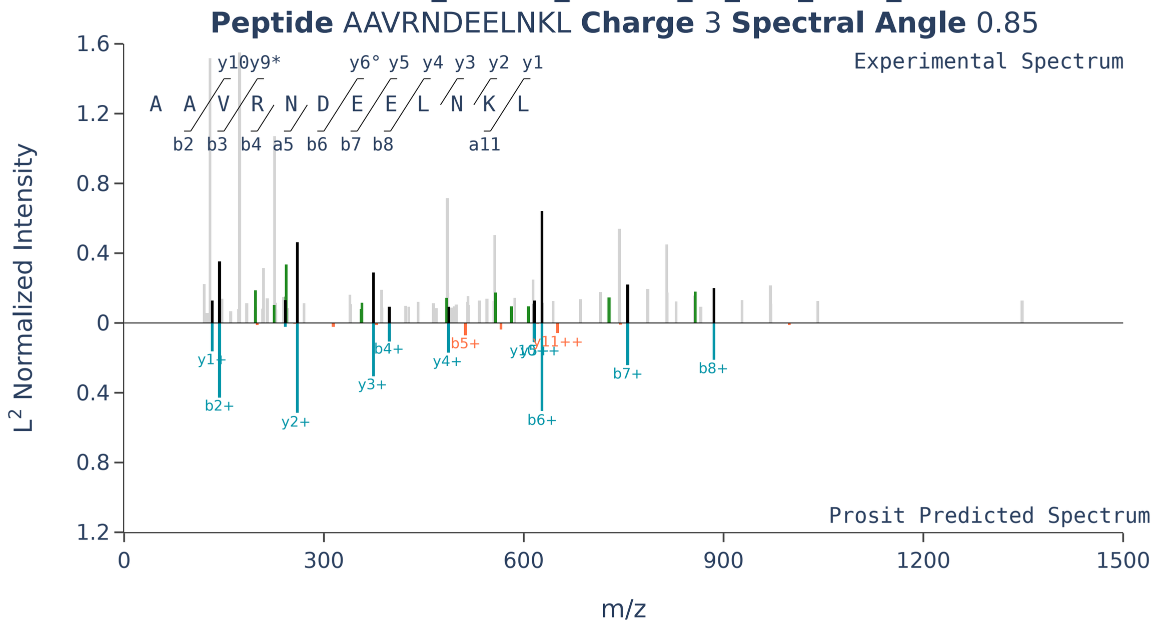

Source HRoetschke_291222_060123_Fu_H2A_b1R1_4h **Scan** 14910
Peptide SSRAGLQFPVGA **Charge** 2 **Spectral Angle** 0.88

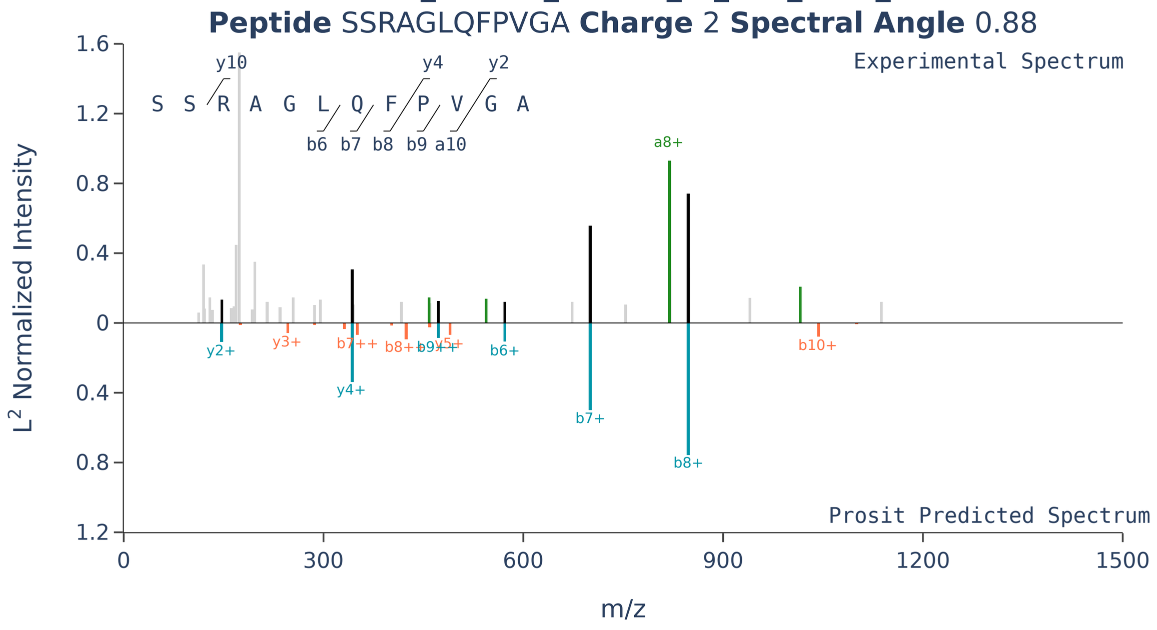

Source HRoetschke_291222_060123_Fu_H2A_b1R1_24h **Scan** 7686
Peptide GNYAERVGAGLR **Charge** 3 **Spectral Angle** 0.81

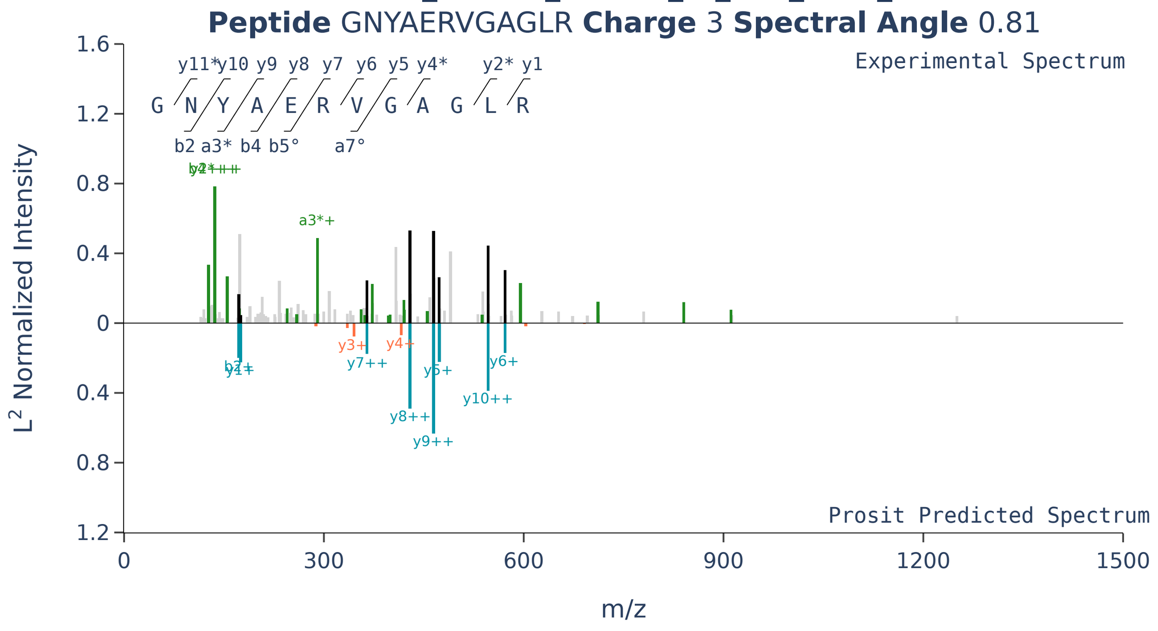

Source HRoetschke_291222_060123_Fu_H2A_b2R2_24h **Scan** 9458
Peptide GNYAERVGQL **Charge** 2 **Spectral Angle** 0.89

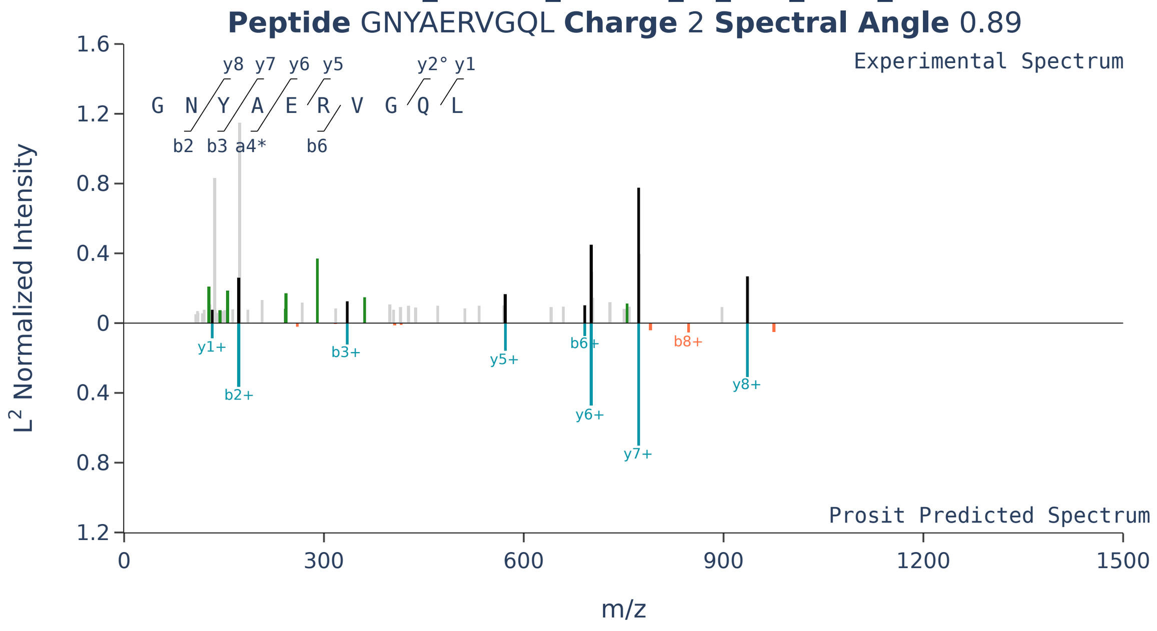

Source HRoetschke_291222_060123_Fu_H2A_b1R1_24h **Scan** 5808
Peptide RAGLQFPVGSGRGKQGGKTR **Charge** 5 **Spectral Angle** 0.83

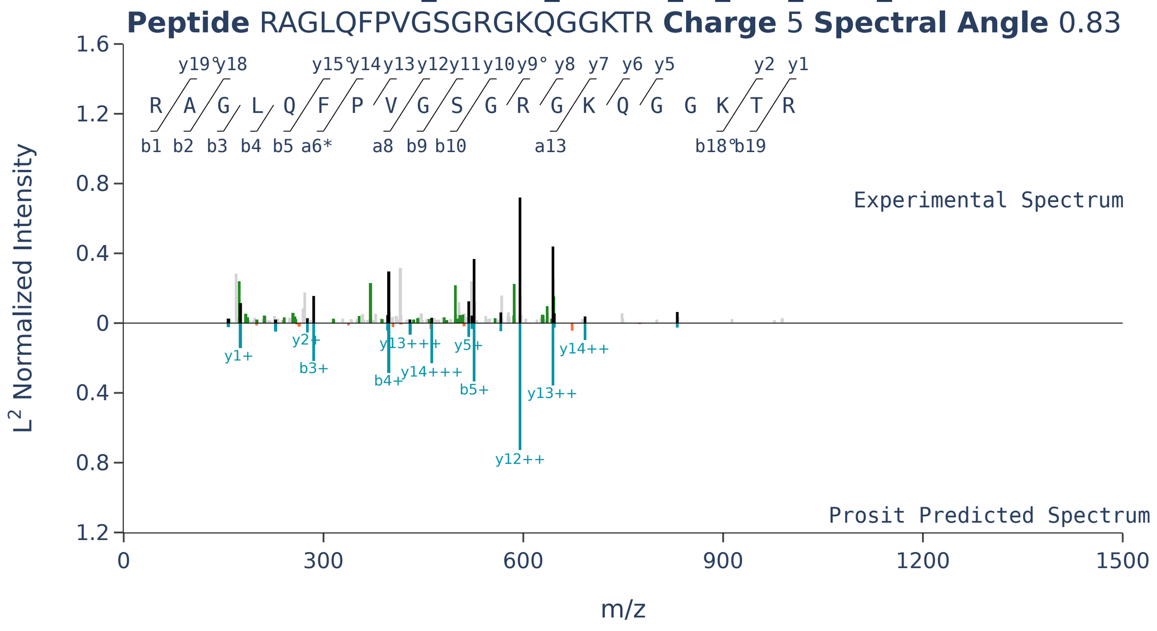

Source HRoetschke_291222_060123_Fu_H2A_b1R1_1h **Scan** 2298
Peptide SVLLPKKTESSKSAKNK **Charge** 3 **Spectral Angle** 0.85

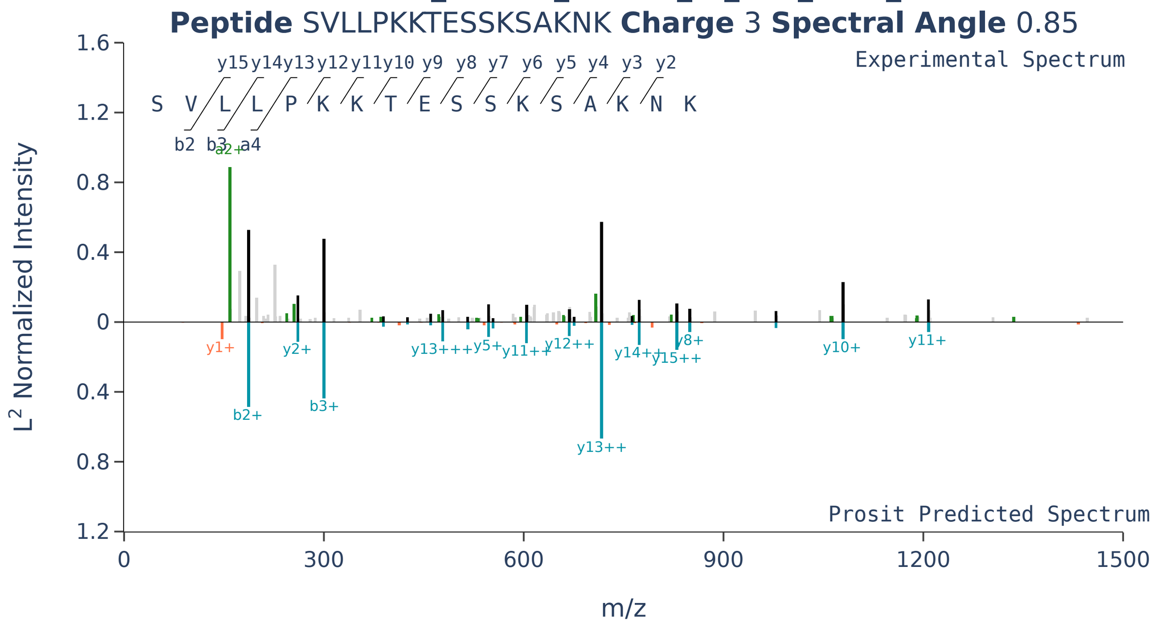

Source HRoetschke_291222_060123_Fu_H2A_b2R1_24h **Scan** 6135
Peptide AGNAAQLAVRND **Charge** 2 **Spectral Angle** 0.89

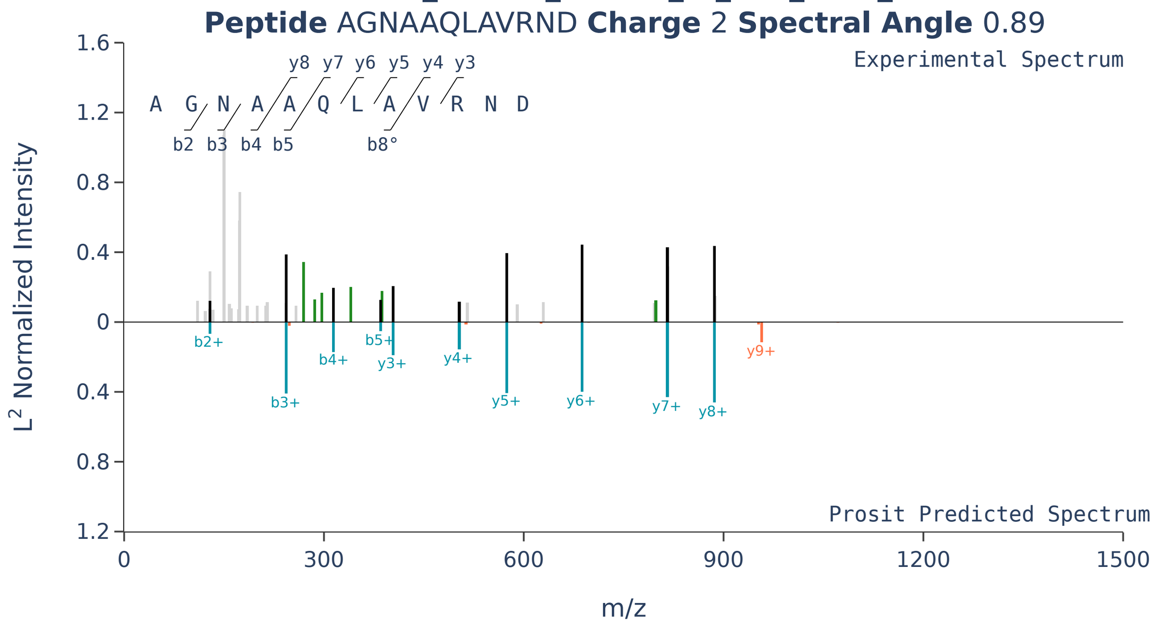

Source HRoetschke_291222_060123_Fu_H2A_b2R1_2h **Scan** 7235
Peptide AQFPVGRVH **Charge** 3 **Spectral Angle** 0.87

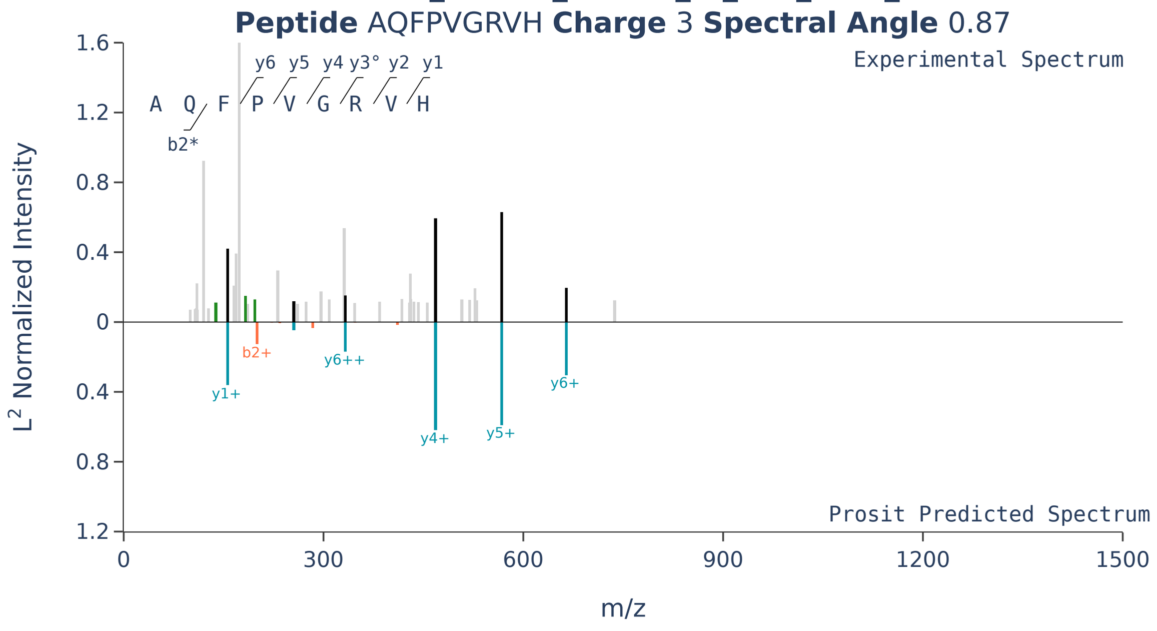

Source HRoetschke_291222_060123_Fu_H2A_b1R1_24h **Scan** 12259
Peptide ERVGAGGGVLPNIQ **Charge** 2 **Spectral Angle** 0.86

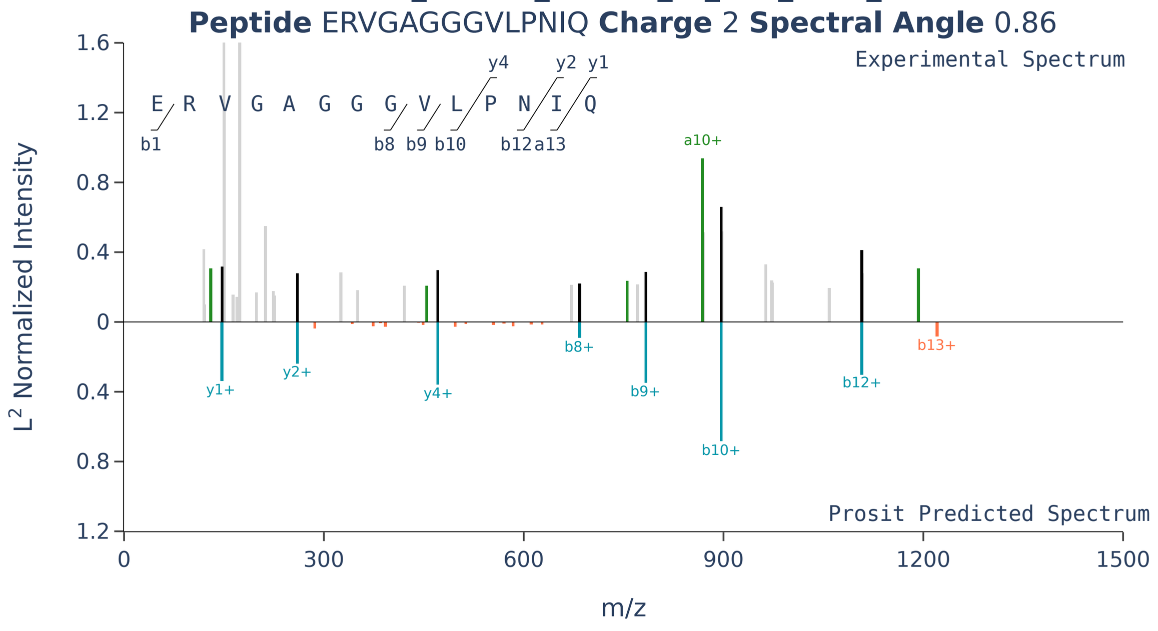

Source HRoetschke_291222_060123_Fu_H2A_b2R1_24h **Scan** 12640
Peptide QAPVYLA **Charge** 2 **Spectral Angle** 0.73

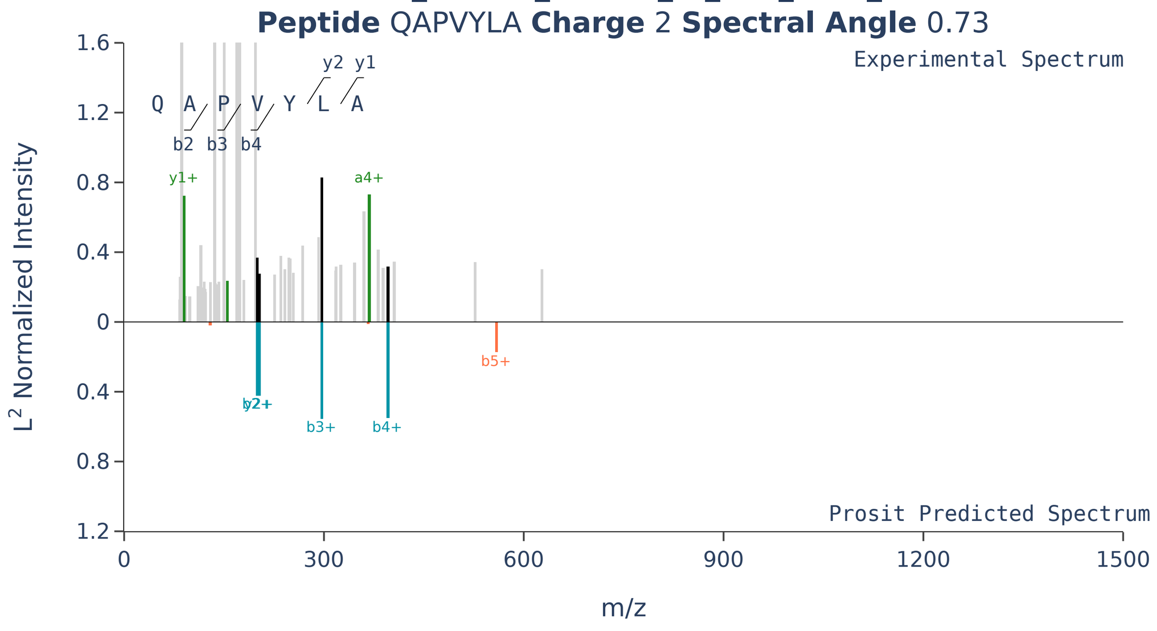

Source HRoetschke_291222_060123_Fu_H2A_b1R2_24h **Scan** 9665
Peptide SSRAGLQFA **Charge** 2 **Spectral Angle** 0.87

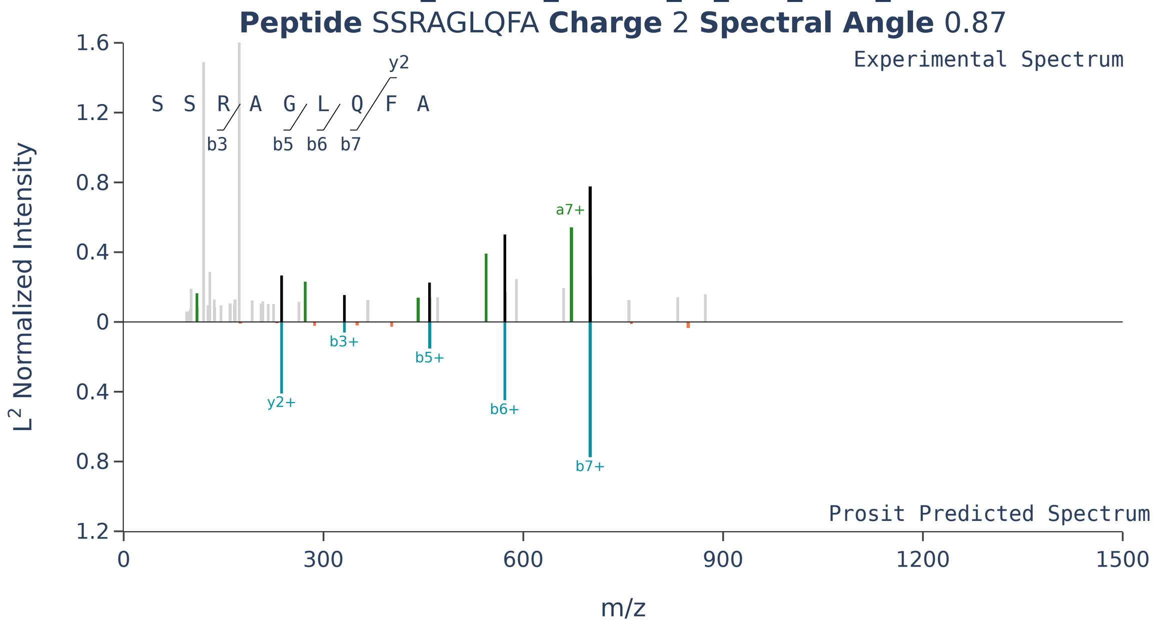

Source HRoetschke_291222_060123_Fu_H2A_b1R1_4h **Scan** 14644
Peptide ARNDEELNKLLGRV **Charge** 4 **Spectral Angle** 0.87

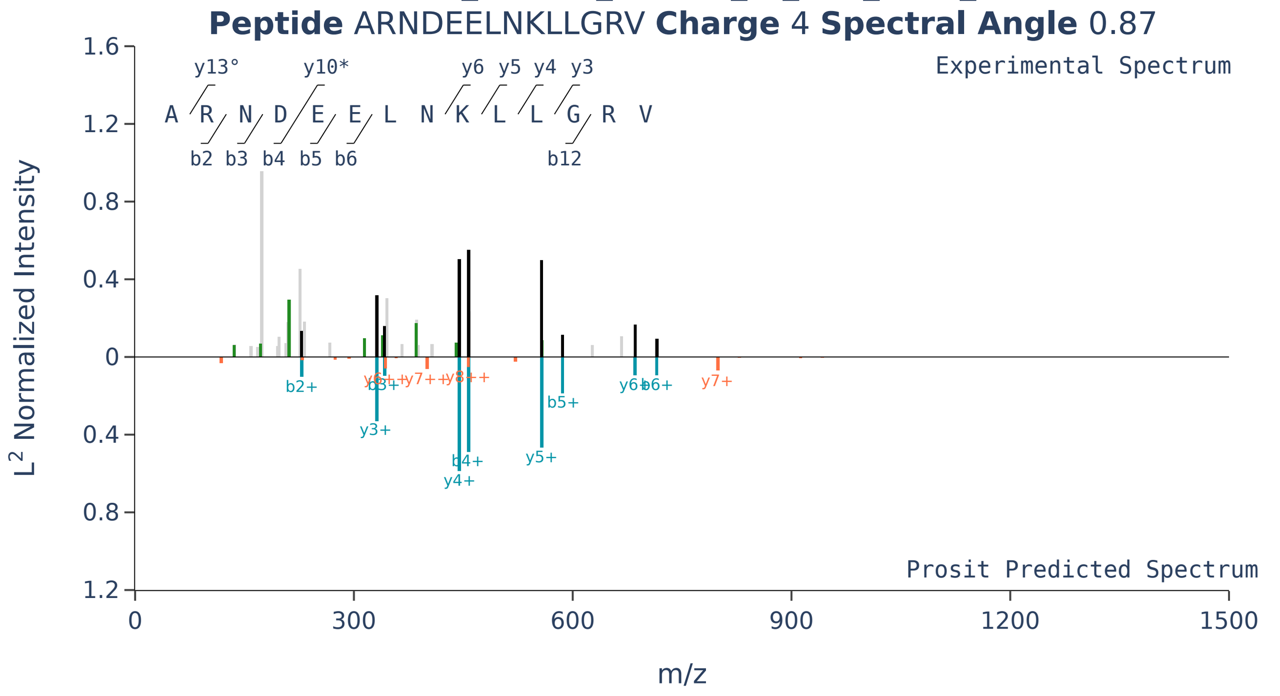

Source HRoetschke_291222_060123_Fu_H2A_b1R2_4h **Scan** 12857
Peptide VRNDEELNKLLGRA **Charge** 4 **Spectral Angle** 0.86

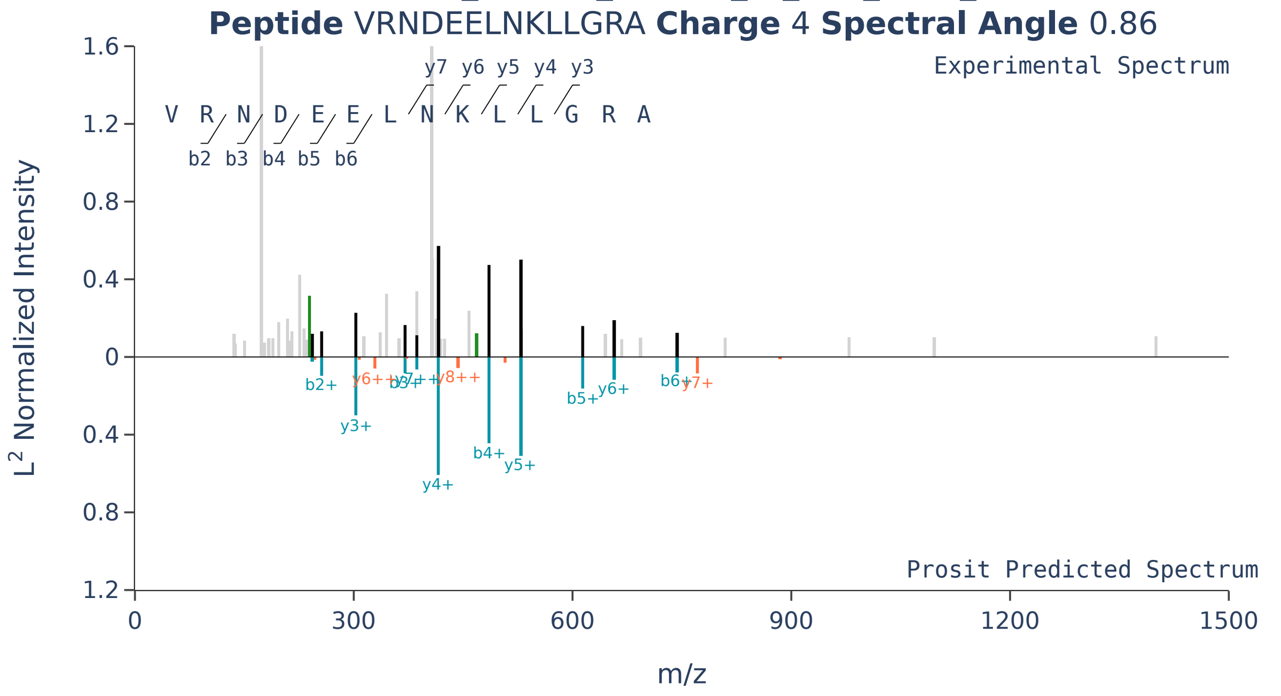

Source HRoetschke_291222_060123_Fu_H2A_b1R2_24h **Scan** 2949
Peptide RLLRKGNYAEQ **Charge** 3 **Spectral Angle** 0.8

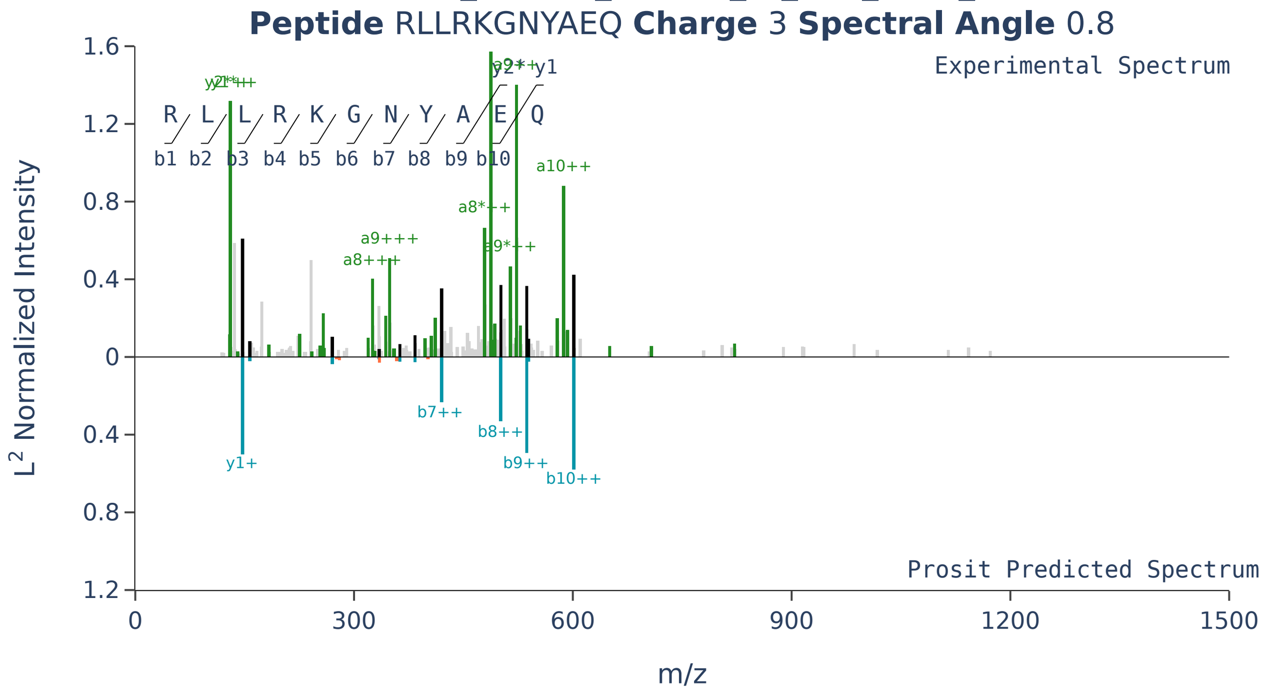

Source HRoetschke_291222_060123_Fu_H2A_b1R1_4h **Scan** 14303
Peptide VRNDEELNKLLGA **Charge** 2 **Spectral Angle** 0.86

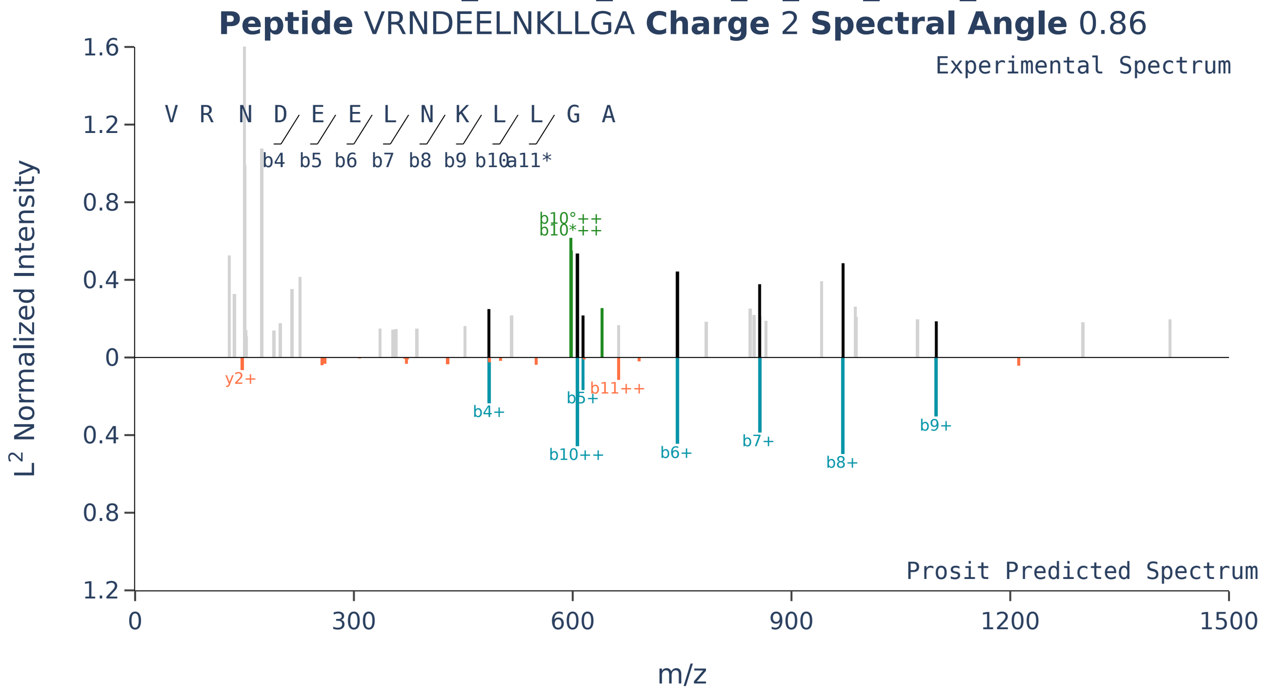

Source HRoetschke_291222_060123_Fu_H2A_b2R2_24h **Scan** 9267
Peptide RSSRAGAGLQFPVGRVH **Charge** 4 **Spectral Angle** 0.83

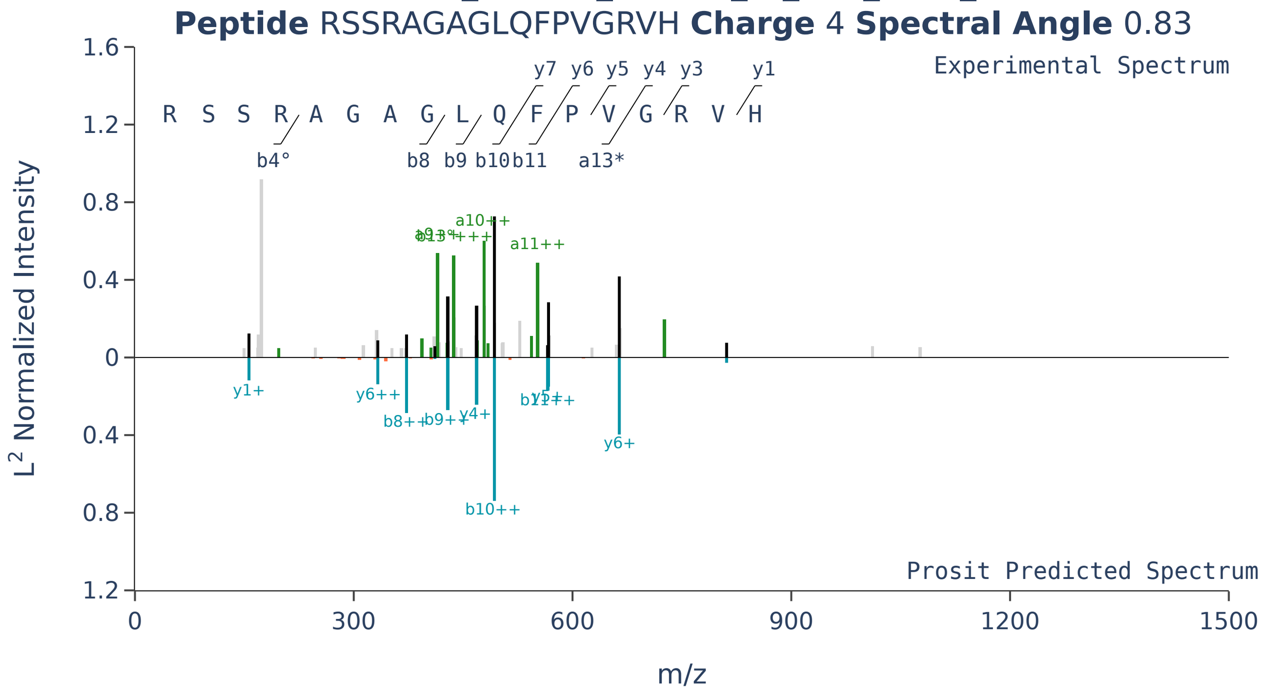

Source HRoetschke_291222_060123_Fu_H2A_b1R2_24h **Scan** 10084
Peptide ASSRAGLQF **Charge** 2 **Spectral Angle** 0.83

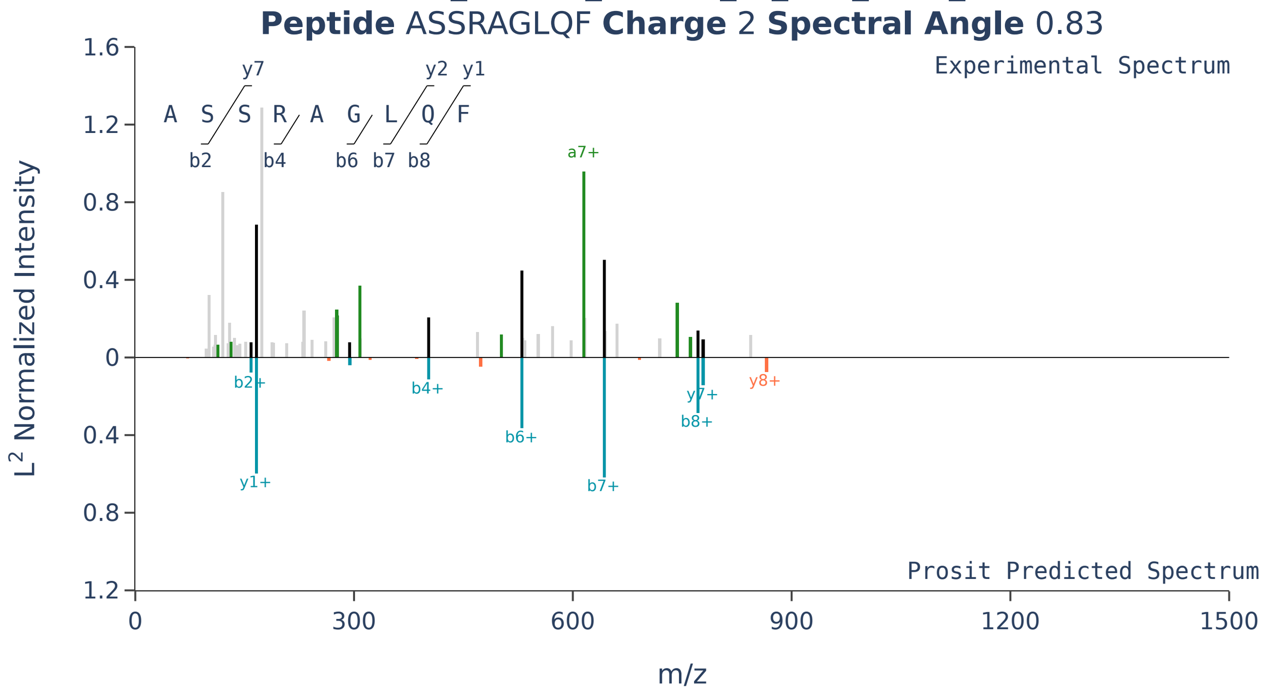

Source HRoetschke_291222_060123_Fu_H2A_b1R1_1h **Scan** 3480
Peptide LQRNDEELN **Charge** 2 **Spectral Angle** 0.93

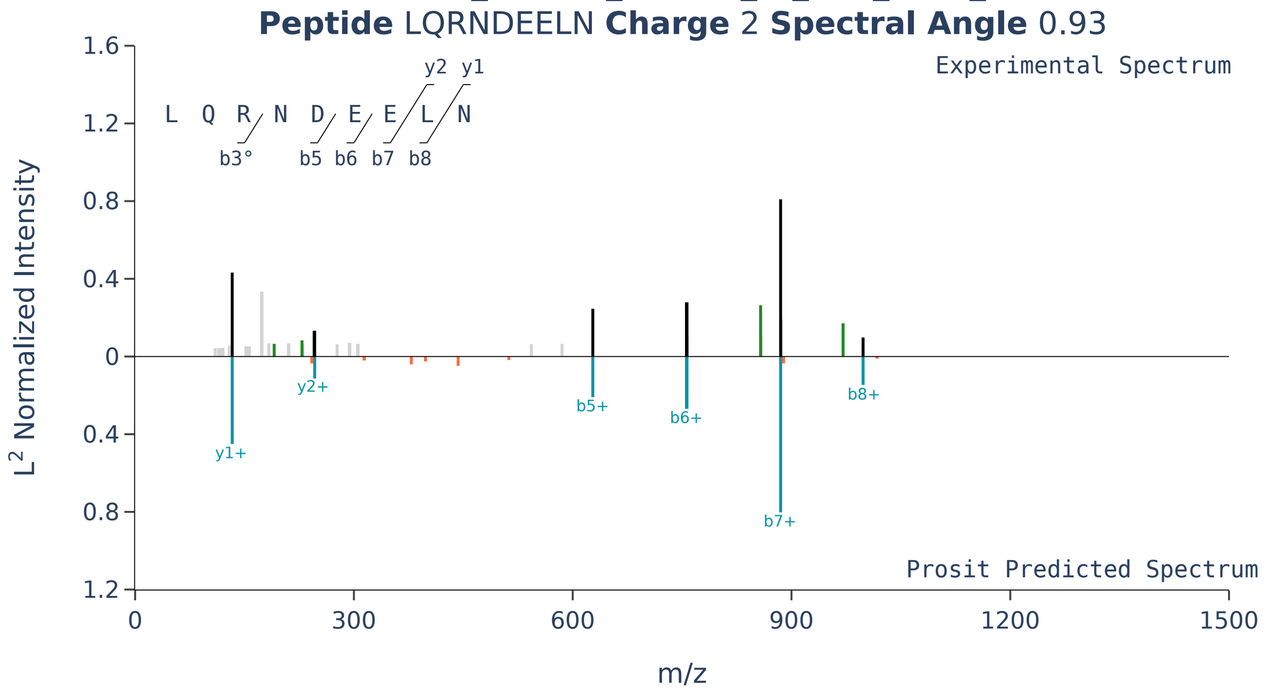

Source HRoetschke_291222_060123_Fu_H2A_b2R1_24h **Scan** 5848
Peptide SSRAGLQFPVGSGRGKQGGKTR **Charge** 4 **Spectral Angle** 0.87

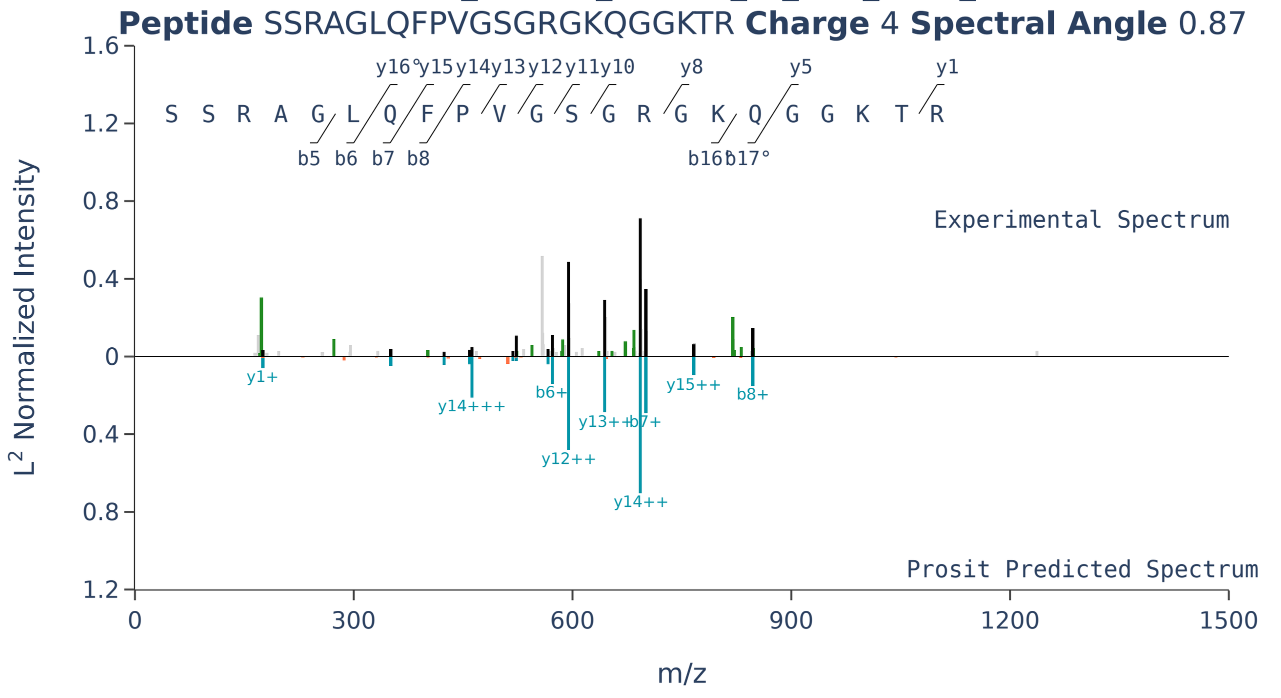

Source HRoetschke_291222_060123_Fu_H2A_b2R2_24h **Scan** 12735
Peptide RNDEELNKLLGA **Charge** 3 **Spectral Angle** 0.85

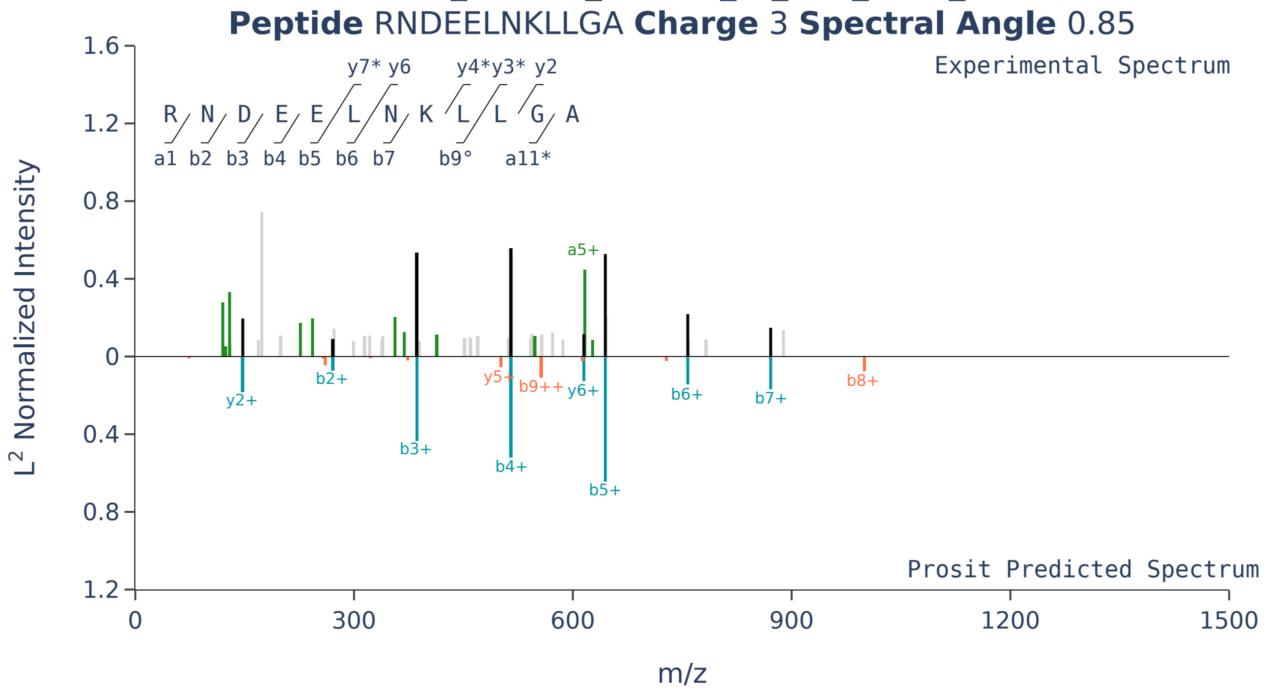

Source HRoetschke_291222_060123_Fu_H2A_b1R1_2h **Scan** 12447
Peptide VRNDEELNKLLRAG **Charge** 4 **Spectral Angle** 0.84

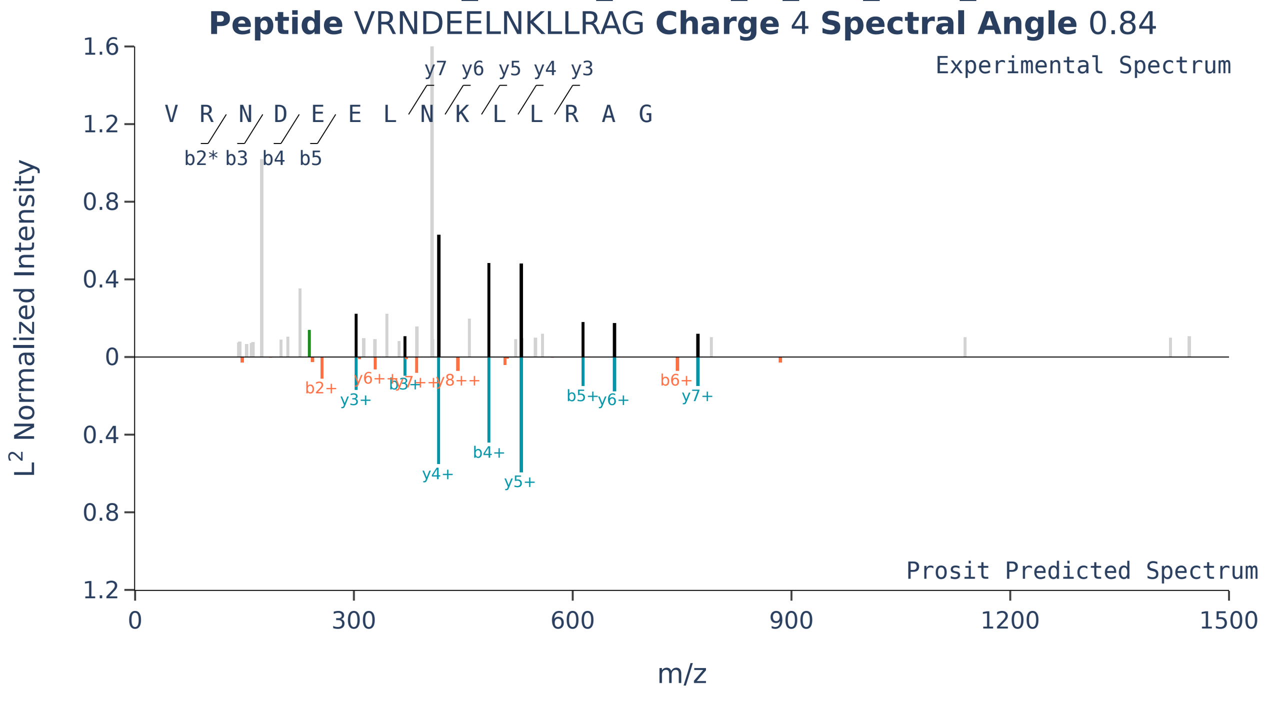

Source HRoetschke_291222_060123_Fu_H2A_b1R1_4h **Scan** 10824
Peptide SSSSRAGLQFPVGRVH **Charge** 3 **Spectral Angle** 0.85

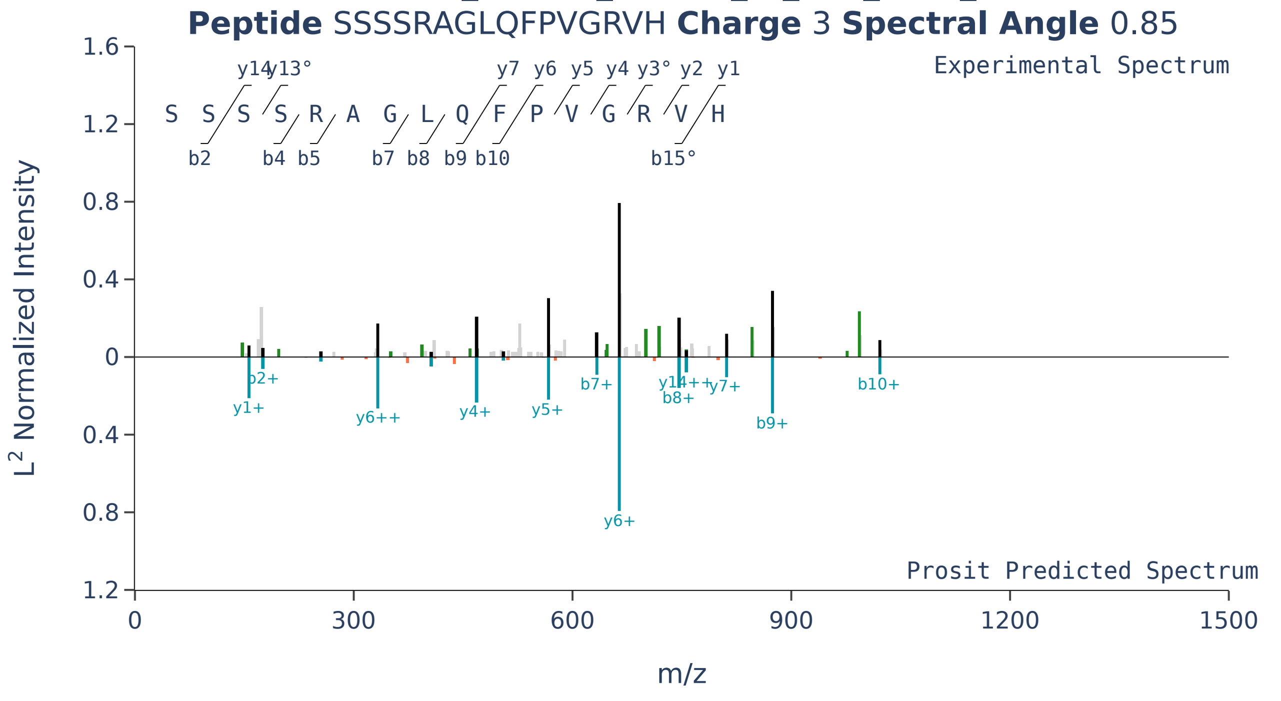

Source HRoetschke_291222_060123_Fu_H2A_b2R2_4h **Scan** 12103
Peptide ERVGQGGVLPNIQ **Charge** 2 **Spectral Angle** 0.86

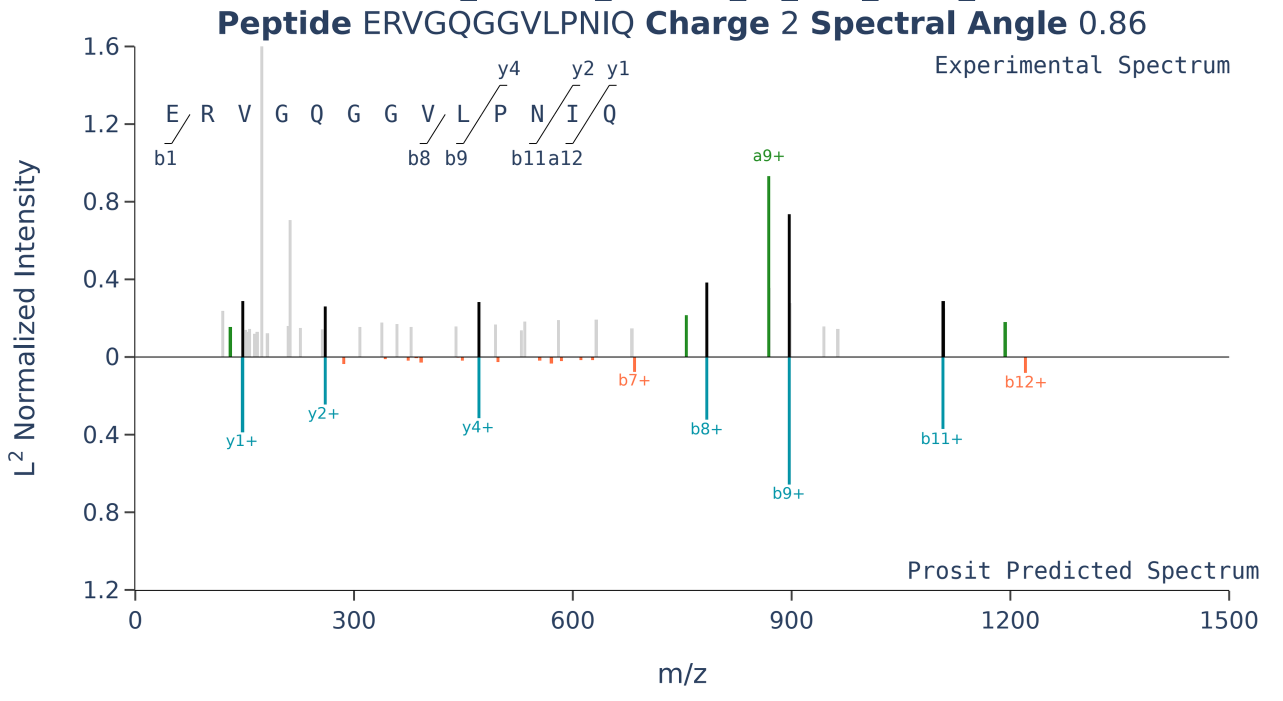

Source HRoetschke_291222_060123_Fu_H2A_b1R1_4h **Scan** 15262
Peptide AVRNDEELNKLLGRAV **Charge** 3 **Spectral Angle** 0.86

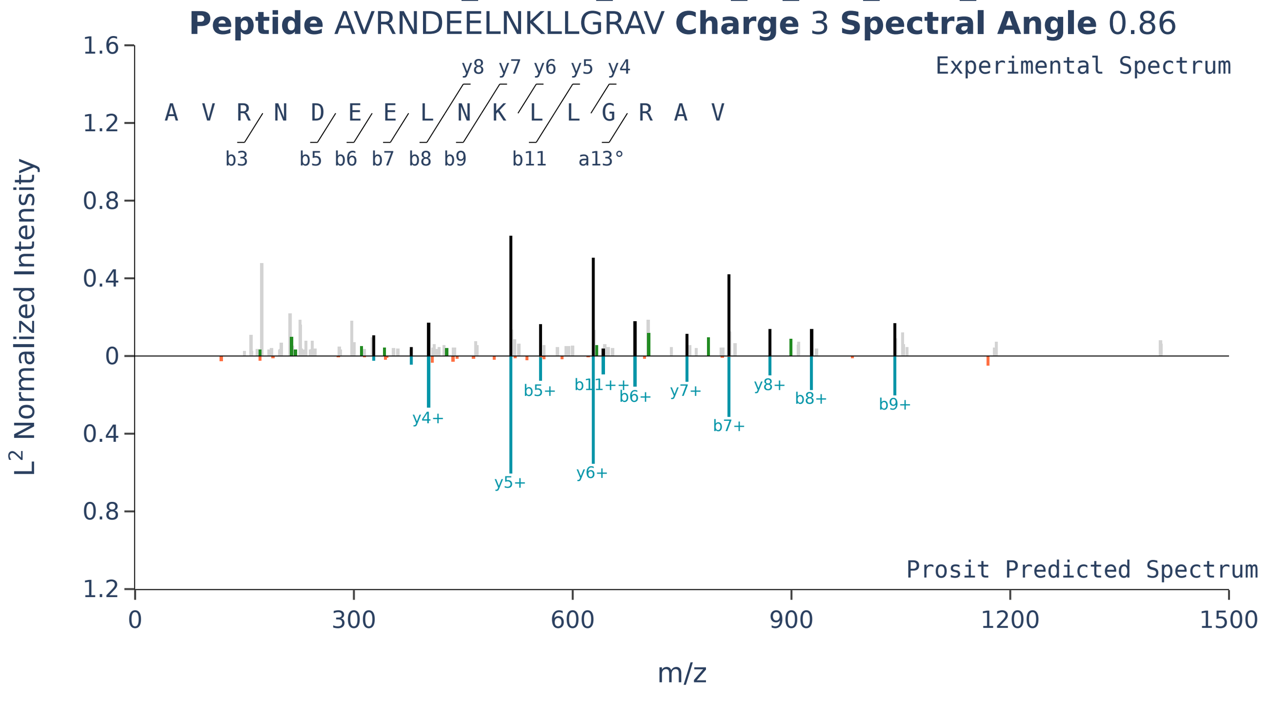

Source HRoetschke_291222_080123_Fu_H2B_b1R2_4h **Scan** 2811
Peptide VHPDTGISSR **Charge** 3 **Spectral Angle** 0.95

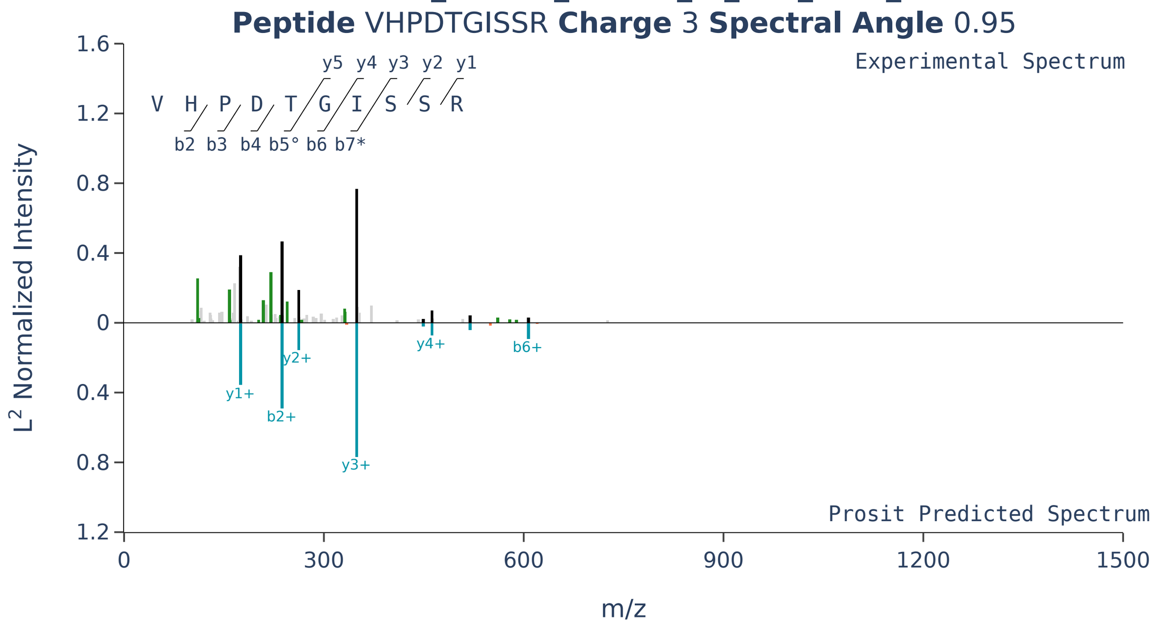

Source HRoetschke_291222_080123_Fu_H2B_b1R1_24h **Scan** 3172
Peptide VHPDTGISRS **Charge** 2 **Spectral Angle** 0.89

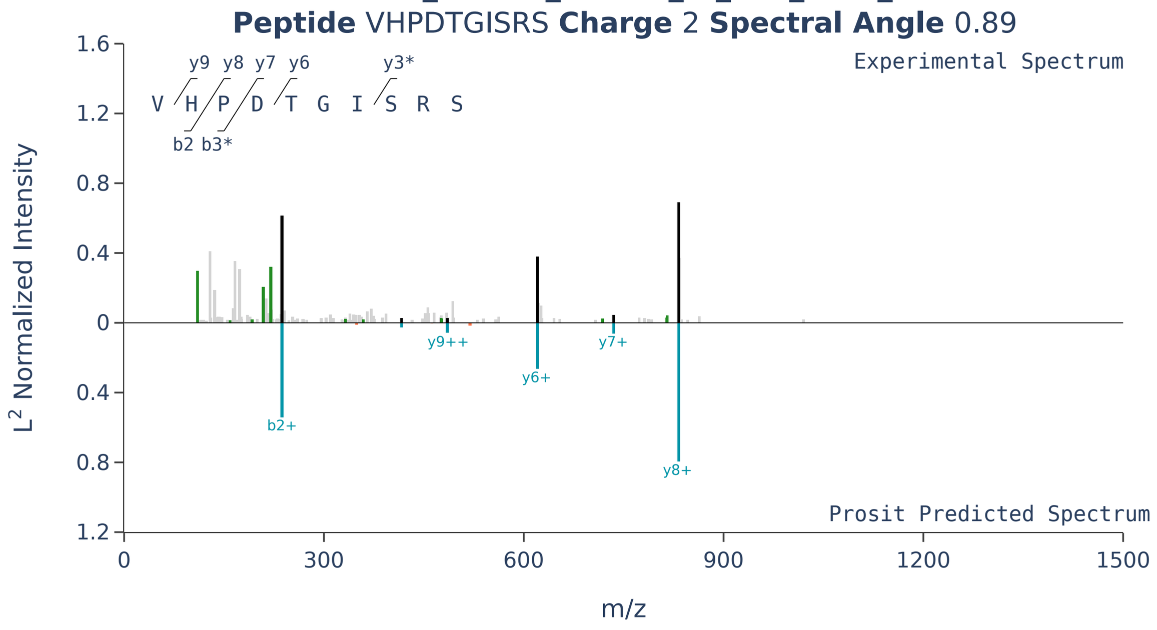

Source HRoetschke_291222_080123_Fu_H2B_b1R2_24h **Scan** 5222
Peptide VHPDTGISTG **Charge** 2 **Spectral Angle** 0.9

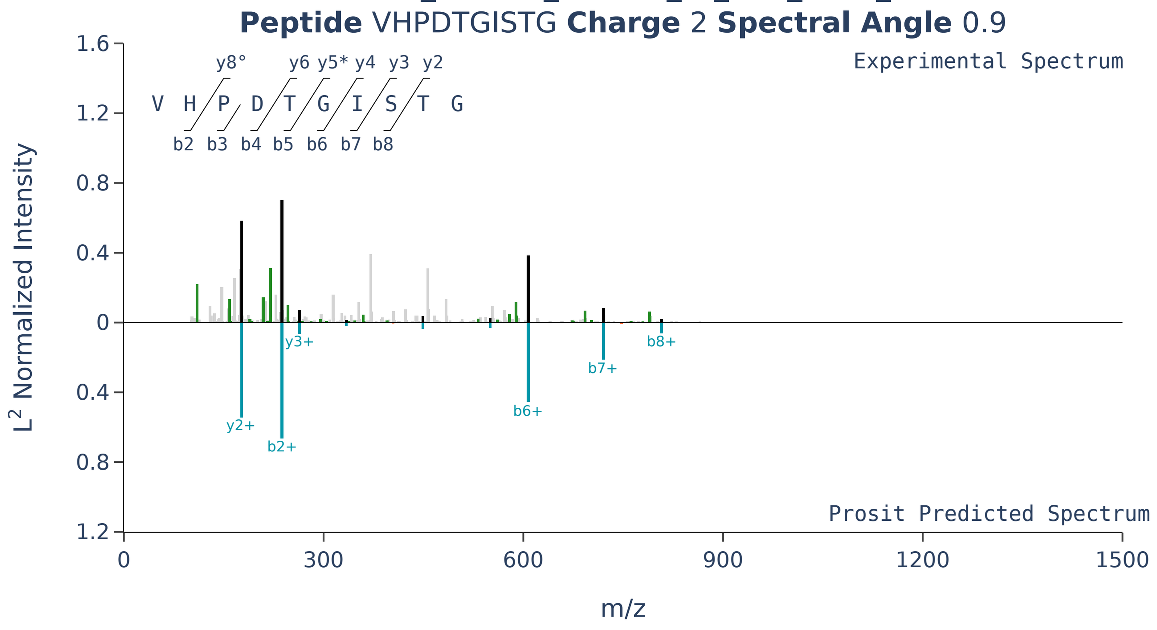

Source HRoetschke_291222_080123_Fu_H2B_b2R2_1h **Scan** 4215
Peptide VHPDTGISSA **Charge** 2 **Spectral Angle** 0.89

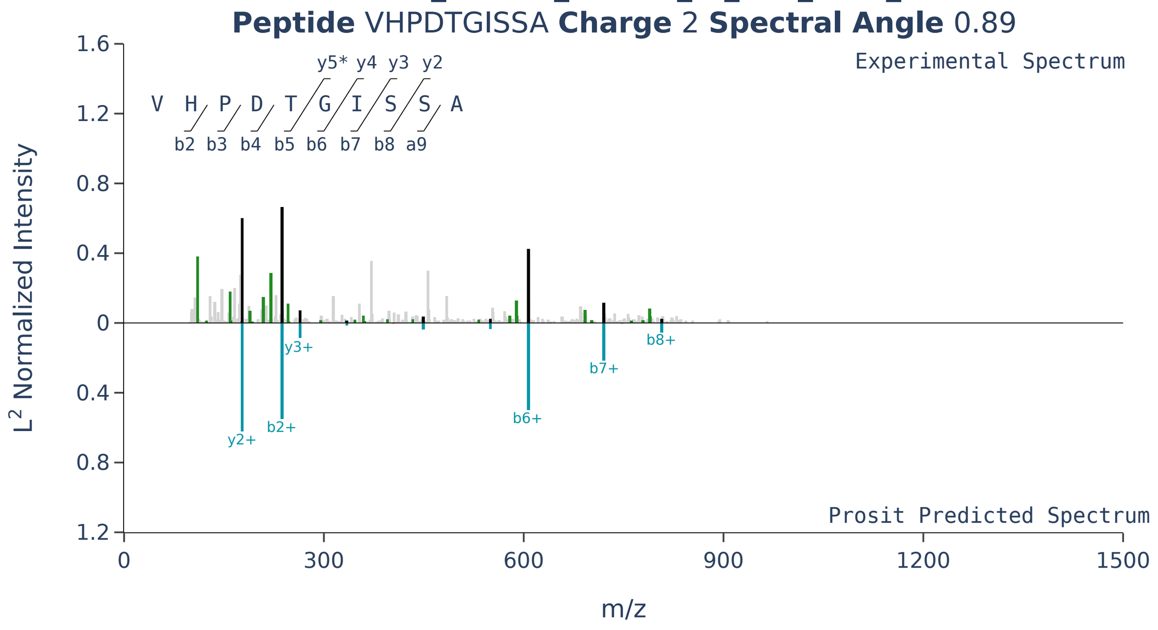

Source HRoetschke_291222_080123_Fu_H2B_b1R1_24h **Scan** 3890
Peptide ERIAGEASVR **Charge** 3 **Spectral Angle** 0.88

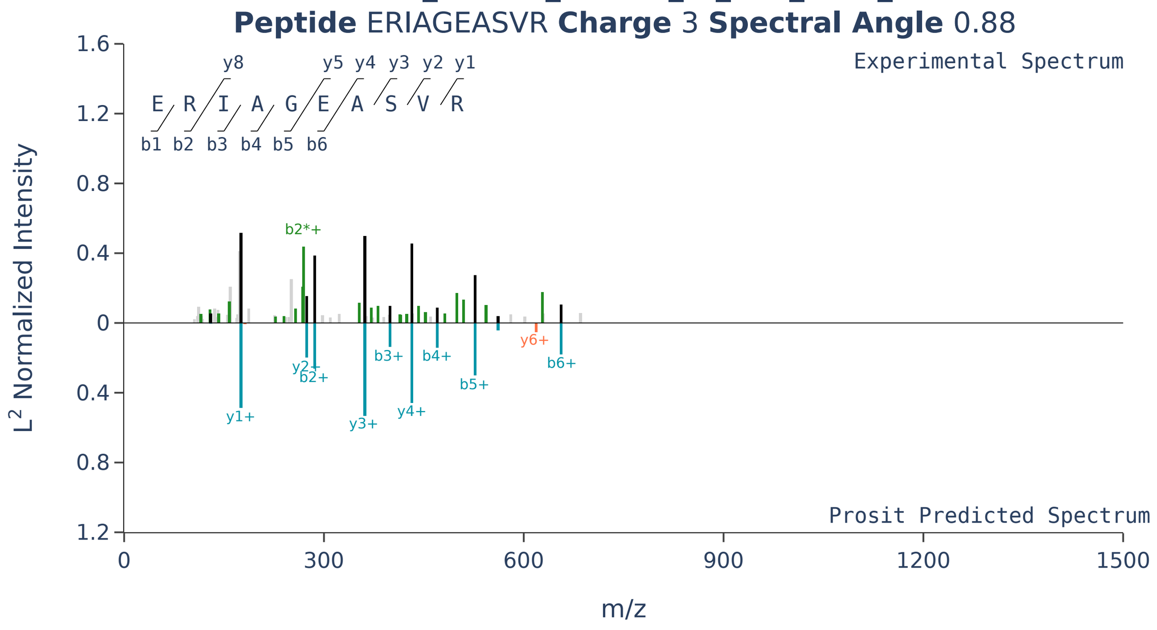

Source HRoetschke_291222_080123_Fu_H2B_b1R2_24h **Scan** 4439
Peptide VHPDTGISSM **Charge** 2 **Spectral Angle** 0.89

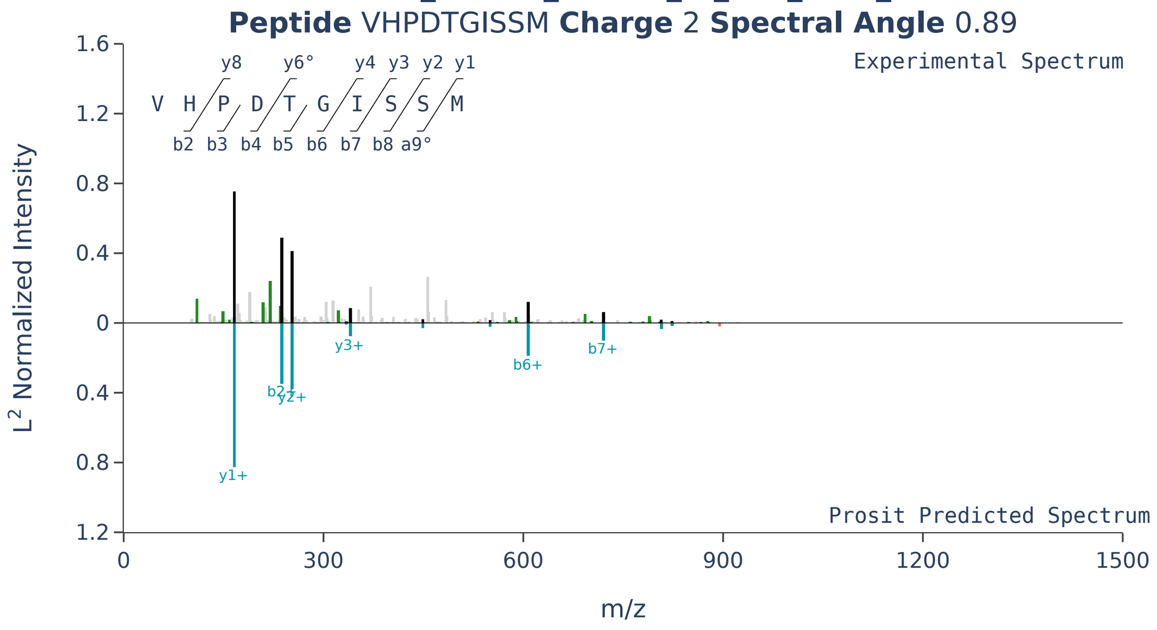

Source HRoetschke_291222_080123_Fu_H2B_b2R1_24h **Scan** 5255
Peptide VHPDTGISSKQV **Charge** 3 **Spectral Angle** 0.88

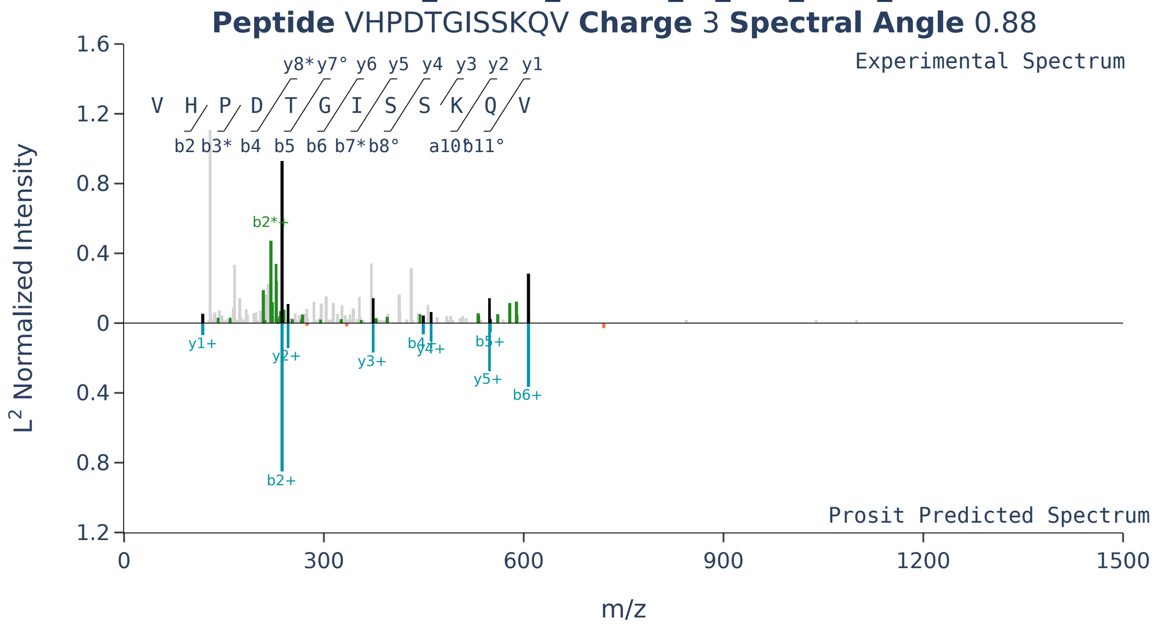

Source HRoetschke_291222_080123_Fu_H2B_b2R1_4h **Scan** 5784
Peptide LKQVHPDTGISTG **Charge** 2 **Spectral Angle** 0.91

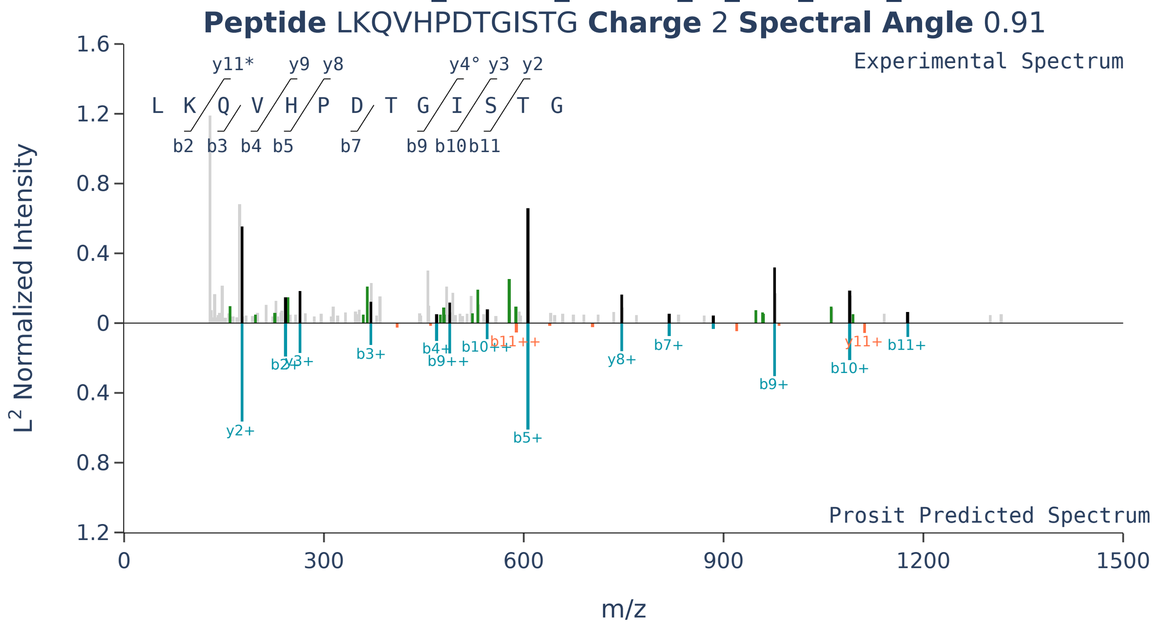

Source HRoetschke_291222_080123_Fu_H2B_b1R1_4h **Scan** 5418
Peptide QVHPDTGISTG **Charge** 2 **Spectral Angle** 0.86

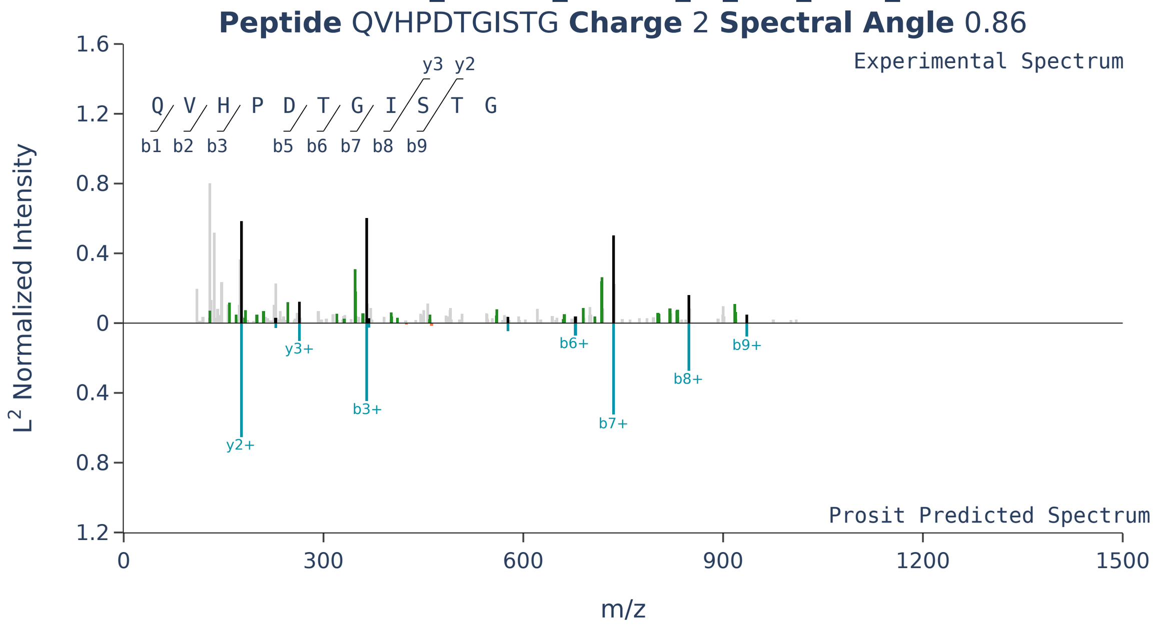

Source HRoetschke_291222_080123_Fu_H2B_b2R1_24h **Scan** 11159
Peptide ERIAGEASAVRL **Charge** 3 **Spectral Angle** 0.89

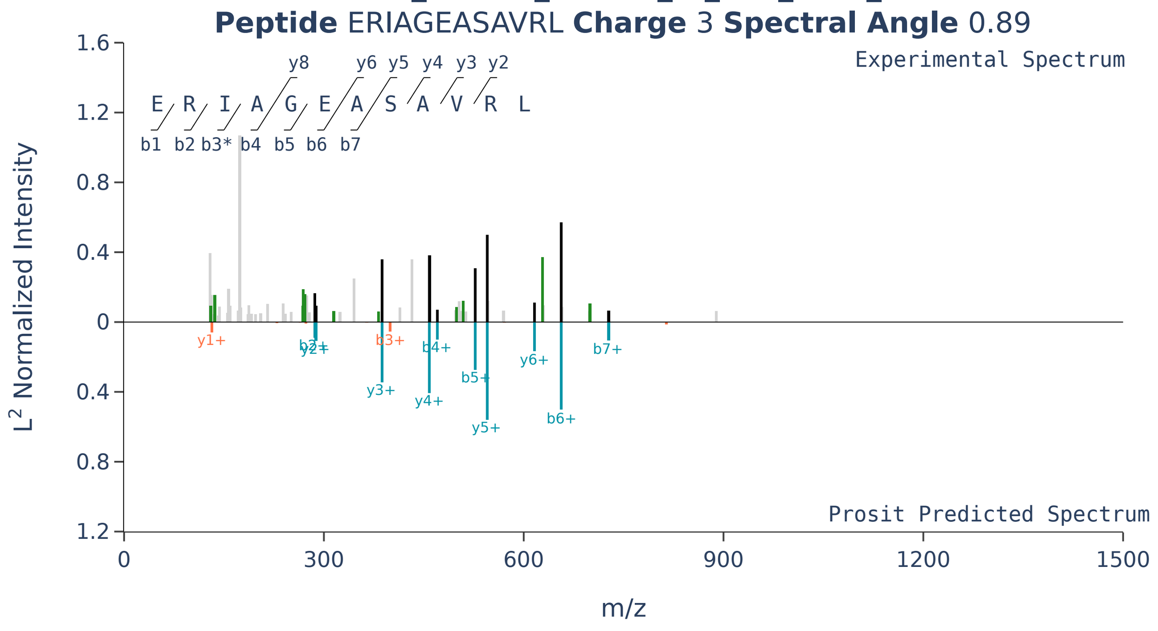

Source HRoetschke_291222_080123_Fu_H2B_b2R2_24h **Scan** 3764
Peptide KQVHPDTGISTG **Charge** 2 **Spectral Angle** 0.87

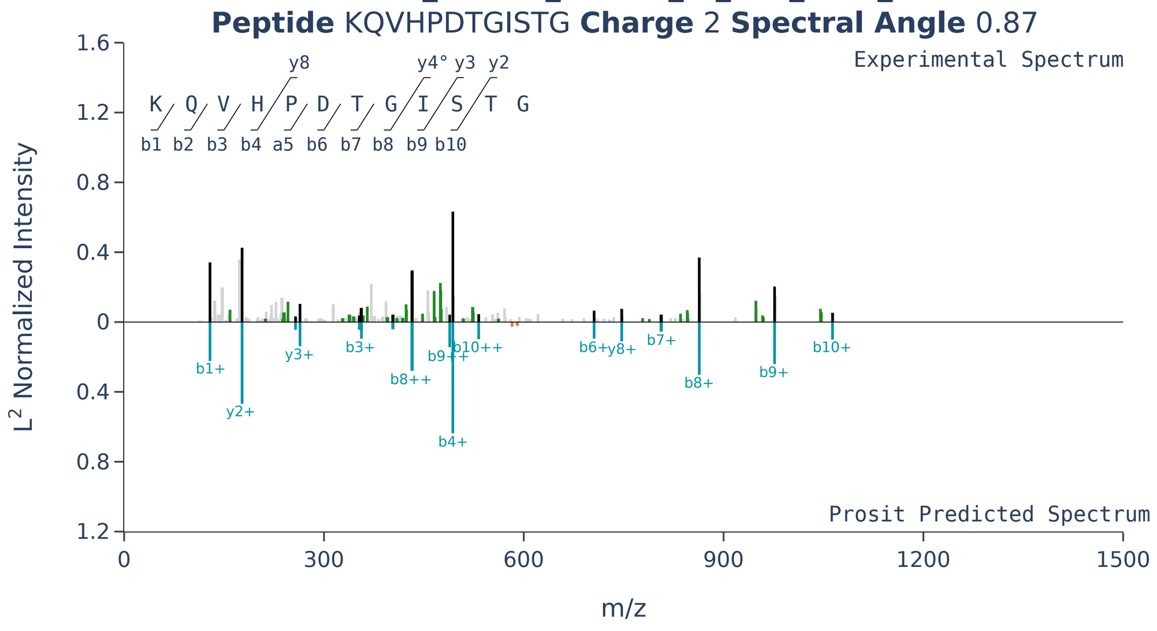

Source HRoetschke_291222_080123_Fu_H2B_b1R1_24h **Scan** 3293
Peptide RIAGEASAHYN **Charge** 2 **Spectral Angle** 0.86

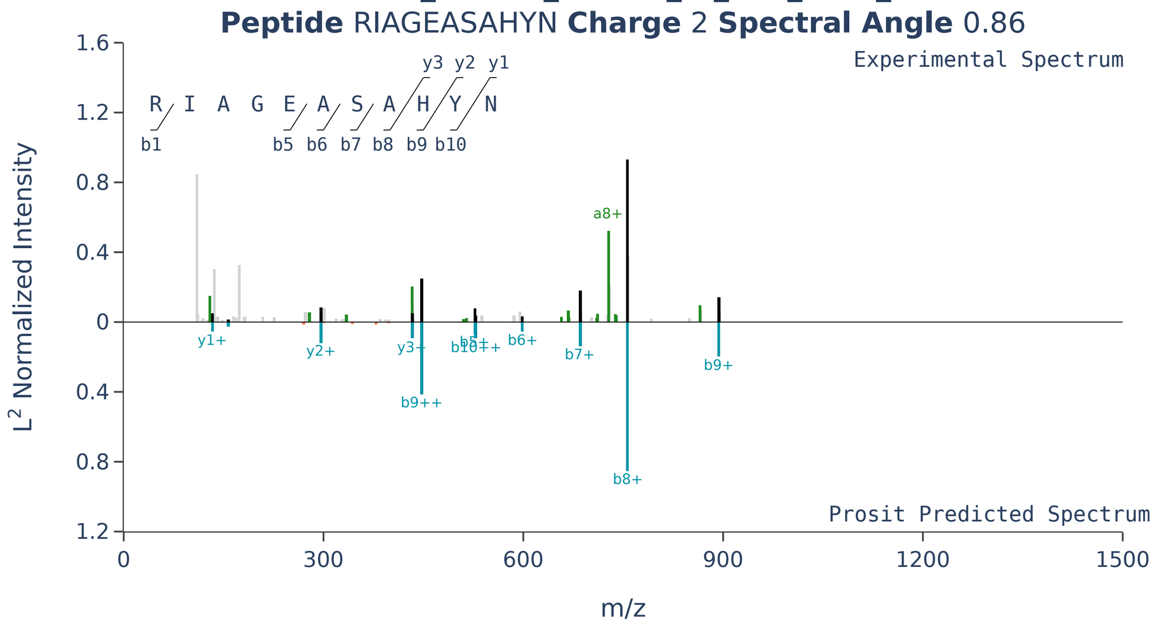

Source HRoetschke_291222_080123_Fu_H2B_b2R1_24h **Scan** 3626
Peptide KQVHPDTGISSA **Charge** 2 **Spectral Angle** 0.82

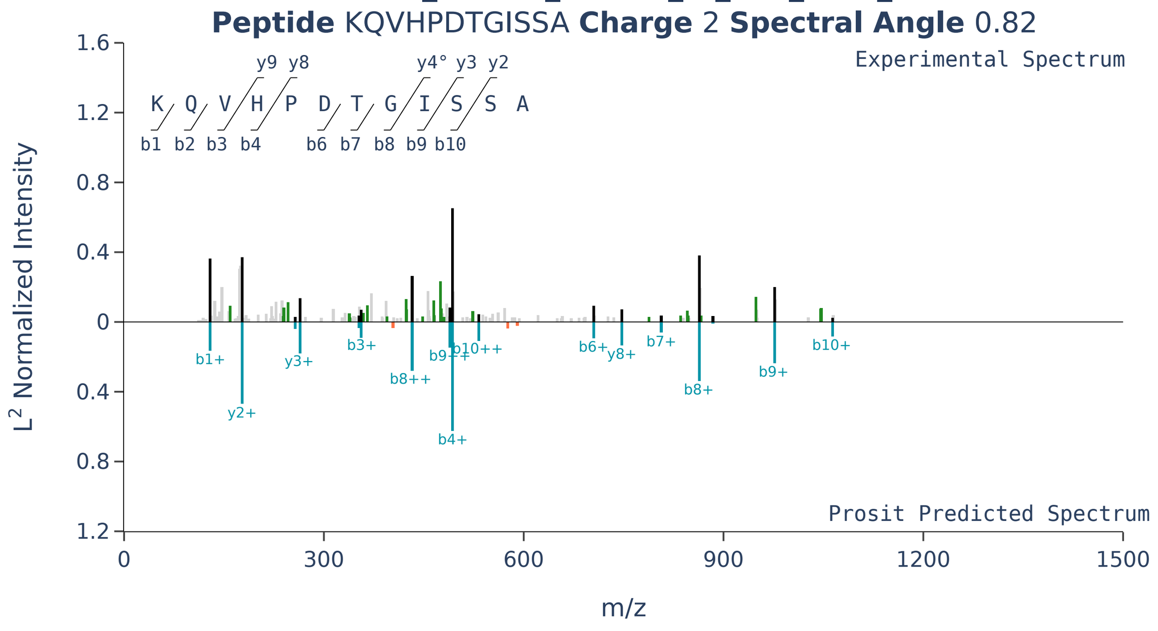

Source HRoetschke_291222_080123_Fu_H2B_b1R1_24h **Scan** 4733
Peptide ITSREIQTM **Charge** 2 **Spectral Angle** 0.91

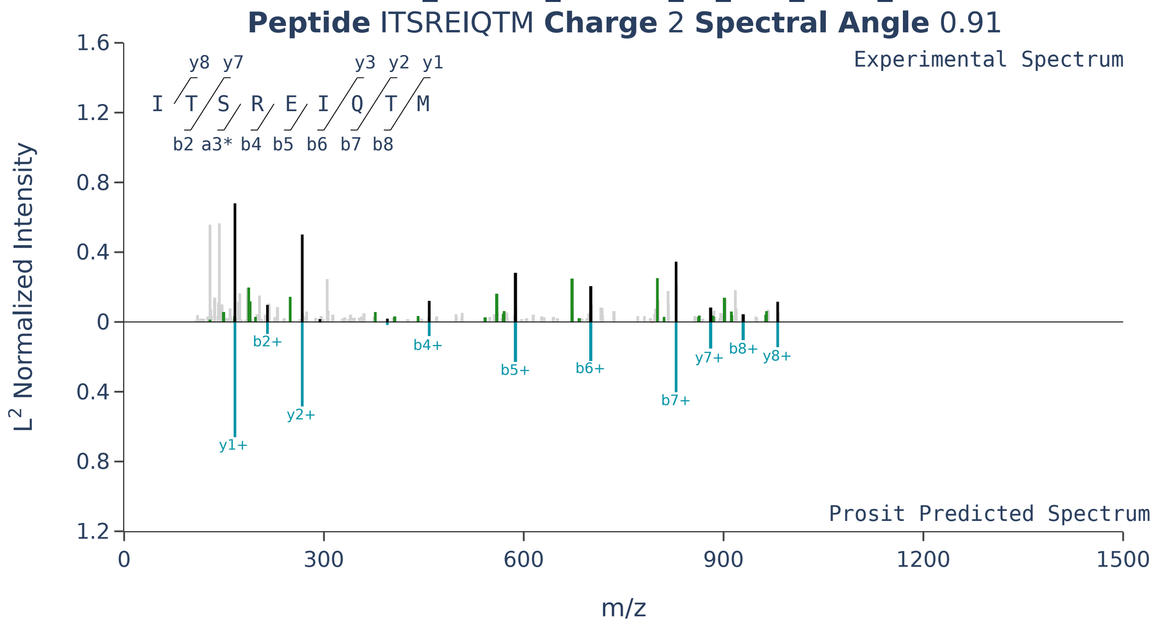

Source HRoetschke_291222_080123_Fu_H2B_b2R2_24h **Scan** 8138
Peptide SASTITSREIQT **Charge** 2 **Spectral Angle** 0.91

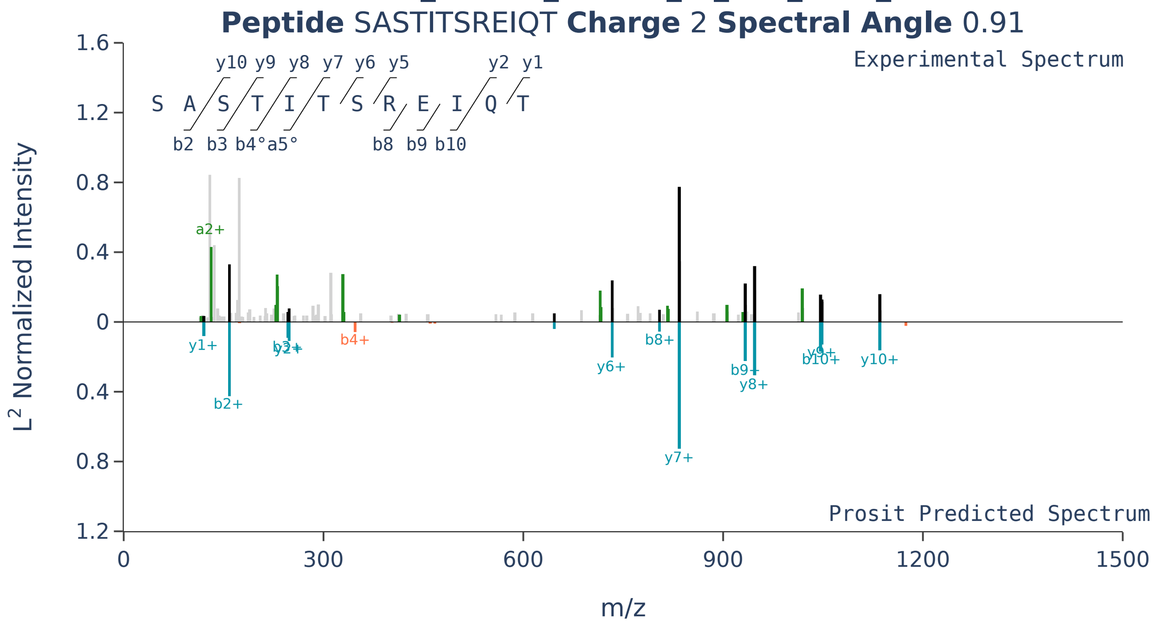

Source HRoetschke_291222_080123_Fu_H2B_b2R1_2h **Scan** 12766
Peptide MAKSIMNSF **Charge** 2 **Spectral Angle** 0.81

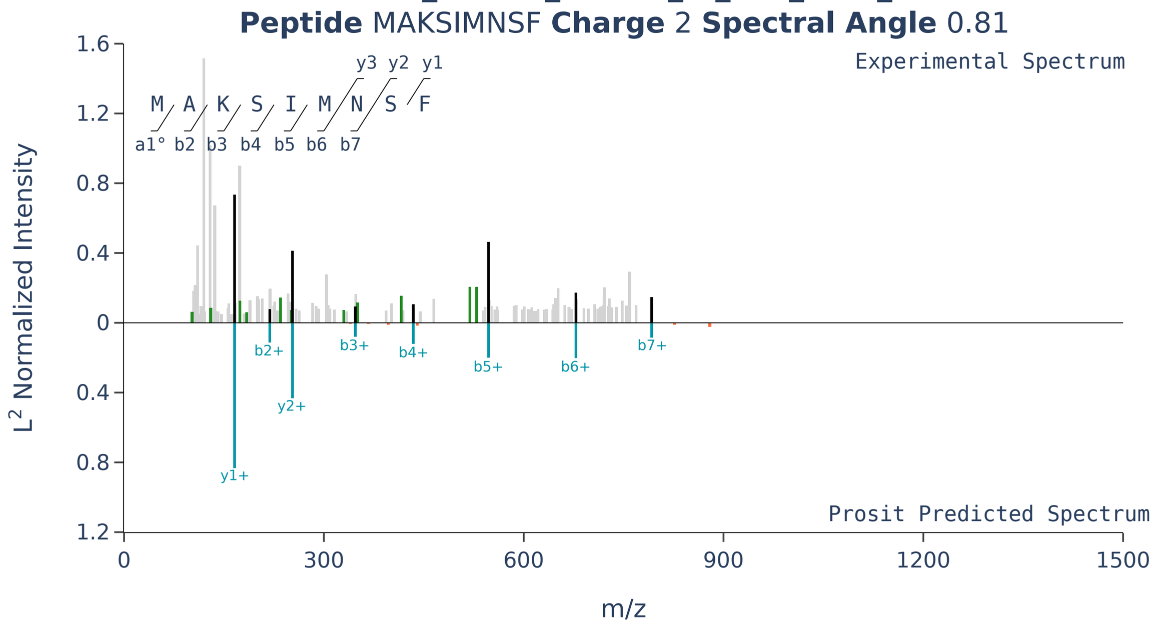

Source HRoetschke_291222_080123_Fu_H2B_b2R2_24h **Scan** 9236
Peptide VHPDTGISSRL **Charge** 3 **Spectral Angle** 0.92

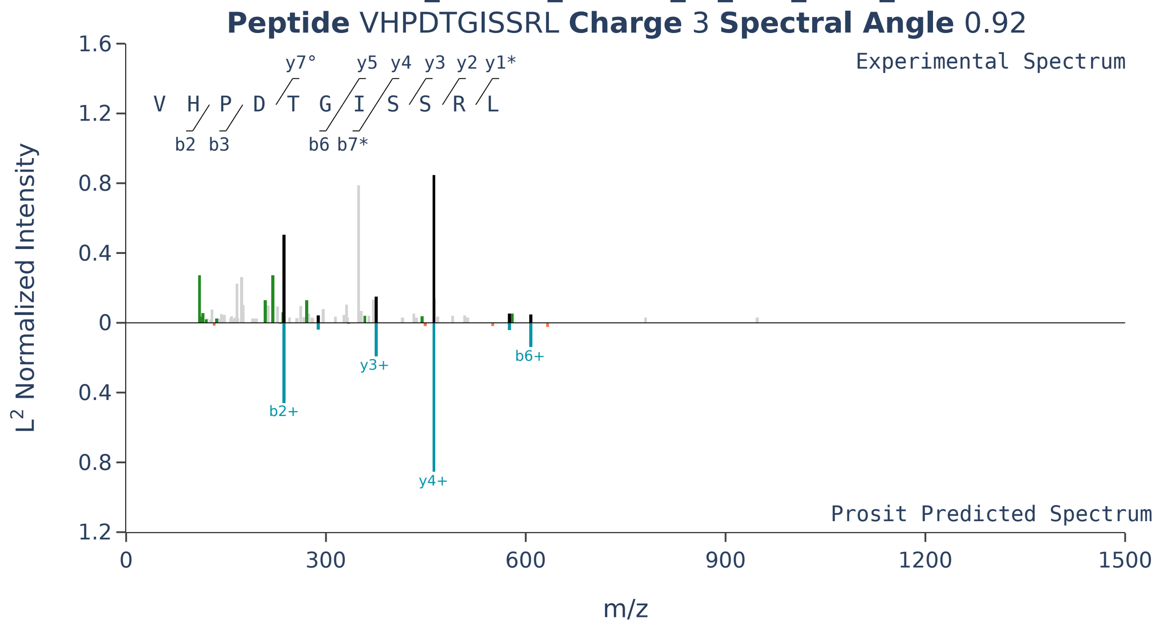

Source HRoetschke_291222_080123_Fu_H2B_b2R2_2h **Scan** 5380
Peptide LKQVHPDTGISSA **Charge** 2 **Spectral Angle** 0.84

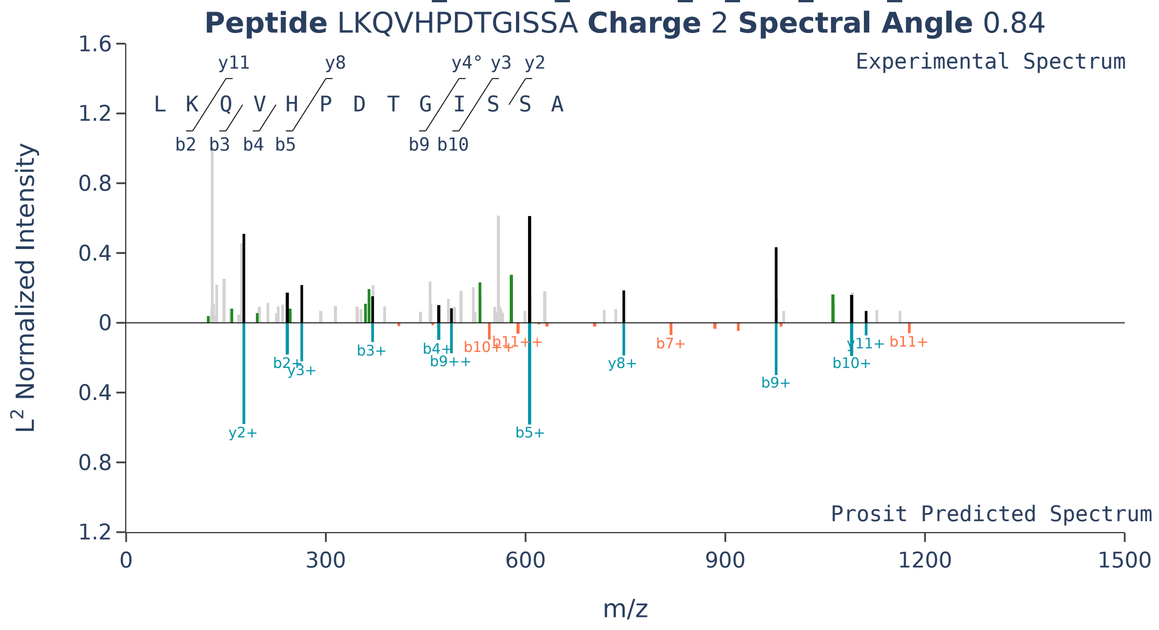

Source HRoetschke_291222_080123_Fu_H2B_b1R2_24h **Scan** 3658
Peptide QVHPDTGISSR **Charge** 3 **Spectral Angle** 0.91

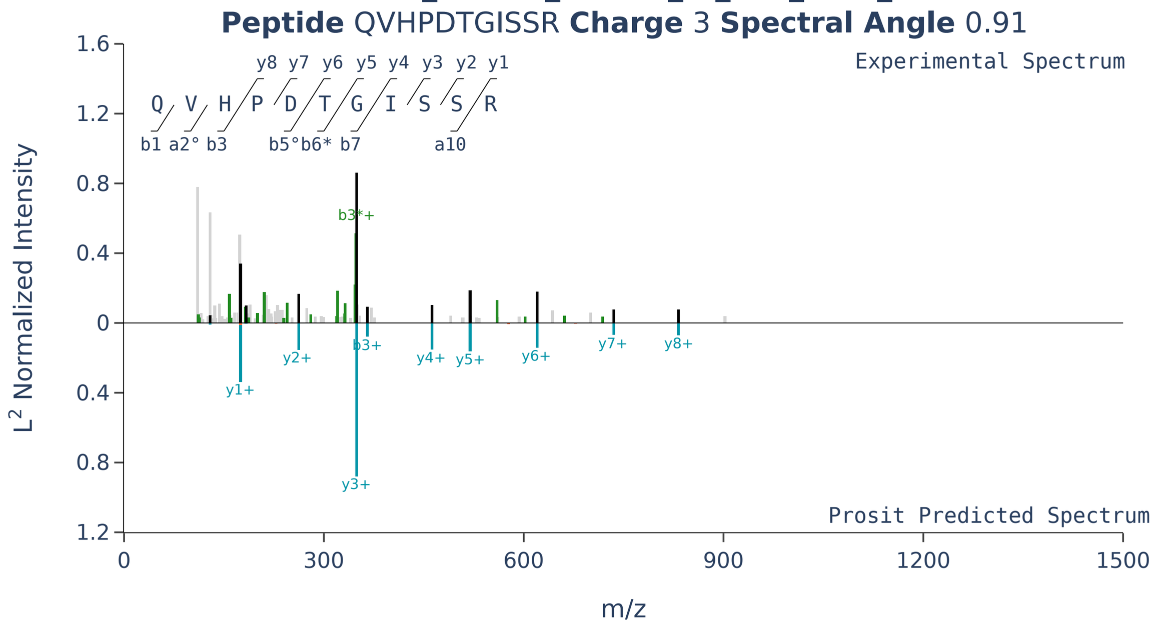

Source HRoetschke_291222_080123_Fu_H2B_b2R1_24h **Scan** 10352
Peptide AMSTITSREIQT **Charge** 2 **Spectral Angle** 0.87

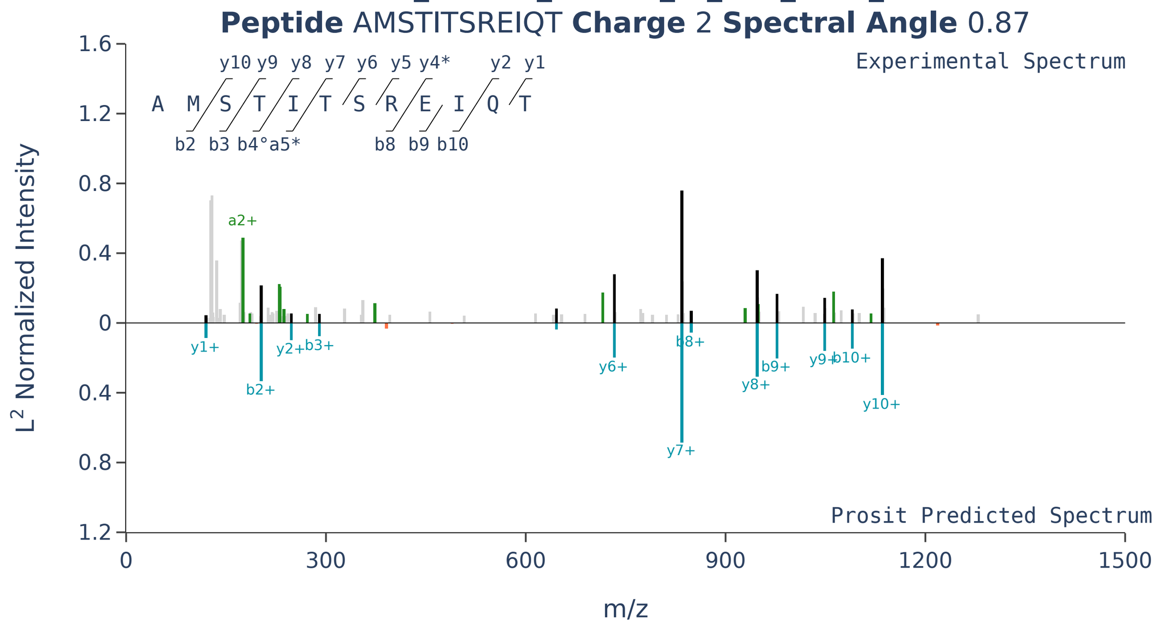

Source HRoetschke_291222_080123_Fu_H2B_b2R1_1h **Scan** 6768
Peptide ASSTITSREIQT **Charge** 2 **Spectral Angle** 0.89

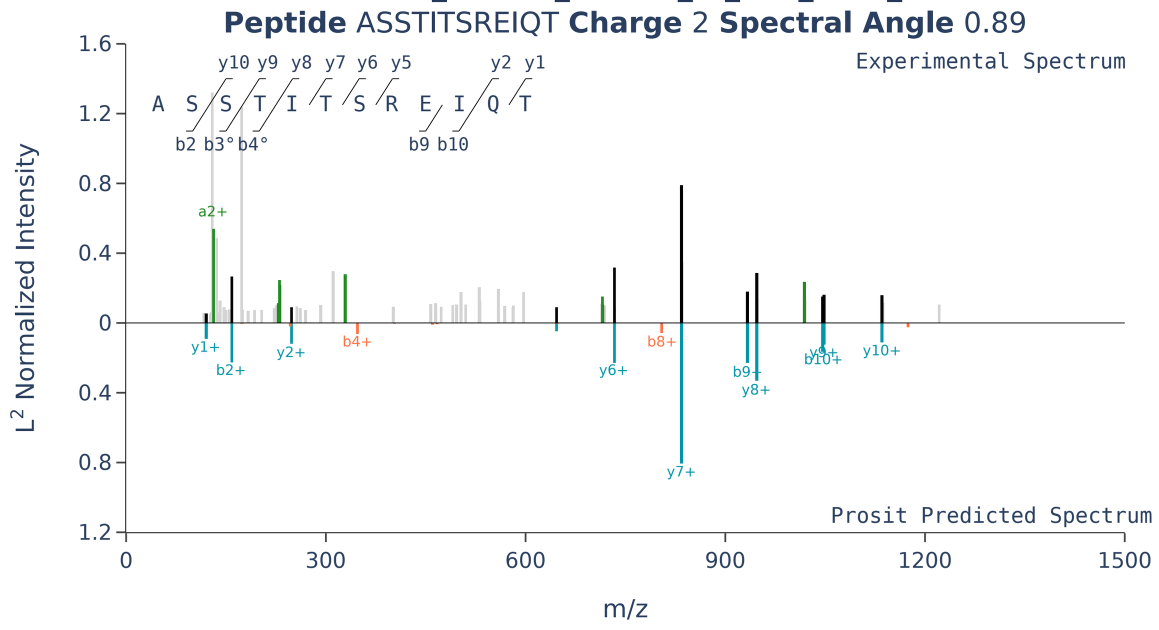

Source HRoetschke_291222_080123_Fu_H2B_b1R1_24h **Scan** 3307
Peptide VHPDTGISSKAMN **Charge** 3 **Spectral Angle** 0.81

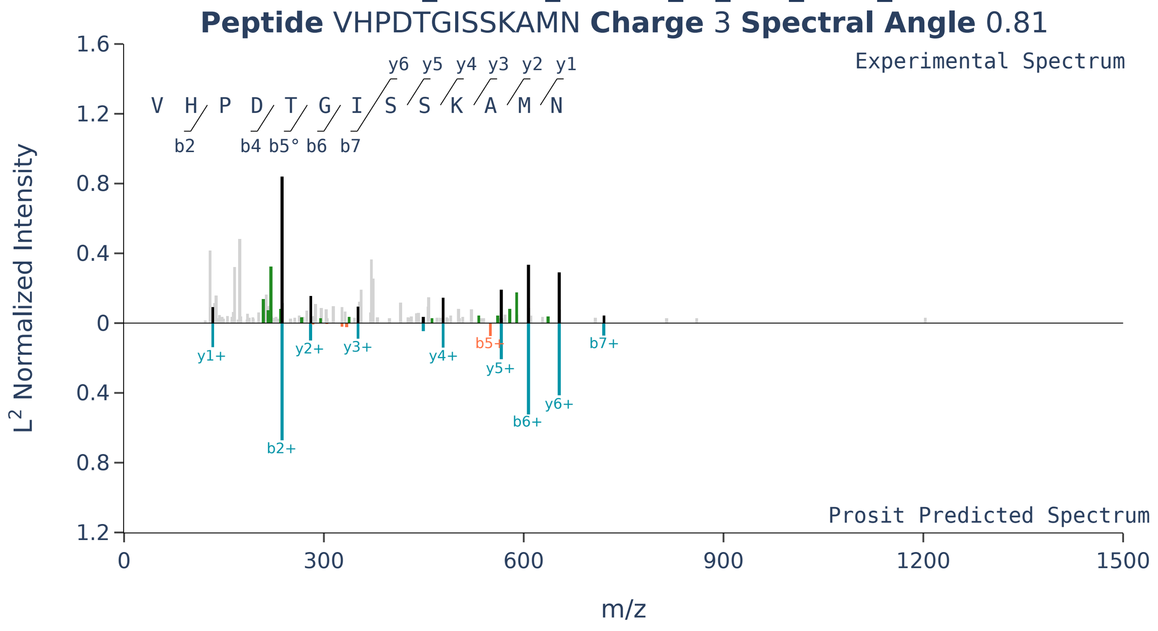

Source HRoetschke_291222_080123_Fu_H2B_b1R2_24h **Scan** 6923
Peptide VHPDTGISSKAMV **Charge** 3 **Spectral Angle** 0.82

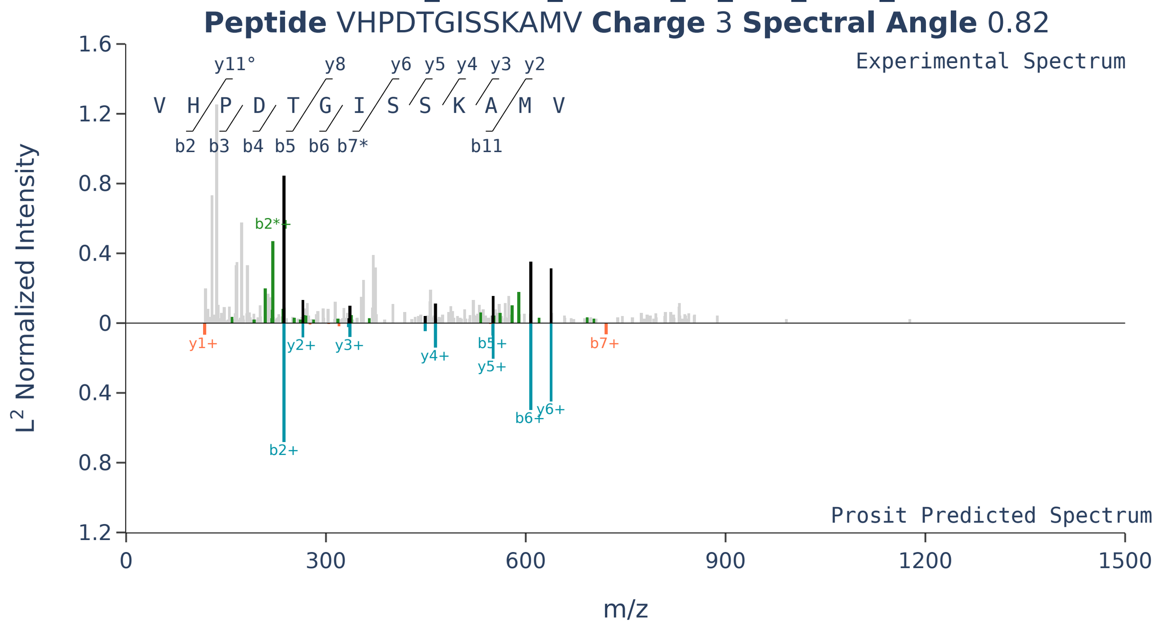

Source HRoetschke_291222_080123_Fu_H2B_b1R1_24h **Scan** 5879
Peptide QVHPDTGISSA **Charge** 2 **Spectral Angle** 0.79

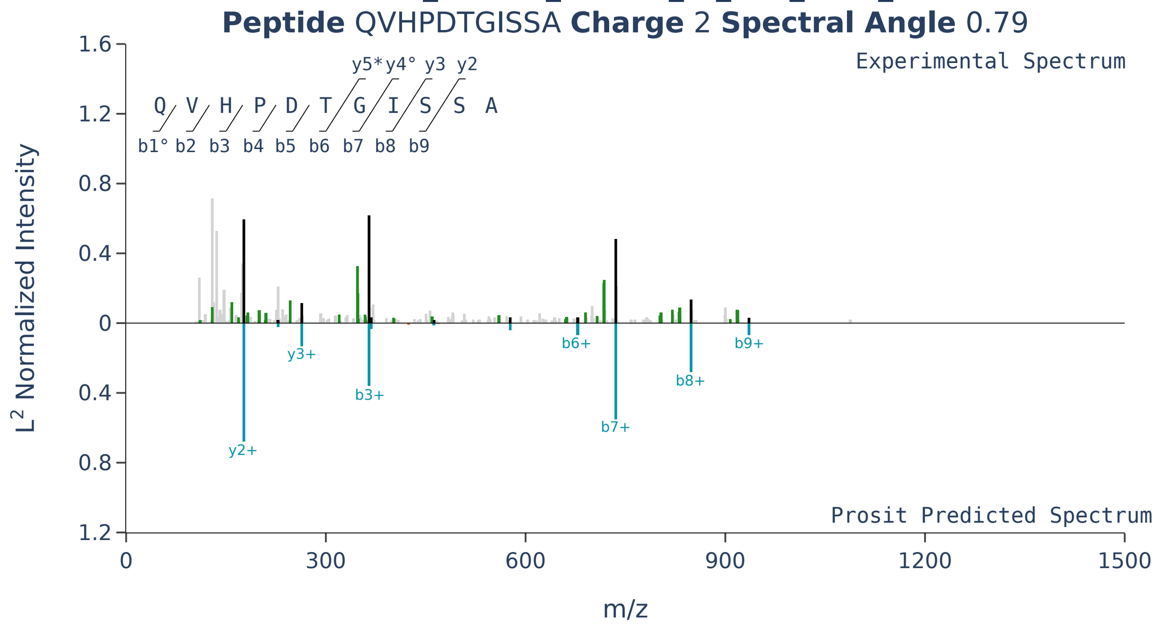

Source HRoetschke_291222_080123_Fu_H2B_b1R2_24h **Scan** 8224
Peptide TGSTITSREIQT **Charge** 2 **Spectral Angle** 0.87

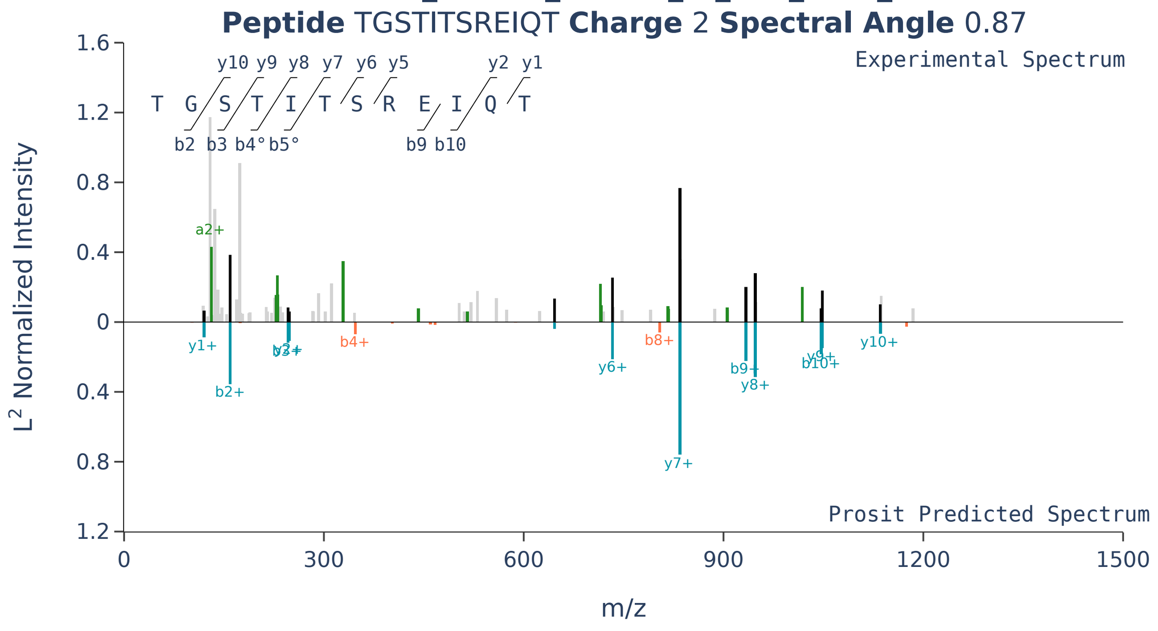

Source HRoetschke_291222_080123_Fu_H2B_b1R2_4h **Scan** 2815
Peptide VHPDTGISSKTS **Charge** 3 **Spectral Angle** 0.78

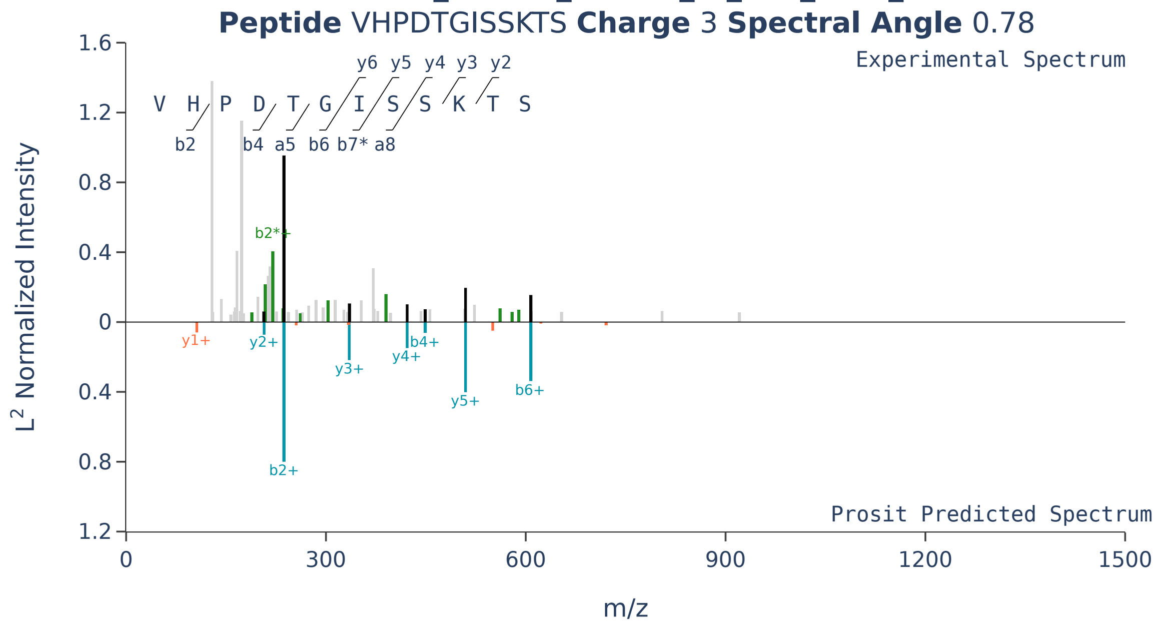

Source HRoetschke_291222_080123_Fu_H2B_b2R2_24h **Scan** 3285
Peptide KQVHPDTGISSM **Charge** 2 **Spectral Angle** 0.84

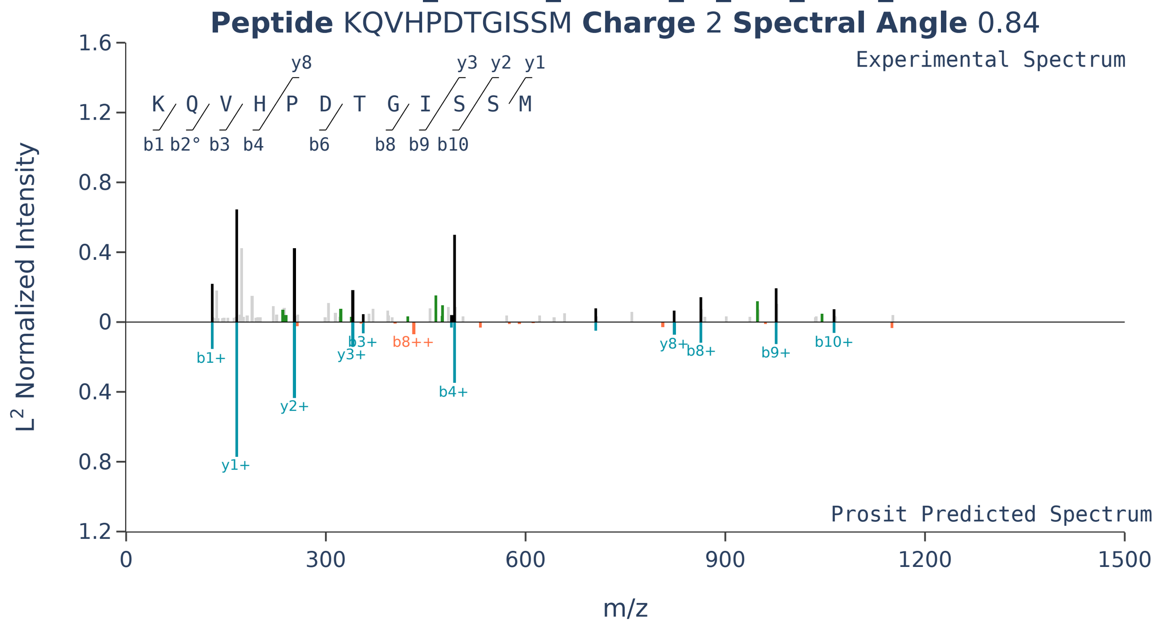

Source HRoetschke_291222_080123_Fu_H2B_b2R2_24h **Scan** 6670
Peptide STITSREIQTM **Charge** 2 **Spectral Angle** 0.89

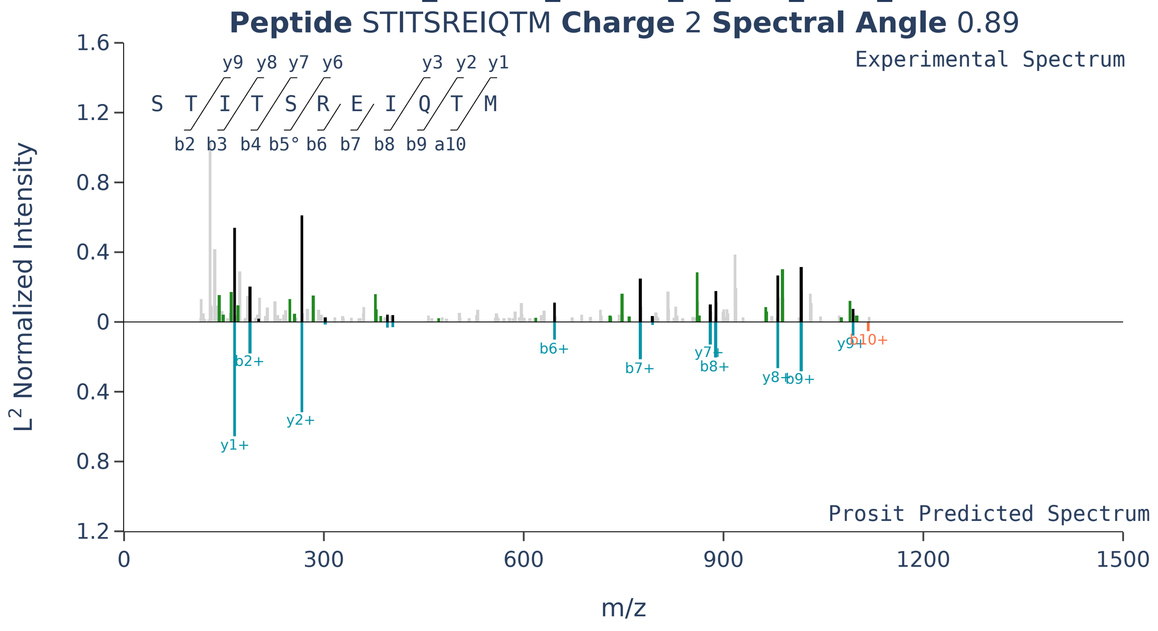

Source HRoetschke_291222_080123_Fu_H2B_b1R2_24h **Scan** 7599
Peptide STITSREIQTAM **Charge** 2 **Spectral Angle** 0.91

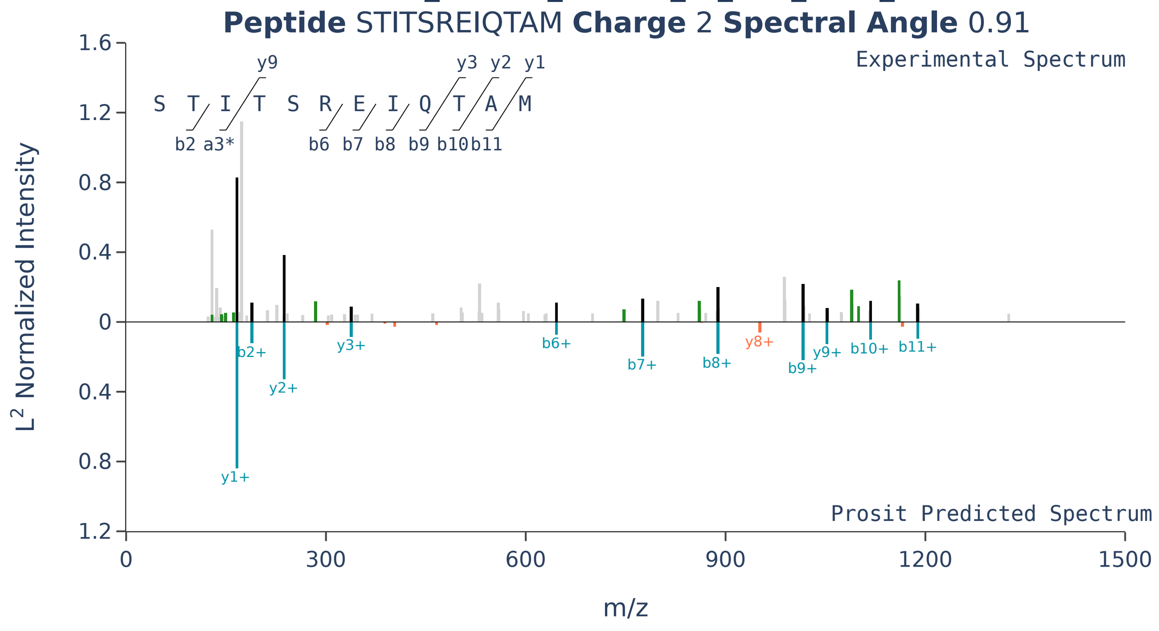

Source HRoetschke_291222_080123_Fu_H2B_b1R2_24h **Scan** 5819
Peptide LKQVHPDTGISSM **Charge** 3 **Spectral Angle** 0.9

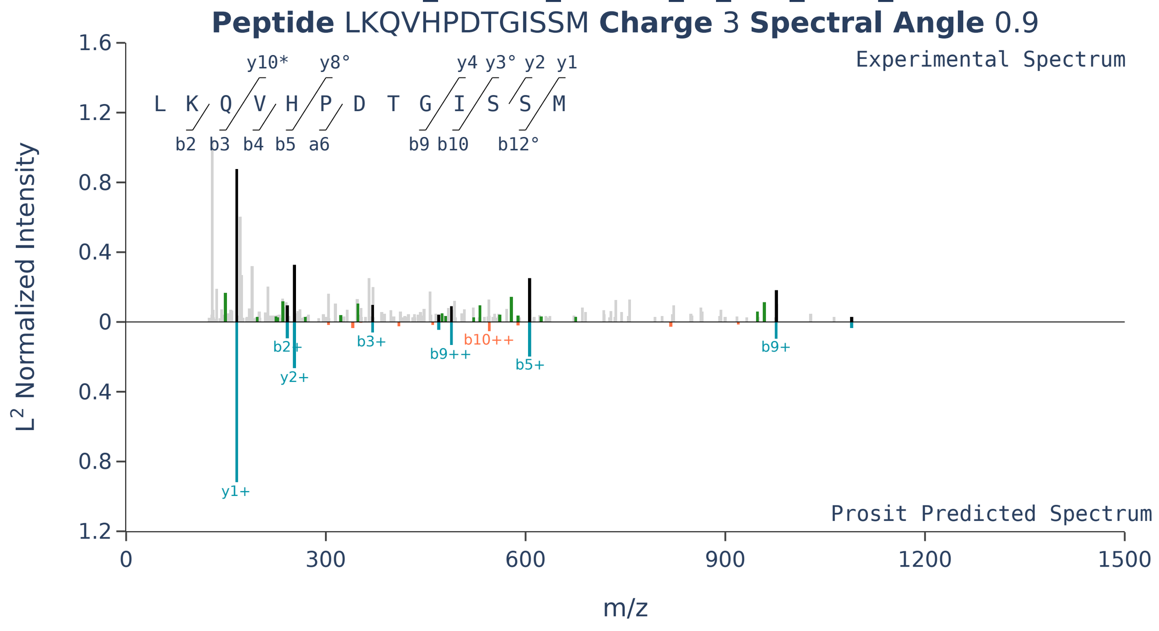

Source HRoetschke_291222_080123_Fu_H2B_b2R1_24h **Scan** 4747
Peptide QVHPDTGISSM **Charge** 2 **Spectral Angle** 0.92

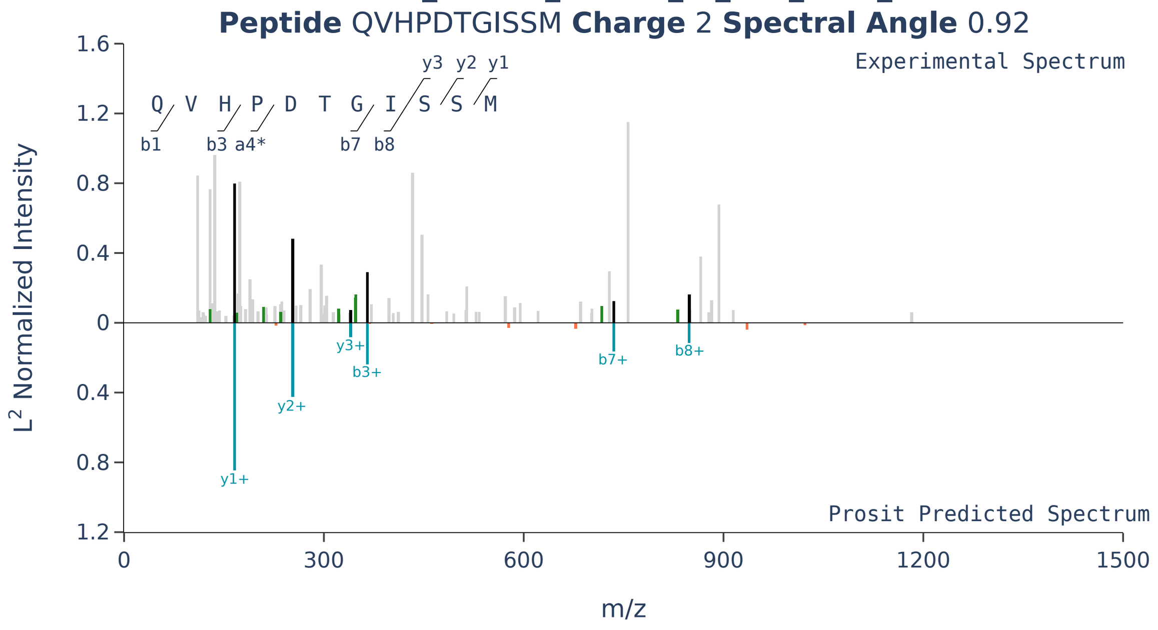

Source HRoetschke_291222_080123_Fu_H2B_b1R1_24h **Scan** 10630
Peptide ERIAGEASVRL **Charge** 3 **Spectral Angle** 0.82

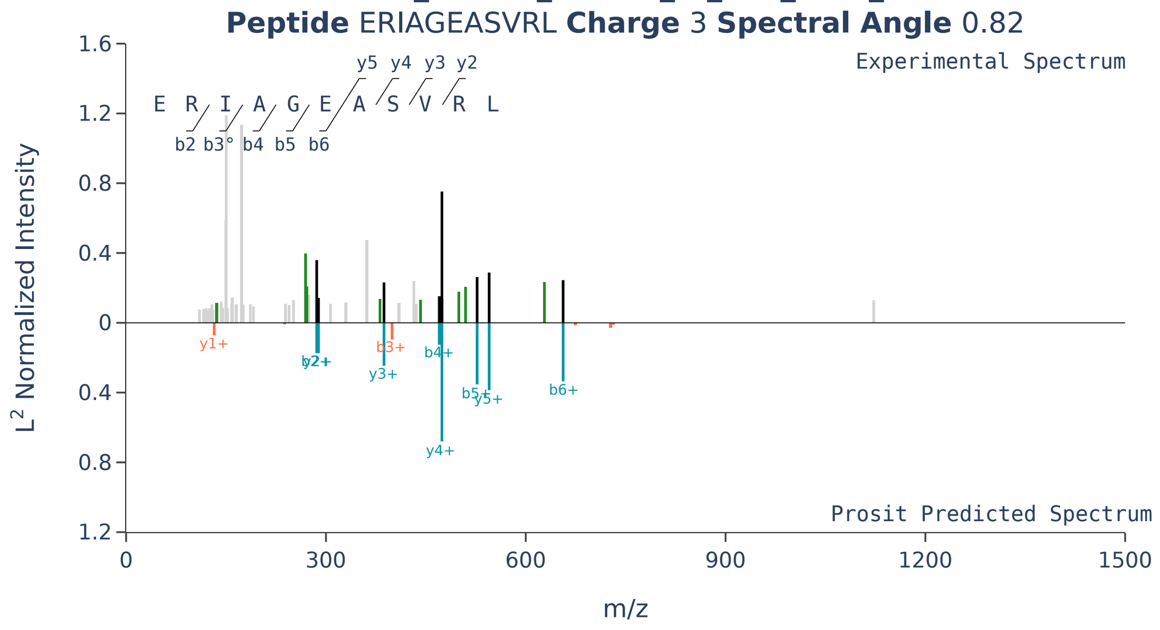

Source HRoetschke_291222_080123_Fu_H2B_b1R1_4h **Scan** 3288
Peptide KTQVTKYTSA **Charge** 3 **Spectral Angle** 0.92

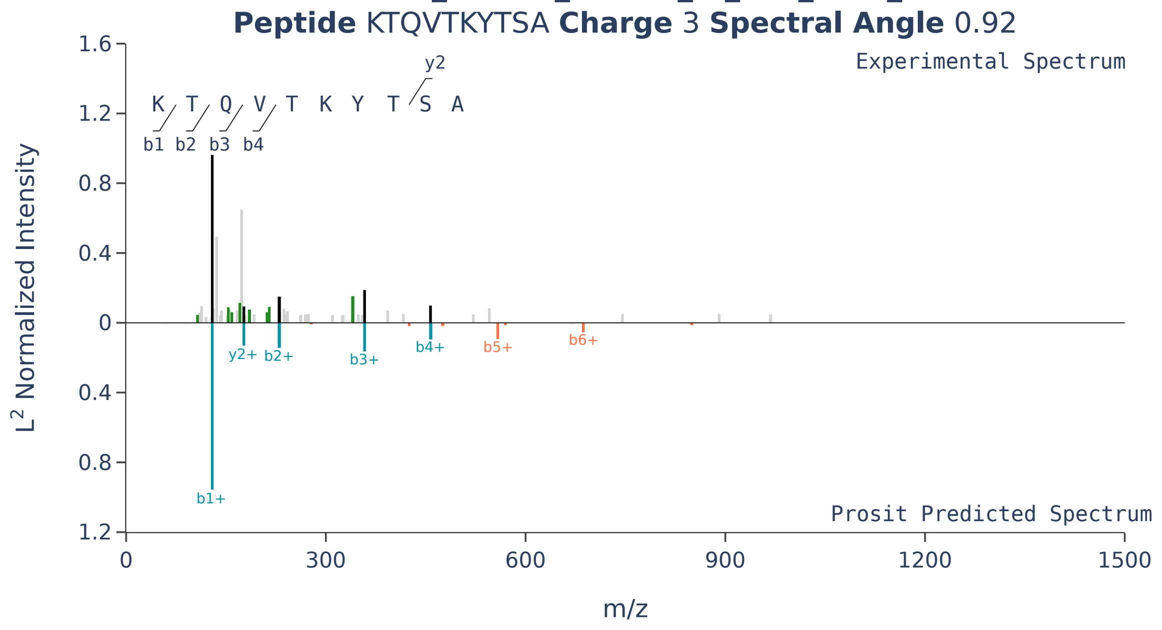

Source HRoetschke_291222_080123_Fu_H2B_b1R2_24h **Scan** 8732
Peptide ERIAGEASMNSF **Charge** 2 **Spectral Angle** 0.9

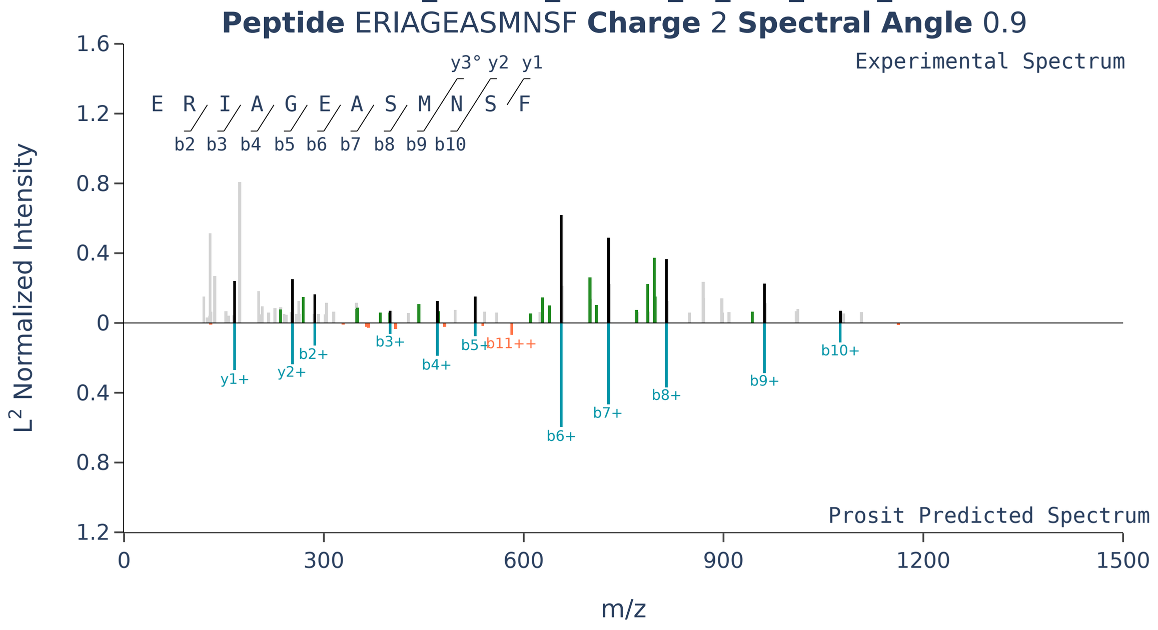

Source HRoetschke_291222_080123_Fu_H2B_b2R1_1h **Scan** 4361
Peptide VHPDTGISSKMN **Charge** 3 **Spectral Angle** 0.88

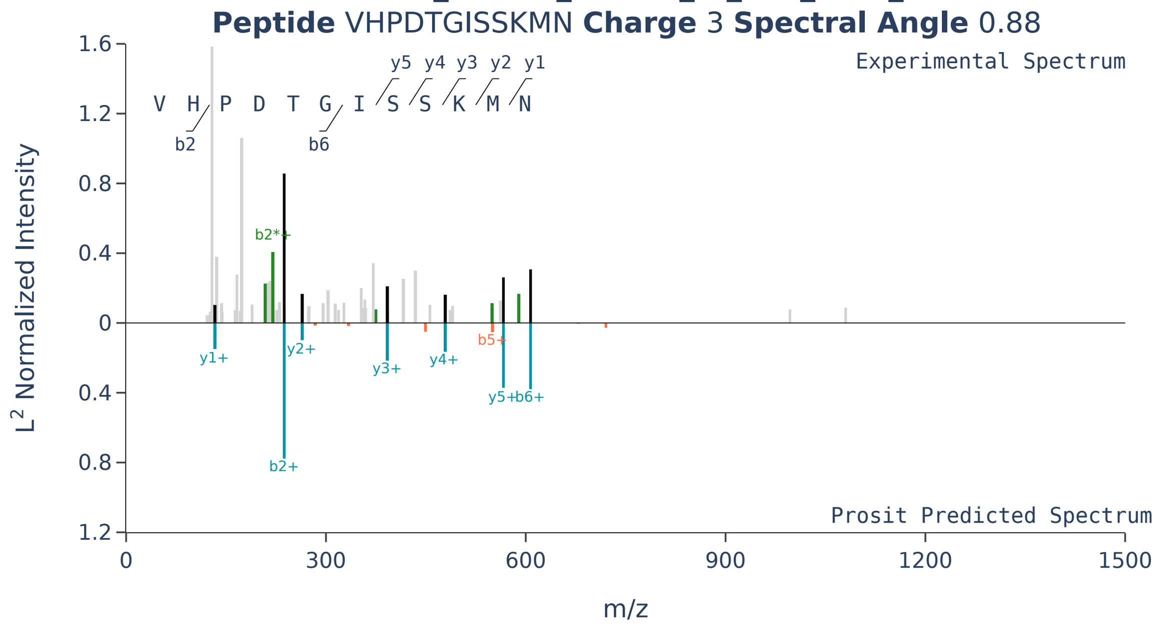

Source HRoetschke_291222_080123_Fu_H2B_b2R2_24h **Scan** 5049
Peptide VHPDTGISSKVND **Charge** 3 **Spectral Angle** 0.8

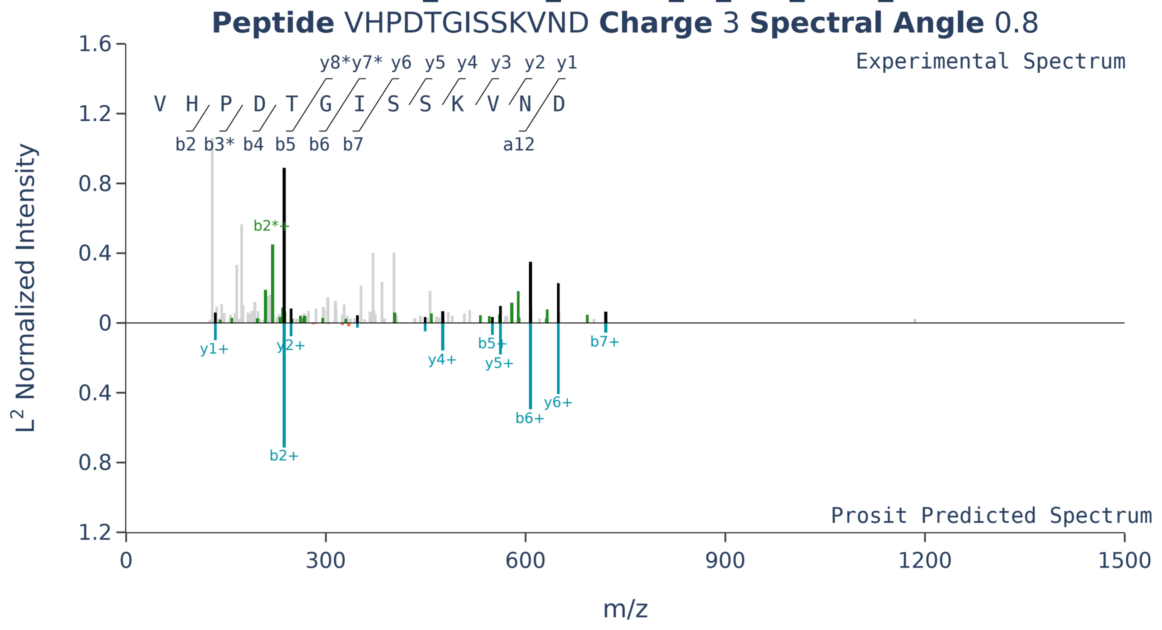

Source HRoetschke_291222_080123_Fu_H2B_b1R1_4h **Scan** 5913
Peptide VHPDTGISSKAMM **Charge** 3 **Spectral Angle** 0.82

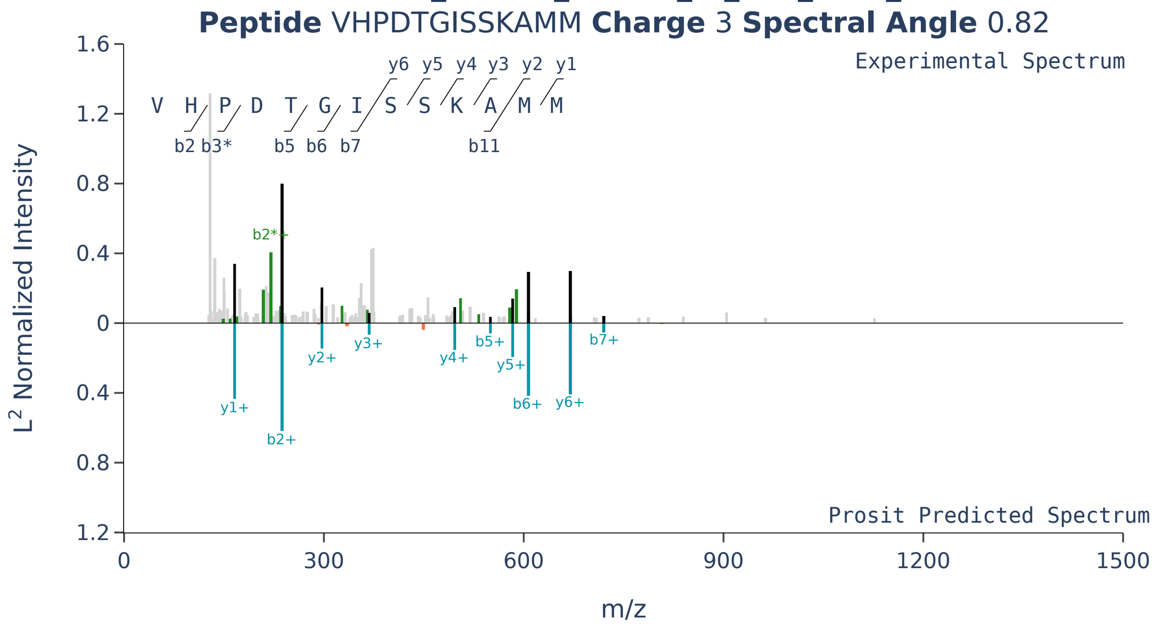

Source HRoetschke_291222_080123_Fu_H2B_b2R1_4h **Scan** 8360
Peptide VSTITSREIQT **Charge** 2 **Spectral Angle** 0.88

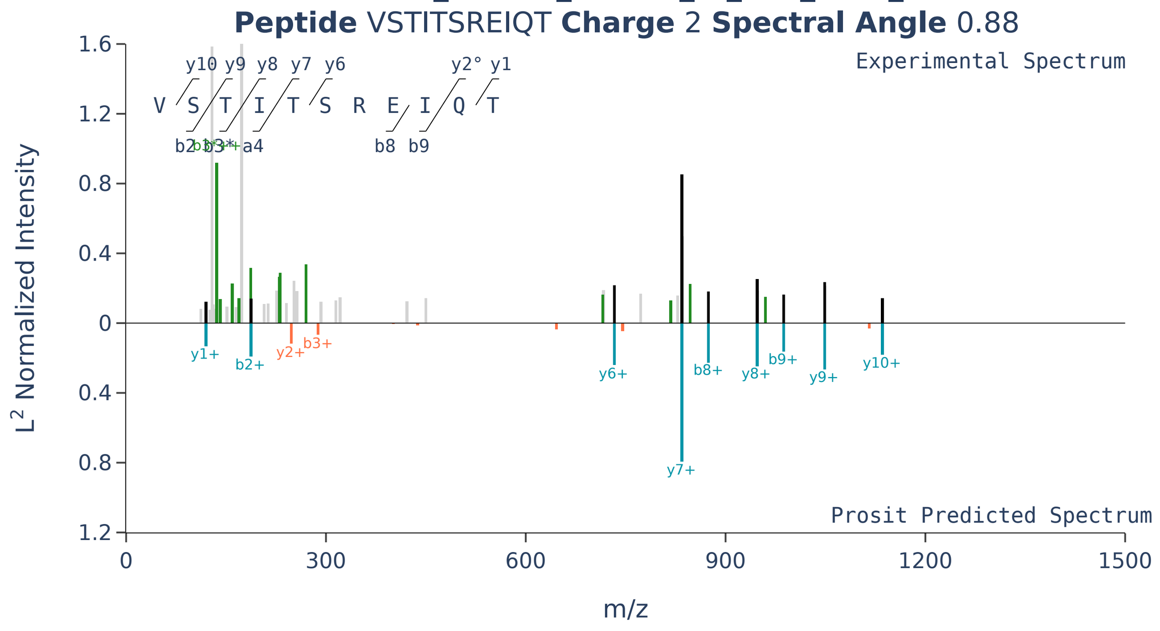

Source HRoetschke_291222_080123_Fu_H2B_b2R2_24h **Scan** 3043
Peptide VHPDTGISSKST **Charge** 3 **Spectral Angle** 0.82

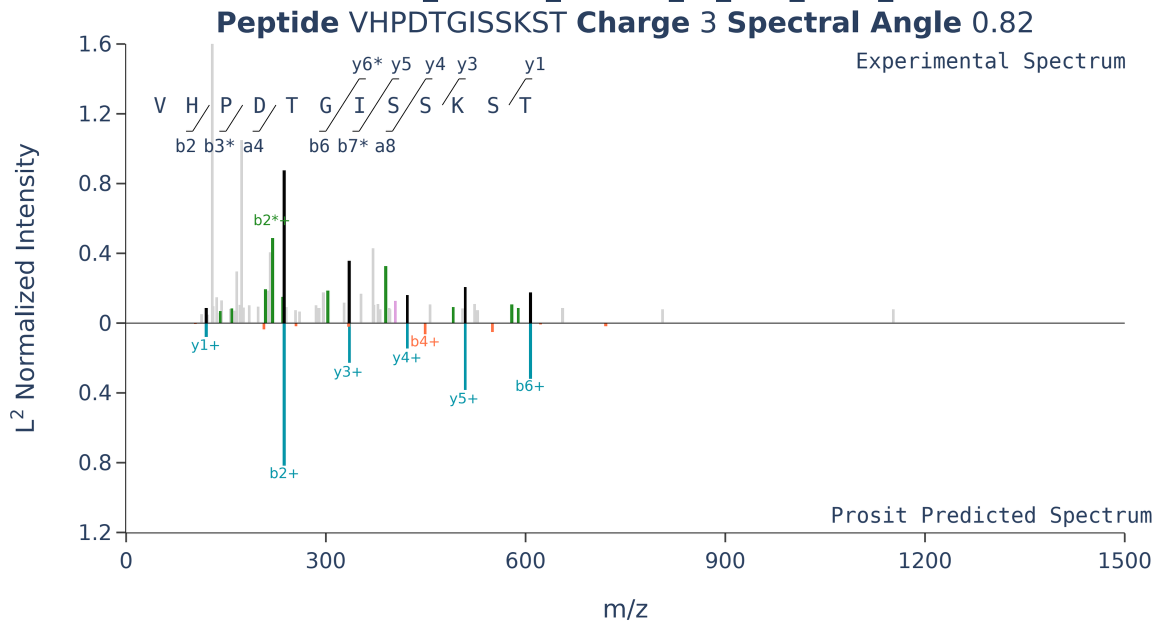

Source HRoetschke_291222_080123_Fu_H2B_b2R1_24h **Scan** 6429
Peptide VHPDTGISSKAMA **Charge** 3 **Spectral Angle** 0.83

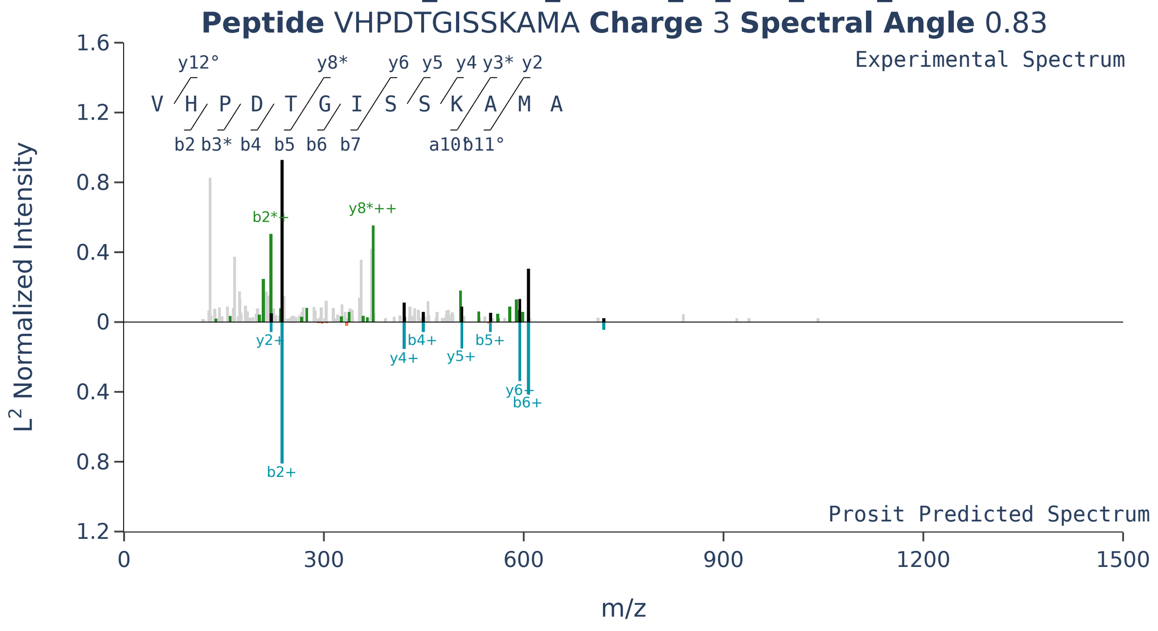

Source HRoetschke_291222_080123_Fu_H2B_b1R2_24h **Scan** 10112
Peptide SREIQTVRL **Charge** 3 **Spectral Angle** 0.82

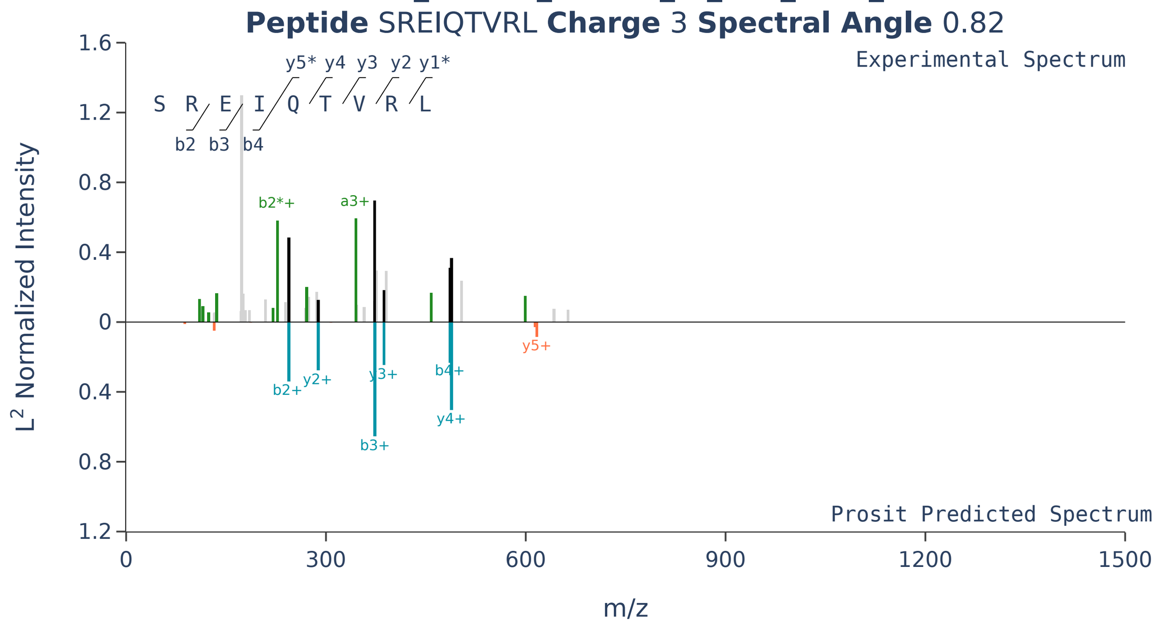

Source HRoetschke_291222_080123_Fu_H2B_b2R2_24h **Scan** 9933
Peptide TSSTITSREIQTAVR **Charge** 3 **Spectral Angle** 0.84

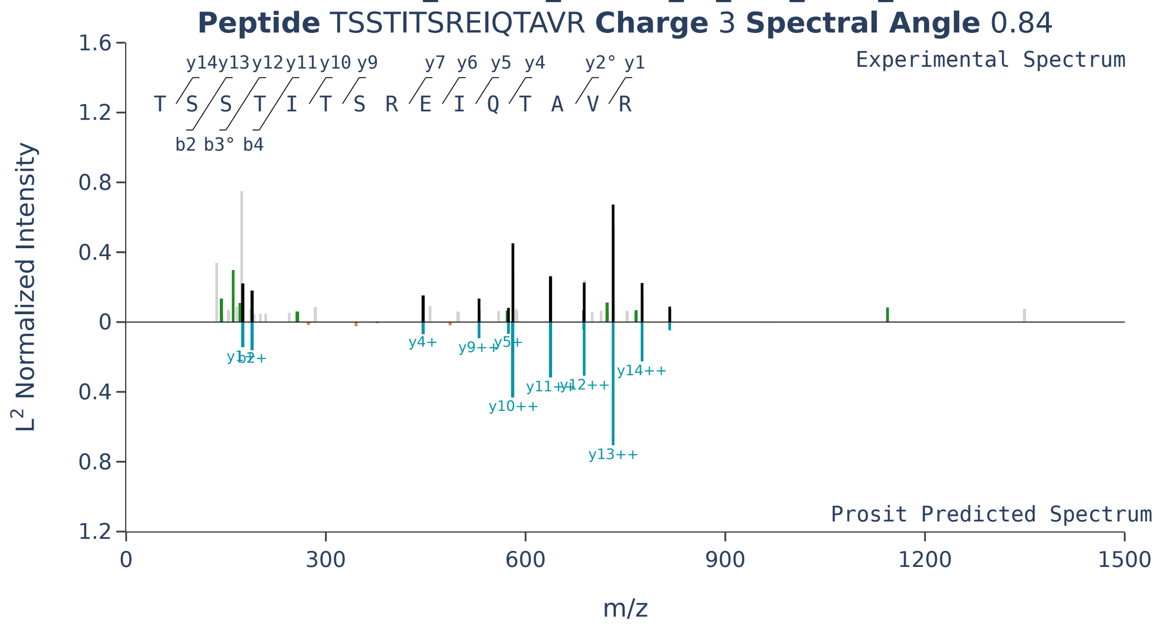

Source HRoetschke_291222_080123_Fu_H2B_b1R1_24h **Scan** 9216
Peptide ERIAGEASL **Charge** 2 **Spectral Angle** 0.87

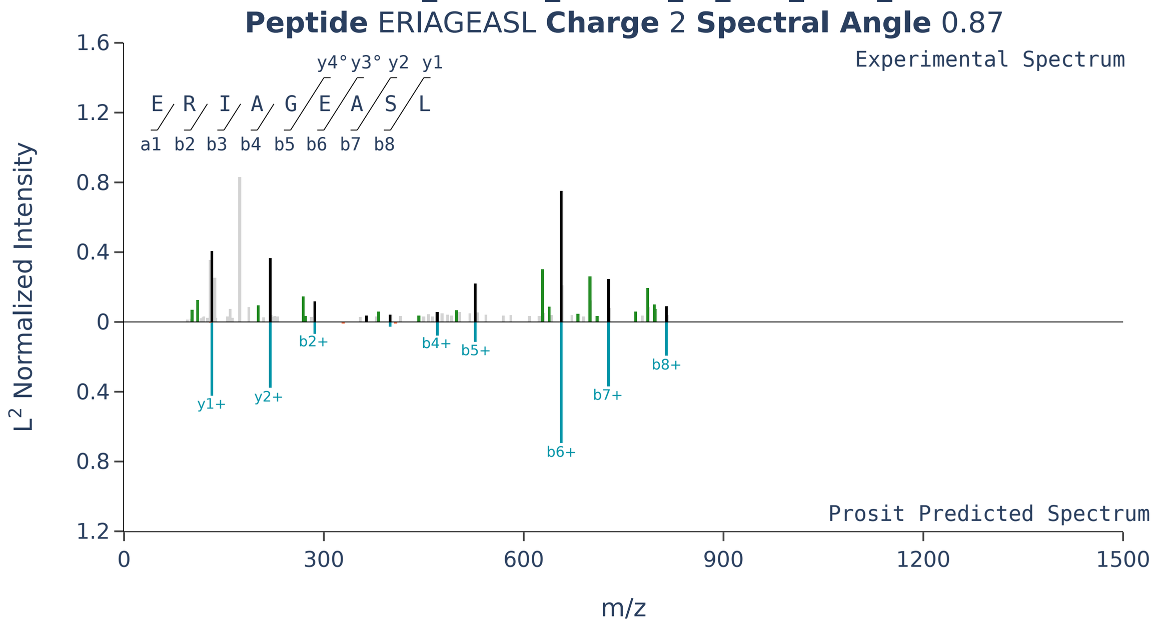

Source HRoetschke_291222_080123_Fu_H2B_b1R2_24h **Scan** 2446
Peptide ERIAGEASAHYNKR **Charge** 3 **Spectral Angle** 0.86

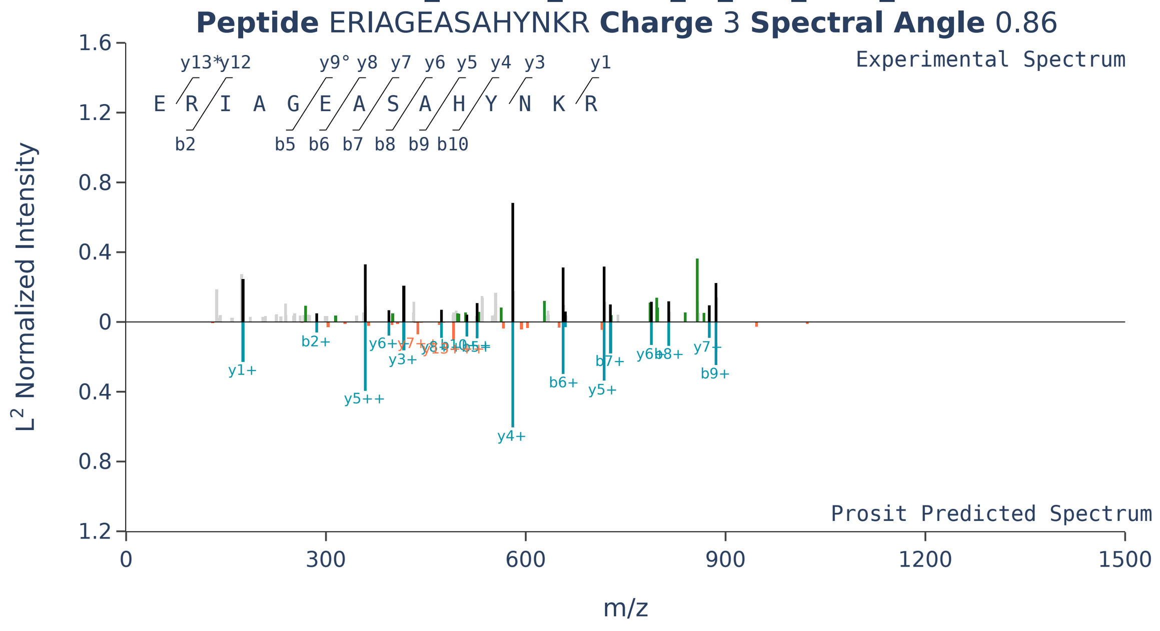

Source HRoetschke_291222_080123_Fu_H2B_b1R2_24h **Scan** 4252
Peptide HPDTGISSKQV **Charge** 3 **Spectral Angle** 0.88

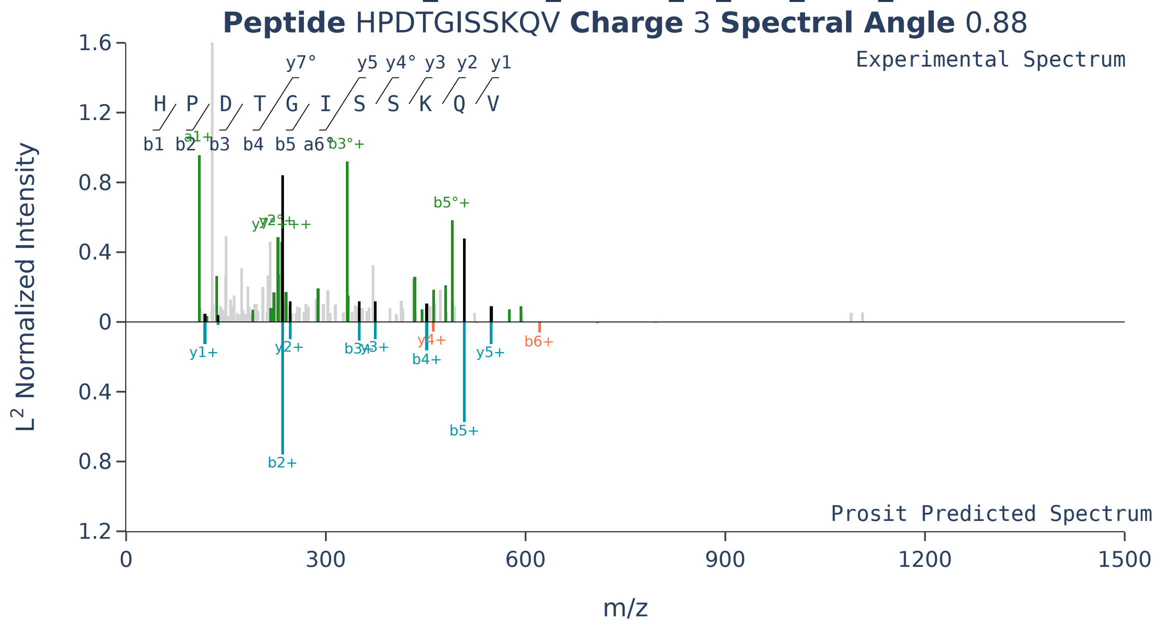

Source HRoetschke_291222_080123_Fu_H2B_b1R2_4h **Scan** 3786
Peptide ERIAGEASKV **Charge** 3 **Spectral Angle** 0.74

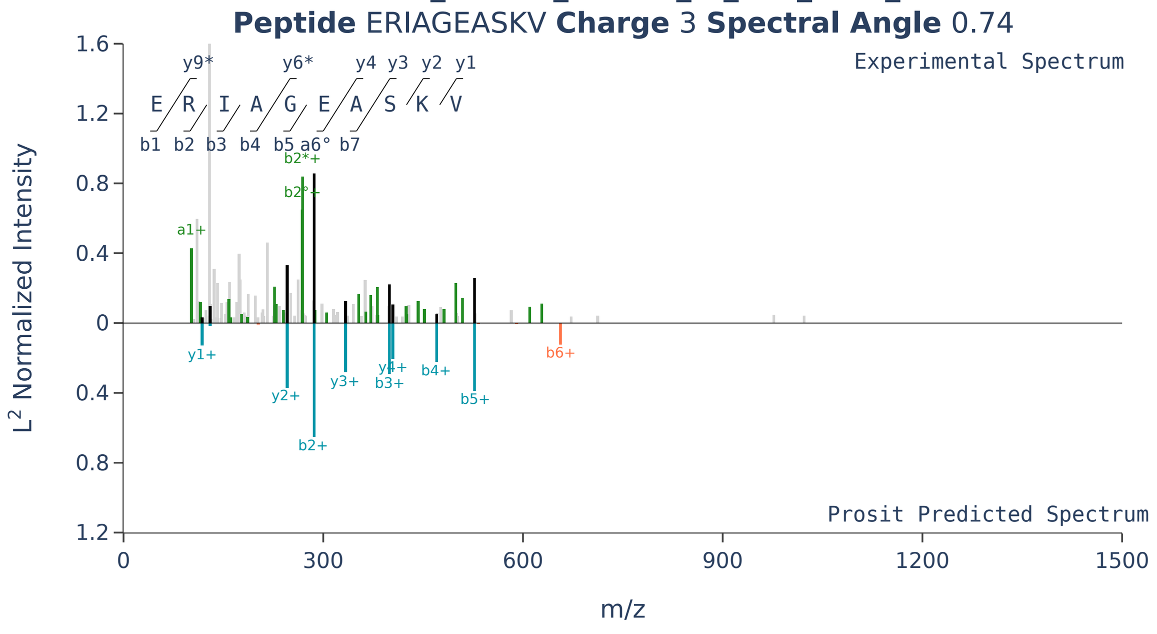

Source HRoetschke_291222_080123_Fu_H2B_b2R2_24h **Scan** 10634
Peptide MASTITSREIQT **Charge** 2 **Spectral Angle** 0.85

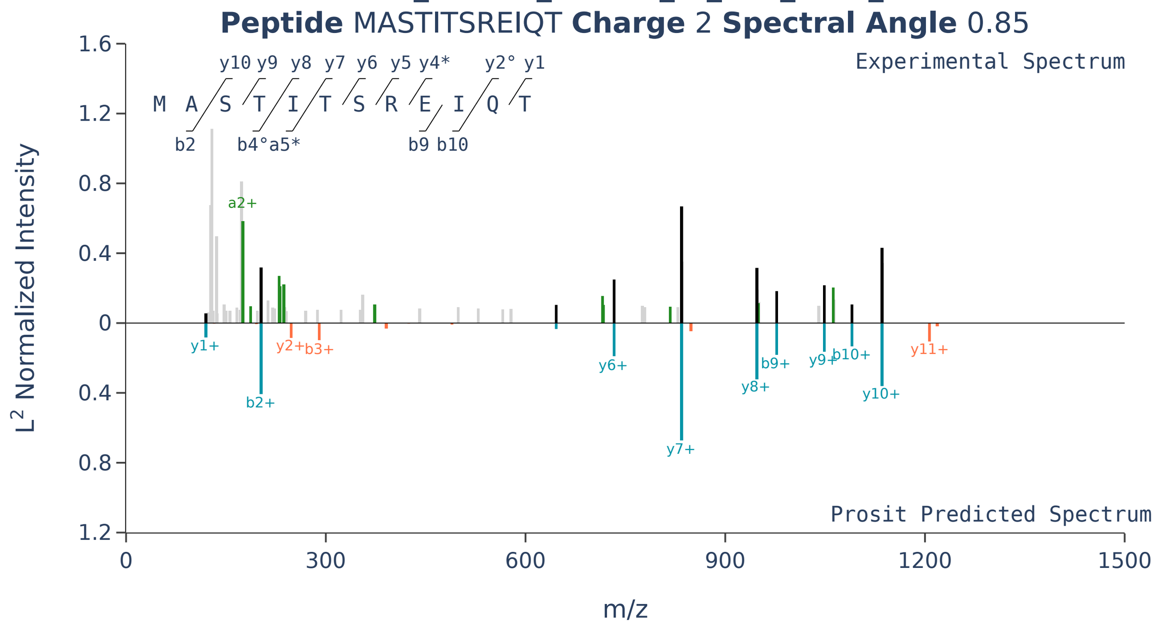

Source HRoetschke_291222_080123_Fu_H2B_b1R2_24h **Scan** 6526
Peptide ERIAGEASM **Charge** 2 **Spectral Angle** 0.85

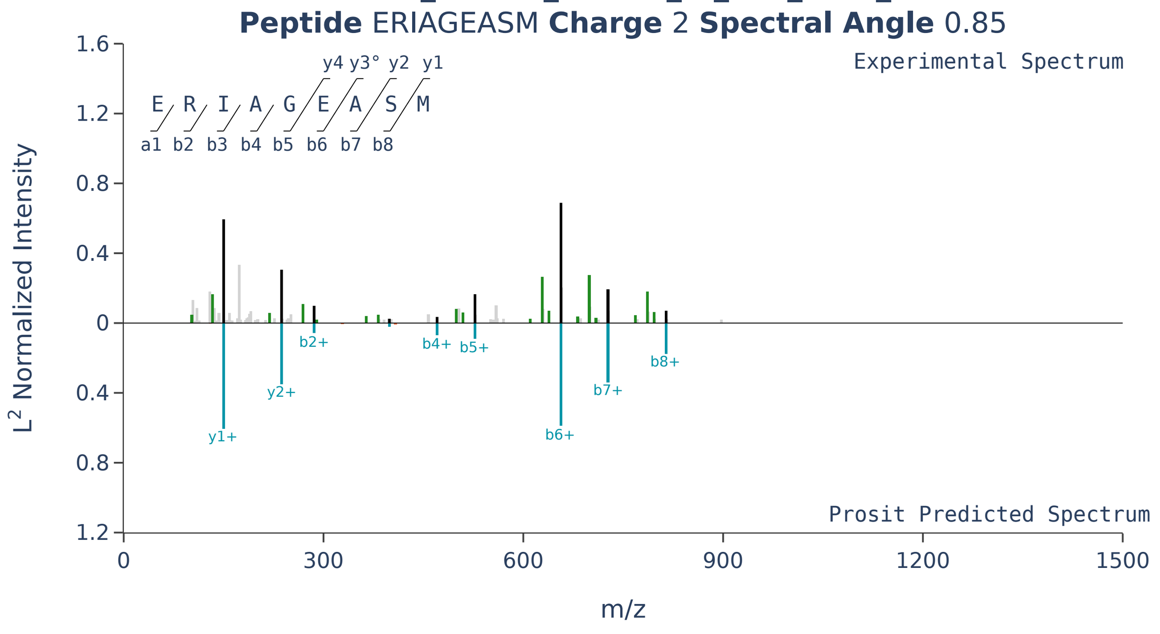

Source HRoetschke_291222_080123_Fu_H2B_b2R1_24h **Scan** 2957
Peptide HPDTGISSM **Charge** 2 **Spectral Angle** 0.87

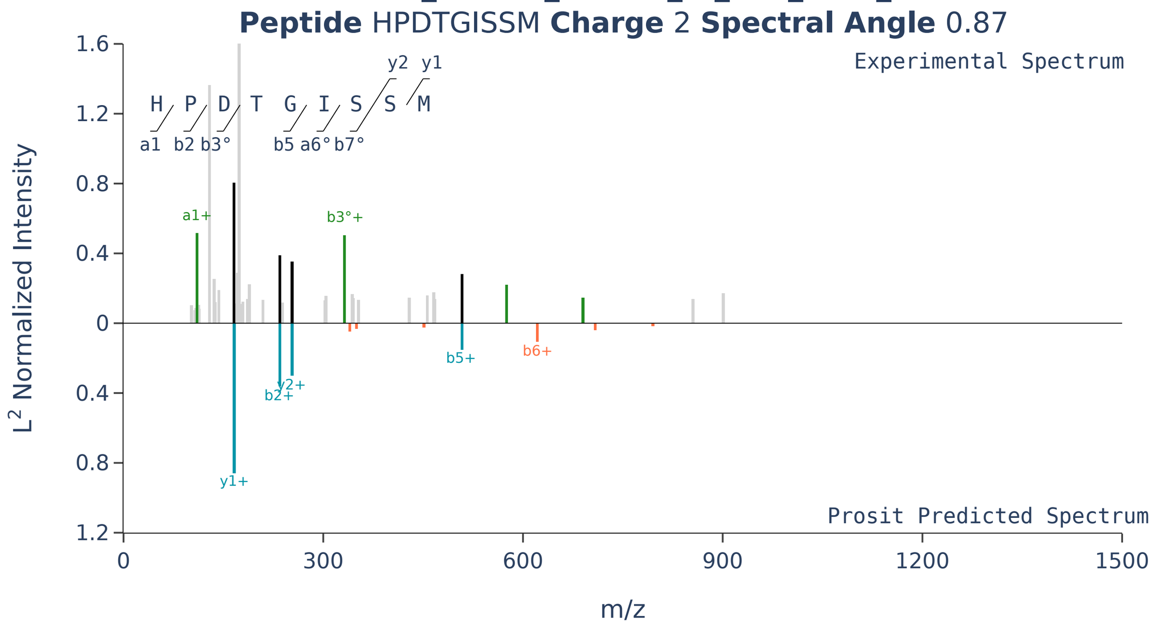

Source HRoetschke_291222_080123_Fu_H2B_b1R2_24h **Scan** 4831
Peptide SVHPDTGISS **Charge** 2 **Spectral Angle** 0.82

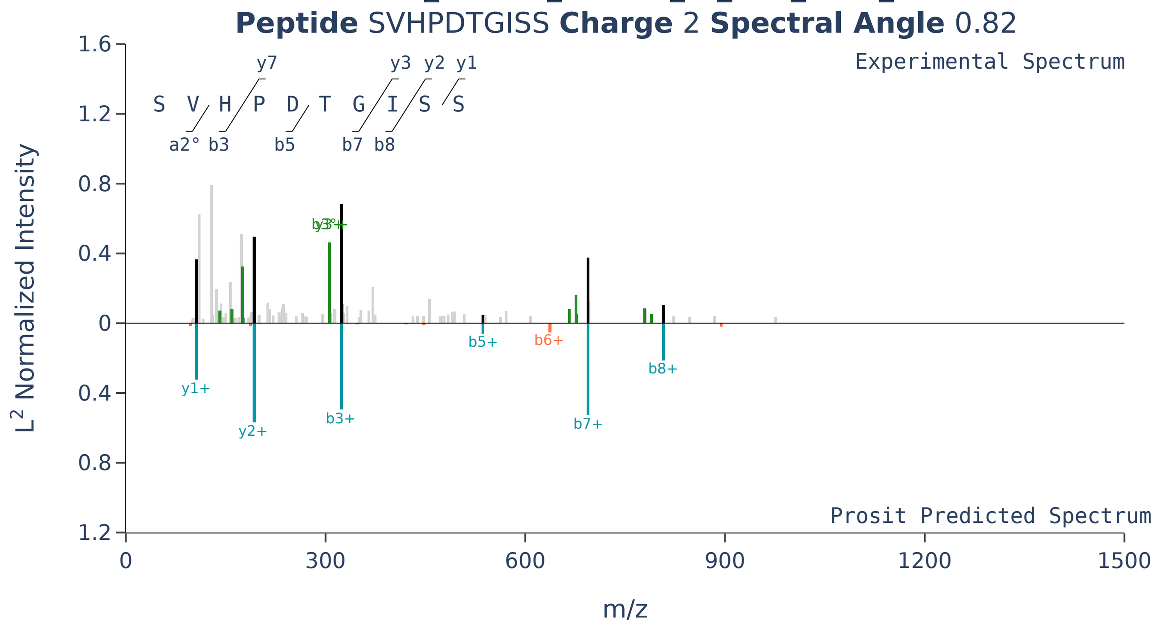

Source HRoetschke_291222_080123_Fu_H2B_b2R2_1h **Scan** 3223
Peptide VHPDTGISSKVH **Charge** 3 **Spectral Angle** 0.86

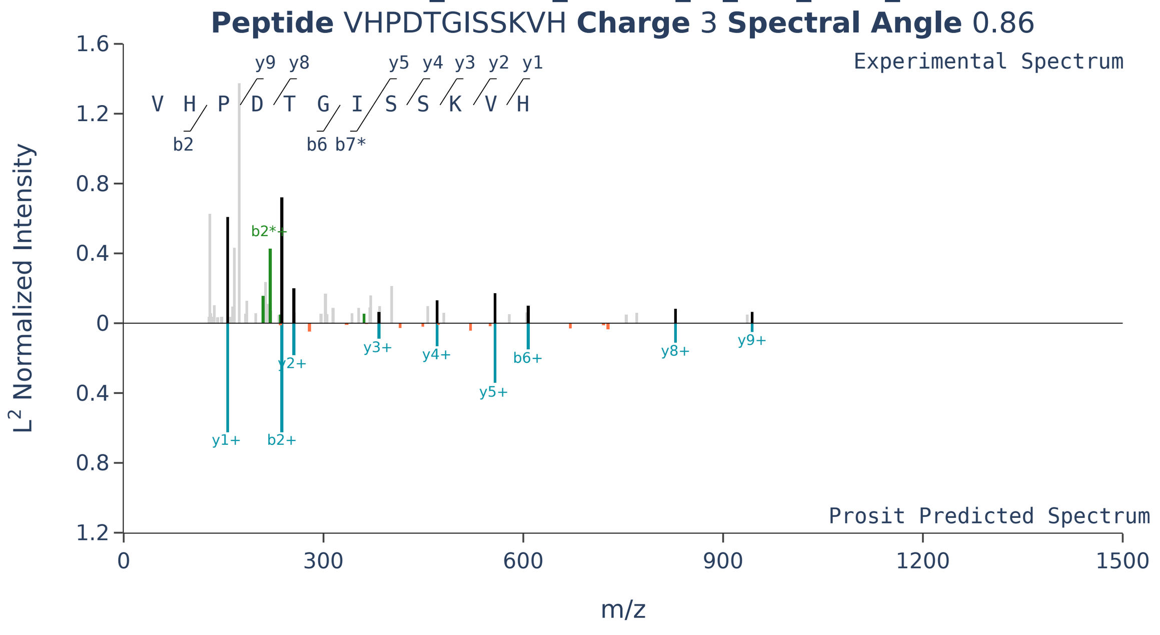

Source HRoetschke_291222_080123_Fu_H2B_b1R1_4h **Scan** 3667
Peptide VHPDTGISSS **Charge** 2 **Spectral Angle** 0.89

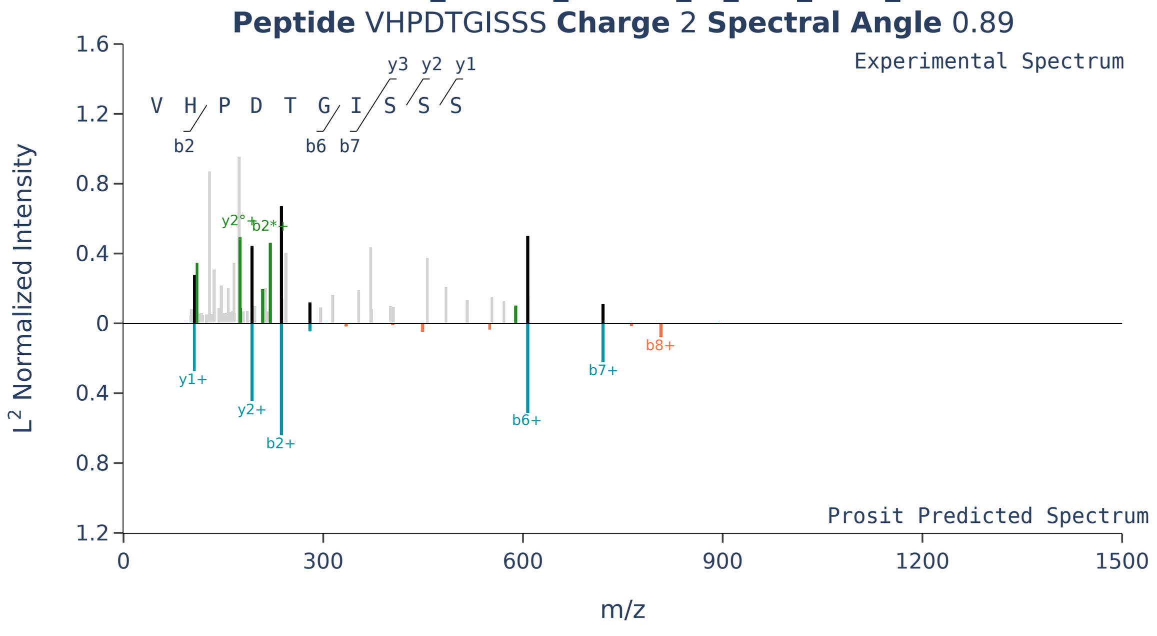

Source HRoetschke_291222_080123_Fu_H2B_b1R1_24h **Scan** 9678
Peptide HPDTGISSNSF **Charge** 2 **Spectral Angle** 0.86

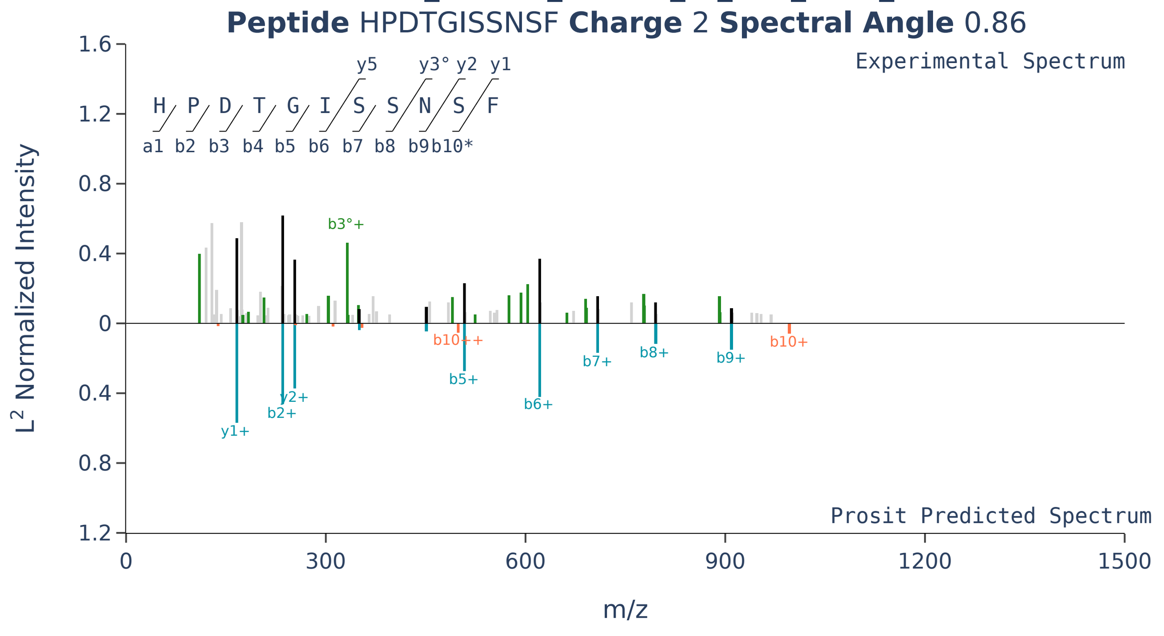

Source HRoetschke_291222_080123_Fu_H2B_b2R2_4h **Scan** 2238
Peptide KQVHPDTGISSR **Charge** 3 **Spectral Angle** 0.82

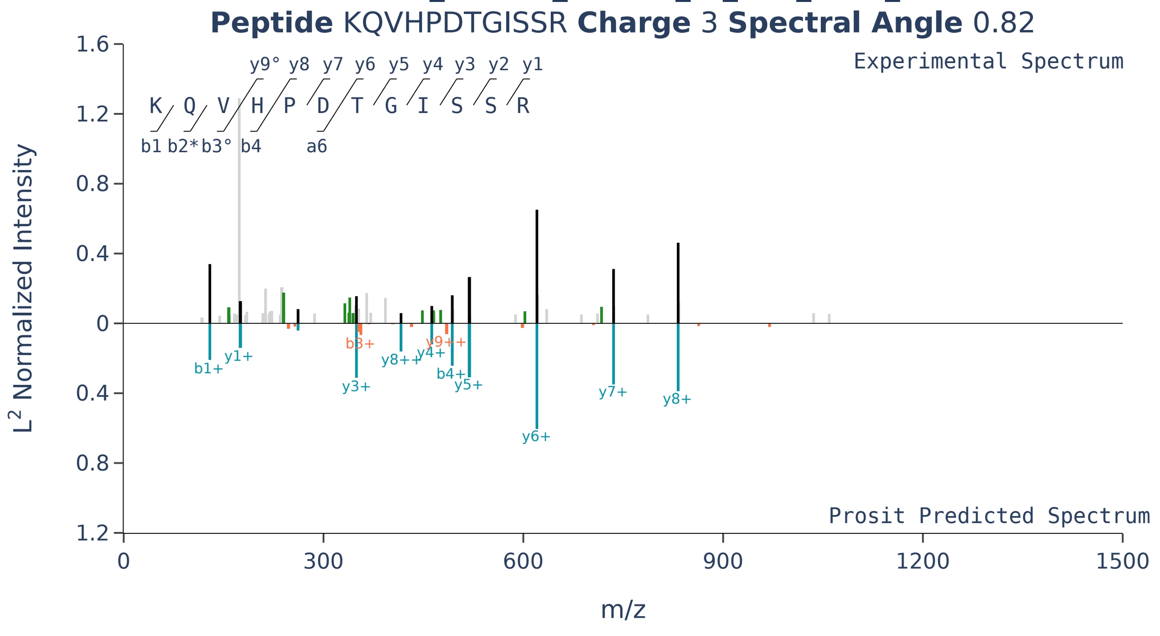

Source HRoetschke_291222_080123_Fu_H2B_b1R1_4h **Scan** 7000
Peptide SSTITSREIQT **Charge** 2 **Spectral Angle** 0.8

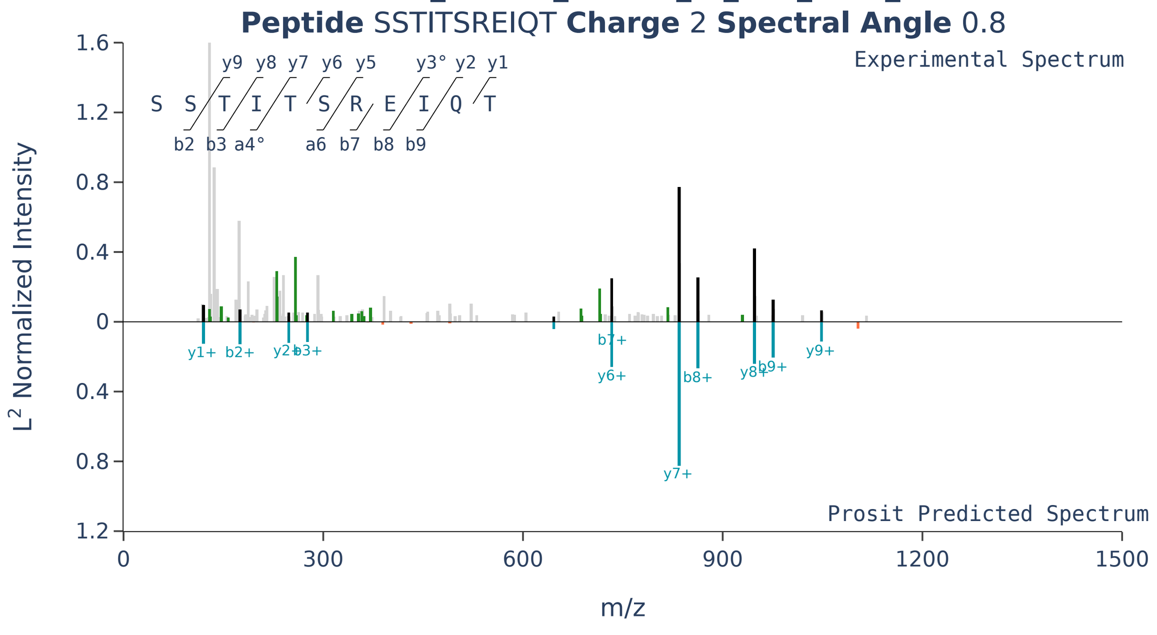

Source HRoetschke_291222_080123_Fu_H2B_b1R2_24h **Scan** 4176
Peptide AGEASREIQTA **Charge** 2 **Spectral Angle** 0.85

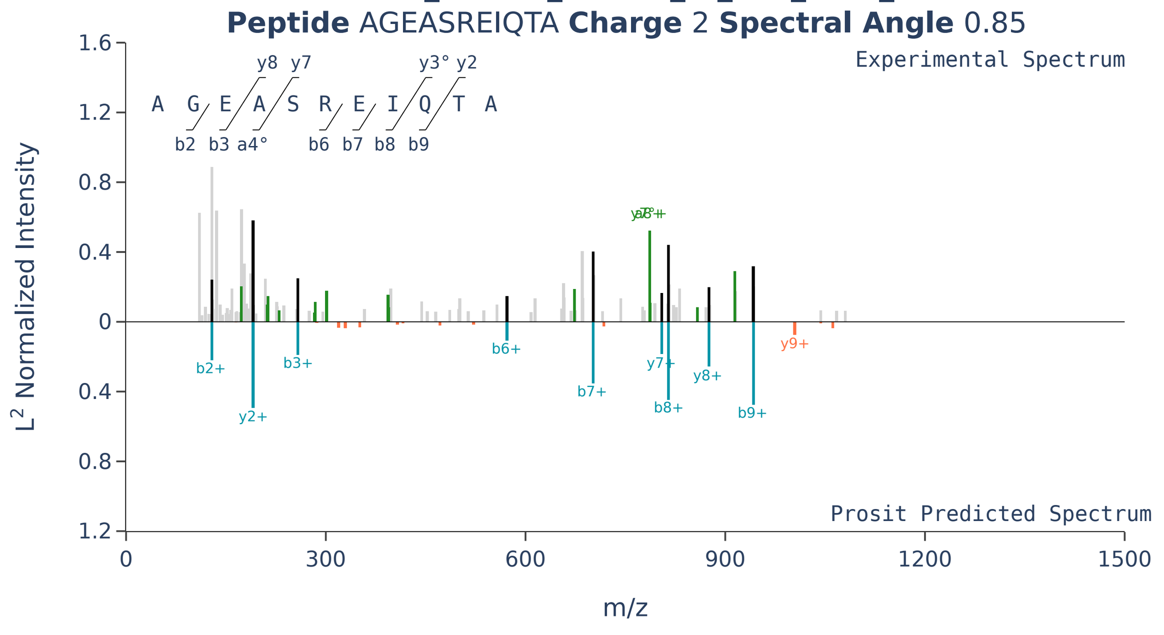

Source HRoetschke_291222_080123_Fu_H2B_b2R2_4h **Scan** 3463
Peptide AHYNKRSTITSREIQTM **Charge** 4 **Spectral Angle** 0.86

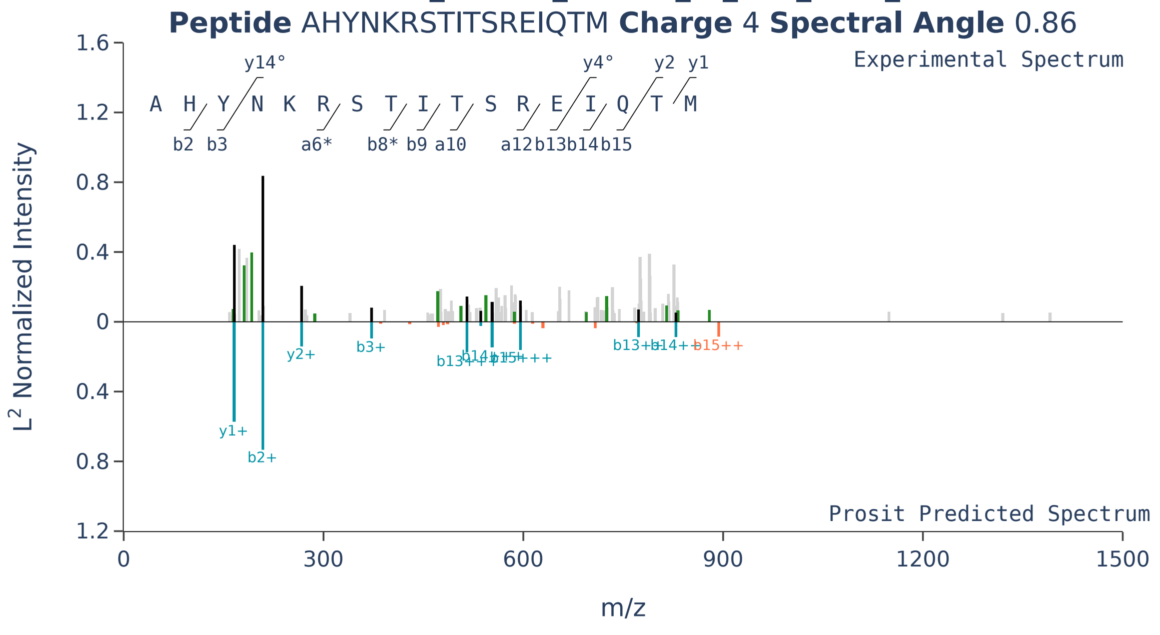

Source HRoetschke_291222_080123_Fu_H2B_b1R1_4h **Scan** 2237
Peptide AHYNKRSTITA **Charge** 2 **Spectral Angle** 0.89

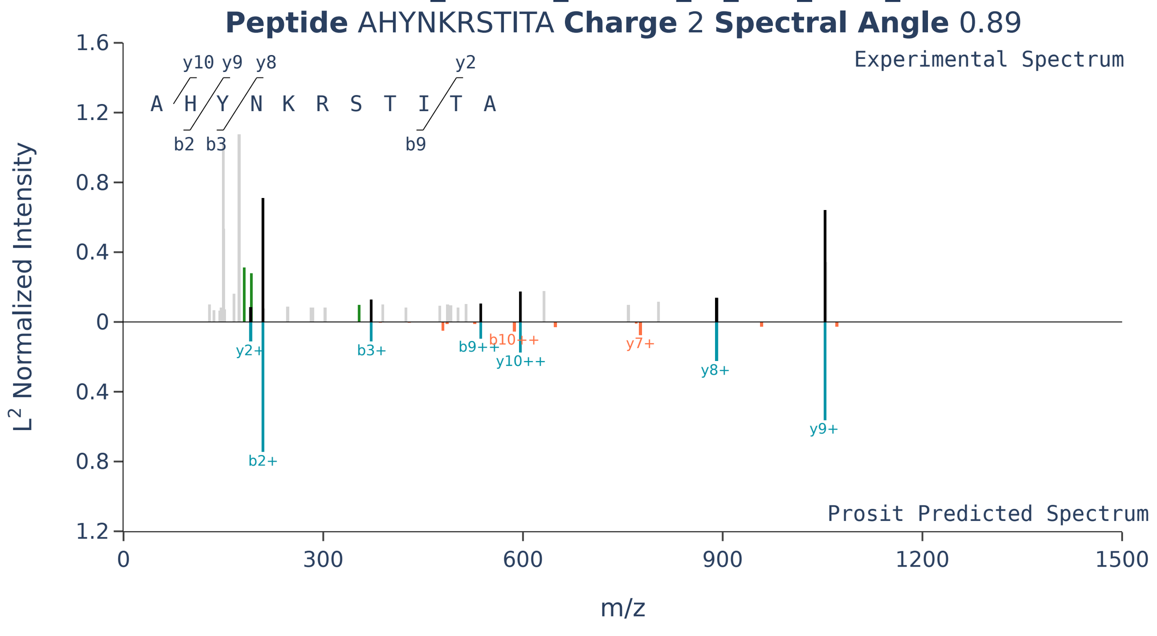

Source HRoetschke_291222_080123_Fu_H2B_b2R1_24h **Scan** 5477
Peptide REIQTRL **Charge** 2 **Spectral Angle** 0.8

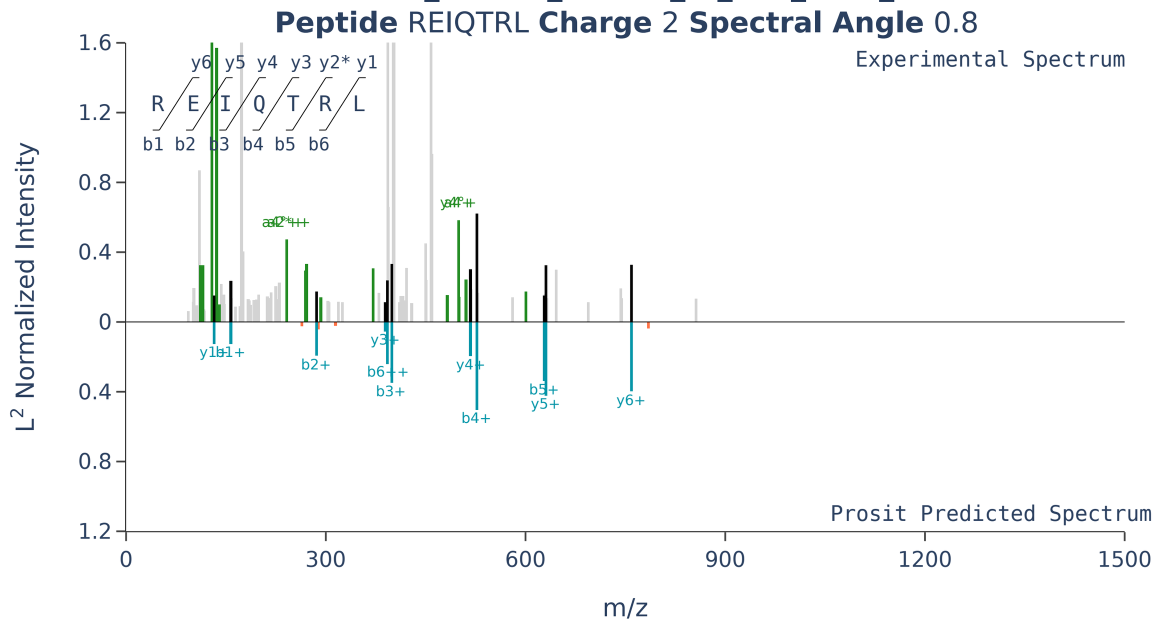

Source HRoetschke_291222_080123_Fu_H2B_b1R1_4h **Scan** 4457
Peptide VSHPDTGISS **Charge** 2 **Spectral Angle** 0.8

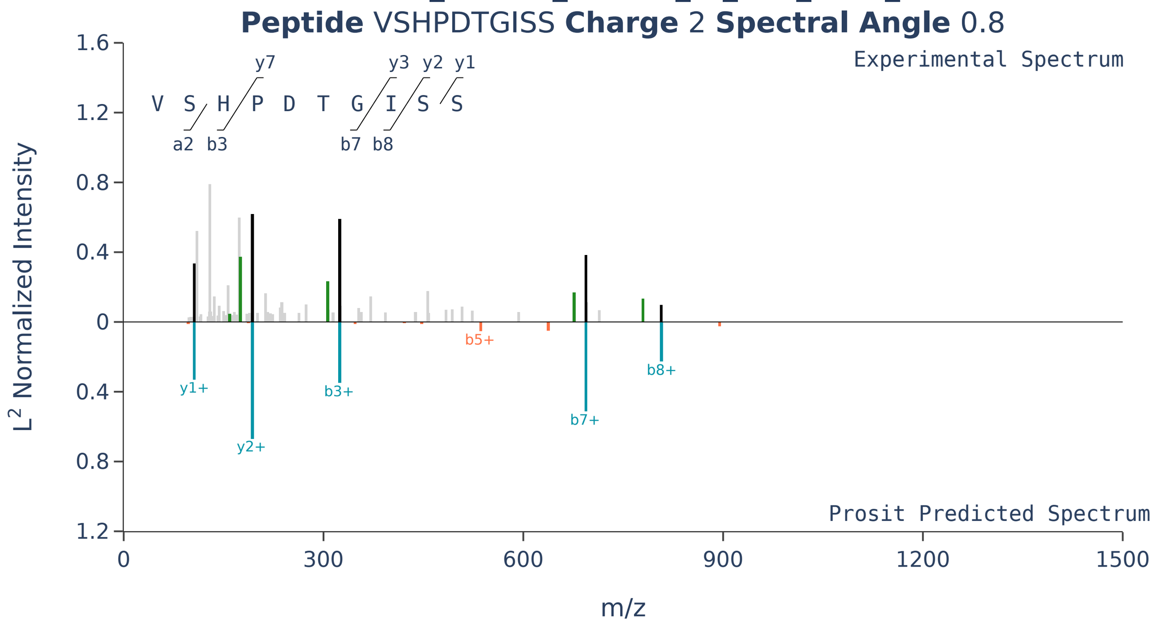

Source HRoetschke_291222_080123_Fu_H2B_b2R2_4h **Scan** 8990
Peptide YSTITSREIQT **Charge** 2 **Spectral Angle** 0.85

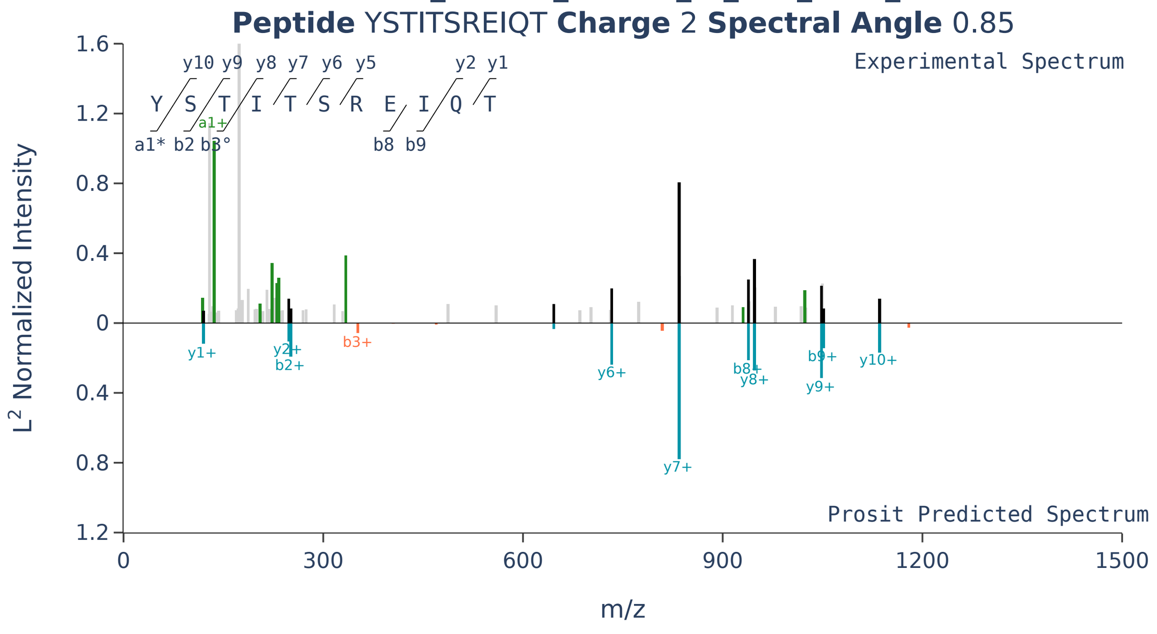

Source HRoetschke_291222_080123_Fu_H2B_b1R1_24h **Scan** 3361
Peptide VHPDTGISSAHYNKR **Charge** 4 **Spectral Angle** 0.82

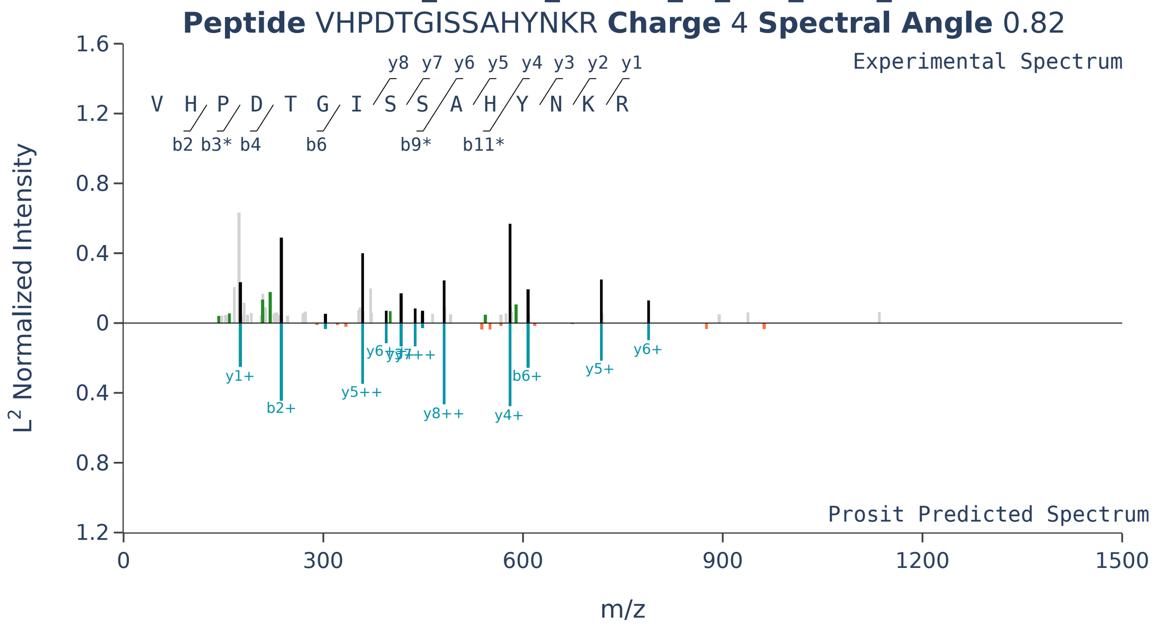

Source HRoetschke_291222_080123_Fu_H2B_b2R2_24h **Scan** 6902
Peptide HPDTGISSRIAG **Charge** 3 **Spectral Angle** 0.86

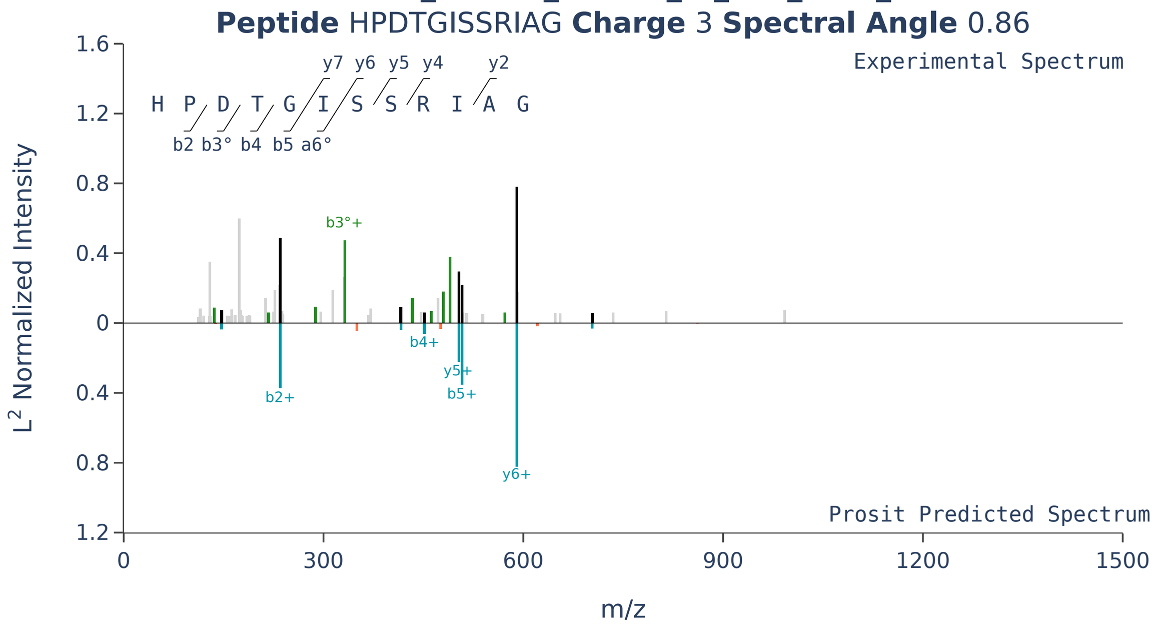

Source HRoetschke_291222_080123_Fu_H2B_b1R2_24h **Scan** 8180
Peptide ERIAGEASAGEASRL **Charge** 3 **Spectral Angle** 0.84

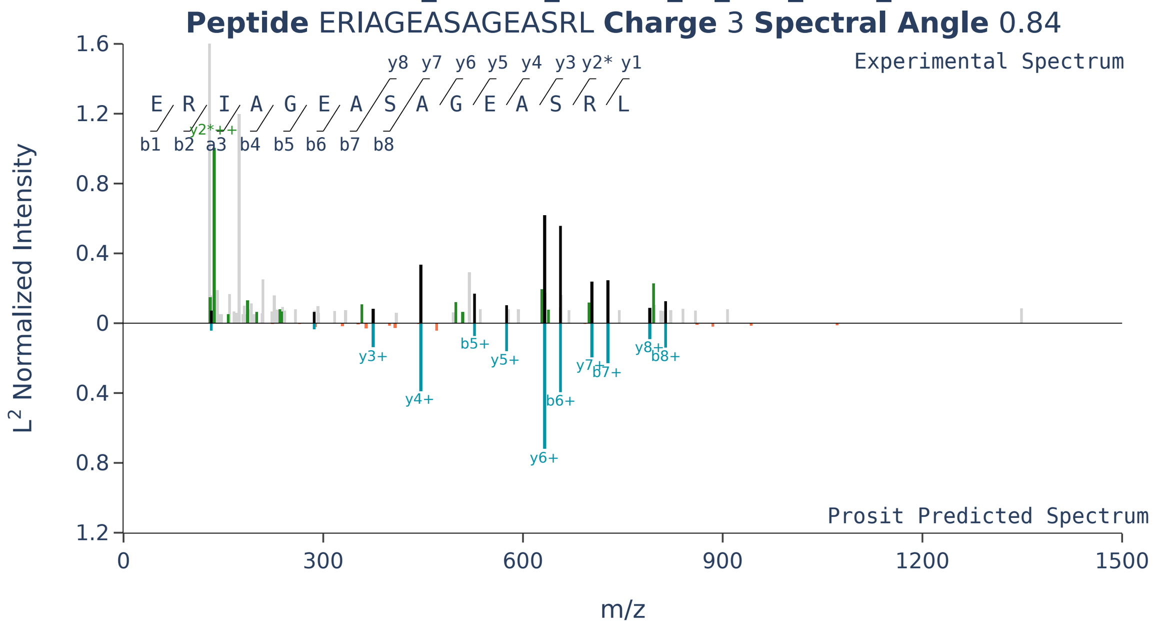

Source HRoetschke_291222_080123_Fu_H2B_b1R2_1h **Scan** 2769
Peptide DTKAVTKYTSAK **Charge** 3 **Spectral Angle** 0.79

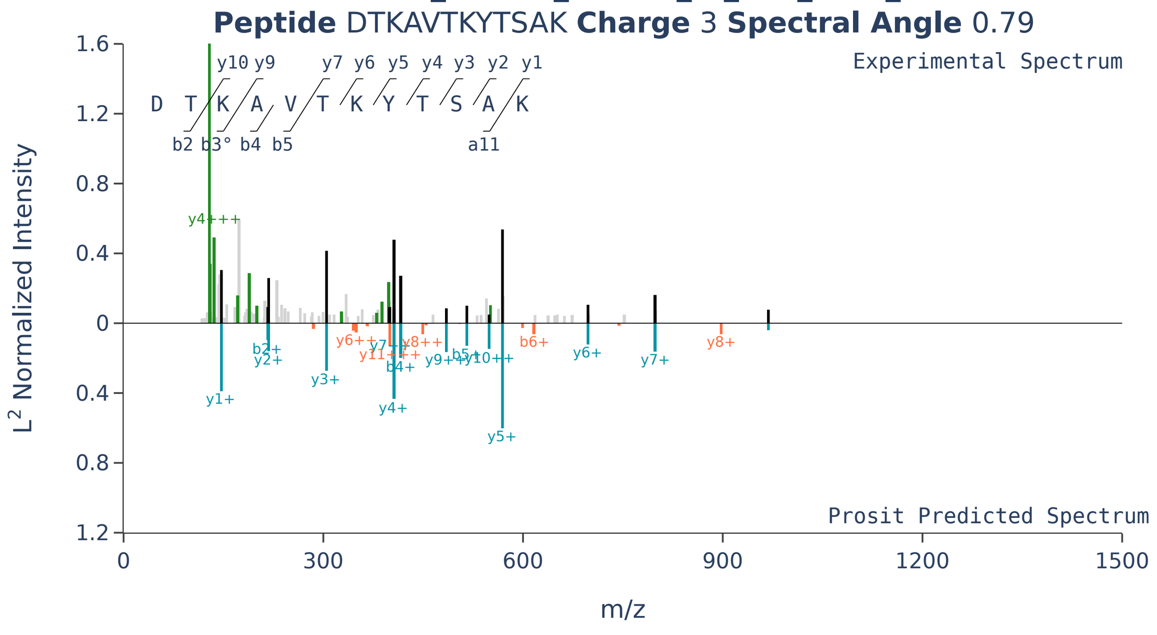

Source HRoetschke_291222_080123_Fu_H2B_b1R2_24h **Scan** 13313
Peptide KSMSIMNSF **Charge** 2 **Spectral Angle** 0.92

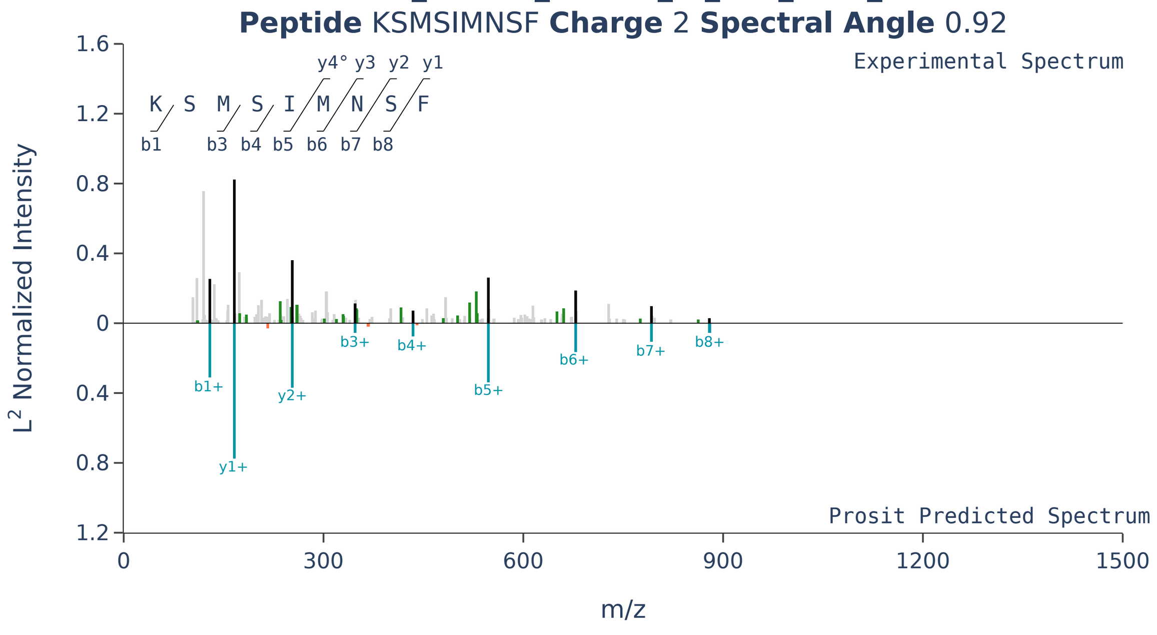

Source HRoetschke_291222_080123_Fu_H2B_b2R2_24h **Scan** 8553
Peptide VHPDTGISSV **Charge** 2 **Spectral Angle** 0.88

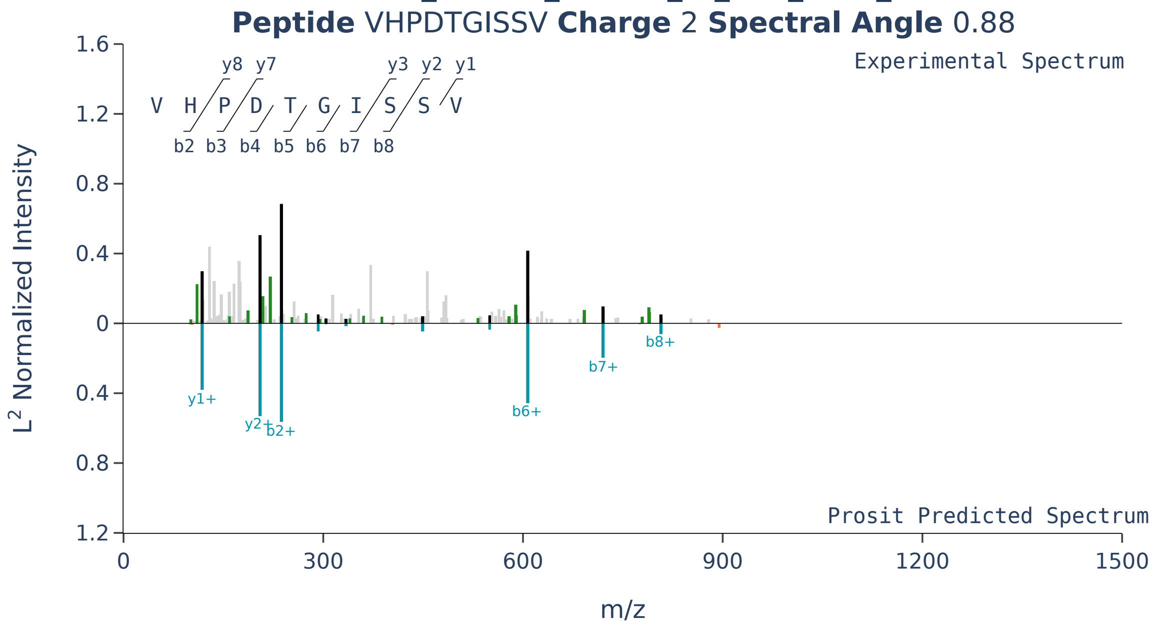

Source HRoetschke_291222_080123_Fu_H2B_b1R1_24h **Scan** 3121
Peptide KESAVTKYTSAK **Charge** 3 **Spectral Angle** 0.87

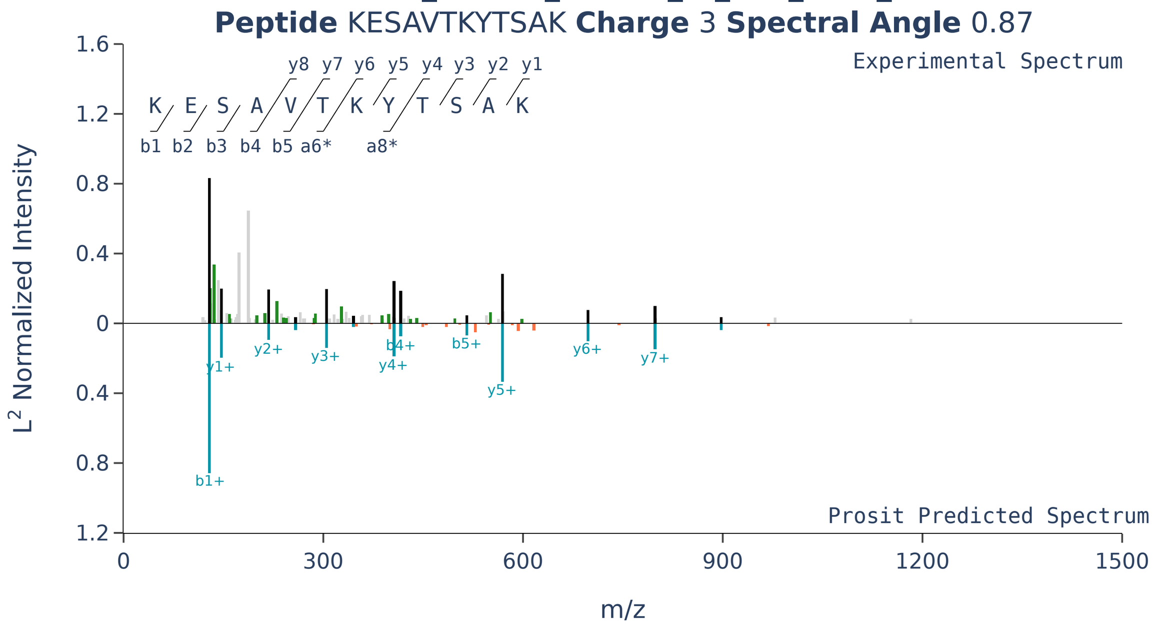

Source HRoetschke_291222_080123_Fu_H2B_b2R1_4h **Scan** 10770
Peptide ELSTITSREIQT **Charge** 2 **Spectral Angle** 0.85

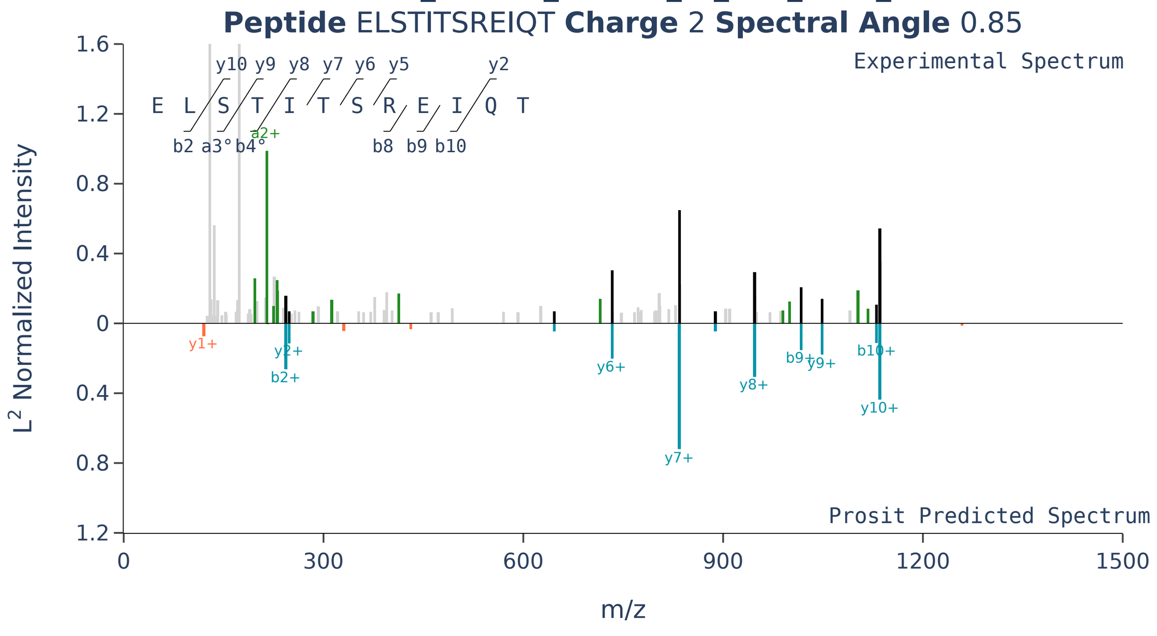

Source HRoetschke_291222_080123_Fu_H2B_b2R2_2h **Scan** 4063
Peptide EIQTTKYTSAK **Charge** 3 **Spectral Angle** 0.81

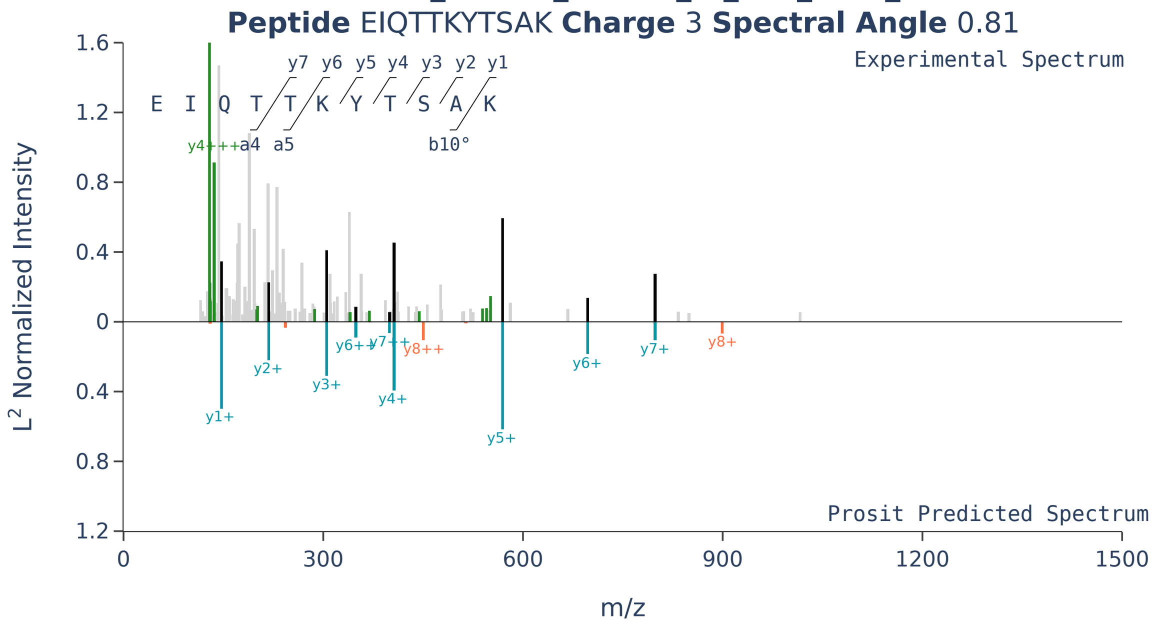

Source HRoetschke_291222_080123_Fu_H2B_b1R1_24h **Scan** 6911
Peptide VHPDTGISSVND **Charge** 2 **Spectral Angle** 0.84

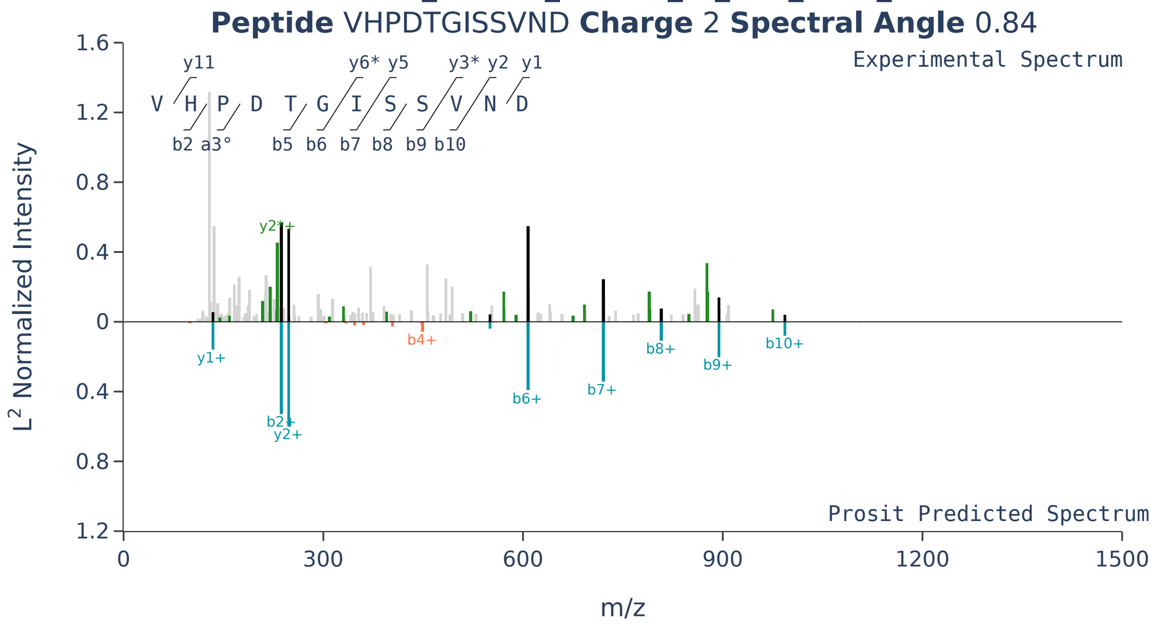

Source HRoetschke_291222_080123_Fu_H2B_b1R2_24h **Scan** 2295
Peptide AHYNKRSTITTG **Charge** 2 **Spectral Angle** 0.91

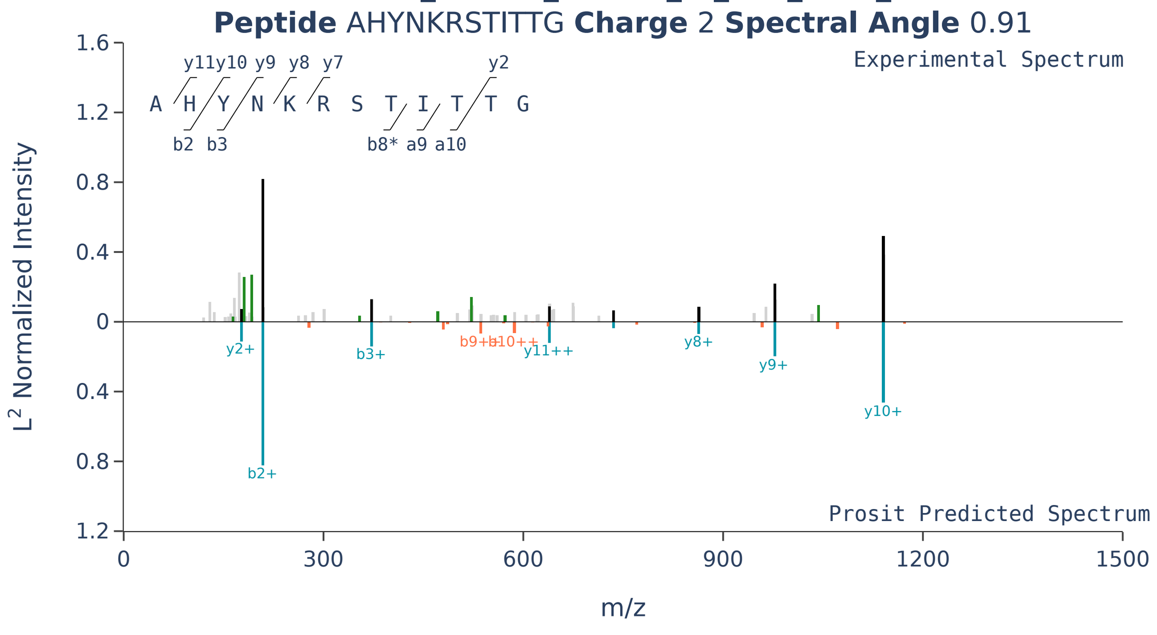

Source HRoetschke_291222_080123_Fu_H2B_b1R2_24h **Scan** 6986
Peptide TSRIAGEASRL **Charge** 3 **Spectral Angle** 0.78

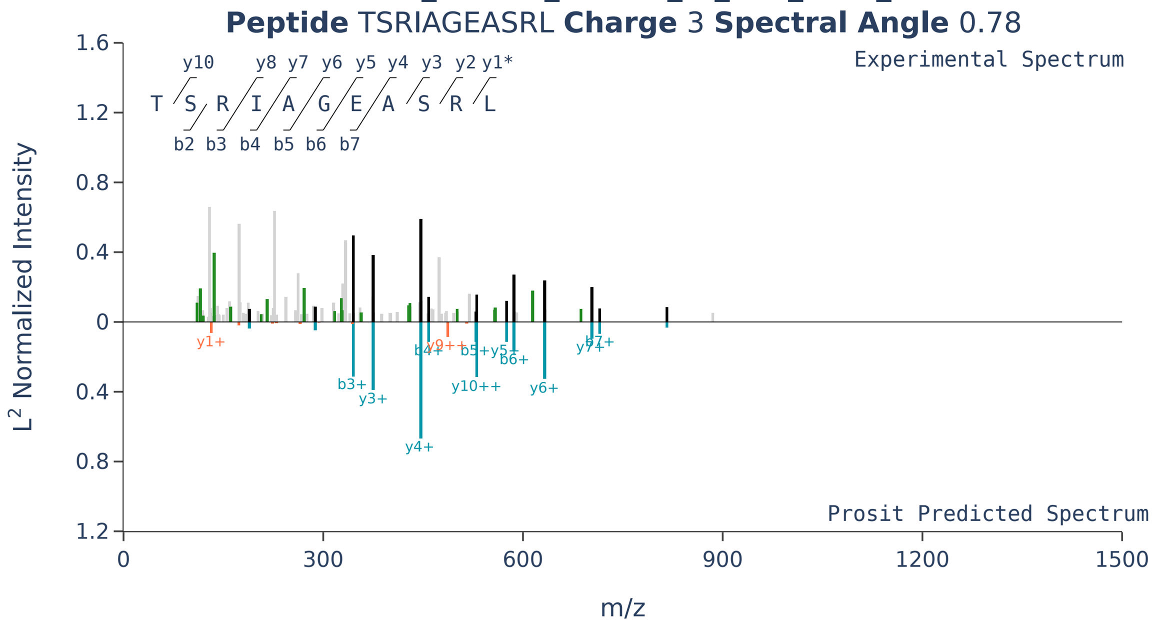

Source HRoetschke_291222_080123_Fu_H2B_b2R2_24h **Scan** 3488
Peptide PGELAKHAVSAG **Charge** 2 **Spectral Angle** 0.79

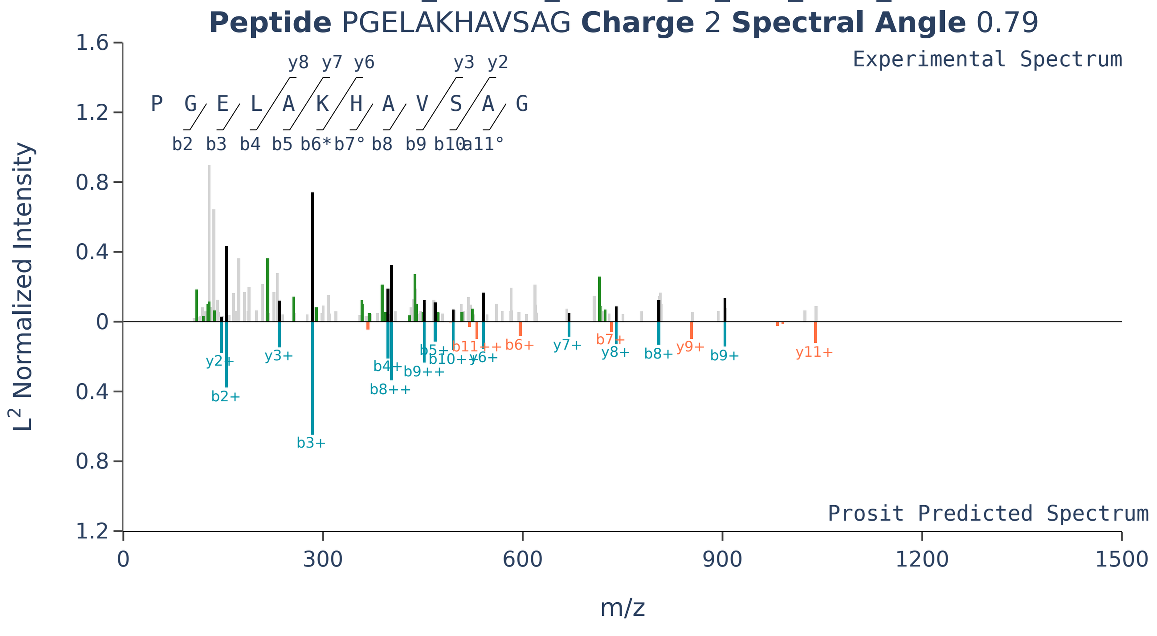

Source HRoetschke_291222_080123_Fu_H2B_b2R1_24h **Scan** 4079
Peptide LKQVHPDTGISSR **Charge** 3 **Spectral Angle** 0.82

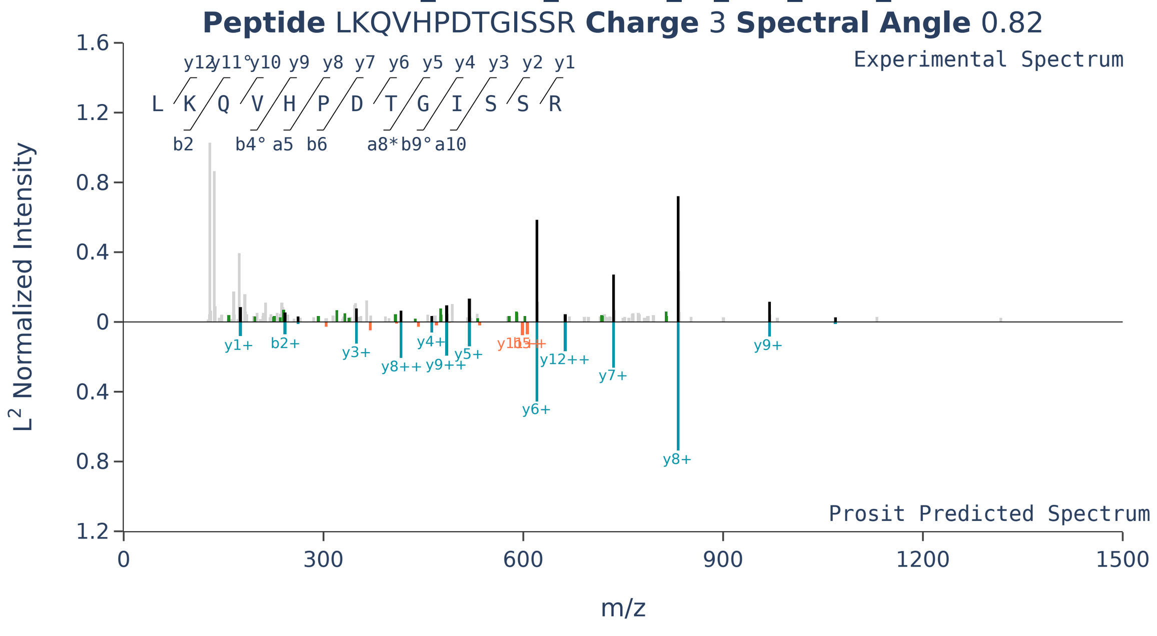

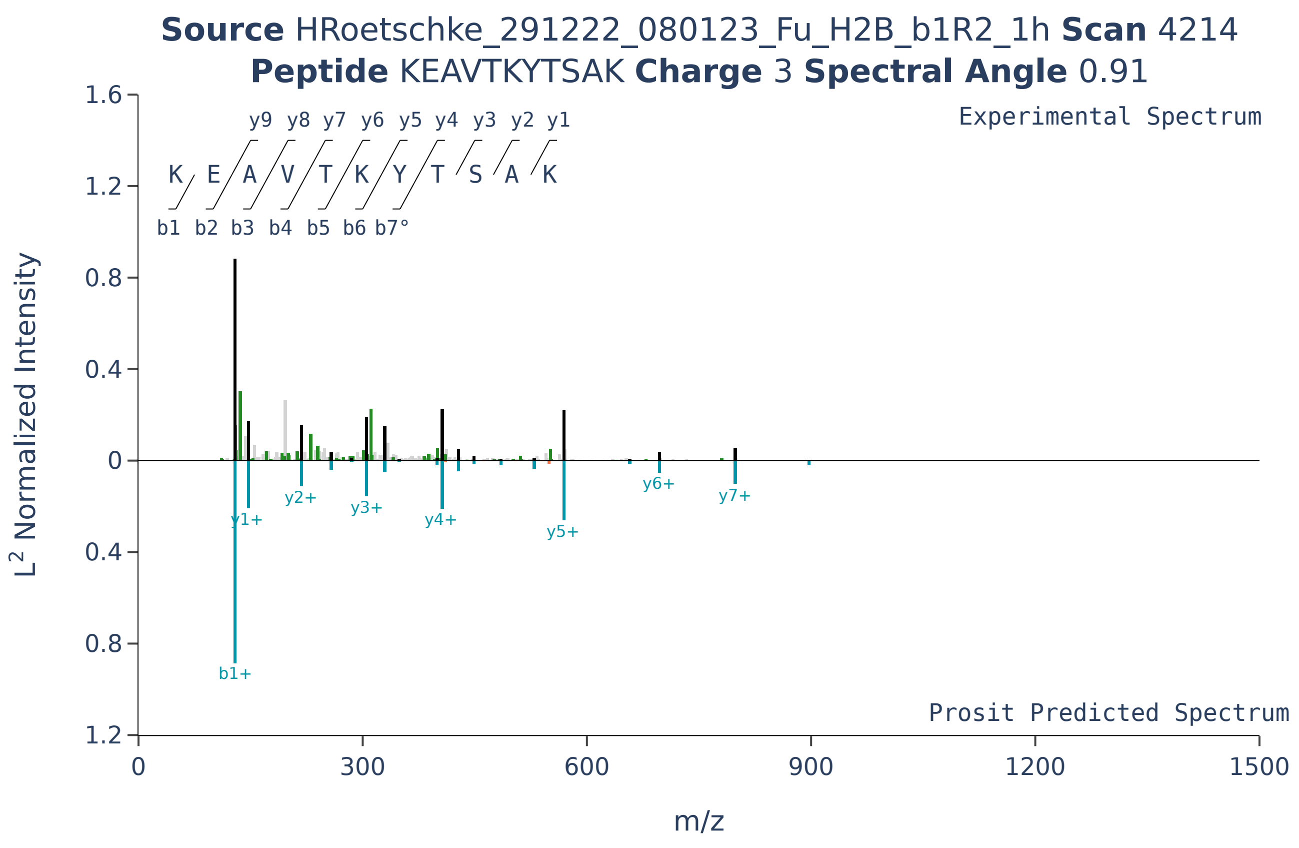
Source HRoetschke_291222_080123_Fu_H2B_b1R2_1h Scan 4214
Peptide KEAVTKYTSAK Charge 3 Spectral Angle 0.91
Experimental Spectrum
Prosit Predicted Spectrum
L² Normalized Intensity
m/z
y1+
y2+
y3+
y4+
y5+
y6+
y7+
b1+

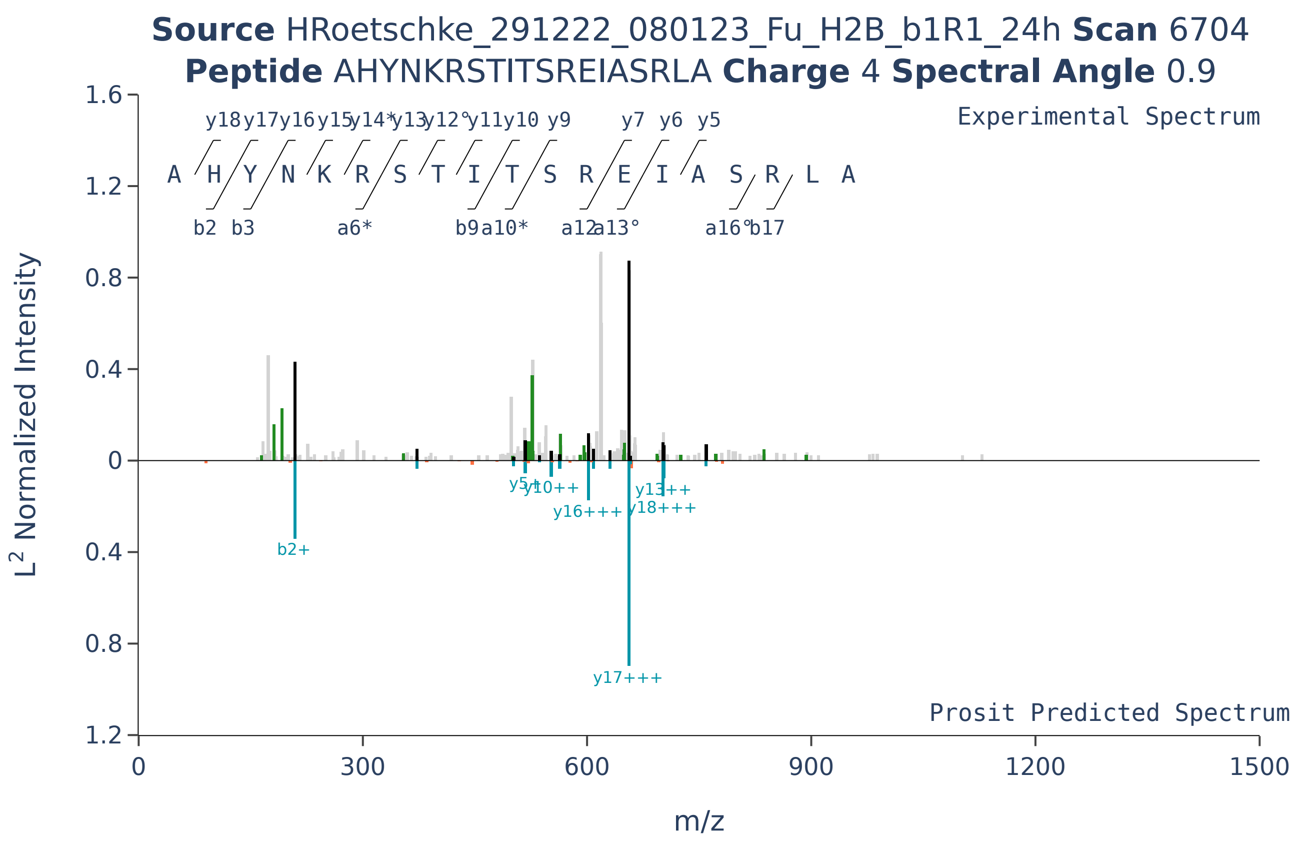
Source HRoetschke_291222_080123_Fu_H2B_b1R1_24h Scan 6704
Peptide AHYNKRSTITSREIASRLA Charge 4 Spectral Angle 0.9
Experimental Spectrum
Prosit Predicted Spectrum
L² Normalized Intensity
m/z
b2+
y17+++
y16+++
y10++
y13++
y18+++

Source HRoetschke_271222_120123_HFGoe_H3_b2R1_24h **Scan** 11023
Peptide SREIAQDFKT **Charge** 3 **Spectral Angle** 0.93

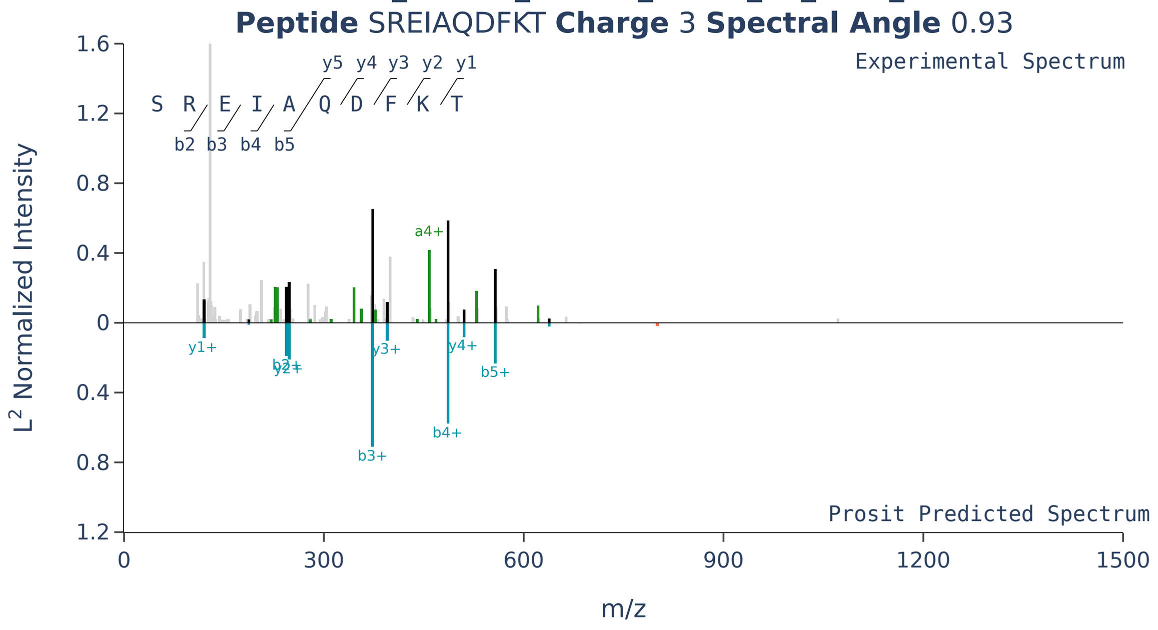

Source HRoetschke_271222_120123_HFGoe_H3_b2R1_2h **Scan** 17832
Peptide FEDTNLLC **Charge** 2 **Spectral Angle** 0.93

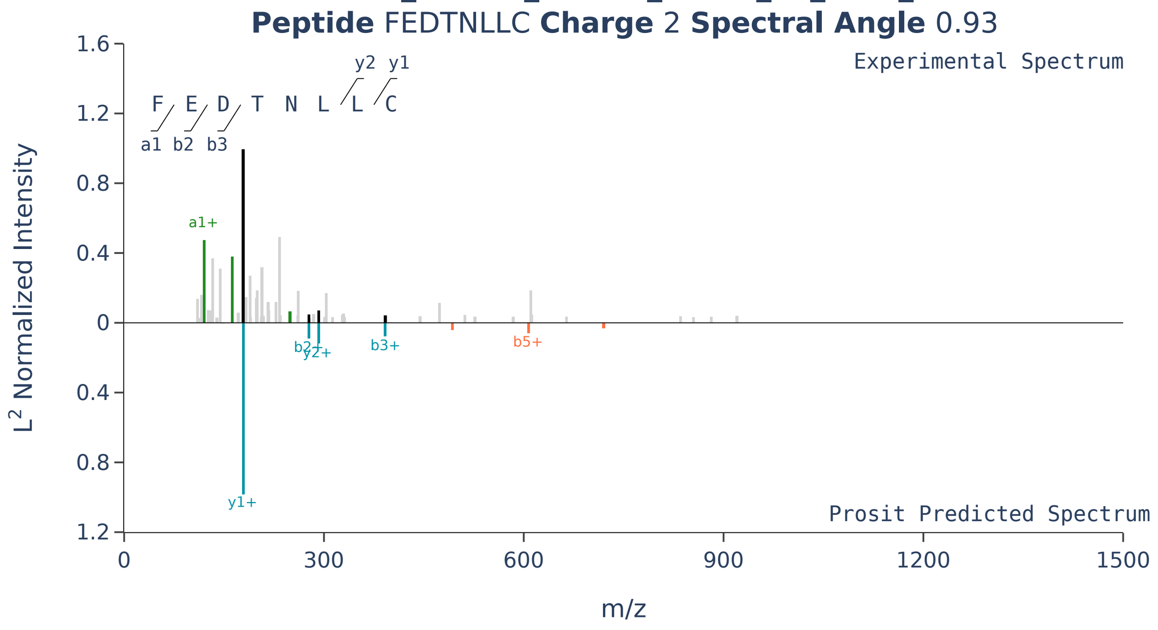

Source HRoetschke_271222_120123_HFGoe_H3_b2R2_24h **Scan** 10974
Peptide KTDLAIHA **Charge** 2 **Spectral Angle** 0.87

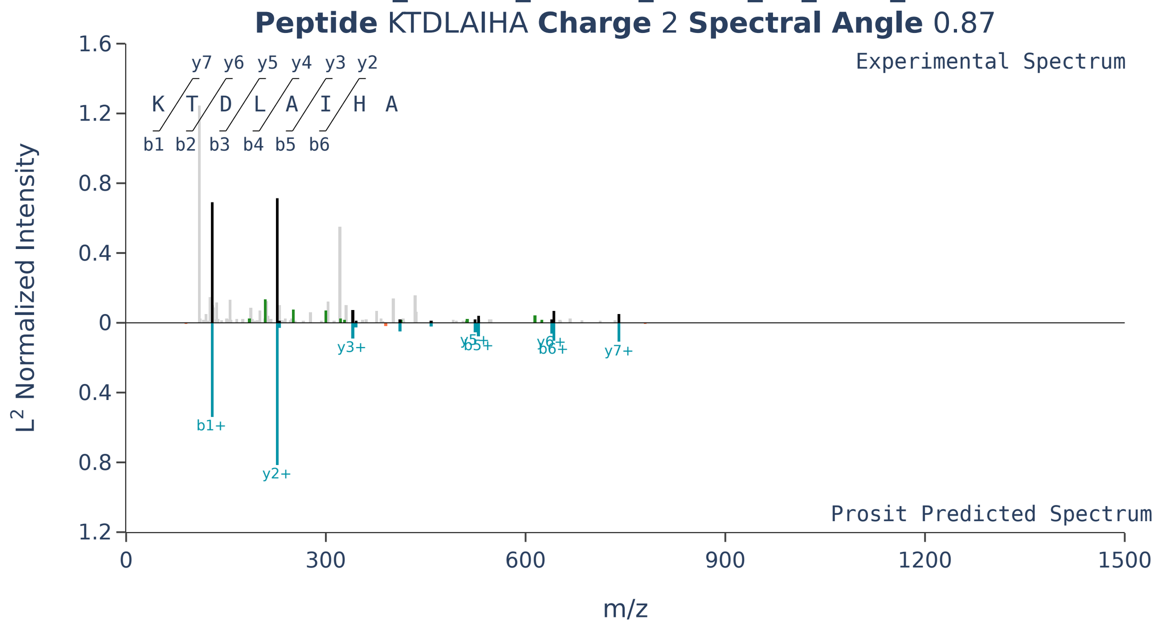

Source HRoetschke_271222_120123_HFGoe_H3_b2R1_2h **Scan** 13325
Peptide RGEAYLVA **Charge** 2 **Spectral Angle** 0.94

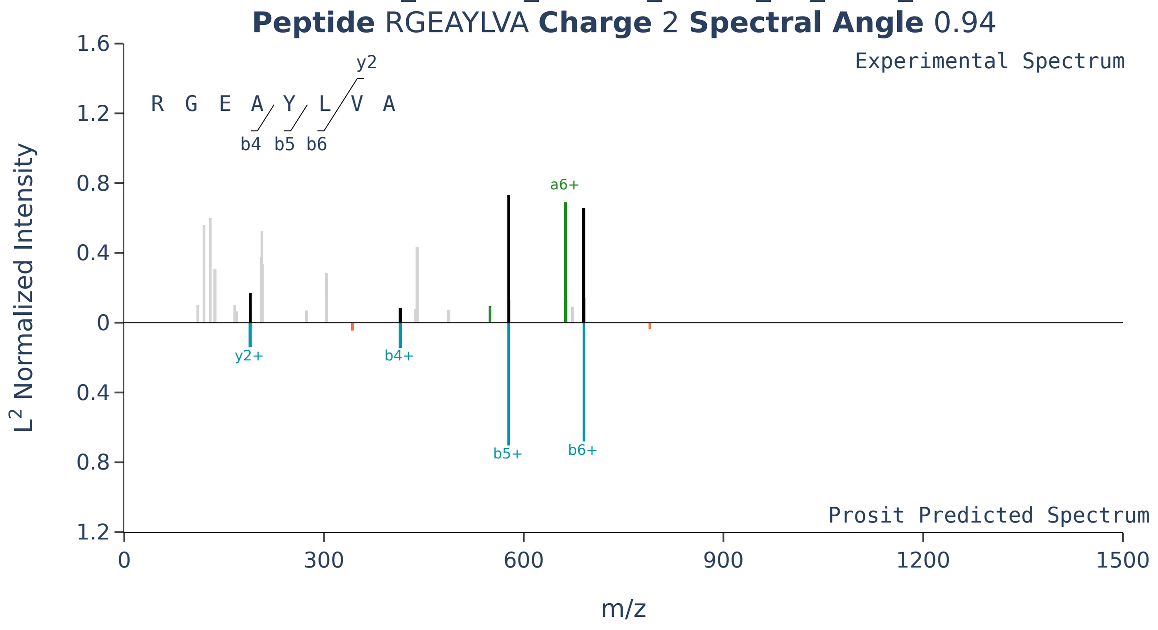

Source HRoetschke_271222_120123_HFGoe_H3_b2R1_24h **Scan** 12071
Peptide LFEDTRRY **Charge** 3 **Spectral Angle** 0.94

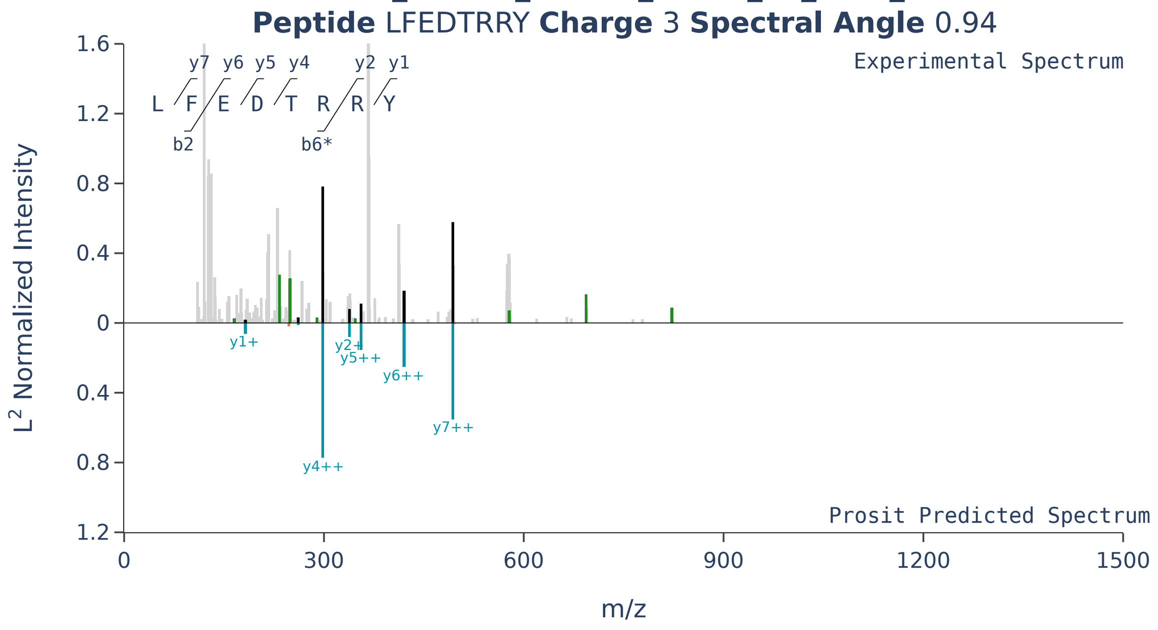

Source HRoetschke_271222_120123_HFGoe_H3_b2R2_4h **Scan** 14042
Peptide AQDFKTDLA **Charge** 1 **Spectral Angle** 0.85

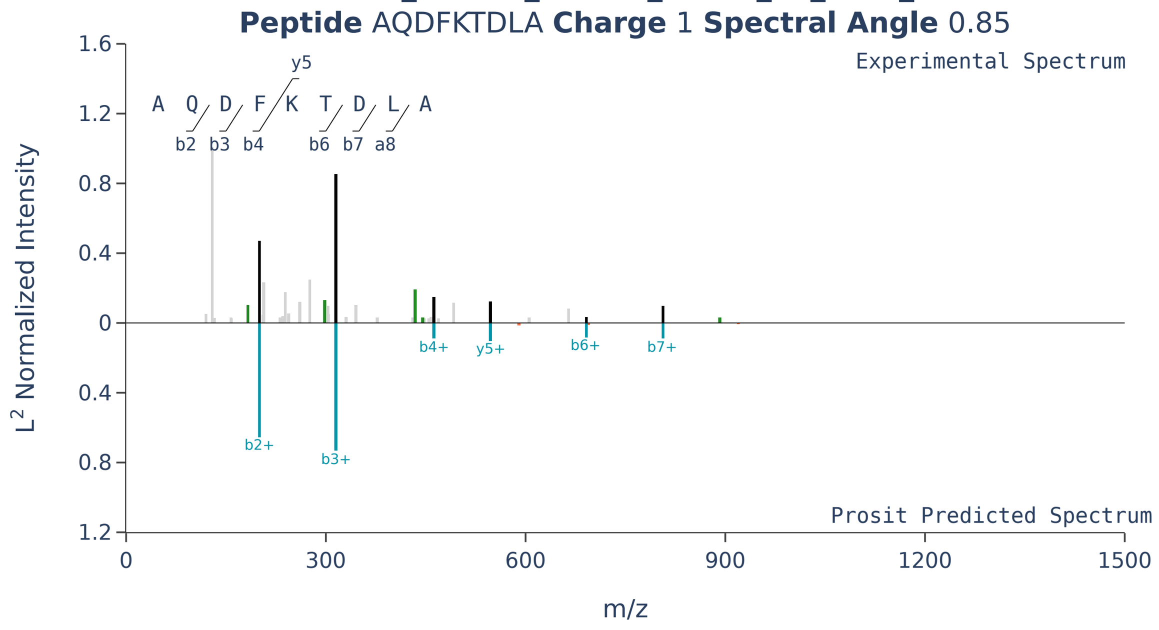

Source HRoetschke_271222_120123_HFGoe_H3_b1R1_24h **Scan** 13868
Peptide GERAYLV **Charge** 2 **Spectral Angle** 0.82

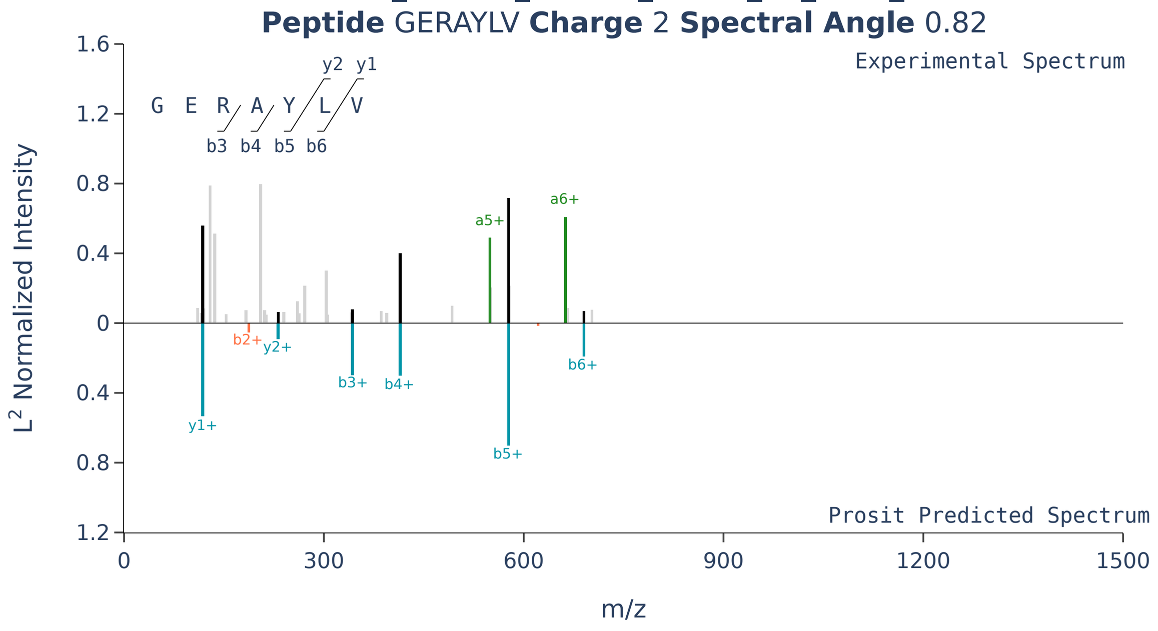

Source HRoetschke_271222_120123_HFGoe_H3_b2R1_4h **Scan** 12837
Peptide VREIAQDFKTA **Charge** 2 **Spectral Angle** 0.9

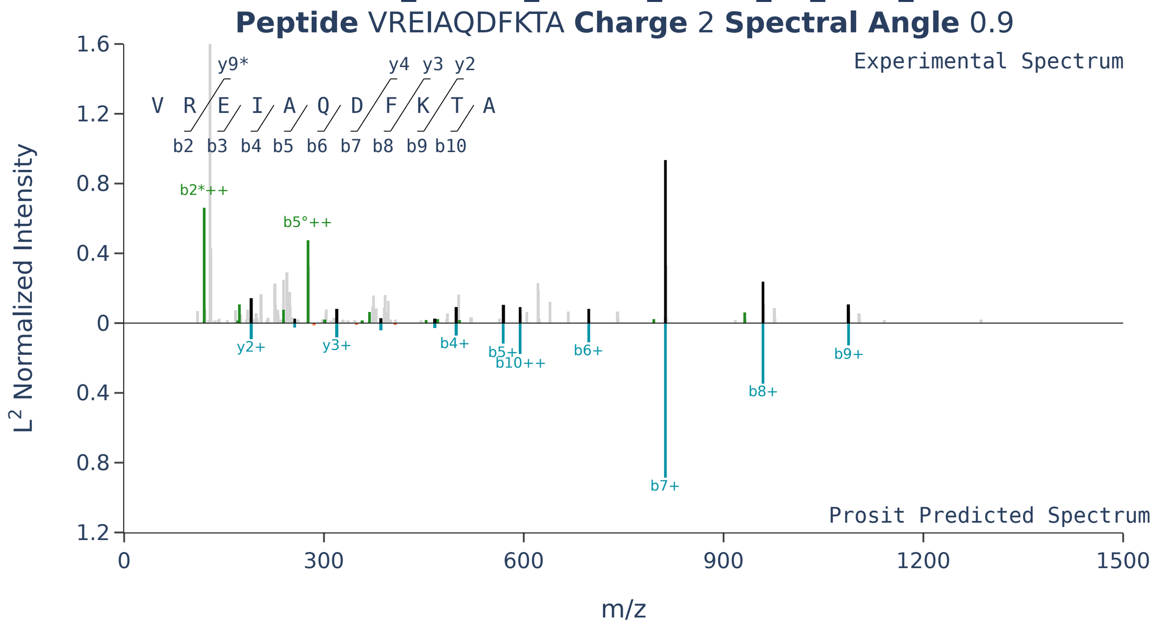

Source HRoetschke_271222_120123_HFGoe_H3_b2R2_24h **Scan** 12098
Peptide ARTKQTALFE **Charge** 3 **Spectral Angle** 0.88

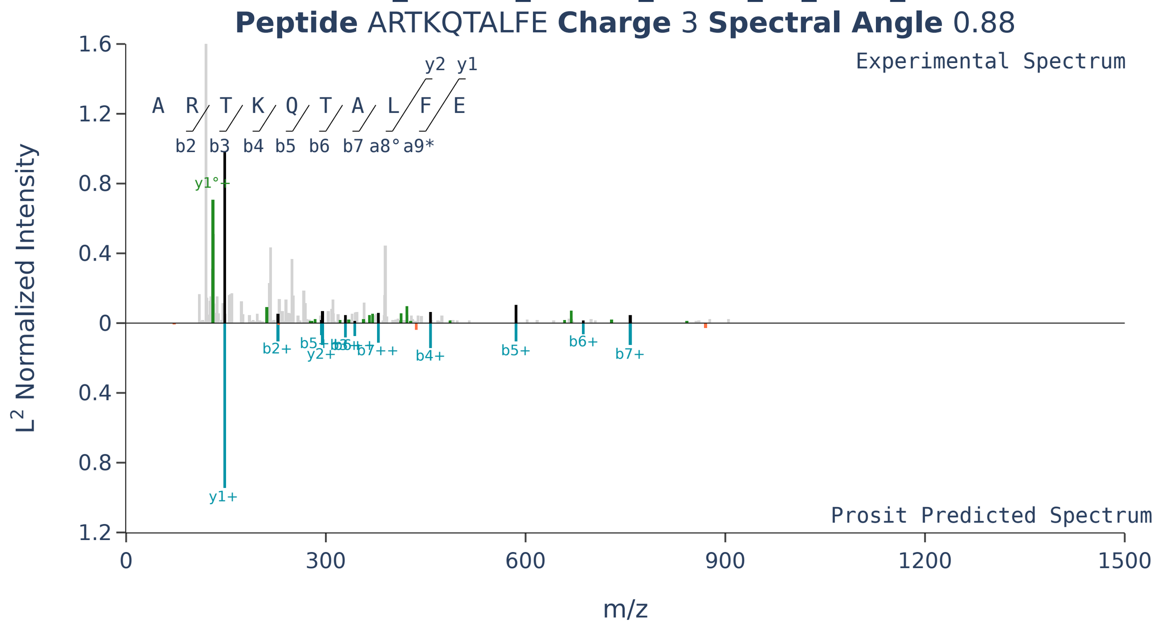

Source HRoetschke_271222_120123_HFGoe_H3_b2R1_4h **Scan** 12245
Peptide LFEDTRYR **Charge** 3 **Spectral Angle** 0.87

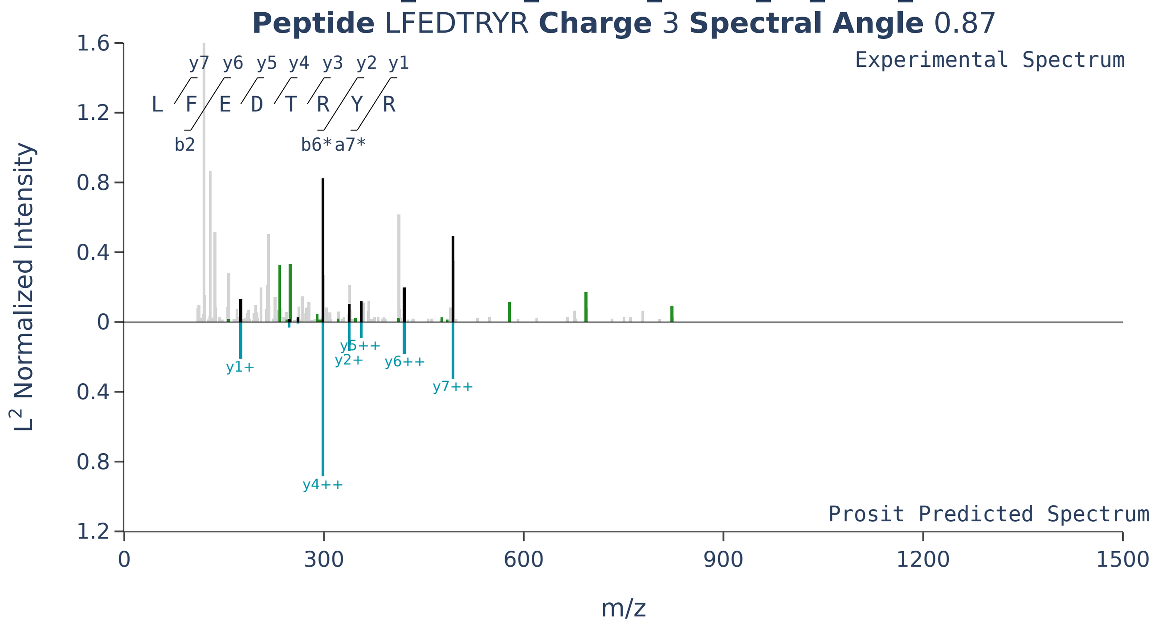

Source HRoetschke_271222_120123_HFGoe_H3_b1R2_2h **Scan** 13813
Peptide GERALFE **Charge** 2 **Spectral Angle** 0.83

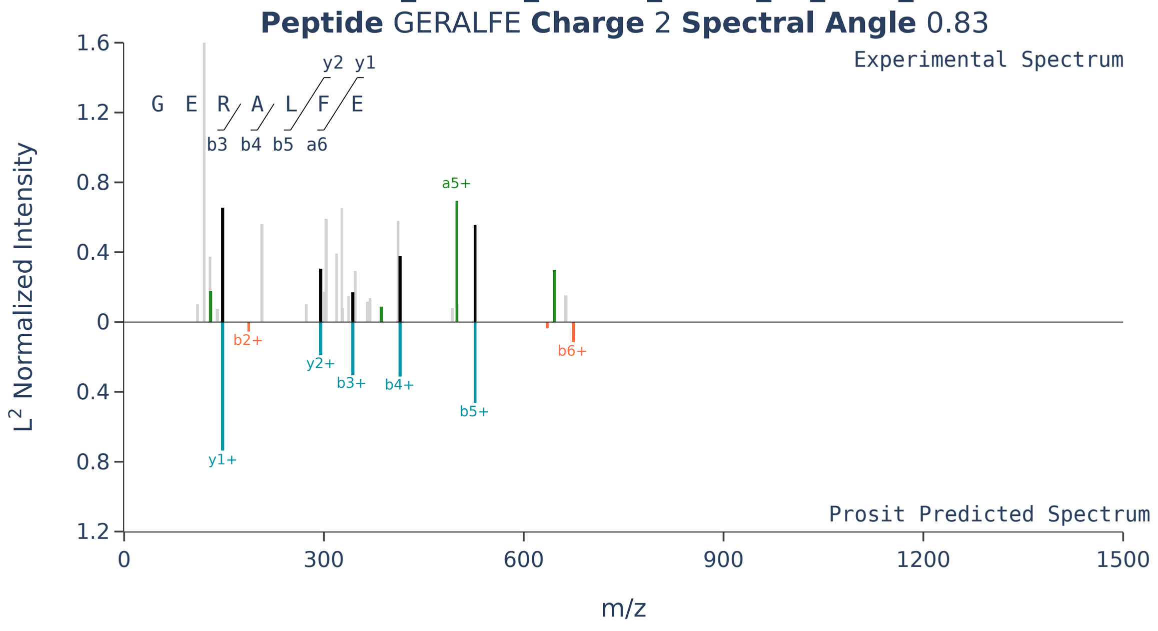

Source HRoetschke_271222_120123_HFGoe_H3_b2R2_2h **Scan** 17215
Peptide DTNLCVREI **Charge** 2 **Spectral Angle** 0.85

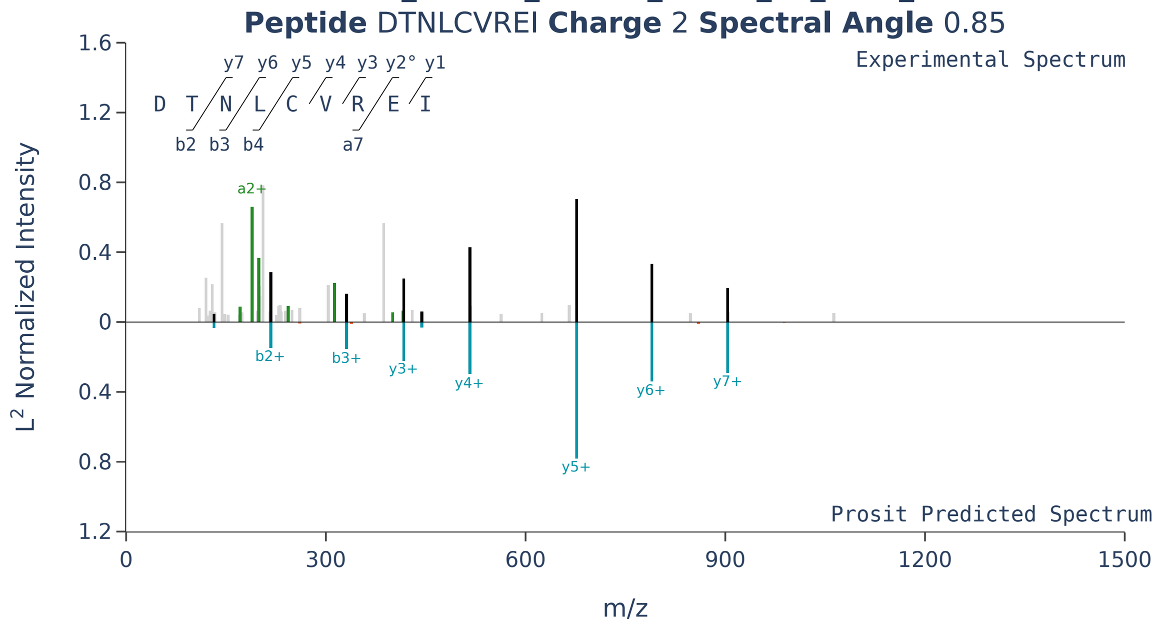

Source HRoetschke_271222_120123_HFGoe_H3_b2R1_4h **Scan** 13950
Peptide DTNLCRFQ **Charge** 2 **Spectral Angle** 0.87

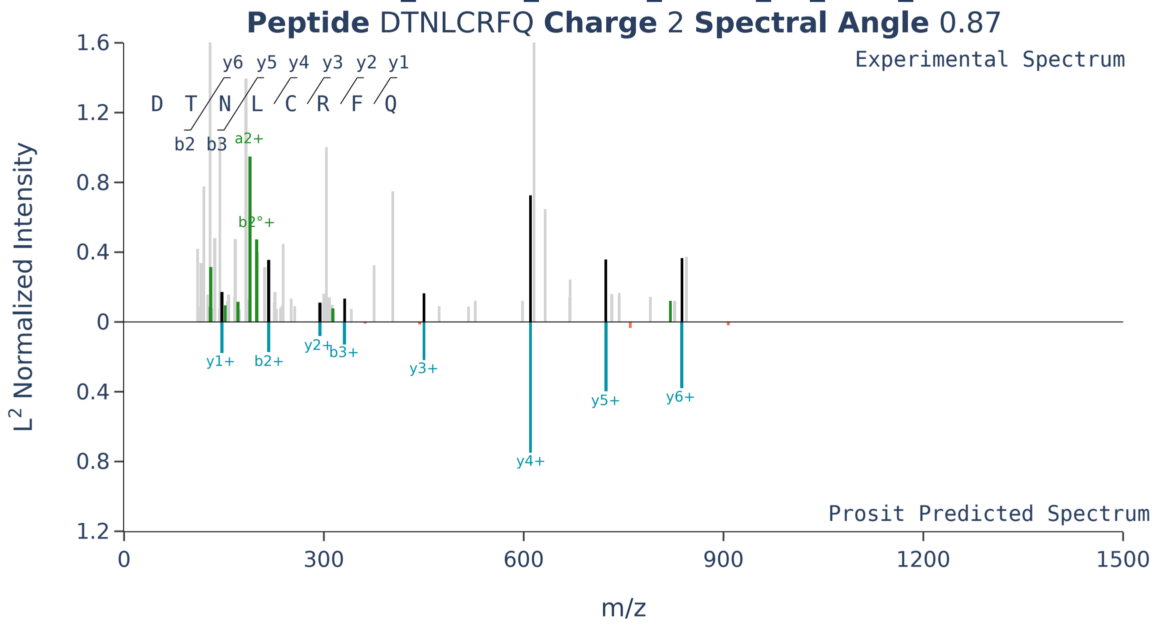

Source HRoetschke_271222_120123_HFGoe_H3_b2R2_2h **Scan** 13314
Peptide AVREIAQDFKT **Charge** 3 **Spectral Angle** 0.85

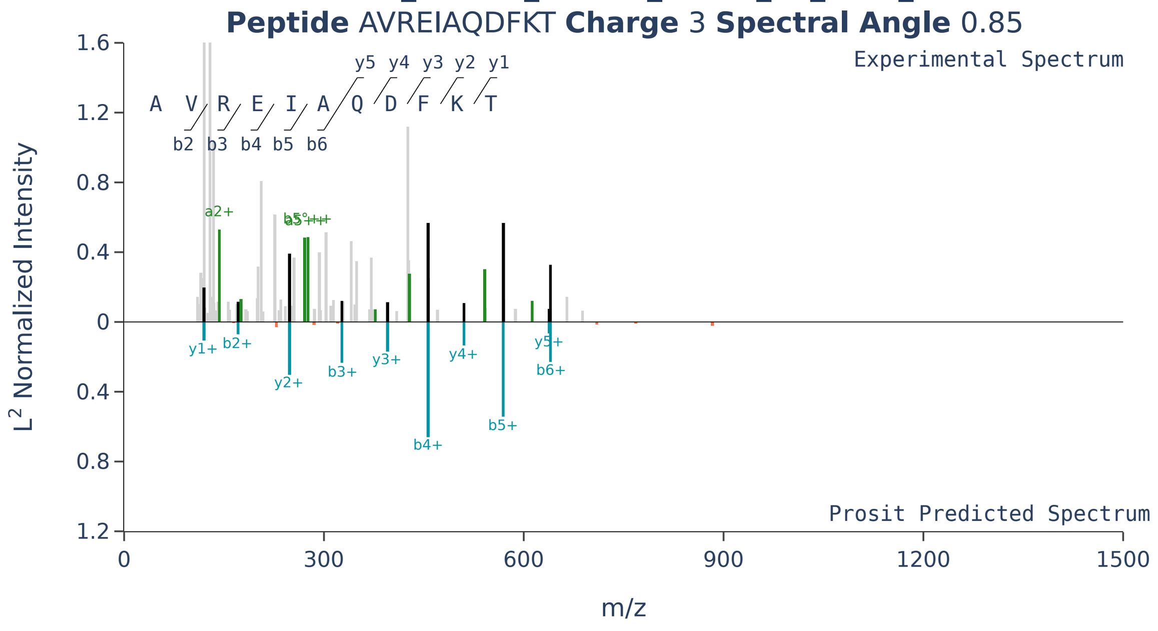

Source HRoetschke_271222_120123_HFGoe_H3_b2R2_2h **Scan** 15120
Peptide RGEALFE **Charge** 2 **Spectral Angle** 0.83

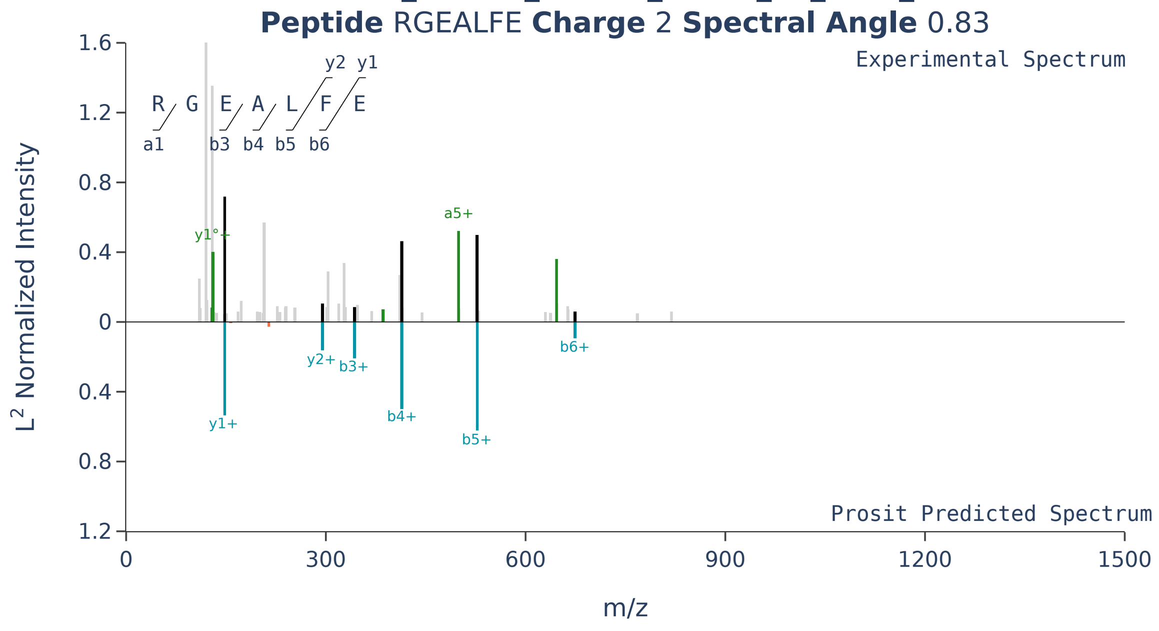

Source HRoetschke_271222_120123_HFGoe_H3_b2R1_4h **Scan** 14659
Peptide AQDFKTDAL **Charge** 1 **Spectral Angle** 0.83

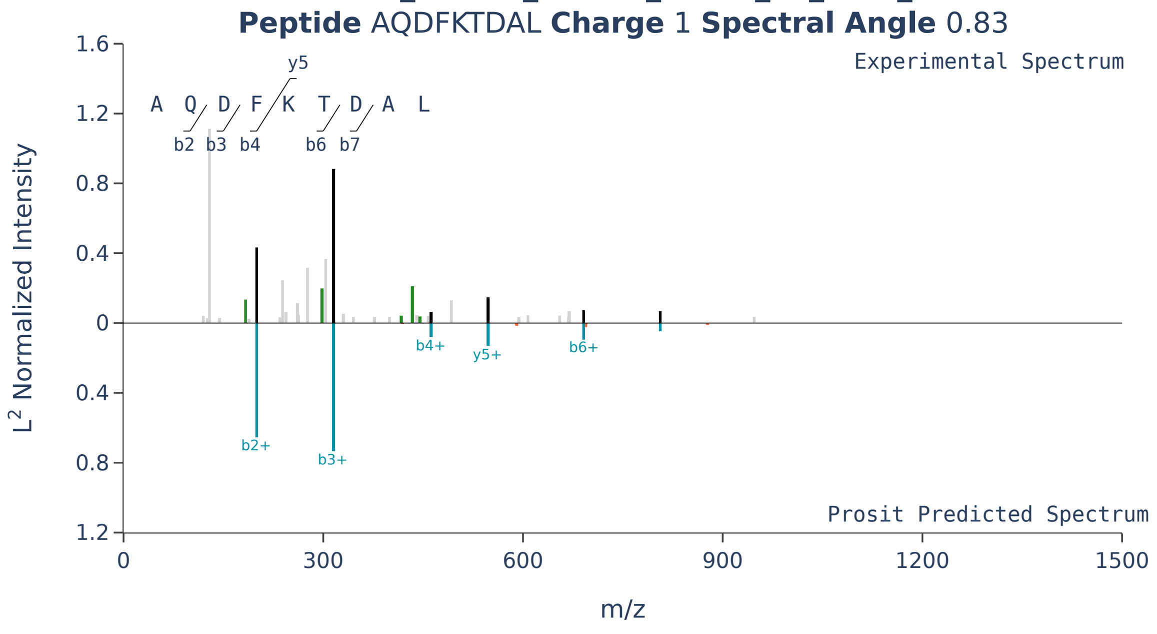

Source HRoetschke_271222_120123_HFGoe_H3_b2R1_24h **Scan** 12008
Peptide RFQSSAVREI **Charge** 3 **Spectral Angle** 0.88

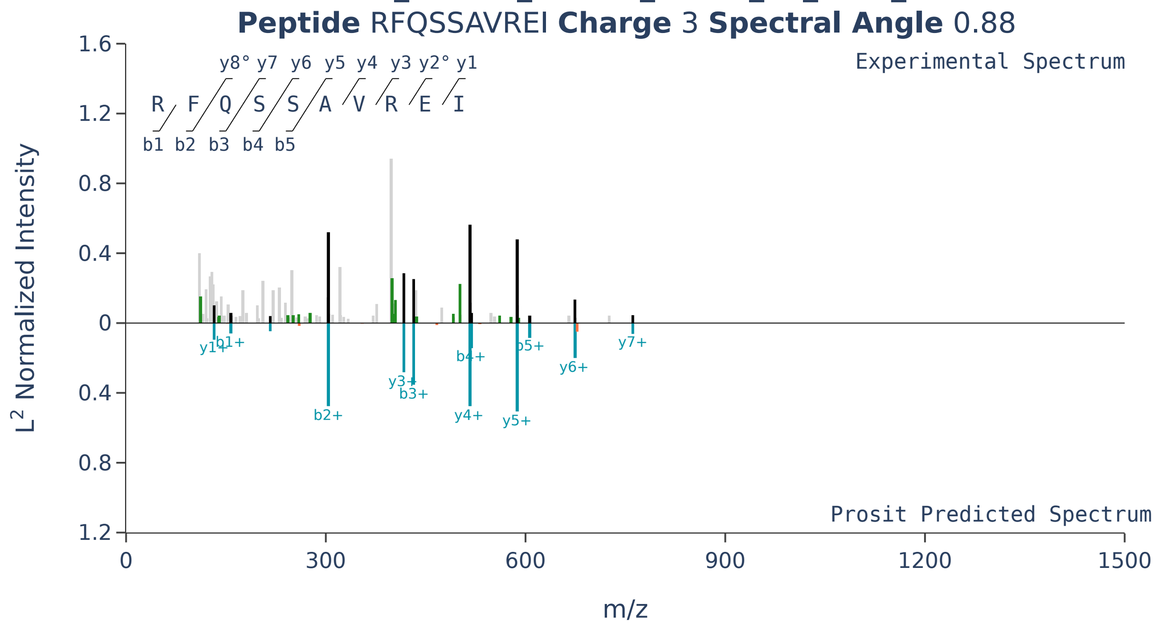

Source HRoetschke_271222_120123_HFGoe_H3_b1R1_24h **Scan** 13802
Peptide AQDFKTDLRY **Charge** 3 **Spectral Angle** 0.87

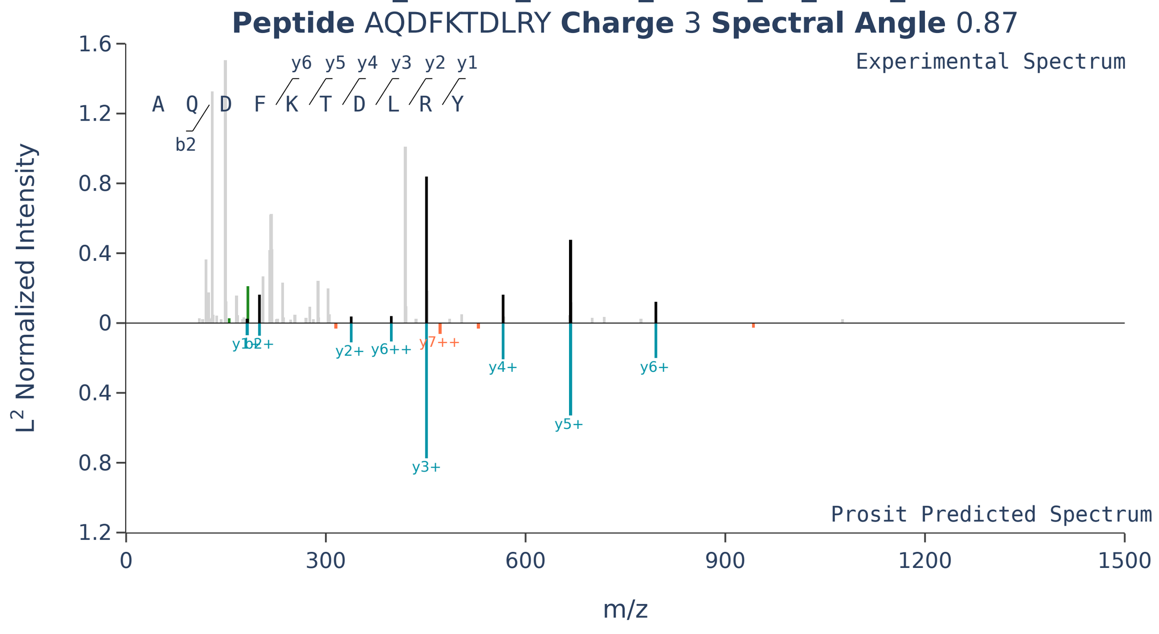

Source HRoetschke_271222_120123_HFGoe_H3_b1R2_24h **Scan** 10798
Peptide DTNLCVRE **Charge** 2 **Spectral Angle** 0.87

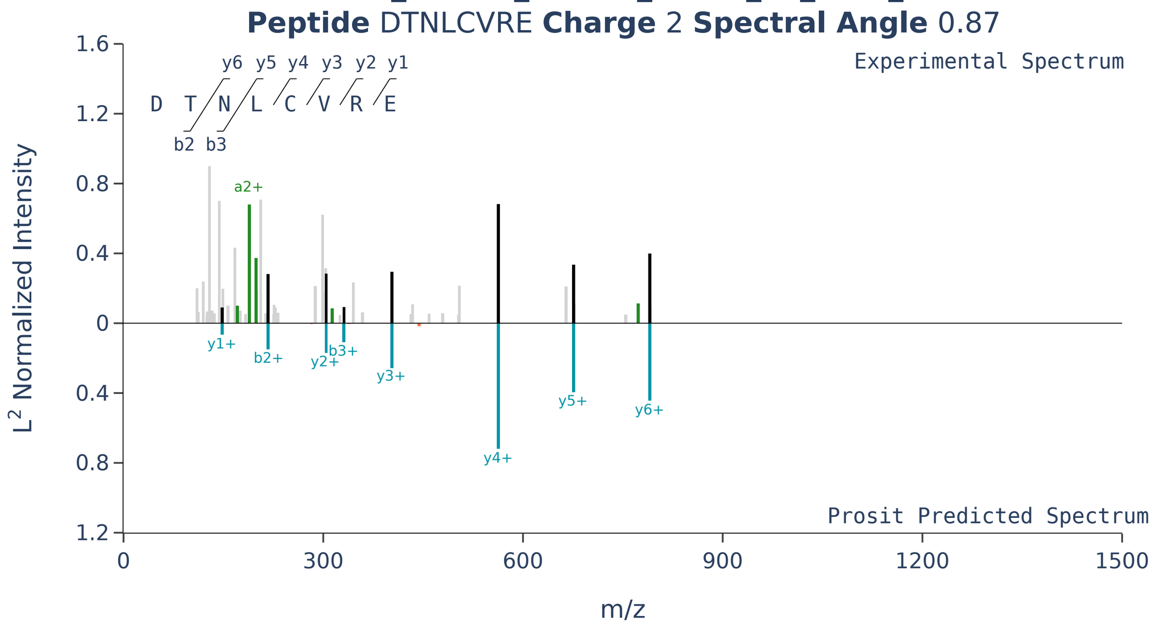

Source HRoetschke_271222_120123_HFGoe_H3_b2R2_24h **Scan** 9423
Peptide LREAIHA **Charge** 2 **Spectral Angle** 0.93

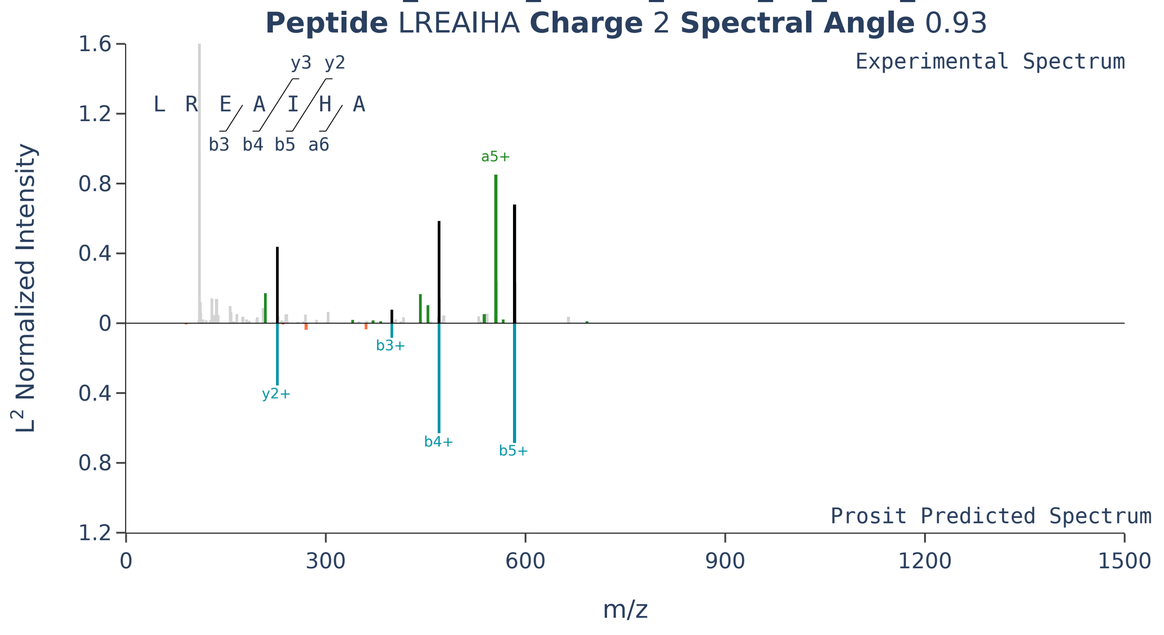

Source HRoetschke_271222_120123_HFGoe_H3_b1R2_24h **Scan** 9482
Peptide EIRAIHA **Charge** 2 **Spectral Angle** 0.84

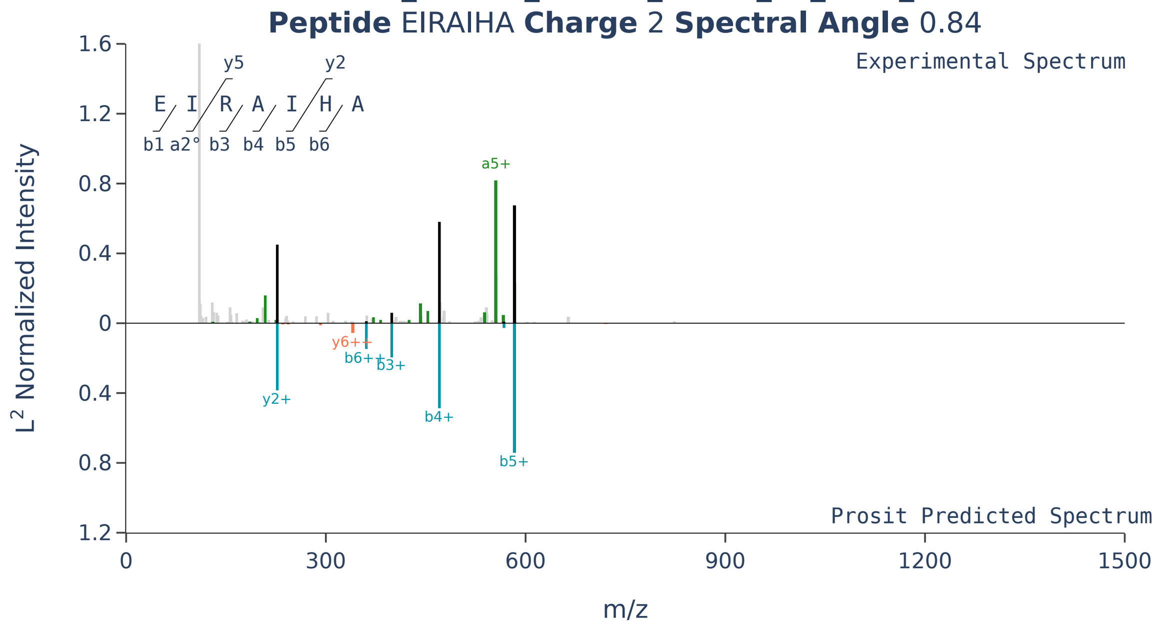

Source HRoetschke_271222_120123_HFGoe_H3_b1R2_2h **Scan** 16251
Peptide SEAAYLVA **Charge** 2 **Spectral Angle** 0.78

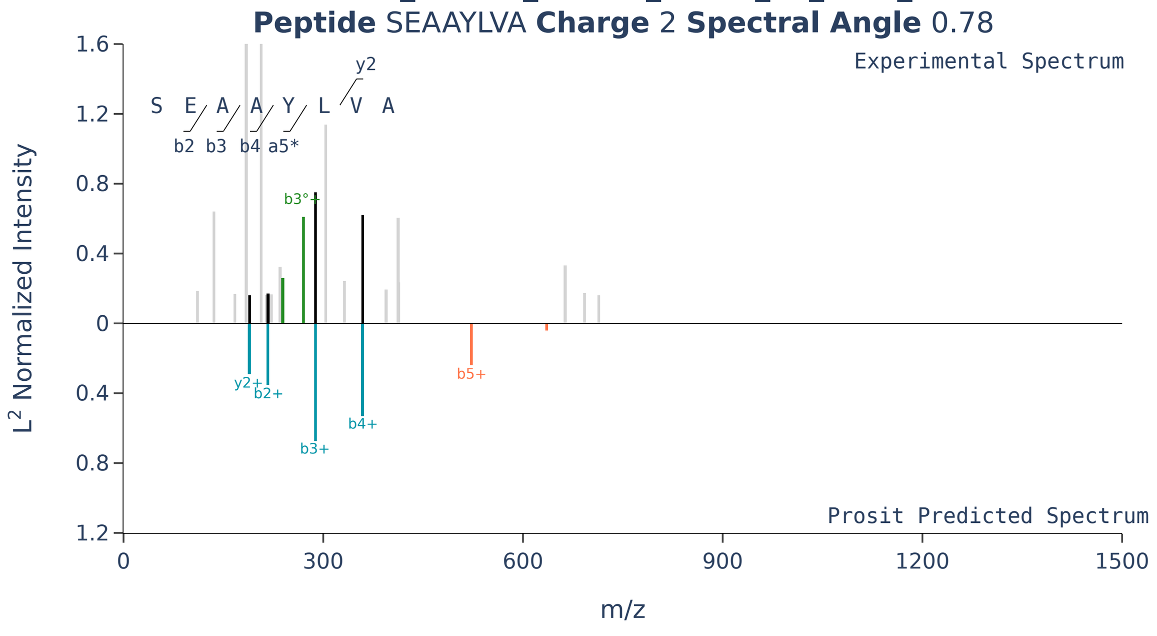

Source HRoetschke_271222_120123_HFGoe_H3_b2R2_2h **Scan** 10913
Peptide RFQSSDL **Charge** 2 **Spectral Angle** 0.83

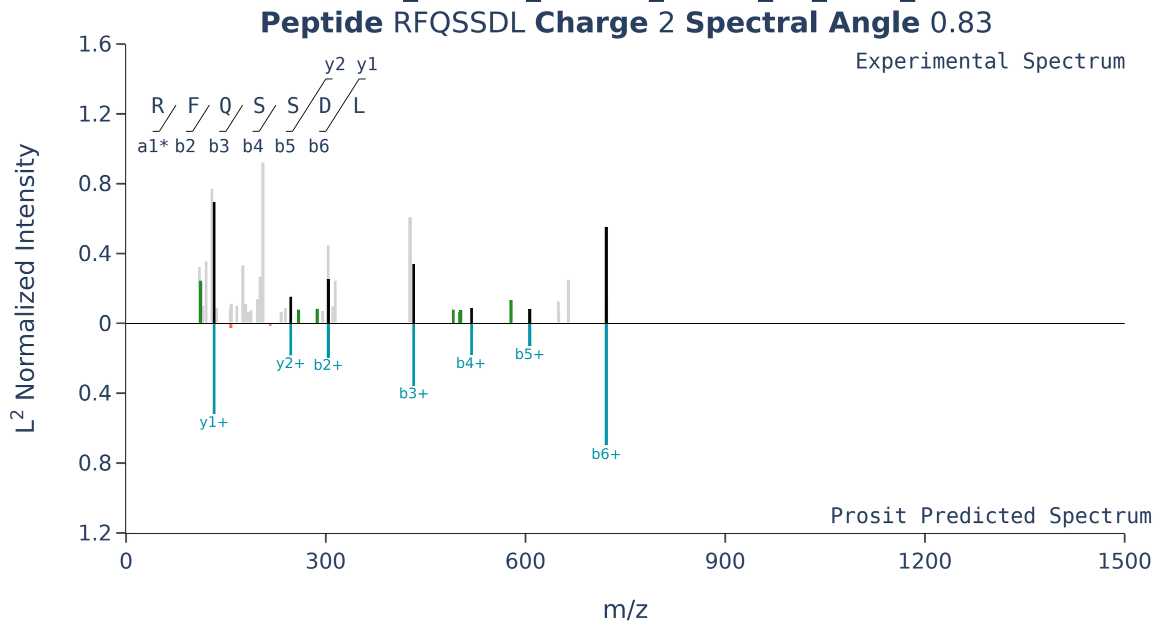

Source HRoetschke_271222_120123_HFGoe_H3_b2R2_4h **Scan** 18845
Peptide DTNFKTDLRF **Charge** 3 **Spectral Angle** 0.87

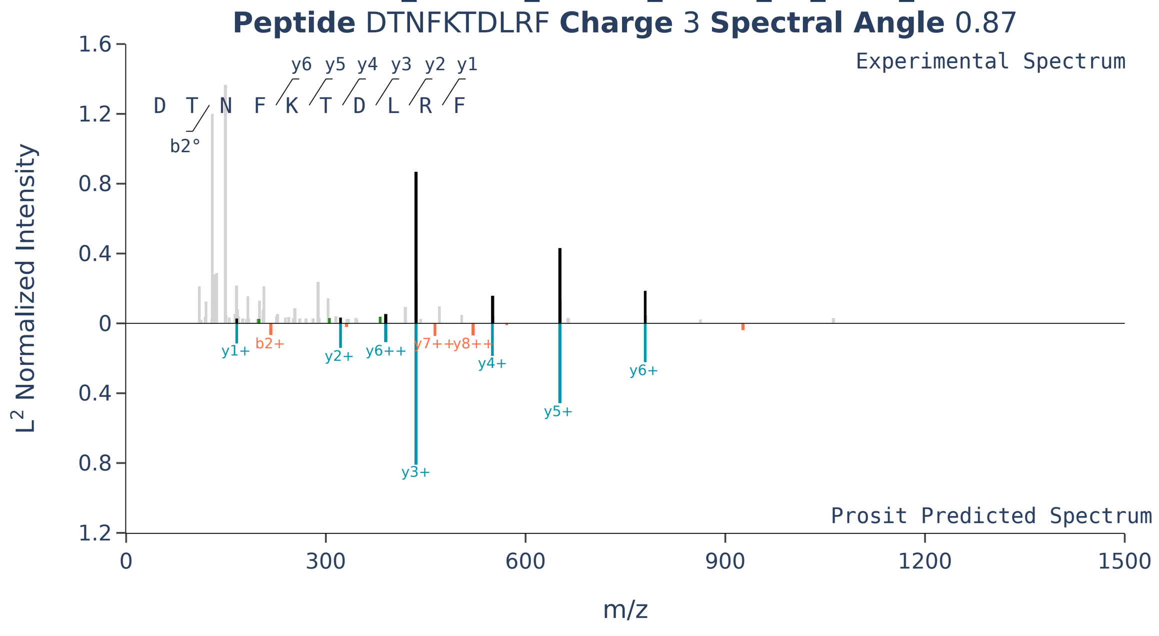

Source HRoetschke_271222_120123_HFGoe_H3_b2R1_4h **Scan** 12516
Peptide RFQSSTIMPKD **Charge** 3 **Spectral Angle** 0.9

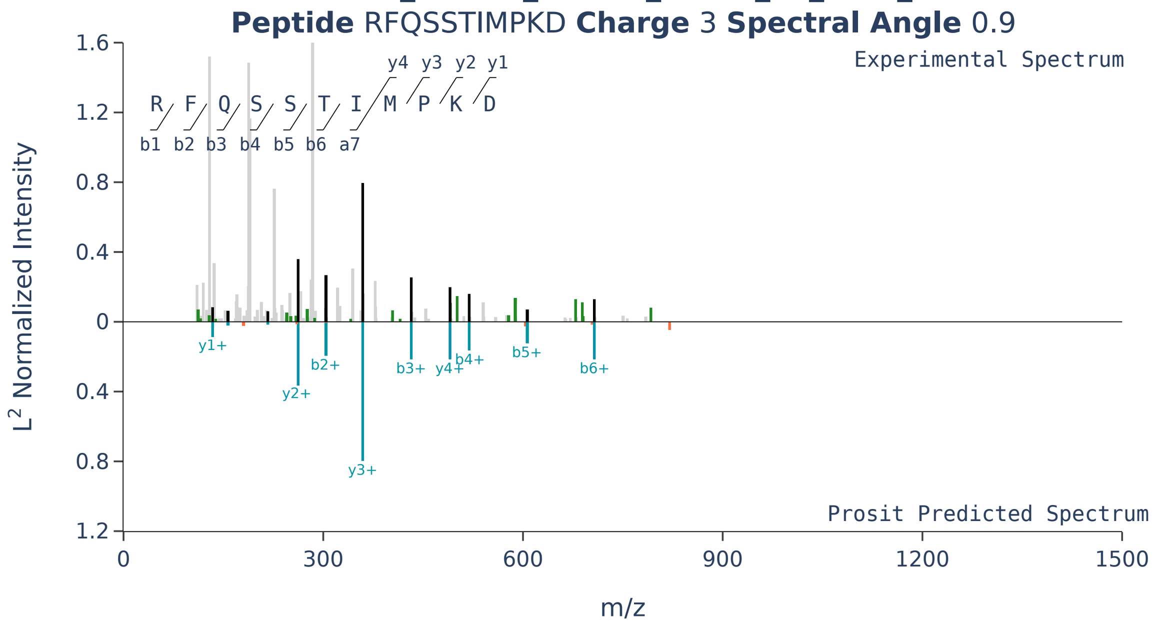

Source HRoetschke_271222_120123_HFGoe_H3_b2R2_24h **Scan** 12518
Peptide RFQSSAQDF **Charge** 2 **Spectral Angle** 0.78

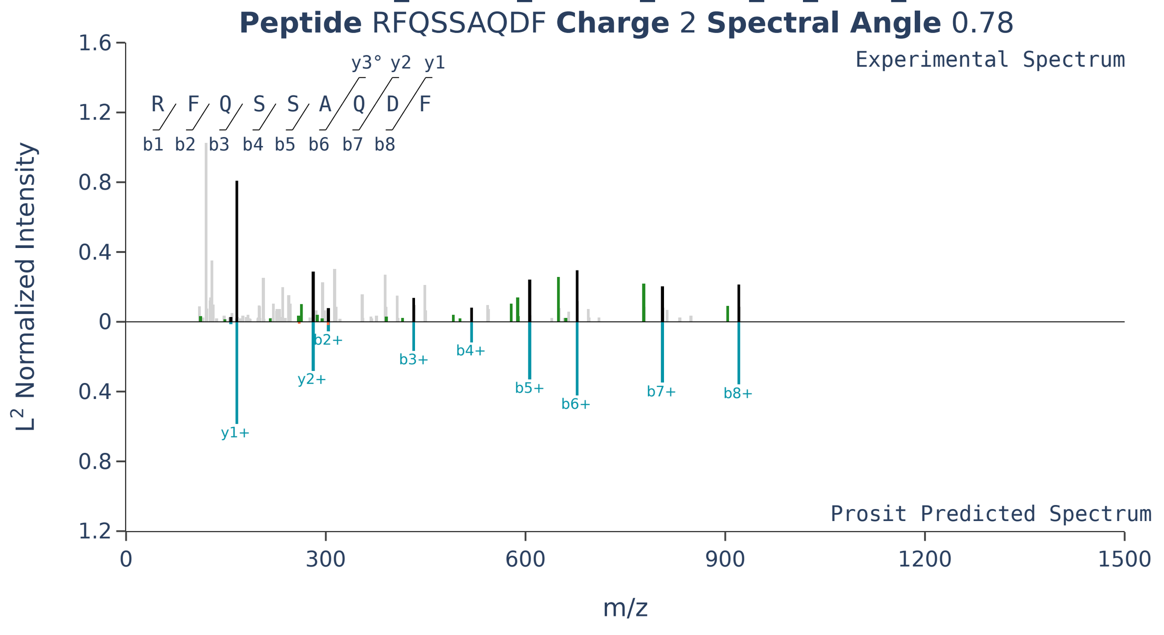

Source HRoetschke_271222_120123_HFGoe_H3_b1R2_24h **Scan** 12258
Peptide AQDFKTAIHA **Charge** 3 **Spectral Angle** 0.94

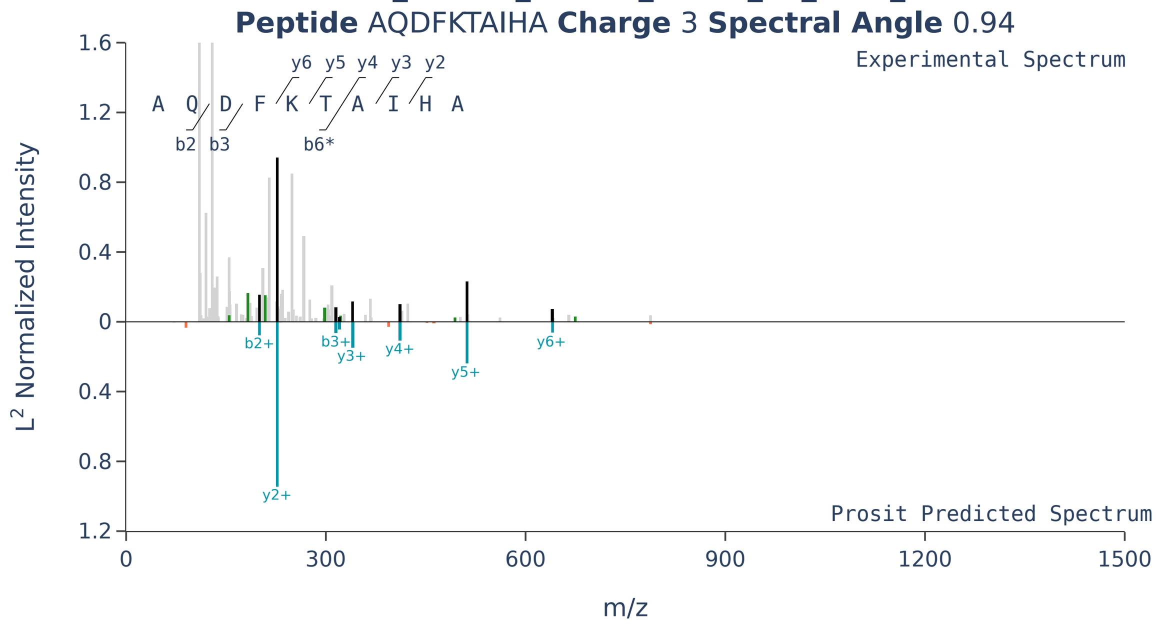

Source HRoetschke_271222_120123_HFGoe_H3_b2R1_24h **Scan** 10770
Peptide RFQSSALQE **Charge** 2 **Spectral Angle** 0.86

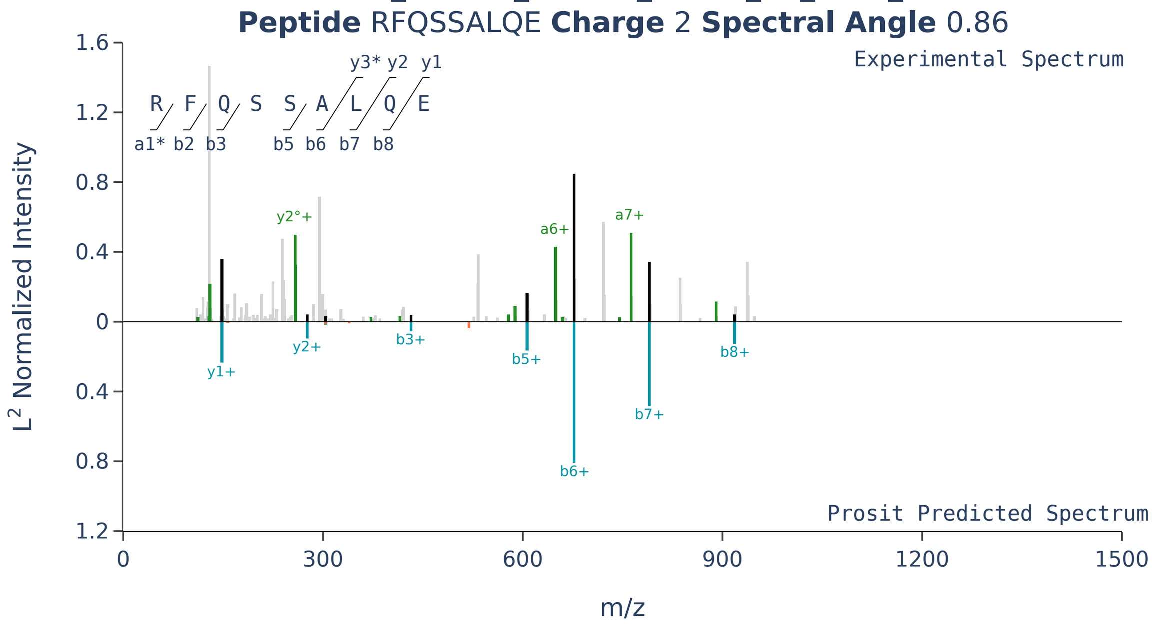

Source HRoetschke_271222_120123_HFGoe_H3_b2R1_4h **Scan** 14654
Peptide SREIAQDF **Charge** 2 **Spectral Angle** 0.83

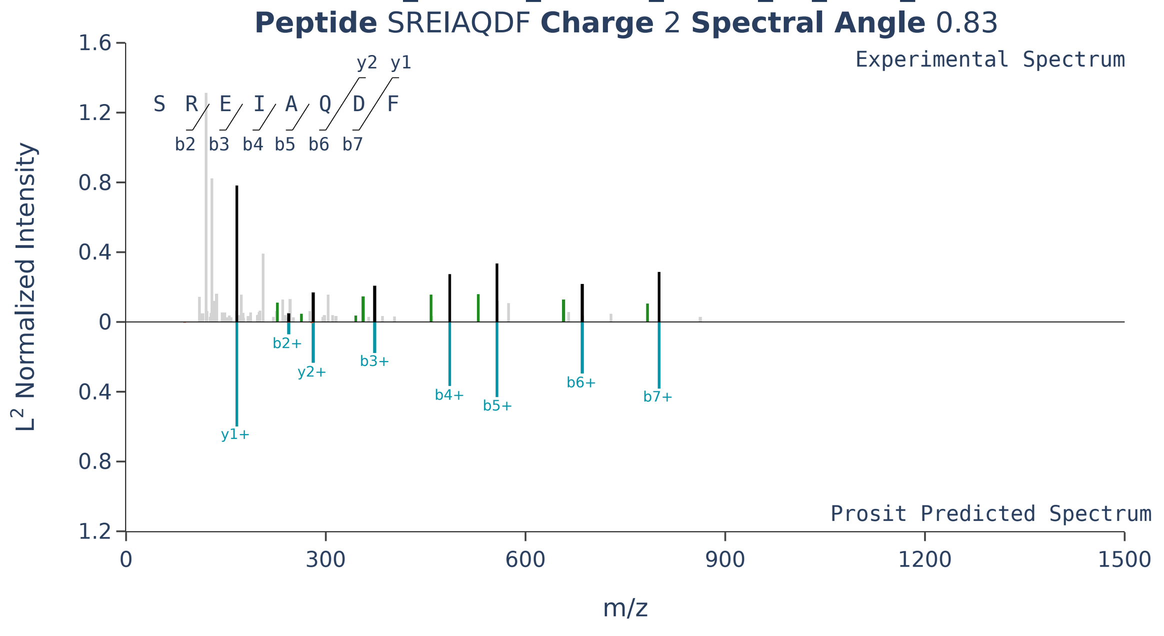

Source HRoetschke_271222_120123_HFGoe_H3_b2R1_4h **Scan** 10677
Peptide IHALVRE **Charge** 2 **Spectral Angle** 0.87

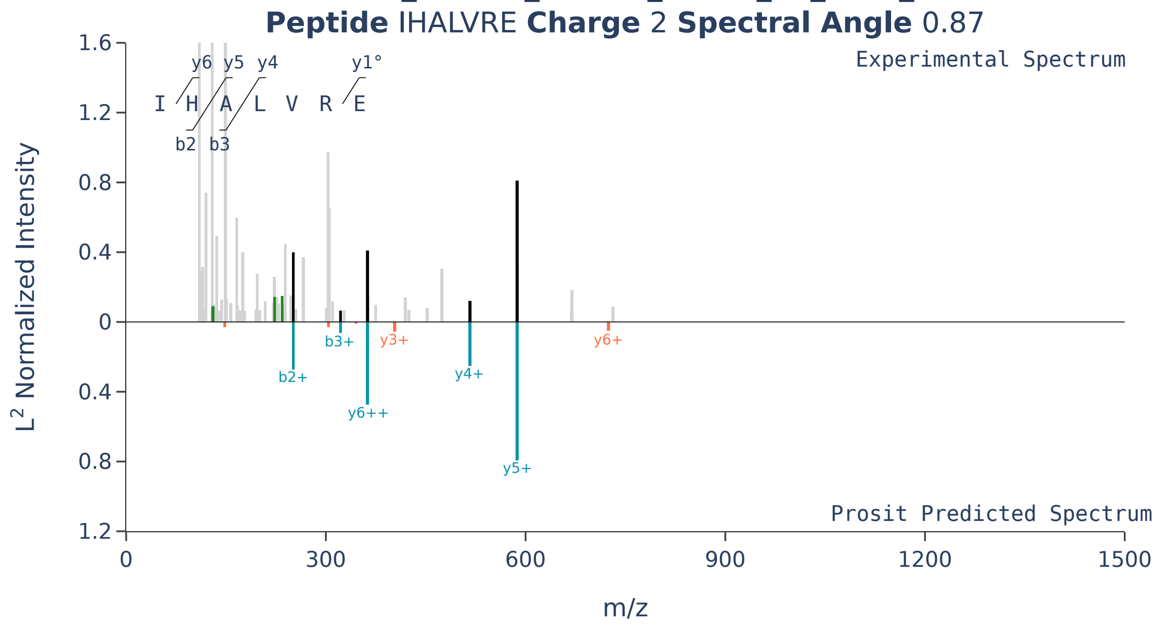

Source HRoetschke_271222_120123_HFGoe_H3_b2R2_2h **Scan** 8494
Peptide IAQDDLR **Charge** 2 **Spectral Angle** 0.8

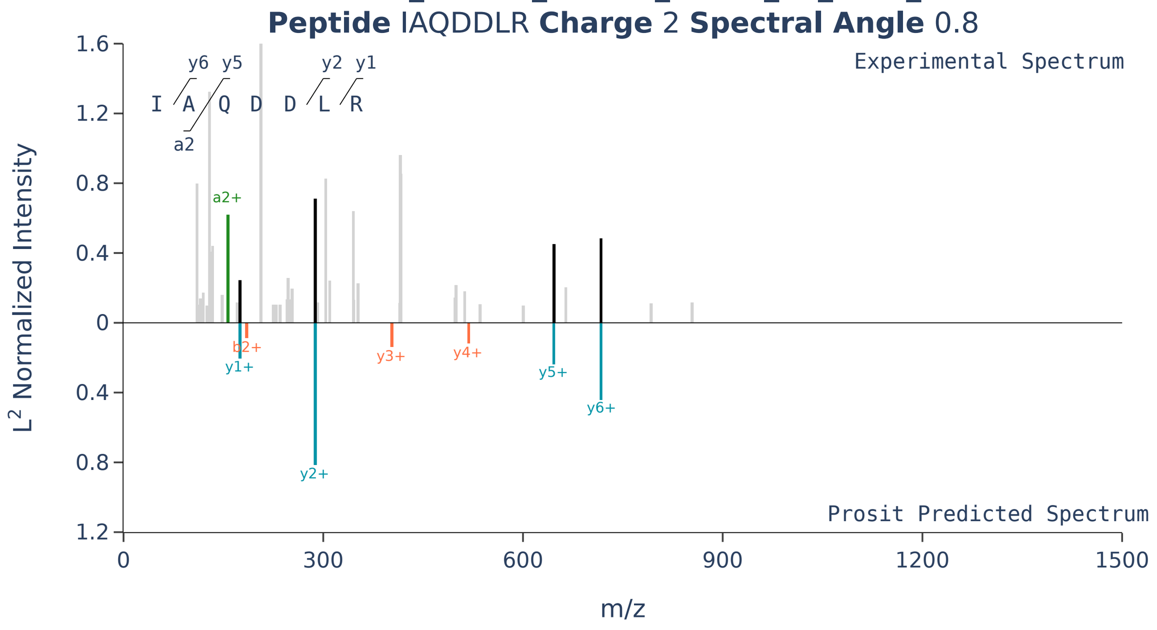

Source HRoetschke_271222_120123_HFGoe_H3_b2R2_1h **Scan** 7873
Peptide RFQSSAY **Charge** 2 **Spectral Angle** 0.9

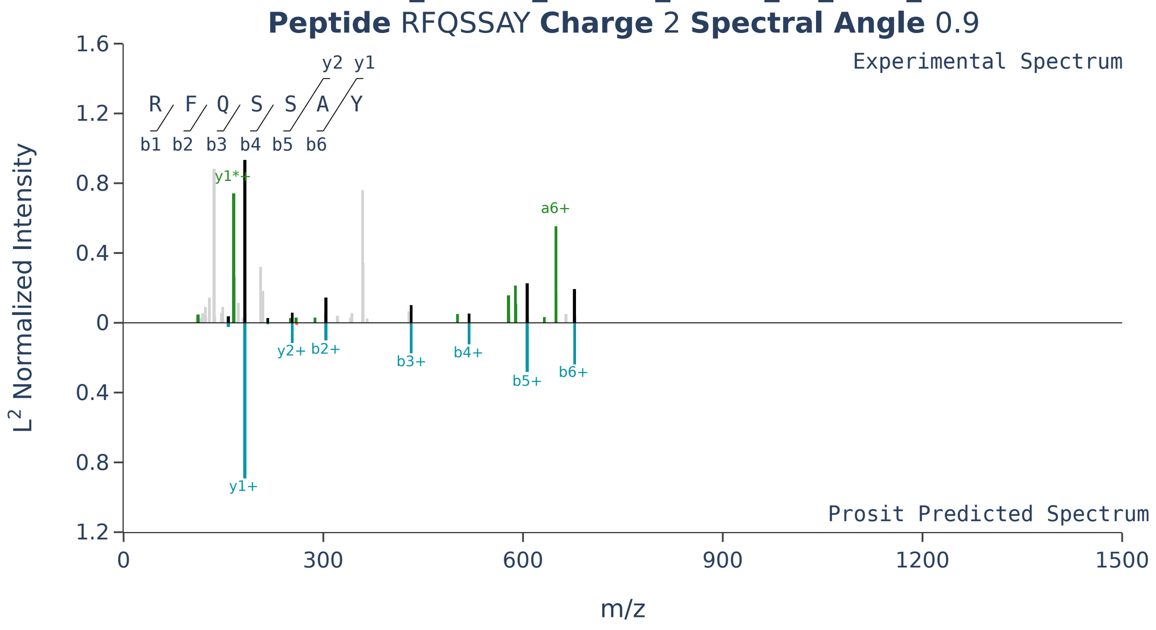

Source HRoetschke_271222_120123_HFGoe_H3_b1R1_4h **Scan** 17738
Peptide SQDFKTDLRF **Charge** 3 **Spectral Angle** 0.86

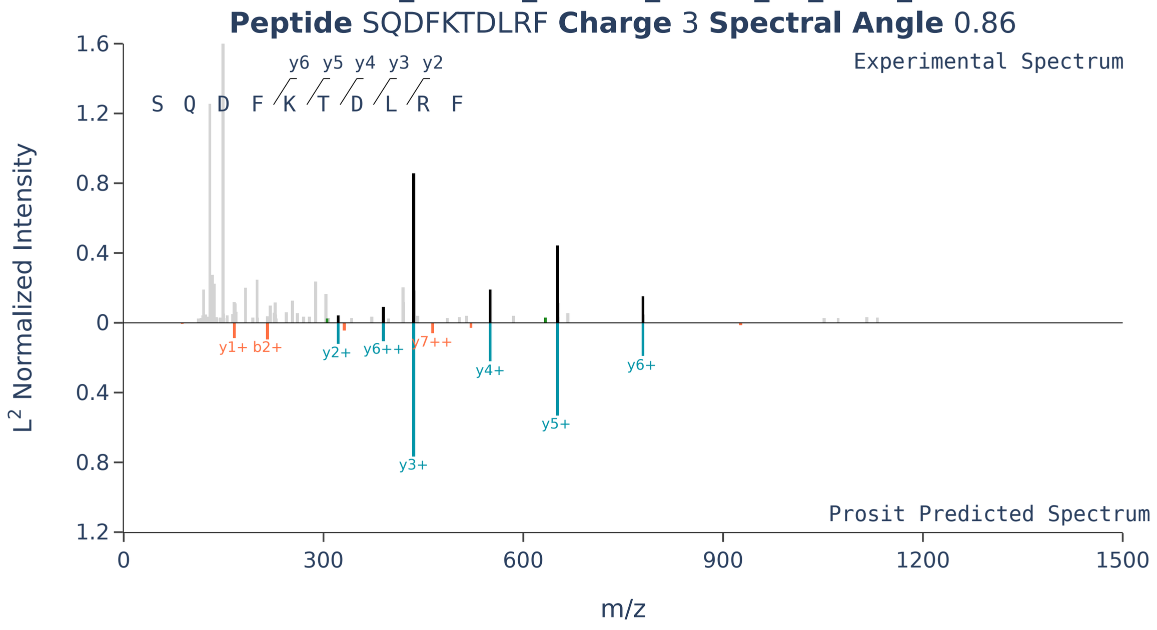

Source HRoetschke_271222_120123_HFGoe_H3_b2R1_4h **Scan** 14462
Peptide DTNLCAIHTA **Charge** 2 **Spectral Angle** 0.81

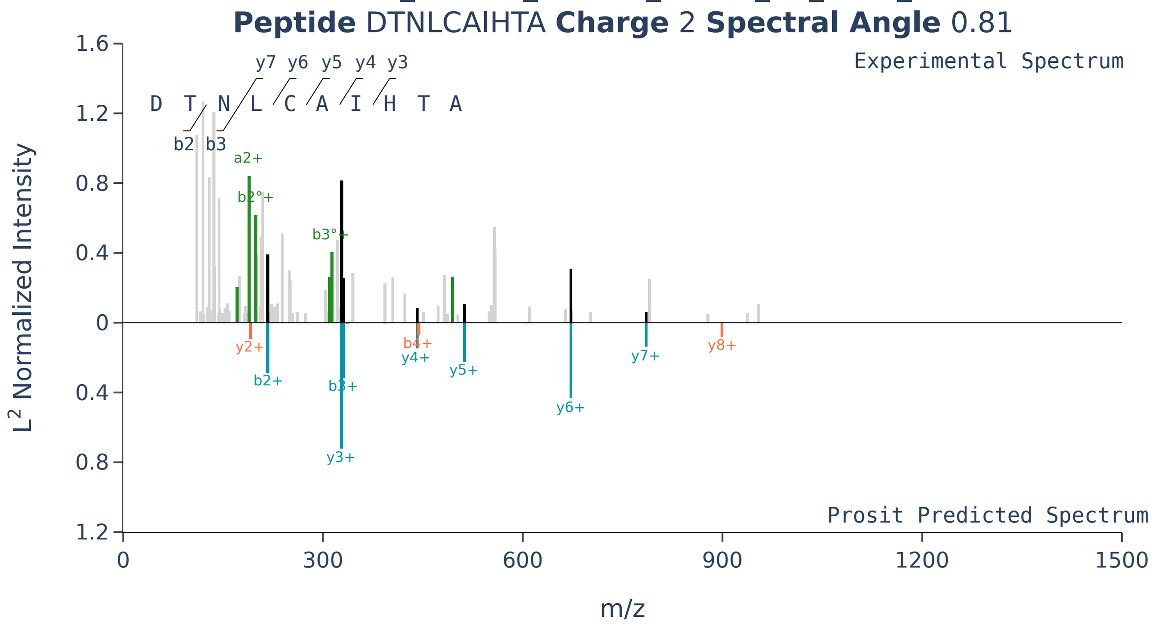

Source HRoetschke_271222_120123_HFGoe_H3_b2R2_2h **Scan** 13922
Peptide PFQRLVQ **Charge** 2 **Spectral Angle** 0.84

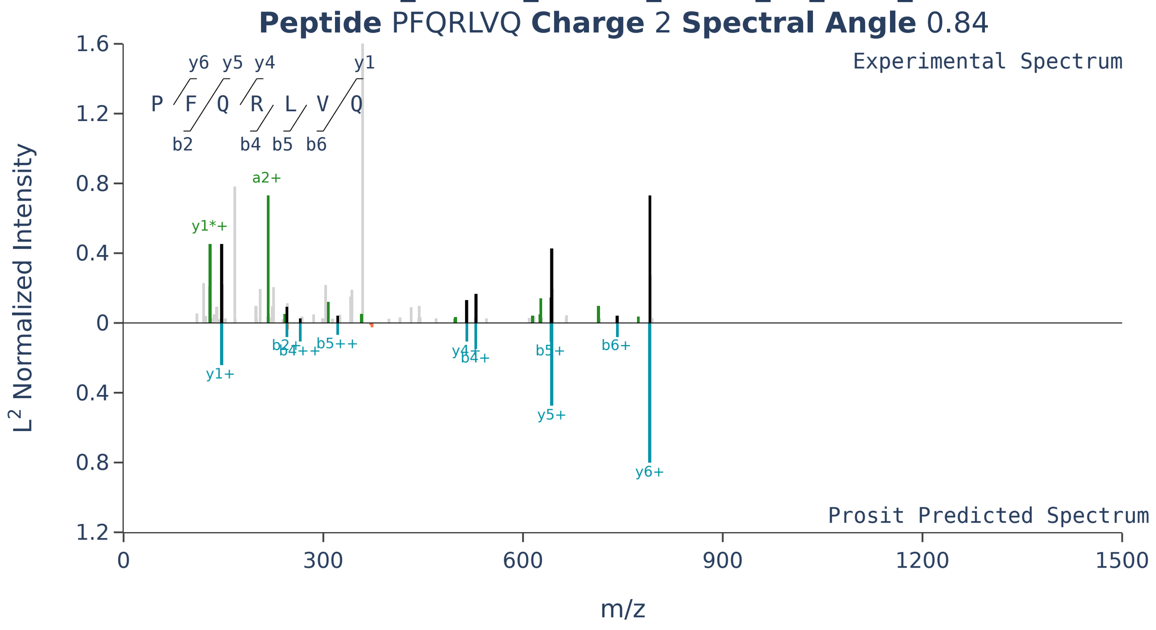

Source HRoetschke_271222_120123_HFGoe_H3_b1R2_4h **Scan** 17449
Peptide RFQSSAYLV **Charge** 2 **Spectral Angle** 0.84

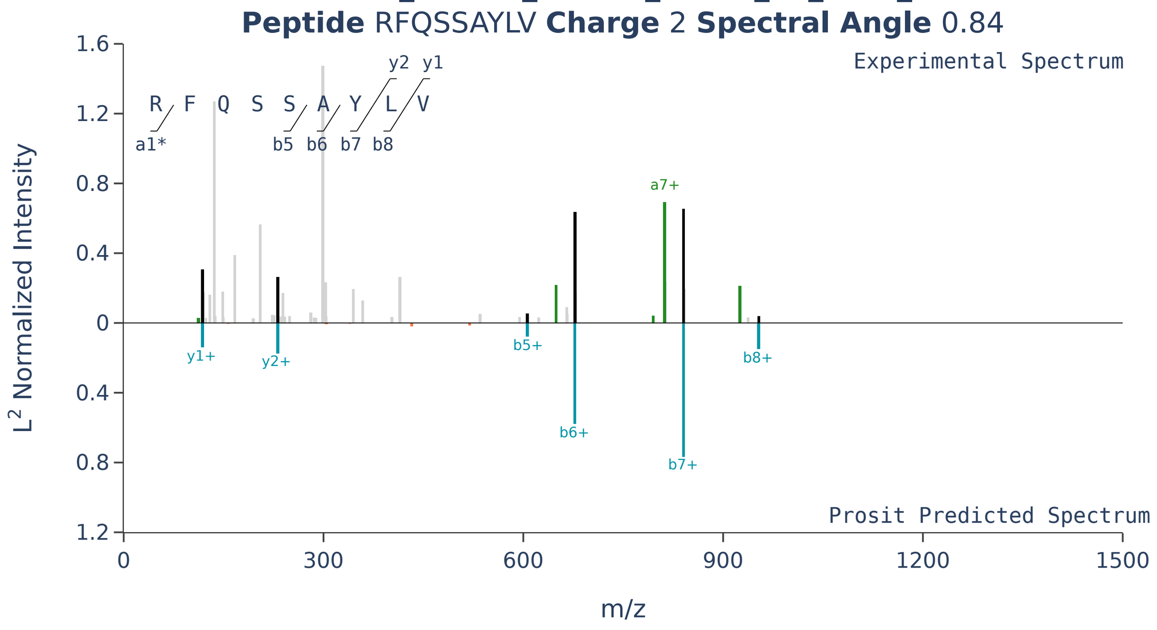

Source HRoetschke_271222_120123_HFGoe_H3_b2R1_4h **Scan** 11399
Peptide RFQSSVREI **Charge** 3 **Spectral Angle** 0.93

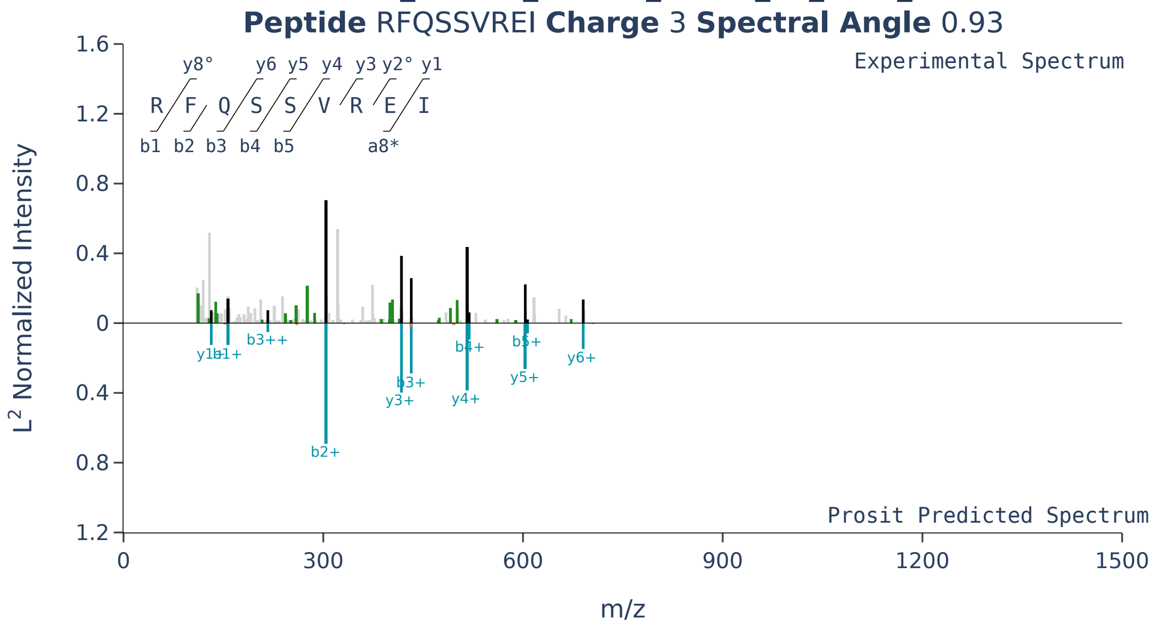

Source HRoetschke_271222_120123_HFGoe_H3_b2R2_4h **Scan** 13410
Peptide AKFEDTNLC **Charge** 2 **Spectral Angle** 0.92

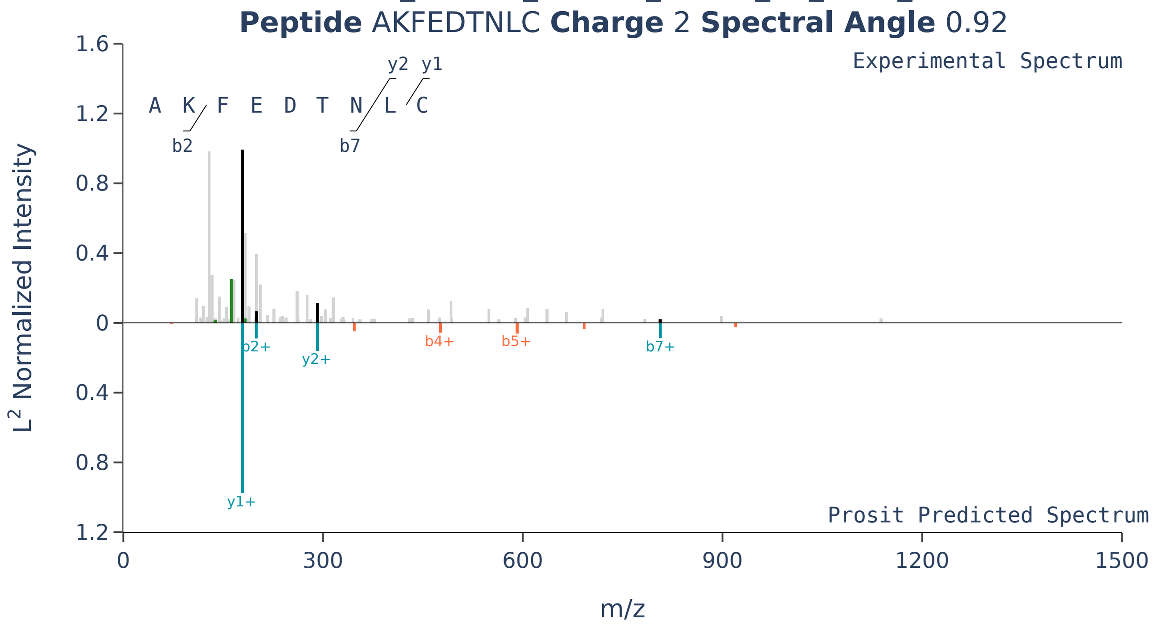

Source HRoetschke_271222_120123_HFGoe_H3_b2R2_24h **Scan** 9743
Peptide SSTIMPKD **Charge** 2 **Spectral Angle** 0.91

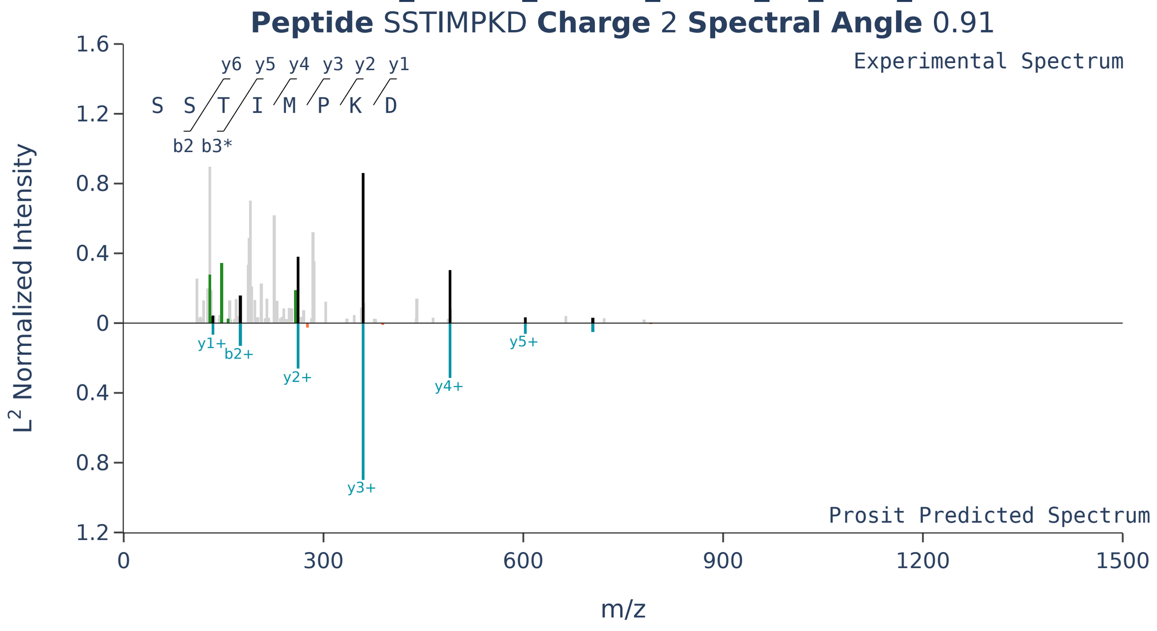

Source HRoetschke_271222_120123_HFGoe_H3_b2R1_24h **Scan** 10591
Peptide REIAQDFKTA **Charge** 2 **Spectral Angle** 0.9

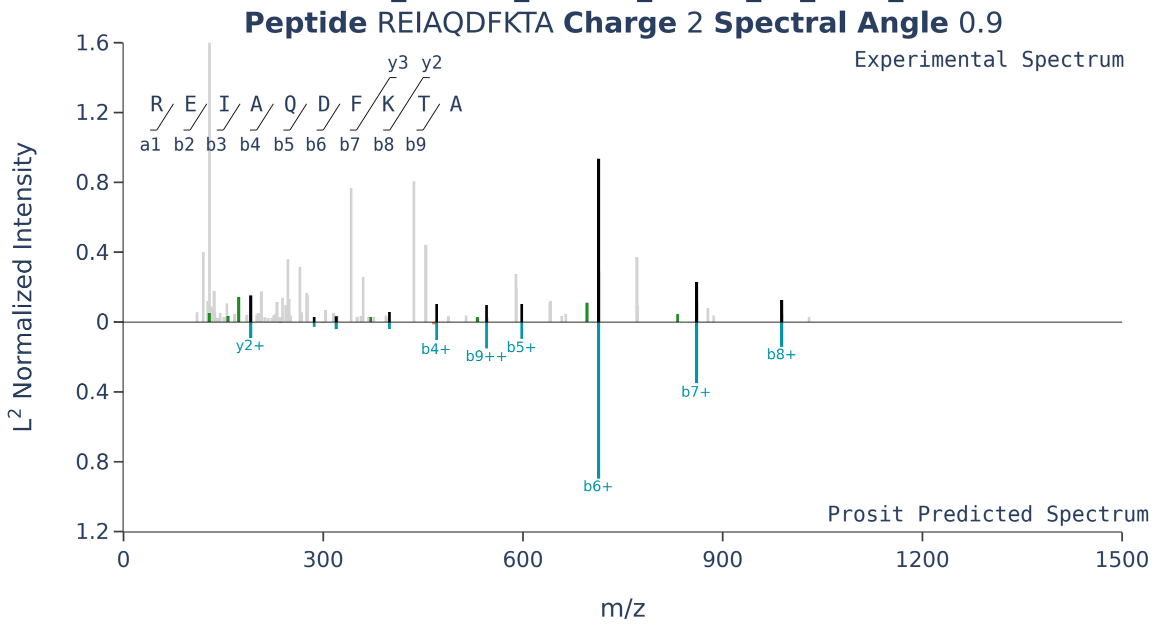

Source HRoetschke_271222_120123_HFGoe_H3_b1R1_24h **Scan** 14611
Peptide AQKSTELLIRKL **Charge** 3 **Spectral Angle** 0.86

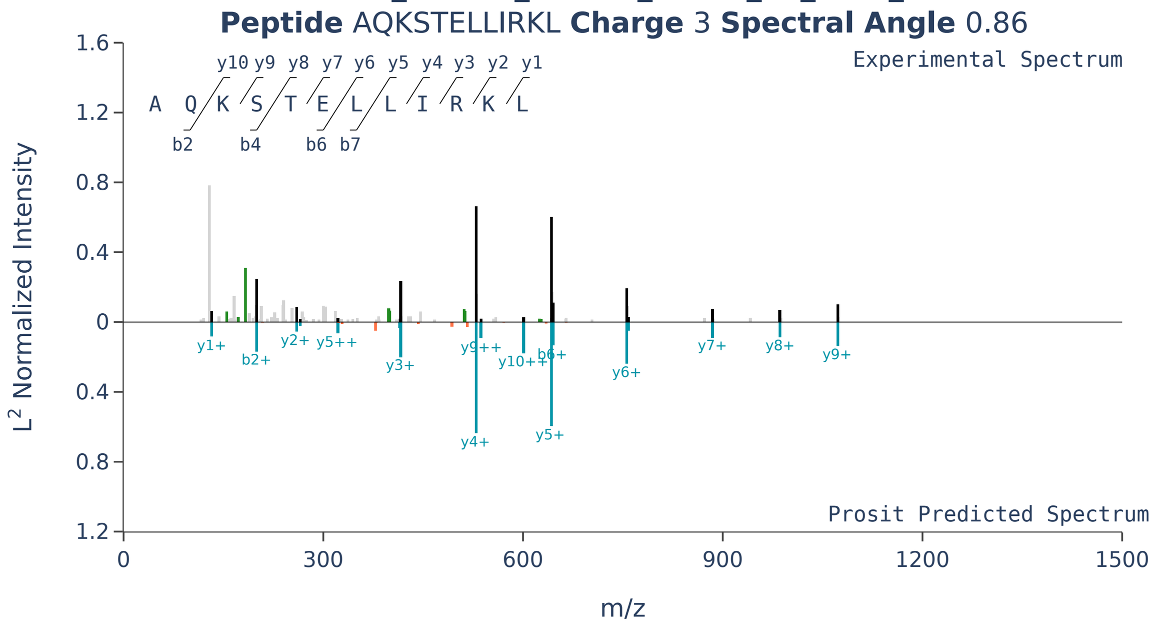

Source HRoetschke_271222_120123_HFGoe_H3_b2R2_2h **Scan** 16734
Peptide RAEIAQDFKTDL **Charge** 3 **Spectral Angle** 0.79

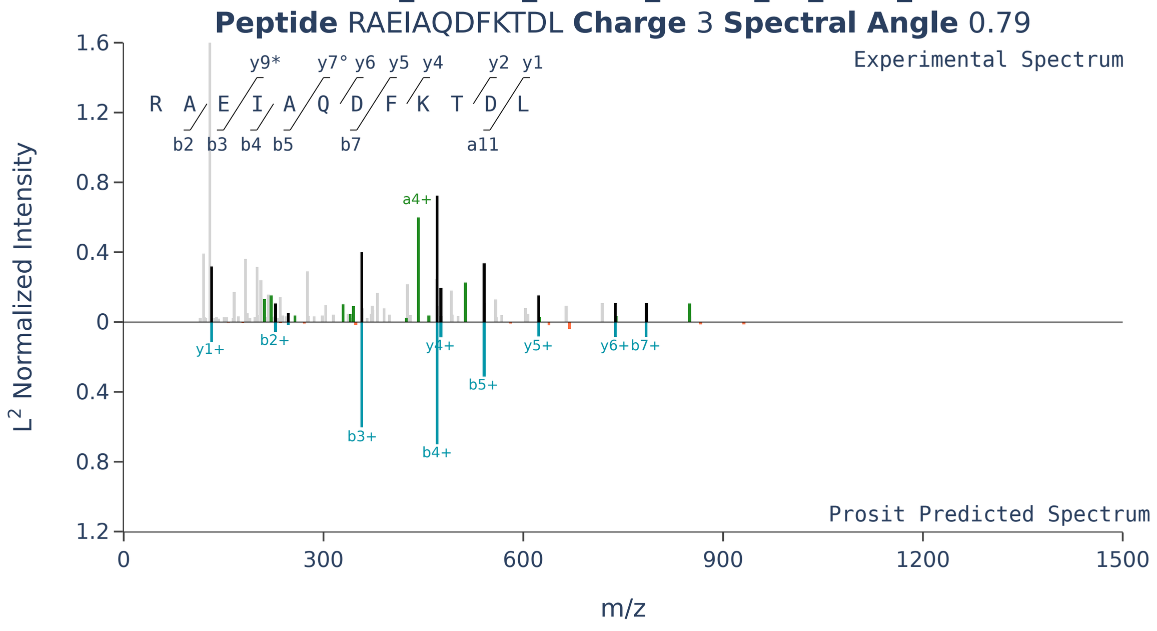

Source HRoetschke_271222_120123_HFGoe_H3_b2R2_4h **Scan** 6850
Peptide YRQSSAVM **Charge** 2 **Spectral Angle** 0.83

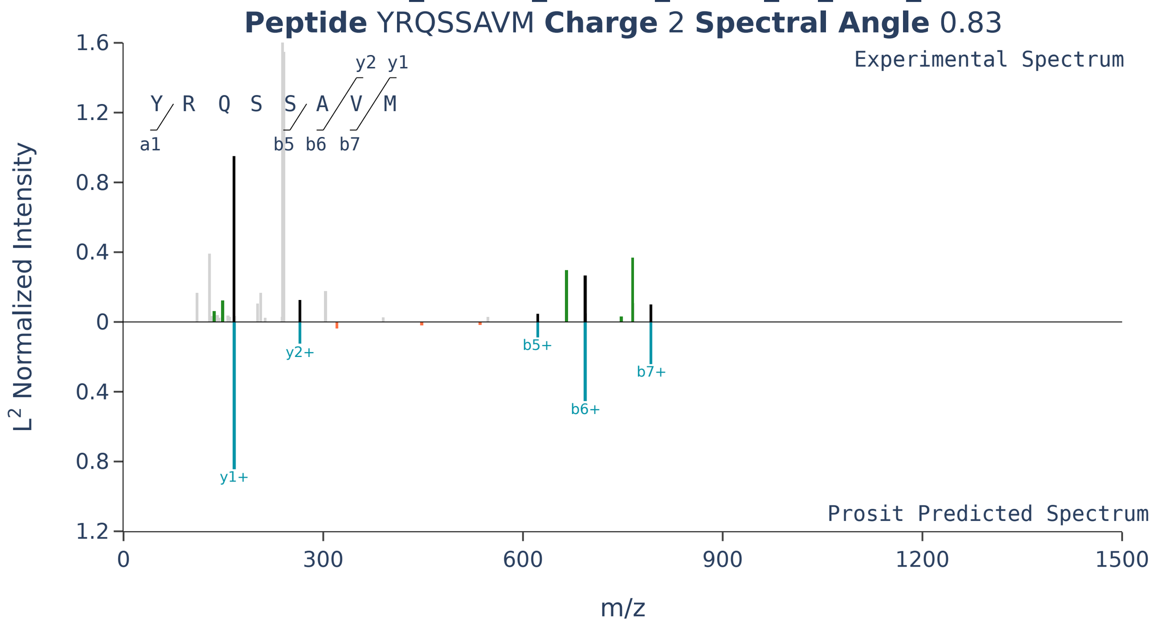

Source HRoetschke_271222_120123_HFGoe_H3_b2R2_2h **Scan** 10268
Peptide AIHAVREI **Charge** 2 **Spectral Angle** 0.87

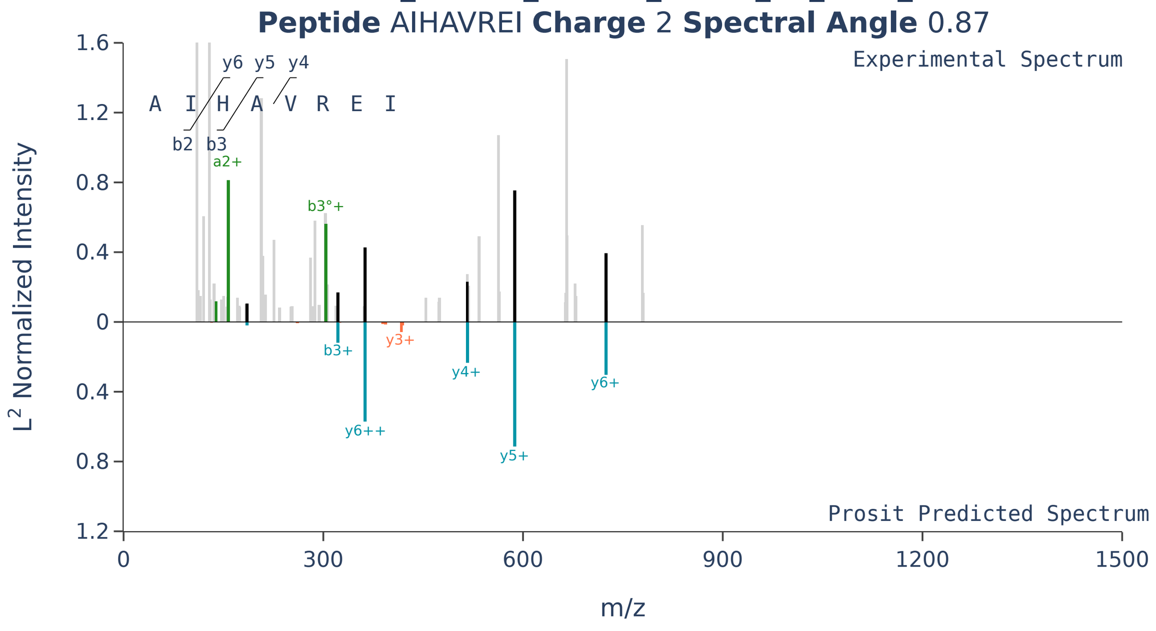

Source HRoetschke_271222_120123_HFGoe_H3_b1R2_24h **Scan** 13483
Peptide ALFEDTKRV **Charge** 3 **Spectral Angle** 0.83

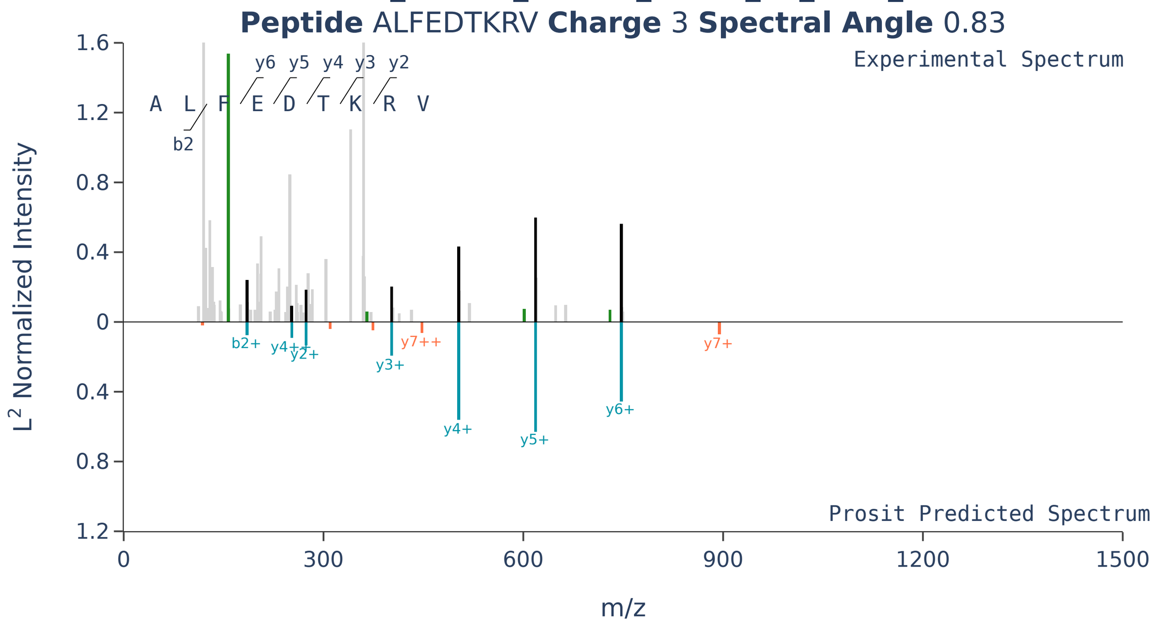

Source HRoetschke_271222_120123_HFGoe_H3_b2R1_4h **Scan** 13057
Peptide AQDFKTDLQ **Charge** 2 **Spectral Angle** 0.79

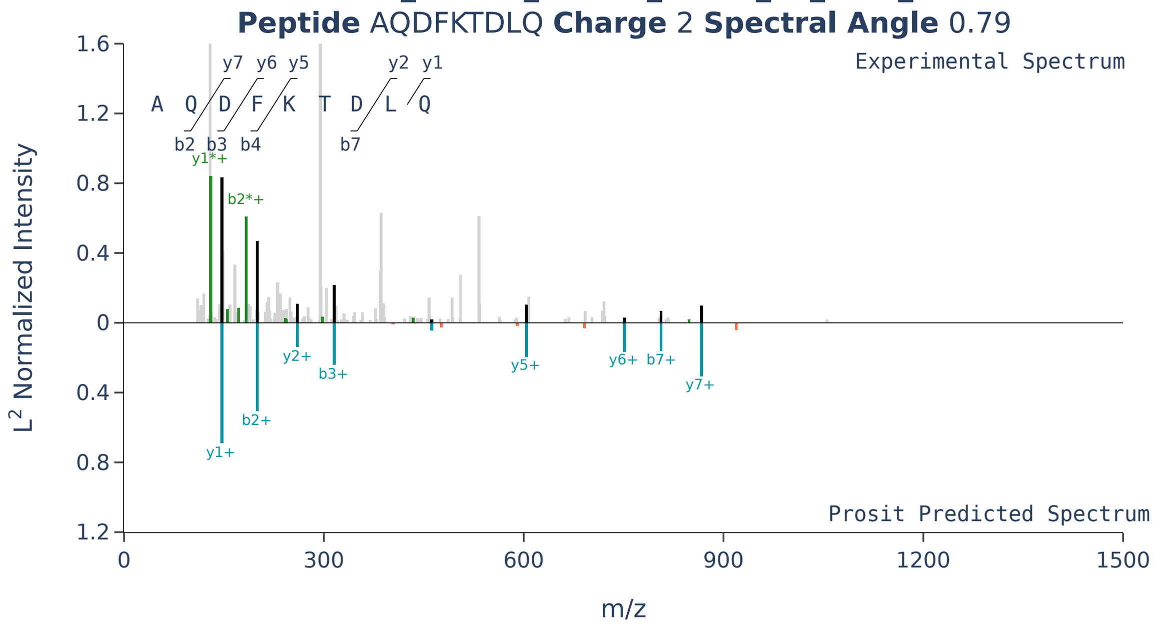

Source HRoetschke_271222_120123_HFGoe_H3_b2R2_24h **Scan** 14367
Peptide DFKTDLA **Charge** 1 **Spectral Angle** 0.82

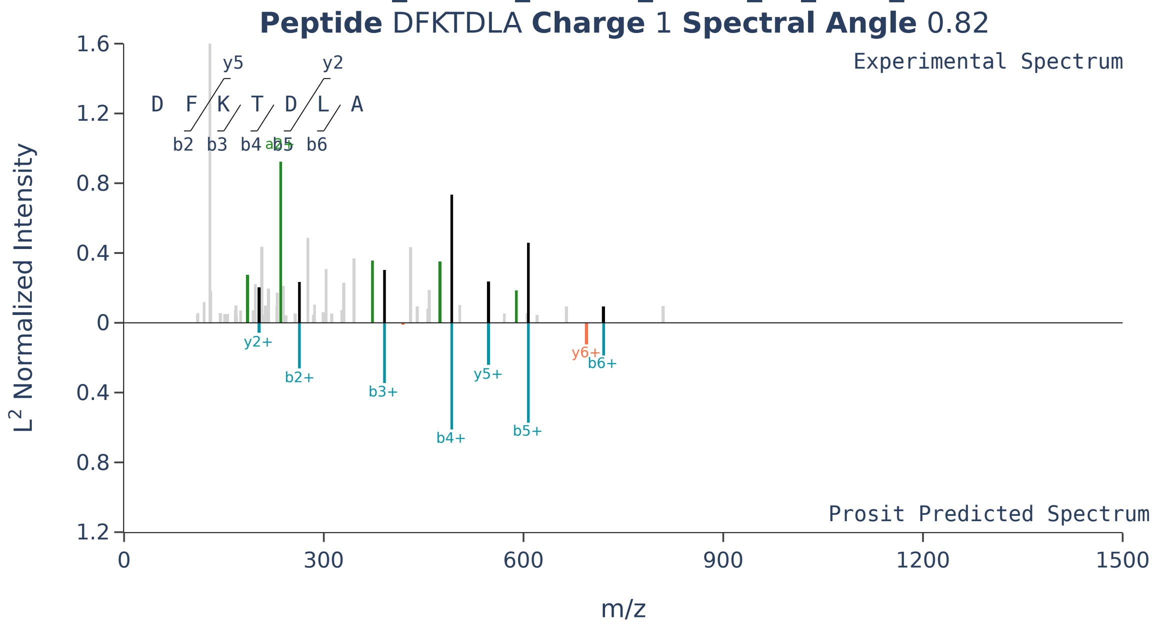

Source HRoetschke_271222_120123_HFGoe_H3_b2R1_24h **Scan** 17051
Peptide STELLRL **Charge** 2 **Spectral Angle** 0.86

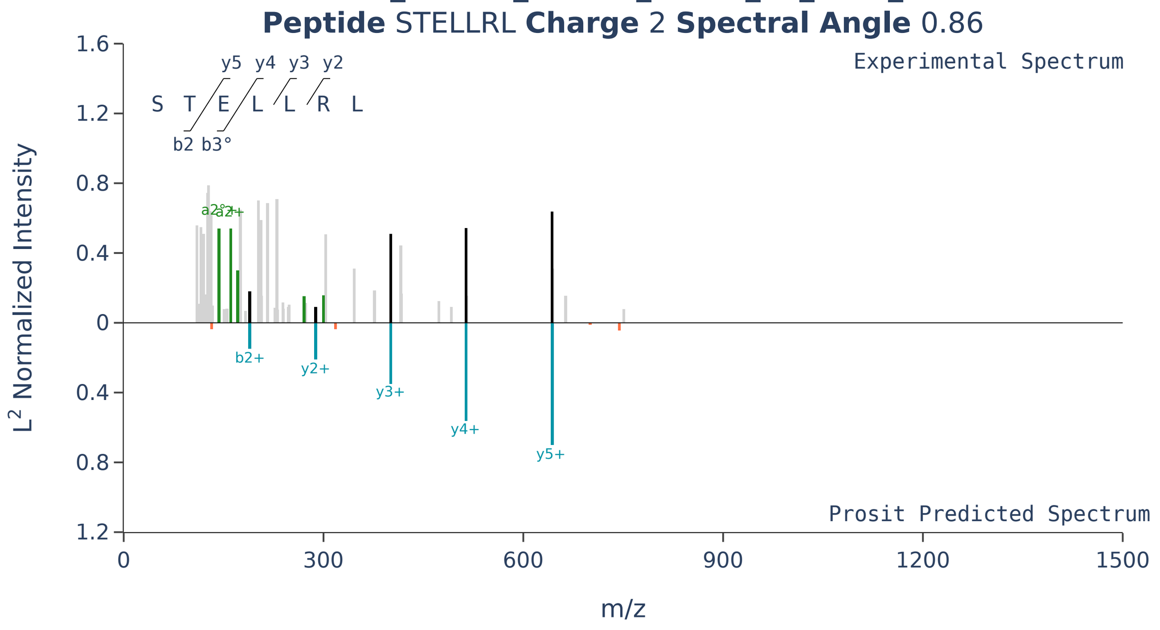

Source HRoetschke_271222_120123_HFGoe_H3_b2R2_2h **Scan** 15873
Peptide REIAQDFKTDAL **Charge** 3 **Spectral Angle** 0.83

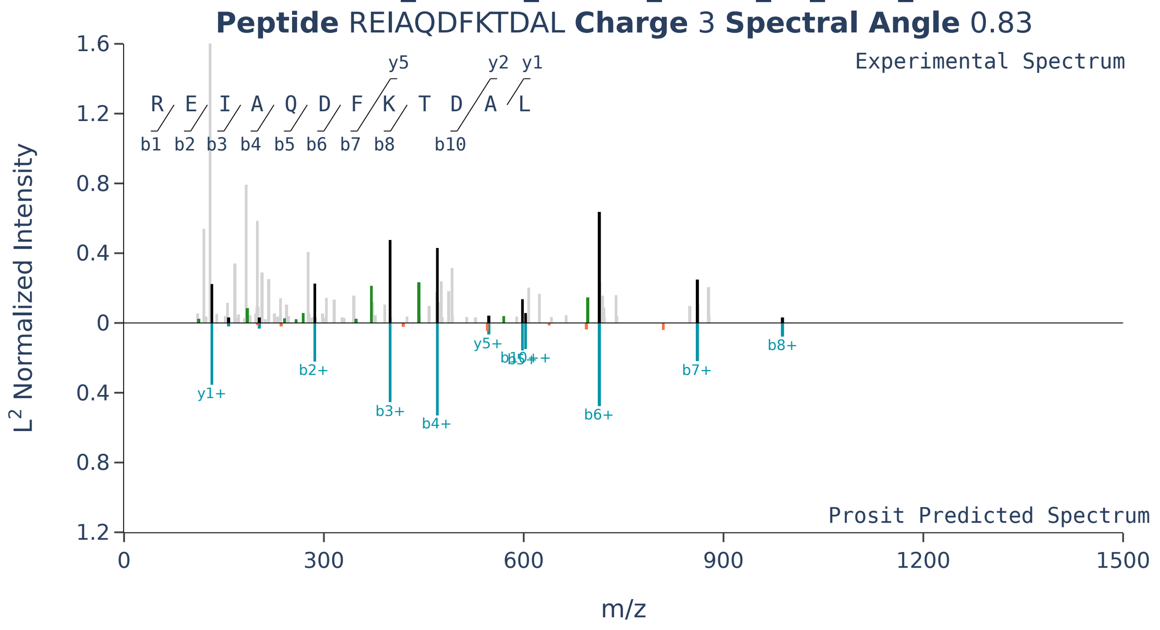

Source HRoetschke_271222_120123_HFGoe_H3_b2R1_24h **Scan** 10972
Peptide RKSALFE **Charge** 2 **Spectral Angle** 0.9

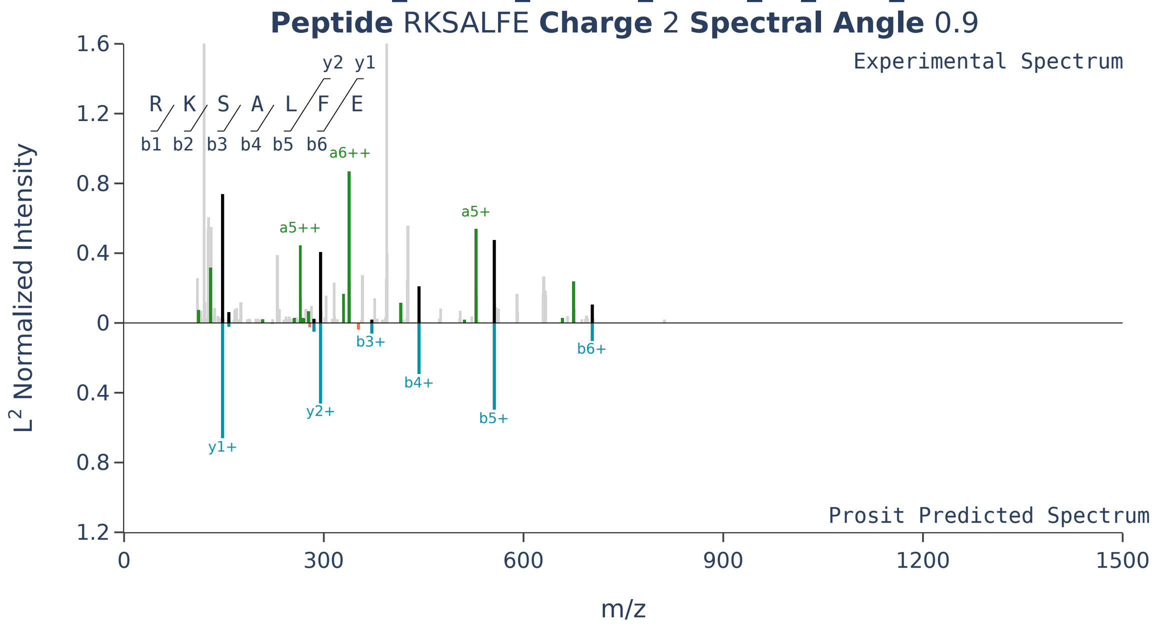

Source HRoetschke_271222_120123_HFGoe_H3_b1R1_24h **Scan** 15787
Peptide SREIAQDFKTDL **Charge** 3 **Spectral Angle** 0.87

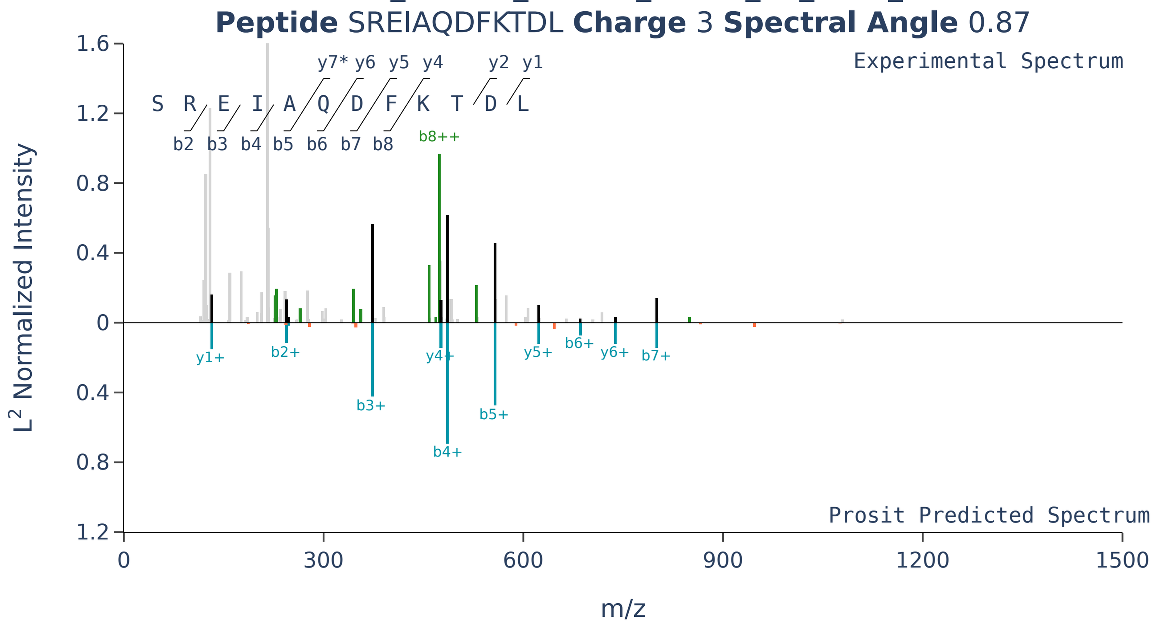

Source HRoetschke_271222_120123_HFGoe_H3_b2R2_4h **Scan** 13693
Peptide AQDFKTDLT **Charge** 2 **Spectral Angle** 0.78

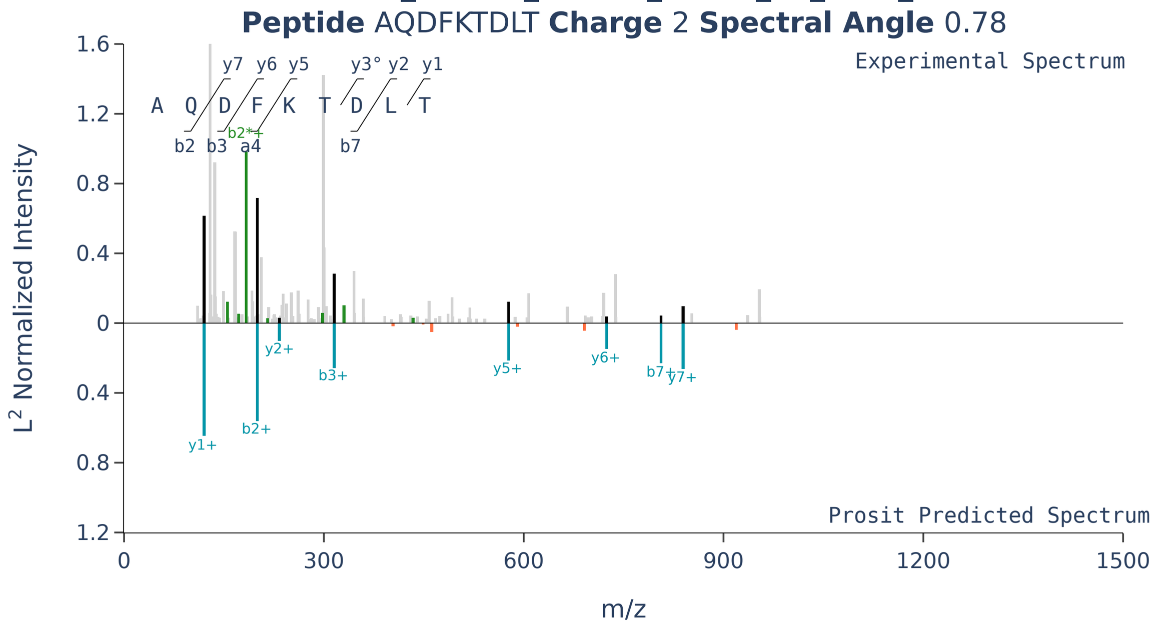

Source HRoetschke_271222_120123_HFGoe_H3_b1R2_4h **Scan** 16577
Peptide AQDFKTDLV **Charge** 2 **Spectral Angle** 0.71

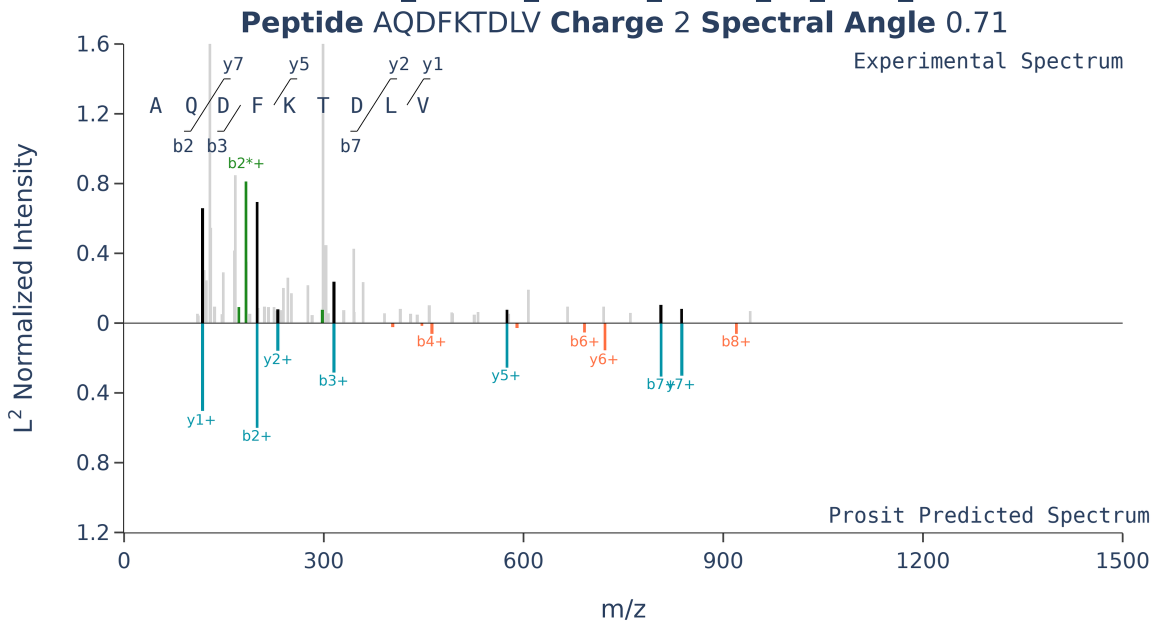

Source HRoetschke_271222_120123_HFGoe_H3_b2R2_4h **Scan** 16186
Peptide RFQSSASEAYL **Charge** 2 **Spectral Angle** 0.82

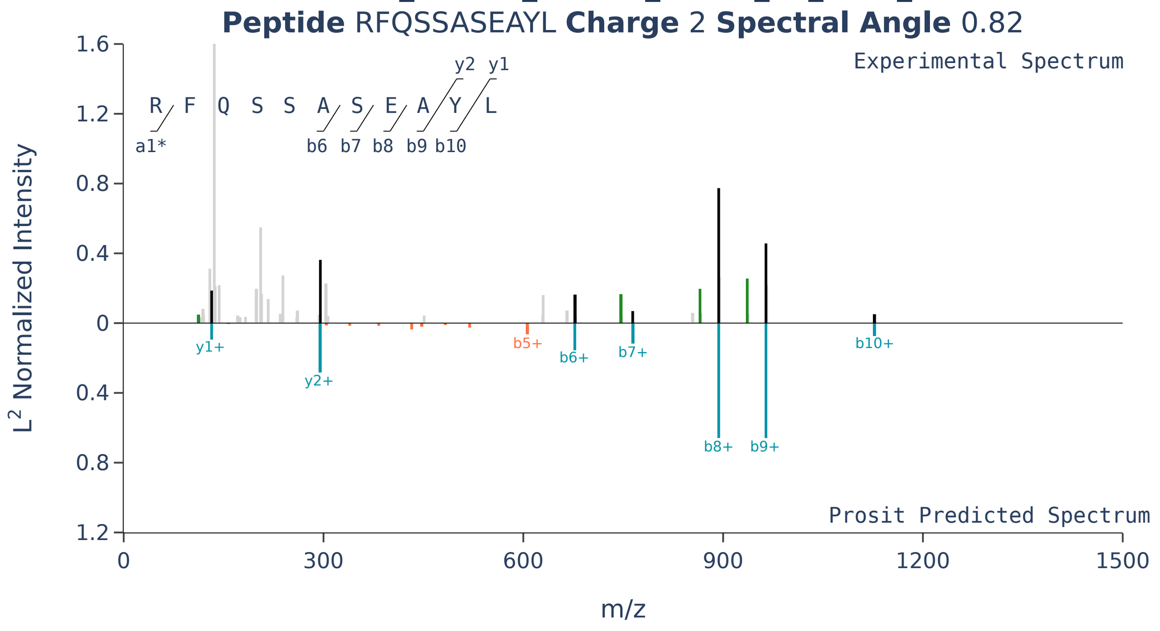

Source HRoetschke_271222_120123_HFGoe_H3_b1R2_24h **Scan** 14211
Peptide ALFEDTRRY **Charge** 3 **Spectral Angle** 0.8

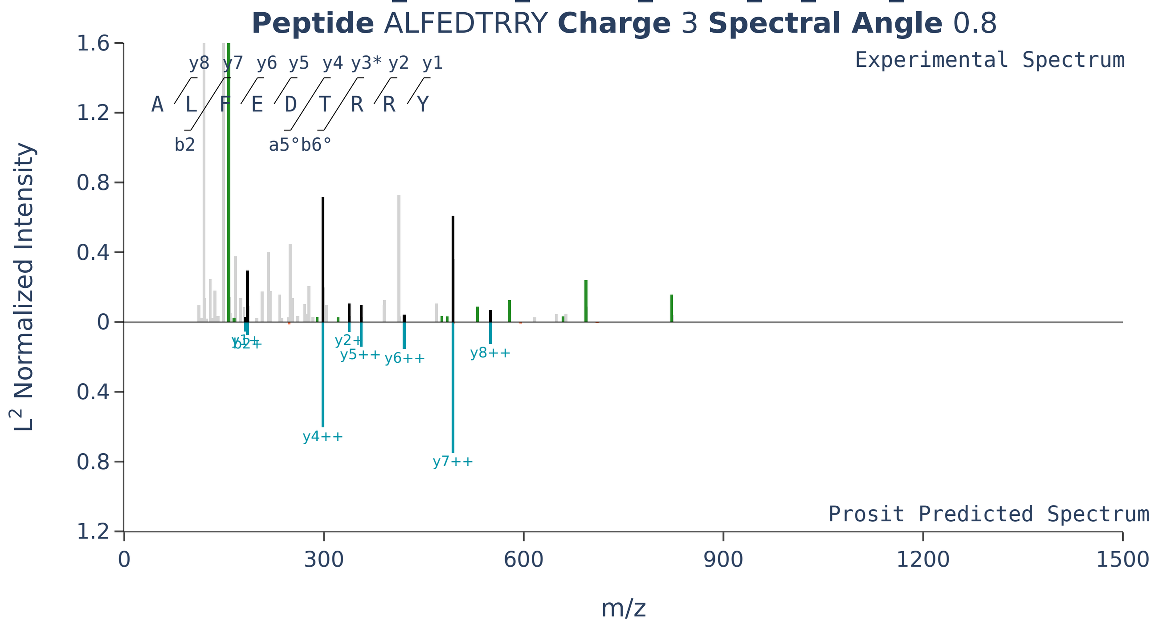

Source HRoetschke_271222_120123_HFGoe_H3_b1R1_24h **Scan** 15859
Peptide AREIAQDFKTDL **Charge** 3 **Spectral Angle** 0.82

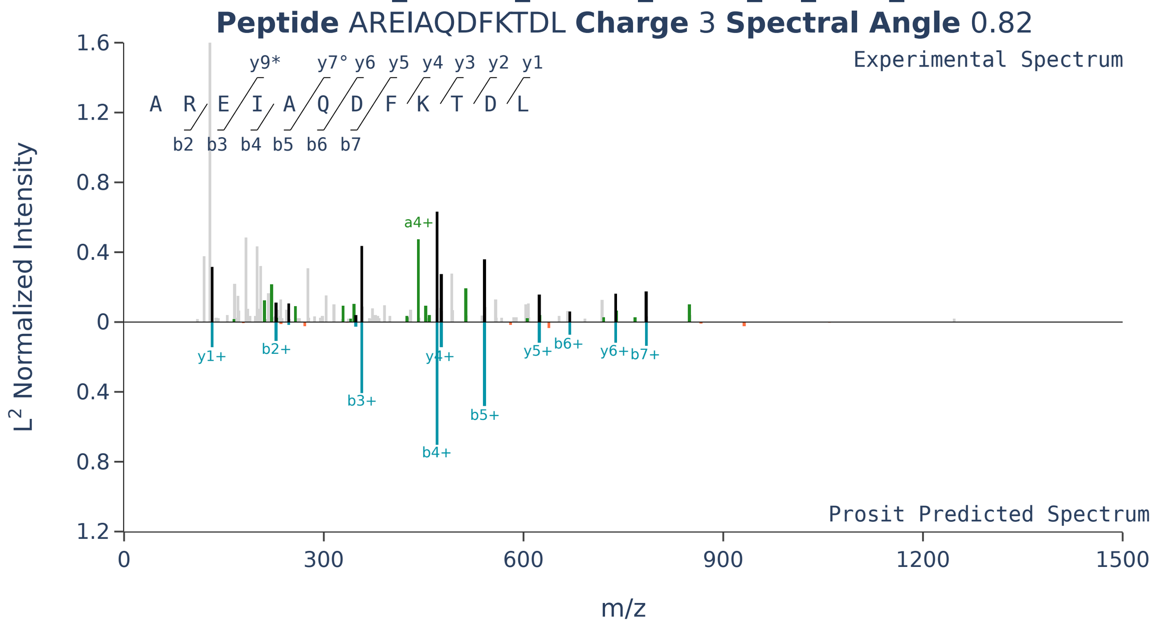

Source HRoetschke_271222_120123_HFGoe_H3_b2R2_4h **Scan** 16849
Peptide AVREIAQDF **Charge** 2 **Spectral Angle** 0.77

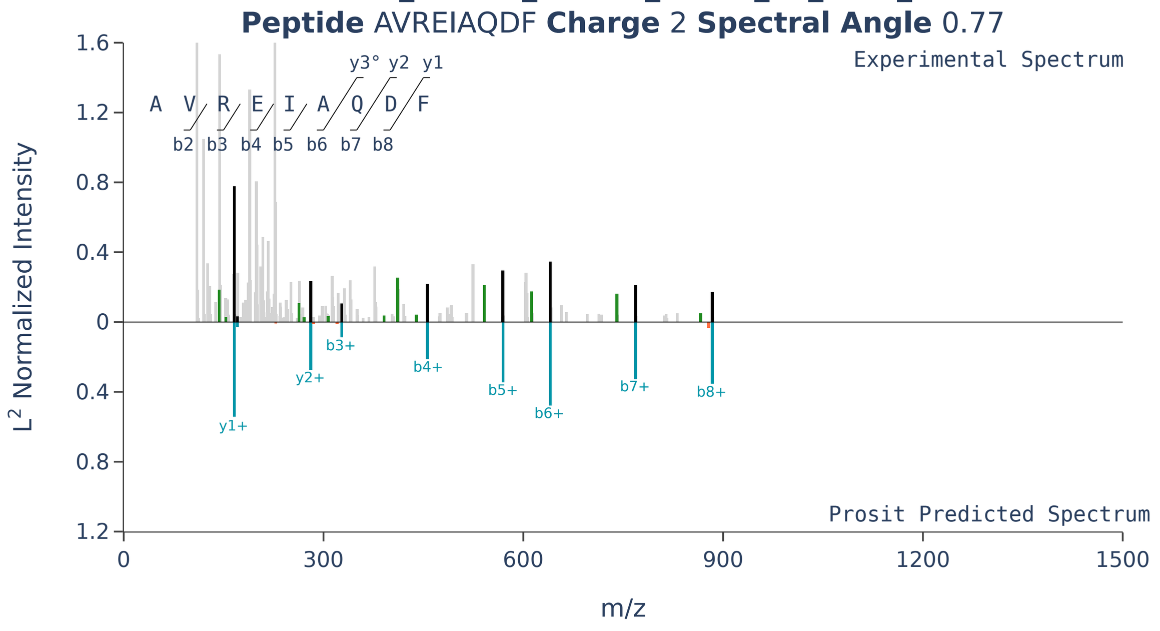

Source HRoetschke_271222_120123_HFGoe_H3_b2R2_24h **Scan** 18082
Peptide ALQDFKTDL **Charge** 2 **Spectral Angle** 0.72

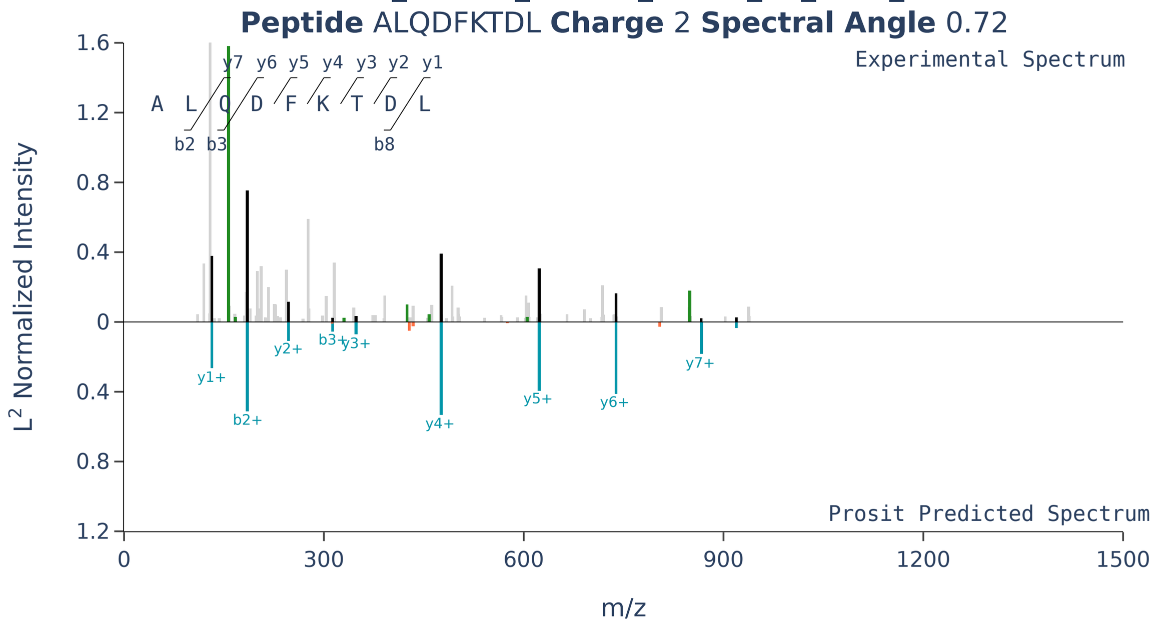

Source HRoetschke_271222_120123_HFGoe_H3_b2R1_24h **Scan** 11142
Peptide RFQSSEL **Charge** 2 **Spectral Angle** 0.79

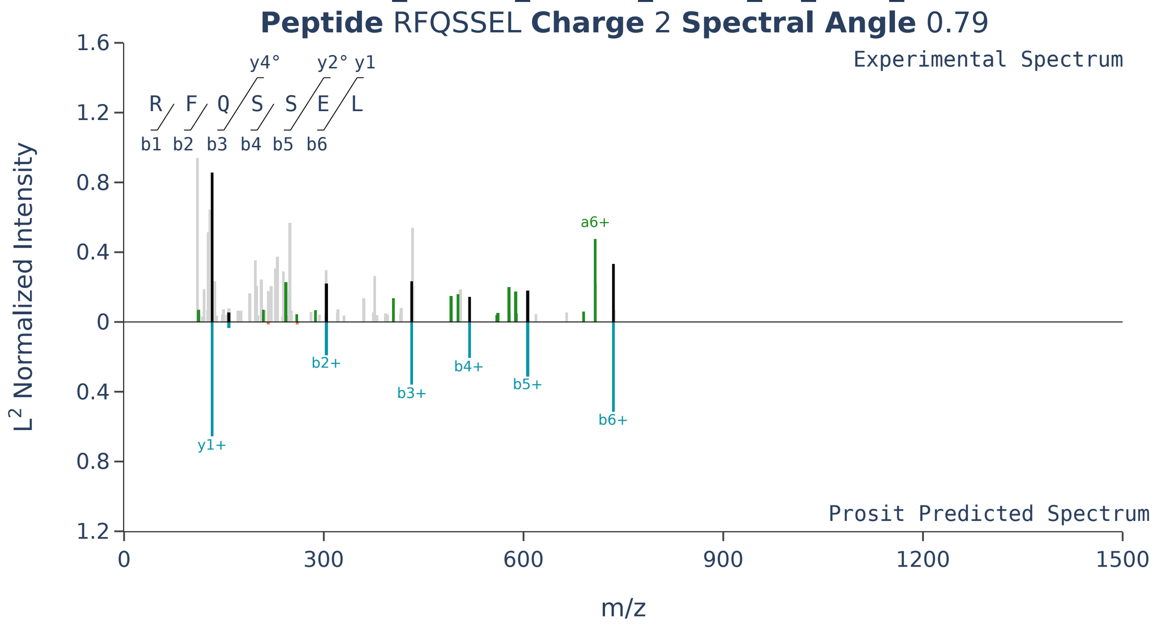

Source HRoetschke_271222_120123_HFGoe_H3_b1R1_24h **Scan** 19144
Peptide EIRALFE **Charge** 2 **Spectral Angle** 0.82

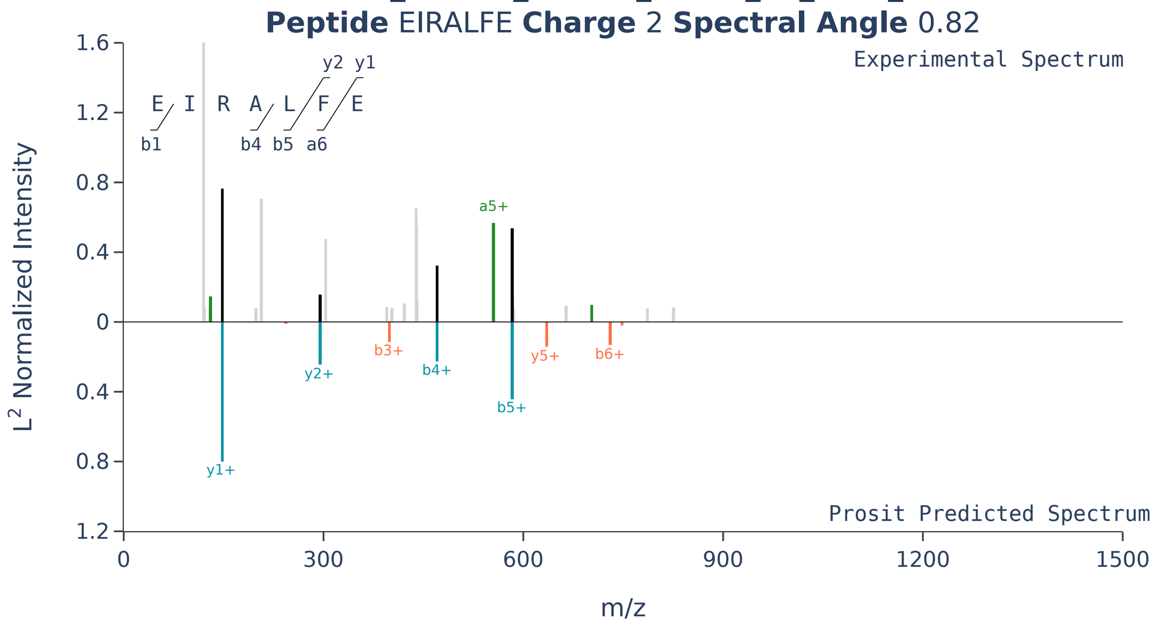

Source HRoetschke_271222_120123_HFGoe_H3_b2R2_2h **Scan** 10966
Peptide VREIAQDFKTN **Charge** 3 **Spectral Angle** 0.86

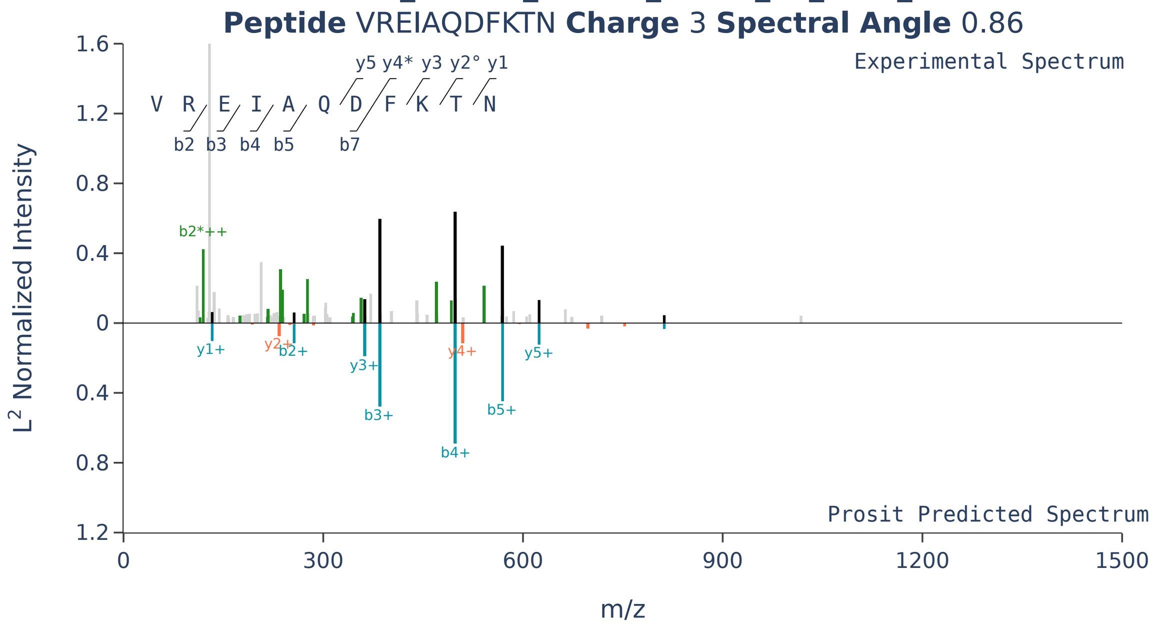

Source HRoetschke_271222_120123_HFGoe_H3_b1R2_24h **Scan** 11989
Peptide AQDFKTDLKRV **Charge** 3 **Spectral Angle** 0.79

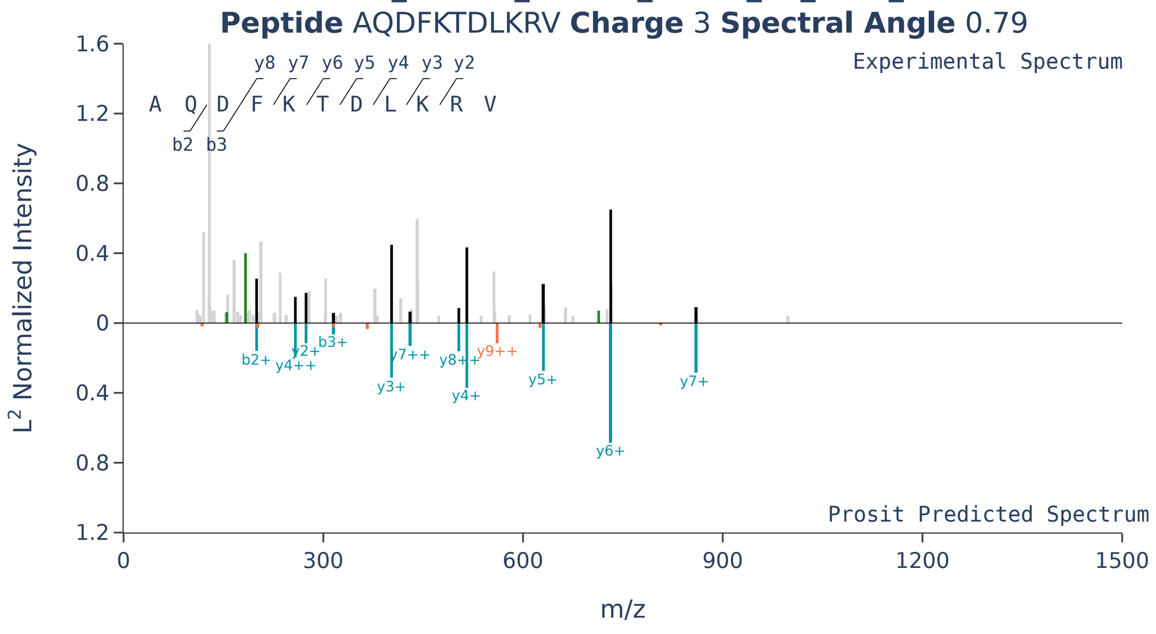

Source HRoetschke_271222_120123_HFGoe_H3_b1R1_24h **Scan** 6226
Peptide REIAIHAKRV **Charge** 3 **Spectral Angle** 0.85

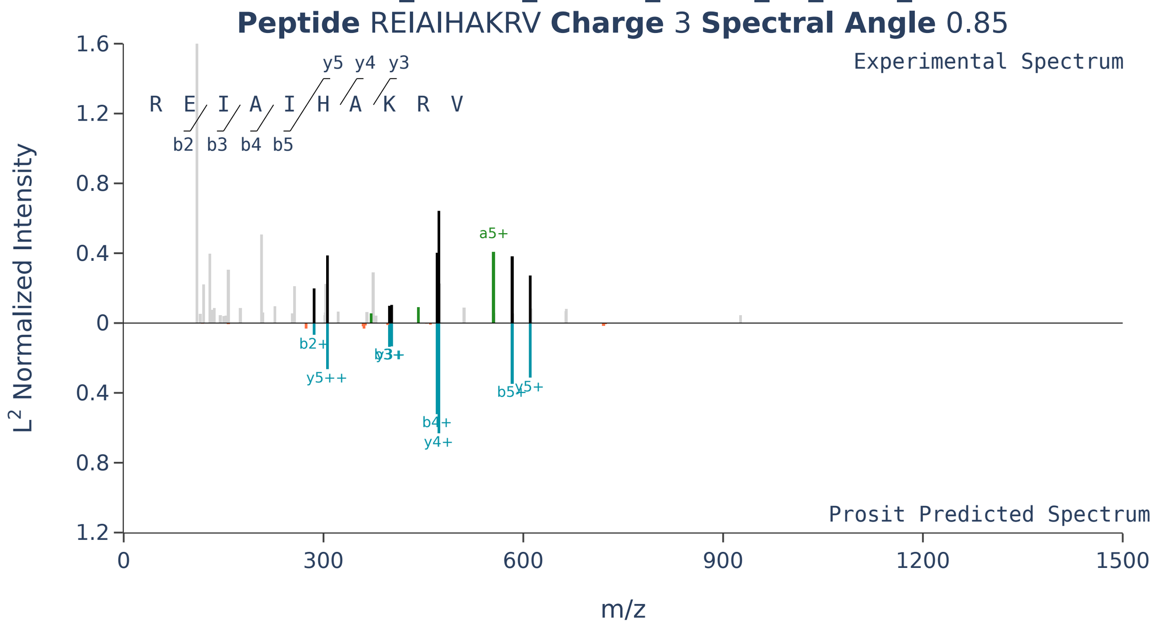

Source HRoetschke_271222_120123_HFGoe_H3_b2R1_4h **Scan** 13959
Peptide AQDFKTLVRE **Charge** 2 **Spectral Angle** 0.8

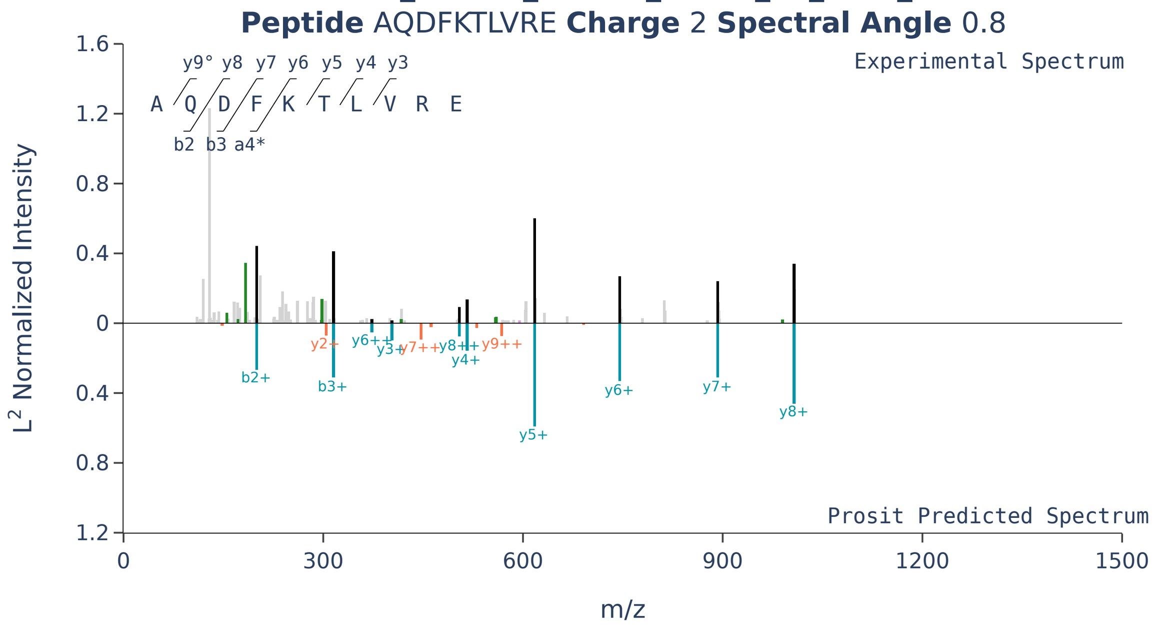

Source HRoetschke_271222_120123_HFGoe_H3_b1R1_24h **Scan** 8797
Peptide REIAIHA **Charge** 2 **Spectral Angle** 0.89

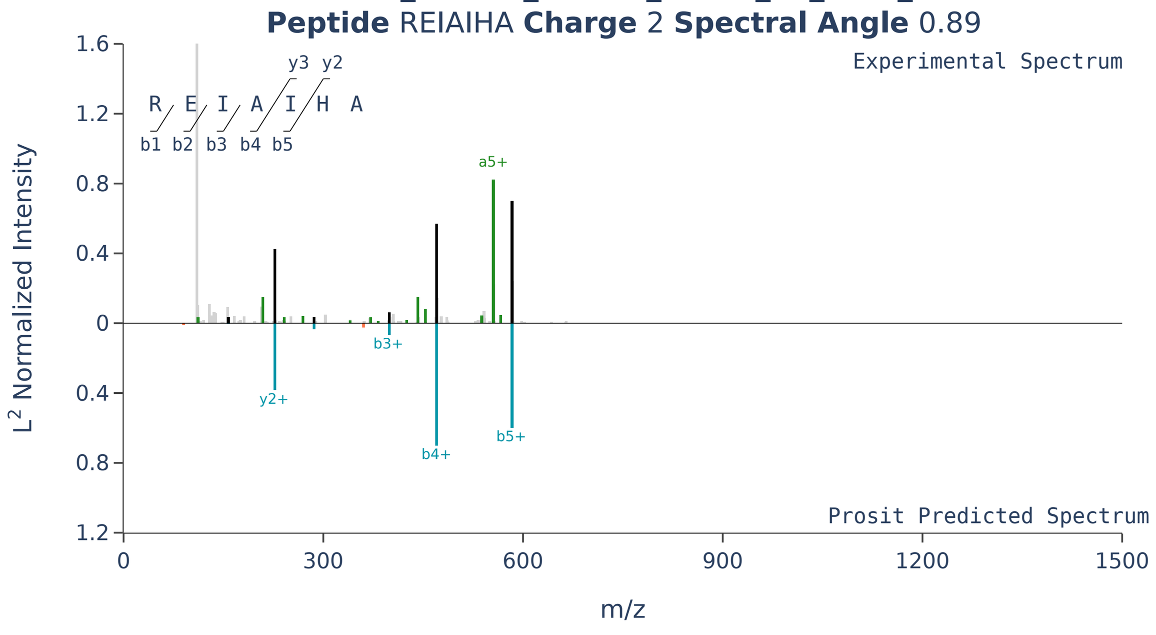

Source HRoetschke_271222_120123_HFGoe_H3_b2R2_2h **Scan** 8971
Peptide TGGSAVMA **Charge** 1 **Spectral Angle** 0.65

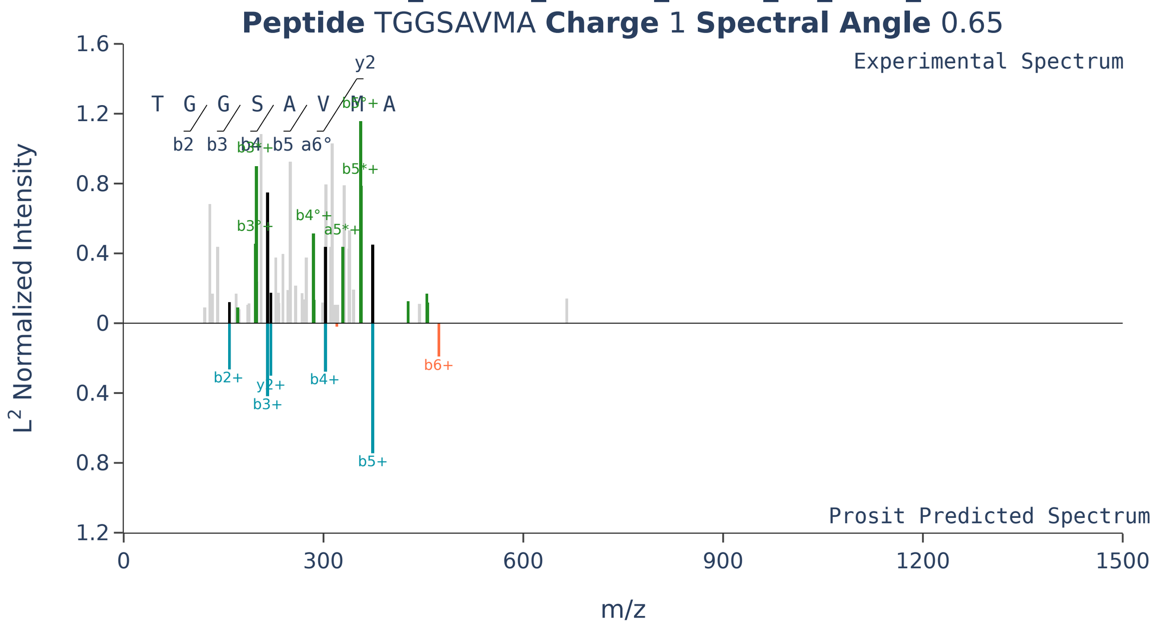

Source HRoetschke_271222_120123_HFGoe_H3_b2R2_24h **Scan** 14578
Peptide SSREIAQDF **Charge** 2 **Spectral Angle** 0.78

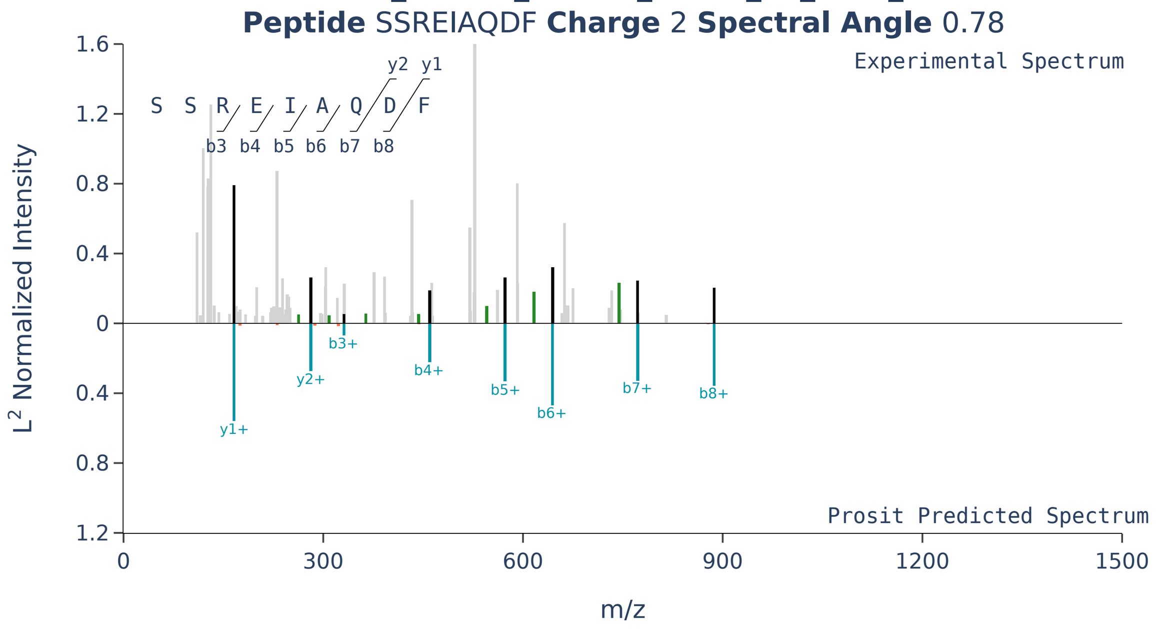

Source HRoetschke_271222_120123_HFGoe_H3_b1R2_4h **Scan** 15214
Peptide AQDFKTDLRFQSSASE **Charge** 2 **Spectral Angle** 0.81

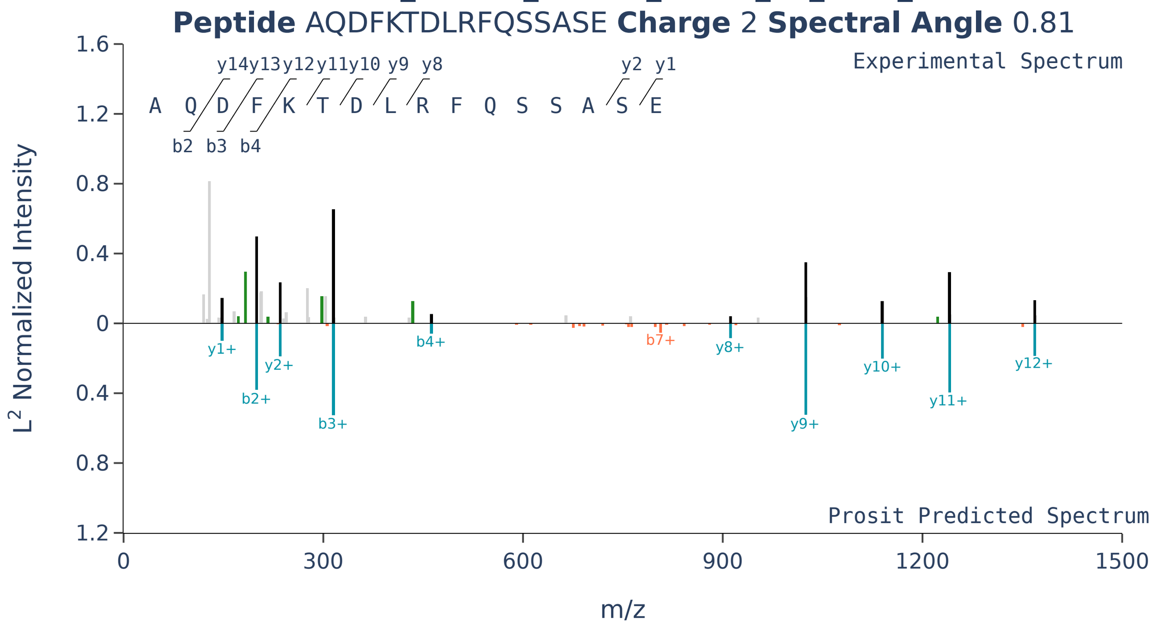

Source HRoetschke_271222_120123_HFGoe_H3_b2R1_4h **Scan** 15210
Peptide DTNLCAIHAA **Charge** 2 **Spectral Angle** 0.79

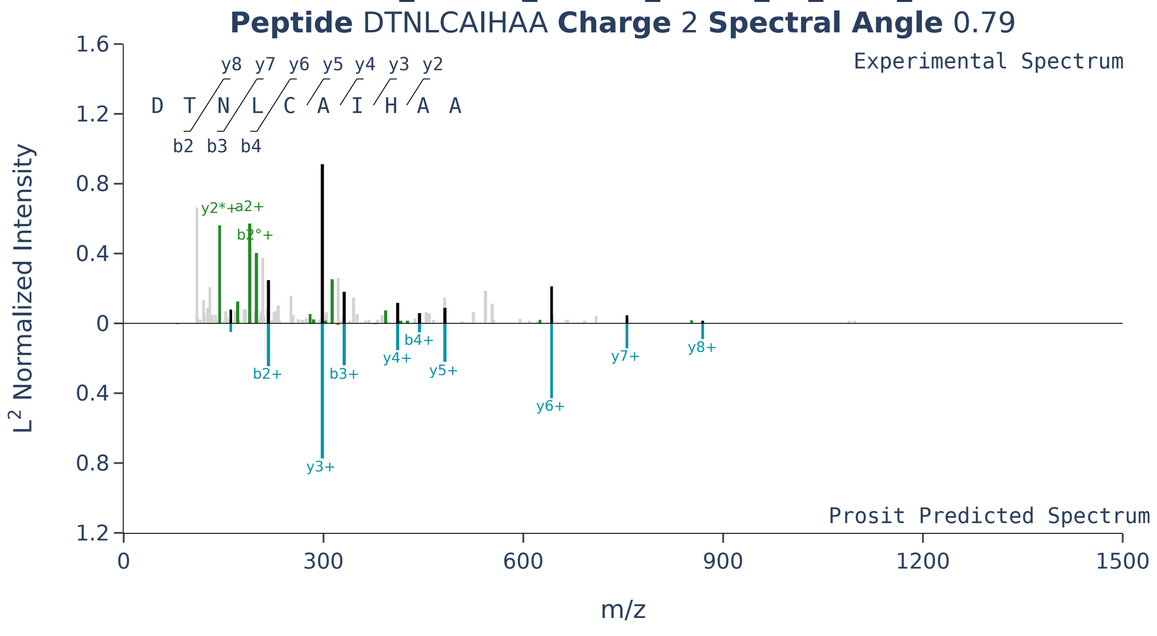

Source HRoetschke_271222_120123_HFGoe_H3_b2R2_4h **Scan** 17775
Peptide SVREIAQDF **Charge** 2 **Spectral Angle** 0.75

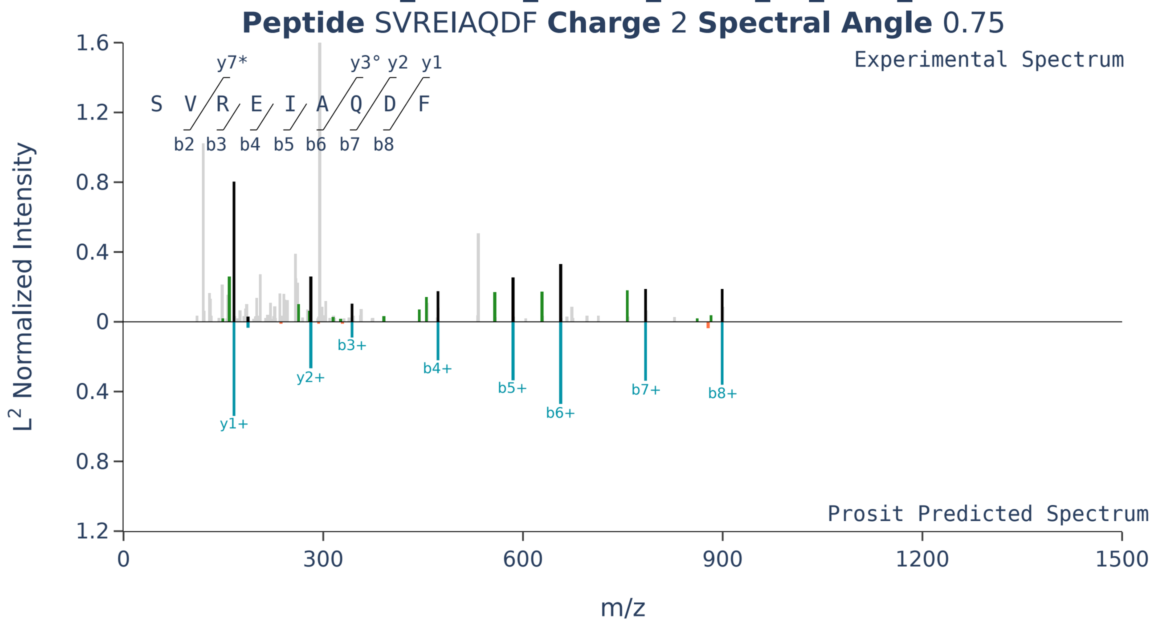

Source HRoetschke_271222_120123_HFGoe_H3_b2R2_2h **Scan** 6391
Peptide ALREIHAKRV **Charge** 3 **Spectral Angle** 0.88

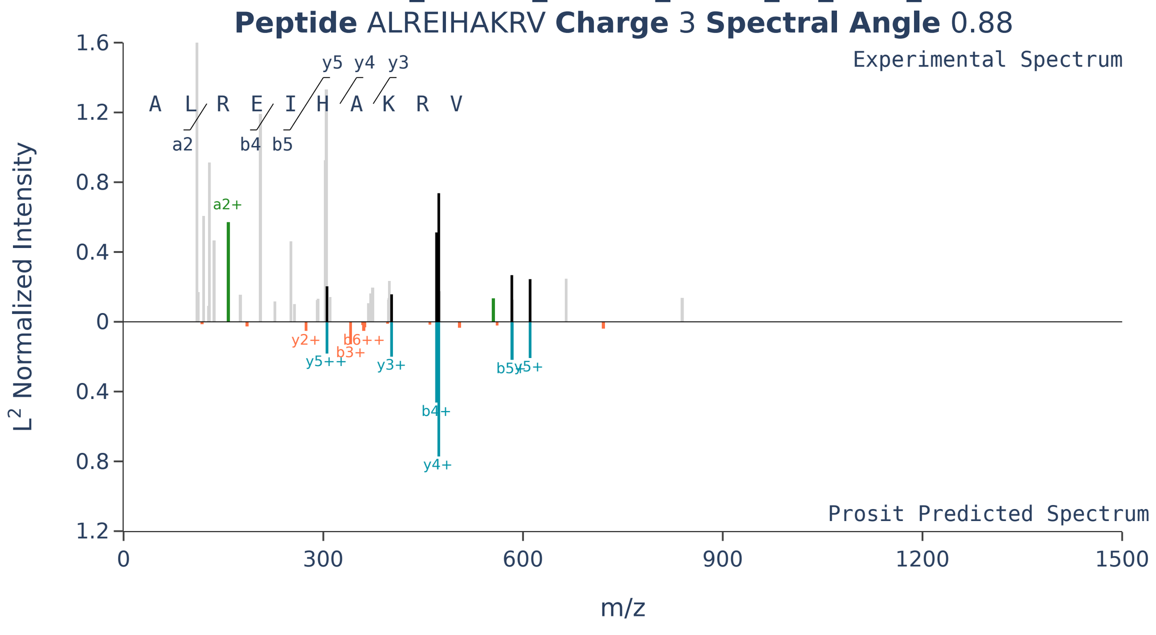

Source HRoetschke_271222_120123_HFGoe_H3_b2R2_4h **Scan** 15861
Peptide SQKSTELLIRKL **Charge** 4 **Spectral Angle** 0.87

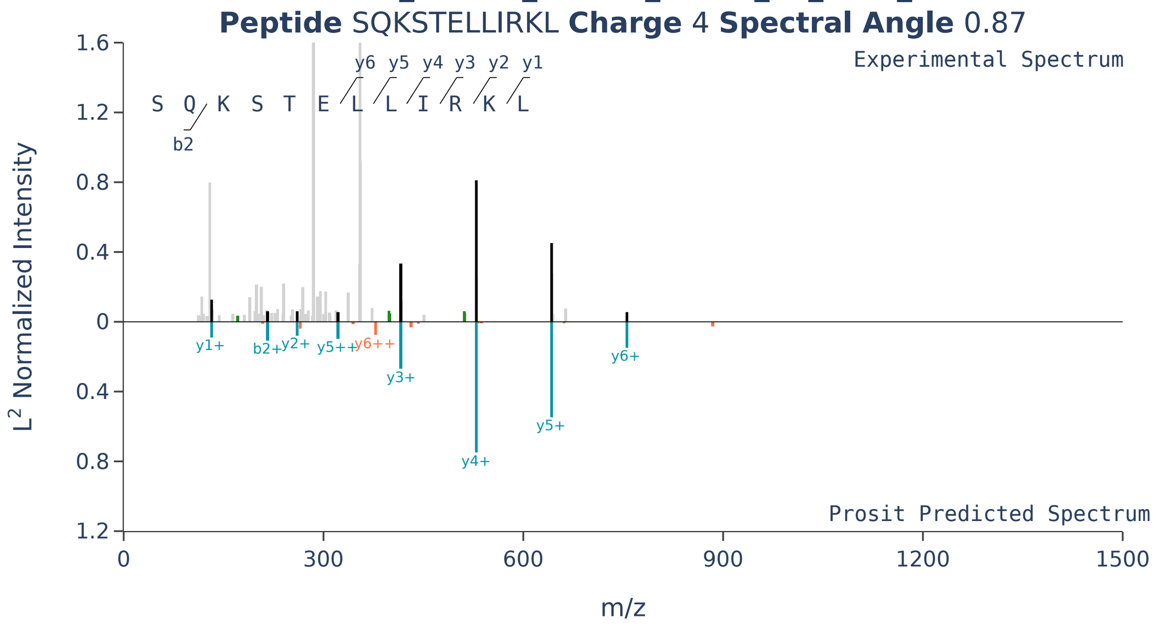

Source HRoetschke_271222_120123_HFGoe_H3_b2R2_4h **Scan** 14036
Peptide ALFEDTRYR **Charge** 3 **Spectral Angle** 0.77

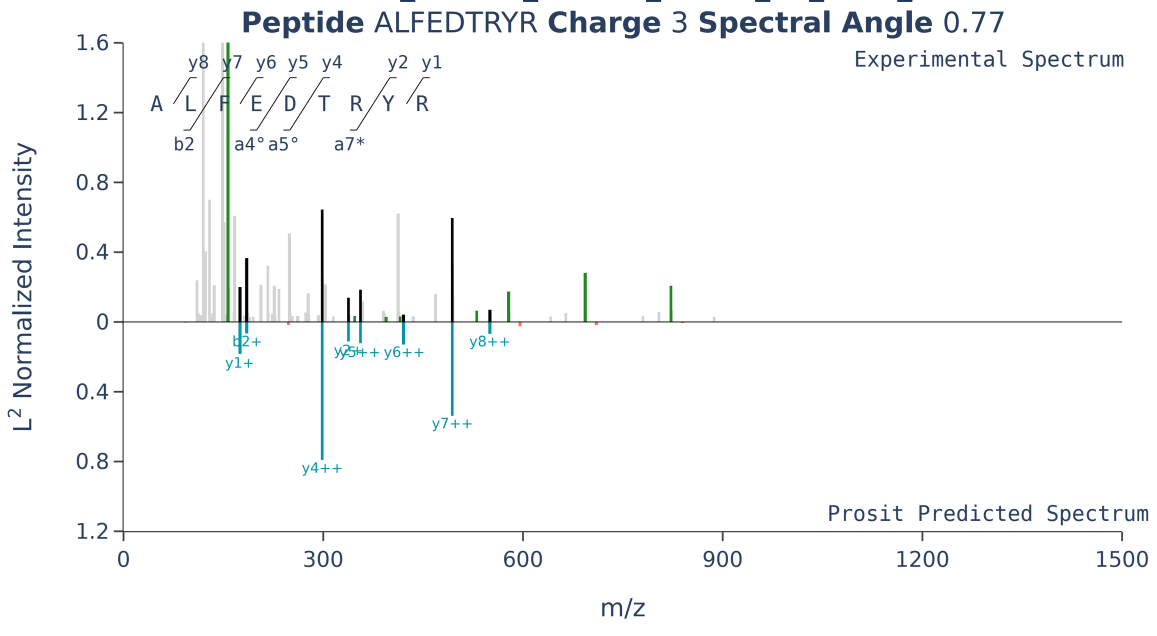

Source HRoetschke_271222_120123_HFGoe_H3_b1R2_2h **Scan** 9926
Peptide ASETIMPKD **Charge** 2 **Spectral Angle** 0.85

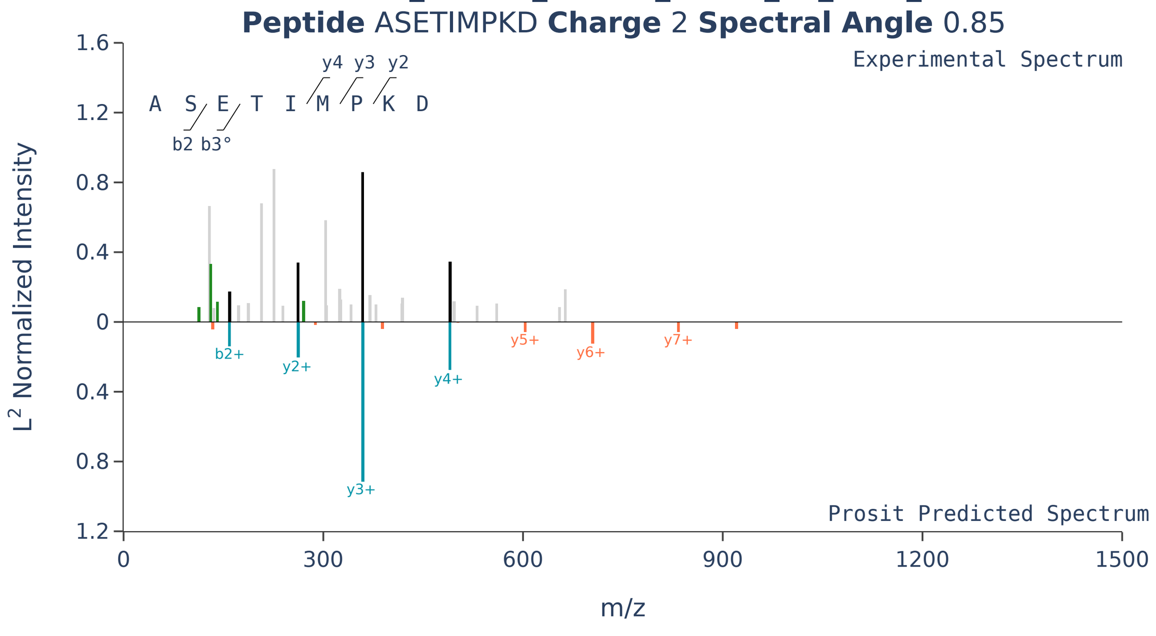

Source HRoetschke_271222_120123_HFGoe_H3_b1R1_24h **Scan** 11204
Peptide AQDFKTDLRYR **Charge** 3 **Spectral Angle** 0.81

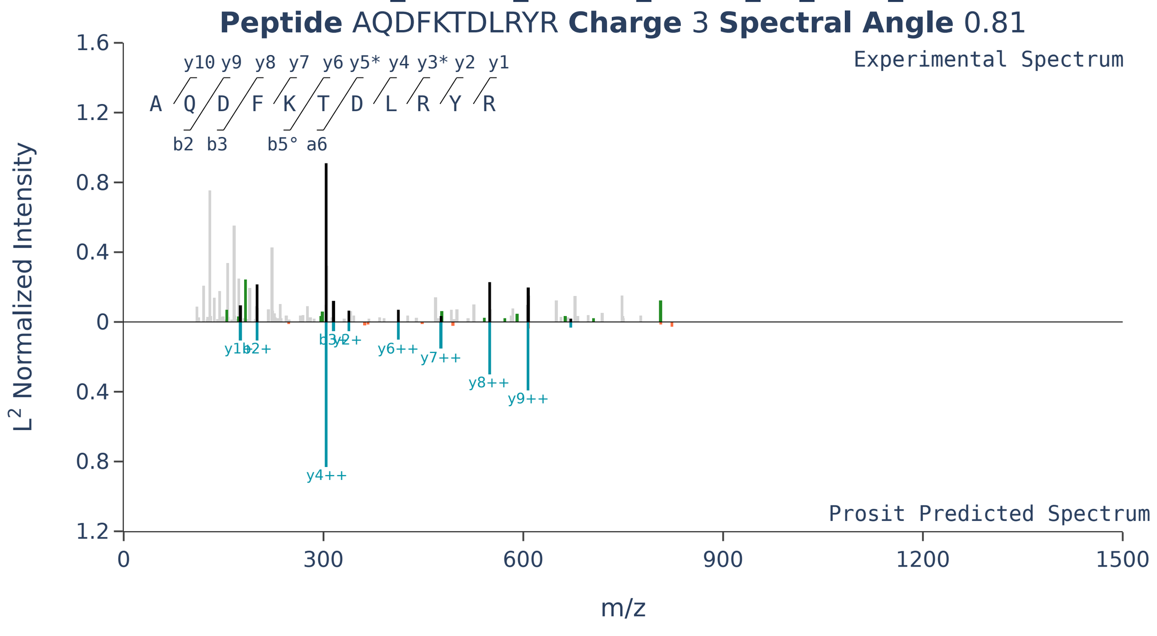

Source HRoetschke_271222_120123_HFGoe_H3_b2R2_2h **Scan** 10469
Peptide VREIAQDFQKS **Charge** 3 **Spectral Angle** 0.86

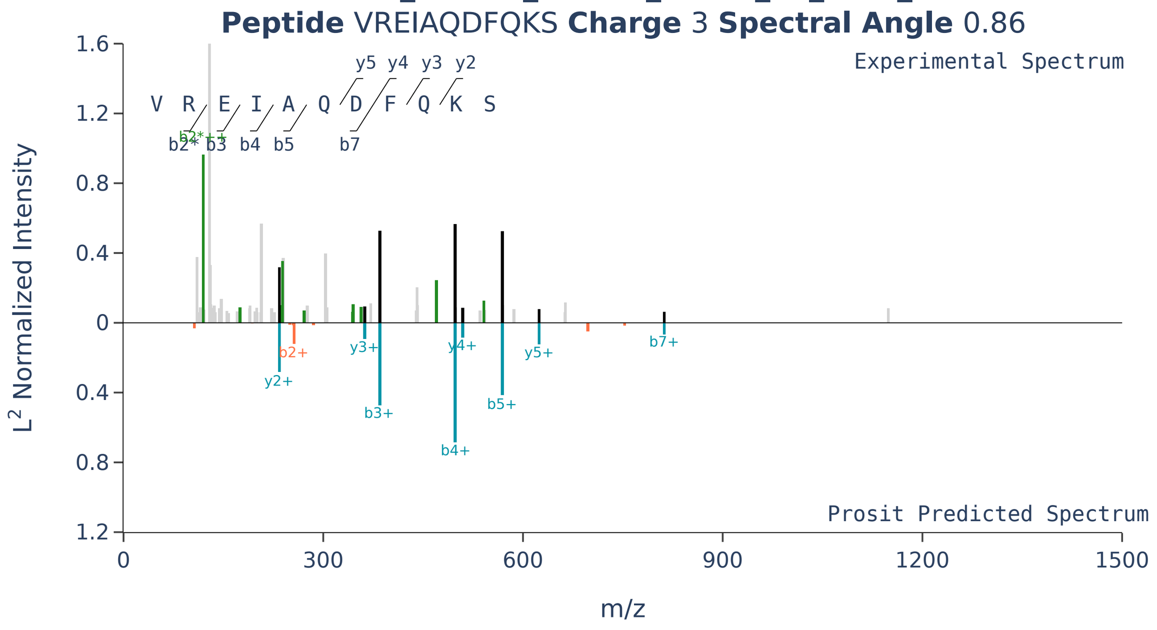

Source HRoetschke_271222_120123_HFGoe_H3_b1R2_24h **Scan** 14437
Peptide PFQQRLV **Charge** 2 **Spectral Angle** 0.9

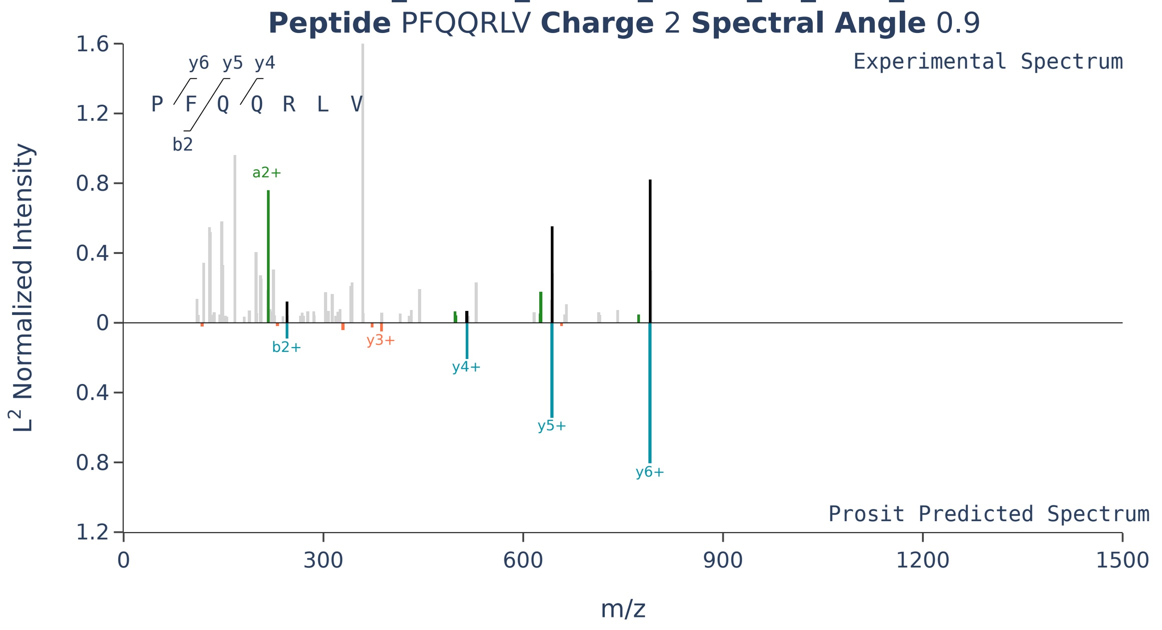

Source HRoetschke_271222_120123_HFGoe_H3_b1R1_4h **Scan** 15131
Peptide REIAQDFKTDLA **Charge** 3 **Spectral Angle** 0.83

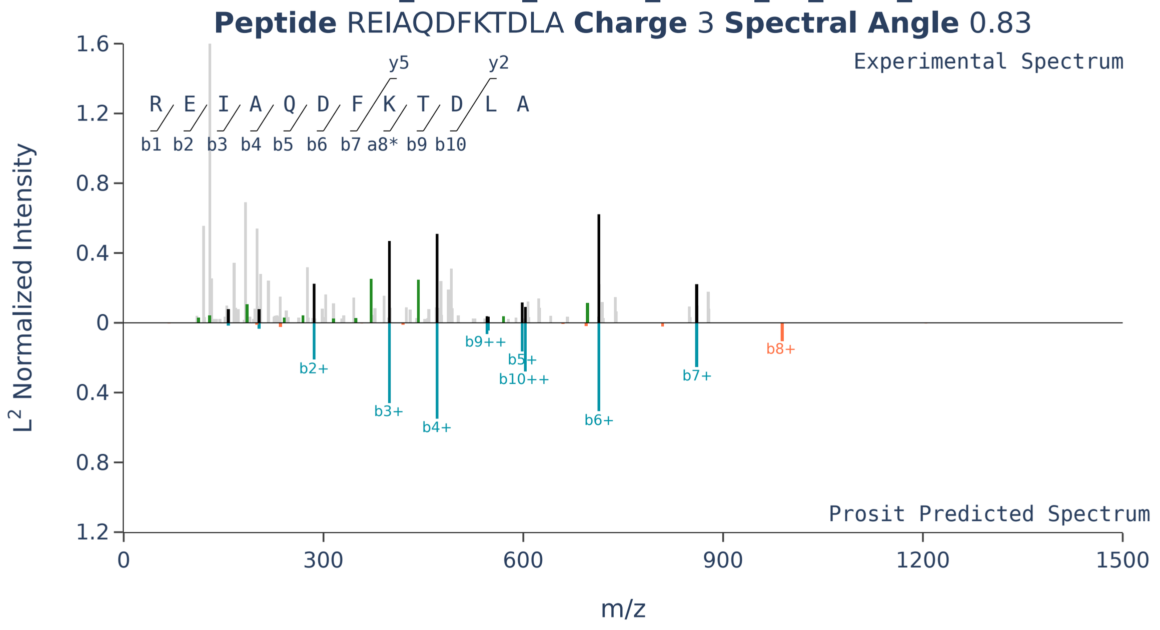

Source HRoetschke_271222_120123_HFGoe_H3_b1R1_24h **Scan** 19728
Peptide AYLVVREI **Charge** 2 **Spectral Angle** 0.82

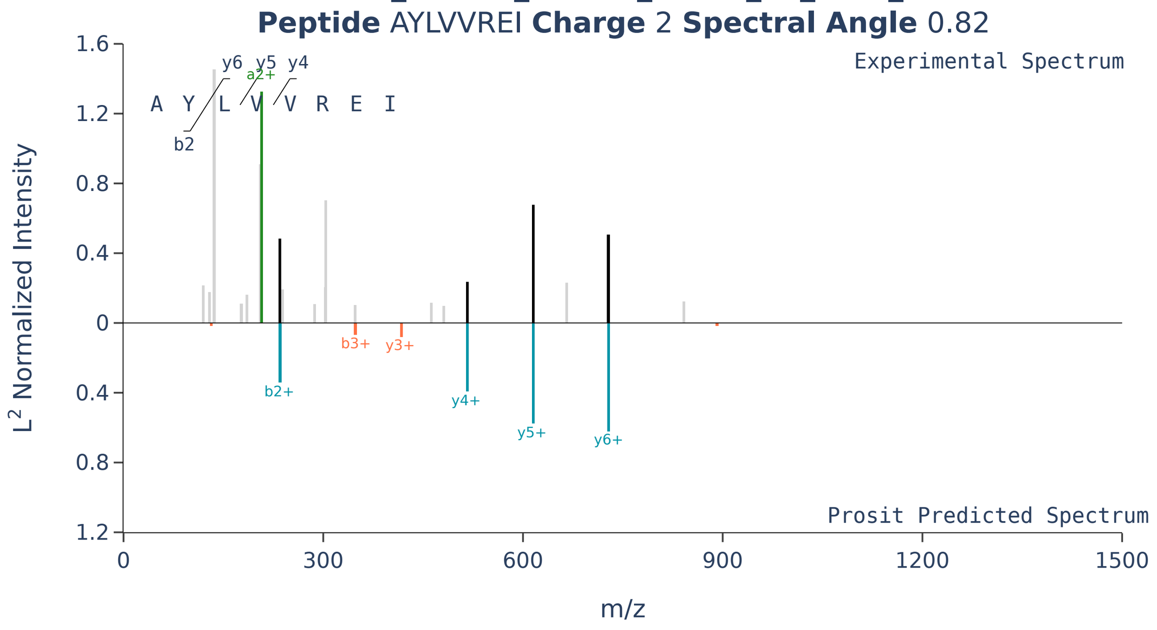

Source HRoetschke_271222_120123_HFGoe_H3_b2R1_4h **Scan** 18080
Peptide QSSAVMTDLRF **Charge** 3 **Spectral Angle** 0.83

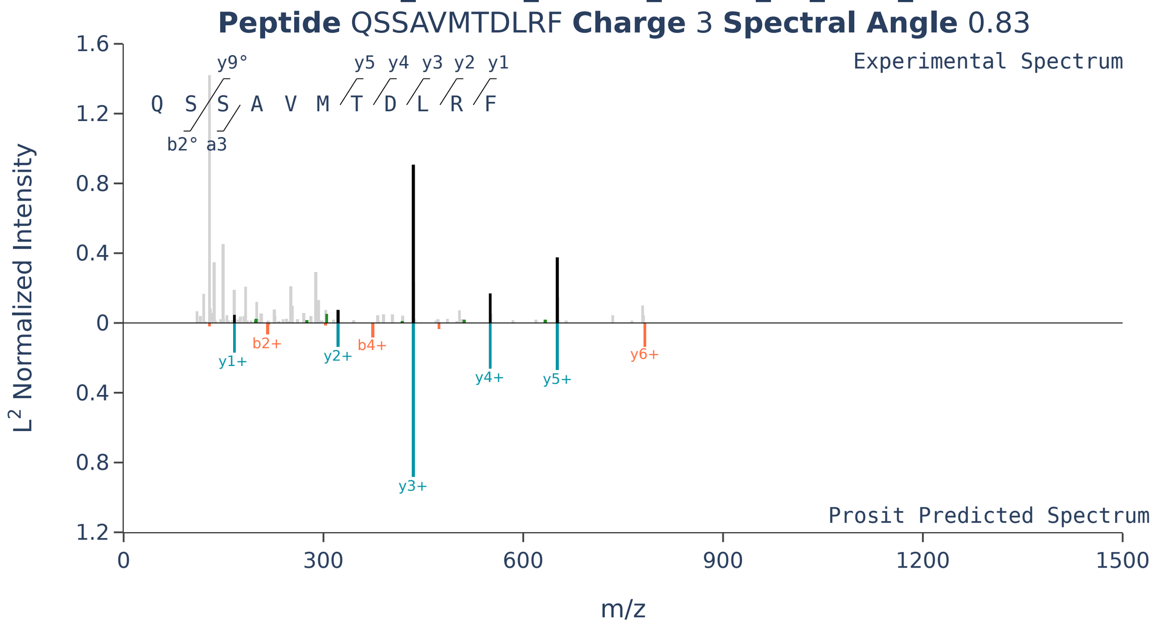

Source HRoetschke_271222_120123_HFGoe_H3_b1R1_24h **Scan** 6835
Peptide REIAQDFRKS **Charge** 3 **Spectral Angle** 0.87

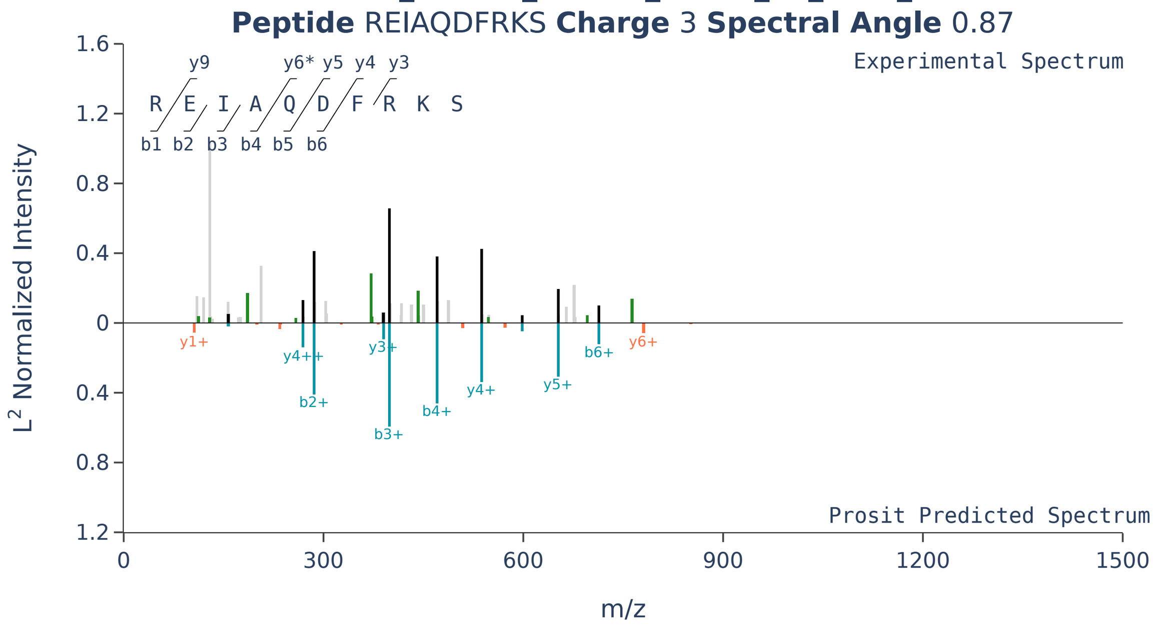

Source HRoetschke_271222_120123_HFGoe_H3_b1R1_1h **Scan** 14953
Peptide VAREIAQDF **Charge** 2 **Spectral Angle** 0.72

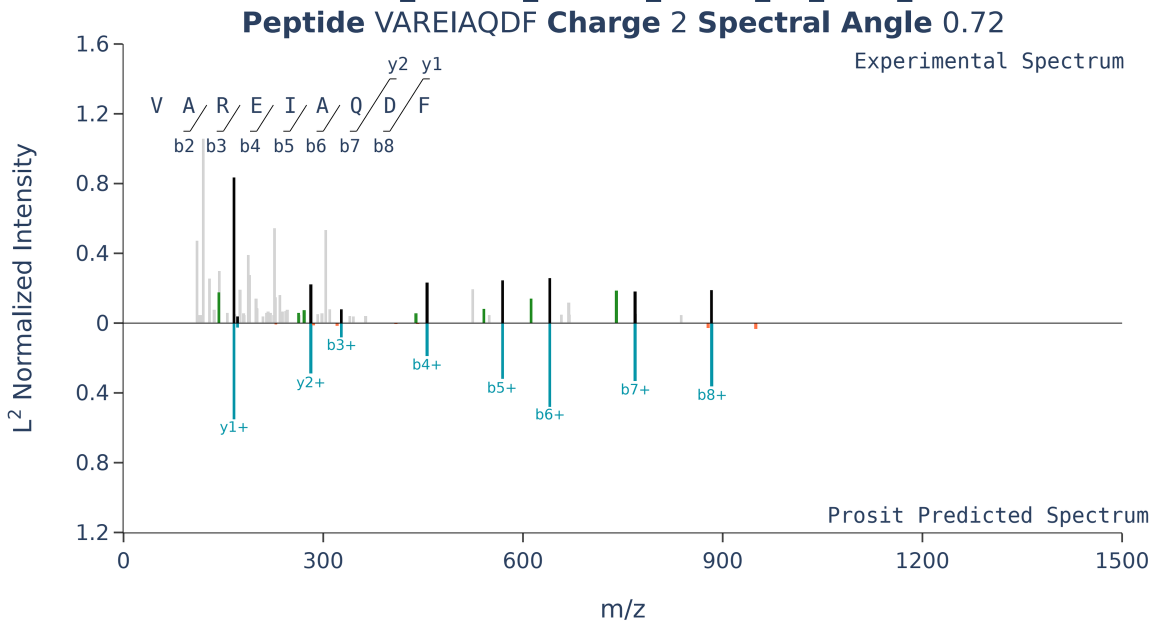

Source HRoetschke_271222_120123_HFGoe_H3_b1R1_24h **Scan** 13347
Peptide FKTDDTNL **Charge** 2 **Spectral Angle** 0.81

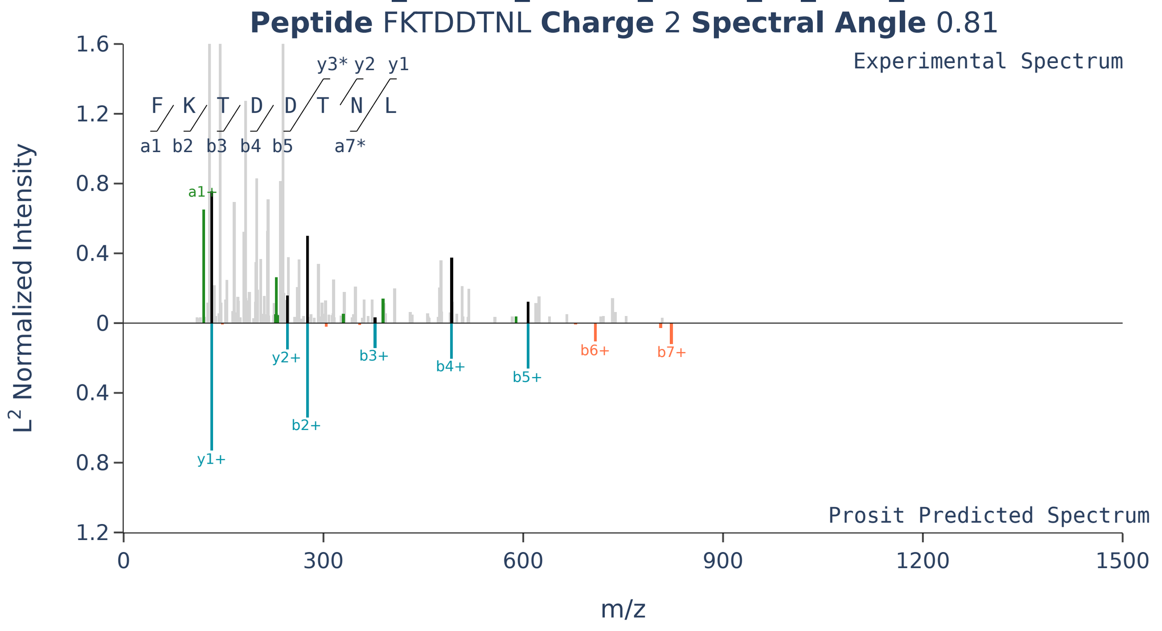

Source HRoetschke_271222_120123_HFGoe_H3_b2R2_4h **Scan** 9233
Peptide RFQSAIHA **Charge** 2 **Spectral Angle** 0.85

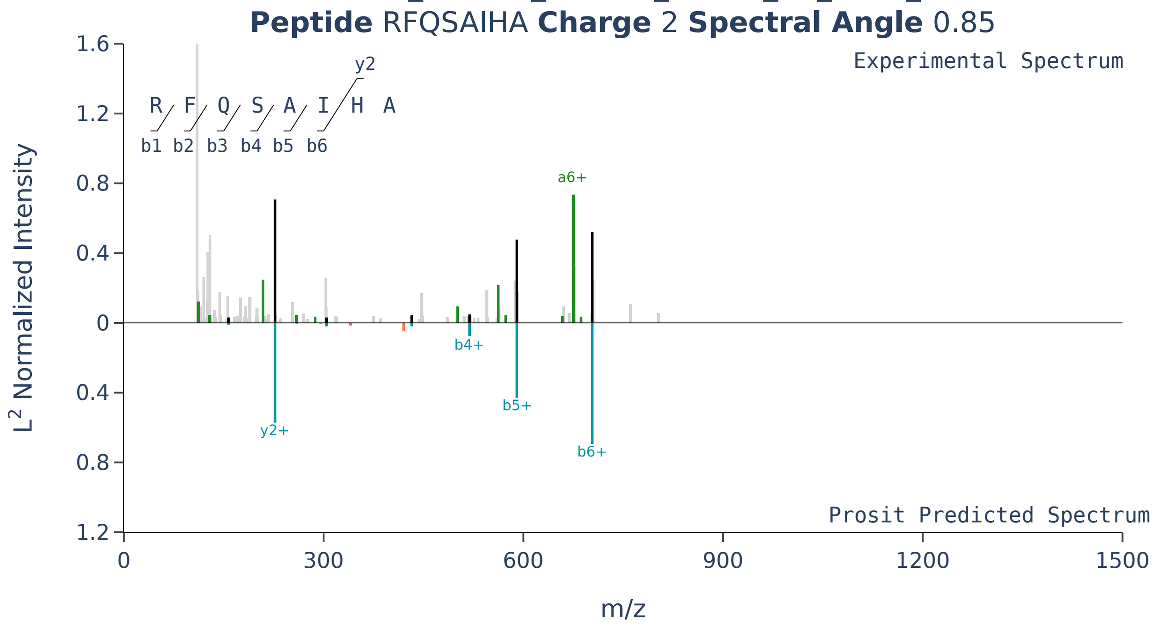

Source HRoetschke_271222_120123_HFGoe_H3_b1R1_1h **Scan** 11387
Peptide EDNLCAIHAKRV **Charge** 3 **Spectral Angle** 0.77

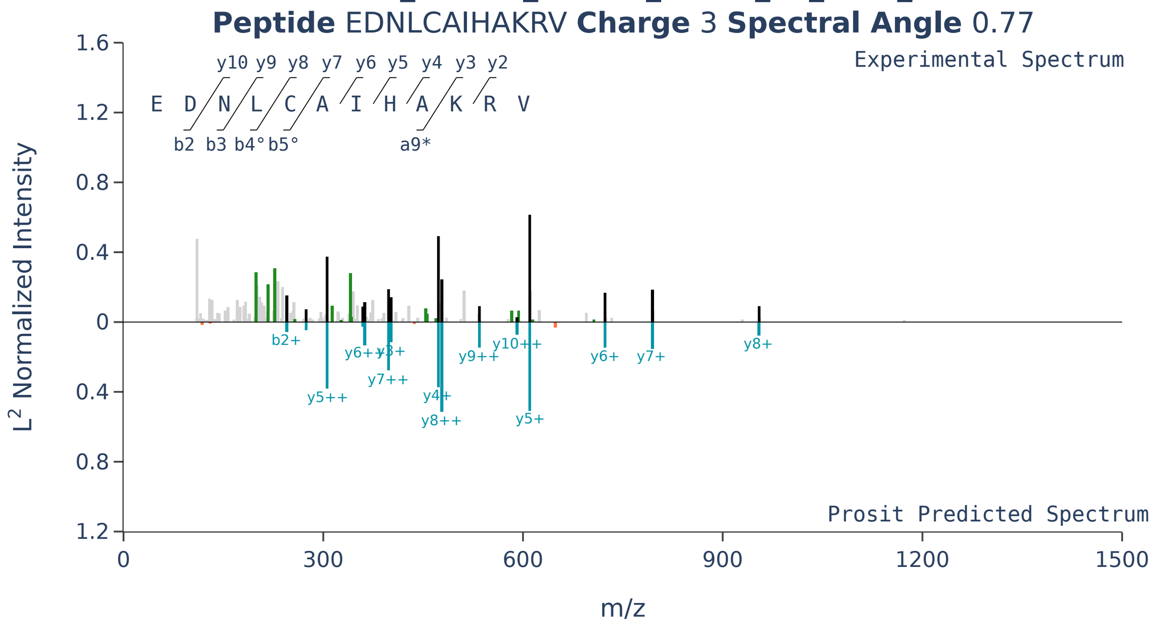

Source HRoetschke_271222_120123_HFGoe_H3_b2R2_24h **Scan** 11530
Peptide QKSTELAIHA **Charge** 3 **Spectral Angle** 0.95

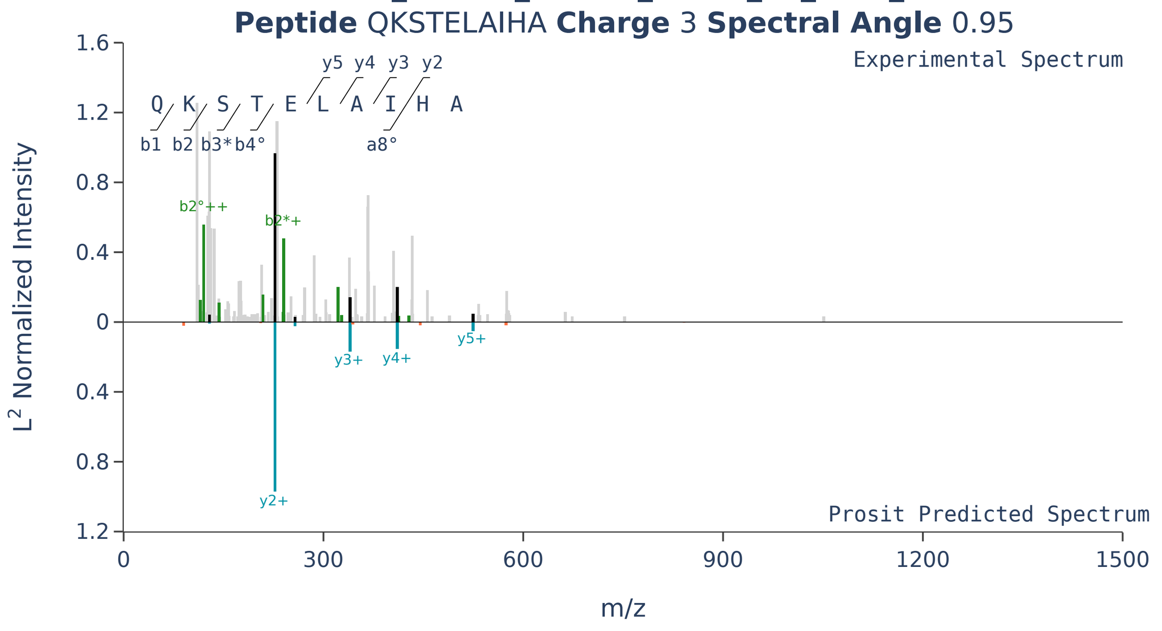

Source HRoetschke_271222_120123_HFGoe_H3_b1R1_4h **Scan** 13346
Peptide SQDFKTDL **Charge** 2 **Spectral Angle** 0.74

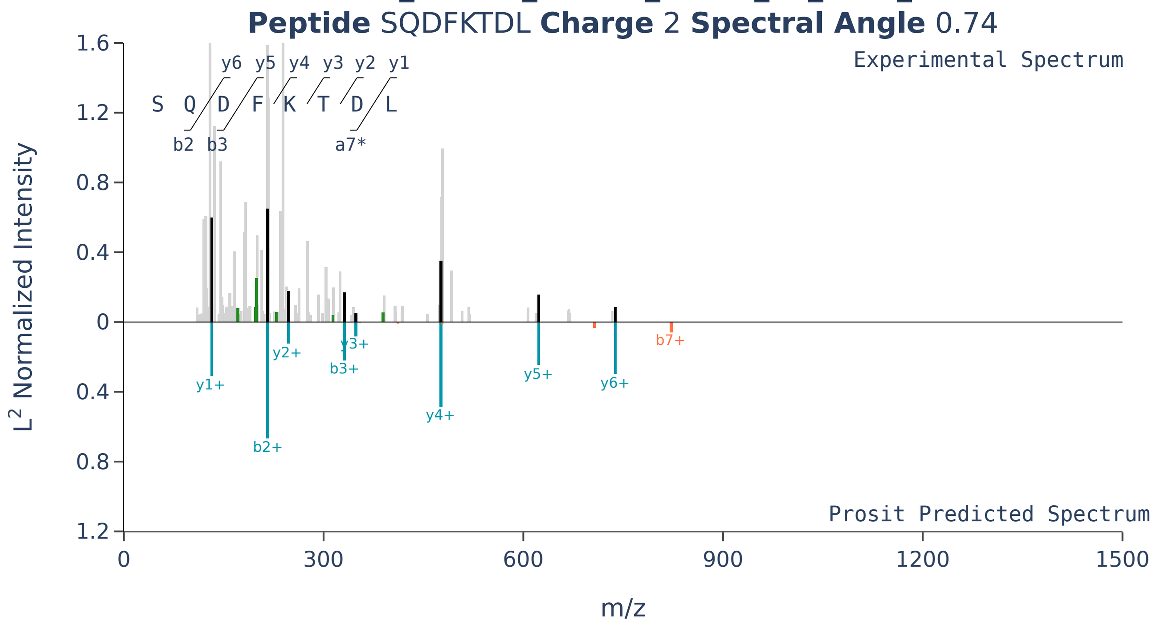

Source HRoetschke_271222_120123_HFGoe_H3_b2R1_4h **Scan** 13305
Peptide ASEAYLVKRV **Charge** 2 **Spectral Angle** 0.74

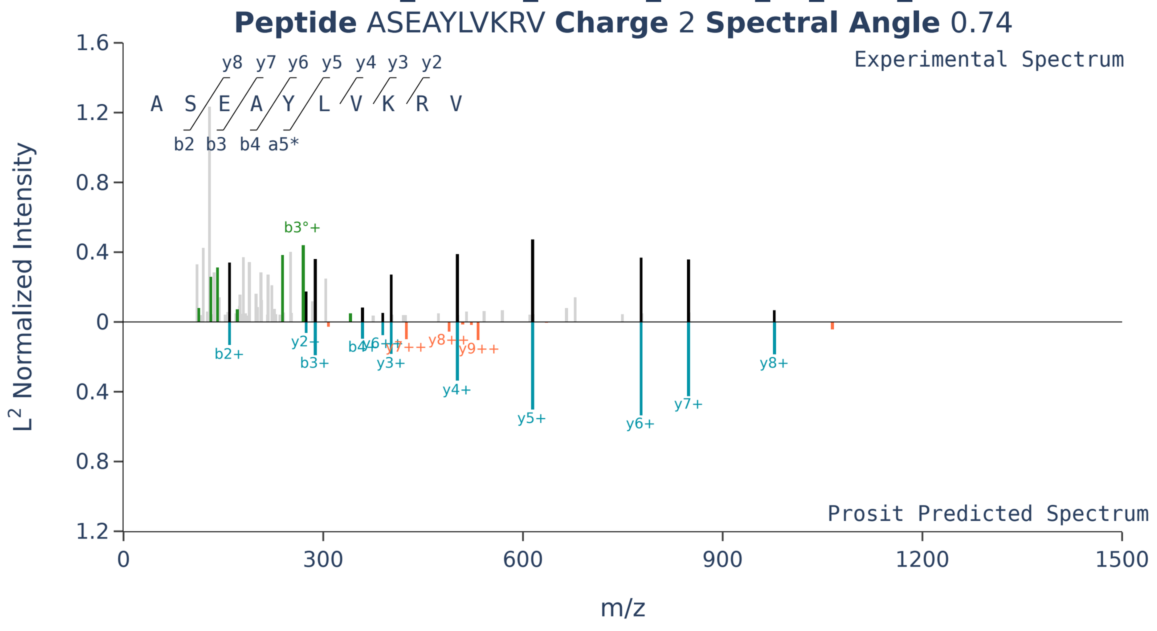

Source HRoetschke_271222_120123_HFGoe_H3_b1R1_24h **Scan** 13971
Peptide AQDFKTEL **Charge** 2 **Spectral Angle** 0.82

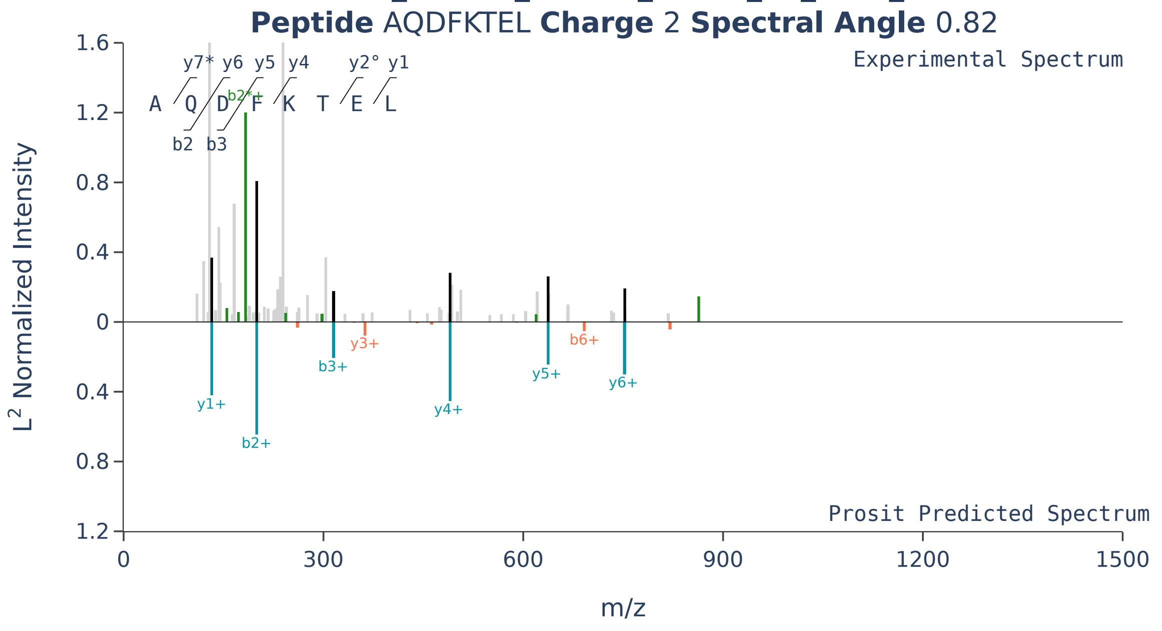

Source HRoetschke_271222_120123_HFGoe_H3_b1R2_24h **Scan** 12278
Peptide AQDFKTDLQSS **Charge** 2 **Spectral Angle** 0.78

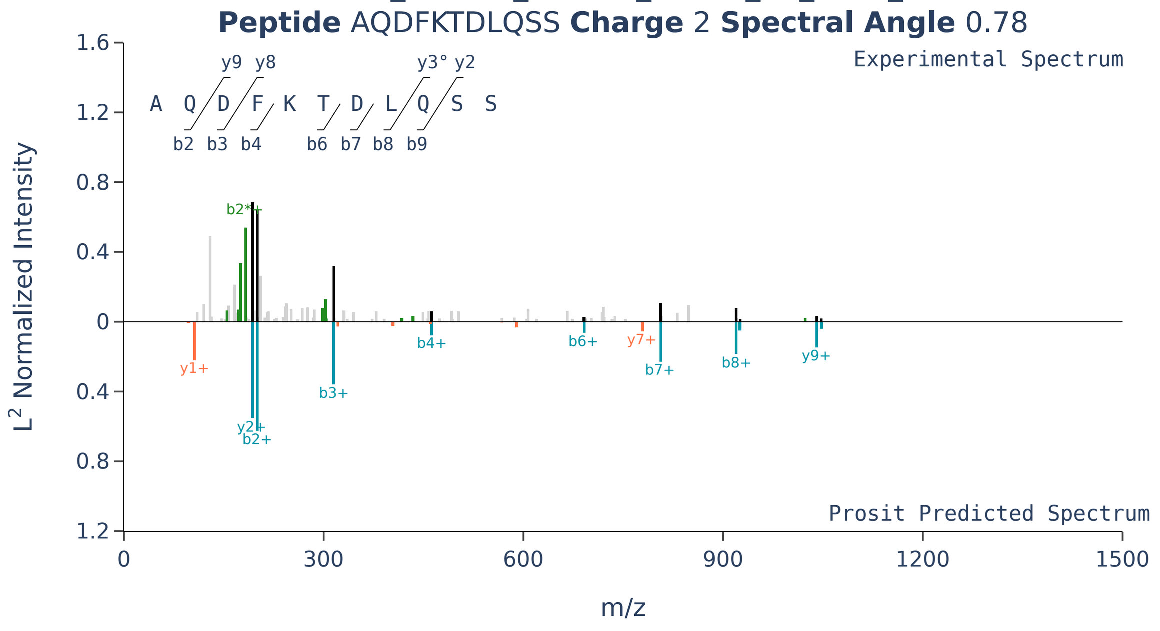

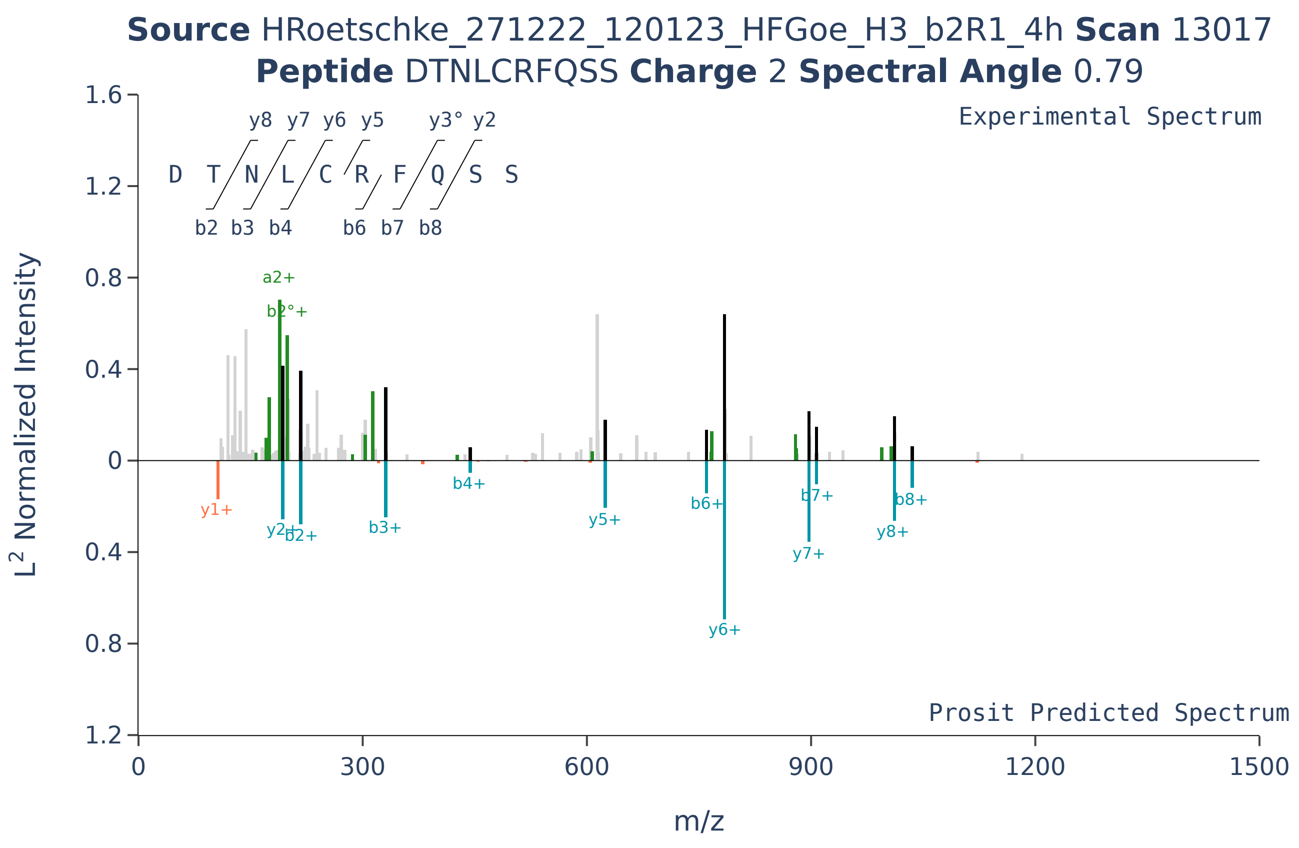
Source HRoetschke_271222_120123_HFGoe_H3_b2R1_4h Scan 13017
Peptide DTNLCRFQSS Charge 2 Spectral Angle 0.79
Experimental Spectrum
Prosit Predicted Spectrum
L² Normalized Intensity
m/z

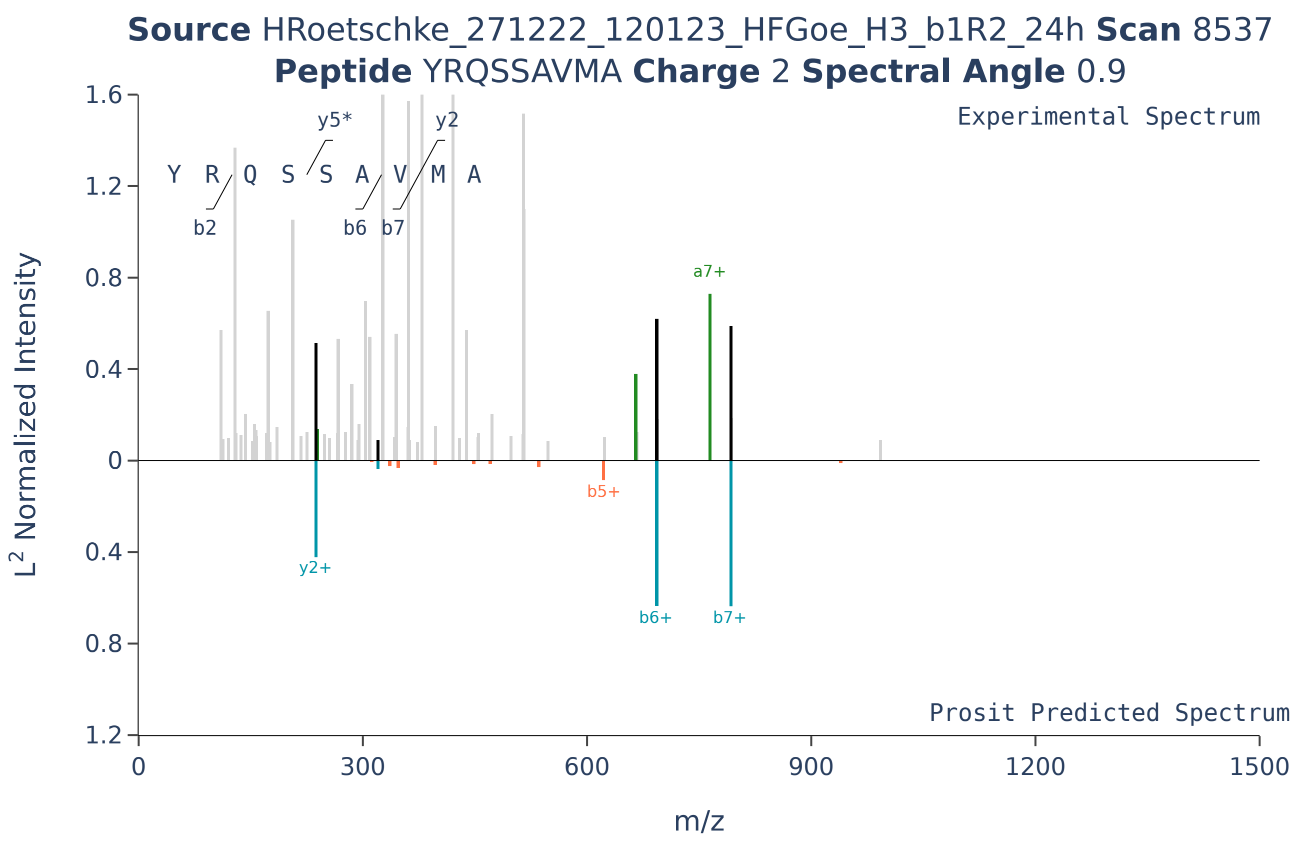
Source HRoetschke_271222_120123_HFGoe_H3_b1R2_24h Scan 8537
Peptide YRQSSAVMA Charge 2 Spectral Angle 0.9
Experimental Spectrum
Prosit Predicted Spectrum
L² Normalized Intensity
m/z

Source HRoetschke_291222_140123_HFGoe_H4_b2R1_4h **Scan** 14023
Peptide SGLIYEETRT **Charge** 2 **Spectral Angle** 0.85

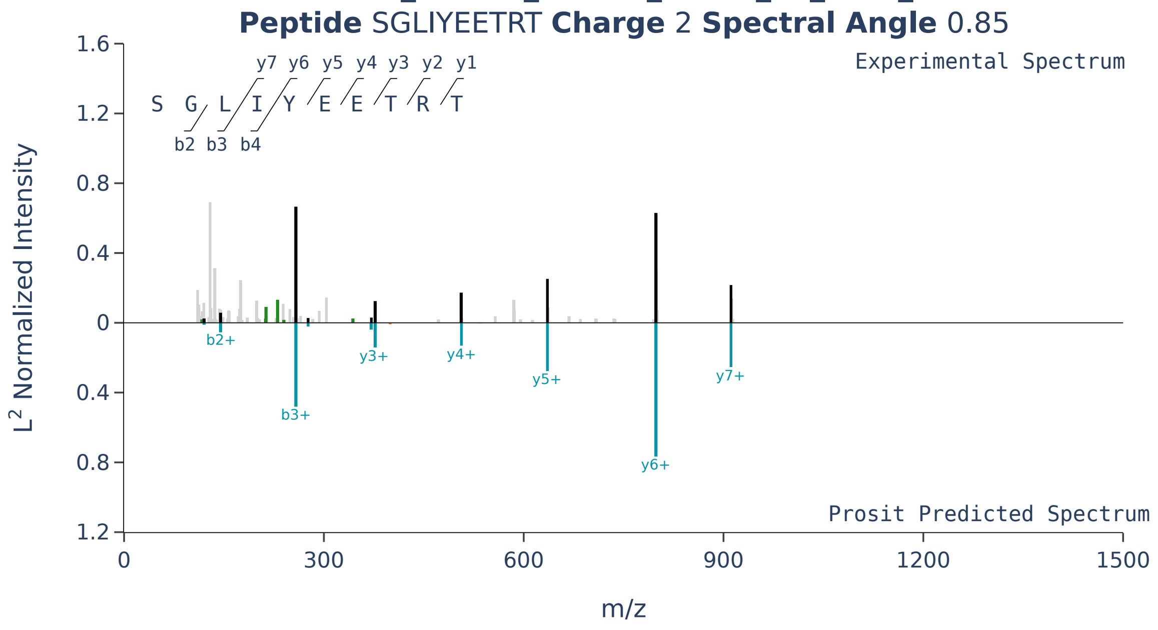

Source HRoetschke_291222_140123_HFGoe_H4_b1R1_24h **Scan** 13960
Peptide SGLIYEETRD **Charge** 1 **Spectral Angle** 0.87

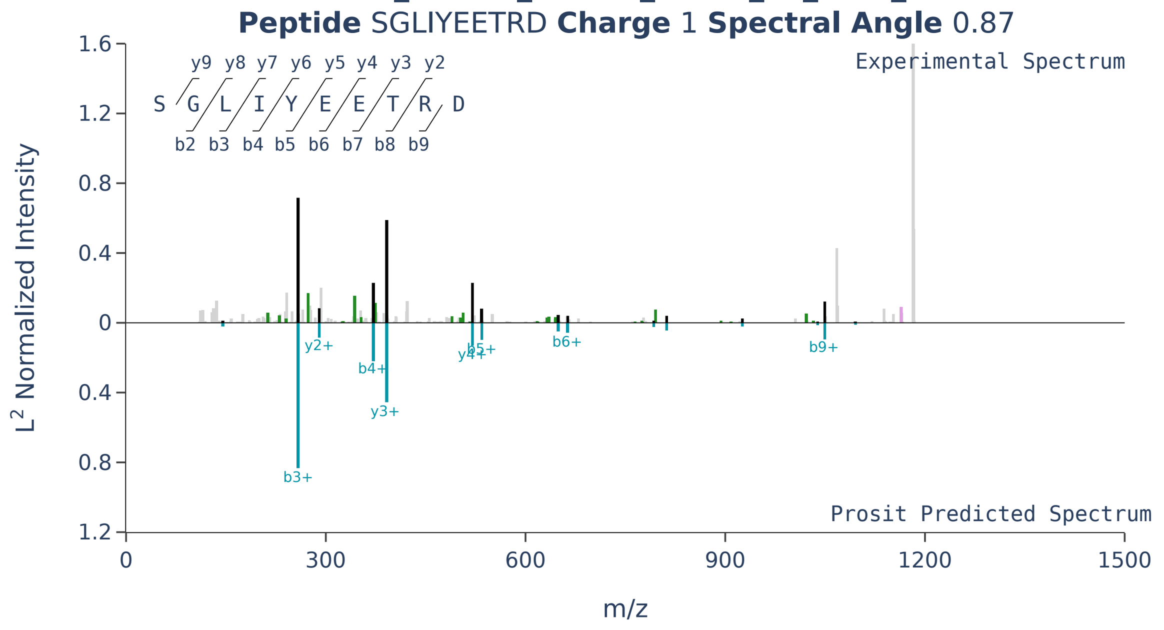

Source HRoetschke_291222_140123_HFGoe_H4_b1R1_0h **Scan** 13964
Peptide TRGVLIYEETR **Charge** 3 **Spectral Angle** 0.86

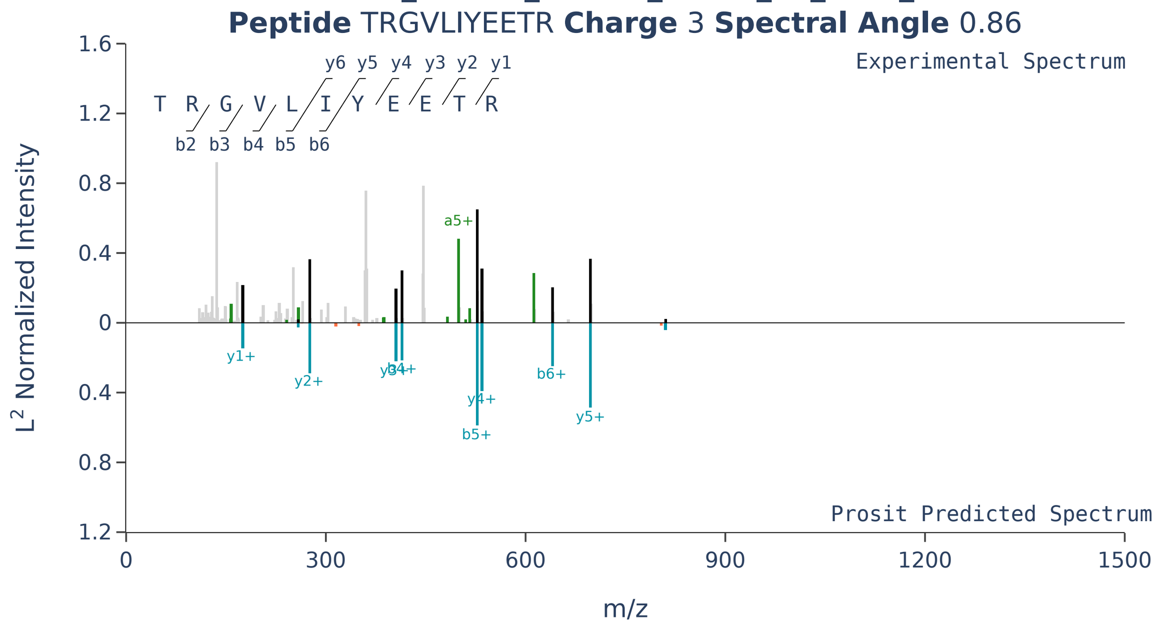

Source HRoetschke_291222_140123_HFGoe_H4_b1R2_1h **Scan** 8743
Peptide VYALKRGA **Charge** 2 **Spectral Angle** 0.88

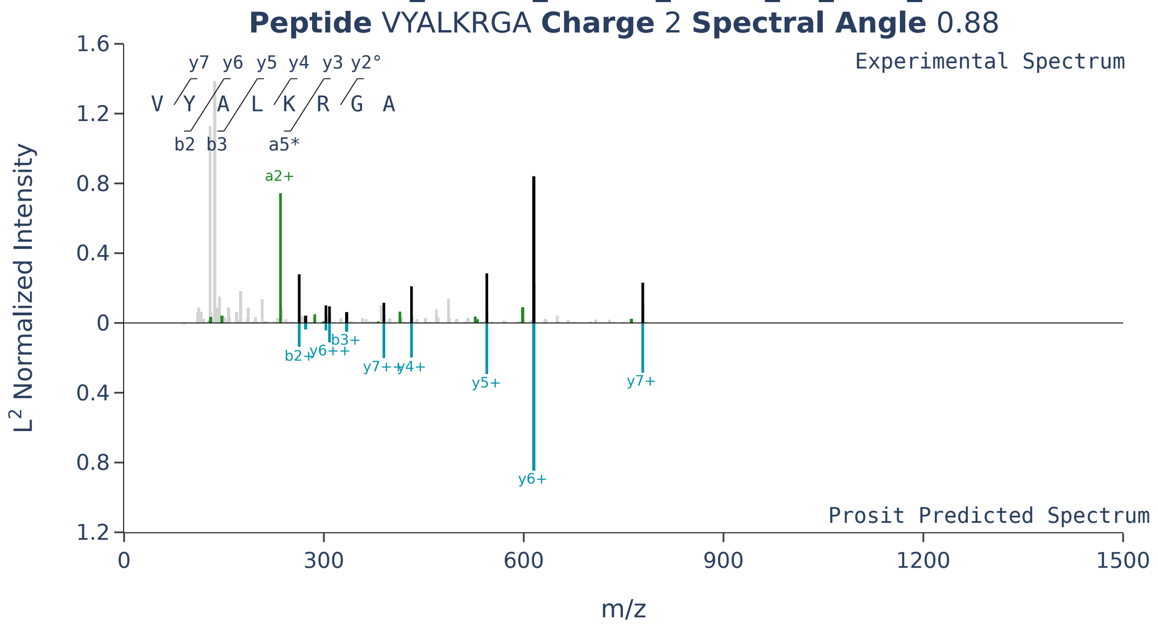

Source HRoetschke_291222_140123_HFGoe_H4_b1R2_24h **Scan** 12793
Peptide SGLIYEERG **Charge** 2 **Spectral Angle** 0.83

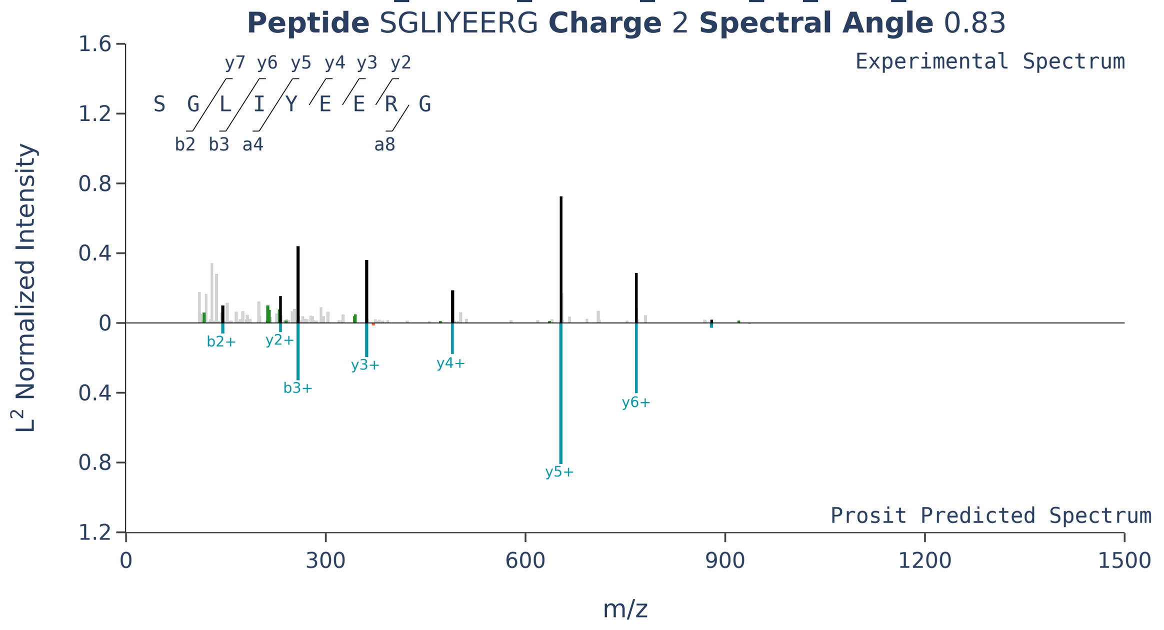

Source HRoetschke_291222_140123_HFGoe_H4_b1R1_24h **Scan** 16355
Peptide TAMDVVVY **Charge** 1 **Spectral Angle** 0.77

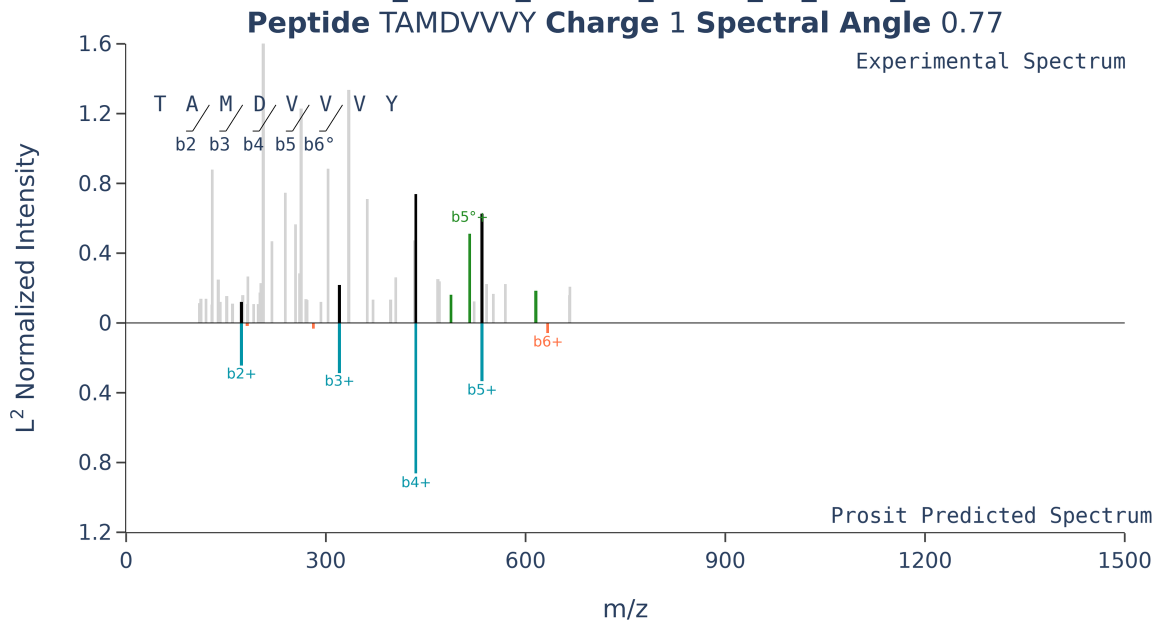

Source HRoetschke_291222_140123_HFGoe_H4_b1R2_24h **Scan** 10608
Peptide AMRGVLKV **Charge** 2 **Spectral Angle** 0.87

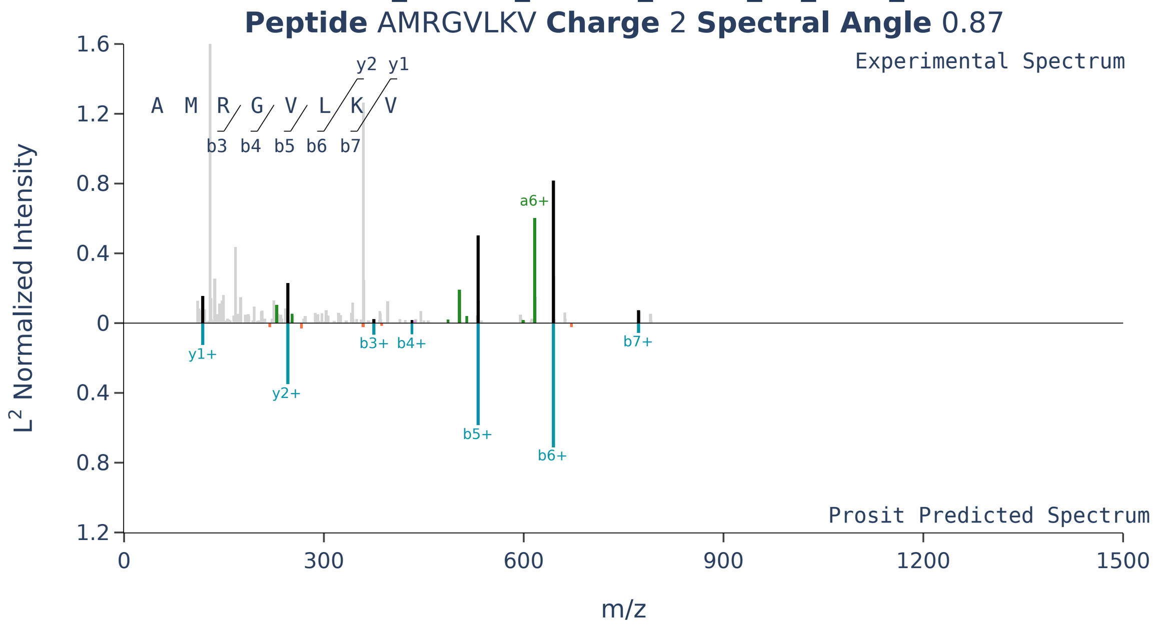

Source HRoetschke_291222_140123_HFGoe_H4_b1R2_24h **Scan** 18414
Peptide AMDVLYG **Charge** 1 **Spectral Angle** 0.92

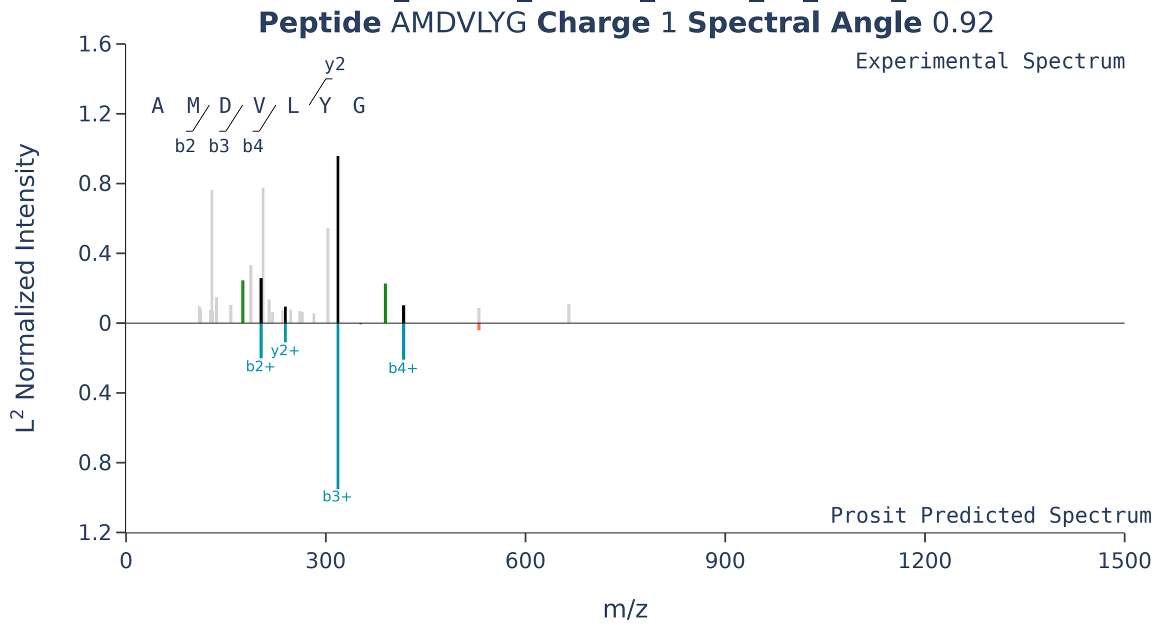

Source HRoetschke_291222_140123_HFGoe_H4_b2R1_1h **Scan** 8501
Peptide VYALGAKR **Charge** 2 **Spectral Angle** 0.87

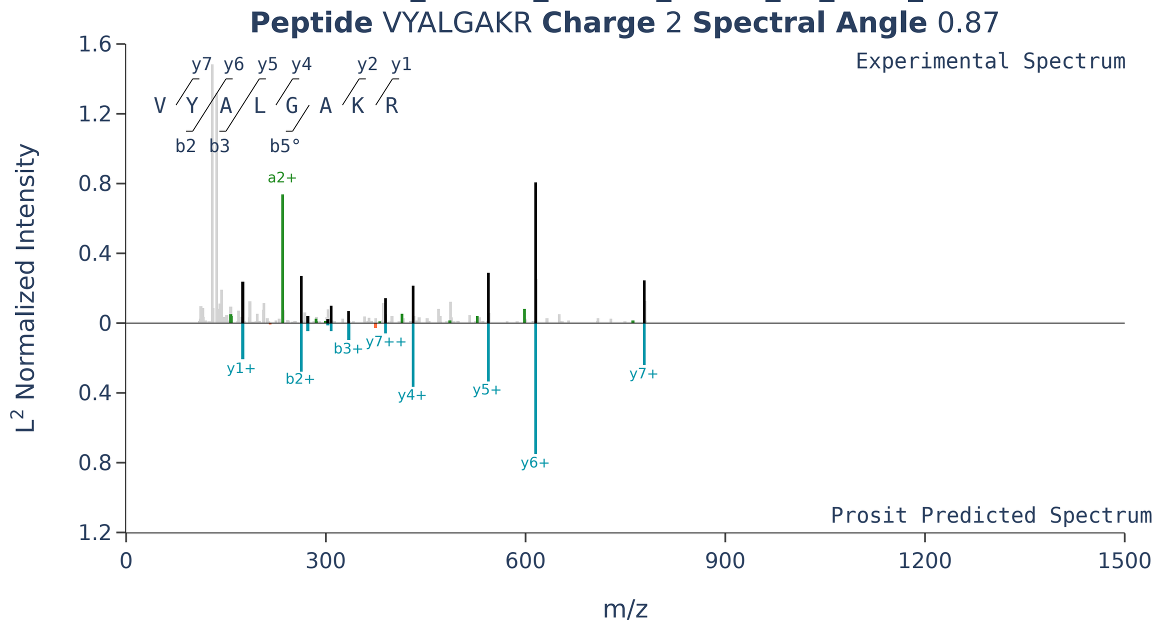

Source HRoetschke_291222_140123_HFGoe_H4_b2R1_4h **Scan** 13389
Peptide GRSGLIYE **Charge** 2 **Spectral Angle** 0.9

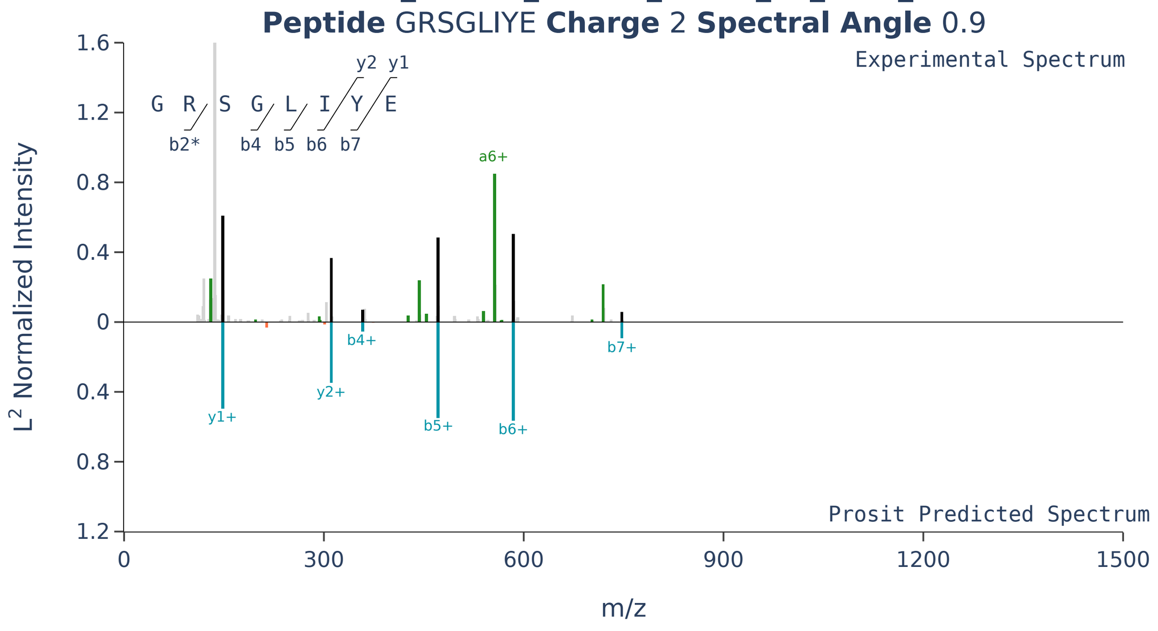

Source HRoetschke_291222_140123_HFGoe_H4_b1R1_24h **Scan** 13322
Peptide RISGLYE **Charge** 2 **Spectral Angle** 0.87

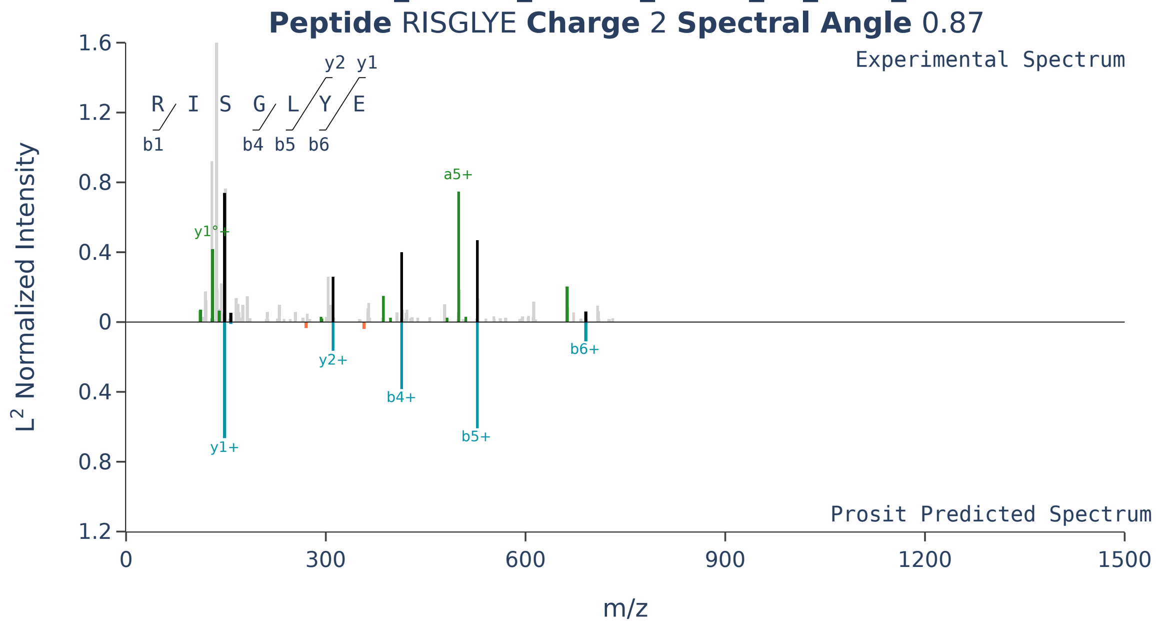

Source HRoetschke_291222_140123_HFGoe_H4_b1R2_1h **Scan** 17416
Peptide TSGLIYEET **Charge** 2 **Spectral Angle** 0.91

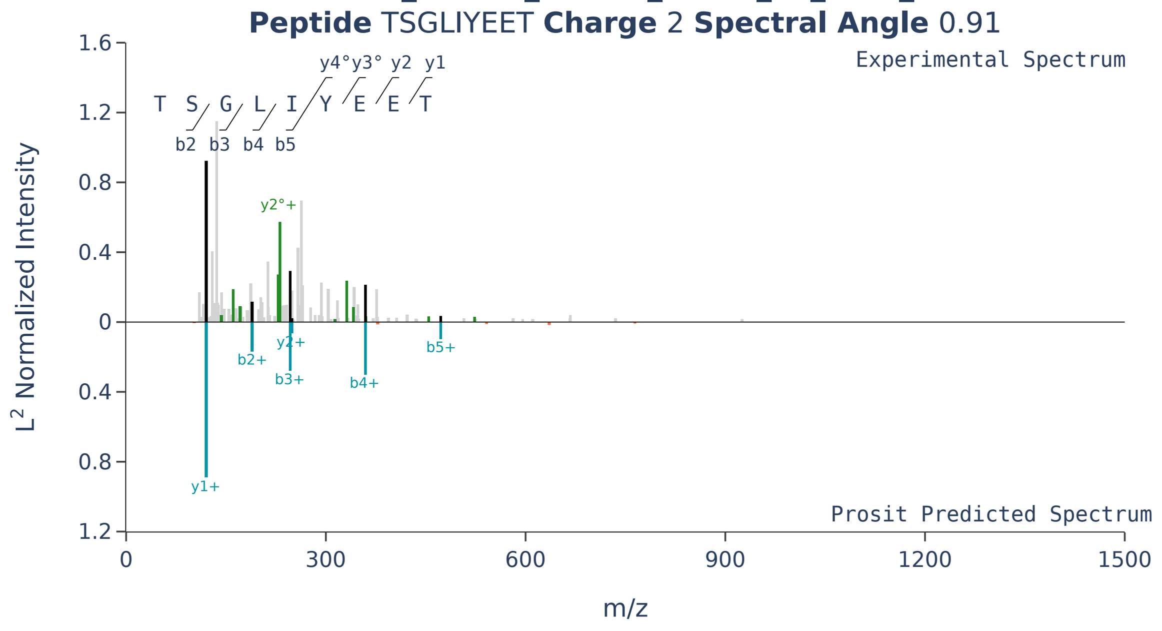

Source HRoetschke_291222_140123_HFGoe_H4_b1R2_24h **Scan** 9145
Peptide YRDNIQGITK **Charge** 3 **Spectral Angle** 0.88

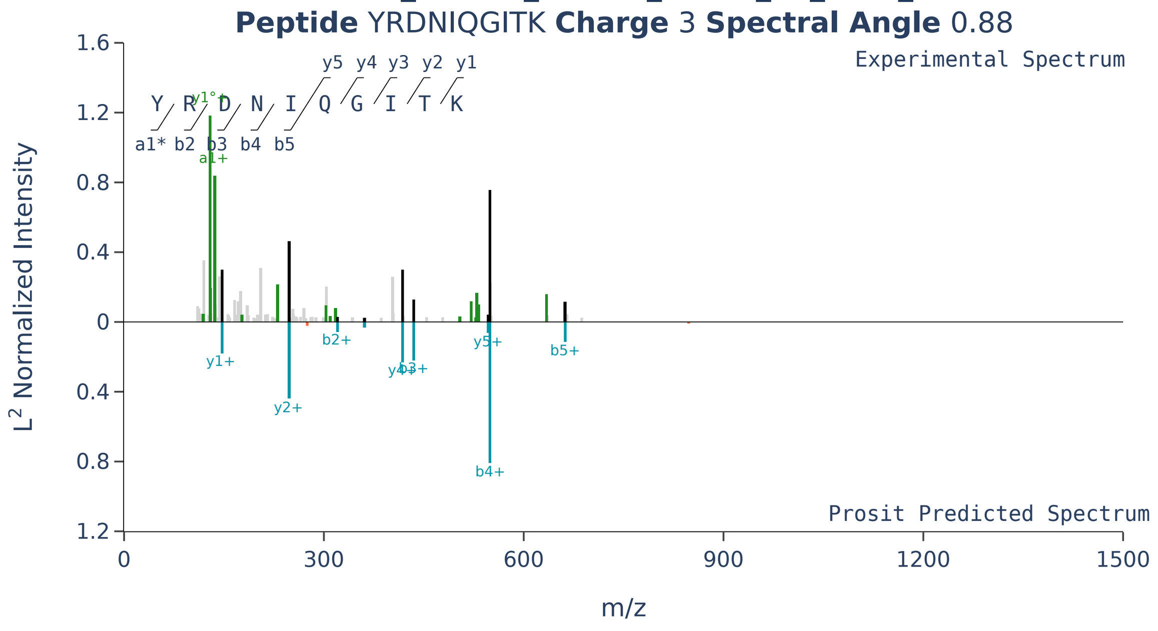

Source HRoetschke_291222_140123_HFGoe_H4_b1R2_24h **Scan** 13784
Peptide RGNIQGITKPAI **Charge** 3 **Spectral Angle** 0.83

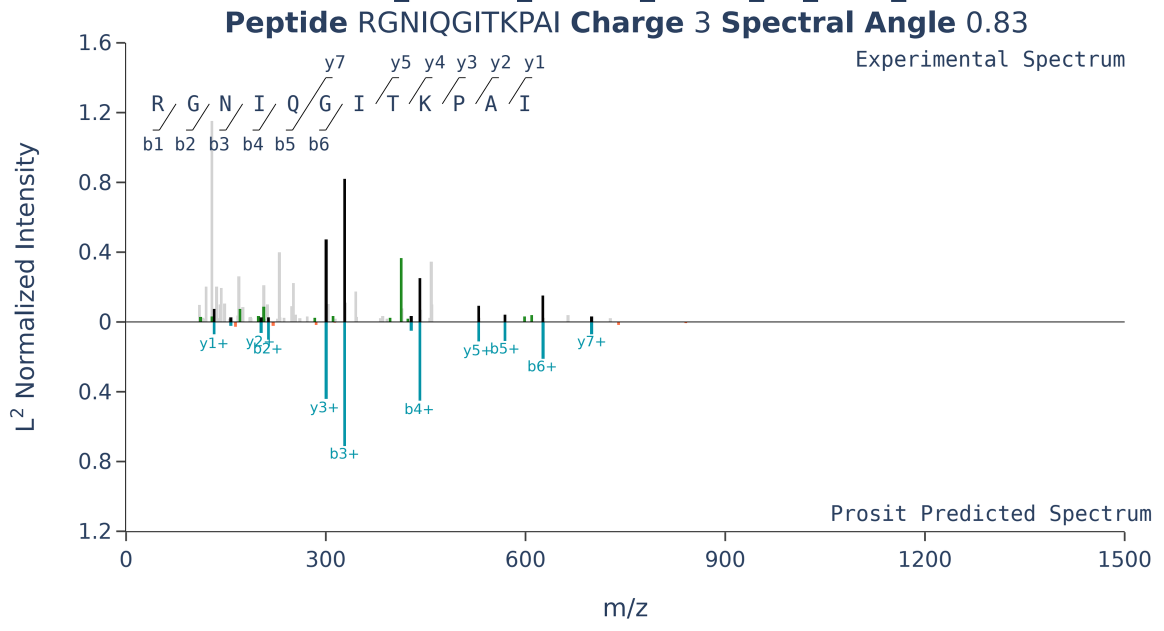

Source HRoetschke_291222_140123_HFGoe_H4_b1R2_4h **Scan** 18811
Peptide SGLIYEETLRD **Charge** 2 **Spectral Angle** 0.77

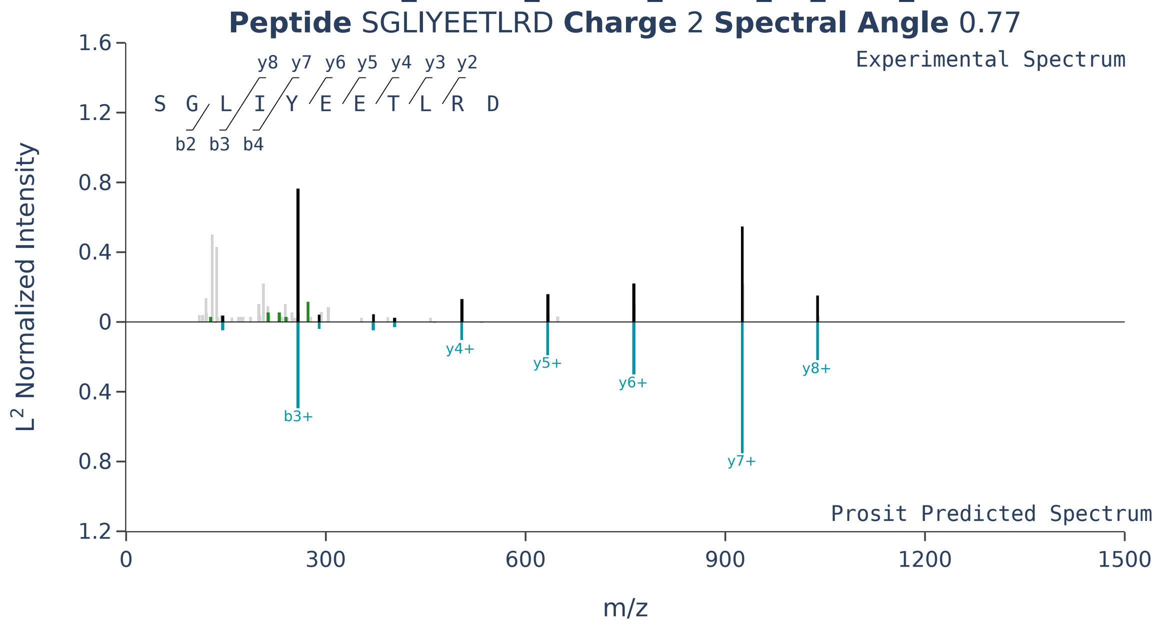

Source HRoetschke_291222_140123_HFGoe_H4_b1R2_24h **Scan** 9127
Peptide TAMDVVYGR **Charge** 2 **Spectral Angle** 0.86

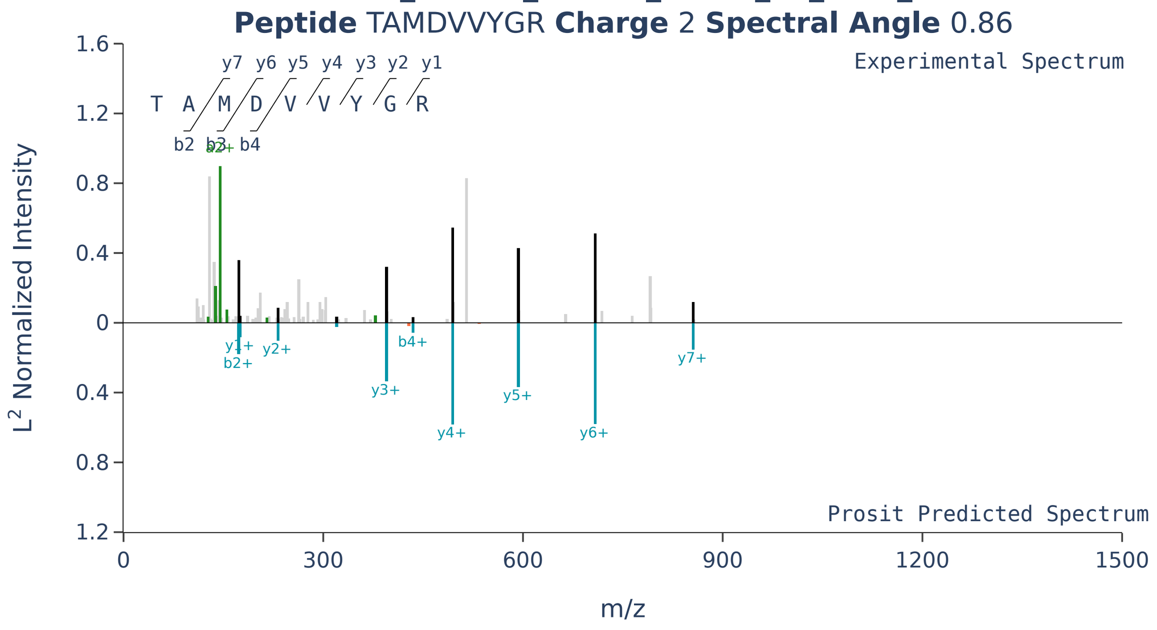

Source HRoetschke_291222_140123_HFGoe_H4_b1R2_24h **Scan** 12310
Peptide YRDNIQGITKPAIR **Charge** 4 **Spectral Angle** 0.88

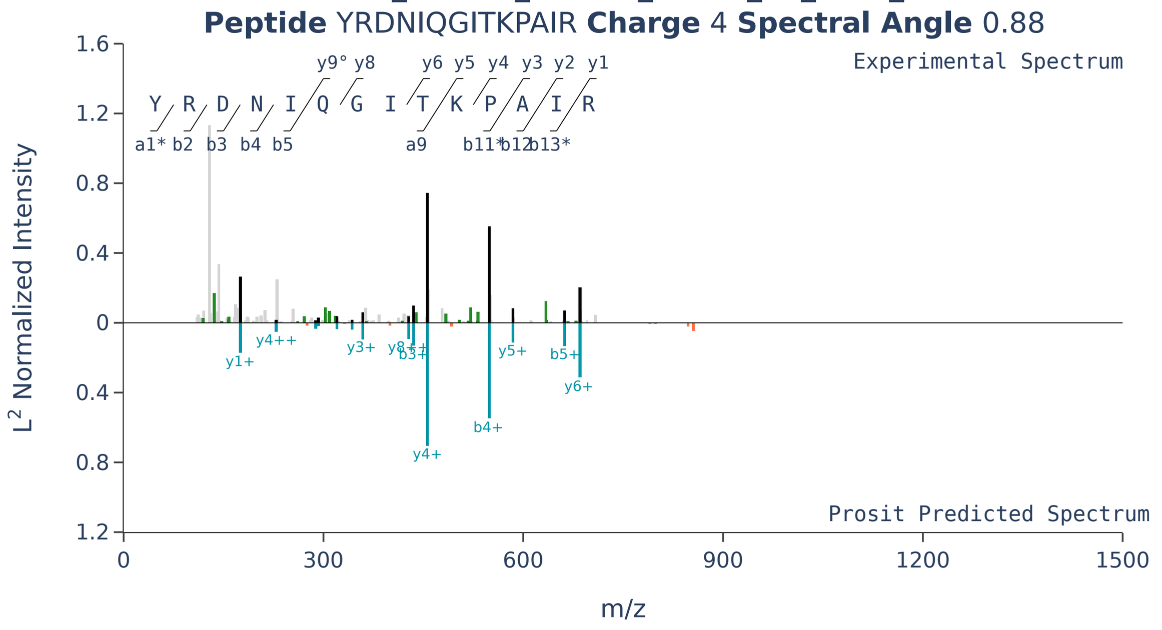

Source HRoetschke_291222_140123_HFGoe_H4_b1R2_24h **Scan** 8371
Peptide AMDVVYRG **Charge** 2 **Spectral Angle** 0.9

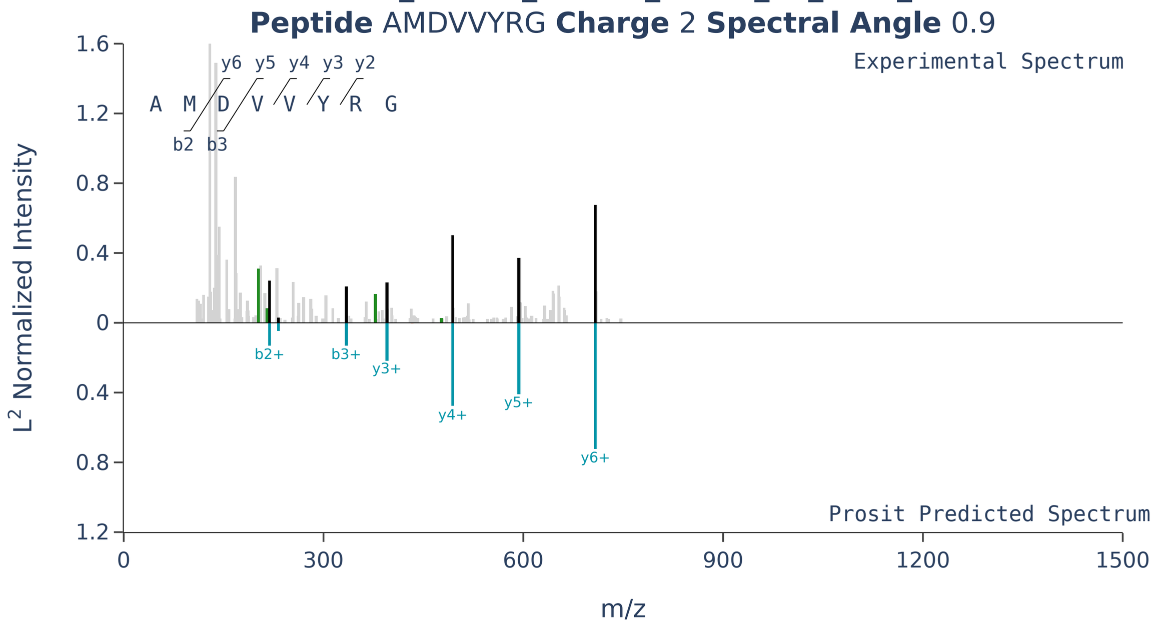

Source HRoetschke_291222_140123_HFGoe_H4_b1R1_2h **Scan** 14573
Peptide SGLIYEETRGGVKRI **Charge** 3 **Spectral Angle** 0.86

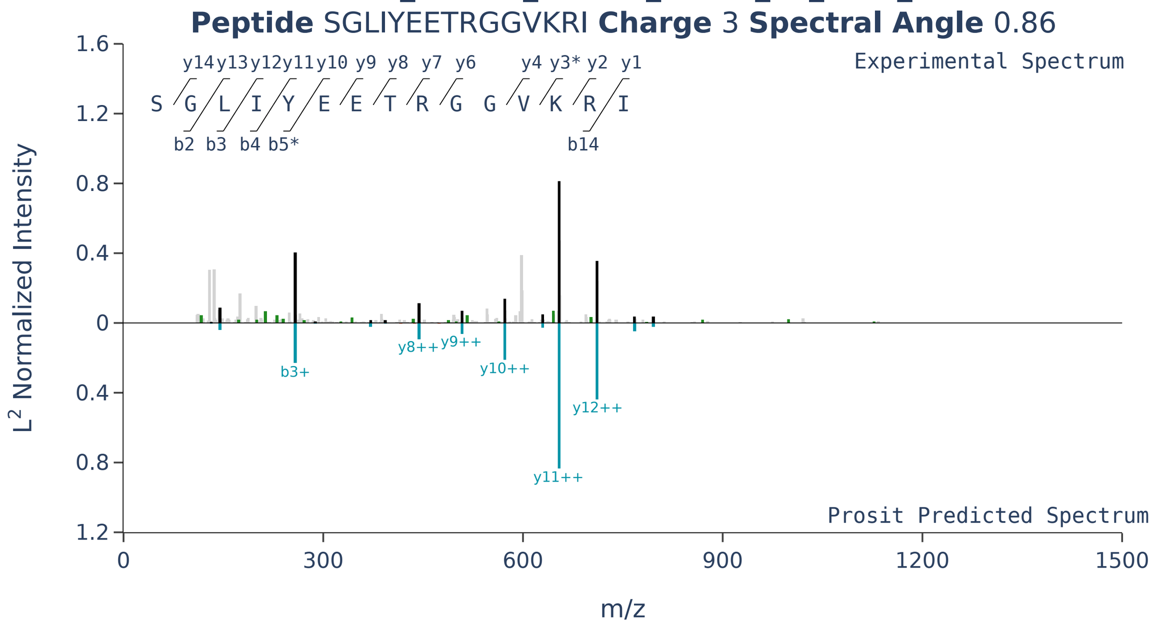

Source HRoetschke_291222_140123_HFGoe_H4_b2R1_4h **Scan** 13850
Peptide TSGLIYEETRG **Charge** 2 **Spectral Angle** 0.83

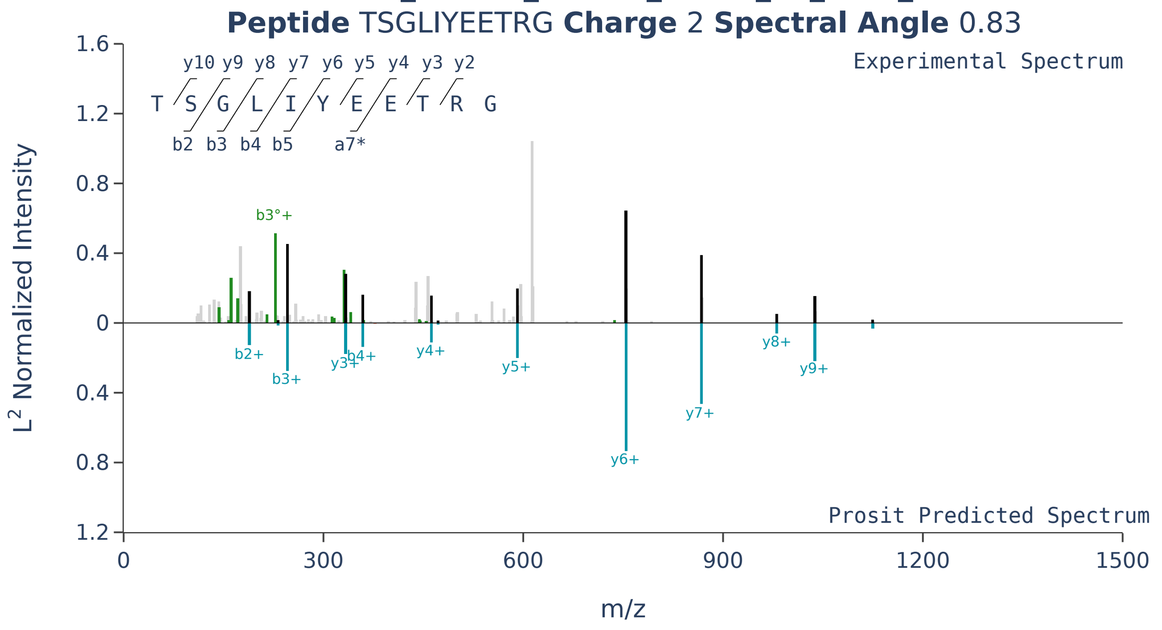

Source HRoetschke_291222_140123_HFGoe_H4_b1R1_24h **Scan** 12628
Peptide SGLIYEETGGVKR **Charge** 2 **Spectral Angle** 0.8

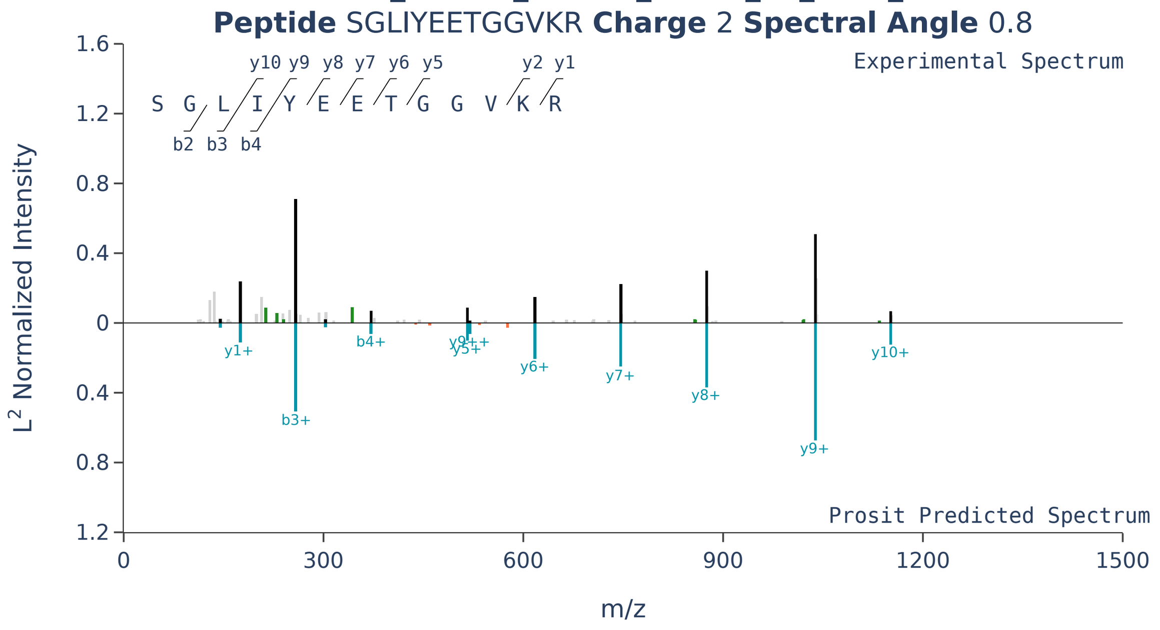

Source HRoetschke_291222_140123_HFGoe_H4_b1R2_1h **Scan** 17476
Peptide ISGLIYEETRD **Charge** 2 **Spectral Angle** 0.84

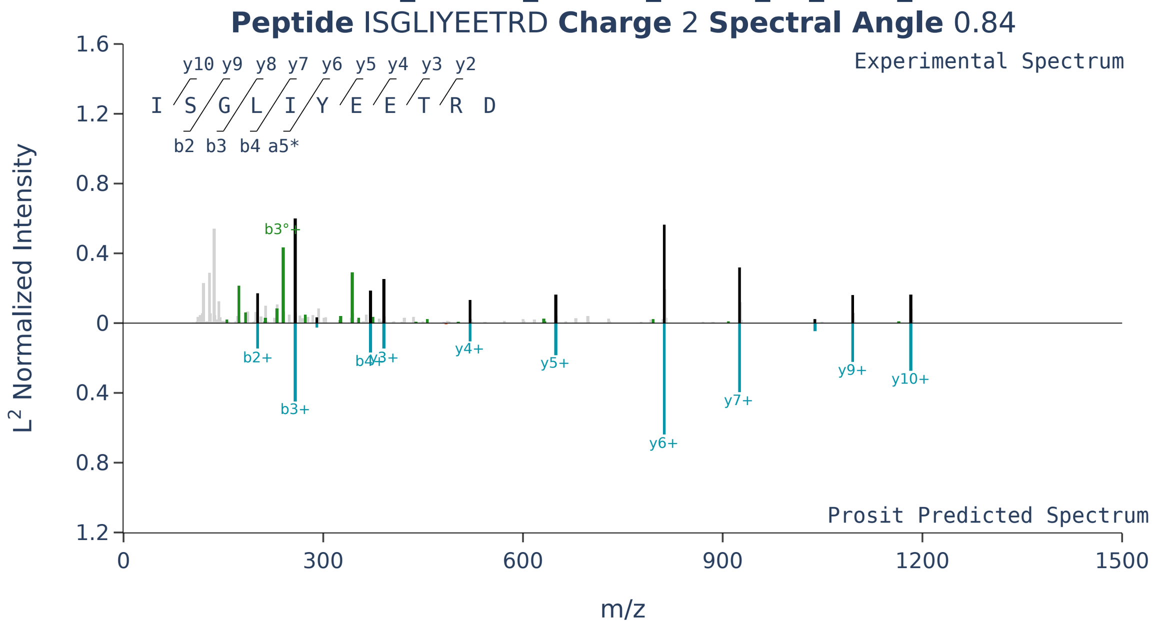

Source HRoetschke_291222_140123_HFGoe_H4_b2R2_24h **Scan** 11048
Peptide ARDNIQGITKPAIR **Charge** 4 **Spectral Angle** 0.86

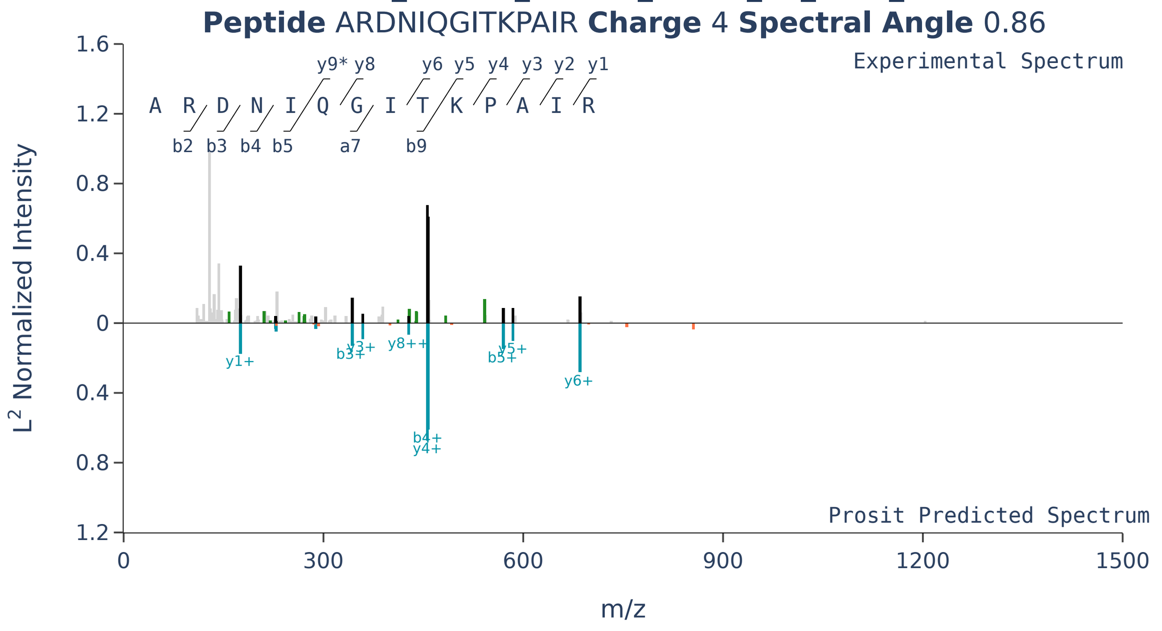

Source HRoetschke_291222_140123_HFGoe_H4_b2R1_4h **Scan** 14163
Peptide TRGSGLIYEET **Charge** 2 **Spectral Angle** 0.8

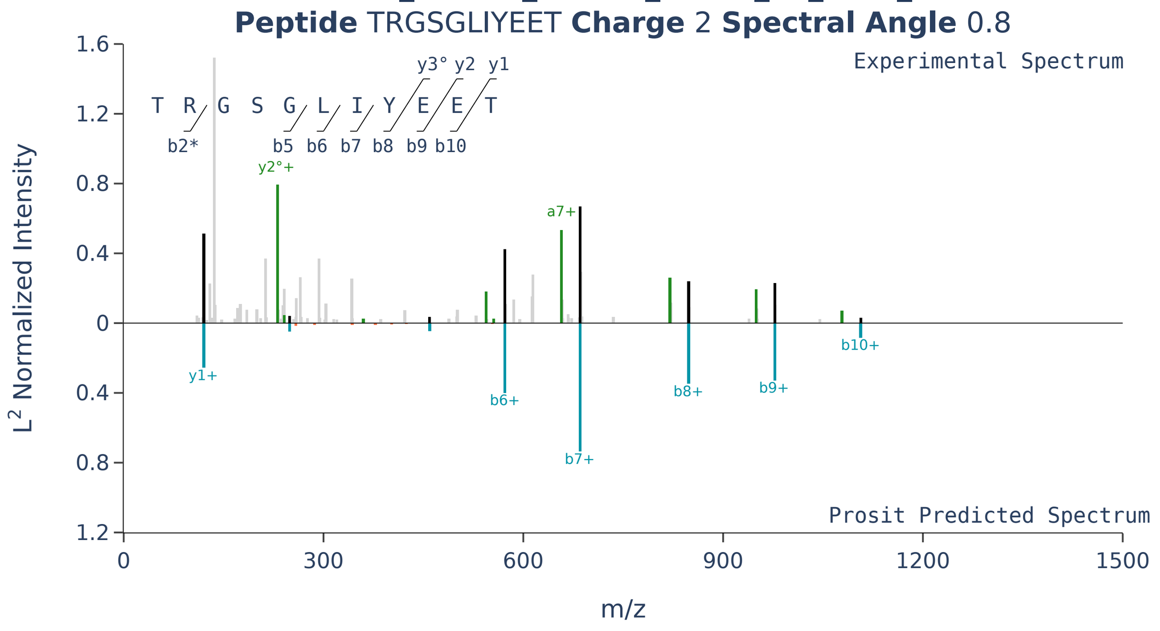

Source HRoetschke_291222_140123_HFGoe_H4_b2R1_24h **Scan** 13166
Peptide GKGGSGLIYE **Charge** 2 **Spectral Angle** 0.78

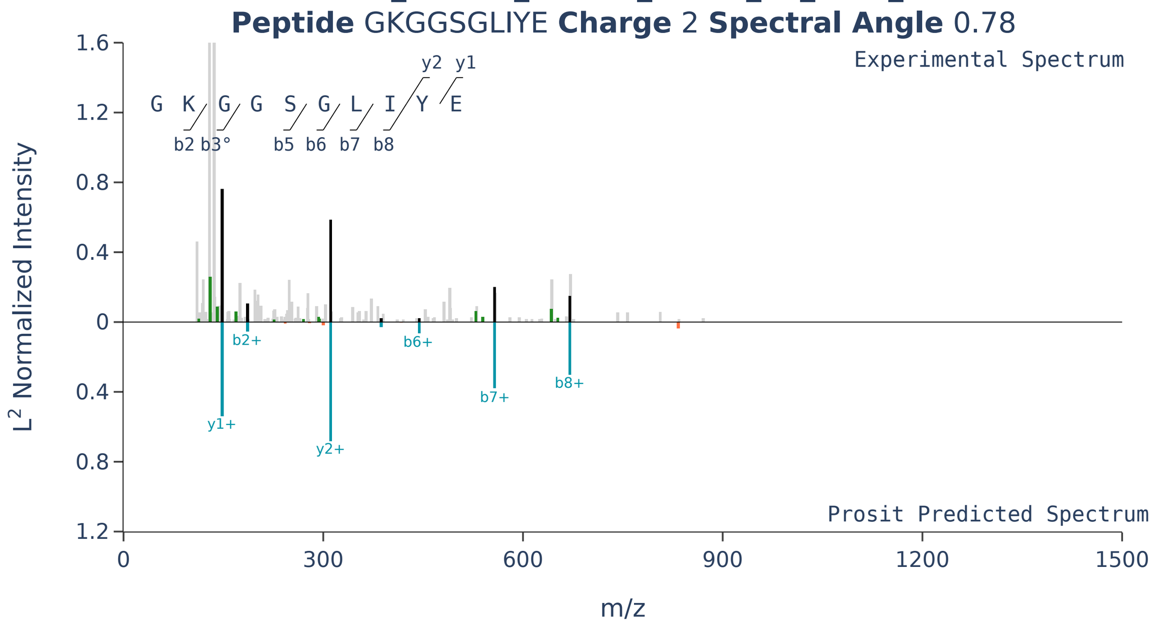

Source HRoetschke_291222_140123_HFGoe_H4_b2R1_24h **Scan** 13538
Peptide SGRGLIYE **Charge** 2 **Spectral Angle** 0.88

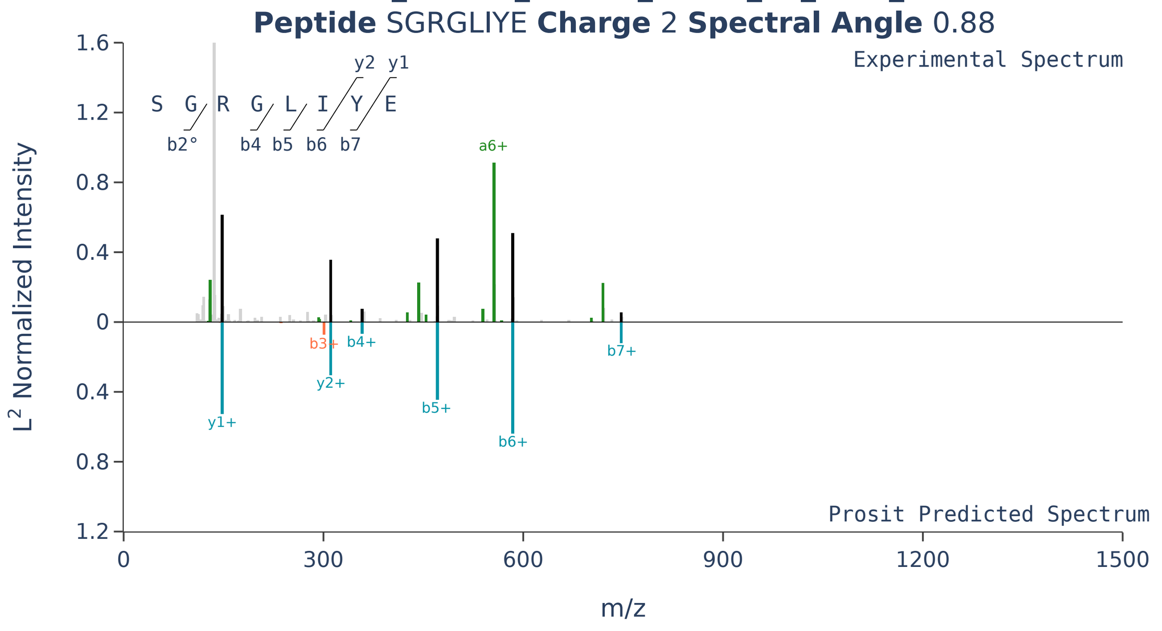

Source HRoetschke_291222_140123_HFGoe_H4_b2R1_4h **Scan** 9874
Peptide TKPAIRLR **Charge** 2 **Spectral Angle** 0.86

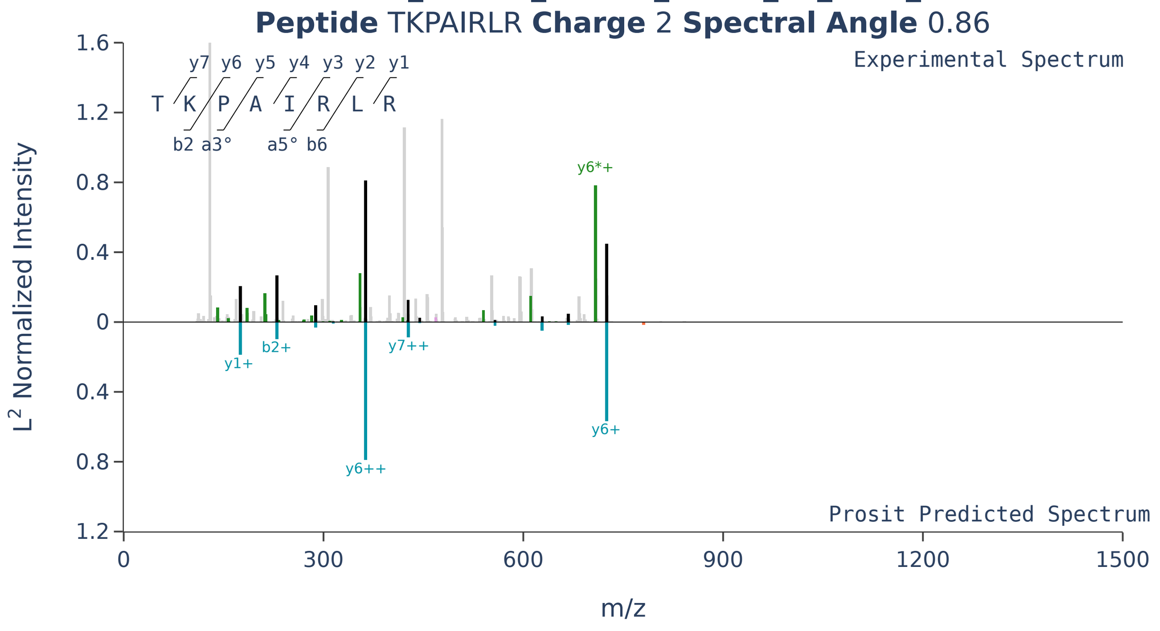

Source HRoetschke_291222_140123_HFGoe_H4_b1R1_1h **Scan** 17243
Peptide LRDSGLIYEET **Charge** 2 **Spectral Angle** 0.79

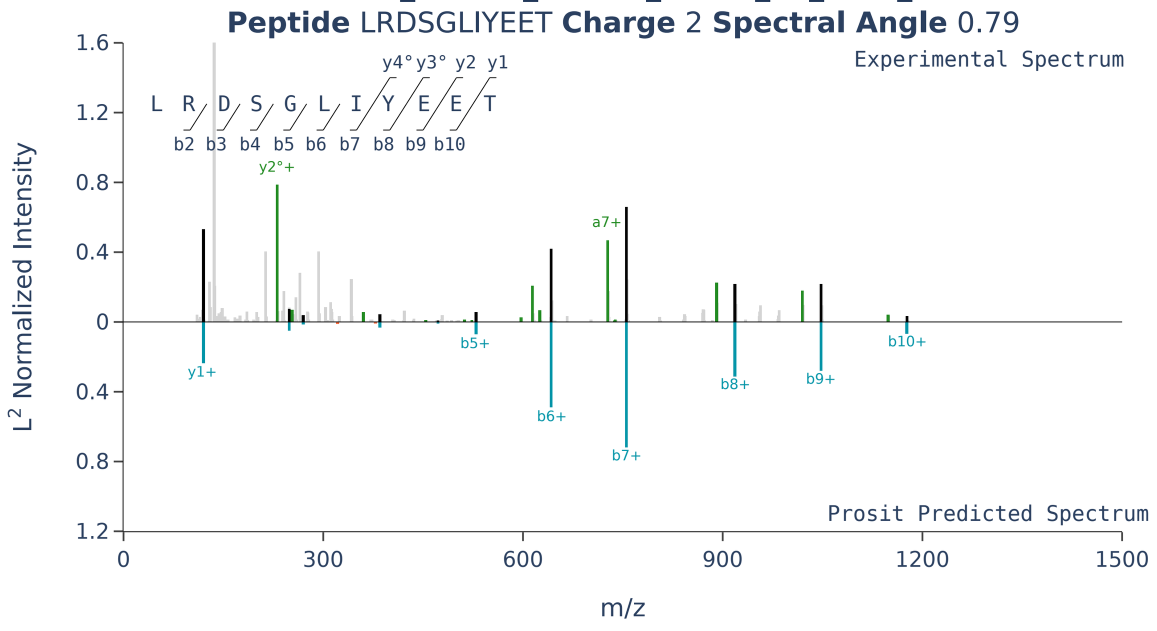

Source HRoetschke_291222_140123_HFGoe_H4_b2R1_1h **Scan** 22589
Peptide SGLIYEETNI **Charge** 2 **Spectral Angle** 0.87

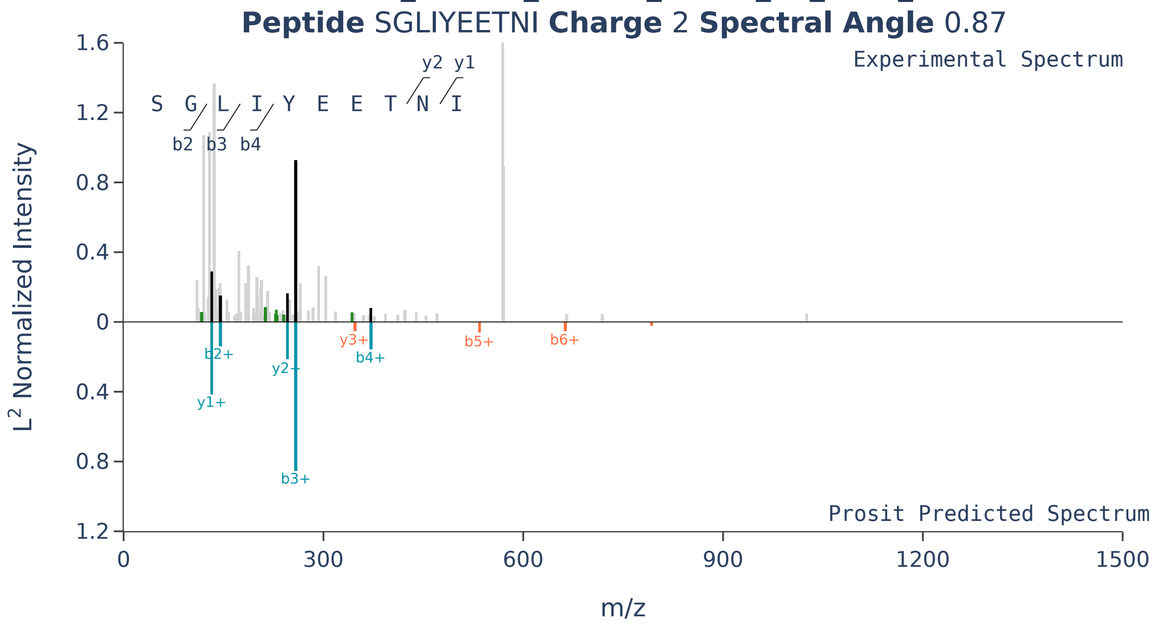

Source HRoetschke_291222_140123_HFGoe_H4_b1R1_2h **Scan** 16148
Peptide ISGLIYEETLKRQGRT **Charge** 3 **Spectral Angle** 0.81

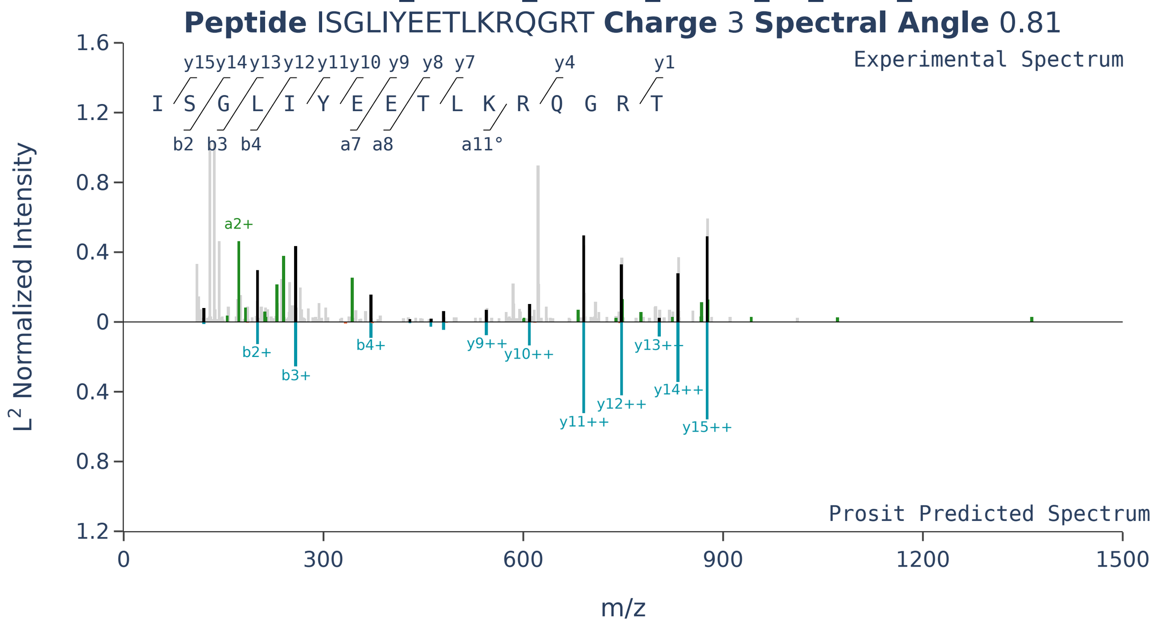

Source HRoetschke_291222_140123_HFGoe_H4_b2R1_4h **Scan** 14733
Peptide RDASGLIYE **Charge** 2 **Spectral Angle** 0.81

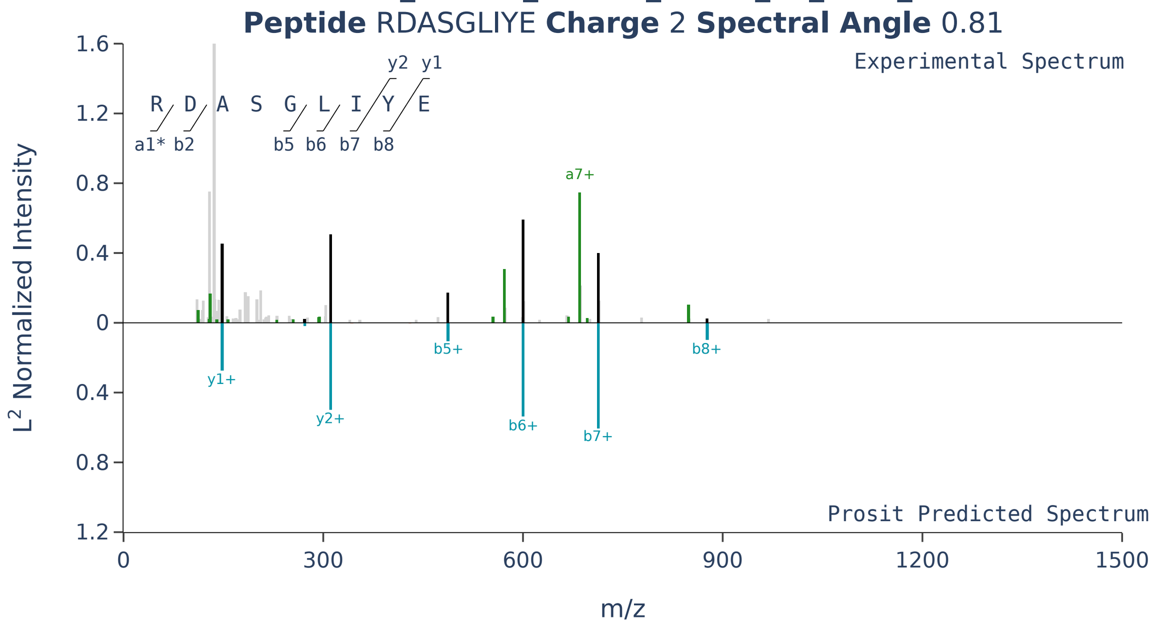

Source HRoetschke_291222_140123_HFGoe_H4_b2R2_24h **Scan** 11712
Peptide RGNIQGITKPAIRRL **Charge** 4 **Spectral Angle** 0.85

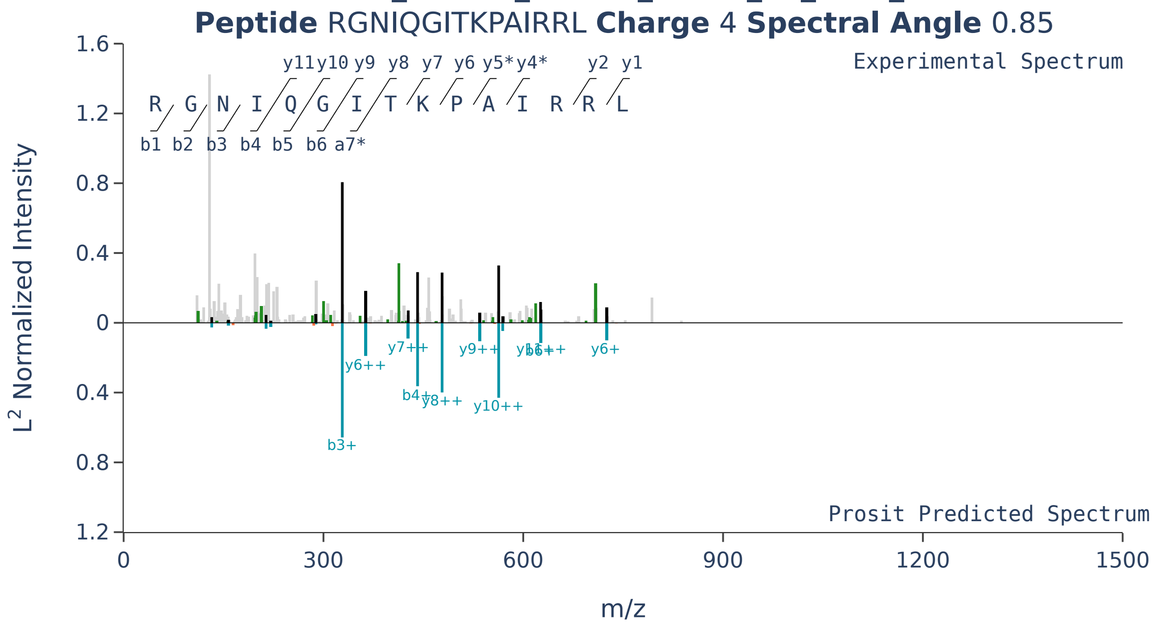

Source HRoetschke_291222_140123_HFGoe_H4_b1R1_24h **Scan** 13434
Peptide TEHANIQGITKPAIRRL **Charge** 3 **Spectral Angle** 0.85

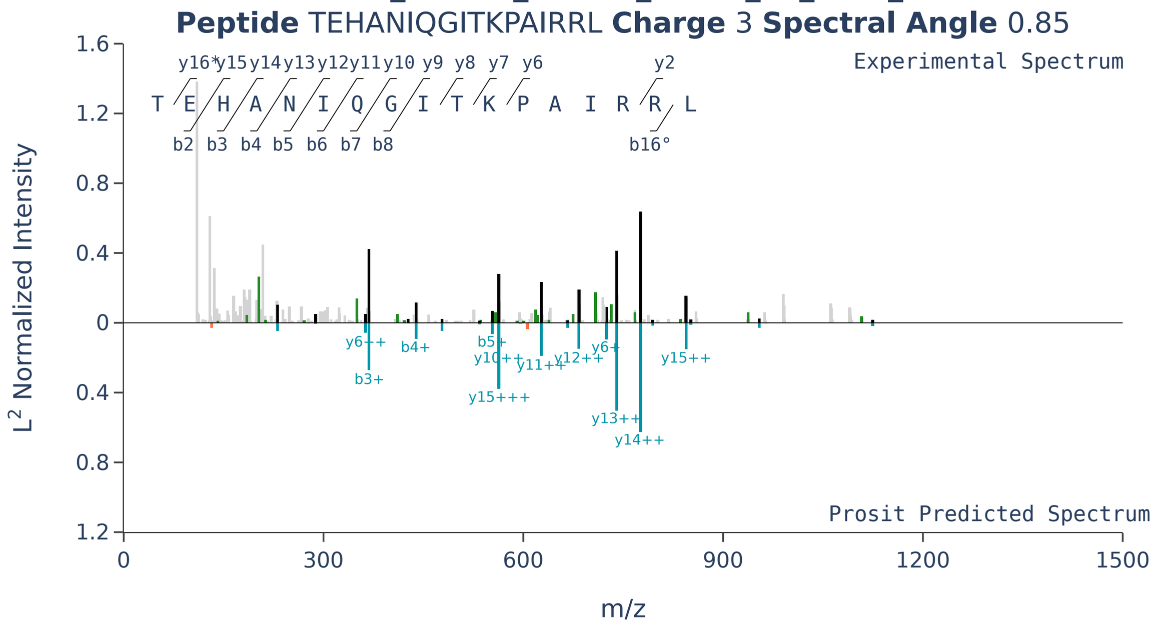

Source HRoetschke_291222_140123_HFGoe_H4_b1R1_24h **Scan** 11366
Peptide DIQGITK **Charge** 2 **Spectral Angle** 0.86

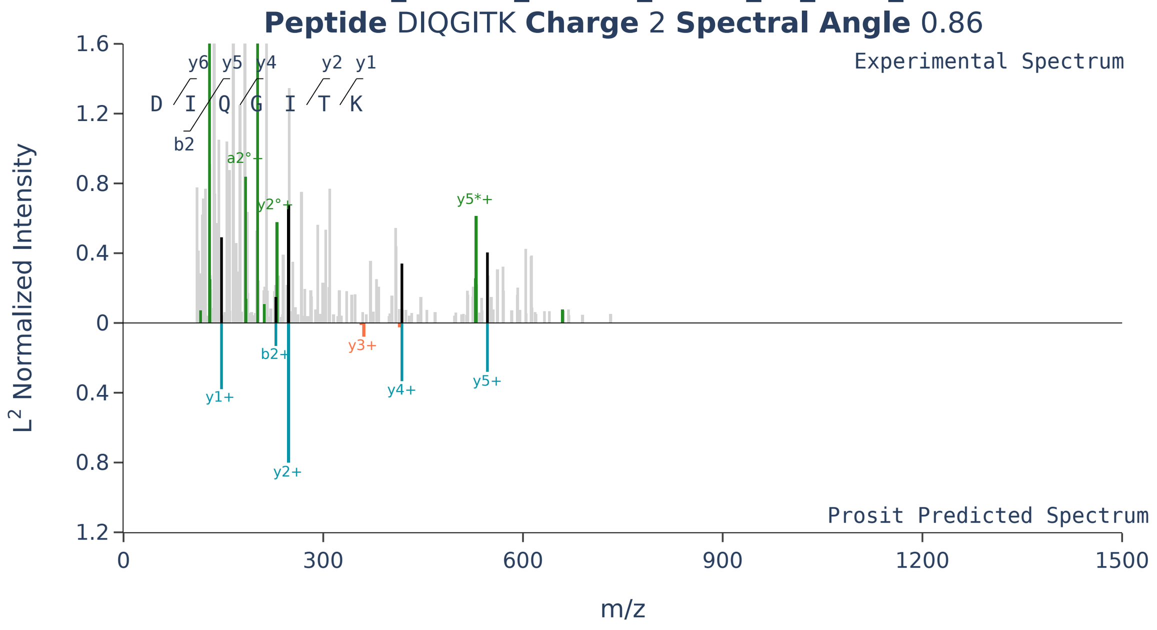

Source HRoetschke_291222_140123_HFGoe_H4_b1R1_24h **Scan** 13782
Peptide GLIYEETRD **Charge** 2 **Spectral Angle** 0.78

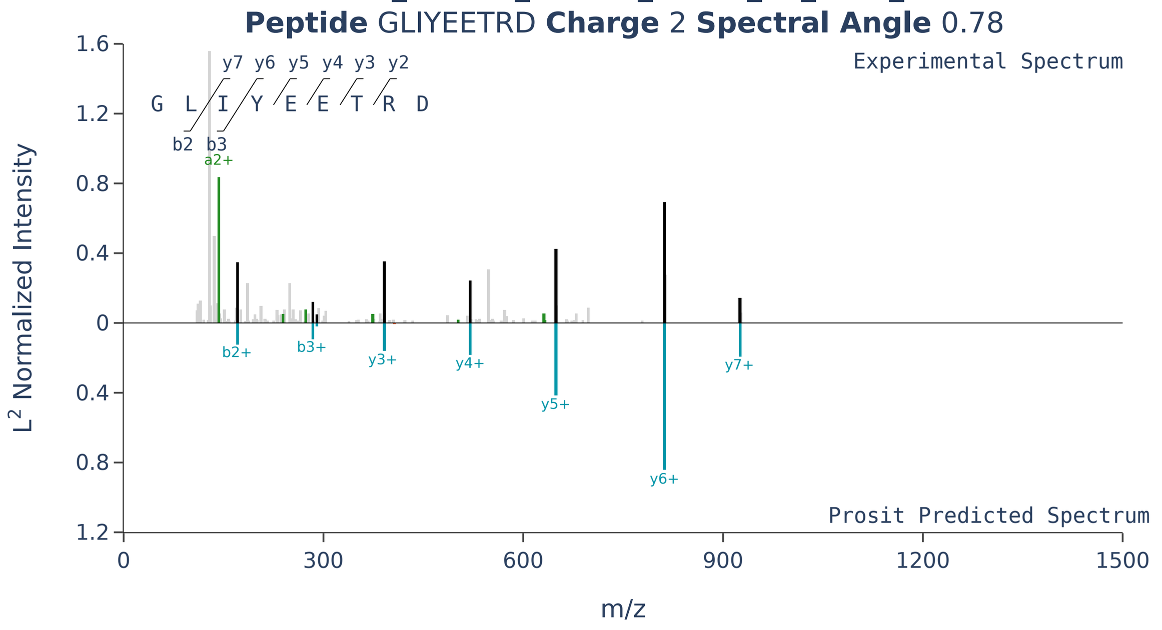

Source HRoetschke_291222_140123_HFGoe_H4_b2R2_24h **Scan** 10296
Peptide YRDAVTY **Charge** 2 **Spectral Angle** 0.88

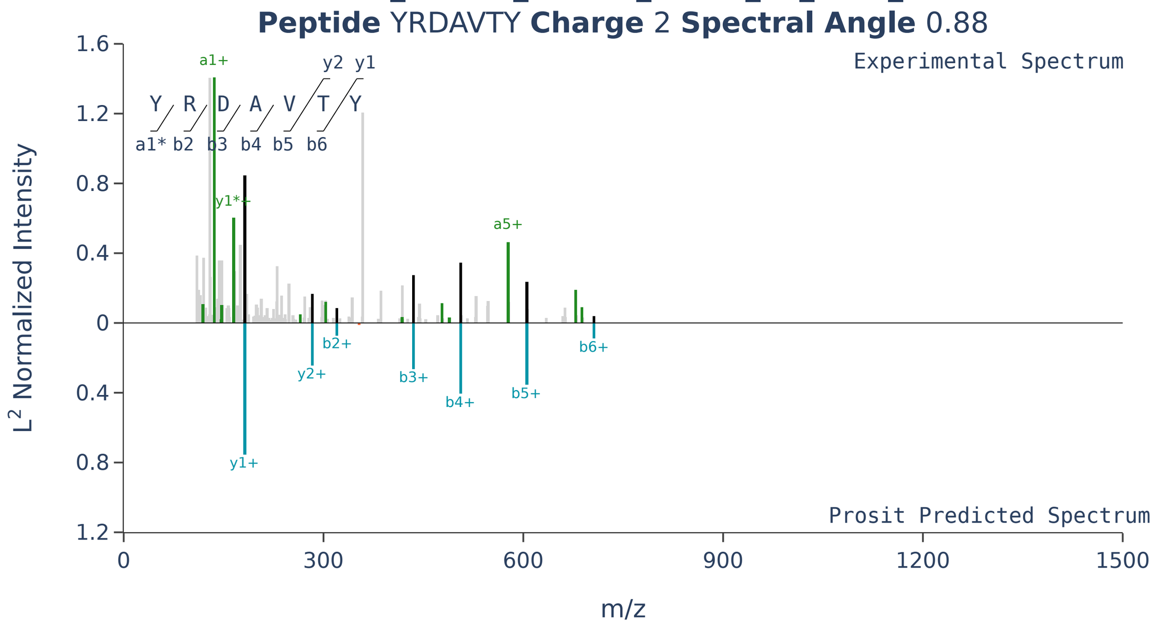

Source HRoetschke_291222_140123_HFGoe_H4_b1R2_24h **Scan** 8882
Peptide ETQGITKPAIR **Charge** 3 **Spectral Angle** 0.94

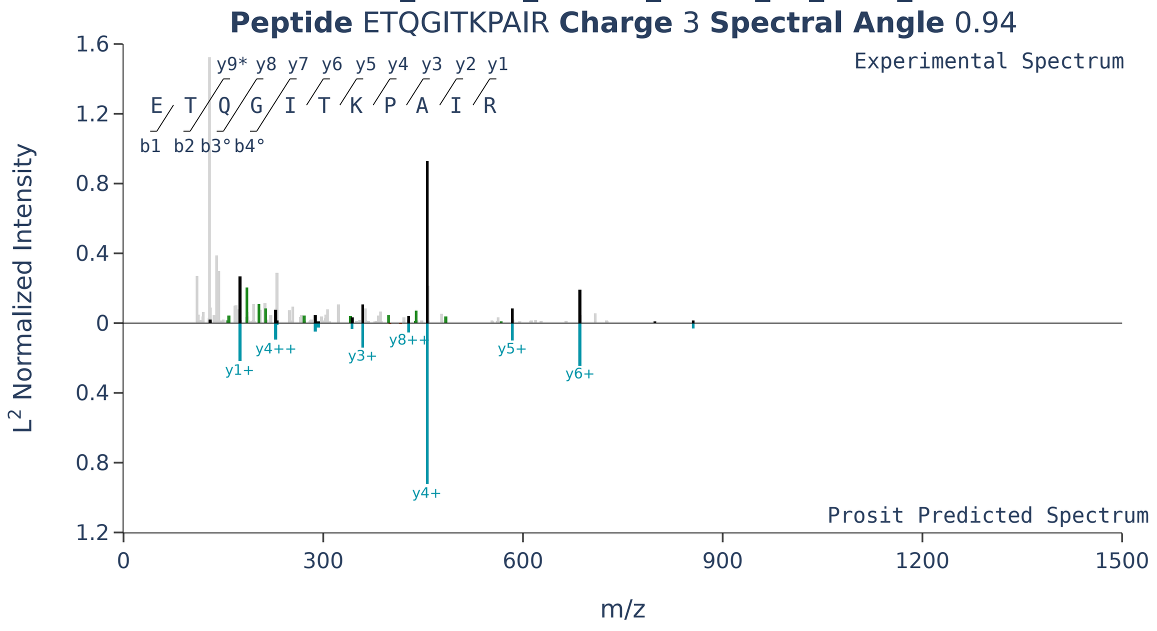

Source HRoetschke_291222_140123_HFGoe_H4_b1R2_24h **Scan** 8748
Peptide YRDAVTYTEHA **Charge** 3 **Spectral Angle** 0.89

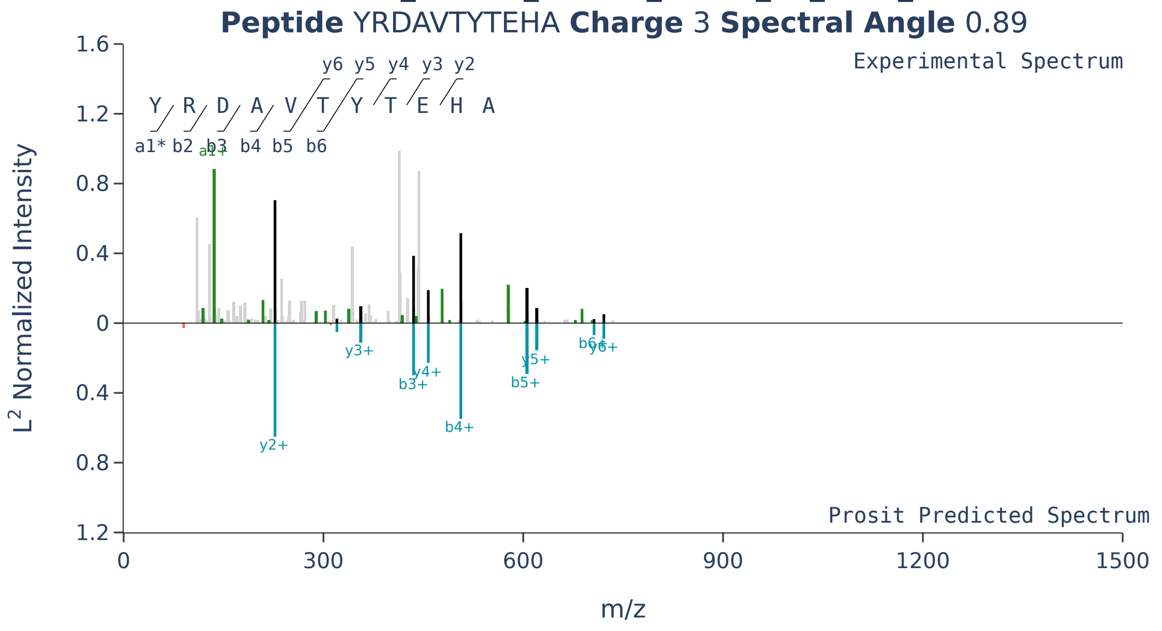

Source HRoetschke_291222_140123_HFGoe_H4_b1R1_24h **Scan** 8309
Peptide AQGITKPAIR **Charge** 3 **Spectral Angle** 0.91

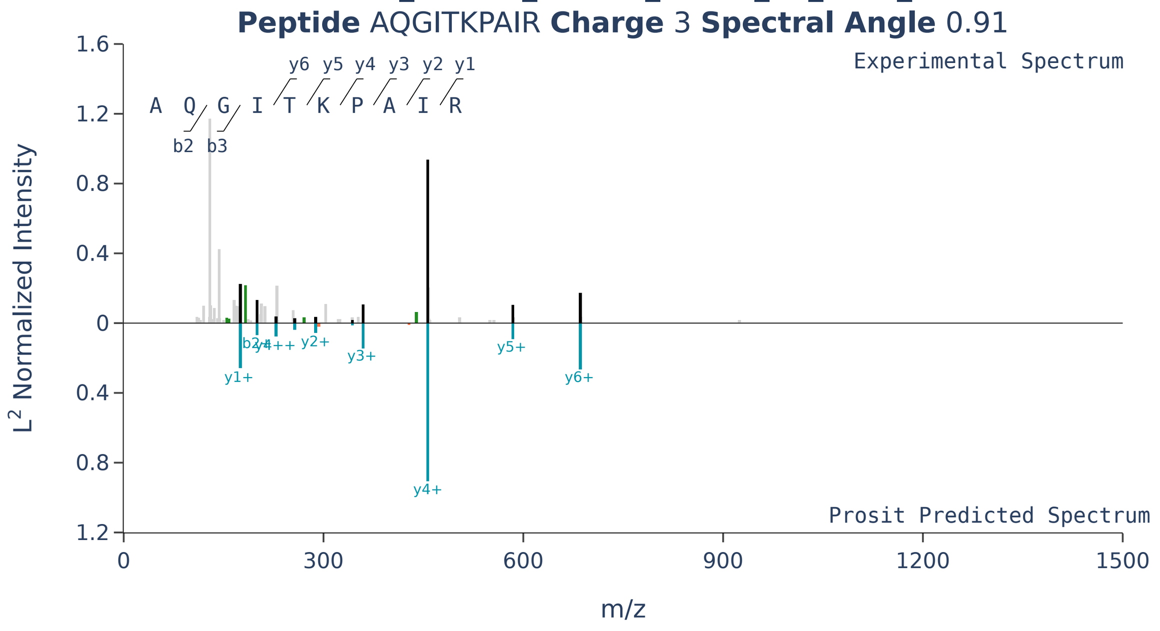

Source HRoetschke_291222_140123_HFGoe_H4_b1R1_1h **Scan** 19423
Peptide SGLIYEETKVLRD **Charge** 2 **Spectral Angle** 0.78

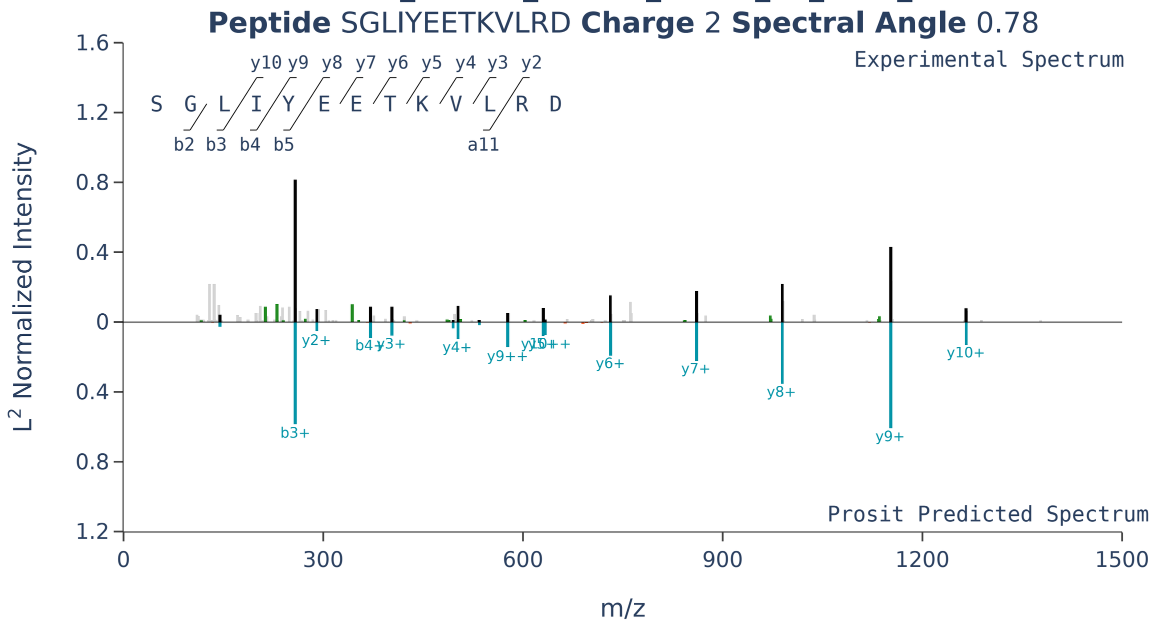

Source HRoetschke_291222_140123_HFGoe_H4_b1R2_1h **Scan** 17357
Peptide TSGLIYE **Charge** 1 **Spectral Angle** 0.77

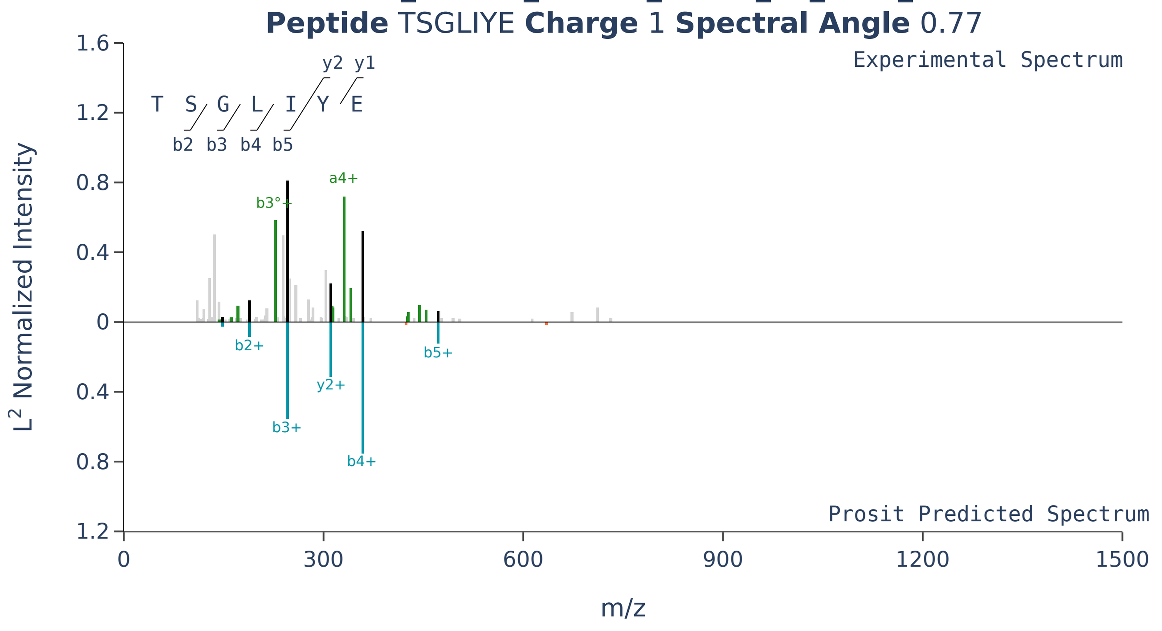

Source HRoetschke_291222_140123_HFGoe_H4_b2R1_2h **Scan** 17531
Peptide GSGLIYEET **Charge** 1 **Spectral Angle** 0.9

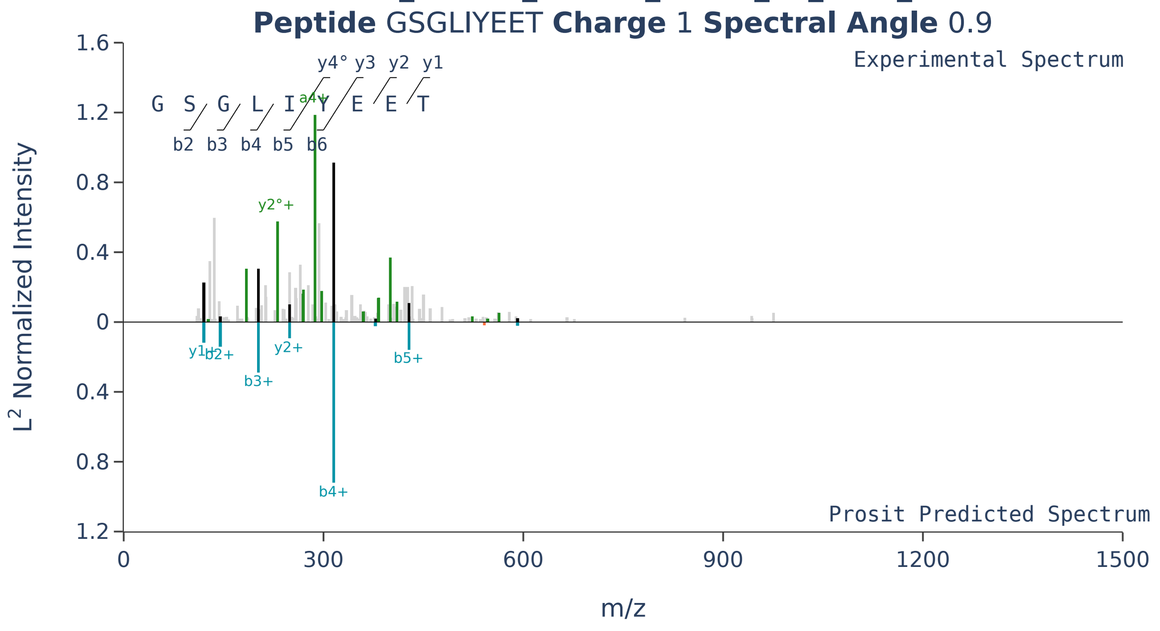

Source HRoetschke_291222_140123_HFGoe_H4_b1R2_24h **Scan** 7296
Peptide ARDAVTYTEHA **Charge** 3 **Spectral Angle** 0.89

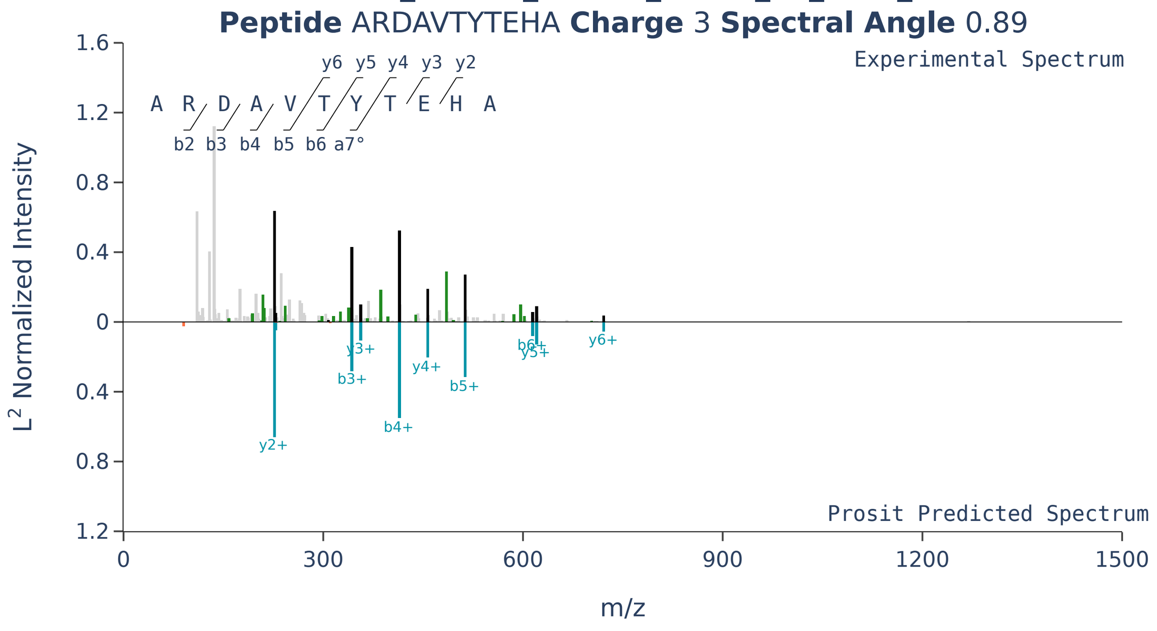

Source HRoetschke_291222_140123_HFGoe_H4_b1R1_2h **Scan** 10951
Peptide KRKTVTAMDVVYRG **Charge** 3 **Spectral Angle** 0.87

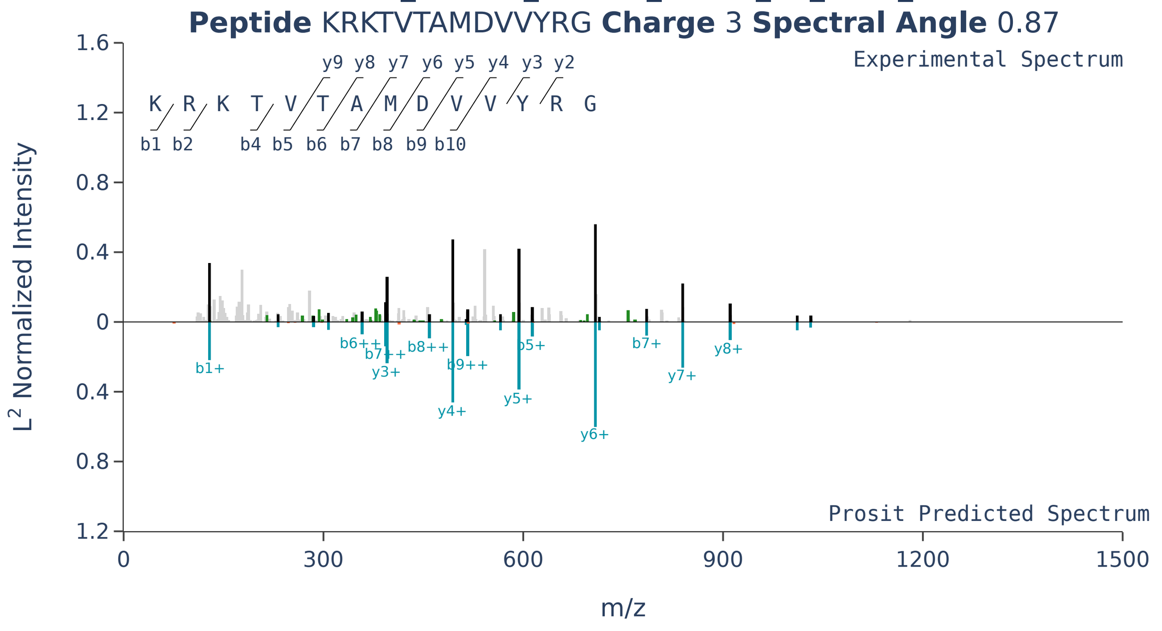

Source HRoetschke_291222_140123_HFGoe_H4_b2R2_24h **Scan** 10371
Peptide LAKRQGRTL **Charge** 2 **Spectral Angle** 0.96

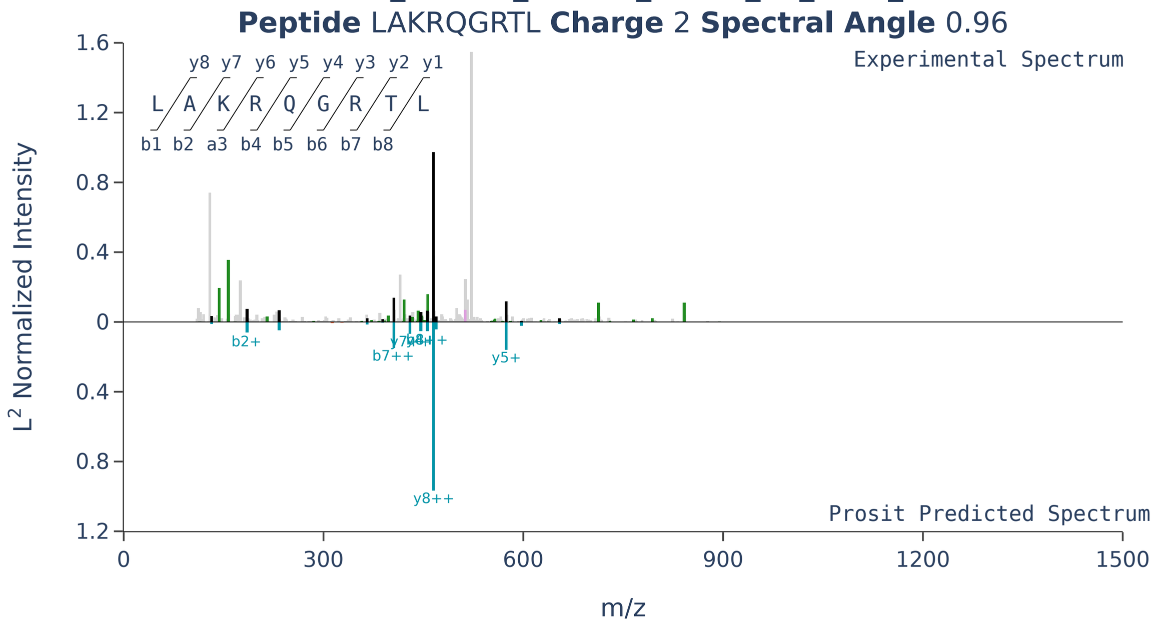

Source HRoetschke_291222_140123_HFGoe_H4_b1R1_1h **Scan** 12803
Peptide SGLIYEETLKRQGRT **Charge** 3 **Spectral Angle** 0.91

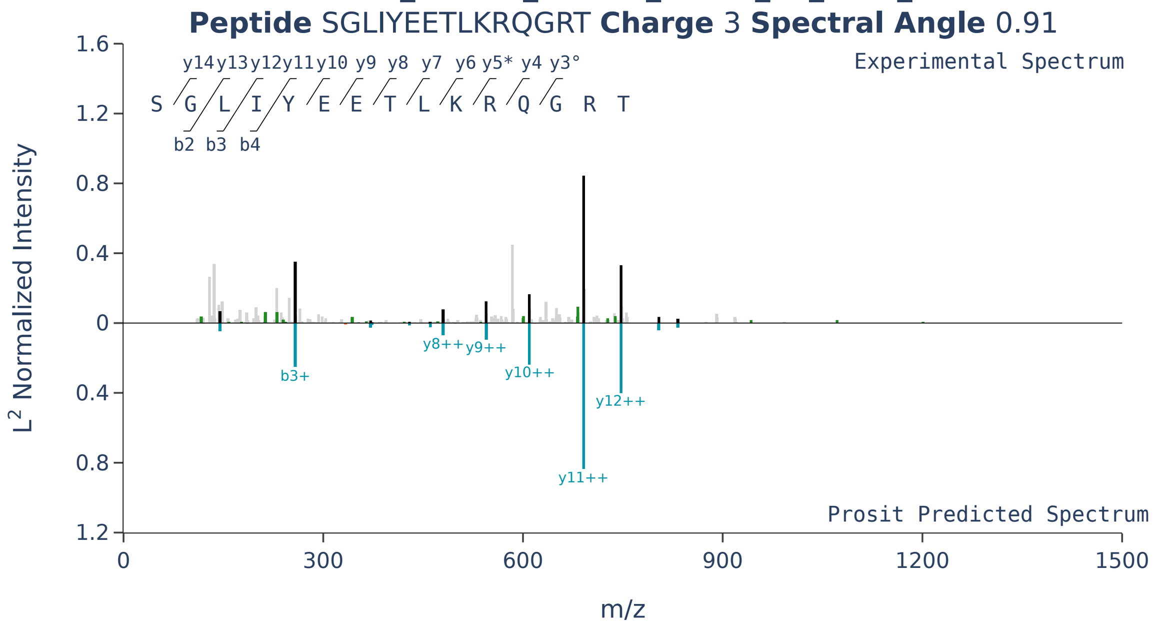

Source HRoetschke_291222_140123_HFGoe_H4_b1R2_2h **Scan** 20854
Peptide DNRISGLIYE **Charge** 2 **Spectral Angle** 0.86

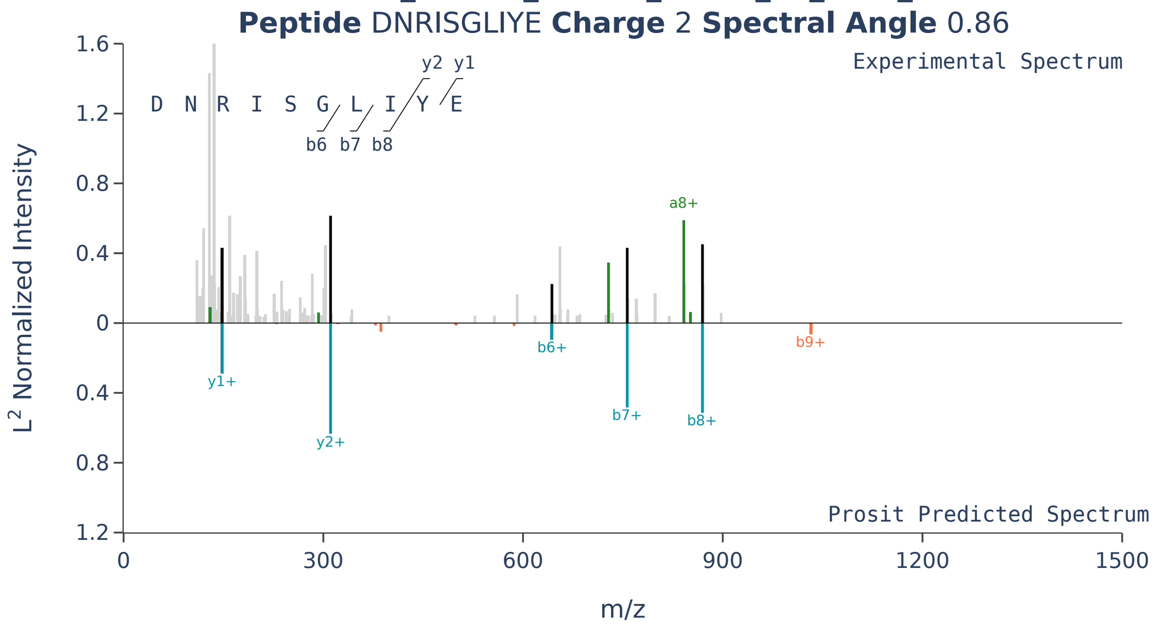

Source HRoetschke_291222_140123_HFGoe_H4_b1R1_2h **Scan** 18576
Peptide SGLIYEETA **Charge** 1 **Spectral Angle** 0.93

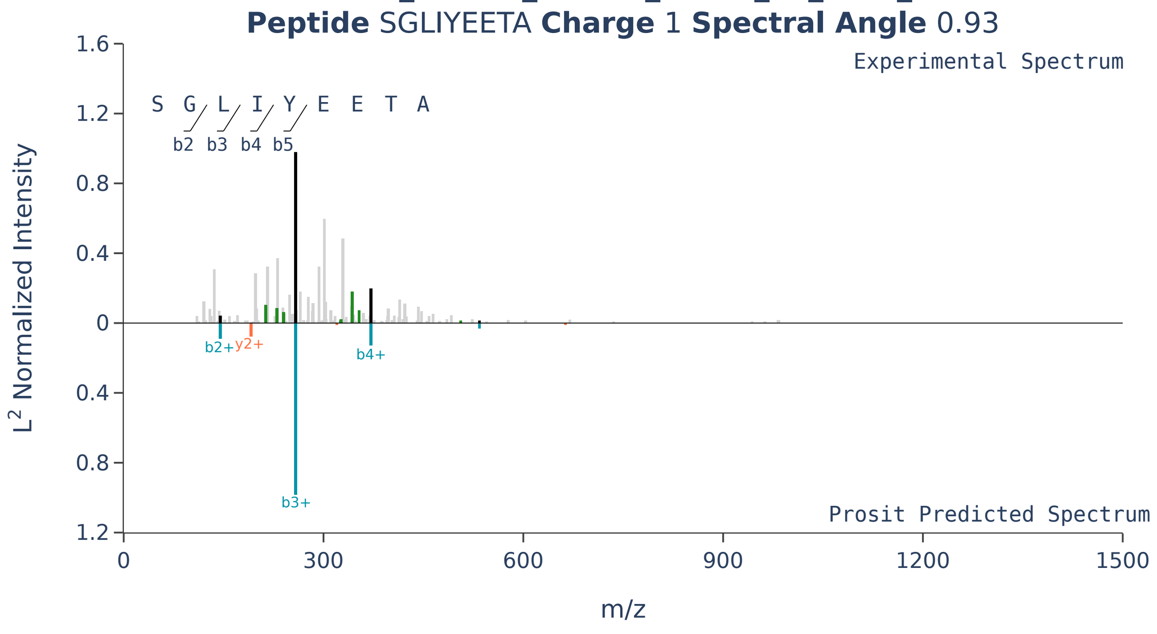

Source HRoetschke_291222_140123_HFGoe_H4_b1R1_2h **Scan** 15117
Peptide MDVVYALKRQGRTA **Charge** 4 **Spectral Angle** 0.89

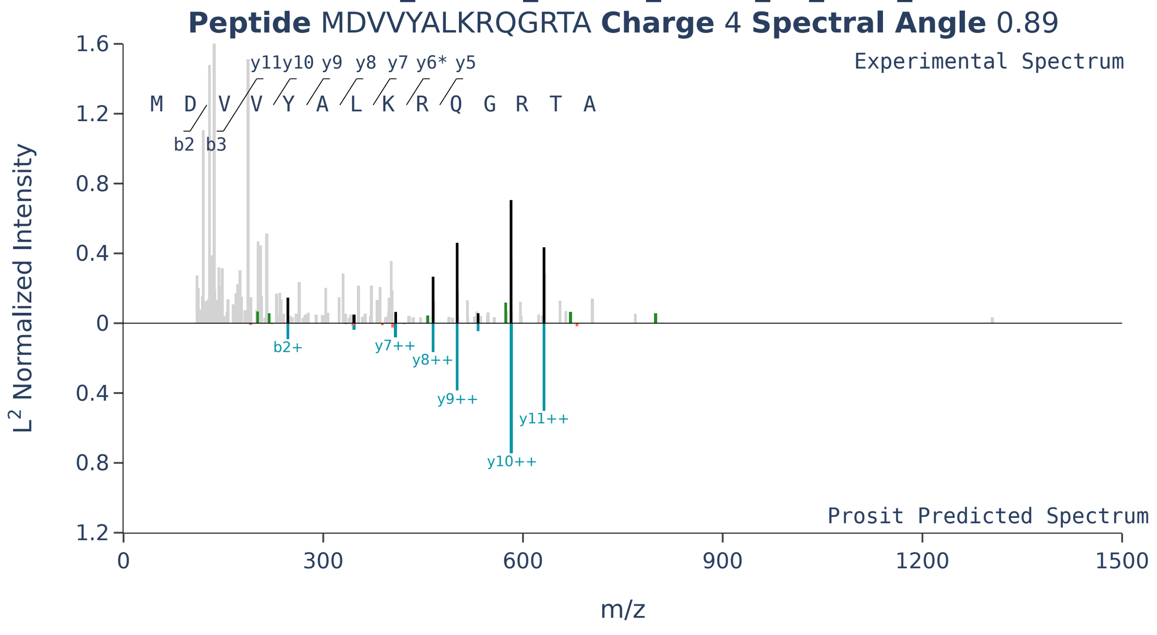

Source HRoetschke_291222_140123_HFGoe_H4_b1R2_24h **Scan** 12809
Peptide ANIQGITKPAIR **Charge** 3 **Spectral Angle** 0.81

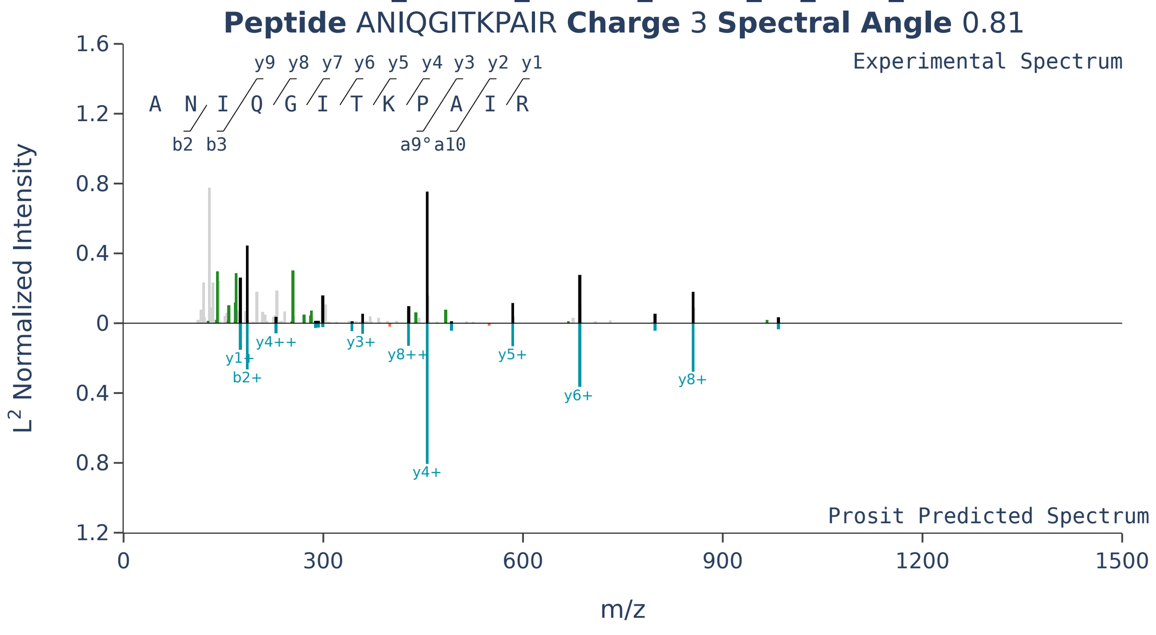

Source HRoetschke_291222_140123_HFGoe_H4_b1R1_4h **Scan** 11993
Peptide YQGITKPAIRRL **Charge** 3 **Spectral Angle** 0.89

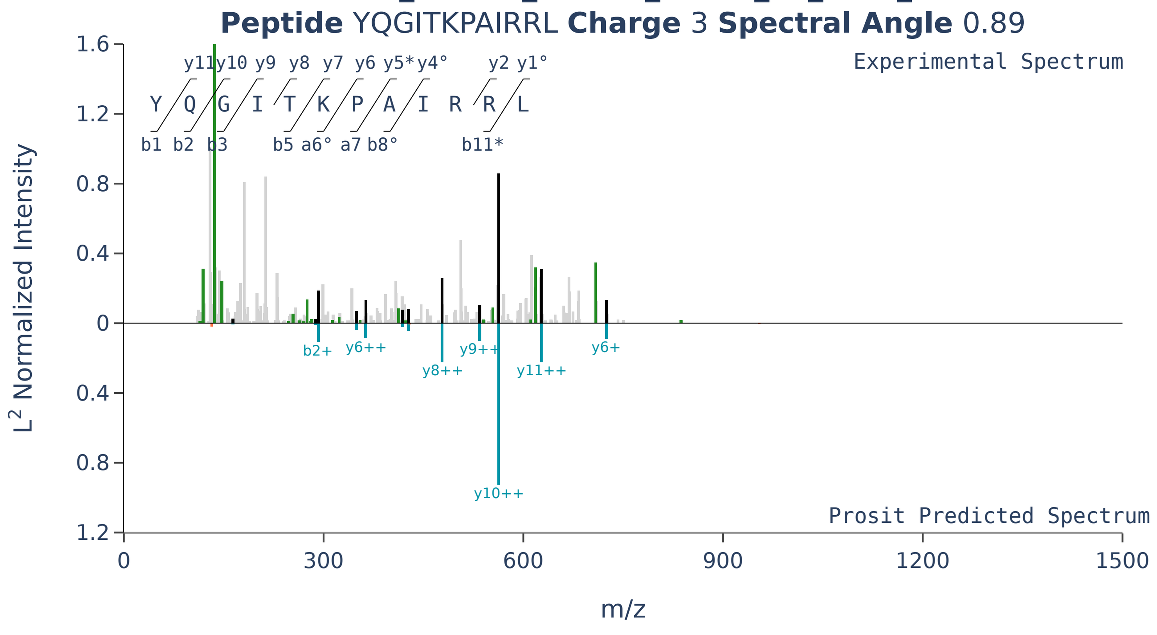

Source HRoetschke_291222_140123_HFGoe_H4_b1R1_1h **Scan** 21287
Peptide DNRISGLIYEET **Charge** 2 **Spectral Angle** 0.8

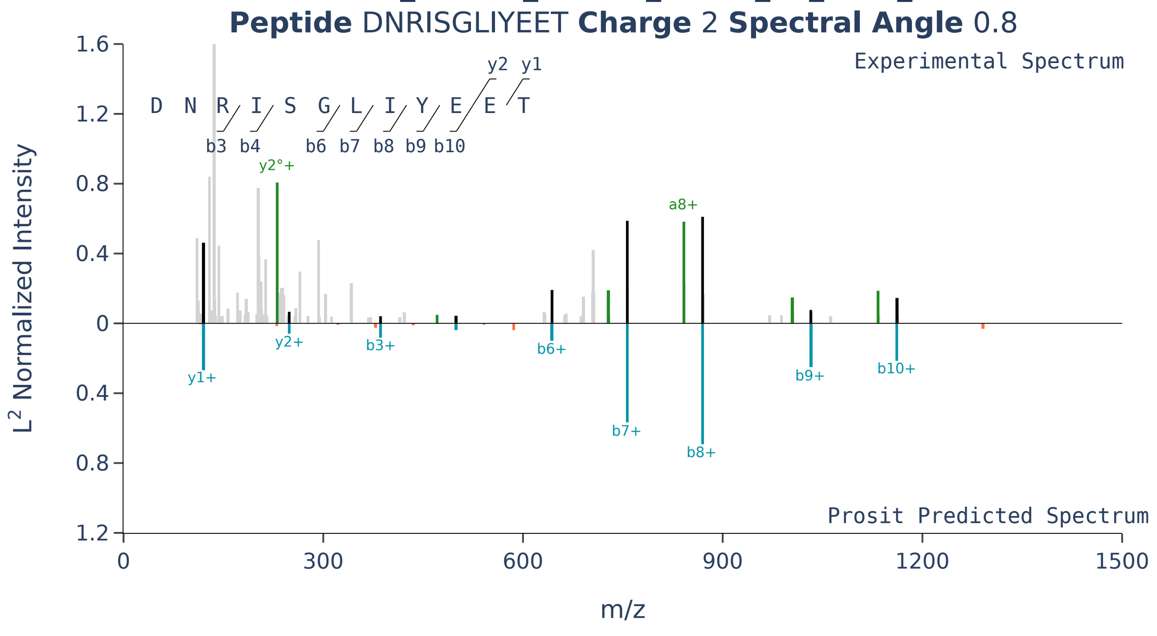

Source HRoetschke_291222_140123_HFGoe_H4_b1R2_24h **Scan** 14652
Peptide RDSGLIYEET **Charge** 2 **Spectral Angle** 0.75

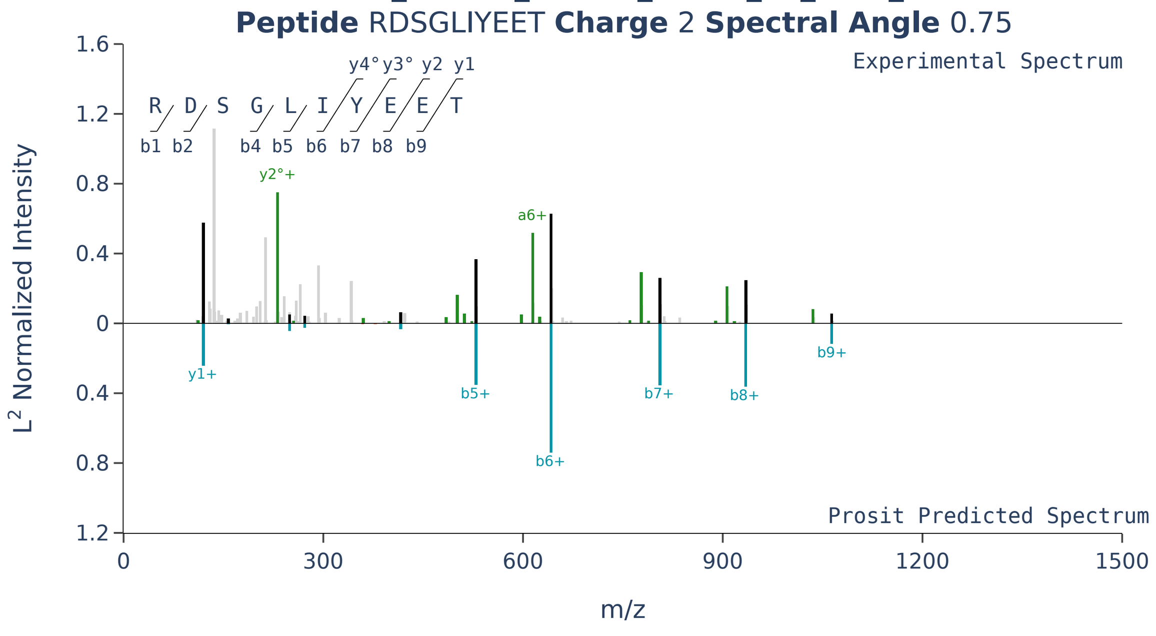

Source HRoetschke_291222_140123_HFGoe_H4_b2R2_4h **Scan** 15084
Peptide ESGLIYEETRG **Charge** 2 **Spectral Angle** 0.84

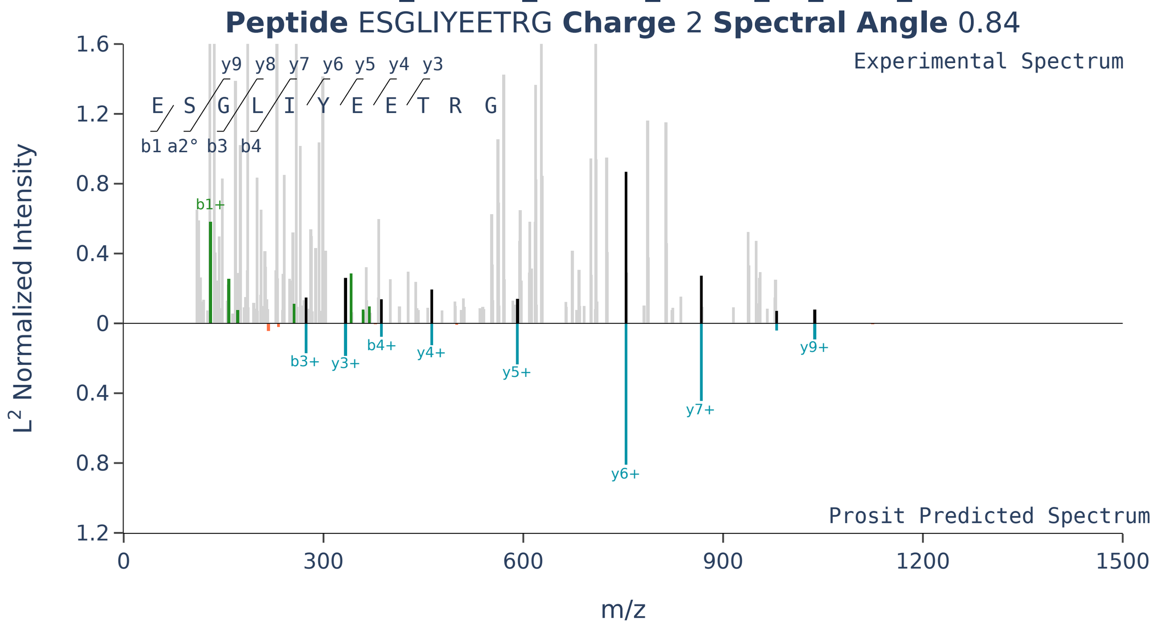

Source HRoetschke_291222_140123_HFGoe_H4_b1R1_2h **Scan** 19779
Peptide TKVTAMDVVY **Charge** 2 **Spectral Angle** 0.8

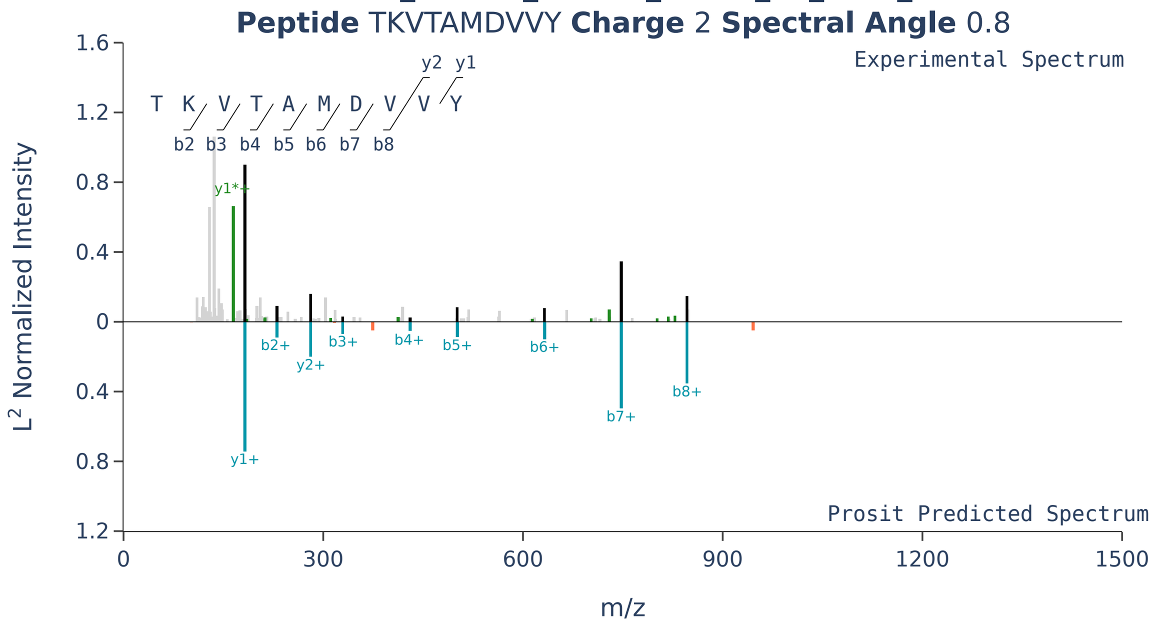

Source HRoetschke_291222_140123_HFGoe_H4_b2R1_4h **Scan** 25106
Peptide SGLILIYE **Charge** 1 **Spectral Angle** 0.88

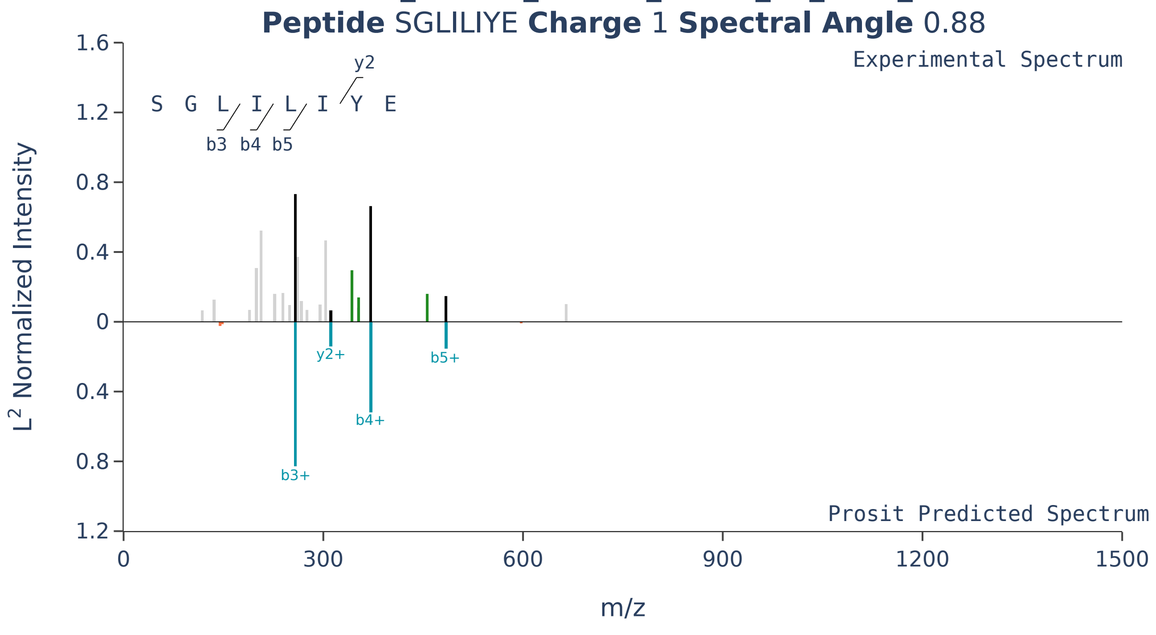

Source HRoetschke_291222_140123_HFGoe_H4_b1R2_24h **Scan** 13969
Peptide YRDNIQGITKPAIRRL **Charge** 4 **Spectral Angle** 0.79

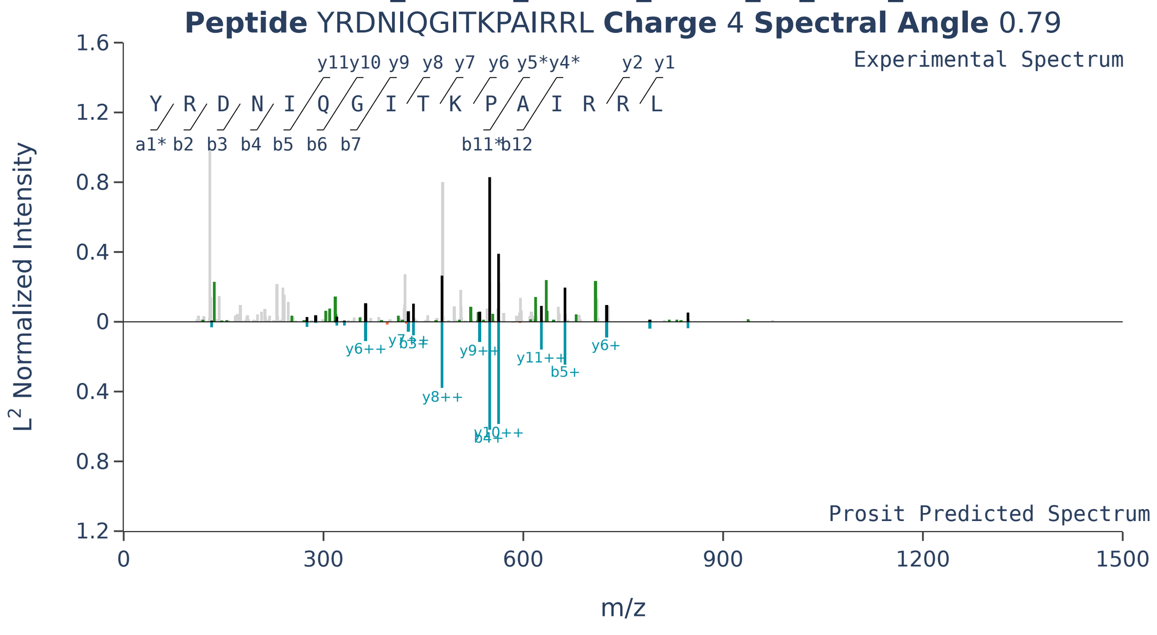

Source HRoetschke_291222_140123_HFGoe_H4_b1R1_1h **Scan** 18326
Peptide SGLIYEETKV **Charge** 2 **Spectral Angle** 0.79

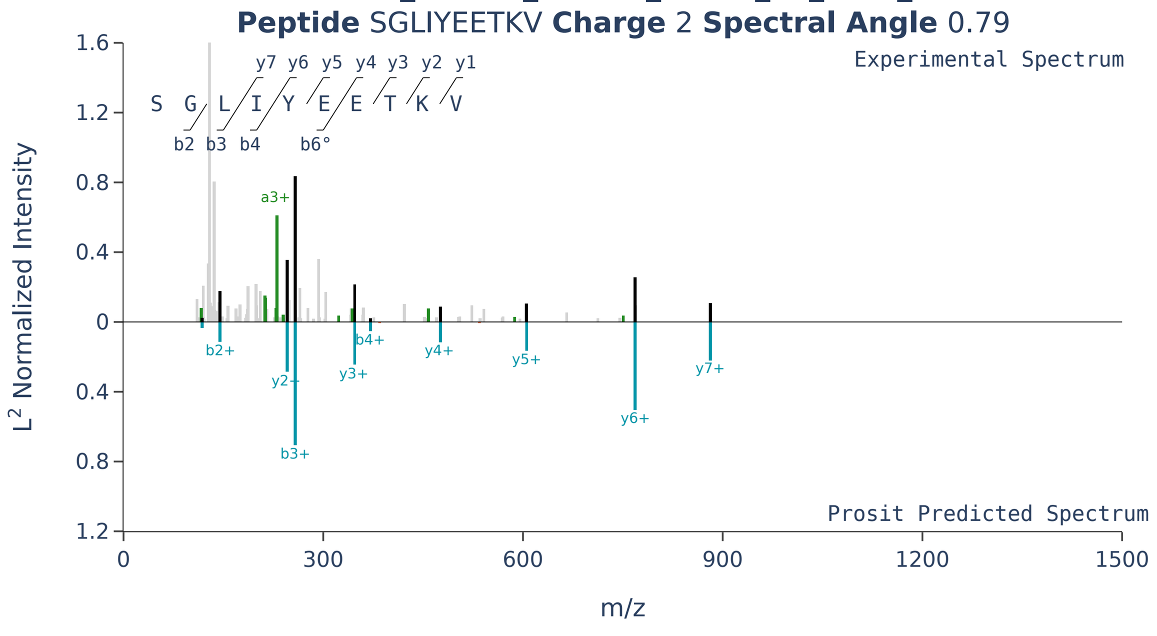

Source HRoetschke_291222_140123_HFGoe_H4_b1R1_24h **Scan** 13917
Peptide ETNIQGITKPAIR **Charge** 3 **Spectral Angle** 0.84

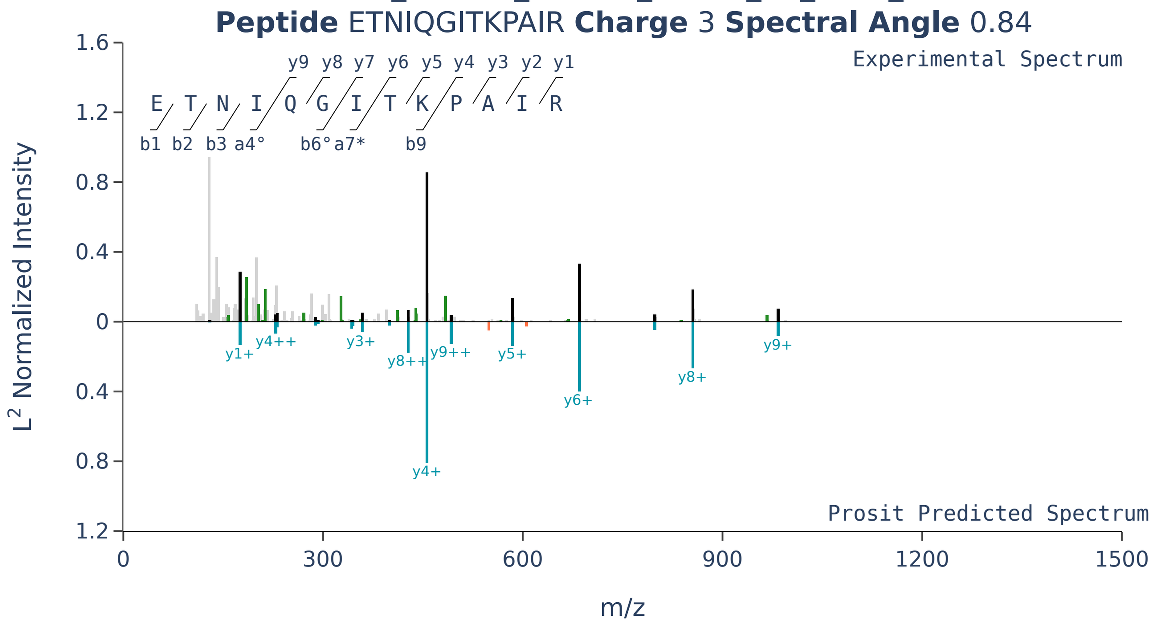

Source HRoetschke_291222_140123_HFGoe_H4_b1R1_4h **Scan** 15643
Peptide ETNIQGITKPAIRRL **Charge** 3 **Spectral Angle** 0.87

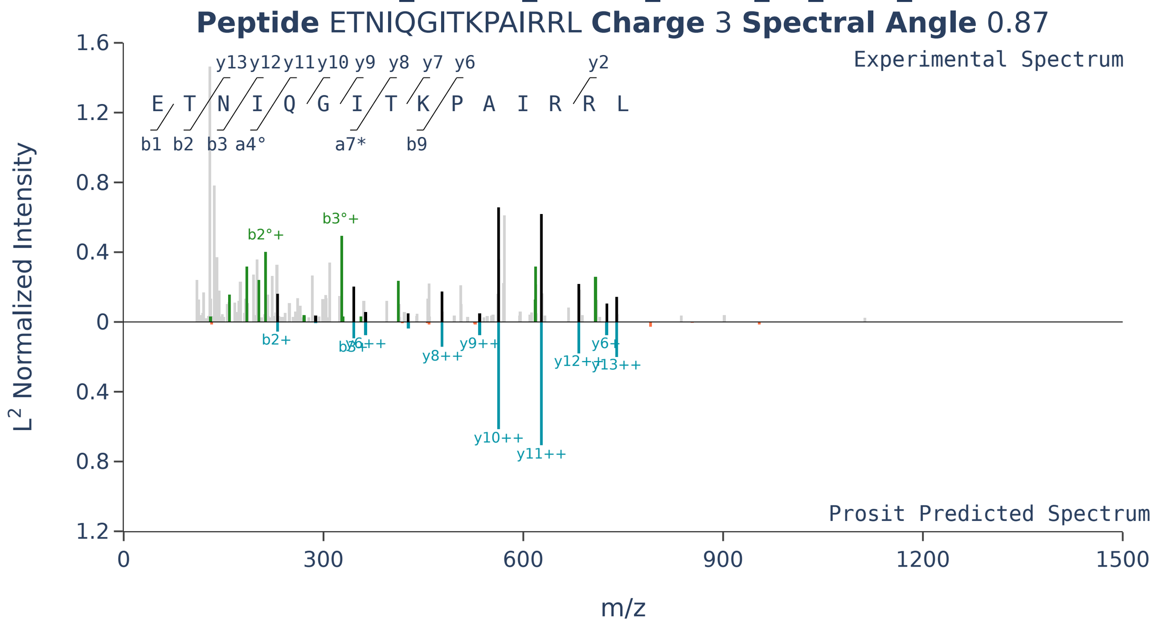

Source HRoetschke_291222_140123_HFGoe_H4_b1R1_2h **Scan** 26613
Peptide ISGLLIYE **Charge** 1 **Spectral Angle** 0.88

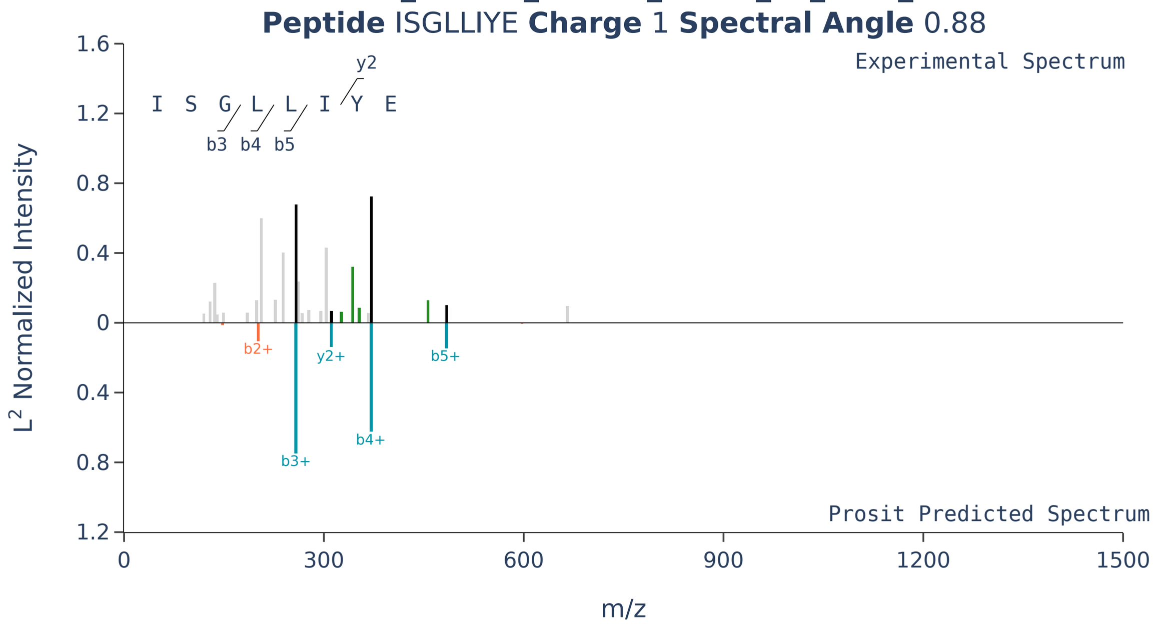

Source HRoetschke_291222_140123_HFGoe_H4_b1R1_24h **Scan** 7850
Peptide FLENKRQGRTL **Charge** 3 **Spectral Angle** 0.89

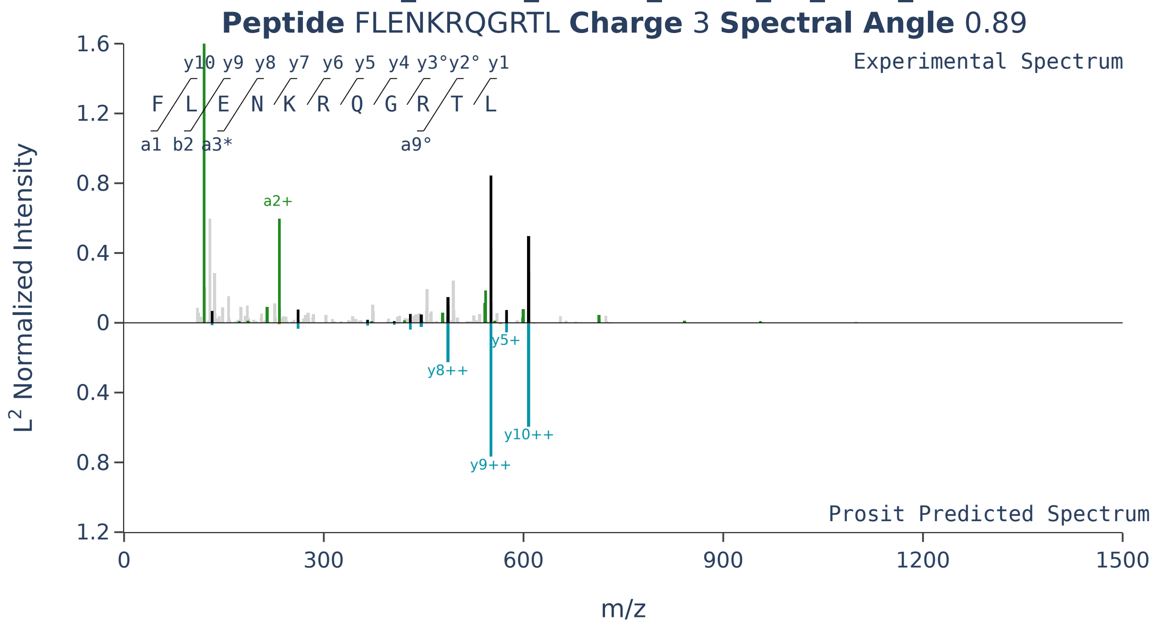

Source HRoetschke_291222_140123_HFGoe_H4_b1R2_24h **Scan** 9635
Peptide GRNIQGITKPAIR **Charge** 3 **Spectral Angle** 0.85

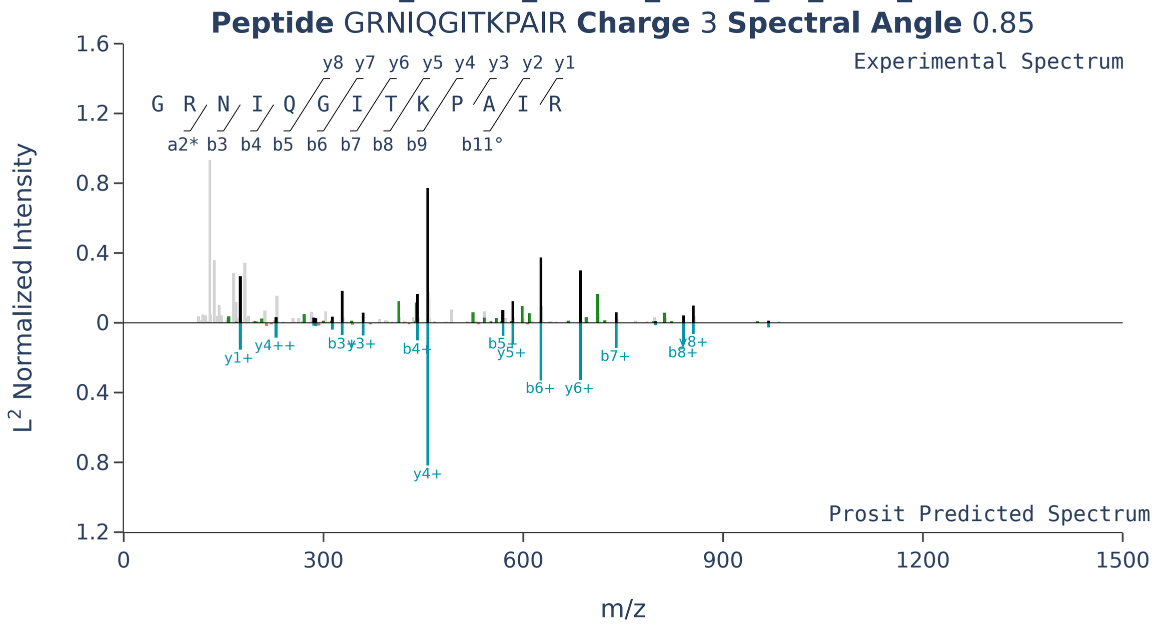

Source HRoetschke_291222_140123_HFGoe_H4_b1R2_24h **Scan** 8587
Peptide SGLIYEETRKRQG **Charge** 4 **Spectral Angle** 0.83

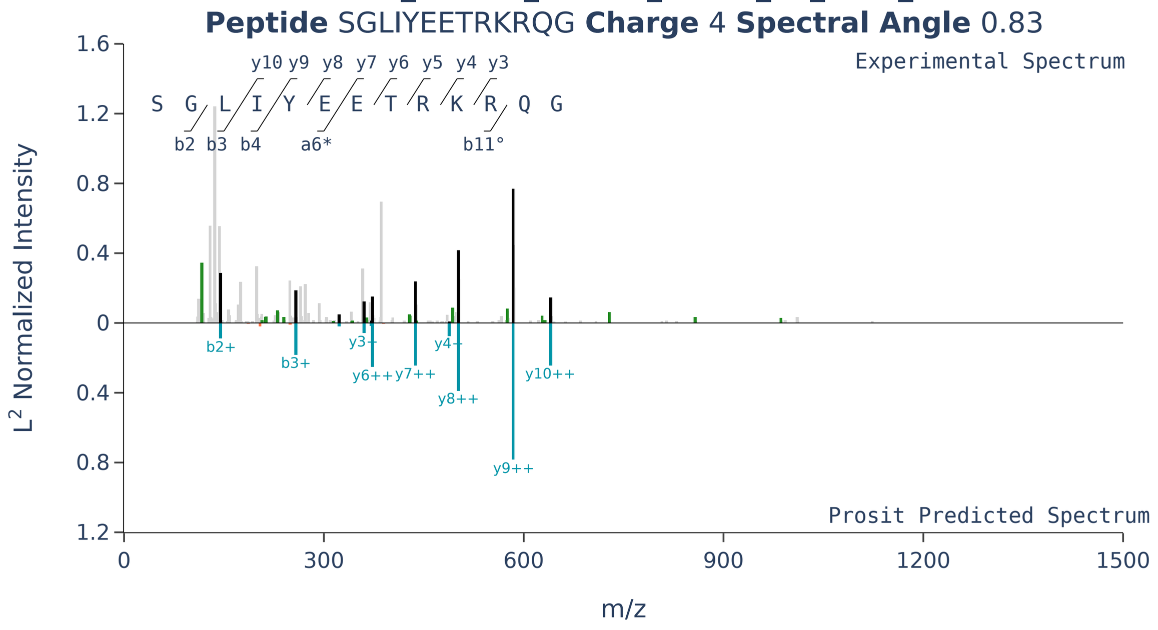

Source HRoetschke_291222_140123_HFGoe_H4_b2R2_4h **Scan** 13907
Peptide QGRSGLIYE **Charge** 2 **Spectral Angle** 0.85

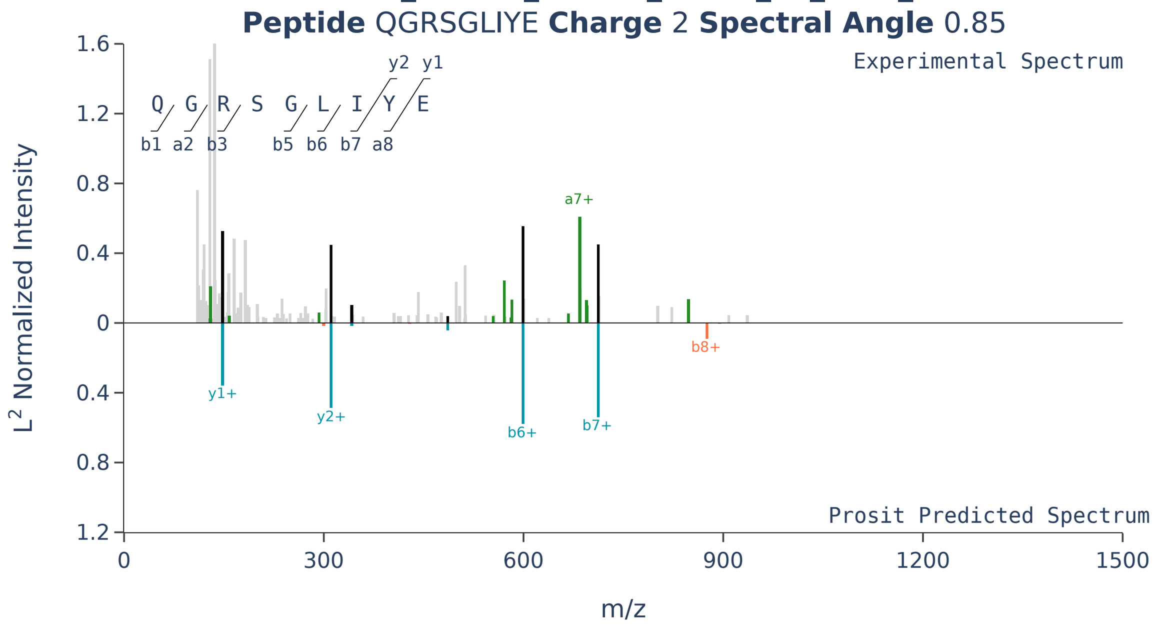

Source HRoetschke_291222_140123_HFGoe_H4_b1R1_24h **Scan** 14088
Peptide SNIQGITKPAIRRL **Charge** 4 **Spectral Angle** 0.8

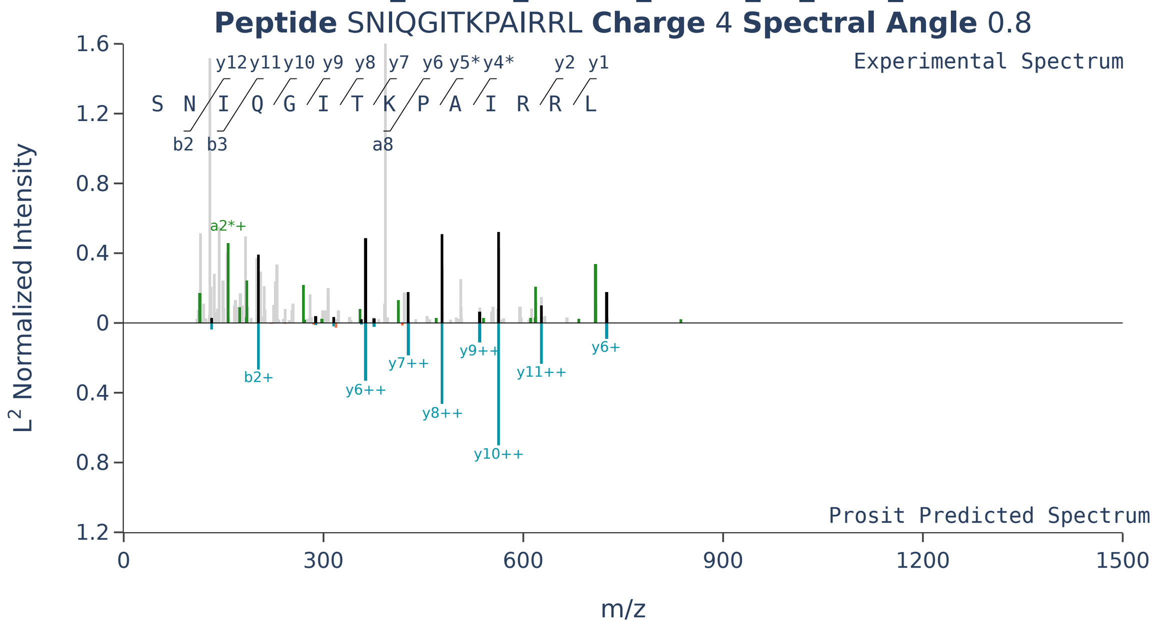

Source HRoetschke_291222_140123_HFGoe_H4_b2R1_24h **Scan** 13903
Peptide RDNSGLIYE **Charge** 2 **Spectral Angle** 0.77

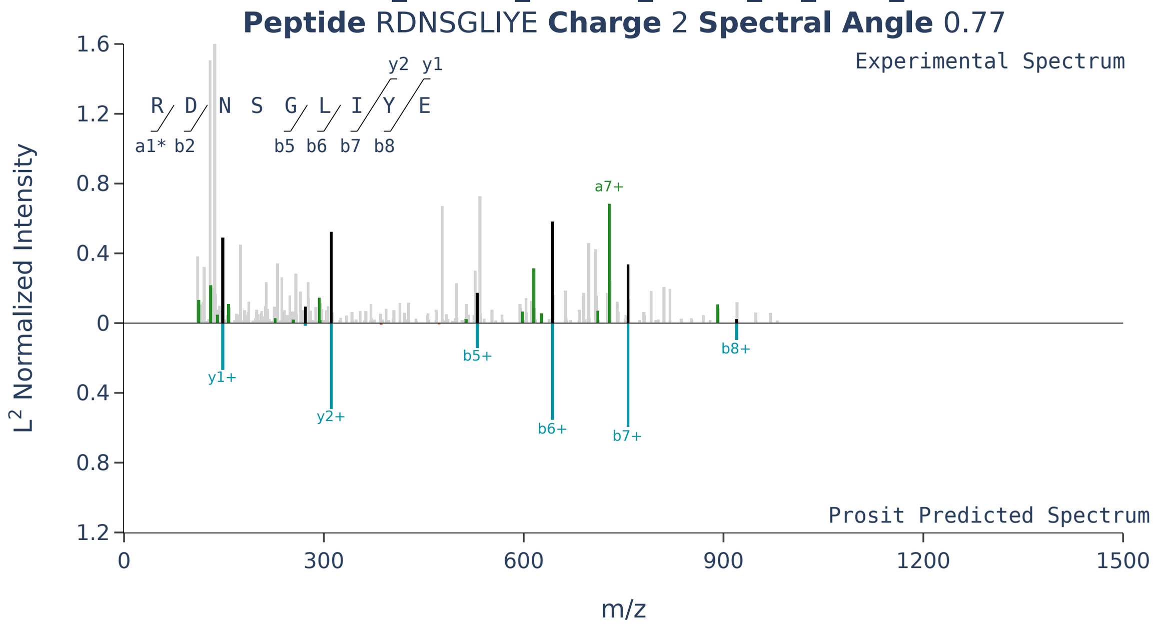

Source HRoetschke_291222_140123_HFGoe_H4_b1R1_24h **Scan** 6651
Peptide GRNIQGITK **Charge** 2 **Spectral Angle** 0.87

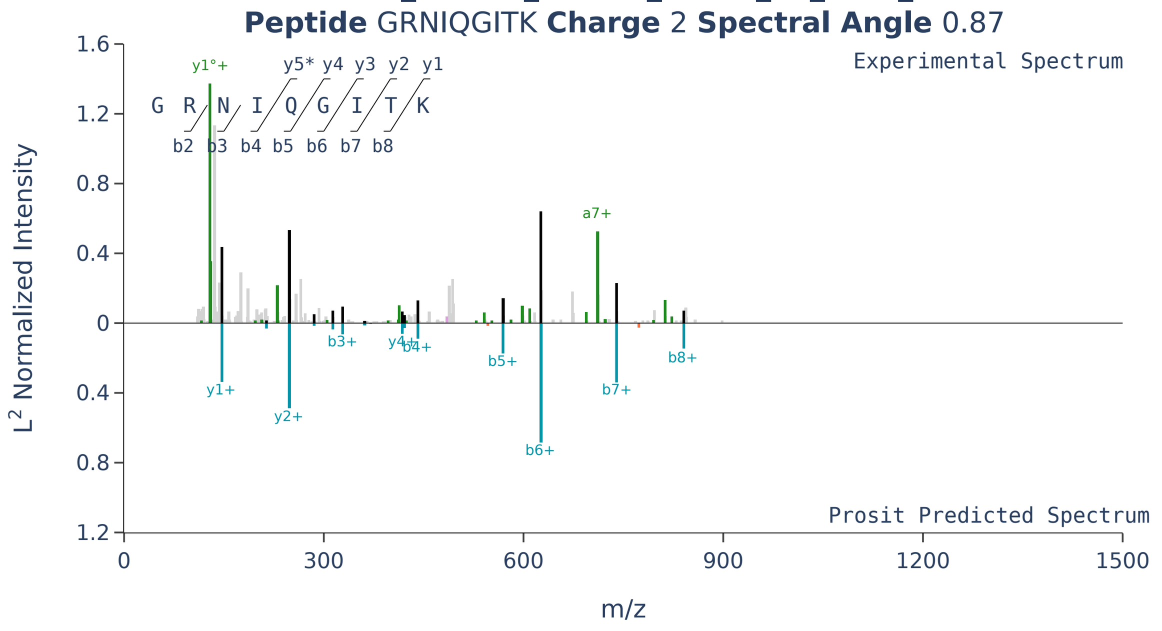

Source HRoetschke_291222_140123_HFGoe_H4_b1R2_1h **Scan** 17859
Peptide TESGLIYEET **Charge** 2 **Spectral Angle** 0.77

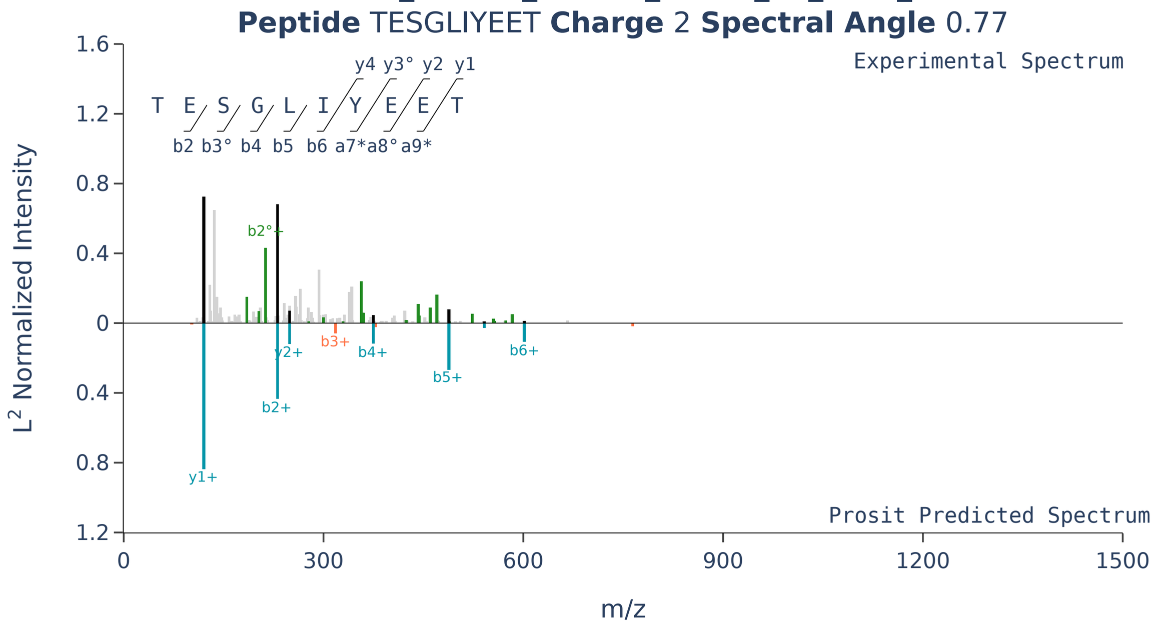

Source HRoetschke_291222_140123_HFGoe_H4_b1R2_1h **Scan** 14764
Peptide ENNIQGITKPAIRRL **Charge** 3 **Spectral Angle** 0.83

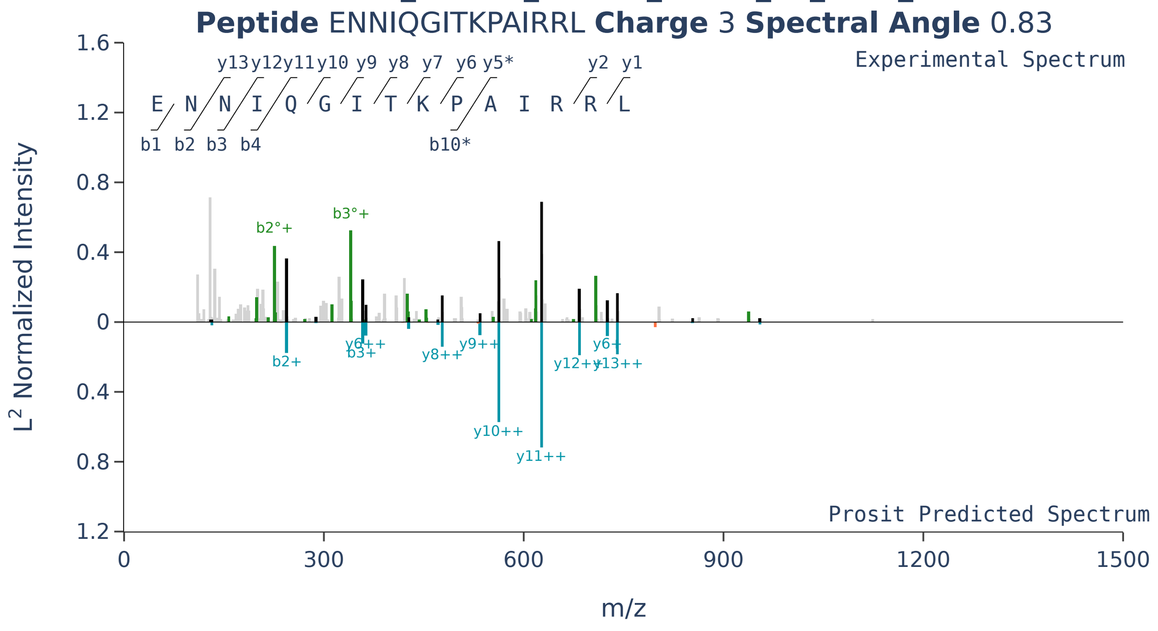

Source HRoetschke_291222_140123_HFGoe_H4_b1R2_24h **Scan** 5718
Peptide RDAVTYTEHRG **Charge** 3 **Spectral Angle** 0.84

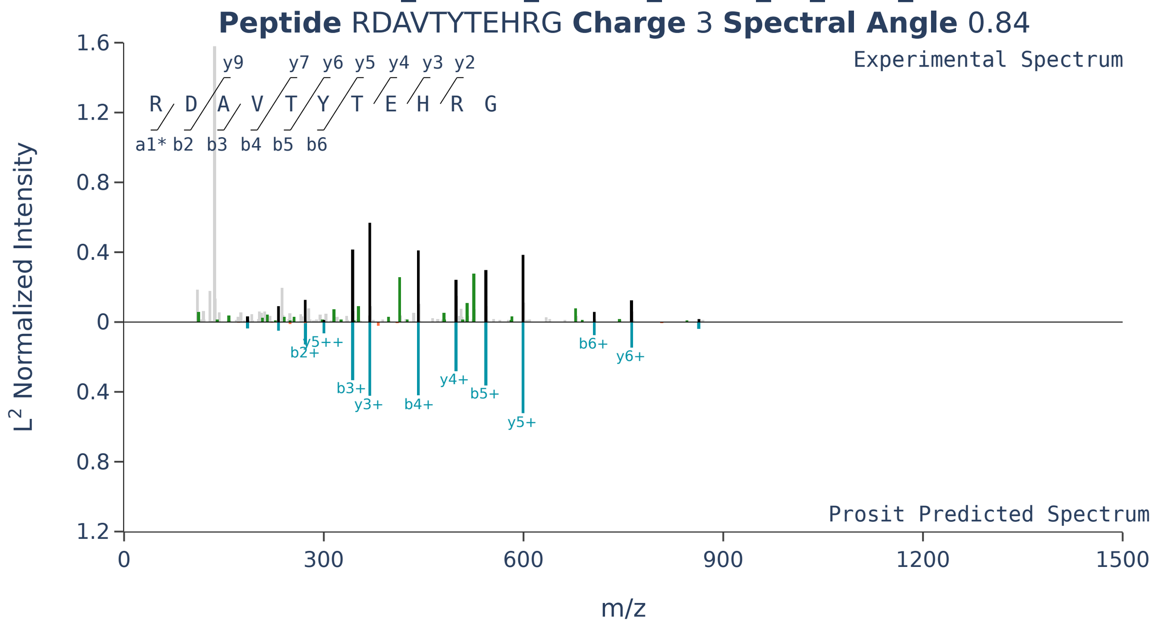

Source HRoetschke_291222_140123_HFGoe_H4_b1R1_24h **Scan** 16100
Peptide RGVLKVFLENRG **Charge** 3 **Spectral Angle** 0.86

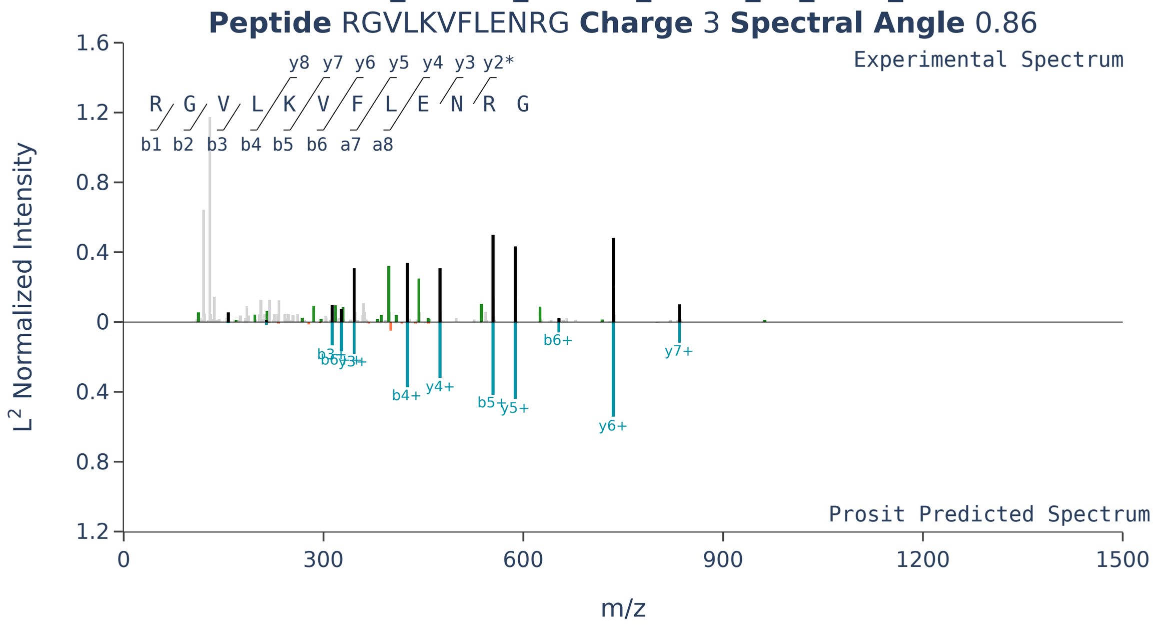

Source HRoetschke_291222_140123_HFGoe_H4_b1R1_2h **Scan** 8698
Peptide SGLIYEETKRK **Charge** 3 **Spectral Angle** 0.86

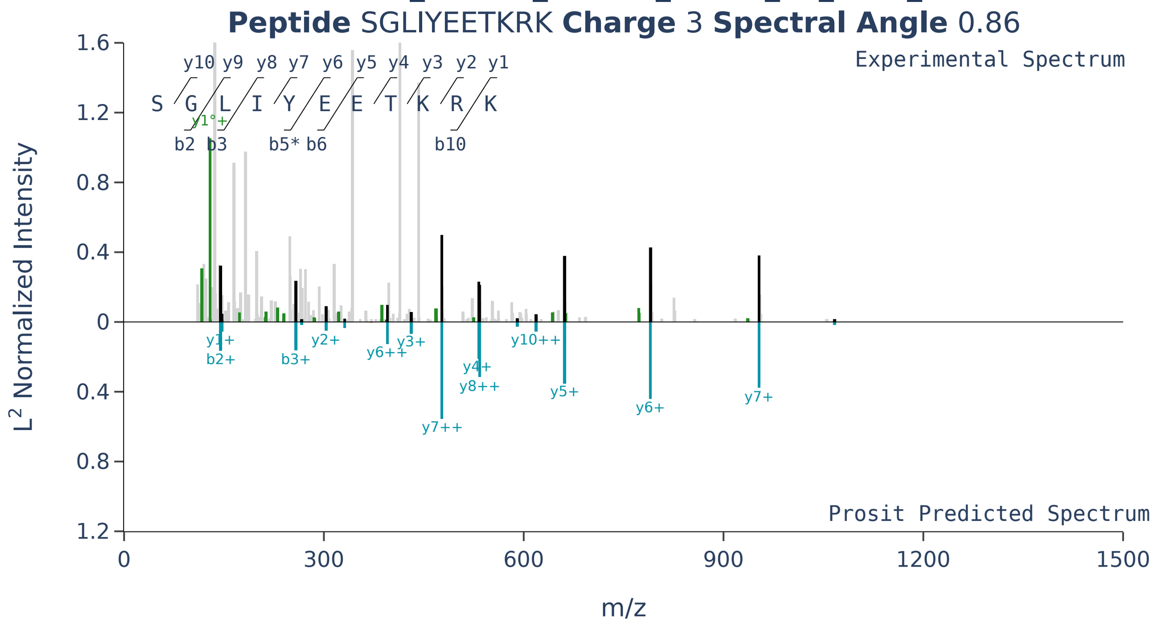

Source HRoetschke_291222_140123_HFGoe_H4_b1R2_24h **Scan** 16013
Peptide RGVLKVFLENGR **Charge** 3 **Spectral Angle** 0.83

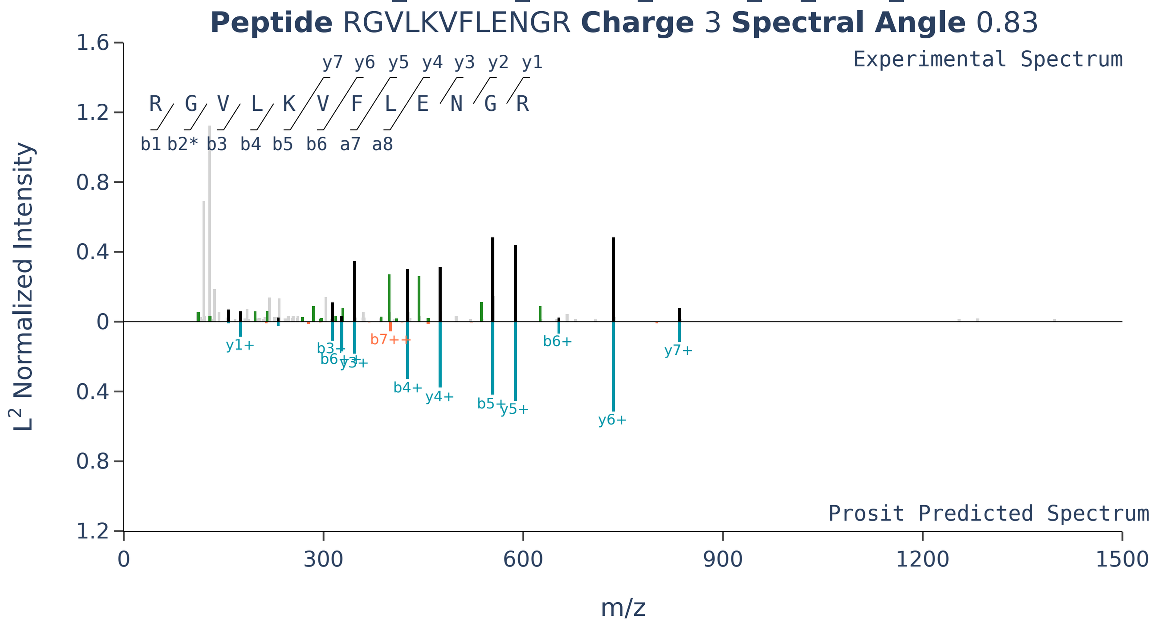

Source HRoetschke_291222_140123_HFGoe_H4_b2R1_4h **Scan** 22903
Peptide LKVVFLE **Charge** 2 **Spectral Angle** 0.9

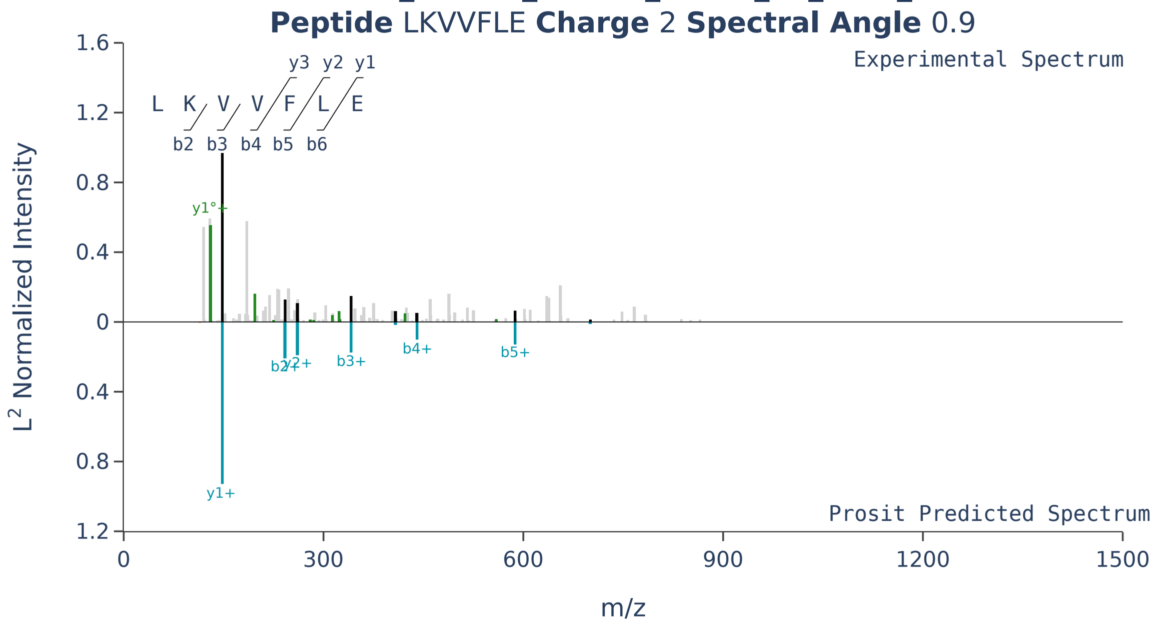

Source HRoetschke_291222_140123_HFGoe_H4_b1R1_2h **Scan** 22550
Peptide AMDVVYALLKRQGRT **Charge** 3 **Spectral Angle** 0.76

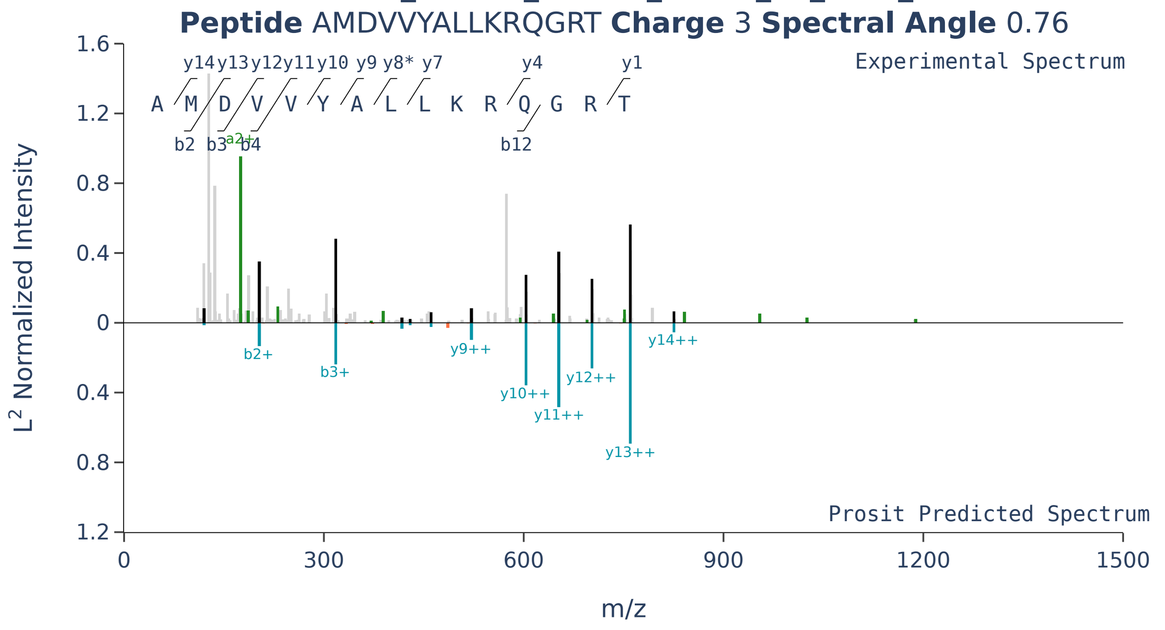

Source HRoetschke_291222_140123_HFGoe_H4_b1R1_24h **Scan** 8166
Peptide GNIQGITK **Charge** 2 **Spectral Angle** 0.86

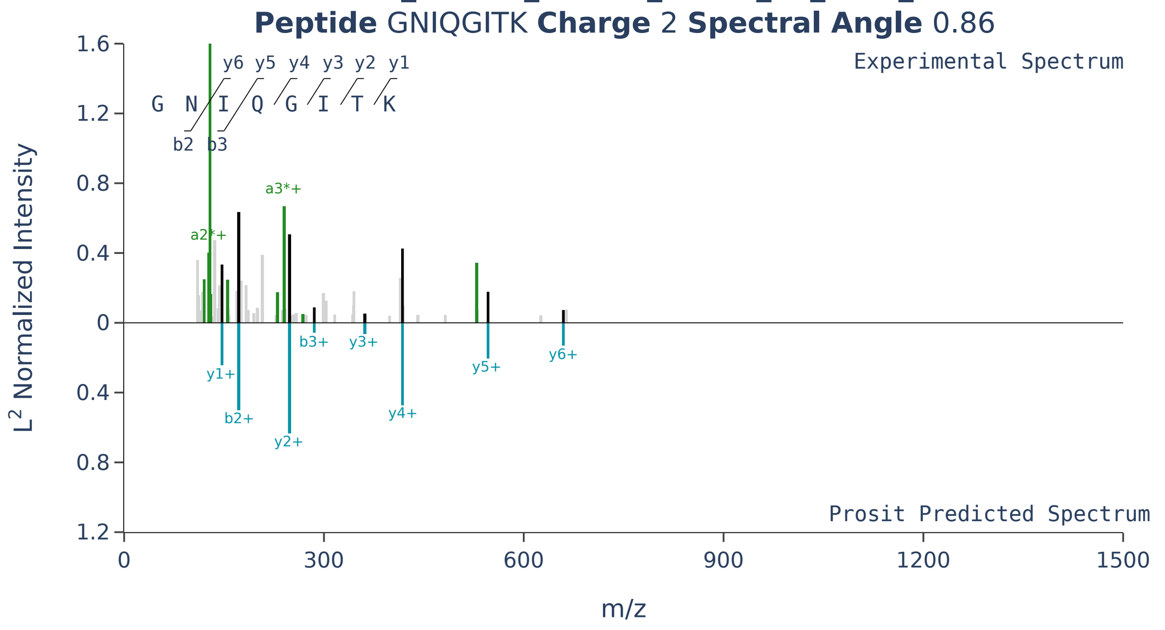

Source HRoetschke_291222_140123_HFGoe_H4_b2R2_24h **Scan** 8279
Peptide MDVVYKRQG **Charge** 3 **Spectral Angle** 0.92

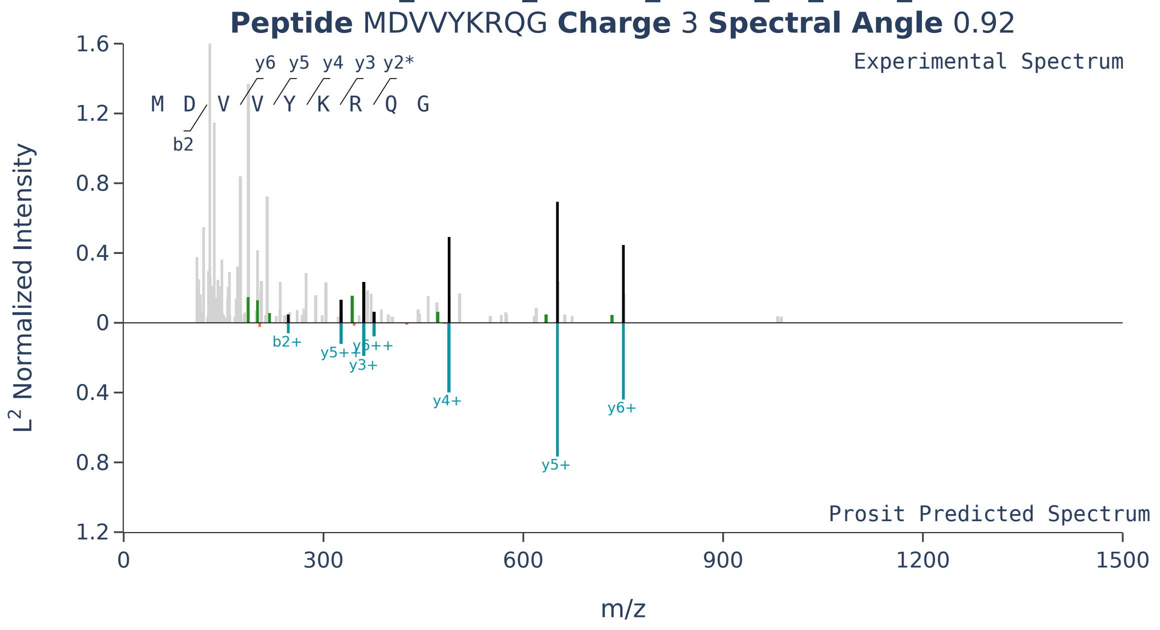

Source HRoetschke_291222_140123_HFGoe_H4_b1R1_24h **Scan** 13962
Peptide RSGLIYEET **Charge** 2 **Spectral Angle** 0.75

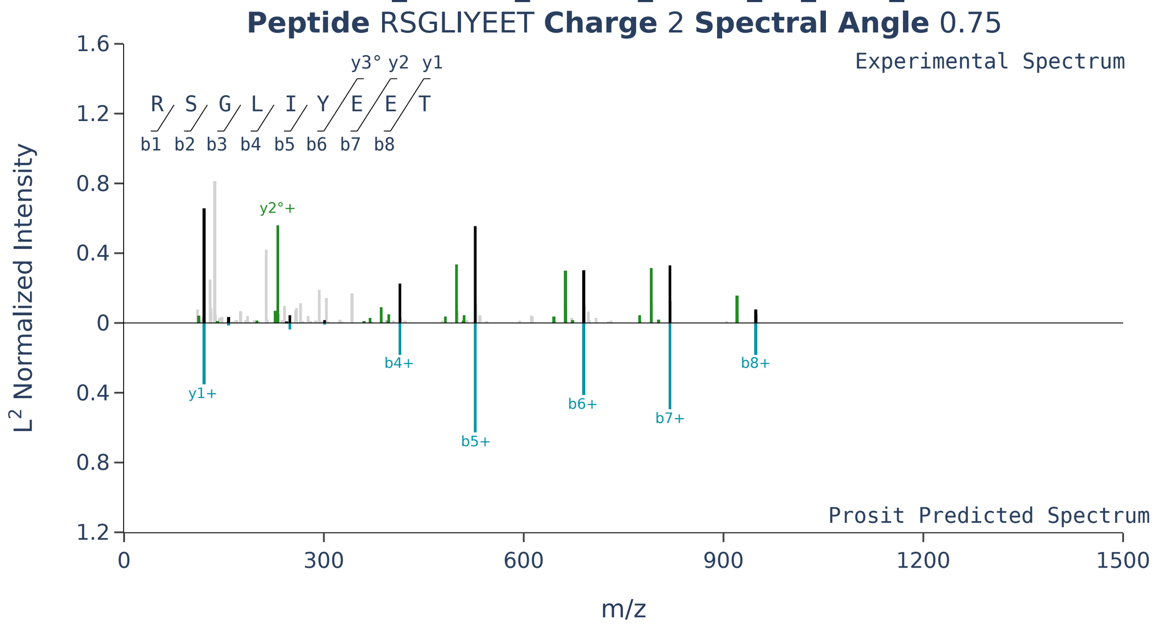

Source HRoetschke_291222_140123_HFGoe_H4_b1R1_24h **Scan** 7898
Peptide YRDAVTYTEH **Charge** 3 **Spectral Angle** 0.89

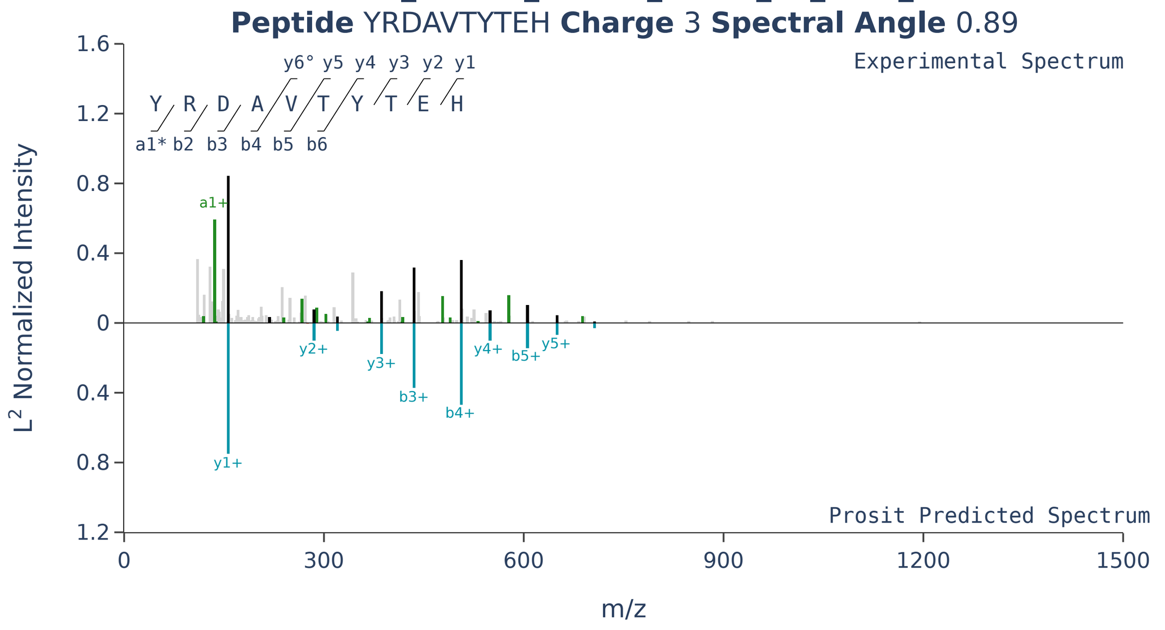

Source HRoetschke_291222_140123_HFGoe_H4_b1R1_2h **Scan** 15108
Peptide SGRGLIYEET **Charge** 1 **Spectral Angle** 0.64

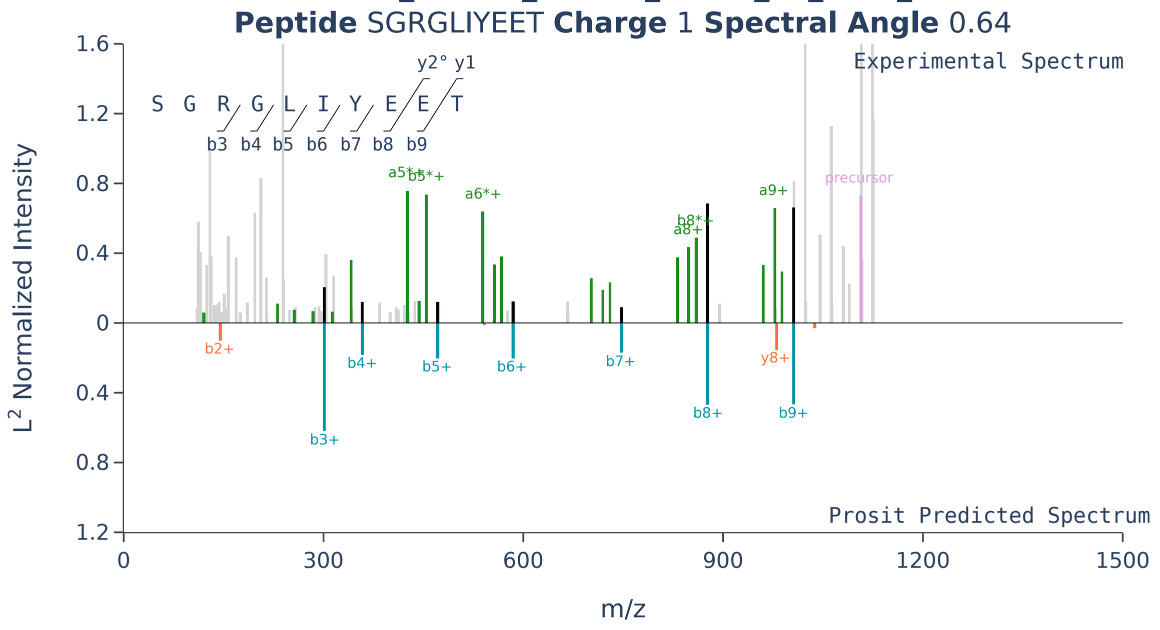

Source HRoetschke_291222_140123_HFGoe_H4_b1R2_2h **Scan** 10829
Peptide GITKPAIAIRR **Charge** 3 **Spectral Angle** 0.87

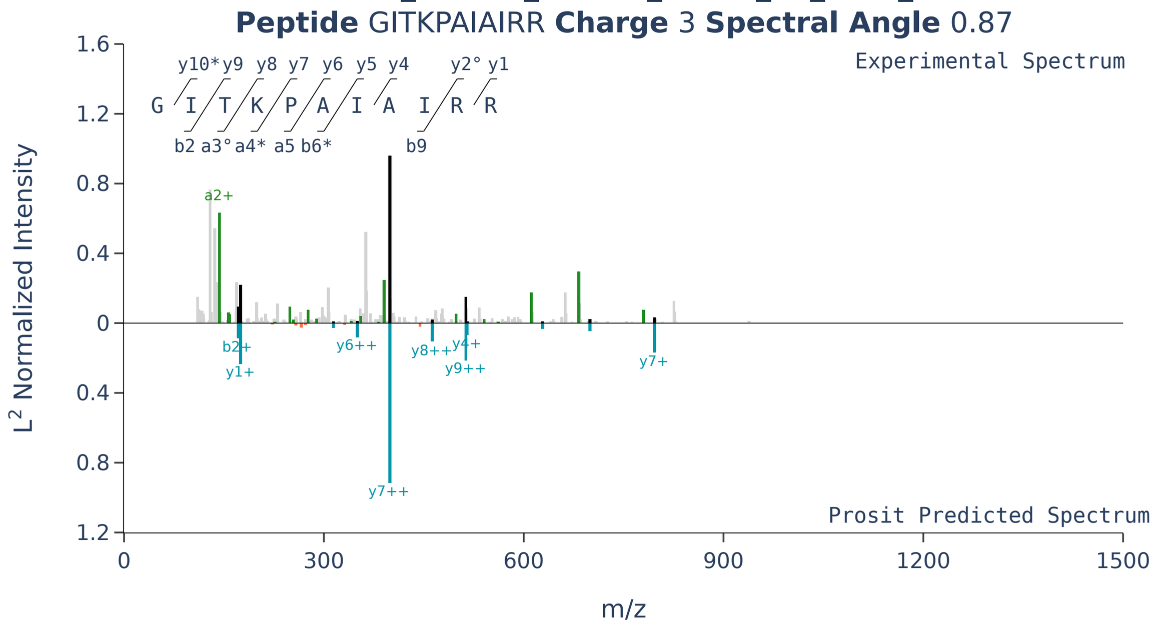

Source HRoetschke_291222_140123_HFGoe_H4_b1R2_24h **Scan** 14040
Peptide RDSGLIYE **Charge** 2 **Spectral Angle** 0.82

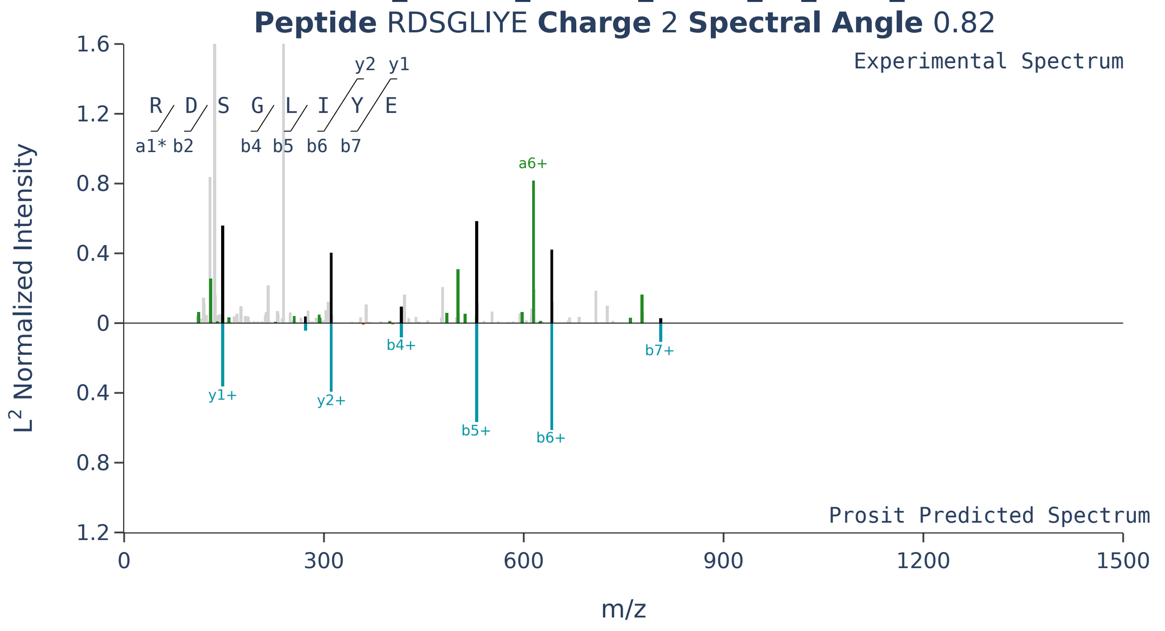

Source HRoetschke_291222_140123_HFGoe_H4_b1R1_24h **Scan** 9691
Peptide RGNIQGITKPAIR **Charge** 3 **Spectral Angle** 0.87

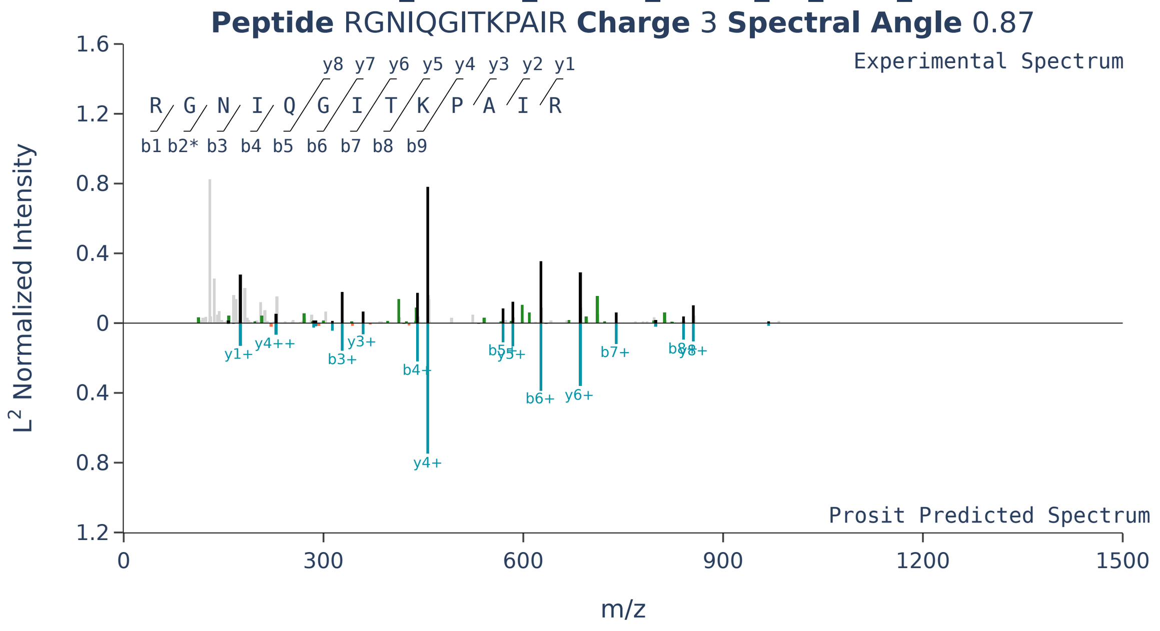

Source HRoetschke_291222_140123_HFGoe_H4_b2R2_4h **Scan** 12951
Peptide SGLIYEETKRQGRTL **Charge** 4 **Spectral Angle** 0.91

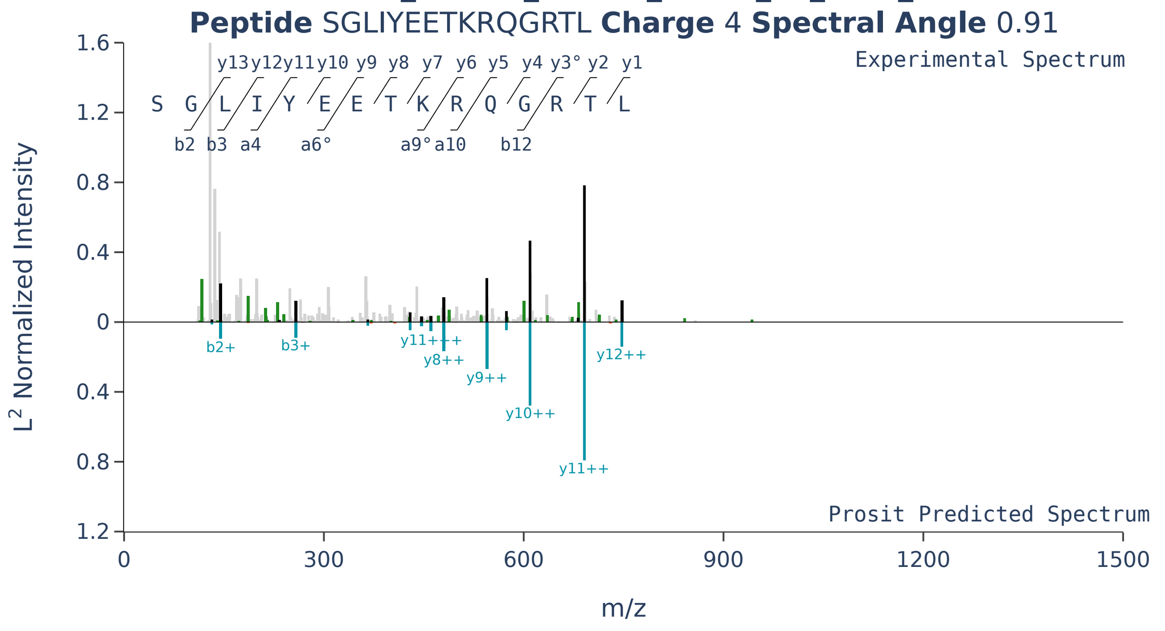

Source HRoetschke_291222_140123_HFGoe_H4_b1R1_2h **Scan** 13556
Peptide RGDNIQGITKPAIRRL **Charge** 4 **Spectral Angle** 0.84

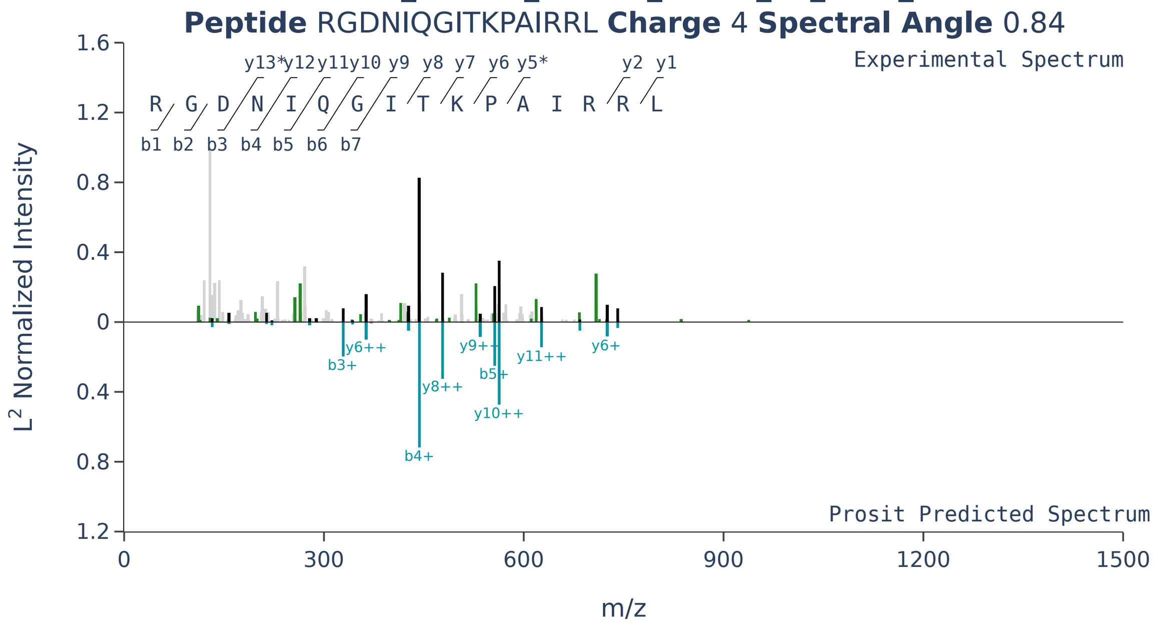

Source HRoetschke_291222_140123_HFGoe_H4_b2R1_4h **Scan** 5355
Peptide HAKRKTVTET **Charge** 3 **Spectral Angle** 0.91

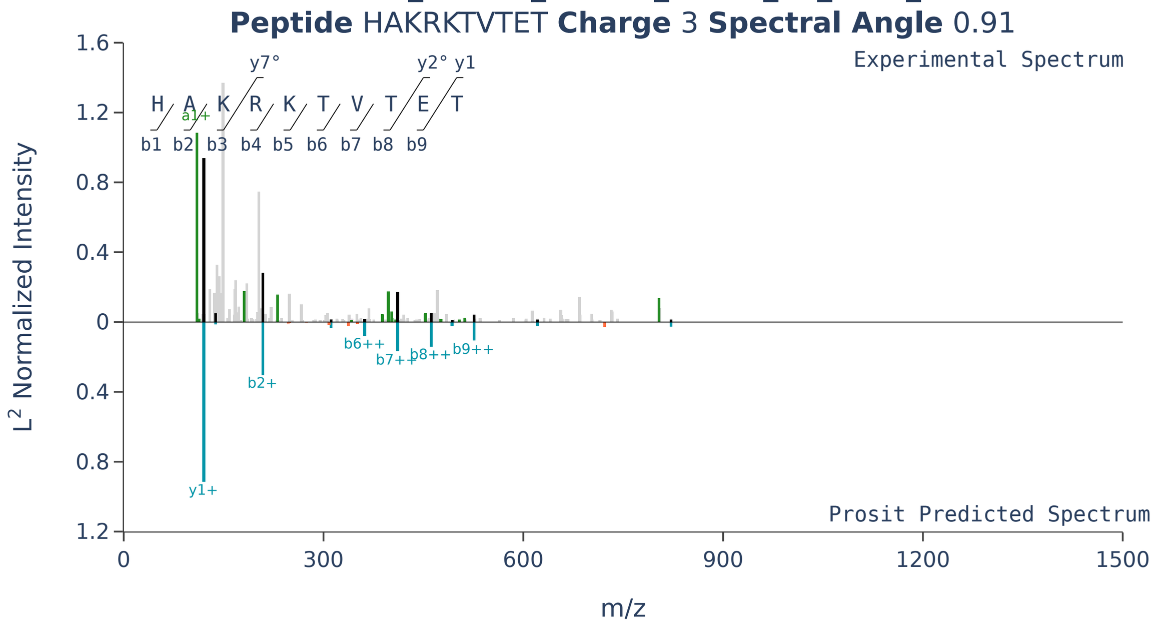

Source HRoetschke_291222_140123_HFGoe_H4_b2R2_24h **Scan** 12360
Peptide GNIQGITKPAIR **Charge** 3 **Spectral Angle** 0.8

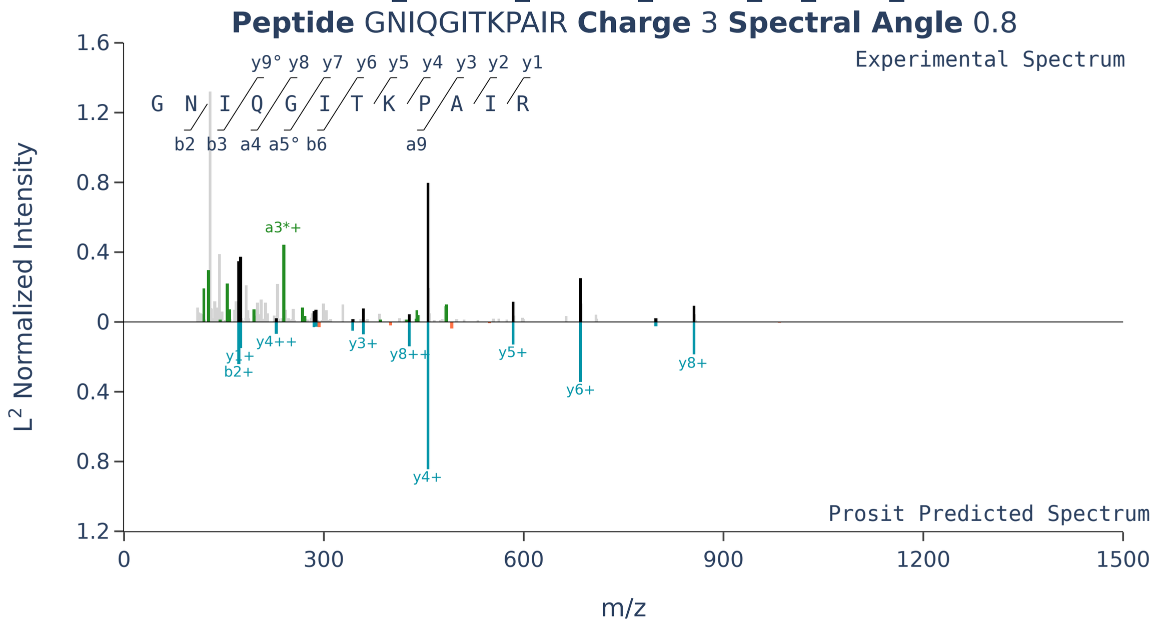

Source HRoetschke_291222_140123_HFGoe_H4_b1R2_4h **Scan** 17834
Peptide ASGLIYEET **Charge** 1 **Spectral Angle** 0.88

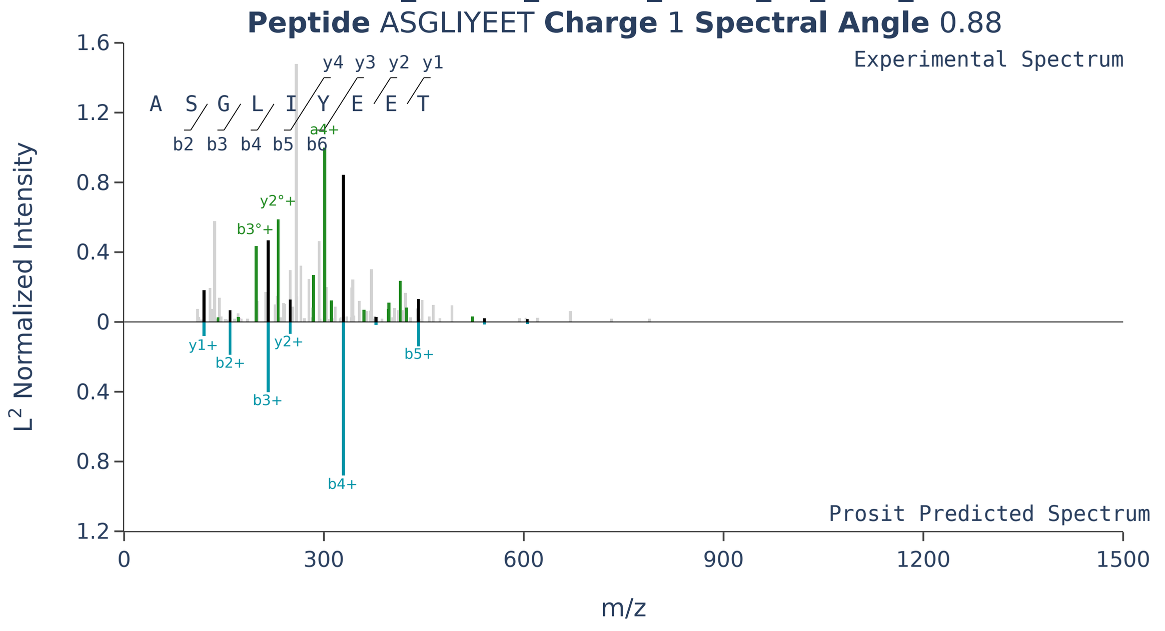

Source HRoetschke_291222_140123_HFGoe_H4_b2R2_24h **Scan** 8828
Peptide SGLIYEETRGKRQ **Charge** 3 **Spectral Angle** 0.8

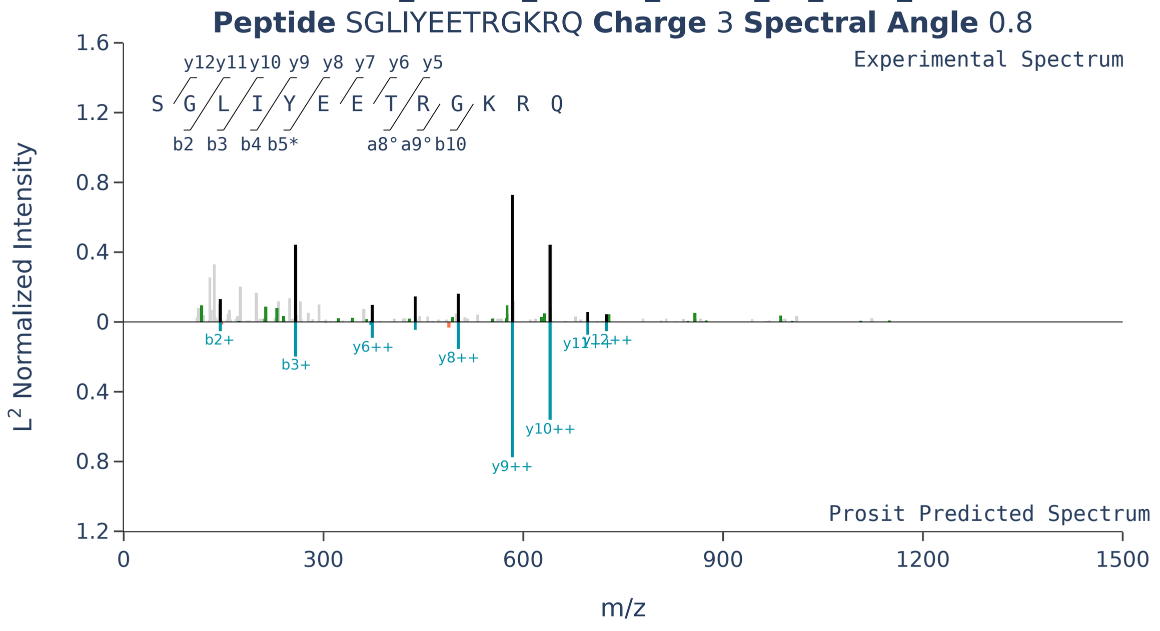

Source HRoetschke_291222_140123_HFGoe_H4_b1R1_4h **Scan** 17281
Peptide YNIQGITKPAIRRL **Charge** 4 **Spectral Angle** 0.89

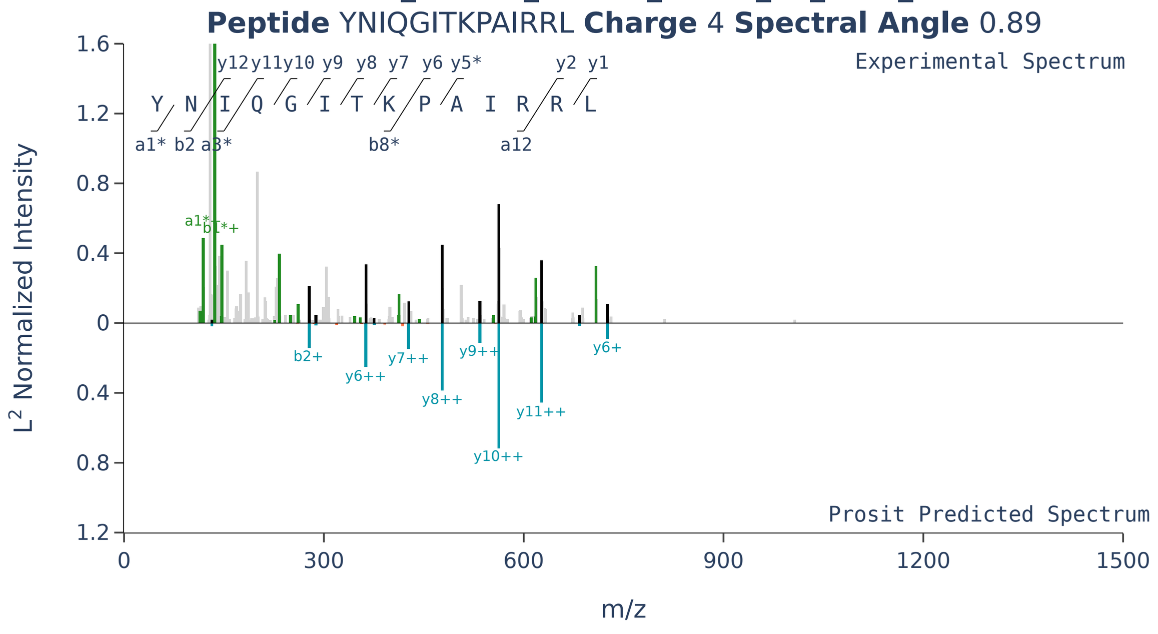

Source HRoetschke_291222_140123_HFGoe_H4_b1R1_4h **Scan** 11800
Peptide ISGLIYEEKRQGRT **Charge** 3 **Spectral Angle** 0.74

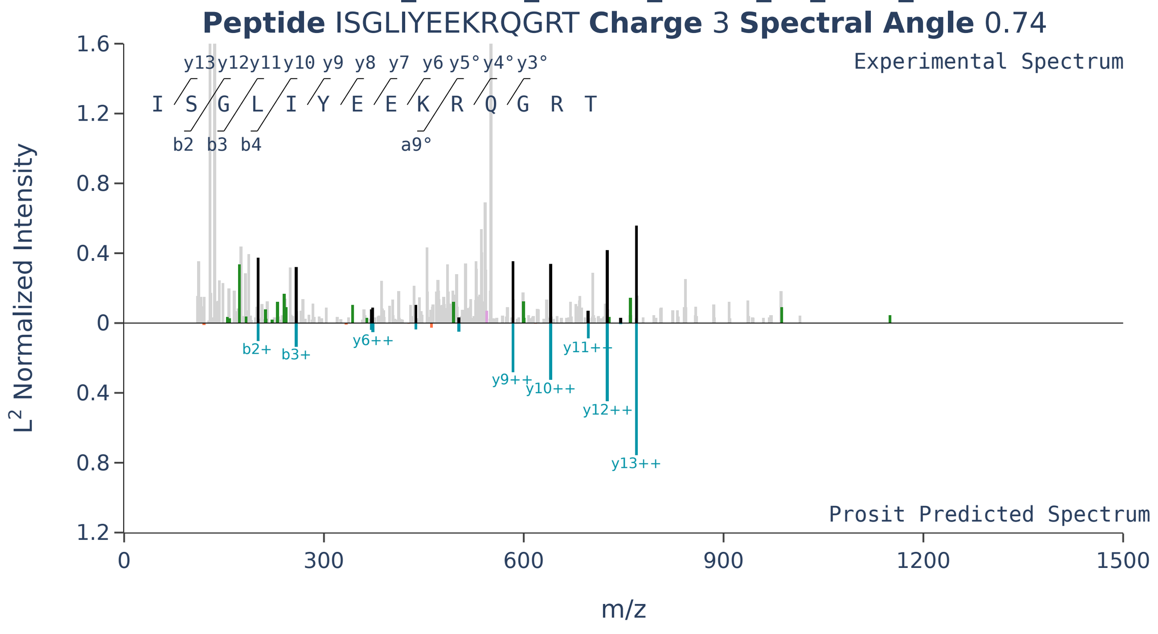

Source HRoetschke_291222_140123_HFGoe_H4_b2R2_2h **Scan** 12733
Peptide QGIQGITKPAIR **Charge** 3 **Spectral Angle** 0.84

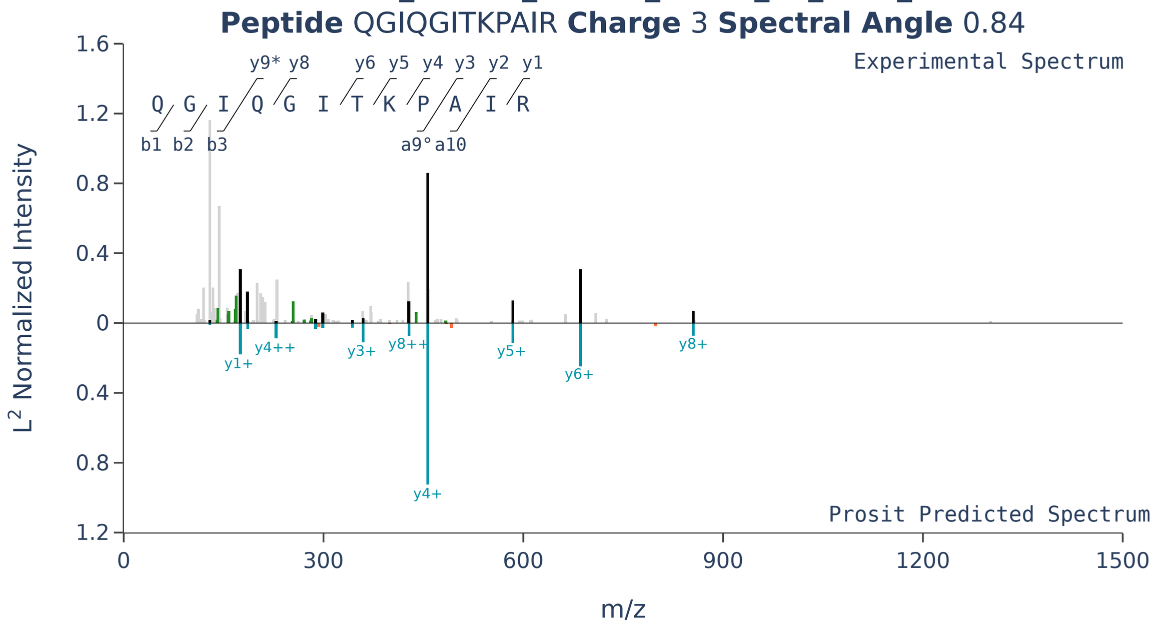

Source HRoetschke_291222_140123_HFGoe_H4_b1R1_2h **Scan** 8681
Peptide ARDAVTY **Charge** 2 **Spectral Angle** 0.92

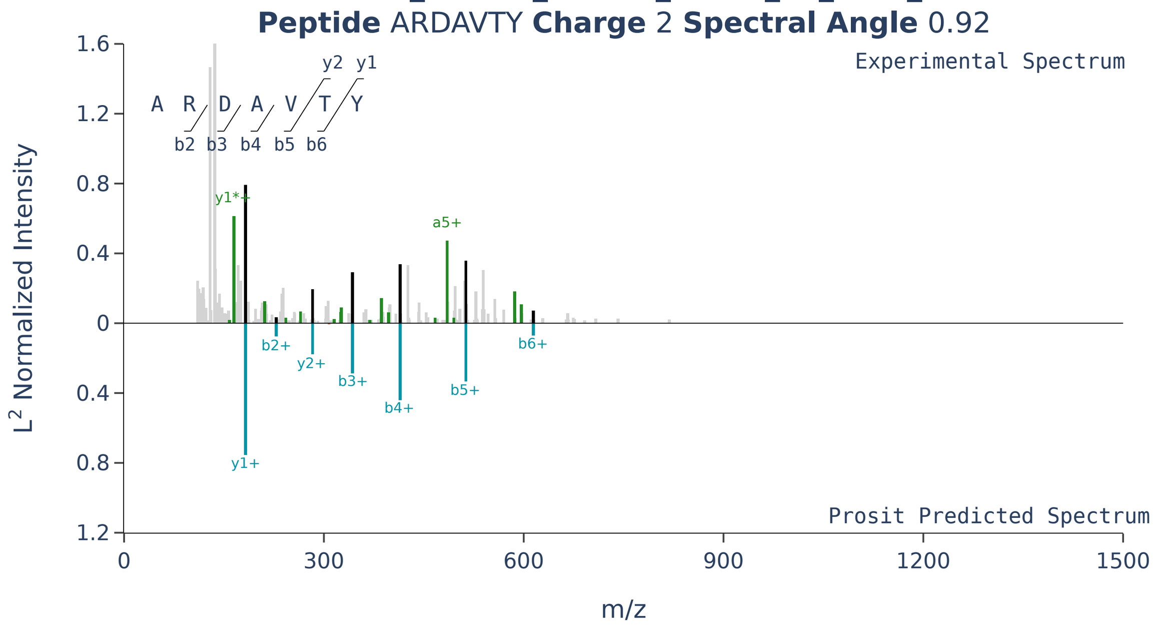

Source HRoetschke_291222_140123_HFGoe_H4_b1R1_1h **Scan** 15448
Peptide TESGLIYEETRG **Charge** 2 **Spectral Angle** 0.71

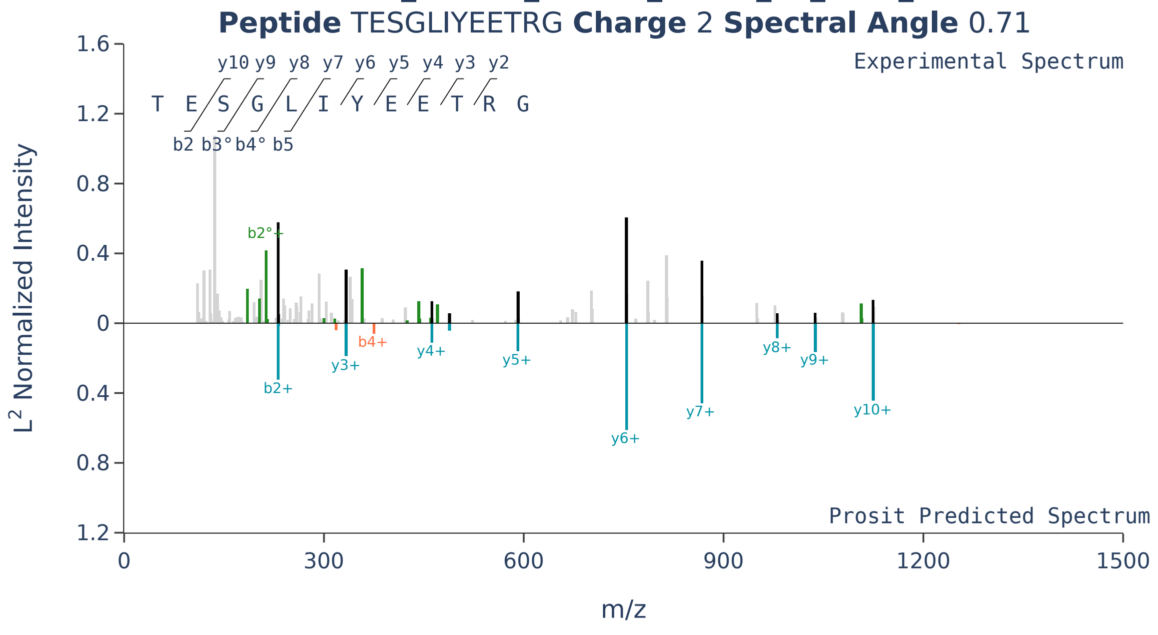

Source HRoetschke_291222_140123_HFGoe_H4_b1R2_2h **Scan** 14494
Peptide ISGLIYEETKRKTVTA **Charge** 4 **Spectral Angle** 0.65

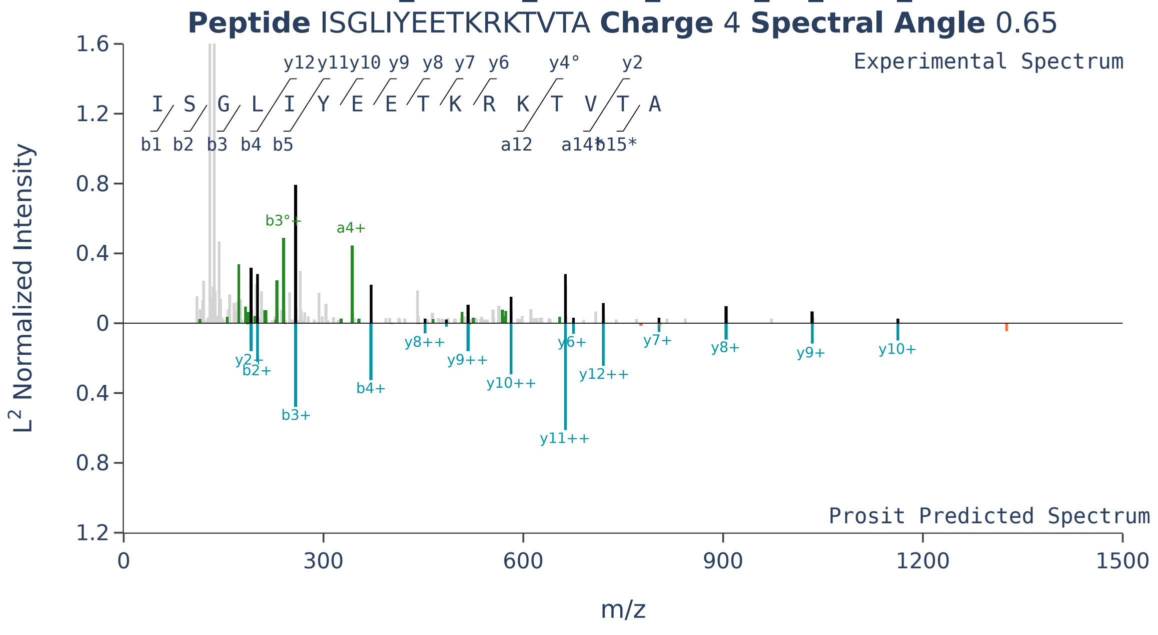

Source HRoetschke_291222_140123_HFGoe_H4_b1R2_2h **Scan** 7560
Peptide VYALKARRGG **Charge** 3 **Spectral Angle** 0.95

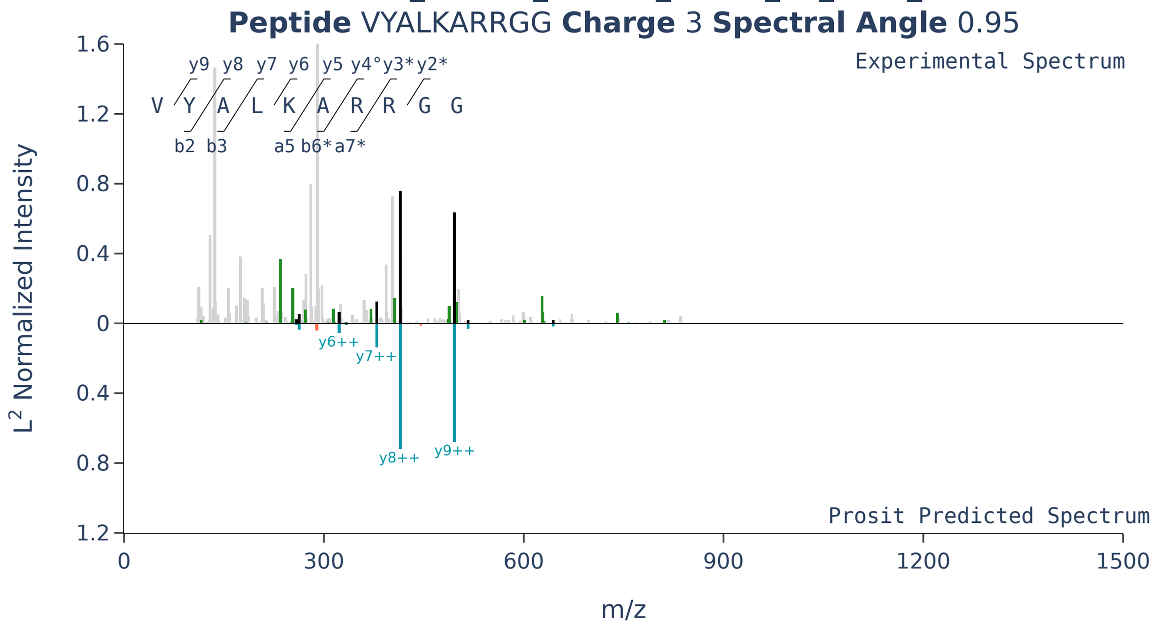

Source HRoetschke_291222_140123_HFGoe_H4_b1R1_4h **Scan** 20256
Peptide TEHAFLENVIRD **Charge** 3 **Spectral Angle** 0.88

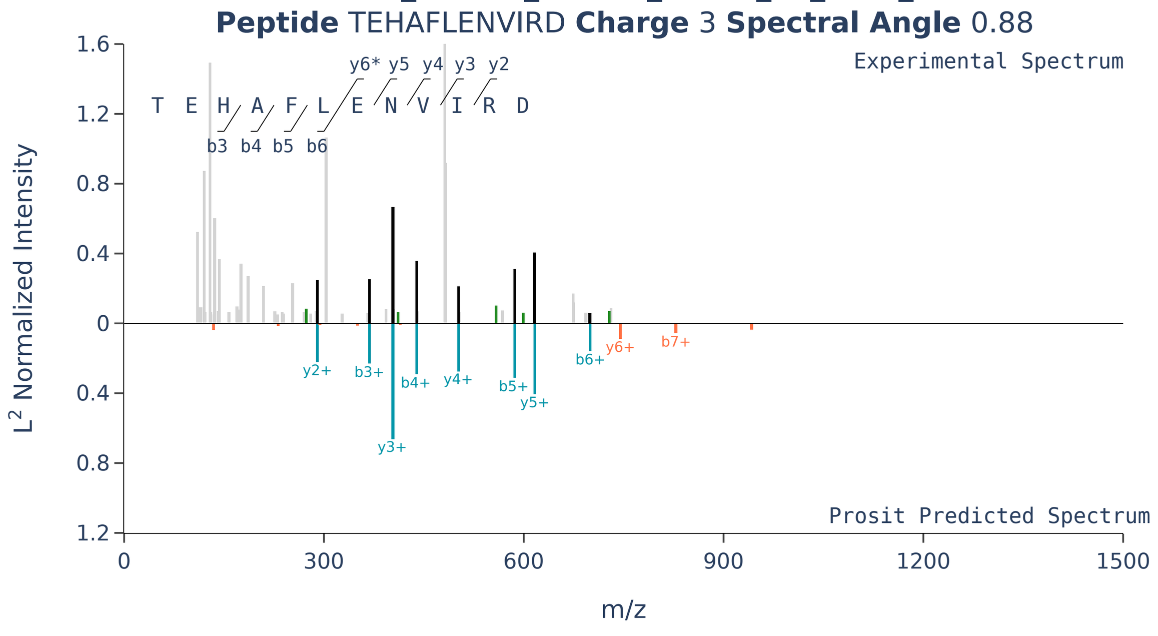

Source HRoetschke_291222_140123_HFGoe_H4_b1R1_4h **Scan** 12289
Peptide GRENVIRDAVTYTEHAKRKT **Charge** 5 **Spectral Angle** 0.82

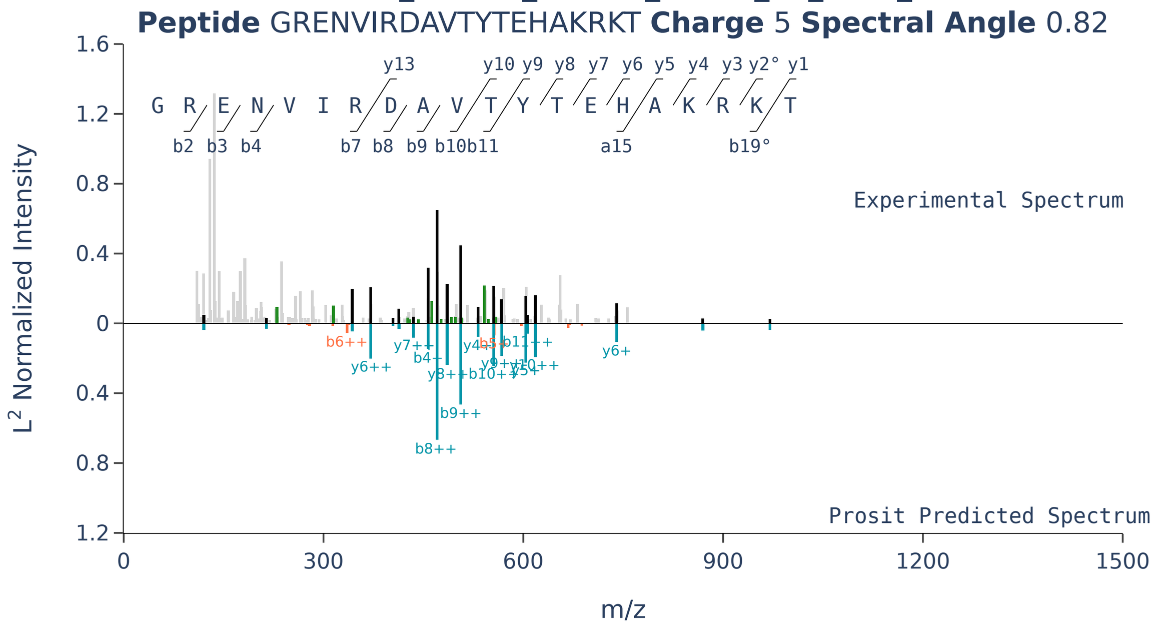

Source HRoetschke_291222_140123_HFGoe_H4_b1R1_4h **Scan** 10180
Peptide TERGVLK **Charge** 2 **Spectral Angle** 0.79

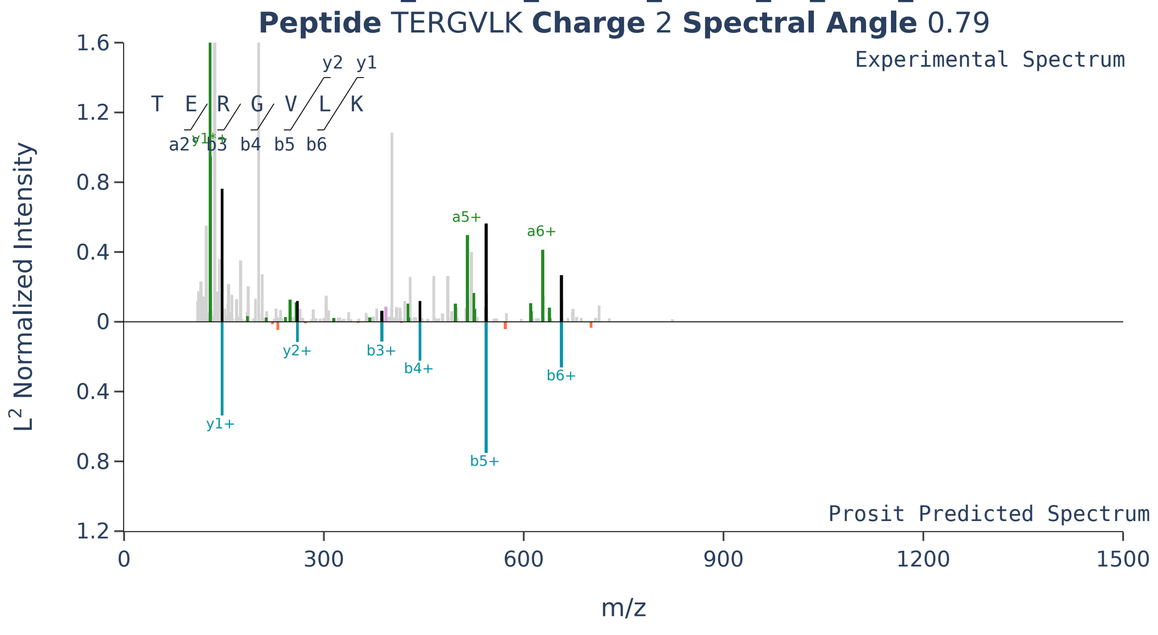

Source HRoetschke_291222_140123_HFGoe_H4_b1R1_2h **Scan** 17376
Peptide ENVIRDAVTYTEHGR **Charge** 3 **Spectral Angle** 0.79

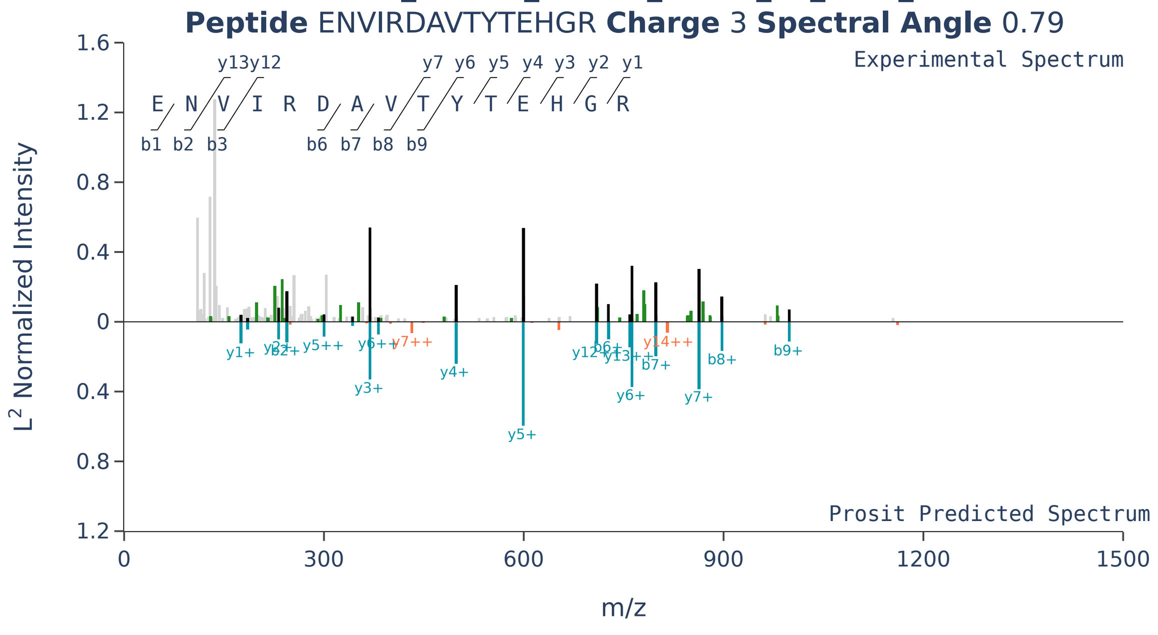

Source HRoetschke_291222_140123_HFGoe_H4_b2R2_24h **Scan** 6939
Peptide YALKRGGA **Charge** 2 **Spectral Angle** 0.84

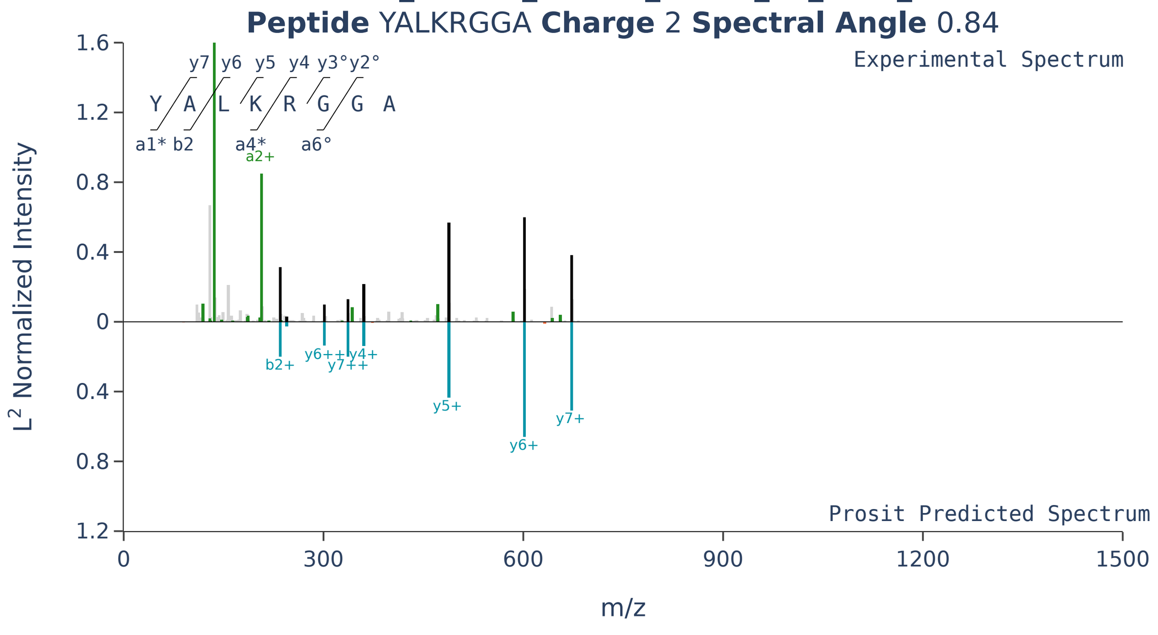

Source HRoetschke_291222_140123_HFGoe_H4_b1R2_24h **Scan** 6369
Peptide VVYLKRQGRT **Charge** 3 **Spectral Angle** 0.9

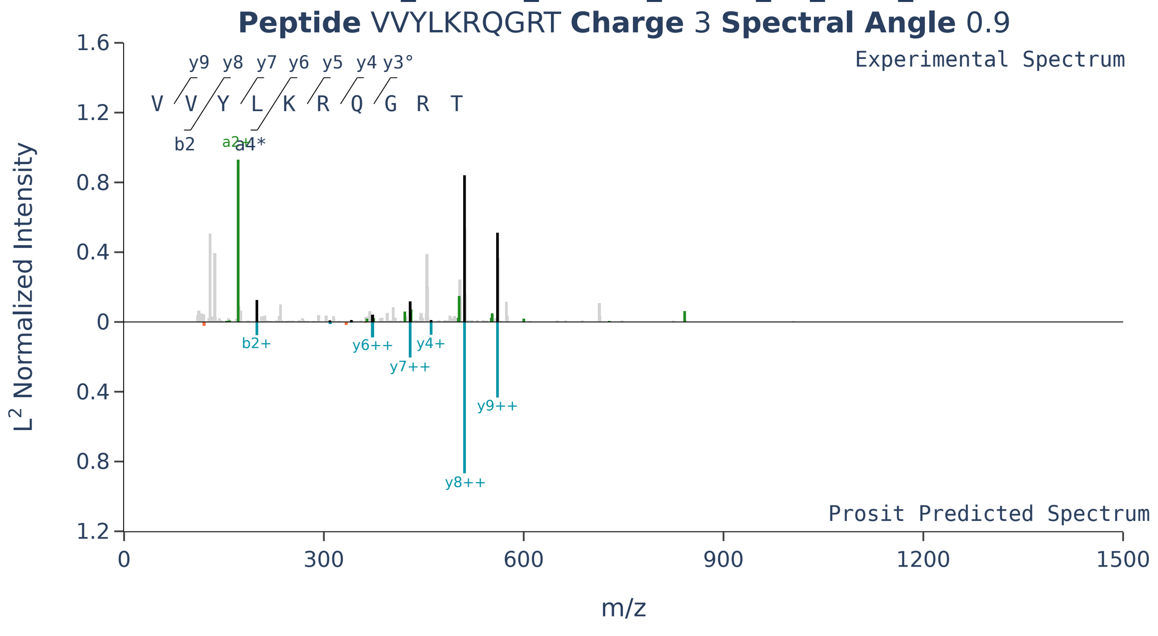

Source HRoetschke_291222_140123_HFGoe_H4_b1R1_4h **Scan** 15754
Peptide RDASGLIYEET **Charge** 2 **Spectral Angle** 0.8

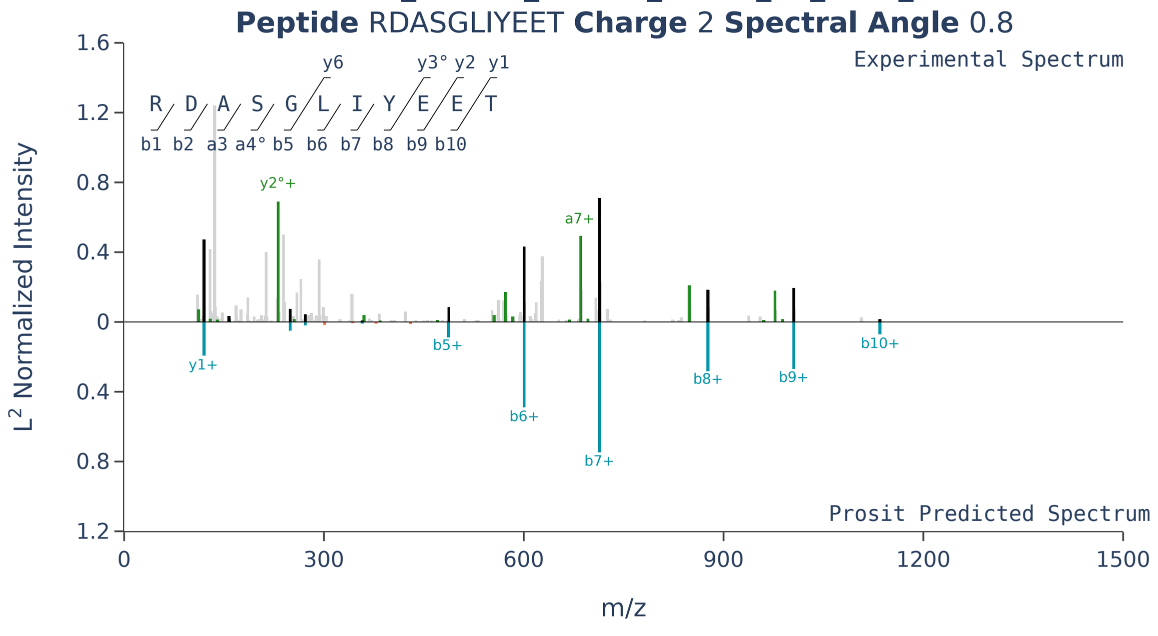

Source HRoetschke_291222_140123_HFGoe_H4_b1R1_2h **Scan** 13105
Peptide LRGVLKV **Charge** 2 **Spectral Angle** 0.87

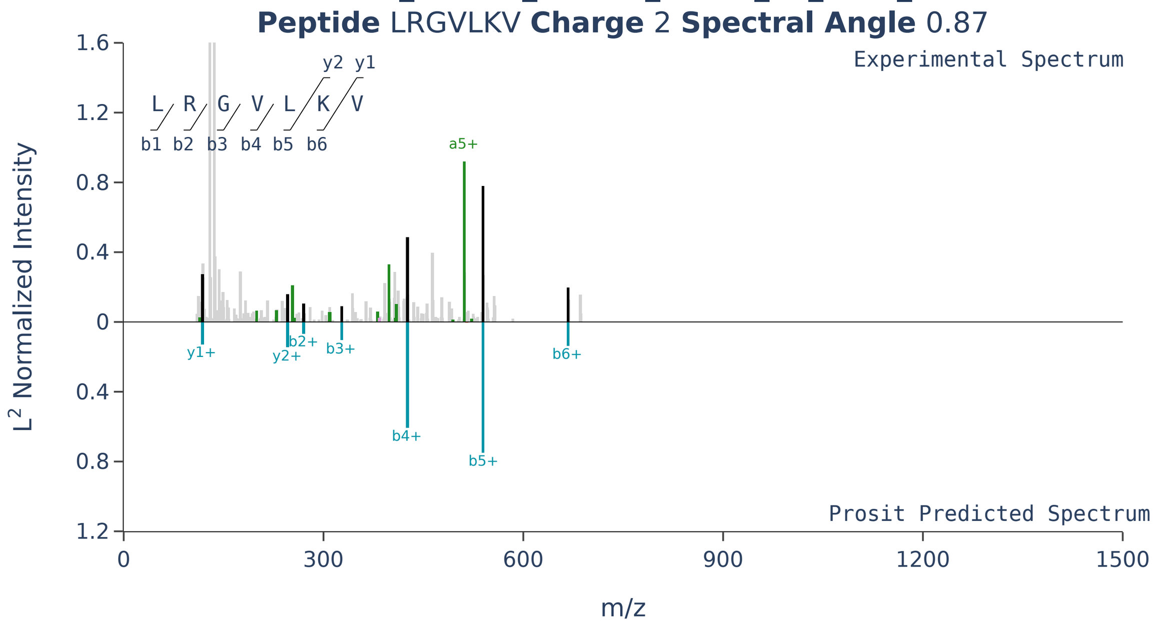

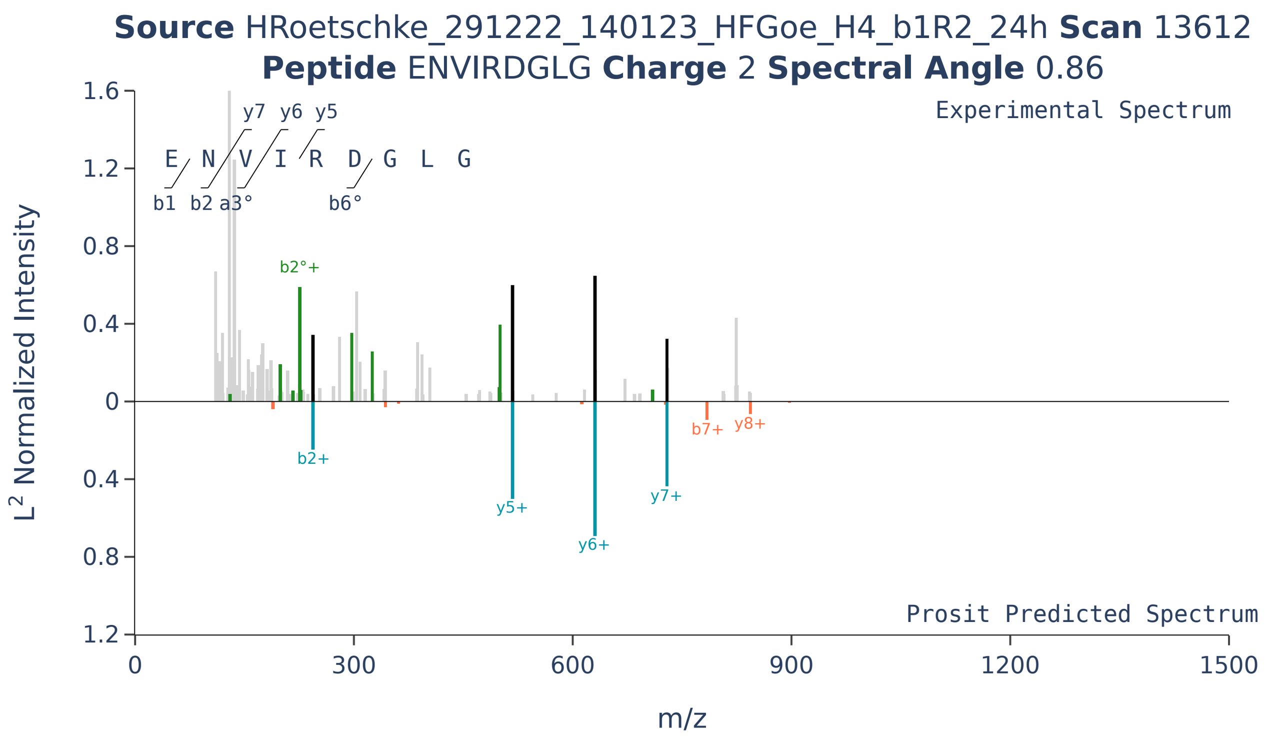
Source HRoetschke_291222_140123_HFGoe_H4_b1R2_24h Scan 13612
Peptide ENVIRDGLG Charge 2 Spectral Angle 0.86
Experimental Spectrum
Prosit Predicted Spectrum
L² Normalized Intensity
m/z

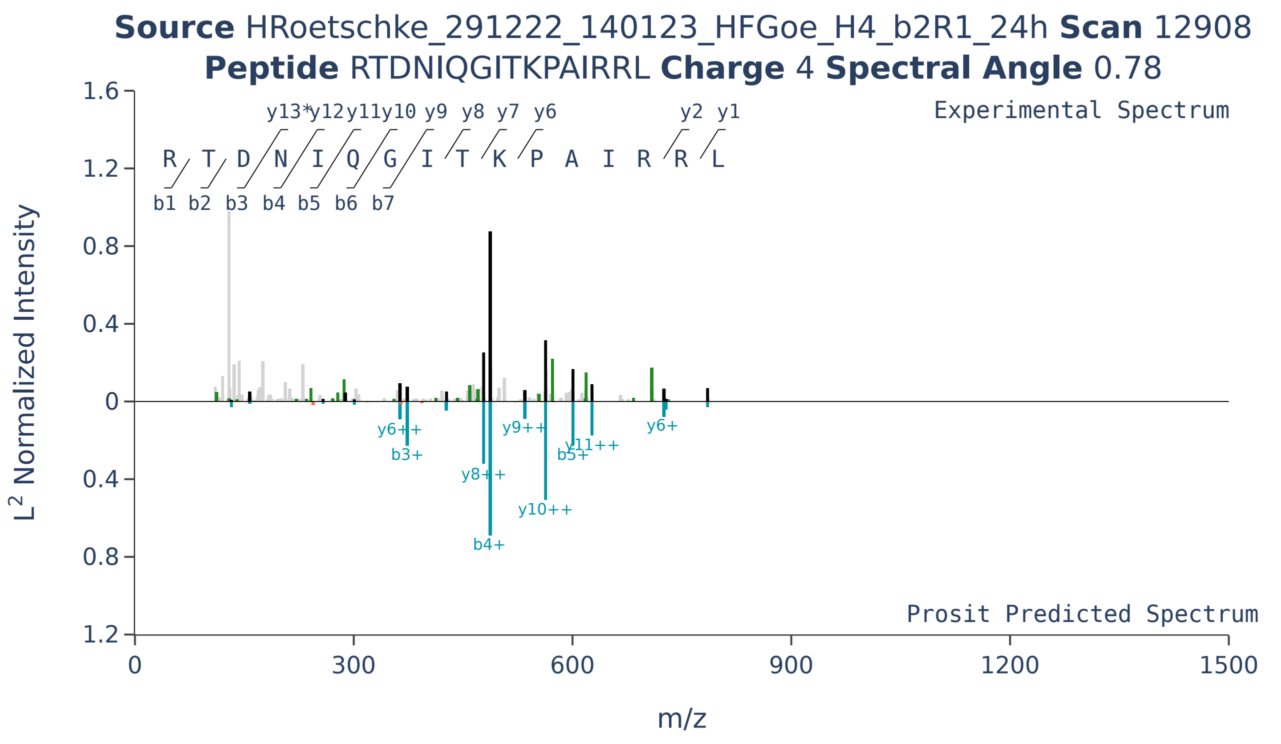
Source HRoetschke_291222_140123_HFGoe_H4_b2R1_24h Scan 12908
Peptide RTDNIQGITKPAIRRL Charge 4 Spectral Angle 0.78
Experimental Spectrum
Prosit Predicted Spectrum
L² Normalized Intensity
m/z

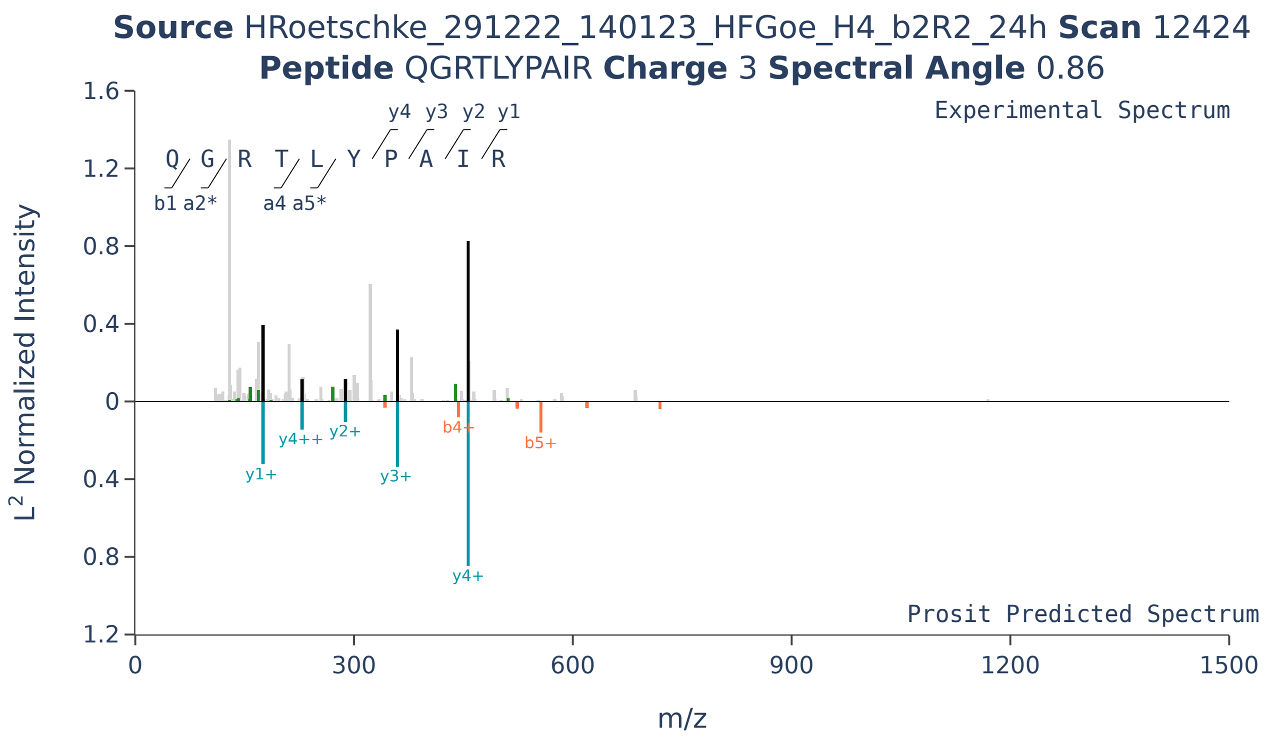
Source HRoetschke_291222_140123_HFGoe_H4_b2R2_24h Scan 12424
Peptide QGRTLYPAIR Charge 3 Spectral Angle 0.86
Experimental Spectrum
Prosit Predicted Spectrum
L² Normalized Intensity
m/z

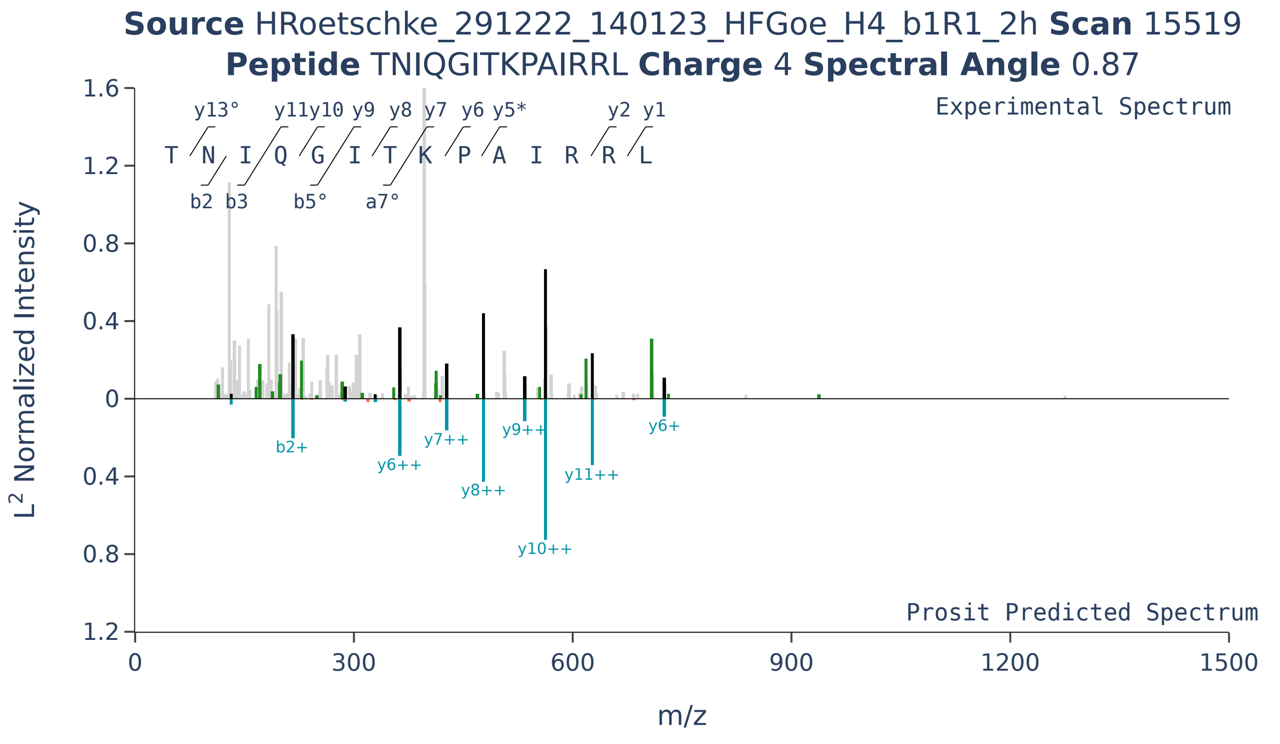
Source HRoetschke_291222_140123_HFGoe_H4_b1R1_2h Scan 15519
Peptide TNIQGITKPAIRRL Charge 4 Spectral Angle 0.87
Experimental Spectrum
Prosit Predicted Spectrum
L² Normalized Intensity
m/z

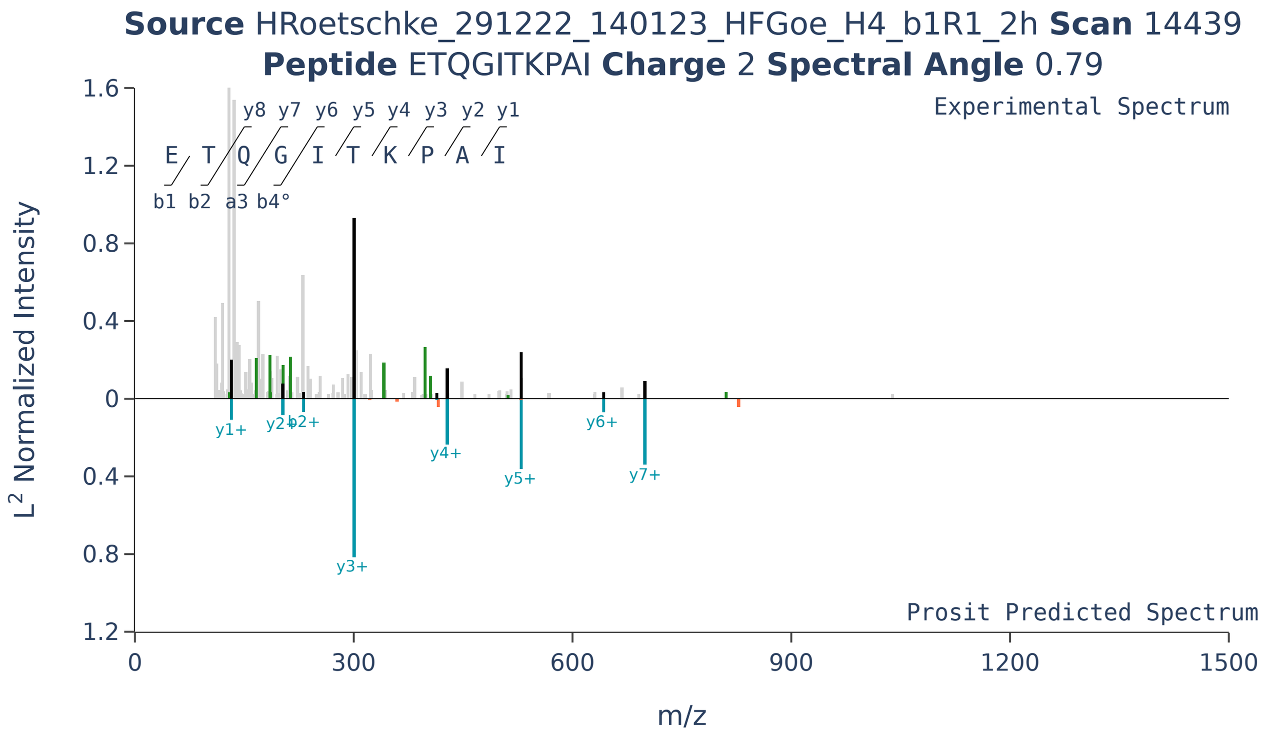
Source HRoetschke_291222_140123_HFGoe_H4_b1R1_2h Scan 14439
Peptide ETQGITKPAI Charge 2 Spectral Angle 0.79
Experimental Spectrum
Prosit Predicted Spectrum
L² Normalized Intensity
m/z

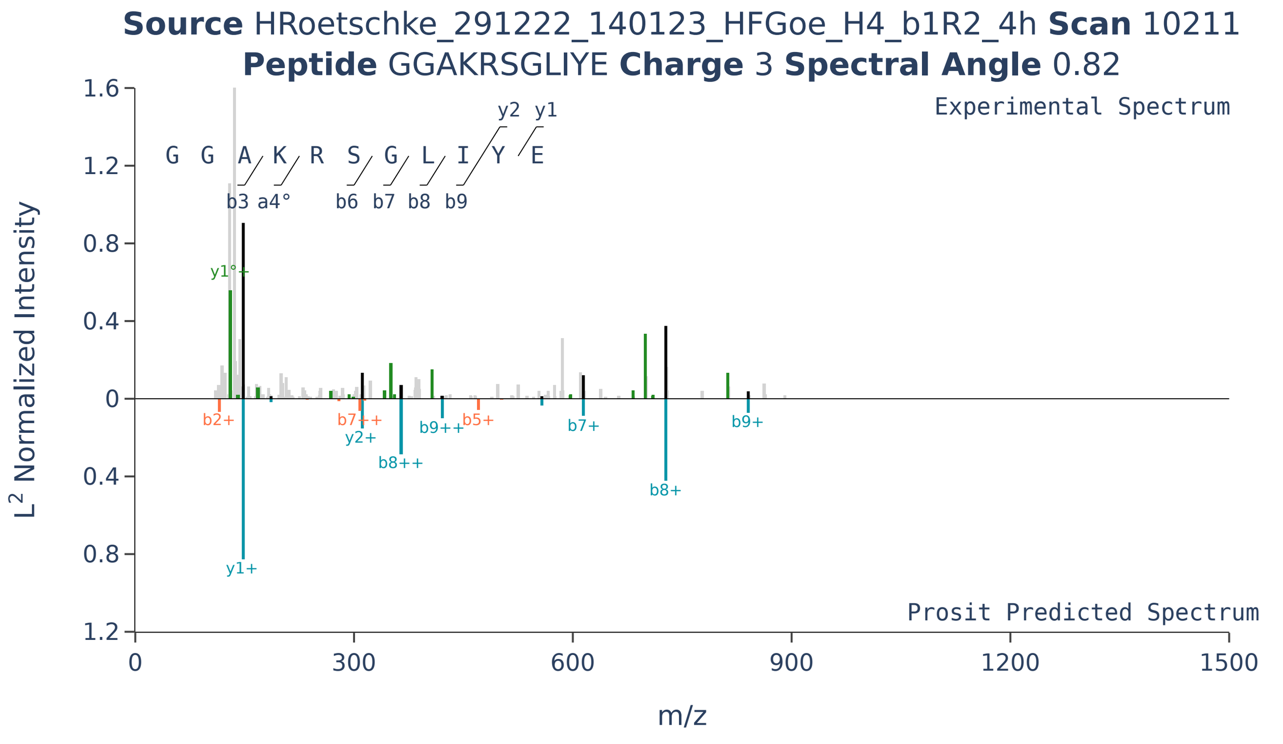
Source HRoetschke_291222_140123_HFGoe_H4_b1R2_4h Scan 10211
Peptide GGAKRSGLIYE Charge 3 Spectral Angle 0.82
Experimental Spectrum
Prosit Predicted Spectrum
L² Normalized Intensity
m/z

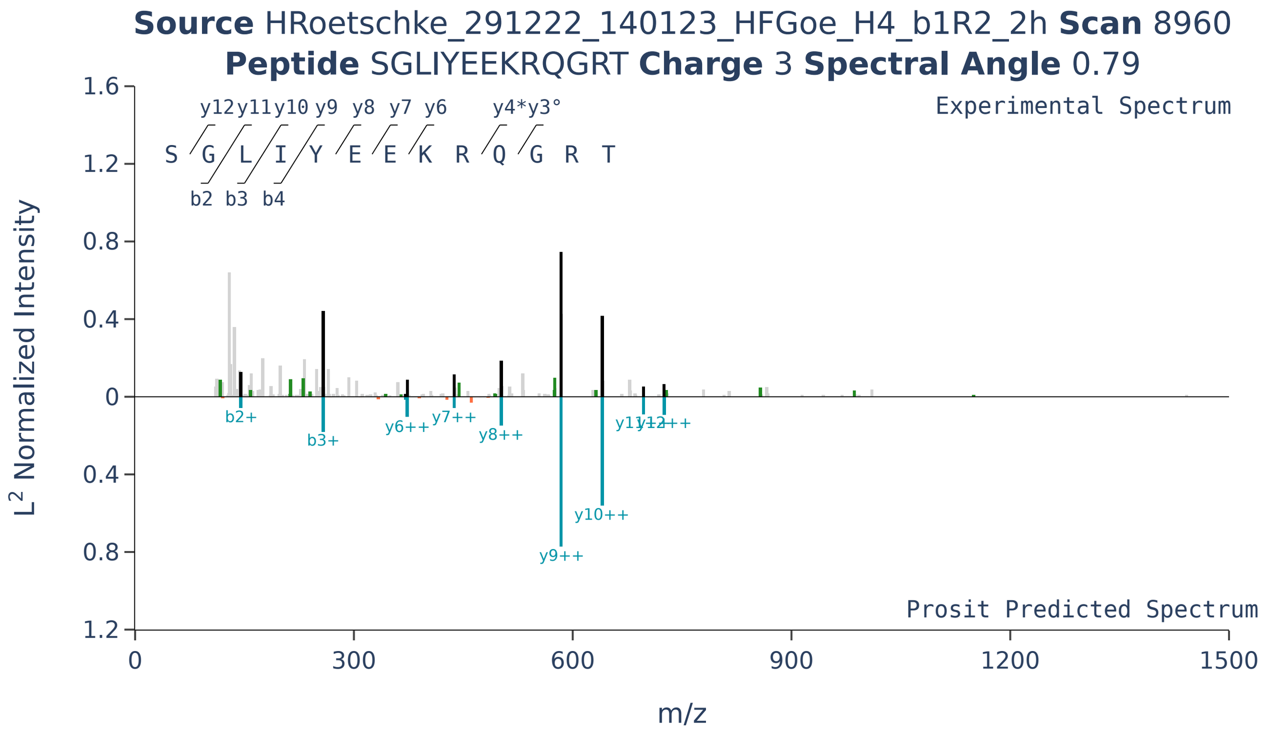
Source HRoetschke_291222_140123_HFGoe_H4_b1R2_2h Scan 8960
Peptide SGLIYEEKRQGRT Charge 3 Spectral Angle 0.79
Experimental Spectrum
Prosit Predicted Spectrum
L² Normalized Intensity
m/z

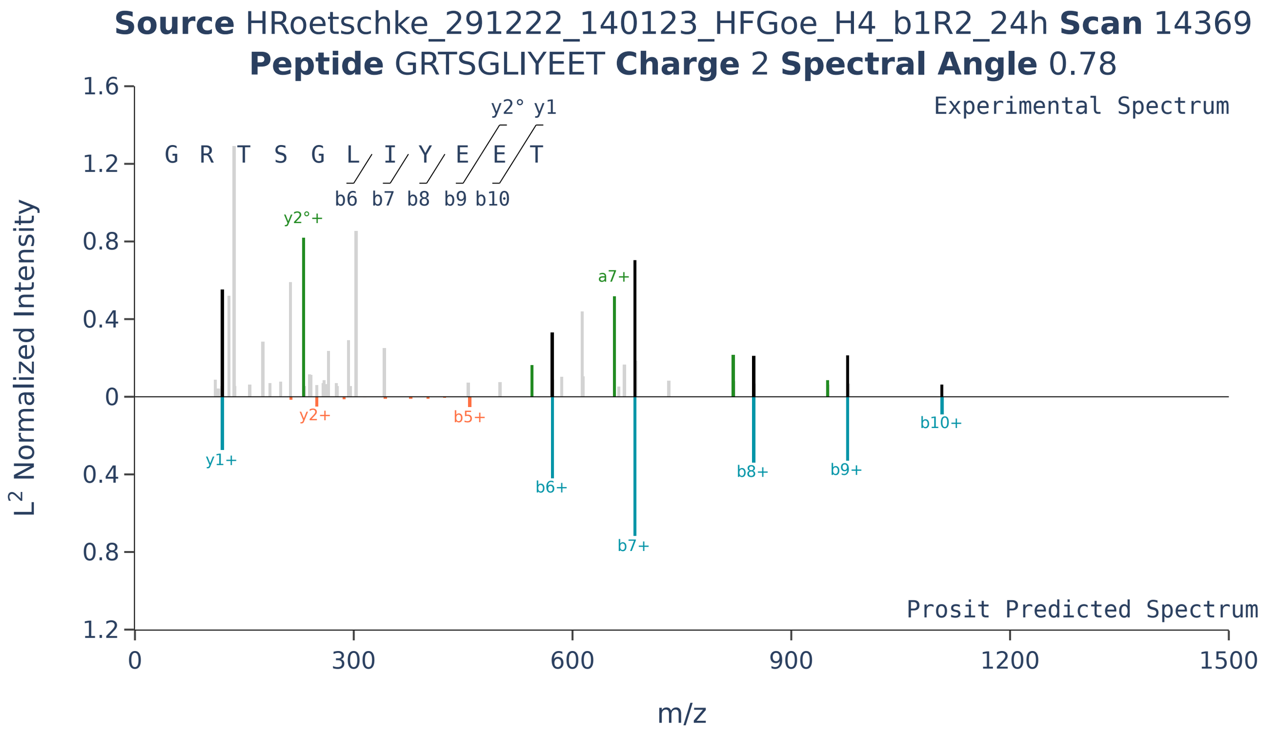
Source HRoetschke_291222_140123_HFGoe_H4_b1R2_24h Scan 14369
Peptide GRTSGLIYEET Charge 2 Spectral Angle 0.78
Experimental Spectrum
Prosit Predicted Spectrum
L² Normalized Intensity
m/z

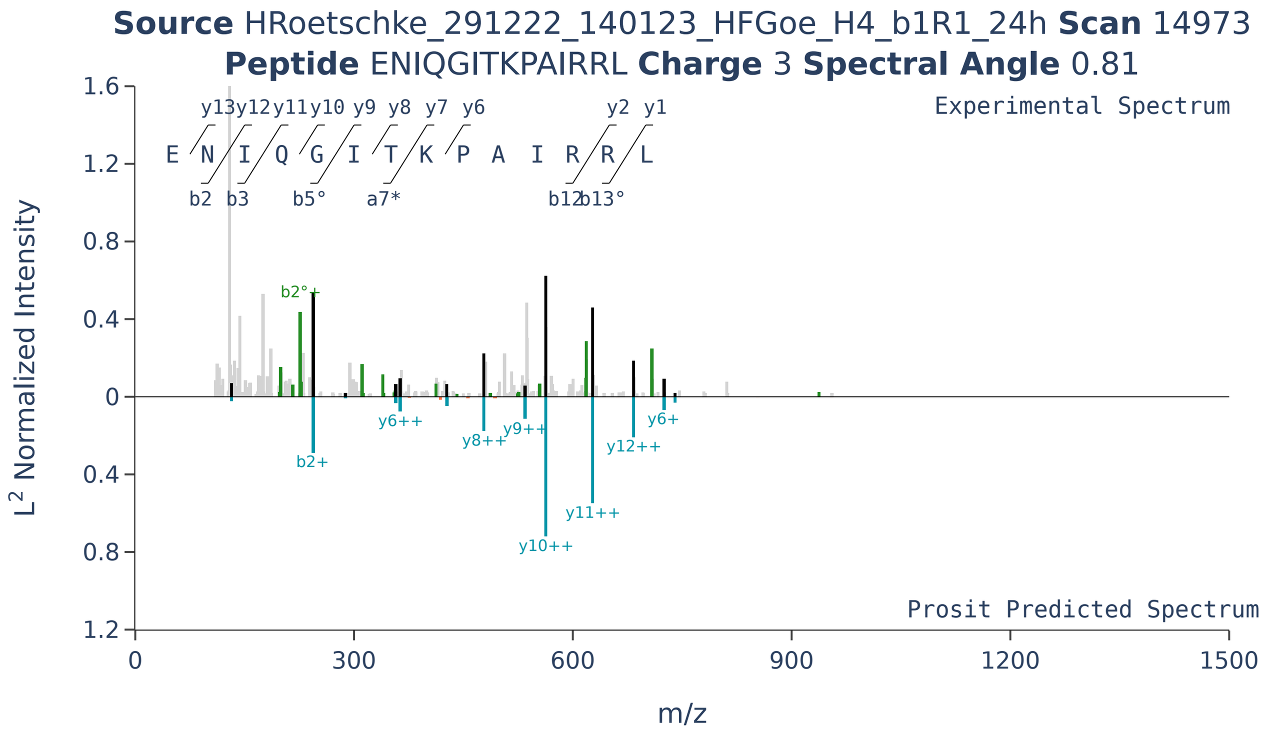
Source HRoetschke_291222_140123_HFGoe_H4_b1R1_24h Scan 14973
Peptide ENIQGITKPAIRRL Charge 3 Spectral Angle 0.81
Experimental Spectrum
Prosit Predicted Spectrum
L² Normalized Intensity
m/z

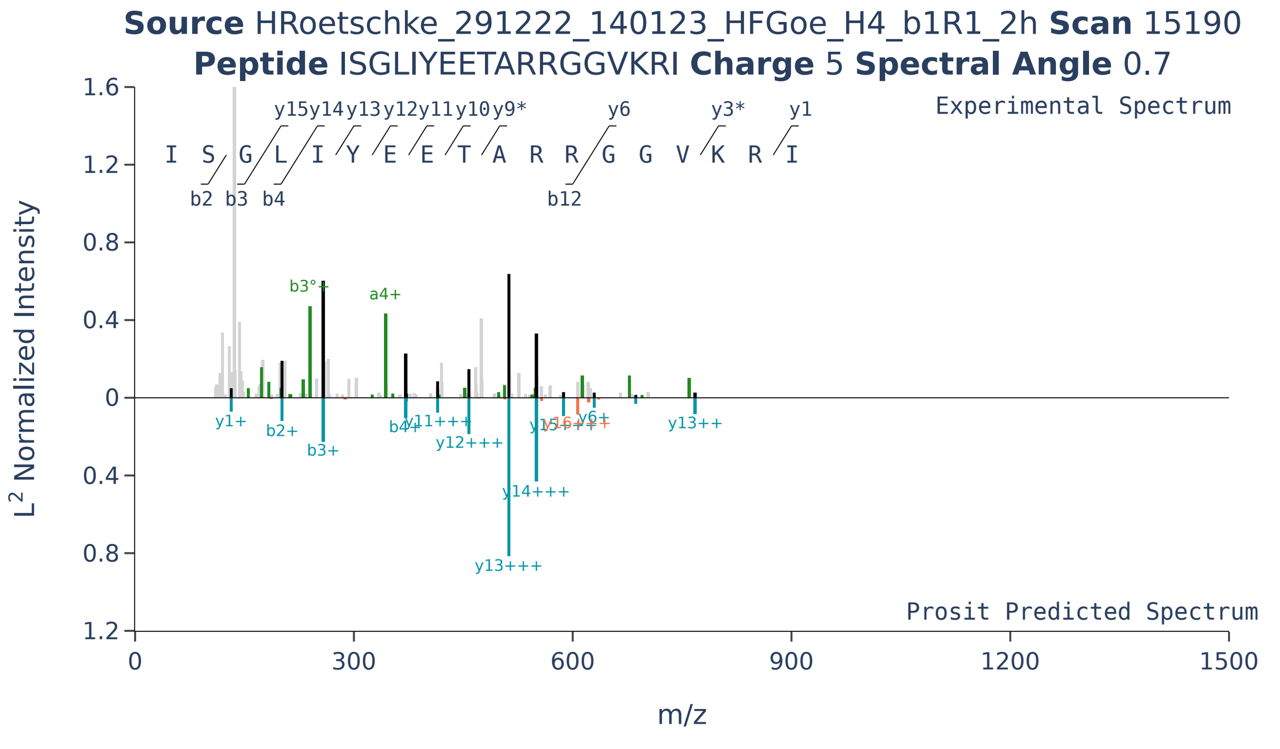
Source HRoetschke_291222_140123_HFGoe_H4_b1R1_2h Scan 15190
Peptide ISGLIYEETARRGGVKRI Charge 5 Spectral Angle 0.7
Experimental Spectrum
Prosit Predicted Spectrum
L² Normalized Intensity
m/z

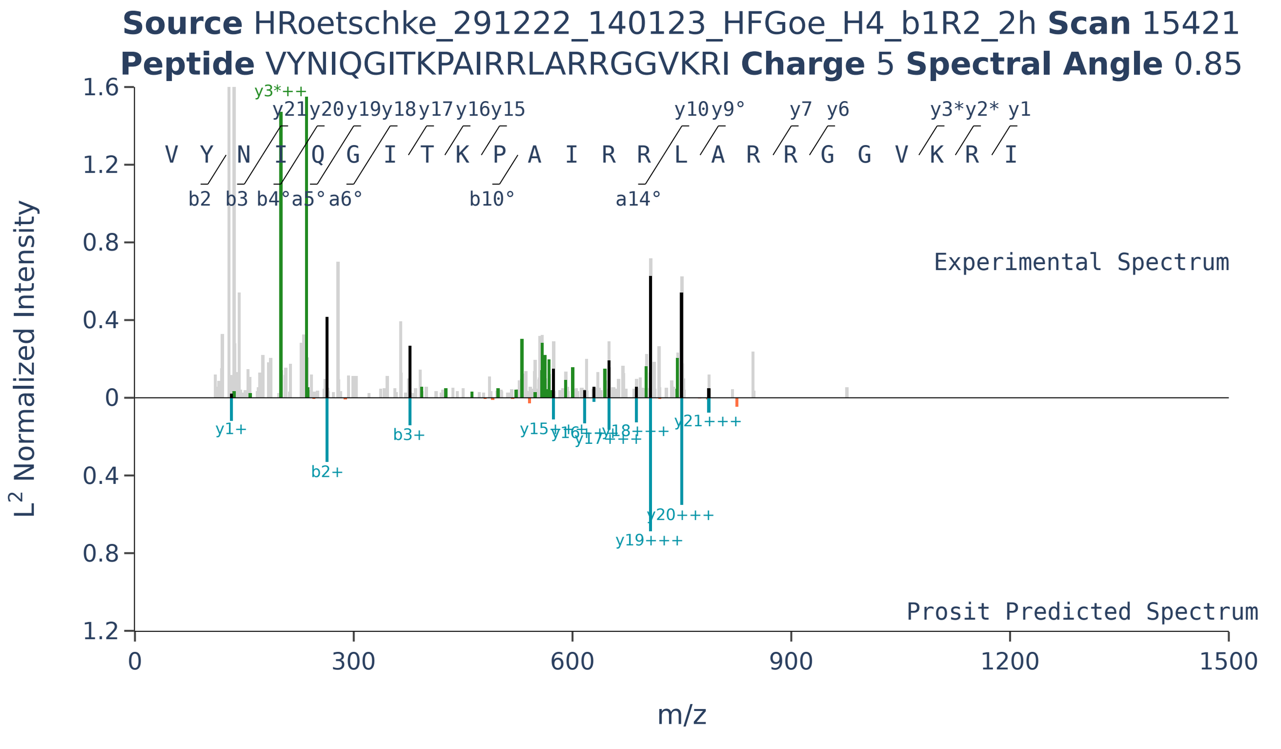
Source HRoetschke_291222_140123_HFGoe_H4_b1R2_2h Scan 15421
Peptide VYNIQGITKPAIRRLARRGGVKRI Charge 5 Spectral Angle 0.85
Experimental Spectrum
Prosit Predicted Spectrum
L² Normalized Intensity
m/z

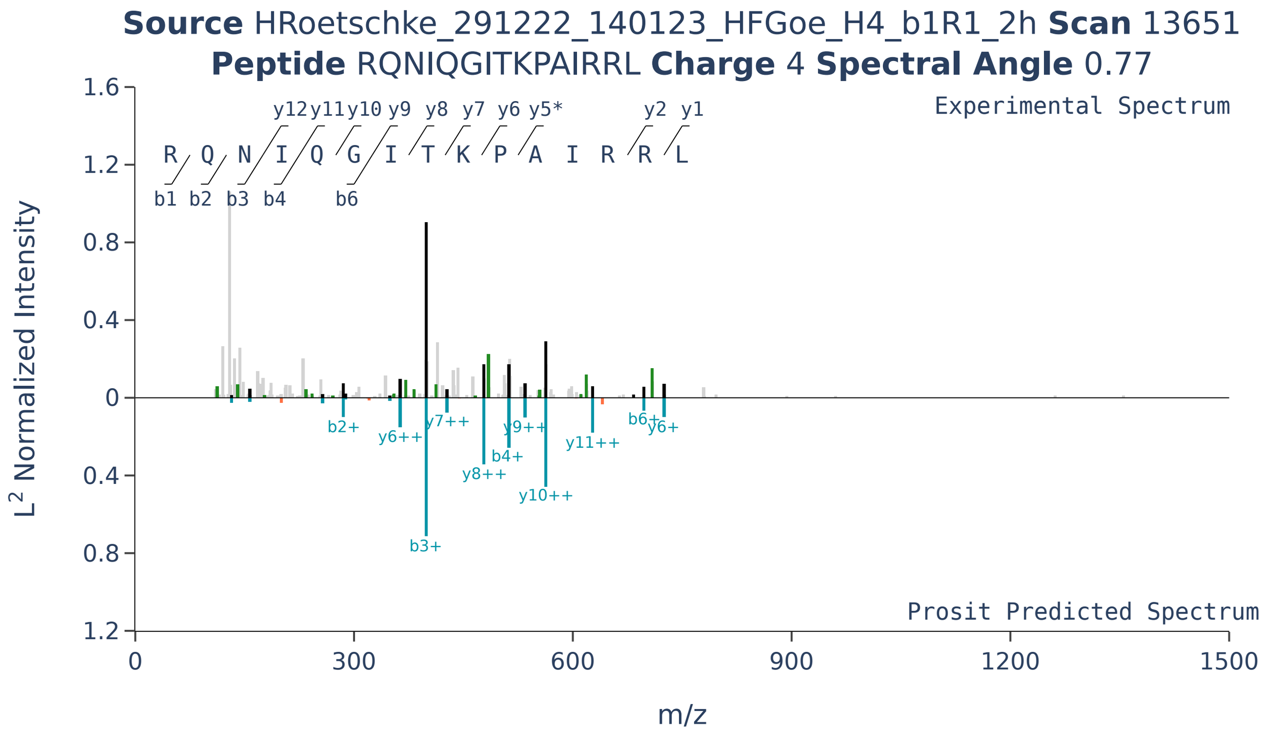
Source HRoetschke_291222_140123_HFGoe_H4_b1R1_2h Scan 13651
Peptide RQNIQGITKPAIRRL Charge 4 Spectral Angle 0.77
Experimental Spectrum
Prosit Predicted Spectrum
L² Normalized Intensity
m/z

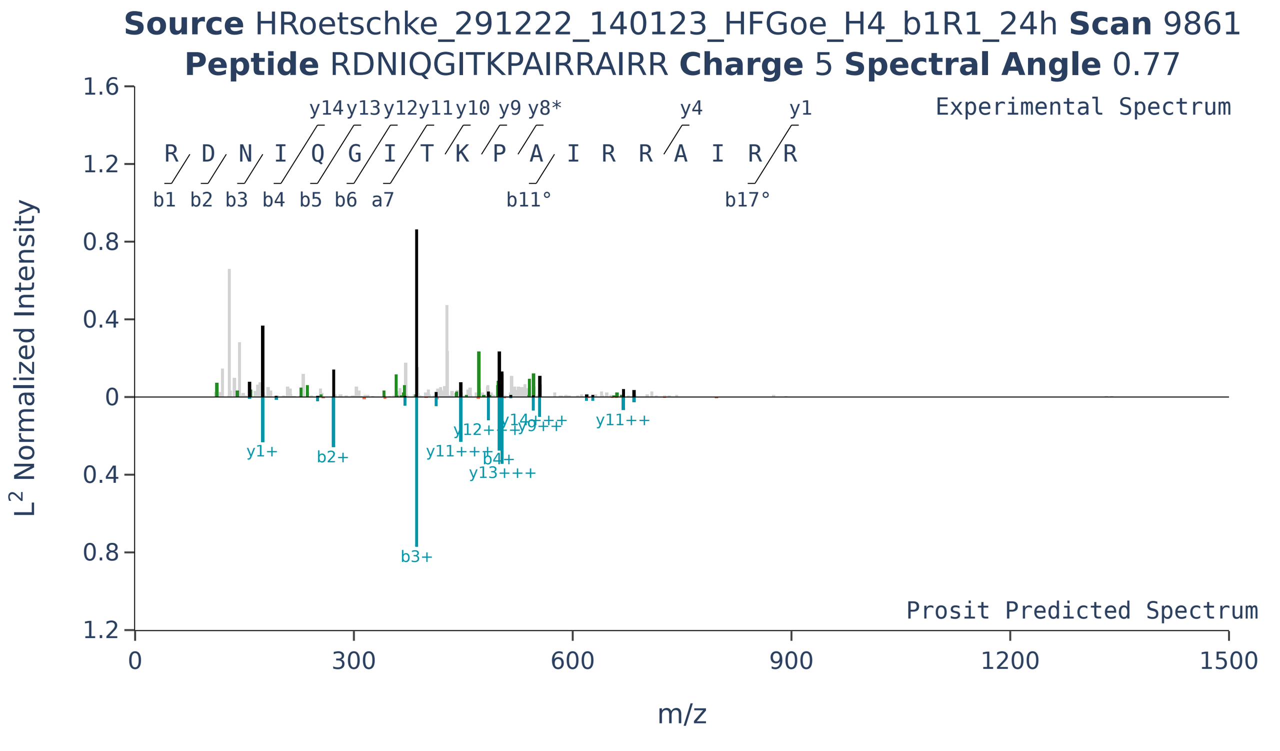
Source HRoetschke_291222_140123_HFGoe_H4_b1R1_24h Scan 9861
Peptide RDNIQGITKPAIRRAIRR Charge 5 Spectral Angle 0.77
Experimental Spectrum
Prosit Predicted Spectrum
L² Normalized Intensity
m/z

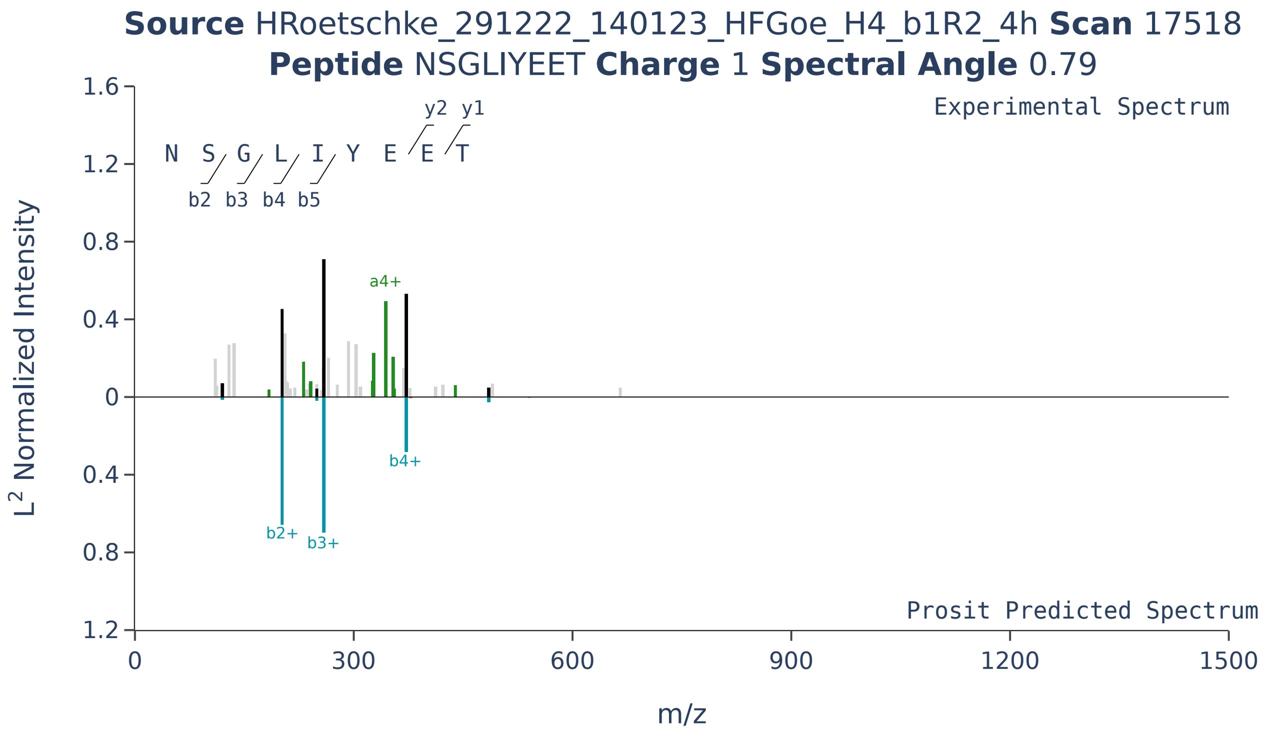
Source HRoetschke_291222_140123_HFGoe_H4_b1R2_4h Scan 17518
Peptide NSGLIYEET Charge 1 Spectral Angle 0.79
Experimental Spectrum
Prosit Predicted Spectrum
L² Normalized Intensity
m/z

Source WSoh_221121_011221_HFGoe_L2_hTau_24h_R1 **Scan** 12463
Peptide PVDLSKVTNK **Charge** 3 **Spectral Angle** 0.92

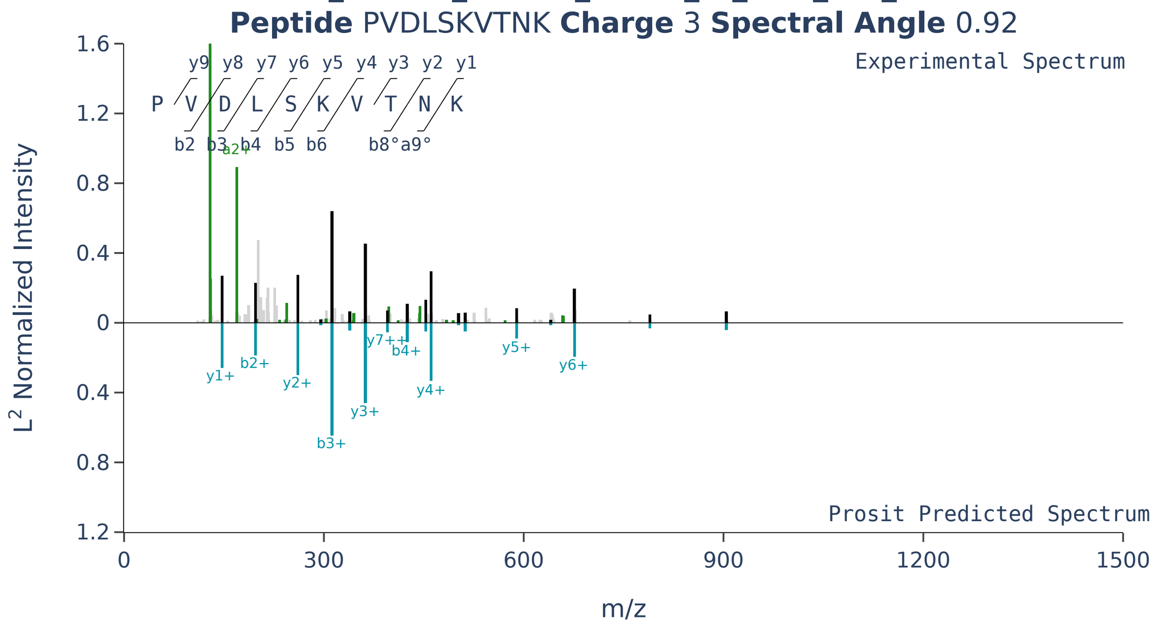

Source WSoh_221121_011221_HFGoe_L1_hTau_24h_R1 **Scan** 6972
Peptide DFKDRVQSH **Charge** 2 **Spectral Angle** 0.95

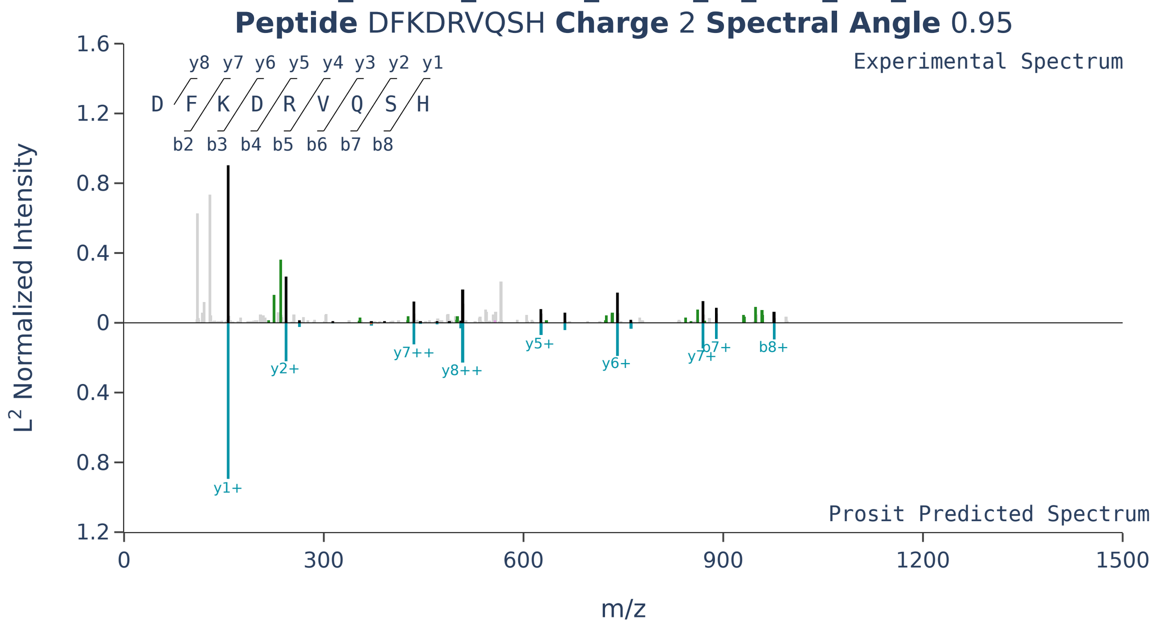

Source WSoh_221121_011221_HFGoe_L2_hTau_24h_R1 **Scan** 18066
Peptide DHAGTYGSPLQTPTE **Charge** 2 **Spectral Angle** 0.96

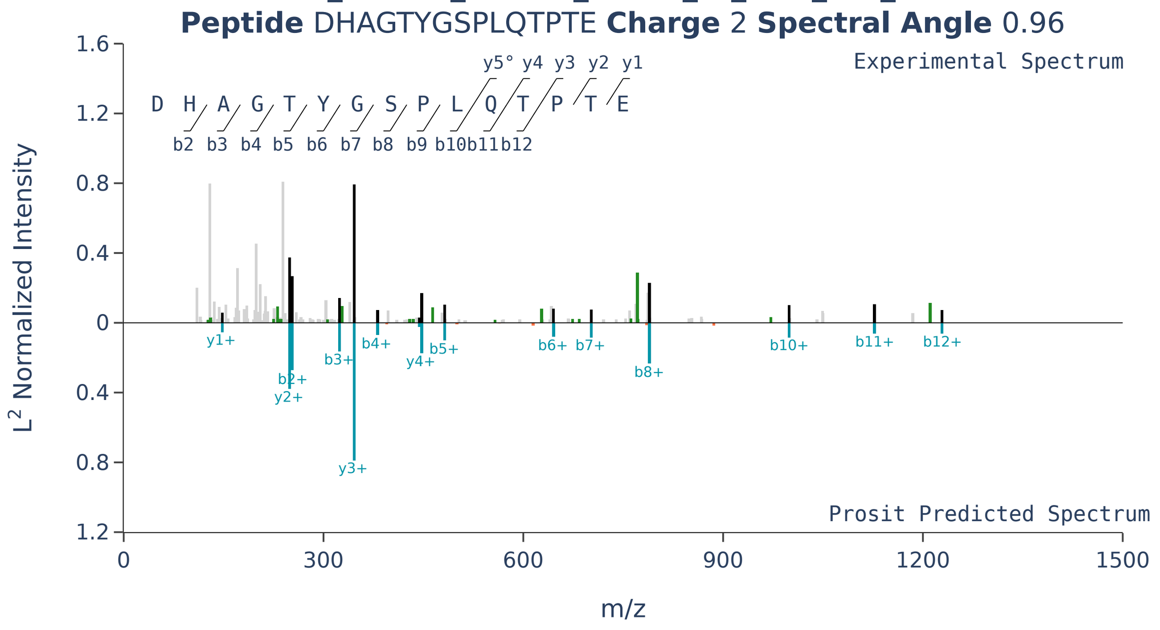

Source WSoh_221121_011221_HFGoe_L2_hTau_24h_R2 **Scan** 11295
Peptide PVDLSKVTSKH **Charge** 3 **Spectral Angle** 0.96

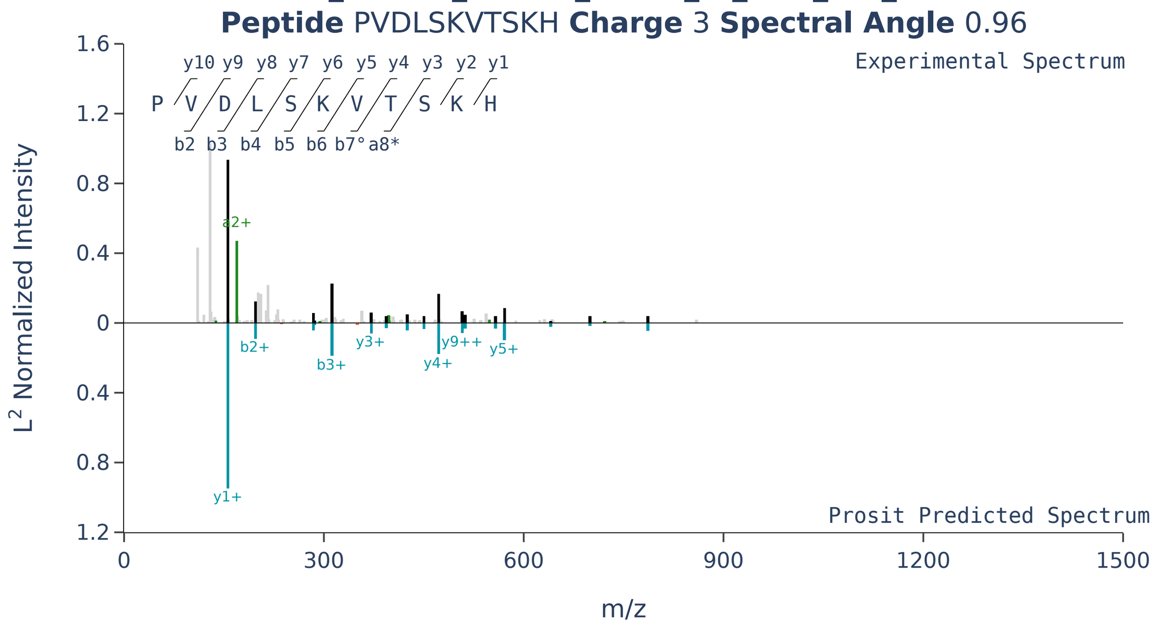

Source WSoh_221121_011221_HFGoe_L1_hTau_24h_R2 **Scan** 15161
Peptide AAQPHTEIPEGTTDAGLKE **Charge** 3 **Spectral Angle** 0.93

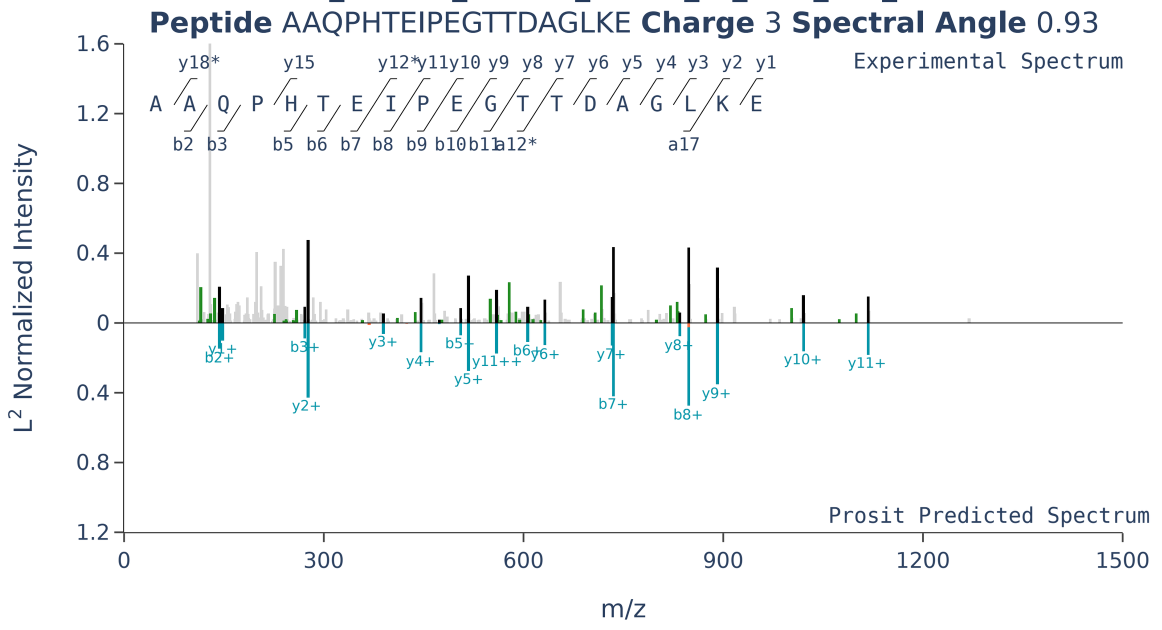

Source WSoh_221121_011221_HFGoe_L1_hTau_24h_R2 **Scan** 18569
Peptide DLSNVQSRL **Charge** 2 **Spectral Angle** 0.94

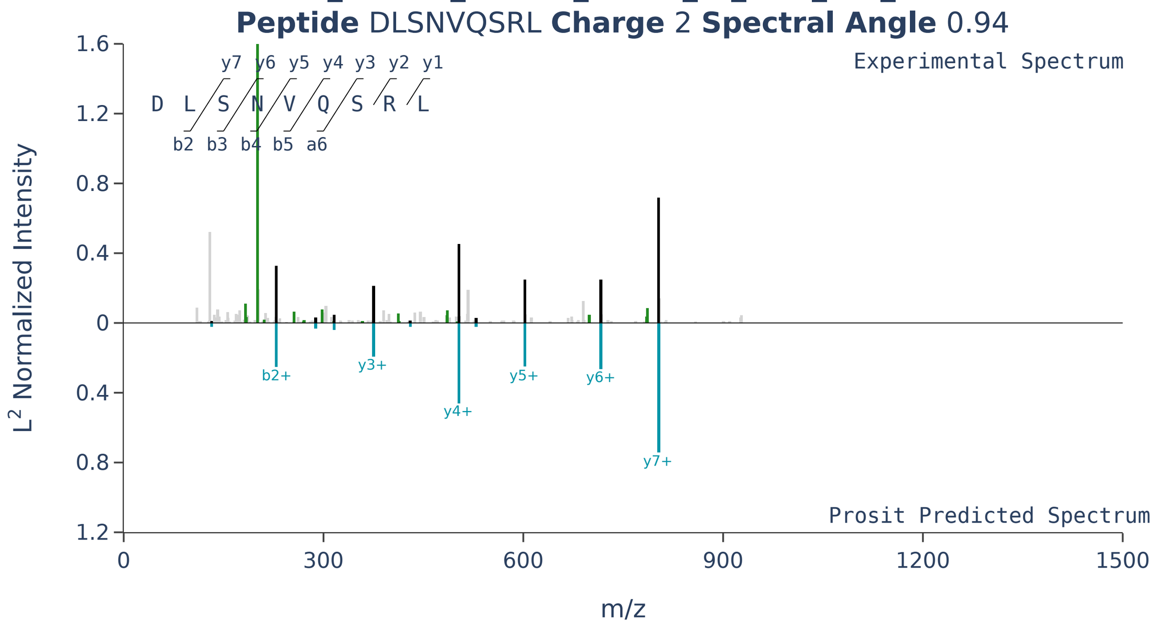

Source WSoh_221121_011221_HFGoe_L2_hTau_24h_R2 **Scan** 21832
Peptide DEAPRQEFEVME **Charge** 2 **Spectral Angle** 0.89

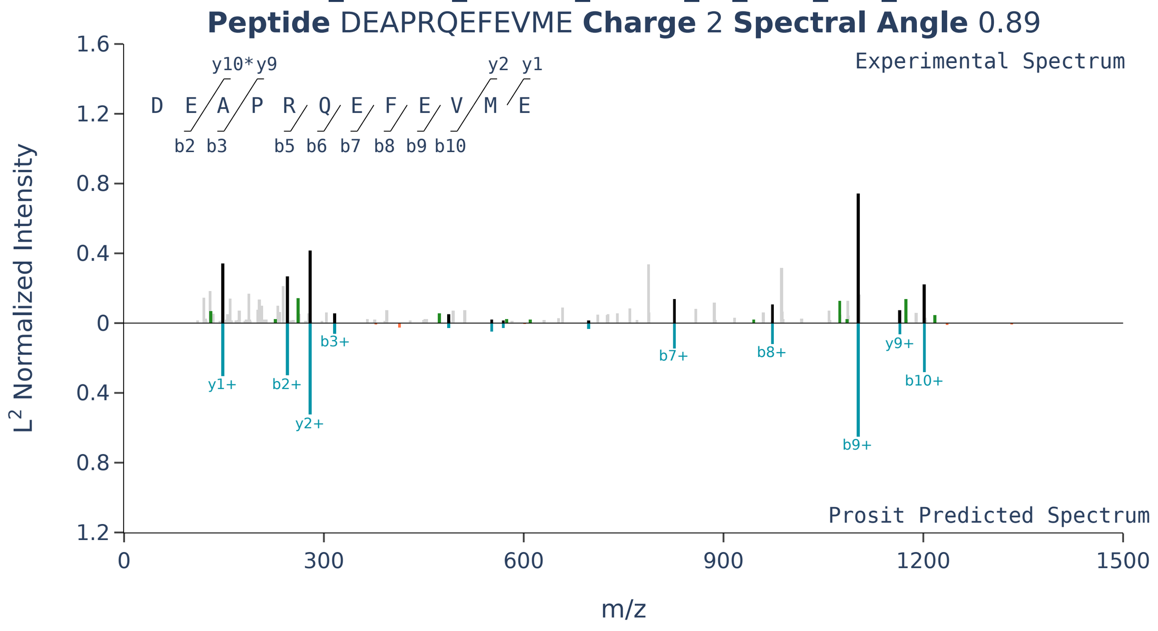

Source WSoh_221121_011221_HFGoe_L2_hTau_24h_R2 **Scan** 22383
Peptide DAEPRQEFEVM **Charge** 2 **Spectral Angle** 0.91

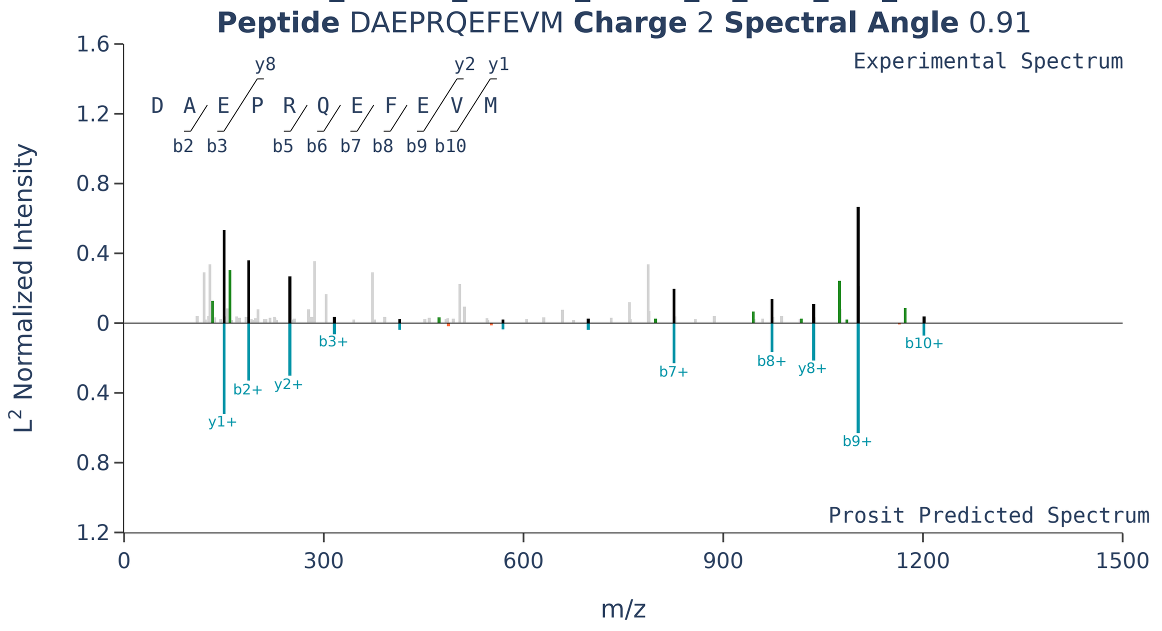

Source WSoh_221121_011221_HFGoe_L1_hTau_24h_R2 **Scan** 19836
Peptide SAEPRQEFEVME **Charge** 2 **Spectral Angle** 0.92

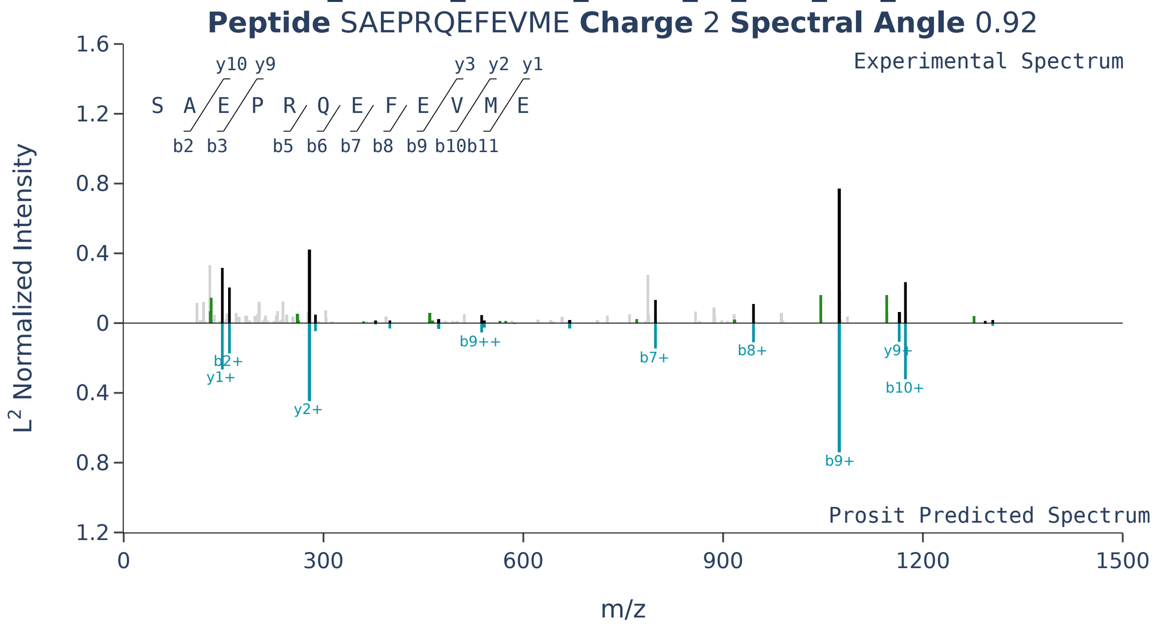

Source WSoh_221121_011221_HFGoe_L2_hTau_2h_R1 **Scan** 8538
Peptide DFKDRVQST **Charge** 2 **Spectral Angle** 0.92

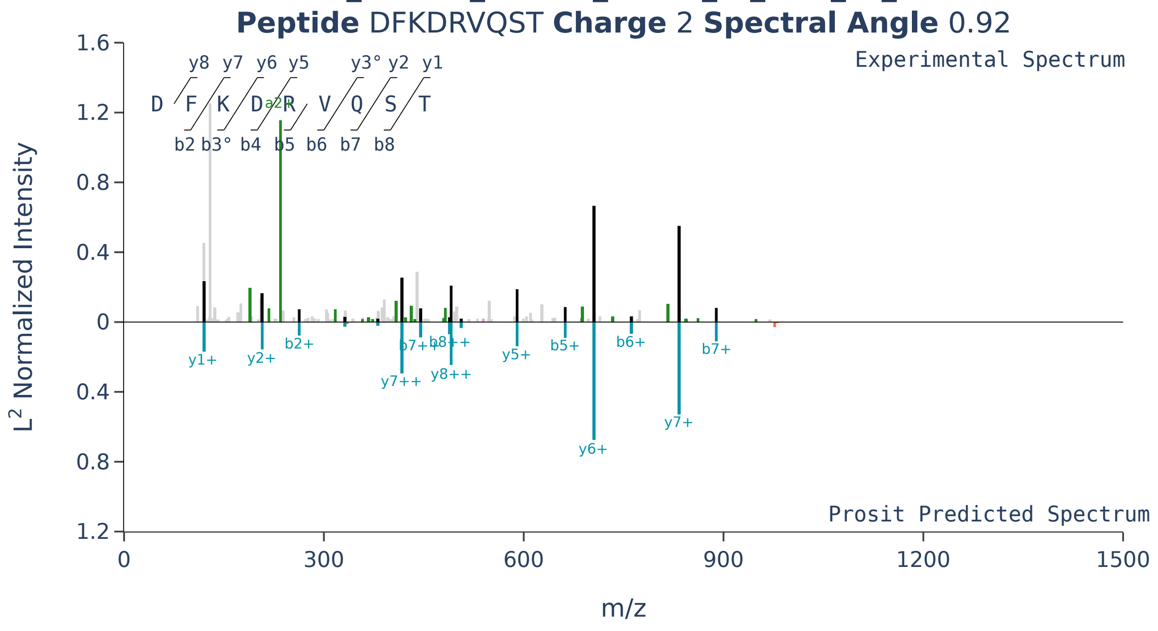

Source WSoh_221121_011221_HFGoe_L2_hTau_2h_R2 **Scan** 16595
Peptide DFKDRVQSVY **Charge** 2 **Spectral Angle** 0.93

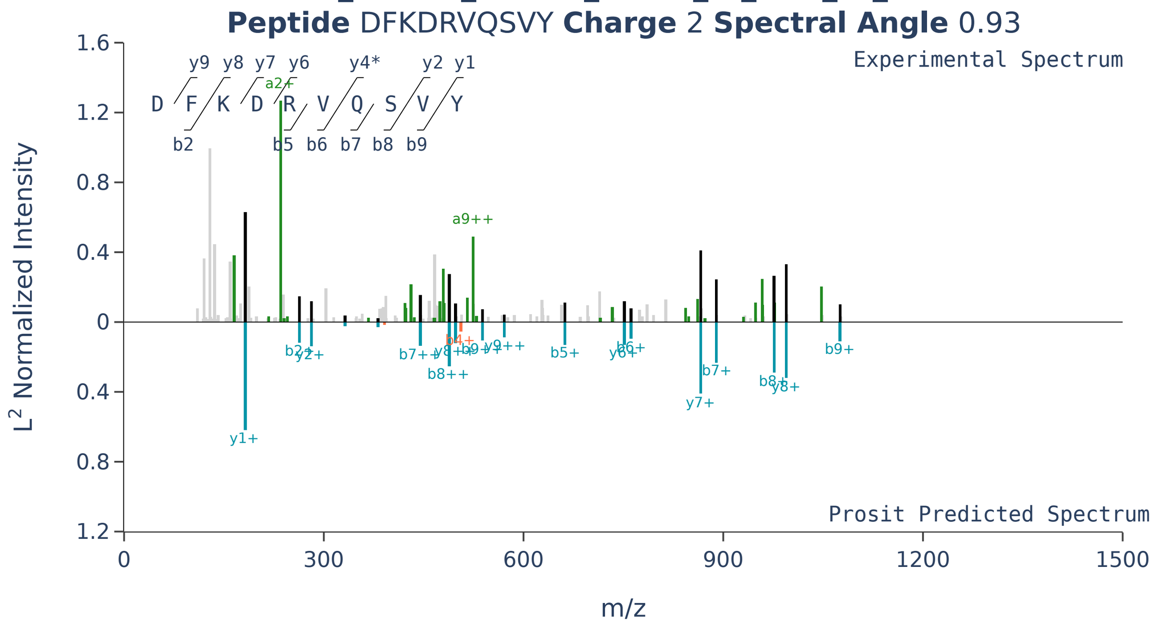

Source WSoh_221121_011221_HFGoe_L2_hTau_24h_R2 **Scan** 9776
Peptide SNVSSTGTDAGLKE **Charge** 2 **Spectral Angle** 0.95

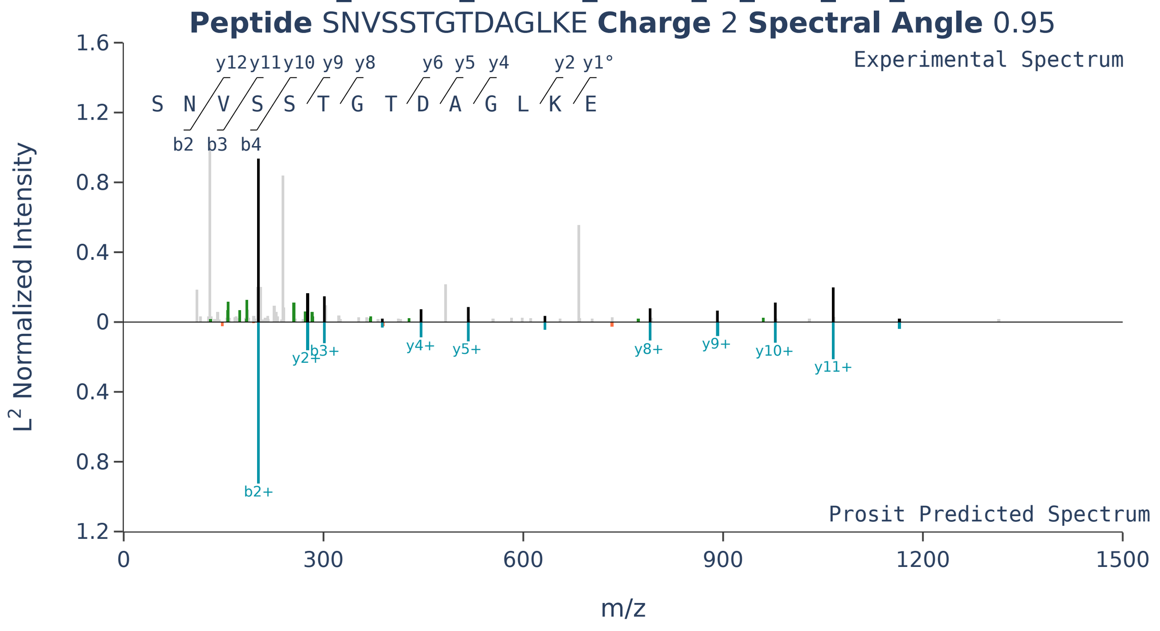

Source WSoh_221121_011221_HFGoe_L2_hTau_24h_R1 **Scan** 18118
Peptide DLSNVQSKL **Charge** 2 **Spectral Angle** 0.87

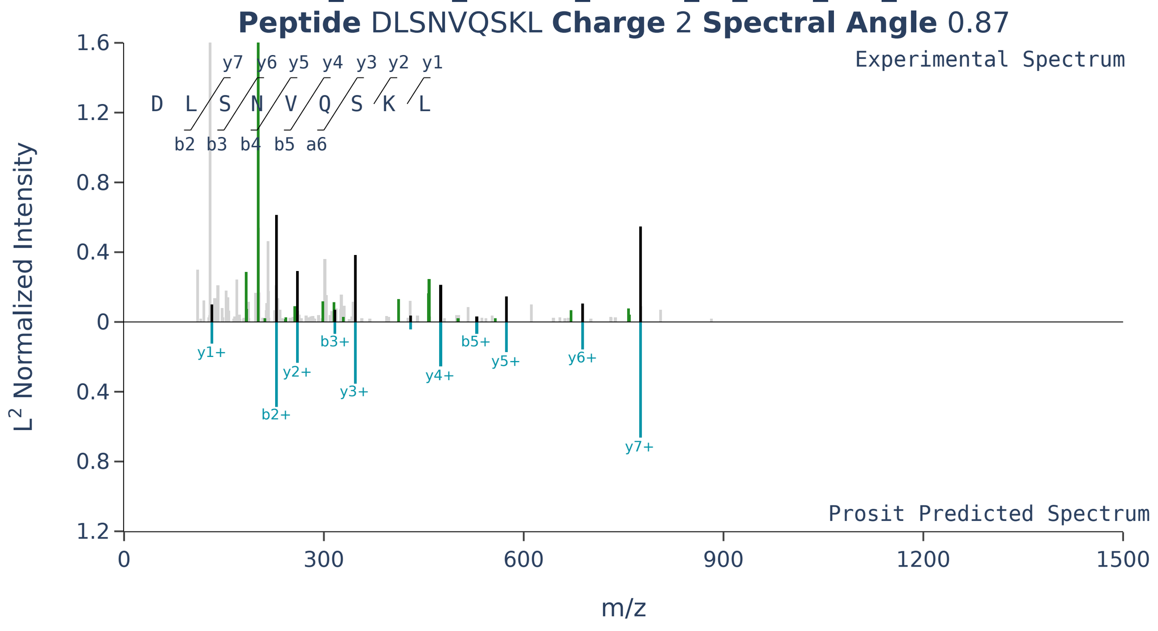

Source WSoh_221121_011221_HFGoe_L1_hTau_4h_R1 **Scan** 13383
Peptide DFKDRVQSRL **Charge** 3 **Spectral Angle** 0.95

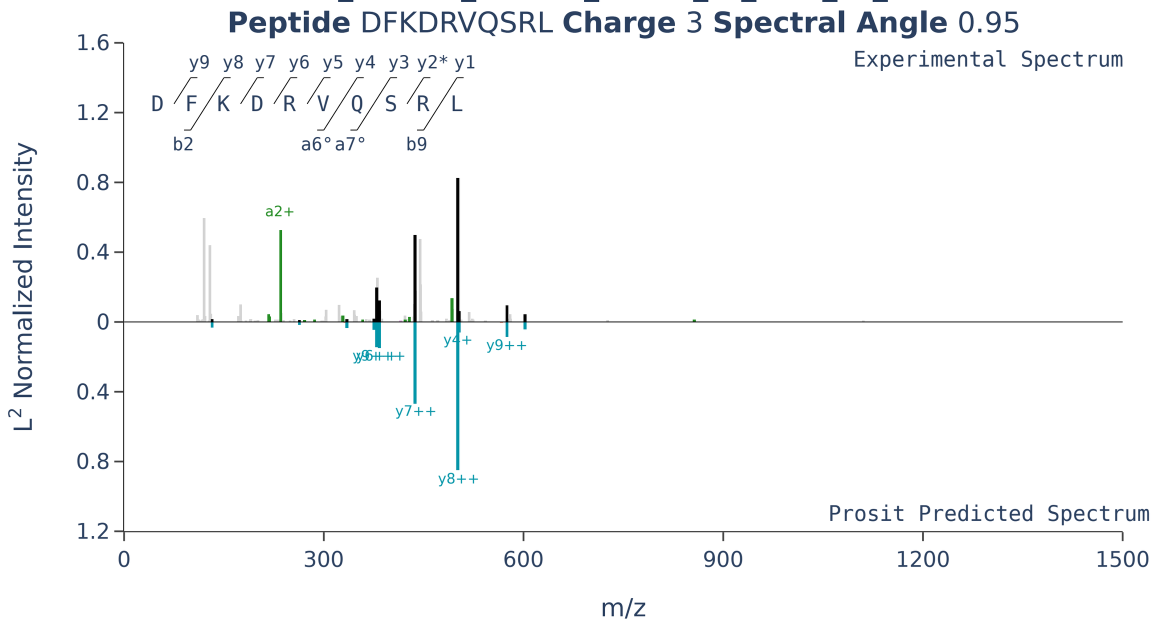

Source WSoh_221121_011221_HFGoe_L1_hTau_24h_R2 **Scan** 20050
Peptide AAEPRQEFEVME **Charge** 2 **Spectral Angle** 0.92

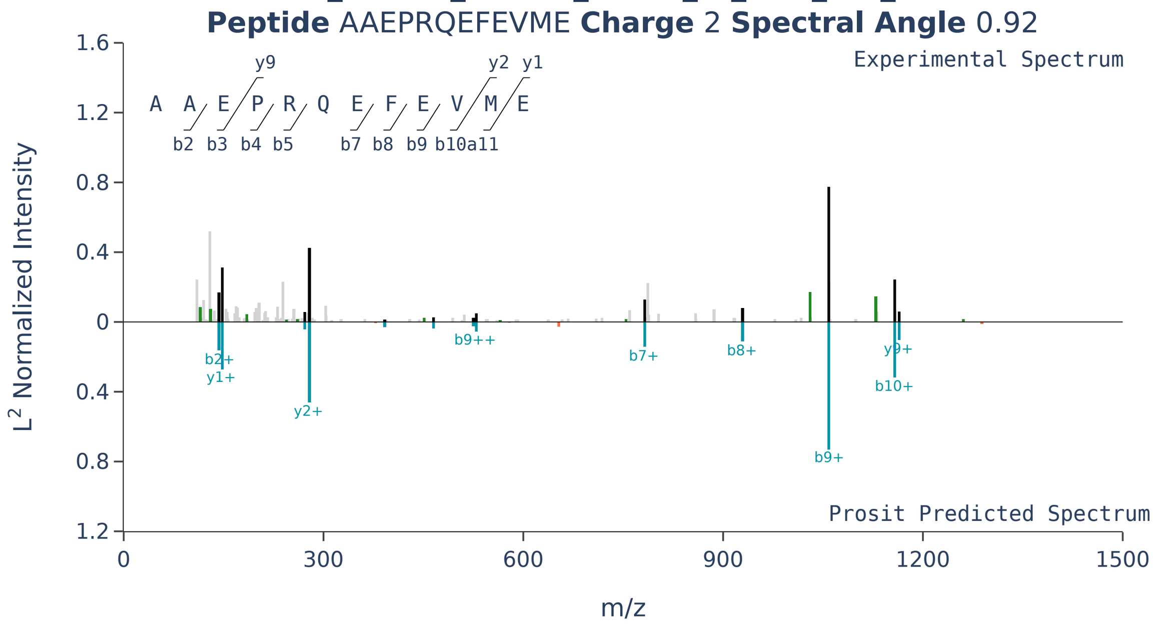

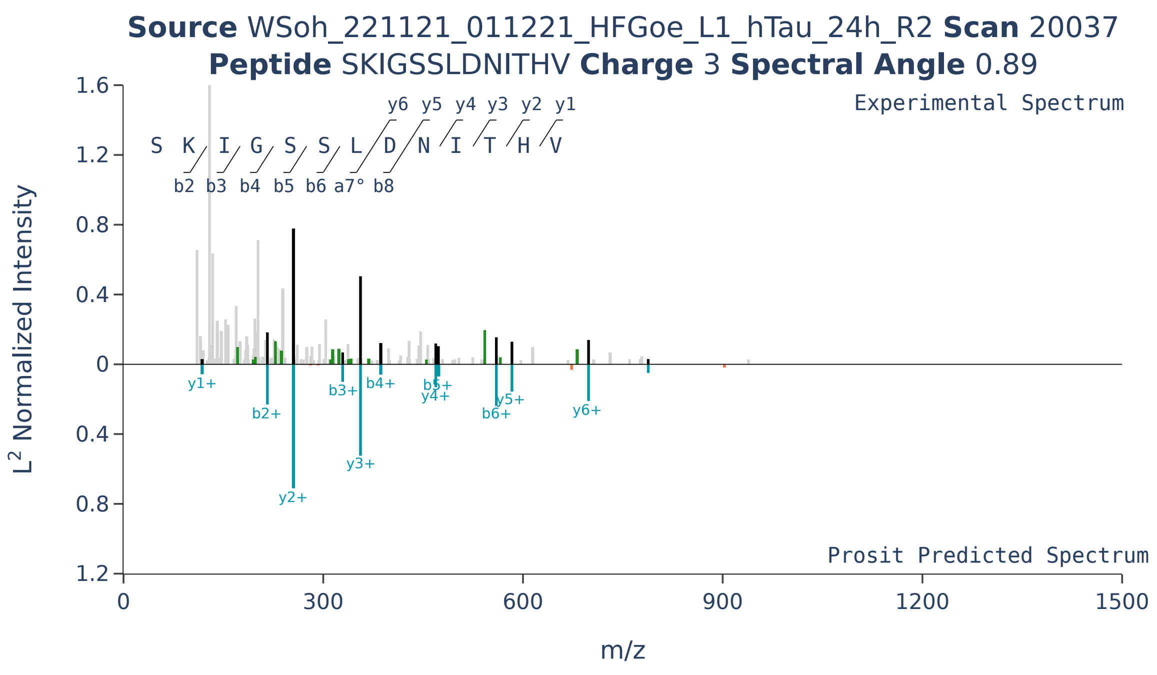
Source WSoh_221121_011221_HFGoe_L1_hTau_24h_R2 **Scan** 20037
Peptide SKIGSSLDNITHV **Charge** 3 **Spectral Angle** 0.89
Experimental Spectrum
Prosit Predicted Spectrum
L² Normalized Intensity
m/z

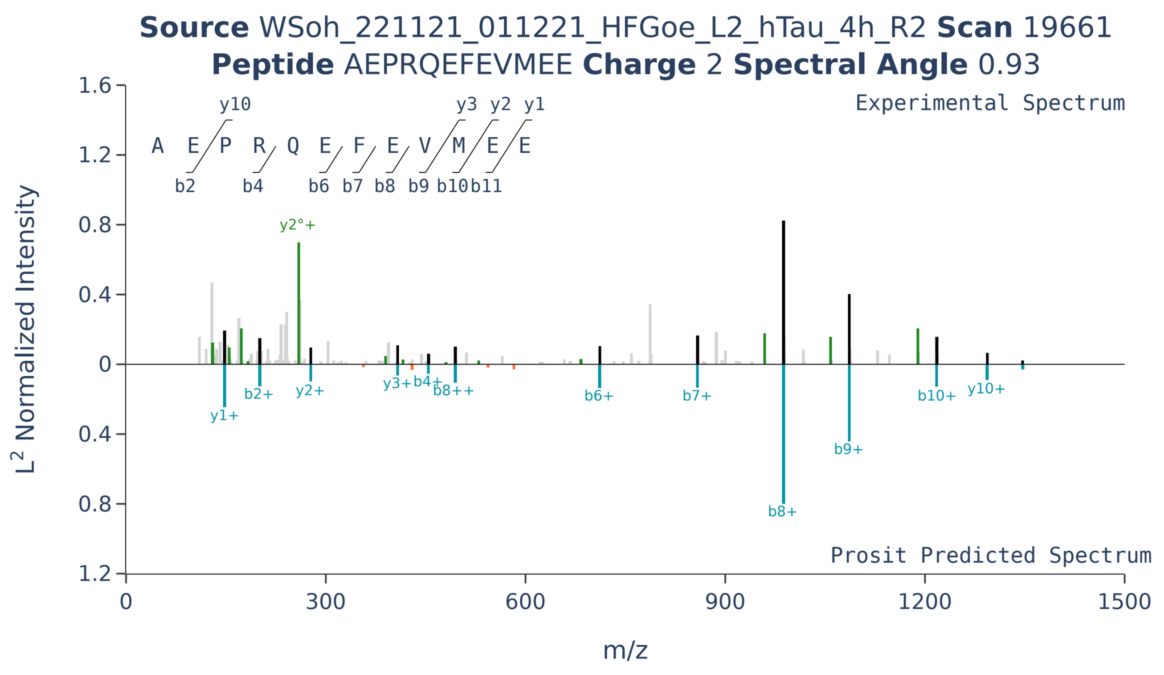
Source WSoh_221121_011221_HFGoe_L2_hTau_4h_R2 **Scan** 19661
Peptide AEPRQEFEVMEE **Charge** 2 **Spectral Angle** 0.93
Experimental Spectrum
Prosit Predicted Spectrum
L² Normalized Intensity
m/z

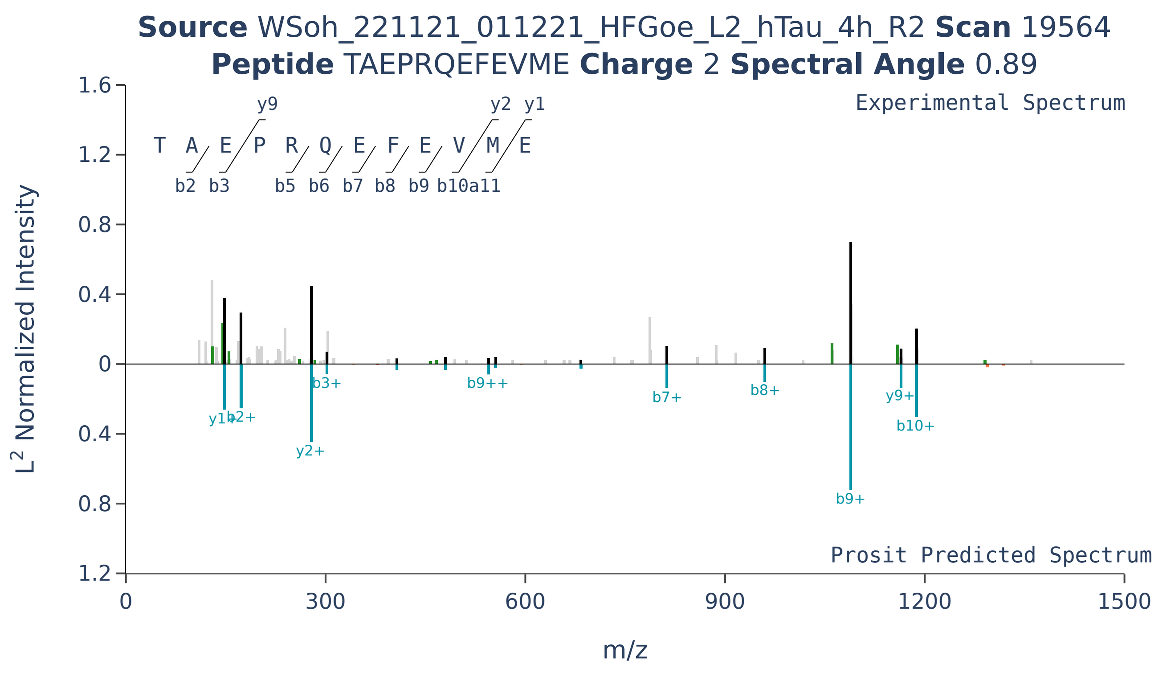
Source WSoh_221121_011221_HFGoe_L2_hTau_4h_R2 **Scan** 19564
Peptide TAEPRQEFEVME **Charge** 2 **Spectral Angle** 0.89
Experimental Spectrum
Prosit Predicted Spectrum
L² Normalized Intensity
m/z

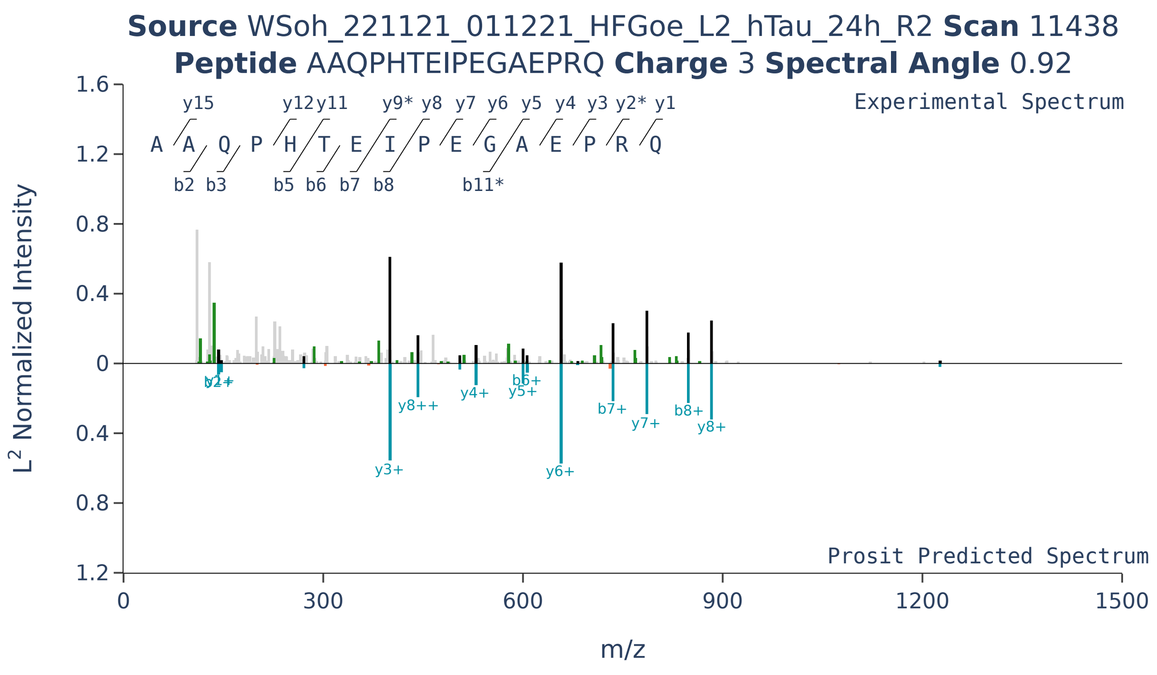
Source WSoh_221121_011221_HFGoe_L2_hTau_24h_R2 **Scan** 11438
Peptide AAQPHTEIPEGAEPRQ **Charge** 3 **Spectral Angle** 0.92
Experimental Spectrum
Prosit Predicted Spectrum
L² Normalized Intensity
m/z

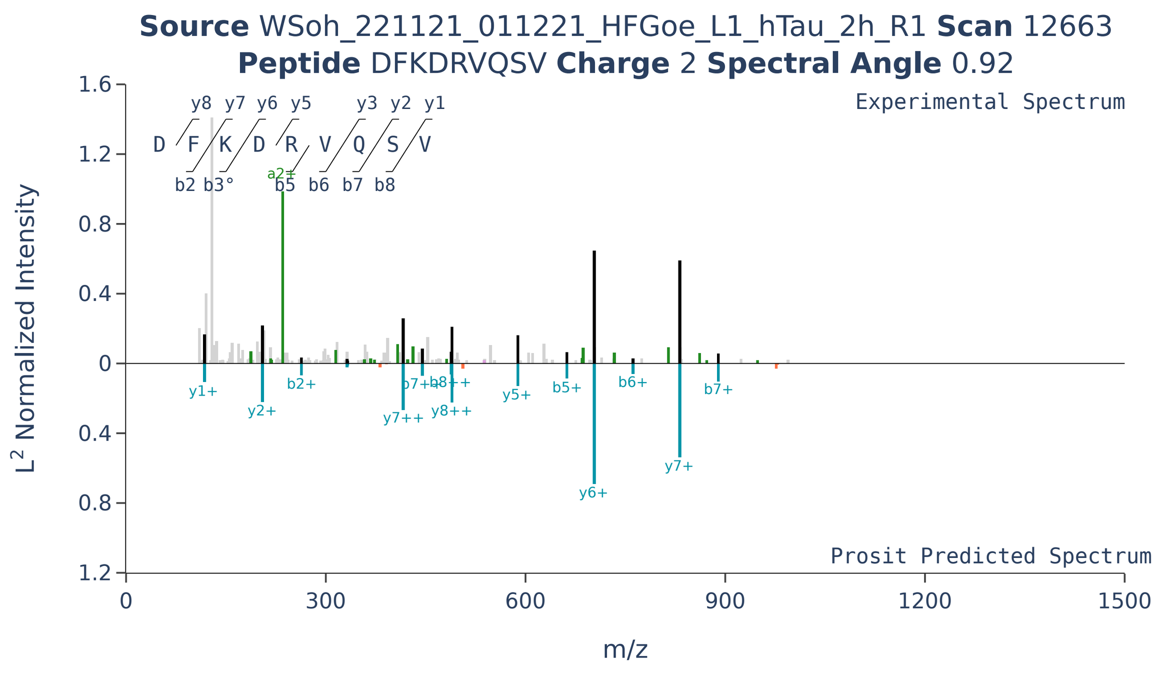
Source WSoh_221121_011221_HFGoe_L1_hTau_2h_R1 **Scan** 12663
Peptide DFKDRVQSV **Charge** 2 **Spectral Angle** 0.92
Experimental Spectrum
Prosit Predicted Spectrum
L² Normalized Intensity
m/z

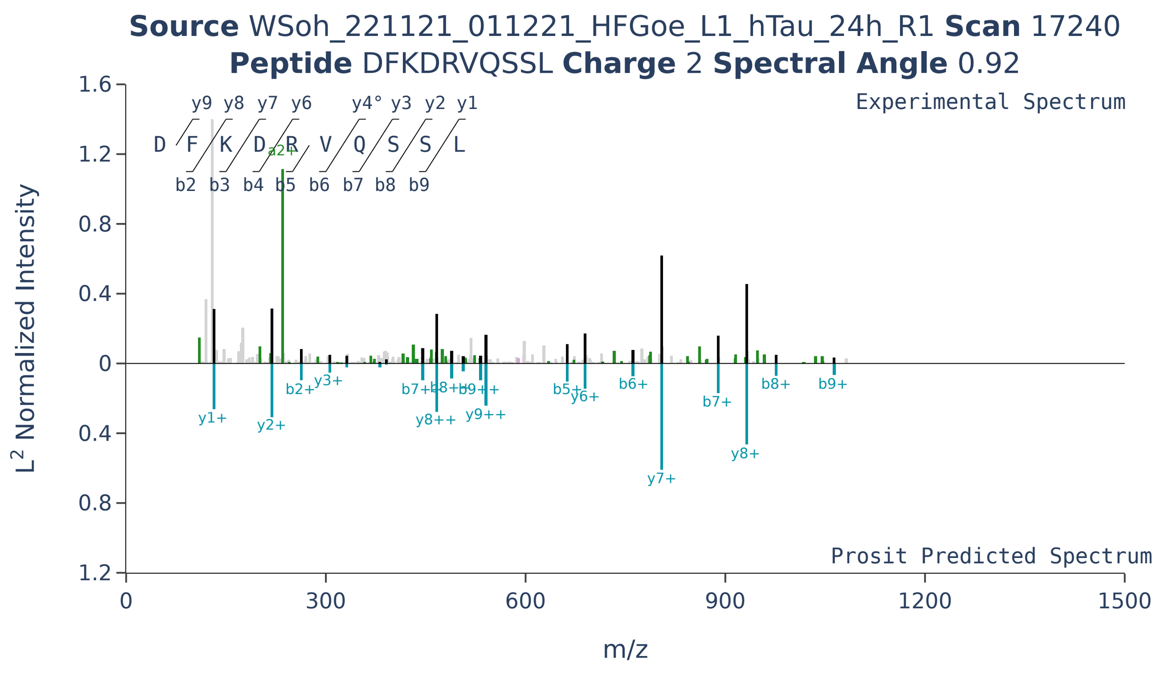
Source WSoh_221121_011221_HFGoe_L1_hTau_24h_R1 **Scan** 17240
Peptide DFKDRVQSSL **Charge** 2 **Spectral Angle** 0.92
Experimental Spectrum
Prosit Predicted Spectrum
L² Normalized Intensity
m/z

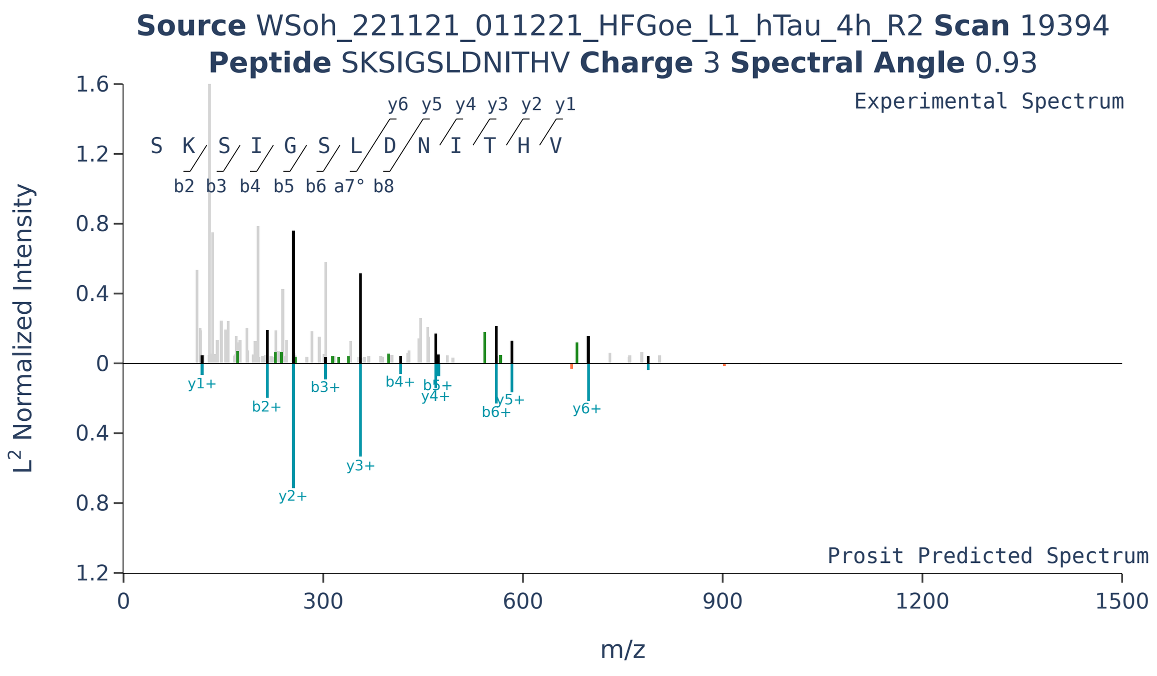
Source WSoh_221121_011221_HFGoe_L1_hTau_4h_R2 **Scan** 19394
Peptide SKSIGSLDNITHV **Charge** 3 **Spectral Angle** 0.93
Experimental Spectrum
Prosit Predicted Spectrum
L² Normalized Intensity
m/z

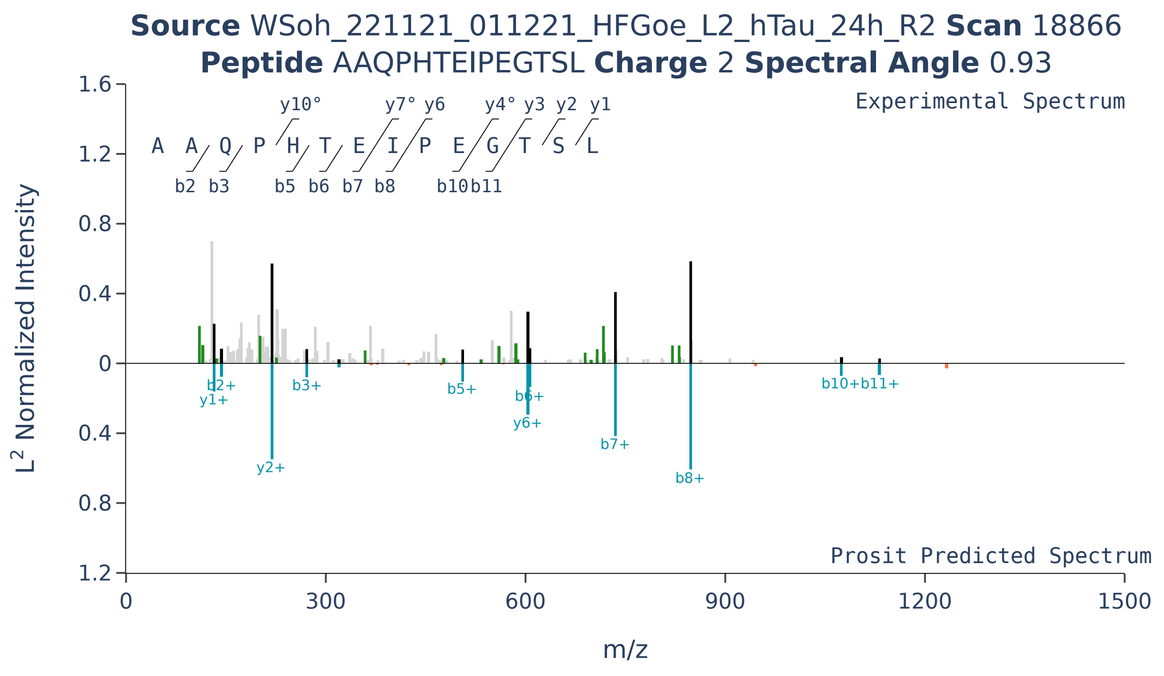
Source WSoh_221121_011221_HFGoe_L2_hTau_24h_R2 **Scan** 18866
Peptide AAQPHTEIPEGTSL **Charge** 2 **Spectral Angle** 0.93
Experimental Spectrum
Prosit Predicted Spectrum
L² Normalized Intensity
m/z

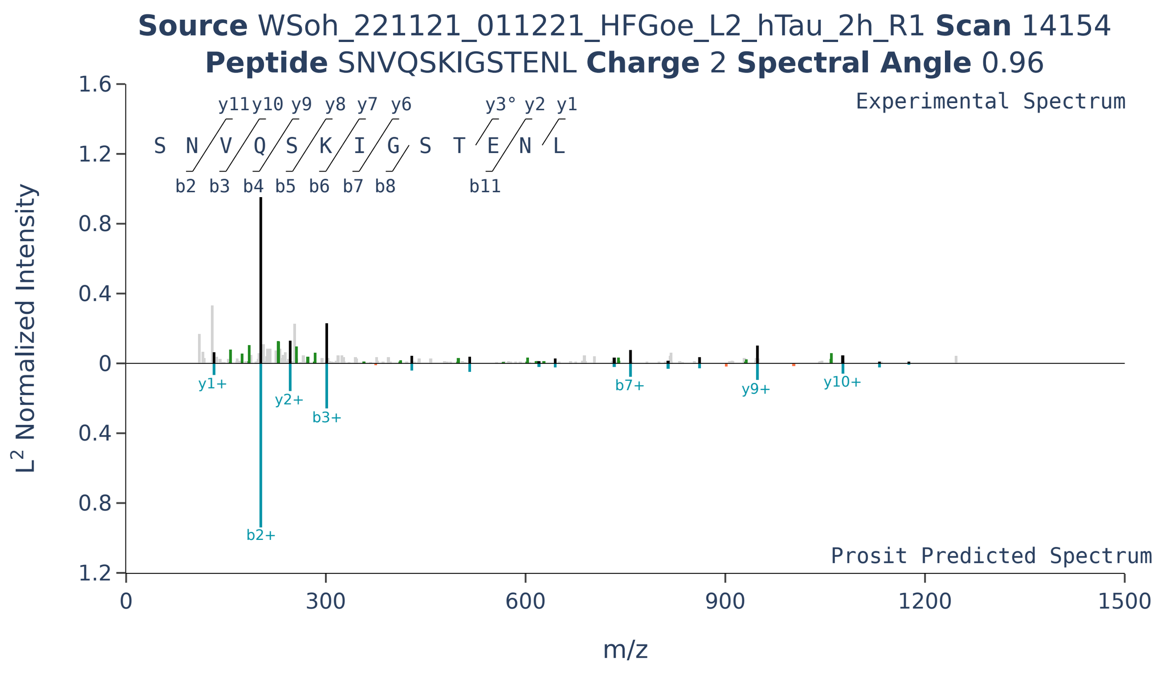
Source WSoh_221121_011221_HFGoe_L2_hTau_2h_R1 **Scan** 14154
Peptide SNVQSKIGSTENL **Charge** 2 **Spectral Angle** 0.96
Experimental Spectrum
Prosit Predicted Spectrum
L² Normalized Intensity
m/z

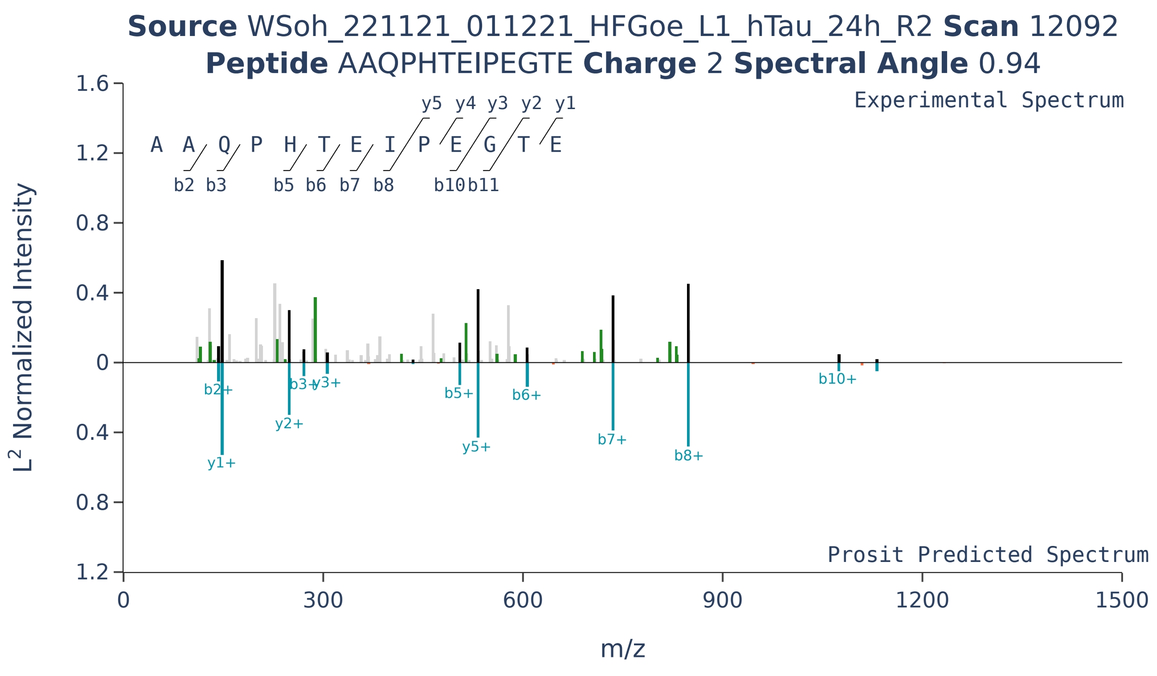
Source WSoh_221121_011221_HFGoe_L1_hTau_24h_R2 **Scan** 12092
Peptide AAQPHTEIPEGTE **Charge** 2 **Spectral Angle** 0.94
Experimental Spectrum
Prosit Predicted Spectrum
L² Normalized Intensity
m/z

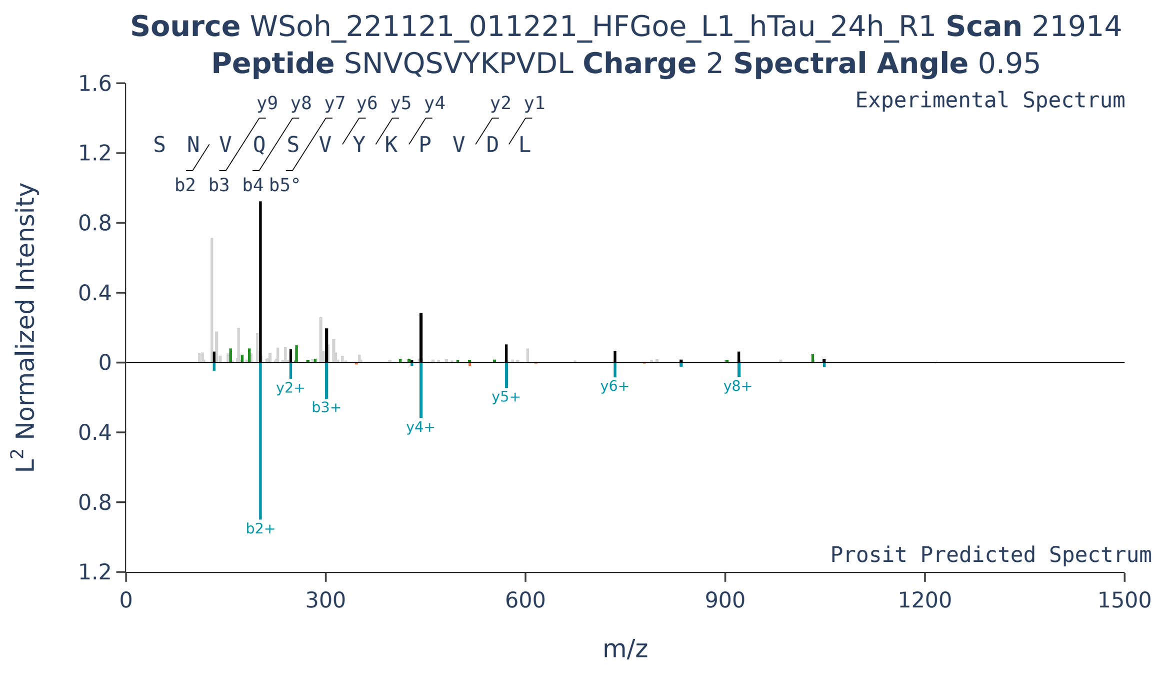
Source WSoh_221121_011221_HFGoe_L1_hTau_24h_R1 **Scan** 21914
Peptide SNVQSVYKPVDL **Charge** 2 **Spectral Angle** 0.95
Experimental Spectrum
Prosit Predicted Spectrum
L² Normalized Intensity
m/z

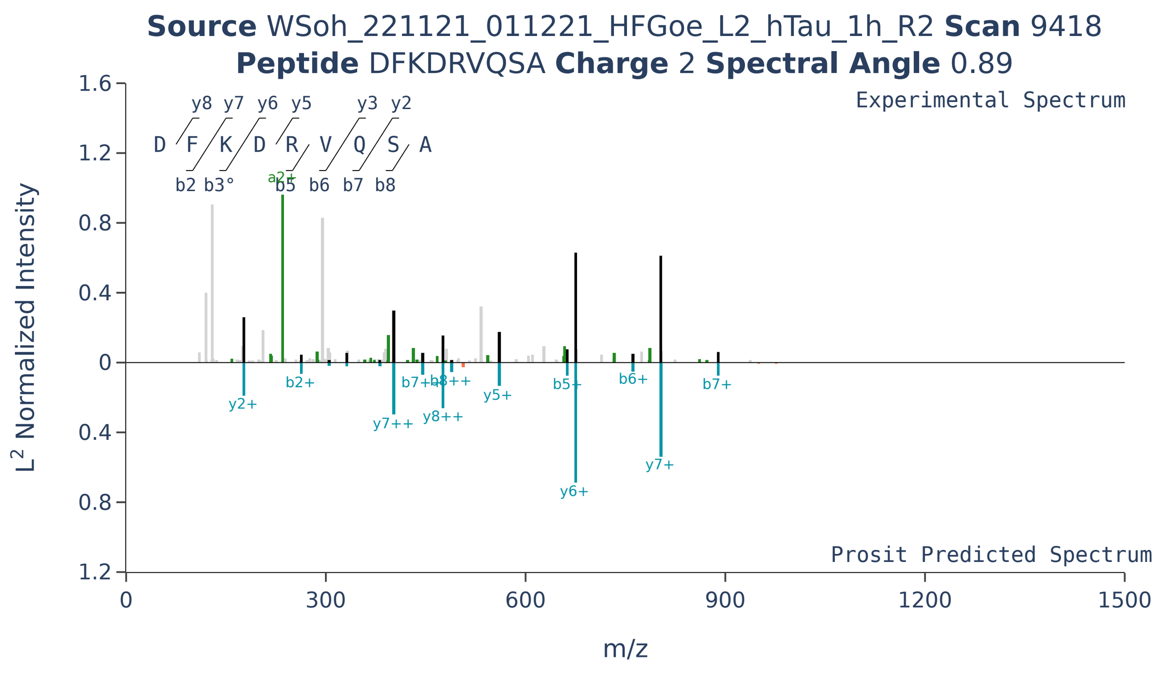
Source WSoh_221121_011221_HFGoe_L2_hTau_1h_R2 **Scan** 9418
Peptide DFKDRVQSA **Charge** 2 **Spectral Angle** 0.89
Experimental Spectrum
Prosit Predicted Spectrum
L² Normalized Intensity
m/z

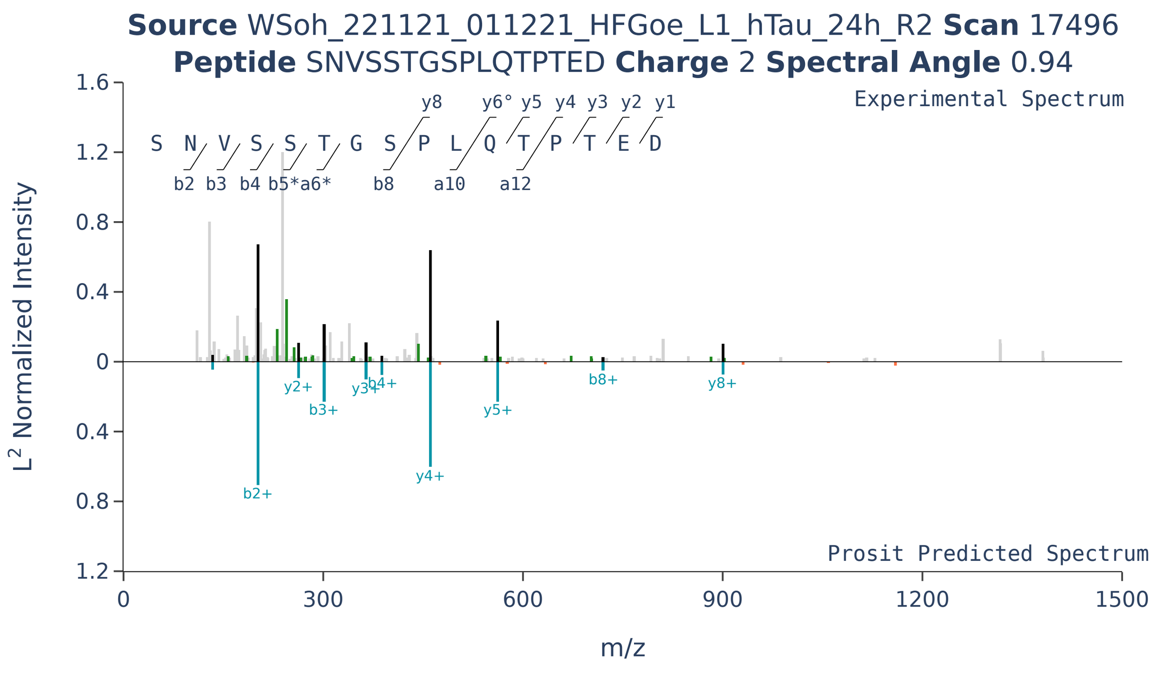
Source WSoh_221121_011221_HFGoe_L1_hTau_24h_R2 **Scan** 17496
Peptide SNVSSTGSPLQTPTED **Charge** 2 **Spectral Angle** 0.94
Experimental Spectrum
Prosit Predicted Spectrum
L² Normalized Intensity
m/z

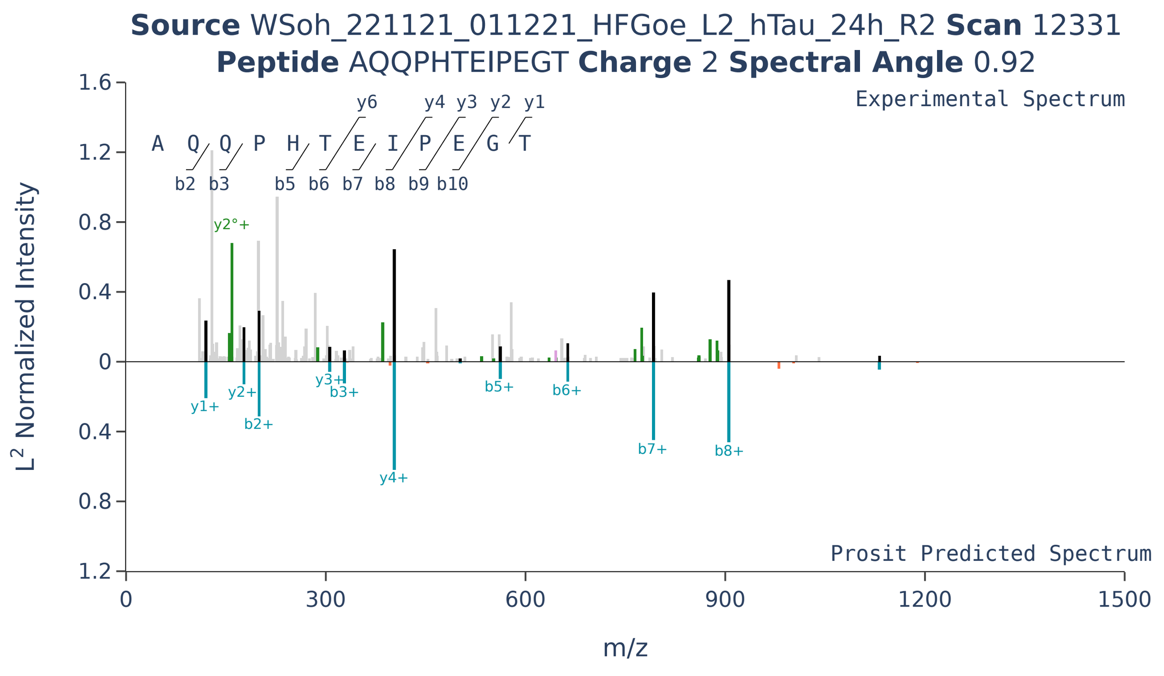
Source WSoh_221121_011221_HFGoe_L2_hTau_24h_R2 **Scan** 12331
Peptide AQQPHTEIPEGT **Charge** 2 **Spectral Angle** 0.92
Experimental Spectrum
Prosit Predicted Spectrum
L² Normalized Intensity
m/z

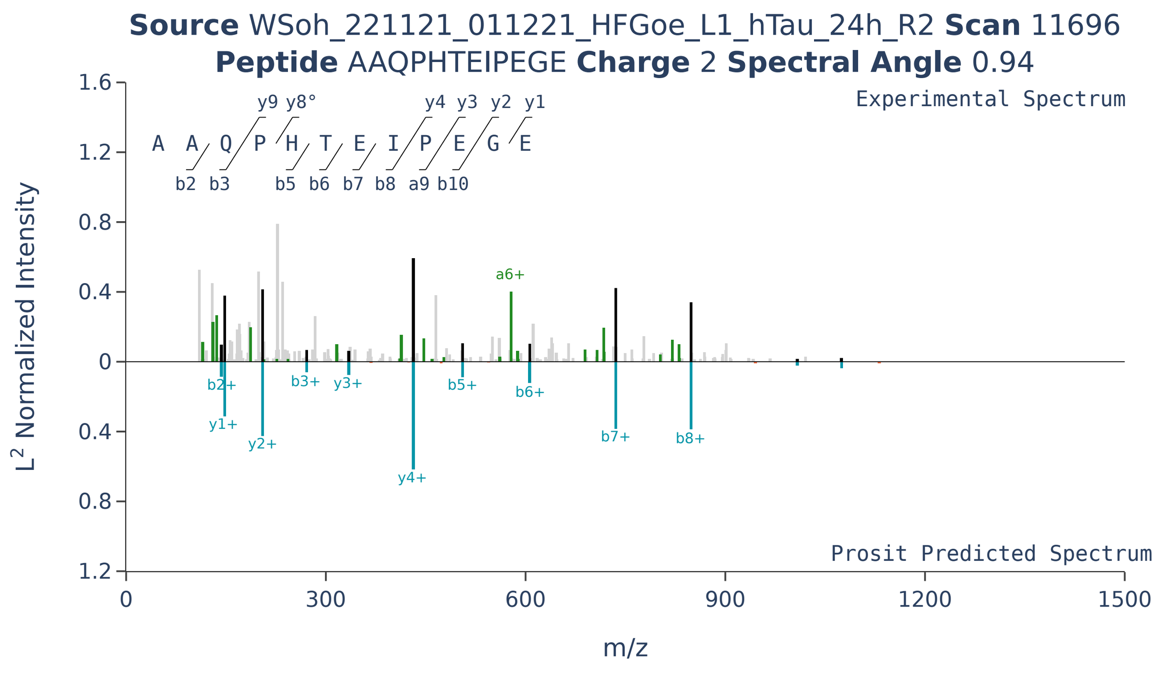
Source WSoh_221121_011221_HFGoe_L1_hTau_24h_R2 **Scan** 11696
Peptide AAQPHTEIPEGE **Charge** 2 **Spectral Angle** 0.94
Experimental Spectrum
Prosit Predicted Spectrum
L² Normalized Intensity
m/z

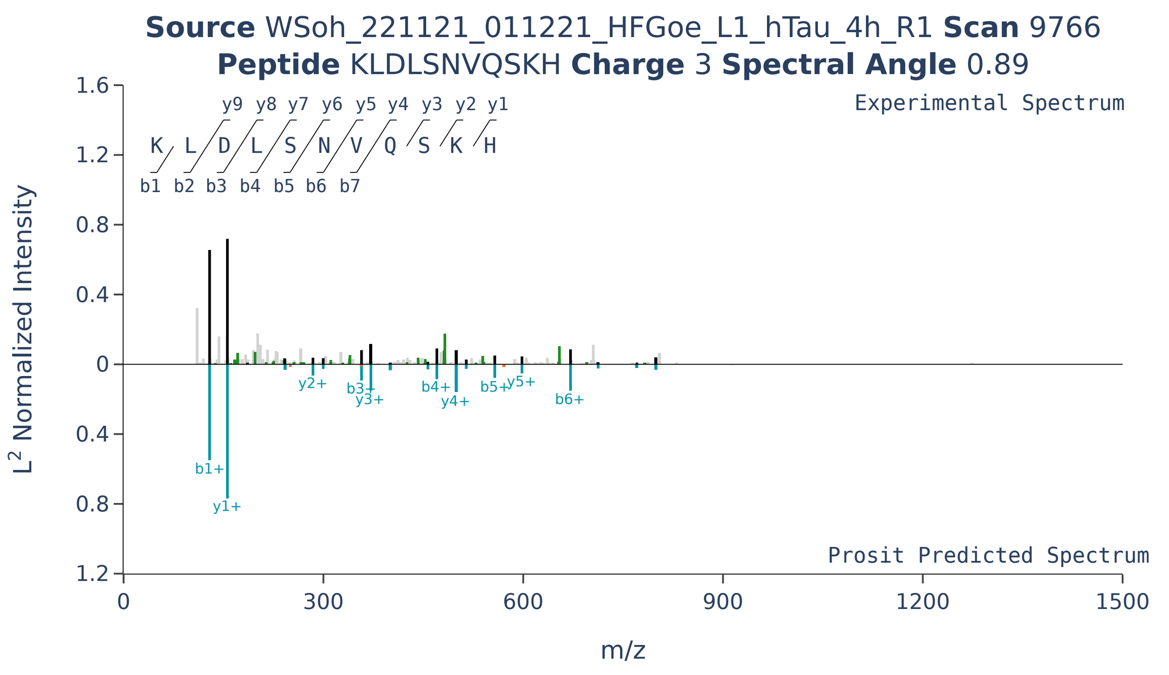
Source WSoh_221121_011221_HFGoe_L1_hTau_4h_R1 Scan 9766
Peptide KLDLSNVQSKH Charge 3 Spectral Angle 0.89
Experimental Spectrum
Prosit Predicted Spectrum
L² Normalized Intensity
m/z

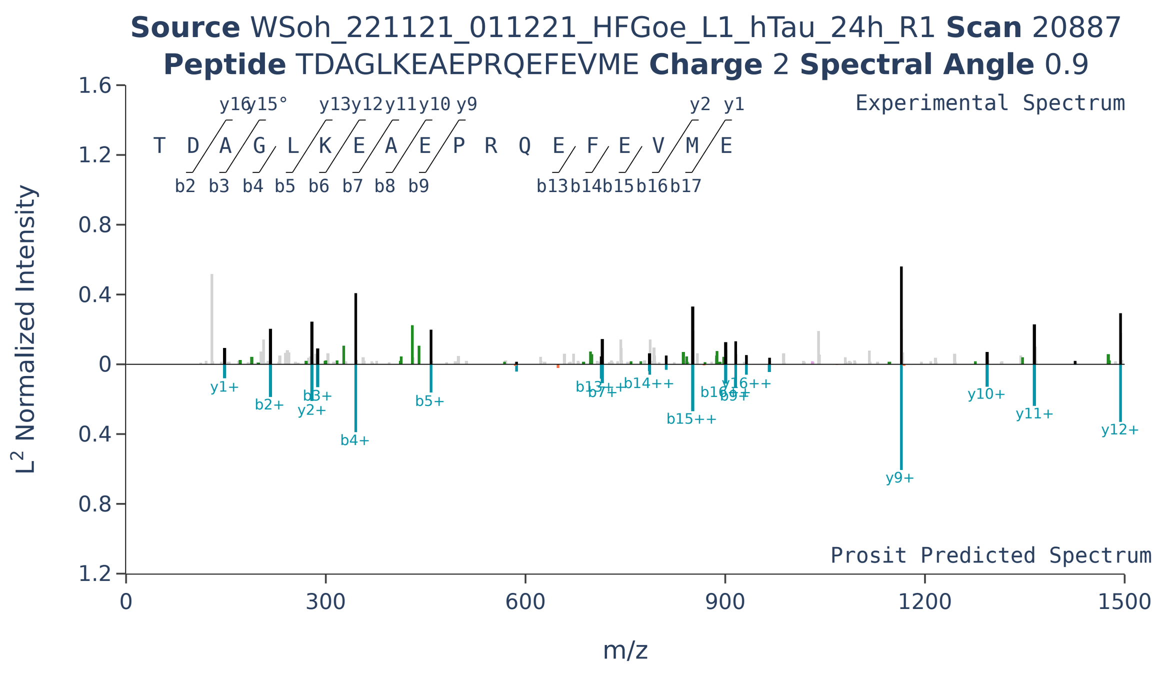
Source WSoh_221121_011221_HFGoe_L1_hTau_24h_R1 Scan 20887
Peptide TDAGLKEAEPRQEFEVME Charge 2 Spectral Angle 0.9
Experimental Spectrum
Prosit Predicted Spectrum
L² Normalized Intensity
m/z

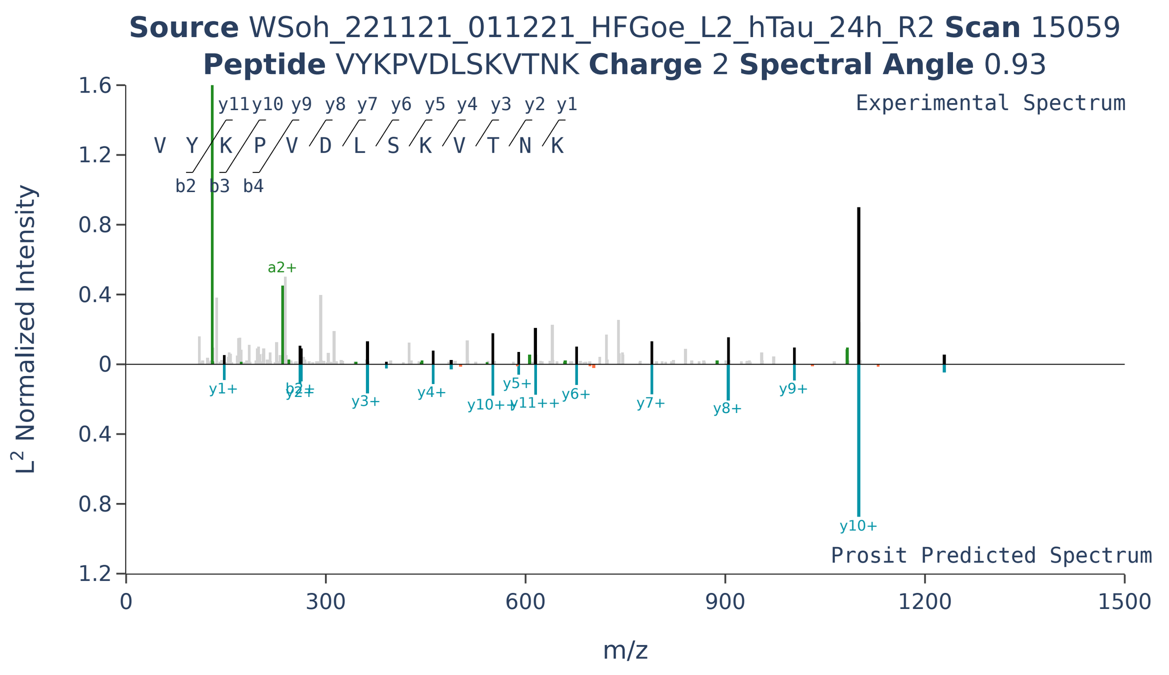
Source WSoh_221121_011221_HFGoe_L2_hTau_24h_R2 Scan 15059
Peptide VYKPVDLSKVTNK Charge 2 Spectral Angle 0.93
Experimental Spectrum
Prosit Predicted Spectrum
L² Normalized Intensity
m/z

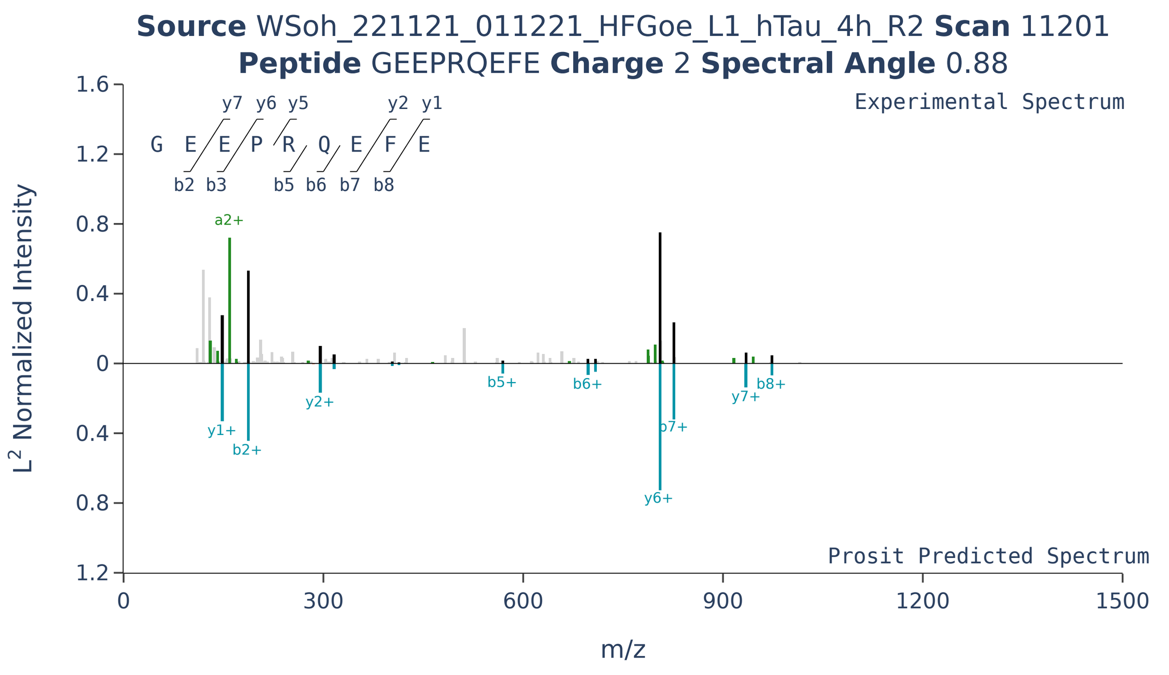
Source WSoh_221121_011221_HFGoe_L1_hTau_4h_R2 Scan 11201
Peptide GEEPRQEFE Charge 2 Spectral Angle 0.88
Experimental Spectrum
Prosit Predicted Spectrum
L² Normalized Intensity
m/z

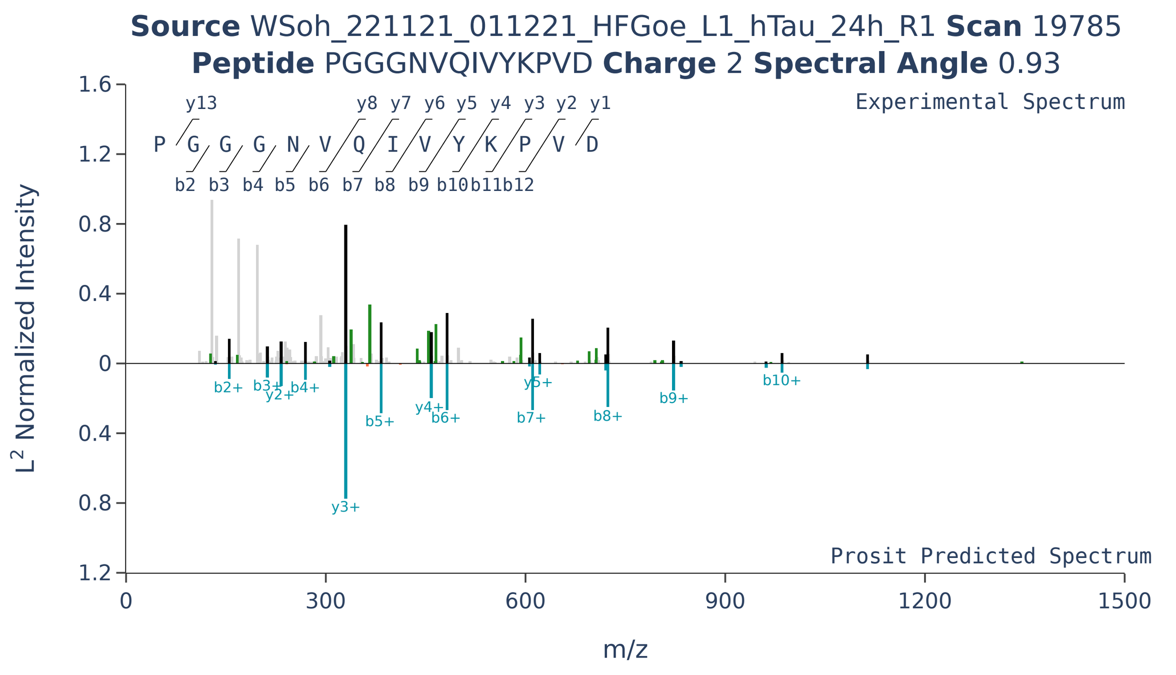
Source WSoh_221121_011221_HFGoe_L1_hTau_24h_R1 Scan 19785
Peptide PGGGNVQIVYKPVD Charge 2 Spectral Angle 0.93
Experimental Spectrum
Prosit Predicted Spectrum
L² Normalized Intensity
m/z

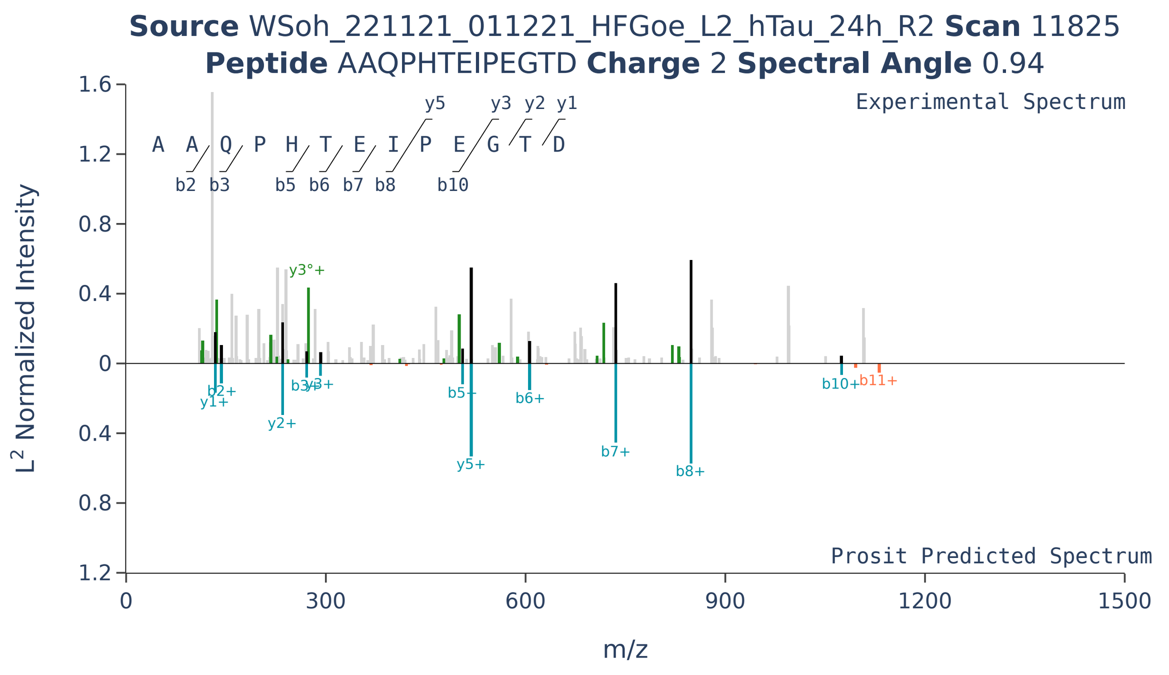
Source WSoh_221121_011221_HFGoe_L2_hTau_24h_R2 Scan 11825
Peptide AAQPHTEIPEGTD Charge 2 Spectral Angle 0.94
Experimental Spectrum
Prosit Predicted Spectrum
L² Normalized Intensity
m/z

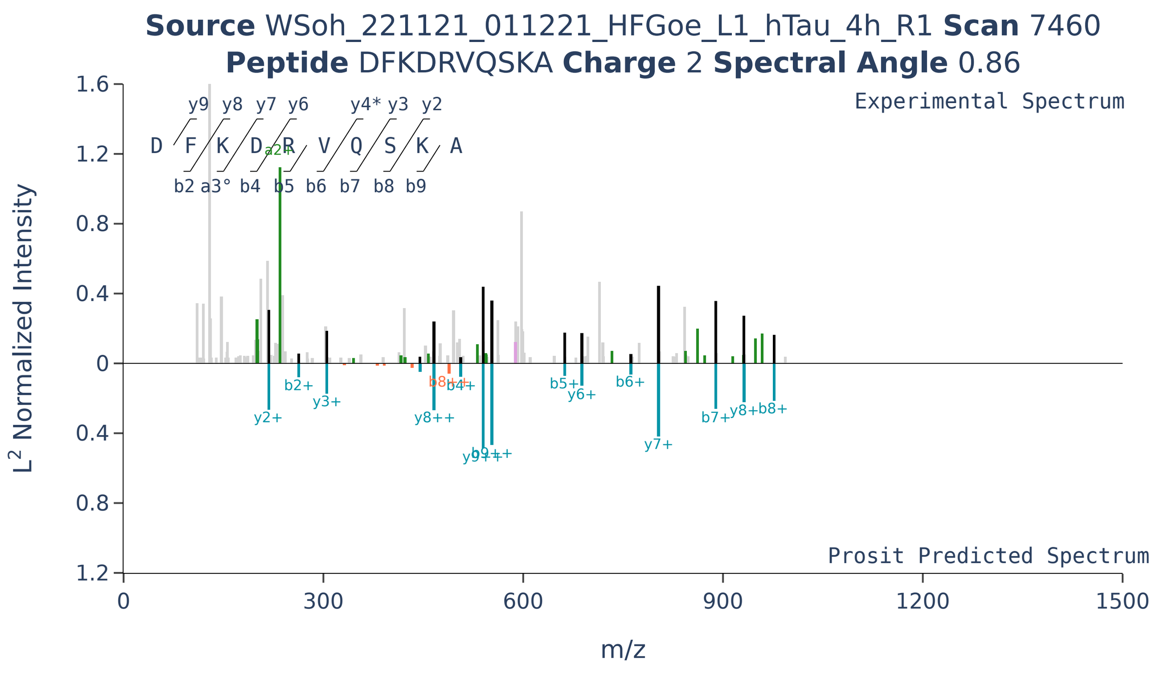
Source WSoh_221121_011221_HFGoe_L1_hTau_4h_R1 Scan 7460
Peptide DFKDRVQSKA Charge 2 Spectral Angle 0.86
Experimental Spectrum
Prosit Predicted Spectrum
L² Normalized Intensity
m/z

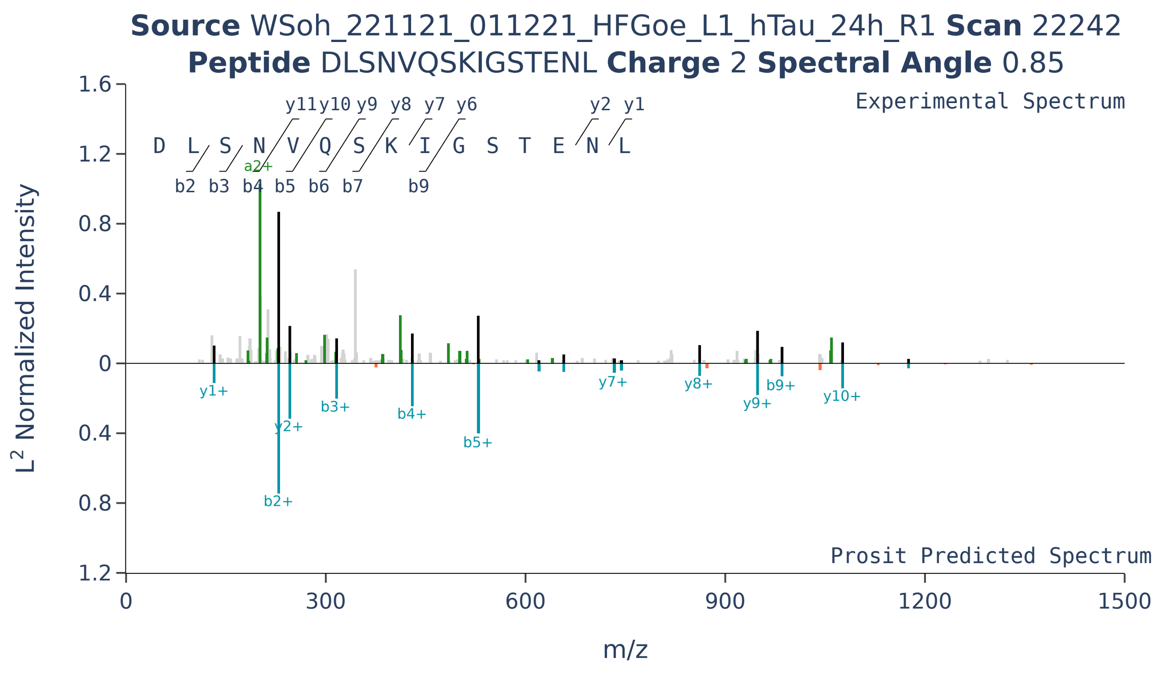
Source WSoh_221121_011221_HFGoe_L1_hTau_24h_R1 Scan 22242
Peptide DLSNVQSKIGSTENL Charge 2 Spectral Angle 0.85
Experimental Spectrum
Prosit Predicted Spectrum
L² Normalized Intensity
m/z

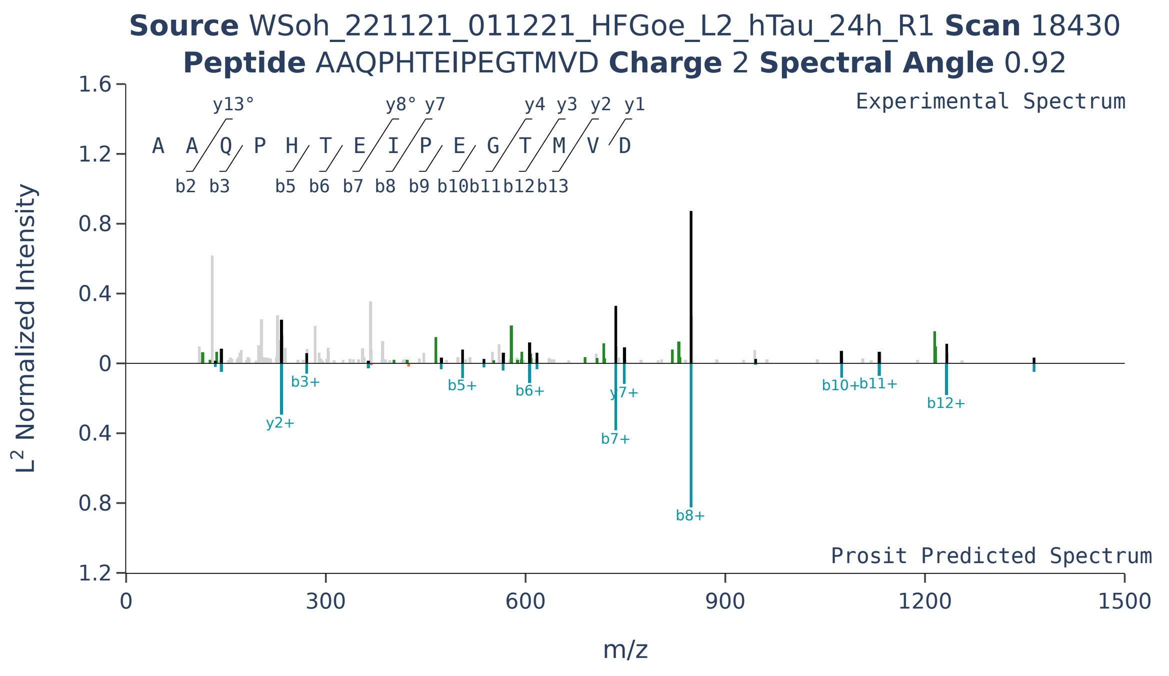
Source WSoh_221121_011221_HFGoe_L2_hTau_24h_R1 Scan 18430
Peptide AAQPHTEIPEGTMVD Charge 2 Spectral Angle 0.92
Experimental Spectrum
Prosit Predicted Spectrum
L² Normalized Intensity
m/z

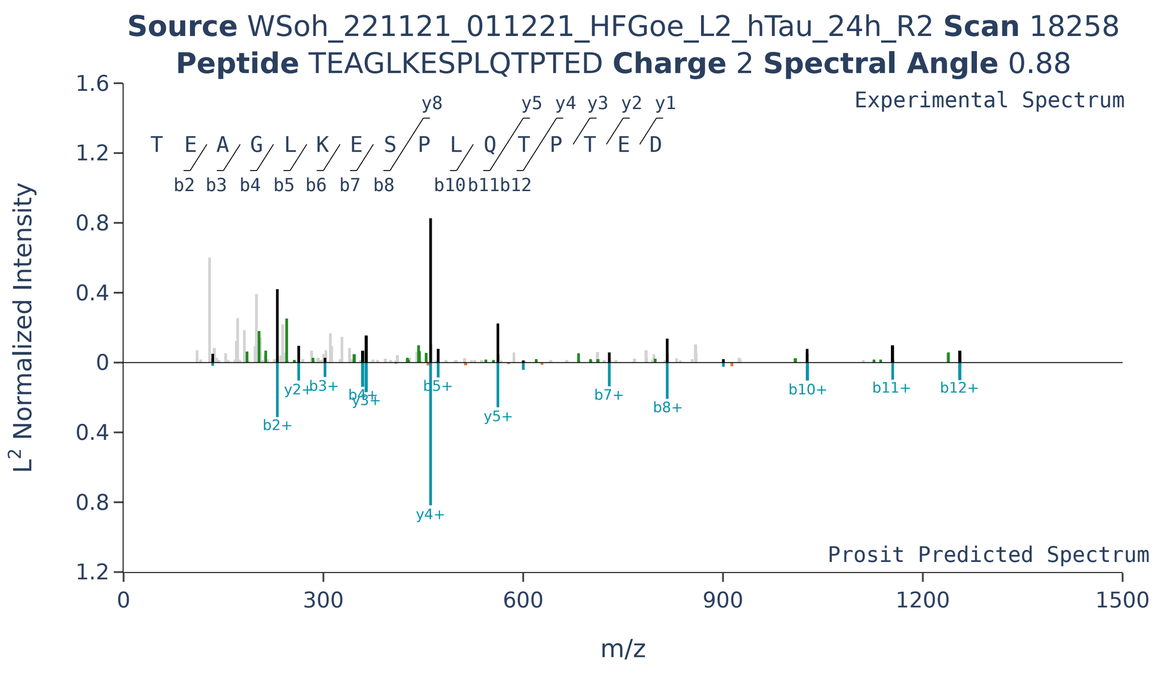
Source WSoh_221121_011221_HFGoe_L2_hTau_24h_R2 Scan 18258
Peptide TEAGLKESPLQTPTED Charge 2 Spectral Angle 0.88
Experimental Spectrum
Prosit Predicted Spectrum
L² Normalized Intensity
m/z

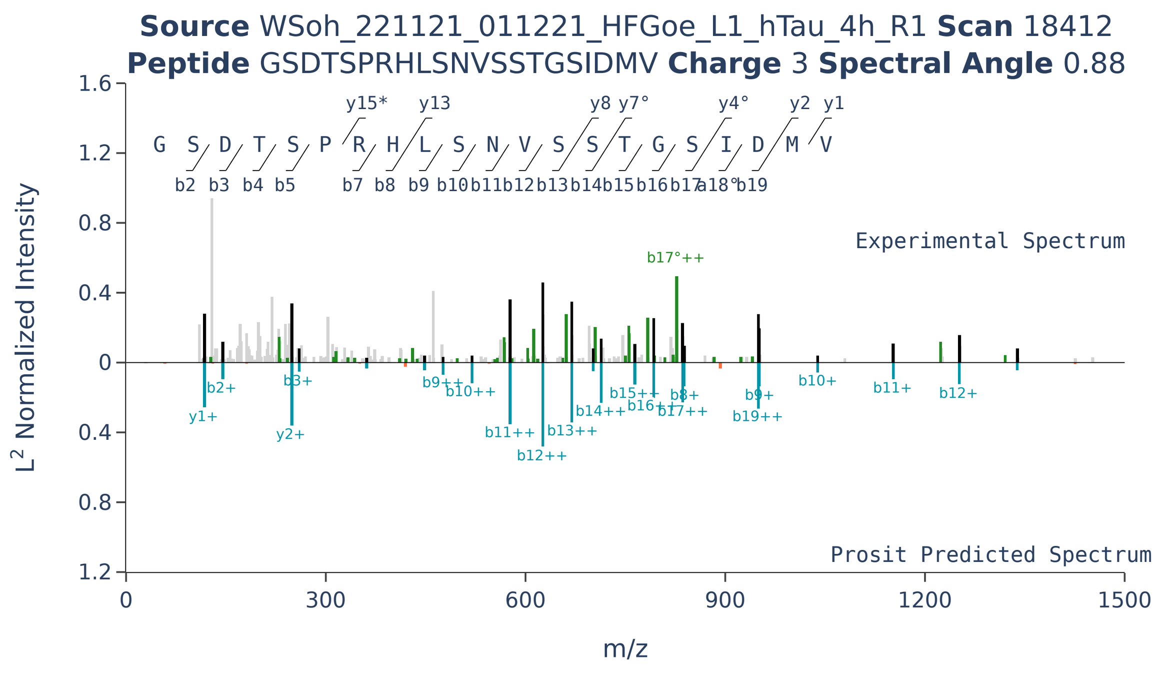
Source WSoh_221121_011221_HFGoe_L1_hTau_4h_R1 Scan 18412
Peptide GSDTSPRHLSNVSSTGSIDMV Charge 3 Spectral Angle 0.88
Experimental Spectrum
Prosit Predicted Spectrum
L² Normalized Intensity
m/z

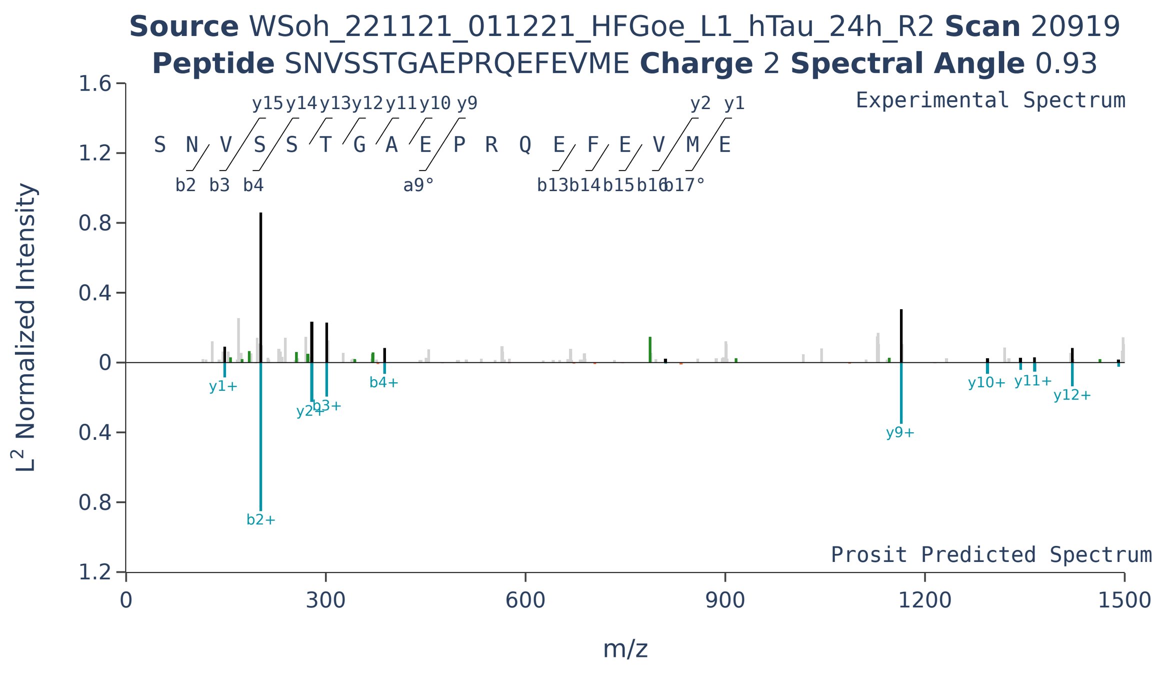
Source WSoh_221121_011221_HFGoe_L1_hTau_24h_R2 Scan 20919
Peptide SNVSSTGAEPRQEFEVME Charge 2 Spectral Angle 0.93
Experimental Spectrum
Prosit Predicted Spectrum
L² Normalized Intensity
m/z

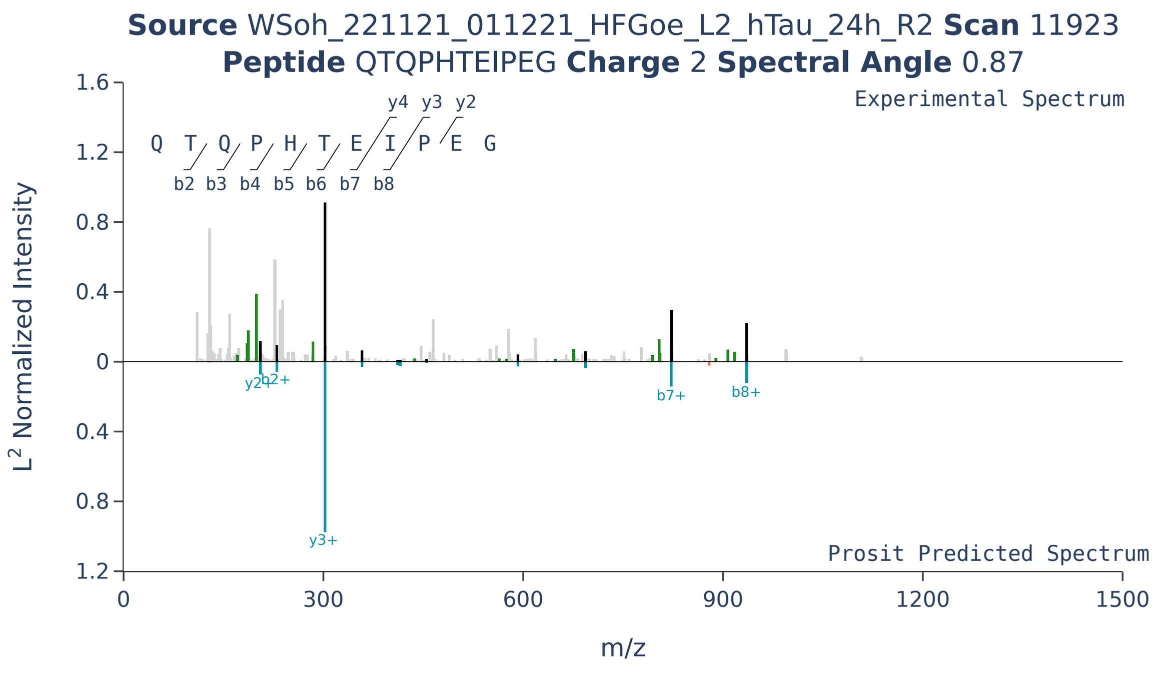
Source WSoh_221121_011221_HFGoe_L2_hTau_24h_R2 Scan 11923
Peptide QTQPHTEIPEG Charge 2 Spectral Angle 0.87
Experimental Spectrum
Prosit Predicted Spectrum
L² Normalized Intensity
m/z

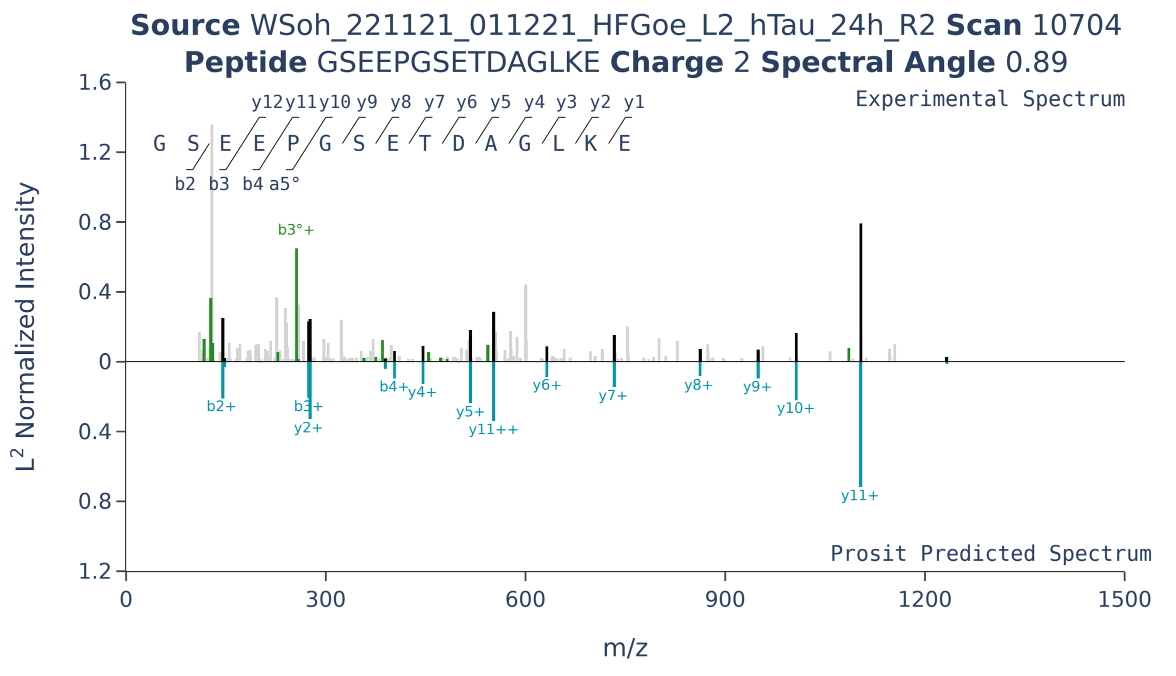
Source WSoh_221121_011221_HFGoe_L2_hTau_24h_R2 Scan 10704
Peptide GSEEPGSETDAGLKE Charge 2 Spectral Angle 0.89
Experimental Spectrum
Prosit Predicted Spectrum
L² Normalized Intensity
m/z

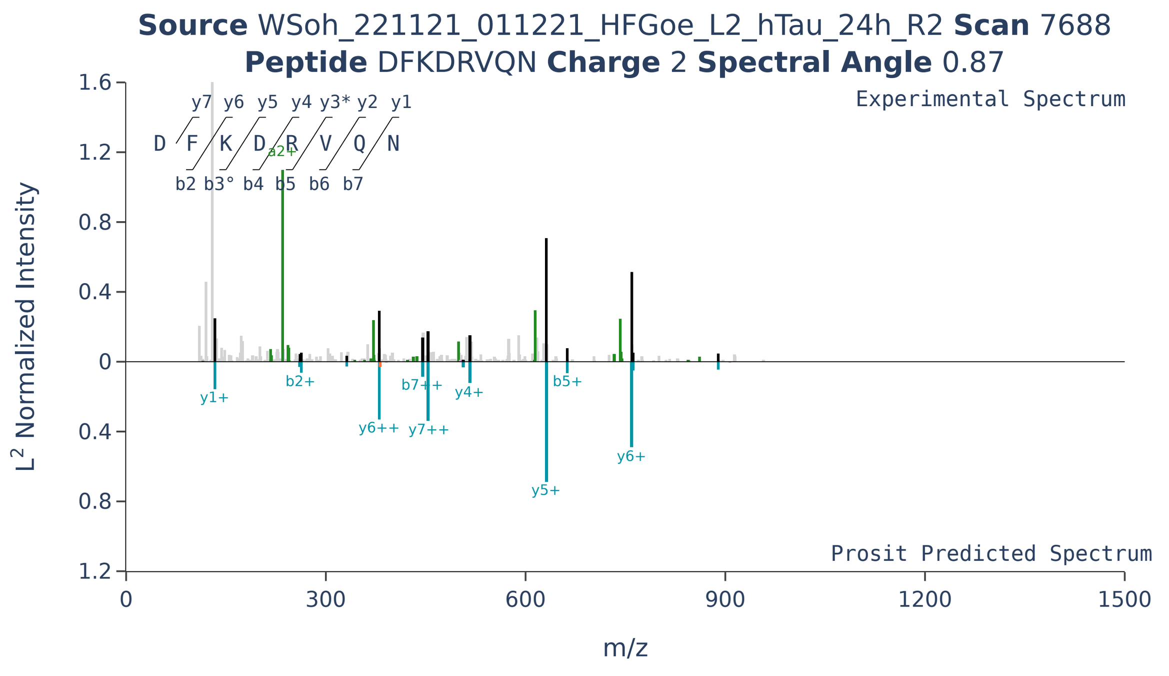
Source WSoh_221121_011221_HFGoe_L2_hTau_24h_R2 Scan 7688
Peptide DFKDRVQN Charge 2 Spectral Angle 0.87
Experimental Spectrum
Prosit Predicted Spectrum
L² Normalized Intensity
m/z

Source WSoh_221121_011221_HFGoe_L2_hTau_4h_R1 **Scan** 11615
Peptide AQQPHTEIPEG **Charge** 2 **Spectral Angle** 0.93

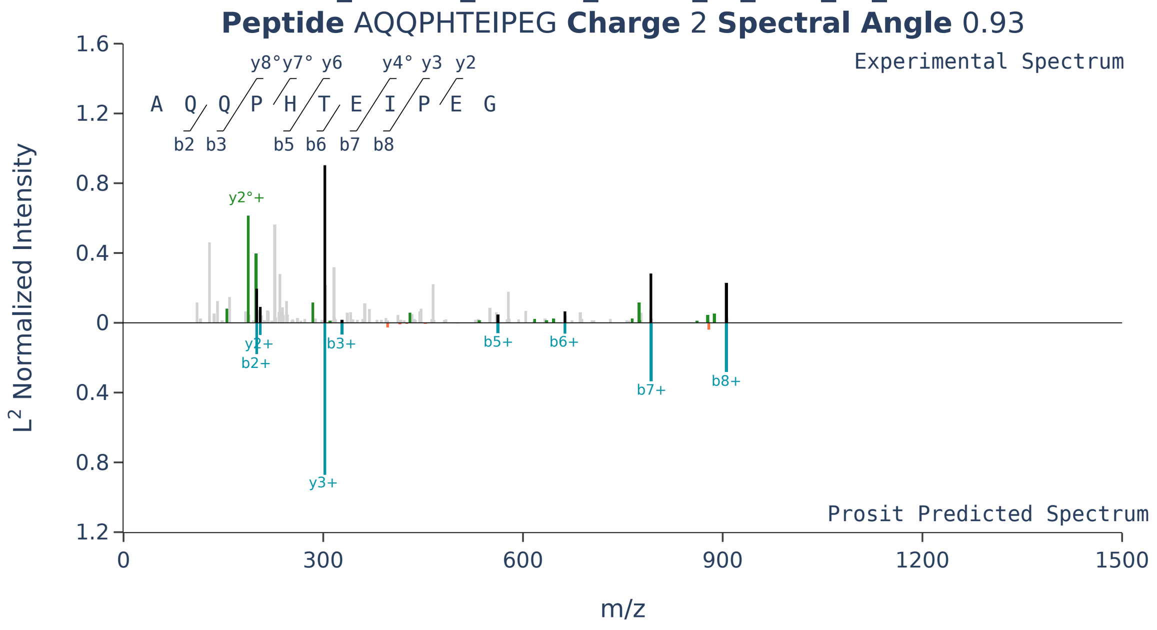

Source WSoh_221121_011221_HFGoe_L1_hTau_24h_R2 **Scan** 18738
Peptide DFKDRVQSTF **Charge** 2 **Spectral Angle** 0.86

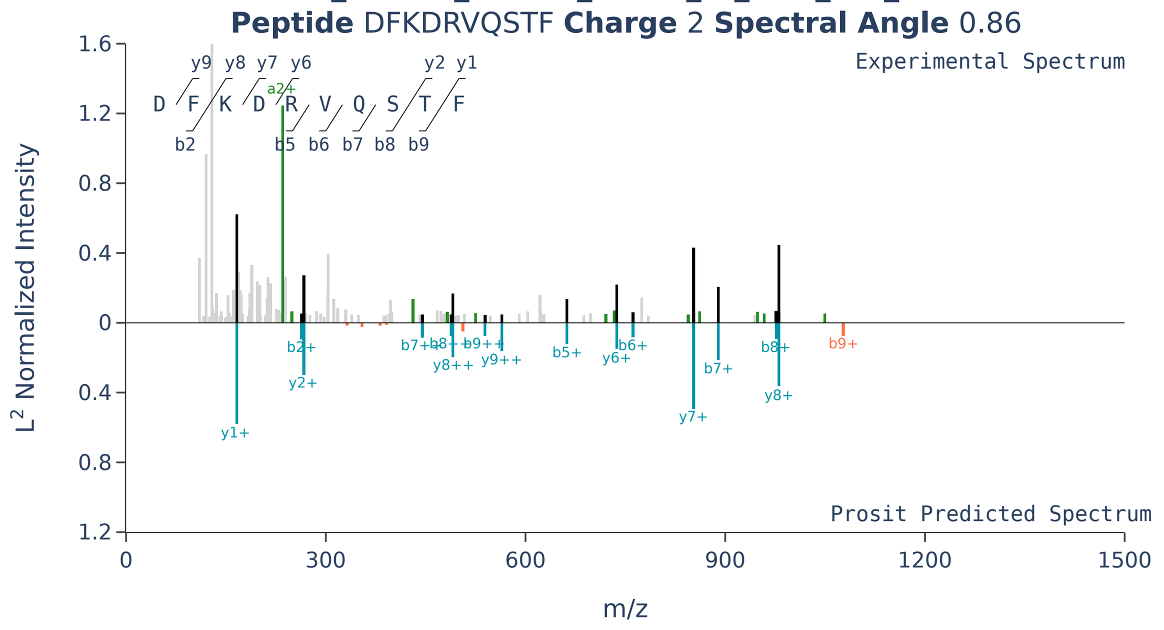

Source WSoh_221121_011221_HFGoe_L1_hTau_4h_R2 **Scan** 20905
Peptide DLSNVQSVYKPVD **Charge** 2 **Spectral Angle** 0.91

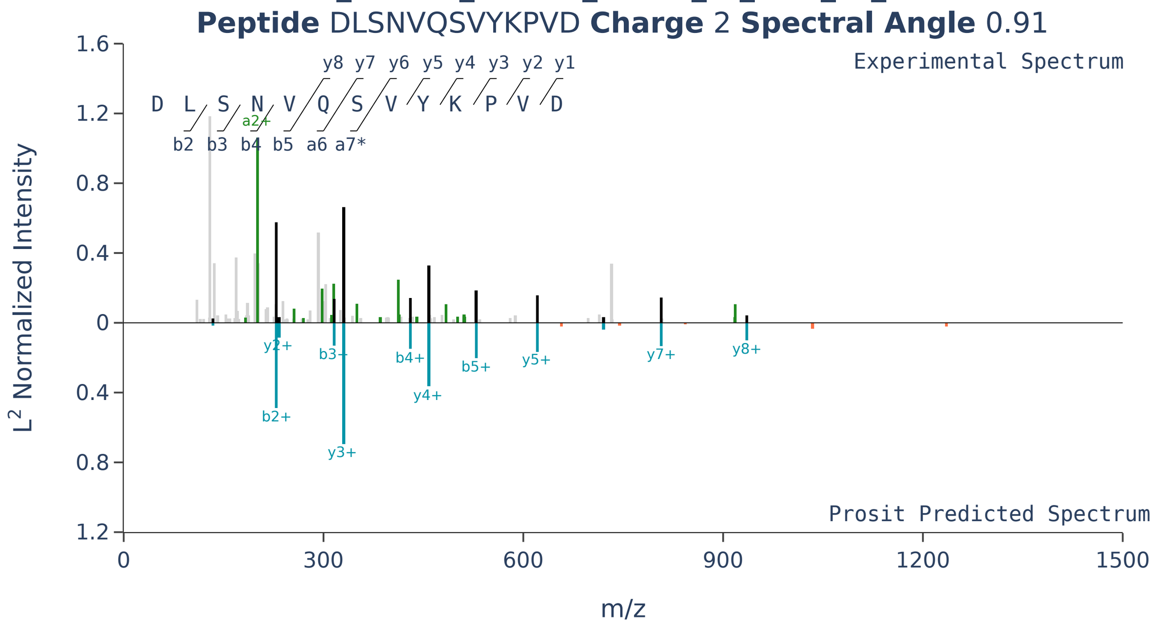

Source WSoh_221121_011221_HFGoe_L1_hTau_24h_R2 **Scan** 17599
Peptide SNVSSTGSPLQTPTE **Charge** 2 **Spectral Angle** 0.94

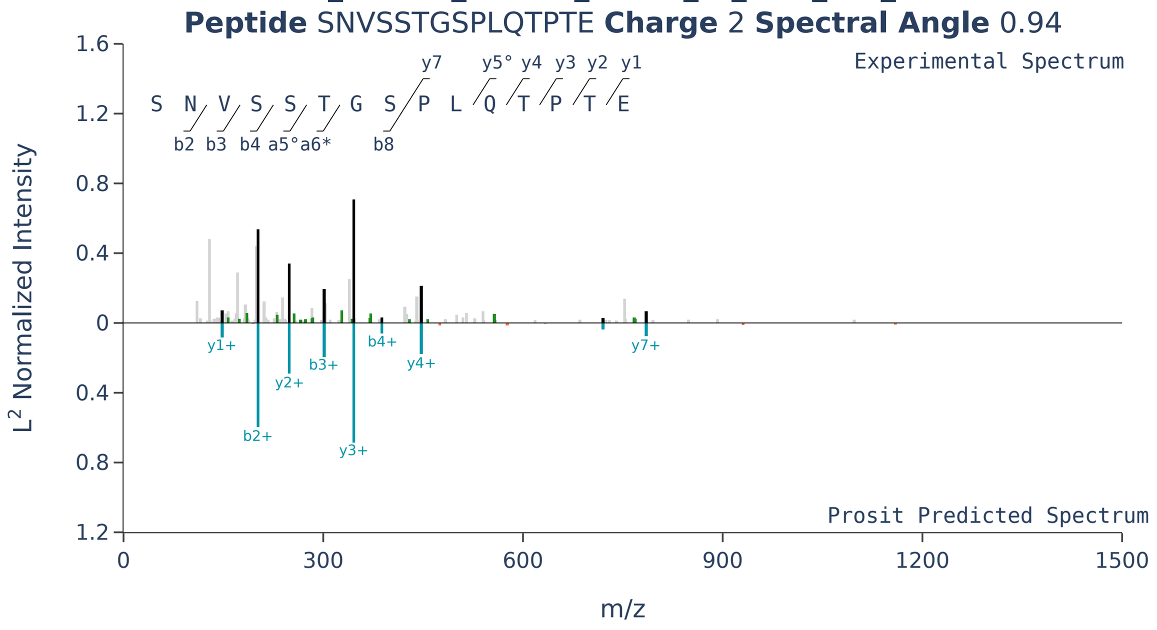

Source WSoh_221121_011221_HFGoe_L2_hTau_24h_R2 **Scan** 12188
Peptide AAQPHTEIPEGTTD **Charge** 2 **Spectral Angle** 0.94

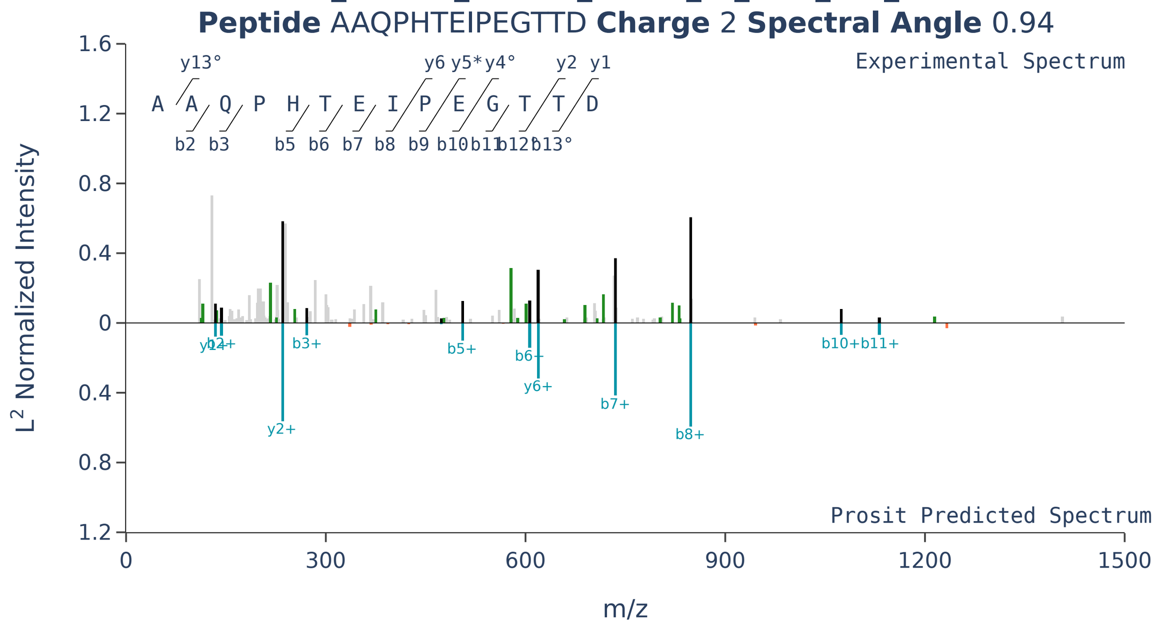

Source WSoh_221121_011221_HFGoe_L2_hTau_24h_R1 **Scan** 19120
Peptide GTDHGAEIVYKSPVV **Charge** 3 **Spectral Angle** 0.88

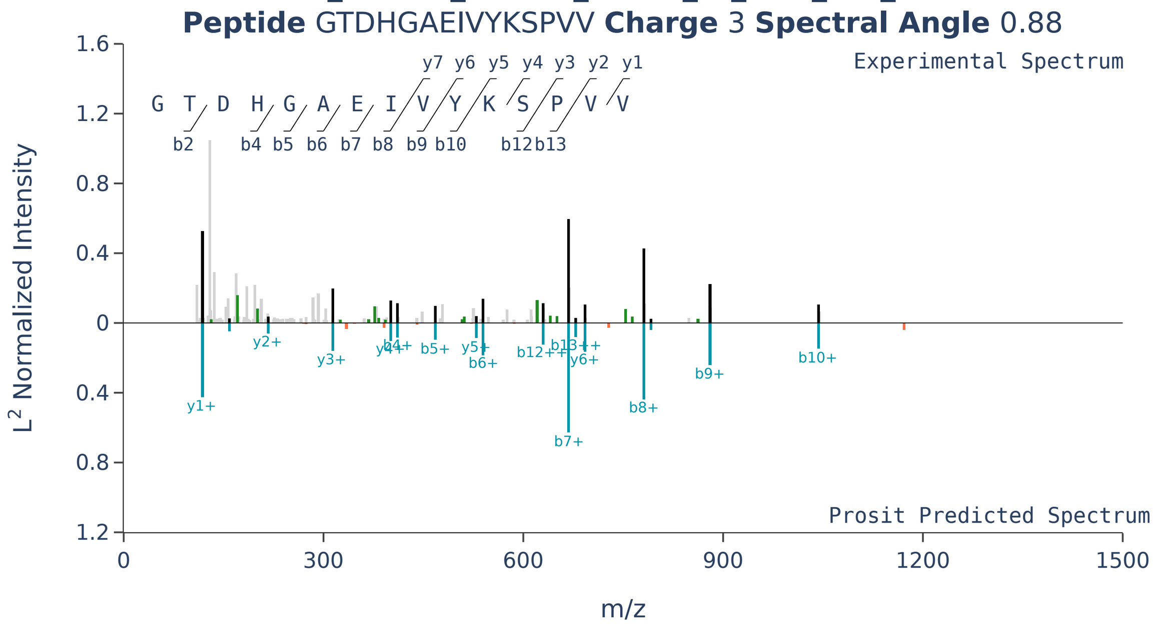

Source WSoh_221121_011221_HFGoe_L2_hTau_1h_R2 **Scan** 12133
Peptide HGAEIKL **Charge** 2 **Spectral Angle** 0.9

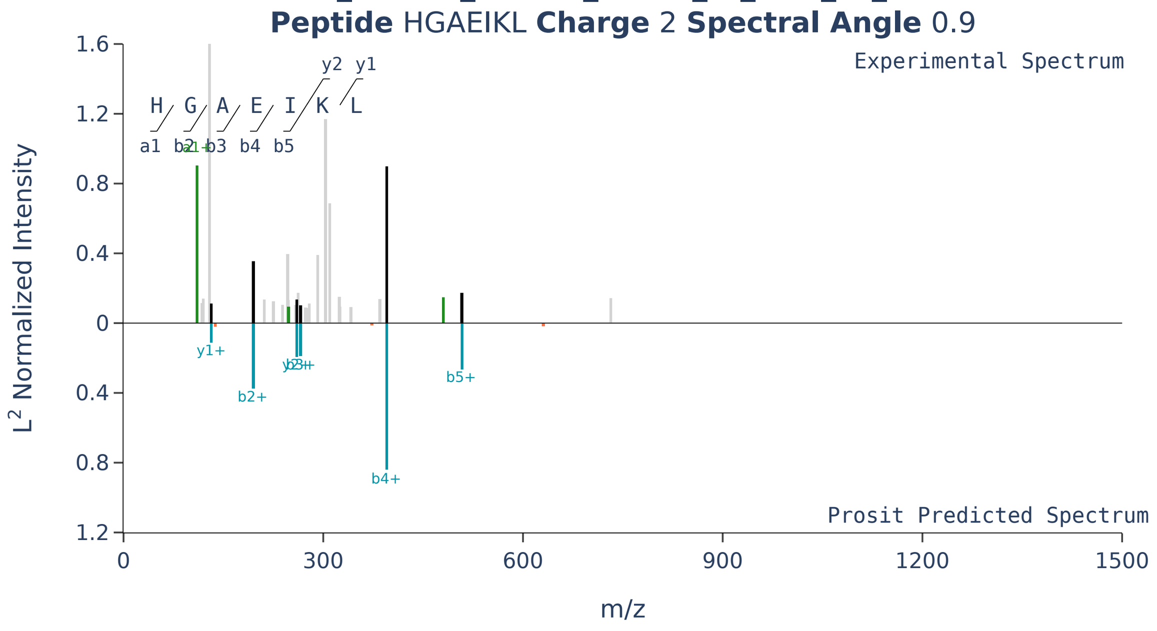

Source WSoh_221121_011221_HFGoe_L1_hTau_24h_R1 **Scan** 19252
Peptide APVPMPDLKNVKN **Charge** 3 **Spectral Angle** 0.88

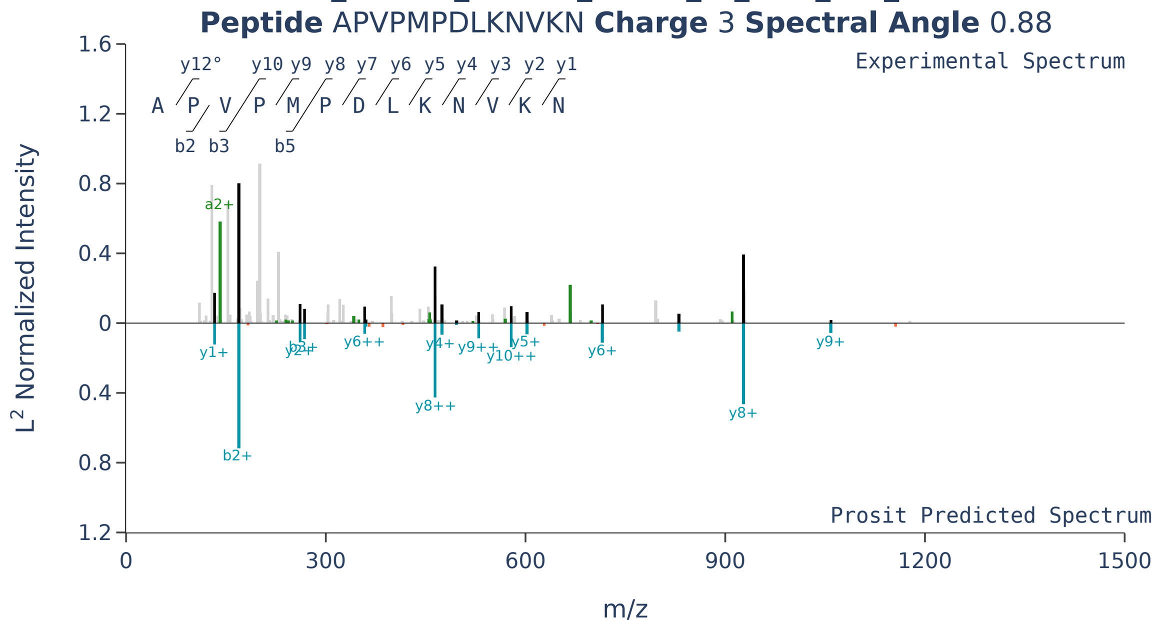

Source WSoh_221121_011221_HFGoe_L2_hTau_4h_R1 **Scan** 17492
Peptide DHAGTYGSPLQTPTED **Charge** 2 **Spectral Angle** 0.88

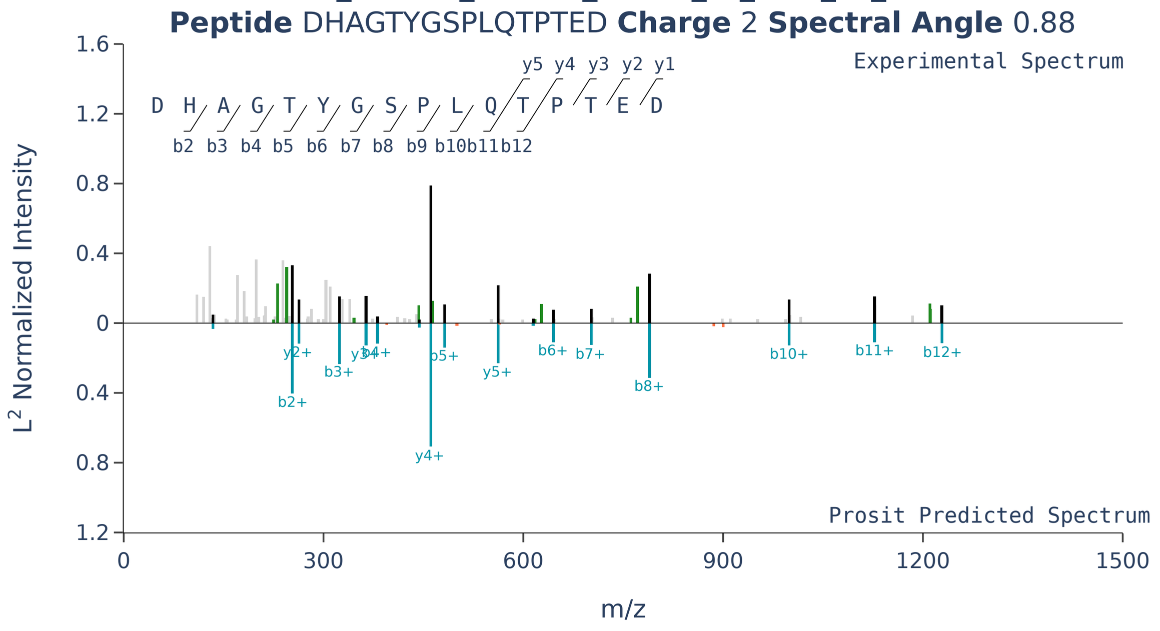

Source WSoh_221121_011221_HFGoe_L1_hTau_4h_R1 **Scan** 13970
Peptide DAAQPHTEIPEGTTAE **Charge** 2 **Spectral Angle** 0.89

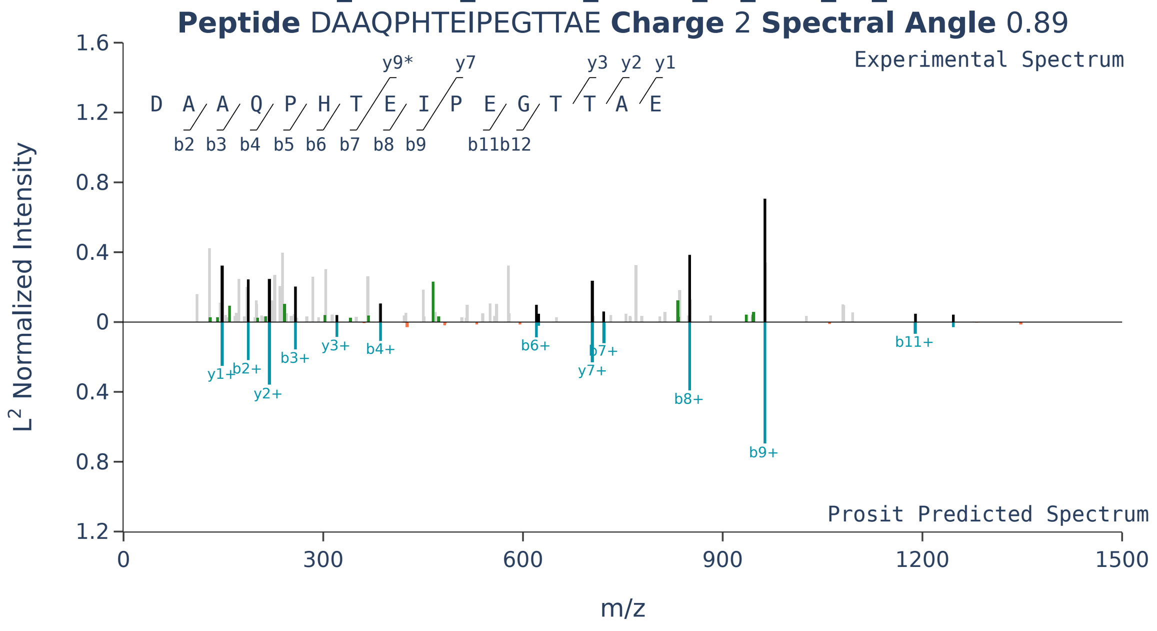

Source WSoh_221121_011221_HFGoe_L1_hTau_24h_R2 **Scan** 13197
Peptide KLDLSNVQSKCSKDNIKHV **Charge** 3 **Spectral Angle** 0.93

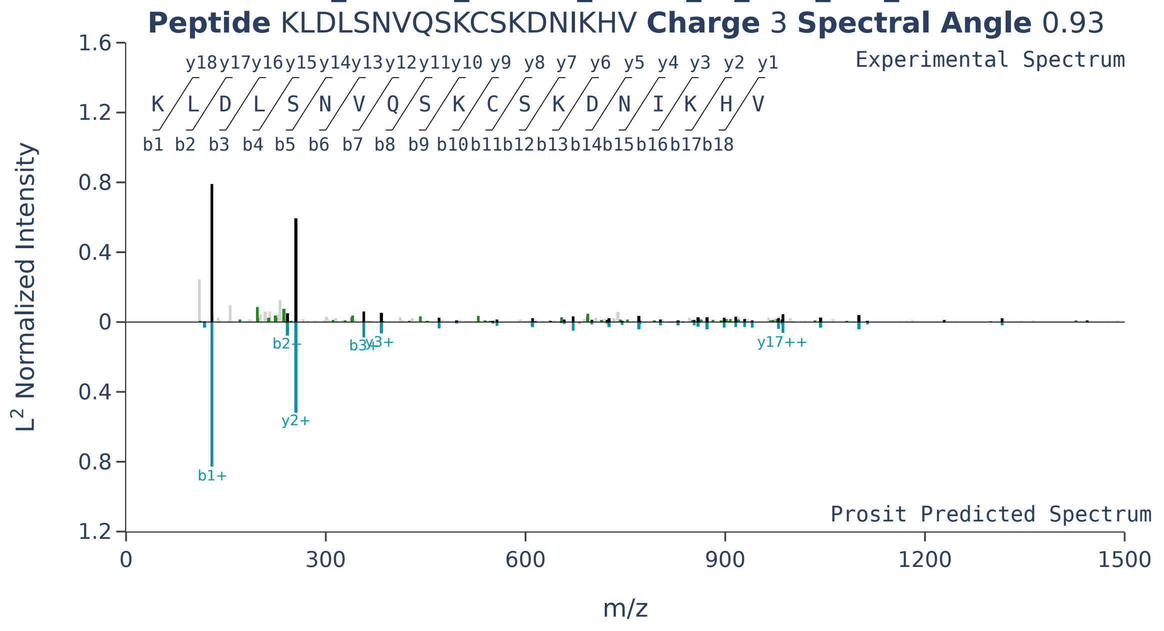

Source WSoh_221121_011221_HFGoe_L2_hTau_24h_R2 **Scan** 11967
Peptide KLDFKDRVQTG **Charge** 2 **Spectral Angle** 0.86

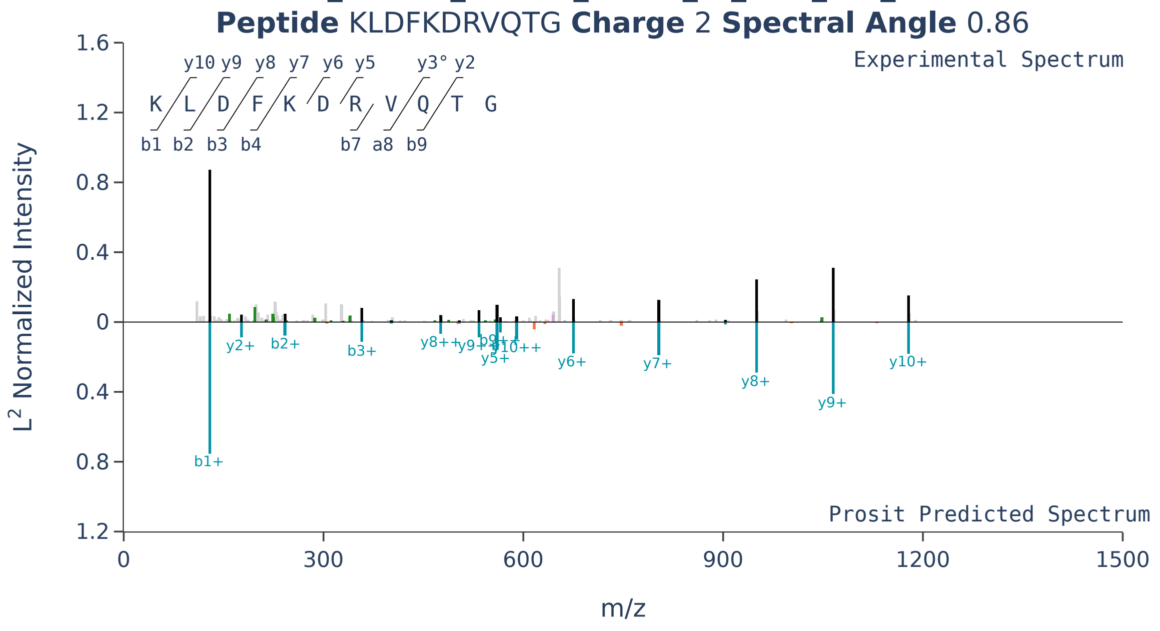

Source WSoh_221121_011221_HFGoe_L2_hTau_2h_R2 **Scan** 11575
Peptide GAAQPHTEIPEG **Charge** 2 **Spectral Angle** 0.91

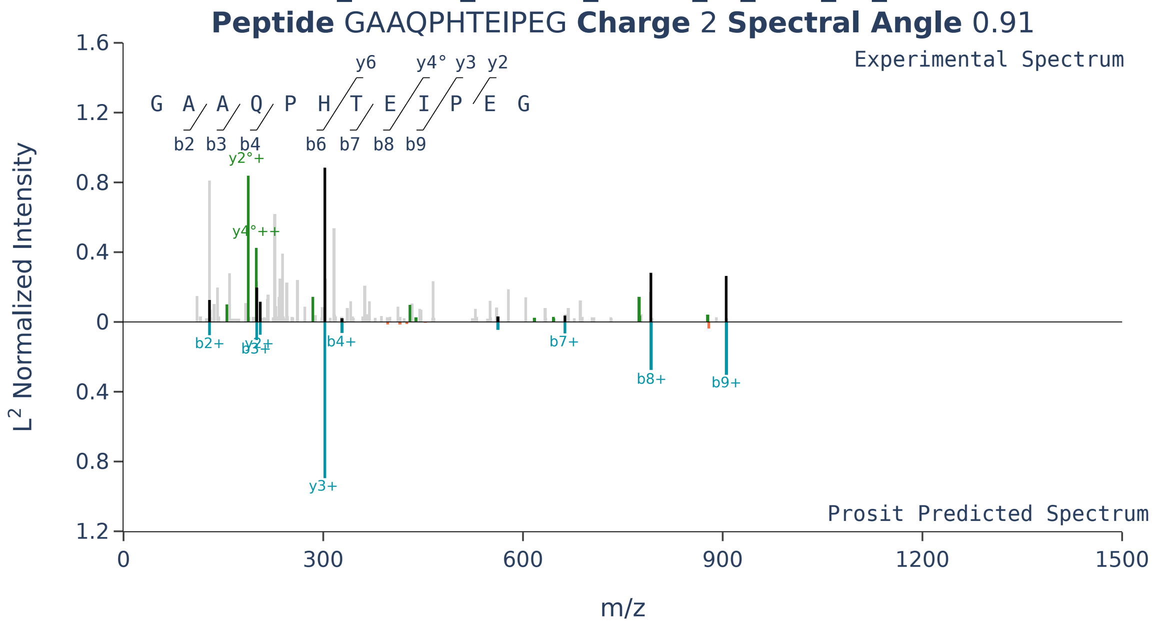

Source WSoh_221121_011221_HFGoe_L2_hTau_4h_R2 **Scan** 15310
Peptide AAQPHTEIPEGRMV **Charge** 3 **Spectral Angle** 0.89

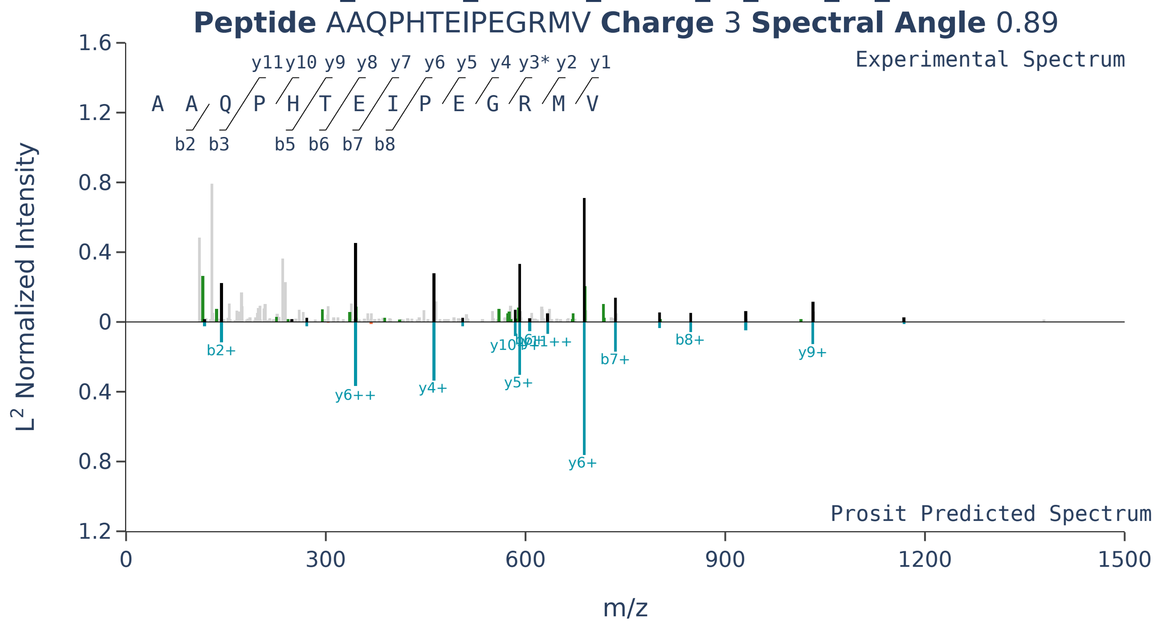

Source WSoh_221121_011221_HFGoe_L1_hTau_24h_R2 **Scan** 11930
Peptide KLDFKDRVQSA **Charge** 2 **Spectral Angle** 0.85

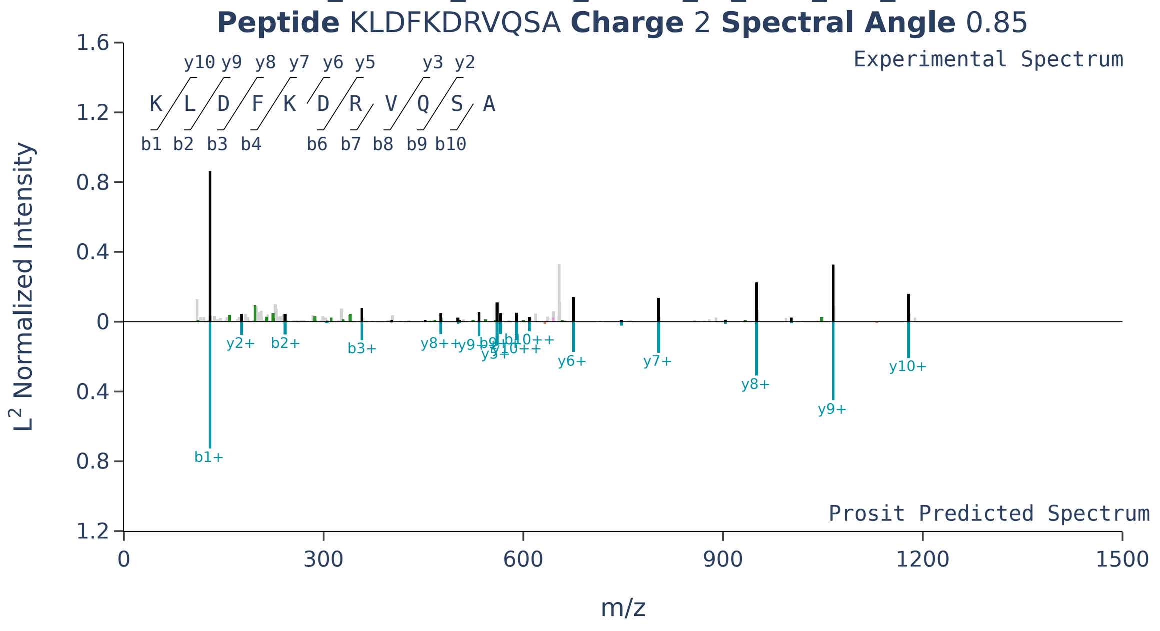

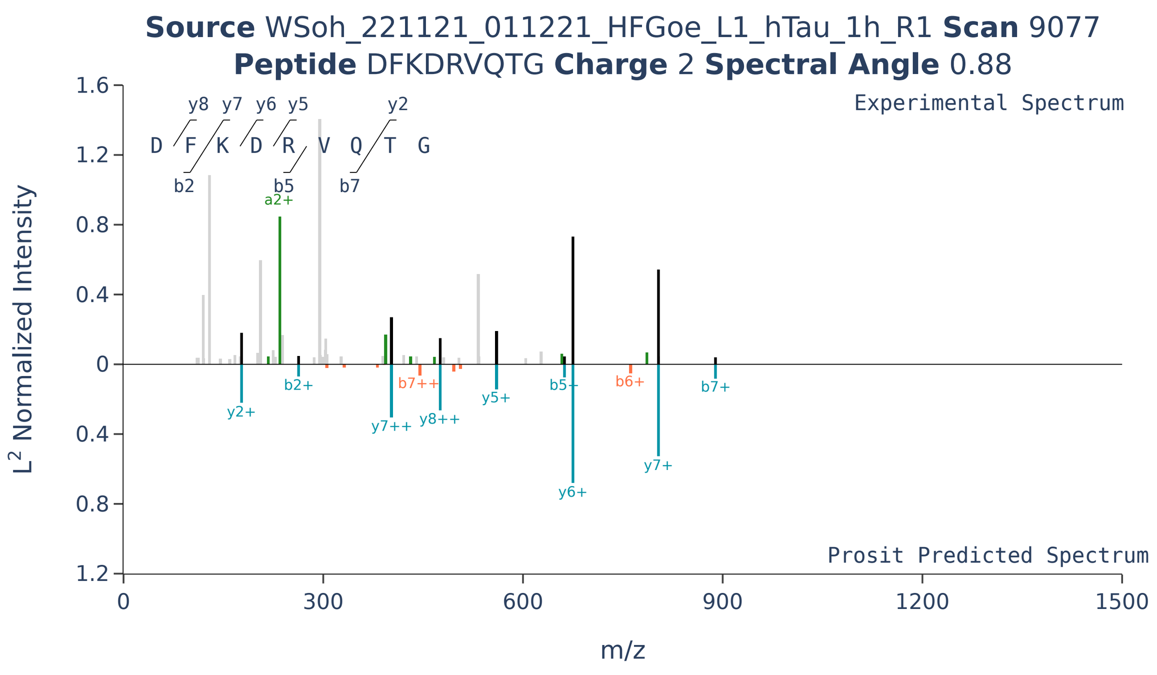
Source WSoh_221121_011221_HFGoe_L1_hTau_1h_R1 Scan 9077
Peptide DFKDRVQTG Charge 2 Spectral Angle 0.88
Experimental Spectrum
Prosit Predicted Spectrum
L² Normalized Intensity
m/z

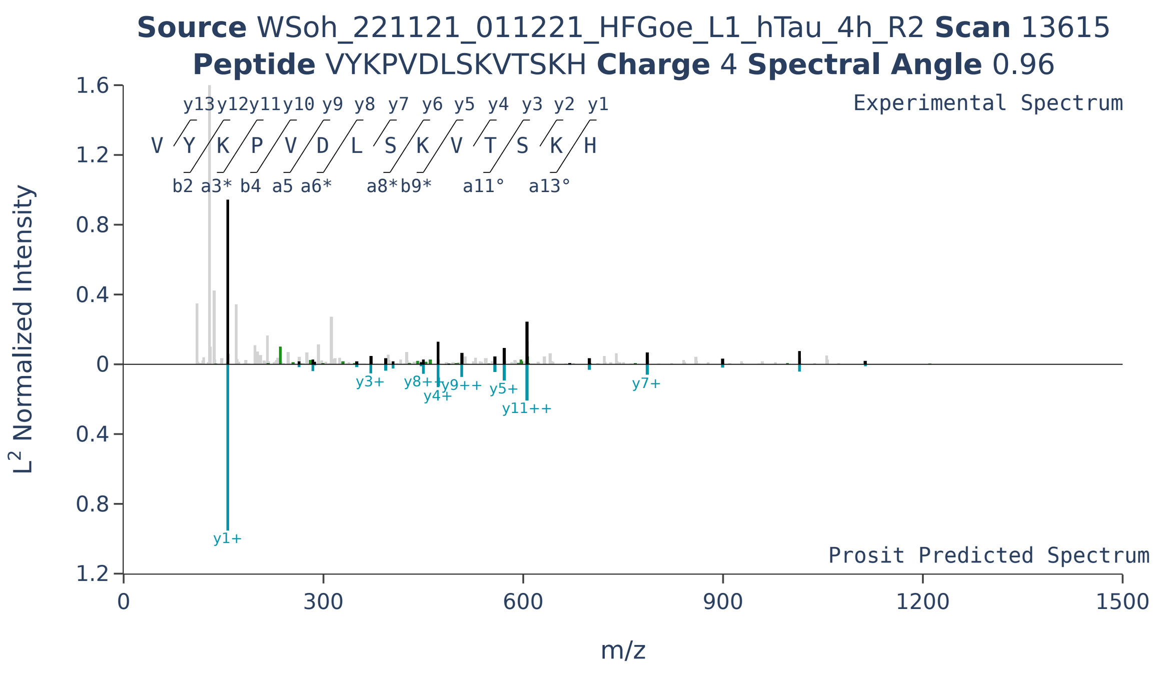
Source WSoh_221121_011221_HFGoe_L1_hTau_4h_R2 Scan 13615
Peptide VYKPVDLSKVTSKH Charge 4 Spectral Angle 0.96
Experimental Spectrum
Prosit Predicted Spectrum
L² Normalized Intensity
m/z

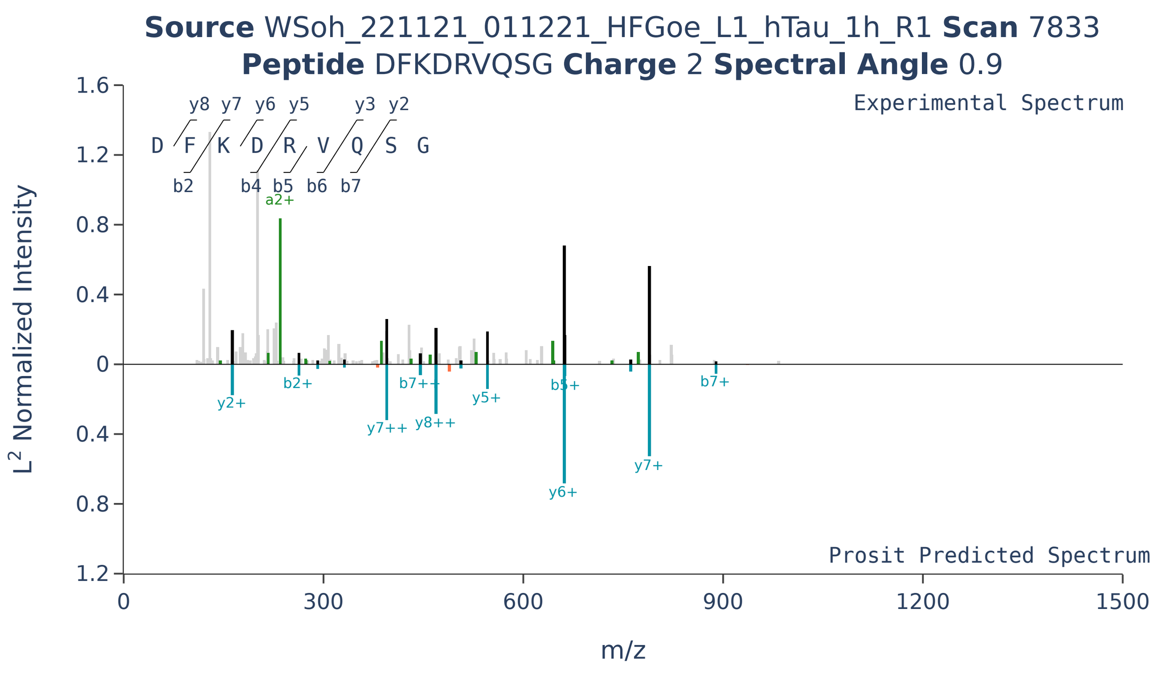
Source WSoh_221121_011221_HFGoe_L1_hTau_1h_R1 Scan 7833
Peptide DFKDRVQSG Charge 2 Spectral Angle 0.9
Experimental Spectrum
Prosit Predicted Spectrum
L² Normalized Intensity
m/z

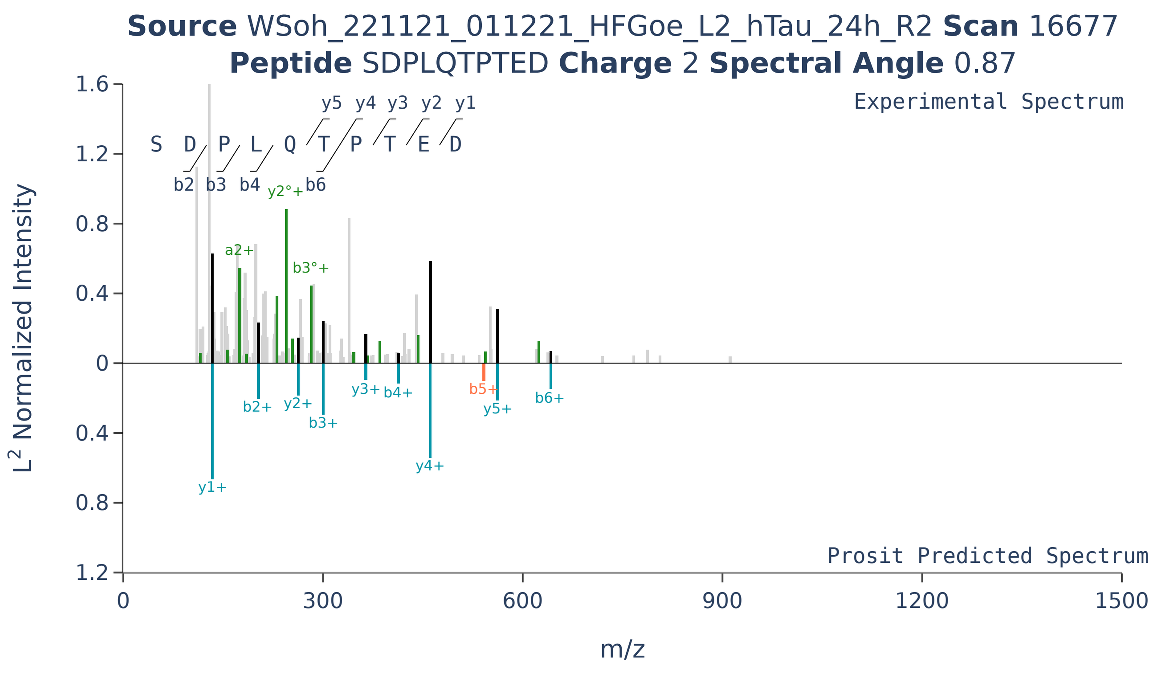
Source WSoh_221121_011221_HFGoe_L2_hTau_24h_R2 Scan 16677
Peptide SDPLQTPTED Charge 2 Spectral Angle 0.87
Experimental Spectrum
Prosit Predicted Spectrum
L² Normalized Intensity
m/z

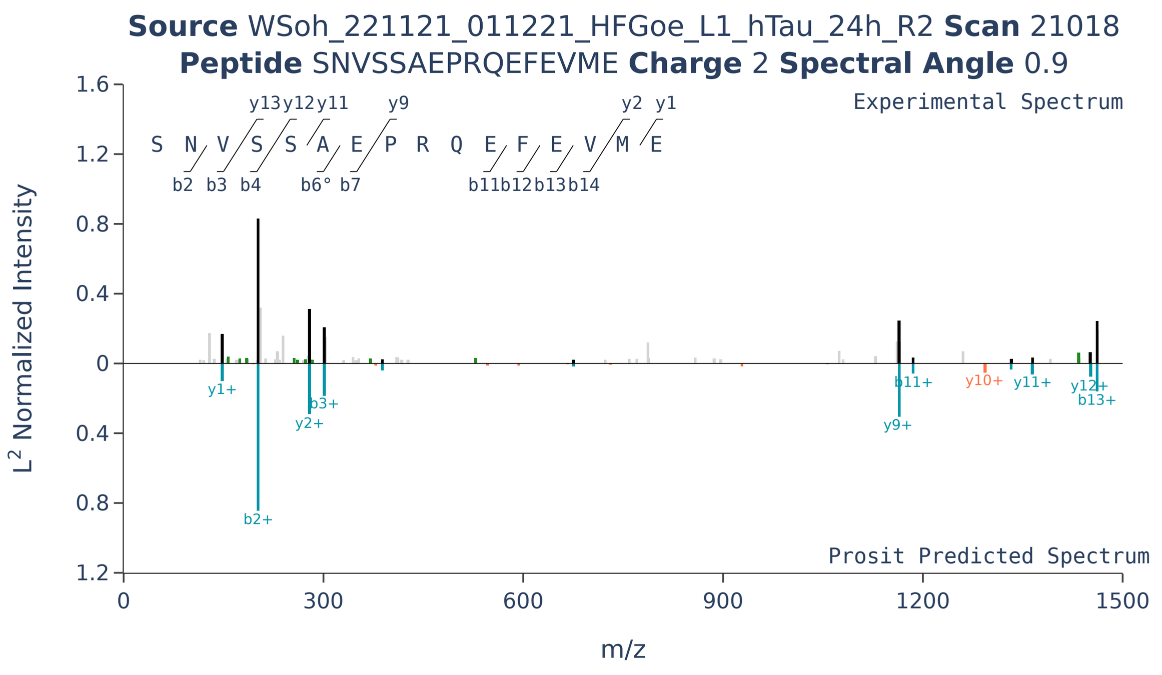
Source WSoh_221121_011221_HFGoe_L1_hTau_24h_R2 Scan 21018
Peptide SNVSSAEPRQEFEVME Charge 2 Spectral Angle 0.9
Experimental Spectrum
Prosit Predicted Spectrum
L² Normalized Intensity
m/z

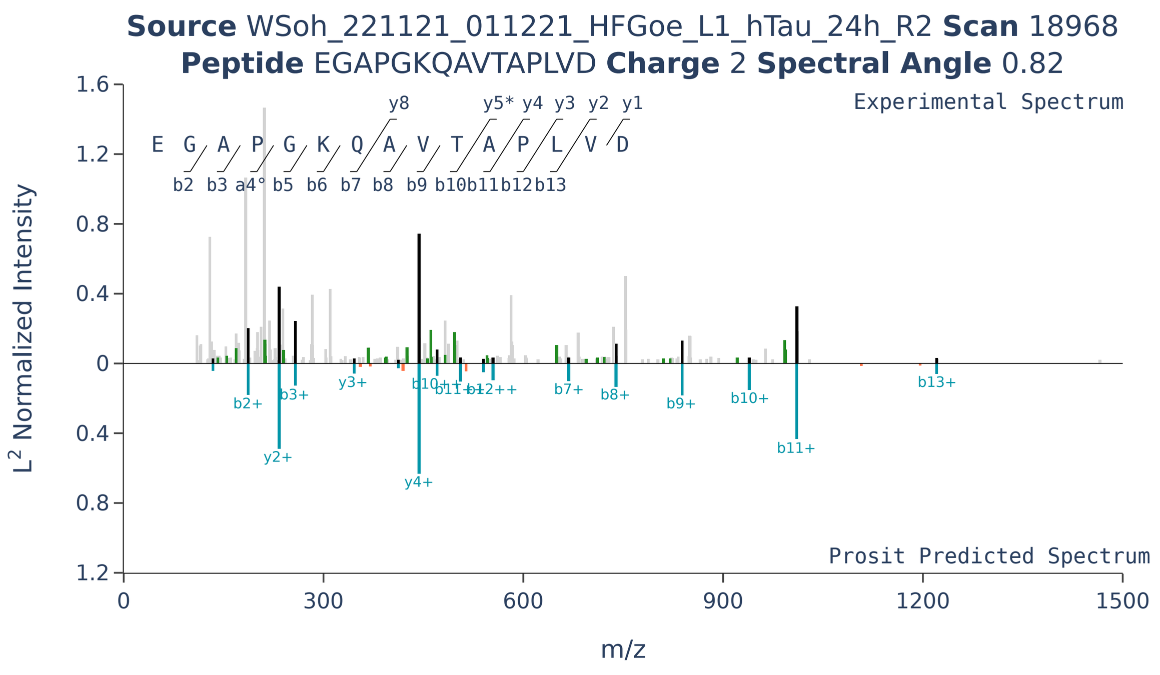
Source WSoh_221121_011221_HFGoe_L1_hTau_24h_R2 Scan 18968
Peptide EGAPGKQAVTAPLVD Charge 2 Spectral Angle 0.82
Experimental Spectrum
Prosit Predicted Spectrum
L² Normalized Intensity
m/z

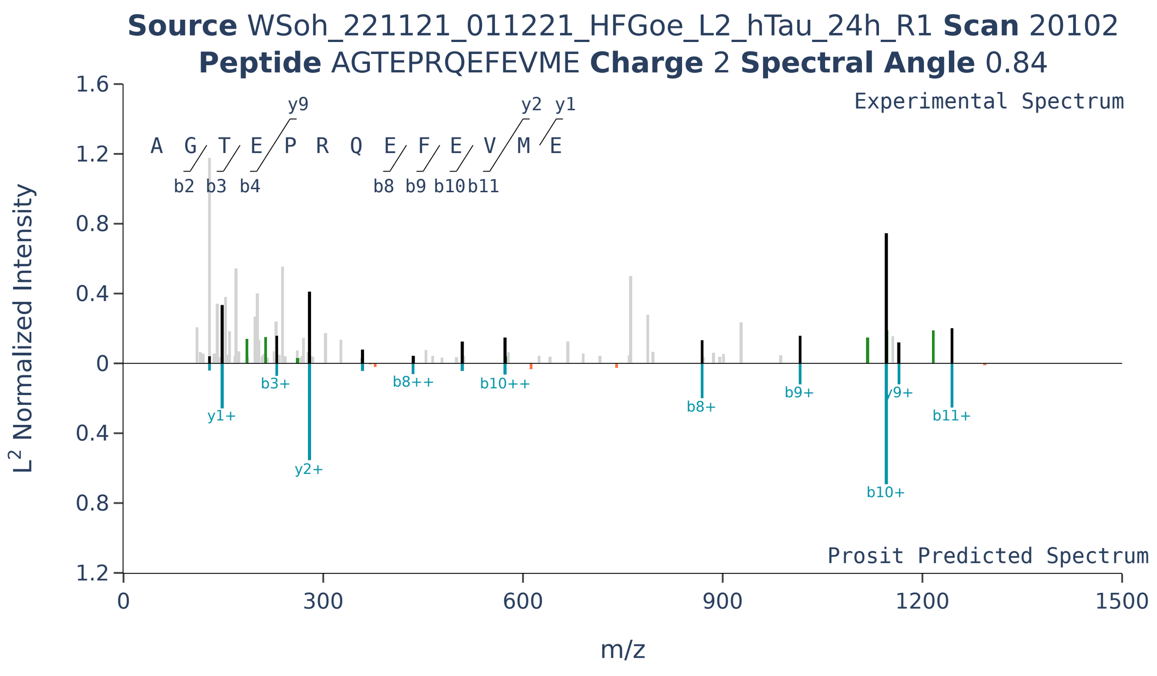
Source WSoh_221121_011221_HFGoe_L2_hTau_24h_R1 Scan 20102
Peptide AGTEPRQEFEVME Charge 2 Spectral Angle 0.84
Experimental Spectrum
Prosit Predicted Spectrum
L² Normalized Intensity
m/z

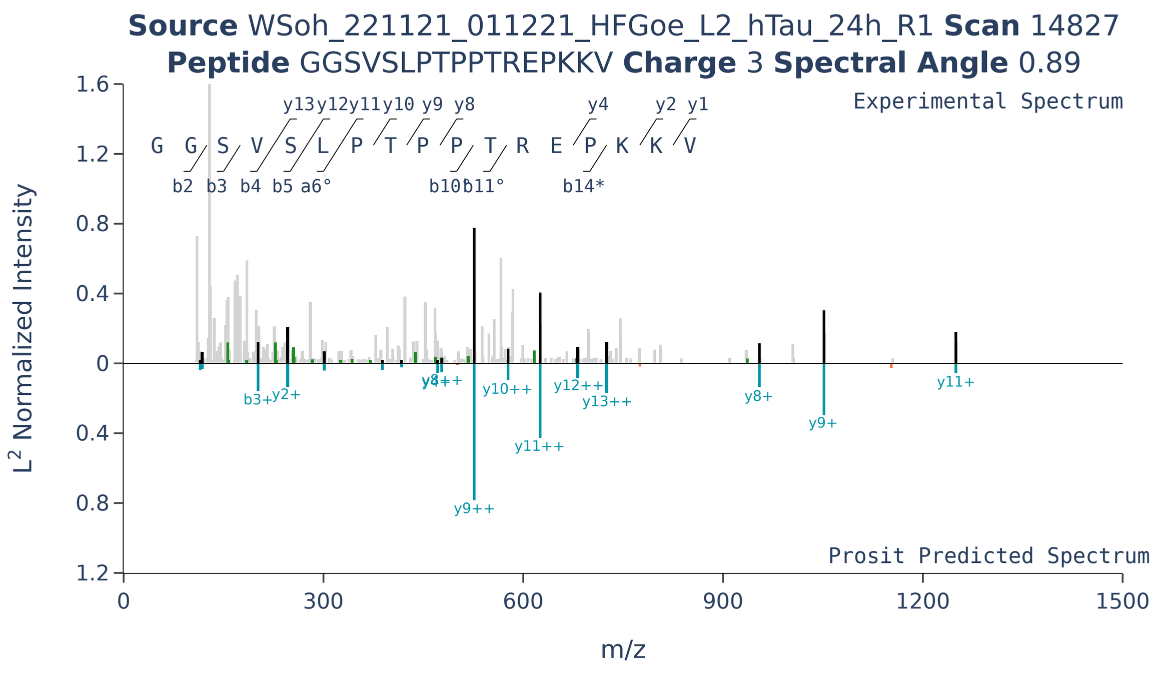
Source WSoh_221121_011221_HFGoe_L2_hTau_24h_R1 Scan 14827
Peptide GGSVSLPTPPTREPKKV Charge 3 Spectral Angle 0.89
Experimental Spectrum
Prosit Predicted Spectrum
L² Normalized Intensity
m/z

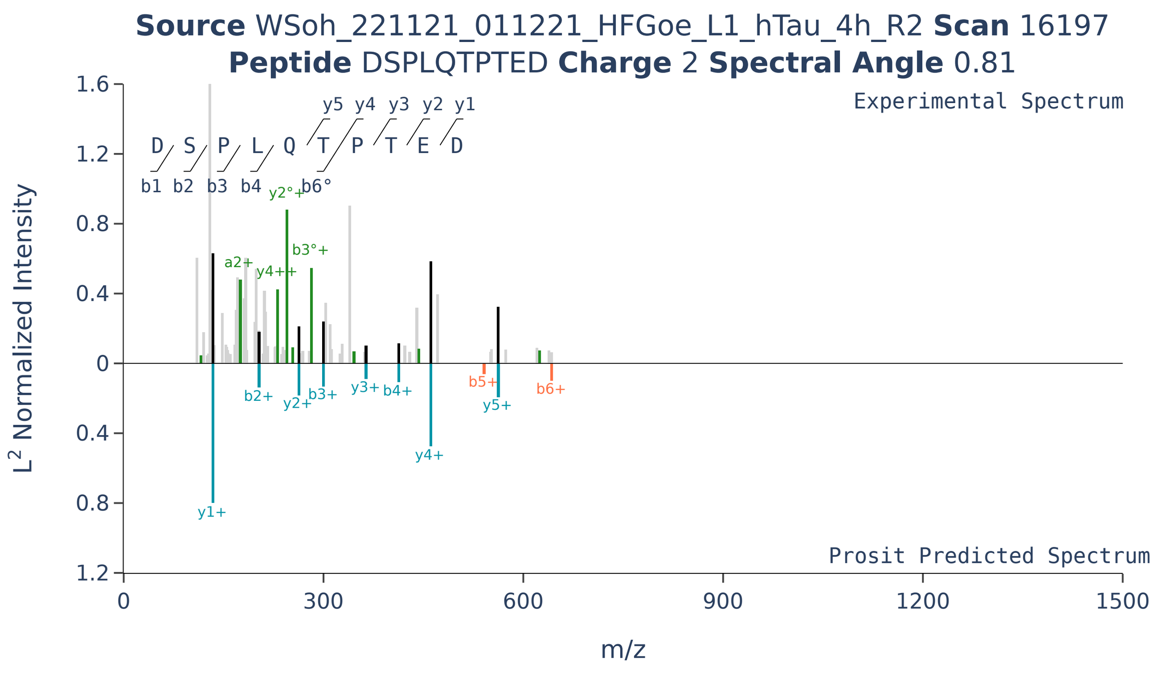
Source WSoh_221121_011221_HFGoe_L1_hTau_4h_R2 Scan 16197
Peptide DSPLQTPTED Charge 2 Spectral Angle 0.81
Experimental Spectrum
Prosit Predicted Spectrum
L² Normalized Intensity
m/z

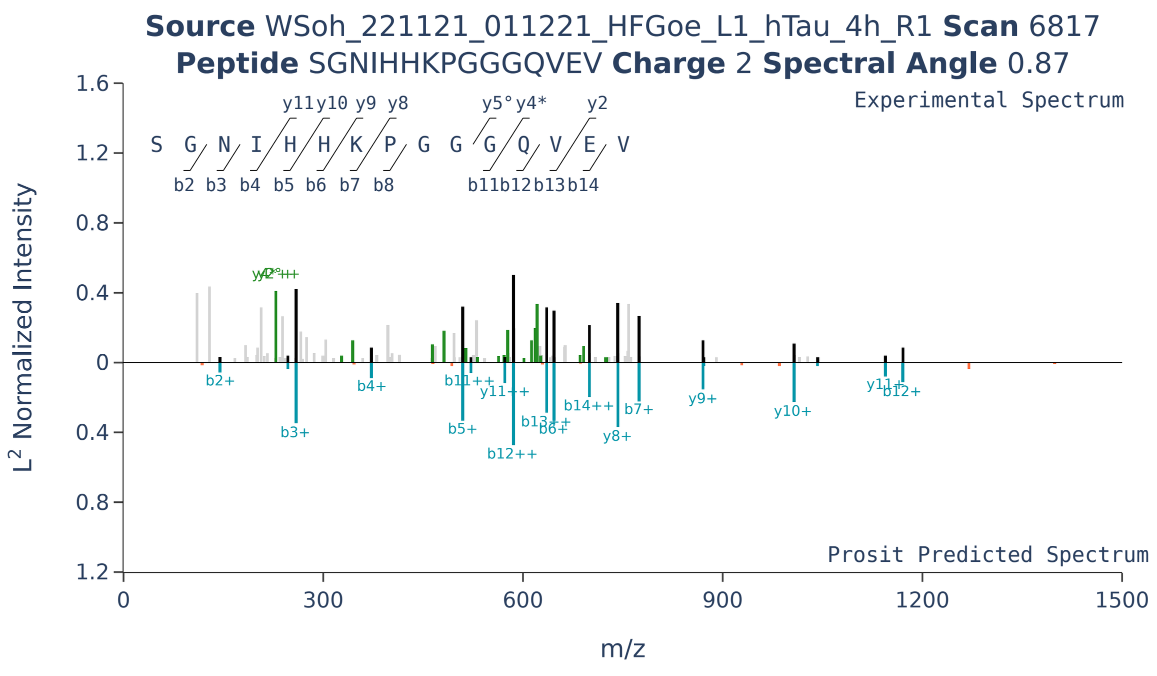
Source WSoh_221121_011221_HFGoe_L1_hTau_4h_R1 Scan 6817
Peptide SGNIHHKPGGGQVEV Charge 2 Spectral Angle 0.87
Experimental Spectrum
Prosit Predicted Spectrum
L² Normalized Intensity
m/z

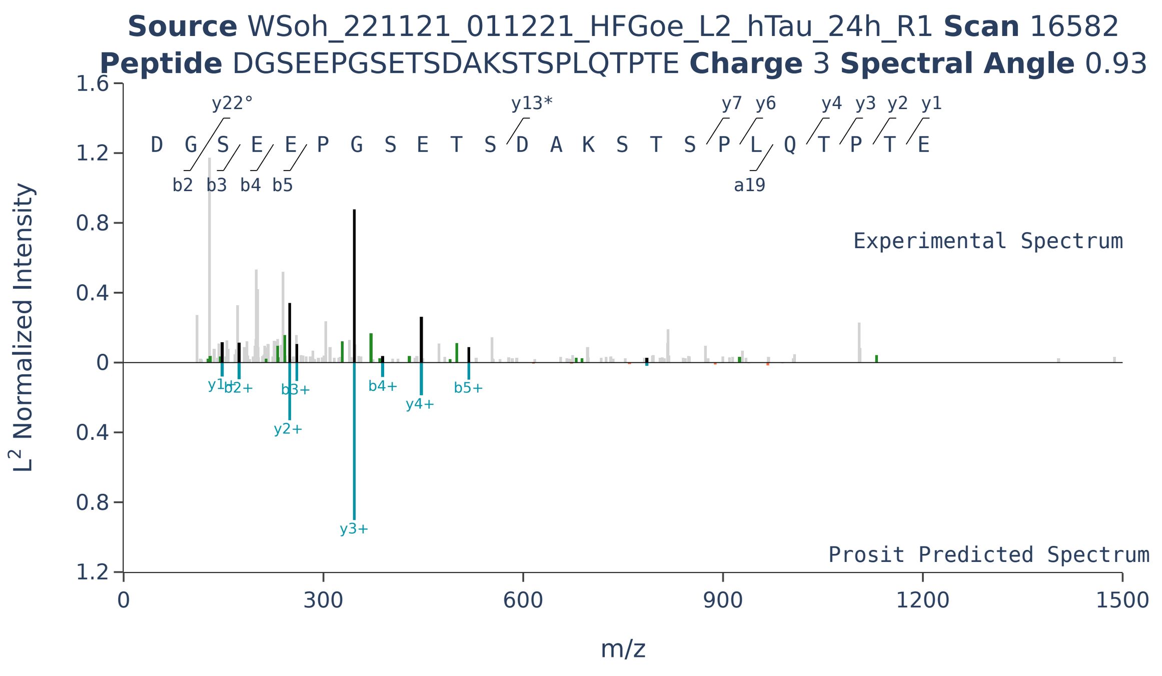
Source WSoh_221121_011221_HFGoe_L2_hTau_24h_R1 Scan 16582
Peptide DGSEEPGSETSDAKSTSPLQTPTE Charge 3 Spectral Angle 0.93
Experimental Spectrum
Prosit Predicted Spectrum
L² Normalized Intensity
m/z

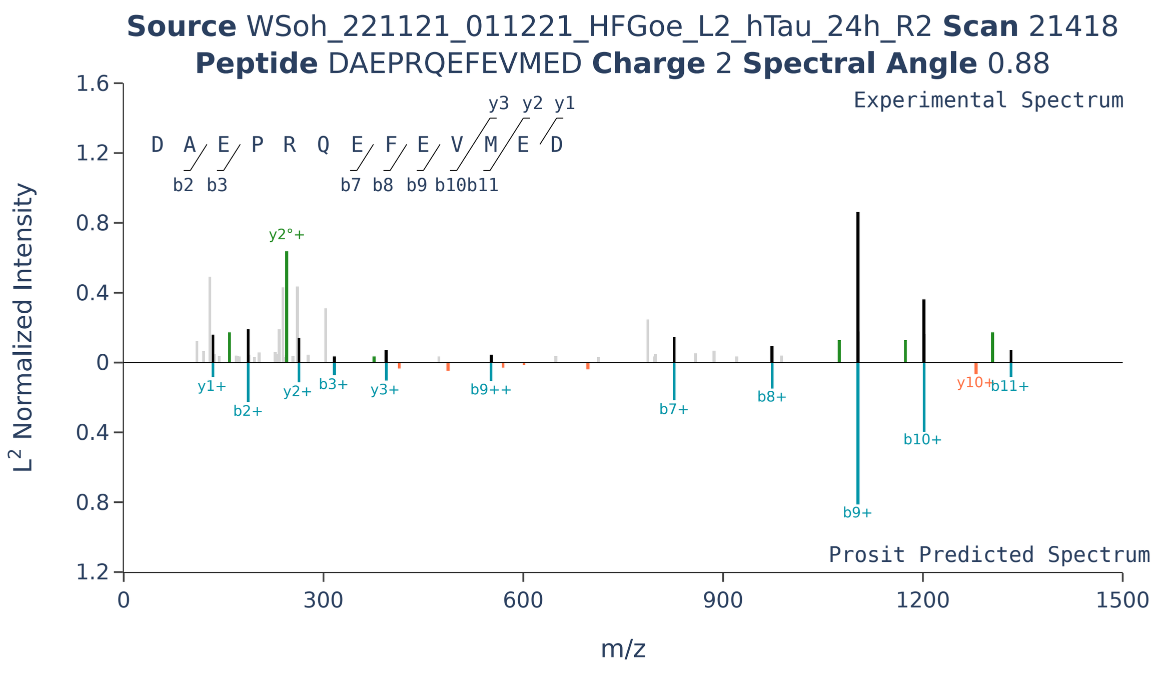
Source WSoh_221121_011221_HFGoe_L2_hTau_24h_R2 Scan 21418
Peptide DAEPRQEFEVMED Charge 2 Spectral Angle 0.88
Experimental Spectrum
Prosit Predicted Spectrum
L² Normalized Intensity
m/z

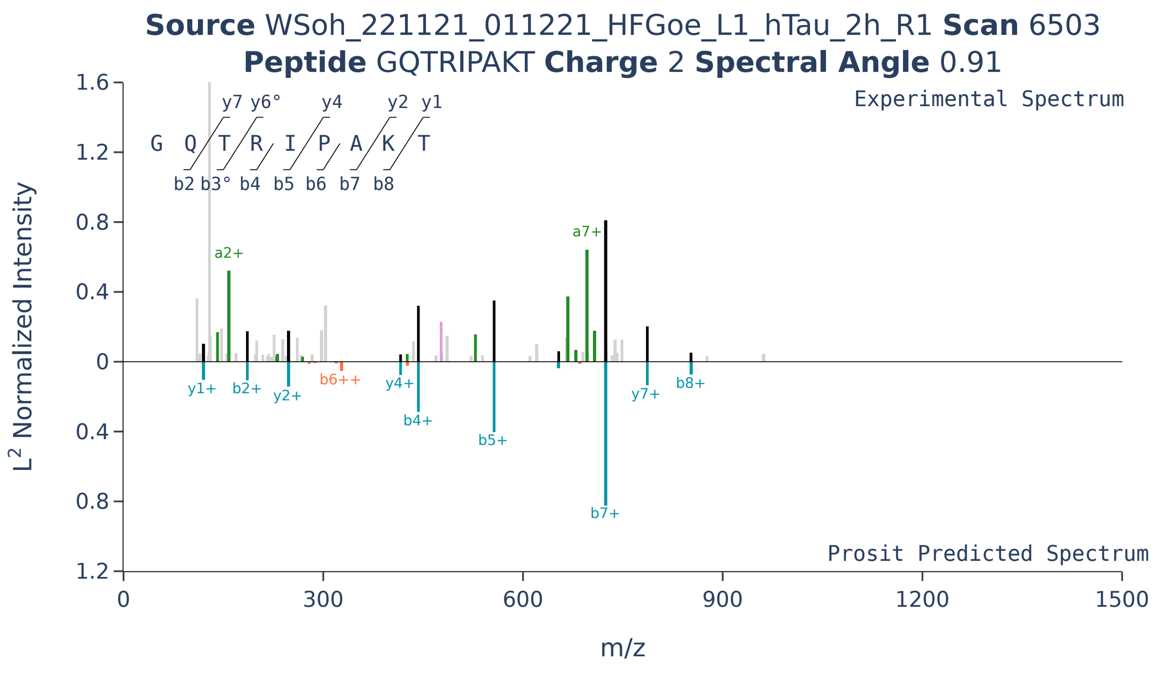
Source WSoh_221121_011221_HFGoe_L1_hTau_2h_R1 Scan 6503
Peptide GQTRIPAKT Charge 2 Spectral Angle 0.91
Experimental Spectrum
Prosit Predicted Spectrum
L² Normalized Intensity
m/z

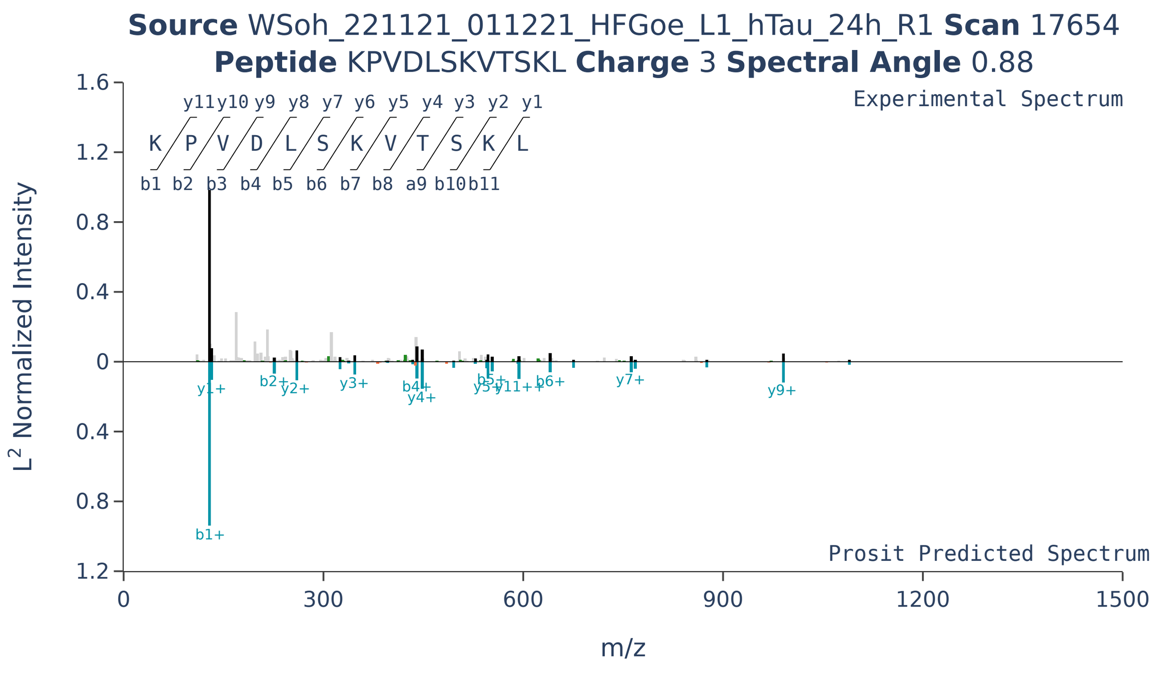
Source WSoh_221121_011221_HFGoe_L1_hTau_24h_R1 Scan 17654
Peptide KPVDLSKVTSKL Charge 3 Spectral Angle 0.88
Experimental Spectrum
Prosit Predicted Spectrum
L² Normalized Intensity
m/z

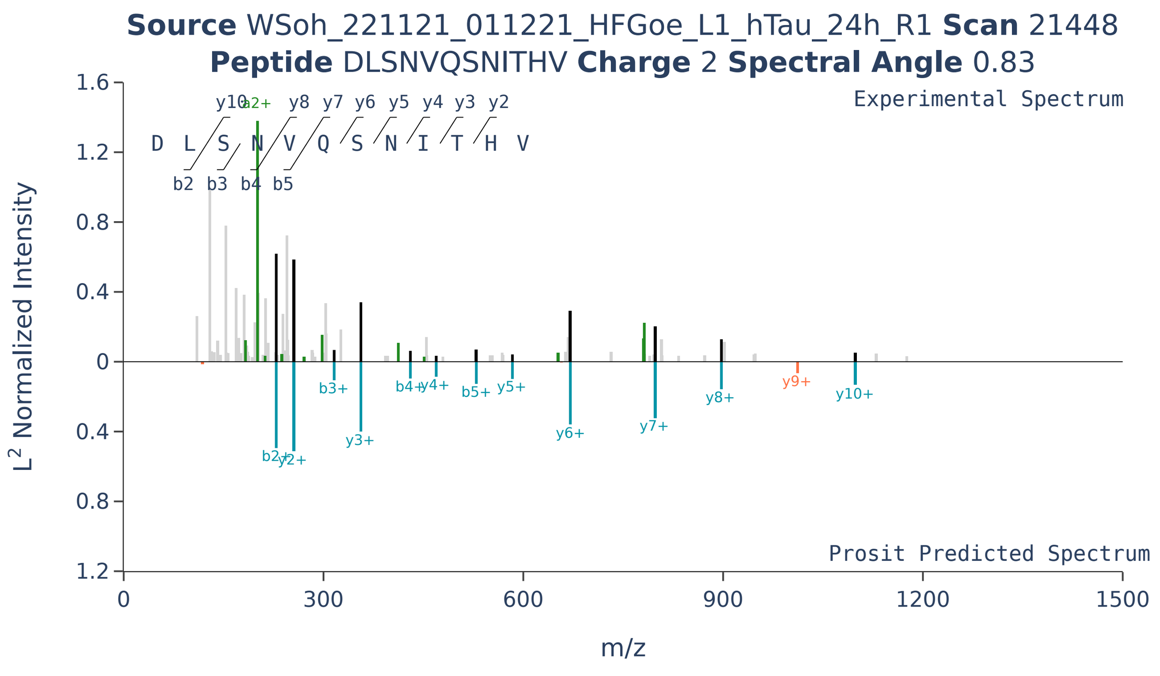
Source WSoh_221121_011221_HFGoe_L1_hTau_24h_R1 Scan 21448
Peptide DLSNVQSNITHV Charge 2 Spectral Angle 0.83
Experimental Spectrum
Prosit Predicted Spectrum
L² Normalized Intensity
m/z

Source WSoh_221121_011221_HFGoe_L1_hTau_24h_R2 **Scan** 18357
Peptide TEAGLKESPLQTPTE **Charge** 2 **Spectral Angle** 0.9

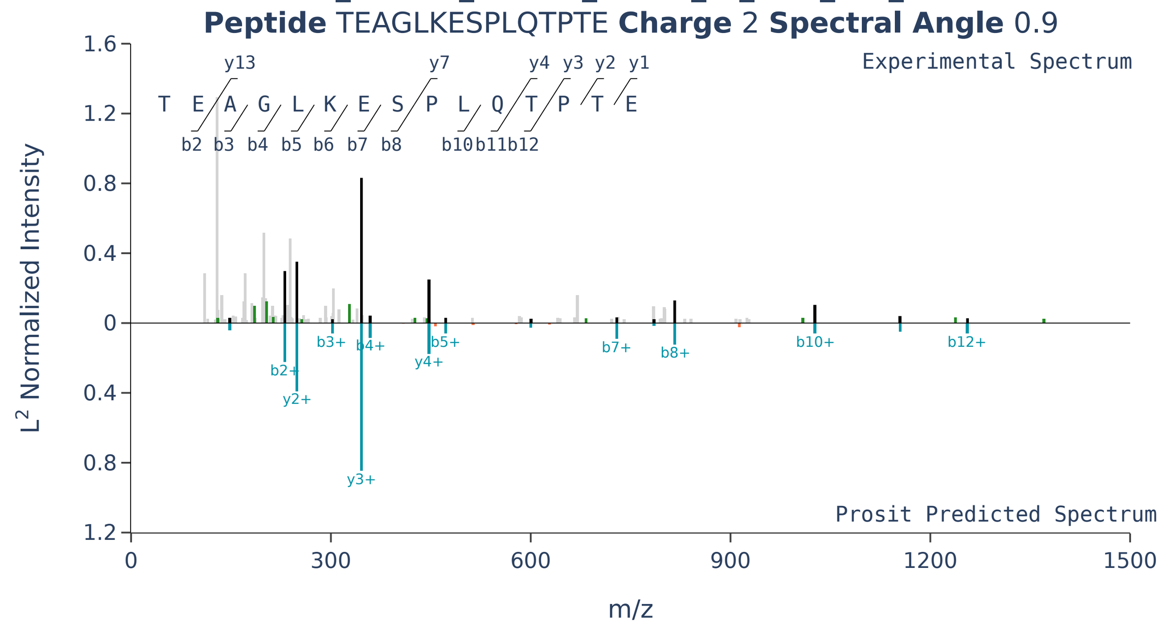

Source WSoh_221121_011221_HFGoe_L2_hTau_4h_R2 **Scan** 19265
Peptide TGEPRQEFEVME **Charge** 2 **Spectral Angle** 0.87

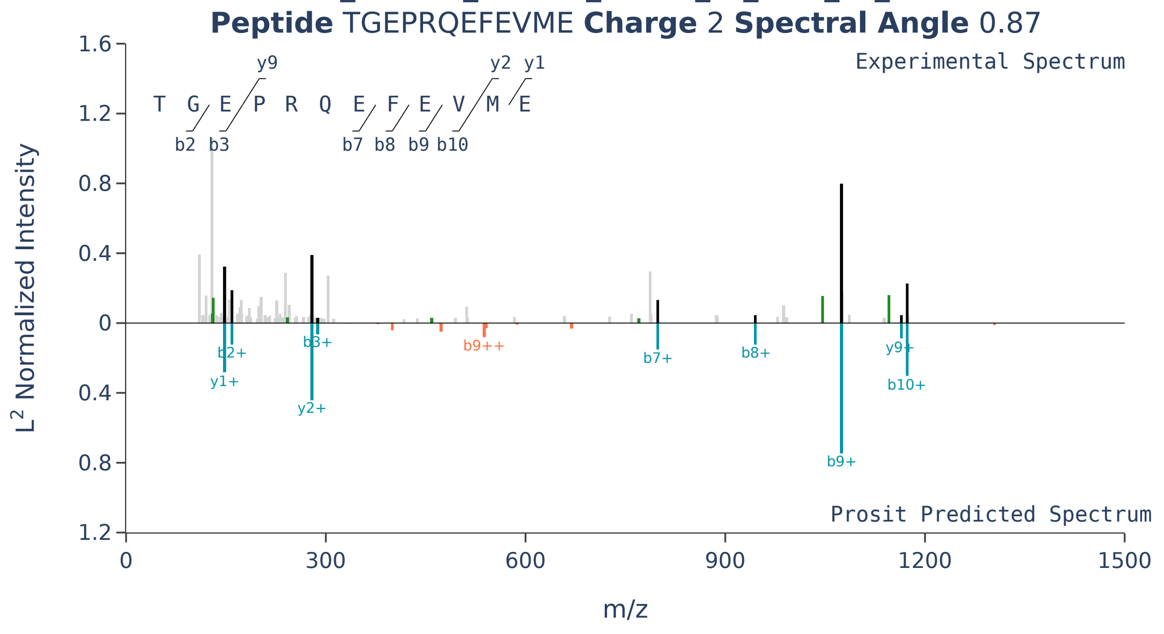

Source WSoh_221121_011221_HFGoe_L2_hTau_24h_R2 **Scan** 13706
Peptide AQQPHTEIPEGTTAEE **Charge** 2 **Spectral Angle** 0.85

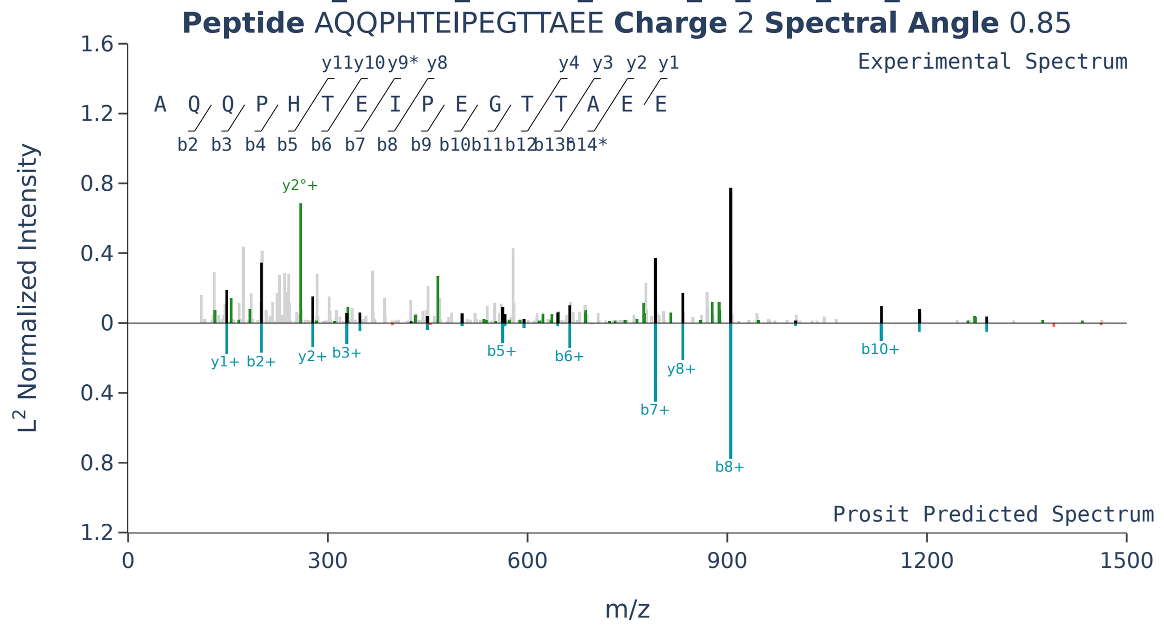

Source WSoh_221121_011221_HFGoe_L1_hTau_4h_R1 **Scan** 8008
Peptide KSEKLDFKDRVQSH **Charge** 4 **Spectral Angle** 0.83

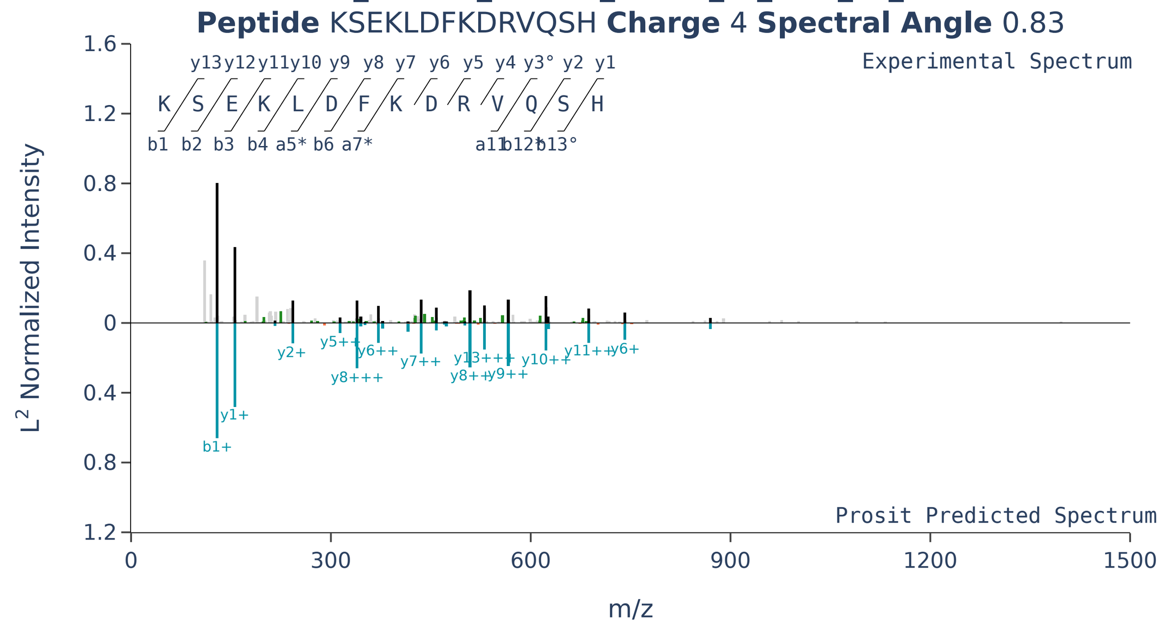

Source WSoh_221121_011221_HFGoe_L1_hTau_2h_R1 **Scan** 17320
Peptide GNLDNITHV **Charge** 2 **Spectral Angle** 0.85

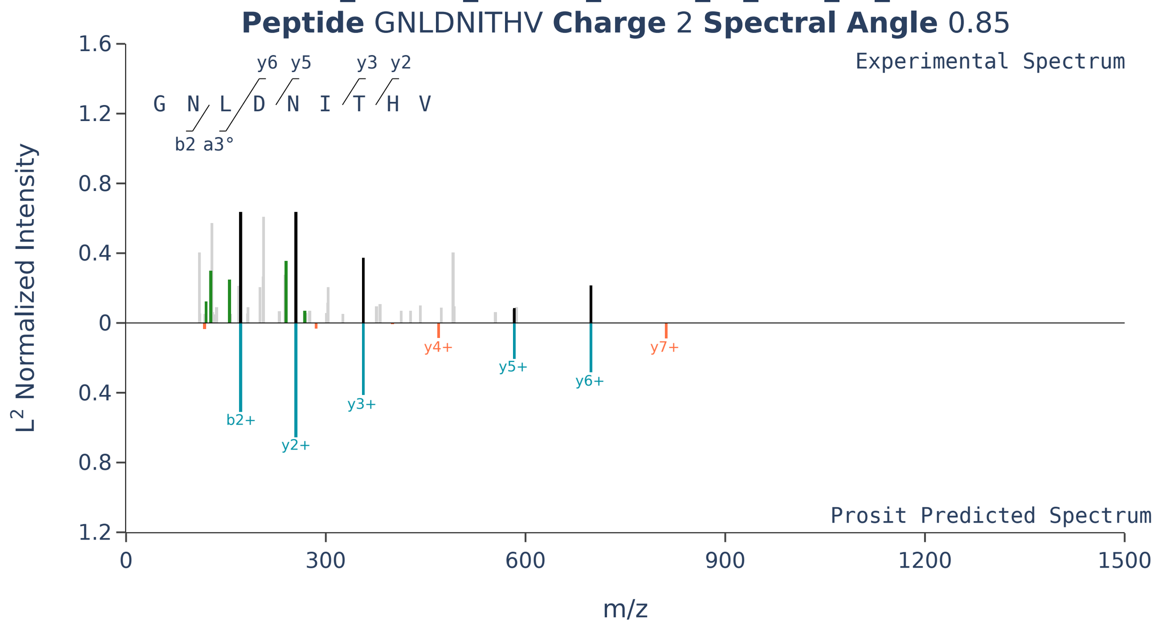

Source WSoh_221121_011221_HFGoe_L2_hTau_24h_R2 **Scan** 8336
Peptide DLSNVQSKCSKD **Charge** 2 **Spectral Angle** 0.82

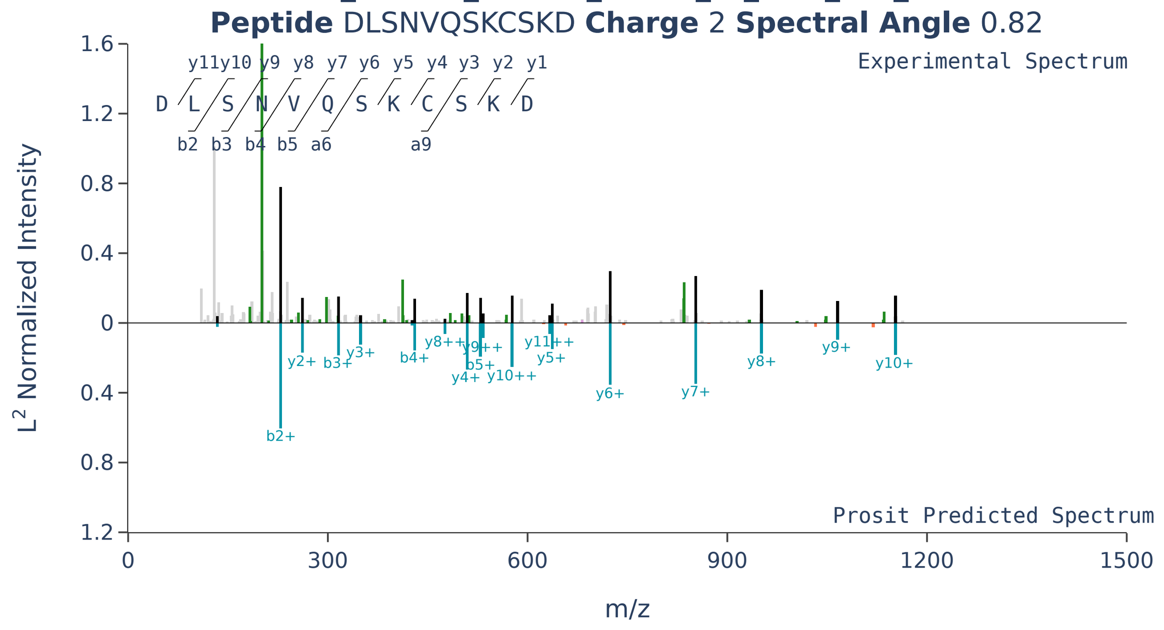

Source WSoh_221121_011221_HFGoe_L1_hTau_24h_R2 **Scan** 11022
Peptide NKKLDLSNVQSKCSKDNIKHV **Charge** 3 **Spectral Angle** 0.93

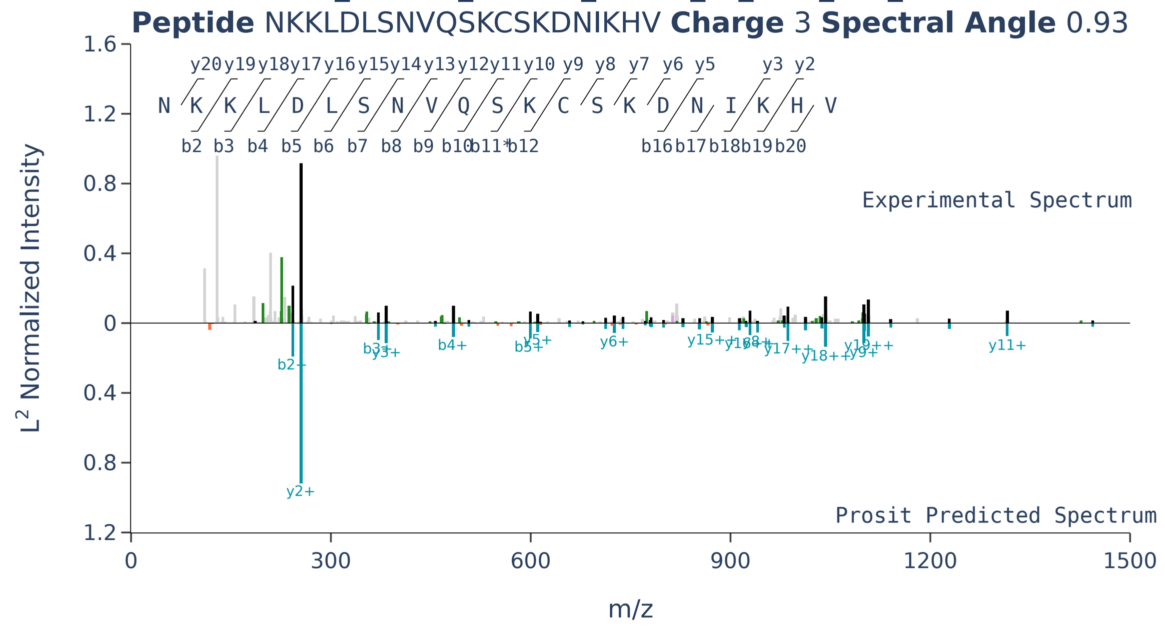

Source WSoh_221121_011221_HFGoe_L1_hTau_4h_R2 **Scan** 14710
Peptide VYKPVDLSKVTGGK **Charge** 2 **Spectral Angle** 0.88

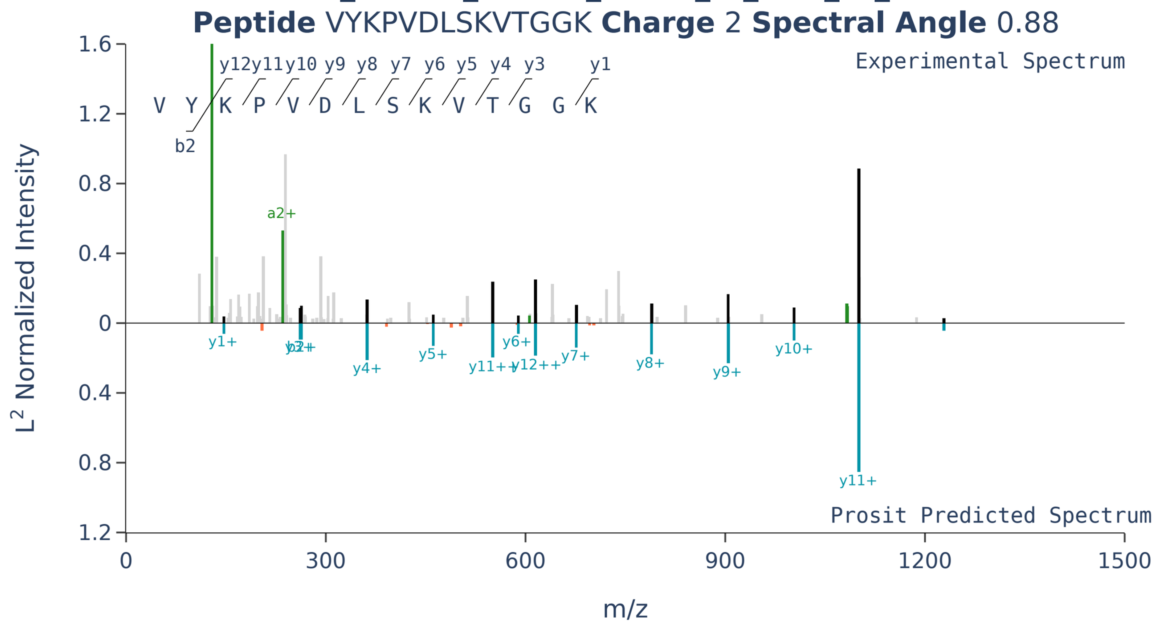

Source WSoh_221121_011221_HFGoe_L1_hTau_24h_R2 **Scan** 10038
Peptide SVYKPVD **Charge** 2 **Spectral Angle** 0.95

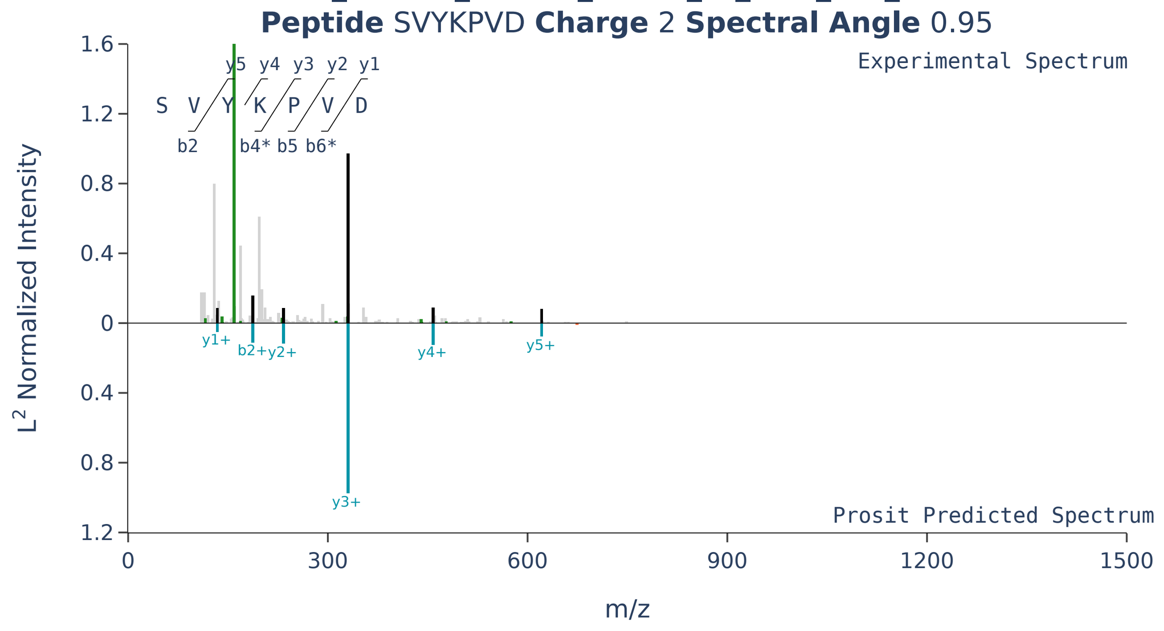

Source WSoh_221121_011221_HFGoe_L1_hTau_4h_R1 **Scan** 19186
Peptide GAEPRQEFEVME **Charge** 2 **Spectral Angle** 0.9

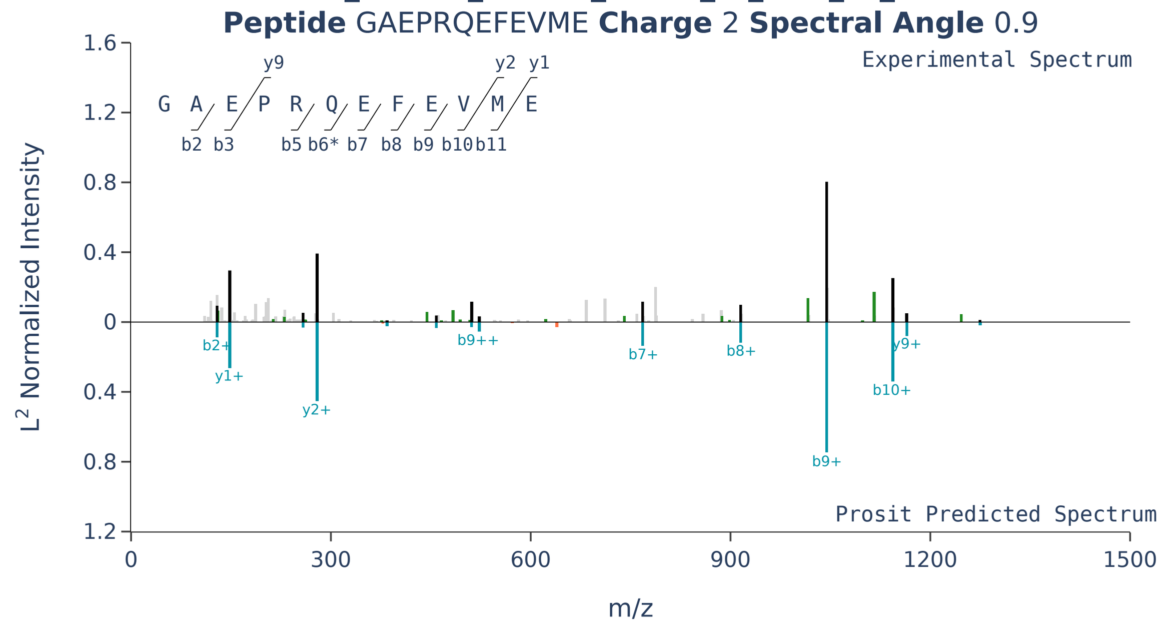

Source WSoh_221121_011221_HFGoe_L1_hTau_24h_R2 **Scan** 8783
Peptide KGSLGNIHHKPGGGQVEV **Charge** 2 **Spectral Angle** 0.85

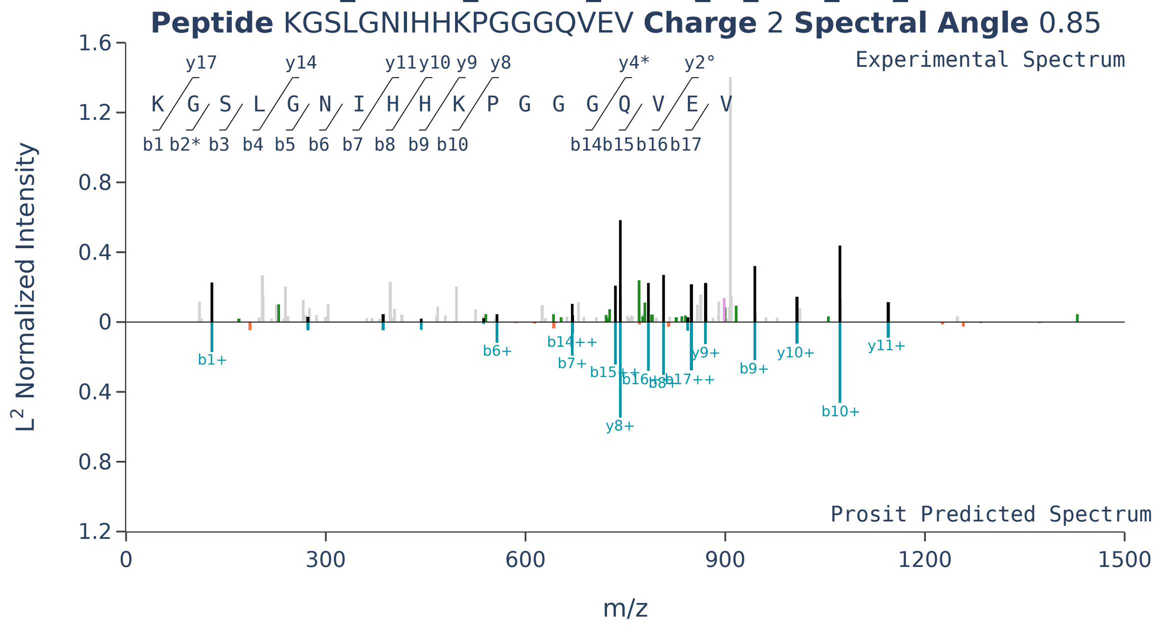

Source WSoh_221121_011221_HFGoe_L1_hTau_4h_R1 **Scan** 10287
Peptide KLDFKDRVQSS **Charge** 2 **Spectral Angle** 0.8

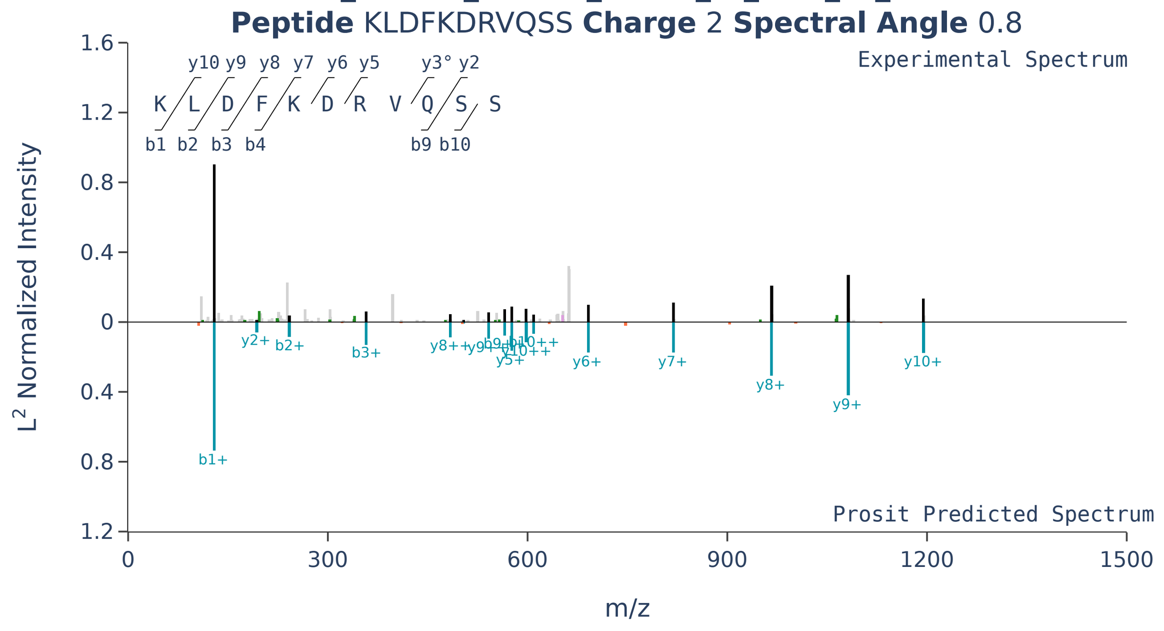

Source WSoh_221121_011221_HFGoe_L2_hTau_24h_R1 **Scan** 8928
Peptide SNVQSRLQT **Charge** 2 **Spectral Angle** 0.85

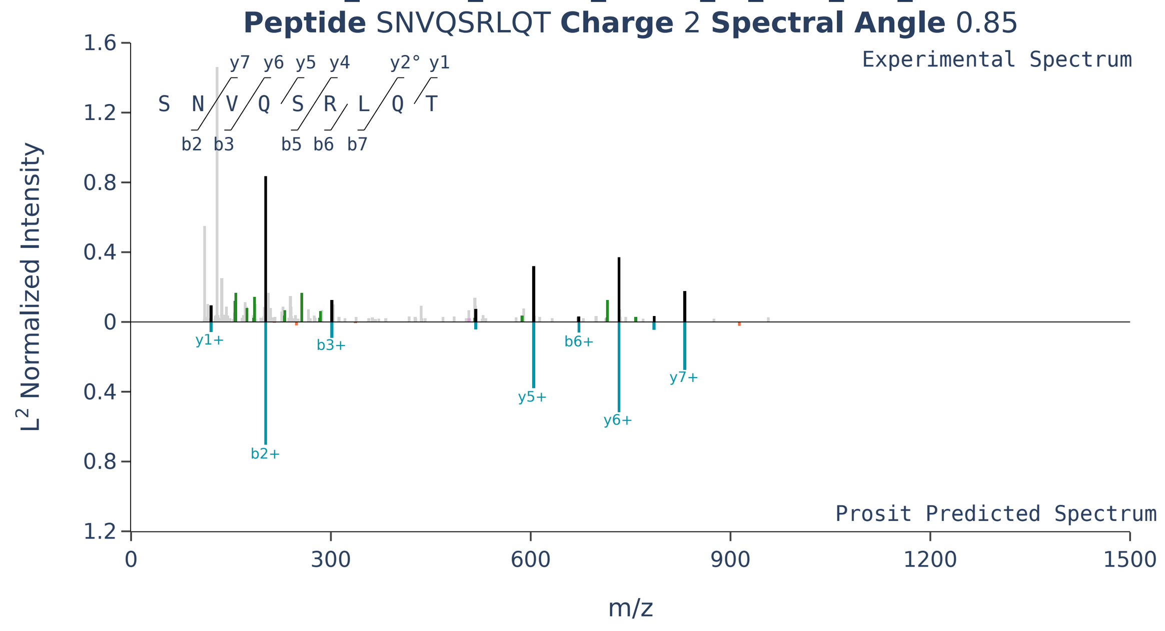

Source WSoh_221121_011221_HFGoe_L2_hTau_4h_R1 **Scan** 11161
Peptide GQLAKQGL **Charge** 2 **Spectral Angle** 0.84

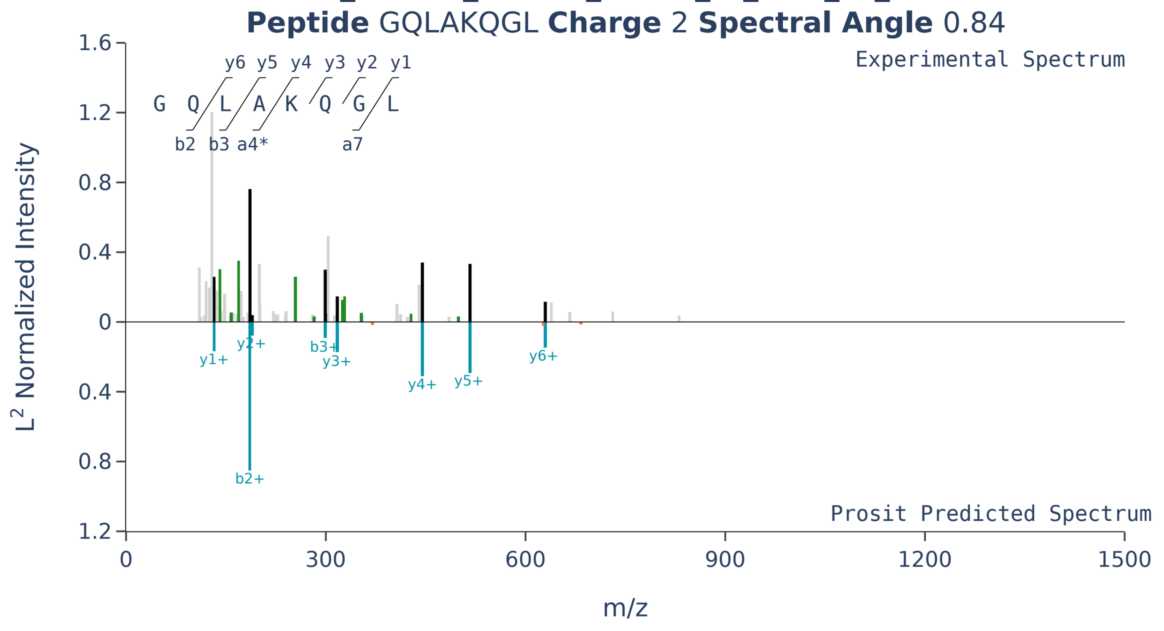

Source WSoh_221121_011221_HFGoe_L2_hTau_4h_R1 **Scan** 19191
Peptide EGTPRQEFEVME **Charge** 2 **Spectral Angle** 0.92

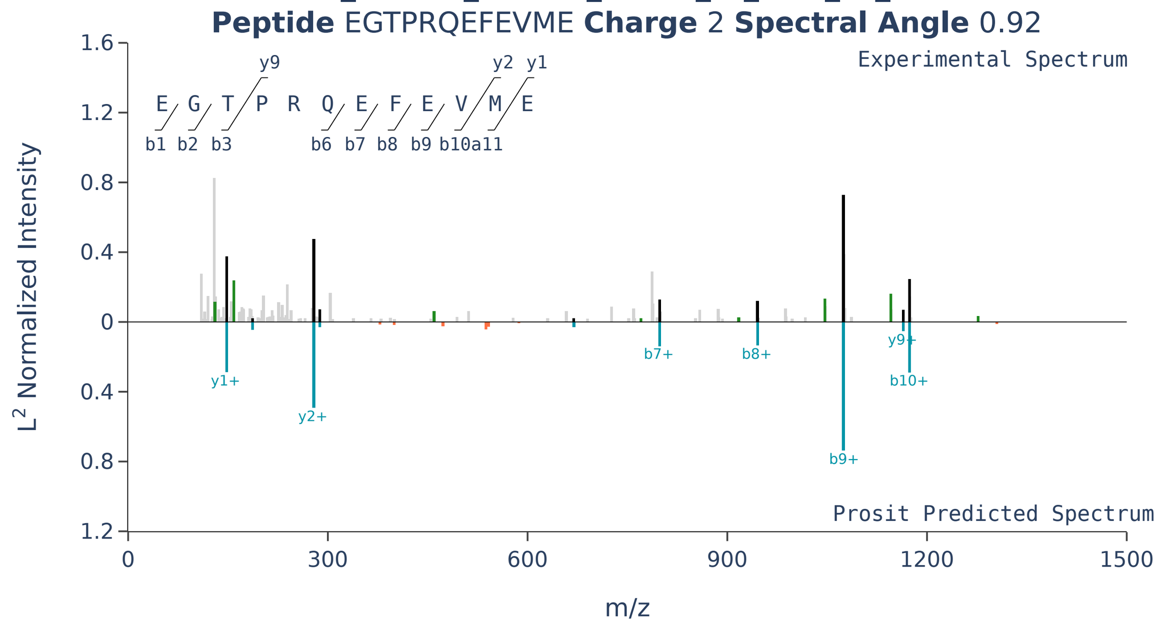

Source WSoh_221121_011221_HFGoe_L1_hTau_24h_R1 **Scan** 14610
Peptide DAAQPHTEIPEGTTAEE **Charge** 2 **Spectral Angle** 0.9

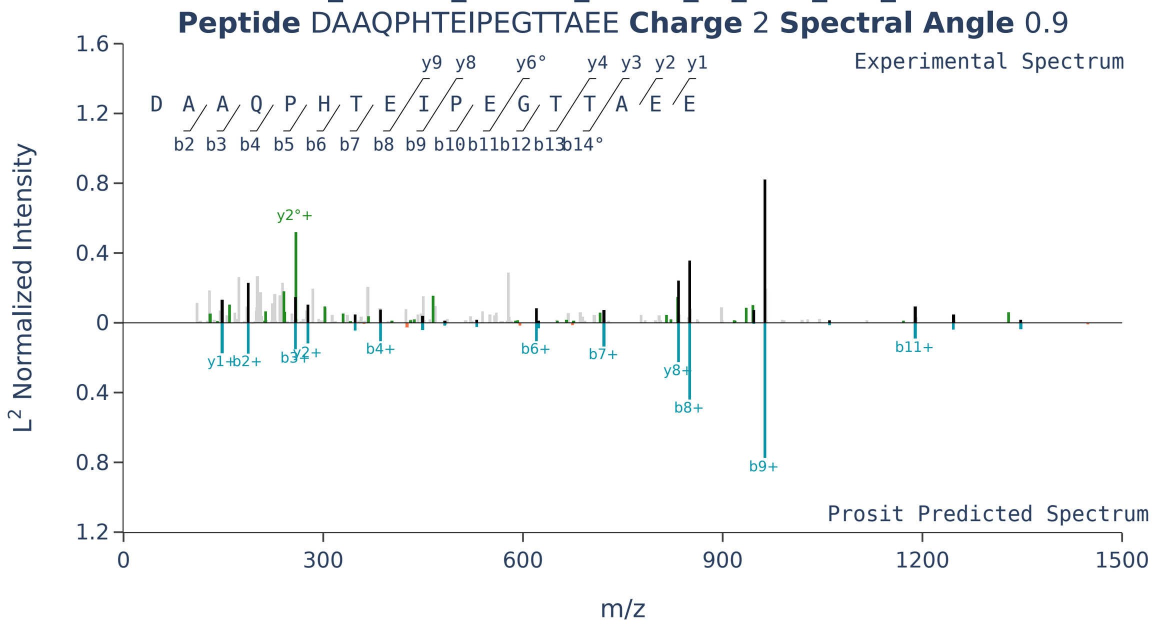

Source WSoh_221121_011221_HFGoe_L1_hTau_24h_R2 **Scan** 14815
Peptide EDAAQPHTEIPEGTTAEE **Charge** 2 **Spectral Angle** 0.89

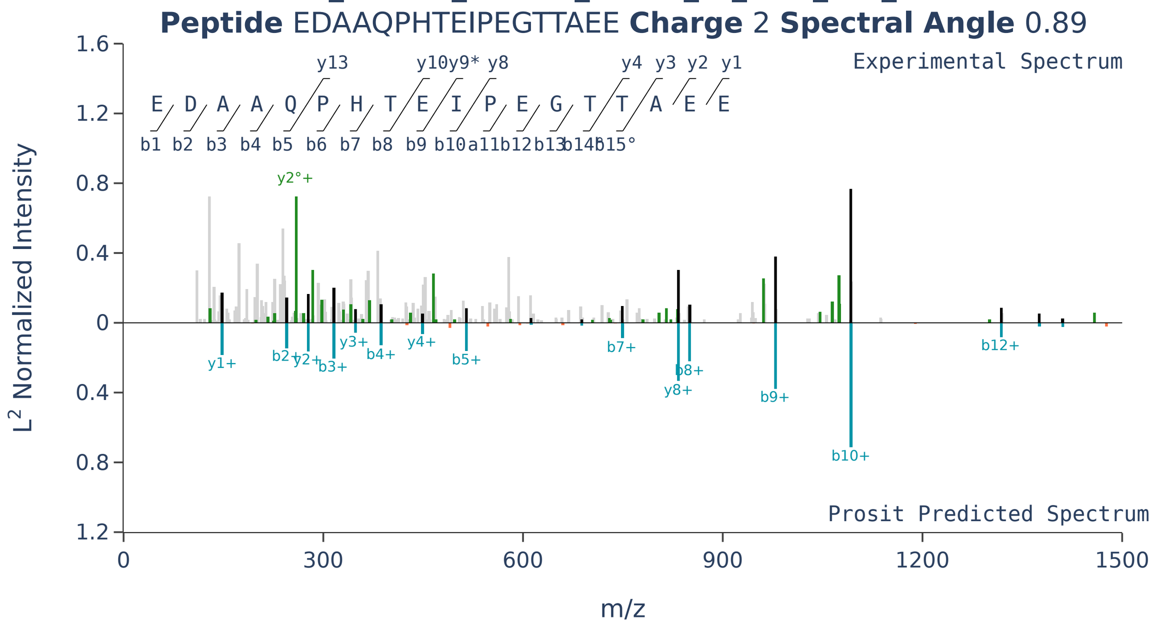

Source WSoh_221121_011221_HFGoe_L2_hTau_24h_R1 **Scan** 22862
Peptide VYKPVDLSKVTSRL **Charge** 3 **Spectral Angle** 0.91

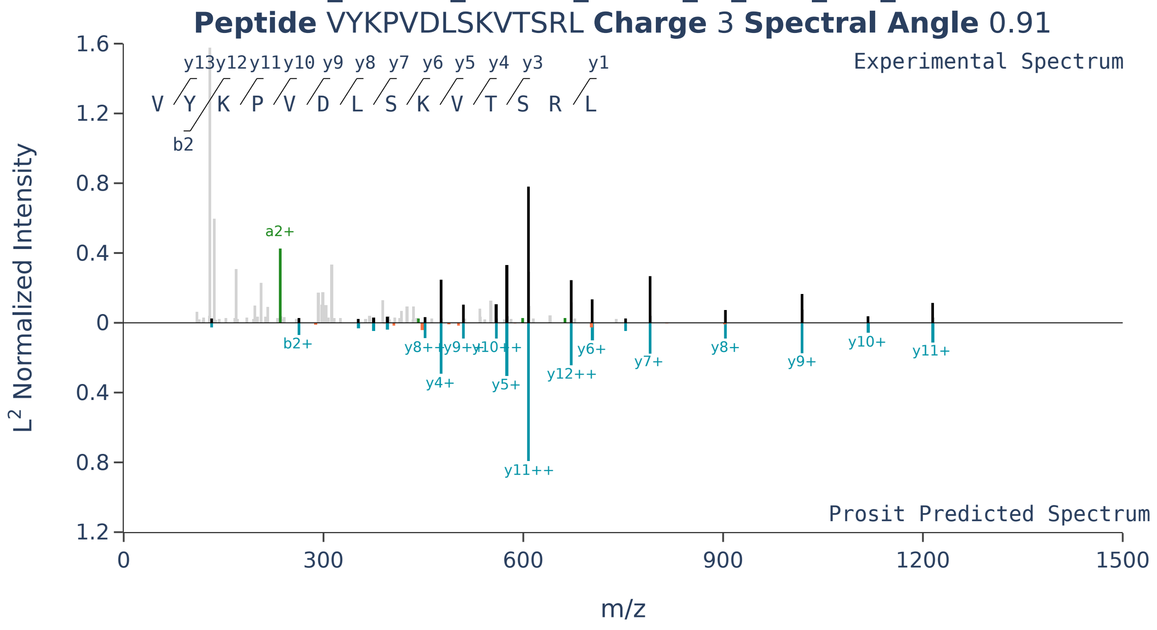

Source WSoh_221121_011221_HFGoe_L1_hTau_24h_R2 **Scan** 12647
Peptide GSDLGNIHHKPGGGQVEV **Charge** 2 **Spectral Angle** 0.84

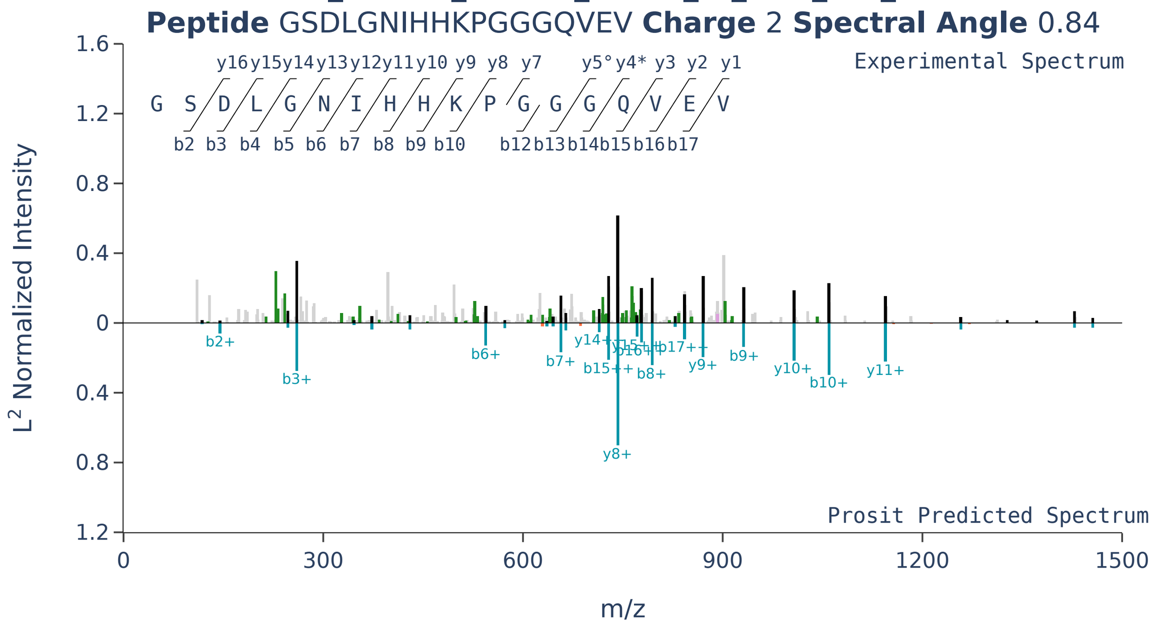

Source WSoh_221121_011221_HFGoe_L2_hTau_24h_R2 **Scan** 19168
Peptide GDTHGAEIVYKSPVV **Charge** 3 **Spectral Angle** 0.86

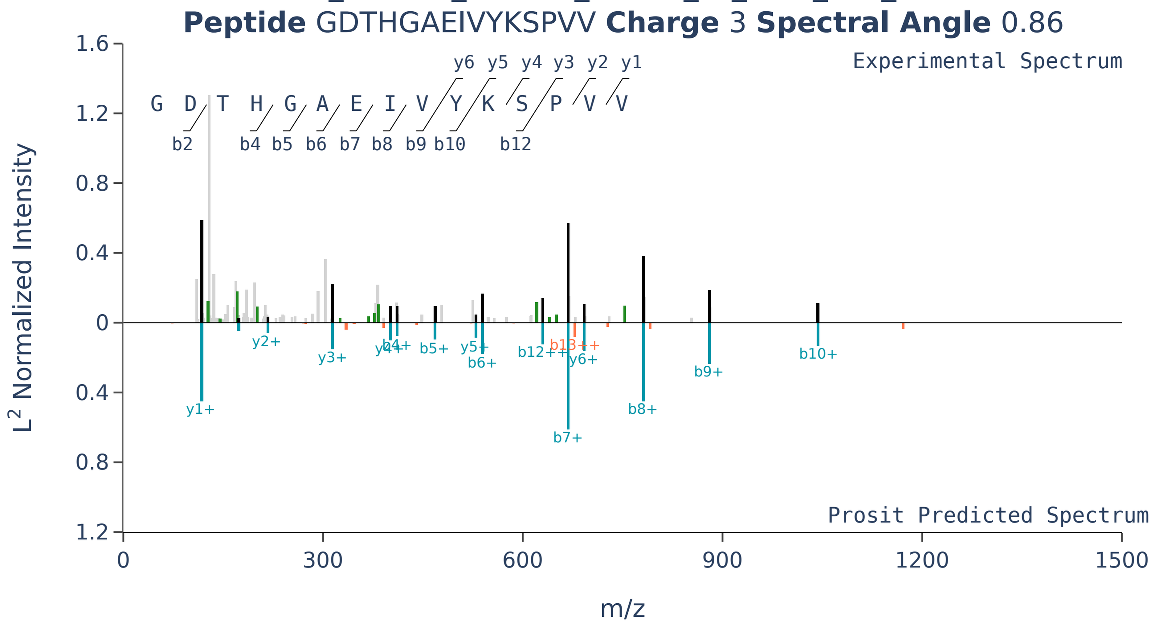

Source WSoh_221121_011221_HFGoe_L2_hTau_24h_R1 **Scan** 11859
Peptide AAQPHTEIPEGTQE **Charge** 2 **Spectral Angle** 0.9

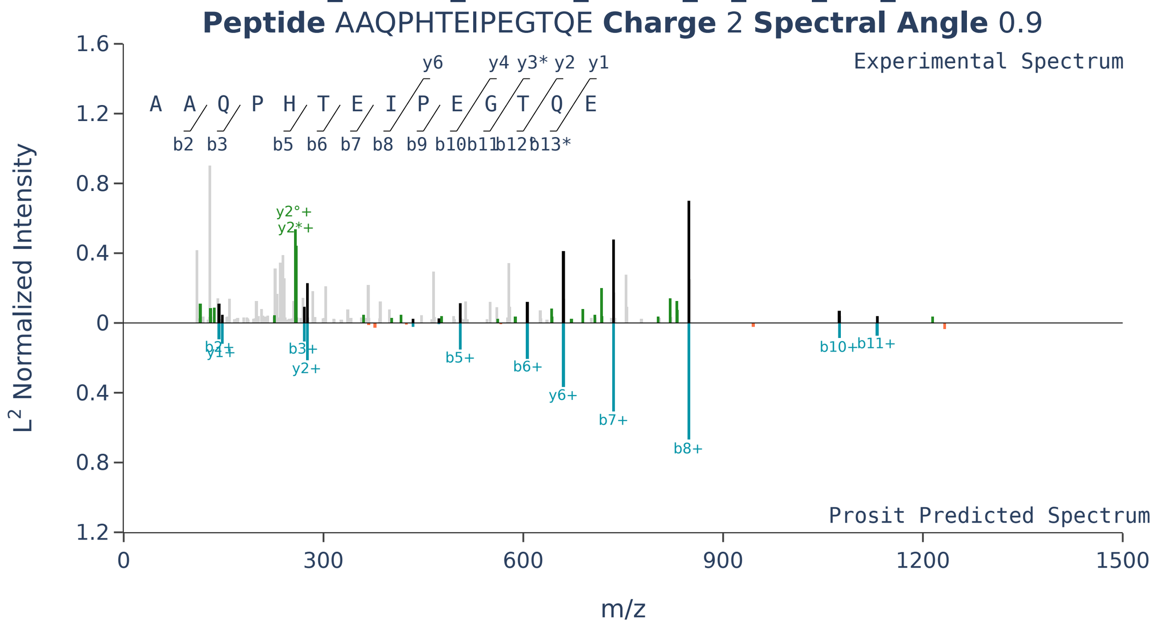

Source WSoh_221121_011221_HFGoe_L2_hTau_24h_R1 **Scan** 16102
Peptide VYKPVDLSKVTSR **Charge** 3 **Spectral Angle** 0.92

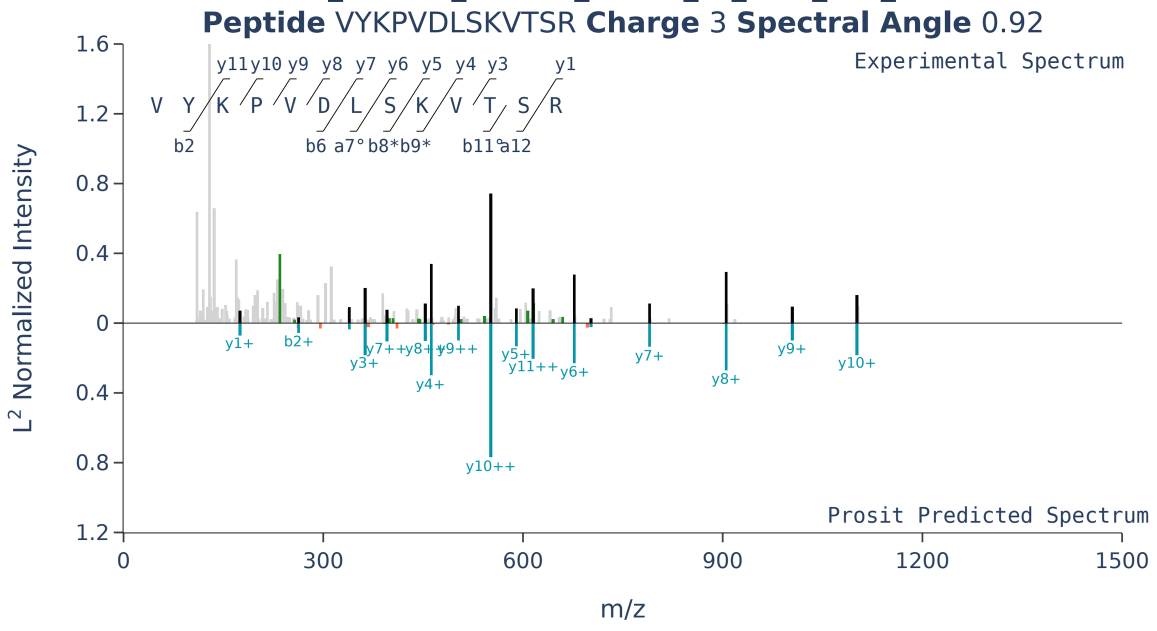

Source WSoh_221121_011221_HFGoe_L2_hTau_4h_R2 **Scan** 8930
Peptide SNVQSTRI **Charge** 2 **Spectral Angle** 0.93

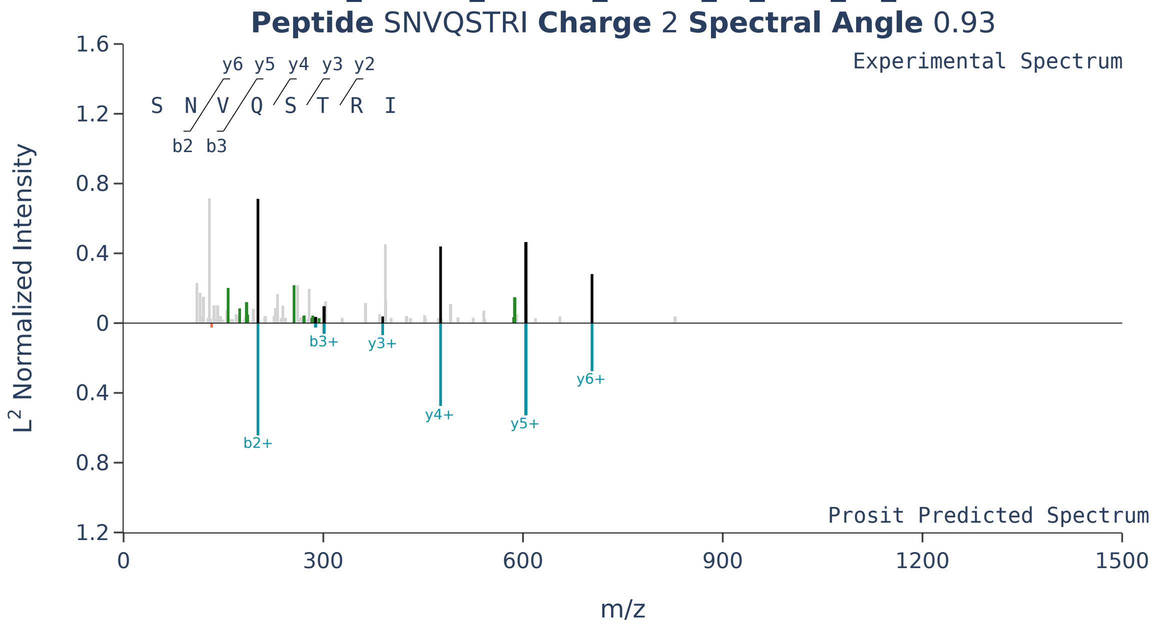

Source WSoh_221121_011221_HFGoe_L1_hTau_24h_R2 **Scan** 12124
Peptide SKTPSLPTPPTREPKKV **Charge** 4 **Spectral Angle** 0.89

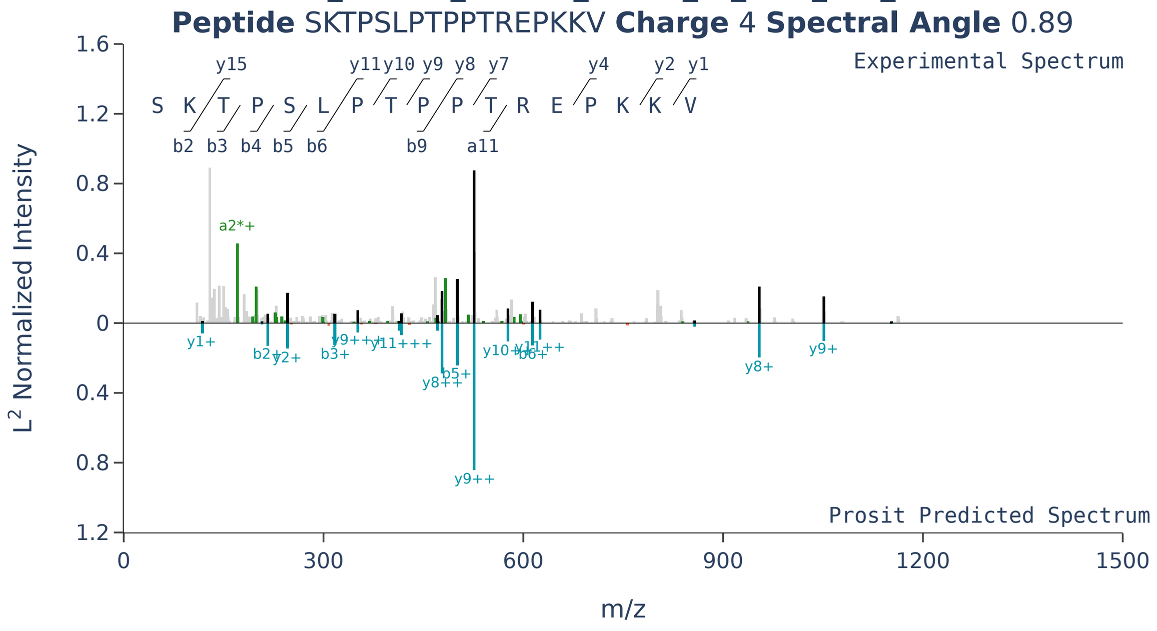

Source WSoh_221121_011221_HFGoe_L2_hTau_24h_R2 **Scan** 7527
Peptide EHAGTYGLGDRKD **Charge** 3 **Spectral Angle** 0.9

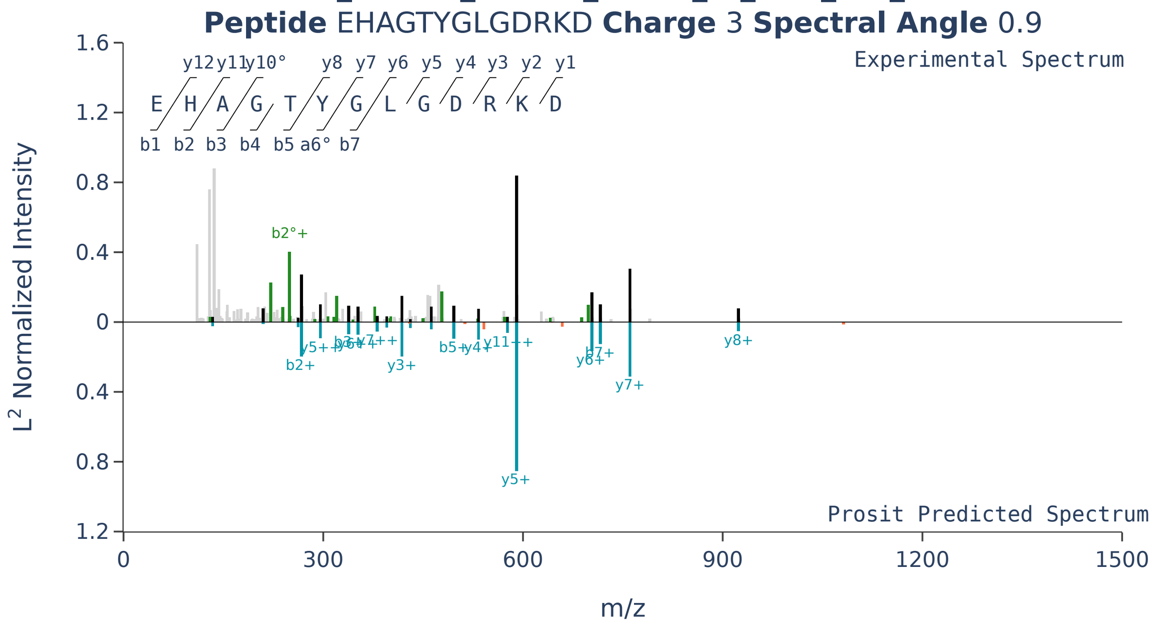

Source WSoh_221121_011221_HFGoe_L2_hTau_24h_R1 **Scan** 19420
Peptide NKKLDLSNVQSL **Charge** 2 **Spectral Angle** 0.84

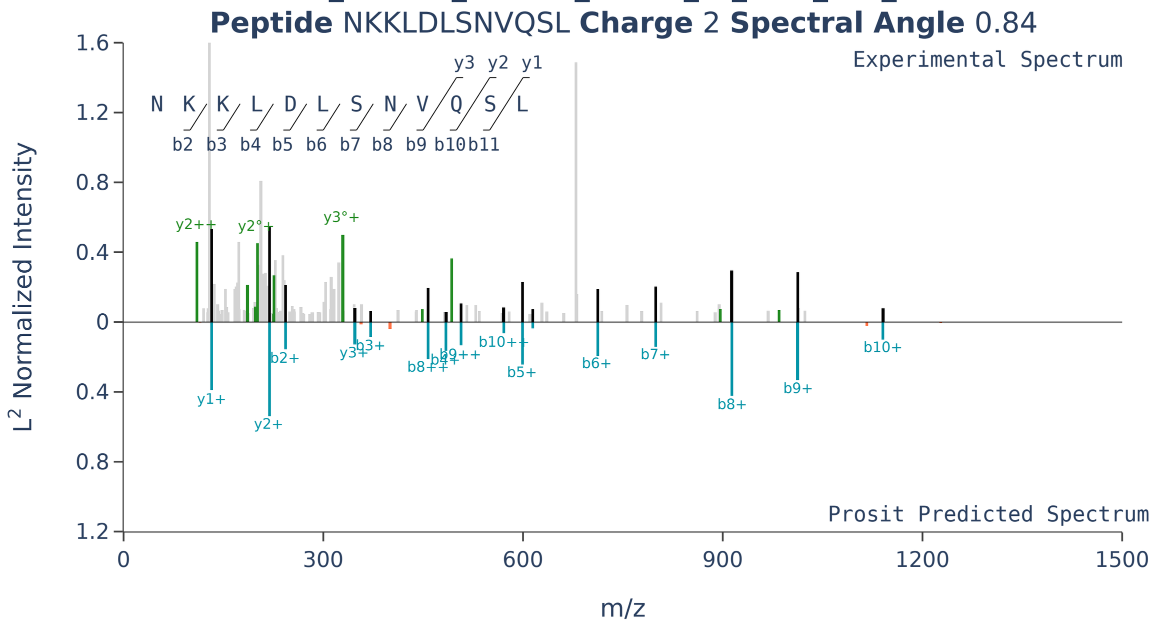

Source WSoh_221121_011221_HFGoe_L2_hTau_24h_R1 **Scan** 8176
Peptide EVSGDTSPRHL **Charge** 2 **Spectral Angle** 0.91

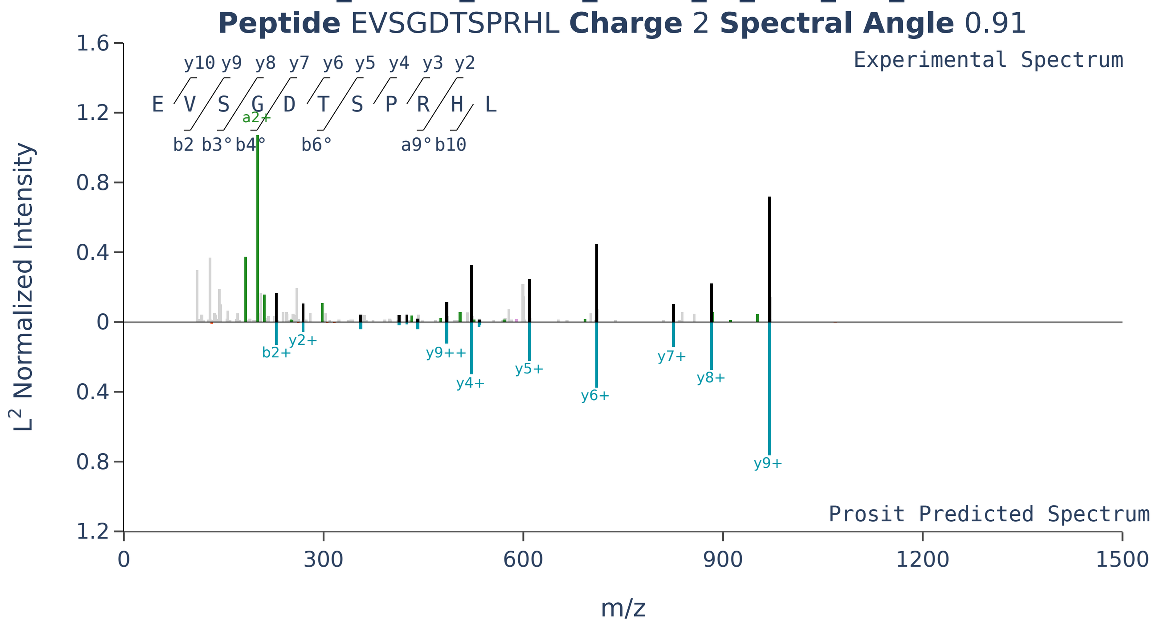

Source WSoh_221121_011221_HFGoe_L1_hTau_24h_R2 **Scan** 19839
Peptide EAAGHVTQAAGIGDTPSLED **Charge** 2 **Spectral Angle** 0.87

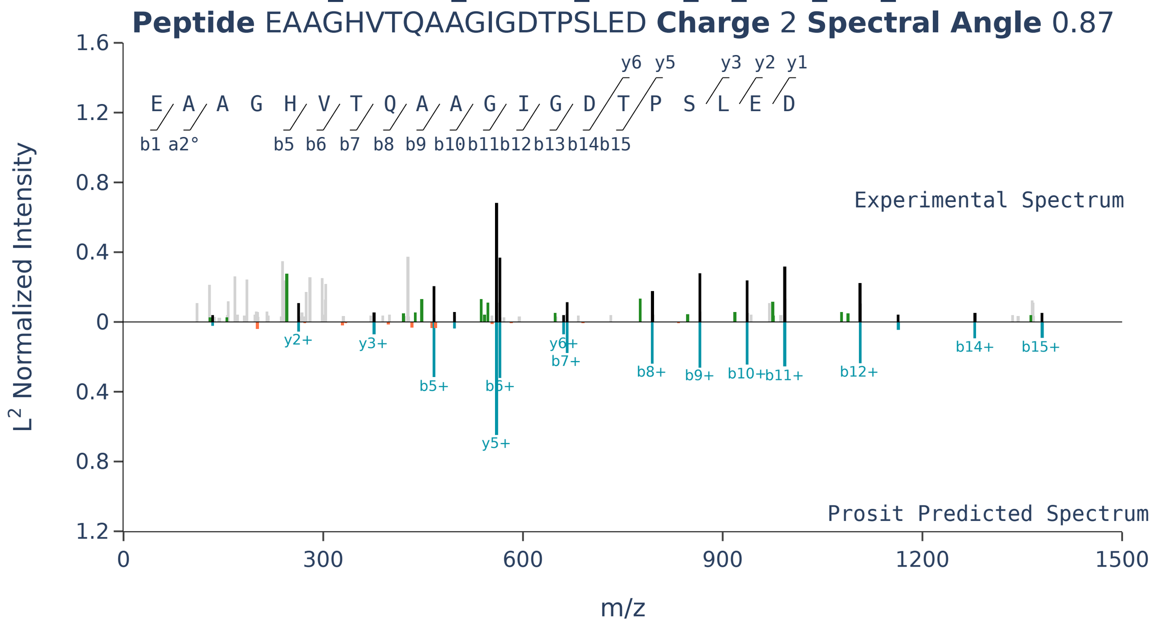

Source WSoh_221121_011221_HFGoe_L2_hTau_4h_R1 **Scan** 16679
Peptide VYKPVDLSKGSLG **Charge** 3 **Spectral Angle** 0.8

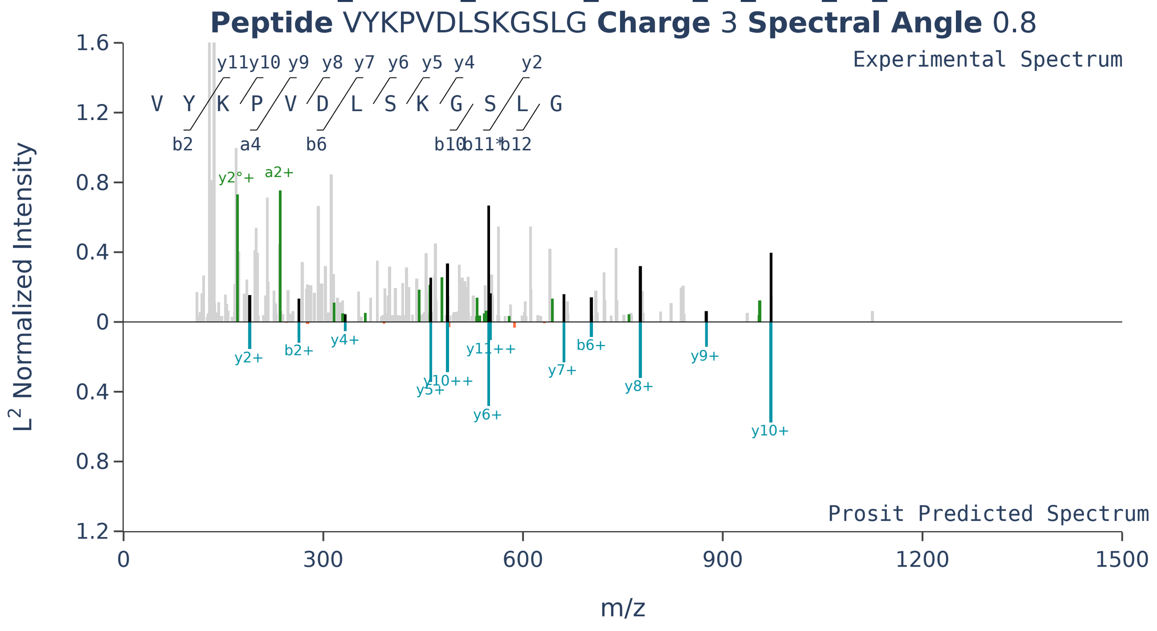

Source WSoh_221121_011221_HFGoe_L1_hTau_24h_R2 **Scan** 11437
Peptide GKIGSTENL **Charge** 2 **Spectral Angle** 0.91

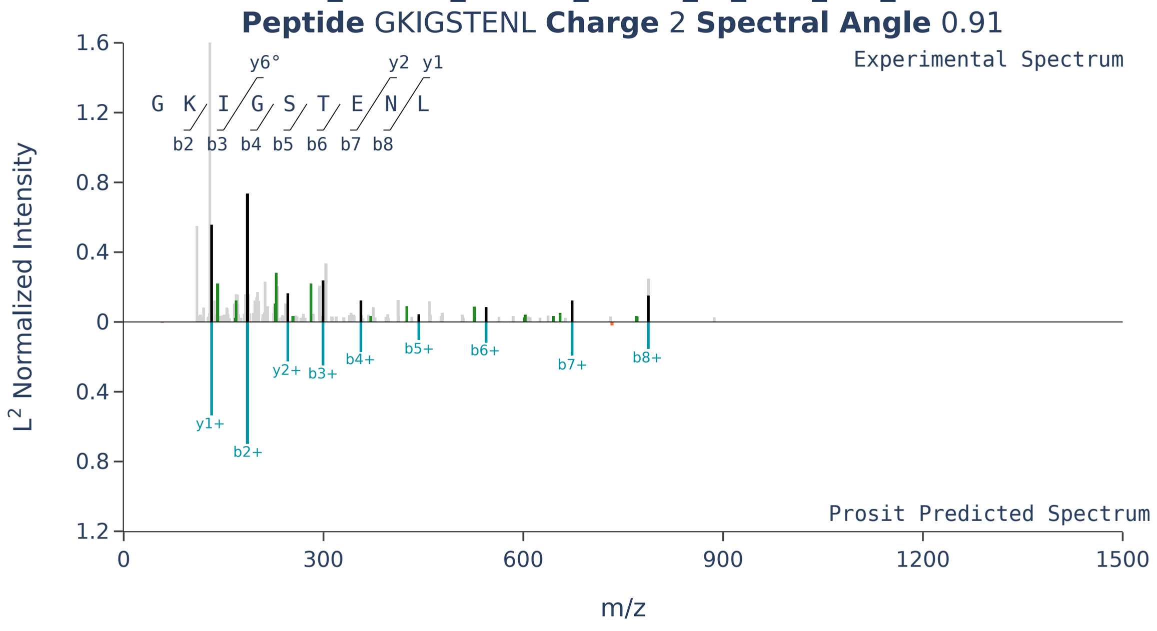

Source WSoh_221121_011221_HFGoe_L2_hTau_24h_R2 **Scan** 8195
Peptide SNVQSKIGSTE **Charge** 2 **Spectral Angle** 0.91

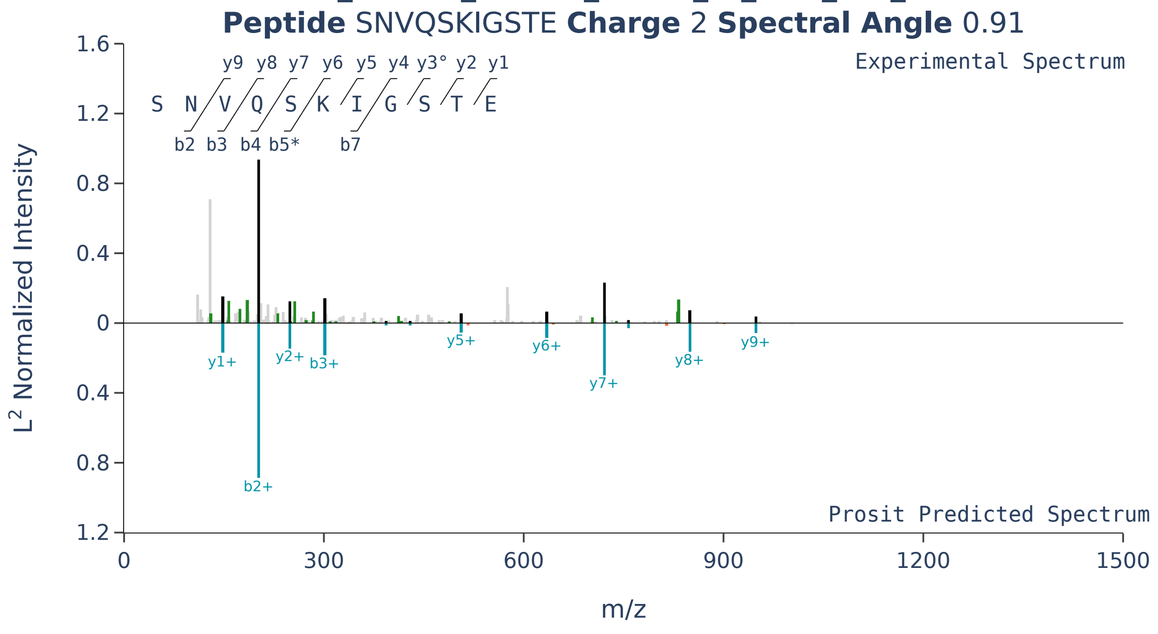

Source WSoh_221121_011221_HFGoe_L2_hTau_4h_R1 **Scan** 10258
Peptide KSEKLDFKDRVQSA **Charge** 2 **Spectral Angle** 0.79

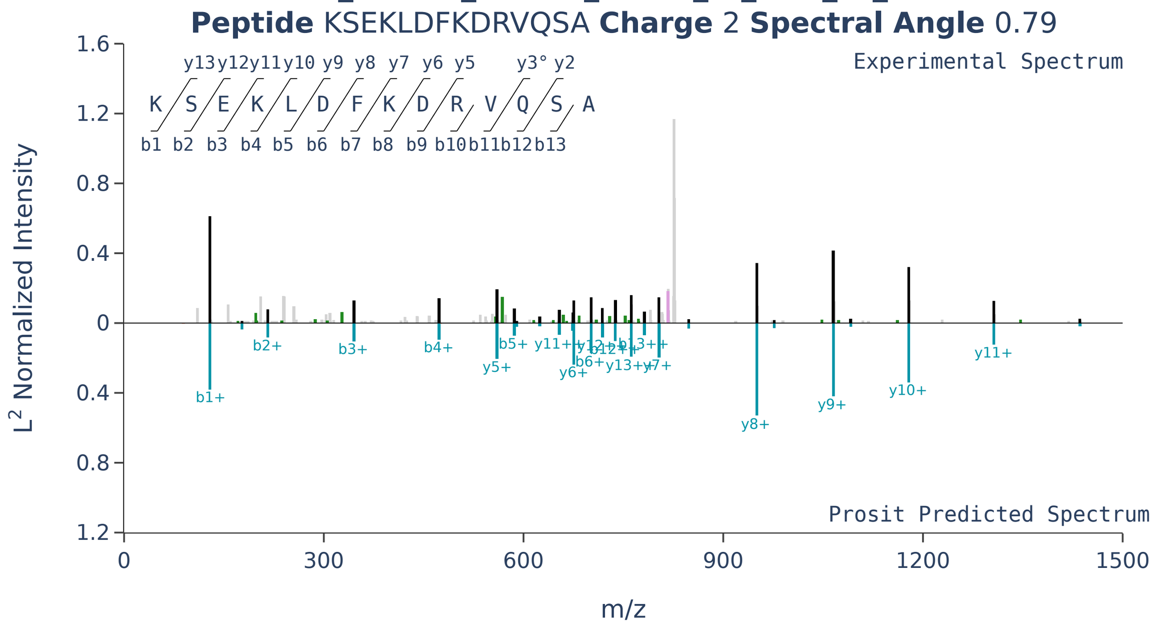

Source WSoh_221121_011221_HFGoe_L1_hTau_24h_R2 **Scan** 13760
Peptide AAQPHTEIPEGTTAEEAGEGAPGKQA **Charge** 3 **Spectral Angle** 0.91

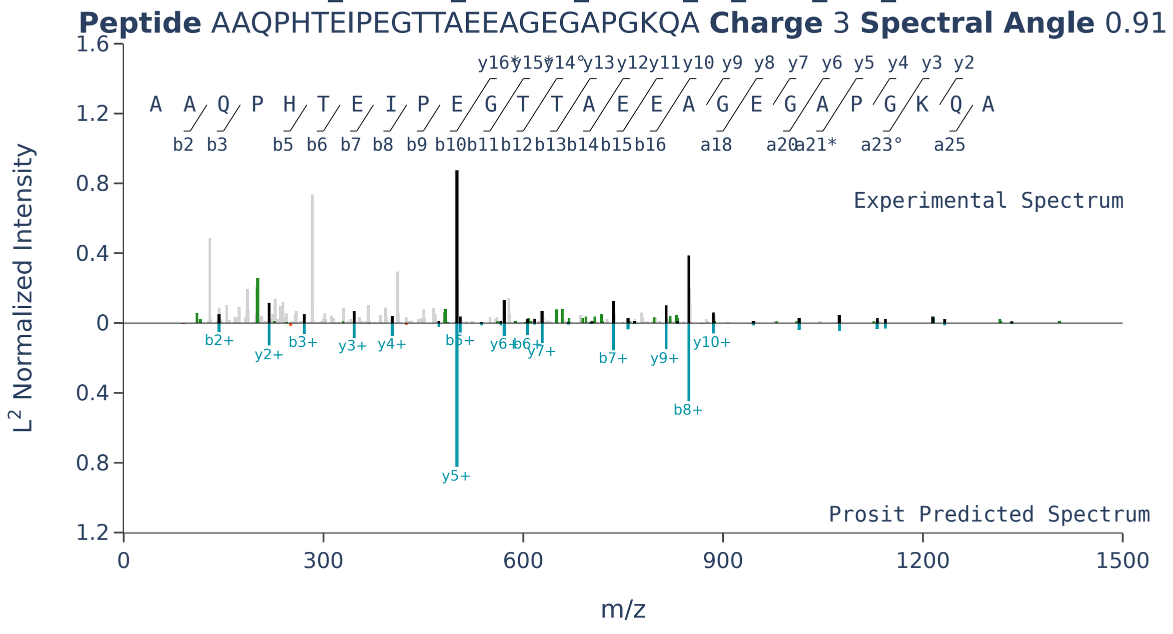

Source WSoh_221121_011221_HFGoe_L2_hTau_4h_R1 **Scan** 9518
Peptide ADEVSASLAKH **Charge** 3 **Spectral Angle** 0.9

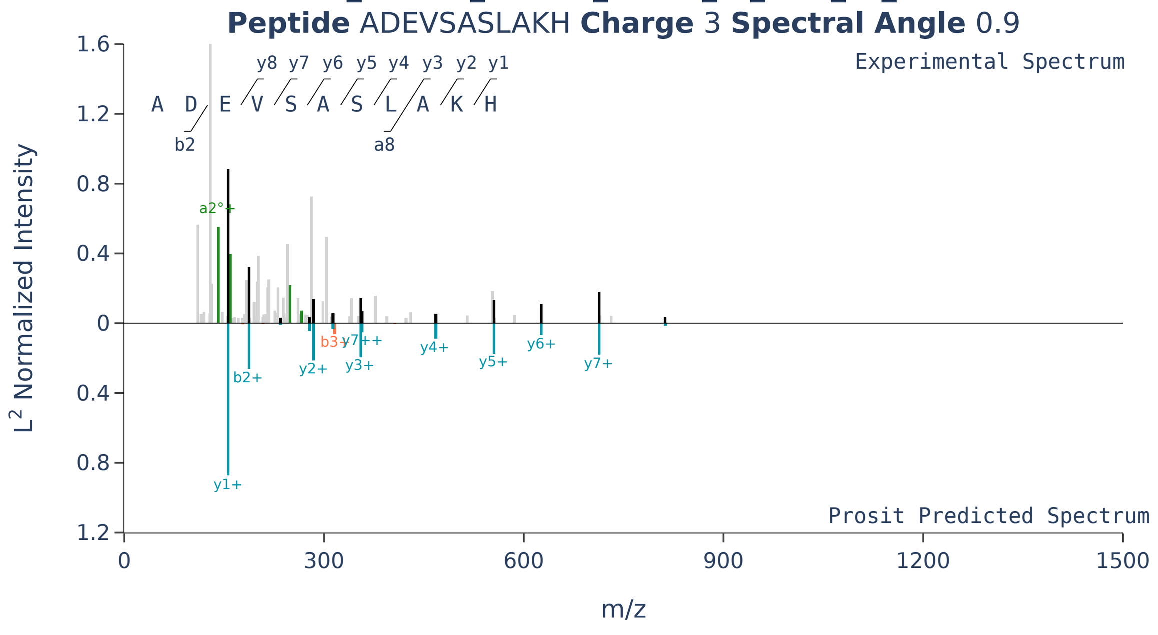

Source WSoh_221121_011221_HFGoe_L1_hTau_1h_R1 **Scan** 15616
Peptide LDNASLAKQGL **Charge** 2 **Spectral Angle** 0.84

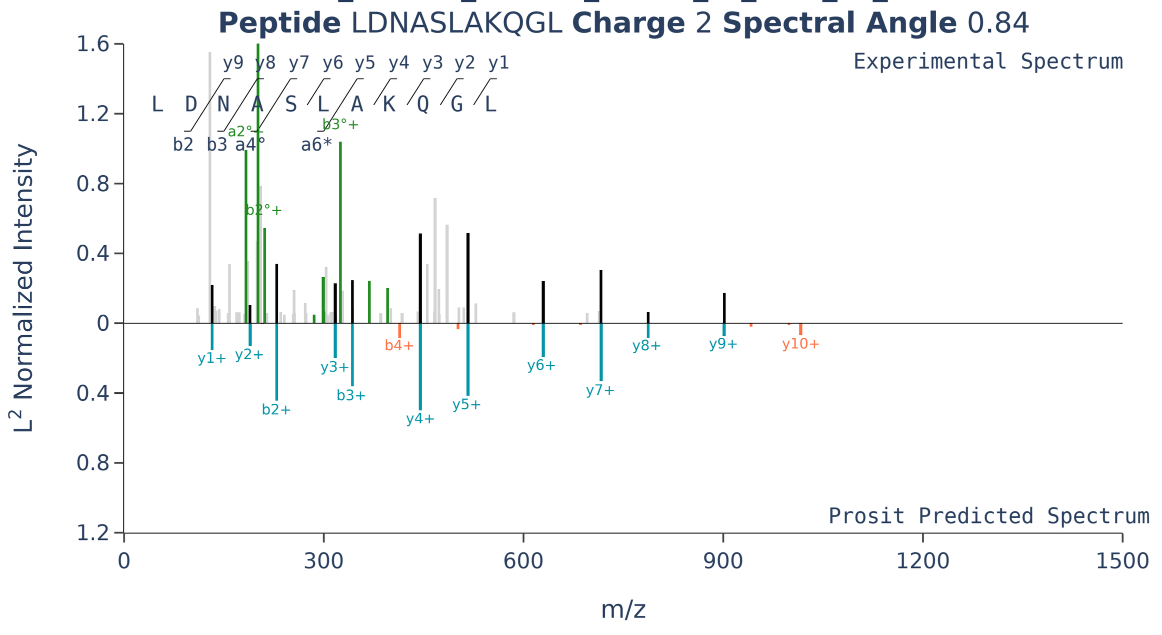

Source WSoh_221121_011221_HFGoe_L2_hTau_4h_R2 **Scan** 11921
Peptide AKIGSTENL **Charge** 2 **Spectral Angle** 0.88

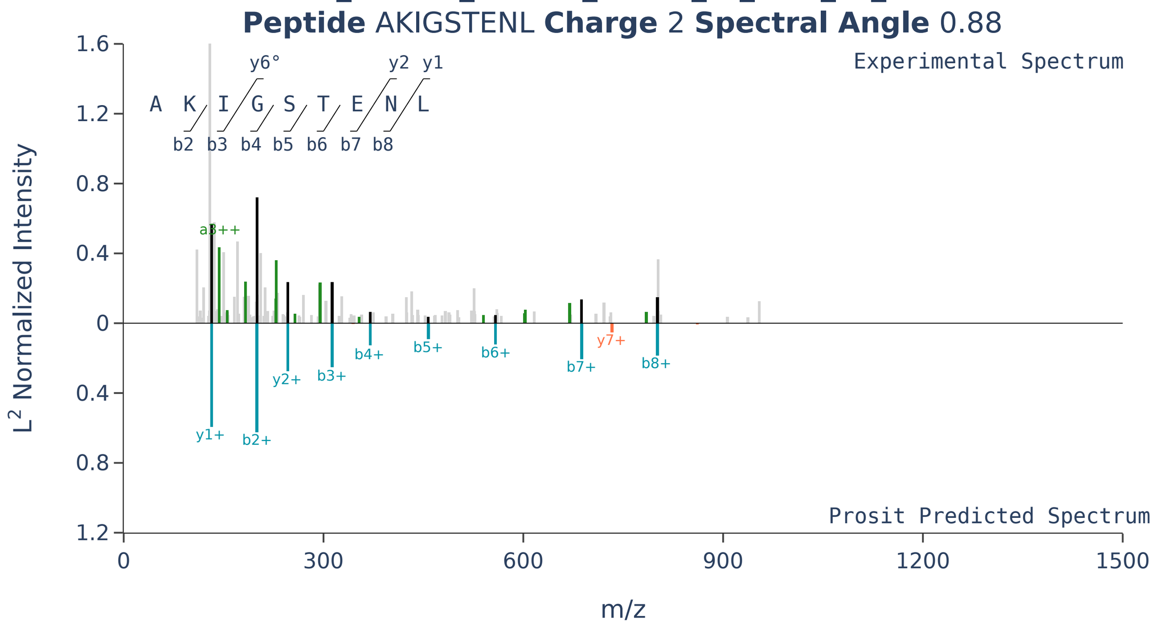

Source WSoh_221121_011221_HFGoe_L1_hTau_2h_R1 **Scan** 8992
Peptide KSEKLDFKDRVQSS **Charge** 2 **Spectral Angle** 0.81

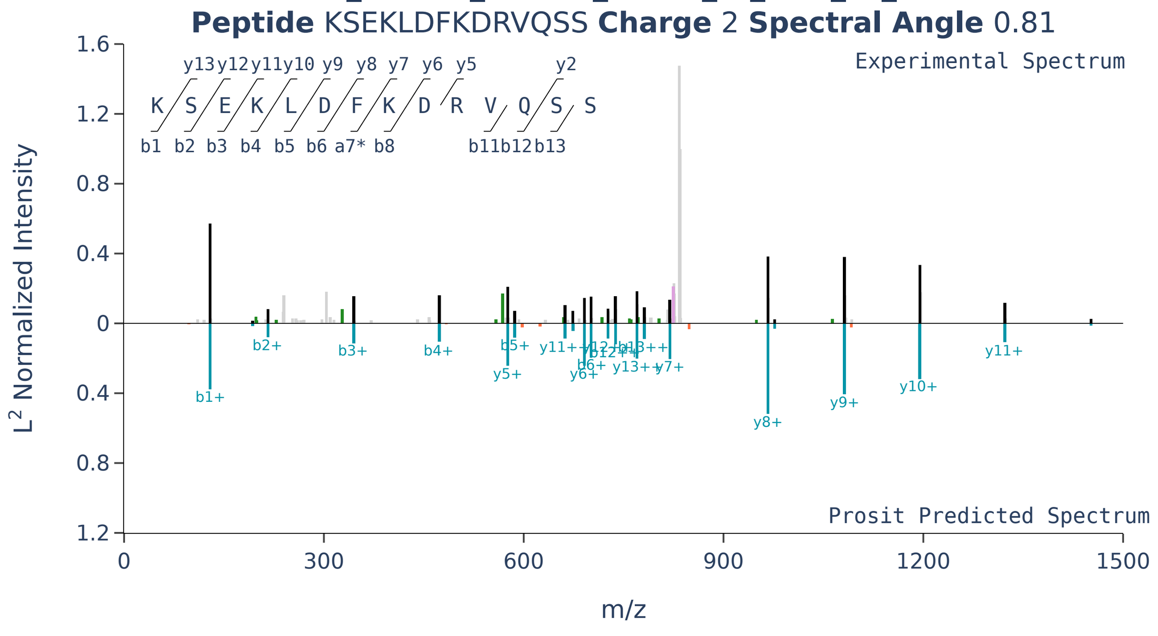

Source WSoh_221121_011221_HFGoe_L1_hTau_24h_R1 **Scan** 18824
Peptide AAQPHTEIPEGSLAKQGL **Charge** 3 **Spectral Angle** 0.86

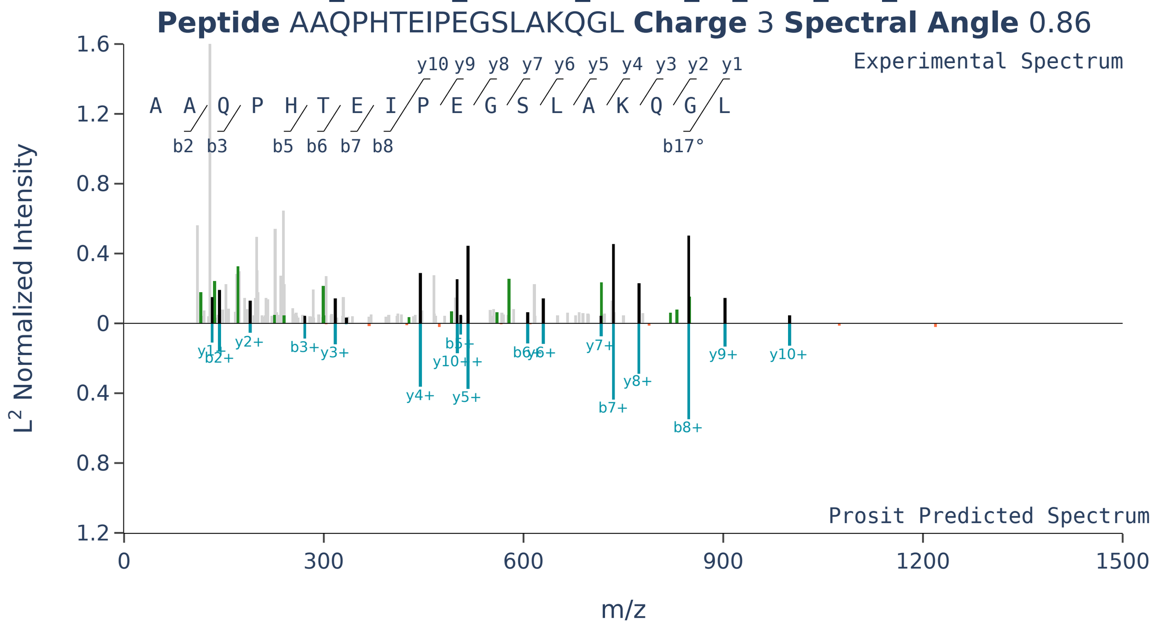

Source WSoh_221121_011221_HFGoe_L1_hTau_24h_R2 **Scan** 11423
Peptide DAEPRQEFE **Charge** 1 **Spectral Angle** 0.89

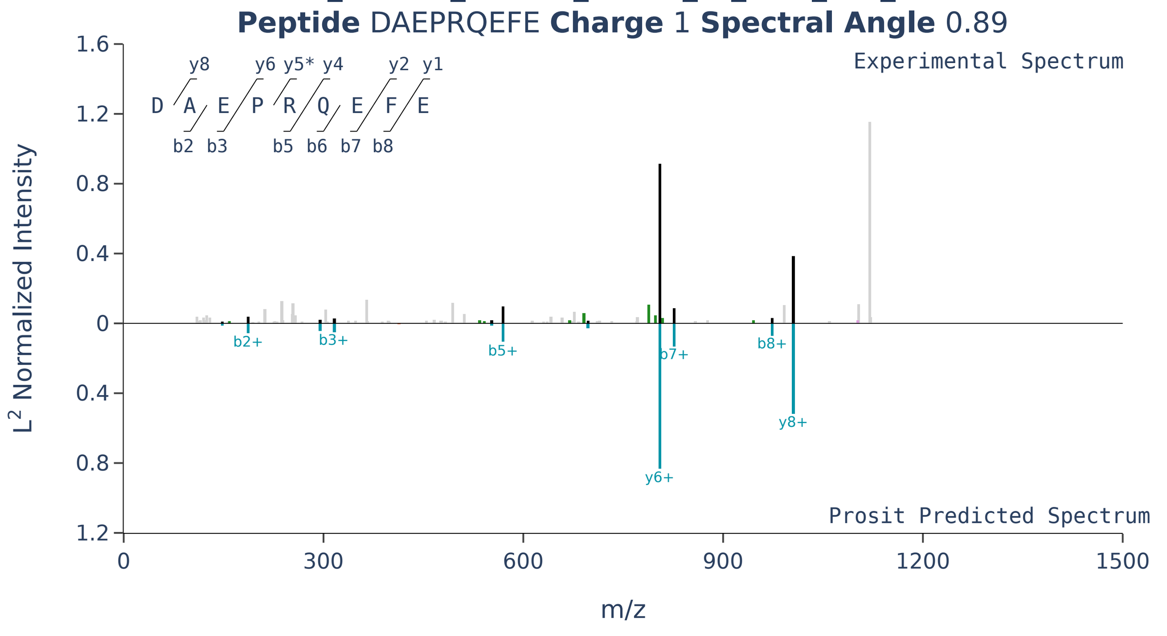

Source WSoh_221121_011221_HFGoe_L1_hTau_4h_R2 **Scan** 9175
Peptide KDRVQSVYKPVD **Charge** 2 **Spectral Angle** 0.77

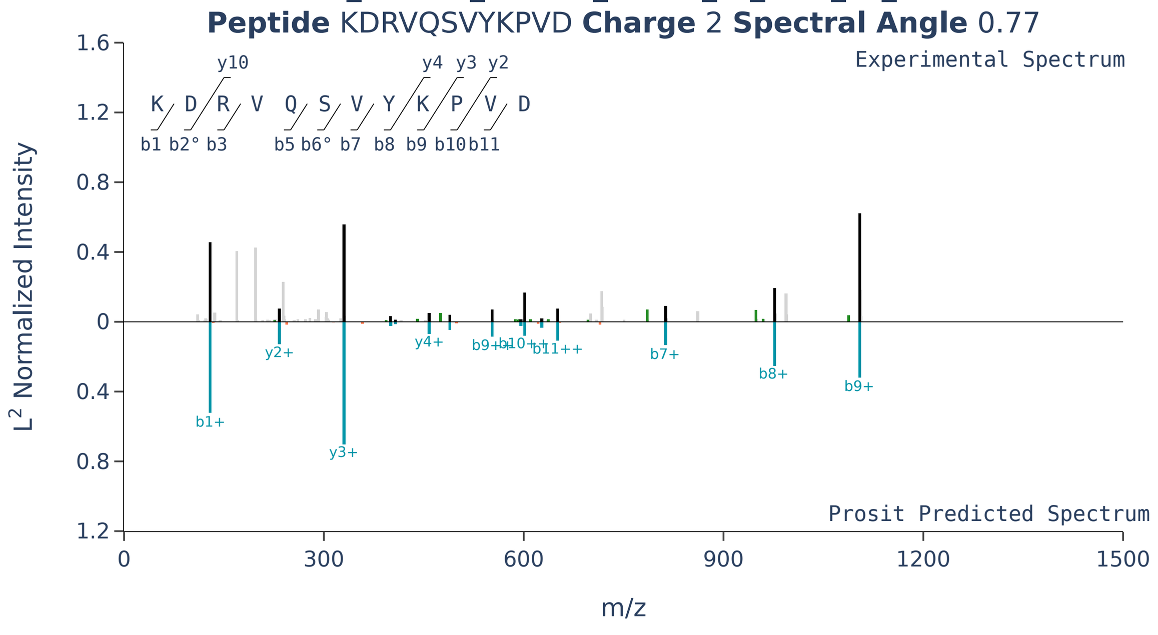

Source WSoh_221121_011221_HFGoe_L2_hTau_24h_R1 **Scan** 8166
Peptide EVSSGDTSPRHL **Charge** 2 **Spectral Angle** 0.89

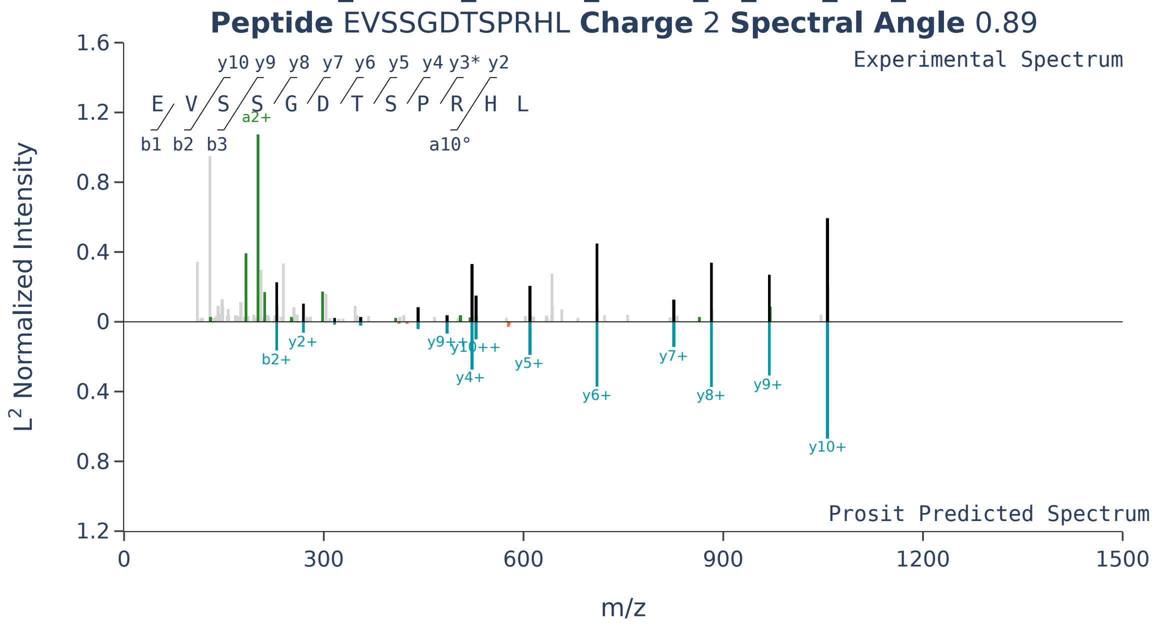

Source WSoh_221121_011221_HFGoe_L1_hTau_24h_R2 **Scan** 18944
Peptide AAQPHTEIPESASL **Charge** 2 **Spectral Angle** 0.9

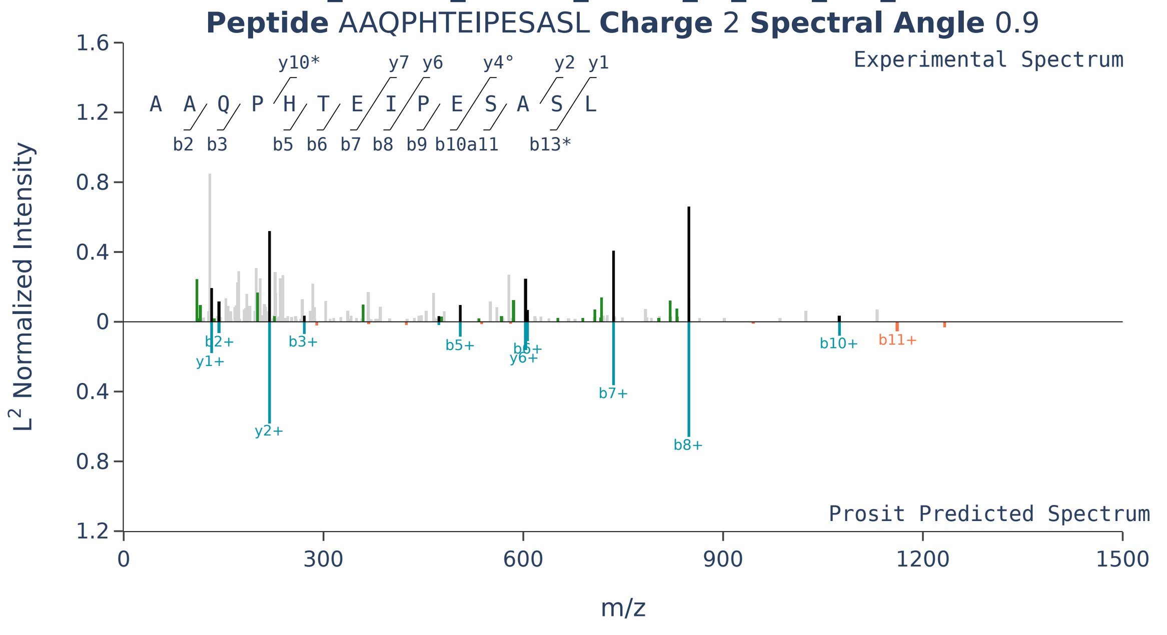

Source WSoh_221121_011221_HFGoe_L1_hTau_24h_R1 **Scan** 12386
Peptide DGSLGNIHHKPGGGQVEV **Charge** 2 **Spectral Angle** 0.87

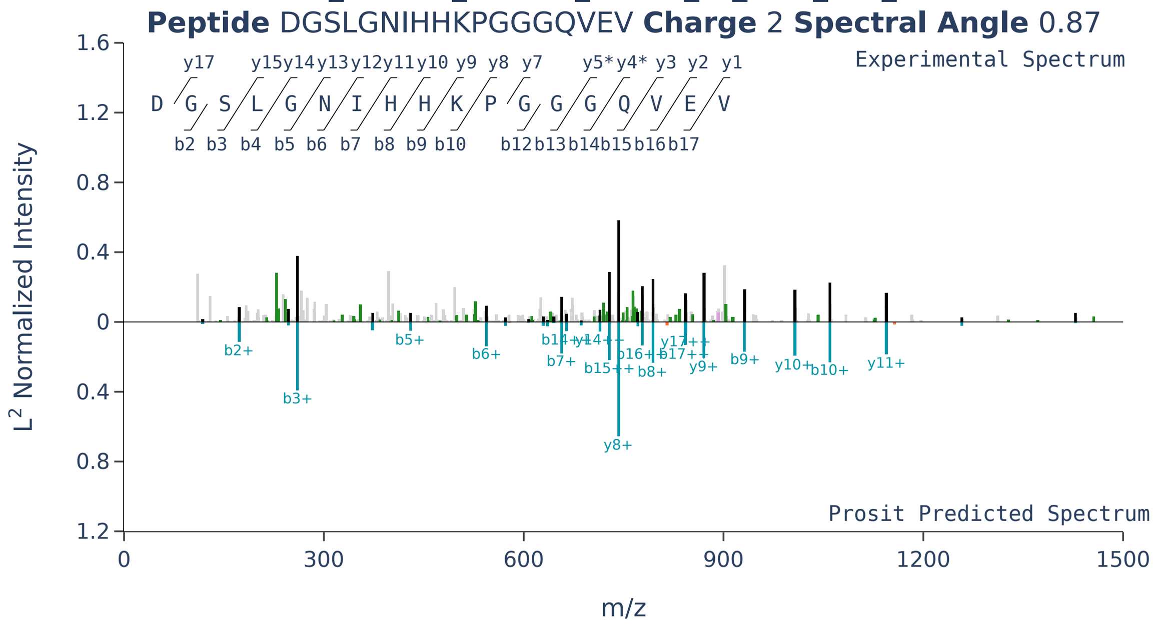

Source WSoh_221121_011221_HFGoe_L2_hTau_2h_R2 **Scan** 6237
Peptide TRIPAKTA **Charge** 2 **Spectral Angle** 0.87

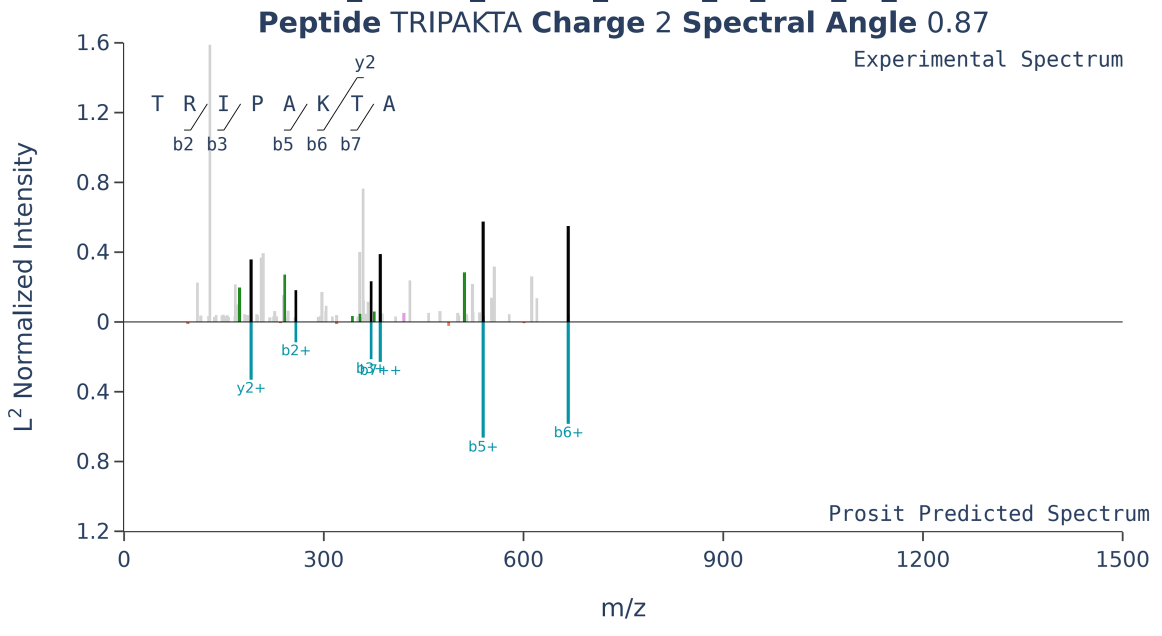

Source WSoh_221121_011221_HFGoe_L1_hTau_24h_R1 **Scan** 21995
Peptide SNVQSKIGSLDNITHV **Charge** 2 **Spectral Angle** 0.9

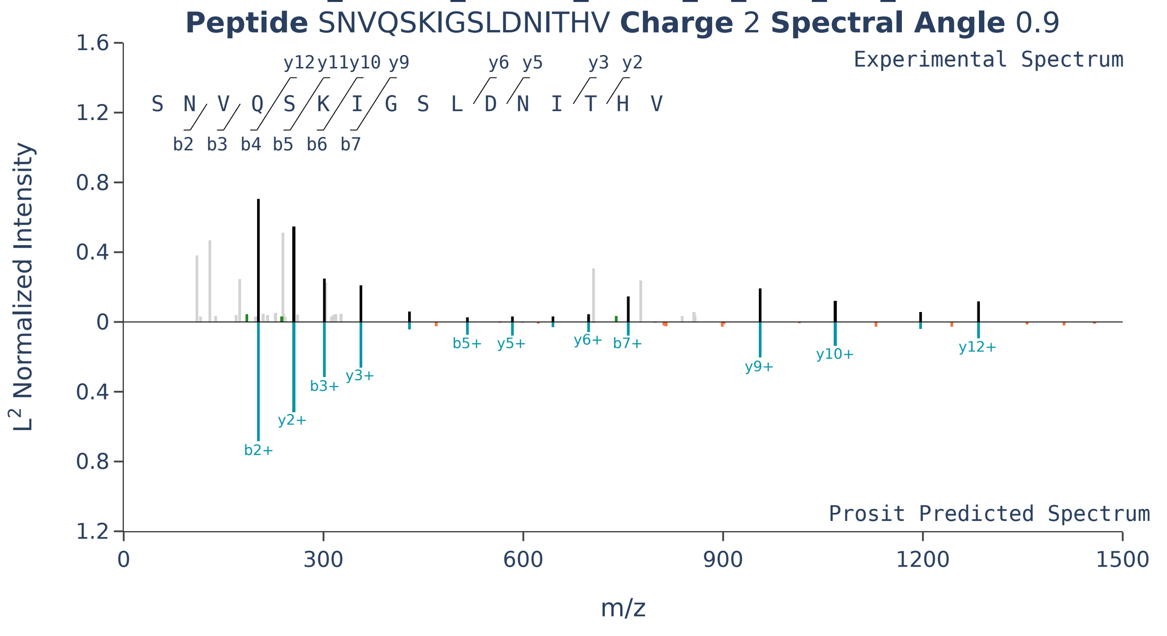

Source WSoh_221121_011221_HFGoe_L2_hTau_24h_R1 **Scan** 19922
Peptide PLVDQPHTEI **Charge** 1 **Spectral Angle** 0.9

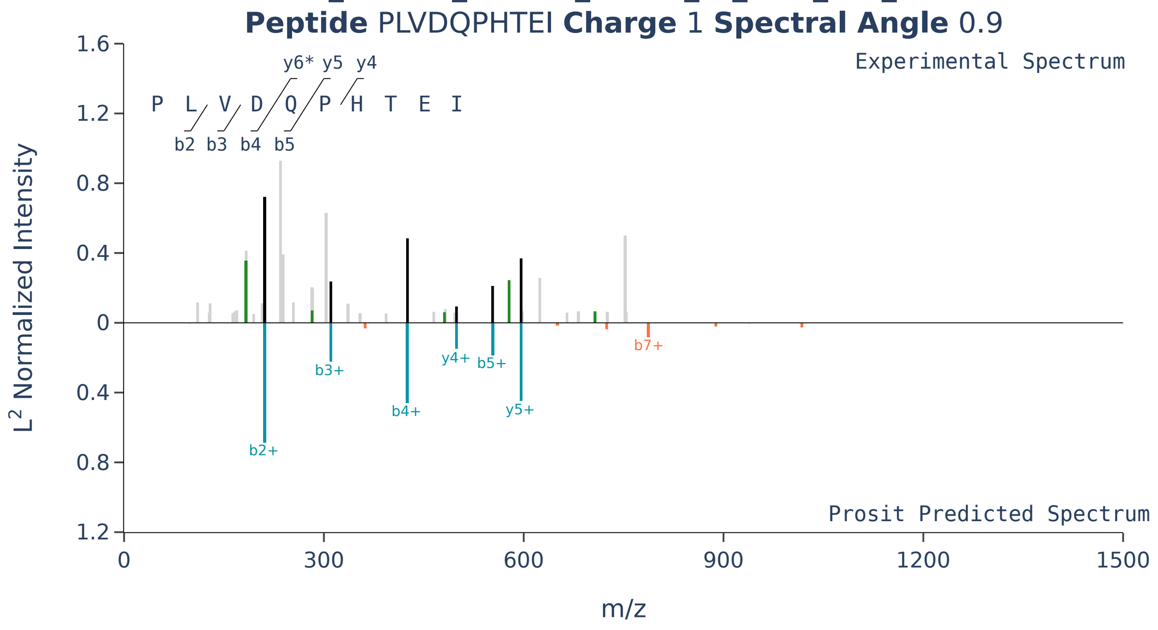

Source WSoh_221121_011221_HFGoe_L2_hTau_4h_R2 **Scan** 11787
Peptide AAQPHTEIPEGTQT **Charge** 2 **Spectral Angle** 0.89

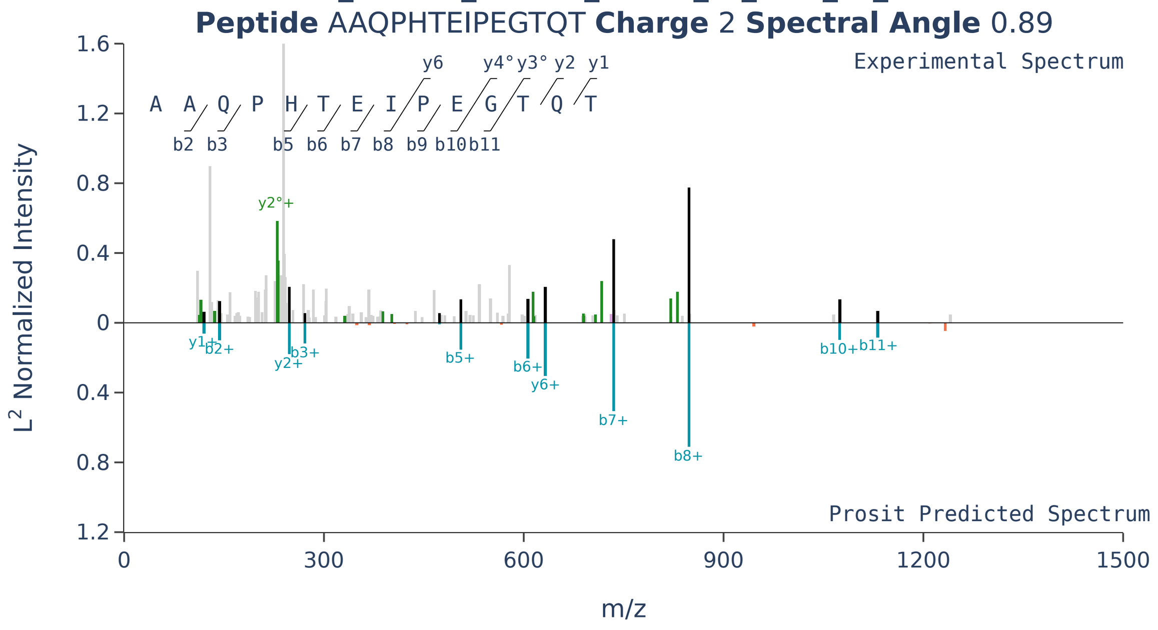

Source WSoh_221121_011221_HFGoe_L1_hTau_24h_R2 **Scan** 16232
Peptide AAQPHTEIPEGTRMV **Charge** 3 **Spectral Angle** 0.92

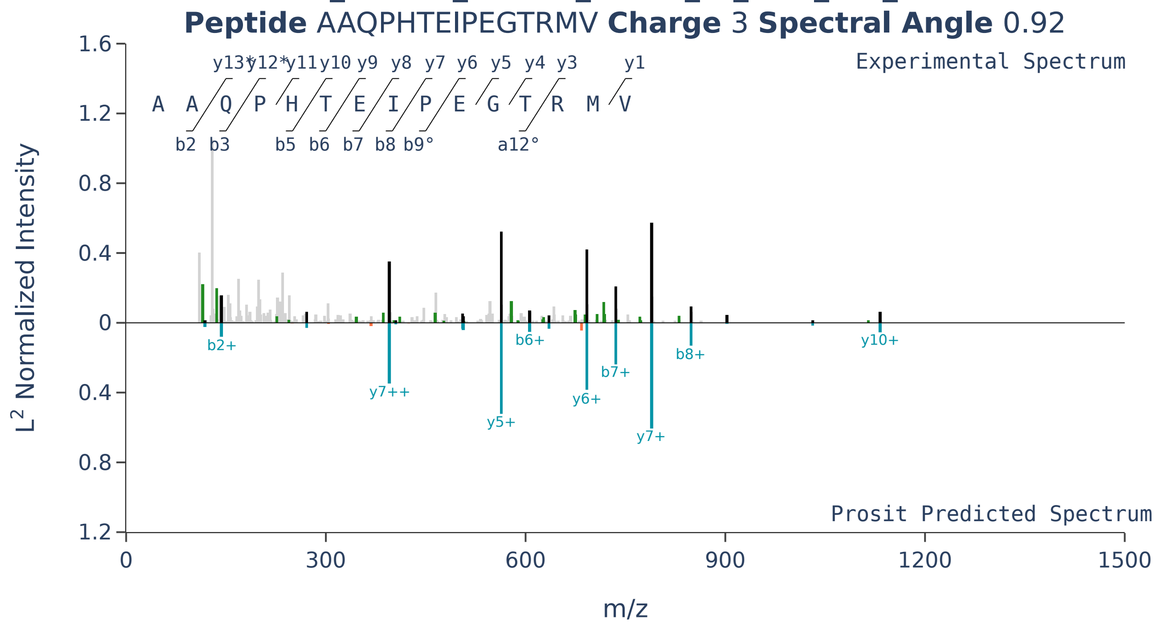

Source WSoh_221121_011221_HFGoe_L1_hTau_24h_R1 **Scan** 5877
Peptide SNVQSKCSKDNIKHV **Charge** 2 **Spectral Angle** 0.95

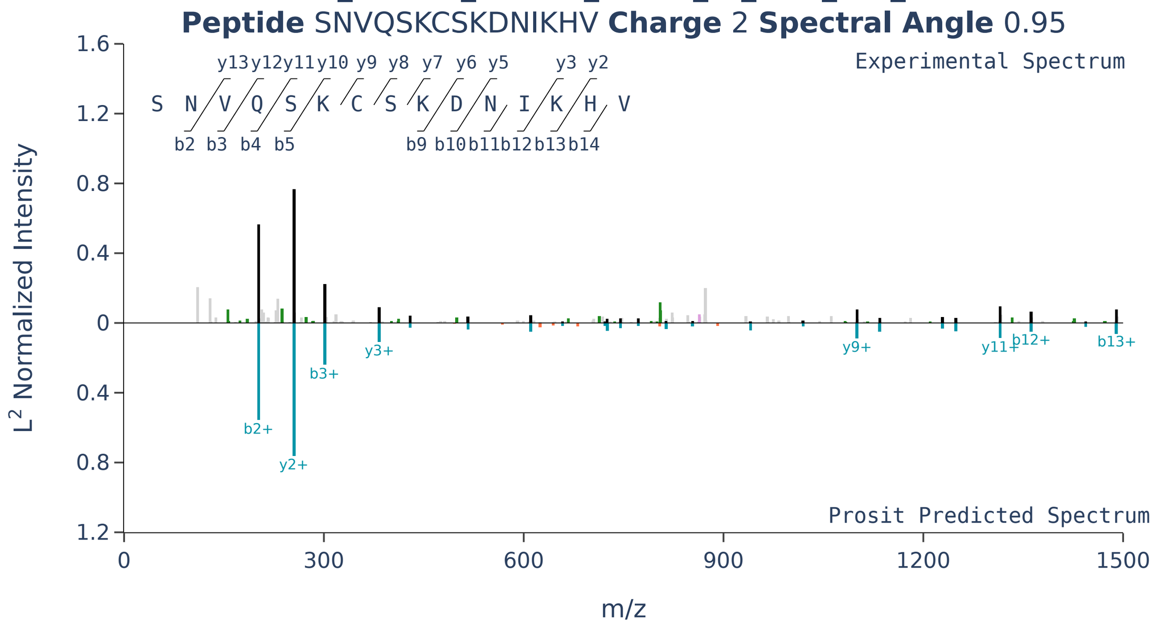

Source WSoh_221121_011221_HFGoe_L1_hTau_24h_R1 **Scan** 20741
Peptide ADEVSASLAKQGLAA **Charge** 2 **Spectral Angle** 0.94

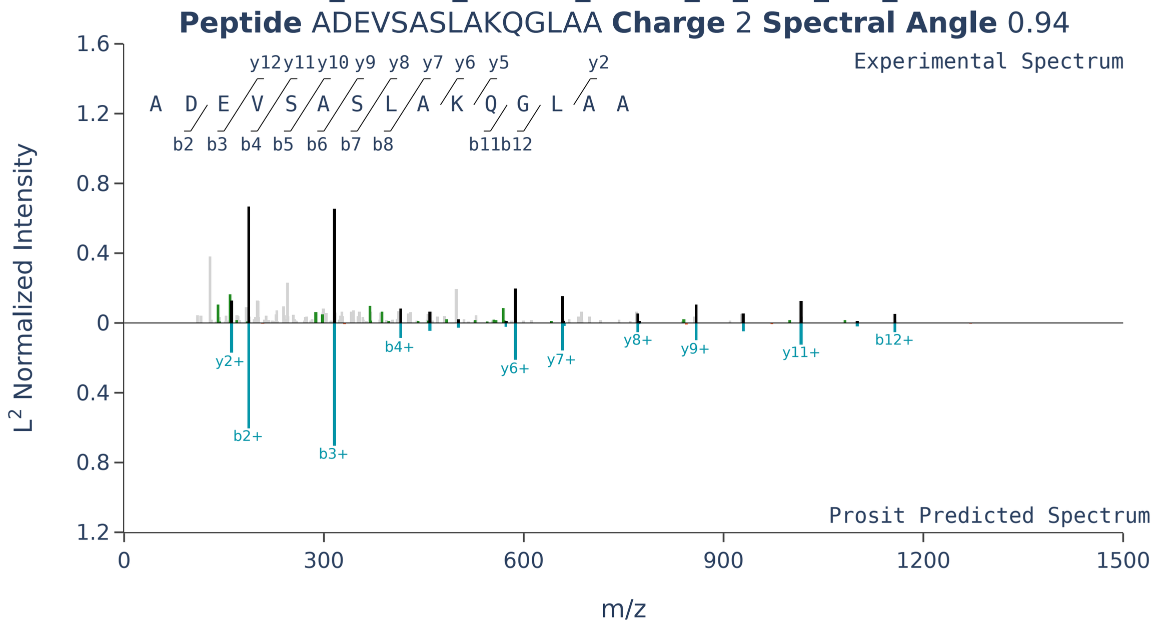

Source WSoh_221121_011221_HFGoe_L1_hTau_24h_R2 **Scan** 7911
Peptide DFKDRVQSN **Charge** 2 **Spectral Angle** 0.78

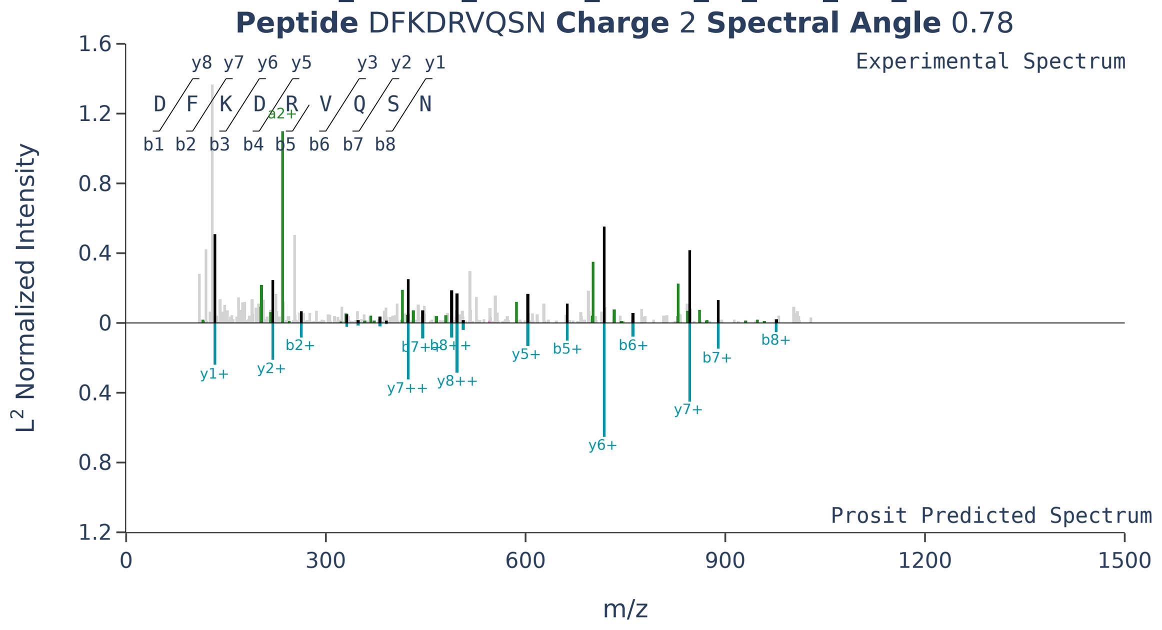

Source WSoh_221121_011221_HFGoe_L1_hTau_24h_R1 **Scan** 20769
Peptide GRLQTAPVPMPDLKNVKS **Charge** 3 **Spectral Angle** 0.87

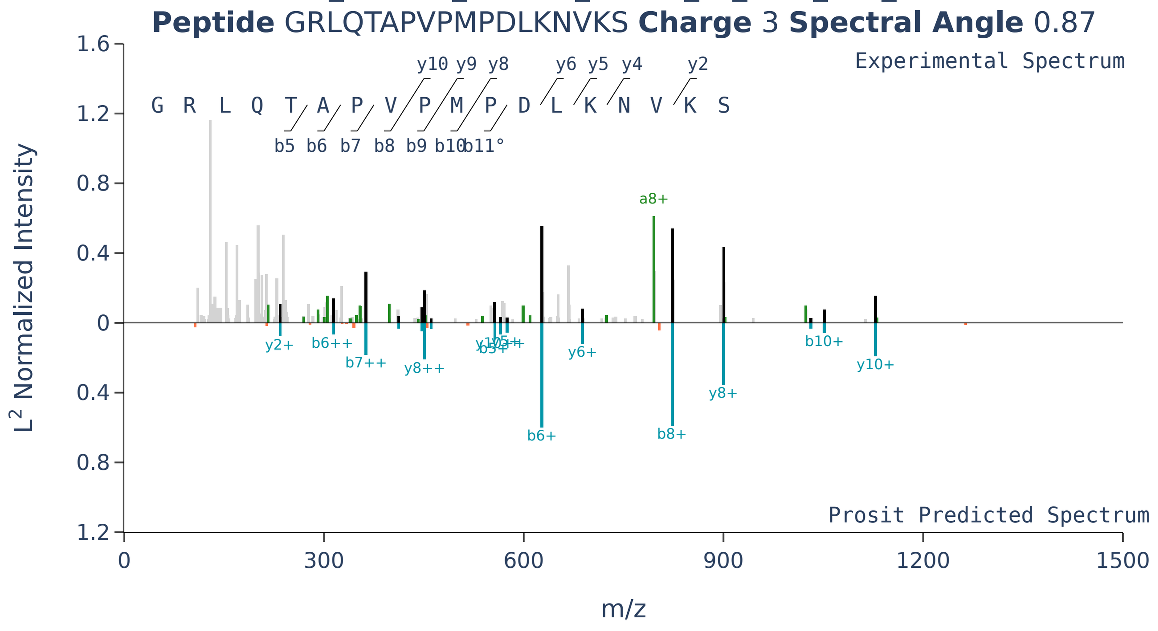

Source WSoh_221121_011221_HFGoe_L1_hTau_24h_R2 **Scan** 9992
Peptide VSLGNIHHKPGGGQVE **Charge** 2 **Spectral Angle** 0.88

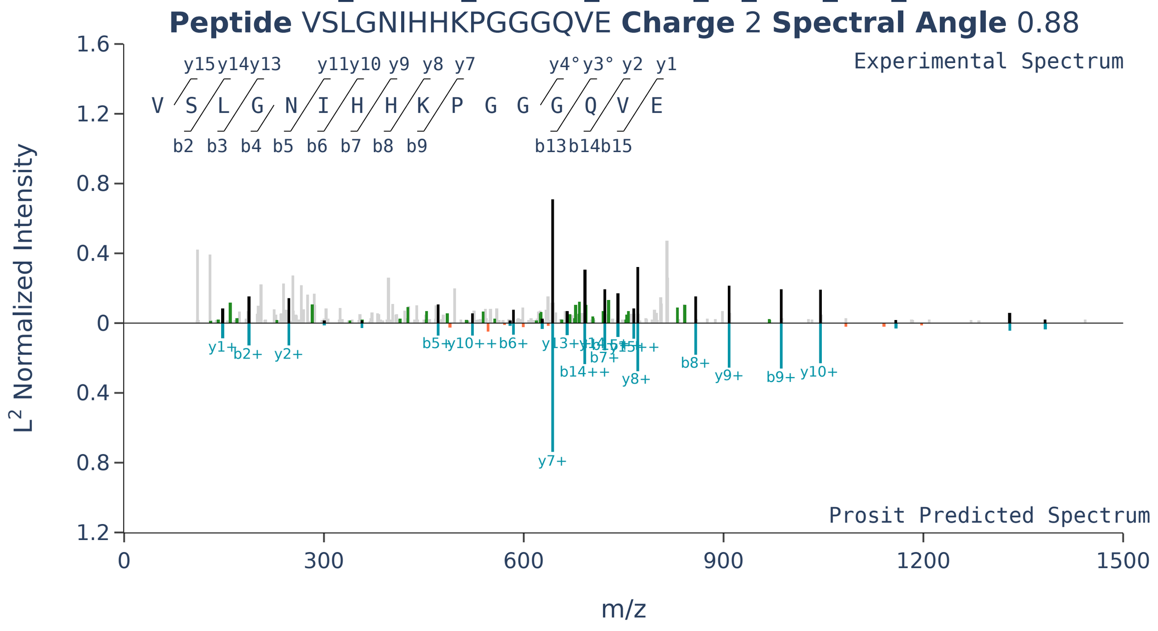

Source WSoh_221121_011221_HFGoe_L1_hTau_1h_R2 **Scan** 10728
Peptide PGSPGTPGSRSRTSLP **Charge** 3 **Spectral Angle** 0.87

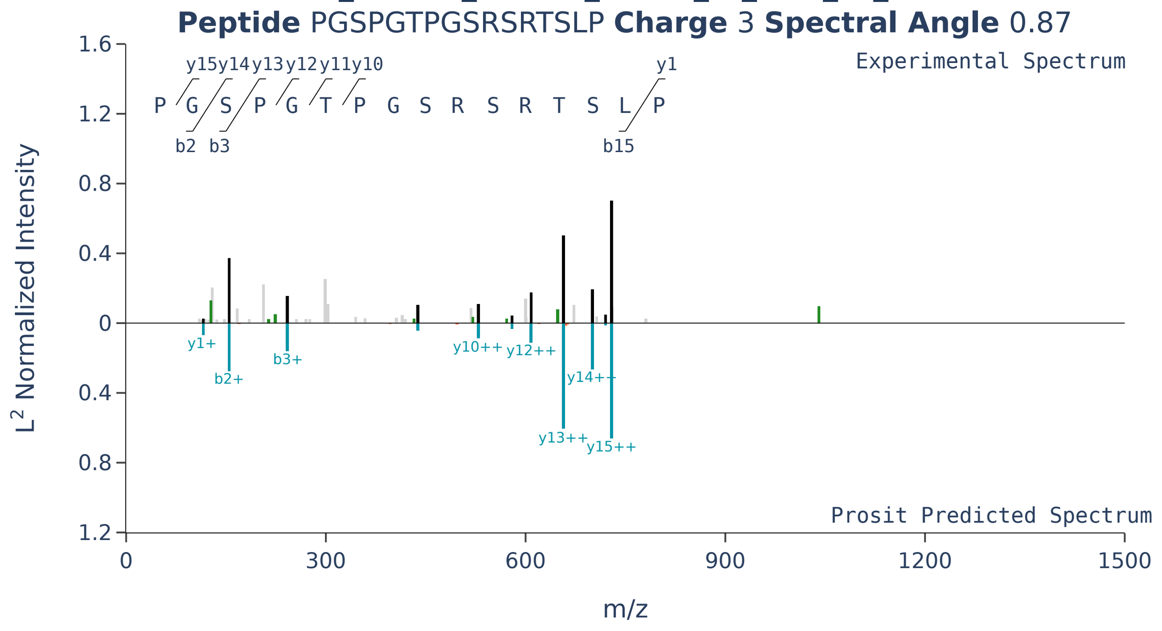

Source WSoh_221121_011221_HFGoe_L1_hTau_2h_R2 **Scan** 10356
Peptide KSEKLDFKDRVQGT **Charge** 2 **Spectral Angle** 0.83

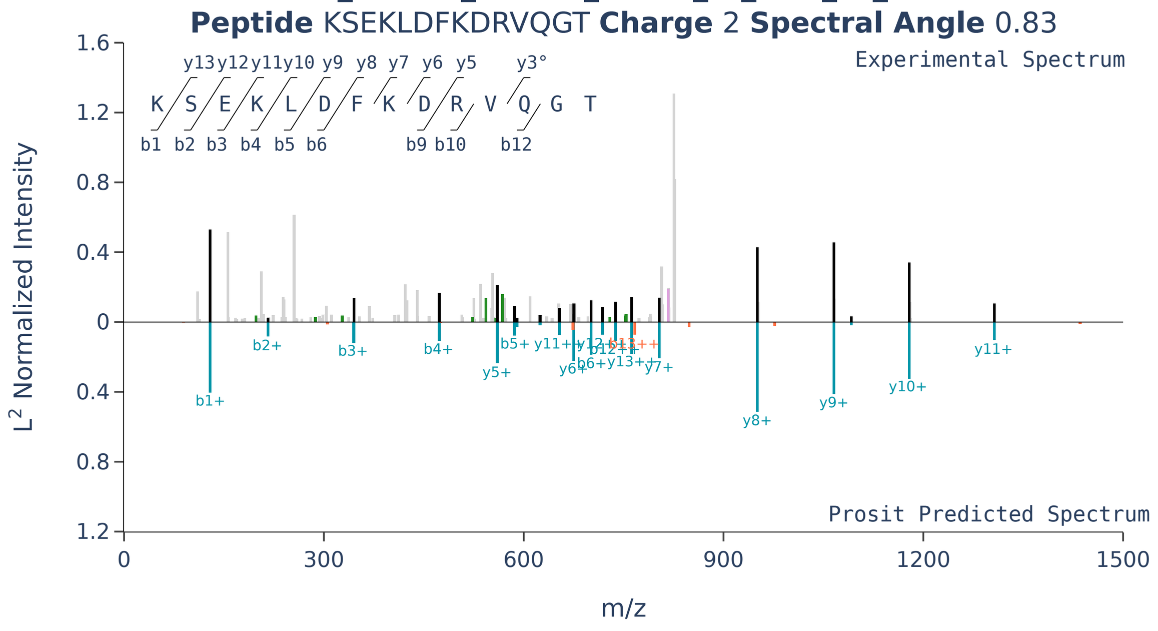

Source WSoh_221121_011221_HFGoe_L2_hTau_24h_R1 **Scan** 20639
Peptide AAQPHTEIPEGTSPLQTPTE **Charge** 3 **Spectral Angle** 0.87

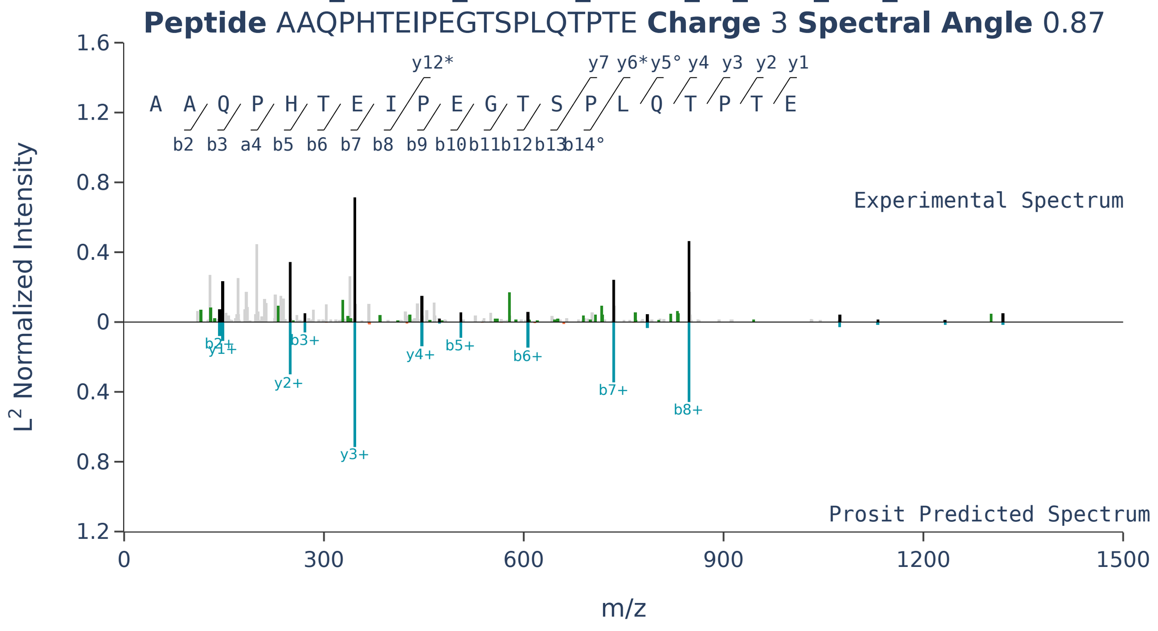

Source WSoh_221121_011221_HFGoe_L1_hTau_24h_R1 **Scan** 8682
Peptide KSSLGNIHHKPGGGQVEV **Charge** 2 **Spectral Angle** 0.84

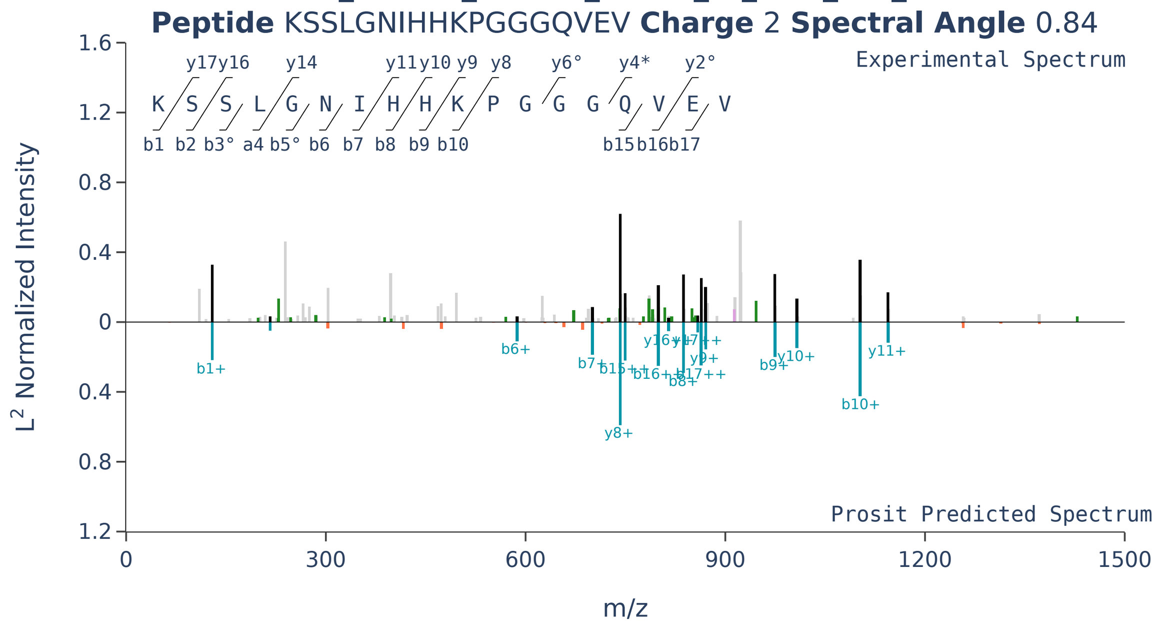

Source WSoh_221121_011221_HFGoe_L1_hTau_24h_R2 **Scan** 16452
Peptide GDTSPRHLSNVSSTGSPLQTPTED **Charge** 3 **Spectral Angle** 0.9

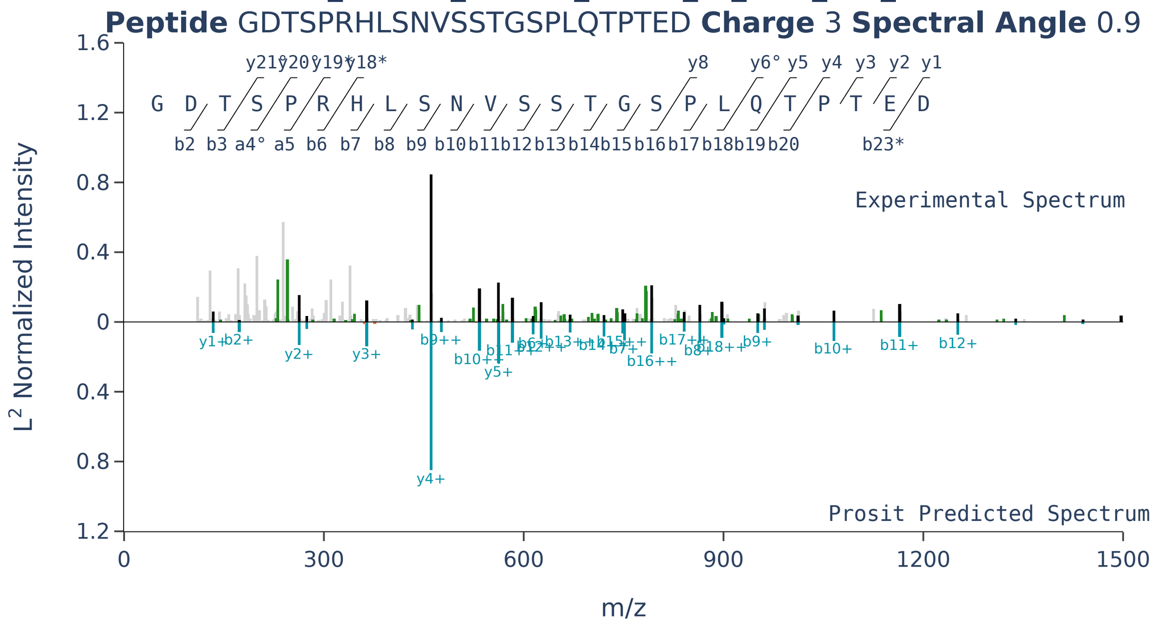

Source WSoh_221121_011221_HFGoe_L2_hTau_4h_R2 **Scan** 6941
Peptide GSNIHHKPGGGQVEV **Charge** 3 **Spectral Angle** 0.82

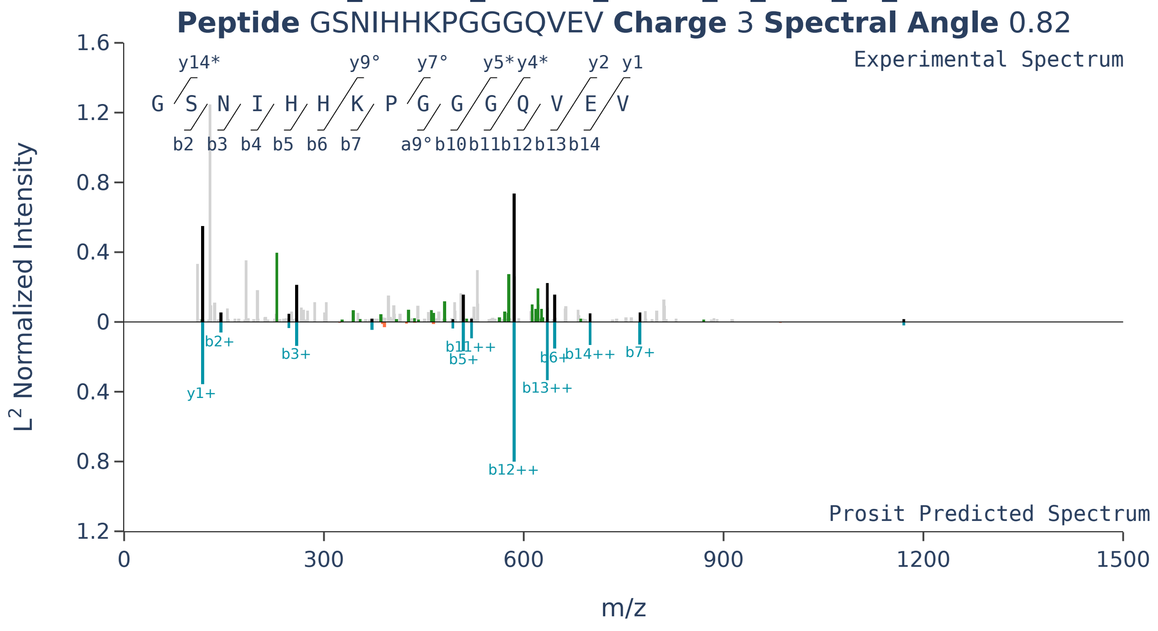

Source WSoh_221121_011221_HFGoe_L1_hTau_24h_R2 **Scan** 16419
Peptide SGDTSPRHLSNVSSTGSPLQTPTE **Charge** 3 **Spectral Angle** 0.9

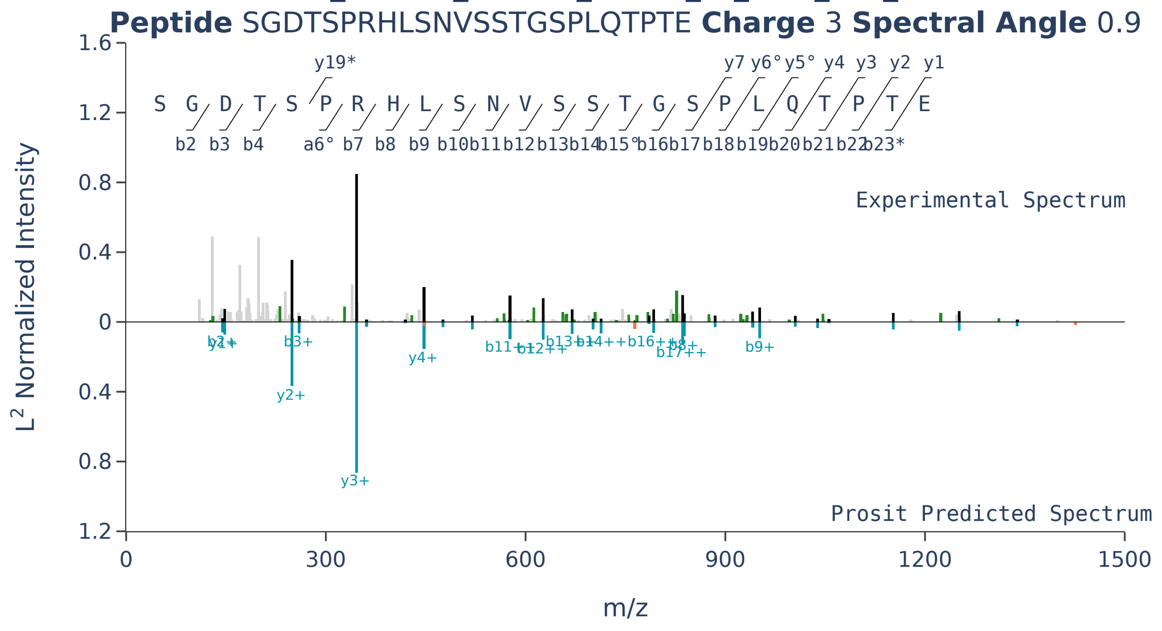

Source WSoh_221121_011221_HFGoe_L2_hTau_24h_R1 **Scan** 9382
Peptide KNEKLDFKDRVQS **Charge** 2 **Spectral Angle** 0.77

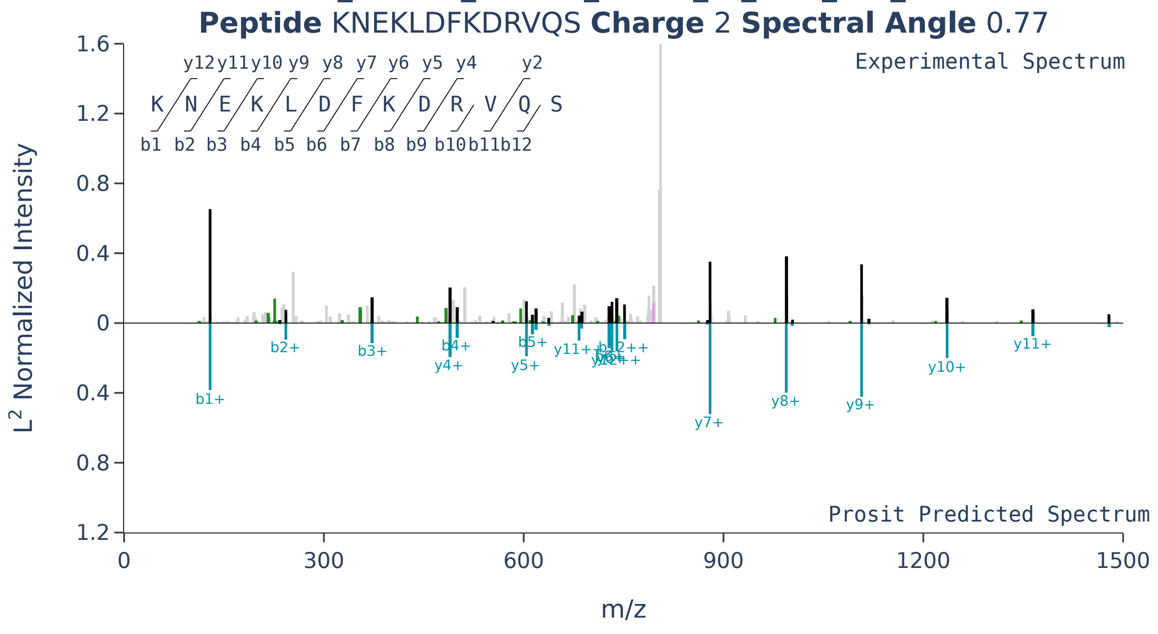

Source WSoh_221121_011221_HFGoe_L1_hTau_4h_R2 **Scan** 16241
Peptide HAGTYGSPLQTPTED **Charge** 2 **Spectral Angle** 0.87

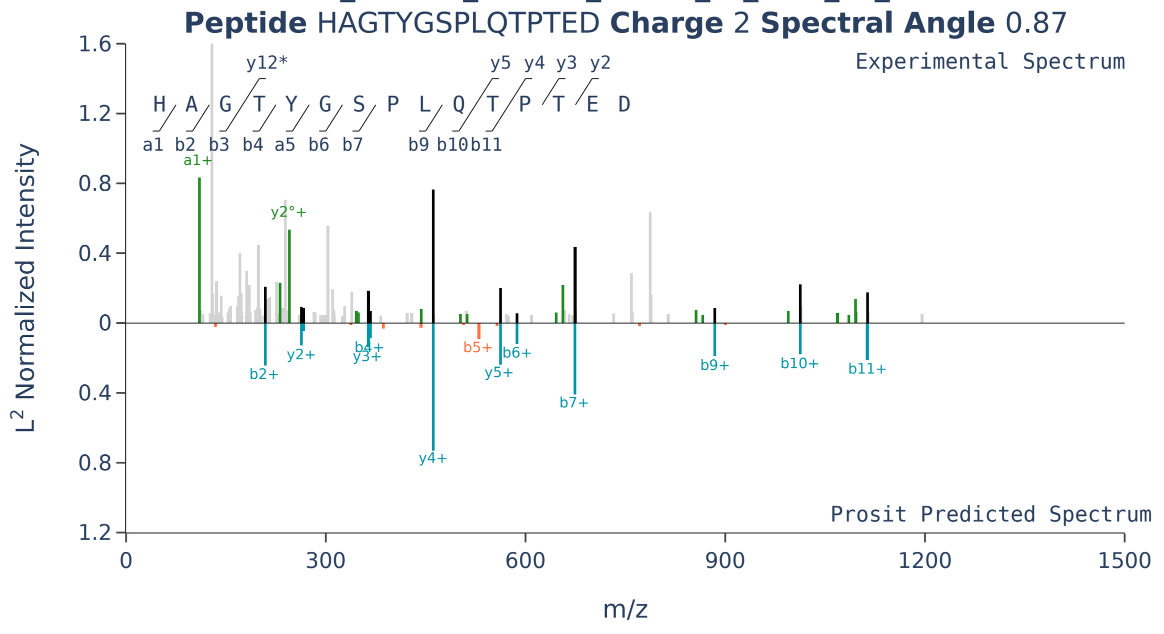

Source WSoh_221121_011221_HFGoe_L1_hTau_24h_R1 **Scan** 20541
Peptide AAQPHTEIPEGTSPLQTPTED **Charge** 2 **Spectral Angle** 0.92

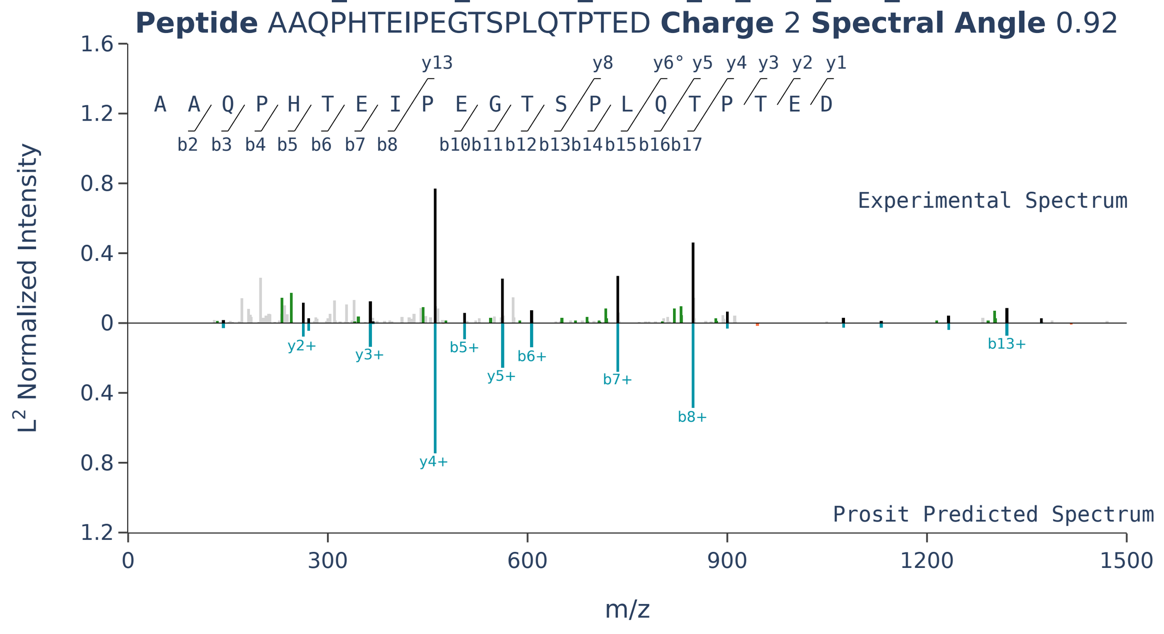

Source WSoh_221121_011221_HFGoe_L1_hTau_4h_R1 **Scan** 9588
Peptide SAEPRQEFE **Charge** 2 **Spectral Angle** 0.89

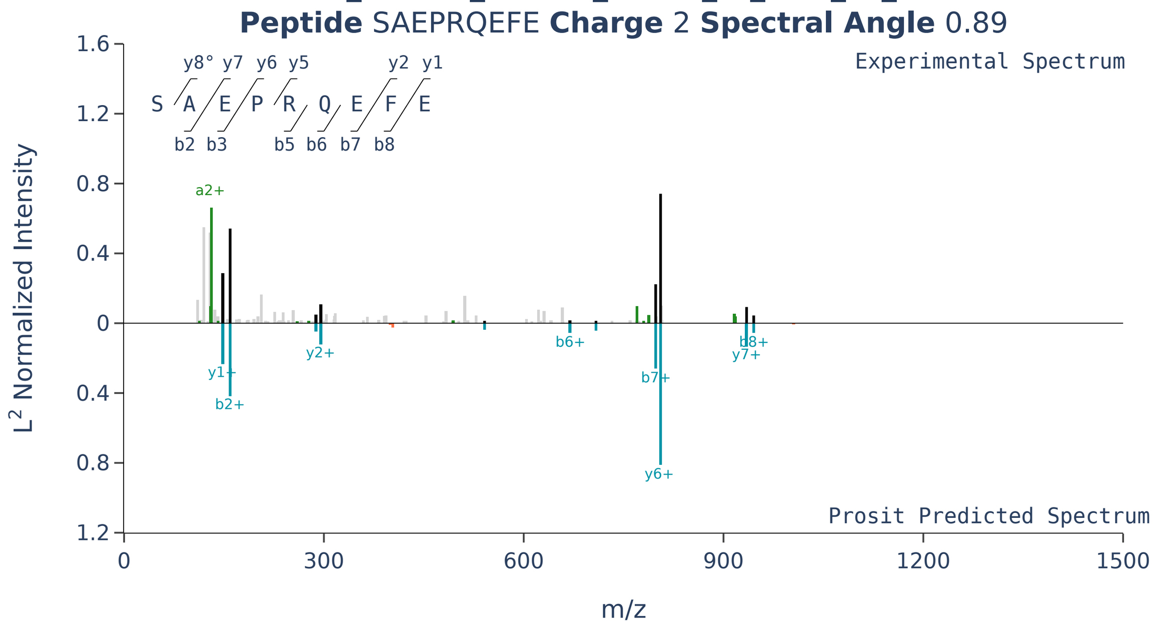

Source WSoh_221121_011221_HFGoe_L1_hTau_4h_R2 **Scan** 10388
Peptide KSEKLDFKDRVQTG **Charge** 3 **Spectral Angle** 0.83

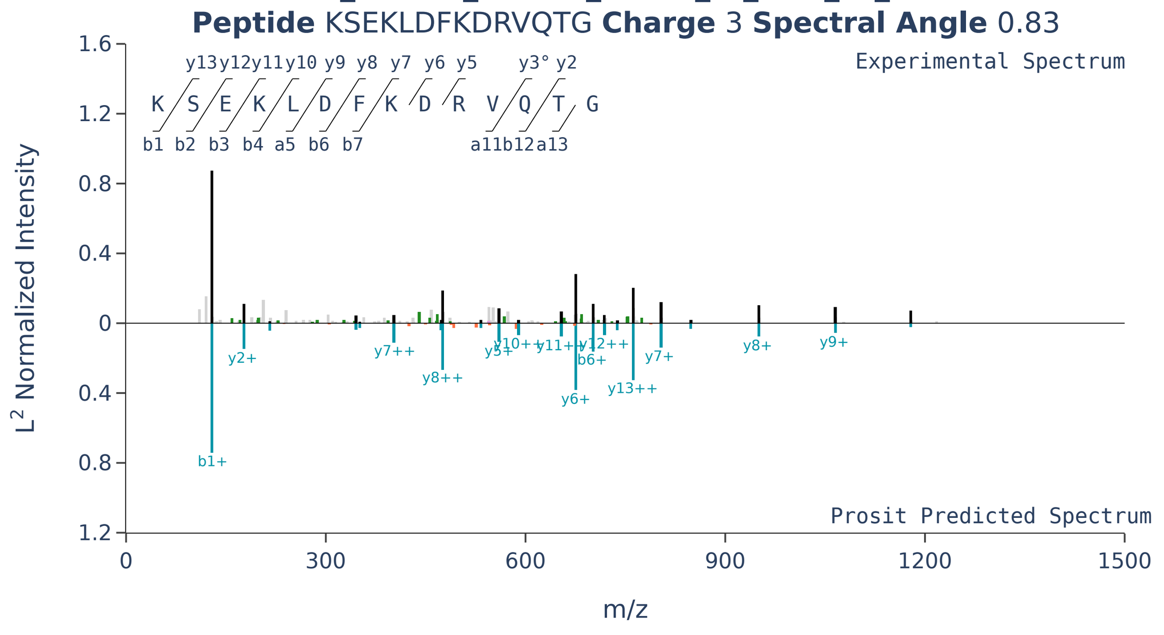

Source WSoh_221121_011221_HFGoe_L2_hTau_4h_R2 **Scan** 15821
Peptide VYKPVDLSKVTSH **Charge** 3 **Spectral Angle** 0.88

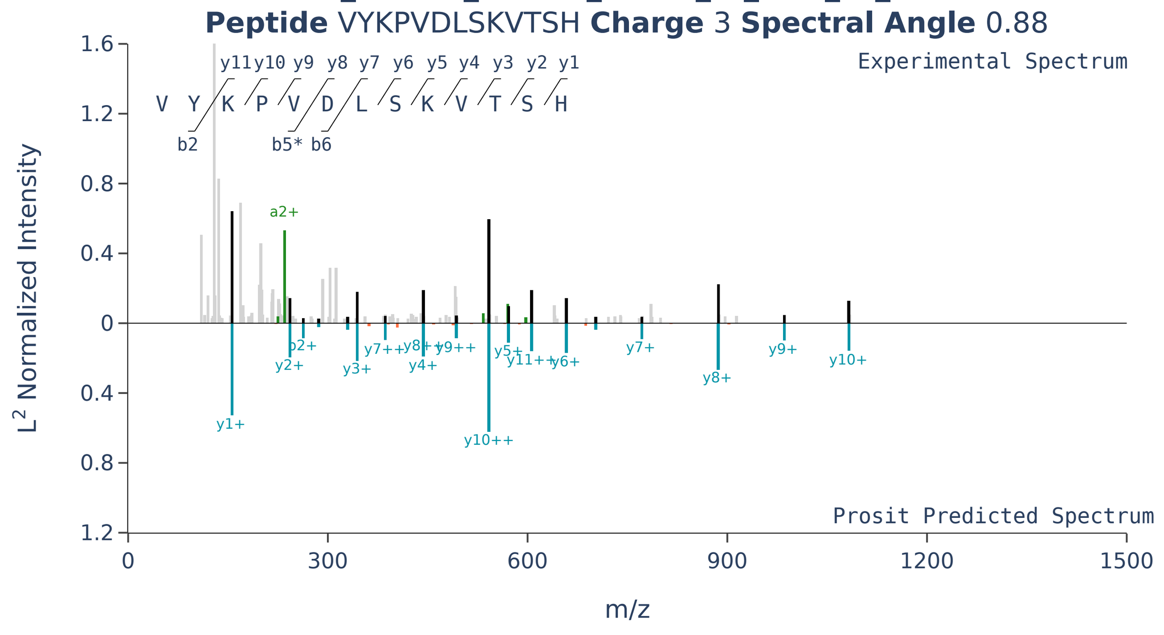

Source WSoh_221121_011221_HFGoe_L2_hTau_4h_R1 **Scan** 11588
Peptide SKVTSKIGSTENL **Charge** 2 **Spectral Angle** 0.91

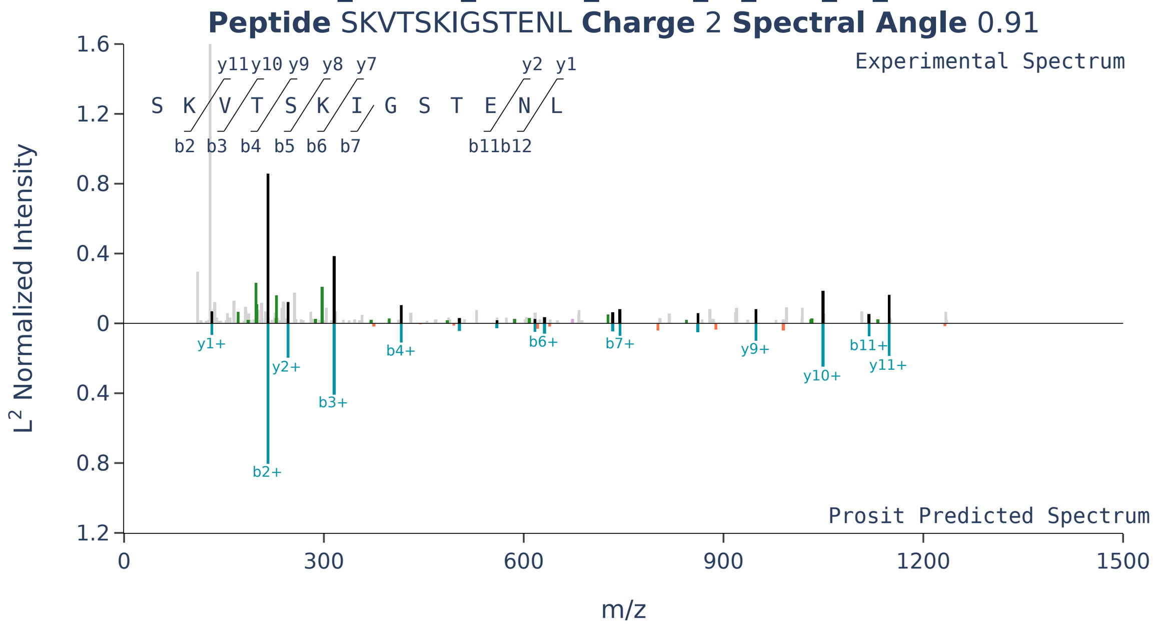

Source WSoh_221121_011221_HFGoe_L1_hTau_24h_R1 **Scan** 20000
Peptide TAPVPMPDLKNVKN **Charge** 3 **Spectral Angle** 0.82

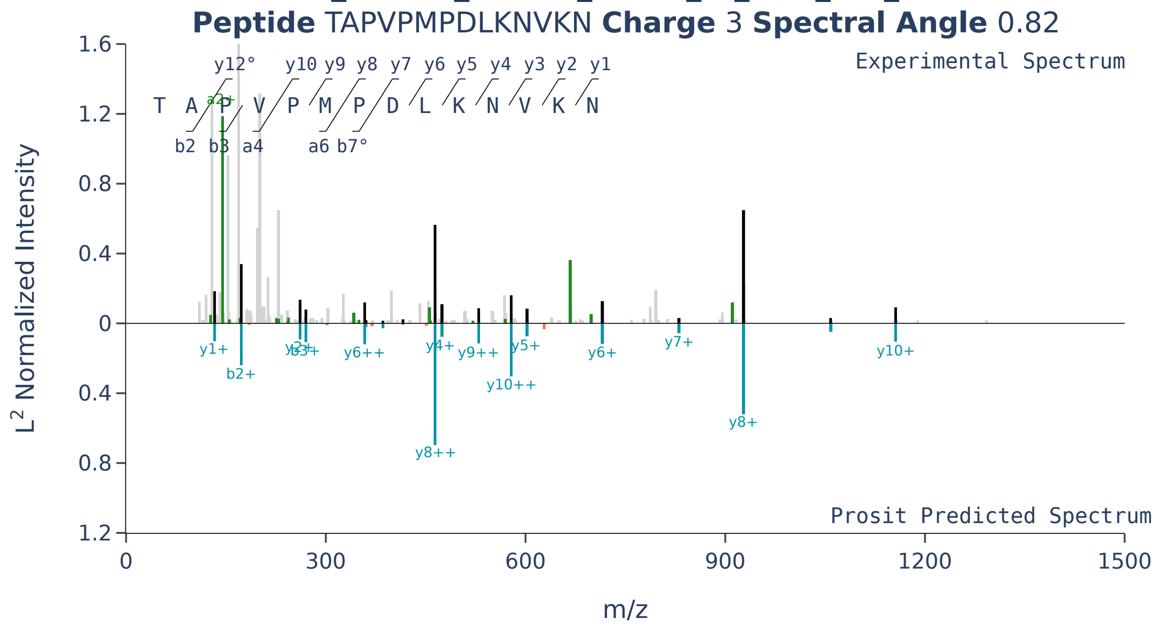

Source WSoh_221121_011221_HFGoe_L1_hTau_24h_R2 **Scan** 16112
Peptide AKSTPTAESPLQTPTE **Charge** 2 **Spectral Angle** 0.93

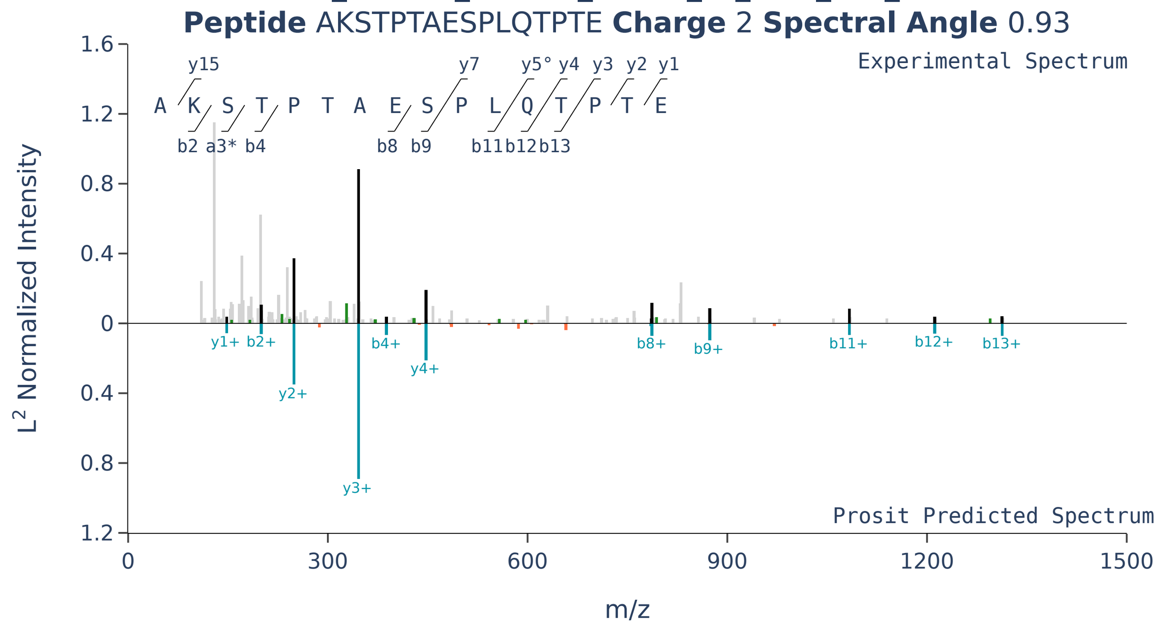

Source WSoh_221121_011221_HFGoe_L2_hTau_24h_R2 **Scan** 20713
Peptide APVPMPDLKNVKTG **Charge** 3 **Spectral Angle** 0.85

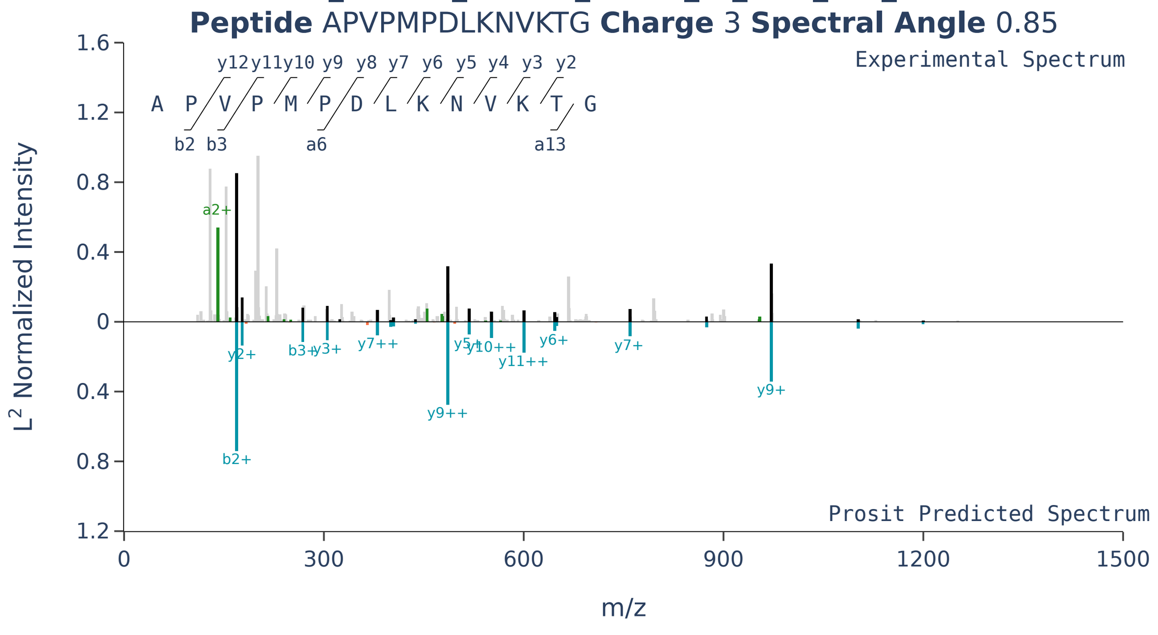

Source WSoh_221121_011221_HFGoe_L2_hTau_24h_R1 **Scan** 20201
Peptide RLQTAPVPMPDLKNKNV **Charge** 3 **Spectral Angle** 0.92

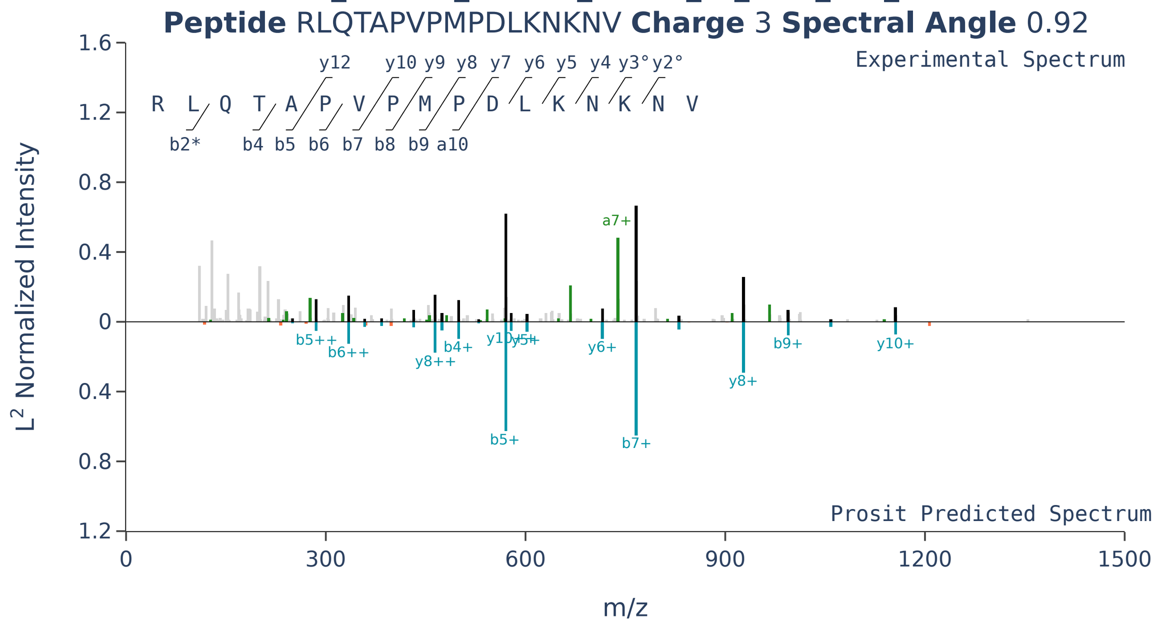

Source WSoh_221121_011221_HFGoe_L1_hTau_24h_R2 **Scan** 7022
Peptide AKHQPGGGKVQIINK **Charge** 2 **Spectral Angle** 0.89

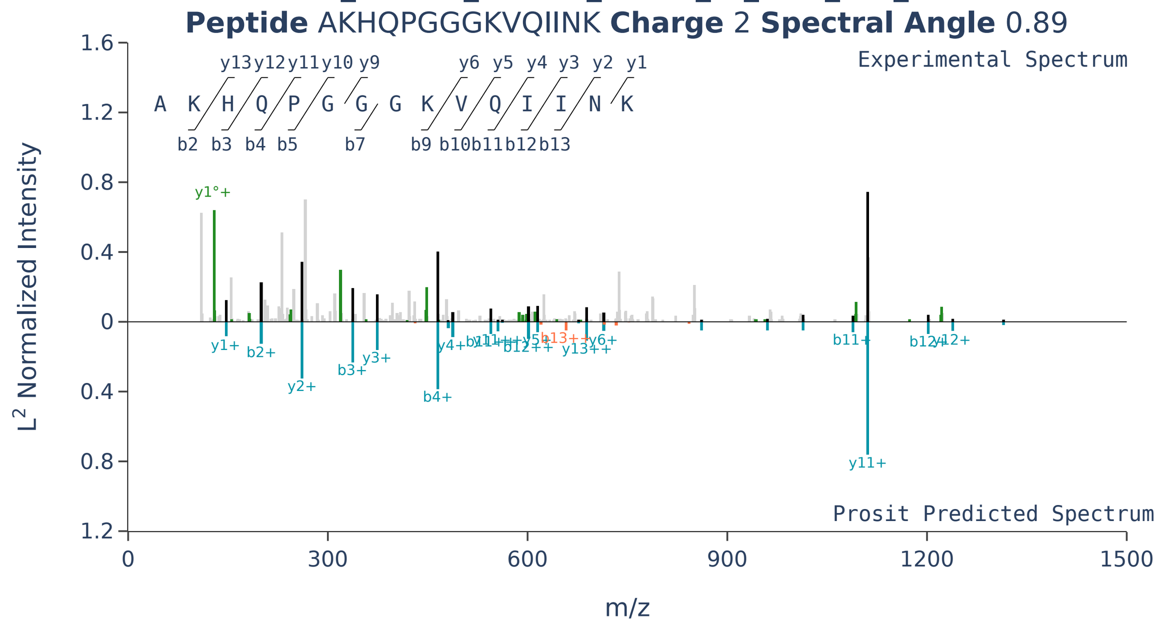

Source WSoh_221121_011221_HFGoe_L1_hTau_24h_R2 **Scan** 22709
Peptide EDEAAGHVTQAAGIGDTPSL **Charge** 2 **Spectral Angle** 0.92

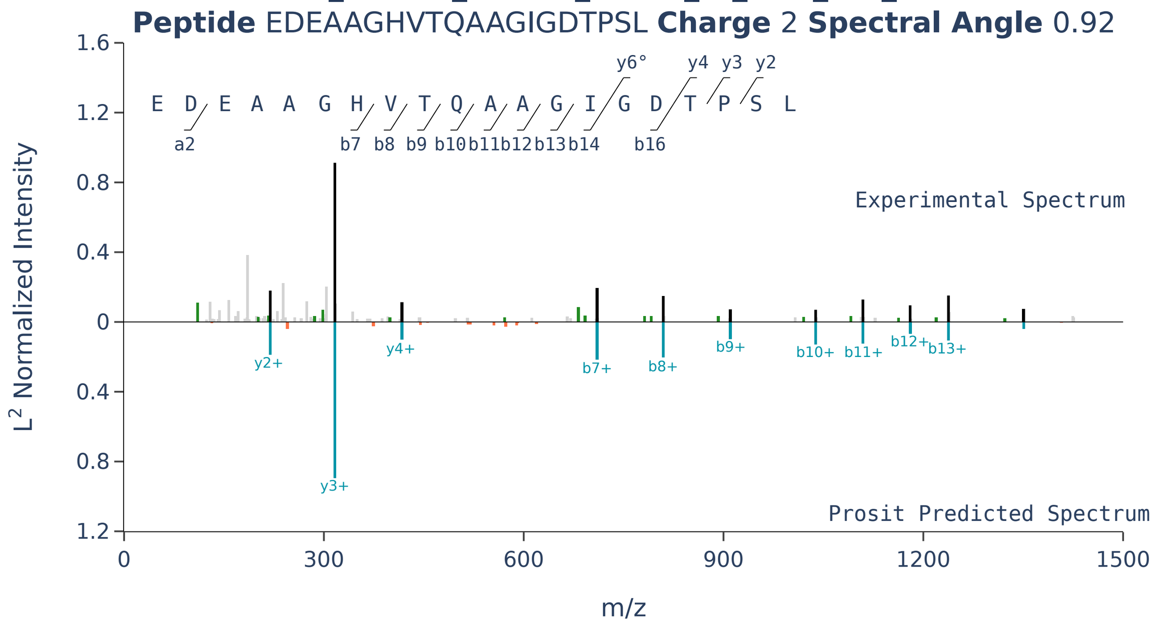

Source WSoh_221121_011221_HFGoe_L2_hTau_2h_R1 **Scan** 9629
Peptide KSEKLDFKDRVQST **Charge** 2 **Spectral Angle** 0.83

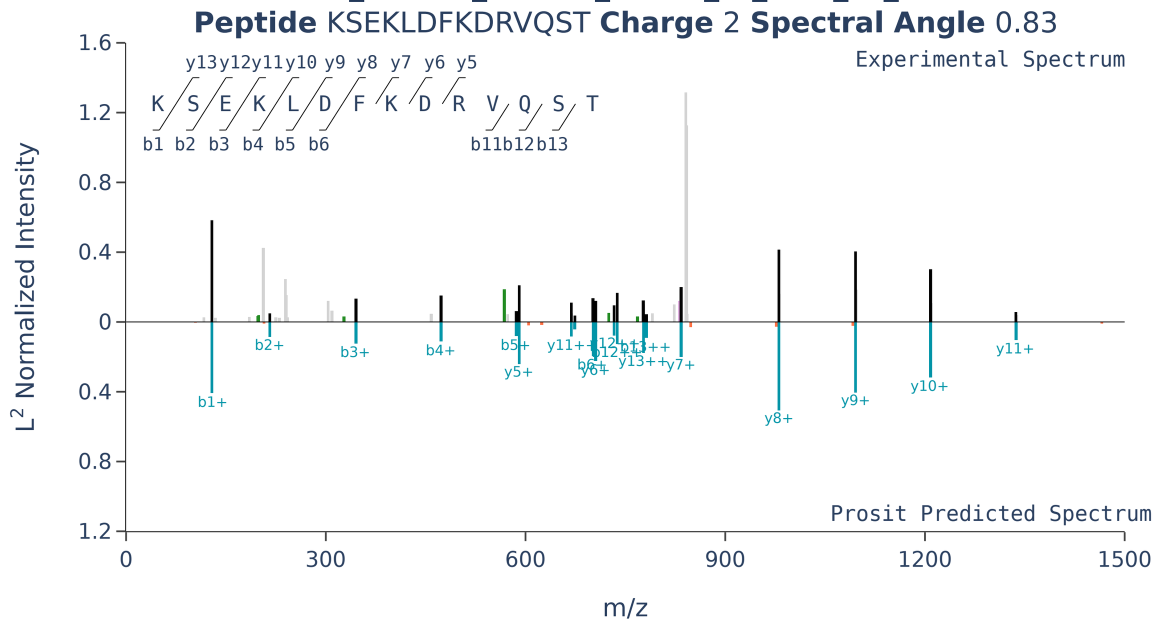

Source WSoh_221121_011221_HFGoe_L1_hTau_4h_R1 **Scan** 15749
Peptide KDRVQSVYKPVDL **Charge** 2 **Spectral Angle** 0.79

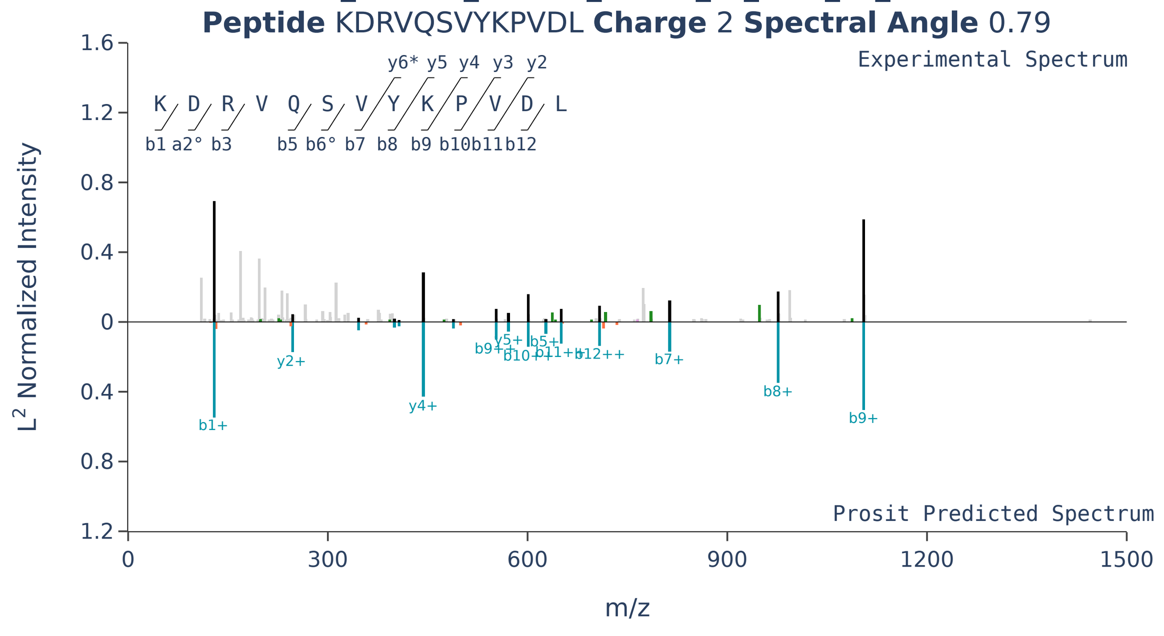

Source WSoh_221121_011221_HFGoe_L2_hTau_24h_R1 **Scan** 14761
Peptide QSTPSLPTPPTREPKKV **Charge** 4 **Spectral Angle** 0.88

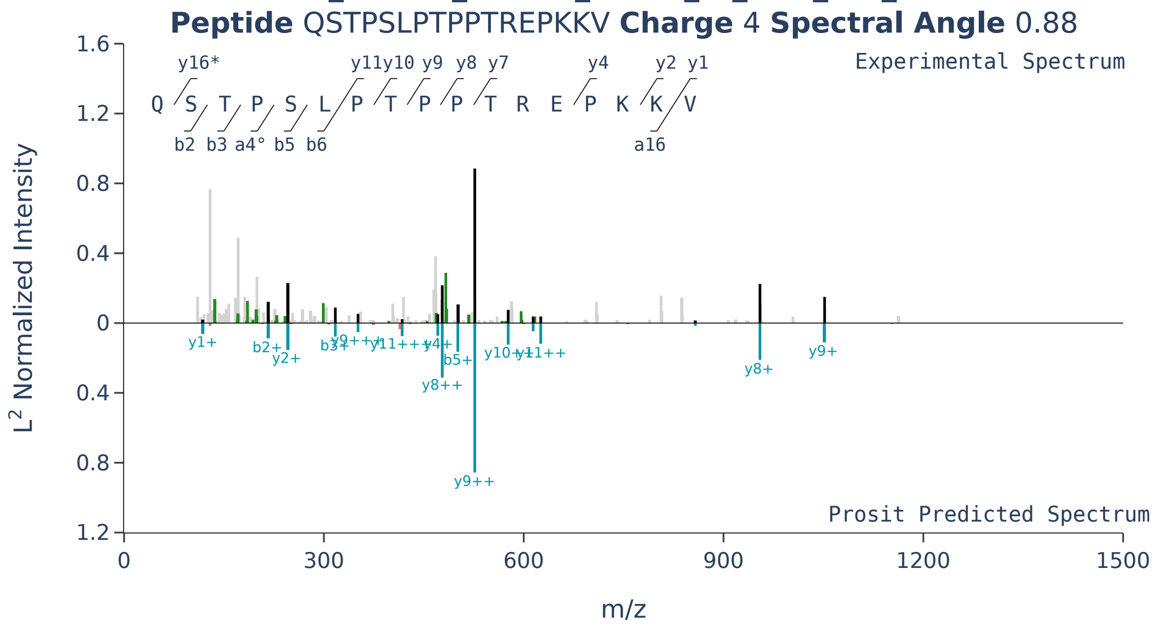

Source WSoh_221121_011221_HFGoe_L1_hTau_24h_R2 **Scan** 12601
Peptide VEVSLGNIHHKPGGGQ **Charge** 3 **Spectral Angle** 0.8

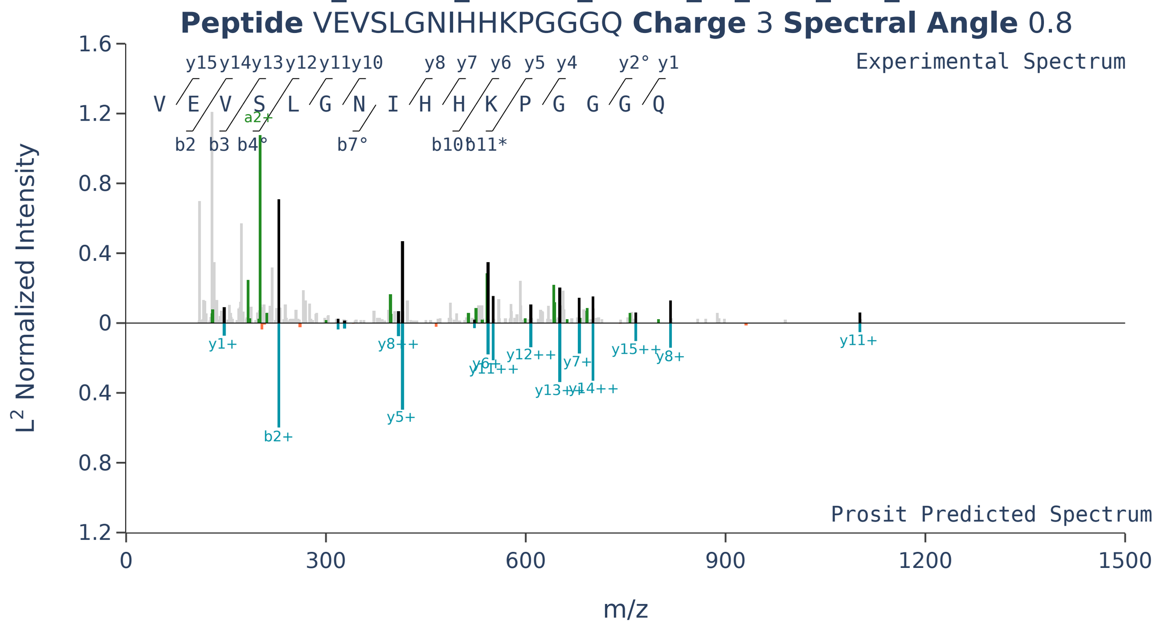

Source WSoh_221121_011221_HFGoe_L1_hTau_2h_R2 **Scan** 18985
Peptide GTEPRQEFEVME **Charge** 2 **Spectral Angle** 0.85

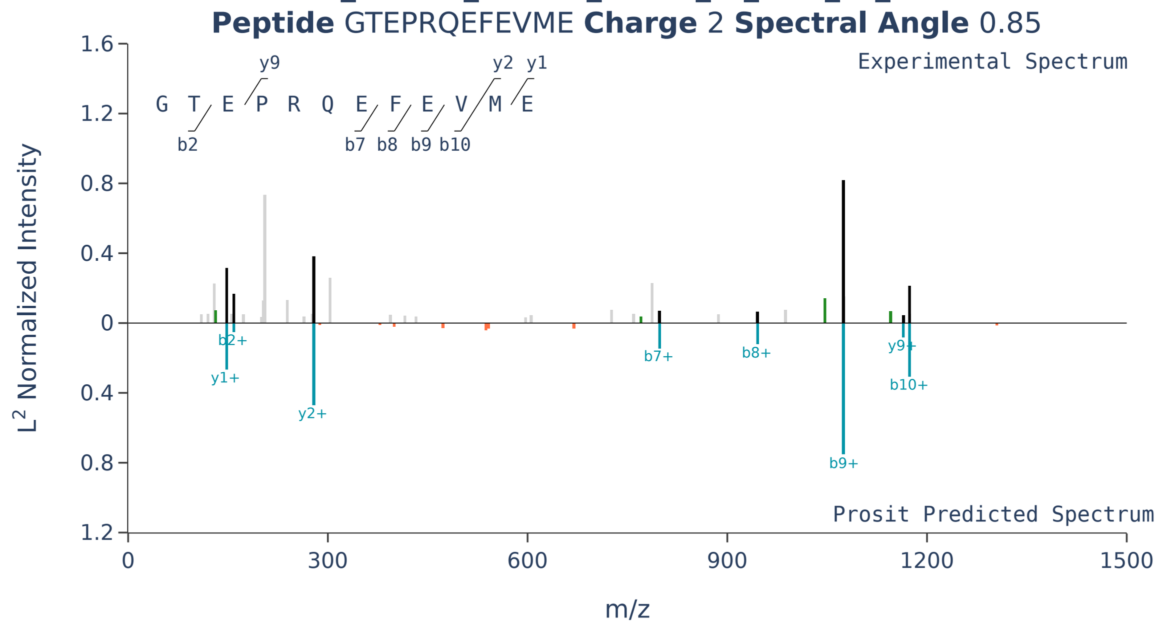

Source WSoh_221121_011221_HFGoe_L1_hTau_24h_R2 **Scan** 7513
Peptide SNVQSKHQPGGGKVQIINK **Charge** 3 **Spectral Angle** 0.89

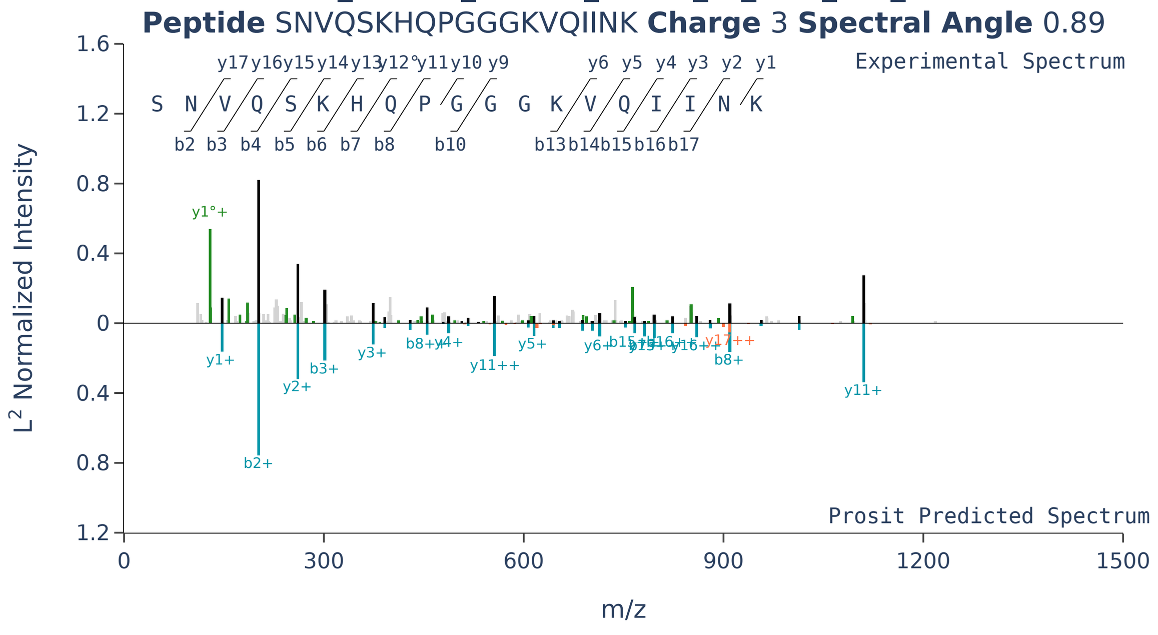

Source WSoh_221121_011221_HFGoe_L2_hTau_24h_R2 **Scan** 21509
Peptide PSLEDEAADNIT **Charge** 1 **Spectral Angle** 0.81

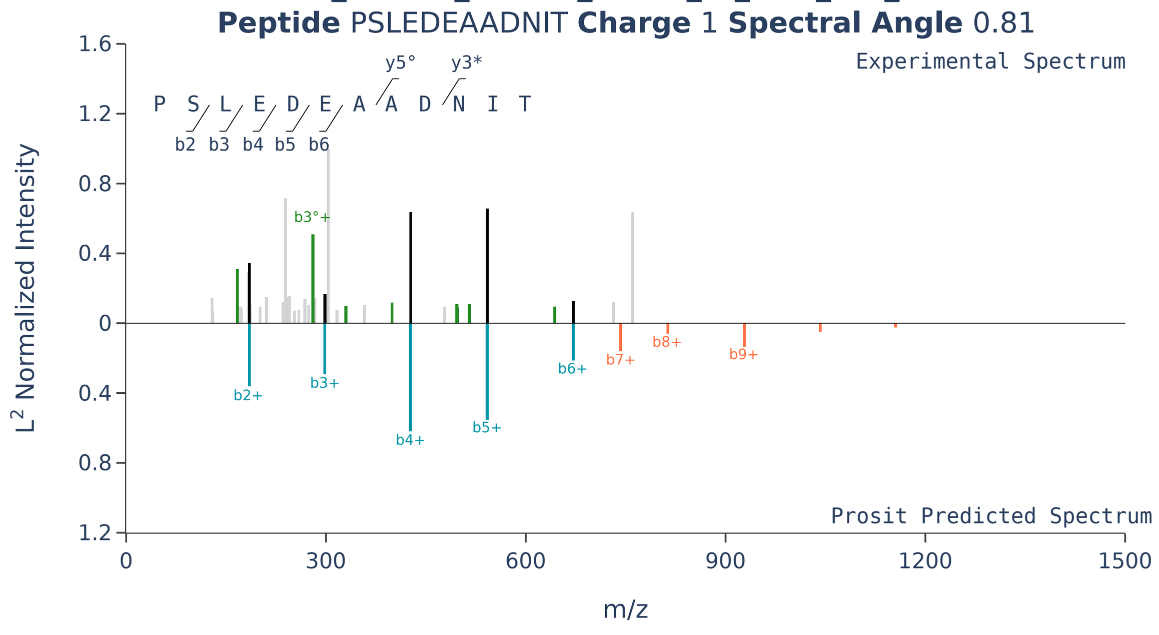

Source WSoh_221121_011221_HFGoe_L2_hTau_4h_R1 **Scan** 12322
Peptide GDSLGNIHHKPGGGQVEV **Charge** 2 **Spectral Angle** 0.83

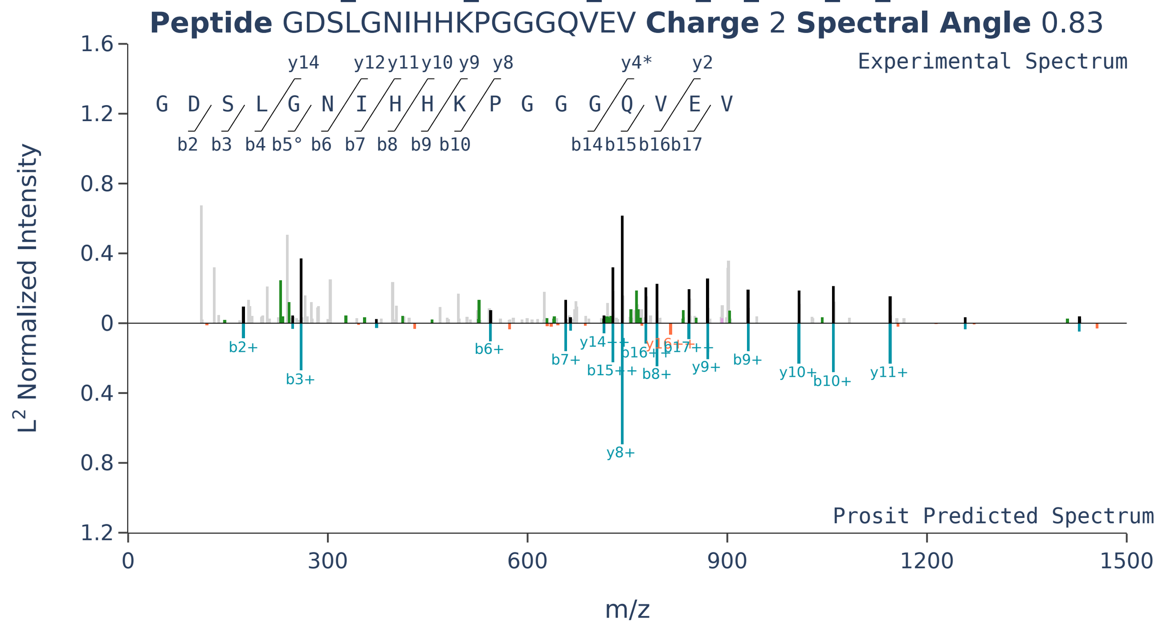

Source WSoh_221121_011221_HFGoe_L1_hTau_4h_R2 **Scan** 19338
Peptide APVPMPDLKNVKSS **Charge** 3 **Spectral Angle** 0.87

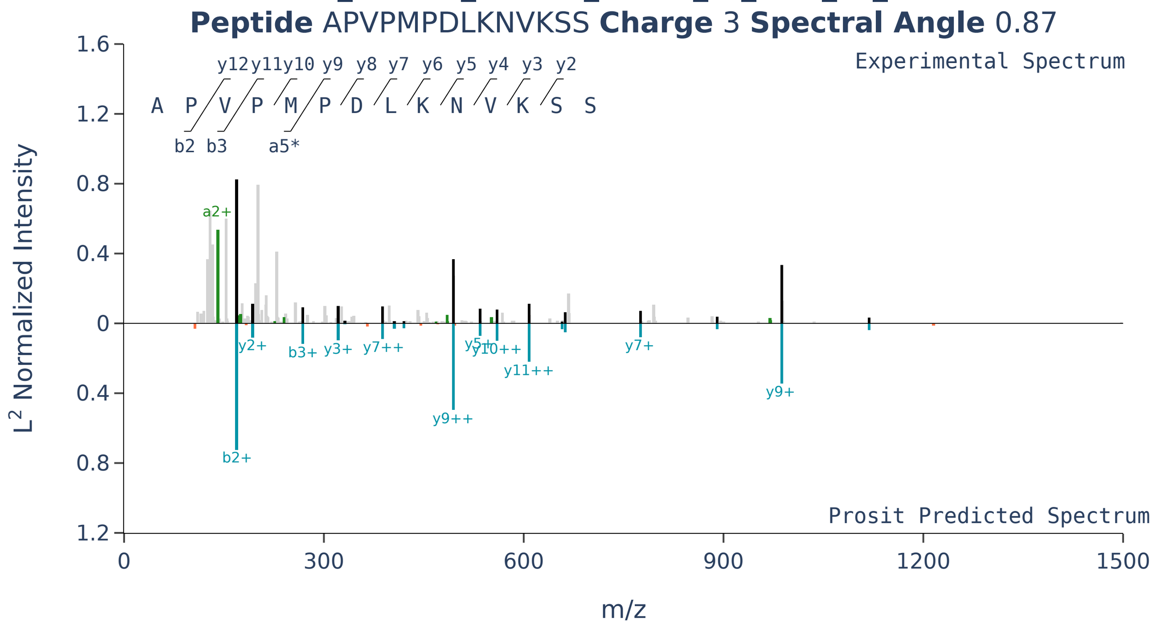

Source WSoh_221121_011221_HFGoe_L1_hTau_24h_R2 **Scan** 11519
Peptide AAQPHTEIPEGTEGAPGKQA **Charge** 2 **Spectral Angle** 0.85

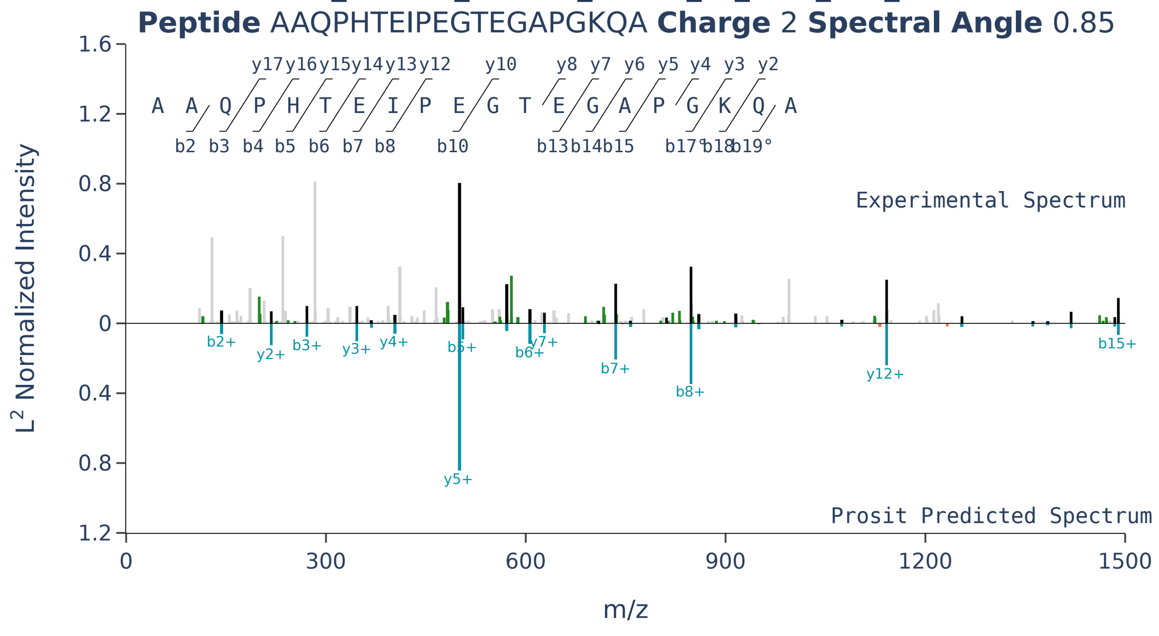

Source WSoh_221121_011221_HFGoe_L2_hTau_24h_R1 **Scan** 11766
Peptide LGGDRKDQGGYTMHQDQEGDTDAGLKE **Charge** 5 **Spectral Angle** 0.78

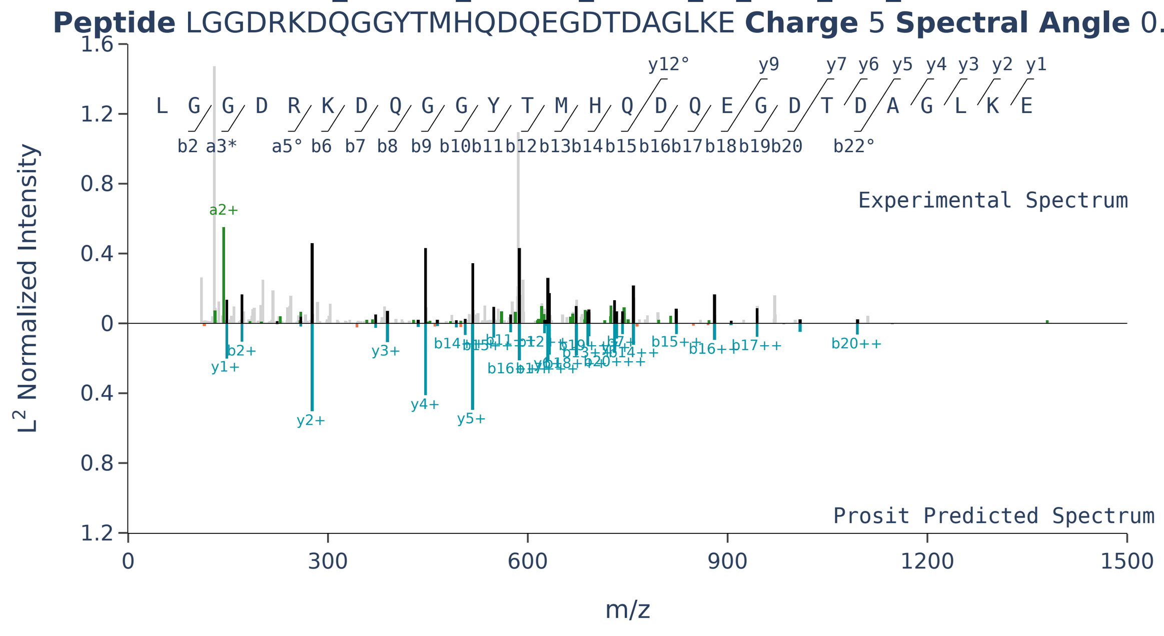

Source WSoh_221121_011221_HFGoe_L1_hTau_24h_R1 **Scan** 22731
Peptide ADEVSASLARMVSKS **Charge** 3 **Spectral Angle** 0.85

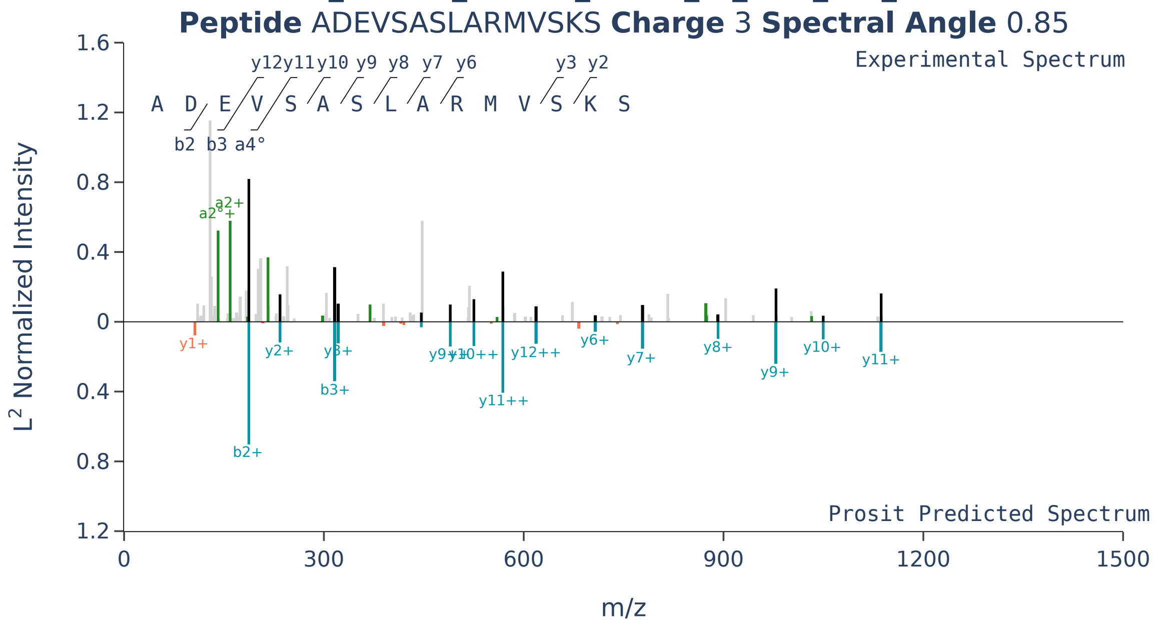

Source WSoh_221121_011221_HFGoe_L1_hTau_24h_R1 **Scan** 10847
Peptide AAQPHTEIPEGEGAPGKQA **Charge** 2 **Spectral Angle** 0.83

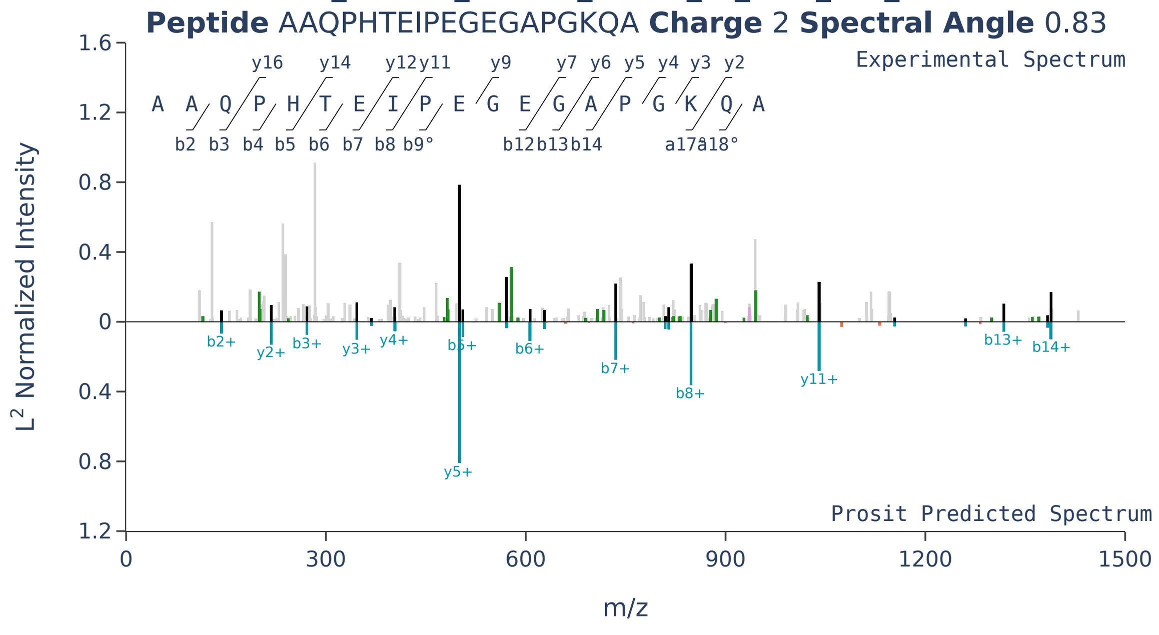

Source WSoh_221121_011221_HFGoe_L2_hTau_24h_R1 **Scan** 19752
Peptide SAEPRQEFEVMED **Charge** 2 **Spectral Angle** 0.89

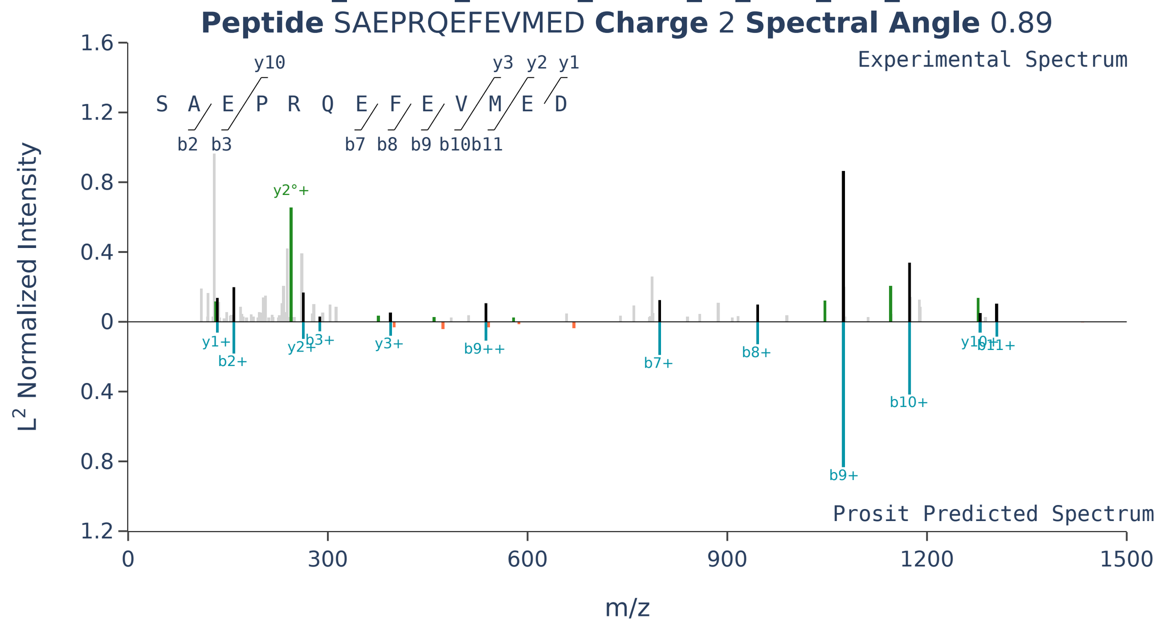

Source WSoh_221121_011221_HFGoe_L1_hTau_4h_R2 **Scan** 19308
Peptide QSVYKPVDL **Charge** 2 **Spectral Angle** 0.85

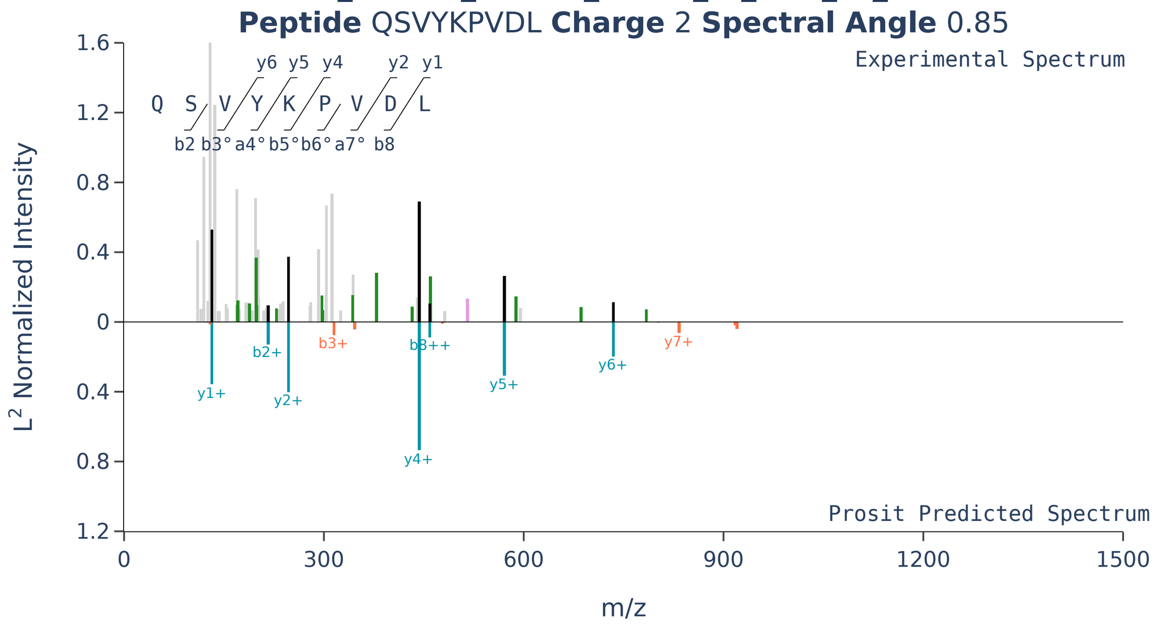

Source WSoh_221121_011221_HFGoe_L1_hTau_4h_R2 **Scan** 11686
Peptide SKHVPGGGSVQI **Charge** 3 **Spectral Angle** 0.89

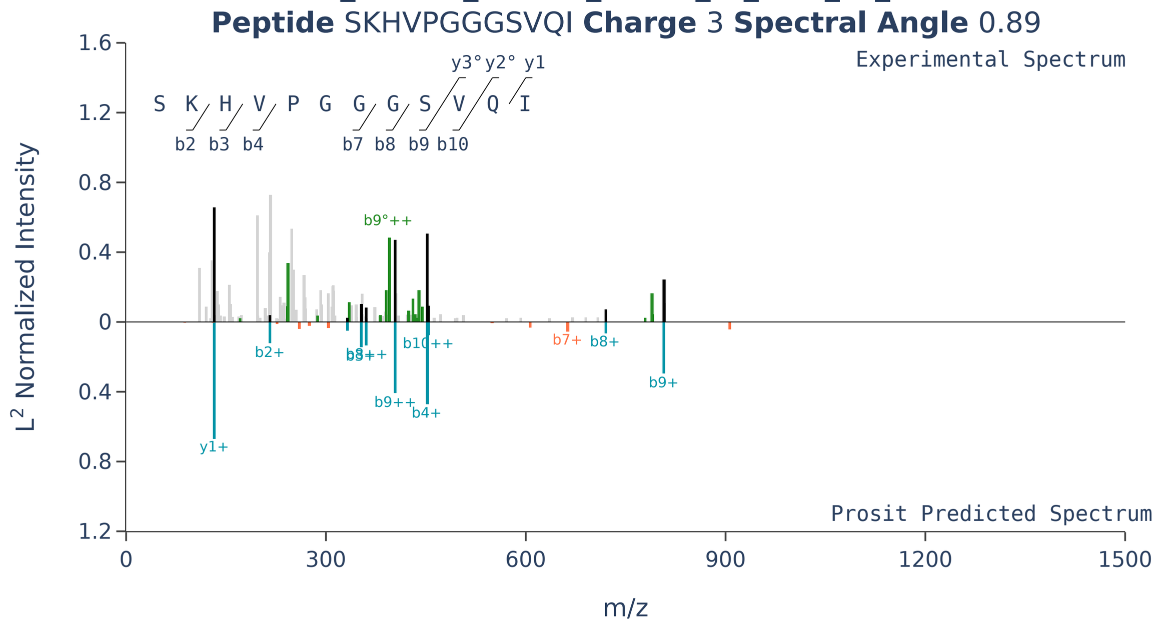

Source WSoh_221121_011221_HFGoe_L2_hTau_4h_R1 **Scan** 9289
Peptide SKPGGGQVEV **Charge** 2 **Spectral Angle** 0.88

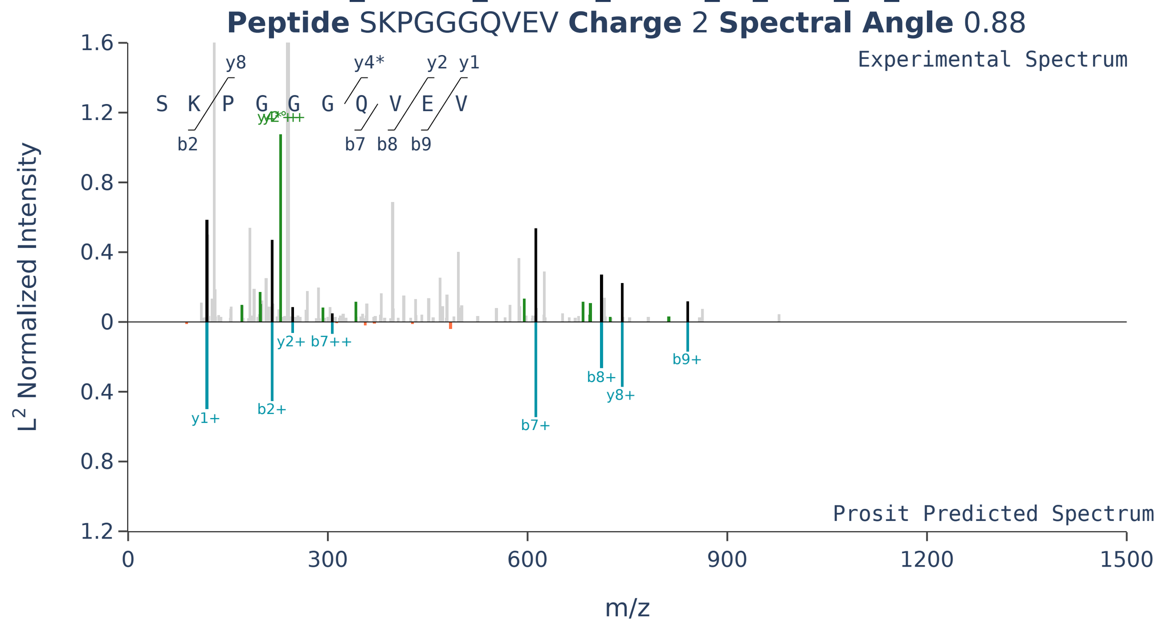

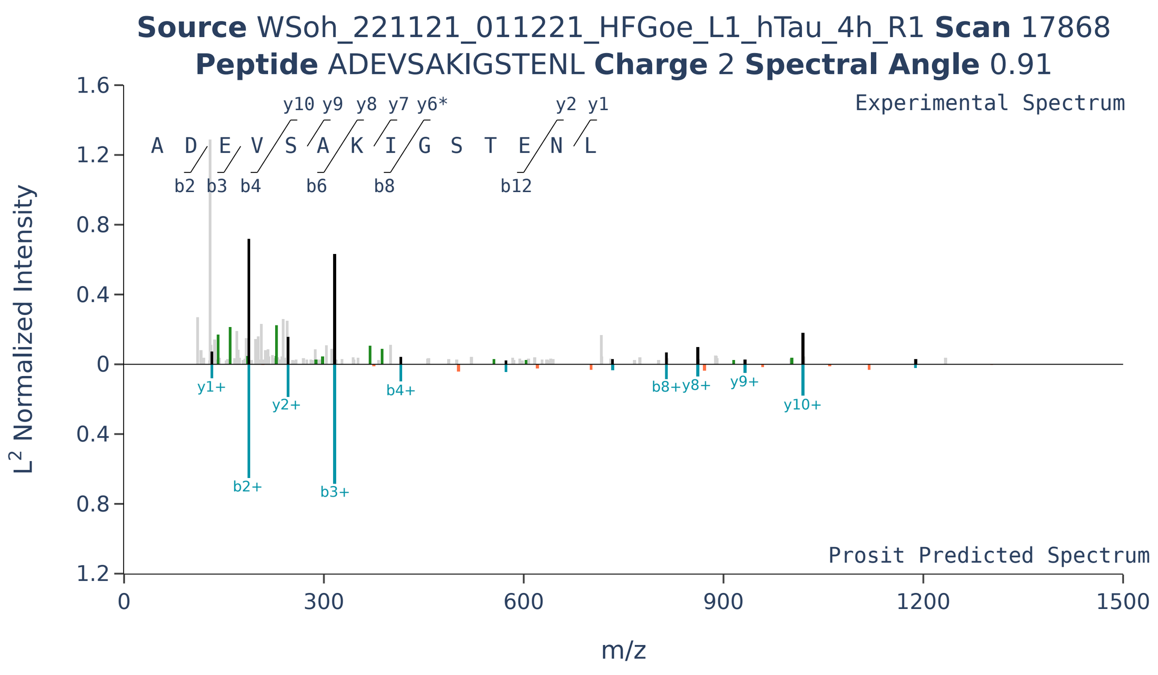
Source WSoh_221121_011221_HFGoe_L1_hTau_4h_R1 **Scan** 17868
Peptide ADEVSAKIGSTENL **Charge** 2 **Spectral Angle** 0.91

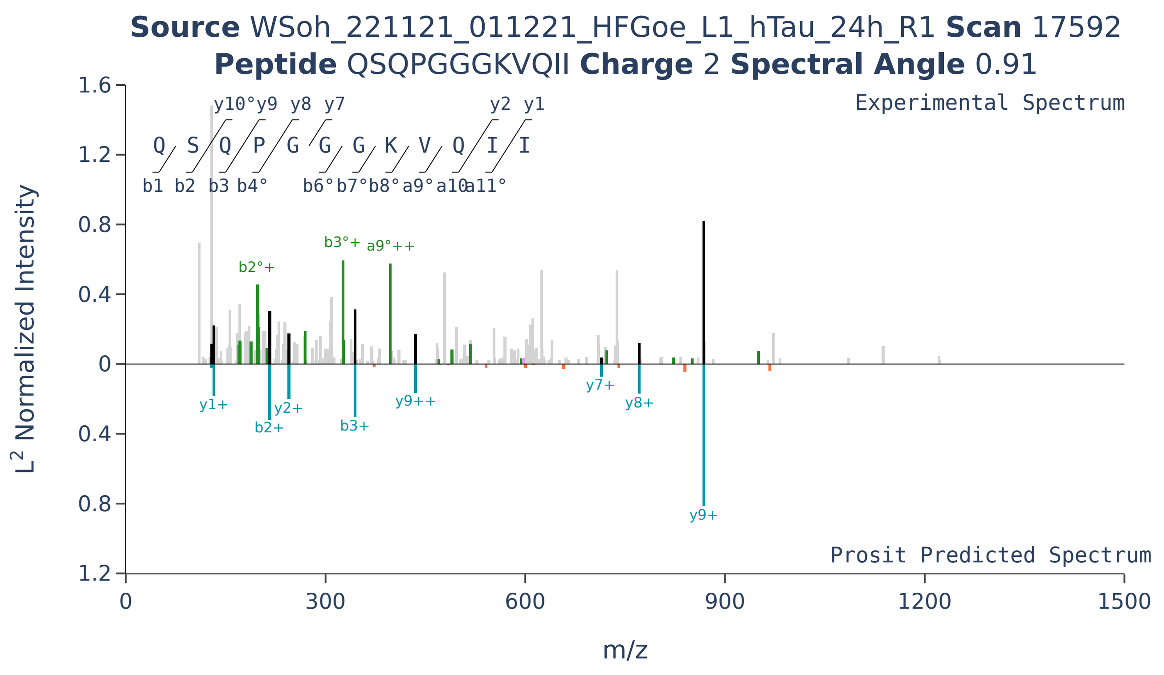
Source WSoh_221121_011221_HFGoe_L1_hTau_24h_R1 **Scan** 17592
Peptide QSQPGGGKVQII **Charge** 2 **Spectral Angle** 0.91

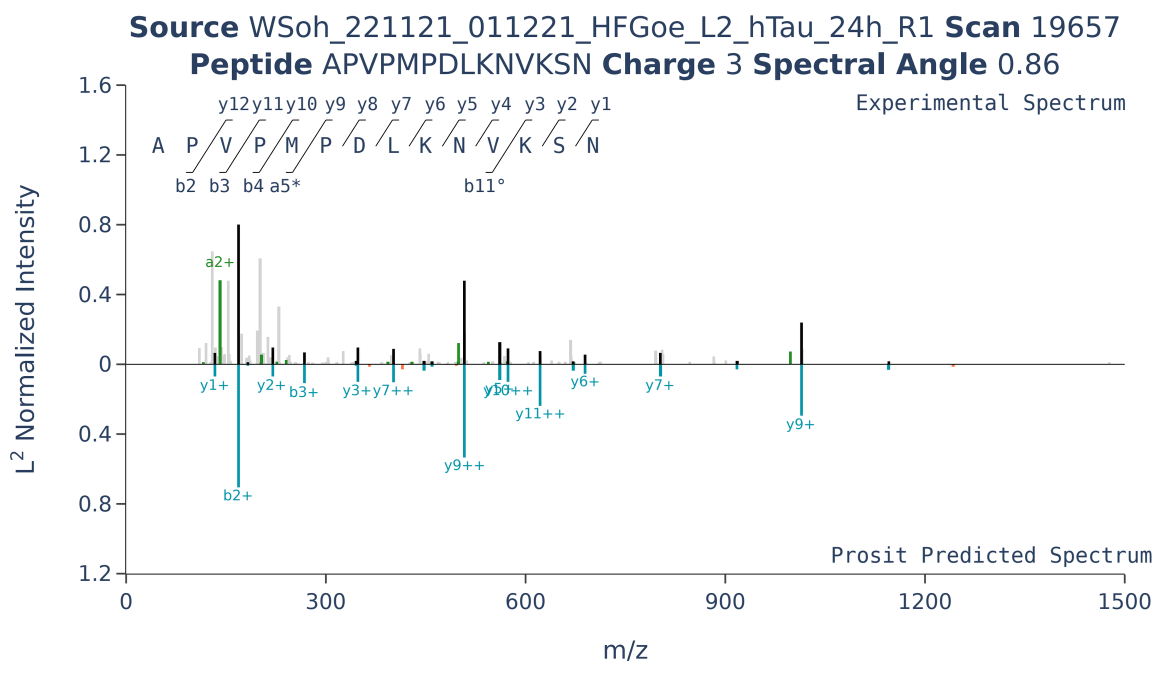
Source WSoh_221121_011221_HFGoe_L2_hTau_24h_R1 **Scan** 19657
Peptide APVPMPDLKNVKSN **Charge** 3 **Spectral Angle** 0.86

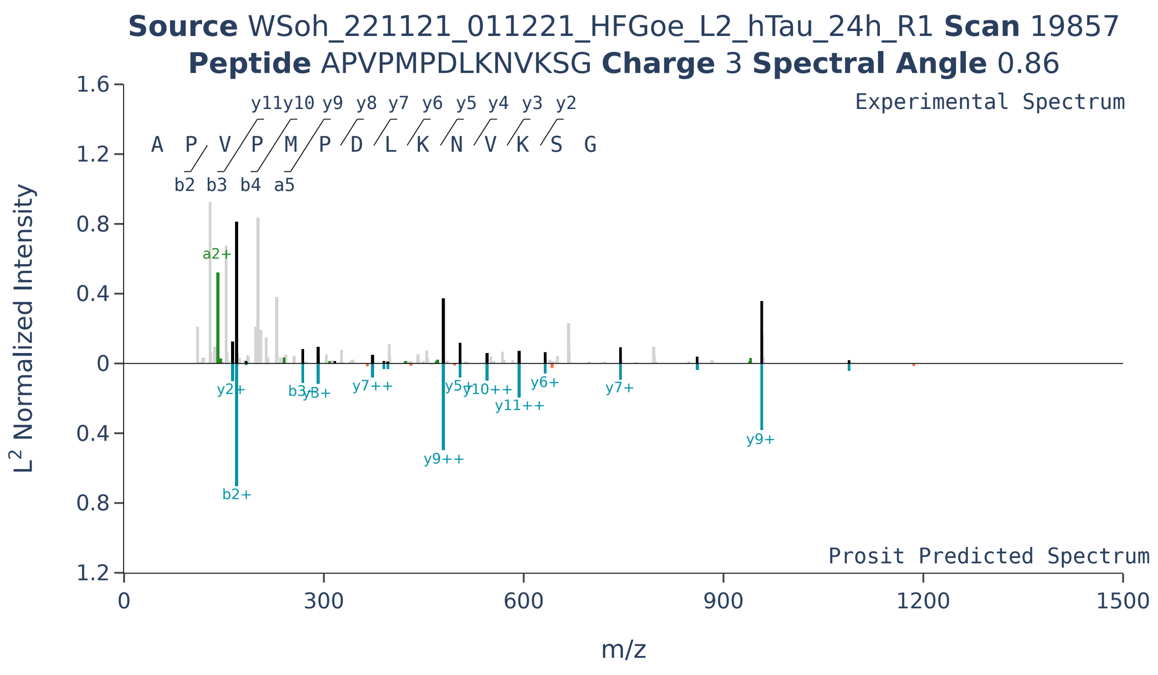
Source WSoh_221121_011221_HFGoe_L2_hTau_24h_R1 **Scan** 19857
Peptide APVPMPDLKNVKSG **Charge** 3 **Spectral Angle** 0.86

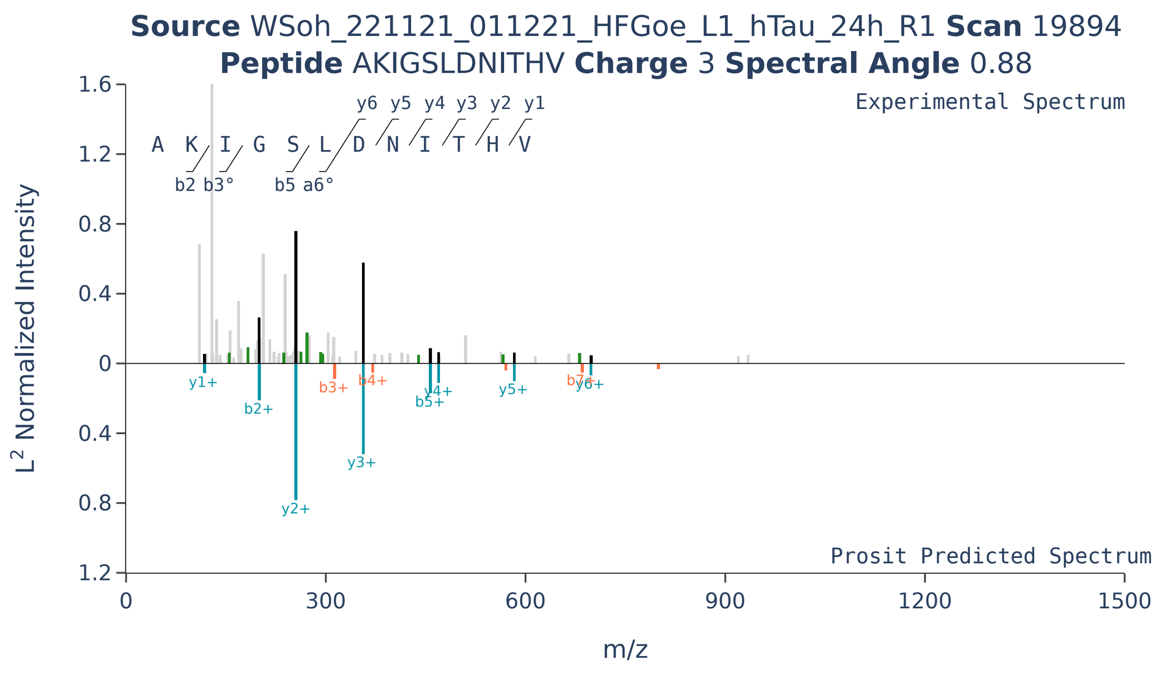
Source WSoh_221121_011221_HFGoe_L1_hTau_24h_R1 **Scan** 19894
Peptide AKIGSLDNITHV **Charge** 2 **Spectral Angle** 0.88

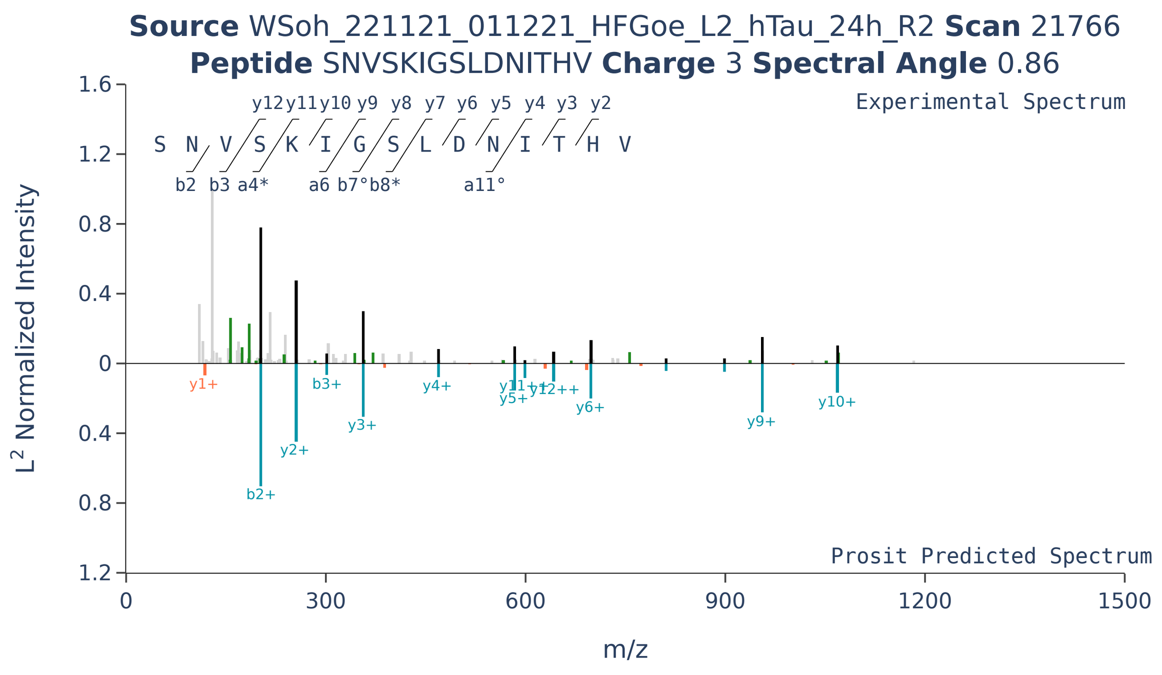
Source WSoh_221121_011221_HFGoe_L2_hTau_24h_R2 **Scan** 21766
Peptide SNVSKIGSLDNITHV **Charge** 3 **Spectral Angle** 0.86

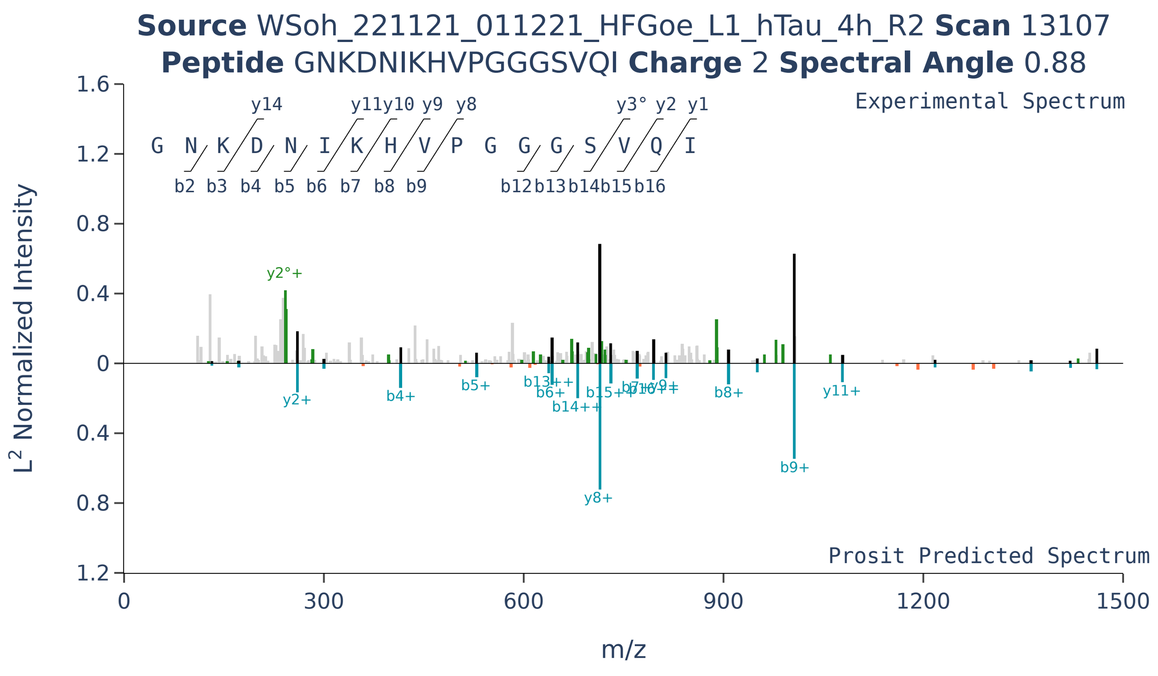
Source WSoh_221121_011221_HFGoe_L1_hTau_4h_R2 **Scan** 13107
Peptide GNKDNIKHVPGGGSVQI **Charge** 2 **Spectral Angle** 0.88

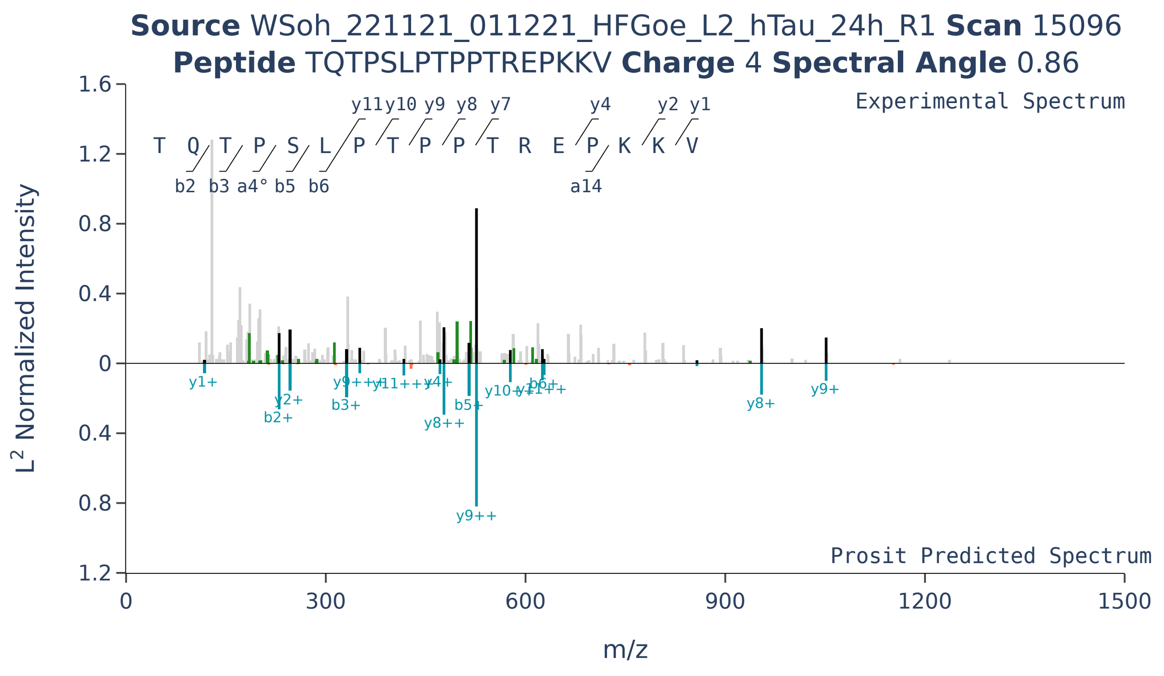
Source WSoh_221121_011221_HFGoe_L2_hTau_24h_R1 **Scan** 15096
Peptide TQTPSLPTPPTREPKKV **Charge** 4 **Spectral Angle** 0.86

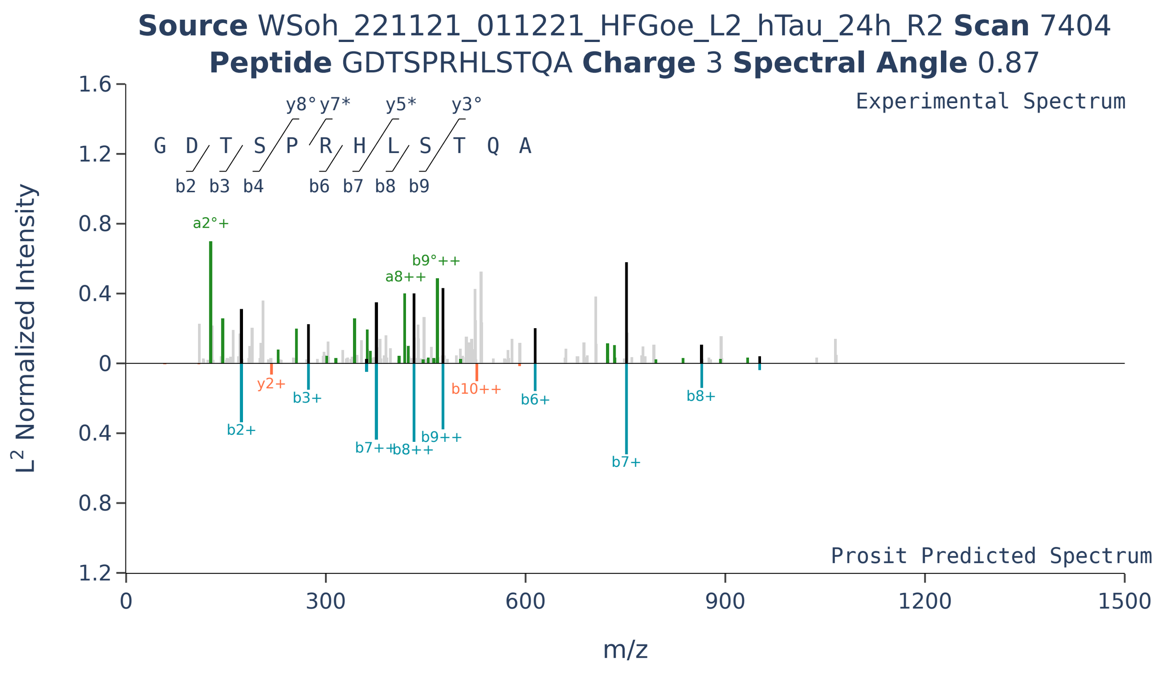
Source WSoh_221121_011221_HFGoe_L2_hTau_24h_R2 **Scan** 7404
Peptide GDTSPRHLSTQA **Charge** 3 **Spectral Angle** 0.87

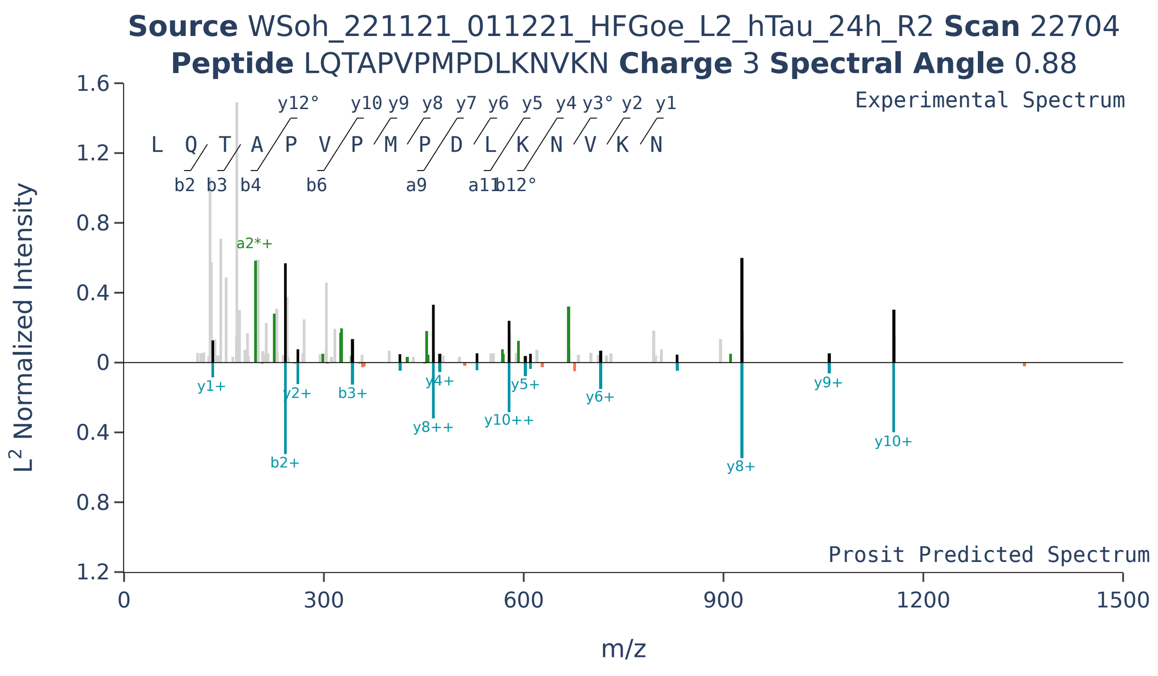
Source WSoh_221121_011221_HFGoe_L2_hTau_24h_R2 **Scan** 22704
Peptide LQTAPVPMPDLKNVKN **Charge** 3 **Spectral Angle** 0.88

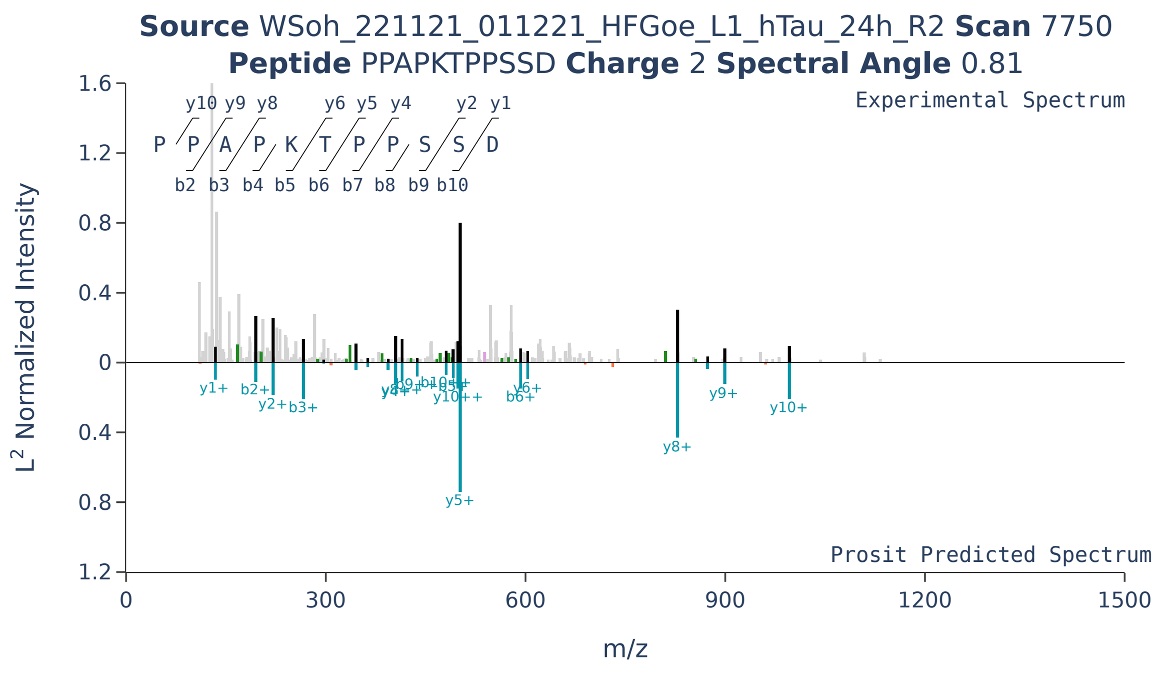
Source WSoh_221121_011221_HFGoe_L1_hTau_24h_R2 **Scan** 7750
Peptide PPAPKTPPSSD **Charge** 2 **Spectral Angle** 0.81

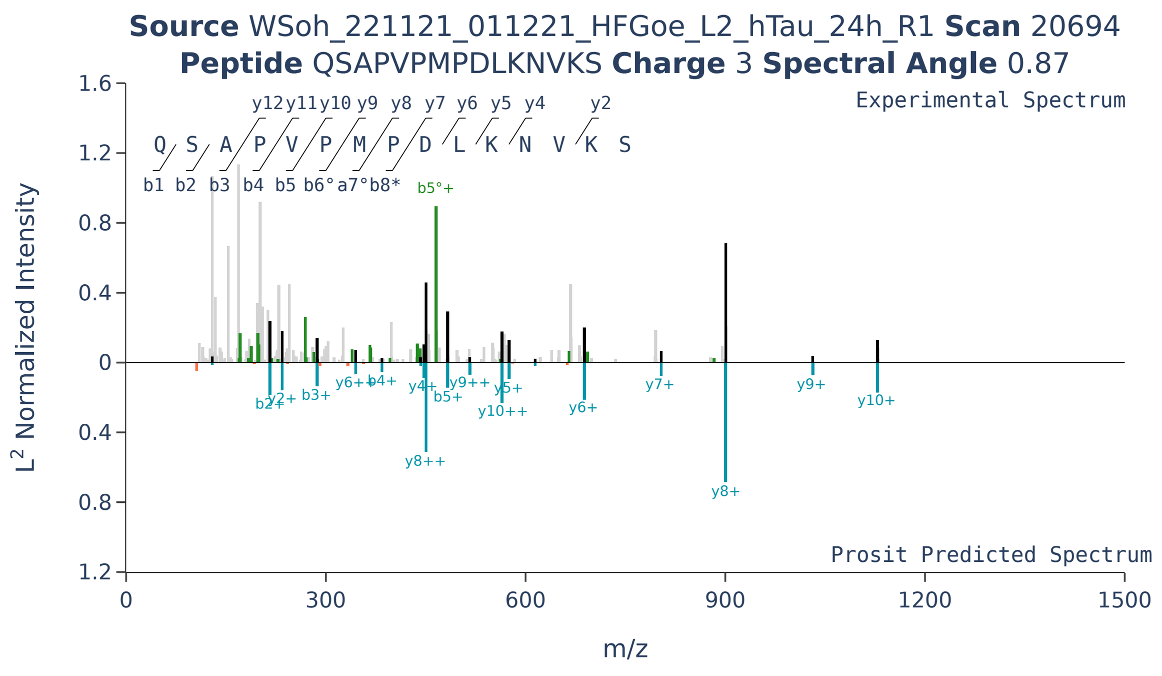
Source WSoh_221121_011221_HFGoe_L2_hTau_24h_R1 **Scan** 20694
Peptide QSAPVPMPDLKNVKS **Charge** 3 **Spectral Angle** 0.87

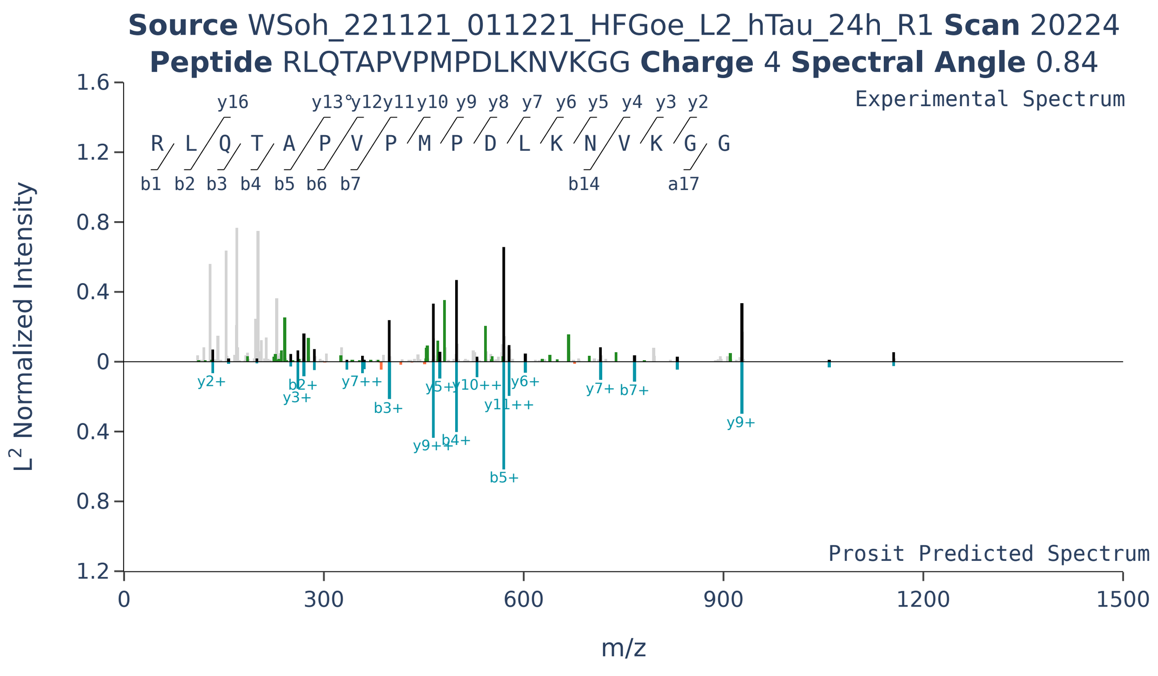
Source WSoh_221121_011221_HFGoe_L2_hTau_24h_R1 **Scan** 20224
Peptide RLQTAPVPMPDLKNVKGG **Charge** 4 **Spectral Angle** 0.84

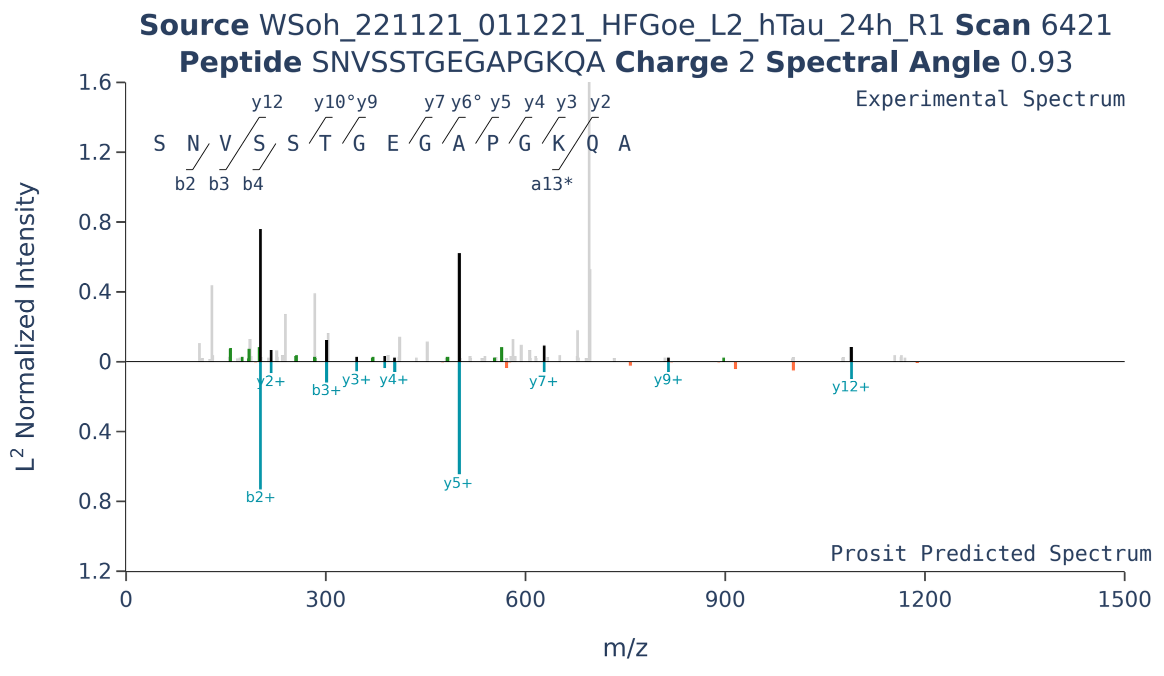
Source WSoh_221121_011221_HFGoe_L2_hTau_24h_R1 **Scan** 6421
Peptide SNVSSTGEGAPGKQA **Charge** 2 **Spectral Angle** 0.93

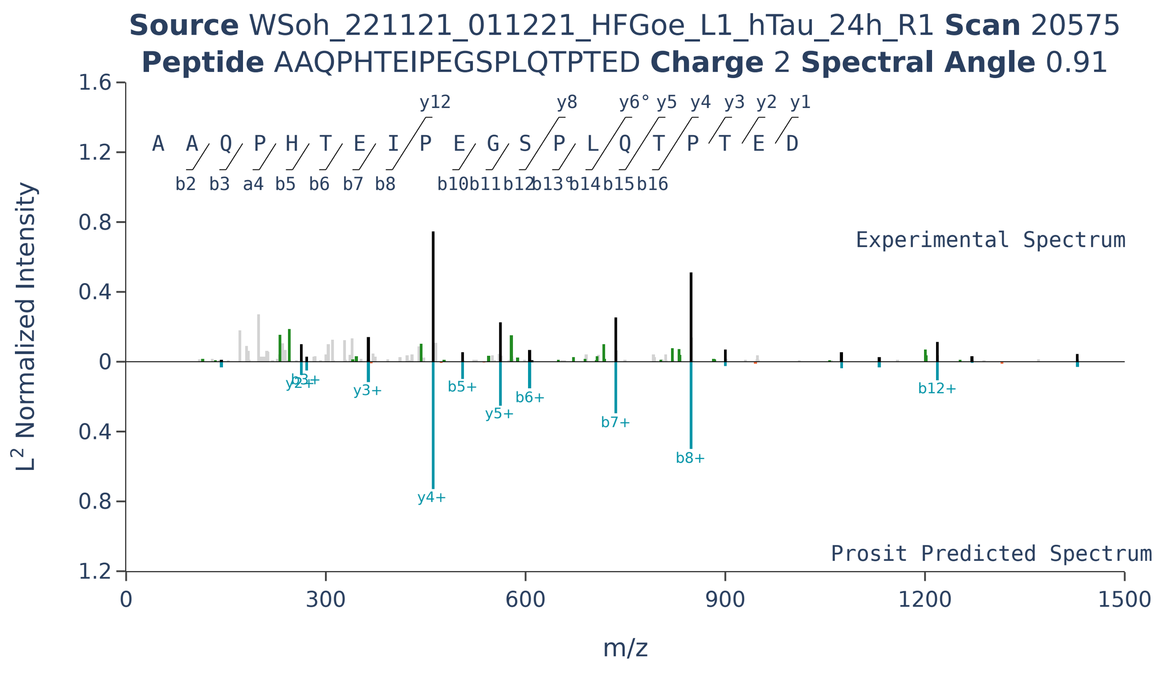
Source WSoh_221121_011221_HFGoe_L1_hTau_24h_R1 **Scan** 20575
Peptide AAQPHTEIPEGSPLQTPTED **Charge** 2 **Spectral Angle** 0.91

Source WSoh_221121_011221_HFGoe_L1_hTau_24h_R1 **Scan** 20388
Peptide QTAPVPMPDLKNVKN **Charge** 3 **Spectral Angle** 0.85

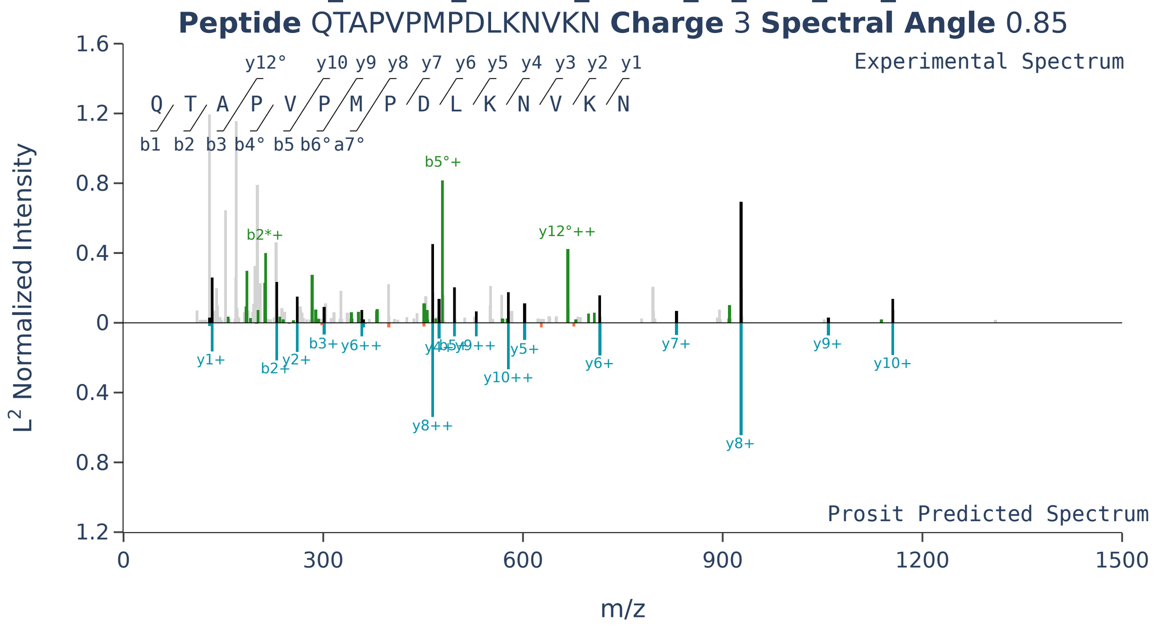

Source WSoh_221121_011221_HFGoe_L1_hTau_24h_R2 **Scan** 20946
Peptide NATRIAPVPMPDLKNVKS **Charge** 3 **Spectral Angle** 0.89

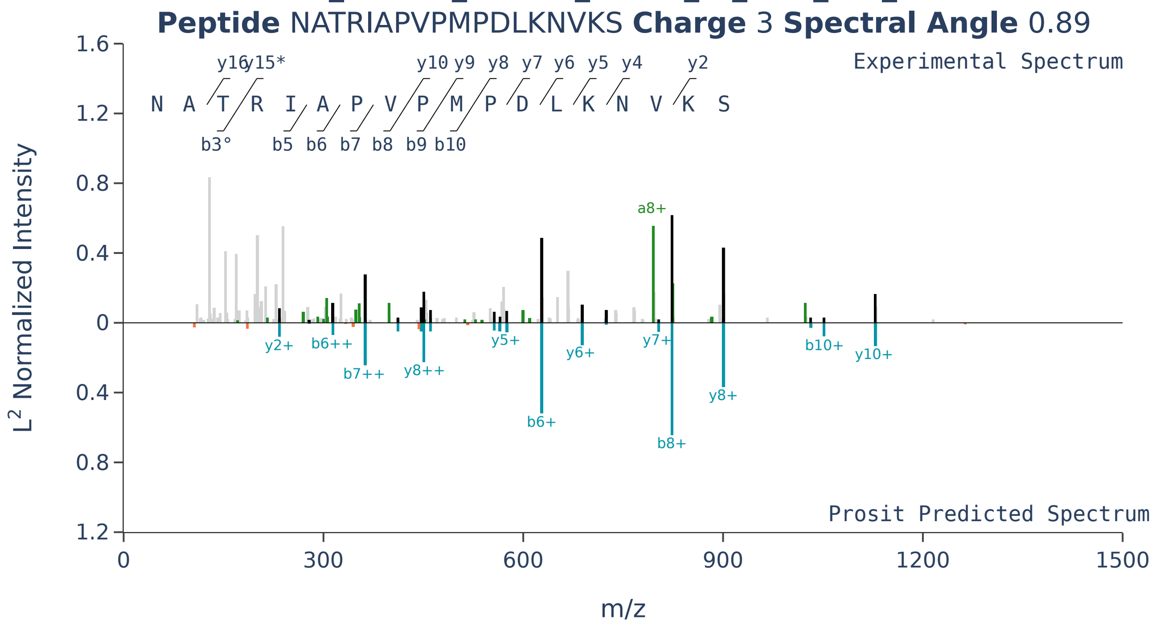

Source WSoh_221121_011221_HFGoe_L1_hTau_24h_R1 **Scan** 17679
Peptide KLDFKDRVQSL **Charge** 2 **Spectral Angle** 0.8

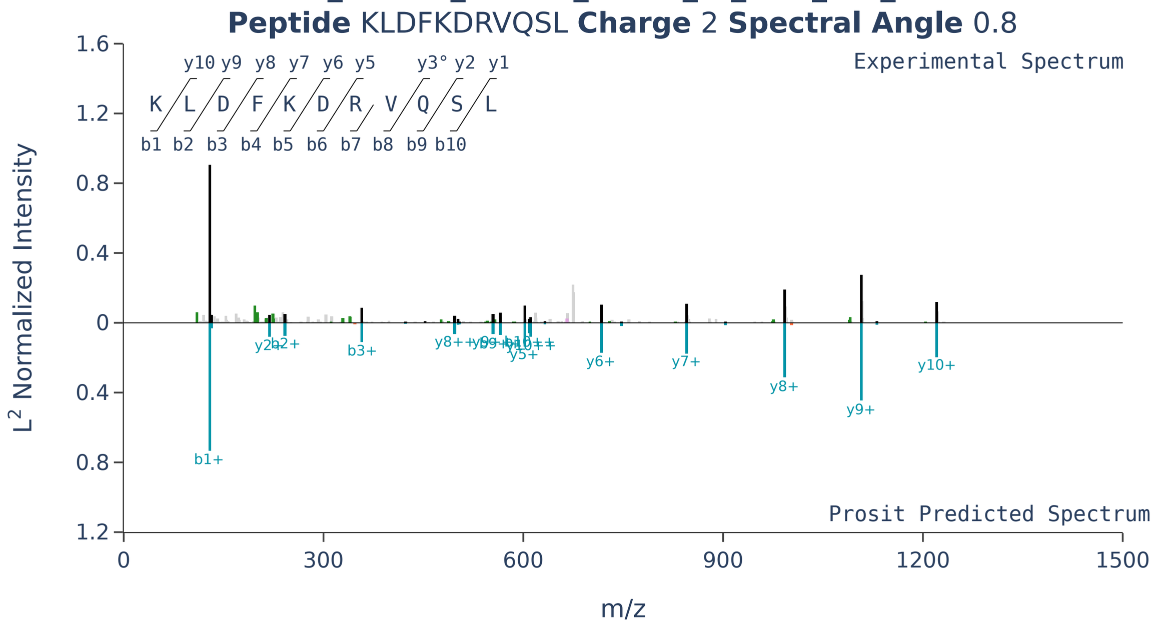

Source WSoh_221121_011221_HFGoe_L2_hTau_24h_R1 **Scan** 20558
Peptide VSSAEPRQEFEVME **Charge** 2 **Spectral Angle** 0.84

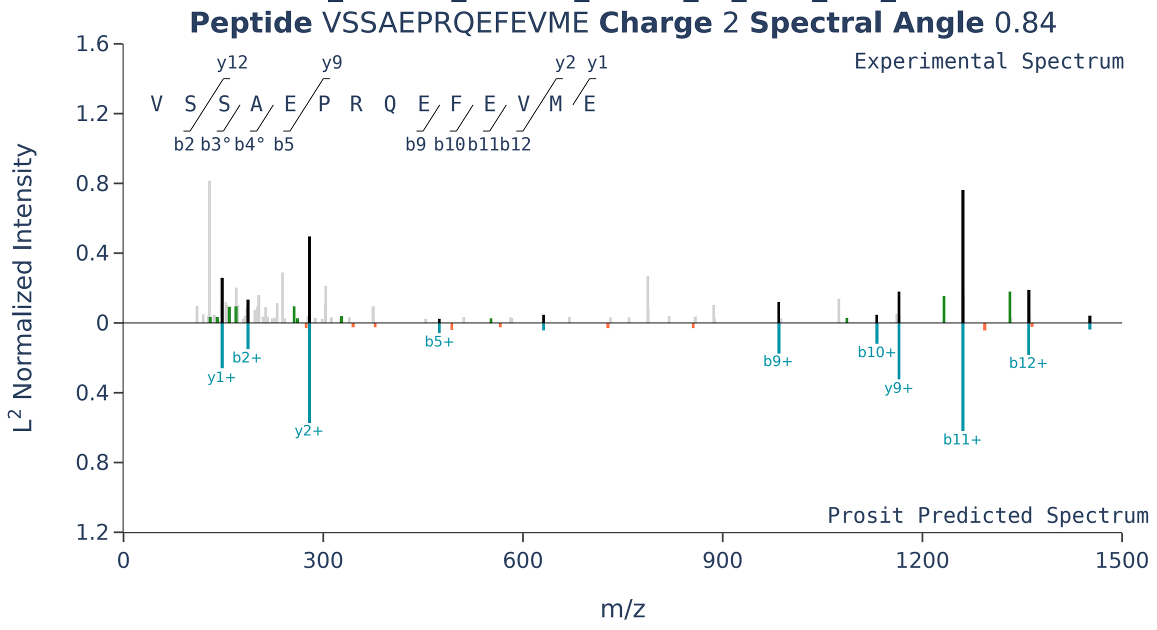

Source WSoh_221121_011221_HFGoe_L2_hTau_24h_R2 **Scan** 10085
Peptide ESGGKVQIINK **Charge** 3 **Spectral Angle** 0.88

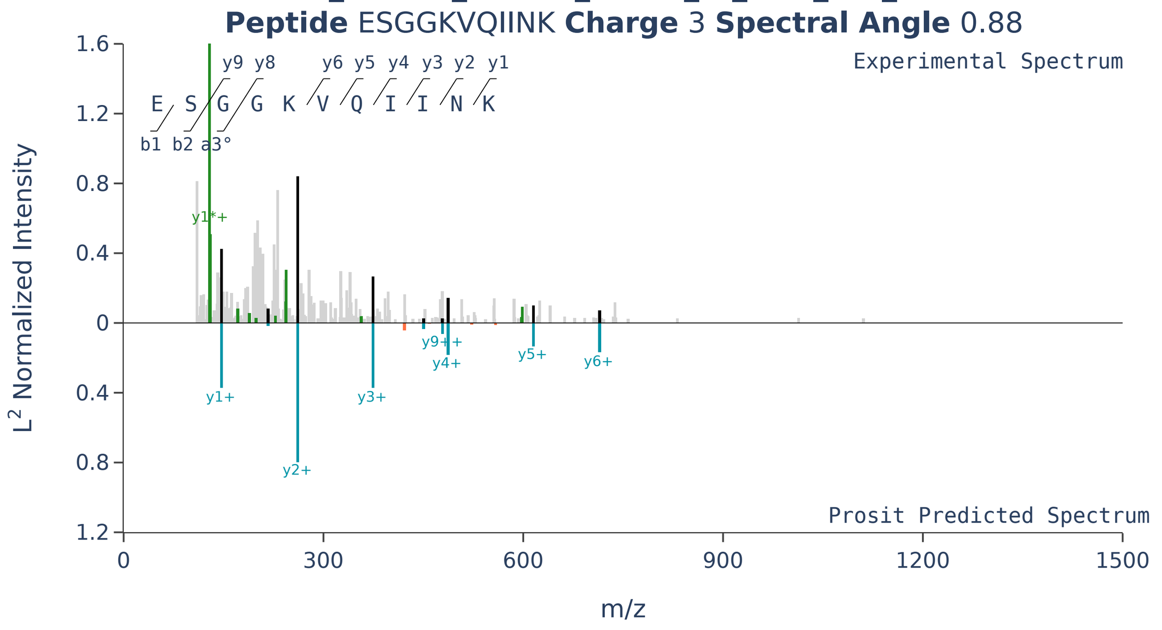

Source WSoh_221121_011221_HFGoe_L1_hTau_24h_R1 **Scan** 6908
Peptide GDTSPRHLSNVSN **Charge** 2 **Spectral Angle** 0.84

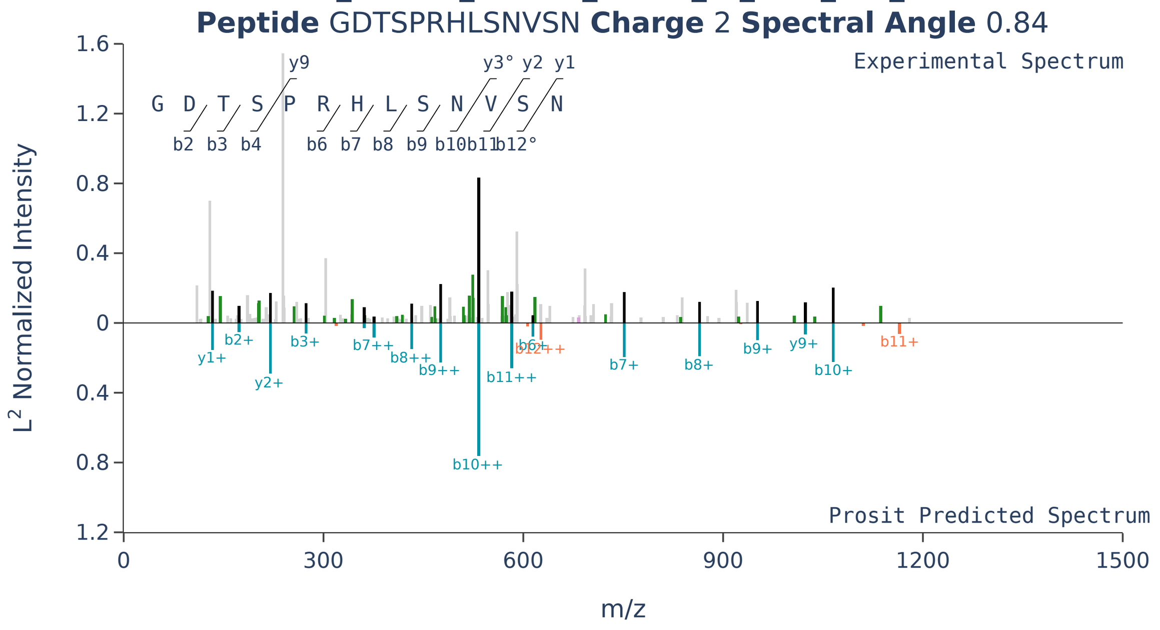

Source WSoh_221121_011221_HFGoe_L1_hTau_2h_R2 **Scan** 19057
Peptide AGEPRQEFEVME **Charge** 2 **Spectral Angle** 0.87

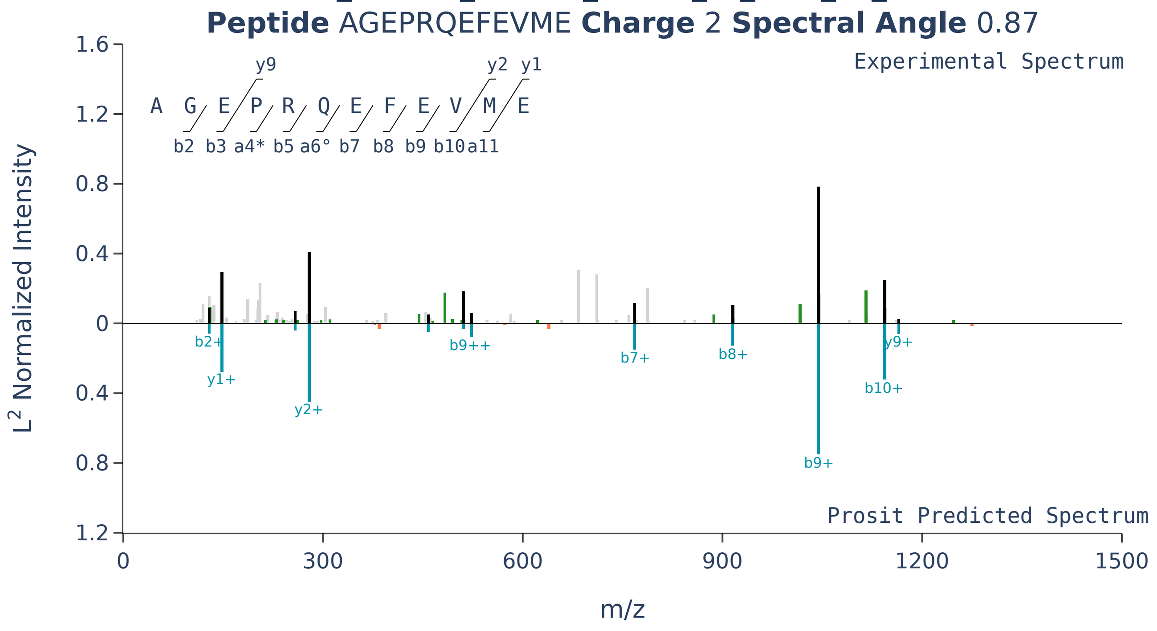

Source WSoh_221121_011221_HFGoe_L1_hTau_24h_R1 **Scan** 14465
Peptide SKTPSLPTPPTREPKKVAV **Charge** 4 **Spectral Angle** 0.86

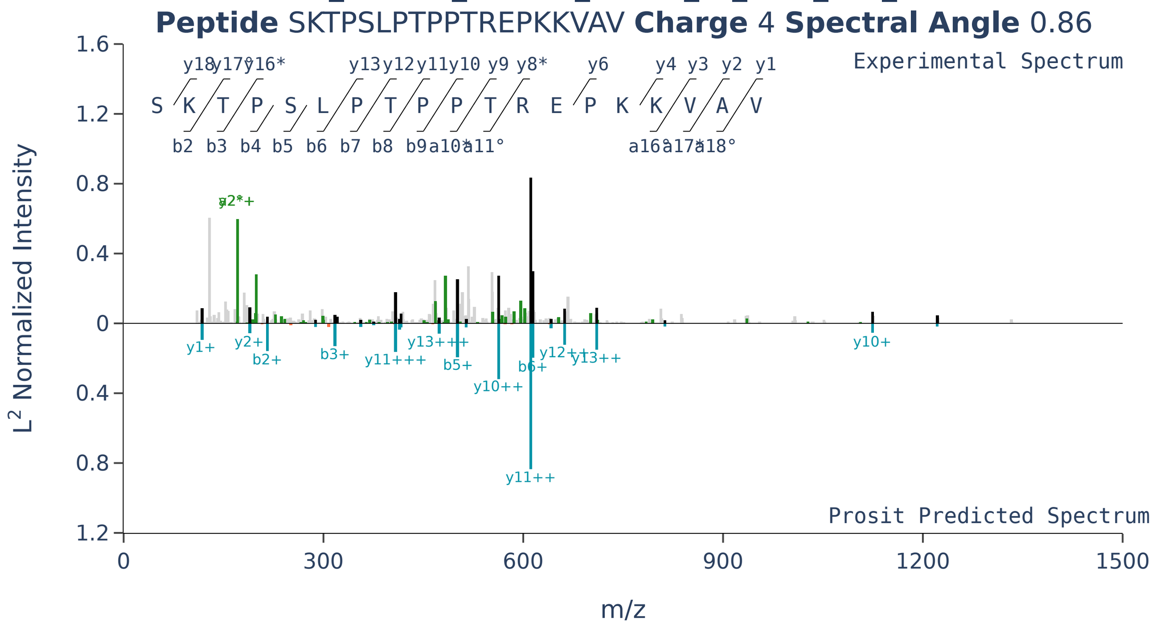

Source WSoh_221121_011221_HFGoe_L1_hTau_4h_R2 **Scan** 15607
Peptide SNVQSKIGSL **Charge** 2 **Spectral Angle** 0.9

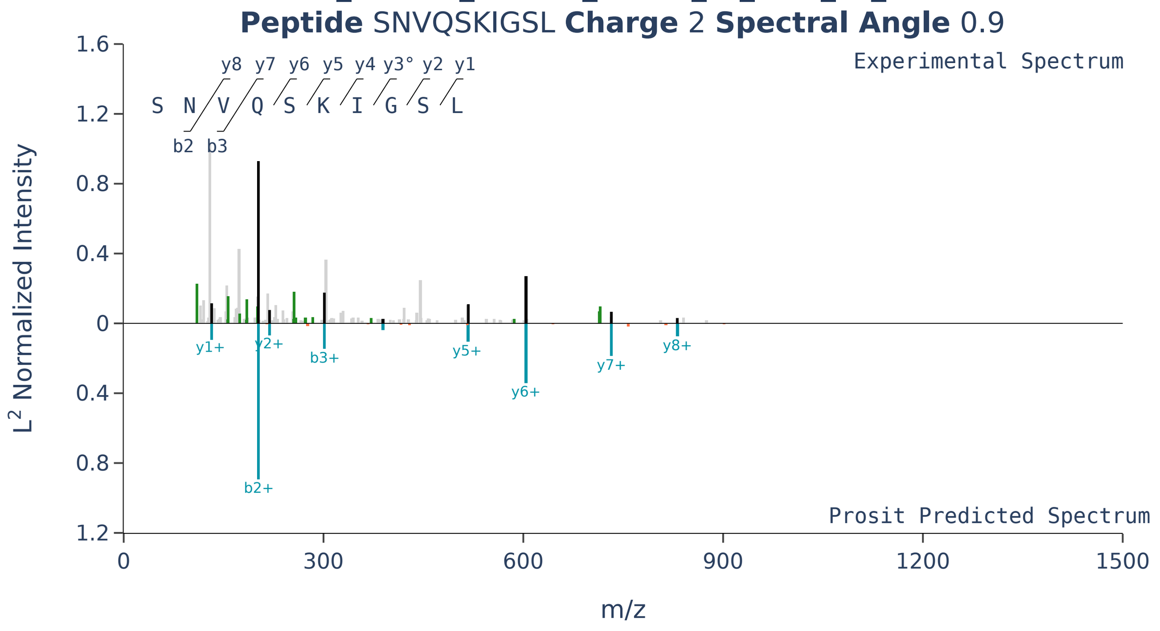

Source WSoh_221121_011221_HFGoe_L1_hTau_4h_R1 **Scan** 12086
Peptide AKSIGSTENL **Charge** 2 **Spectral Angle** 0.82

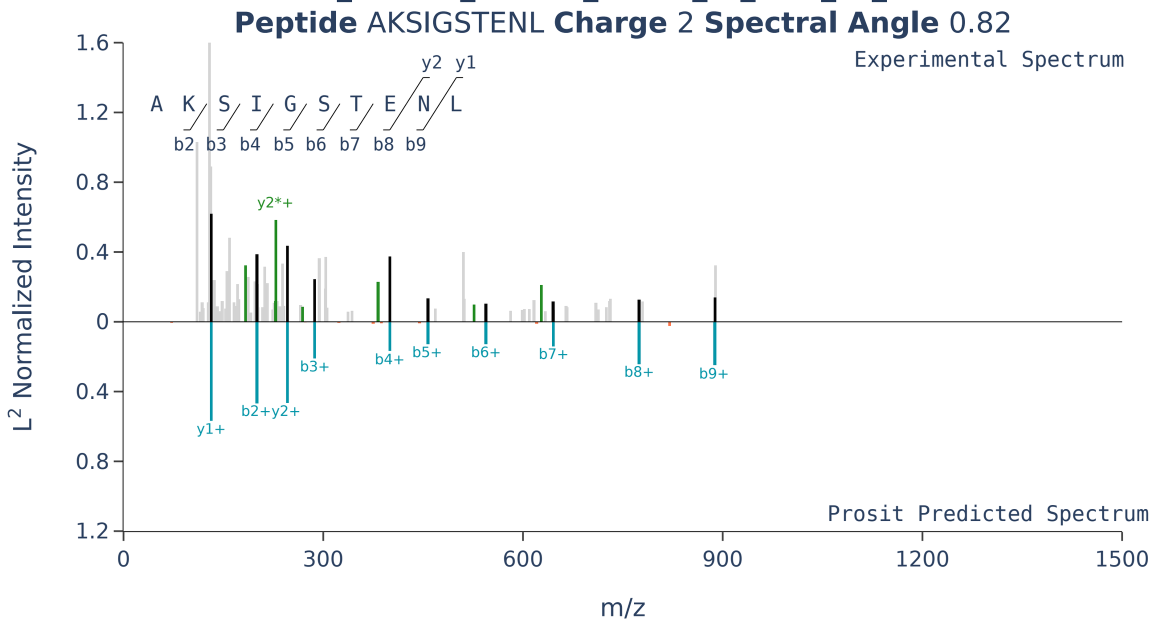

Source WSoh_221121_011221_HFGoe_L2_hTau_24h_R2 **Scan** 13194
Peptide SNVQSKLTFREN **Charge** 3 **Spectral Angle** 0.86

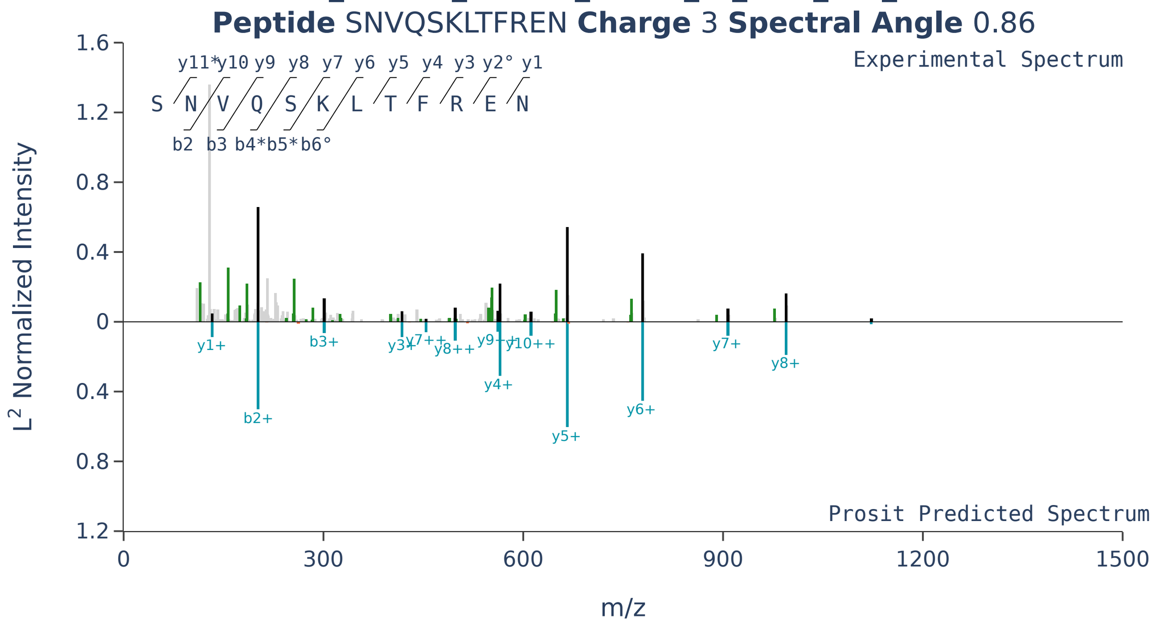

Source WSoh_221121_011221_HFGoe_L2_hTau_24h_R2 **Scan** 16941
Peptide VYKPVDLSKVTSKA **Charge** 3 **Spectral Angle** 0.86

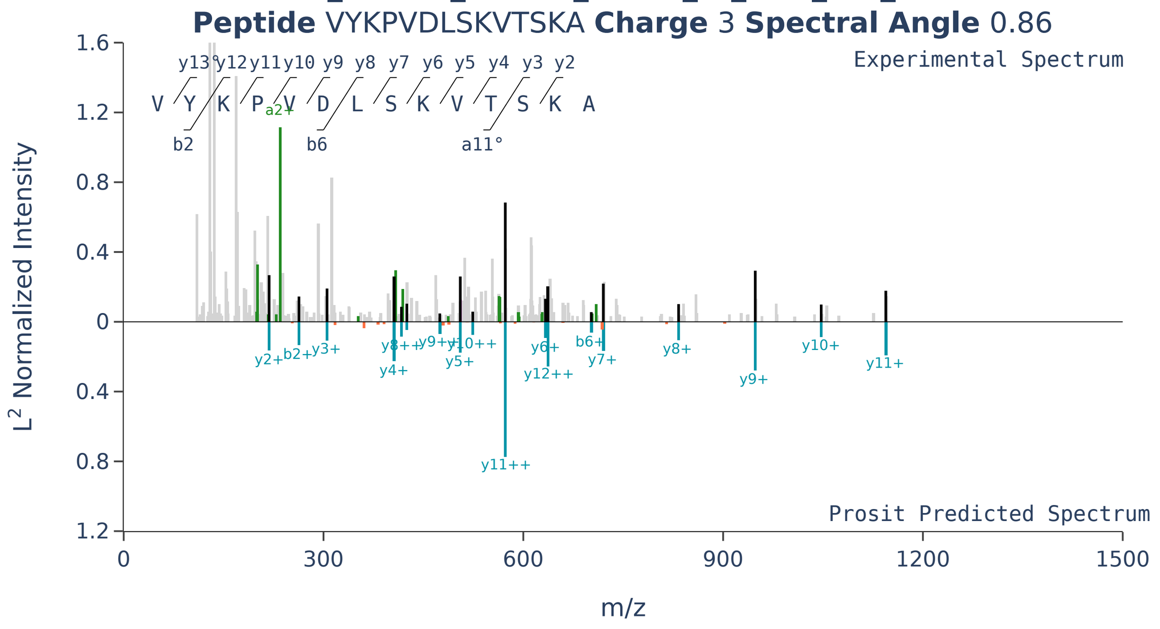

Source WSoh_221121_011221_HFGoe_L2_hTau_24h_R2 **Scan** 20868
Peptide RGLQTAPVPMPDLKNVKS **Charge** 3 **Spectral Angle** 0.83

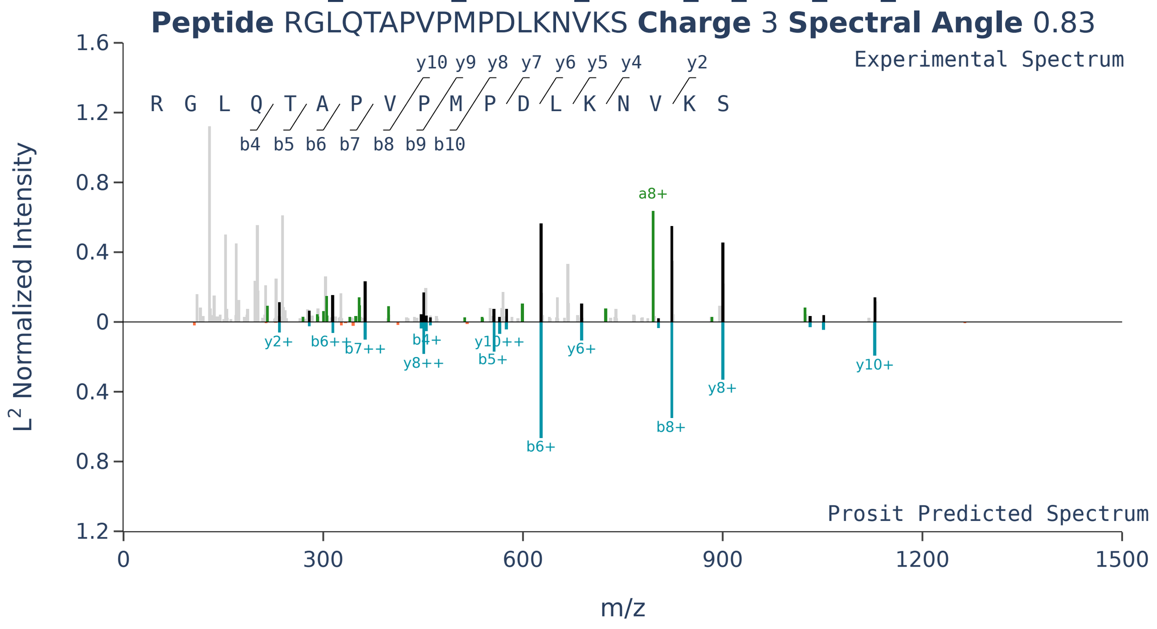

Source WSoh_221121_011221_HFGoe_L1_hTau_4h_R2 **Scan** 11290
Peptide AKLDFKDRVQS **Charge** 3 **Spectral Angle** 0.82

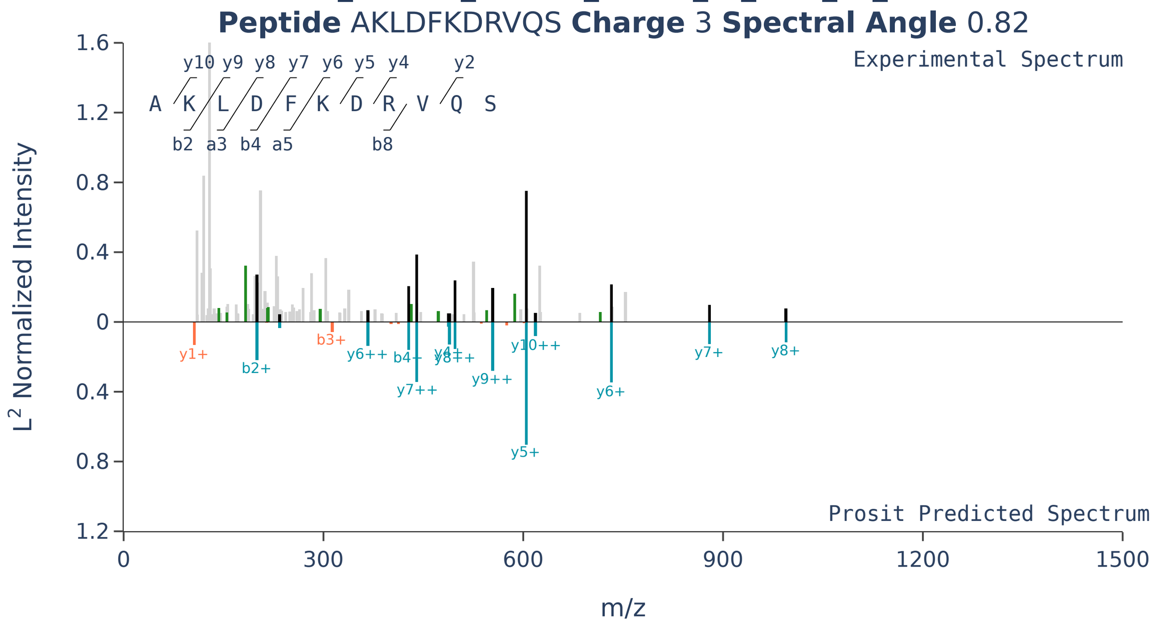

Source WSoh_221121_011221_HFGoe_L1_hTau_24h_R2 **Scan** 8310
Peptide KSEKLDFKDRVQSR **Charge** 3 **Spectral Angle** 0.86

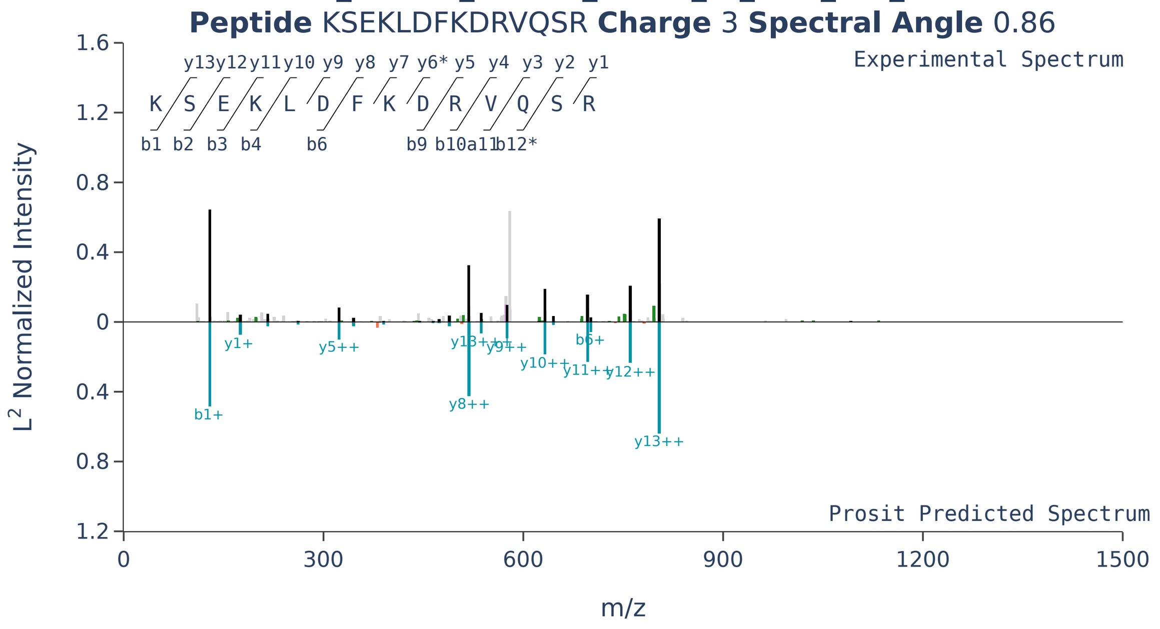

Source WSoh_221121_011221_HFGoe_L1_hTau_4h_R2 **Scan** 20160
Peptide APVPMPDLKNVAKS **Charge** 3 **Spectral Angle** 0.84

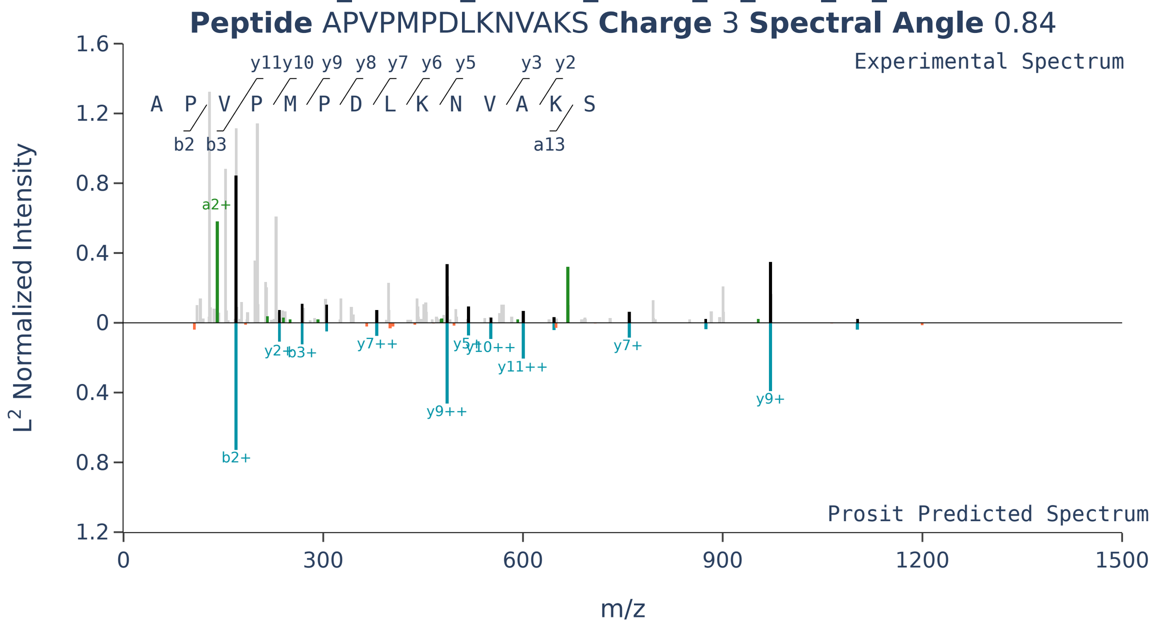

Source WSoh_221121_011221_HFGoe_L1_hTau_24h_R1 **Scan** 18487
Peptide PVPMPDLKNVKN **Charge** 3 **Spectral Angle** 0.81

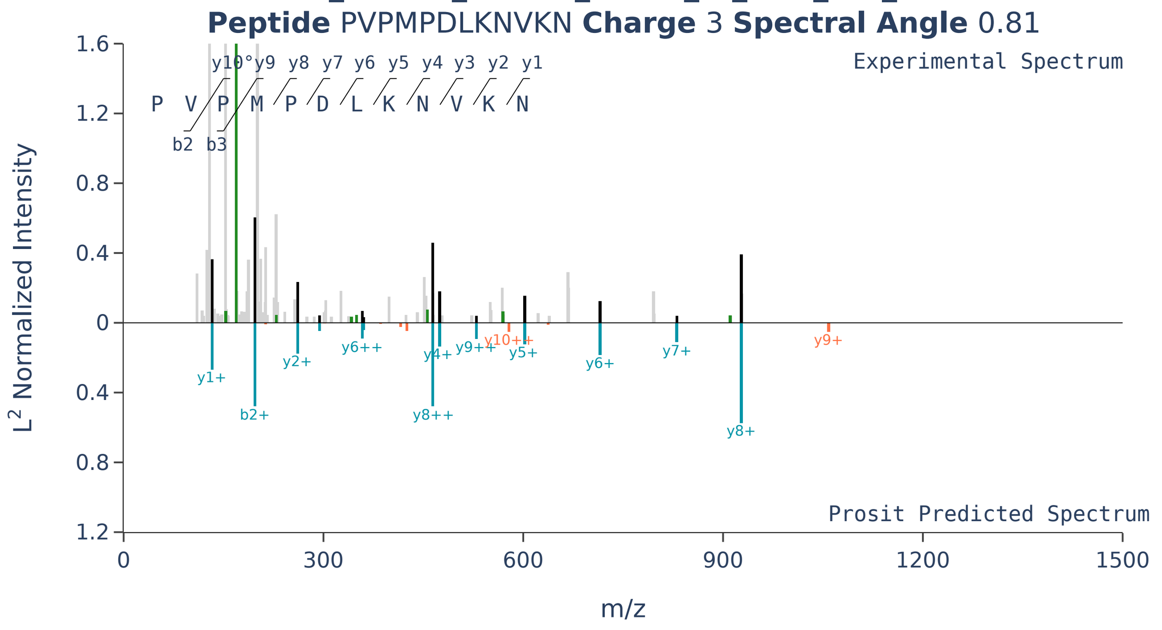

Source WSoh_221121_011221_HFGoe_L1_hTau_4h_R1 **Scan** 10265
Peptide QSKSEKLDFKDRV **Charge** 4 **Spectral Angle** 0.84

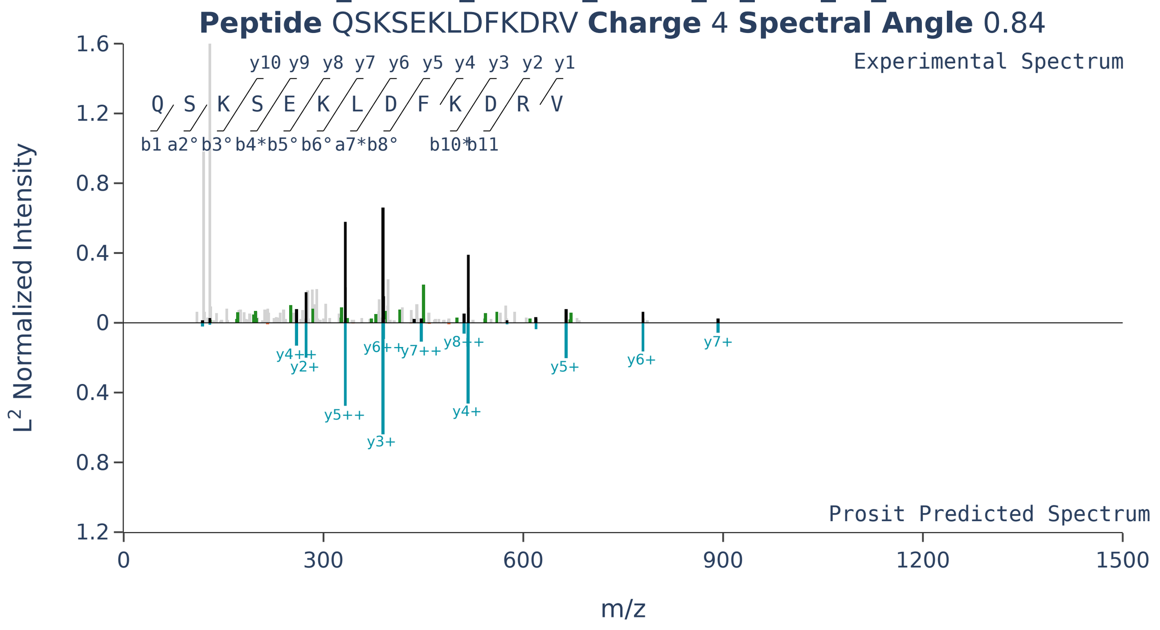

Source WSoh_221121_011221_HFGoe_L1_hTau_24h_R1 **Scan** 21981
Peptide AAQPHTEIPEGAEPRQEFEVME **Charge** 3 **Spectral Angle** 0.9

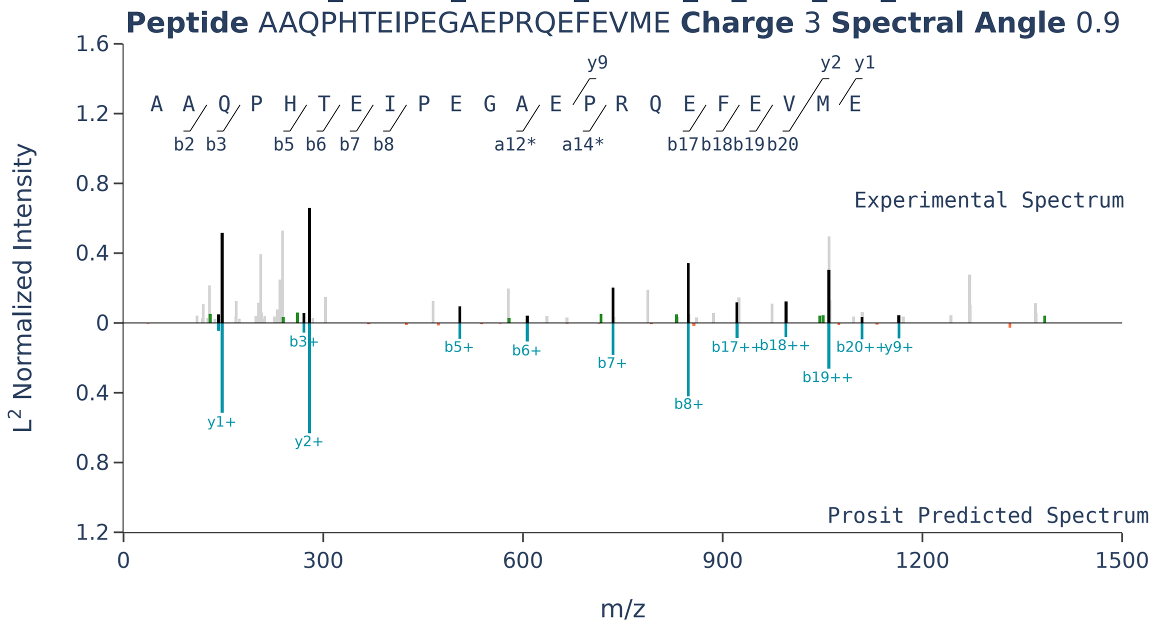

Source WSoh_221121_011221_HFGoe_L1_hTau_24h_R1 **Scan** 16198
Peptide SVYKPVDLSKVTSK **Charge** 3 **Spectral Angle** 0.81

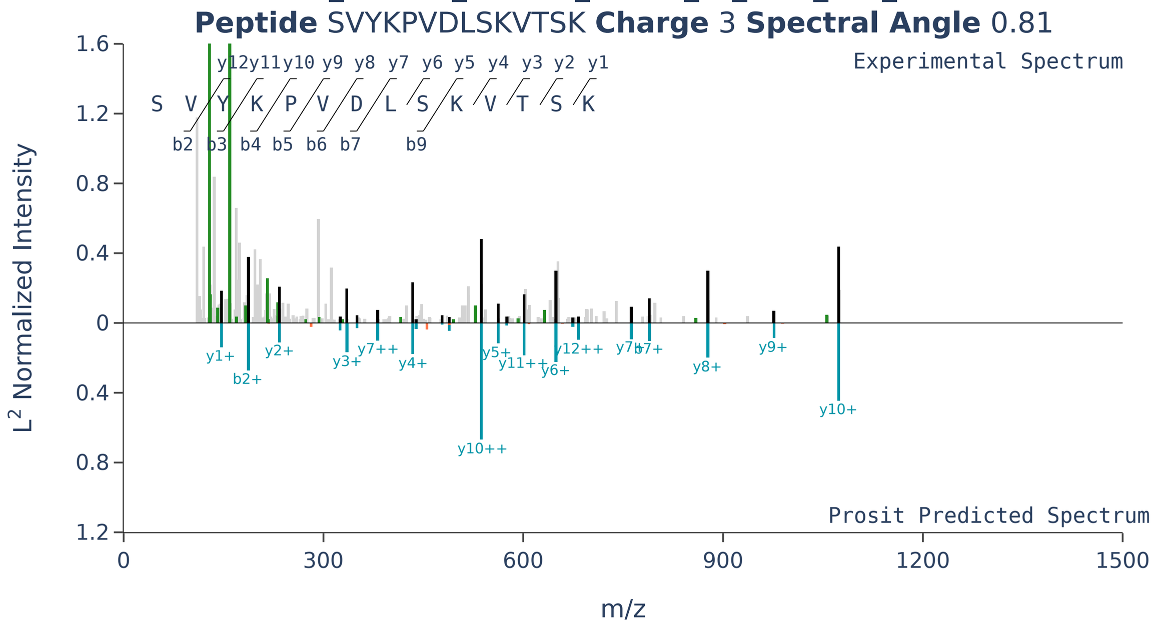

Source WSoh_221121_011221_HFGoe_L1_hTau_4h_R1 **Scan** 9291
Peptide SKLTFREN **Charge** 2 **Spectral Angle** 0.93

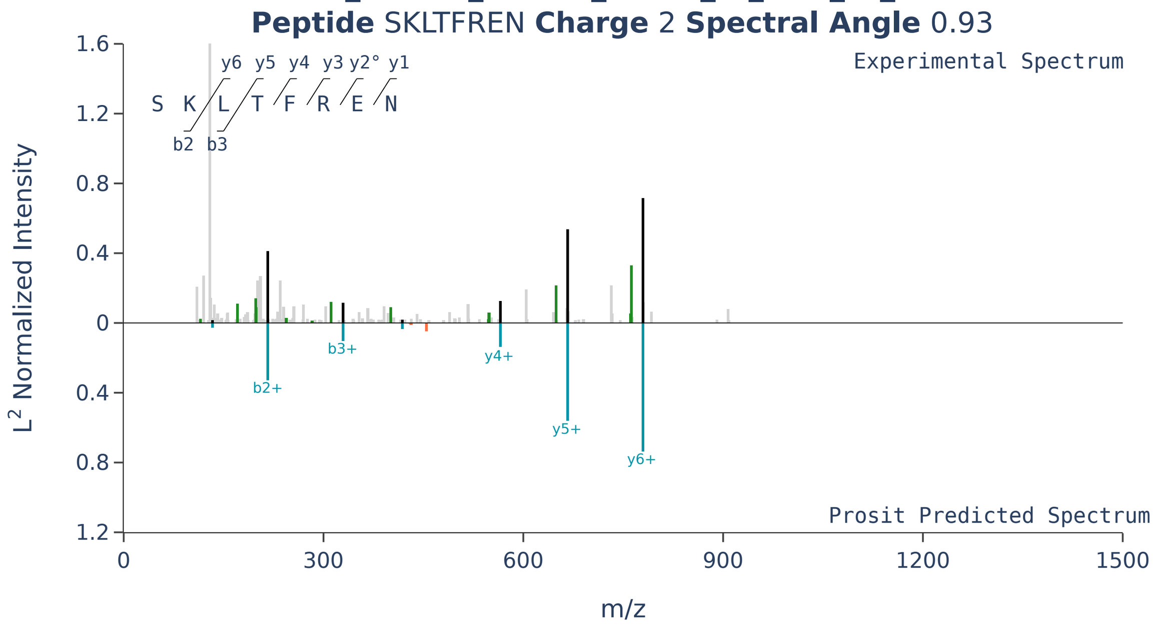

Source WSoh_221121_011221_HFGoe_L2_hTau_24h_R1 **Scan** 20072
Peptide TAPVPMPDLKNVNK **Charge** 3 **Spectral Angle** 0.8

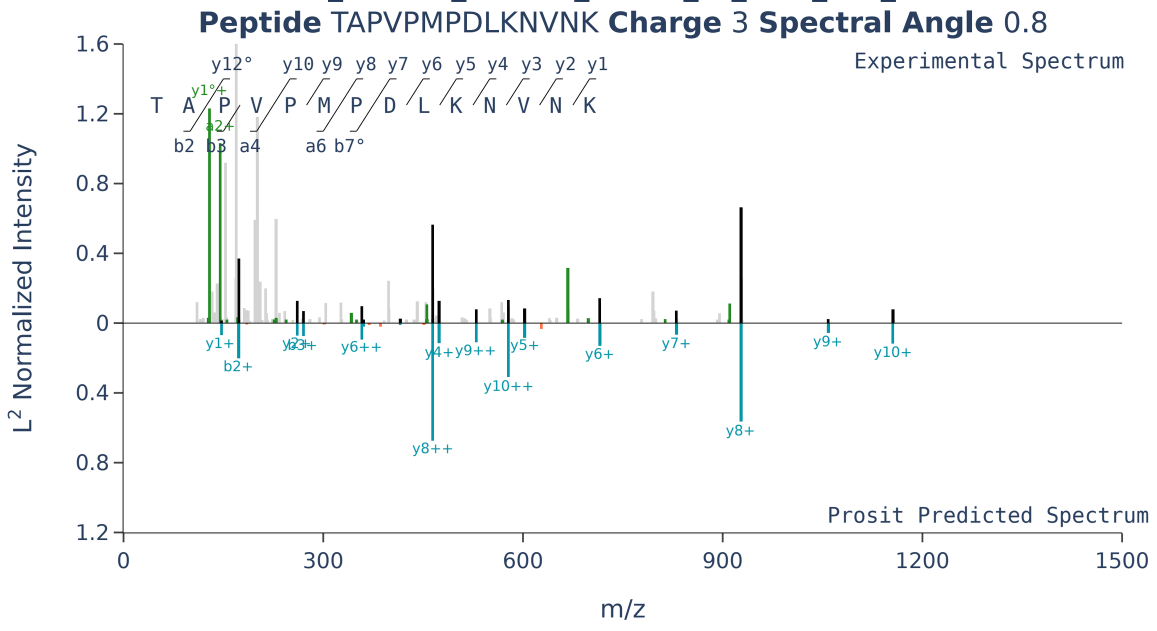

Source WSoh_221121_011221_HFGoe_L1_hTau_4h_R2 **Scan** 7857
Peptide GGGKDNIKHVPGGGSV **Charge** 2 **Spectral Angle** 0.91

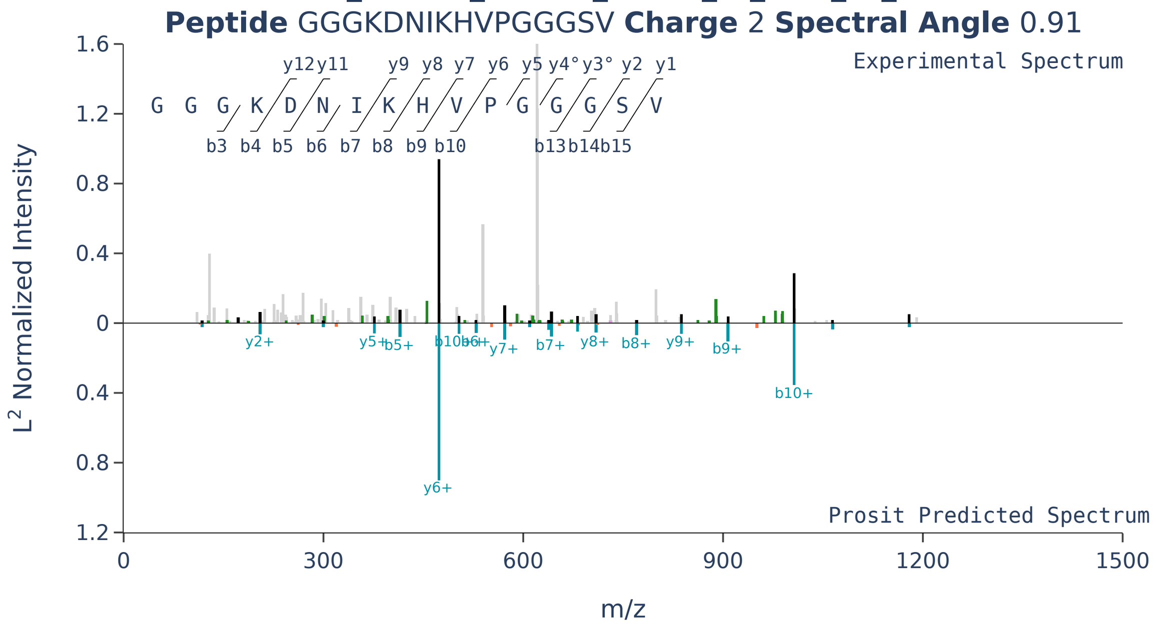

Source WSoh_221121_011221_HFGoe_L2_hTau_4h_R1 **Scan** 8799
Peptide KSEKLDFKDRVQN **Charge** 2 **Spectral Angle** 0.77

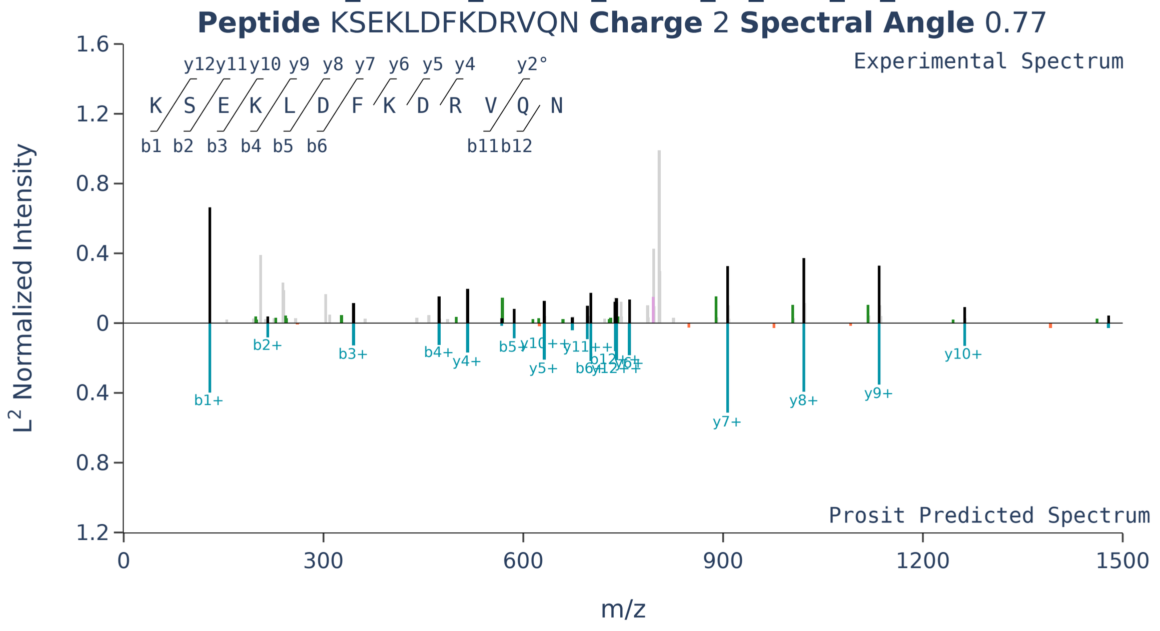

Source WSoh_221121_011221_HFGoe_L2_hTau_24h_R1 **Scan** 17367
Peptide GNKDNIKHVPGGGSVQIVY **Charge** 3 **Spectral Angle** 0.86

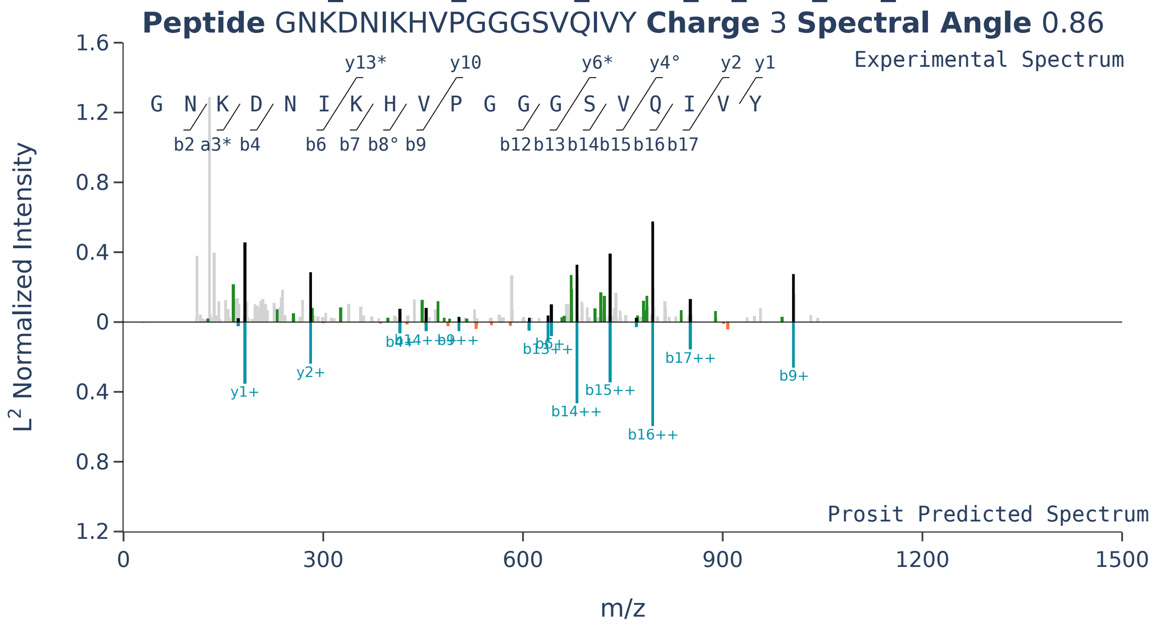

Source WSoh_221121_011221_HFGoe_L1_hTau_4h_R2 **Scan** 17979
Peptide GDTAGLKESPLQTPTE **Charge** 2 **Spectral Angle** 0.9

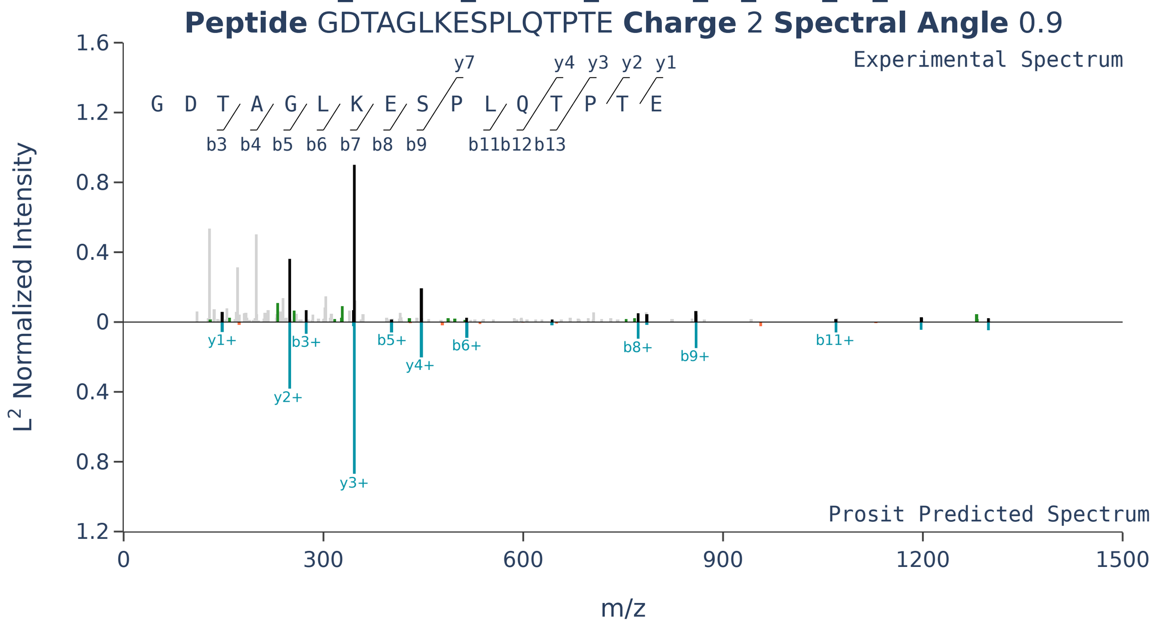

Source WSoh_221121_011221_HFGoe_L2_hTau_24h_R2 **Scan** 18964
Peptide SKVTSKIGSLDNITHV **Charge** 3 **Spectral Angle** 0.82

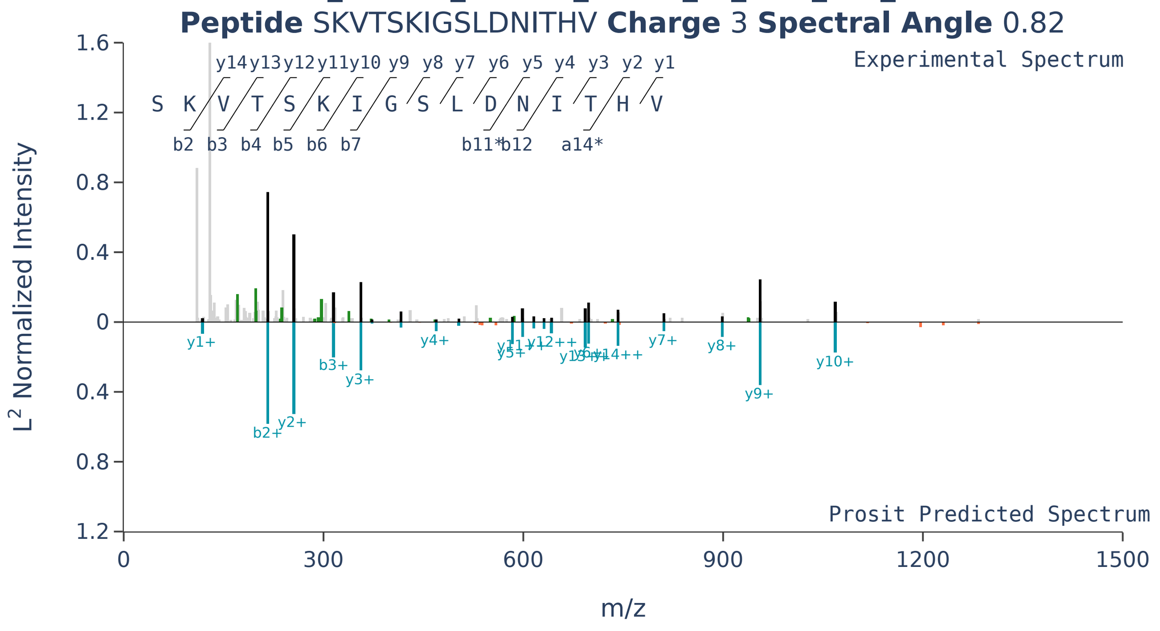

Source WSoh_221121_011221_HFGoe_L2_hTau_2h_R1 **Scan** 17801
Peptide DLSNVNIT **Charge** 1 **Spectral Angle** 0.84

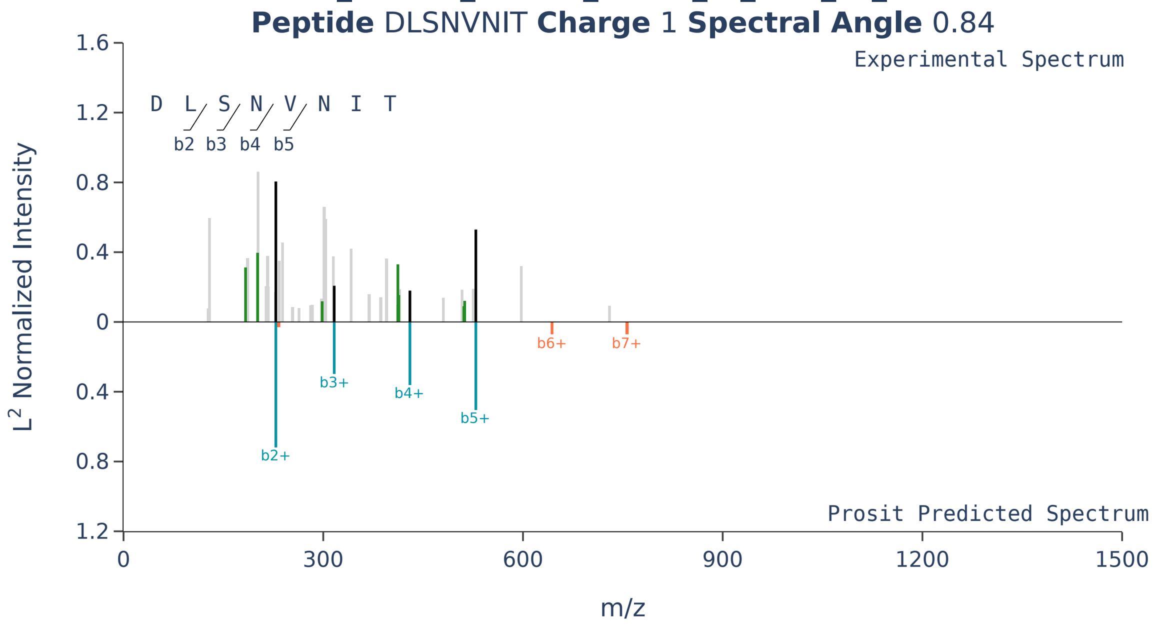

Source WSoh_221121_011221_HFGoe_L1_hTau_24h_R1 **Scan** 7612
Peptide PGGGNKKIETHVY **Charge** 3 **Spectral Angle** 0.83

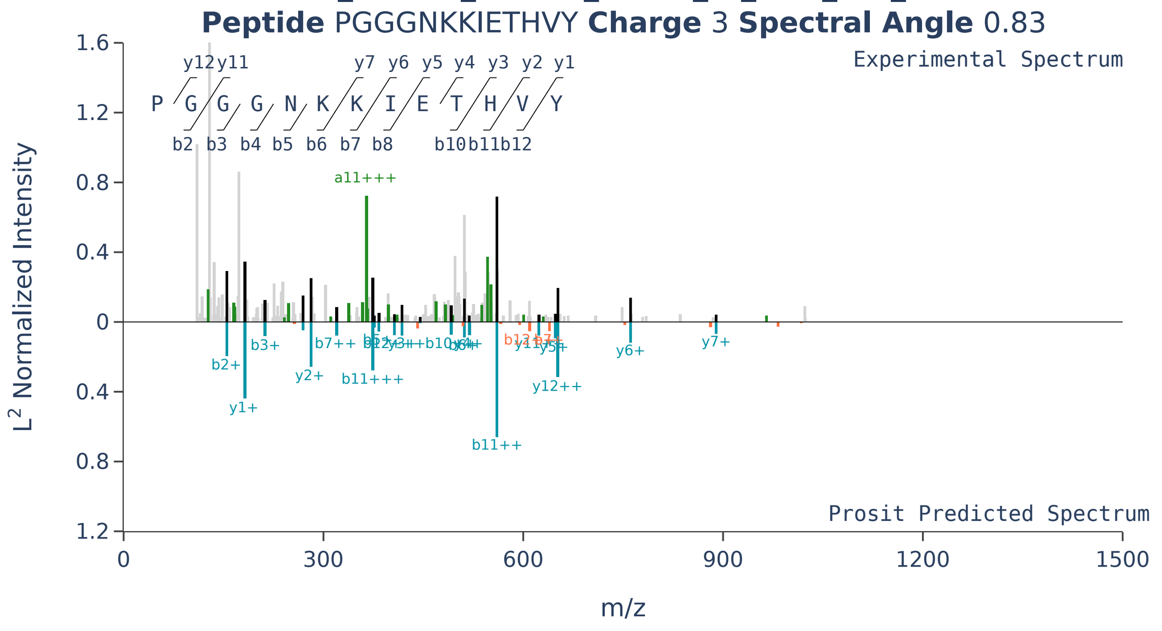

Source WSoh_221121_011221_HFGoe_L2_hTau_4h_R2 **Scan** 16999
Peptide GGGKDNIKHVPGGGSVQIVY **Charge** 3 **Spectral Angle** 0.89

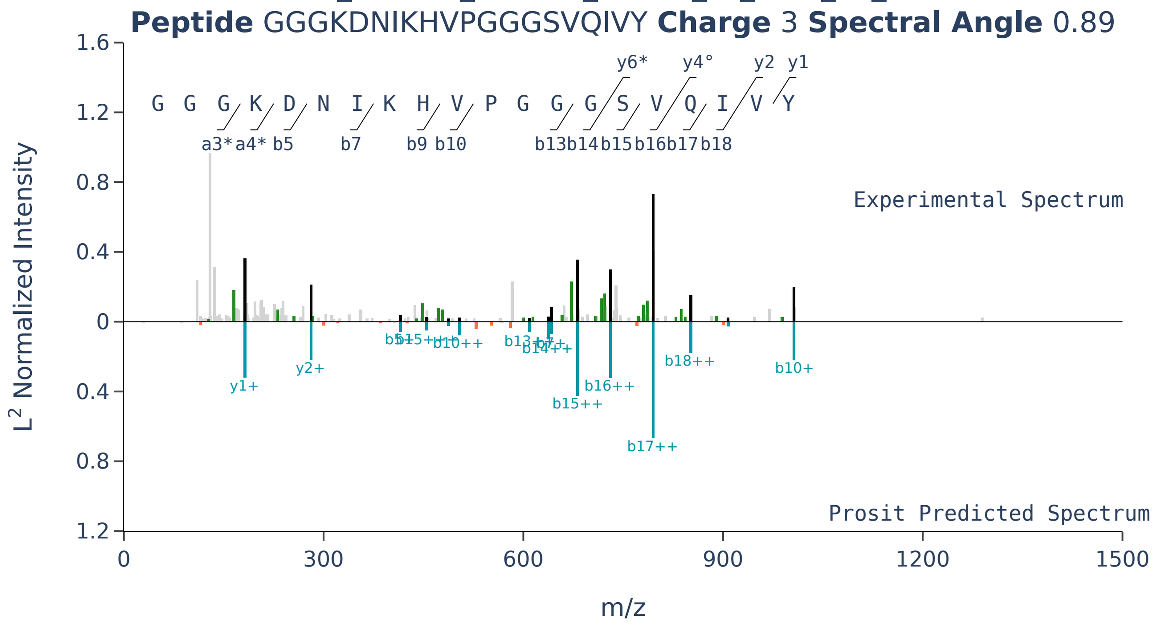

Source WSoh_221121_011221_HFGoe_L1_hTau_24h_R1 **Scan** 15399
Peptide DTPSLPTPPTREPKKV **Charge** 3 **Spectral Angle** 0.79

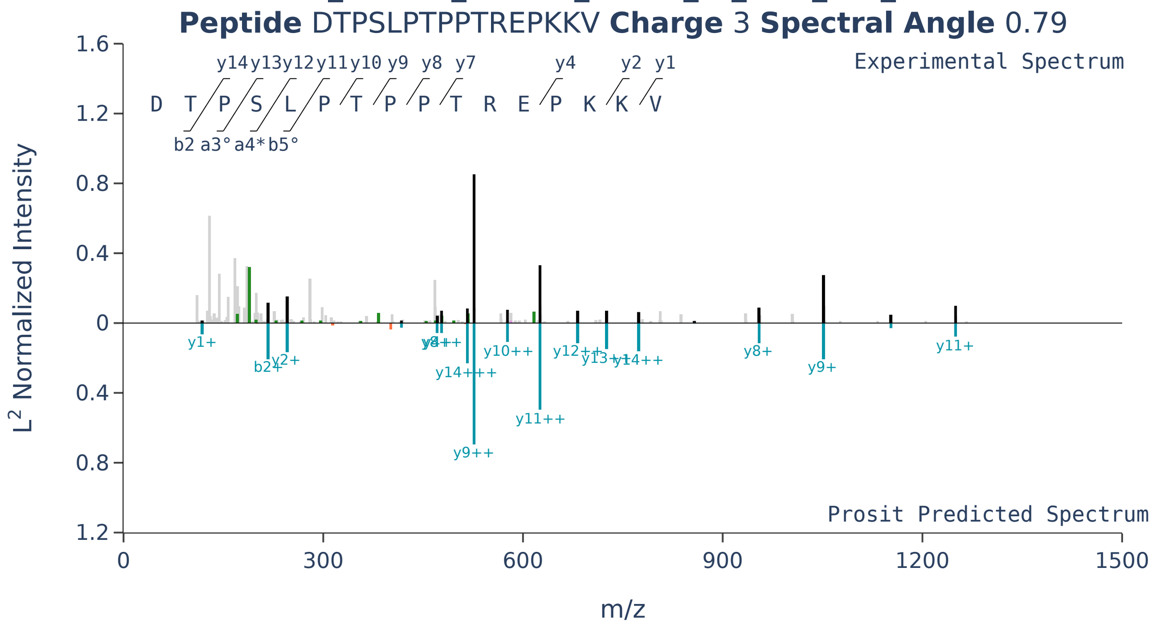

Source WSoh_221121_011221_HFGoe_L1_hTau_24h_R2 **Scan** 17097
Peptide NKKLDLSNVQSRL **Charge** 3 **Spectral Angle** 0.93

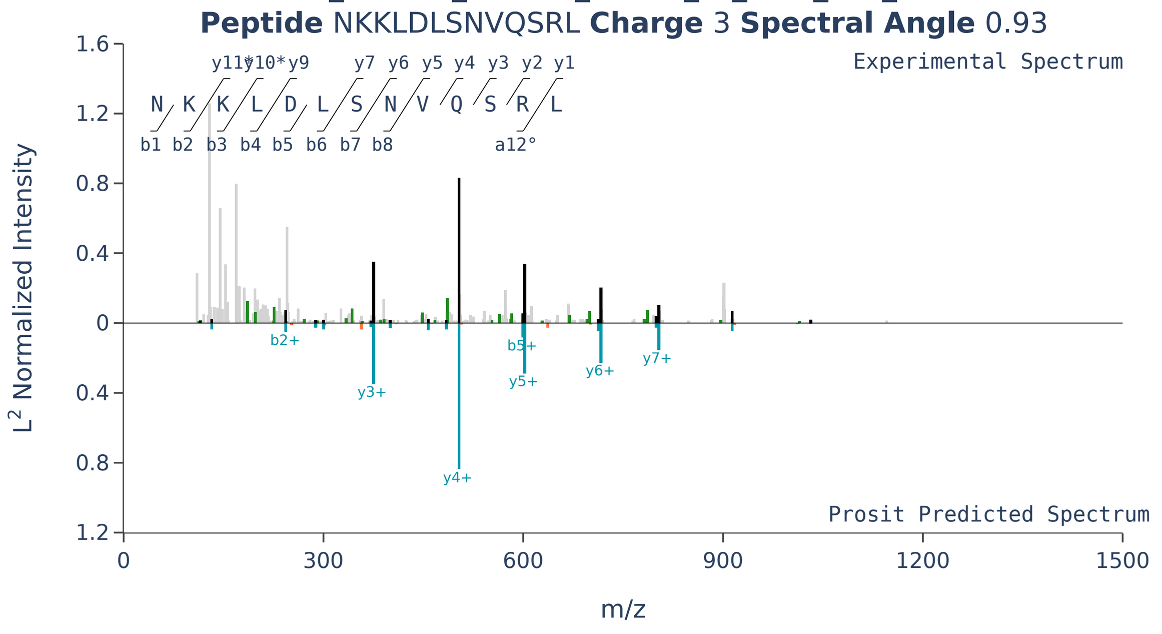

Source WSoh_221121_011221_HFGoe_L1_hTau_24h_R1 **Scan** 8566
Peptide KPVDLSKVTSKH **Charge** 3 **Spectral Angle** 0.82

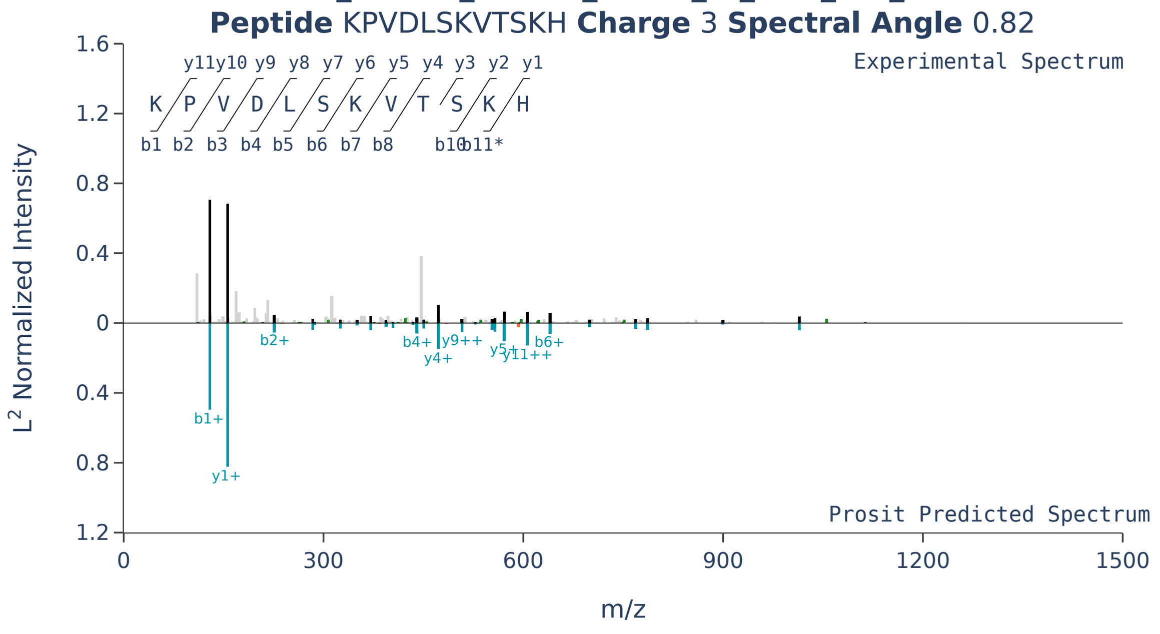

Source WSoh_221121_011221_HFGoe_L2_hTau_2h_R1 **Scan** 7861
Peptide DFKDRVQSS **Charge** 2 **Spectral Angle** 0.88

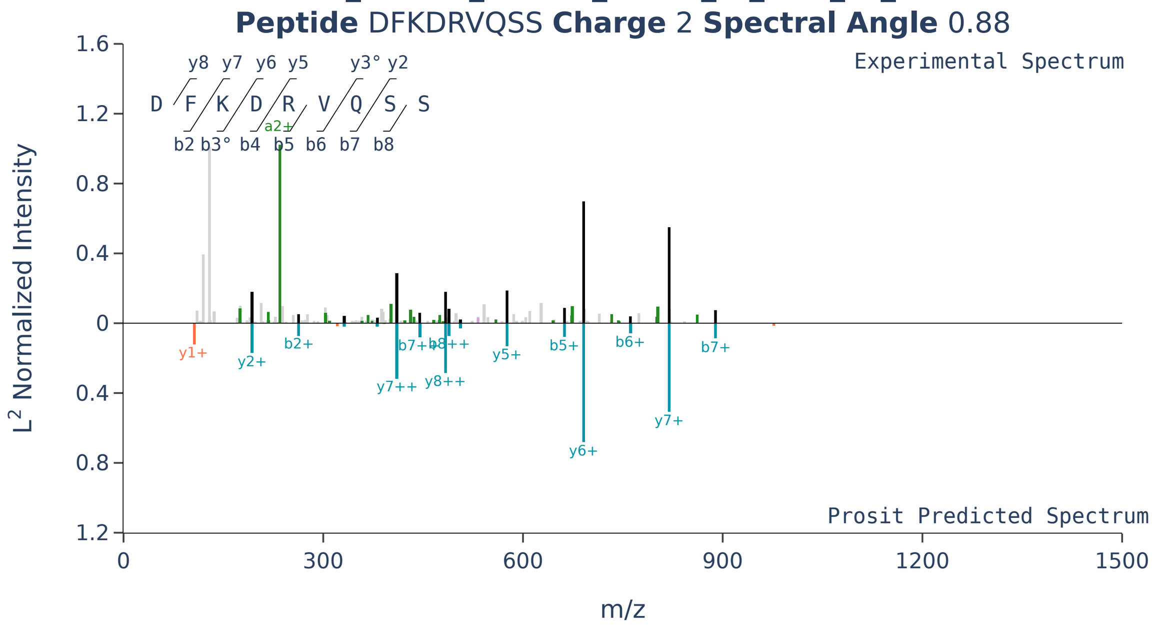

Source WSoh_221121_011221_HFGoe_L1_hTau_24h_R1 **Scan** 17621
Peptide APVPMPDLKNVKSH **Charge** 3 **Spectral Angle** 0.85

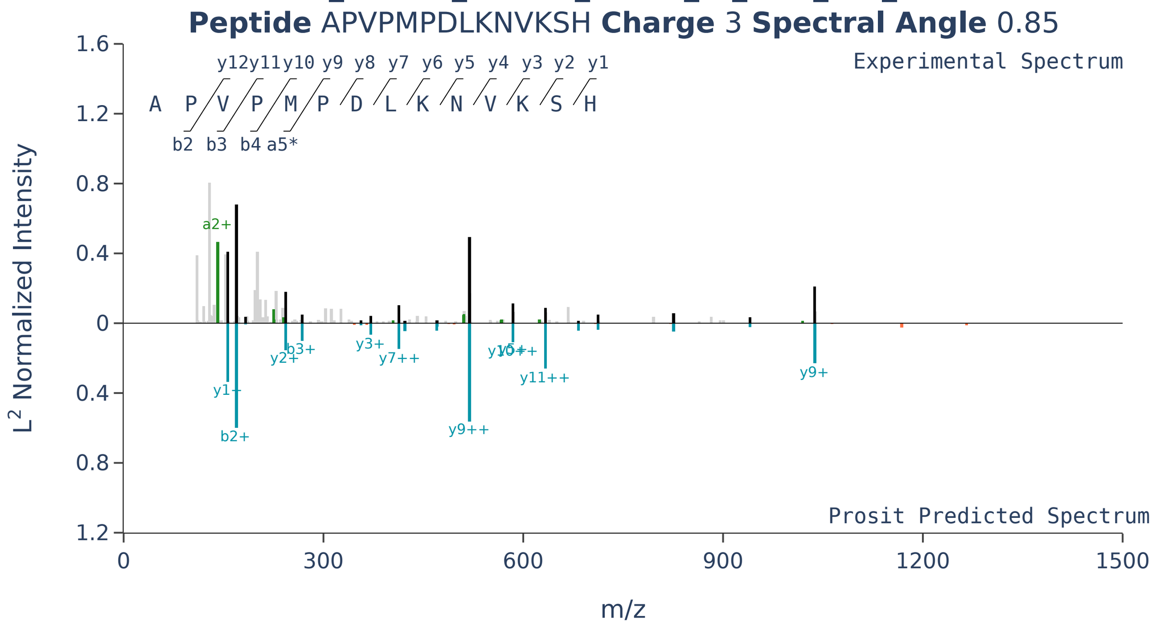

Source WSoh_221121_011221_HFGoe_L1_hTau_24h_R2 **Scan** 20865
Peptide ADAEPRQEFEVME **Charge** 2 **Spectral Angle** 0.85

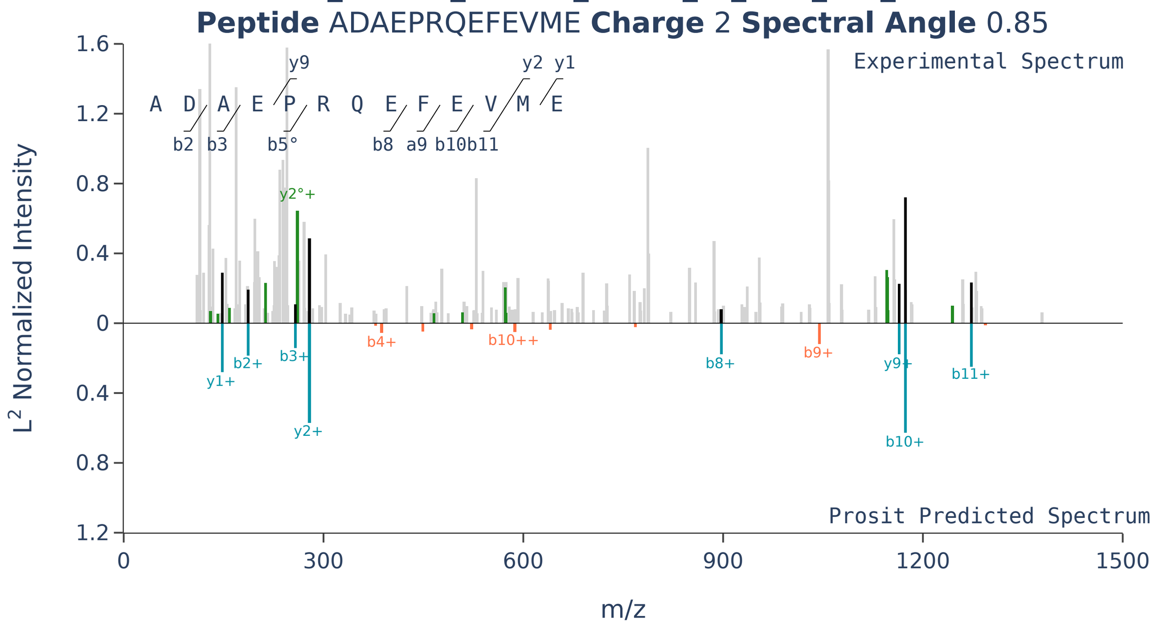

Source WSoh_221121_011221_HFGoe_L1_hTau_24h_R1 **Scan** 21653
Peptide DLKIGSTENL **Charge** 2 **Spectral Angle** 0.73

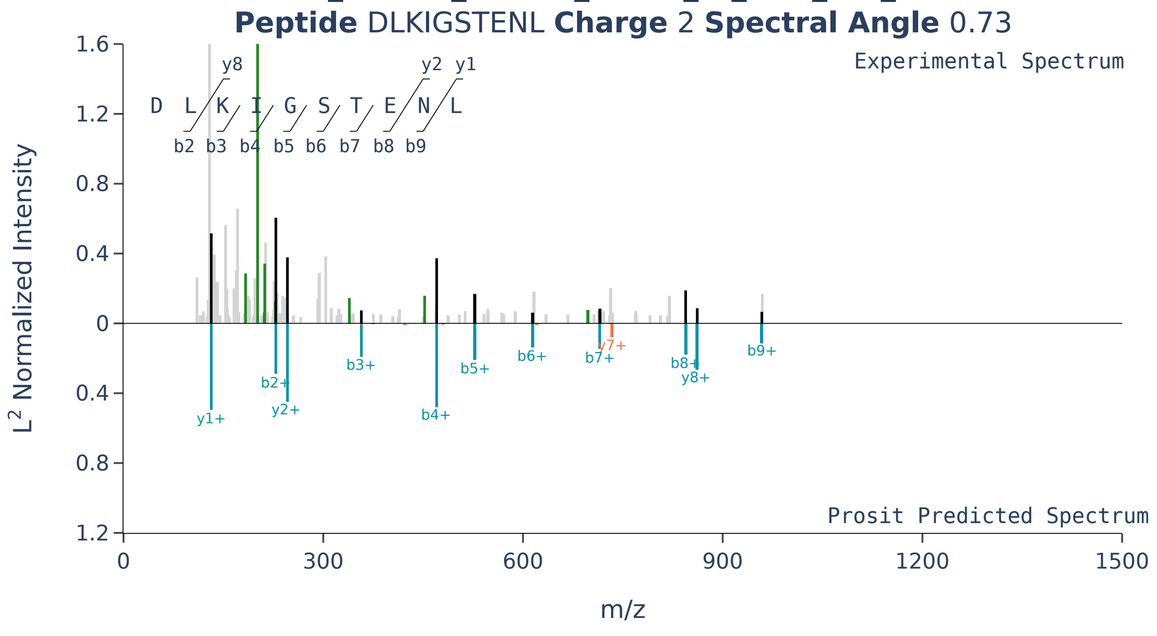

Source WSoh_221121_011221_HFGoe_L2_hTau_24h_R1 **Scan** 19702
Peptide APVPMPDLKNVKSQ **Charge** 3 **Spectral Angle** 0.87

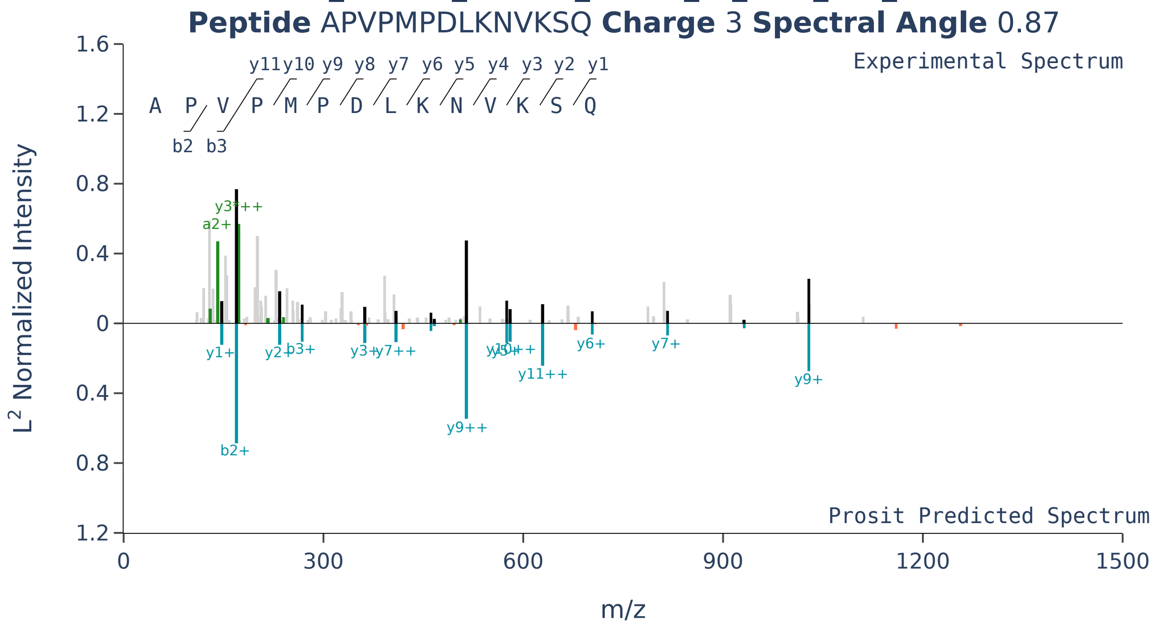

Source WSoh_221121_011221_HFGoe_L2_hTau_1h_R2 **Scan** 13623
Peptide SNVSVYKPVD **Charge** 2 **Spectral Angle** 0.9

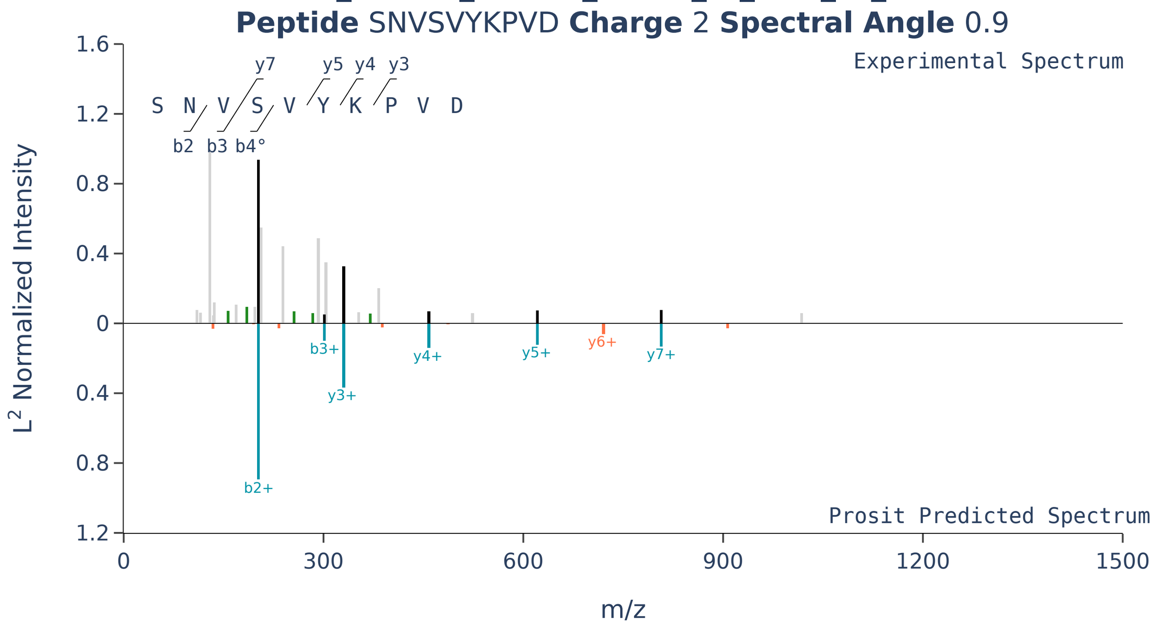

Source WSoh_221121_011221_HFGoe_L2_hTau_4h_R1 **Scan** 12550
Peptide EKLDFKDRVQSA **Charge** 3 **Spectral Angle** 0.83

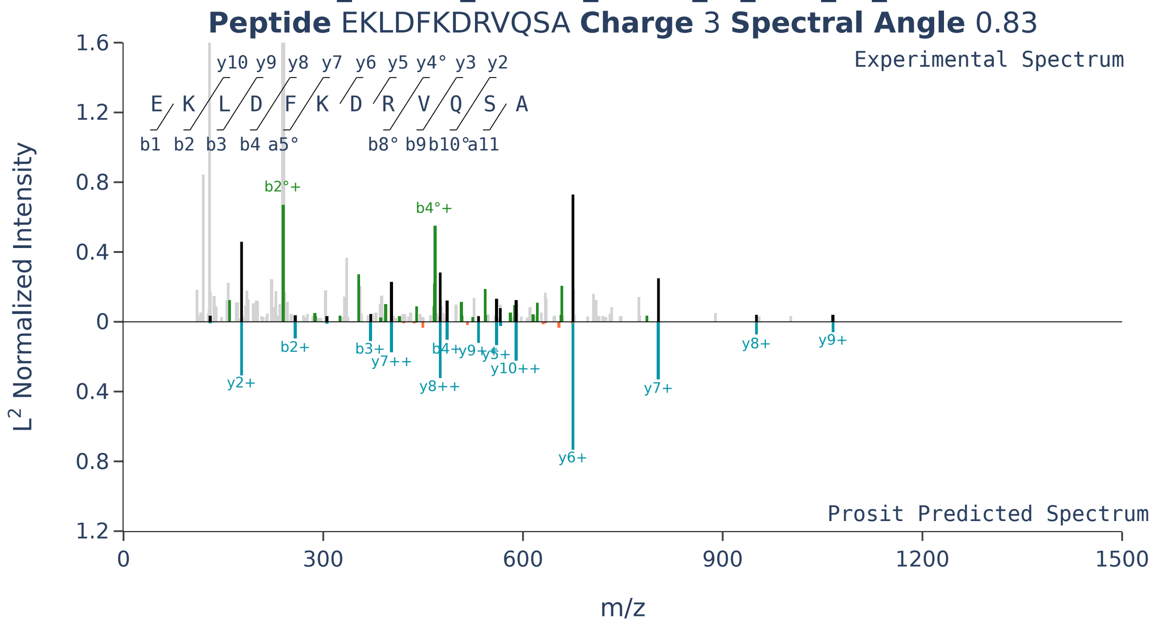

Source WSoh_221121_011221_HFGoe_L2_hTau_24h_R2 **Scan** 12350
Peptide PGKQAAAQQPHTEIPEGTTAEE **Charge** 3 **Spectral Angle** 0.82

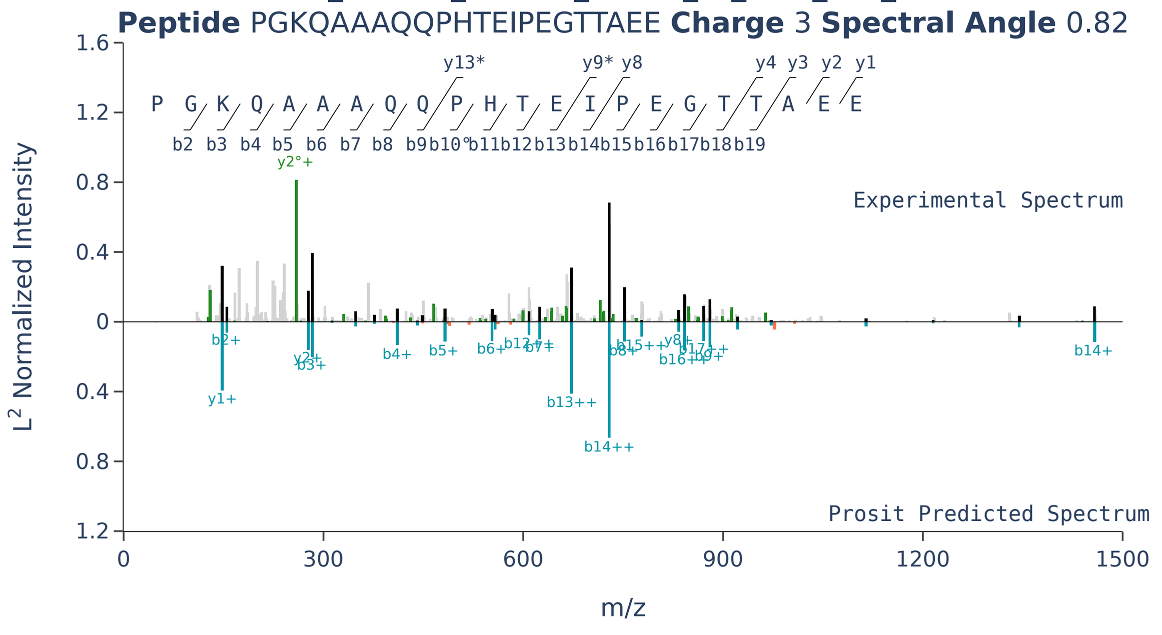

Source WSoh_221121_011221_HFGoe_L1_hTau_4h_R2 **Scan** 6057
Peptide DFKDRVQSKH **Charge** 3 **Spectral Angle** 0.95

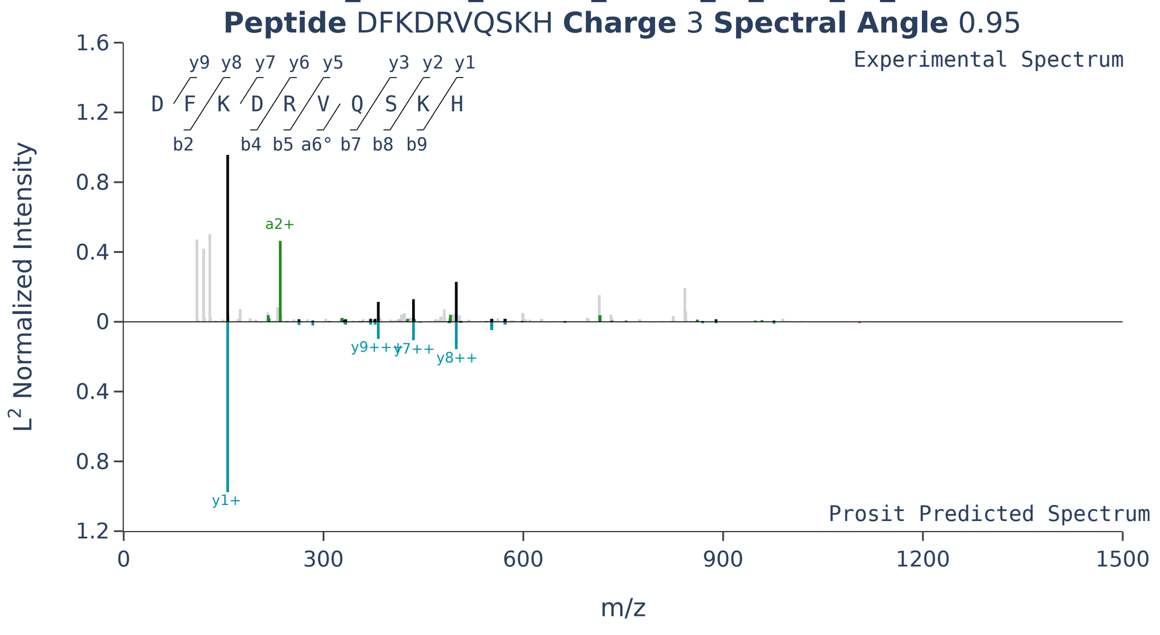

Source WSoh_221121_011221_HFGoe_L2_hTau_24h_R1 **Scan** 11432
Peptide NITHVPGGGNKKIETHKLTFREGG **Charge** 3 **Spectral Angle** 0.79

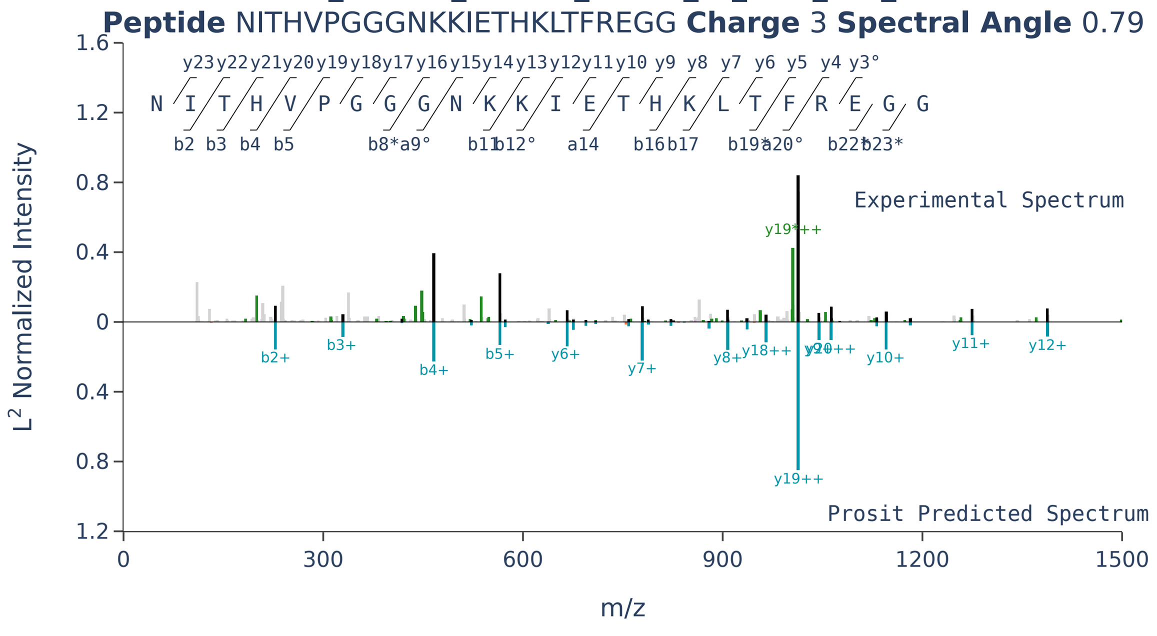

Source WSoh_221121_011221_HFGoe_L1_hTau_2h_R2 **Scan** 7855
Peptide GNKDNIKHVPGGGSV **Charge** 3 **Spectral Angle** 0.84

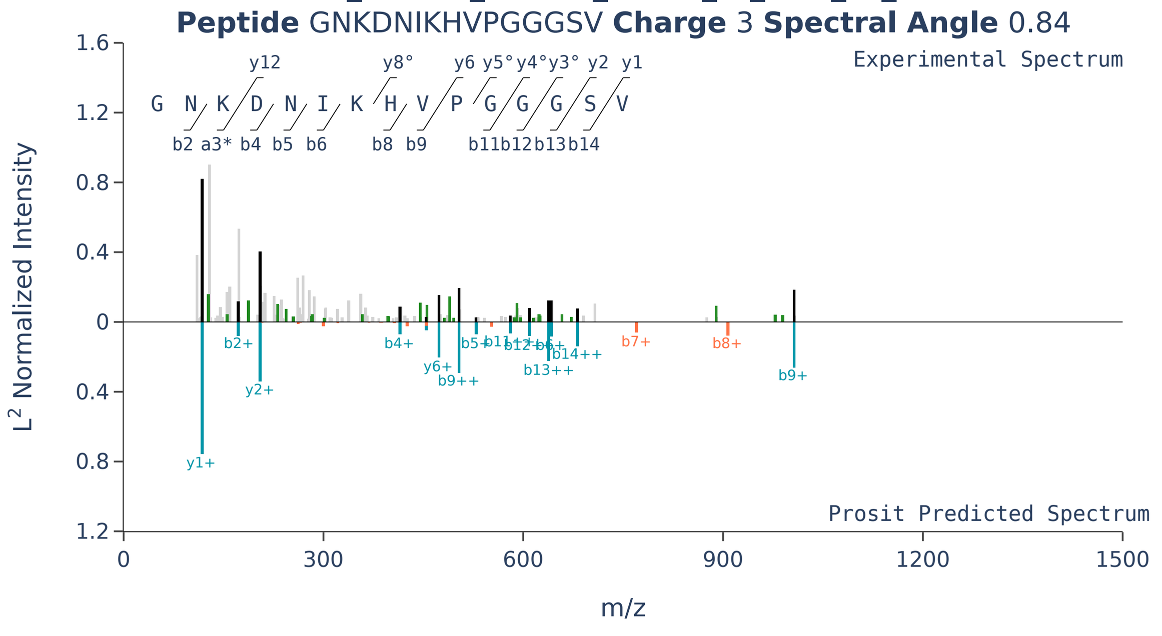

Source WSoh_221121_011221_HFGoe_L1_hTau_4h_R2 **Scan** 11381
Peptide NITHVPGGGNKKIETHKLTFRDAG **Charge** 3 **Spectral Angle** 0.8

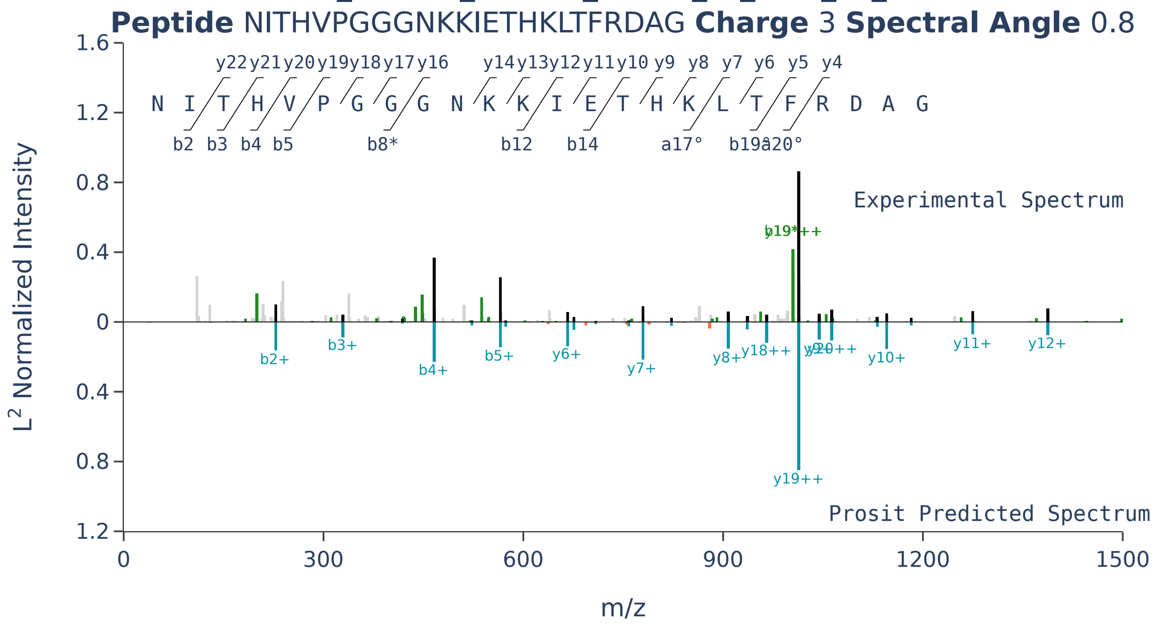

Source WSoh_221121_011221_HFGoe_L2_hTau_24h_R1 **Scan** 7296
Peptide SKHQPGGGKVQI **Charge** 2 **Spectral Angle** 0.86

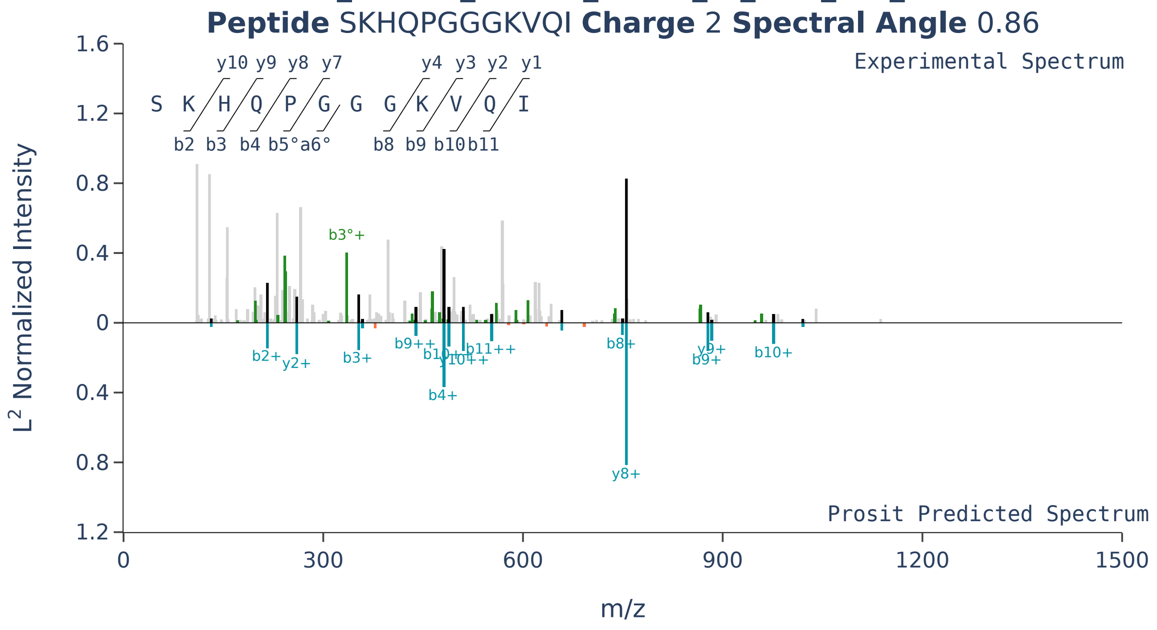

Source WSoh_221121_011221_HFGoe_L1_hTau_2h_R2 **Scan** 7298
Peptide SGSLGNIHH **Charge** 2 **Spectral Angle** 0.8

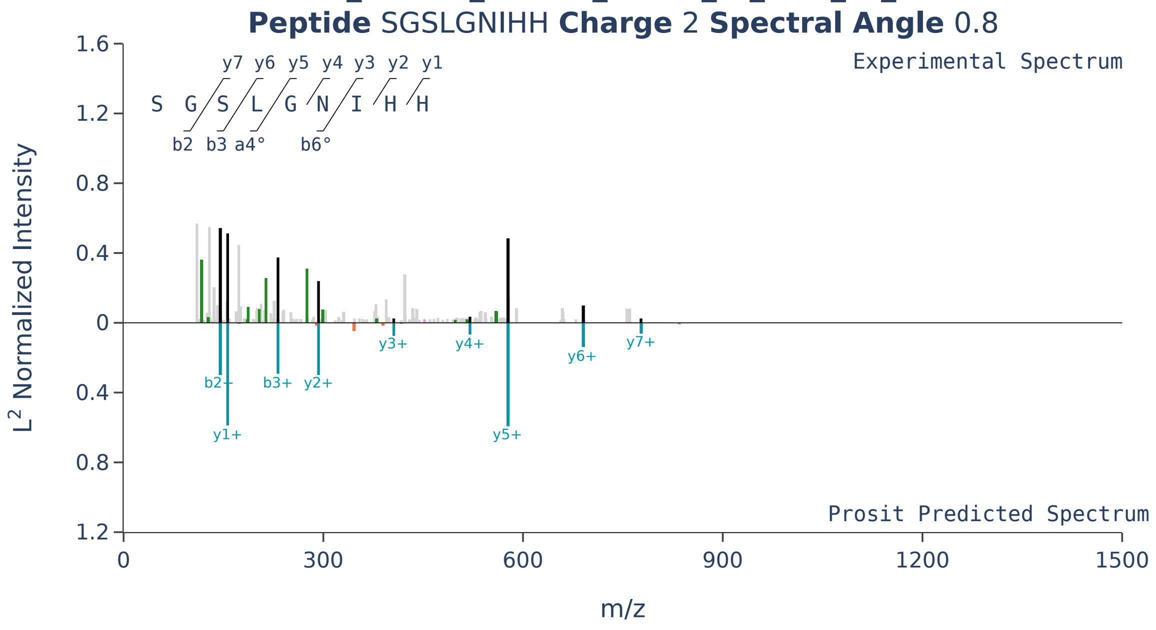

Source WSoh_221121_011221_HFGoe_L2_hTau_24h_R1 **Scan** 21553
Peptide QTAPVPMPDLKNVKTG **Charge** 3 **Spectral Angle** 0.84

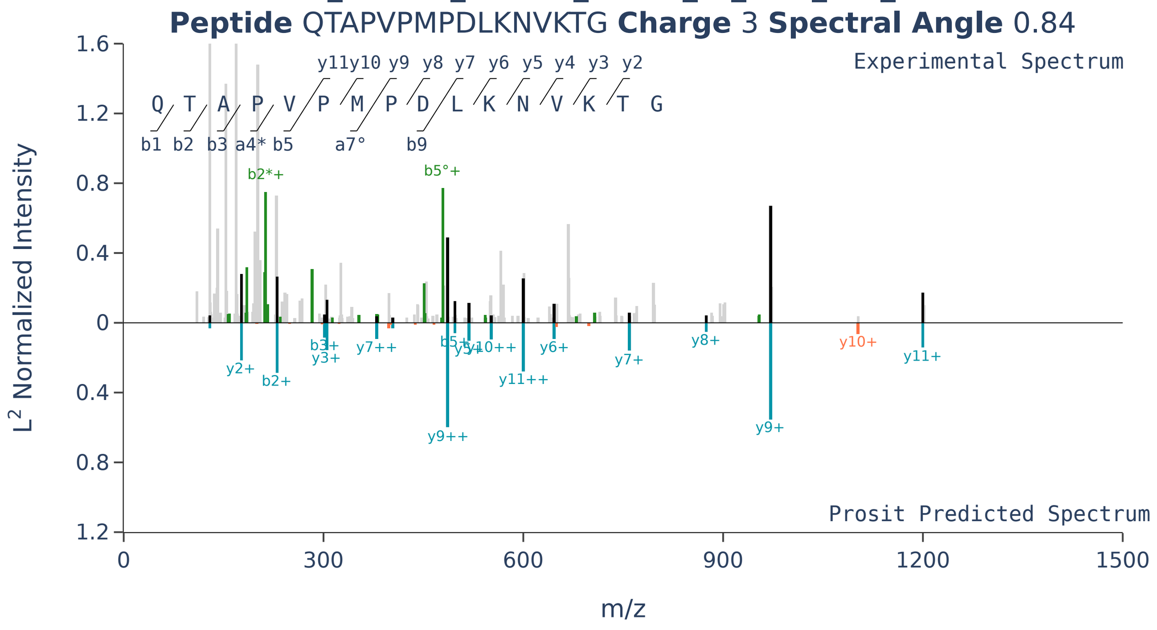

Source WSoh_221121_011221_HFGoe_L1_hTau_4h_R2 **Scan** 11667
Peptide NLDRKDQGGYTMHQDQEGDTDAGLKE **Charge** 5 **Spectral Angle** 0.78

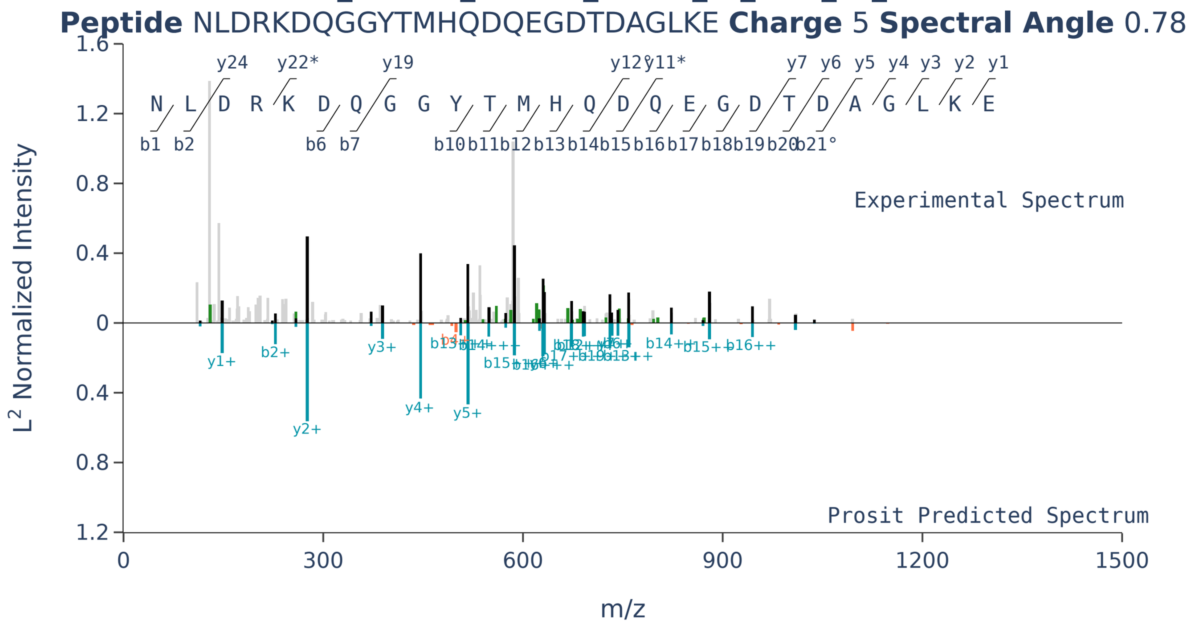

Source WSoh_221121_011221_HFGoe_L1_hTau_24h_R2 **Scan** 18279
Peptide KSQTAPVPMPDLKNVKS **Charge** 2 **Spectral Angle** 0.83

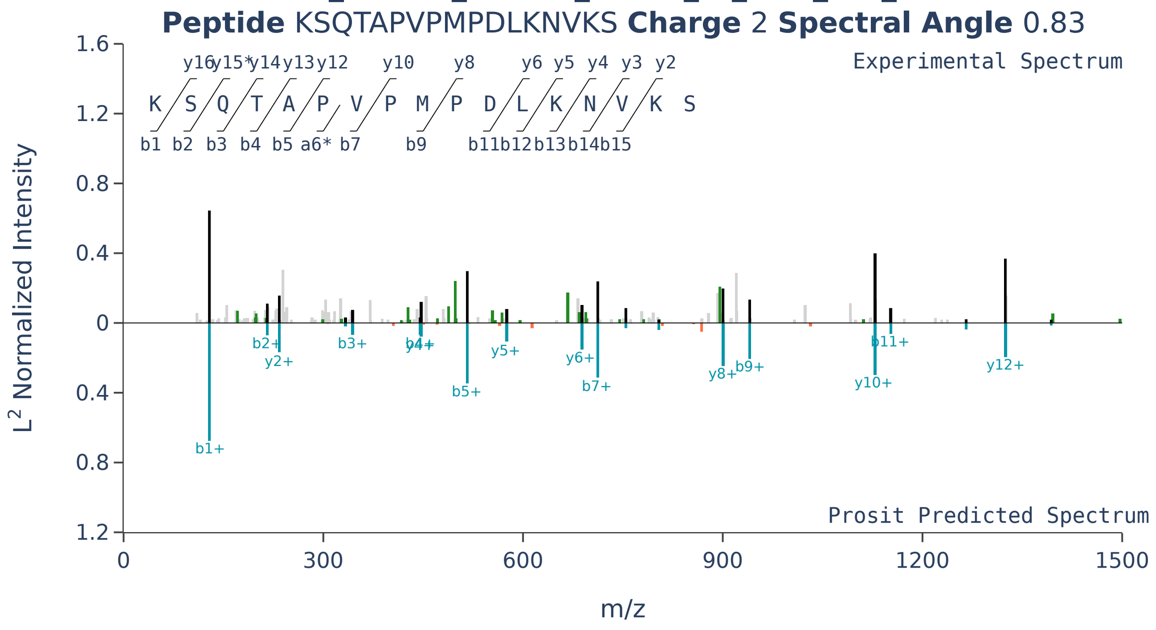

Source WSoh_221121_011221_HFGoe_L2_hTau_2h_R2 **Scan** 11561
Peptide ADEVSASLAKQS **Charge** 2 **Spectral Angle** 0.85

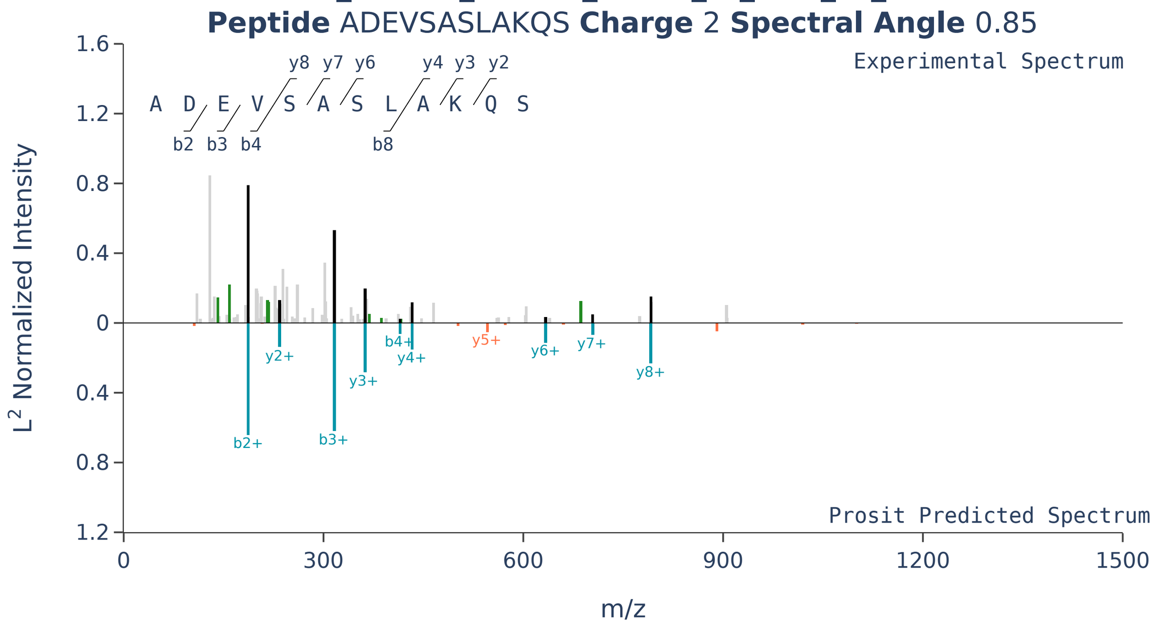

Source WSoh_221121_011221_HFGoe_L2_hTau_4h_R1 **Scan** 9305
Peptide KSEKLDFKDRVQGS **Charge** 2 **Spectral Angle** 0.79

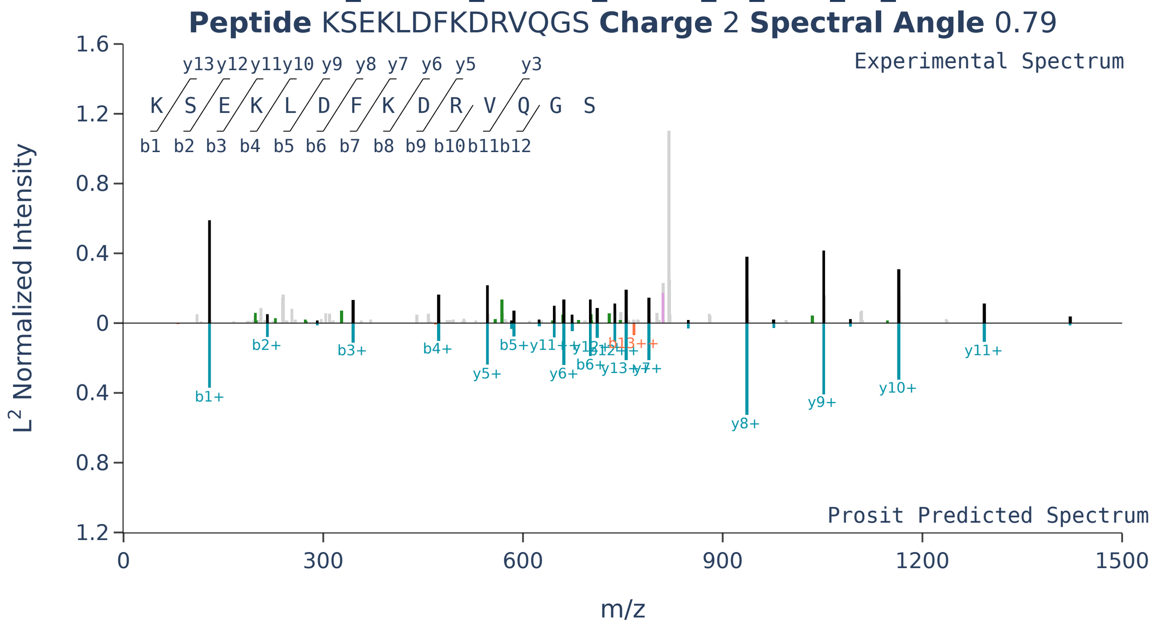

Source WSoh_221121_011221_HFGoe_L2_hTau_1h_R1 **Scan** 11374
Peptide KNEKLDF **Charge** 2 **Spectral Angle** 0.87

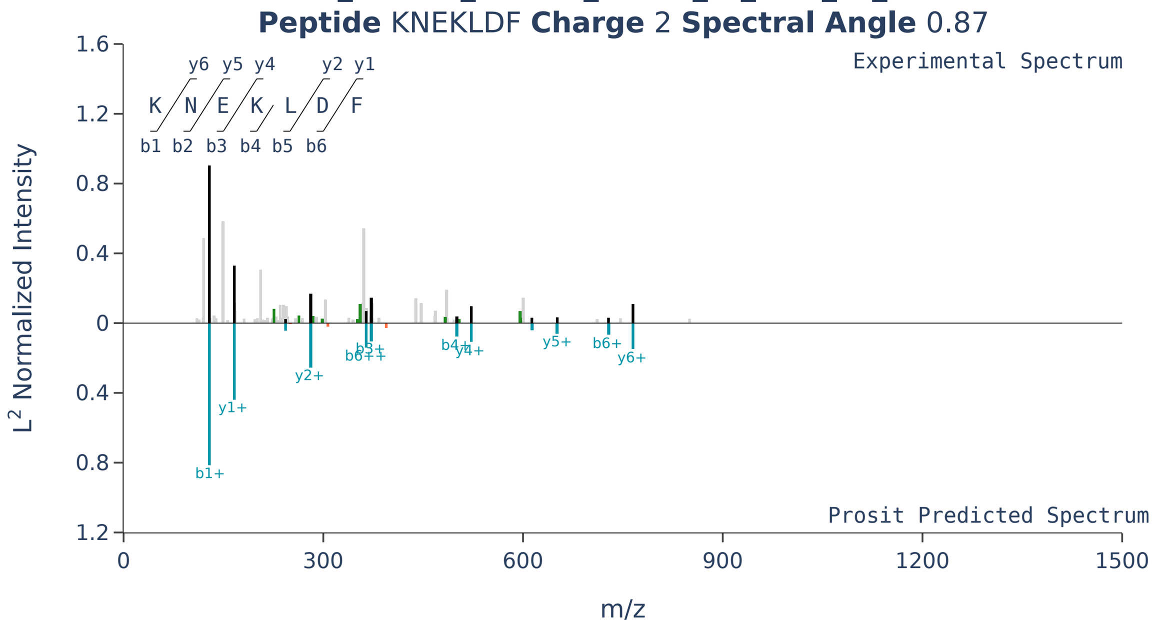

Source WSoh_221121_011221_HFGoe_L1_hTau_24h_R1 **Scan** 13807
Peptide TPRGAAPLP **Charge** 2 **Spectral Angle** 0.78

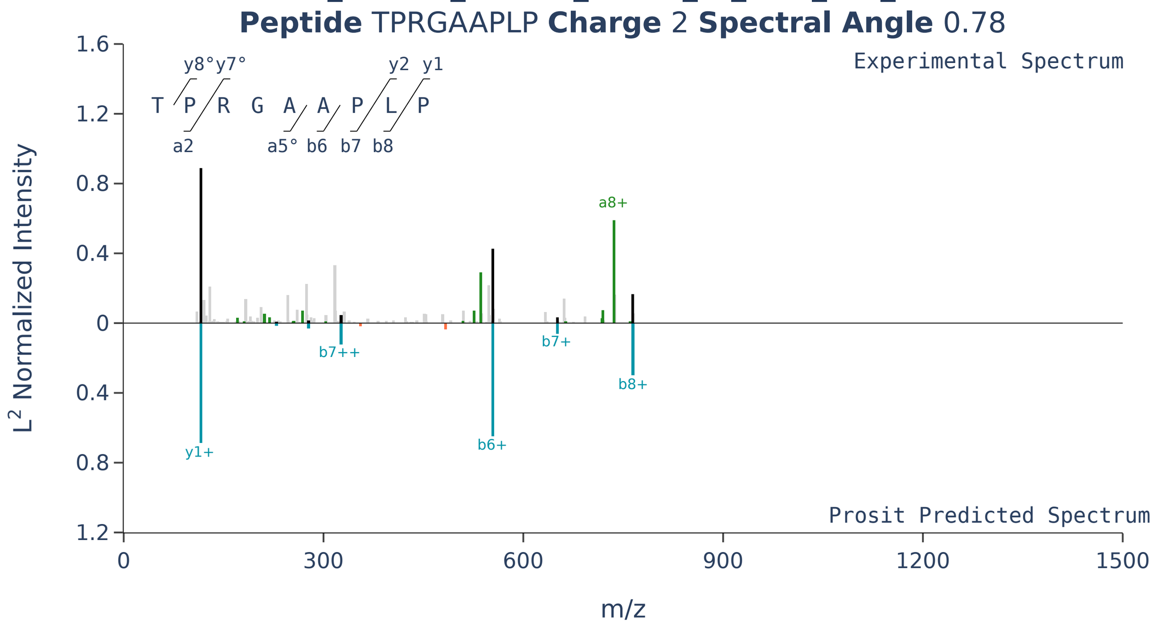

Source WSoh_221121_011221_HFGoe_L2_hTau_24h_R1 **Scan** 20881
Peptide TDAGLKEAEPRQEFEVMED **Charge** 3 **Spectral Angle** 0.82

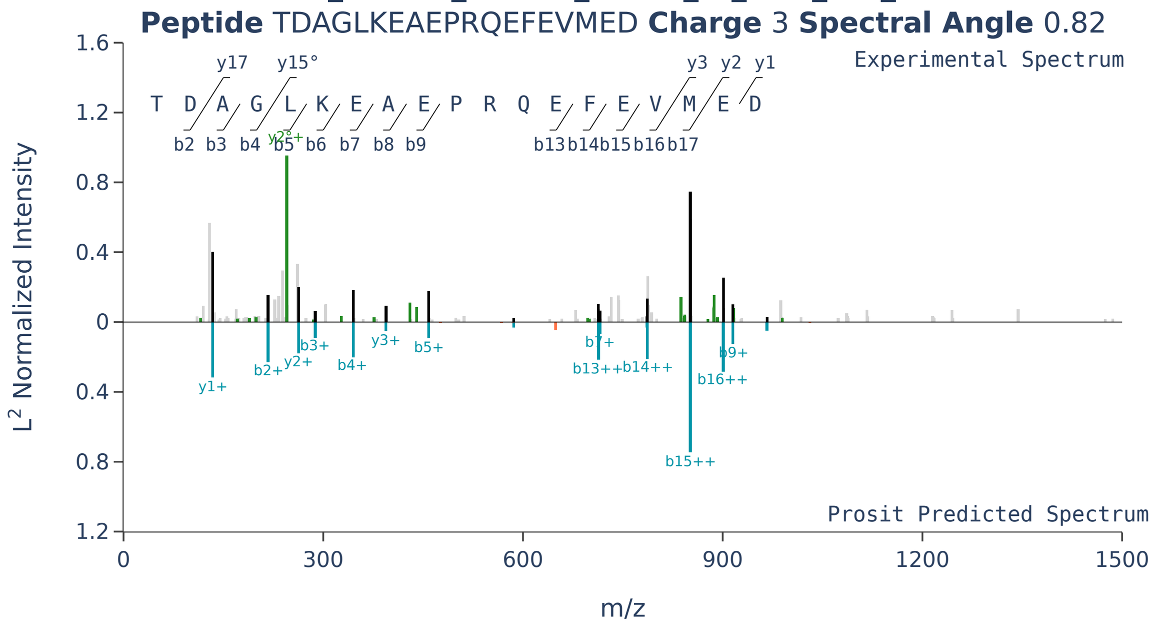

Source WSoh_221121_011221_HFGoe_L2_hTau_24h_R2 **Scan** 10200
Peptide DLSNVQSKCSKDNIKHV **Charge** 5 **Spectral Angle** 0.89

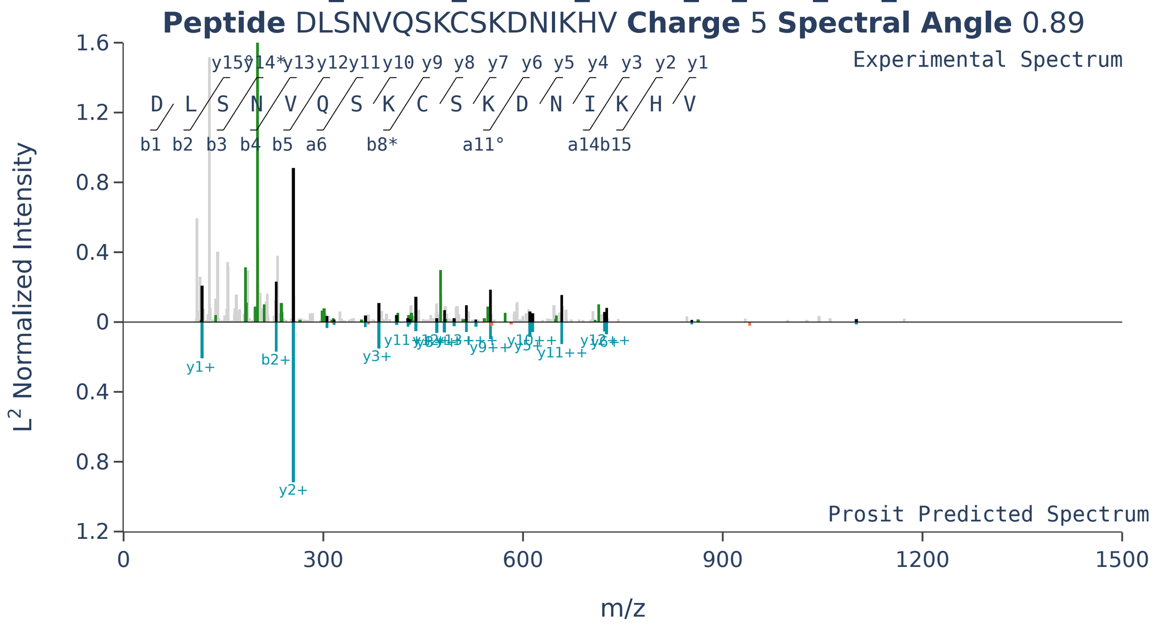

Source WSoh_221121_011221_HFGoe_L1_hTau_24h_R1 **Scan** 9957
Peptide AAEPRQEFE **Charge** 2 **Spectral Angle** 0.86

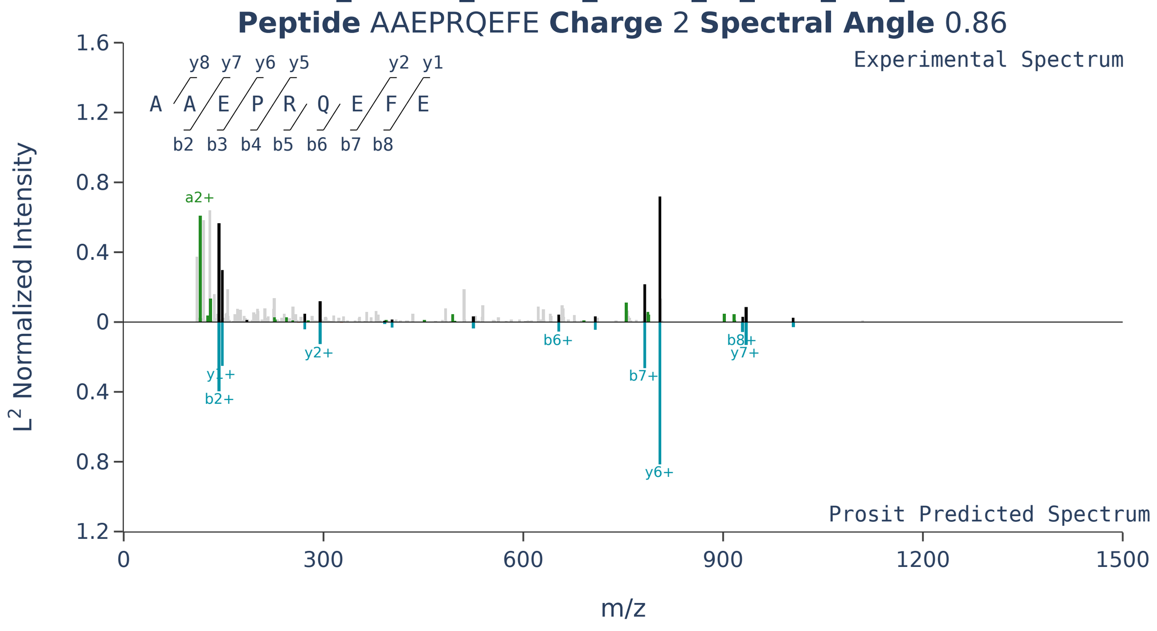

Source WSoh_221121_011221_HFGoe_L1_hTau_2h_R2 **Scan** 13514
Peptide SNVQSVYKPVD **Charge** 2 **Spectral Angle** 0.92

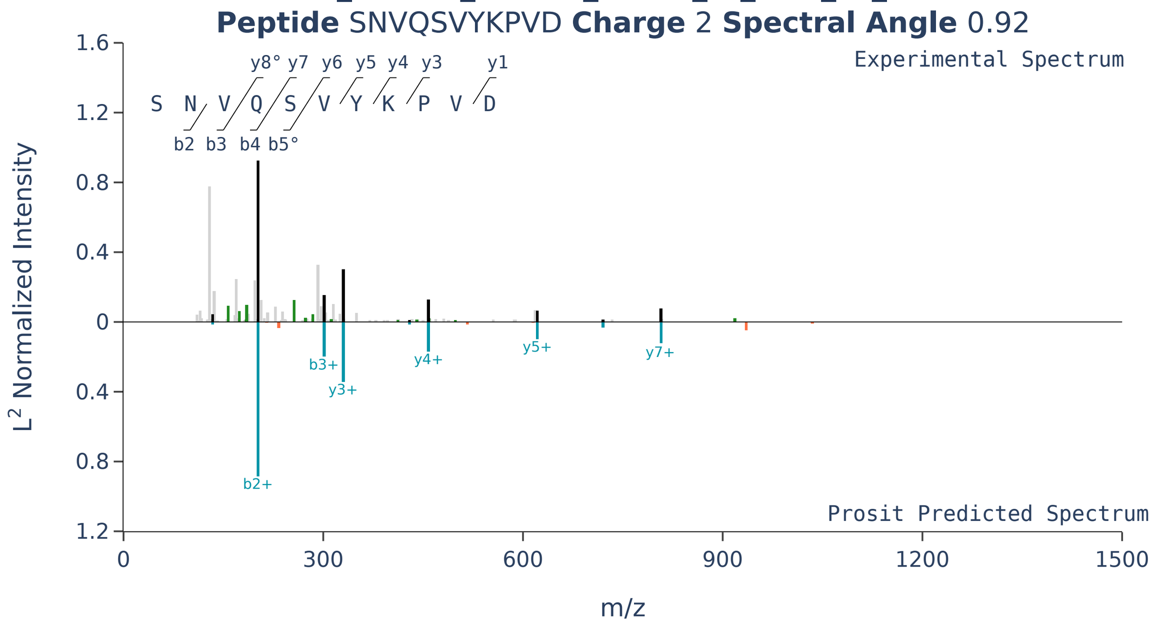

Source WSoh_221121_011221_HFGoe_L2_hTau_24h_R1 **Scan** 21500
Peptide PSLEDEAAGIGDT **Charge** 1 **Spectral Angle** 0.81

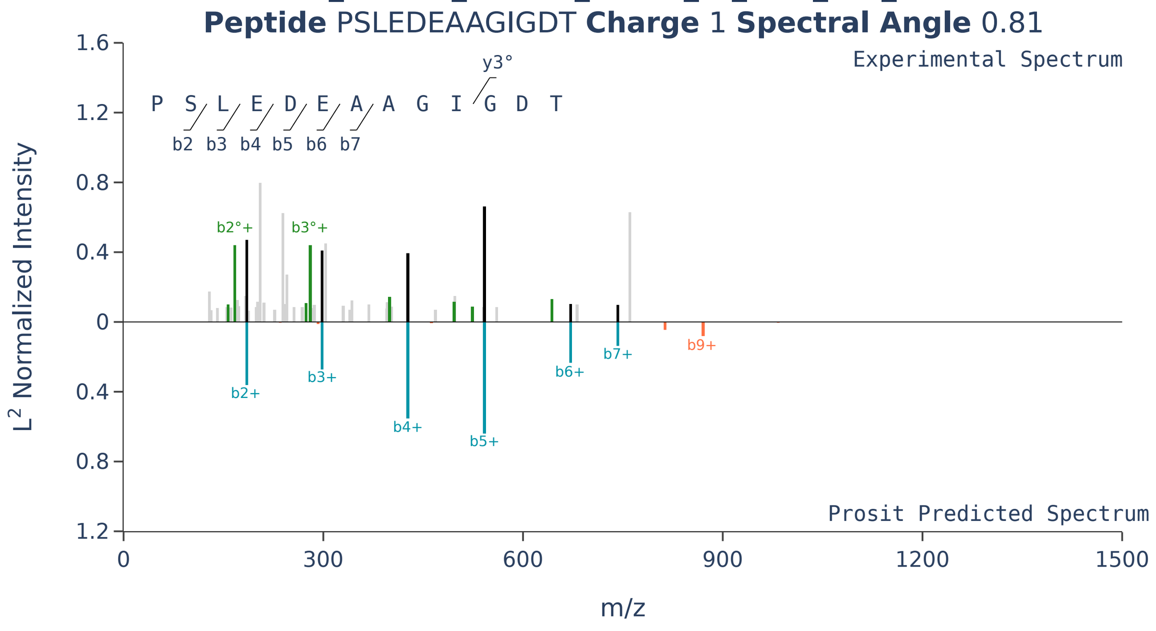

Source WSoh_221121_011221_HFGoe_L2_hTau_24h_R1 **Scan** 20770
Peptide QTAPVPMPDLKNVKSS **Charge** 3 **Spectral Angle** 0.82

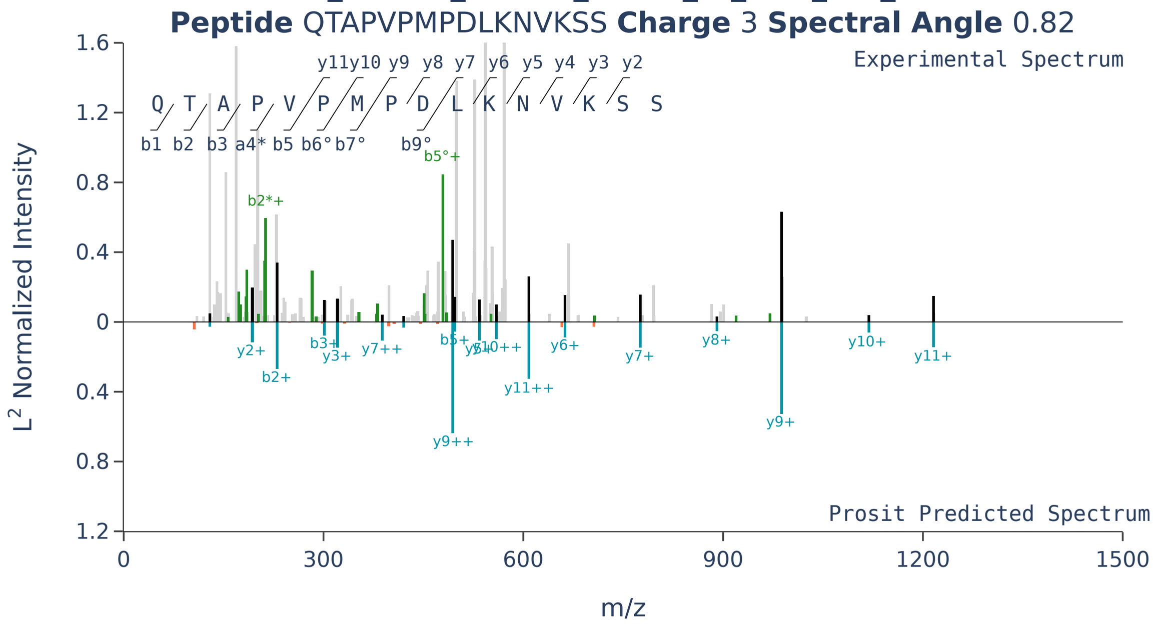

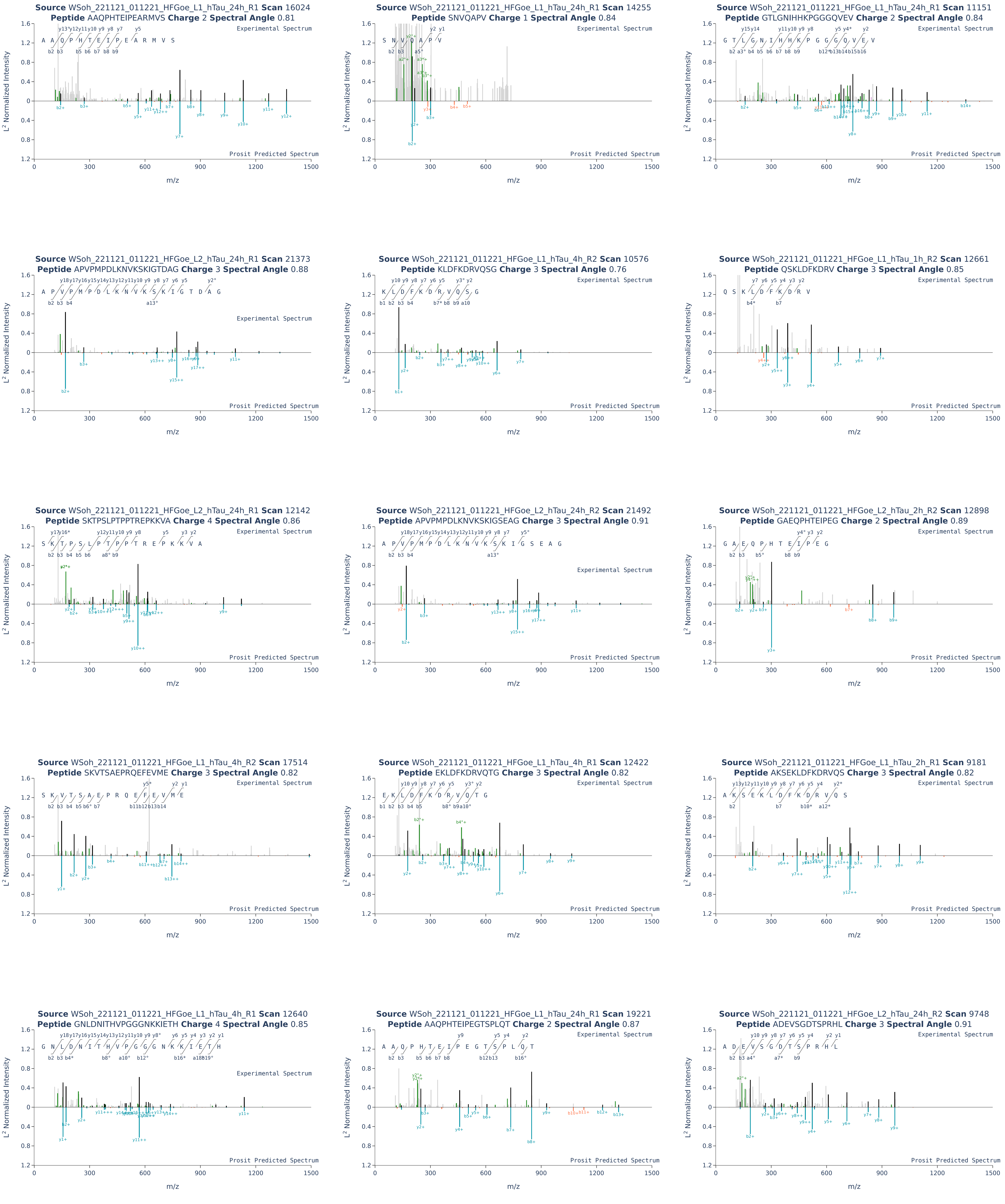
Source WSoh_221121_011221_HFGoe_L1_hTau_24h_R1 Scan 16024
Peptide AAQPHTEIPEARMVS Charge 2 Spectral Angle 0.81
Source WSoh_221121_011221_HFGoe_L1_hTau_24h_R1 Scan 14255
Peptide SNVQAPV Charge 1 Spectral Angle 0.84
Source WSoh_221121_011221_HFGoe_L1_hTau_24h_R1 Scan 11151
Peptide GTLGNIHHKPGGGQVEV Charge 2 Spectral Angle 0.84
Source WSoh_221121_011221_HFGoe_L2_hTau_24h_R1 Scan 21373
Peptide APVPMPDLKNVKSKIGTDAG Charge 3 Spectral Angle 0.88
Source WSoh_221121_011221_HFGoe_L1_hTau_4h_R2 Scan 10576
Peptide KLDFKDRVQSG Charge 3 Spectral Angle 0.76
Source WSoh_221121_011221_HFGoe_L1_hTau_1h_R2 Scan 12661
Peptide QSKLDFKDRV Charge 3 Spectral Angle 0.85
Source WSoh_221121_011221_HFGoe_L2_hTau_24h_R1 Scan 12142
Peptide SKTPSLPTPPTREPKKVA Charge 4 Spectral Angle 0.86
Source WSoh_221121_011221_HFGoe_L2_hTau_24h_R2 Scan 21492
Peptide APVPMPDLKNVKSKIGSEAG Charge 3 Spectral Angle 0.91
Source WSoh_221121_011221_HFGoe_L2_hTau_2h_R2 Scan 12898
Peptide GAEQPHTEIPEG Charge 2 Spectral Angle 0.89
Source WSoh_221121_011221_HFGoe_L1_hTau_4h_R2 Scan 17514
Peptide SKVTSAEPRQEFEVME Charge 3 Spectral Angle 0.82
Source WSoh_221121_011221_HFGoe_L1_hTau_4h_R1 Scan 12422
Peptide EKLDFKDRVQTG Charge 3 Spectral Angle 0.82
Source WSoh_221121_011221_HFGoe_L1_hTau_2h_R1 Scan 9181
Peptide AKSEKLDFKDRVQS Charge 3 Spectral Angle 0.82
Source WSoh_221121_011221_HFGoe_L1_hTau_4h_R1 Scan 12640
Peptide GNLDNITHVPGGGNKKIETH Charge 4 Spectral Angle 0.85
Source WSoh_221121_011221_HFGoe_L1_hTau_24h_R1 Scan 19221
Peptide AAQPHTEIPEGTSPLQT Charge 2 Spectral Angle 0.87
Source WSoh_221121_011221_HFGoe_L2_hTau_24h_R2 Scan 9748
Peptide ADEVSGDTSPRHL Charge 3 Spectral Angle 0.91
Experimental Spectrum
Prosit Predicted Spectrum
m/z
L² Normalized Intensity

Source WSoh_221121_011221_HFGoe_L1_hTau_24h_R2 **Scan** 20652
Peptide TAPVPMPDLKNVSKS **Charge** 3 **Spectral Angle** 0.8

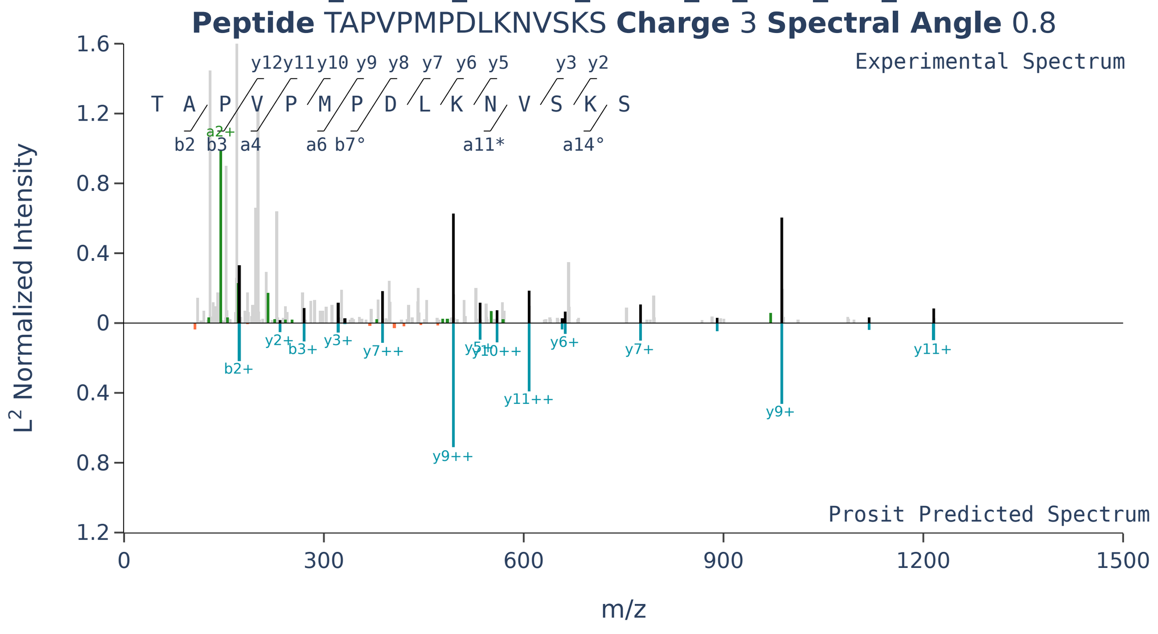

Source WSoh_221121_011221_HFGoe_L1_hTau_24h_R1 **Scan** 8074
Peptide VRTPPKSPSSARMVS **Charge** 3 **Spectral Angle** 0.83

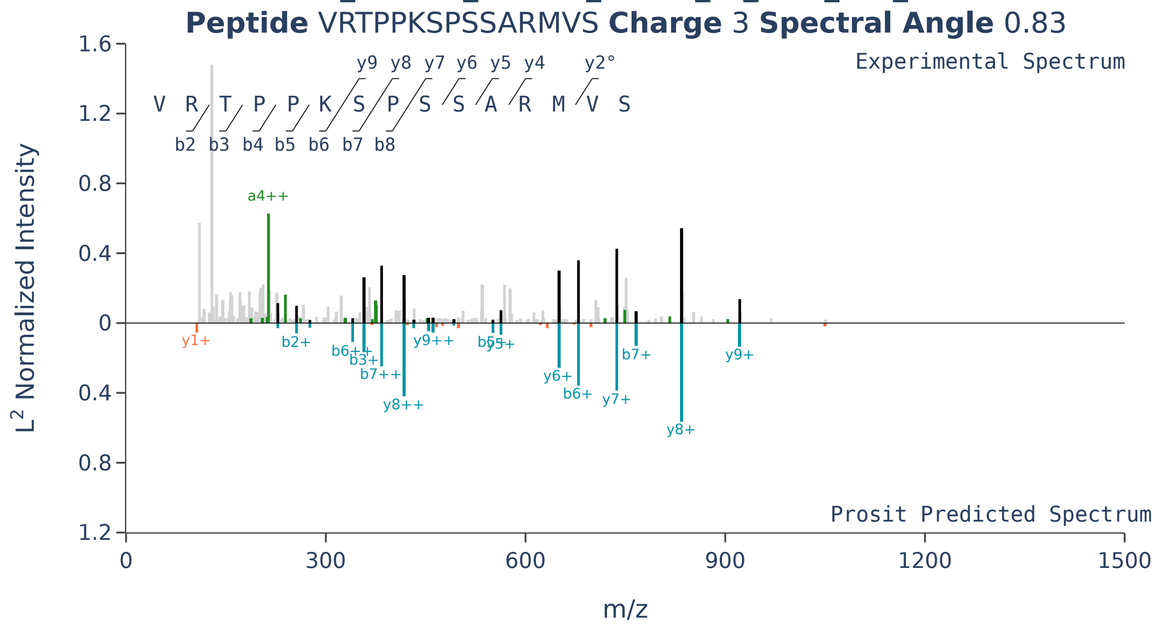

Source WSoh_221121_011221_HFGoe_L2_hTau_24h_R2 **Scan** 6502
Peptide ITHVPGGGNKKIETHGG **Charge** 3 **Spectral Angle** 0.91

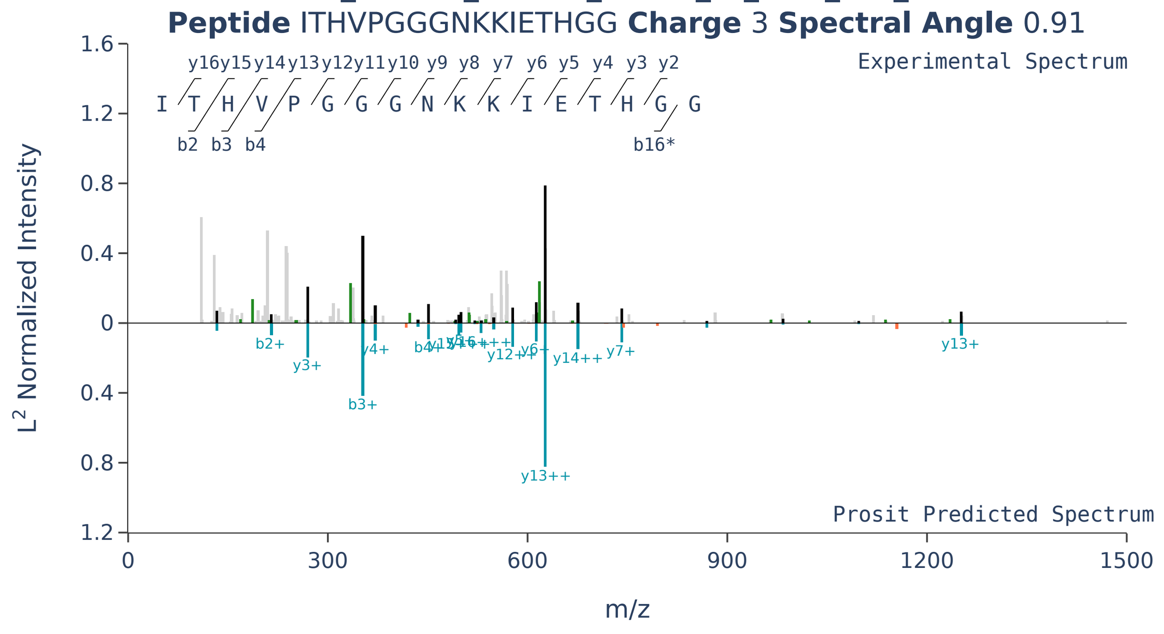

Source WSoh_221121_011221_HFGoe_L1_hTau_4h_R1 **Scan** 16229
Peptide RLQTSPLQTPTED **Charge** 2 **Spectral Angle** 0.87

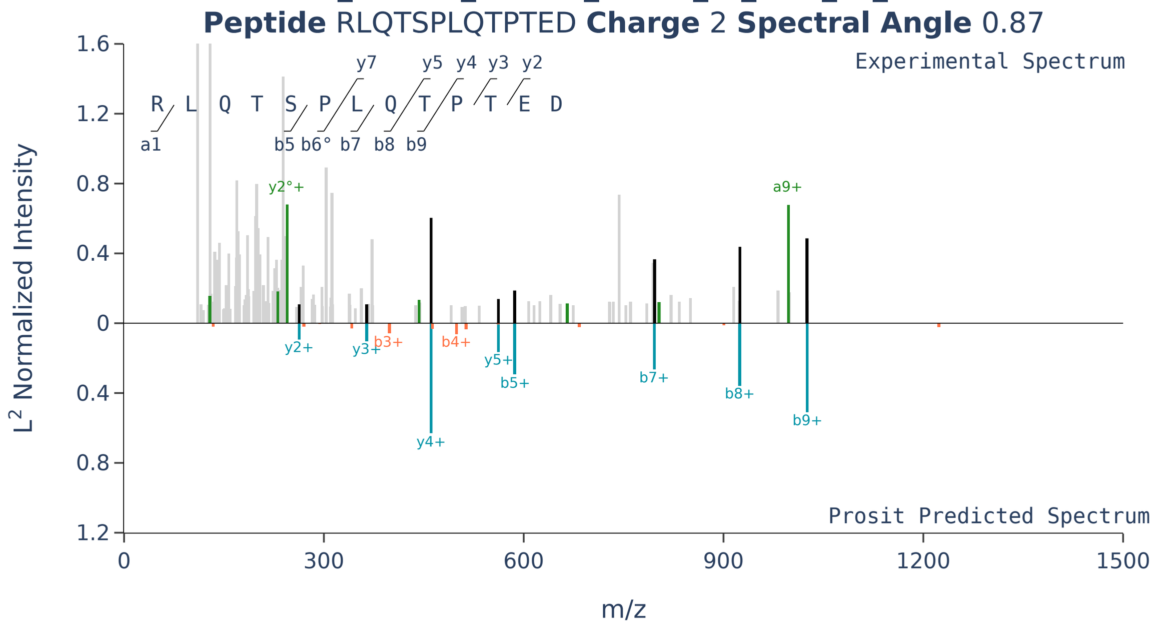

Source WSoh_221121_011221_HFGoe_L1_hTau_24h_R1 **Scan** 9560
Peptide DLTHVPGGGNKKIETH **Charge** 3 **Spectral Angle** 0.84

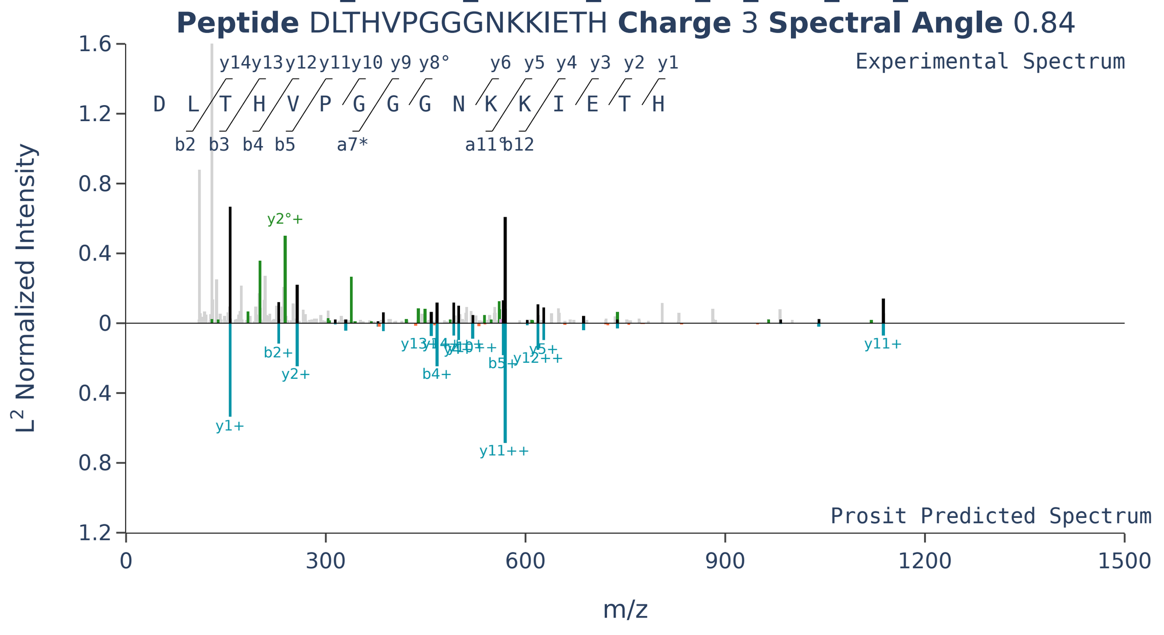

Source WSoh_221121_011221_HFGoe_L2_hTau_0h_R2 **Scan** 8690
Peptide KVDLSKV **Charge** 2 **Spectral Angle** 0.94

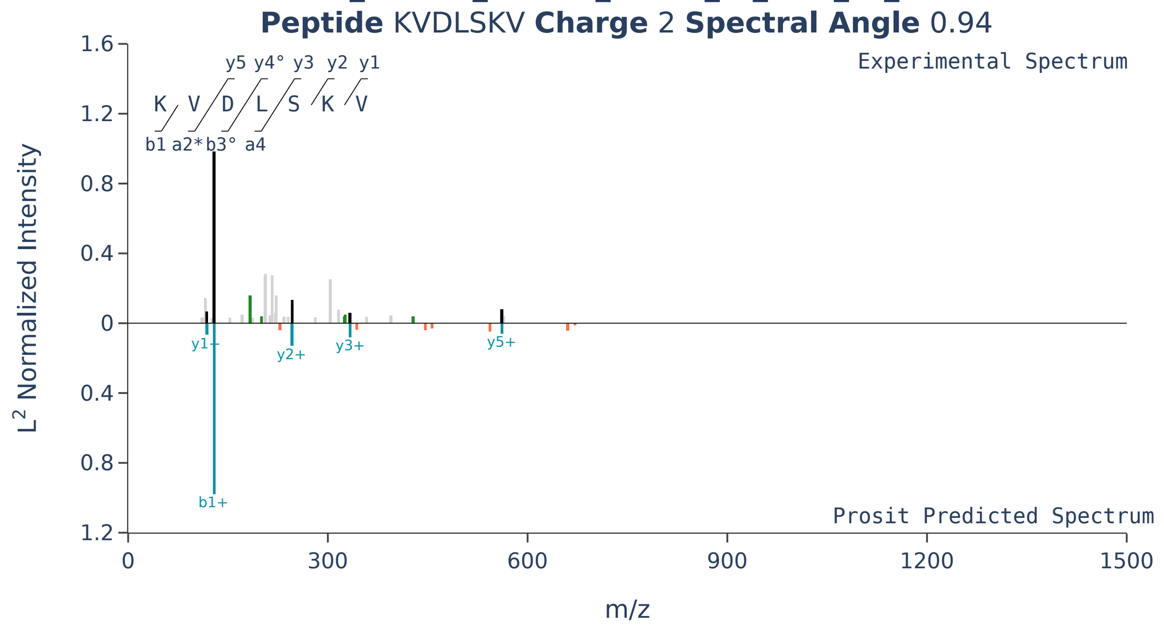

Source WSoh_221121_011221_HFGoe_L1_hTau_24h_R1 **Scan** 16481
Peptide VYKPVDLNVK **Charge** 3 **Spectral Angle** 0.81

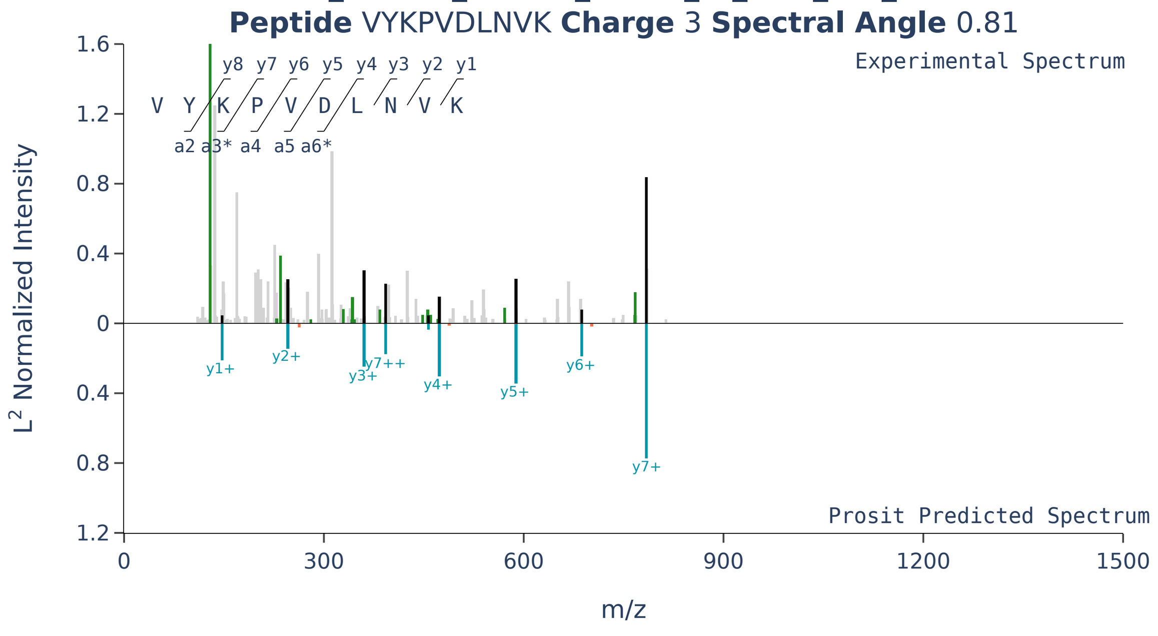

Source WSoh_221121_011221_HFGoe_L2_hTau_24h_R1 **Scan** 18049
Peptide AAQPHTEIPEGTNL **Charge** 2 **Spectral Angle** 0.88

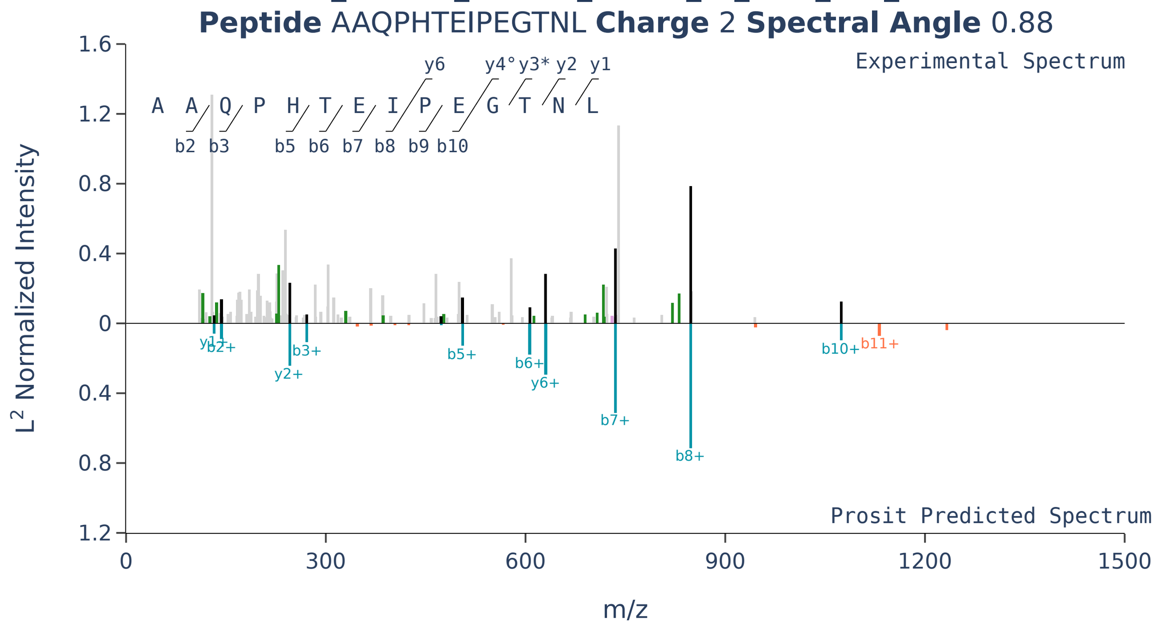

Source WSoh_221121_011221_HFGoe_L1_hTau_24h_R2 **Scan** 16837
Peptide APVPMPDLKNVKGGK **Charge** 3 **Spectral Angle** 0.75

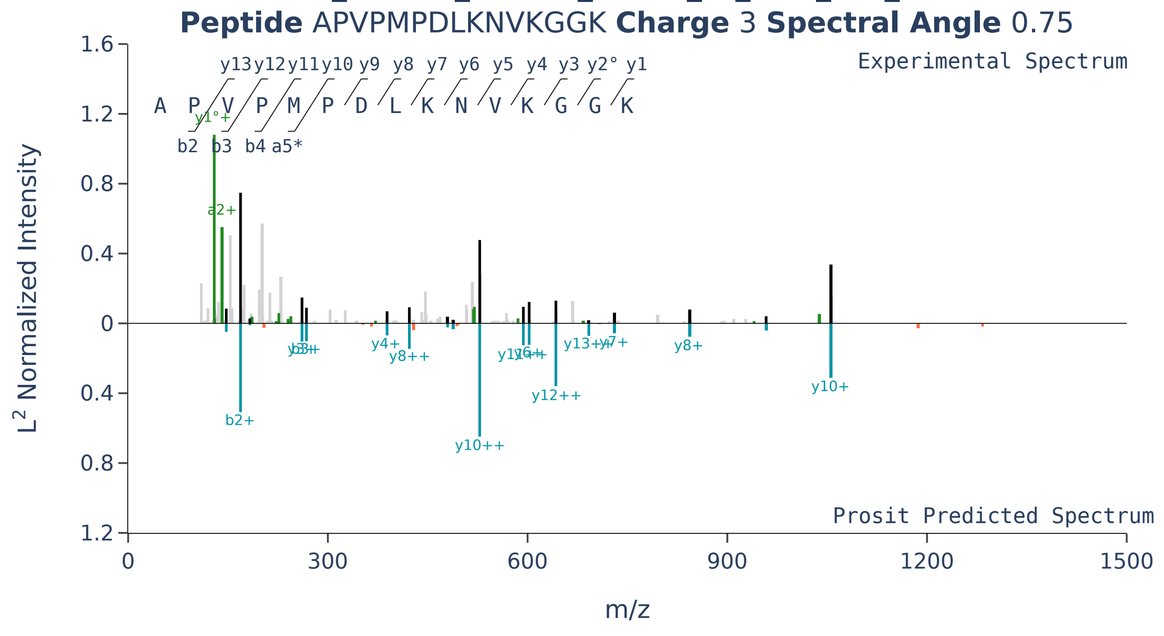

Source WSoh_221121_011221_HFGoe_L1_hTau_24h_R1 **Scan** 14605
Peptide LDLSNVQSKCSKDNIKHV **Charge** 3 **Spectral Angle** 0.91

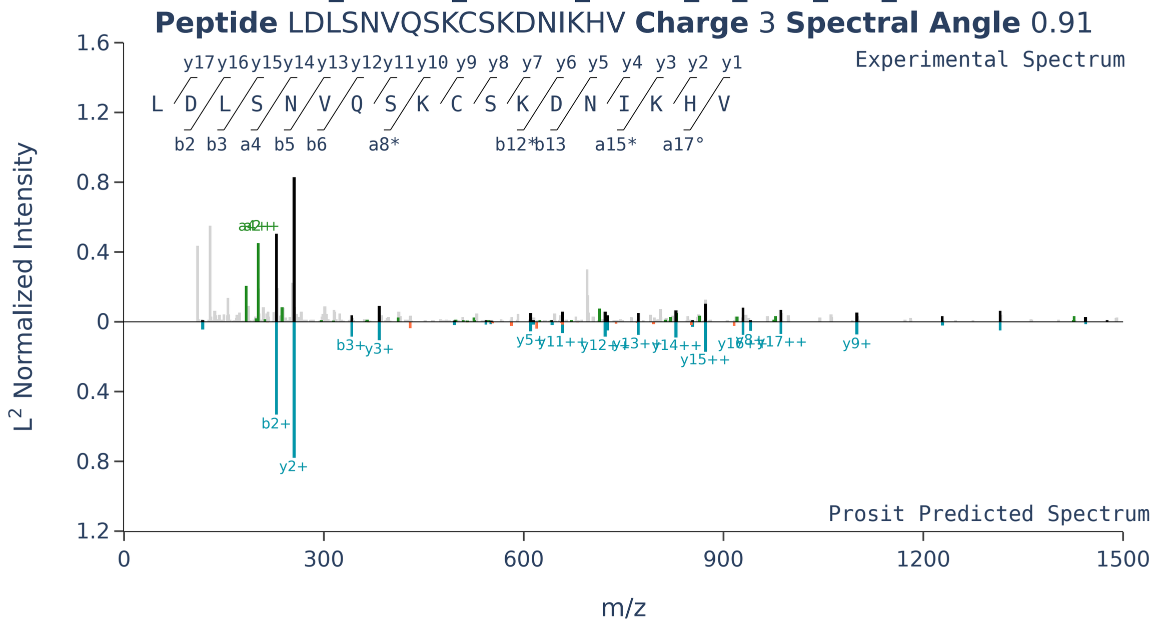

Source WSoh_221121_011221_HFGoe_L2_hTau_2h_R1 **Scan** 12718
Peptide KSEKLDFKDRVQSV **Charge** 4 **Spectral Angle** 0.87

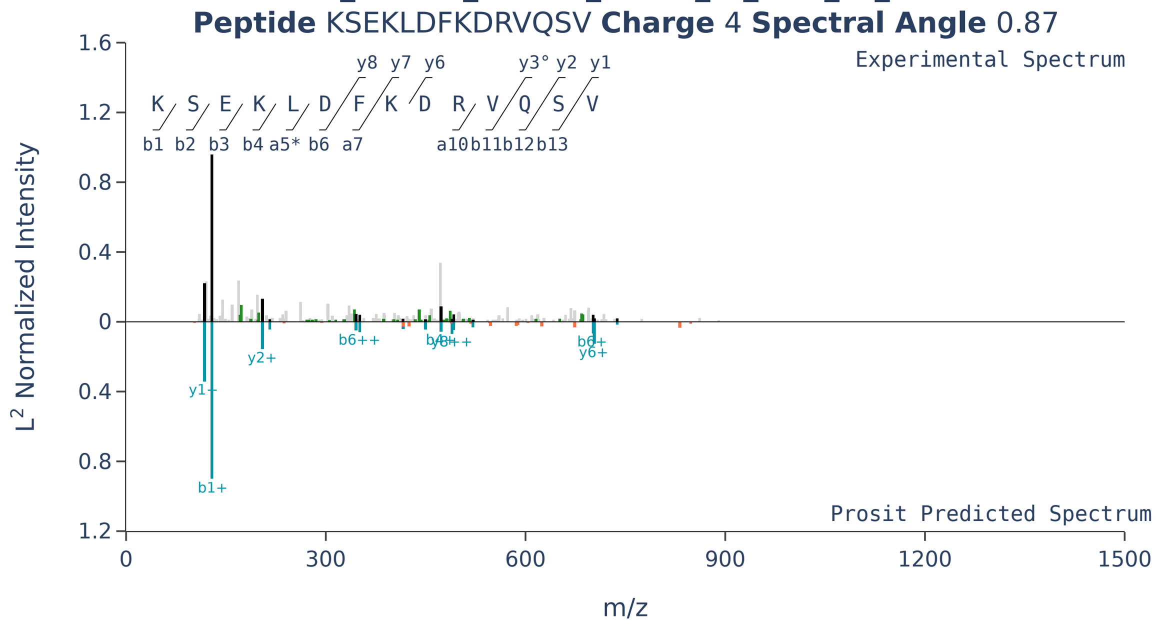

Source WSoh_221121_011221_HFGoe_L1_hTau_24h_R2 **Scan** 7045
Peptide KTHQPGGGKVQIINK **Charge** 4 **Spectral Angle** 0.89

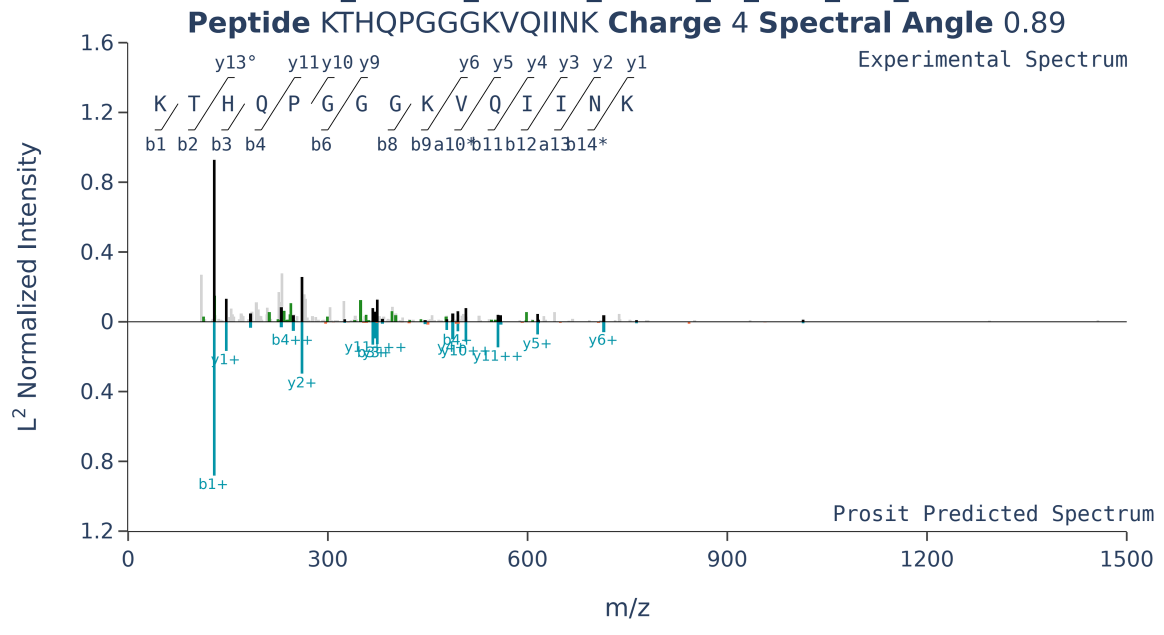

Source WSoh_221121_011221_HFGoe_L2_hTau_24h_R2 **Scan** 18068
Peptide NKQTAPVPMPDLKNVKS **Charge** 3 **Spectral Angle** 0.84

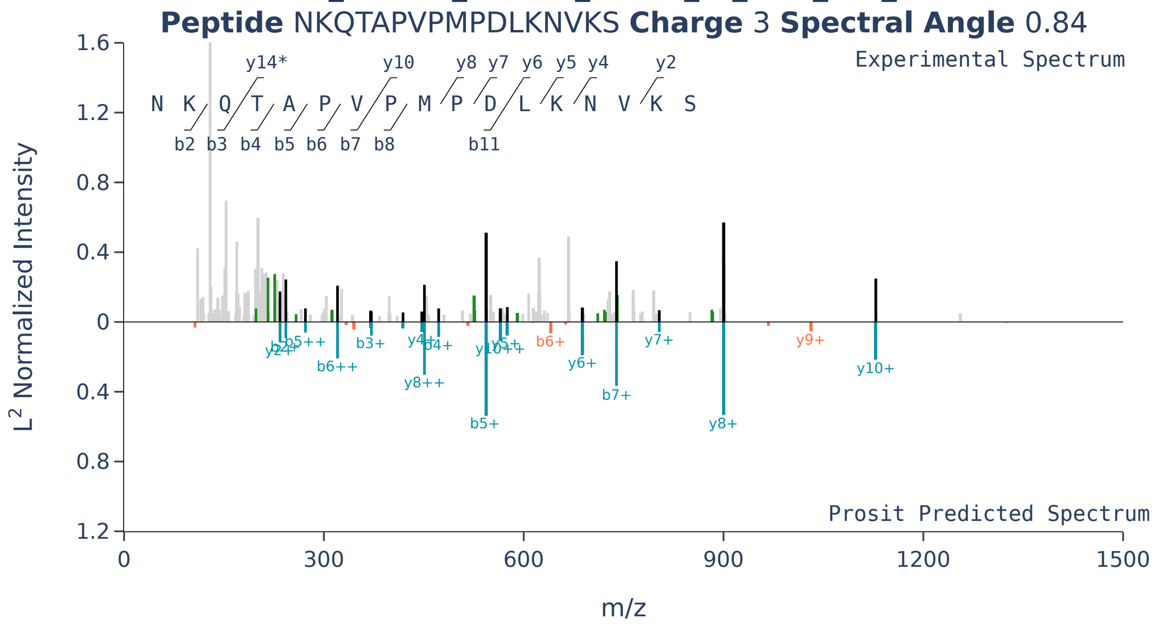

Source WSoh_221121_011221_HFGoe_L1_hTau_4h_R1 **Scan** 8190
Peptide SKKIGSTENL **Charge** 1 **Spectral Angle** 0.81

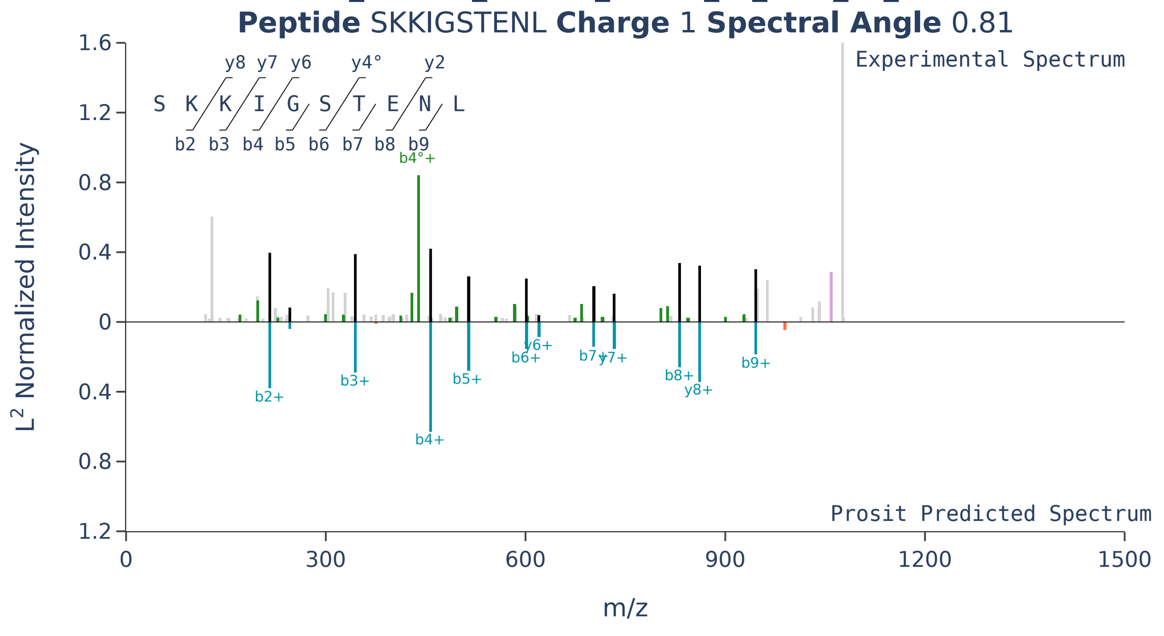

Source WSoh_221121_011221_HFGoe_L1_hTau_24h_R1 **Scan** 11192
Peptide QEGDAEPRQEFE **Charge** 2 **Spectral Angle** 0.92

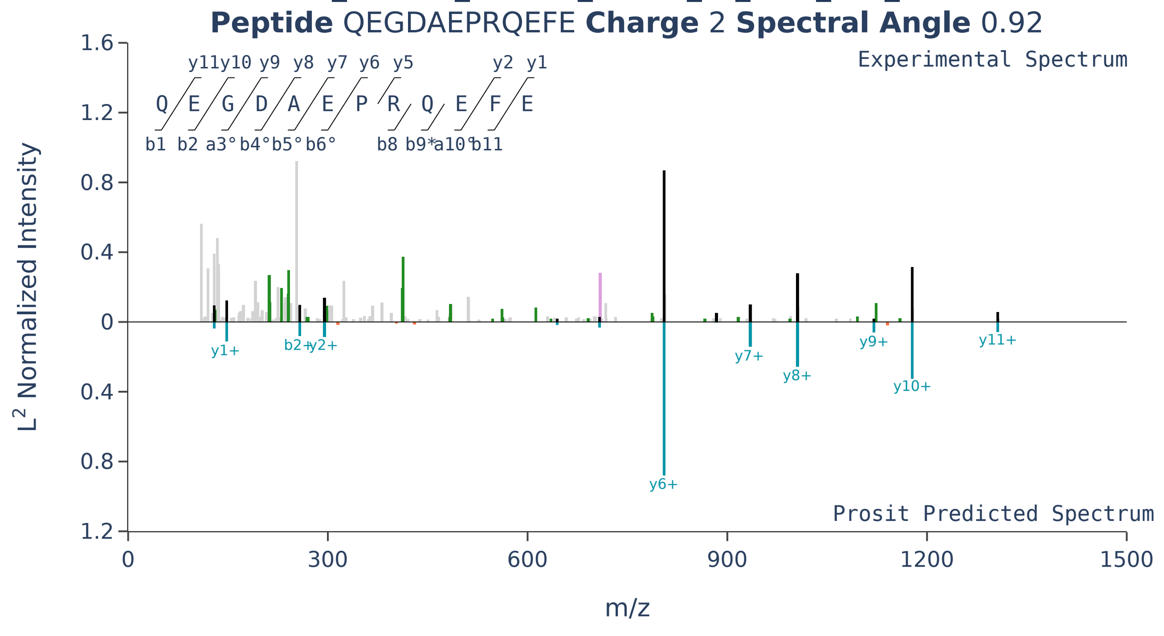

Source WSoh_221121_011221_HFGoe_L1_hTau_4h_R2 **Scan** 13076
Peptide DLSNVQSKCSKDNIKHVPGGGSV **Charge** 3 **Spectral Angle** 0.94

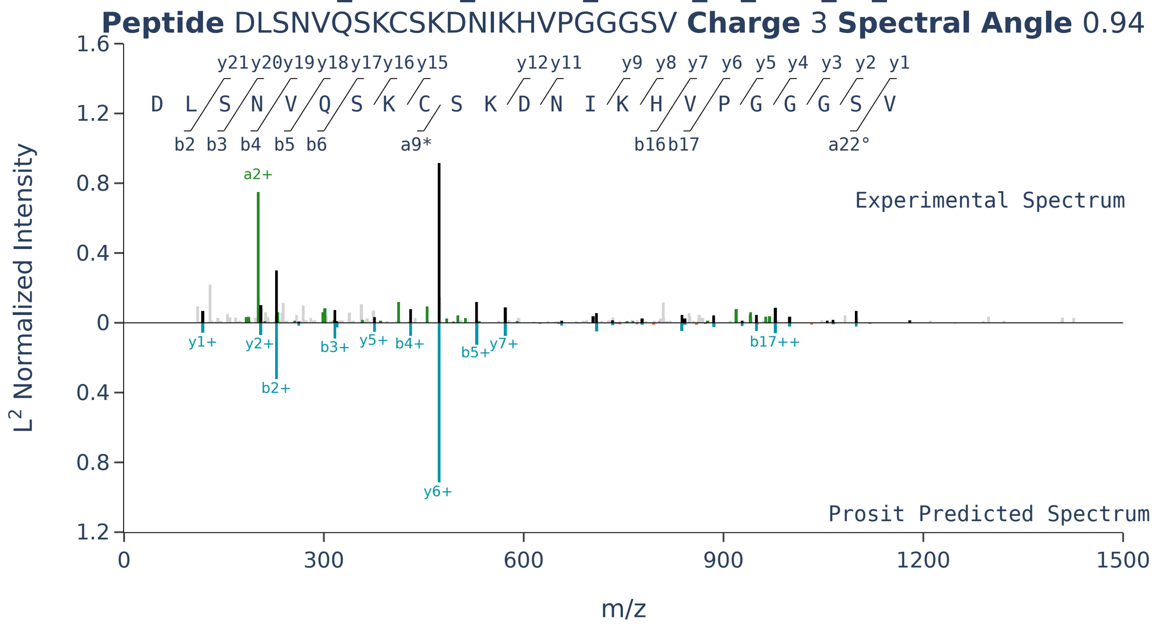

Source WSoh_221121_011221_HFGoe_L2_hTau_4h_R2 **Scan** 19485
Peptide AGIGDTPSLEDEAAGHVTQARMVKS **Charge** 4 **Spectral Angle** 0.85

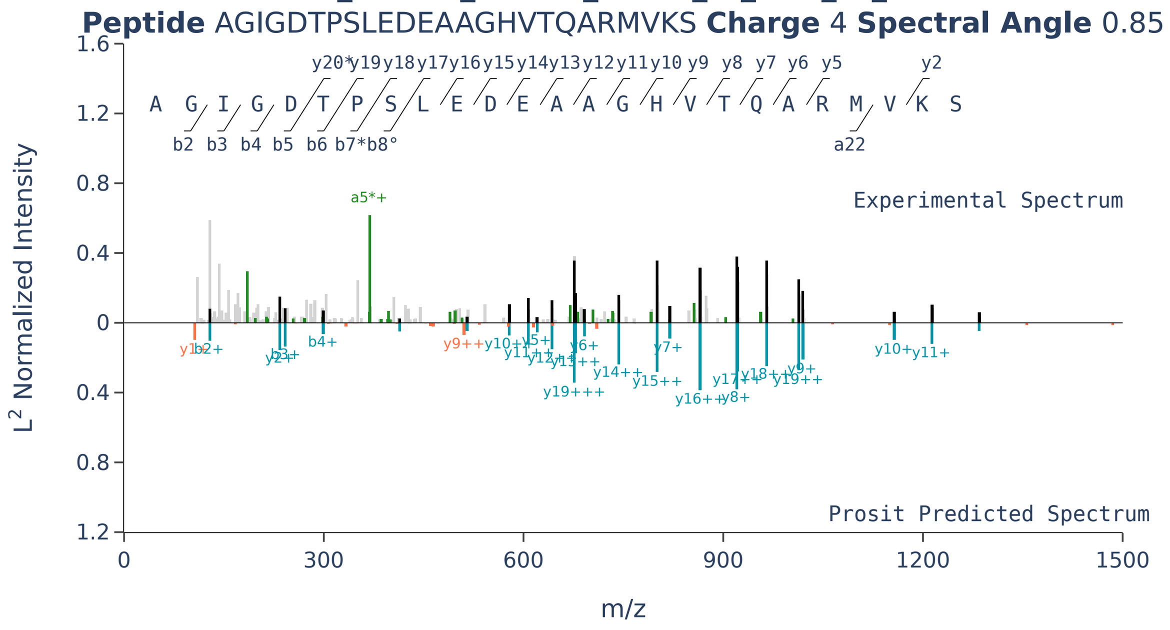

Source WSoh_221121_011221_HFGoe_L1_hTau_24h_R1 **Scan** 20907
Peptide QPGPVPMPDLKNVKS **Charge** 2 **Spectral Angle** 0.88

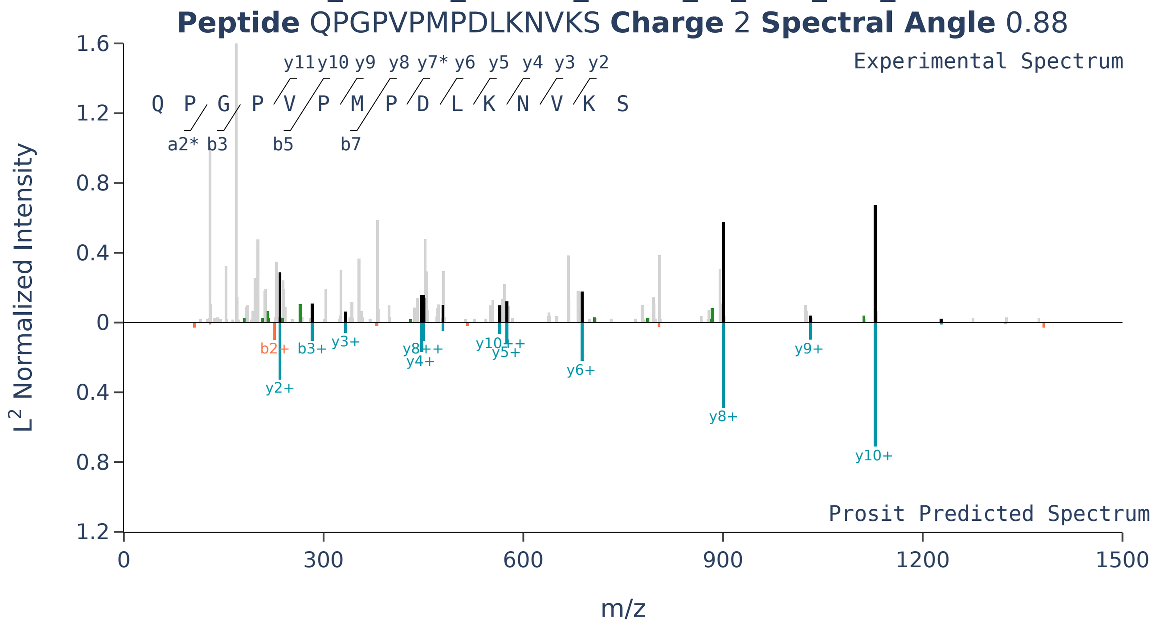

Source WSoh_221121_011221_HFGoe_L2_hTau_24h_R1 **Scan** 22749
Peptide LQTAPVPMPDLKNVKGG **Charge** 2 **Spectral Angle** 0.88

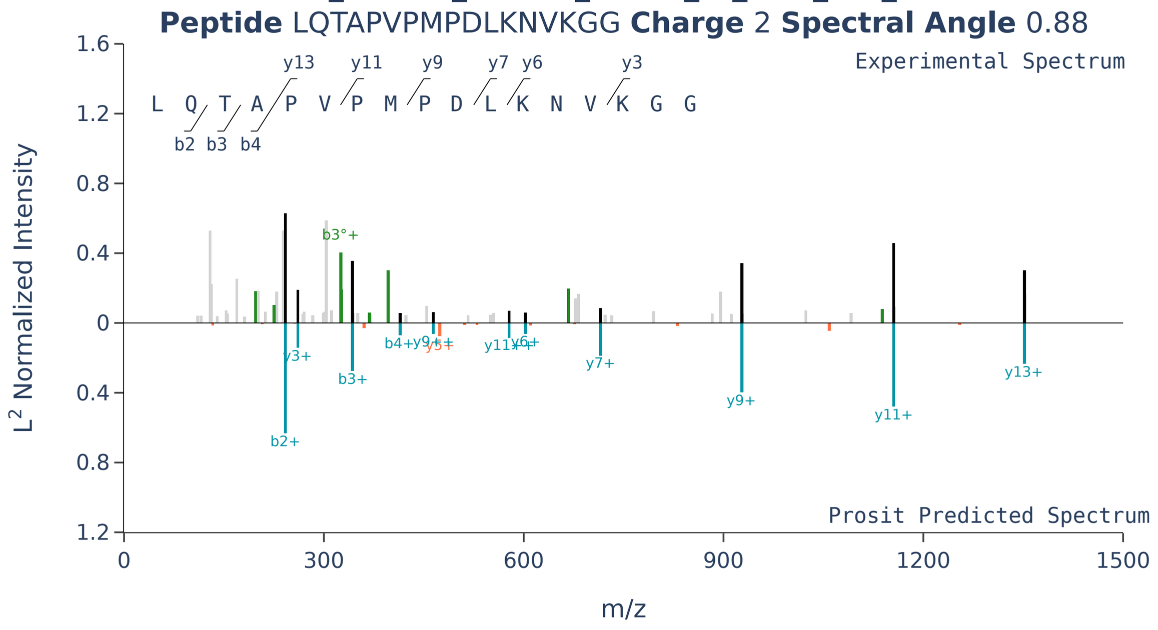

Source WSoh_221121_011221_HFGoe_L1_hTau_24h_R1 **Scan** 8667
Peptide VGSLGNIHHKPGGGQVE **Charge** 2 **Spectral Angle** 0.83

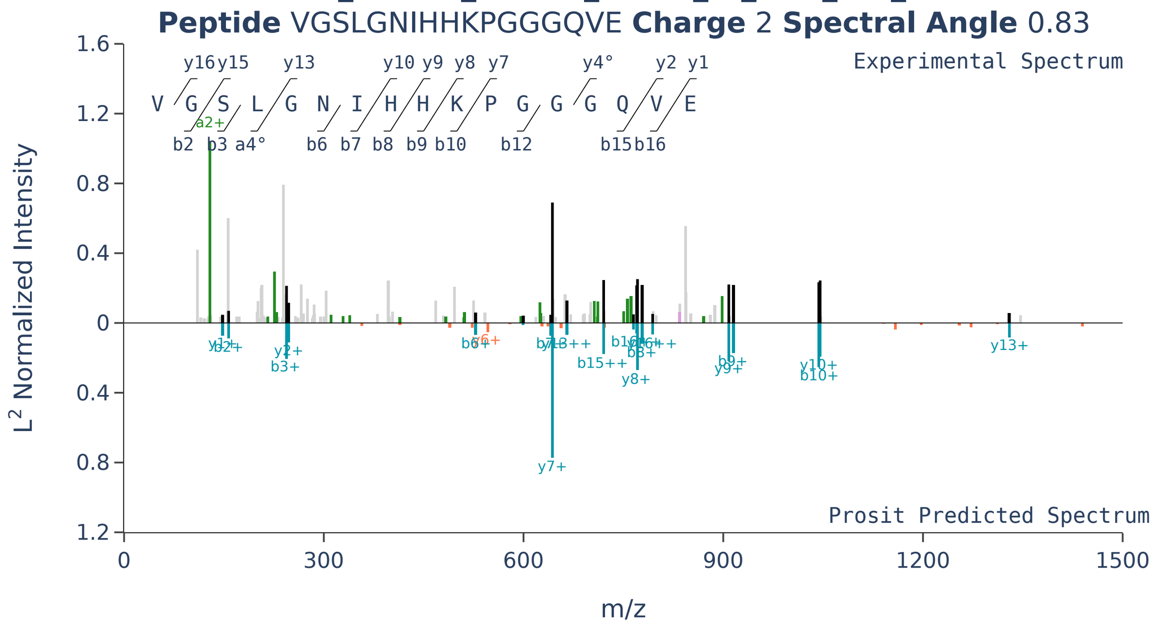

Source WSoh_221121_011221_HFGoe_L1_hTau_2h_R1 **Scan** 12535
Peptide DAAQPHTEIPEG **Charge** 2 **Spectral Angle** 0.86

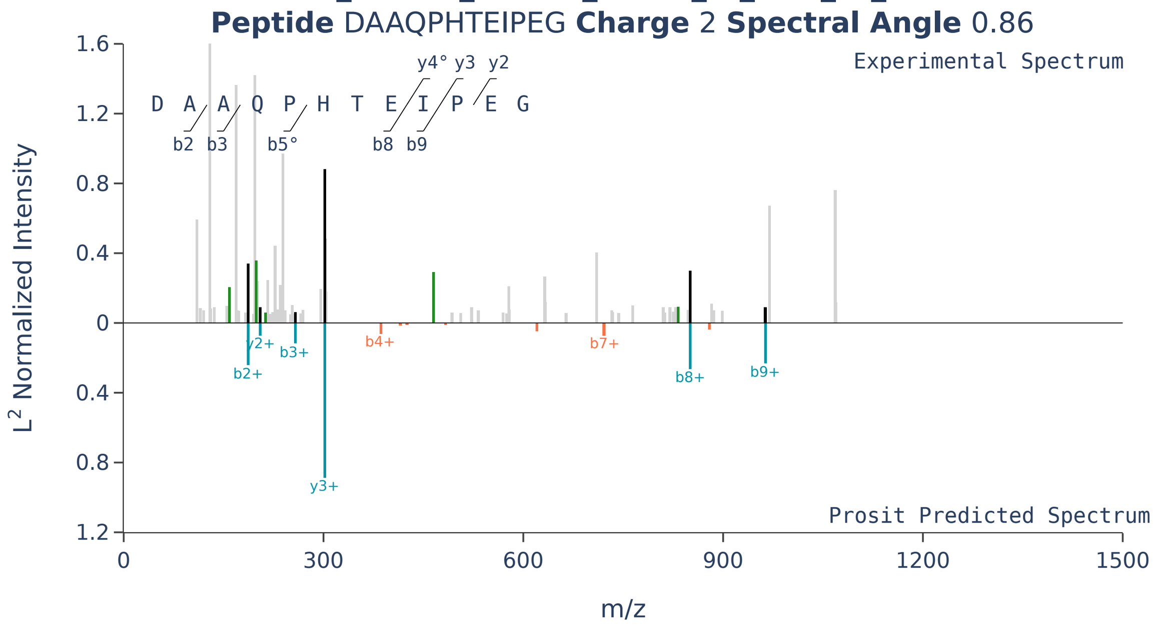

Source WSoh_221121_011221_HFGoe_L2_hTau_24h_R1 **Scan** 14026
Peptide TPSLPTPPTREPKKVG **Charge** 4 **Spectral Angle** 0.81

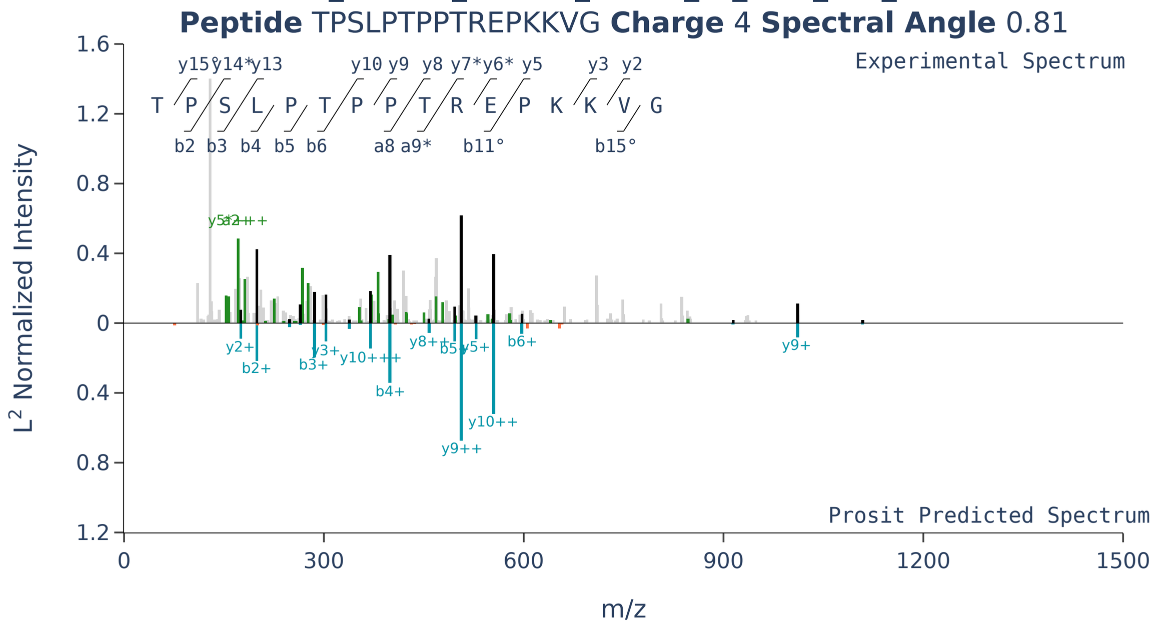

Source WSoh_221121_011221_HFGoe_L2_hTau_4h_R1 **Scan** 7474
Peptide KLDFKDRVQSKH **Charge** 3 **Spectral Angle** 0.81

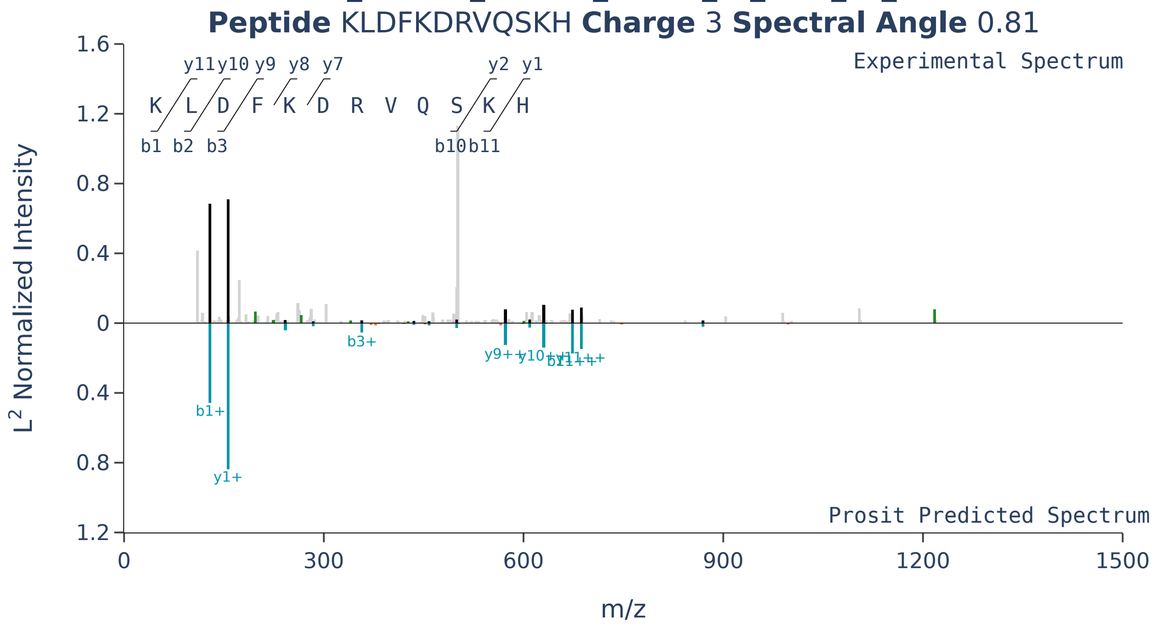

Source WSoh_221121_011221_HFGoe_L1_hTau_24h_R2 **Scan** 7021
Peptide QSKHQPGGGKVQIINK **Charge** 2 **Spectral Angle** 0.91

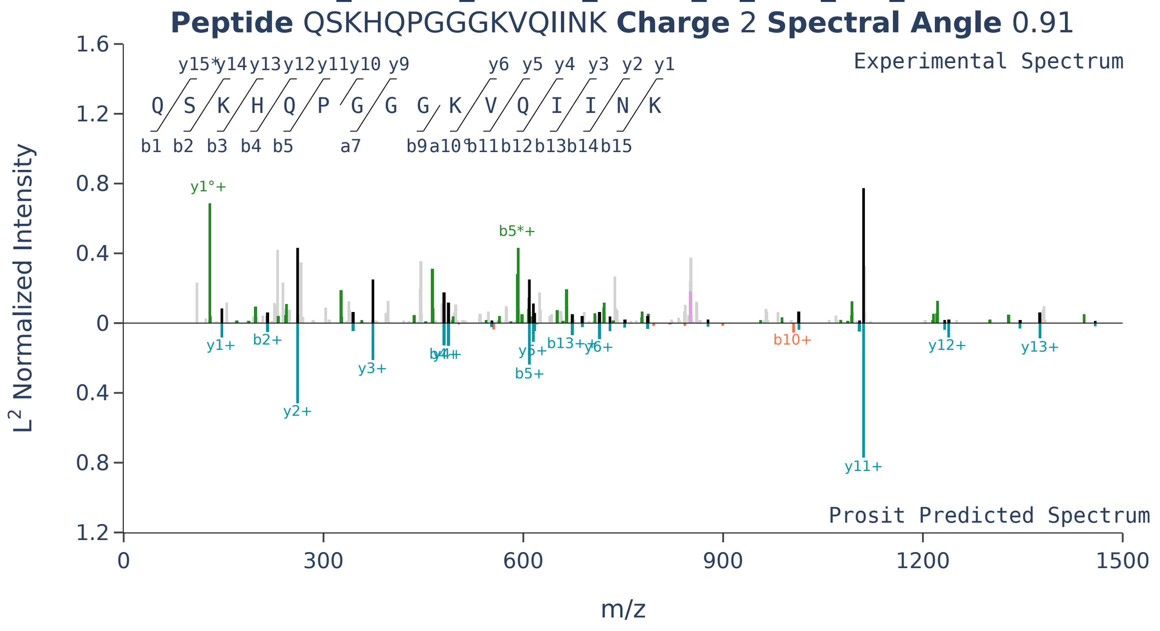

Source WSoh_221121_011221_HFGoe_L1_hTau_4h_R2 **Scan** 13029
Peptide DLSNVQSKCSKDNI **Charge** 2 **Spectral Angle** 0.84

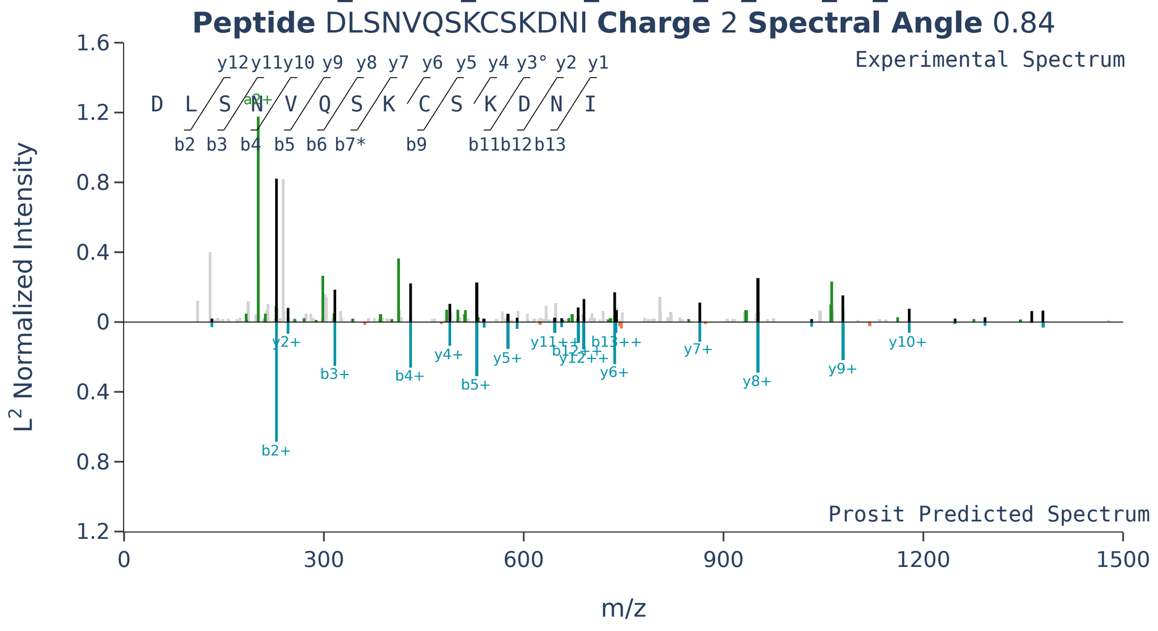

Source WSoh_221121_011221_HFGoe_L1_hTau_24h_R2 **Scan** 16445
Peptide GDTSPRHLSNVSSTGSPLQTPTE **Charge** 3 **Spectral Angle** 0.91

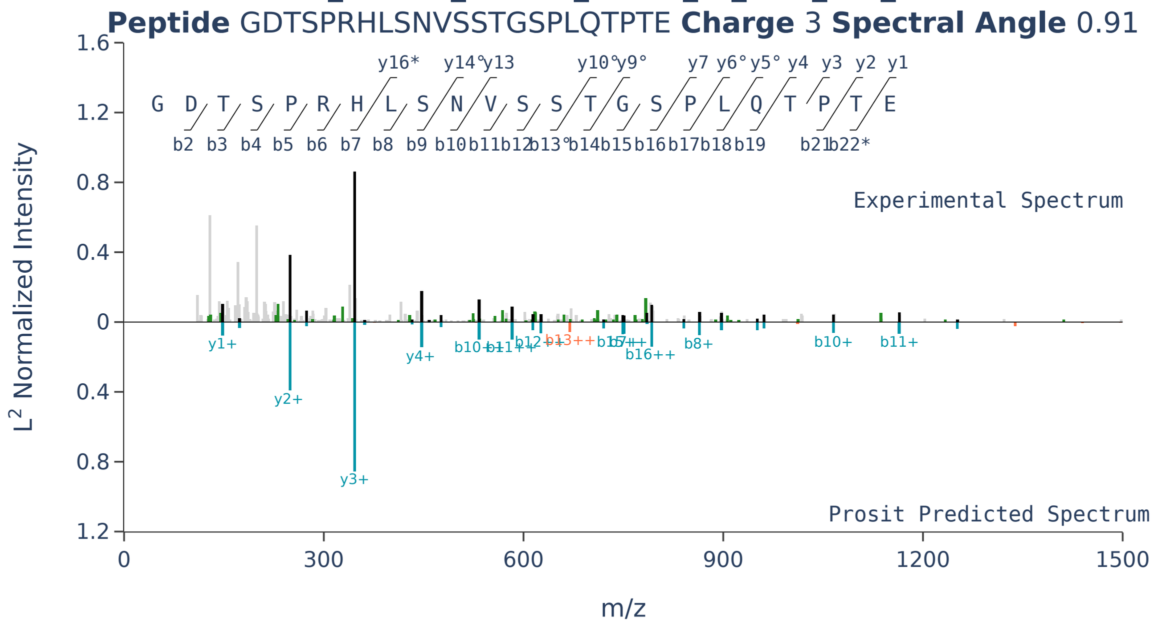

Source WSoh_221121_011221_HFGoe_L2_hTau_4h_R1 **Scan** 17752
Peptide PVDLSEAAG **Charge** 1 **Spectral Angle** 0.86

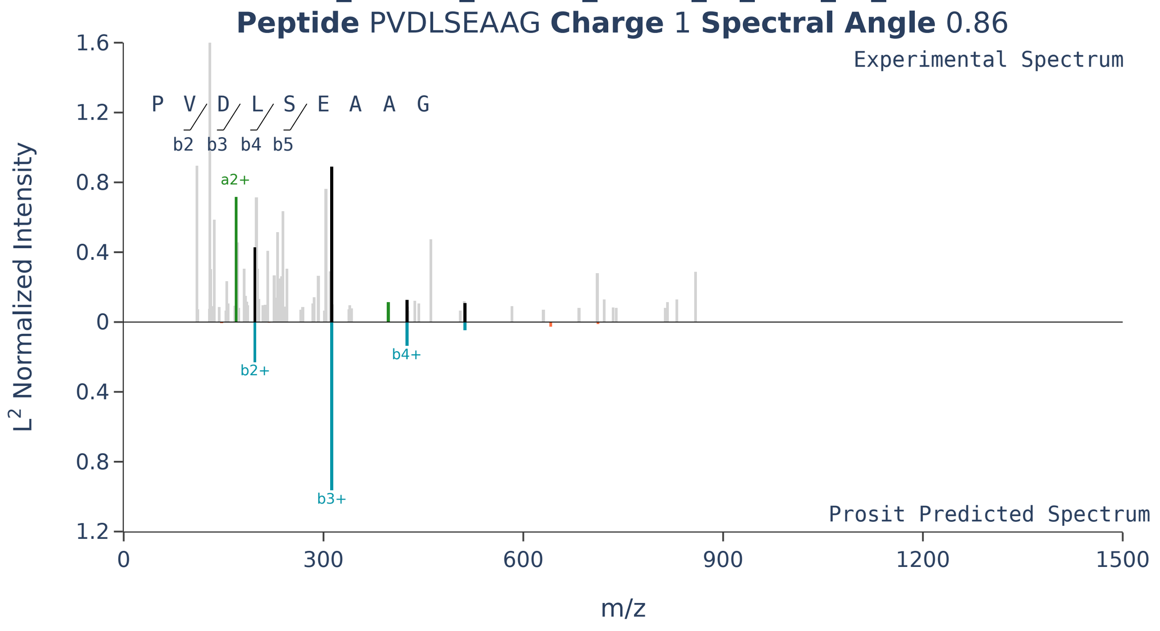

Source WSoh_221121_011221_HFGoe_L2_hTau_24h_R1 **Scan** 7309
Peptide ENKIETH **Charge** 2 **Spectral Angle** 0.91

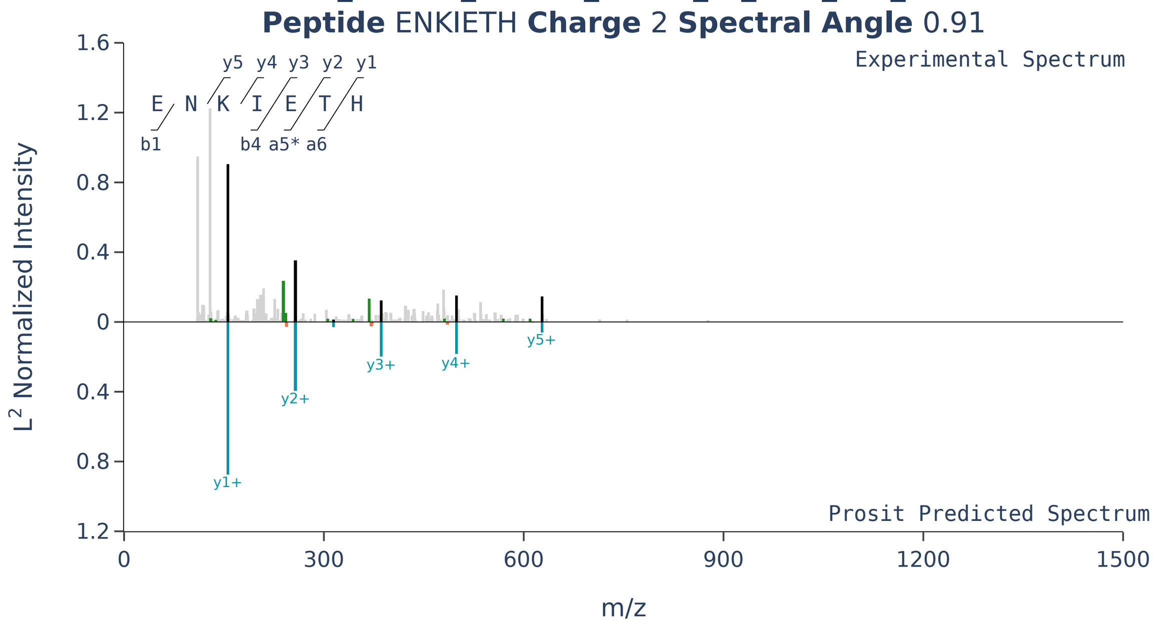

Source WSoh_221121_011221_HFGoe_L2_hTau_4h_R2 **Scan** 16743
Peptide DFKDRVQSDL **Charge** 3 **Spectral Angle** 0.8

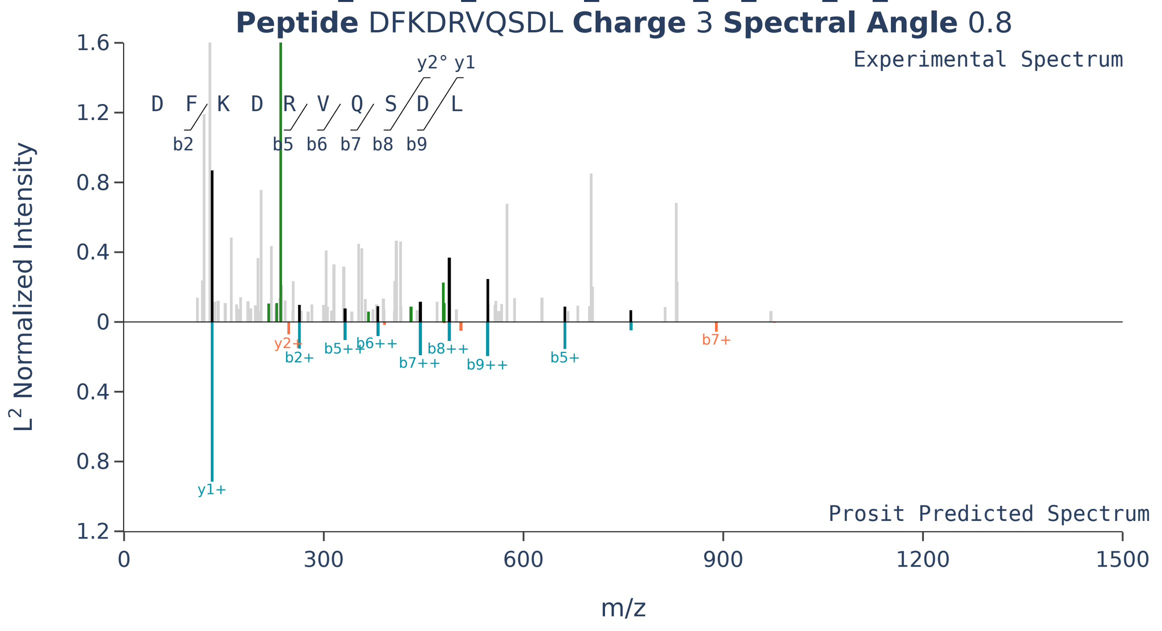

Source WSoh_221121_011221_HFGoe_L2_hTau_24h_R1 **Scan** 7692
Peptide ANITHVPGGGNKKIETH **Charge** 3 **Spectral Angle** 0.83

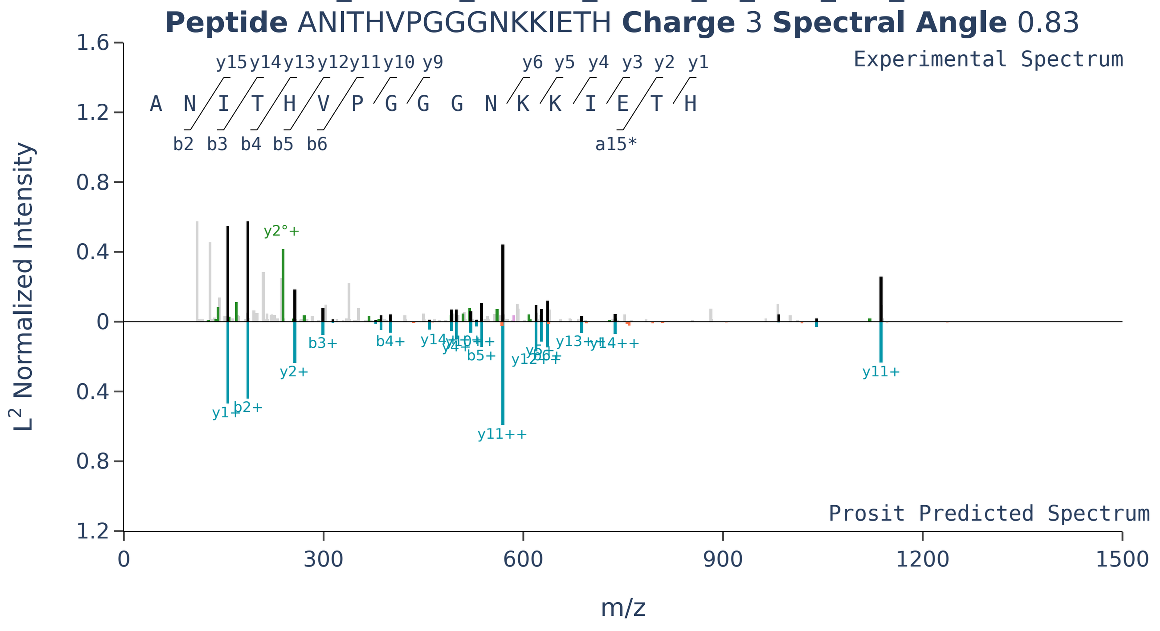

Source WSoh_221121_011221_HFGoe_L2_hTau_24h_R2 **Scan** 13499
Peptide KSEKLDFKDRVQSRL **Charge** 5 **Spectral Angle** 0.82

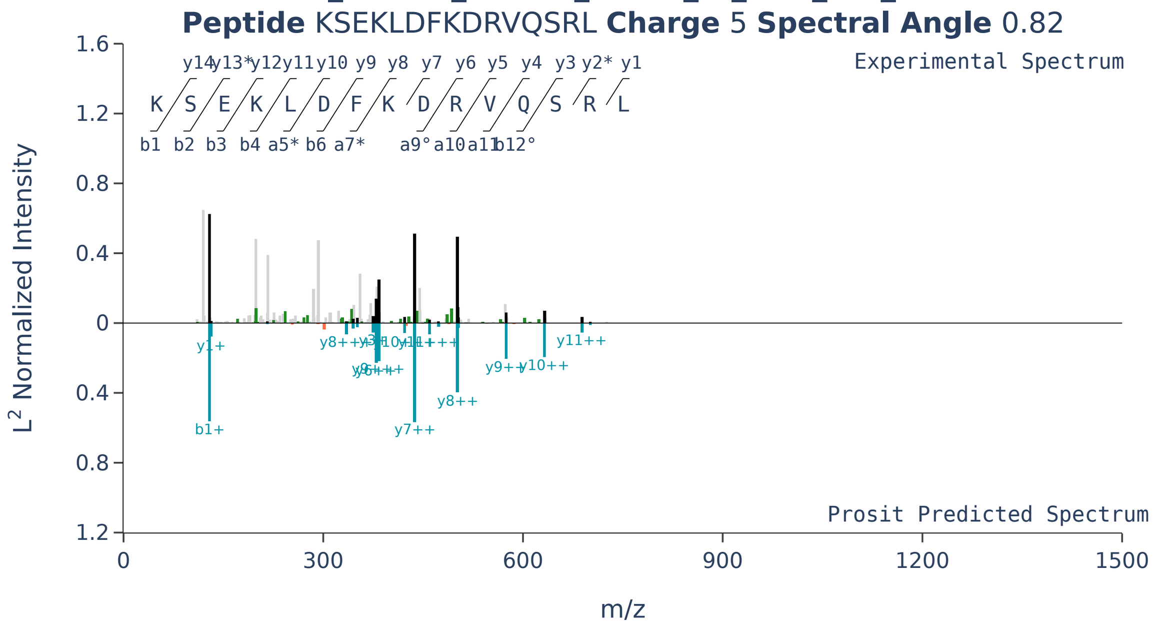

Source WSoh_221121_011221_HFGoe_L2_hTau_24h_R2 **Scan** 10988
Peptide SGSLGNIHHKPGGGQVEV **Charge** 2 **Spectral Angle** 0.78

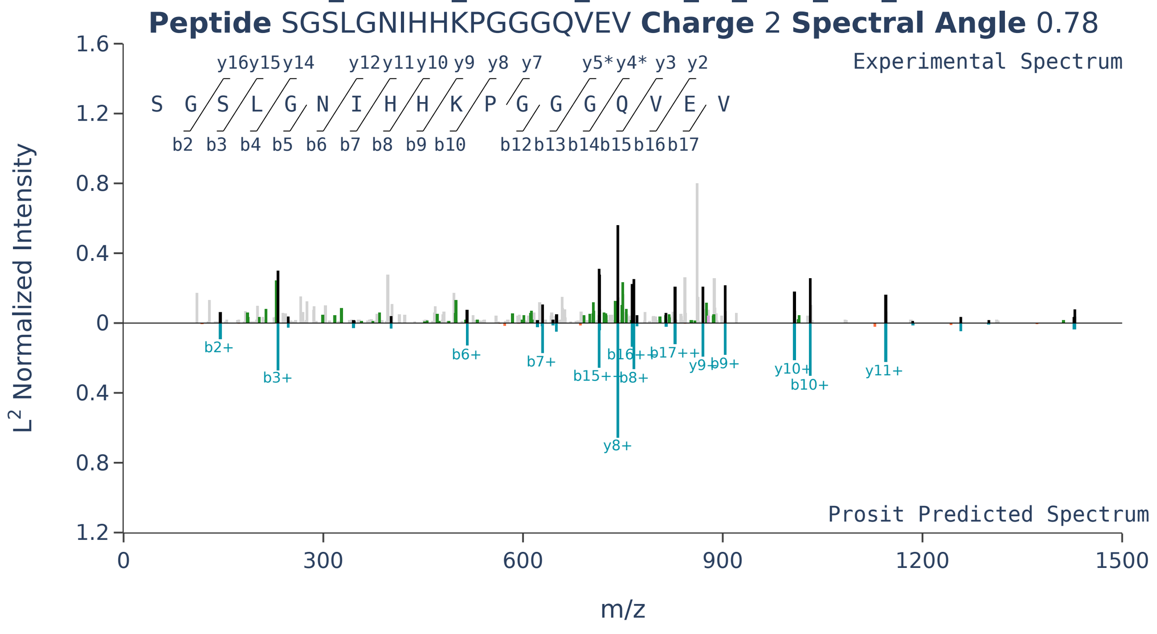

Source WSoh_221121_011221_HFGoe_L1_hTau_24h_R2 **Scan** 16318
Peptide SNVSSTGHGAEIVY **Charge** 2 **Spectral Angle** 0.89

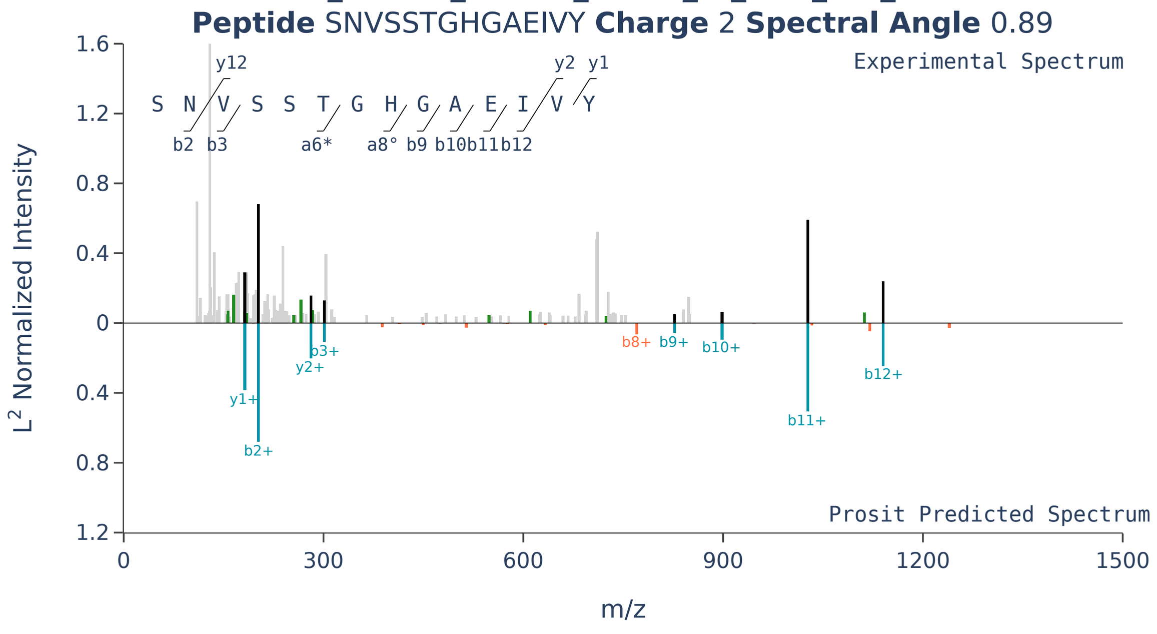

Source WSoh_221121_011221_HFGoe_L2_hTau_24h_R2 **Scan** 12252
Peptide AAQPHTEIPEGTED **Charge** 2 **Spectral Angle** 0.86

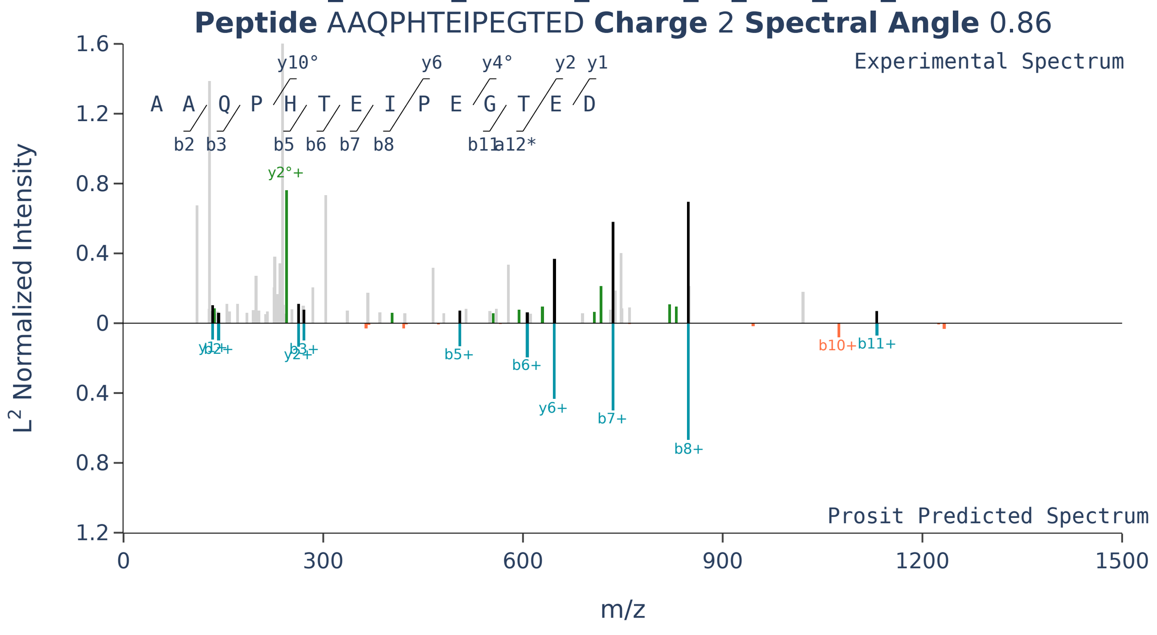

Source WSoh_221121_011221_HFGoe_L1_hTau_24h_R1 **Scan** 13238
Peptide PHTEIVE **Charge** 1 **Spectral Angle** 0.78

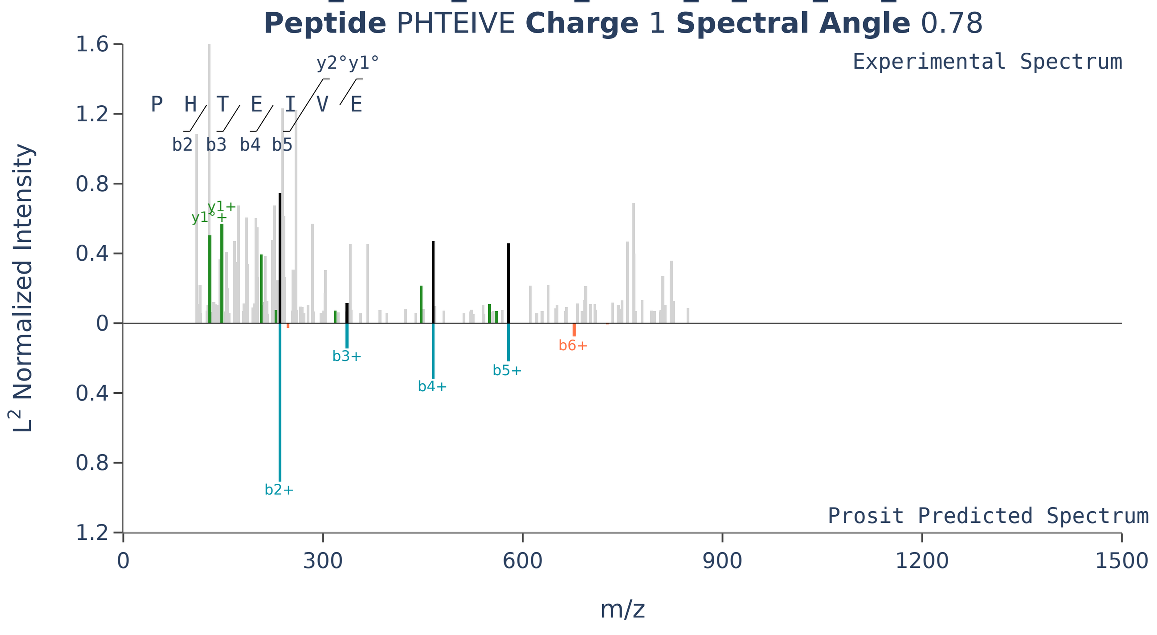

Source WSoh_221121_011221_HFGoe_L2_hTau_2h_R1 **Scan** 15270
Peptide KSEKLDFKDRVQSVY **Charge** 3 **Spectral Angle** 0.84

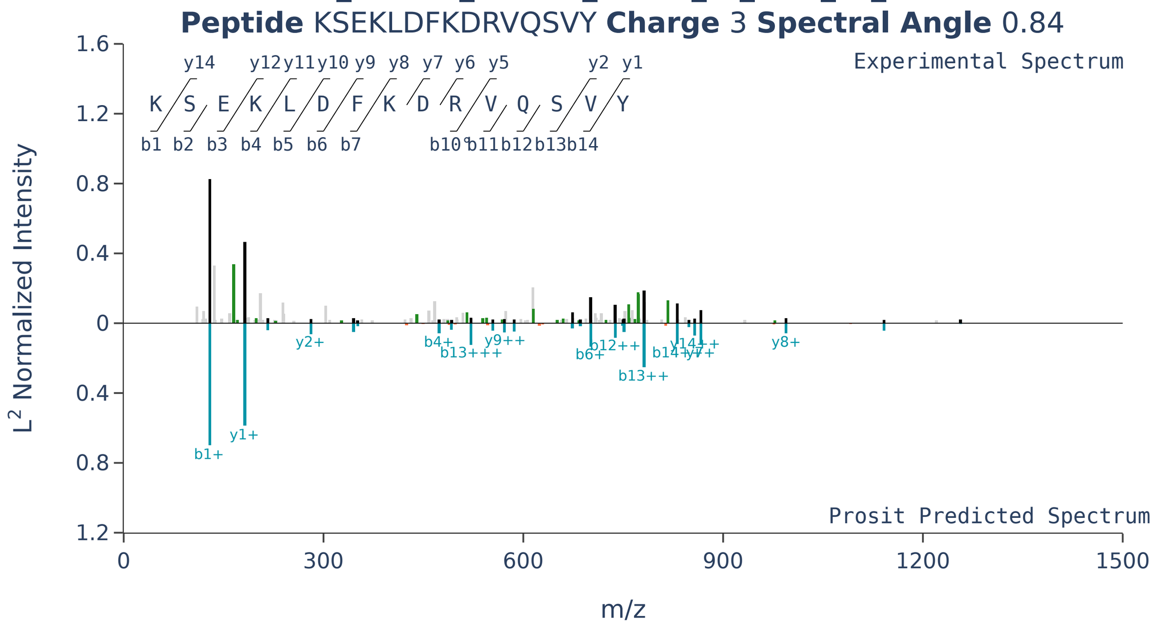

Source WSoh_221121_011221_HFGoe_L2_hTau_1h_R1 **Scan** 12361
Peptide ADEVSAKL **Charge** 2 **Spectral Angle** 0.83

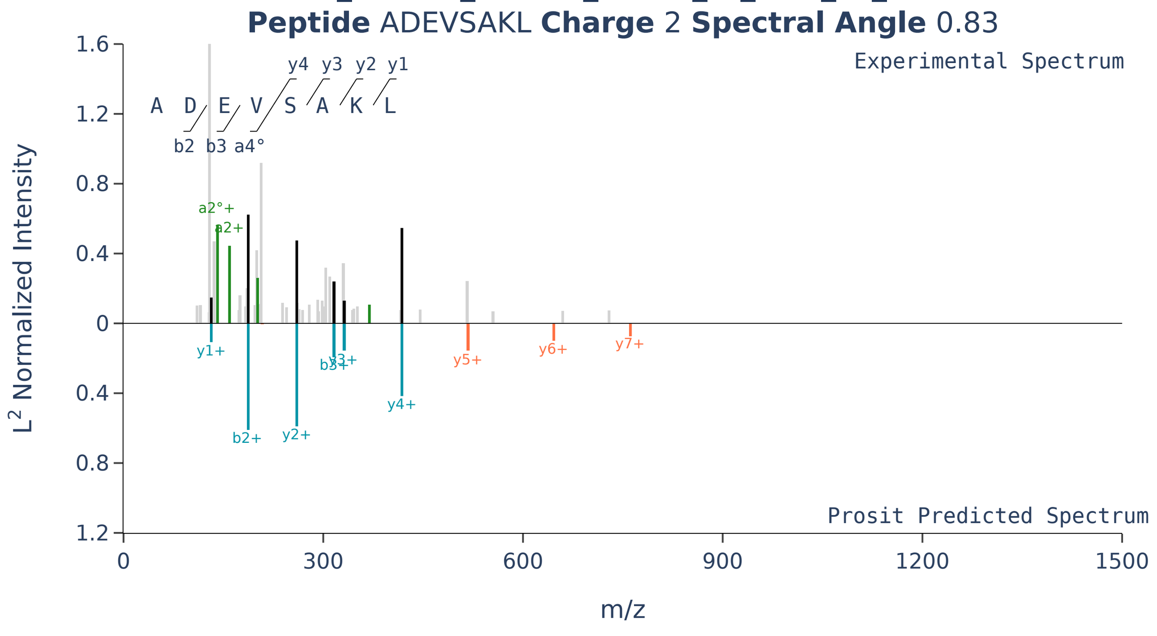

Source WSoh_221121_011221_HFGoe_L2_hTau_24h_R1 **Scan** 20397
Peptide AEPRQEEFEVME **Charge** 2 **Spectral Angle** 0.81

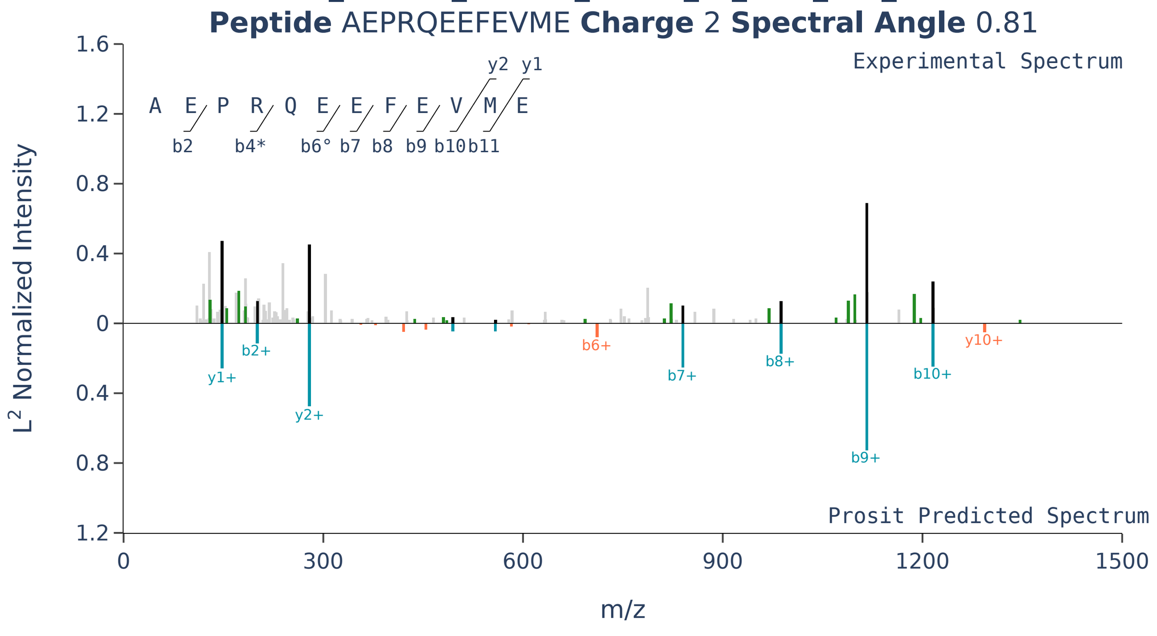

Source WSoh_221121_011221_HFGoe_L1_hTau_24h_R2 **Scan** 7130
Peptide VVRTPPKSPSSAKSKL **Charge** 4 **Spectral Angle** 0.79

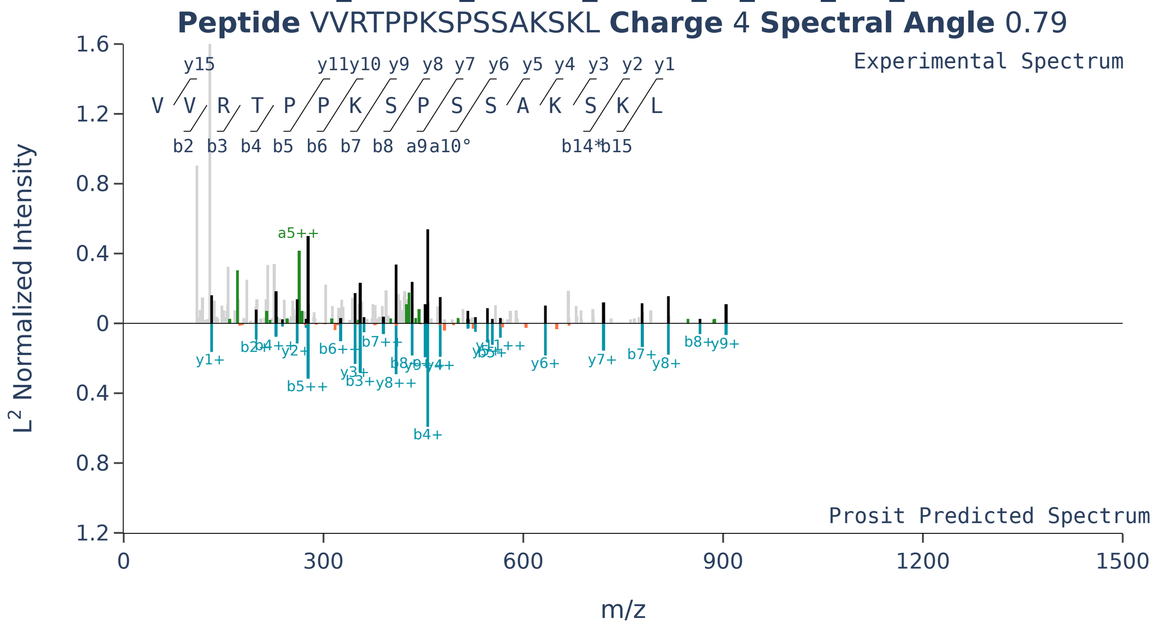

Source WSoh_221121_011221_HFGoe_L1_hTau_24h_R1 **Scan** 8305
Peptide SKKHQPGGGKVQII **Charge** 2 **Spectral Angle** 0.82

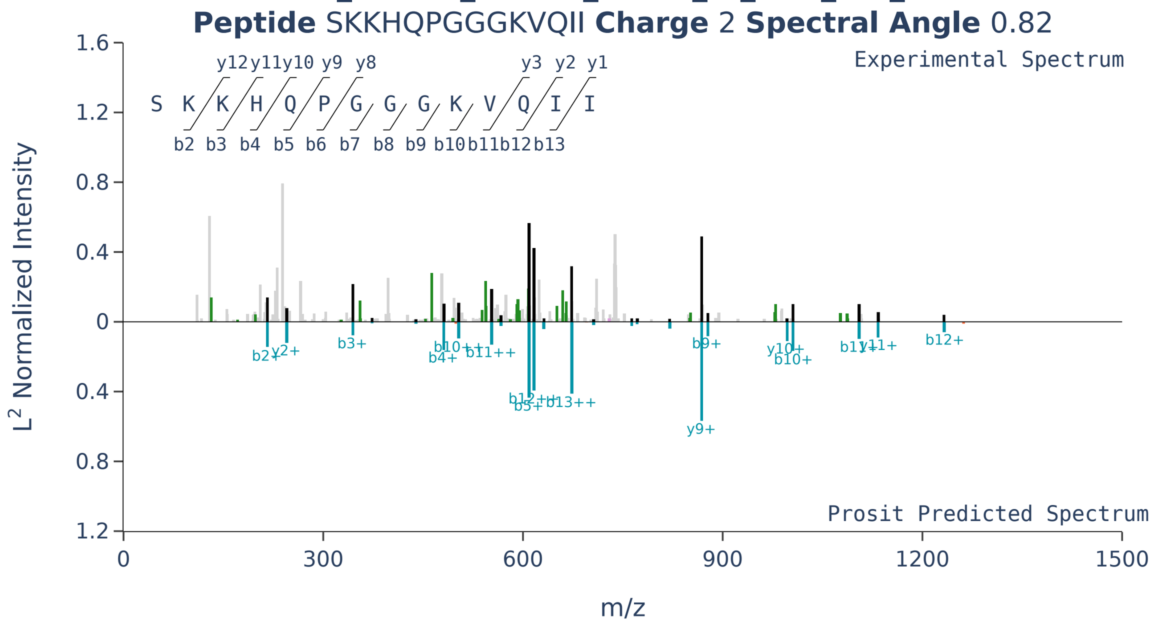

Source WSoh_221121_011221_HFGoe_L1_hTau_4h_R2 **Scan** 14702
Peptide SNVSSKIGSTENL **Charge** 2 **Spectral Angle** 0.92

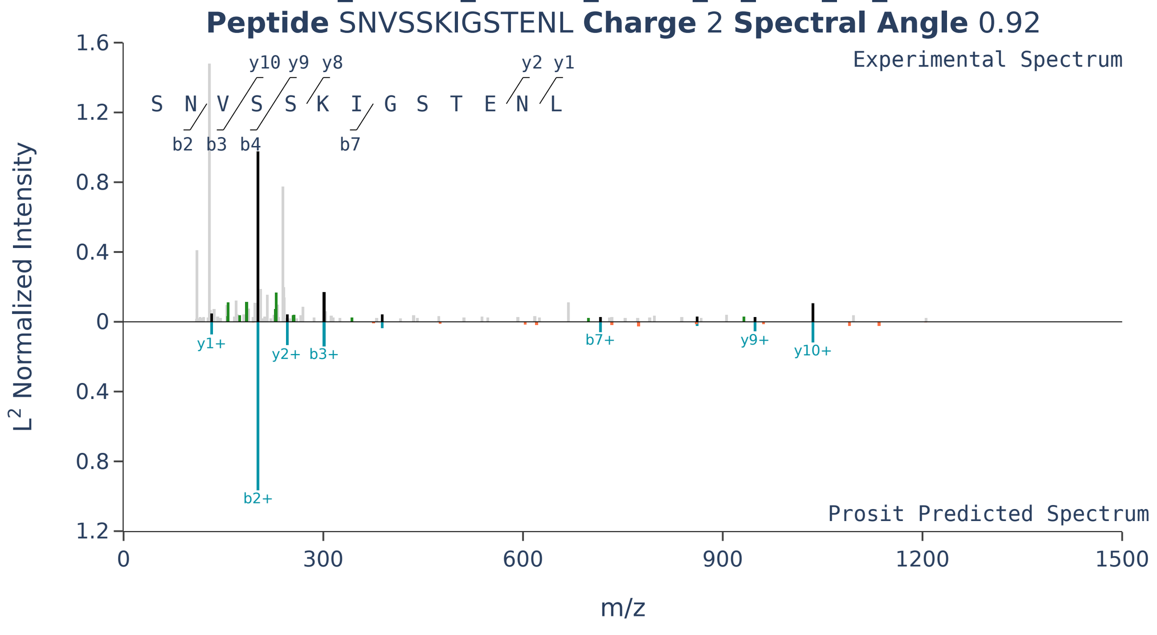

Source WSoh_221121_011221_HFGoe_L1_hTau_24h_R1 **Scan** 20603
Peptide AAQPHTEIPEGSPLQTPTE **Charge** 2 **Spectral Angle** 0.91

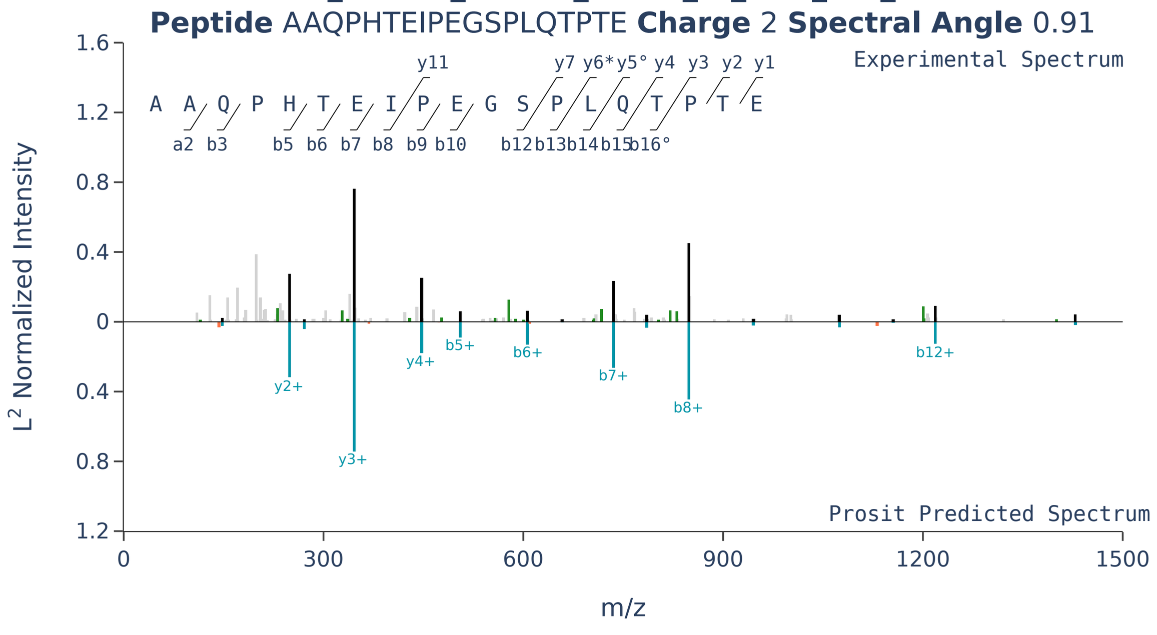

Source WSoh_221121_011221_HFGoe_L2_hTau_24h_R1 **Scan** 21928
Peptide VYKPVDLSKVTSKL **Charge** 3 **Spectral Angle** 0.83

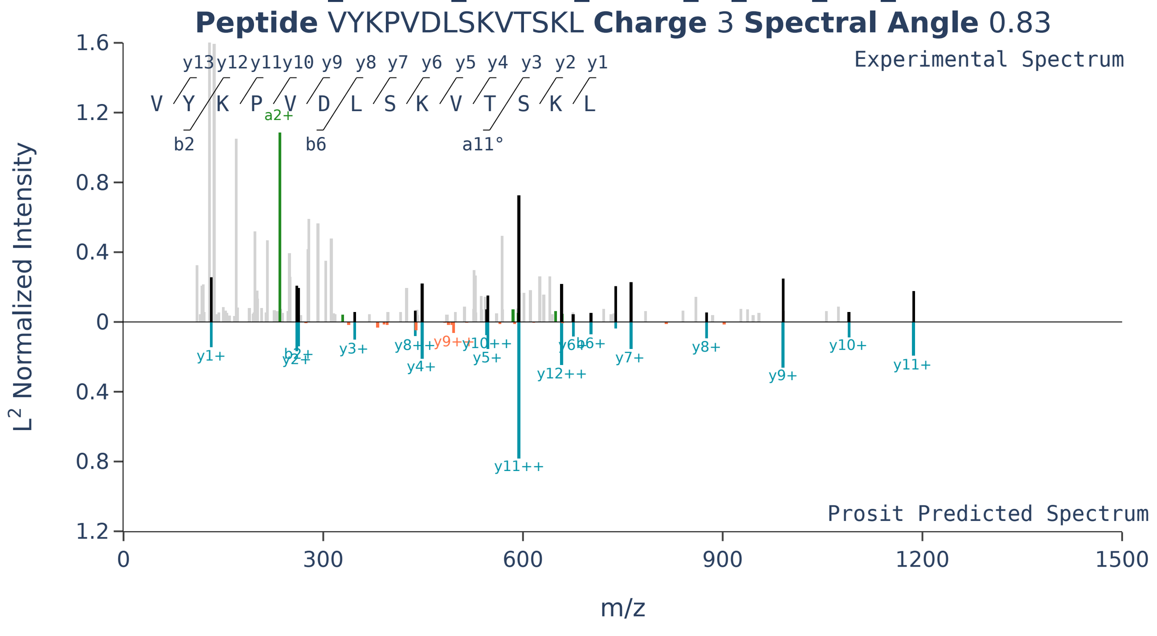

Source WSoh_221121_011221_HFGoe_L1_hTau_24h_R1 **Scan** 21759
Peptide QTAPVPMPDLKNVKTDA **Charge** 3 **Spectral Angle** 0.88

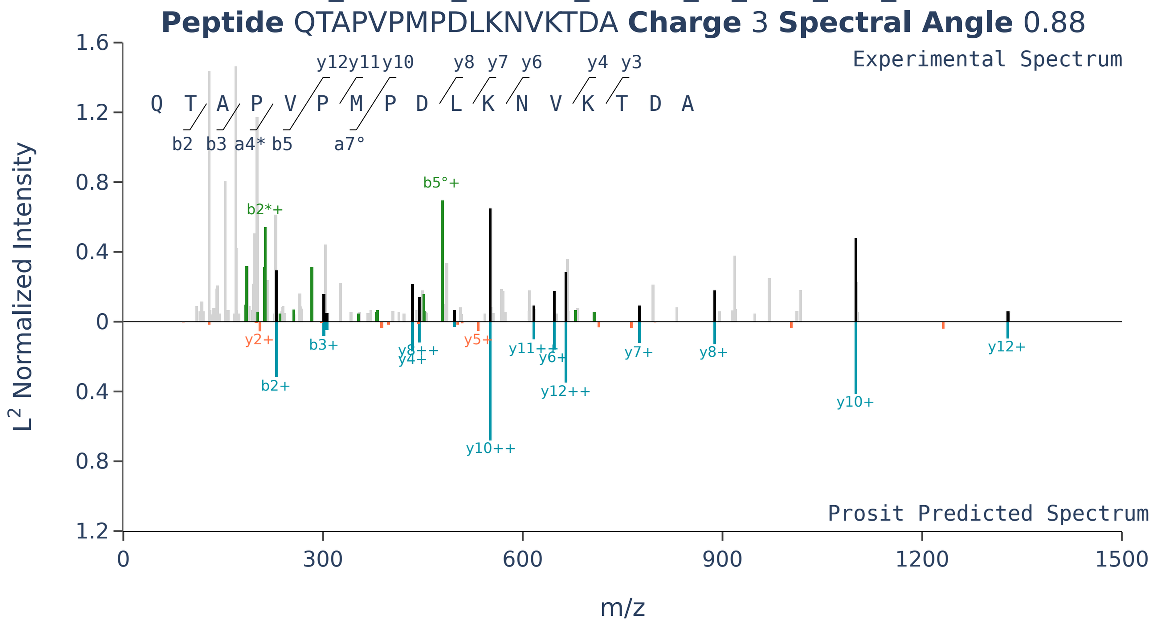

Source WSoh_221121_011221_HFGoe_L1_hTau_4h_R1 **Scan** 6430
Peptide SKVTSKHQPGGGKVQIINK **Charge** 3 **Spectral Angle** 0.83

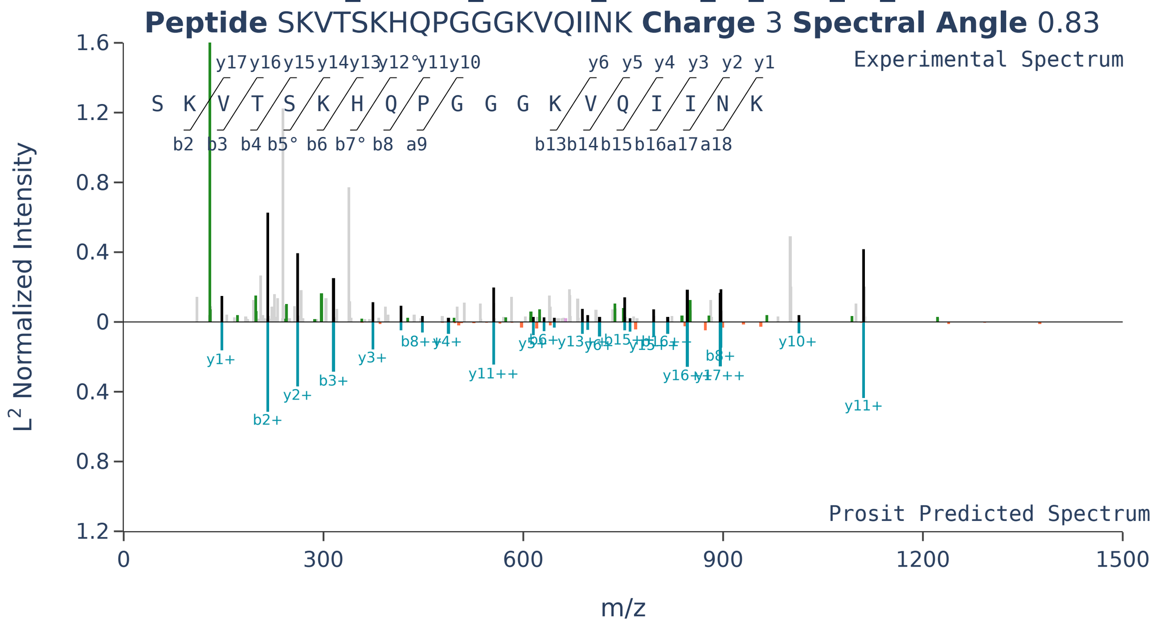

Source WSoh_221121_011221_HFGoe_L1_hTau_24h_R2 **Scan** 11517
Peptide KCSKDNIKHVPGGGSVQI **Charge** 2 **Spectral Angle** 0.88

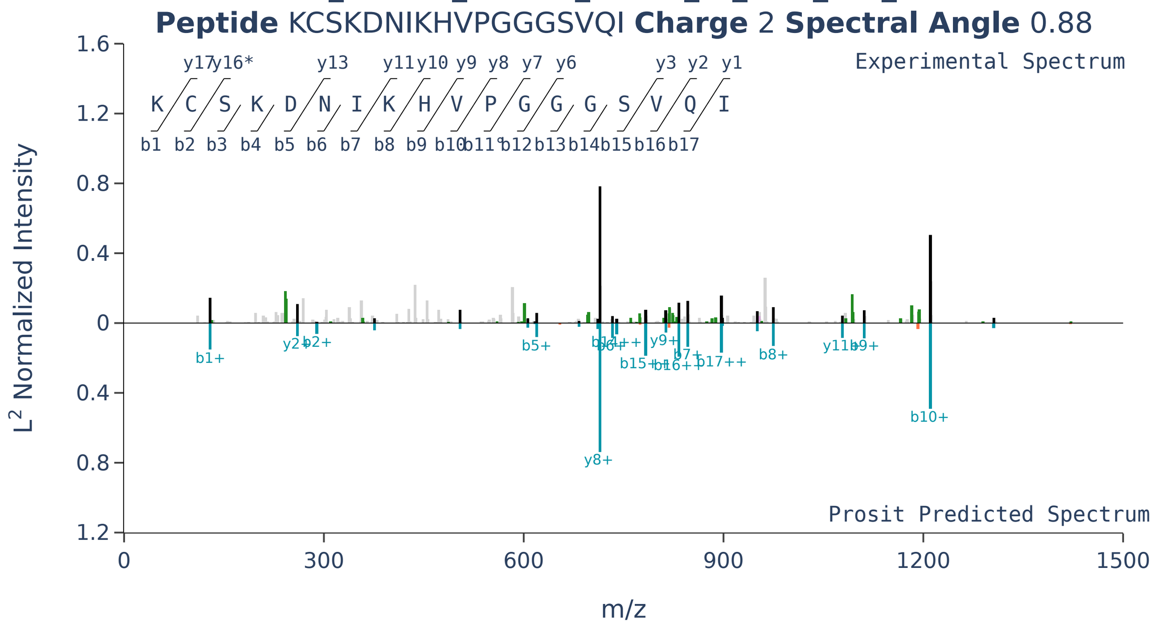

Source WSoh_221121_011221_HFGoe_L1_hTau_24h_R1 **Scan** 9264
Peptide KSEKLDFKDRVQSG **Charge** 2 **Spectral Angle** 0.75

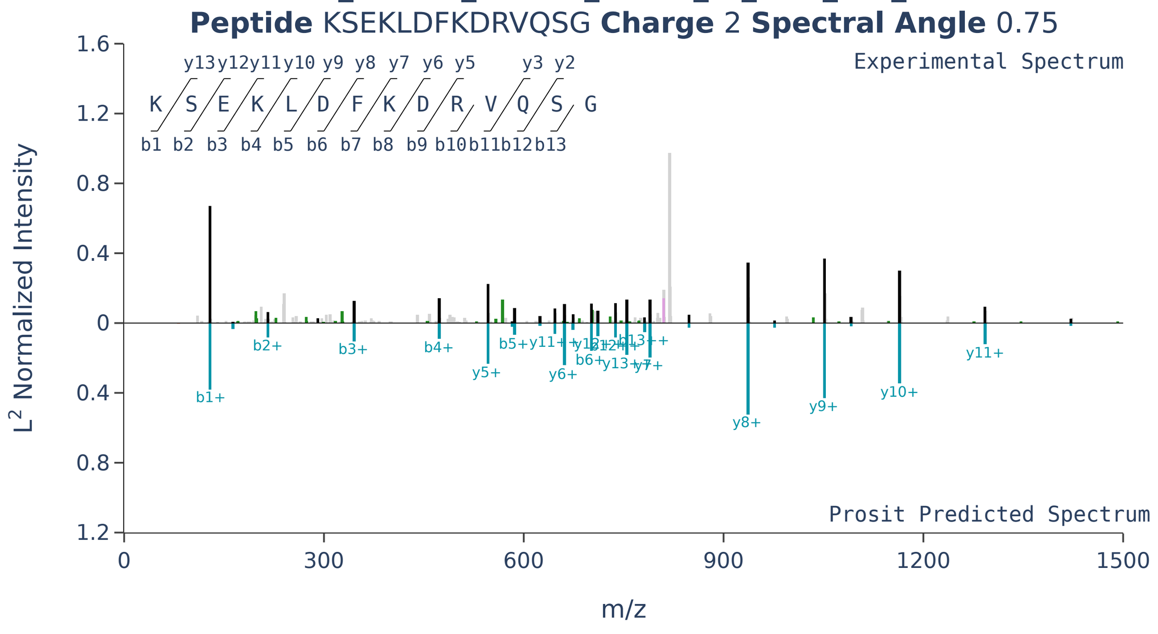

Source WSoh_221121_011221_HFGoe_L2_hTau_24h_R1 **Scan** 19879
Peptide SSKIGSLDNITHV **Charge** 2 **Spectral Angle** 0.88

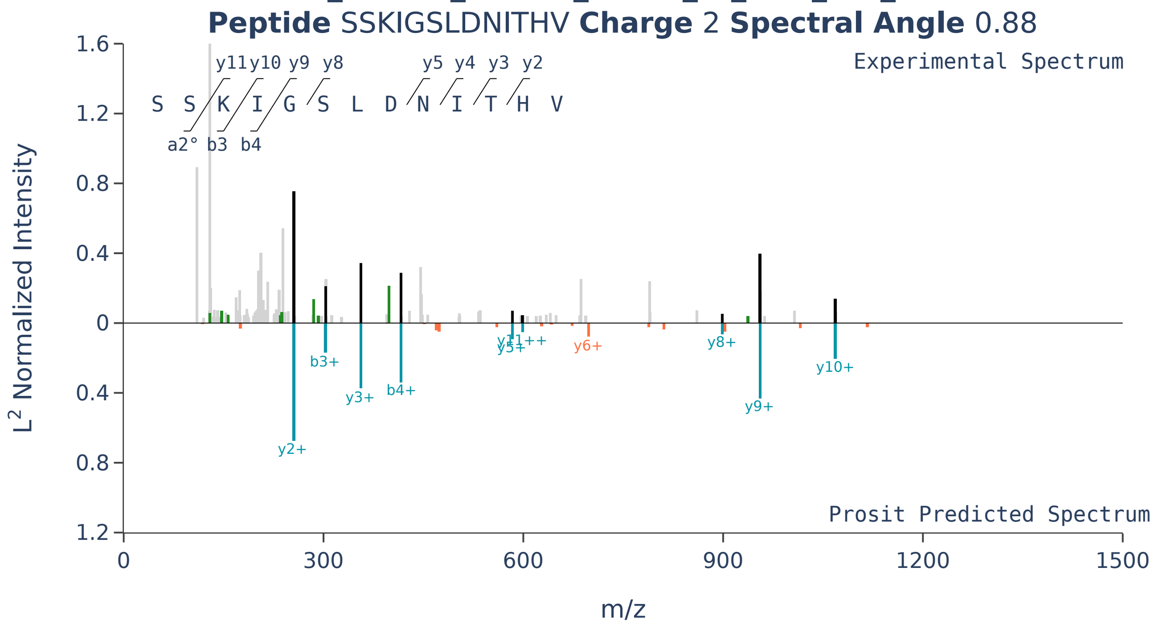

Source WSoh_221121_011221_HFGoe_L2_hTau_24h_R2 **Scan** 20938
Peptide QSQTAPVPMPDLKNVKS **Charge** 3 **Spectral Angle** 0.88

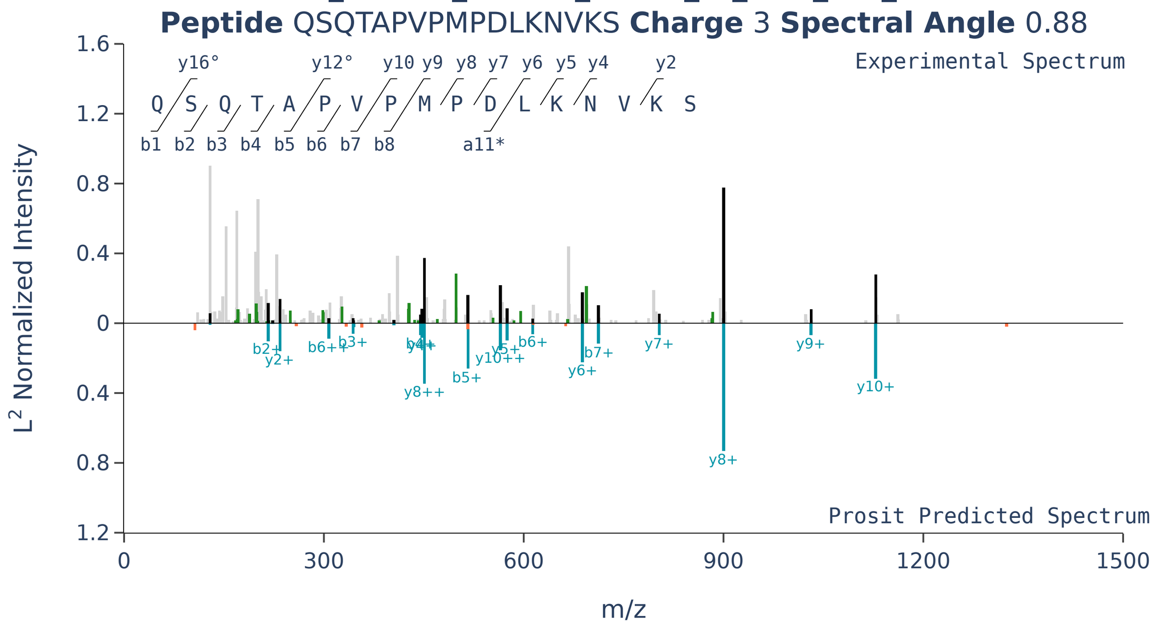

Source WSoh_221121_011221_HFGoe_L2_hTau_24h_R1 **Scan** 17075
Peptide PGGGSVKIGSTENL **Charge** 2 **Spectral Angle** 0.77

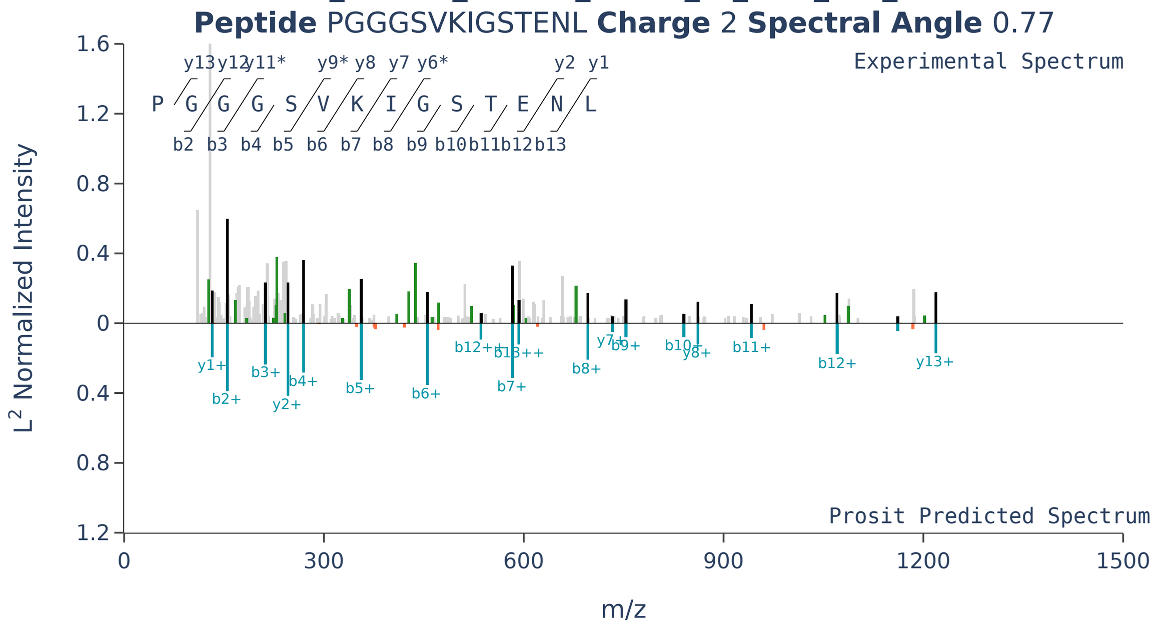

Source WSoh_221121_011221_HFGoe_L2_hTau_2h_R1 **Scan** 19085
Peptide STGKIGSLDNITHV **Charge** 3 **Spectral Angle** 0.87

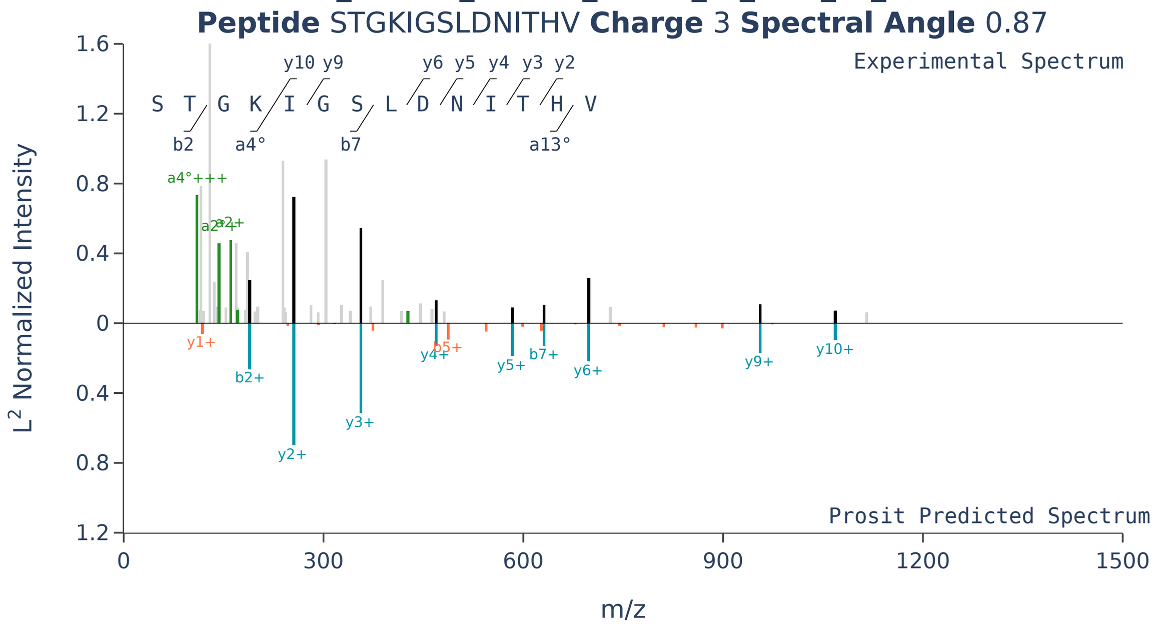

Source WSoh_221121_011221_HFGoe_L1_hTau_24h_R1 **Scan** 21229
Peptide AQTAPVPMPDLKNVKS **Charge** 3 **Spectral Angle** 0.88

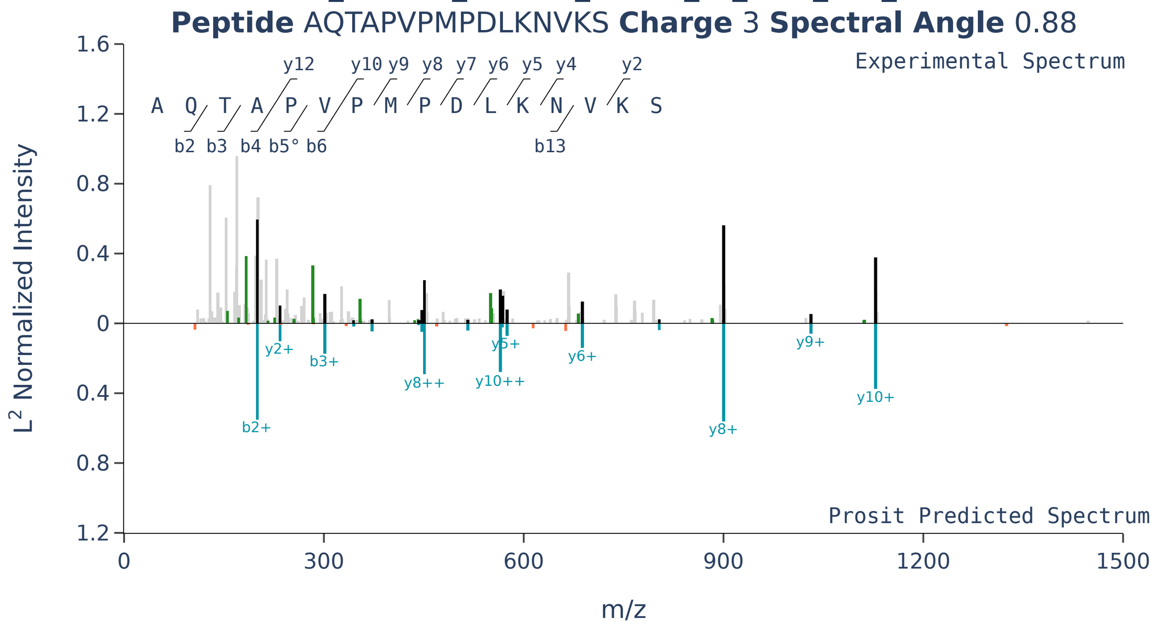

Source WSoh_221121_011221_HFGoe_L1_hTau_24h_R1 **Scan** 18813
Peptide KPVDLSKVTSRL **Charge** 3 **Spectral Angle** 0.69

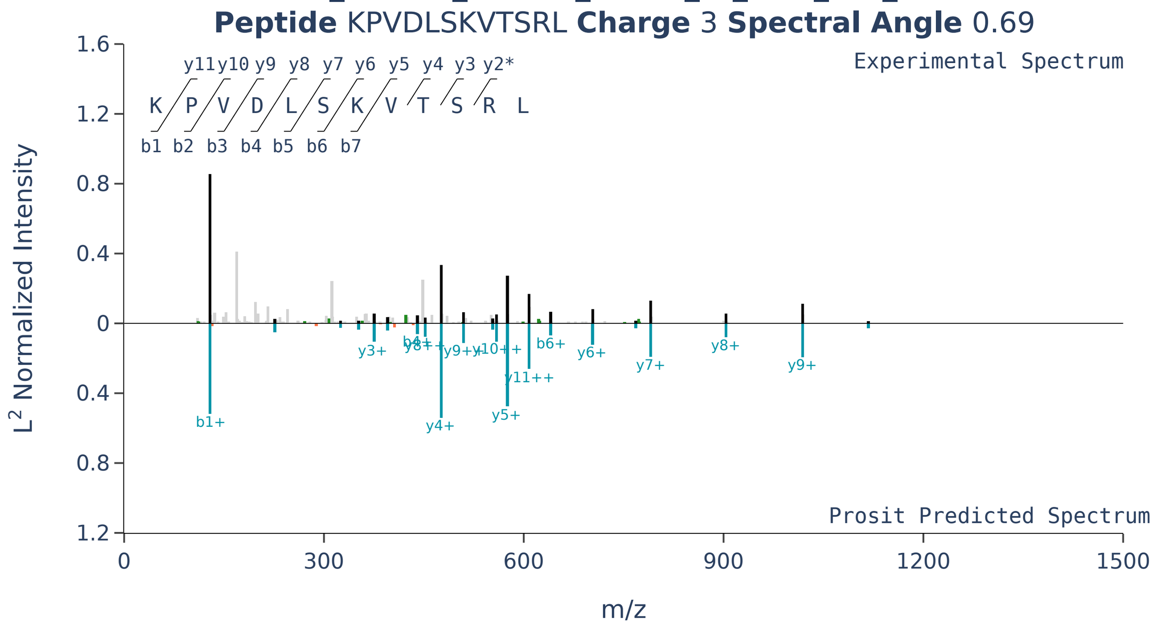

Source WSoh_221121_011221_HFGoe_L2_hTau_4h_R2 **Scan** 11362
Peptide NITHVPGGGNKKIETHKLTFRADG **Charge** 3 **Spectral Angle** 0.82

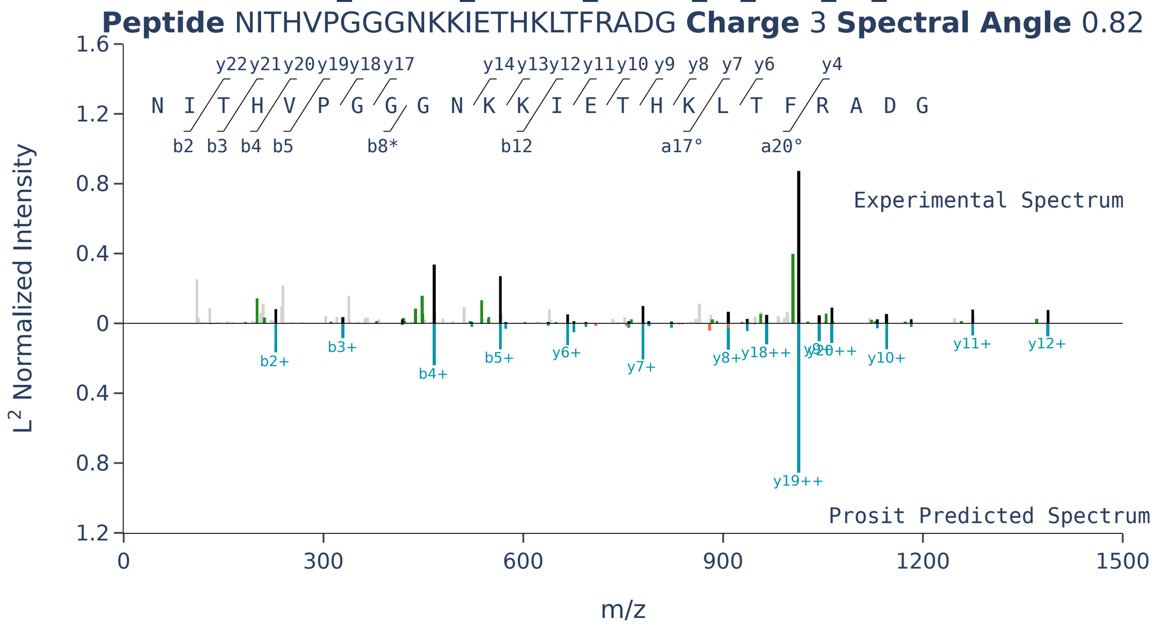

Source WSoh_221121_011221_HFGoe_L1_hTau_4h_R1 **Scan** 8274
Peptide RVQSKPGGGQVEV **Charge** 3 **Spectral Angle** 0.82

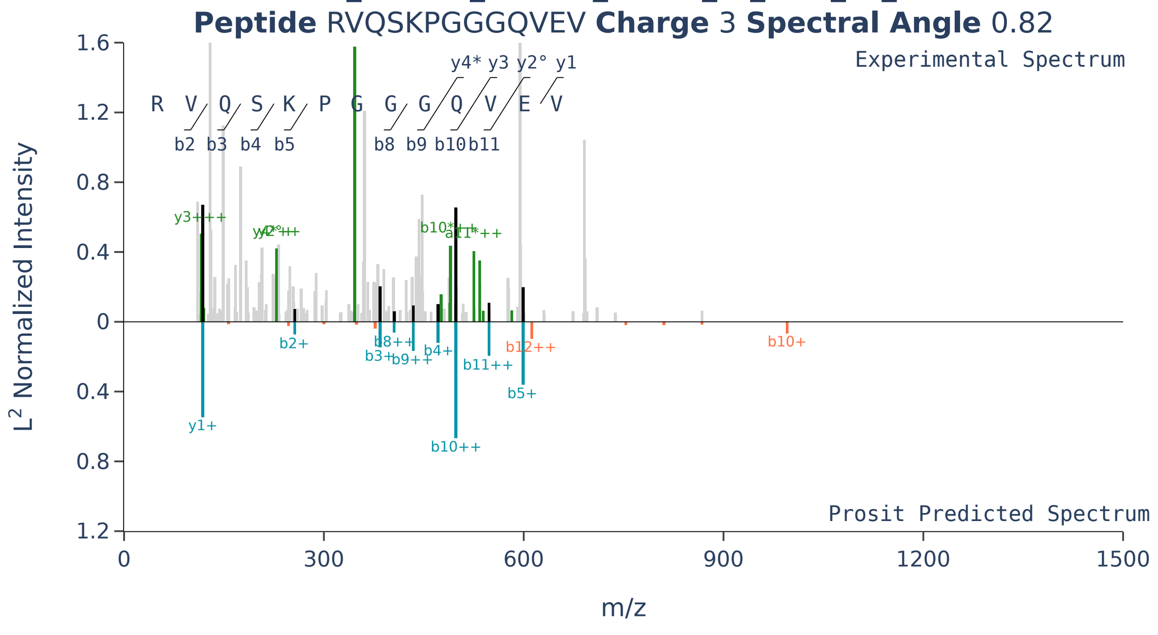

Source WSoh_221121_011221_HFGoe_L1_hTau_2h_R2 **Scan** 6750
Peptide KAHQPGGGKVQIINK **Charge** 4 **Spectral Angle** 0.84

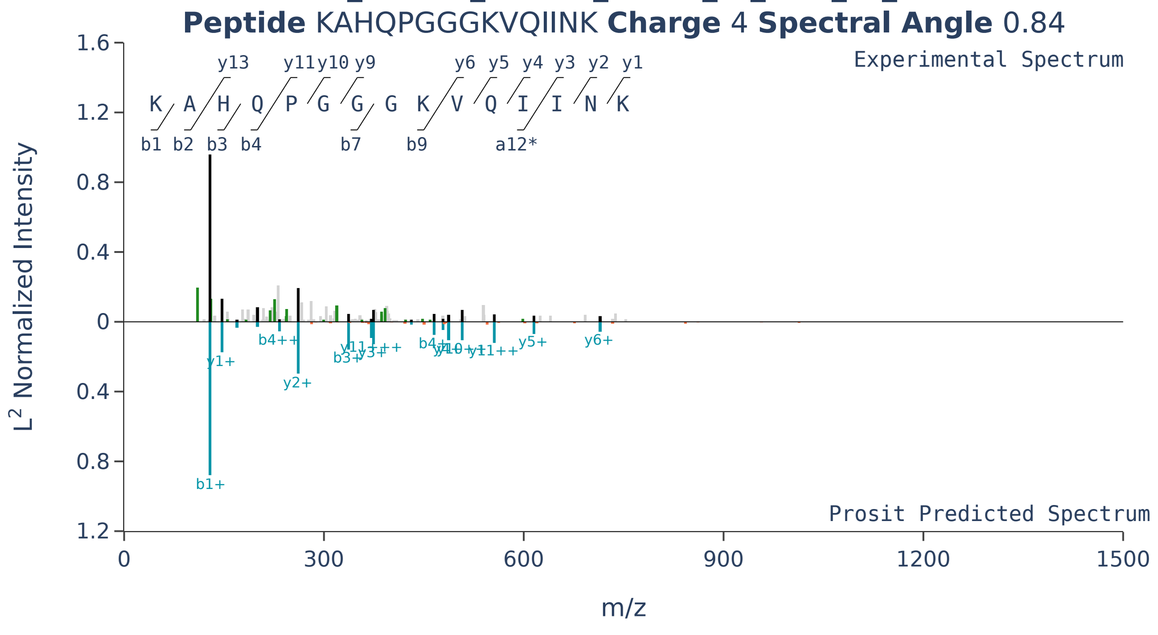

Source WSoh_221121_011221_HFGoe_L1_hTau_24h_R1 **Scan** 10596
Peptide KKLDLSNVQSKCSKDNIKHV **Charge** 5 **Spectral Angle** 0.91

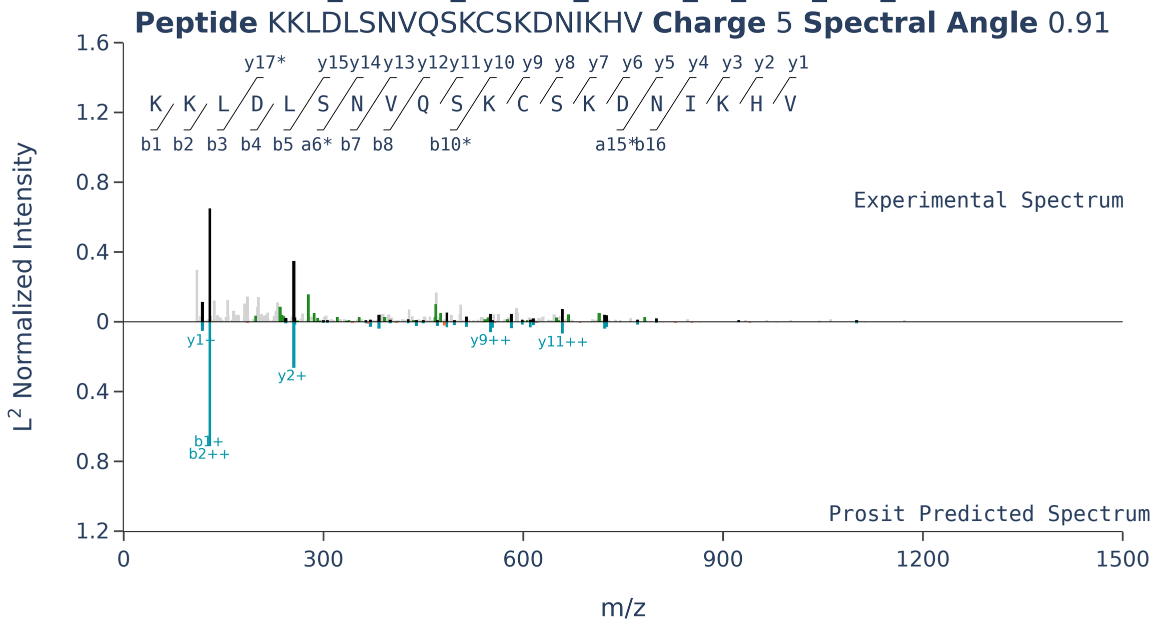

Source WSoh_221121_011221_HFGoe_L1_hTau_24h_R2 **Scan** 8457
Peptide PGGGKVTA **Charge** 1 **Spectral Angle** 0.89

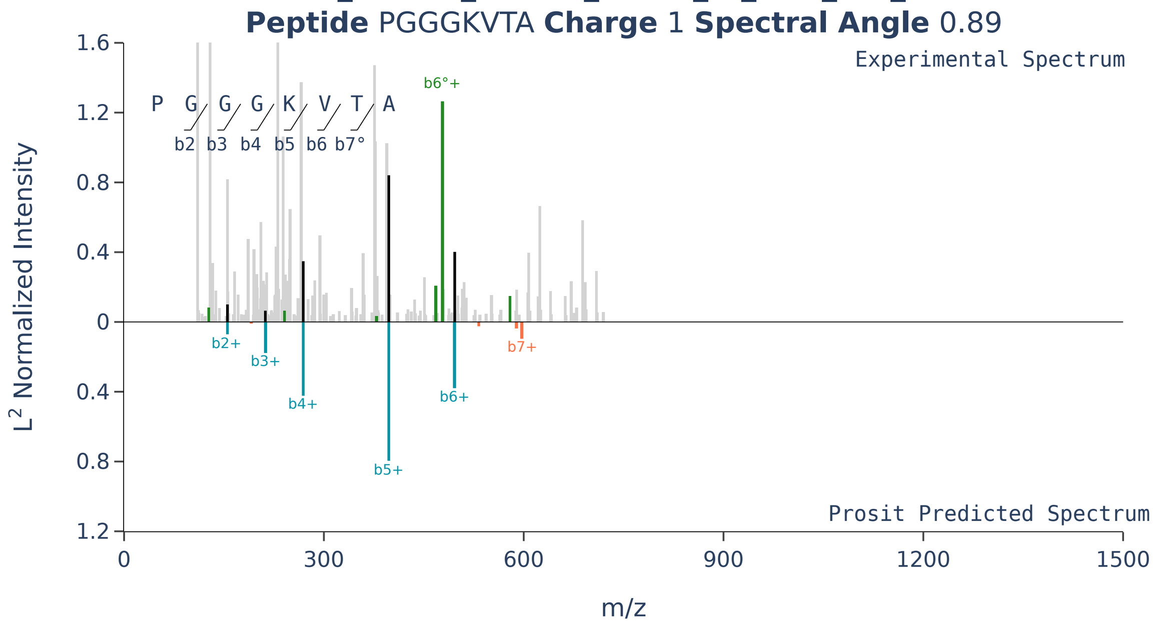

Source WSoh_221121_011221_HFGoe_L1_hTau_24h_R2 **Scan** 14450
Peptide QEGKAKTDHGAEIVYKSPVV **Charge** 2 **Spectral Angle** 0.82

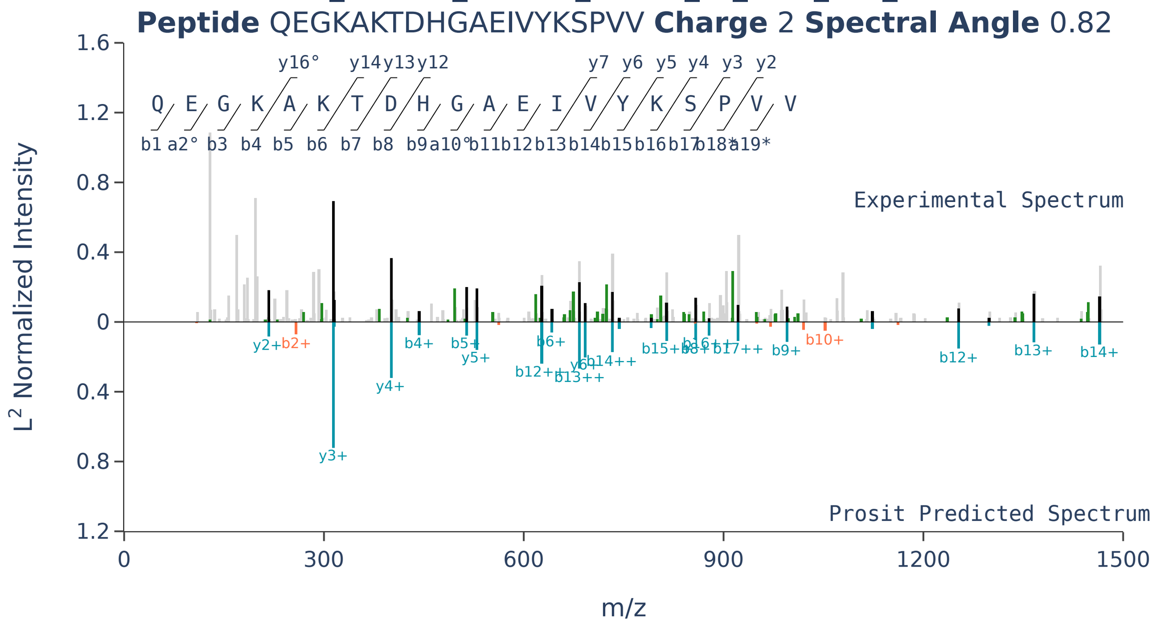

Source WSoh_221121_011221_HFGoe_L1_hTau_4h_R2 **Scan** 7347
Peptide PGGGNKKIETHDL **Charge** 3 **Spectral Angle** 0.81

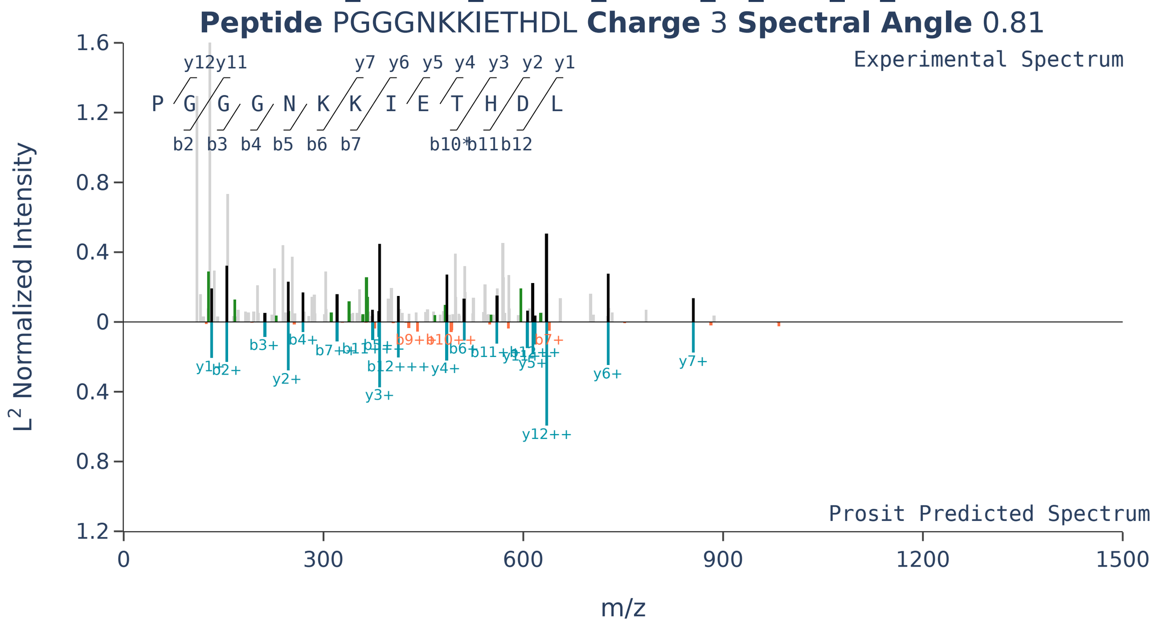

Source WSoh_221121_011221_HFGoe_L1_hTau_24h_R1 **Scan** 8757
Peptide HGSLGNIHHKPGGGQVEV **Charge** 3 **Spectral Angle** 0.76

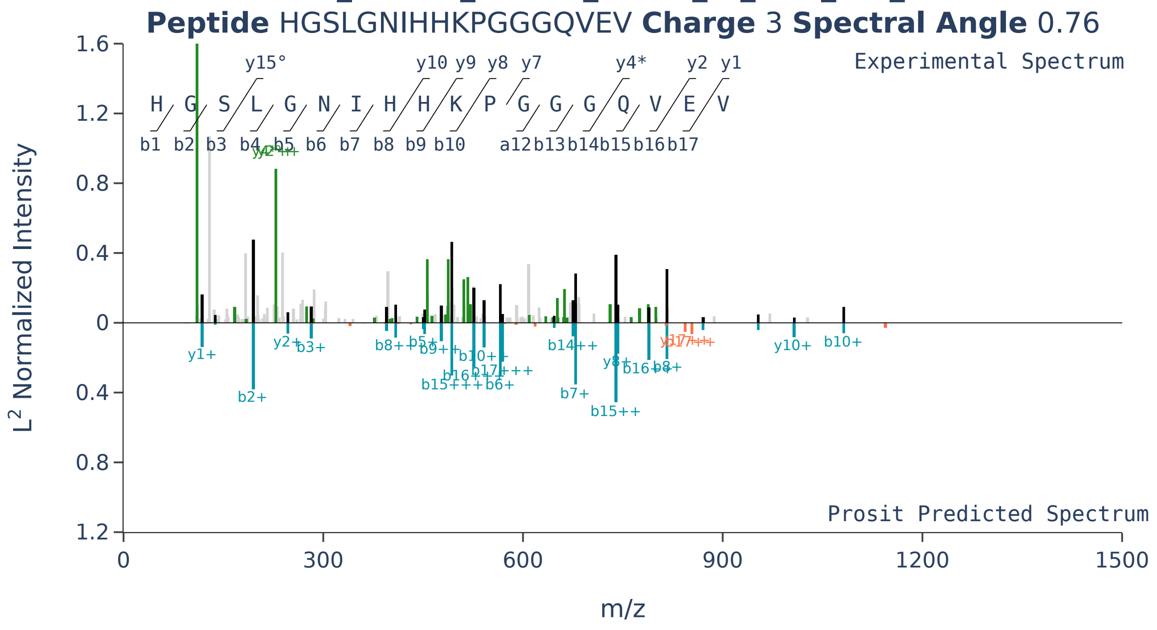

Source WSoh_221121_011221_HFGoe_L2_hTau_24h_R1 **Scan** 9214
Peptide GTTHKLTFR **Charge** 2 **Spectral Angle** 0.75

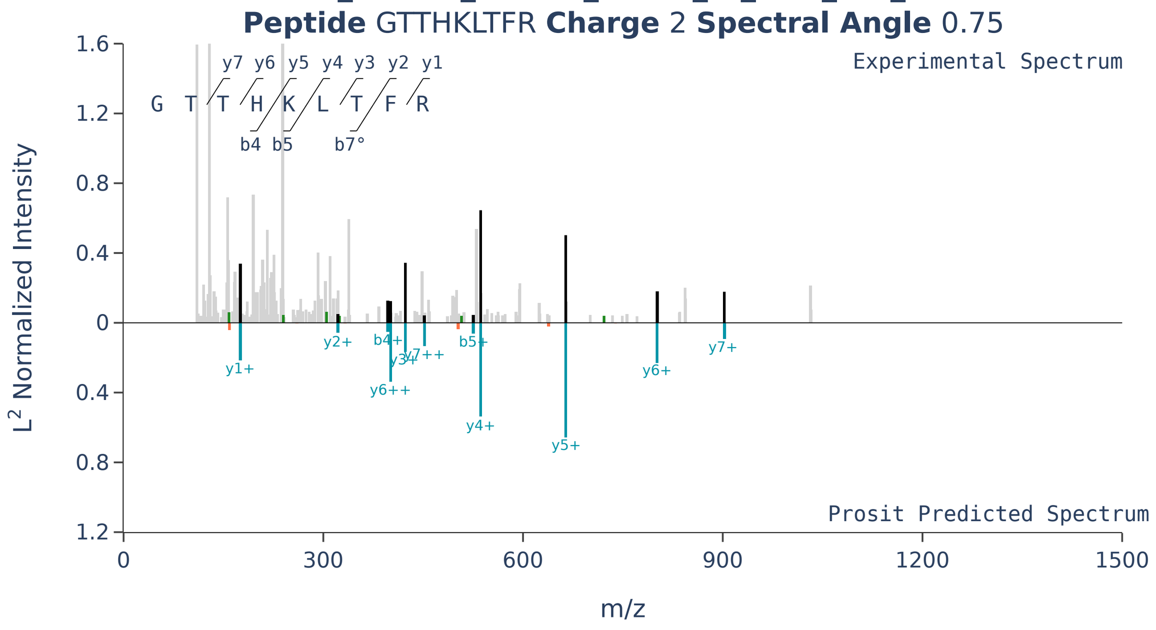

Source WSoh_221121_011221_HFGoe_L2_hTau_24h_R1 **Scan** 17366
Peptide KSAPVPMPDLKNVKS **Charge** 3 **Spectral Angle** 0.79

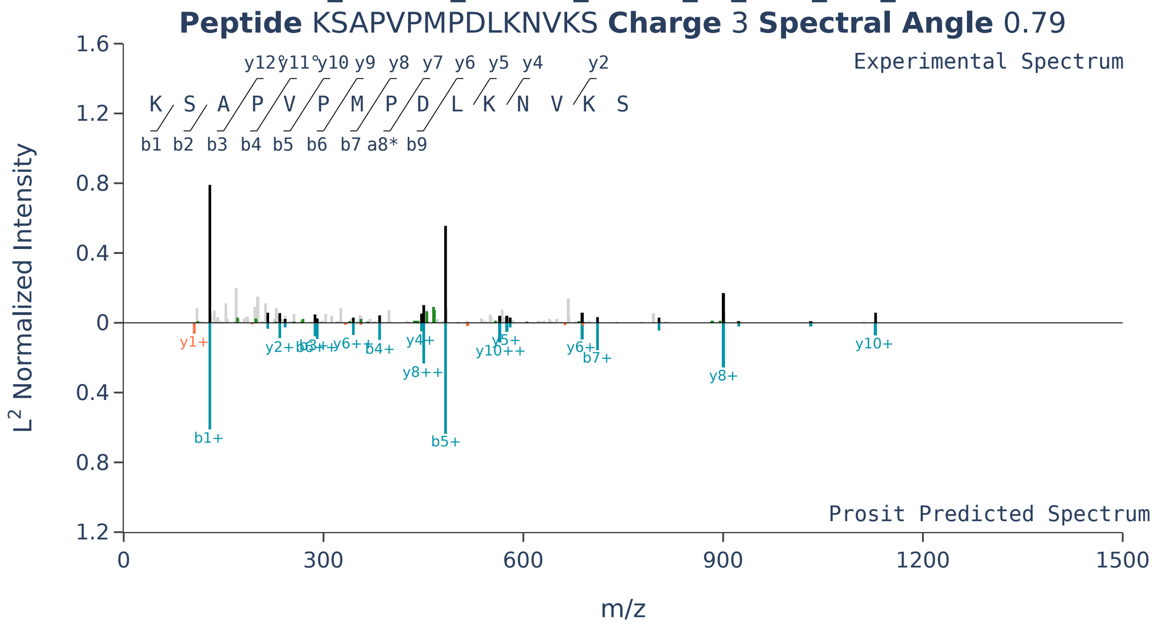

Source WSoh_221121_011221_HFGoe_L1_hTau_24h_R2 **Scan** 22323
Peptide SNVSSKIGSLDNITHV **Charge** 3 **Spectral Angle** 0.88

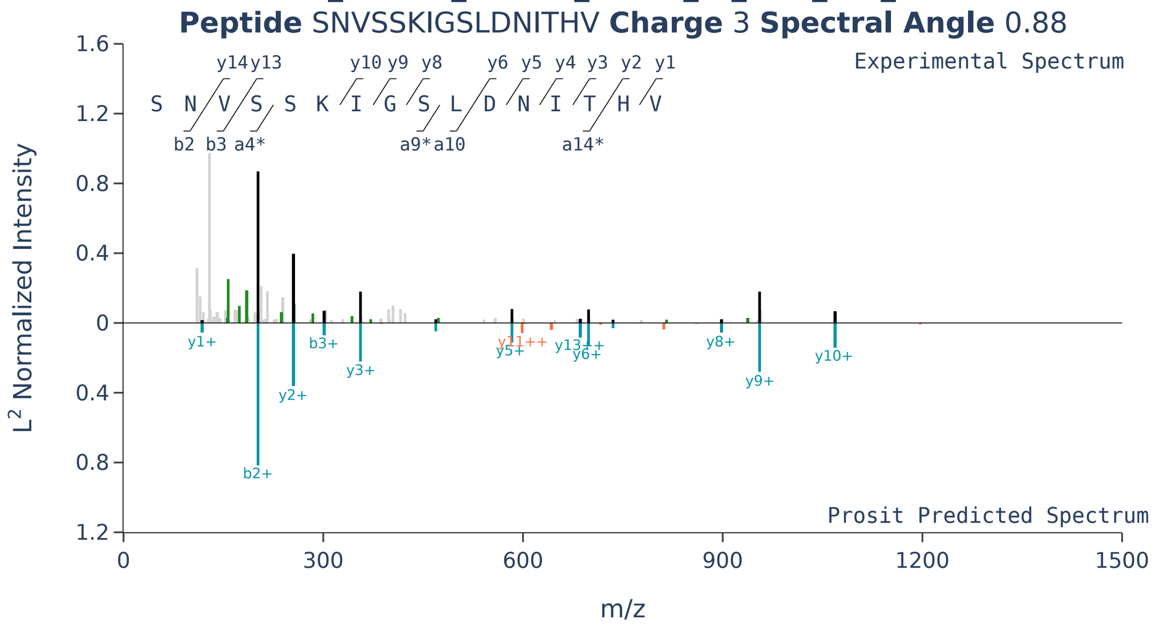

Source WSoh_221121_011221_HFGoe_L1_hTau_24h_R1 **Scan** 17970
Peptide EPGPVPMPDLKNVKSK **Charge** 4 **Spectral Angle** 0.78

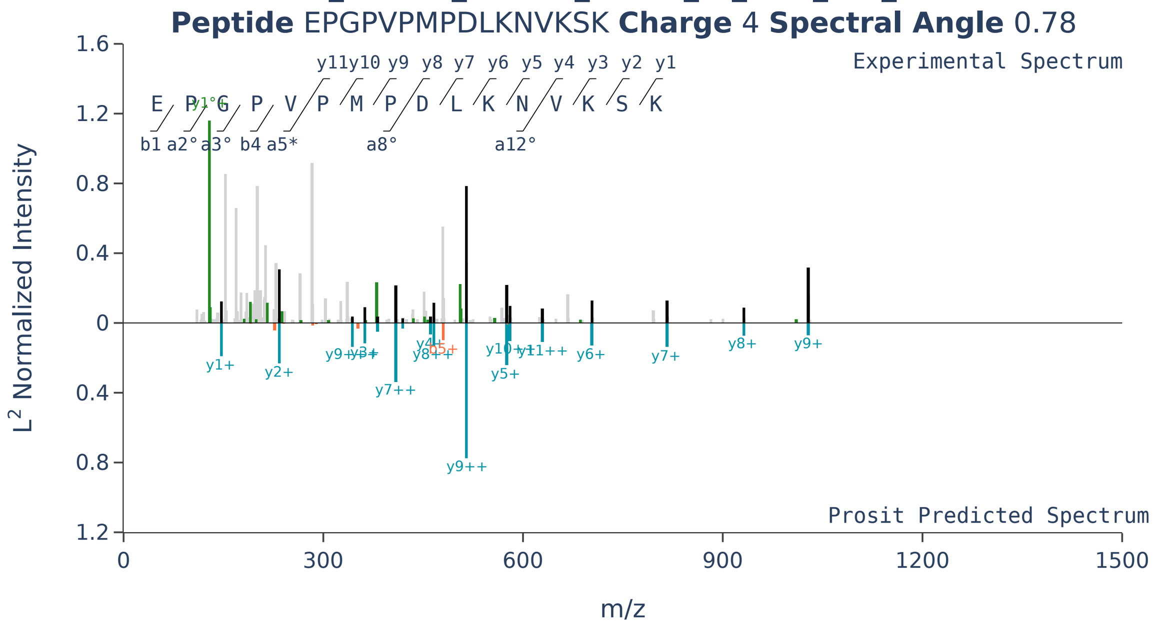

Source WSoh_221121_011221_HFGoe_L2_hTau_4h_R2 **Scan** 19624
Peptide VRTPPKSPSSAKSQTAPVPMPDL **Charge** 3 **Spectral Angle** 0.94

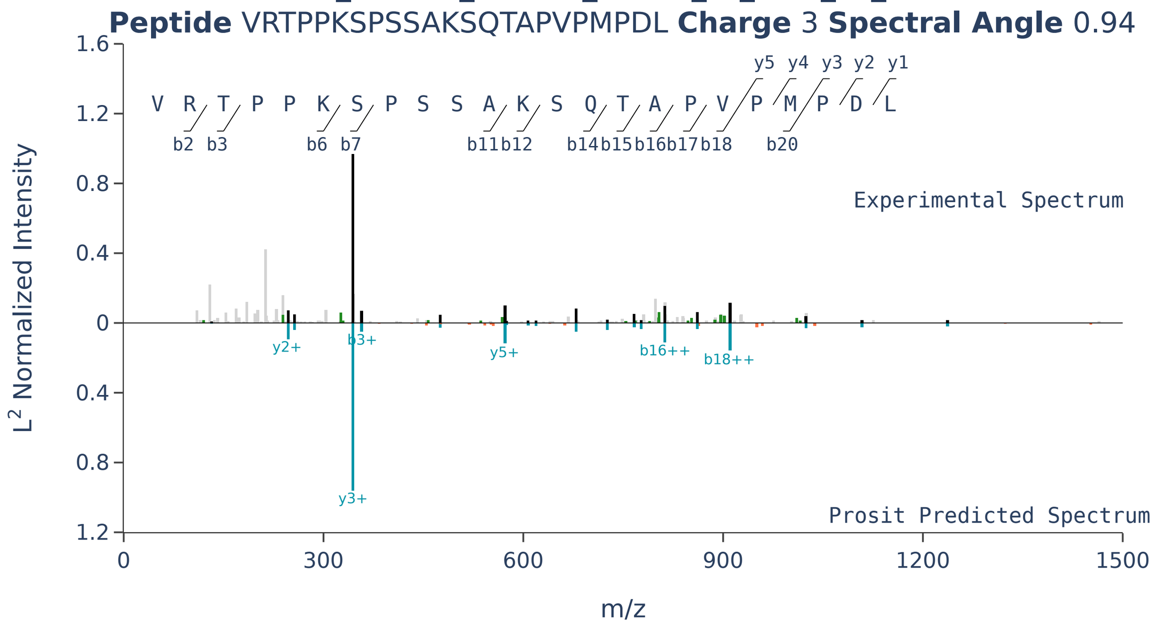

Source WSoh_221121_011221_HFGoe_L1_hTau_24h_R2 **Scan** 18006
Peptide EGAPGKQAAEPRQEFEVME **Charge** 3 **Spectral Angle** 0.9

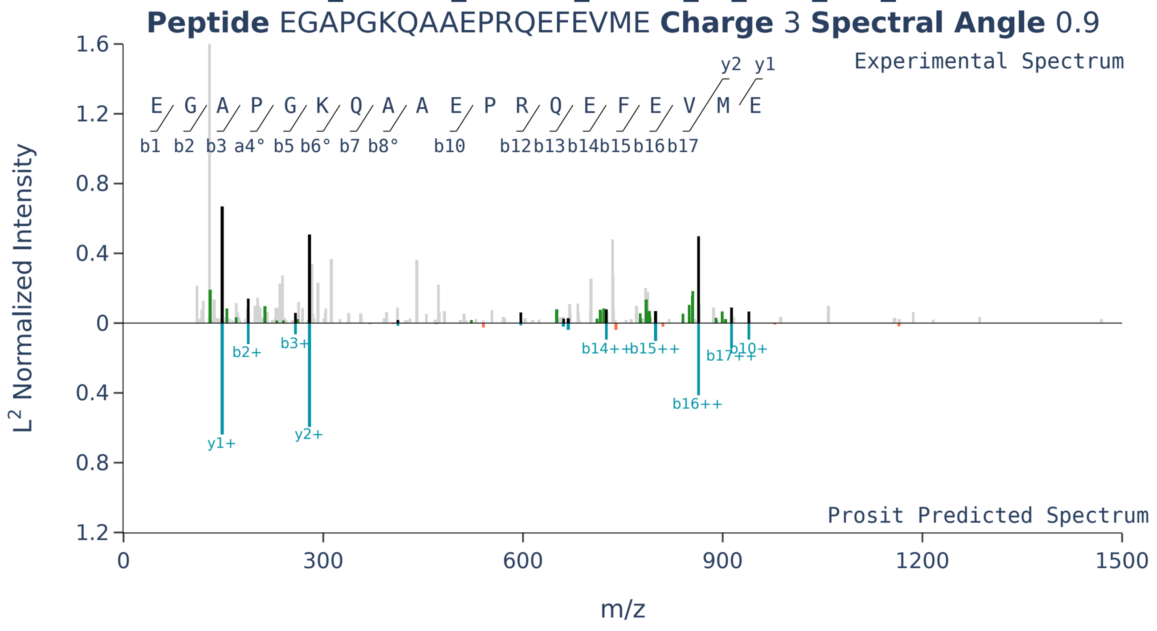

Source WSoh_221121_011221_HFGoe_L1_hTau_24h_R2 **Scan** 11632
Peptide DLSNVQSKHQPGGGKVQIINK **Charge** 3 **Spectral Angle** 0.87

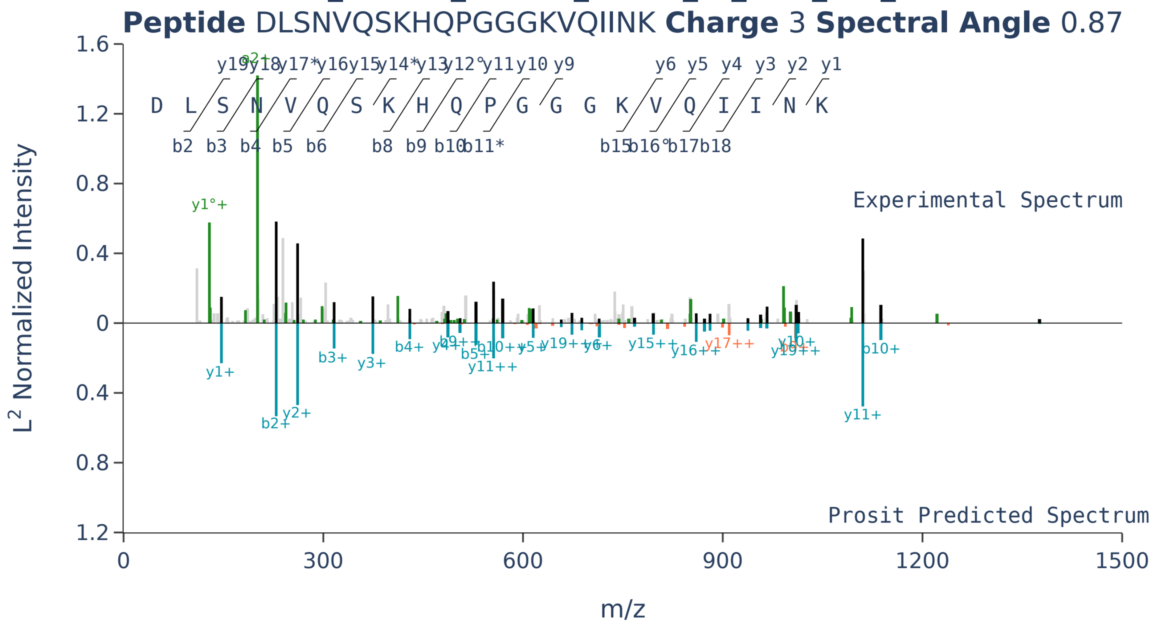

Source WSoh_221121_011221_HFGoe_L2_hTau_4h_R2 **Scan** 7997
Peptide DGNIHHKPGGGQVEV **Charge** 2 **Spectral Angle** 0.86

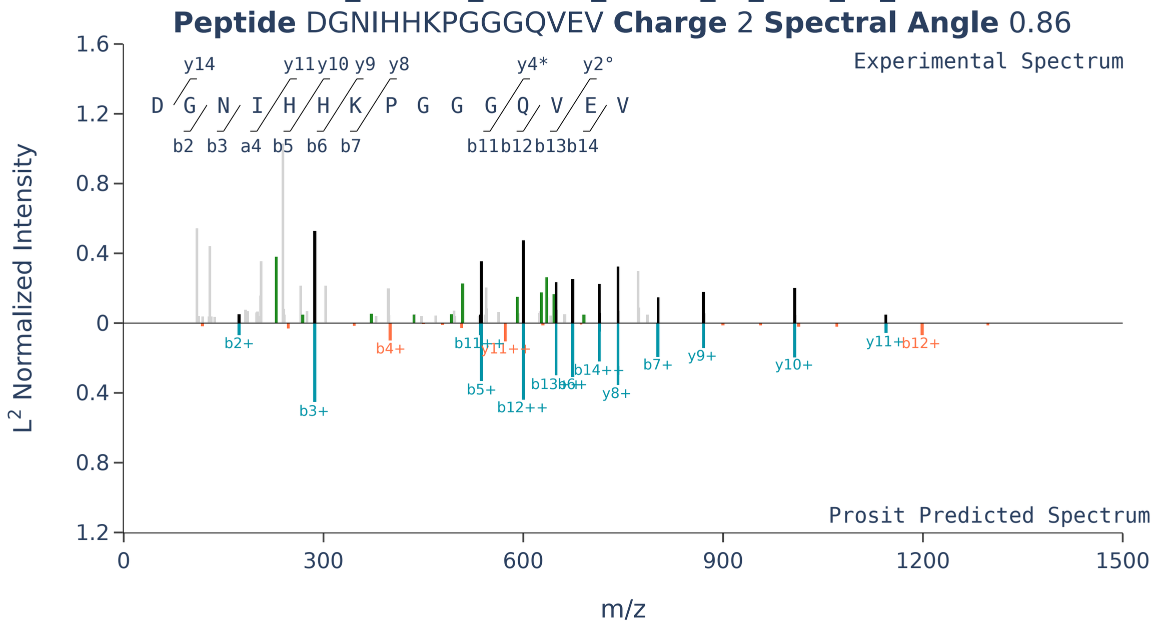

Source WSoh_221121_011221_HFGoe_L2_hTau_24h_R2 **Scan** 17148
Peptide DLSNVQSKCSKDNIKHVPGGGSVQI **Charge** 4 **Spectral Angle** 0.84

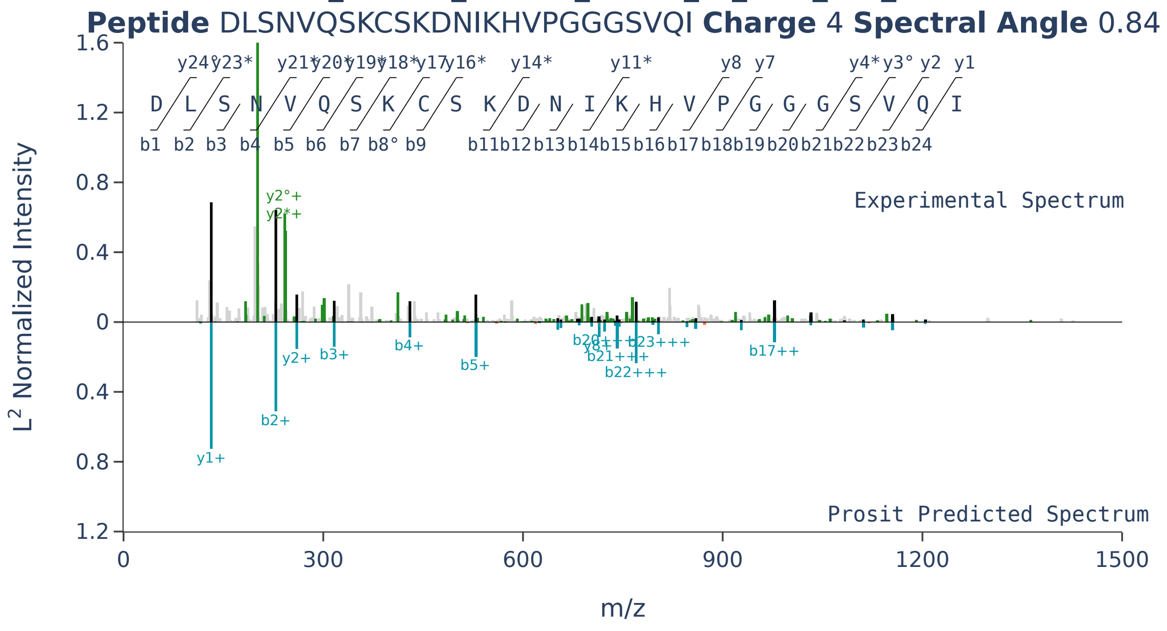

Source WSoh_221121_011221_HFGoe_L2_hTau_2h_R1 **Scan** 17584
Peptide ADEVKIGSTENL **Charge** 2 **Spectral Angle** 0.86

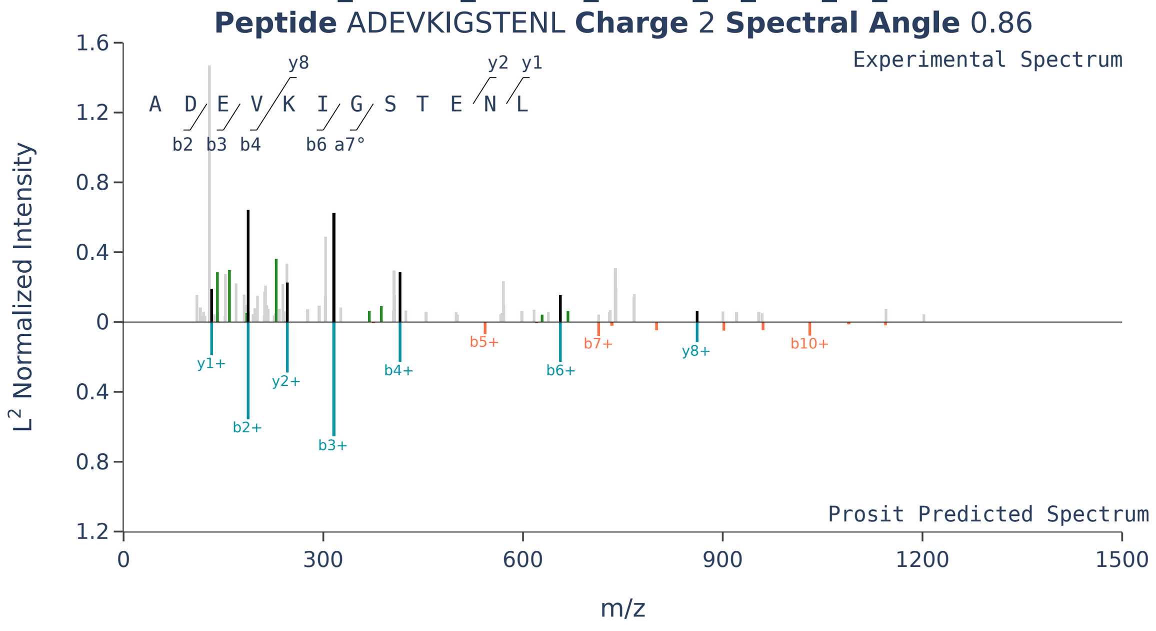

Source WSoh_221121_011221_HFGoe_L1_hTau_2h_R1 **Scan** 9482
Peptide QSKLTFREN **Charge** 3 **Spectral Angle** 0.9

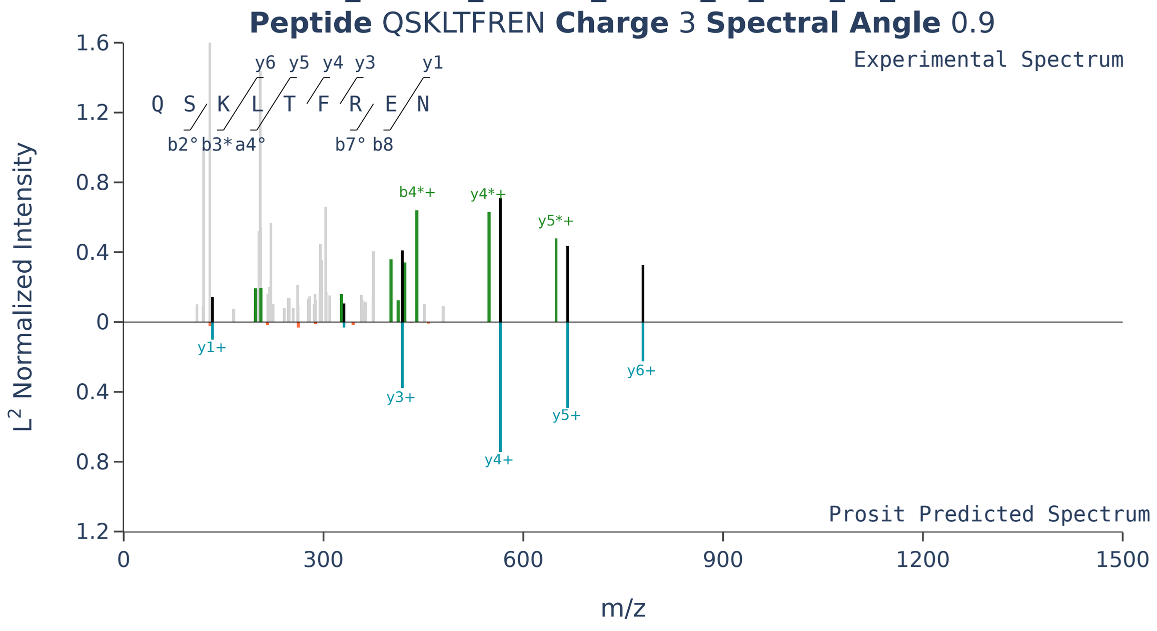

Source WSoh_221121_011221_HFGoe_L1_hTau_4h_R1 **Scan** 16511
Peptide VYKPVDLSKVTN **Charge** 2 **Spectral Angle** 0.8

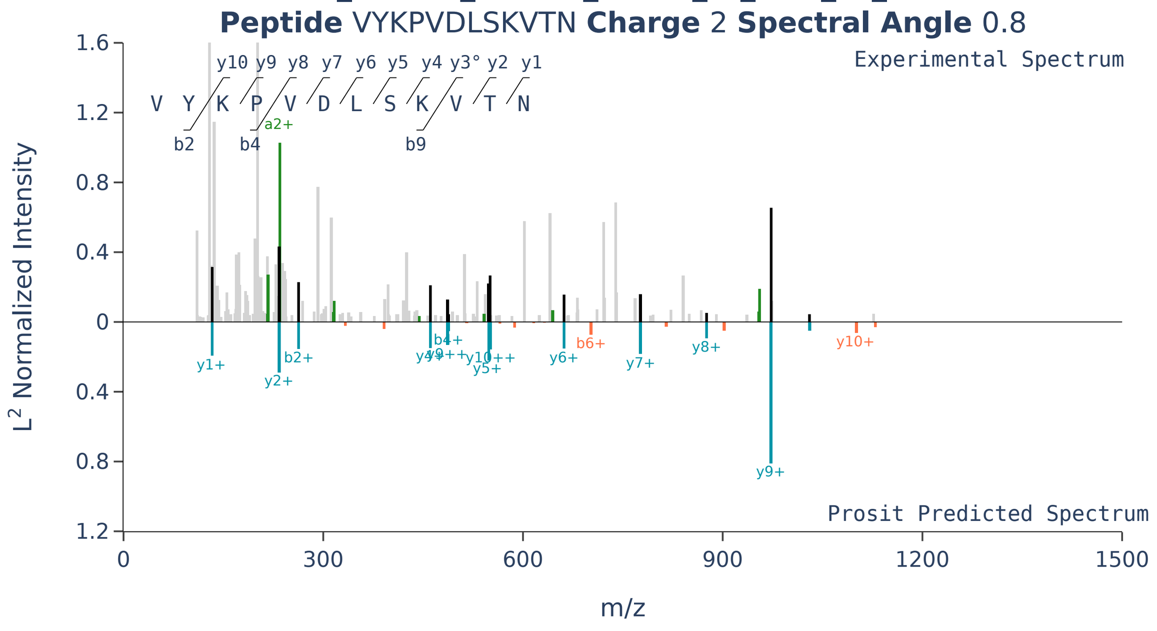

Source WSoh_221121_011221_HFGoe_L1_hTau_24h_R1 **Scan** 19327
Peptide ADEVSASLAKQSL **Charge** 2 **Spectral Angle** 0.89

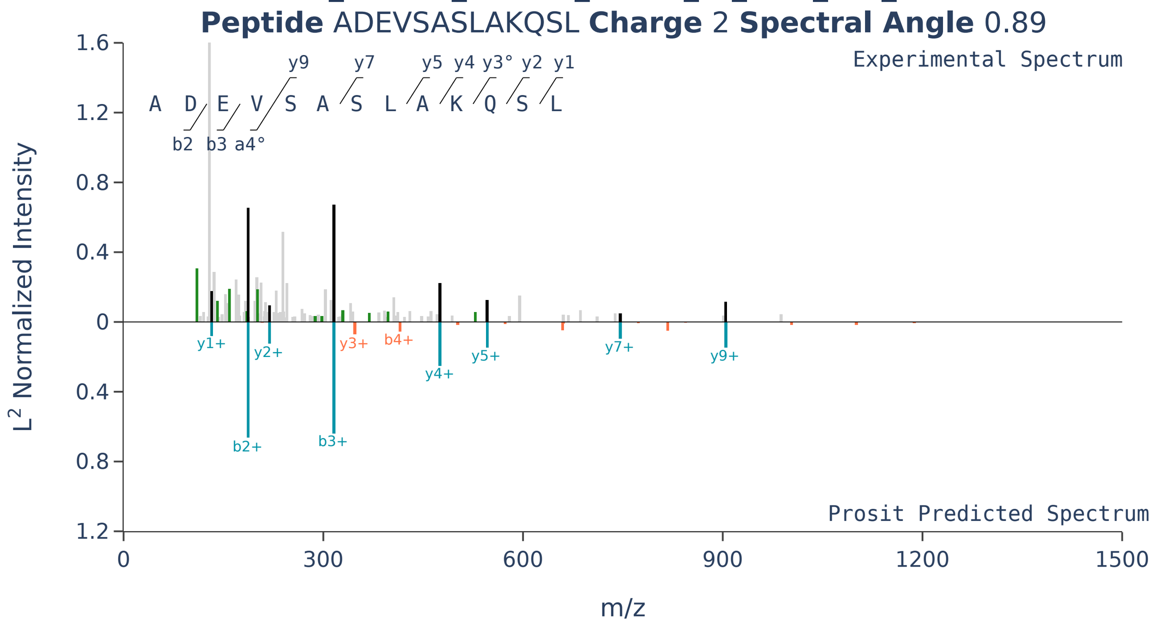

Source WSoh_221121_011221_HFGoe_L1_hTau_24h_R1 **Scan** 18222
Peptide GAIGDTPSLEDEAAGHVTQARMVSKSKD **Charge** 4 **Spectral Angle** 0.77

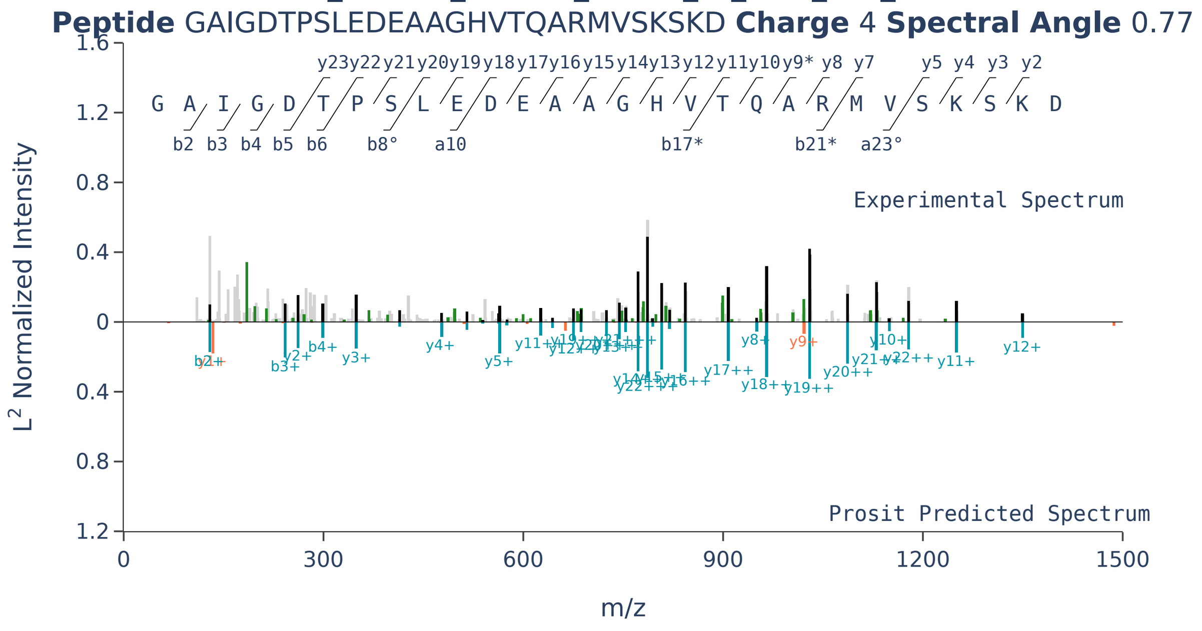

Source WSoh_221121_011221_HFGoe_L1_hTau_4h_R2 **Scan** 15619
Peptide KSEKLDFKDRSVQI **Charge** 2 **Spectral Angle** 0.67

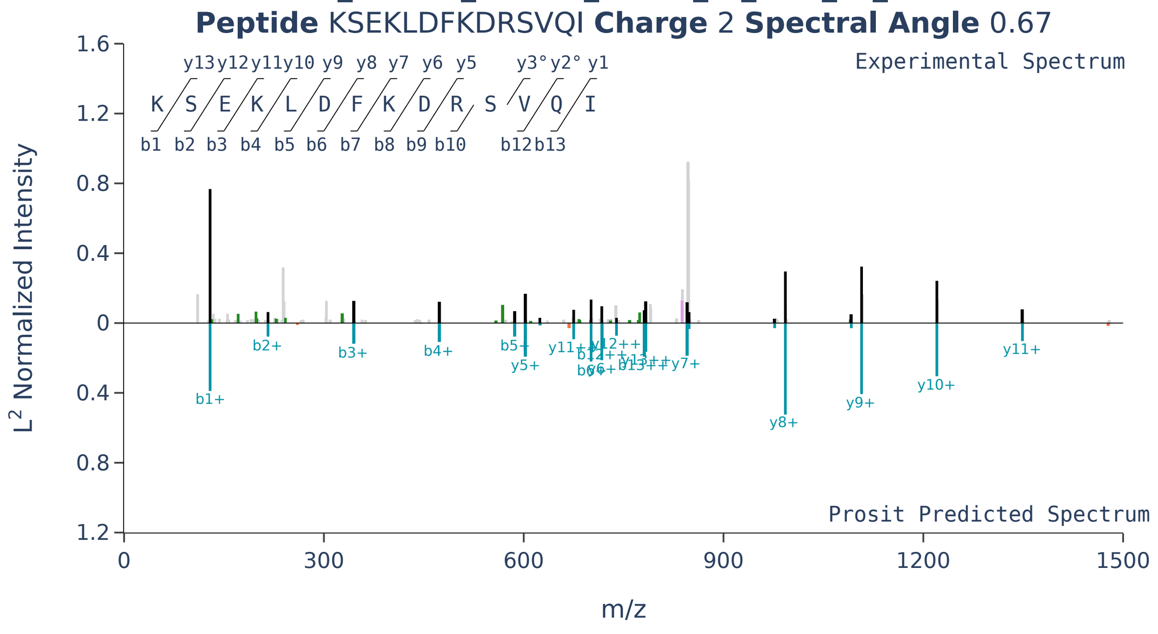

Source WSoh_221121_011221_HFGoe_L2_hTau_24h_R1 **Scan** 20274
Peptide EGAIGDTPSLEDEAAGHVTQARMVSK **Charge** 4 **Spectral Angle** 0.81

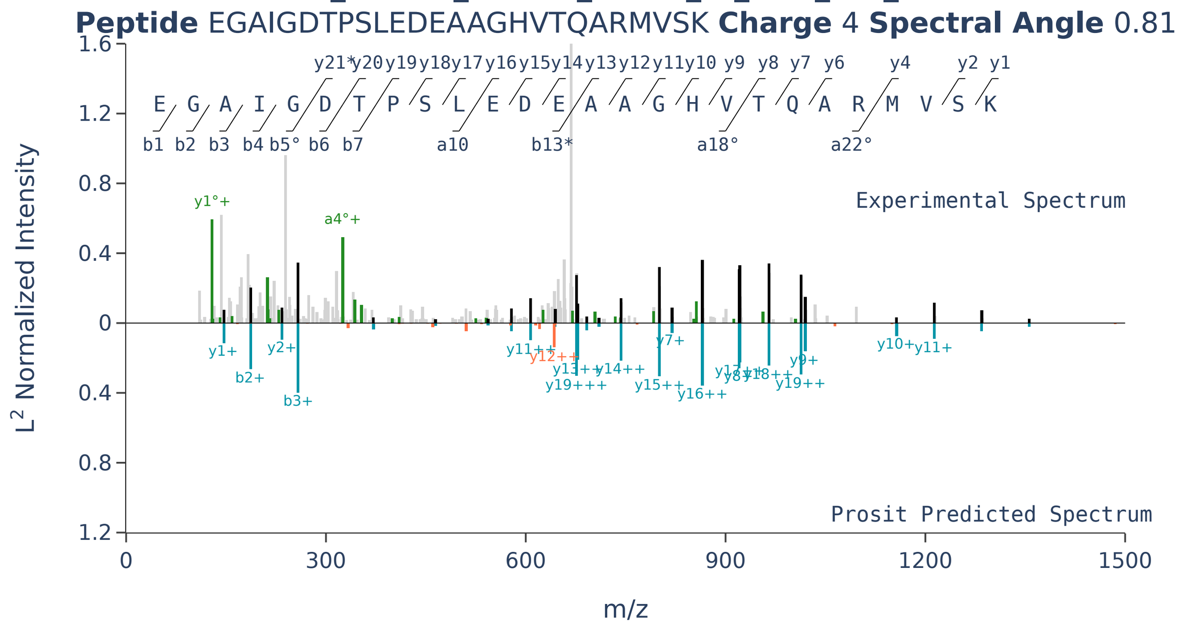

Source WSoh_221121_011221_HFGoe_L2_hTau_24h_R1 **Scan** 9058
Peptide KLDFKDRVQSH **Charge** 3 **Spectral Angle** 0.71

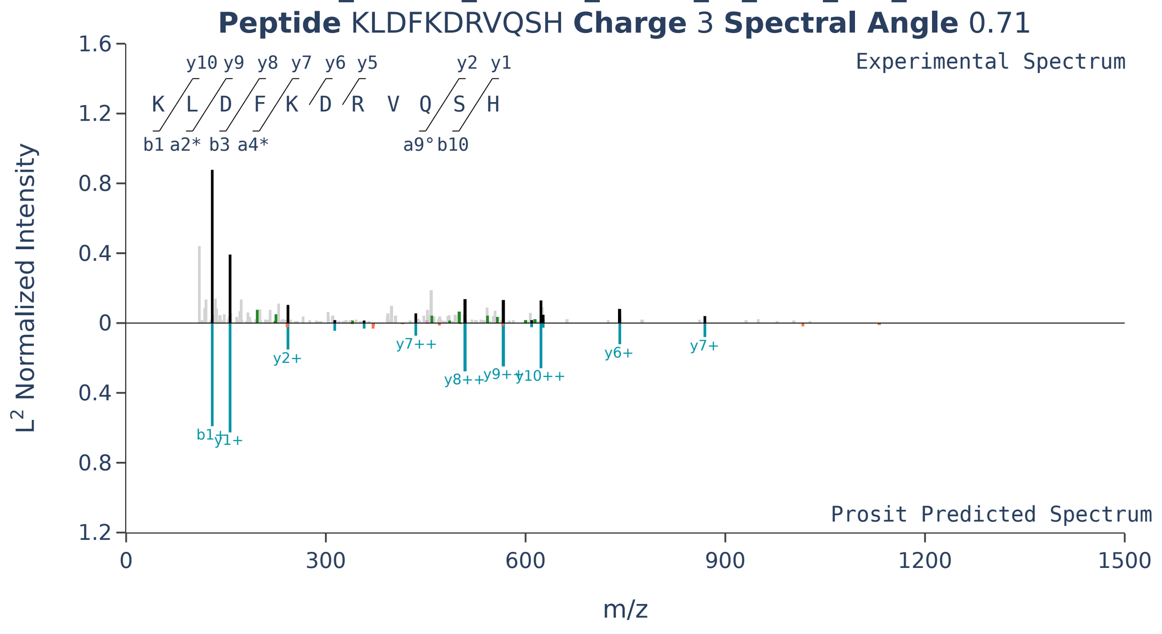

Source WSoh_221121_011221_HFGoe_L2_hTau_24h_R2 **Scan** 22032
Peptide QTAPVPMPDLKNVKSKIGSEAG **Charge** 4 **Spectral Angle** 0.8

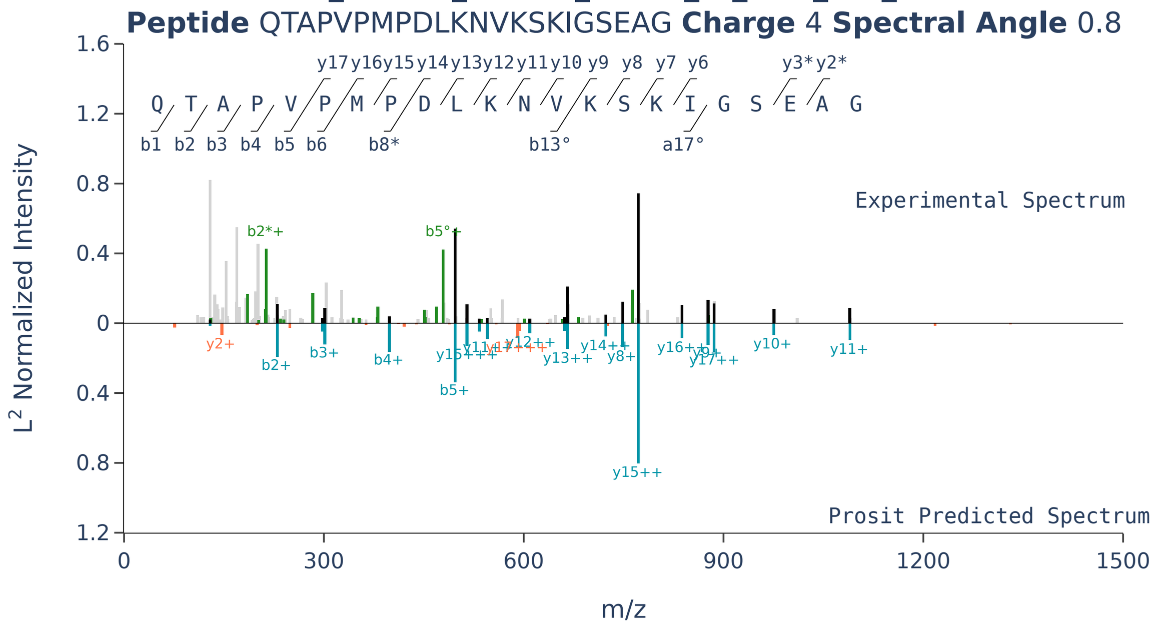

Source WSoh_221121_011221_HFGoe_L1_hTau_4h_R2 **Scan** 12211
Peptide DNITHVPGGGNKKIETHSL **Charge** 3 **Spectral Angle** 0.86

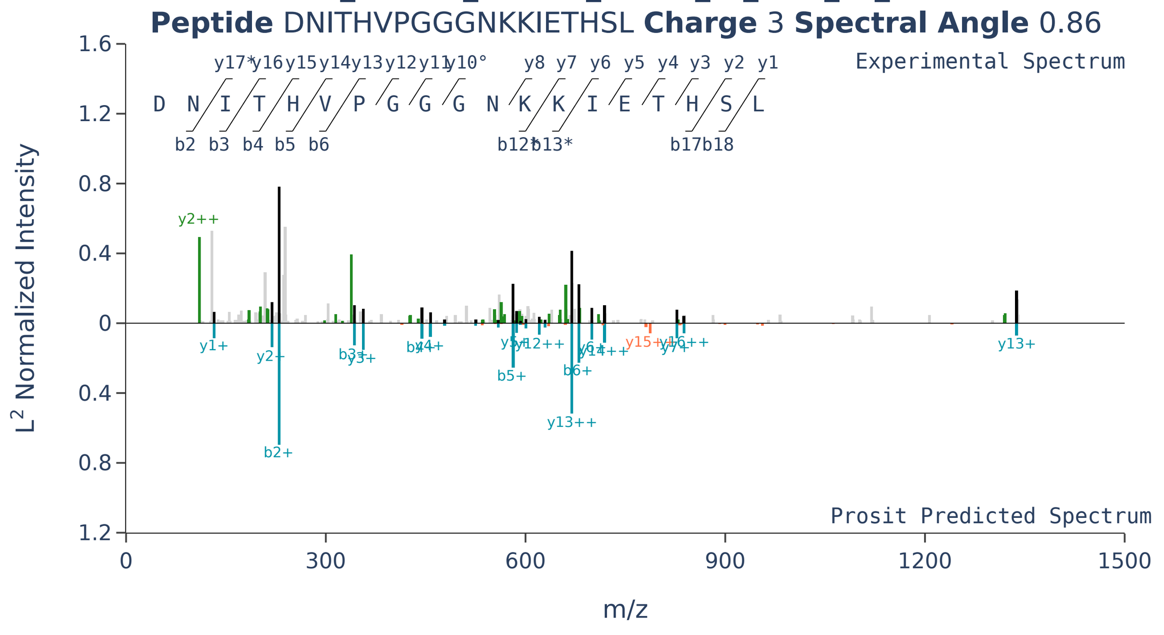

Source WSoh_221121_011221_HFGoe_L2_hTau_24h_R1 **Scan** 13828
Peptide SPQLPTPPTREPKKV **Charge** 4 **Spectral Angle** 0.87

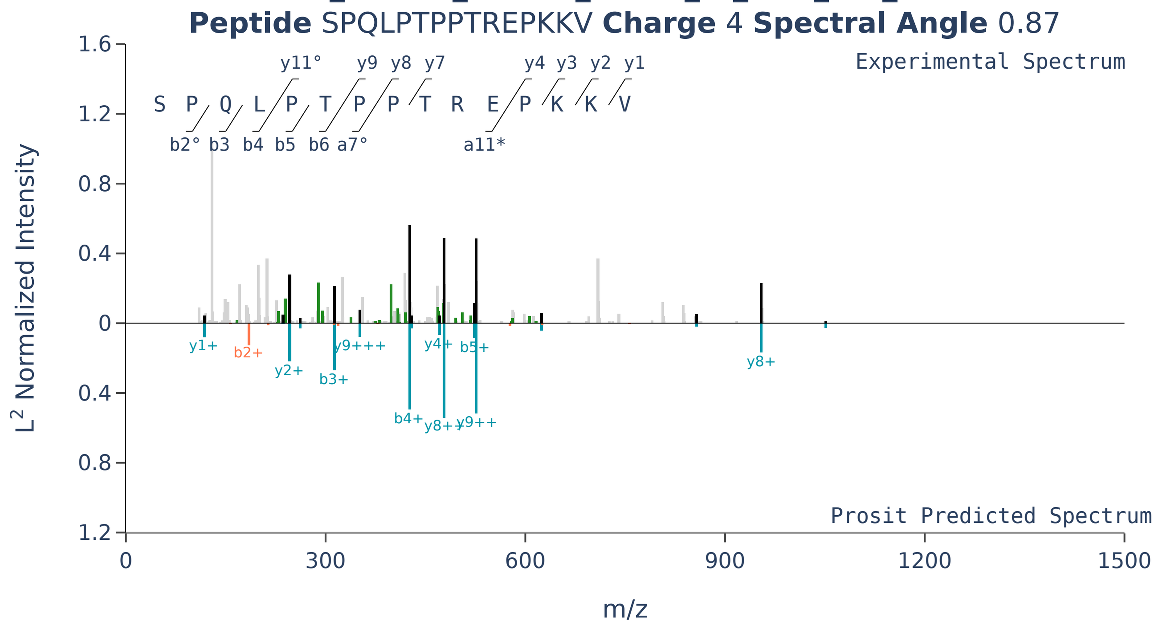

Source WSoh_221121_011221_HFGoe_L1_hTau_4h_R2 **Scan** 6861
Peptide GDTSPRHLSNVSGG **Charge** 2 **Spectral Angle** 0.84

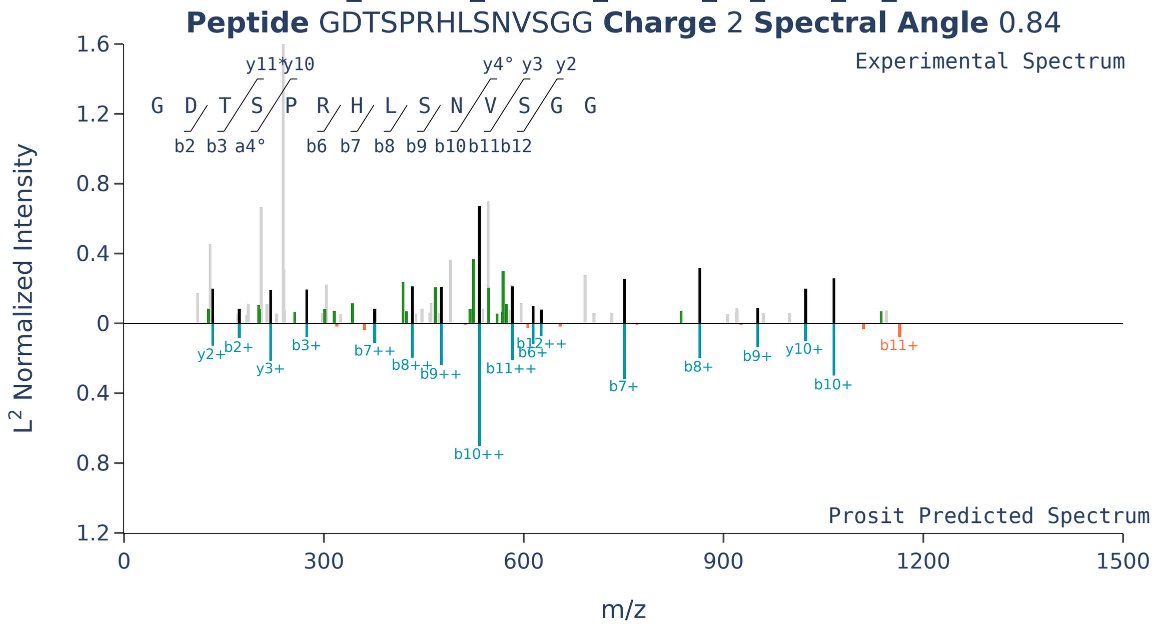

Source WSoh_221121_011221_HFGoe_L1_hTau_24h_R2 **Scan** 16779
Peptide DKNIKHVPGGGSVQIVYKPVD **Charge** 3 **Spectral Angle** 0.85

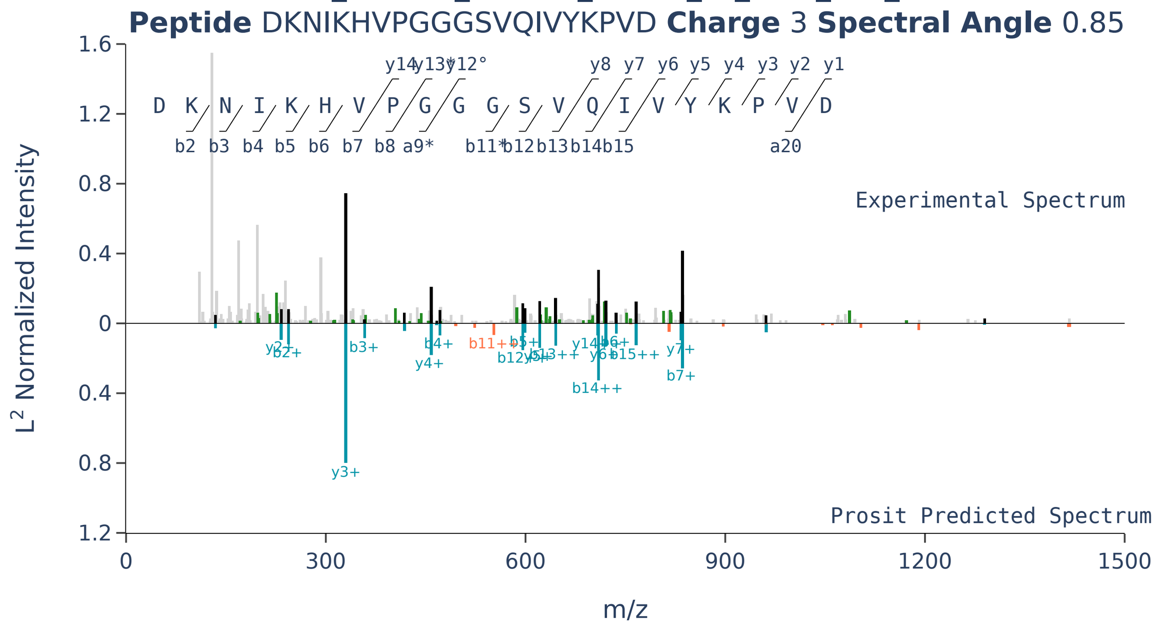

Source WSoh_221121_011221_HFGoe_L2_hTau_24h_R2 **Scan** 23114
Peptide APVPMPDLKNVKSV **Charge** 3 **Spectral Angle** 0.86

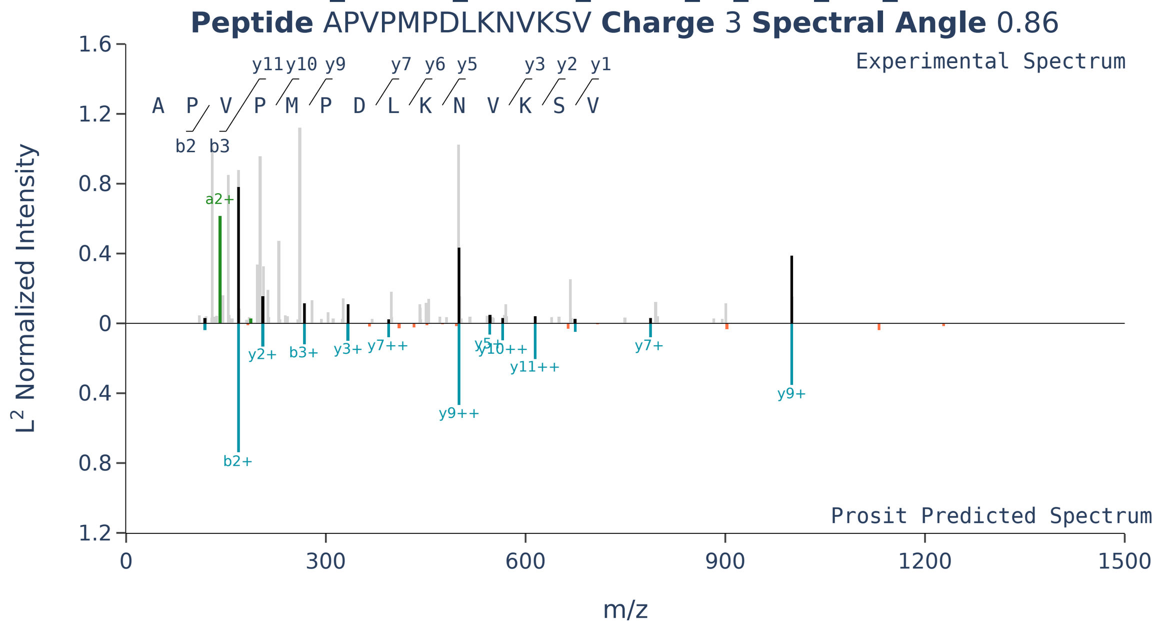

Source WSoh_221121_011221_HFGoe_L2_hTau_24h_R2 **Scan** 13236
Peptide DLSNVQSKCGSKDLG **Charge** 3 **Spectral Angle** 0.79

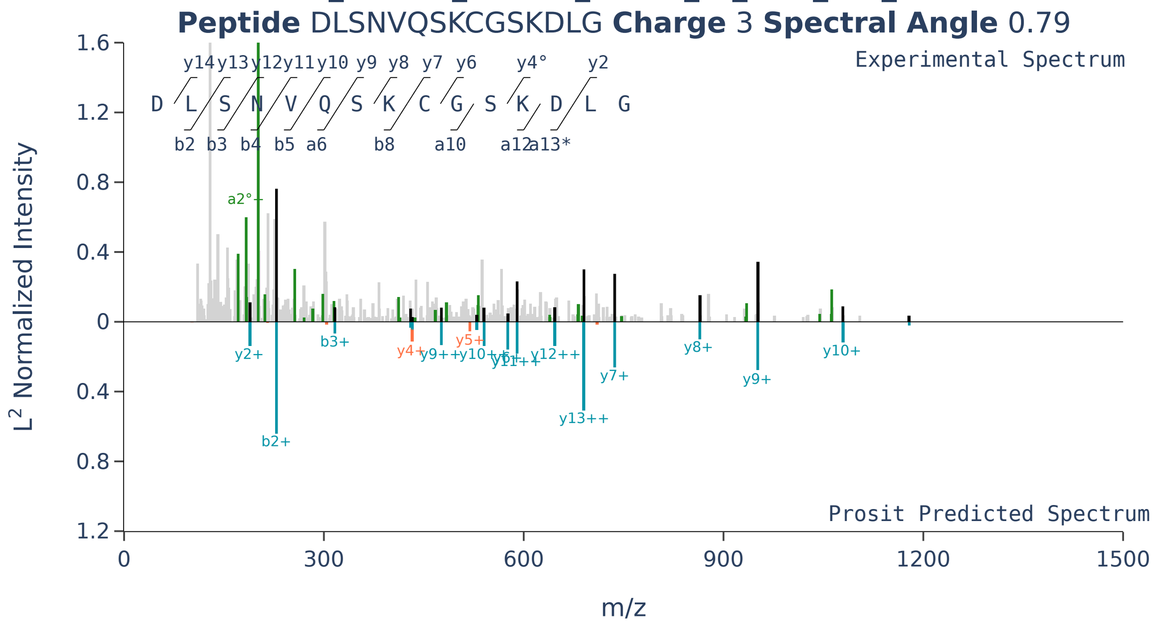

Source WSoh_221121_011221_HFGoe_L2_hTau_24h_R2 **Scan** 21692
Peptide SNVQSAPVPMPDLKNVKS **Charge** 3 **Spectral Angle** 0.89

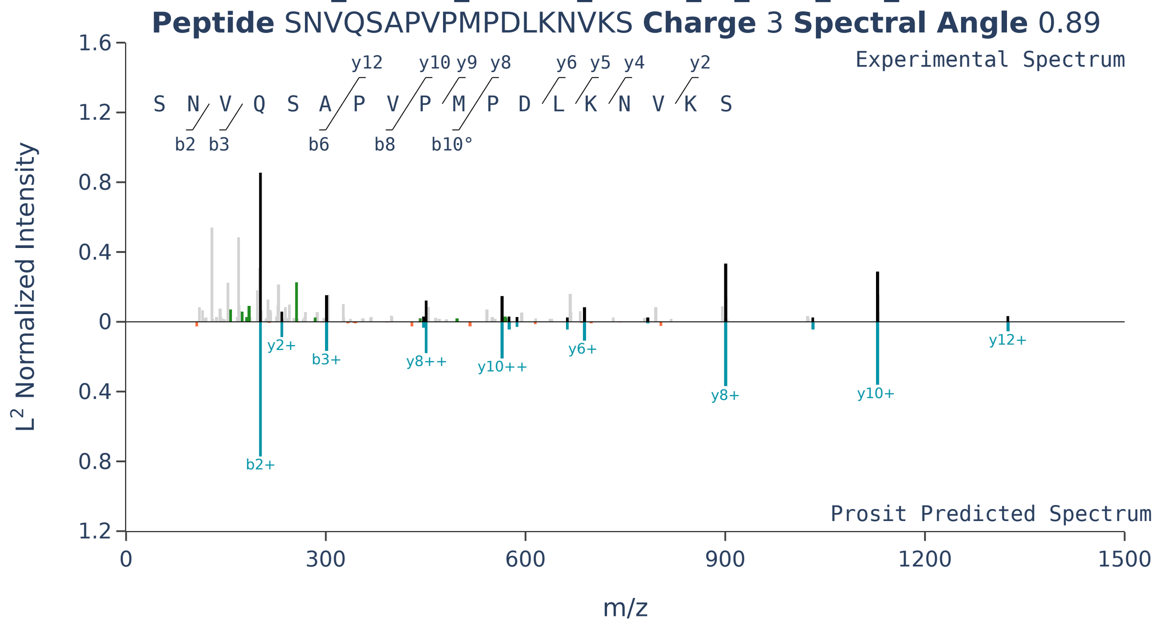

Source WSoh_221121_011221_HFGoe_L1_hTau_24h_R2 **Scan** 19891
Peptide LDFAEEAG **Charge** 2 **Spectral Angle** 0.69

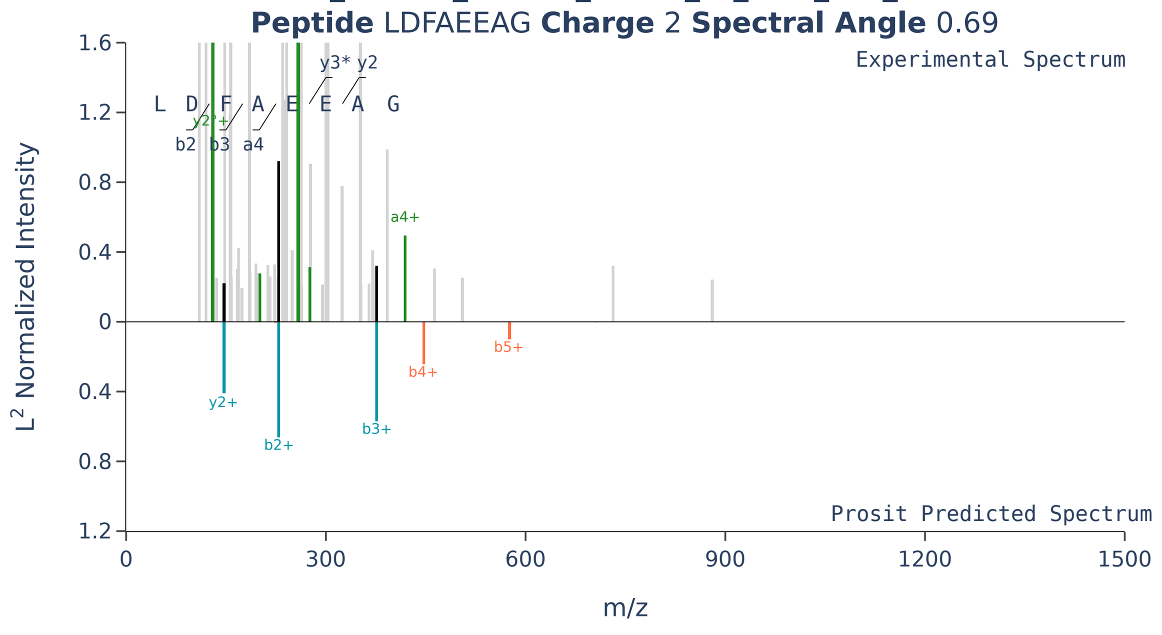

Source WSoh_221121_011221_HFGoe_L1_hTau_24h_R1 **Scan** 22945
Peptide LQTAPVPMPDLKNVKSS **Charge** 3 **Spectral Angle** 0.88

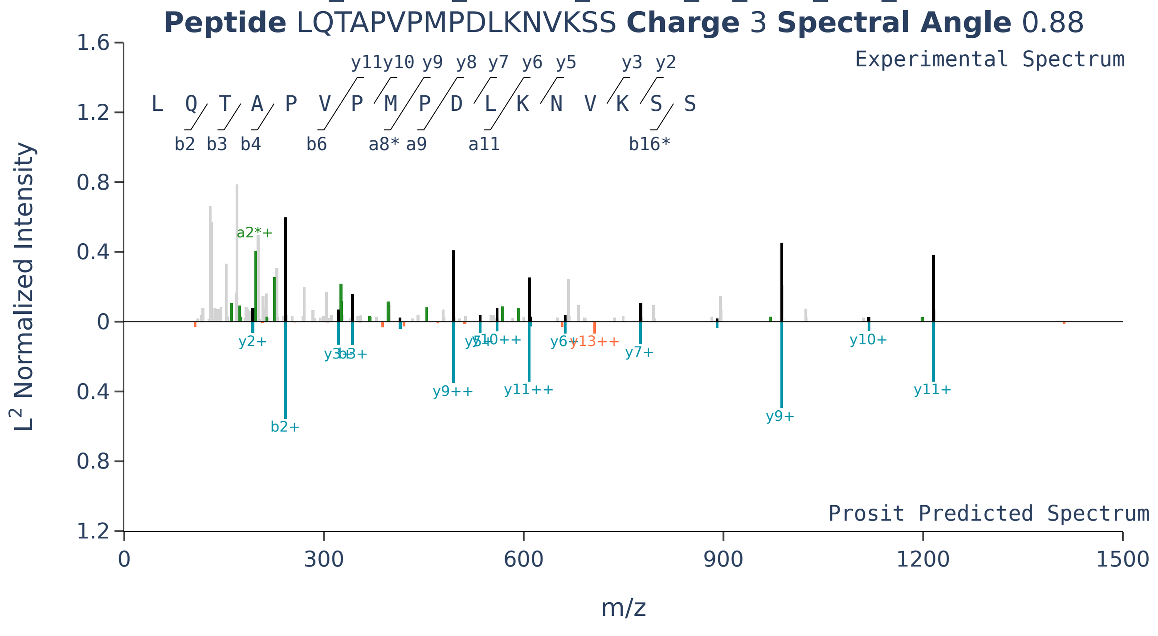

Source WSoh_221121_011221_HFGoe_L2_hTau_24h_R2 **Scan** 12721
Peptide GEAPGKQAAAQPHTEIPEGTTAE **Charge** 3 **Spectral Angle** 0.79

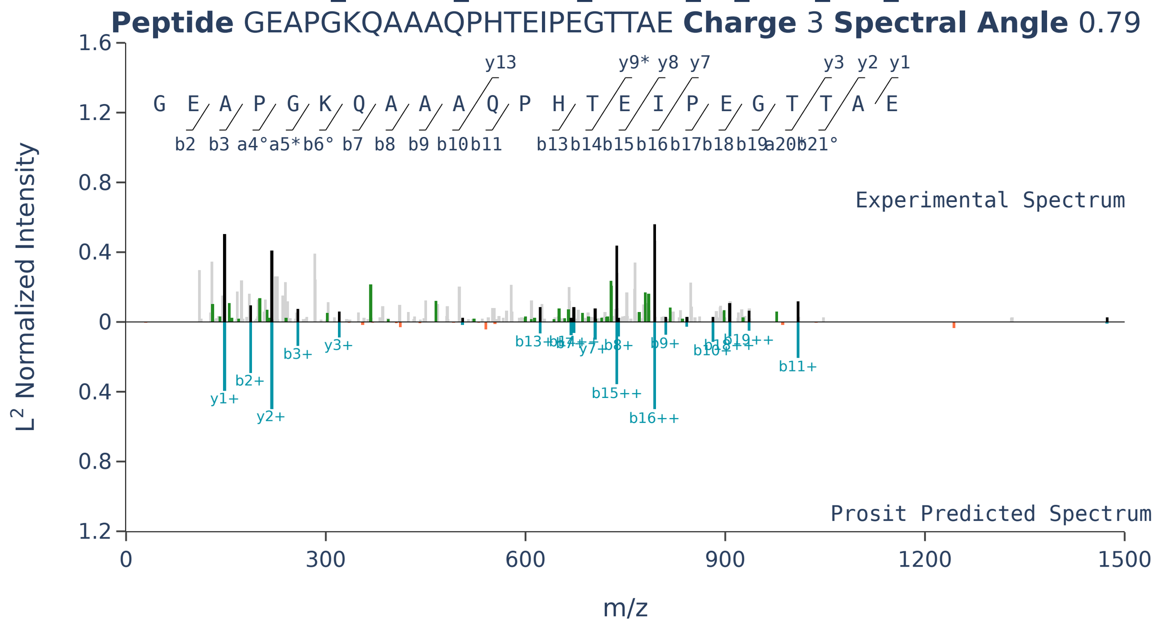

Source WSoh_221121_011221_HFGoe_L2_hTau_24h_R1 **Scan** 10452
Peptide AKQKLDFKDRV **Charge** 3 **Spectral Angle** 0.78

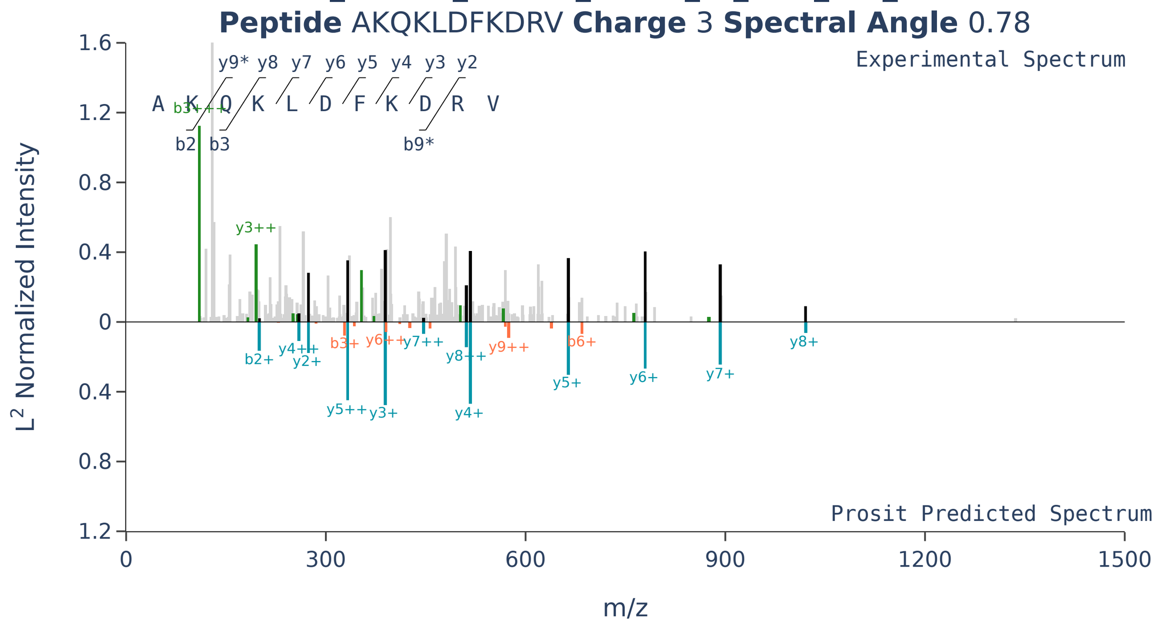

Source WSoh_221121_011221_HFGoe_L2_hTau_4h_R1 **Scan** 19289
Peptide APVPMPDLKNVSKS **Charge** 3 **Spectral Angle** 0.84

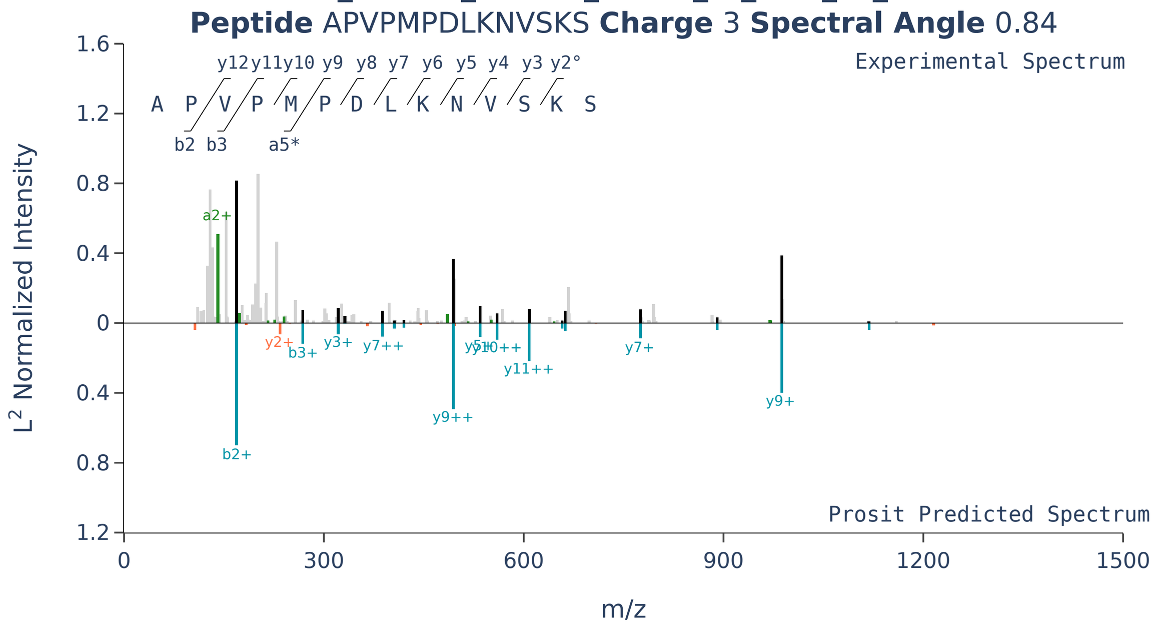

Source WSoh_221121_011221_HFGoe_L1_hTau_24h_R2 **Scan** 14908
Peptide TTPSLPTPPTREPKKV **Charge** 3 **Spectral Angle** 0.76

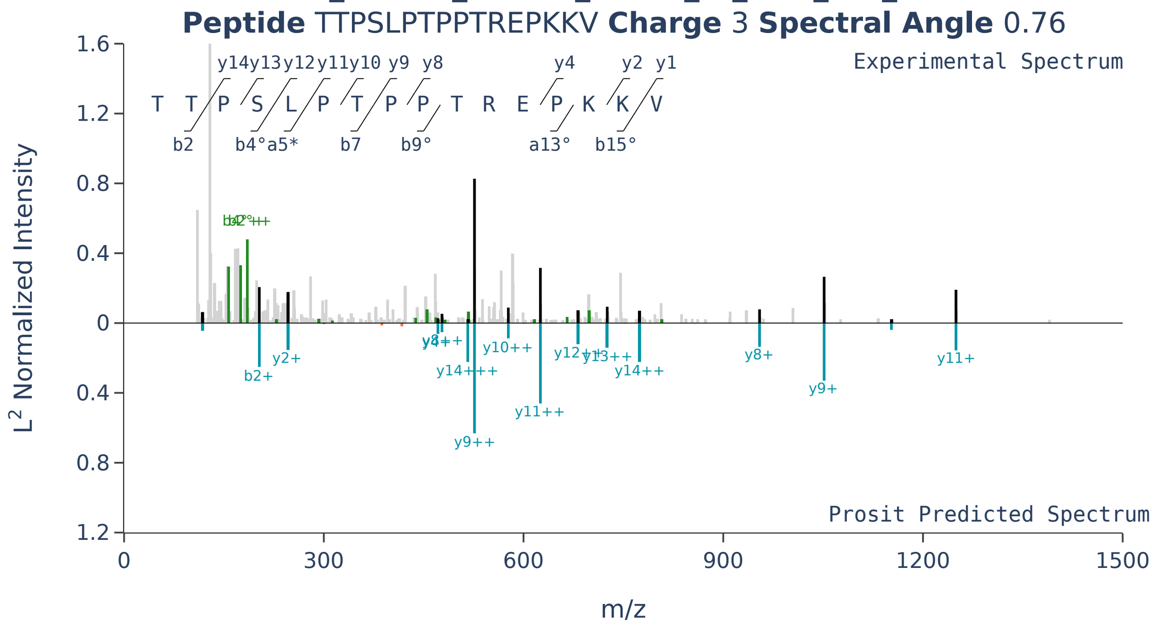

Source WSoh_221121_011221_HFGoe_L2_hTau_24h_R1 **Scan** 20816
Peptide QTAPVPMPDLKNVKSG **Charge** 3 **Spectral Angle** 0.76

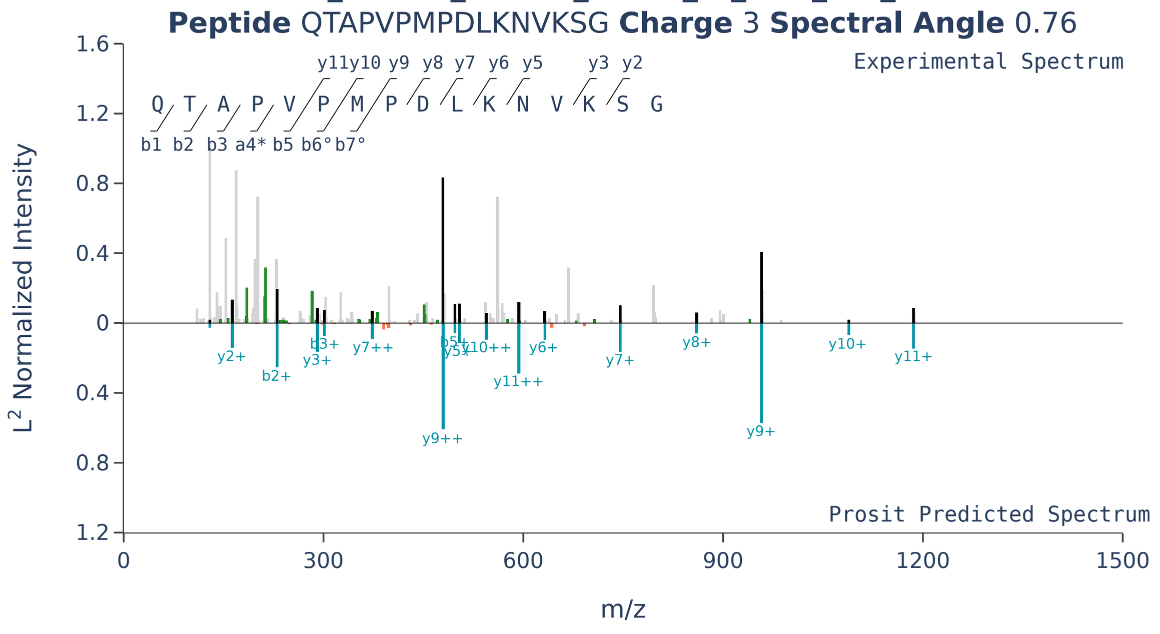

Source WSoh_221121_011221_HFGoe_L1_hTau_24h_R1 **Scan** 18033
Peptide QSQIINKKLDL **Charge** 3 **Spectral Angle** 0.78

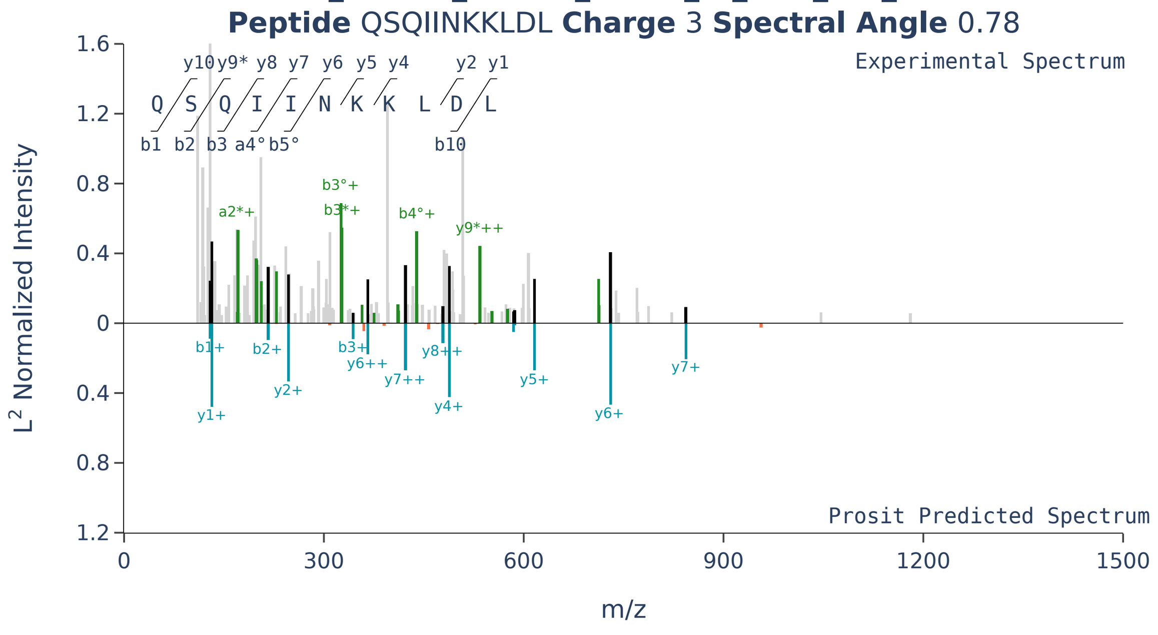

Source WSoh_221121_011221_HFGoe_L1_hTau_4h_R1 **Scan** 20267
Peptide PSLEDEADNIT **Charge** 1 **Spectral Angle** 0.77

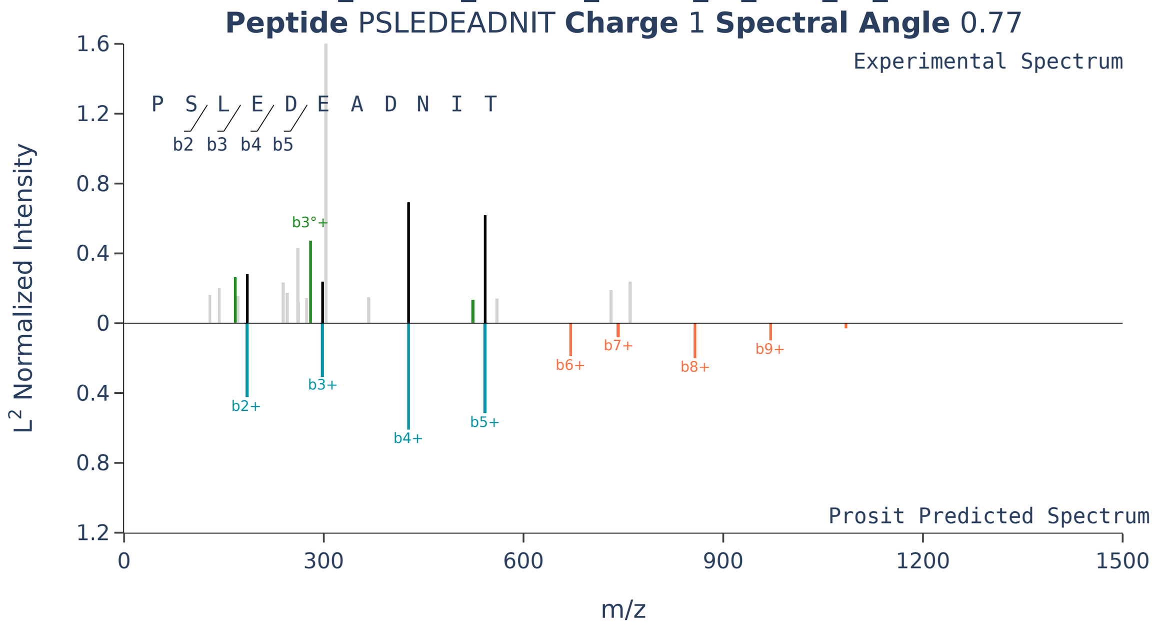

Source WSoh_221121_011221_HFGoe_L2_hTau_24h_R2 **Scan** 11319
Peptide KLDLSNVQSKCSKDNIKH **Charge** 3 **Spectral Angle** 0.86

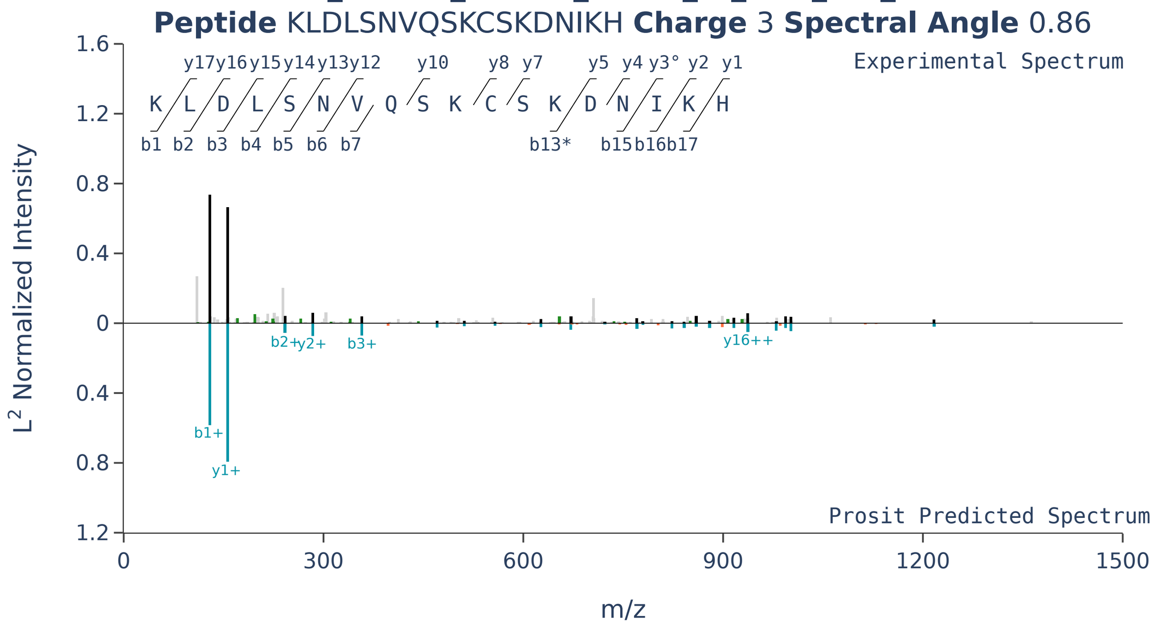

Source WSoh_221121_011221_HFGoe_L2_hTau_24h_R1 **Scan** 6884
Peptide AVVRTPPKSPSSAKSA **Charge** 2 **Spectral Angle** 0.8

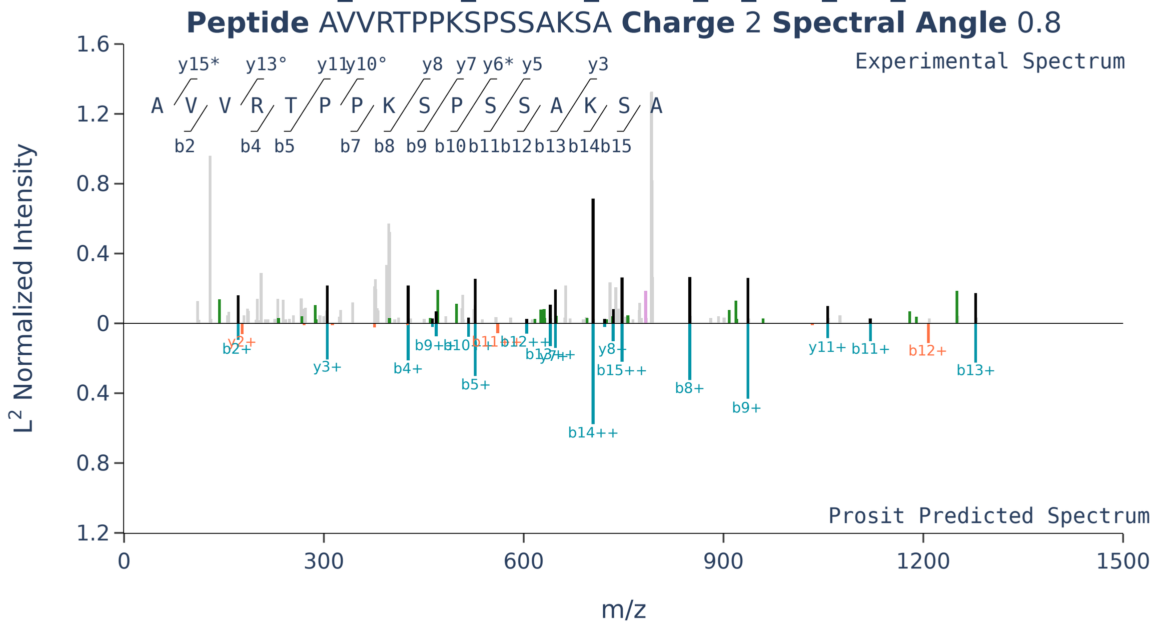

Source WSoh_221121_011221_HFGoe_L1_hTau_24h_R1 **Scan** 23589
Peptide LQTAPVPMPDLKNVKSKIGSEAG **Charge** 4 **Spectral Angle** 0.89

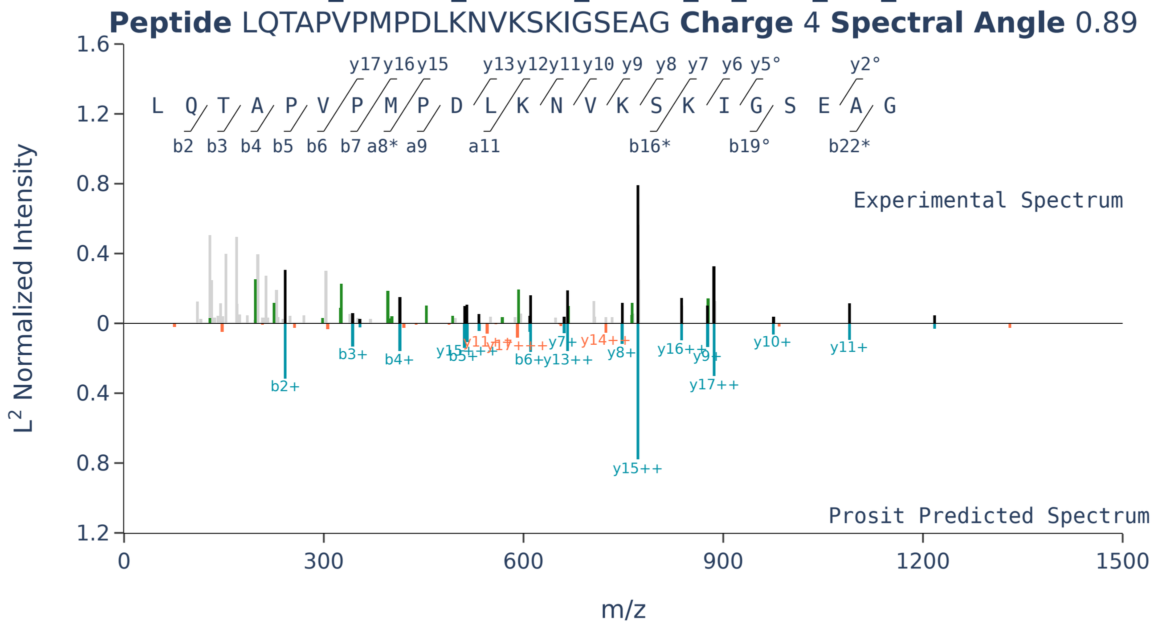

Source WSoh_221121_011221_HFGoe_L2_hTau_24h_R2 **Scan** 8194
Peptide VTKVTSKCGSLGNIHHKPGGGQVE **Charge** 3 **Spectral Angle** 0.84

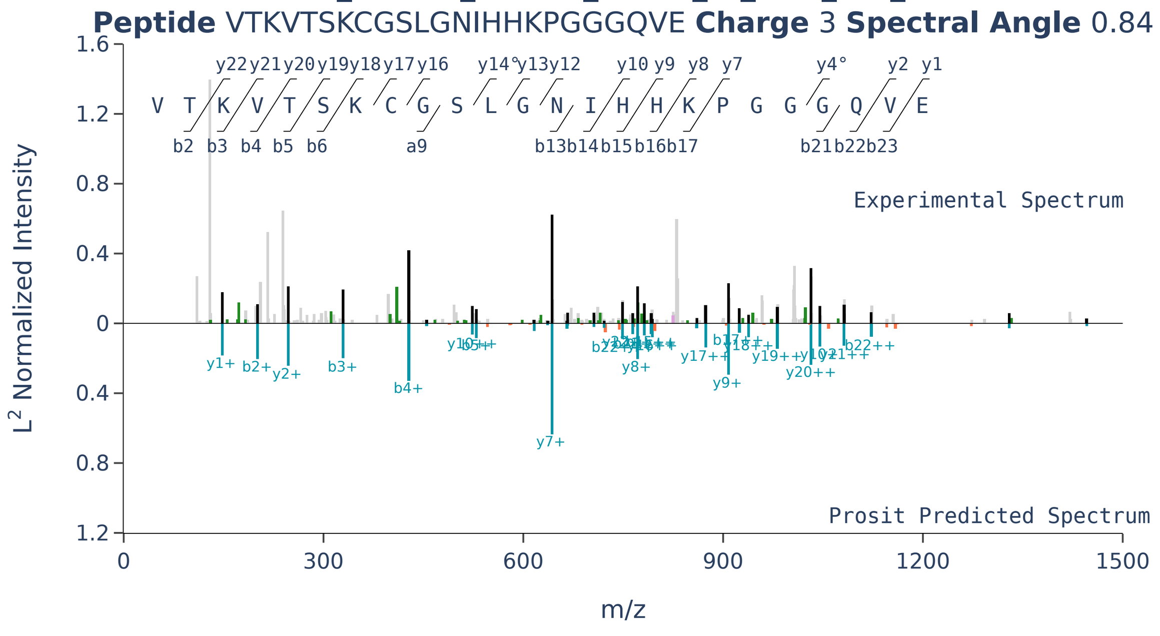

Source WSoh_221121_011221_HFGoe_L2_hTau_24h_R1 **Scan** 14450
Peptide KSTPTKTDHGAEIVYKSPVVSGDTSPRHL **Charge** 4 **Spectral Angle** 0.86

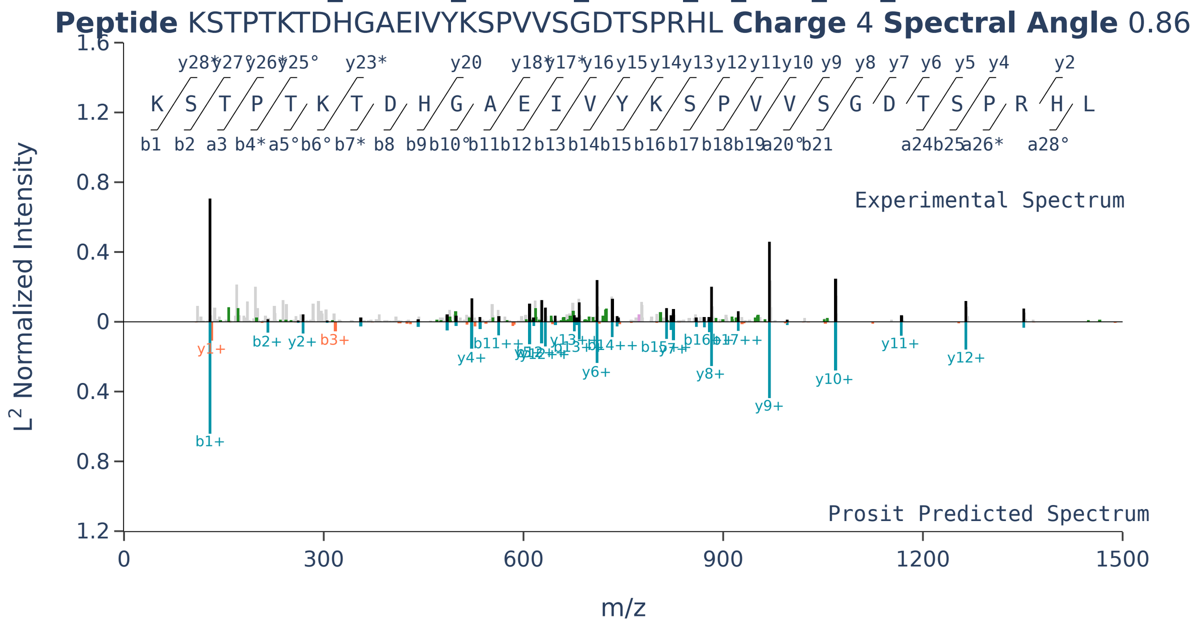

Source WSoh_221121_011221_HFGoe_L2_hTau_24h_R2 **Scan** 17639
Peptide APVPMPDLKNVKH **Charge** 3 **Spectral Angle** 0.86

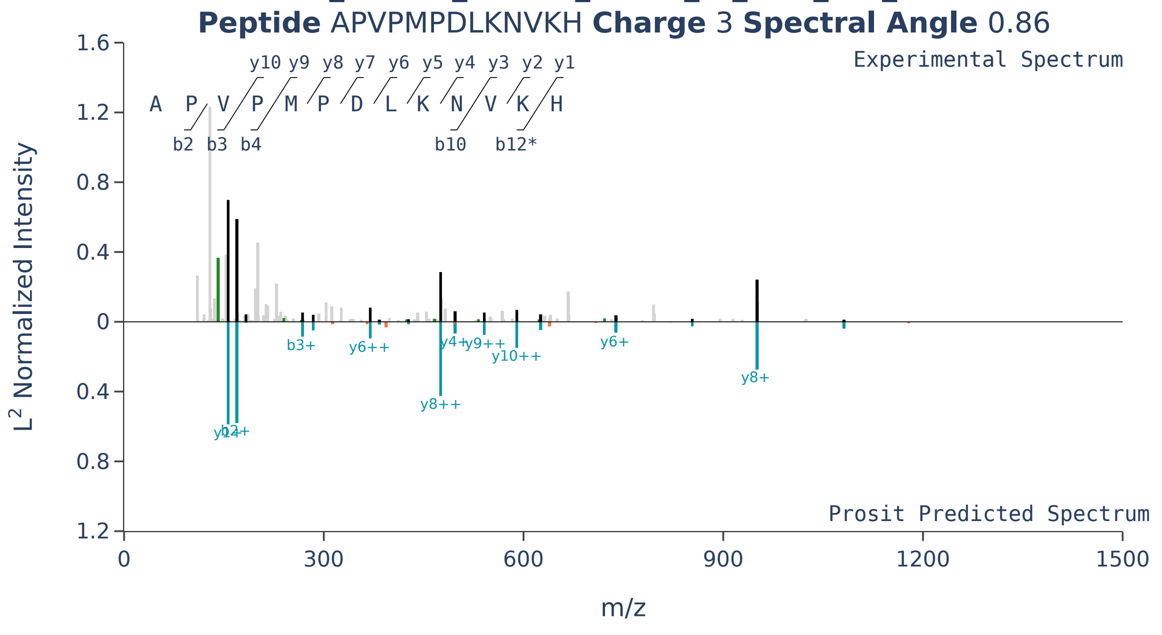

Source WSoh_221121_011221_HFGoe_L2_hTau_4h_R2 **Scan** 8188
Peptide PRHLSNVSLK **Charge** 3 **Spectral Angle** 0.72

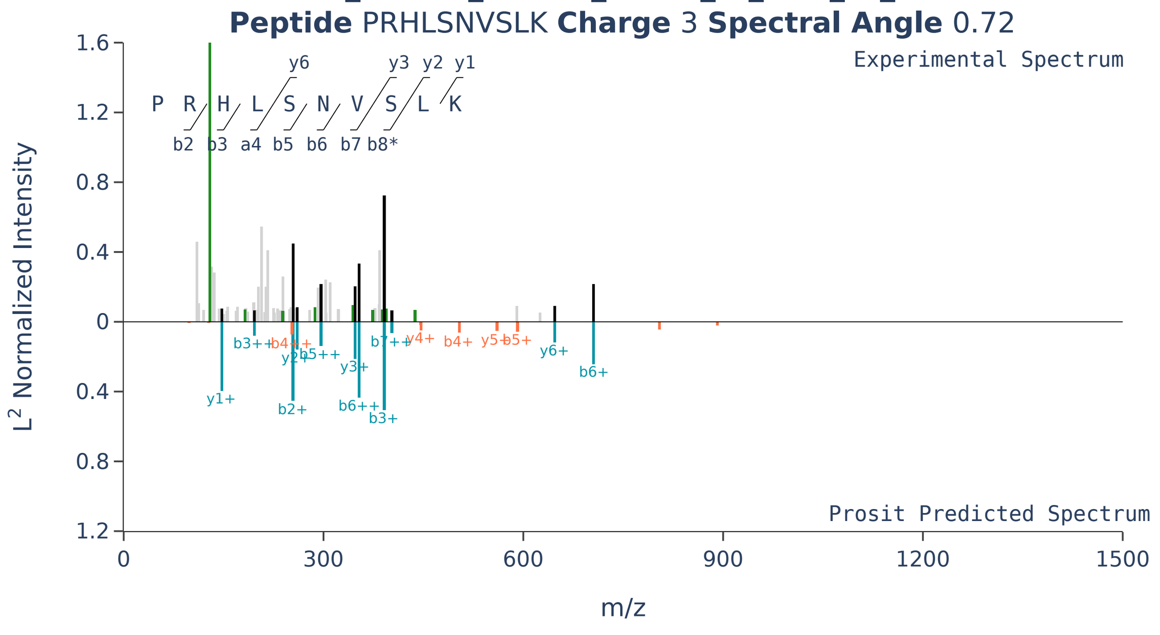

Source WSoh_221121_011221_HFGoe_L2_hTau_24h_R2 **Scan** 12000
Peptide GDTSPRHLSNVVDEG **Charge** 2 **Spectral Angle** 0.9

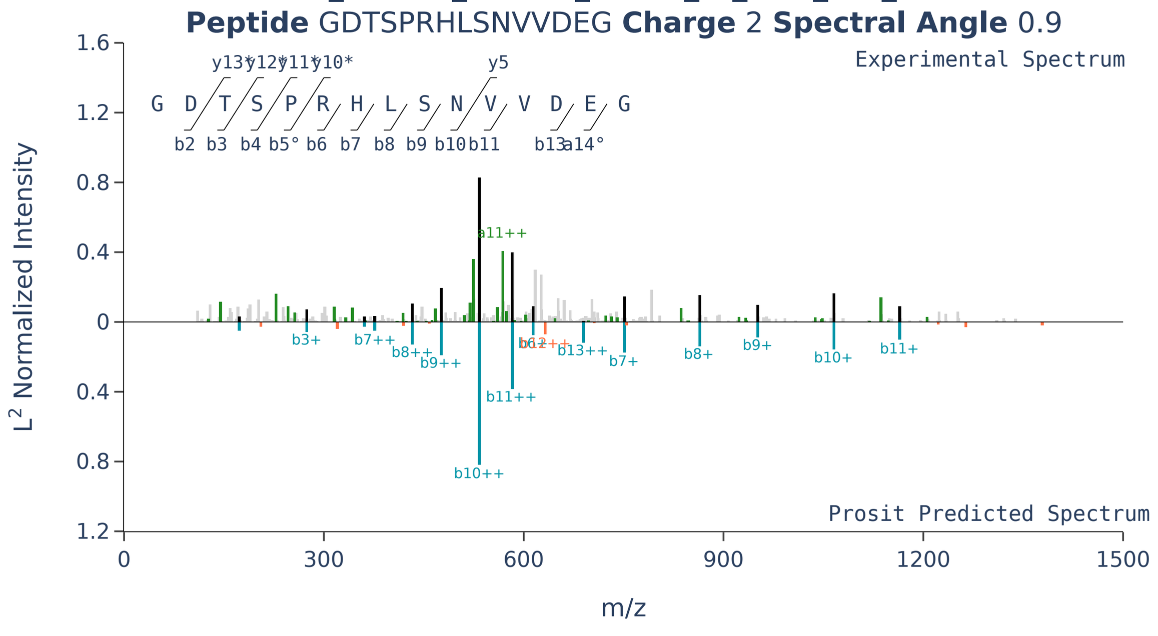

Source WSoh_221121_011221_HFGoe_L2_hTau_4h_R2 **Scan** 11529
Peptide KHLTFRENAKAKTDHGAEIVY **Charge** 4 **Spectral Angle** 0.88

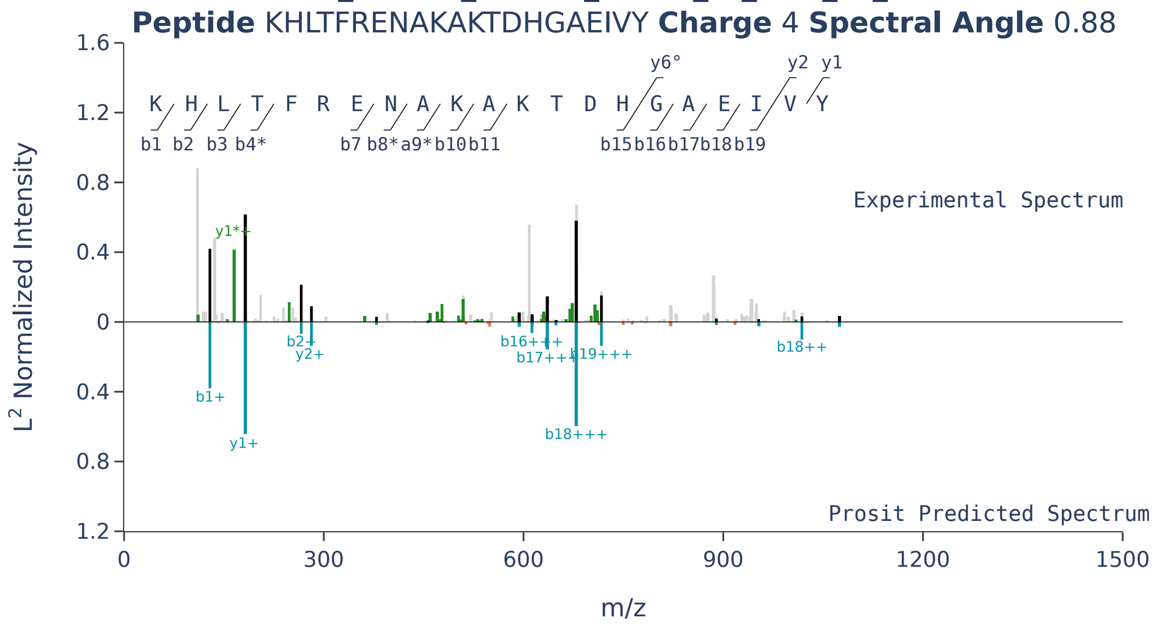

Source WSoh_221121_011221_HFGoe_L2_hTau_24h_R2 **Scan** 23617
Peptide TAGIGDTPSLEDEAAGHVTQARMV **Charge** 3 **Spectral Angle** 0.87

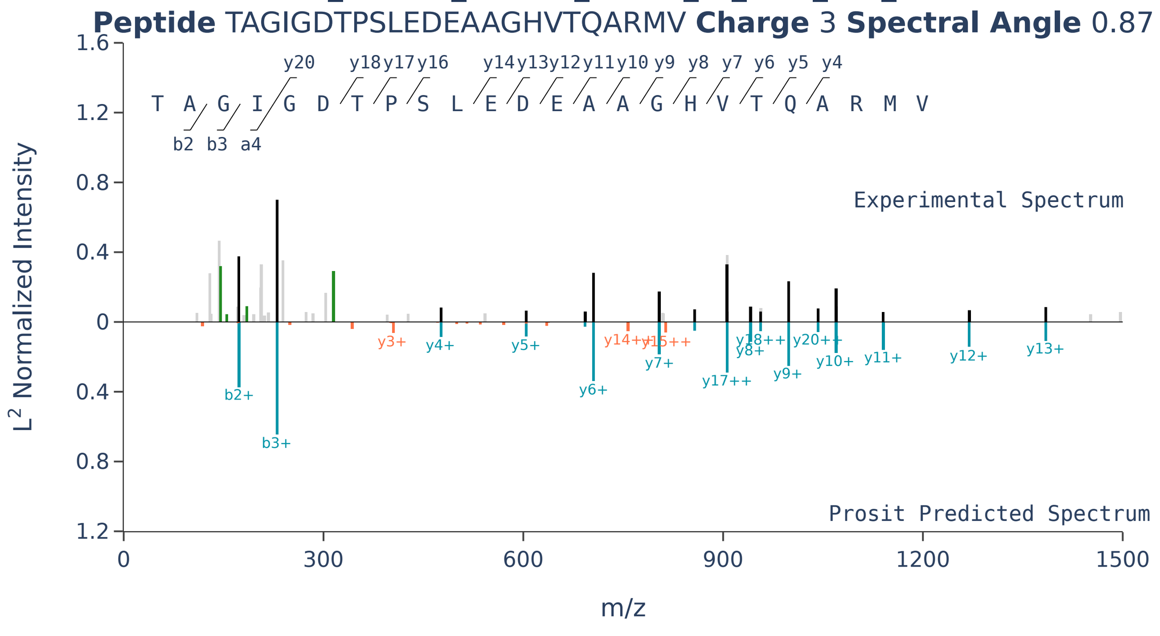

Source WSoh_221121_011221_HFGoe_L2_hTau_2h_R1 **Scan** 6678
Peptide GTKHQPGGGKVQIINK **Charge** 4 **Spectral Angle** 0.84

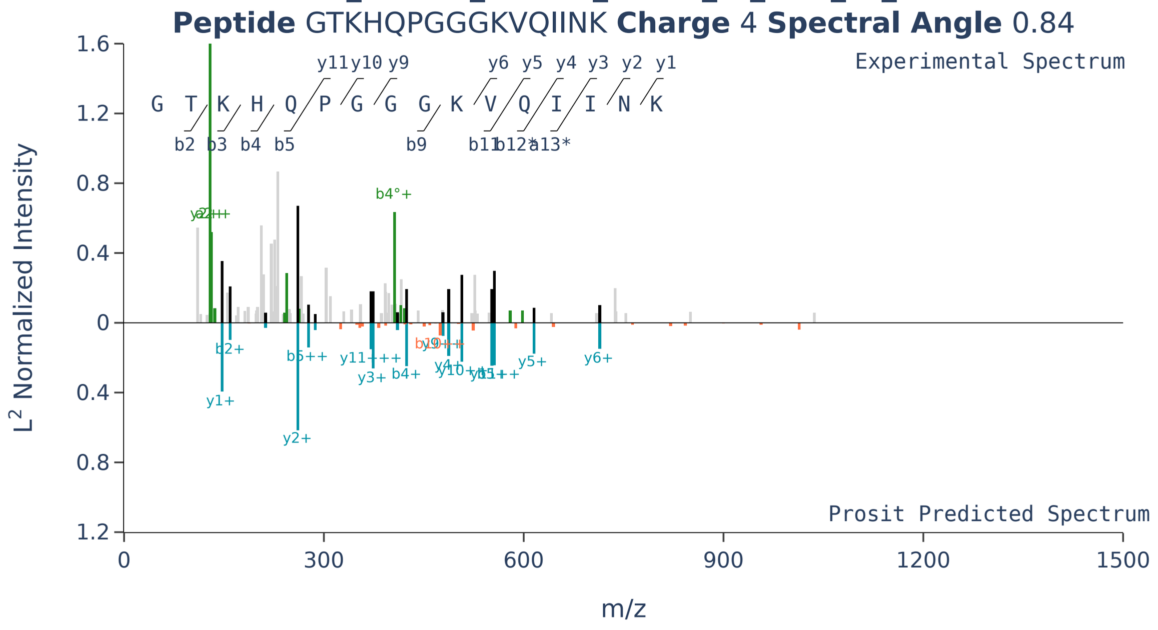

Source WSoh_221121_011221_HFGoe_L2_hTau_1h_R1 **Scan** 6505
Peptide SKHQPGGGKVQIINK **Charge** 2 **Spectral Angle** 0.87

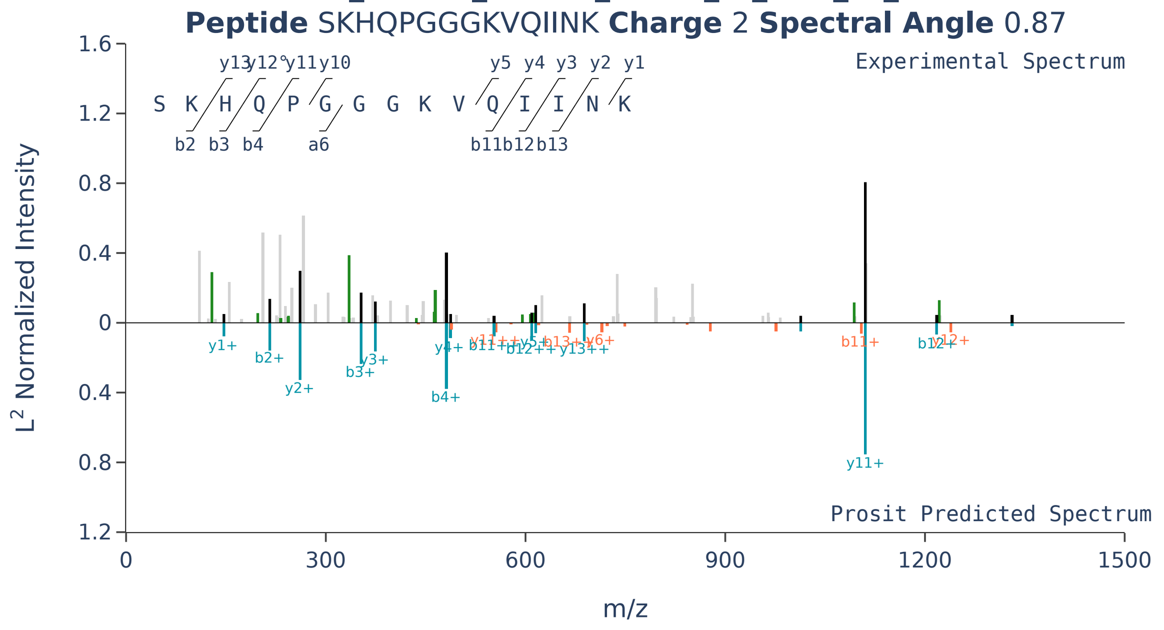

Source WSoh_221121_011221_HFGoe_L2_hTau_24h_R2 **Scan** 19113
Peptide DHAGTYGLGDRKDAEPRQEFEVME **Charge** 3 **Spectral Angle** 0.85

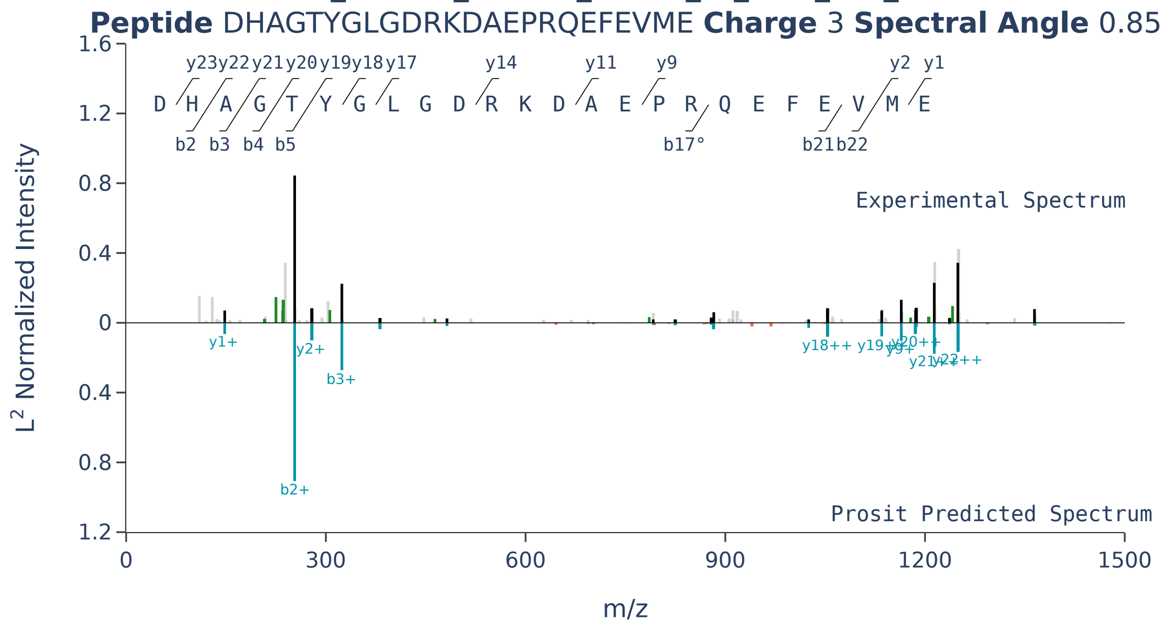

Source WSoh_221121_011221_HFGoe_L1_hTau_4h_R2 **Scan** 10081
Peptide KLDFKDRVQN **Charge** 3 **Spectral Angle** 0.72

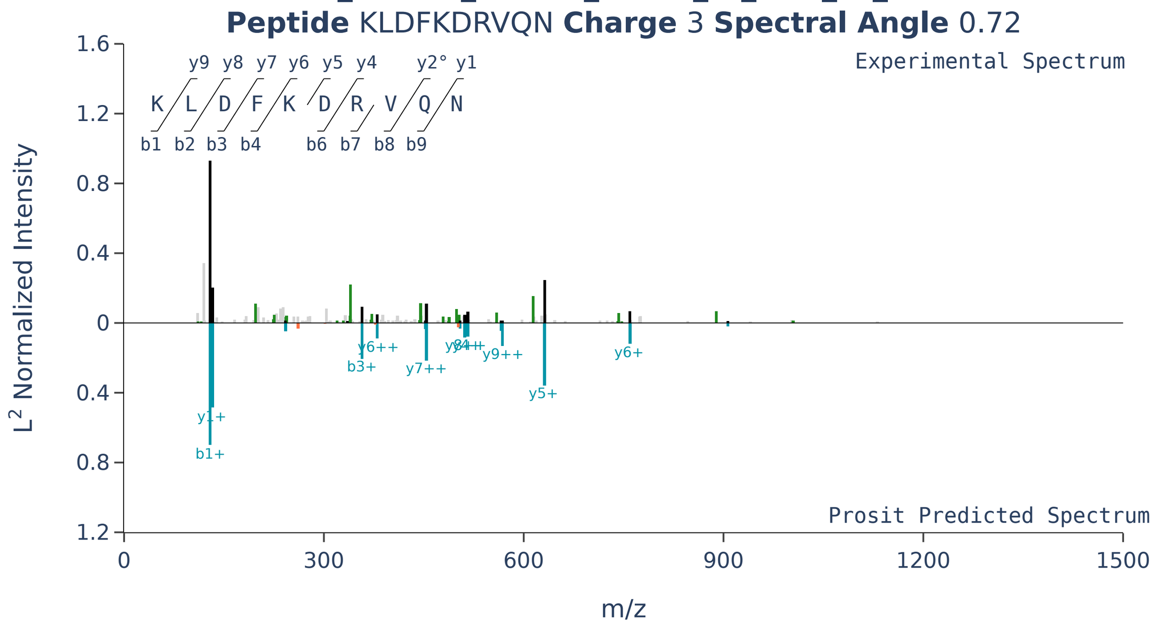

Source WSoh_221121_011221_HFGoe_L1_hTau_24h_R2 **Scan** 8693
Peptide AKHQPGGGKVQIINKKL **Charge** 2 **Spectral Angle** 0.8

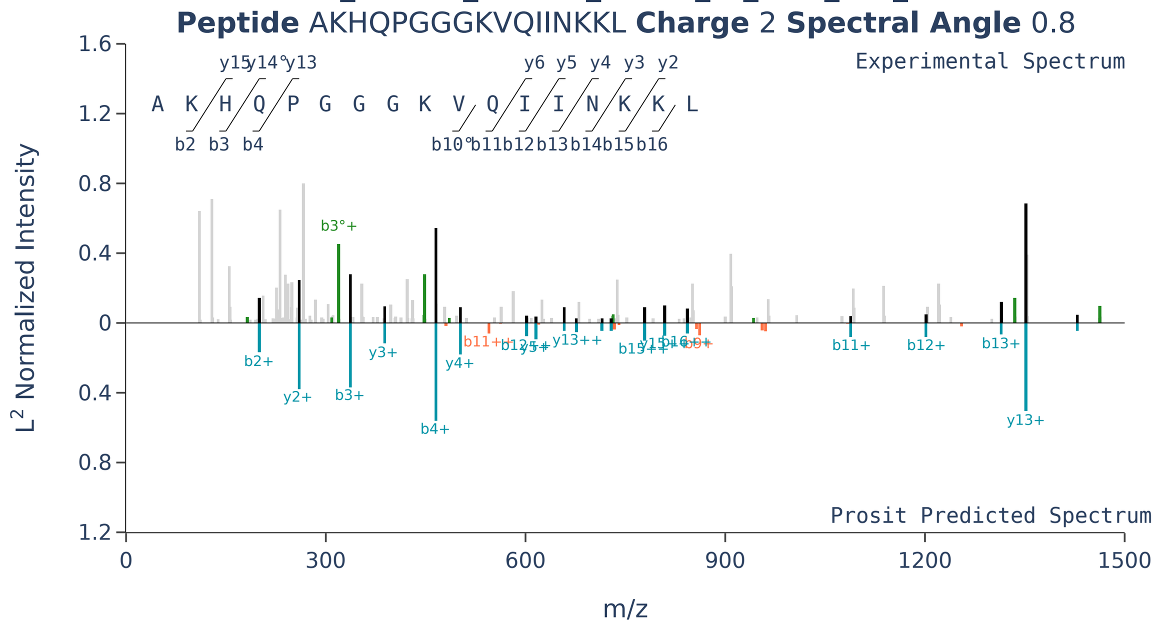

Source WSoh_221121_011221_HFGoe_L1_hTau_24h_R2 **Scan** 15201
Peptide GTPGSRDNIKHVPGGGSVQI **Charge** 3 **Spectral Angle** 0.82

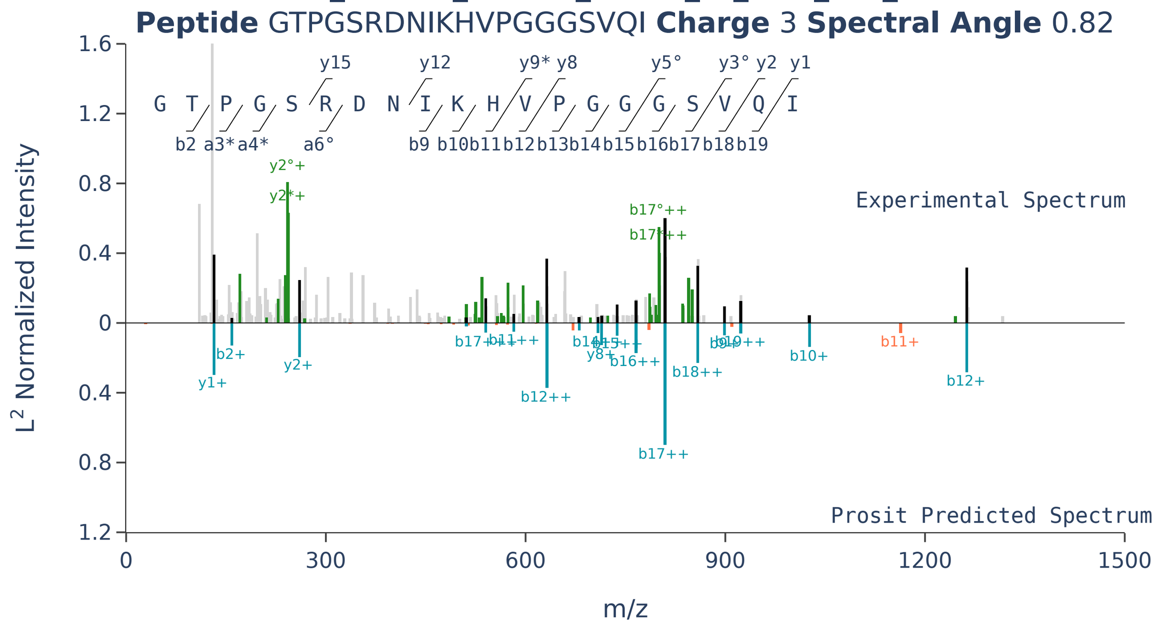

Source WSoh_221121_011221_HFGoe_L2_hTau_2h_R1 **Scan** 8428
Peptide KSEKLDFKDRVQSKA **Charge** 3 **Spectral Angle** 0.84

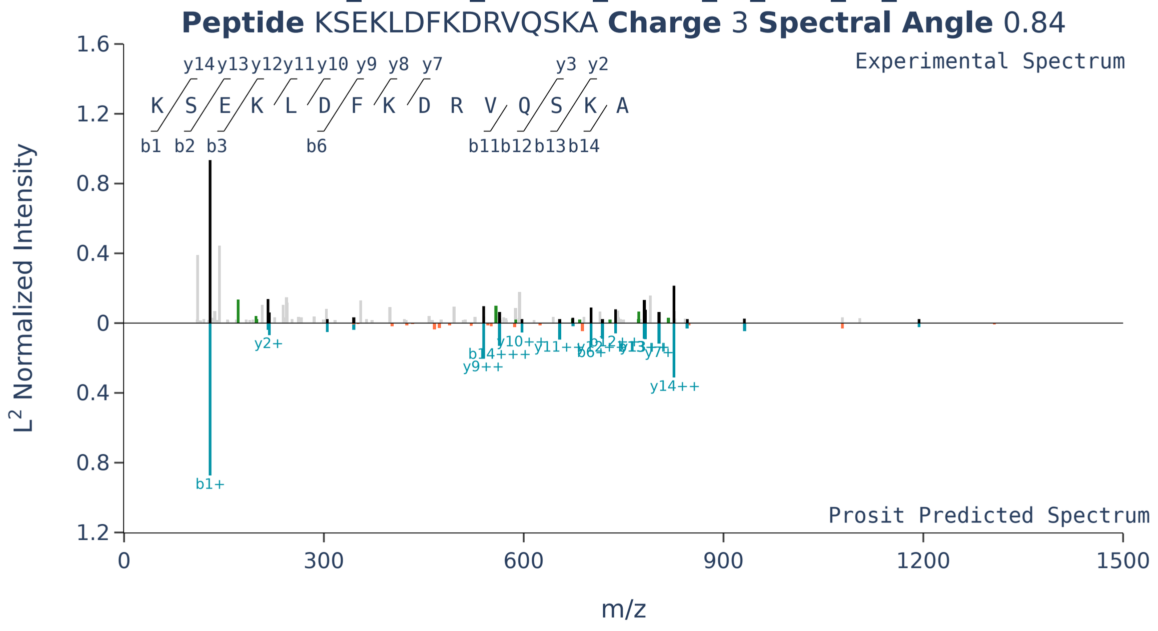

Source WSoh_221121_011221_HFGoe_L2_hTau_4h_R1 **Scan** 15953
Peptide SKKIGSLDNITHV **Charge** 3 **Spectral Angle** 0.83

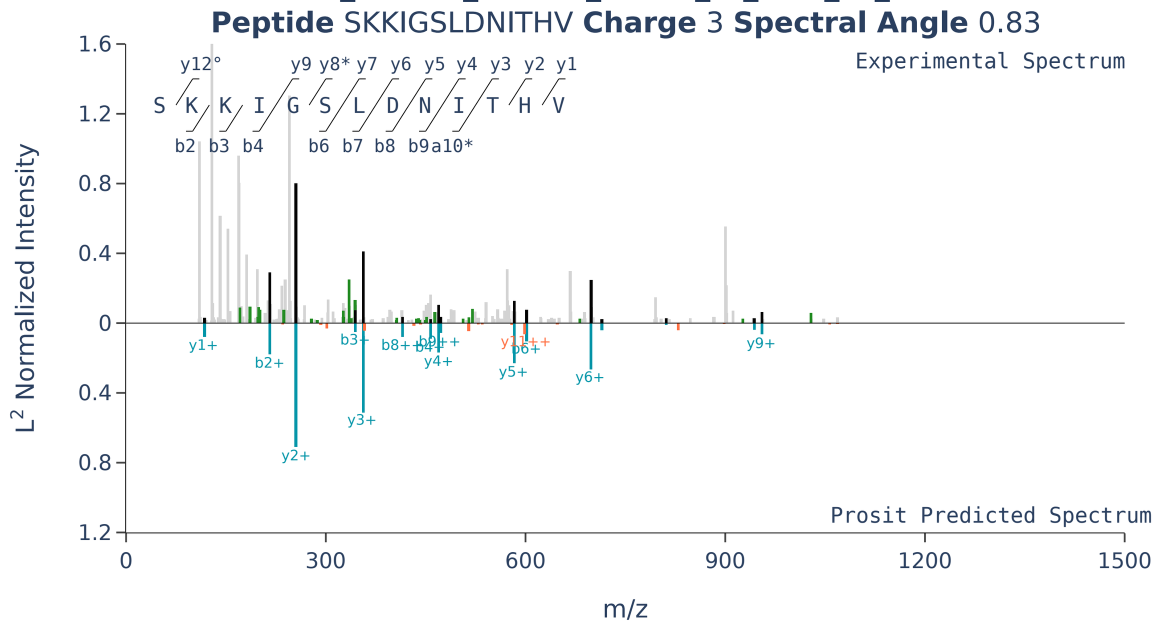

Source WSoh_221121_011221_HFGoe_L1_hTau_24h_R2 **Scan** 15758
Peptide EDTPSLPTPPTREPKKV **Charge** 4 **Spectral Angle** 0.87

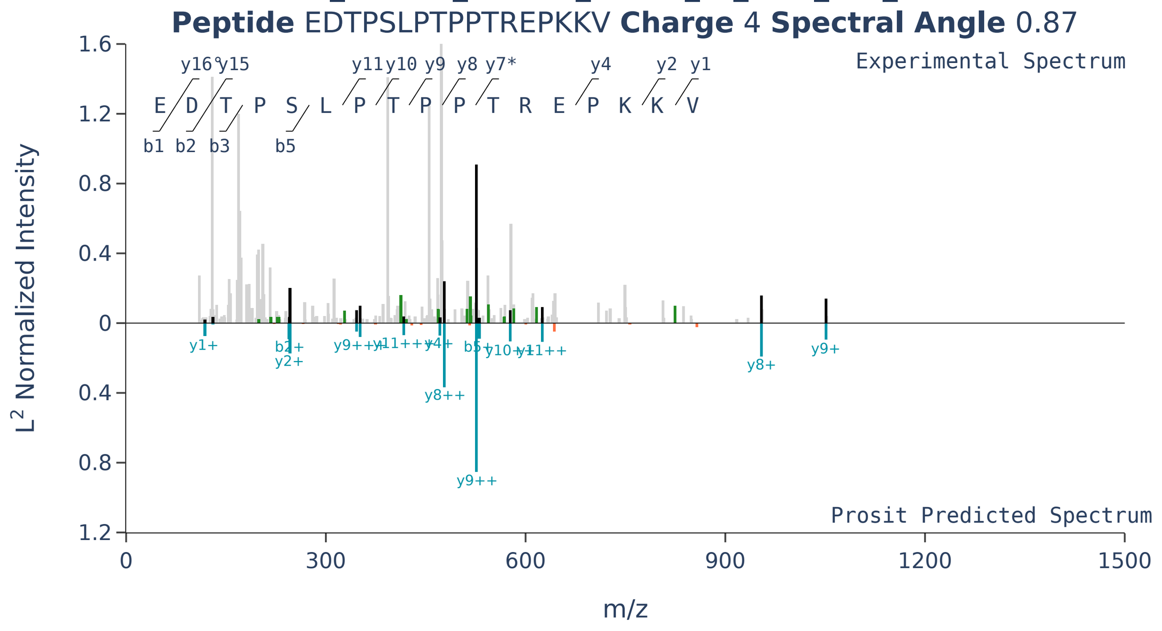

Source WSoh_221121_011221_HFGoe_L2_hTau_4h_R2 **Scan** 13931
Peptide KSQIINKKLDL **Charge** 3 **Spectral Angle** 0.79

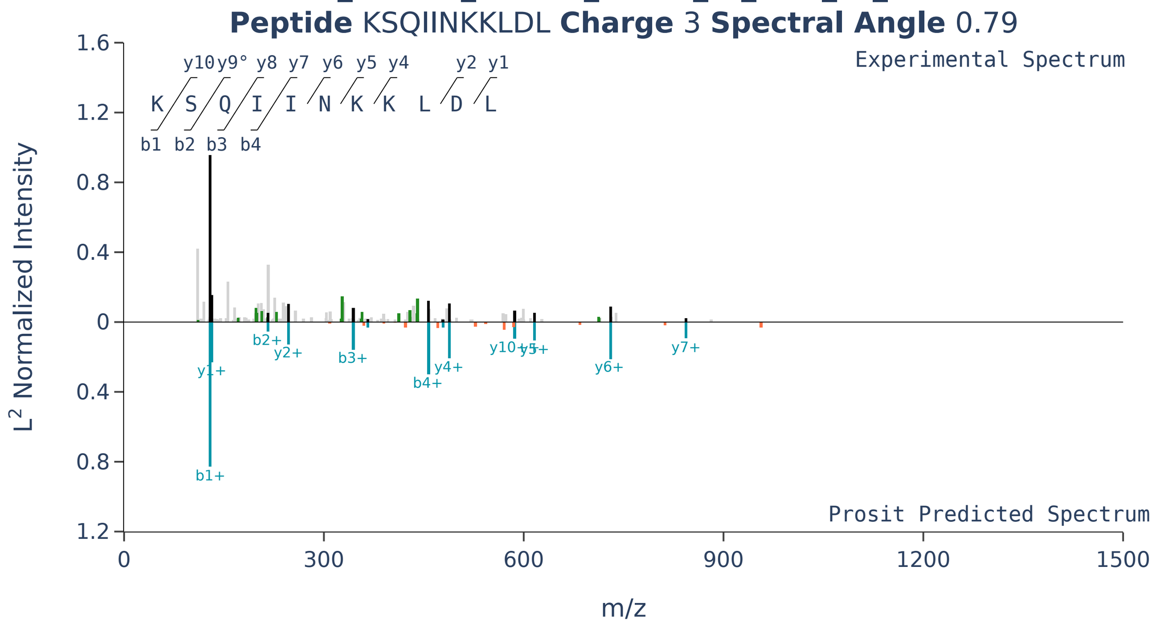

Source WSoh_221121_011221_HFGoe_L2_hTau_24h_R1 **Scan** 8937
Peptide SNVSSTGNIHHKPGGGQVEV **Charge** 3 **Spectral Angle** 0.9

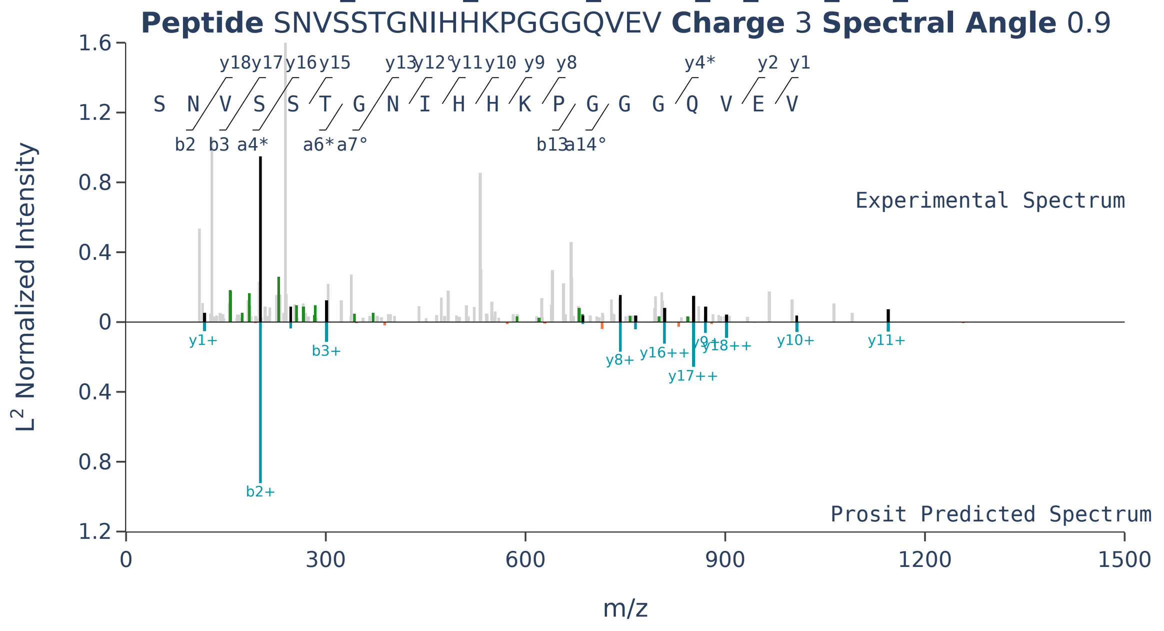

Source WSoh_221121_011221_HFGoe_L2_hTau_4h_R2 **Scan** 7177
Peptide GSDDTSPRHL **Charge** 3 **Spectral Angle** 0.85

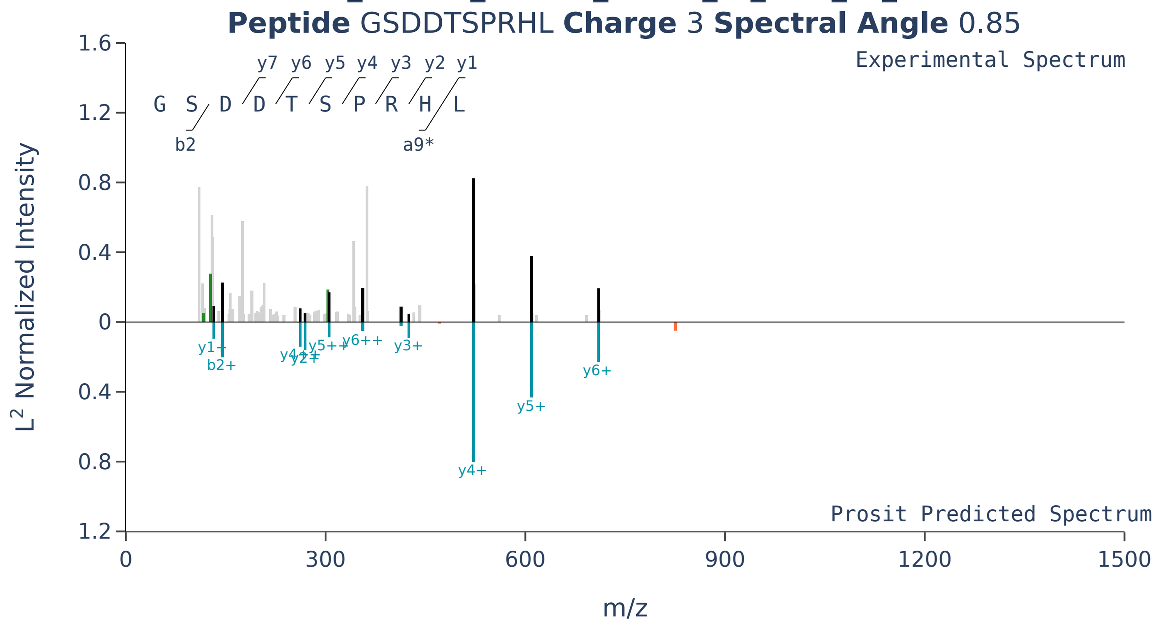

Source WSoh_221121_011221_HFGoe_L1_hTau_24h_R1 **Scan** 22861
Peptide KSQTAPVPMPDLKNV **Charge** 3 **Spectral Angle** 0.86

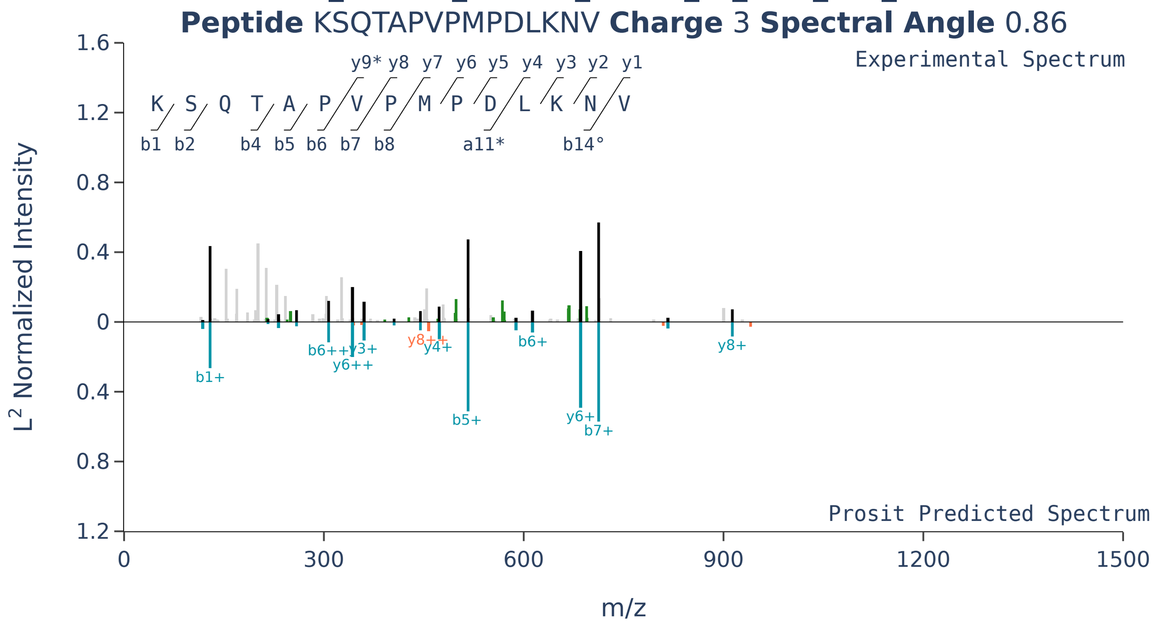

Source WSoh_221121_011221_HFGoe_L2_hTau_24h_R2 **Scan** 16443
Peptide SGDTSPRHLSNVSSTGSPLQTPTED **Charge** 3 **Spectral Angle** 0.88

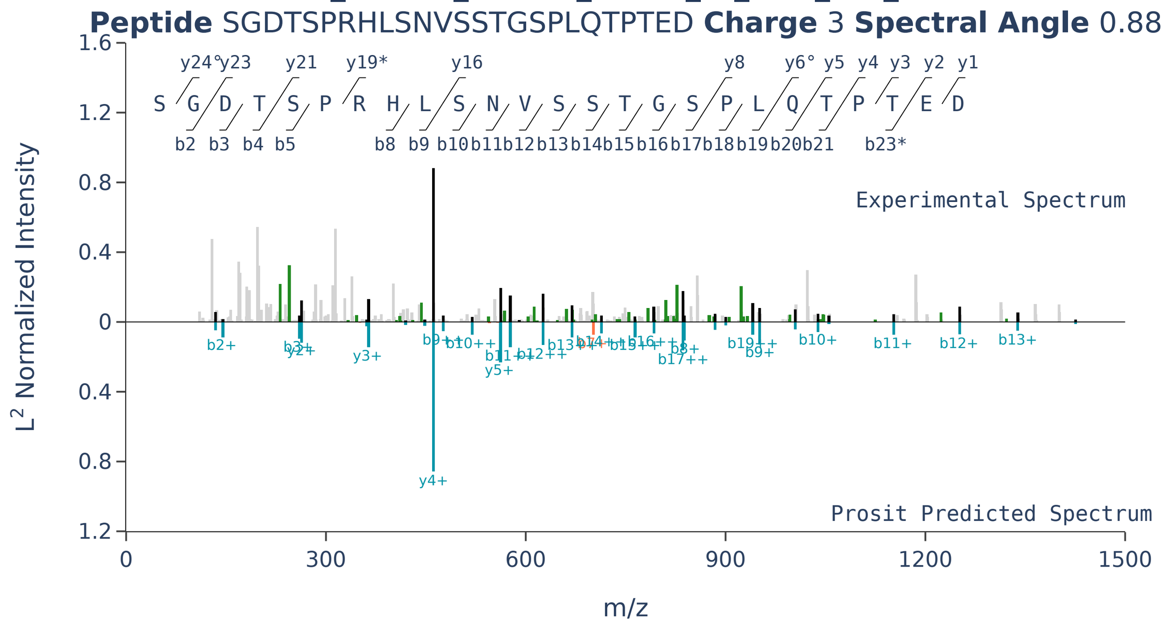

Source WSoh_221121_011221_HFGoe_L2_hTau_24h_R1 **Scan** 21445
Peptide TDAGLKEAEPRQEFEVM **Charge** 3 **Spectral Angle** 0.88

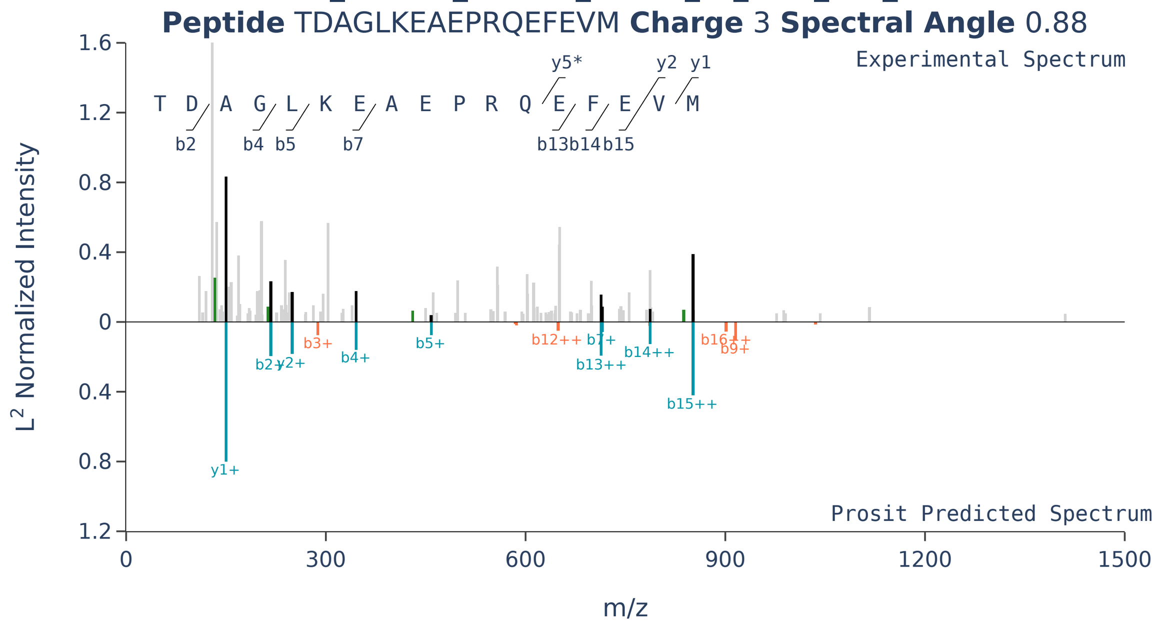

Source WSoh_221121_011221_HFGoe_L2_hTau_24h_R2 **Scan** 12139
Peptide LSKVTSKCGSLNIHHKPGGGQVEV **Charge** 3 **Spectral Angle** 0.79

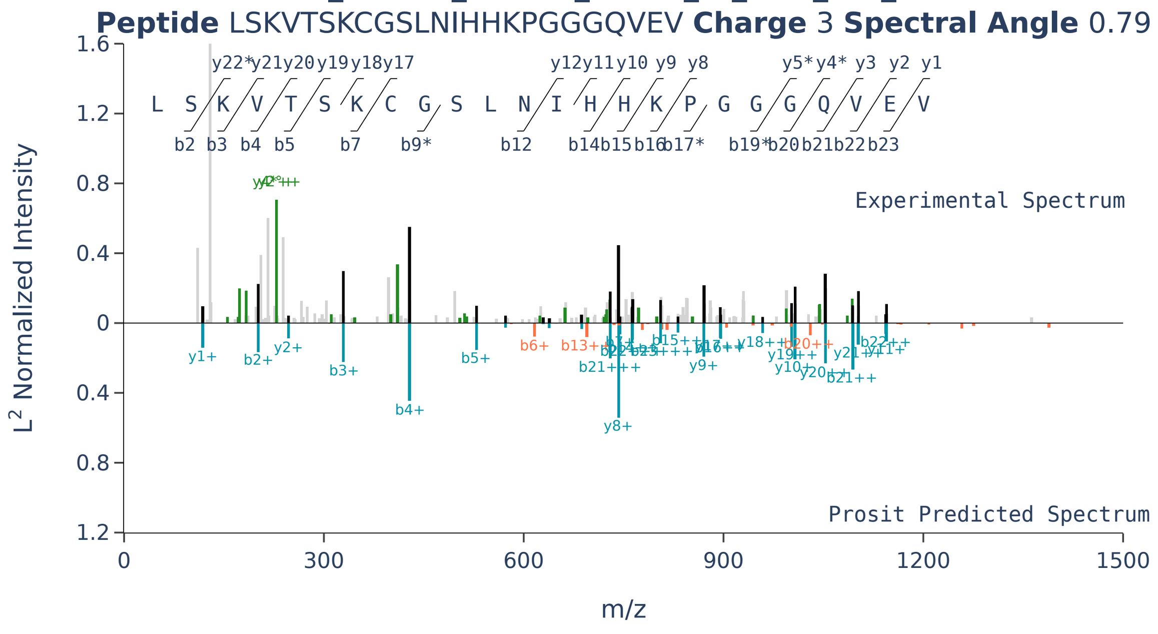

Source WSoh_221121_011221_HFGoe_L2_hTau_2h_R2 **Scan** 7450
Peptide QSGSLGNIHH **Charge** 3 **Spectral Angle** 0.8

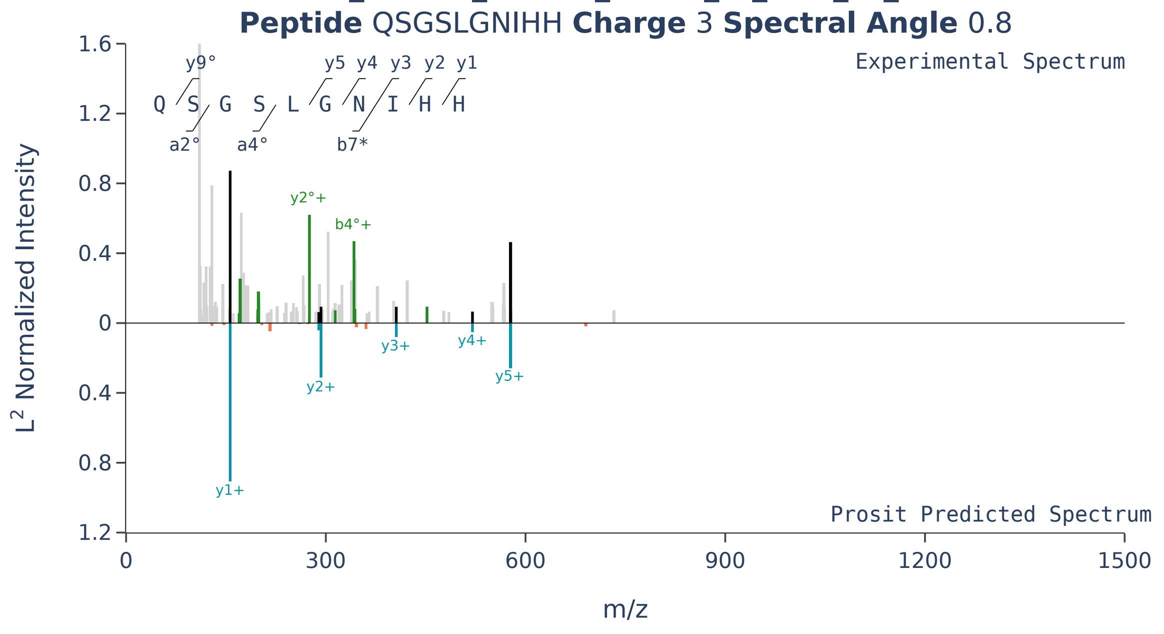

Source WSoh_221121_011221_HFGoe_L1_hTau_24h_R2 **Scan** 11178
Peptide SSSLGNIHHKPGGGQVEV **Charge** 2 **Spectral Angle** 0.81

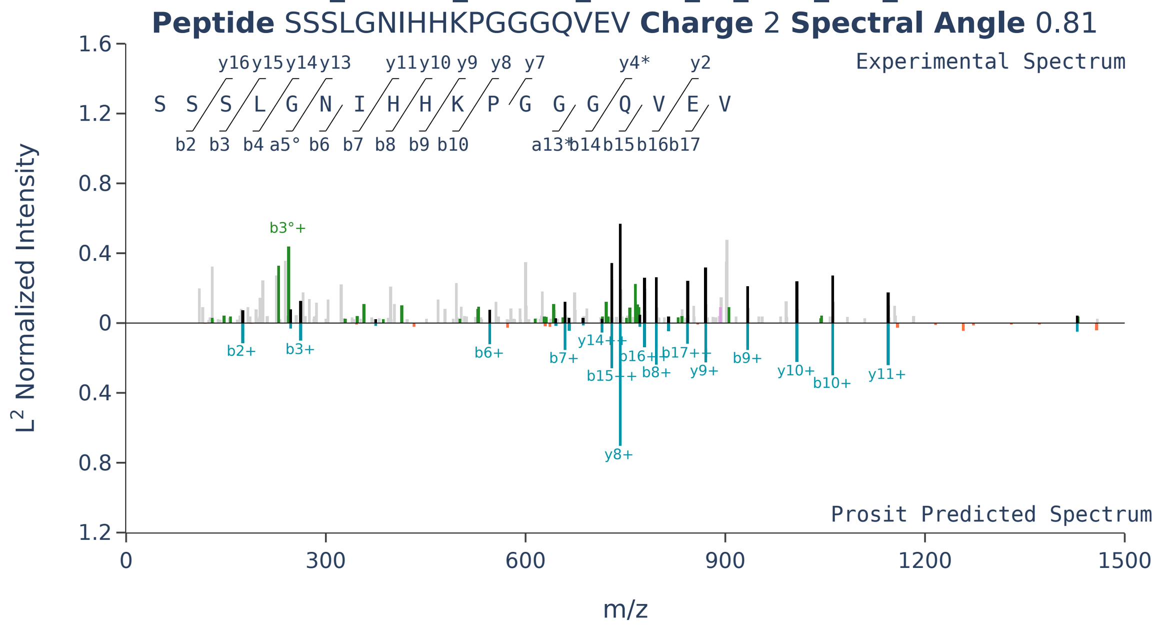

Source WSoh_221121_011221_HFGoe_L1_hTau_24h_R1 **Scan** 8385
Peptide VRTPPKSPSSARL **Charge** 3 **Spectral Angle** 0.85

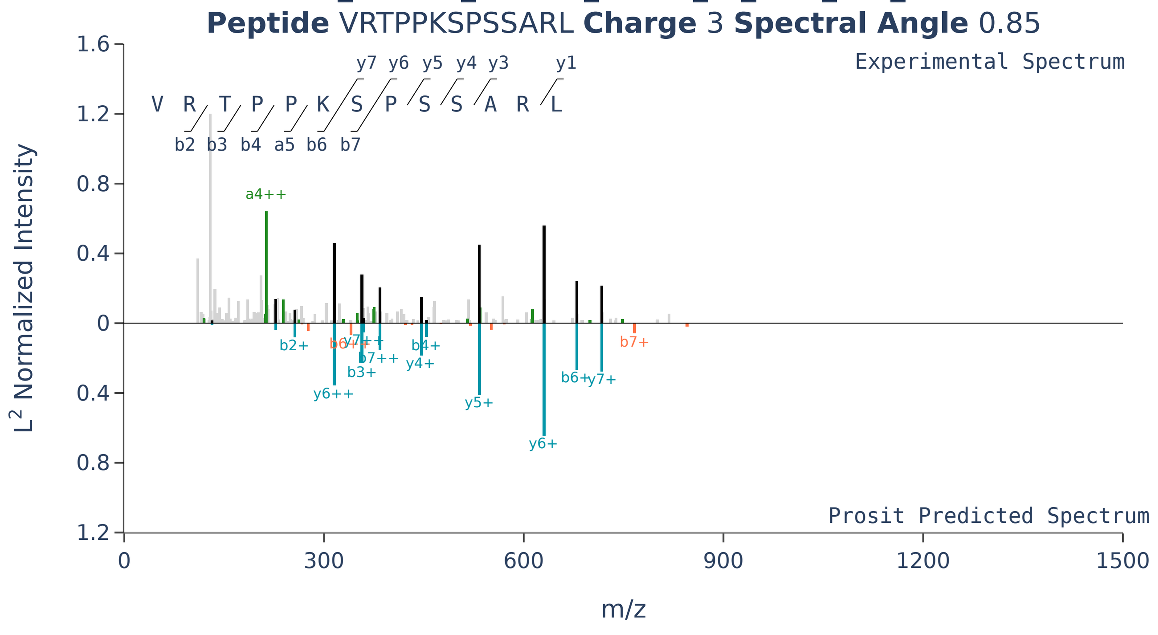

Source WSoh_221121_011221_HFGoe_L1_hTau_24h_R1 **Scan** 12150
Peptide KLDLSNVQSKCSKD **Charge** 2 **Spectral Angle** 0.84

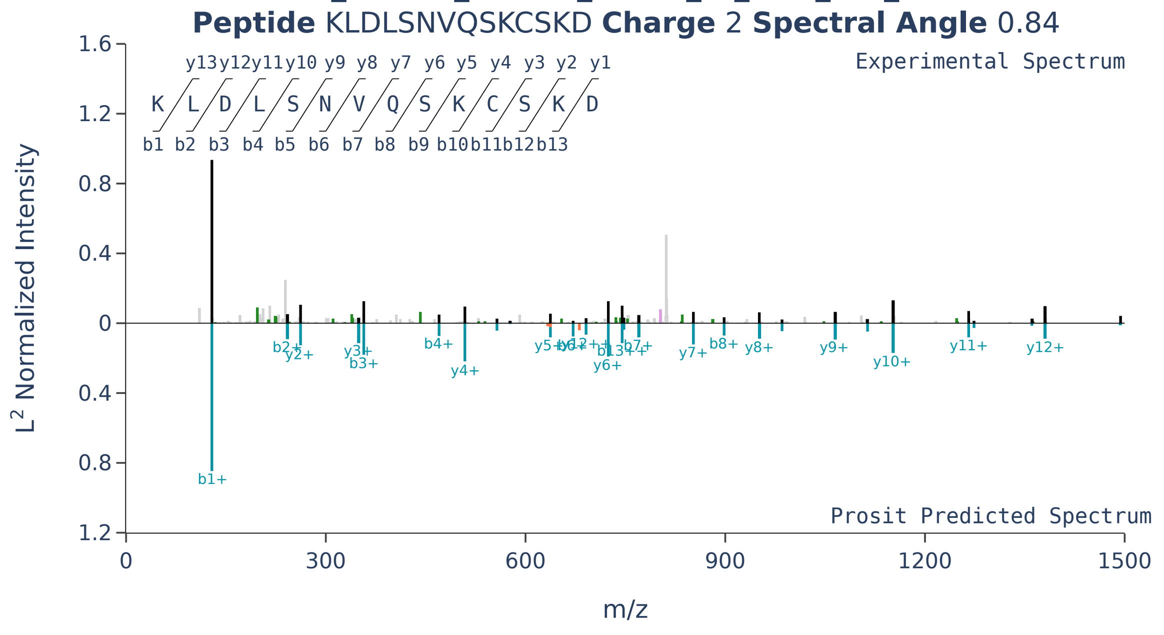

Source WSoh_221121_011221_HFGoe_L1_hTau_4h_R2 **Scan** 6830
Peptide SAKHQPGGGKVQIINK **Charge** 4 **Spectral Angle** 0.83

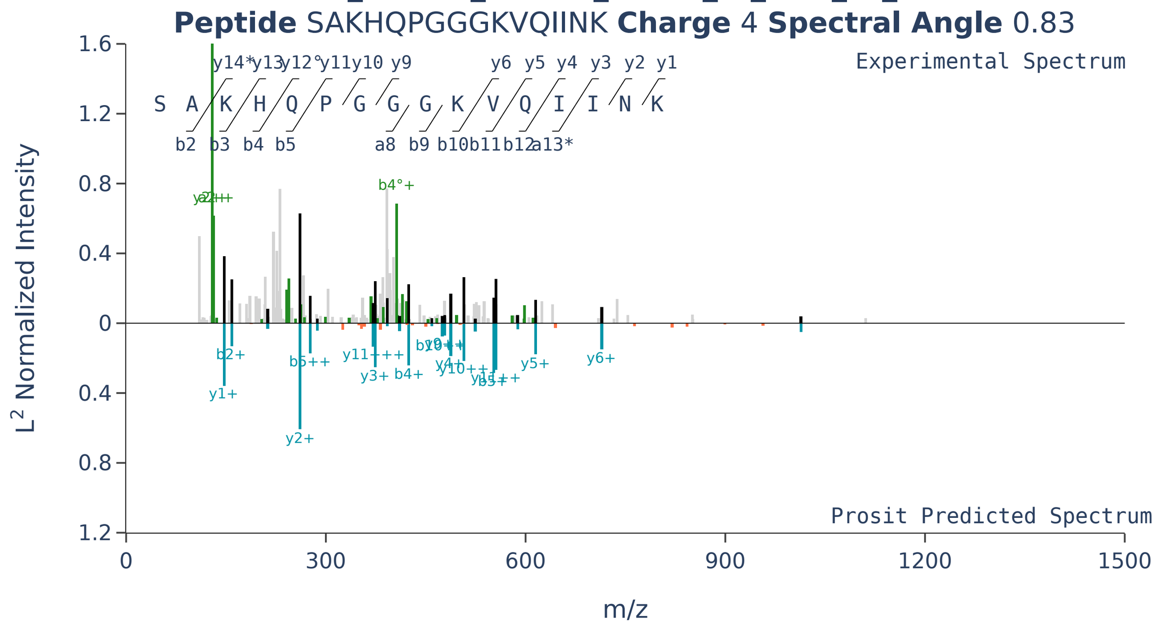

Source WSoh_221121_011221_HFGoe_L1_hTau_4h_R2 **Scan** 5604
Peptide VRTPPKSPSSAKSA **Charge** 2 **Spectral Angle** 0.87

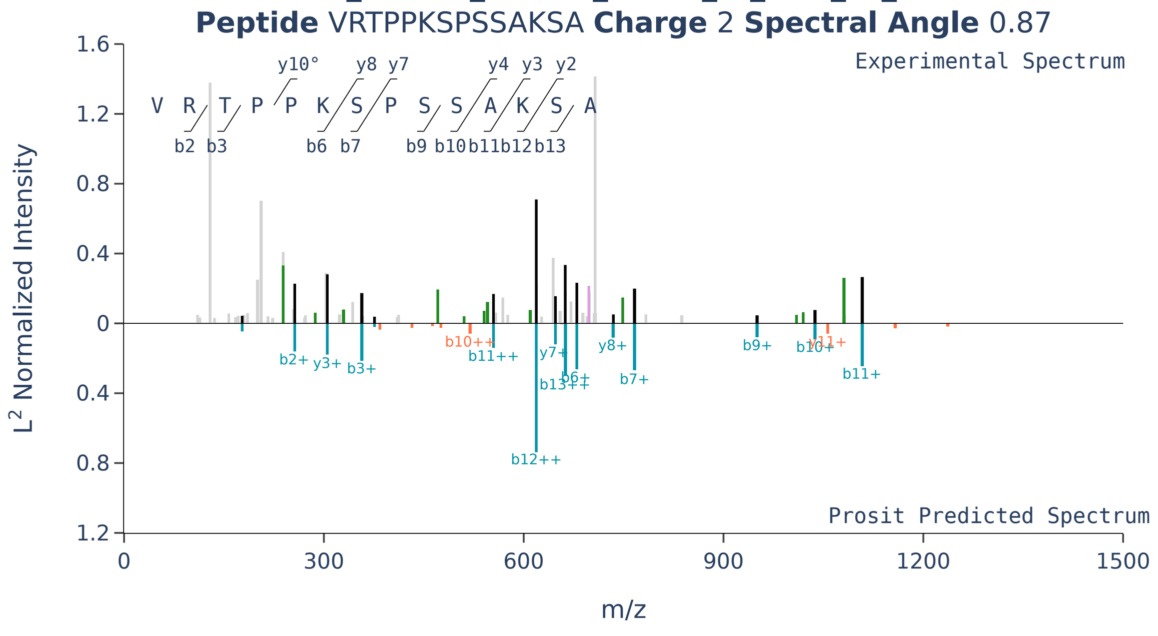

Source WSoh_221121_011221_HFGoe_L1_hTau_24h_R1 **Scan** 24406
Peptide DAGLGDTPSLEDEAAGHVTQARMV **Charge** 3 **Spectral Angle** 0.85

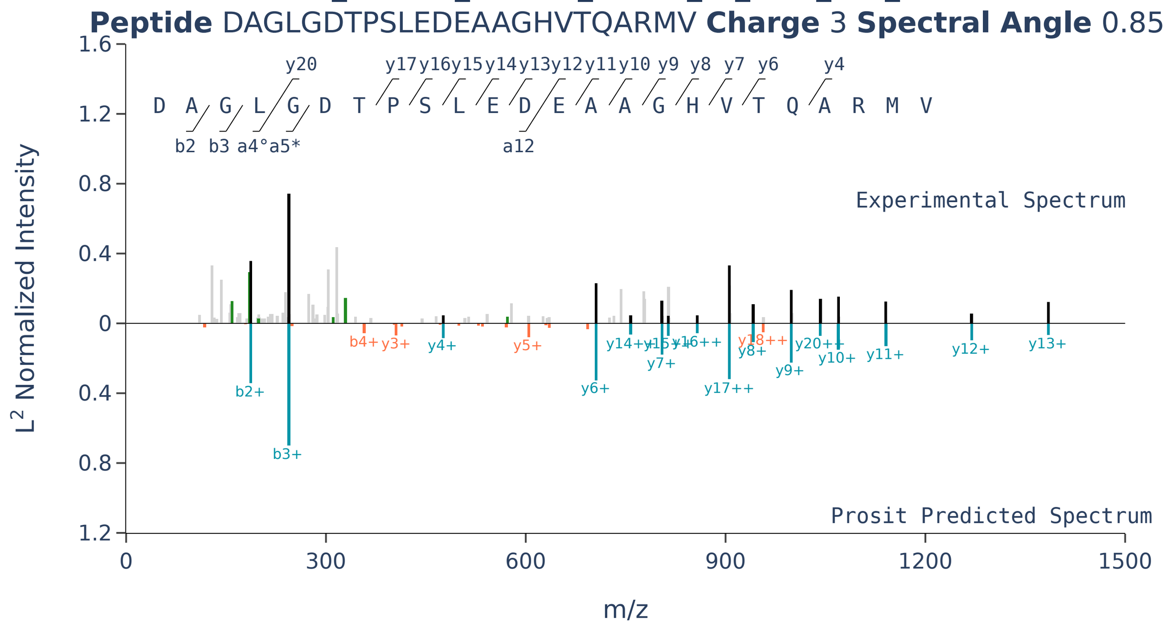

Source WSoh_221121_011221_HFGoe_L1_hTau_24h_R1 **Scan** 7026
Peptide SNIHHKPGGGQVEV **Charge** 3 **Spectral Angle** 0.7

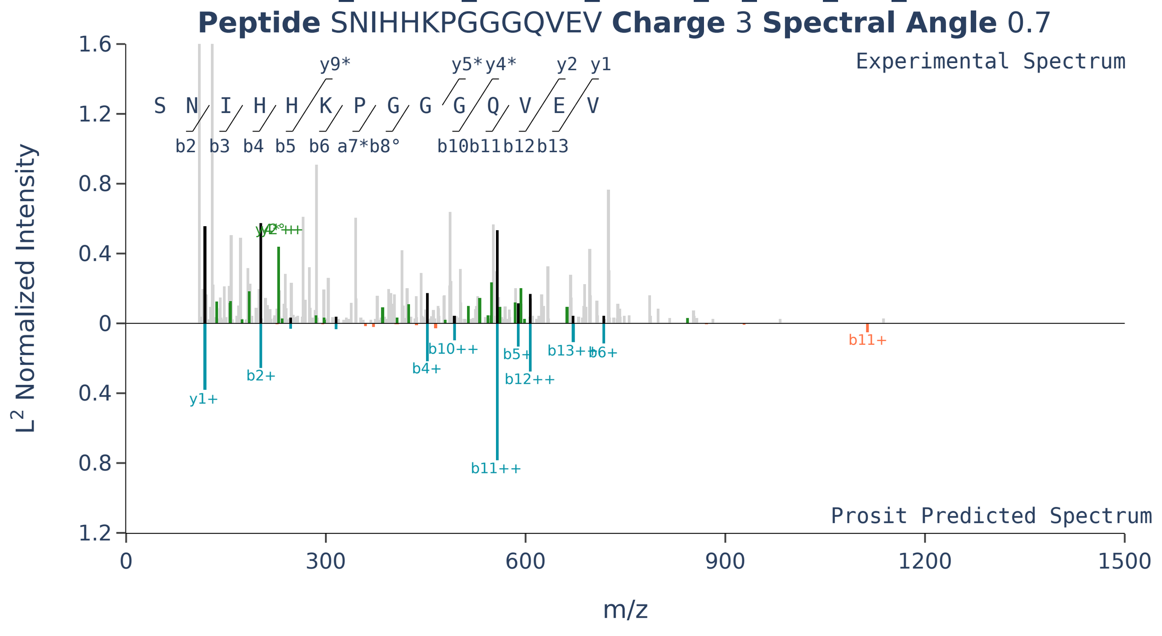

Source WSoh_221121_011221_HFGoe_L1_hTau_24h_R1 **Scan** 10623
Peptide DLSNVQST **Charge** 1 **Spectral Angle** 0.78

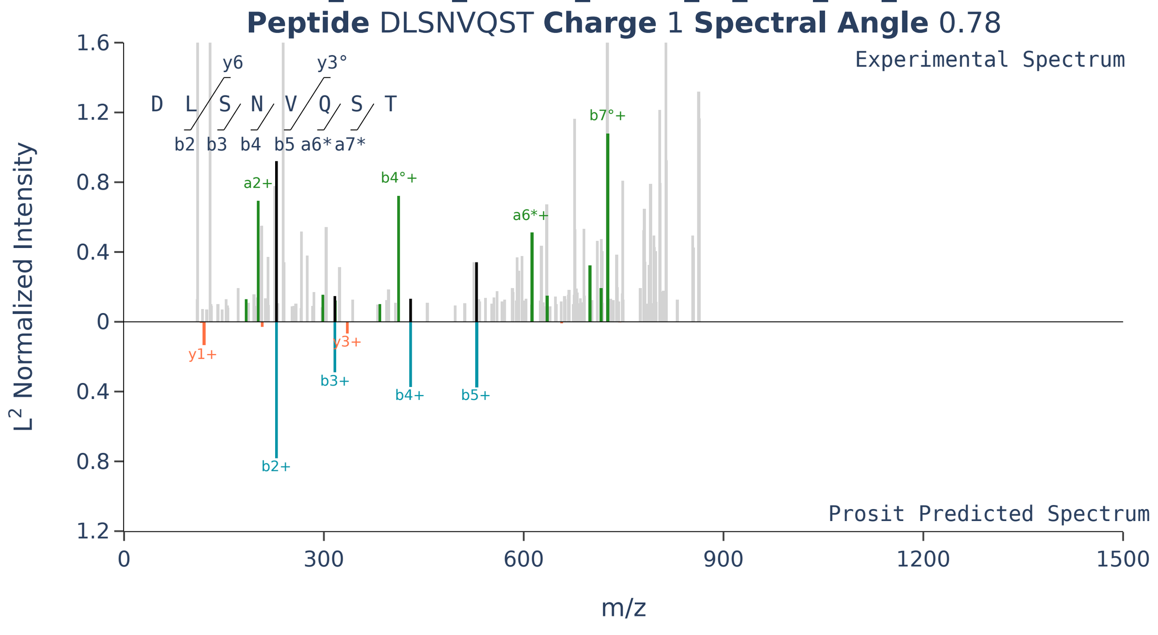

Source WSoh_221121_011221_HFGoe_L1_hTau_24h_R1 **Scan** 12489
Peptide DSLGNIHHKPGGGQVEV **Charge** 2 **Spectral Angle** 0.83

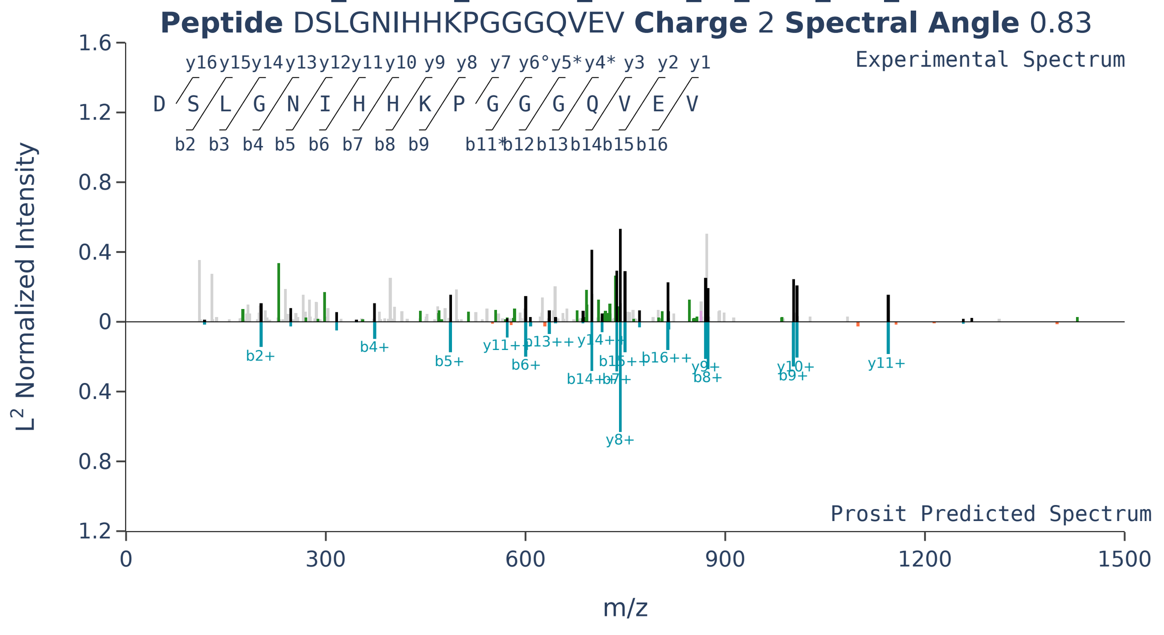

Source WSoh_221121_011221_HFGoe_L1_hTau_24h_R1 **Scan** 8915
Peptide RIPAKTPPAPKTPPSGG **Charge** 3 **Spectral Angle** 0.77

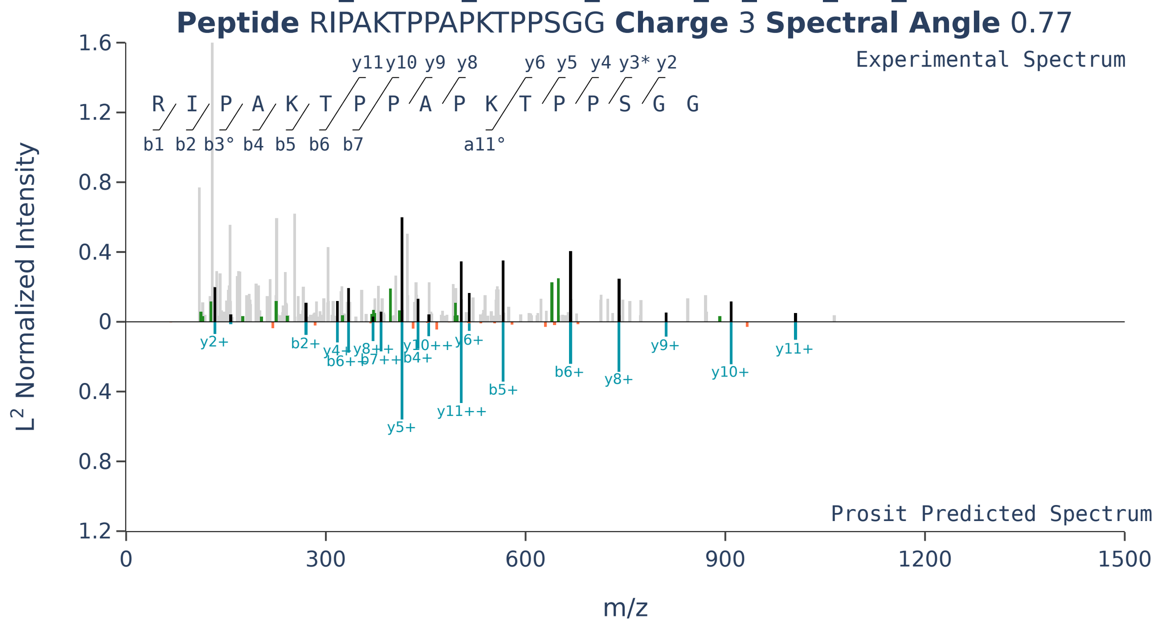

Source WSoh_221121_011221_HFGoe_L2_hTau_2h_R2 **Scan** 16032
Peptide NVSSTGSIDMVDEPGSE **Charge** 2 **Spectral Angle** 0.89

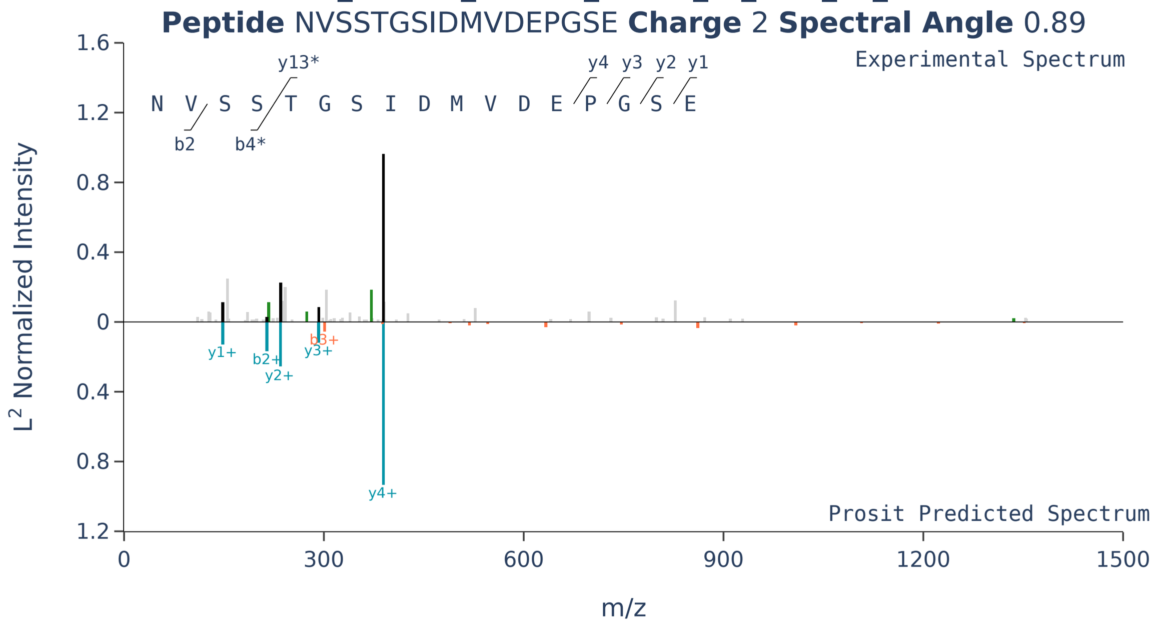

Source WSoh_221121_011221_HFGoe_L2_hTau_24h_R1 **Scan** 5702
Peptide RQEKIETH **Charge** 2 **Spectral Angle** 0.86

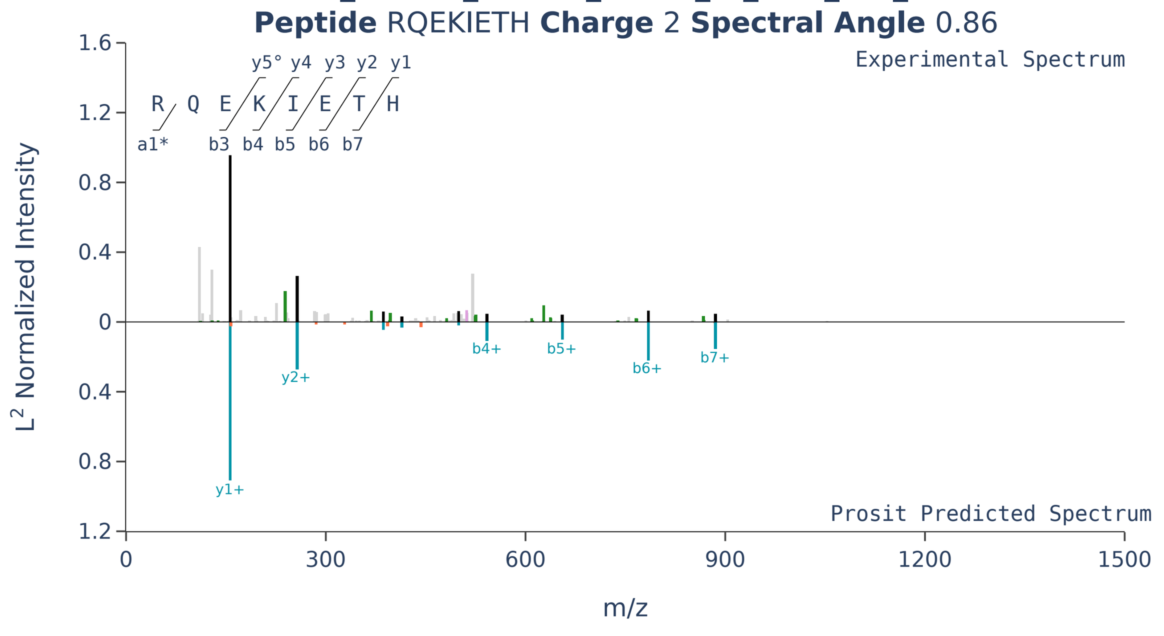

Source WSoh_221121_011221_HFGoe_L2_hTau_24h_R1 **Scan** 20648
Peptide DFKDRVQSKLTF **Charge** 3 **Spectral Angle** 0.87

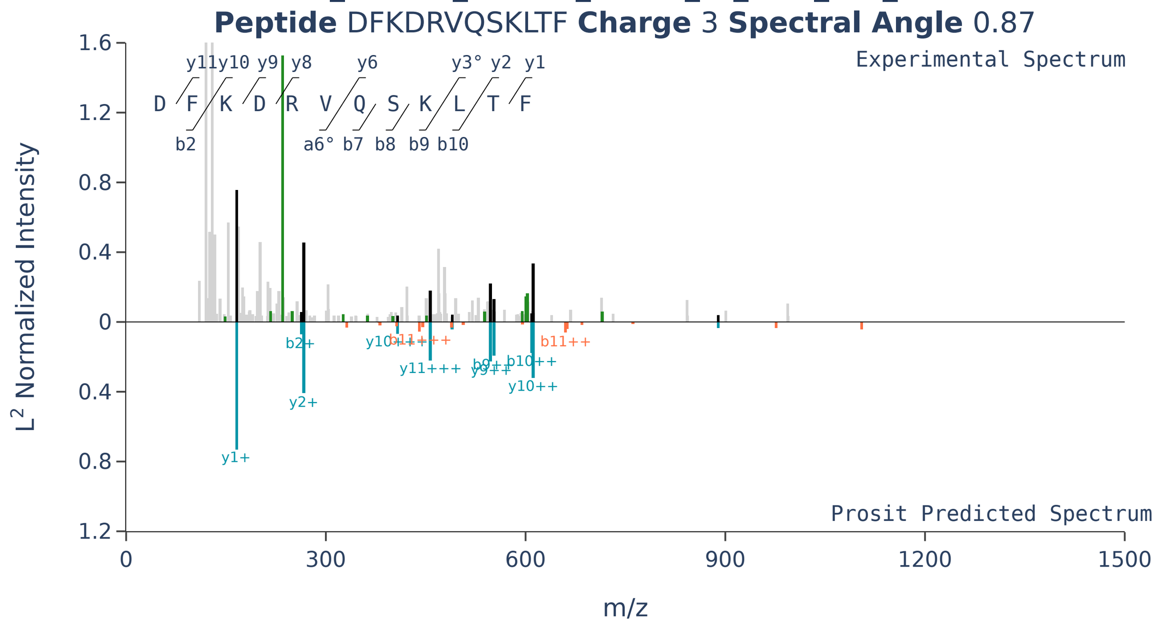

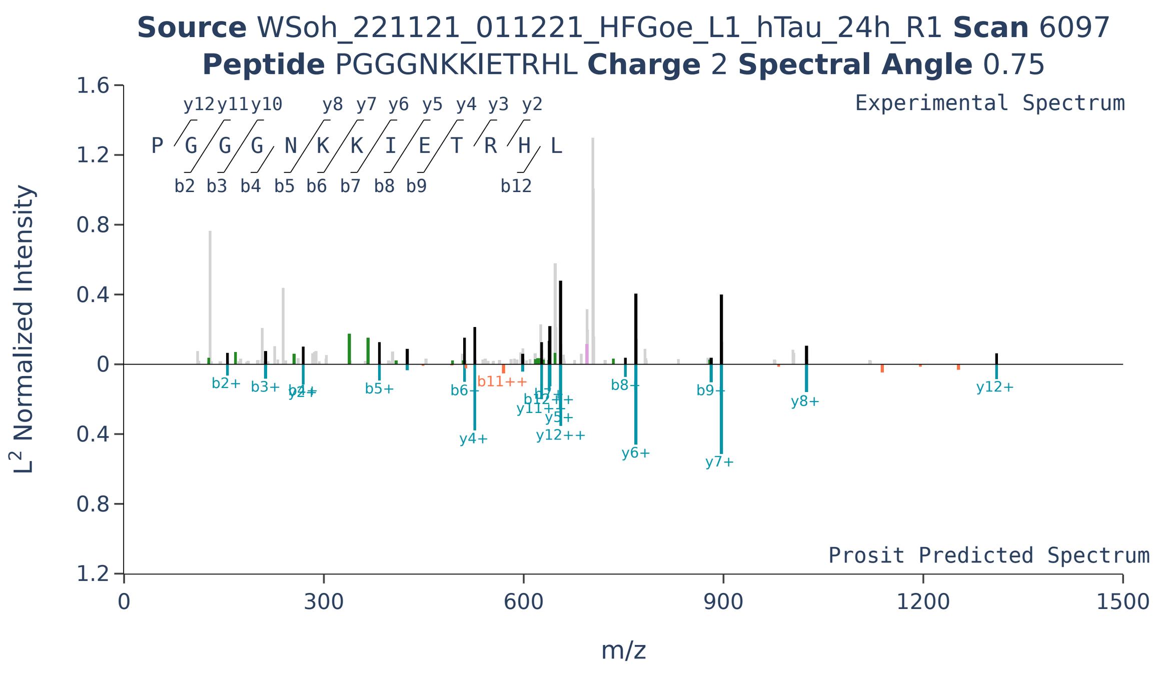
Source WSoh_221121_011221_HFGoe_L1_hTau_24h_R1 Scan 6097
Peptide PGGGNKKIETRHL Charge 2 Spectral Angle 0.75
Experimental Spectrum
Prosit Predicted Spectrum
L² Normalized Intensity
m/z

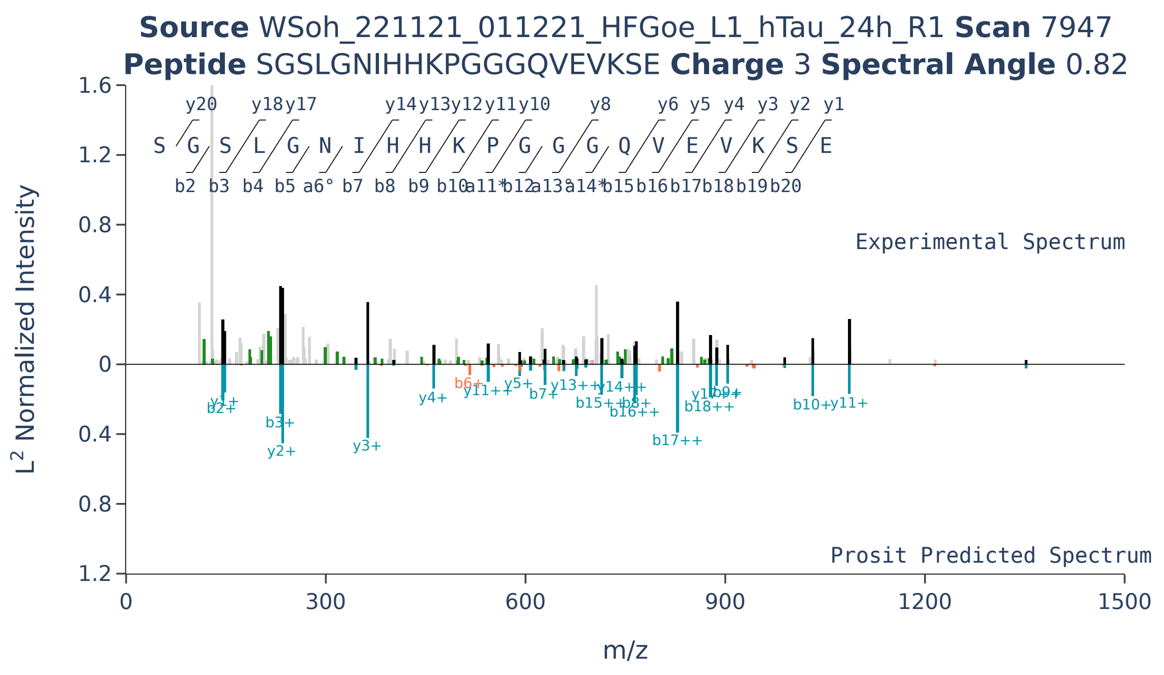
Source WSoh_221121_011221_HFGoe_L1_hTau_24h_R1 Scan 7947
Peptide SGSLGNIHHKPGGGQVEVKSE Charge 3 Spectral Angle 0.82
Experimental Spectrum
Prosit Predicted Spectrum
L² Normalized Intensity
m/z

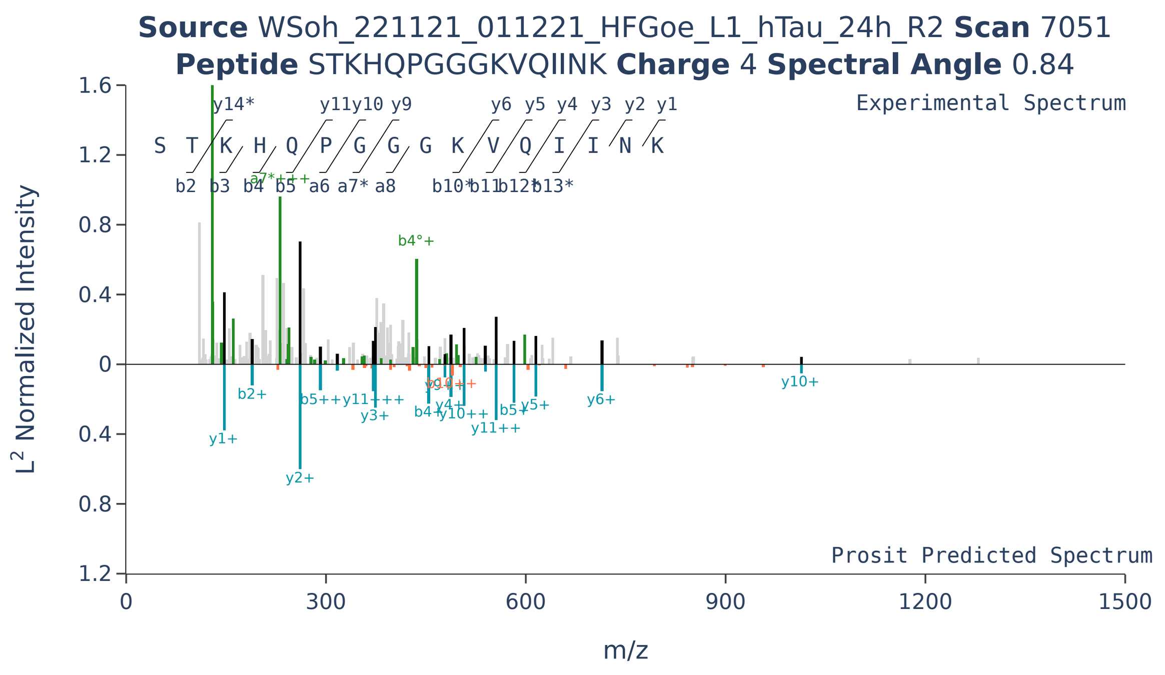
Source WSoh_221121_011221_HFGoe_L1_hTau_24h_R2 Scan 7051
Peptide STKHQPGGGKVQIINK Charge 4 Spectral Angle 0.84
Experimental Spectrum
Prosit Predicted Spectrum
L² Normalized Intensity
m/z

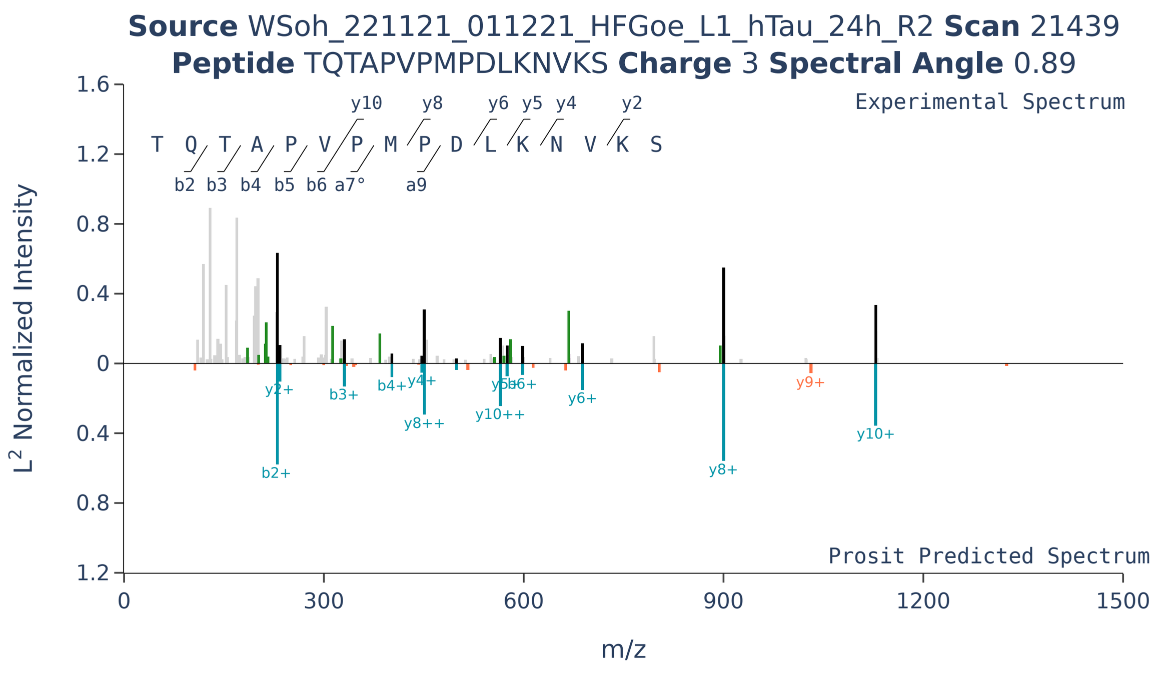
Source WSoh_221121_011221_HFGoe_L1_hTau_24h_R2 Scan 21439
Peptide TQTAPVPMPDLKNVKS Charge 3 Spectral Angle 0.89
Experimental Spectrum
Prosit Predicted Spectrum
L² Normalized Intensity
m/z

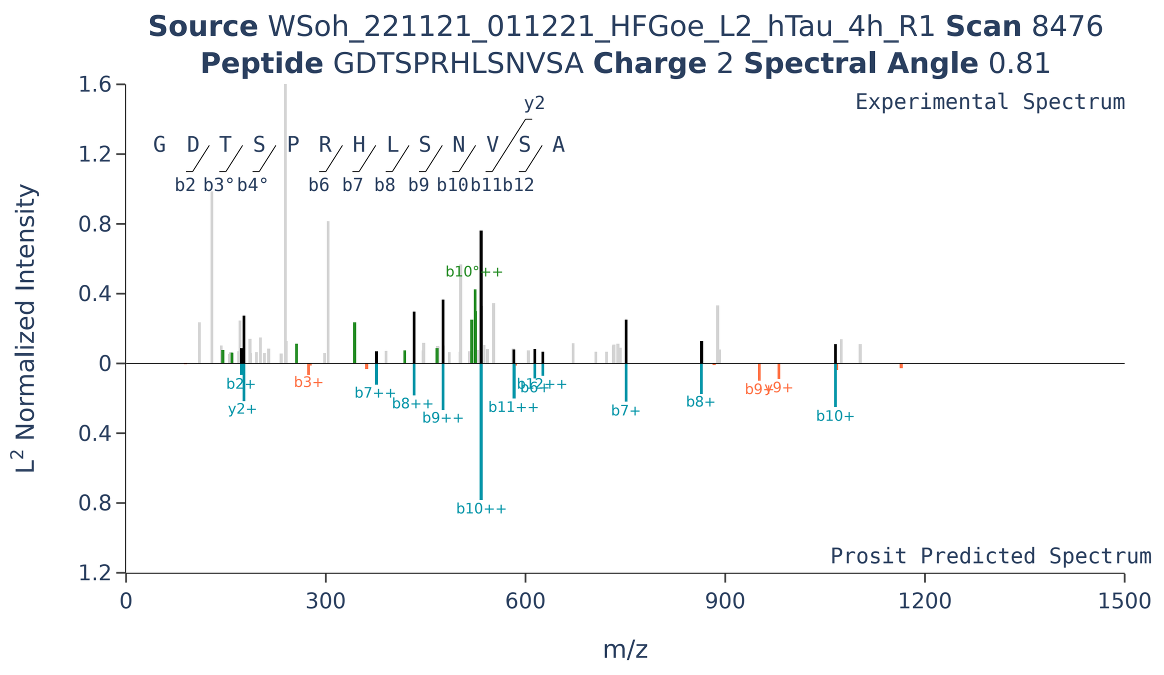
Source WSoh_221121_011221_HFGoe_L2_hTau_4h_R1 Scan 8476
Peptide GDTSPRHLSNVSA Charge 2 Spectral Angle 0.81
Experimental Spectrum
Prosit Predicted Spectrum
L² Normalized Intensity
m/z

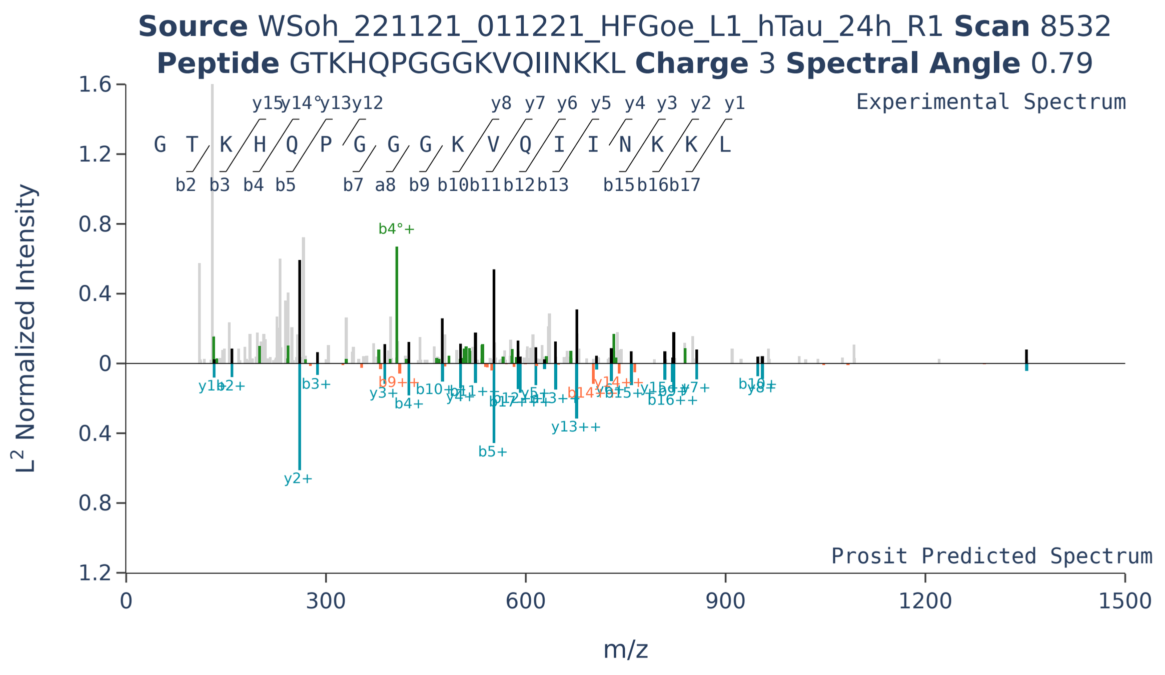
Source WSoh_221121_011221_HFGoe_L1_hTau_24h_R1 Scan 8532
Peptide GTKHQPGGGKVQIINKKL Charge 3 Spectral Angle 0.79
Experimental Spectrum
Prosit Predicted Spectrum
L² Normalized Intensity
m/z

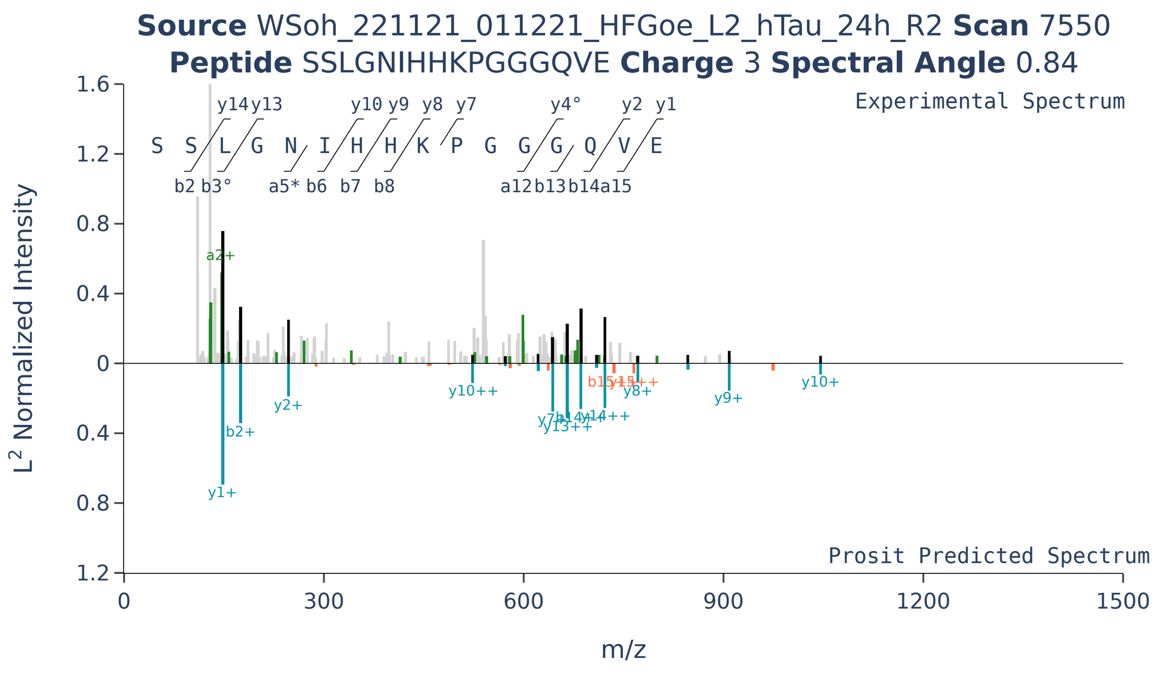
Source WSoh_221121_011221_HFGoe_L2_hTau_24h_R2 Scan 7550
Peptide SSLGNIHHKPGGGQVE Charge 3 Spectral Angle 0.84
Experimental Spectrum
Prosit Predicted Spectrum
L² Normalized Intensity
m/z

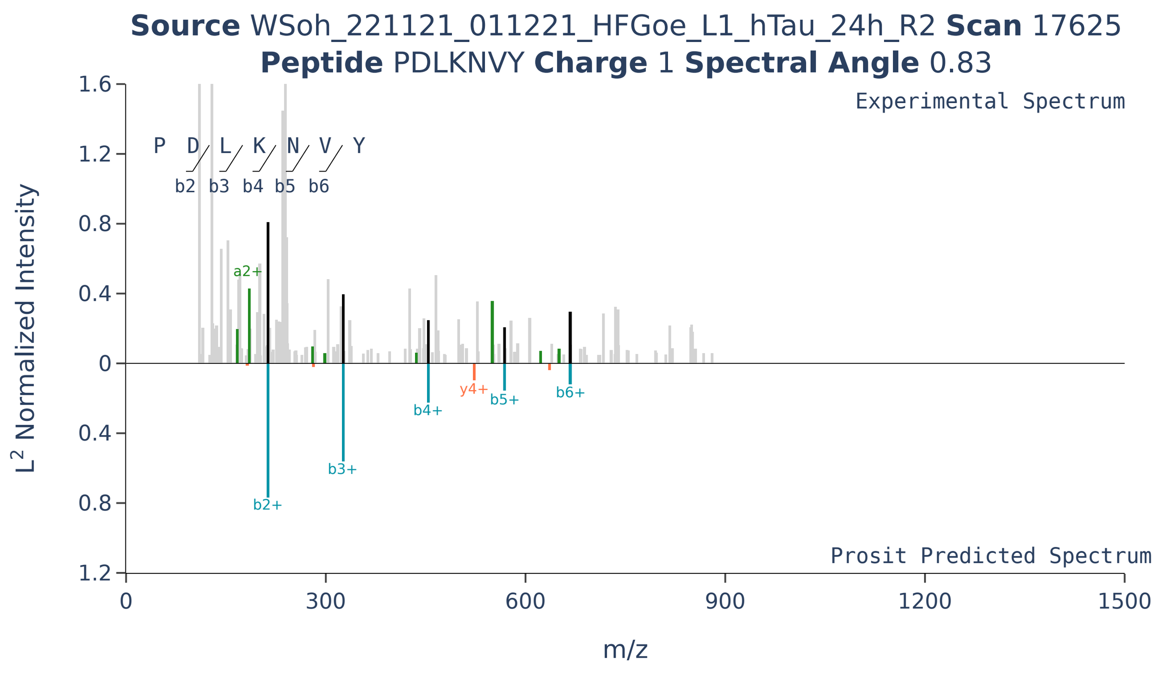
Source WSoh_221121_011221_HFGoe_L1_hTau_24h_R2 Scan 17625
Peptide PDLKNVY Charge 1 Spectral Angle 0.83
Experimental Spectrum
Prosit Predicted Spectrum
L² Normalized Intensity
m/z

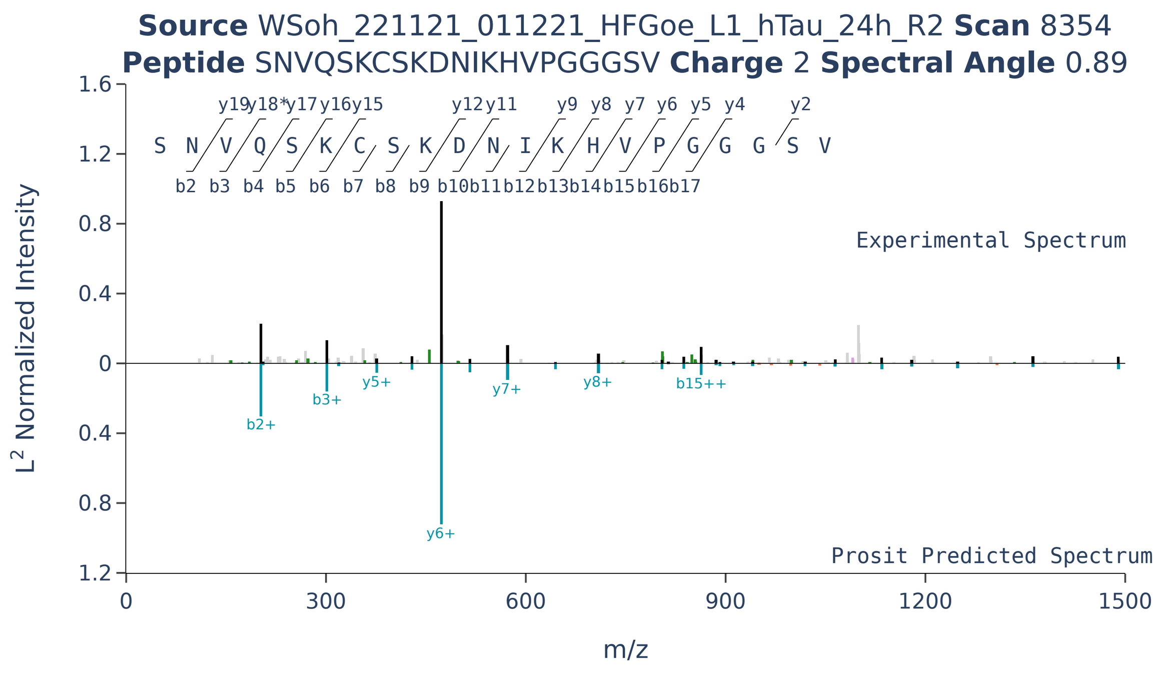
Source WSoh_221121_011221_HFGoe_L1_hTau_24h_R2 Scan 8354
Peptide SNVQSKCSKDNIKHVPGGGSV Charge 2 Spectral Angle 0.89
Experimental Spectrum
Prosit Predicted Spectrum
L² Normalized Intensity
m/z

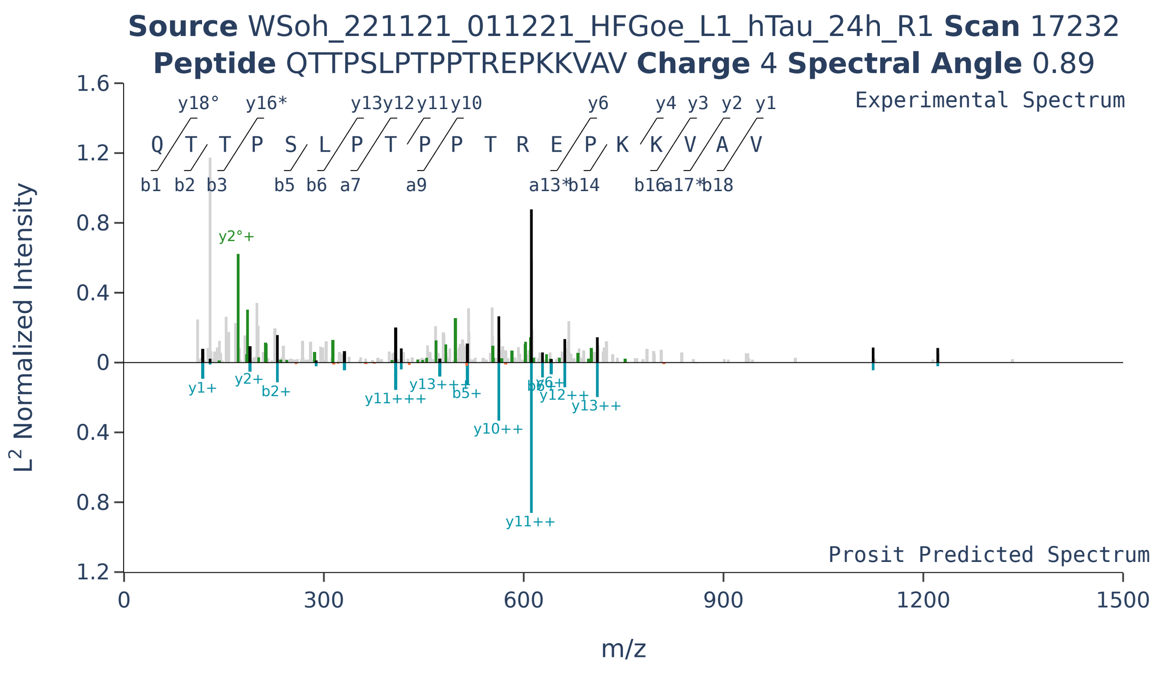
Source WSoh_221121_011221_HFGoe_L1_hTau_24h_R1 Scan 17232
Peptide QTTPSLPTPPTREPKKVAV Charge 4 Spectral Angle 0.89
Experimental Spectrum
Prosit Predicted Spectrum
L² Normalized Intensity
m/z

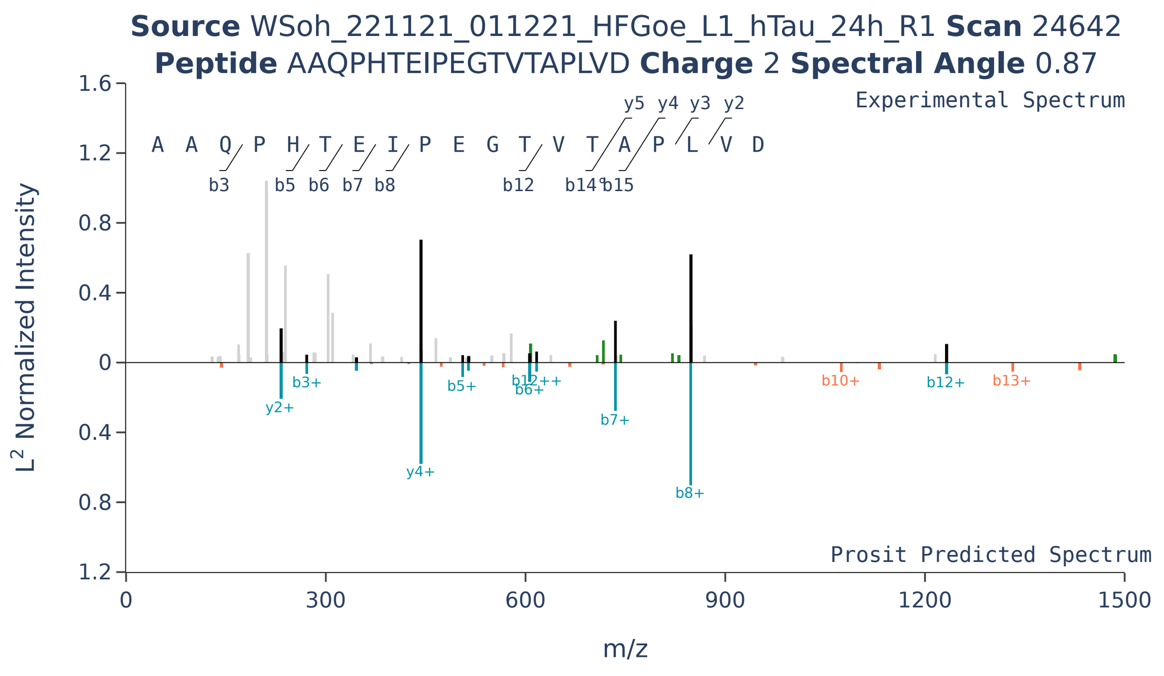
Source WSoh_221121_011221_HFGoe_L1_hTau_24h_R1 Scan 24642
Peptide AAQPHTEIPEGTVTAPLVD Charge 2 Spectral Angle 0.87
Experimental Spectrum
Prosit Predicted Spectrum
L² Normalized Intensity
m/z

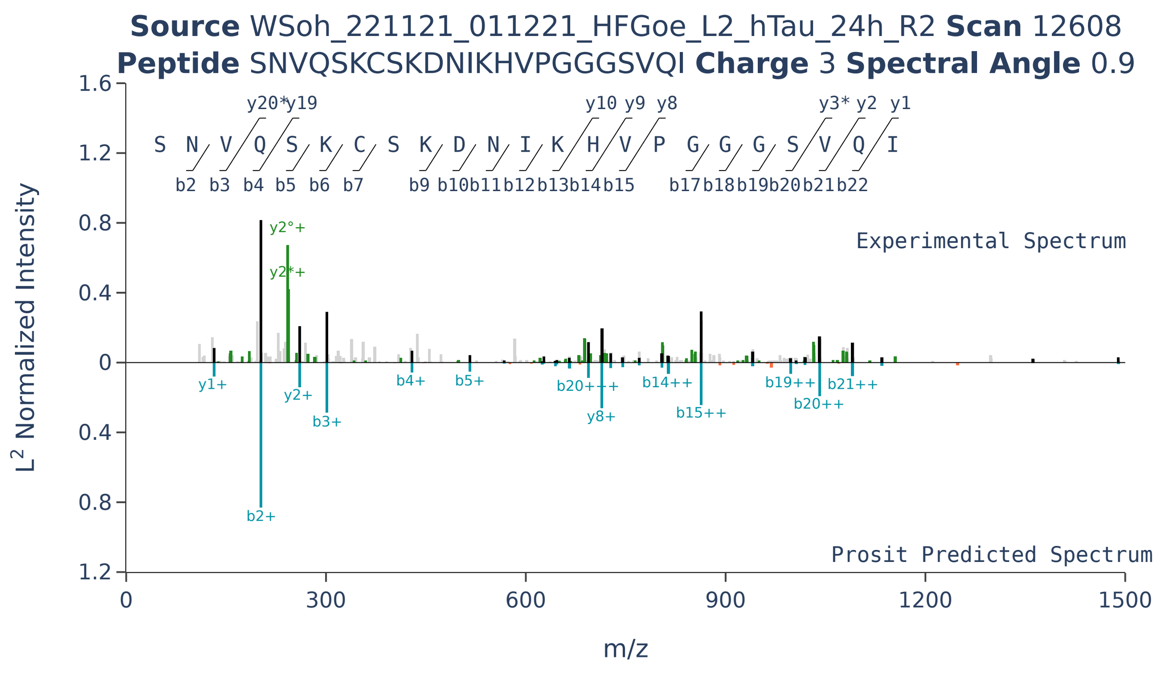
Source WSoh_221121_011221_HFGoe_L2_hTau_24h_R2 Scan 12608
Peptide SNVQSKCSKDNIKHVPGGGSVQI Charge 3 Spectral Angle 0.9
Experimental Spectrum
Prosit Predicted Spectrum
L² Normalized Intensity
m/z

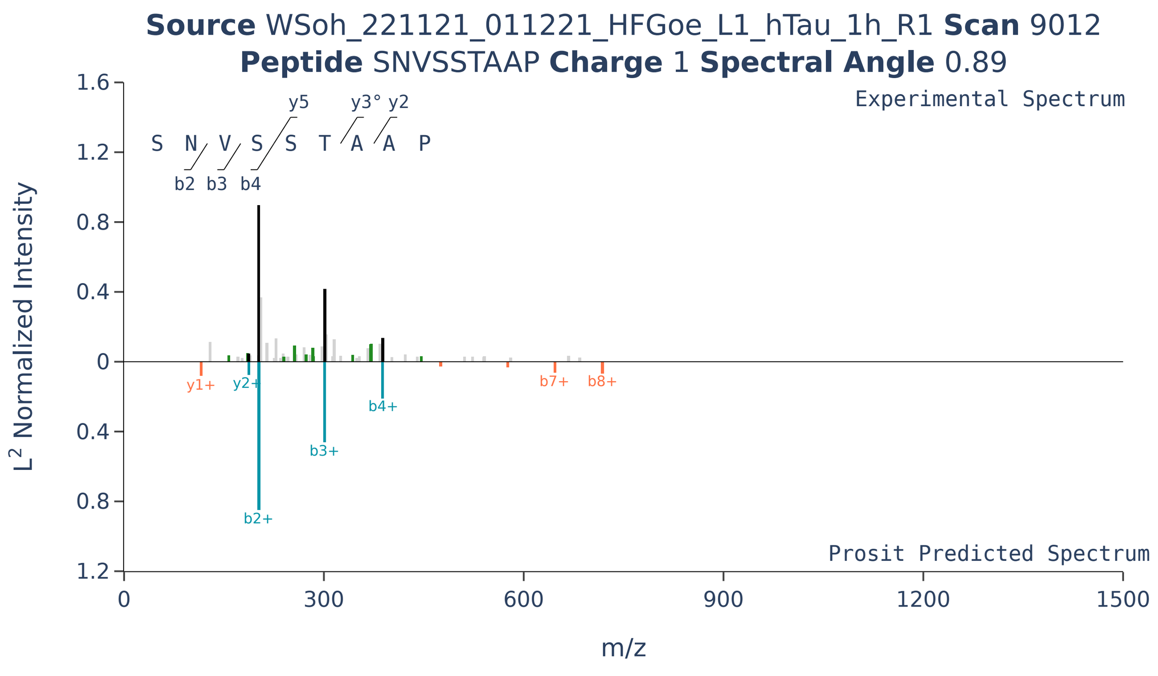
Source WSoh_221121_011221_HFGoe_L1_hTau_1h_R1 Scan 9012
Peptide SNVSSTAAP Charge 1 Spectral Angle 0.89
Experimental Spectrum
Prosit Predicted Spectrum
L² Normalized Intensity
m/z

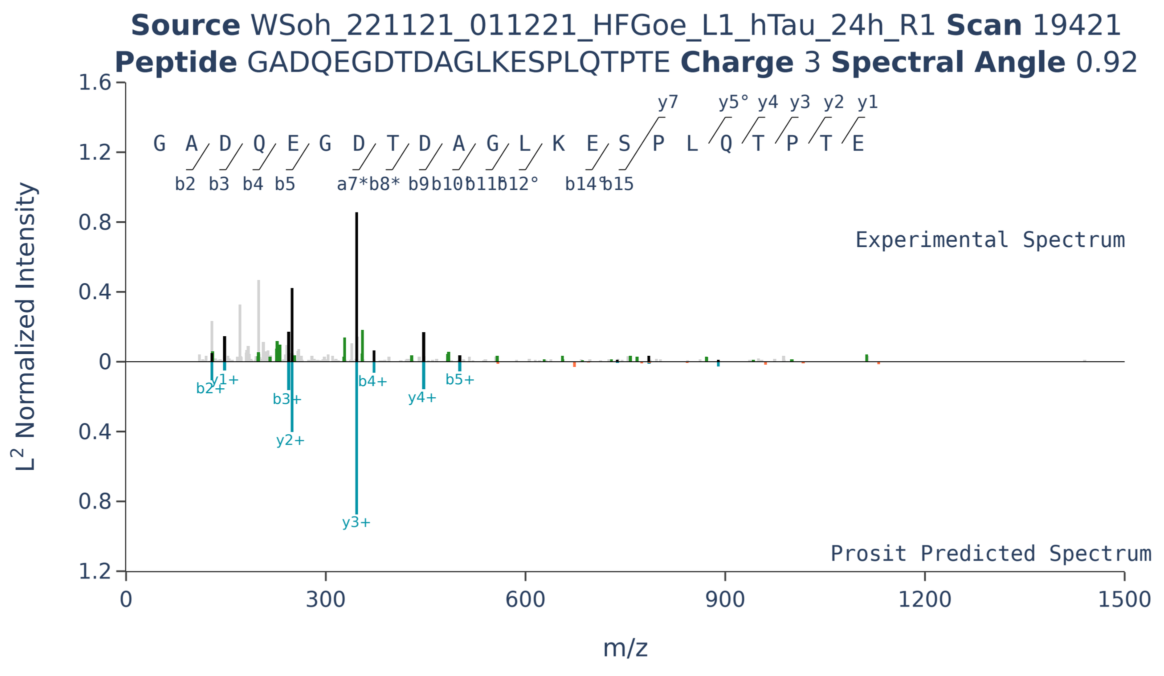
Source WSoh_221121_011221_HFGoe_L1_hTau_24h_R1 Scan 19421
Peptide GADQEGDTDAGLKESPLQTPTE Charge 3 Spectral Angle 0.92
Experimental Spectrum
Prosit Predicted Spectrum
L² Normalized Intensity
m/z

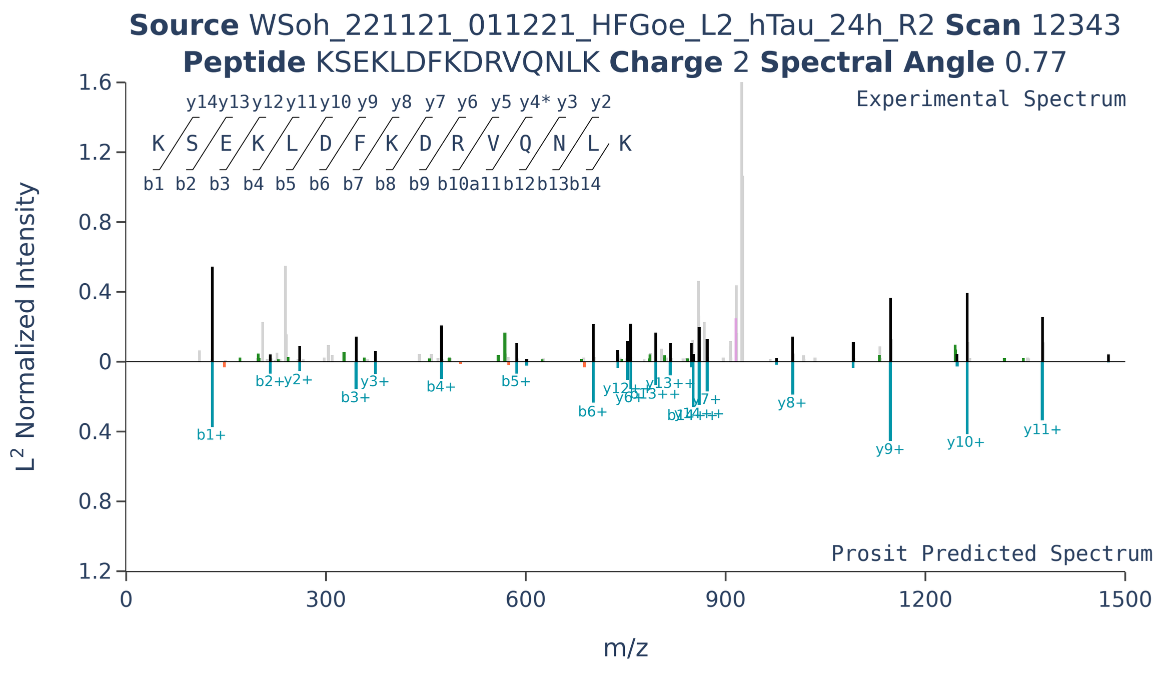
Source WSoh_221121_011221_HFGoe_L2_hTau_24h_R2 Scan 12343
Peptide KSEKLDFKDRVQNLK Charge 2 Spectral Angle 0.77
Experimental Spectrum
Prosit Predicted Spectrum
L² Normalized Intensity
m/z

Source WSoh_221121_011221_HFGoe_L1_hTau_24h_R1 **Scan** 20537
Peptide ENAEPRQEFEVME **Charge** 2 **Spectral Angle** 0.83

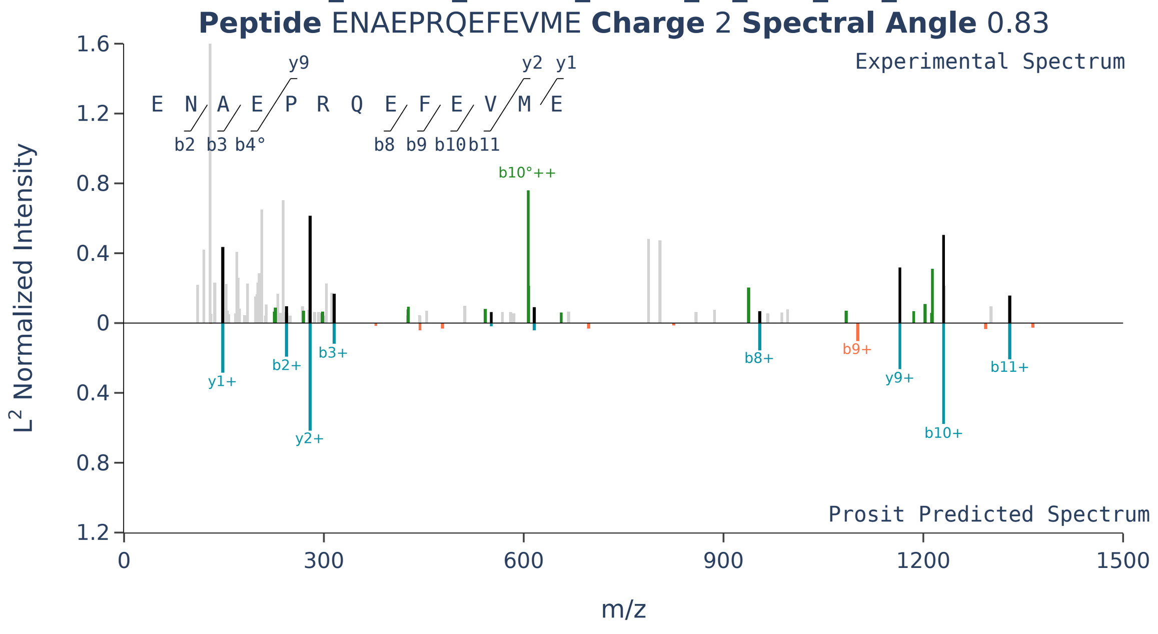

Source WSoh_221121_011221_HFGoe_L1_hTau_24h_R1 **Scan** 13985
Peptide SNVQSKHQPGGGKVQIINKKLDL **Charge** 3 **Spectral Angle** 0.86

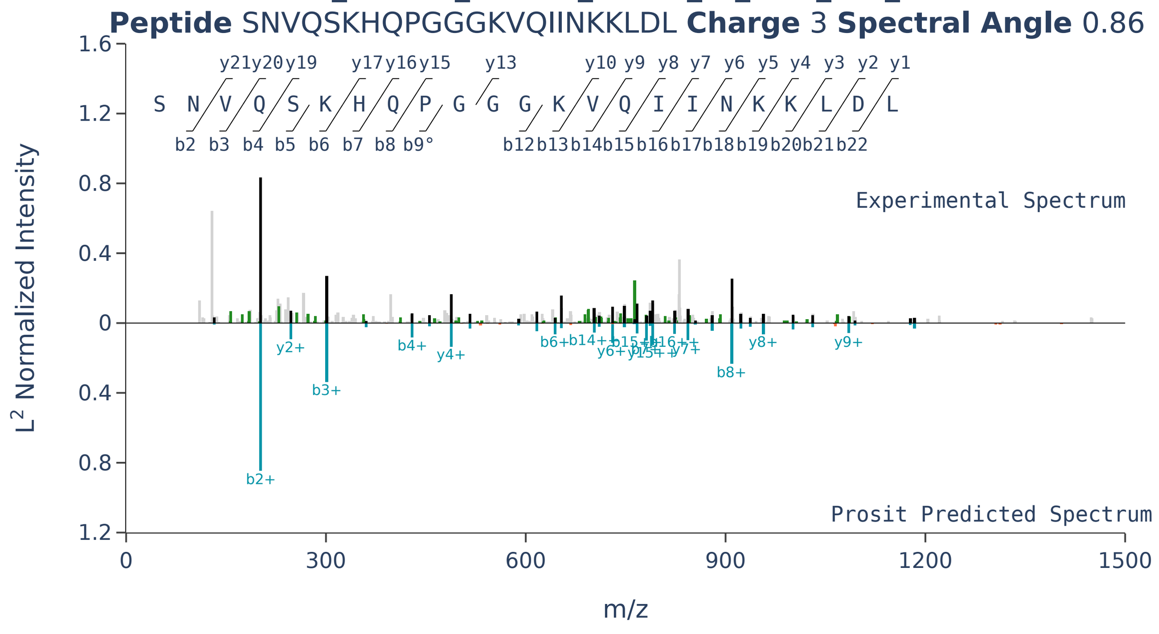

Source WSoh_221121_011221_HFGoe_L1_hTau_24h_R1 **Scan** 16326
Peptide APVPMPDLKHQPGGGKVQ **Charge** 4 **Spectral Angle** 0.88

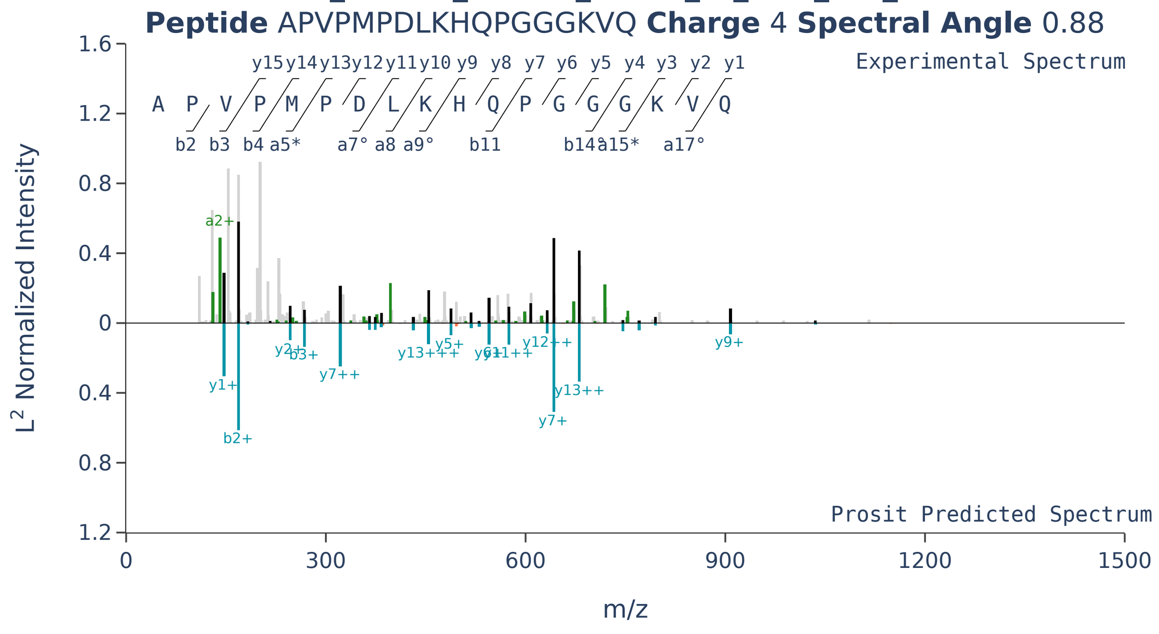

Source WSoh_221121_011221_HFGoe_L1_hTau_24h_R1 **Scan** 13863
Peptide KHLTFRENAKAKTDHGAEIVYKSPVV **Charge** 4 **Spectral Angle** 0.83

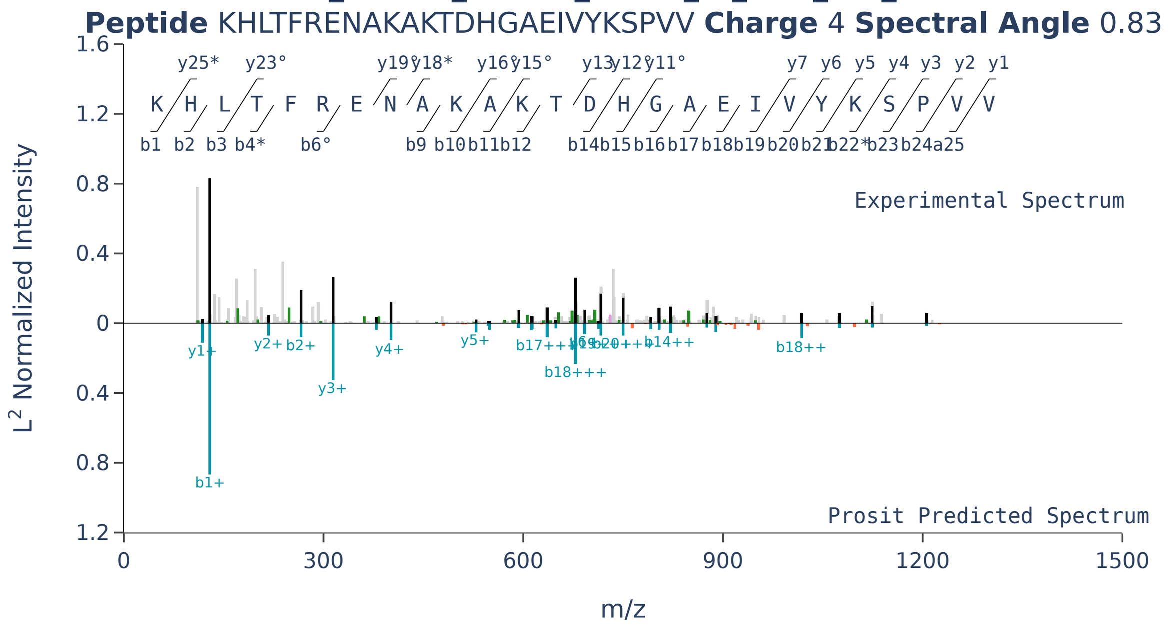

Source WSoh_221121_011221_HFGoe_L1_hTau_4h_R1 **Scan** 19686
Peptide AEEPRQEFEVME **Charge** 2 **Spectral Angle** 0.76

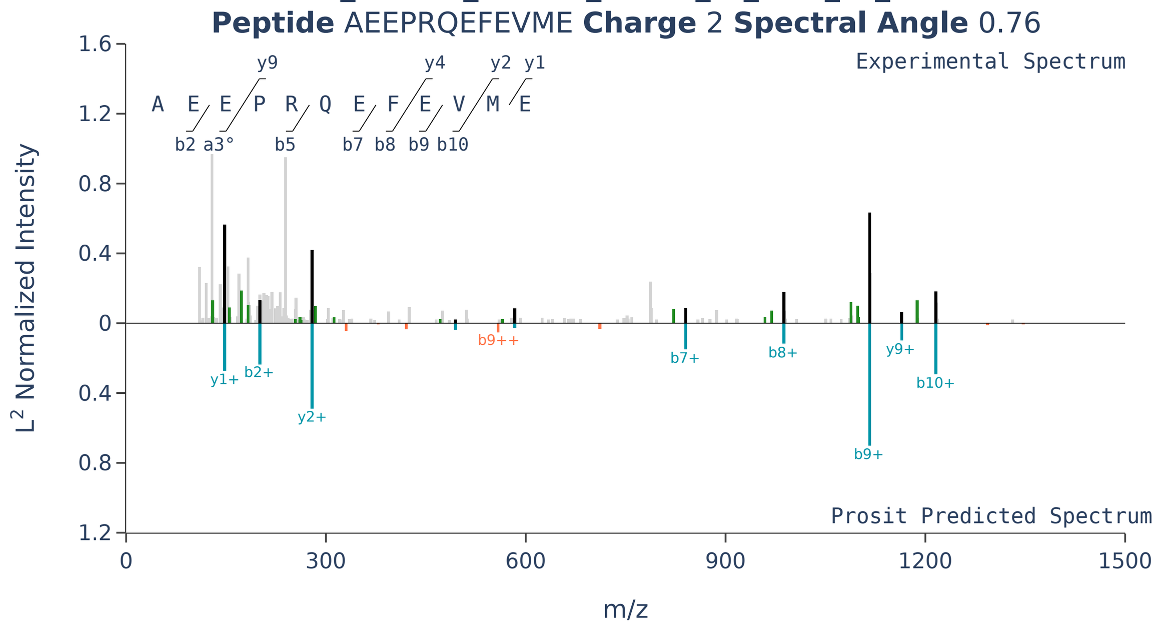

Source WSoh_221121_011221_HFGoe_L2_hTau_24h_R2 **Scan** 20776
Peptide QEGDAEPRQEFEVME **Charge** 2 **Spectral Angle** 0.85

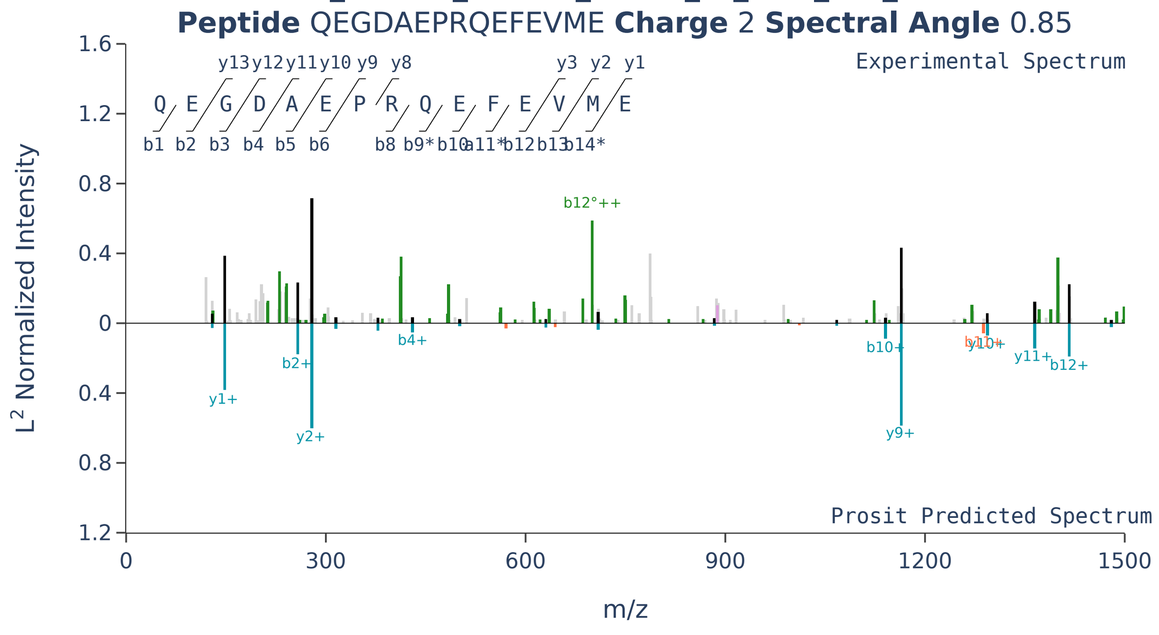

Source WSoh_221121_011221_HFGoe_L1_hTau_4h_R1 **Scan** 13074
Peptide KNVKSVYKPVDL **Charge** 3 **Spectral Angle** 0.81

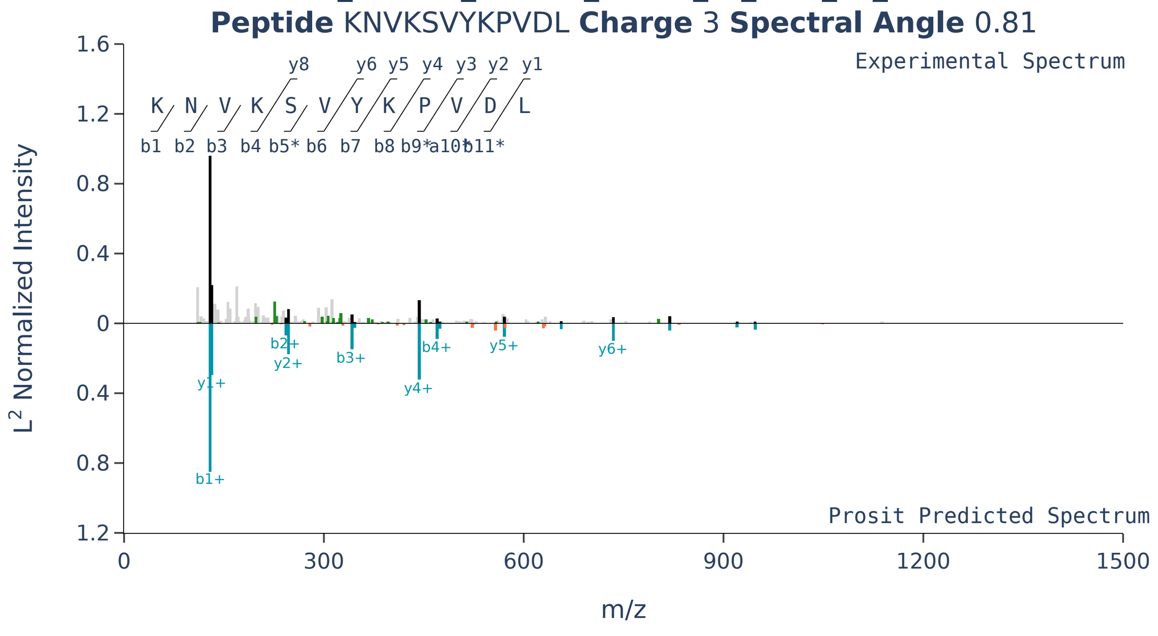

Source WSoh_221121_011221_HFGoe_L2_hTau_24h_R2 **Scan** 7174
Peptide KCSKDNIKHVPGGGSV **Charge** 2 **Spectral Angle** 0.87

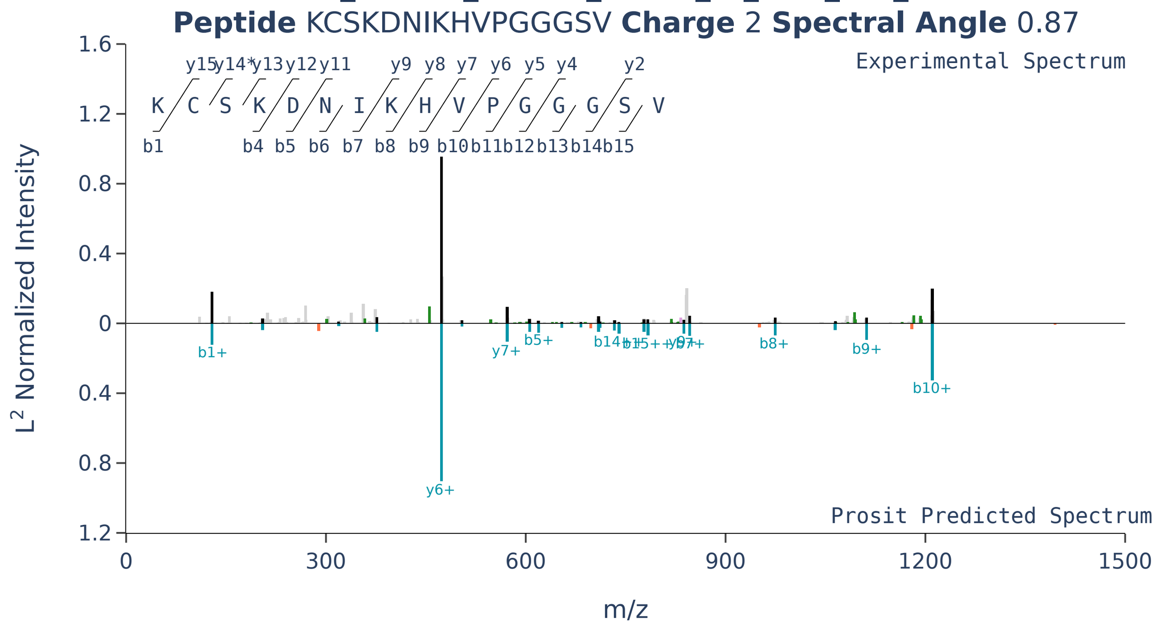

Source WSoh_221121_011221_HFGoe_L2_hTau_24h_R2 **Scan** 12911
Peptide SPGSPGKQAAAQPHTEIPEGTTAEE **Charge** 3 **Spectral Angle** 0.82

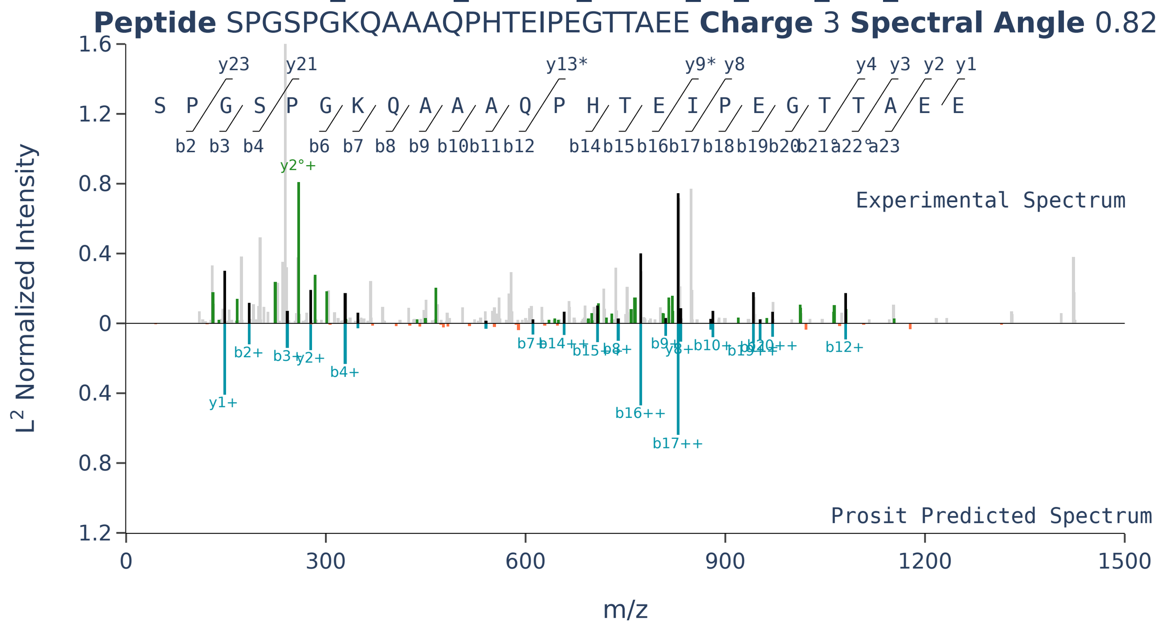

Source WSoh_221121_011221_HFGoe_L1_hTau_24h_R2 **Scan** 9082
Peptide AKAKTDHGAEIVYG **Charge** 2 **Spectral Angle** 0.78

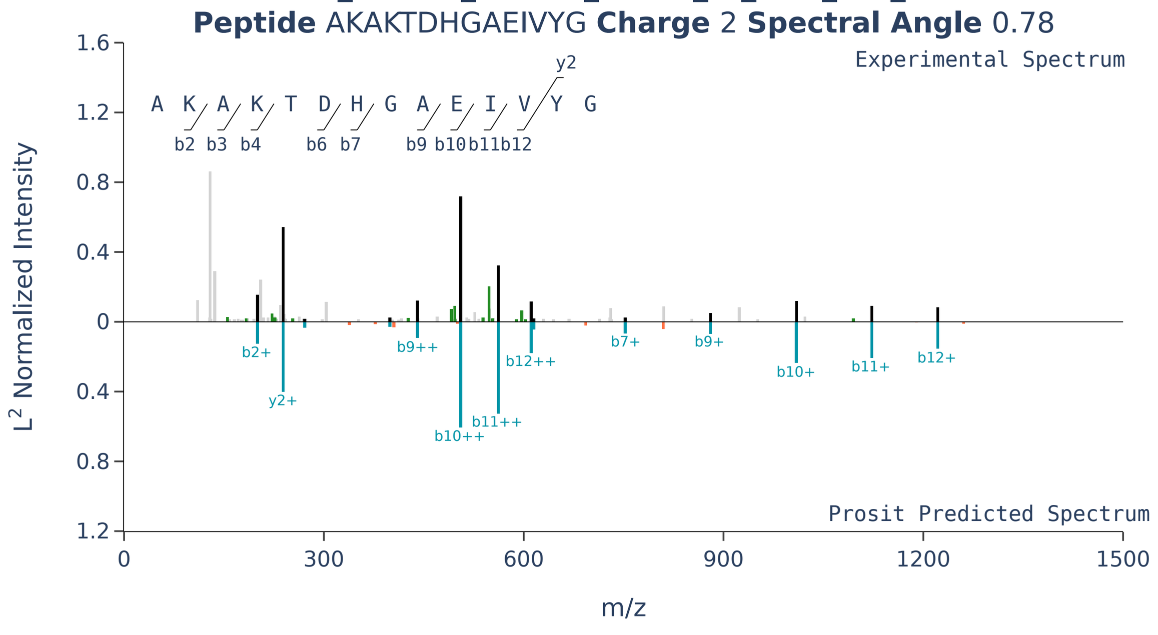

Source WSoh_221121_011221_HFGoe_L1_hTau_24h_R1 **Scan** 9118
Peptide PVVSGDTSGTT **Charge** 1 **Spectral Angle** 0.91

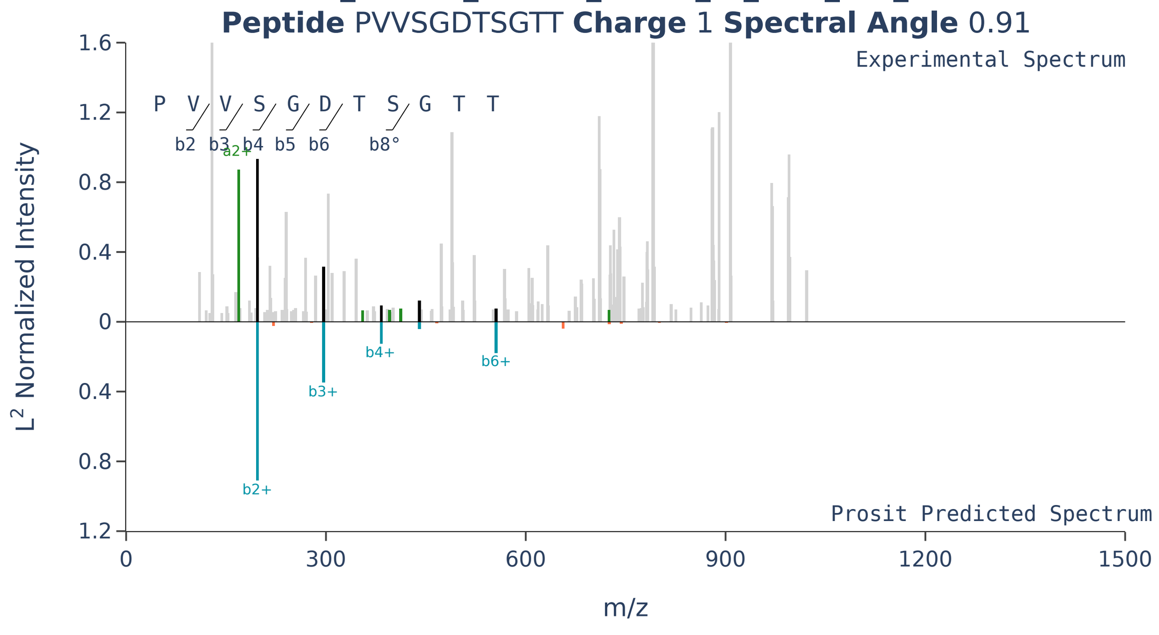

Source WSoh_221121_011221_HFGoe_L1_hTau_24h_R2 **Scan** 9017
Peptide VRTPPKSPSSAKSVYKPVD **Charge** 3 **Spectral Angle** 0.82

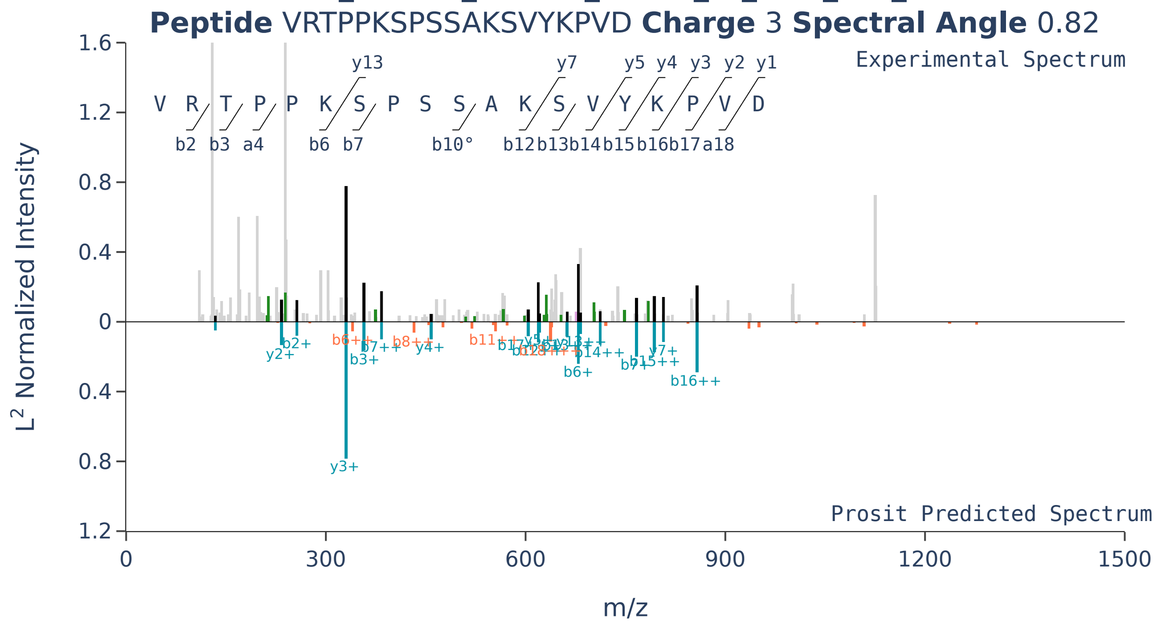

Source WSoh_221121_011221_HFGoe_L1_hTau_4h_R1 **Scan** 8028
Peptide PRHLSNVSKL **Charge** 3 **Spectral Angle** 0.85

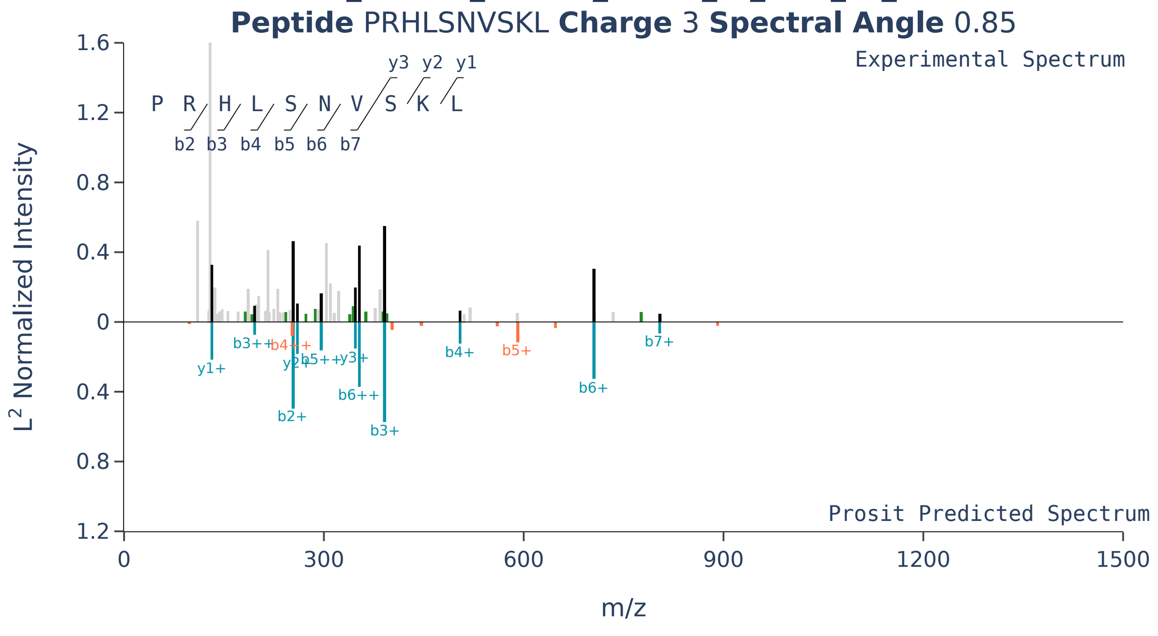

Source WSoh_221121_011221_HFGoe_L1_hTau_4h_R1 **Scan** 11910
Peptide GAAQPHTEIPEGT **Charge** 2 **Spectral Angle** 0.78

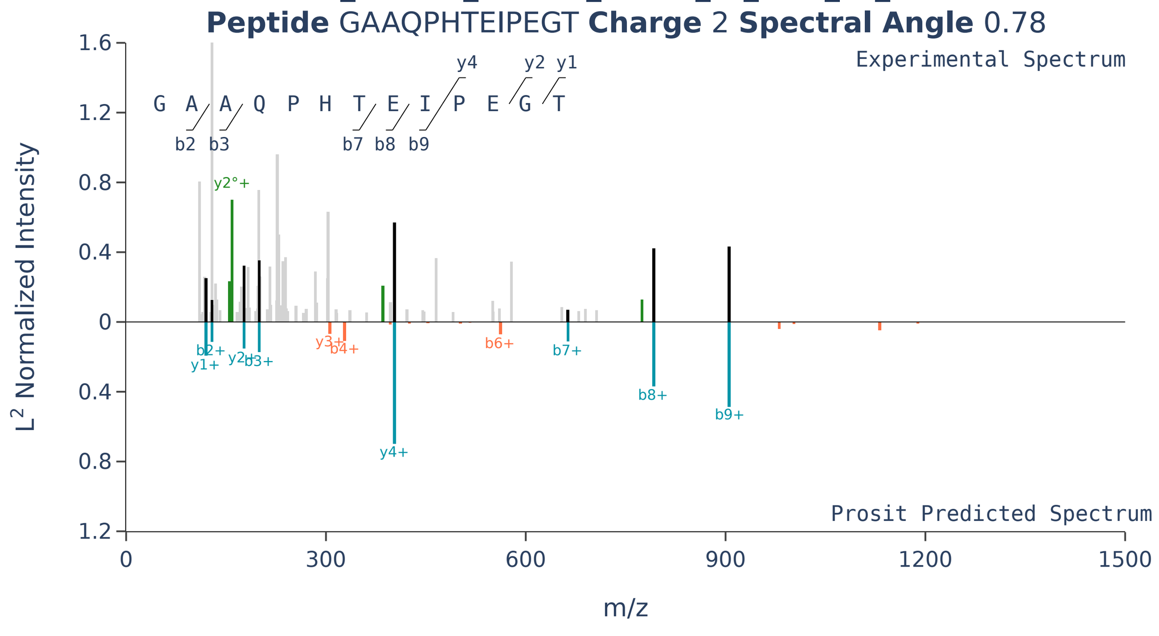

Source WSoh_221121_011221_HFGoe_L2_hTau_4h_R2 **Scan** 13927
Peptide SKHQPGGGKVQIINKKLDL **Charge** 3 **Spectral Angle** 0.81

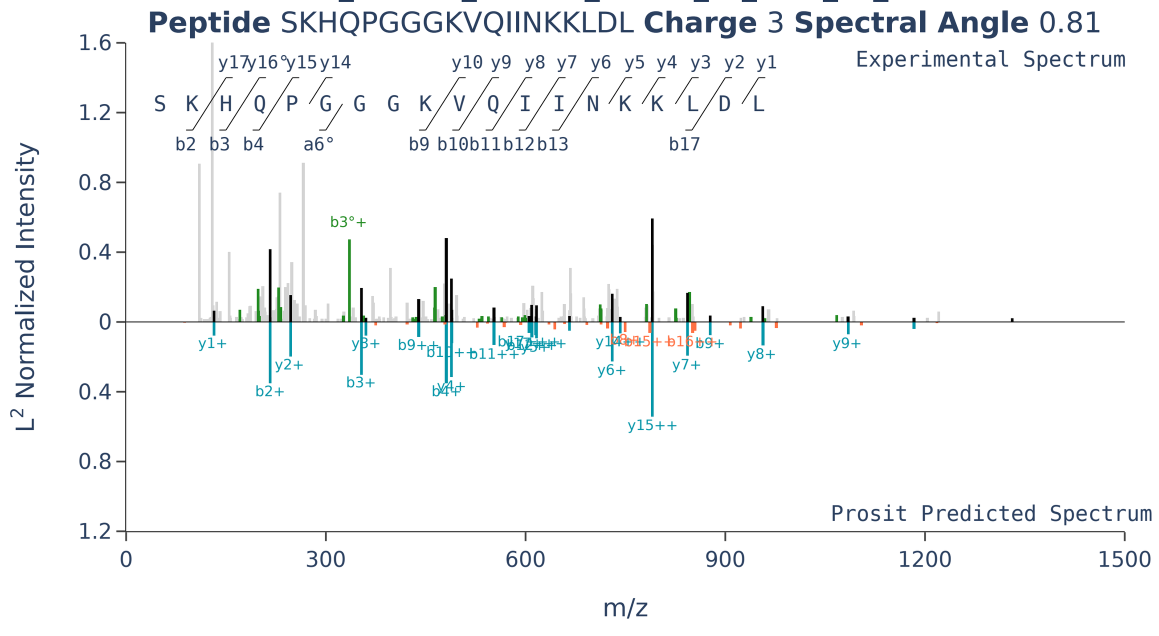

Source WSoh_221121_011221_HFGoe_L1_hTau_4h_R1 **Scan** 11746
Peptide EKLDFKDRVQST **Charge** 3 **Spectral Angle** 0.78

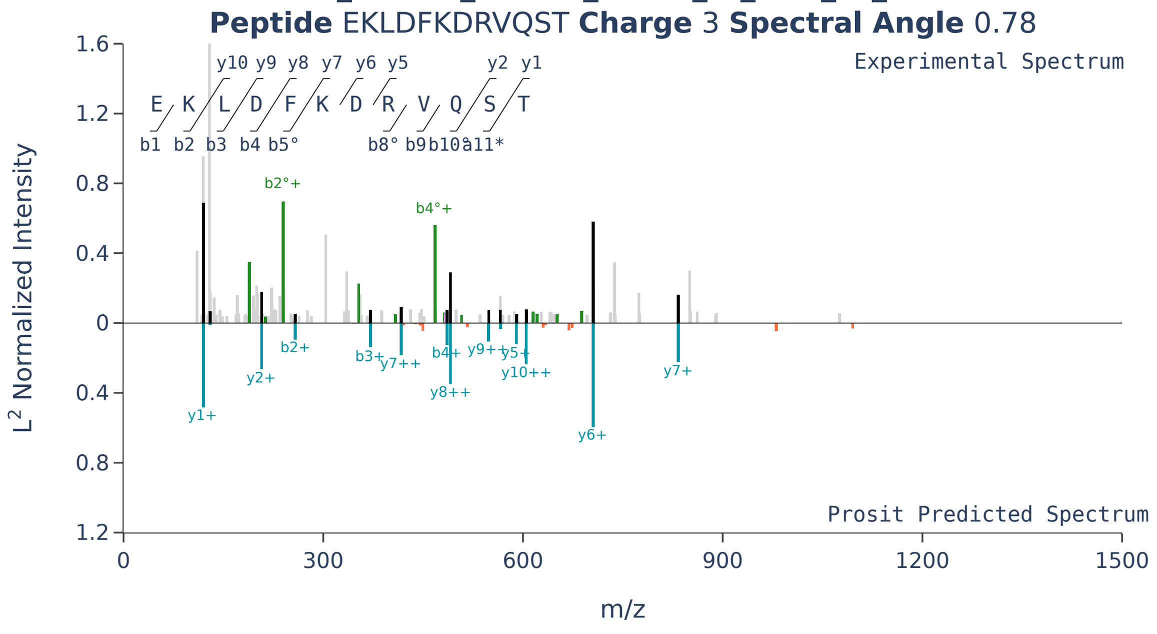

Source WSoh_221121_011221_HFGoe_L2_hTau_24h_R2 **Scan** 7824
Peptide THVPGGGNKKIETHGLG **Charge** 2 **Spectral Angle** 0.92

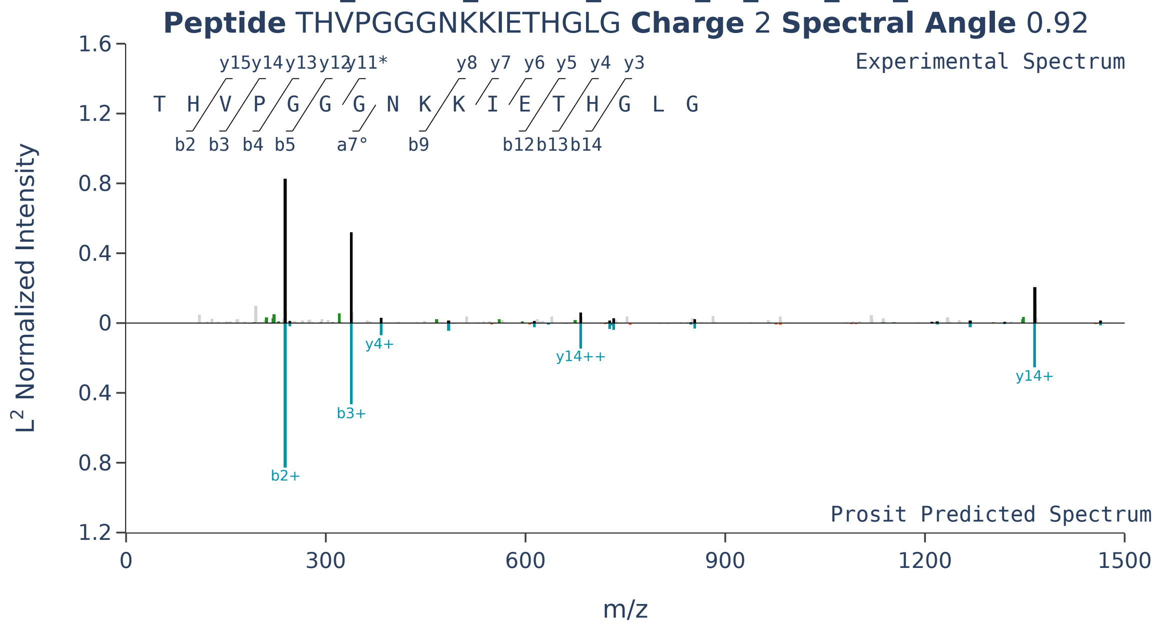

Source WSoh_221121_011221_HFGoe_L1_hTau_2h_R1 **Scan** 9071
Peptide SNVSQEG **Charge** 1 **Spectral Angle** 0.97

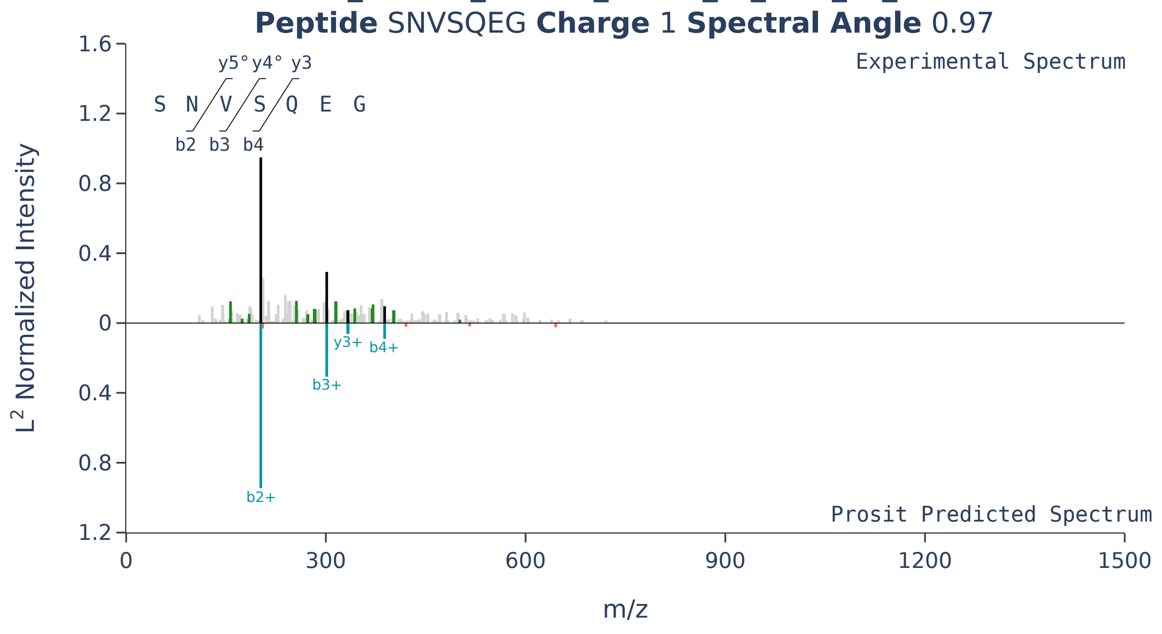

Source WSoh_221121_011221_HFGoe_L2_hTau_4h_R1 **Scan** 8292
Peptide KSKHQPGGGKVQII **Charge** 2 **Spectral Angle** 0.8

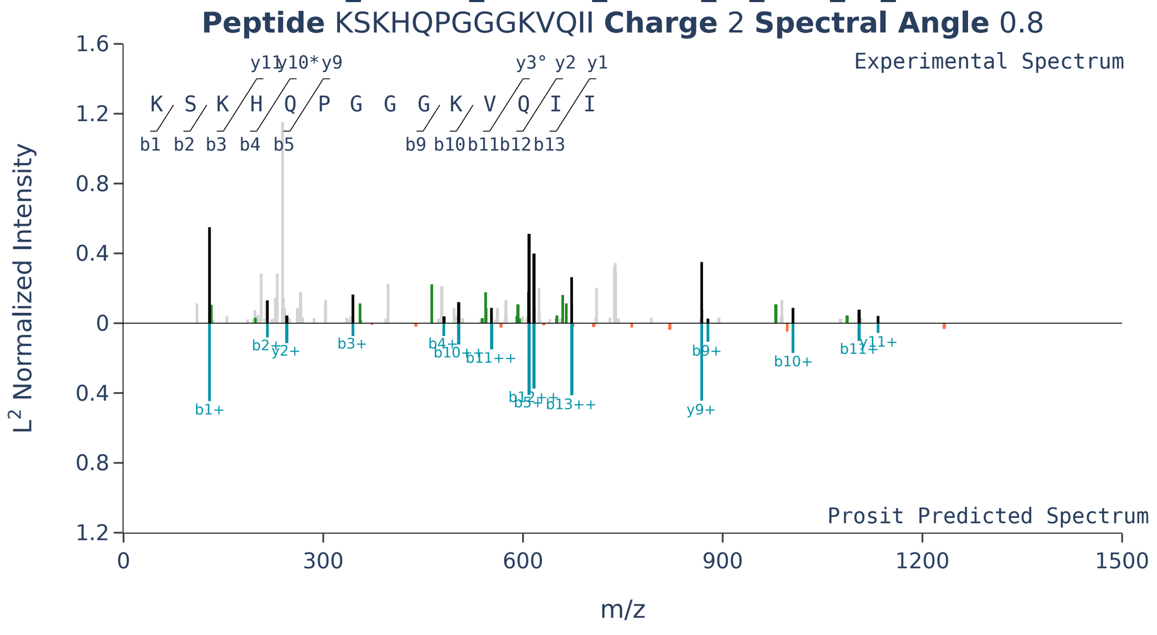

Source WSoh_221121_011221_HFGoe_L2_hTau_4h_R1 **Scan** 15356
Peptide TDHGAEIVVDEG **Charge** 2 **Spectral Angle** 0.76

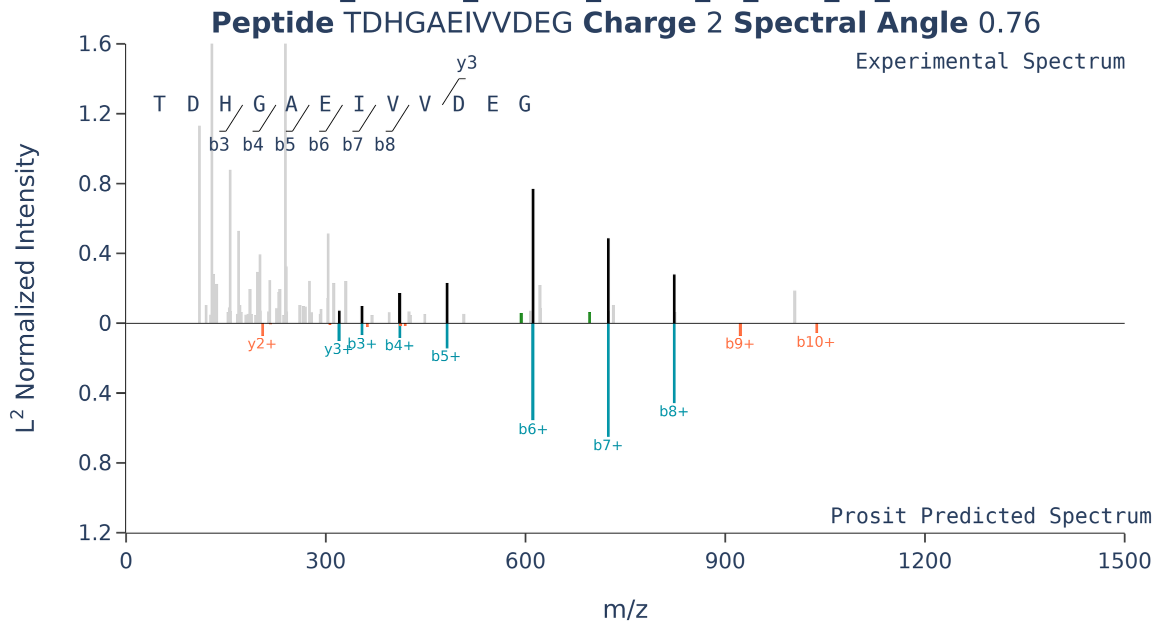

Source WSoh_221121_011221_HFGoe_L2_hTau_1h_R2 **Scan** 12321
Peptide SNVQSNITHV **Charge** 2 **Spectral Angle** 0.84

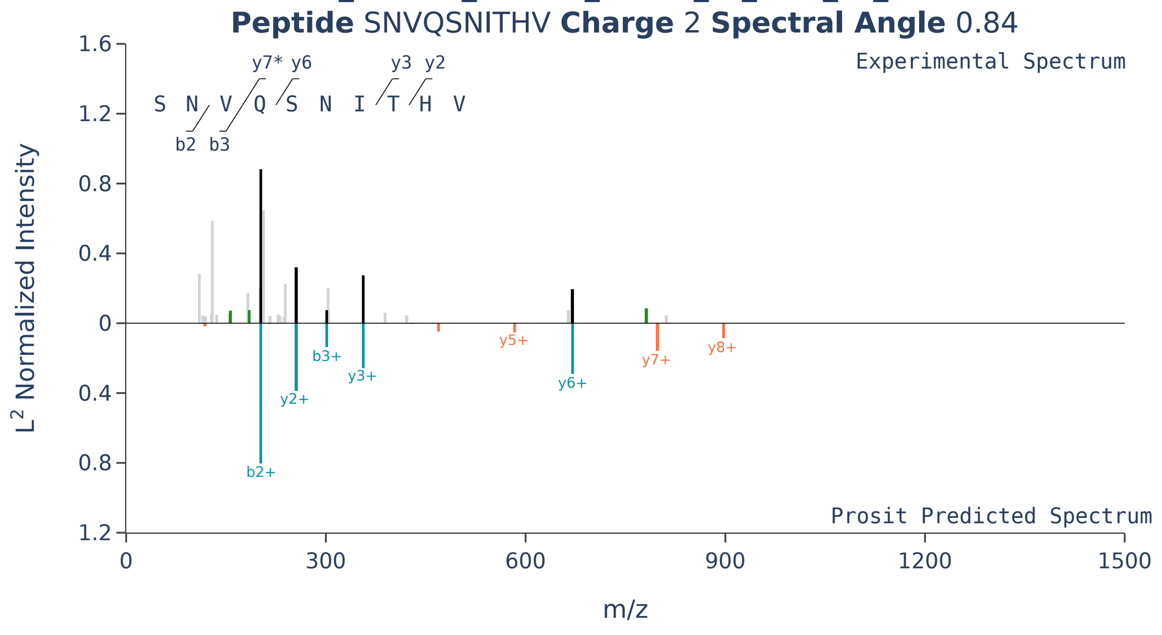

Source WSoh_221121_011221_HFGoe_L1_hTau_24h_R2 **Scan** 14558
Peptide LDLSNVQSKCSKD **Charge** 3 **Spectral Angle** 0.72

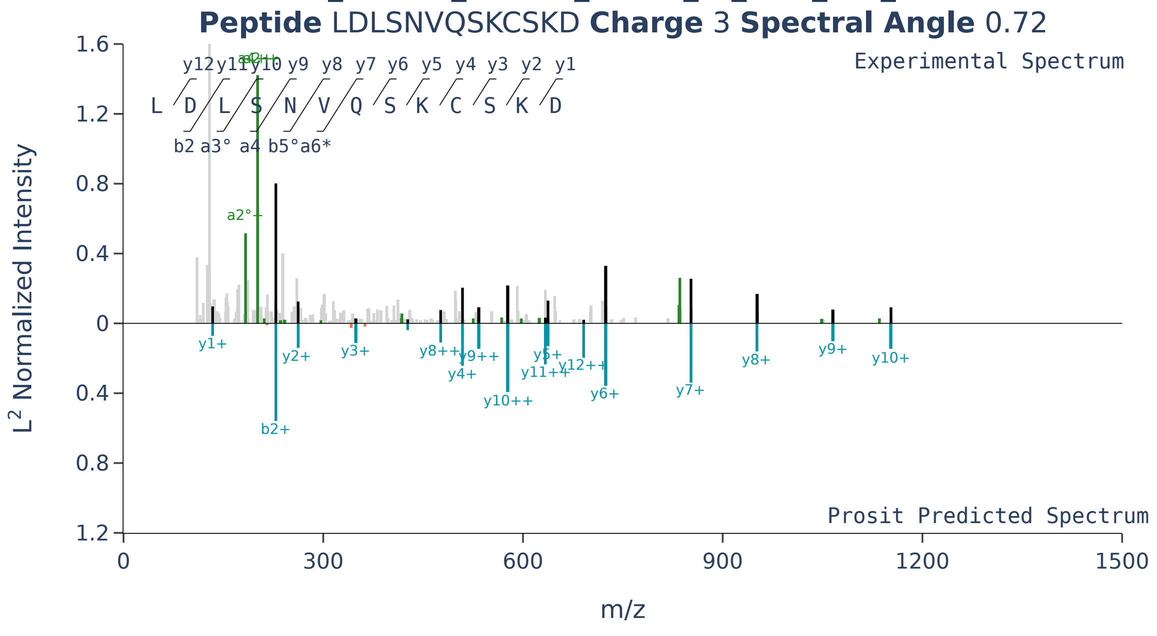

Source WSoh_221121_011221_HFGoe_L1_hTau_24h_R1 **Scan** 9313
Peptide SKSEKLDFKDRVQS **Charge** 3 **Spectral Angle** 0.8

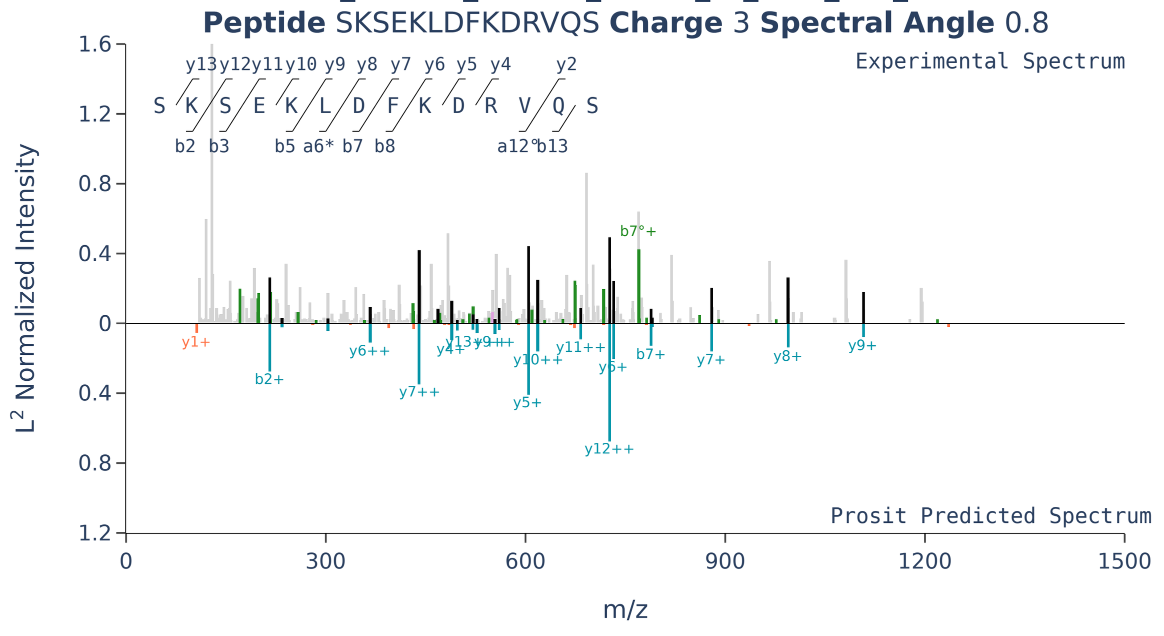

Source WSoh_221121_011221_HFGoe_L1_hTau_24h_R1 **Scan** 11872
Peptide LSKVTSKCSLGNIHHKPGGGQVEV **Charge** 3 **Spectral Angle** 0.81

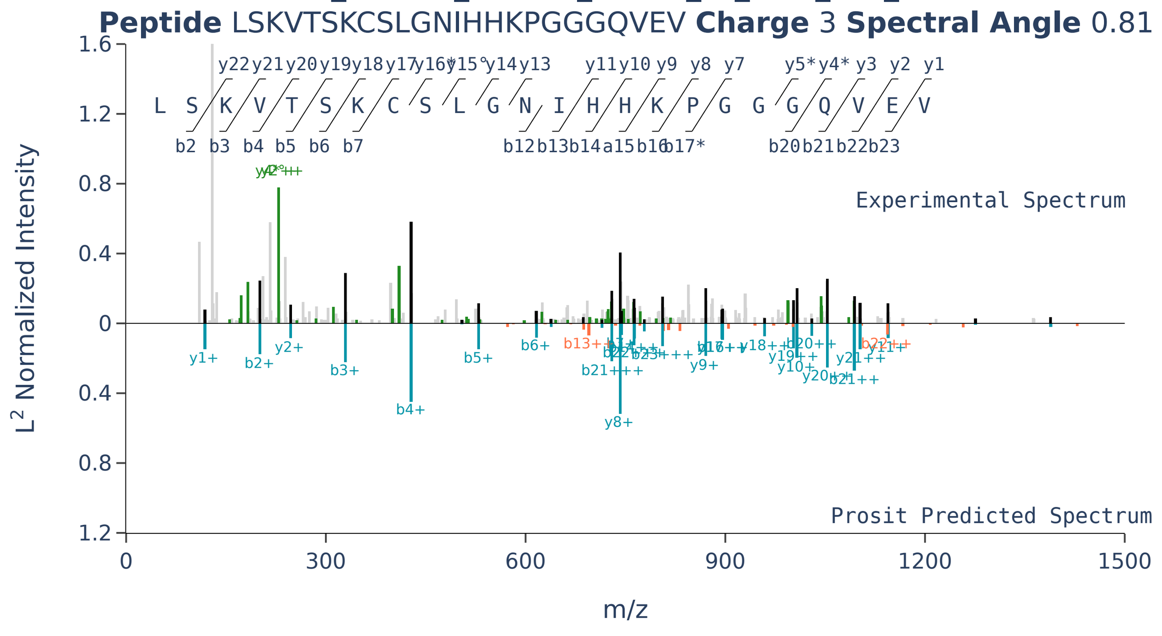

Source WSoh_221121_011221_HFGoe_L1_hTau_1h_R1 **Scan** 11321
Peptide LDFKDRVQGGK **Charge** 3 **Spectral Angle** 0.8

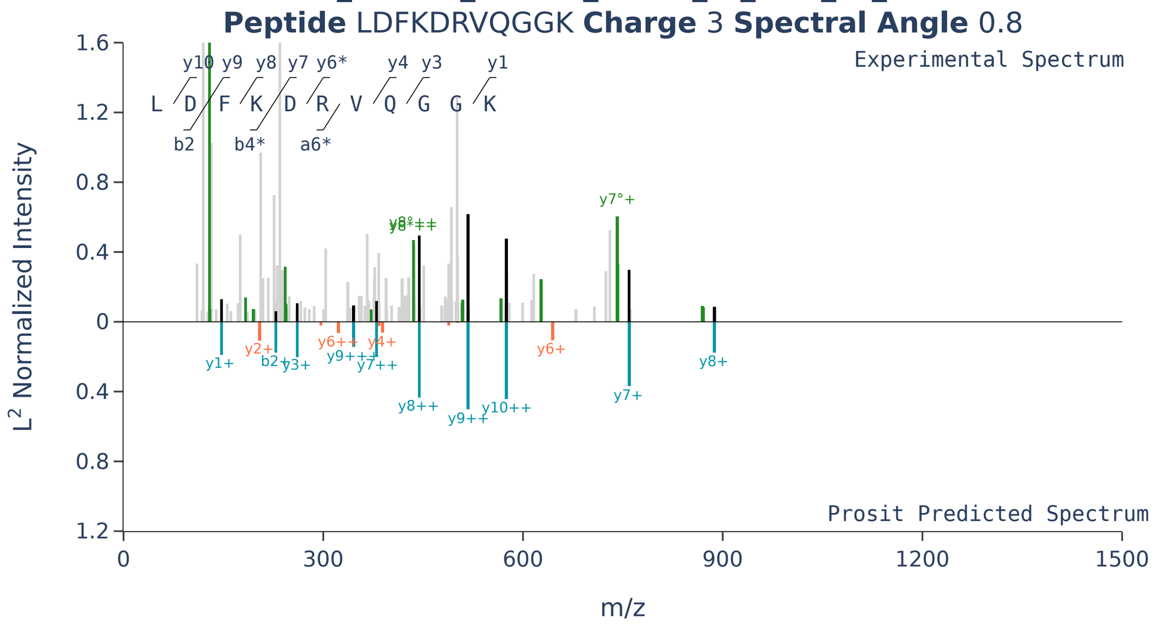

Source WSoh_221121_011221_HFGoe_L1_hTau_24h_R2 **Scan** 21970
Peptide TAPVPMPDLKNVKSKIGSEAG **Charge** 3 **Spectral Angle** 0.89

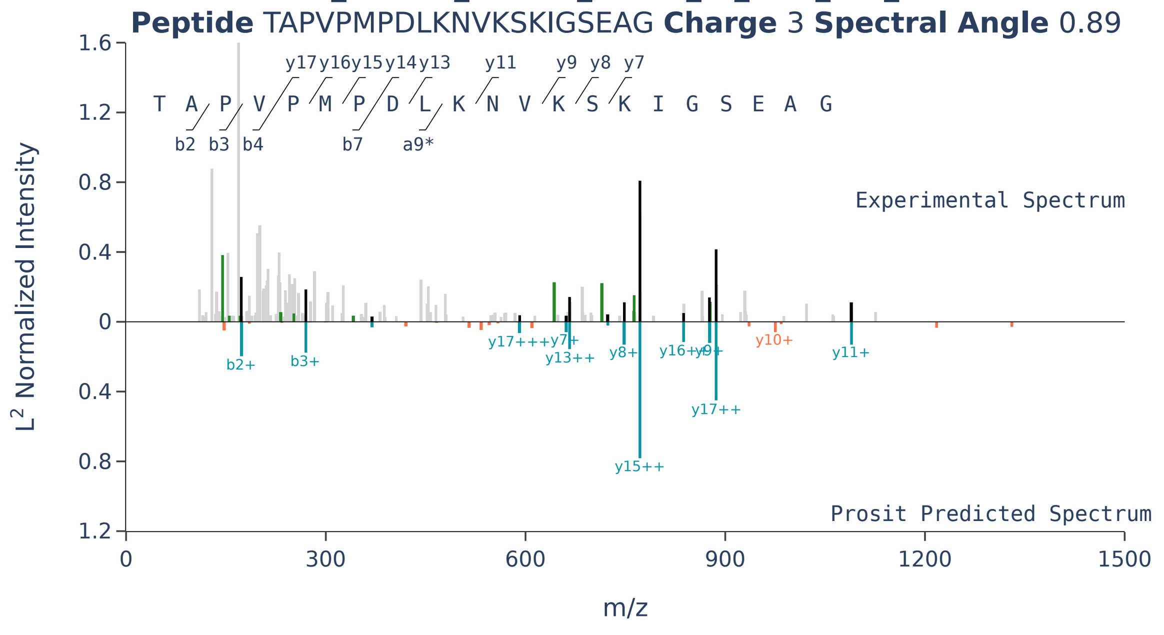

Source WSoh_221121_011221_HFGoe_L2_hTau_2h_R1 **Scan** 11913
Peptide DNITHVPGGGNKKIETHLS **Charge** 3 **Spectral Angle** 0.83

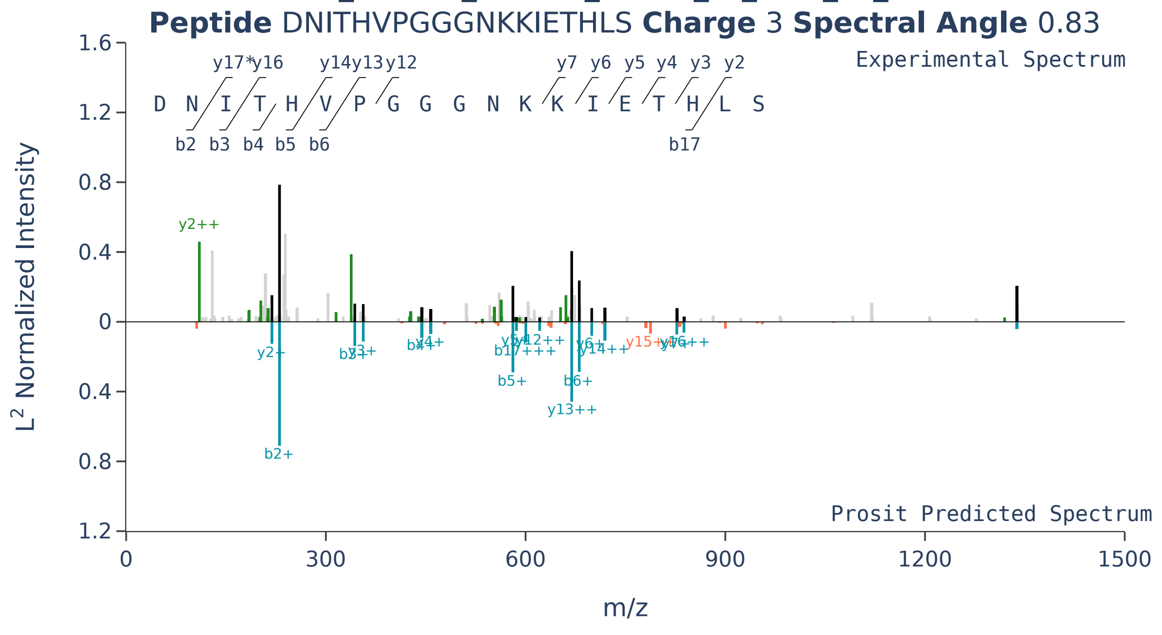

Source WSoh_221121_011221_HFGoe_L1_hTau_4h_R1 **Scan** 12430
Peptide QEAPGKQAAAQPHTEIPEGTTAEE **Charge** 3 **Spectral Angle** 0.67

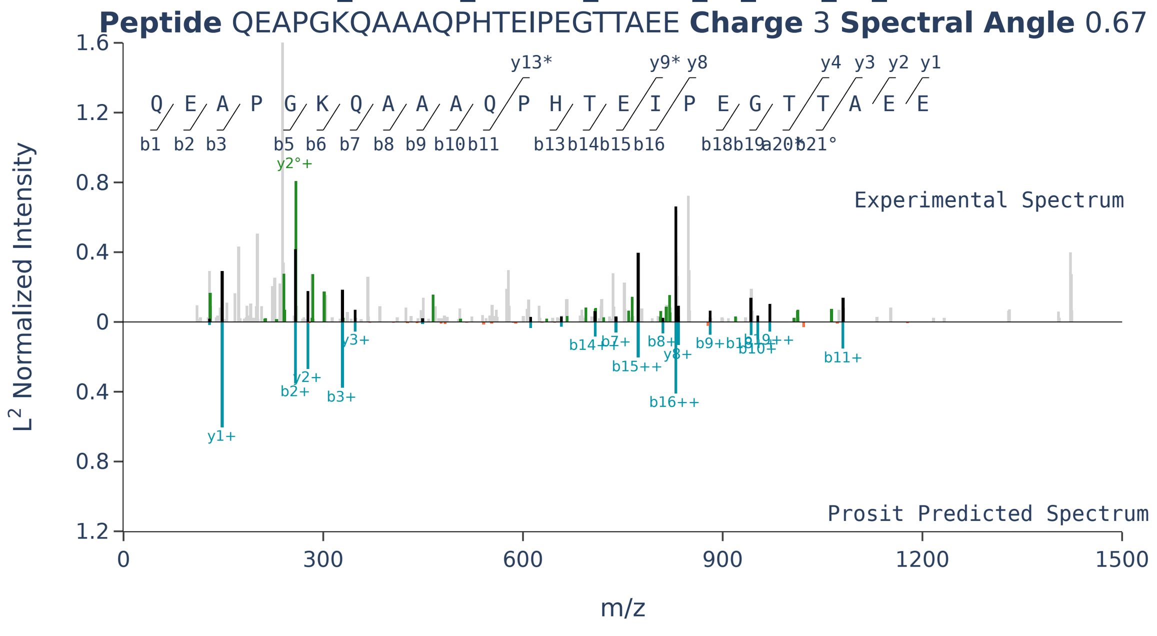

Source WSoh_221121_011221_HFGoe_L1_hTau_24h_R1 **Scan** 22281
Peptide PGSETSDAGSIDMV **Charge** 1 **Spectral Angle** 0.77

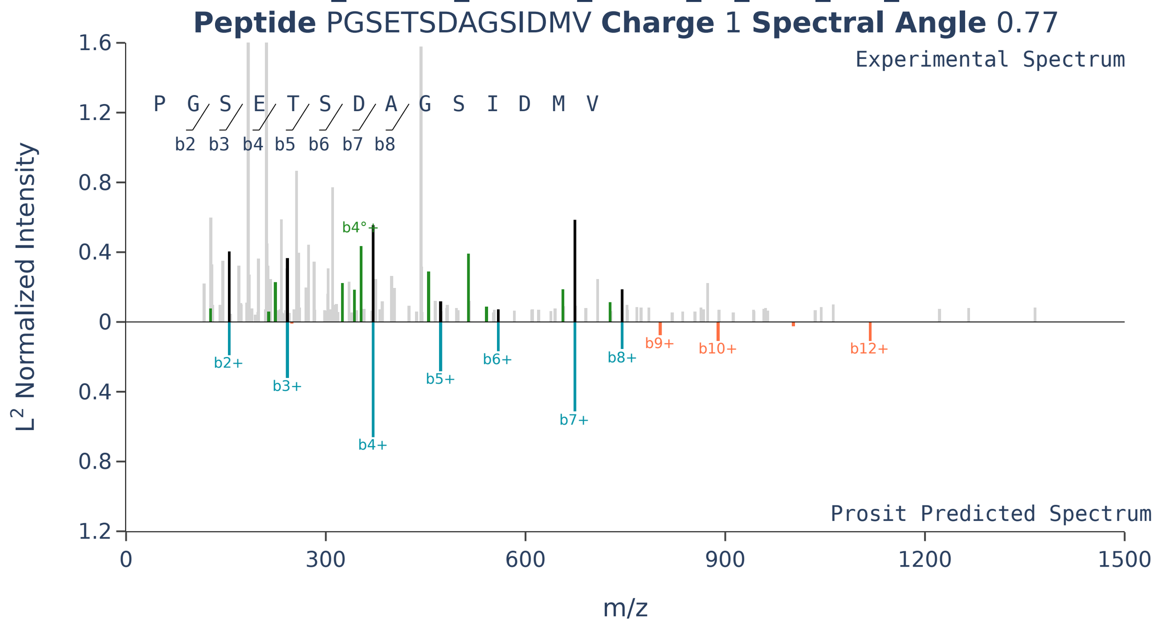

Source WSoh_221121_011221_HFGoe_L2_hTau_24h_R2 **Scan** 8145
Peptide NSLGNIHHKPGGGQVEVKSE **Charge** 3 **Spectral Angle** 0.78

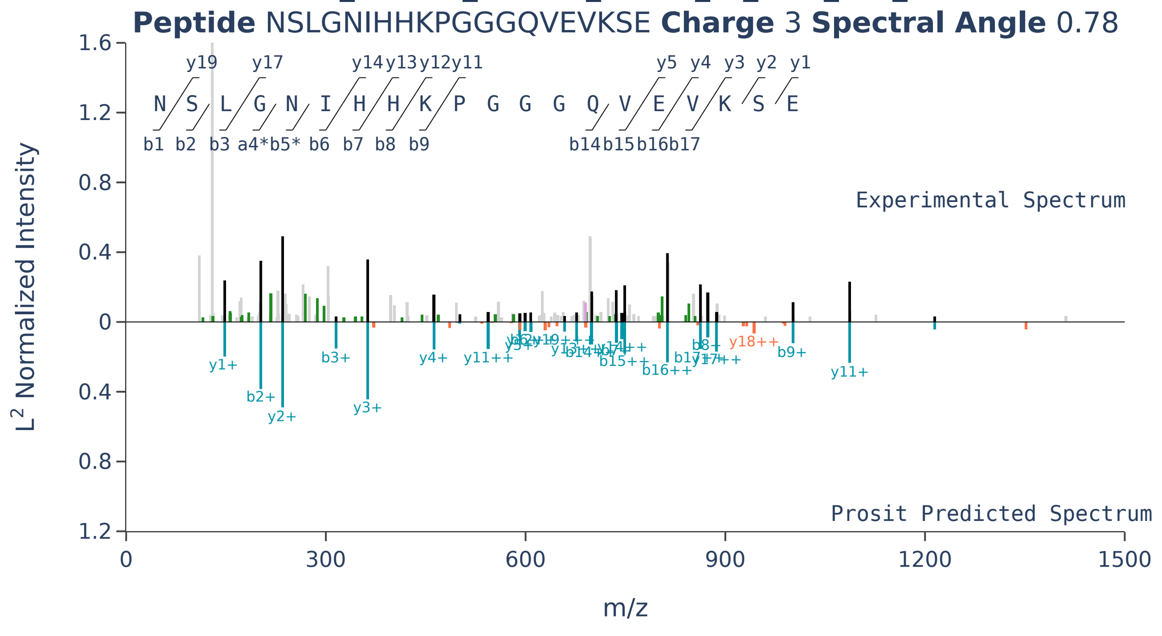

Source WSoh_221121_011221_HFGoe_L1_hTau_24h_R2 **Scan** 7582
Peptide KSKLTFREN **Charge** 3 **Spectral Angle** 0.82

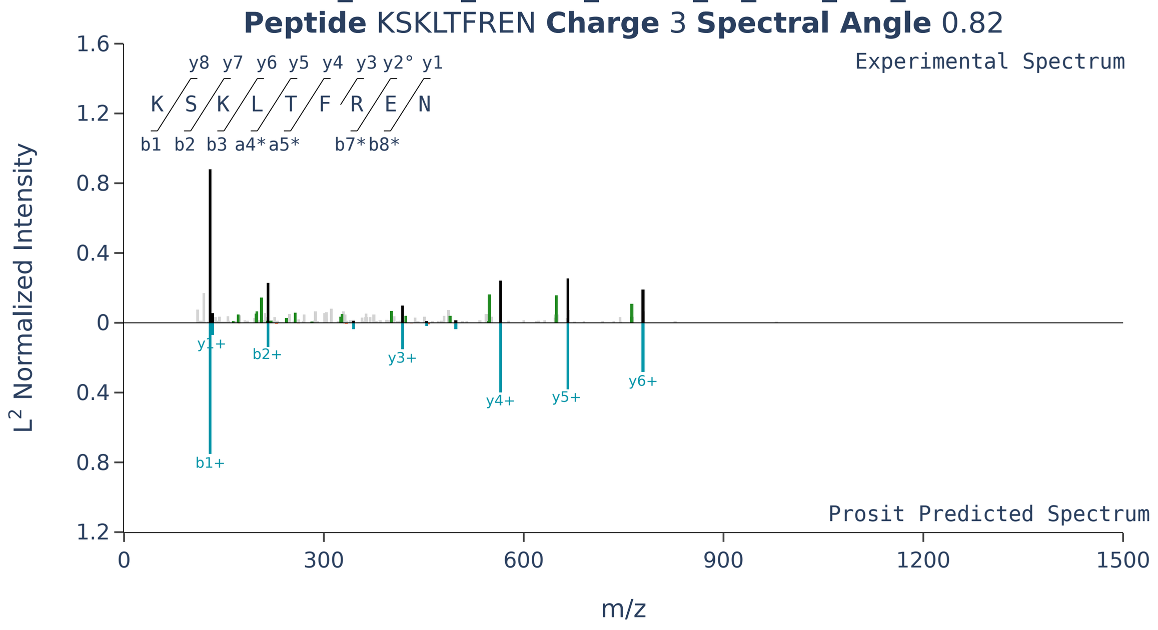

Source WSoh_221121_011221_HFGoe_L2_hTau_4h_R2 **Scan** 9836
Peptide GAEPRQEFE **Charge** 2 **Spectral Angle** 0.83

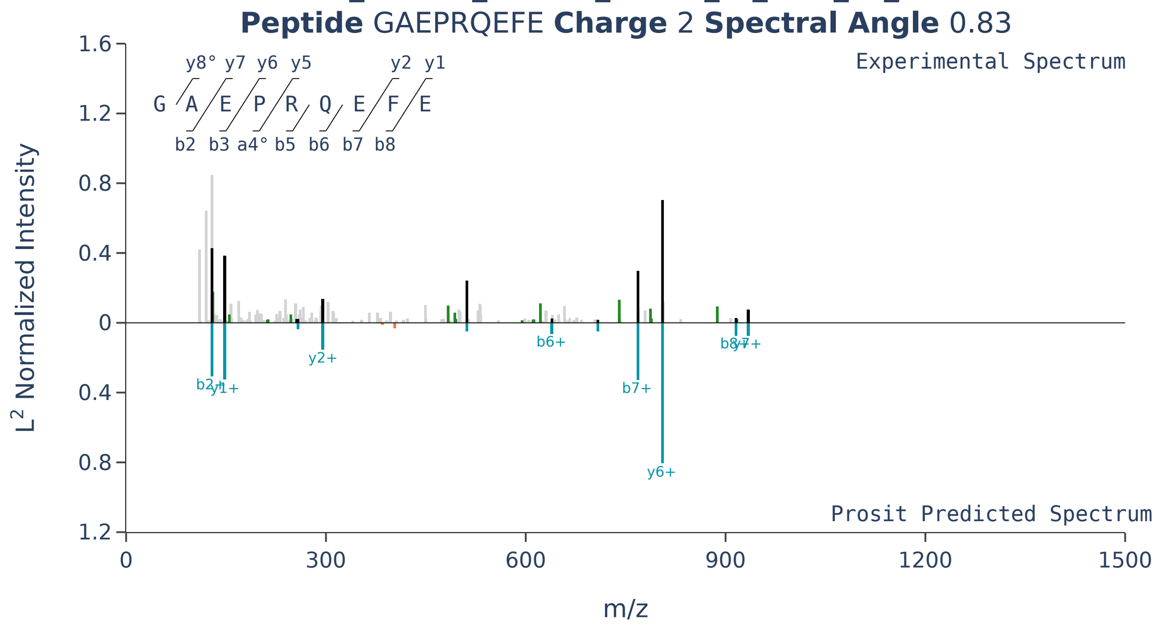

Source WSoh_221121_011221_HFGoe_L1_hTau_24h_R2 **Scan** 6412
Peptide HGNIHHKPGGGQVEV **Charge** 2 **Spectral Angle** 0.82

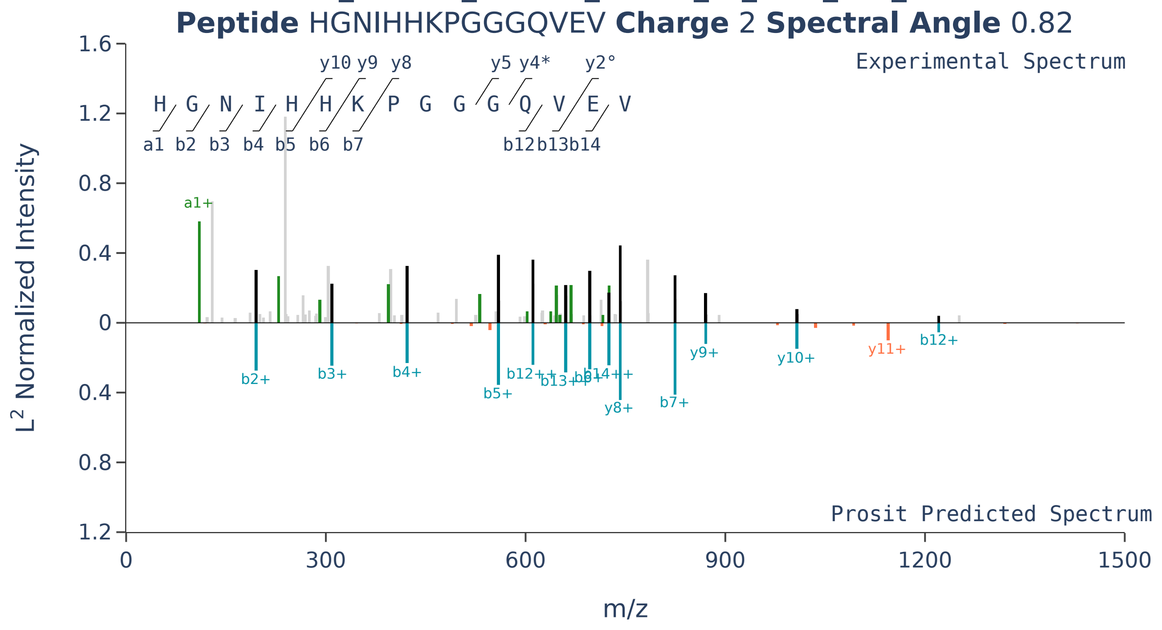

Source WSoh_221121_011221_HFGoe_L2_hTau_24h_R2 **Scan** 7352
Peptide DLSNVQSKHQPGGGKVQ **Charge** 3 **Spectral Angle** 0.86

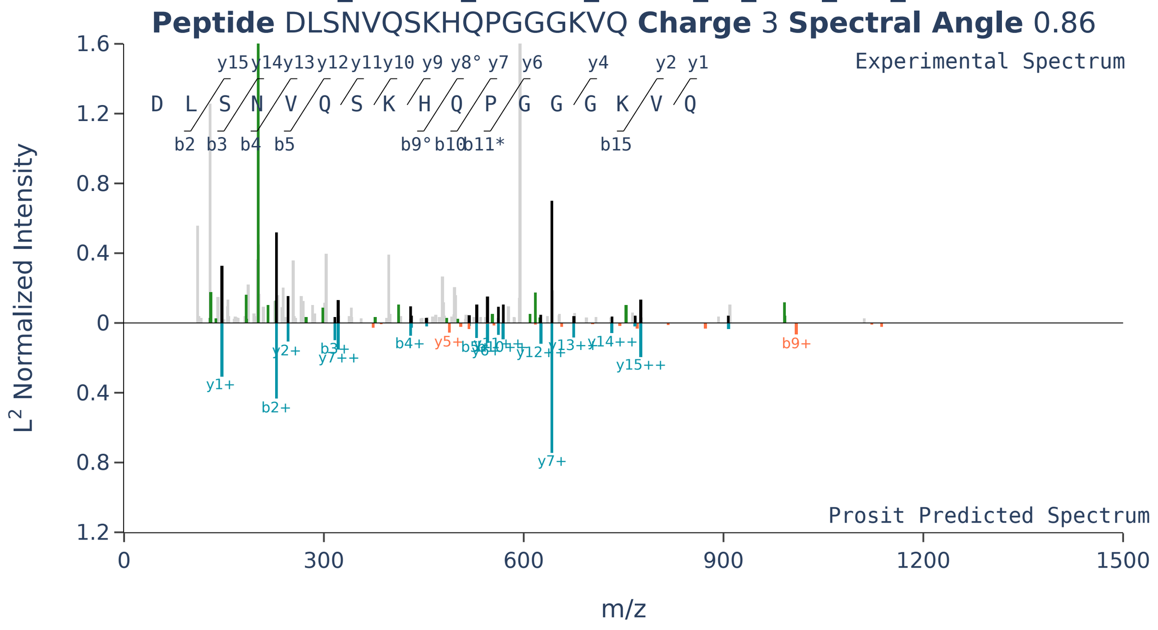

Source WSoh_221121_011221_HFGoe_L1_hTau_4h_R2 **Scan** 8615
Peptide KTHQPGGGKVQIINKKL **Charge** 4 **Spectral Angle** 0.85

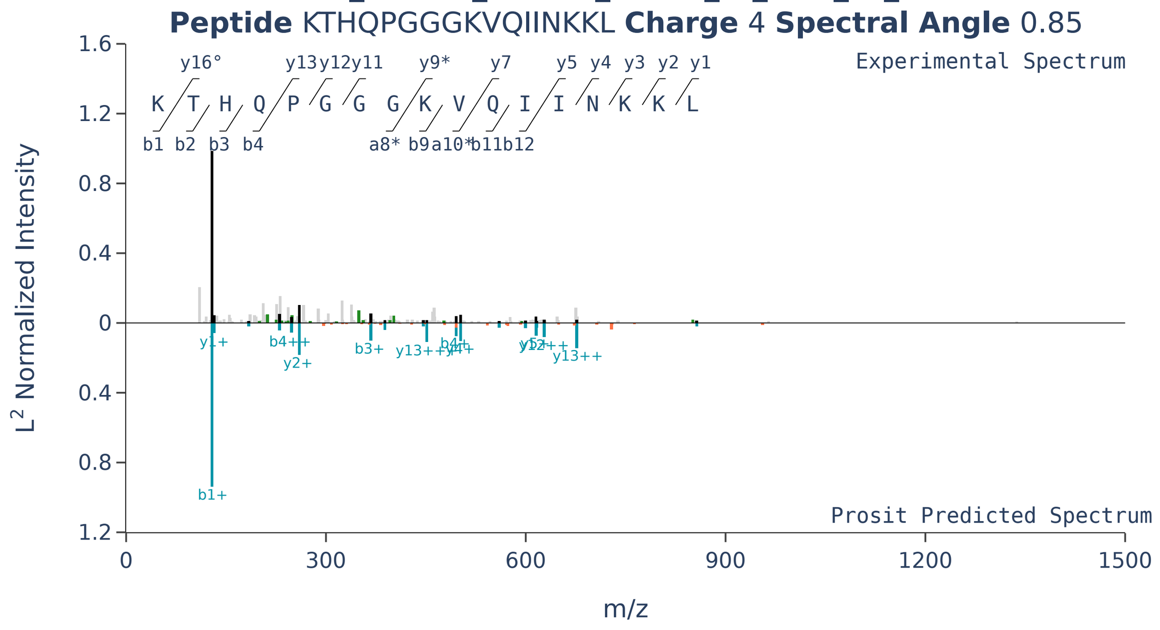

Source WSoh_221121_011221_HFGoe_L2_hTau_24h_R2 **Scan** 24616
Peptide DAAQPHTEIPEGTTAEEAGIGDTPSLE **Charge** 3 **Spectral Angle** 0.9

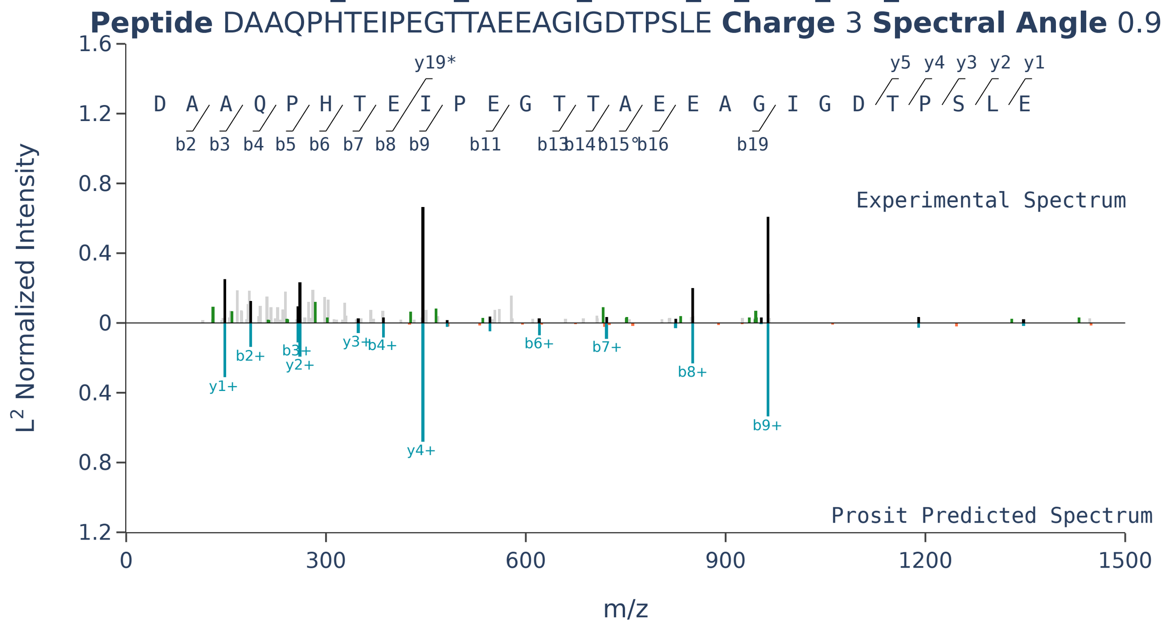

Source WSoh_221121_011221_HFGoe_L1_hTau_24h_R2 **Scan** 12130
Peptide KSTPSLPTPPTREPKKV **Charge** 3 **Spectral Angle** 0.74

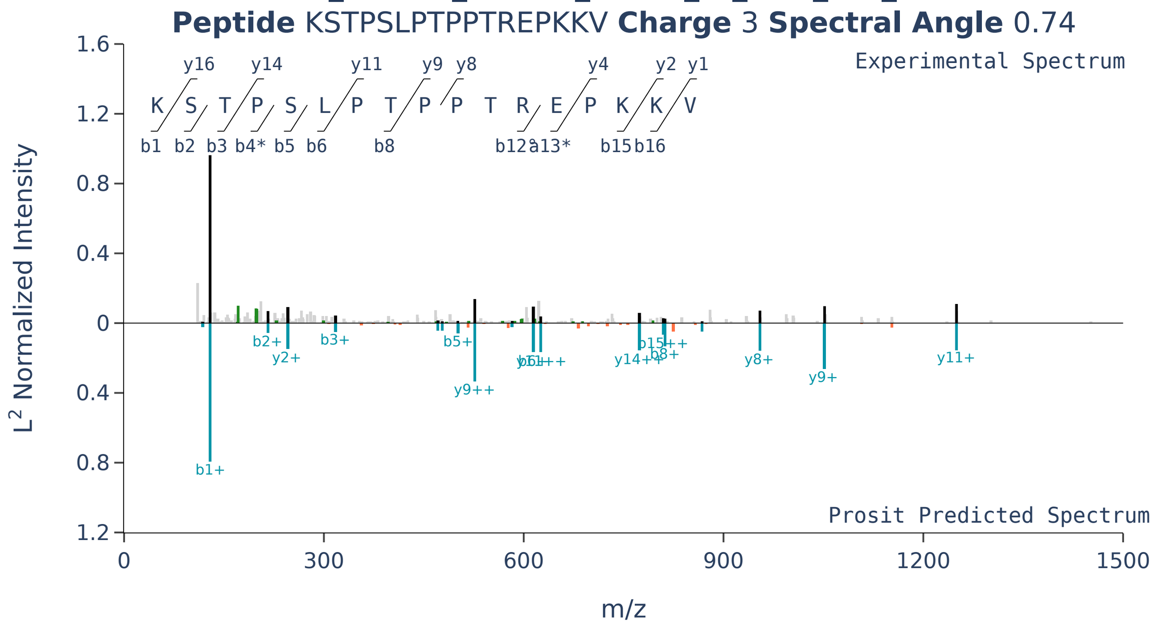

Source WSoh_221121_011221_HFGoe_L1_hTau_24h_R2 **Scan** 9205
Peptide KSEKLDFKDRVQSN **Charge** 2 **Spectral Angle** 0.68

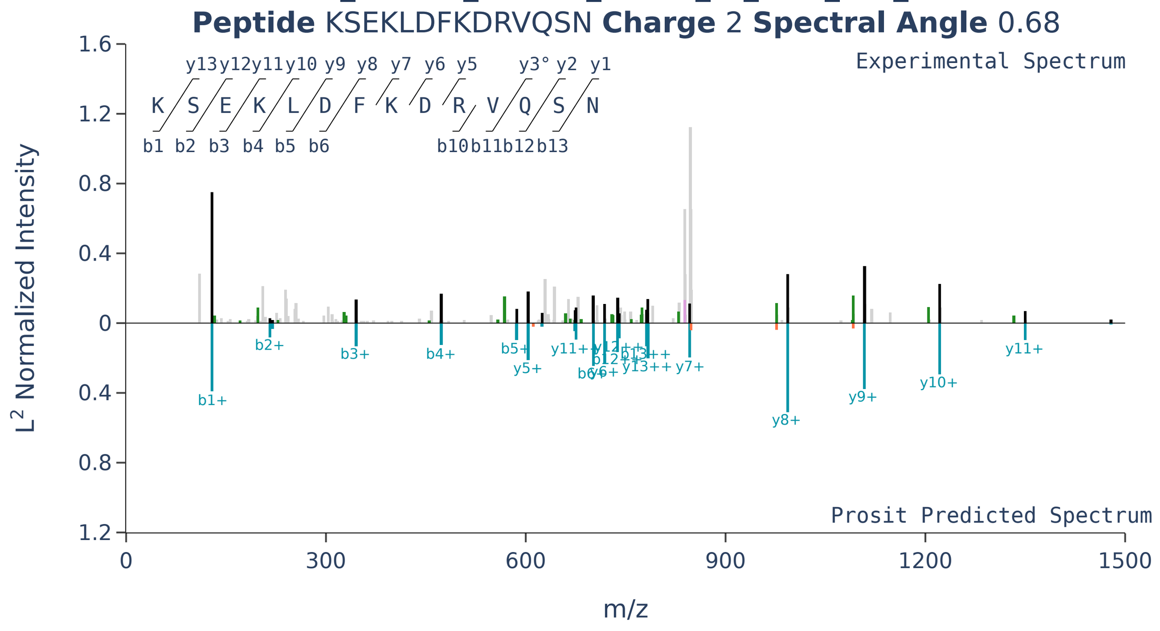

Source WSoh_221121_011221_HFGoe_L1_hTau_2h_R1 **Scan** 8259
Peptide KSHQPGGGKVQIINKKL **Charge** 4 **Spectral Angle** 0.87

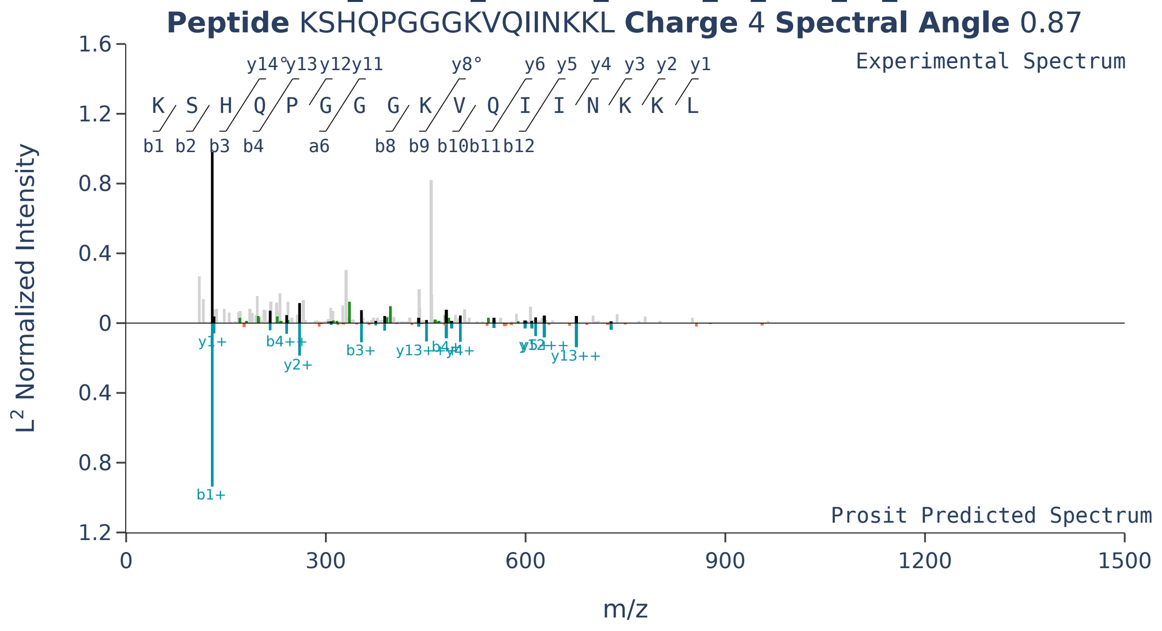

Source WSoh_221121_011221_HFGoe_L1_hTau_4h_R1 **Scan** 10264
Peptide SNVQSKSEKLDFKDRVQS **Charge** 4 **Spectral Angle** 0.83

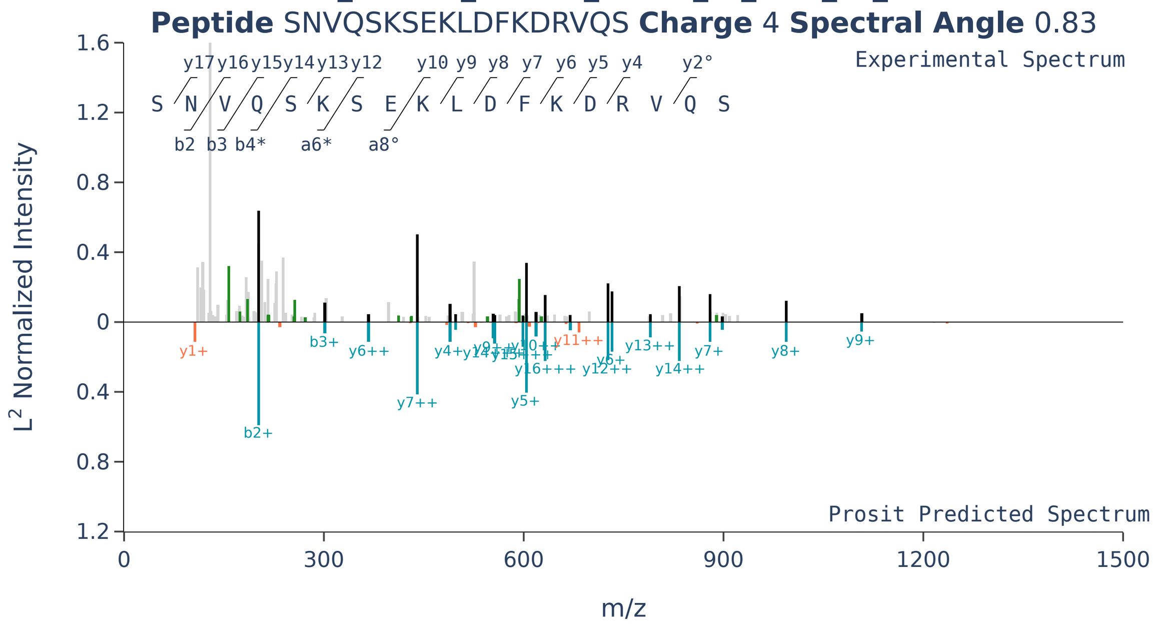

Source WSoh_221121_011221_HFGoe_L1_hTau_24h_R1 **Scan** 10932
Peptide AGSLGNIHHKPGGGQVEV **Charge** 2 **Spectral Angle** 0.81

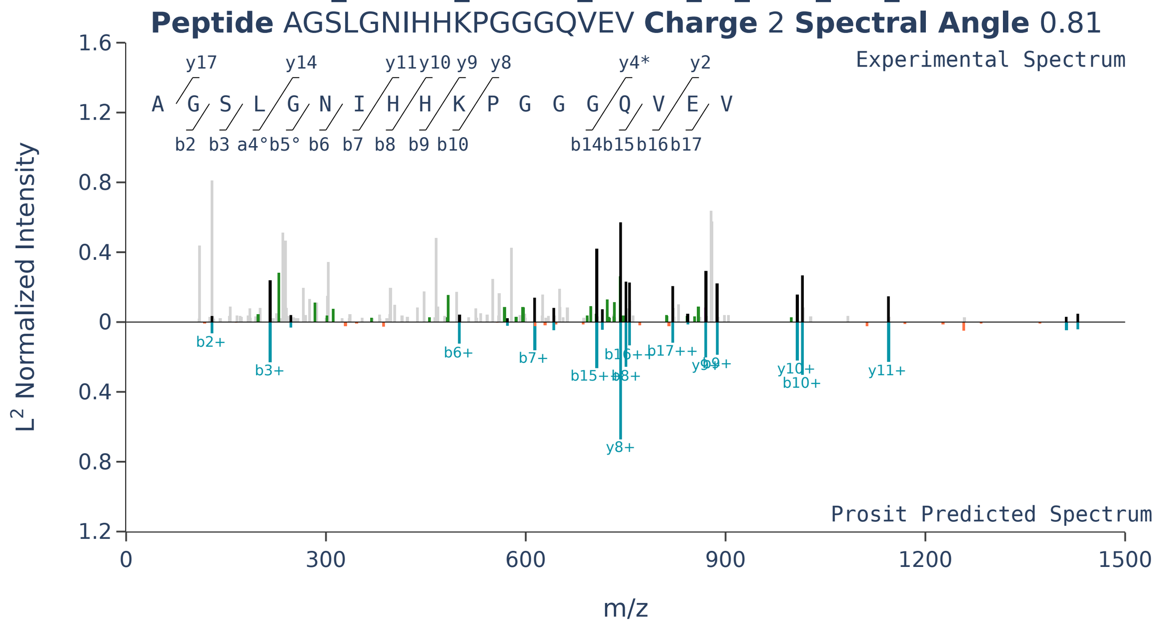

Source WSoh_221121_011221_HFGoe_L1_hTau_4h_R1 **Scan** 10193
Peptide GKHQPGGGKVQII **Charge** 3 **Spectral Angle** 0.82

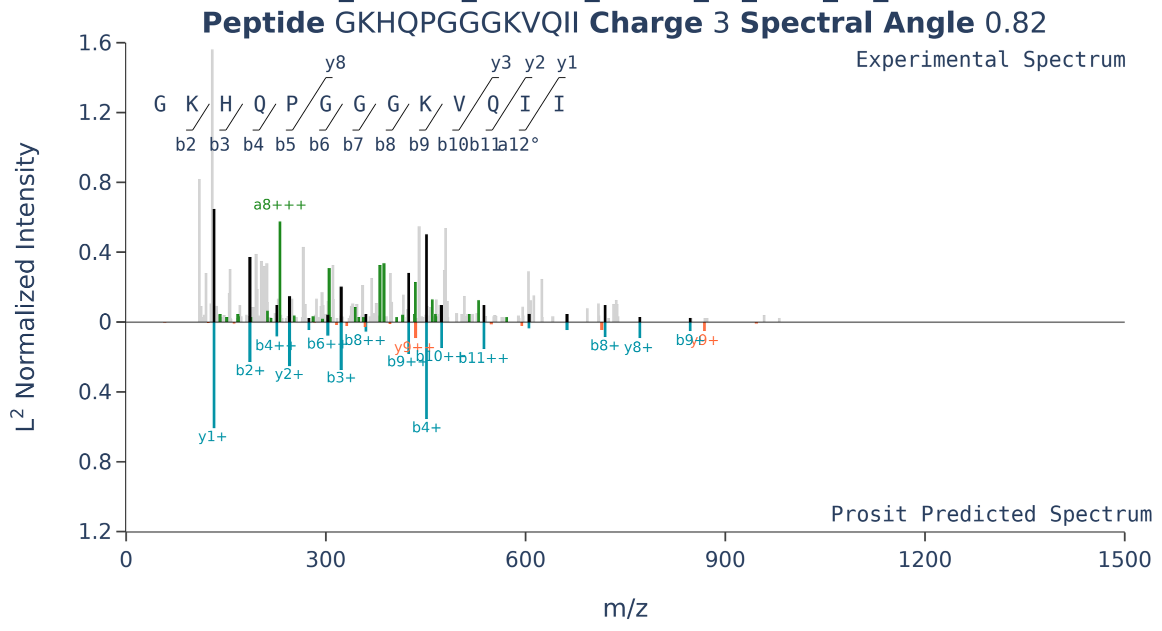

Source WSoh_221121_011221_HFGoe_L2_hTau_24h_R1 **Scan** 10872
Peptide SSLGNIHHKPGGGQVEV **Charge** 2 **Spectral Angle** 0.84

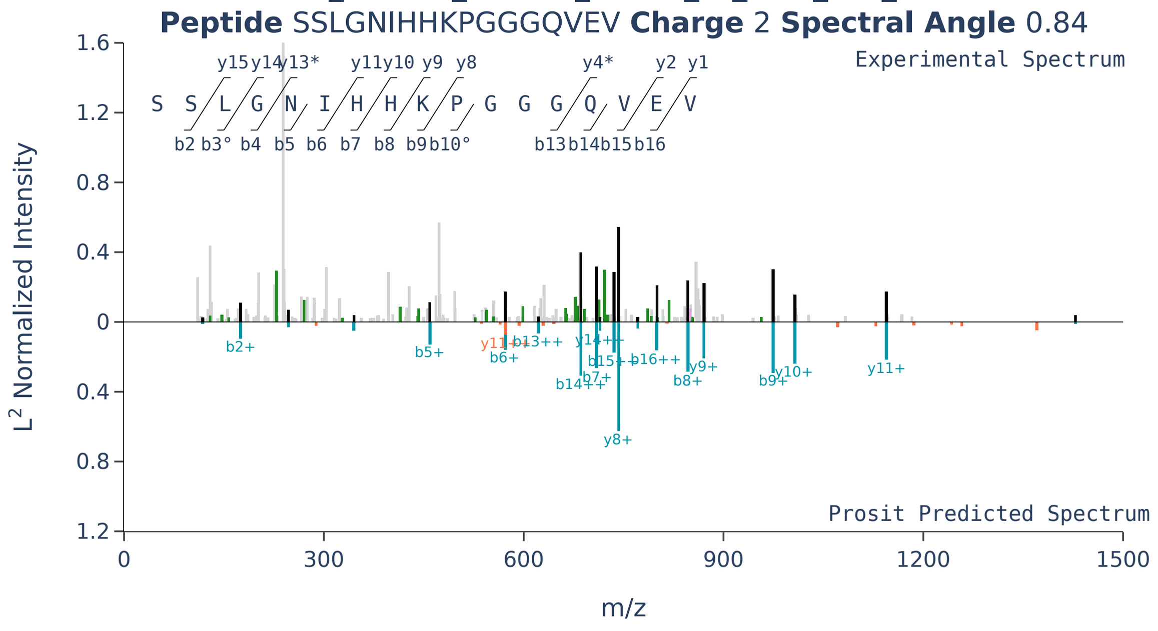

Source WSoh_221121_011221_HFGoe_L2_hTau_24h_R2 **Scan** 7006
Peptide TKHQPGGGKVQIINK **Charge** 3 **Spectral Angle** 0.77

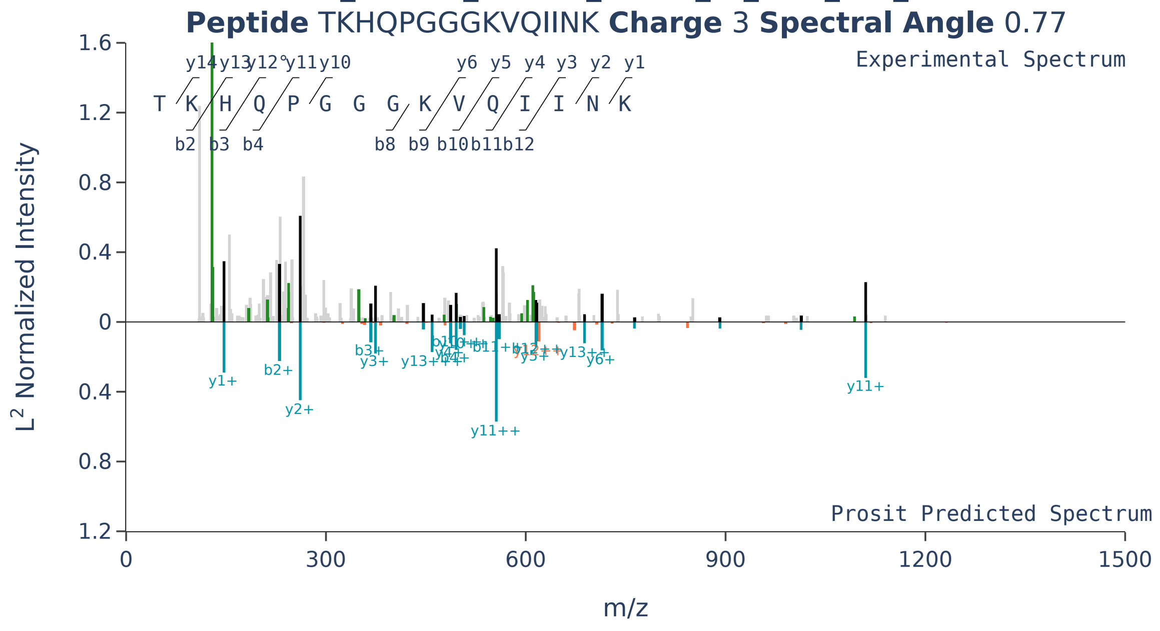

Source WSoh_221121_011221_HFGoe_L1_hTau_24h_R1 **Scan** 18485
Peptide QTAPVPMPDLKNVKH **Charge** 4 **Spectral Angle** 0.84

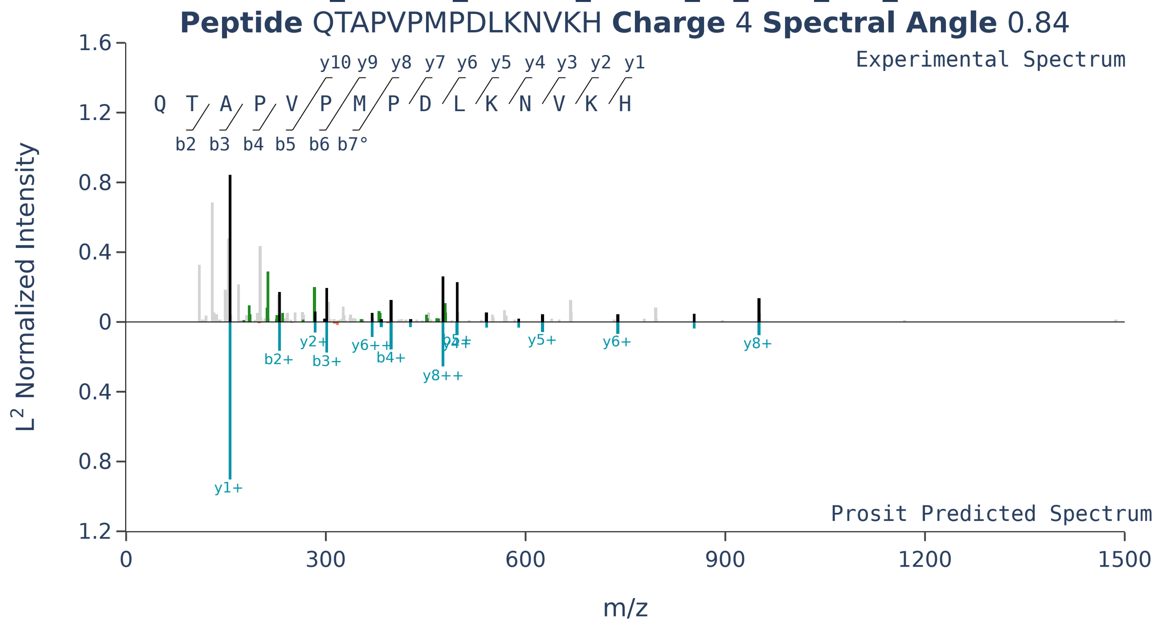

Source WSoh_221121_011221_HFGoe_L1_hTau_4h_R2 **Scan** 14792
Peptide KSVYKPVDL **Charge** 2 **Spectral Angle** 0.75

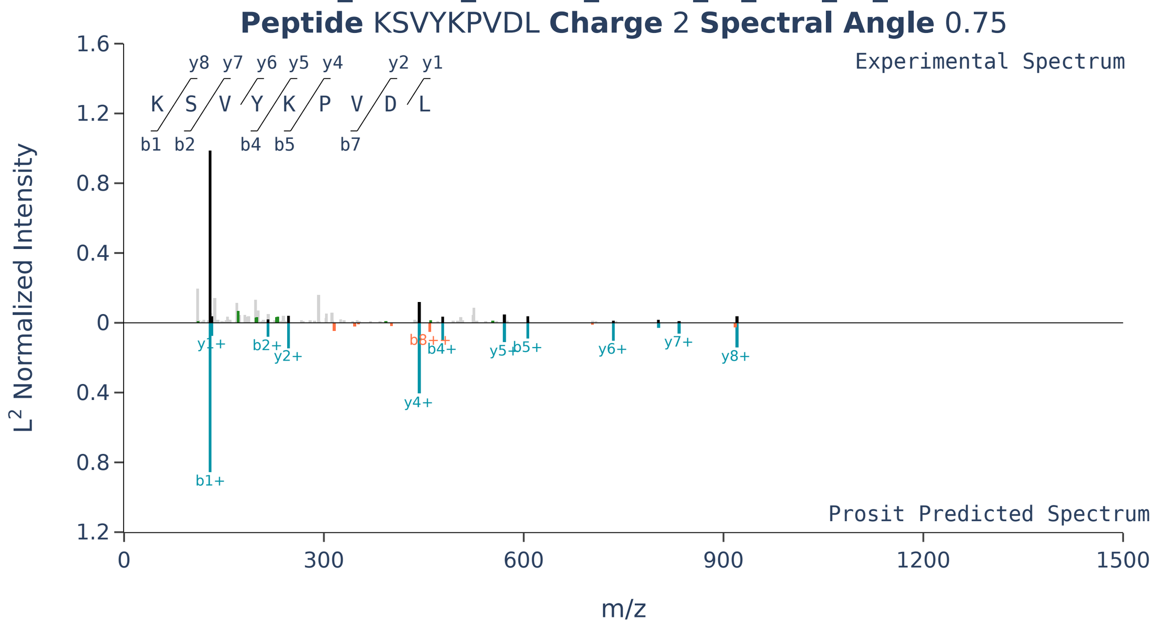

Source WSoh_221121_011221_HFGoe_L1_hTau_24h_R1 **Scan** 21925
Peptide QTAPVPMPDLKNVKSKIGTDAG **Charge** 4 **Spectral Angle** 0.8

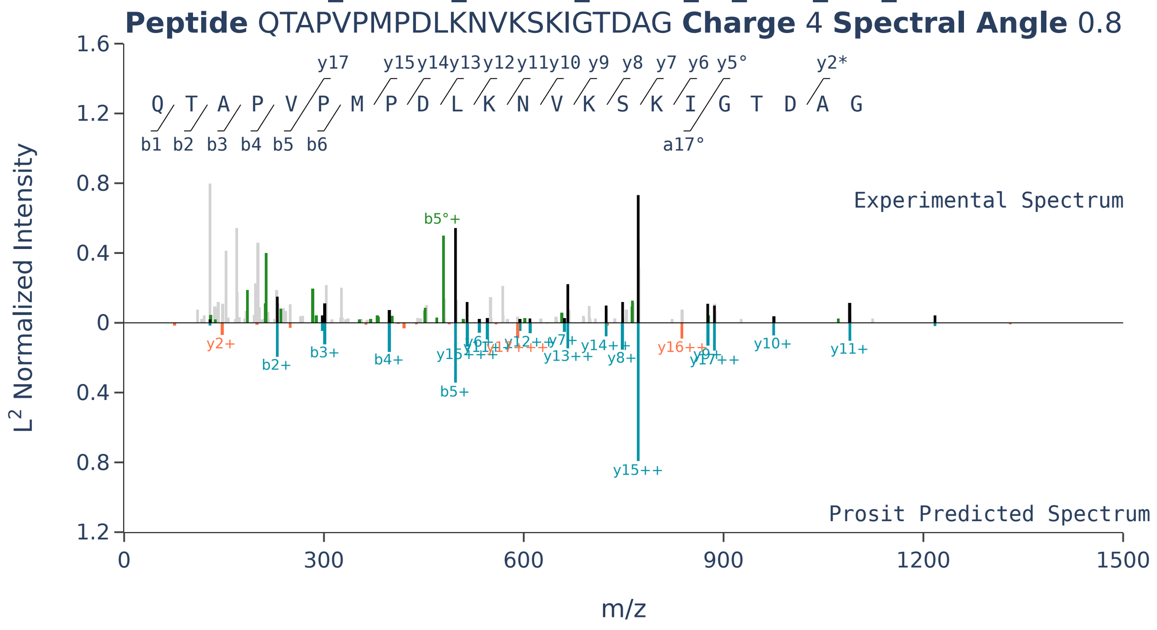

Source WSoh_221121_011221_HFGoe_L1_hTau_4h_R2 **Scan** 14502
Peptide VRQSKIGSLDNITHVPGGGNKKIETHKL **Charge** 5 **Spectral Angle** 0.82

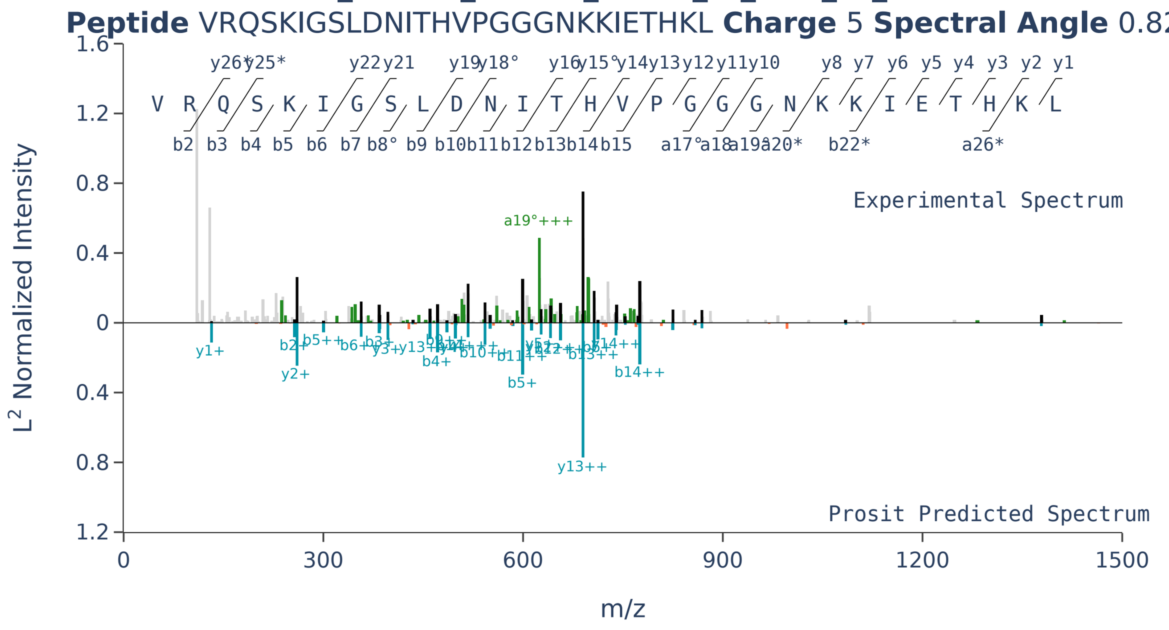

Source WSoh_221121_011221_HFGoe_L1_hTau_4h_R1 **Scan** 6927
Peptide KSEKLDFKDRVQSKH **Charge** 3 **Spectral Angle** 0.85

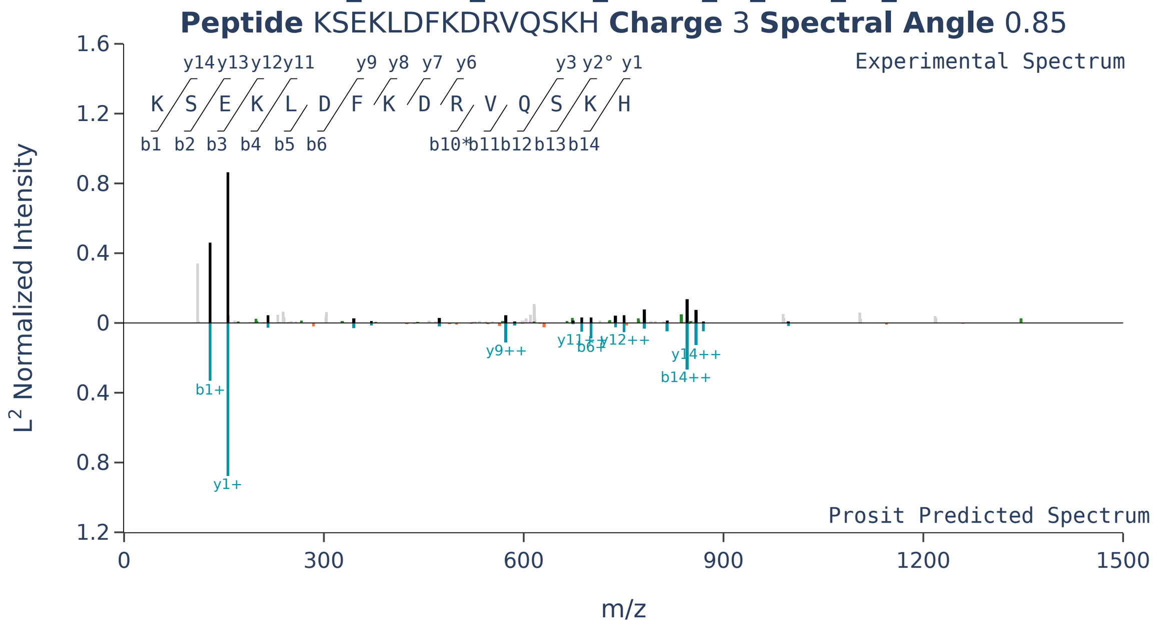

Source WSoh_221121_011221_HFGoe_L1_hTau_2h_R1 **Scan** 6117
Peptide SKHVPGGGSV **Charge** 2 **Spectral Angle** 0.86

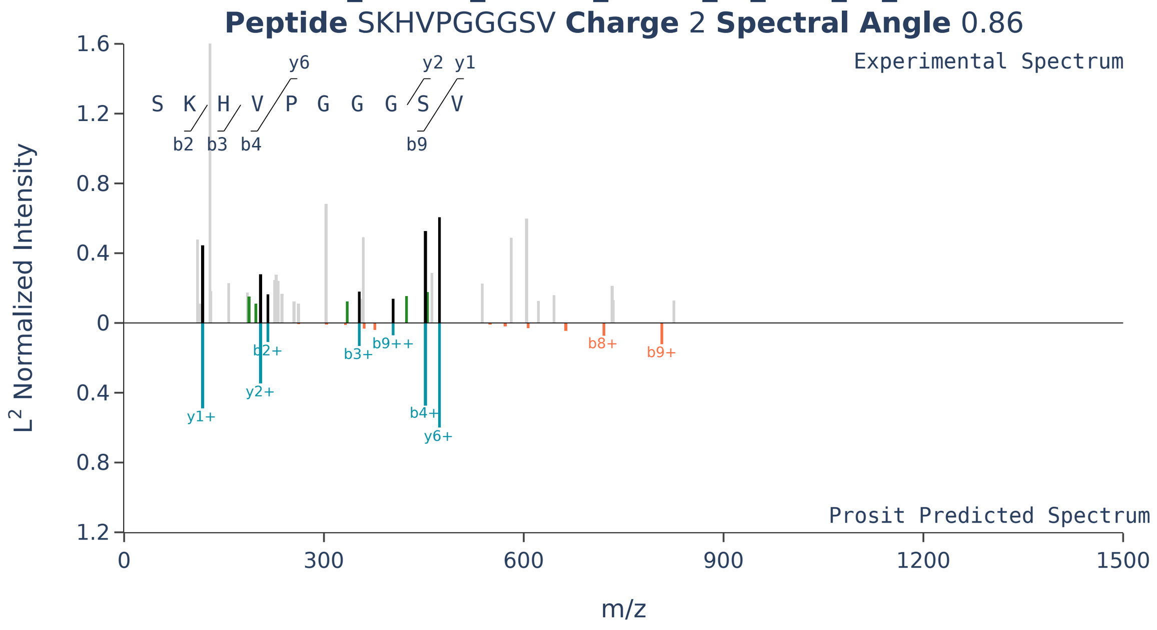

Source WSoh_221121_011221_HFGoe_L2_hTau_24h_R1 **Scan** 16429
Peptide HGAEIVYQTP **Charge** 1 **Spectral Angle** 0.76

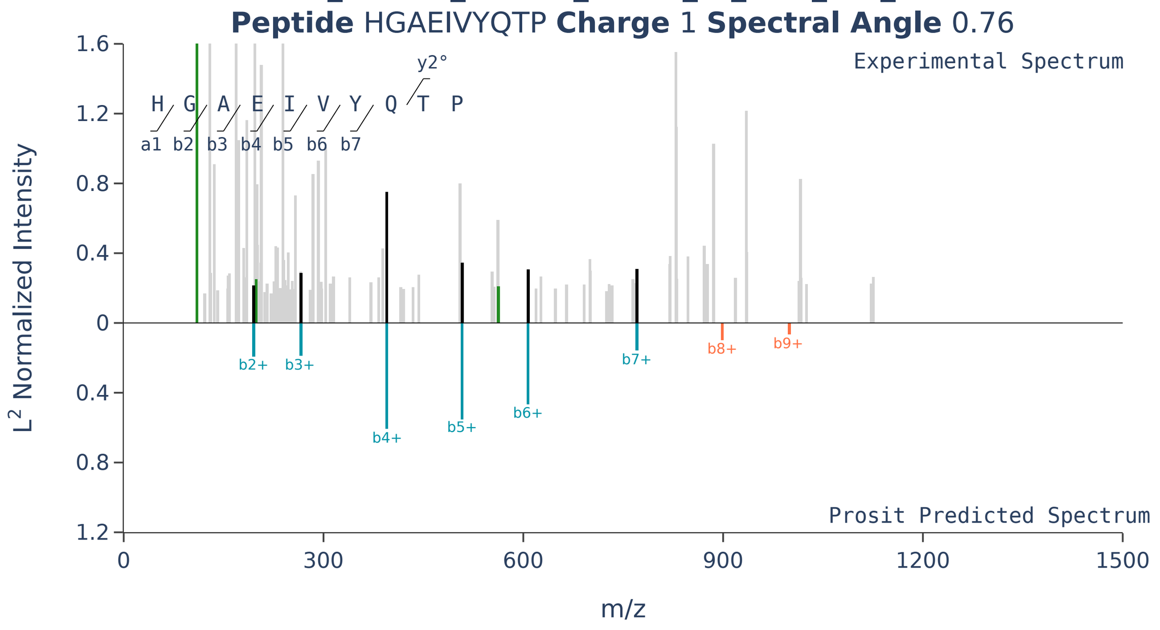

Source WSoh_221121_011221_HFGoe_L1_hTau_24h_R1 **Scan** 21763
Peptide TAPVPMPDLKNVKSKIGTDAG **Charge** 3 **Spectral Angle** 0.88

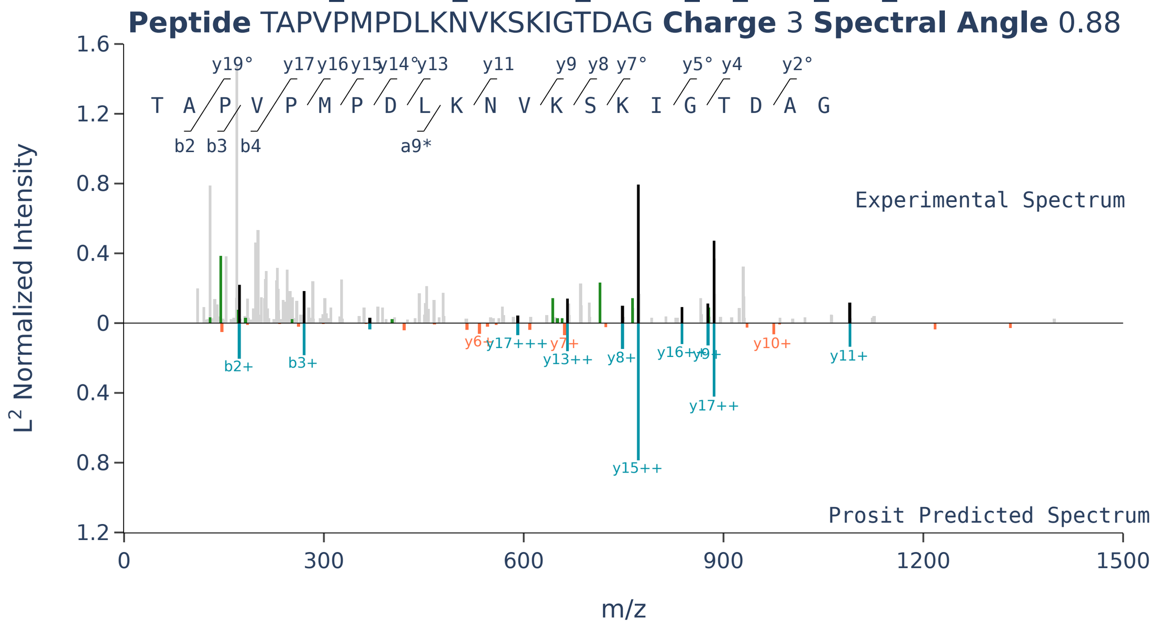

Source WSoh_221121_011221_HFGoe_L2_hTau_4h_R1 **Scan** 11250
Peptide NITHVPGGGNKKIETHKLTFRQD **Charge** 3 **Spectral Angle** 0.81

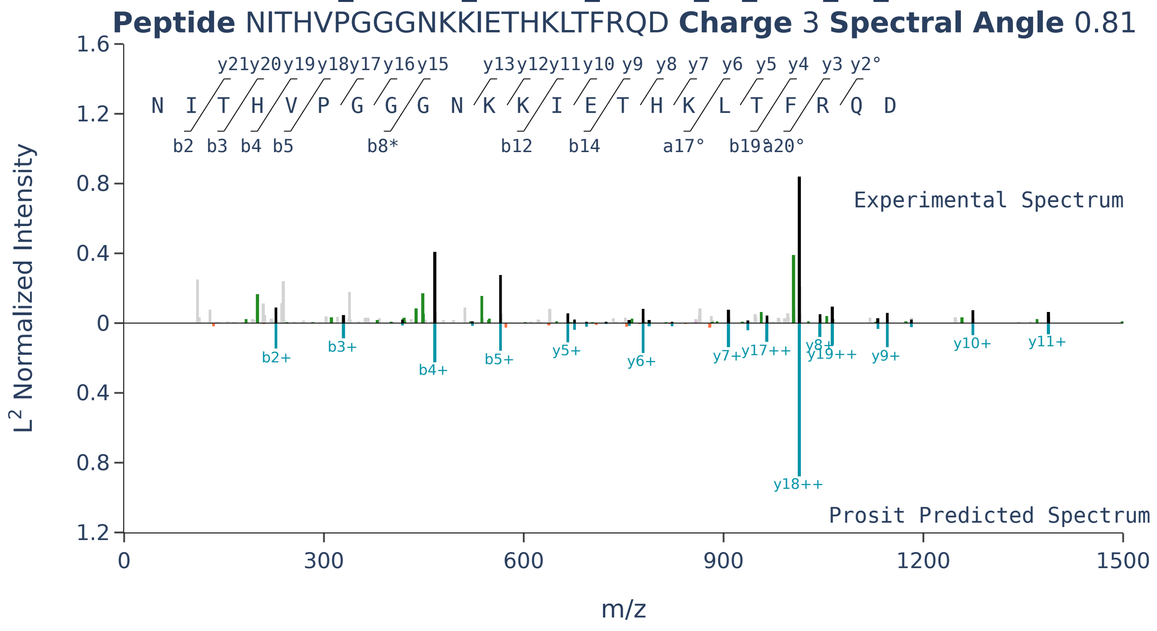

Source WSoh_221121_011221_HFGoe_L2_hTau_24h_R1 **Scan** 21175
Peptide RLQTAPVPMPDLKNSKIG **Charge** 3 **Spectral Angle** 0.86

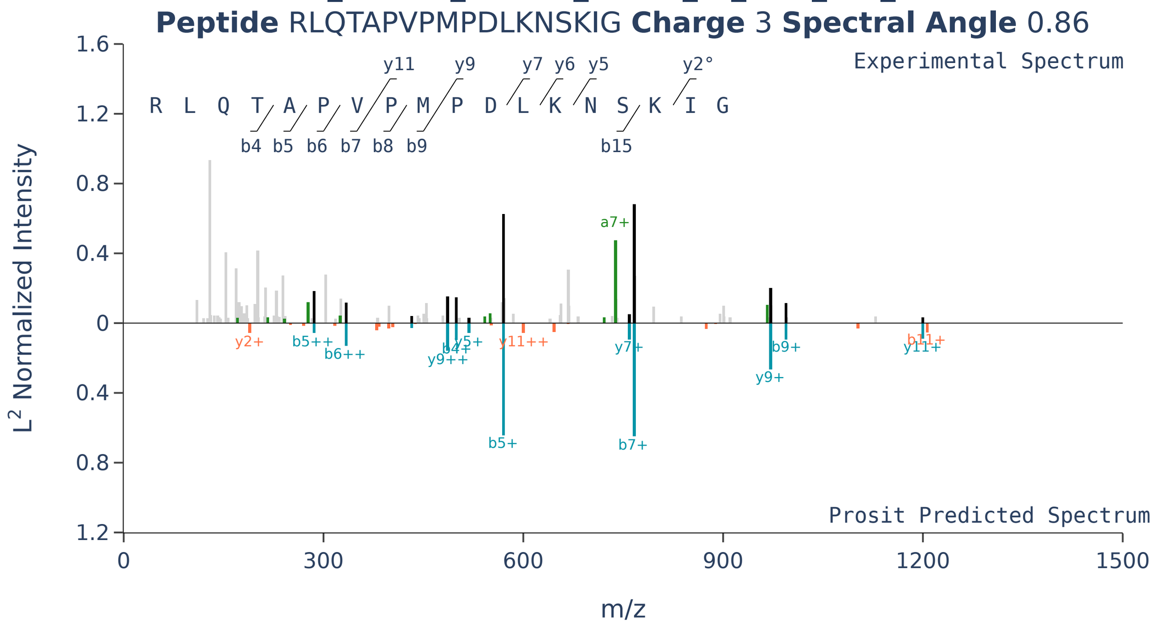

Source WSoh_221121_011221_HFGoe_L1_hTau_0h_R1 **Scan** 5795
Peptide FREPRGA **Charge** 2 **Spectral Angle** 0.85

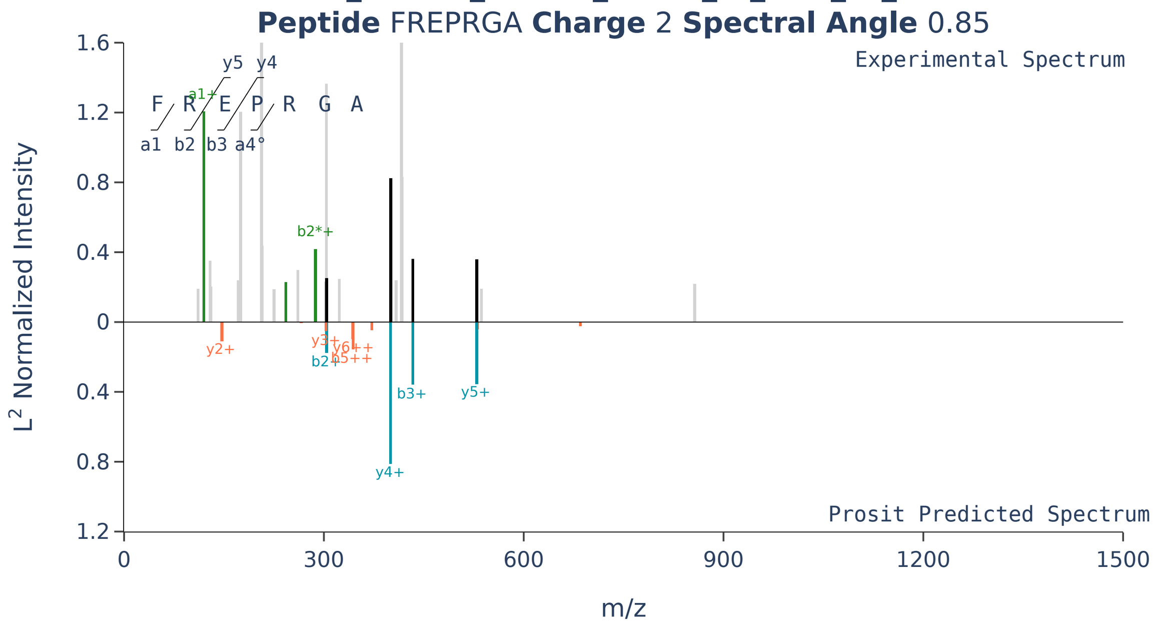

Source WSoh_221121_011221_HFGoe_L1_hTau_24h_R1 **Scan** 18673
Peptide QTAPVPMPDLKNVKSH **Charge** 4 **Spectral Angle** 0.81

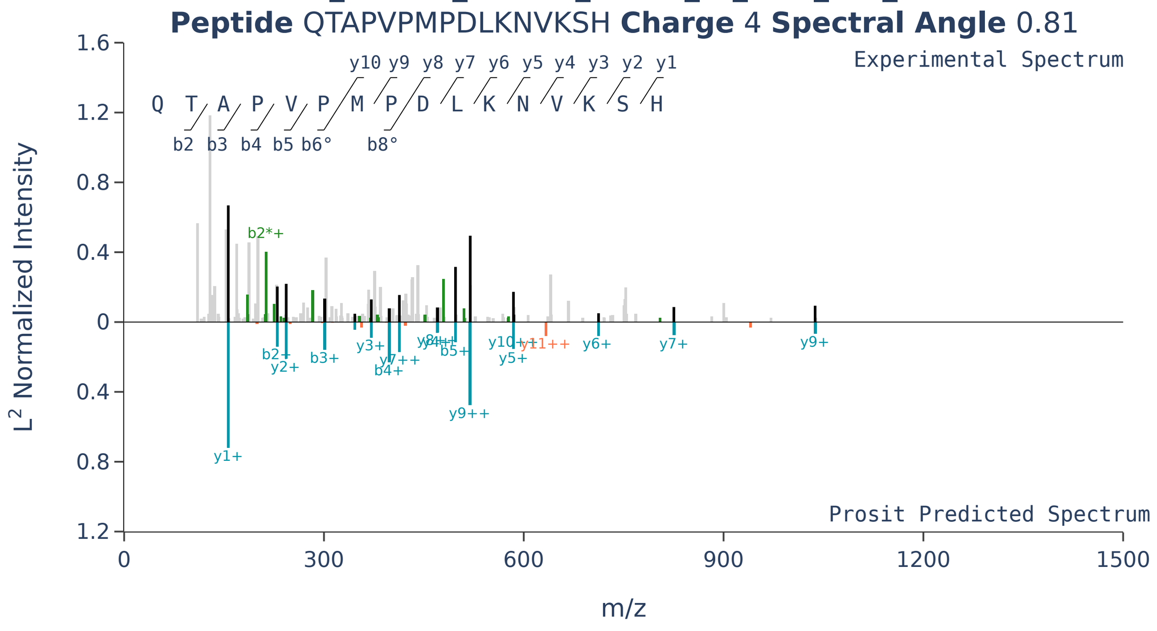

Source WSoh_221121_011221_HFGoe_L1_hTau_24h_R1 **Scan** 16513
Peptide EVMEITHVPGGGNKKIETHKLTFRE **Charge** 5 **Spectral Angle** 0.78

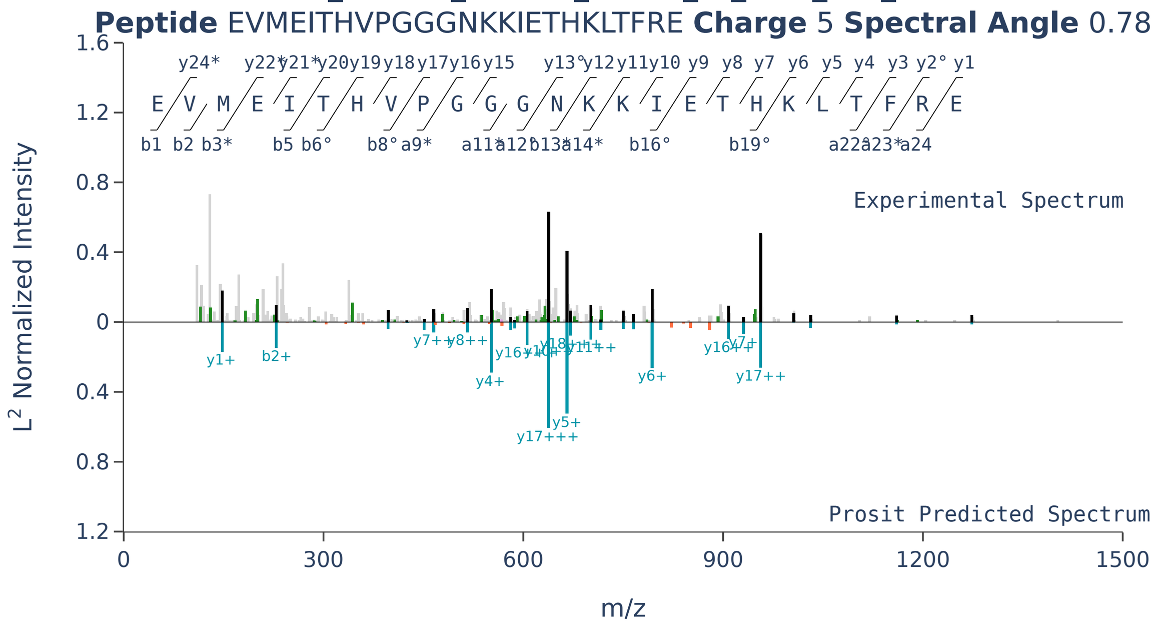

Source WSoh_221121_011221_HFGoe_L2_hTau_1h_R1 **Scan** 11282
Peptide GDTSPRHLSNVAEEA **Charge** 2 **Spectral Angle** 0.83

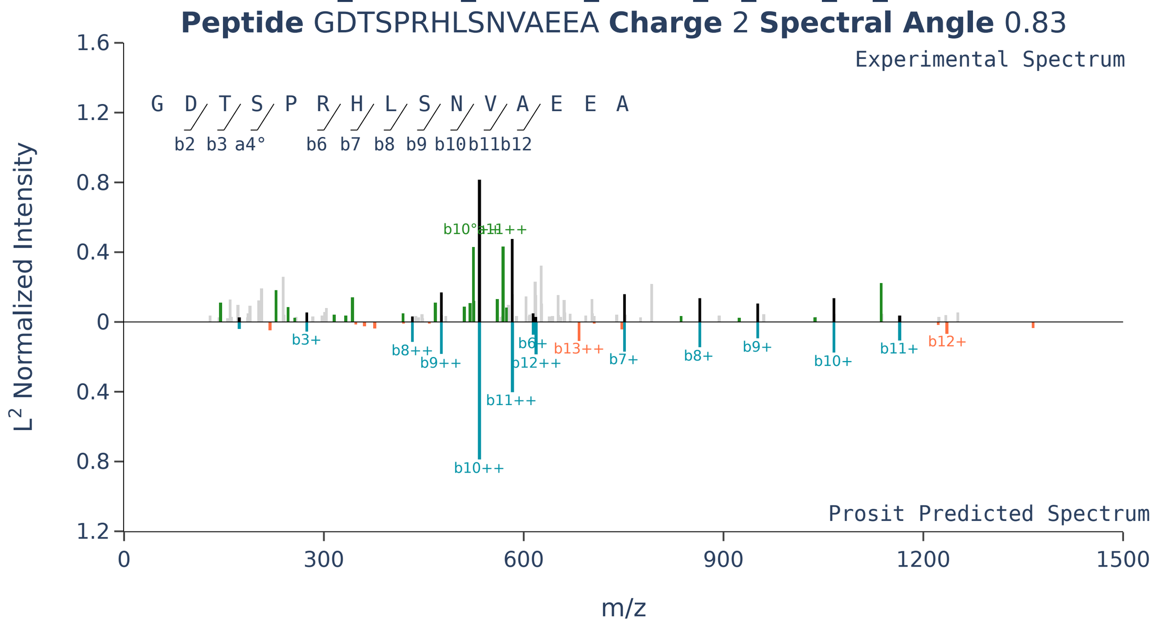

Source WSoh_221121_011221_HFGoe_L2_hTau_24h_R2 **Scan** 25303
Peptide QSKTAPVPMPDL **Charge** 2 **Spectral Angle** 0.87

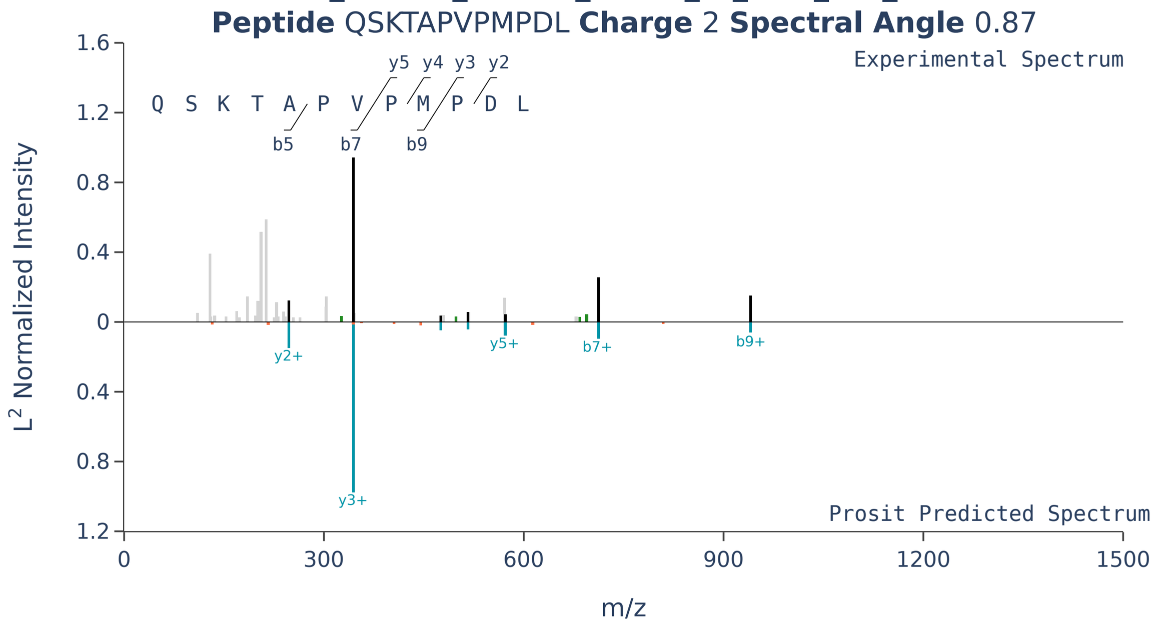

Source WSoh_221121_011221_HFGoe_L1_hTau_1h_R1 **Scan** 11193
Peptide SGDTSPRHLSNVAEEA **Charge** 2 **Spectral Angle** 0.82

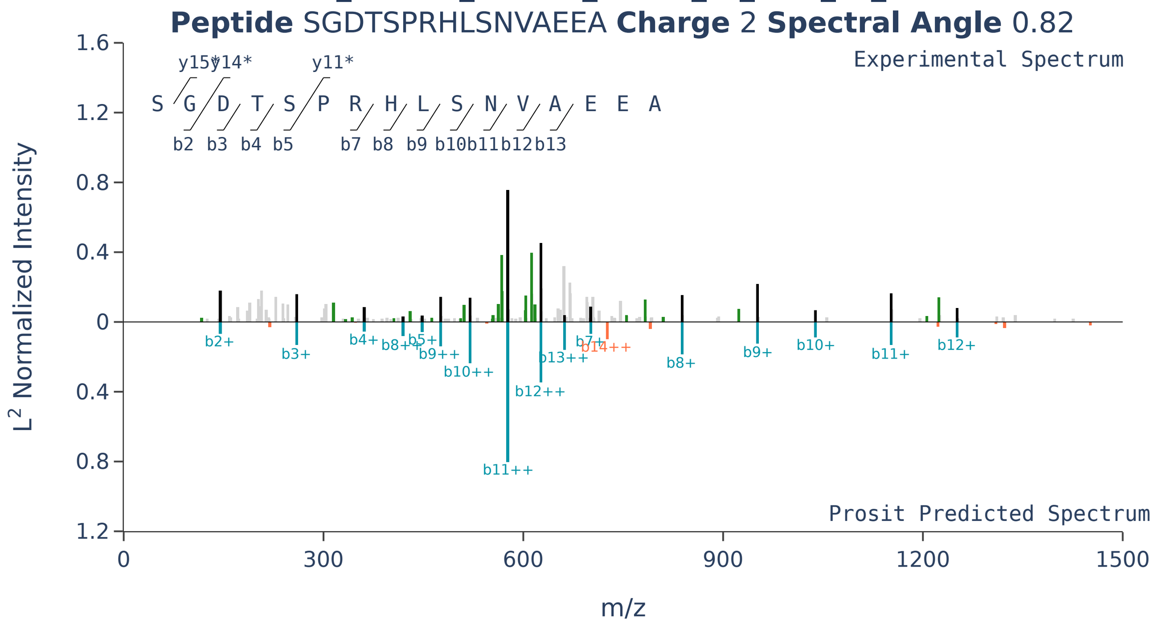

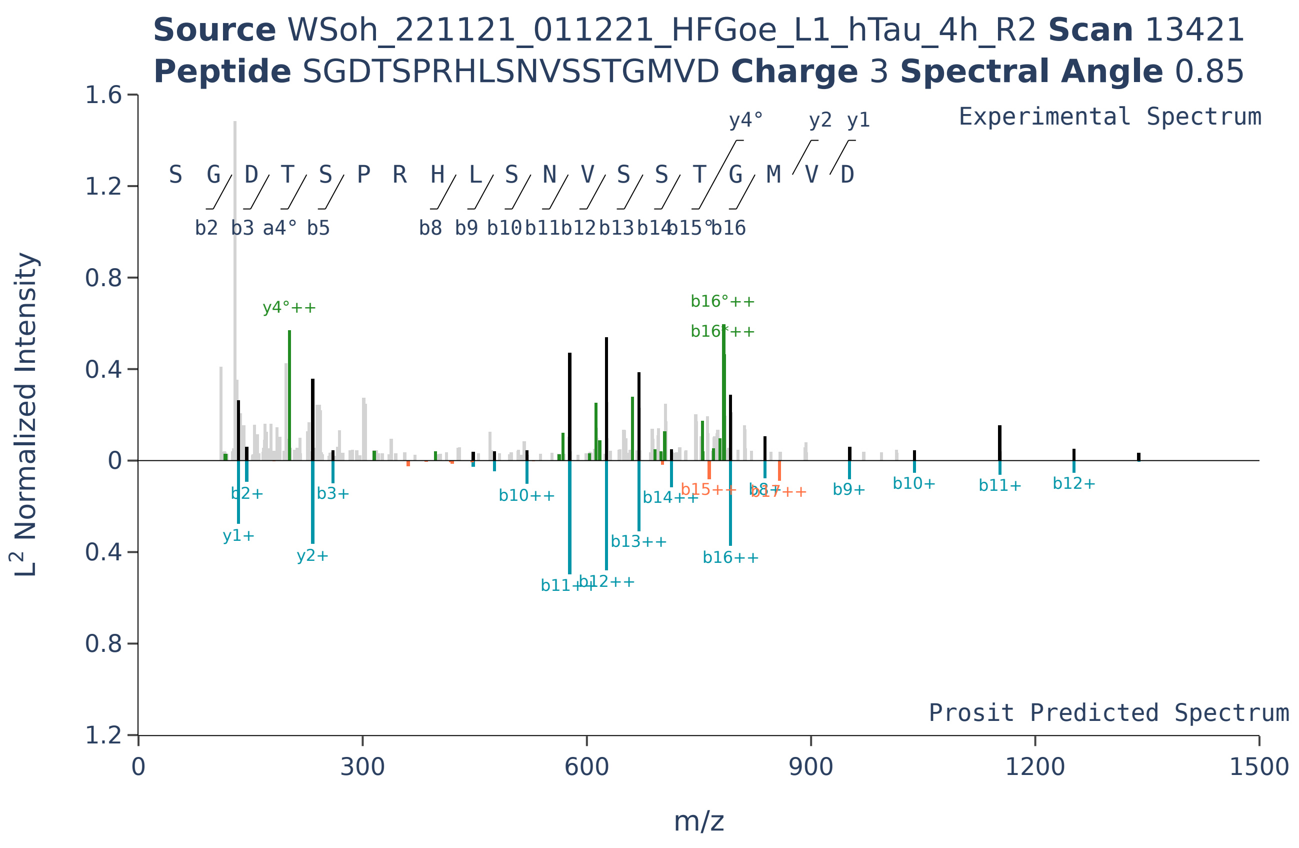

Source WSoh_221121_011221_HFGoe_L1_hTau_4h_R2 **Scan** 13421
Peptide SGDTSPRHLSNVSSTGMVD **Charge** 3 **Spectral Angle** 0.85
Experimental Spectrum
Prosit Predicted Spectrum
L² Normalized Intensity
m/z

Source HRoetschke_211222_160123_HFGoe_IF2_b2R1_2h **Scan** 13795
Peptide DPQAQLVA **Charge** 1 **Spectral Angle** 0.92

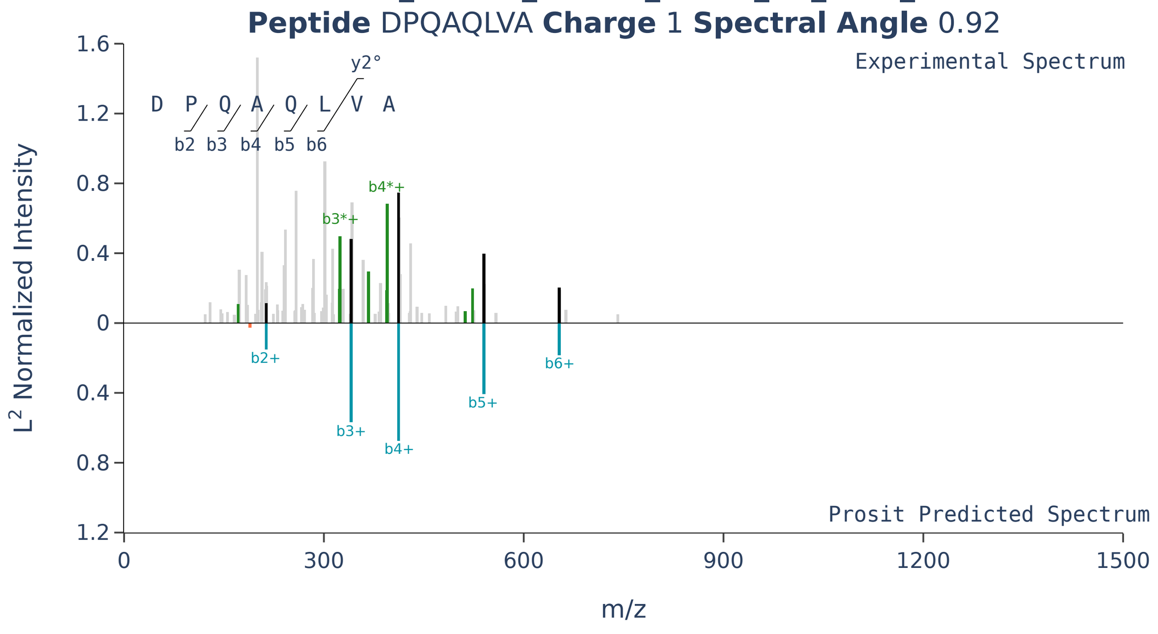

Source HRoetschke_211222_160123_HFGoe_IF2_b1R2_1h **Scan** 14378
Peptide DPQAQLVAE **Charge** 1 **Spectral Angle** 0.95

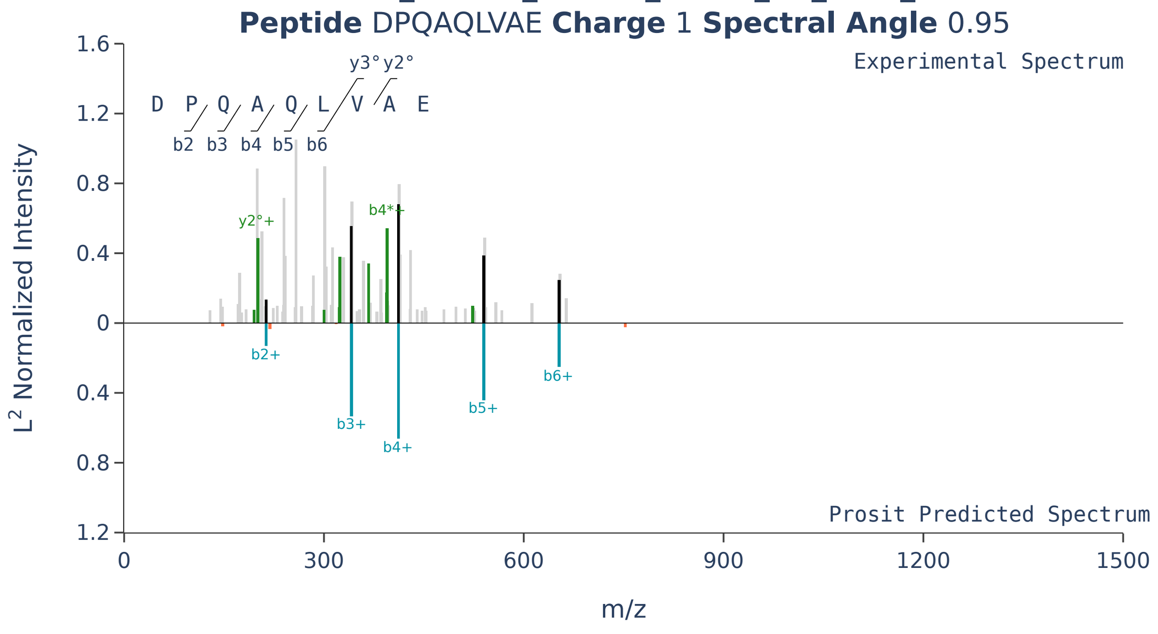

Source HRoetschke_211222_160123_HFGoe_IF2_b1R1_4h **Scan** 18555
Peptide LDAQEVLE **Charge** 2 **Spectral Angle** 0.91

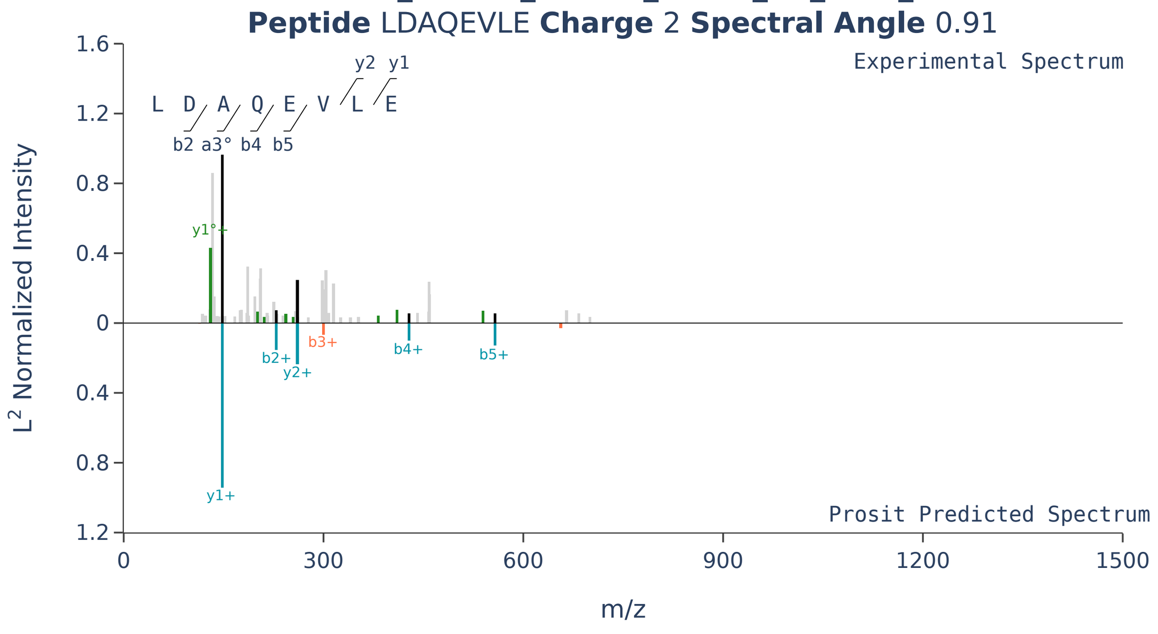

Source HRoetschke_211222_160123_HFGoe_IF2_b2R1_24h **Scan** 11427
Peptide GRDVVIGE **Charge** 2 **Spectral Angle** 0.89

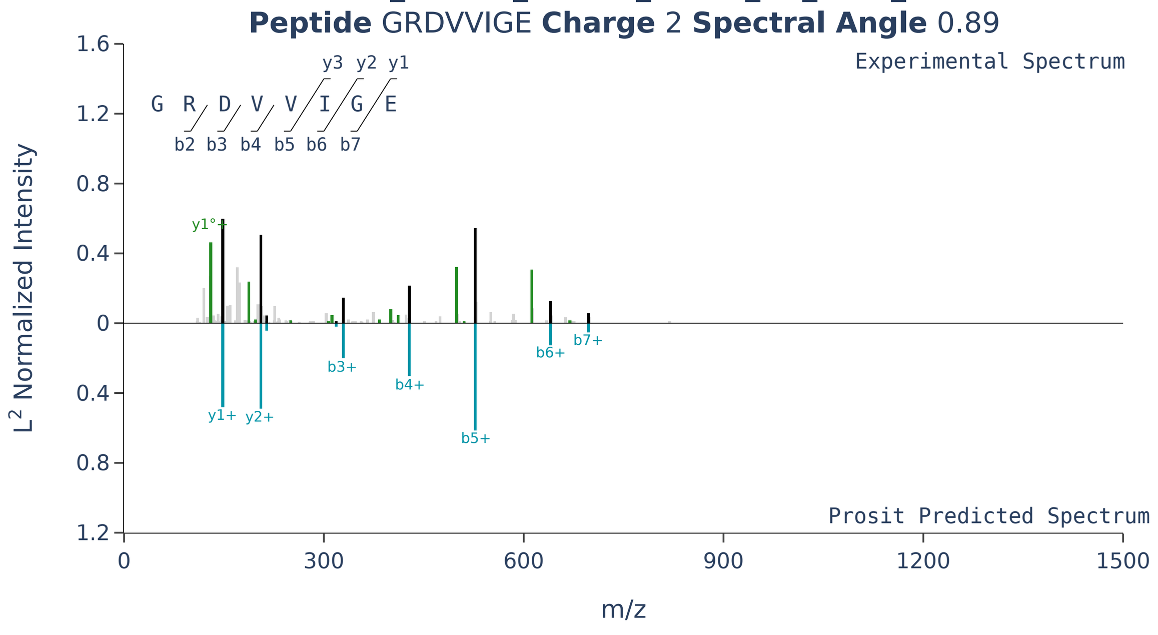

Source HRoetschke_211222_160123_HFGoe_IF2_b1R1_24h **Scan** 16974
Peptide NQVIPVA **Charge** 1 **Spectral Angle** 0.86

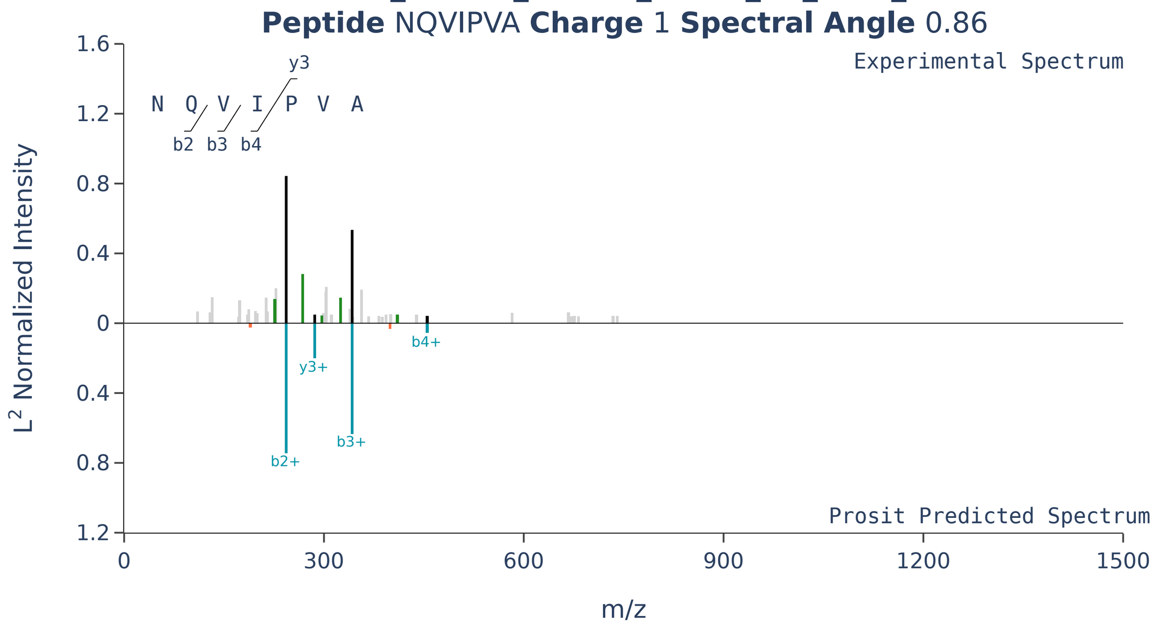

Source HRoetschke_211222_160123_HFGoe_IF2_b2R1_24h **Scan** 11295
Peptide NIPGTIRST **Charge** 2 **Spectral Angle** 0.86

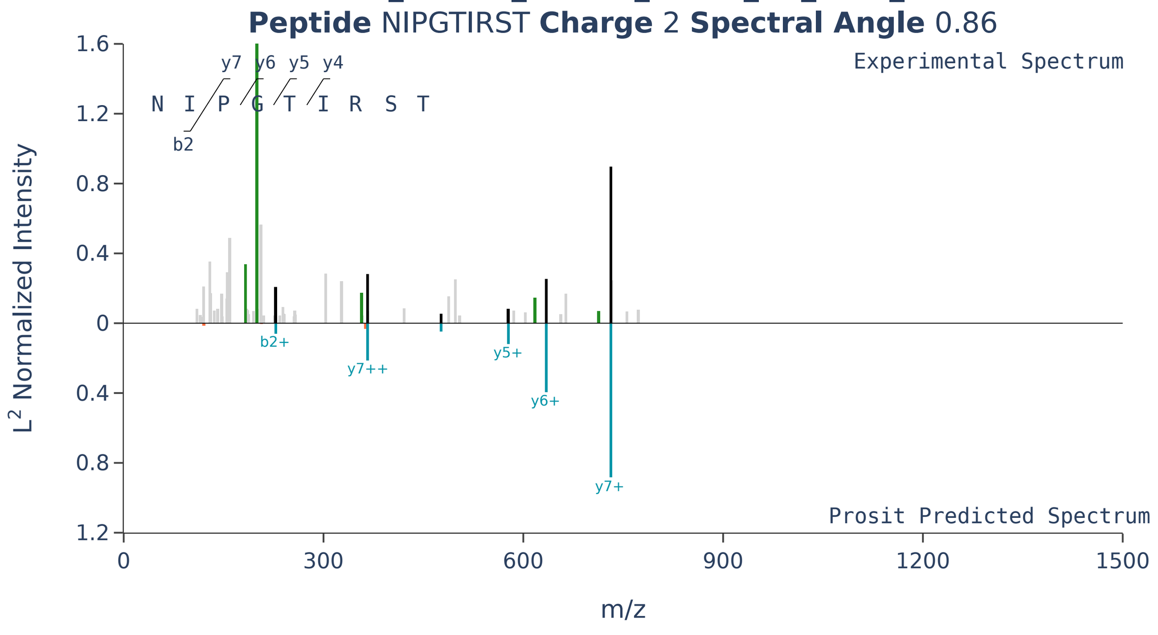

Source HRoetschke_211222_160123_HFGoe_IF2_b1R1_4h **Scan** 6729
Peptide ASGEAGGITQRD **Charge** 2 **Spectral Angle** 0.89

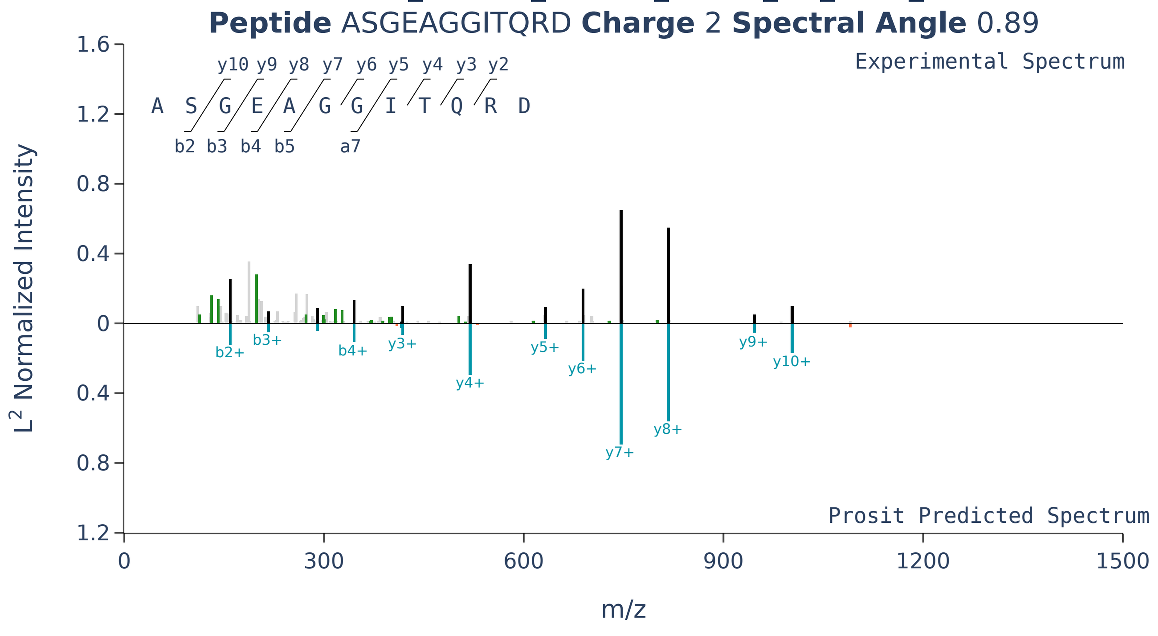

Source HRoetschke_211222_160123_HFGoe_IF2_b1R2_4h **Scan** 13667
Peptide SFLDKGRGPVAD **Charge** 3 **Spectral Angle** 0.89

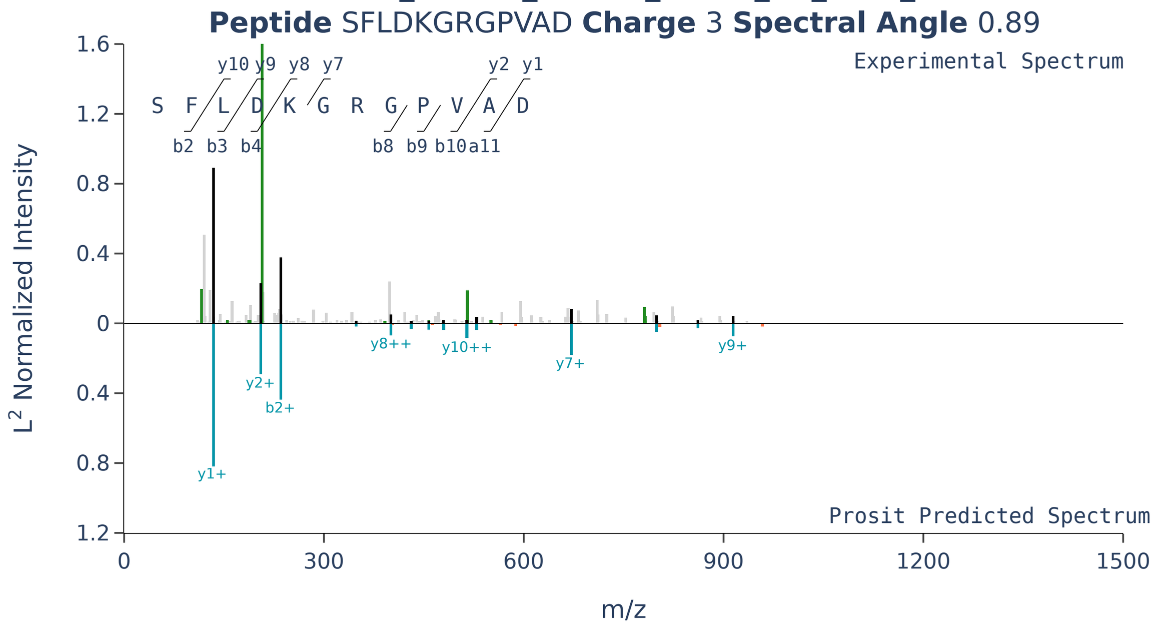

Source HRoetschke_211222_160123_HFGoe_IF2_b2R1_24h **Scan** 6039
Peptide KVASGEAGGITQK **Charge** 3 **Spectral Angle** 0.84

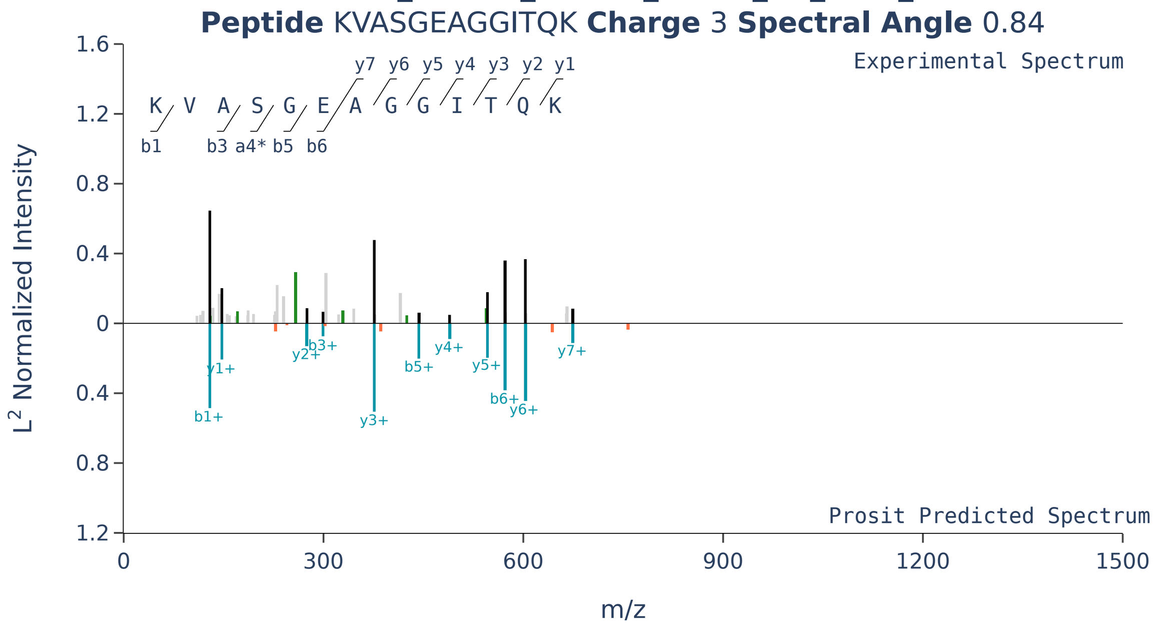

Source HRoetschke_211222_160123_HFGoe_IF2_b1R1_2h **Scan** 8288
Peptide IGAYHVERD **Charge** 3 **Spectral Angle** 0.92

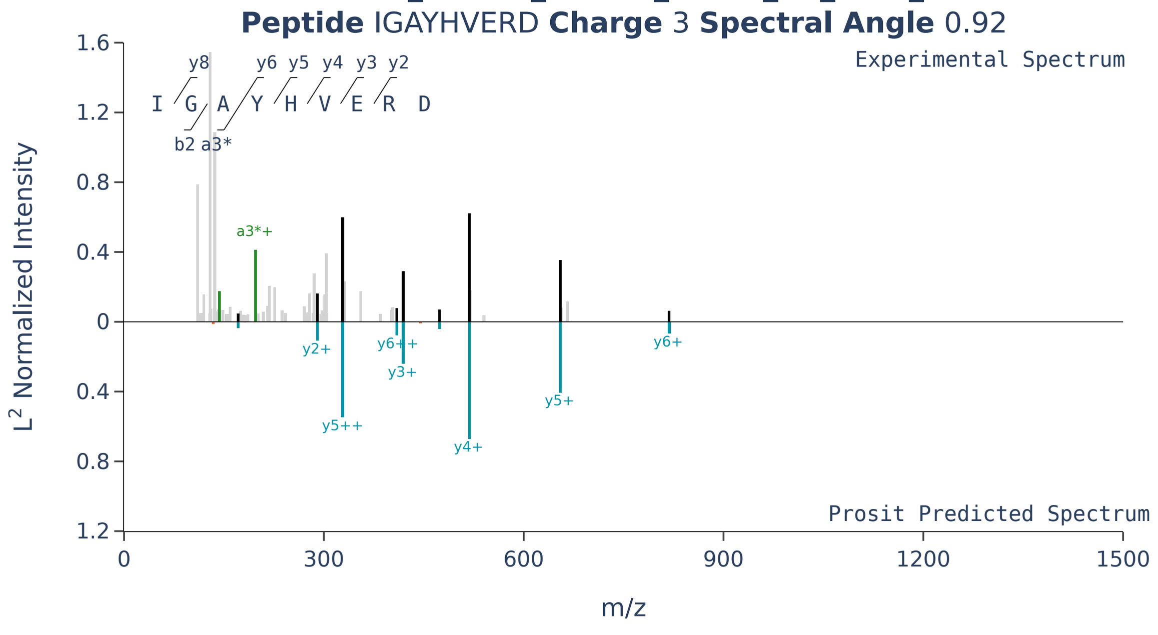

Source HRoetschke_211222_160123_HFGoe_IF2_b2R2_24h **Scan** 18162
Peptide GQEVLELE **Charge** 2 **Spectral Angle** 0.89

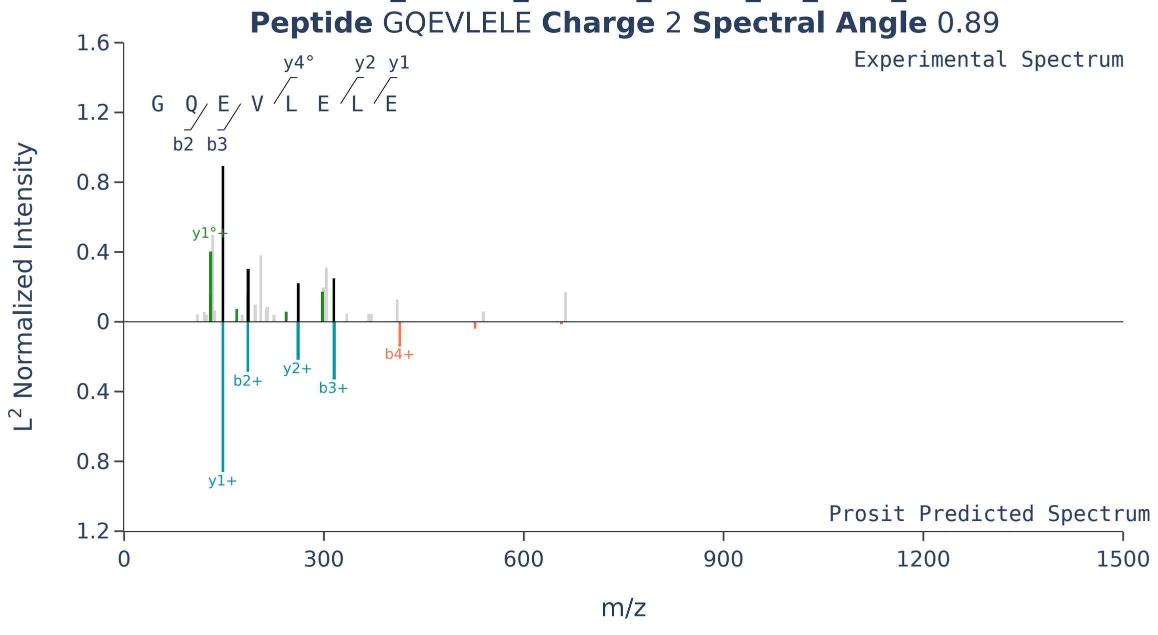

Source HRoetschke_211222_160123_HFGoe_IF2_b1R1_2h **Scan** 6509
Peptide ASGEAGGITRQ **Charge** 2 **Spectral Angle** 0.88

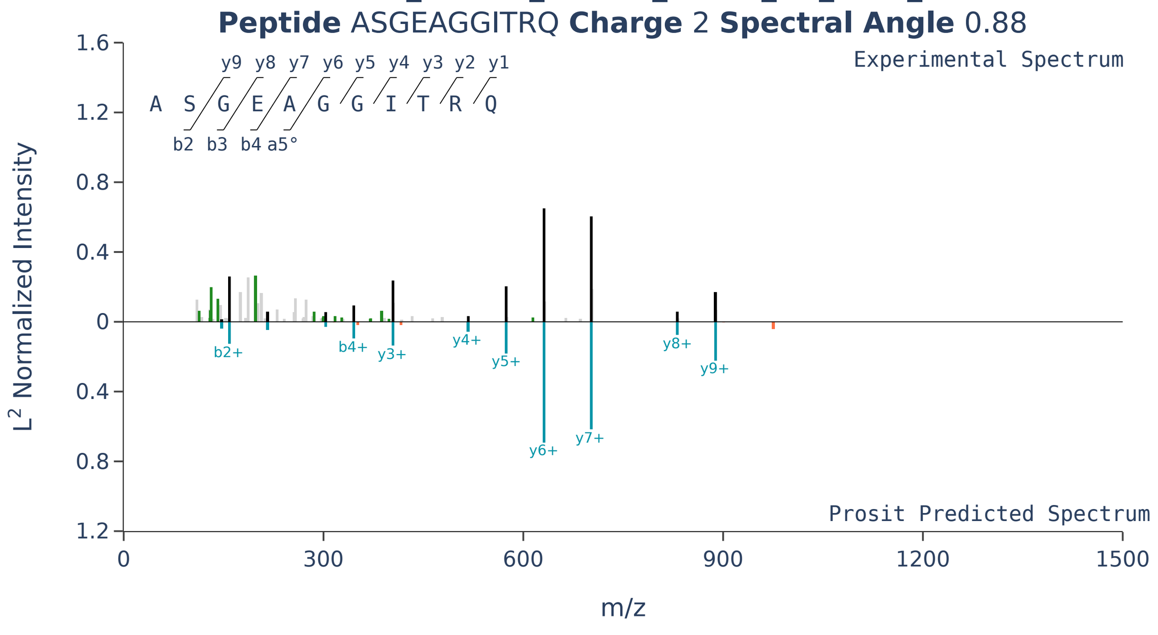

Source HRoetschke_211222_160123_HFGoe_IF2_b1R1_4h **Scan** 21204
Peptide PVVVAVNGGI **Charge** 1 **Spectral Angle** 0.93

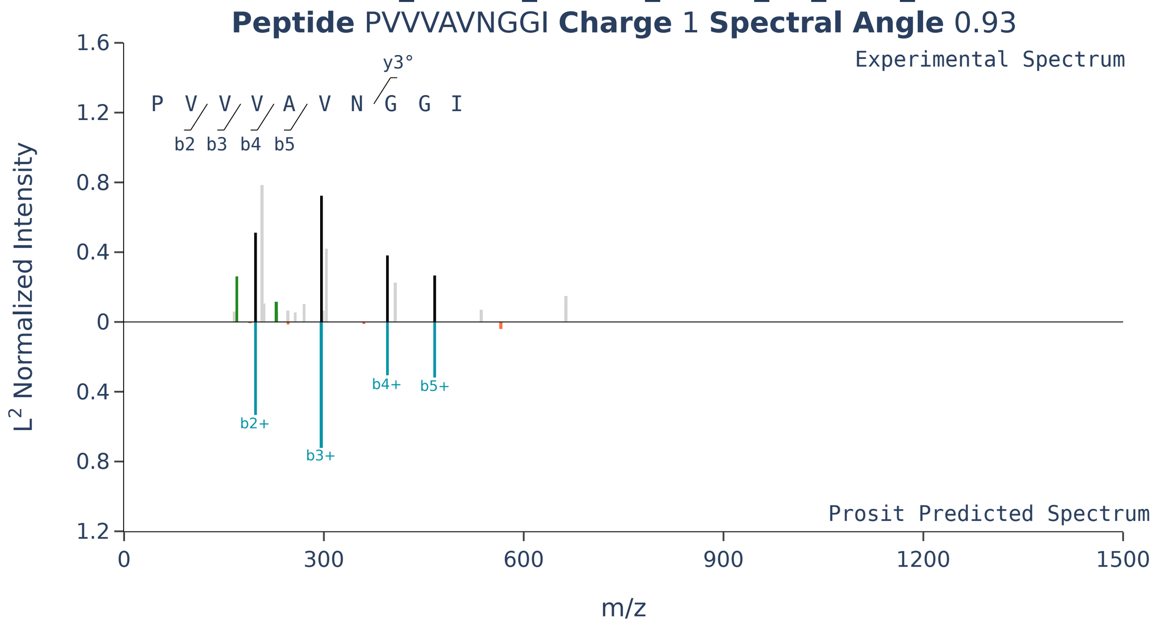

Source HRoetschke_211222_160123_HFGoe_IF2_b1R1_1h **Scan** 11636
Peptide VNRDVVLD **Charge** 2 **Spectral Angle** 0.92

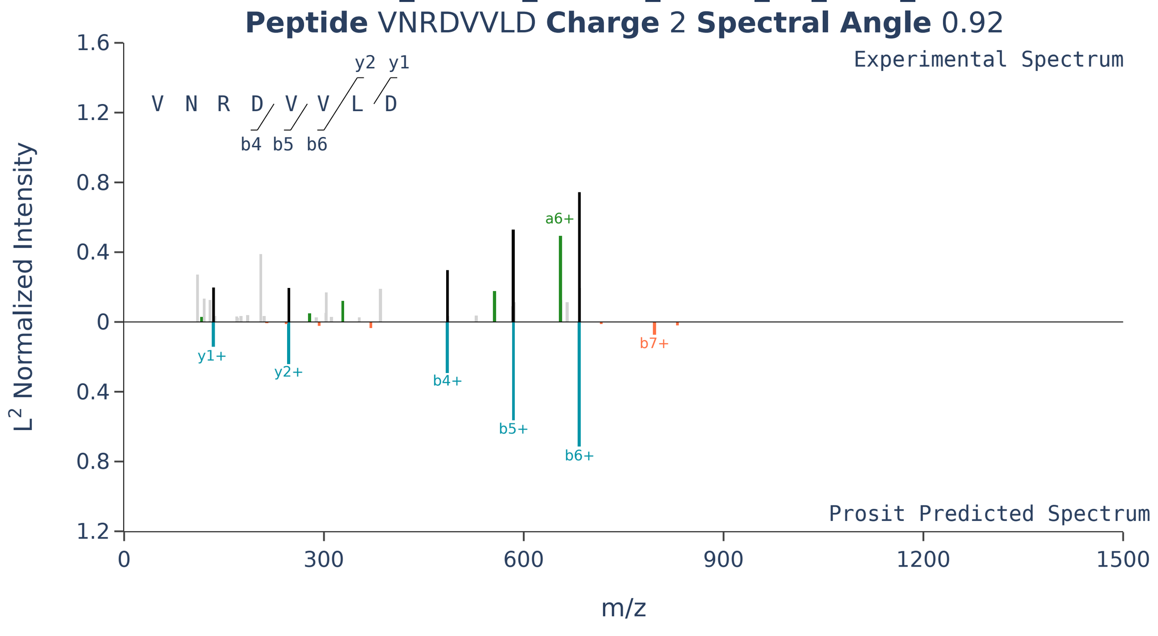

Source HRoetschke_211222_160123_HFGoe_IF2_b1R1_4h **Scan** 23662
Peptide LQAEVLL **Charge** 1 **Spectral Angle** 0.86

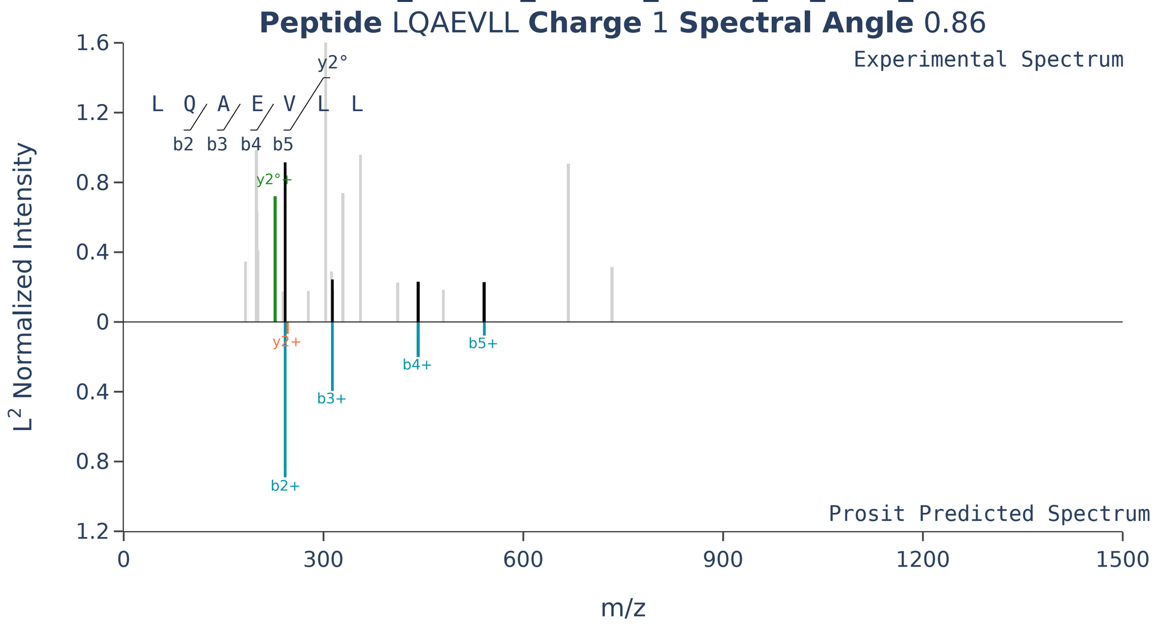

Source HRoetschke_211222_160123_HFGoe_IF2_b1R1_4h **Scan** 6475
Peptide ASGEAGGITQR **Charge** 2 **Spectral Angle** 0.85

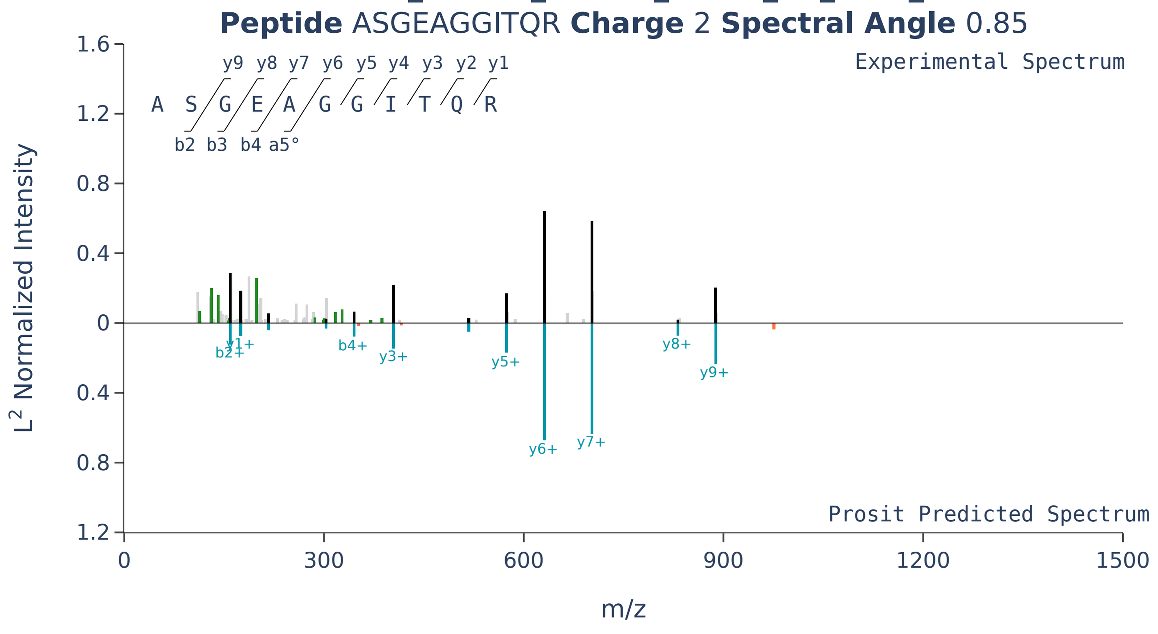

Source HRoetschke_211222_160123_HFGoe_IF2_b2R2_24h **Scan** 15464
Peptide PQTIEAMTD **Charge** 1 **Spectral Angle** 0.91

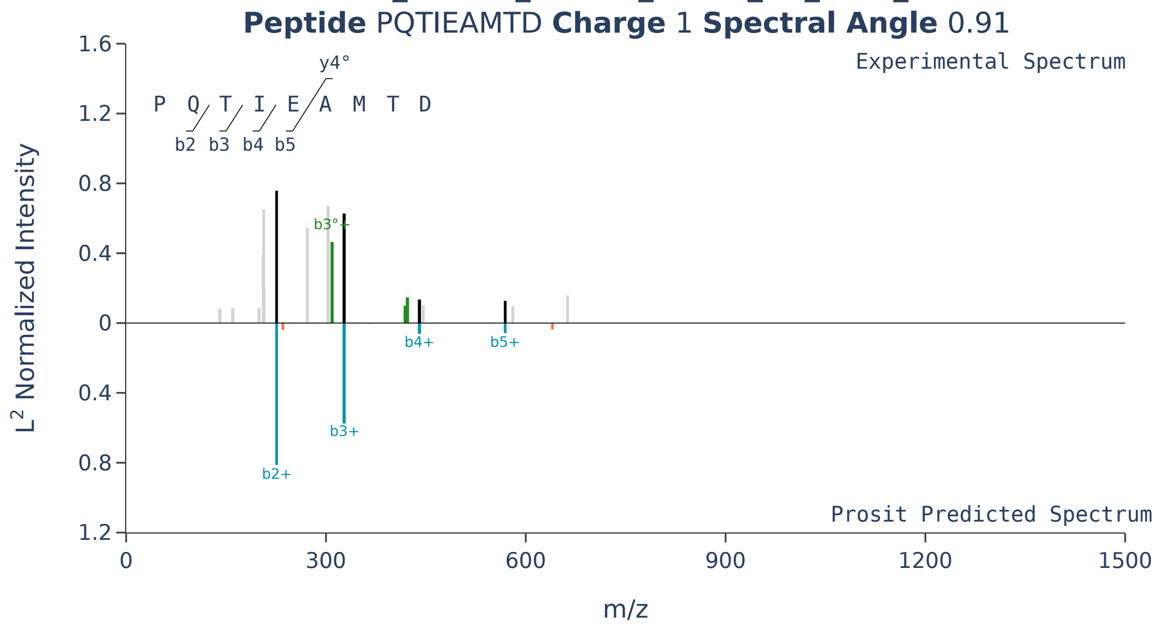

Source HRoetschke_211222_160123_HFGoe_IF2_b1R2_24h **Scan** 17142
Peptide NQVIAPV **Charge** 1 **Spectral Angle** 0.84

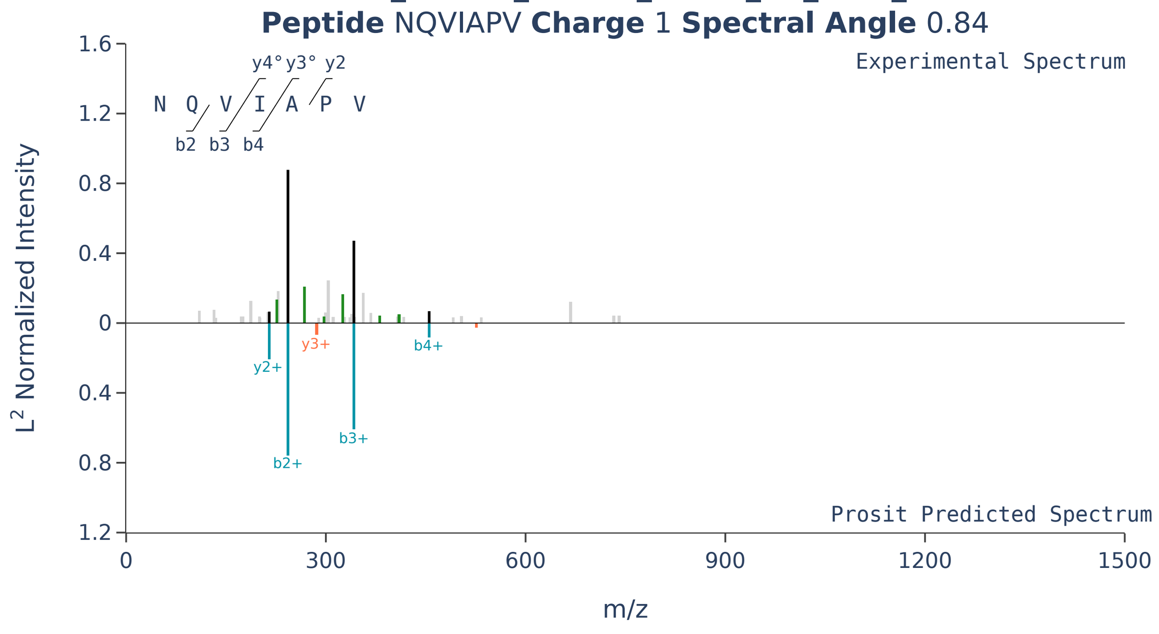

Source HRoetschke_211222_160123_HFGoe_IF2_b1R1_2h **Scan** 7932
Peptide SGVPAAGE **Charge** 1 **Spectral Angle** 0.89

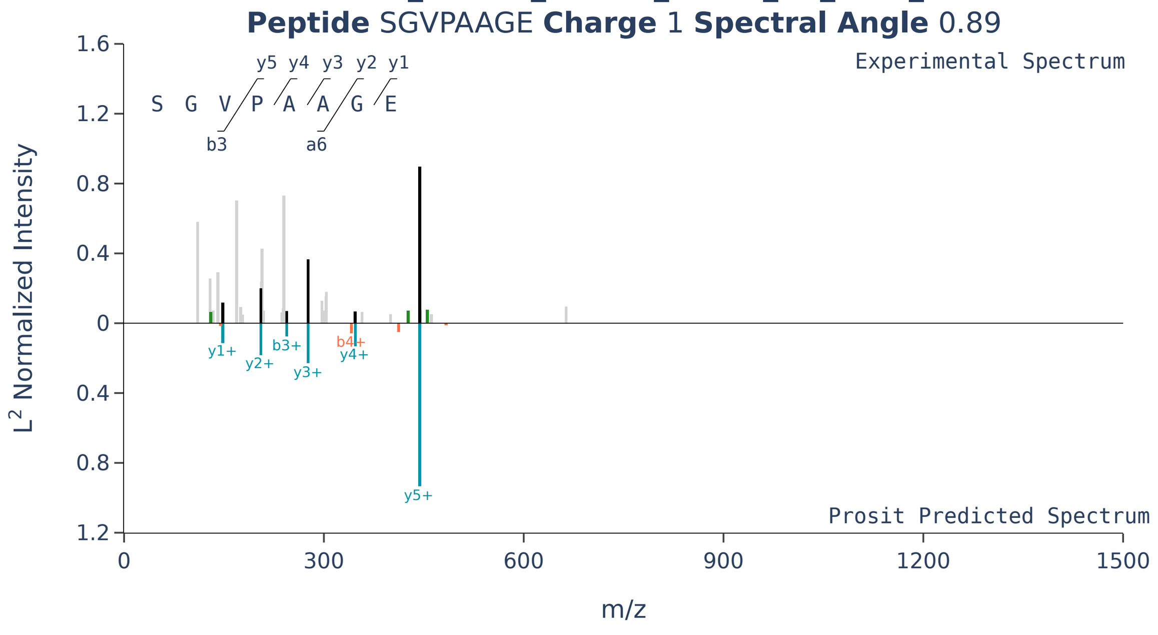

Source HRoetschke_211222_160123_HFGoe_IF2_b2R2_2h **Scan** 11704
Peptide AQLVEAE **Charge** 1 **Spectral Angle** 0.93

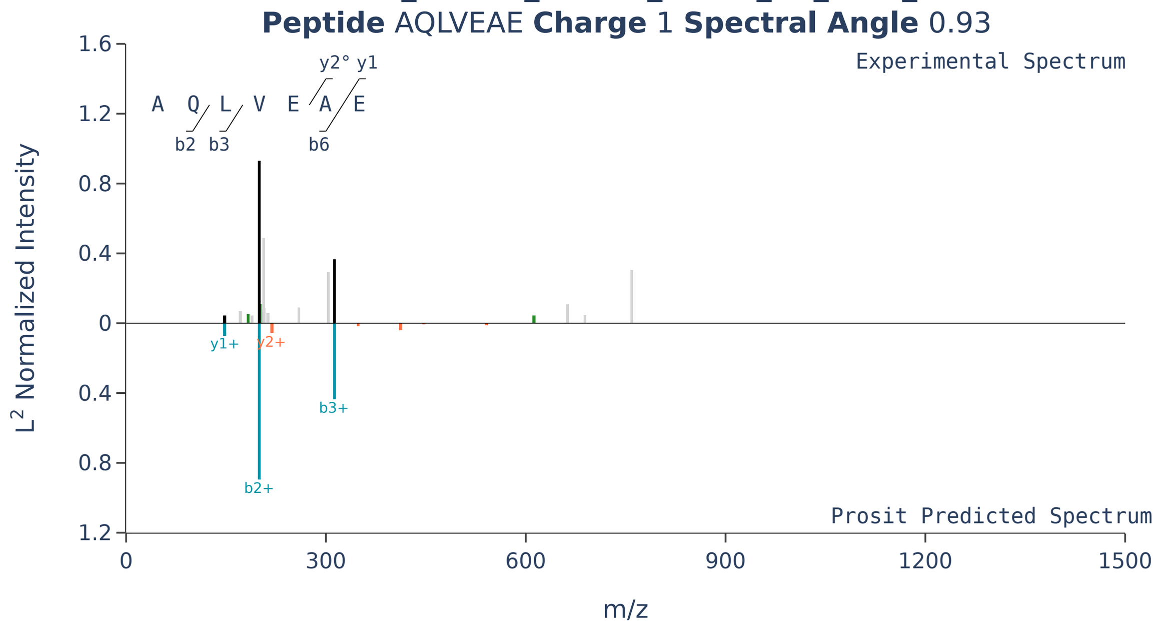

Source HRoetschke_211222_160123_HFGoe_IF2_b1R2_1h **Scan** 9280
Peptide PAQAVNG **Charge** 1 **Spectral Angle** 0.9

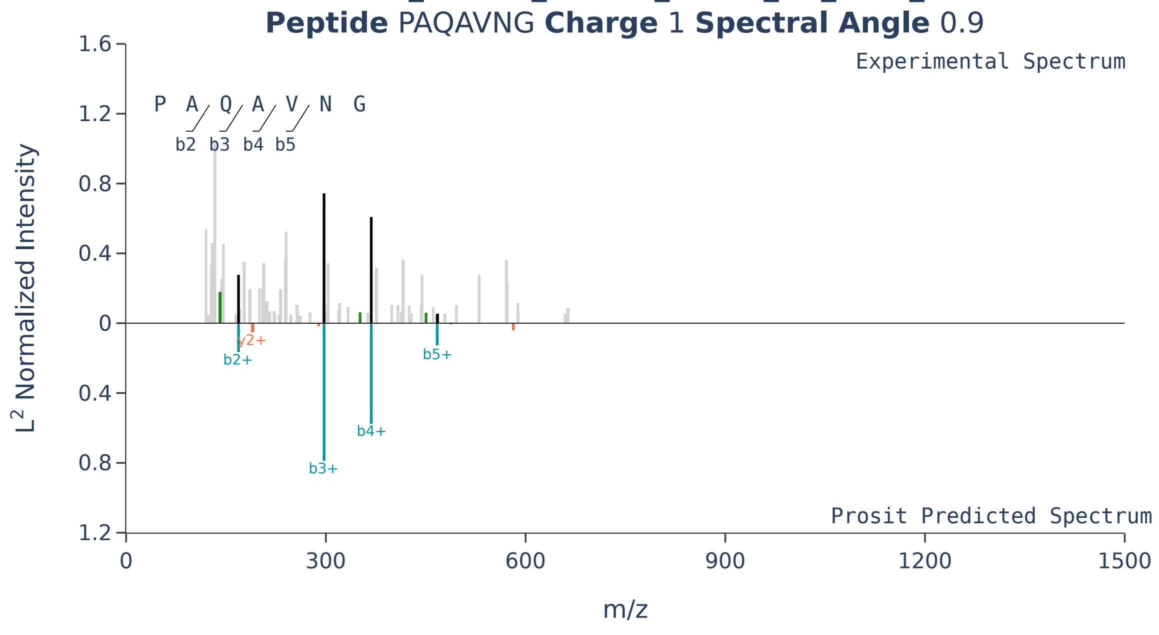

Source HRoetschke_211222_160123_HFGoe_IF2_b1R1_24h **Scan** 17837
Peptide GQEVEIIE **Charge** 2 **Spectral Angle** 0.86

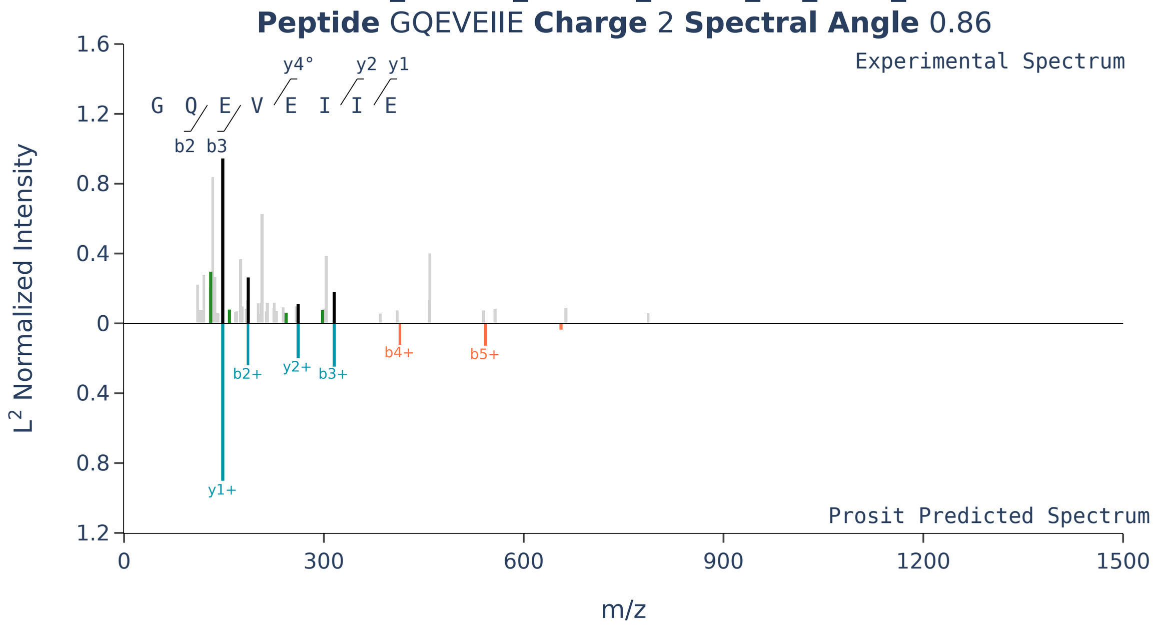

Source HRoetschke_211222_160123_HFGoe_IF2_b1R2_2h **Scan** 9918
Peptide ASGAVITD **Charge** 1 **Spectral Angle** 0.88

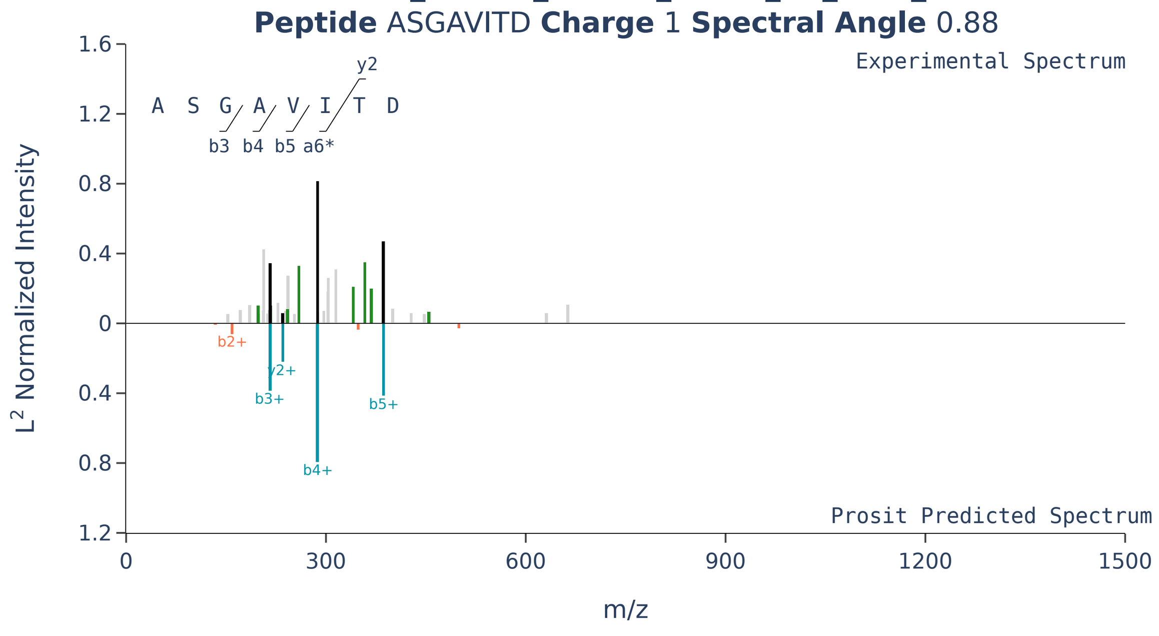

Source HRoetschke_211222_160123_HFGoe_IF2_b2R2_24h **Scan** 12276
Peptide SGVPAAGRNEL **Charge** 2 **Spectral Angle** 0.9

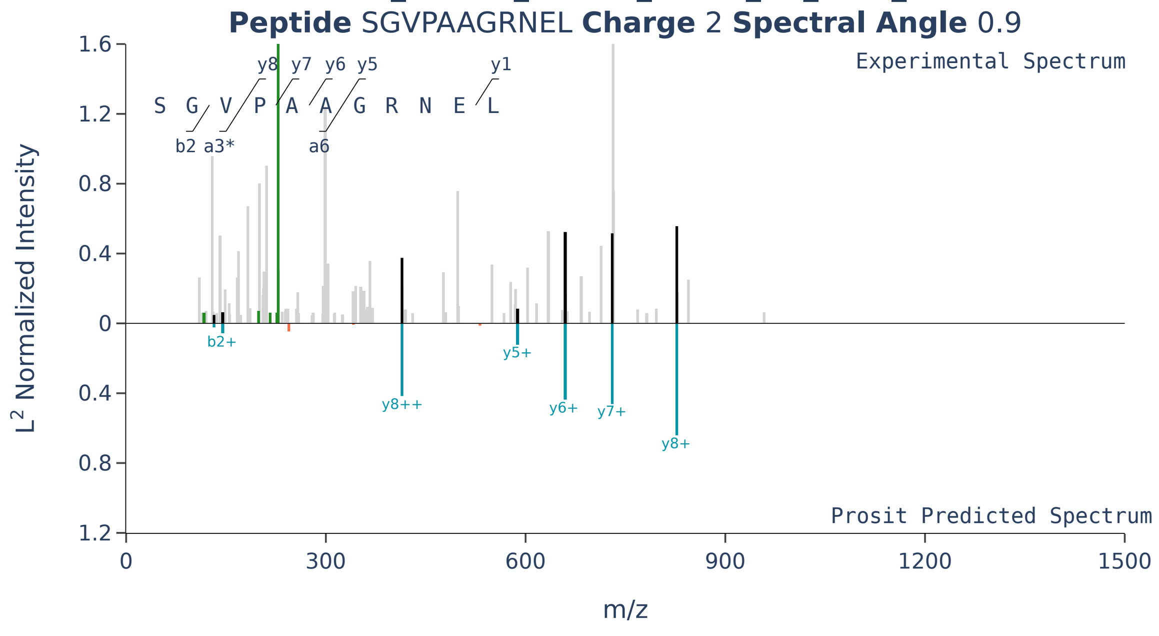

Source HRoetschke_211222_160123_HFGoe_IF2_b1R1_1h **Scan** 6627
Peptide ASGEAGGITREN **Charge** 2 **Spectral Angle** 0.86

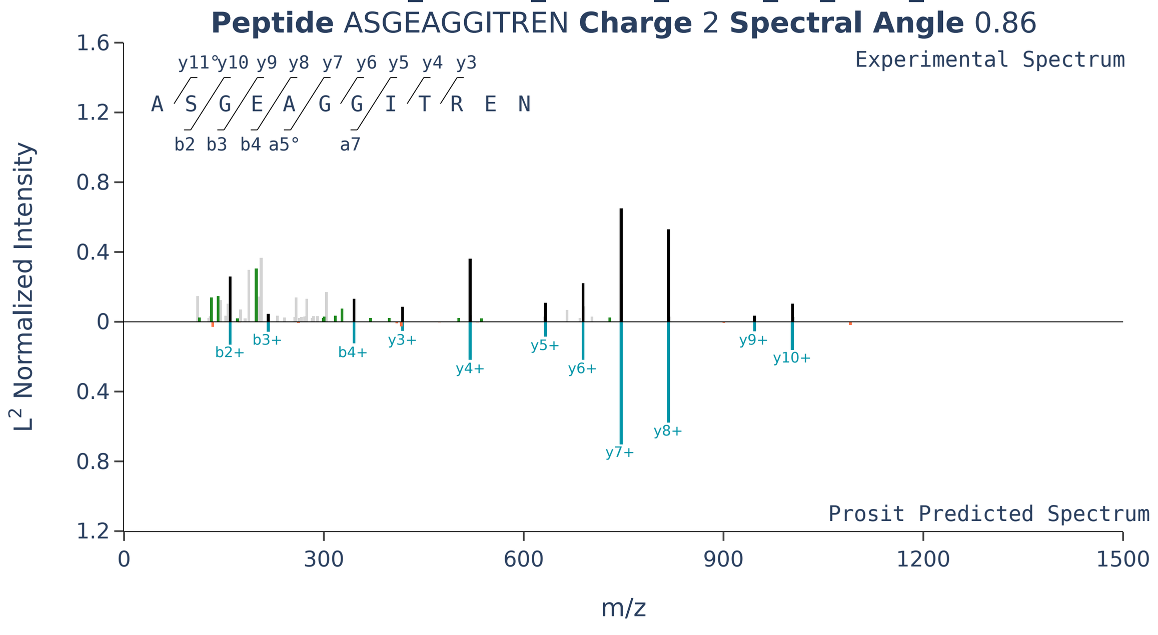

Source HRoetschke_211222_160123_HFGoe_IF2_b1R1_1h **Scan** 6417
Peptide ASGEAGGTLQR **Charge** 2 **Spectral Angle** 0.86

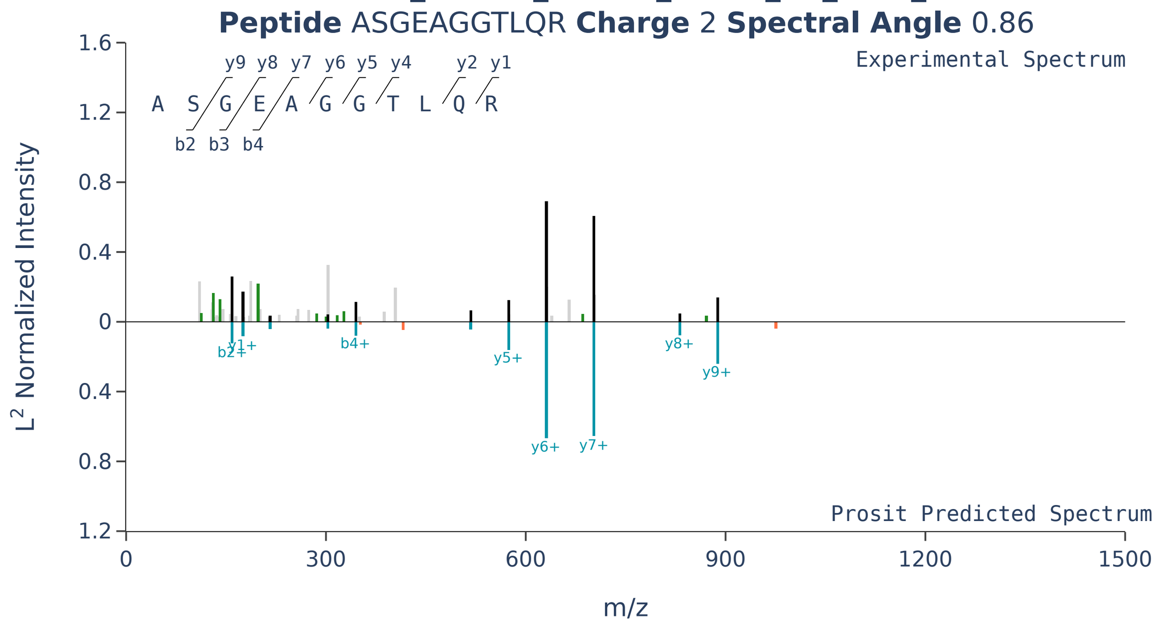

Source HRoetschke_211222_160123_HFGoe_IF2_b2R1_24h **Scan** 7141
Peptide DHLNQKNSAA **Charge** 3 **Spectral Angle** 0.94

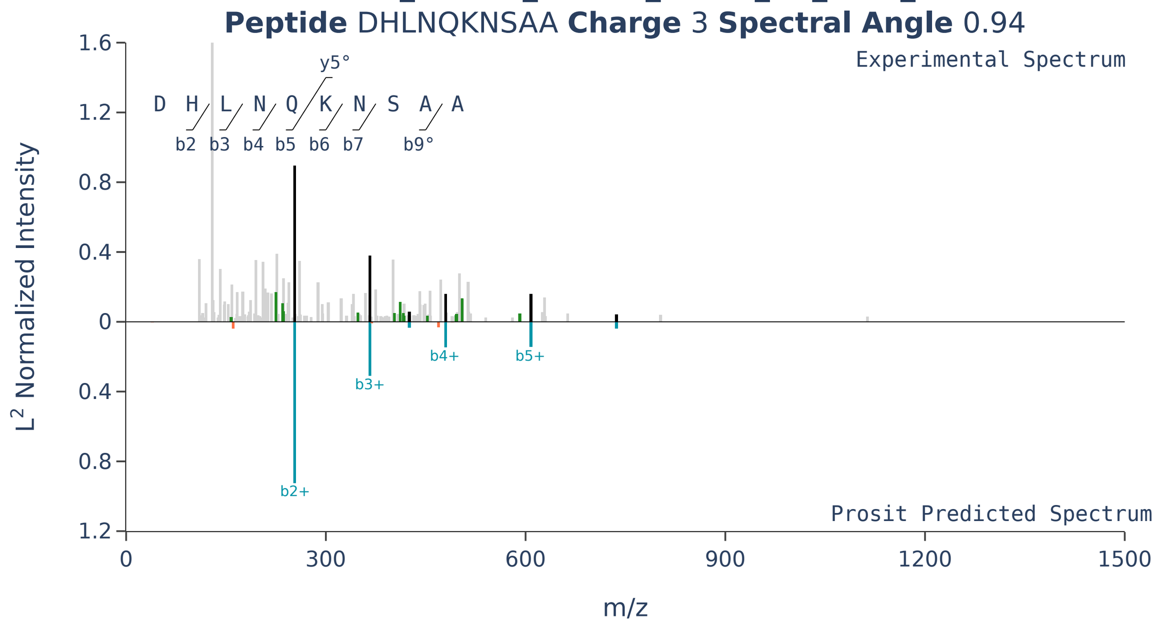

Source HRoetschke_211222_160123_HFGoe_IF2_b1R1_24h **Scan** 9186
Peptide SGEAGGITQA **Charge** 2 **Spectral Angle** 0.84

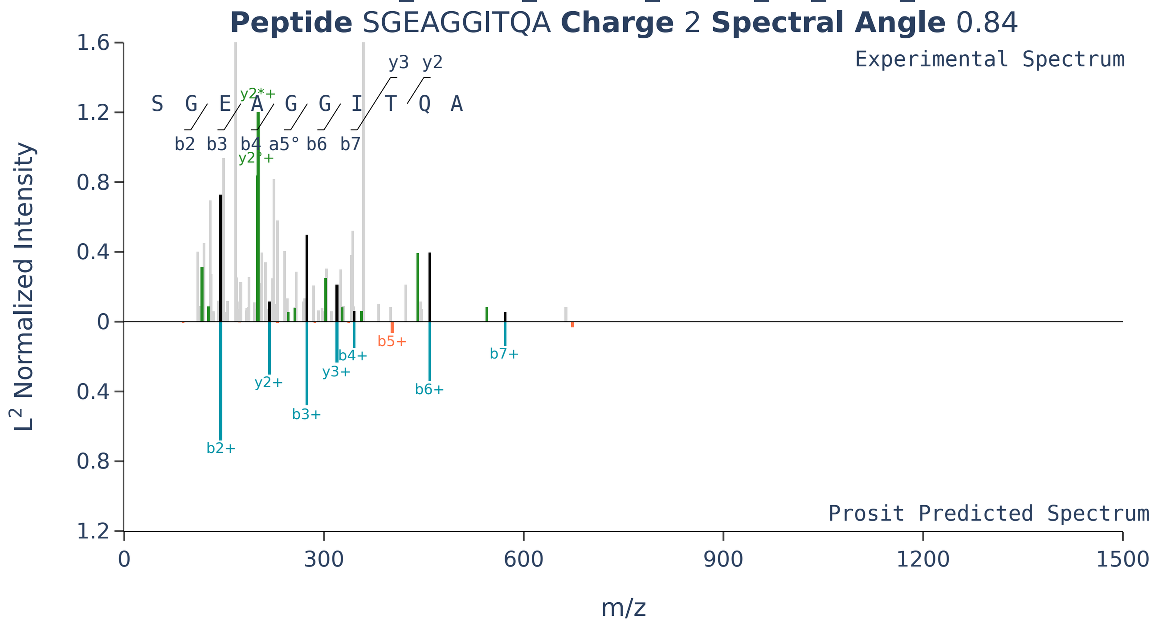

Source HRoetschke_211222_160123_HFGoe_IF2_b2R1_1h **Scan** 11307
Peptide VNRDVVIQE **Charge** 2 **Spectral Angle** 0.85

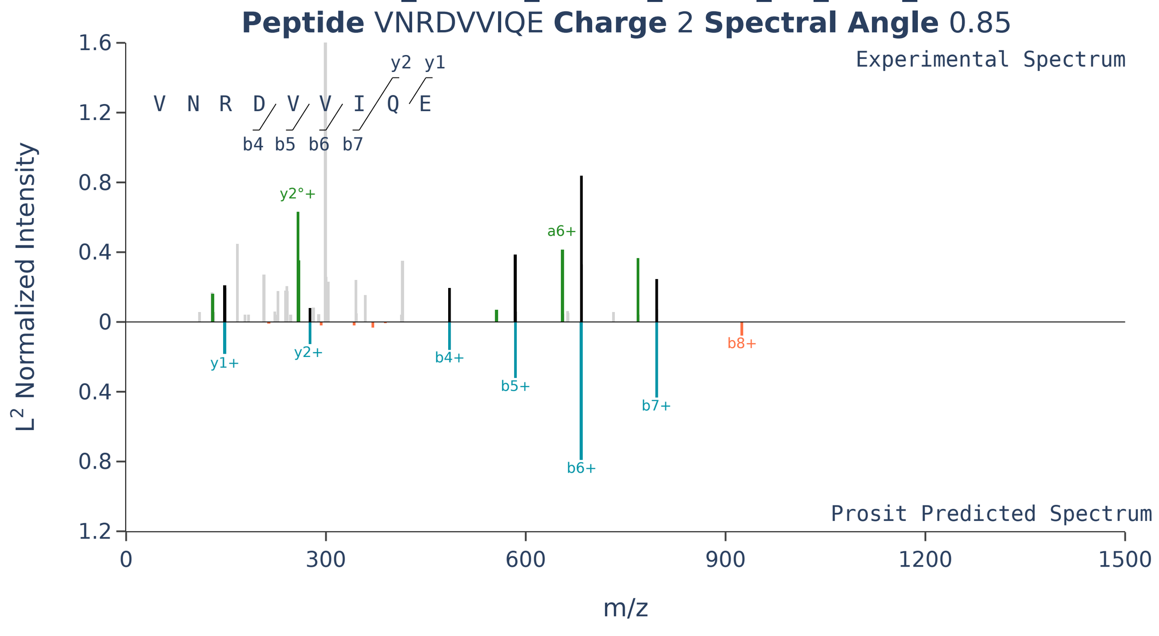

Source HRoetschke_211222_160123_HFGoe_IF2_b2R2_1h **Scan** 11552
Peptide NIPGTRSTL **Charge** 2 **Spectral Angle** 0.88

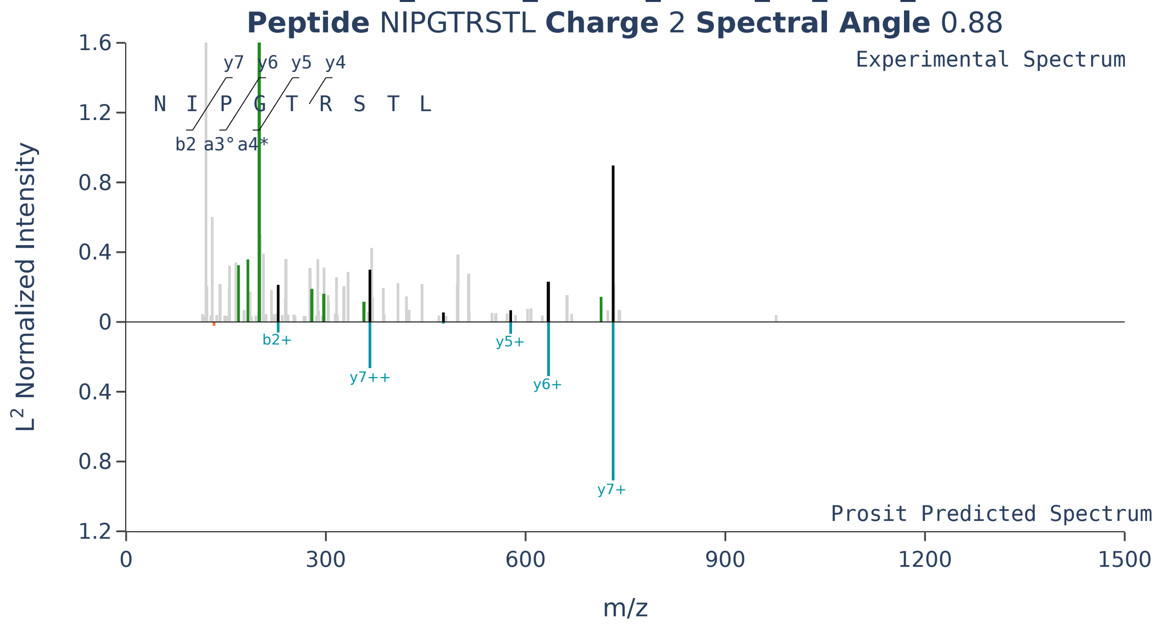

Source HRoetschke_211222_160123_HFGoe_IF2_b2R2_2h **Scan** 13663
Peptide QATQEVLEA **Charge** 1 **Spectral Angle** 0.78

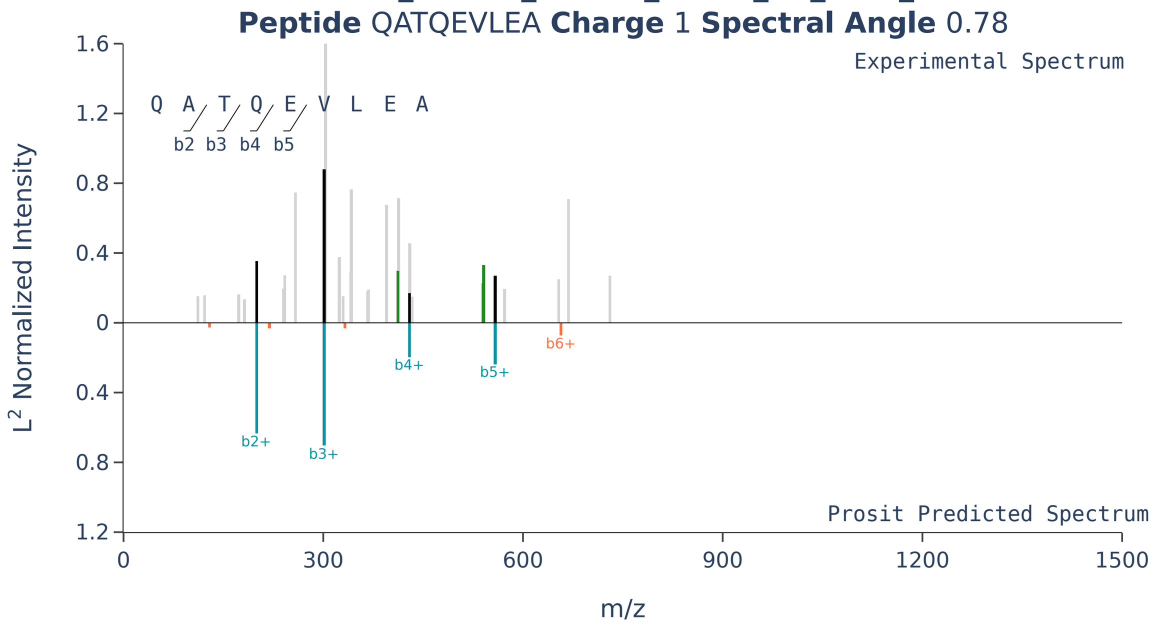

Source HRoetschke_211222_160123_HFGoe_IF2_b2R1_24h **Scan** 9910
Peptide ASGAVEVT **Charge** 1 **Spectral Angle** 0.81

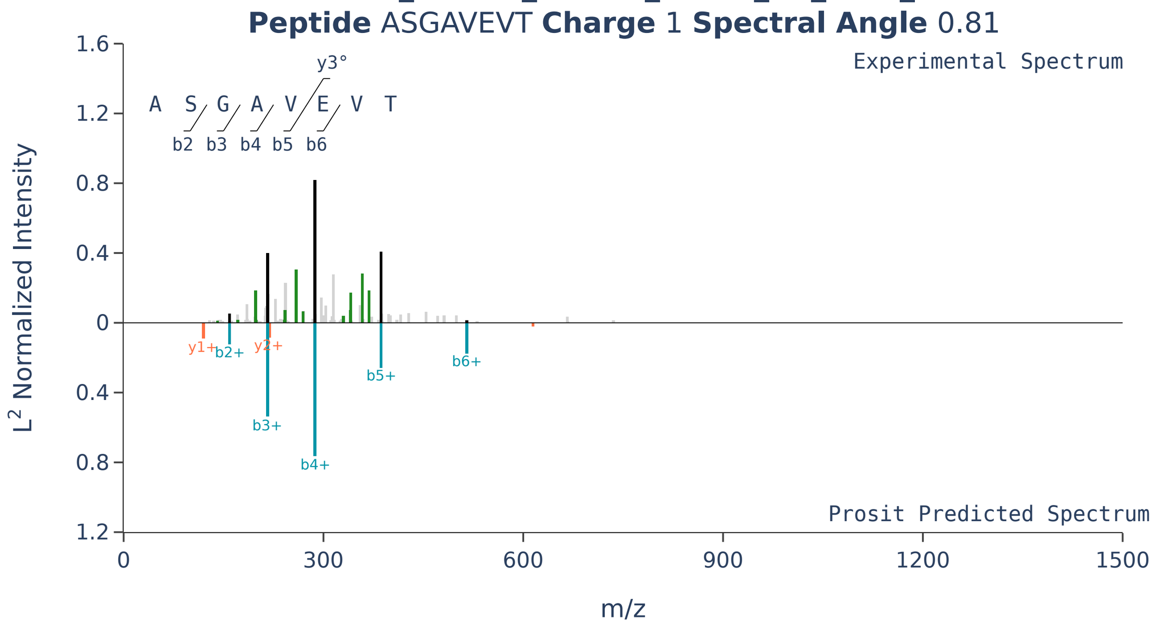

Source HRoetschke_211222_160123_HFGoe_IF2_b2R1_24h **Scan** 14622
Peptide DPQAQLV **Charge** 1 **Spectral Angle** 0.82

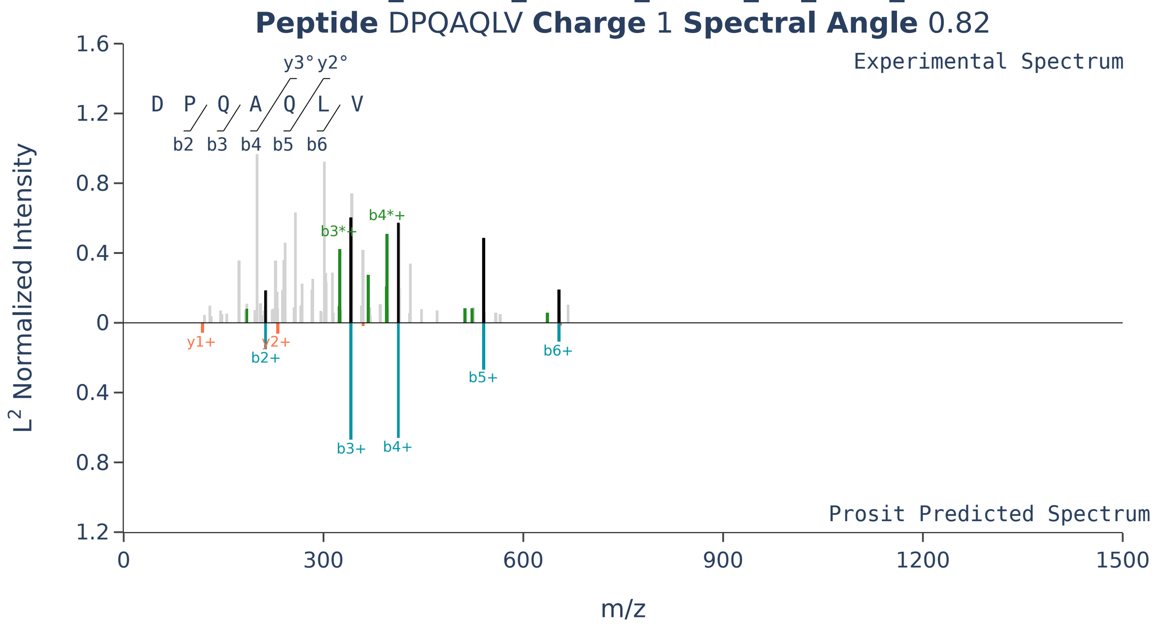

Source HRoetschke_211222_160123_HFGoe_IF2_b1R2_24h **Scan** 11482
Peptide GNRDVVIGE **Charge** 2 **Spectral Angle** 0.79

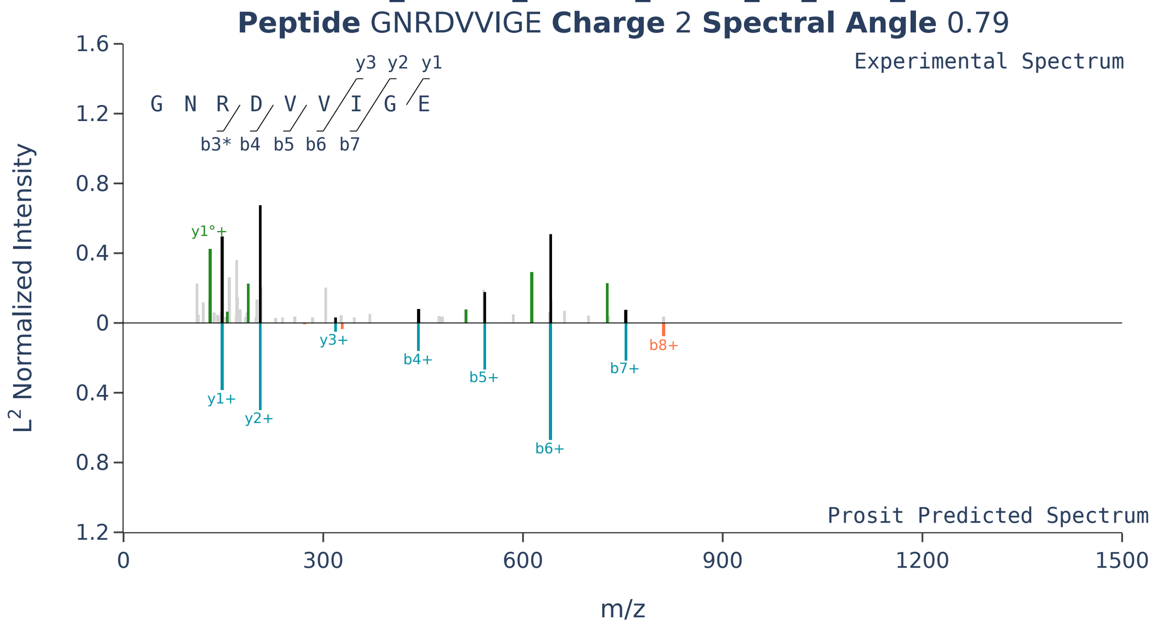

Source HRoetschke_211222_160123_HFGoe_IF2_b1R1_4h **Scan** 6524
Peptide SGEAGGITREN **Charge** 2 **Spectral Angle** 0.81

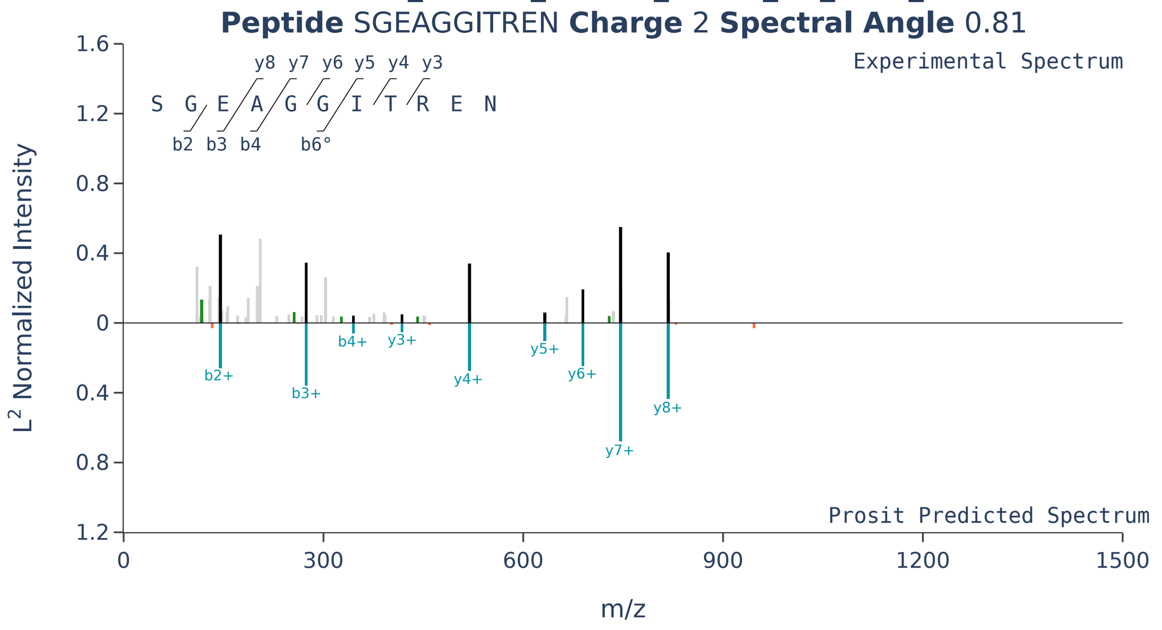

Source HRoetschke_211222_160123_HFGoe_IF2_b2R2_4h **Scan** 7691
Peptide KVASGEAGGITQSD **Charge** 2 **Spectral Angle** 0.81

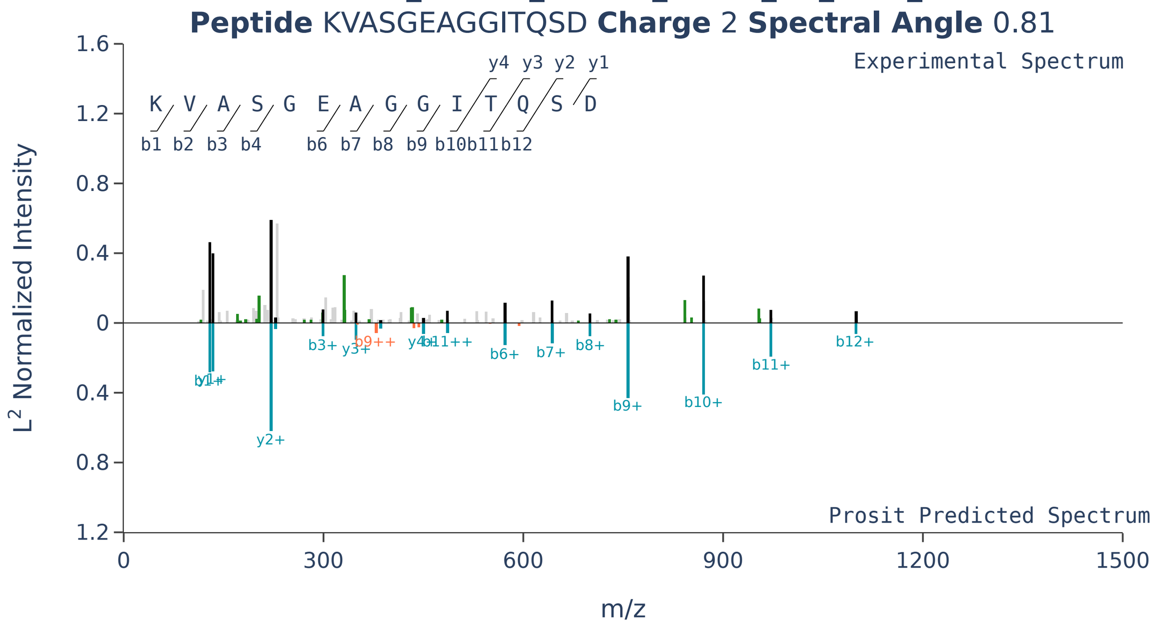

Source HRoetschke_211222_160123_HFGoe_IF2_b1R1_24h **Scan** 9435
Peptide IGAYHVETEDR **Charge** 3 **Spectral Angle** 0.88

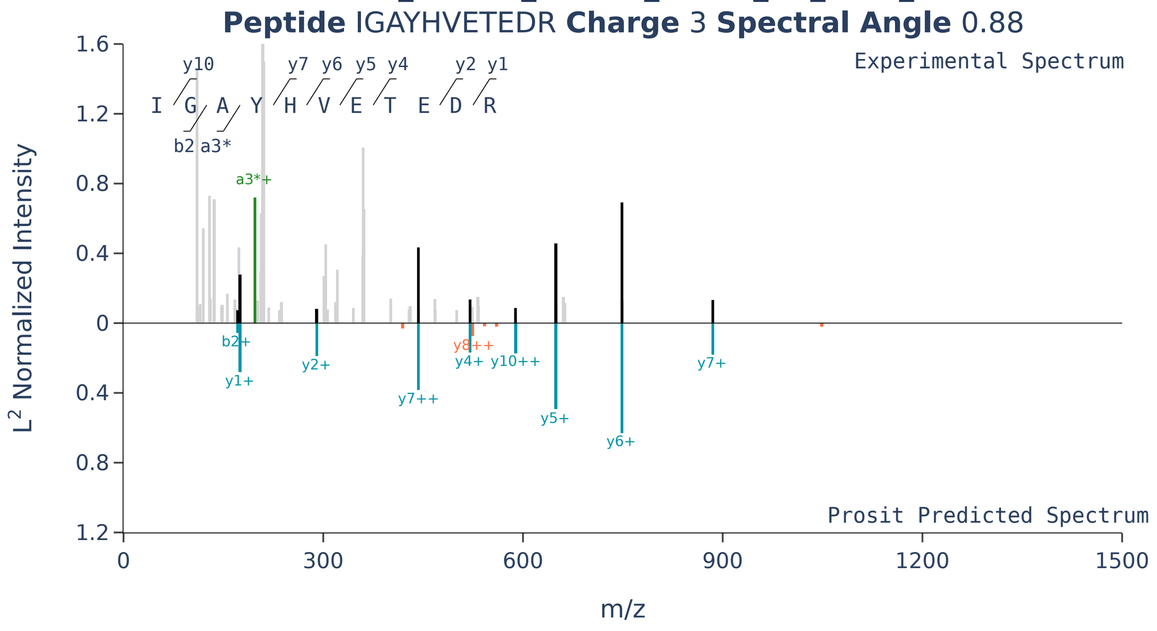

Source HRoetschke_211222_160123_HFGoe_IF2_b1R1_2h **Scan** 10601
Peptide QETAQLVREG **Charge** 2 **Spectral Angle** 0.84

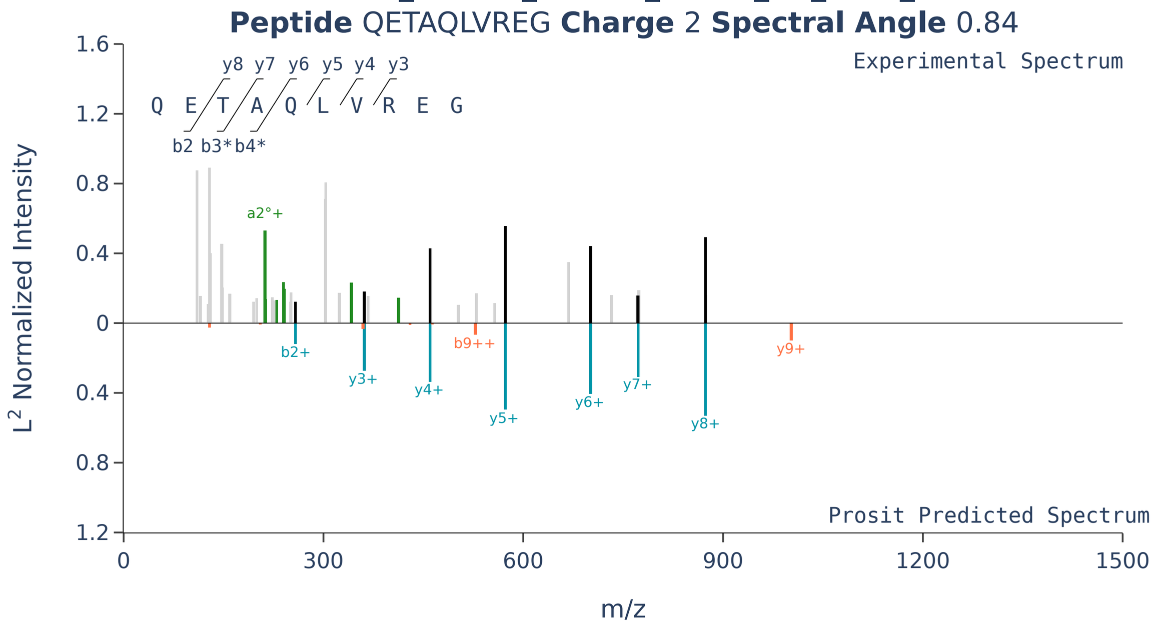

Source HRoetschke_211222_160123_HFGoe_IF2_b1R2_2h **Scan** 15713
Peptide DYLVAEE **Charge** 2 **Spectral Angle** 0.82

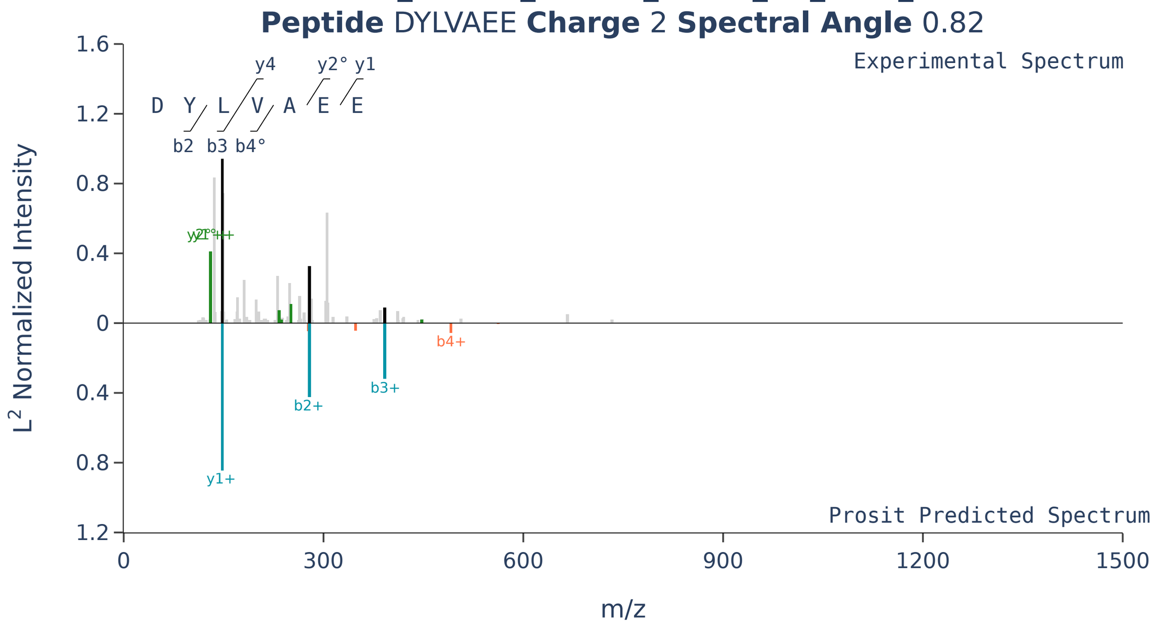

Source HRoetschke_211222_160123_HFGoe_IF2_b1R1_4h **Scan** 16241
Peptide GRNELGQEVLE **Charge** 2 **Spectral Angle** 0.81

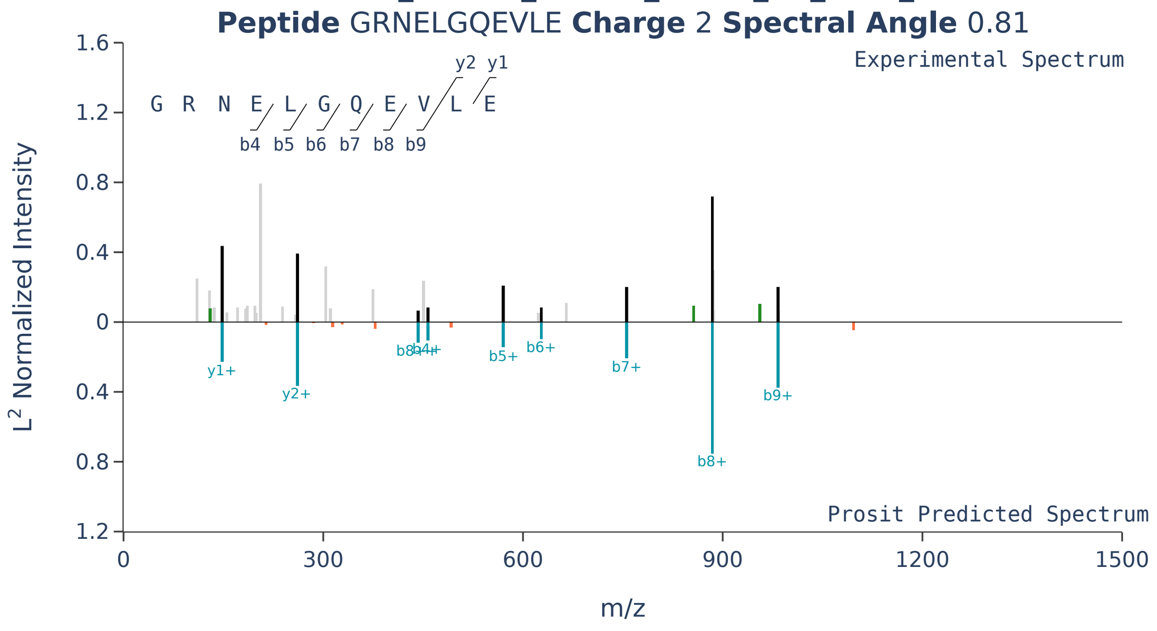

Source HRoetschke_211222_160123_HFGoe_IF2_b1R1_2h **Scan** 9854
Peptide TVLVRSADD **Charge** 2 **Spectral Angle** 0.87

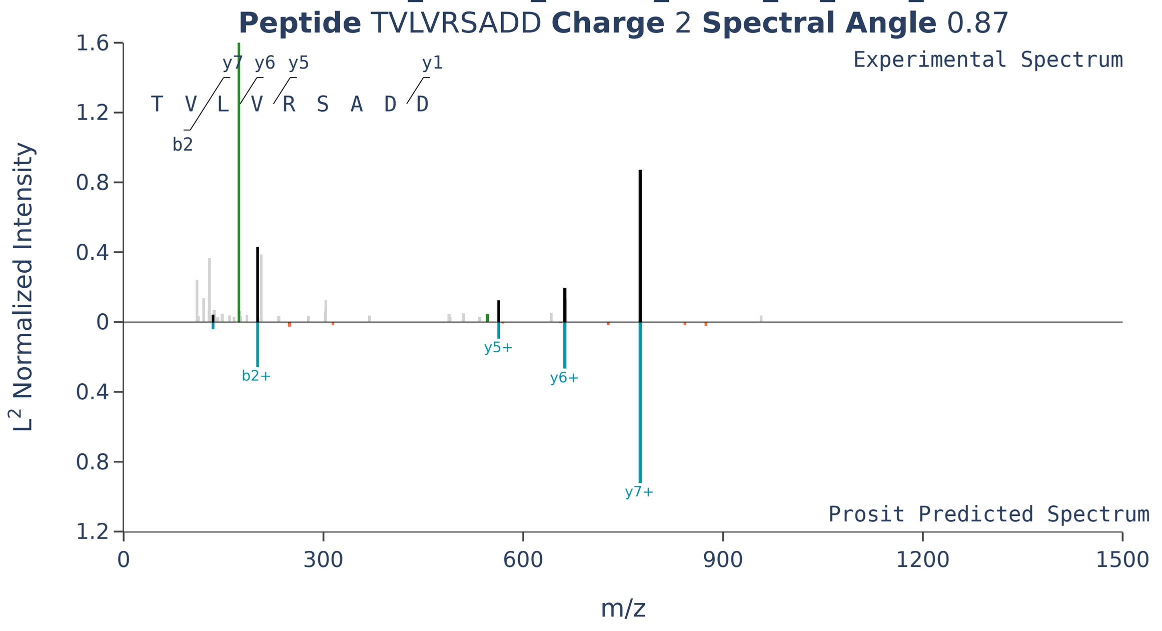

Source HRoetschke_211222_160123_HFGoe_IF2_b1R1_2h **Scan** 14047
Peptide VREGRNELGQEVLE **Charge** 3 **Spectral Angle** 0.78

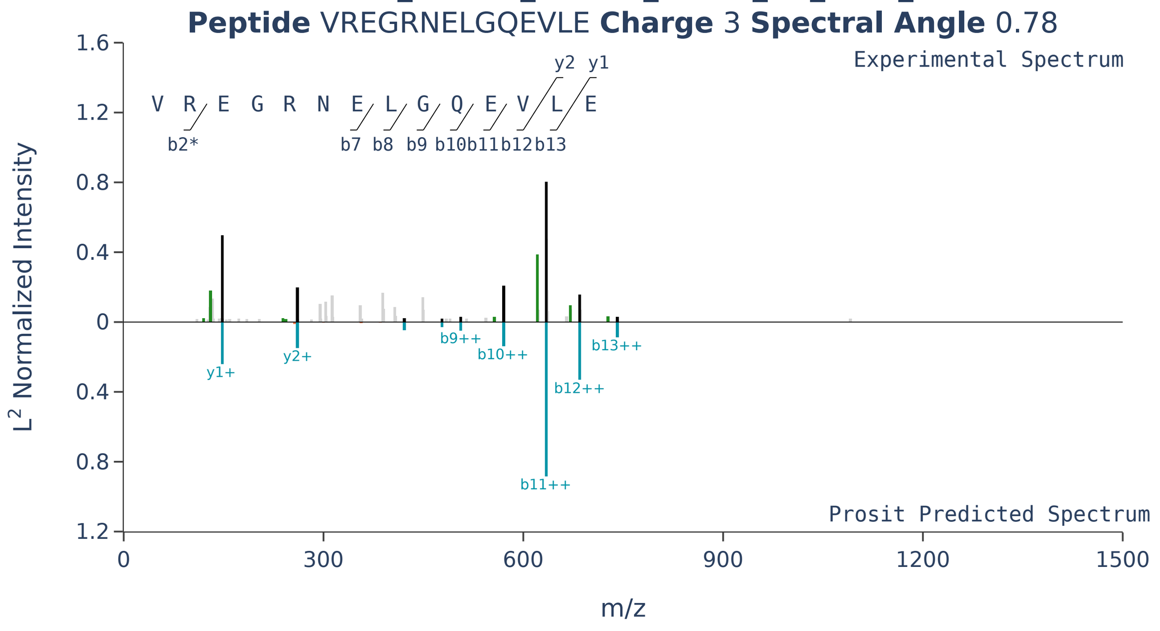

Source HRoetschke_211222_160123_HFGoe_IF2_b1R2_1h **Scan** 14568
Peptide EQTAQLV **Charge** 2 **Spectral Angle** 0.84

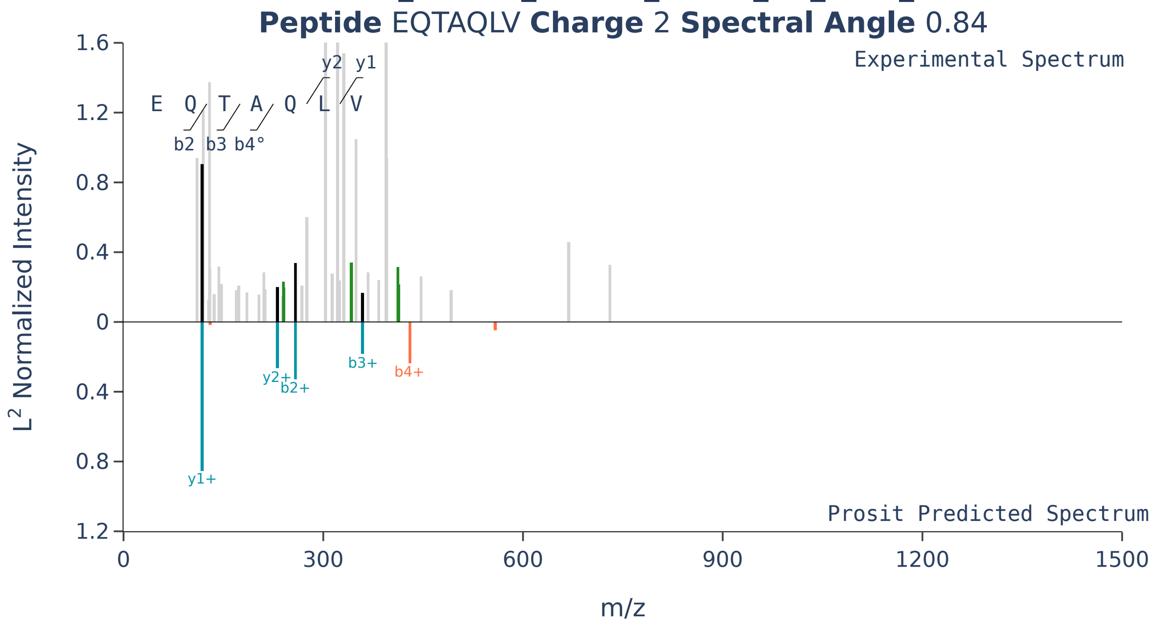

Source HRoetschke_211222_160123_HFGoe_IF2_b1R2_2h **Scan** 6670
Peptide KVASGEAGGITQRD **Charge** 2 **Spectral Angle** 0.82

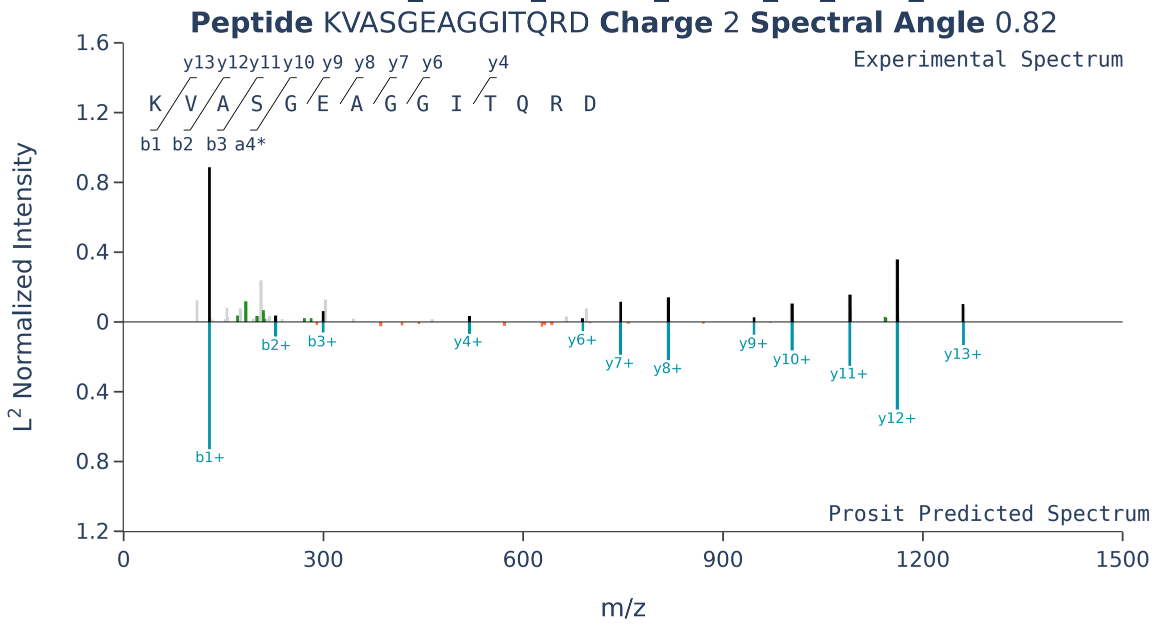

Source HRoetschke_211222_160123_HFGoe_IF2_b1R1_4h **Scan** 11623
Peptide SLQQGFQKPANE **Charge** 2 **Spectral Angle** 0.88

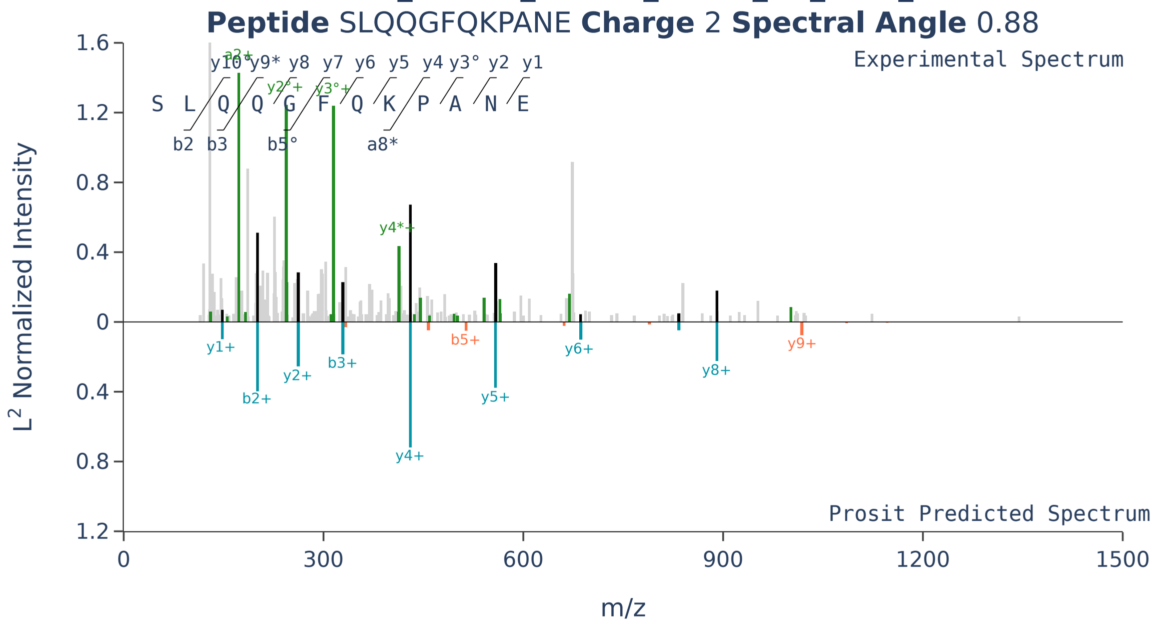

Source HRoetschke_211222_160123_HFGoe_IF2_b1R1_24h **Scan** 15830
Peptide VLVVAVRTG **Charge** 2 **Spectral Angle** 0.84

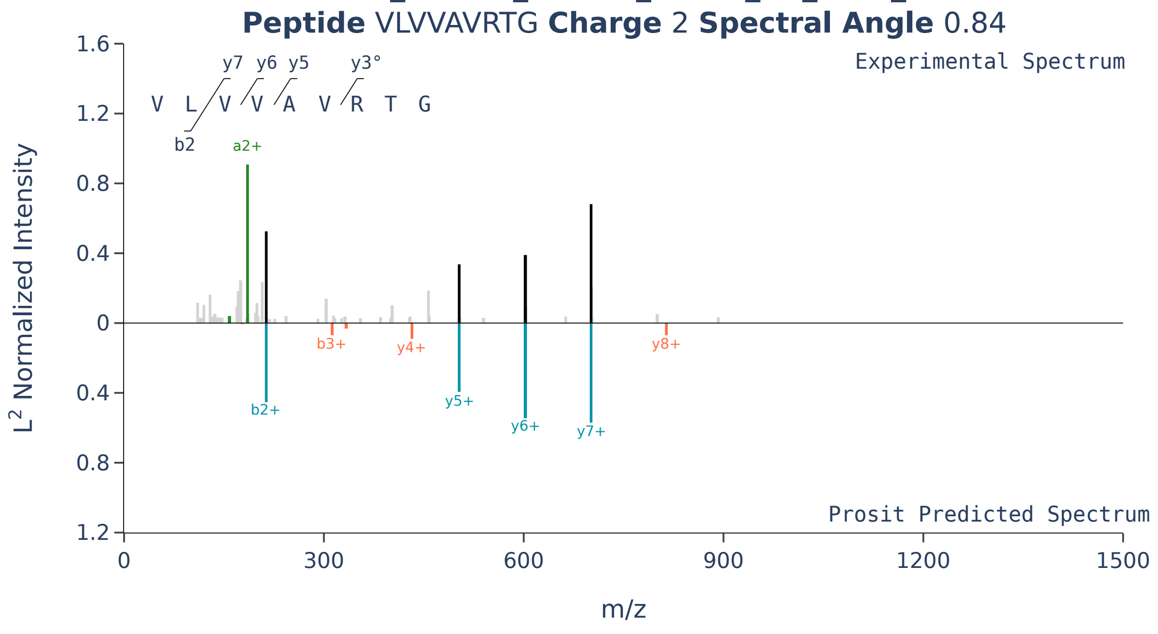

Source HRoetschke_211222_160123_HFGoe_IF2_b1R2_1h **Scan** 11112
Peptide VNRDVVISD **Charge** 2 **Spectral Angle** 0.81

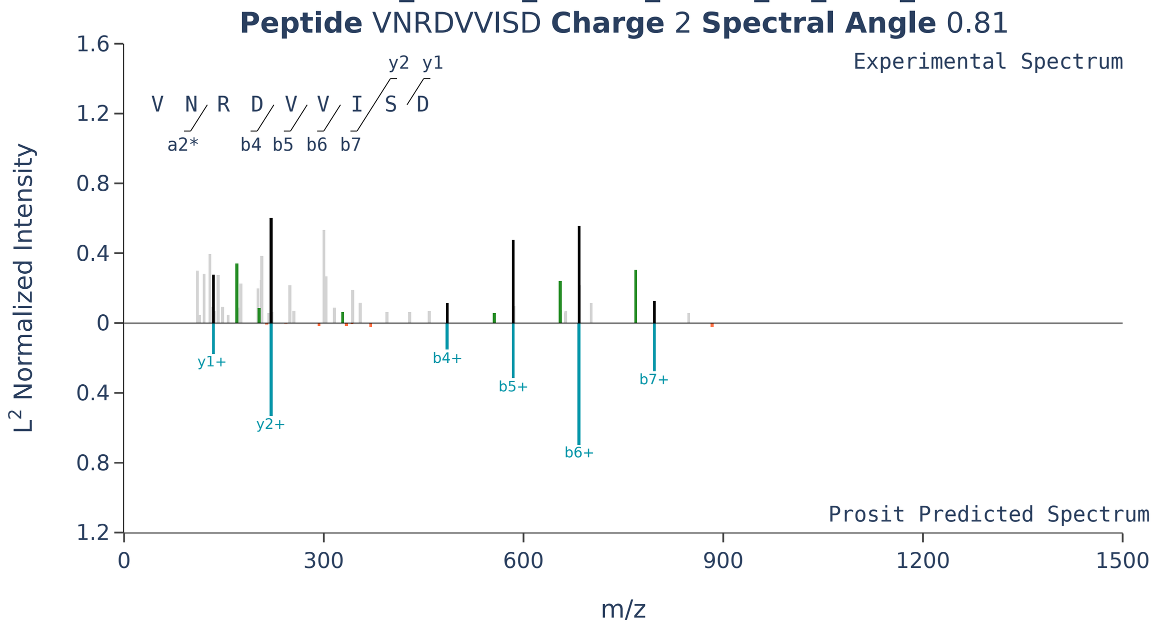

Source HRoetschke_211222_160123_HFGoe_IF2_b2R1_4h **Scan** 6746
Peptide GFQKPAQD **Charge** 2 **Spectral Angle** 0.84

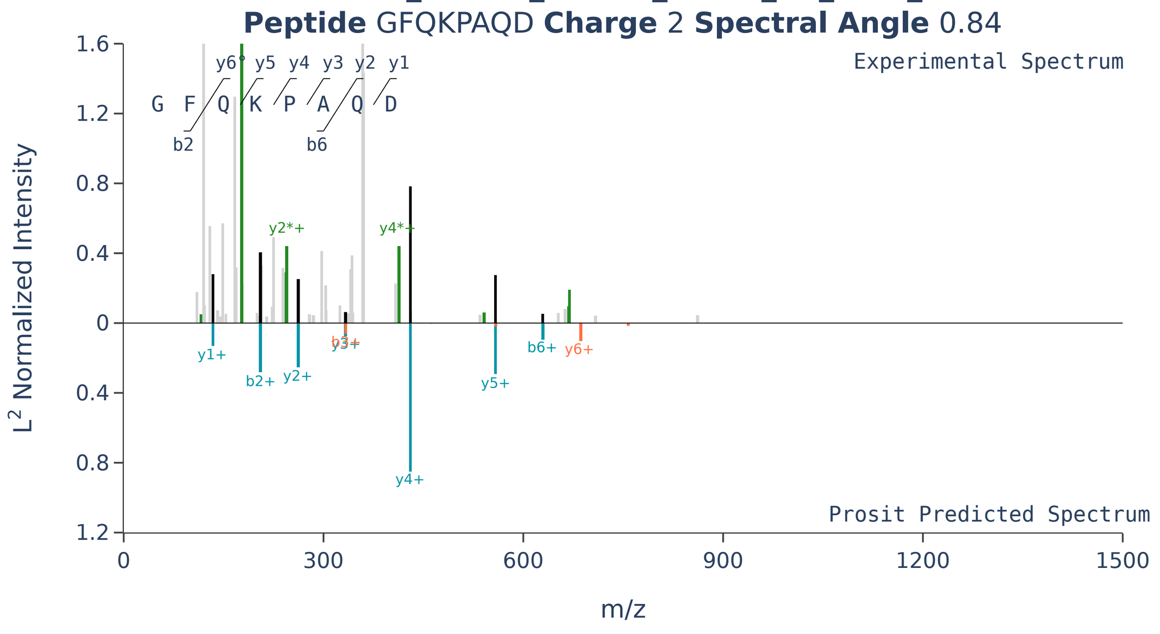

Source HRoetschke_211222_160123_HFGoe_IF2_b2R1_24h **Scan** 11424
Peptide NGRDVVIGE **Charge** 2 **Spectral Angle** 0.75

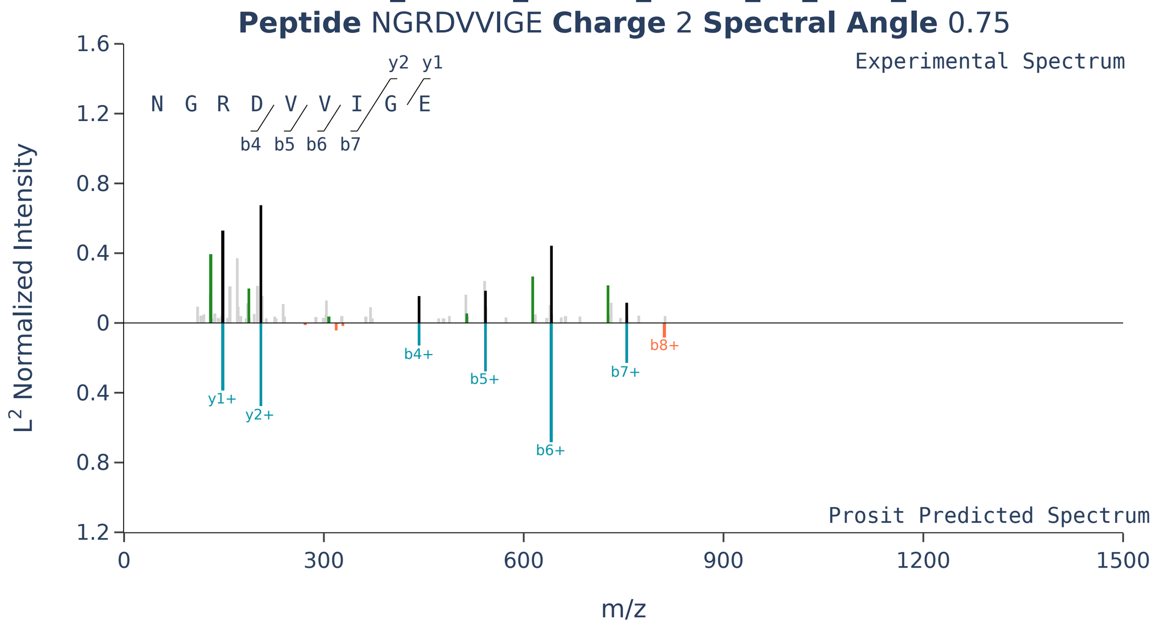

Source HRoetschke_211222_160123_HFGoe_IF2_b1R1_4h **Scan** 13396
Peptide GLGAYHVETE **Charge** 2 **Spectral Angle** 0.75

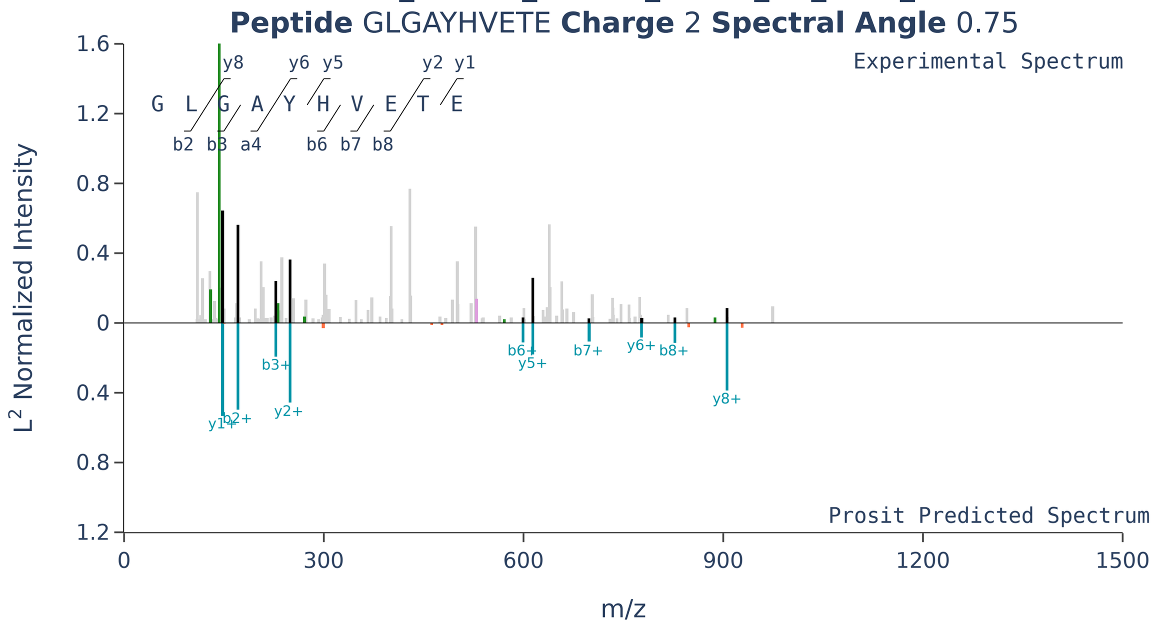

Source HRoetschke_211222_160123_HFGoe_IF2_b1R1_2h **Scan** 9691
Peptide RVAAYHVETE **Charge** 2 **Spectral Angle** 0.89

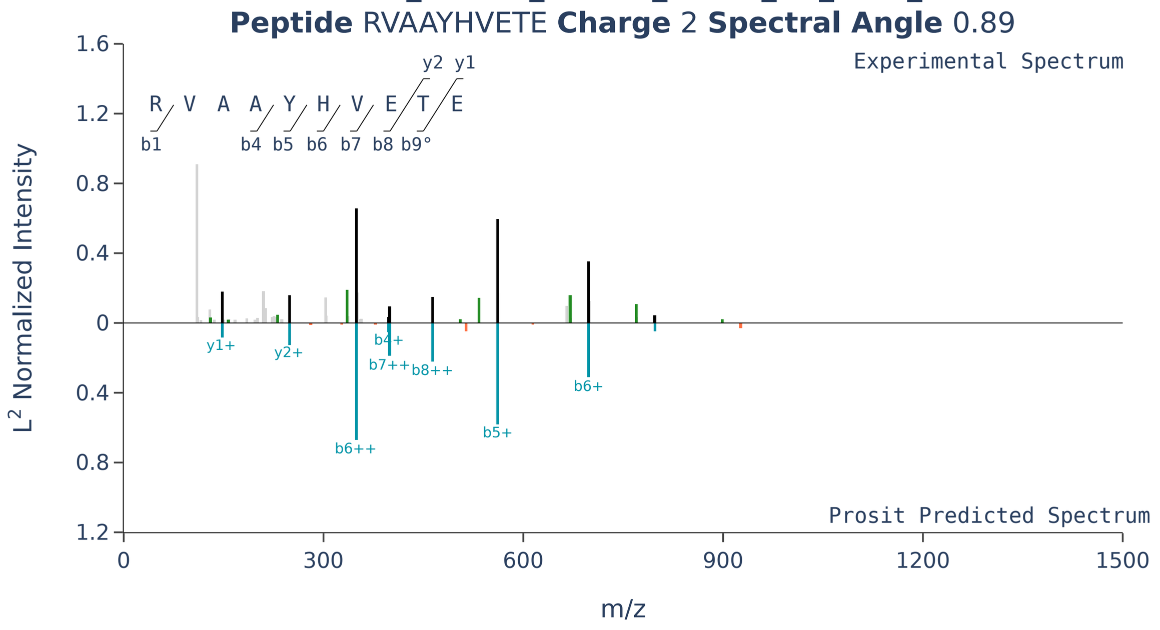

Source HRoetschke_211222_160123_HFGoe_IF2_b1R2_4h **Scan** 9656
Peptide TVLVREGSD **Charge** 2 **Spectral Angle** 0.87

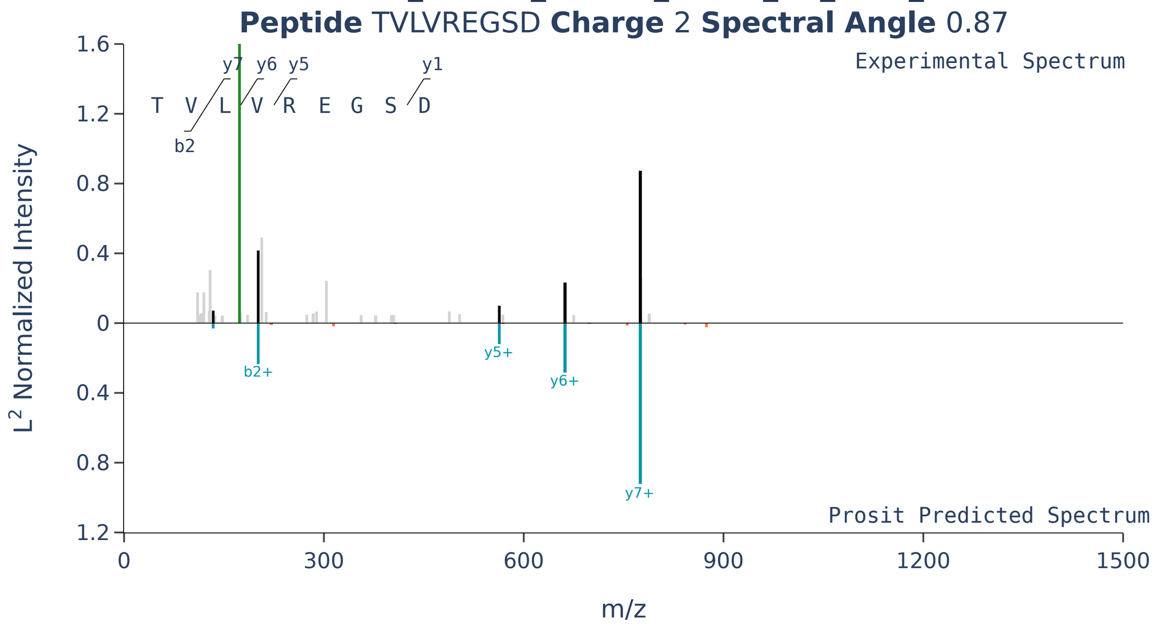

Source HRoetschke_211222_160123_HFGoe_IF2_b1R1_4h **Scan** 21761
Peptide PVVVAVNLAN **Charge** 1 **Spectral Angle** 0.86

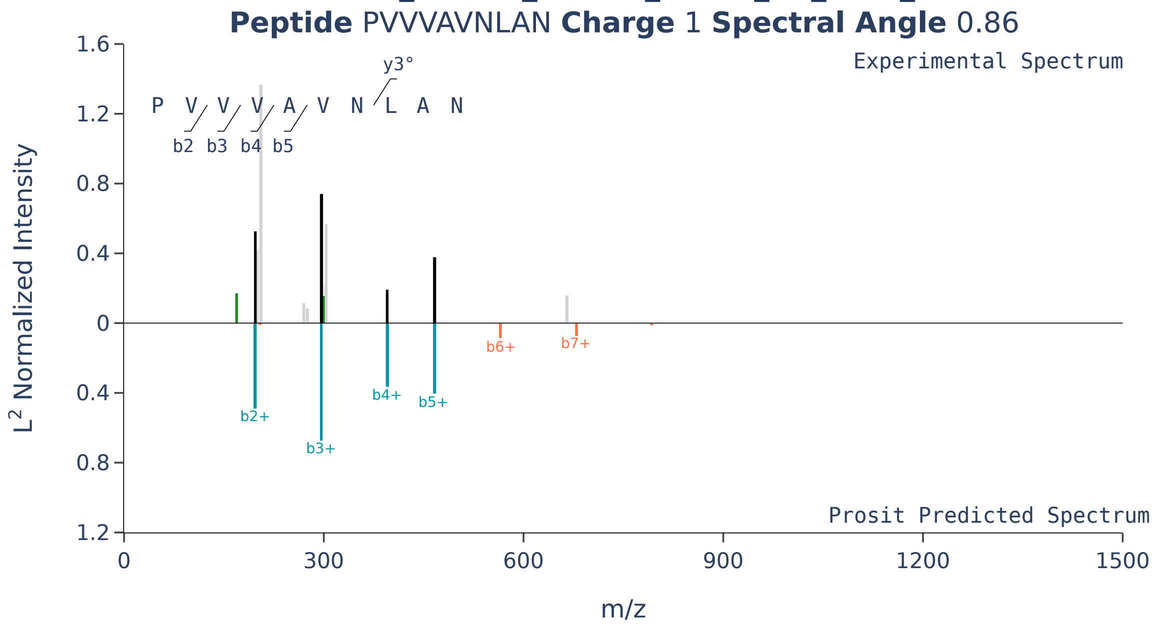

Source HRoetschke_211222_160123_HFGoe_IF2_b1R1_4h **Scan** 11733
Peptide ADRIGAYHVETE **Charge** 2 **Spectral Angle** 0.85

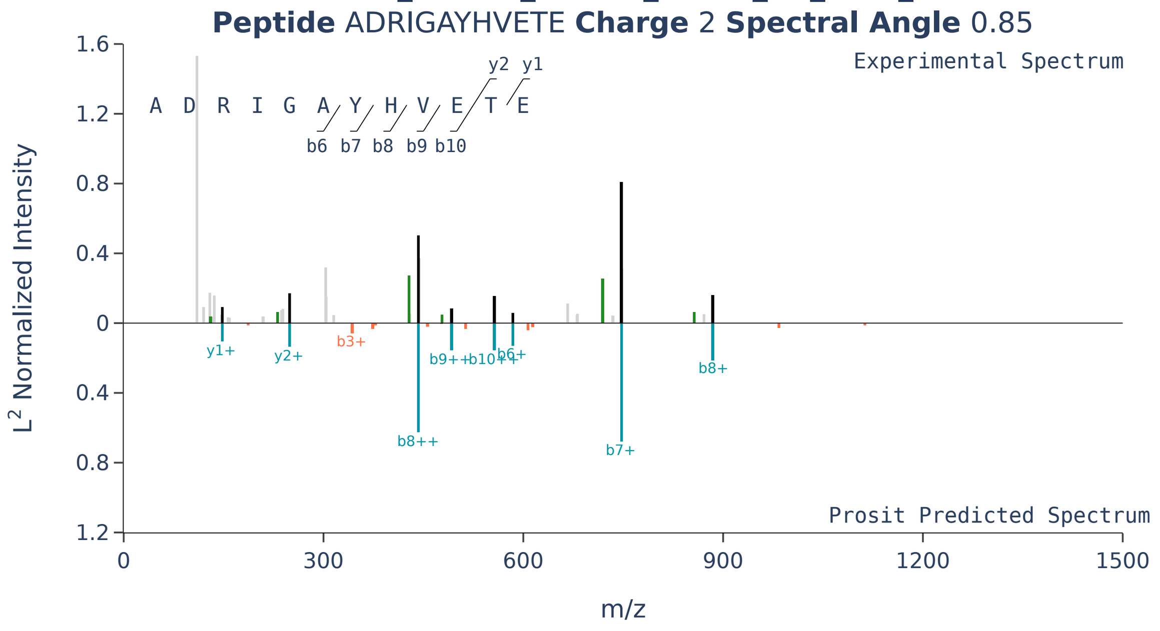

Source HRoetschke_211222_160123_HFGoe_IF2_b1R2_2h **Scan** 16247
Peptide HAIIGLAE **Charge** 2 **Spectral Angle** 0.8

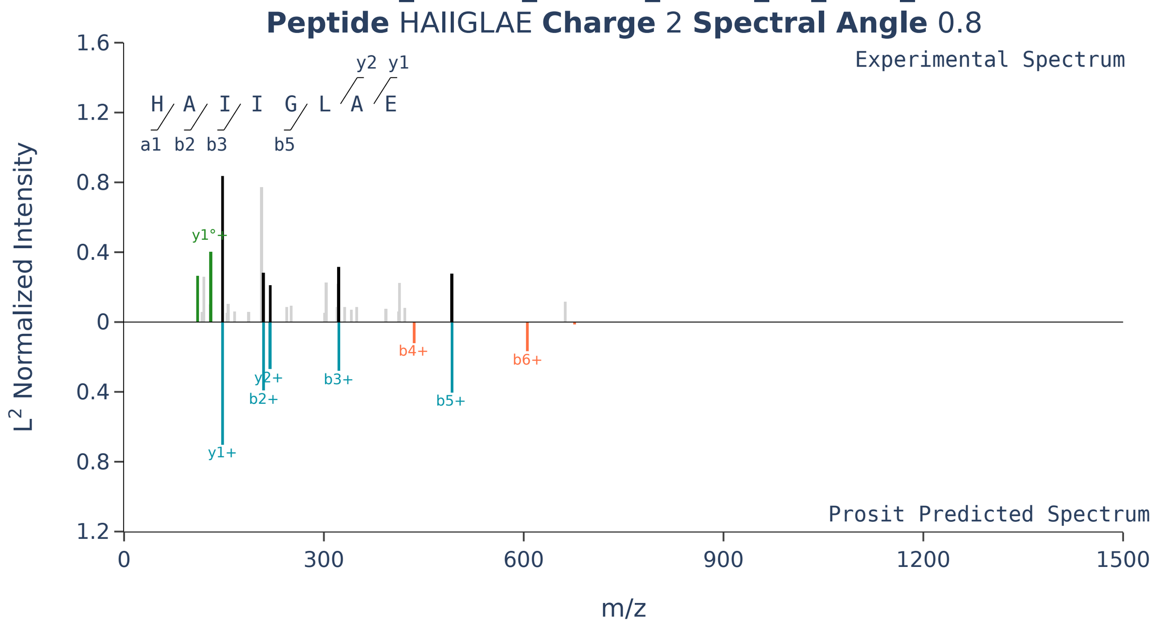

Source HRoetschke_211222_160123_HFGoe_IF2_b1R1_4h **Scan** 13644
Peptide ADRRNELGQEVLE **Charge** 3 **Spectral Angle** 0.76

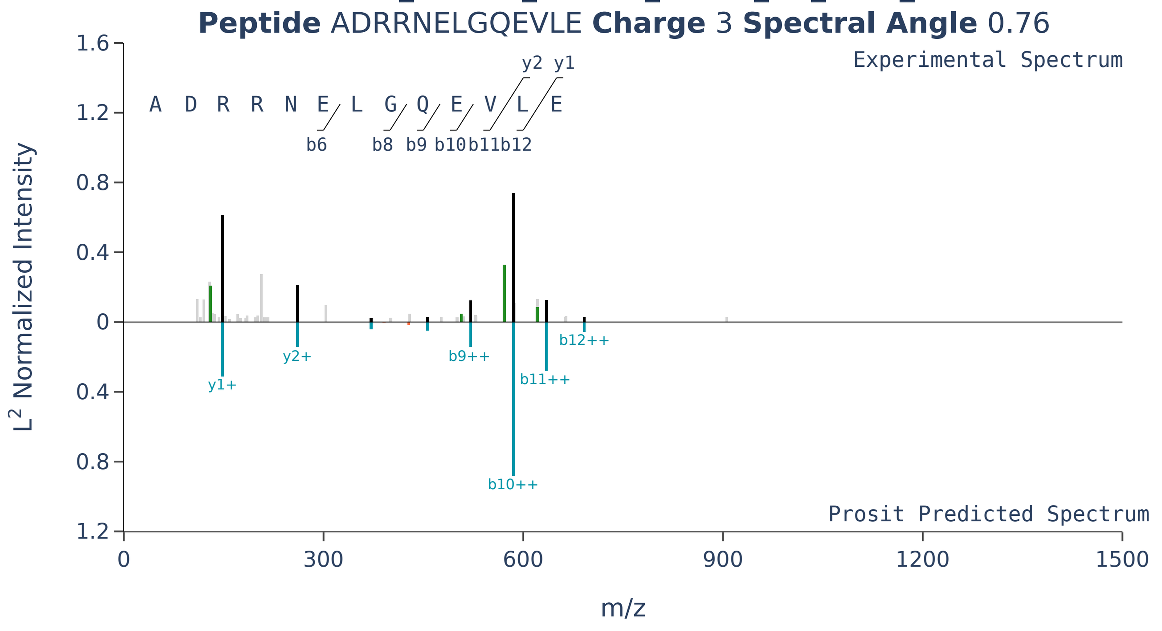

Source HRoetschke_211222_160123_HFGoe_IF2_b2R1_24h **Scan** 6639
Peptide KVASGEAGGITREN **Charge** 2 **Spectral Angle** 0.79

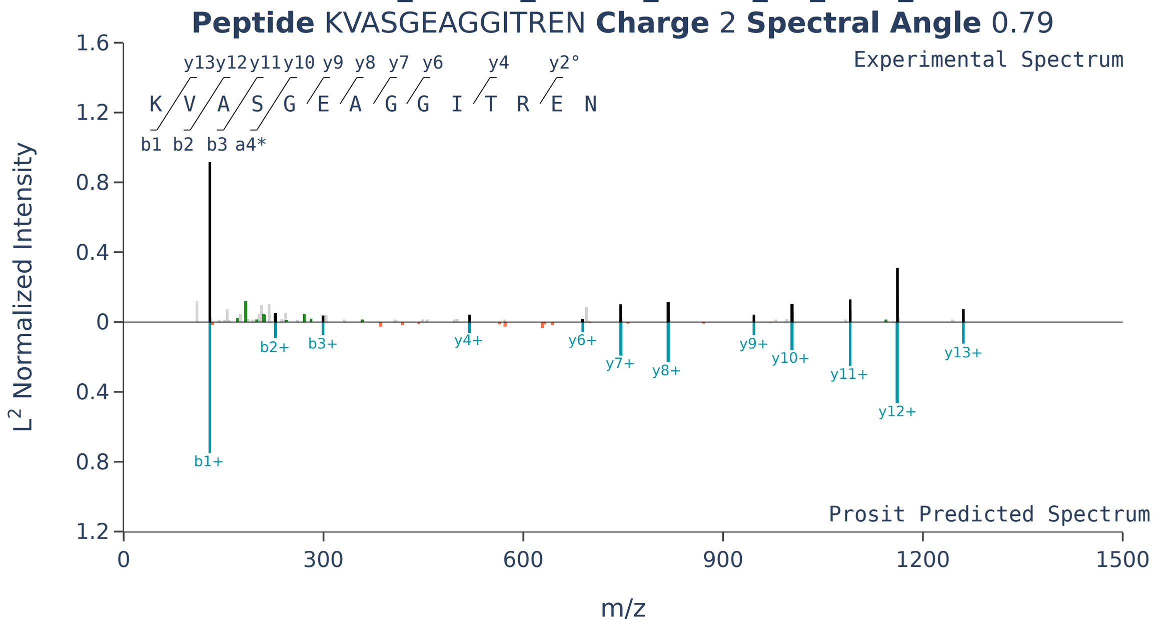

Source HRoetschke_211222_160123_HFGoe_IF2_b1R1_24h **Scan** 9116
Peptide IGAYHVETENDR **Charge** 3 **Spectral Angle** 0.83

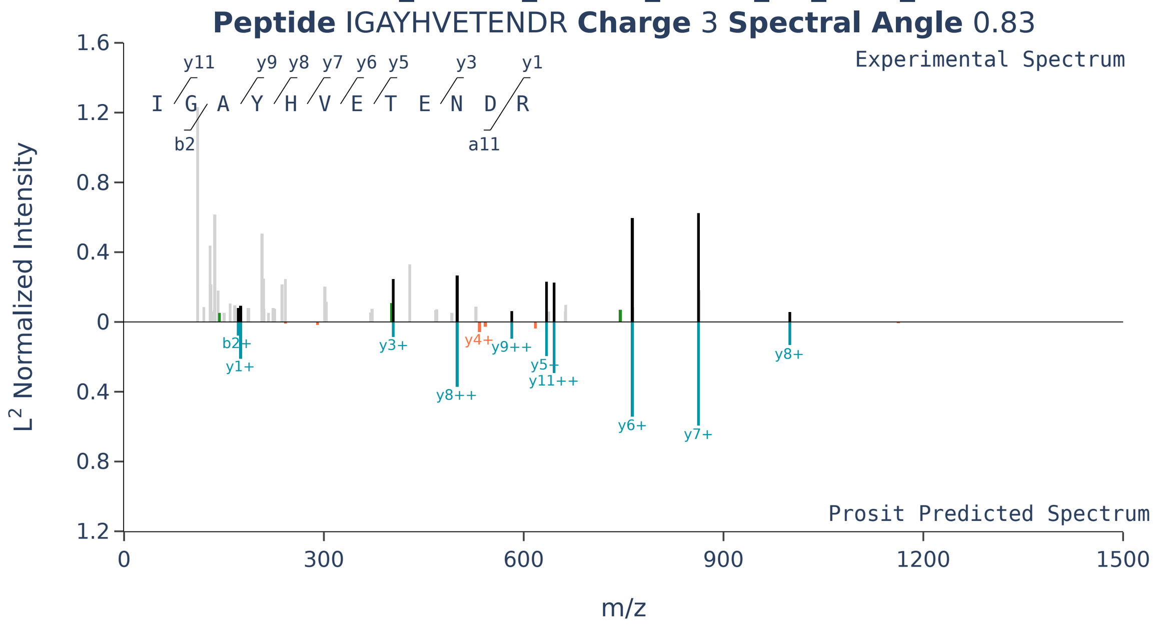

Source HRoetschke_211222_160123_HFGoe_IF2_b1R2_4h **Scan** 6643
Peptide KVASGEAGGITRNE **Charge** 2 **Spectral Angle** 0.81

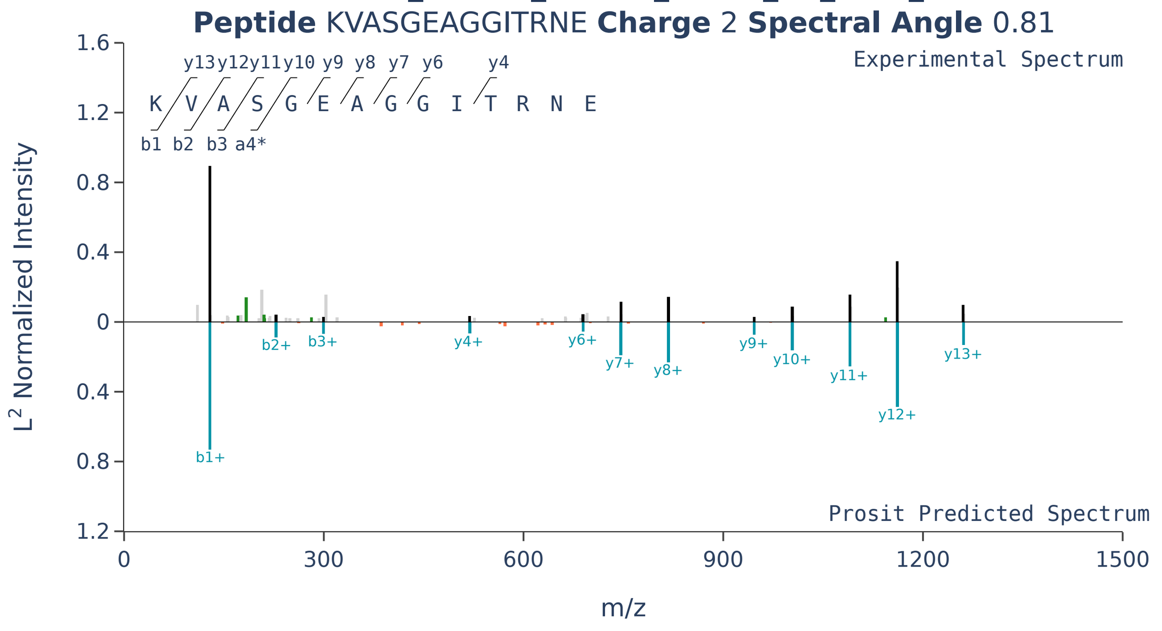

Source HRoetschke_211222_160123_HFGoe_IF2_b2R1_24h **Scan** 9248
Peptide TDVTREV **Charge** 2 **Spectral Angle** 0.81

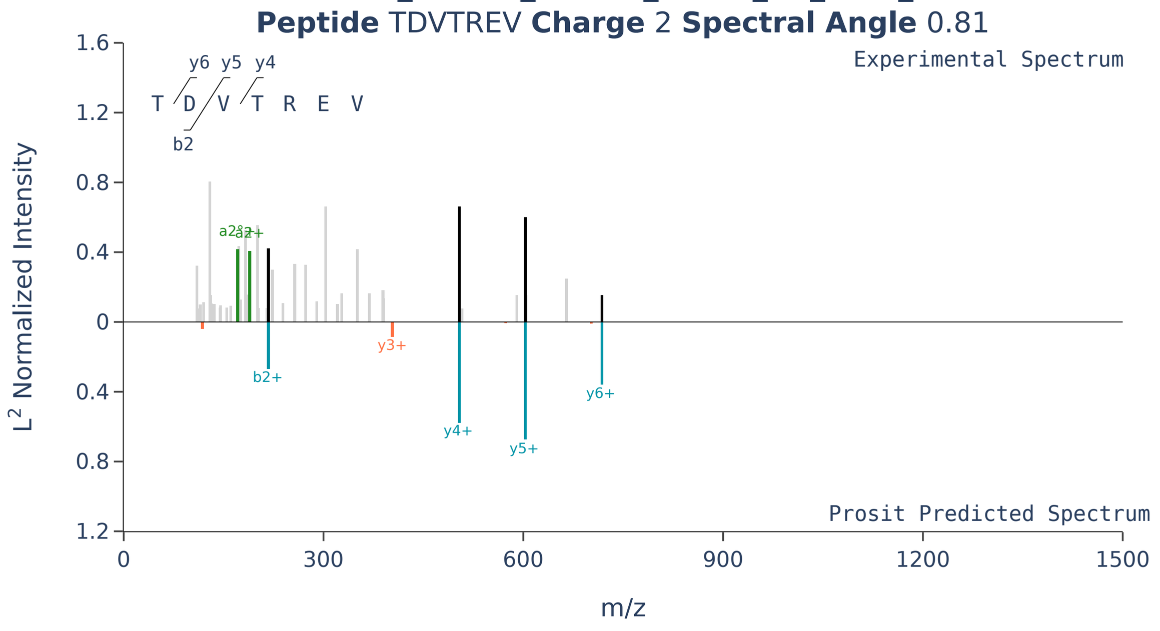

Source HRoetschke_211222_160123_HFGoe_IF2_b1R2_24h **Scan** 8837
Peptide VVKIDKPEAD **Charge** 3 **Spectral Angle** 0.89

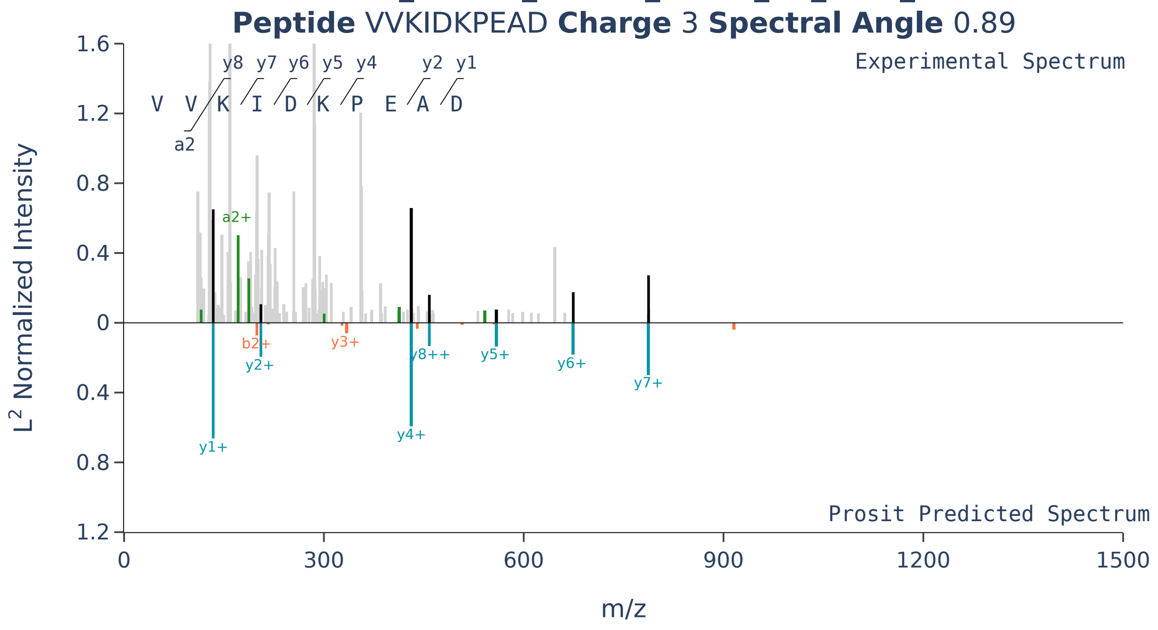

Source HRoetschke_211222_160123_HFGoe_IF2_b2R2_24h **Scan** 6458
Peptide KVASGEAGGIRQT **Charge** 2 **Spectral Angle** 0.83

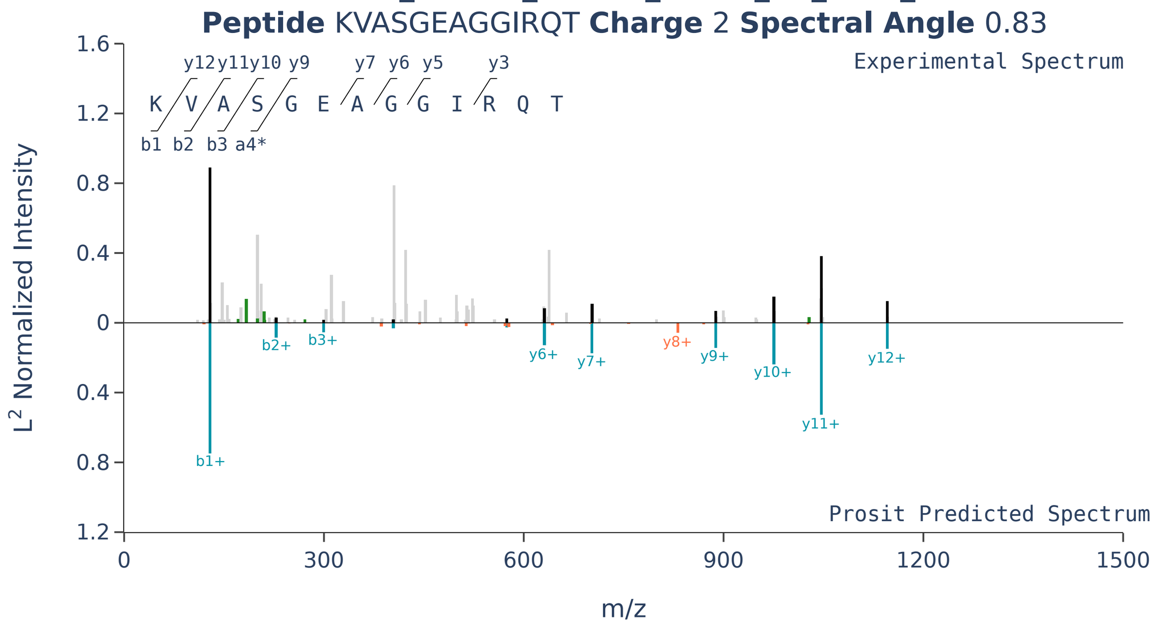

Source HRoetschke_211222_160123_HFGoe_IF2_b1R1_4h **Scan** 15023
Peptide PGHAAFTELS **Charge** 1 **Spectral Angle** 0.86

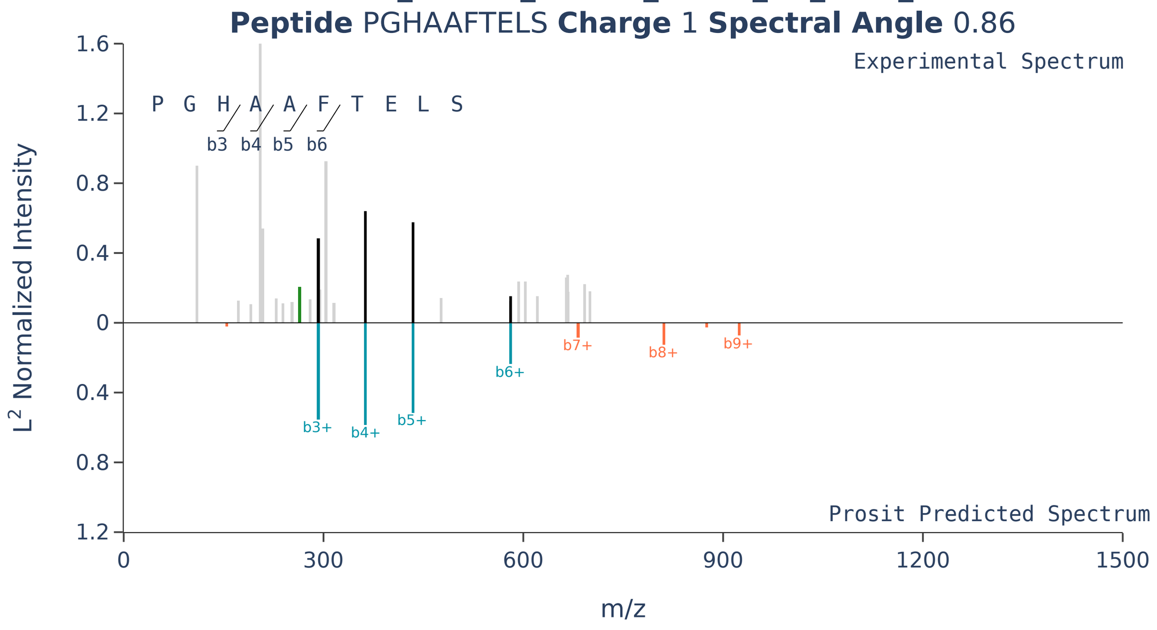

Source HRoetschke_211222_160123_HFGoe_IF2_b1R2_2h **Scan** 12128
Peptide SSLQQGFQKPQAE **Charge** 2 **Spectral Angle** 0.87

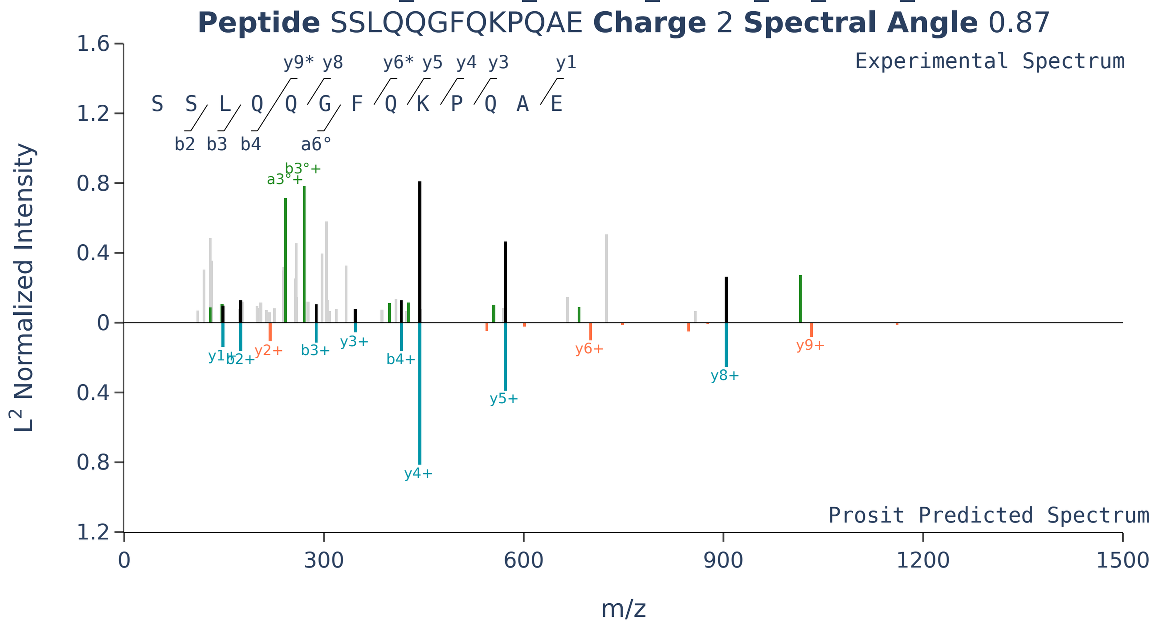

Source HRoetschke_211222_160123_HFGoe_IF2_b1R1_4h **Scan** 15865
Peptide RDNLGQEVLE **Charge** 2 **Spectral Angle** 0.83

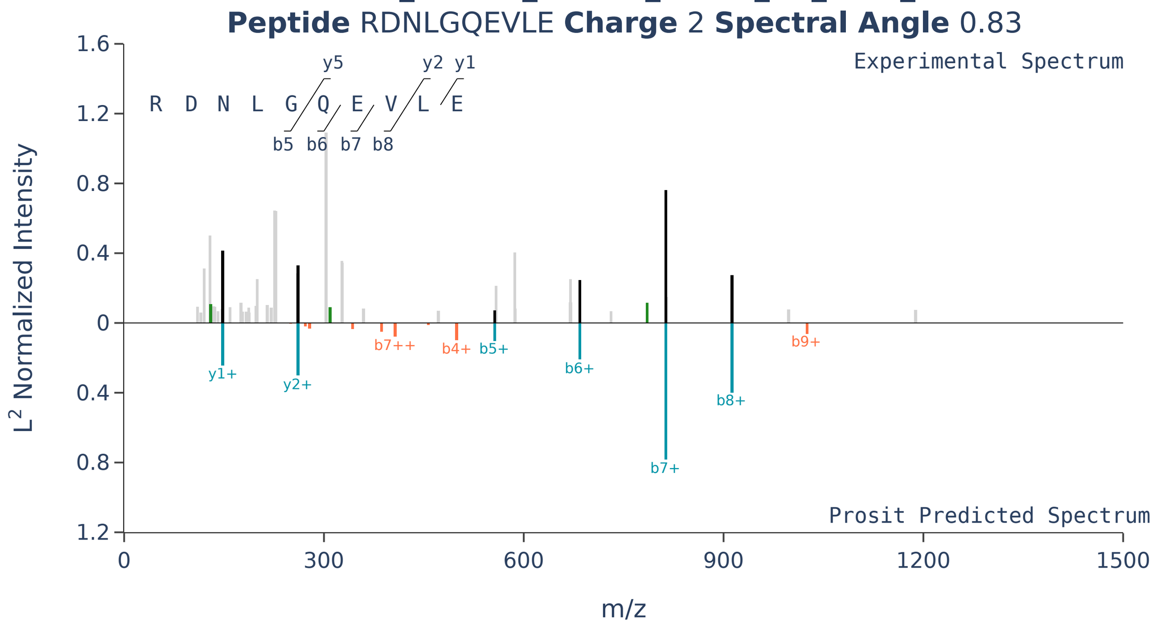

Source HRoetschke_211222_160123_HFGoe_IF2_b1R2_2h **Scan** 8312
Peptide AVKGSAVMSD **Charge** 2 **Spectral Angle** 0.88

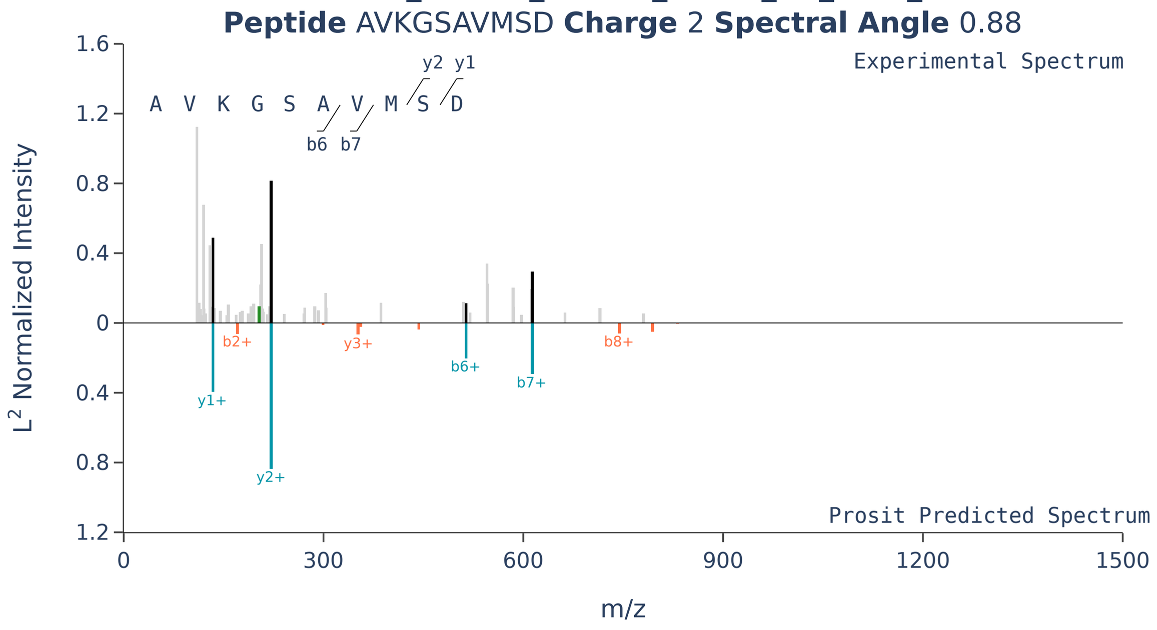

Source HRoetschke_211222_160123_HFGoe_IF2_b1R1_4h **Scan** 15301
Peptide LDYIDNAE **Charge** 2 **Spectral Angle** 0.84

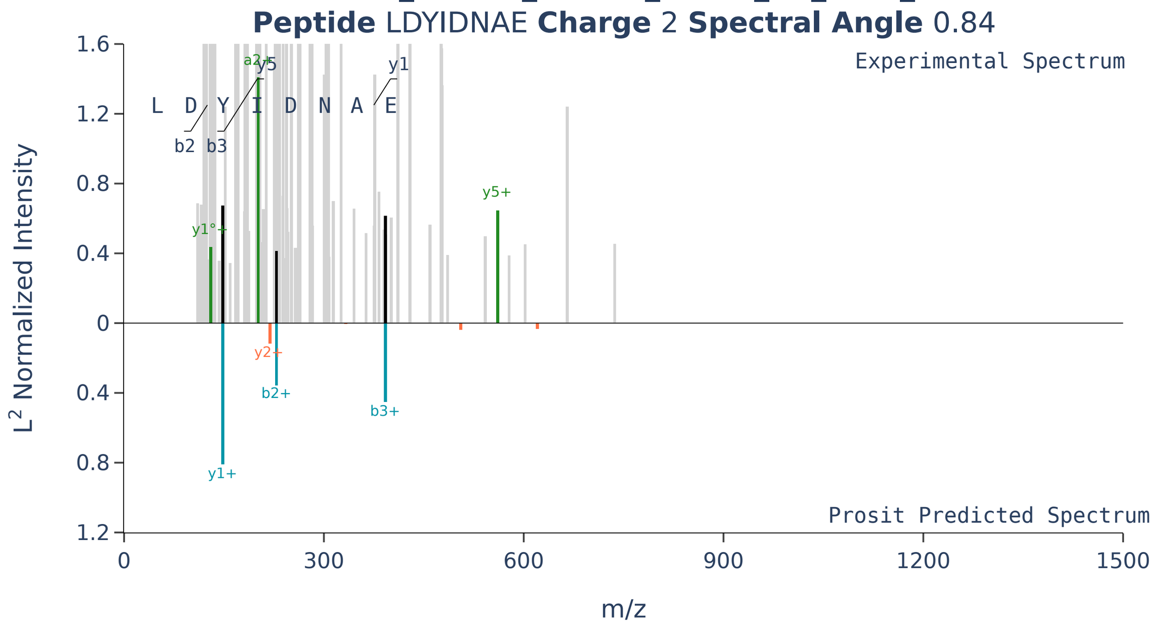

Source HRoetschke_211222_160123_HFGoe_IF2_b1R2_2h **Scan** 14279
Peptide PAQAVNRDVVLD **Charge** 2 **Spectral Angle** 0.81

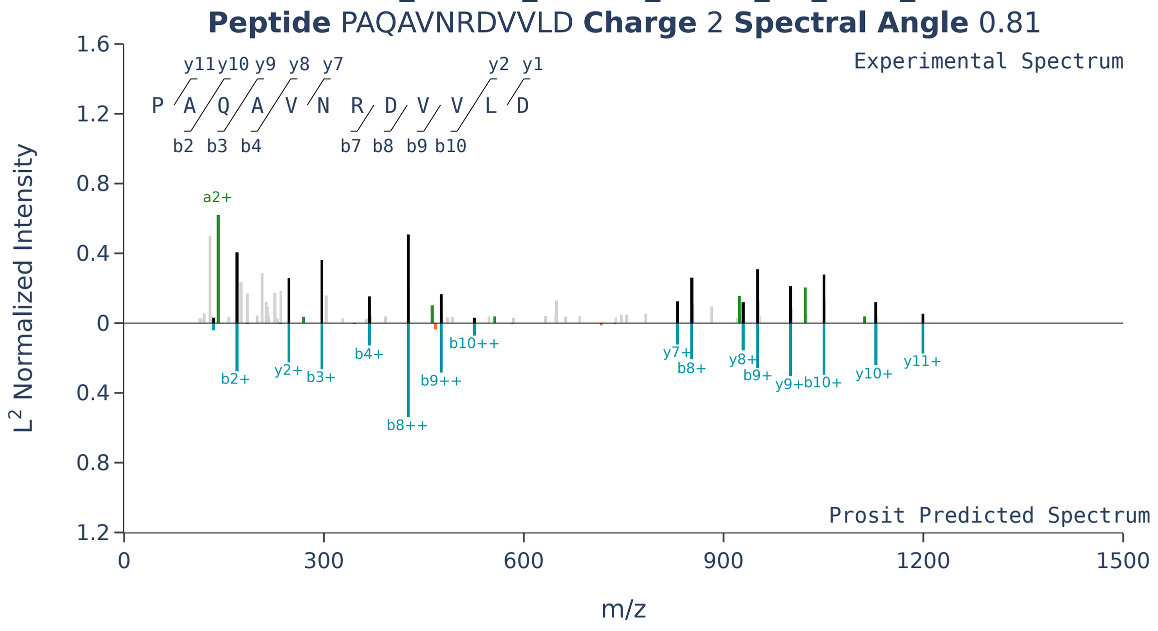

Source HRoetschke_211222_160123_HFGoe_IF2_b1R2_4h **Scan** 7287
Peptide HVETSGEAG **Charge** 1 **Spectral Angle** 0.88

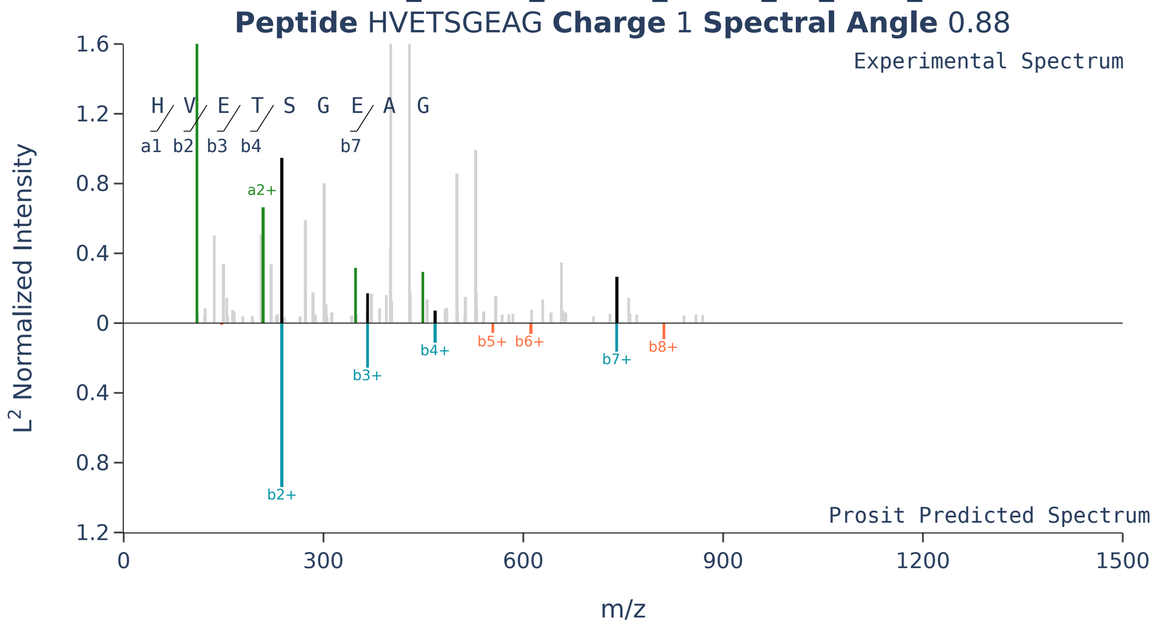

Source HRoetschke_211222_160123_HFGoe_IF2_b2R1_24h **Scan** 11589
Peptide EESNKWTDNAEPTE **Charge** 2 **Spectral Angle** 0.94

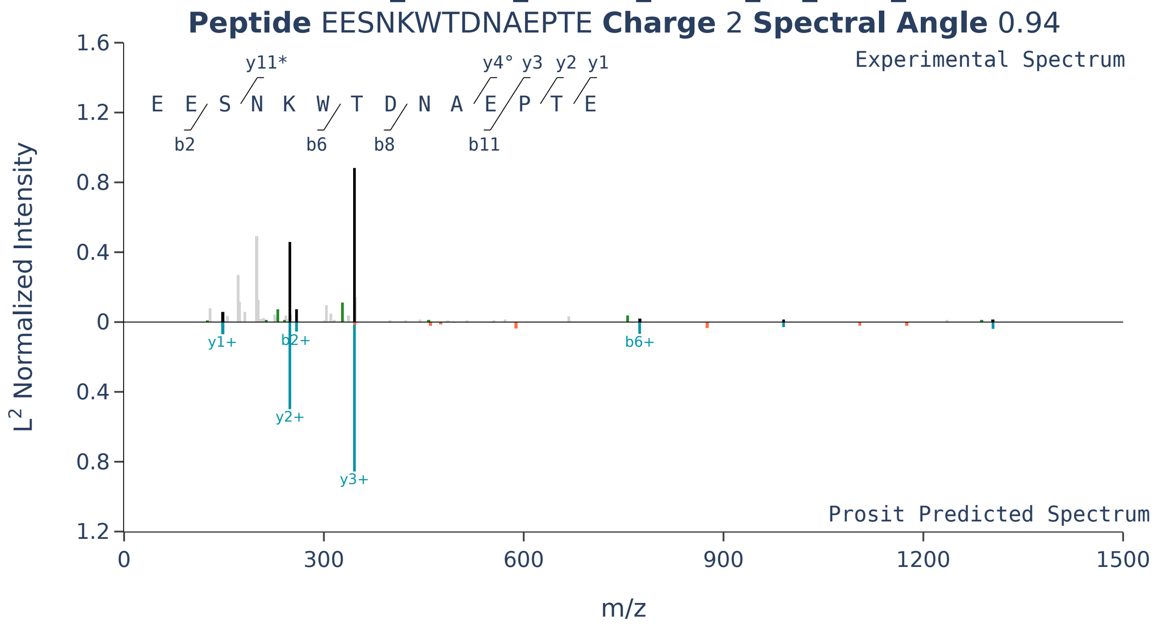

Source HRoetschke_211222_160123_HFGoe_IF2_b1R1_4h **Scan** 16466
Peptide PVVVAVDN **Charge** 1 **Spectral Angle** 0.95

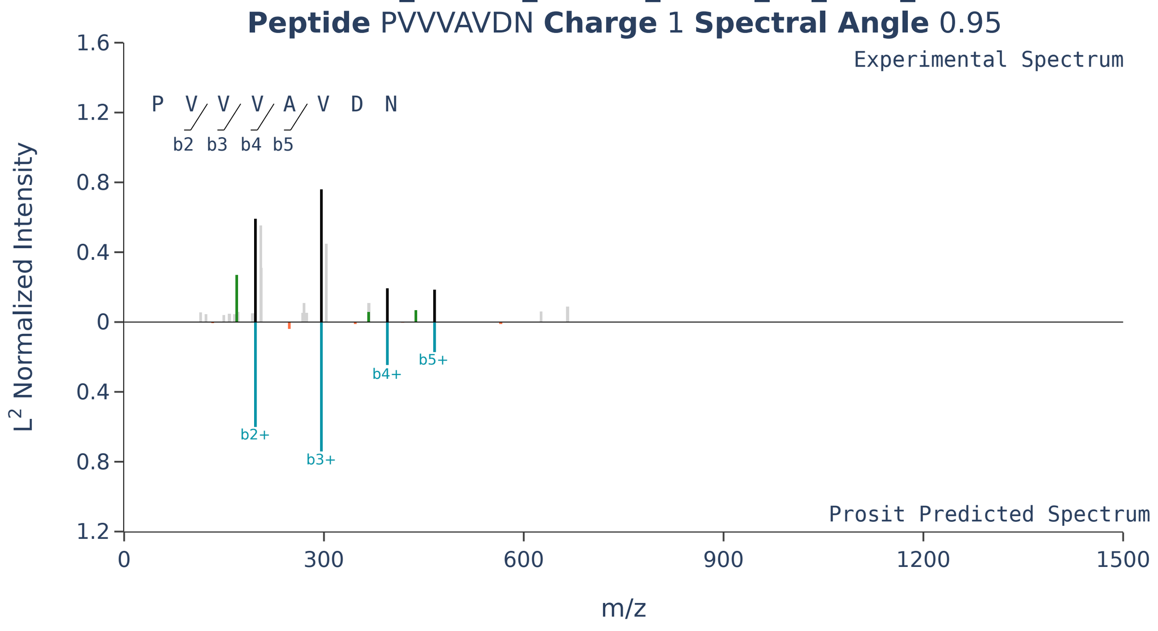

Source HRoetschke_211222_160123_HFGoe_IF2_b2R2_24h **Scan** 16051
Peptide QAVEVLE **Charge** 1 **Spectral Angle** 0.86

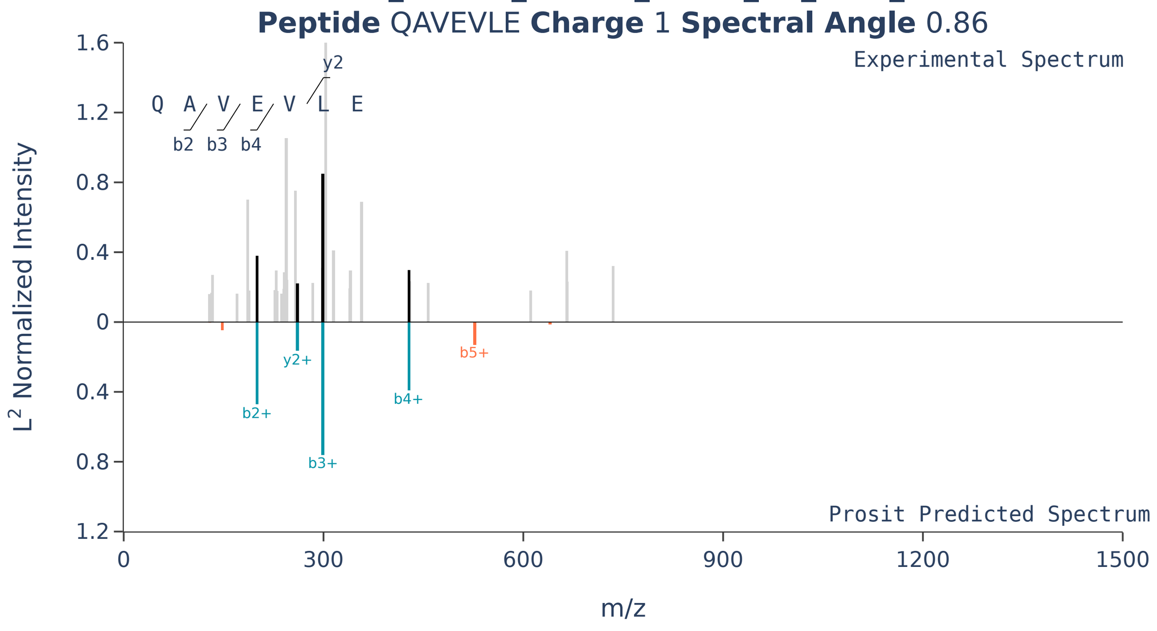

Source HRoetschke_211222_160123_HFGoe_IF2_b1R2_4h **Scan** 12248
Peptide LDYIRSTSD **Charge** 2 **Spectral Angle** 0.87

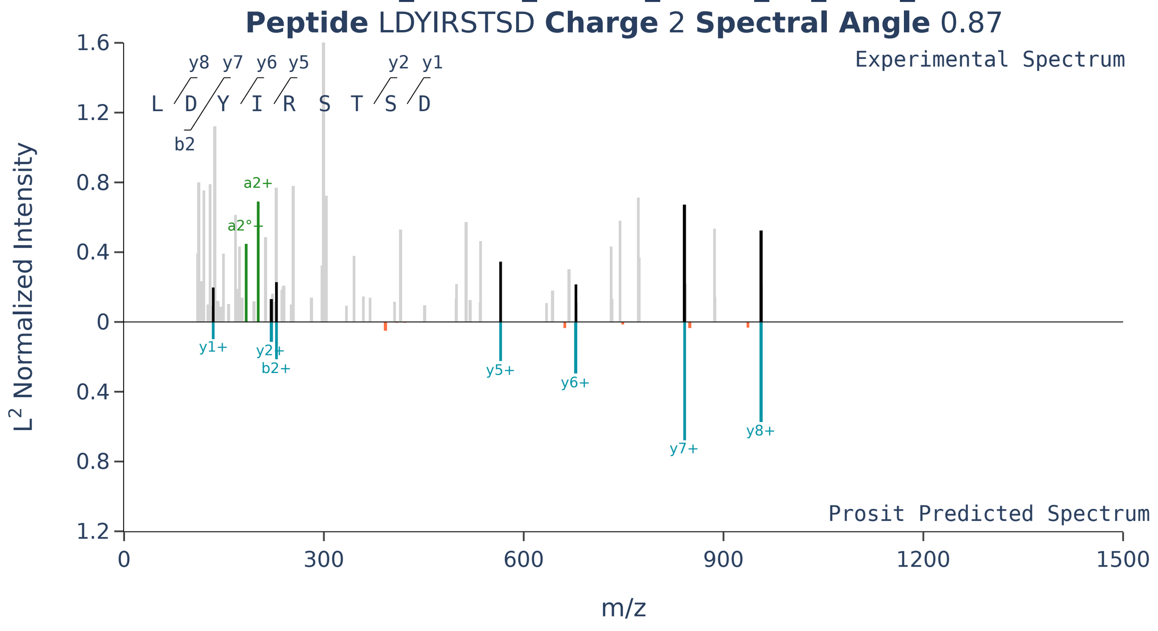

Source HRoetschke_211222_160123_HFGoe_IF2_b1R1_4h **Scan** 17793
Peptide GVGVIYE **Charge** 1 **Spectral Angle** 0.79

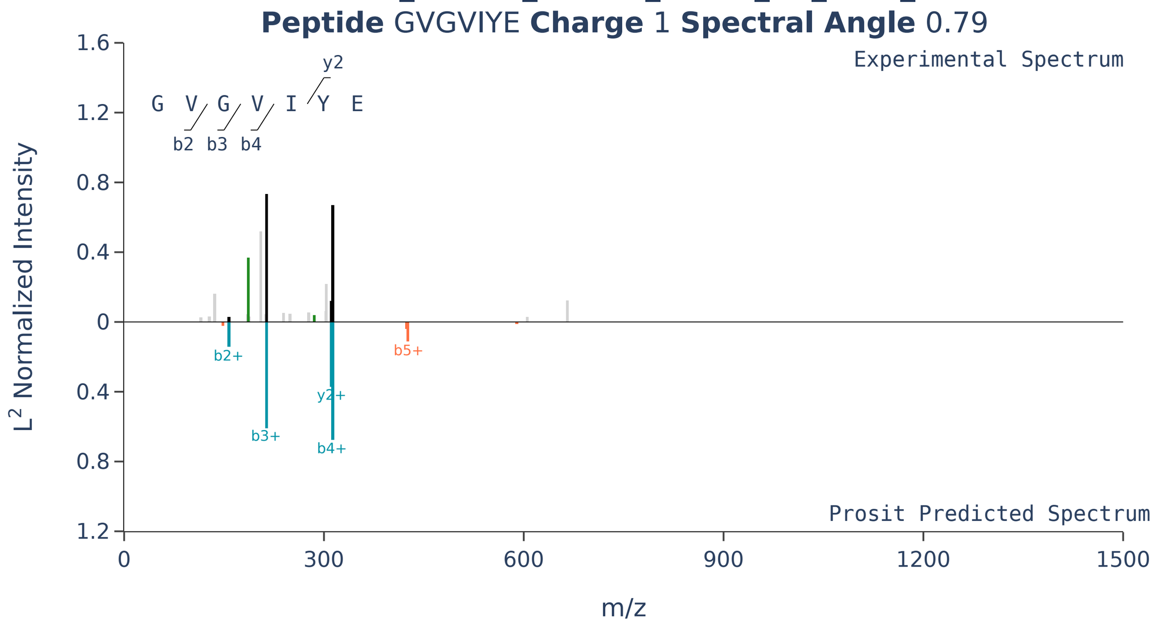

Source HRoetschke_211222_160123_HFGoe_IF2_b1R2_24h **Scan** 15549
Peptide RNGELGQEVLE **Charge** 2 **Spectral Angle** 0.76

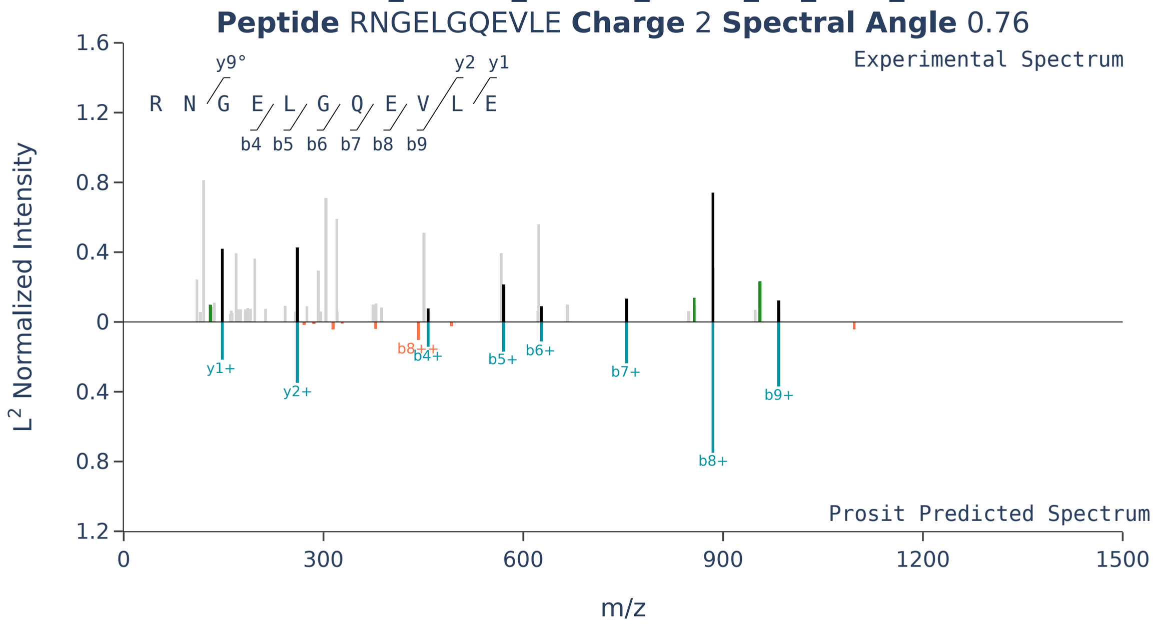

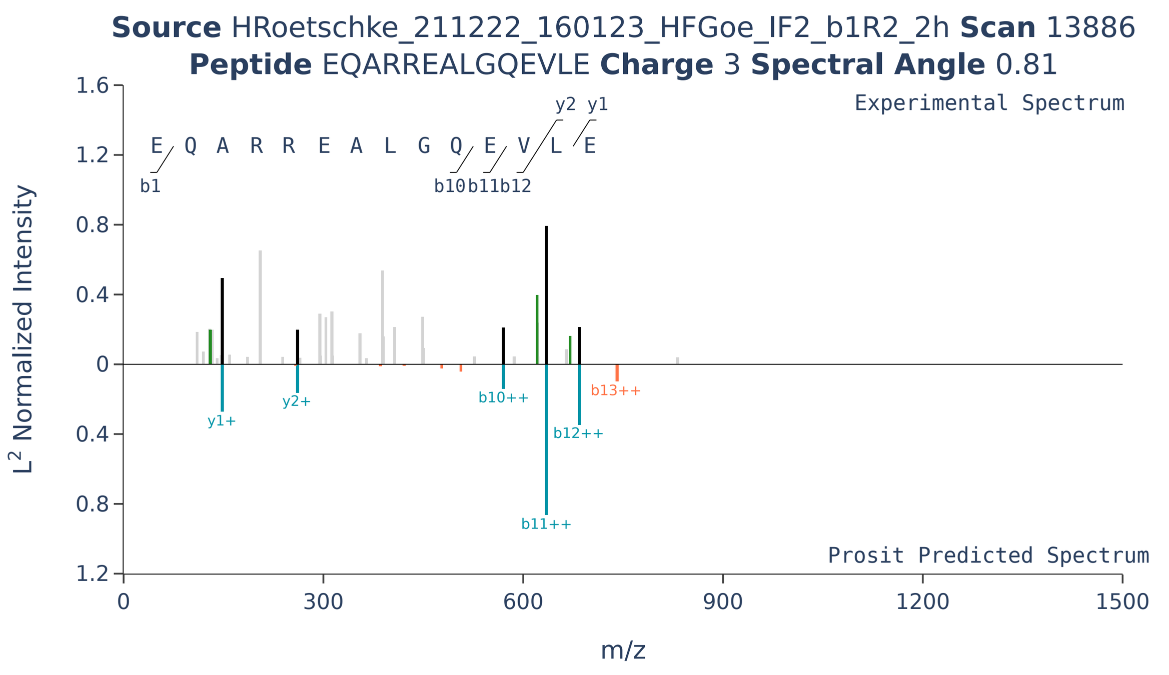
Source HRoetschke_211222_160123_HFGoe_IF2_b1R2_2h Scan 13886
Peptide EQARREALGQEVLE Charge 3 Spectral Angle 0.81
Experimental Spectrum
Prosit Predicted Spectrum
L² Normalized Intensity
m/z

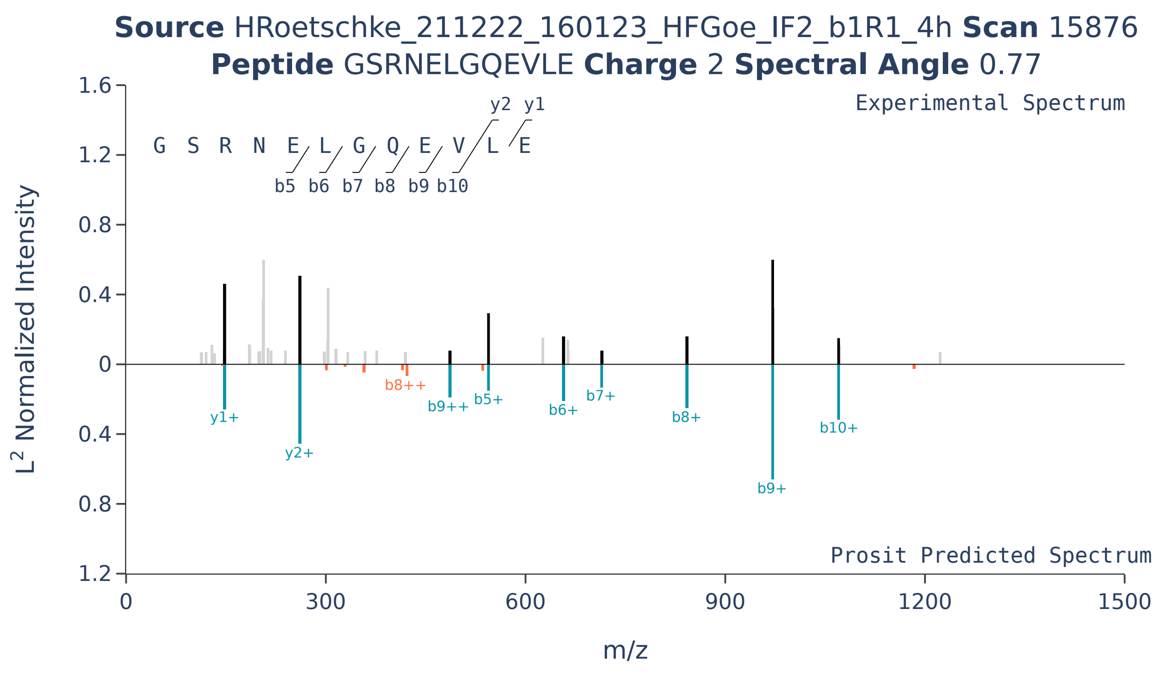
Source HRoetschke_211222_160123_HFGoe_IF2_b1R1_4h Scan 15876
Peptide GSRNELGQEVLE Charge 2 Spectral Angle 0.77
Experimental Spectrum
Prosit Predicted Spectrum
L² Normalized Intensity
m/z

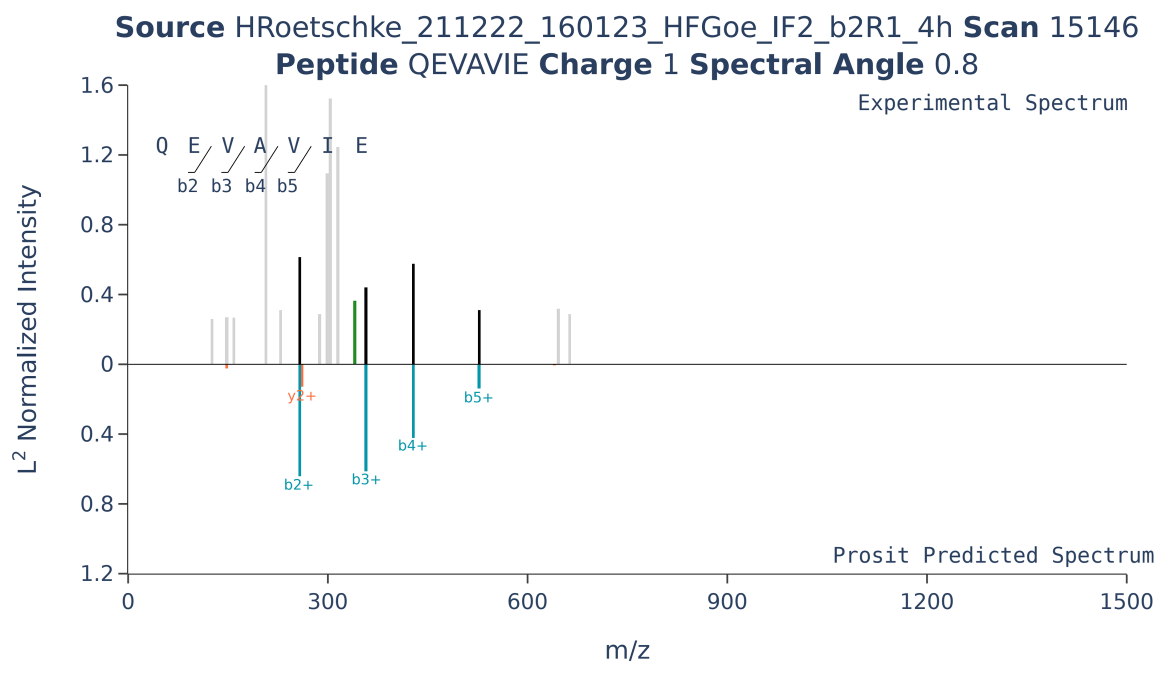
Source HRoetschke_211222_160123_HFGoe_IF2_b2R1_4h Scan 15146
Peptide QEVAVIE Charge 1 Spectral Angle 0.8
Experimental Spectrum
Prosit Predicted Spectrum
L² Normalized Intensity
m/z

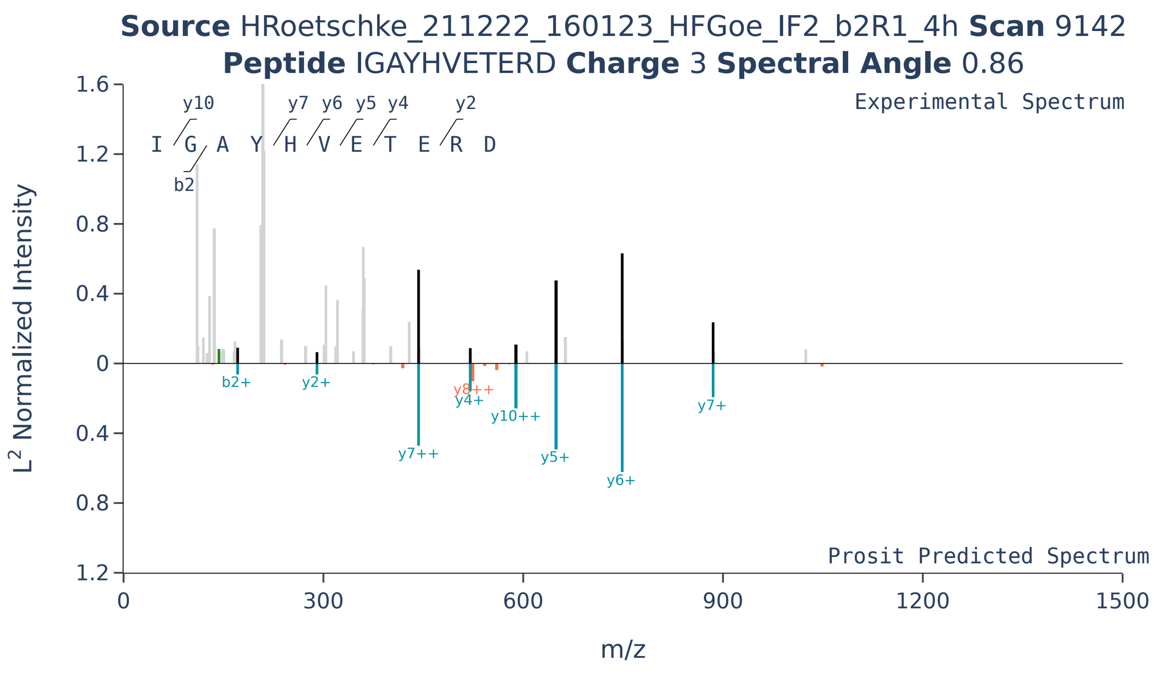
Source HRoetschke_211222_160123_HFGoe_IF2_b2R1_4h Scan 9142
Peptide IGAYHVETERD Charge 3 Spectral Angle 0.86
Experimental Spectrum
Prosit Predicted Spectrum
L² Normalized Intensity
m/z

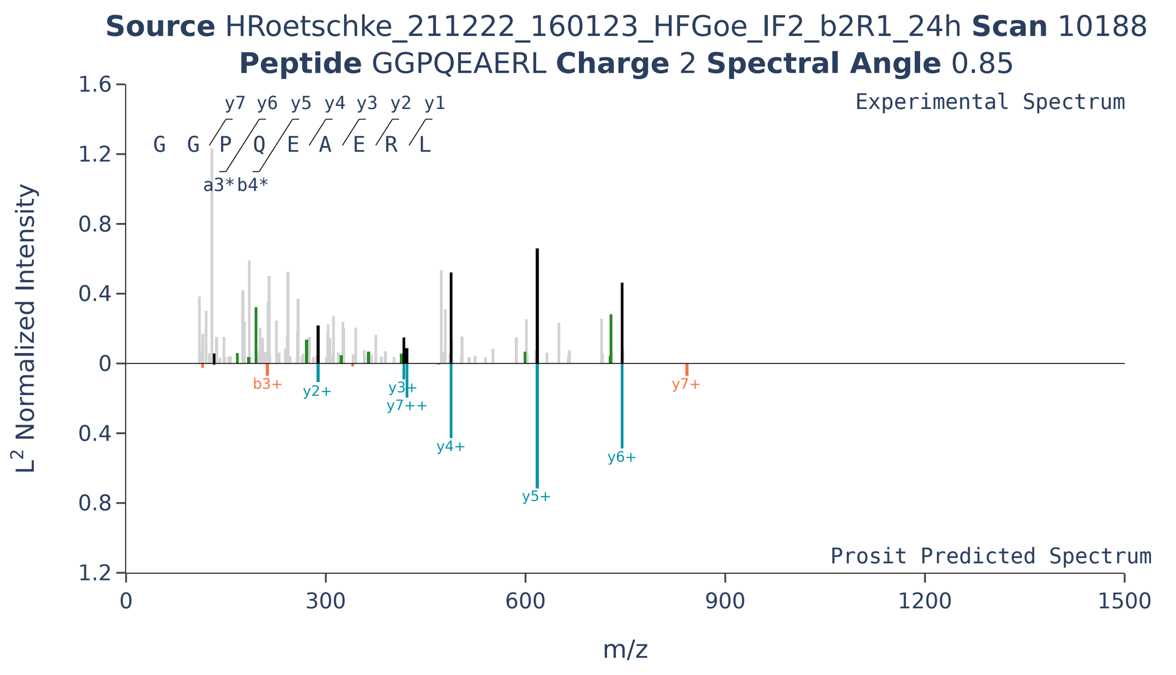
Source HRoetschke_211222_160123_HFGoe_IF2_b2R1_24h Scan 10188
Peptide GGPQEAERL Charge 2 Spectral Angle 0.85
Experimental Spectrum
Prosit Predicted Spectrum
L² Normalized Intensity
m/z

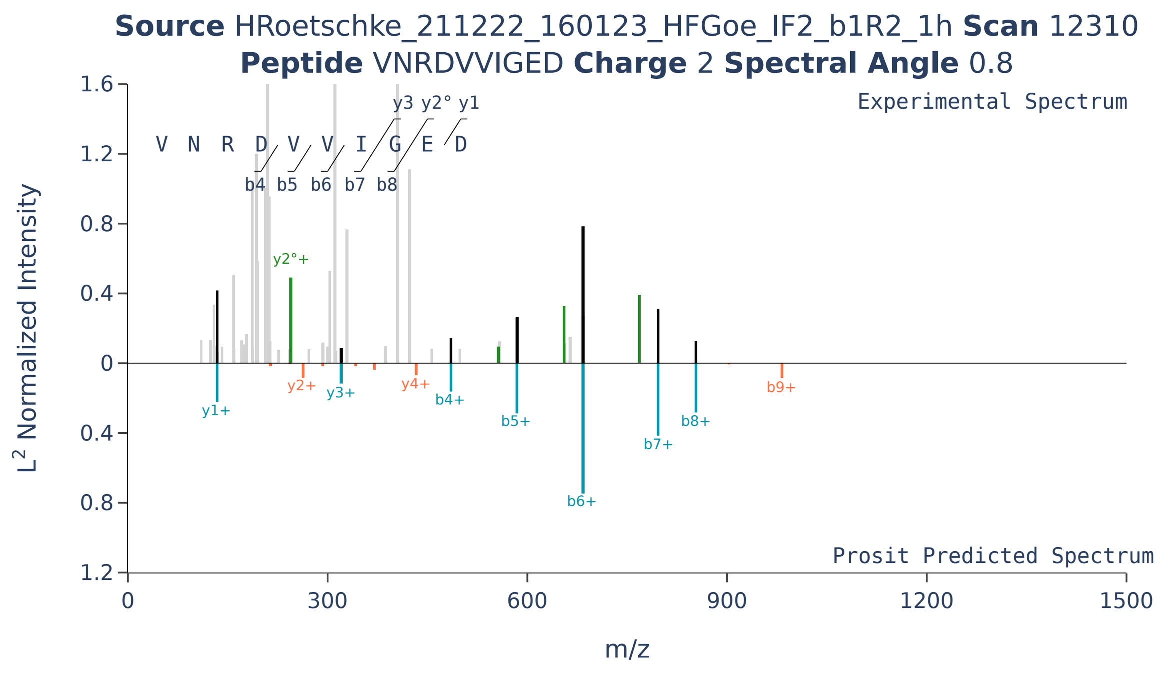
Source HRoetschke_211222_160123_HFGoe_IF2_b1R2_1h Scan 12310
Peptide VNRDVVIGED Charge 2 Spectral Angle 0.8
Experimental Spectrum
Prosit Predicted Spectrum
L² Normalized Intensity
m/z

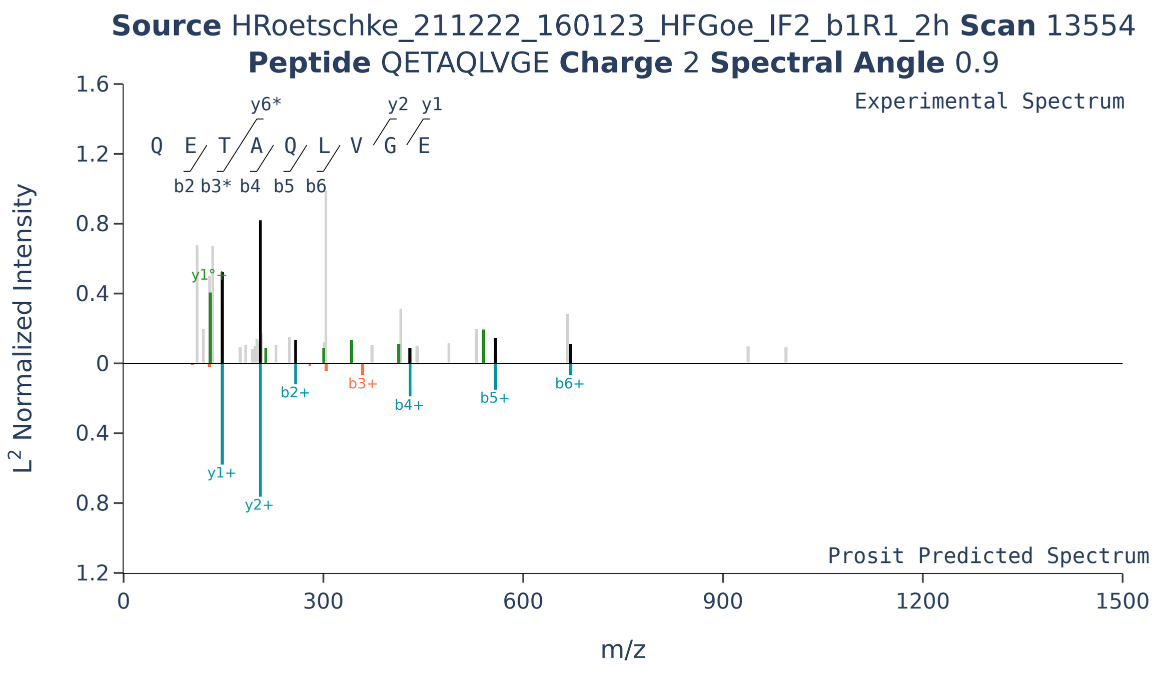
Source HRoetschke_211222_160123_HFGoe_IF2_b1R2_2h Scan 13554
Peptide QETAQLVGE Charge 2 Spectral Angle 0.9
Experimental Spectrum
Prosit Predicted Spectrum
L² Normalized Intensity
m/z

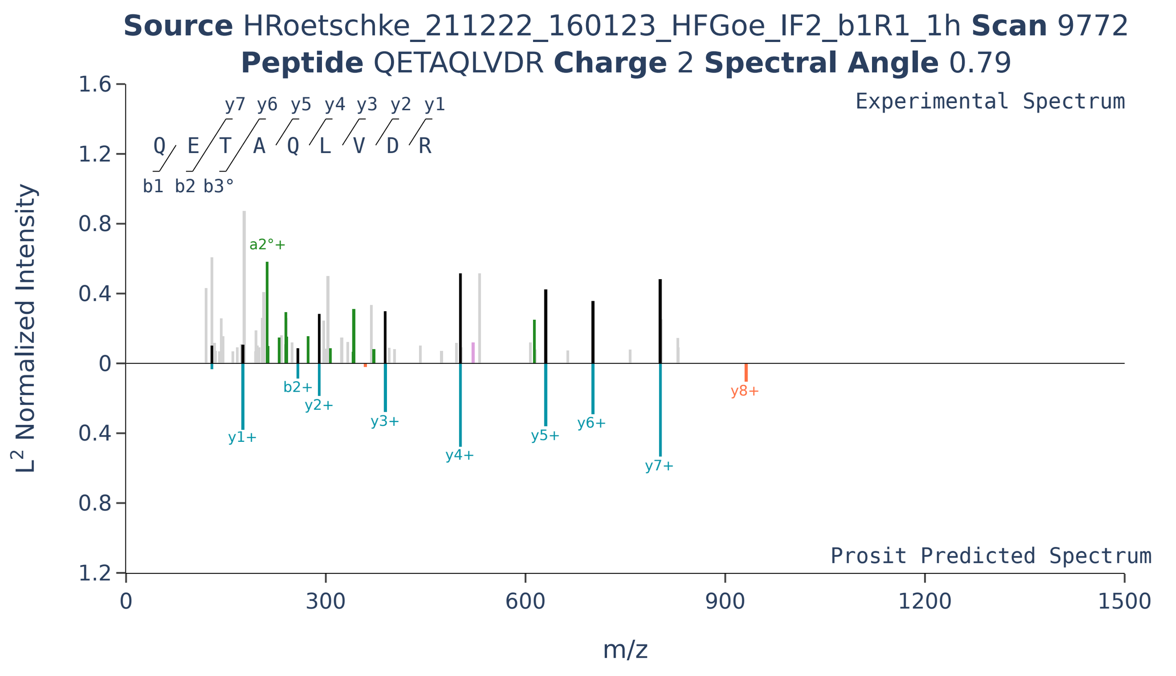
Source HRoetschke_211222_160123_HFGoe_IF2_b1R1_1h Scan 9772
Peptide QETAQLVDR Charge 2 Spectral Angle 0.79
Experimental Spectrum
Prosit Predicted Spectrum
L² Normalized Intensity
m/z

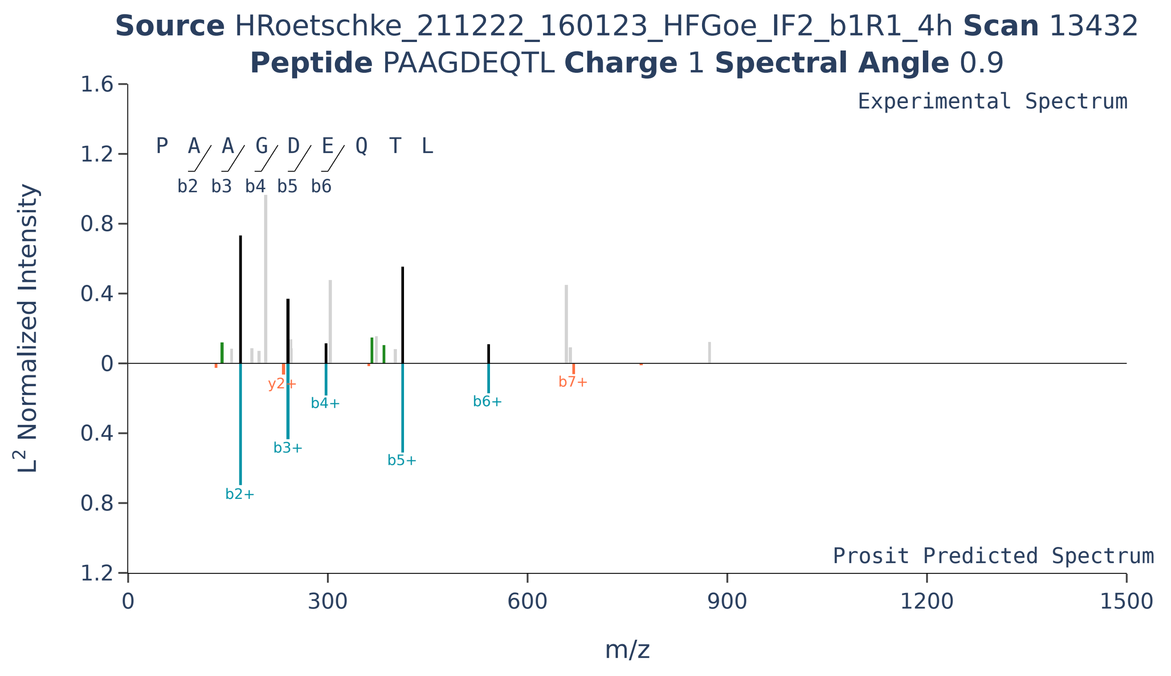
Source HRoetschke_211222_160123_HFGoe_IF2_b1R1_4h Scan 13432
Peptide PAAGDEQTL Charge 1 Spectral Angle 0.9
Experimental Spectrum
Prosit Predicted Spectrum
L² Normalized Intensity
m/z

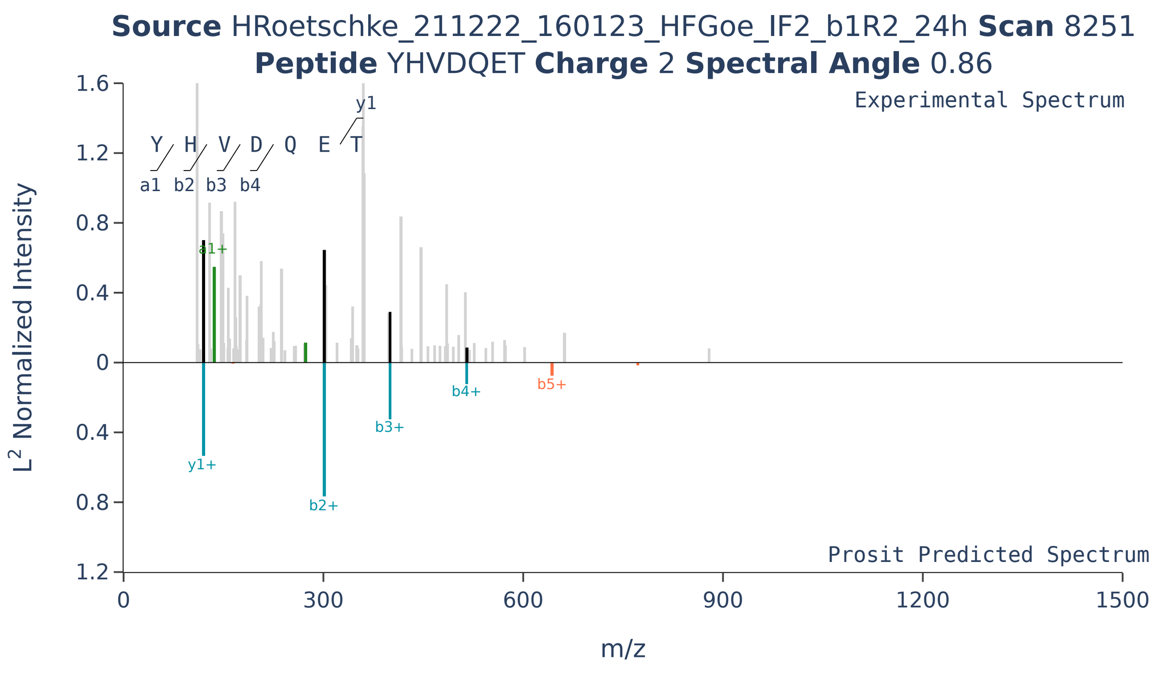
Source HRoetschke_211222_160123_HFGoe_IF2_b1R2_24h Scan 8251
Peptide YHVDQET Charge 2 Spectral Angle 0.86
Experimental Spectrum
Prosit Predicted Spectrum
L² Normalized Intensity
m/z

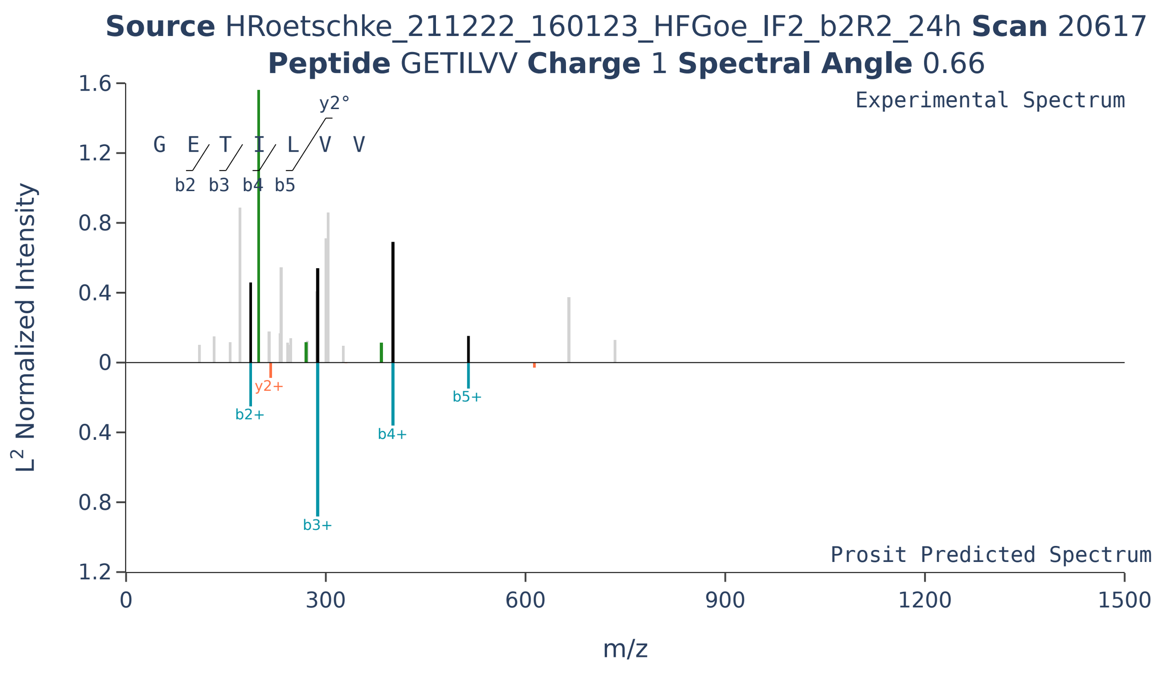
Source HRoetschke_211222_160123_HFGoe_IF2_b2R2_24h Scan 20617
Peptide GETILVV Charge 1 Spectral Angle 0.66
Experimental Spectrum
Prosit Predicted Spectrum
L² Normalized Intensity
m/z

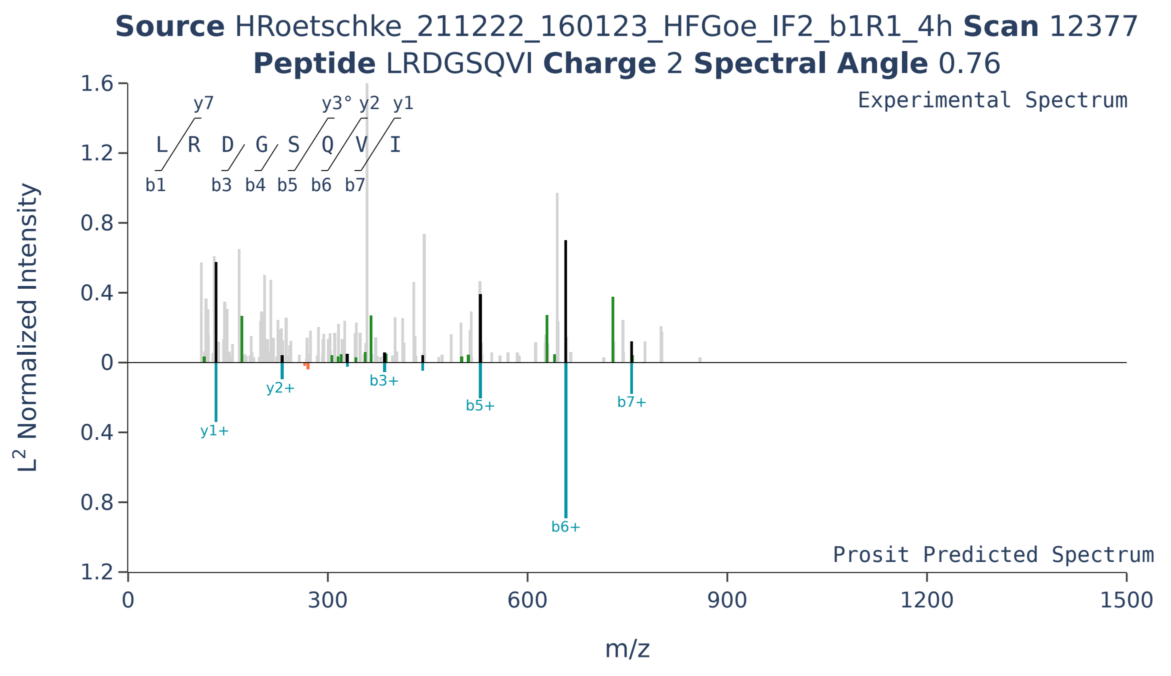
Source HRoetschke_211222_160123_HFGoe_IF2_b1R1_4h Scan 12377
Peptide LRDGSQVI Charge 2 Spectral Angle 0.76
Experimental Spectrum
Prosit Predicted Spectrum
L² Normalized Intensity
m/z

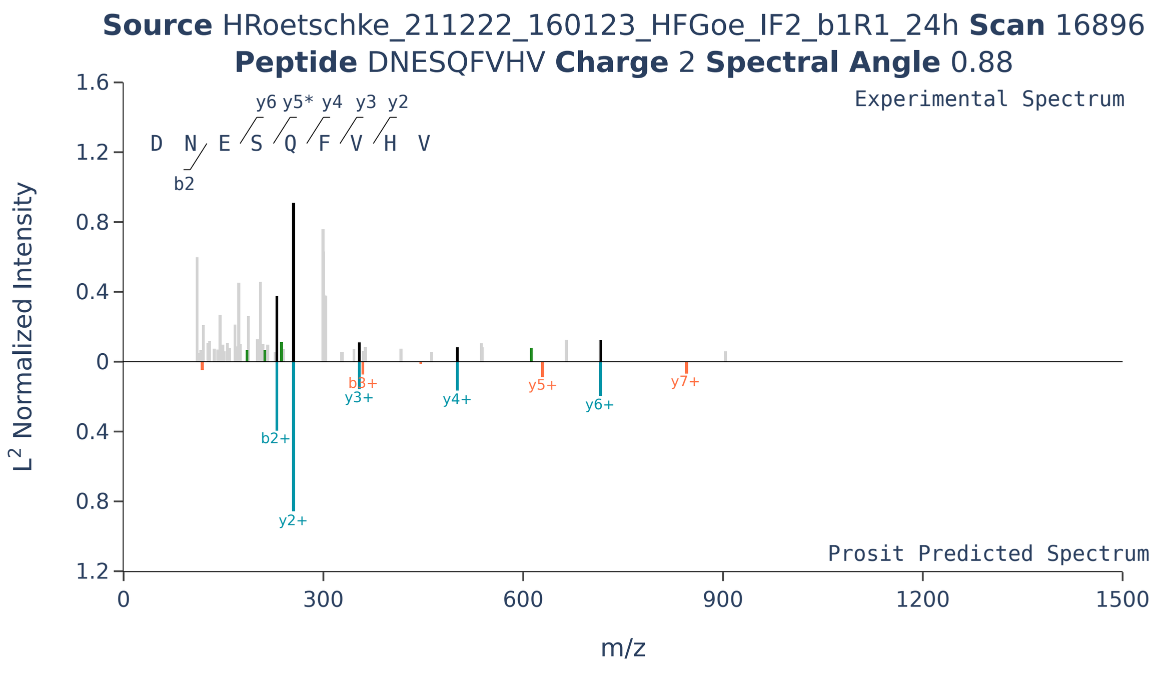
Source HRoetschke_211222_160123_HFGoe_IF2_b1R1_24h Scan 16896
Peptide DNESQFVHV Charge 2 Spectral Angle 0.88
Experimental Spectrum
Prosit Predicted Spectrum
L² Normalized Intensity
m/z

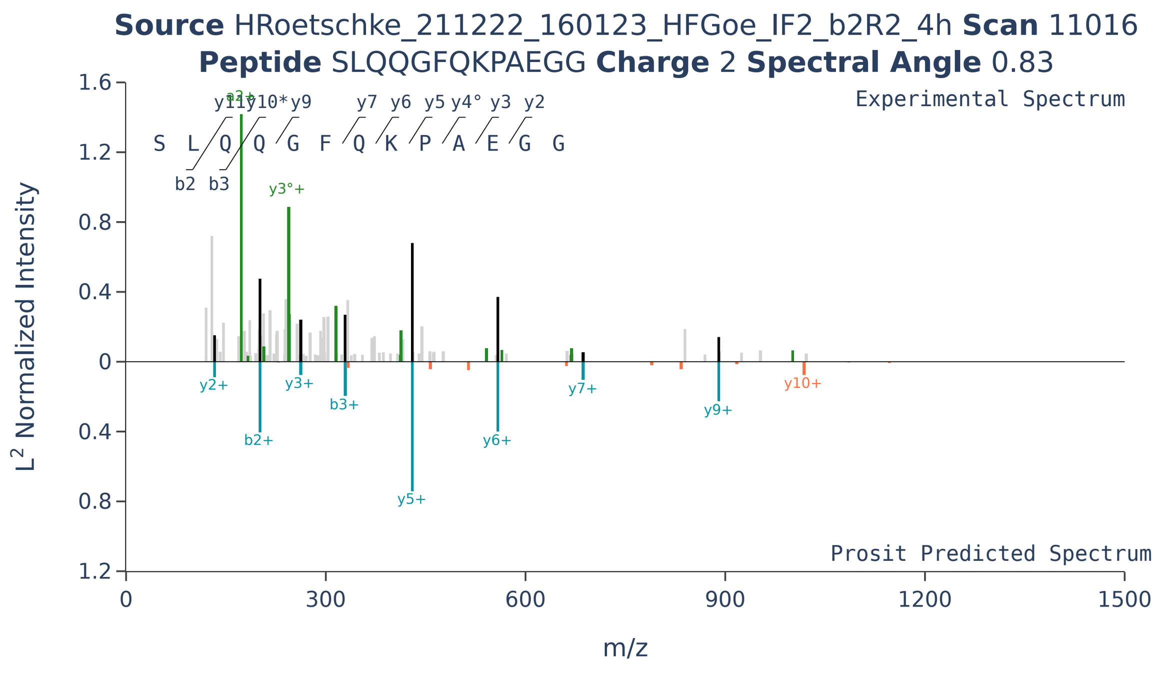
Source HRoetschke_211222_160123_HFGoe_IF2_b2R2_4h Scan 11016
Peptide SLQQGFQKPAEGG Charge 2 Spectral Angle 0.83
Experimental Spectrum
Prosit Predicted Spectrum
L² Normalized Intensity
m/z

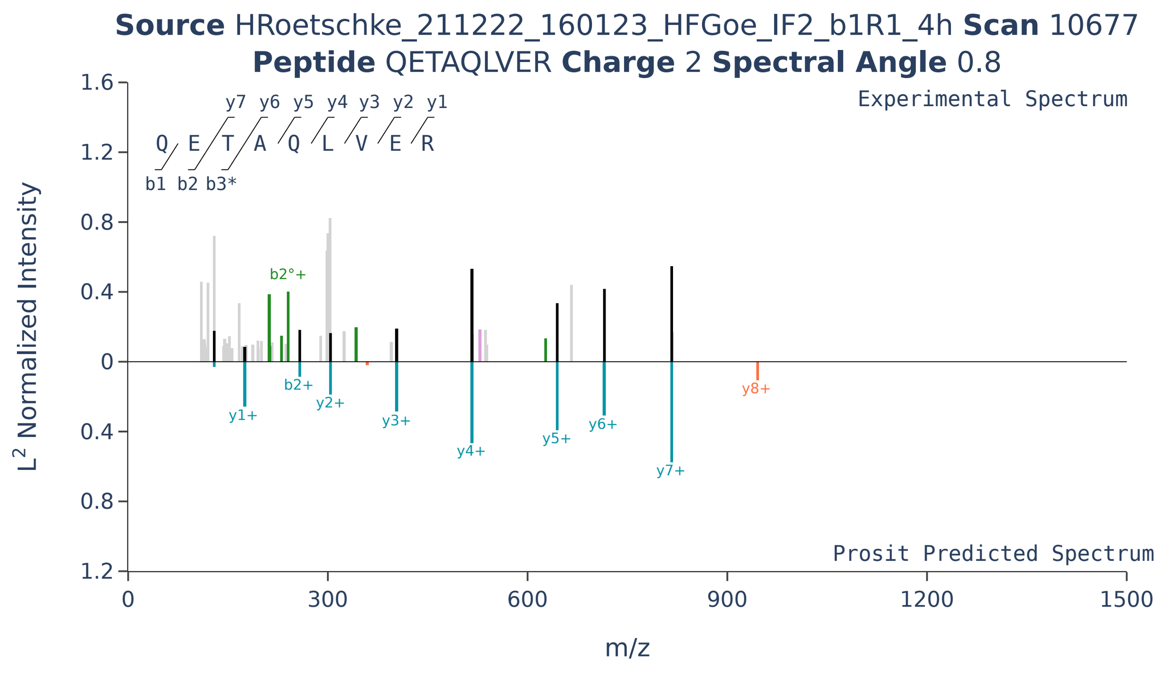
Source HRoetschke_211222_160123_HFGoe_IF2_b1R1_4h Scan 10677
Peptide QETAQLVER Charge 2 Spectral Angle 0.8
Experimental Spectrum
Prosit Predicted Spectrum
L² Normalized Intensity
m/z

Source HRoetschke_211222_160123_HFGoe_IF2_b1R1_4h **Scan** 11469
Peptide GLSGVPAAGDR **Charge** 2 **Spectral Angle** 0.87

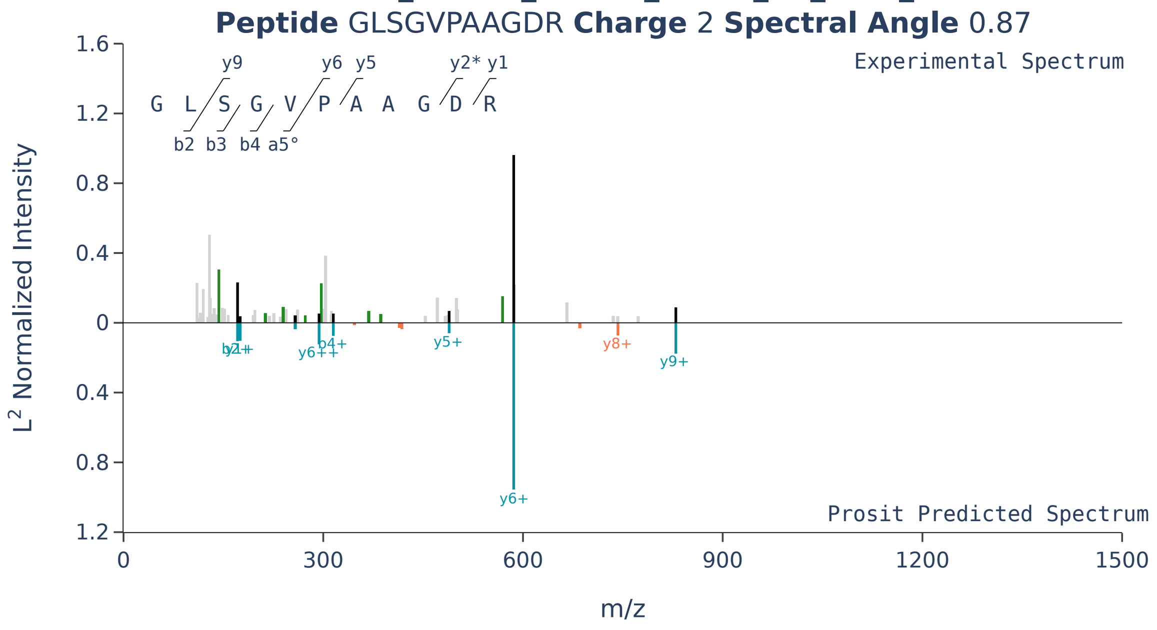

Source HRoetschke_211222_160123_HFGoe_IF2_b1R1_4h **Scan** 16250
Peptide ADRNELGQEVLE **Charge** 2 **Spectral Angle** 0.75

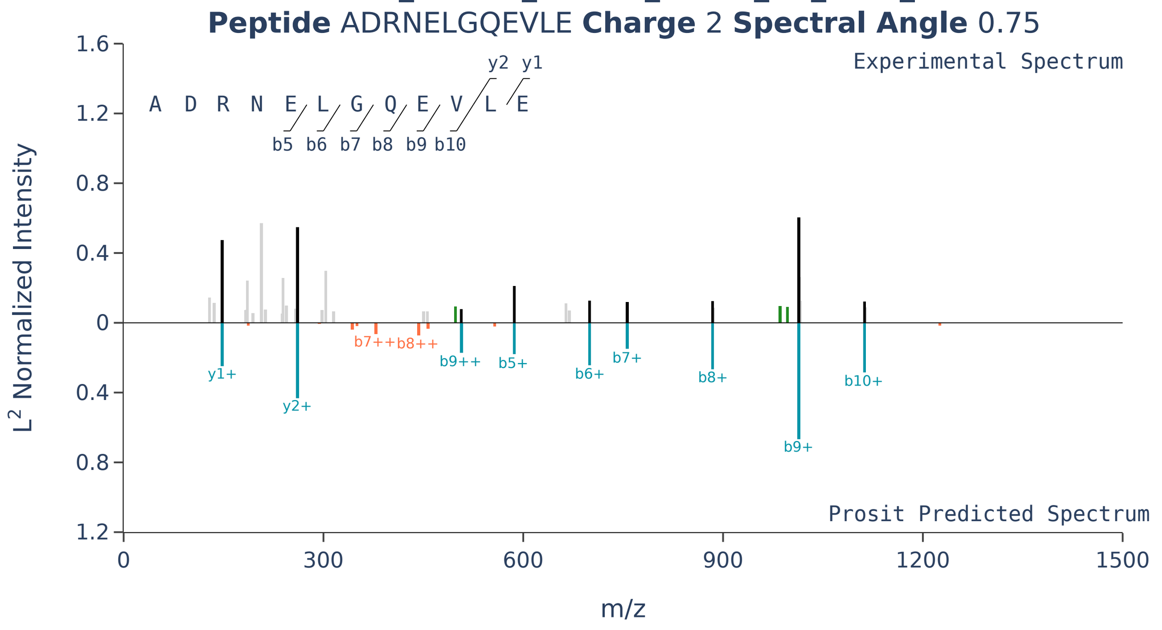

Source HRoetschke_211222_160123_HFGoe_IF2_b2R2_2h **Scan** 7754
Peptide HVETQGS **Charge** 1 **Spectral Angle** 0.9

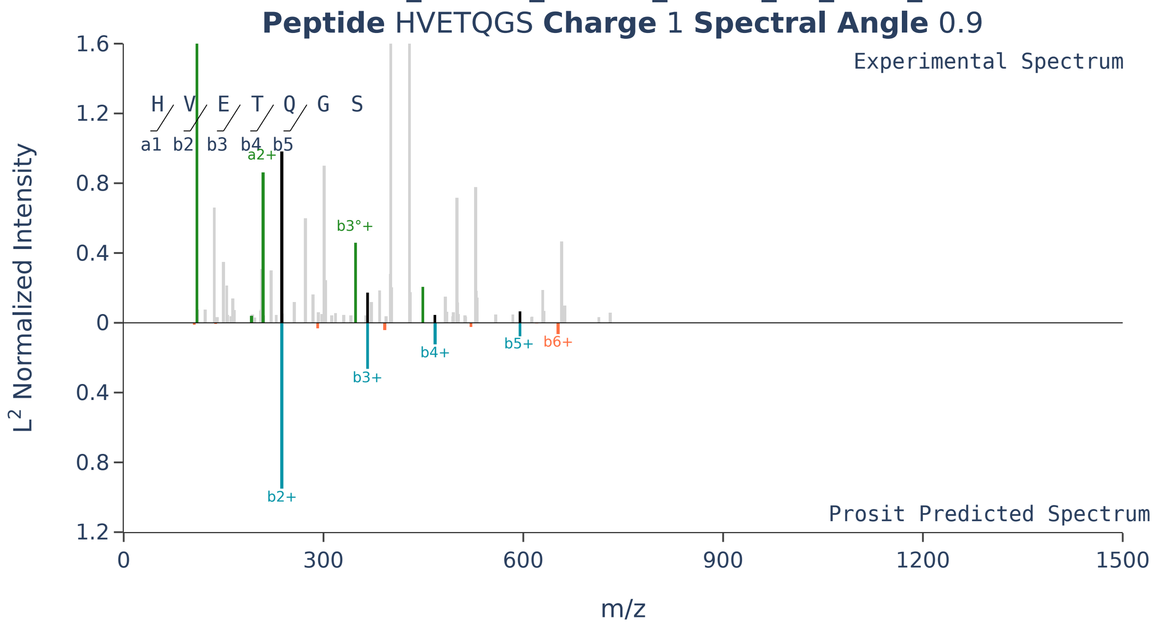

Source HRoetschke_211222_160123_HFGoe_IF2_b1R1_4h **Scan** 14123
Peptide KNAQRNELGQEVLE **Charge** 3 **Spectral Angle** 0.84

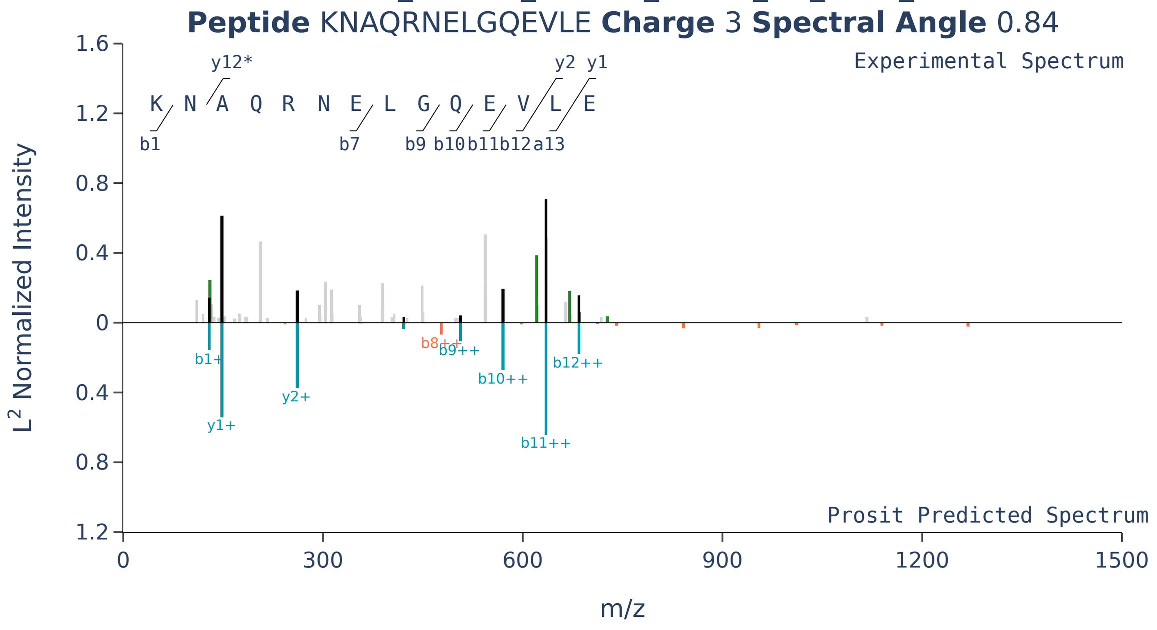

Source HRoetschke_211222_160123_HFGoe_IF2_b1R2_24h **Scan** 11643
Peptide TEDNKWTDNAEPTE **Charge** 2 **Spectral Angle** 0.9

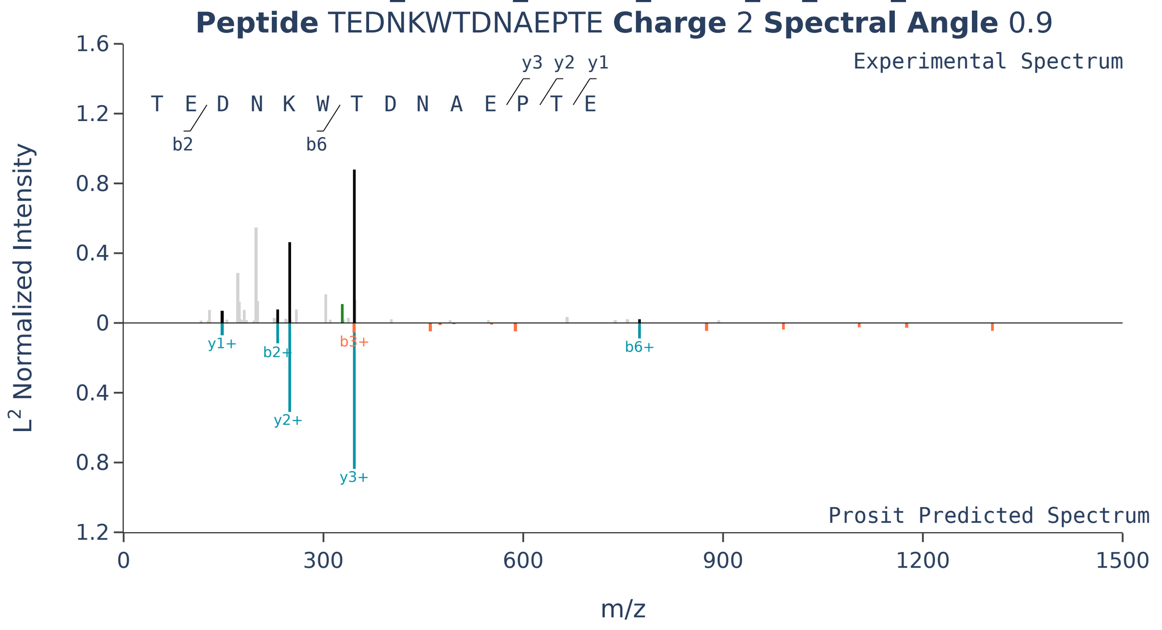

Source HRoetschke_211222_160123_HFGoe_IF2_b1R2_4h **Scan** 15199
Peptide ITVGEGID **Charge** 2 **Spectral Angle** 0.82

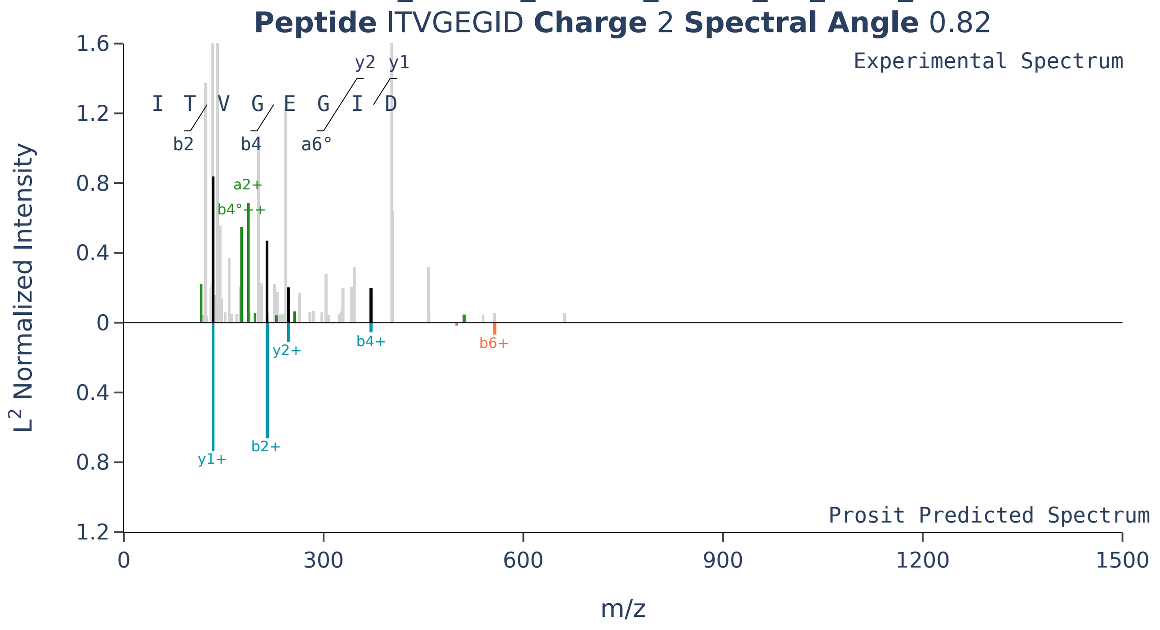

Source HRoetschke_211222_160123_HFGoe_IF2_b1R2_2h **Scan** 11015
Peptide NRDVVLE **Charge** 2 **Spectral Angle** 0.79

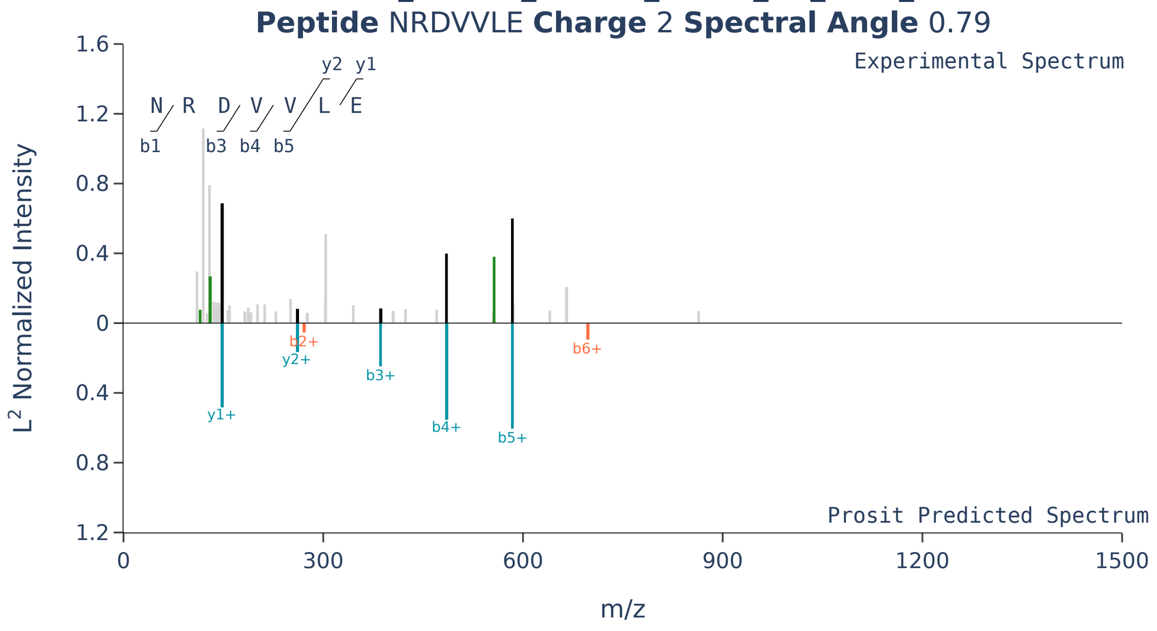

Source HRoetschke_211222_160123_HFGoe_IF2_b2R1_4h **Scan** 9850
Peptide VTEGISD **Charge** 2 **Spectral Angle** 0.84

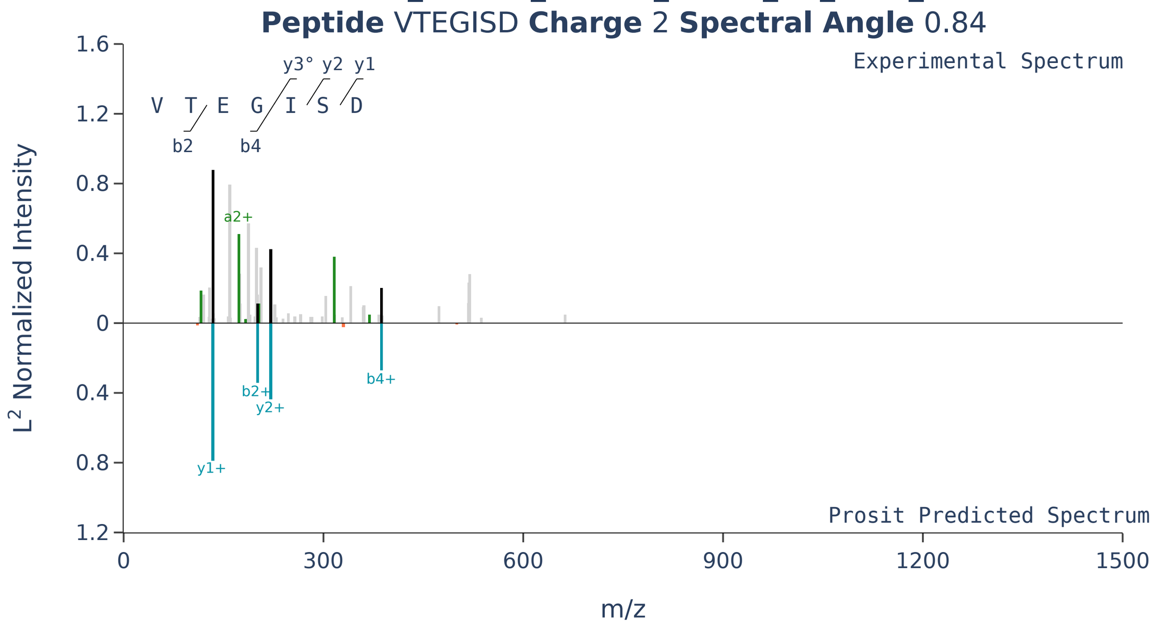

Source HRoetschke_211222_160123_HFGoe_IF2_b1R1_1h **Scan** 7266
Peptide HVETENTG **Charge** 1 **Spectral Angle** 0.84

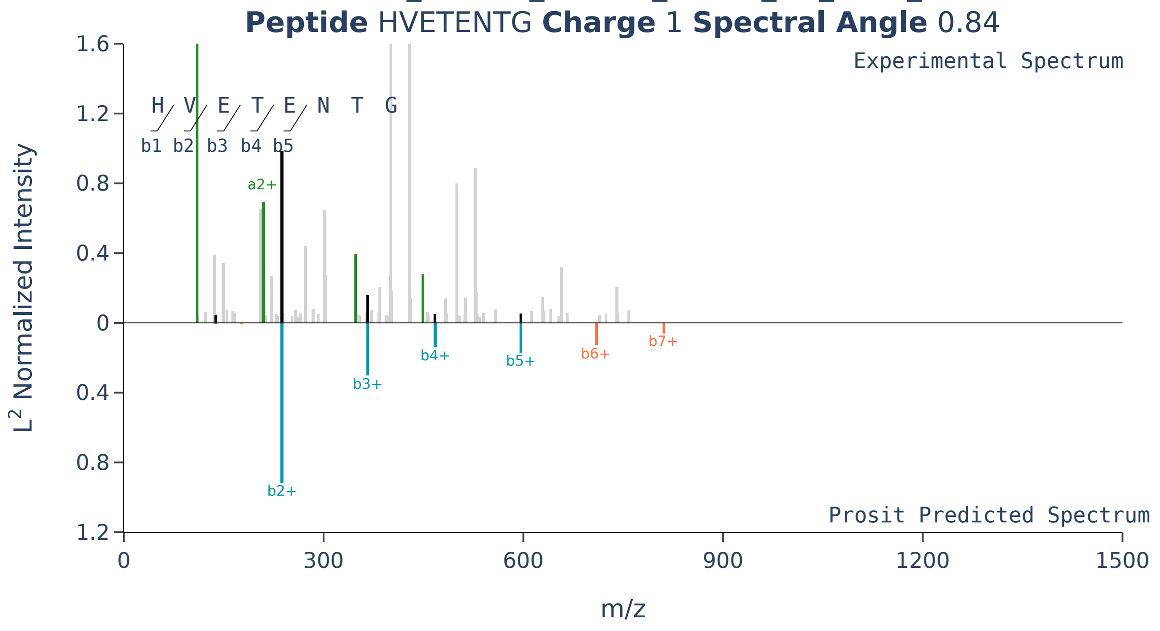

Source HRoetschke_211222_160123_HFGoe_IF2_b1R1_4h **Scan** 11079
Peptide WTDNAVHVSAK **Charge** 3 **Spectral Angle** 0.82

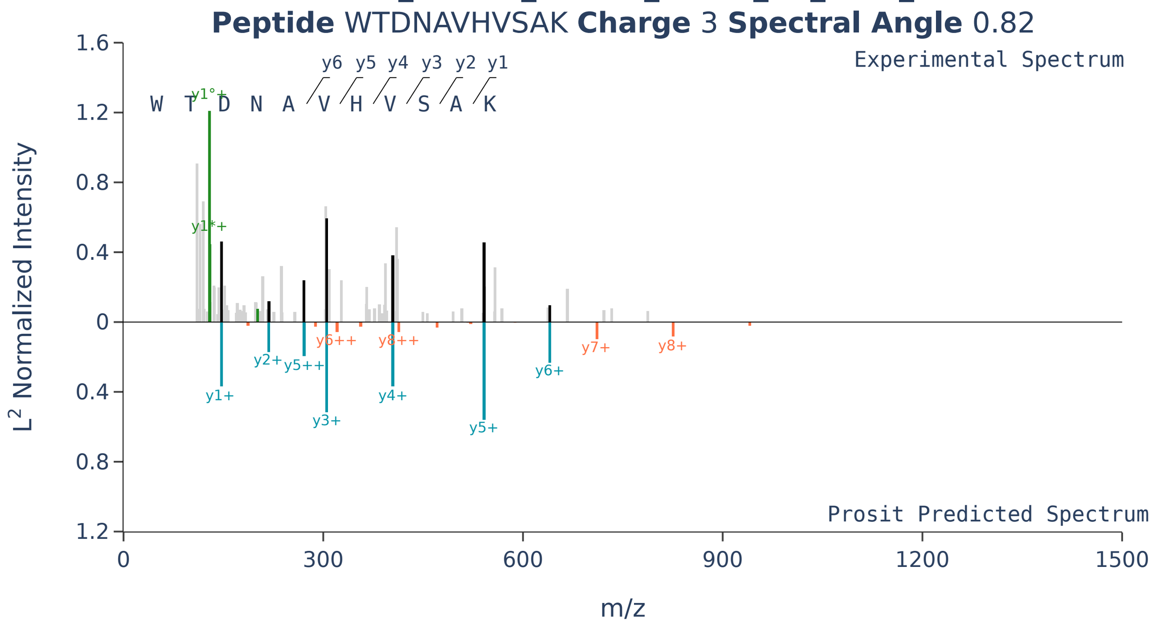

Source HRoetschke_211222_160123_HFGoe_IF2_b1R2_2h **Scan** 11205
Peptide VVTIREAAE **Charge** 2 **Spectral Angle** 0.9

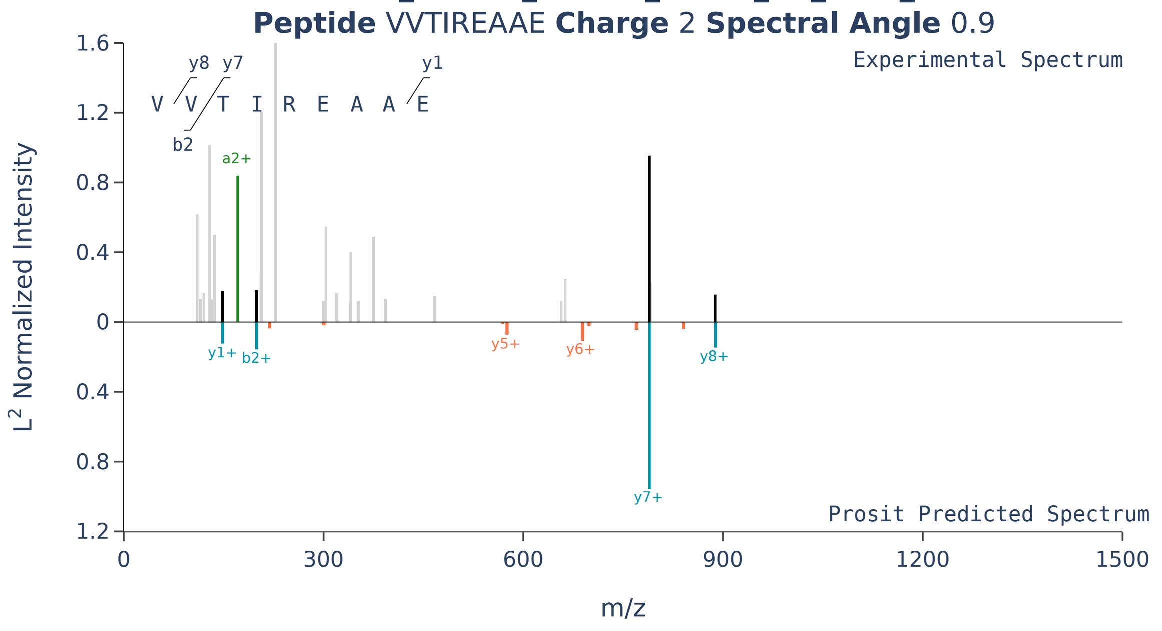

Source HRoetschke_211222_160123_HFGoe_IF2_b1R2_4h **Scan** 20322
Peptide PVVVAVNNL **Charge** 1 **Spectral Angle** 0.88

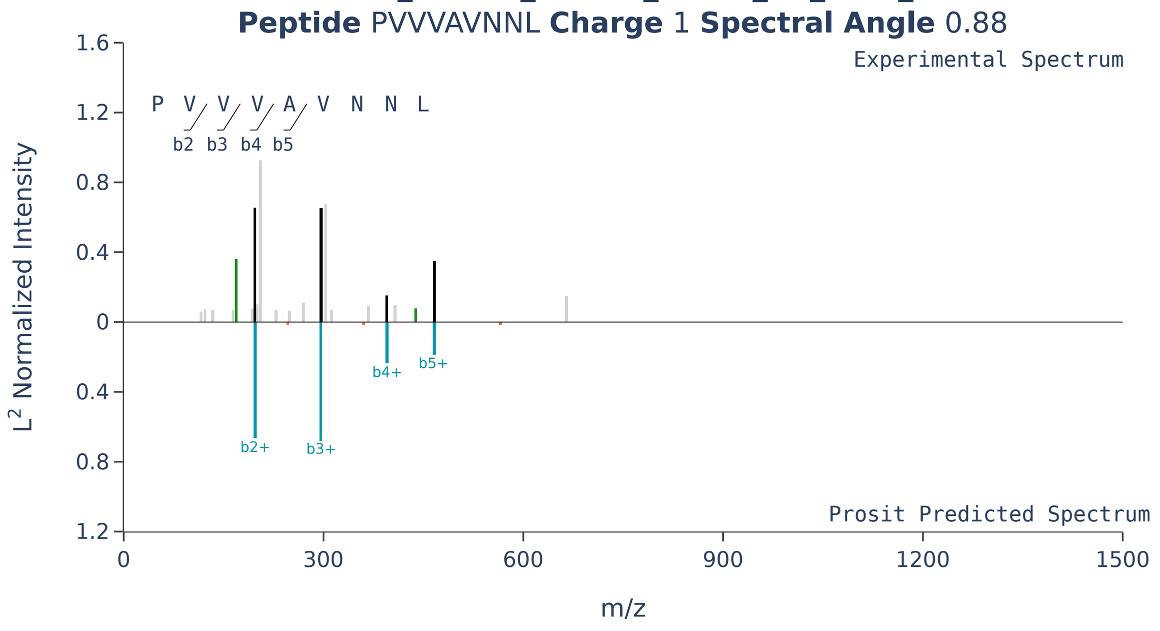

Source HRoetschke_211222_160123_HFGoe_IF2_b2R2_24h **Scan** 14104
Peptide PAAGDQLVAE **Charge** 2 **Spectral Angle** 0.7

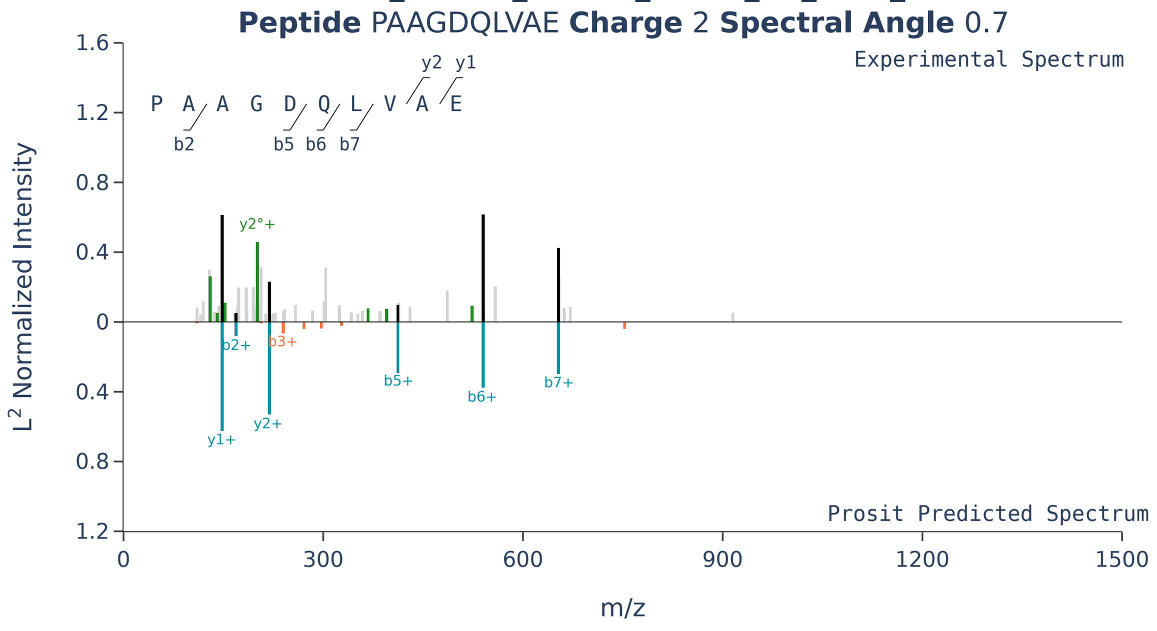

Source HRoetschke_211222_160123_HFGoe_IF2_b1R1_24h **Scan** 13133
Peptide PAAGDEVGSV **Charge** 1 **Spectral Angle** 0.83

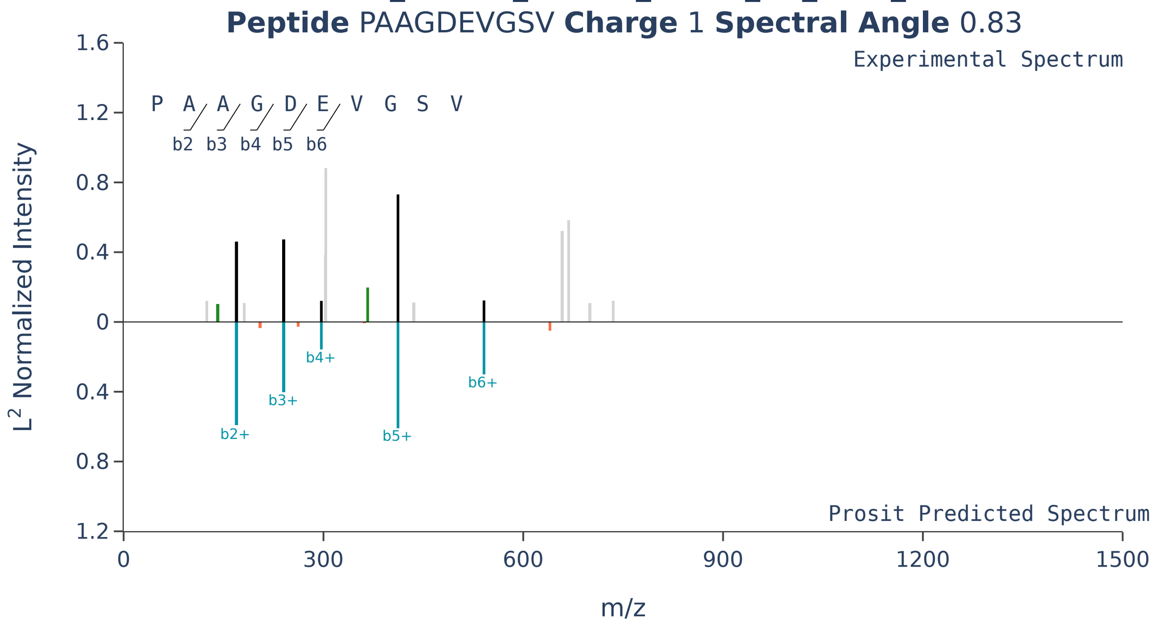

Source HRoetschke_211222_160123_HFGoe_IF2_b1R1_2h **Scan** 15702
Peptide SRNELGQEVLE **Charge** 2 **Spectral Angle** 0.82

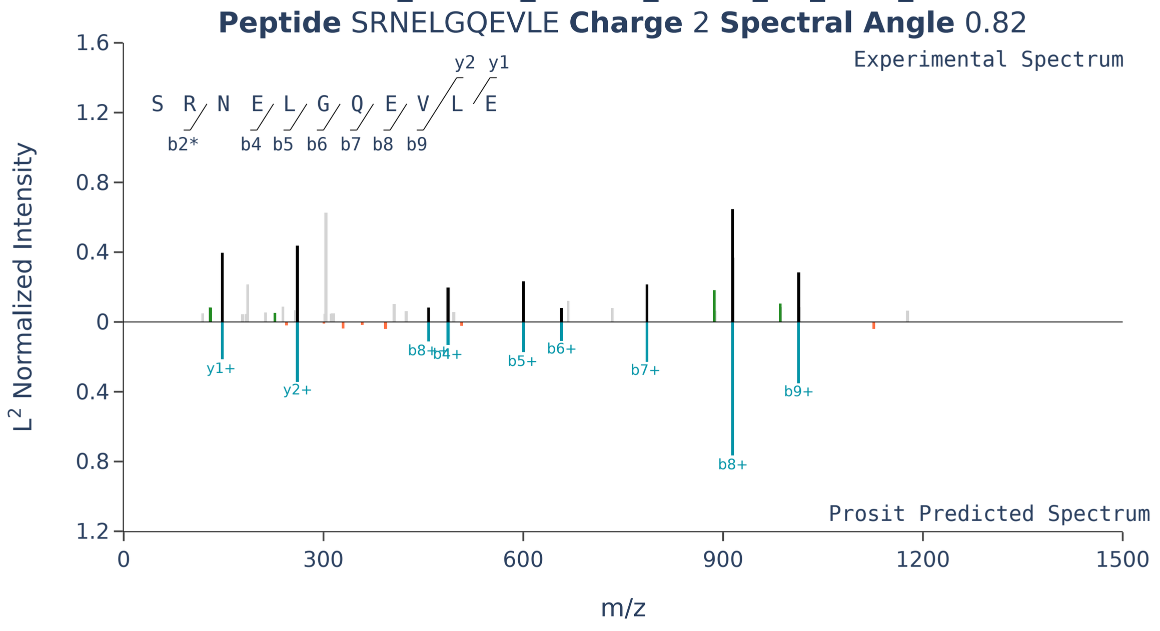

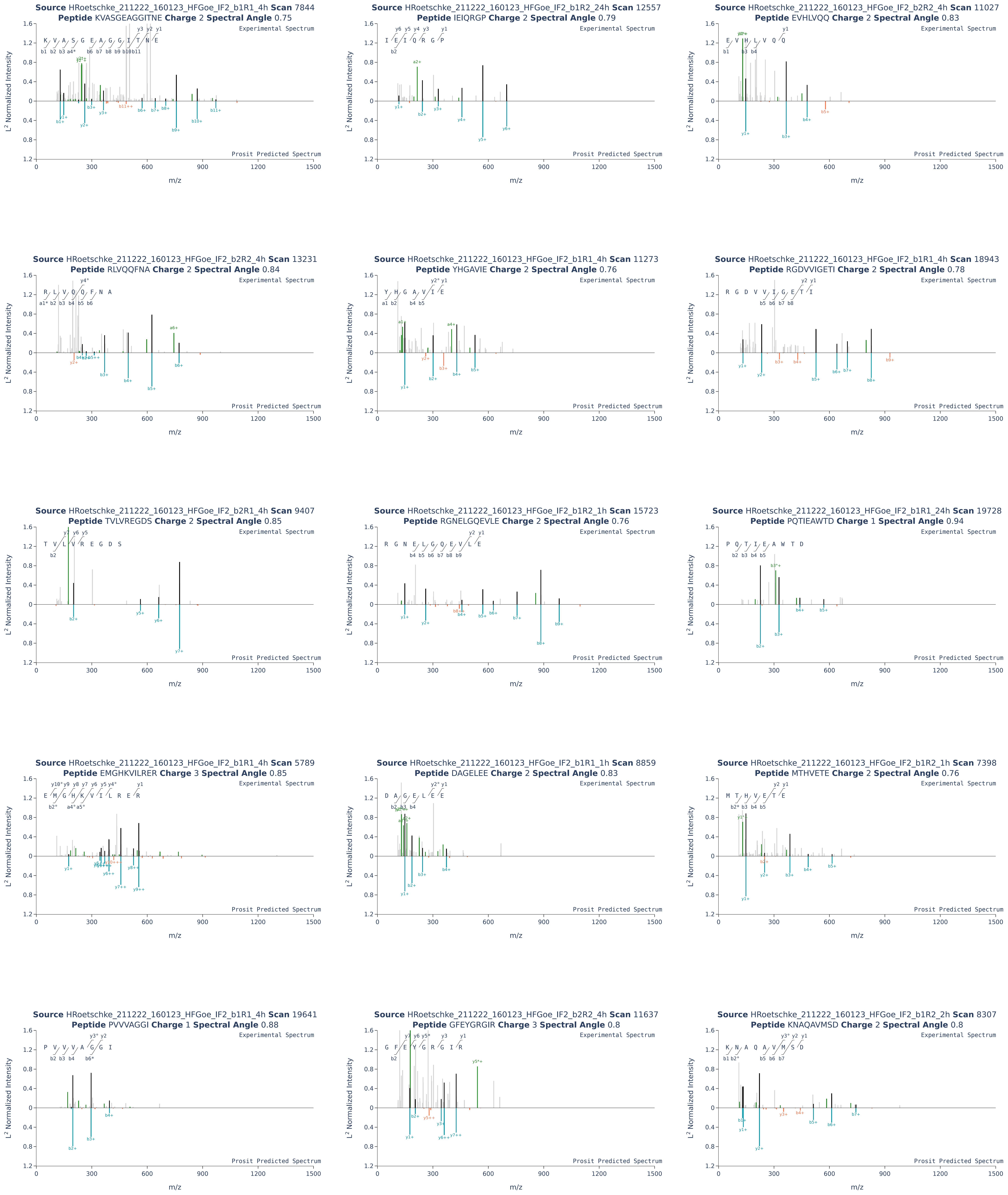
Source HRoetschke_211222_160123_HFGoe_IF2_b1R1_4h Scan 7844
Peptide KVASGEAGGITNE Charge 2 Spectral Angle 0.75
Source HRoetschke_211222_160123_HFGoe_IF2_b1R2_24h Scan 12557
Peptide IEIQRGP Charge 2 Spectral Angle 0.79
Source HRoetschke_211222_160123_HFGoe_IF2_b2R2_4h Scan 11027
Peptide EVHLVQQ Charge 2 Spectral Angle 0.83
Source HRoetschke_211222_160123_HFGoe_IF2_b2R2_4h Scan 13231
Peptide RLVQQFNA Charge 2 Spectral Angle 0.84
Source HRoetschke_211222_160123_HFGoe_IF2_b1R1_4h Scan 11273
Peptide YHGAVIE Charge 2 Spectral Angle 0.76
Source HRoetschke_211222_160123_HFGoe_IF2_b1R1_4h Scan 18943
Peptide RGDVVIGETI Charge 2 Spectral Angle 0.78
Source HRoetschke_211222_160123_HFGoe_IF2_b2R1_4h Scan 9407
Peptide TVLVREGDS Charge 2 Spectral Angle 0.85
Source HRoetschke_211222_160123_HFGoe_IF2_b1R2_1h Scan 15723
Peptide RGNELGQEVLE Charge 2 Spectral Angle 0.76
Source HRoetschke_211222_160123_HFGoe_IF2_b1R1_24h Scan 19728
Peptide PQTIEAWTD Charge 1 Spectral Angle 0.94
Source HRoetschke_211222_160123_HFGoe_IF2_b1R1_4h Scan 5789
Peptide EMGHKVILRER Charge 3 Spectral Angle 0.85
Source HRoetschke_211222_160123_HFGoe_IF2_b1R1_1h Scan 8859
Peptide DAGELEE Charge 2 Spectral Angle 0.83
Source HRoetschke_211222_160123_HFGoe_IF2_b1R2_1h Scan 7398
Peptide MTHVETE Charge 2 Spectral Angle 0.76
Source HRoetschke_211222_160123_HFGoe_IF2_b1R1_4h Scan 19641
Peptide PVVVAGGI Charge 1 Spectral Angle 0.88
Source HRoetschke_211222_160123_HFGoe_IF2_b2R2_4h Scan 11637
Peptide GFEYGRGIR Charge 3 Spectral Angle 0.8
Source HRoetschke_211222_160123_HFGoe_IF2_b1R2_2h Scan 8307
Peptide KNAQAVMSD Charge 2 Spectral Angle 0.8
Experimental Spectrum
Prosit Predicted Spectrum
L² Normalized Intensity
m/z

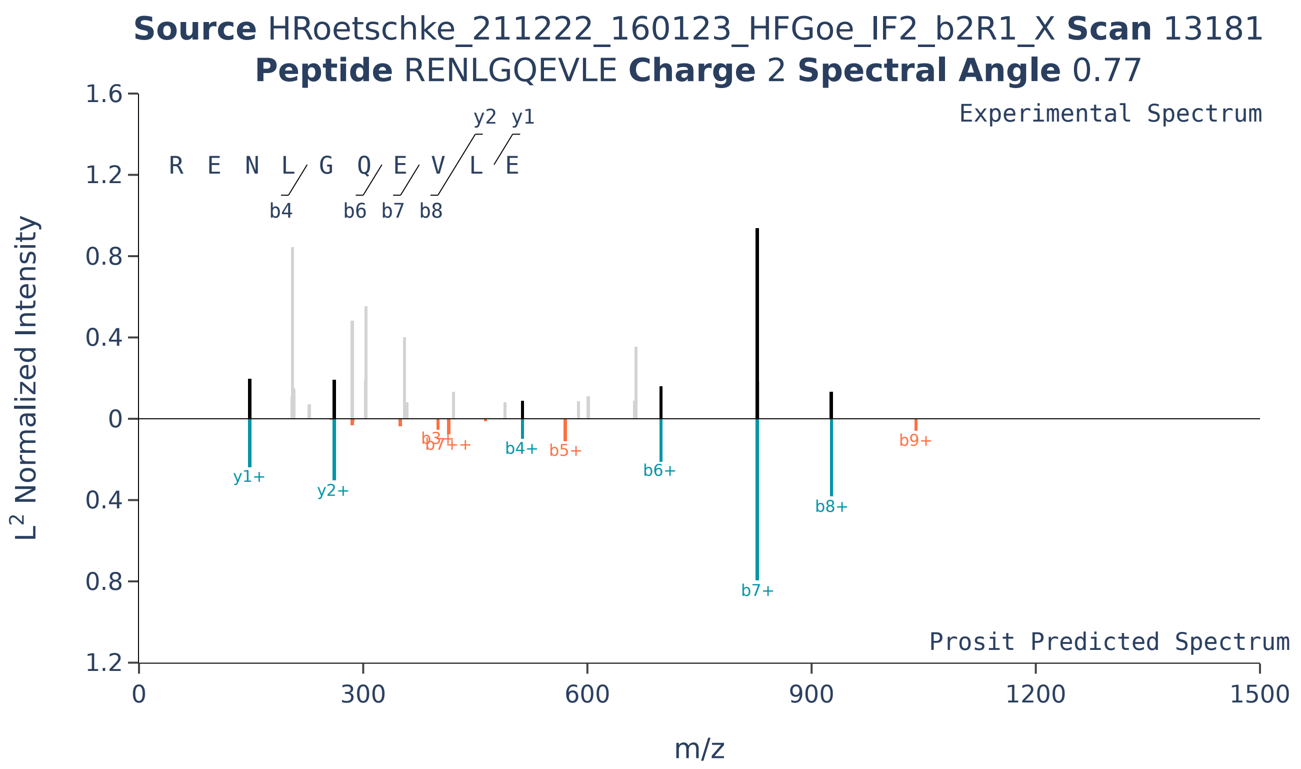
Source HRoetschke_211222_160123_HFGoe_IF2_b2R1_X **Scan** 13181
Peptide RENLGQEVLE **Charge** 2 **Spectral Angle** 0.77
Experimental Spectrum
Prosit Predicted Spectrum
L² Normalized Intensity
m/z

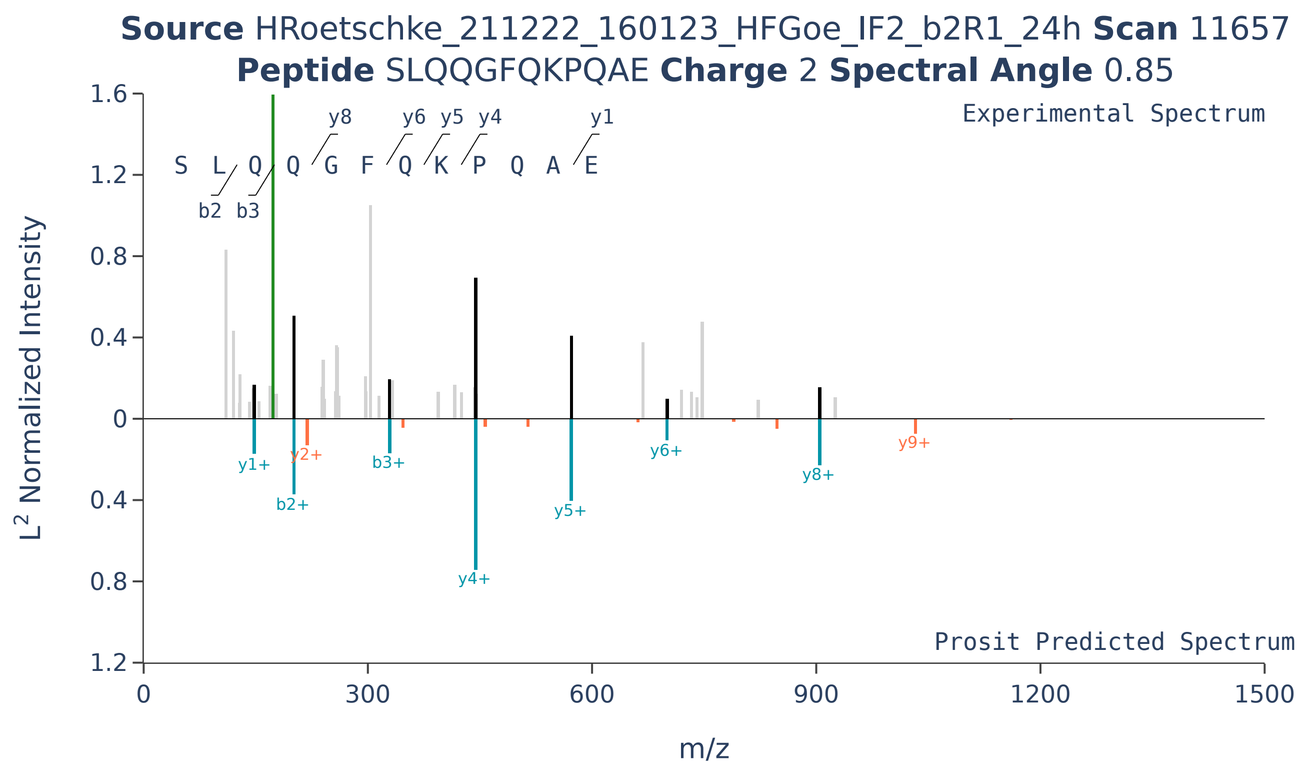
Source HRoetschke_211222_160123_HFGoe_IF2_b2R1_24h **Scan** 11657
Peptide SLQQGFQKPQAE **Charge** 2 **Spectral Angle** 0.85
Experimental Spectrum
Prosit Predicted Spectrum
L² Normalized Intensity
m/z

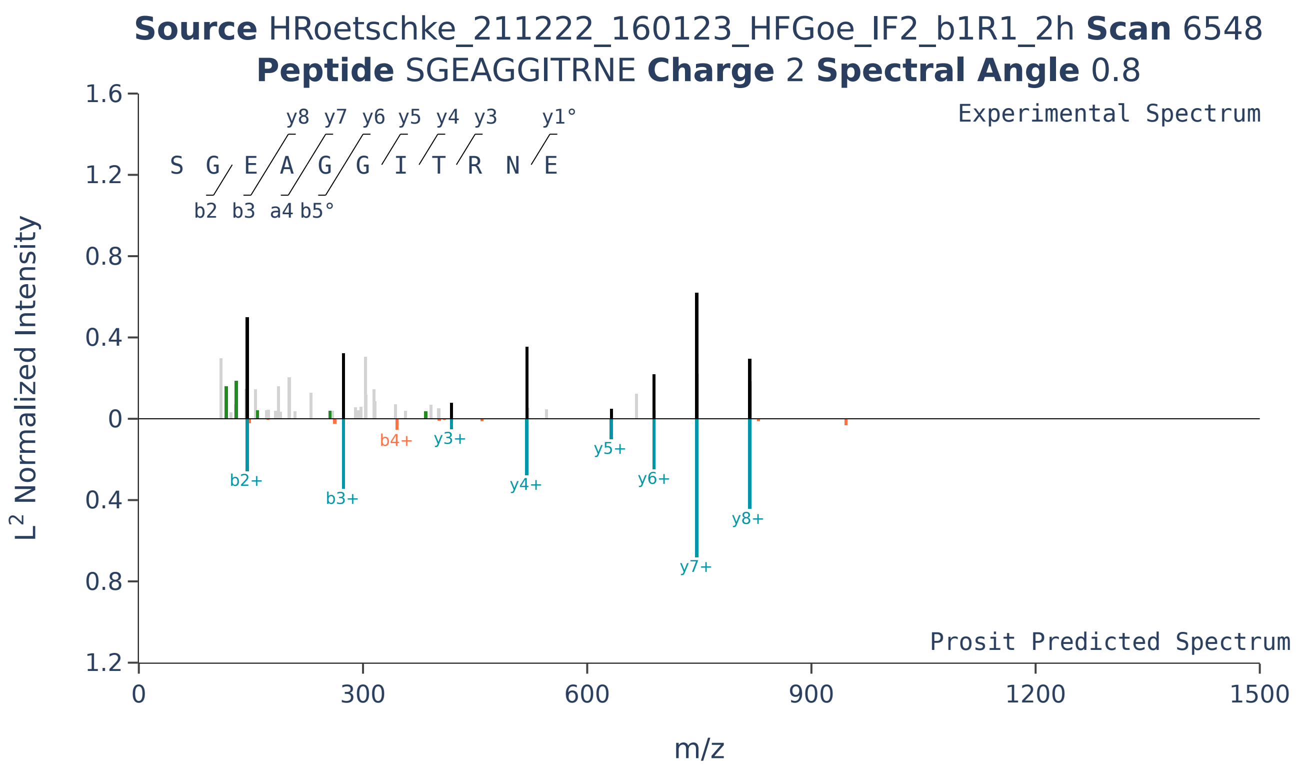
Source HRoetschke_211222_160123_HFGoe_IF2_b1R1_2h **Scan** 6548
Peptide SGEAGGITRNE **Charge** 2 **Spectral Angle** 0.8
Experimental Spectrum
Prosit Predicted Spectrum
L² Normalized Intensity
m/z

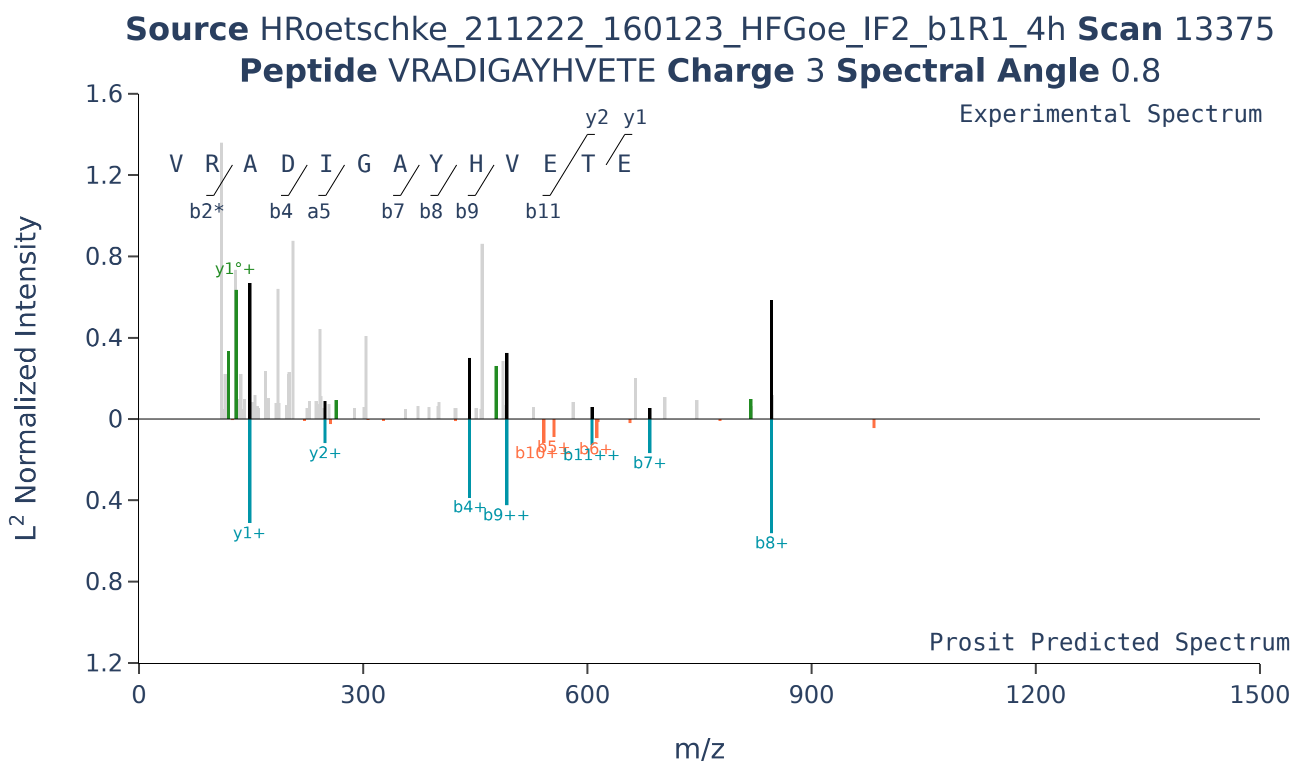
Source HRoetschke_211222_160123_HFGoe_IF2_b1R1_4h **Scan** 13375
Peptide VRADIGAYHVETE **Charge** 3 **Spectral Angle** 0.8
Experimental Spectrum
Prosit Predicted Spectrum
L² Normalized Intensity
m/z

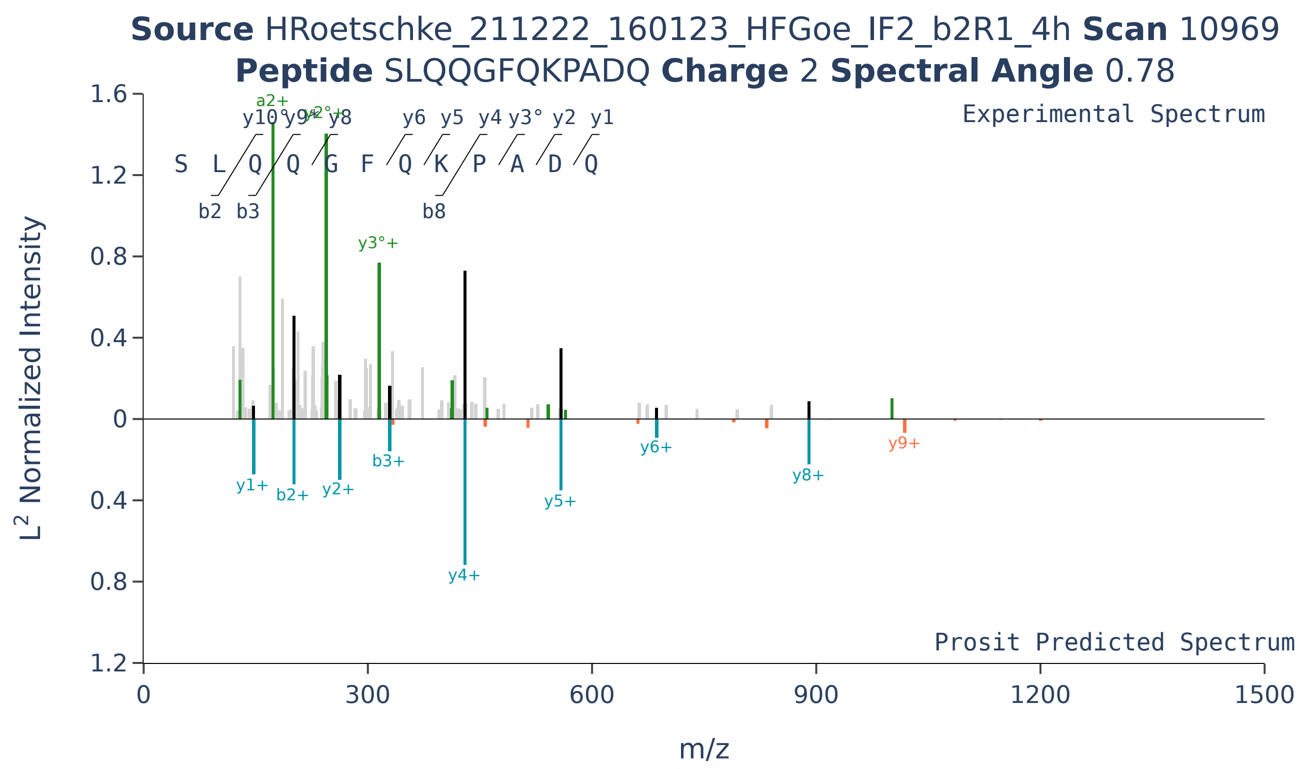
Source HRoetschke_211222_160123_HFGoe_IF2_b2R1_4h **Scan** 10969
Peptide SLQQGFQKPADQ **Charge** 2 **Spectral Angle** 0.78
Experimental Spectrum
Prosit Predicted Spectrum
L² Normalized Intensity
m/z

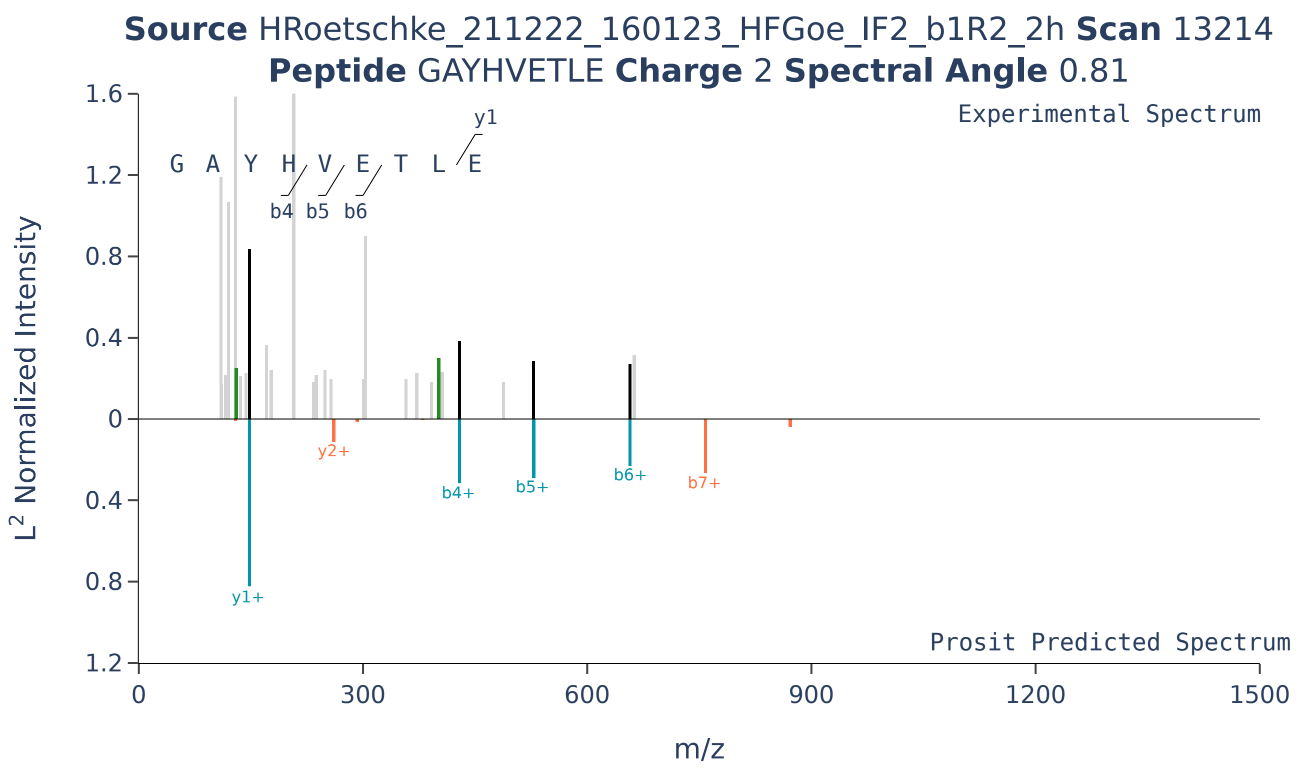
Source HRoetschke_211222_160123_HFGoe_IF2_b1R2_2h **Scan** 13214
Peptide GAYHVETLE **Charge** 2 **Spectral Angle** 0.81
Experimental Spectrum
Prosit Predicted Spectrum
L² Normalized Intensity
m/z

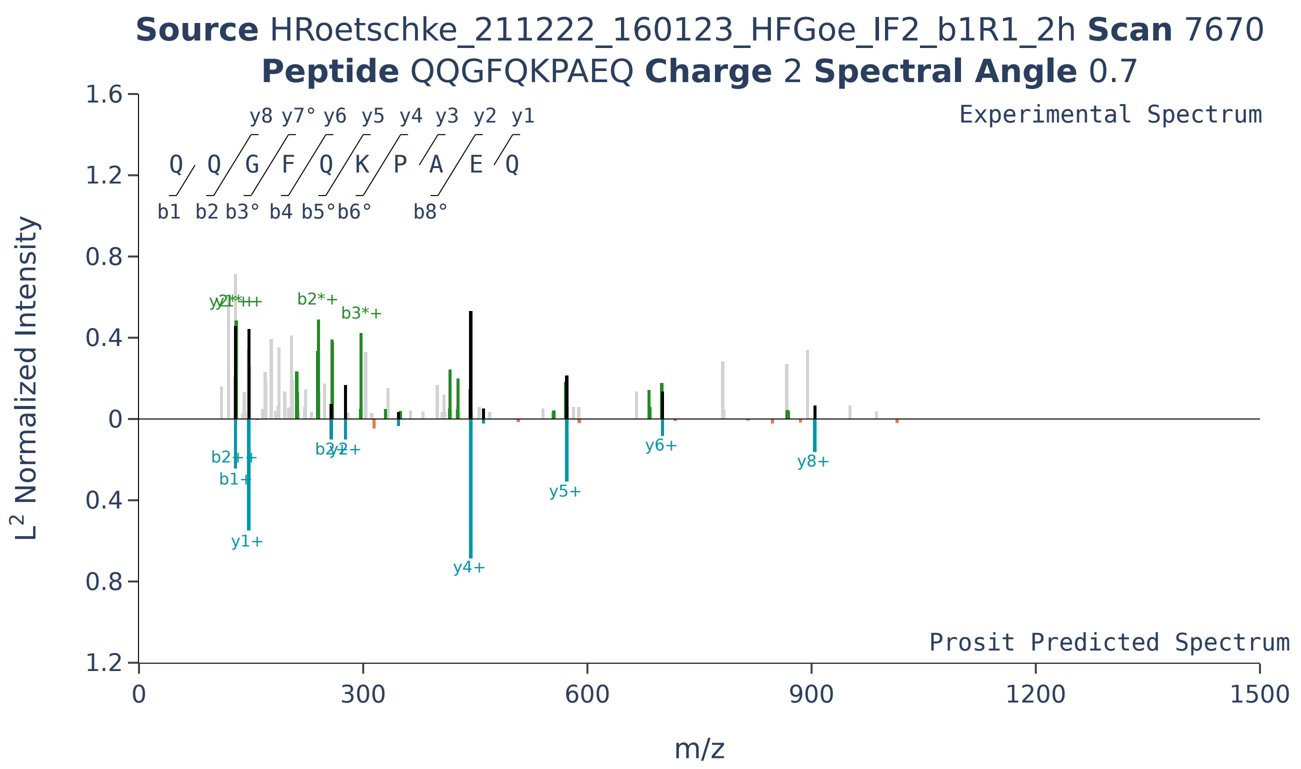
Source HRoetschke_211222_160123_HFGoe_IF2_b1R1_2h **Scan** 7670
Peptide QQGFQKPAEQ **Charge** 2 **Spectral Angle** 0.7
Experimental Spectrum
Prosit Predicted Spectrum
L² Normalized Intensity
m/z

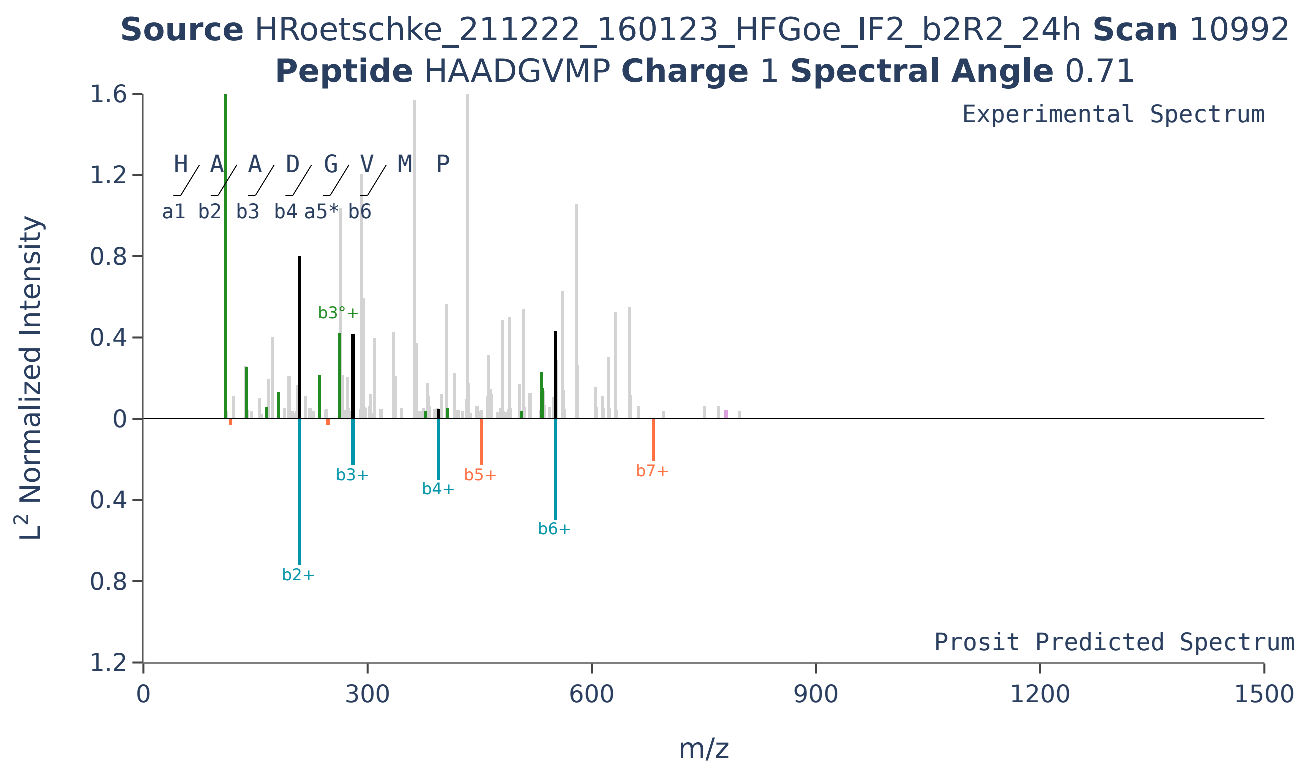
Source HRoetschke_211222_160123_HFGoe_IF2_b2R2_24h **Scan** 10992
Peptide HAADGVMP **Charge** 1 **Spectral Angle** 0.71
Experimental Spectrum
Prosit Predicted Spectrum
L² Normalized Intensity
m/z

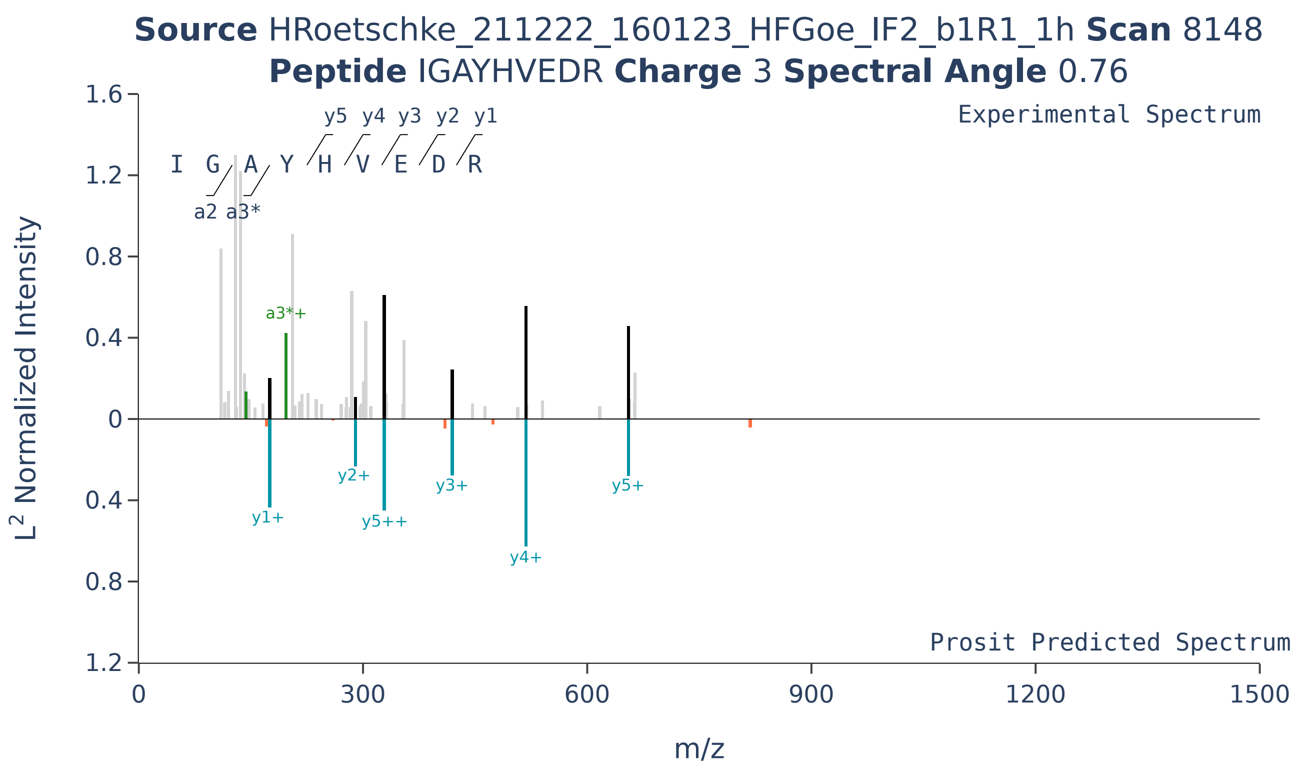
Source HRoetschke_211222_160123_HFGoe_IF2_b1R1_1h **Scan** 8148
Peptide IGAYHVEDR **Charge** 3 **Spectral Angle** 0.76
Experimental Spectrum
Prosit Predicted Spectrum
L² Normalized Intensity
m/z

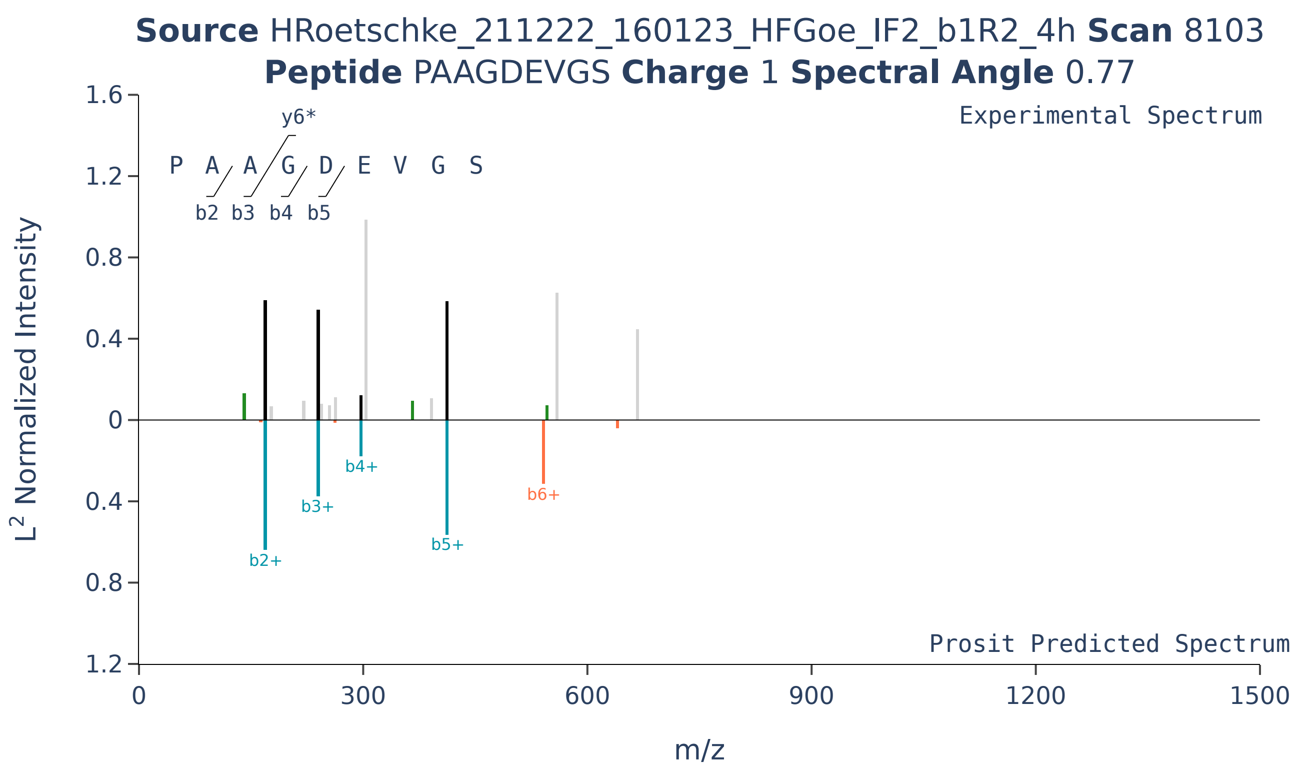
Source HRoetschke_211222_160123_HFGoe_IF2_b1R2_4h **Scan** 8103
Peptide PAAGDEVGS **Charge** 1 **Spectral Angle** 0.77
Experimental Spectrum
Prosit Predicted Spectrum
L² Normalized Intensity
m/z

Source WSoh_101121_151121_HFGoe_G1_IL37b_24h_R2 **Scan** 16220
Peptide SANLIAVPDKN **Charge** 2 **Spectral Angle** 0.92

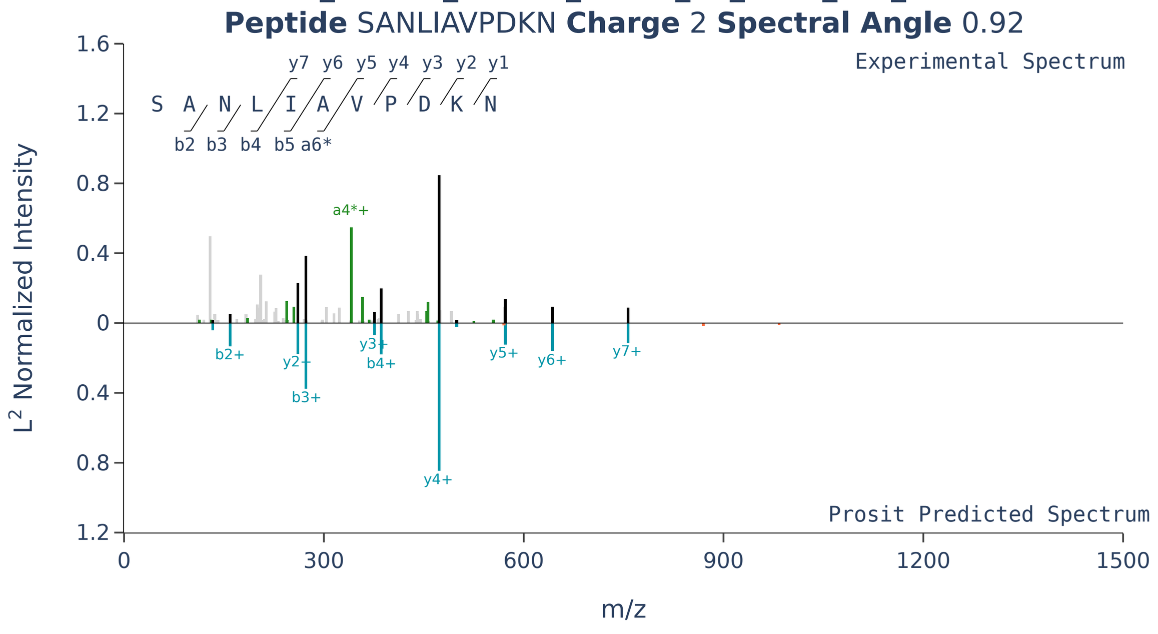

Source WSoh_101121_151121_HFGoe_G2_IL37b_24h_R2 **Scan** 9492
Peptide ANLNPKKF **Charge** 2 **Spectral Angle** 0.85

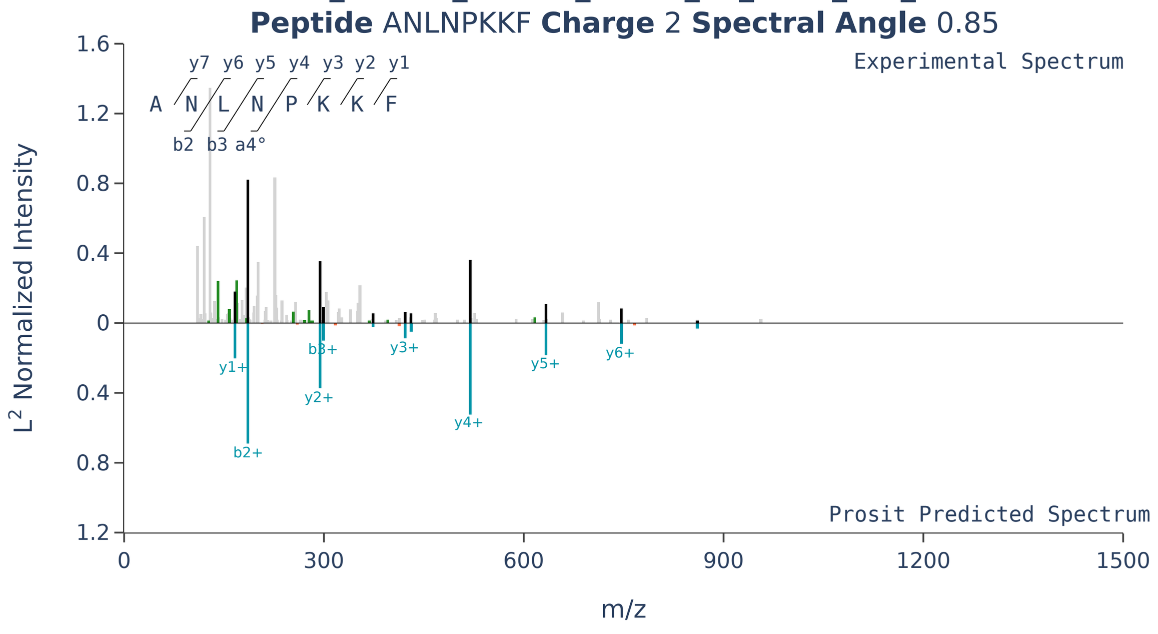

Source WSoh_101121_151121_HFGoe_G1_IL37b_24h_R2 **Scan** 7085
Peptide MVHTSPKV **Charge** 2 **Spectral Angle** 0.91

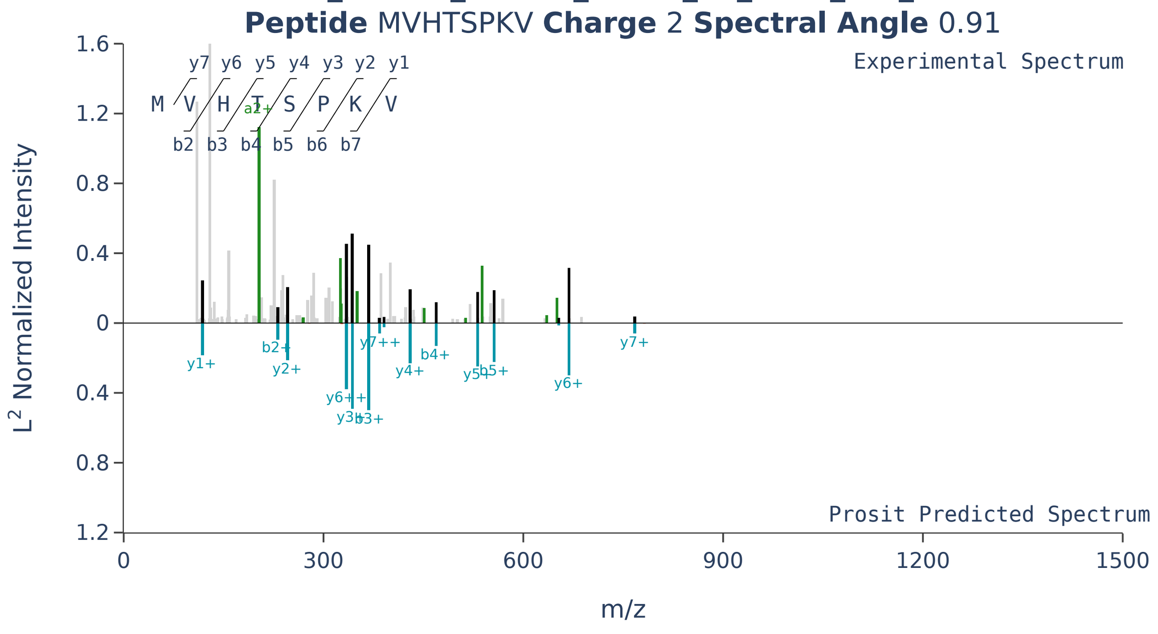

Source WSoh_101121_151121_HFGoe_G1_IL37b_24h_R1 **Scan** 16078
Peptide SNLIAVPDKN **Charge** 2 **Spectral Angle** 0.89

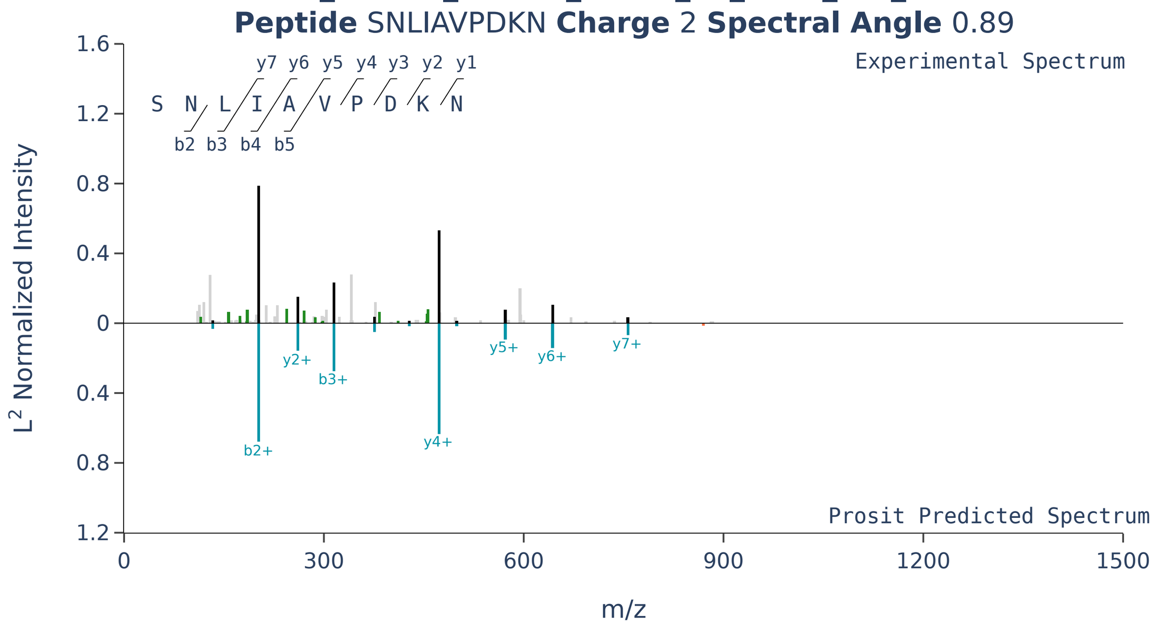

Source WSoh_101121_151121_HFGoe_G1_IL37b_4h_R1 **Scan** 11115
Peptide DSGNLNPKKF **Charge** 2 **Spectral Angle** 0.88

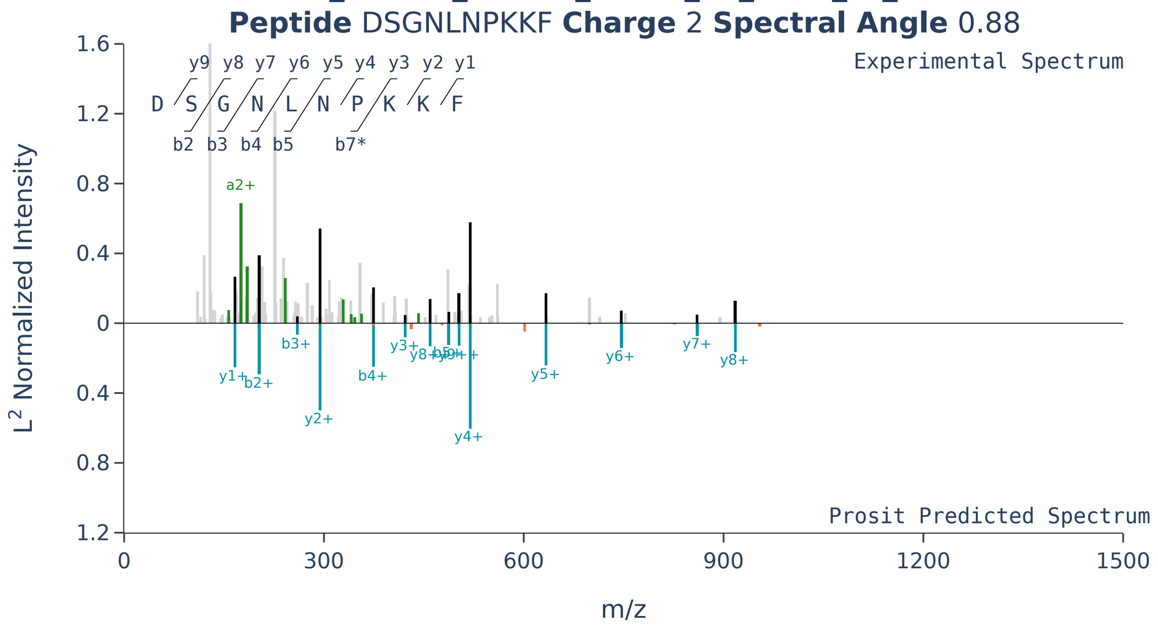

Source WSoh_101121_151121_HFGoe_G2_IL37b_24h_R1 **Scan** 6163
Peptide AHTSPKVKNL **Charge** 2 **Spectral Angle** 0.9

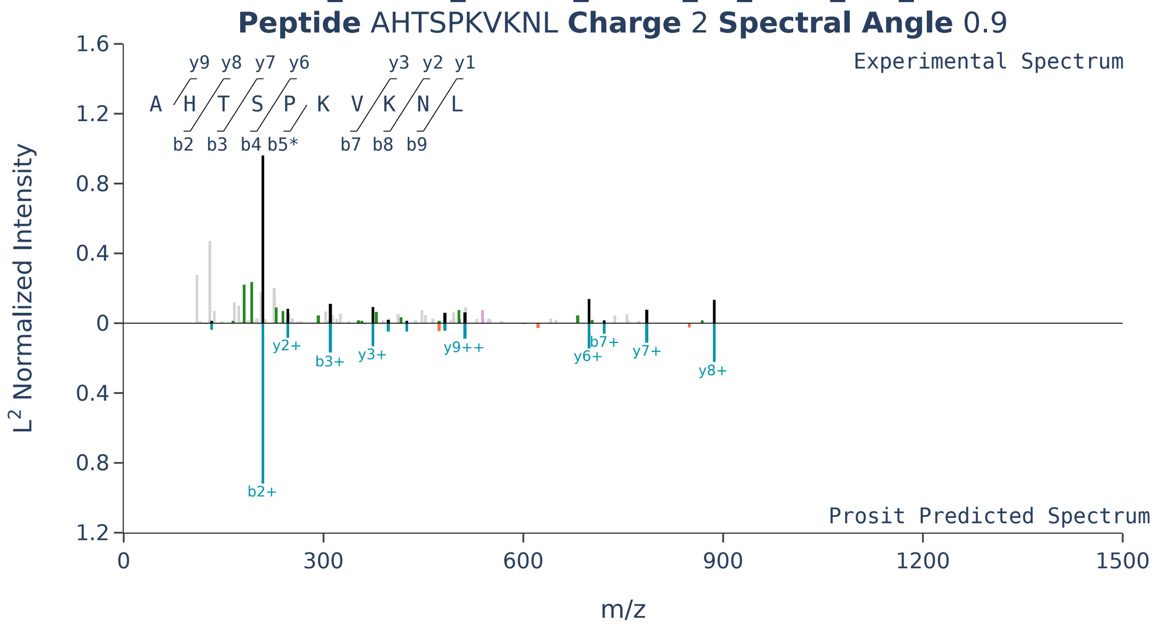

Source WSoh_101121_151121_HFGoe_G2_IL37b_2h_R1 **Scan** 5600
Peptide MVHTSPKVK **Charge** 2 **Spectral Angle** 0.89

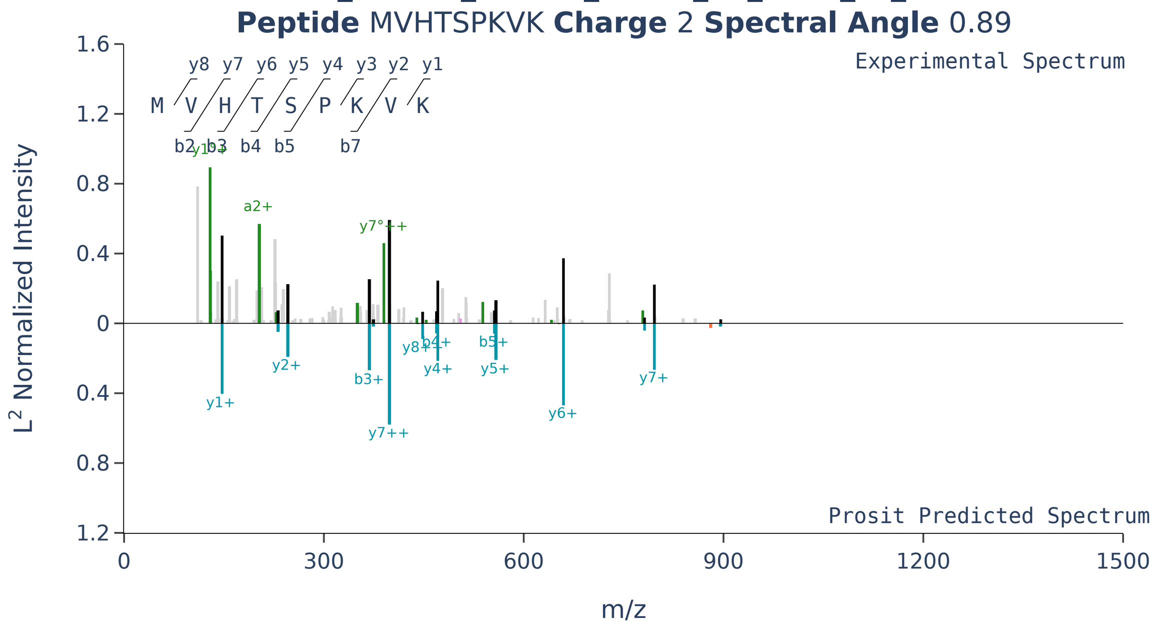

Source WSoh_101121_151121_HFGoe_G1_IL37b_24h_R2 **Scan** 12486
Peptide DLNPKKF **Charge** 2 **Spectral Angle** 0.9

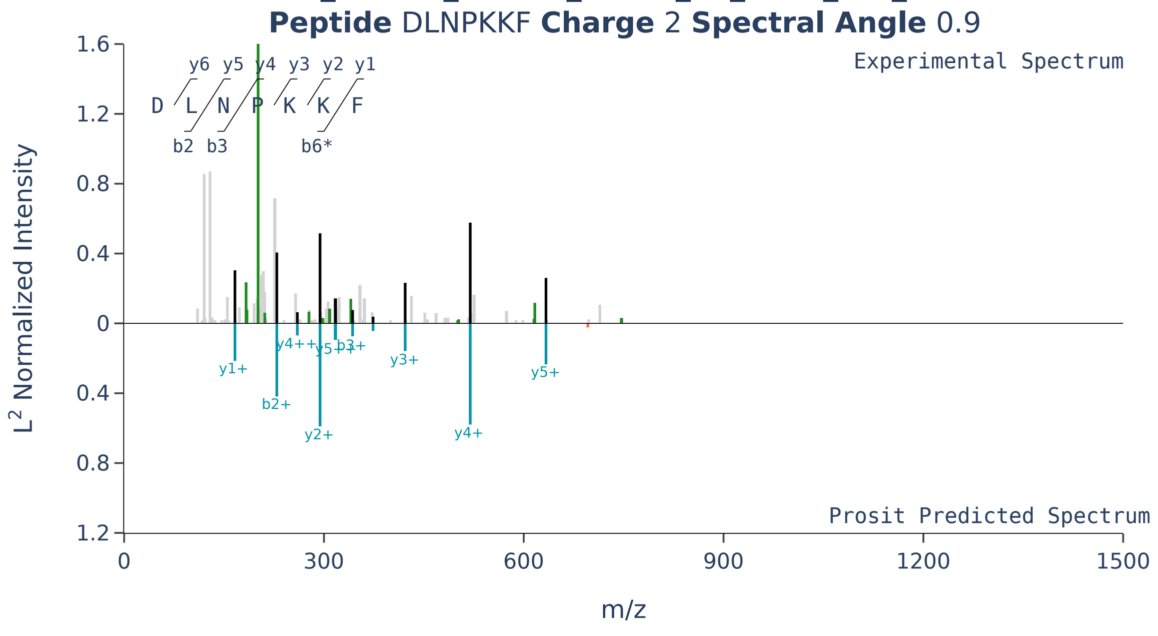

Source WSoh_101121_151121_HFGoe_G2_IL37b_24h_R1 **Scan** 16705
Peptide ANLIAVPDKN **Charge** 2 **Spectral Angle** 0.88

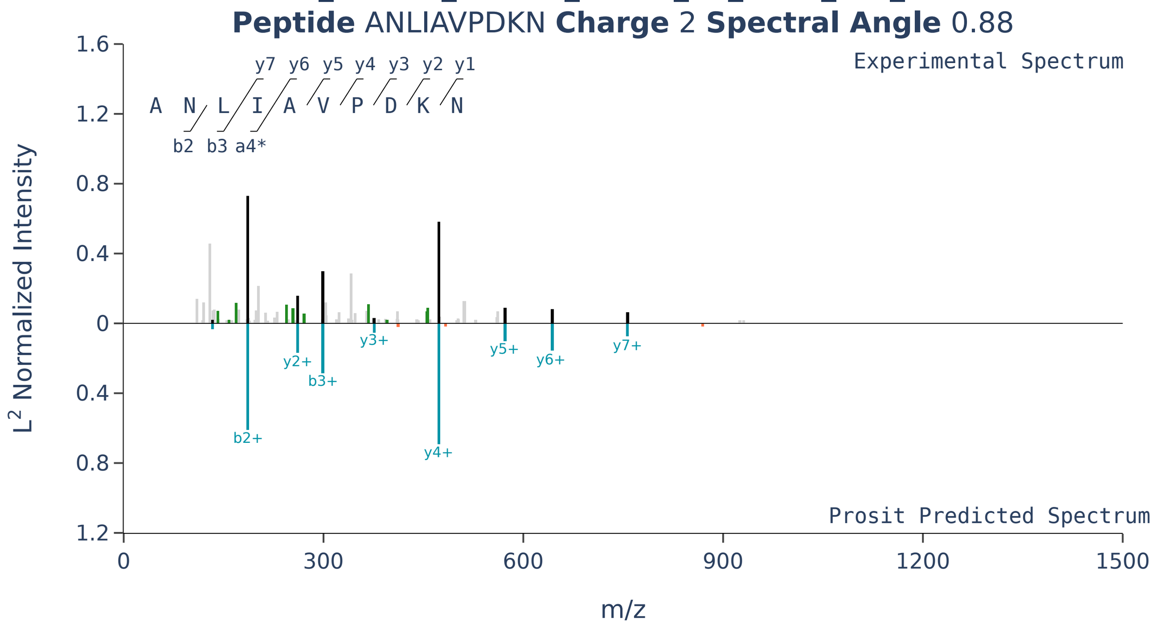

Source WSoh_101121_151121_HFGoe_G2_IL37b_24h_R1 **Scan** 6874
Peptide DSGHTSPKVKNL **Charge** 3 **Spectral Angle** 0.85

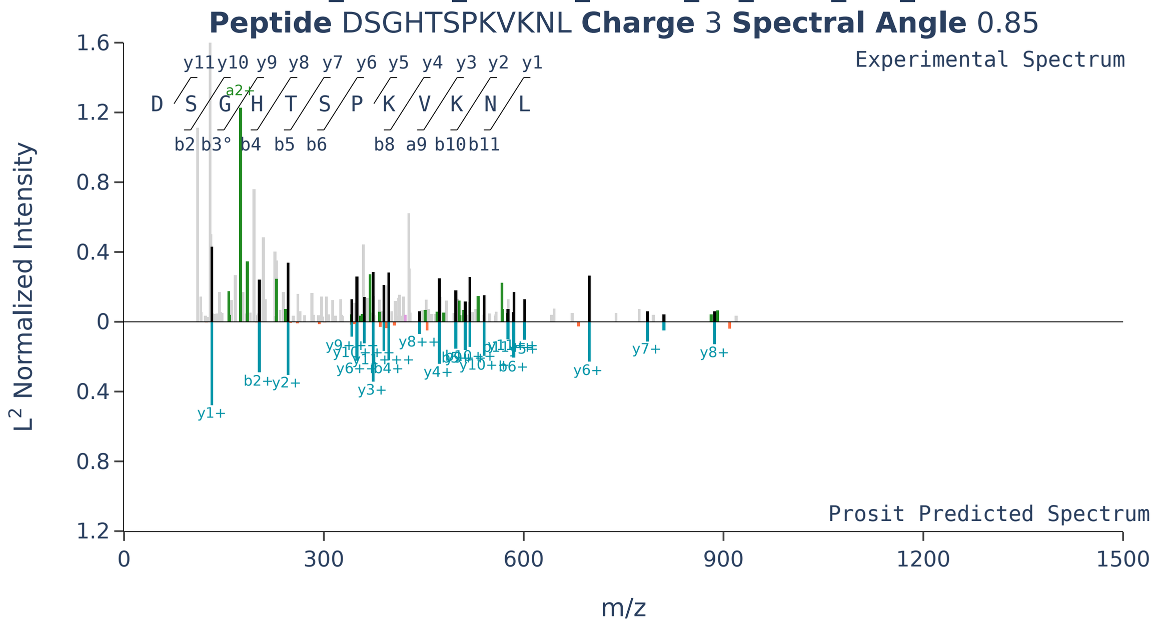

Source WSoh_101121_151121_HFGoe_G2_IL37b_4h_R2 **Scan** 7176
Peptide SAVHTSPKVKNL **Charge** 3 **Spectral Angle** 0.86

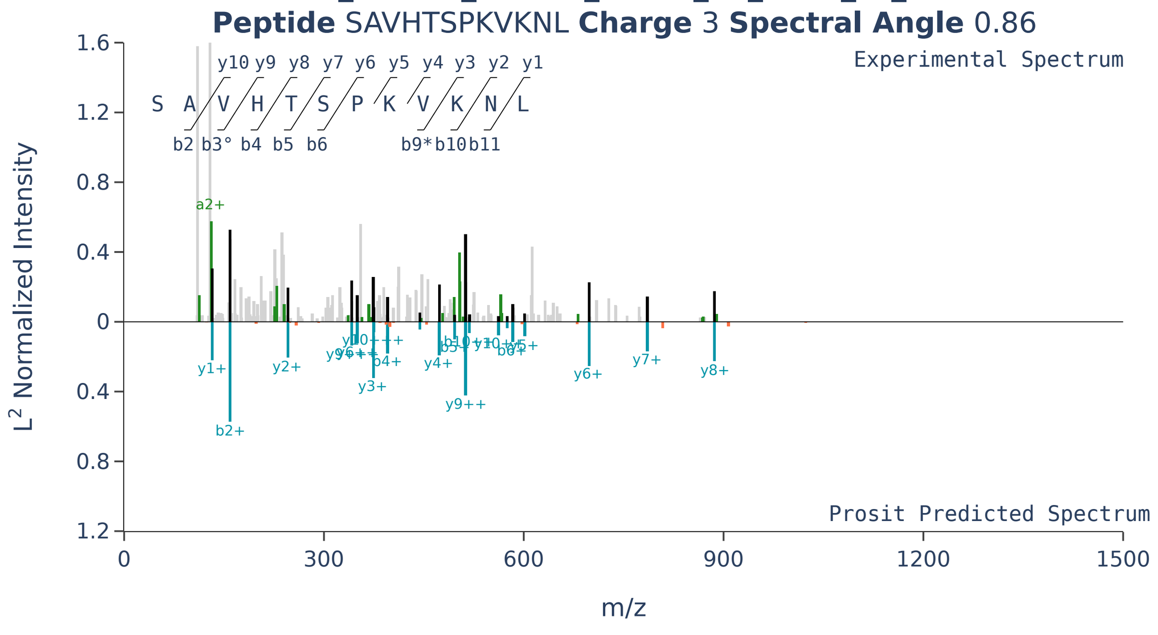

Source WSoh_101121_151121_HFGoe_G1_IL37b_4h_R2 **Scan** 6337
Peptide GSVHTSPKV **Charge** 2 **Spectral Angle** 0.81

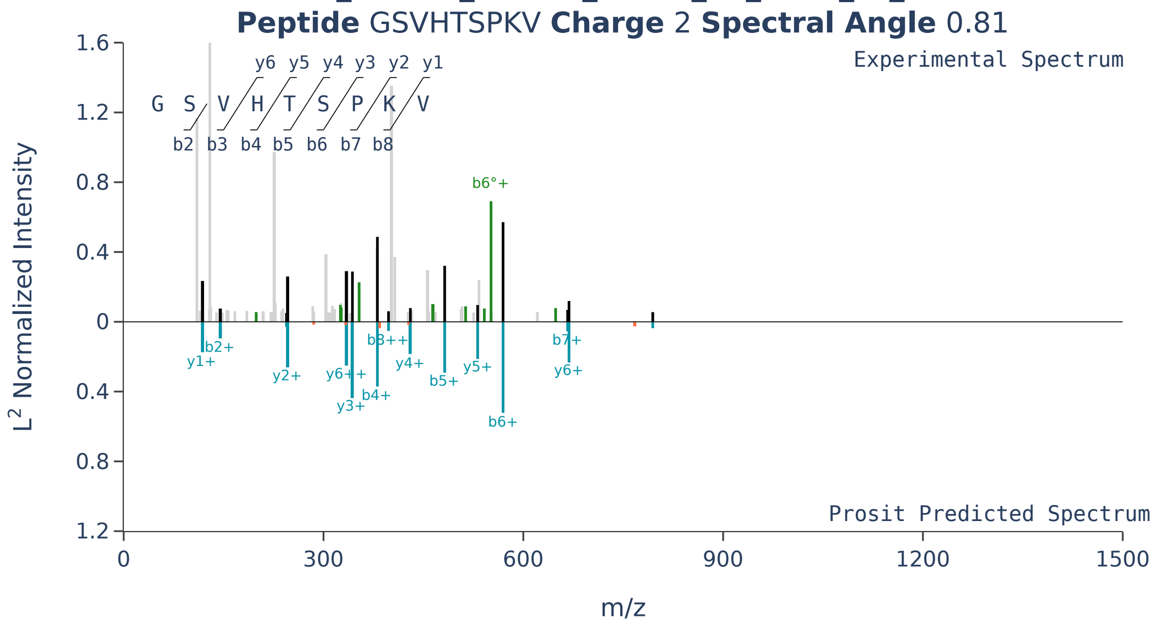

Source WSoh_101121_151121_HFGoe_G1_IL37b_24h_R1 **Scan** 9232
Peptide ASNLNPKKF **Charge** 2 **Spectral Angle** 0.85

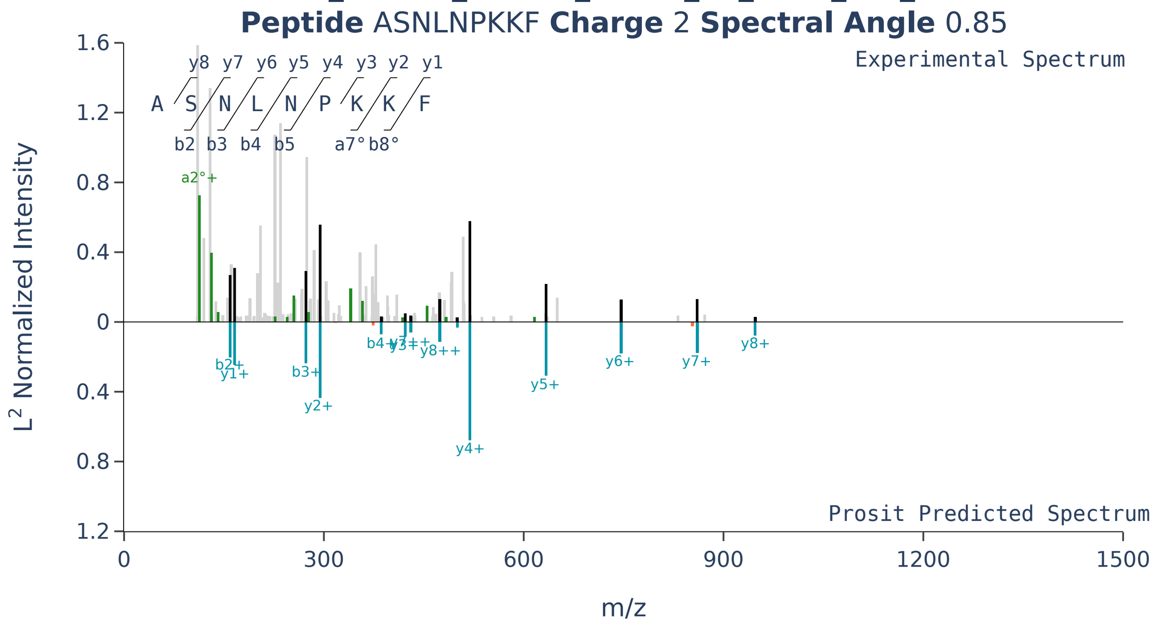

Source WSoh_101121_151121_HFGoe_G1_IL37b_24h_R1 **Scan** 9688
Peptide TNLNPKKF **Charge** 2 **Spectral Angle** 0.86

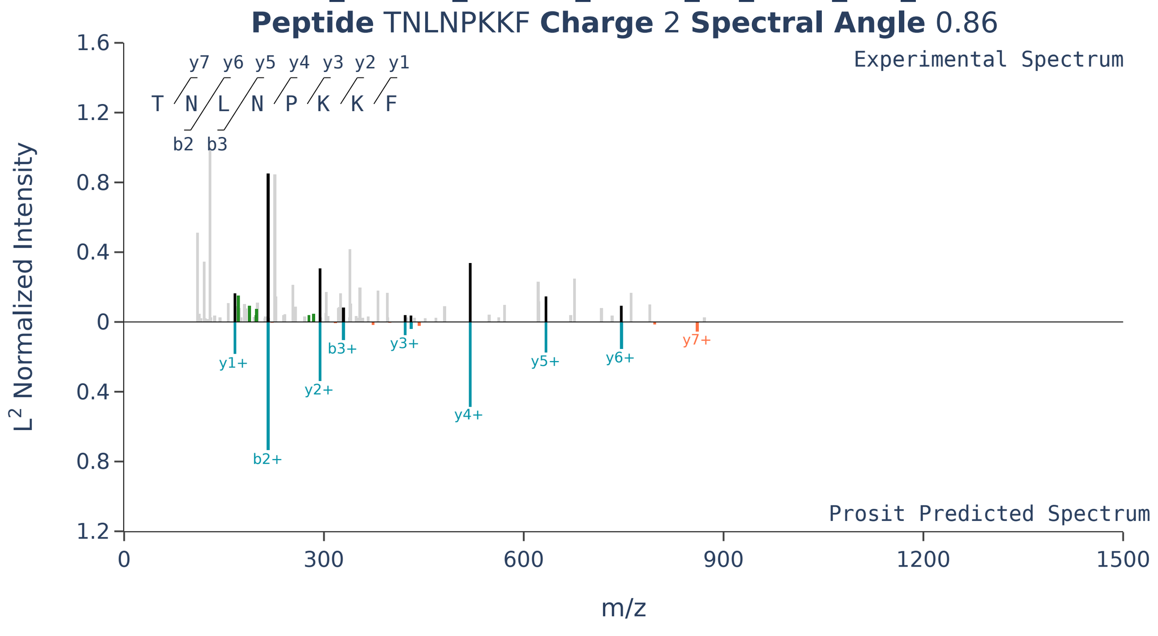

Source WSoh_101121_151121_HFGoe_G2_IL37b_24h_R1 **Scan** 16171
Peptide ASNLIAVPDKN **Charge** 2 **Spectral Angle** 0.88

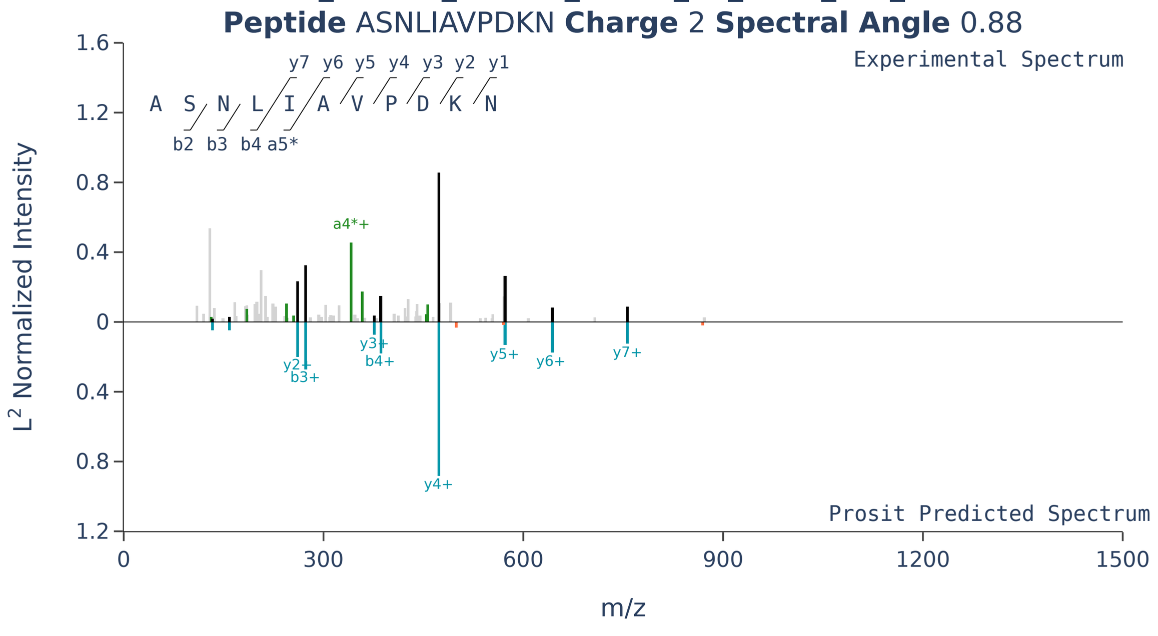

Source WSoh_101121_151121_HFGoe_G1_IL37b_24h_R2 **Scan** 6438
Peptide SCVHTSPKV **Charge** 2 **Spectral Angle** 0.87

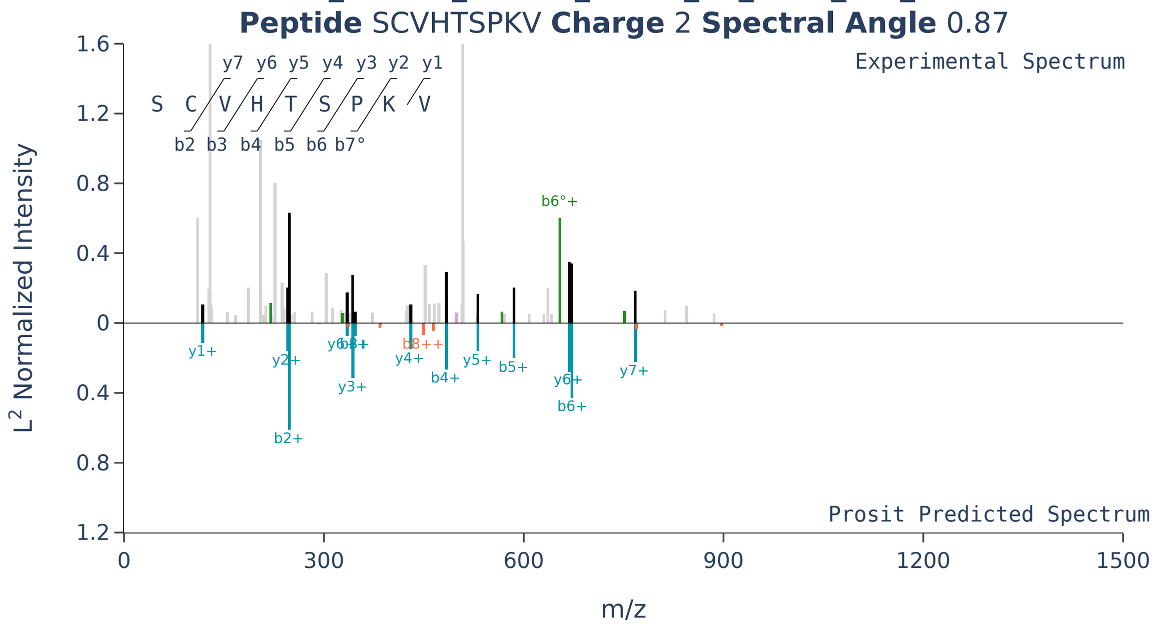

Source WSoh_101121_151121_HFGoe_G1_IL37b_4h_R2 **Scan** 8398
Peptide DSGVHTSPKVKNL **Charge** 2 **Spectral Angle** 0.84

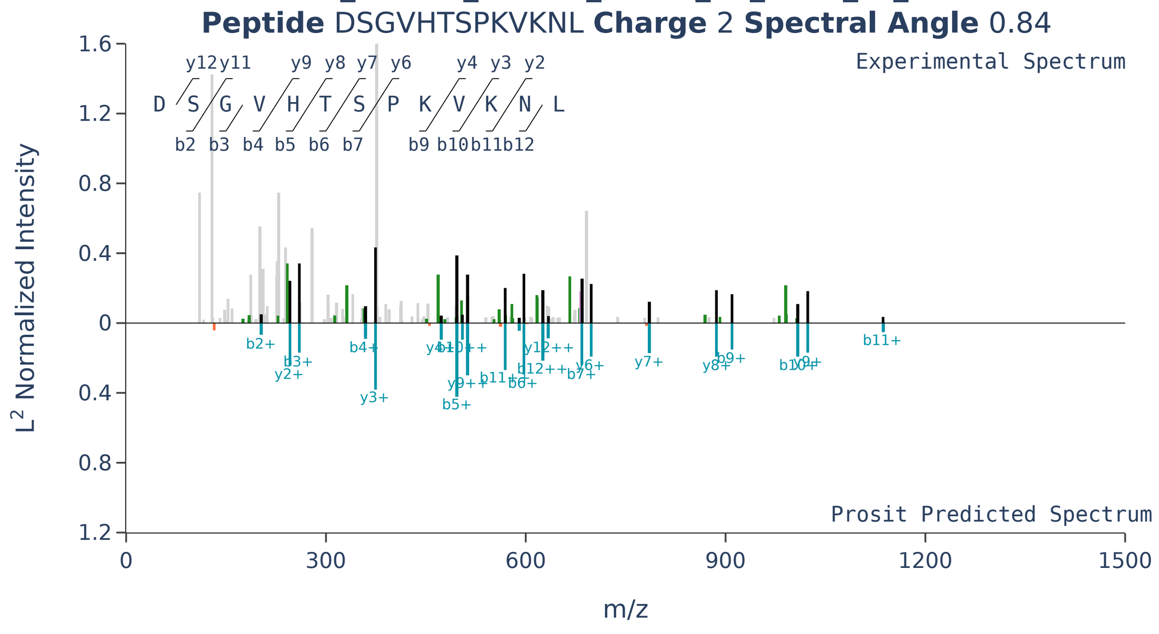

Source WSoh_101121_151121_HFGoe_G1_IL37b_1h_R1 **Scan** 17823
Peptide GCNEPVGVTDKF **Charge** 2 **Spectral Angle** 0.92

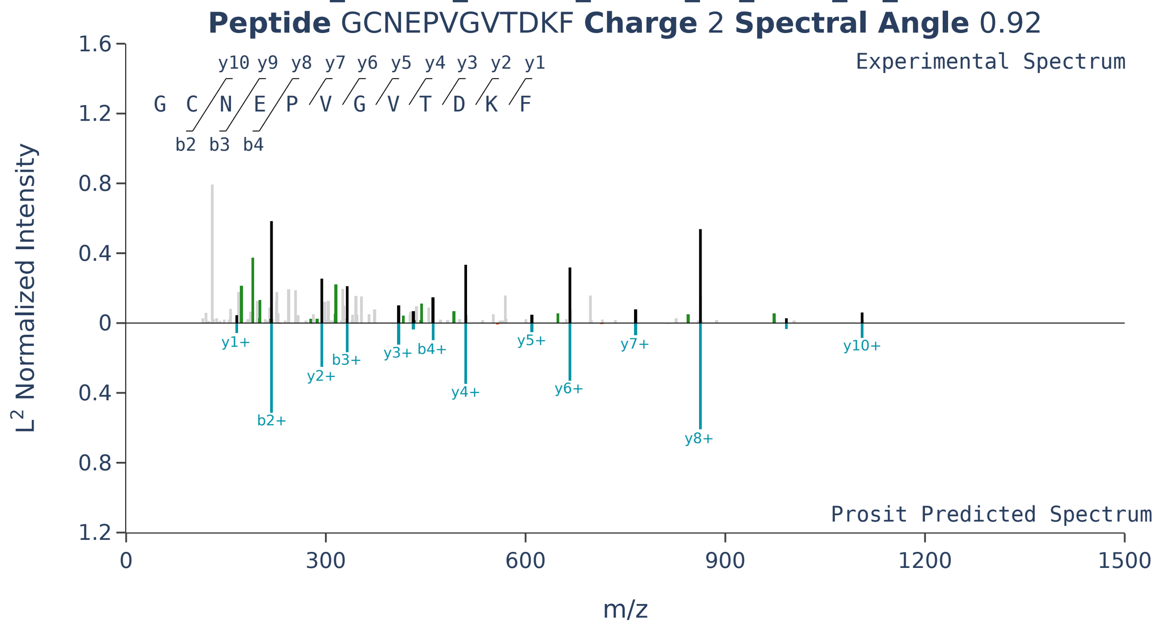

Source WSoh_101121_151121_HFGoe_G1_IL37b_4h_R1 **Scan** 9622
Peptide GQLNPKKF **Charge** 2 **Spectral Angle** 0.88

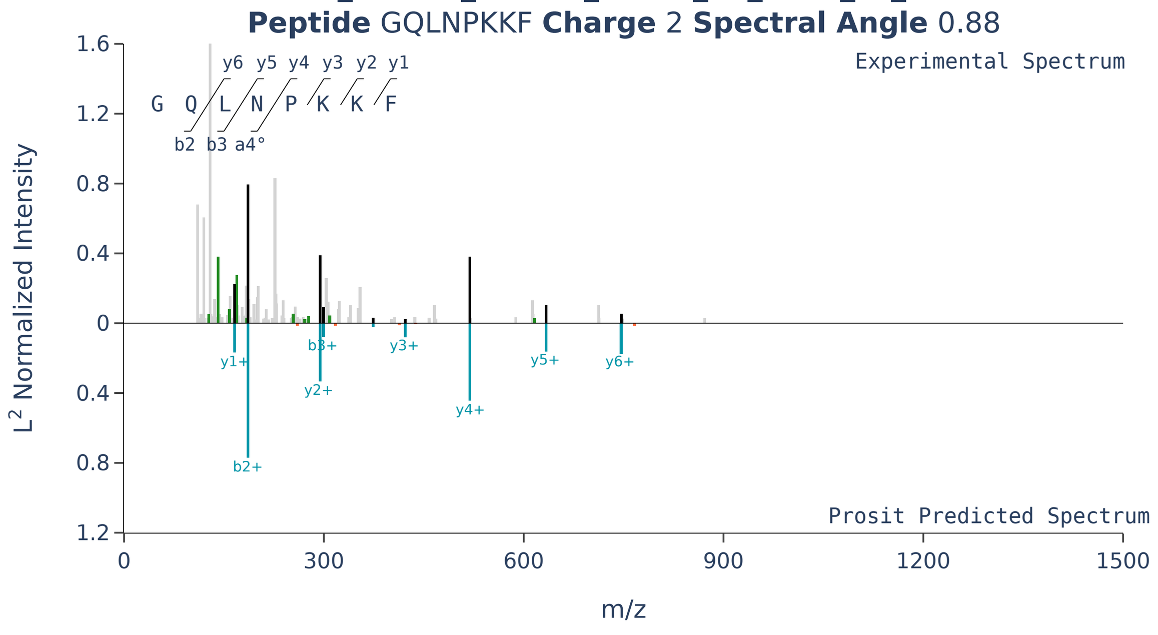

Source WSoh_101121_151121_HFGoe_G2_IL37b_24h_R1 **Scan** 13996
Peptide GCNEPVGVTDKFENR **Charge** 3 **Spectral Angle** 0.89

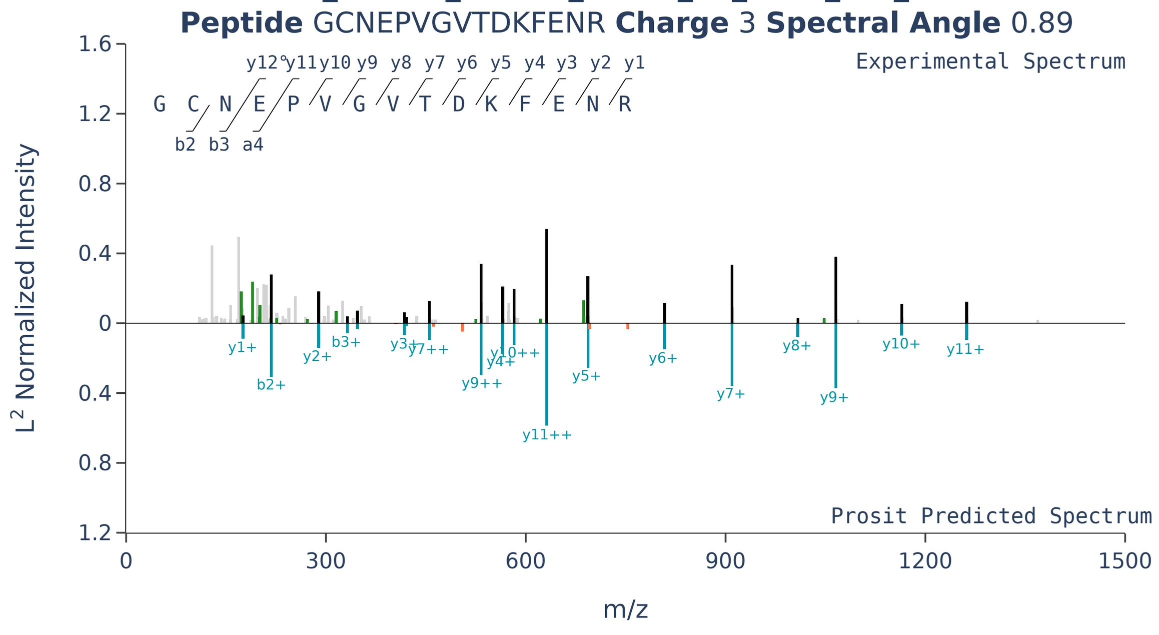

Source WSoh_101121_151121_HFGoe_G2_IL37b_24h_R2 **Scan** 6921
Peptide TVHTSPKVKNL **Charge** 2 **Spectral Angle** 0.87

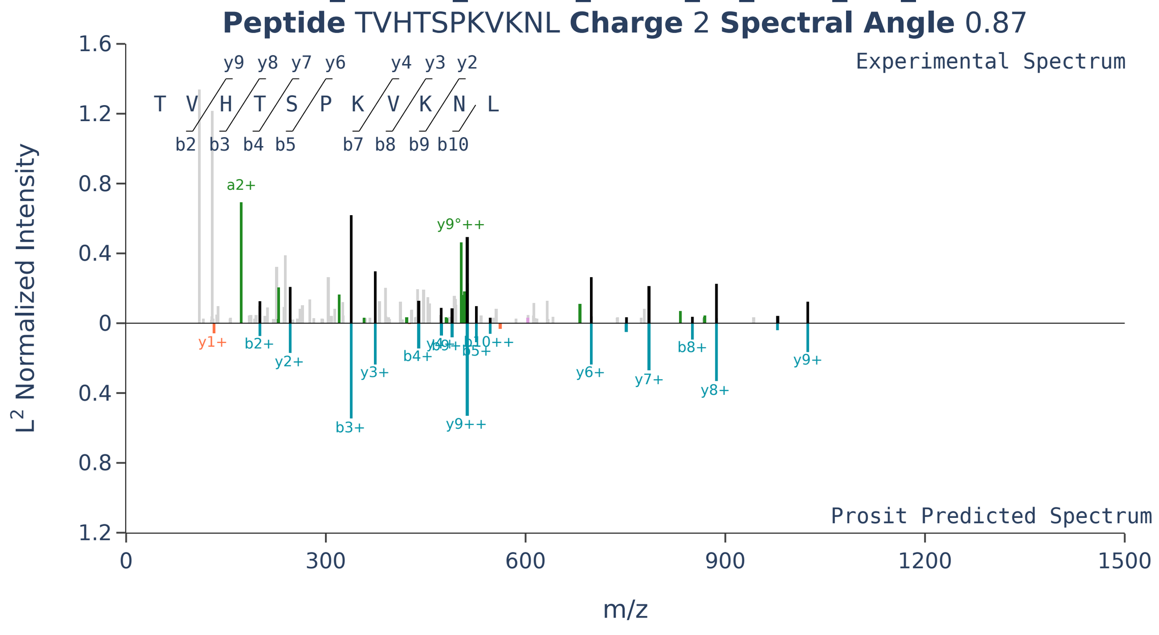

Source WSoh_101121_151121_HFGoe_G1_IL37b_24h_R1 **Scan** 7058
Peptide GSVHTSPKVKNL **Charge** 2 **Spectral Angle** 0.85

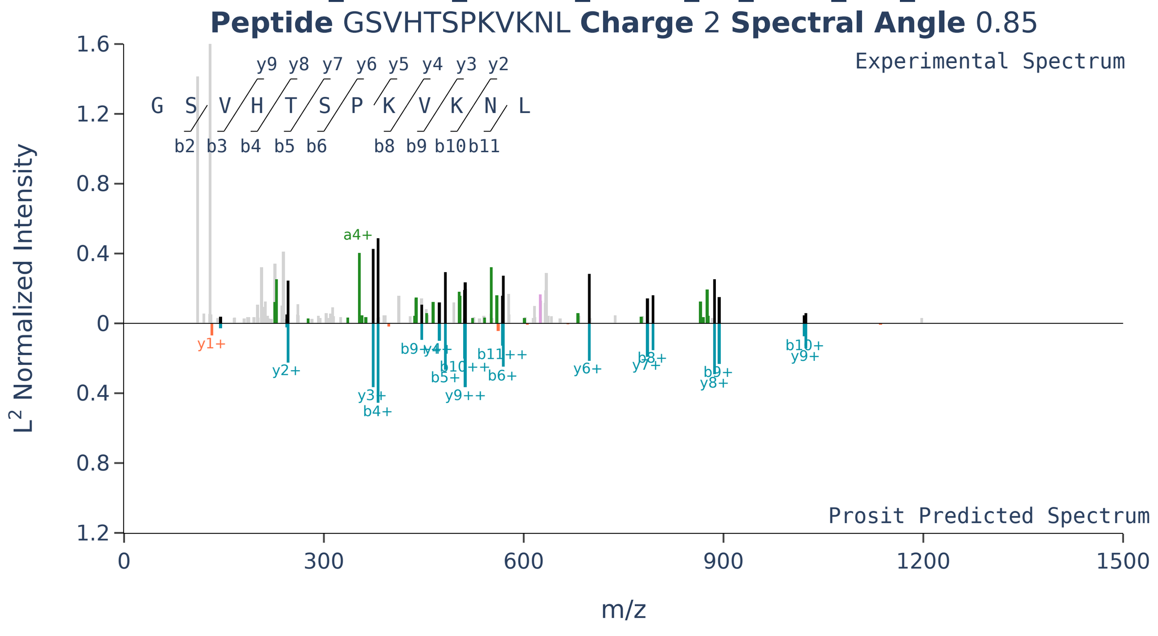

Source WSoh_101121_151121_HFGoe_G1_IL37b_24h_R2 **Scan** 8935
Peptide GNLNPKKF **Charge** 2 **Spectral Angle** 0.81

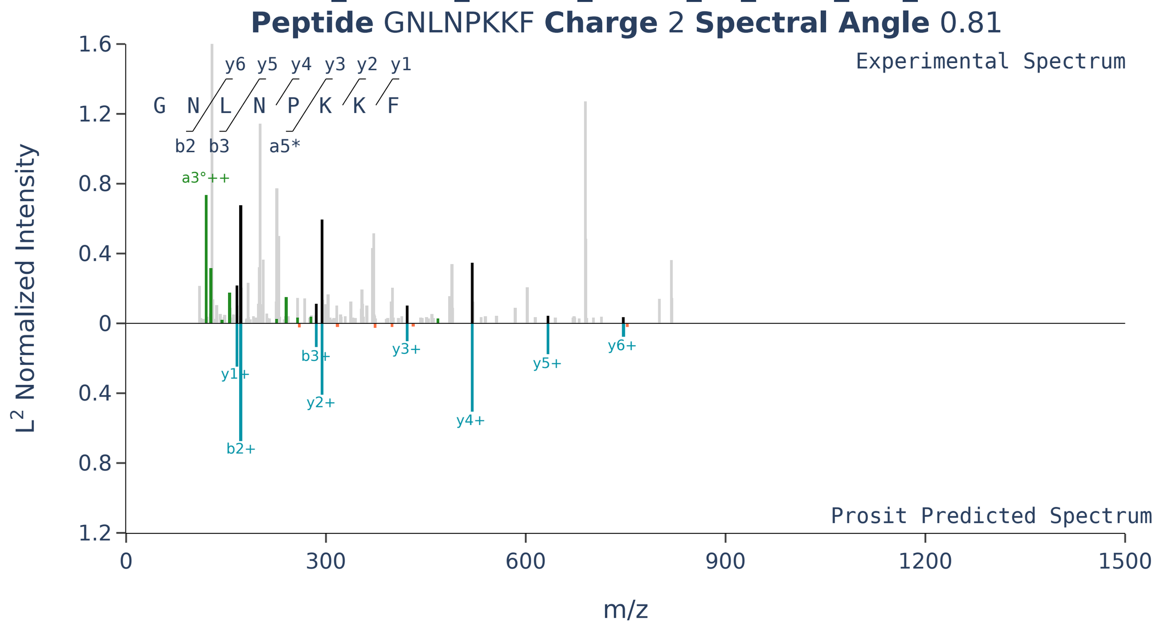

Source WSoh_101121_151121_HFGoe_G1_IL37b_24h_R1 **Scan** 7489
Peptide ESVHTSPKVKNL **Charge** 2 **Spectral Angle** 0.84

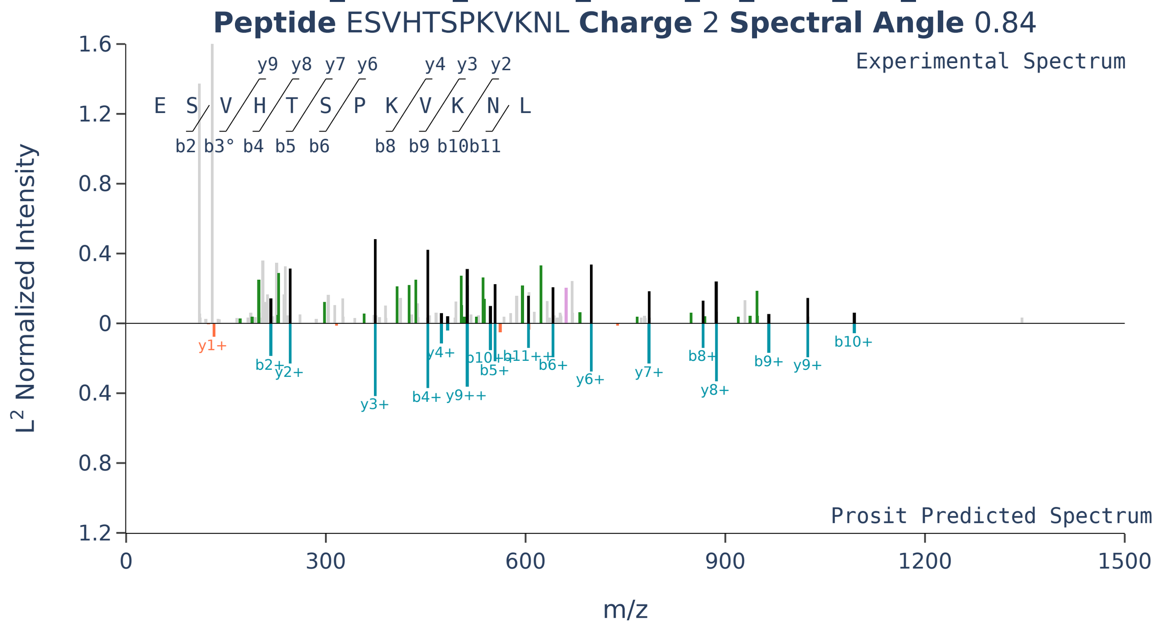

Source WSoh_101121_151121_HFGoe_G2_IL37b_24h_R1 **Scan** 14140
Peptide GCNEPVGV **Charge** 2 **Spectral Angle** 0.9

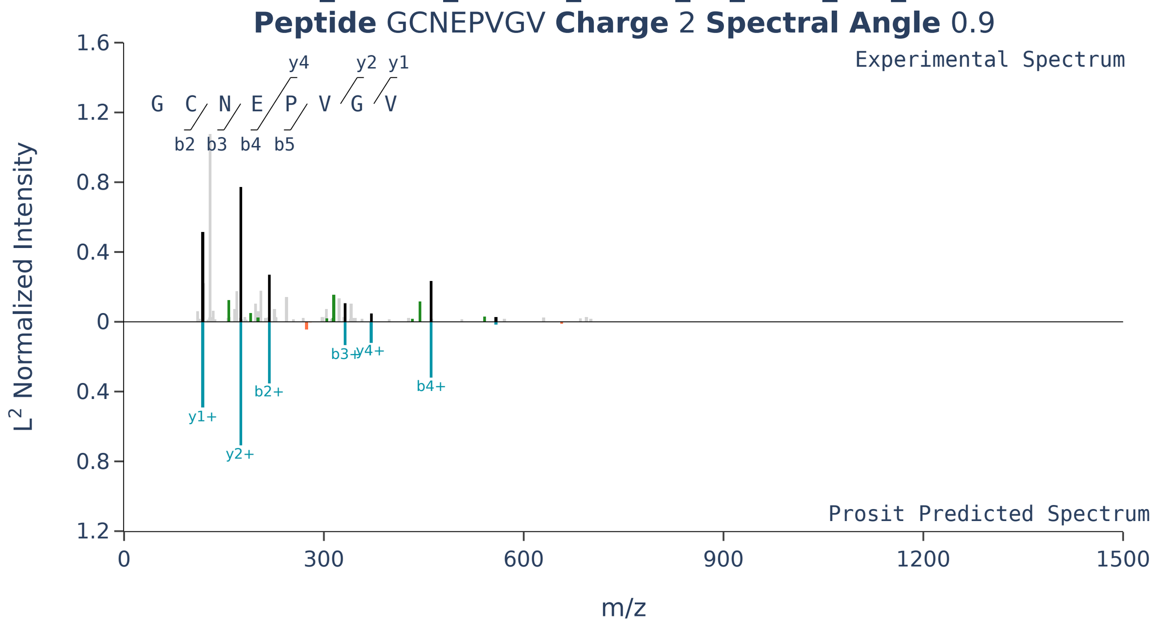

Source WSoh_101121_151121_HFGoe_G1_IL37b_1h_R2 **Scan** 15307
Peptide SGNLIAVPKN **Charge** 2 **Spectral Angle** 0.92

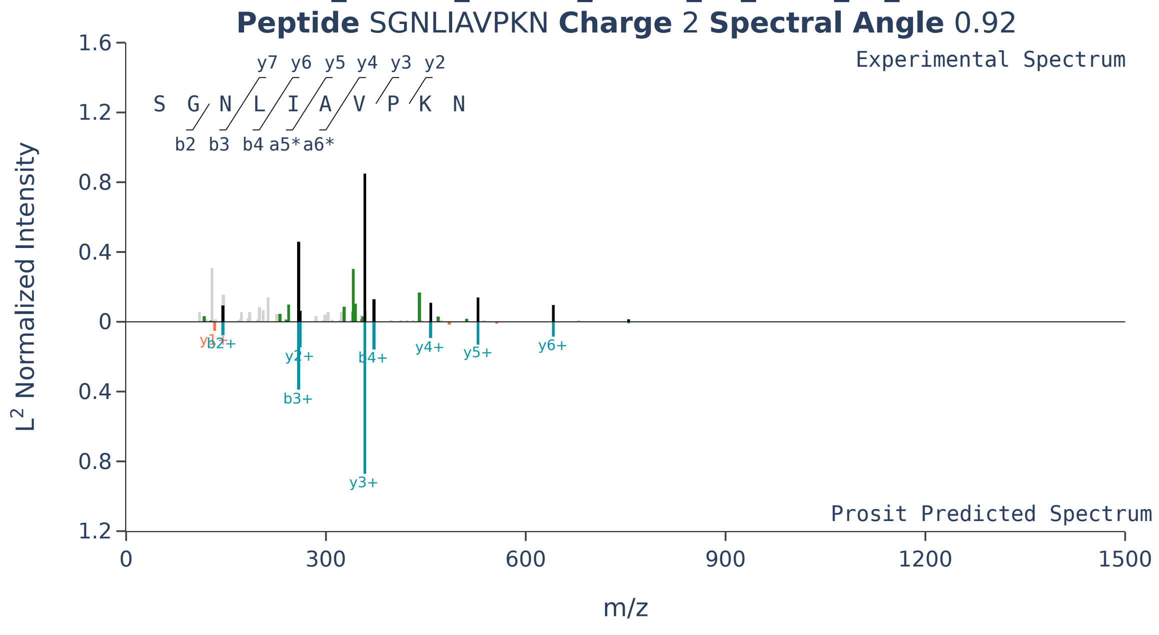

Source WSoh_101121_151121_HFGoe_G2_IL37b_24h_R2 **Scan** 6491
Peptide ESHTSPKVKNL **Charge** 2 **Spectral Angle** 0.85

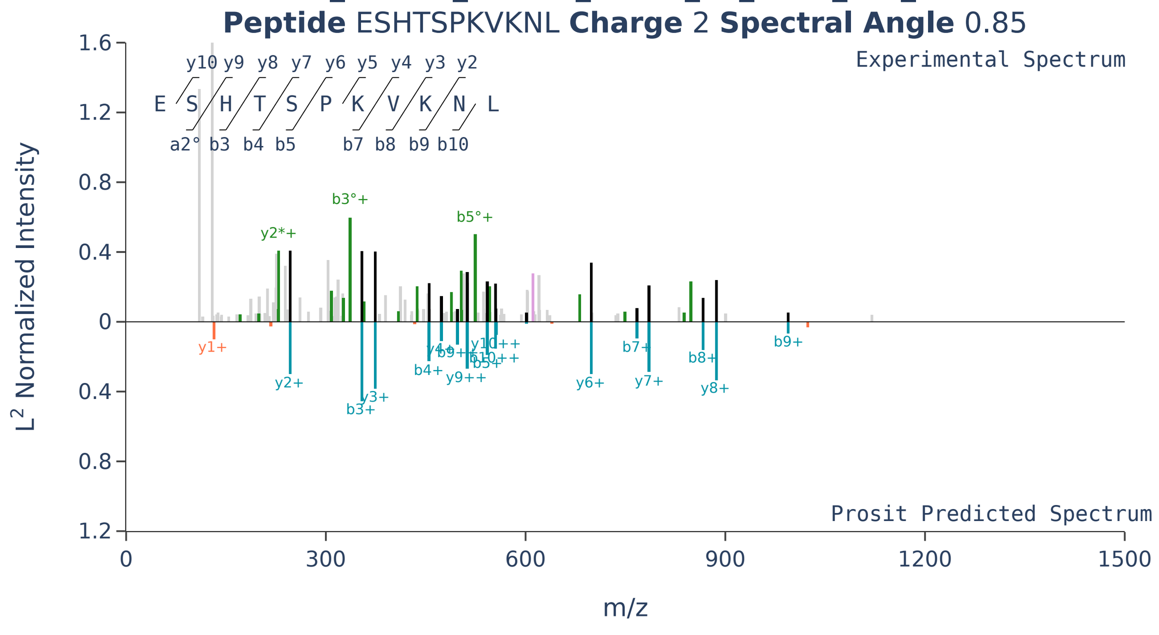

Source WSoh_101121_151121_HFGoe_G2_IL37b_24h_R2 **Scan** 12436
Peptide DSKDKGQSHPSLQL **Charge** 3 **Spectral Angle** 0.85

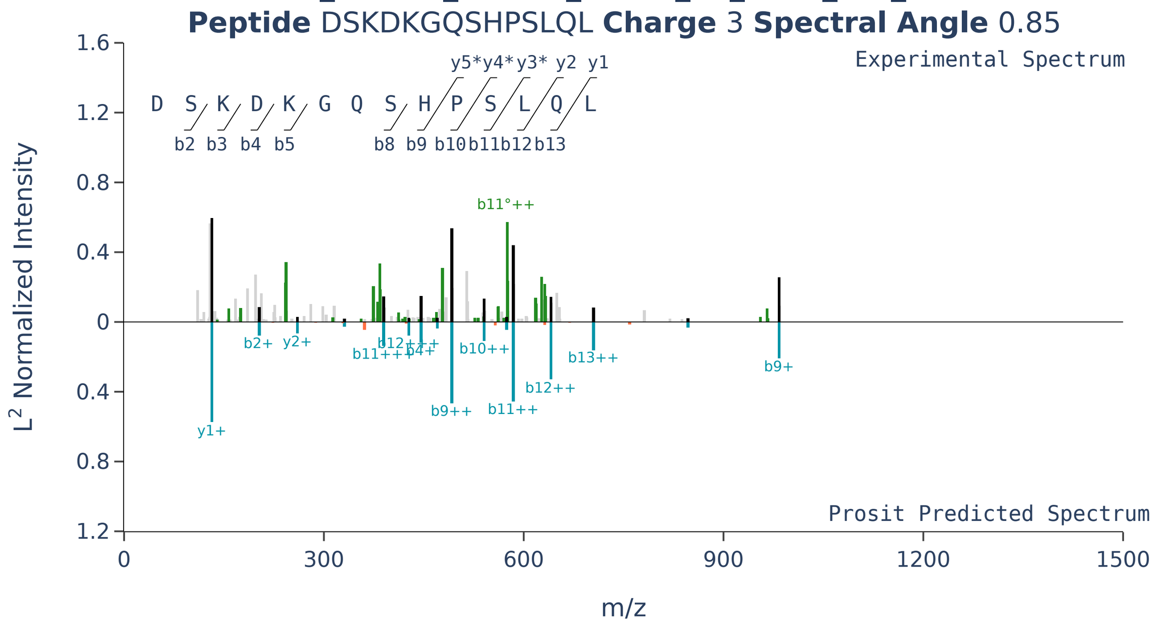

Source WSoh_101121_151121_HFGoe_G2_IL37b_24h_R1 **Scan** 17228
Peptide SSKGEFCL **Charge** 2 **Spectral Angle** 0.9

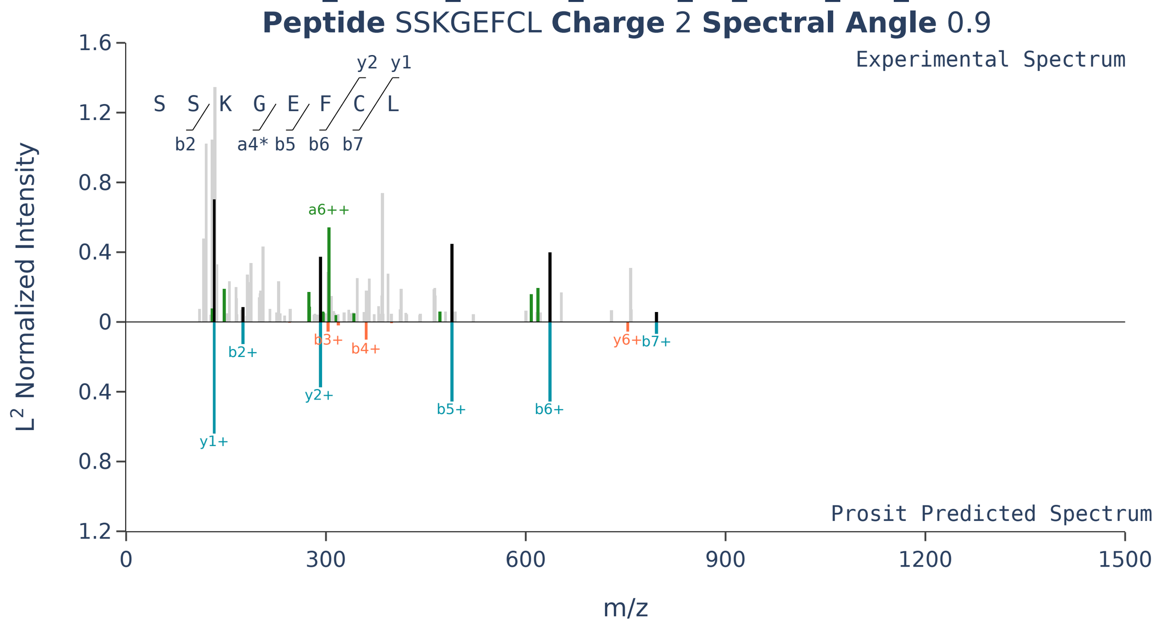

Source WSoh_101121_151121_HFGoe_G1_IL37b_24h_R1 **Scan** 10176
Peptide GCNEPVGVTDKFENRKH **Charge** 4 **Spectral Angle** 0.89

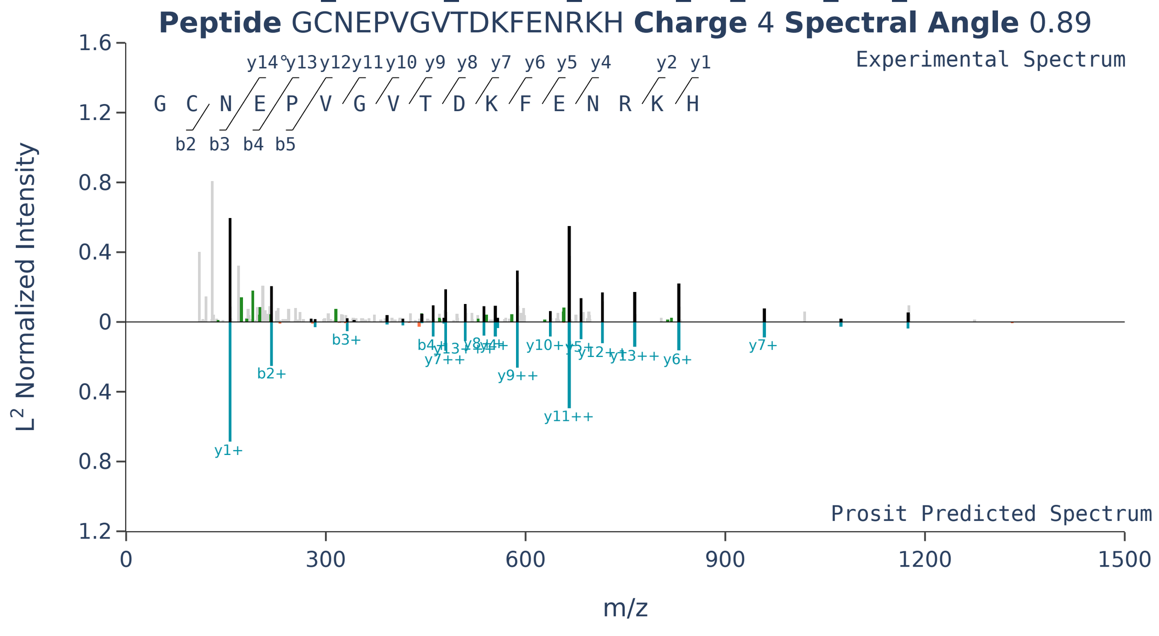

Source WSoh_101121_151121_HFGoe_G2_IL37b_24h_R2 **Scan** 7136
Peptide ASVHTSPKVKNL **Charge** 2 **Spectral Angle** 0.79

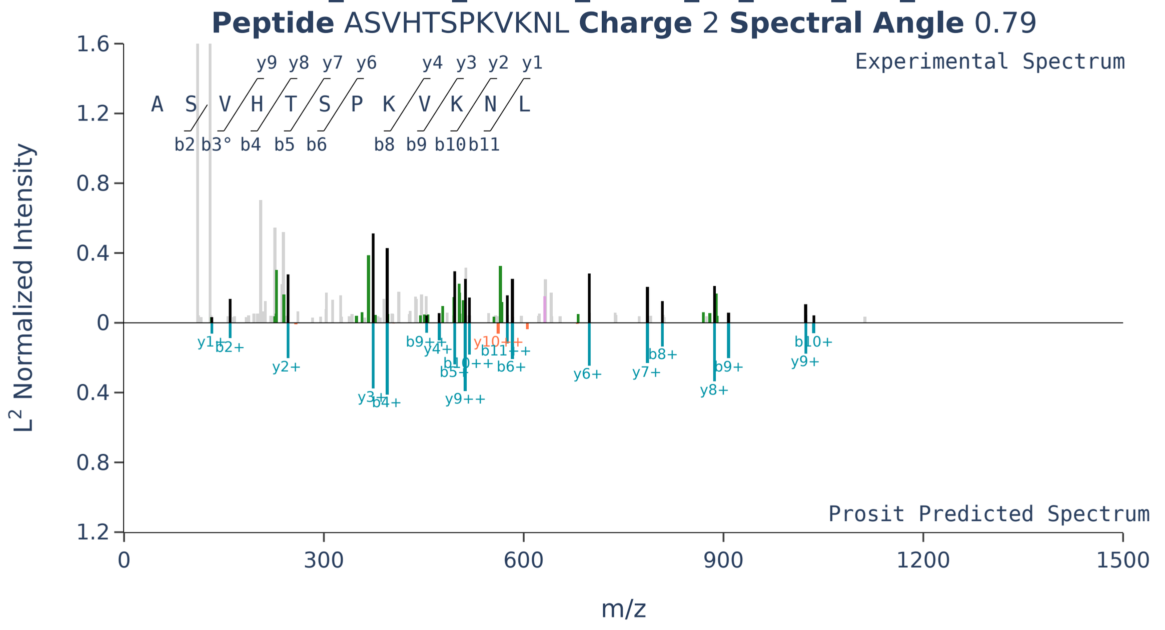

Source WSoh_101121_151121_HFGoe_G1_IL37b_24h_R2 **Scan** 13237
Peptide SFQPVCN **Charge** 1 **Spectral Angle** 0.85

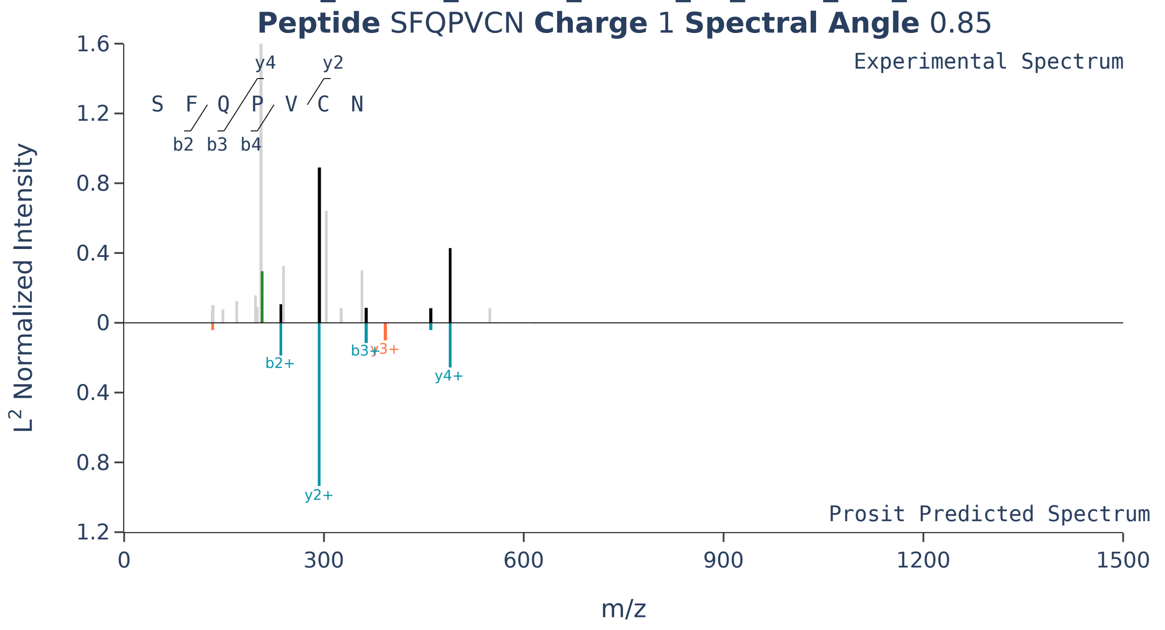

Source WSoh_101121_151121_HFGoe_G2_IL37b_24h_R2 **Scan** 9869
Peptide ESNLNPKKF **Charge** 2 **Spectral Angle** 0.85

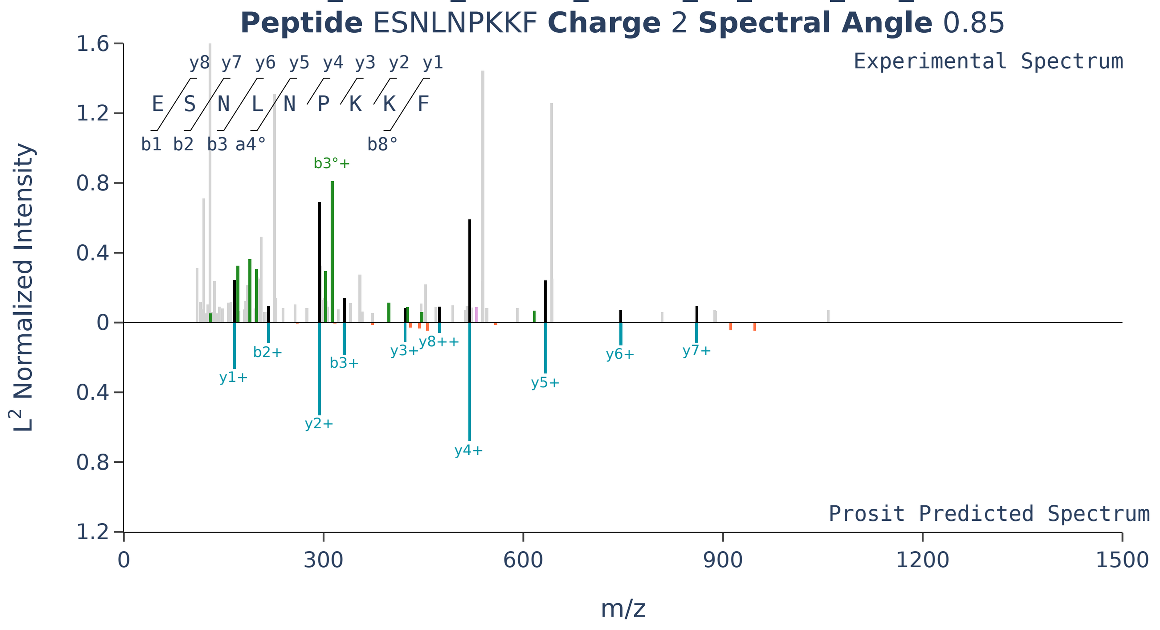

Source WSoh_101121_151121_HFGoe_G1_IL37b_24h_R1 **Scan** 6855
Peptide AVHTSPKVKNL **Charge** 2 **Spectral Angle** 0.86

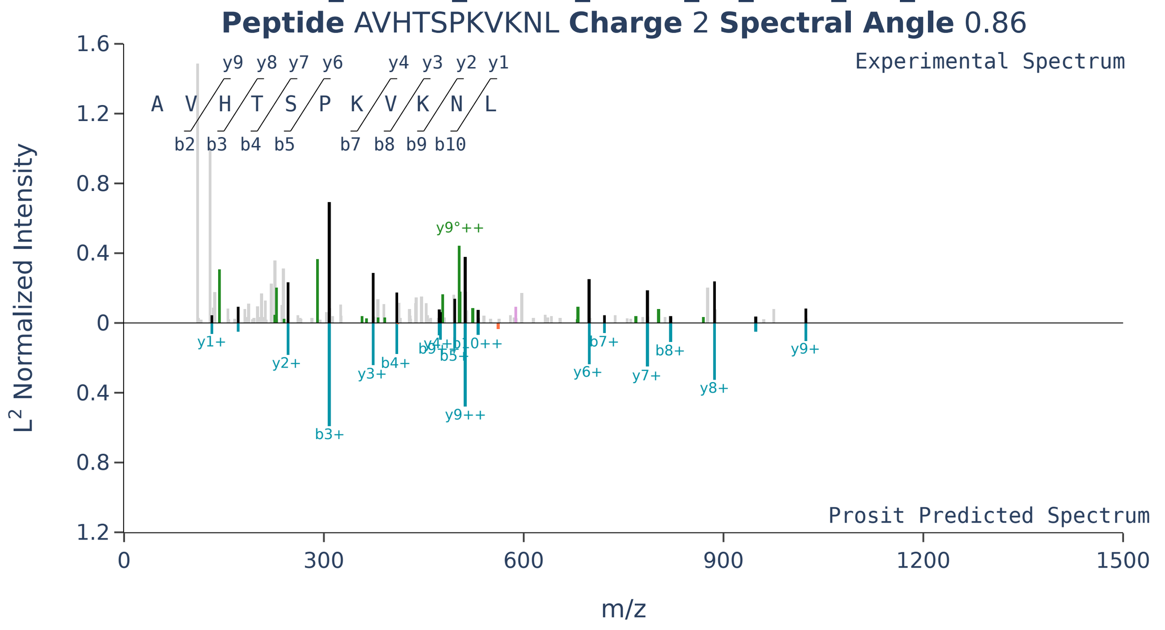

Source WSoh_101121_151121_HFGoe_G1_IL37b_2h_R1 **Scan** 7090
Peptide DSGVHTSPKV **Charge** 2 **Spectral Angle** 0.88

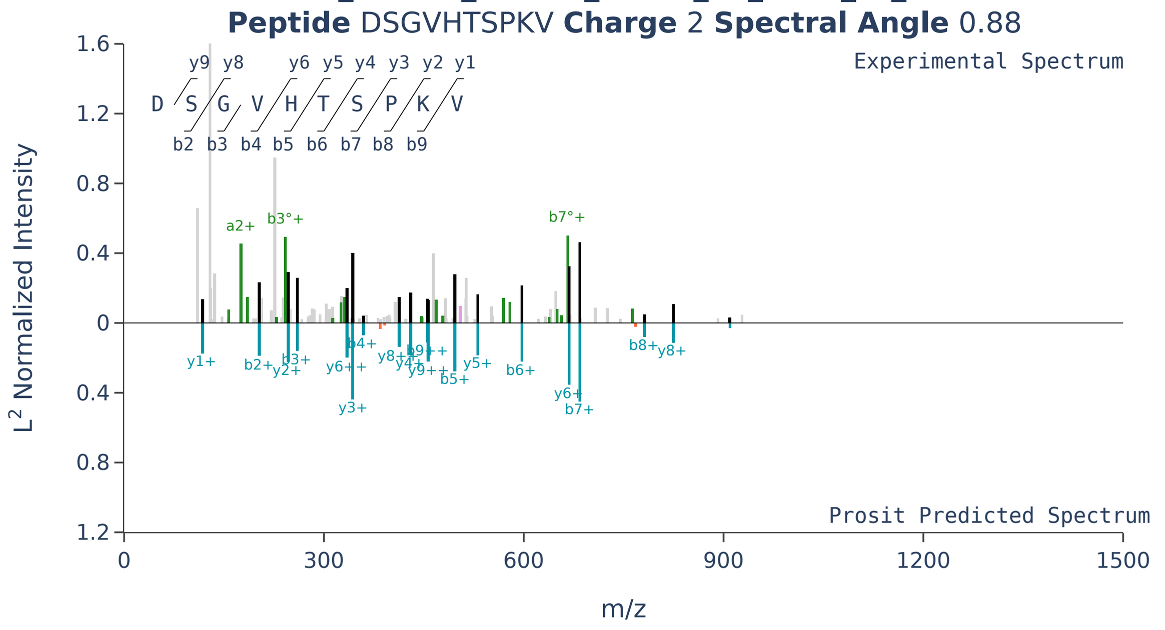

Source WSoh_101121_151121_HFGoe_G2_IL37b_24h_R2 **Scan** 10381
Peptide ALSSLSS **Charge** 1 **Spectral Angle** 0.88

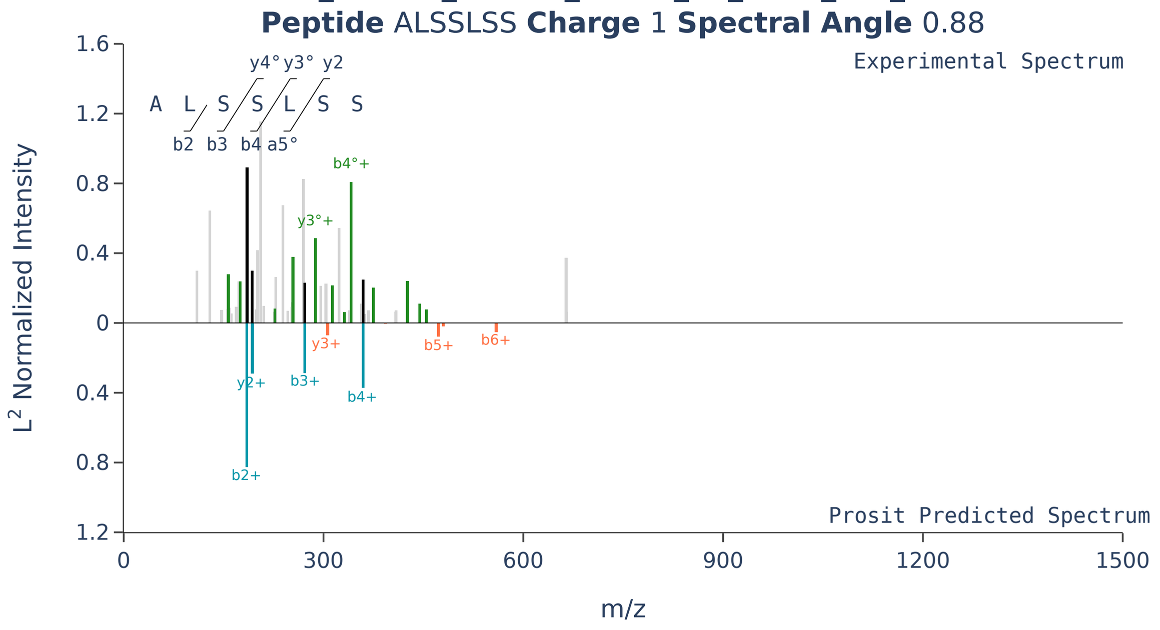

Source WSoh_101121_151121_HFGoe_G2_IL37b_24h_R1 **Scan** 7889
Peptide MVHTSPKVKNL **Charge** 2 **Spectral Angle** 0.85

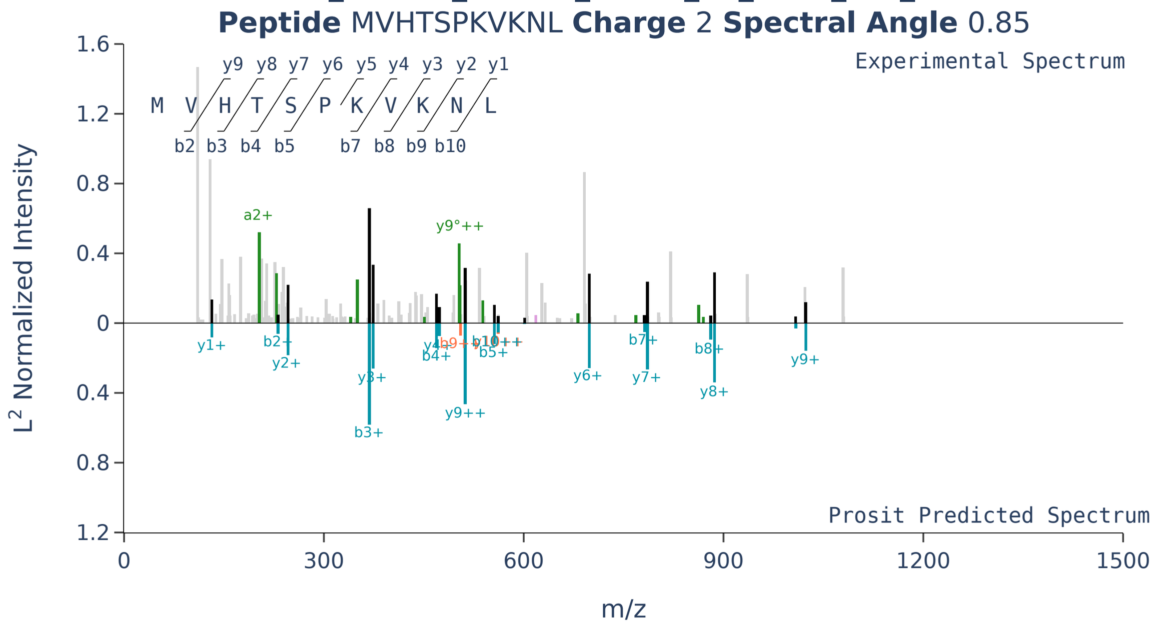

Source WSoh_101121_151121_HFGoe_G1_IL37b_24h_R2 **Scan** 12581
Peptide GCNEPVGVTD **Charge** 2 **Spectral Angle** 0.91

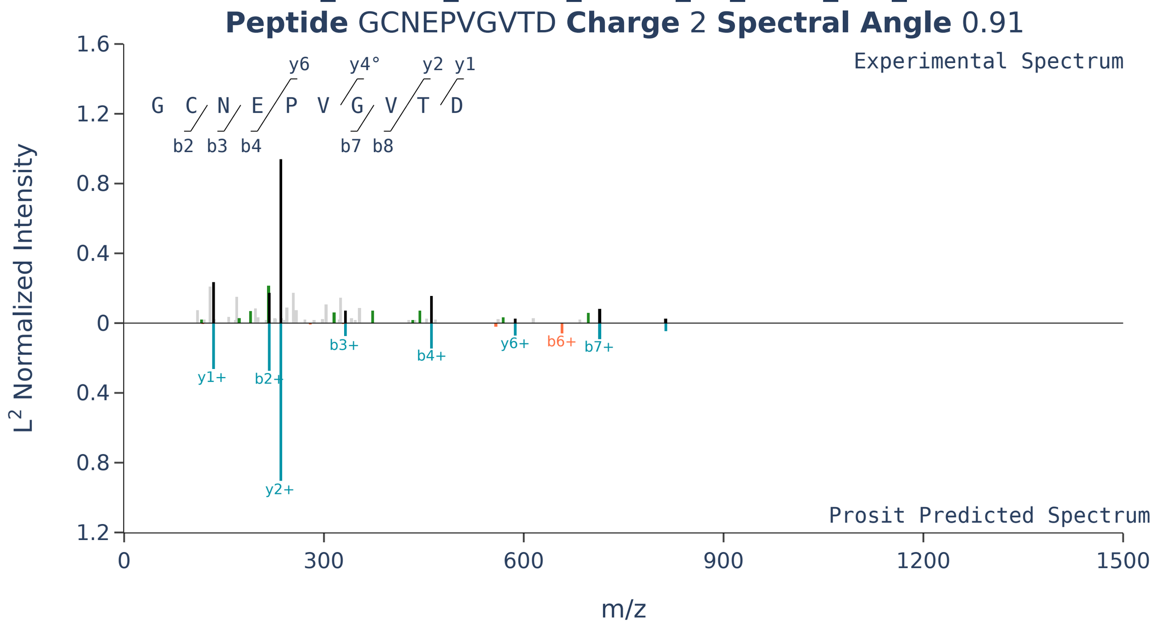

Source WSoh_101121_151121_HFGoe_G1_IL37b_24h_R2 **Scan** 11723
Peptide VLDSGVHTSPKVKNL **Charge** 3 **Spectral Angle** 0.85

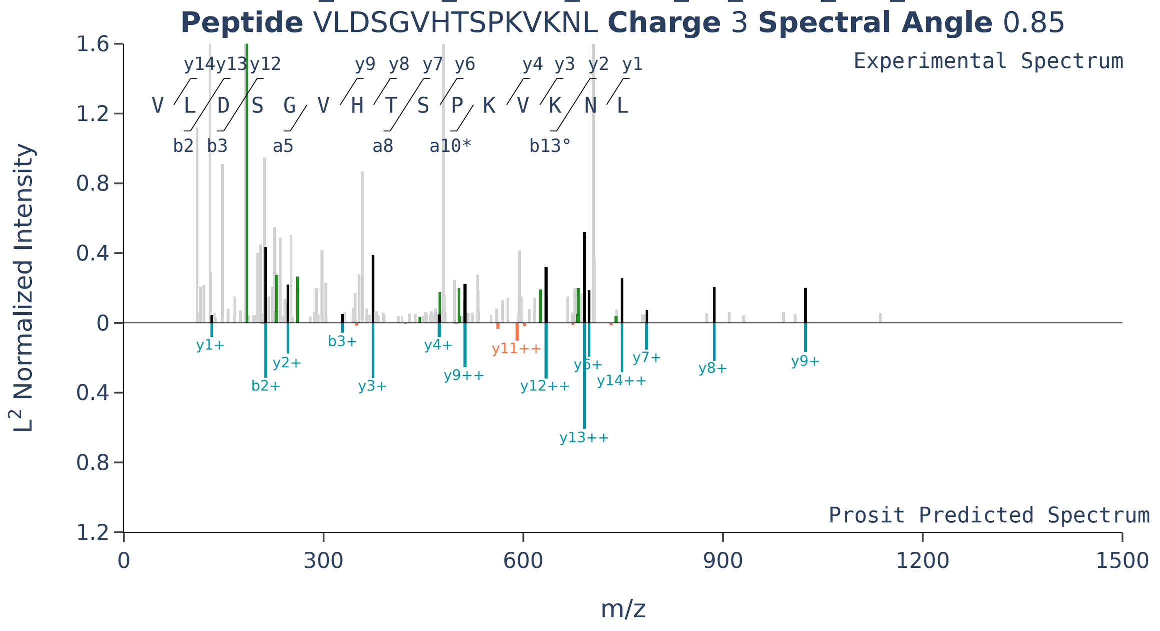

Source WSoh_101121_151121_HFGoe_G1_IL37b_4h_R2 **Scan** 9785
Peptide PSEVSDMS **Charge** 1 **Spectral Angle** 0.87

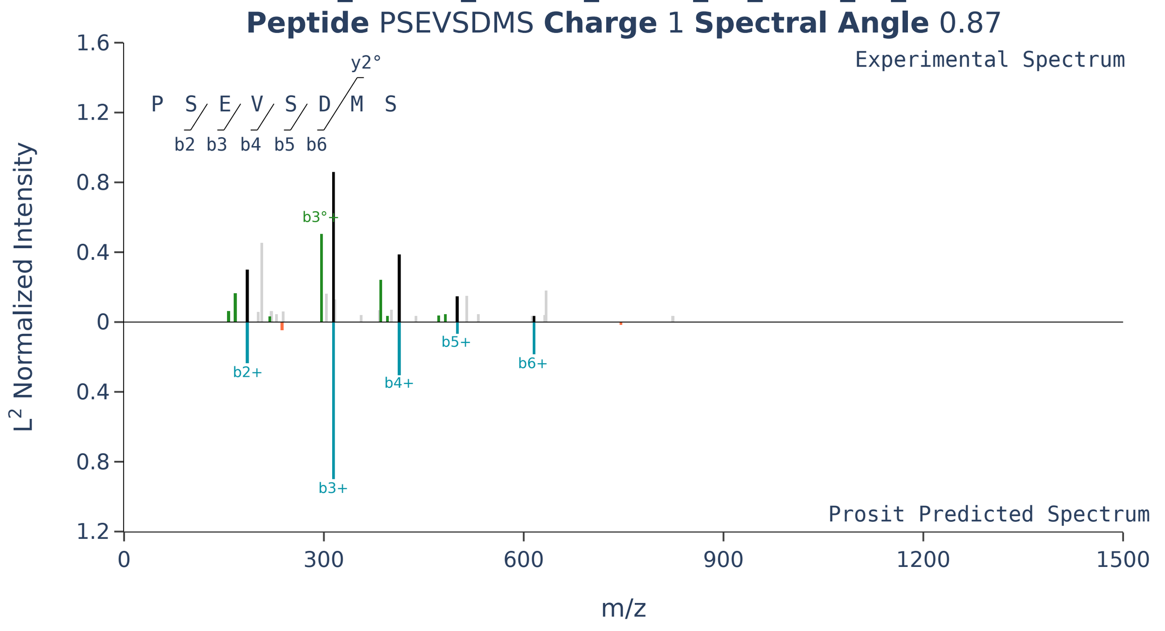

Source WSoh_101121_151121_HFGoe_G2_IL37b_24h_R2 **Scan** 21651
Peptide GEFVGSW **Charge** 2 **Spectral Angle** 0.74

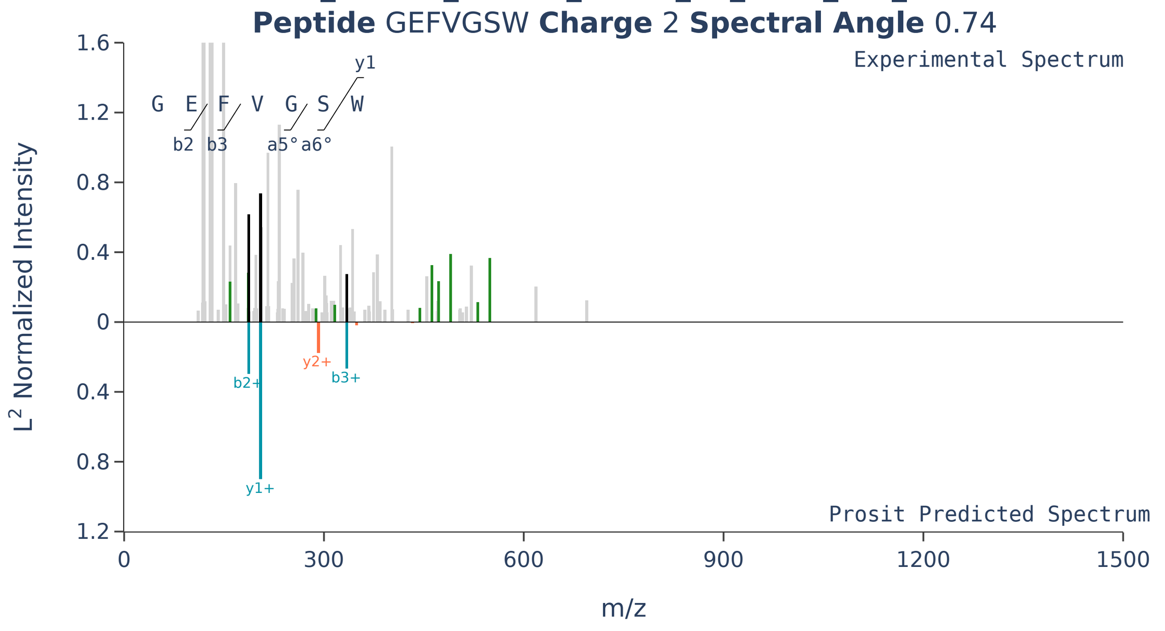

Source WSoh_101121_151121_HFGoe_G2_IL37b_24h_R1 **Scan** 7162
Peptide SCVHTSPKVKNL **Charge** 2 **Spectral Angle** 0.81

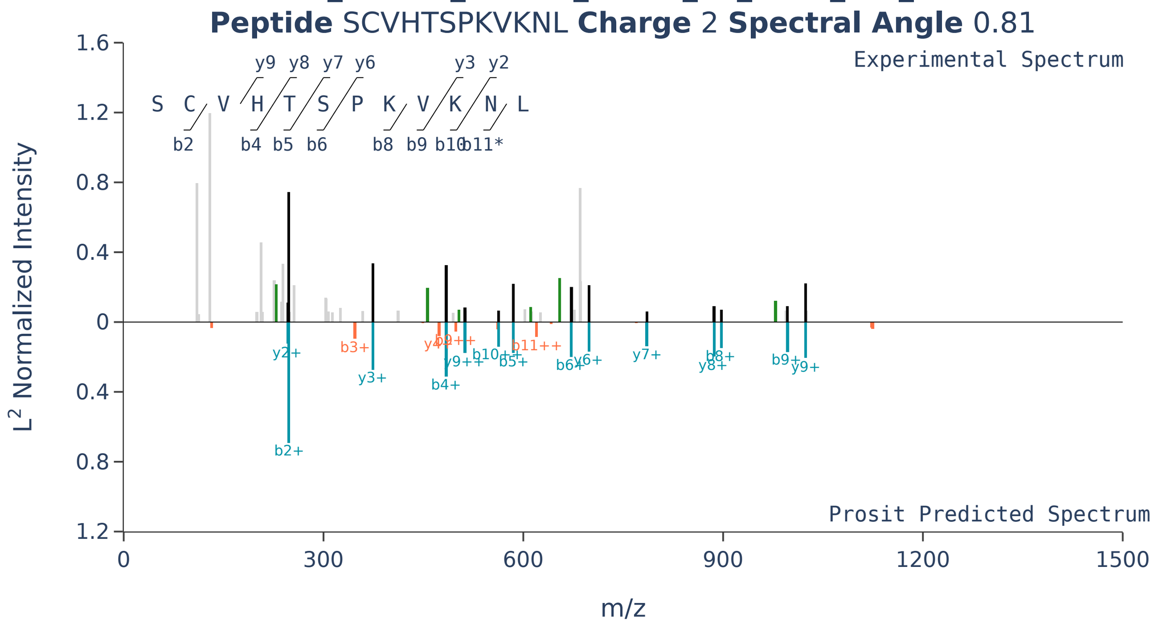

Source WSoh_101121_151121_HFGoe_G1_IL37b_24h_R2 **Scan** 16765
Peptide SKGEFSL **Charge** 2 **Spectral Angle** 0.91

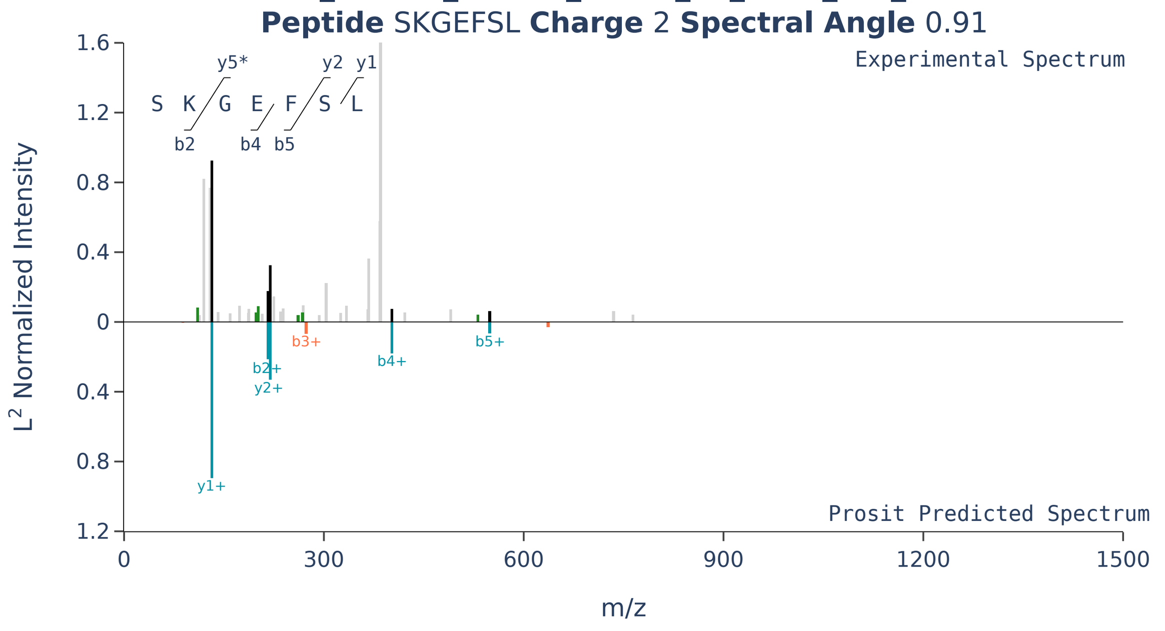

Source WSoh_101121_151121_HFGoe_G1_IL37b_4h_R1 **Scan** 7108
Peptide SGVHTSPKVKNL **Charge** 2 **Spectral Angle** 0.84

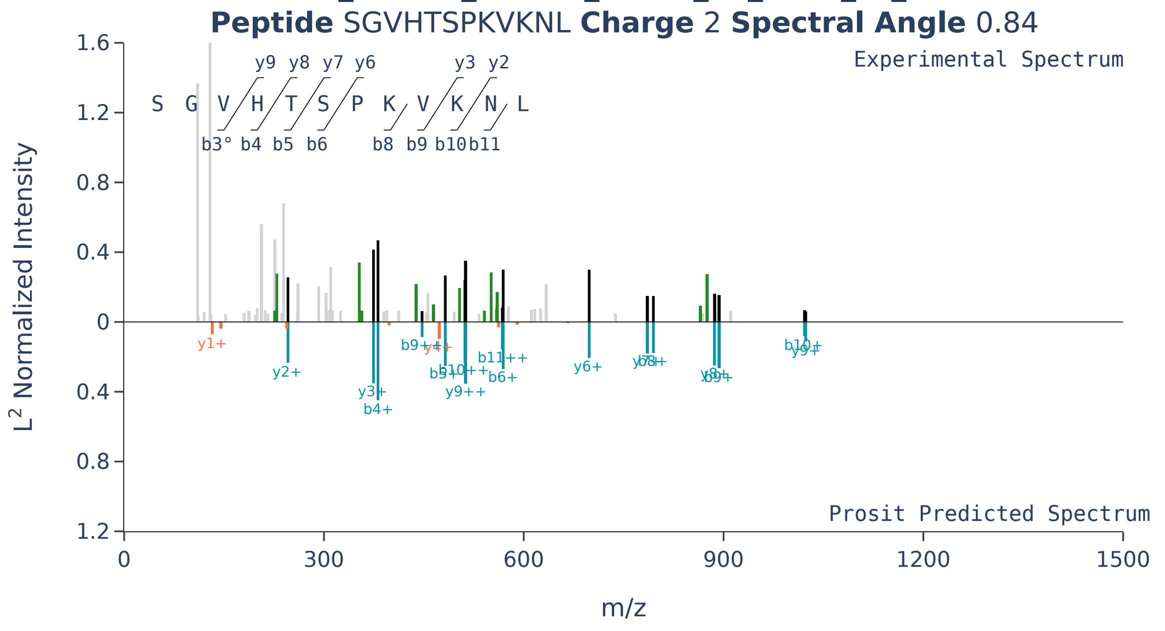

Source WSoh_101121_151121_HFGoe_G1_IL37b_1h_R1 **Scan** 7768
Peptide ASSLSSSAE **Charge** 2 **Spectral Angle** 0.96

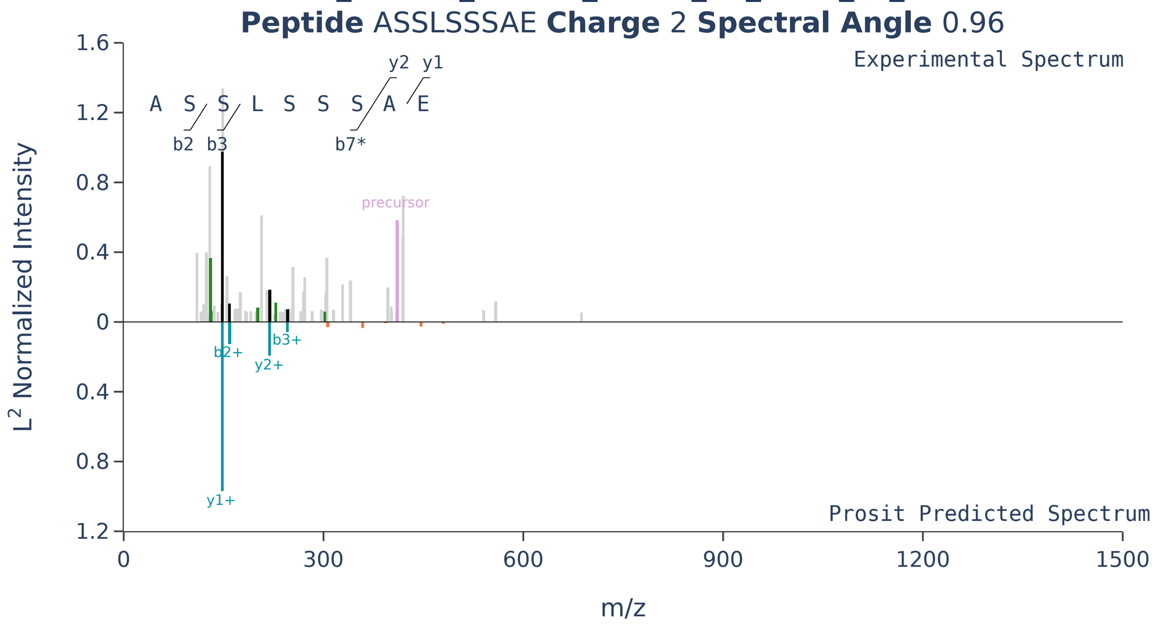

Source WSoh_101121_151121_HFGoe_G2_IL37b_1h_R2 **Scan** 6267
Peptide SGVHTSPKV **Charge** 2 **Spectral Angle** 0.82

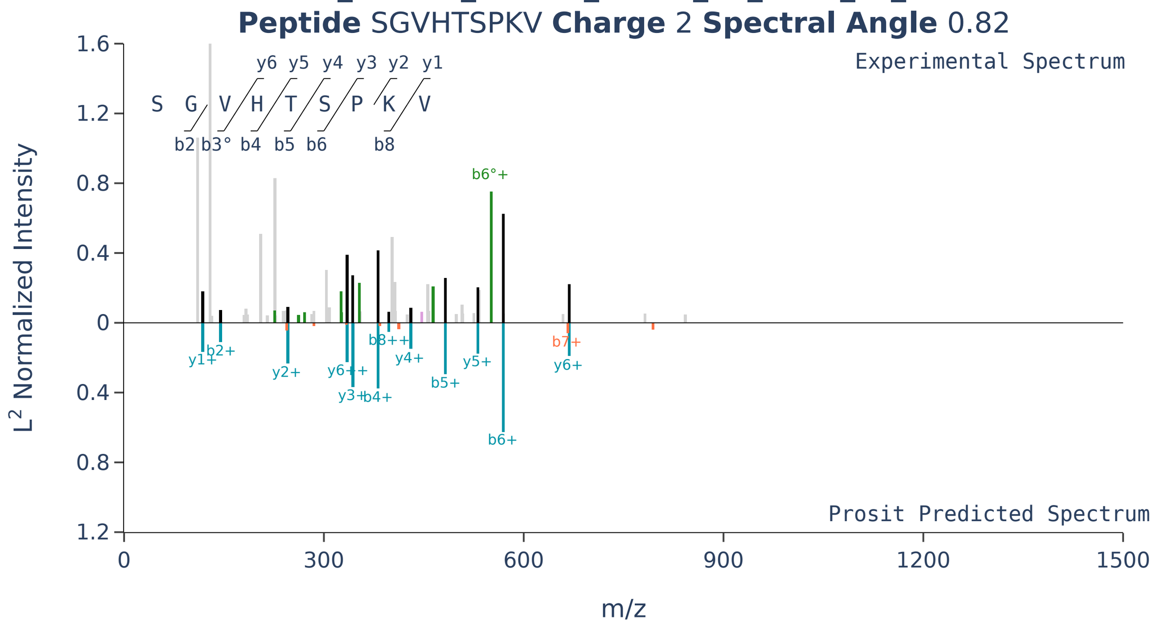

Source WSoh_101121_151121_HFGoe_G2_IL37b_24h_R1 **Scan** 12092
Peptide PSEVSDAEMS **Charge** 1 **Spectral Angle** 0.87

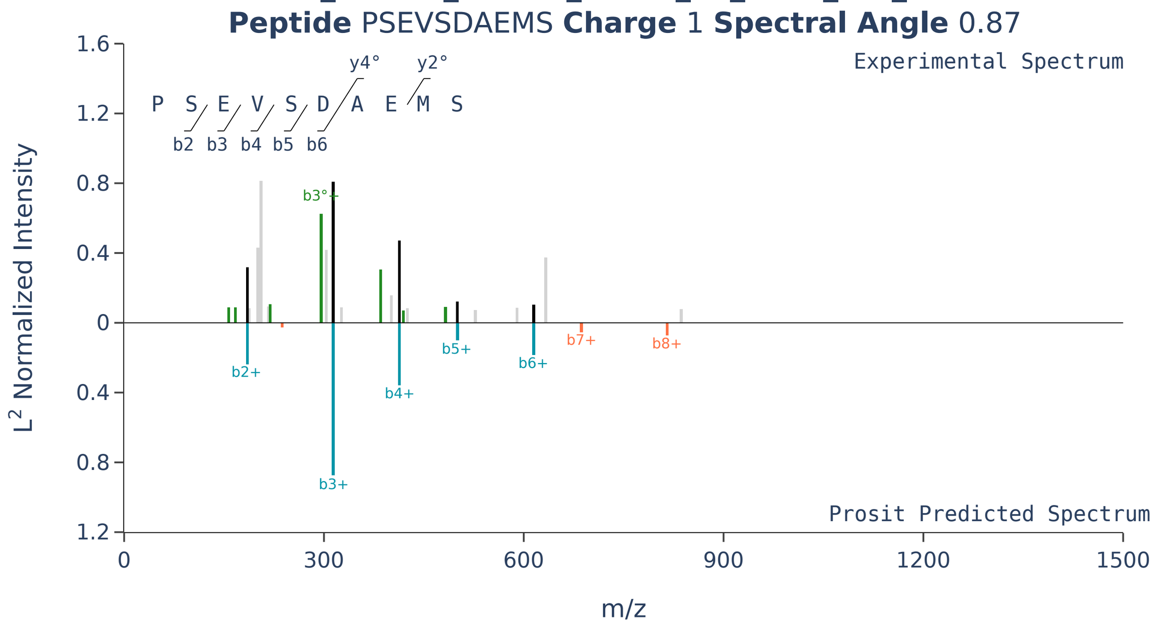

Source WSoh_101121_151121_HFGoe_G2_IL37b_24h_R2 **Scan** 11308
Peptide AVPDKNYM **Charge** 2 **Spectral Angle** 0.82

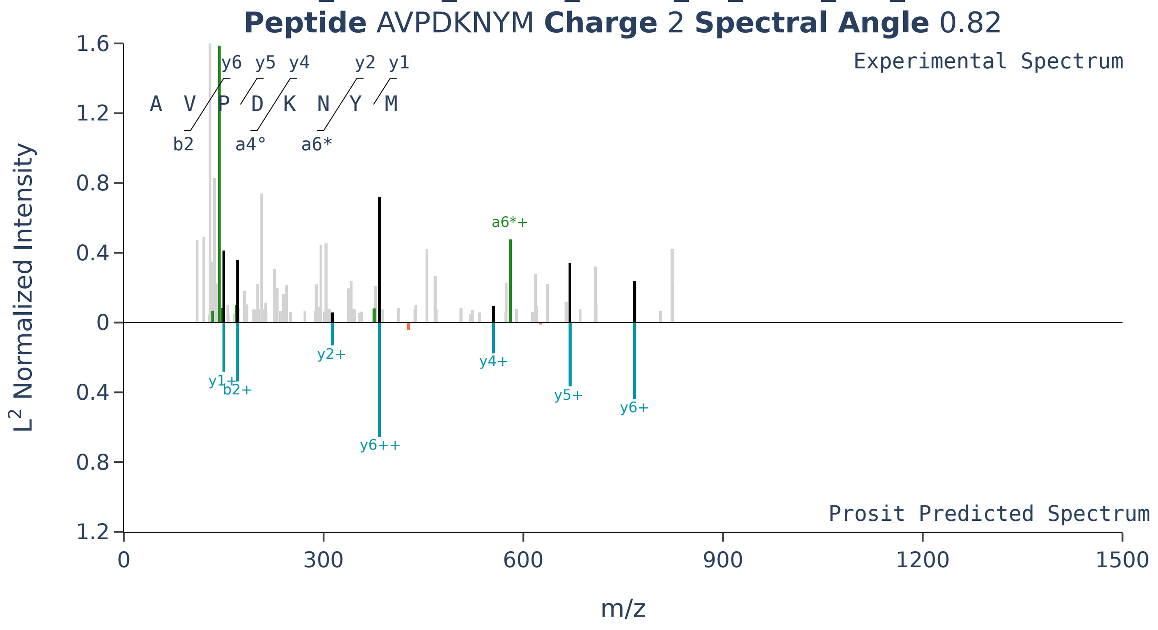

Source WSoh_101121_151121_HFGoe_G1_IL37b_24h_R2 **Scan** 6396
Peptide LASVHTSPKVK **Charge** 3 **Spectral Angle** 0.87

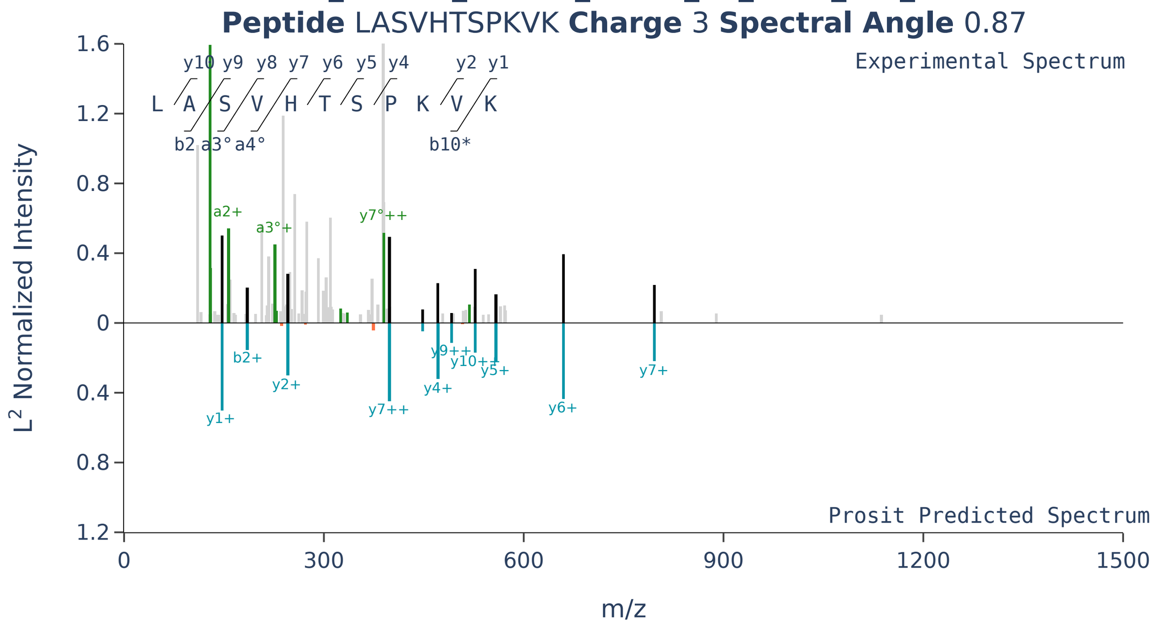

Source WSoh_101121_151121_HFGoe_G1_IL37b_4h_R2 **Scan** 20305
Peptide SKEFCLYC **Charge** 2 **Spectral Angle** 0.92

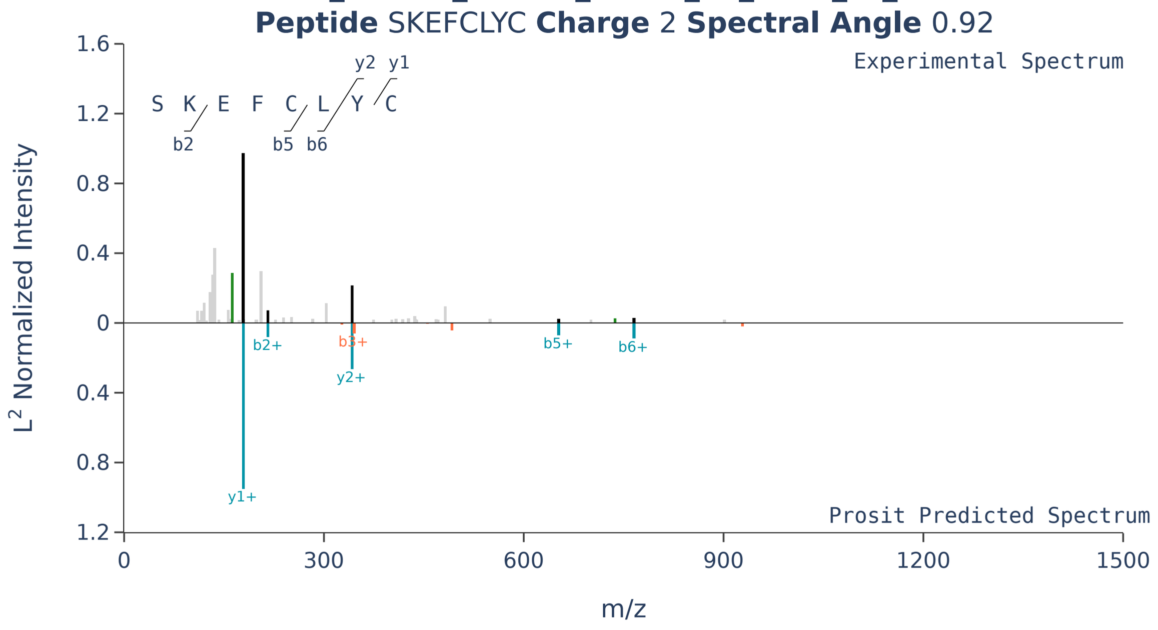

Source WSoh_101121_151121_HFGoe_G1_IL37b_4h_R2 **Scan** 15578
Peptide DKDKGQSHPSLQLV **Charge** 3 **Spectral Angle** 0.84

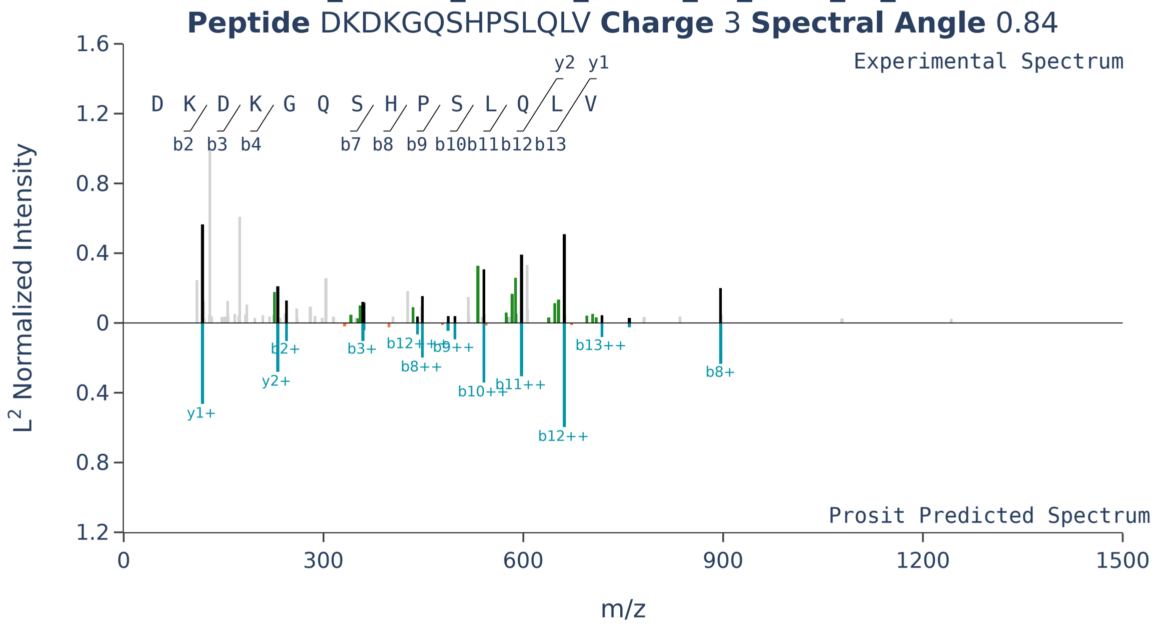

Source WSoh_101121_151121_HFGoe_G2_IL37b_24h_R2 **Scan** 8657
Peptide VGVMSPSE **Charge** 2 **Spectral Angle** 0.74

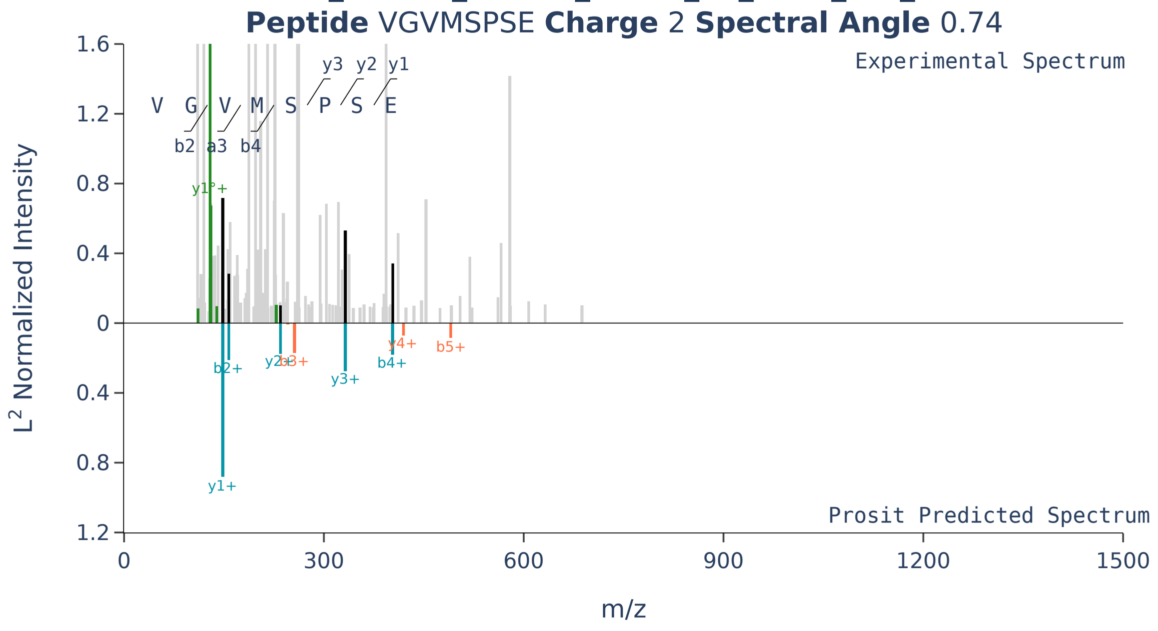

Source WSoh_101121_151121_HFGoe_G2_IL37b_24h_R2 **Scan** 11825
Peptide SSLSKGEF **Charge** 2 **Spectral Angle** 0.87

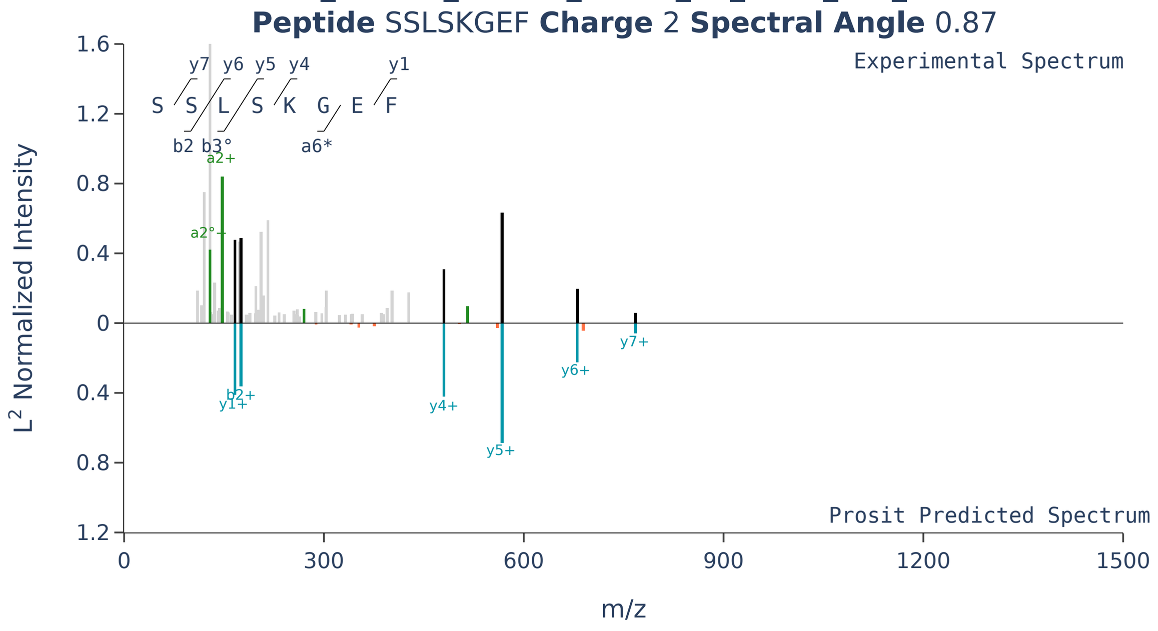

Source WSoh_101121_151121_HFGoe_G1_IL37b_24h_R2 **Scan** 12597
Peptide NCNEPVGVVHT **Charge** 2 **Spectral Angle** 0.85

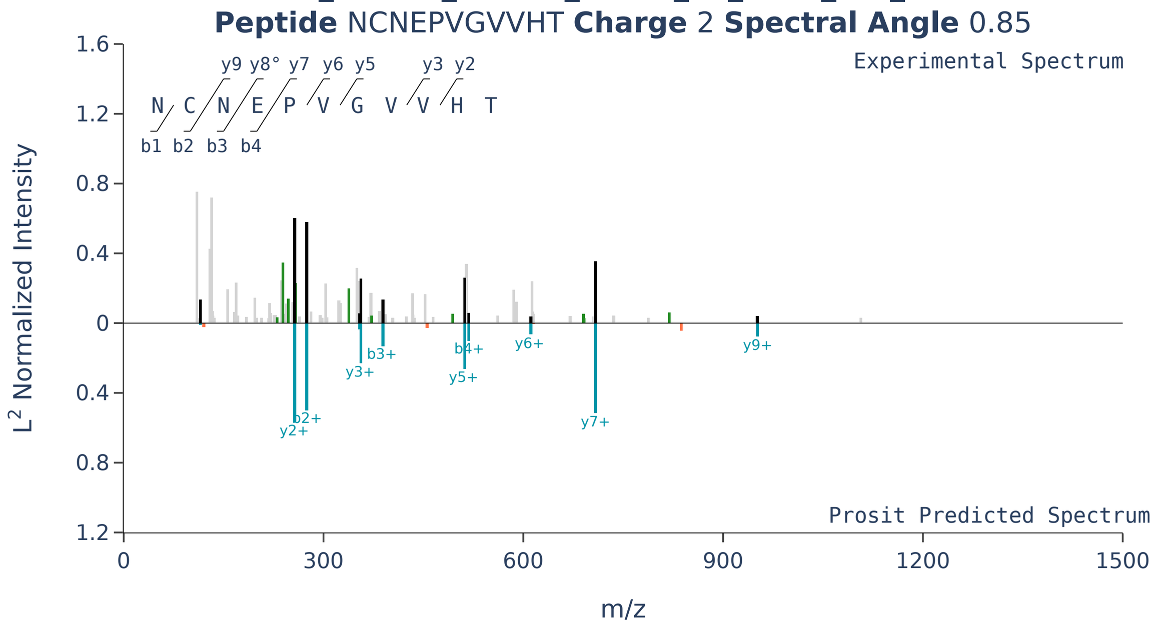

Source WSoh_101121_151121_HFGoe_G2_IL37b_24h_R1 **Scan** 17175
Peptide DSGNLIAVPKN **Charge** 2 **Spectral Angle** 0.92

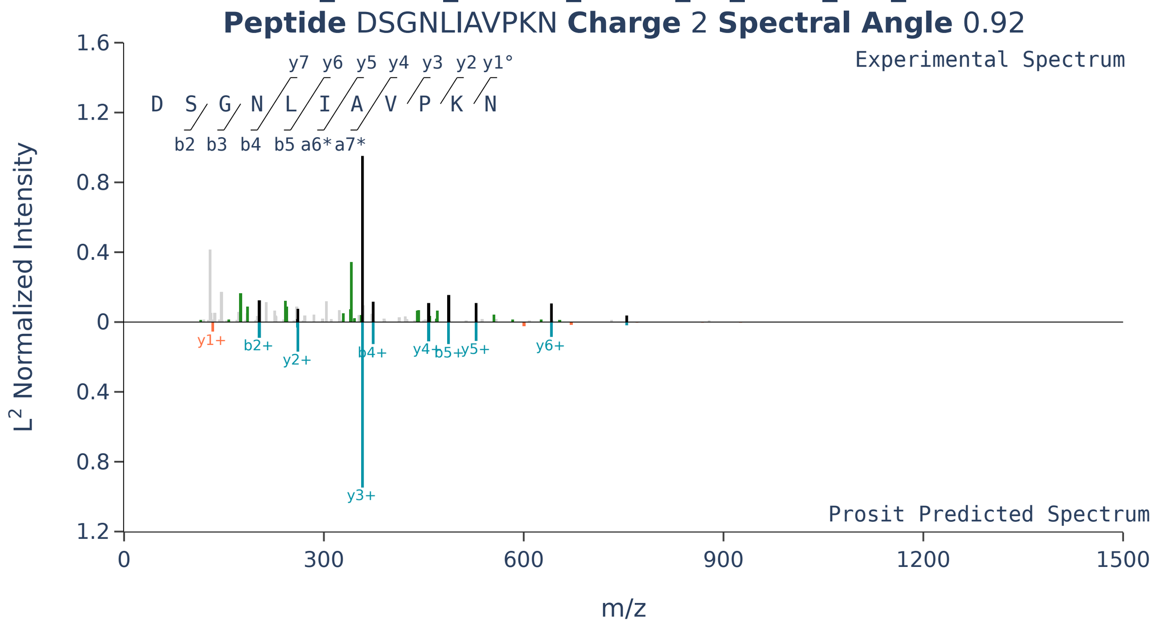

Source WSoh_101121_151121_HFGoe_G2_IL37b_24h_R1 **Scan** 14177
Peptide SHDQDHKVLVL **Charge** 3 **Spectral Angle** 0.82

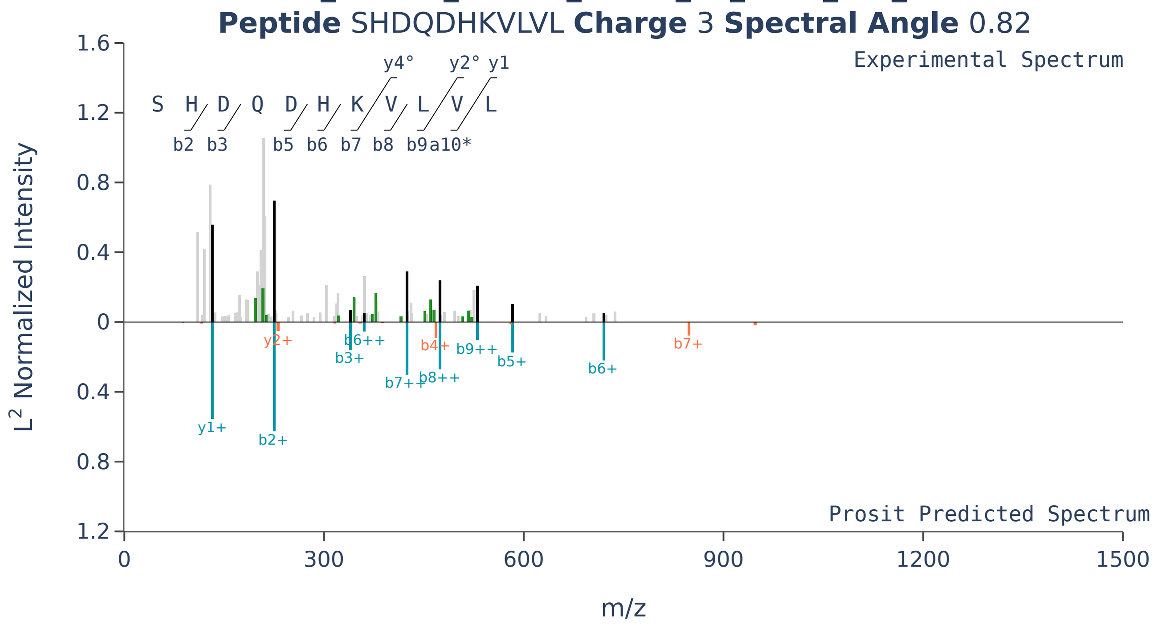

Source WSoh_101121_151121_HFGoe_G2_IL37b_24h_R2 **Scan** 6743
Peptide DKDKGQSHPSLVHT **Charge** 2 **Spectral Angle** 0.86

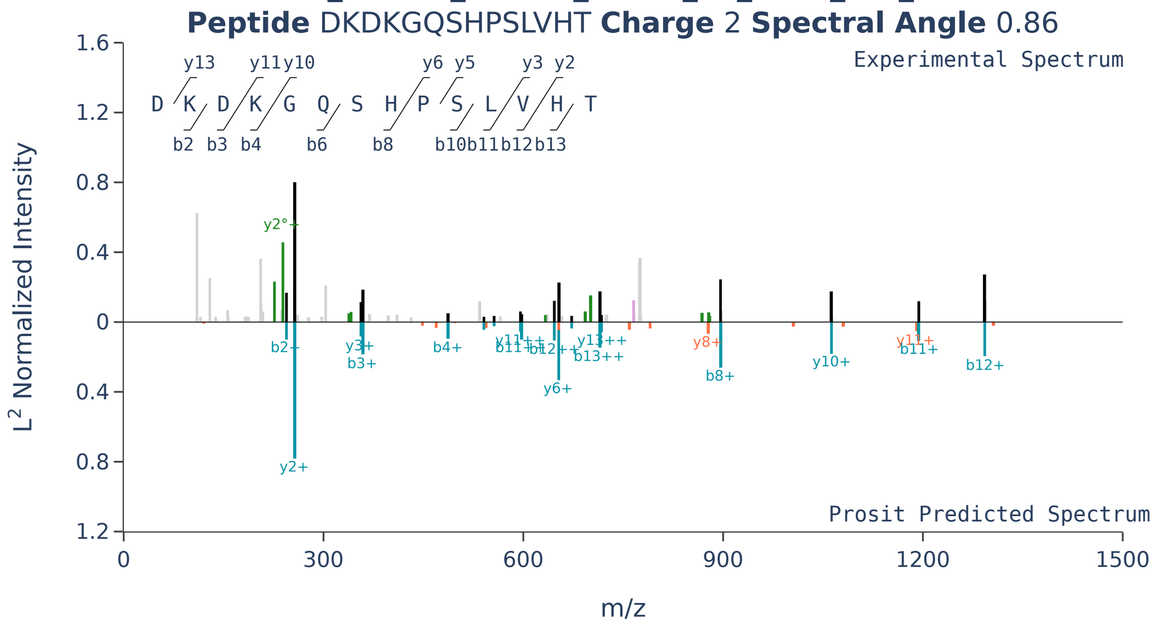

Source WSoh_101121_151121_HFGoe_G1_IL37b_24h_R1 **Scan** 17582
Peptide DKDKGQSHPSLNLI **Charge** 3 **Spectral Angle** 0.88

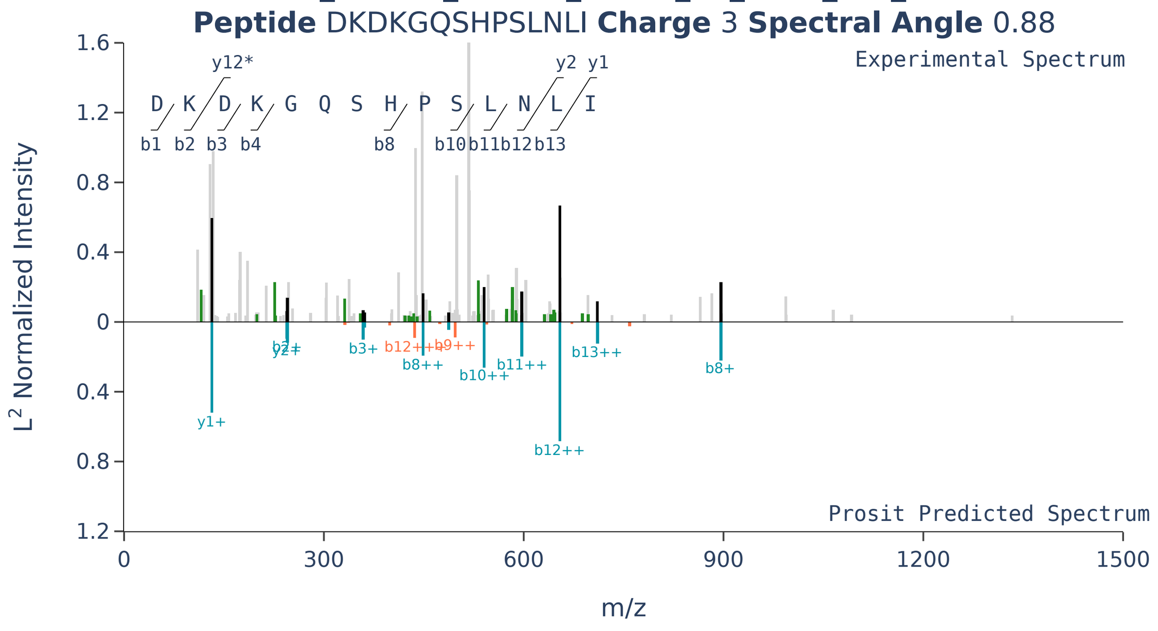

Source WSoh_101121_151121_HFGoe_G2_IL37b_24h_R1 **Scan** 14868
Peptide SQDHKVLVLD **Charge** 2 **Spectral Angle** 0.79

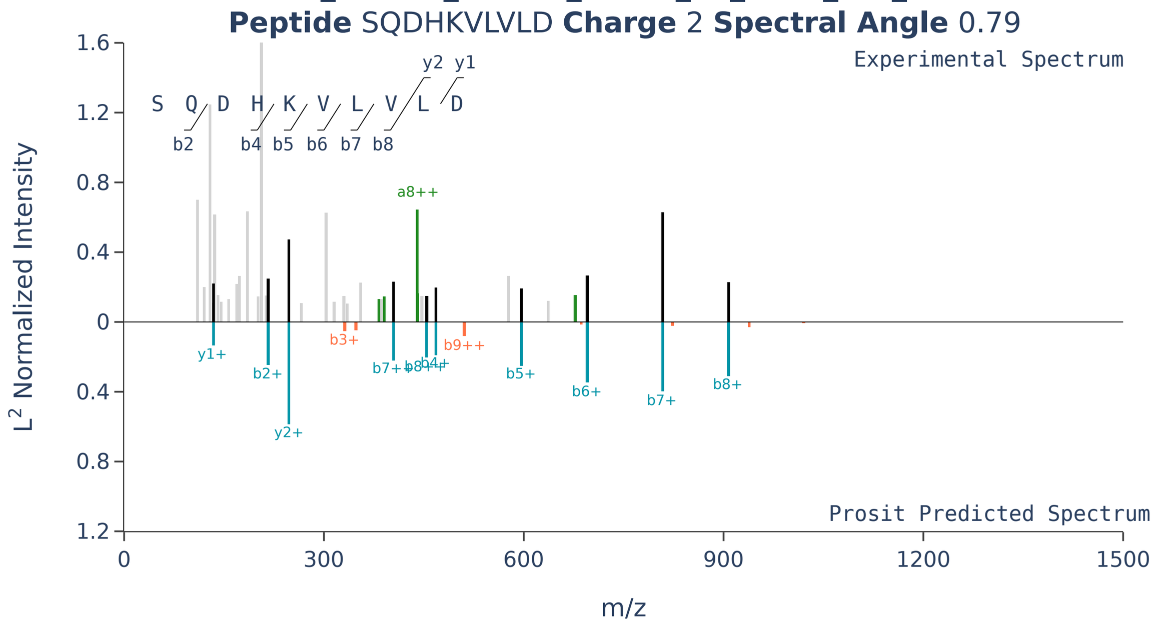

Source WSoh_101121_151121_HFGoe_G2_IL37b_24h_R2 **Scan** 16525
Peptide SNLNPKKFSI **Charge** 2 **Spectral Angle** 0.88

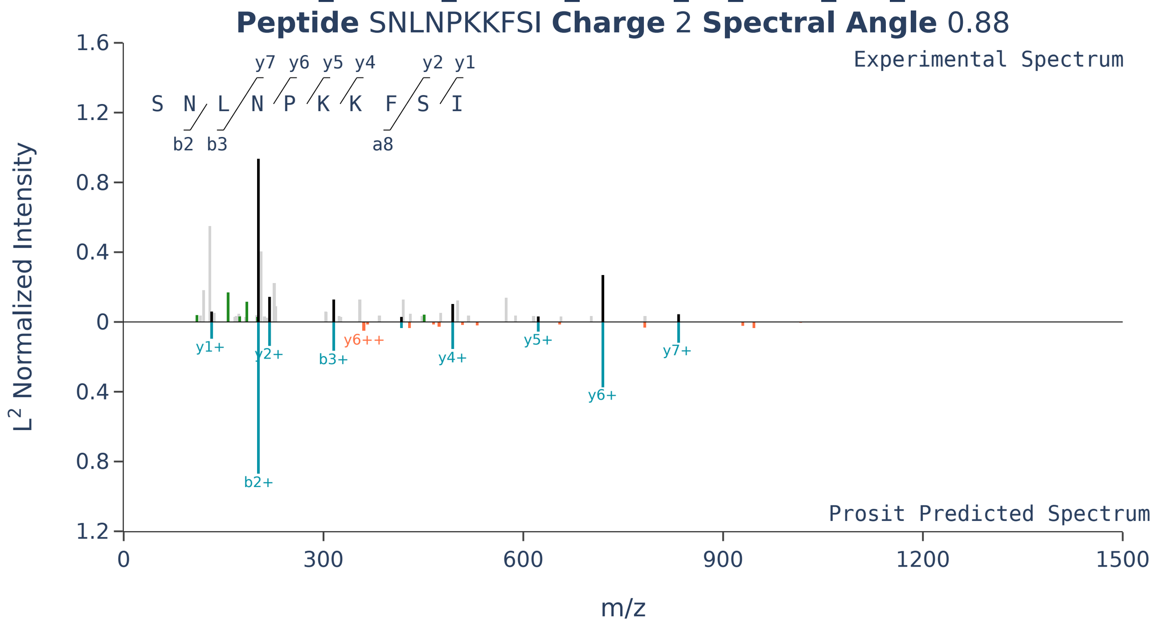

Source WSoh_101121_151121_HFGoe_G1_IL37b_24h_R1 **Scan** 9369
Peptide DSGVHTSPKVKNLNPKKF **Charge** 3 **Spectral Angle** 0.76

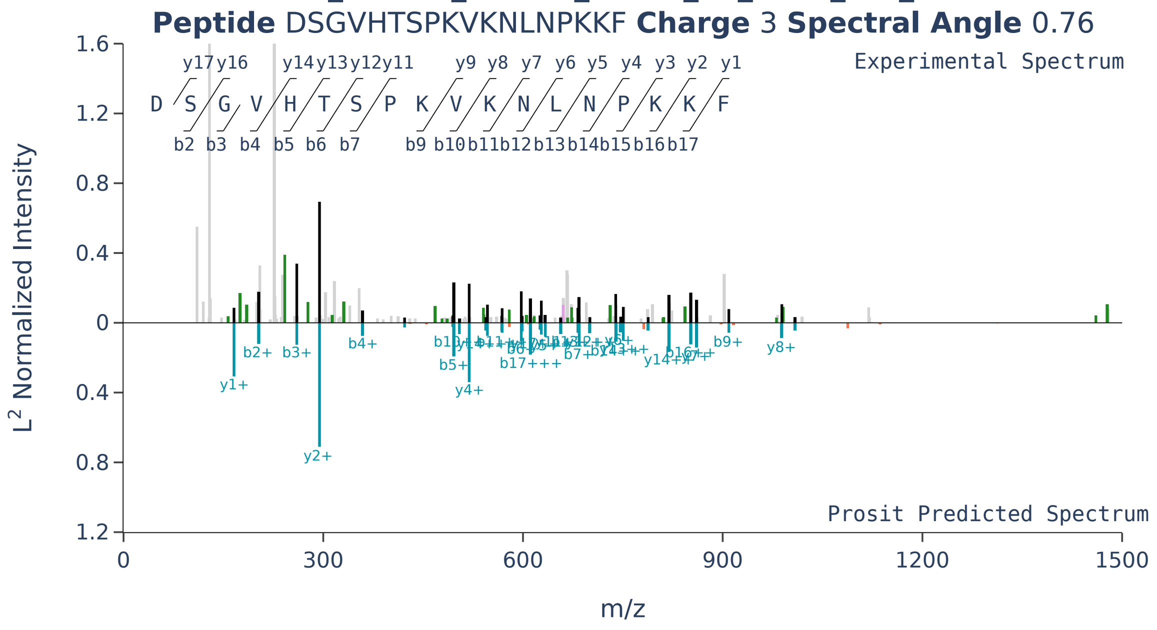

Source WSoh_101121_151121_HFGoe_G1_IL37b_24h_R1 **Scan** 12333
Peptide ADKDKGQSHPSLQL **Charge** 3 **Spectral Angle** 0.85

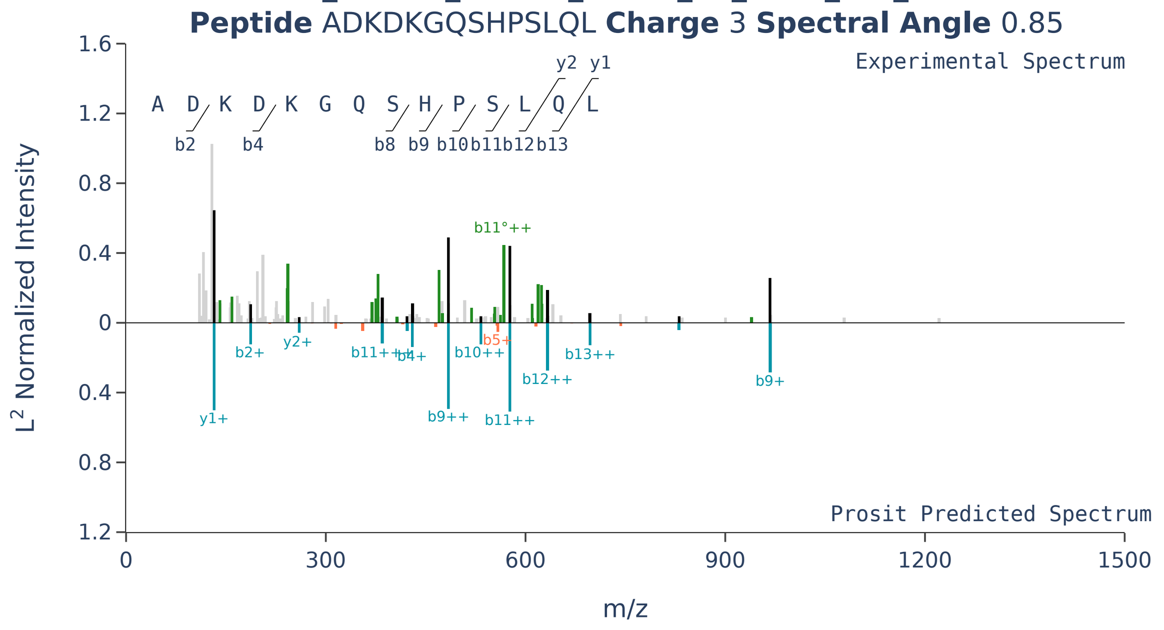

Source WSoh_101121_151121_HFGoe_G1_IL37b_4h_R2 **Scan** 9127
Peptide MVHTSPKVKNLNPKKF **Charge** 4 **Spectral Angle** 0.81

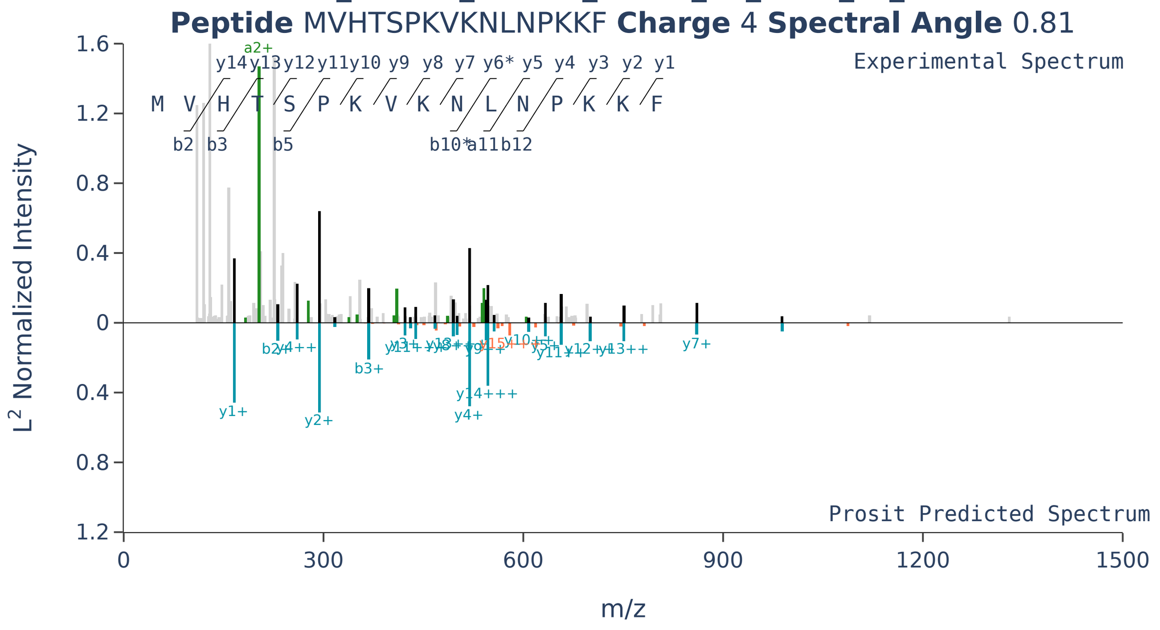

Source WSoh_101121_151121_HFGoe_G2_IL37b_24h_R1 **Scan** 16207
Peptide GCNEPVGVTDKFEN **Charge** 2 **Spectral Angle** 0.89

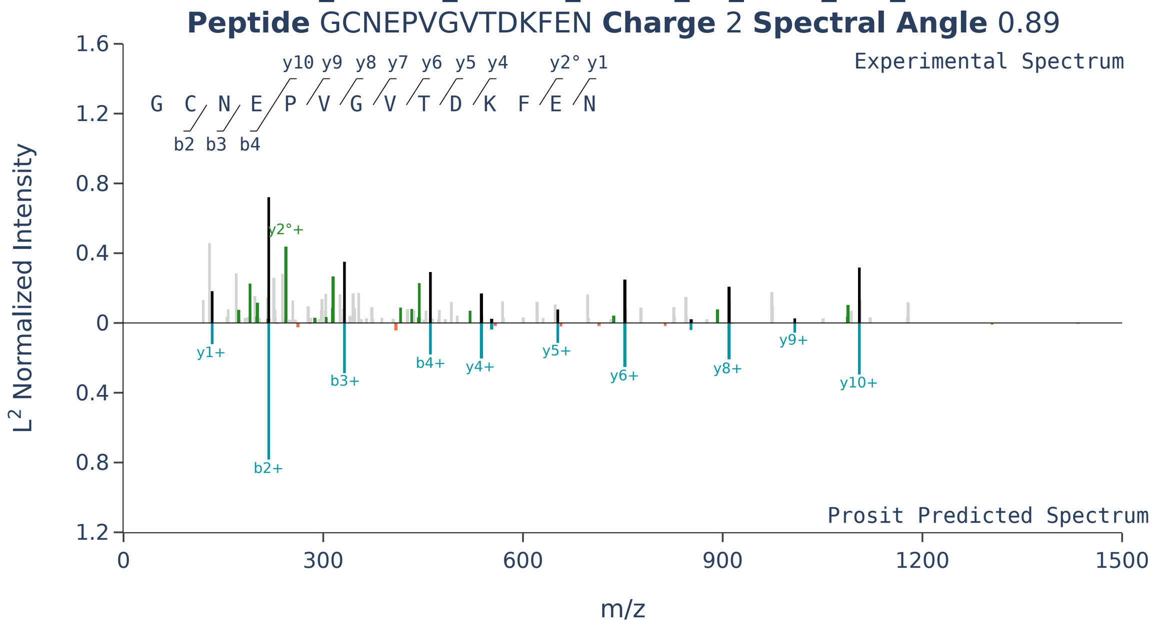

Source WSoh_101121_151121_HFGoe_G1_IL37b_4h_R1 **Scan** 9347
Peptide SSLSSAVHTS **Charge** 2 **Spectral Angle** 0.84

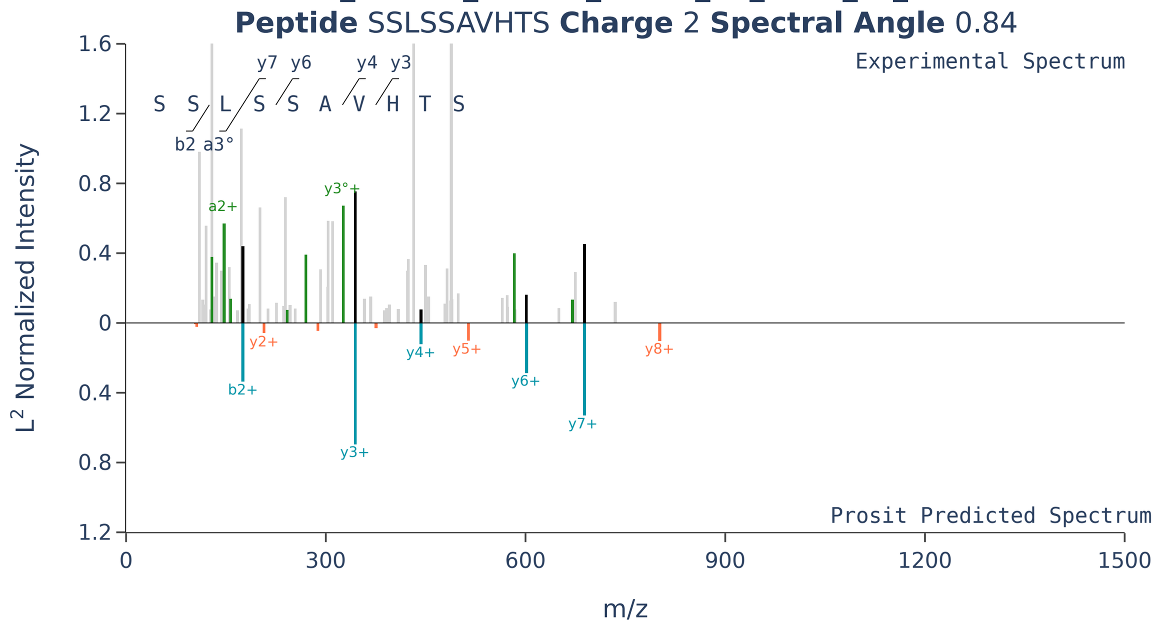

Source WSoh_101121_151121_HFGoe_G2_IL37b_24h_R2 **Scan** 7275
Peptide SSAVHTSPKVKNL **Charge** 3 **Spectral Angle** 0.82

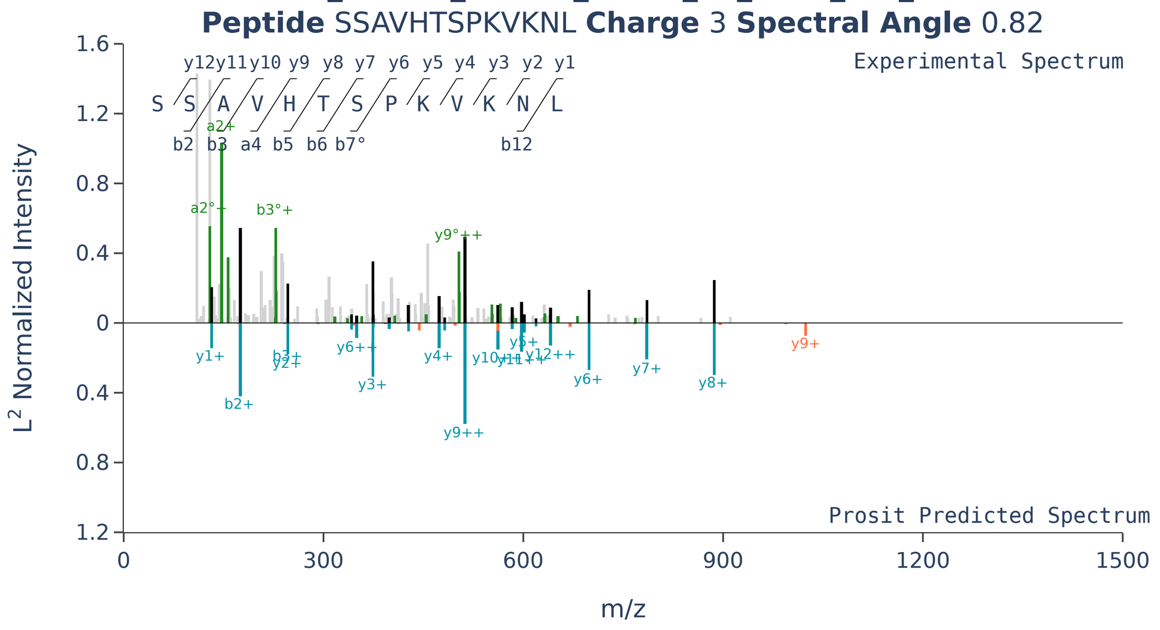

Source WSoh_101121_151121_HFGoe_G2_IL37b_24h_R2 **Scan** 17284
Peptide ASSKGEFCL **Charge** 2 **Spectral Angle** 0.83

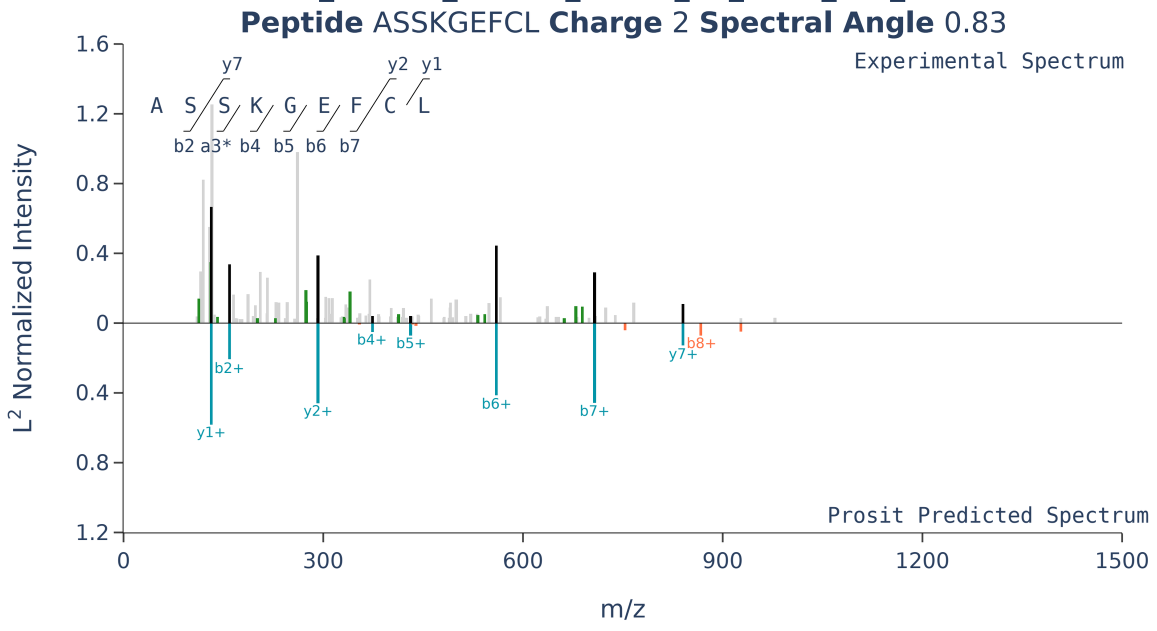

Source WSoh_101121_151121_HFGoe_G1_IL37b_24h_R1 **Scan** 16864
Peptide TNLIAVPDKN **Charge** 2 **Spectral Angle** 0.88

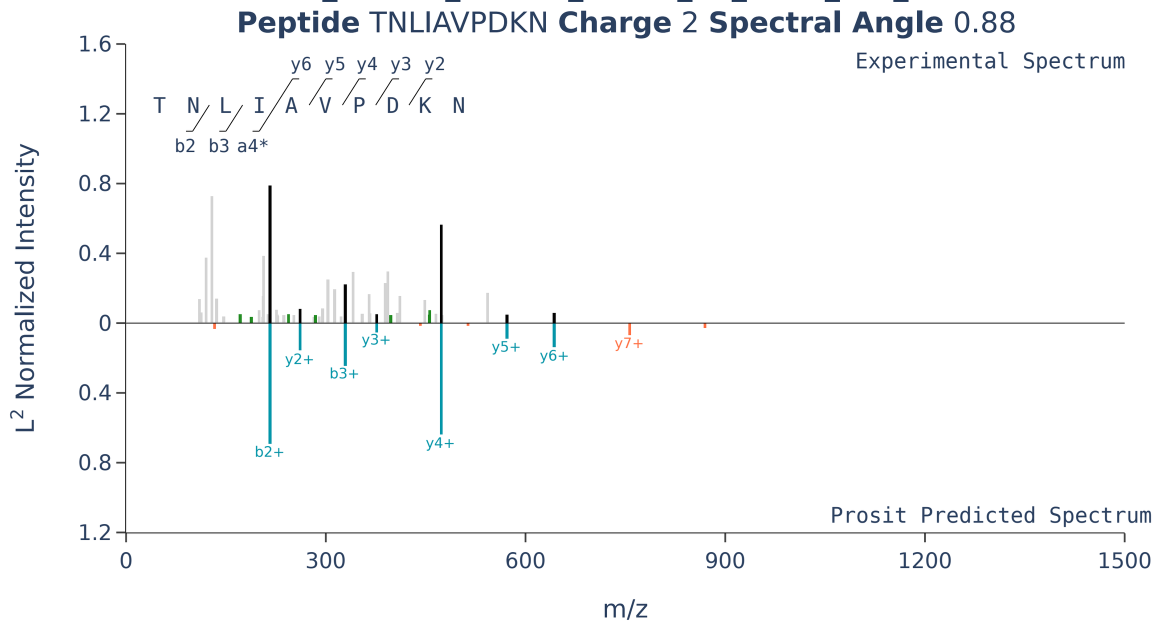

Source WSoh_101121_151121_HFGoe_G1_IL37b_24h_R2 **Scan** 17315
Peptide ANCNEPVGVTDKF **Charge** 2 **Spectral Angle** 0.84

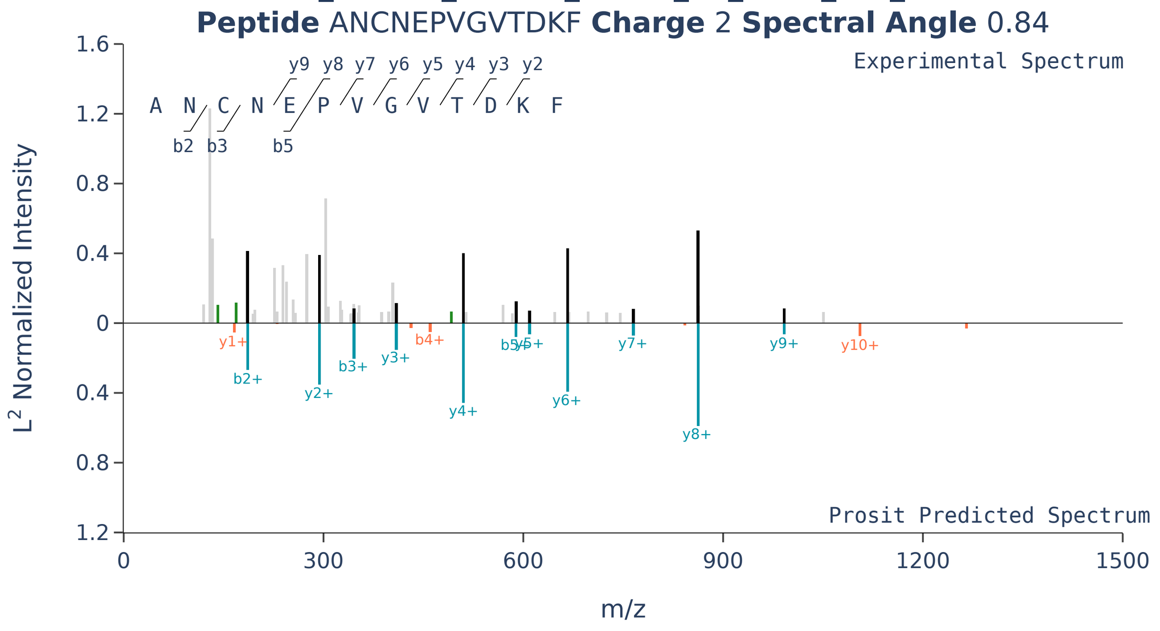

Source WSoh_101121_151121_HFGoe_G1_IL37b_24h_R1 **Scan** 6606
Peptide ESVHTSPKV **Charge** 2 **Spectral Angle** 0.81

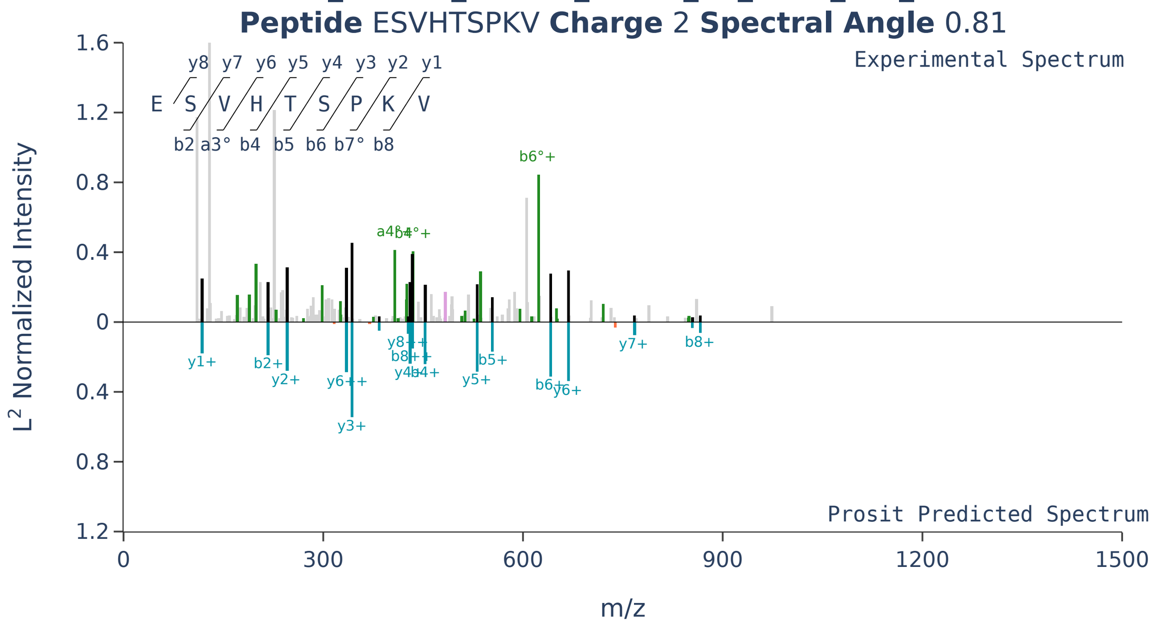

Source WSoh_101121_151121_HFGoe_G2_IL37b_24h_R1 **Scan** 10937
Peptide PSEVSDEMS **Charge** 1 **Spectral Angle** 0.84

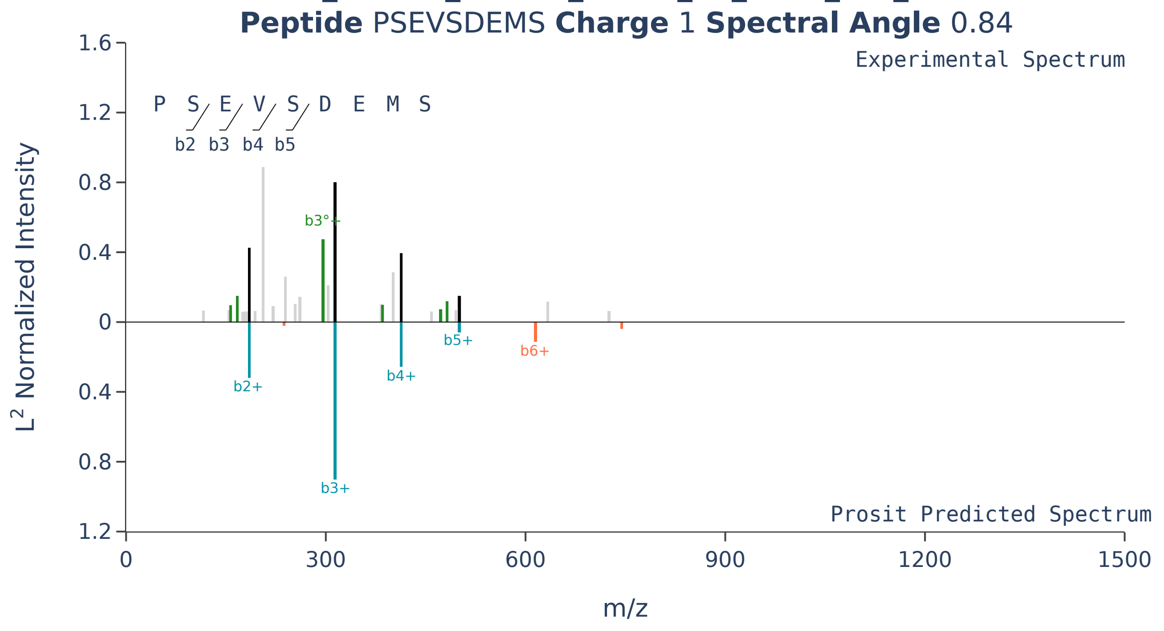

Source WSoh_101121_151121_HFGoe_G1_IL37b_24h_R1 **Scan** 13642
Peptide KEGSPIL **Charge** 1 **Spectral Angle** 0.79

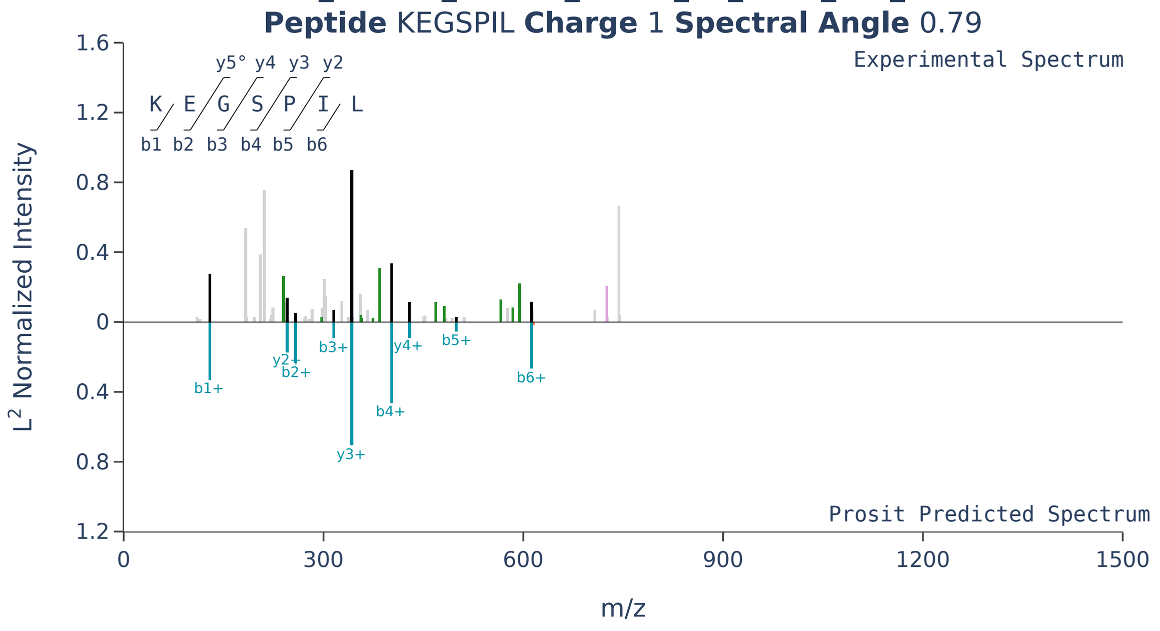

Source WSoh_101121_151121_HFGoe_G1_IL37b_24h_R1 **Scan** 21272
Peptide RPEIFFN **Charge** 2 **Spectral Angle** 0.76

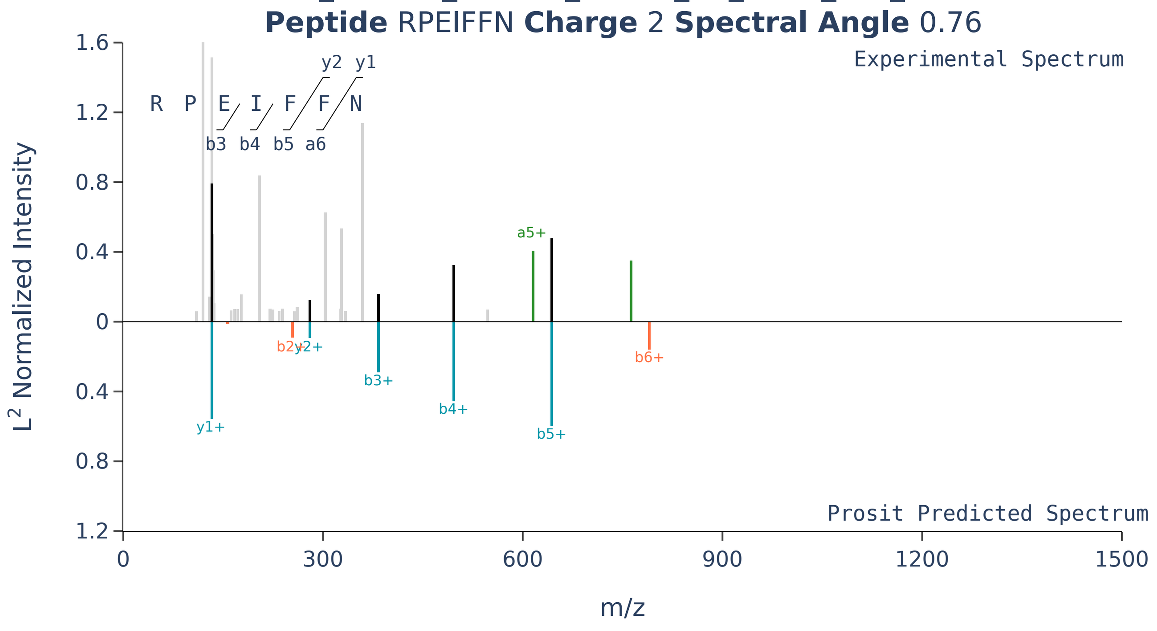

Source WSoh_101121_151121_HFGoe_G2_IL37b_2h_R1 **Scan** 17048
Peptide SAEKGSFCL **Charge** 2 **Spectral Angle** 0.82

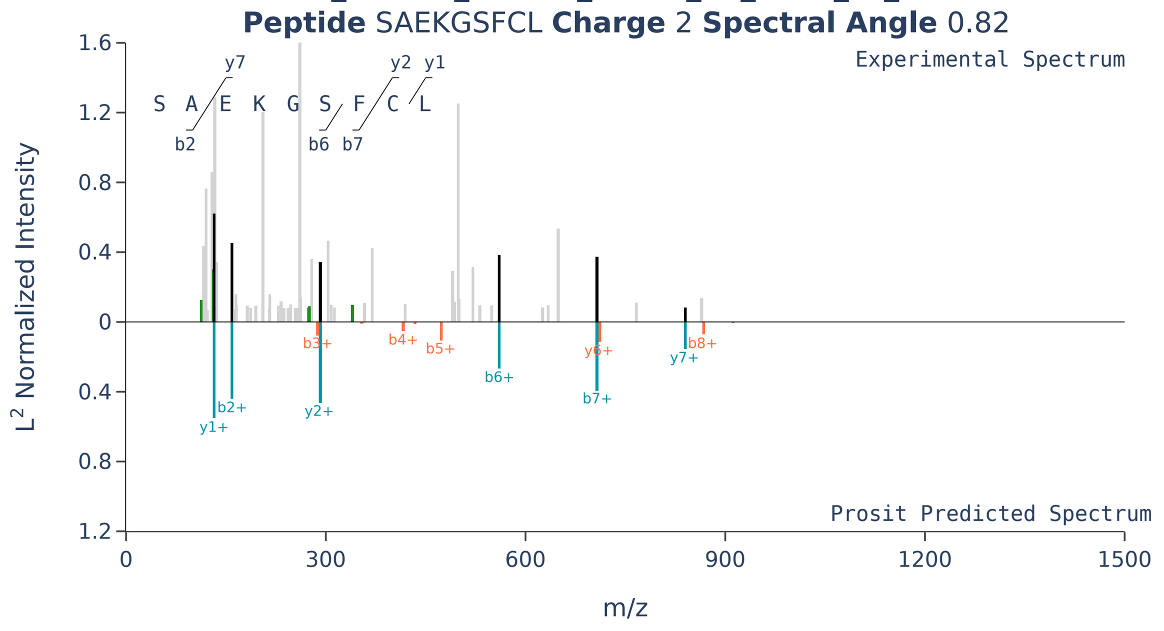

Source WSoh_101121_151121_HFGoe_G1_IL37b_4h_R1 **Scan** 20329
Peptide TDKFCLYC **Charge** 2 **Spectral Angle** 0.91

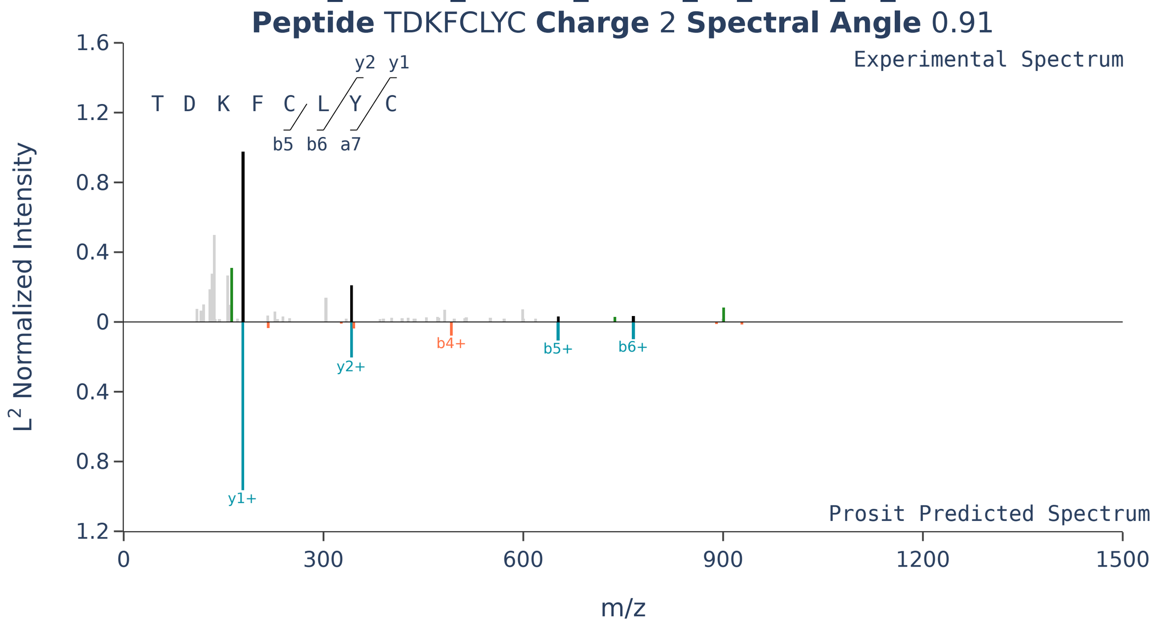

Source WSoh_101121_151121_HFGoe_G1_IL37b_24h_R2 **Scan** 16588
Peptide SENLIAVPDKN **Charge** 2 **Spectral Angle** 0.81

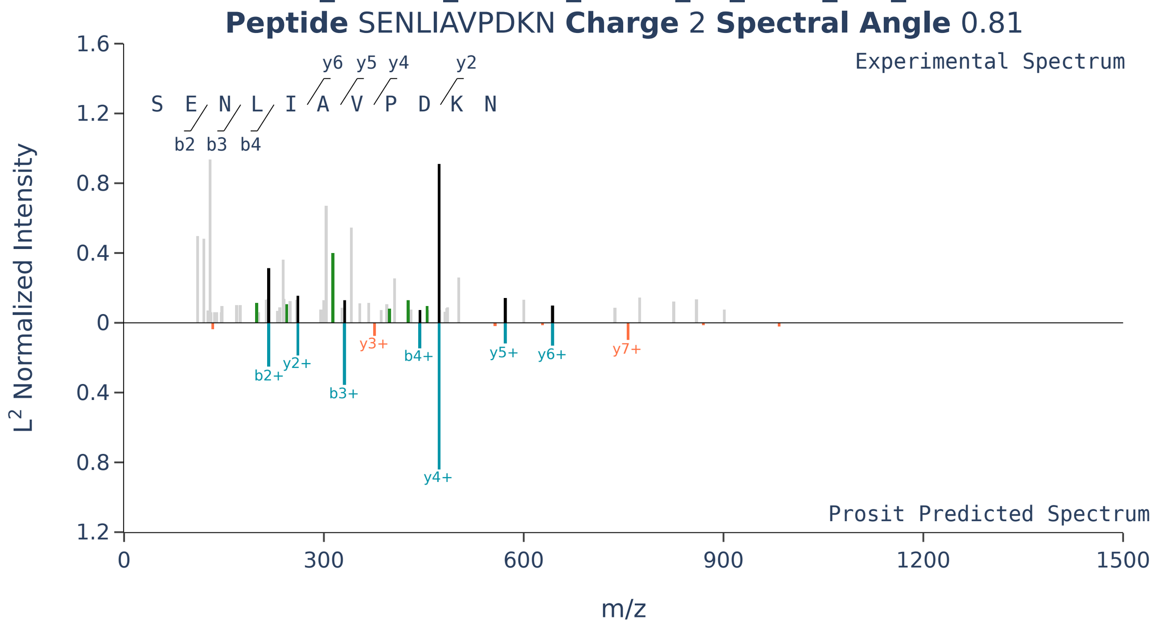

Source WSoh_101121_151121_HFGoe_G2_IL37b_24h_R1 **Scan** 8848
Peptide QVGSKGEF **Charge** 2 **Spectral Angle** 0.85

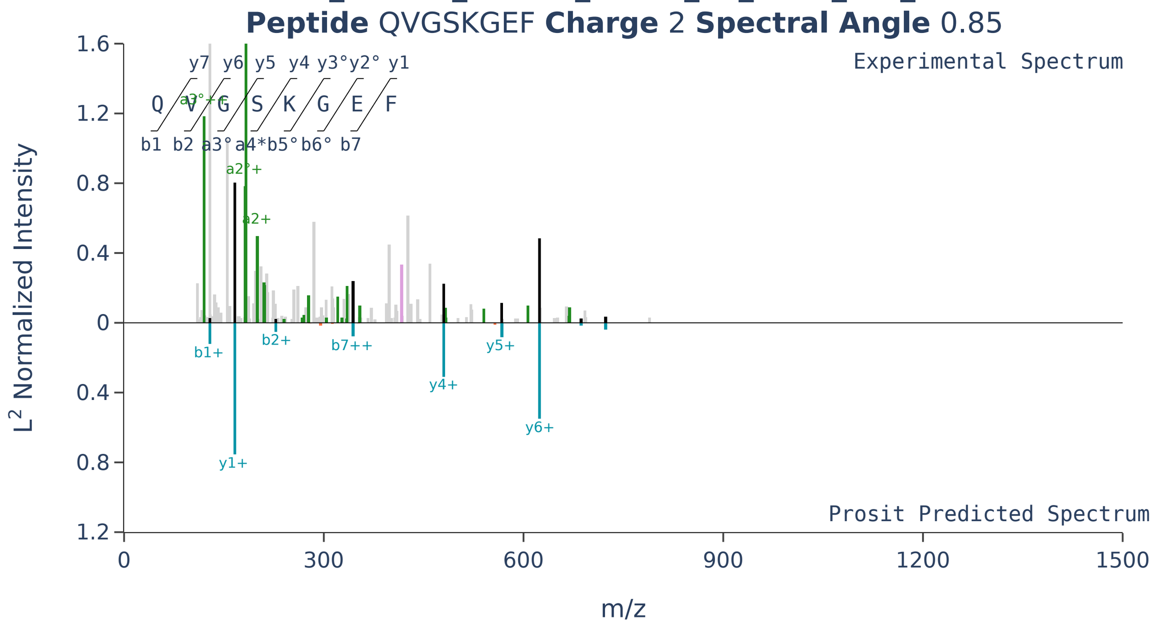

Source WSoh_101121_151121_HFGoe_G2_IL37b_24h_R1 **Scan** 17298
Peptide TNCNEPVGVTDKF **Charge** 2 **Spectral Angle** 0.85

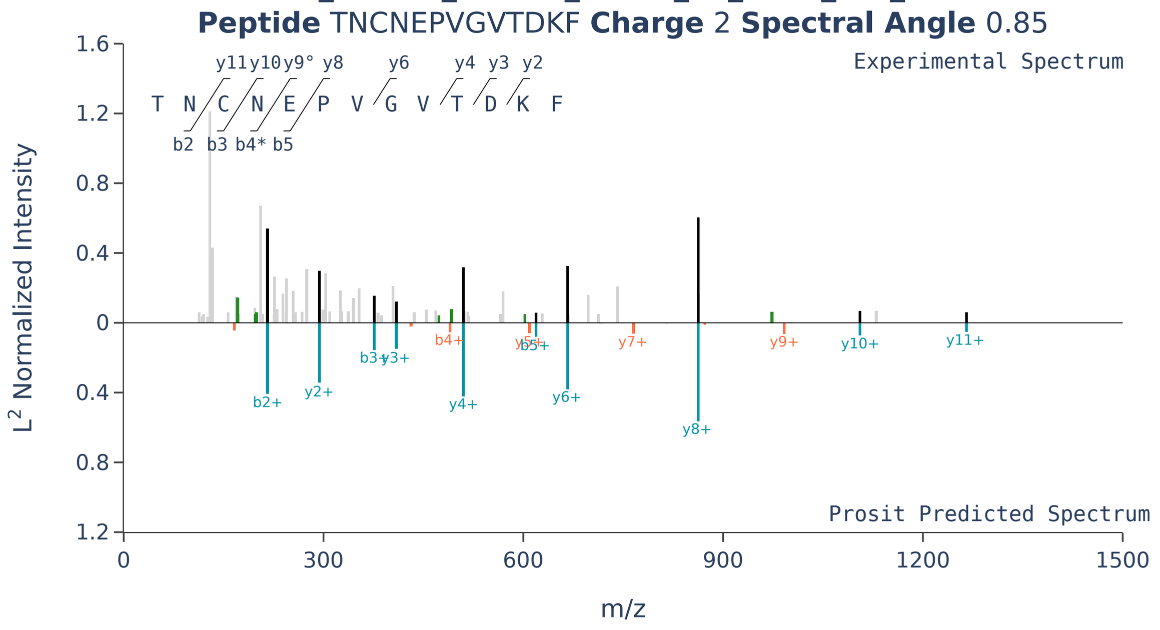

Source WSoh_101121_151121_HFGoe_G2_IL37b_4h_R2 **Scan** 8969
Peptide SNLNPKKF **Charge** 2 **Spectral Angle** 0.87

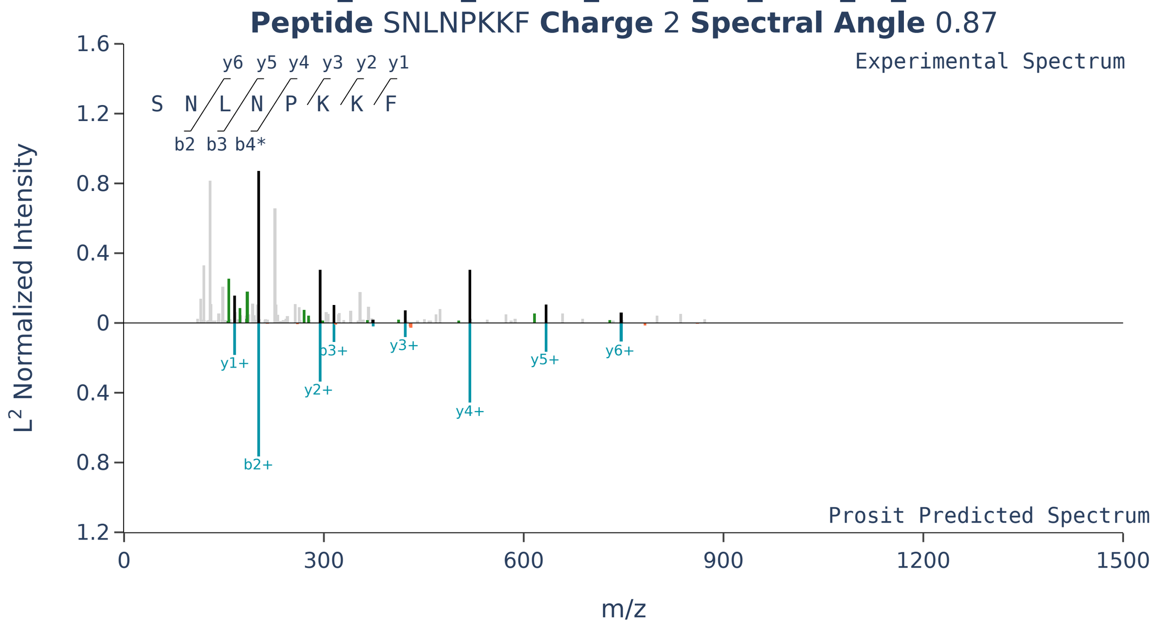

Source WSoh_101121_151121_HFGoe_G2_IL37b_24h_R2 **Scan** 10697
Peptide KKEKLMKLAV **Charge** 3 **Spectral Angle** 0.89

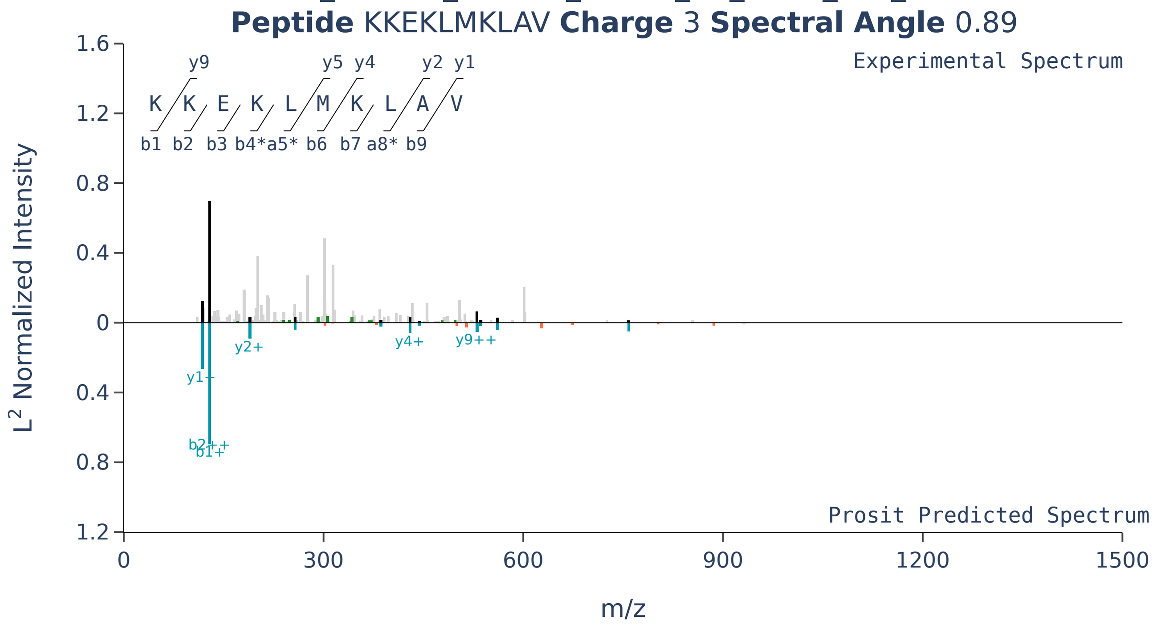

Source WSoh_101121_151121_HFGoe_G1_IL37b_24h_R1 **Scan** 14487
Peptide SAVPDKNYI **Charge** 2 **Spectral Angle** 0.8

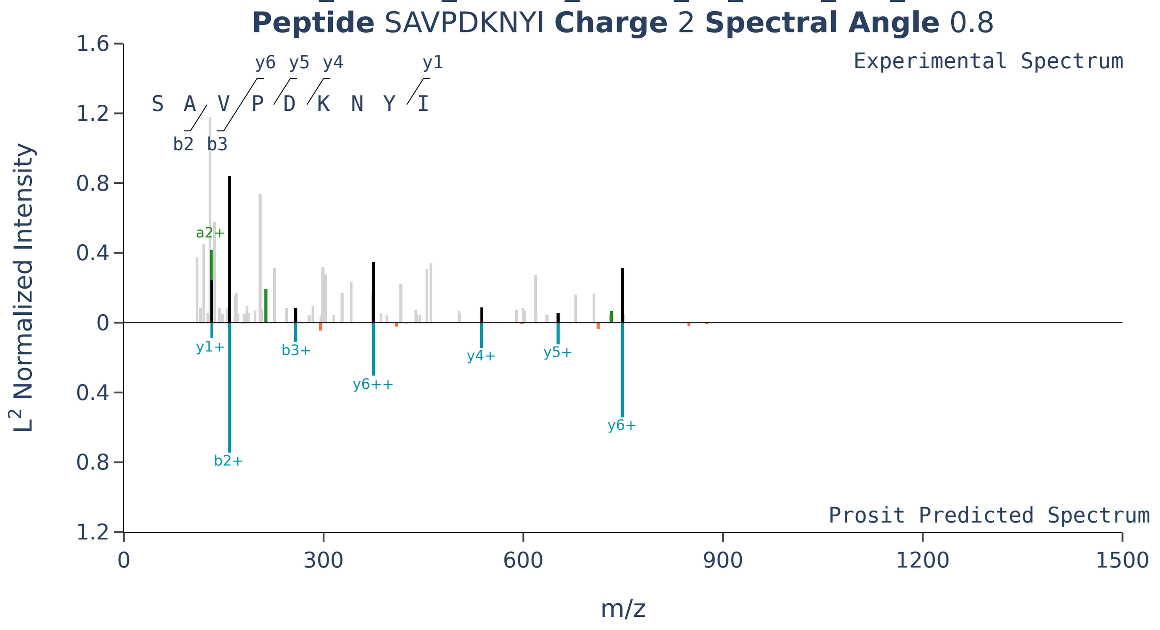

Source WSoh_101121_151121_HFGoe_G2_IL37b_2h_R1 **Scan** 6837
Peptide SVHTSPKVKNL **Charge** 2 **Spectral Angle** 0.84

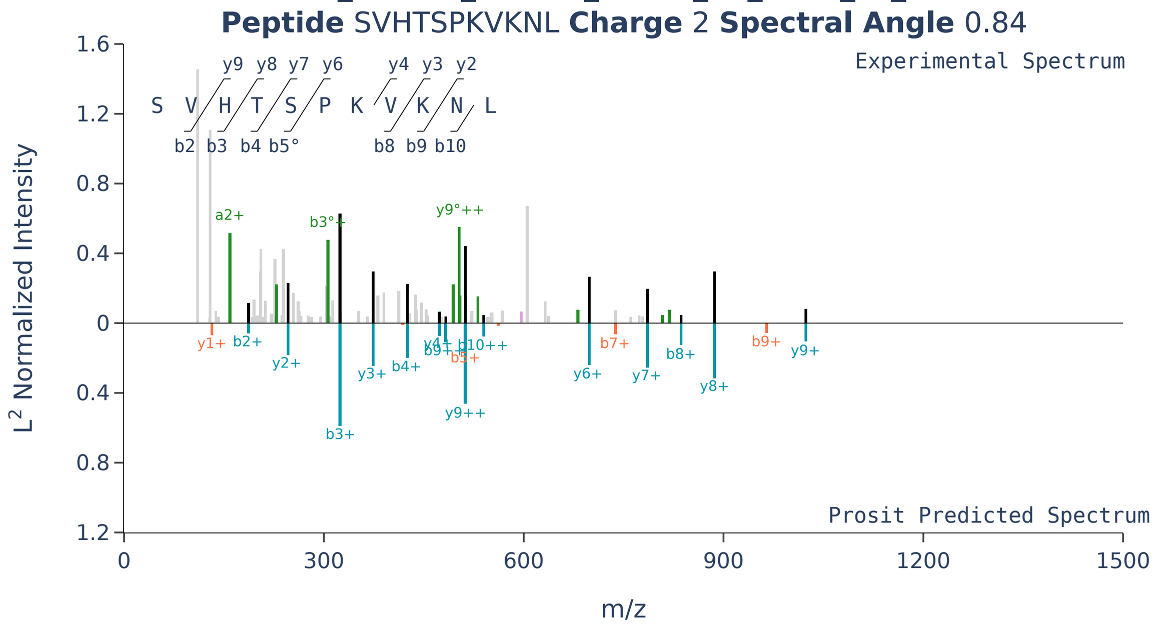

Source WSoh_101121_151121_HFGoe_G1_IL37b_24h_R1 **Scan** 12205
Peptide ASSLSKGEF **Charge** 2 **Spectral Angle** 0.83

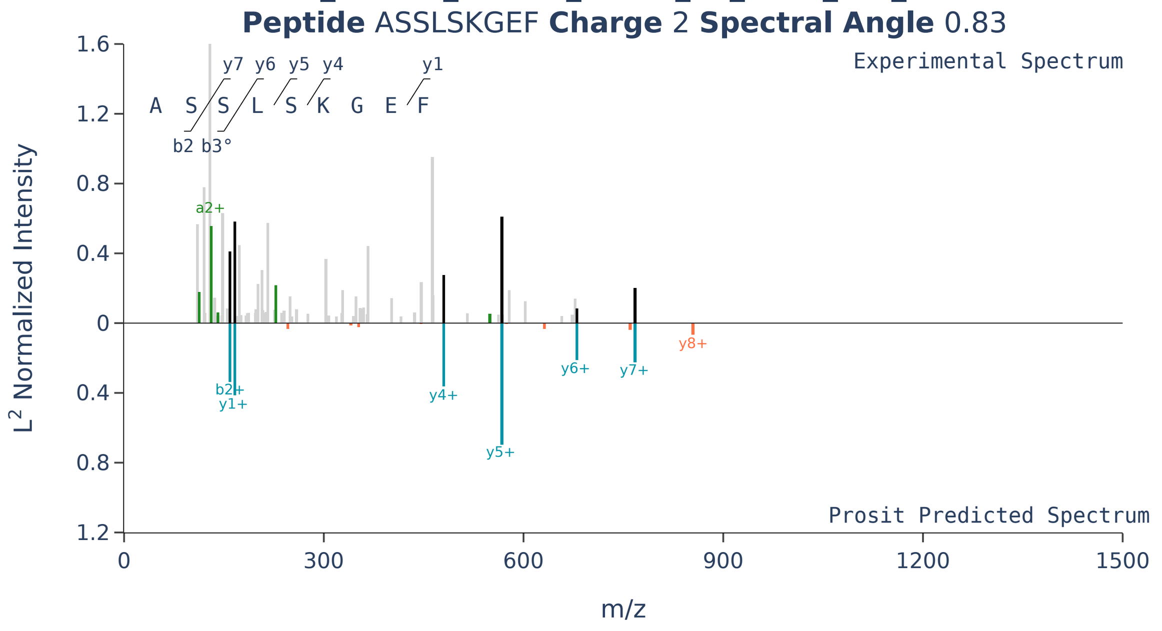

Source WSoh_101121_151121_HFGoe_G1_IL37b_24h_R1 **Scan** 6752
Peptide DSGDKDKGQSHPSL **Charge** 2 **Spectral Angle** 0.86

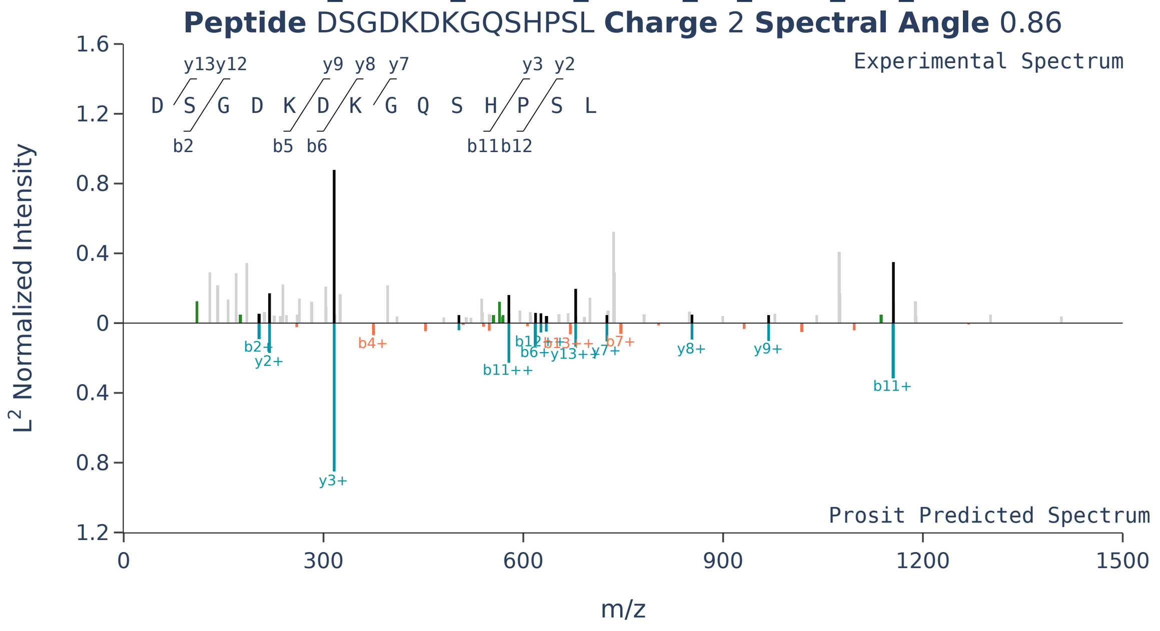

Source WSoh_101121_151121_HFGoe_G1_IL37b_24h_R1 **Scan** 7950
Peptide DSGKNLNPKKF **Charge** 2 **Spectral Angle** 0.79

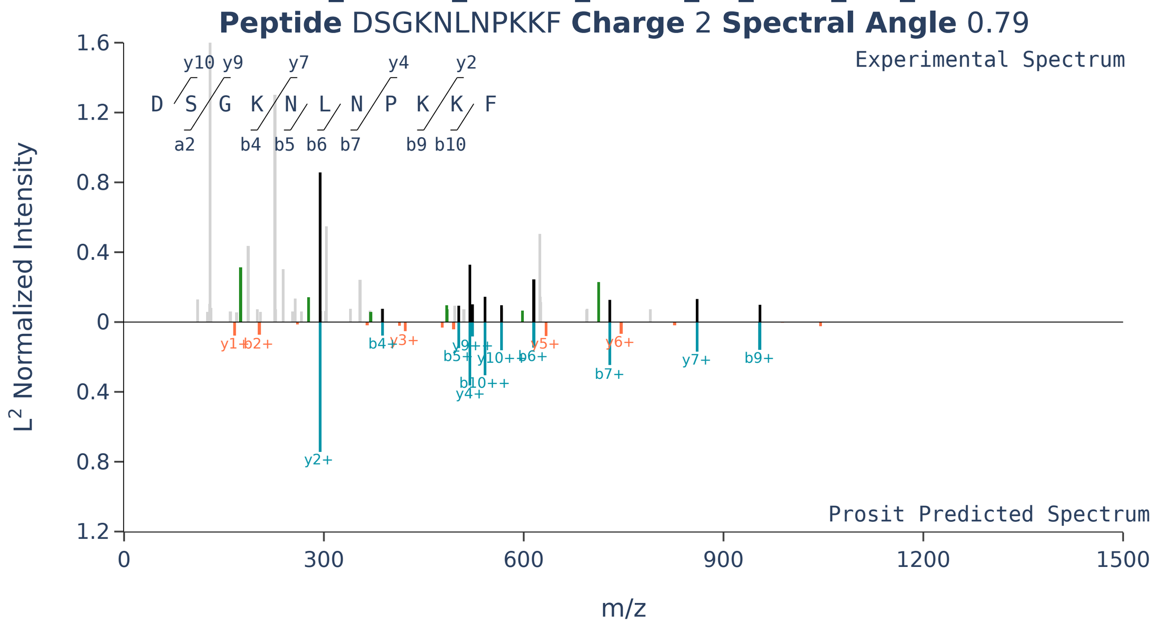

Source WSoh_101121_151121_HFGoe_G2_IL37b_2h_R2 **Scan** 15702
Peptide NLIAVPKN **Charge** 2 **Spectral Angle** 0.93

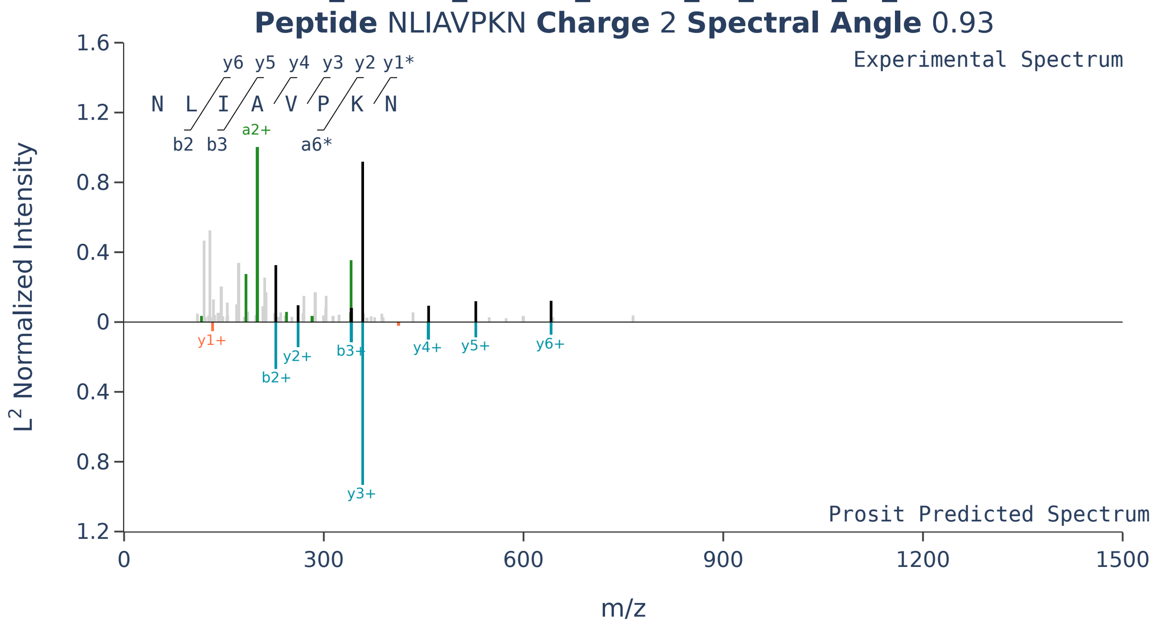

Source WSoh_101121_151121_HFGoe_G2_IL37b_1h_R1 **Scan** 8306
Peptide VGAEMSPSEVSD **Charge** 2 **Spectral Angle** 0.9

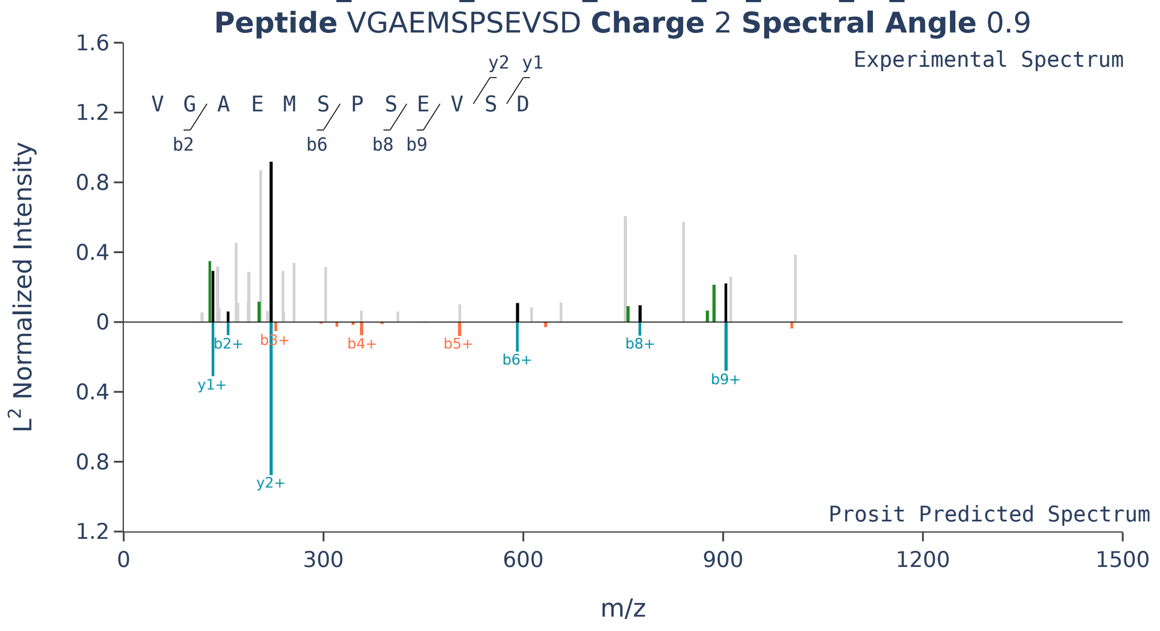

Source WSoh_101121_151121_HFGoe_G2_IL37b_24h_R2 **Scan** 8058
Peptide DVHTSPKVKNL **Charge** 3 **Spectral Angle** 0.82

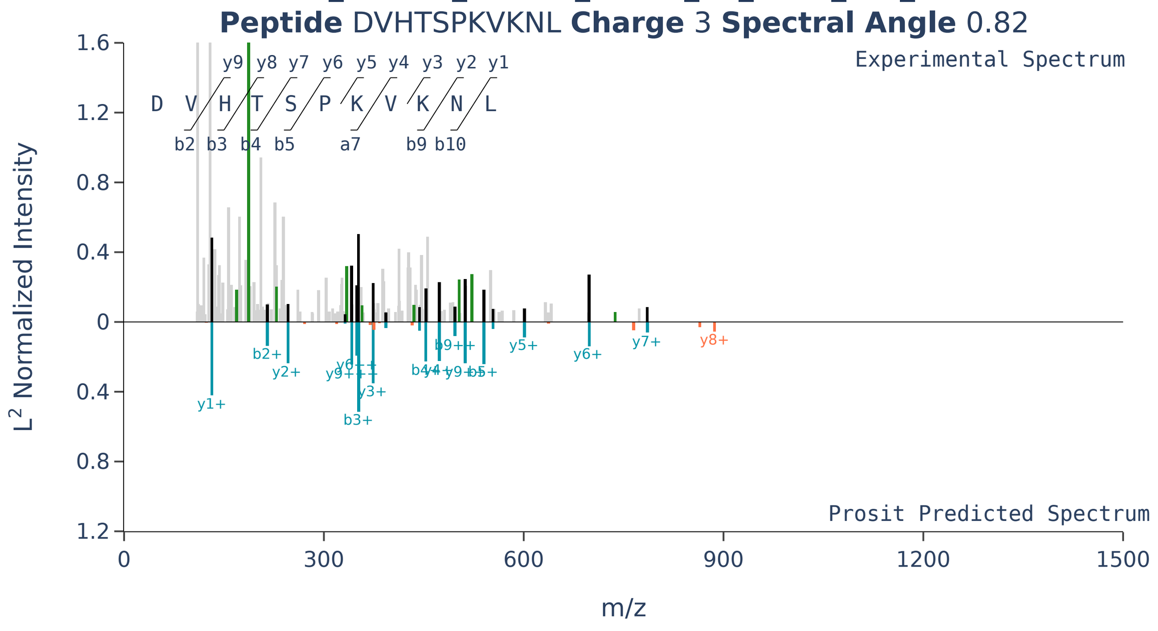

Source WSoh_101121_151121_HFGoe_G2_IL37b_24h_R1 **Scan** 6883
Peptide SSVHTSPKVKNL **Charge** 3 **Spectral Angle** 0.78

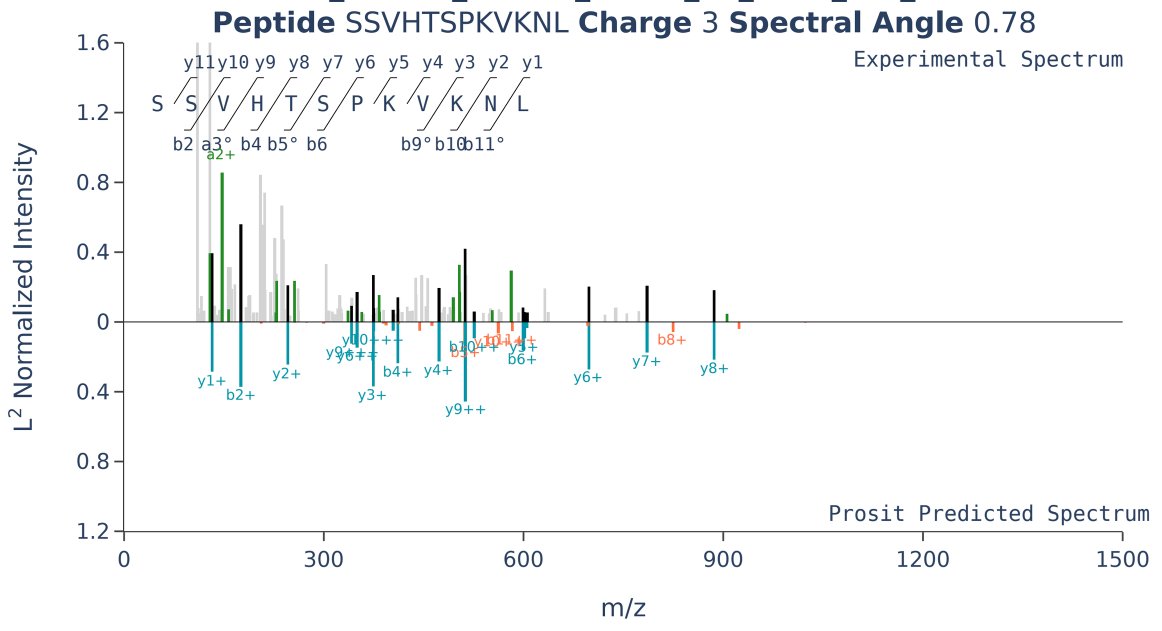

Source WSoh_101121_151121_HFGoe_G1_IL37b_24h_R1 **Scan** 9539
Peptide KLAIRPE **Charge** 2 **Spectral Angle** 0.87

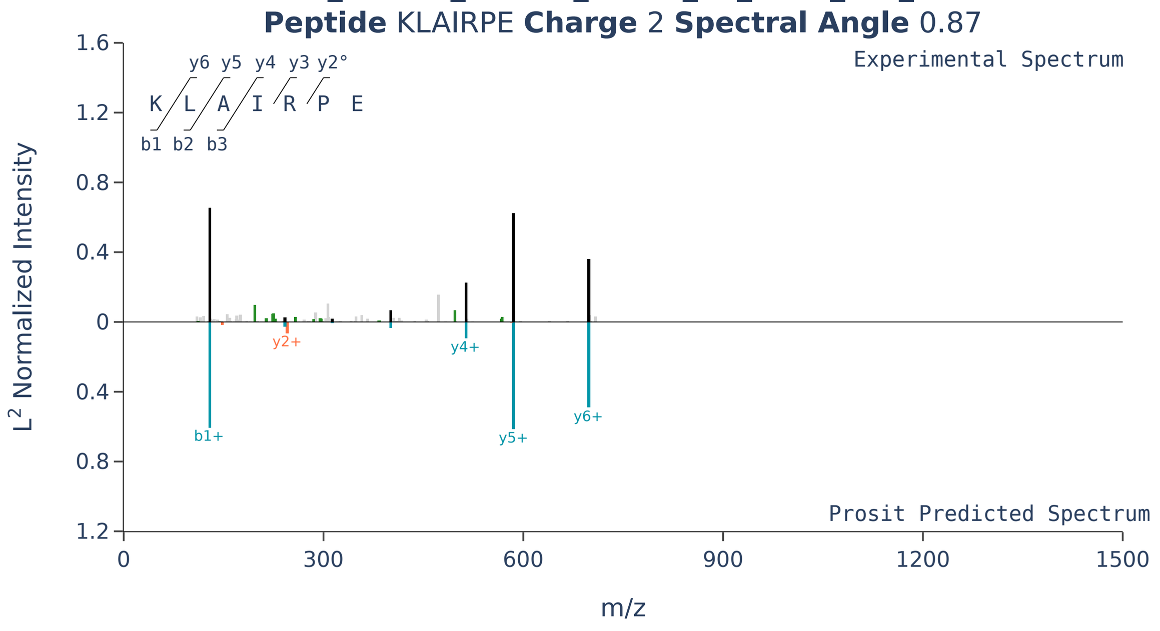

Source WSoh_101121_151121_HFGoe_G1_IL37b_1h_R1 **Scan** 15420
Peptide SSLHDQDHKVLVL **Charge** 3 **Spectral Angle** 0.86

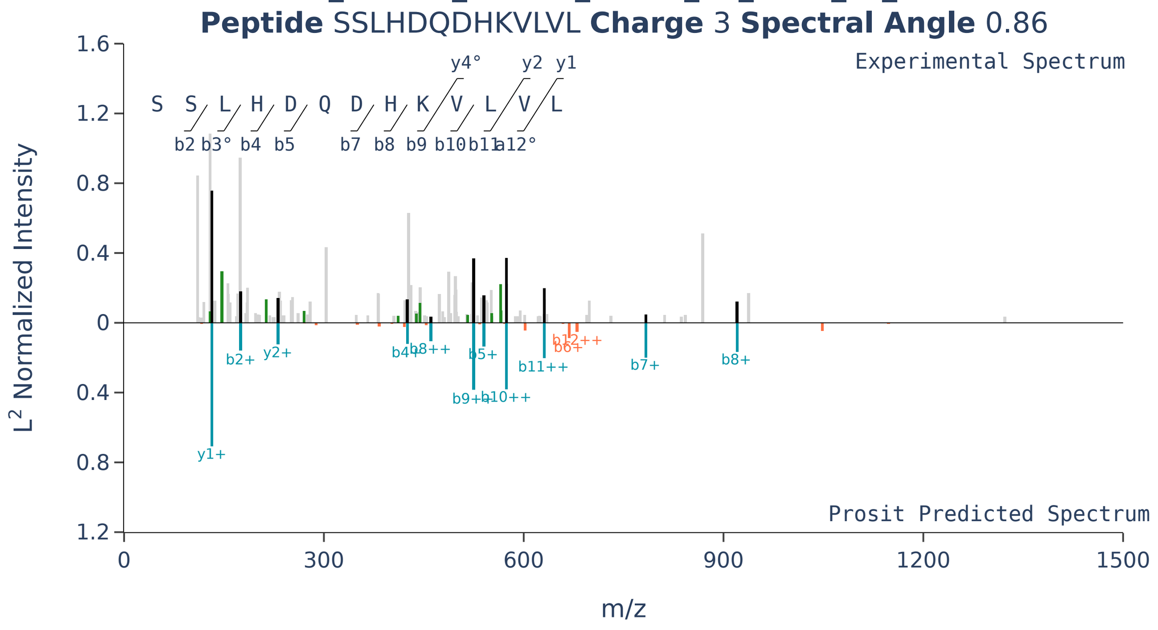

Source WSoh_101121_151121_HFGoe_G1_IL37b_24h_R1 **Scan** 17164
Peptide SNCNEPVGVTDKF **Charge** 2 **Spectral Angle** 0.85

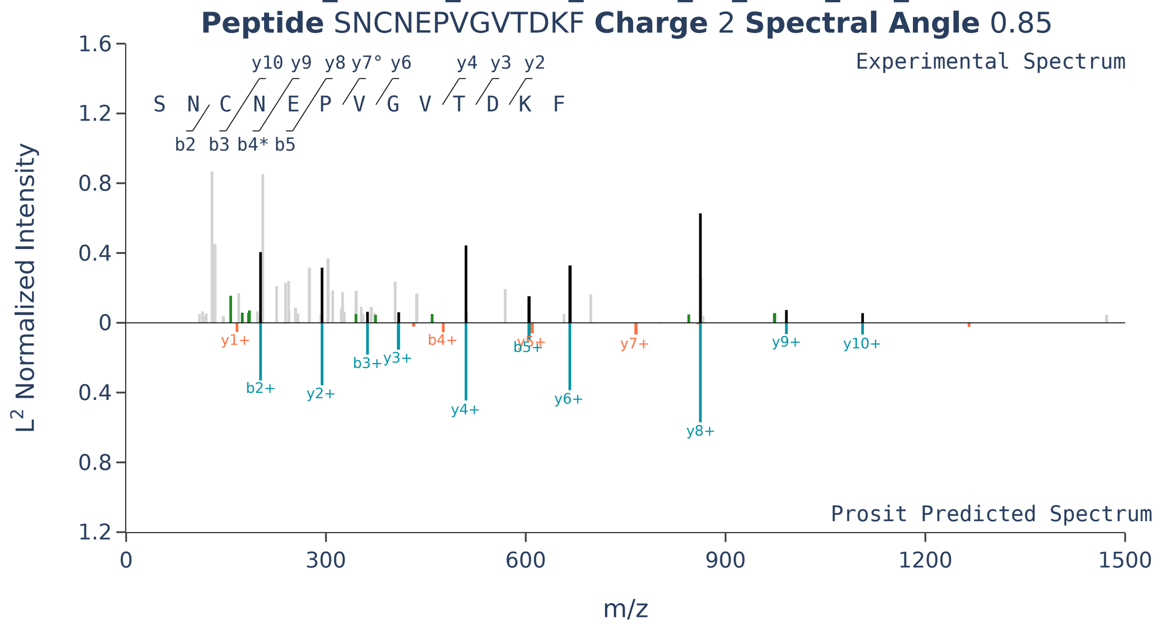

Source WSoh_101121_151121_HFGoe_G2_IL37b_24h_R2 **Scan** 6952
Peptide ASSLVHTSPKVK **Charge** 3 **Spectral Angle** 0.78

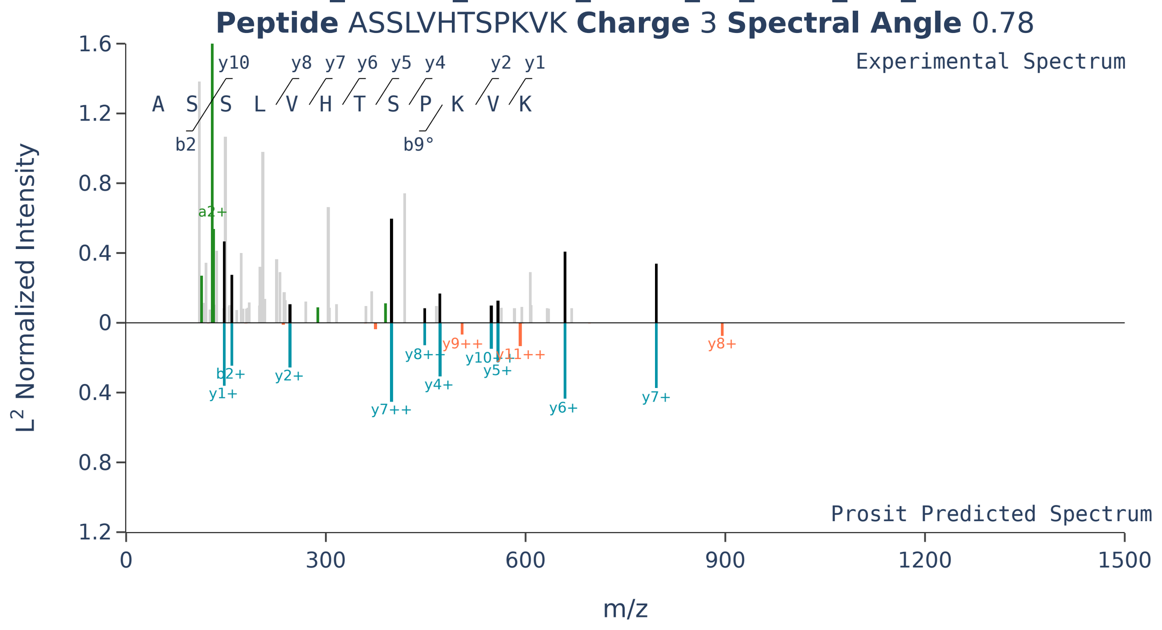

Source WSoh_101121_151121_HFGoe_G1_IL37b_24h_R1 **Scan** 19838
Peptide SAKGEFCLY **Charge** 2 **Spectral Angle** 0.81

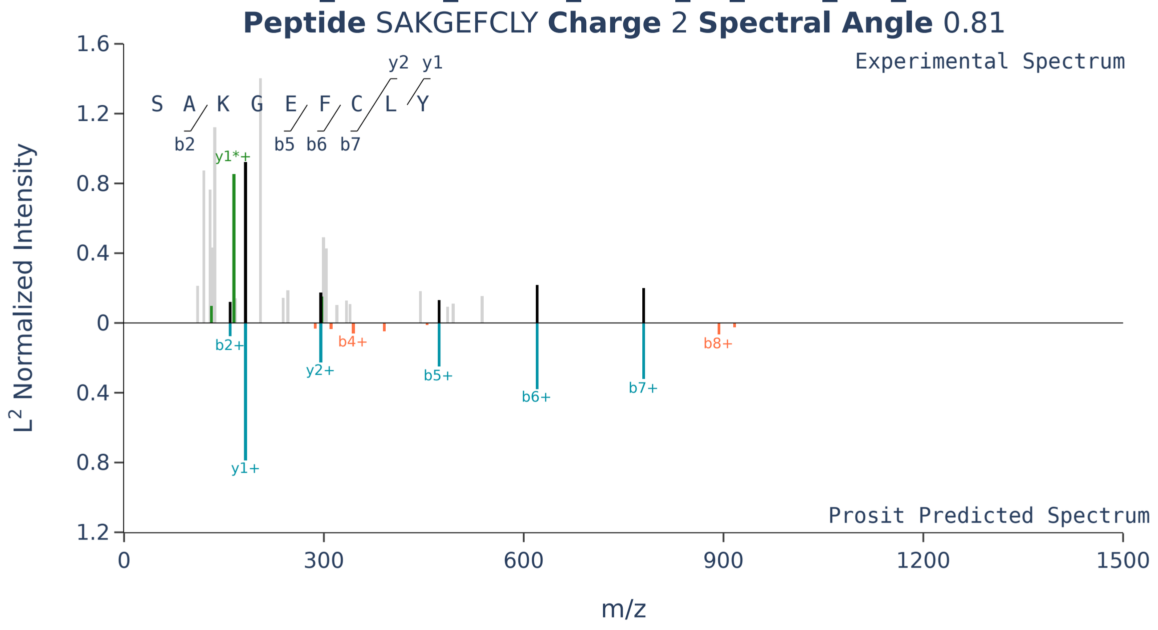

Source WSoh_101121_151121_HFGoe_G2_IL37b_24h_R2 **Scan** 20609
Peptide ASSEKGSPILL **Charge** 2 **Spectral Angle** 0.81

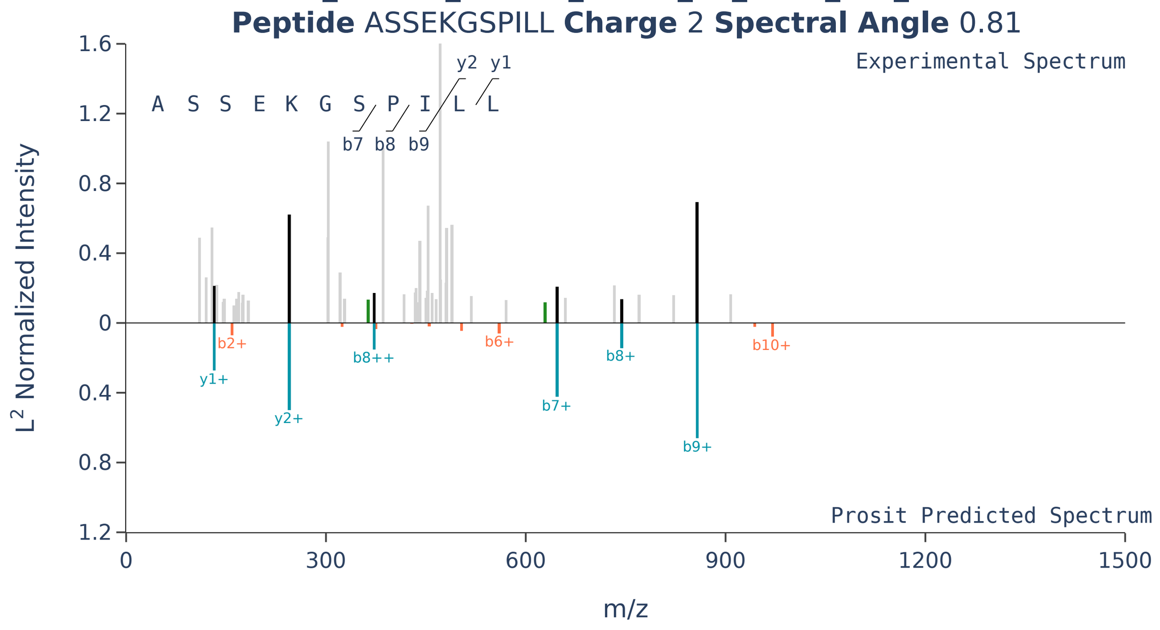

Source WSoh_101121_151121_HFGoe_G2_IL37b_24h_R1 **Scan** 8207
Peptide ESMSPSEVSD **Charge** 2 **Spectral Angle** 0.9

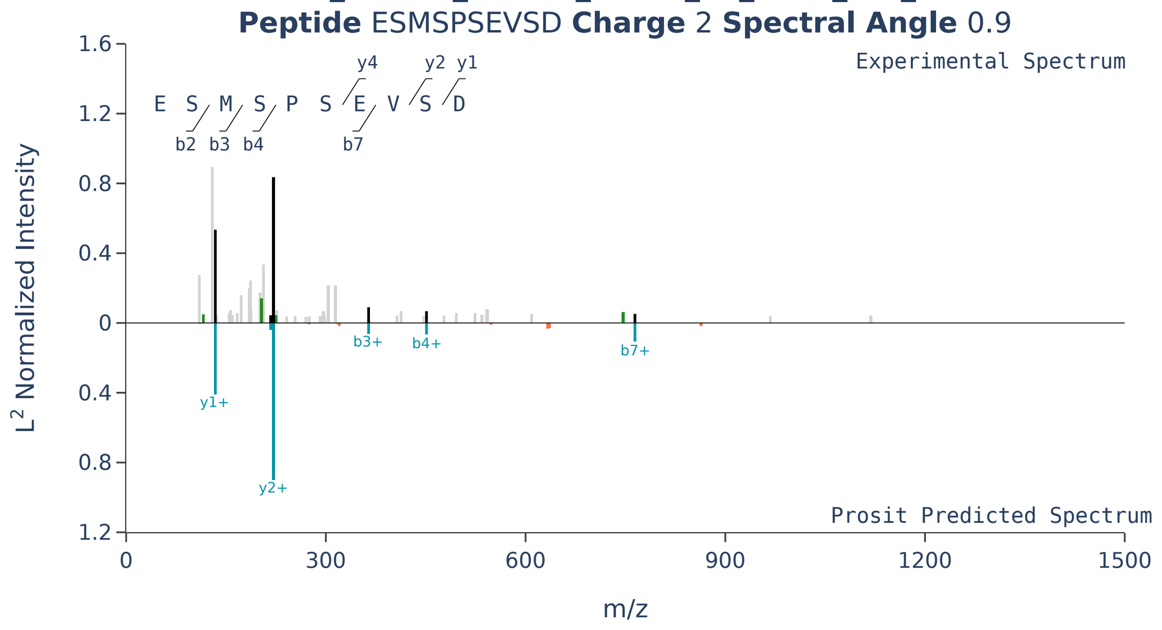

Source WSoh_101121_151121_HFGoe_G2_IL37b_24h_R2 **Scan** 7244
Peptide AAVHTSPKVKNL **Charge** 3 **Spectral Angle** 0.79

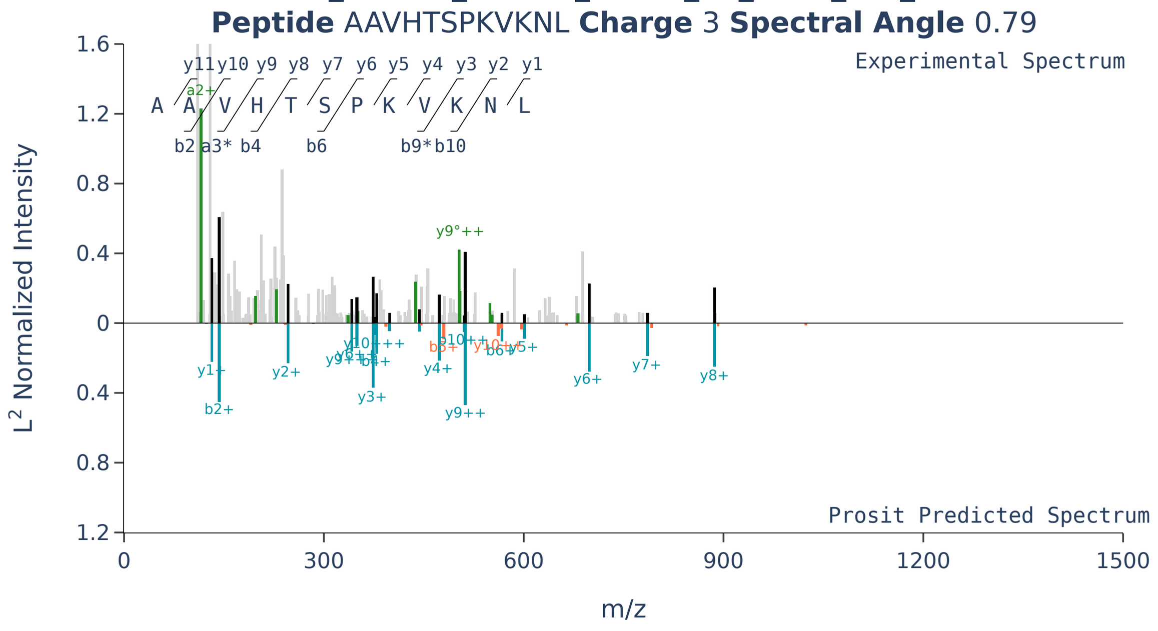

Source WSoh_101121_151121_HFGoe_G1_IL37b_24h_R1 **Scan** 17129
Peptide YCDKDKGQSHPSLNLI **Charge** 3 **Spectral Angle** 0.82

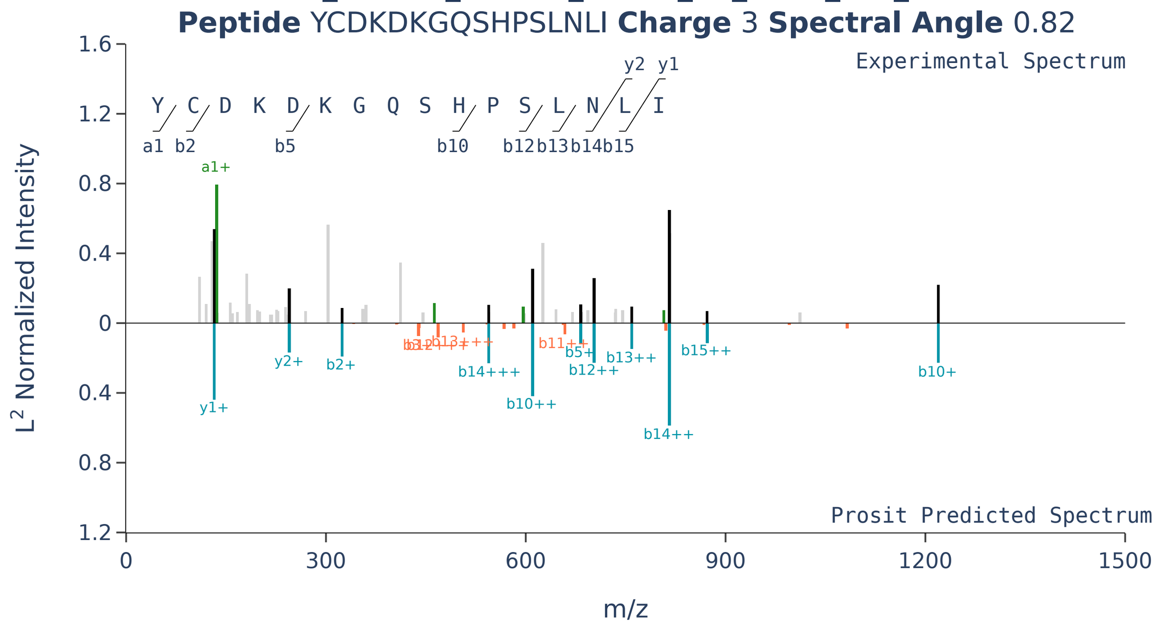

Source WSoh_101121_151121_HFGoe_G2_IL37b_24h_R2 **Scan** 11975
Peptide GCNEPVGVTDKFENRKHIE **Charge** 4 **Spectral Angle** 0.78

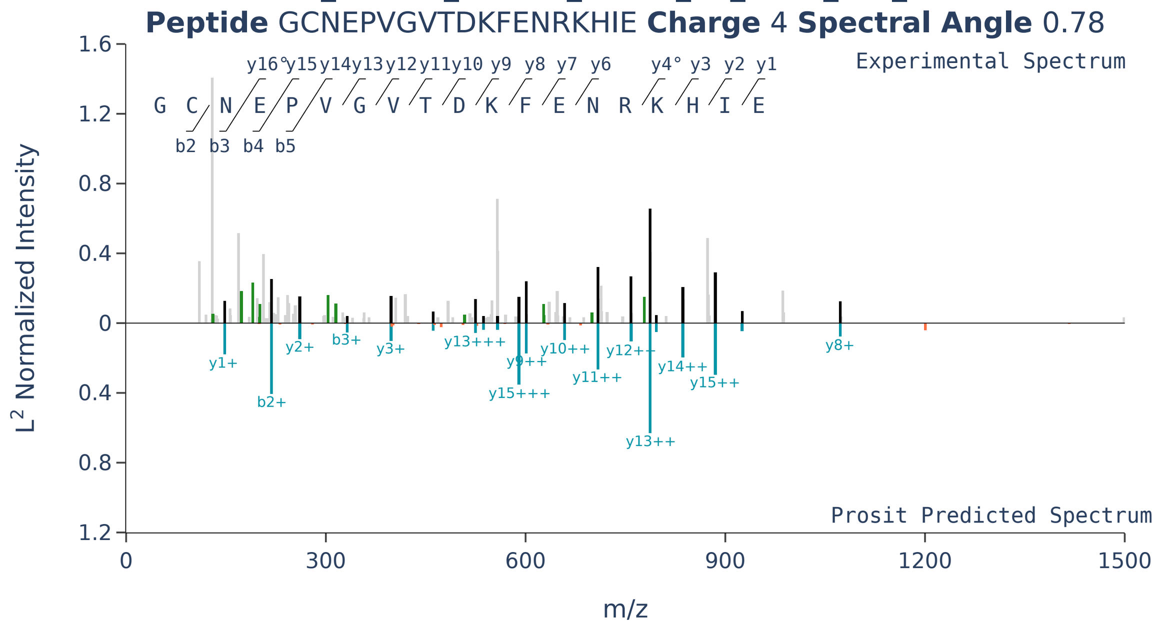

Source WSoh_101121_151121_HFGoe_G2_IL37b_24h_R2 **Scan** 7459
Peptide SSLHDQDHKVL **Charge** 2 **Spectral Angle** 0.85

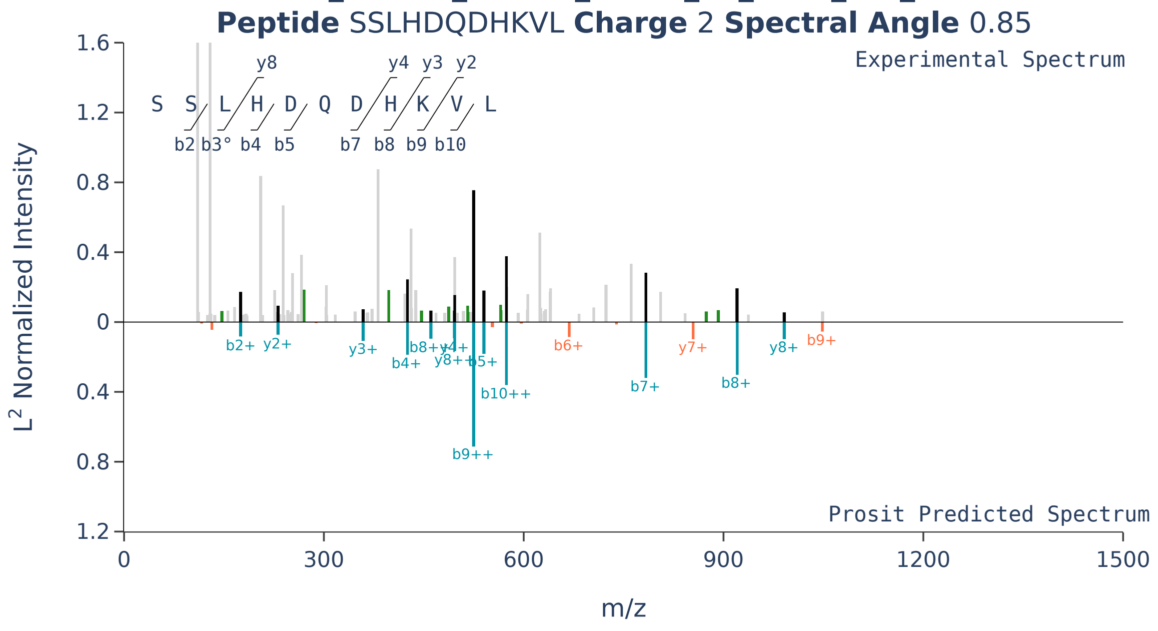

Source WSoh_101121_151121_HFGoe_G2_IL37b_24h_R2 **Scan** 9872
Peptide LESVHTSPKVKNL **Charge** 3 **Spectral Angle** 0.86

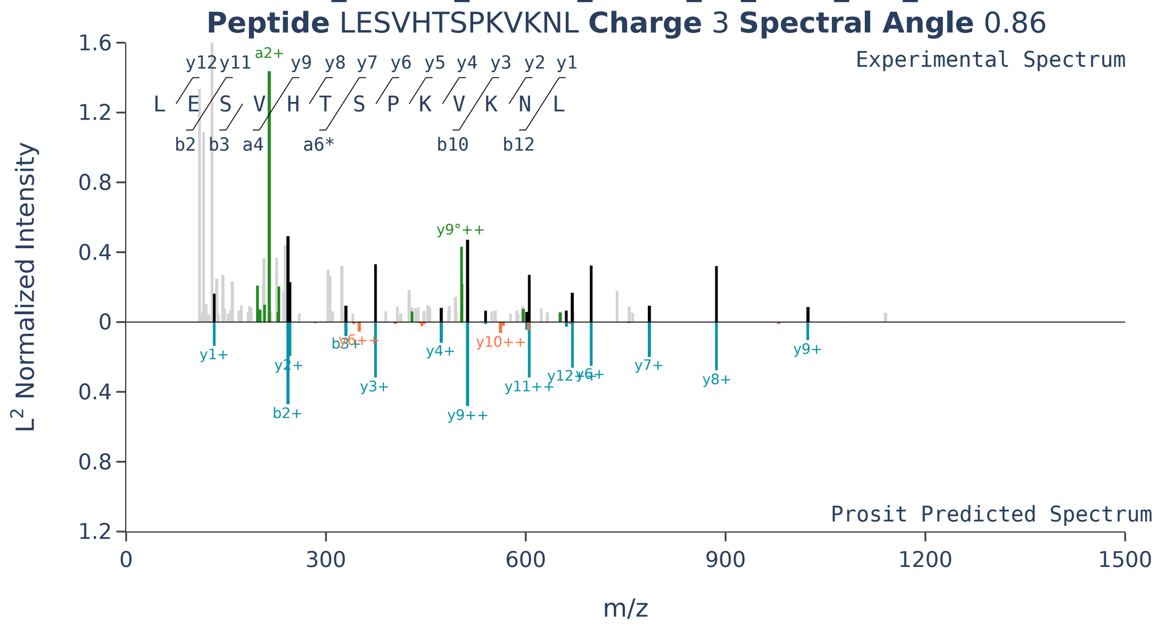

Source WSoh_101121_151121_HFGoe_G2_IL37b_24h_R1 **Scan** 15443
Peptide ASSLHDQDHKVLVL **Charge** 3 **Spectral Angle** 0.86

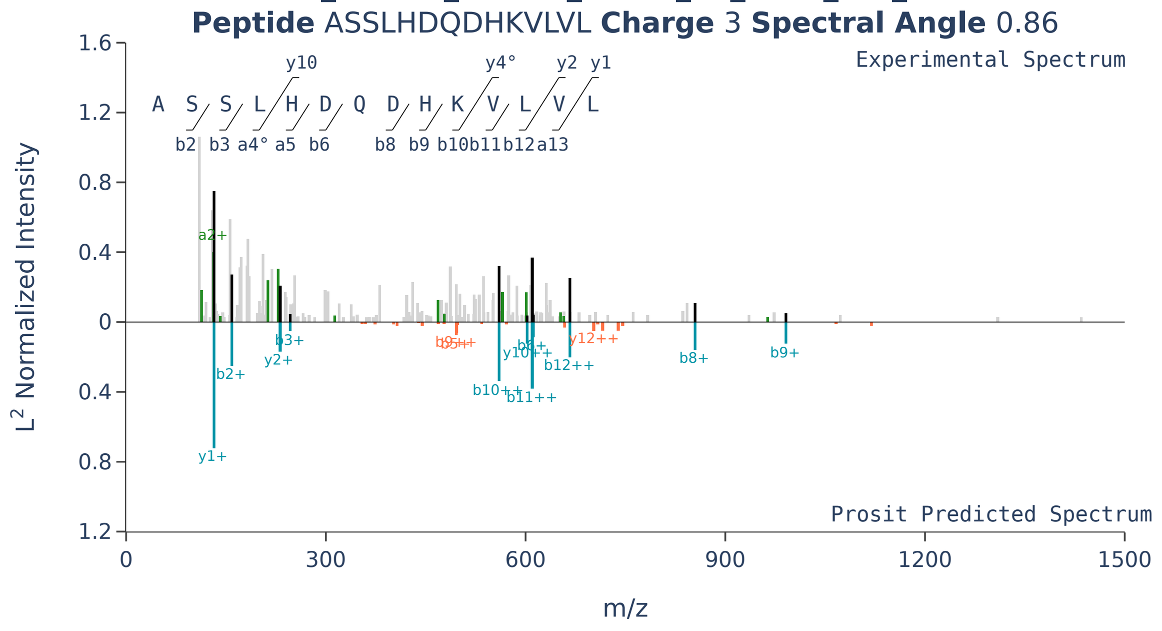

Source WSoh_101121_151121_HFGoe_G1_IL37b_24h_R2 **Scan** 9047
Peptide LESVHTSPKV **Charge** 3 **Spectral Angle** 0.8

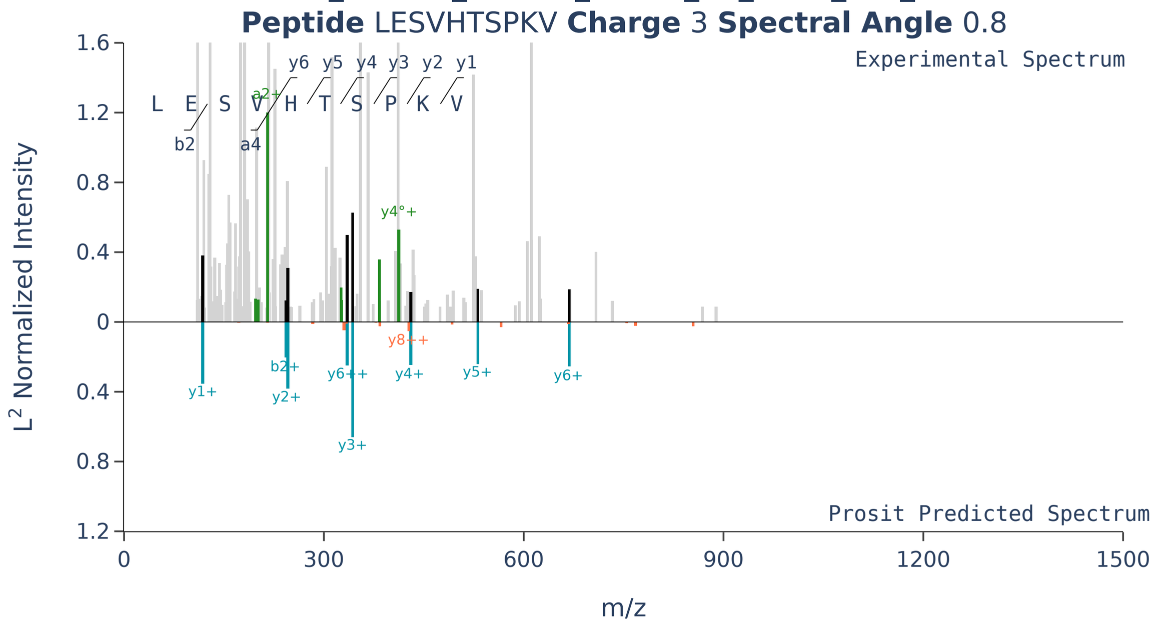

Source WSoh_101121_151121_HFGoe_G2_IL37b_24h_R1 **Scan** 7907
Peptide AVHTSPKVKNLNPKKF **Charge** 3 **Spectral Angle** 0.83

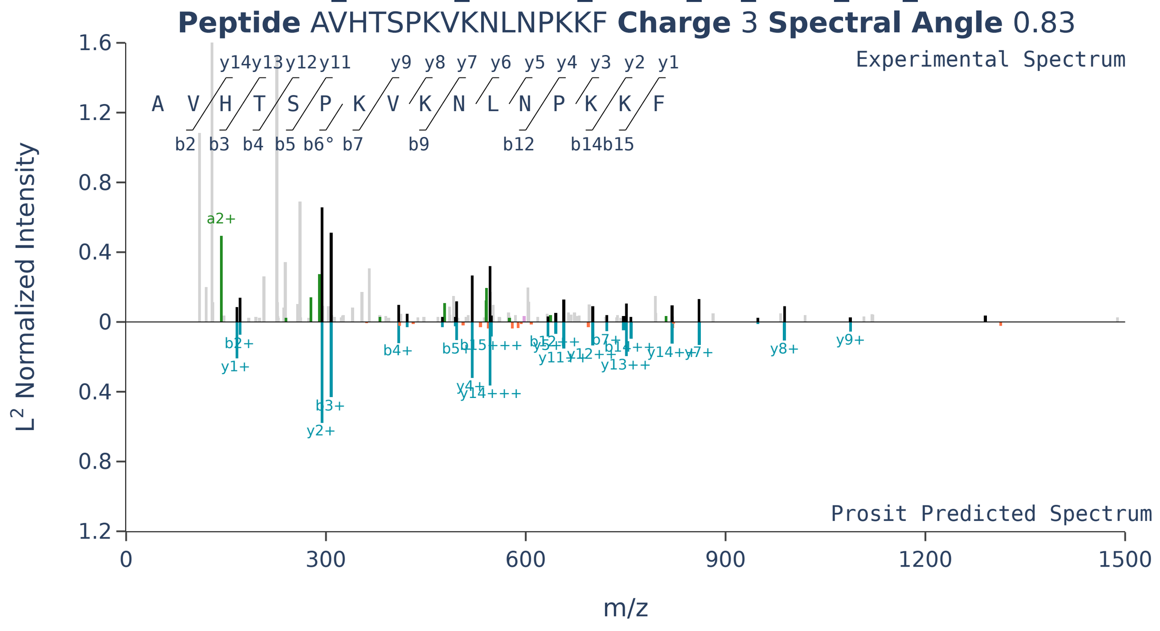

Source WSoh_101121_151121_HFGoe_G2_IL37b_1h_R2 **Scan** 6585
Peptide KESMSPSEVSD **Charge** 2 **Spectral Angle** 0.87

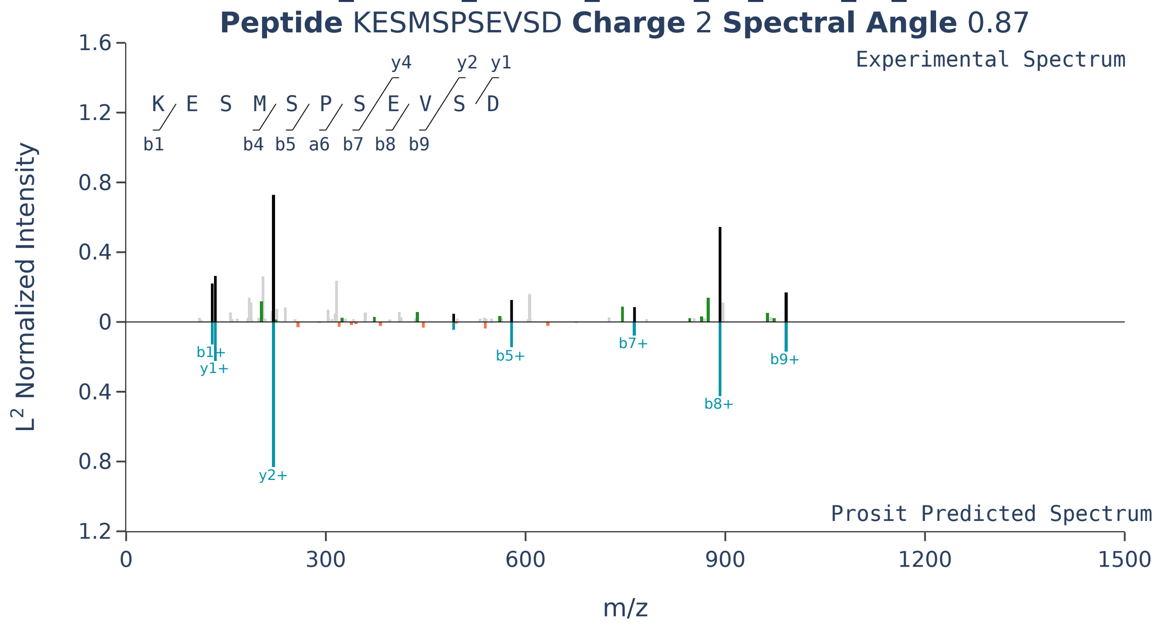

Source HRoetschke_271222_090123_HFGoe_LEDGF_b1R2_2h **Scan** 27596
Peptide NEGLWIEA **Charge** 1 **Spectral Angle** 0.96

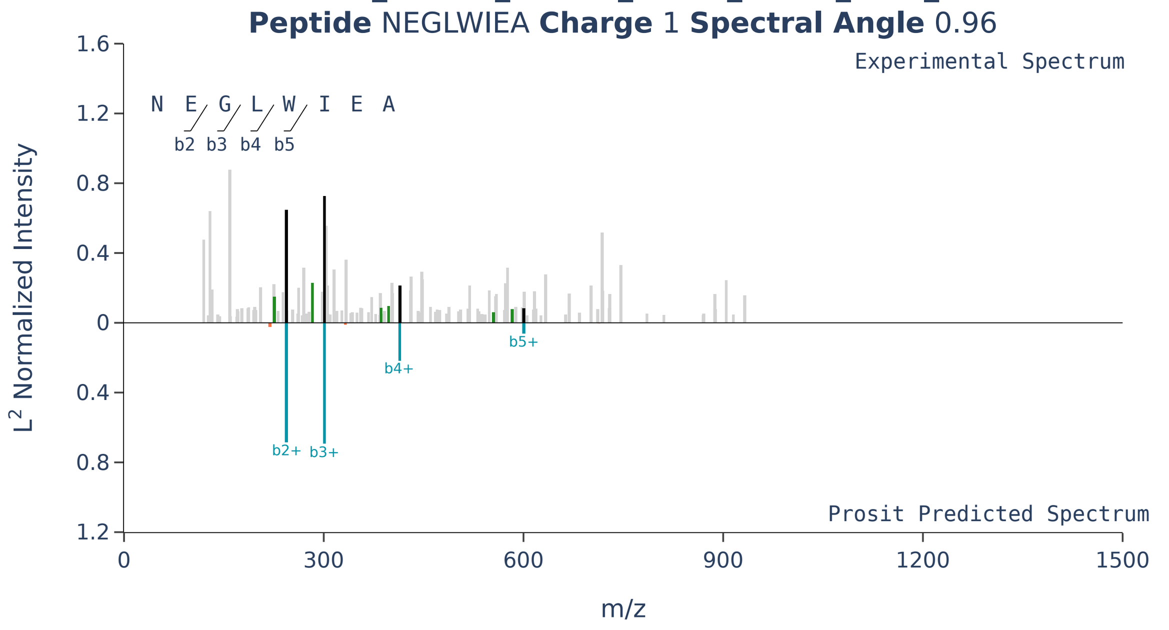

Source HRoetschke_271222_090123_HFGoe_LEDGF_b2R2_2h **Scan** 7344
Peptide DAVKPPTN **Charge** 2 **Spectral Angle** 0.93

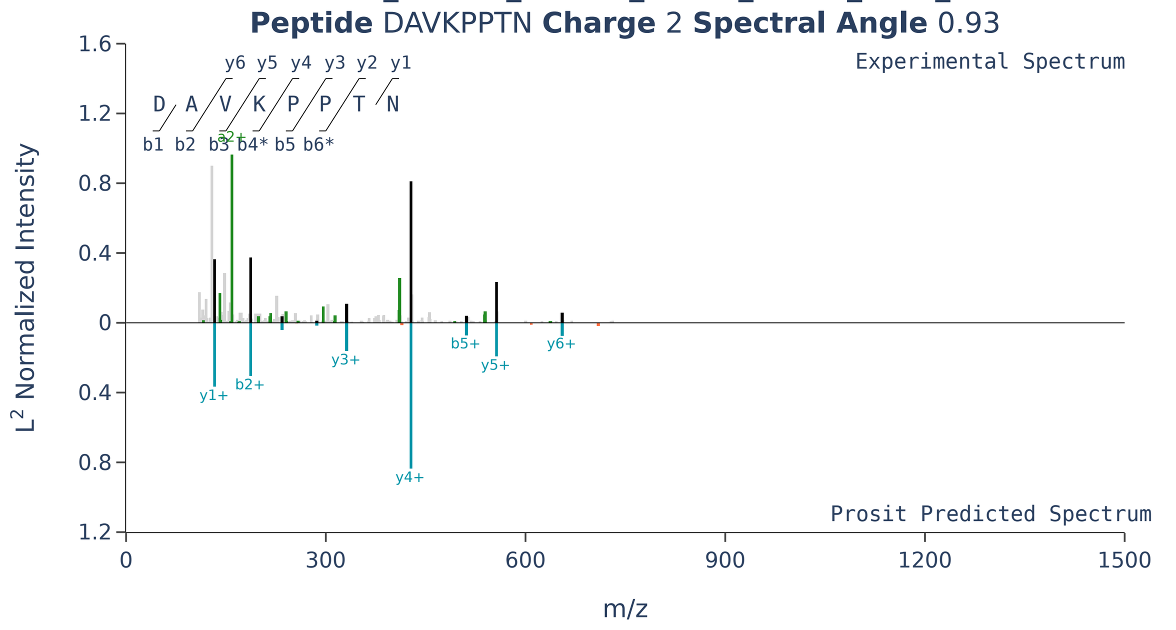

Source HRoetschke_271222_090123_HFGoe_LEDGF_b1R2_2h **Scan** 24045
Peptide GPKDLIFA **Charge** 2 **Spectral Angle** 0.89

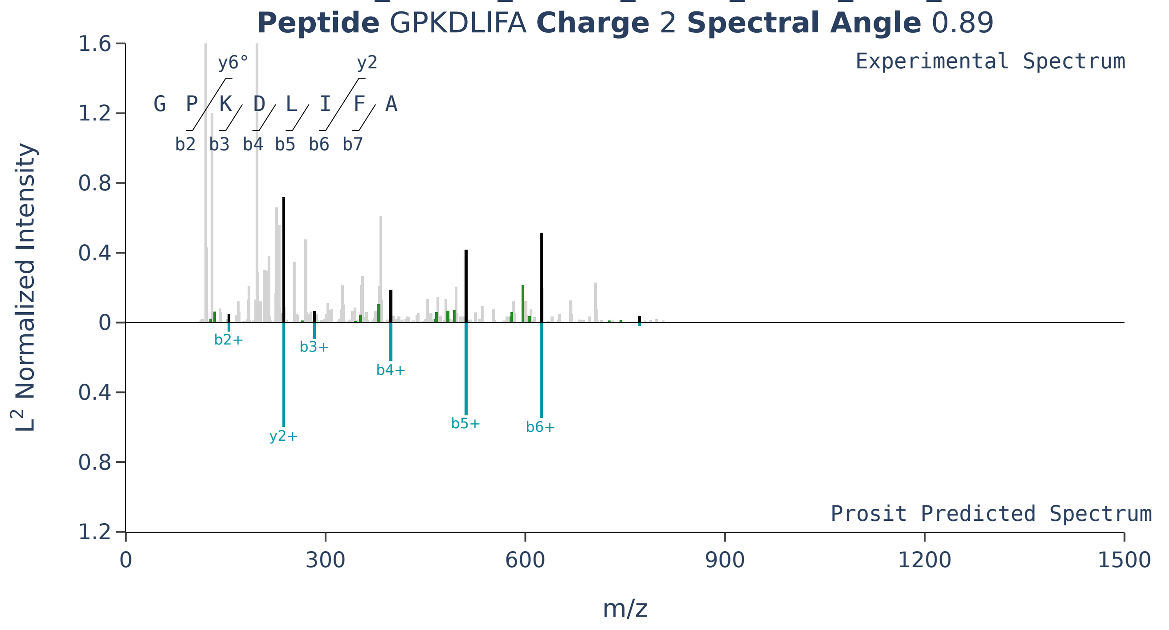

Source HRoetschke_271222_090123_HFGoe_LEDGF_b2R1_4h **Scan** 7020
Peptide TAVKPPTN **Charge** 2 **Spectral Angle** 0.95

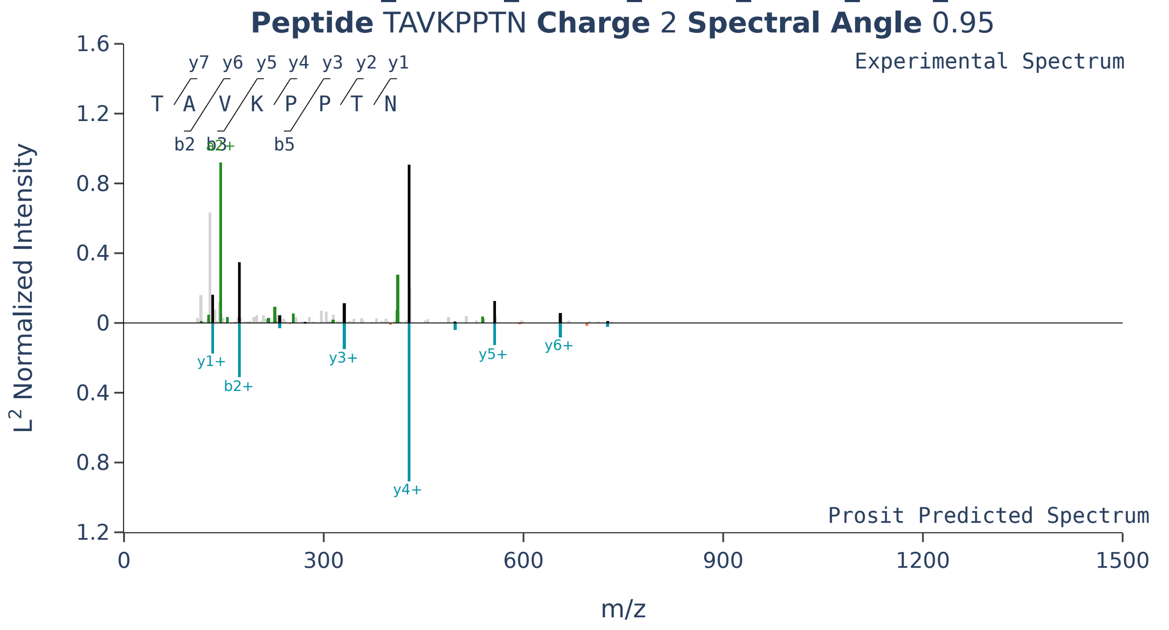

Source HRoetschke_271222_090123_HFGoe_LEDGF_b1R2_24h **Scan** 6526
Peptide ALDKETSVSKE **Charge** 3 **Spectral Angle** 0.89

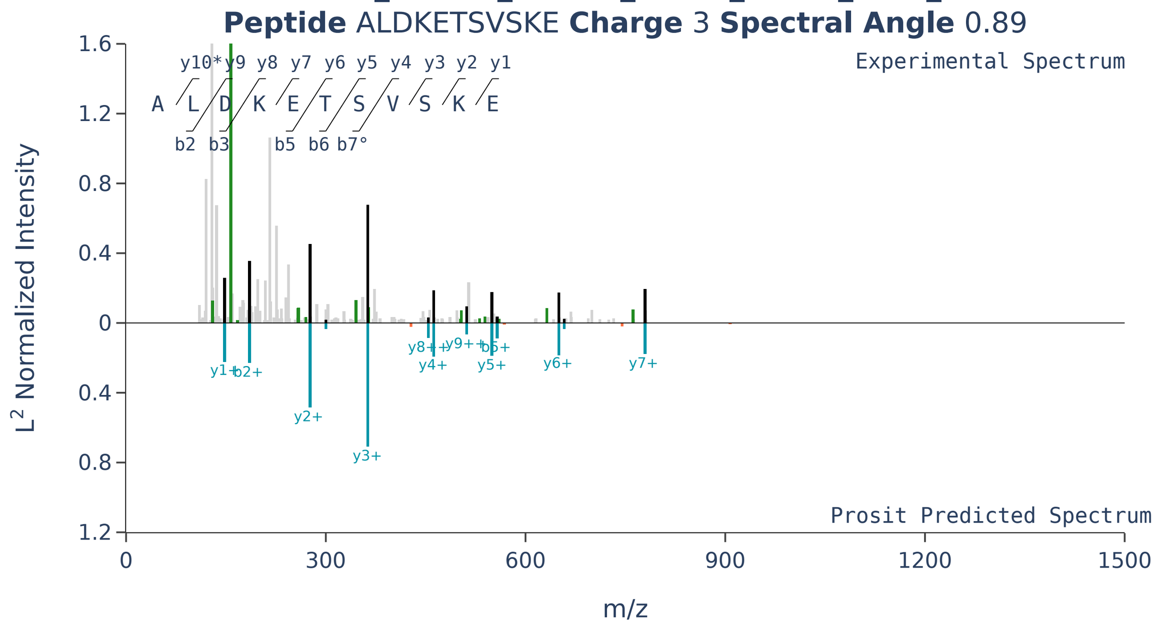

Source HRoetschke_271222_090123_HFGoe_LEDGF_b2R1_4h **Scan** 18185
Peptide TSMPHWPARV **Charge** 3 **Spectral Angle** 0.95

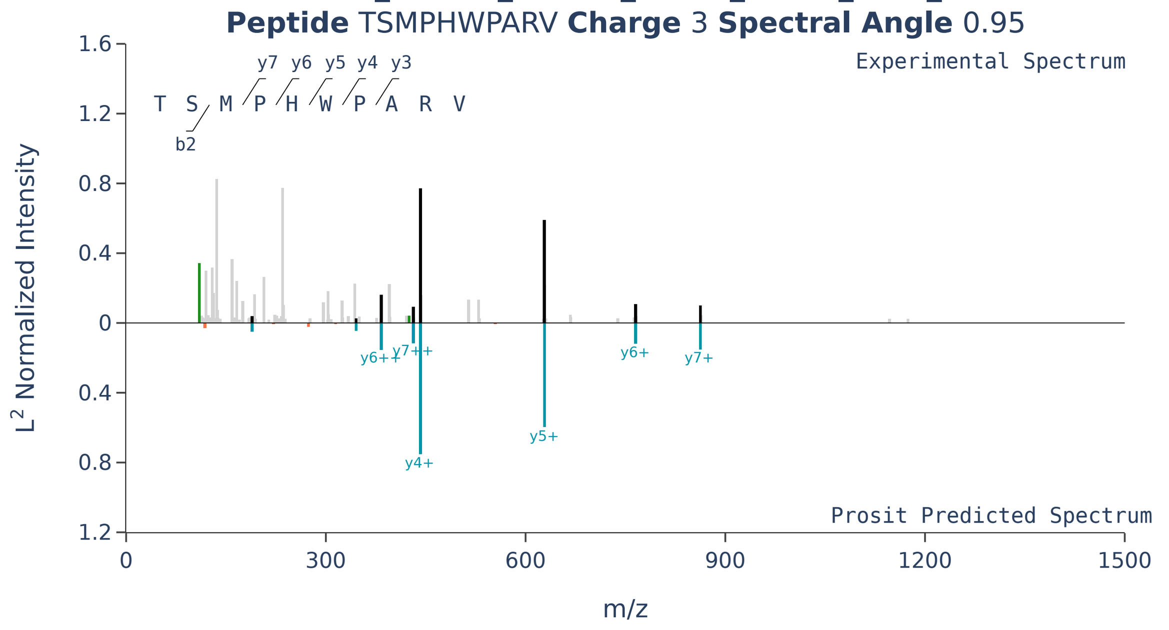

Source HRoetschke_271222_090123_HFGoe_LEDGF_b1R1_24h **Scan** 14138
Peptide FLVGKET **Charge** 2 **Spectral Angle** 0.9

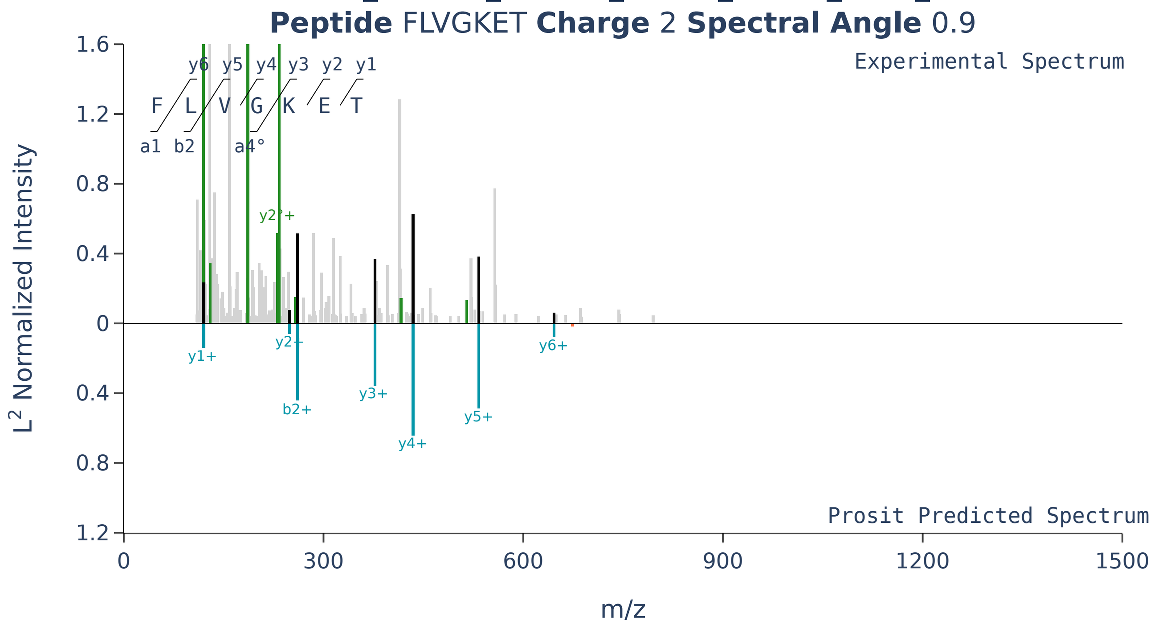

Source HRoetschke_271222_090123_HFGoe_LEDGF_b2R1_4h **Scan** 7004
Peptide AAVKPPTN **Charge** 2 **Spectral Angle** 0.92

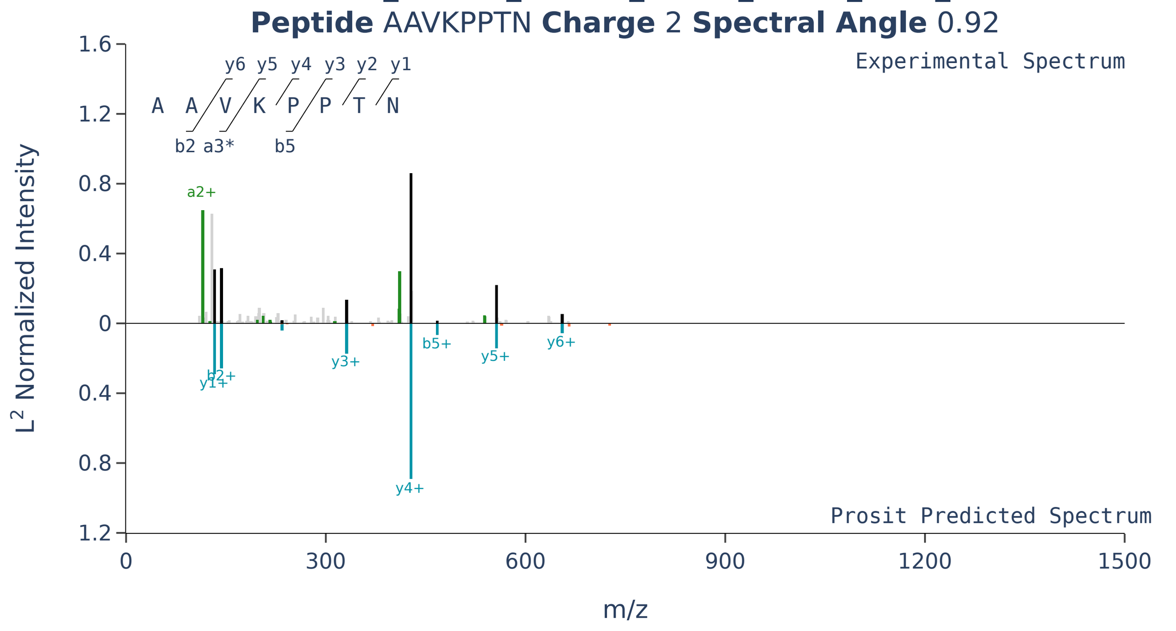

Source HRoetschke_271222_090123_HFGoe_LEDGF_b1R1_4h **Scan** 20479
Peptide GYGYPHWPARV **Charge** 3 **Spectral Angle** 0.91

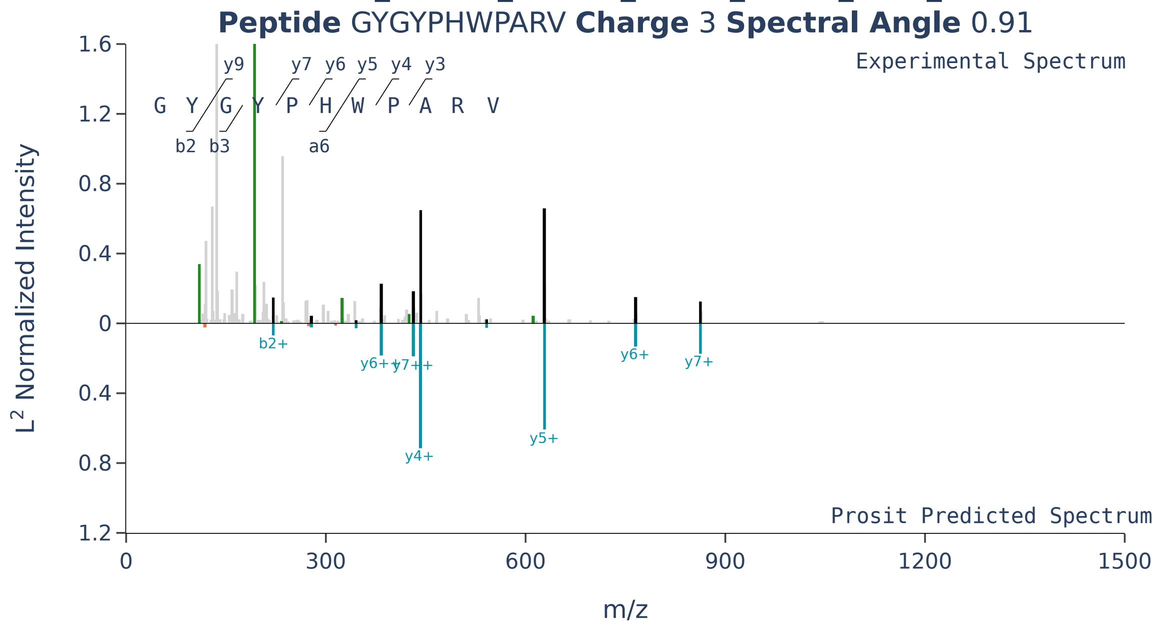

Source HRoetschke_271222_090123_HFGoe_LEDGF_b1R1_24h **Scan** 14036
Peptide TPHWPARVD **Charge** 3 **Spectral Angle** 0.94

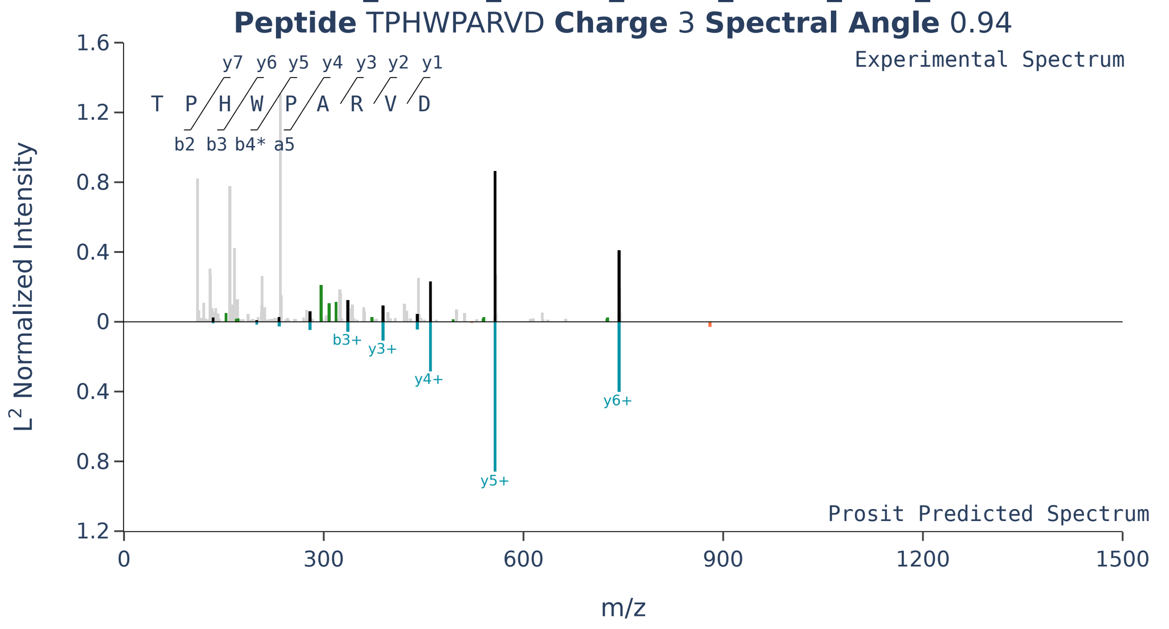

Source HRoetschke_271222_090123_HFGoe_LEDGF_b2R1_4h **Scan** 13075
Peptide TGSKTLALD **Charge** 2 **Spectral Angle** 0.89

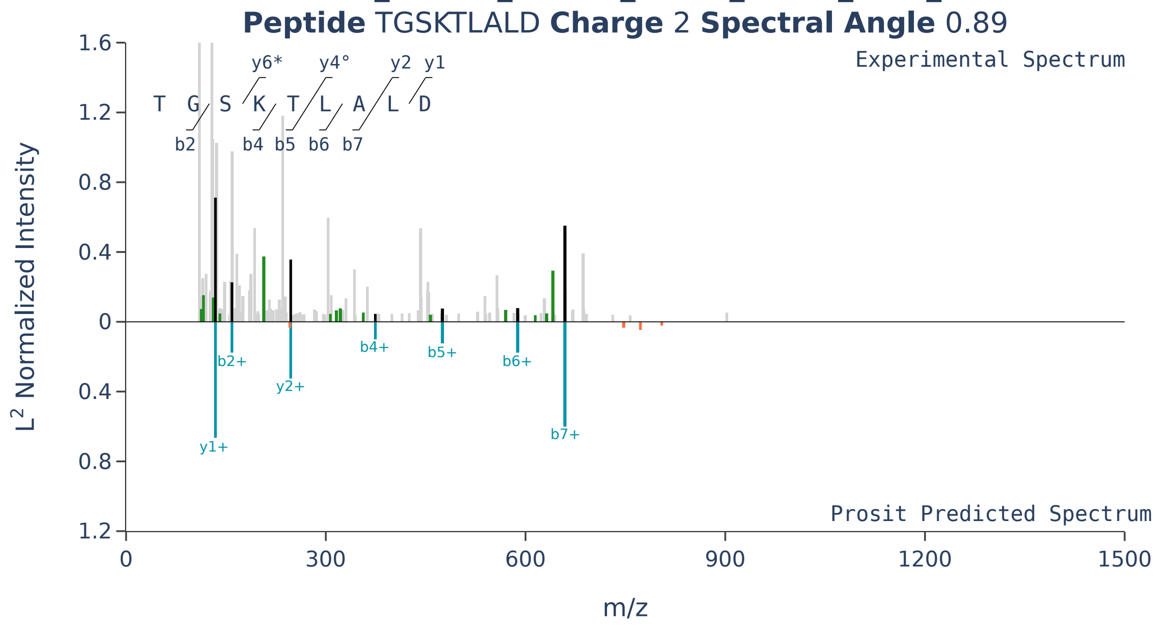

Source HRoetschke_271222_090123_HFGoe_LEDGF_b2R1_24h **Scan** 10744
Peptide TAVKPPTNKL **Charge** 3 **Spectral Angle** 0.84

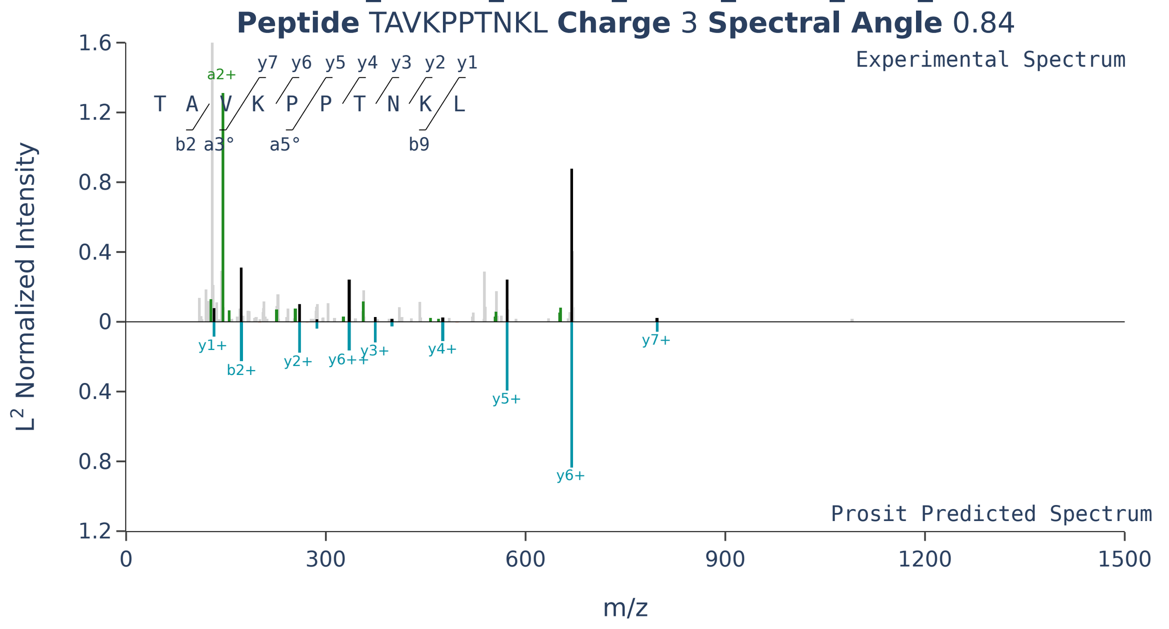

Source HRoetschke_271222_090123_HFGoe_LEDGF_b1R1_24h **Scan** 14858
Peptide ATKQSYPHWPARV **Charge** 4 **Spectral Angle** 0.95

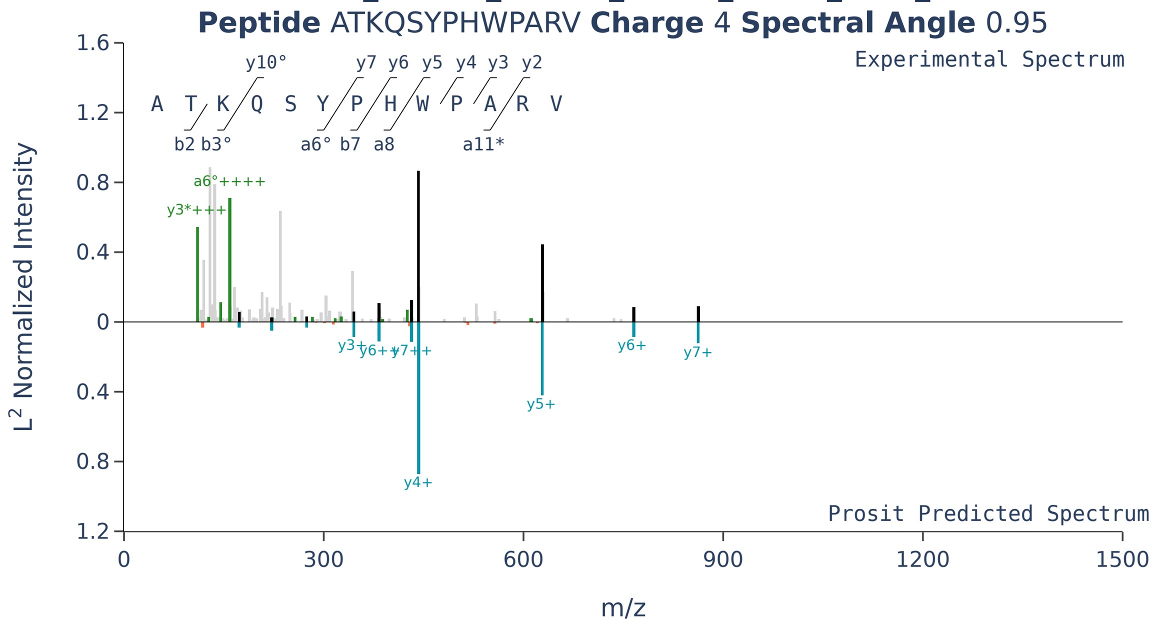

Source HRoetschke_271222_090123_HFGoe_LEDGF_b2R2_1h **Scan** 12812
Peptide TEISLKE **Charge** 2 **Spectral Angle** 0.94

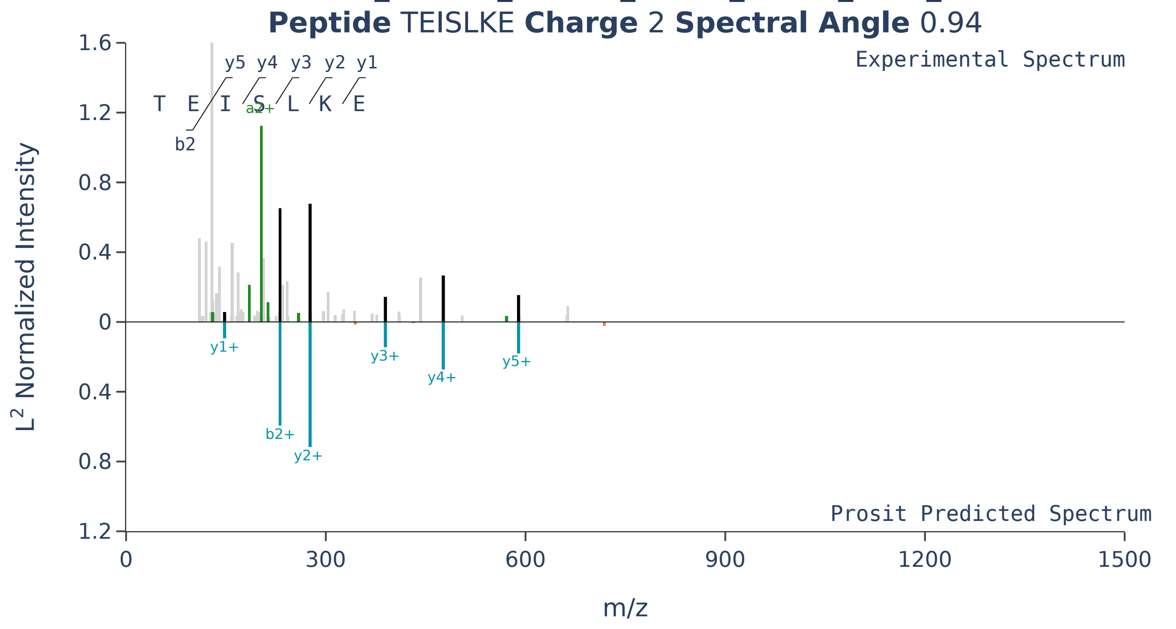

Source HRoetschke_271222_090123_HFGoe_LEDGF_b2R2_24h **Scan** 6702
Peptide FPYSENKEKYGKPNKRTA **Charge** 5 **Spectral Angle** 0.91

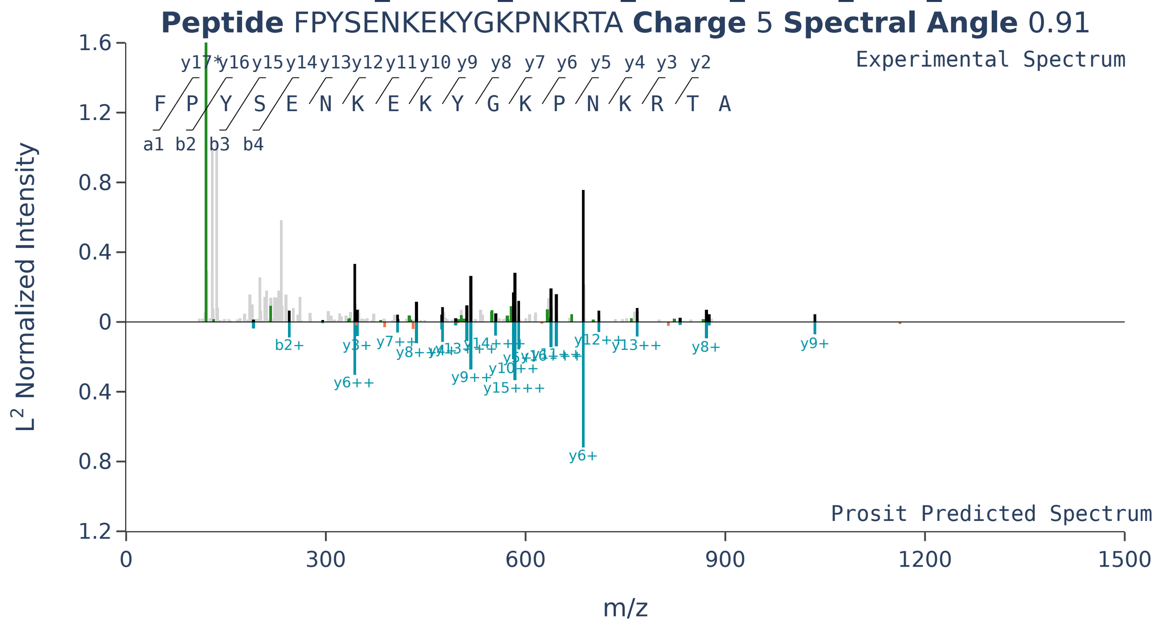

Source HRoetschke_271222_090123_HFGoe_LEDGF_b1R1_1h **Scan** 7896
Peptide KETSVSKEEL **Charge** 3 **Spectral Angle** 0.87

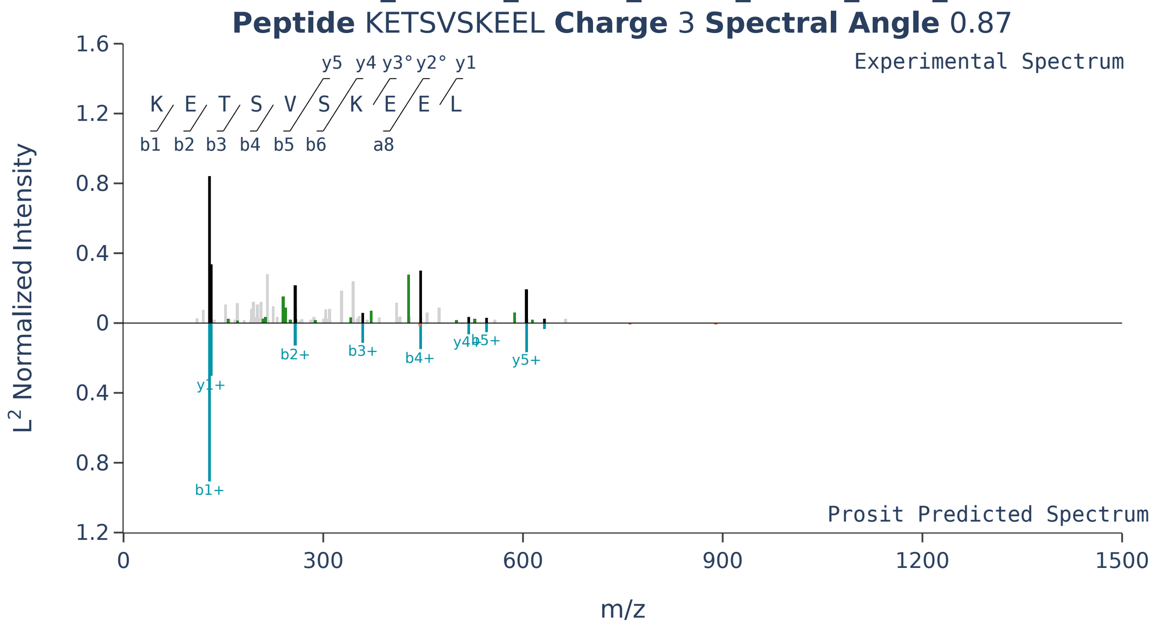

Source HRoetschke_271222_090123_HFGoe_LEDGF_b1R1_24h **Scan** 9927
Peptide KGTEISLKD **Charge** 2 **Spectral Angle** 0.93

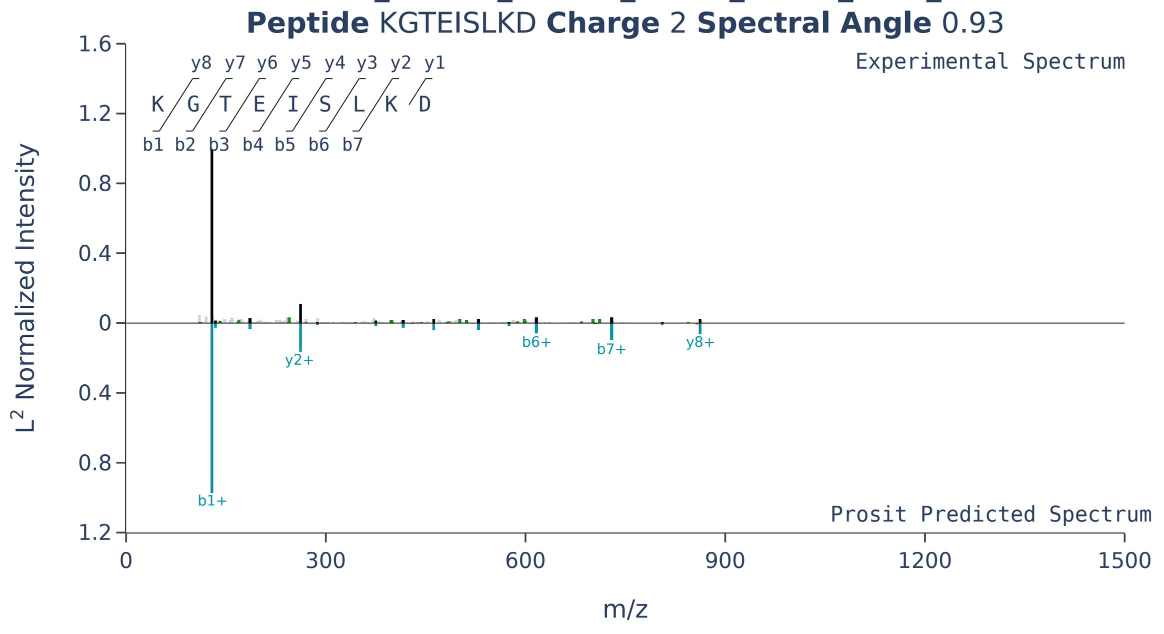

Source HRoetschke_271222_090123_HFGoe_LEDGF_b1R2_4h **Scan** 21069
Peptide NGGSDELASL **Charge** 1 **Spectral Angle** 0.84

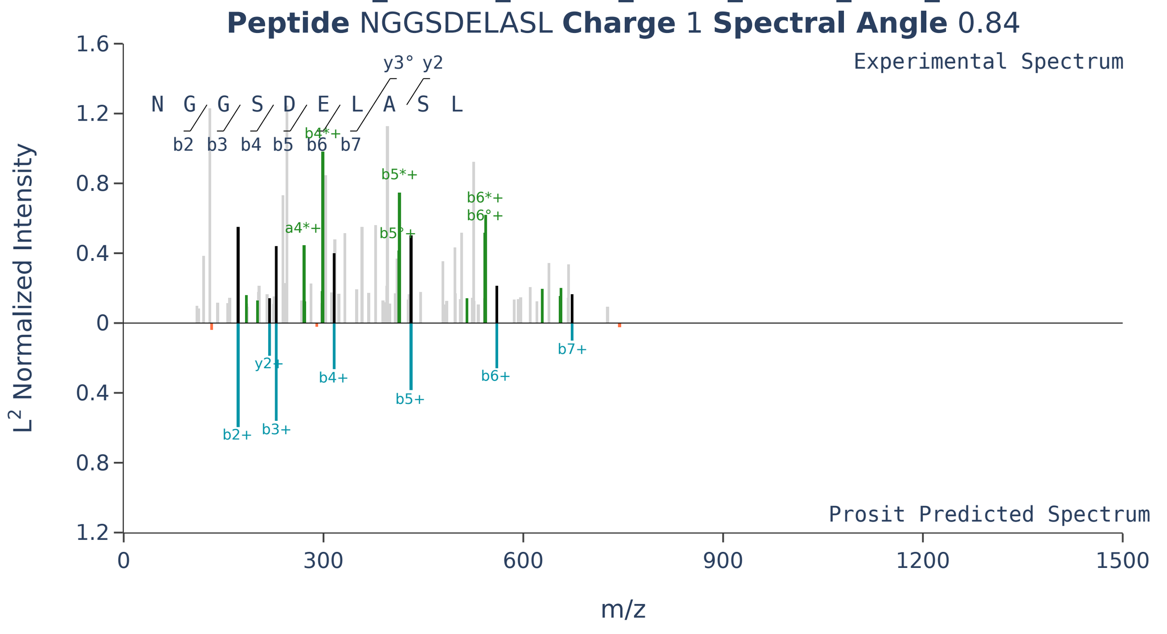

Source HRoetschke_271222_090123_HFGoe_LEDGF_b1R1_24h **Scan** 13697
Peptide GDEVPDGAVKPPTN **Charge** 2 **Spectral Angle** 0.85

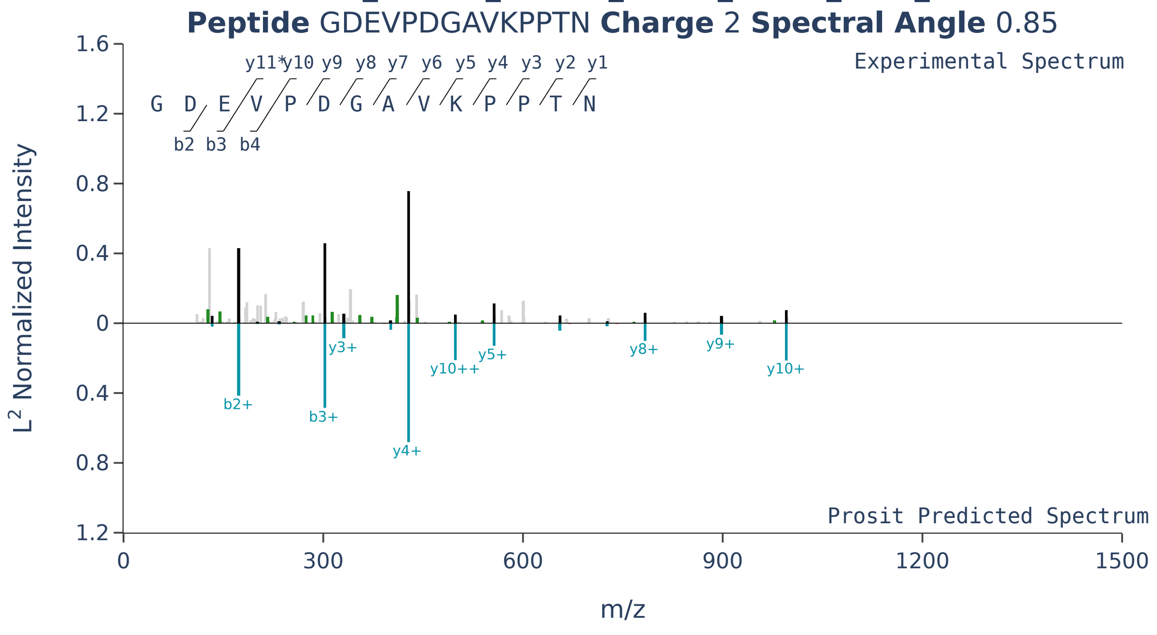

Source HRoetschke_271222_090123_HFGoe_LEDGF_b1R2_24h **Scan** 13635
Peptide TTEISLKD **Charge** 2 **Spectral Angle** 0.85

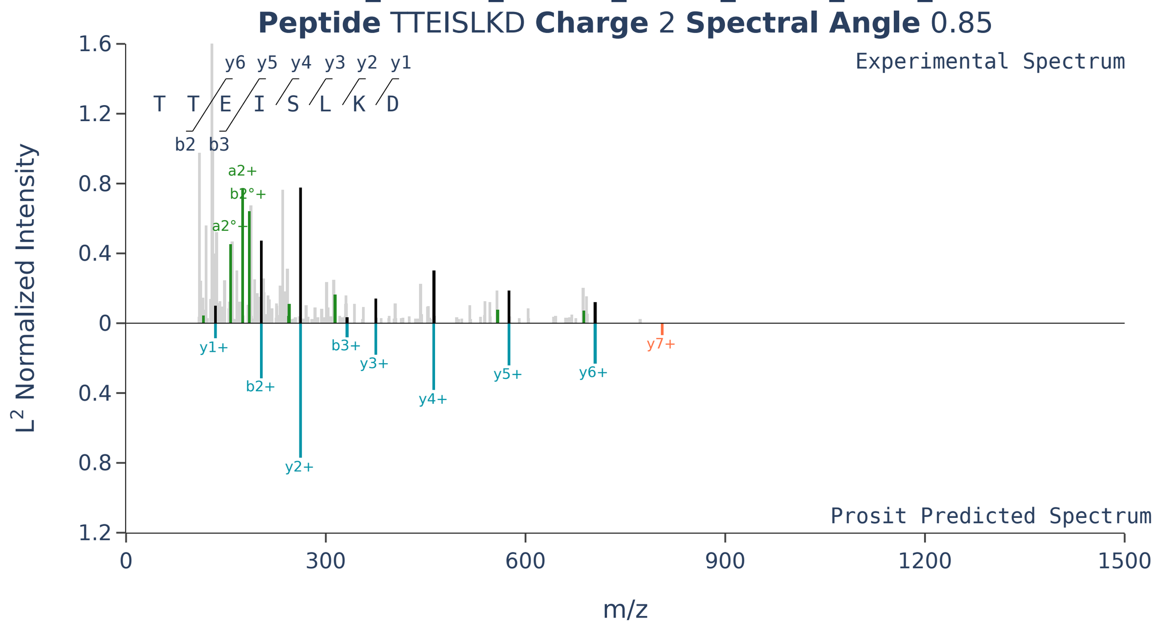

Source HRoetschke_271222_090123_HFGoe_LEDGF_b2R2_4h **Scan** 10493
Peptide FLVGQQAQKH **Charge** 3 **Spectral Angle** 0.93

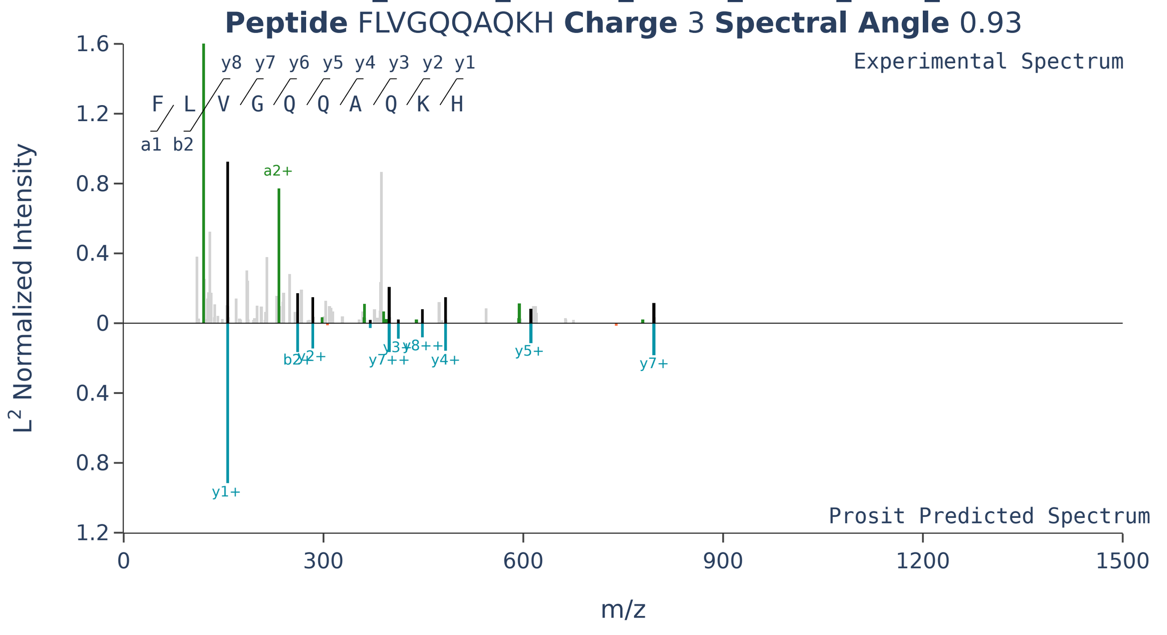

Source HRoetschke_271222_090123_HFGoe_LEDGF_b2R1_24h **Scan** 8153
Peptide RIHAEIKNV **Charge** 3 **Spectral Angle** 0.89

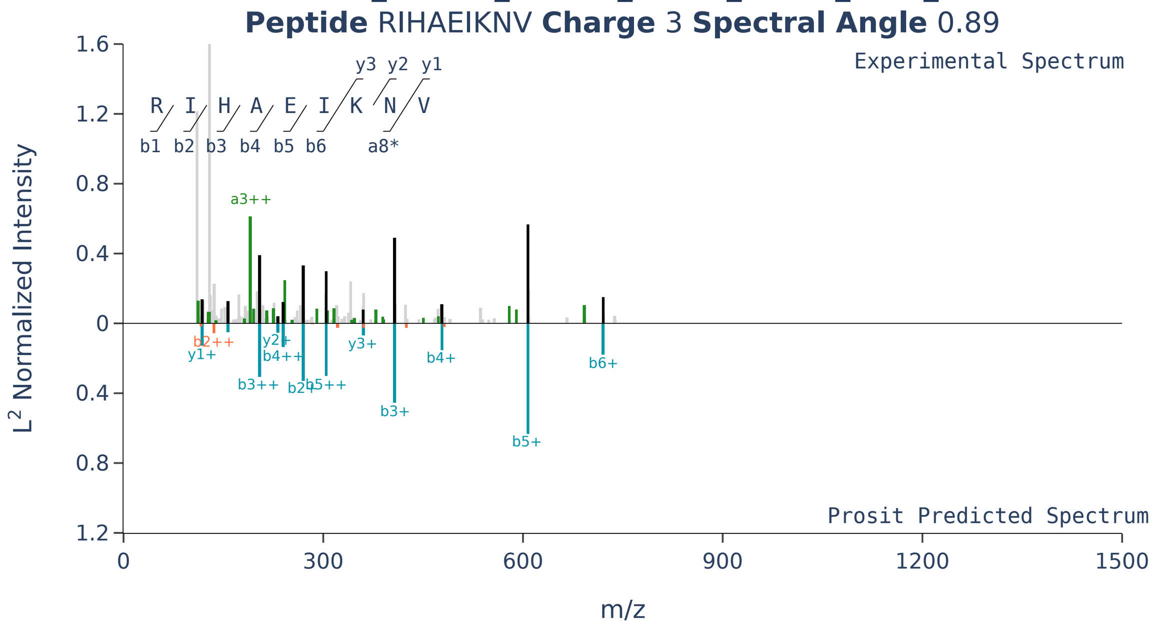

Source HRoetschke_271222_090123_HFGoe_LEDGF_b2R1_24h **Scan** 20377
Peptide ASLKIDNL **Charge** 2 **Spectral Angle** 0.77

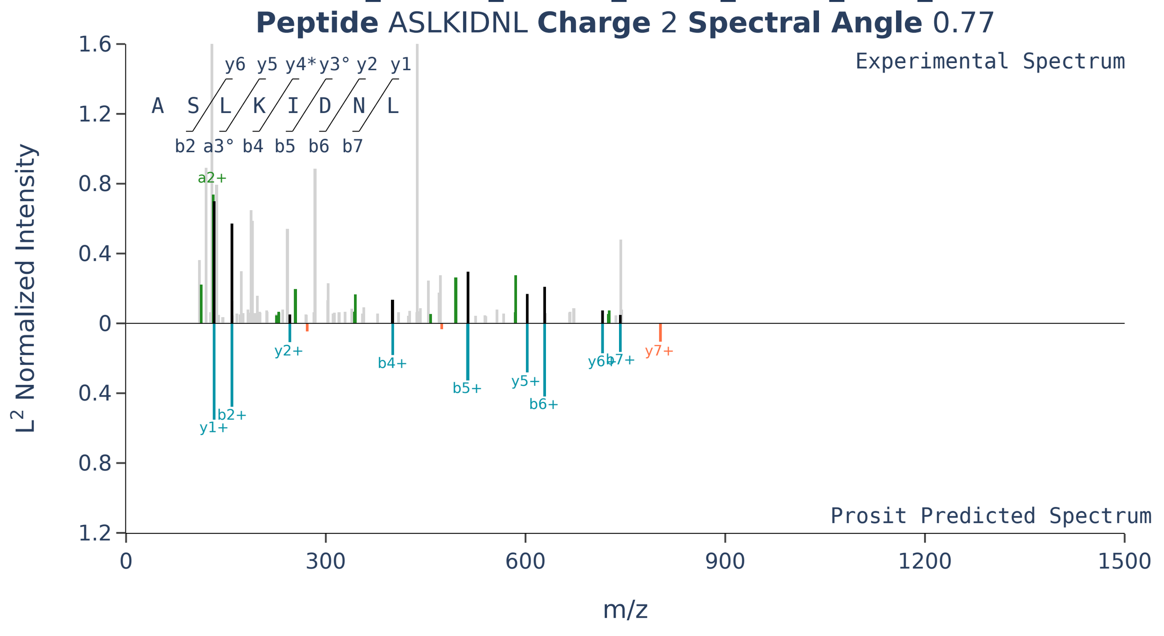

Source HRoetschke_271222_090123_HFGoe_LEDGF_b2R1_4h **Scan** 11714
Peptide GSPGMTRDFKPGD **Charge** 3 **Spectral Angle** 0.9

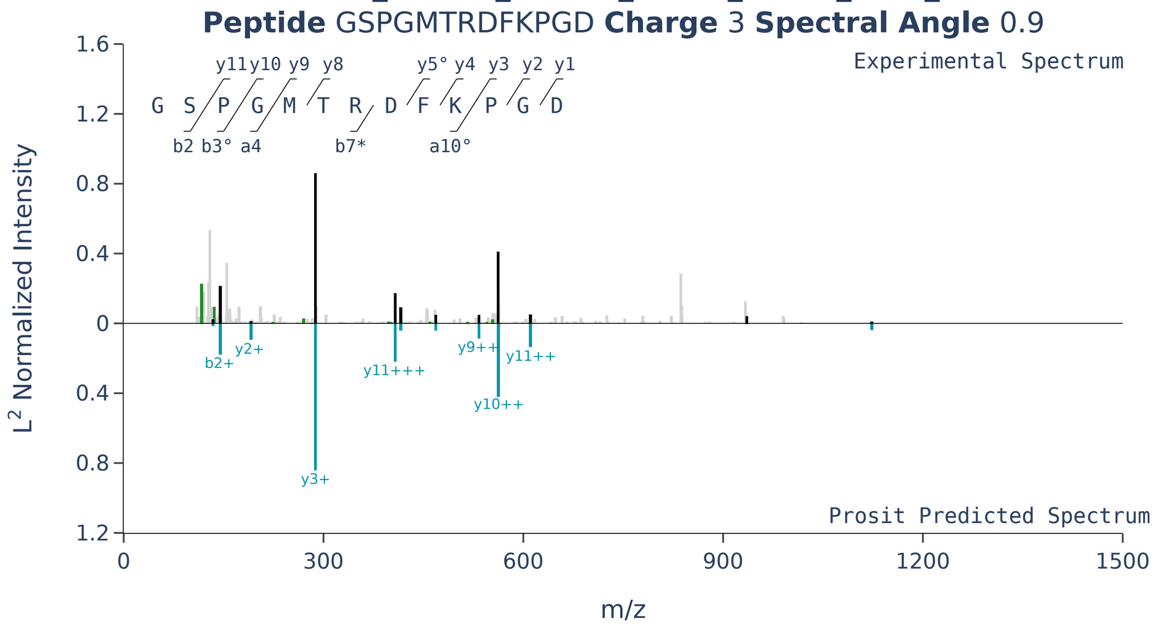

Source HRoetschke_271222_090123_HFGoe_LEDGF_b1R1_24h **Scan** 13619
Peptide GTHETTAFLGPKD **Charge** 3 **Spectral Angle** 0.91

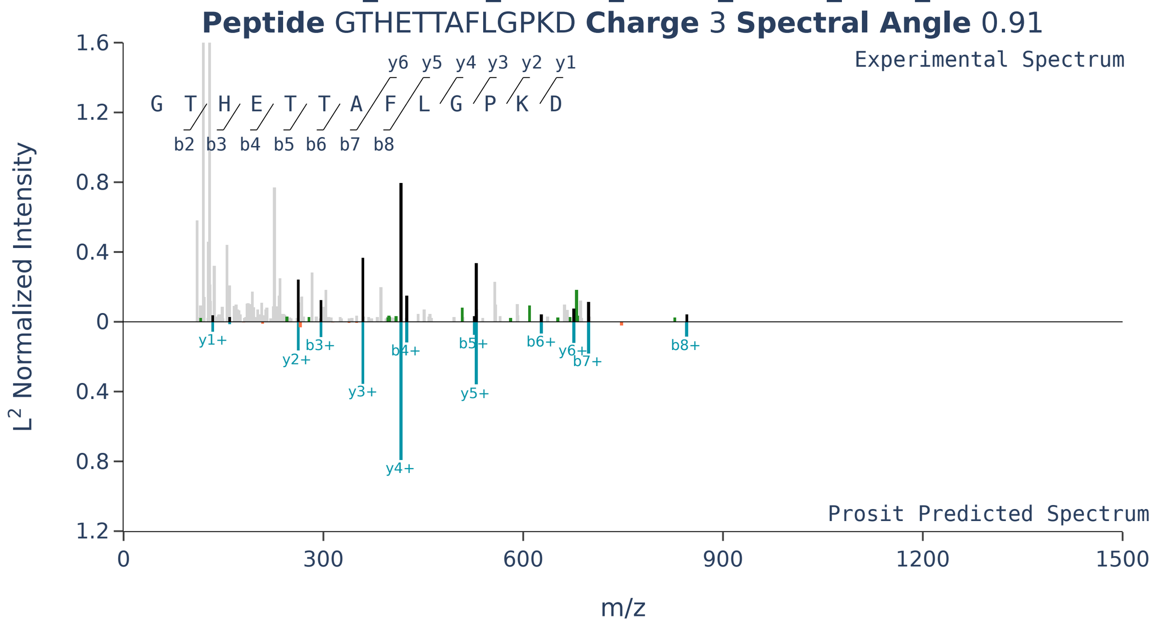

Source HRoetschke_271222_090123_HFGoe_LEDGF_b1R2_4h **Scan** 17592
Peptide ETAFHWPAR **Charge** 3 **Spectral Angle** 0.82

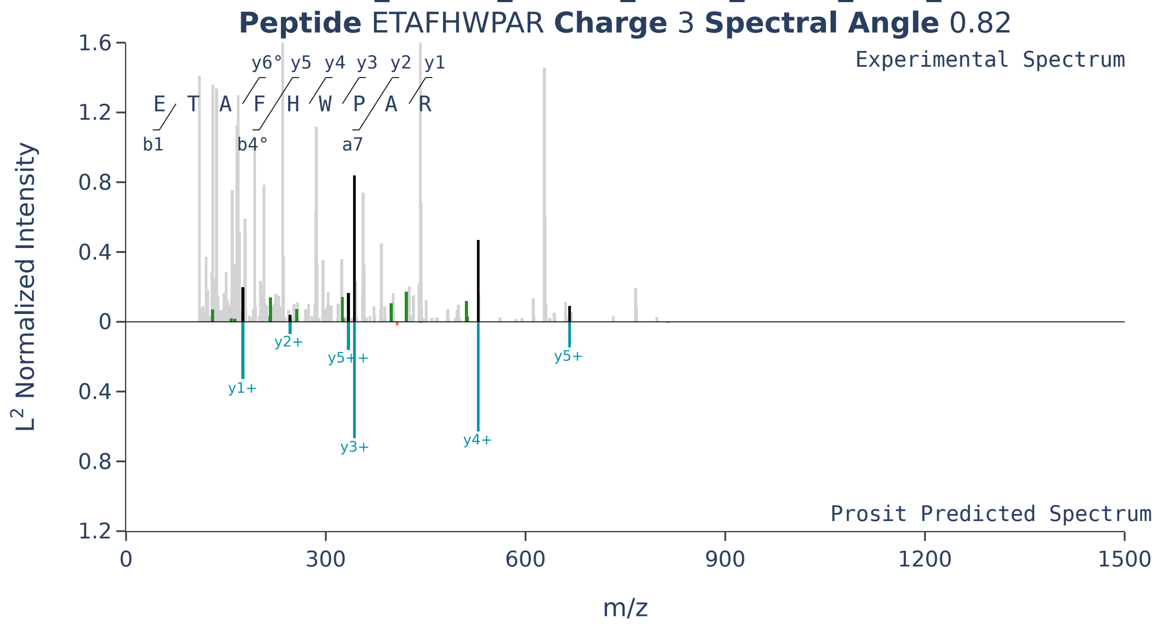

Source HRoetschke_271222_090123_HFGoe_LEDGF_b1R1_24h **Scan** 12390
Peptide GTHETGPGMTRDF **Charge** 3 **Spectral Angle** 0.88

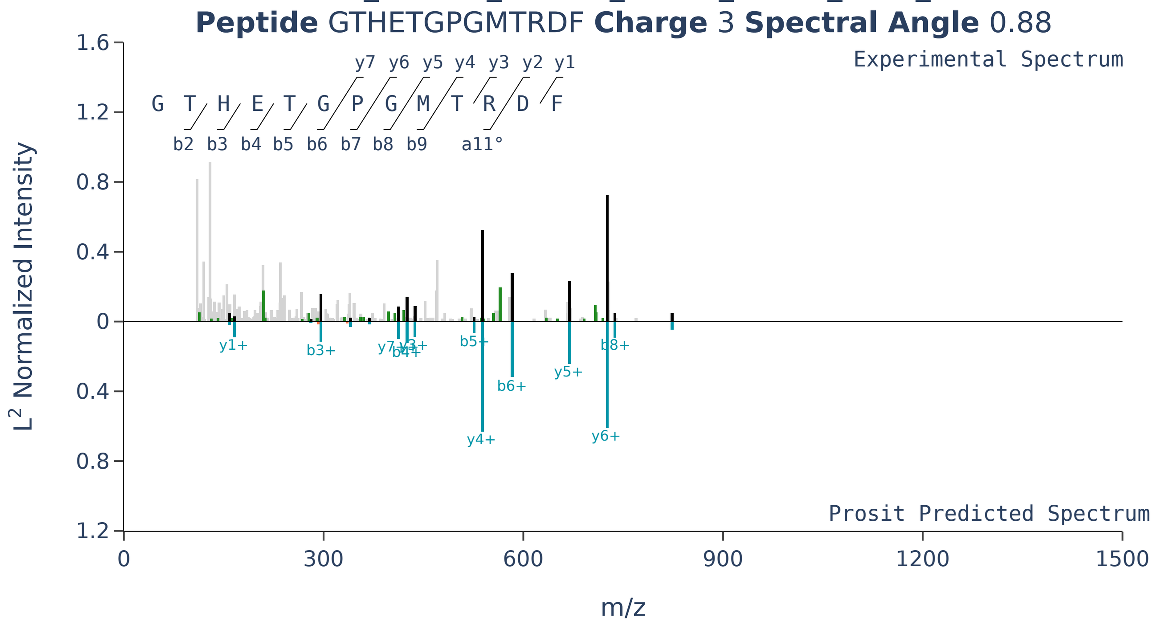

Source HRoetschke_271222_090123_HFGoe_LEDGF_b2R1_24h **Scan** 12083
Peptide GTPGMTRDFKPGD **Charge** 3 **Spectral Angle** 0.89

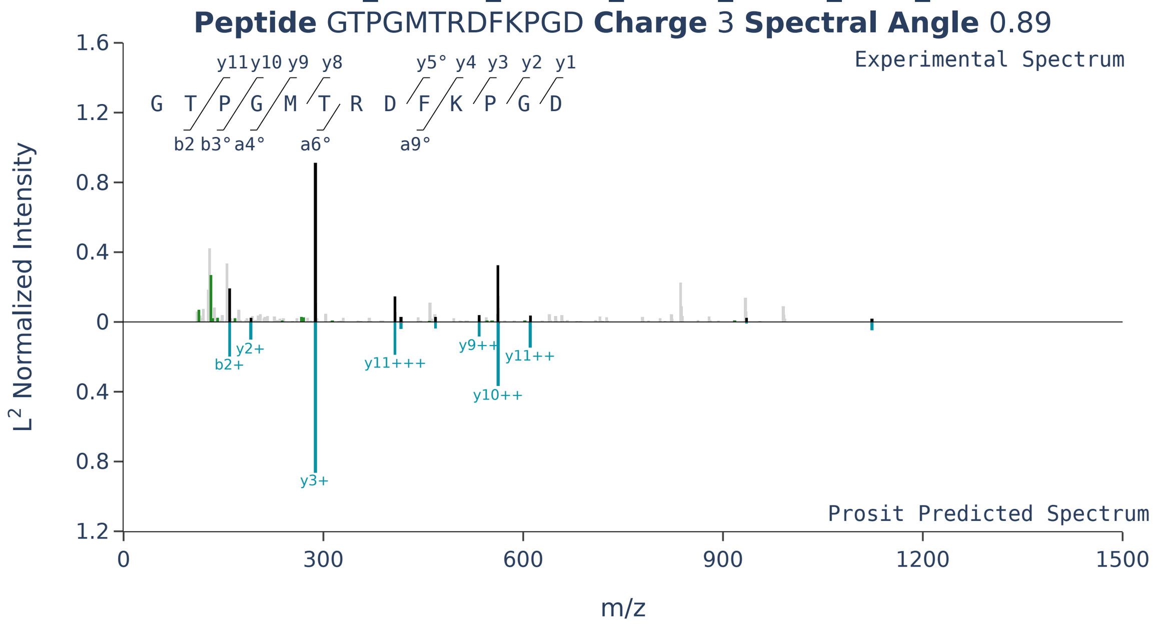

Source HRoetschke_271222_090123_HFGoe_LEDGF_b1R2_4h **Scan** 23625
Peptide GTHETLIFA **Charge** 2 **Spectral Angle** 0.81

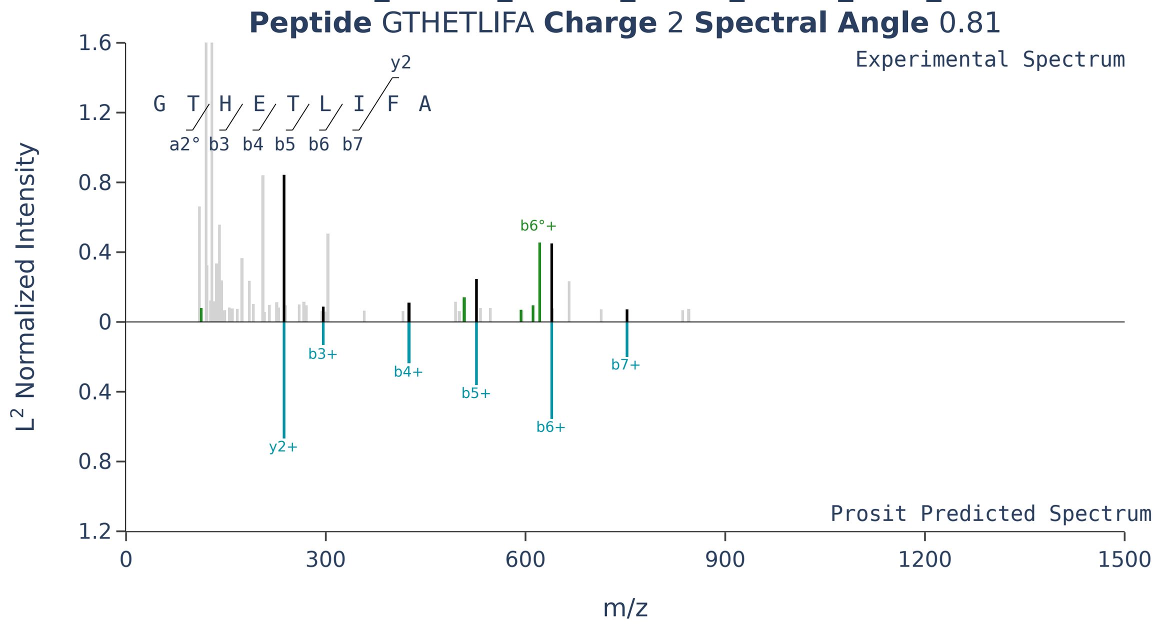

Source HRoetschke_271222_090123_HFGoe_LEDGF_b2R2_4h **Scan** 7088
Peptide ATVKPPTN **Charge** 2 **Spectral Angle** 0.85

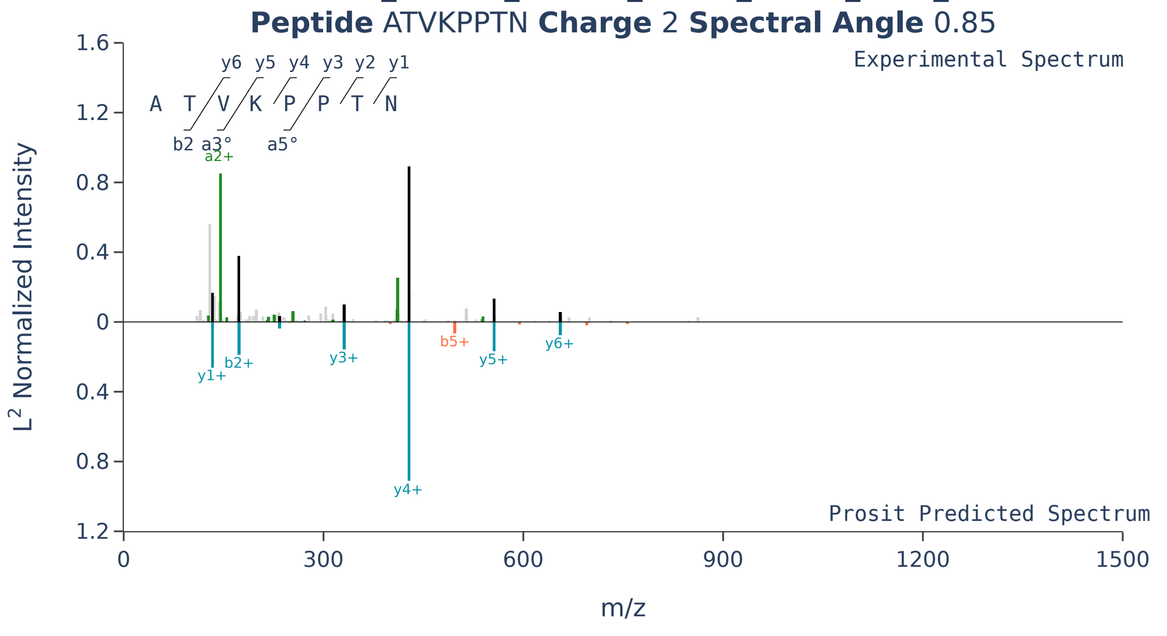

Source HRoetschke_271222_090123_HFGoe_LEDGF_b1R1_24h **Scan** 6873
Peptide SVNLKVSPKRGRAT **Charge** 4 **Spectral Angle** 0.92

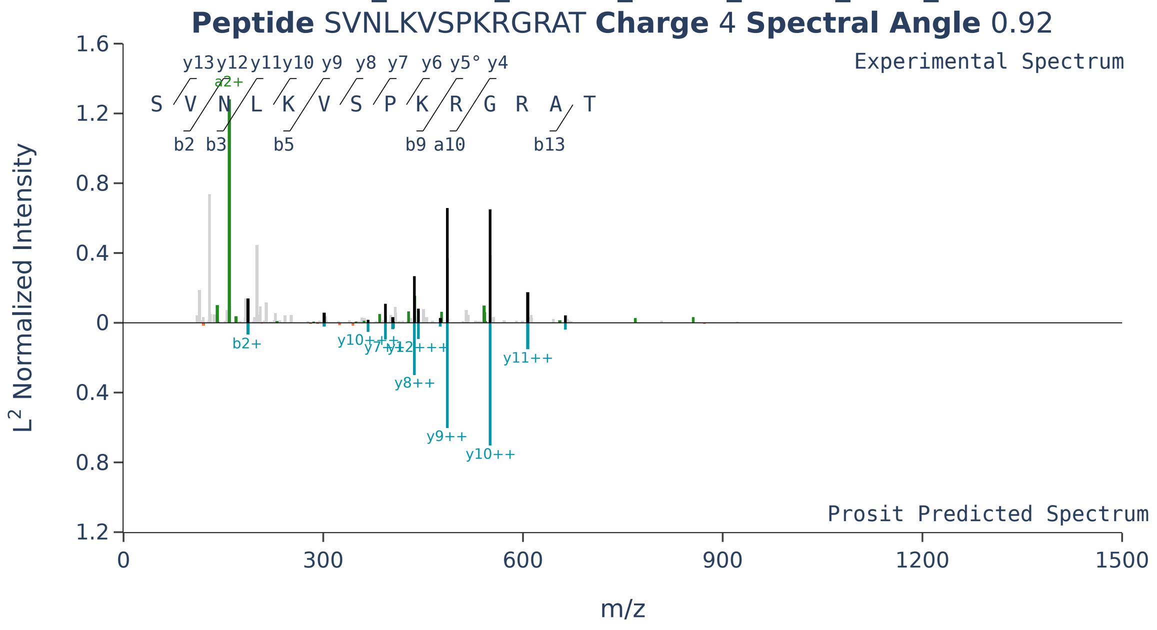

Source HRoetschke_271222_090123_HFGoe_LEDGF_b1R2_24h **Scan** 17923
Peptide TGYPHWPARVD **Charge** 3 **Spectral Angle** 0.9

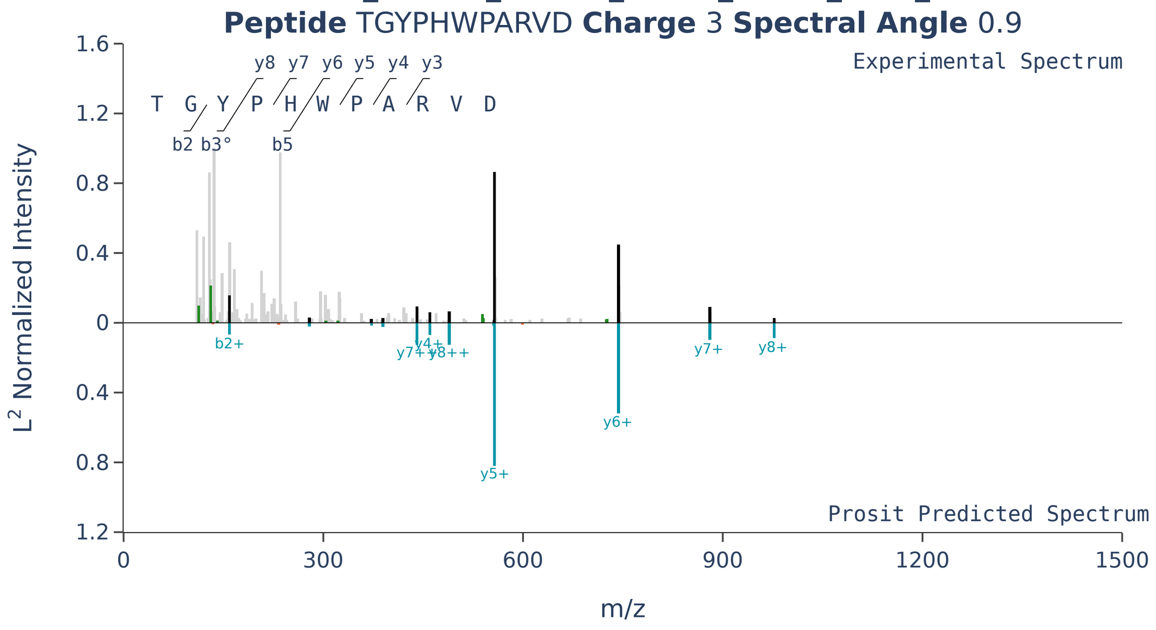

Source HRoetschke_271222_090123_HFGoe_LEDGF_b1R1_4h **Scan** 12702
Peptide FLVGKETSVSKE **Charge** 3 **Spectral Angle** 0.87

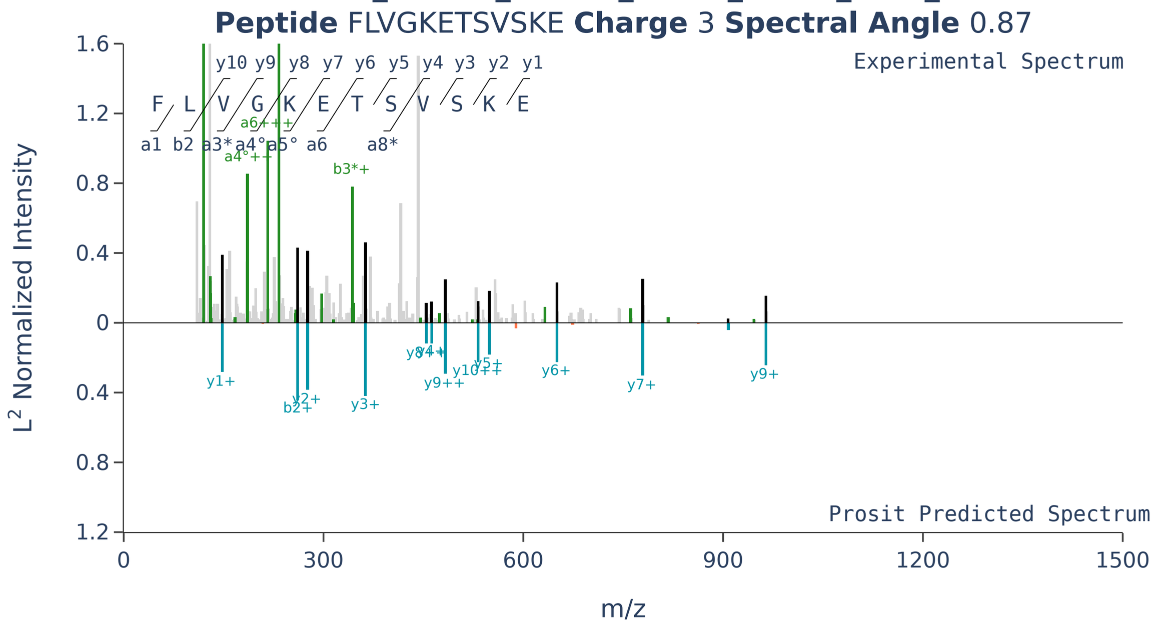

Source HRoetschke_271222_090123_HFGoe_LEDGF_b2R1_2h **Scan** 6640
Peptide FPYSENKEKYGKPNKRAT **Charge** 3 **Spectral Angle** 0.79

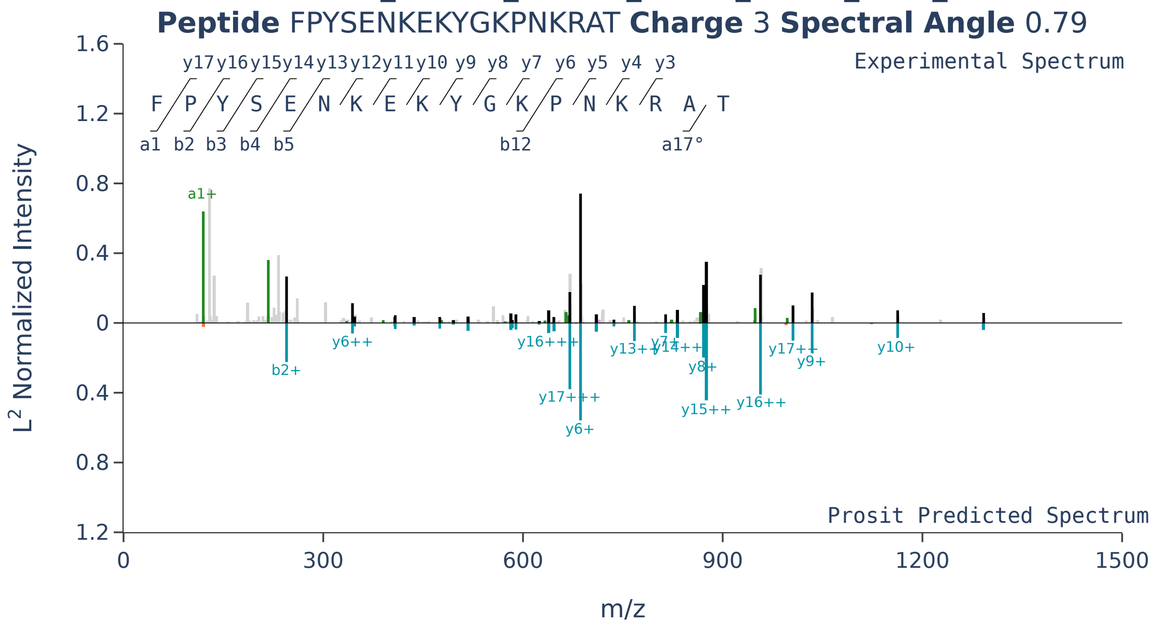

Source HRoetschke_271222_090123_HFGoe_LEDGF_b1R2_24h **Scan** 15491
Peptide TNVPDGAVKPPTNKL **Charge** 2 **Spectral Angle** 0.91

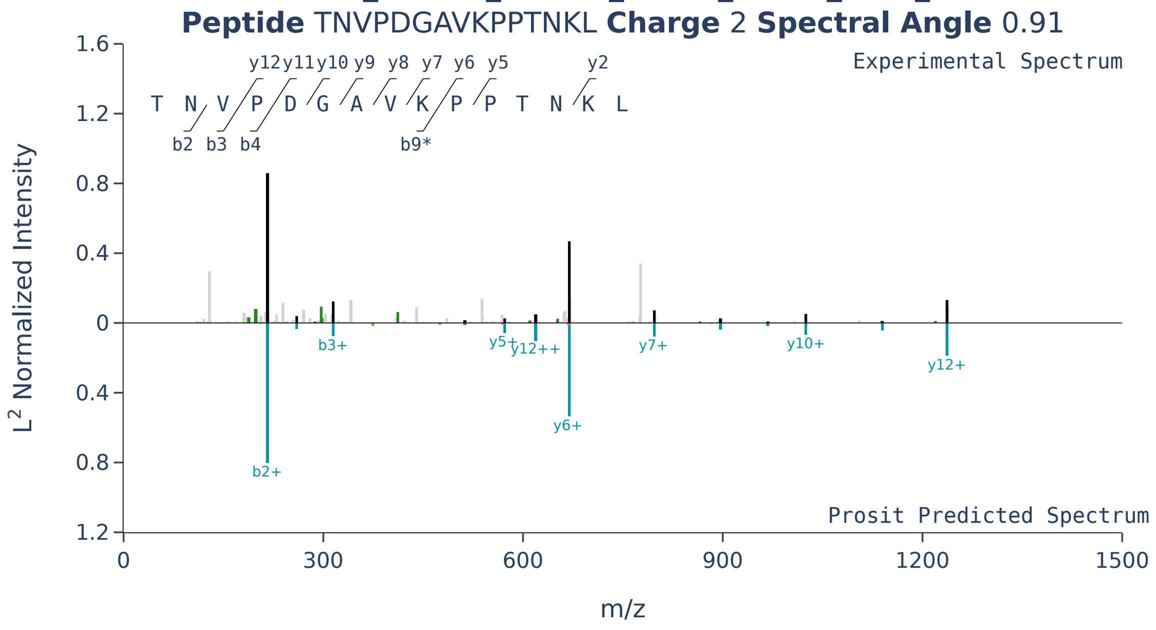

Source HRoetschke_271222_090123_HFGoe_LEDGF_b2R1_2h **Scan** 8218
Peptide GKNASSDVEVEE **Charge** 2 **Spectral Angle** 0.86

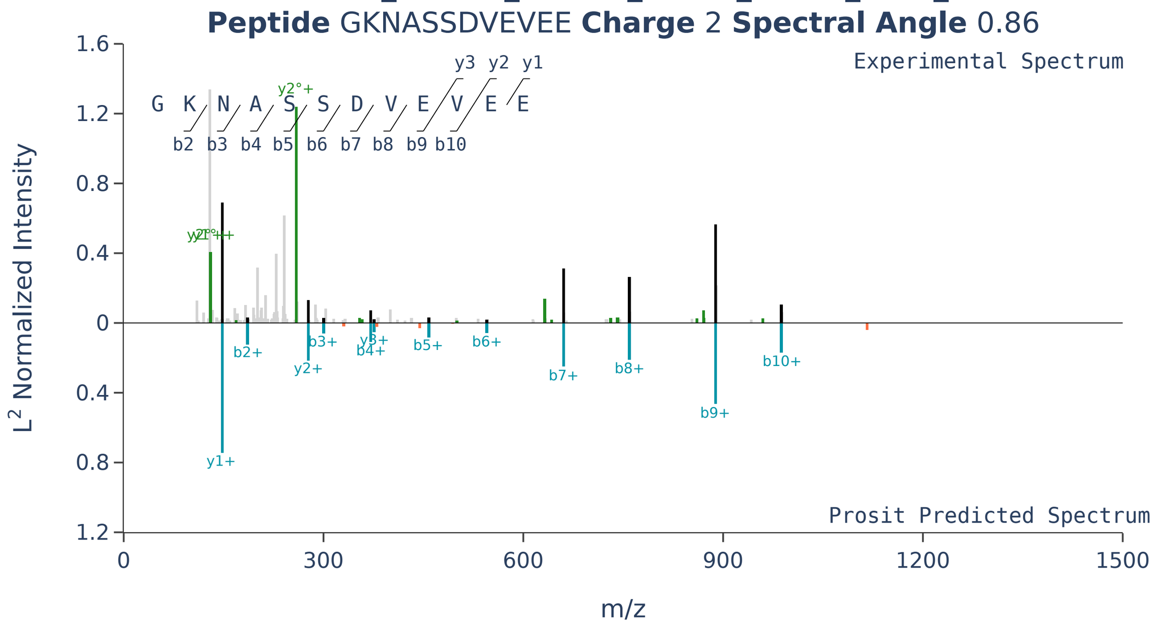

Source HRoetschke_271222_090123_HFGoe_LEDGF_b2R2_2h **Scan** 17841
Peptide TAYPHWPARVD **Charge** 3 **Spectral Angle** 0.9

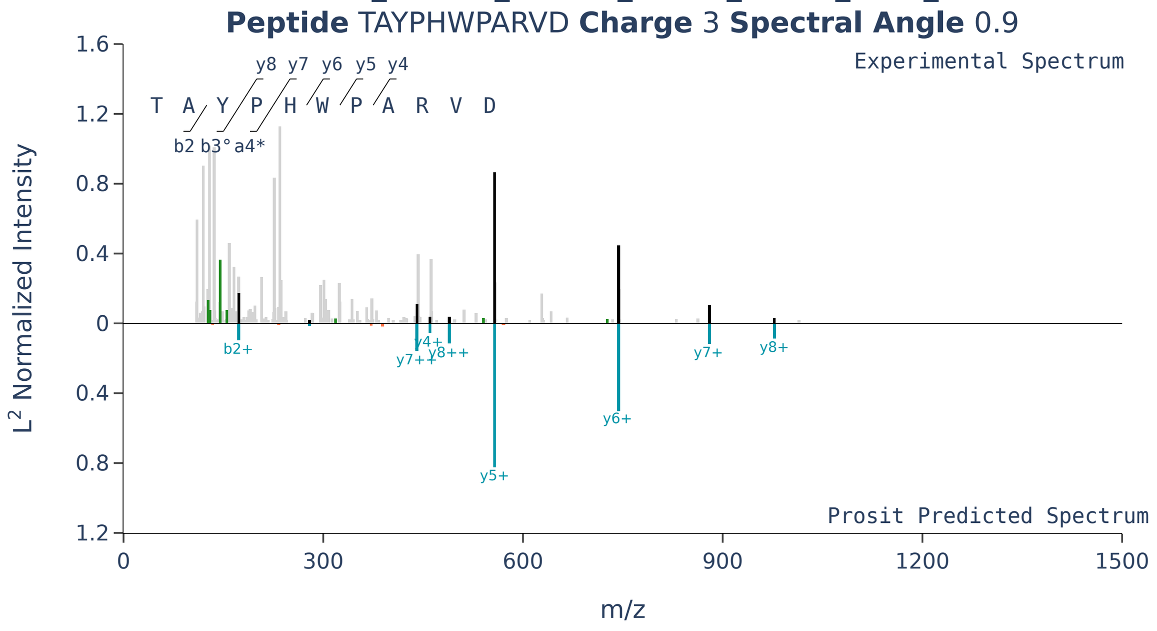

Source HRoetschke_271222_090123_HFGoe_LEDGF_b2R1_4h **Scan** 5427
Peptide NHEASTKKKPSSEEREEL **Charge** 3 **Spectral Angle** 0.84

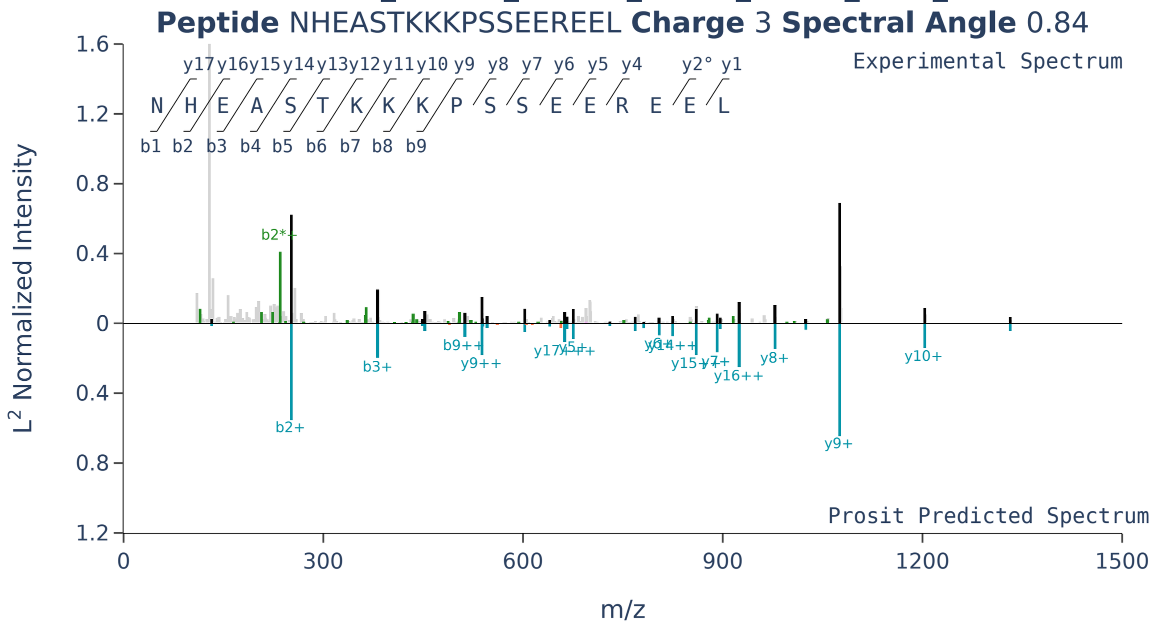

Source HRoetschke_271222_090123_HFGoe_LEDGF_b2R1_2h **Scan** 11805
Peptide AGPGMTRDFKPGD **Charge** 3 **Spectral Angle** 0.9

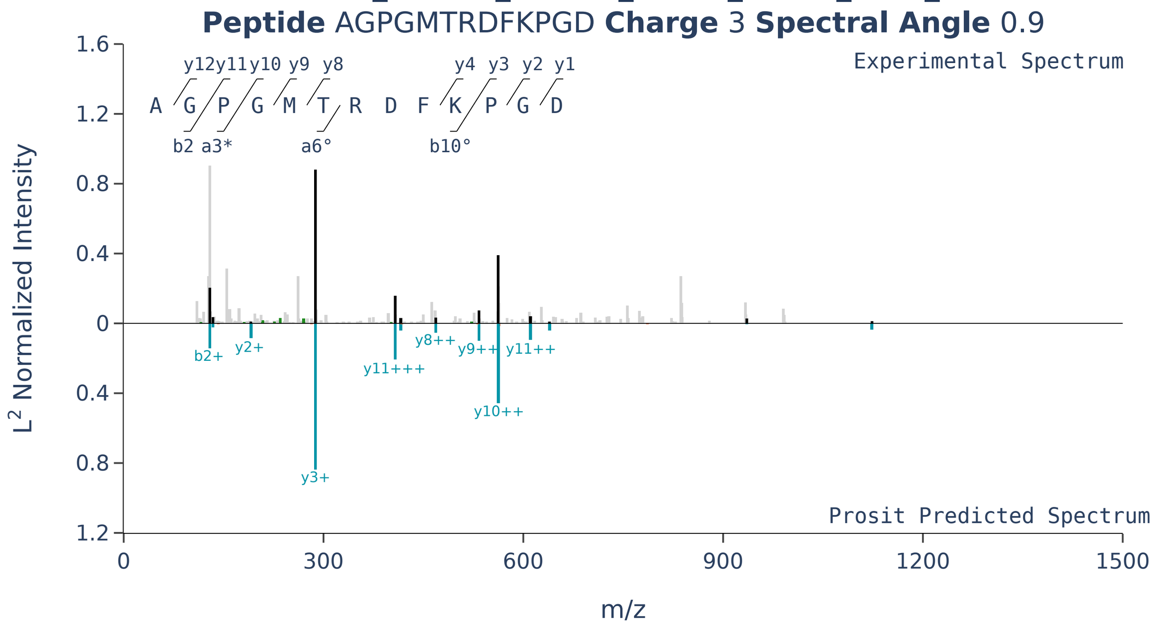

Source HRoetschke_271222_090123_HFGoe_LEDGF_b1R2_4h **Scan** 12326
Peptide EGPGMTRDFKPGD **Charge** 3 **Spectral Angle** 0.89

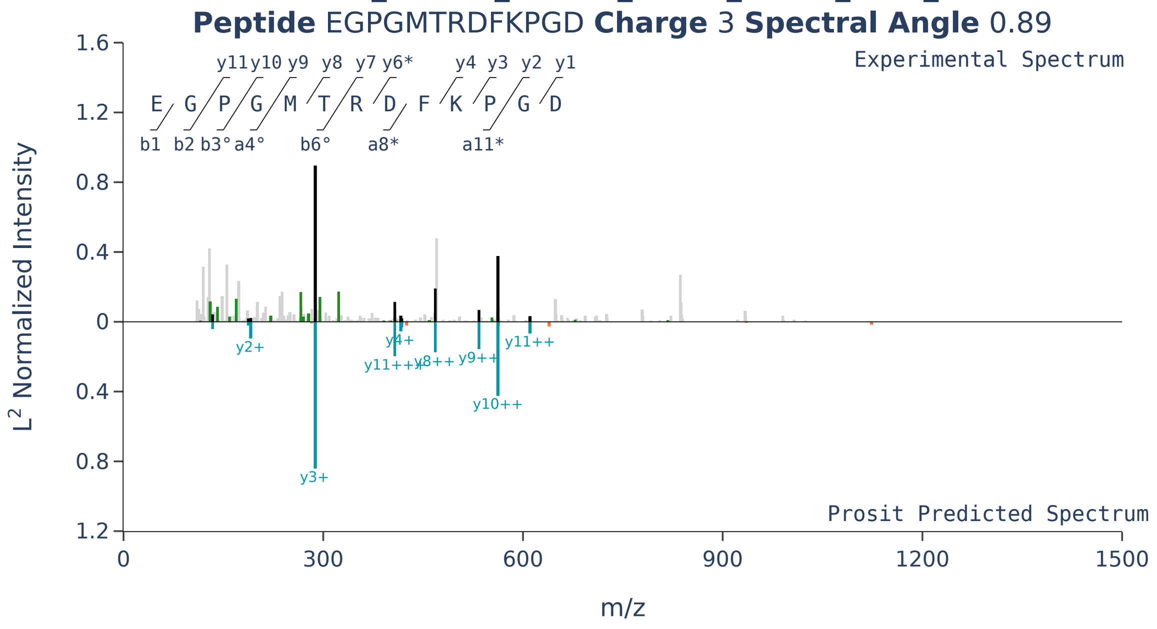

Source HRoetschke_271222_090123_HFGoe_LEDGF_b1R1_24h **Scan** 16907
Peptide GVQQAQKHTEMITTL **Charge** 3 **Spectral Angle** 0.86

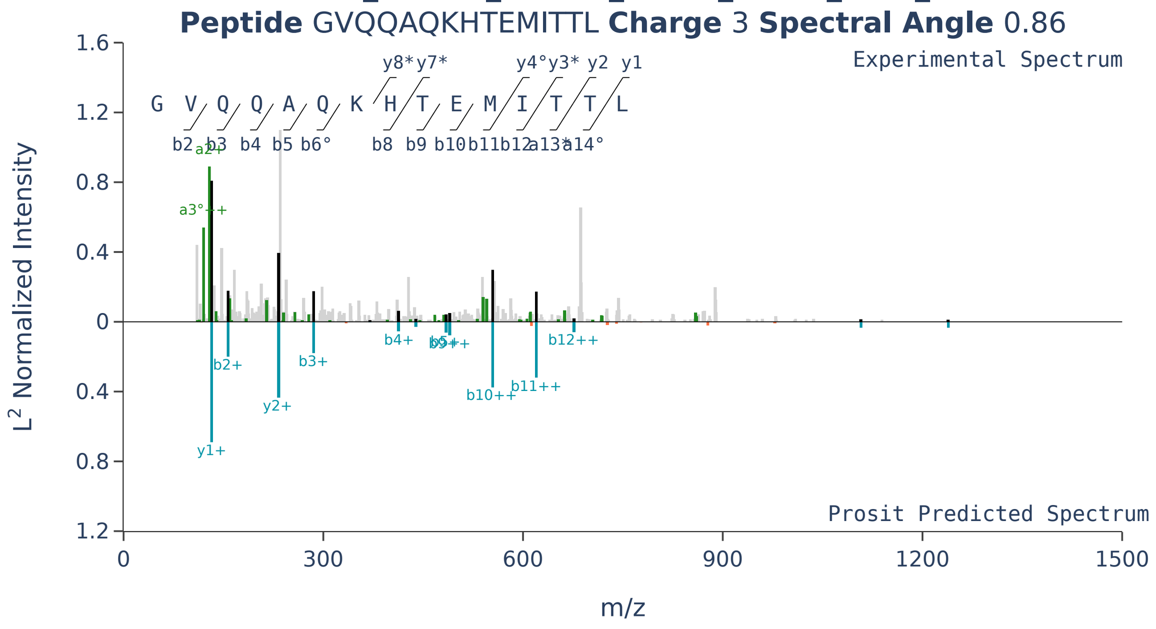

Source HRoetschke_271222_090123_HFGoe_LEDGF_b2R1_2h **Scan** 21421
Peptide TNKGFNEGLWE **Charge** 2 **Spectral Angle** 0.75

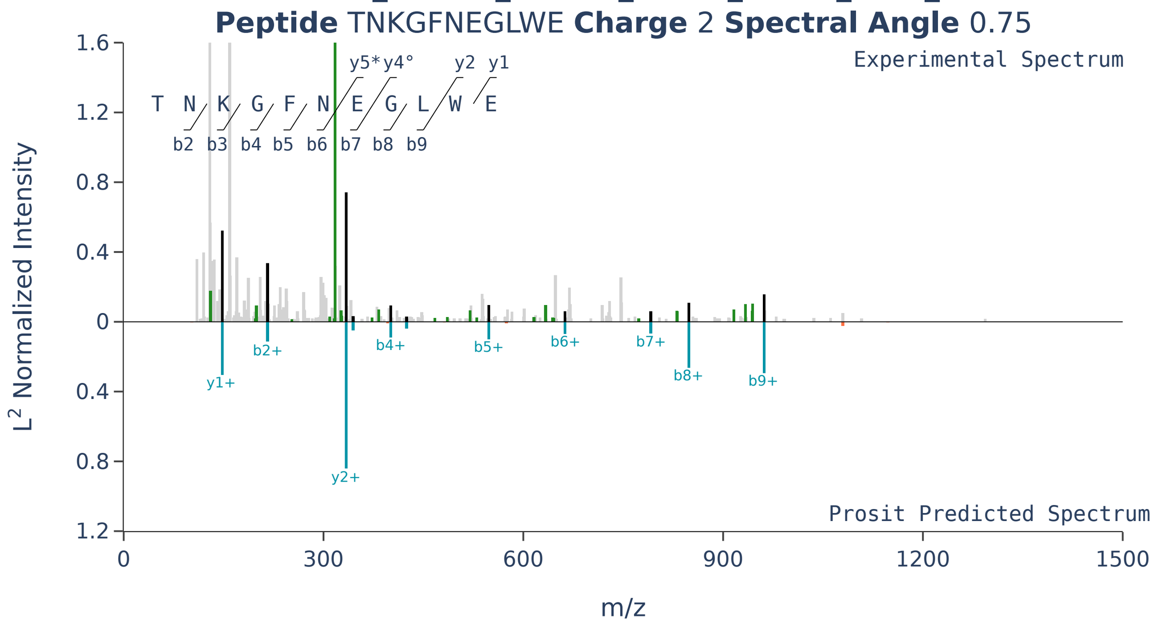

Source HRoetschke_271222_090123_HFGoe_LEDGF_b1R1_24h **Scan** 9778
Peptide GTHETAVKPPTNKL **Charge** 2 **Spectral Angle** 0.88

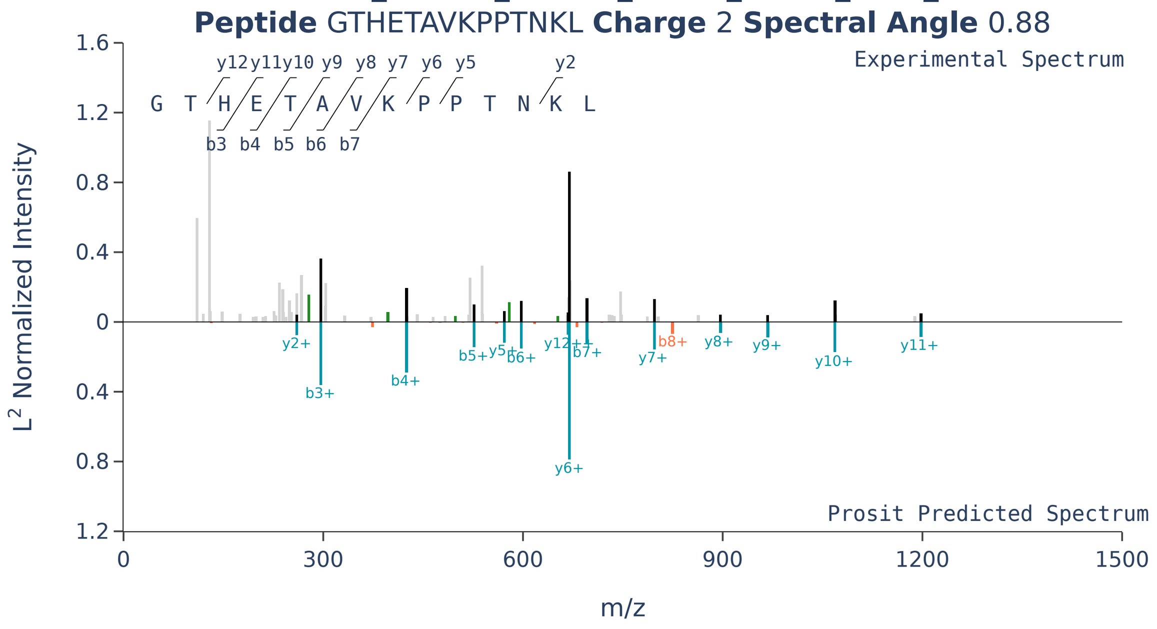

Source HRoetschke_271222_090123_HFGoe_LEDGF_b2R2_2h **Scan** 17185
Peptide FLGPKDIFPYSENKEKYGKPNKRTA **Charge** 5 **Spectral Angle** 0.8

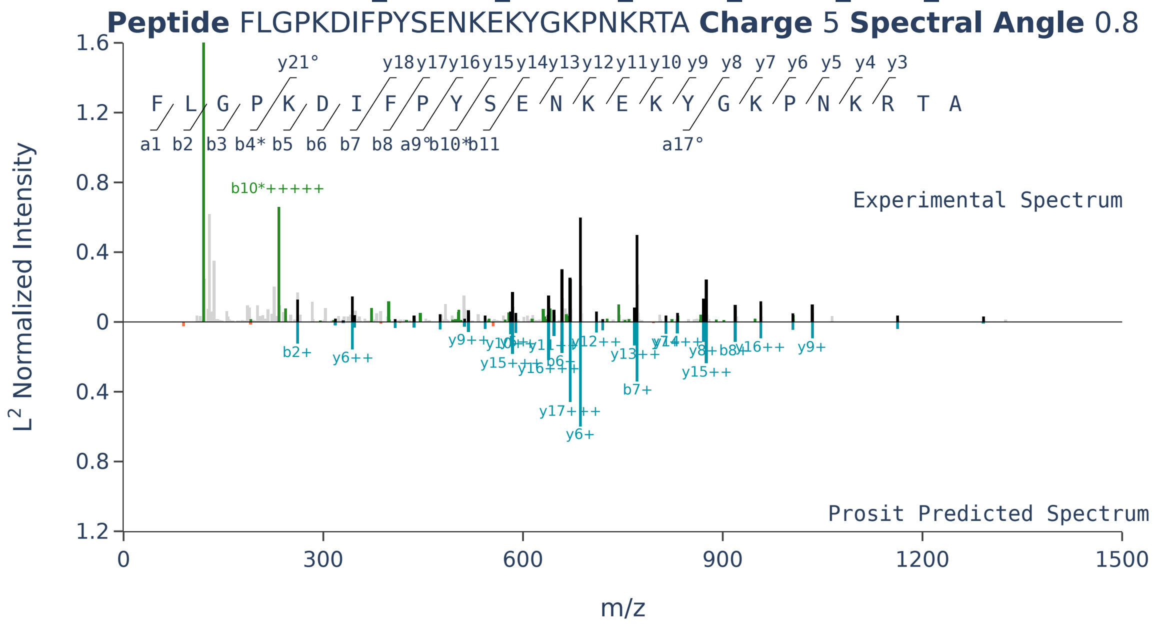

Source HRoetschke_271222_090123_HFGoe_LEDGF_b1R2_4h **Scan** 15906
Peptide MLEKSTMLYNKFKNM **Charge** 2 **Spectral Angle** 0.81

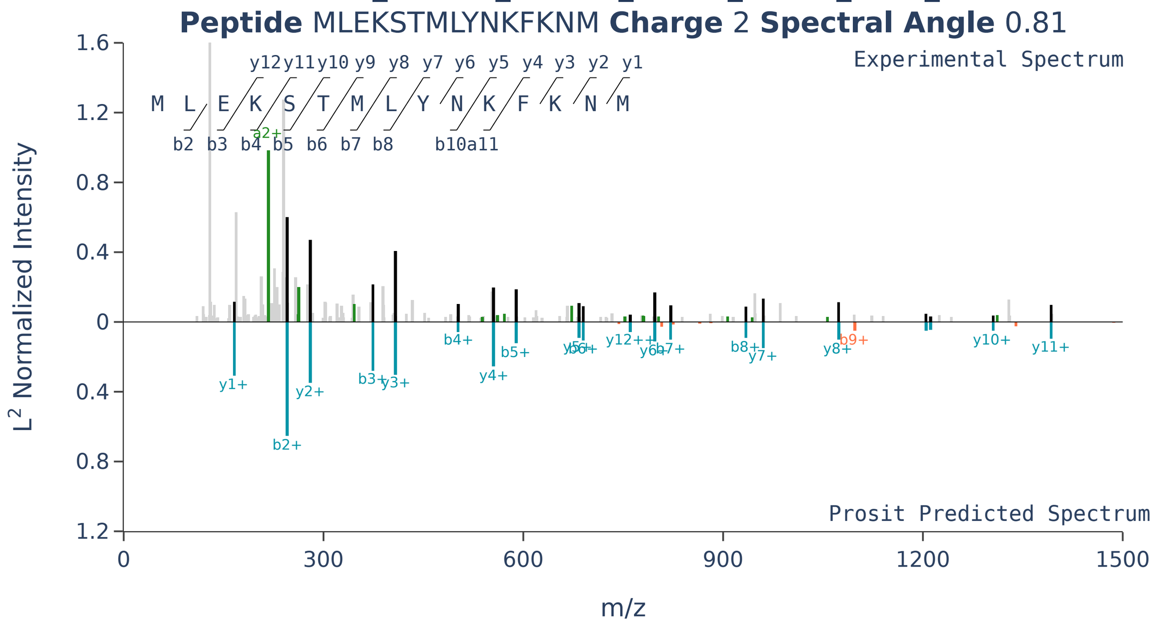

Source HRoetschke_271222_090123_HFGoe_LEDGF_b1R2_1h **Scan** 11974
Peptide TNGPGMTRDFKPGD **Charge** 2 **Spectral Angle** 0.86

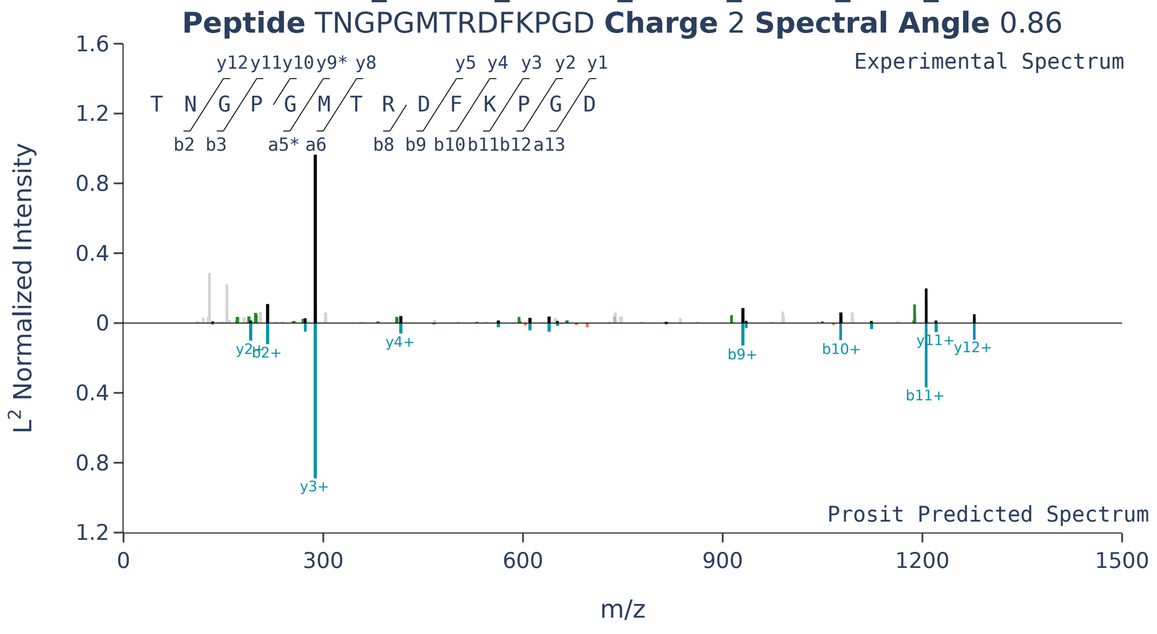

Source HRoetschke_271222_090123_HFGoe_LEDGF_b1R1_24h **Scan** 8716
Peptide TEEAGVVTTKE **Charge** 2 **Spectral Angle** 0.86

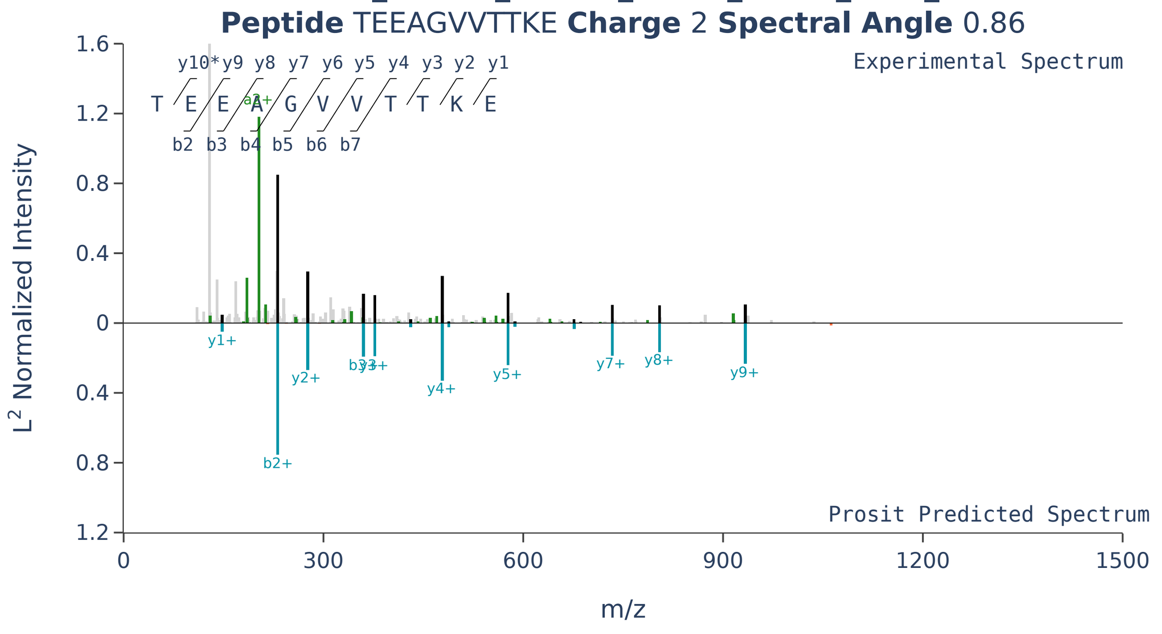

Source HRoetschke_271222_090123_HFGoe_LEDGF_b1R2_2h **Scan** 17238
Peptide QSGYPHWPARVDE **Charge** 3 **Spectral Angle** 0.96

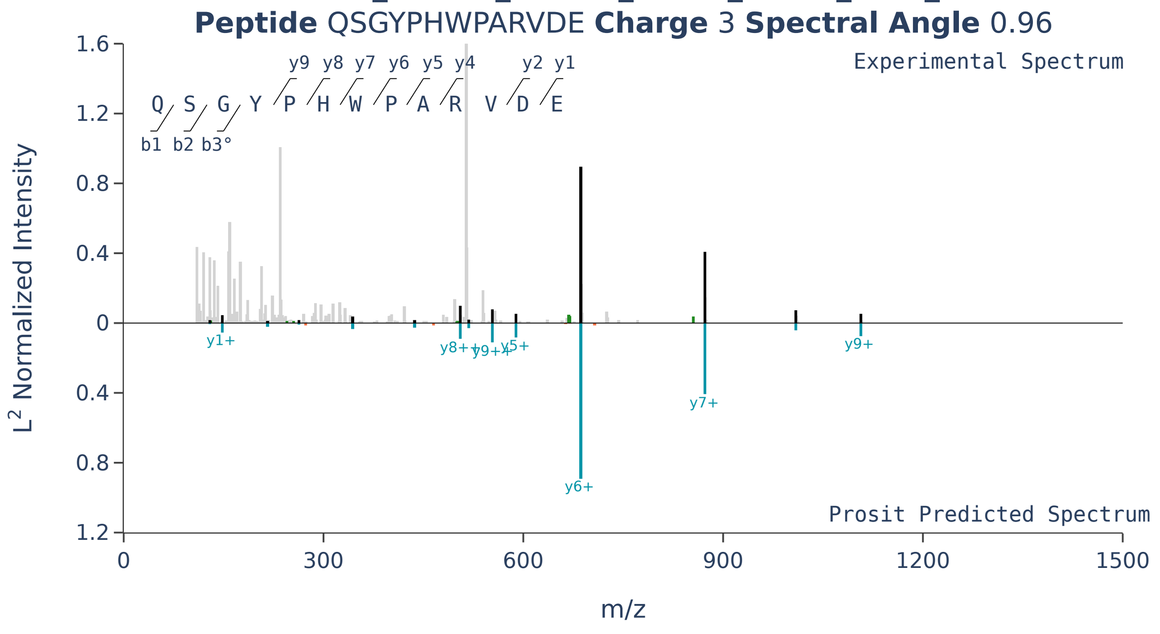

Source HRoetschke_271222_090123_HFGoe_LEDGF_b1R2_24h **Scan** 25047
Peptide TQVLSVNL **Charge** 1 **Spectral Angle** 0.87

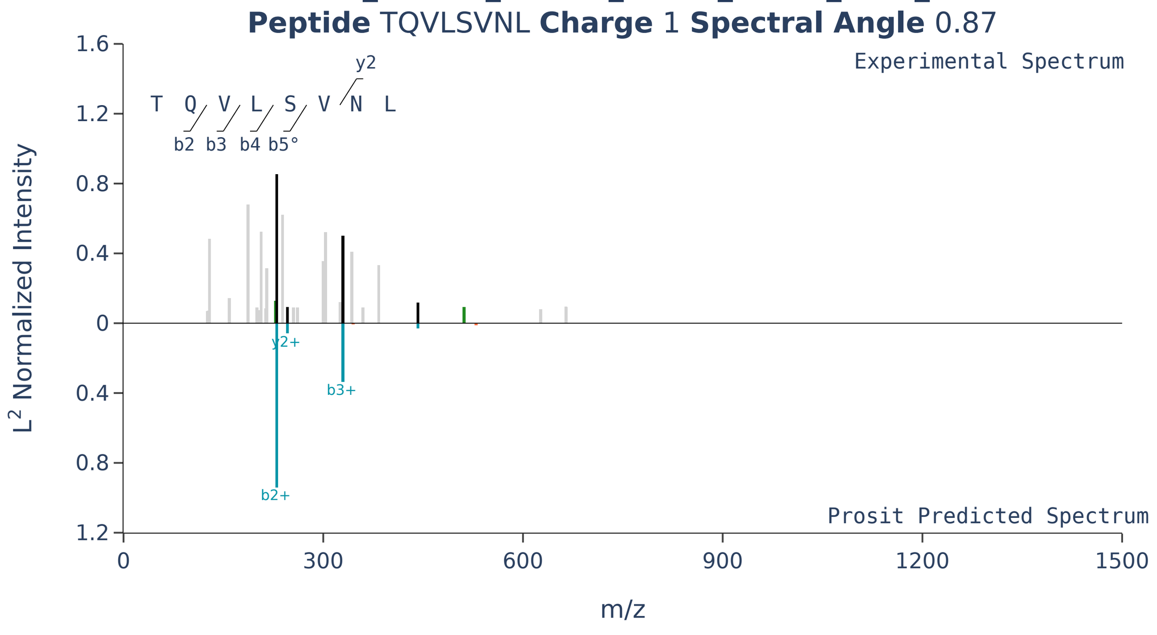

Source HRoetschke_271222_090123_HFGoe_LEDGF_b1R2_24h **Scan** 13188
Peptide TGEISLKD **Charge** 2 **Spectral Angle** 0.84

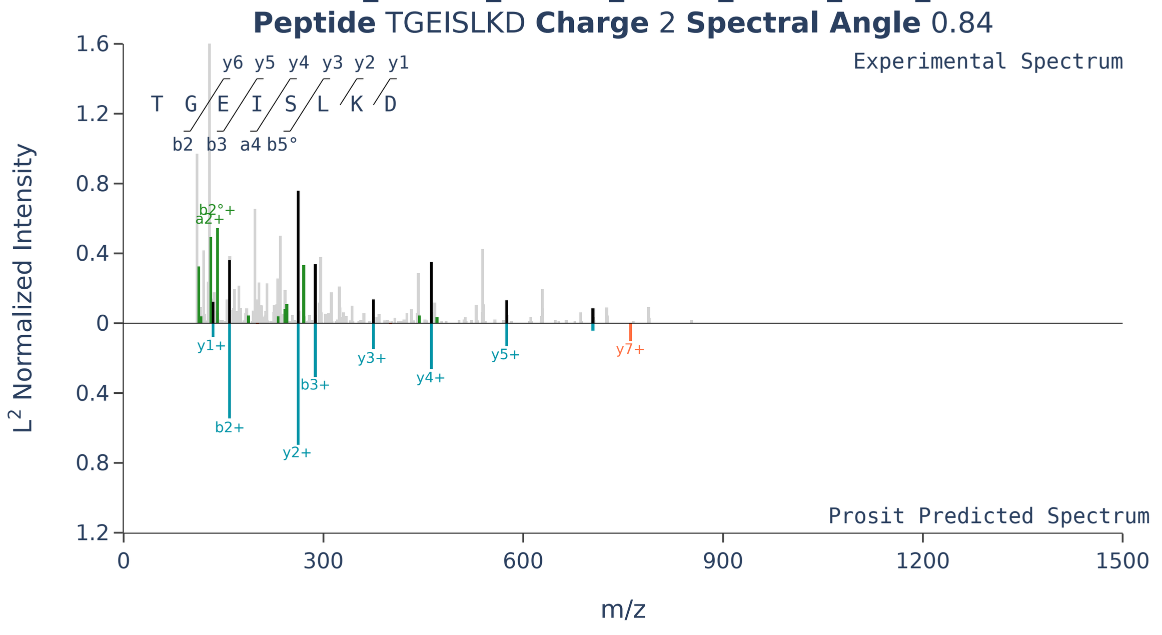

Source HRoetschke_271222_090123_HFGoe_LEDGF_b1R2_24h **Scan** 21741
Peptide TGLKIDNLDVNRCIE **Charge** 2 **Spectral Angle** 0.87

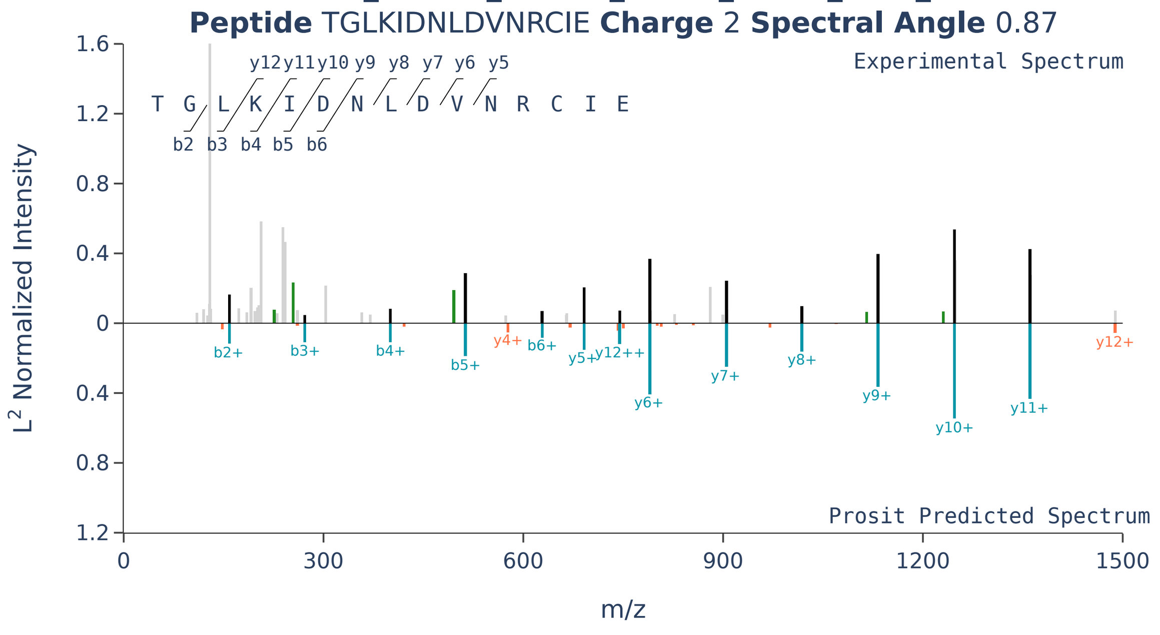

Source HRoetschke_271222_090123_HFGoe_LEDGF_b2R1_2h **Scan** 9293
Peptide SQVIMEKSQQA **Charge** 2 **Spectral Angle** 0.89

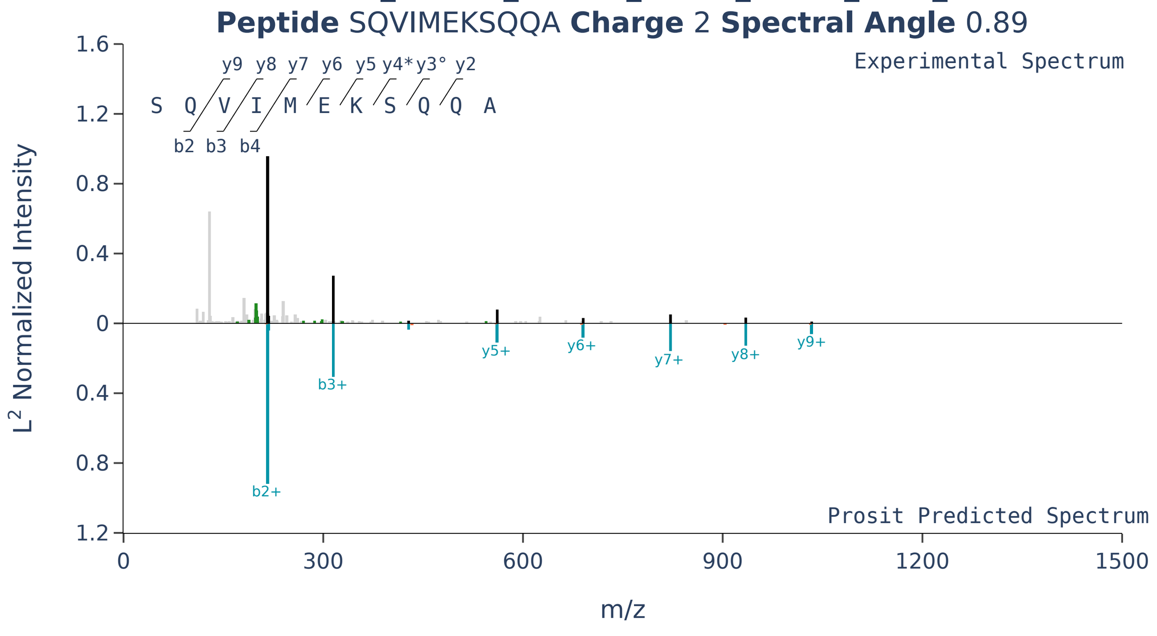

Source HRoetschke_271222_090123_HFGoe_LEDGF_b2R1_4h **Scan** 18251
Peptide NASYPHWPARV **Charge** 3 **Spectral Angle** 0.94

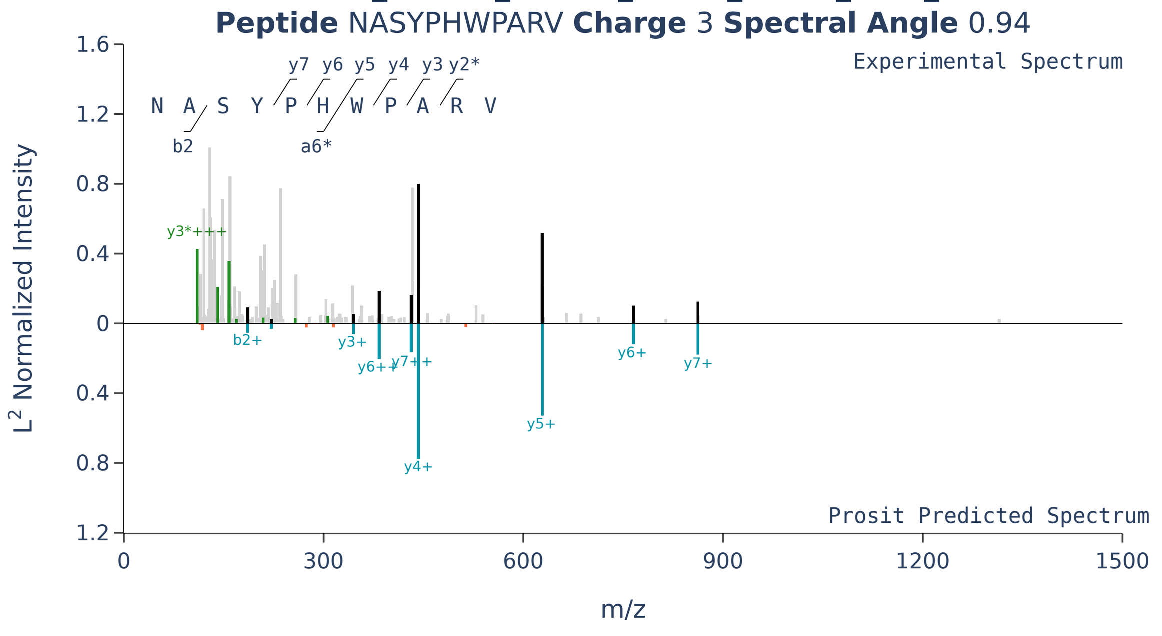

Source HRoetschke_271222_090123_HFGoe_LEDGF_b2R2_2h **Scan** 18208
Peptide TGYPHWPARV **Charge** 3 **Spectral Angle** 0.93

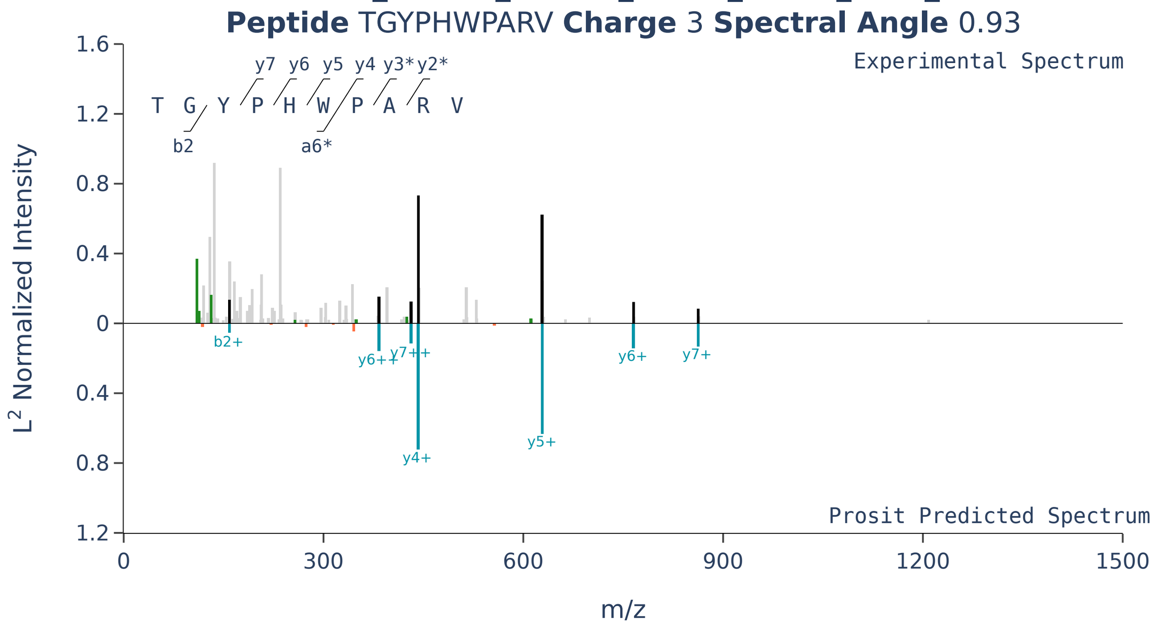

Source HRoetschke_271222_090123_HFGoe_LEDGF_b2R2_2h **Scan** 21938
Peptide SQKGFNEGLWE **Charge** 2 **Spectral Angle** 0.78

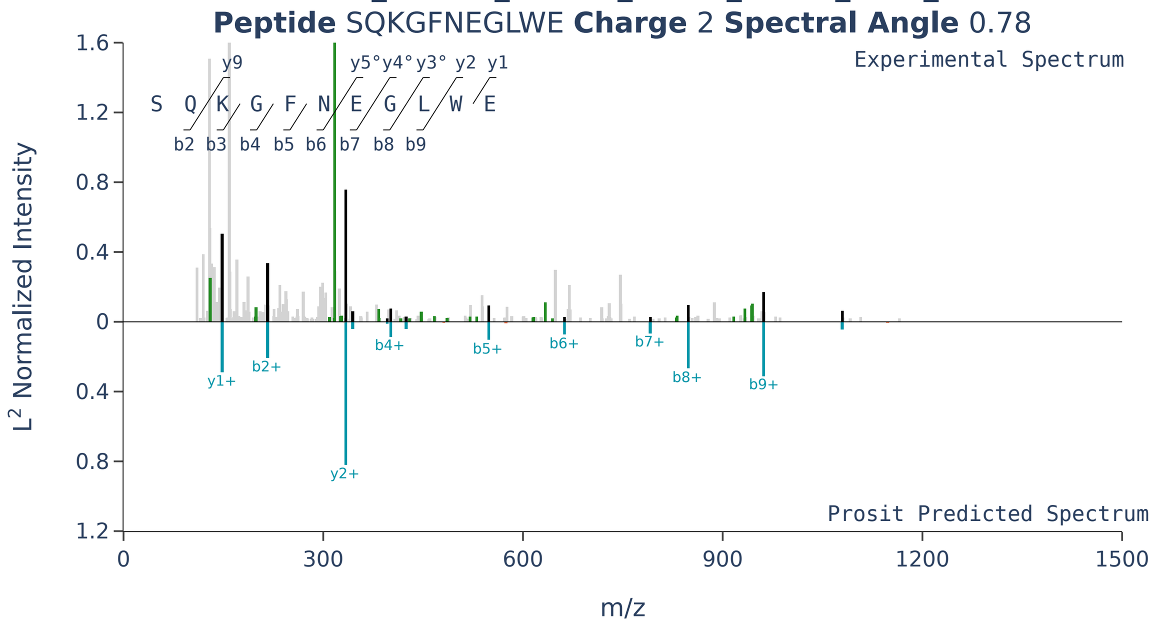

Source HRoetschke_271222_090123_HFGoe_LEDGF_b2R1_2h **Scan** 14740
Peptide GYGPGMTRDFKPGD **Charge** 3 **Spectral Angle** 0.83

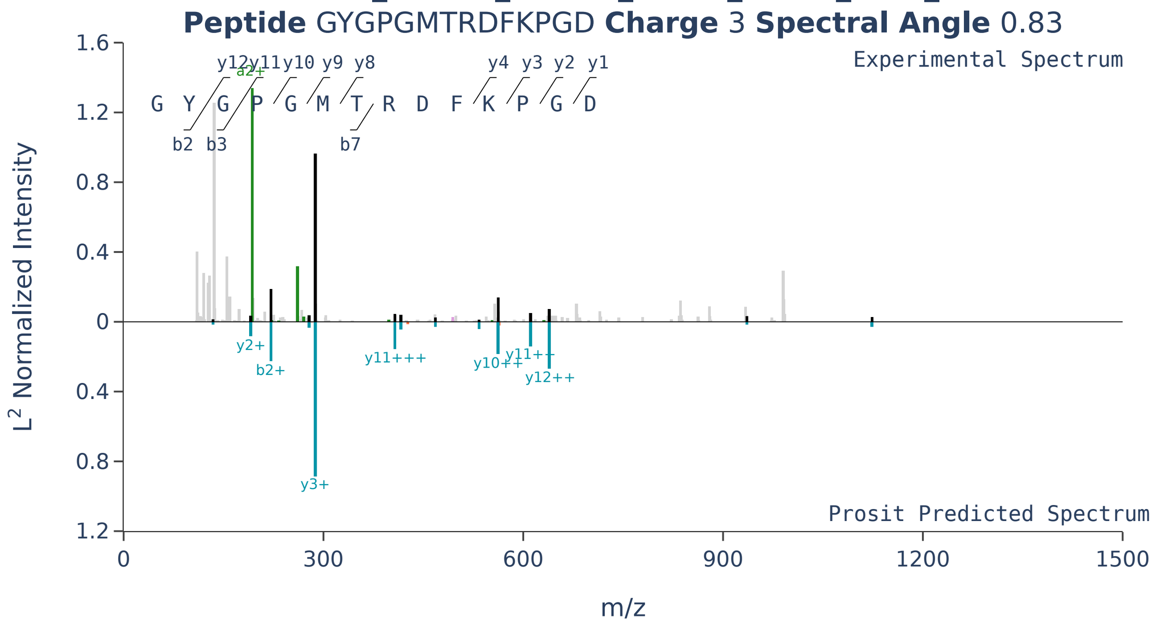

Source HRoetschke_271222_090123_HFGoe_LEDGF_b1R1_24h **Scan** 13905
Peptide GTHETKVSQVIME **Charge** 2 **Spectral Angle** 0.83

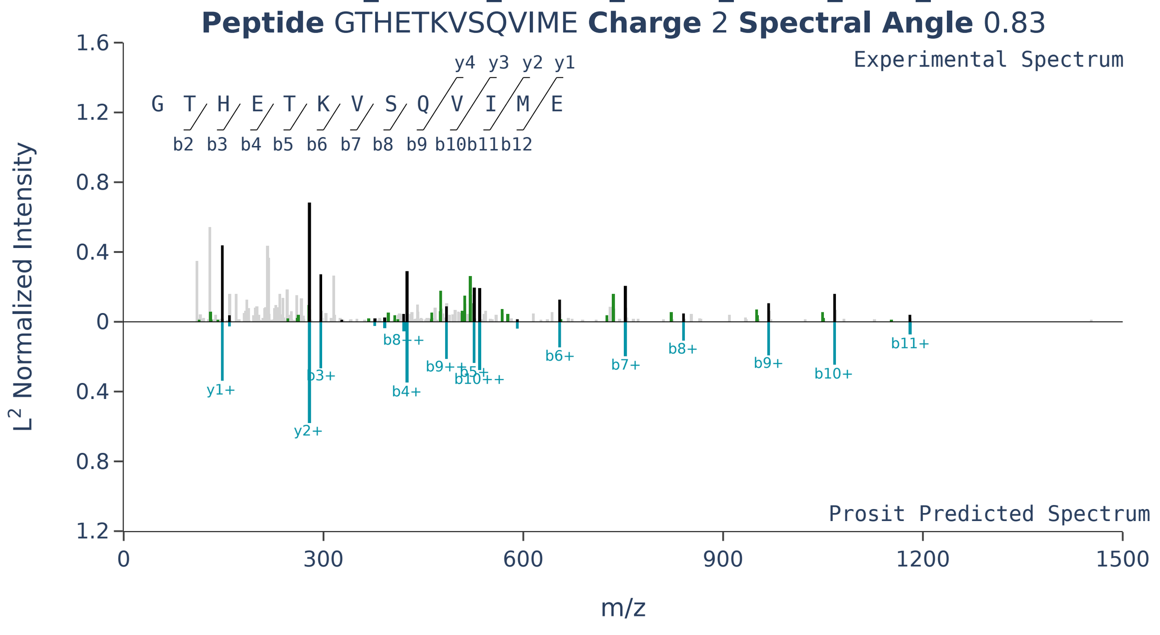

Source HRoetschke_271222_090123_HFGoe_LEDGF_b1R1_24h **Scan** 13466
Peptide SQQAATKQSVPDGAVKPPTNKL **Charge** 3 **Spectral Angle** 0.87

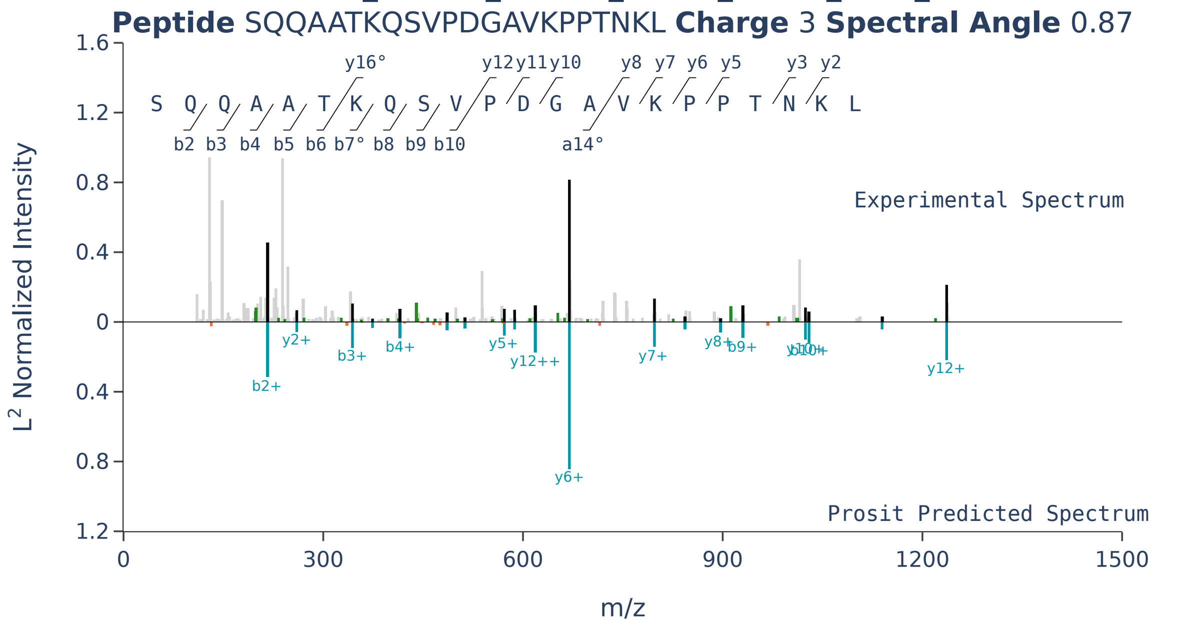

Source HRoetschke_271222_090123_HFGoe_LEDGF_b1R1_24h **Scan** 11102
Peptide ALDKPPTNKL **Charge** 3 **Spectral Angle** 0.88

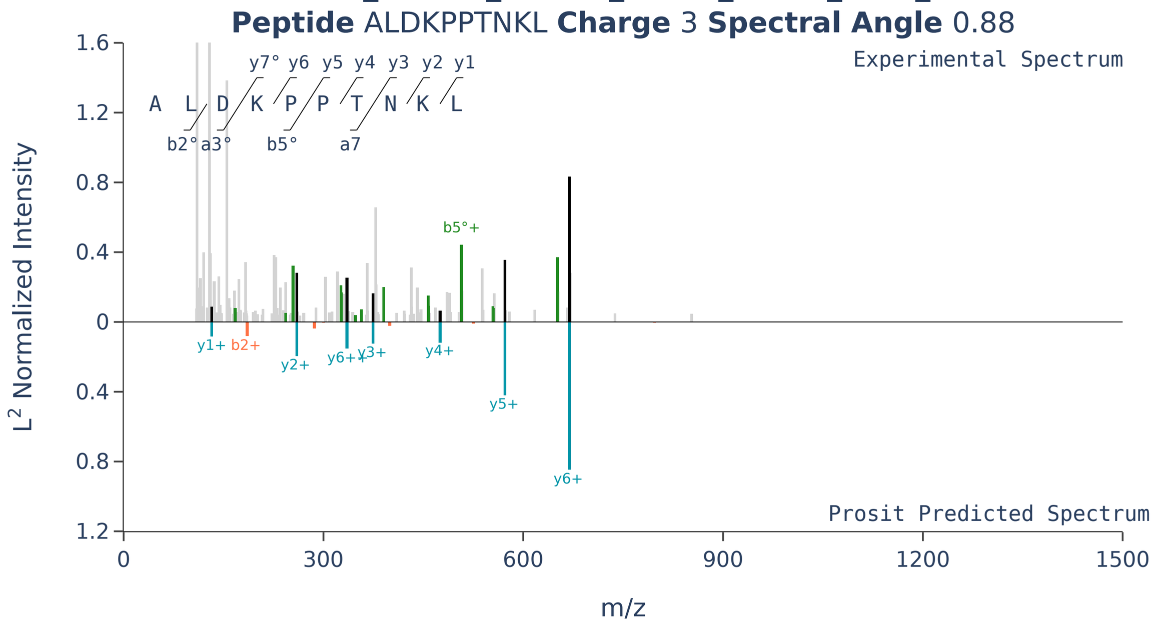

Source HRoetschke_271222_090123_HFGoe_LEDGF_b2R2_24h **Scan** 8733
Peptide GTMDSRLQ **Charge** 2 **Spectral Angle** 0.84

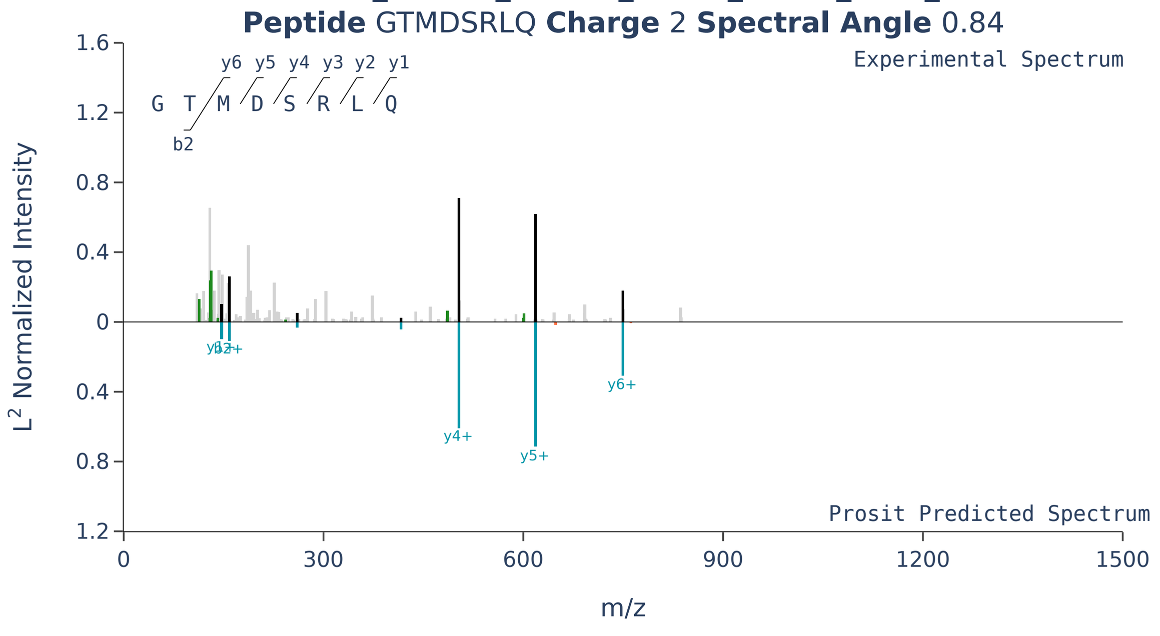

Source HRoetschke_271222_090123_HFGoe_LEDGF_b1R1_24h **Scan** 13428
Peptide SPHWPARVD **Charge** 3 **Spectral Angle** 0.91

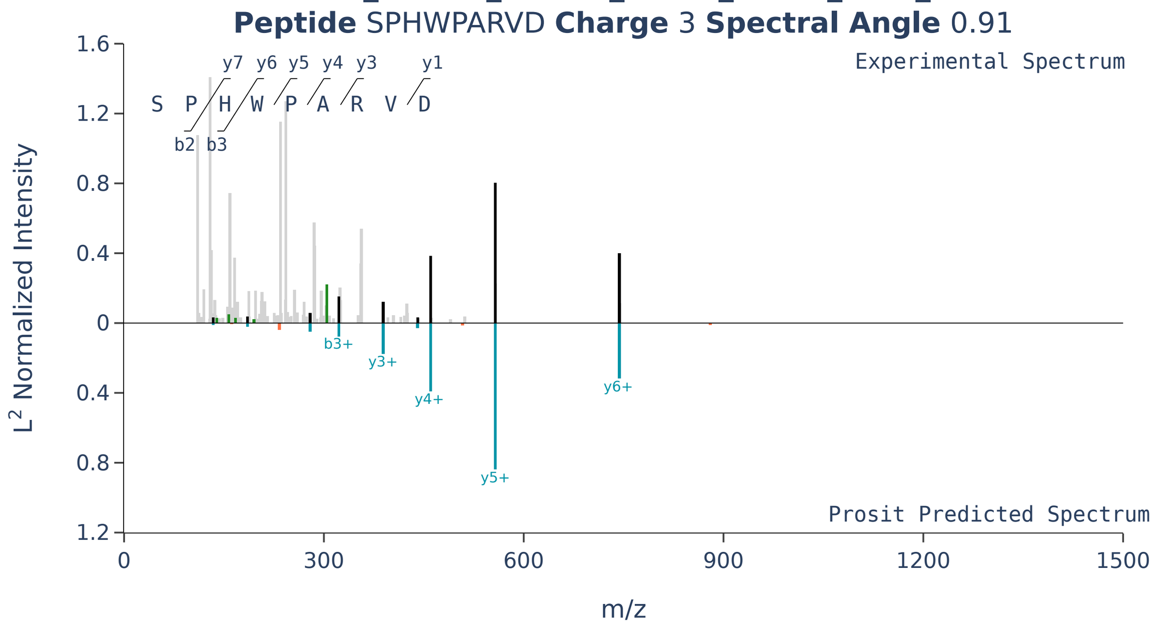

Source HRoetschke_271222_090123_HFGoe_LEDGF_b2R2_2h **Scan** 6799
Peptide PKMVKQPCTEE **Charge** 3 **Spectral Angle** 0.89

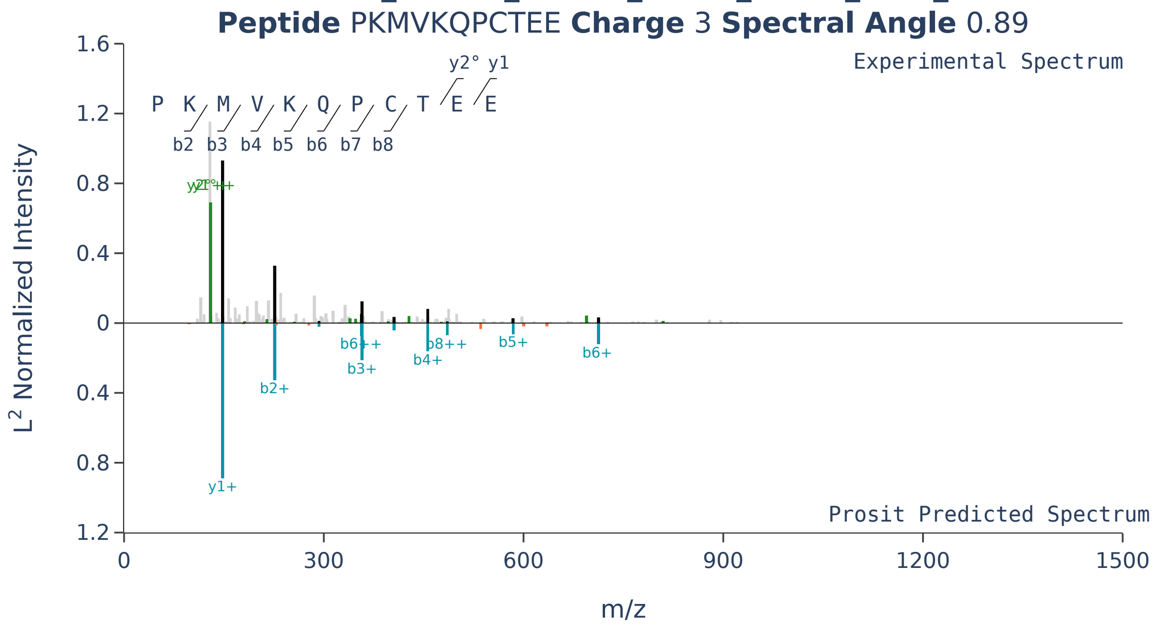

Source HRoetschke_271222_090123_HFGoe_LEDGF_b1R2_24h **Scan** 18899
Peptide TAYPHWPARV **Charge** 3 **Spectral Angle** 0.91

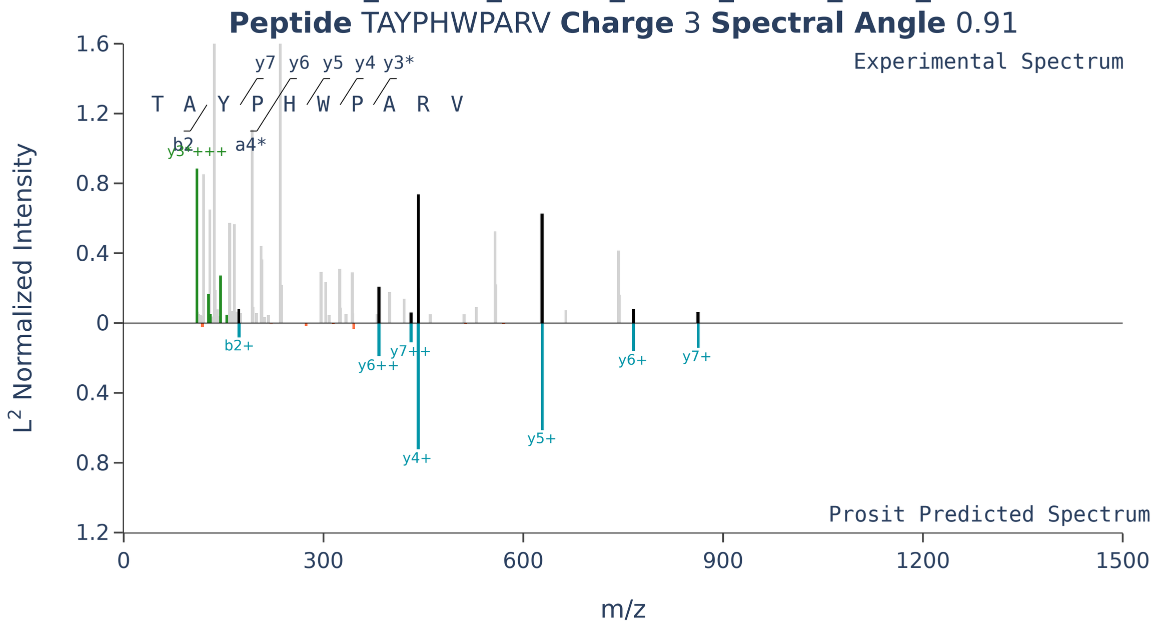

Source HRoetschke_271222_090123_HFGoe_LEDGF_b1R1_24h **Scan** 10081
Peptide KVSQVIMEKSMT **Charge** 2 **Spectral Angle** 0.89

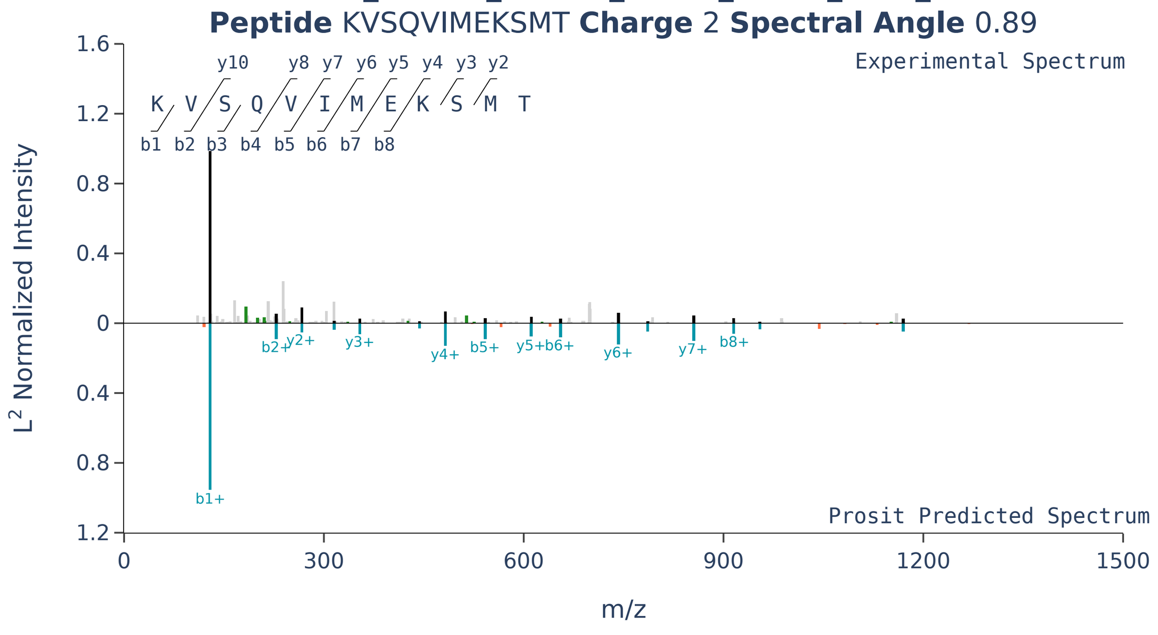

Source HRoetschke_271222_090123_HFGoe_LEDGF_b1R1_24h **Scan** 17684
Peptide FSSPHWPARVD **Charge** 3 **Spectral Angle** 0.94

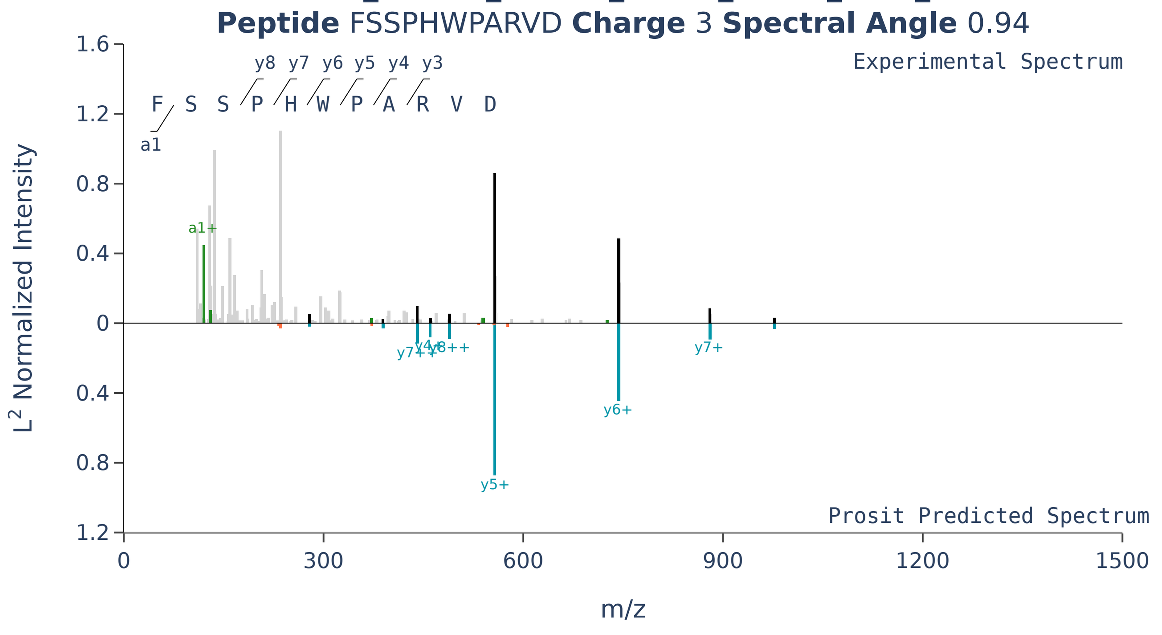

Source HRoetschke_271222_090123_HFGoe_LEDGF_b1R2_24h **Scan** 11973
Peptide TEEAGVVTTT **Charge** 2 **Spectral Angle** 0.91

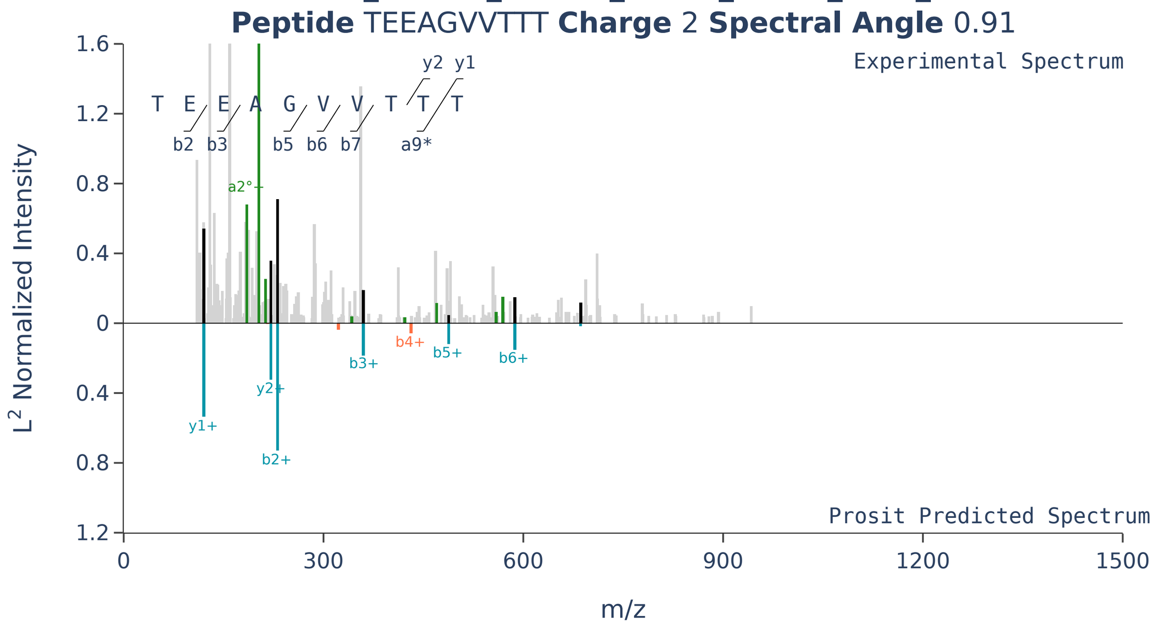

Source HRoetschke_271222_090123_HFGoe_LEDGF_b1R2_24h **Scan** 18266
Peptide ELYPHWPAR **Charge** 3 **Spectral Angle** 0.78

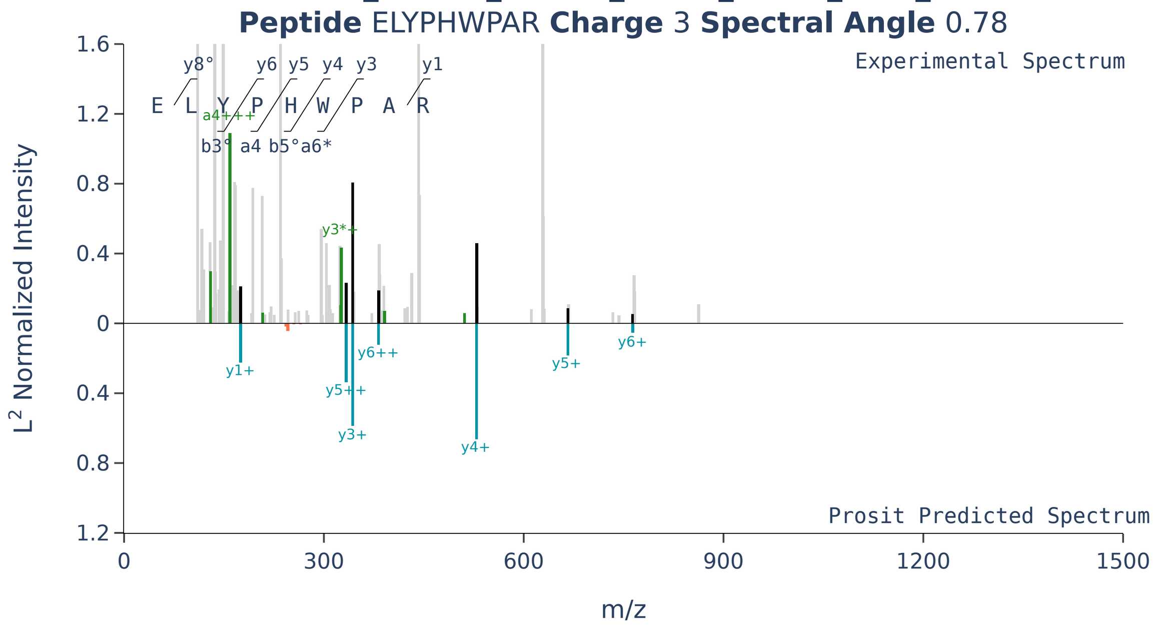

Source HRoetschke_271222_090123_HFGoe_LEDGF_b1R1_1h **Scan** 11816
Peptide GAPGMTRDFKPGD **Charge** 3 **Spectral Angle** 0.86

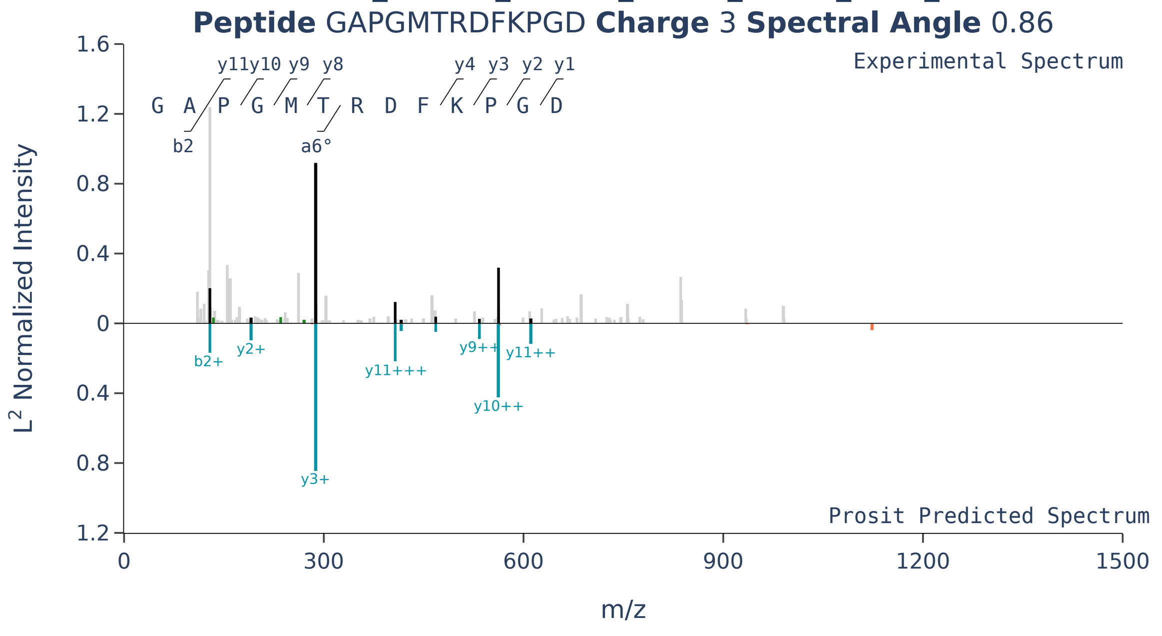

Source HRoetschke_271222_090123_HFGoe_LEDGF_b1R1_4h **Scan** 11952
Peptide LIFAKMKGKET **Charge** 3 **Spectral Angle** 0.84

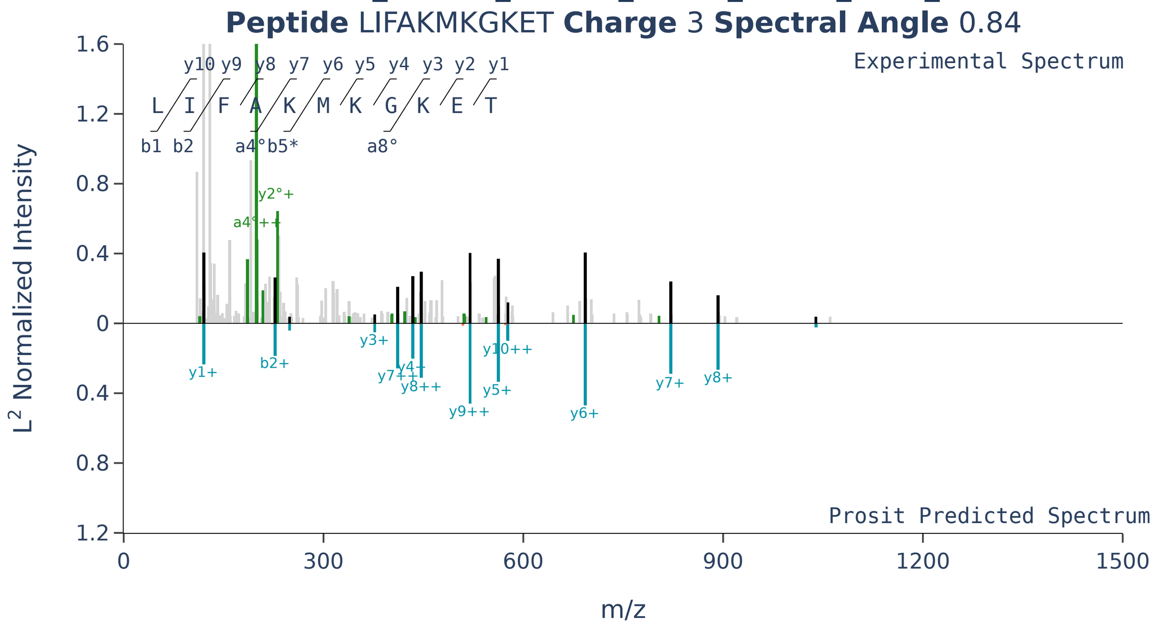

Source HRoetschke_271222_090123_HFGoe_LEDGF_b2R2_1h **Scan** 17350
Peptide SQGYPHWPARV **Charge** 3 **Spectral Angle** 0.91

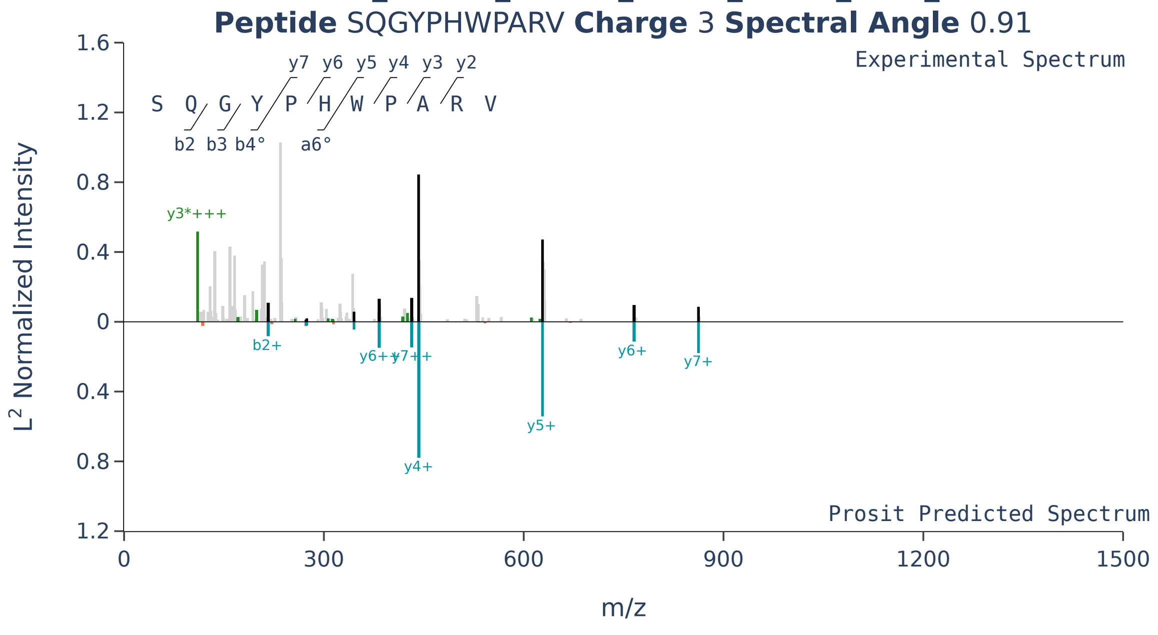

Source HRoetschke_271222_090123_HFGoe_LEDGF_b2R2_2h **Scan** 11486
Peptide GGPGMTRDFKPGD **Charge** 3 **Spectral Angle** 0.86

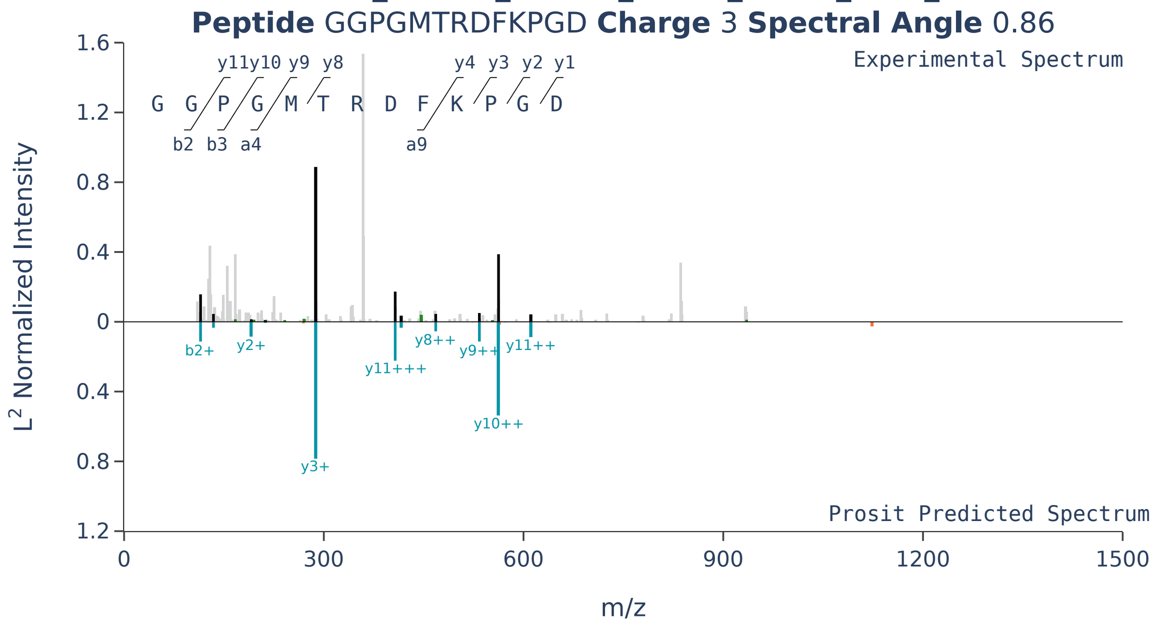

Source HRoetschke_271222_090123_HFGoe_LEDGF_b2R2_4h **Scan** 8390
Peptide GKSNASSDVEVEE **Charge** 2 **Spectral Angle** 0.9

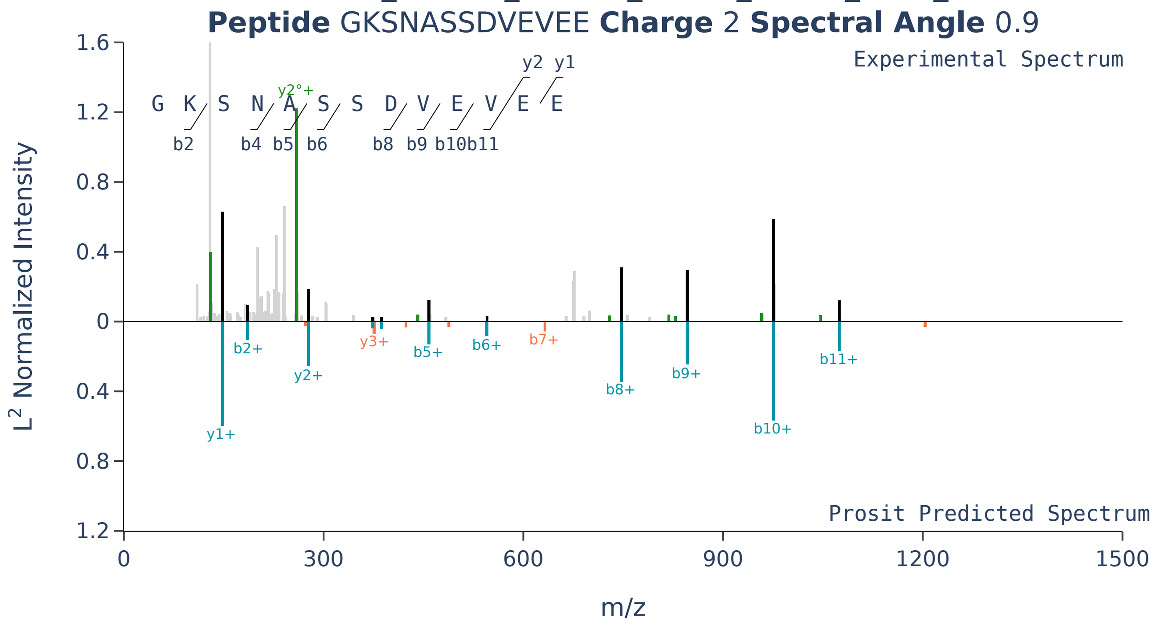

Source HRoetschke_271222_090123_HFGoe_LEDGF_b2R1_24h **Scan** 11661
Peptide TEEAGVVTTQQA **Charge** 2 **Spectral Angle** 0.89

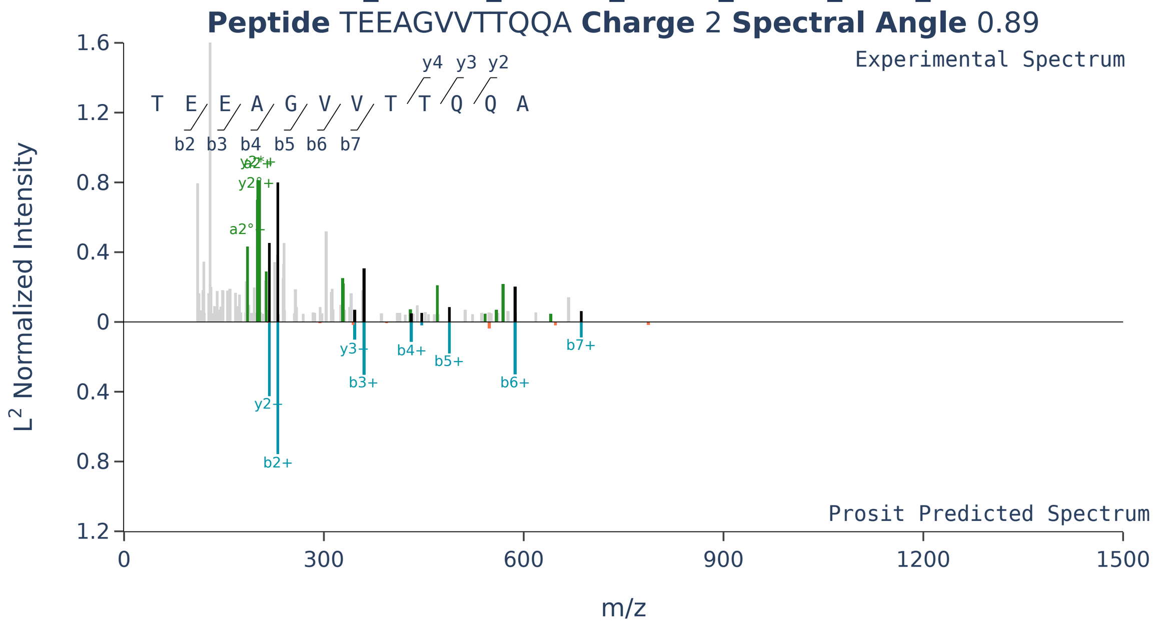

Source HRoetschke_271222_090123_HFGoe_LEDGF_b2R1_2h **Scan** 6874
Peptide PKMVKQPCPNE **Charge** 3 **Spectral Angle** 0.82

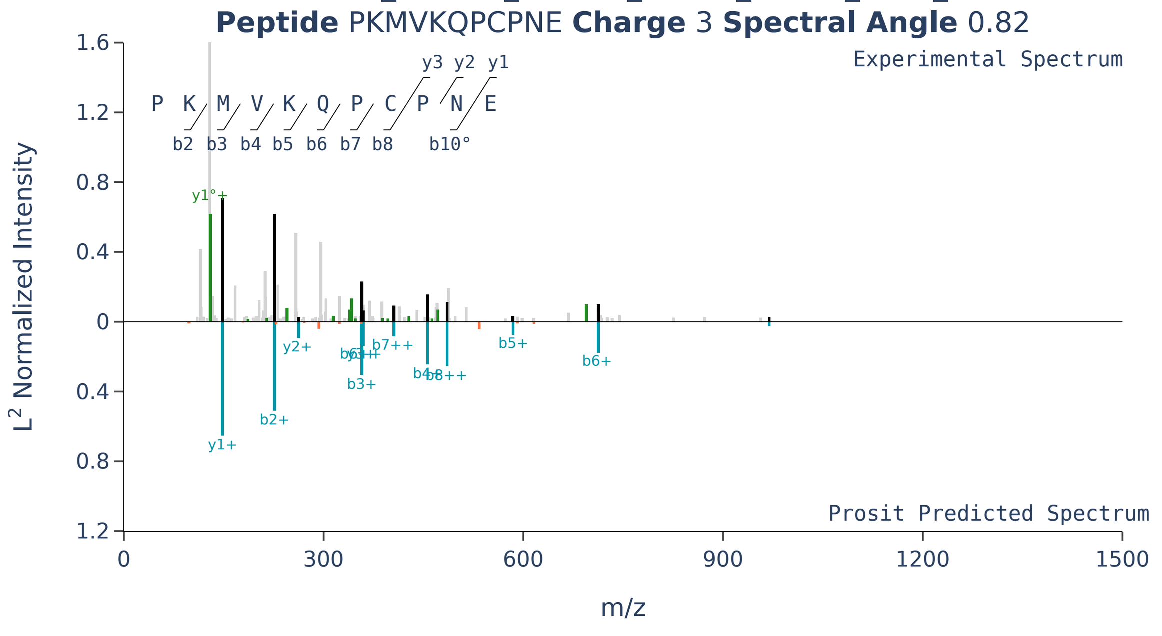

Source HRoetschke_271222_090123_HFGoe_LEDGF_b2R2_4h **Scan** 8866
Peptide PSSEERELE **Charge** 2 **Spectral Angle** 0.89

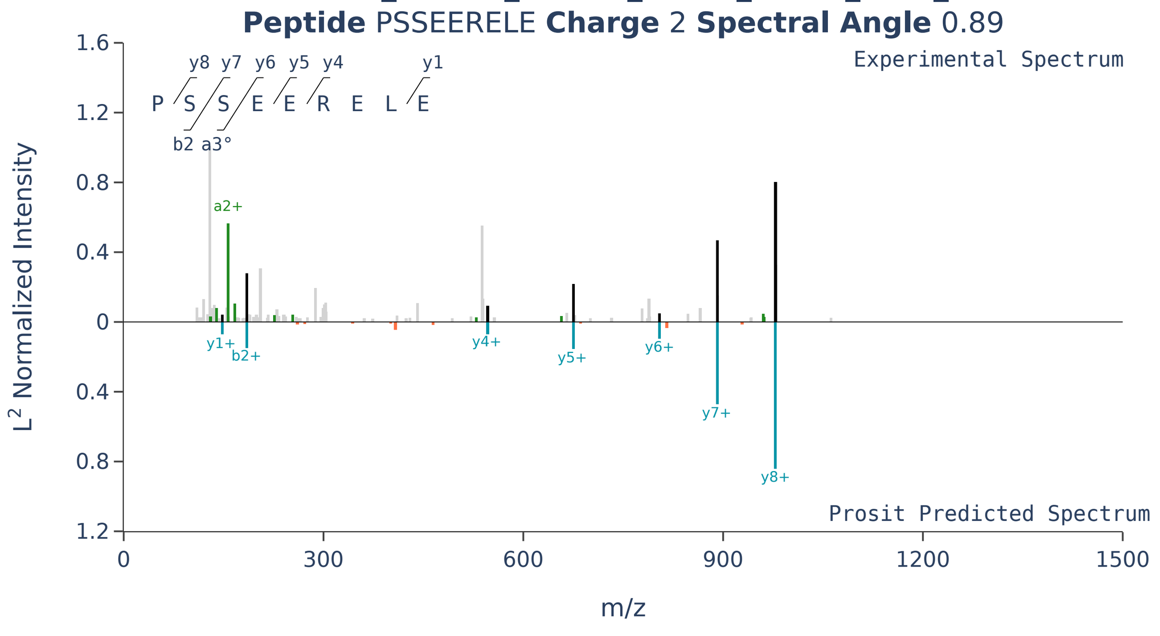

Source HRoetschke_271222_090123_HFGoe_LEDGF_b1R2_24h **Scan** 15376
Peptide KGFNEEL **Charge** 2 **Spectral Angle** 0.82

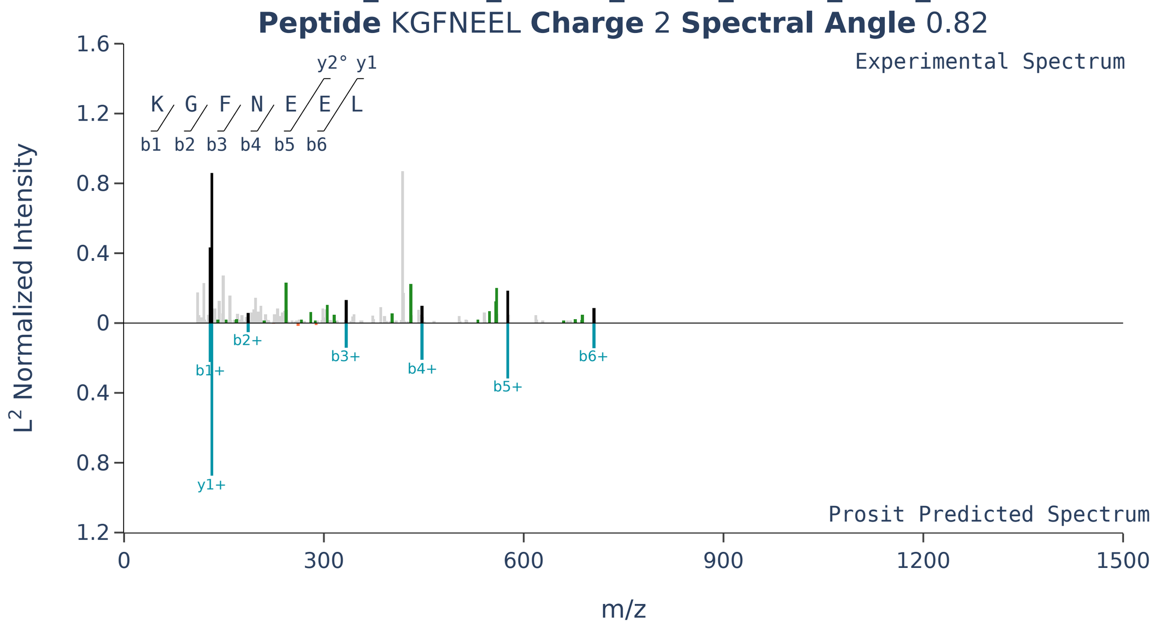

Source HRoetschke_271222_090123_HFGoe_LEDGF_b1R1_24h **Scan** 16408
Peptide KGFNEGTAF **Charge** 2 **Spectral Angle** 0.8

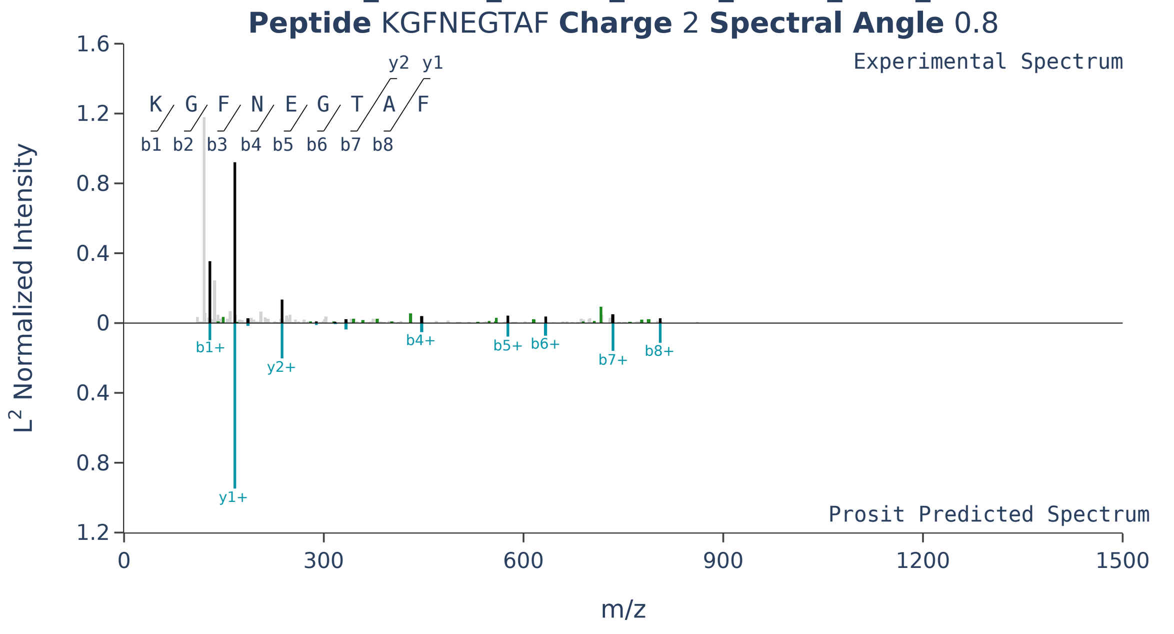

Source HRoetschke_271222_090123_HFGoe_LEDGF_b1R1_24h **Scan** 17187
Peptide SQVIMEKSEL **Charge** 2 **Spectral Angle** 0.88

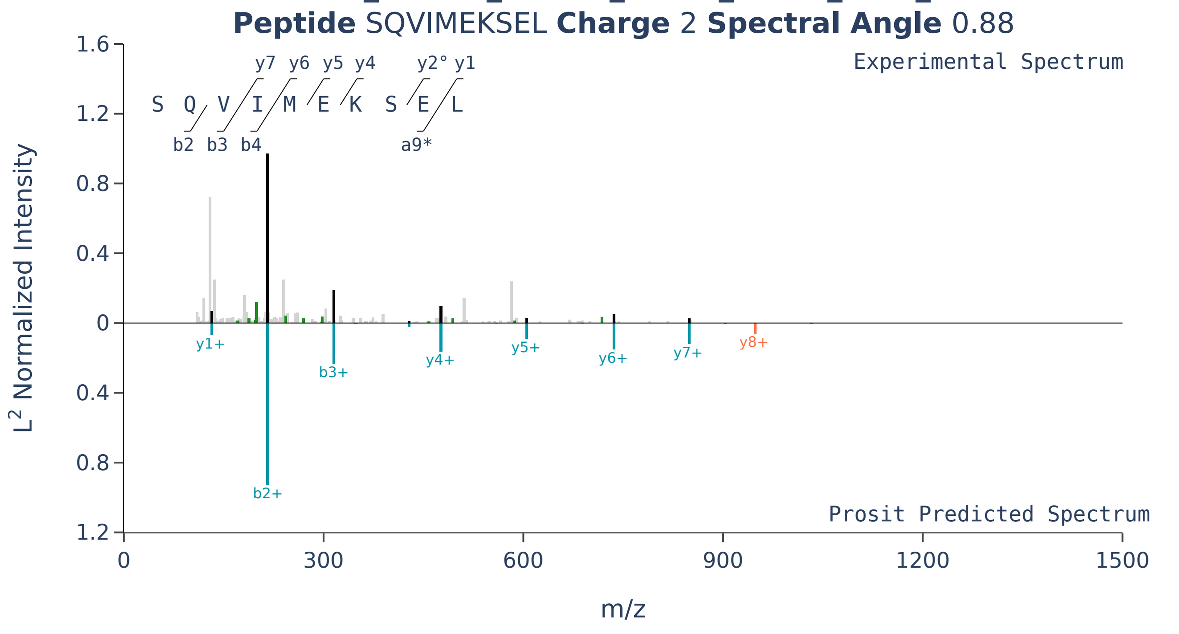

Source HRoetschke_271222_090123_HFGoe_LEDGF_b2R2_2h **Scan** 6940
Peptide SQQAATKQSGPGM **Charge** 2 **Spectral Angle** 0.83

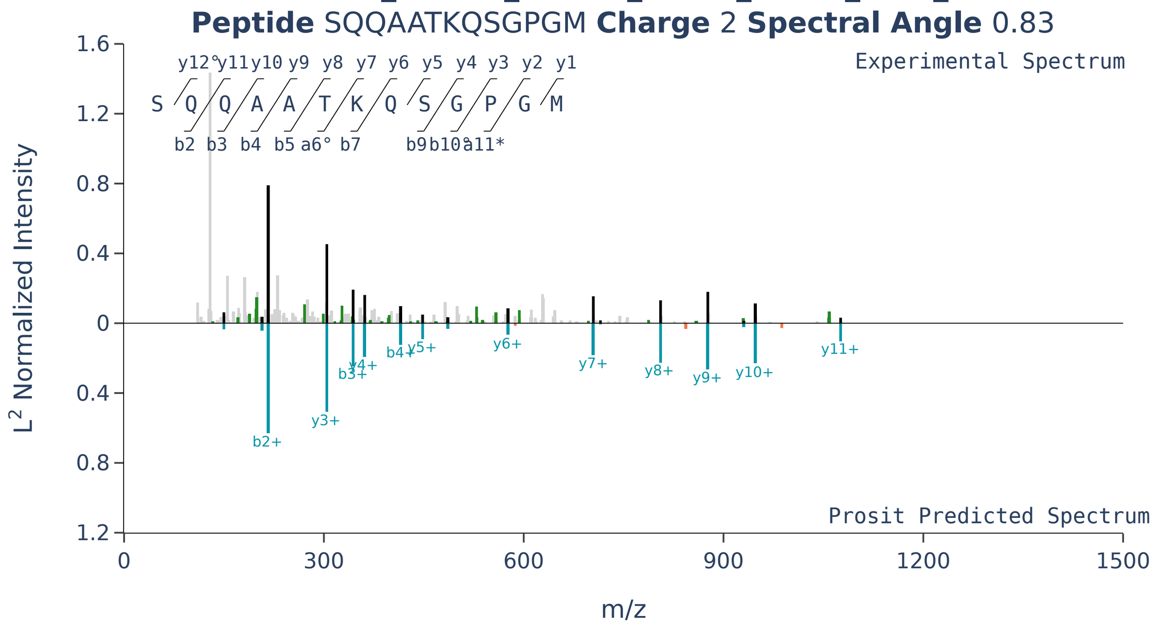

Source HRoetschke_271222_090123_HFGoe_LEDGF_b1R2_24h **Scan** 8841
Peptide TEEAGVVTKET **Charge** 2 **Spectral Angle** 0.91

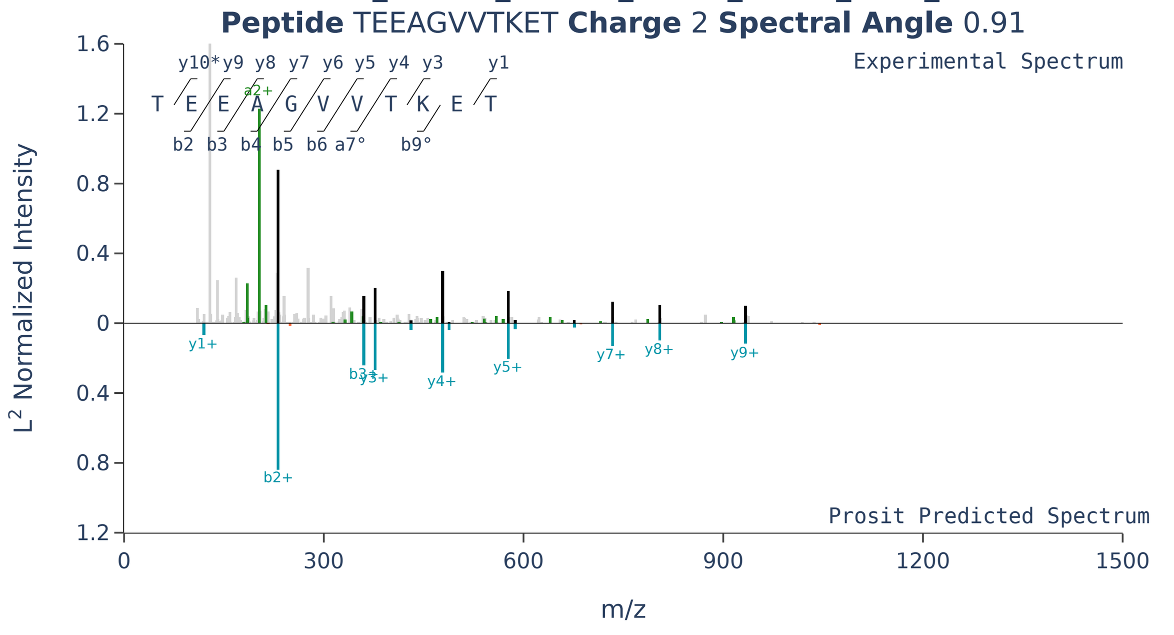

Source HRoetschke_271222_090123_HFGoe_LEDGF_b1R2_24h **Scan** 16535
Peptide GTHETKNMFL **Charge** 3 **Spectral Angle** 0.83

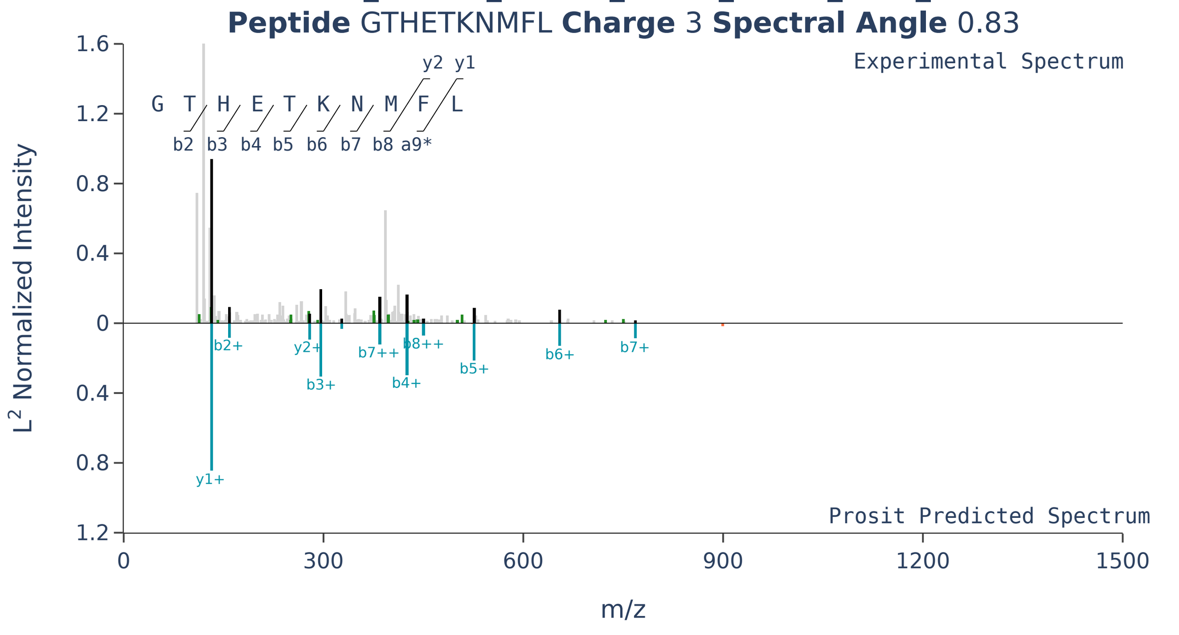

Source HRoetschke_271222_090123_HFGoe_LEDGF_b2R1_4h **Scan** 19287
Peptide EDGYPHWPARV **Charge** 3 **Spectral Angle** 0.93

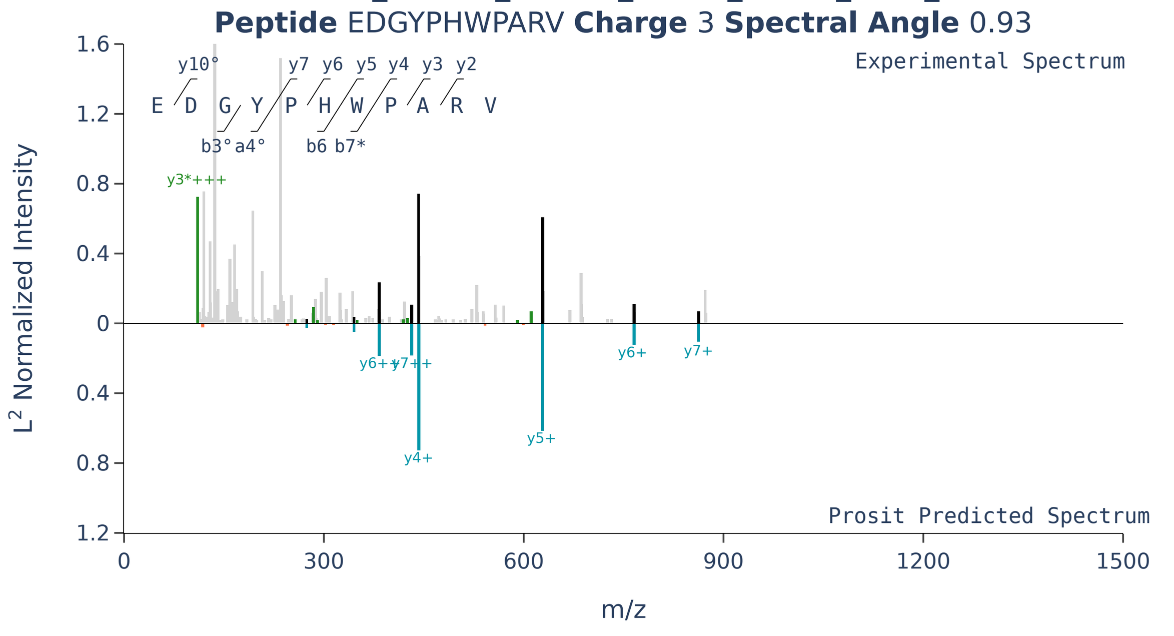

Source HRoetschke_271222_090123_HFGoe_LEDGF_b2R2_2h **Scan** 17407
Peptide TGYPHWPARVDE **Charge** 3 **Spectral Angle** 0.87

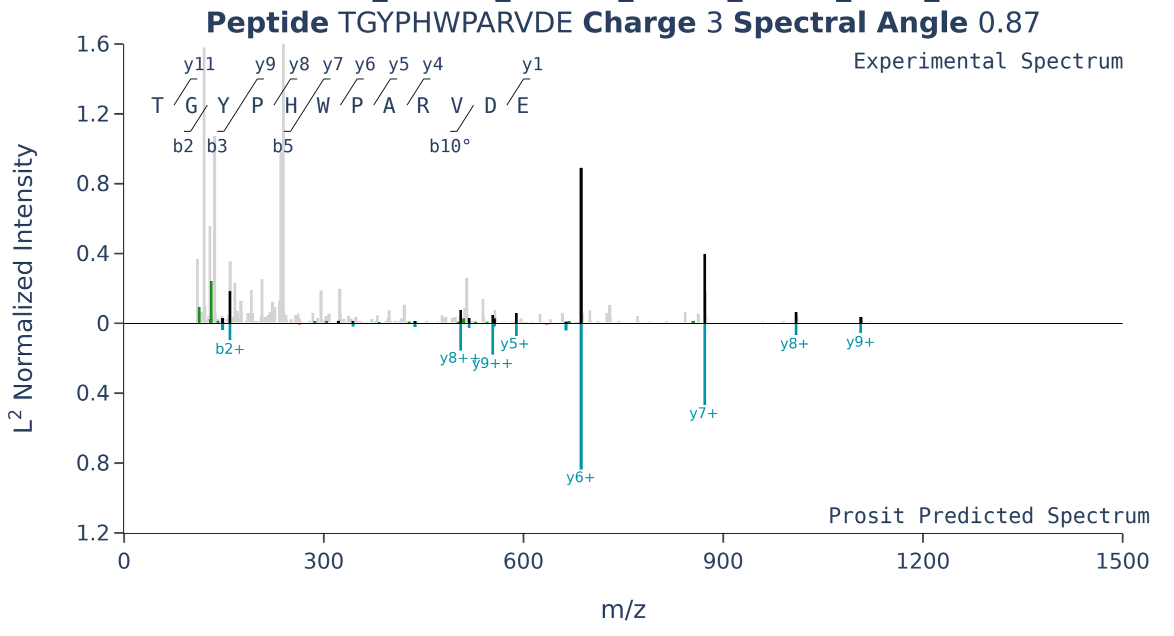

Source HRoetschke_271222_090123_HFGoe_LEDGF_b1R1_24h **Scan** 17942
Peptide SGYPHWPARV **Charge** 3 **Spectral Angle** 0.86

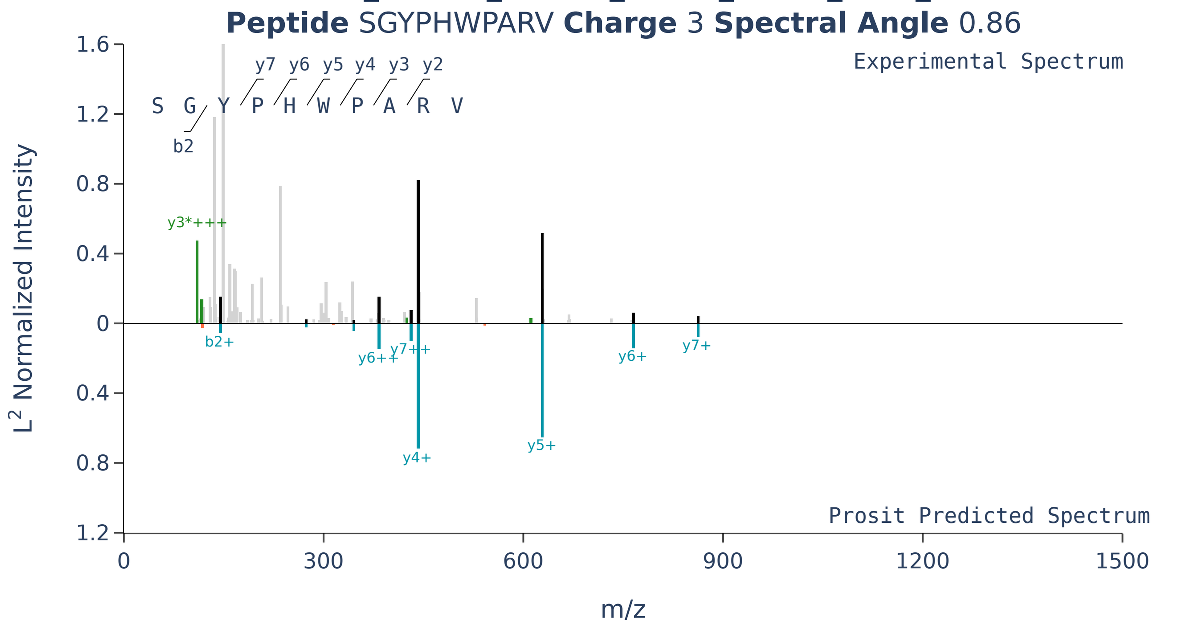

Source HRoetschke_271222_090123_HFGoe_LEDGF_b1R2_4h **Scan** 29870
Peptide NEGLWELA **Charge** 1 **Spectral Angle** 0.94

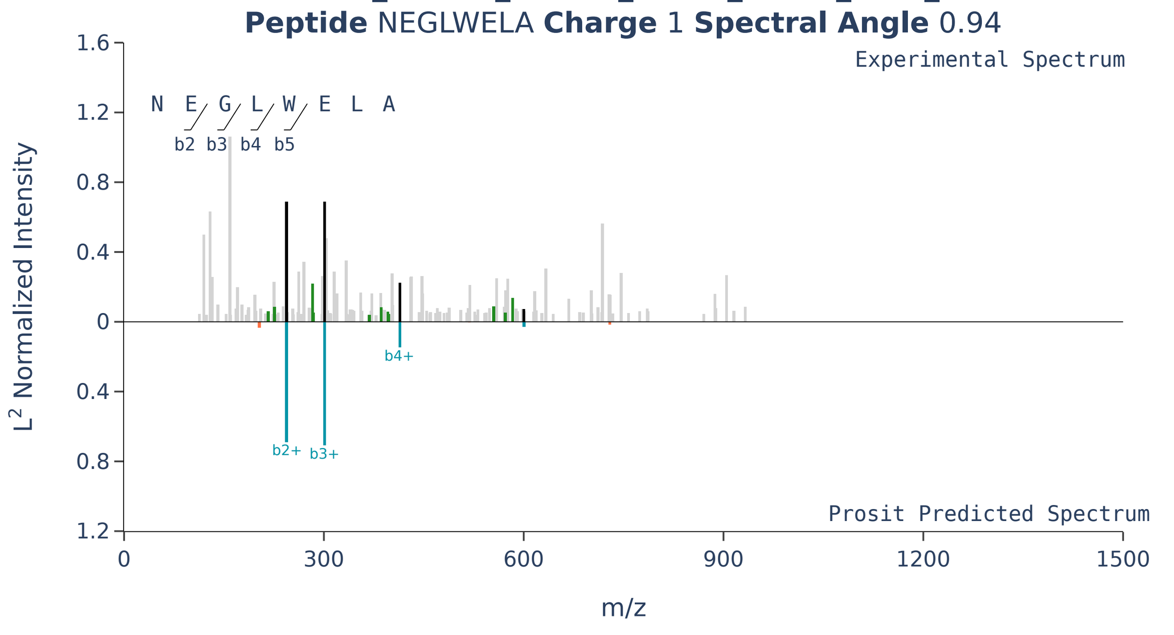

Source HRoetschke_271222_090123_HFGoe_LEDGF_b1R2_2h **Scan** 9015
Peptide PSSEEREEL **Charge** 2 **Spectral Angle** 0.91

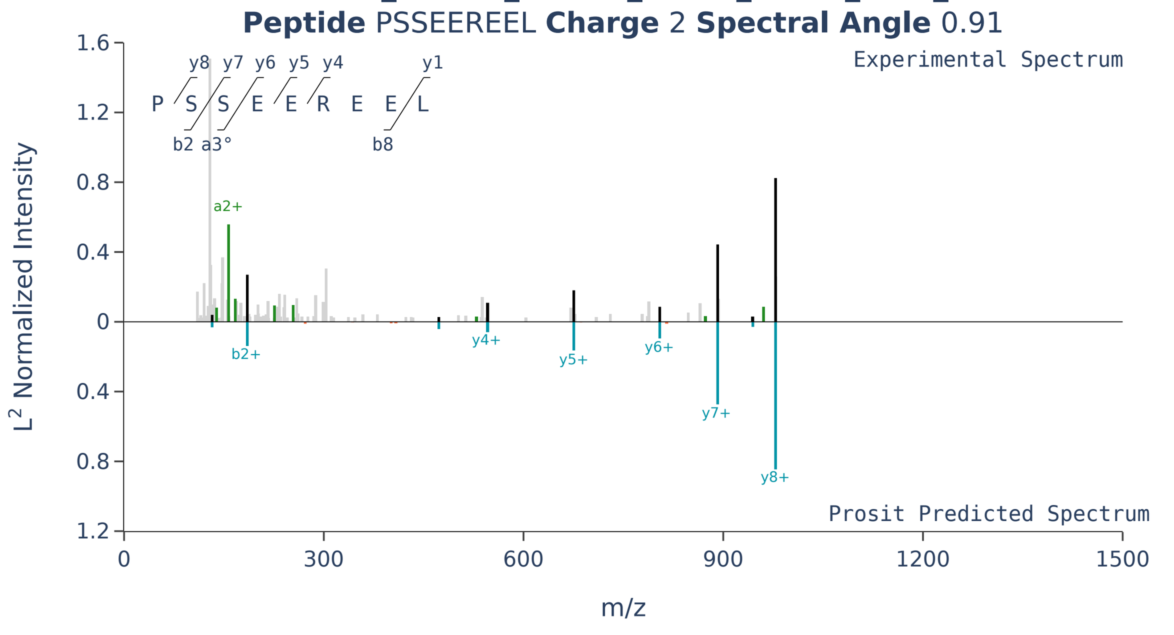

Source HRoetschke_271222_090123_HFGoe_LEDGF_b1R2_4h **Scan** 9261
Peptide GTHETAEL **Charge** 2 **Spectral Angle** 0.86

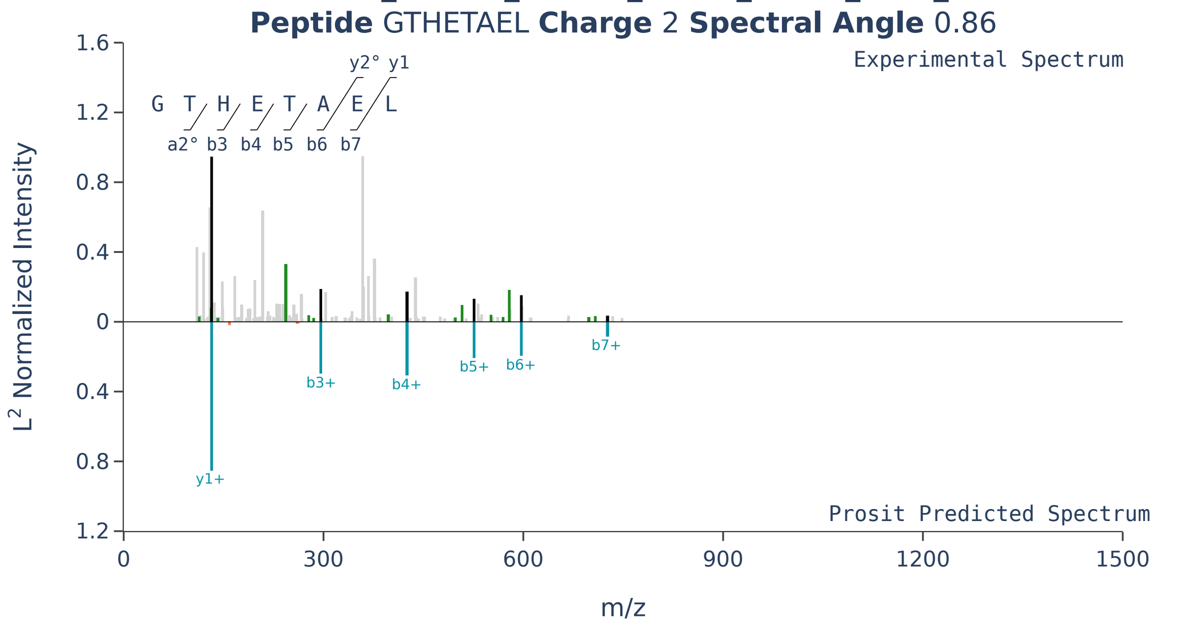

Source HRoetschke_271222_090123_HFGoe_LEDGF_b1R1_2h **Scan** 19991
Peptide GEKLASLQV **Charge** 2 **Spectral Angle** 0.84

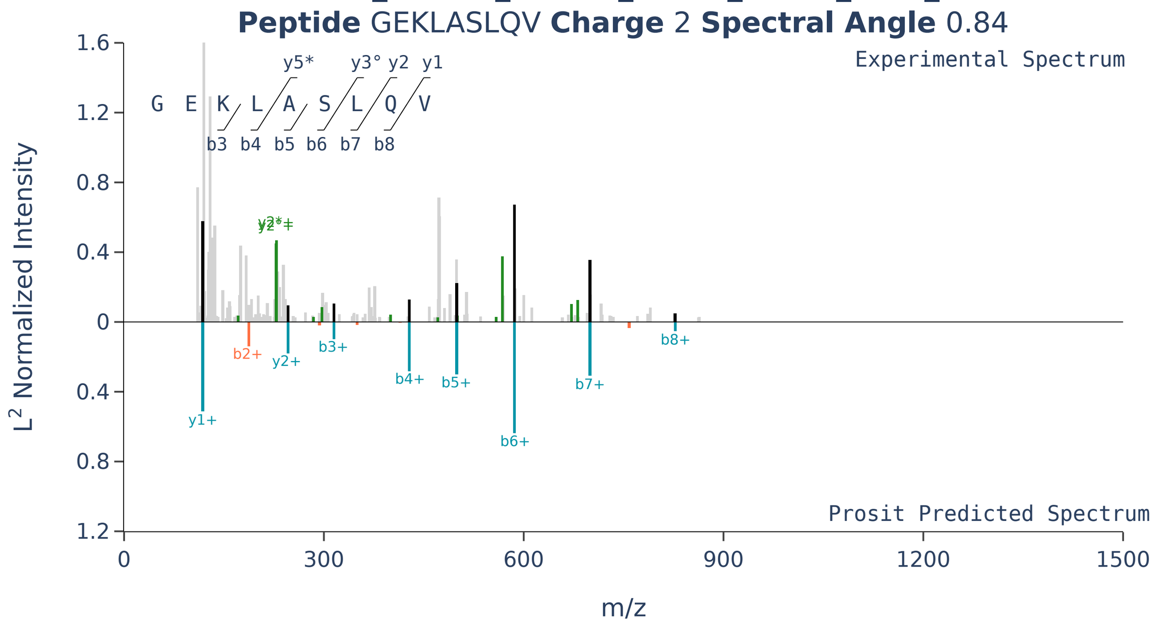

Source HRoetschke_271222_090123_HFGoe_LEDGF_b2R1_2h **Scan** 10119
Peptide GTHETAAVKPPTNKL **Charge** 4 **Spectral Angle** 0.78

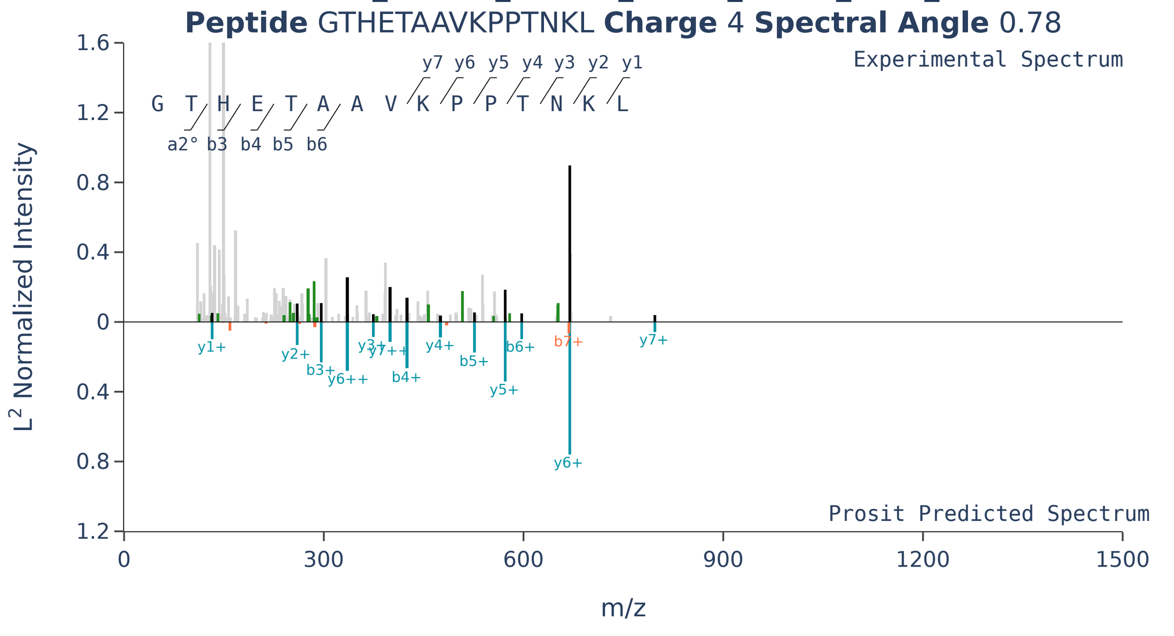

Source HRoetschke_271222_090123_HFGoe_LEDGF_b1R1_24h **Scan** 15654
Peptide DFKGFNEG **Charge** 2 **Spectral Angle** 0.84

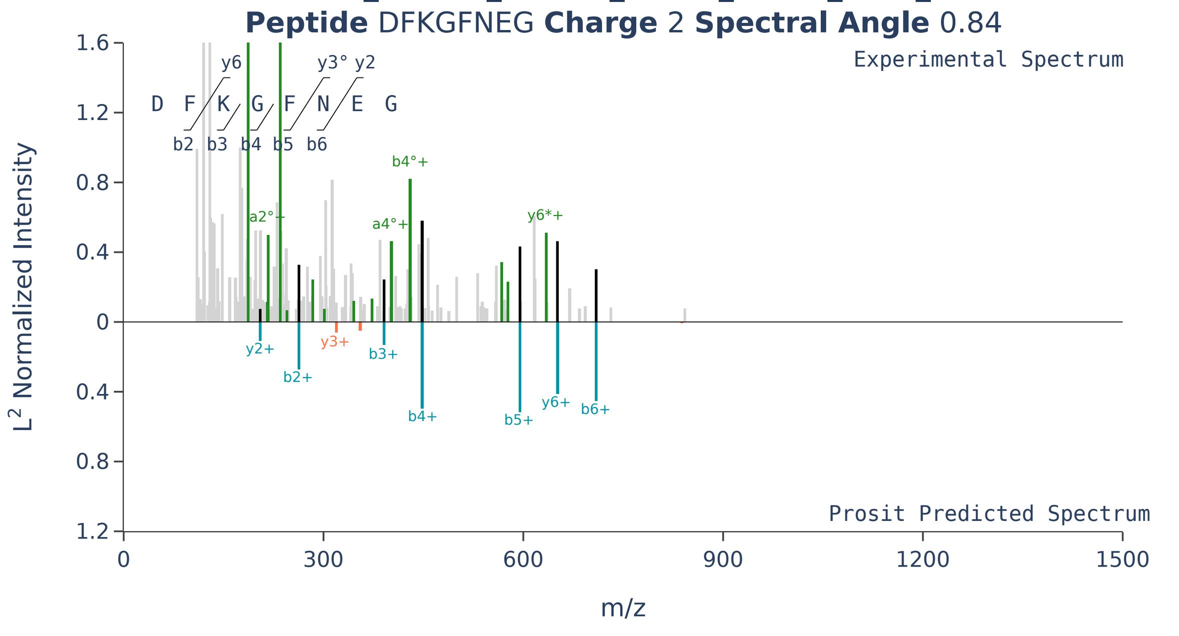

Source HRoetschke_271222_090123_HFGoe_LEDGF_b2R2_24h **Scan** 10890
Peptide AKTGVTITE **Charge** 2 **Spectral Angle** 0.83

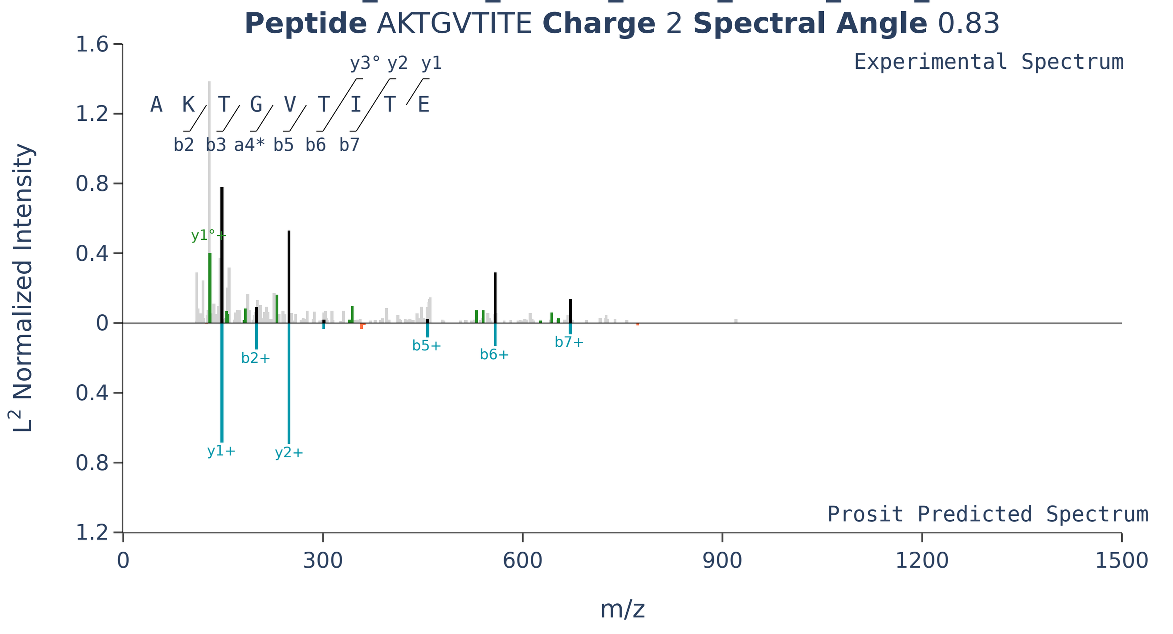

Source HRoetschke_271222_090123_HFGoe_LEDGF_b1R2_24h **Scan** 12400
Peptide RIHAEIKNTAF **Charge** 3 **Spectral Angle** 0.83

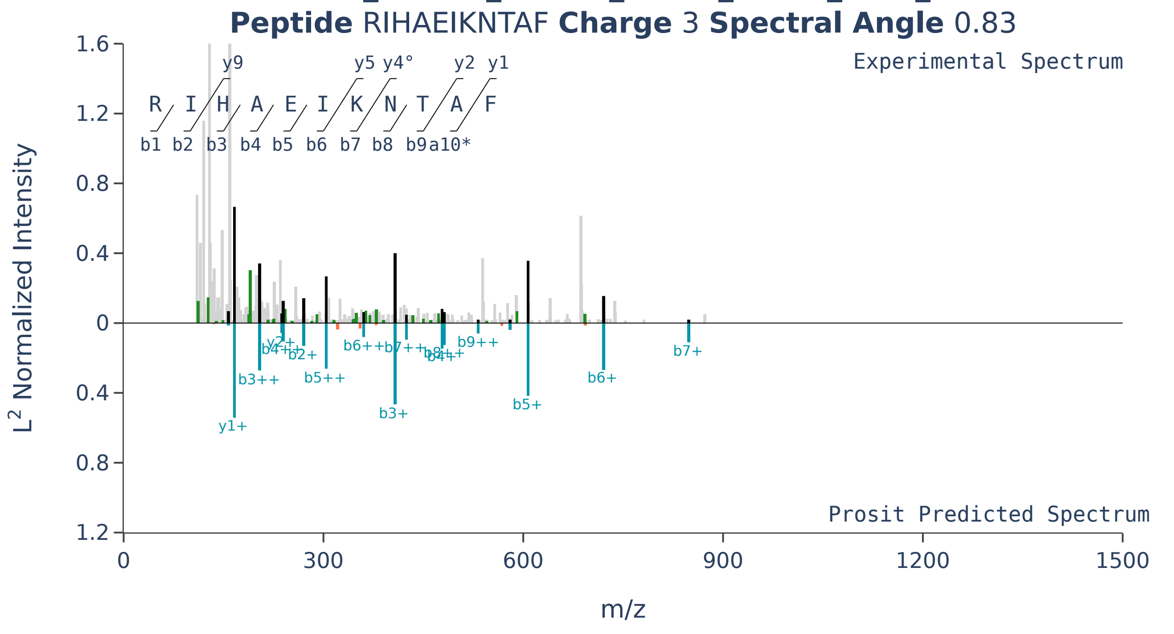

Source HRoetschke_271222_090123_HFGoe_LEDGF_b1R2_2h **Scan** 7763
Peptide GNQPQHEL **Charge** 2 **Spectral Angle** 0.78

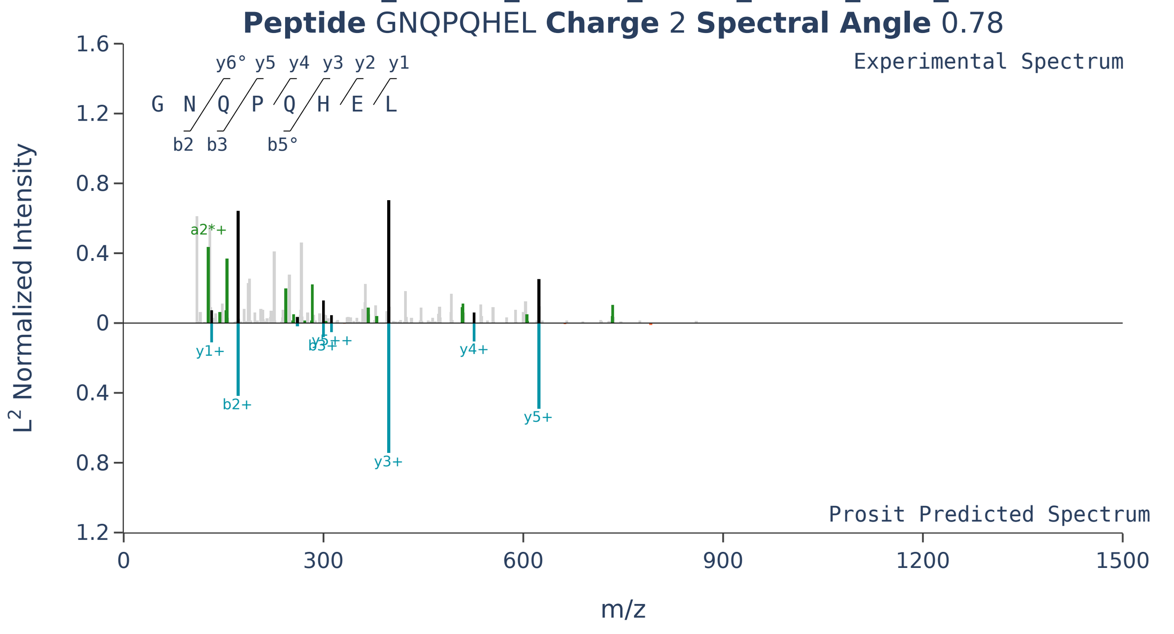

Source HRoetschke_271222_090123_HFGoe_LEDGF_b2R1_2h **Scan** 11844
Peptide ASPGMTRDFKPGD **Charge** 3 **Spectral Angle** 0.86

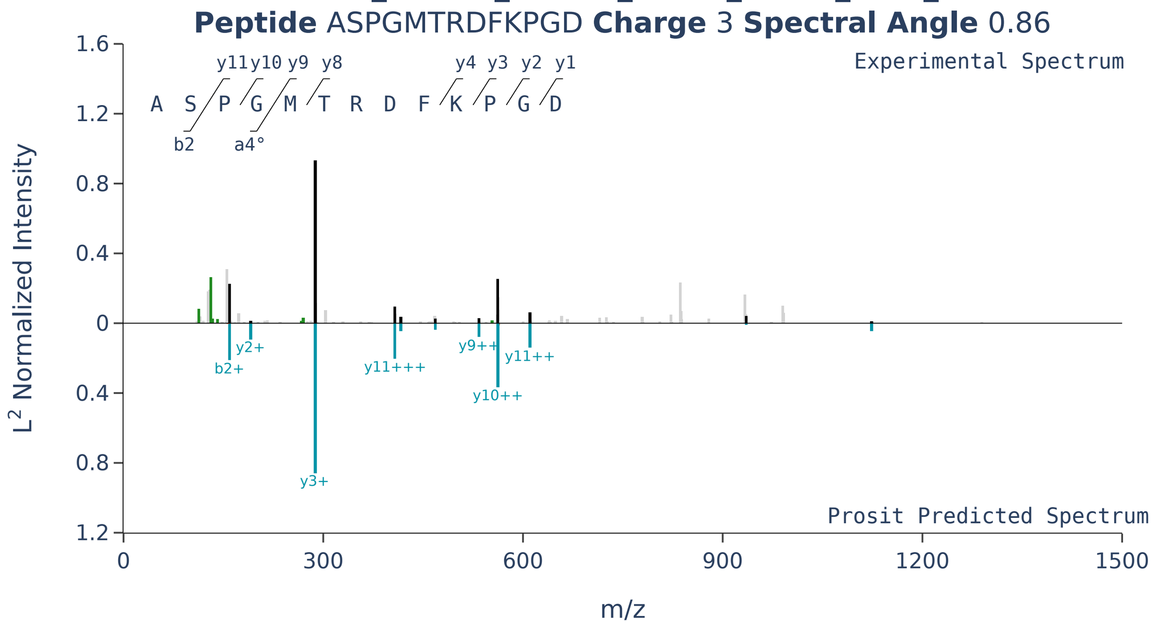

Source HRoetschke_271222_090123_HFGoe_LEDGF_b1R2_4h **Scan** 16243
Peptide MTQQAQKHTEMITTL **Charge** 3 **Spectral Angle** 0.87

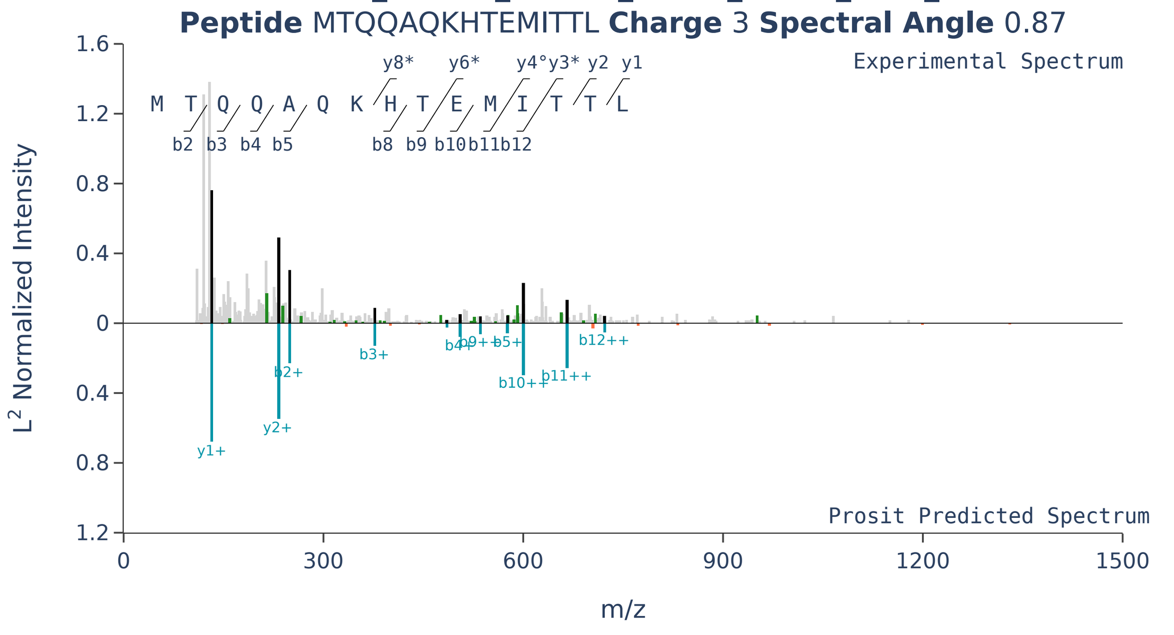

Source HRoetschke_271222_090123_HFGoe_LEDGF_b1R2_24h **Scan** 18239
Peptide ATYPHWPARVD **Charge** 3 **Spectral Angle** 0.88

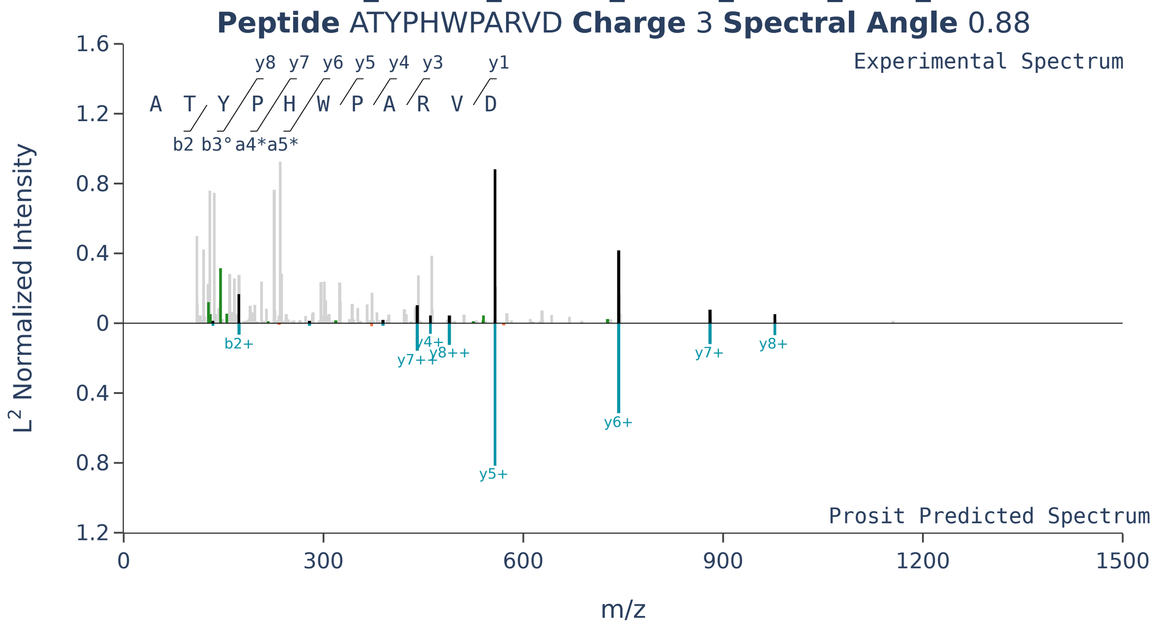

Source HRoetschke_271222_090123_HFGoe_LEDGF_b2R2_24h **Scan** 10495
Peptide DNLDVNTAS **Charge** 1 **Spectral Angle** 0.93

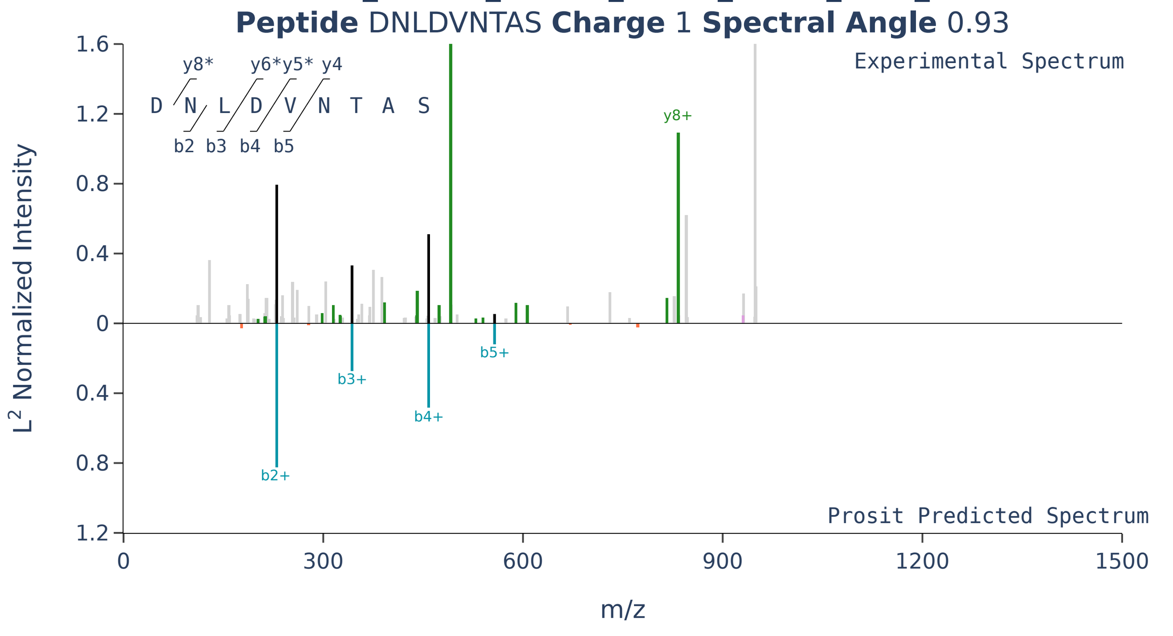

Source HRoetschke_271222_090123_HFGoe_LEDGF_b2R1_24h **Scan** 13419
Peptide ASMDSRLQRI **Charge** 3 **Spectral Angle** 0.9

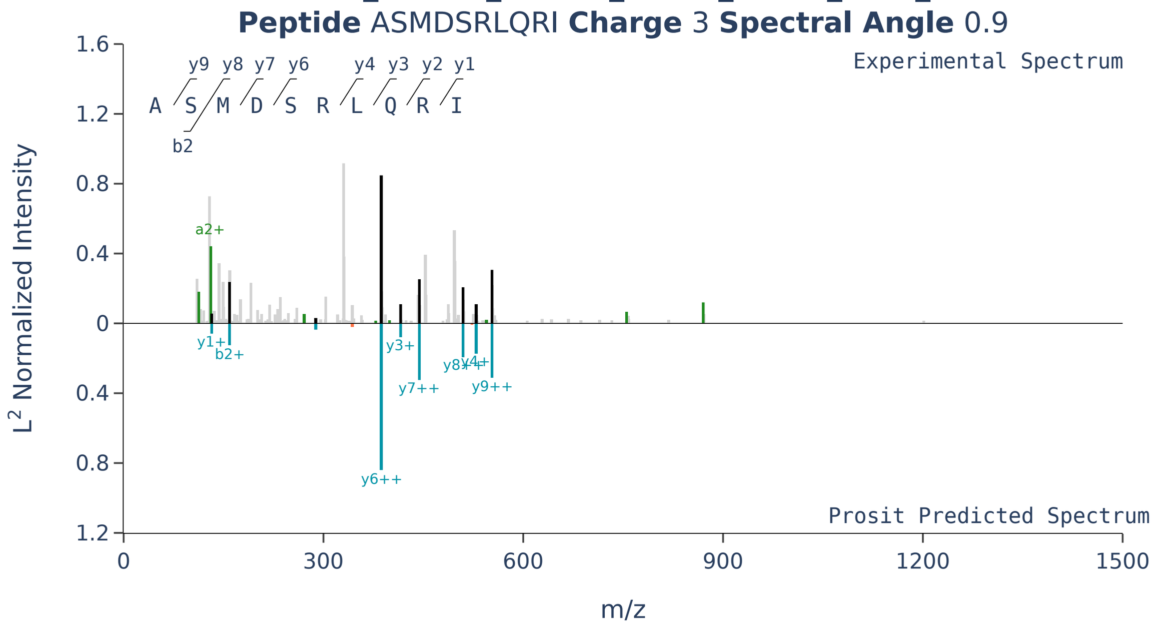

Source HRoetschke_271222_090123_HFGoe_LEDGF_b1R2_24h **Scan** 9577
Peptide TEEAGVVTTAKE **Charge** 2 **Spectral Angle** 0.87

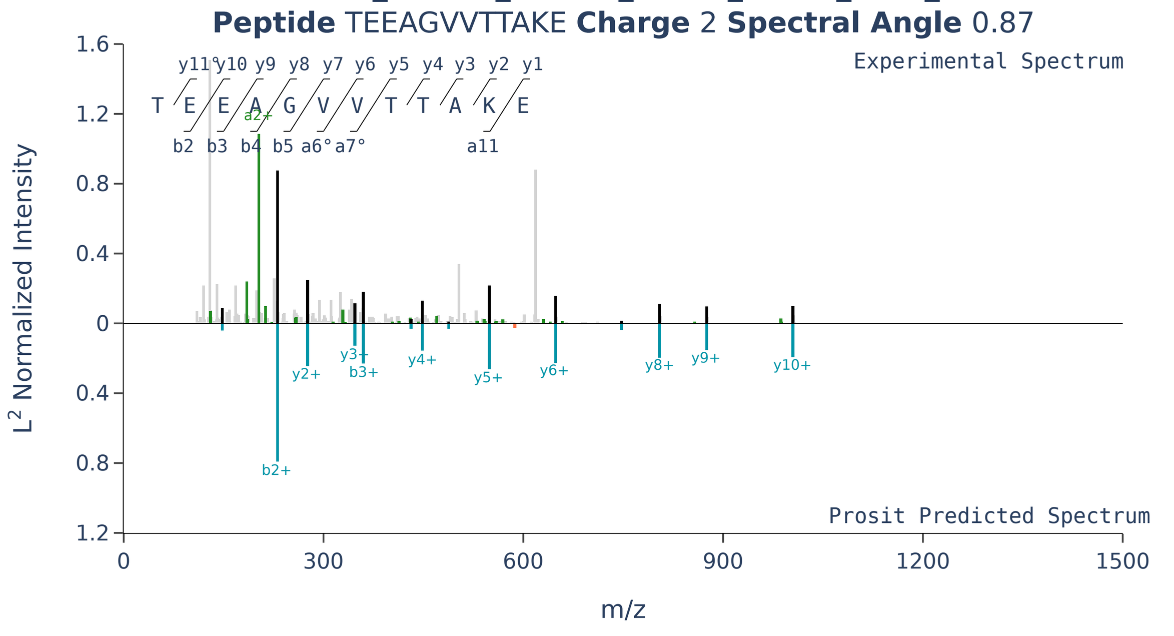

Source HRoetschke_271222_090123_HFGoe_LEDGF_b2R2_2h **Scan** 12081
Peptide SQGPGMTRDFKPGD **Charge** 2 **Spectral Angle** 0.81

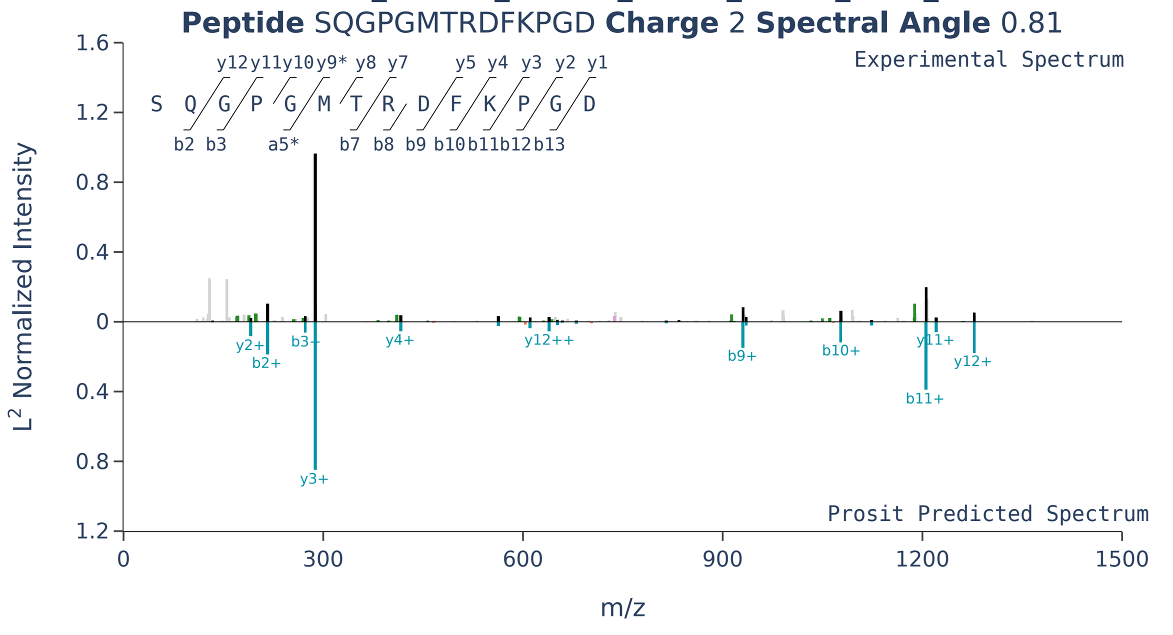

Source HRoetschke_271222_090123_HFGoe_LEDGF_b2R2_4h **Scan** 12550
Peptide GTHETKGYPHWPARV **Charge** 4 **Spectral Angle** 0.93

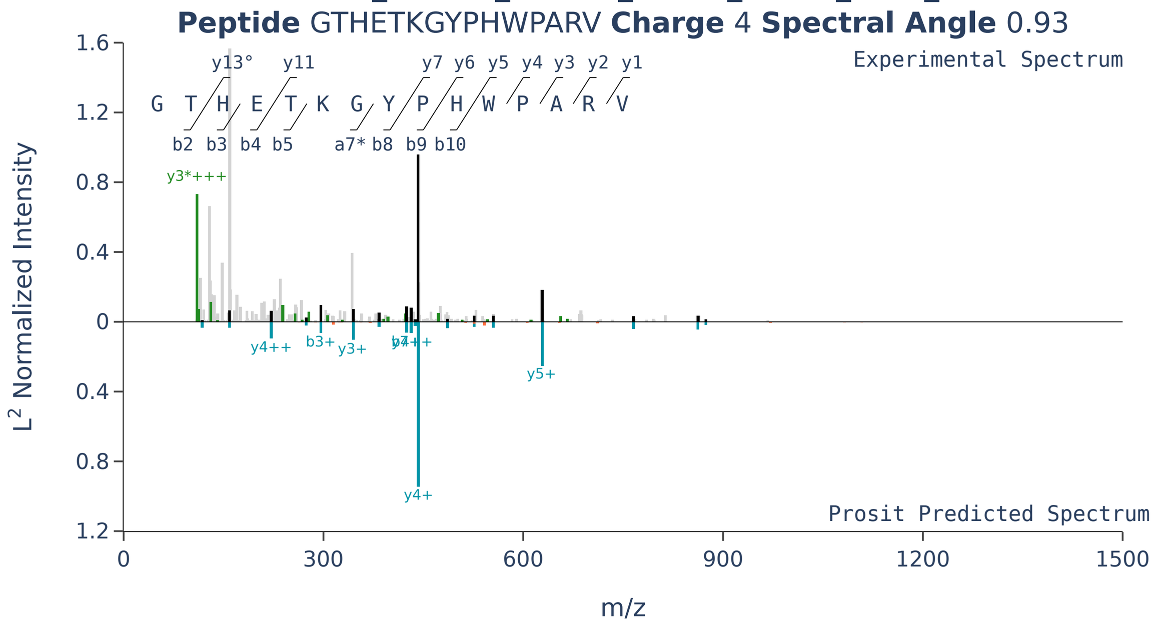

Source HRoetschke_271222_090123_HFGoe_LEDGF_b1R2_24h **Scan** 7185
Peptide KMKGYGPGMTR **Charge** 3 **Spectral Angle** 0.83

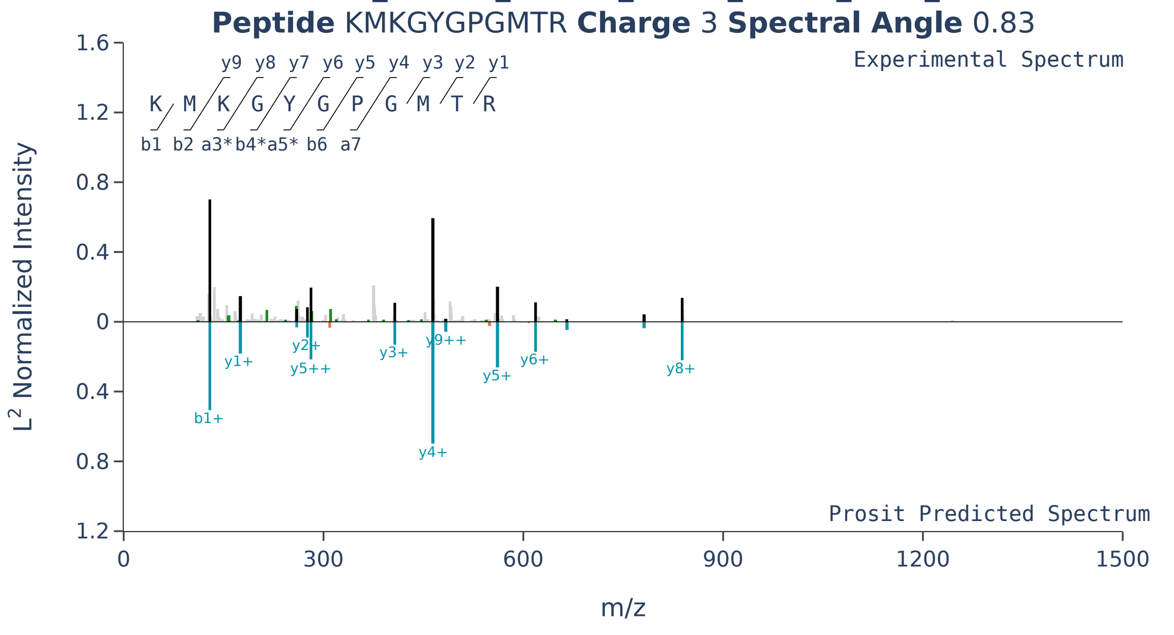

Source HRoetschke_271222_090123_HFGoe_LEDGF_b1R1_24h **Scan** 17843
Peptide TGFLGPKDIFPYSENKEKYGKPNKR **Charge** 5 **Spectral Angle** 0.78

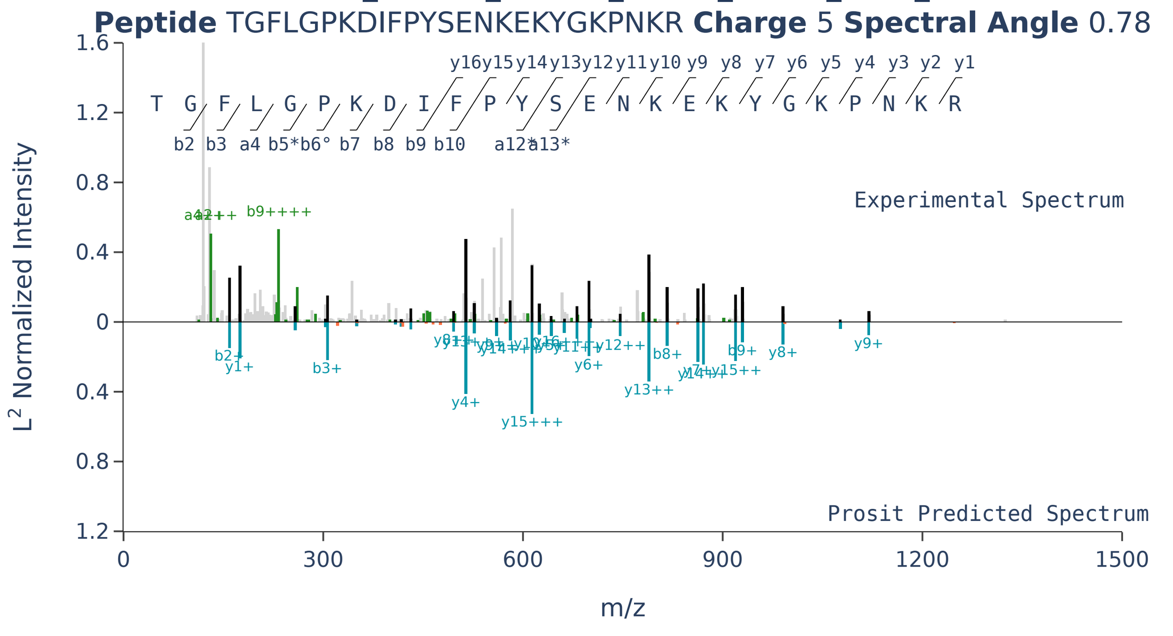

Source HRoetschke_271222_090123_HFGoe_LEDGF_b1R1_24h **Scan** 14597
Peptide TEEAGVVTTATQQA **Charge** 2 **Spectral Angle** 0.9

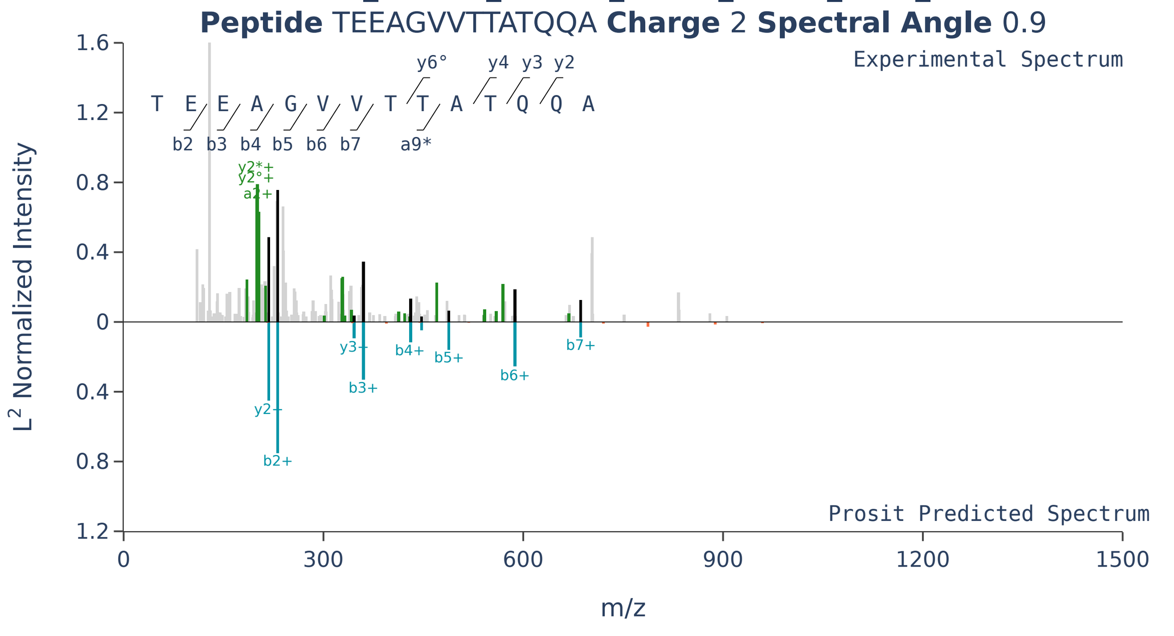

Source HRoetschke_271222_090123_HFGoe_LEDGF_b1R1_4h **Scan** 14323
Peptide ATKQSYPHWPARVD **Charge** 4 **Spectral Angle** 0.93

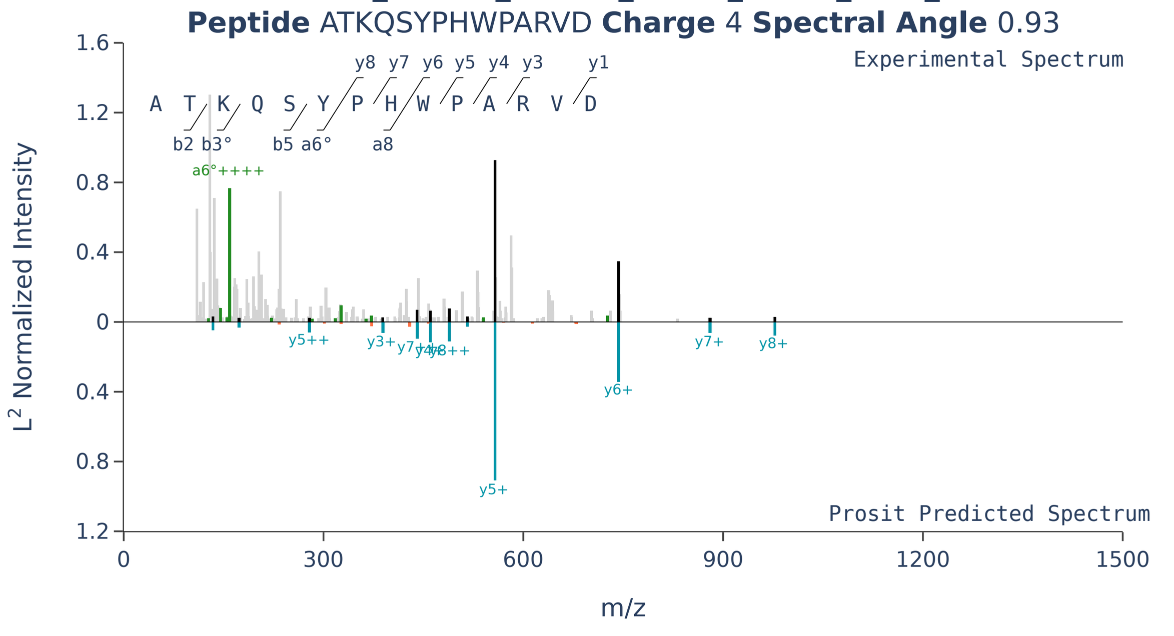

Source HRoetschke_271222_090123_HFGoe_LEDGF_b1R1_24h **Scan** 9446
Peptide SQVIMEKSQAQ **Charge** 2 **Spectral Angle** 0.89

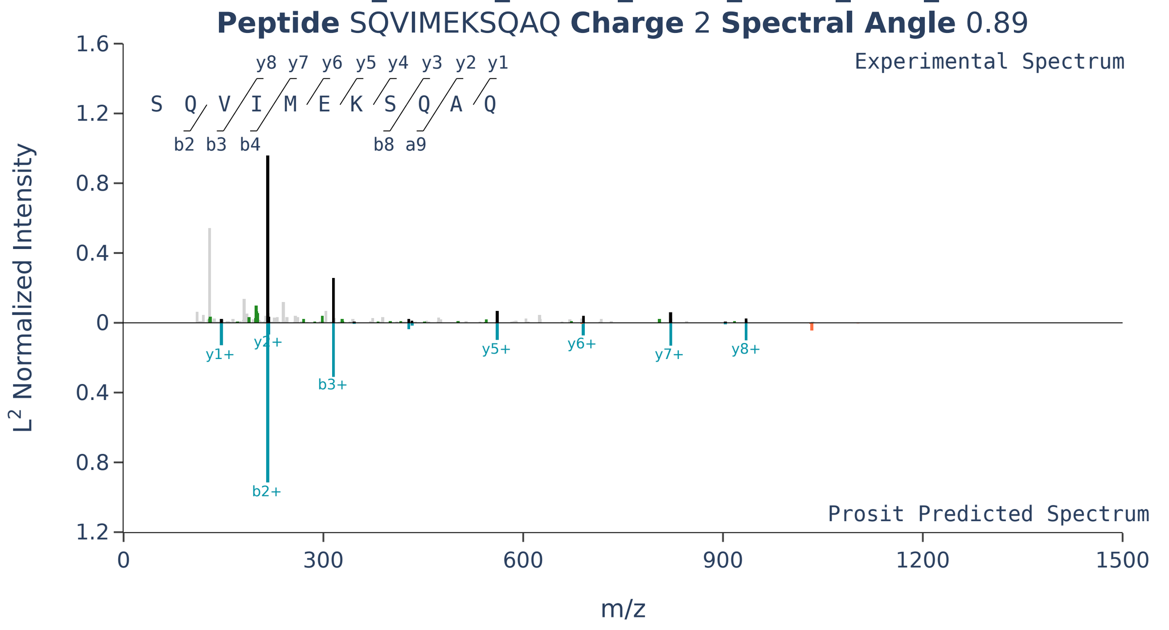

Source HRoetschke_271222_090123_HFGoe_LEDGF_b1R2_24h **Scan** 12144
Peptide TKVSQVIMEKS **Charge** 2 **Spectral Angle** 0.81

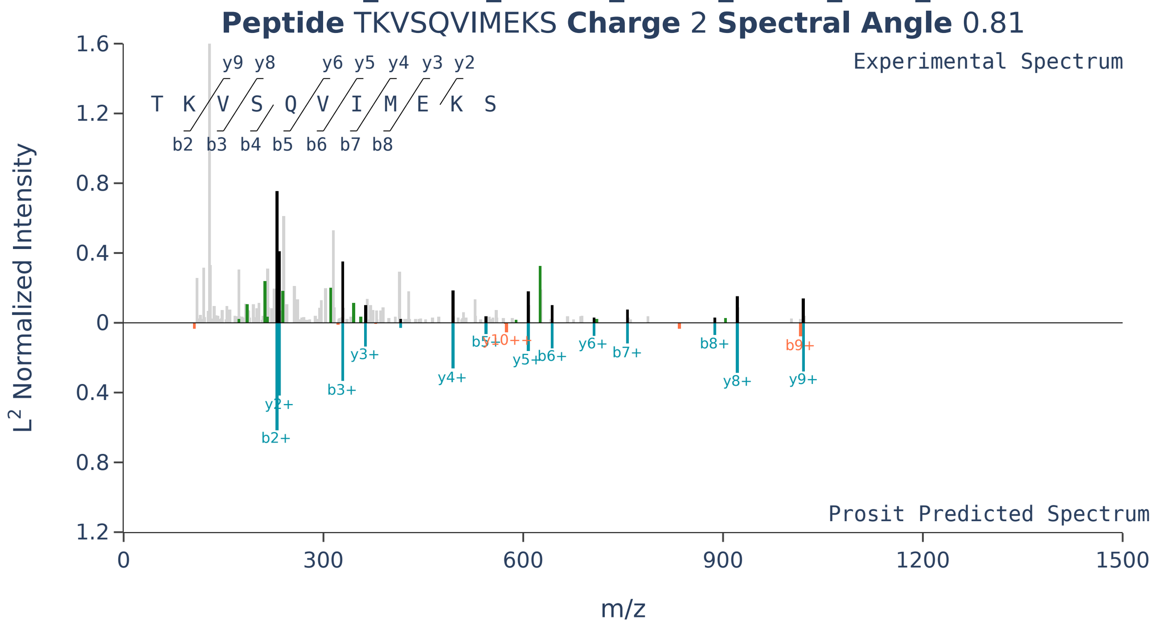

Source HRoetschke_271222_090123_HFGoe_LEDGF_b2R1_4h **Scan** 17851
Peptide TAFLGPKDIFPYSENKEKYGKPNKRTA **Charge** 5 **Spectral Angle** 0.81

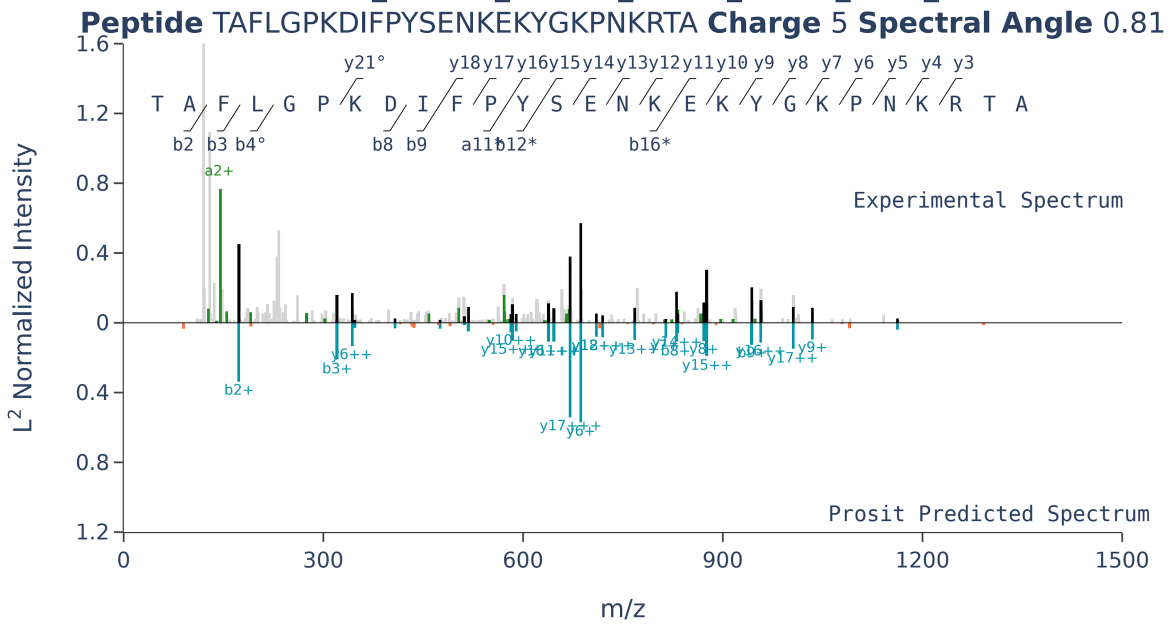

Source HRoetschke_271222_090123_HFGoe_LEDGF_b1R2_24h **Scan** 23039
Peptide NEGLWVET **Charge** 1 **Spectral Angle** 0.92

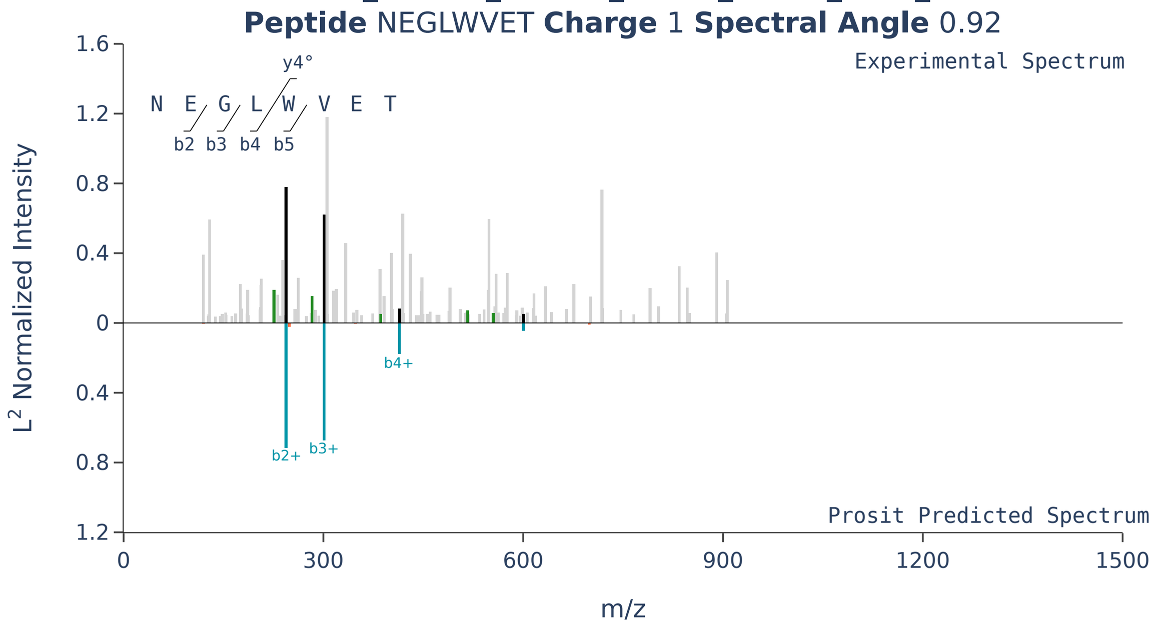

Source HRoetschke_271222_090123_HFGoe_LEDGF_b2R1_2h **Scan** 10376
Peptide FLVGQQAQKHTE **Charge** 2 **Spectral Angle** 0.83

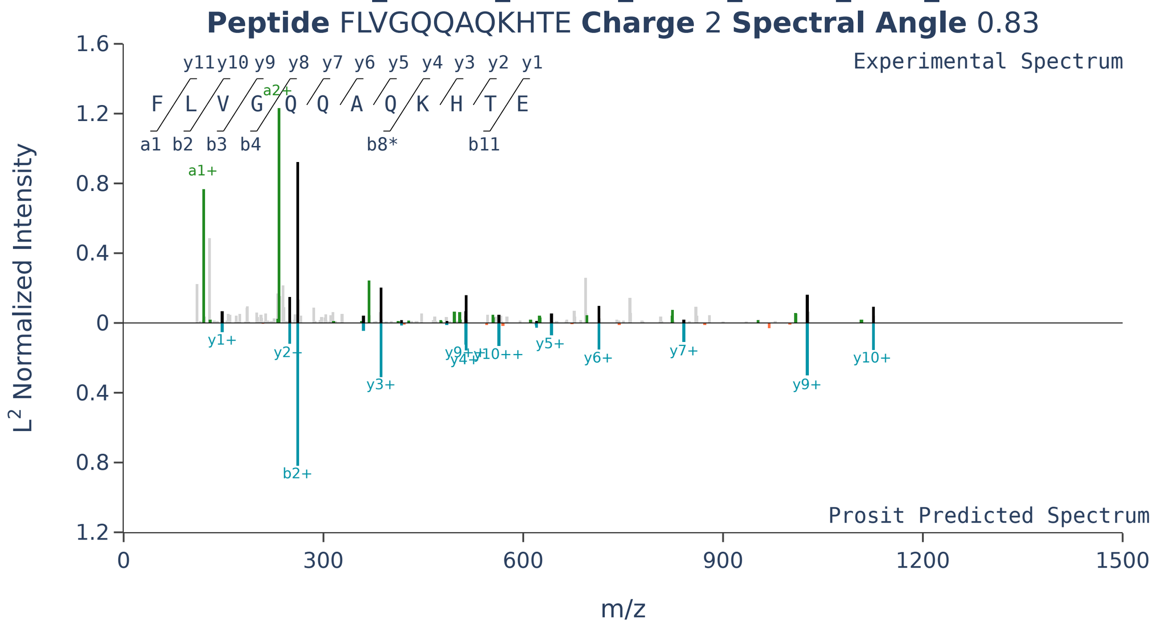

Source HRoetschke_271222_090123_HFGoe_LEDGF_b1R2_24h **Scan** 16677
Peptide KVSQVIMEKSEL **Charge** 2 **Spectral Angle** 0.89

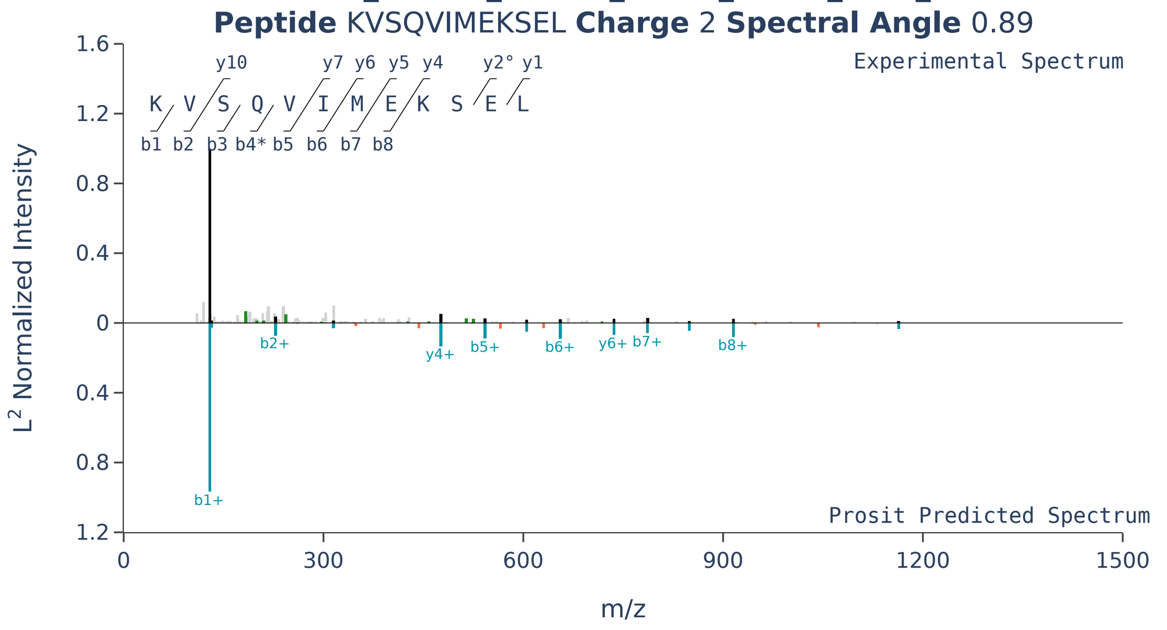

Source HRoetschke_271222_090123_HFGoe_LEDGF_b2R1_1h **Scan** 23665
Peptide GNQPQHELASLQVTM **Charge** 2 **Spectral Angle** 0.81

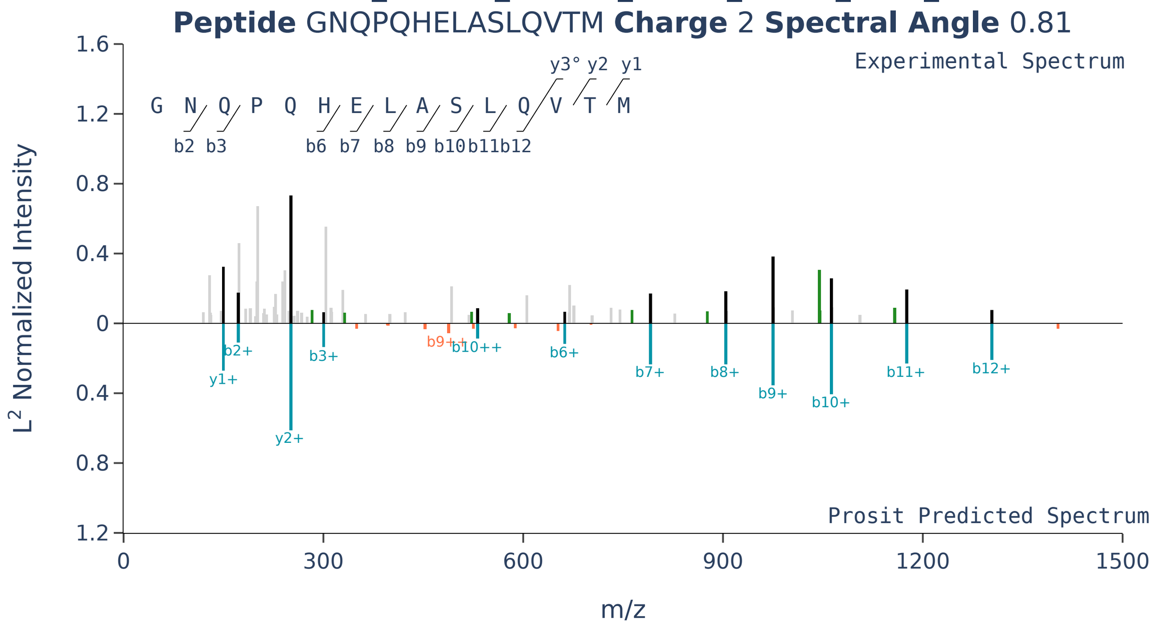

Source HRoetschke_271222_090123_HFGoe_LEDGF_b1R2_24h **Scan** 17371
Peptide TEEAGVVTTKL **Charge** 2 **Spectral Angle** 0.81

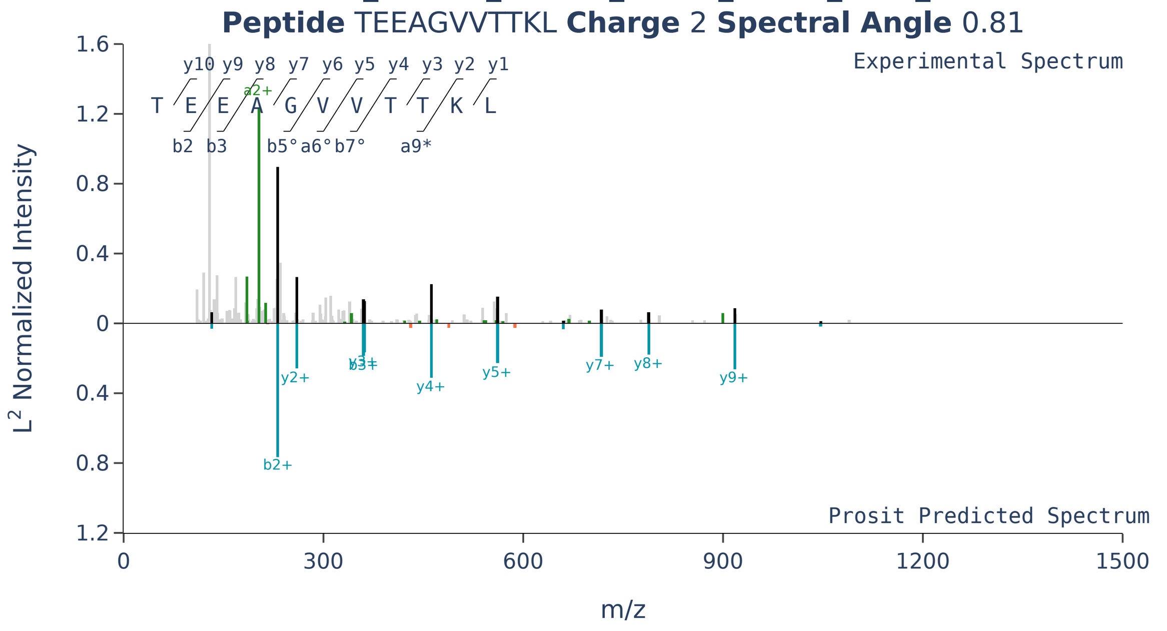

Source HRoetschke_271222_090123_HFGoe_LEDGF_b1R2_24h **Scan** 6609
Peptide RIHAEIKNTA **Charge** 3 **Spectral Angle** 0.85

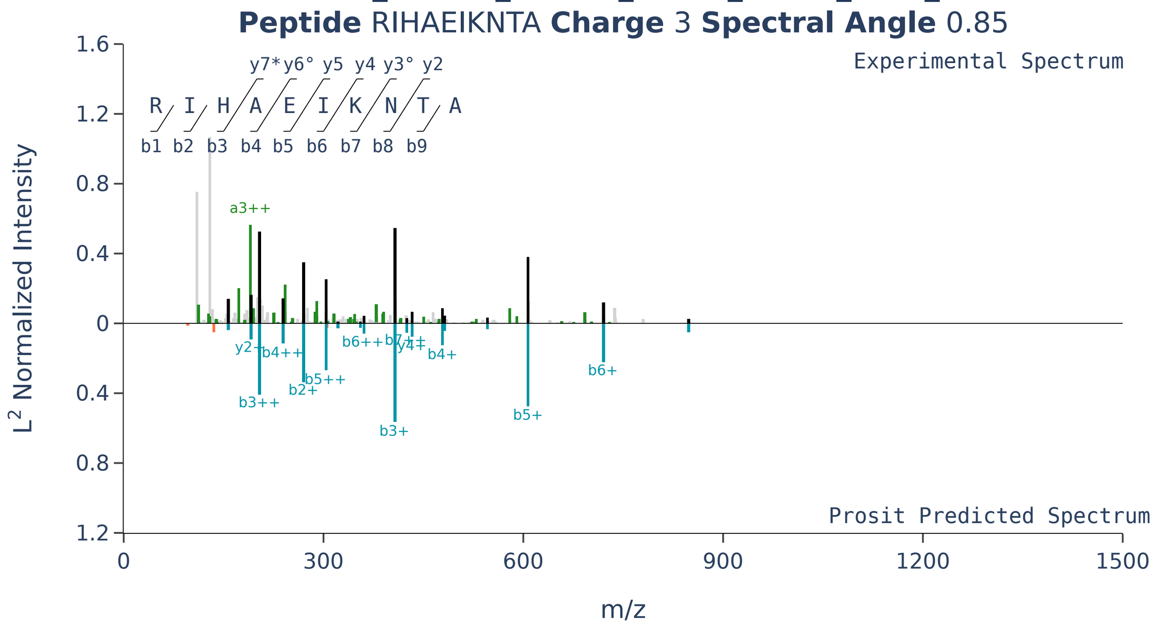

Source HRoetschke_271222_090123_HFGoe_LEDGF_b1R1_24h **Scan** 14900
Peptide ATKQSGYPHWPARV **Charge** 4 **Spectral Angle** 0.94

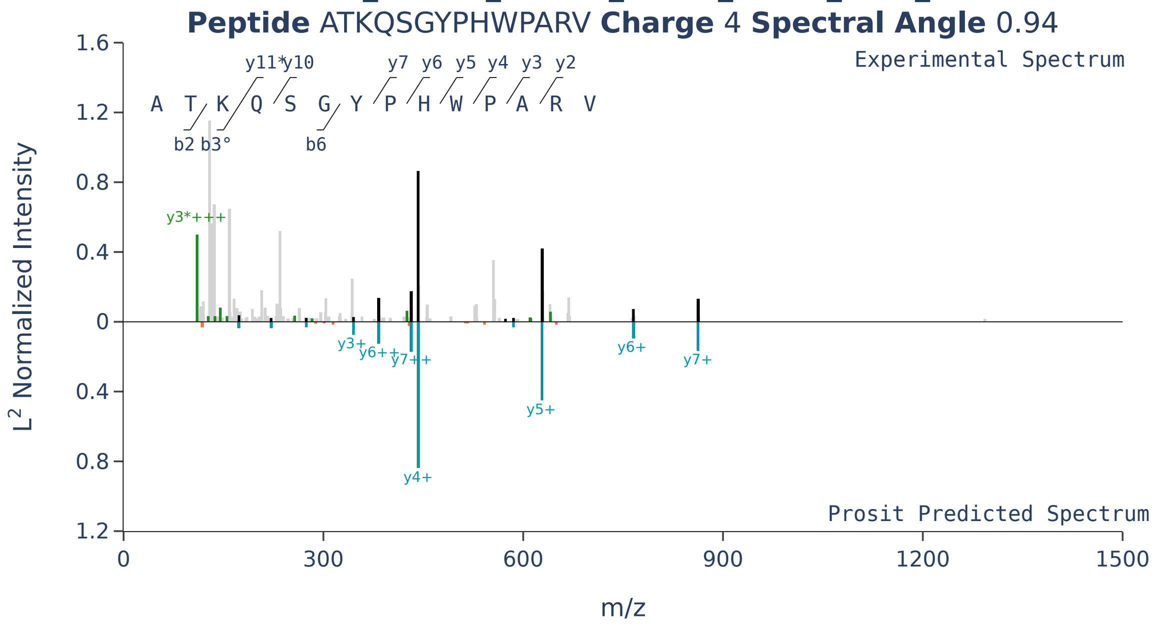

Source HRoetschke_271222_090123_HFGoe_LEDGF_b1R2_1h **Scan** 23547
Peptide KEGLASLQVTM **Charge** 2 **Spectral Angle** 0.79

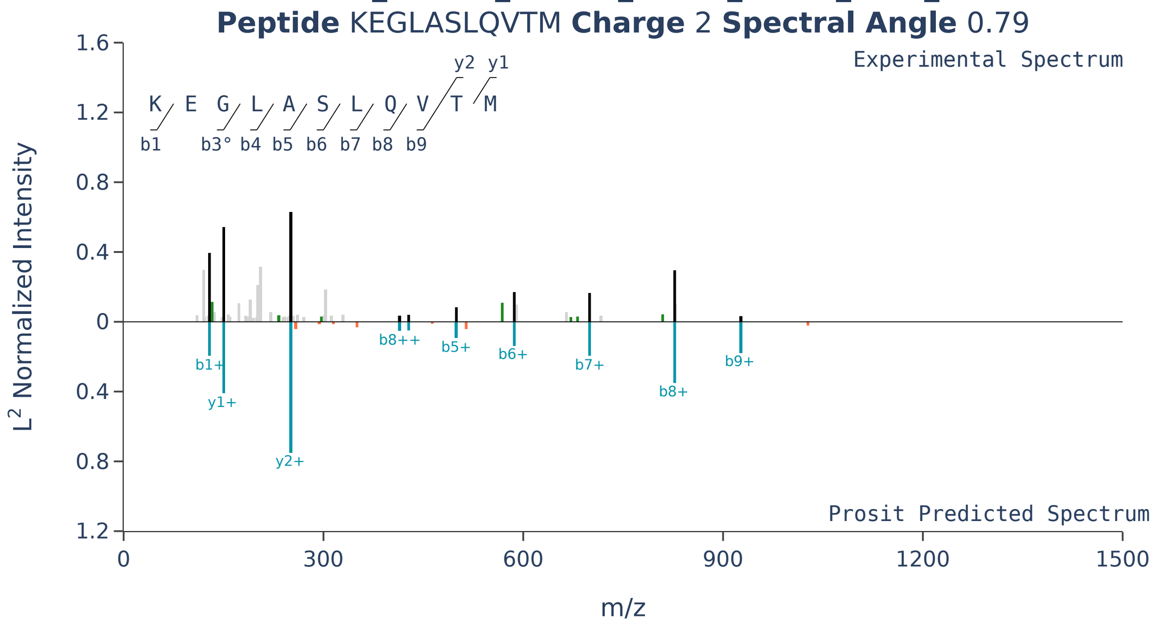

Source HRoetschke_271222_090123_HFGoe_LEDGF_b1R2_2h **Scan** 12065
Peptide IDNNPKVKFSTA **Charge** 3 **Spectral Angle** 0.76

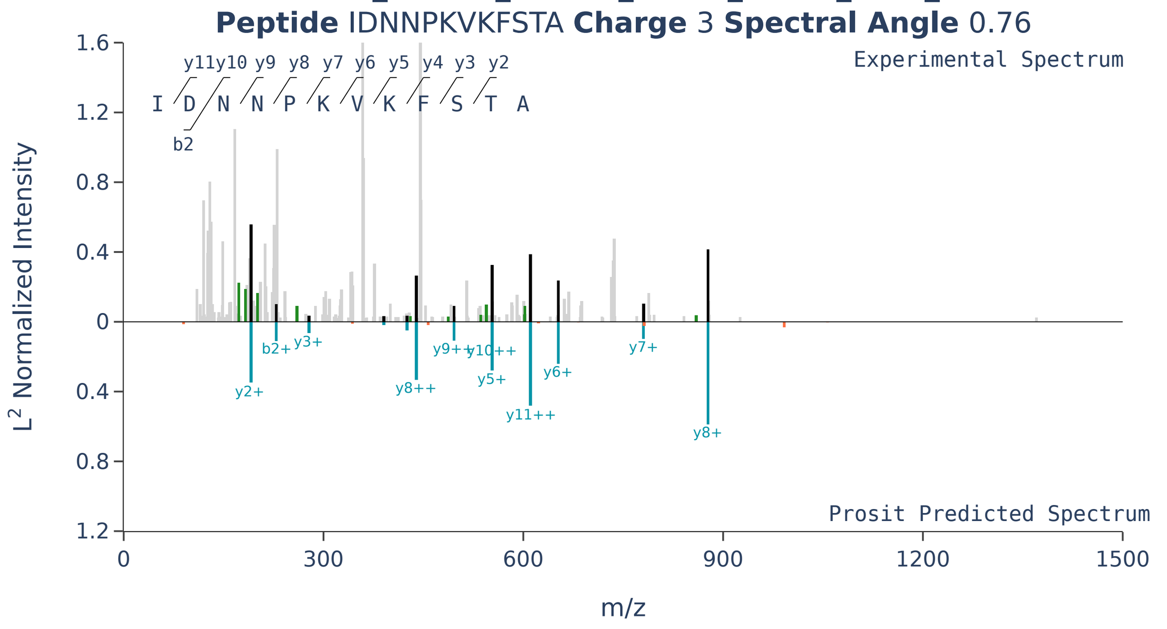

Source HRoetschke_271222_090123_HFGoe_LEDGF_b2R2_2h **Scan** 12608
Peptide QAGPGMTRDFKPGD **Charge** 2 **Spectral Angle** 0.89

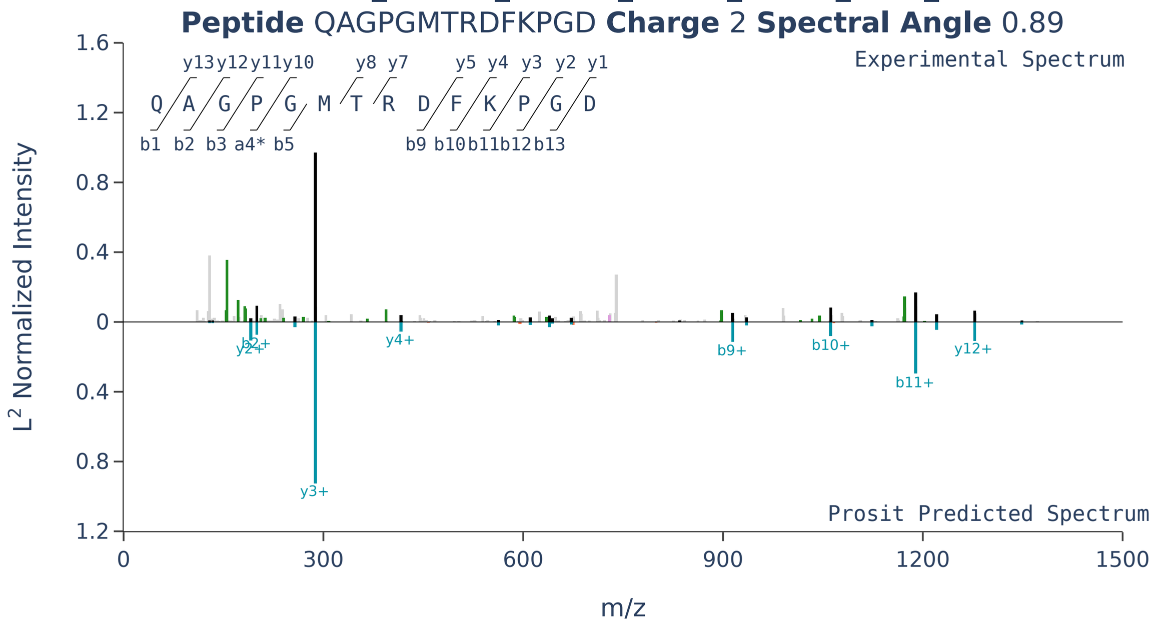

Source HRoetschke_271222_090123_HFGoe_LEDGF_b1R1_4h **Scan** 21983
Peptide KMKGELASLQVTM **Charge** 3 **Spectral Angle** 0.85

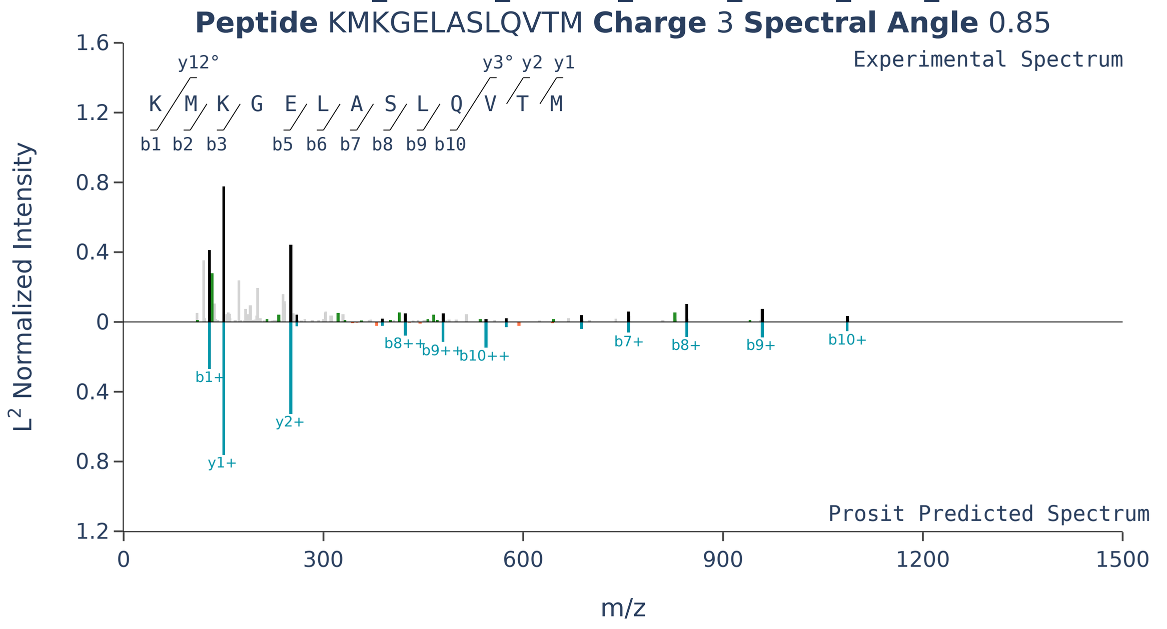

Source HRoetschke_271222_090123_HFGoe_LEDGF_b2R1_2h **Scan** 13330
Peptide QSNPHWPARVDE **Charge** 3 **Spectral Angle** 0.88

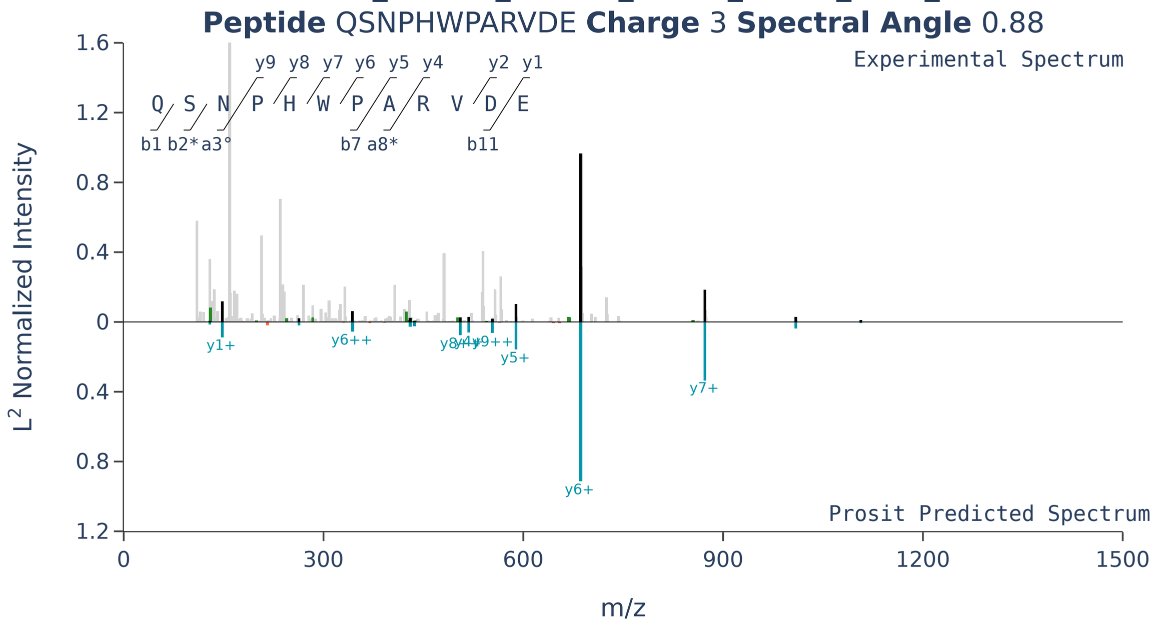

Source HRoetschke_271222_090123_HFGoe_LEDGF_b1R1_24h **Scan** 7734
Peptide QPQHNMITTLKKIRR **Charge** 5 **Spectral Angle** 0.81

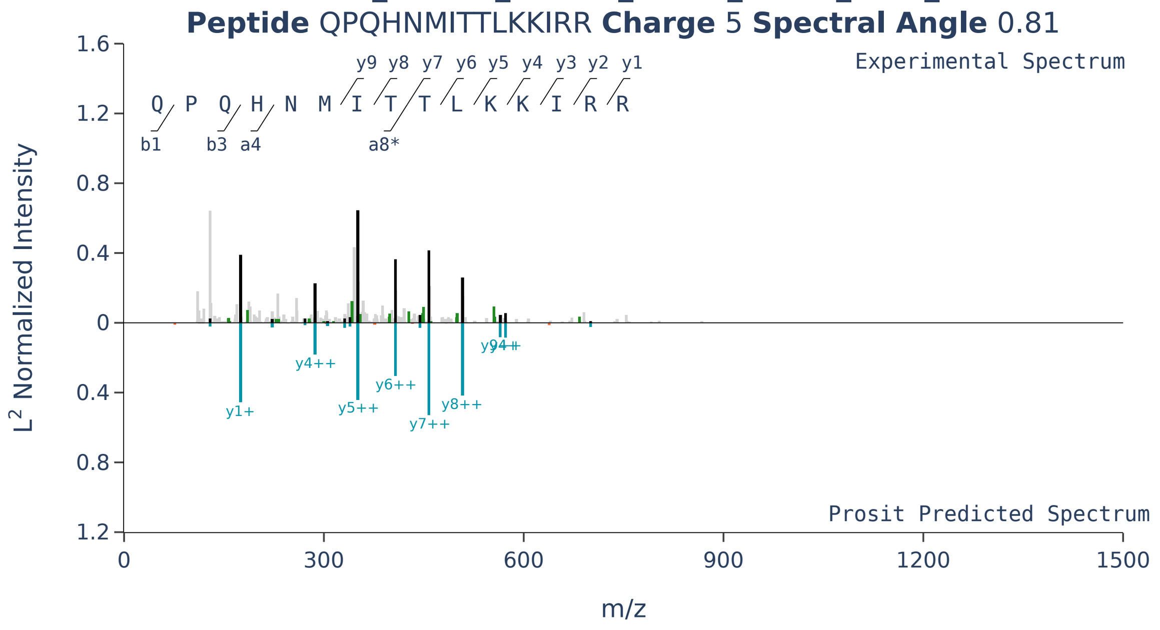

Source HRoetschke_271222_090123_HFGoe_LEDGF_b1R1_24h **Scan** 17501
Peptide NASYPHWPARVDE **Charge** 3 **Spectral Angle** 0.87

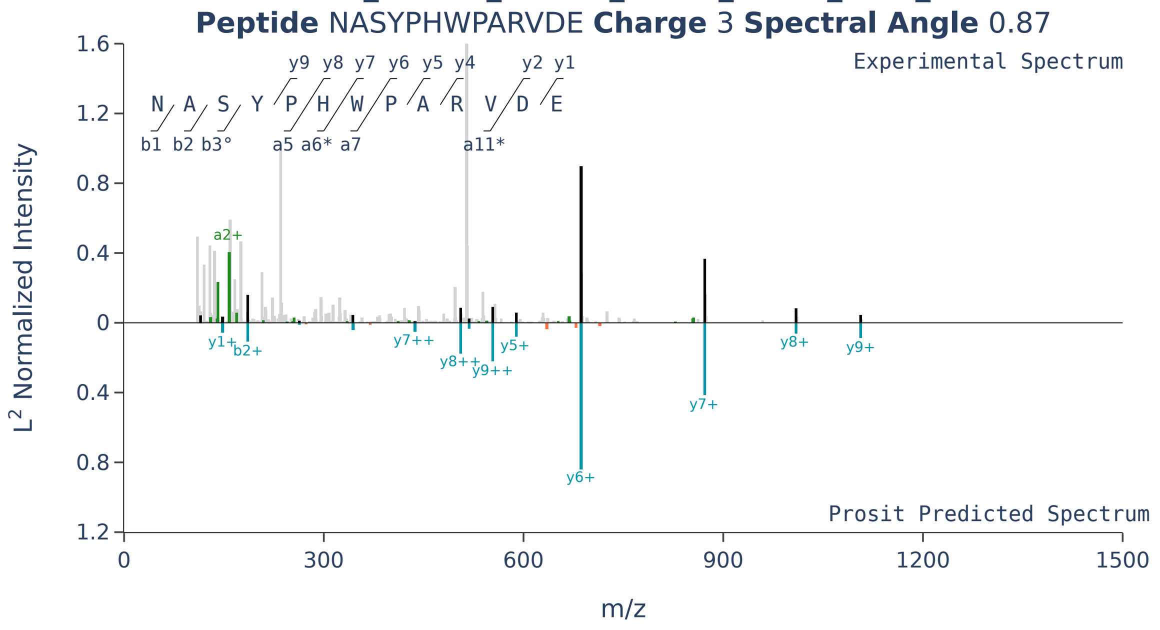

Source HRoetschke_271222_090123_HFGoe_LEDGF_b2R1_4h **Scan** 11970
Peptide QSGPGMTRDFKPGD **Charge** 2 **Spectral Angle** 0.89

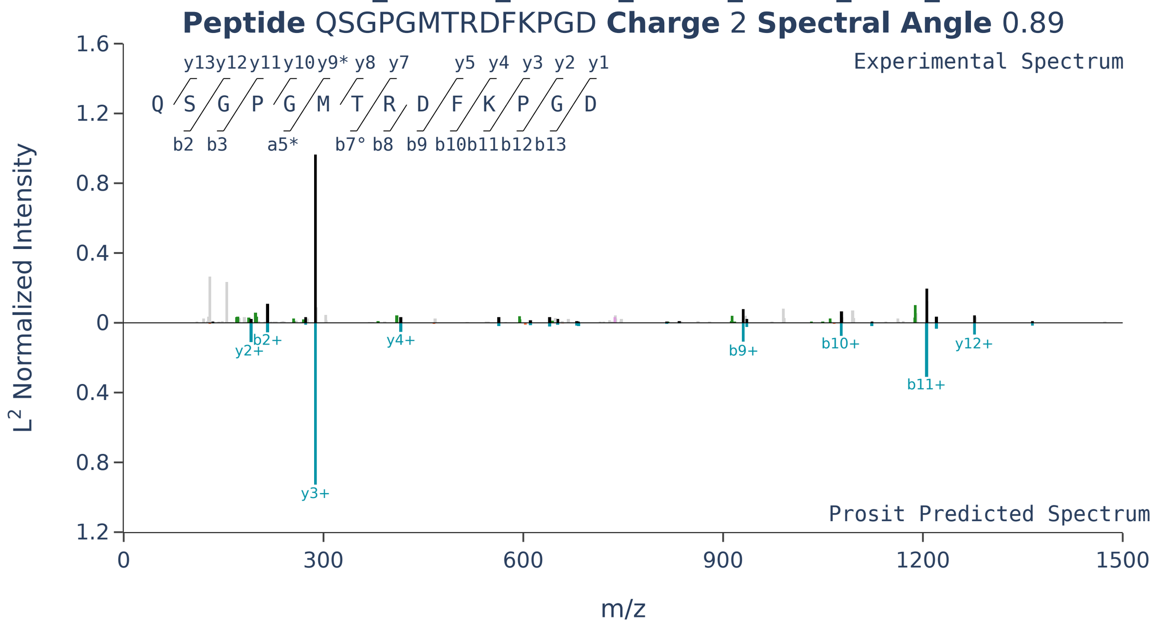

Source HRoetschke_271222_090123_HFGoe_LEDGF_b2R2_2h **Scan** 10143
Peptide KVSQVIMEKSQQA **Charge** 2 **Spectral Angle** 0.81

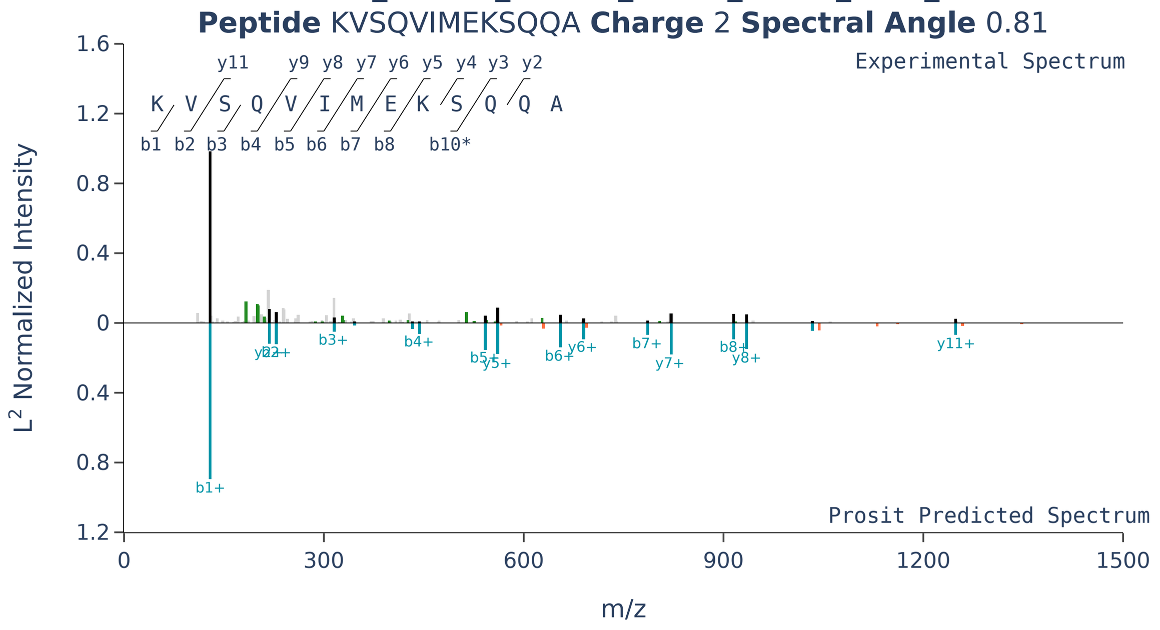

Source HRoetschke_271222_090123_HFGoe_LEDGF_b1R1_24h **Scan** 17038
Peptide DLDVNRCIE **Charge** 2 **Spectral Angle** 0.87

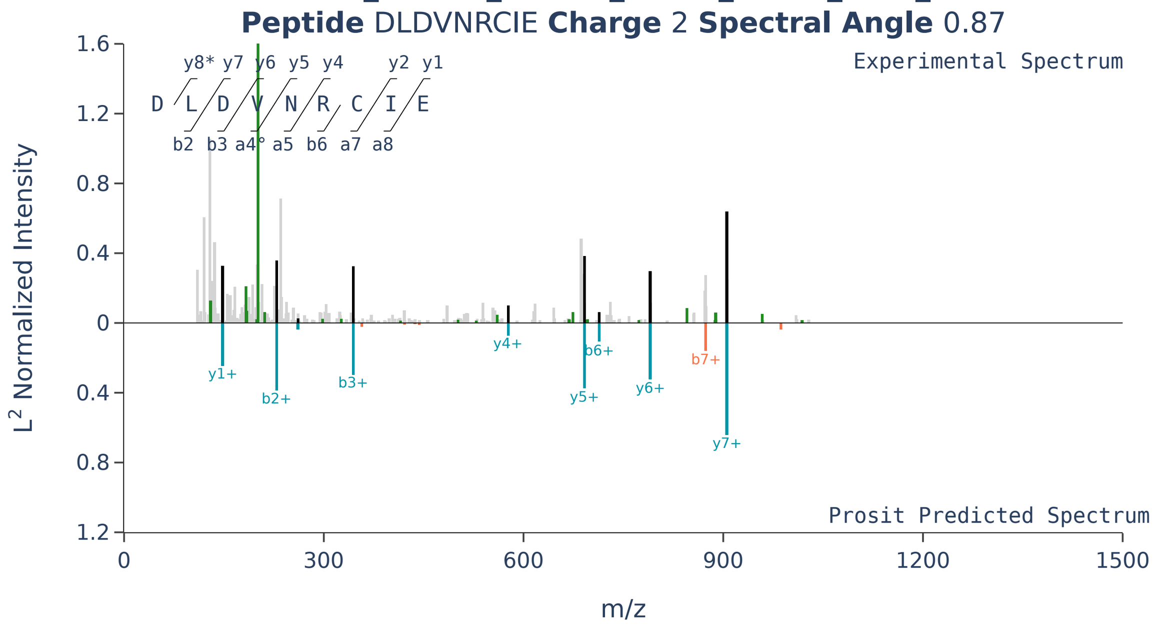

Source HRoetschke_271222_090123_HFGoe_LEDGF_b1R1_4h **Scan** 18977
Peptide GTHETKGFNEGLWE **Charge** 2 **Spectral Angle** 0.87

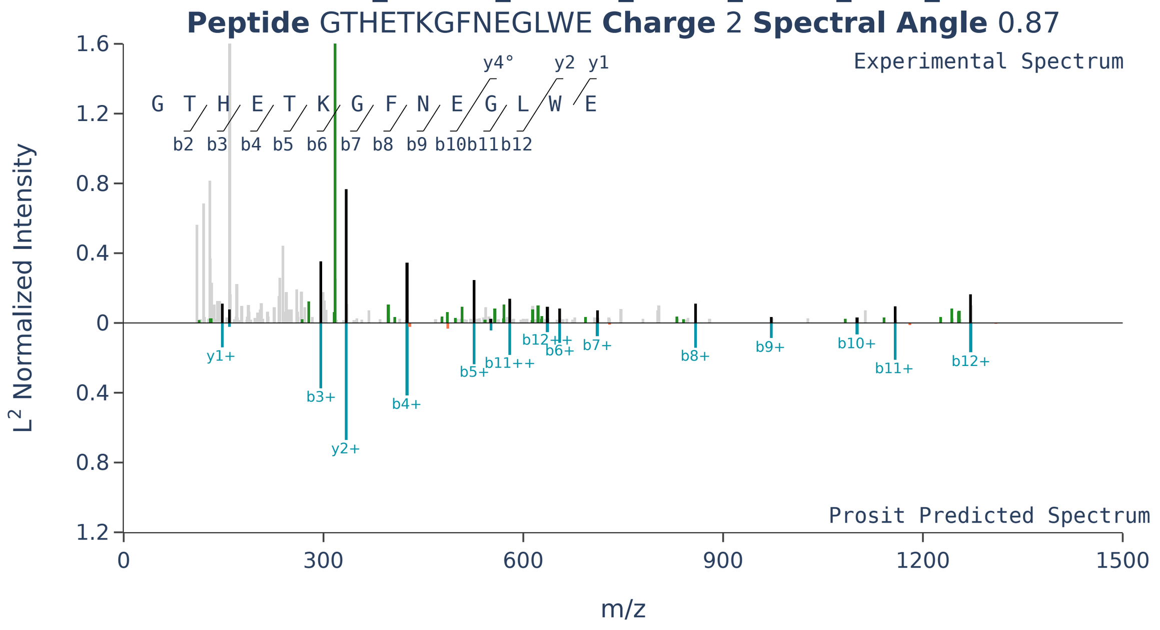

Source HRoetschke_271222_090123_HFGoe_LEDGF_b2R1_24h **Scan** 17782
Peptide GTFLGPKDIFPYSENKEKYGKPNKR **Charge** 5 **Spectral Angle** 0.78

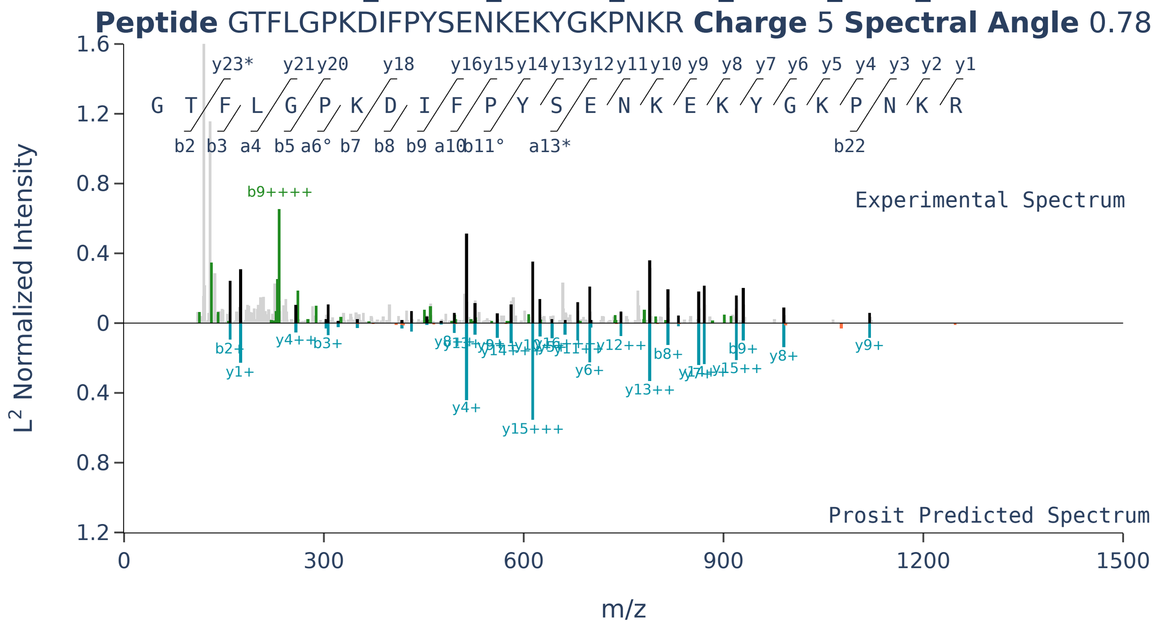

Source HRoetschke_271222_090123_HFGoe_LEDGF_b1R1_24h **Scan** 17859
Peptide WYPHWPAR **Charge** 3 **Spectral Angle** 0.81

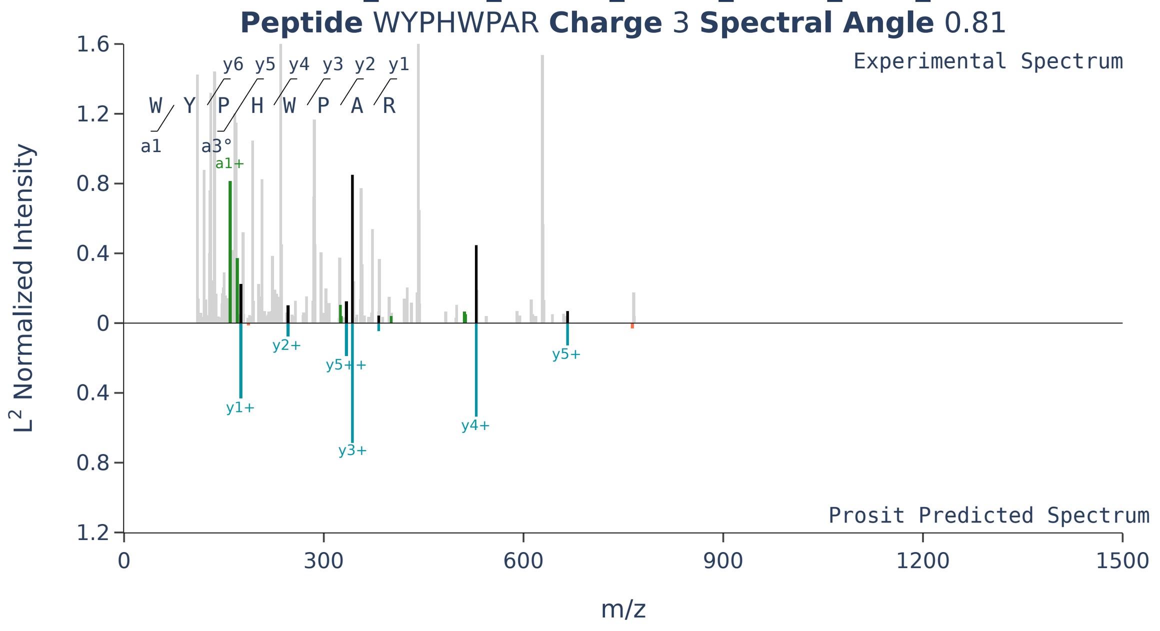

Source HRoetschke_271222_090123_HFGoe_LEDGF_b1R1_24h **Scan** 16049
Peptide KNMFLVGKE **Charge** 2 **Spectral Angle** 0.88

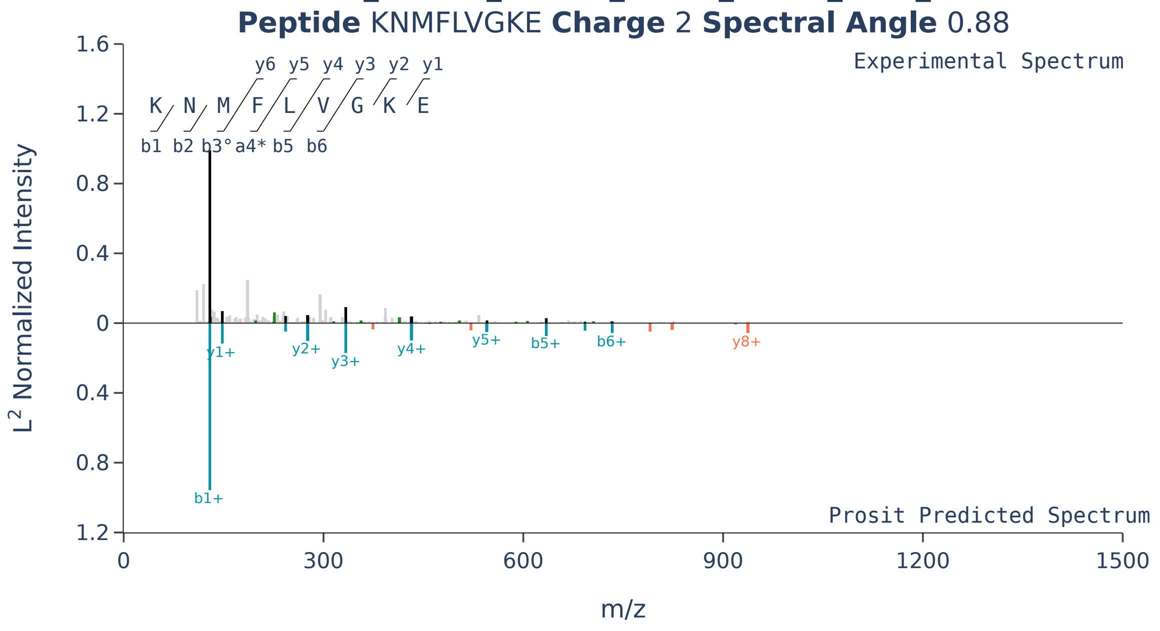

Source HRoetschke_271222_090123_HFGoe_LEDGF_b2R2_2h **Scan** 11378
Peptide IDNNPKVKFSSQQAATE **Charge** 2 **Spectral Angle** 0.82

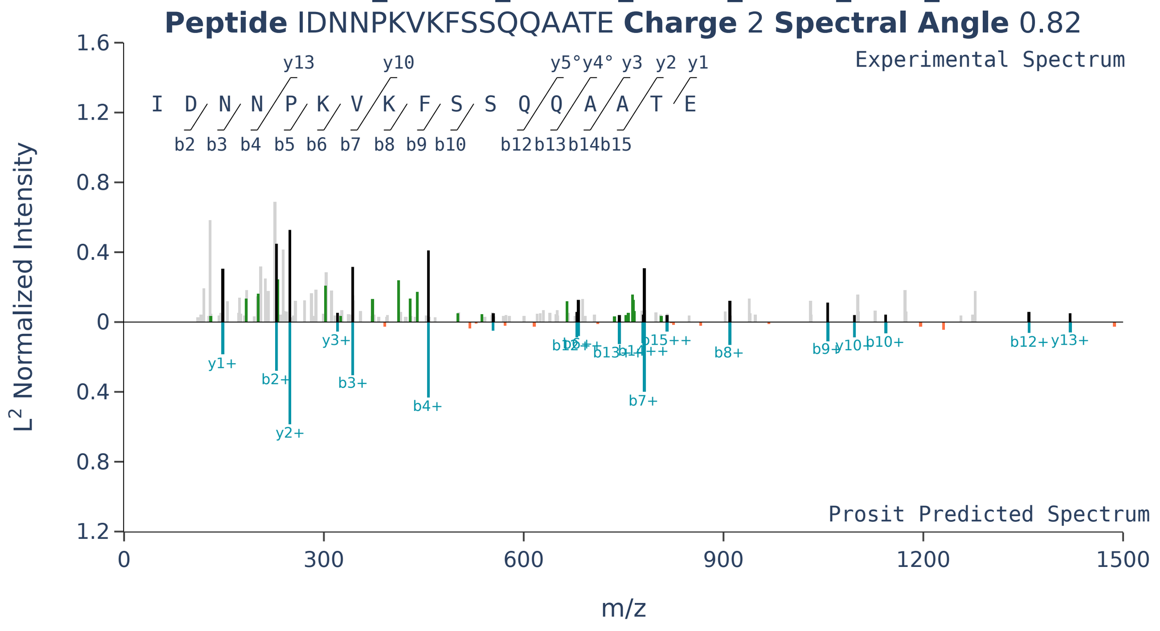

Source HRoetschke_271222_090123_HFGoe_LEDGF_b1R2_24h **Scan** 5897
Peptide AKVSPKRGRPAATEV **Charge** 3 **Spectral Angle** 0.85

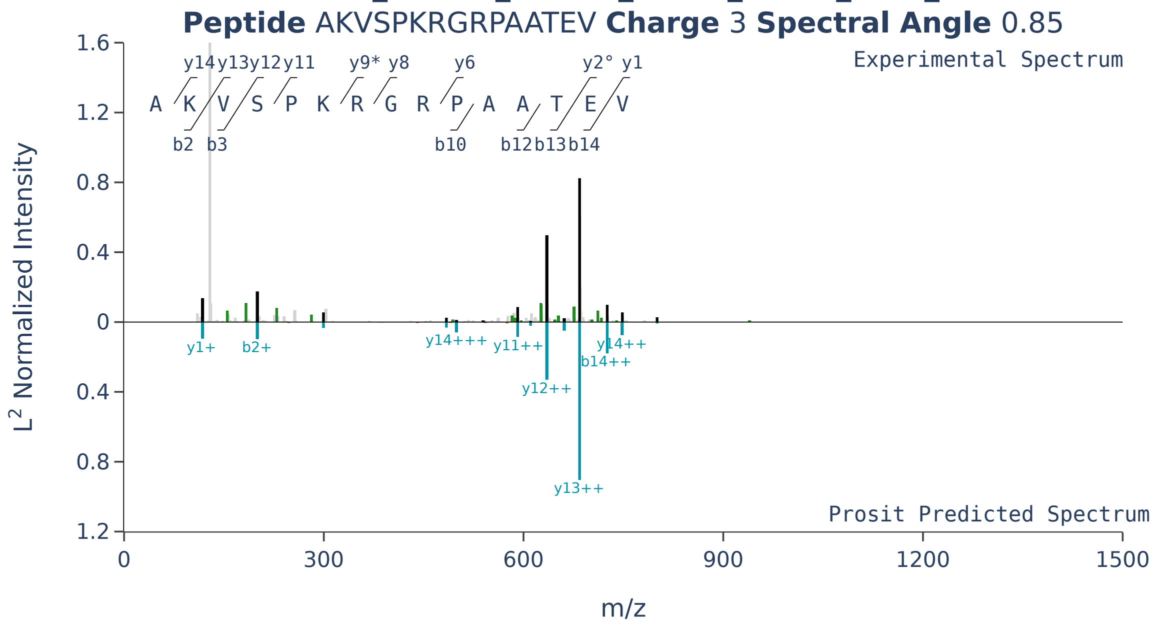

Source HRoetschke_271222_090123_HFGoe_LEDGF_b1R2_4h **Scan** 18359
Peptide GPGMTPHWPARVDE **Charge** 3 **Spectral Angle** 0.85

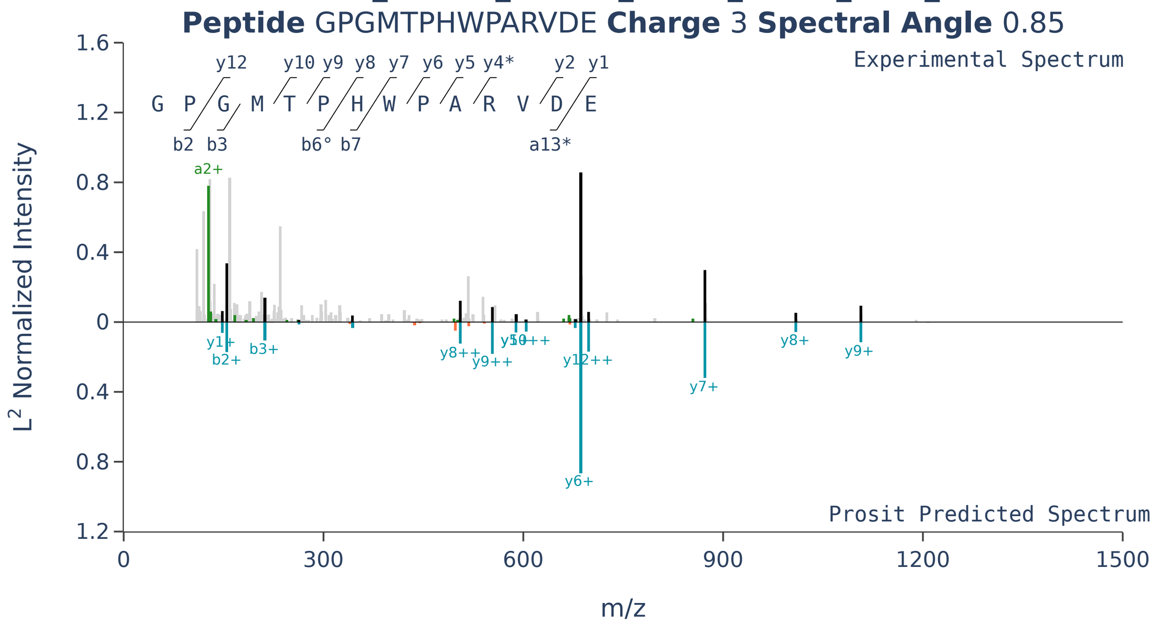

Source HRoetschke_271222_090123_HFGoe_LEDGF_b2R1_4h **Scan** 15415
Peptide GTHETYPHWPARVDE **Charge** 3 **Spectral Angle** 0.91

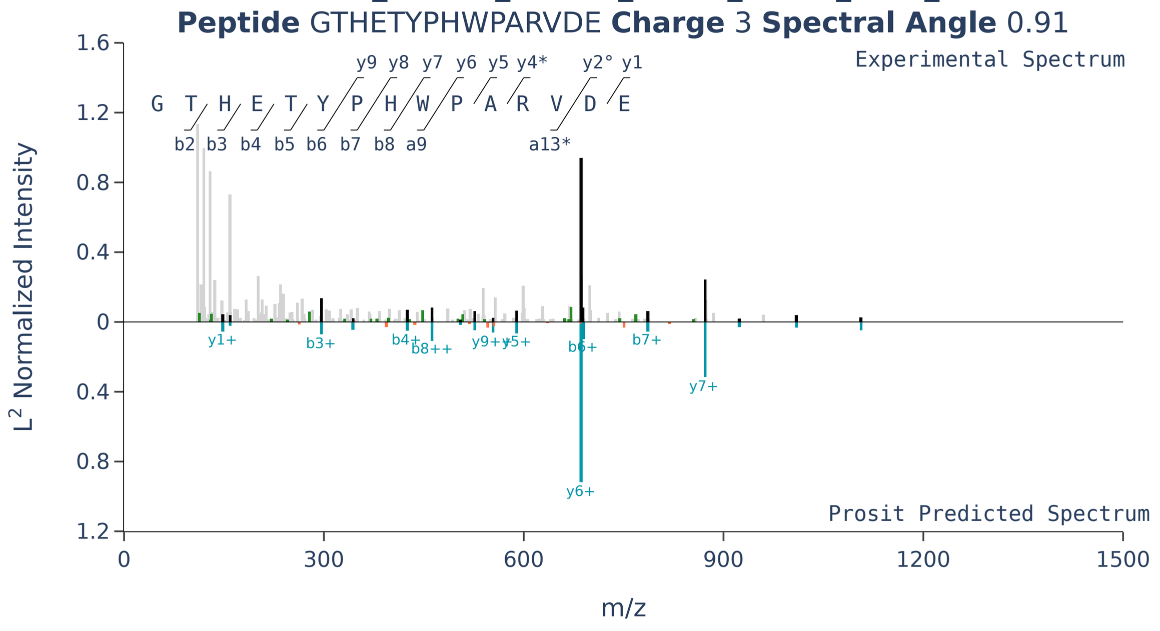

Source HRoetschke_271222_090123_HFGoe_LEDGF_b2R2_24h **Scan** 17536
Peptide IFGPKDIFPYSENKEKYGKPNKR **Charge** 6 **Spectral Angle** 0.81

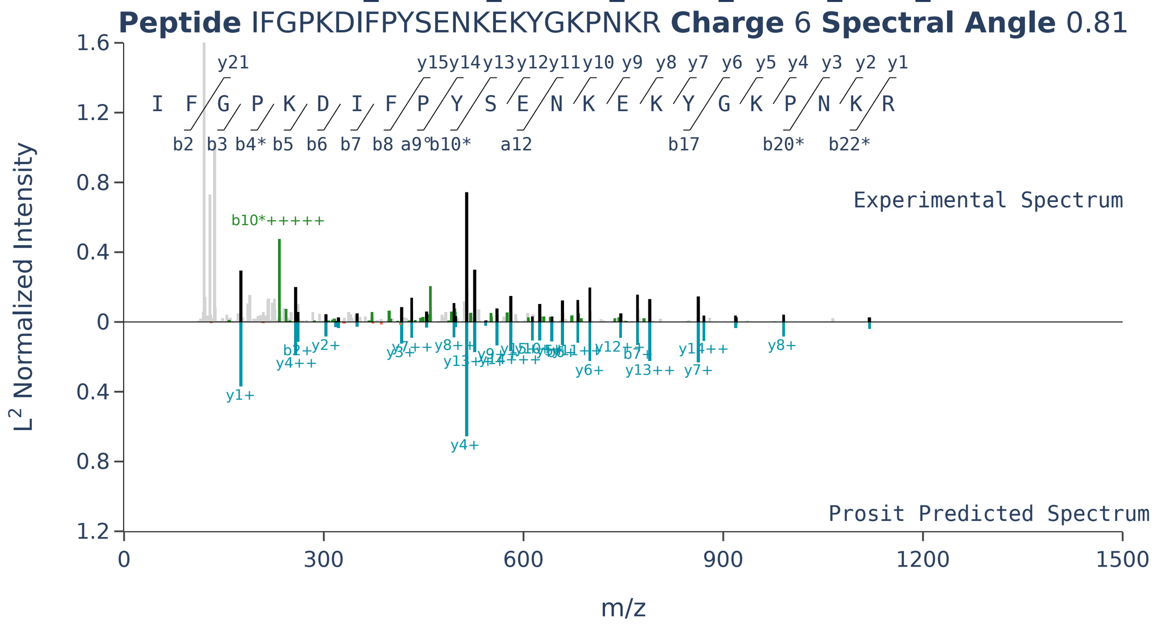

Source HRoetschke_271222_090123_HFGoe_LEDGF_b1R2_24h **Scan** 9974
Peptide PKHWPARVDE **Charge** 3 **Spectral Angle** 0.85

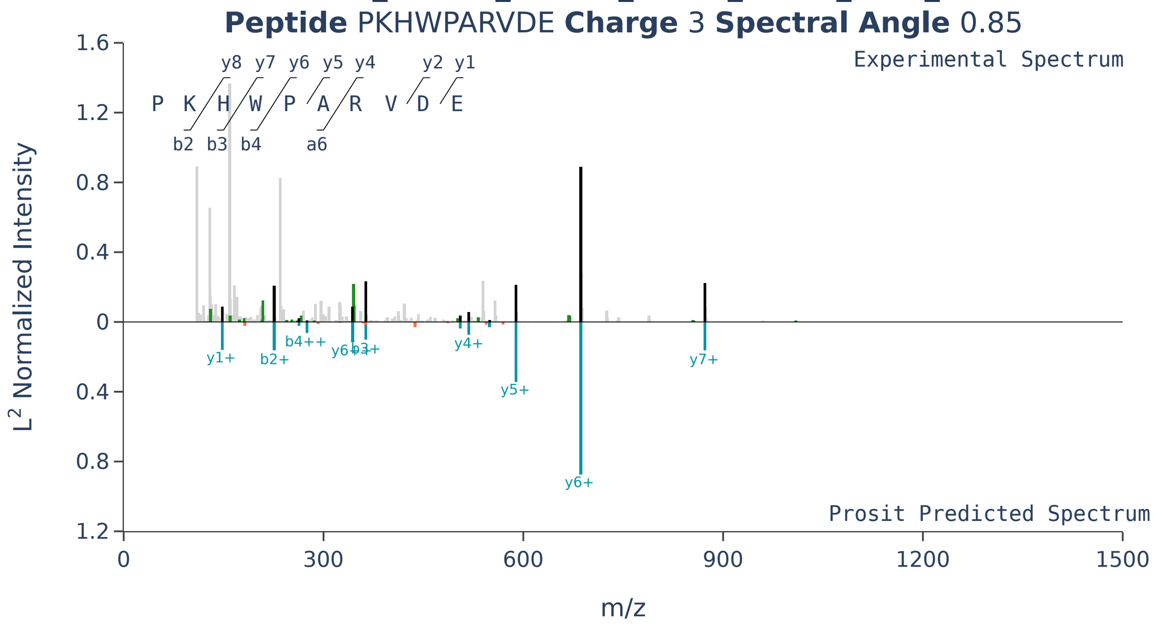

Source HRoetschke_271222_090123_HFGoe_LEDGF_b1R2_24h **Scan** 12197
Peptide VPDGAVKPPTNTA **Charge** 2 **Spectral Angle** 0.85

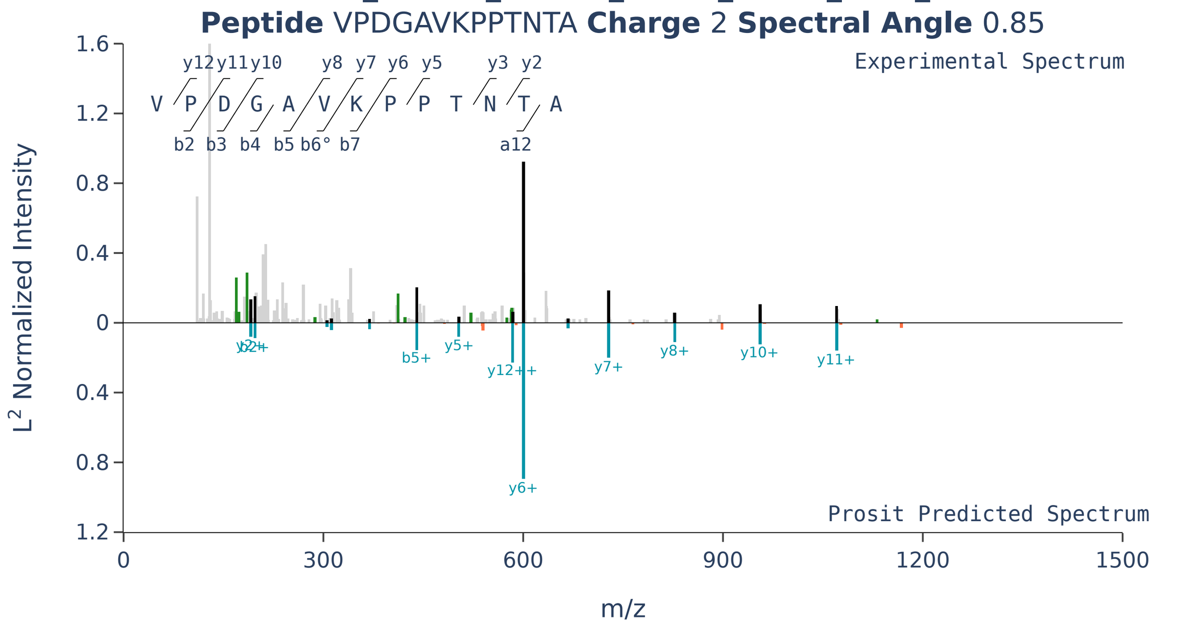

Source HRoetschke_271222_090123_HFGoe_LEDGF_b1R1_24h **Scan** 14296
Peptide LGPKDIFPYSENKEKYGKPNKRTA **Charge** 5 **Spectral Angle** 0.8

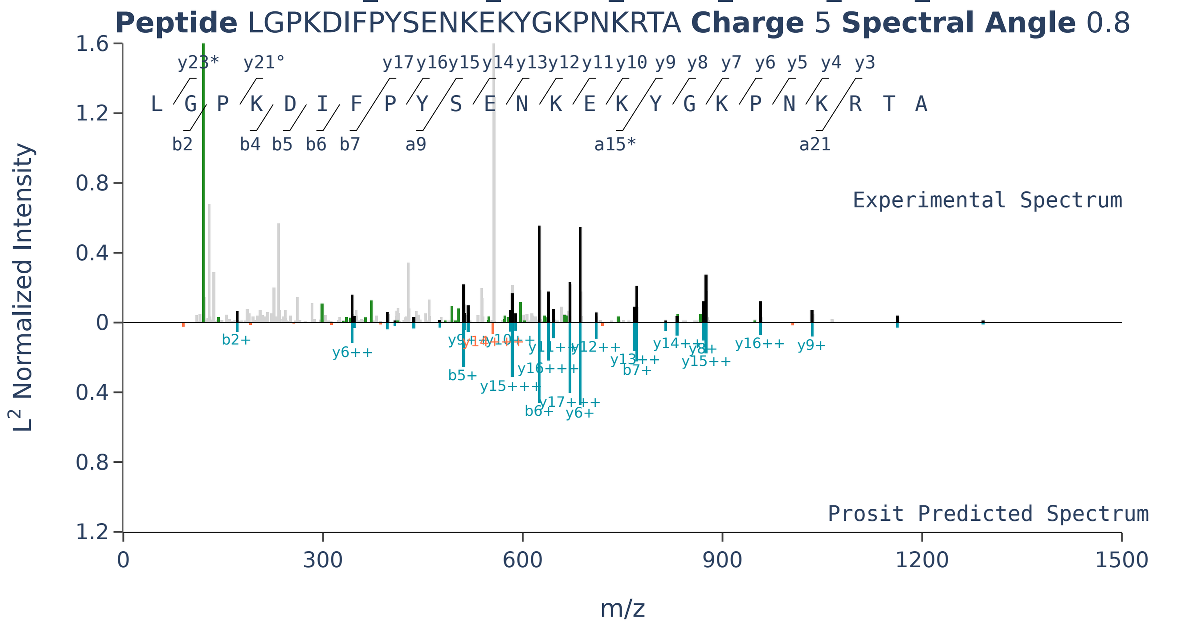

Source HRoetschke_271222_090123_HFGoe_LEDGF_b2R1_4h **Scan** 21199
Peptide ASLKIDNLDVNRCIE **Charge** 3 **Spectral Angle** 0.81

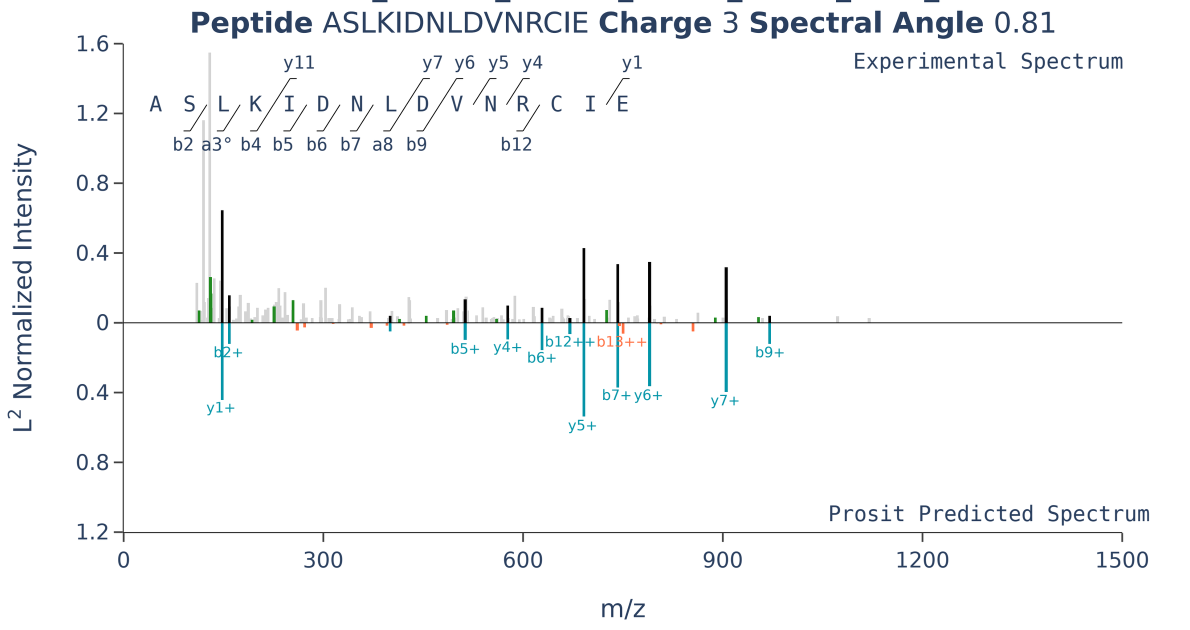

Source HRoetschke_271222_090123_HFGoe_LEDGF_b1R1_24h **Scan** 18392
Peptide KSTKGFNEGLWE **Charge** 3 **Spectral Angle** 0.88

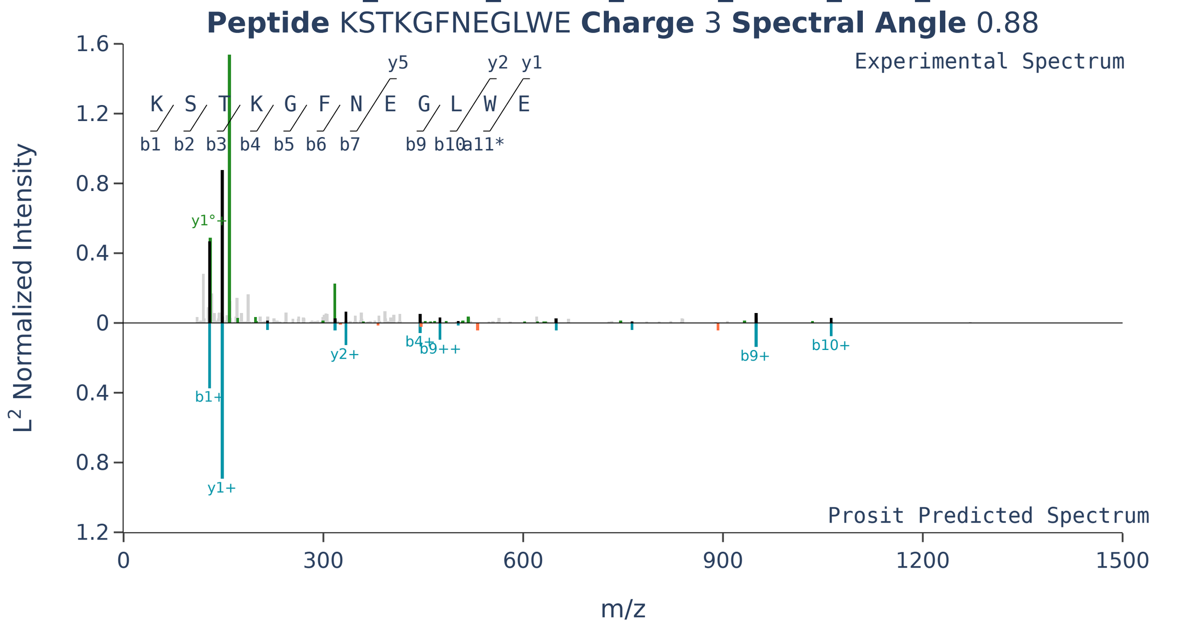

Source HRoetschke_271222_090123_HFGoe_LEDGF_b2R1_2h **Scan** 24675
Peptide EPMTRDFKPGDLIFAKMKGY **Charge** 5 **Spectral Angle** 0.84

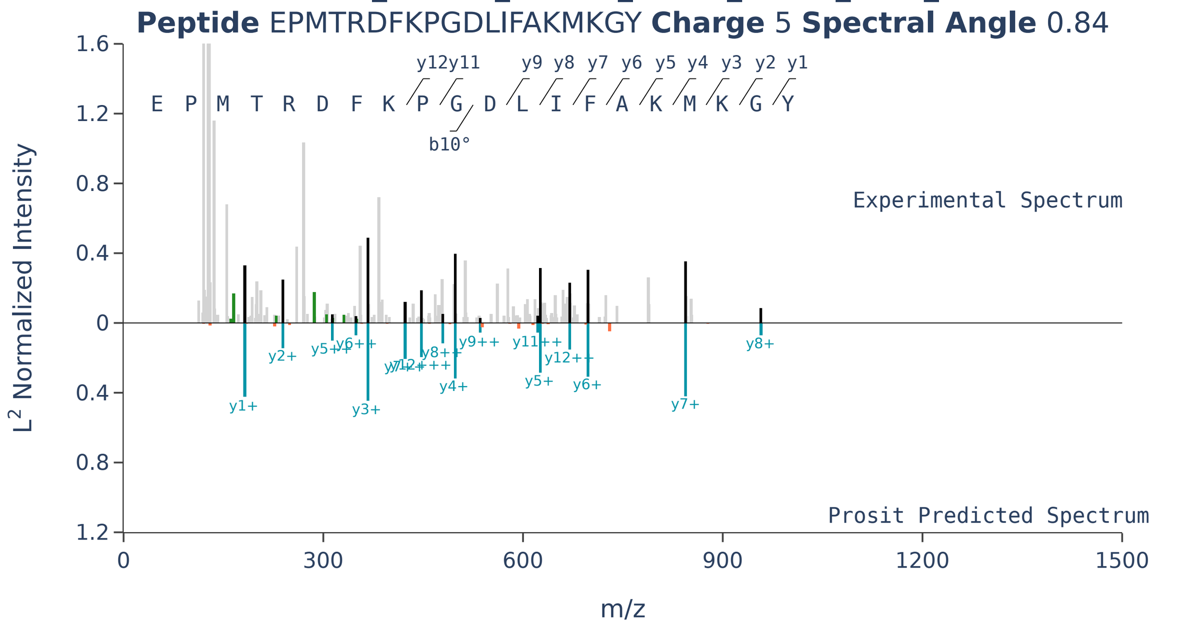

Source HRoetschke_271222_090123_HFGoe_LEDGF_b1R2_24h **Scan** 15521
Peptide EGKLASL **Charge** 2 **Spectral Angle** 0.85

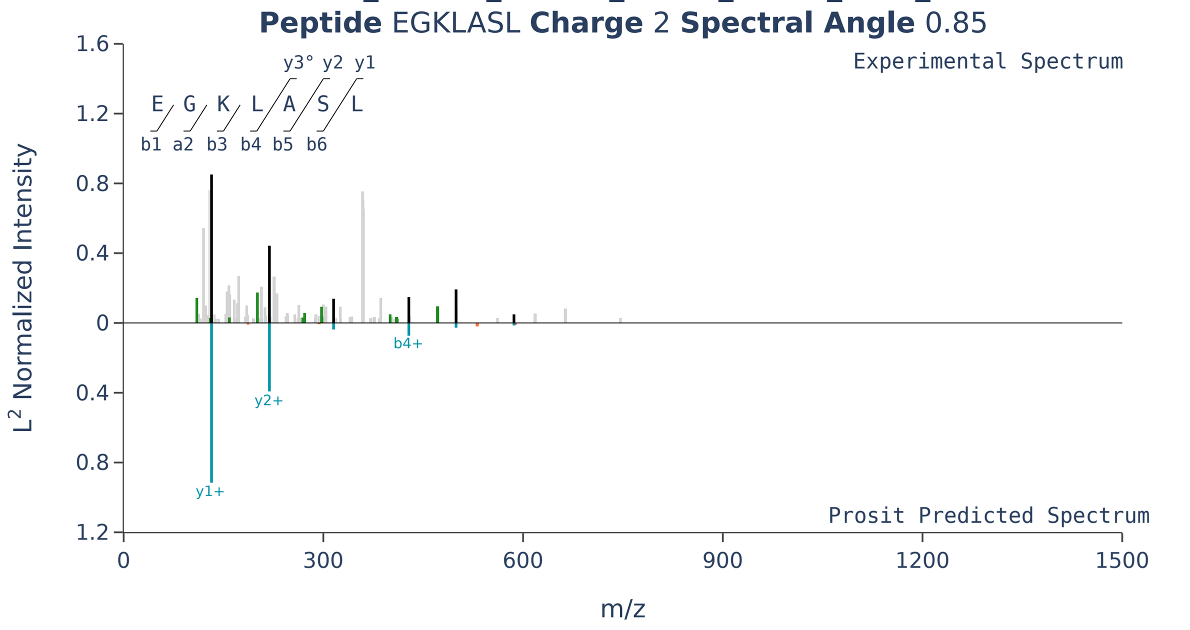

Source HRoetschke_271222_090123_HFGoe_LEDGF_b1R1_24h **Scan** 9955
Peptide VTFPYSENKEKYGKPNKR **Charge** 5 **Spectral Angle** 0.81

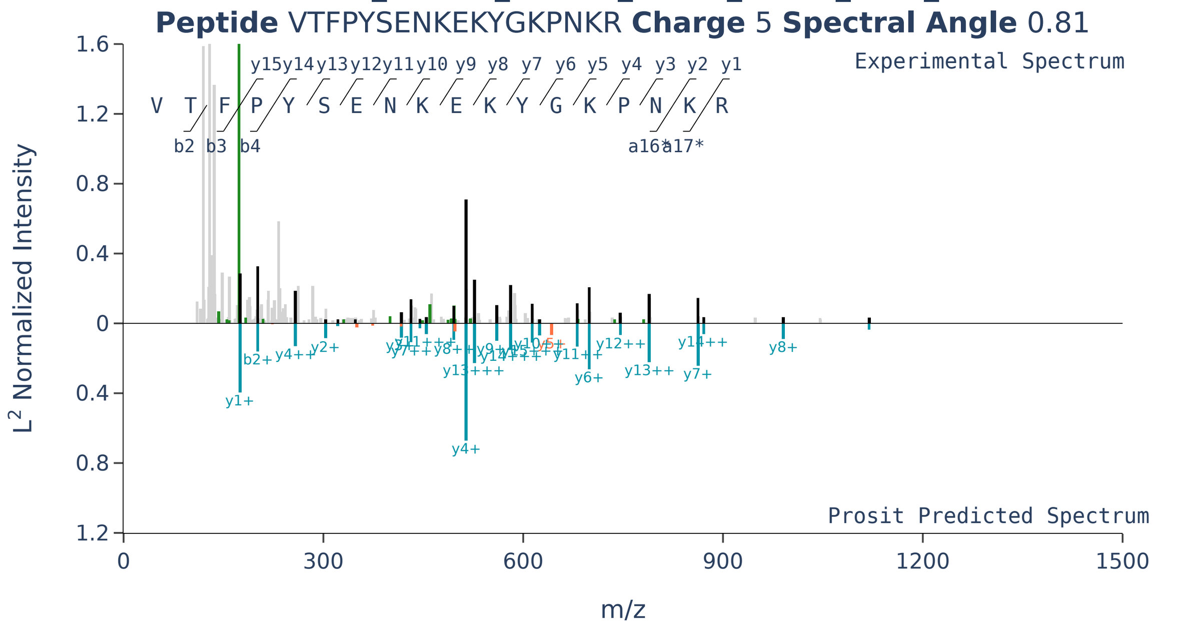

Source HRoetschke_271222_090123_HFGoe_LEDGF_b1R2_24h **Scan** 18131
Peptide TNGYPHWPARV **Charge** 3 **Spectral Angle** 0.82

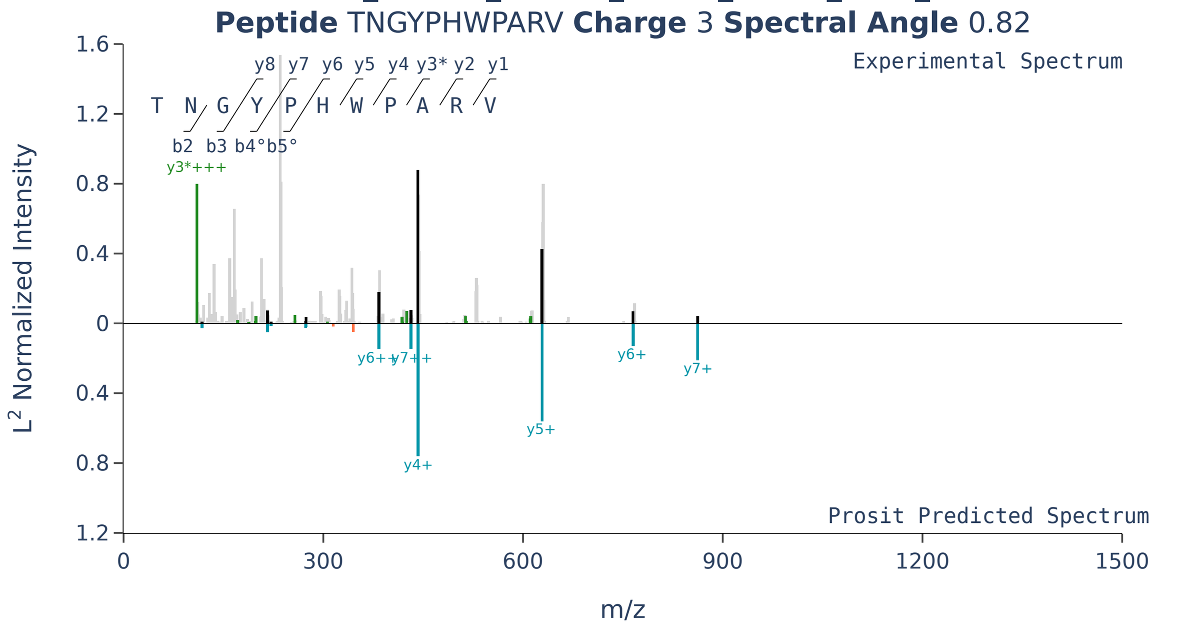

Source HRoetschke_271222_090123_HFGoe_LEDGF_b1R1_2h **Scan** 18047
Peptide SQQAGYPHWPARV **Charge** 3 **Spectral Angle** 0.85

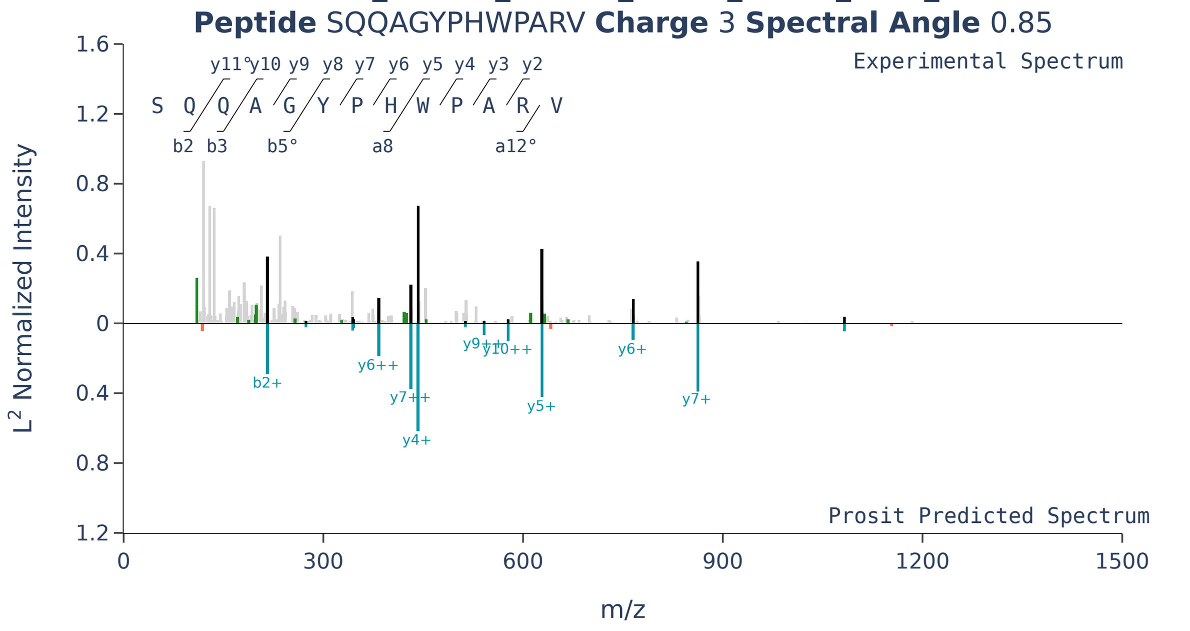

Source HRoetschke_271222_090123_HFGoe_LEDGF_b1R2_24h **Scan** 6263
Peptide RIHAEIKNQQA **Charge** 3 **Spectral Angle** 0.9

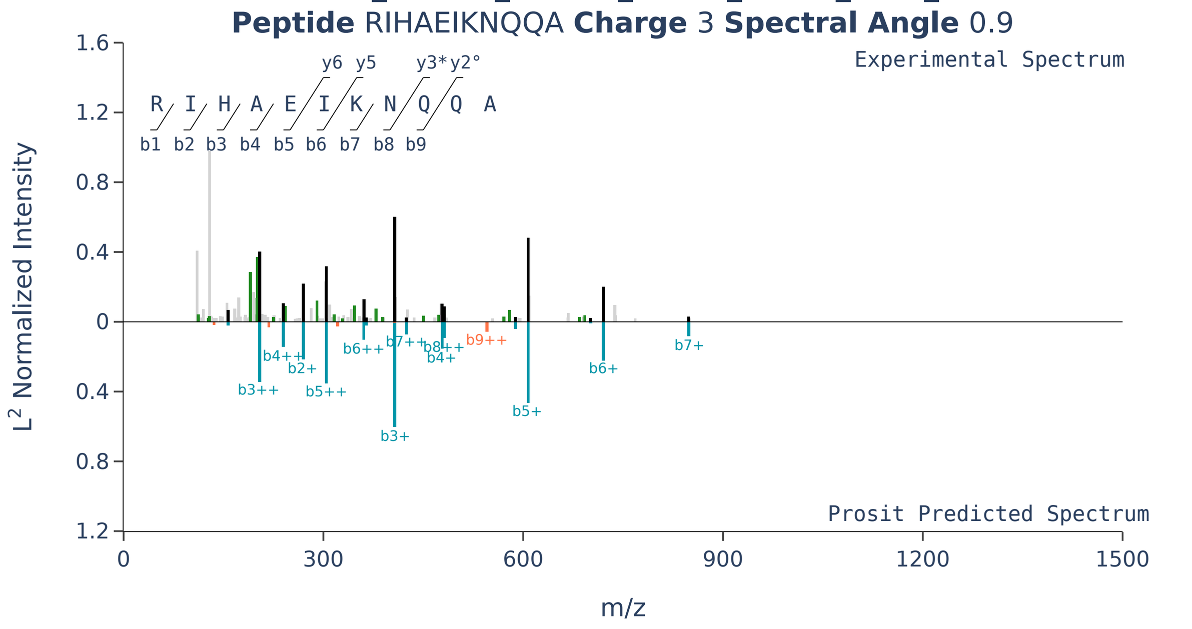

Source HRoetschke_271222_090123_HFGoe_LEDGF_b2R2_4h **Scan** 9089
Peptide GTHETSMDSRL **Charge** 3 **Spectral Angle** 0.92

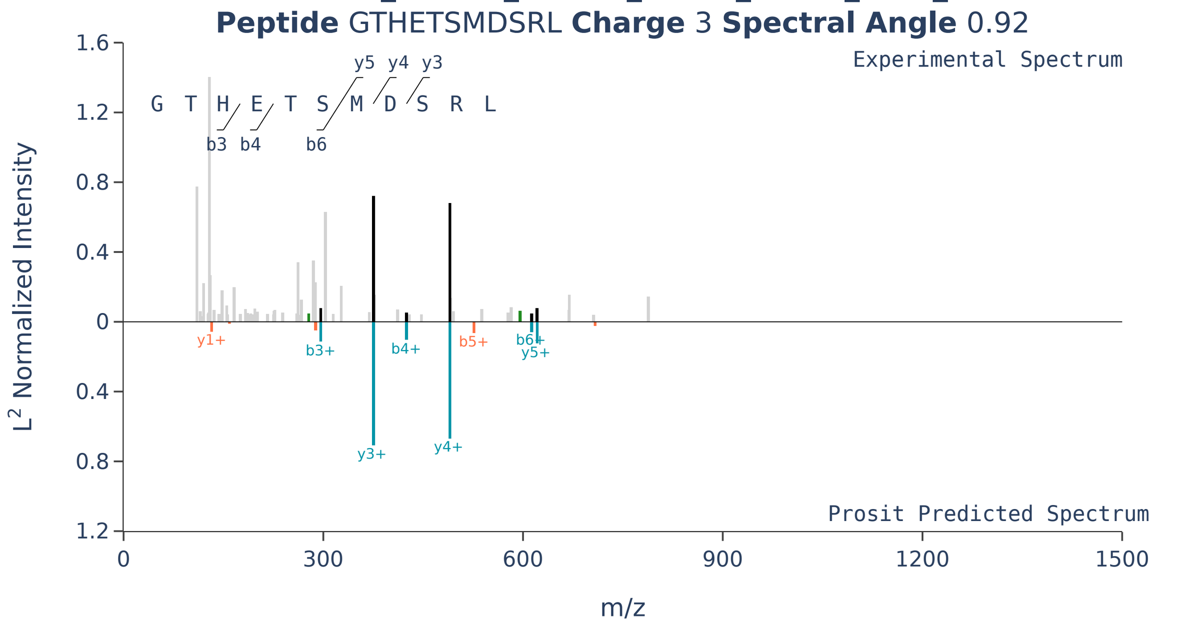

Source HRoetschke_271222_090123_HFGoe_LEDGF_b2R1_4h **Scan** 13534
Peptide TNPHWPARVDE **Charge** 2 **Spectral Angle** 0.84

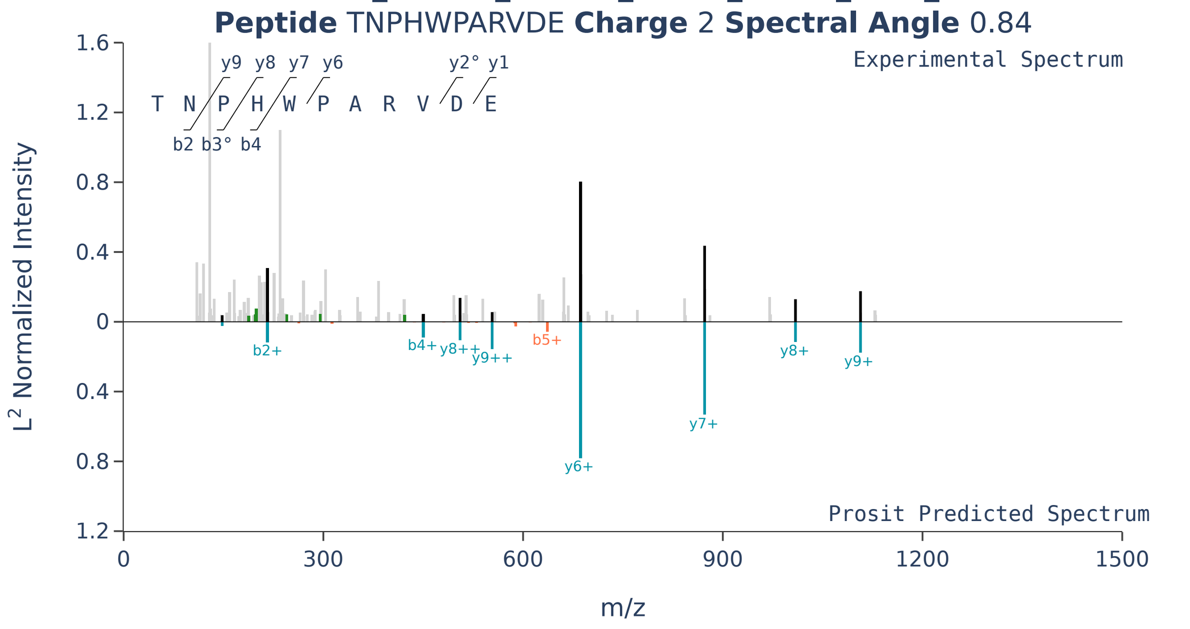

Source HRoetschke_271222_090123_HFGoe_LEDGF_b2R1_2h **Scan** 12378
Peptide SQQAGPGMTRDFKPGD **Charge** 3 **Spectral Angle** 0.82

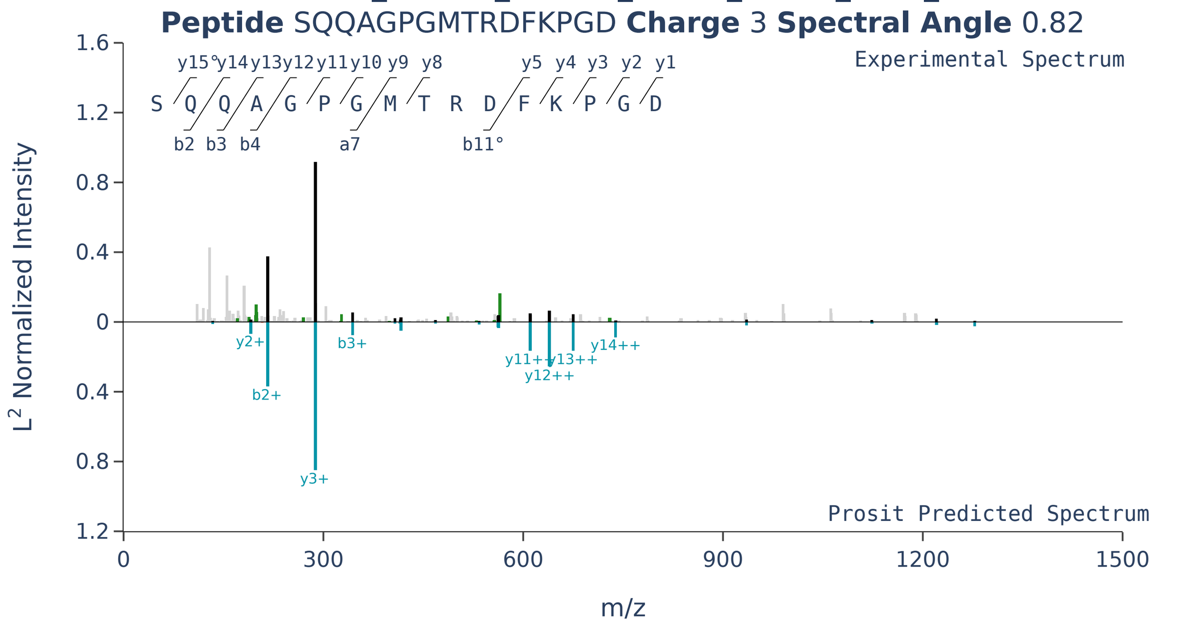

Source HRoetschke_271222_090123_HFGoe_LEDGF_b2R2_24h **Scan** 11033
Peptide DNNPKVKFSTA **Charge** 2 **Spectral Angle** 0.84

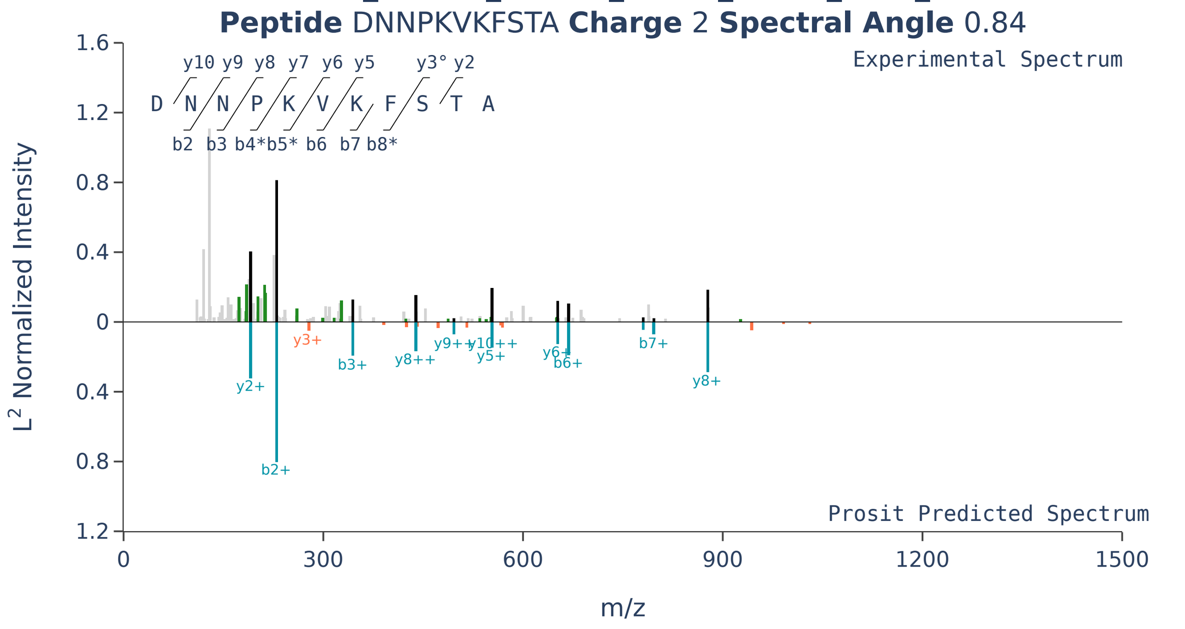

Source HRoetschke_271222_090123_HFGoe_LEDGF_b2R2_24h **Scan** 18961
Peptide GTHETSLKIDNLDVNRCIE **Charge** 3 **Spectral Angle** 0.89

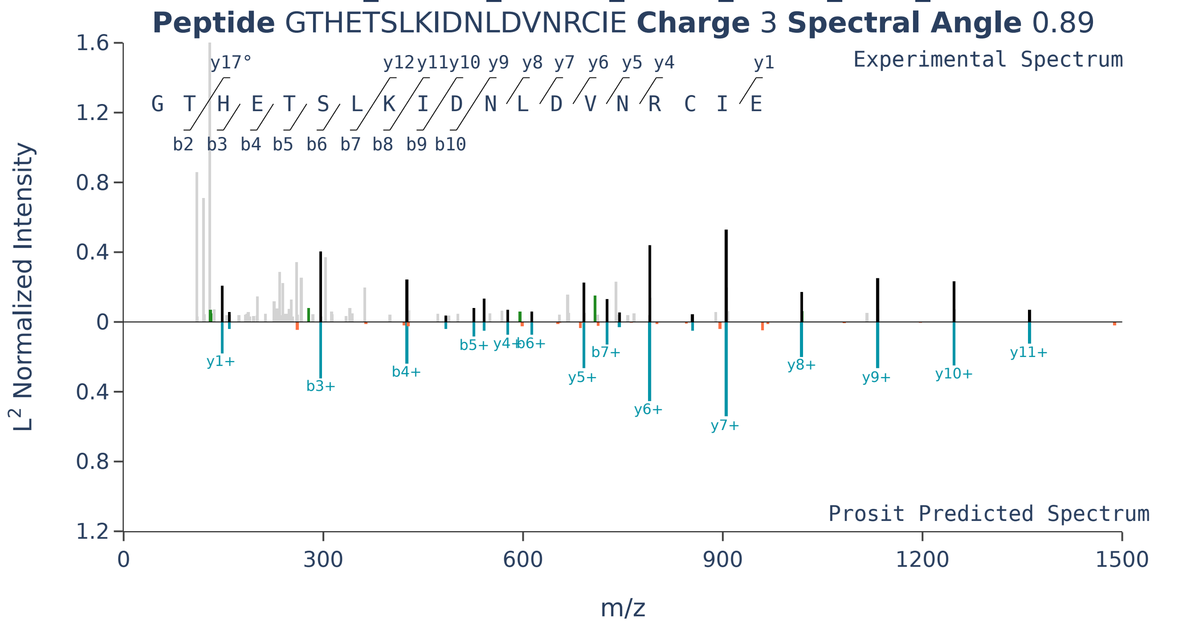

Source HRoetschke_271222_090123_HFGoe_LEDGF_b1R1_4h **Scan** 17687
Peptide GAFLGPKDIFPYSENKEKYGKPNKR **Charge** 4 **Spectral Angle** 0.84

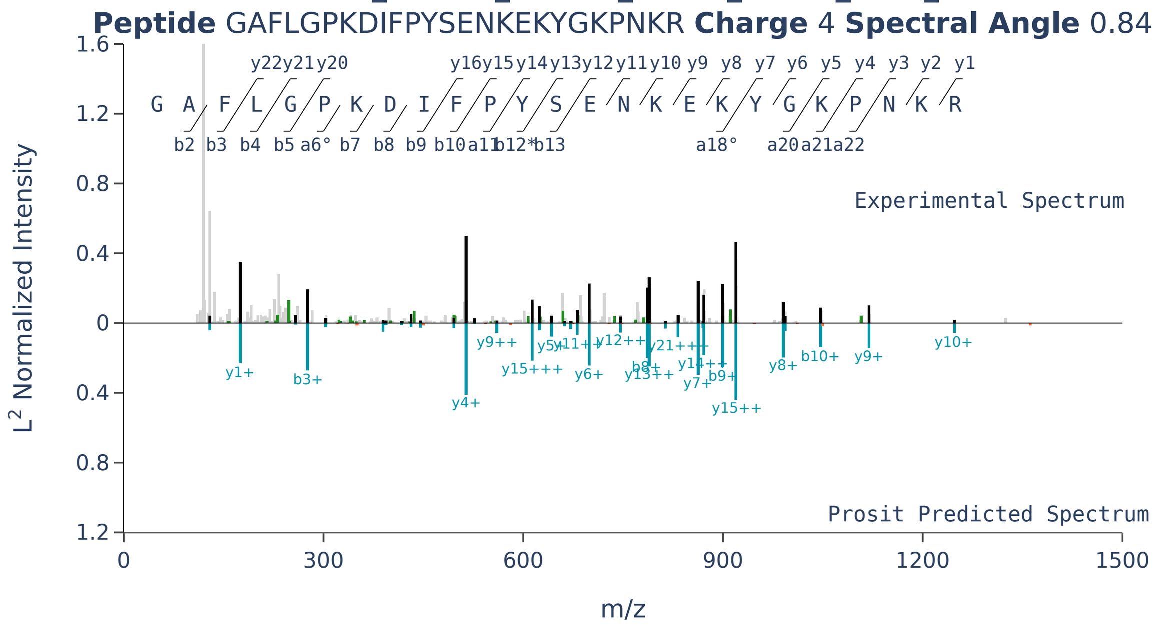

Source HRoetschke_271222_090123_HFGoe_LEDGF_b1R2_1h **Scan** 6860
Peptide GNQPQHNGEALD **Charge** 2 **Spectral Angle** 0.72

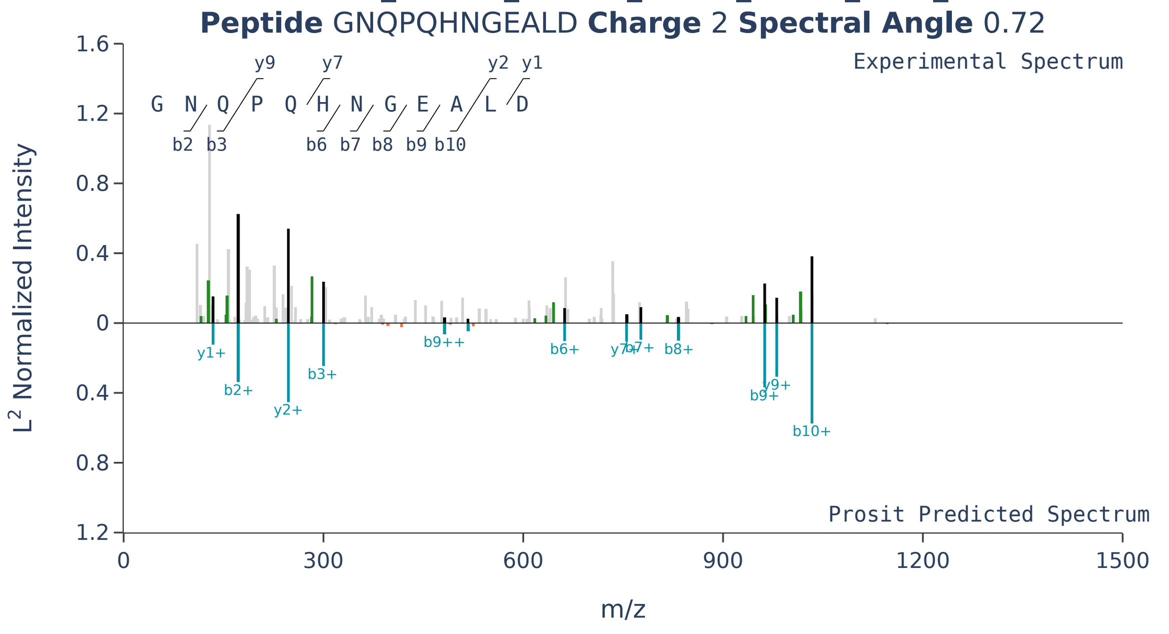

Source HRoetschke_271222_090123_HFGoe_LEDGF_b2R1_24h **Scan** 17068
Peptide FLGPKDIFPYSENKEKYGKPNRKA **Charge** 5 **Spectral Angle** 0.81

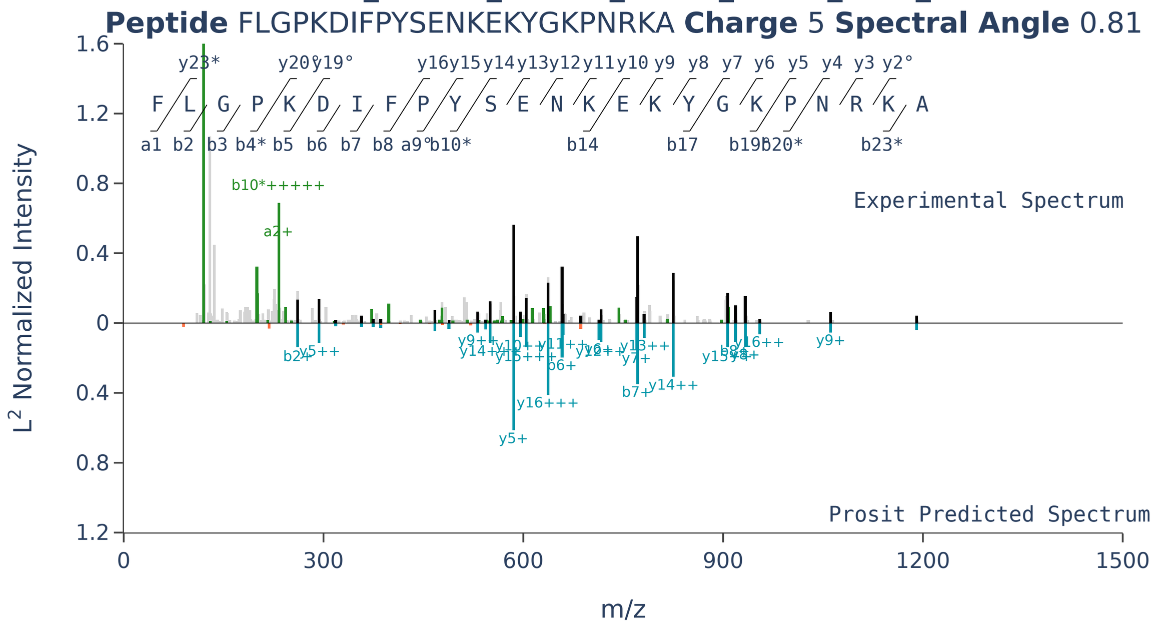

Source HRoetschke_271222_090123_HFGoe_LEDGF_b2R1_24h **Scan** 20873
Peptide SMDSRLQRIEL **Charge** 3 **Spectral Angle** 0.89

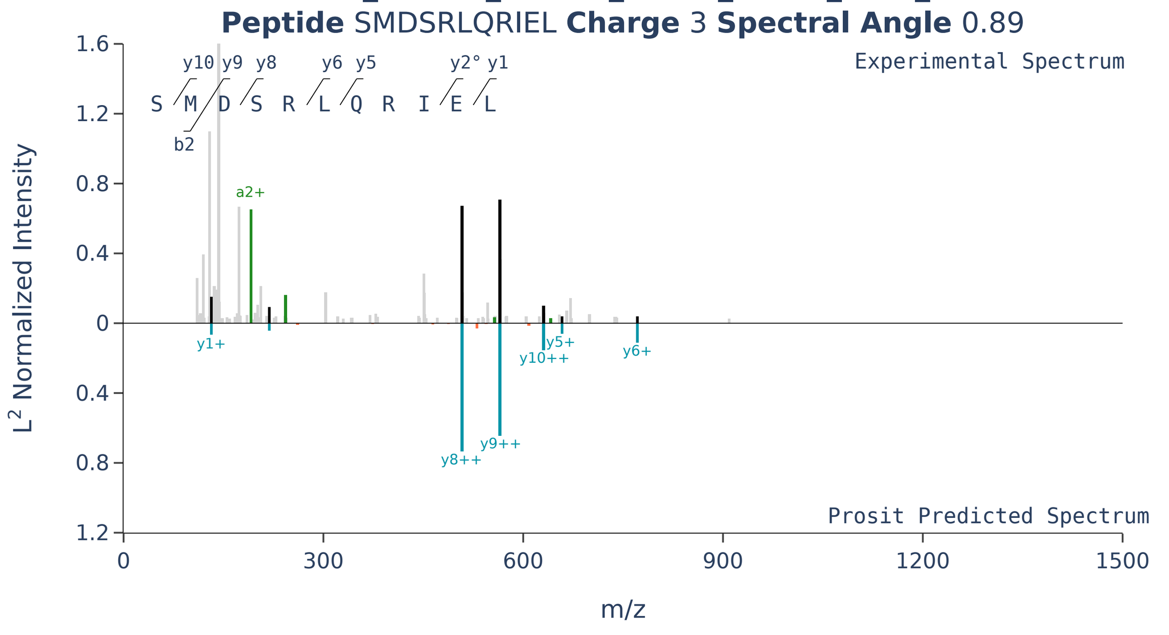

Source HRoetschke_271222_090123_HFGoe_LEDGF_b1R2_24h **Scan** 12947
Peptide AQDGPGMTRDFKPGD **Charge** 3 **Spectral Angle** 0.85

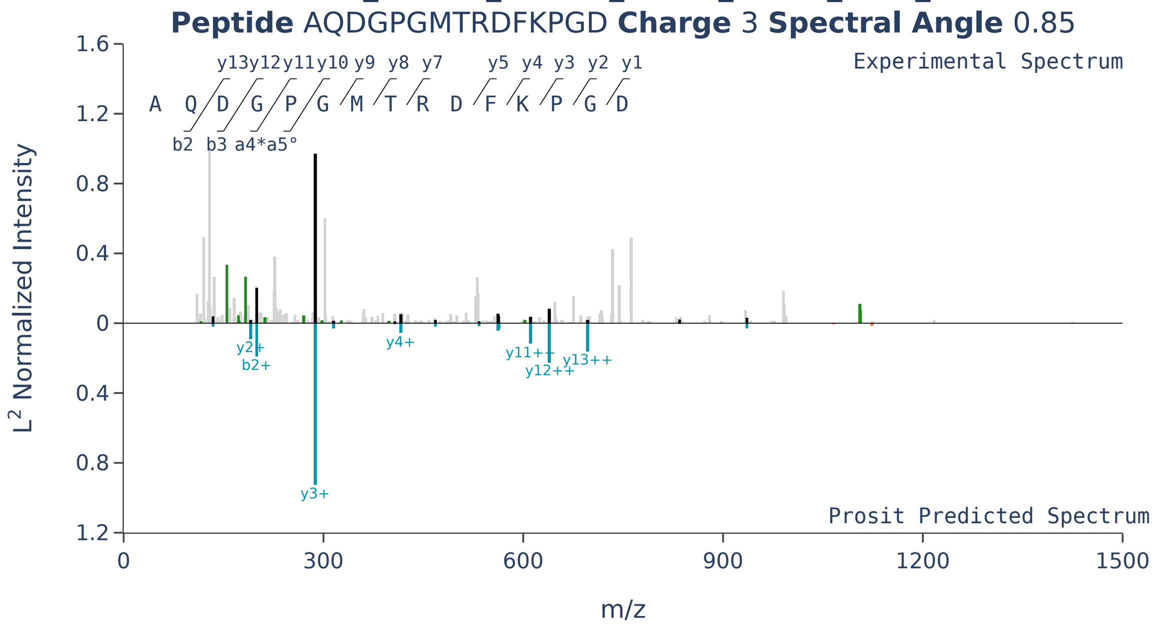

Source HRoetschke_271222_090123_HFGoe_LEDGF_b1R1_4h **Scan** 21859
Peptide NKTGFNEGLWE **Charge** 2 **Spectral Angle** 0.79

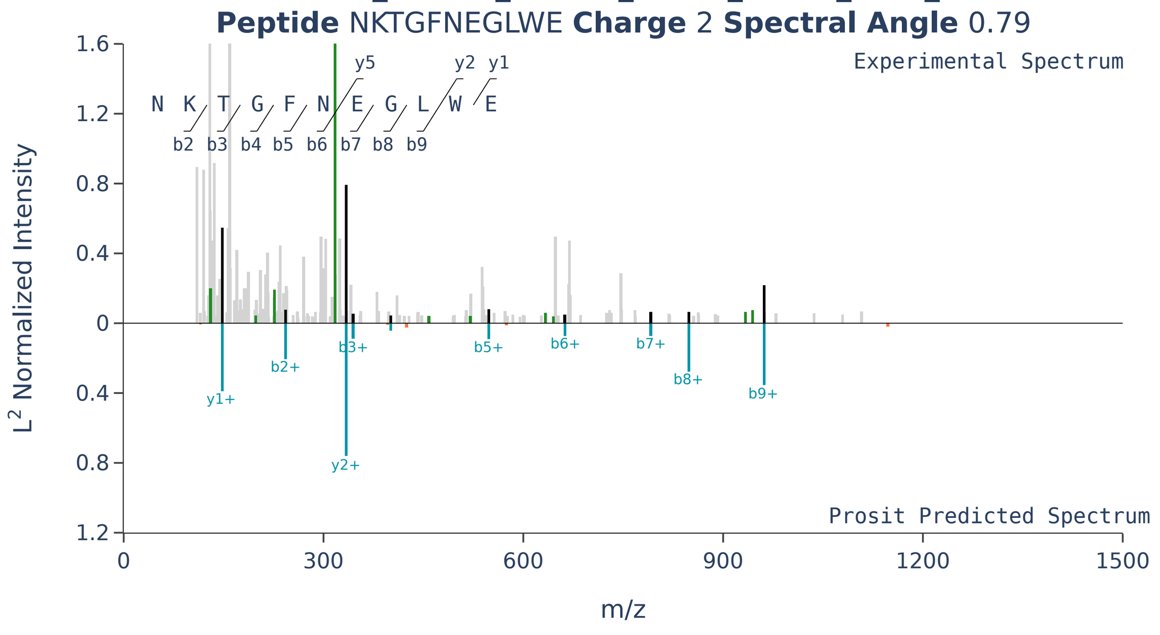

Source HRoetschke_271222_090123_HFGoe_LEDGF_b1R2_24h **Scan** 10768
Peptide AAVKPPTNKL **Charge** 2 **Spectral Angle** 0.83

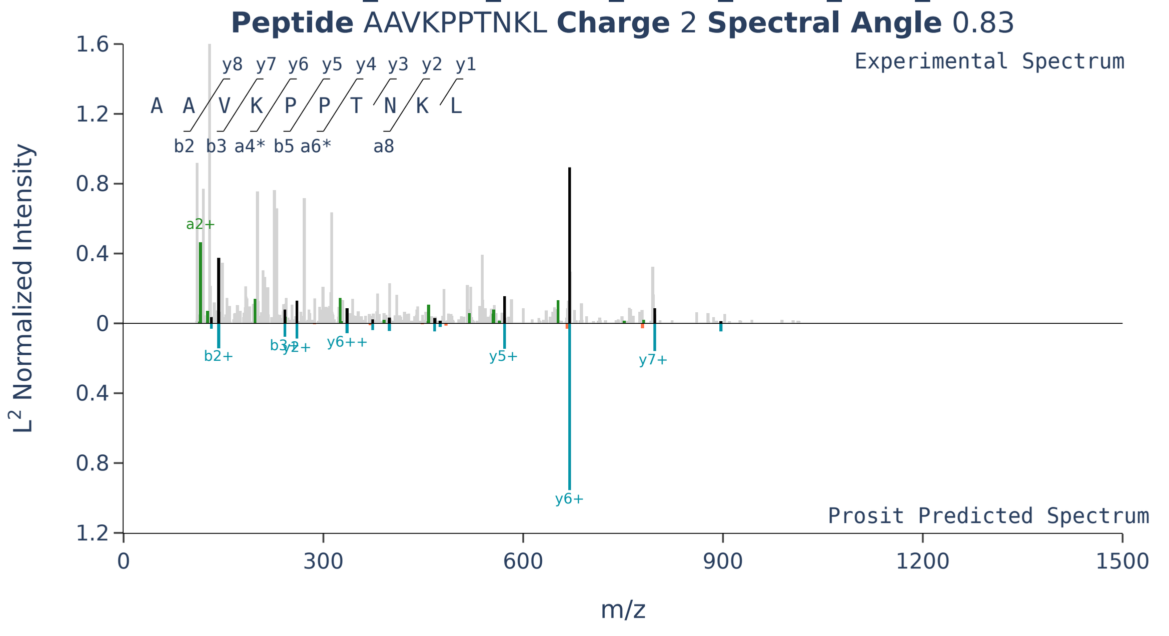

Source HRoetschke_271222_090123_HFGoe_LEDGF_b2R2_2h **Scan** 11129
Peptide STKPHWPARV **Charge** 3 **Spectral Angle** 0.92

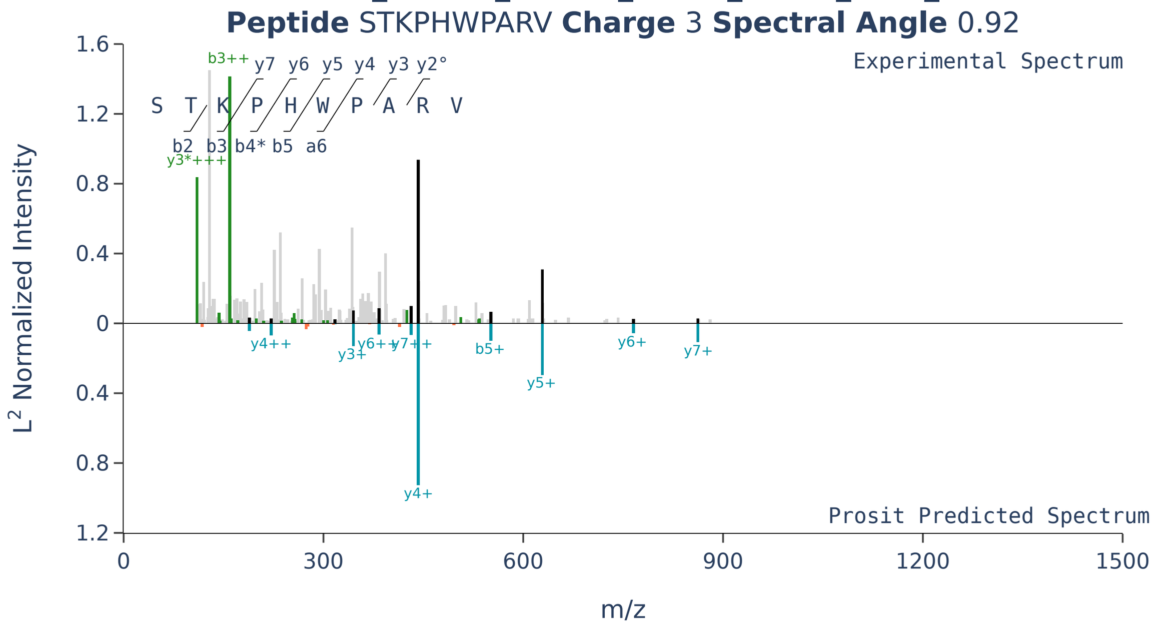

Source HRoetschke_271222_090123_HFGoe_LEDGF_b1R1_24h **Scan** 16050
Peptide KGTAFLGPKDIFPYSENKEKYGKPNKR **Charge** 4 **Spectral Angle** 0.84

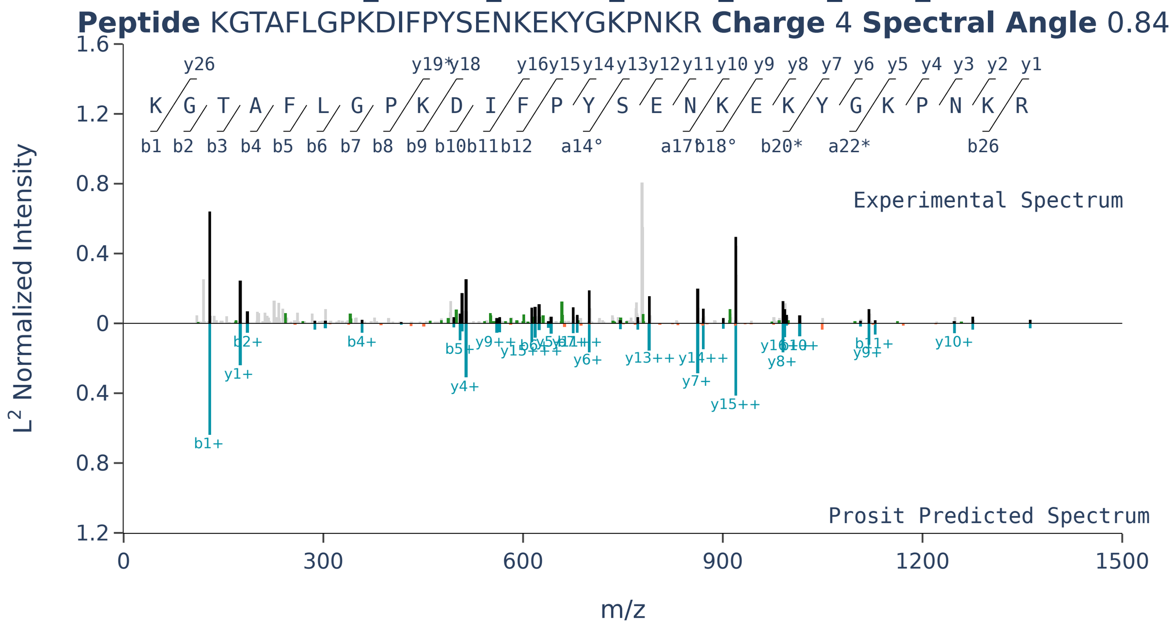

Source HRoetschke_271222_090123_HFGoe_LEDGF_b2R1_4h **Scan** 7512
Peptide TNVIMEKS **Charge** 2 **Spectral Angle** 0.81

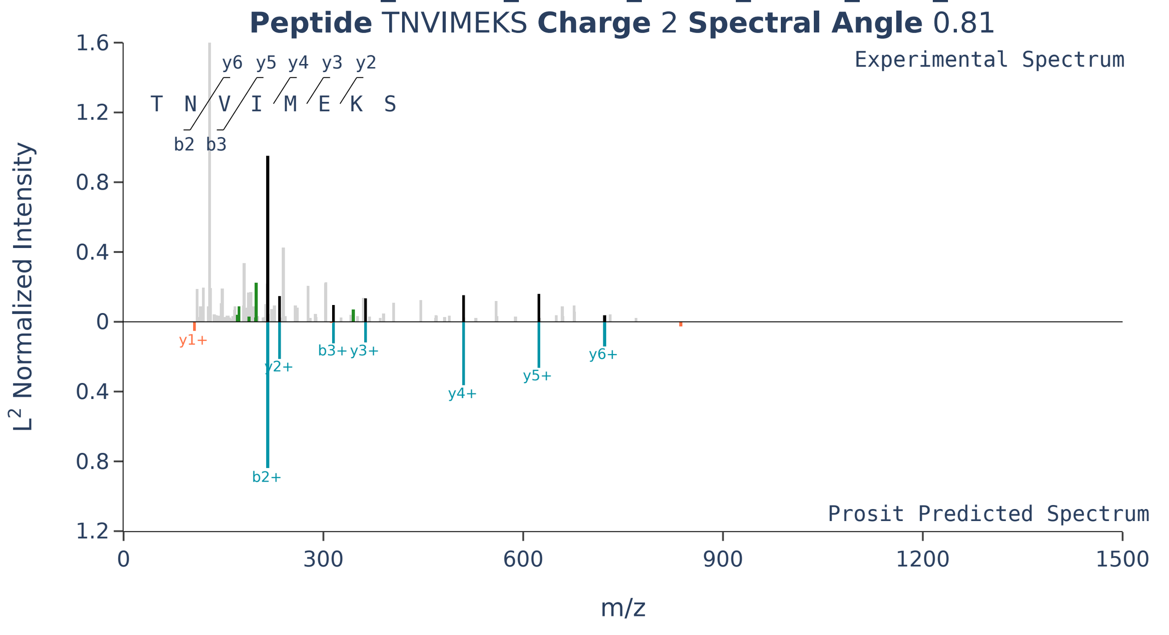

Source HRoetschke_271222_090123_HFGoe_LEDGF_b2R1_2h **Scan** 12324
Peptide ATGPGMTRDFKPGD **Charge** 3 **Spectral Angle** 0.83

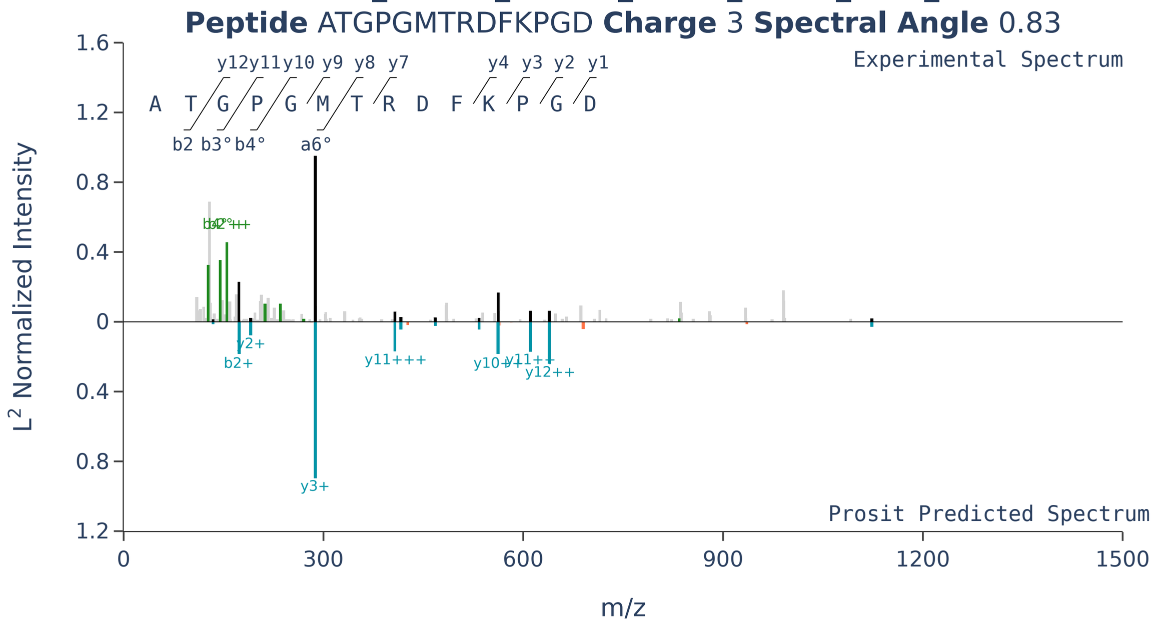

Source HRoetschke_271222_090123_HFGoe_LEDGF_b2R1_4h **Scan** 16845
Peptide QSNYPHWPARVDE **Charge** 3 **Spectral Angle** 0.89

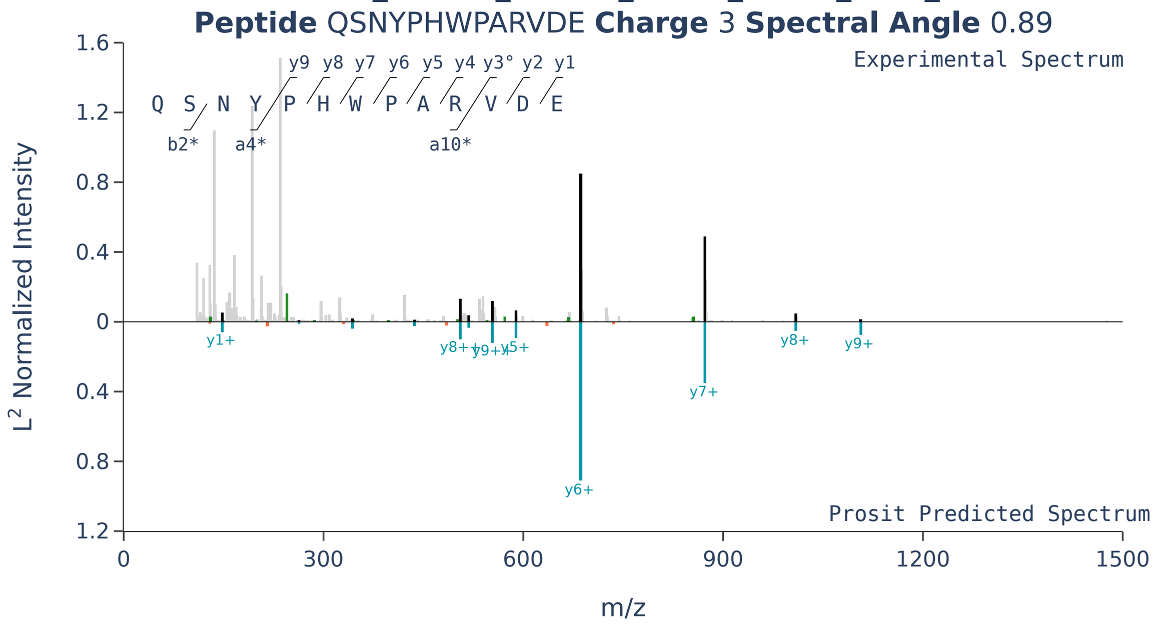

Source HRoetschke_271222_090123_HFGoe_LEDGF_b1R1_2h **Scan** 16425
Peptide DNNPKVKFSSQQAF **Charge** 3 **Spectral Angle** 0.94

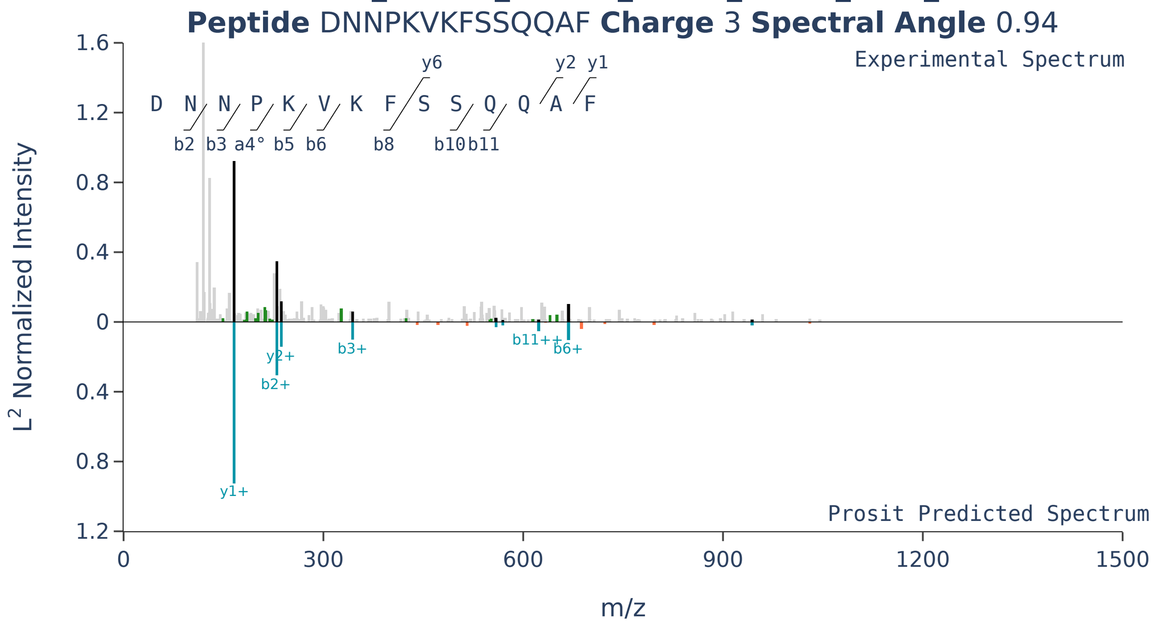

Source HRoetschke_271222_090123_HFGoe_LEDGF_b1R2_4h **Scan** 23475
Peptide EPMTRDFKPGDLIFAKMKG **Charge** 5 **Spectral Angle** 0.86

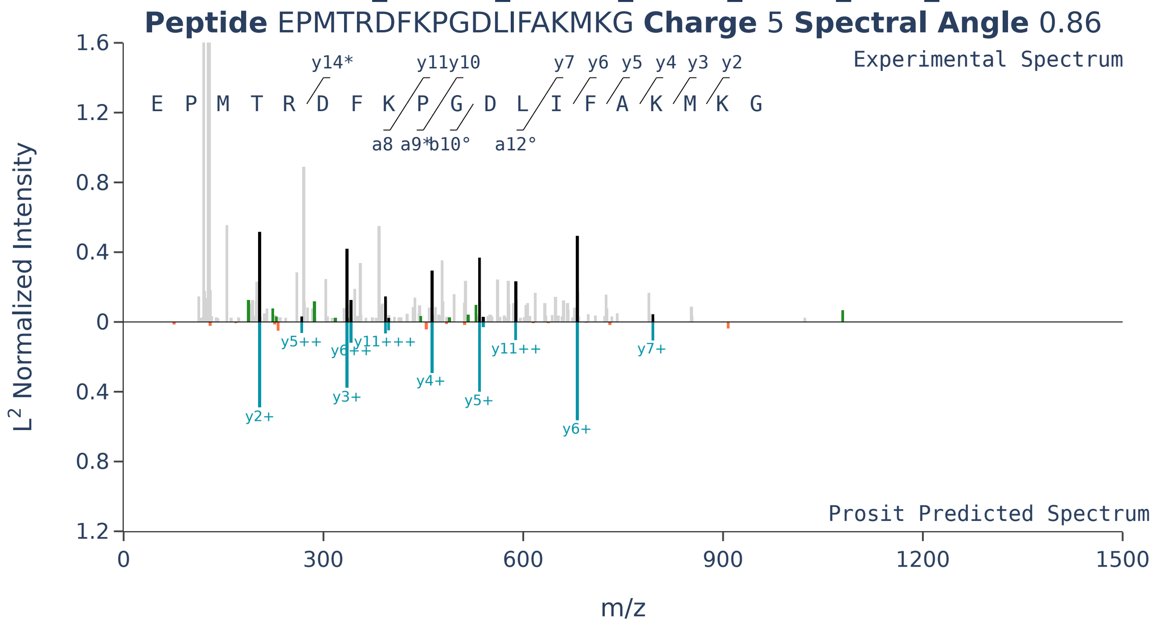

Source HRoetschke_271222_090123_HFGoe_LEDGF_b2R2_2h **Scan** 9474
Peptide GTHETNASSDVEVEE **Charge** 2 **Spectral Angle** 0.83

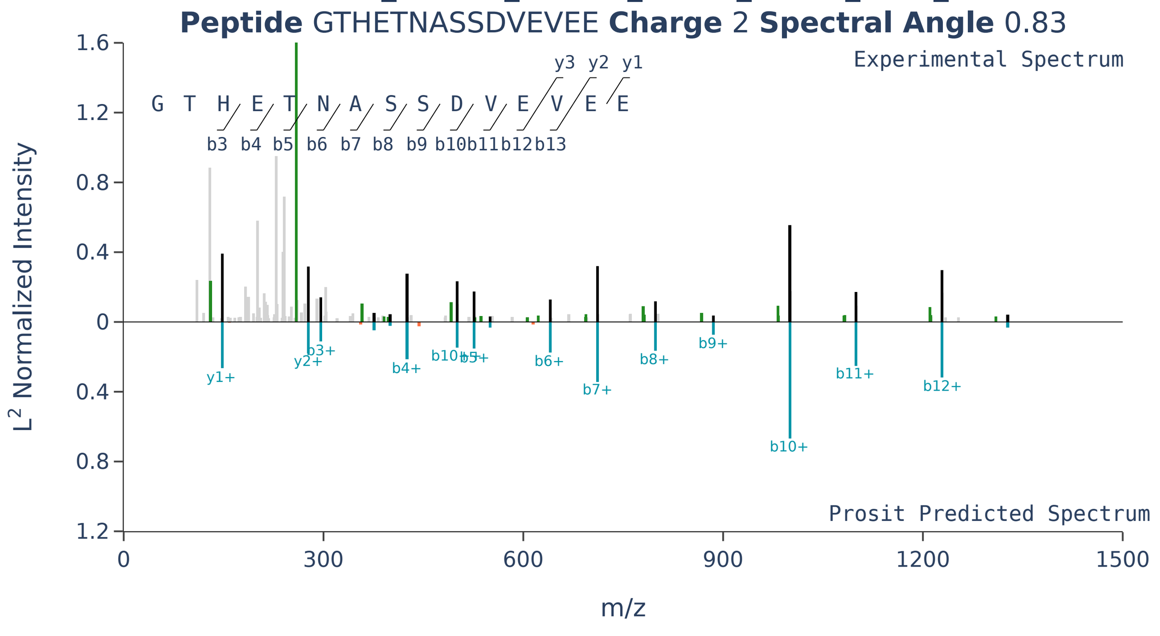

Source HRoetschke_271222_090123_HFGoe_LEDGF_b1R2_1h **Scan** 31571
Peptide PGDLIFVTMQ **Charge** 1 **Spectral Angle** 0.9

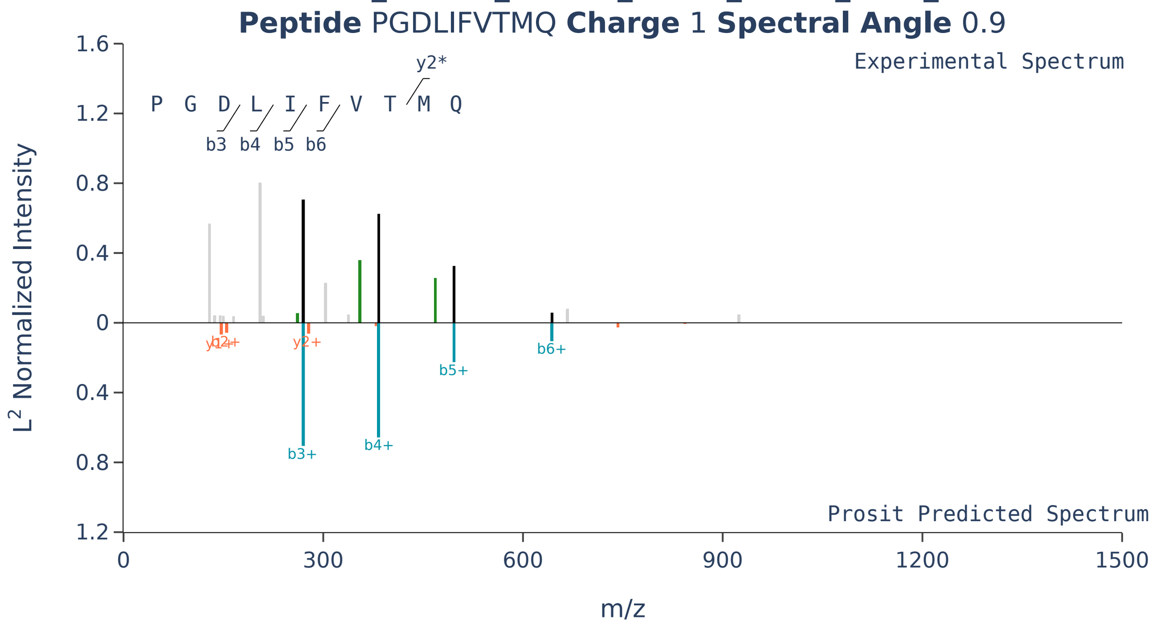

Source HRoetschke_271222_090123_HFGoe_LEDGF_b2R1_24h **Scan** 21303
Peptide STLKIDNLDVNRCIE **Charge** 3 **Spectral Angle** 0.82

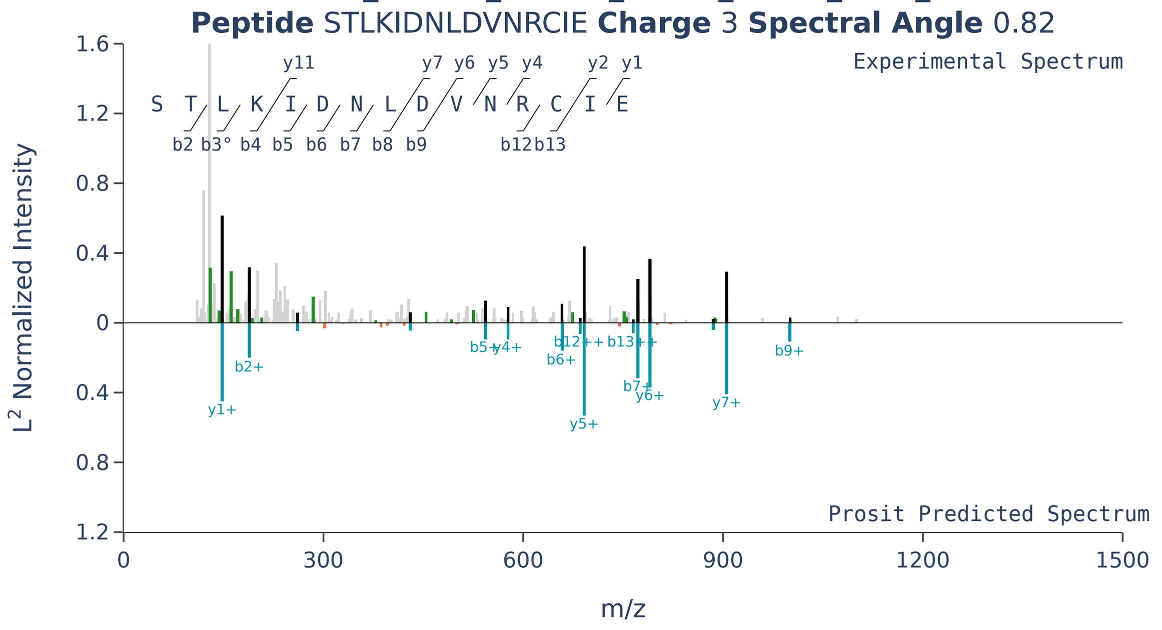

Source HRoetschke_271222_090123_HFGoe_LEDGF_b1R1_24h **Scan** 16892
Peptide SQYPHWPARVDE **Charge** 3 **Spectral Angle** 0.86

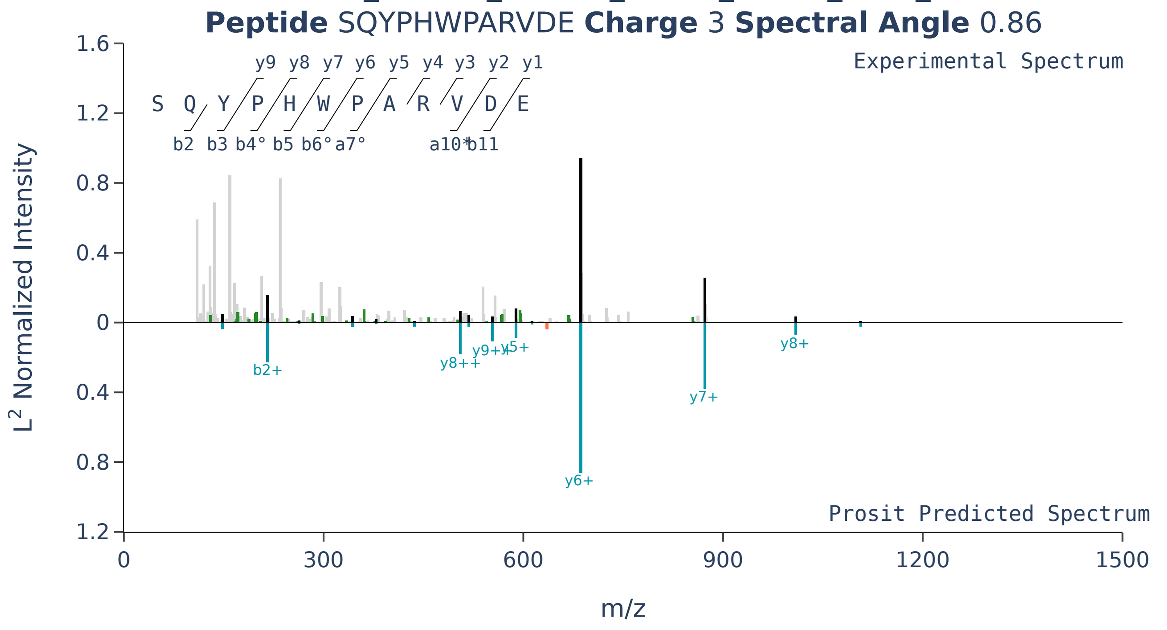

Source HRoetschke_271222_090123_HFGoe_LEDGF_b2R1_1h **Scan** 27742
Peptide PGDLIFEA **Charge** 1 **Spectral Angle** 0.82

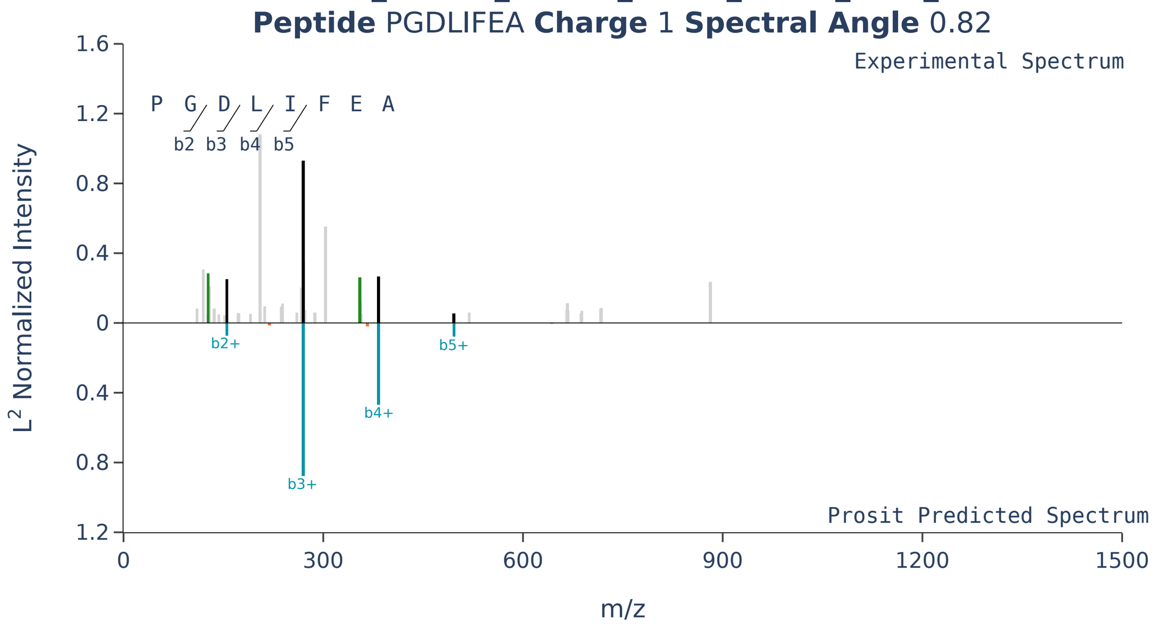

Source HRoetschke_271222_090123_HFGoe_LEDGF_b2R2_2h **Scan** 11881
Peptide EVPDGRDFKPGD **Charge** 3 **Spectral Angle** 0.86

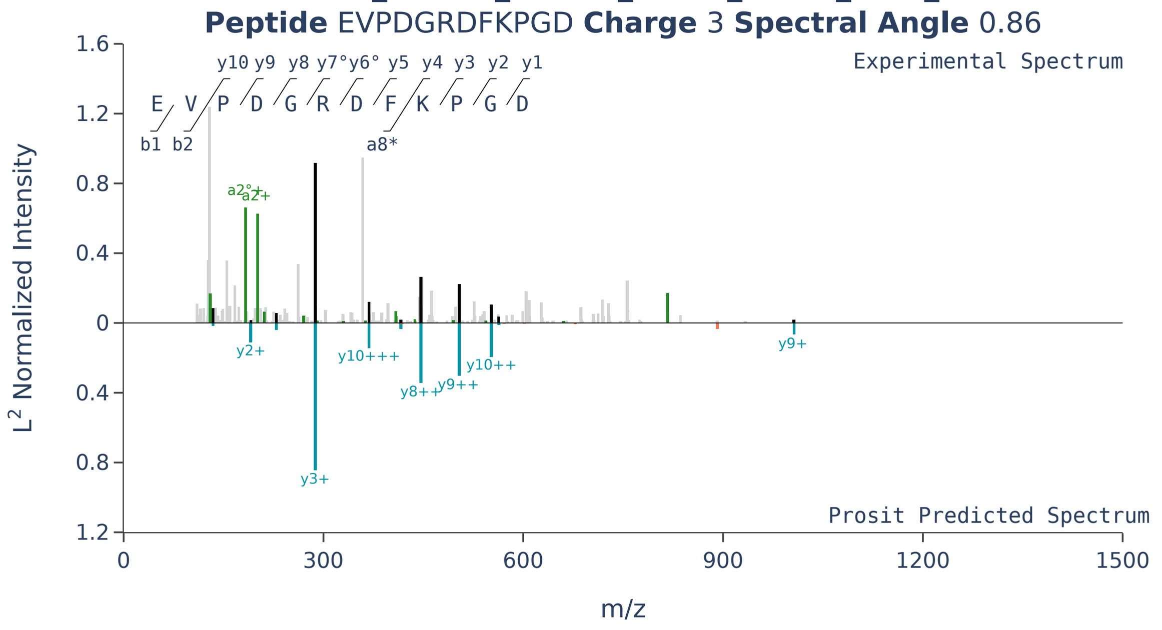

Source HRoetschke_271222_090123_HFGoe_LEDGF_b1R1_2h **Scan** 11036
Peptide GEDSVITQ **Charge** 2 **Spectral Angle** 0.84

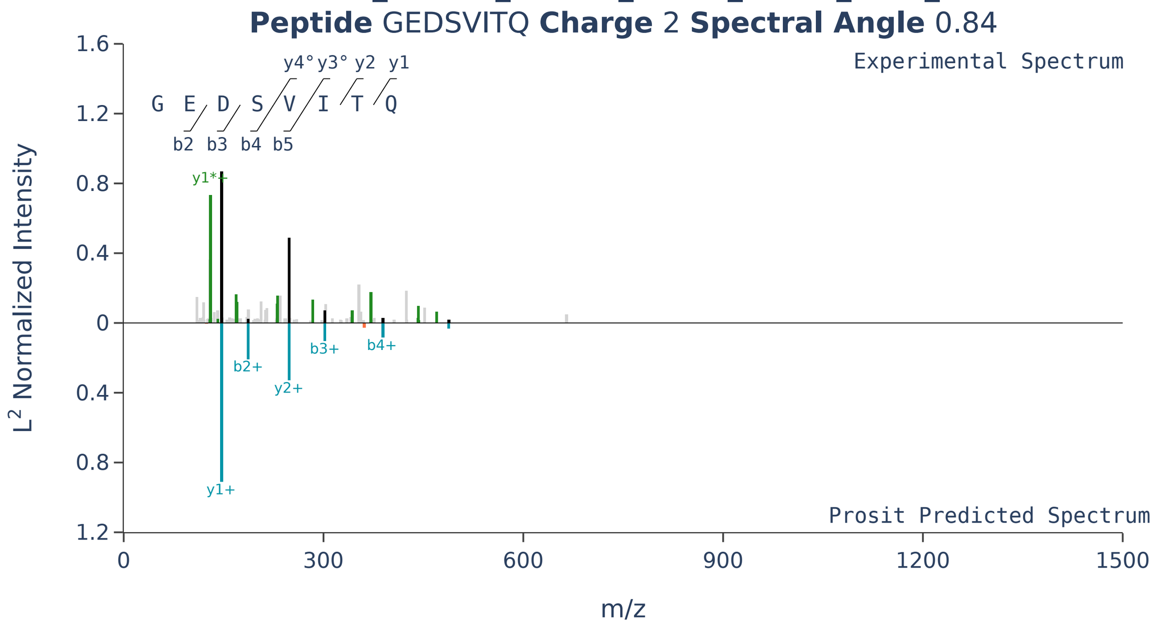

Source HRoetschke_271222_090123_HFGoe_LEDGF_b1R2_24h **Scan** 12023
Peptide QGPGMTRDFKPGD **Charge** 3 **Spectral Angle** 0.9

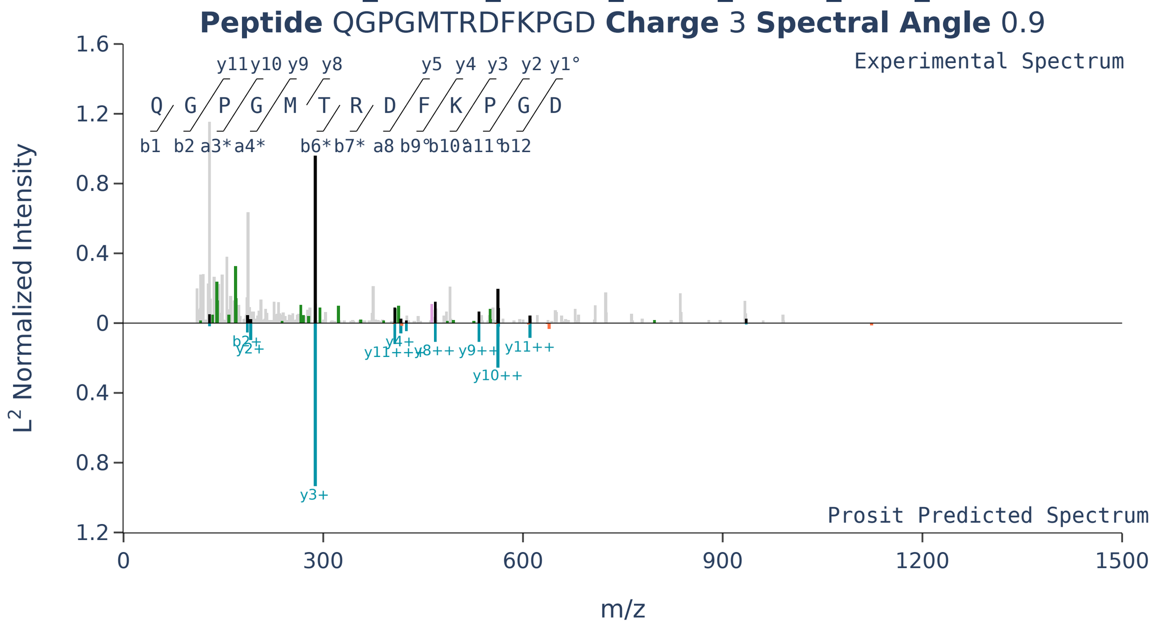

Source HRoetschke_271222_090123_HFGoe_LEDGF_b1R2_4h **Scan** 25121
Peptide LNKSELASLQVTM **Charge** 2 **Spectral Angle** 0.86

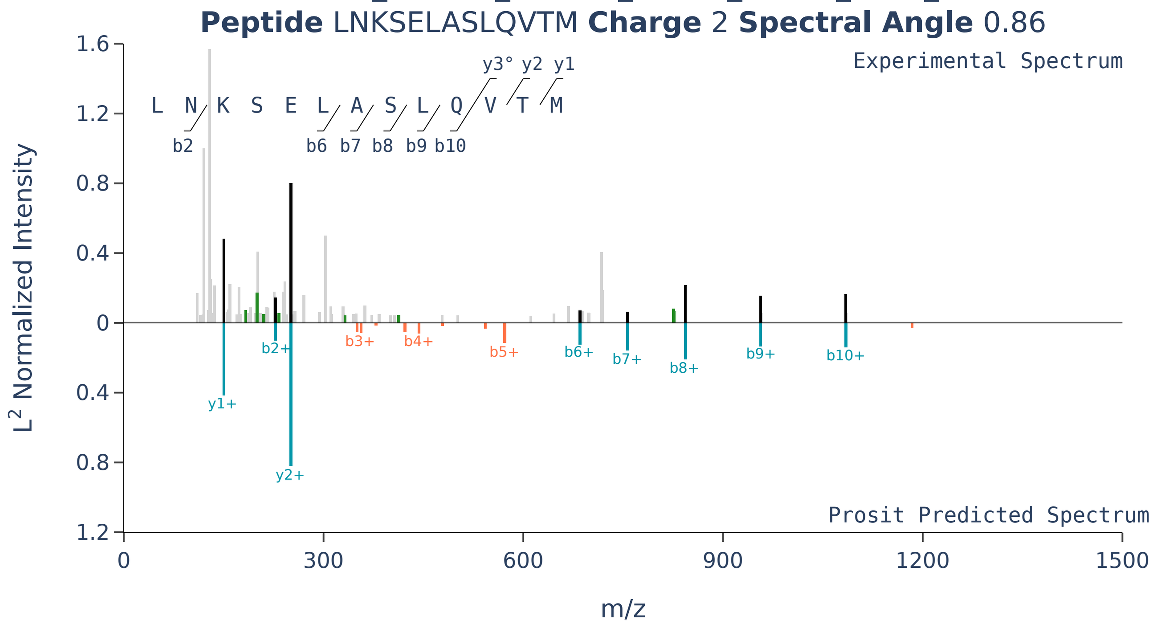

Source HRoetschke_271222_090123_HFGoe_LEDGF_b1R1_4h **Scan** 11012
Peptide IMEKSTALD **Charge** 2 **Spectral Angle** 0.76

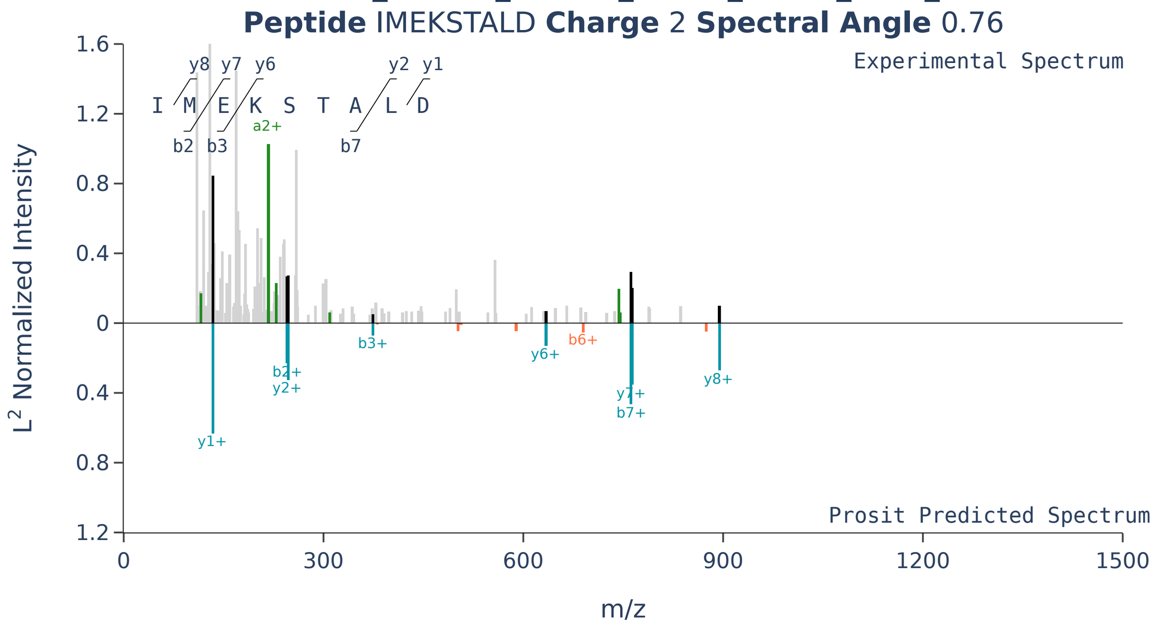

Source HRoetschke_271222_090123_HFGoe_LEDGF_b1R2_24h **Scan** 15687
Peptide THEGYPHWPARV **Charge** 4 **Spectral Angle** 0.82

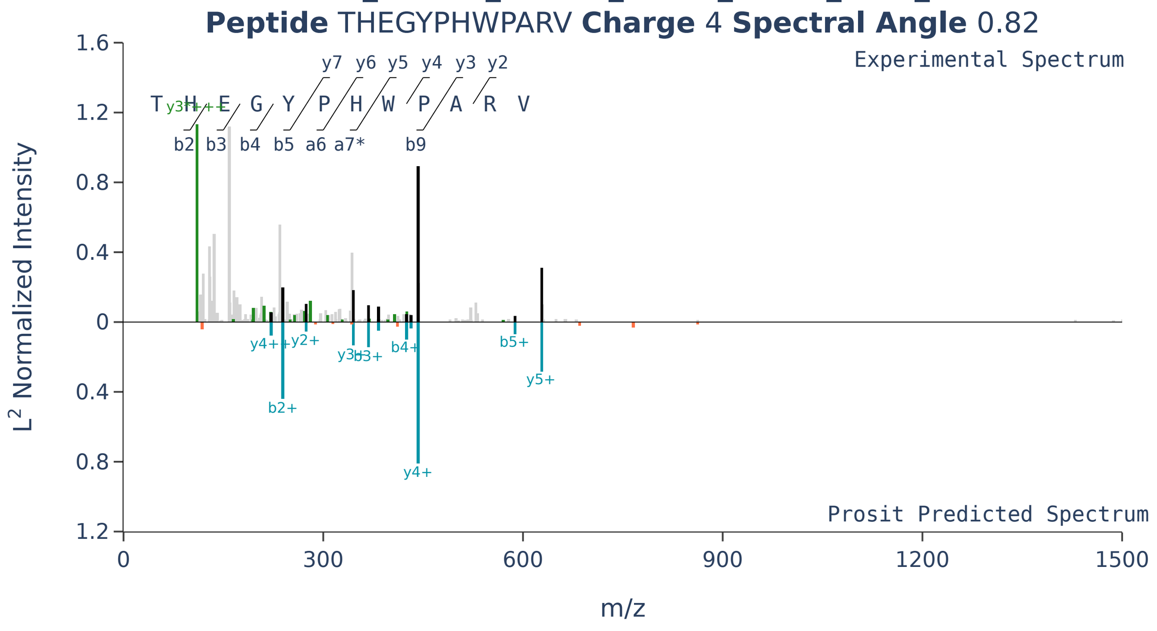

Source HRoetschke_271222_090123_HFGoe_LEDGF_b1R2_24h **Scan** 18138
Peptide QTGFLGPKDIFPYSENKEKYGKPNKR **Charge** 5 **Spectral Angle** 0.83

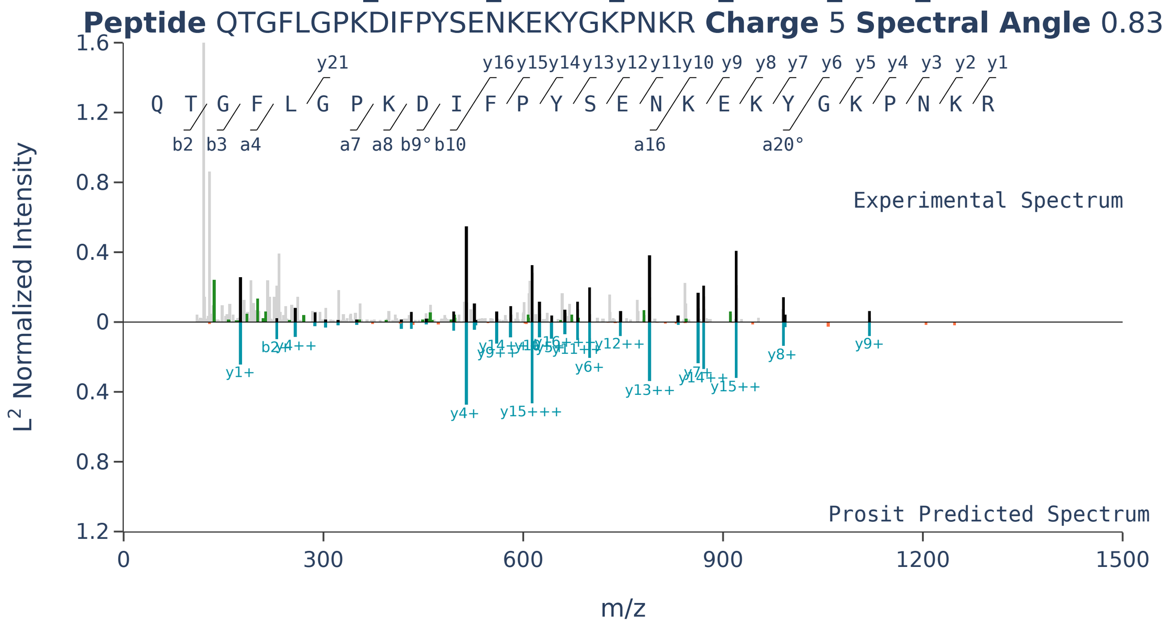

Source HRoetschke_271222_090123_HFGoe_LEDGF_b1R2_24h **Scan** 11276
Peptide TAAVKPPTNKL **Charge** 3 **Spectral Angle** 0.82

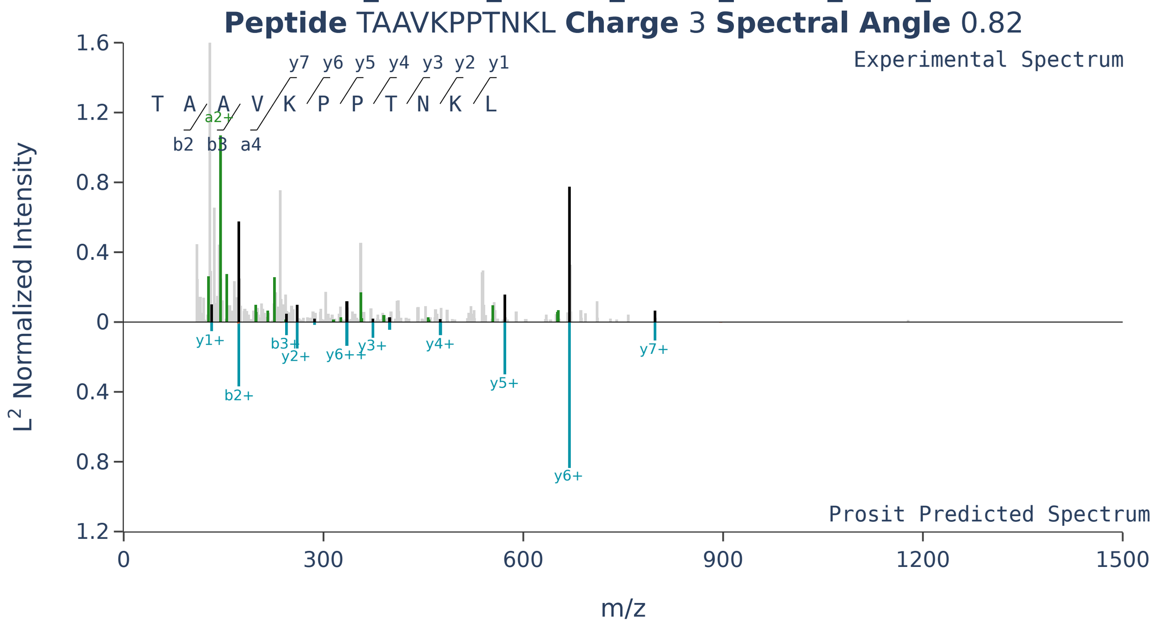

Source HRoetschke_271222_090123_HFGoe_LEDGF_b2R1_4h **Scan** 5635
Peptide PCPSEKETSVSKE **Charge** 2 **Spectral Angle** 0.86

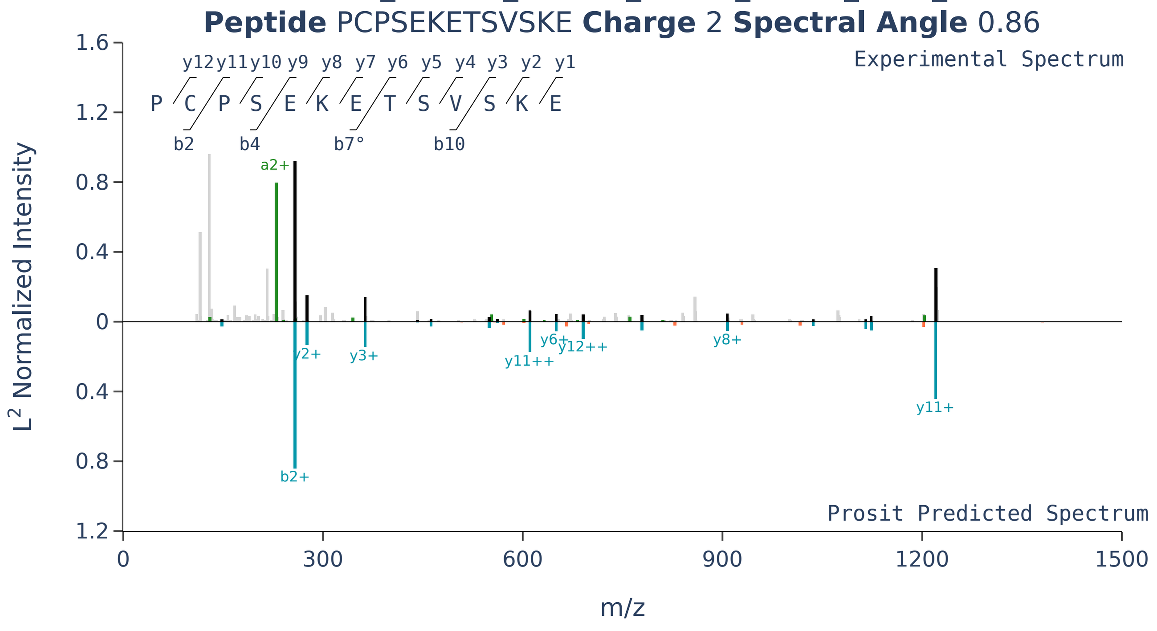

Source HRoetschke_271222_090123_HFGoe_LEDGF_b2R1_2h **Scan** 11924
Peptide QHNGAKMKGYPHWPARVD **Charge** 5 **Spectral Angle** 0.94

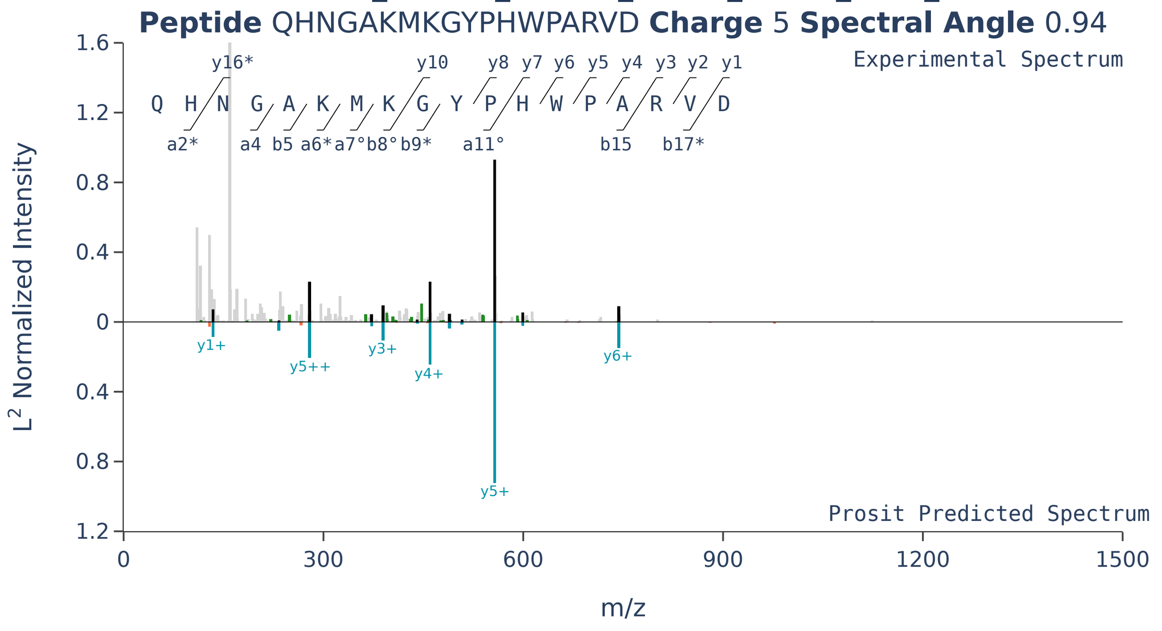

Source HRoetschke_271222_090123_HFGoe_LEDGF_b1R1_24h **Scan** 17382
Peptide SMDSRLQRIHAEIQQA **Charge** 3 **Spectral Angle** 0.87

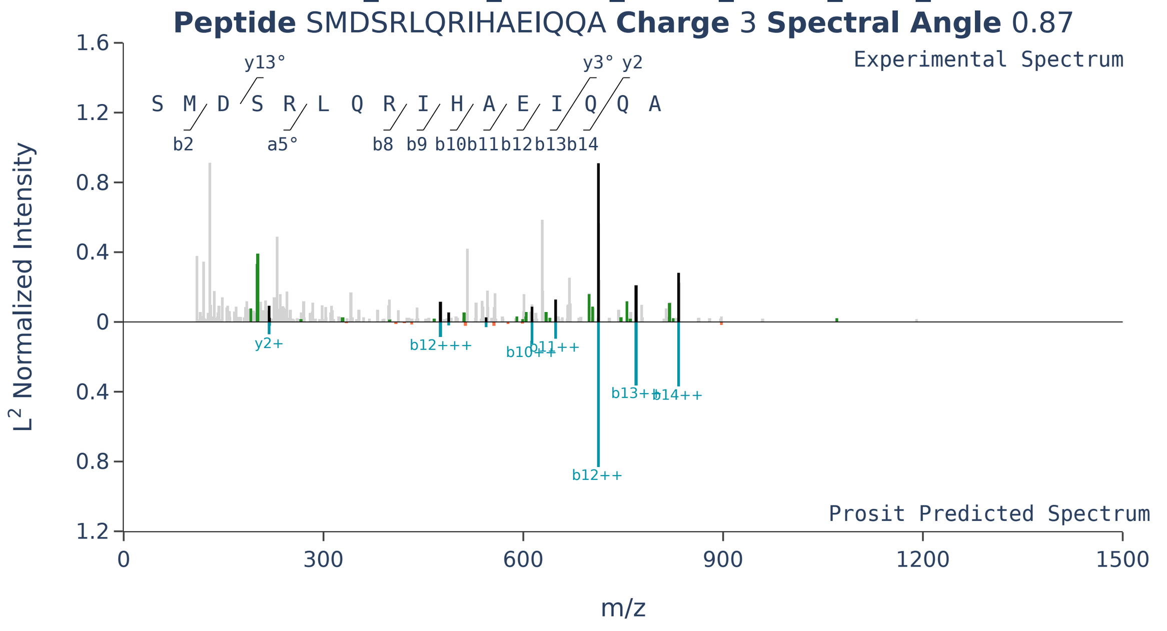

Source HRoetschke_271222_090123_HFGoe_LEDGF_b2R2_24h **Scan** 20711
Peptide QNNSLKIDNLDVNRCIE **Charge** 3 **Spectral Angle** 0.91

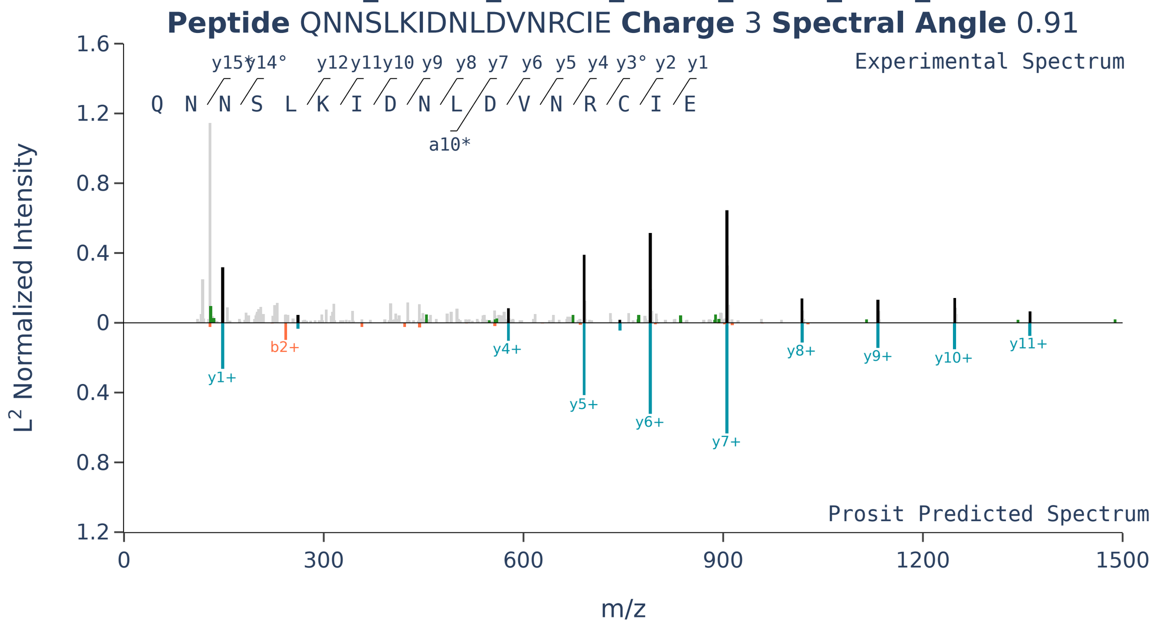

Source HRoetschke_271222_090123_HFGoe_LEDGF_b2R2_4h **Scan** 6358
Peptide RIHAEIKQG **Charge** 3 **Spectral Angle** 0.86

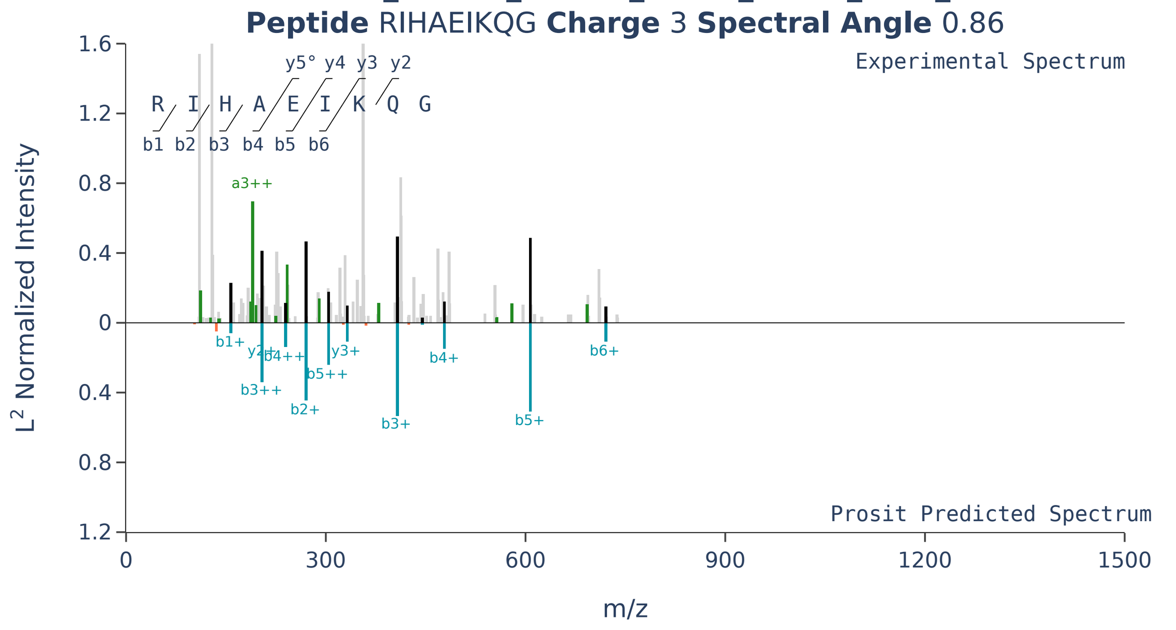

Source HRoetschke_271222_090123_HFGoe_LEDGF_b1R1_2h **Scan** 24510
Peptide QNKDEDFKPGDLIFAKMKGY **Charge** 5 **Spectral Angle** 0.86

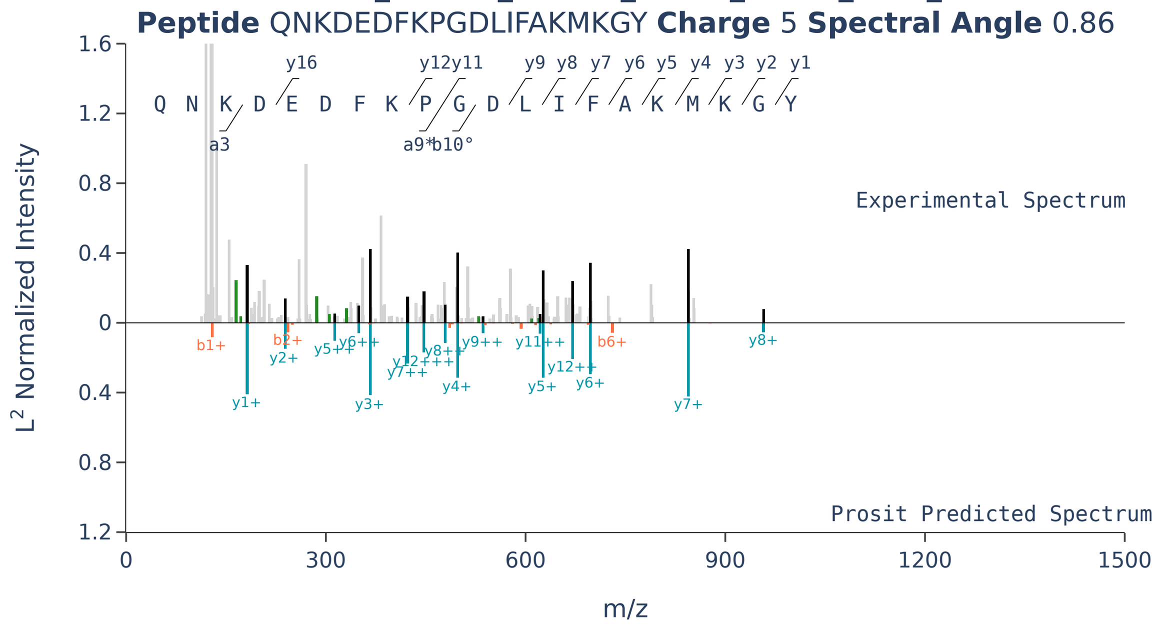

Source HRoetschke_271222_090123_HFGoe_LEDGF_b2R2_2h **Scan** 12564
Peptide GTHETKGYPHWPARVDE **Charge** 3 **Spectral Angle** 0.92

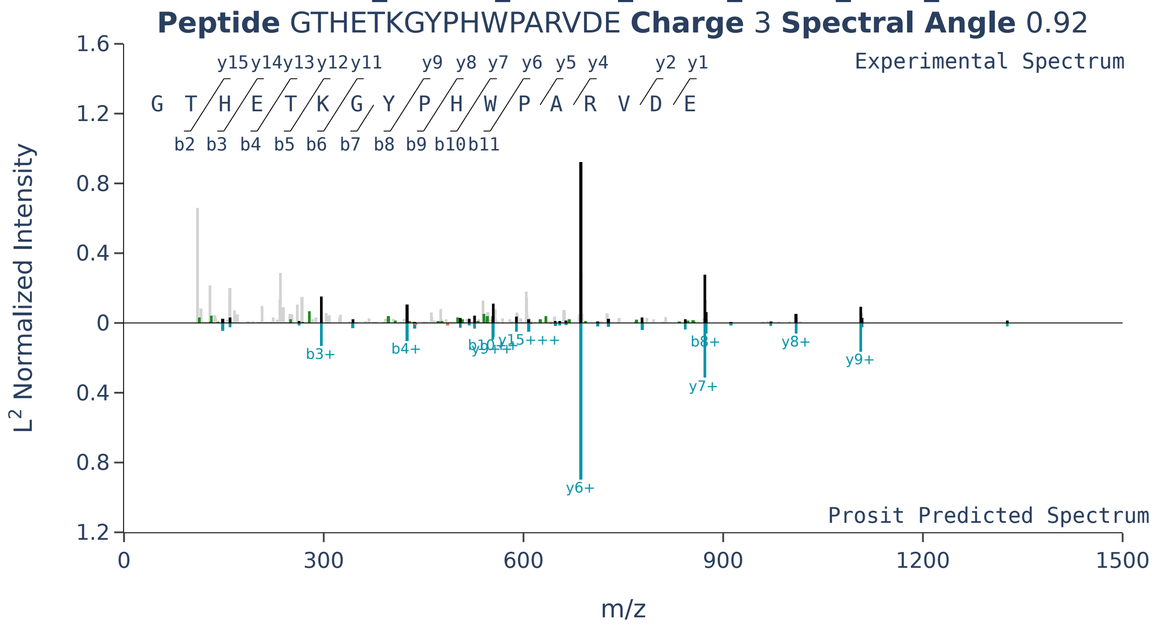

Source HRoetschke_271222_090123_HFGoe_LEDGF_b1R2_4h **Scan** 21102
Peptide GPGMTYPHWPARVDE **Charge** 3 **Spectral Angle** 0.84

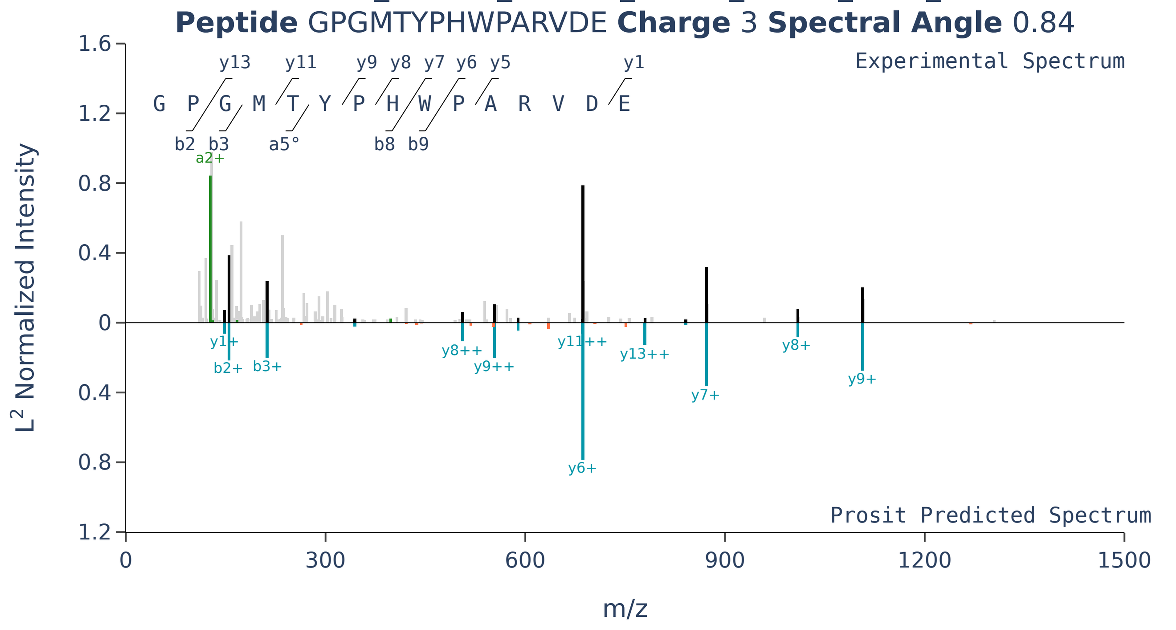

Source HRoetschke_271222_090123_HFGoe_LEDGF_b2R1_24h **Scan** 20879
Peptide SSLKIDNLDVNRCIE **Charge** 2 **Spectral Angle** 0.85

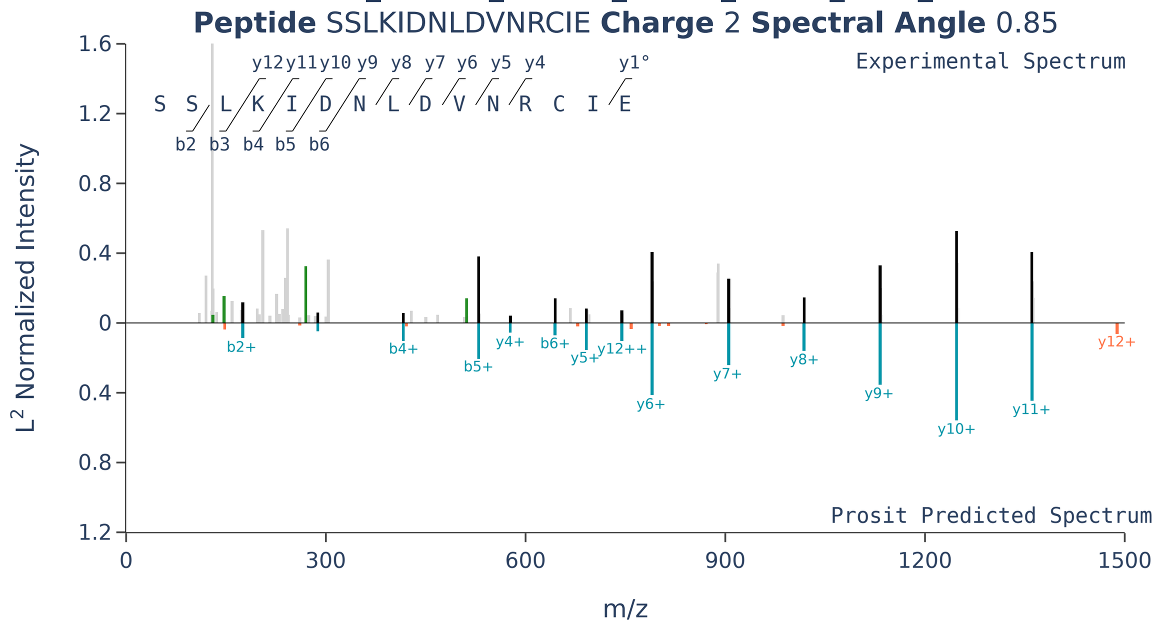

Source HRoetschke_271222_090123_HFGoe_LEDGF_b2R2_2h **Scan** 16974
Peptide SQQYPHWPARVDE **Charge** 3 **Spectral Angle** 0.88

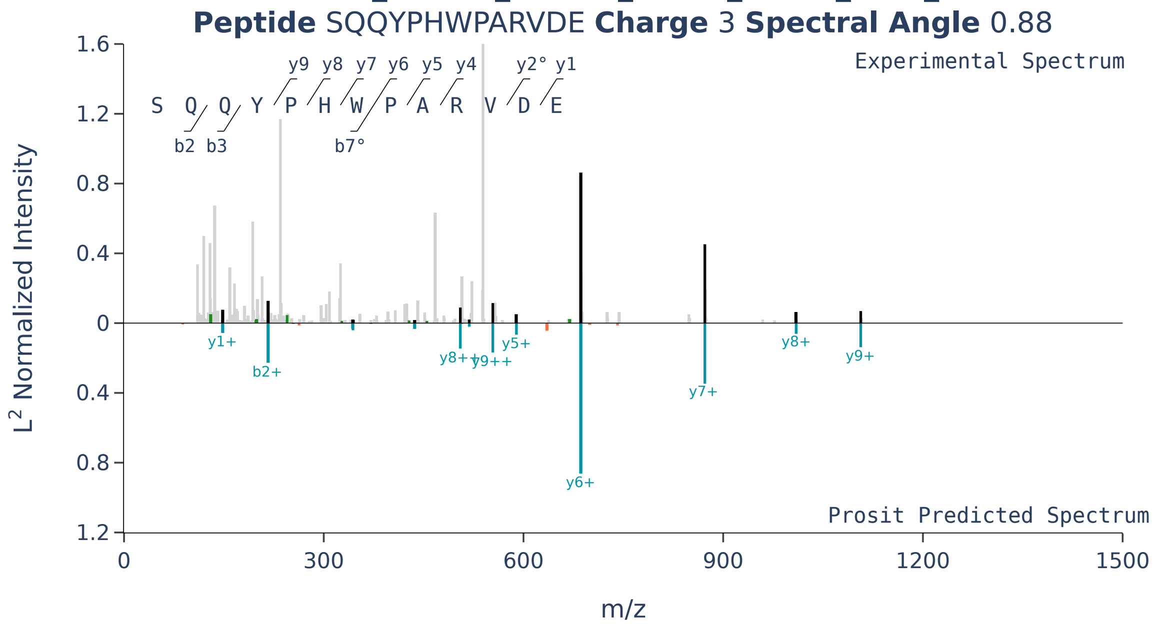

Source HRoetschke_271222_090123_HFGoe_LEDGF_b2R1_2h **Scan** 15068
Peptide KGFNEGF **Charge** 2 **Spectral Angle** 0.71

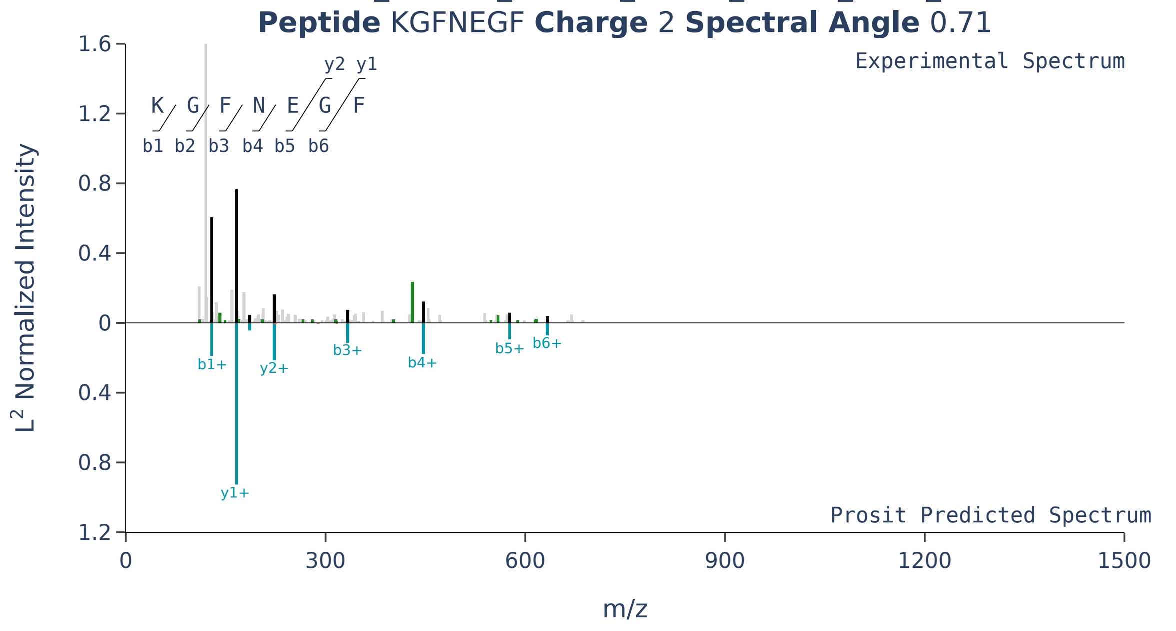

Source HRoetschke_271222_090123_HFGoe_LEDGF_b1R2_24h **Scan** 16736
Peptide TAFLGPKDIFPYSENKEKYGKPKRKGG **Charge** 5 **Spectral Angle** 0.81

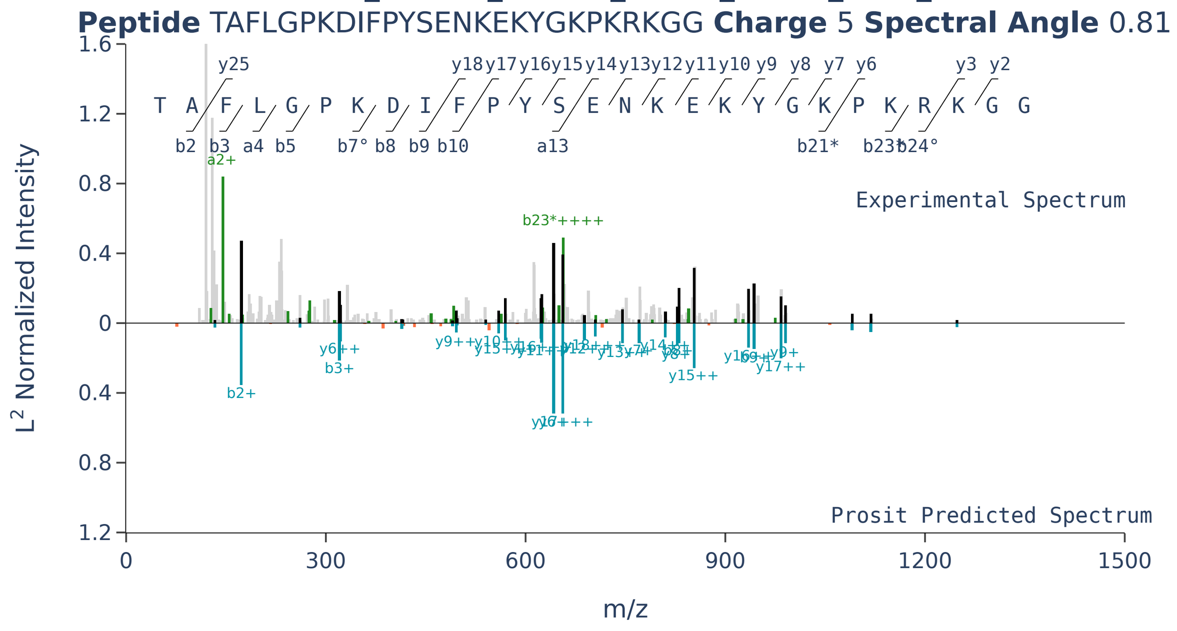

Source HRoetschke_271222_090123_HFGoe_LEDGF_b1R1_24h **Scan** 16318
Peptide TQQAQKHTEMITTL **Charge** 3 **Spectral Angle** 0.82

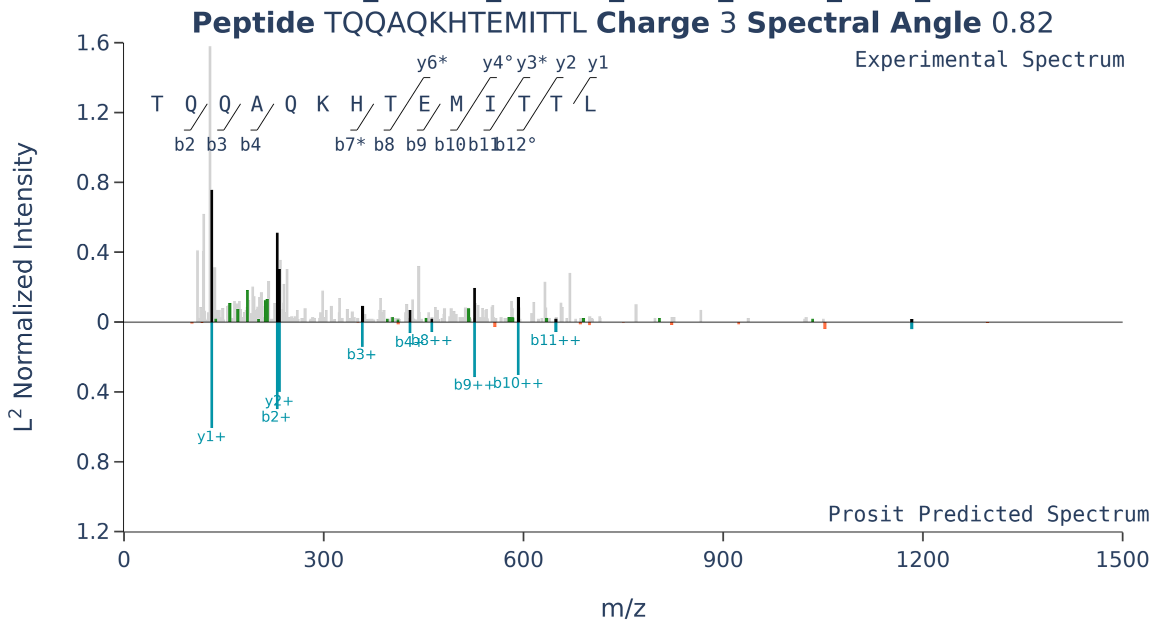

Source HRoetschke_271222_090123_HFGoe_LEDGF_b1R1_24h **Scan** 7177
Peptide LIPYSENKEKYGKPNKR **Charge** 4 **Spectral Angle** 0.76

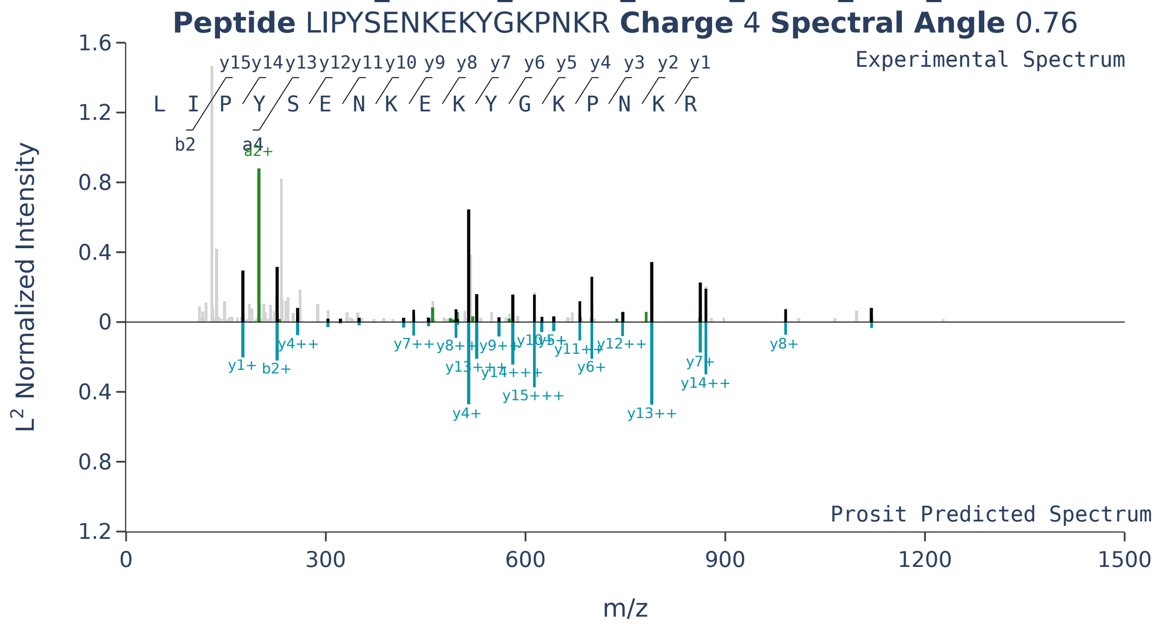

Source HRoetschke_271222_090123_HFGoe_LEDGF_b1R1_24h **Scan** 14266
Peptide GKDEVPDGAVKPPTNKL **Charge** 4 **Spectral Angle** 0.84

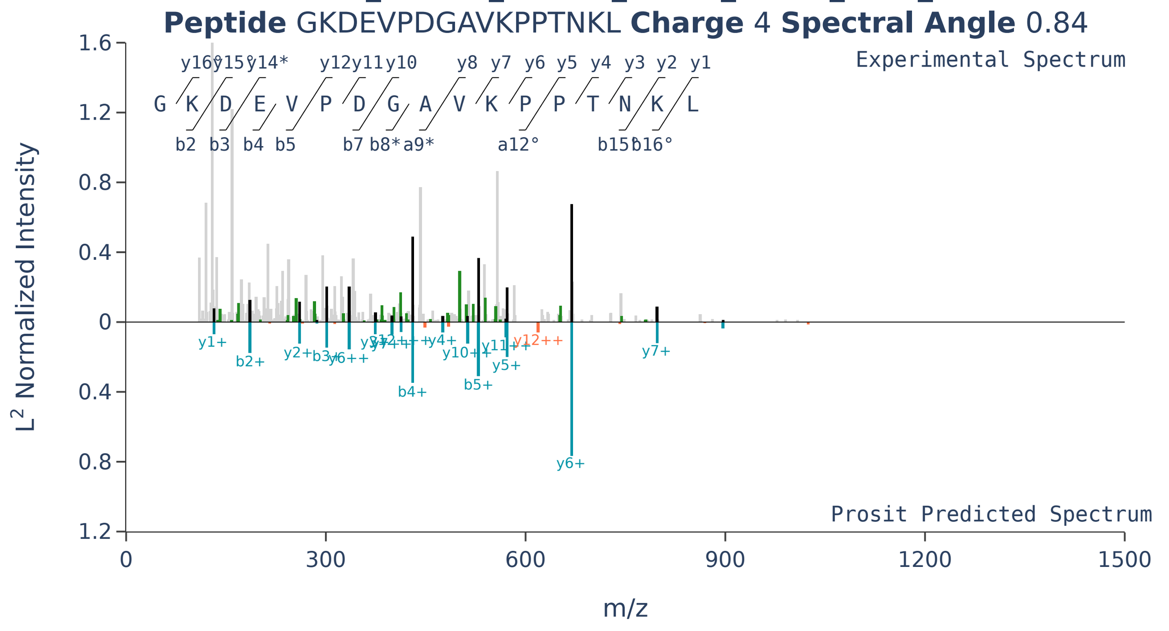

Source HRoetschke_271222_090123_HFGoe_LEDGF_b1R2_4h **Scan** 25016
Peptide GPGMTKGFNEGLWE **Charge** 2 **Spectral Angle** 0.86

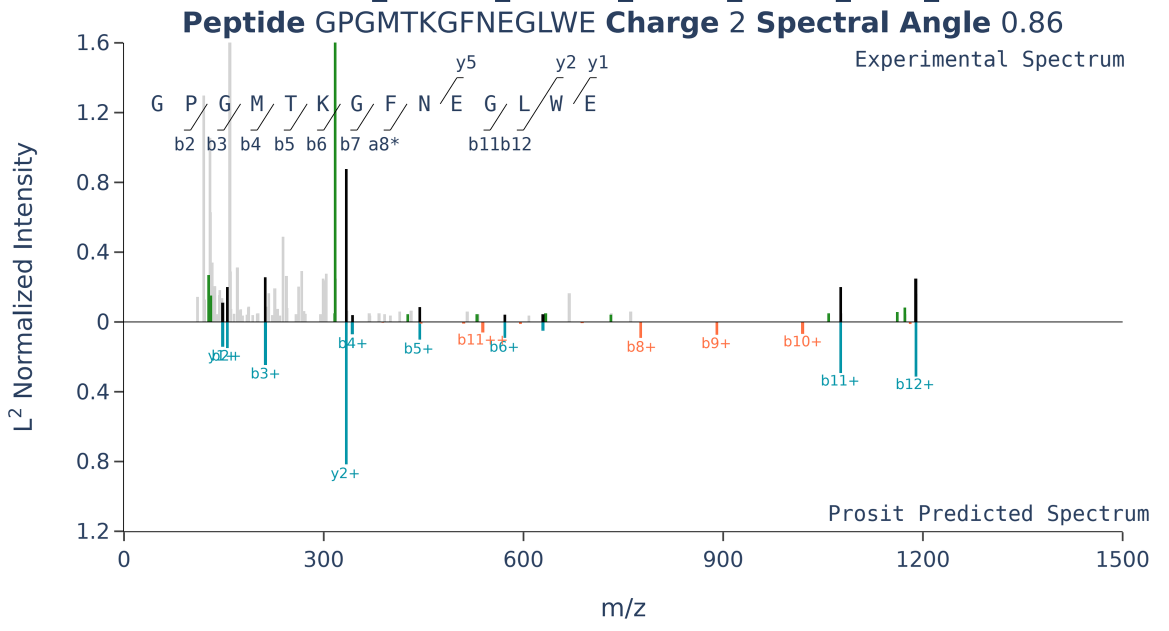

Source HRoetschke_271222_090123_HFGoe_LEDGF_b1R2_24h **Scan** 17119
Peptide TNYPHWPARVDE **Charge** 3 **Spectral Angle** 0.86

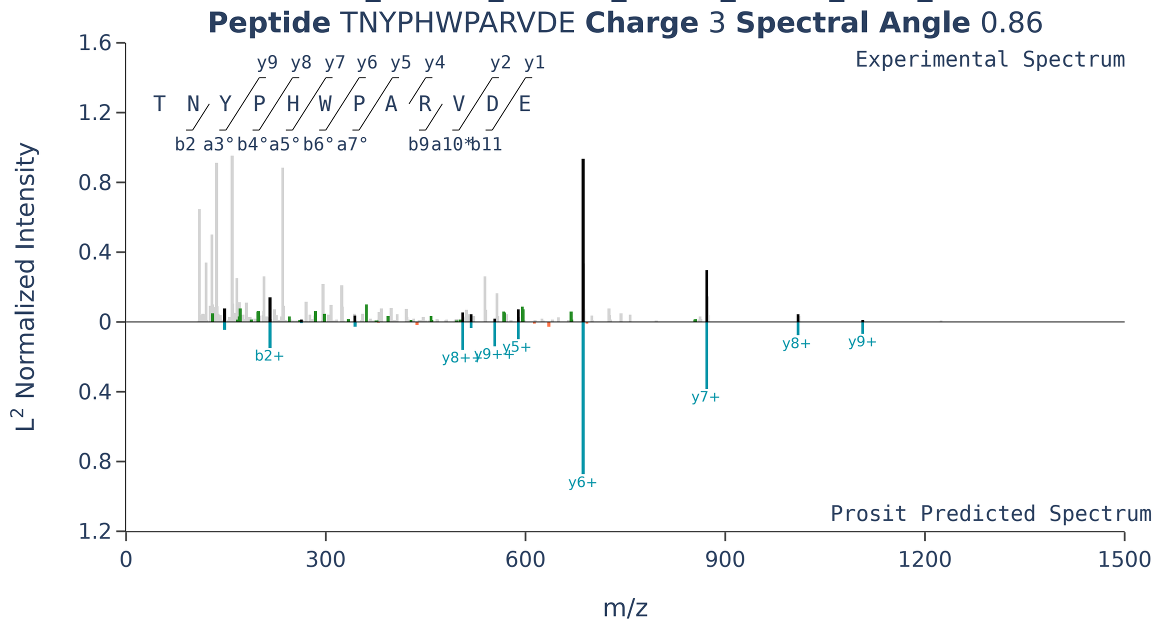

Source HRoetschke_271222_090123_HFGoe_LEDGF_b1R1_24h **Scan** 6743
Peptide GTHETTRDFKPGD **Charge** 4 **Spectral Angle** 0.86

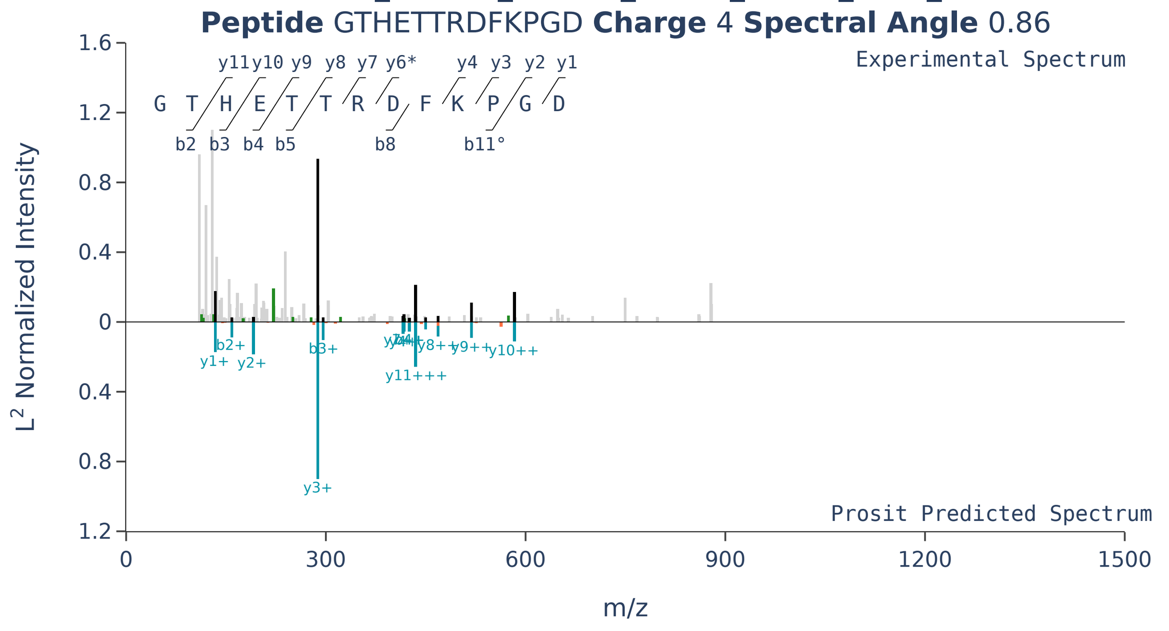

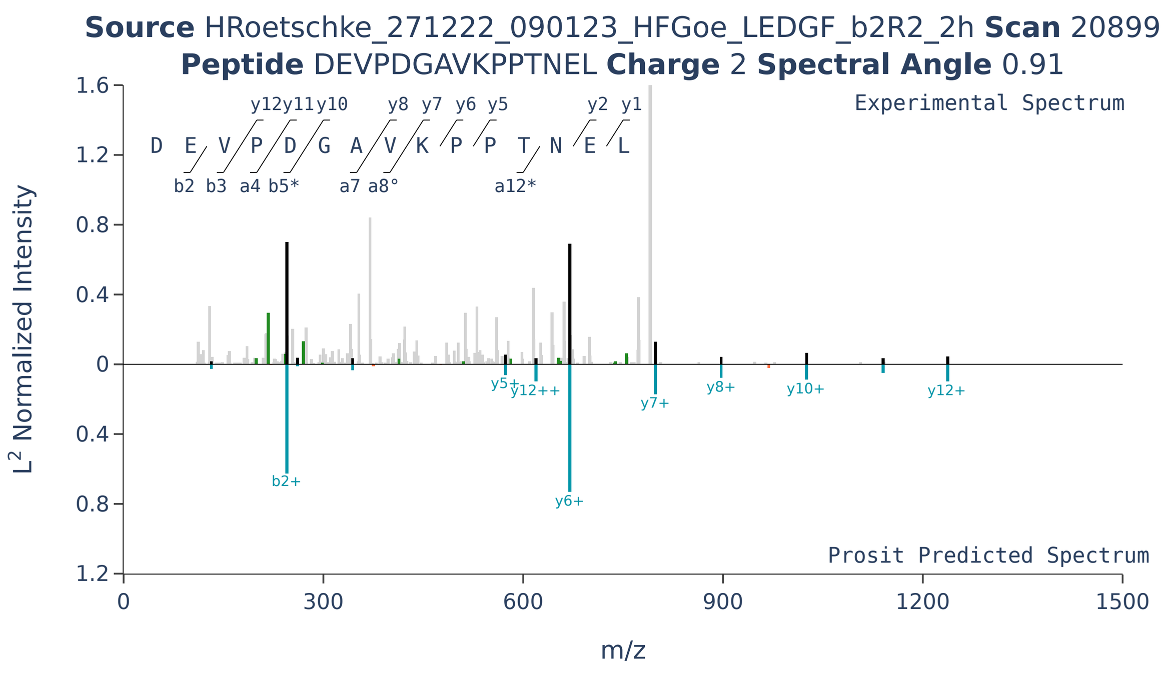
Source HRoetschke_271222_090123_HFGoe_LEDGF_b2R2_2h Scan 20899
Peptide DEVPDGAVKPPTNEL Charge 2 Spectral Angle 0.91
Experimental Spectrum
Prosit Predicted Spectrum
L^2 Normalized Intensity
m/z

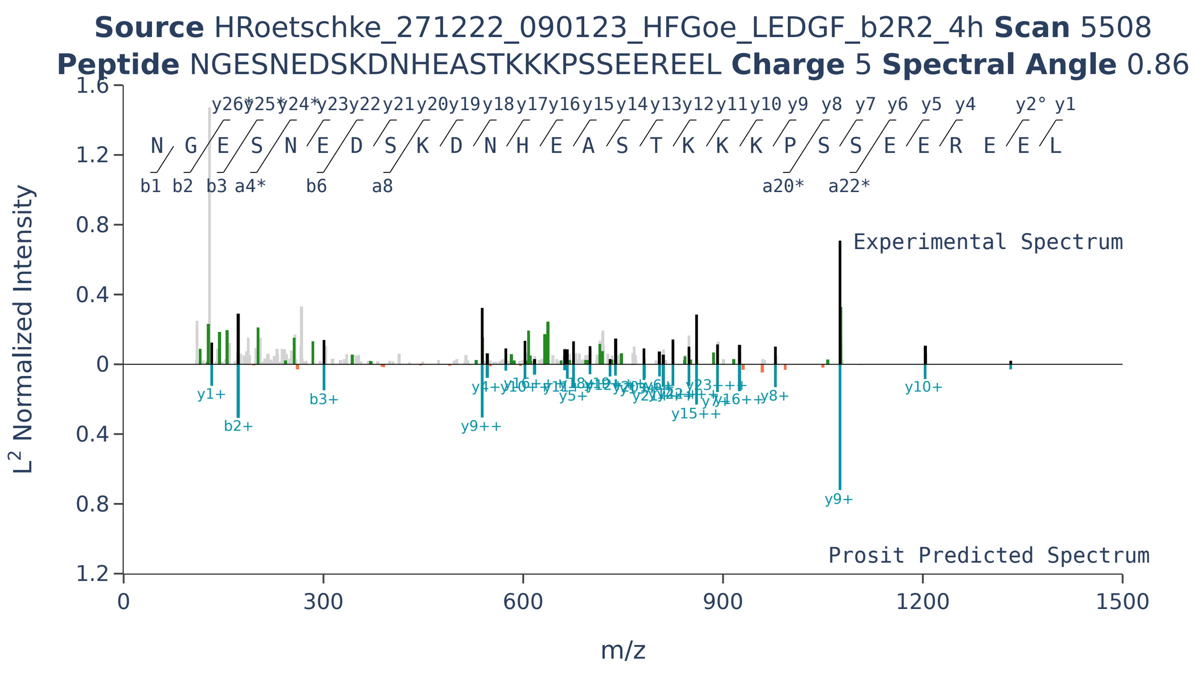
Source HRoetschke_271222_090123_HFGoe_LEDGF_b2R2_4h Scan 5508
Peptide NGESNEDSKDNHEASTKKKPSSEEREEL Charge 5 Spectral Angle 0.86
Experimental Spectrum
Prosit Predicted Spectrum
L^2 Normalized Intensity
m/z

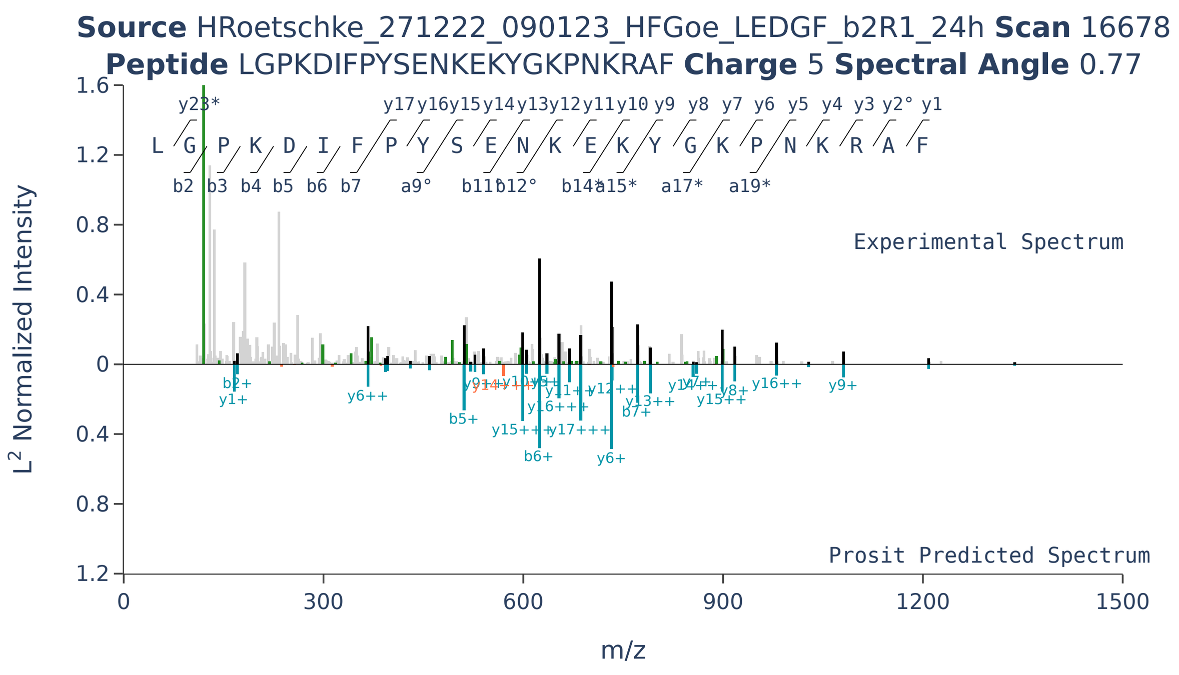
Source HRoetschke_271222_090123_HFGoe_LEDGF_b2R1_24h Scan 16678
Peptide LGPKDIFPYSENKEKYGKPNKRAF Charge 5 Spectral Angle 0.77
Experimental Spectrum
Prosit Predicted Spectrum
L^2 Normalized Intensity
m/z

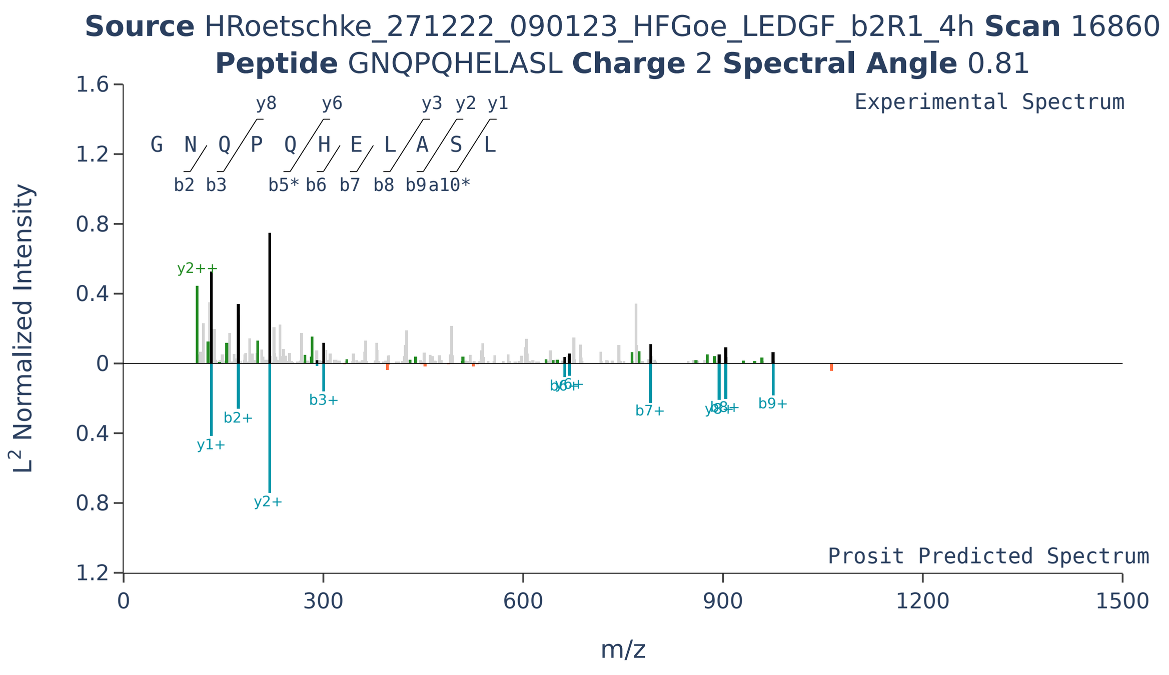
Source HRoetschke_271222_090123_HFGoe_LEDGF_b2R1_4h Scan 16860
Peptide GNQPQHELASL Charge 2 Spectral Angle 0.81
Experimental Spectrum
Prosit Predicted Spectrum
L^2 Normalized Intensity
m/z

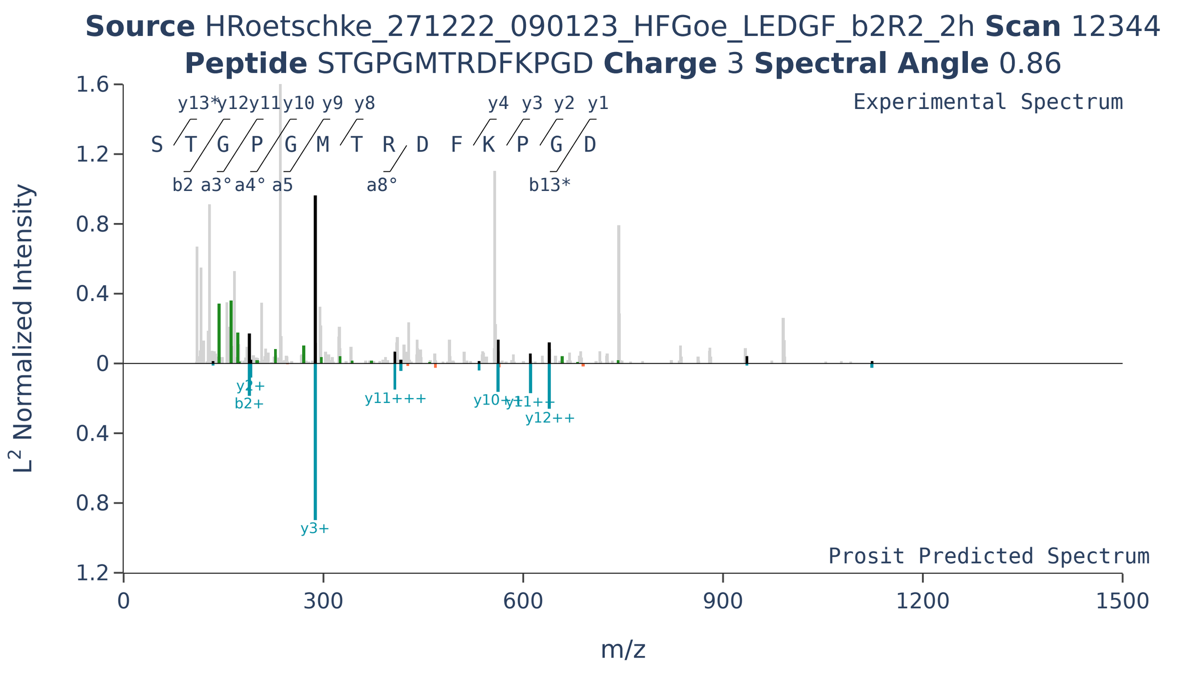
Source HRoetschke_271222_090123_HFGoe_LEDGF_b2R2_2h Scan 12344
Peptide STGPGMTRDFKPGD Charge 3 Spectral Angle 0.86
Experimental Spectrum
Prosit Predicted Spectrum
L^2 Normalized Intensity
m/z

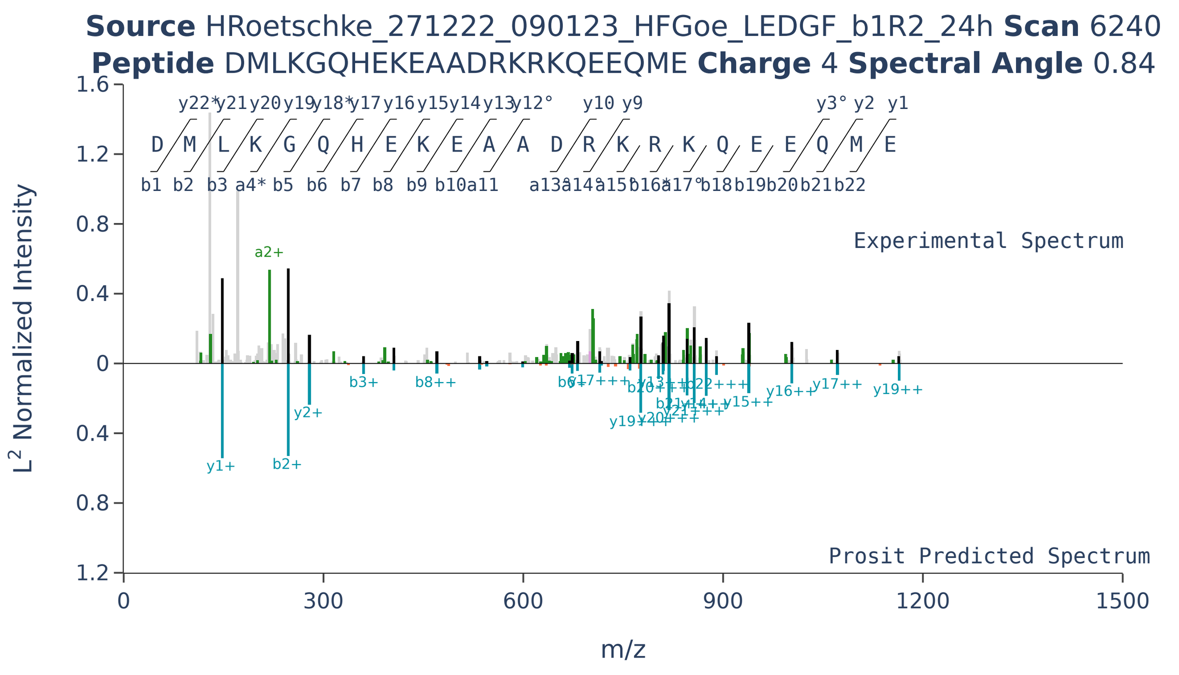
Source HRoetschke_271222_090123_HFGoe_LEDGF_b1R2_24h Scan 6240
Peptide DMLKGQHEKEAADRKRKQEEQME Charge 4 Spectral Angle 0.84
Experimental Spectrum
Prosit Predicted Spectrum
L^2 Normalized Intensity
m/z

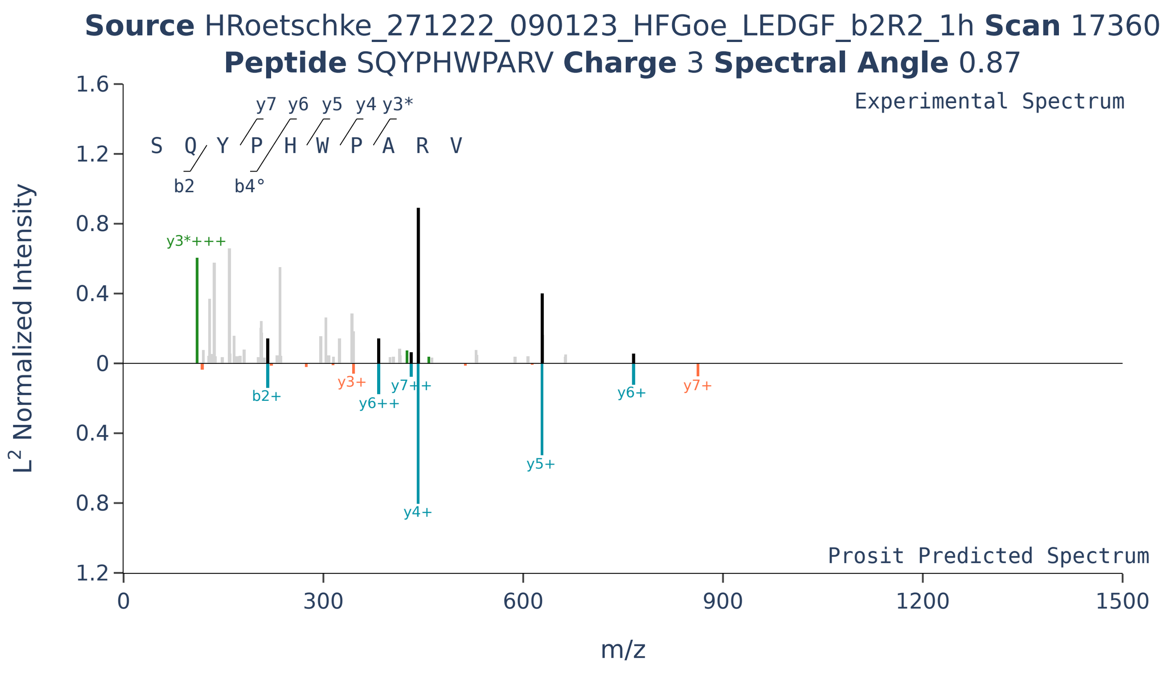
Source HRoetschke_271222_090123_HFGoe_LEDGF_b2R2_1h Scan 17360
Peptide SQYPHWPARV Charge 3 Spectral Angle 0.87
Experimental Spectrum
Prosit Predicted Spectrum
L^2 Normalized Intensity
m/z

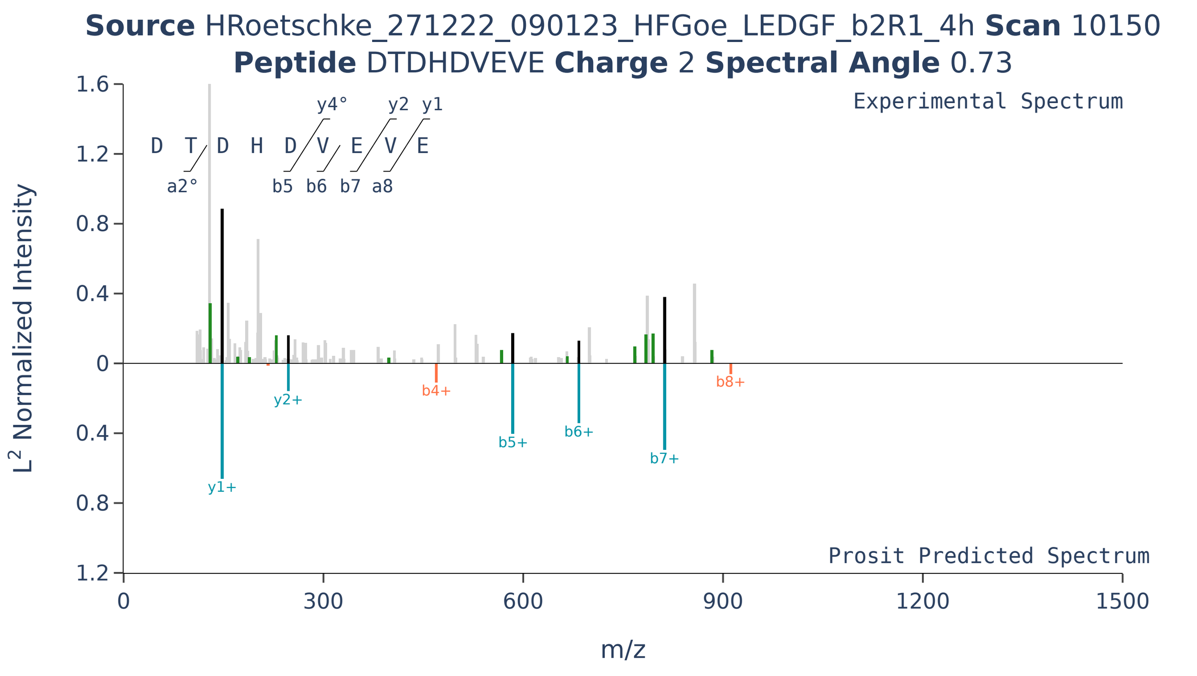
Source HRoetschke_271222_090123_HFGoe_LEDGF_b2R1_4h Scan 10150
Peptide DTDHDVEVE Charge 2 Spectral Angle 0.73
Experimental Spectrum
Prosit Predicted Spectrum
L^2 Normalized Intensity
m/z

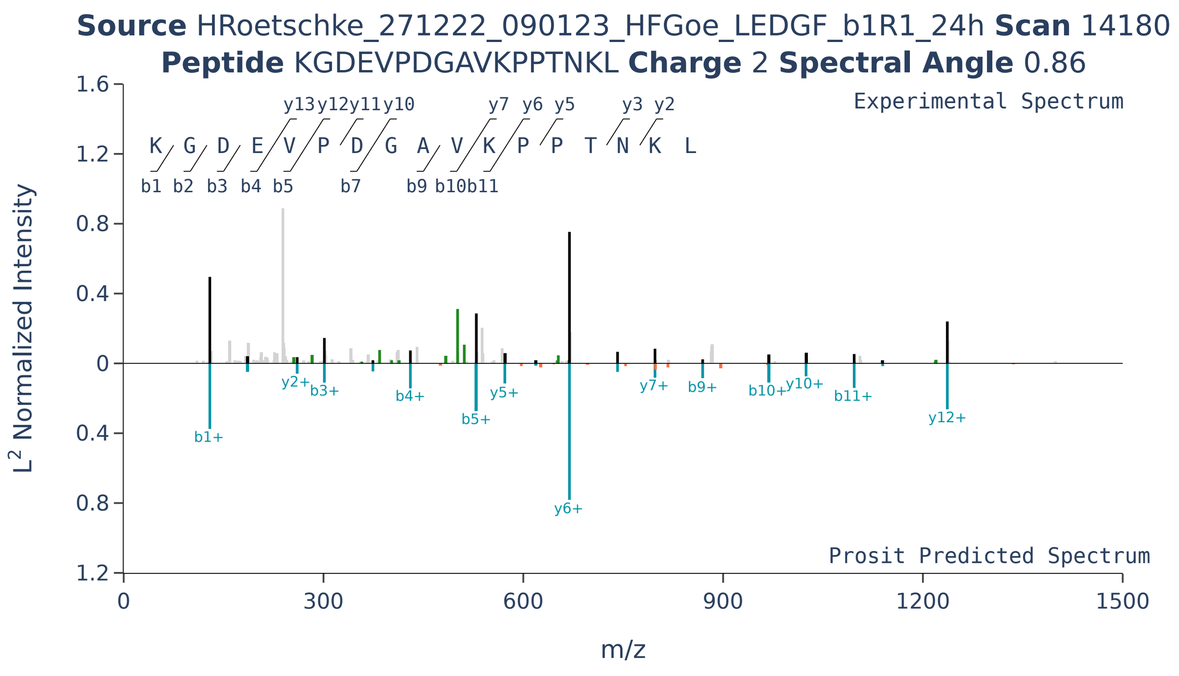
Source HRoetschke_271222_090123_HFGoe_LEDGF_b1R1_24h Scan 14180
Peptide KGDEVPDGAVKPPTNKL Charge 2 Spectral Angle 0.86
Experimental Spectrum
Prosit Predicted Spectrum
L^2 Normalized Intensity
m/z

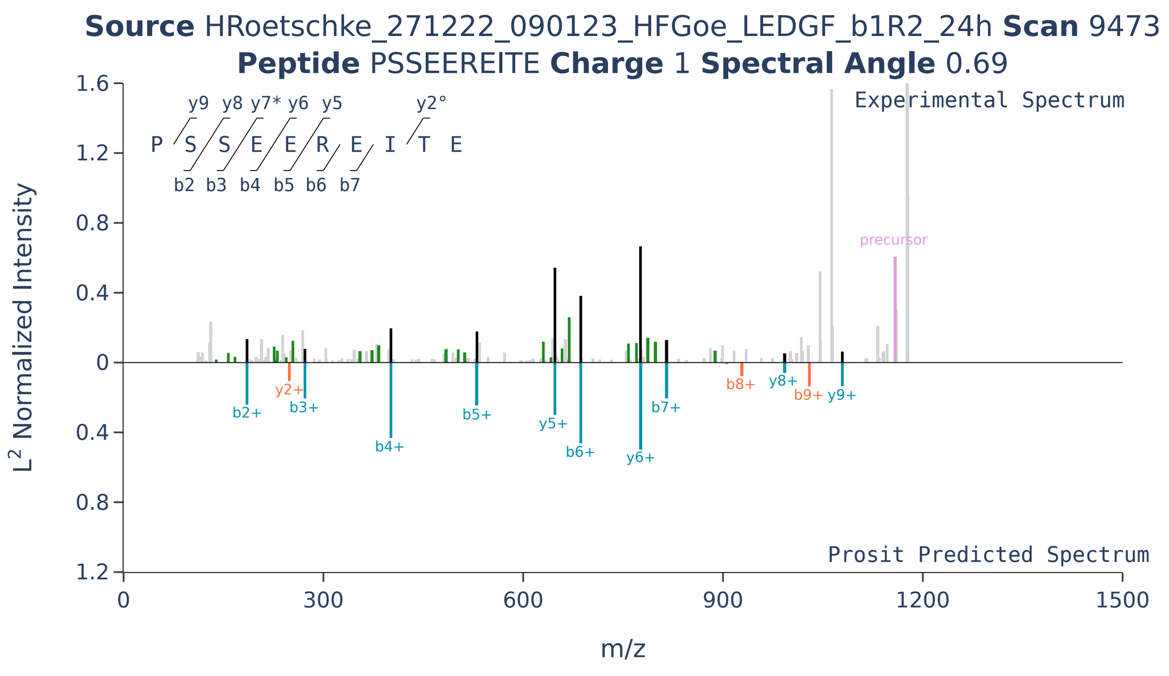
Source HRoetschke_271222_090123_HFGoe_LEDGF_b1R2_24h Scan 9473
Peptide PSSEEREITE Charge 1 Spectral Angle 0.69
Experimental Spectrum
precursor
Prosit Predicted Spectrum
L^2 Normalized Intensity
m/z

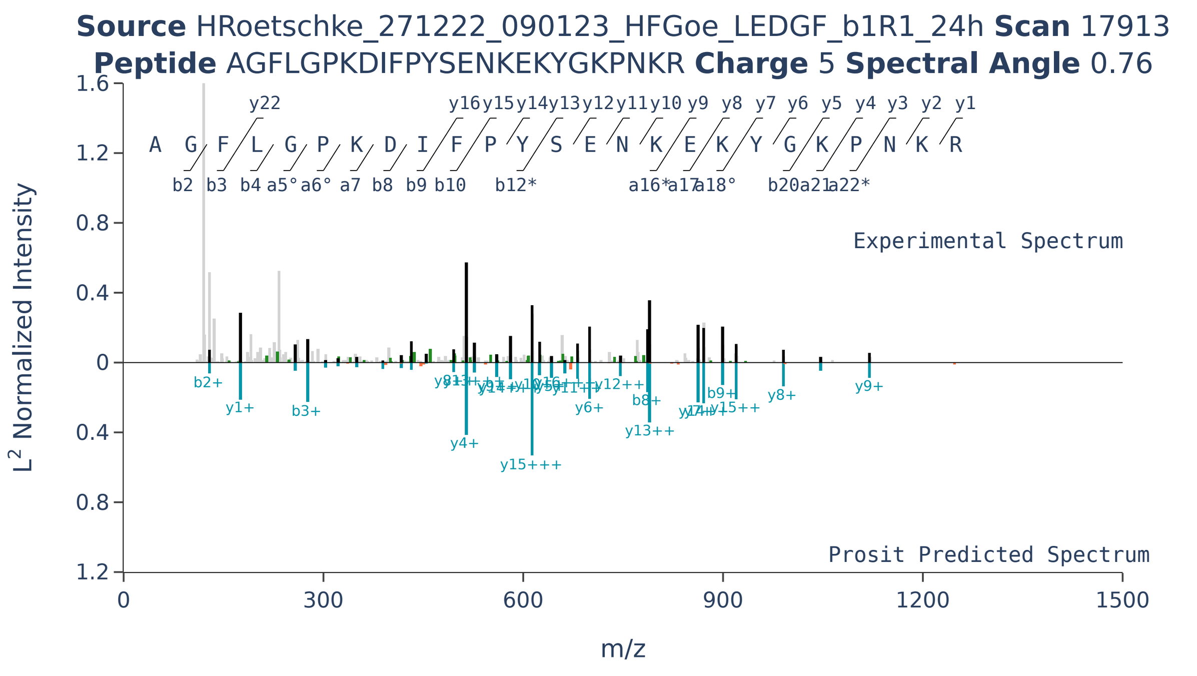
Source HRoetschke_271222_090123_HFGoe_LEDGF_b1R1_24h Scan 17913
Peptide AGFLGPKDIFPYSENKEKYGKPNKR Charge 5 Spectral Angle 0.76
Experimental Spectrum
Prosit Predicted Spectrum
L^2 Normalized Intensity
m/z

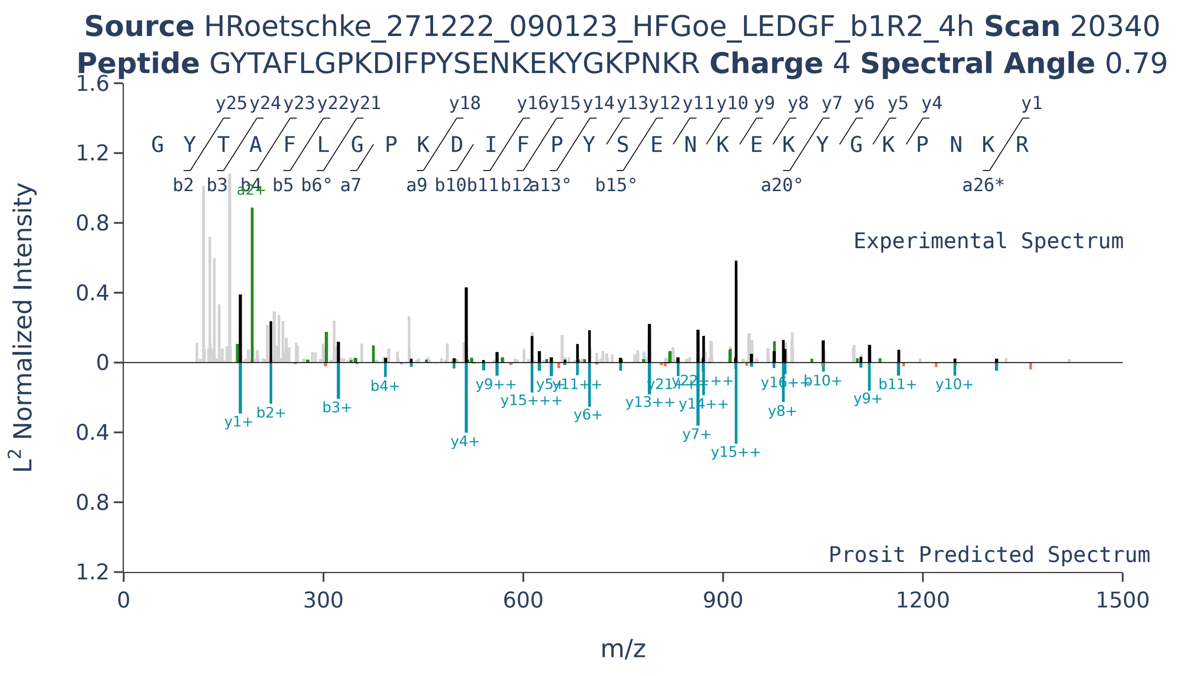
Source HRoetschke_271222_090123_HFGoe_LEDGF_b1R2_4h Scan 20340
Peptide GYTAFLGPKDIFPYSENKEKYGKPNKR Charge 4 Spectral Angle 0.79
Experimental Spectrum
Prosit Predicted Spectrum
L^2 Normalized Intensity
m/z

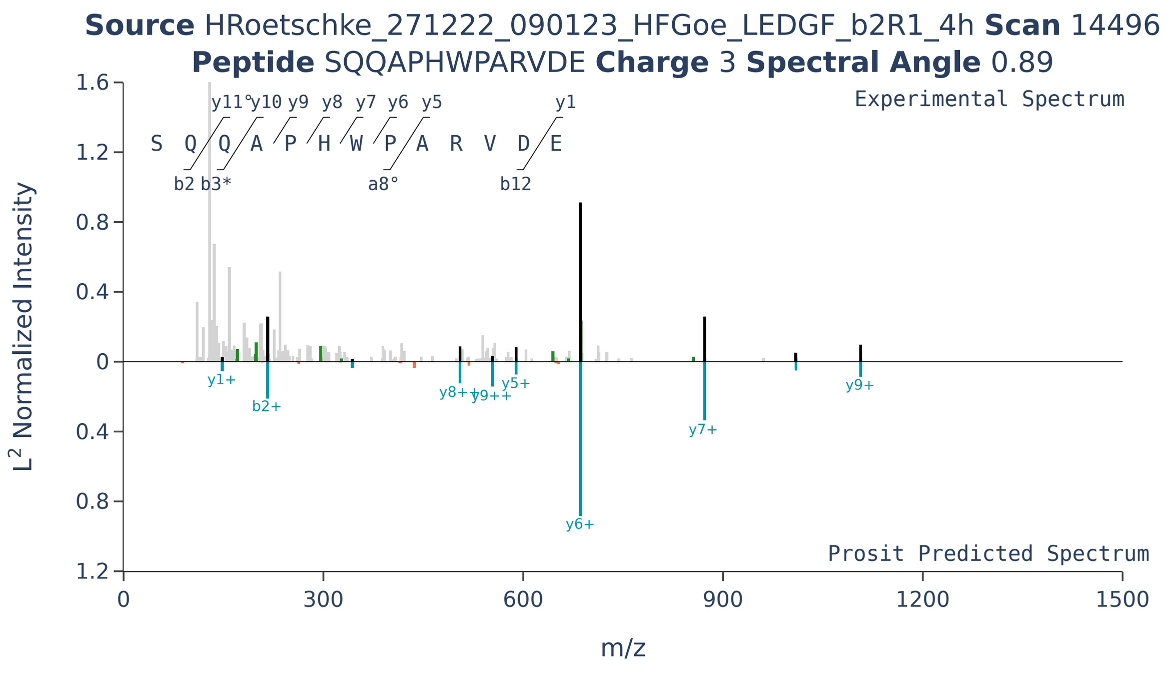
Source HRoetschke_271222_090123_HFGoe_LEDGF_b2R1_4h Scan 14496
Peptide SQQAPHWPARVDE Charge 3 Spectral Angle 0.89
Experimental Spectrum
Prosit Predicted Spectrum
L^2 Normalized Intensity
m/z

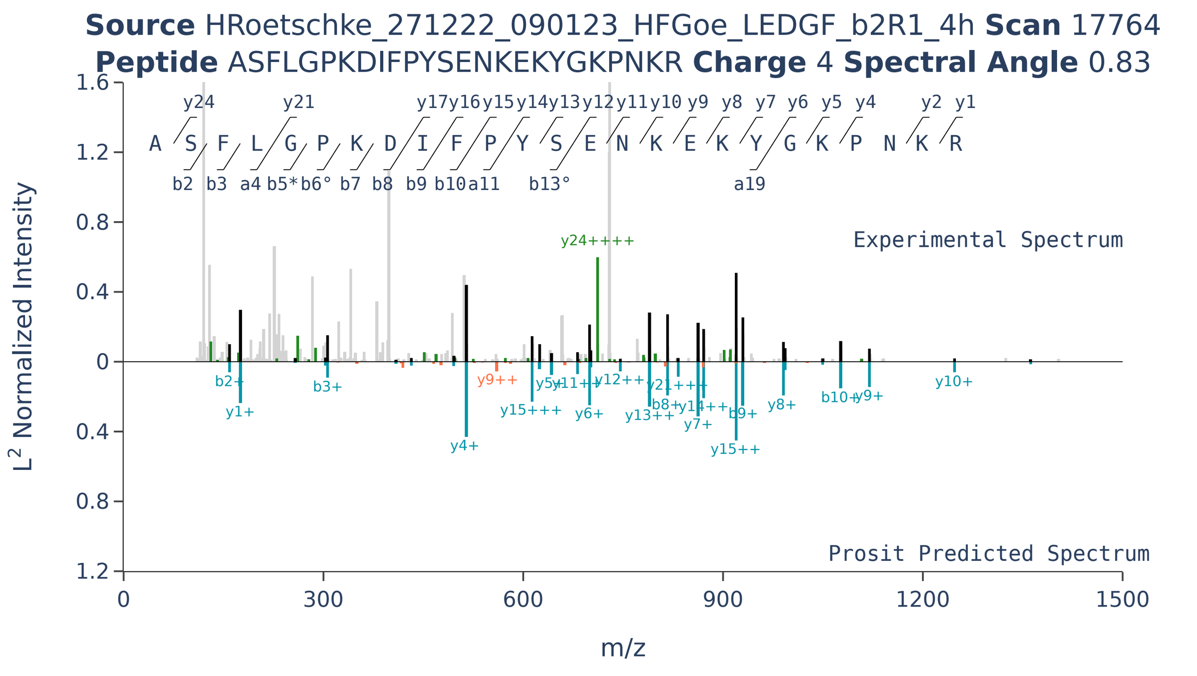
Source HRoetschke_271222_090123_HFGoe_LEDGF_b2R1_4h Scan 17764
Peptide ASFLGPKDIFPYSENKEKYGKPNKR Charge 4 Spectral Angle 0.83
Experimental Spectrum
Prosit Predicted Spectrum
L^2 Normalized Intensity
m/z

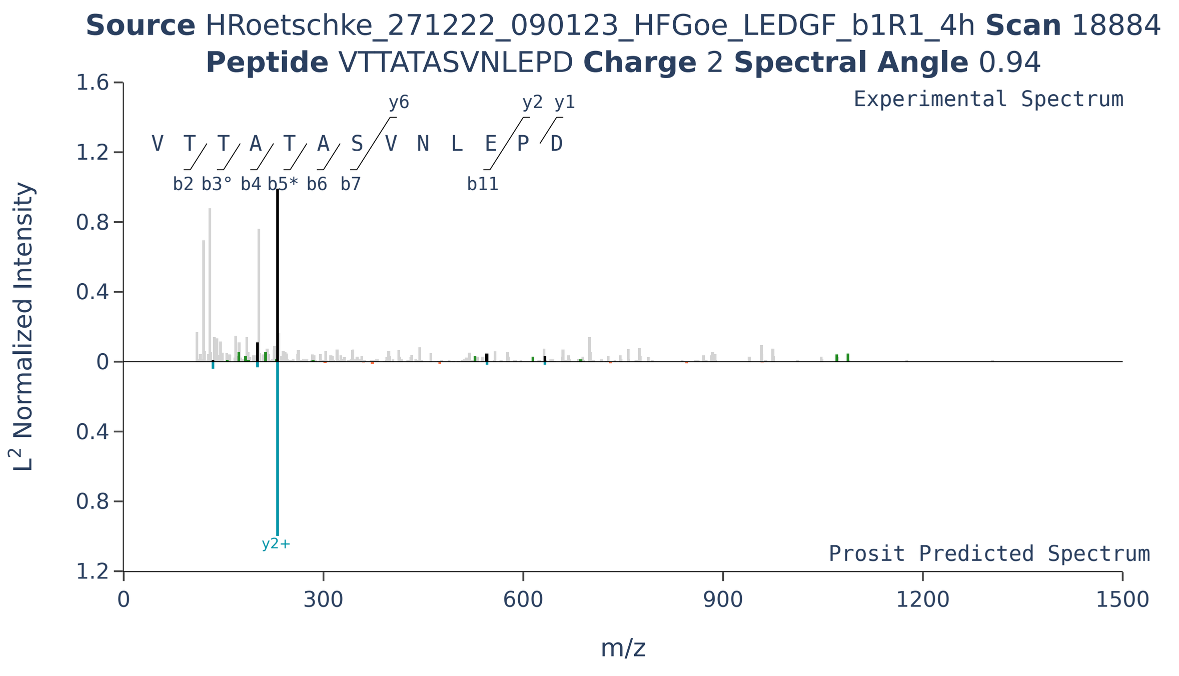
Source HRoetschke_271222_090123_HFGoe_LEDGF_b1R1_4h Scan 18884
Peptide VTTATASVNLEPD Charge 2 Spectral Angle 0.94
Experimental Spectrum
Prosit Predicted Spectrum
L^2 Normalized Intensity
m/z

Source HRoetschke_271222_090123_HFGoe_LEDGF_b2R1_24h **Scan** 19898
Peptide FLGPKDIIFPYSENKEKYGKPNKR **Charge** 5 **Spectral Angle** 0.77

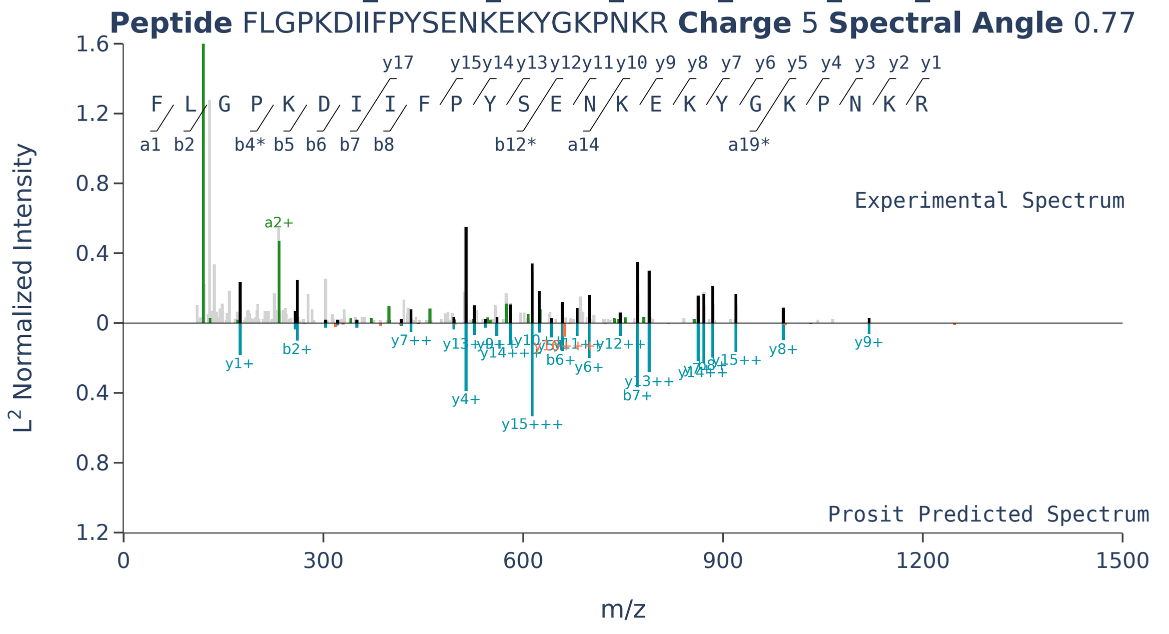

Source HRoetschke_271222_090123_HFGoe_LEDGF_b2R2_24h **Scan** 17576
Peptide AAFLGPKDIFPYSENKEKYGKPNKR **Charge** 4 **Spectral Angle** 0.84

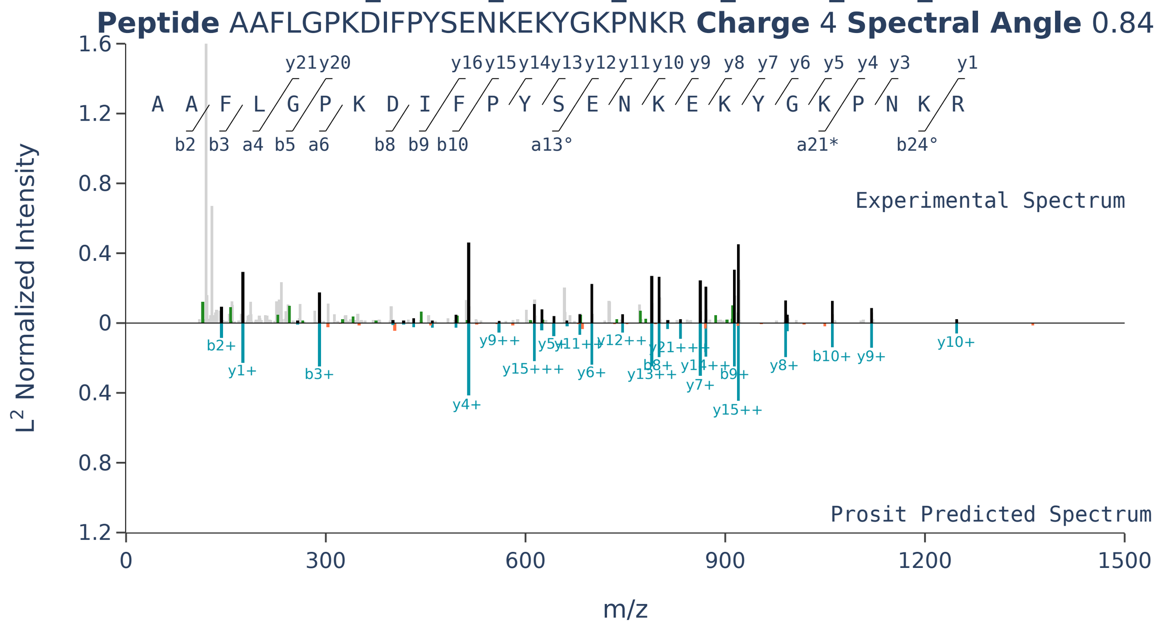

Source HRoetschke_271222_090123_HFGoe_LEDGF_b2R1_24h **Scan** 18809
Peptide LGPKDIFFPYSENKEKYGKPNKR **Charge** 4 **Spectral Angle** 0.77

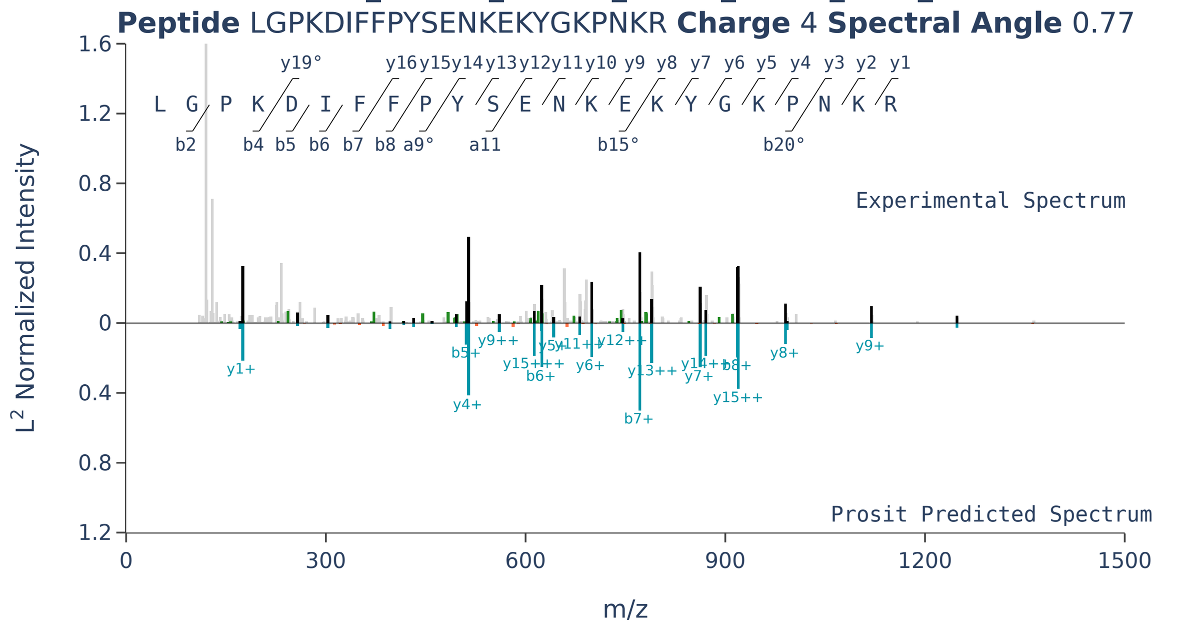

Source HRoetschke_271222_090123_HFGoe_LEDGF_b1R2_24h **Scan** 14923
Peptide KGFNEGFKNM **Charge** 3 **Spectral Angle** 0.9

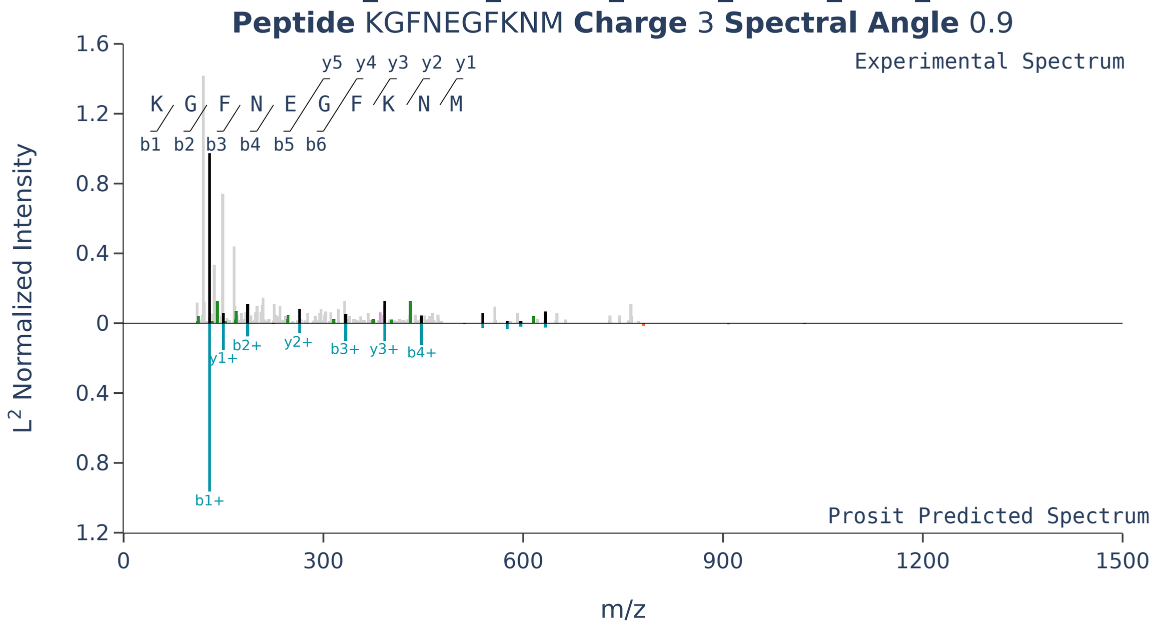

Source HRoetschke_271222_090123_HFGoe_LEDGF_b2R1_4h **Scan** 17472
Peptide AFLGPKDIFPYSENKEKYGKPNKRTA **Charge** 5 **Spectral Angle** 0.74

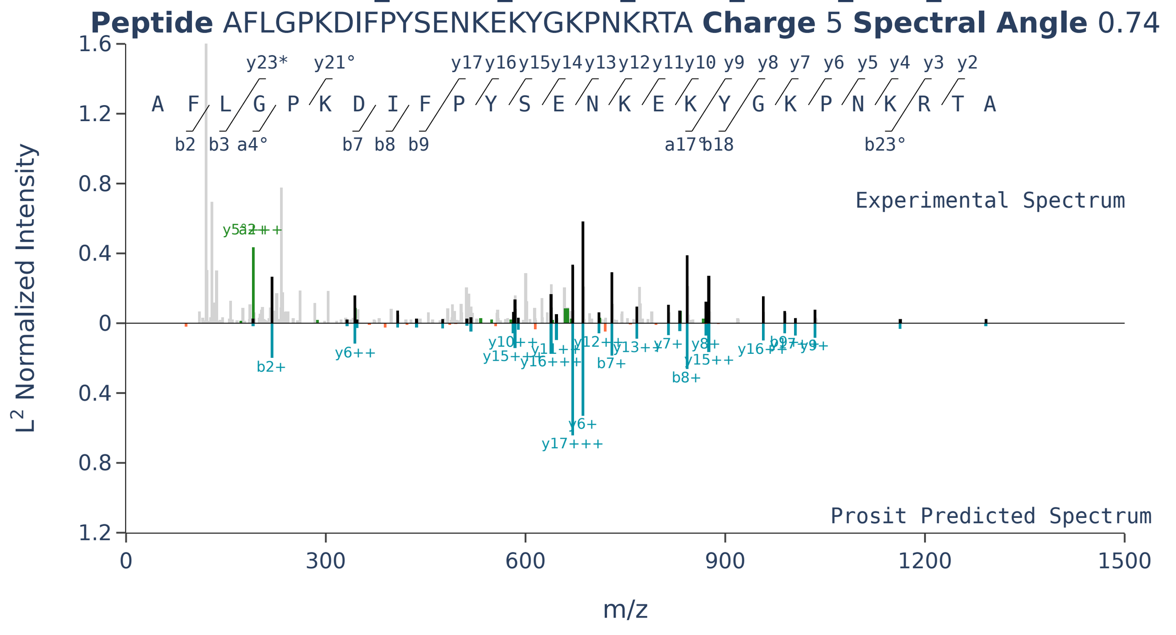

Source HRoetschke_271222_090123_HFGoe_LEDGF_b1R2_24h **Scan** 10959
Peptide SKTMLYNKFKNM **Charge** 4 **Spectral Angle** 0.76

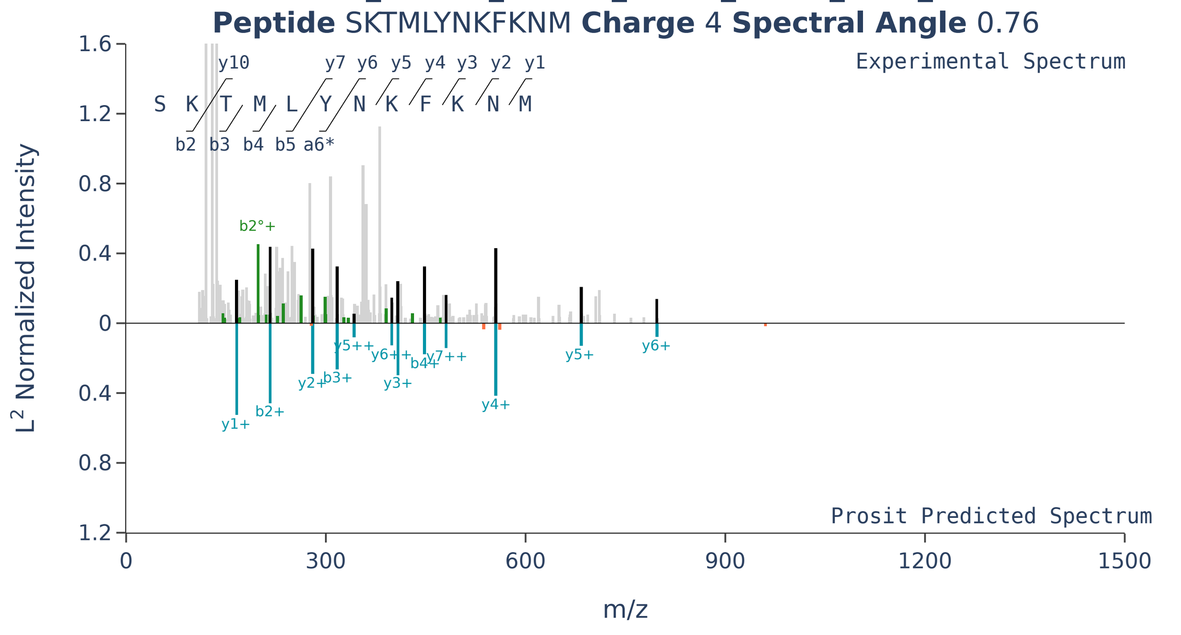

Source HRoetschke_271222_090123_HFGoe_LEDGF_b2R1_2h **Scan** 16940
Peptide GTYPHWPARVDE **Charge** 3 **Spectral Angle** 0.89

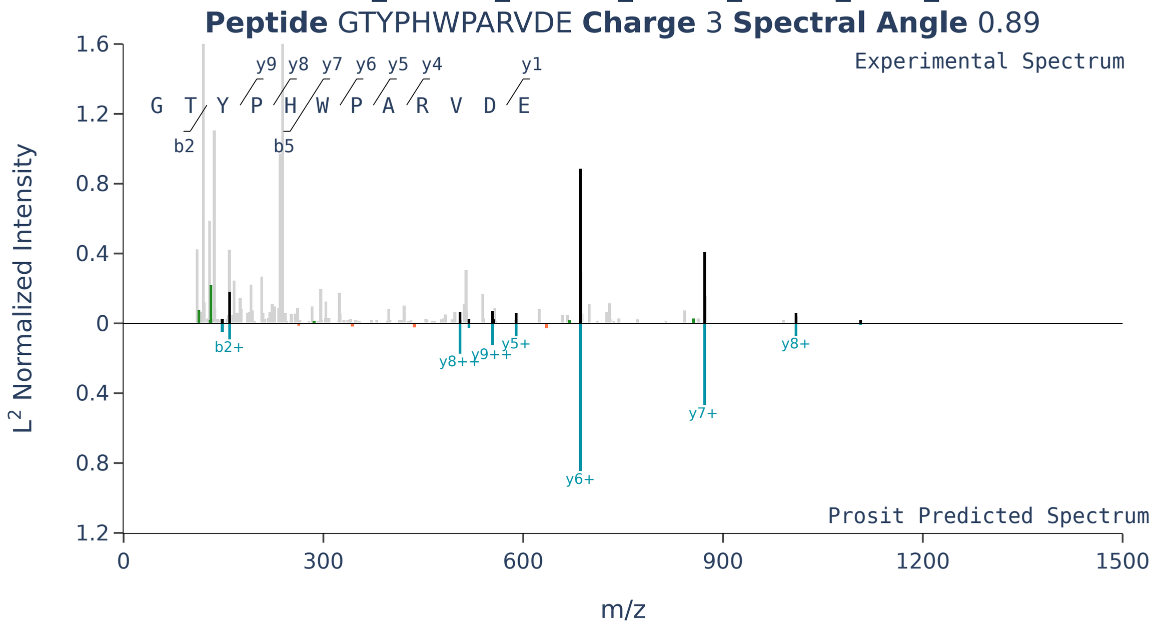

Source HRoetschke_271222_090123_HFGoe_LEDGF_b2R1_24h **Scan** 25803
Peptide ELSLKIDNLDVNRCIE **Charge** 2 **Spectral Angle** 0.86

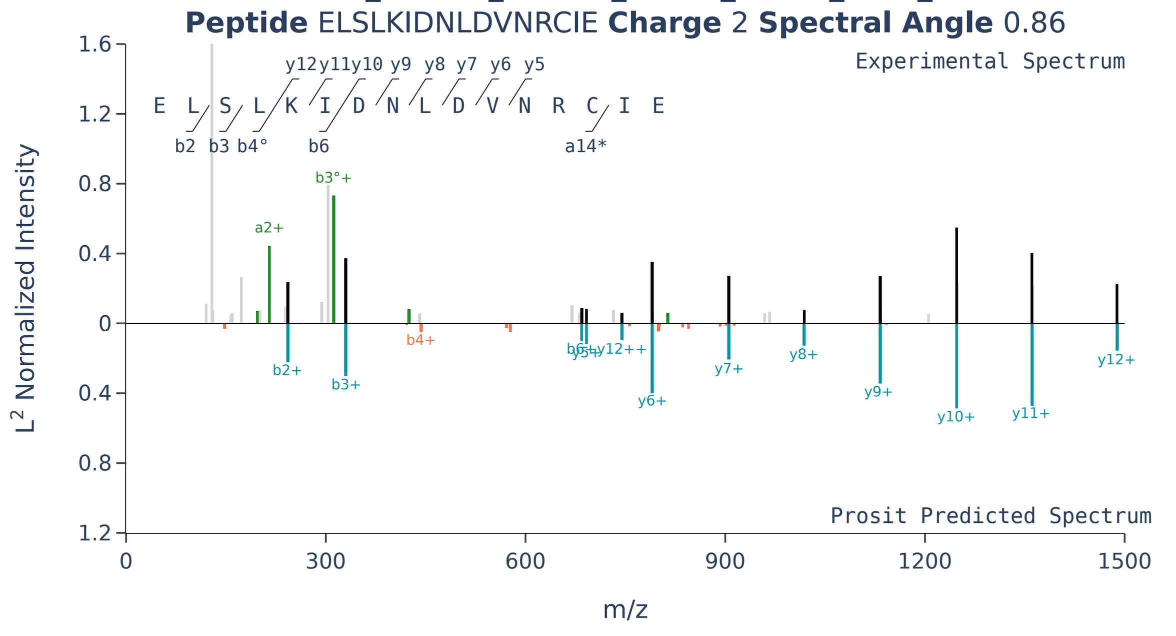

Source HRoetschke_271222_090123_HFGoe_LEDGF_b2R2_24h **Scan** 16666
Peptide FNEHWPARVDE **Charge** 3 **Spectral Angle** 0.89

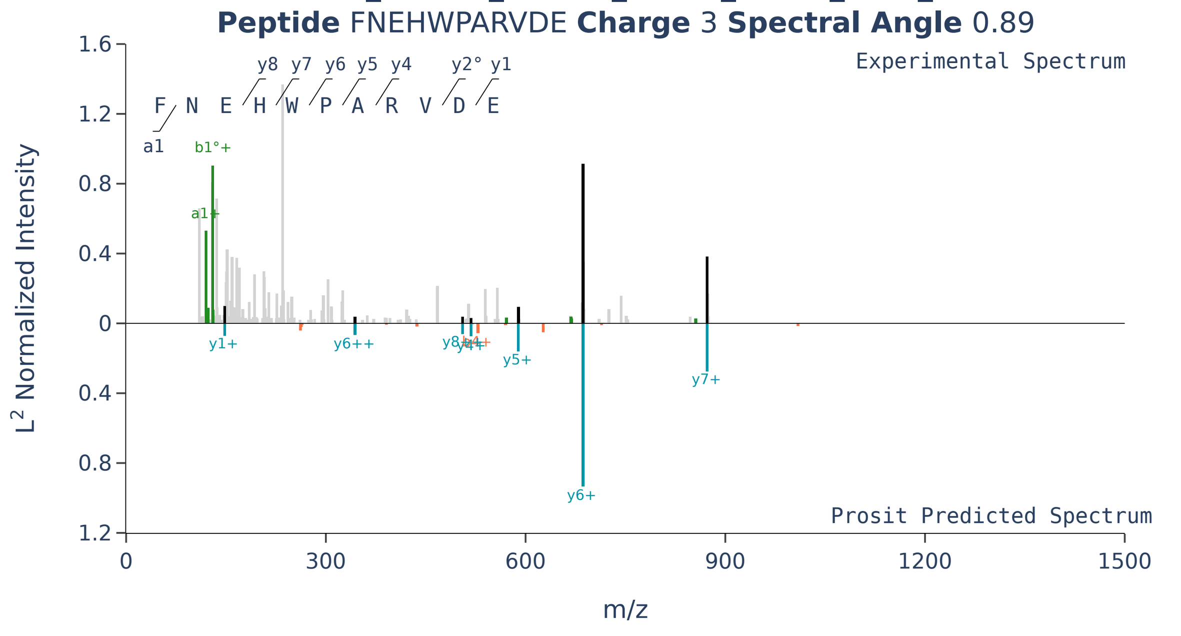

Source HRoetschke_271222_090123_HFGoe_LEDGF_b2R2_4h **Scan** 6202
Peptide PKMVKQPCPSEKE **Charge** 4 **Spectral Angle** 0.81

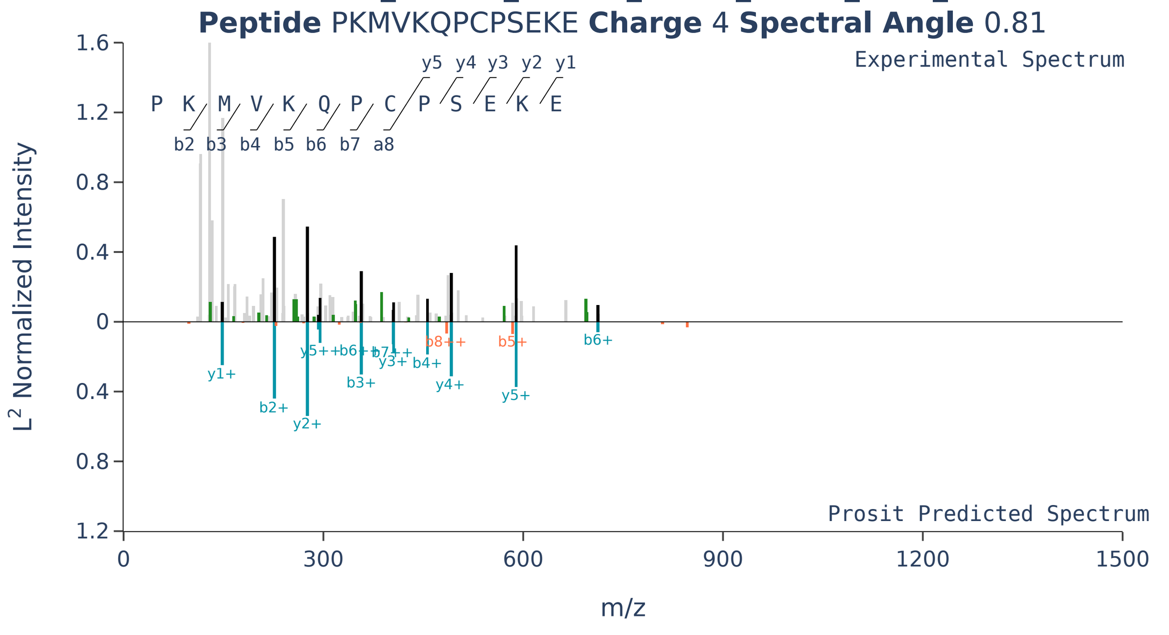

Source HRoetschke_271222_090123_HFGoe_LEDGF_b1R1_2h **Scan** 8447
Peptide GPKDIFPYSENKEKYGKPNKKR **Charge** 6 **Spectral Angle** 0.79

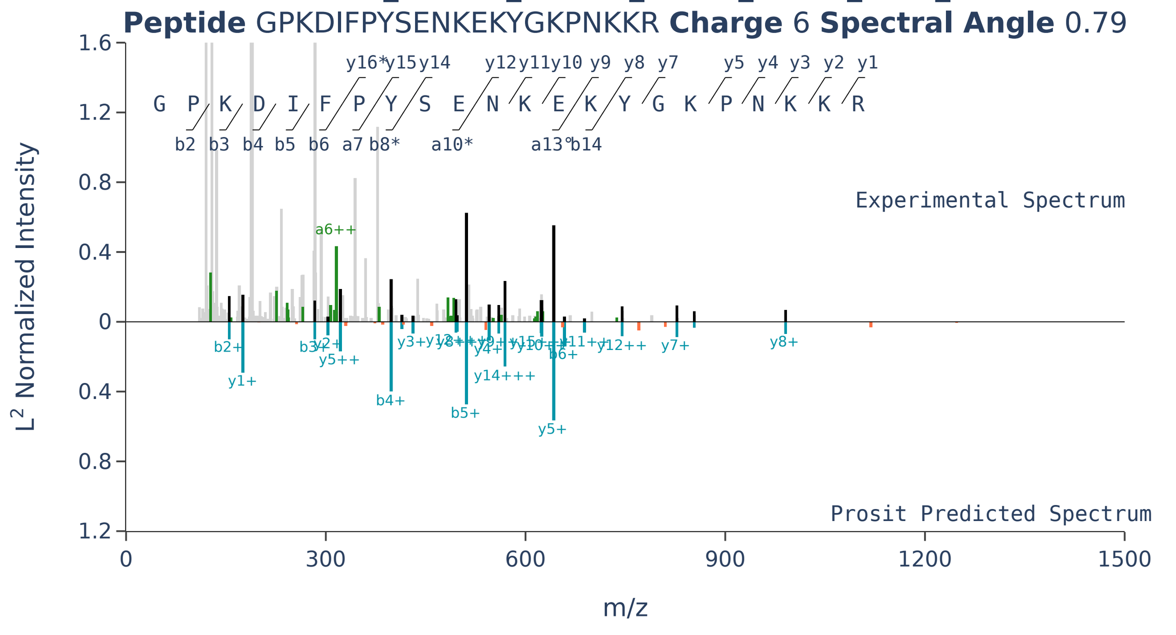

Source HRoetschke_271222_090123_HFGoe_LEDGF_b1R1_1h **Scan** 10547
Peptide GTHETGPGMTRDFKPGD **Charge** 4 **Spectral Angle** 0.8

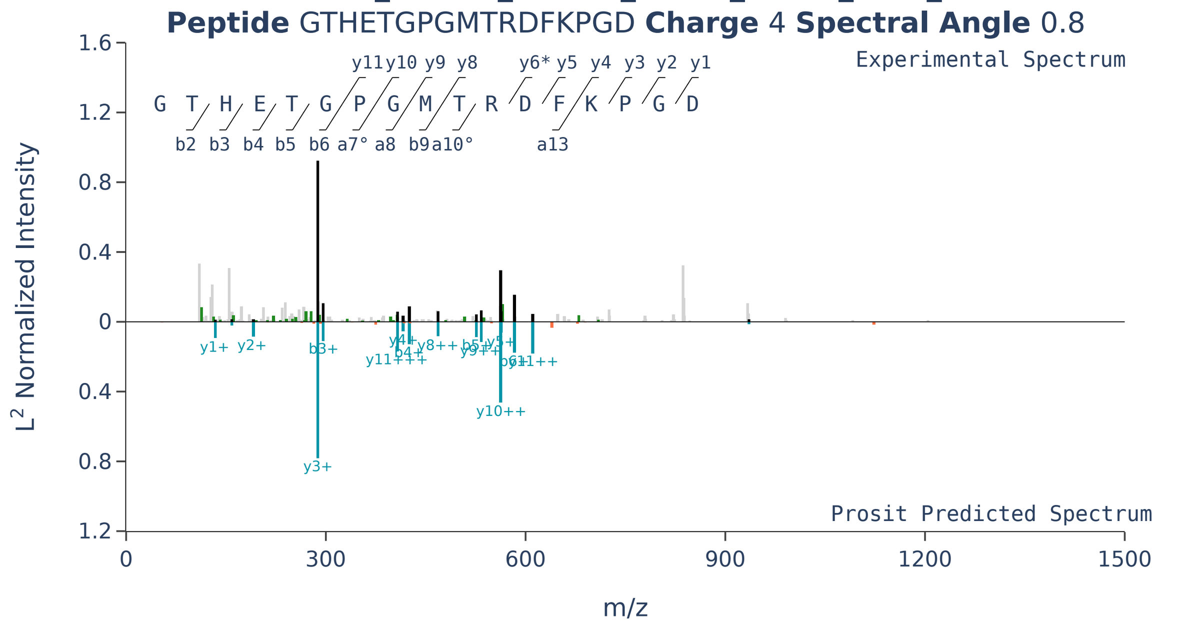

Source HRoetschke_271222_090123_HFGoe_LEDGF_b1R2_24h **Scan** 12123
Peptide TASMDSRLQRIHA **Charge** 2 **Spectral Angle** 0.87

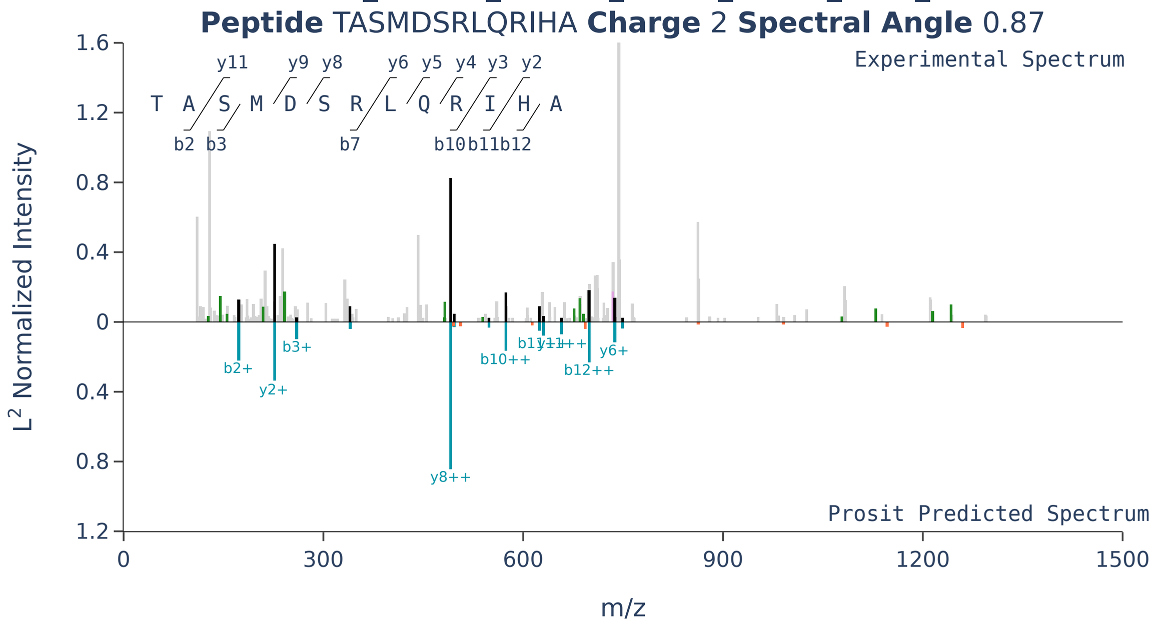

Source HRoetschke_271222_090123_HFGoe_LEDGF_b1R1_2h **Scan** 19202
Peptide TAFLGPKDIFPYSENKEKYGKPNKRLE **Charge** 5 **Spectral Angle** 0.81

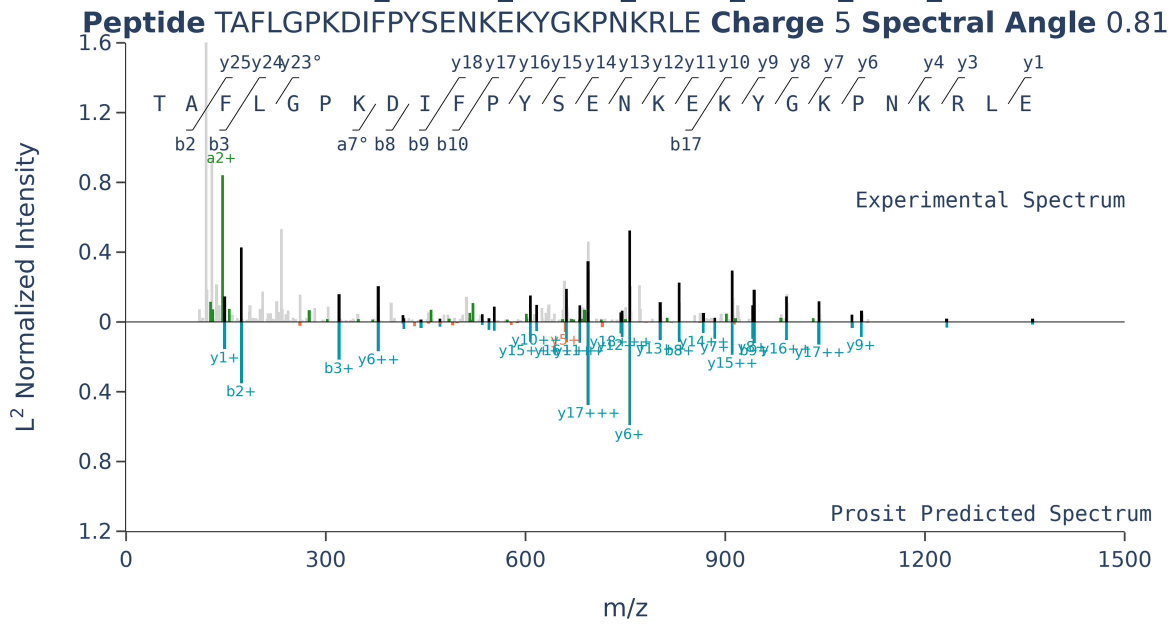

Source HRoetschke_271222_090123_HFGoe_LEDGF_b1R1_24h **Scan** 13270
Peptide DGPGMTRDFKPGD **Charge** 3 **Spectral Angle** 0.79

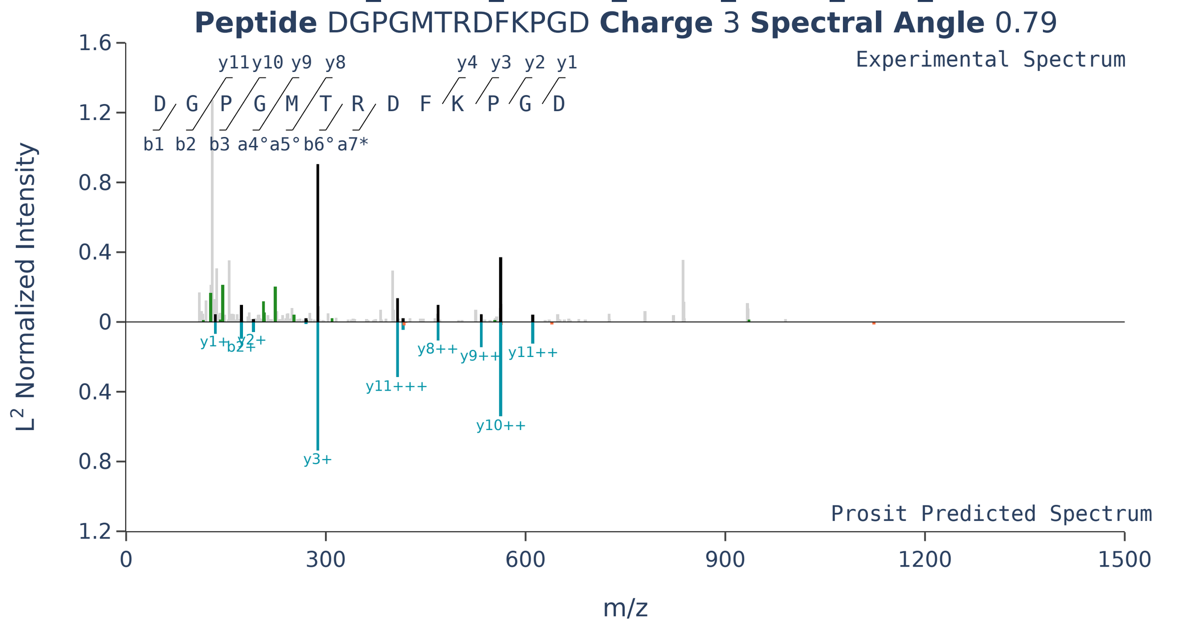

Source HRoetschke_271222_090123_HFGoe_LEDGF_b1R1_24h **Scan** 12975
Peptide ATKQSVPDGAVKPPTNKL **Charge** 3 **Spectral Angle** 0.85

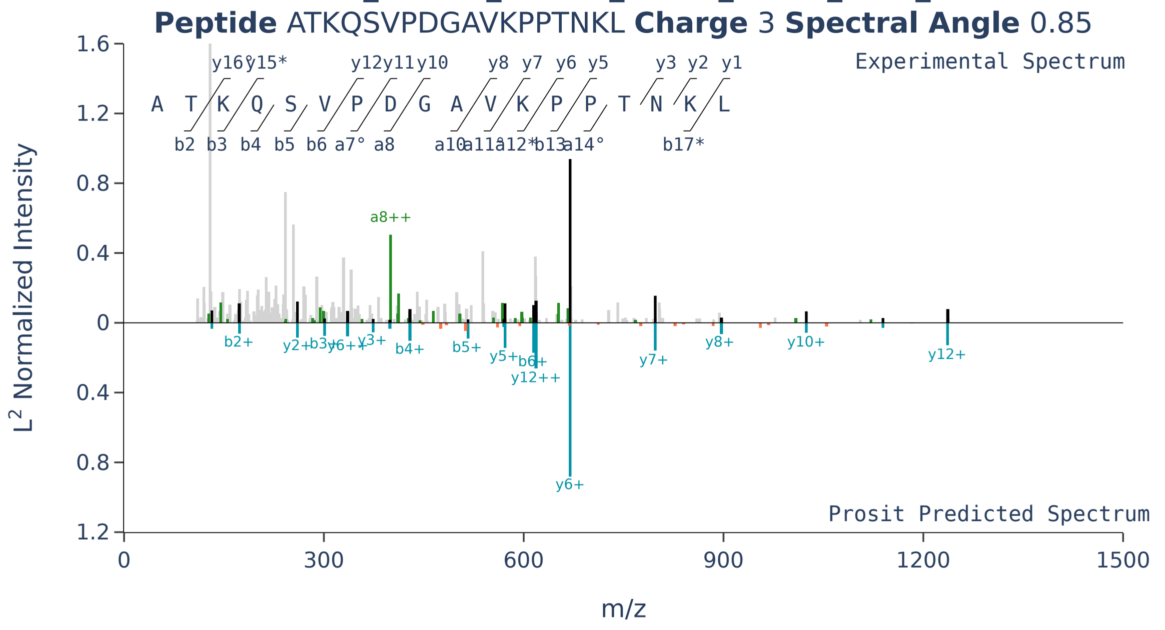

Source HRoetschke_271222_090123_HFGoe_LEDGF_b2R2_24h **Scan** 23517
Peptide GKELASLQVTM **Charge** 2 **Spectral Angle** 0.87

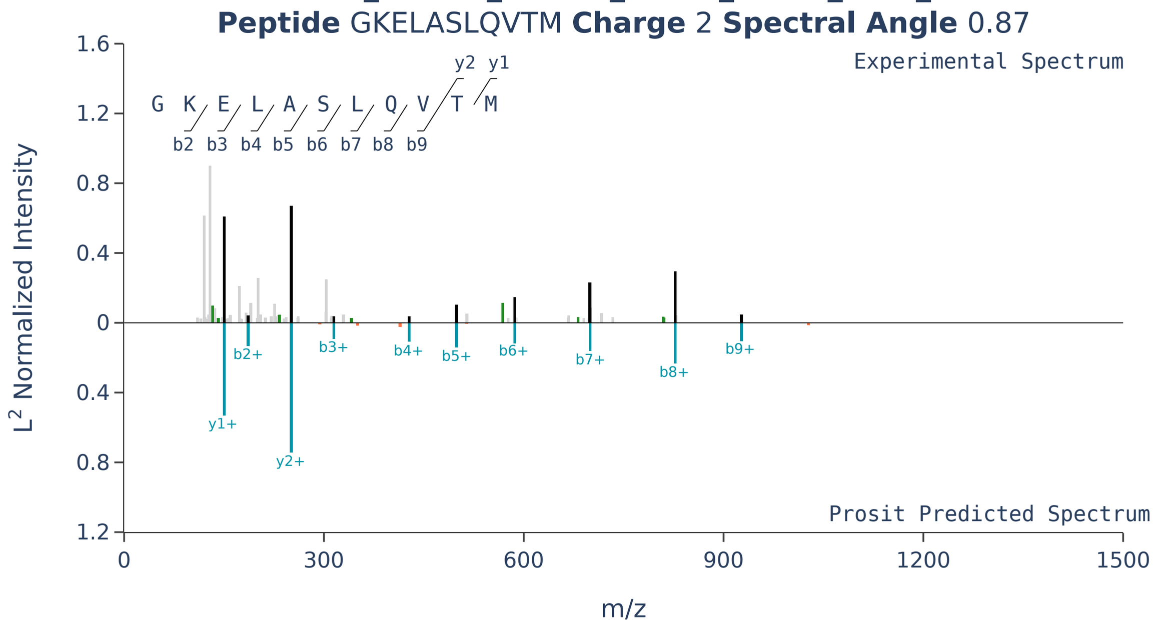

Source HRoetschke_271222_090123_HFGoe_LEDGF_b1R1_24h **Scan** 6553
Peptide LEKEQTGSKTLD **Charge** 3 **Spectral Angle** 0.75

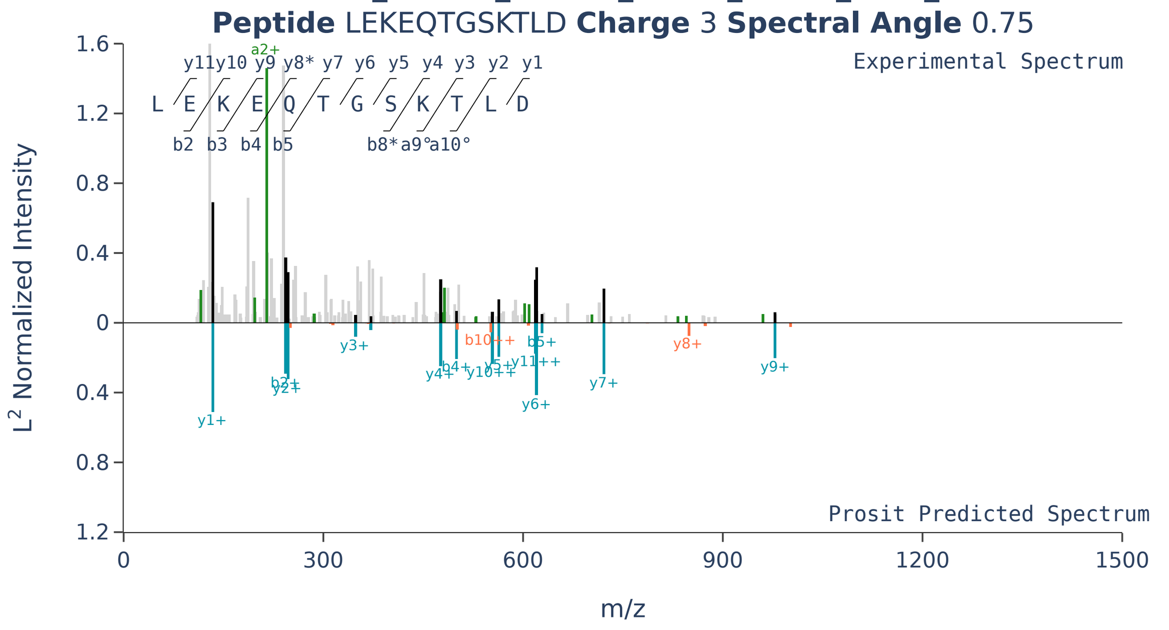

Source HRoetschke_271222_090123_HFGoe_LEDGF_b1R1_4h **Scan** 24323
Peptide REELASLQVTM **Charge** 2 **Spectral Angle** 0.81

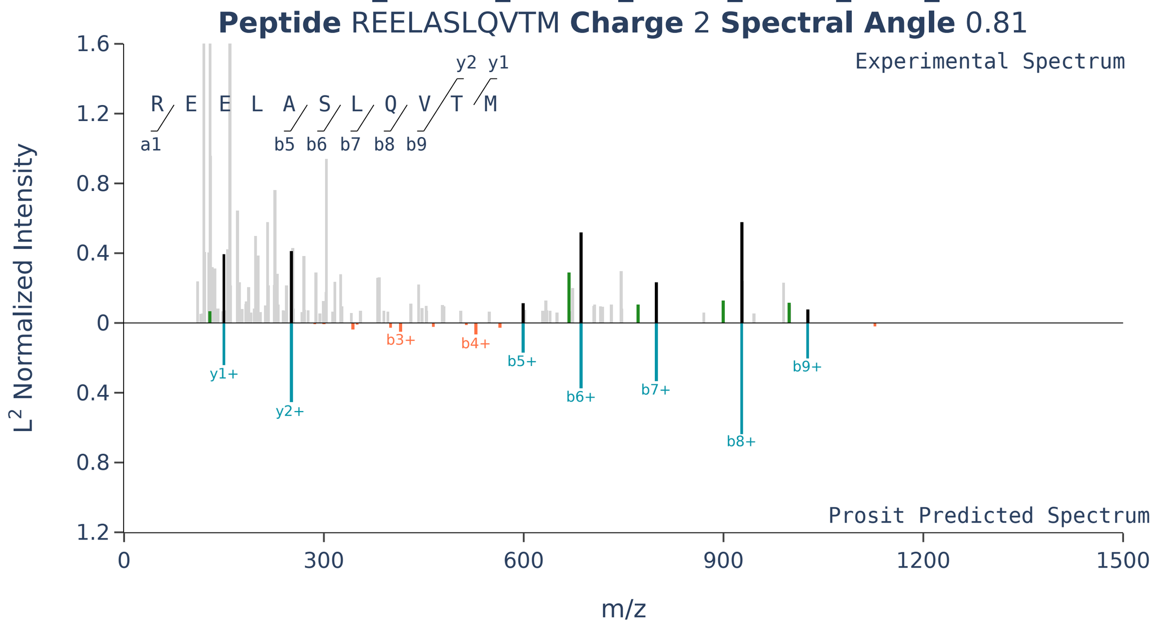

Source HRoetschke_271222_090123_HFGoe_LEDGF_b1R1_24h **Scan** 10544
Peptide LDNVETEE **Charge** 2 **Spectral Angle** 0.91

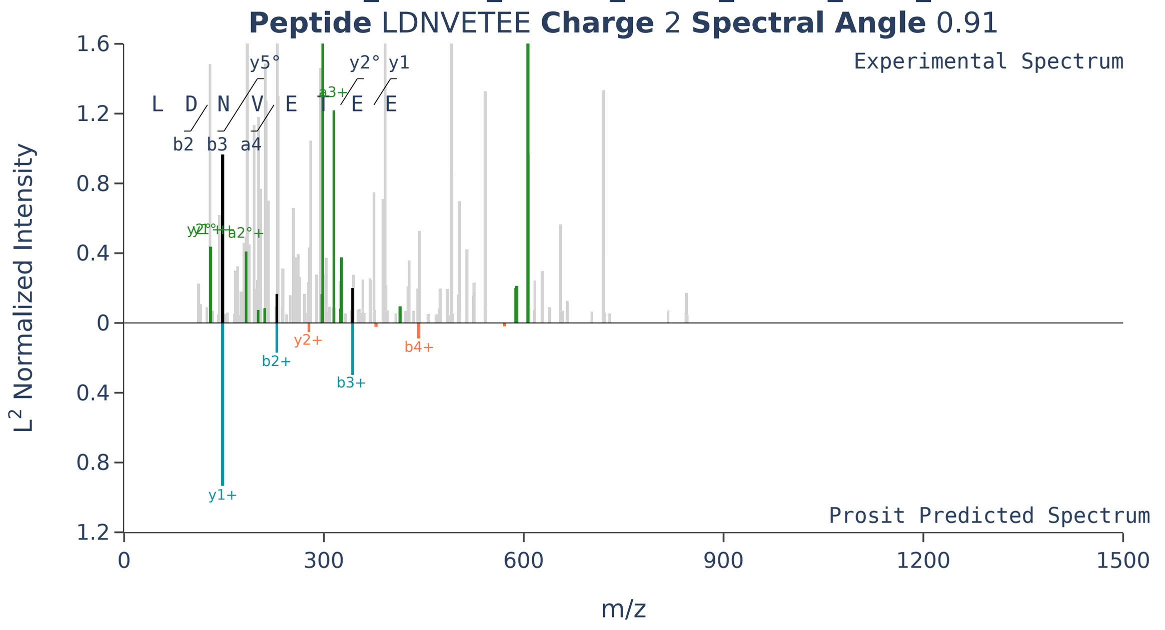

Source HRoetschke_271222_090123_HFGoe_LEDGF_b1R2_24h **Scan** 6419
Peptide GTHETRDFKPGD **Charge** 3 **Spectral Angle** 0.82

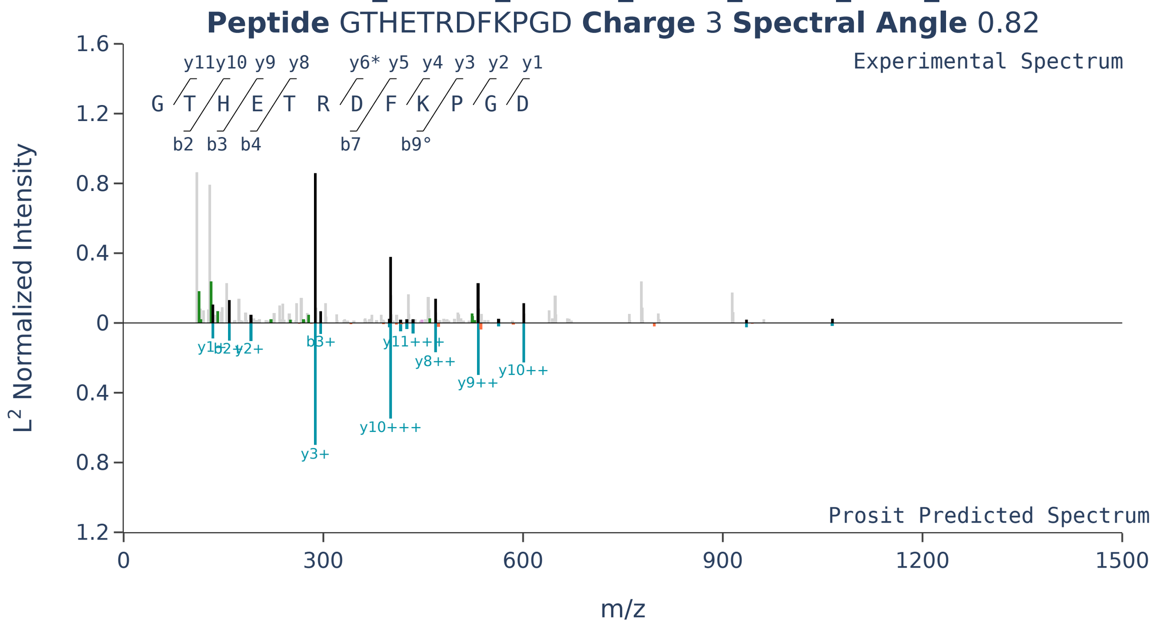

Source HRoetschke_271222_090123_HFGoe_LEDGF_b1R2_24h **Scan** 6011
Peptide VPDGKETSVSKE **Charge** 2 **Spectral Angle** 0.74

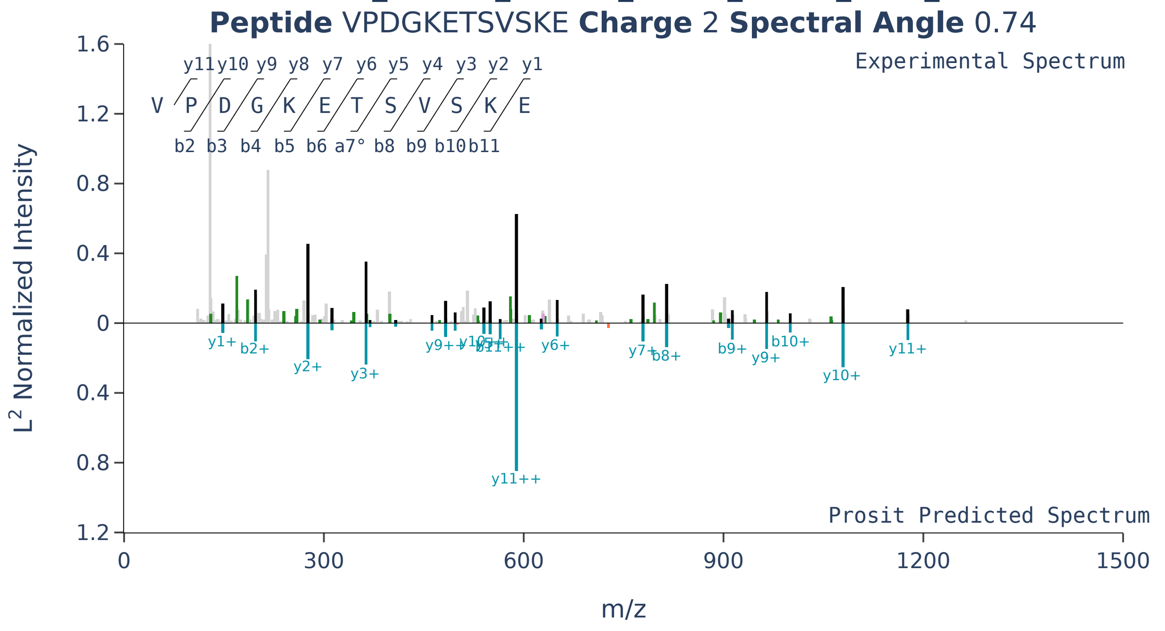

Source HRoetschke_271222_090123_HFGoe_LEDGF_b2R1_24h **Scan** 10751
Peptide RIHAEIKNALD **Charge** 3 **Spectral Angle** 0.83

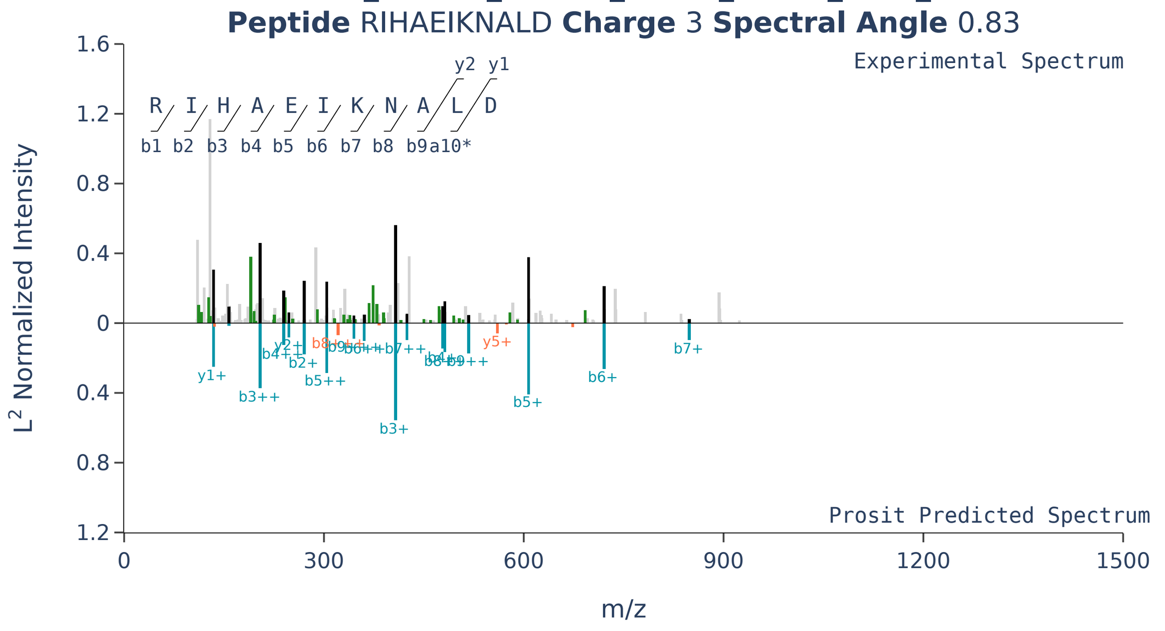

Source HRoetschke_271222_090123_HFGoe_LEDGF_b2R1_24h **Scan** 26000
Peptide TEEAGVVTTSLKIDNLDVNRCIE **Charge** 3 **Spectral Angle** 0.85

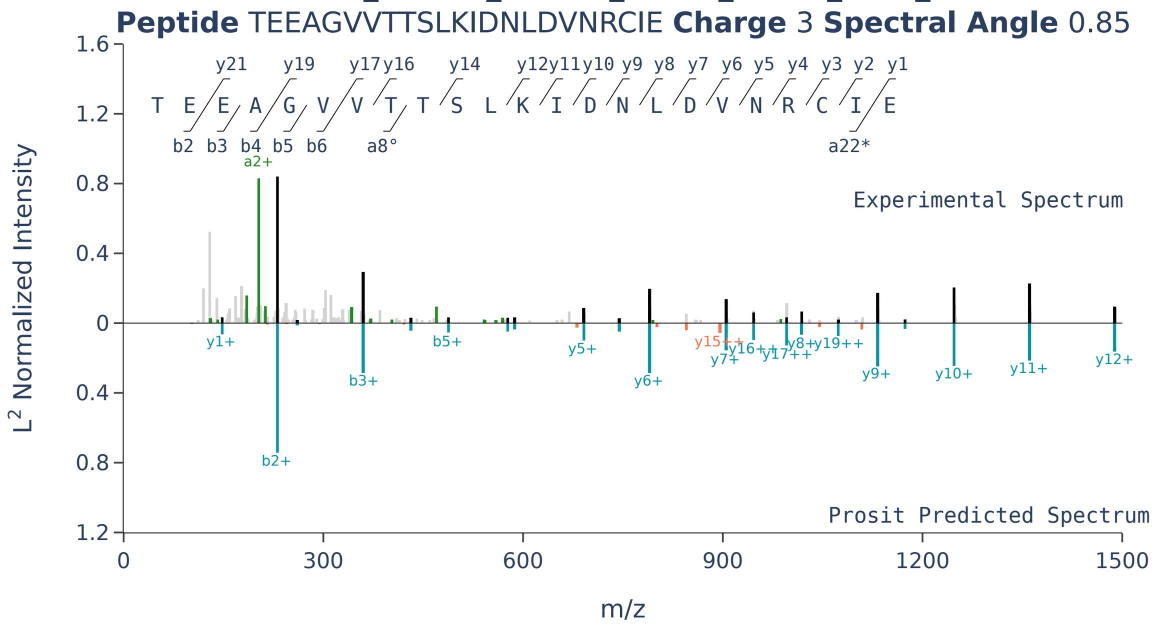

Source HRoetschke_271222_090123_HFGoe_LEDGF_b2R1_2h **Scan** 11982
Peptide PHWPARVDD **Charge** 3 **Spectral Angle** 0.88

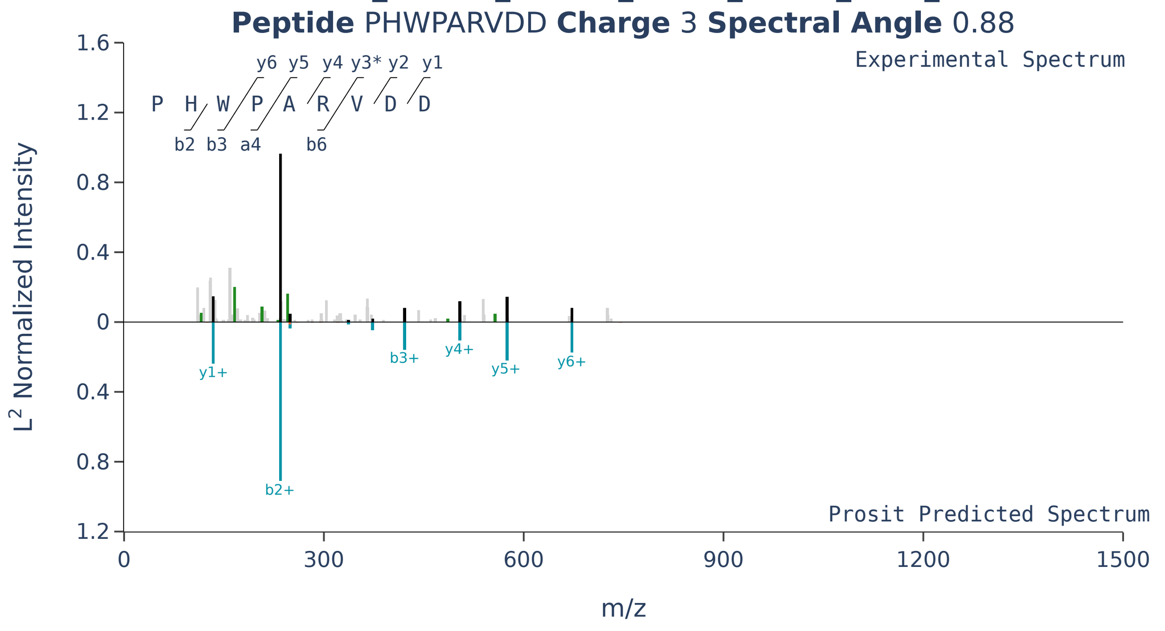

Source HRoetschke_271222_090123_HFGoe_LEDGF_b2R1_24h **Scan** 13603
Peptide QPQHNMITTLK **Charge** 3 **Spectral Angle** 0.93

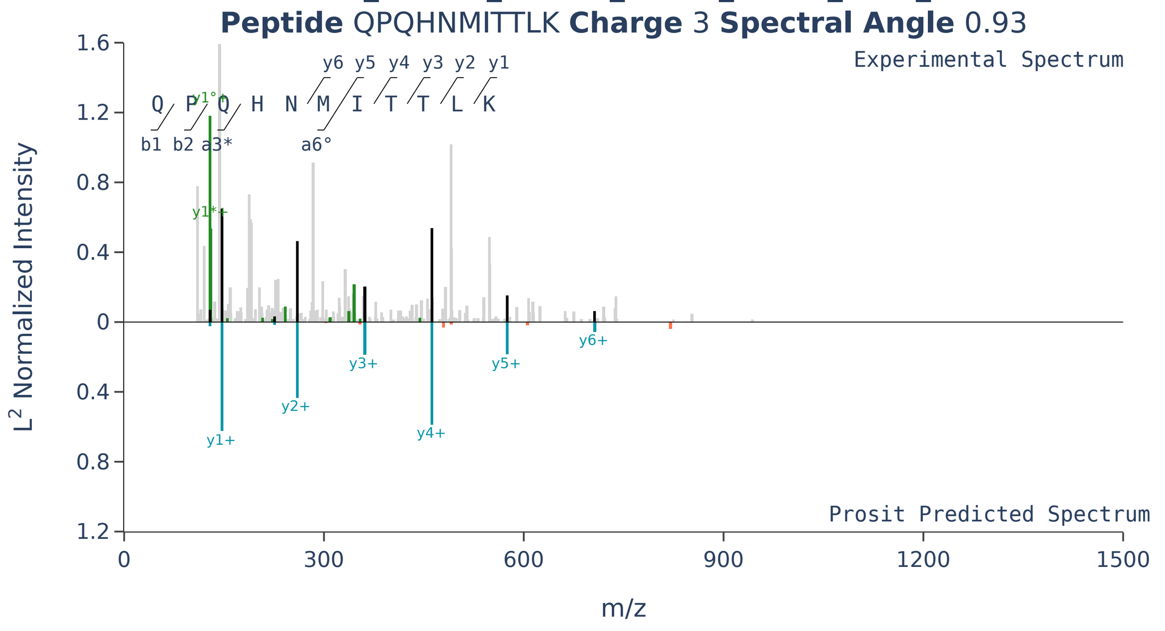

Source HRoetschke_271222_090123_HFGoe_LEDGF_b2R1_24h **Scan** 17457
Peptide TFLGPKDIFPYSENKEKYGKPNKR **Charge** 5 **Spectral Angle** 0.75

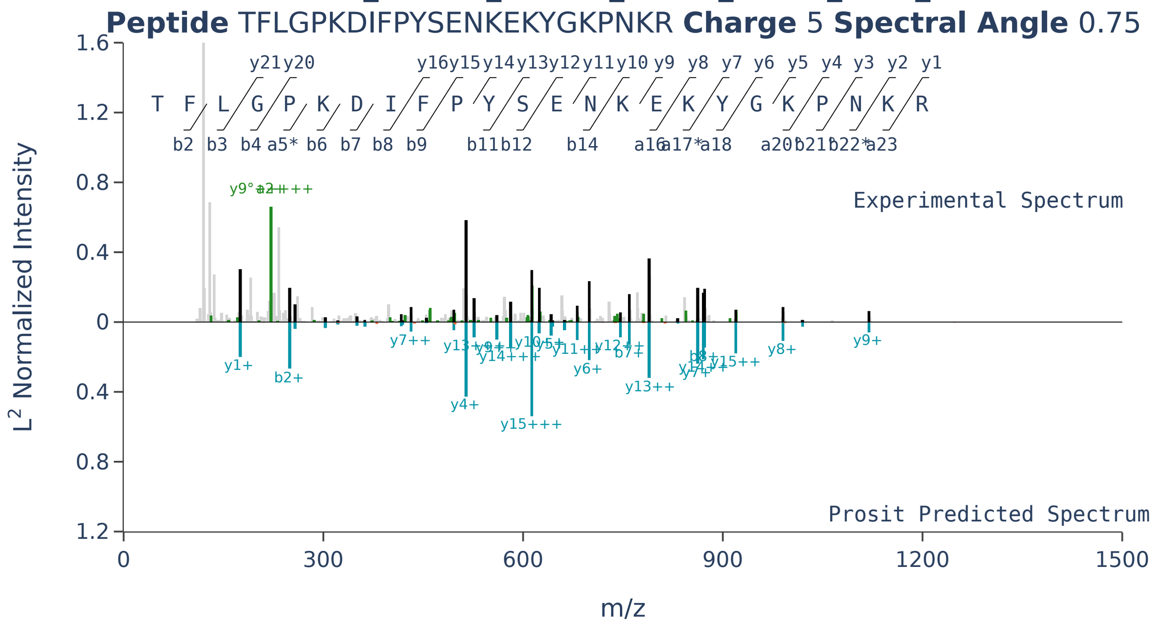

Source HRoetschke_271222_090123_HFGoe_LEDGF_b1R1_4h **Scan** 9168
Peptide GGNPKVKF **Charge** 1 **Spectral Angle** 0.84

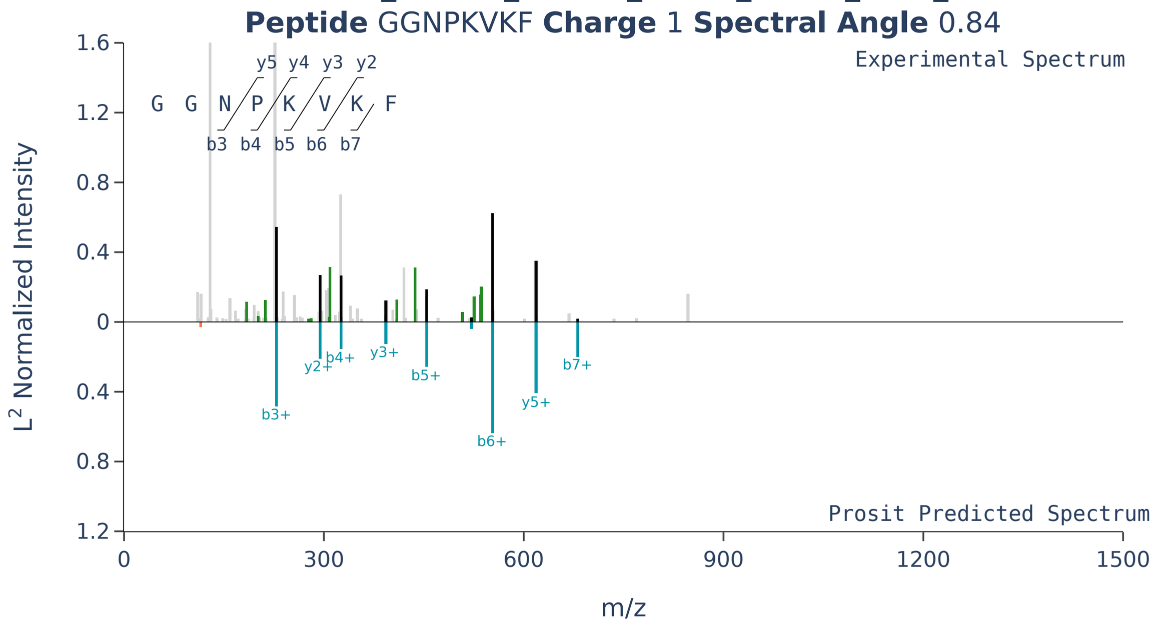

Source HRoetschke_271222_090123_HFGoe_LEDGF_b1R1_2h **Scan** 17236
Peptide VVKIDNLDVNRC **Charge** 2 **Spectral Angle** 0.83

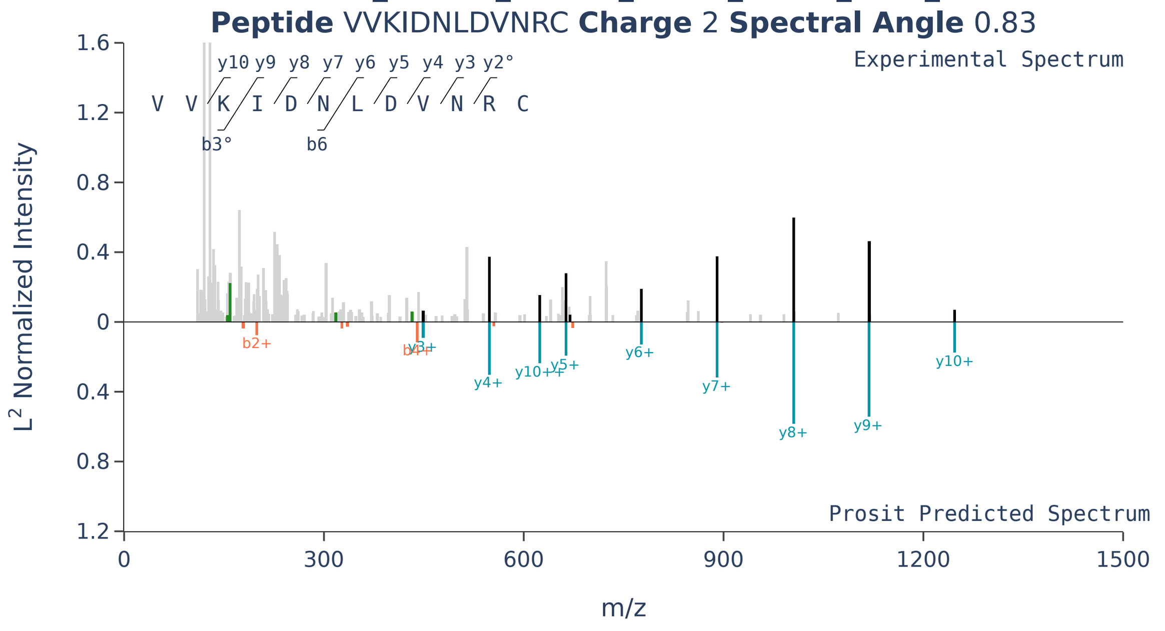

Source HRoetschke_271222_090123_HFGoe_LEDGF_b1R1_4h **Scan** 13247
Peptide GAWPARVD **Charge** 2 **Spectral Angle** 0.94

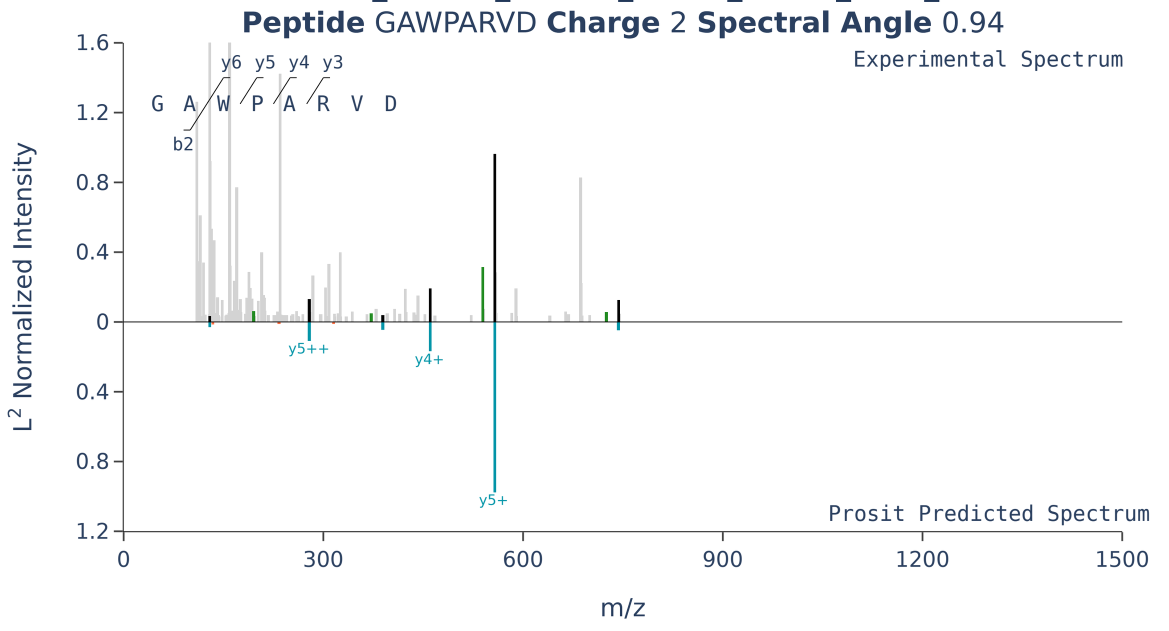

Source HRoetschke_271222_090123_HFGoe_LEDGF_b1R1_4h **Scan** 10645
Peptide IDNNPKVKFN **Charge** 3 **Spectral Angle** 0.66

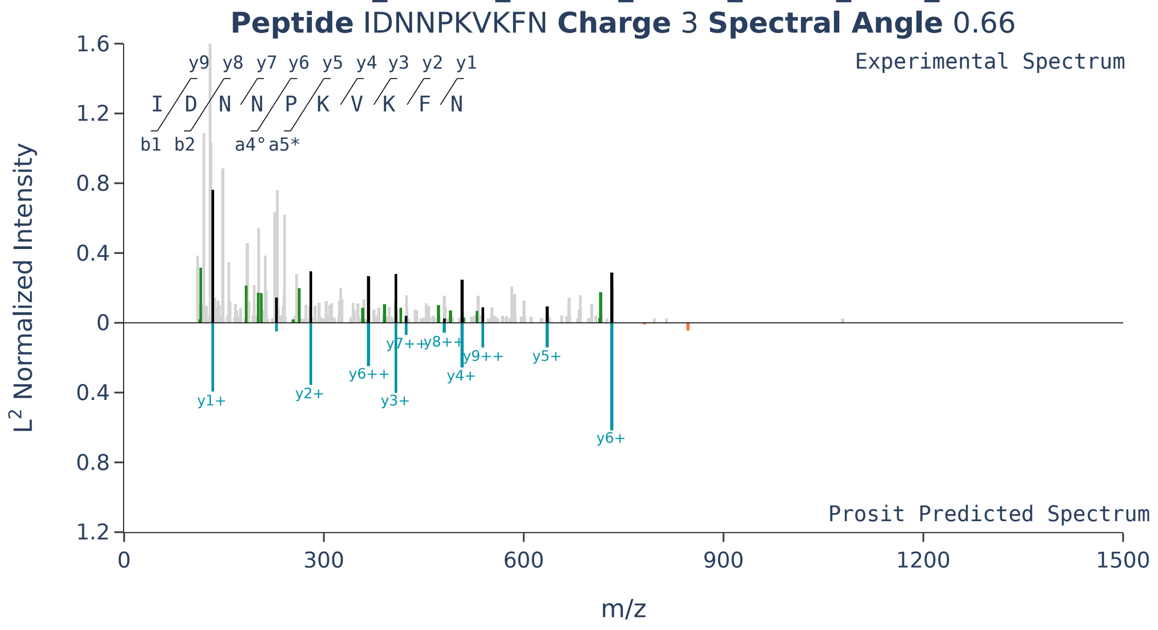

Source HRoetschke_271222_090123_HFGoe_LEDGF_b2R1_24h **Scan** 18039
Peptide SQTAFLGPKDIFPYSENKEKYGKPNKR **Charge** 4 **Spectral Angle** 0.82

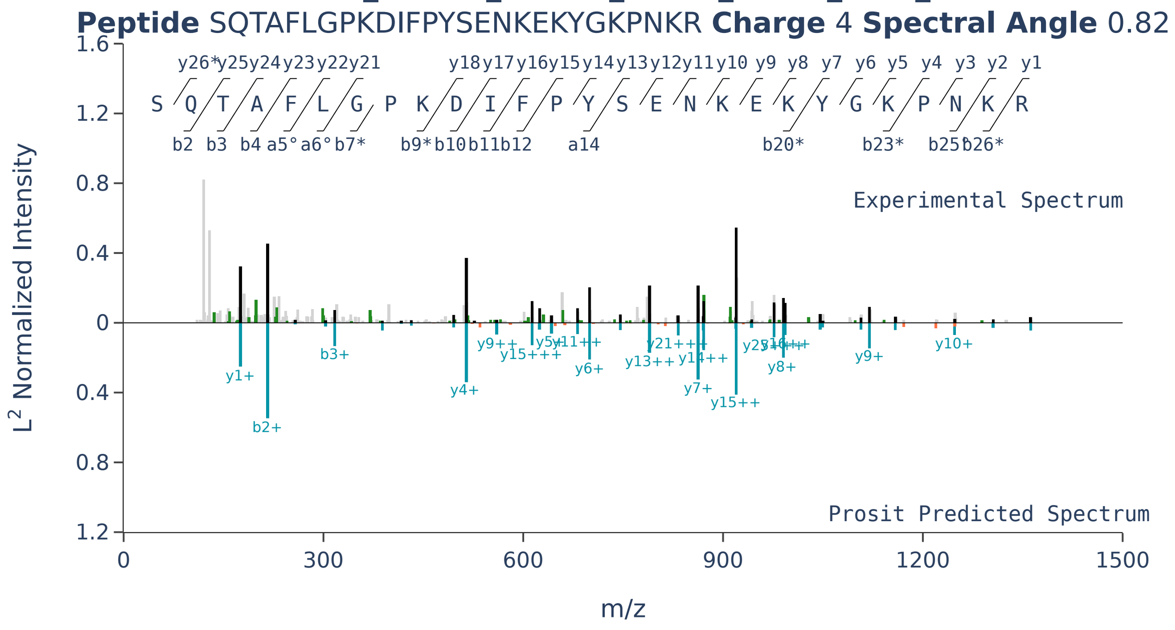

Source HRoetschke_271222_090123_HFGoe_LEDGF_b2R2_24h **Scan** 9419
Peptide TEEAGVVTTKA **Charge** 2 **Spectral Angle** 0.81

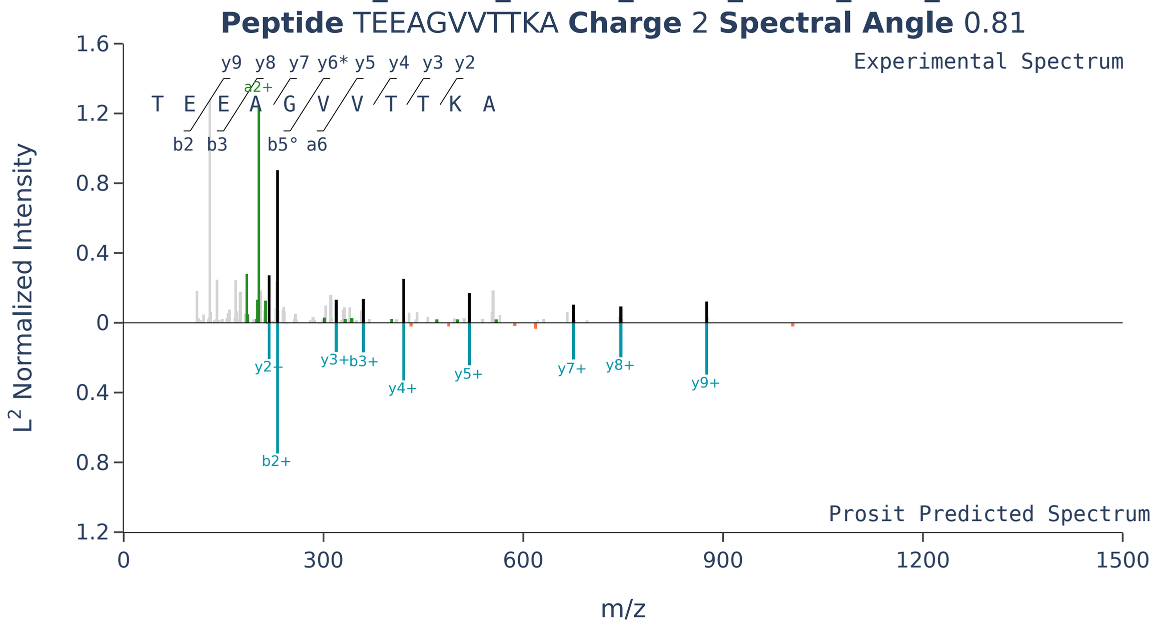

Source HRoetschke_271222_090123_HFGoe_LEDGF_b2R1_24h **Scan** 21287
Peptide GTLKIDNLDVNRCIE **Charge** 2 **Spectral Angle** 0.87

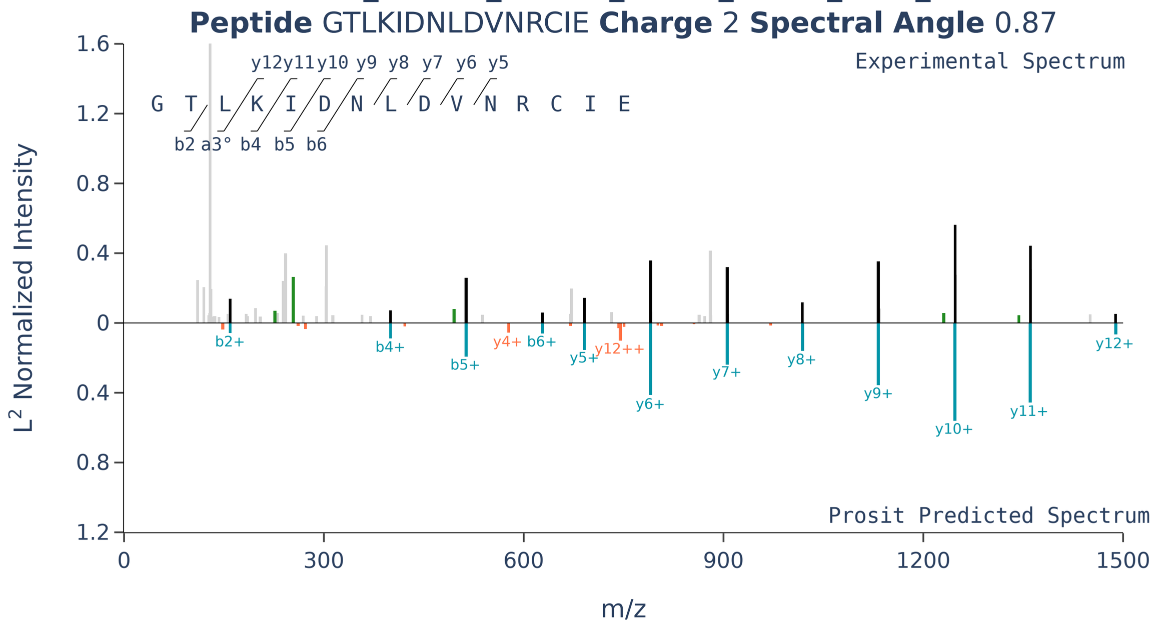

Source HRoetschke_271222_090123_HFGoe_LEDGF_b2R1_24h **Scan** 21941
Peptide GEKLASLQVTMQ **Charge** 2 **Spectral Angle** 0.88

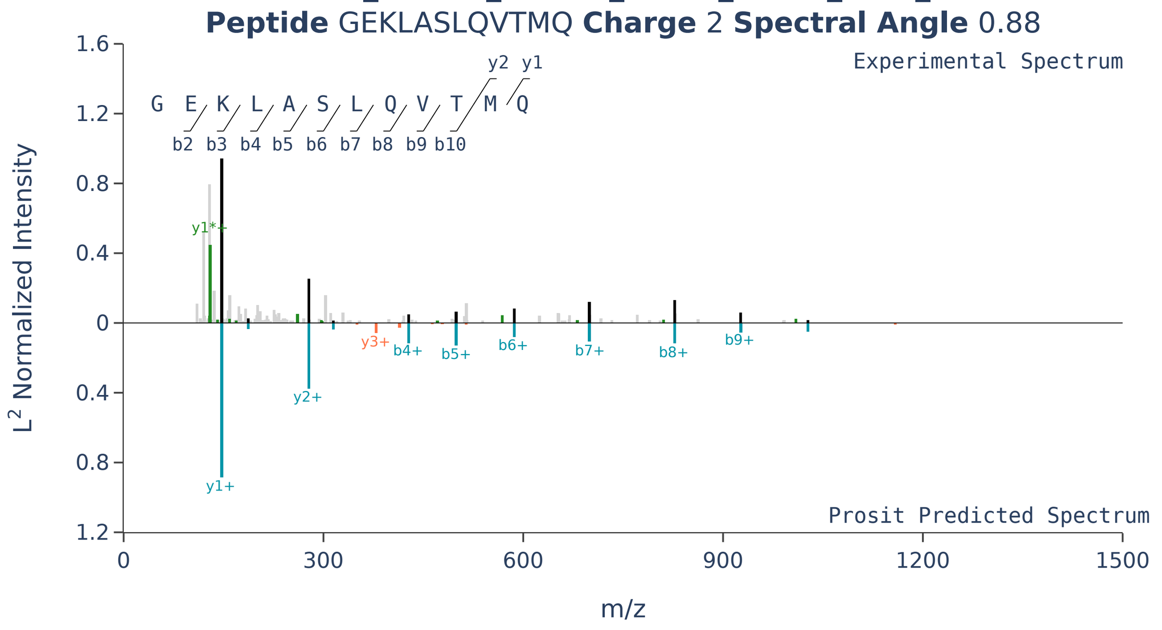

Source HRoetschke_271222_090123_HFGoe_LEDGF_b1R1_4h **Scan** 21258
Peptide SLAKIDNLDVNRCIE **Charge** 3 **Spectral Angle** 0.75

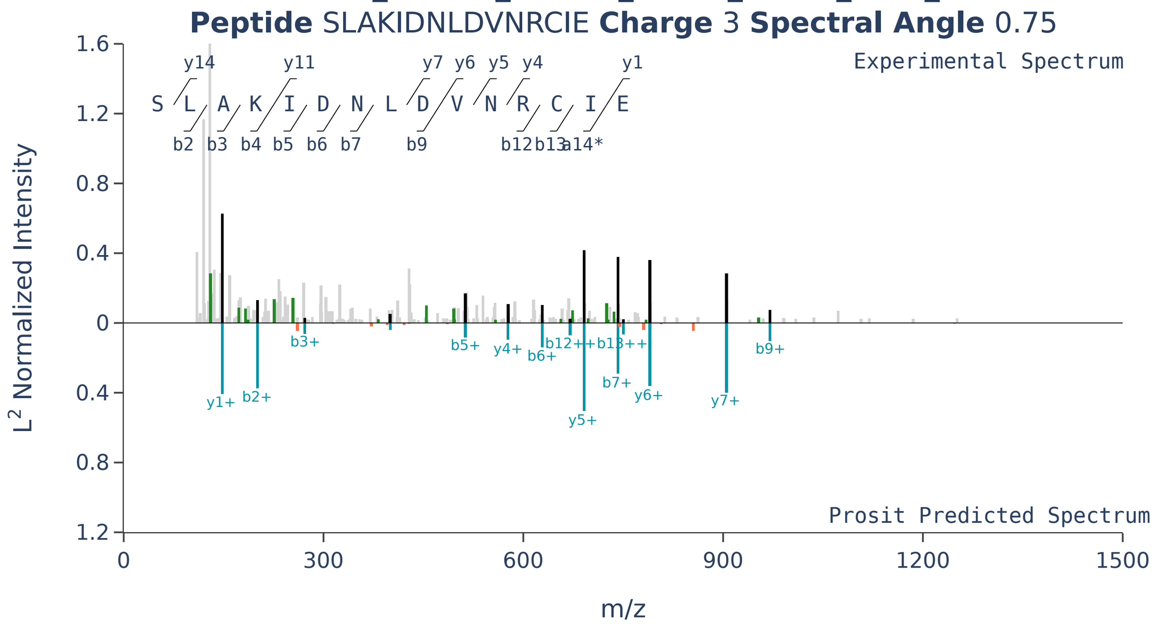

Source HRoetschke_271222_090123_HFGoe_LEDGF_b1R1_4h **Scan** 18073
Peptide GPKDIFIFPYSENKEKYGKPNKR **Charge** 4 **Spectral Angle** 0.79

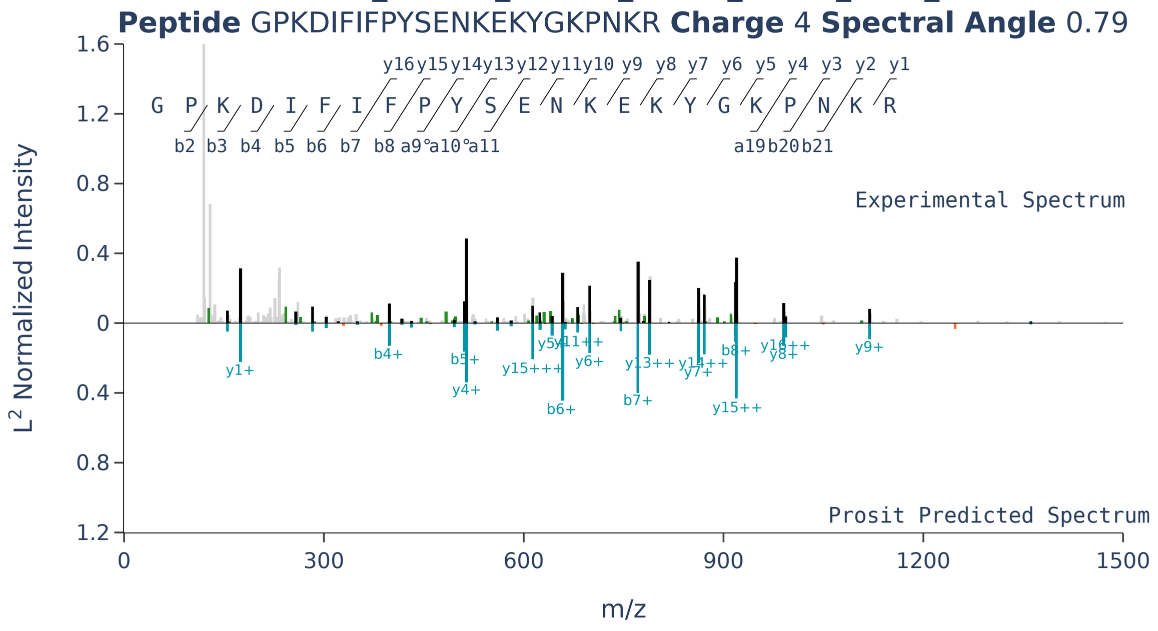

Source HRoetschke_271222_090123_HFGoe_LEDGF_b1R1_4h **Scan** 9807
Peptide FGTHETTRDFKPGD **Charge** 4 **Spectral Angle** 0.84

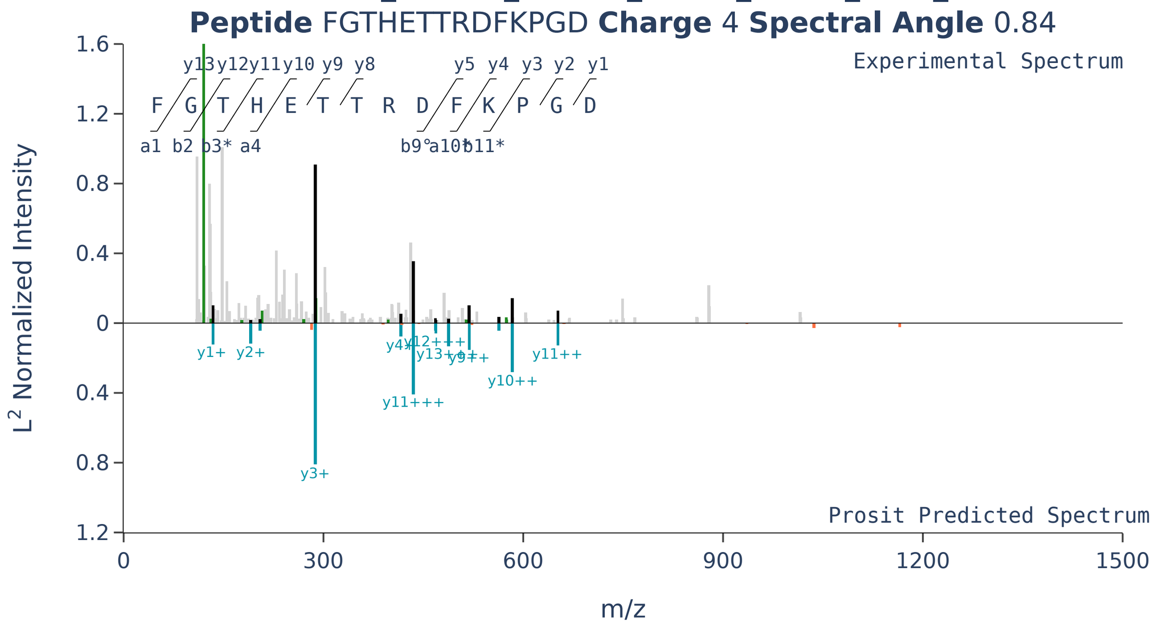

Source HRoetschke_271222_090123_HFGoe_LEDGF_b1R2_24h **Scan** 16143
Peptide AQDVPDGAVKPPTNKL **Charge** 2 **Spectral Angle** 0.93

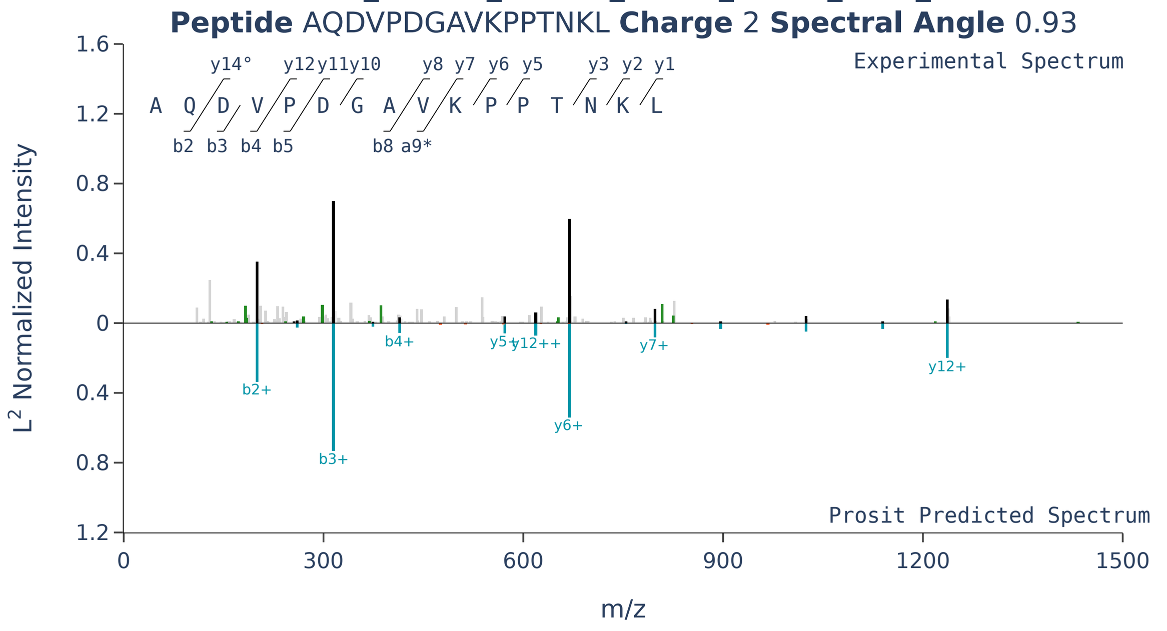

Source HRoetschke_271222_090123_HFGoe_LEDGF_b1R2_24h **Scan** 6733
Peptide FPYSENKEKYGKPNRKA **Charge** 5 **Spectral Angle** 0.87

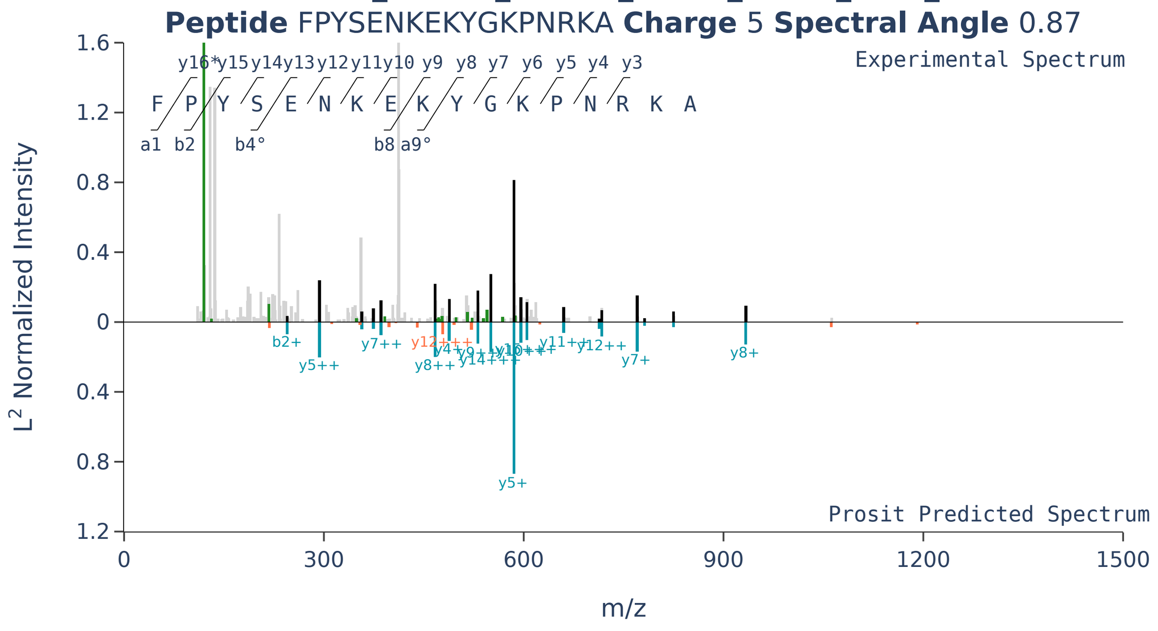

Source HRoetschke_271222_090123_HFGoe_LEDGF_b1R1_2h **Scan** 15410
Peptide GTHETYPHWPARVD **Charge** 3 **Spectral Angle** 0.93

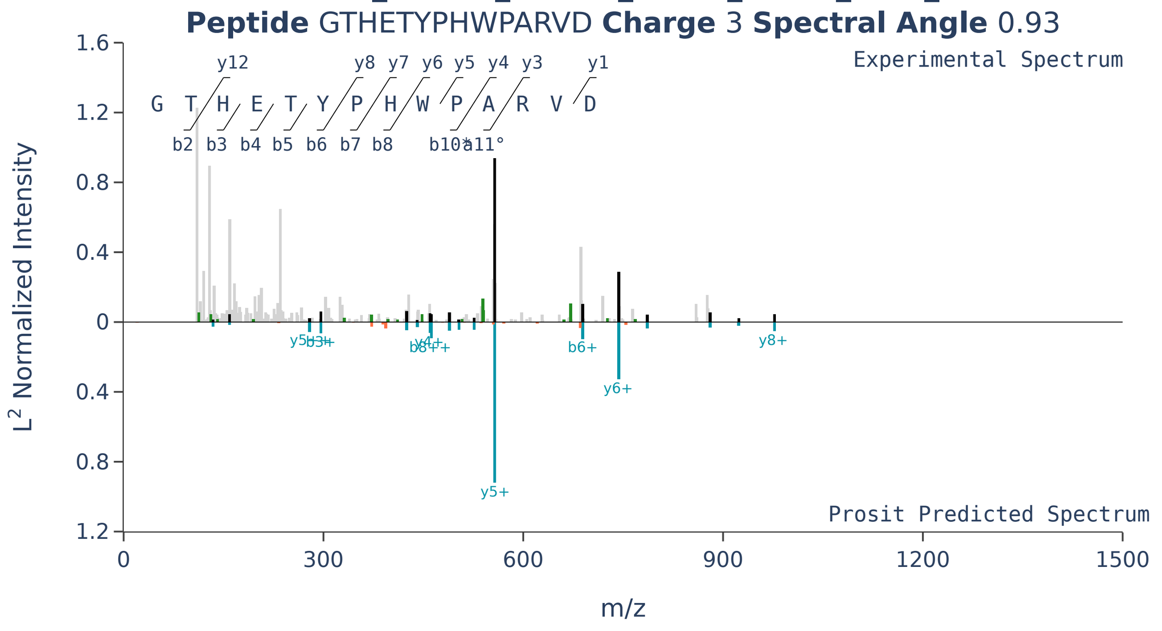

Source HRoetschke_271222_090123_HFGoe_LEDGF_b2R1_24h **Scan** 22597
Peptide EGKLASLQVTMQQA **Charge** 2 **Spectral Angle** 0.82

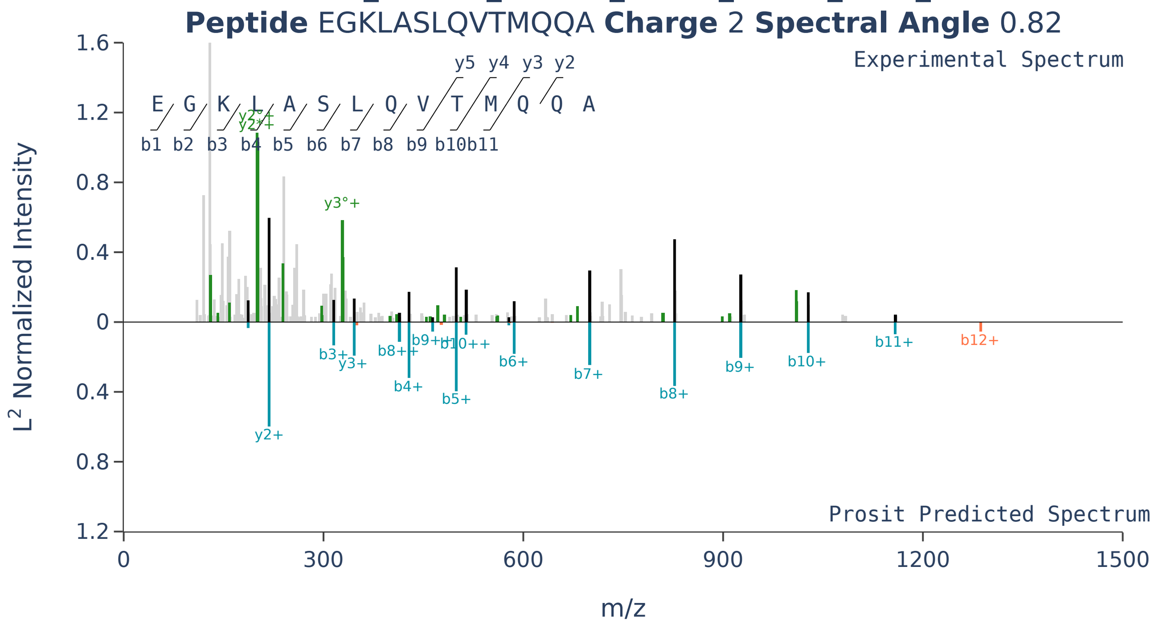

Source HRoetschke_271222_090123_HFGoe_LEDGF_b2R1_2h **Scan** 17574
Peptide STAFLGPKDIFPYSENKEKYGKPNKR **Charge** 5 **Spectral Angle** 0.77

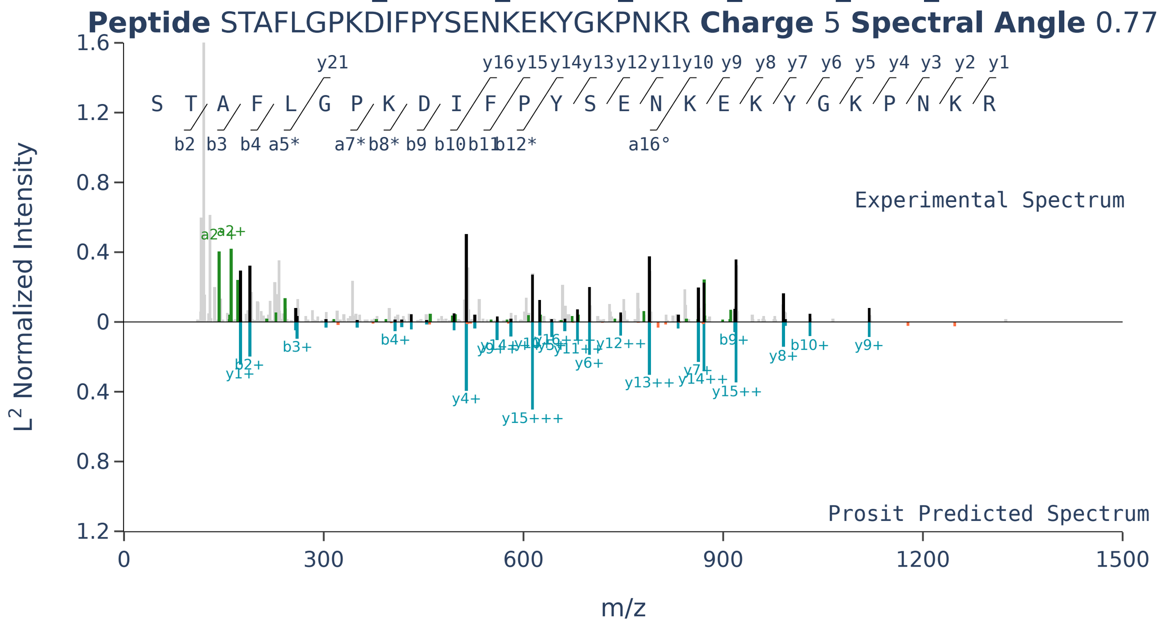

Source HRoetschke_271222_090123_HFGoe_LEDGF_b2R2_2h **Scan** 18885
Peptide VGYPHWPARVDE **Charge** 3 **Spectral Angle** 0.84

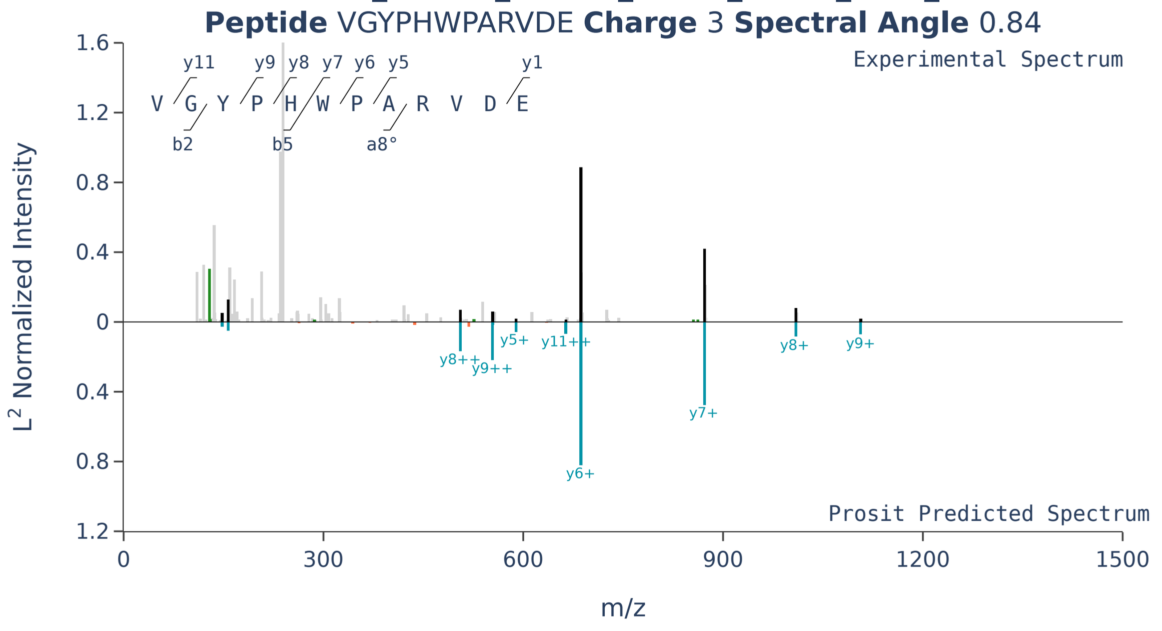

Source HRoetschke_271222_090123_HFGoe_LEDGF_b2R1_24h **Scan** 15682
Peptide GGSDVPDGAVKPPTNKL **Charge** 3 **Spectral Angle** 0.74

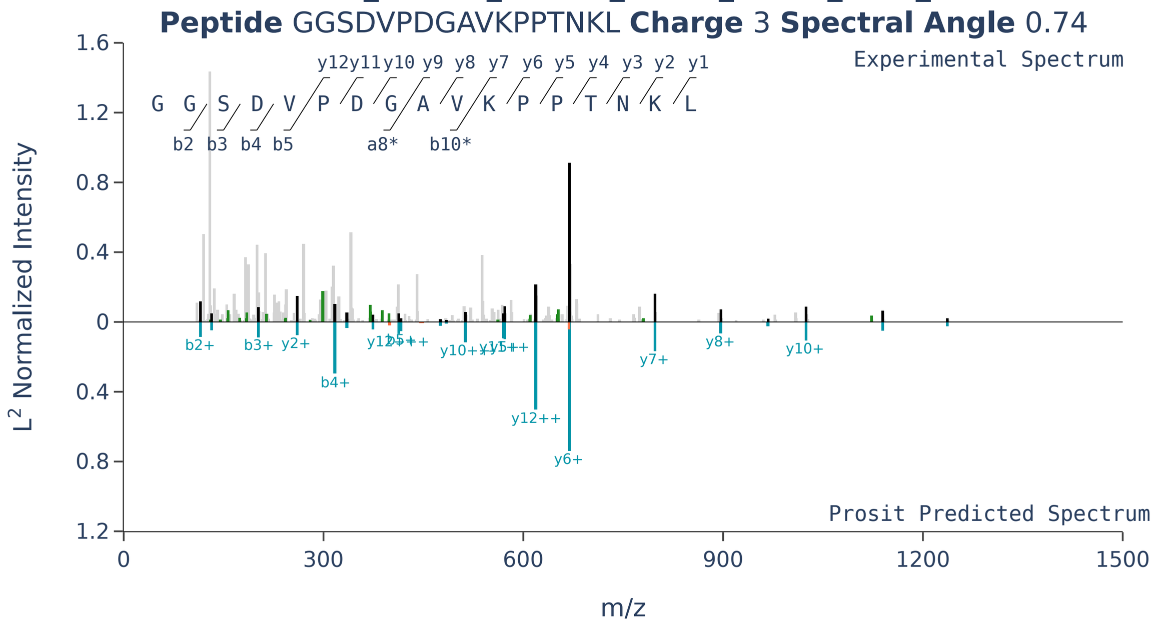

Source HRoetschke_271222_090123_HFGoe_LEDGF_b1R2_2h **Scan** 23194
Peptide EPMTRDFKPGDLIFAKMK **Charge** 5 **Spectral Angle** 0.88

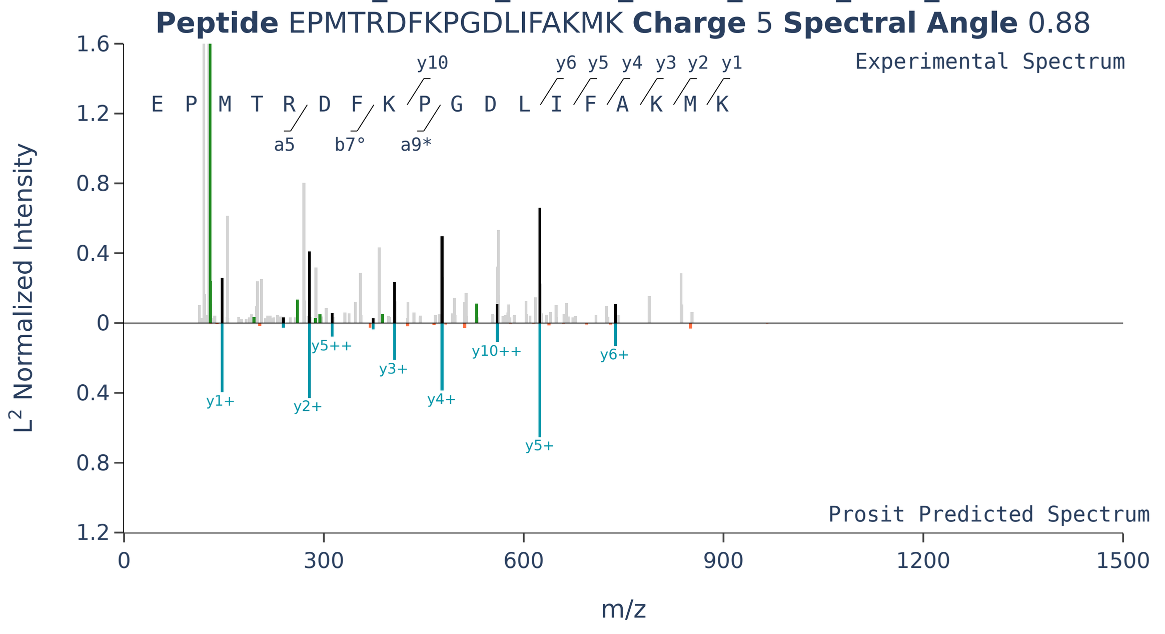

Source HRoetschke_271222_090123_HFGoe_LEDGF_b1R2_1h **Scan** 6581
Peptide SVITQQAQKH **Charge** 3 **Spectral Angle** 0.88

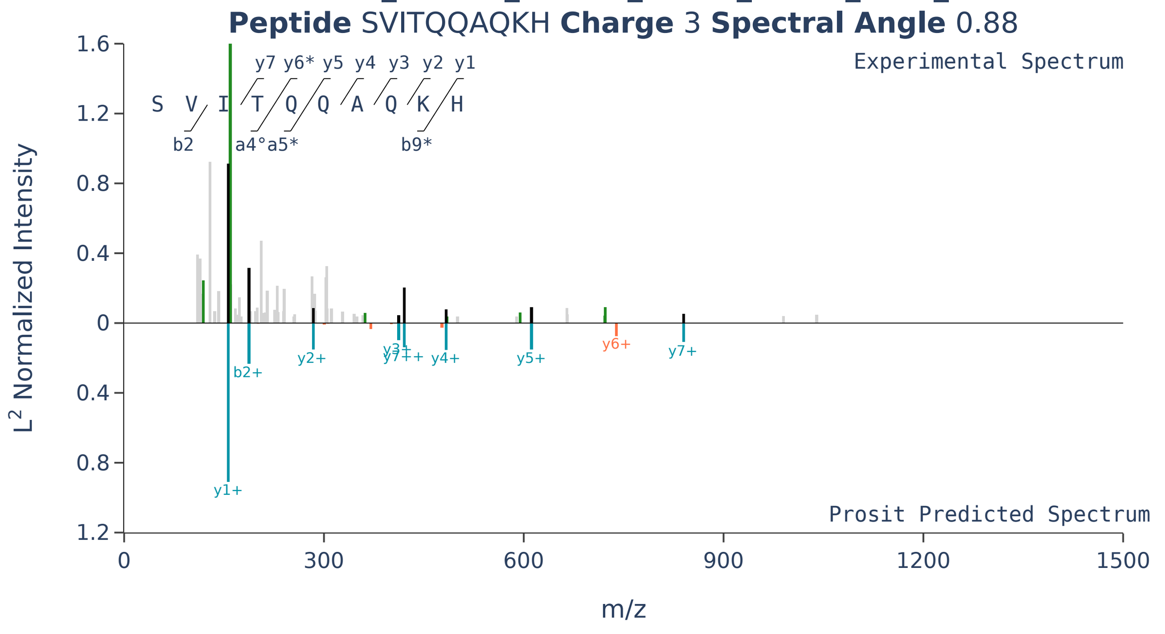

Source HRoetschke_271222_090123_HFGoe_LEDGF_b2R2_2h **Scan** 17706
Peptide ATYPHWPARVDE **Charge** 3 **Spectral Angle** 0.86

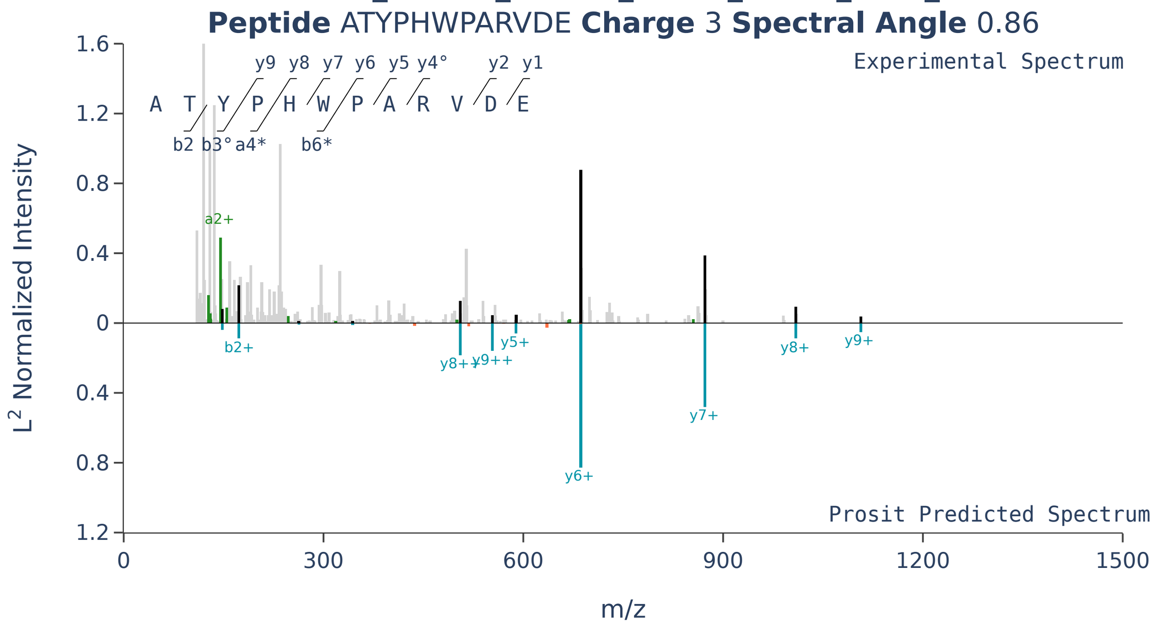

Source HRoetschke_271222_090123_HFGoe_LEDGF_b1R1_4h **Scan** 19007
Peptide AFLGPKDIFPYSENKEKYGKPNKRLE **Charge** 4 **Spectral Angle** 0.85

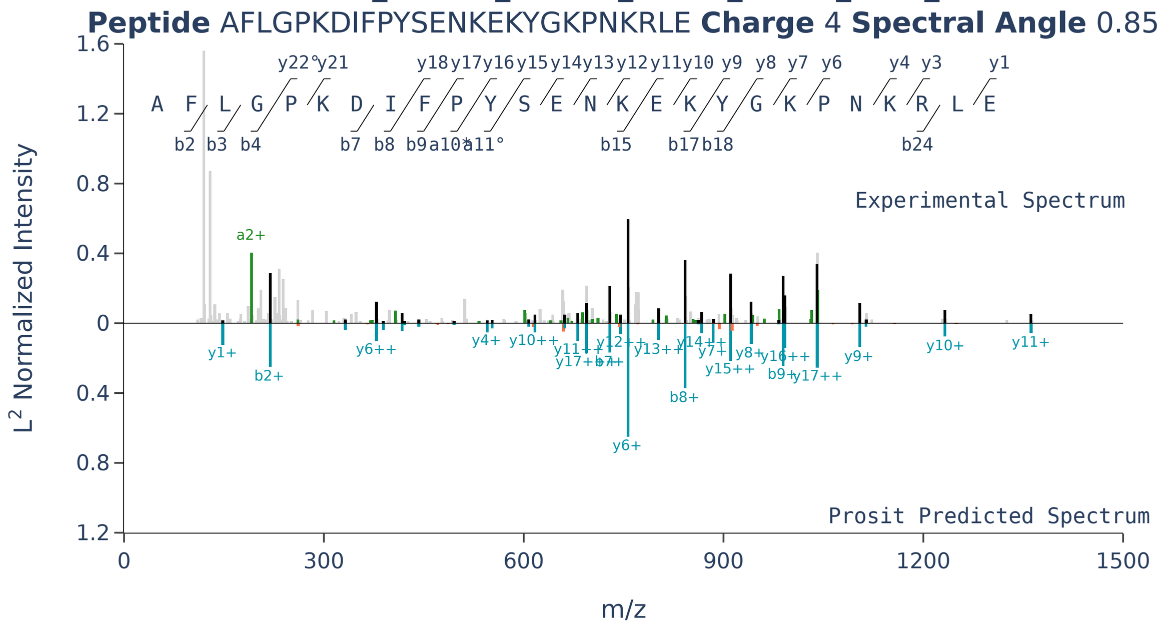

Source HRoetschke_271222_090123_HFGoe_LEDGF_b2R2_24h **Scan** 18882
Peptide VGFLGPKDIFPYSENKEKYGKPNKR **Charge** 5 **Spectral Angle** 0.74

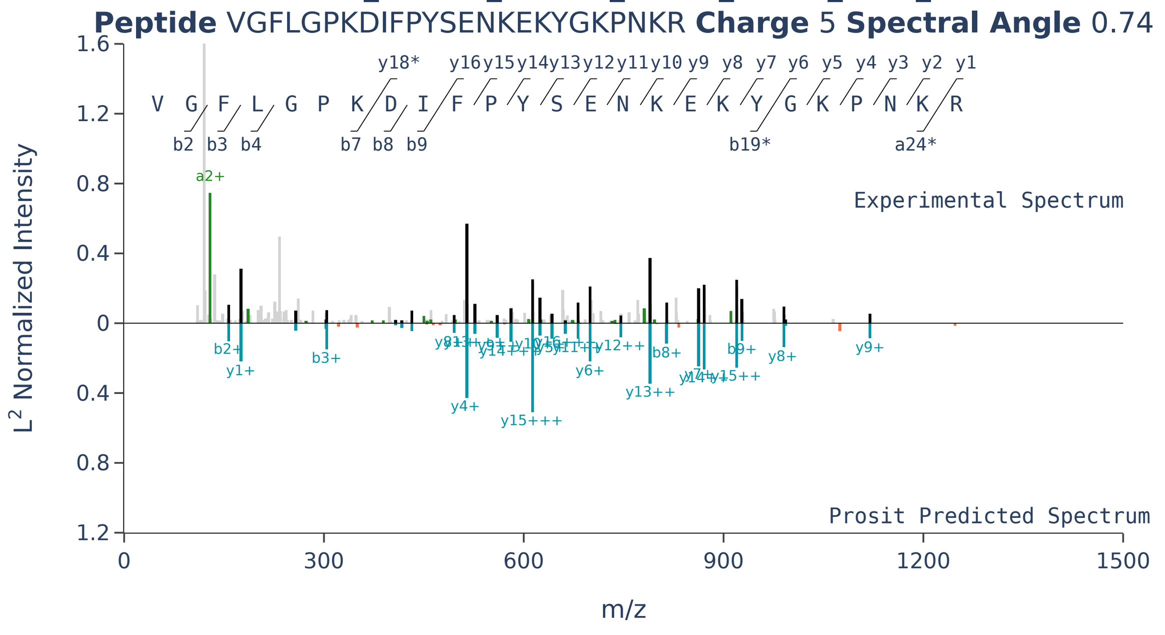

Source HRoetschke_271222_090123_HFGoe_LEDGF_b1R2_2h **Scan** 16239
Peptide GPGMTGPGMTRDFKPGD **Charge** 3 **Spectral Angle** 0.78

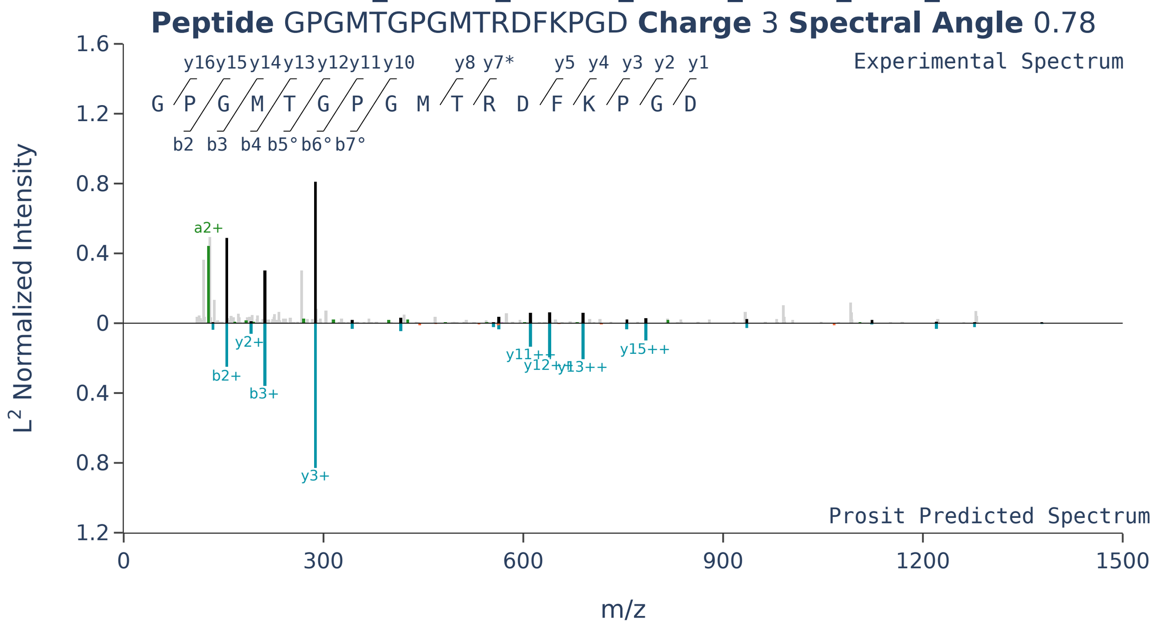

Source HRoetschke_271222_090123_HFGoe_LEDGF_b1R1_4h **Scan** 16428
Peptide QVETEVTTATASVNLKVSPKRGRPAAT **Charge** 4 **Spectral Angle** 0.82

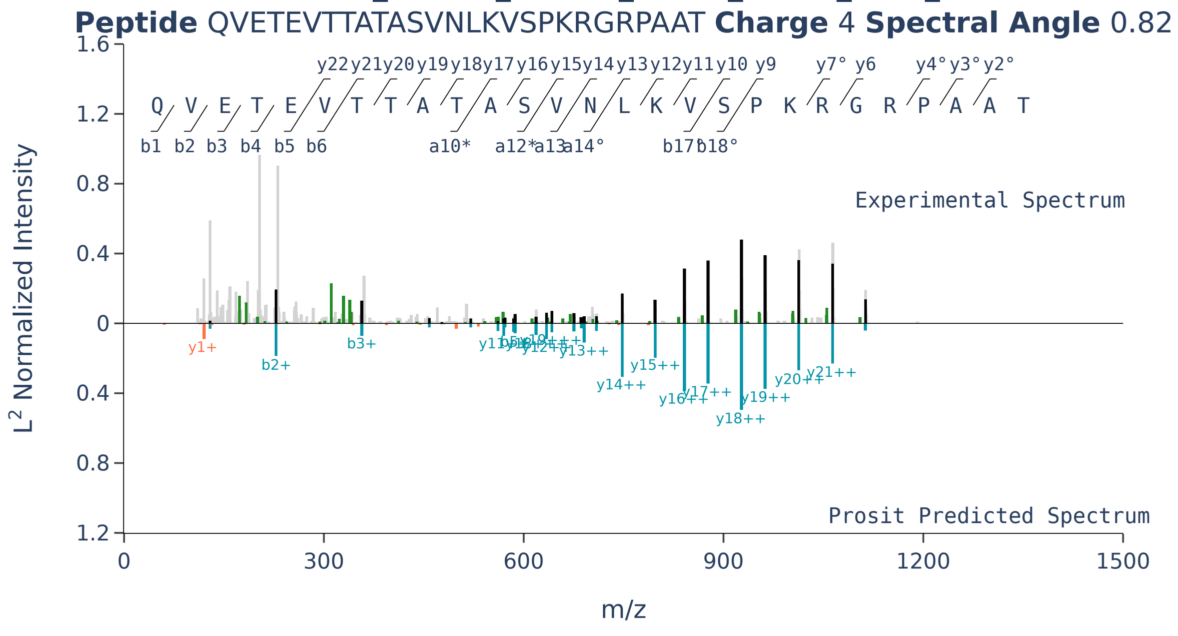

Source HRoetschke_271222_090123_HFGoe_LEDGF_b1R2_24h **Scan** 10824
Peptide NASSDVEVEEG **Charge** 1 **Spectral Angle** 0.73

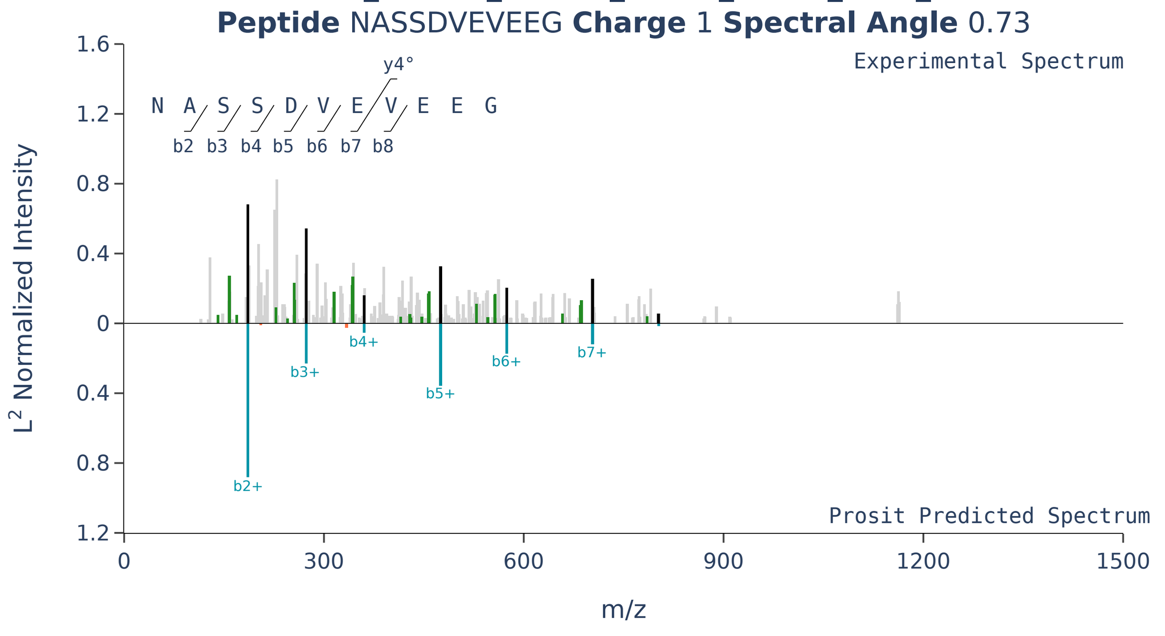

Source HRoetschke_271222_090123_HFGoe_LEDGF_b2R1_2h **Scan** 21567
Peptide NKGFNEGLWE **Charge** 2 **Spectral Angle** 0.75

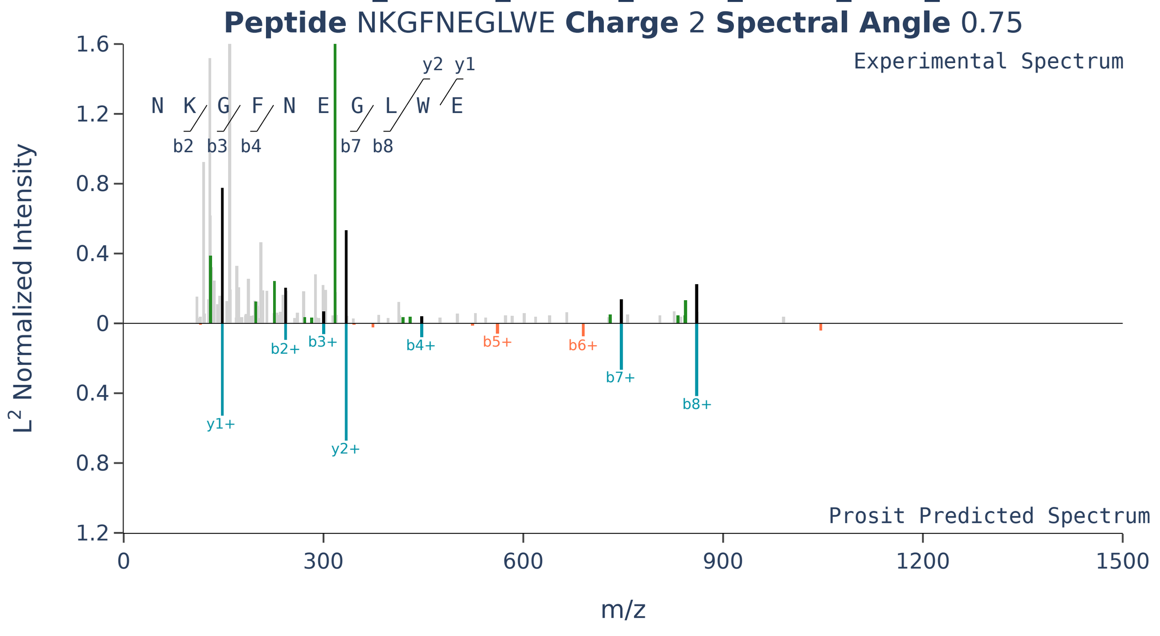

Source HRoetschke_271222_090123_HFGoe_LEDGF_b1R1_24h **Scan** 17347
Peptide SQFLGPKDIFPYSENKEKYGKPNKR **Charge** 5 **Spectral Angle** 0.77

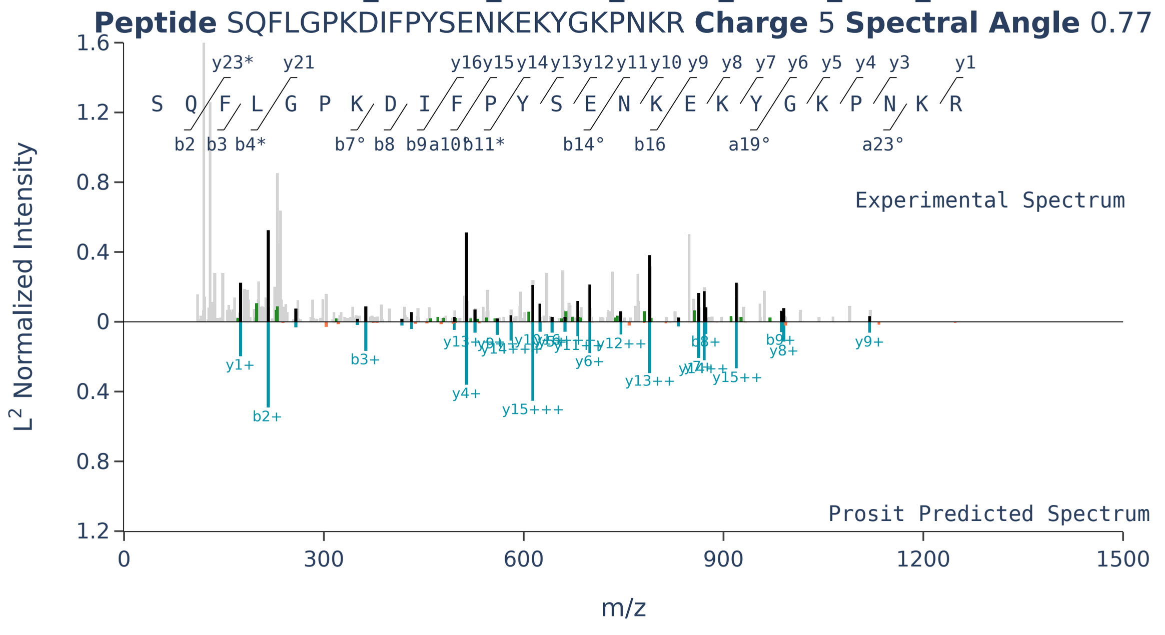

Source HRoetschke_271222_090123_HFGoe_LEDGF_b1R1_24h **Scan** 12126
Peptide ETSMDSRLQRIHAA **Charge** 3 **Spectral Angle** 0.83

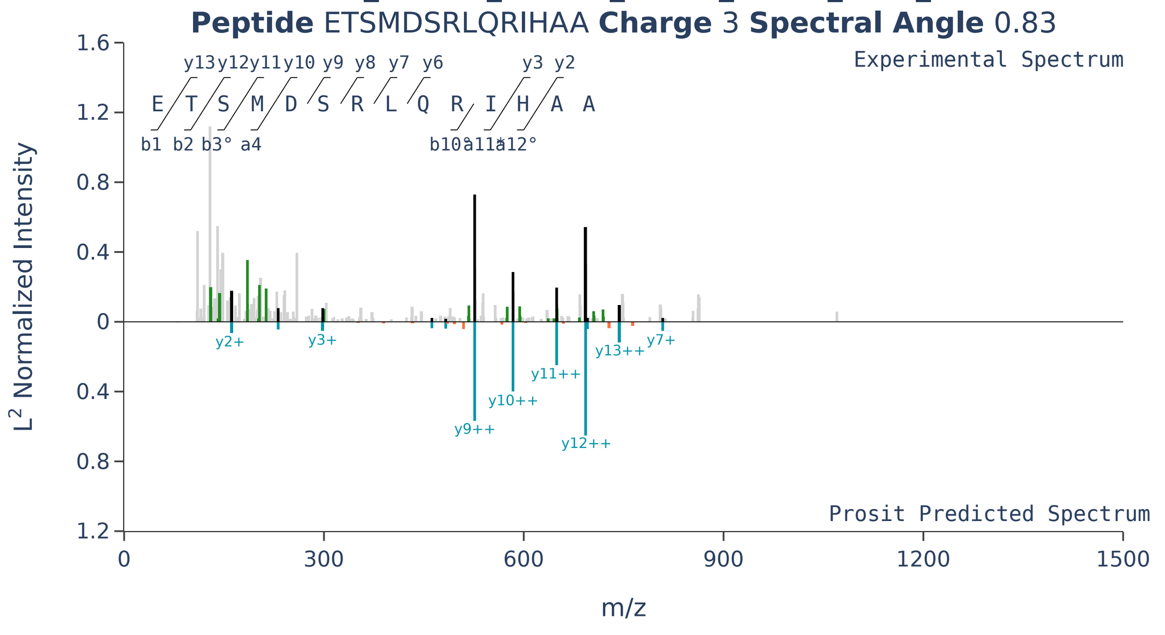

Source HRoetschke_271222_090123_HFGoe_LEDGF_b2R1_24h **Scan** 9526
Peptide ESKPPTNK **Charge** 2 **Spectral Angle** 0.86

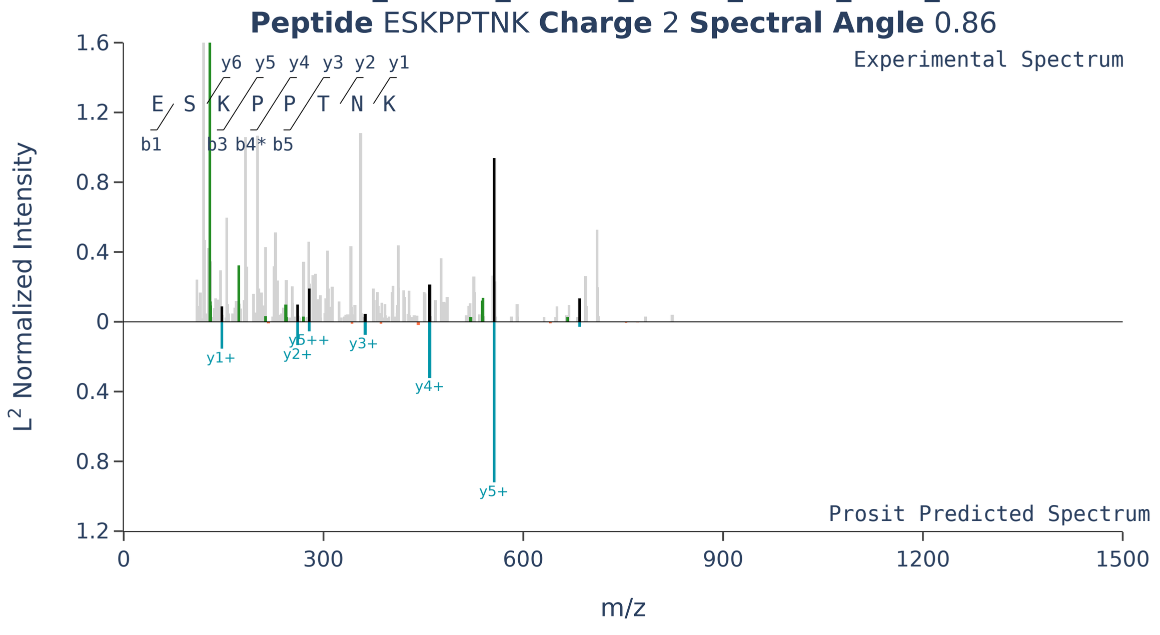

Source HRoetschke_271222_090123_HFGoe_LEDGF_b1R2_24h **Scan** 18399
Peptide TASFLGPKDIFPYSENKEKYGKPNKR **Charge** 5 **Spectral Angle** 0.77

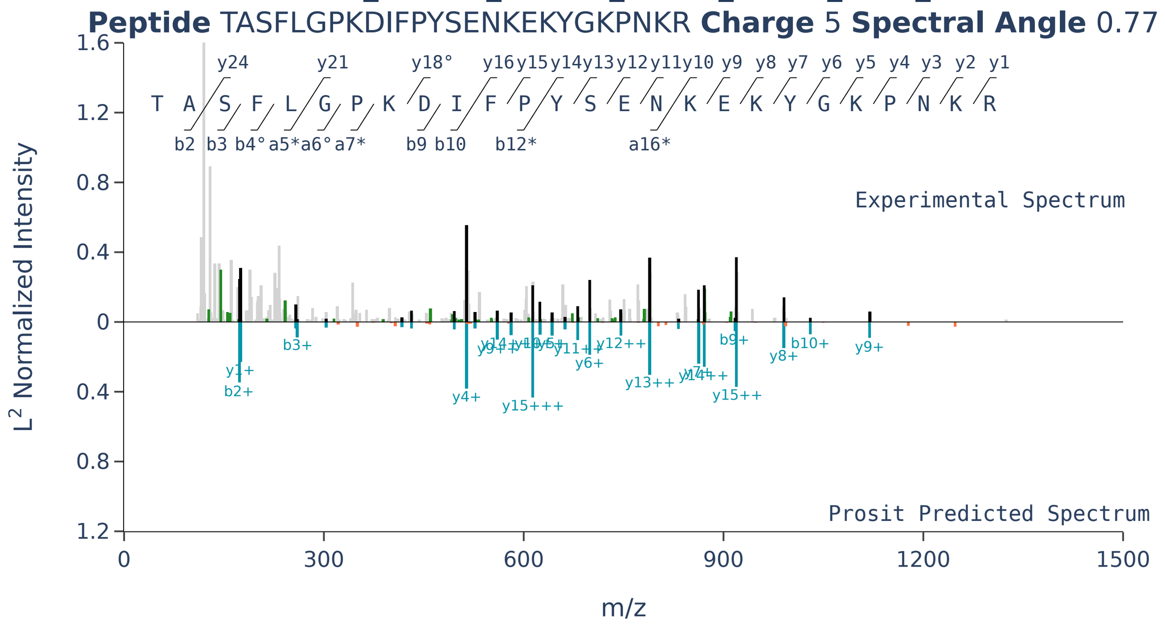

Source HRoetschke_271222_090123_HFGoe_LEDGF_b1R1_1h **Scan** 17401
Peptide TAFLGPKDIFPYSENKEKYGKPNKRAT **Charge** 5 **Spectral Angle** 0.83

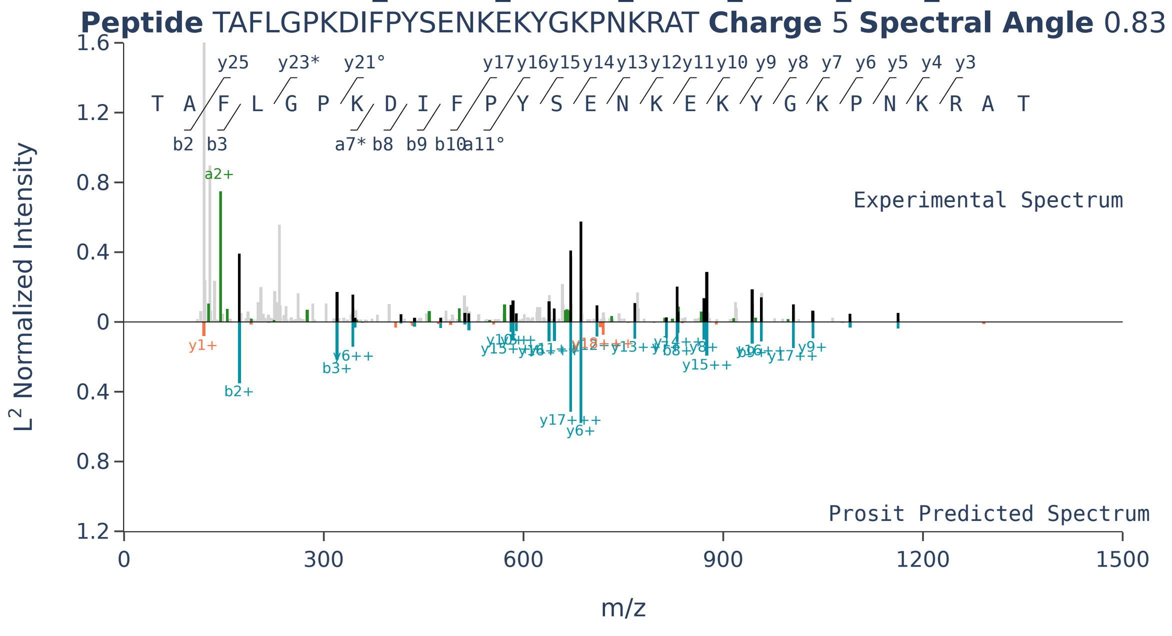

Source HRoetschke_271222_090123_HFGoe_LEDGF_b2R1_24h **Scan** 22356
Peptide GSKTFNEGLWE **Charge** 2 **Spectral Angle** 0.78

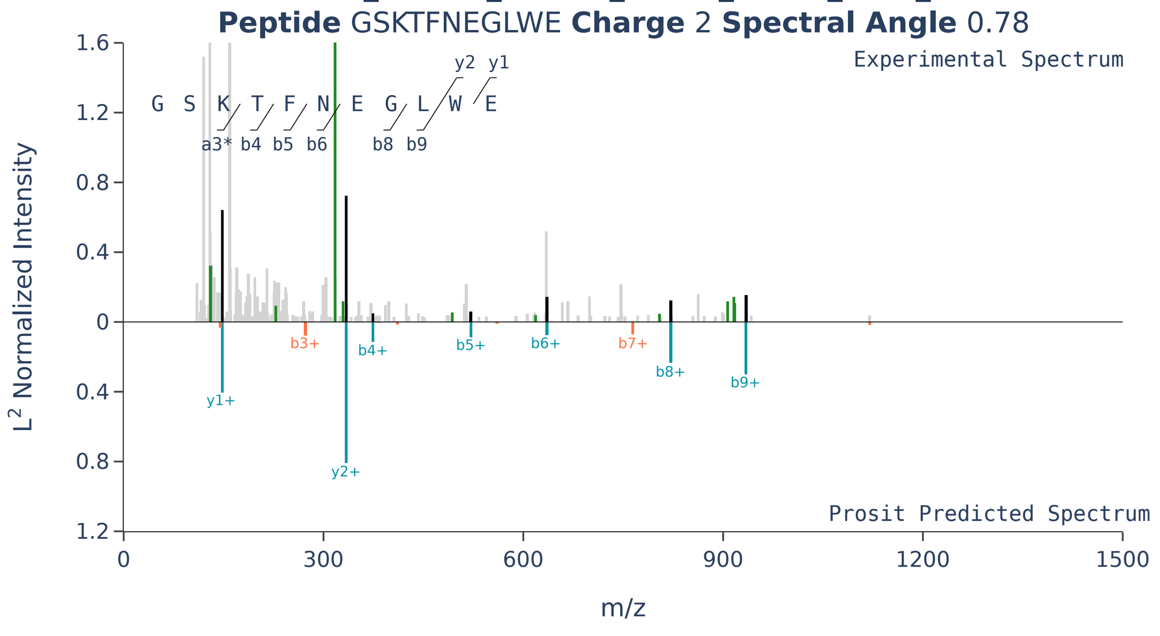

Source HRoetschke_271222_090123_HFGoe_LEDGF_b1R2_24h **Scan** 19279
Peptide EYPHWPARV **Charge** 3 **Spectral Angle** 0.88

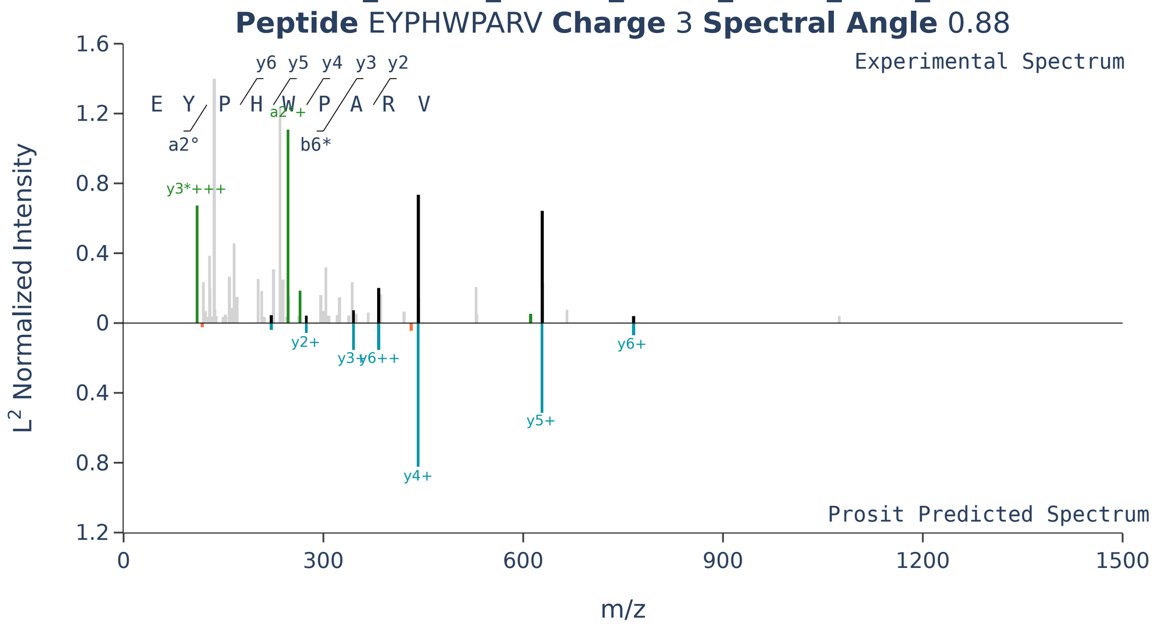

Source HRoetschke_271222_090123_HFGoe_LEDGF_b2R1_24h **Scan** 17050
Peptide FLGPKDIFPYSENKEKYGKPNKRAT **Charge** 4 **Spectral Angle** 0.86

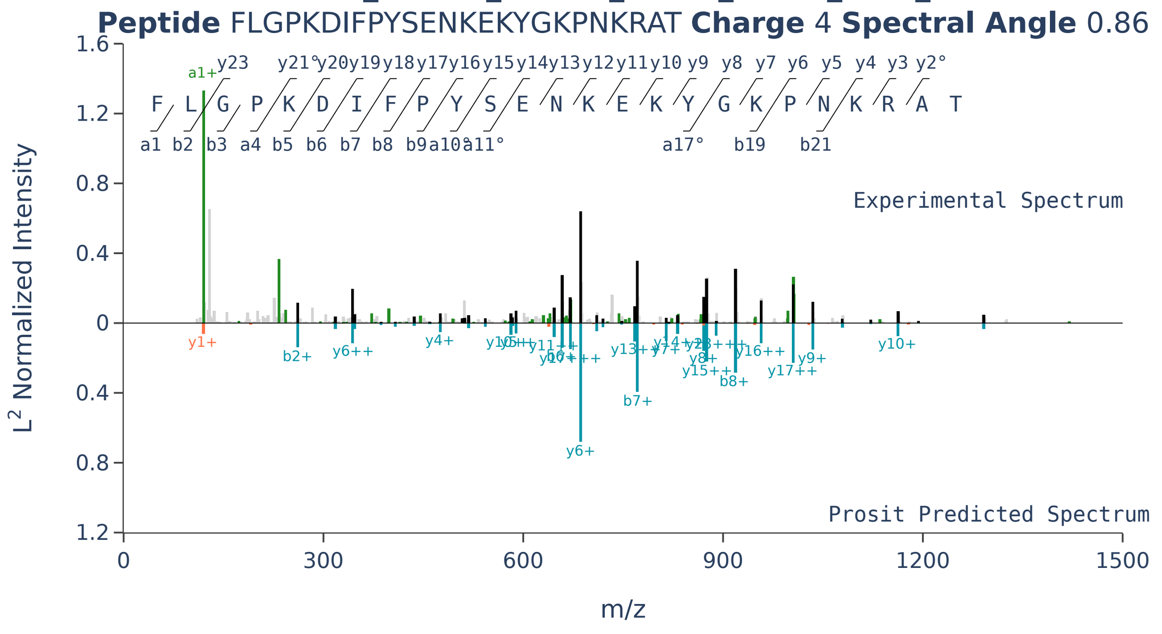

Source HRoetschke_271222_090123_HFGoe_LEDGF_b1R2_2h **Scan** 17307
Peptide AFLGPKDIFPYSENKEKYGKPNKRKFG **Charge** 5 **Spectral Angle** 0.83

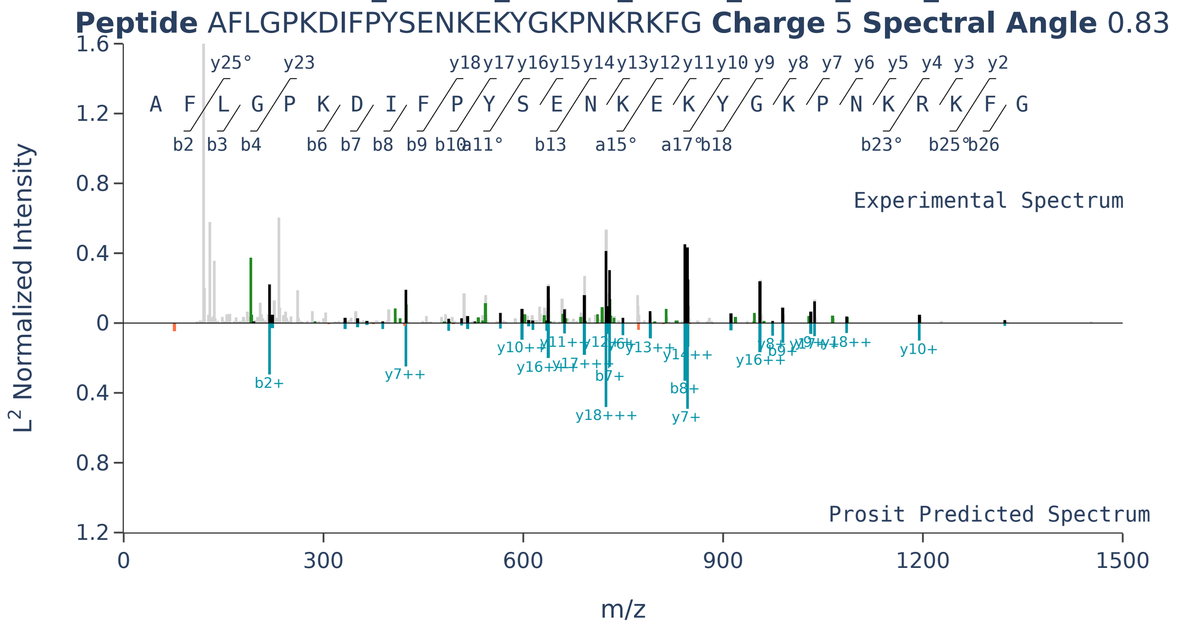

Source HRoetschke_271222_090123_HFGoe_LEDGF_b2R2_2h **Scan** 22422
Peptide VGELASLQVTMQQAQKH **Charge** 3 **Spectral Angle** 0.79

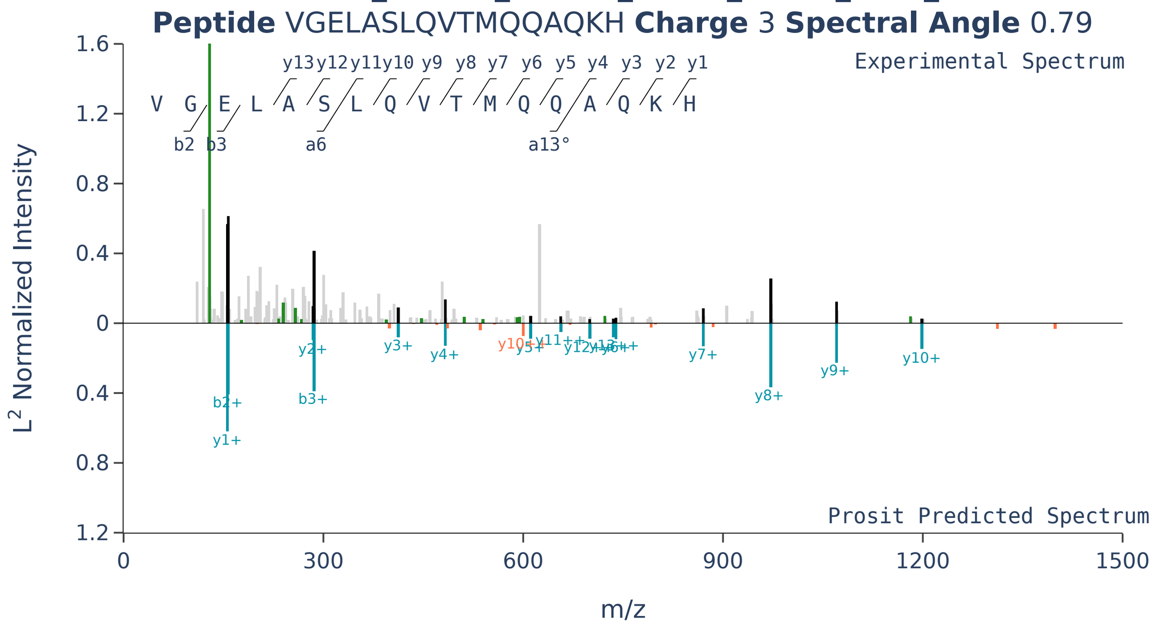

Source HRoetschke_271222_090123_HFGoe_LEDGF_b1R2_24h **Scan** 8547
Peptide HTAQKHTEMI **Charge** 3 **Spectral Angle** 0.81

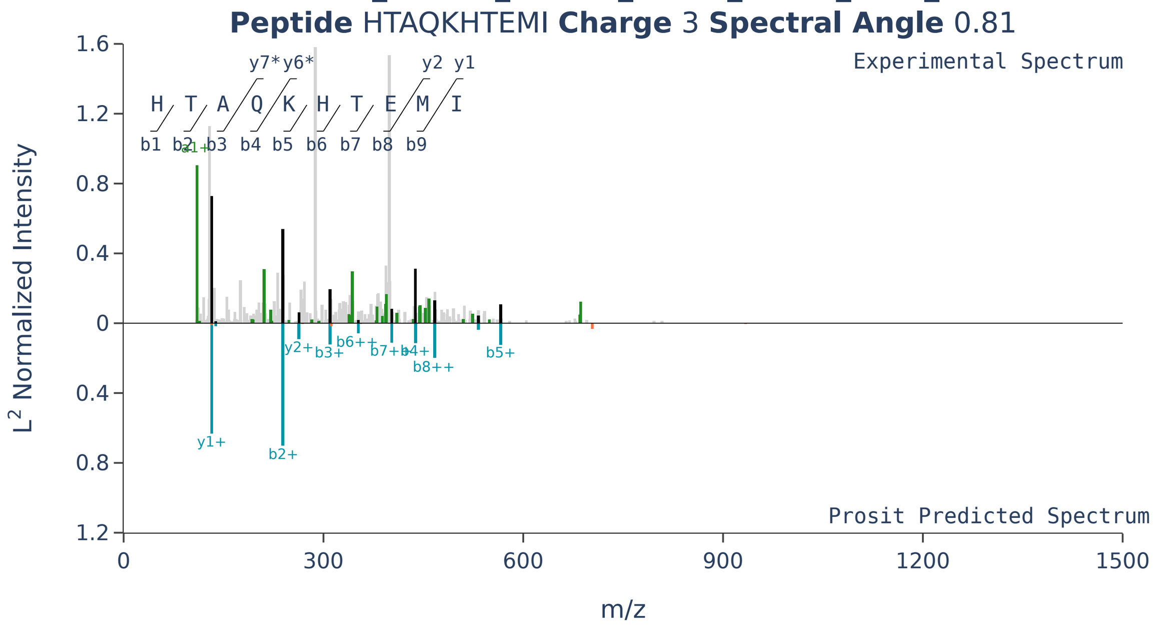

Source HRoetschke_271222_090123_HFGoe_LEDGF_b1R2_24h **Scan** 13193
Peptide FLVGKETSVSKED **Charge** 2 **Spectral Angle** 0.85

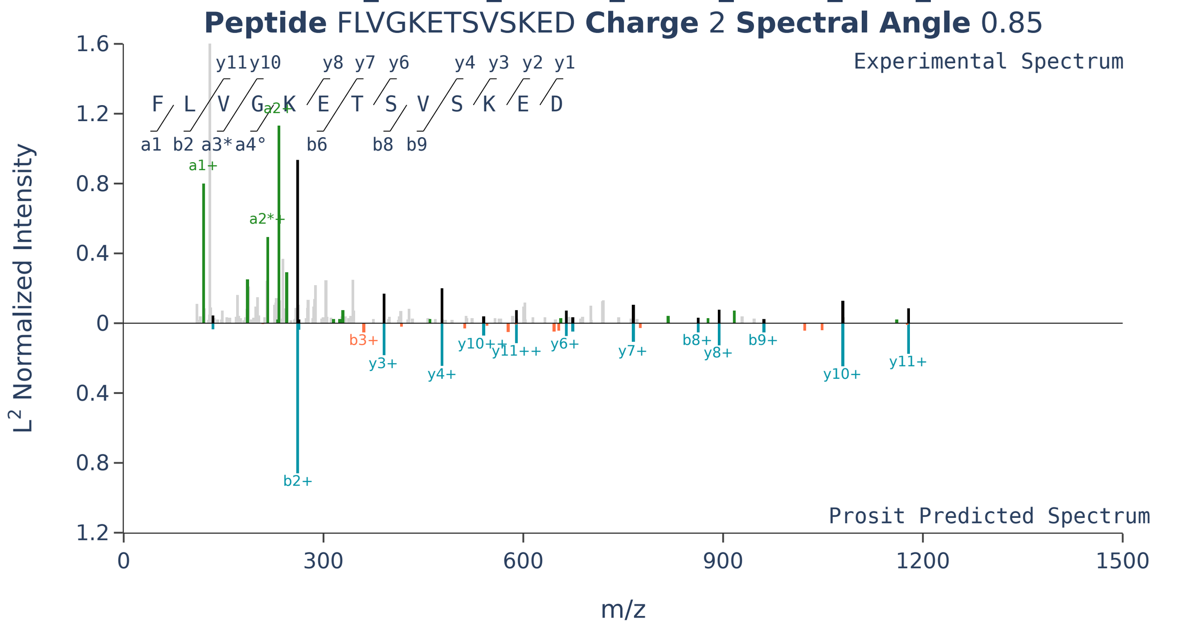

Source HRoetschke_271222_090123_HFGoe_LEDGF_b1R1_24h **Scan** 16179
Peptide AQQAQKHTEMITTL **Charge** 3 **Spectral Angle** 0.82

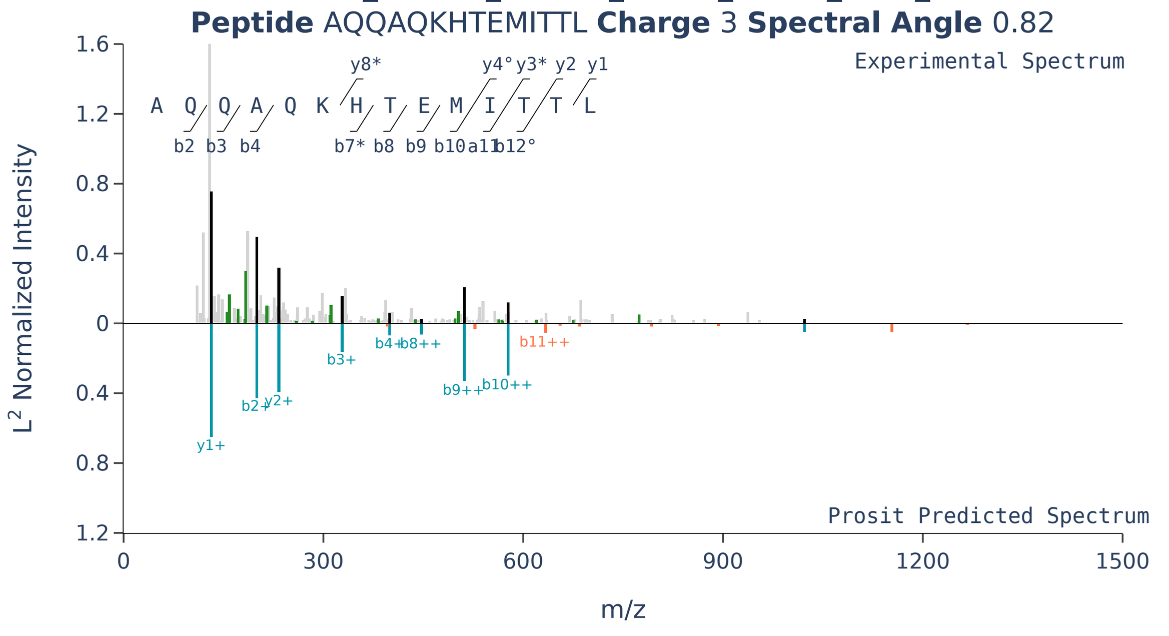

Source HRoetschke_271222_090123_HFGoe_LEDGF_b1R2_24h **Scan** 21246
Peptide GSLKIDNLDVNRCIE **Charge** 3 **Spectral Angle** 0.75

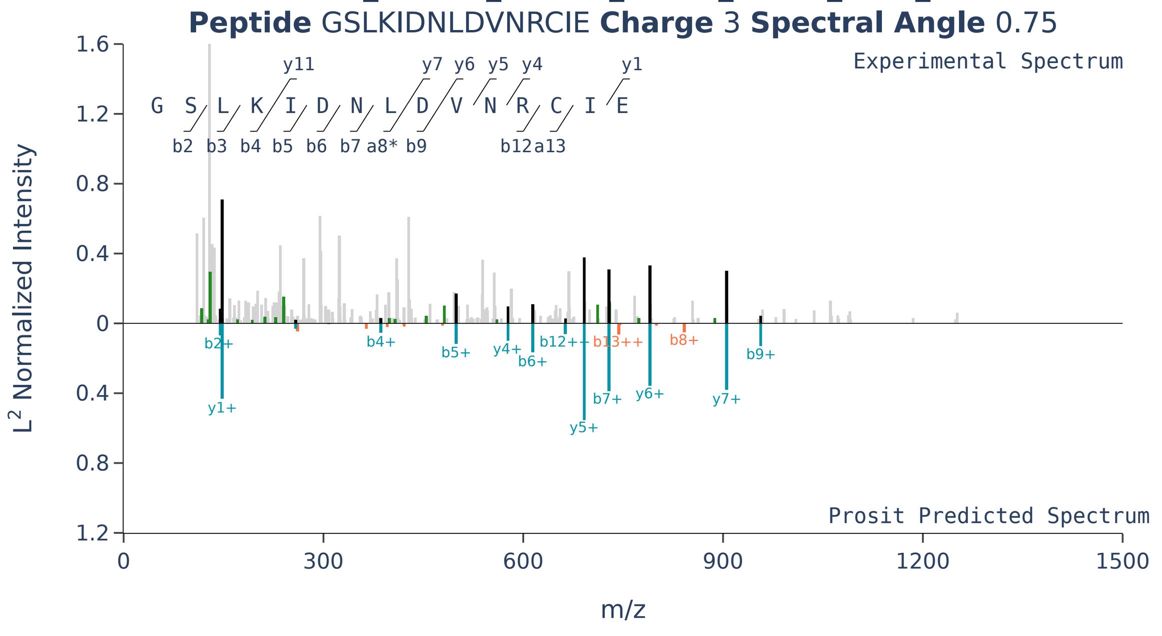

Source HRoetschke_271222_090123_HFGoe_LEDGF_b1R1_2h **Scan** 22306
Peptide VTNEGLWE **Charge** 2 **Spectral Angle** 0.82

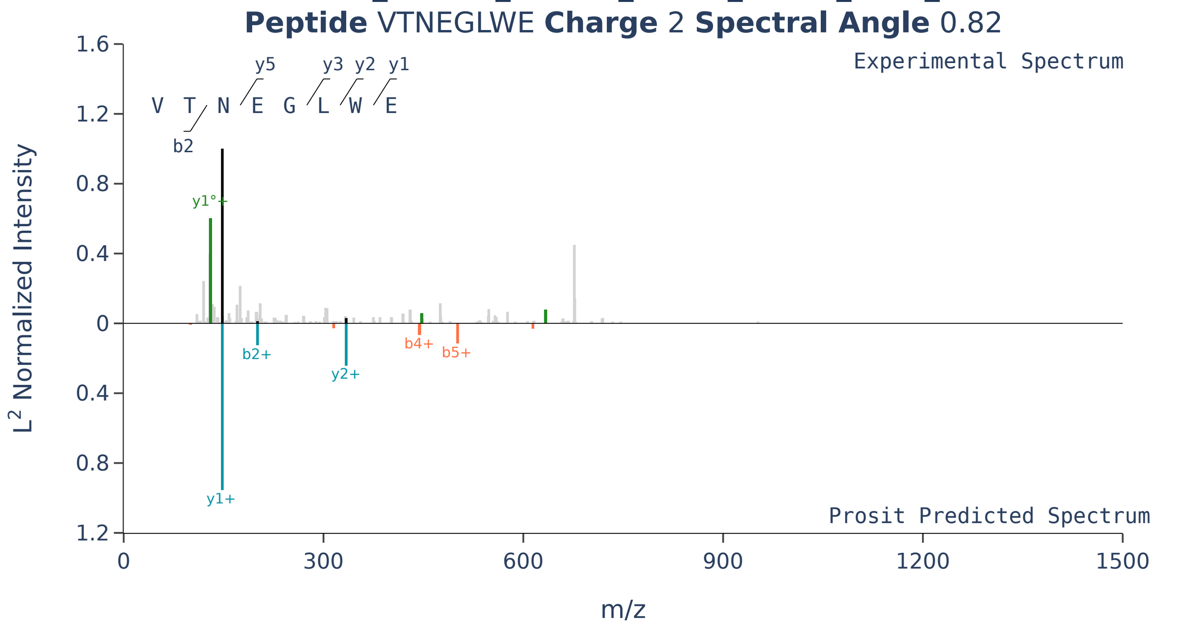

Source HRoetschke_271222_090123_HFGoe_LEDGF_b2R1_2h **Scan** 13226
Peptide VGGPGMTRDFKPGD **Charge** 3 **Spectral Angle** 0.82

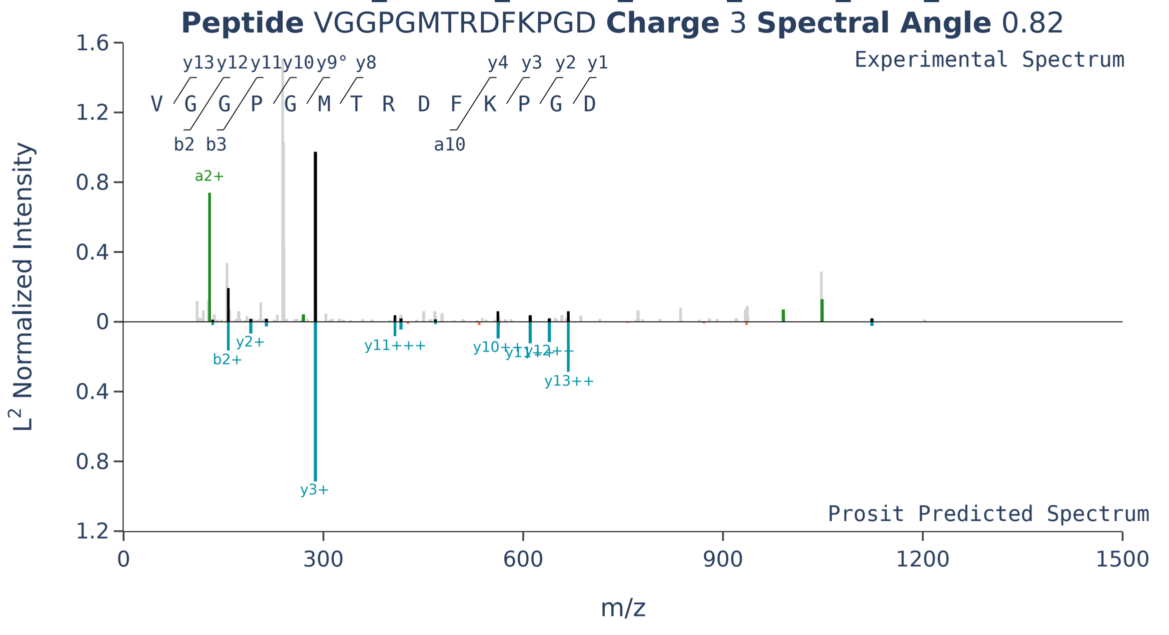

Source HRoetschke_271222_090123_HFGoe_LEDGF_b2R1_4h **Scan** 6824
Peptide FGTHETQQA **Charge** 2 **Spectral Angle** 0.74

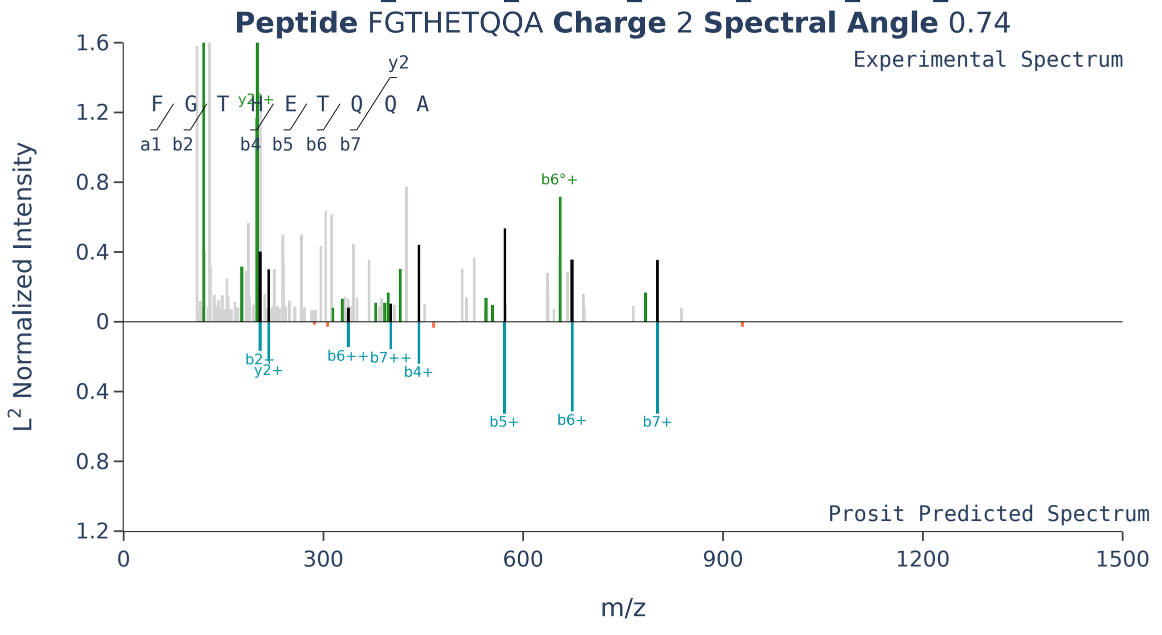

Source HRoetschke_271222_090123_HFGoe_LEDGF_b2R1_24h **Scan** 25472
Peptide AGVVTTATSLKIDNLDVNRCIE **Charge** 3 **Spectral Angle** 0.76

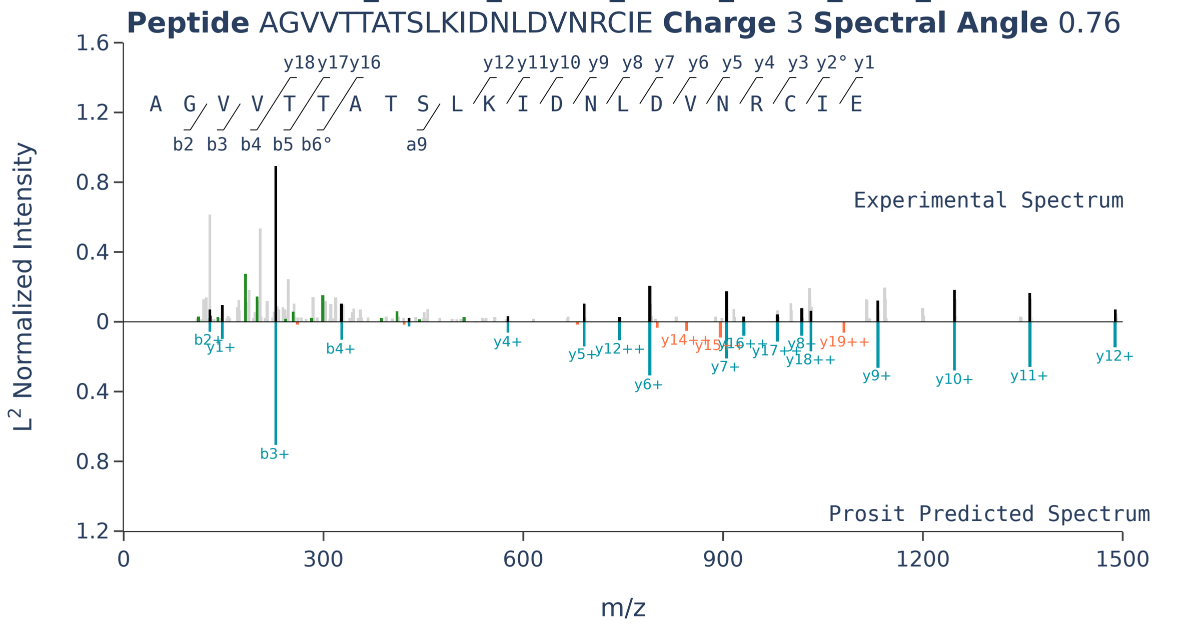

Source HRoetschke_271222_090123_HFGoe_LEDGF_b1R2_24h **Scan** 11696
Peptide ASMDSRLQRIHA **Charge** 3 **Spectral Angle** 0.74

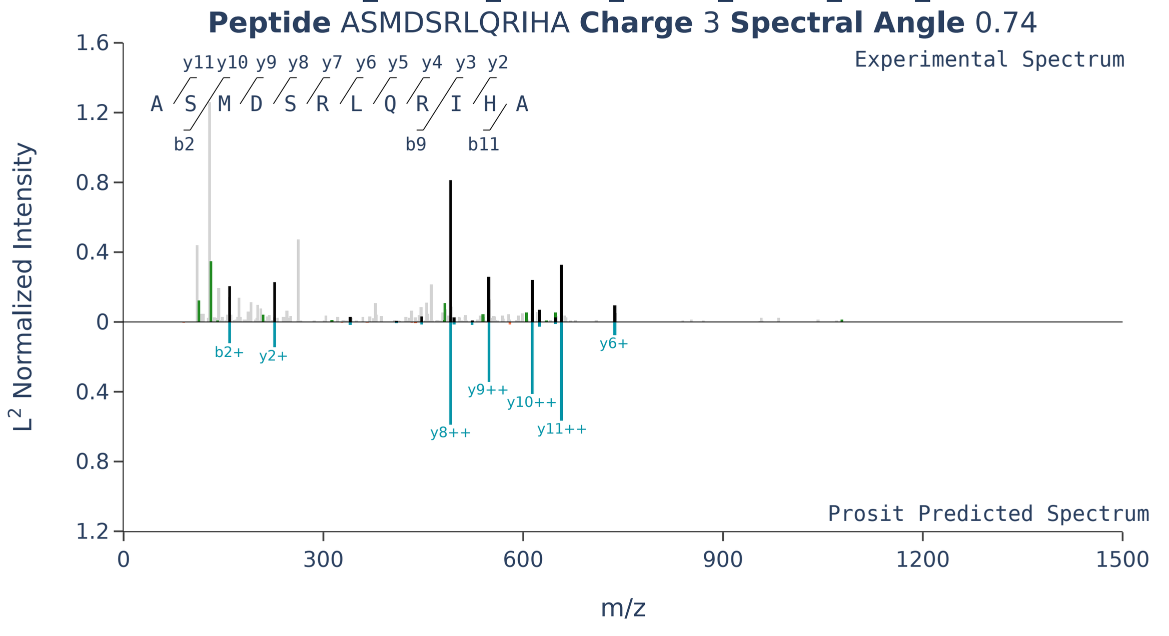

Source HRoetschke_271222_090123_HFGoe_LEDGF_b1R1_24h **Scan** 6117
Peptide RLQHAEIKN **Charge** 2 **Spectral Angle** 0.78

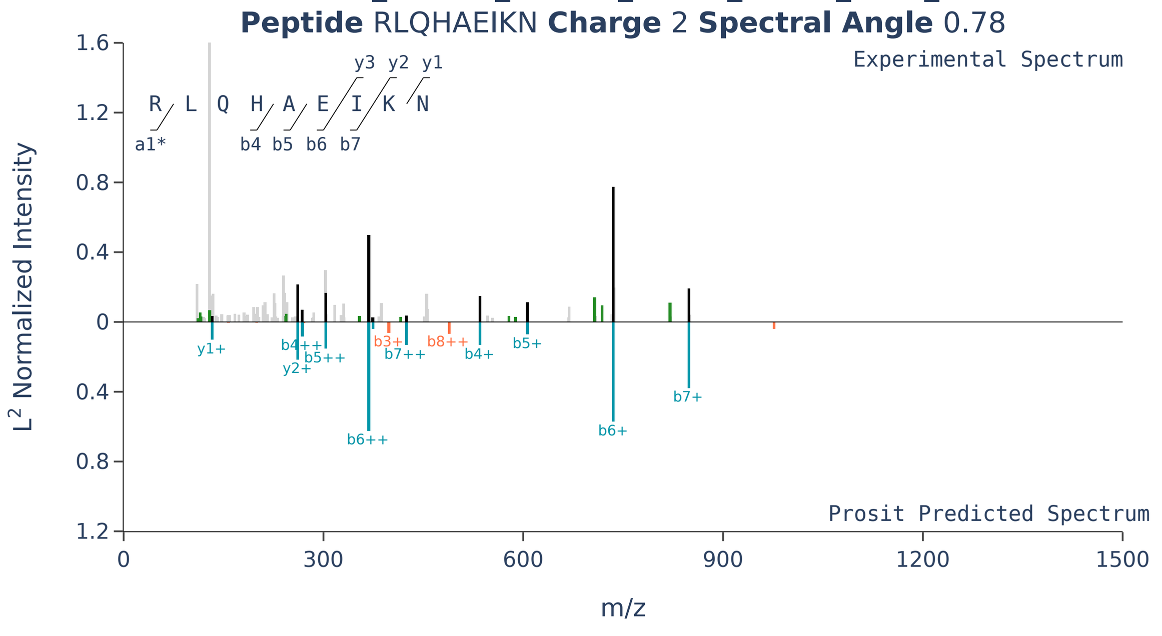

Source HRoetschke_271222_090123_HFGoe_LEDGF_b1R2_24h **Scan** 10132
Peptide QPQHNMITTLKK **Charge** 3 **Spectral Angle** 0.89

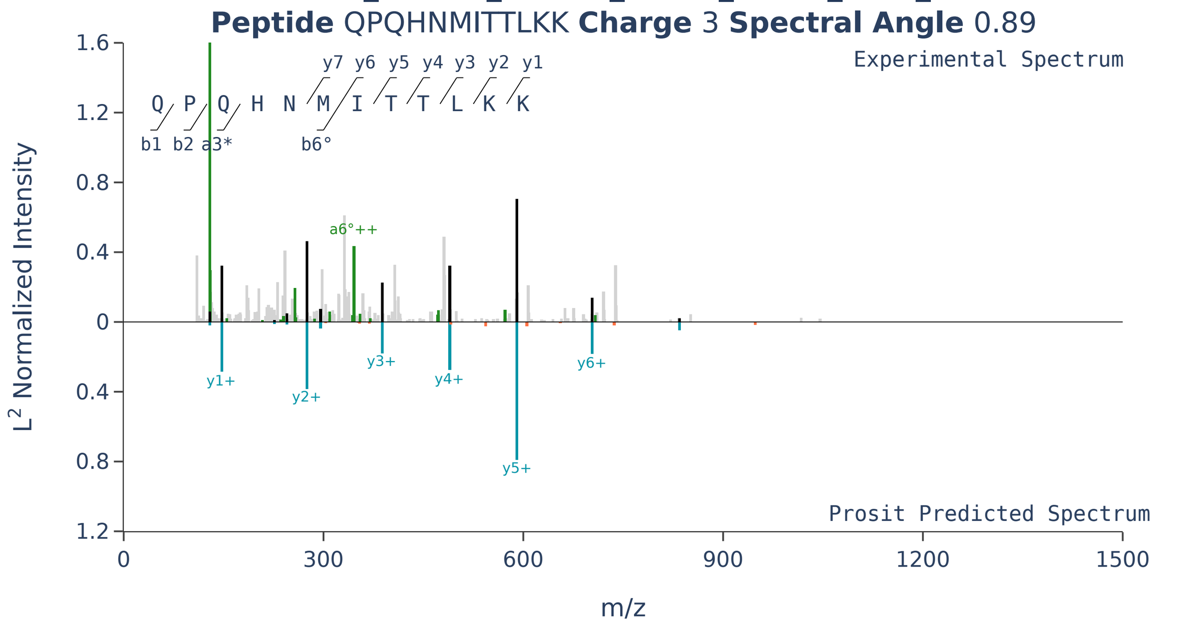

Source HRoetschke_271222_090123_HFGoe_LEDGF_b2R1_24h **Scan** 14851
Peptide RGIHAEIKNSLKI **Charge** 4 **Spectral Angle** 0.78

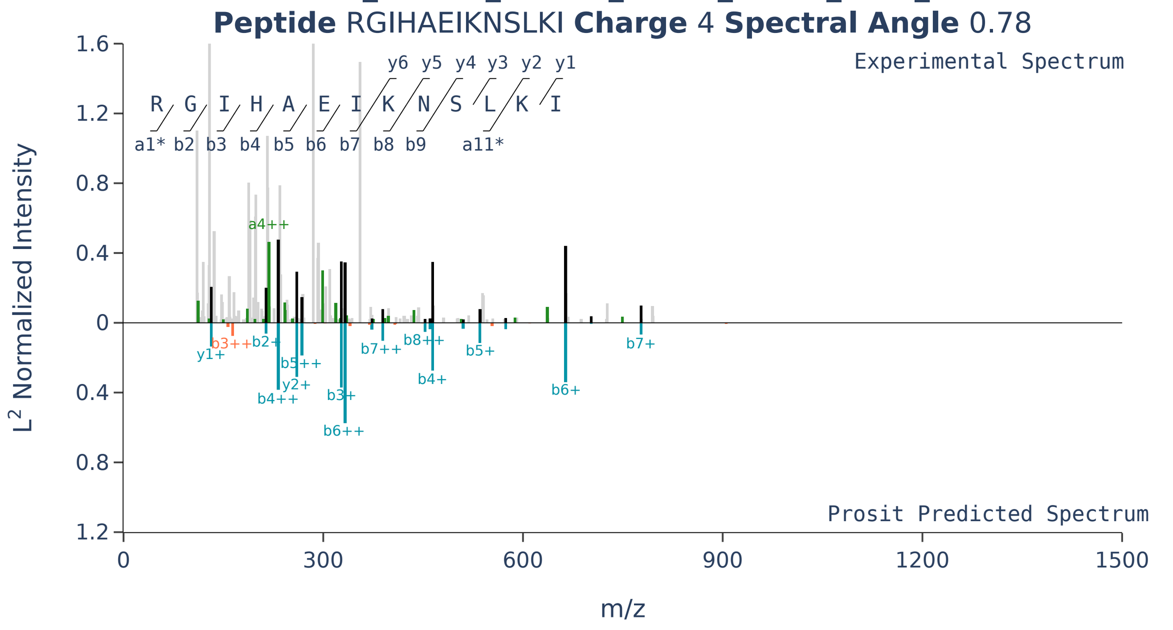

Source HRoetschke_271222_090123_HFGoe_LEDGF_b2R1_24h **Scan** 19445
Peptide KLENSLKIDNL **Charge** 3 **Spectral Angle** 0.86

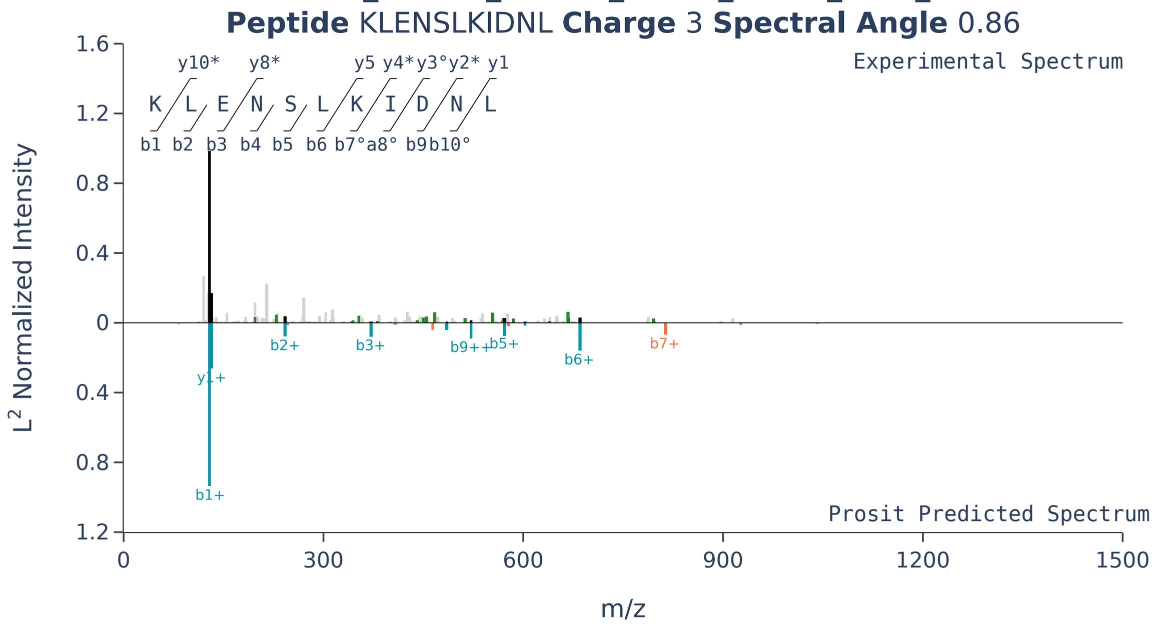

Source HRoetschke_271222_090123_HFGoe_LEDGF_b1R1_24h **Scan** 20285
Peptide PAAHTEMITTL **Charge** 1 **Spectral Angle** 0.71

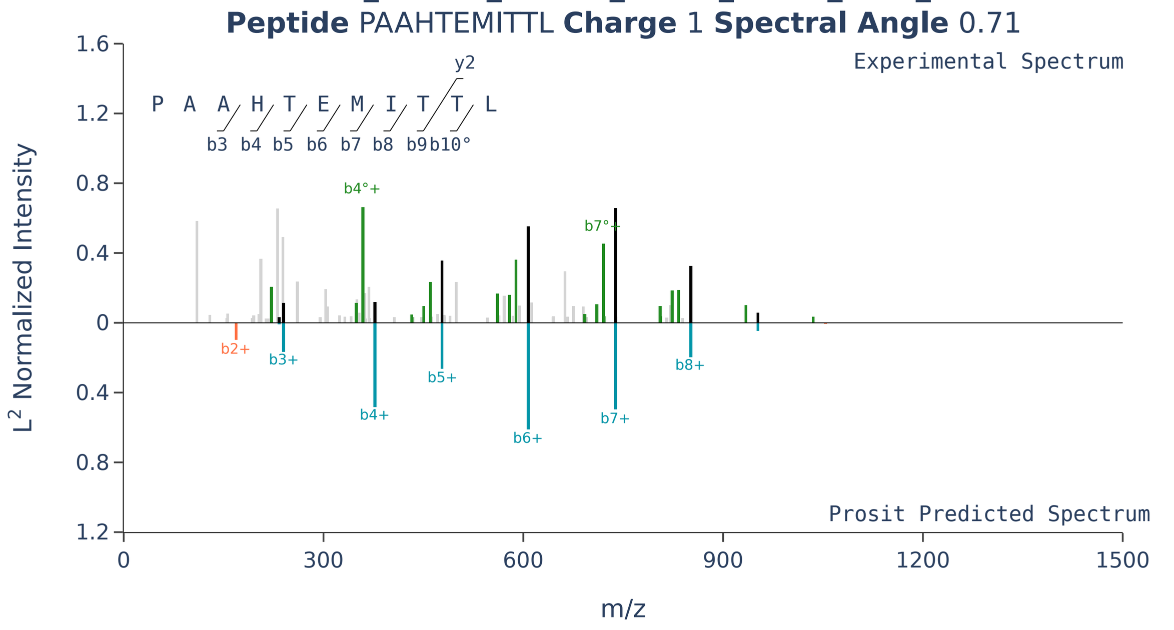

Source HRoetschke_271222_090123_HFGoe_LEDGF_b2R1_24h **Scan** 18285
Peptide EISLMDSRLQR **Charge** 3 **Spectral Angle** 0.79

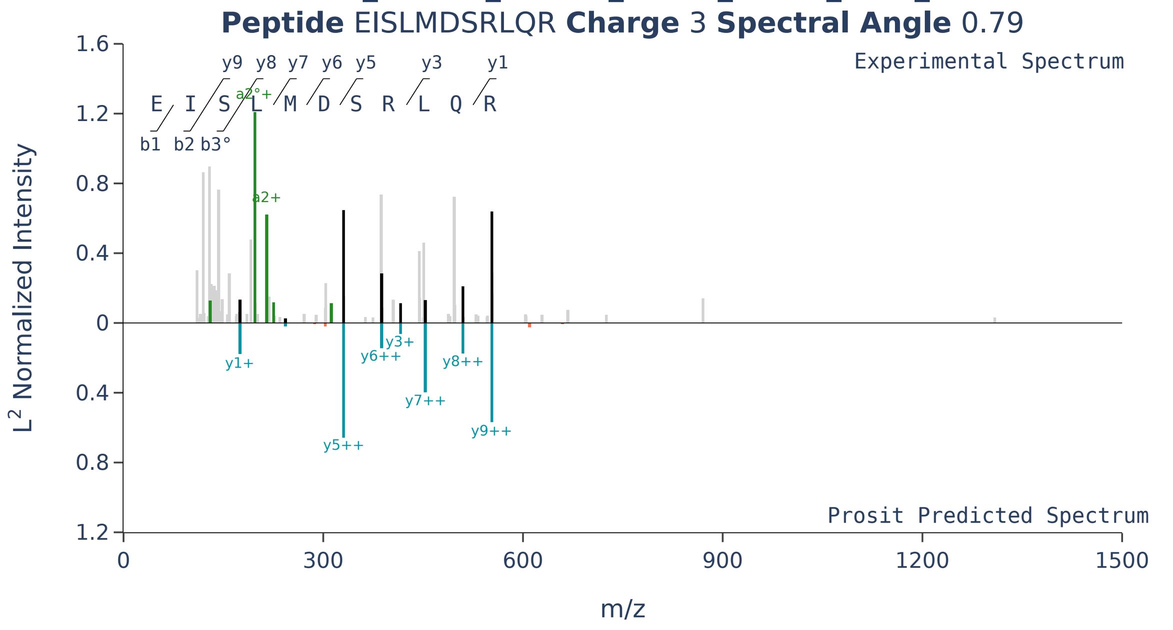

Source HRoetschke_271222_090123_HFGoe_LEDGF_b2R1_4h **Scan** 13184
Peptide GPHWPARVDE **Charge** 2 **Spectral Angle** 0.87

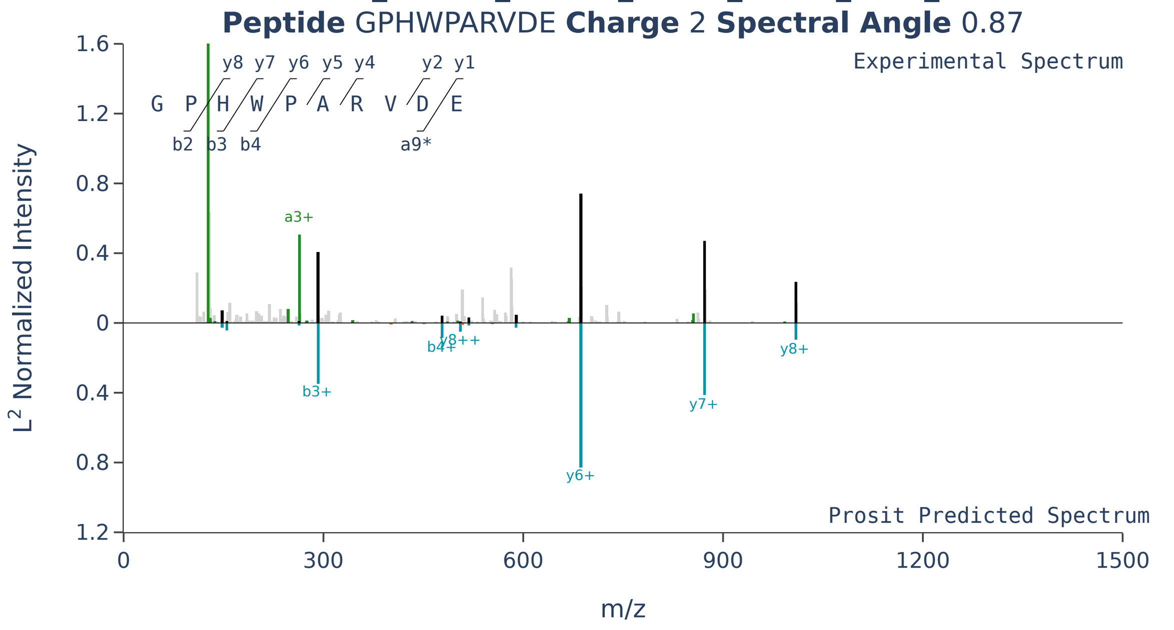

Source HRoetschke_271222_090123_HFGoe_LEDGF_b1R1_4h **Scan** 18915
Peptide QVFLGPKDIFPYSENKEKYGKPNKR **Charge** 4 **Spectral Angle** 0.82

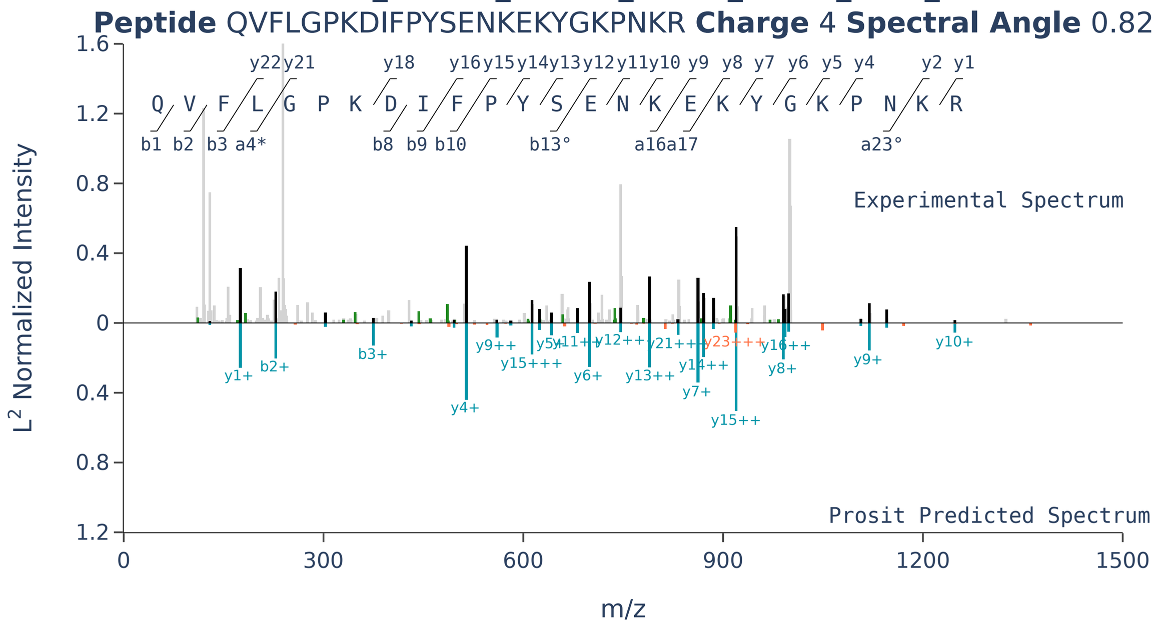

Source HRoetschke_271222_090123_HFGoe_LEDGF_b1R2_24h **Scan** 16291
Peptide ELSMDSRLQRIHA **Charge** 4 **Spectral Angle** 0.89

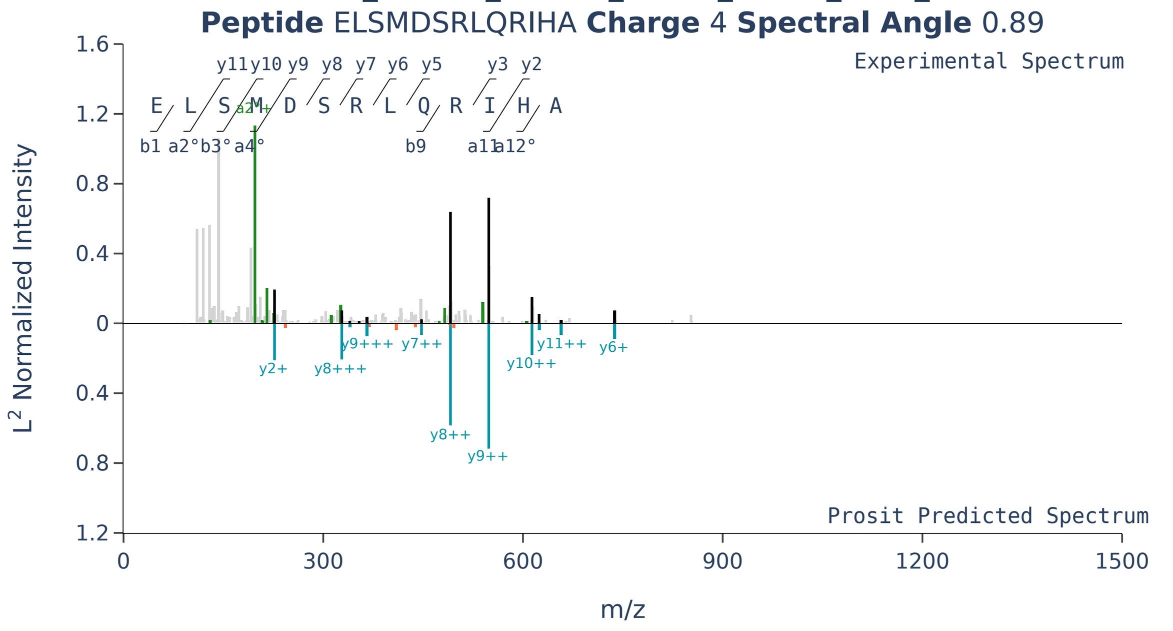

Source HRoetschke_271222_090123_HFGoe_LEDGF_b1R1_24h **Scan** 16533
Peptide ALDELKKIRRF **Charge** 3 **Spectral Angle** 0.86

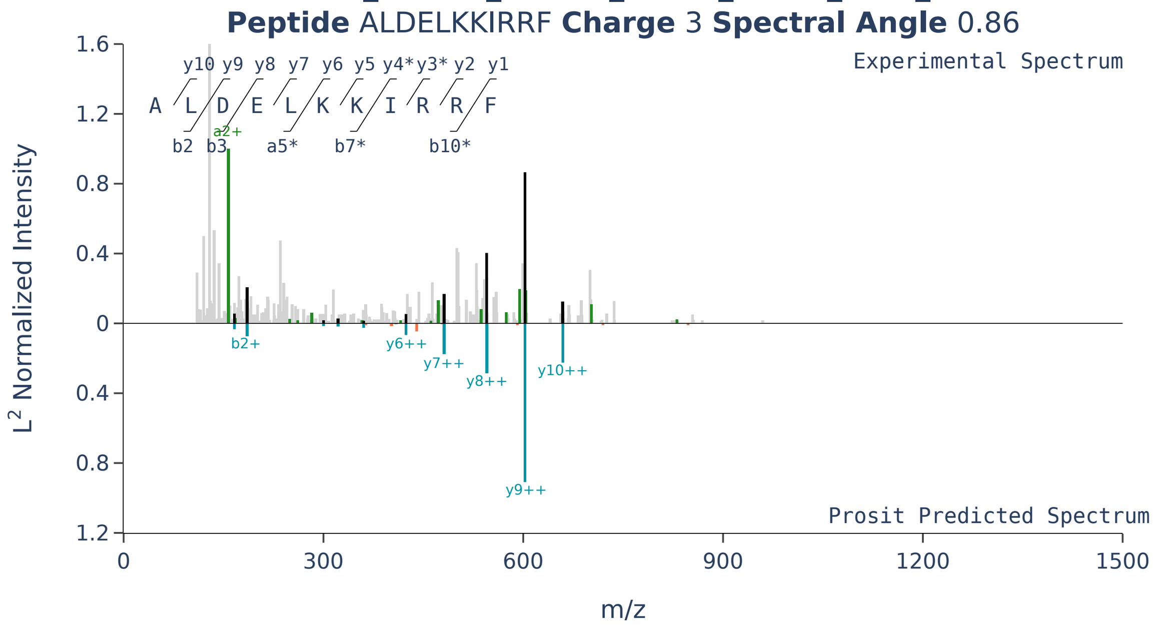

Source HRoetschke_271222_090123_HFGoe_LEDGF_b1R2_4h **Scan** 12670
Peptide LGPKDIFPYSENKEKYGKPNKKR **Charge** 4 **Spectral Angle** 0.75

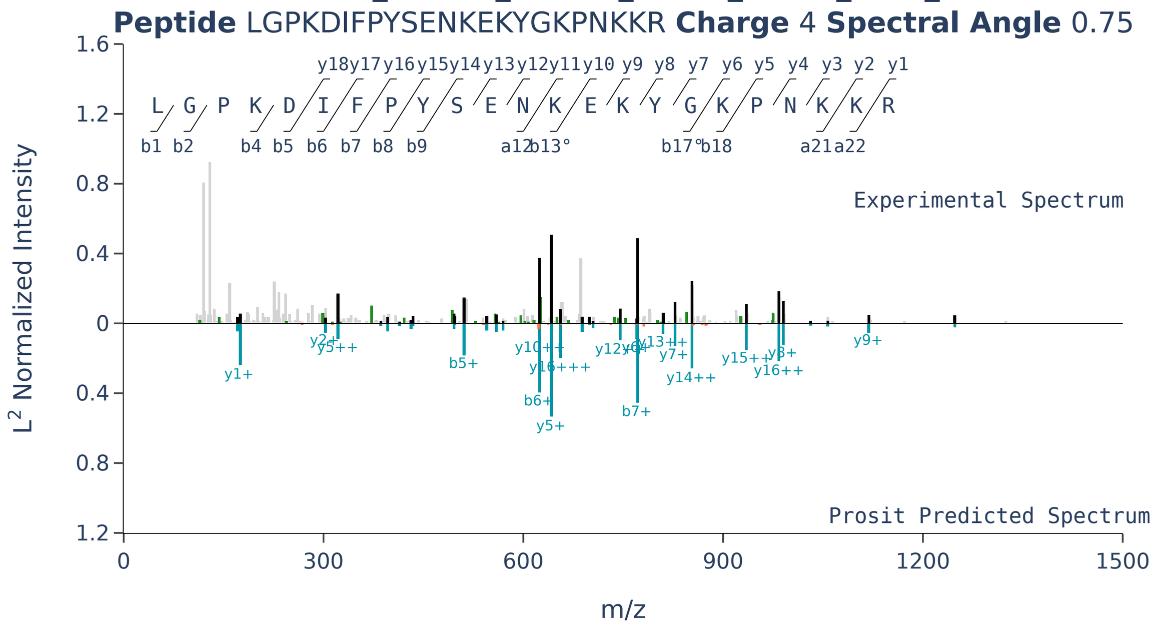

Source HRoetschke_271222_090123_HFGoe_LEDGF_b1R2_24h **Scan** 13214
Peptide KMKGVPDGAVKPPTNKL **Charge** 3 **Spectral Angle** 0.86

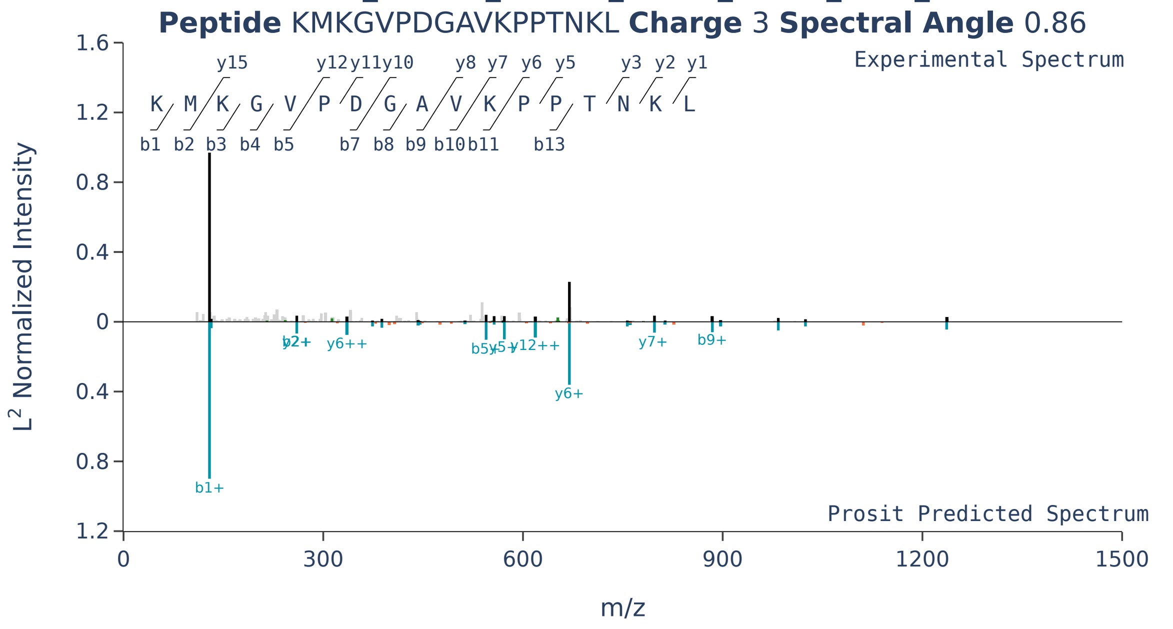

Source HRoetschke_271222_090123_HFGoe_LEDGF_b2R2_1h **Scan** 12768
Peptide KLENSLKID **Charge** 3 **Spectral Angle** 0.94

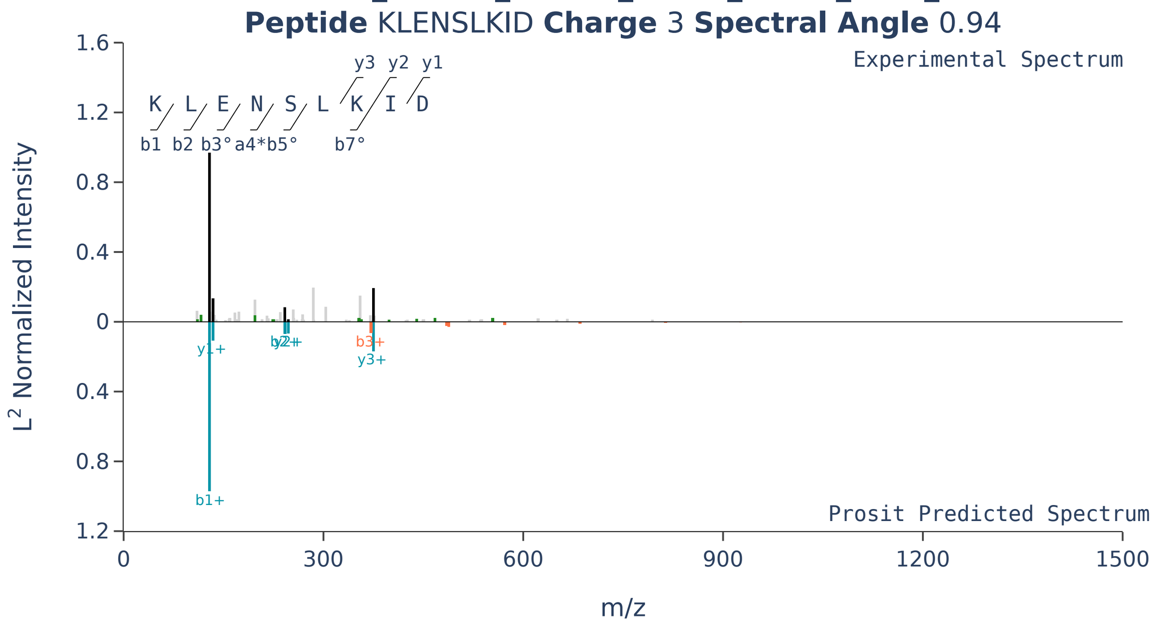

Source HRoetschke_271222_090123_HFGoe_LEDGF_b1R2_24h **Scan** 21539
Peptide QEGELKIDNLDVNRCIE **Charge** 3 **Spectral Angle** 0.88

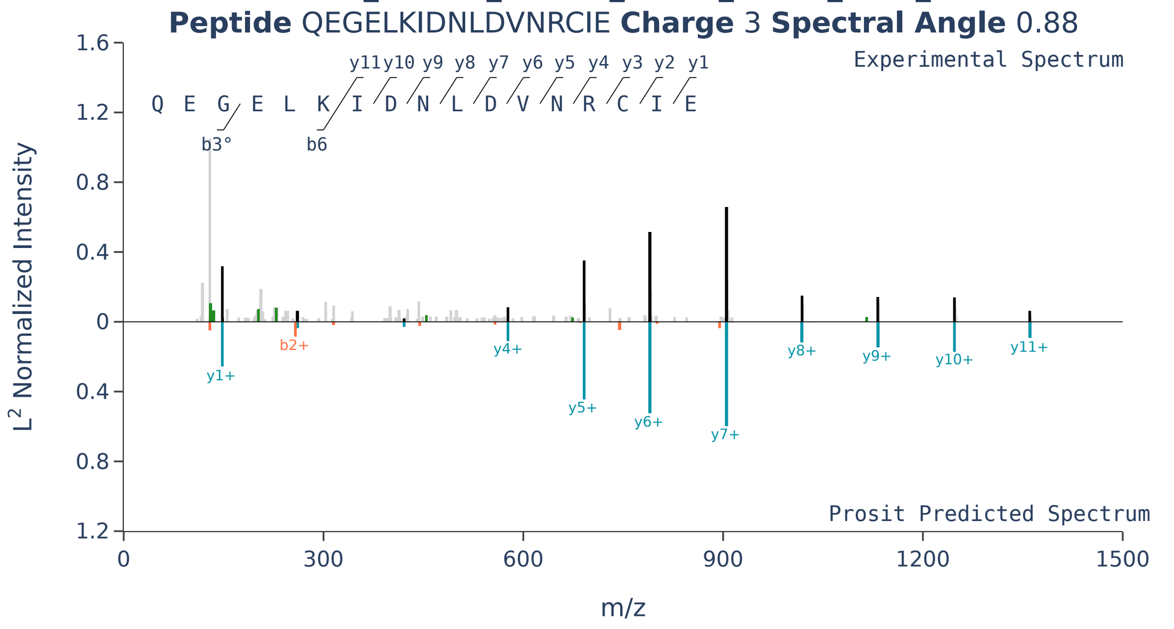

Source HRoetschke_271222_090123_HFGoe_LEDGF_b2R1_4h **Scan** 17529
Peptide AFLGPKDIFPYSENKEKYGKPNKRAT **Charge** 4 **Spectral Angle** 0.87

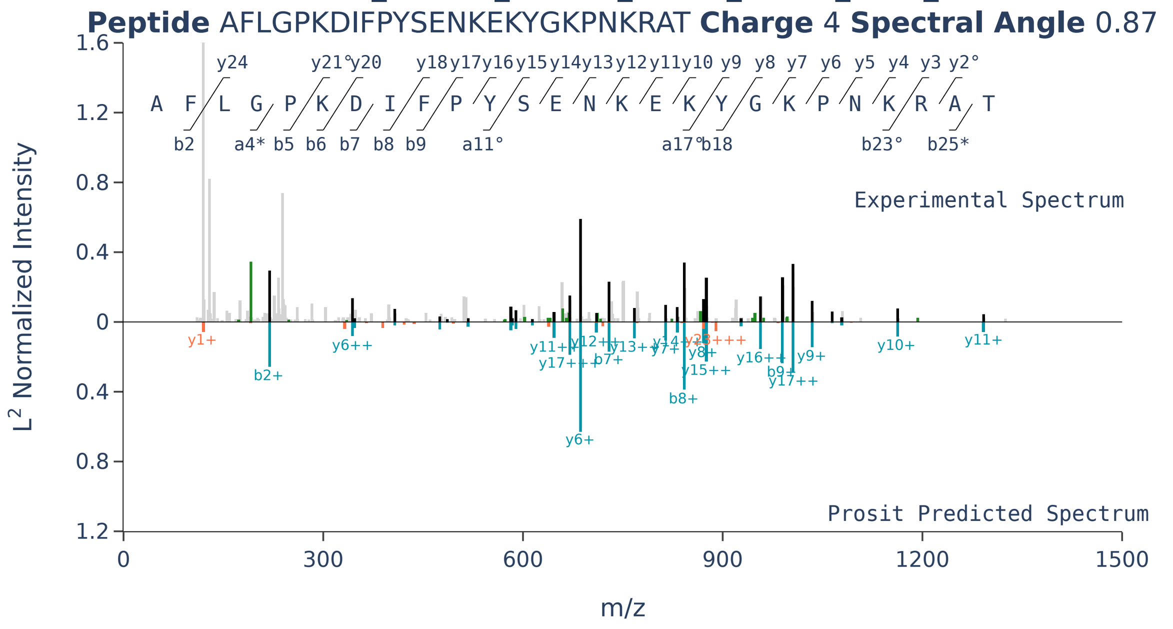

Source HRoetschke_271222_090123_HFGoe_LEDGF_b2R1_4h **Scan** 21236
Peptide TSLKIDNLDVNRCIE **Charge** 2 **Spectral Angle** 0.84

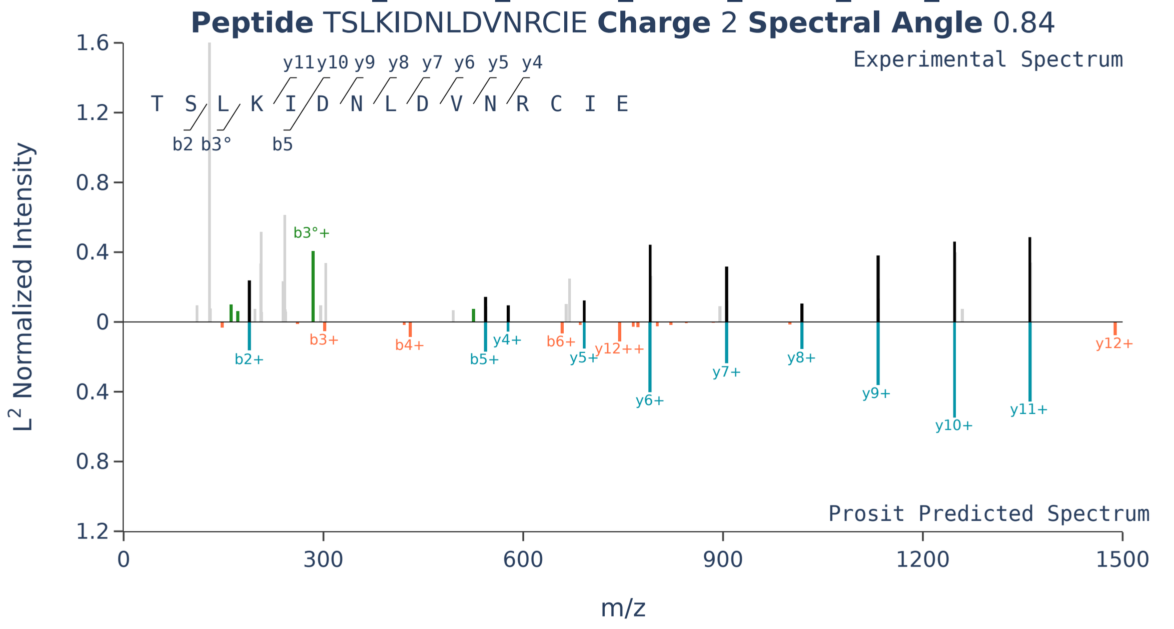

Source HRoetschke_271222_090123_HFGoe_LEDGF_b1R1_4h **Scan** 10790
Peptide SVNLKVSPKRGRPAATATEV **Charge** 5 **Spectral Angle** 0.86

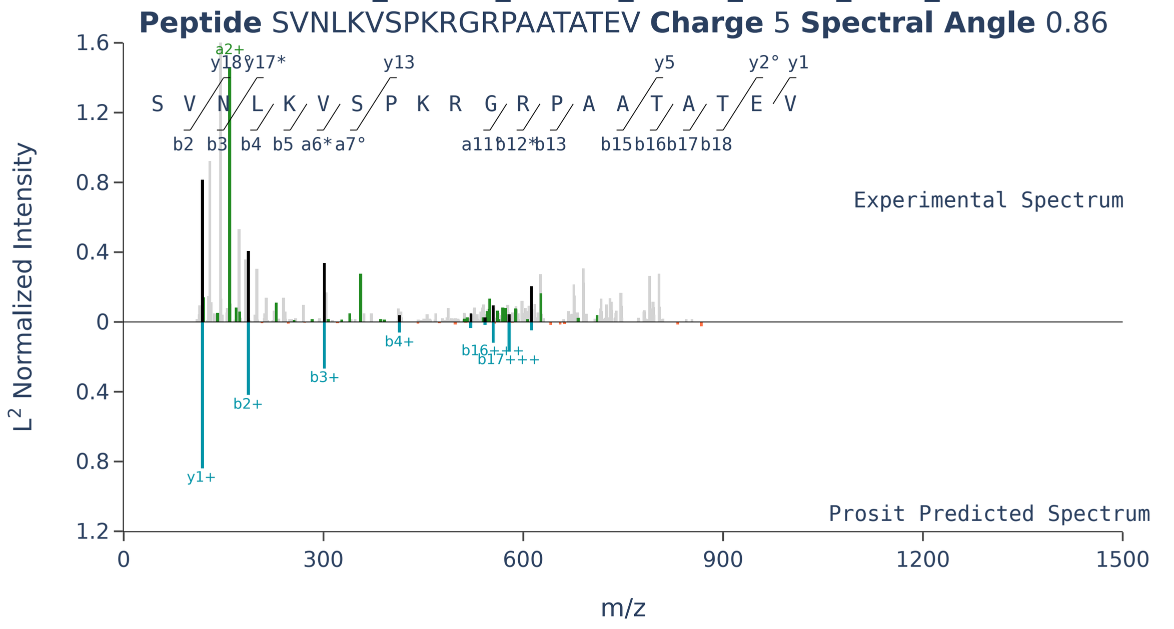

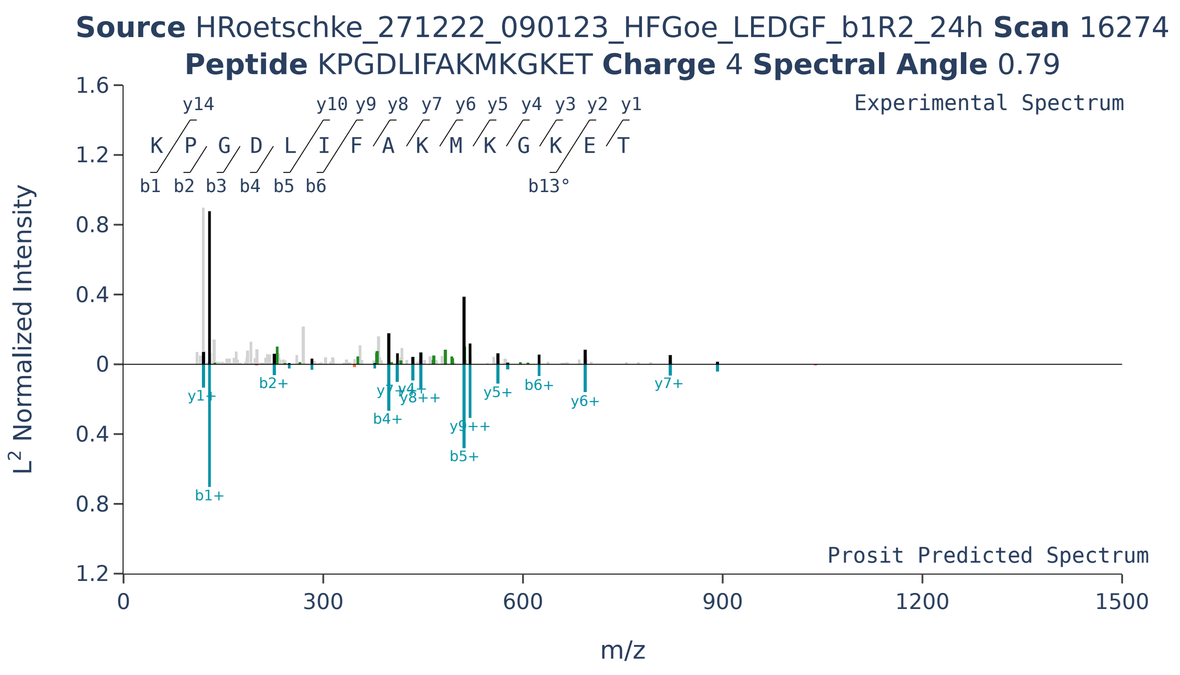
Source HRoetschke_271222_090123_HFGoe_LEDGF_b1R2_24h **Scan** 16274
Peptide KPGDLIFAKMKGKET **Charge** 4 **Spectral Angle** 0.79
Experimental Spectrum
Prosit Predicted Spectrum
L² Normalized Intensity
m/z

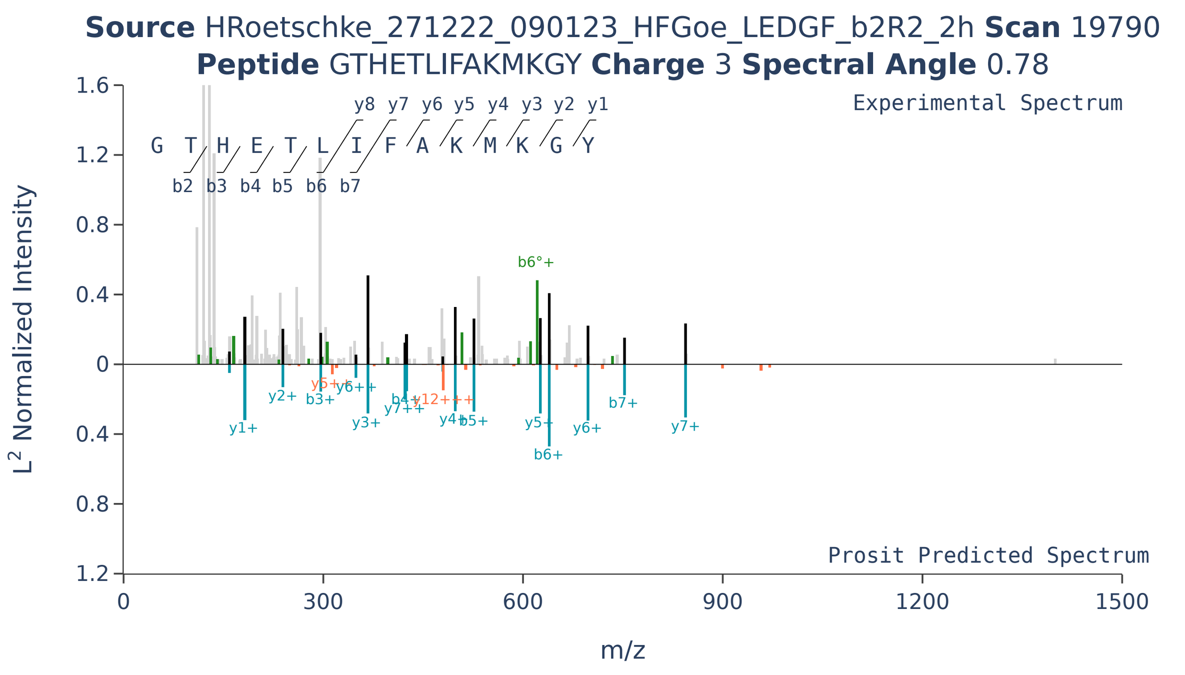
Source HRoetschke_271222_090123_HFGoe_LEDGF_b2R2_2h **Scan** 19790
Peptide GTHETLIFAKMKGY **Charge** 3 **Spectral Angle** 0.78
Experimental Spectrum
Prosit Predicted Spectrum
L² Normalized Intensity
m/z

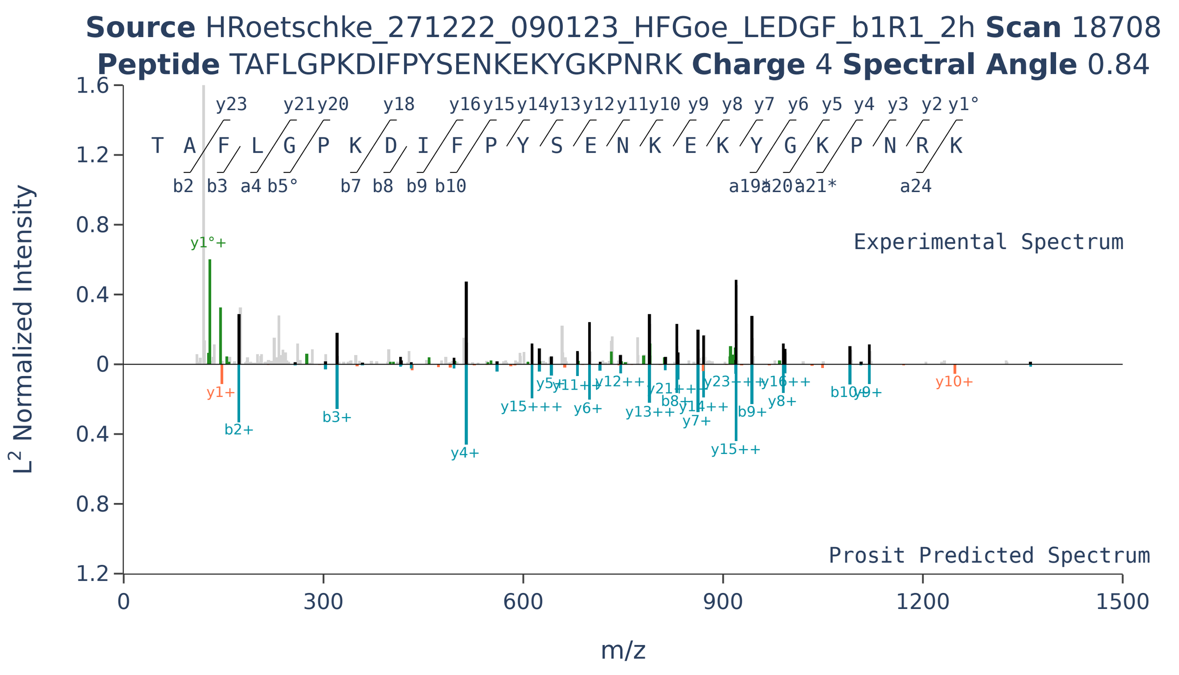
Source HRoetschke_271222_090123_HFGoe_LEDGF_b1R1_2h **Scan** 18708
Peptide TAFLGPKDIFPYSENKEKYGKPNRK **Charge** 4 **Spectral Angle** 0.84
Experimental Spectrum
Prosit Predicted Spectrum
L² Normalized Intensity
m/z

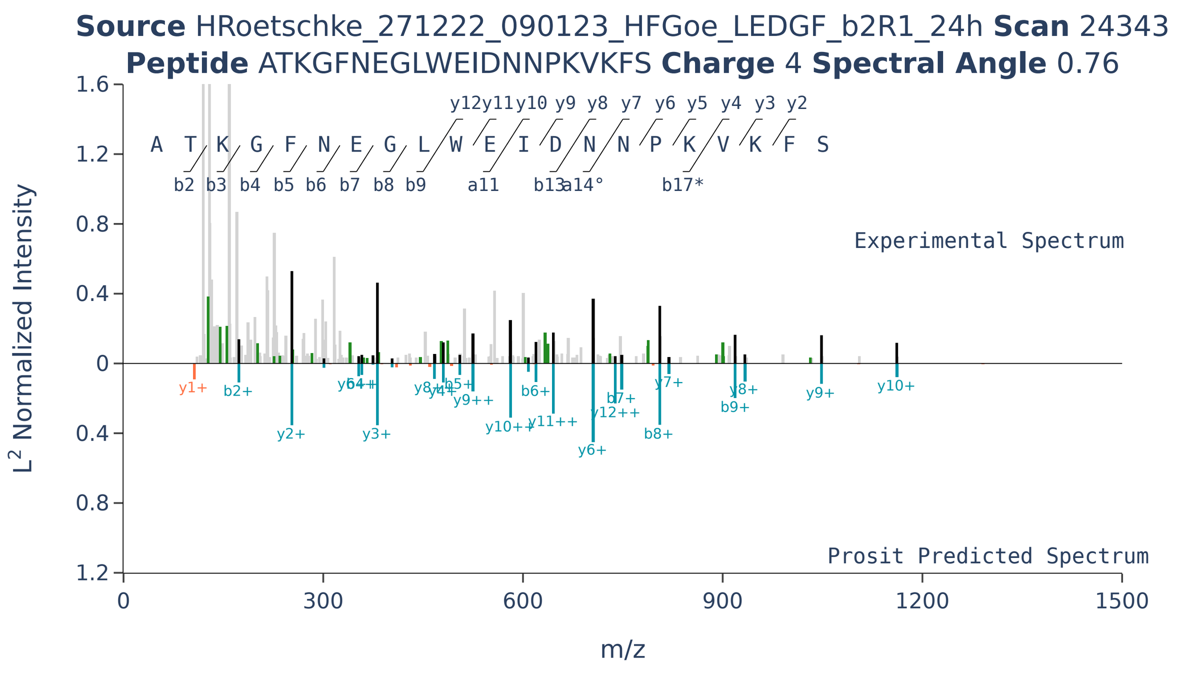
Source HRoetschke_271222_090123_HFGoe_LEDGF_b2R1_24h **Scan** 24343
Peptide ATKGFNEGLWEIDNNPKVKFS **Charge** 4 **Spectral Angle** 0.76
Experimental Spectrum
Prosit Predicted Spectrum
L² Normalized Intensity
m/z

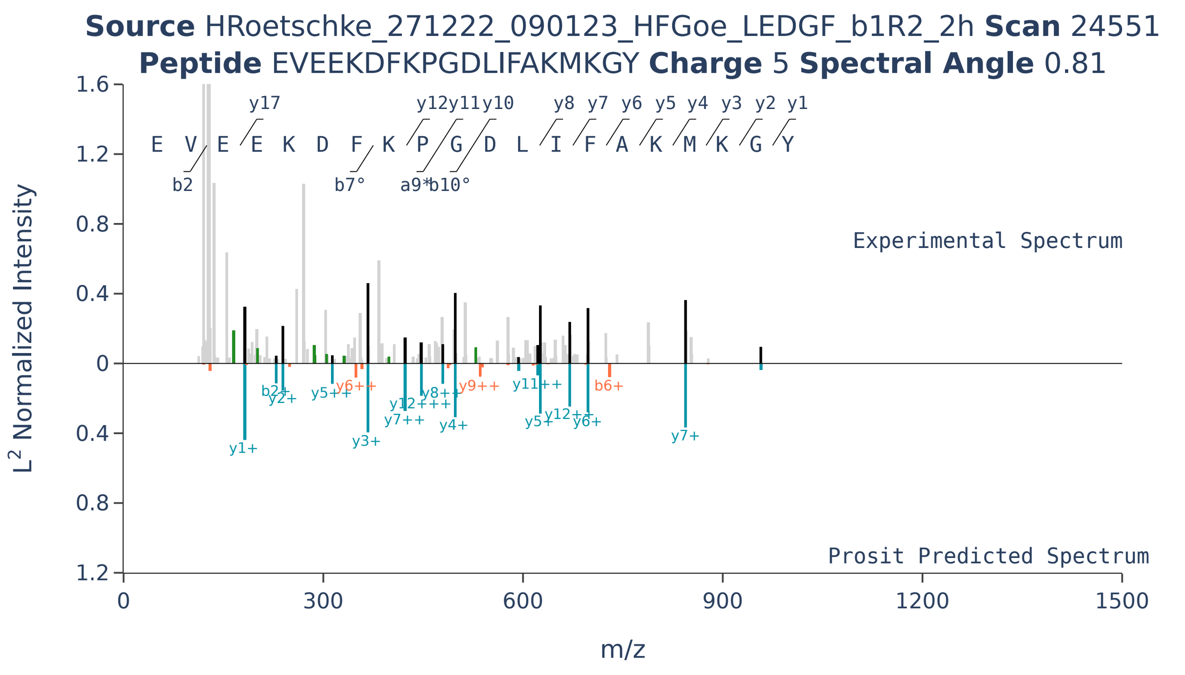
Source HRoetschke_271222_090123_HFGoe_LEDGF_b1R2_2h **Scan** 24551
Peptide EVEEKDFKPGDLIFAKMKGY **Charge** 5 **Spectral Angle** 0.81
Experimental Spectrum
Prosit Predicted Spectrum
L² Normalized Intensity
m/z

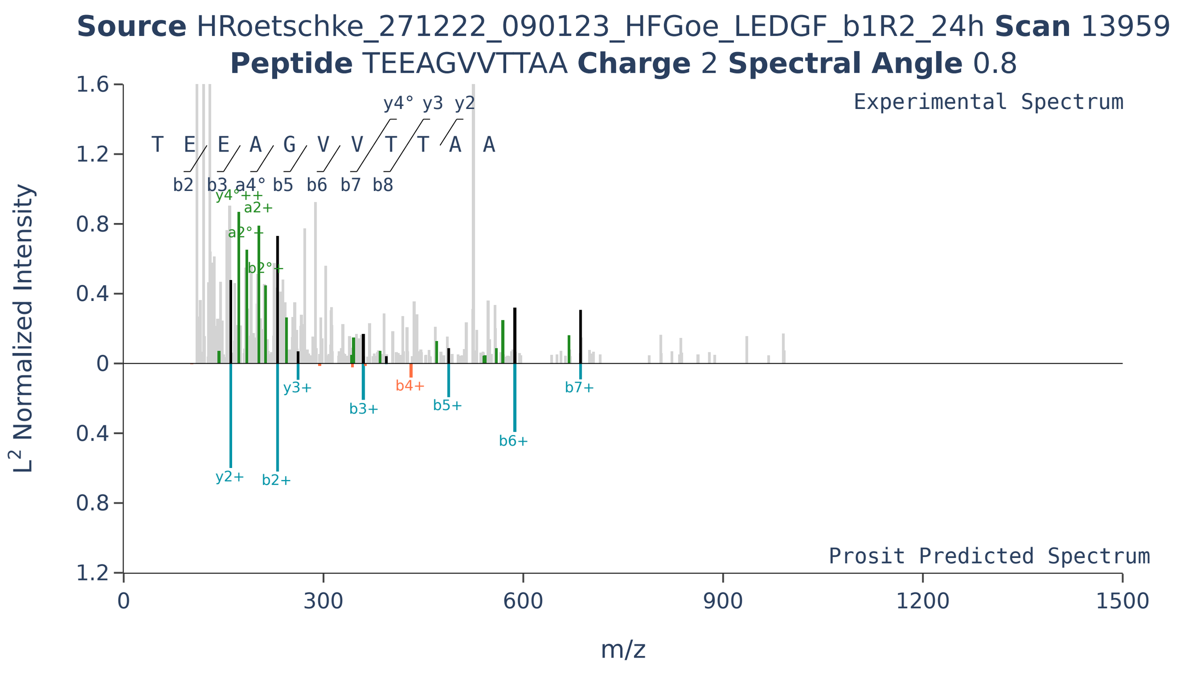
Source HRoetschke_271222_090123_HFGoe_LEDGF_b1R2_24h **Scan** 13959
Peptide TEEAGVVTTAA **Charge** 2 **Spectral Angle** 0.8
Experimental Spectrum
Prosit Predicted Spectrum
L² Normalized Intensity
m/z

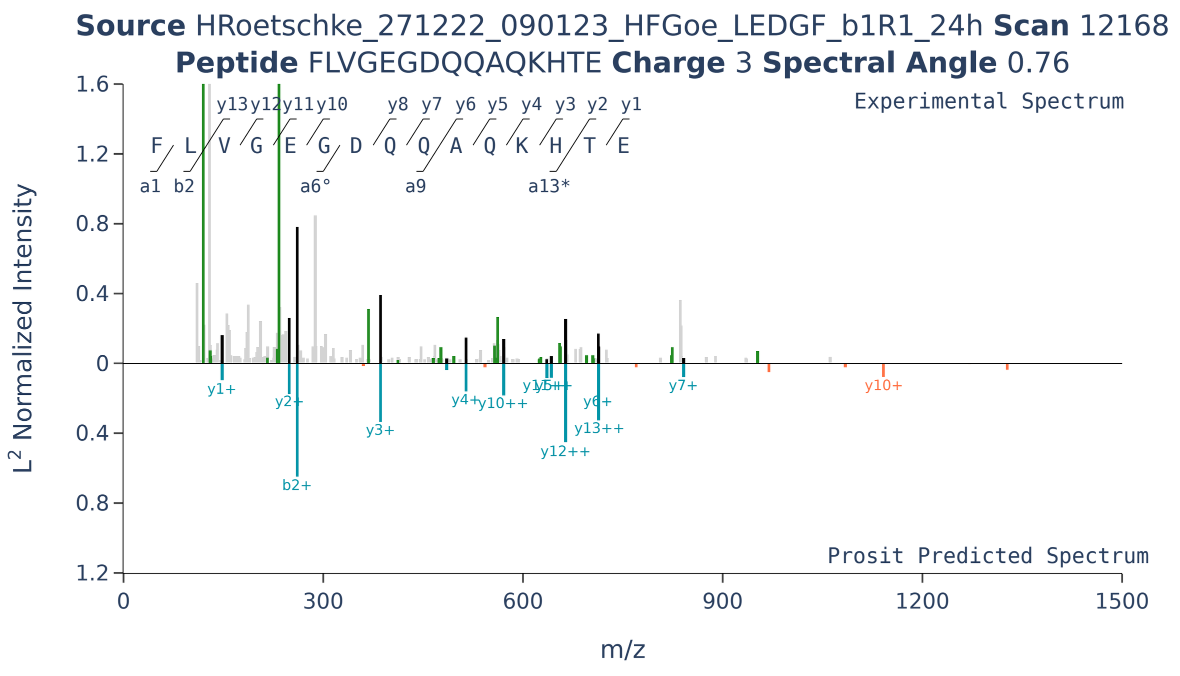
Source HRoetschke_271222_090123_HFGoe_LEDGF_b1R1_24h **Scan** 12168
Peptide FLVGEGDQQAQKHTE **Charge** 3 **Spectral Angle** 0.76
Experimental Spectrum
Prosit Predicted Spectrum
L² Normalized Intensity
m/z

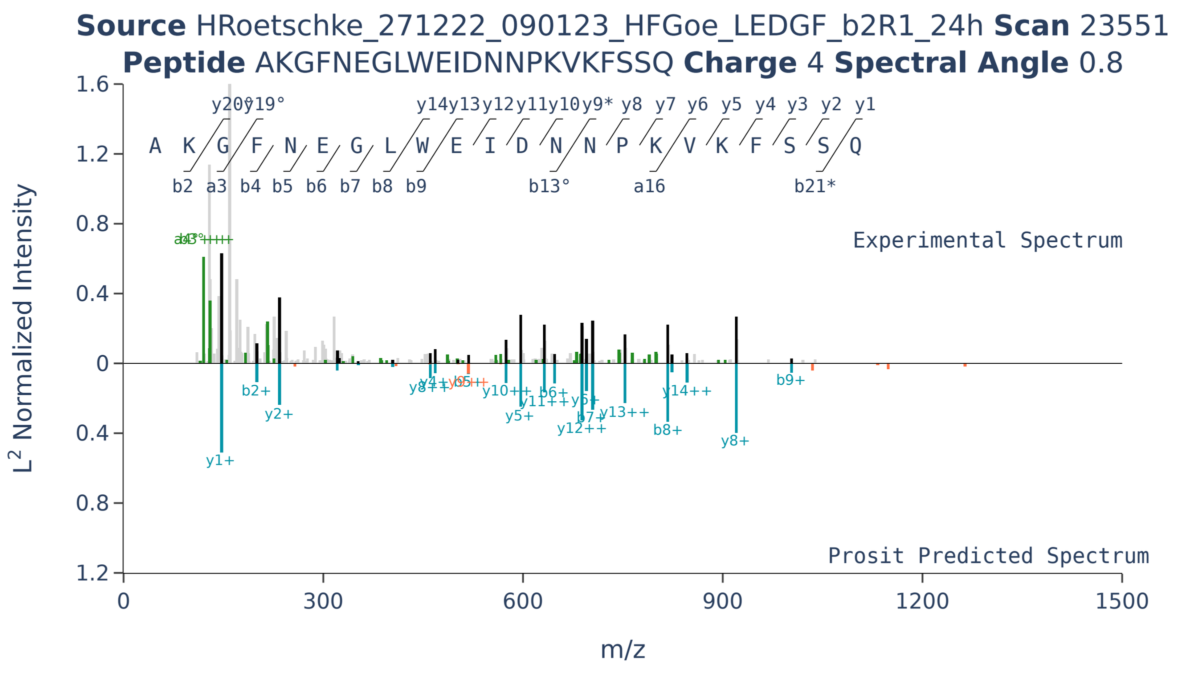
Source HRoetschke_271222_090123_HFGoe_LEDGF_b2R1_24h **Scan** 23551
Peptide AKGFNEGLWEIDNNPKVKFSSQ **Charge** 4 **Spectral Angle** 0.8
Experimental Spectrum
Prosit Predicted Spectrum
L² Normalized Intensity
m/z

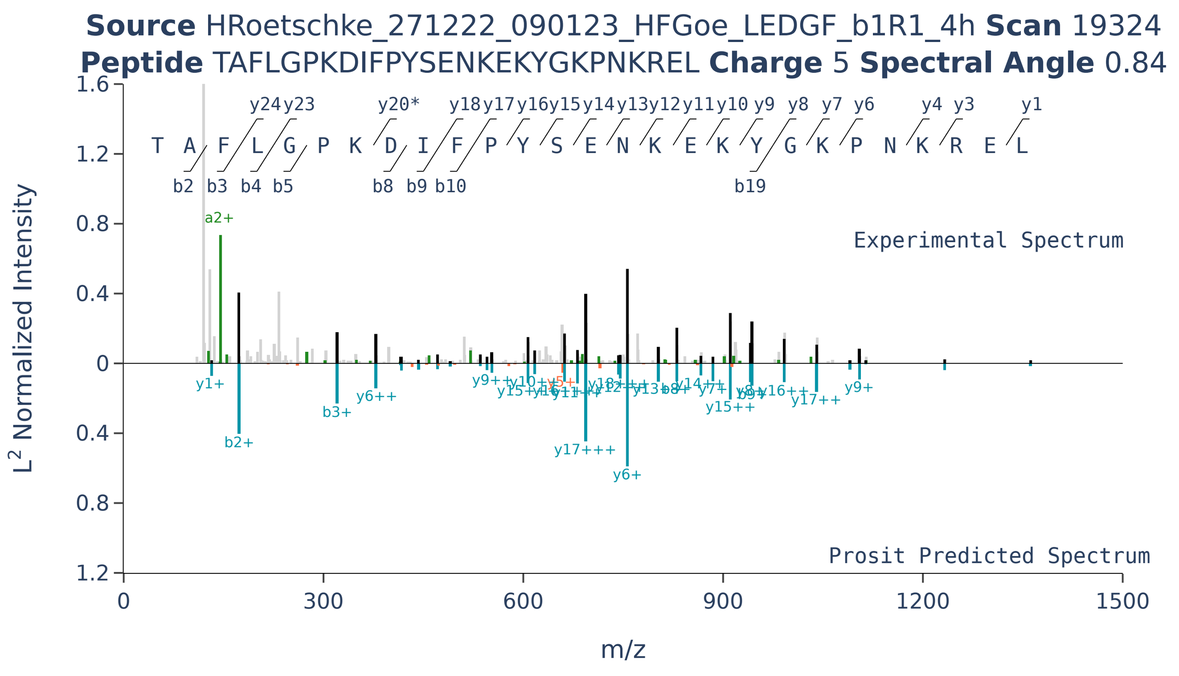
Source HRoetschke_271222_090123_HFGoe_LEDGF_b1R1_4h **Scan** 19324
Peptide TAFLGPKDIFPYSENKEKYGKPNKREL **Charge** 5 **Spectral Angle** 0.84
Experimental Spectrum
Prosit Predicted Spectrum
L² Normalized Intensity
m/z

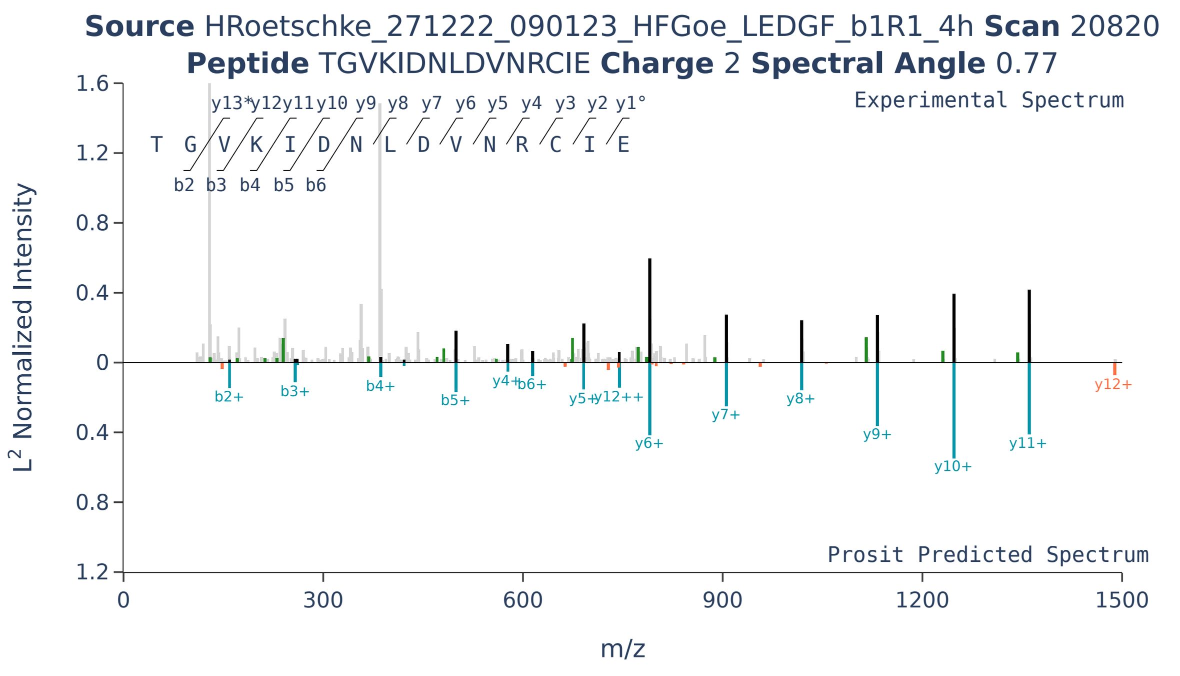
Source HRoetschke_271222_090123_HFGoe_LEDGF_b1R1_4h **Scan** 20820
Peptide TGVKIDNLDVNRCIE **Charge** 2 **Spectral Angle** 0.77
Experimental Spectrum
Prosit Predicted Spectrum
L² Normalized Intensity
m/z

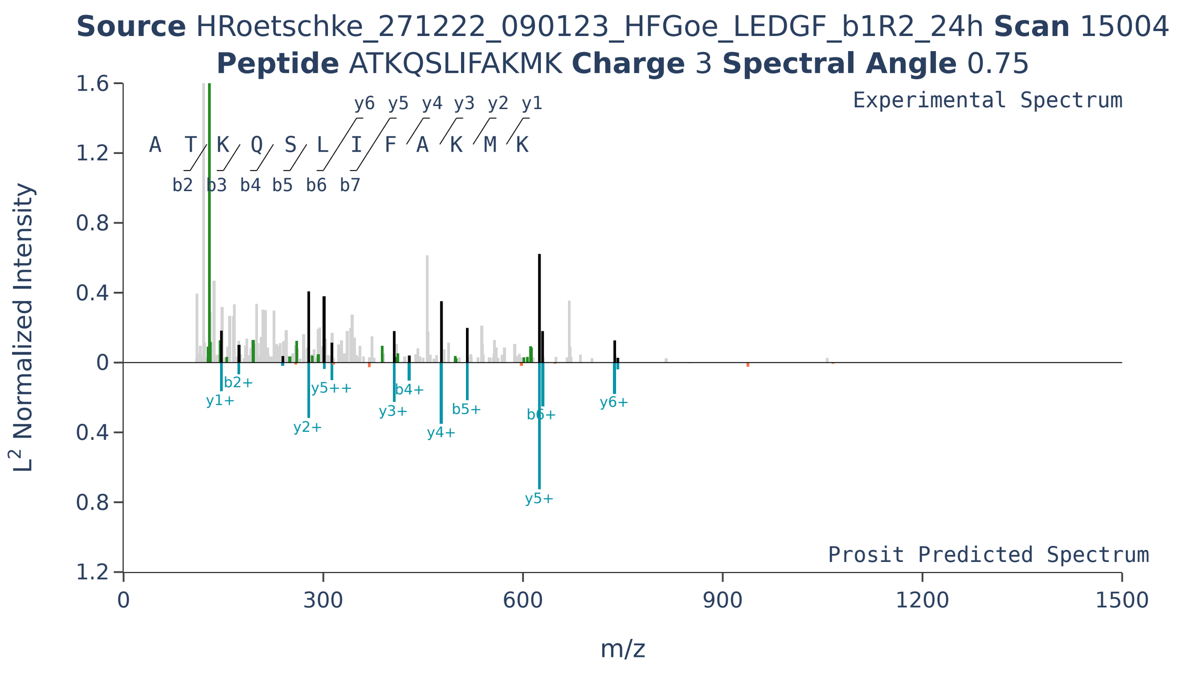
Source HRoetschke_271222_090123_HFGoe_LEDGF_b1R2_24h **Scan** 15004
Peptide ATKQSLIFAKMK **Charge** 3 **Spectral Angle** 0.75
Experimental Spectrum
Prosit Predicted Spectrum
L² Normalized Intensity
m/z

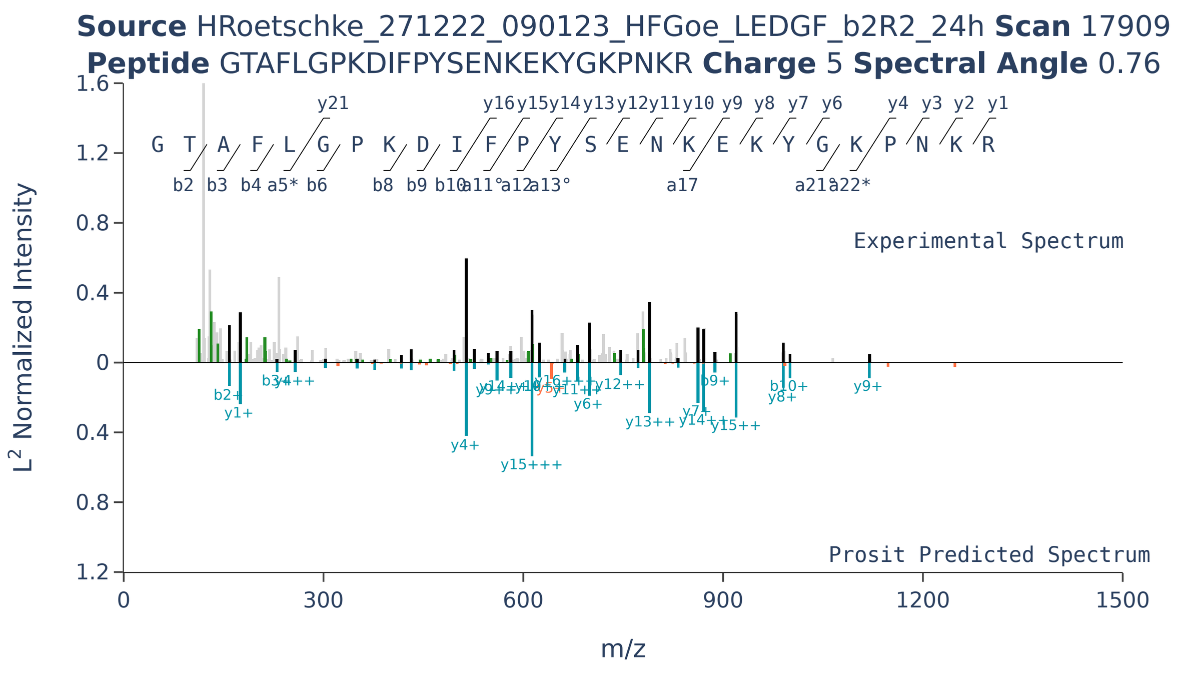
Source HRoetschke_271222_090123_HFGoe_LEDGF_b2R2_24h **Scan** 17909
Peptide GTAFLGPKDIFPYSENKEKYGKPNKR **Charge** 5 **Spectral Angle** 0.76
Experimental Spectrum
Prosit Predicted Spectrum
L² Normalized Intensity
m/z

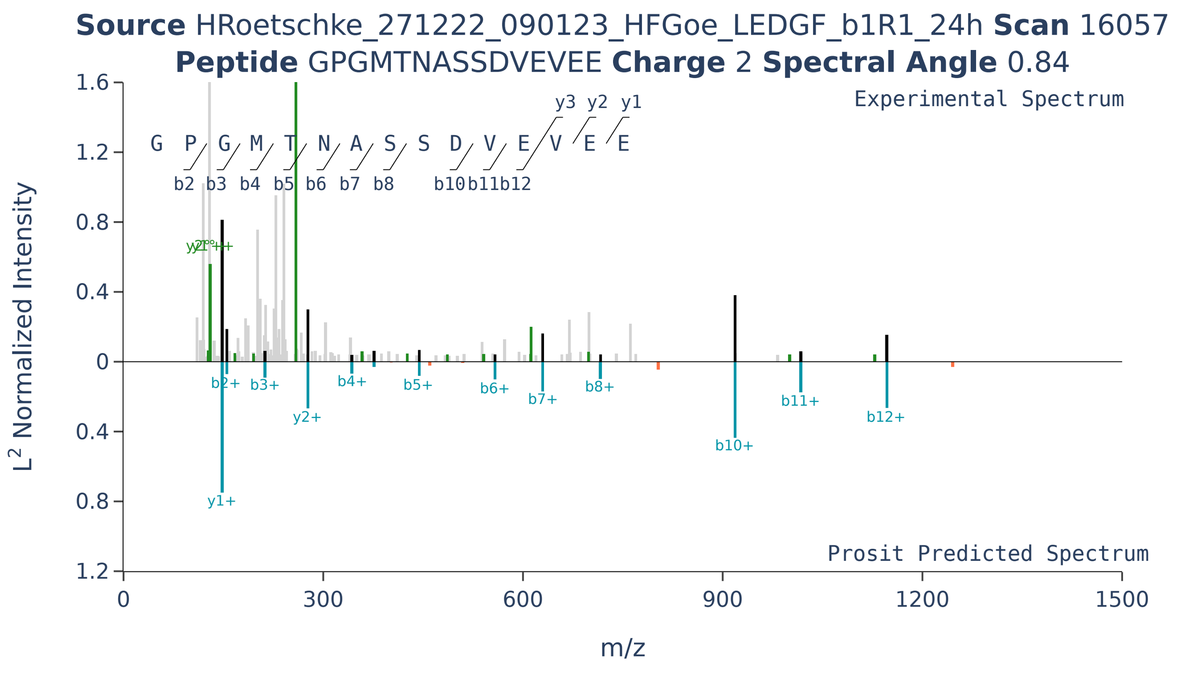
Source HRoetschke_271222_090123_HFGoe_LEDGF_b1R1_24h **Scan** 16057
Peptide GPGMTNASSDVEVEE **Charge** 2 **Spectral Angle** 0.84
Experimental Spectrum
Prosit Predicted Spectrum
L² Normalized Intensity
m/z

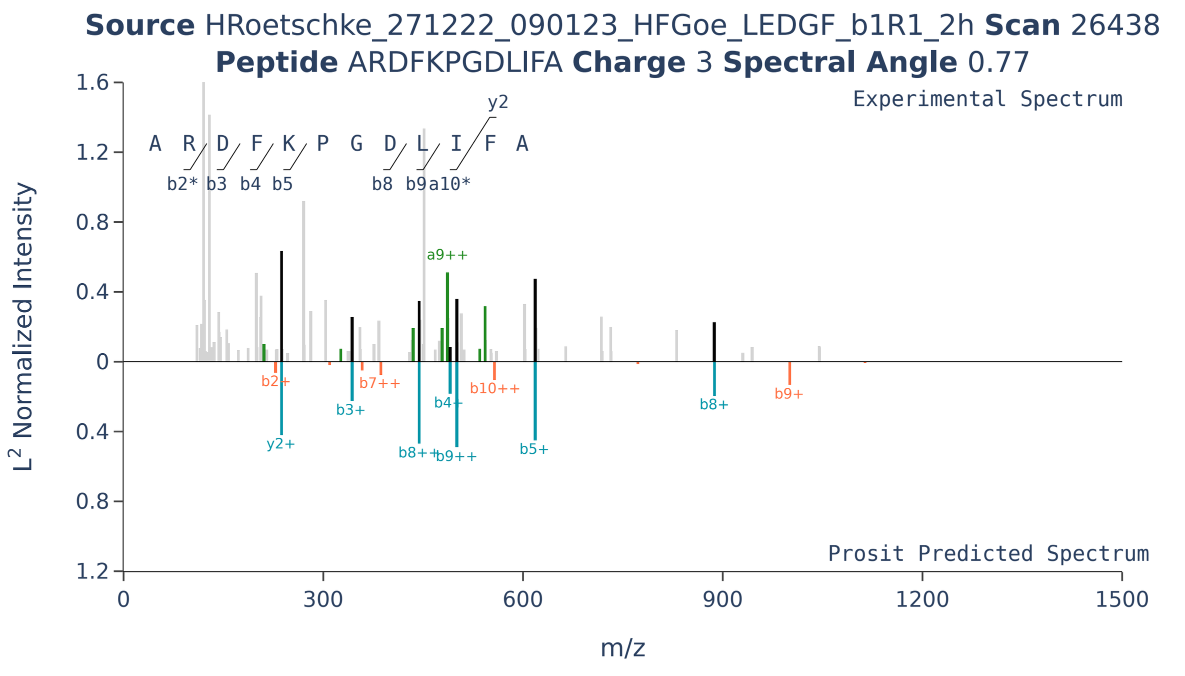
Source HRoetschke_271222_090123_HFGoe_LEDGF_b1R1_2h **Scan** 26438
Peptide ARDFKPGDLIFA **Charge** 3 **Spectral Angle** 0.77
Experimental Spectrum
Prosit Predicted Spectrum
L² Normalized Intensity
m/z

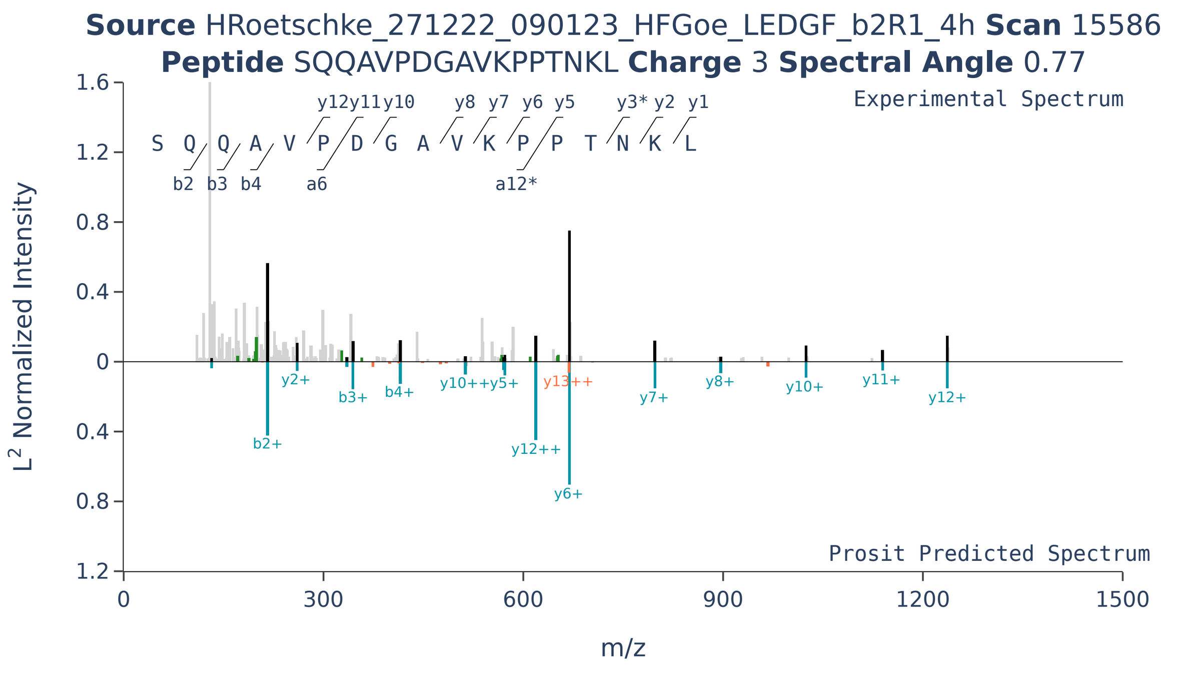
Source HRoetschke_271222_090123_HFGoe_LEDGF_b2R1_4h **Scan** 15586
Peptide SQQAVPDGAVKPPTNKL **Charge** 3 **Spectral Angle** 0.77
Experimental Spectrum
Prosit Predicted Spectrum
L² Normalized Intensity
m/z

Source HRoetschke_271222_090123_HFGoe_LEDGF_b1R1_4h **Scan** 18025
Peptide QVETEVTTATASVNLKVSPKRGRPAATEV **Charge** 5 **Spectral Angle** 0.77

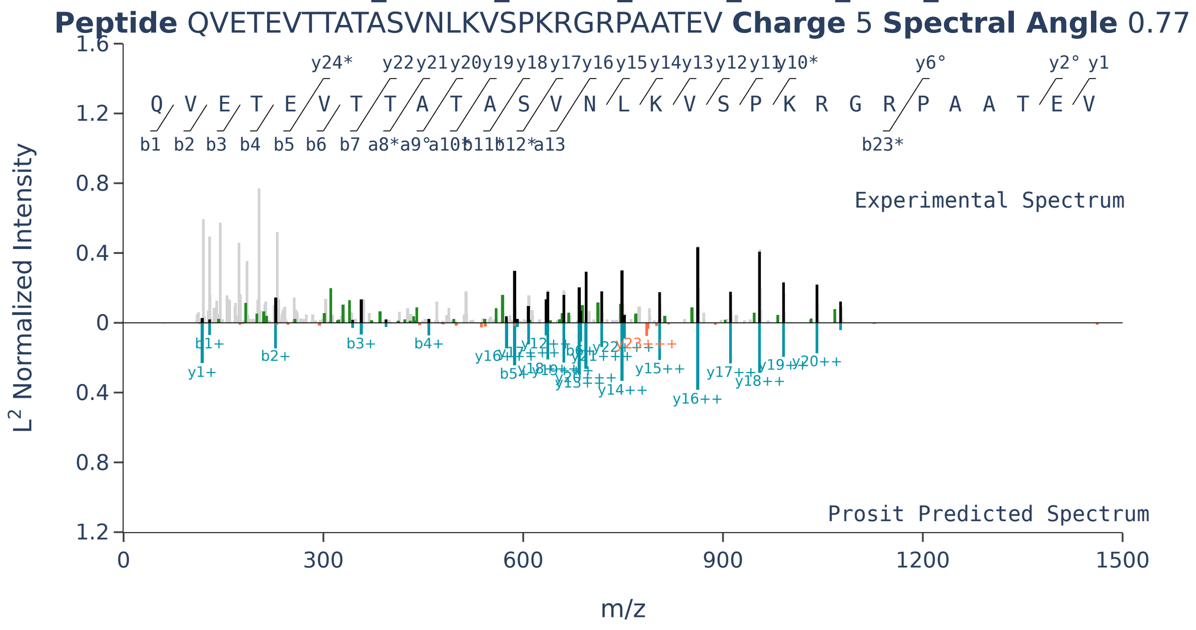

Source HRoetschke_271222_090123_HFGoe_LEDGF_b1R2_24h **Scan** 13442
Peptide GDPGMTRDFKPGD **Charge** 3 **Spectral Angle** 0.82

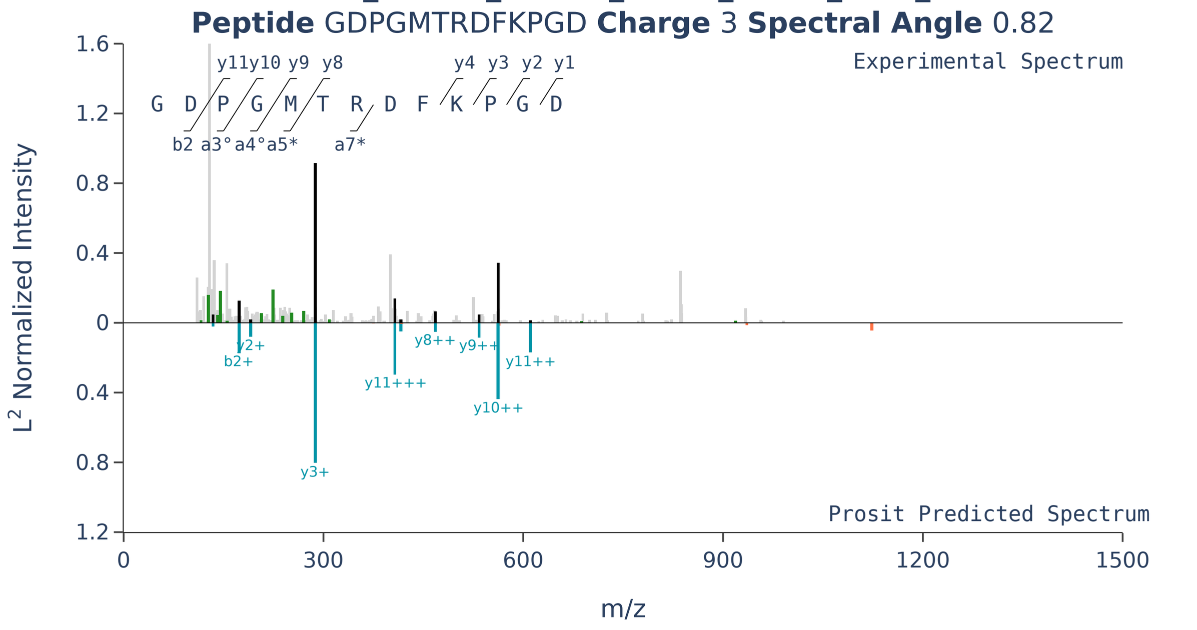

Source HRoetschke_271222_090123_HFGoe_LEDGF_b2R2_2h **Scan** 15054
Peptide EVPDPDGAVKPPTN **Charge** 2 **Spectral Angle** 0.69

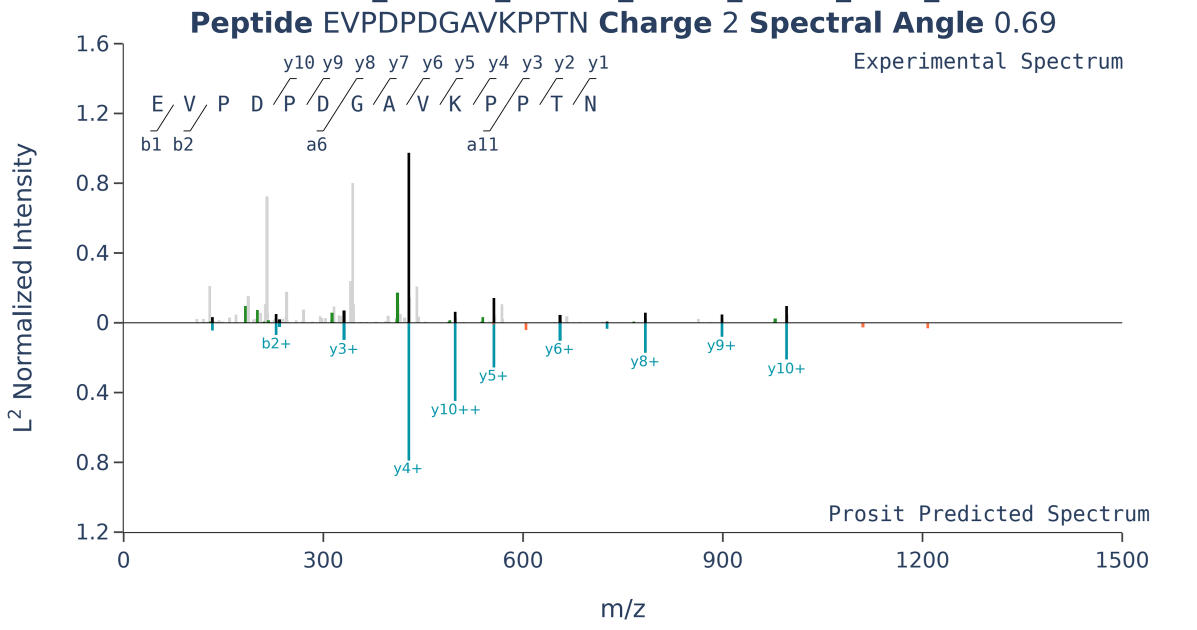

Source HRoetschke_271222_090123_HFGoe_LEDGF_b2R1_24h **Scan** 17146
Peptide FLGPKDIFPYSENKEKYGKPNKRAA **Charge** 5 **Spectral Angle** 0.77

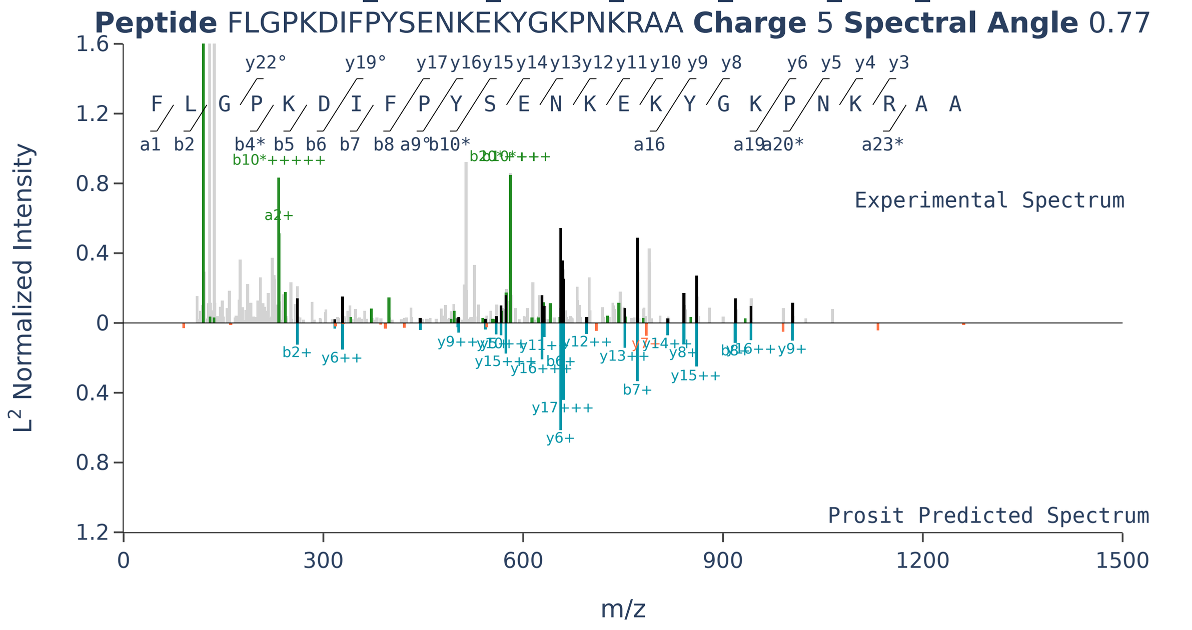

Source HRoetschke_271222_090123_HFGoe_LEDGF_b1R1_2h **Scan** 21180
Peptide KNSLVIMEKSTMLYNKFKNM **Charge** 4 **Spectral Angle** 0.82

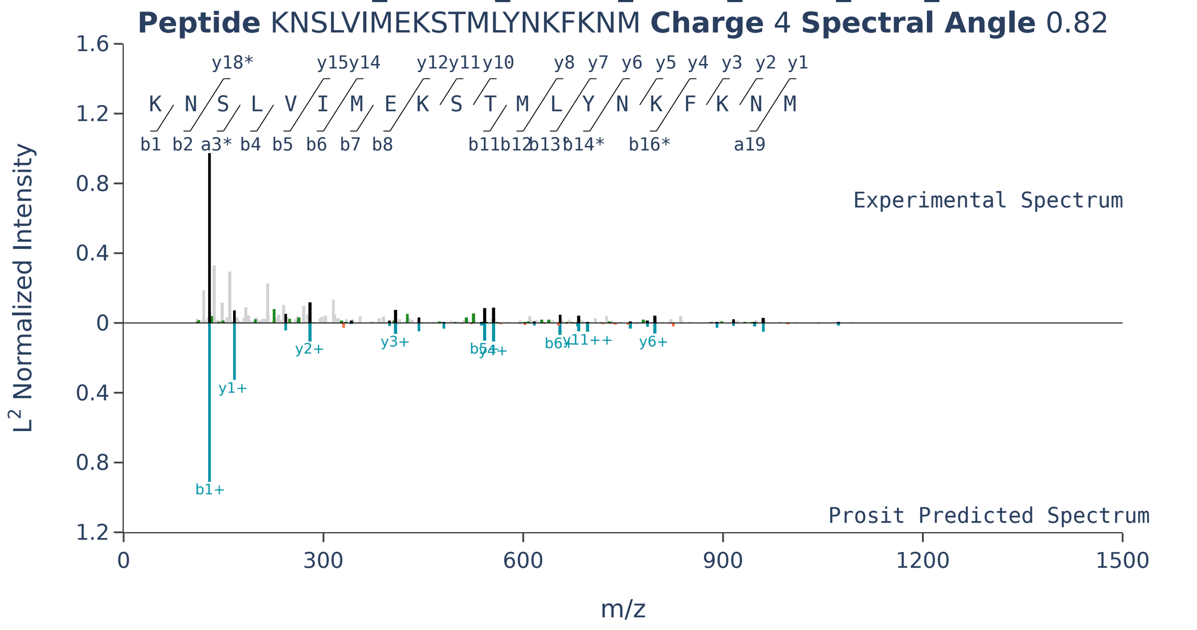

Source HRoetschke_271222_090123_HFGoe_LEDGF_b2R1_24h **Scan** 23922
Peptide EDKLASLQVTMQQA **Charge** 2 **Spectral Angle** 0.76

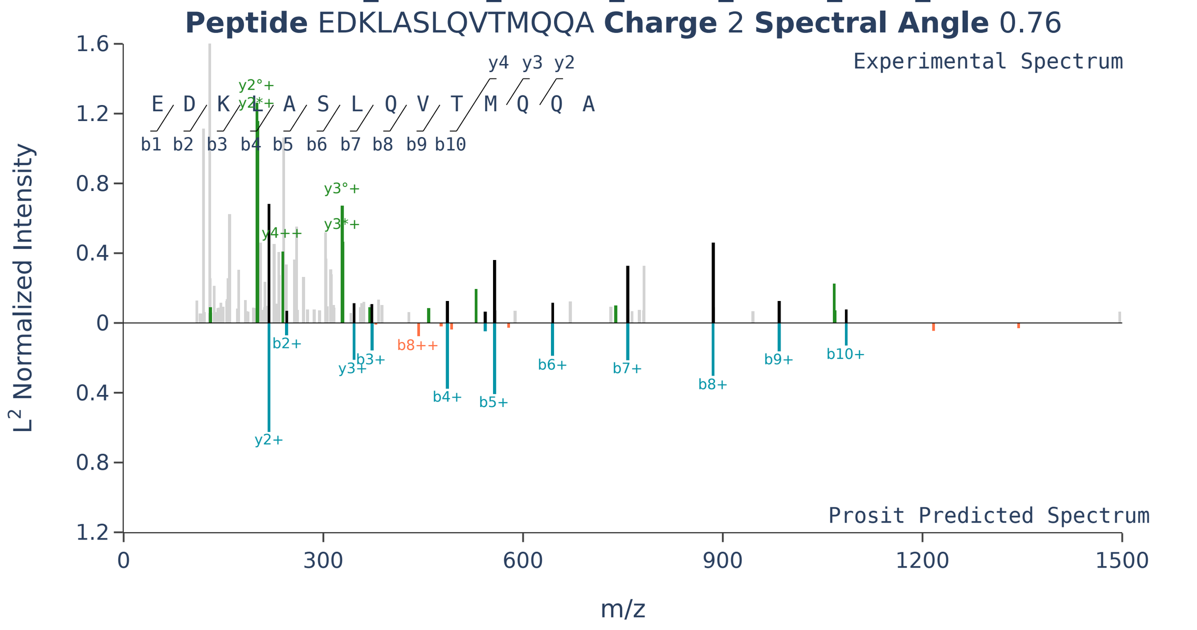

Source HRoetschke_271222_090123_HFGoe_LEDGF_b2R1_24h **Scan** 18806
Peptide FLGPKDIFPYSENKEKYGKPNKREL **Charge** 5 **Spectral Angle** 0.81

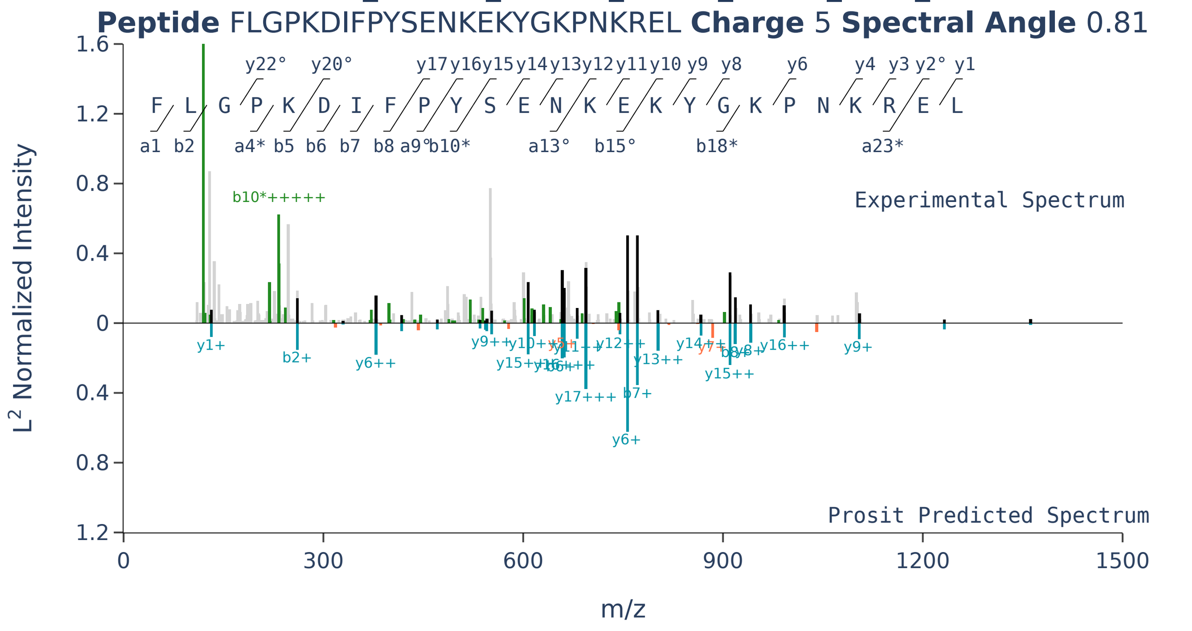

Source HRoetschke_271222_090123_HFGoe_LEDGF_b2R2_4h **Scan** 8917
Peptide NHEASTKSVNL **Charge** 1 **Spectral Angle** 0.74

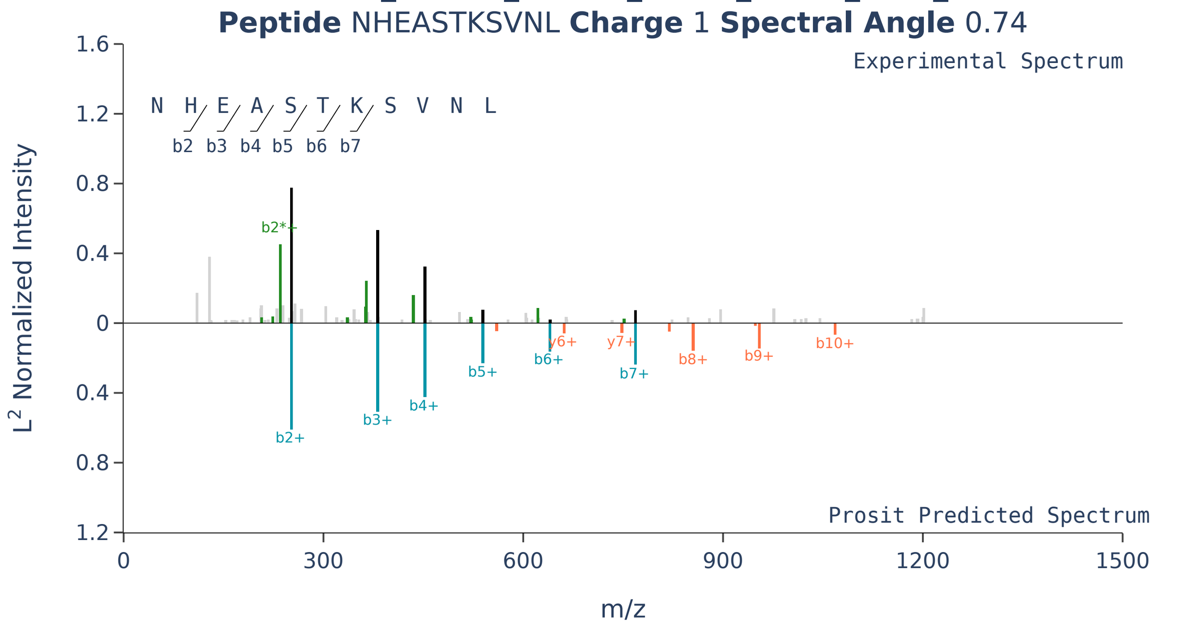

Source HRoetschke_271222_090123_HFGoe_LEDGF_b1R1_4h **Scan** 23753
Peptide SMDSRLQKGFNEGLWE **Charge** 3 **Spectral Angle** 0.83

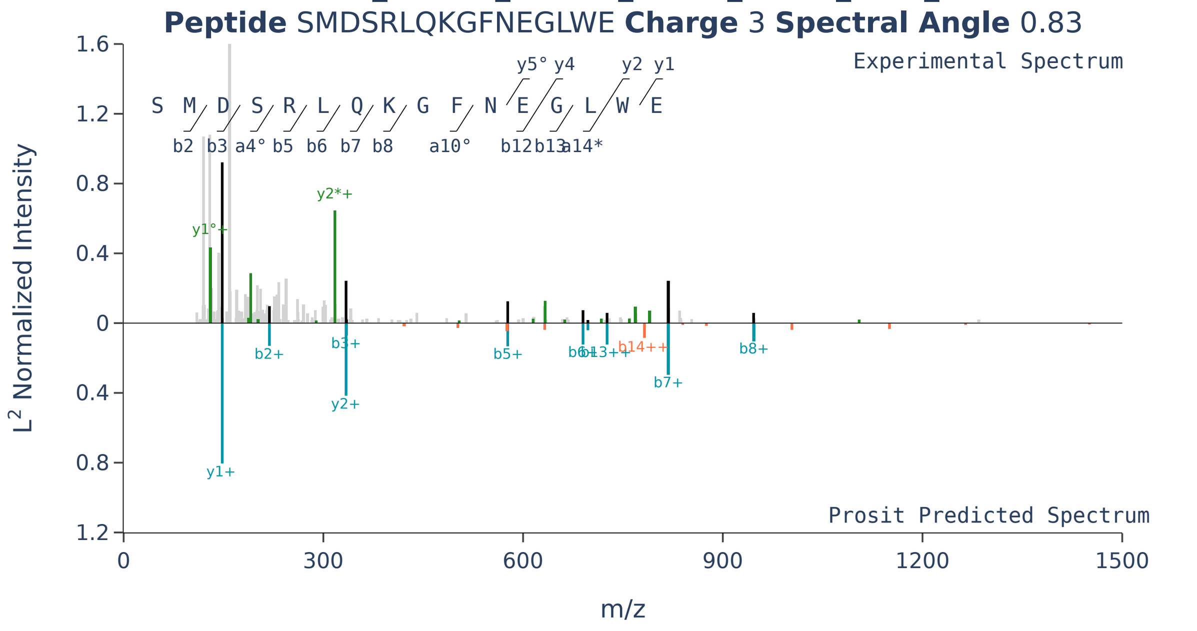

Source HRoetschke_271222_090123_HFGoe_LEDGF_b2R1_4h **Scan** 11531
Peptide IMEKSTHWPARVDE **Charge** 4 **Spectral Angle** 0.93

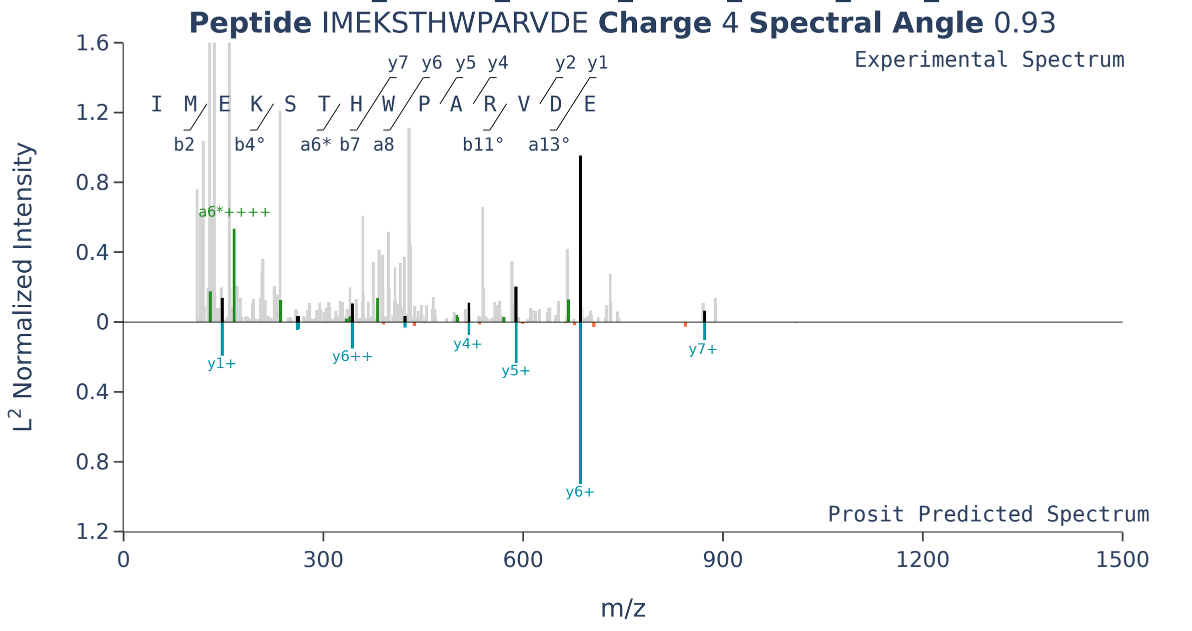

Source HRoetschke_271222_090123_HFGoe_LEDGF_b1R1_4h **Scan** 24843
Peptide NPMTRDFKPGDLIFAKMKGY **Charge** 3 **Spectral Angle** 0.82

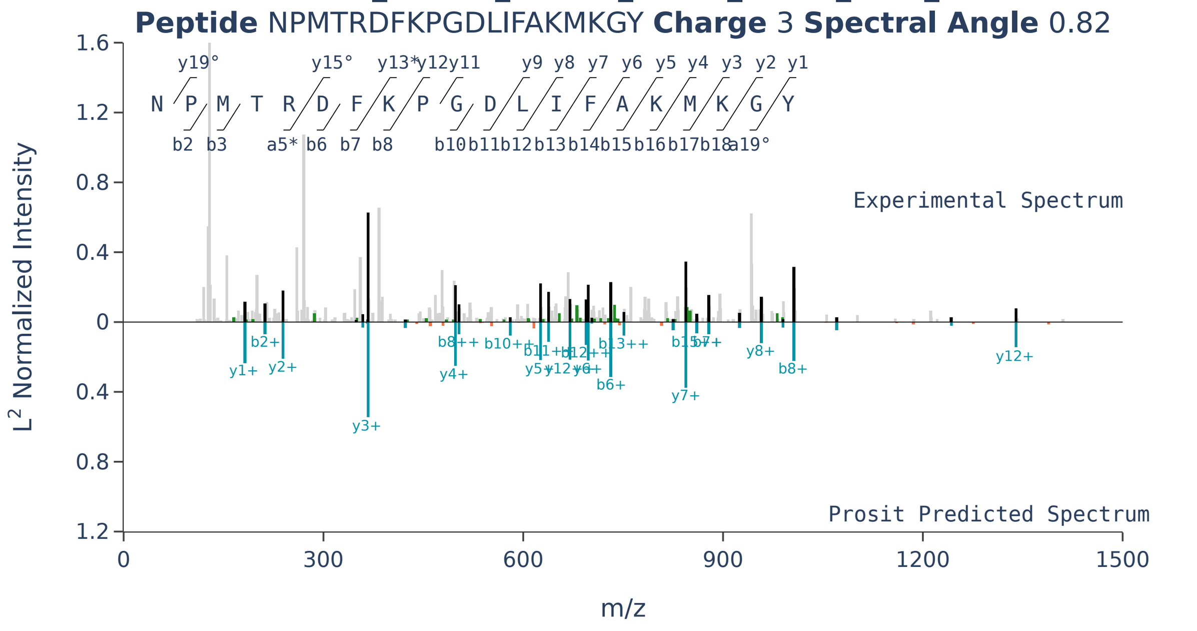

Source HRoetschke_271222_090123_HFGoe_LEDGF_b1R2_24h **Scan** 18484
Peptide ATKQSKGFNEGLWE **Charge** 2 **Spectral Angle** 0.84

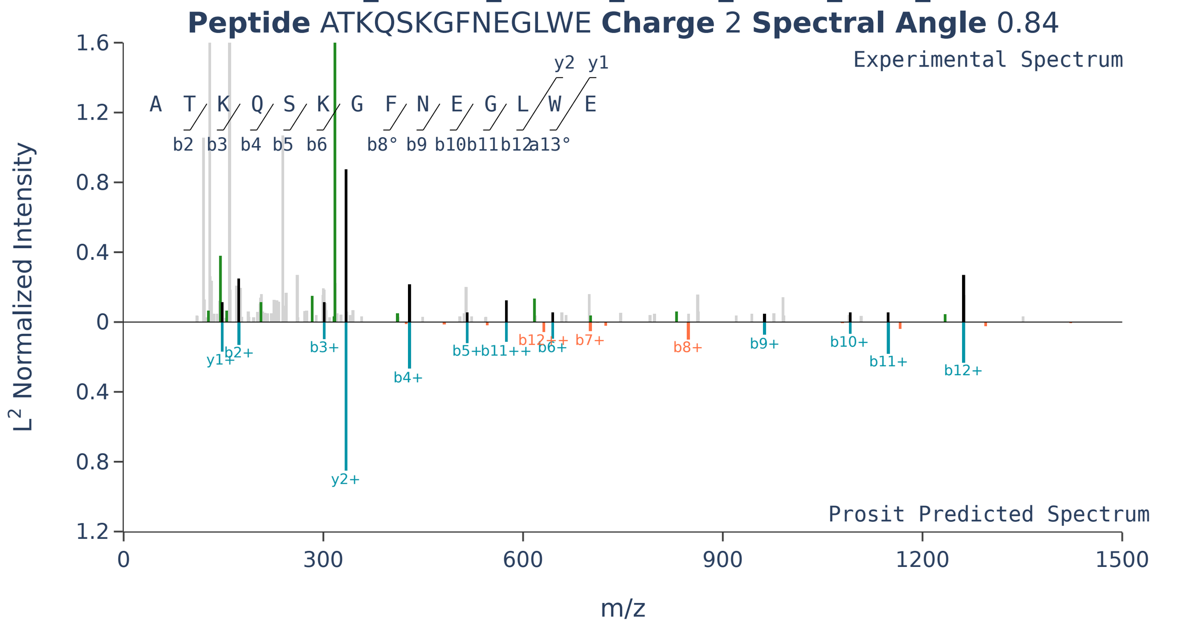

Source HRoetschke_271222_090123_HFGoe_LEDGF_b2R1_24h **Scan** 23945
Peptide RETEISLELA **Charge** 2 **Spectral Angle** 0.85

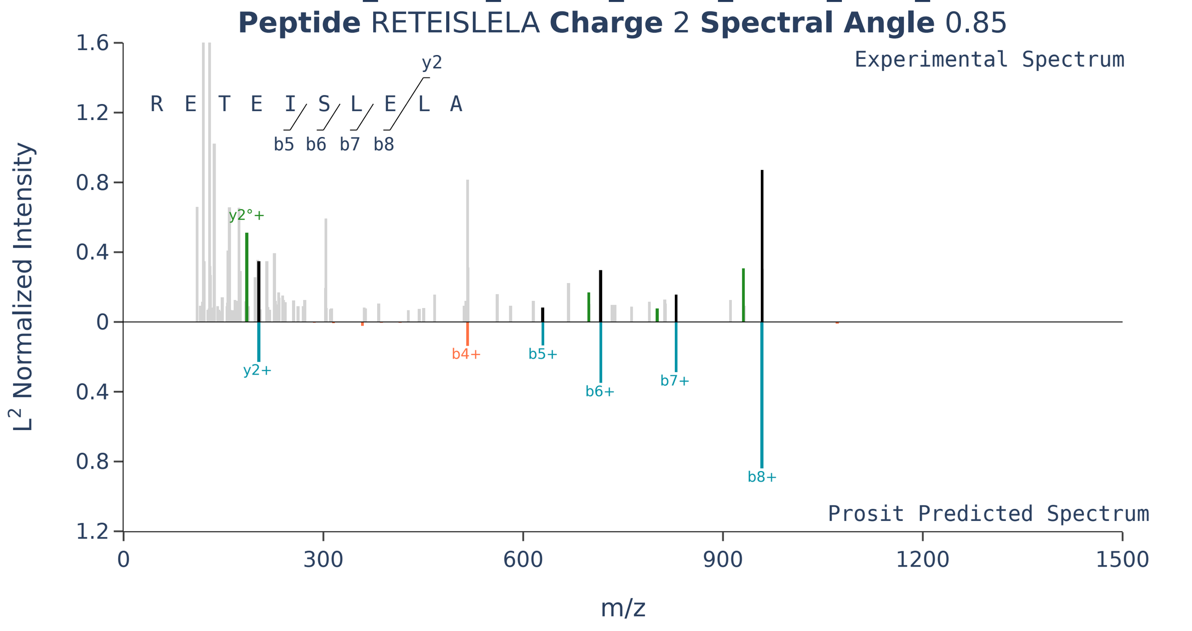

Source HRoetschke_271222_090123_HFGoe_LEDGF_b1R2_24h **Scan** 21561
Peptide AKGFNEGLWE **Charge** 2 **Spectral Angle** 0.81

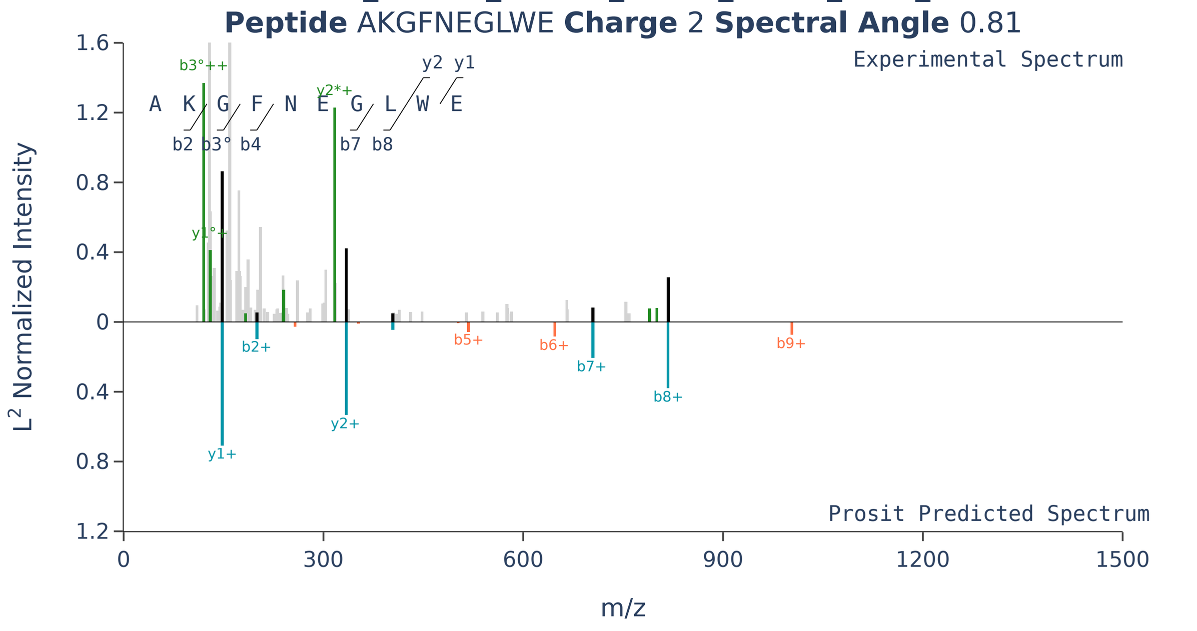

Source HRoetschke_271222_090123_HFGoe_LEDGF_b1R2_1h **Scan** 5452
Peptide KFSSQQAATKQSSEEE **Charge** 2 **Spectral Angle** 0.76

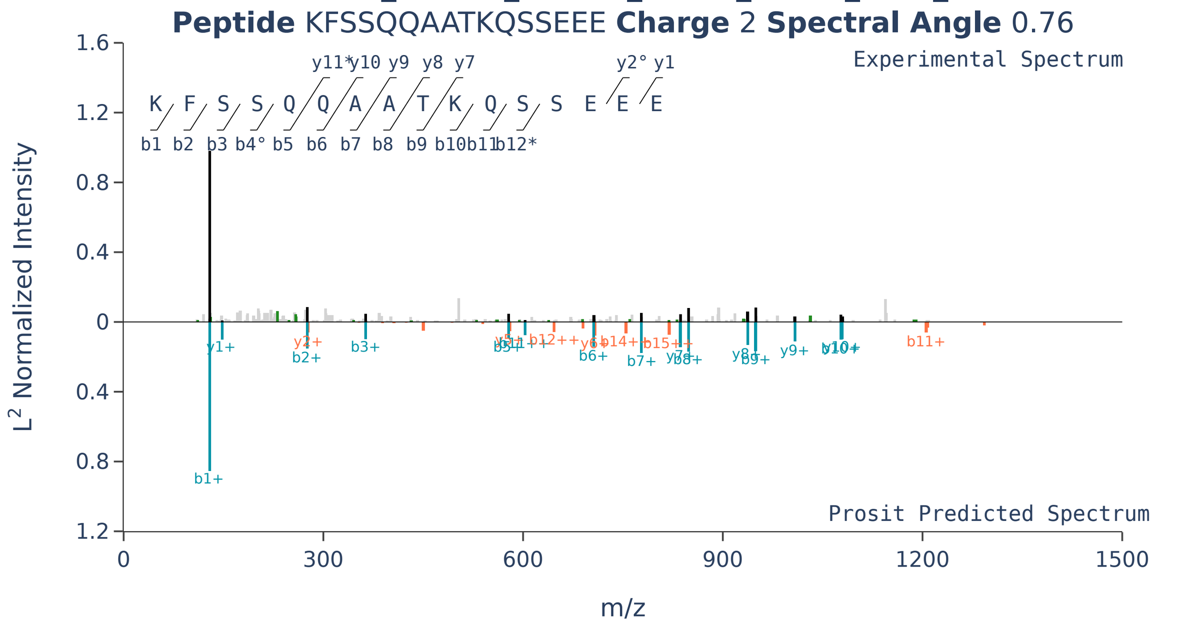

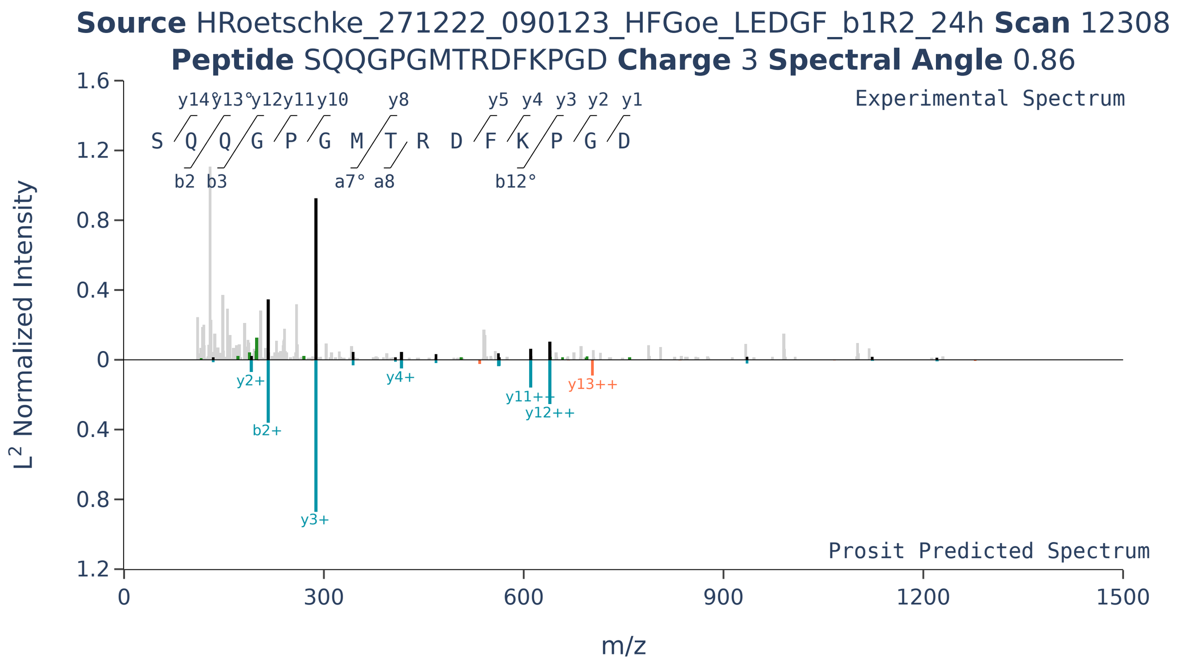
Source HRoetschke_271222_090123_HFGoe_LEDGF_b1R2_24h **Scan** 12308
Peptide SQQGPGMTRDFKPGD **Charge** 3 **Spectral Angle** 0.86
Experimental Spectrum
Prosit Predicted Spectrum
L² Normalized Intensity
m/z

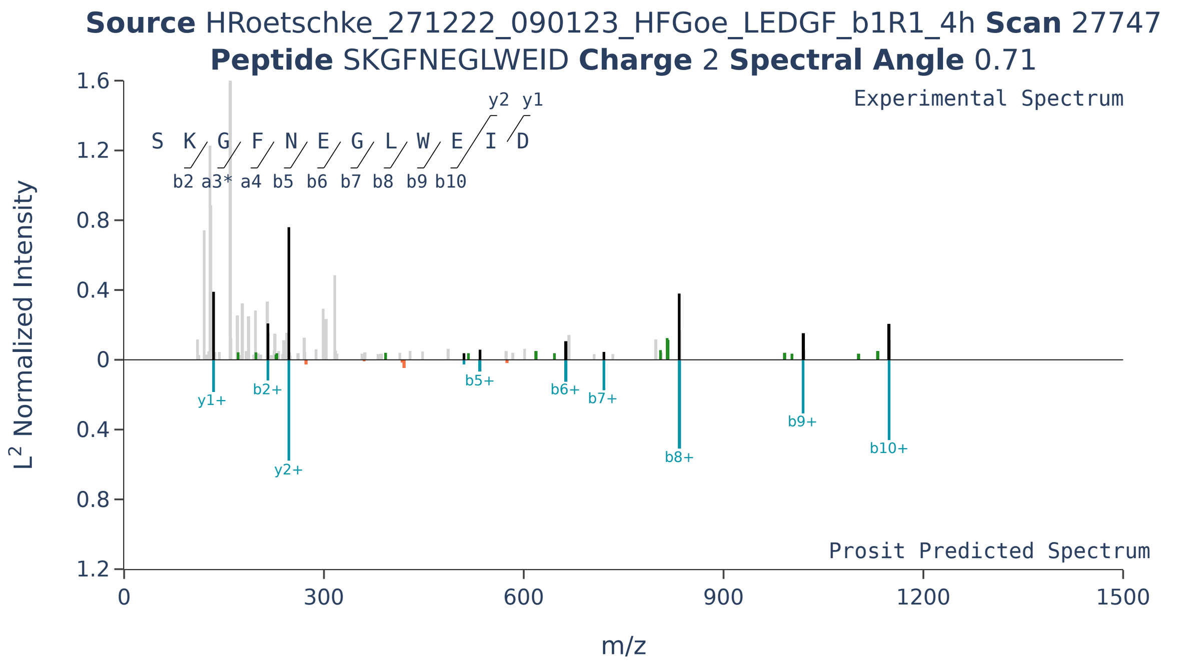
Source HRoetschke_271222_090123_HFGoe_LEDGF_b1R1_4h **Scan** 27747
Peptide SKGFNEGLWEID **Charge** 2 **Spectral Angle** 0.71
Experimental Spectrum
Prosit Predicted Spectrum
L² Normalized Intensity
m/z

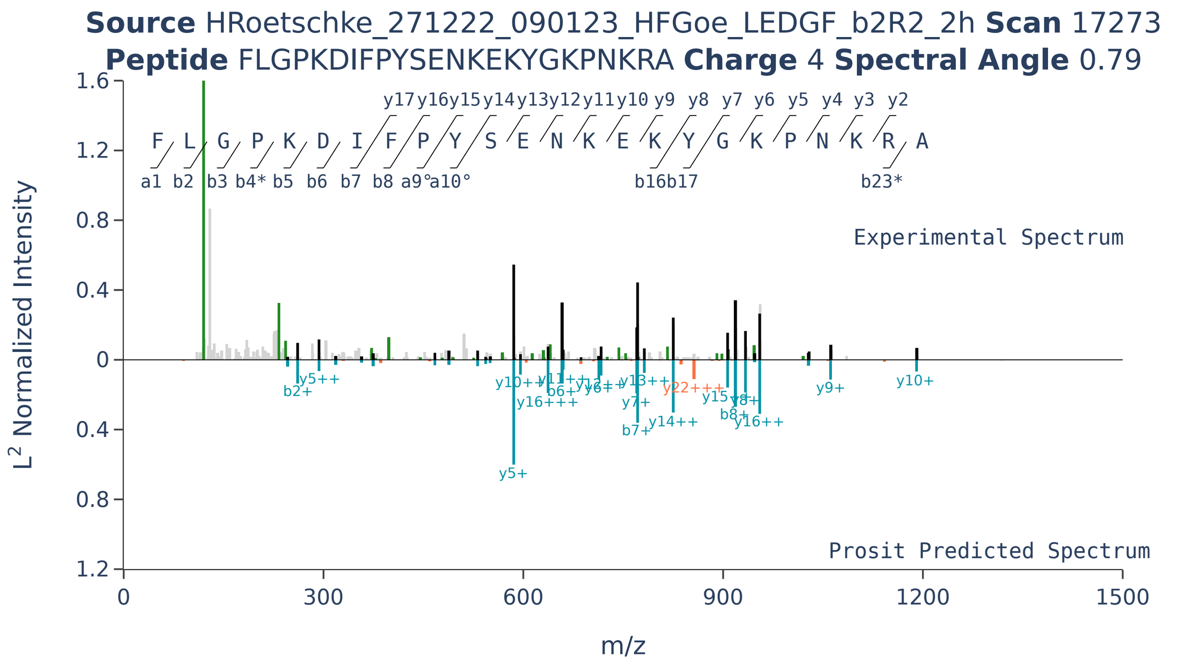
Source HRoetschke_271222_090123_HFGoe_LEDGF_b2R2_2h **Scan** 17273
Peptide FLGPKDIFPYSENKEKYGKPNKRA **Charge** 4 **Spectral Angle** 0.79
Experimental Spectrum
Prosit Predicted Spectrum
L² Normalized Intensity
m/z

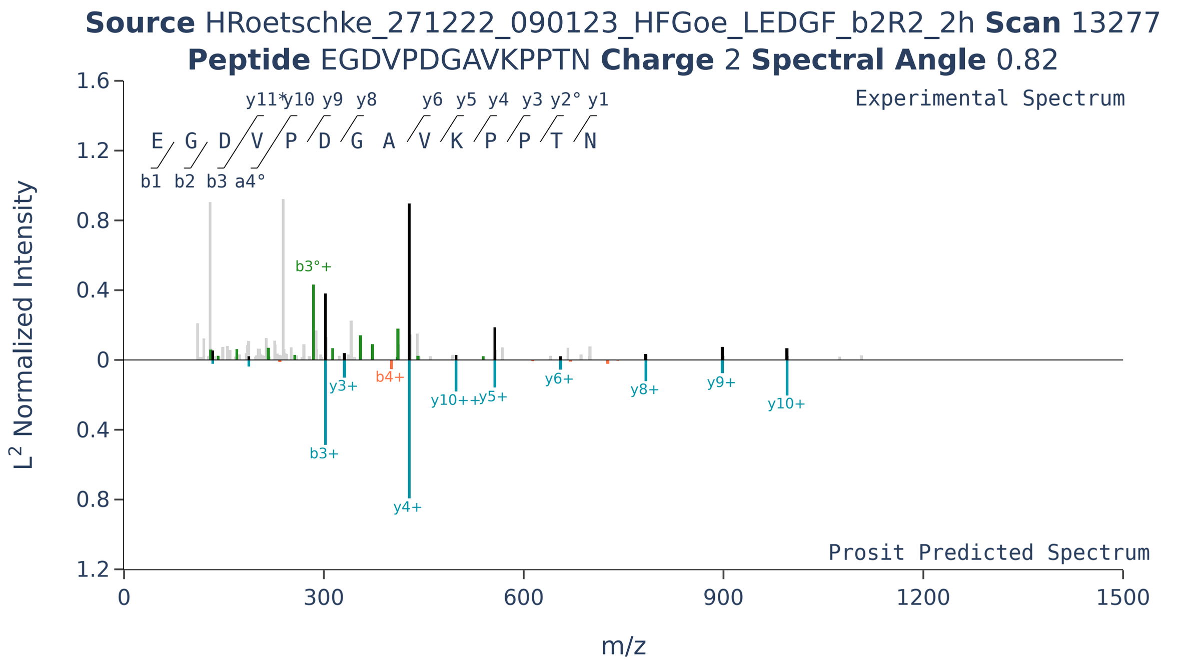
Source HRoetschke_271222_090123_HFGoe_LEDGF_b2R2_2h **Scan** 13277
Peptide EGDVPDGAVKPPTN **Charge** 2 **Spectral Angle** 0.82
Experimental Spectrum
Prosit Predicted Spectrum
L² Normalized Intensity
m/z

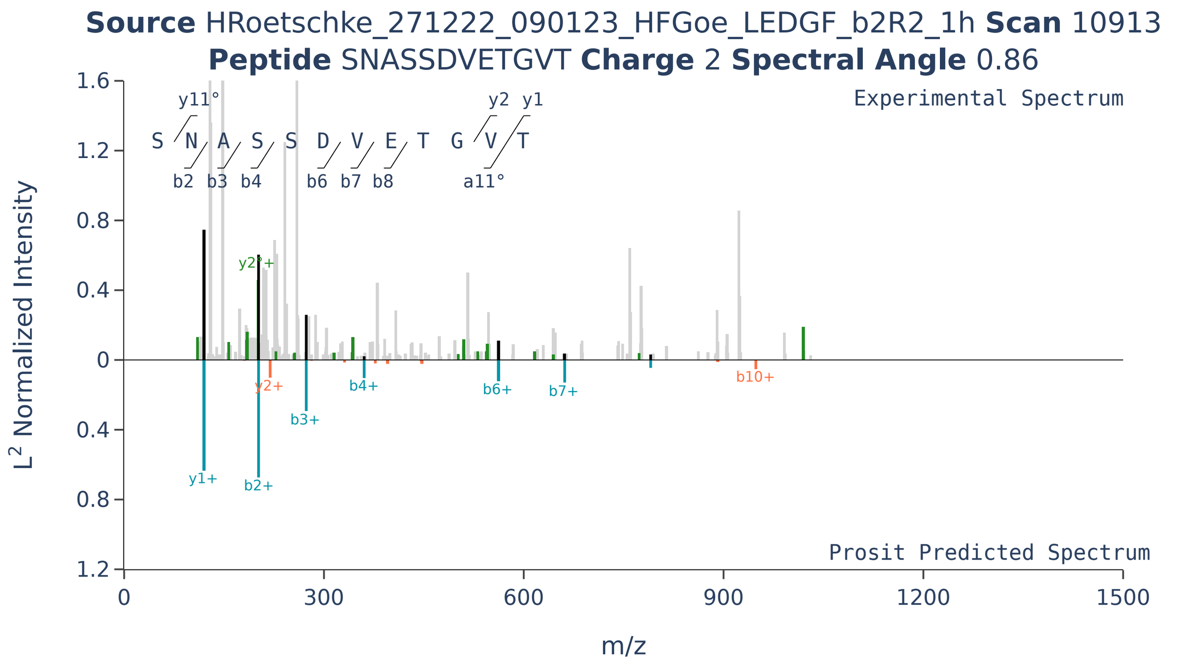
Source HRoetschke_271222_090123_HFGoe_LEDGF_b2R2_1h **Scan** 10913
Peptide SNASSDVETGVT **Charge** 2 **Spectral Angle** 0.86
Experimental Spectrum
Prosit Predicted Spectrum
L² Normalized Intensity
m/z

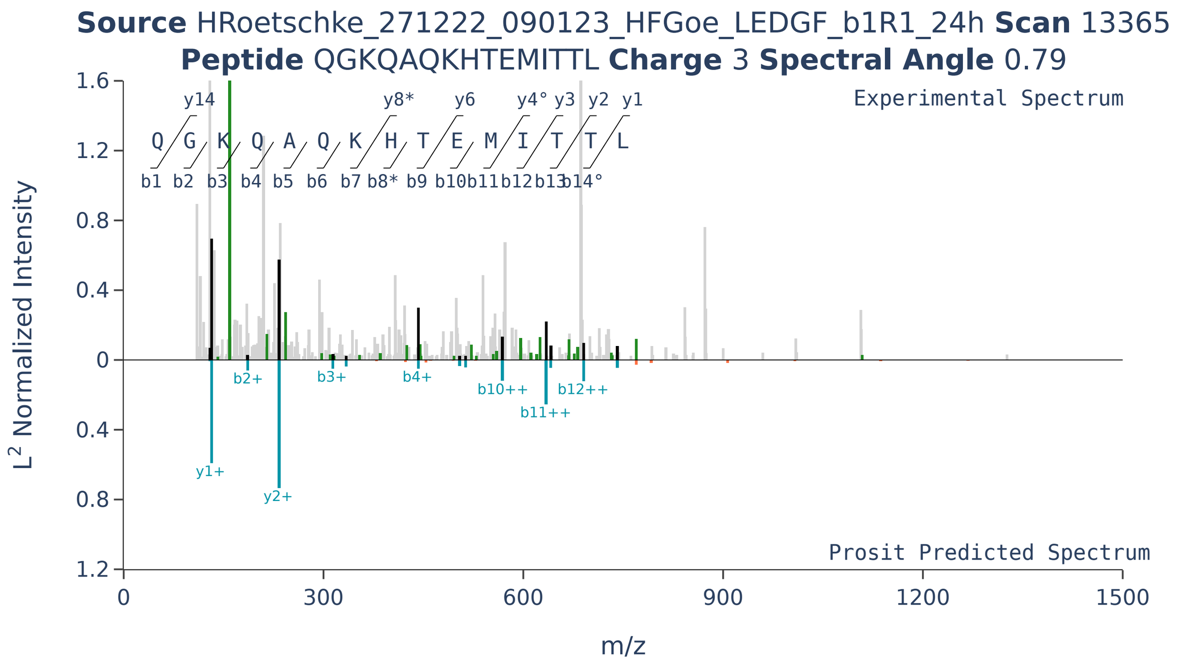
Source HRoetschke_271222_090123_HFGoe_LEDGF_b1R1_24h **Scan** 13365
Peptide QGKQAQKHTEMITTL **Charge** 3 **Spectral Angle** 0.79
Experimental Spectrum
Prosit Predicted Spectrum
L² Normalized Intensity
m/z

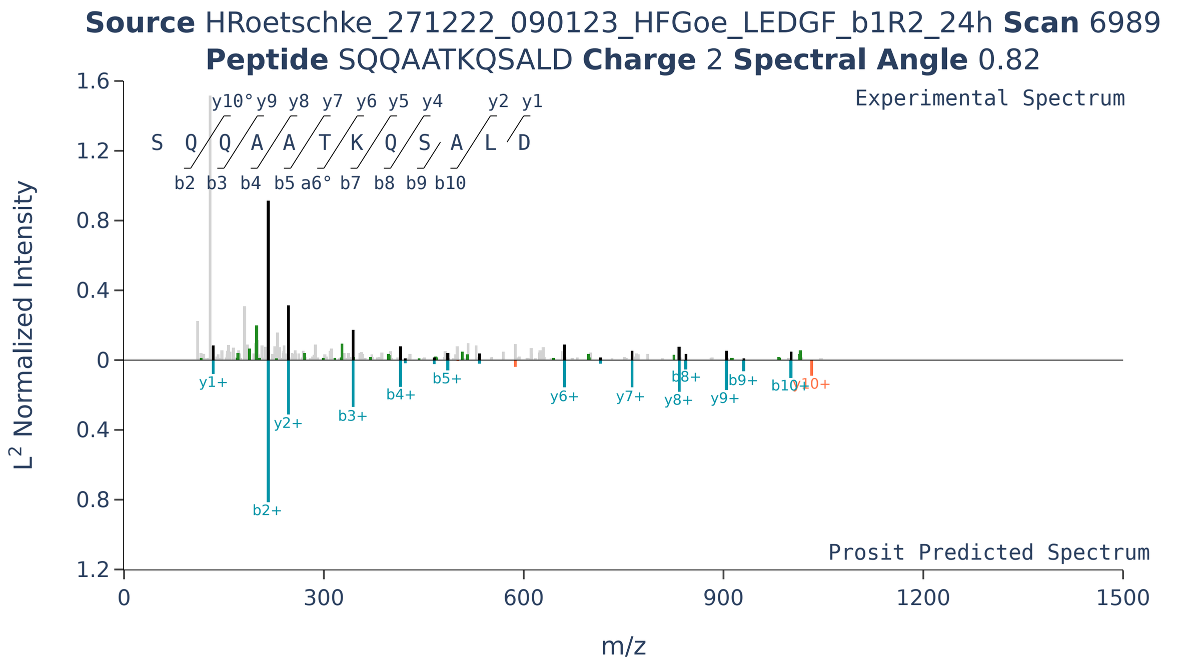
Source HRoetschke_271222_090123_HFGoe_LEDGF_b1R2_24h **Scan** 6989
Peptide SQQAATKQSALD **Charge** 2 **Spectral Angle** 0.82
Experimental Spectrum
Prosit Predicted Spectrum
L² Normalized Intensity
m/z

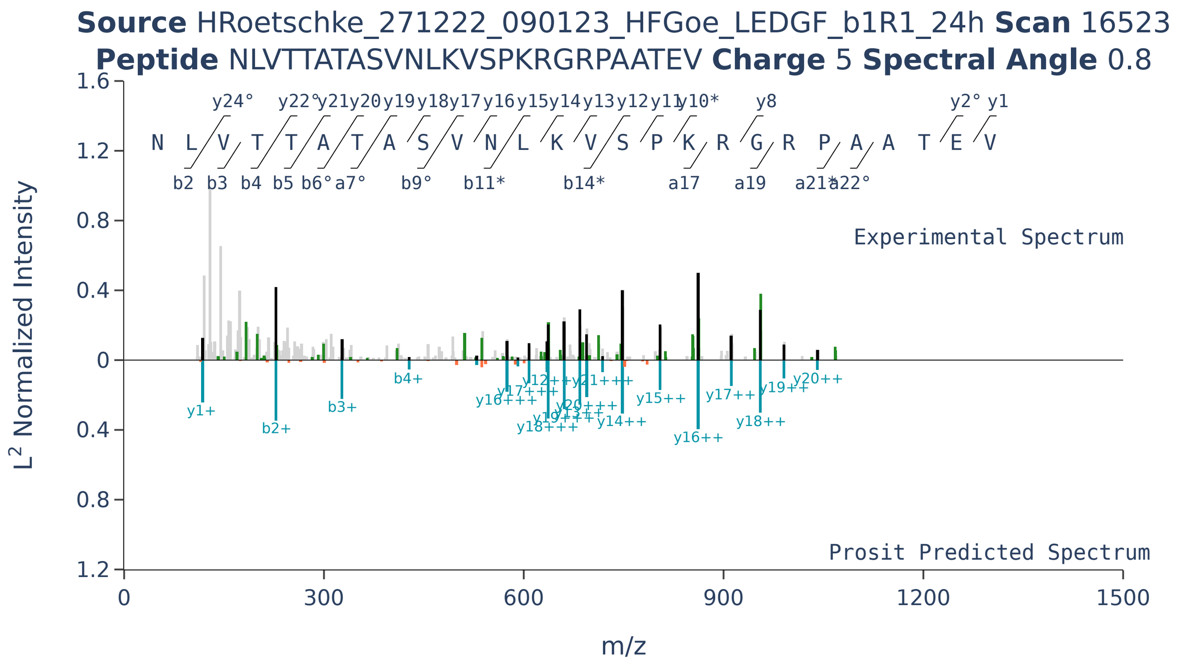
Source HRoetschke_271222_090123_HFGoe_LEDGF_b1R1_24h **Scan** 16523
Peptide NLVTTATASVNLKVSPKRGRPAATEV **Charge** 5 **Spectral Angle** 0.8
Experimental Spectrum
Prosit Predicted Spectrum
L² Normalized Intensity
m/z

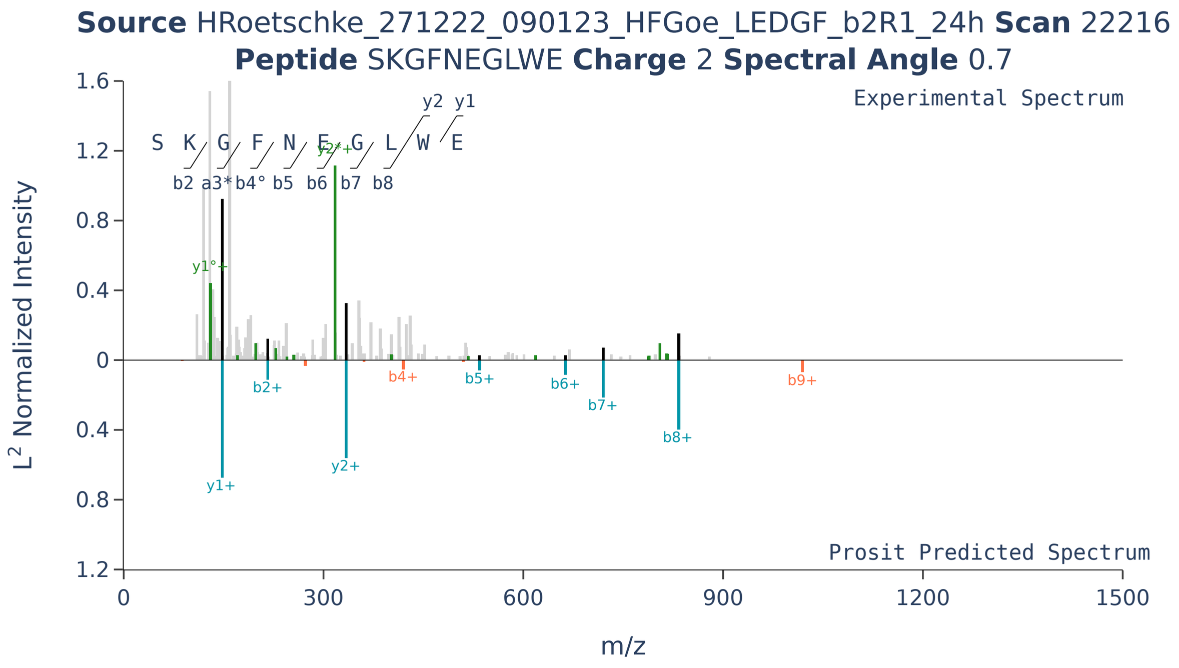
Source HRoetschke_271222_090123_HFGoe_LEDGF_b2R1_24h **Scan** 22216
Peptide SKGFNEGLWE **Charge** 2 **Spectral Angle** 0.7
Experimental Spectrum
Prosit Predicted Spectrum
L² Normalized Intensity
m/z

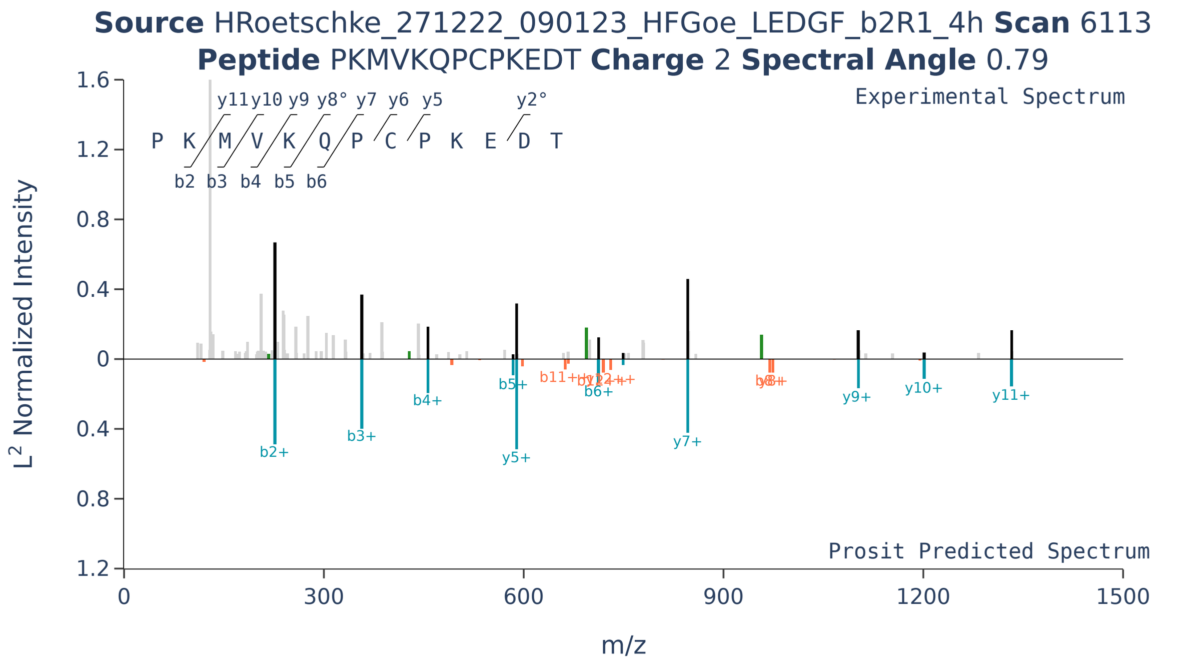
Source HRoetschke_271222_090123_HFGoe_LEDGF_b2R1_4h **Scan** 6113
Peptide PKMVKQPCPKEDT **Charge** 2 **Spectral Angle** 0.79
Experimental Spectrum
Prosit Predicted Spectrum
L² Normalized Intensity
m/z

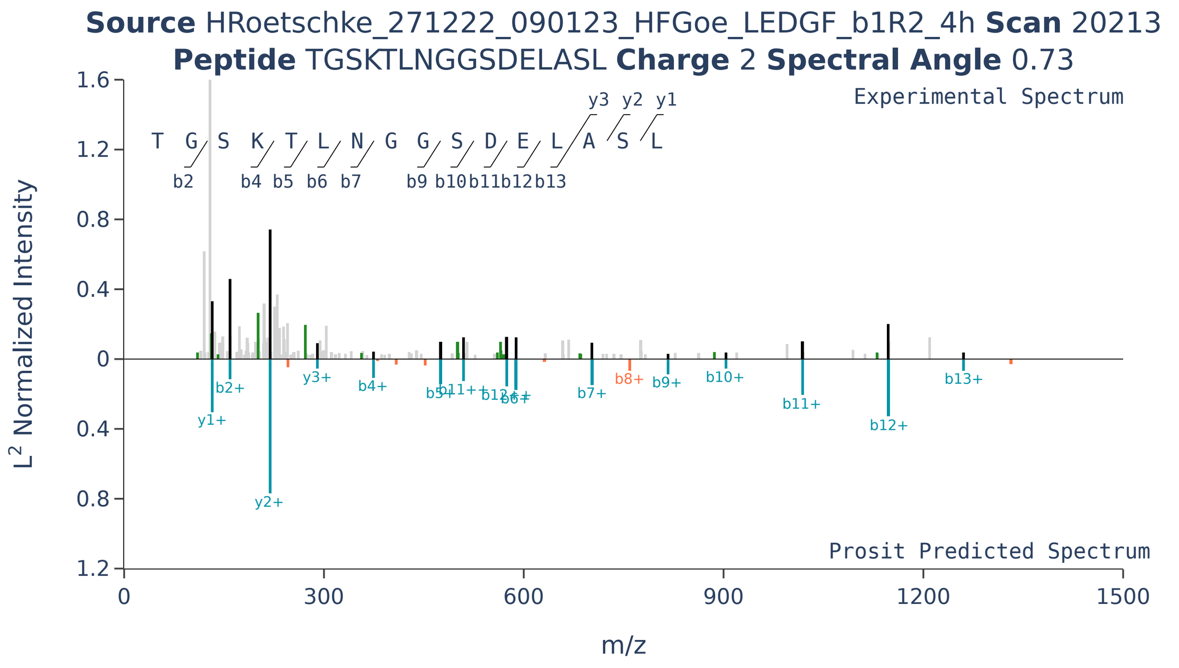
Source HRoetschke_271222_090123_HFGoe_LEDGF_b1R2_4h **Scan** 20213
Peptide TGSKTLNGGSDELASL **Charge** 2 **Spectral Angle** 0.73
Experimental Spectrum
Prosit Predicted Spectrum
L² Normalized Intensity
m/z

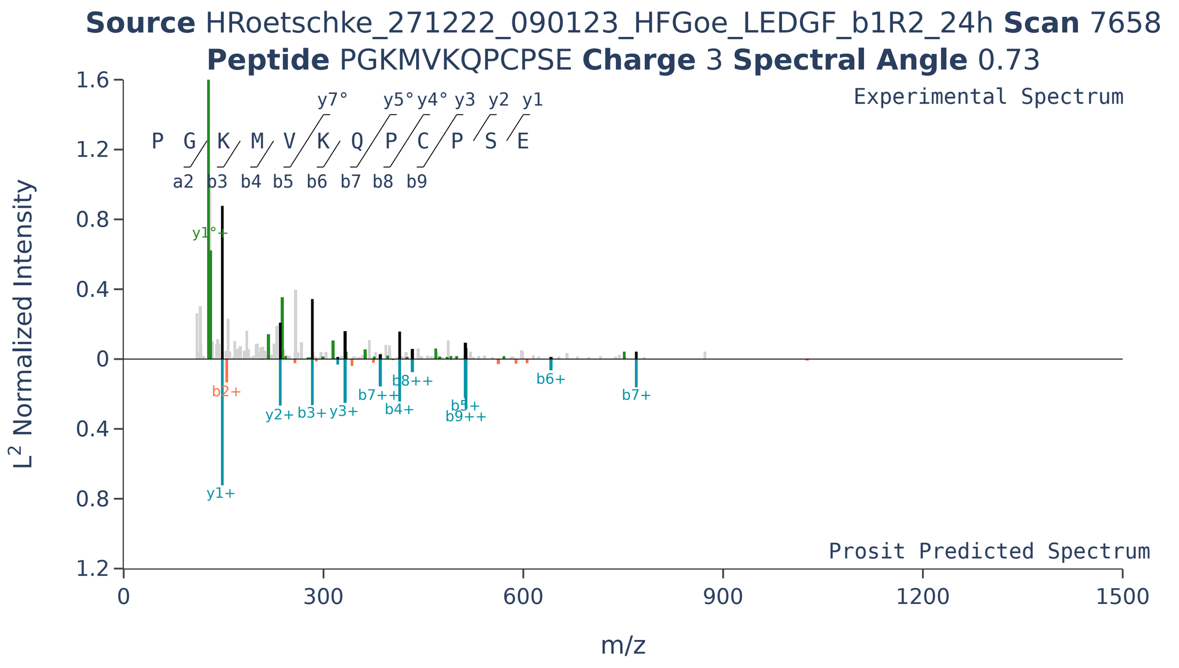
Source HRoetschke_271222_090123_HFGoe_LEDGF_b1R2_24h **Scan** 7658
Peptide PGKMVKQPCPSE **Charge** 3 **Spectral Angle** 0.73
Experimental Spectrum
Prosit Predicted Spectrum
L² Normalized Intensity
m/z

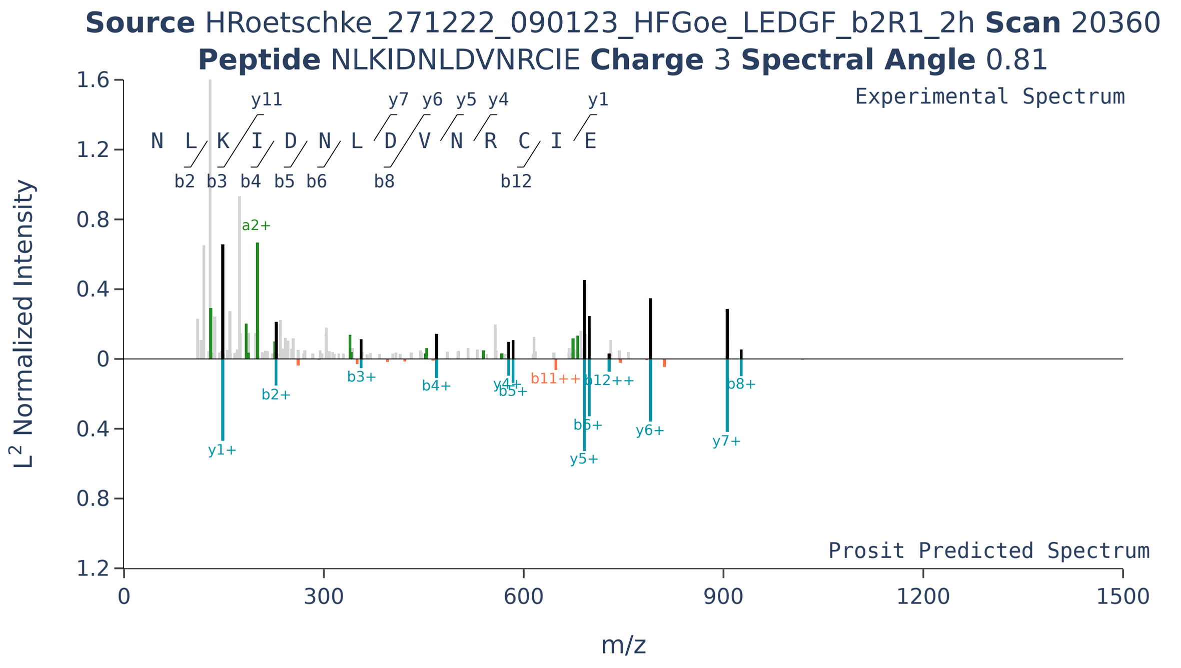
Source HRoetschke_271222_090123_HFGoe_LEDGF_b2R1_2h **Scan** 20360
Peptide NLKIDNLDVNRCIE **Charge** 3 **Spectral Angle** 0.81
Experimental Spectrum
Prosit Predicted Spectrum
L² Normalized Intensity
m/z

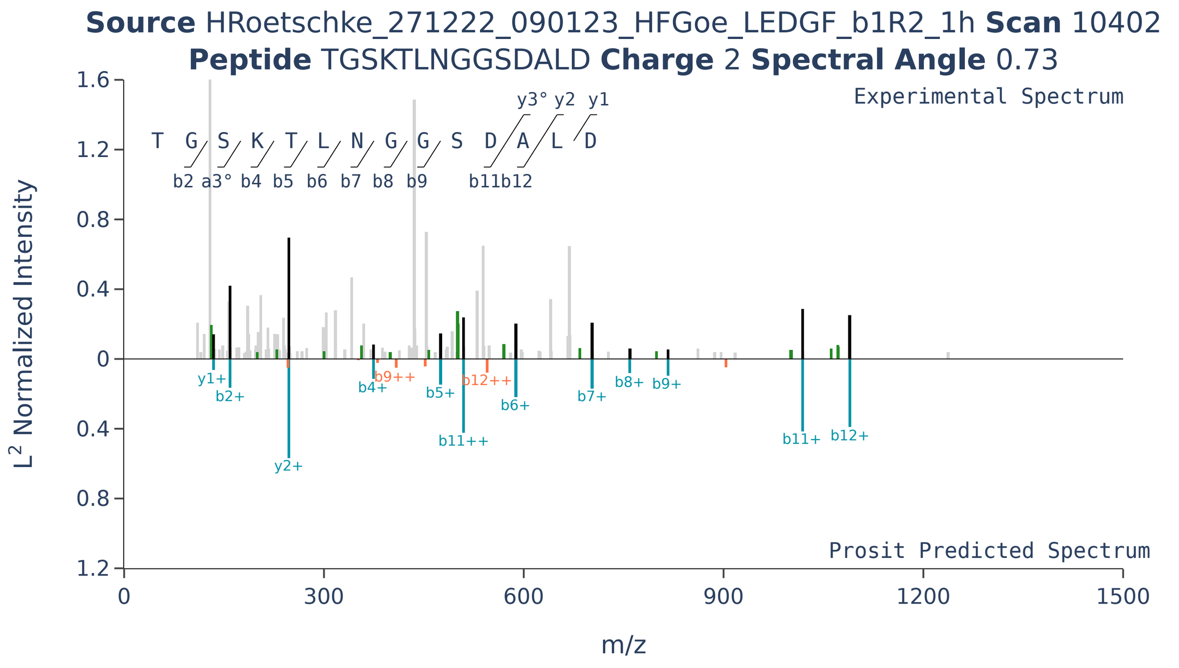
Source HRoetschke_271222_090123_HFGoe_LEDGF_b1R2_1h **Scan** 10402
Peptide TGSKTLNGGSDALD **Charge** 2 **Spectral Angle** 0.73
Experimental Spectrum
Prosit Predicted Spectrum
L² Normalized Intensity
m/z

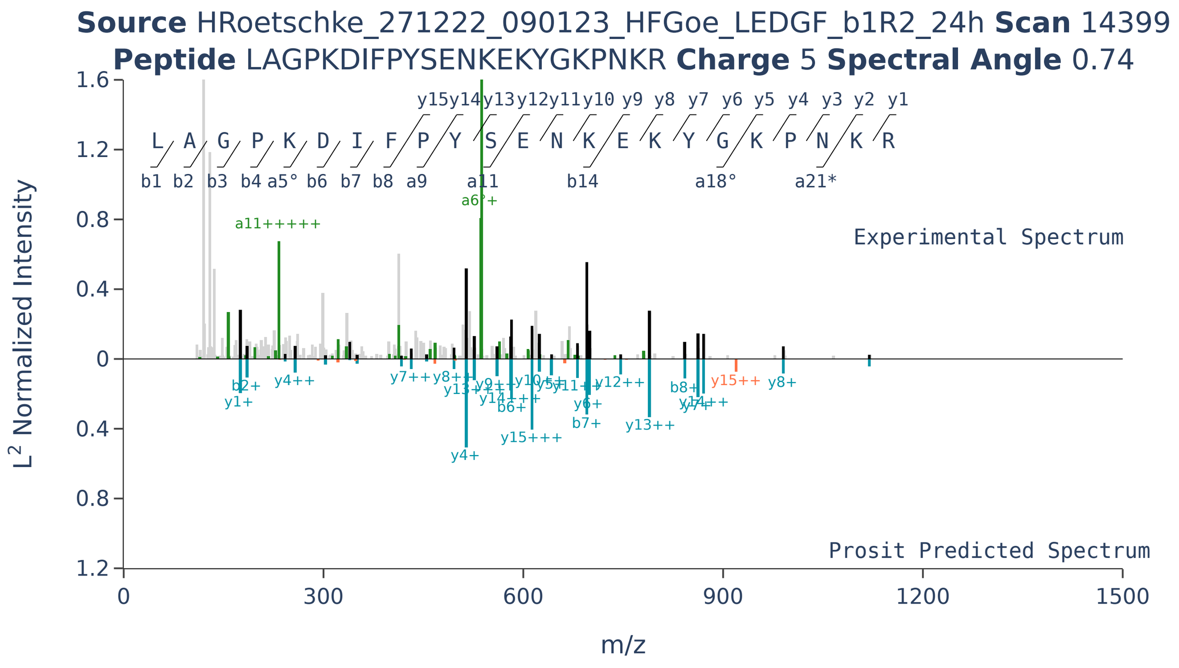
Source HRoetschke_271222_090123_HFGoe_LEDGF_b1R2_24h **Scan** 14399
Peptide LAGPKDIFPYSENKEKYGKPNKR **Charge** 5 **Spectral Angle** 0.74
Experimental Spectrum
Prosit Predicted Spectrum
L² Normalized Intensity
m/z

Source HRoetschke_271222_090123_HFGoe_LEDGF_b1R1_24h **Scan** 27943
Peptide DELALASL **Charge** 2 **Spectral Angle** 0.77

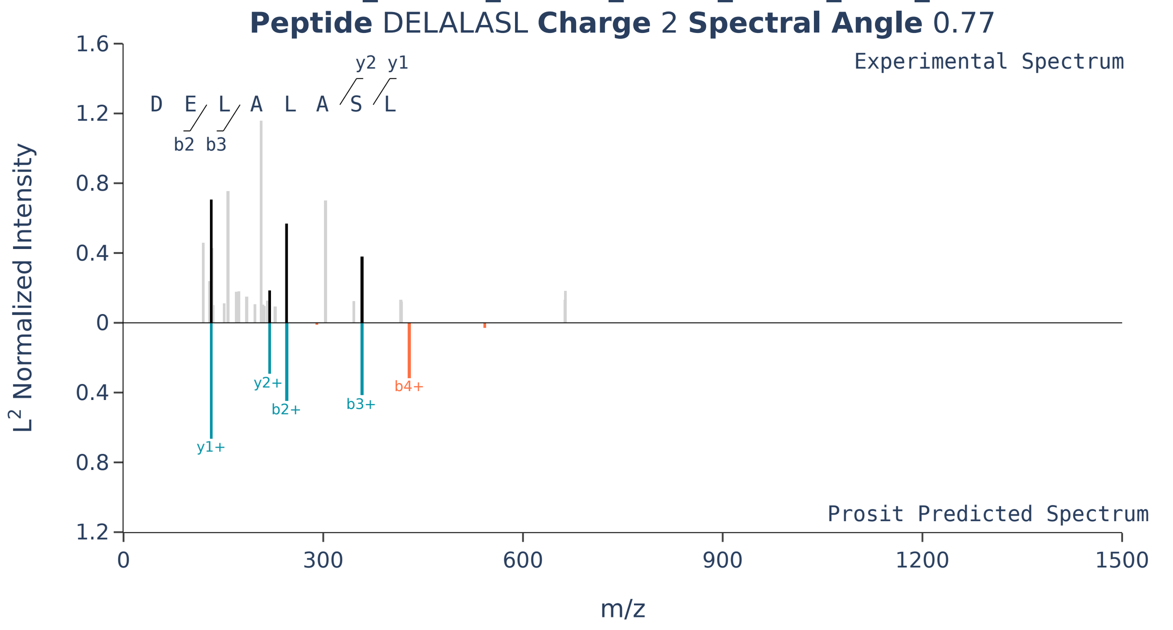

Source HRoetschke_271222_090123_HFGoe_LEDGF_b1R2_24h **Scan** 14122
Peptide NKYPHWPARV **Charge** 3 **Spectral Angle** 0.92

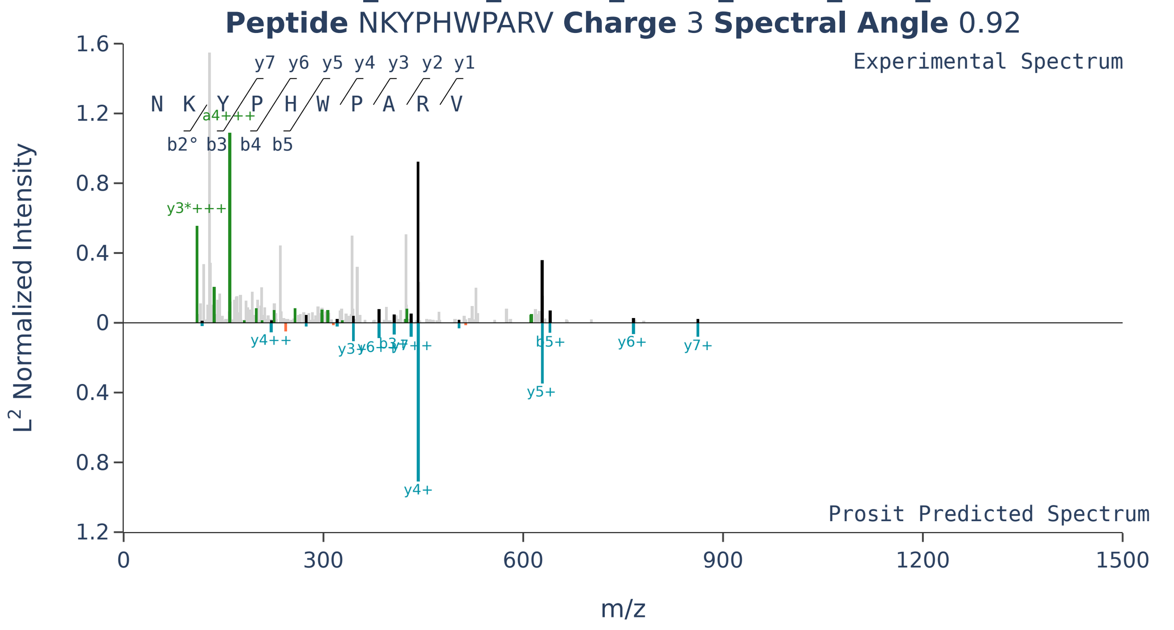

Source HRoetschke_271222_090123_HFGoe_LEDGF_b2R1_24h **Scan** 26223
Peptide AELASLQVTMQQA **Charge** 2 **Spectral Angle** 0.77

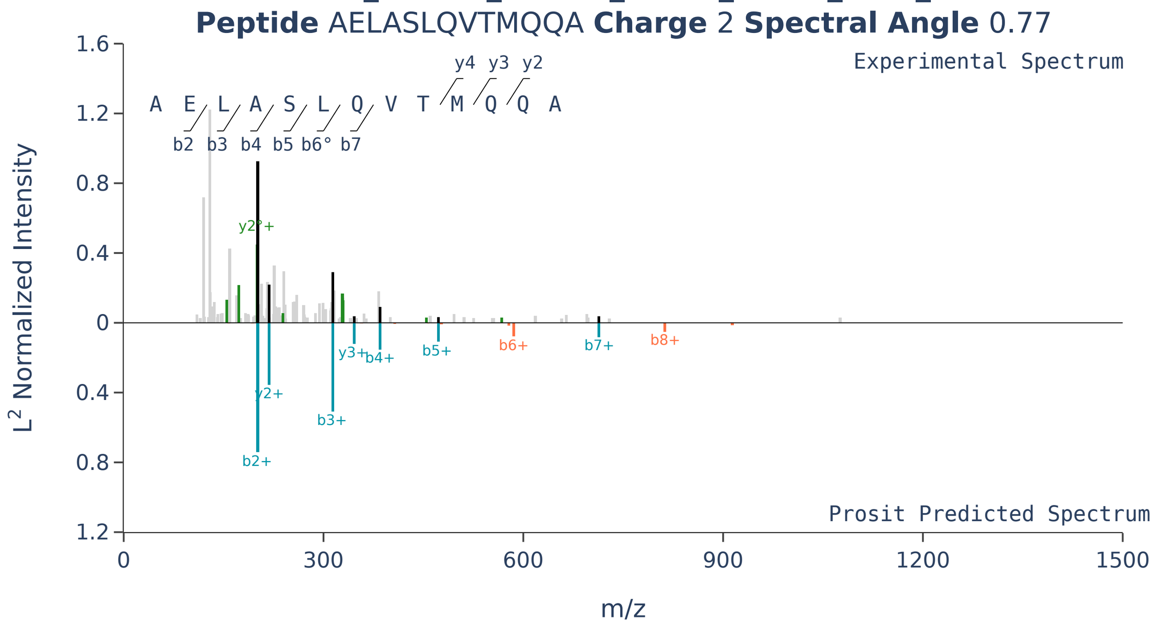

Source HRoetschke_271222_090123_HFGoe_LEDGF_b1R1_4h **Scan** 19813
Peptide DNNPKVKFSAF **Charge** 3 **Spectral Angle** 0.93

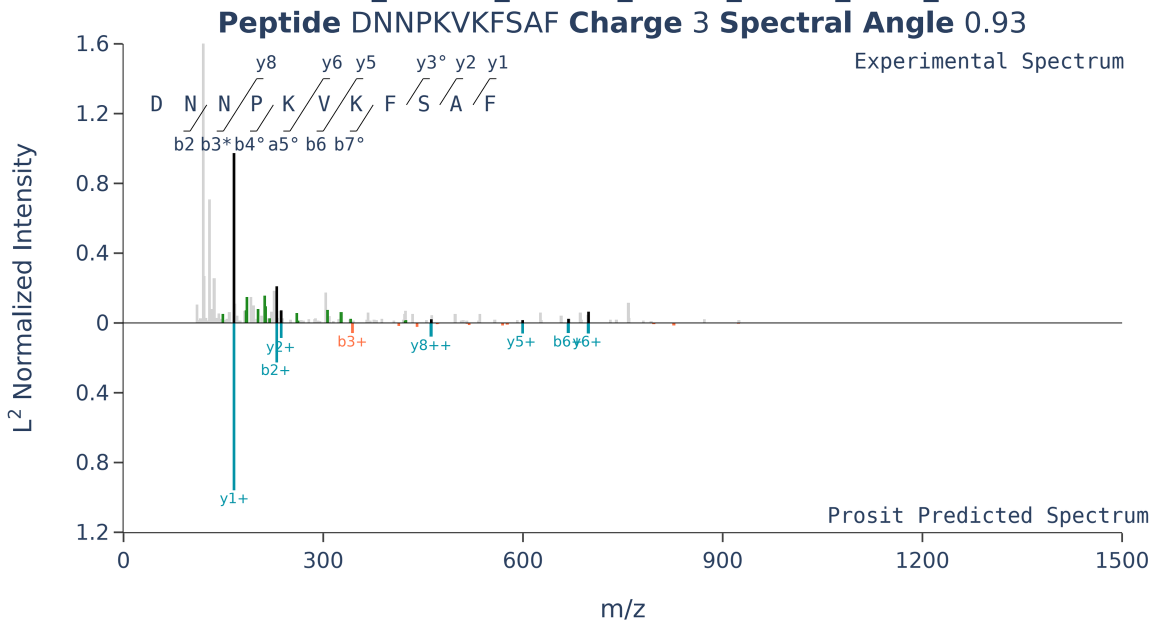

Source HRoetschke_271222_090123_HFGoe_LEDGF_b2R2_2h **Scan** 15389
Peptide SQQAPHWPARV **Charge** 3 **Spectral Angle** 0.9

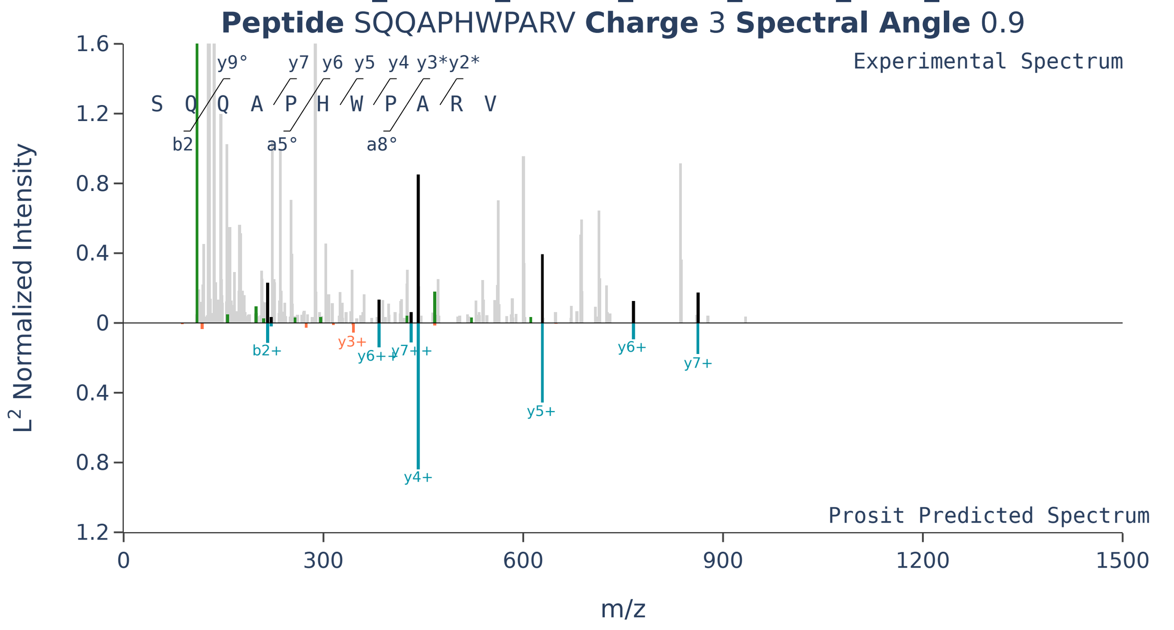

Source HRoetschke_271222_090123_HFGoe_LEDGF_b2R2_2h **Scan** 18327
Peptide EGDYPHWPARVDE **Charge** 3 **Spectral Angle** 0.87

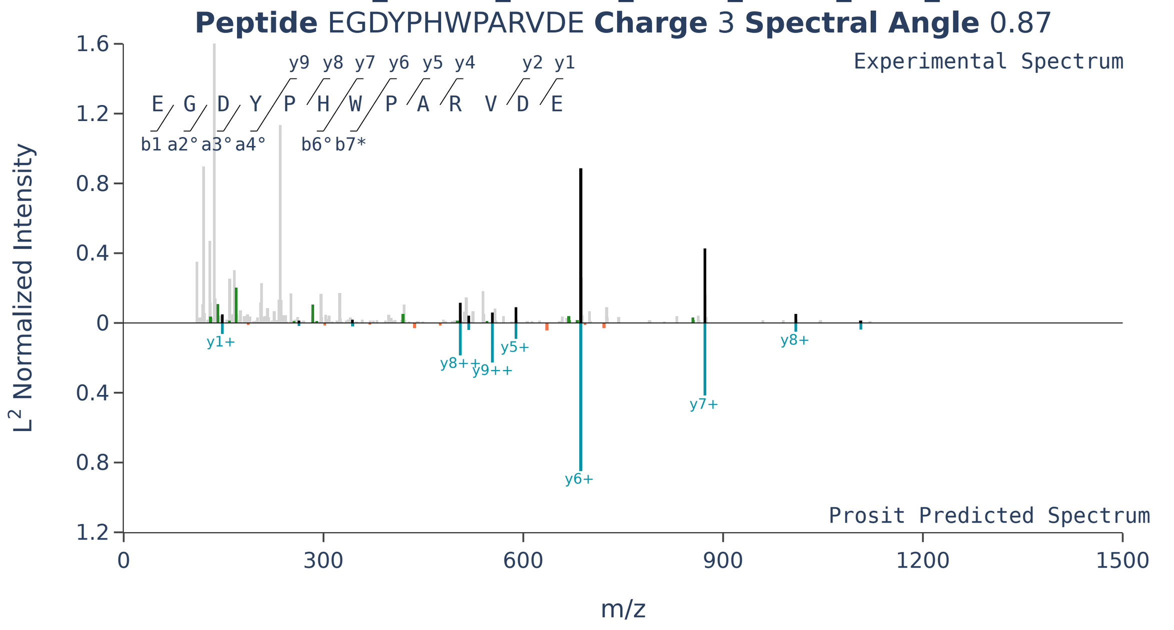

Source HRoetschke_271222_090123_HFGoe_LEDGF_b1R2_24h **Scan** 14150
Peptide KNMFLVGKETSVSKE **Charge** 3 **Spectral Angle** 0.86

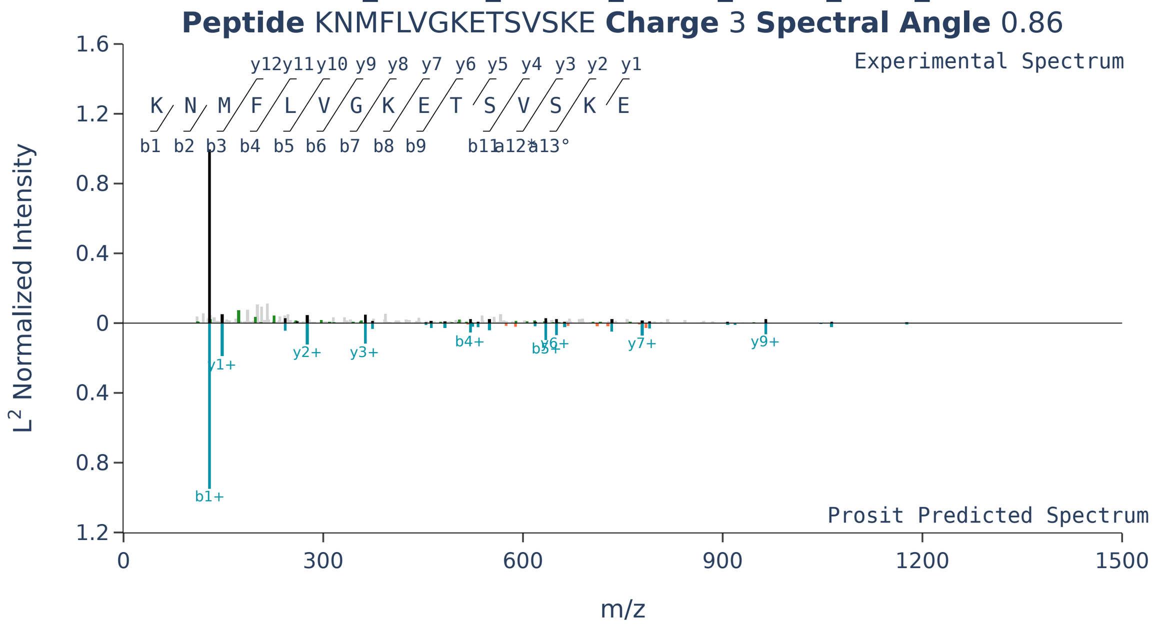

Source HRoetschke_271222_090123_HFGoe_LEDGF_b2R1_4h **Scan** 18290
Peptide ATAFLGPKDIFPYSENKEKYGKPNKR **Charge** 4 **Spectral Angle** 0.79

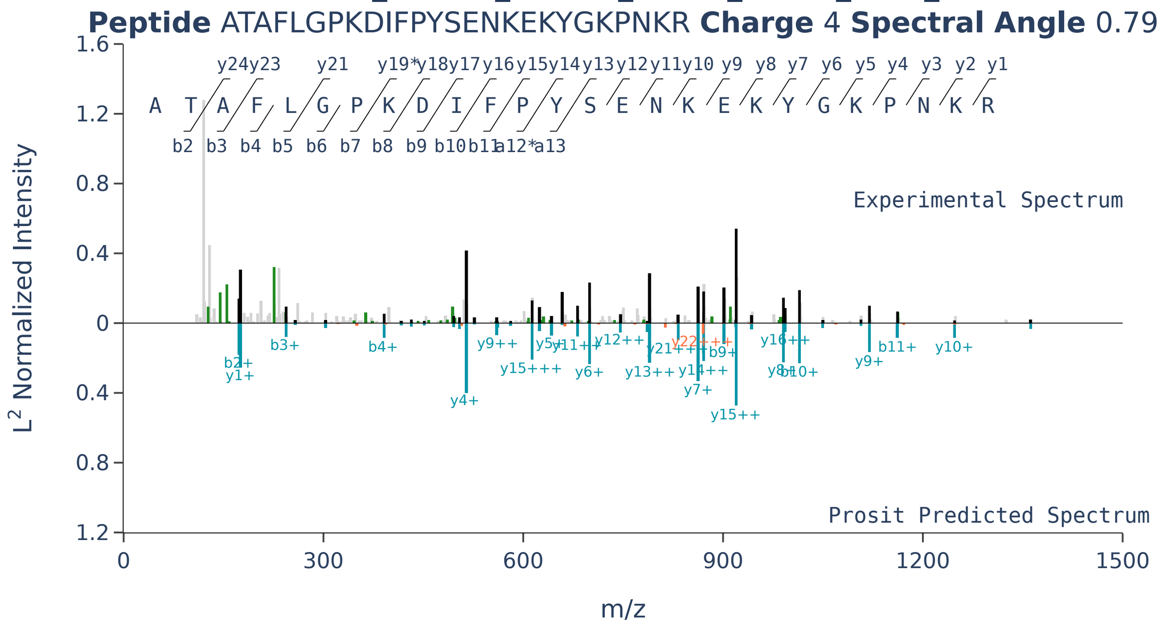

Source HRoetschke_271222_090123_HFGoe_LEDGF_b1R2_24h **Scan** 17255
Peptide SQGYPHWPARVDE **Charge** 3 **Spectral Angle** 0.86

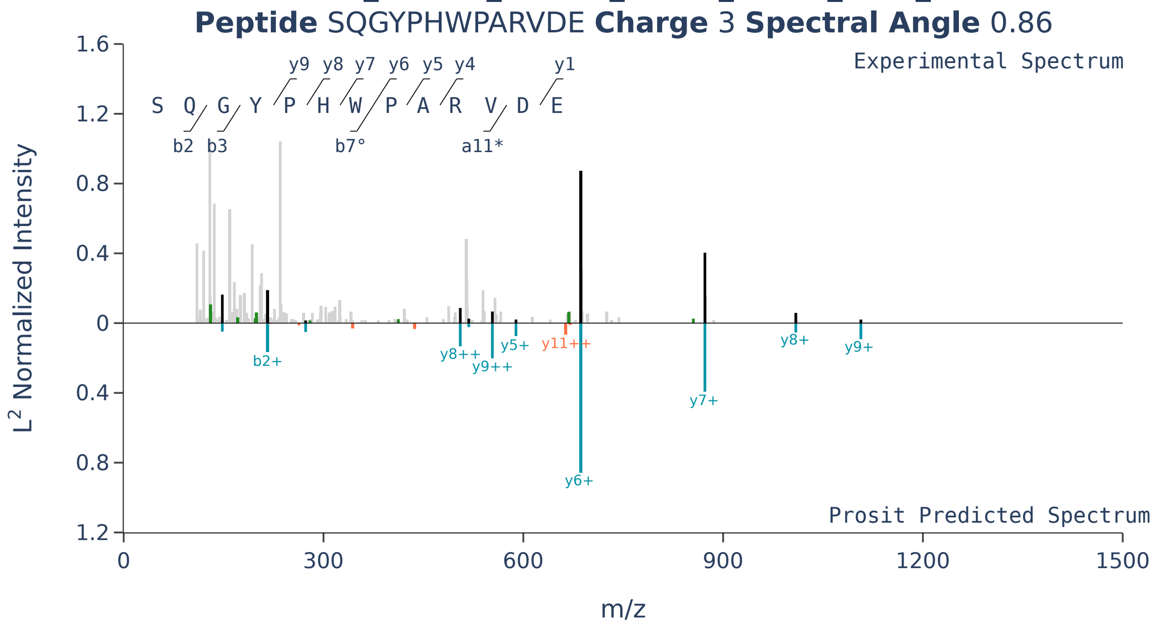

Source HRoetschke_271222_090123_HFGoe_LEDGF_b2R1_1h **Scan** 9621
Peptide KMKGTEISLKD **Charge** 3 **Spectral Angle** 0.93

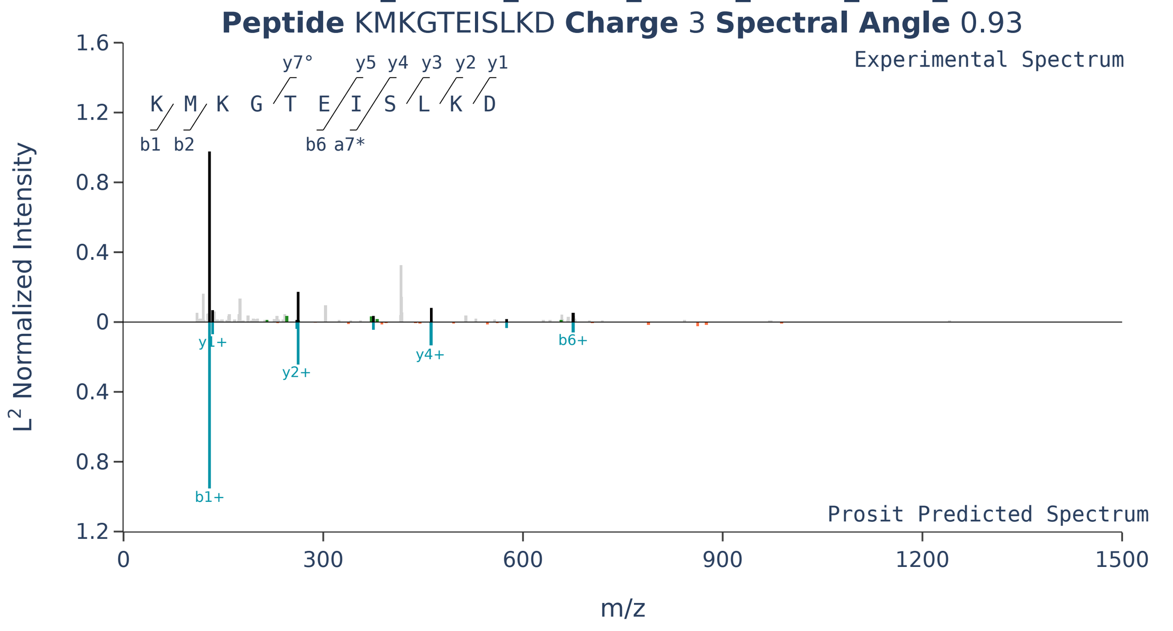

Source HRoetschke_271222_090123_HFGoe_LEDGF_b1R2_4h **Scan** 20216
Peptide YSFLGPKDIFPYSENKEKYGKPNKR **Charge** 5 **Spectral Angle** 0.79

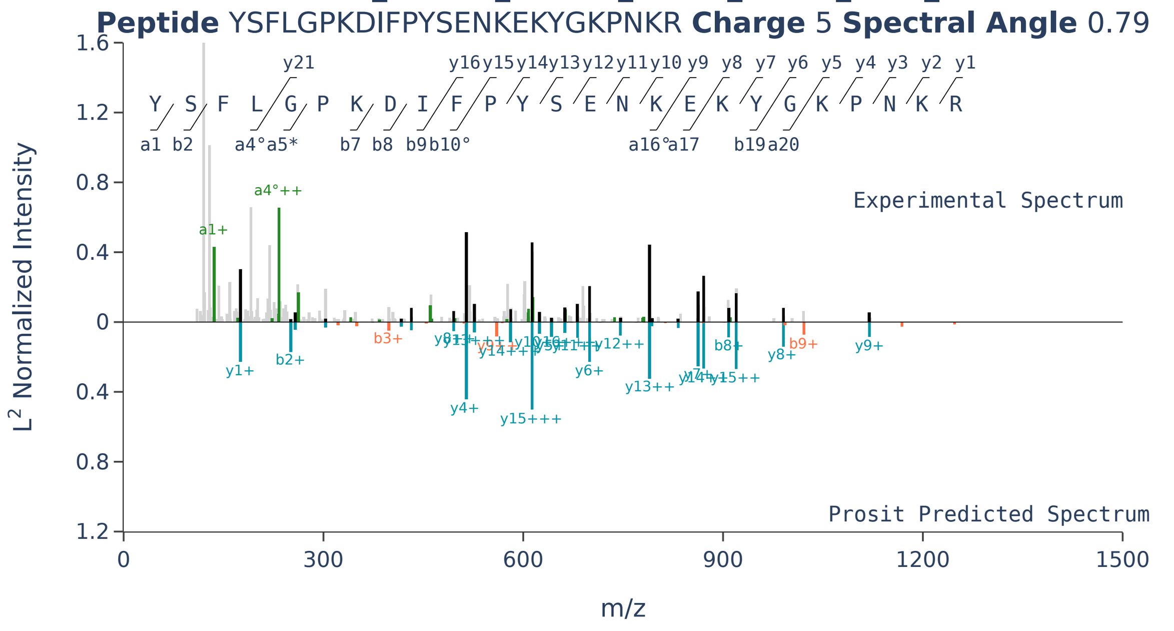

Source HRoetschke_271222_090123_HFGoe_LEDGF_b2R1_24h **Scan** 17977
Peptide TSAFLGPKDIFPYSENKEKYGKPNKR **Charge** 4 **Spectral Angle** 0.79

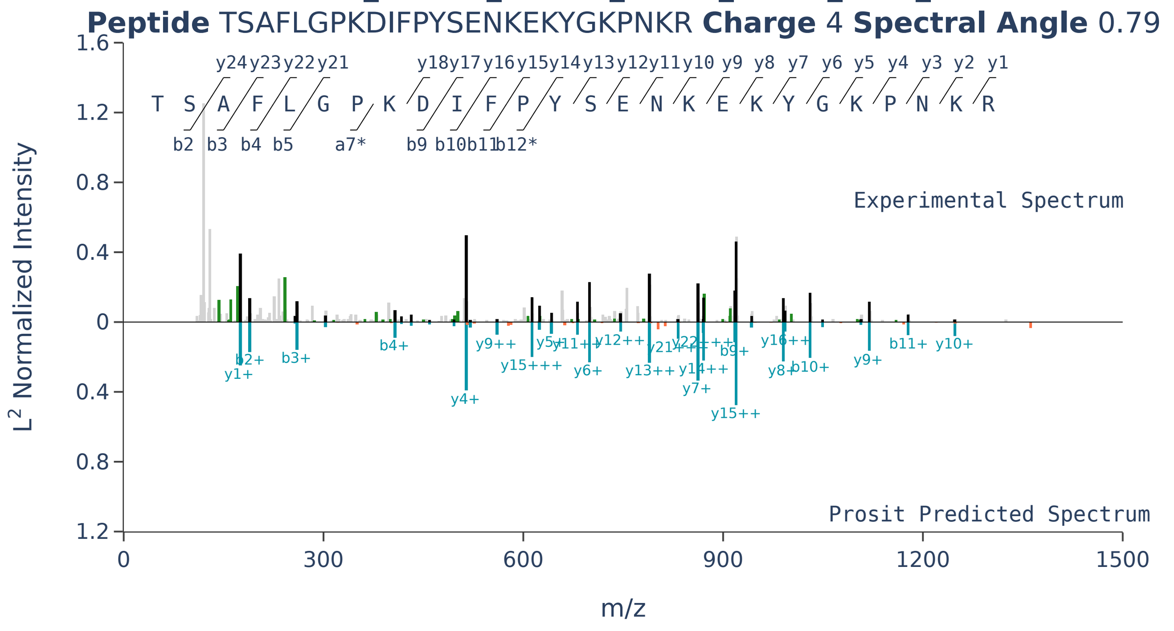

Source HRoetschke_271222_090123_HFGoe_LEDGF_b1R2_24h **Scan** 15975
Peptide NGGSDVPDGAVKPPTNKL **Charge** 3 **Spectral Angle** 0.74

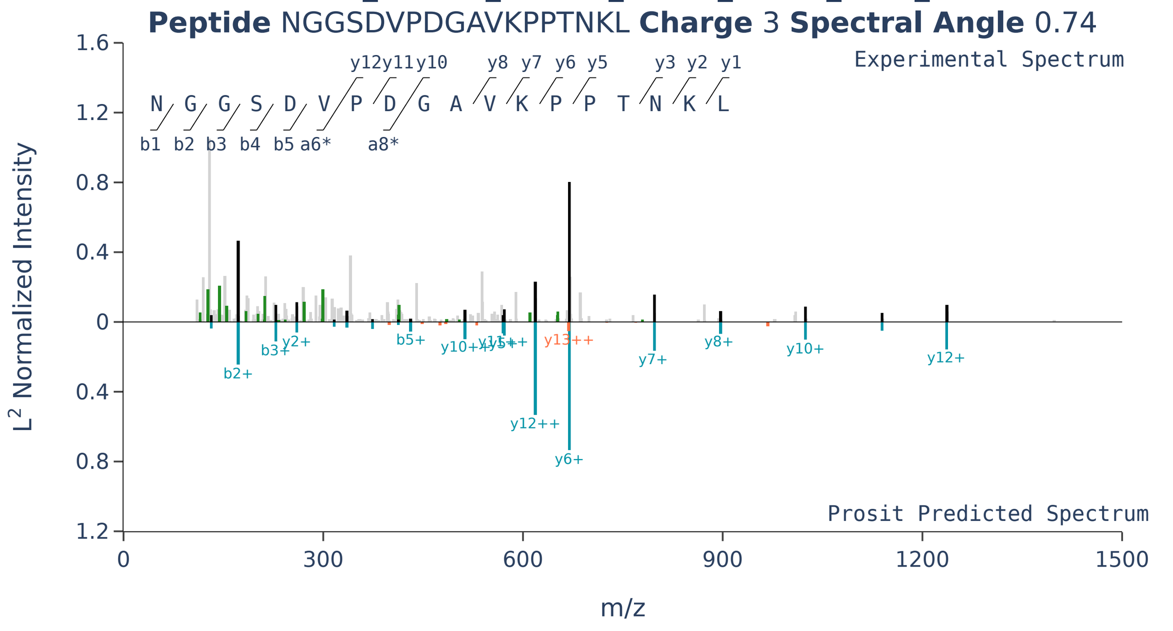

Source HRoetschke_271222_090123_HFGoe_LEDGF_b1R2_24h **Scan** 7583
Peptide NLKVSPKRGRPAAPD **Charge** 3 **Spectral Angle** 0.89

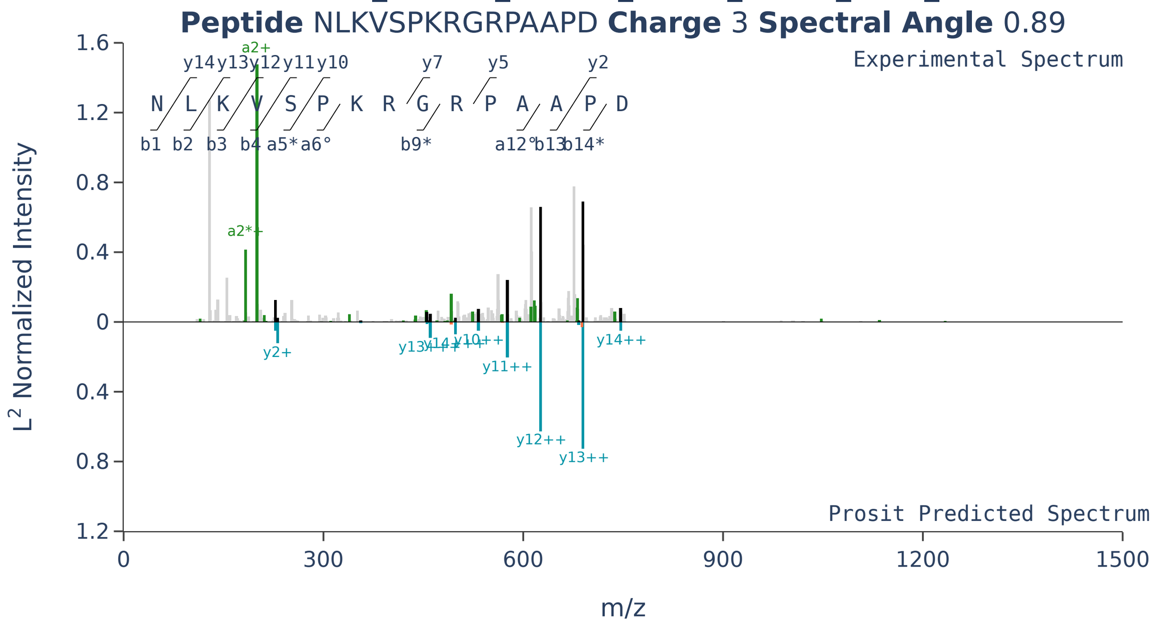

Source HRoetschke_271222_090123_HFGoe_LEDGF_b2R1_2h **Scan** 8326
Peptide AQDGNQPQHEL **Charge** 2 **Spectral Angle** 0.71

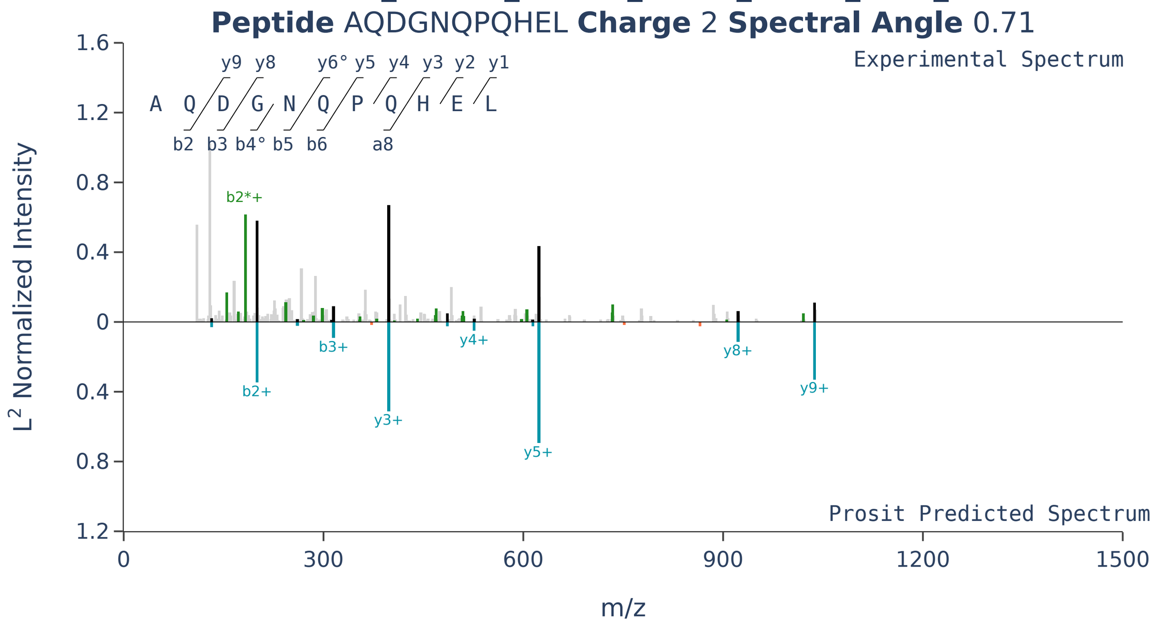

Source HRoetschke_271222_090123_HFGoe_LEDGF_b2R2_4h **Scan** 12003
Peptide EKSTMLFPYSENKEKYGKPNKR **Charge** 5 **Spectral Angle** 0.74

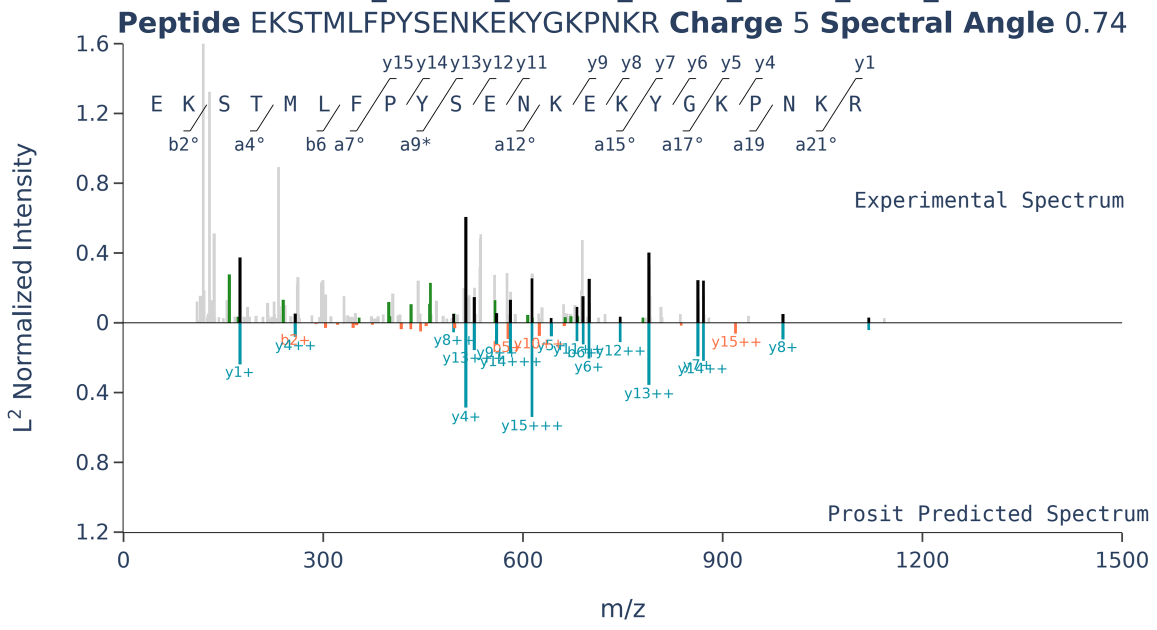

Source HRoetschke_271222_090123_HFGoe_LEDGF_b1R1_1h **Scan** 17691
Peptide GPGVTTA **Charge** 1 **Spectral Angle** 0.76

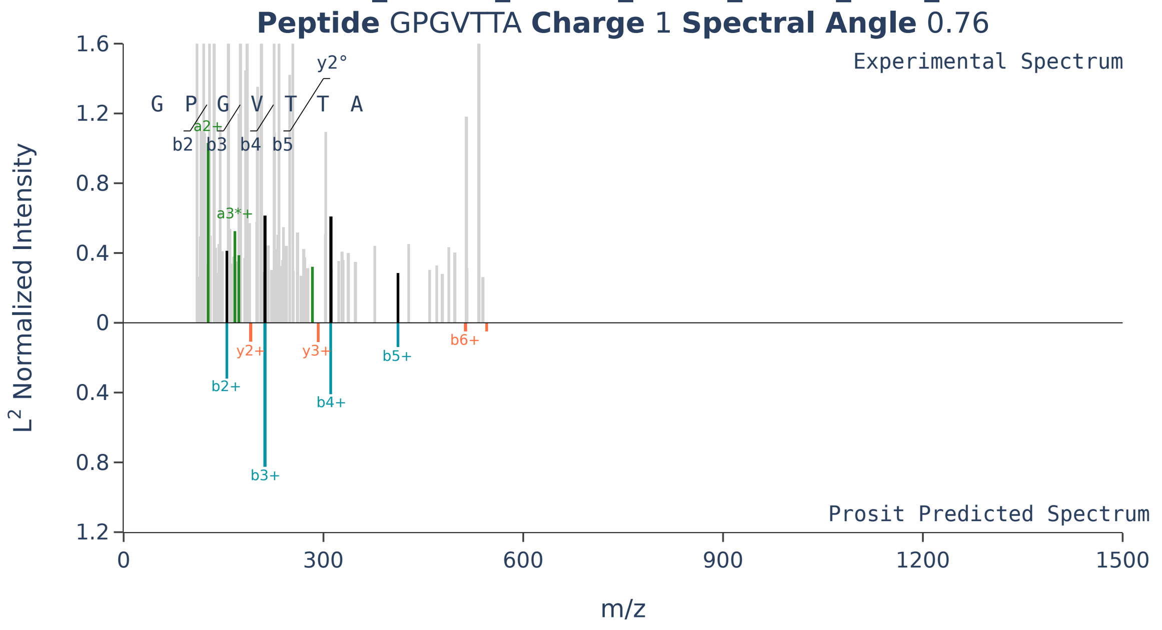

Source HRoetschke_271222_090123_HFGoe_LEDGF_b1R2_4h **Scan** 17508
Peptide TAYPHWPARVDE **Charge** 3 **Spectral Angle** 0.87

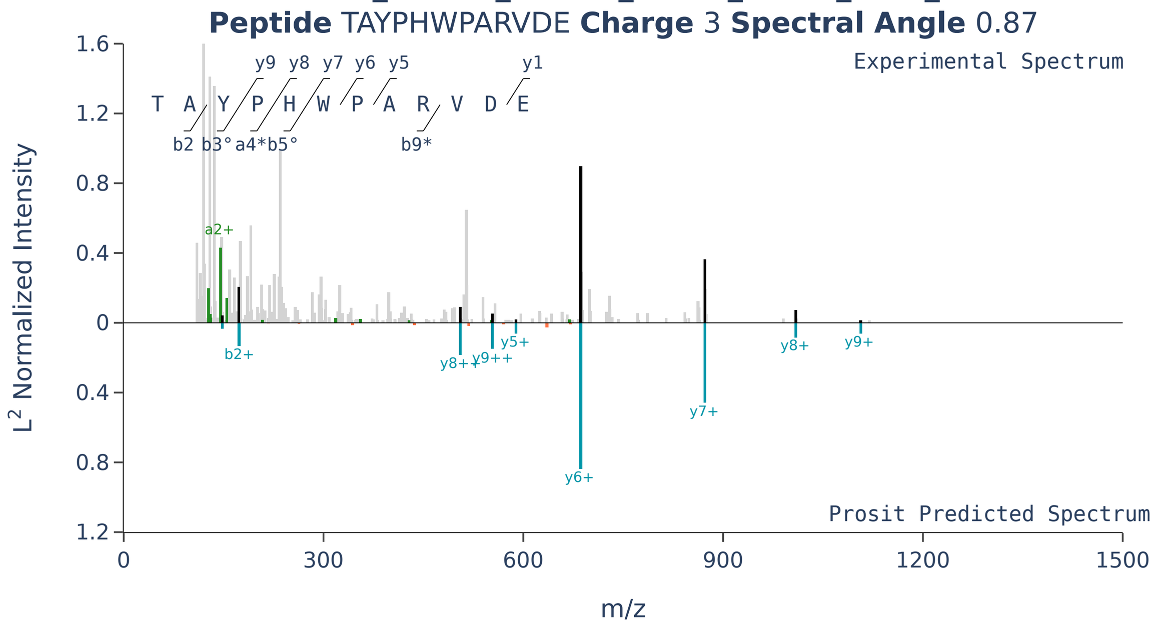

Source HRoetschke_271222_090123_HFGoe_LEDGF_b1R1_2h **Scan** 18526
Peptide LWEIDNNPKVKFSSQQAATKSQ **Charge** 3 **Spectral Angle** 0.89

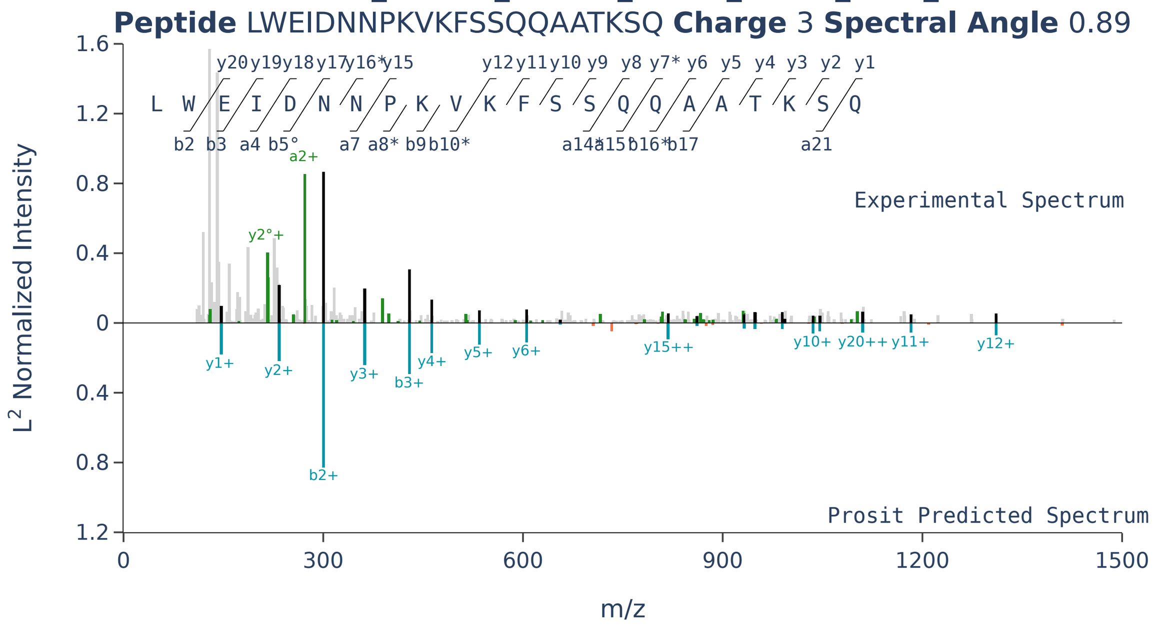

Source HRoetschke_271222_090123_HFGoe_LEDGF_b2R2_24h **Scan** 12327
Peptide NLDVPAR **Charge** 2 **Spectral Angle** 0.81

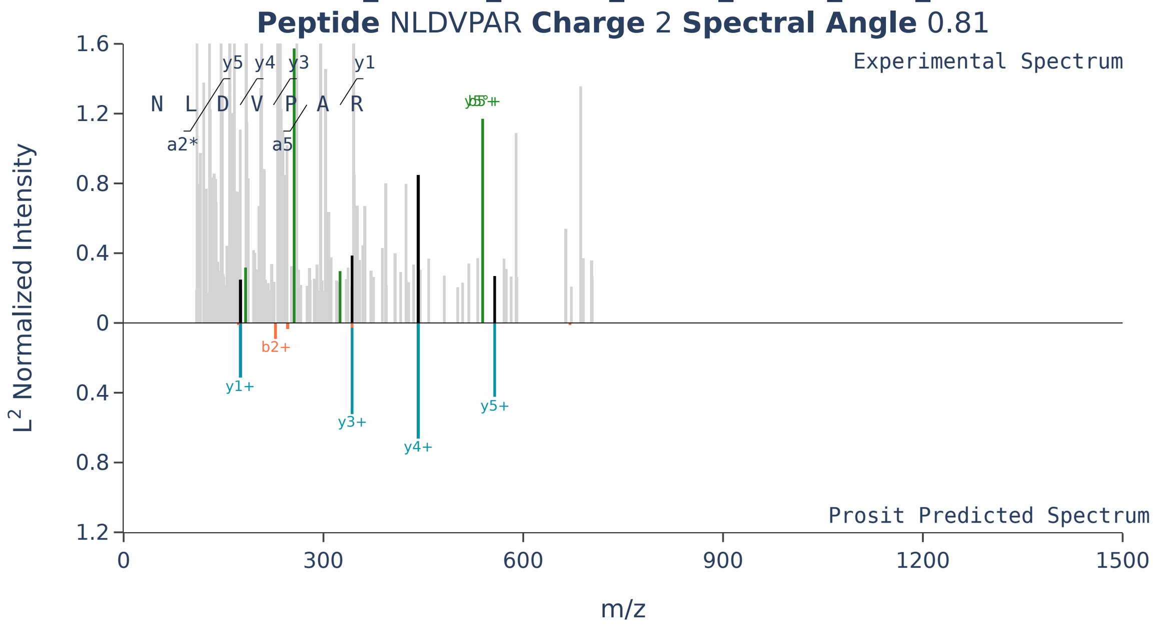

Source HRoetschke_271222_090123_HFGoe_LEDGF_b1R1_2h **Scan** 25879
Peptide NEGLWEY **Charge** 1 **Spectral Angle** 0.89

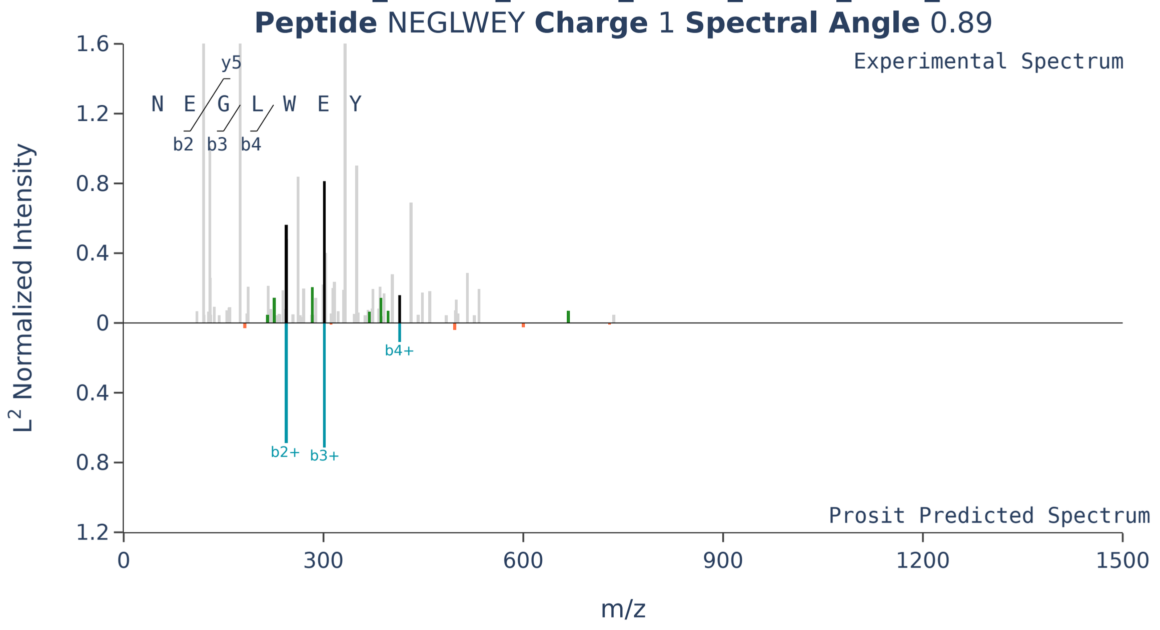

Source HRoetschke_271222_090123_HFGoe_LEDGF_b2R1_24h **Scan** 18083
Peptide ASHETAFLGPKDIFPYSENKEKYGKPNK **Charge** 3 **Spectral Angle** 0.84

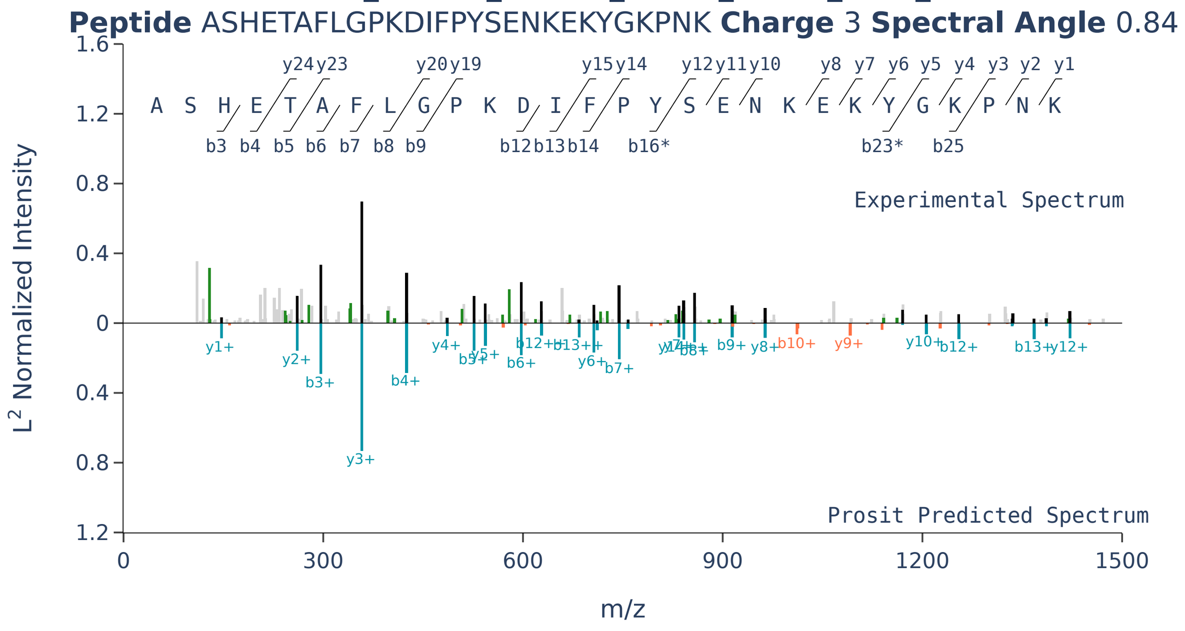

Source HRoetschke_271222_090123_HFGoe_LEDGF_b1R1_4h **Scan** 18055
Peptide TGHETAFLGPKDIFPYSENKEKYGKPNK **Charge** 3 **Spectral Angle** 0.84

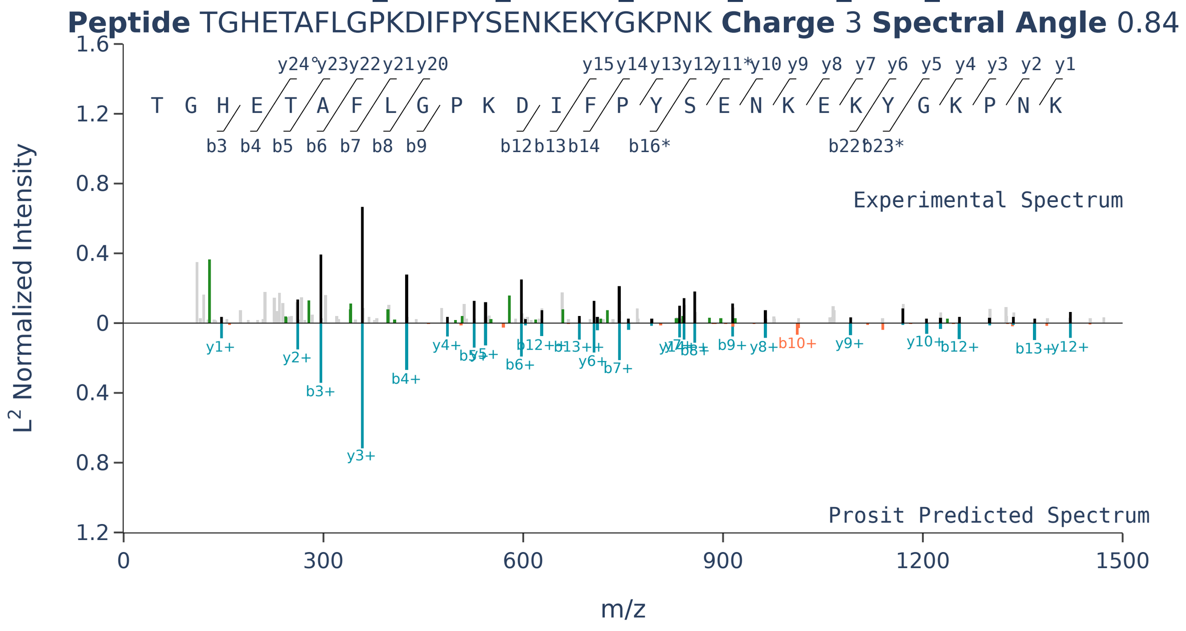

Source HRoetschke_271222_090123_HFGoe_LEDGF_b1R2_24h **Scan** 19481
Peptide KETSLKIDNLDVNRCIE **Charge** 3 **Spectral Angle** 0.85

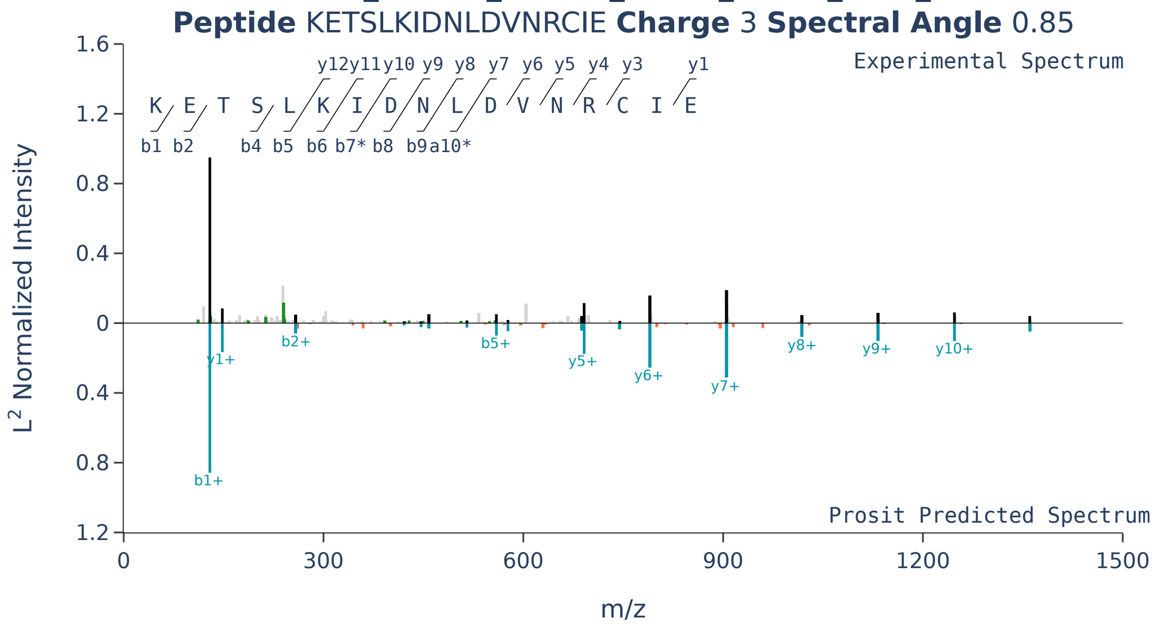

Source HRoetschke_271222_090123_HFGoe_LEDGF_b2R1_2h **Scan** 9542
Peptide PKHWPARVD **Charge** 3 **Spectral Angle** 0.83

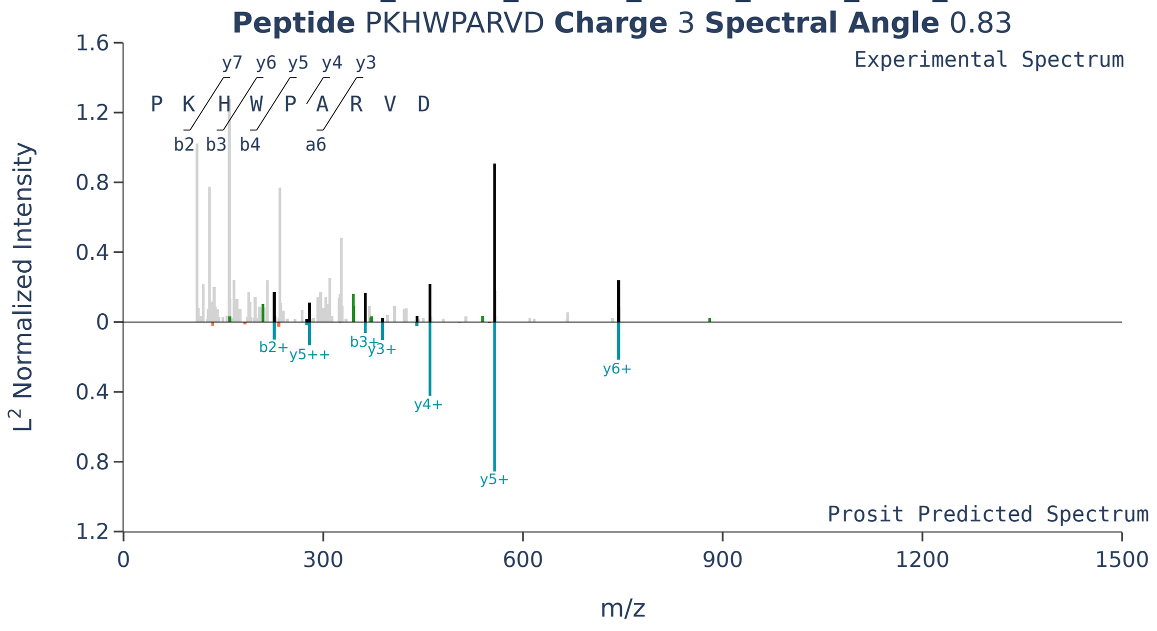

Source HRoetschke_271222_090123_HFGoe_LEDGF_b1R1_24h **Scan** 14576
Peptide GTHETATASVNL **Charge** 2 **Spectral Angle** 0.85

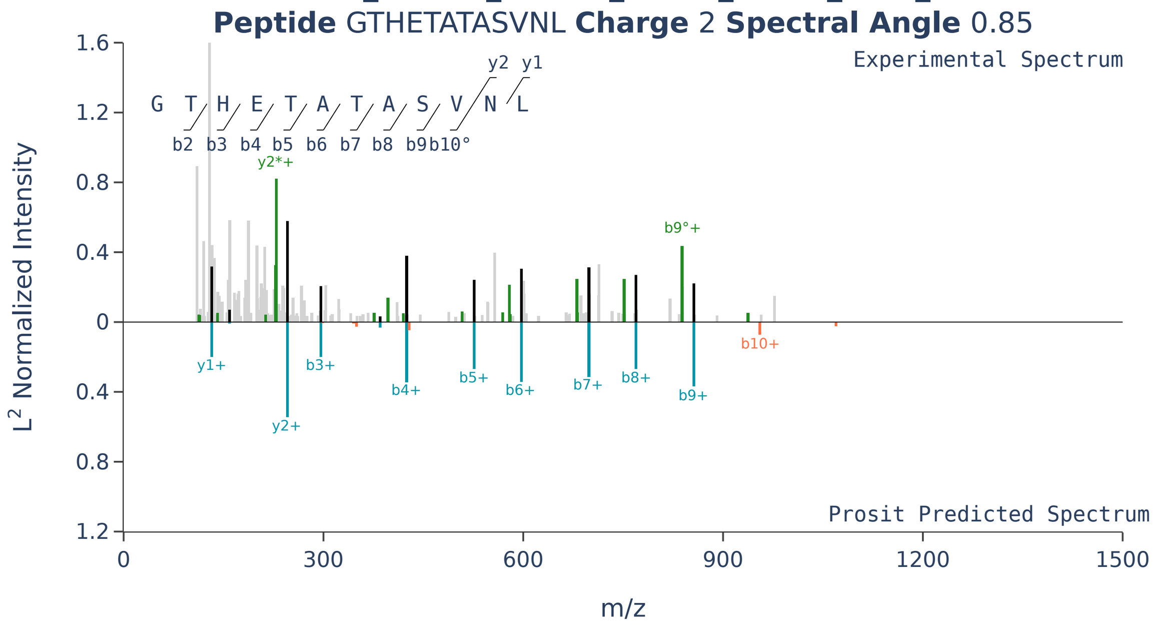

Source HRoetschke_271222_090123_HFGoe_LEDGF_b2R1_4h **Scan** 18121
Peptide SQQAAFLGPKDIFPYSENKEKYGKPNKR **Charge** 5 **Spectral Angle** 0.79

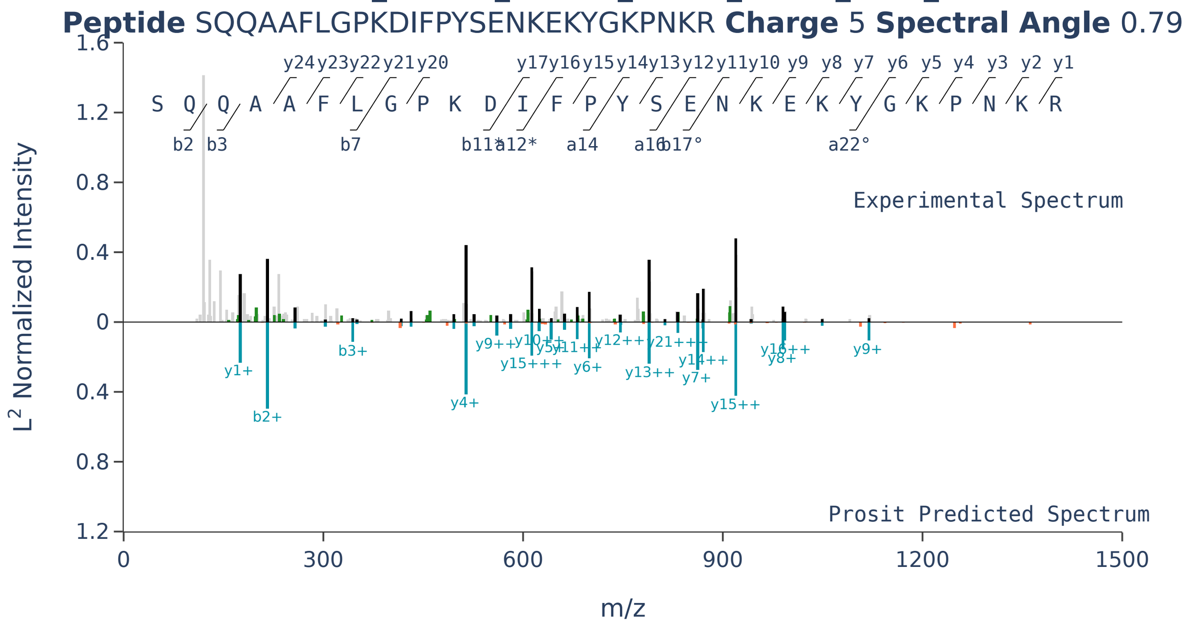

Source HRoetschke_271222_090123_HFGoe_LEDGF_b1R1_2h **Scan** 17919
Peptide TASVNLKDIFPYSENKEKYGKPNKR **Charge** 5 **Spectral Angle** 0.8

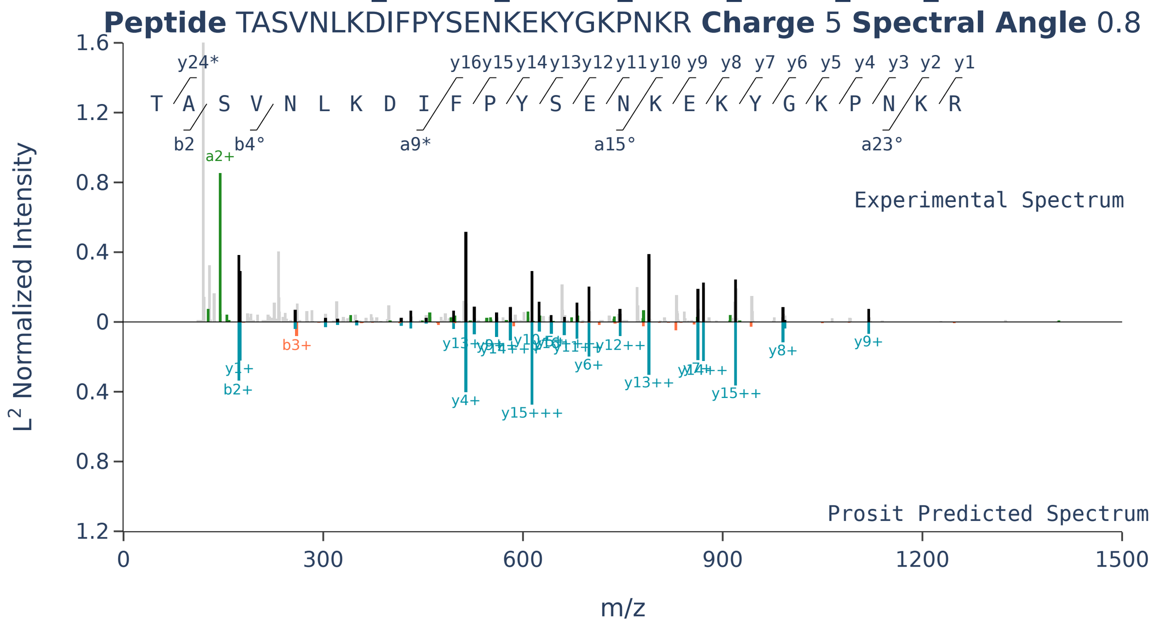

Source HRoetschke_271222_090123_HFGoe_LEDGF_b1R1_4h **Scan** 18094
Peptide QTFLGPKDIFPYSENKEKYGKPNKR **Charge** 5 **Spectral Angle** 0.72

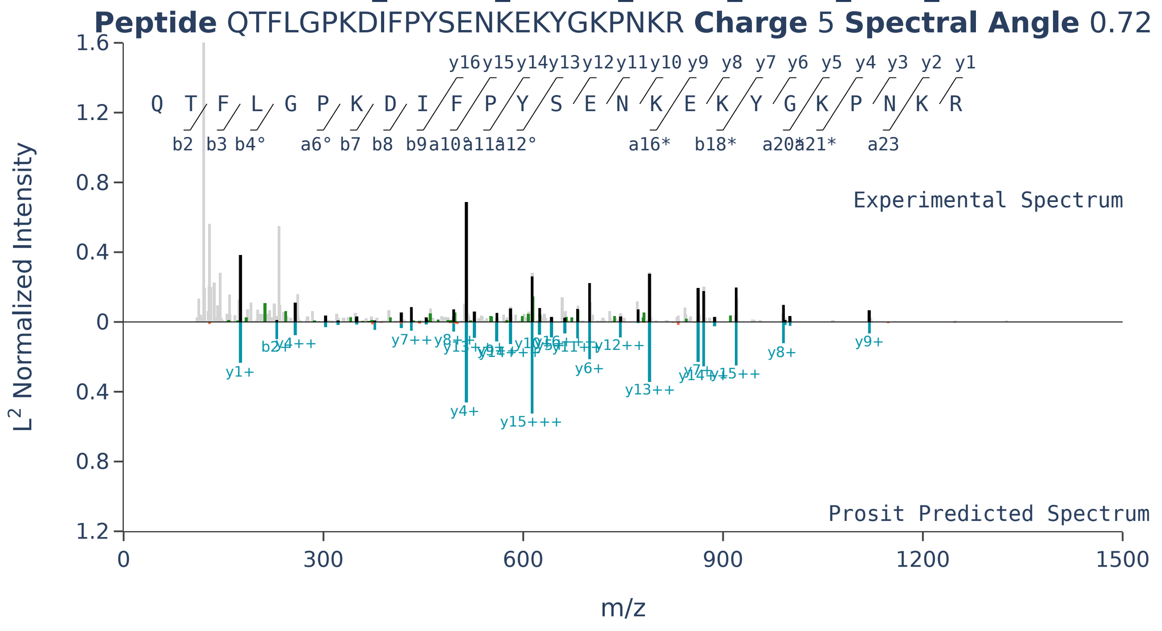

Source HRoetschke_271222_090123_HFGoe_LEDGF_b1R1_24h **Scan** 14223
Peptide GTHETVPDGAVKPPTNKL **Charge** 3 **Spectral Angle** 0.88

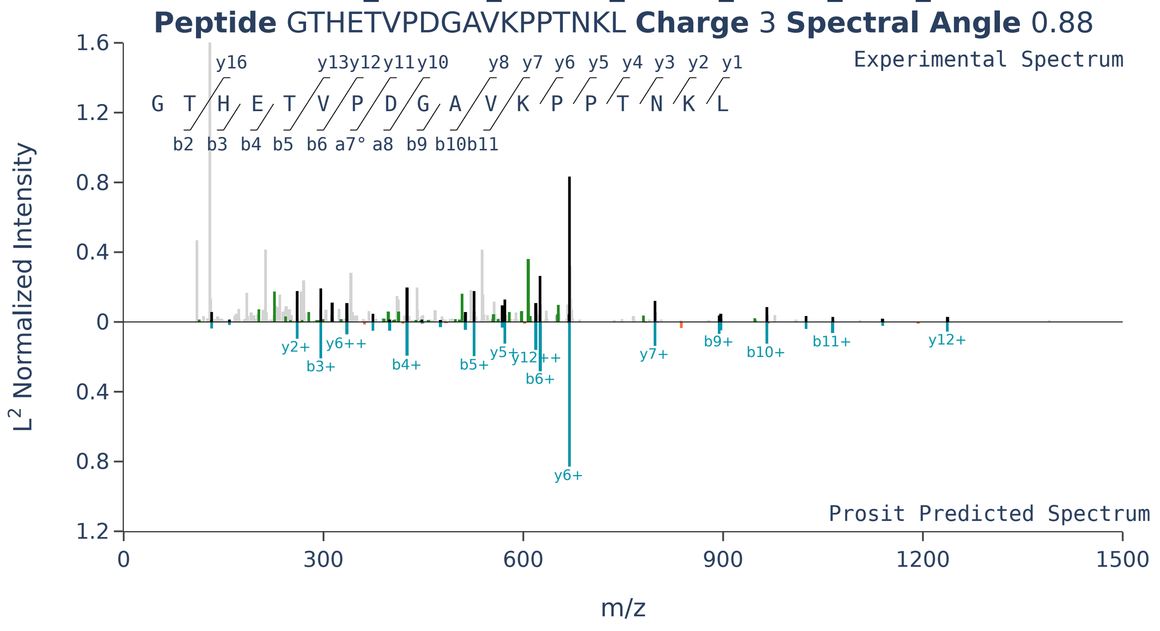

Source HRoetschke_271222_090123_HFGoe_LEDGF_b2R1_24h **Scan** 21146
Peptide LAFLGPKDIFPYSENKEKYGKPNKR **Charge** 5 **Spectral Angle** 0.79

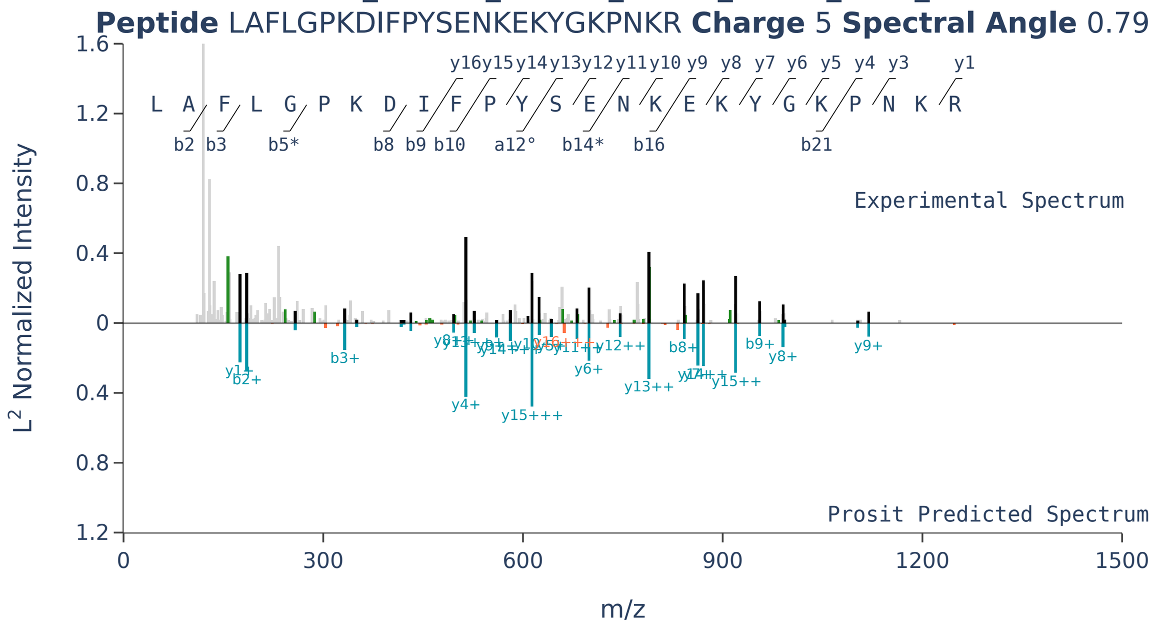

Source HRoetschke_271222_090123_HFGoe_LEDGF_b1R2_24h **Scan** 9306
Peptide ATSMDSRLQ **Charge** 2 **Spectral Angle** 0.8

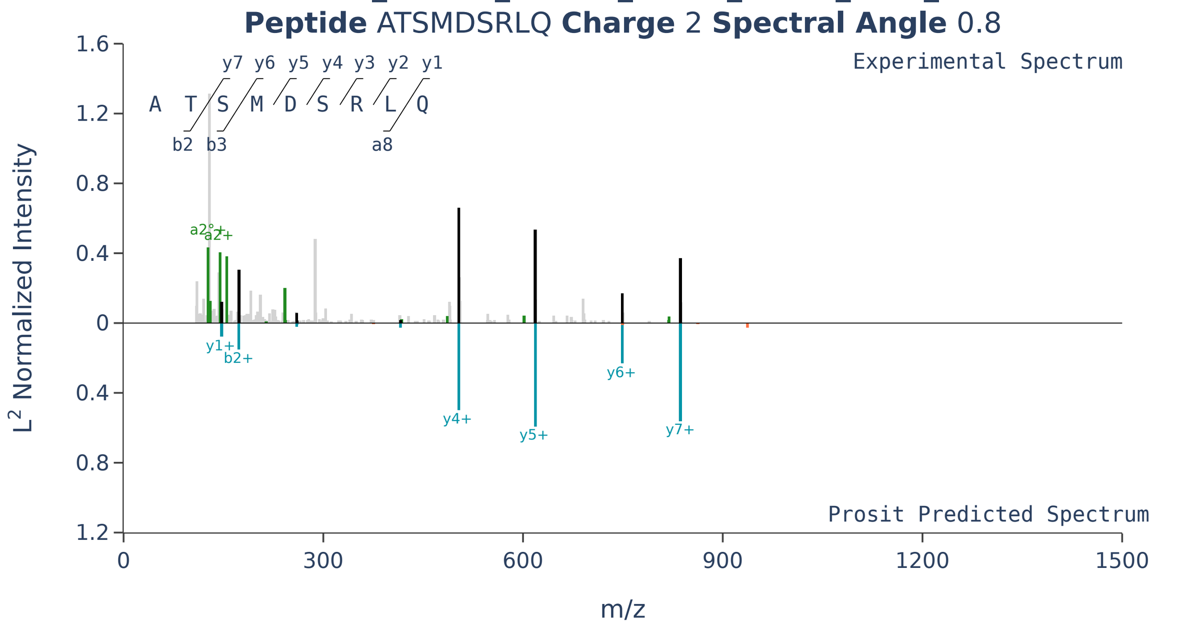

Source HRoetschke_271222_090123_HFGoe_LEDGF_b1R2_4h **Scan** 7756
Peptide SQQAATKQEL **Charge** 2 **Spectral Angle** 0.79

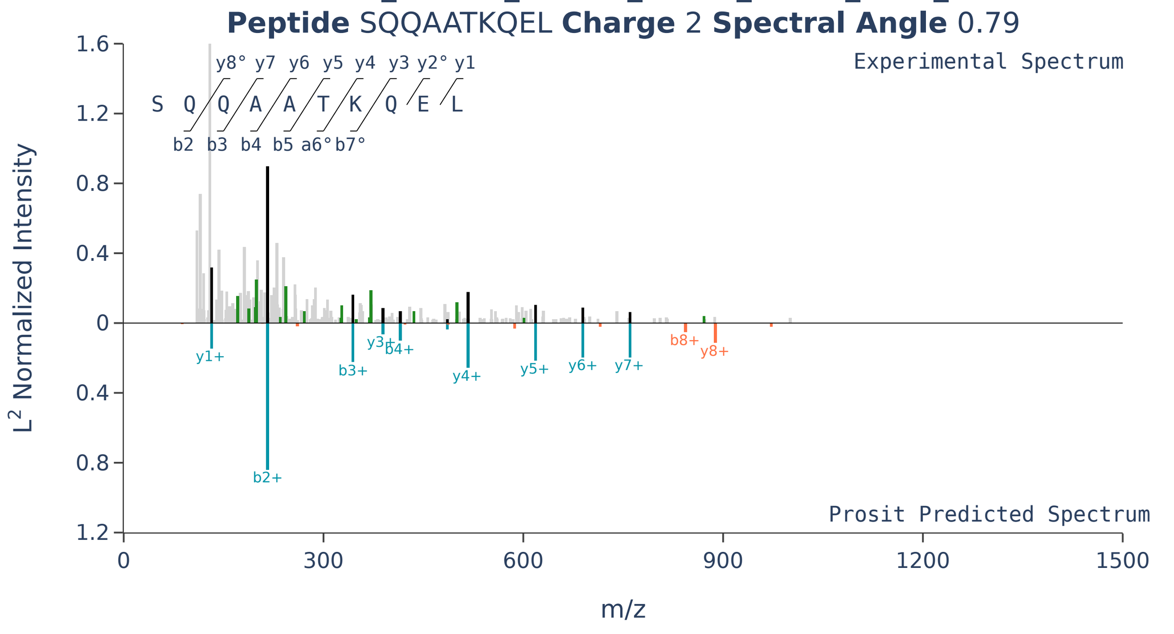

Source HRoetschke_271222_090123_HFGoe_LEDGF_b1R1_1h **Scan** 16231
Peptide YPHWPAQQAAT **Charge** 1 **Spectral Angle** 0.79

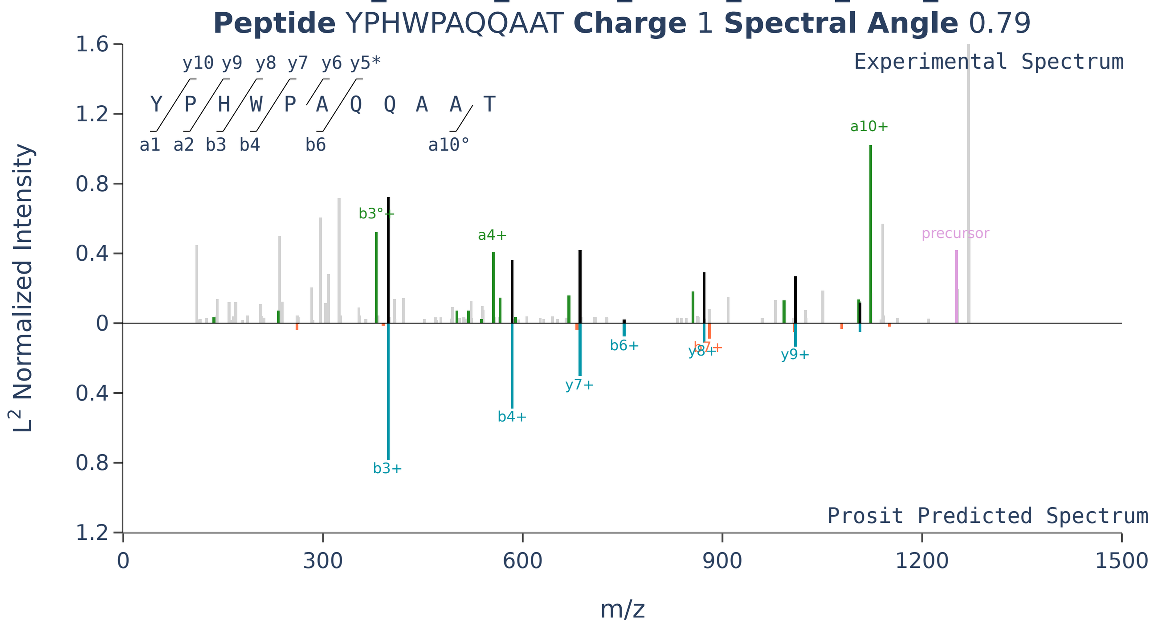

Source HRoetschke_271222_090123_HFGoe_LEDGF_b2R2_4h **Scan** 7920
Peptide DTDHEEKASNELD **Charge** 3 **Spectral Angle** 0.72

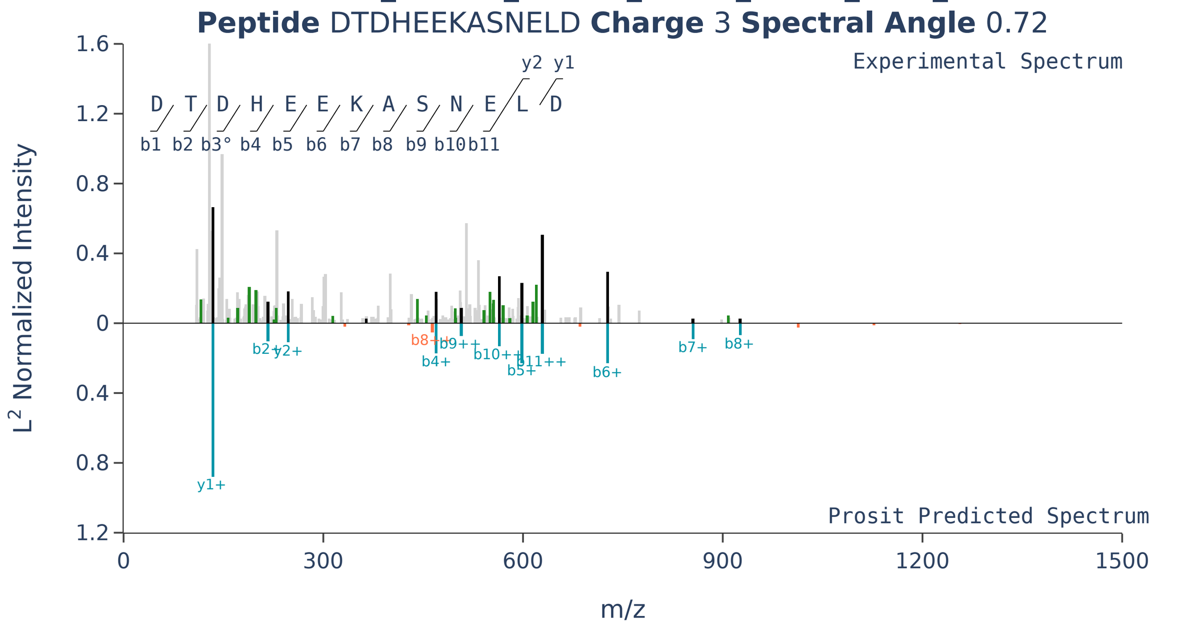

Source HRoetschke_271222_090123_HFGoe_LEDGF_b2R2_2h **Scan** 19454
Peptide DEYPHWPARVDE **Charge** 3 **Spectral Angle** 0.8

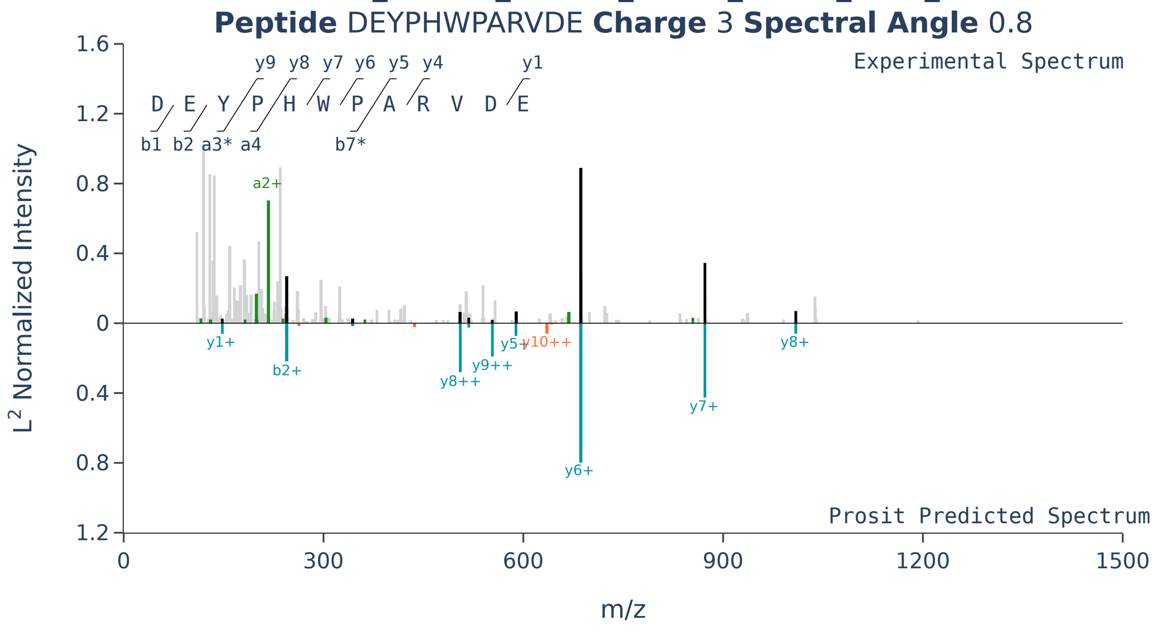

Source HRoetschke_271222_090123_HFGoe_LEDGF_b1R1_4h **Scan** 15738
Peptide FLGPKDIFPYSENKEKYGKPNKKR **Charge** 4 **Spectral Angle** 0.76

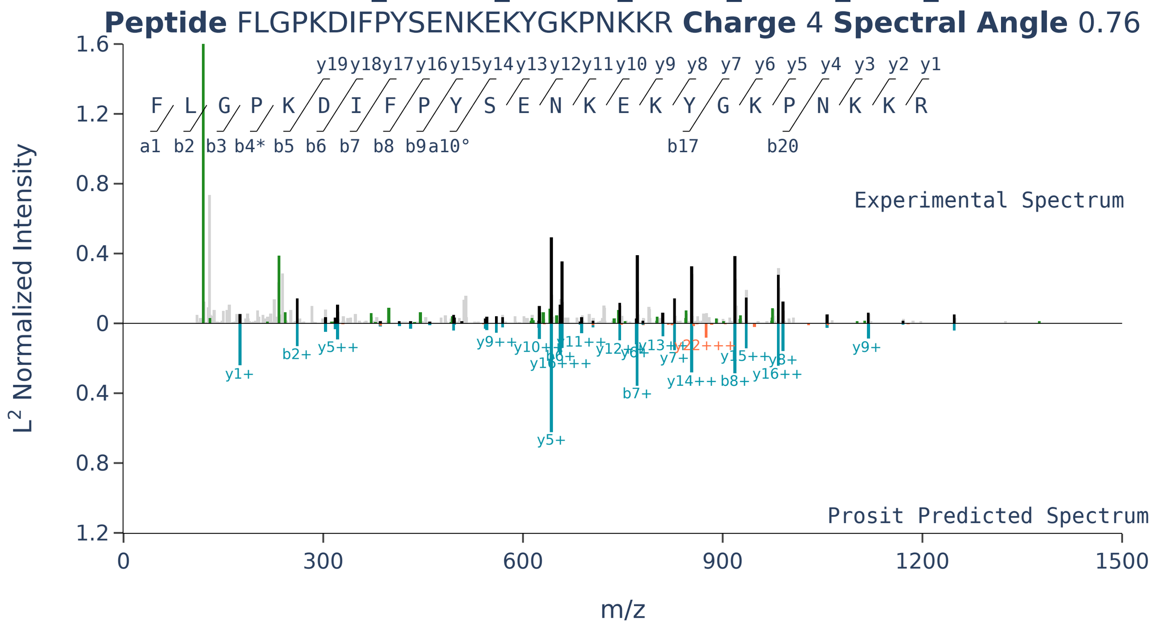

Source HRoetschke_271222_090123_HFGoe_LEDGF_b1R2_4h **Scan** 18769
Peptide KETKGFNEGLWE **Charge** 3 **Spectral Angle** 0.82

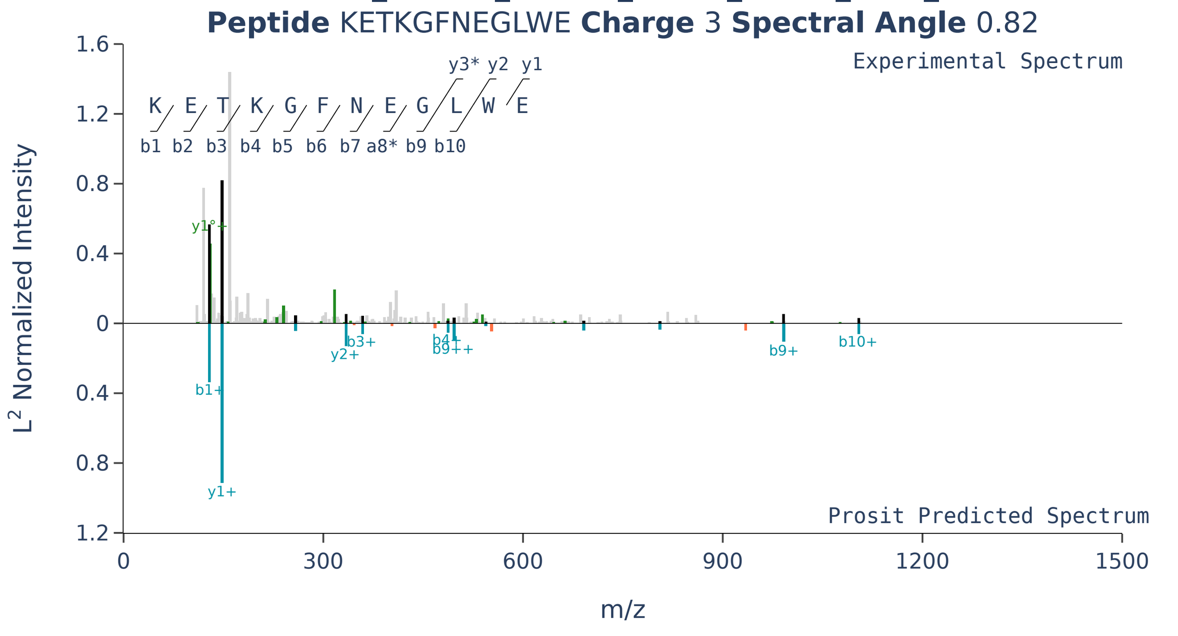

Source HRoetschke_271222_090123_HFGoe_LEDGF_b2R1_1h **Scan** 17893
Peptide TAFLGPKDIFPYSENKEKYGKPRKGG **Charge** 3 **Spectral Angle** 0.86

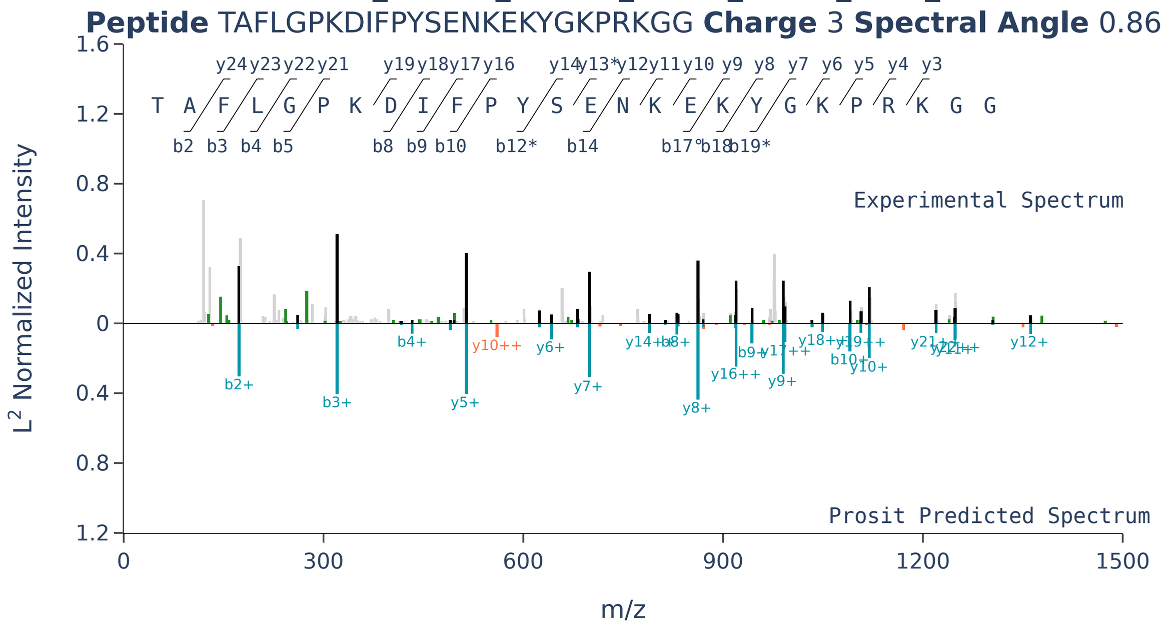

Source HRoetschke_271222_090123_HFGoe_LEDGF_b1R1_4h **Scan** 19950
Peptide LFLGPKDIFPYSENKEKYGKPNKR **Charge** 5 **Spectral Angle** 0.72

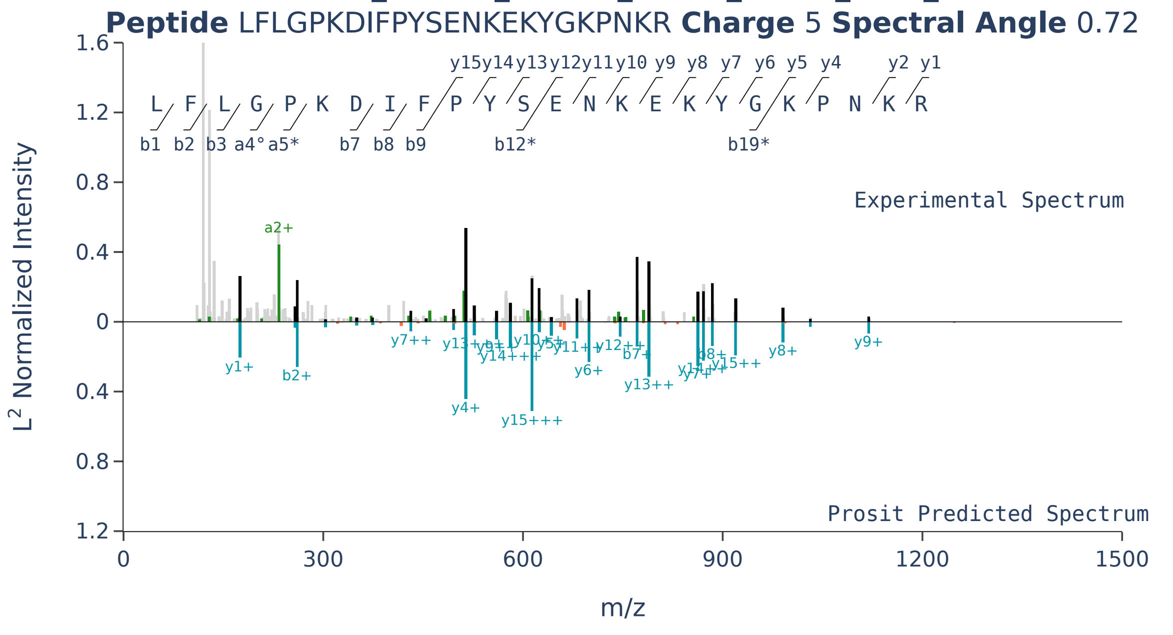

Source HRoetschke_271222_090123_HFGoe_LEDGF_b1R1_2h **Scan** 8406
Peptide SQQAAEDTD **Charge** 1 **Spectral Angle** 0.89

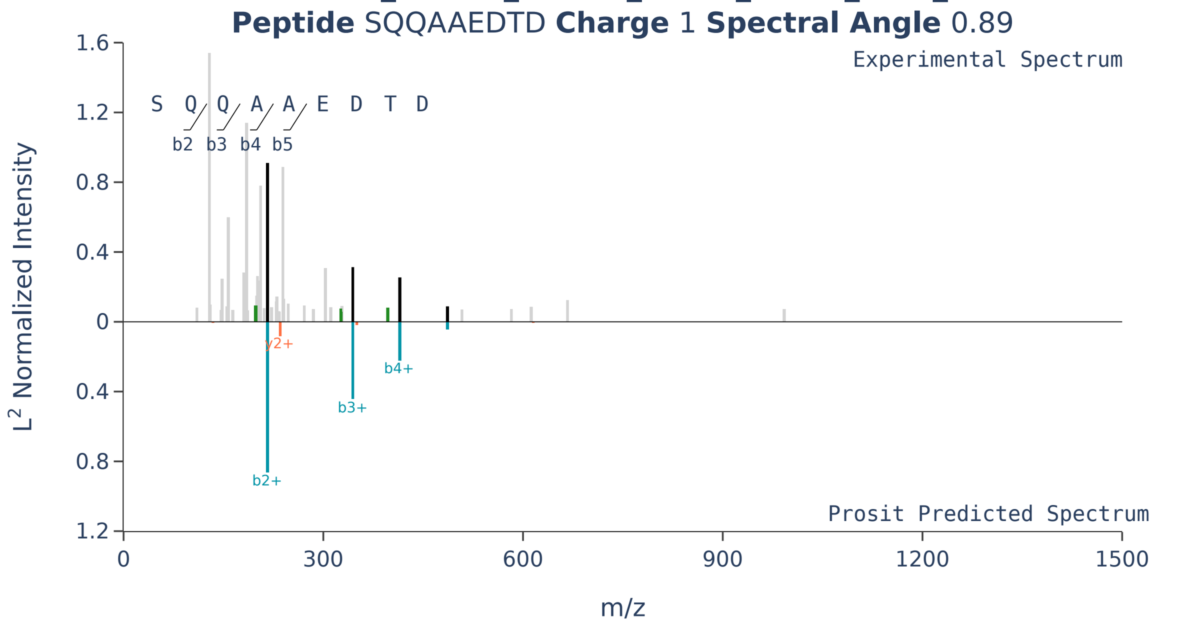

Source HRoetschke_271222_090123_HFGoe_LEDGF_b1R1_24h **Scan** 16807
Peptide PYEKSTMLY **Charge** 2 **Spectral Angle** 0.75

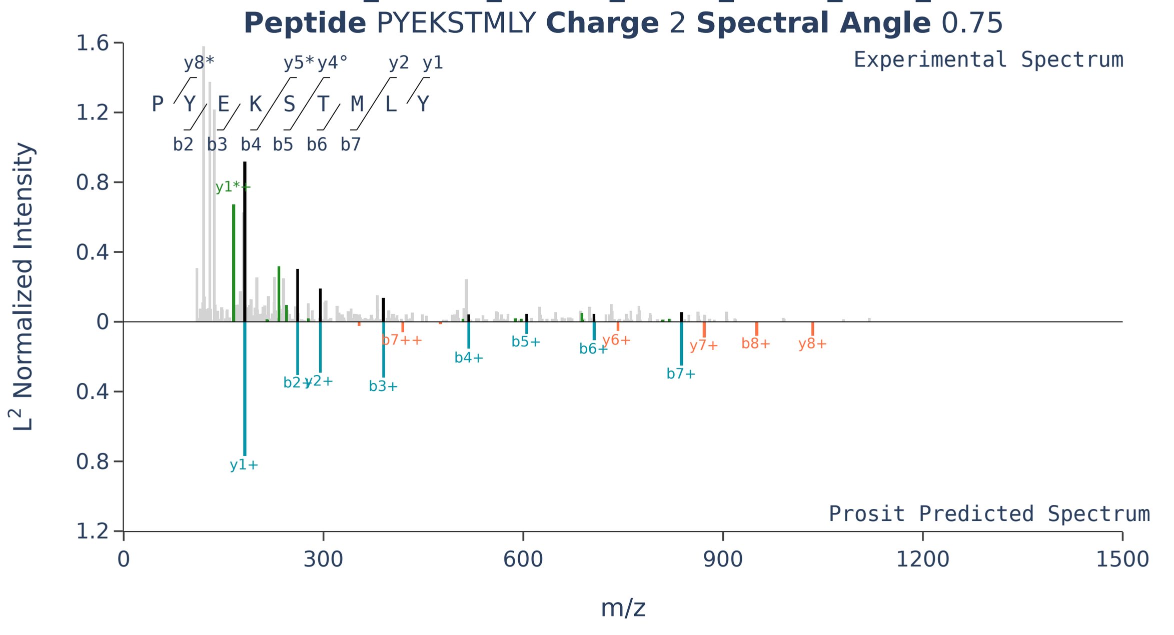

Source HRoetschke_271222_090123_HFGoe_LEDGF_b1R1_4h **Scan** 26848
Peptide QKFNEGLWEID **Charge** 2 **Spectral Angle** 0.78

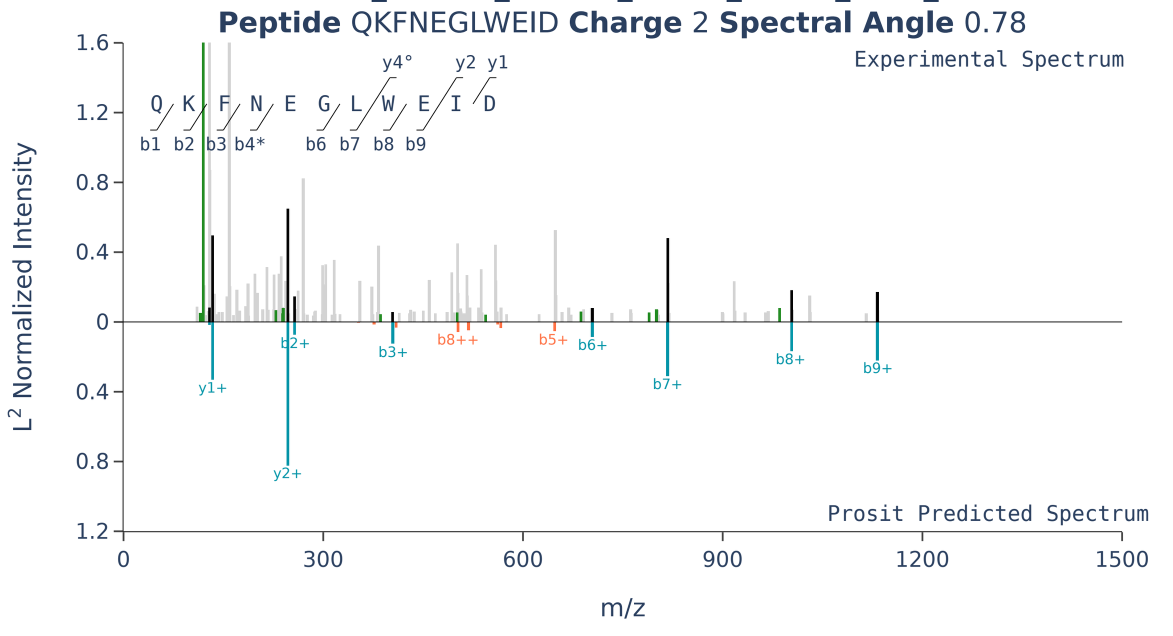

Source HRoetschke_271222_090123_HFGoe_LEDGF_b2R2_2h **Scan** 7439
Peptide PKMVKQPCPSEE **Charge** 2 **Spectral Angle** 0.8

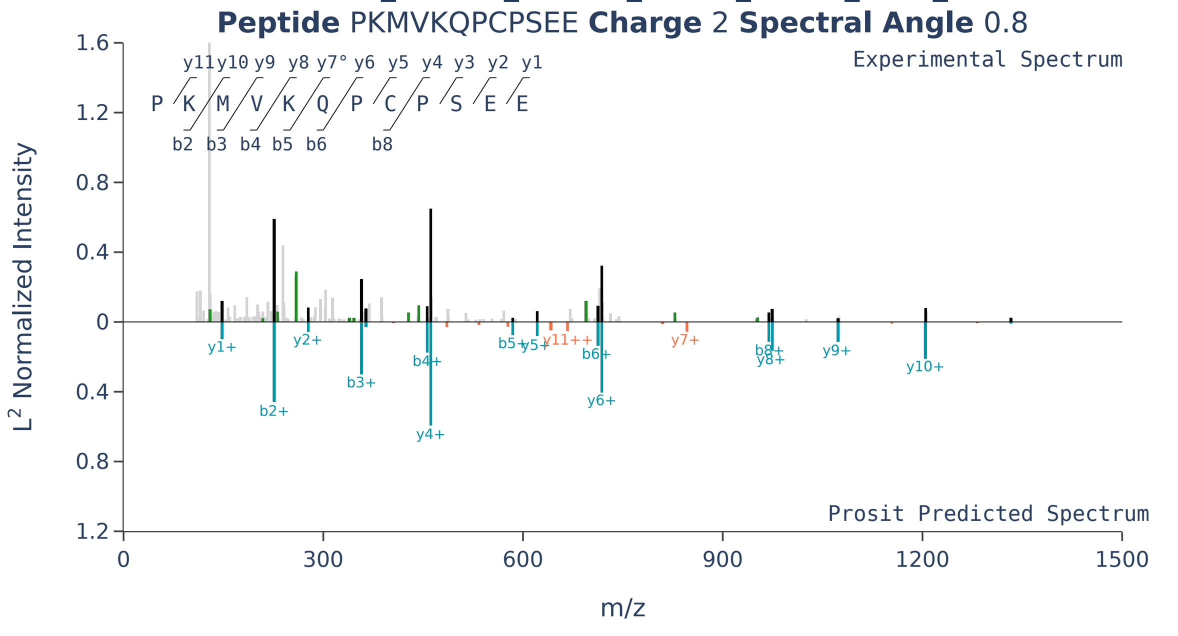

Source HRoetschke_271222_090123_HFGoe_LEDGF_b2R2_2h **Scan** 9151
Peptide IFPYSENKEKYGKPNKREKSTM **Charge** 3 **Spectral Angle** 0.76

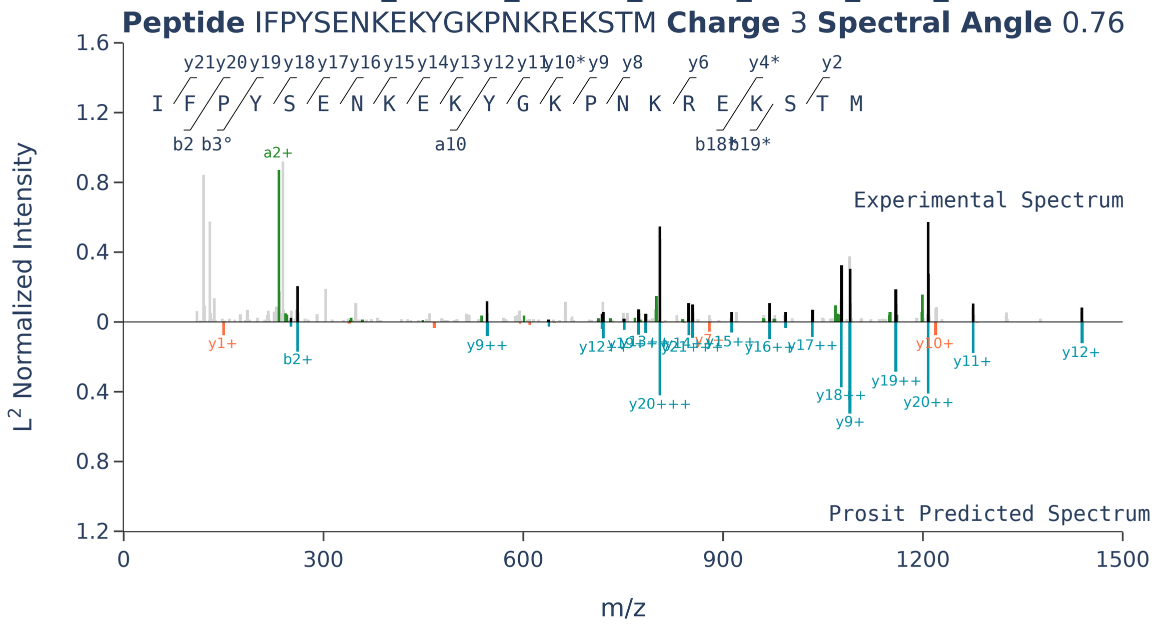

Source HRoetschke_271222_090123_HFGoe_LEDGF_b2R1_2h **Scan** 13805
Peptide LGPKDIFPYSENKEKYGKPNKRKGFNAD **Charge** 5 **Spectral Angle** 0.83

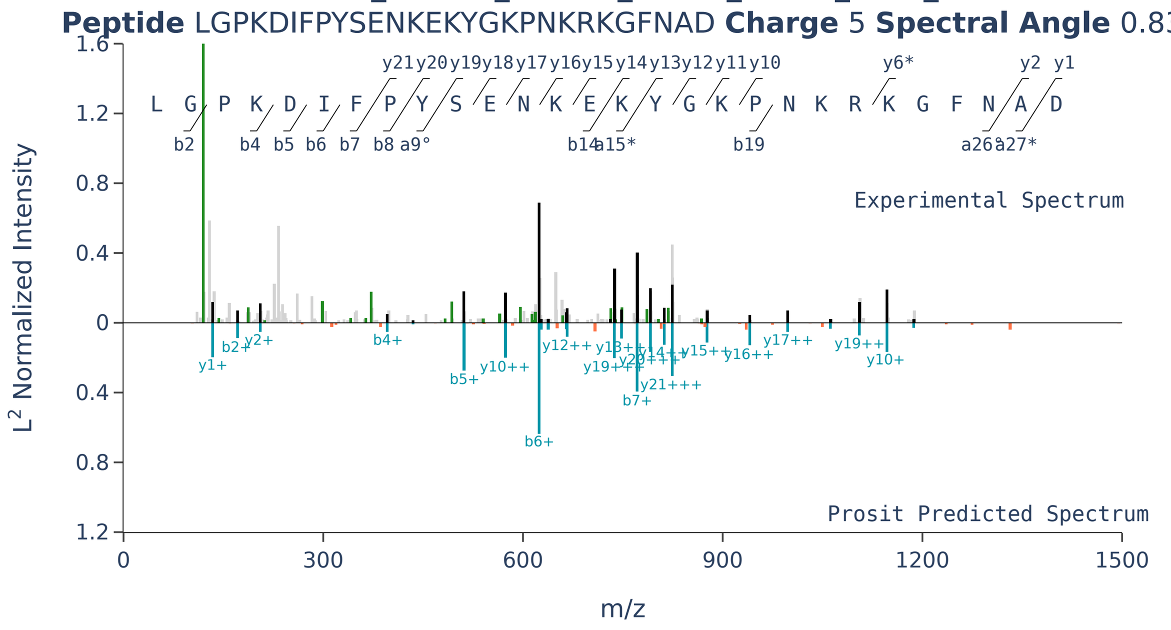

Source HRoetschke_271222_090123_HFGoe_LEDGF_b2R1_24h **Scan** 19839
Peptide TMFLGPKDIFPYSENKEKYGKPNKR **Charge** 5 **Spectral Angle** 0.78

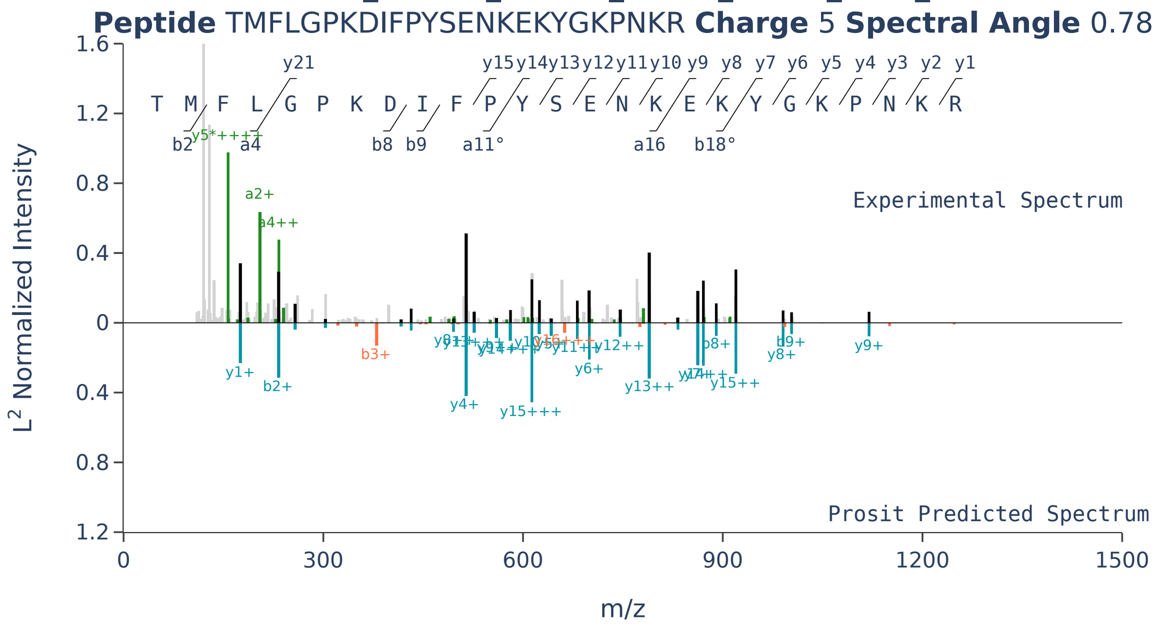

Source HRoetschke_271222_090123_HFGoe_LEDGF_b2R2_2h **Scan** 15613
Peptide QVETEEAHWPARVD **Charge** 4 **Spectral Angle** 0.9

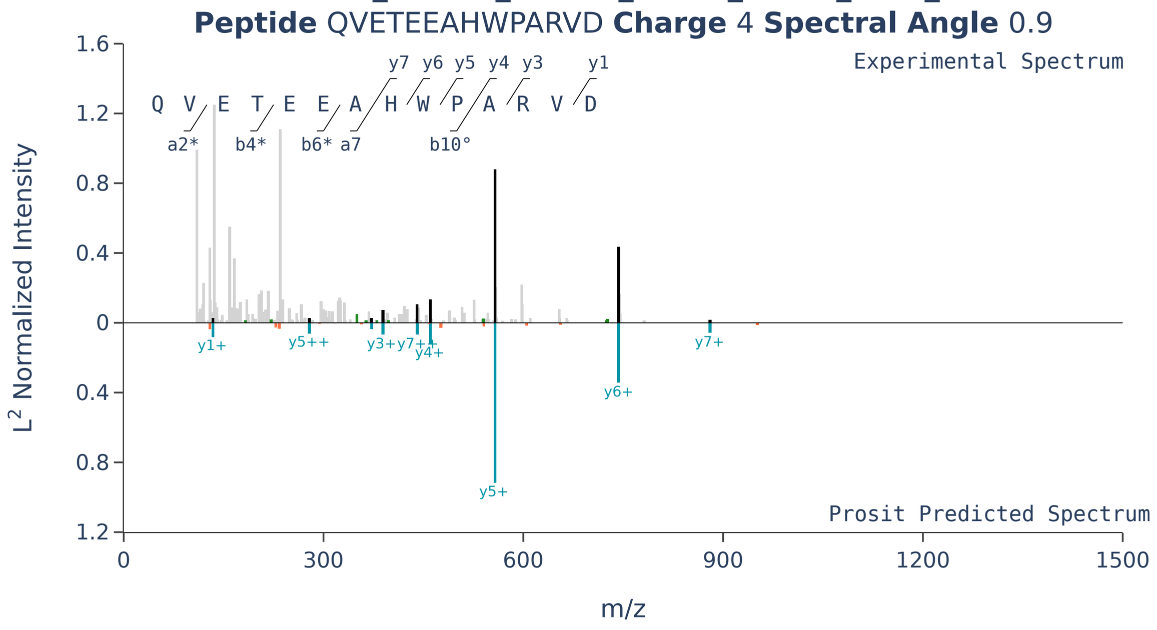

Source HRoetschke_271222_090123_HFGoe_LEDGF_b1R1_24h **Scan** 17050
Peptide TEEAGWPARVD **Charge** 3 **Spectral Angle** 0.76

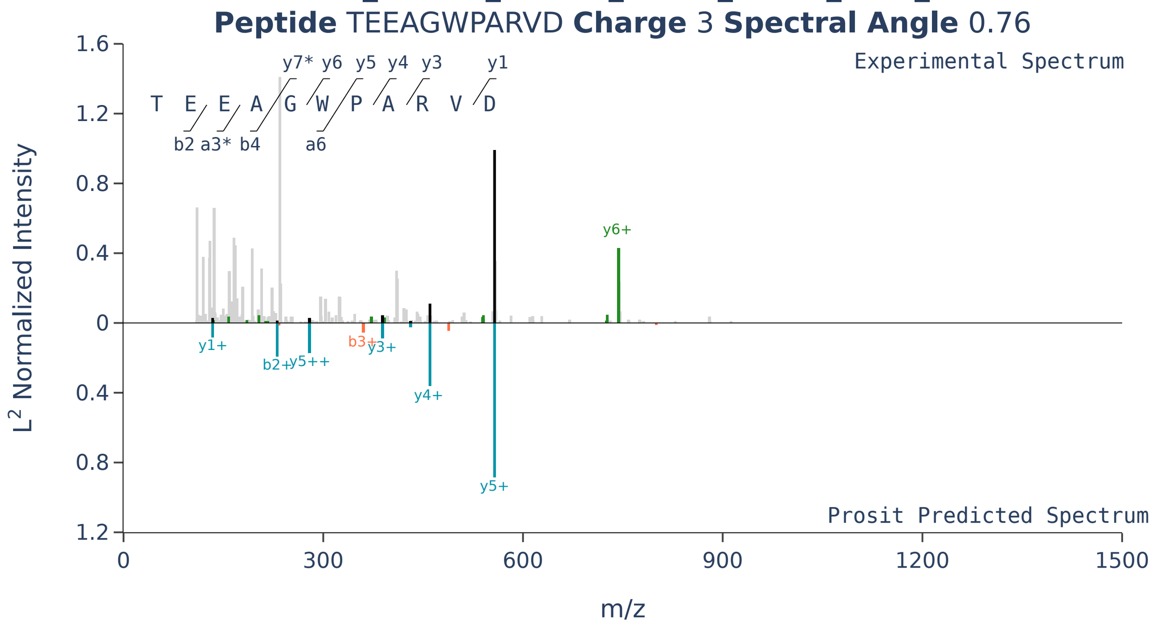

Source HRoetschke_271222_090123_HFGoe_LEDGF_b2R2_24h **Scan** 5941
Peptide FPYSENKEKYGKPNKKR **Charge** 3 **Spectral Angle** 0.83

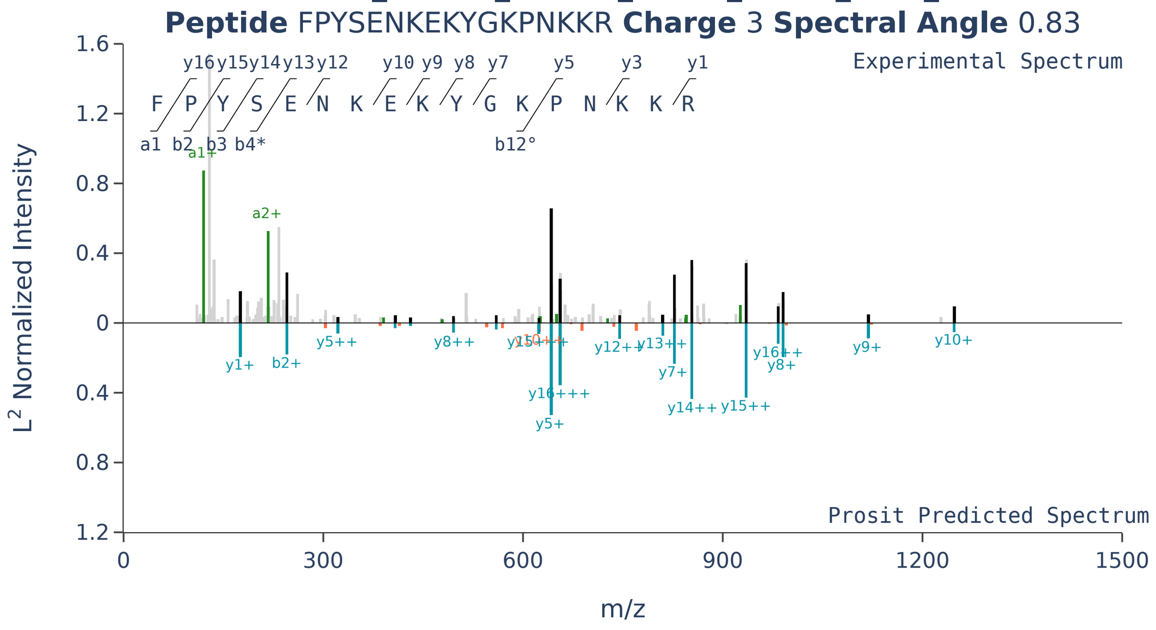

Source HRoetschke_271222_090123_HFGoe_LEDGF_b2R1_24h **Scan** 23405
Peptide GALWEIDNNPKVKFSSQQA **Charge** 3 **Spectral Angle** 0.75

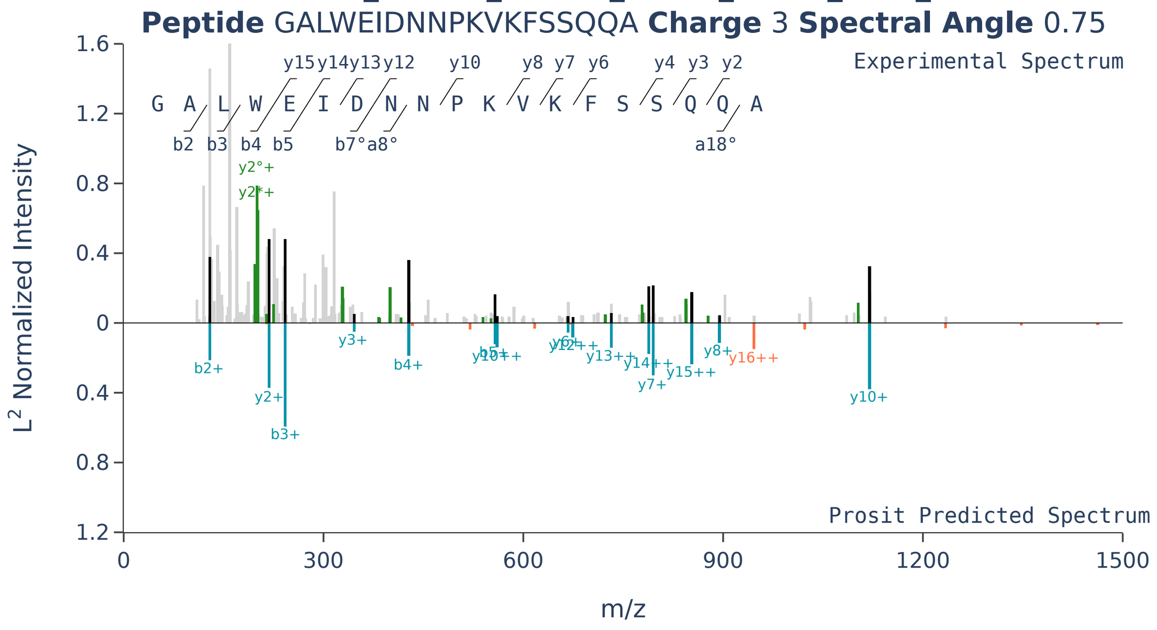

Source HRoetschke_271222_090123_HFGoe_LEDGF_b1R2_2h **Scan** 6122
Peptide RIHAEINKT **Charge** 3 **Spectral Angle** 0.8

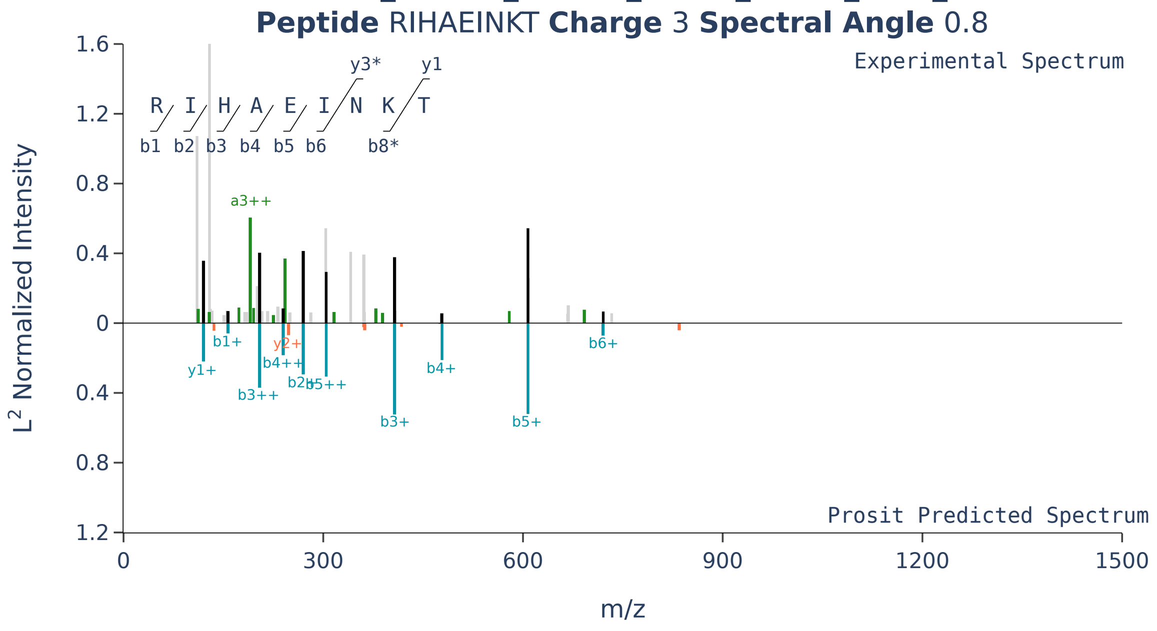

Source HRoetschke_271222_090123_HFGoe_LEDGF_b1R2_24h **Scan** 6986
Peptide ATRDFKPGD **Charge** 2 **Spectral Angle** 0.82

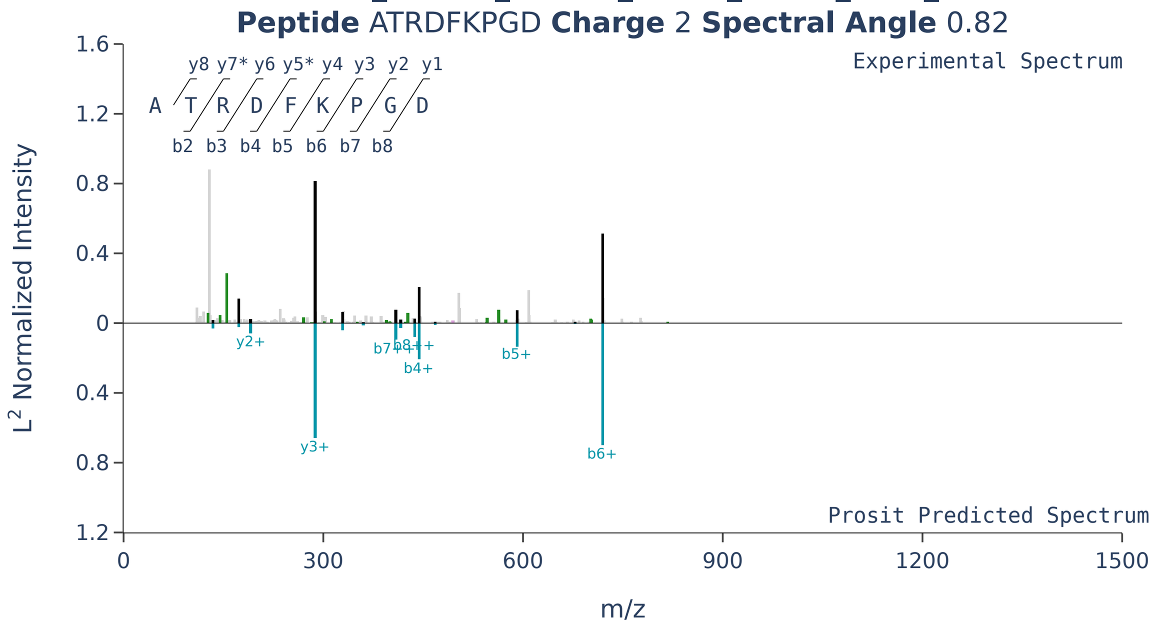

Source HRoetschke_271222_090123_HFGoe_LEDGF_b1R1_4h **Scan** 20161
Peptide LIFAKMKGDEVPDGAVKPPTN **Charge** 3 **Spectral Angle** 0.88

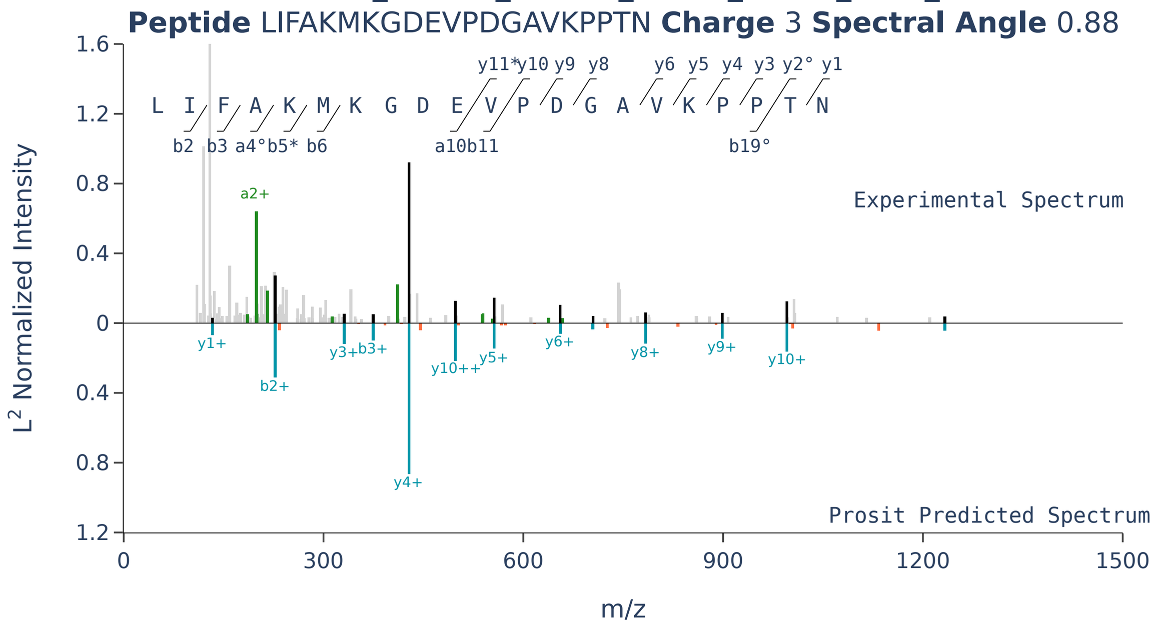

Source HRoetschke_271222_090123_HFGoe_LEDGF_b1R2_4h **Scan** 18897
Peptide AFLGPKDIFPYSENKEKYGKPNKRALD **Charge** 5 **Spectral Angle** 0.75

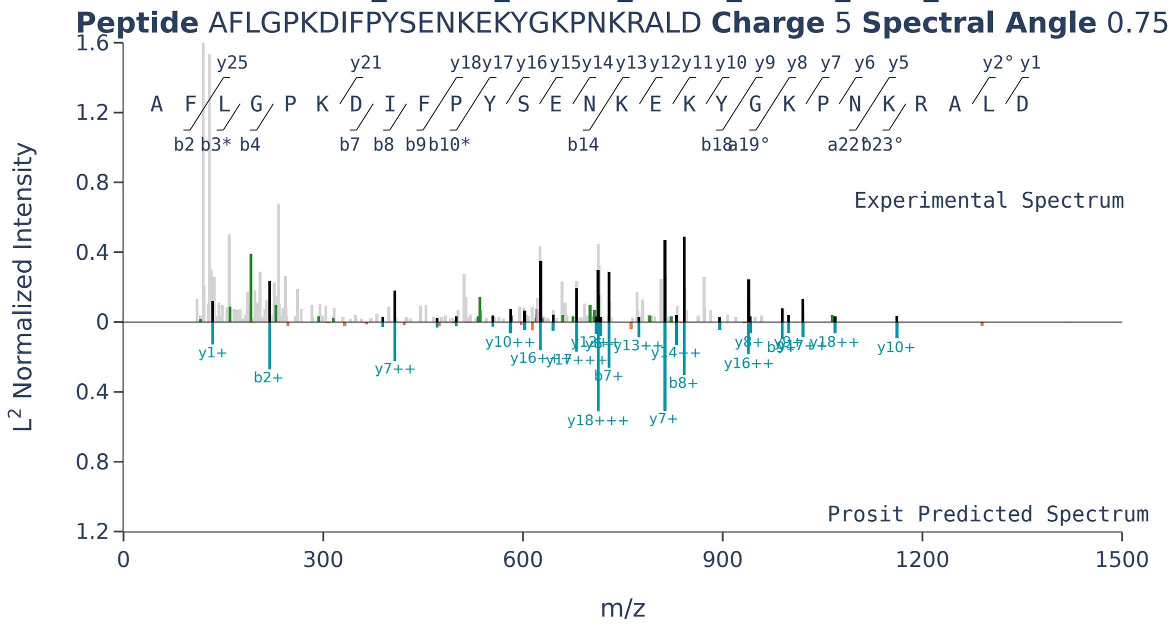

Source HRoetschke_271222_090123_HFGoe_LEDGF_b2R1_24h **Scan** 19146
Peptide GVFLGPKDIFPYSENKEKYGKPNKR **Charge** 5 **Spectral Angle** 0.72

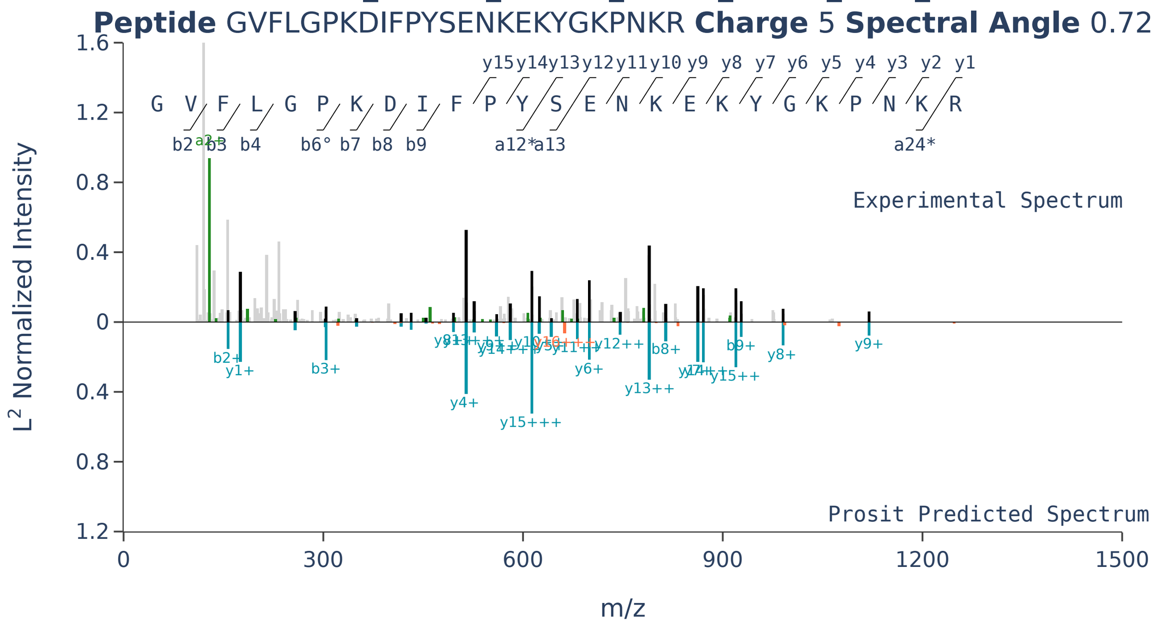

Source HRoetschke_271222_090123_HFGoe_LEDGF_b2R1_24h **Scan** 15286
Peptide KNSLKIGG **Charge** 2 **Spectral Angle** 0.88

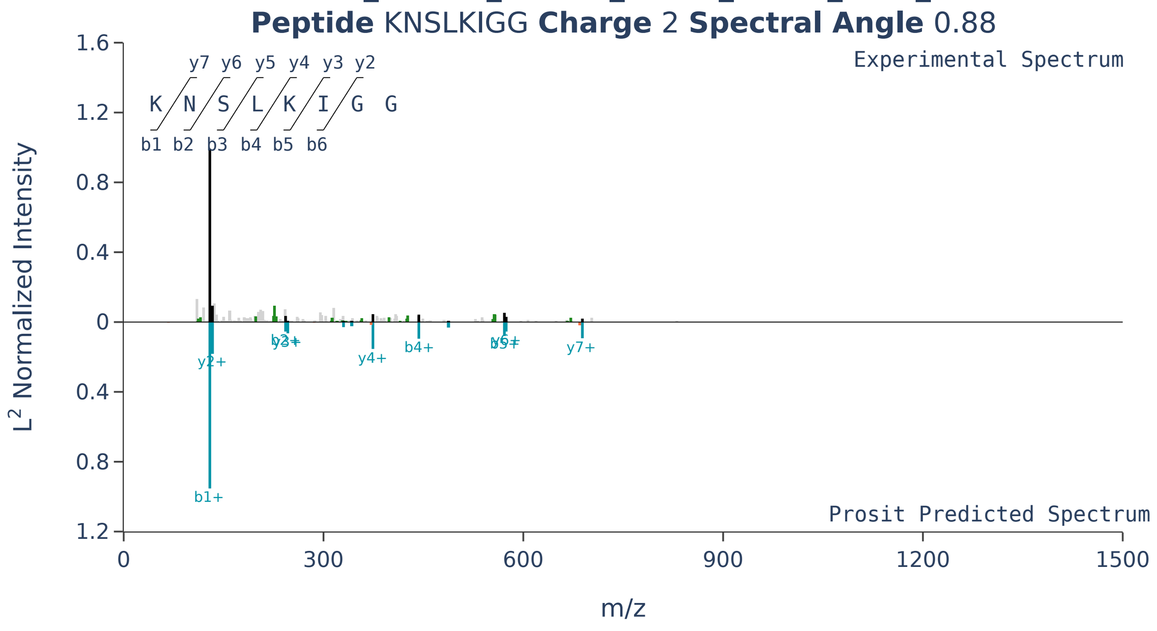

Source HRoetschke_271222_090123_HFGoe_LEDGF_b1R1_24h **Scan** 8868
Peptide FPYSENKEKYGKPNKRLE **Charge** 5 **Spectral Angle** 0.78

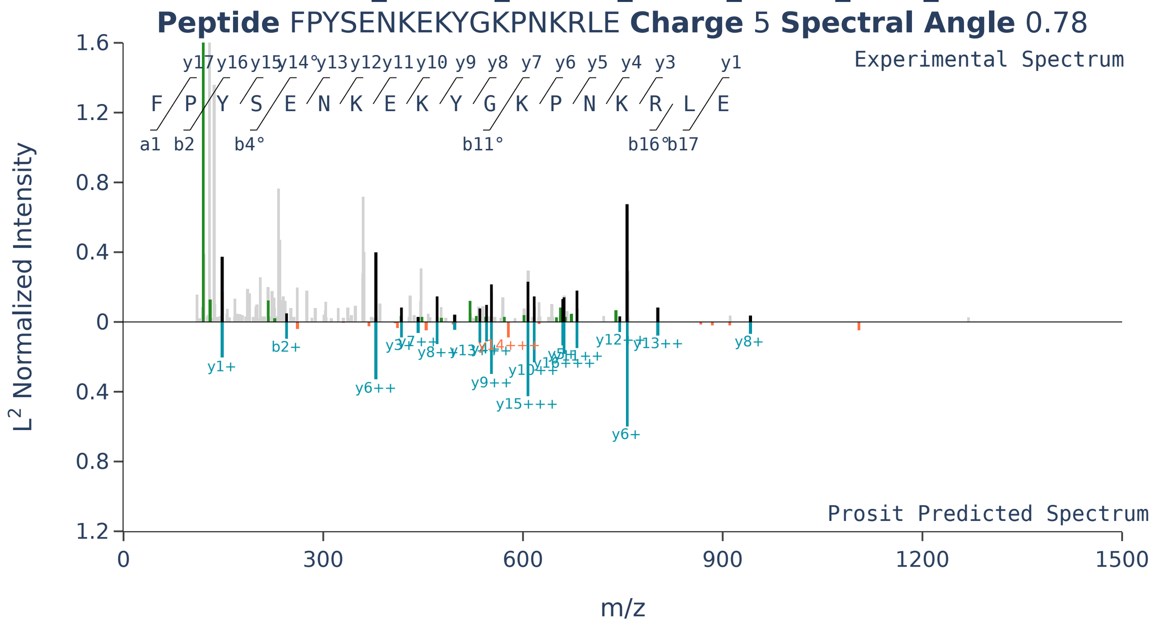

Source HRoetschke_271222_090123_HFGoe_LEDGF_b1R1_2h **Scan** 25132
Peptide DLKIDNLDVNRCIE **Charge** 2 **Spectral Angle** 0.78

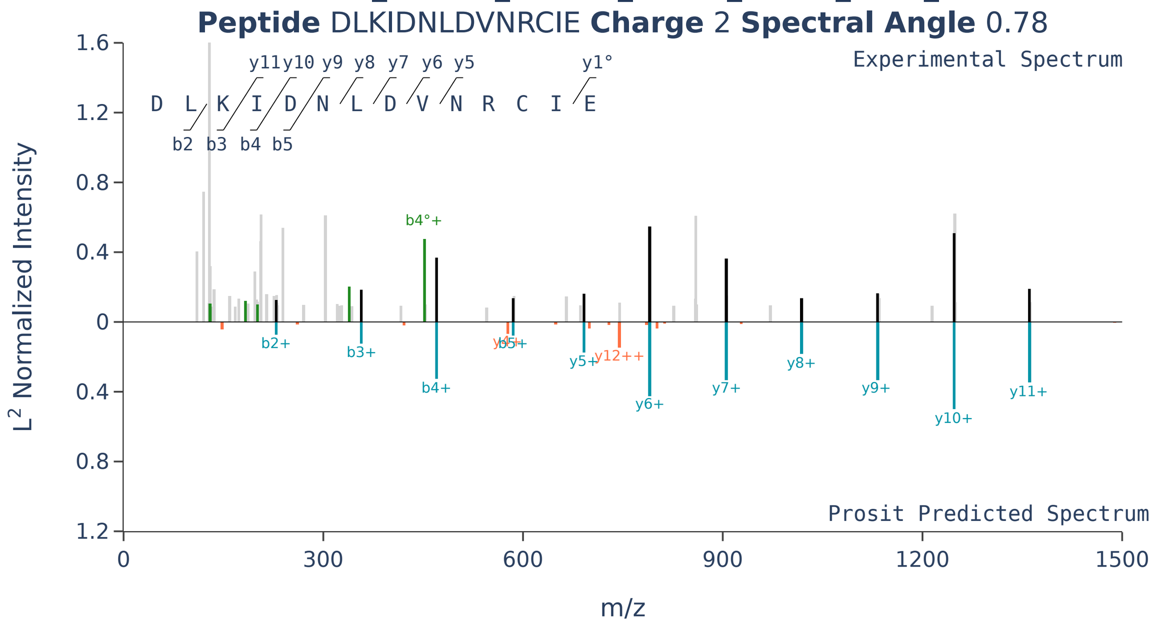

Source HRoetschke_271222_090123_HFGoe_LEDGF_b1R1_4h **Scan** 22272
Peptide STKGFNEGLWE **Charge** 2 **Spectral Angle** 0.7

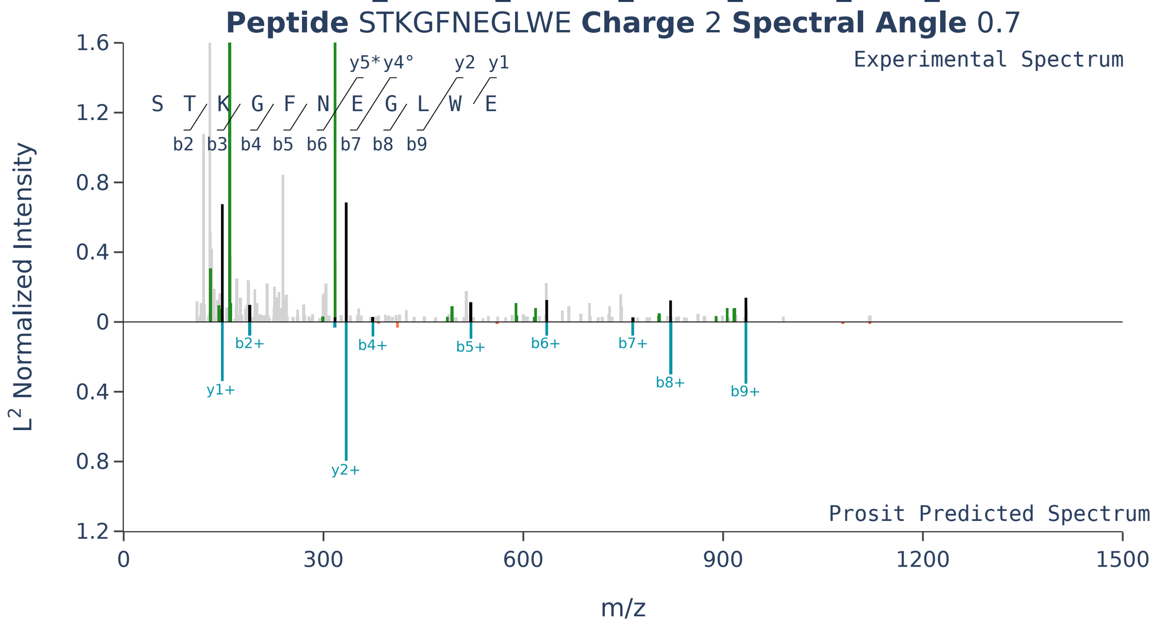

Source HRoetschke_271222_090123_HFGoe_LEDGF_b1R2_4h **Scan** 9934
Peptide ITEEVEE **Charge** 2 **Spectral Angle** 0.88

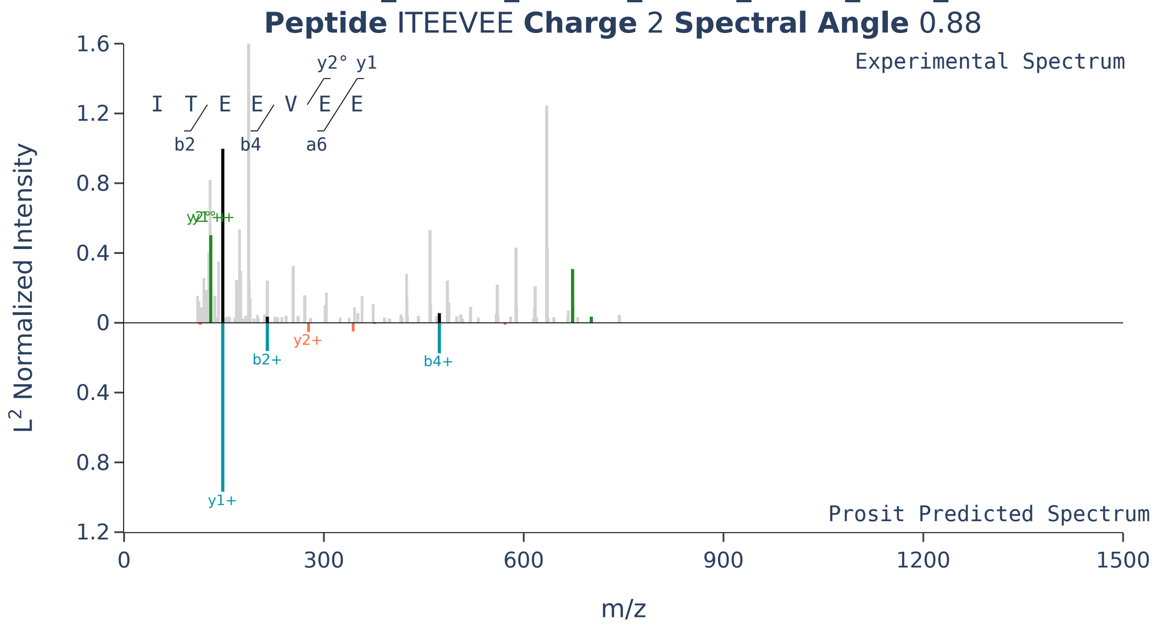

Source HRoetschke_271222_090123_HFGoe_LEDGF_b1R1_24h **Scan** 9355
Peptide PSSEERETLE **Charge** 1 **Spectral Angle** 0.75

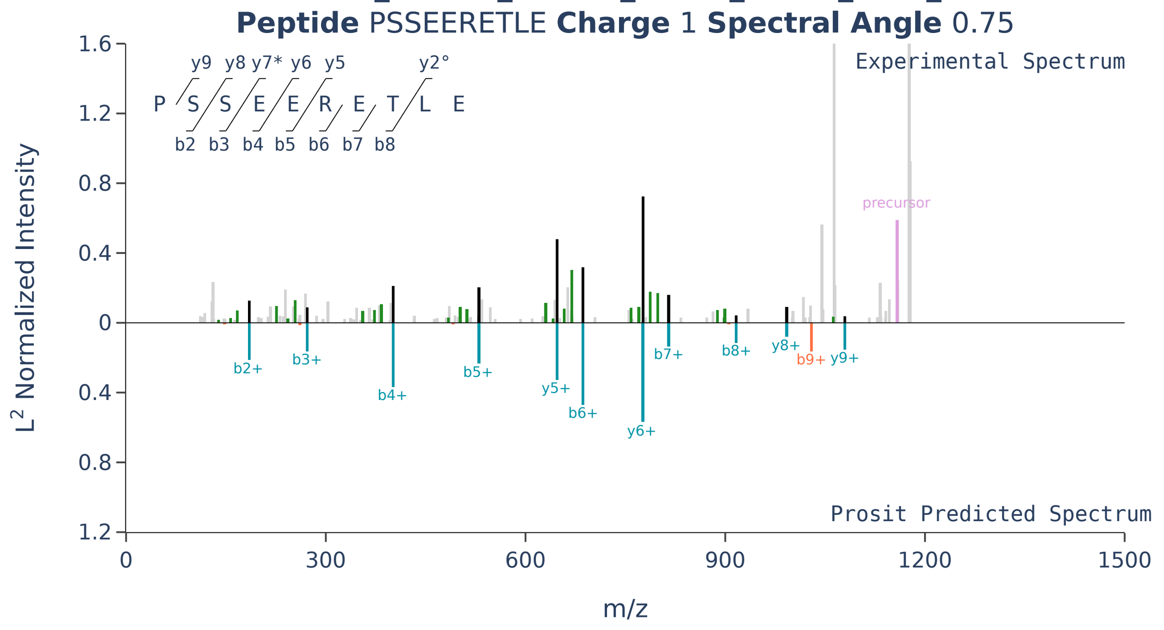

Source HRoetschke_271222_090123_HFGoe_LEDGF_b1R1_24h **Scan** 7533
Peptide PGSVNLKVSPKRGR **Charge** 4 **Spectral Angle** 0.86

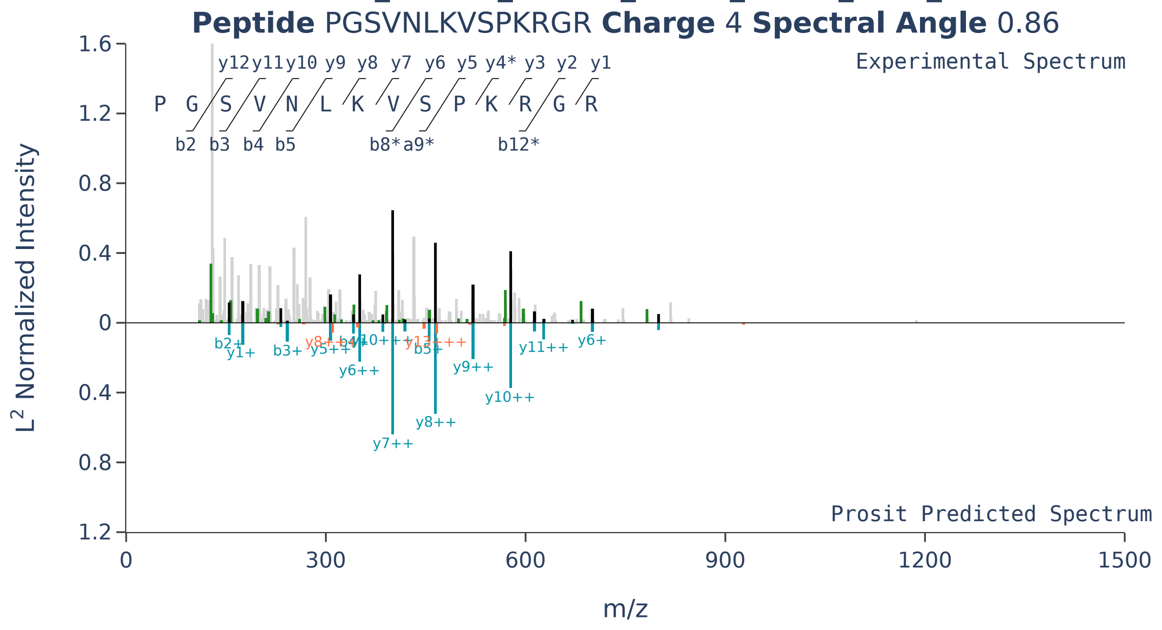

Source HRoetschke_271222_090123_HFGoe_LEDGF_b1R2_1h **Scan** 18009
Peptide STMPHWPARV **Charge** 3 **Spectral Angle** 0.9

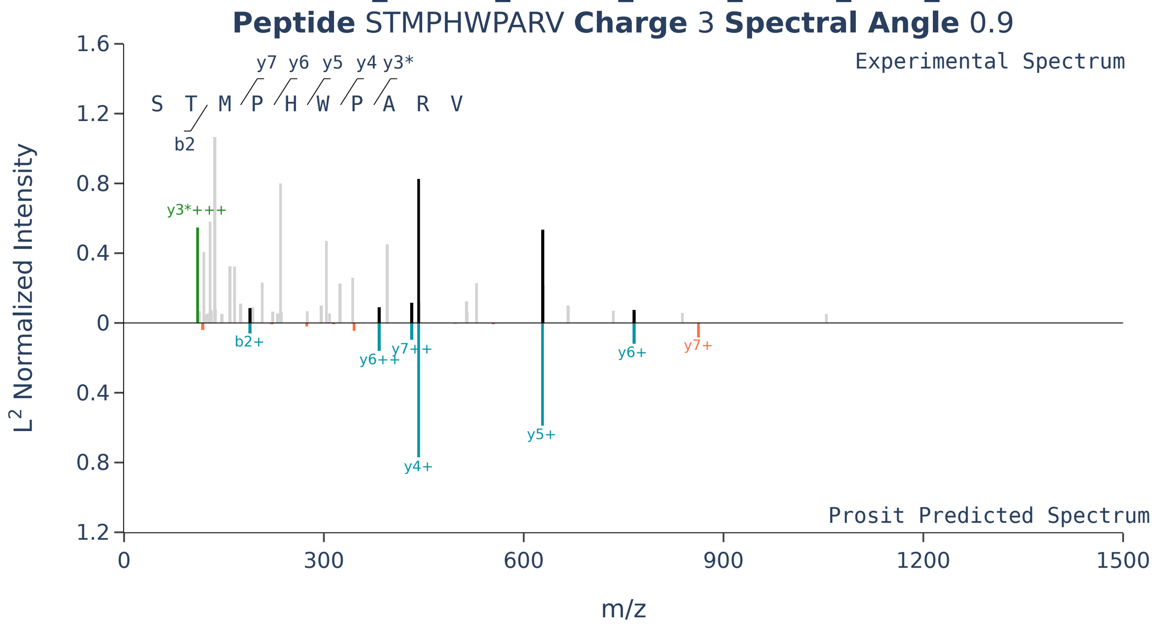

Source HRoetschke_271222_090123_HFGoe_LEDGF_b2R2_2h **Scan** 15833
Peptide FLVGKDIFPYSENKEKYGKPNKRKG **Charge** 6 **Spectral Angle** 0.78

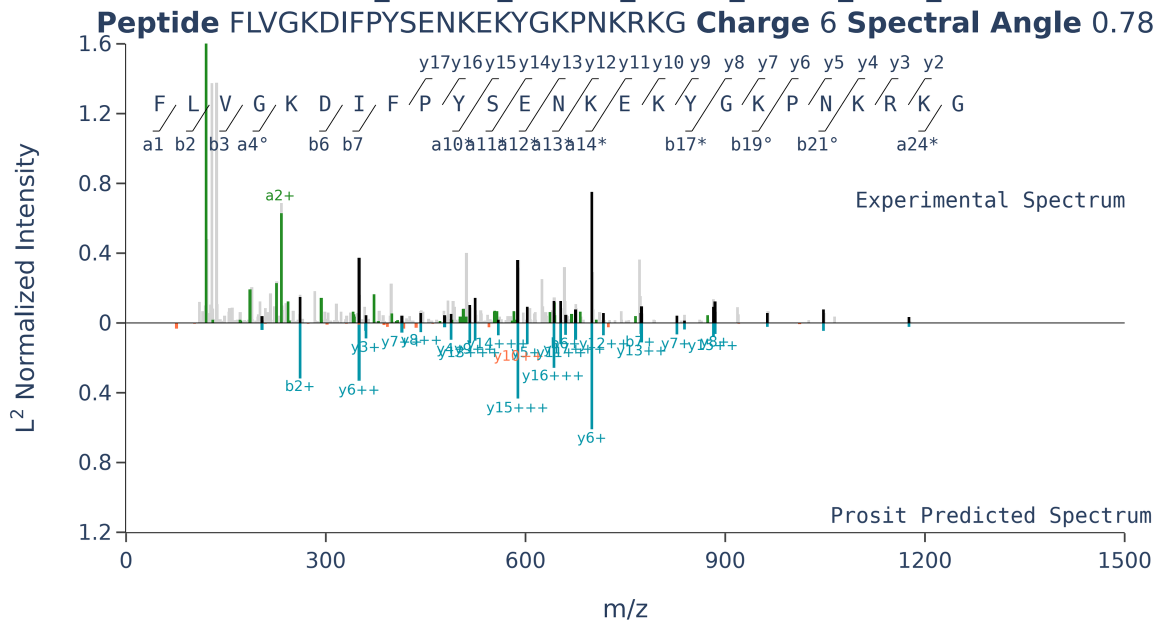

Source HRoetschke_271222_090123_HFGoe_LEDGF_b2R2_2h **Scan** 26766
Peptide AKGFNEGLWEID **Charge** 2 **Spectral Angle** 0.74

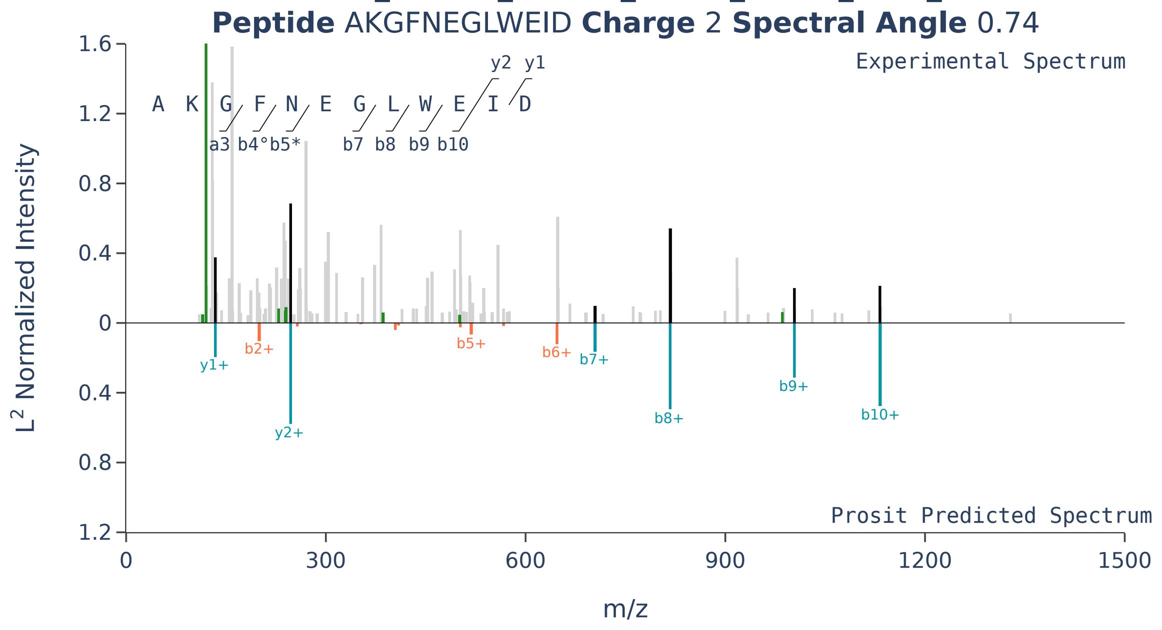

Source HRoetschke_271222_090123_HFGoe_LEDGF_b1R1_24h **Scan** 9262
Peptide QPQHNMITTLKKIR **Charge** 3 **Spectral Angle** 0.84

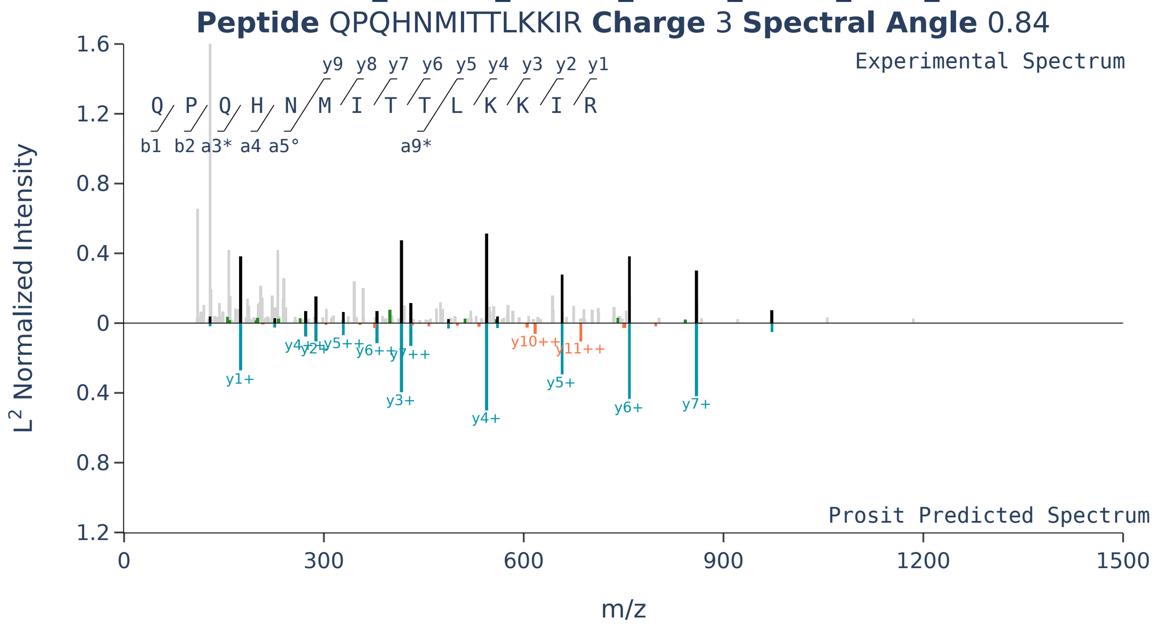

Source HRoetschke_271222_090123_HFGoe_LEDGF_b1R2_24h **Scan** 12323
Peptide TGPGMTRDFKPGD **Charge** 2 **Spectral Angle** 0.86

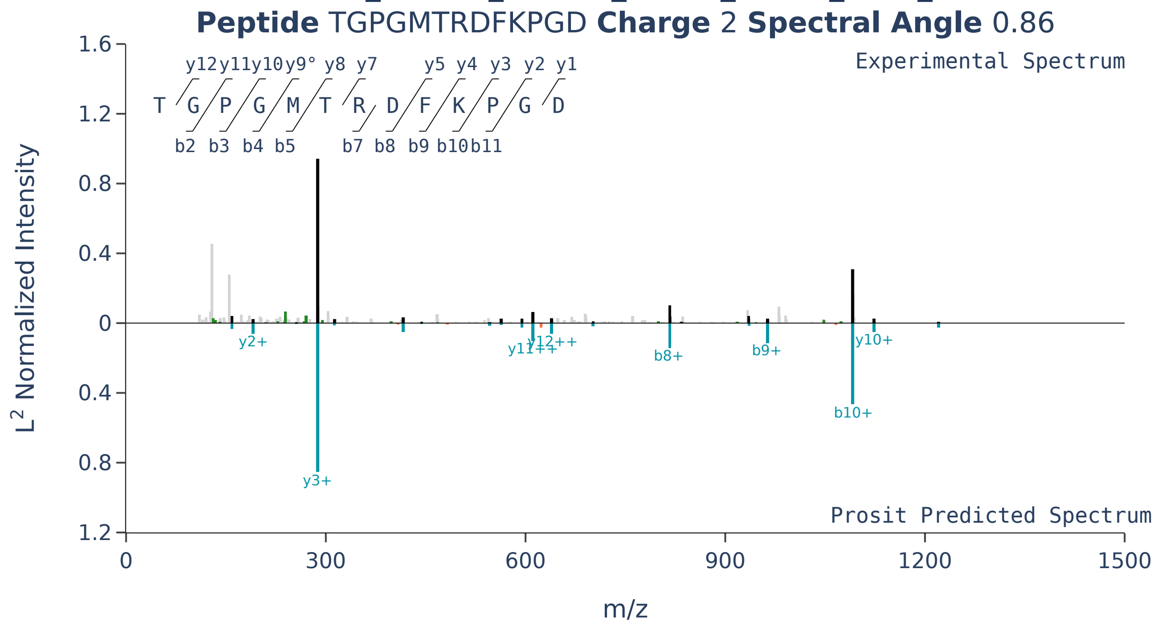

Source HRoetschke_271222_090123_HFGoe_LEDGF_b1R1_24h **Scan** 16044
Peptide TAQKHTEMITTL **Charge** 3 **Spectral Angle** 0.75

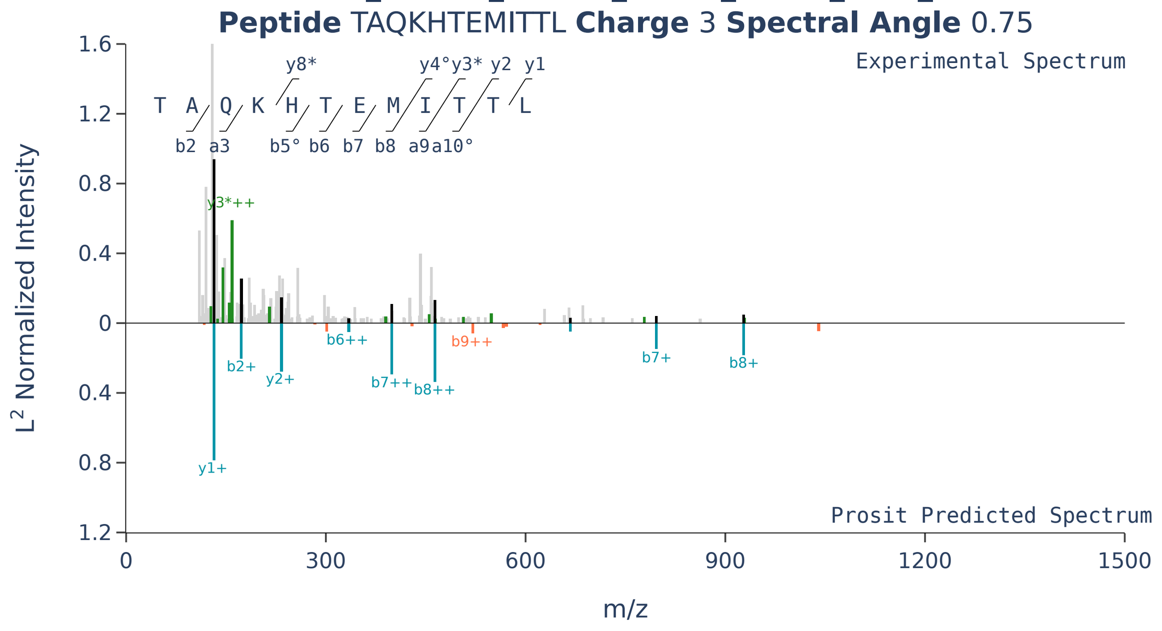

Source HRoetschke_271222_090123_HFGoe_LEDGF_b1R1_24h **Scan** 16164
Peptide LGPKDIFPYSENKEKYGKPNKREL **Charge** 5 **Spectral Angle** 0.77

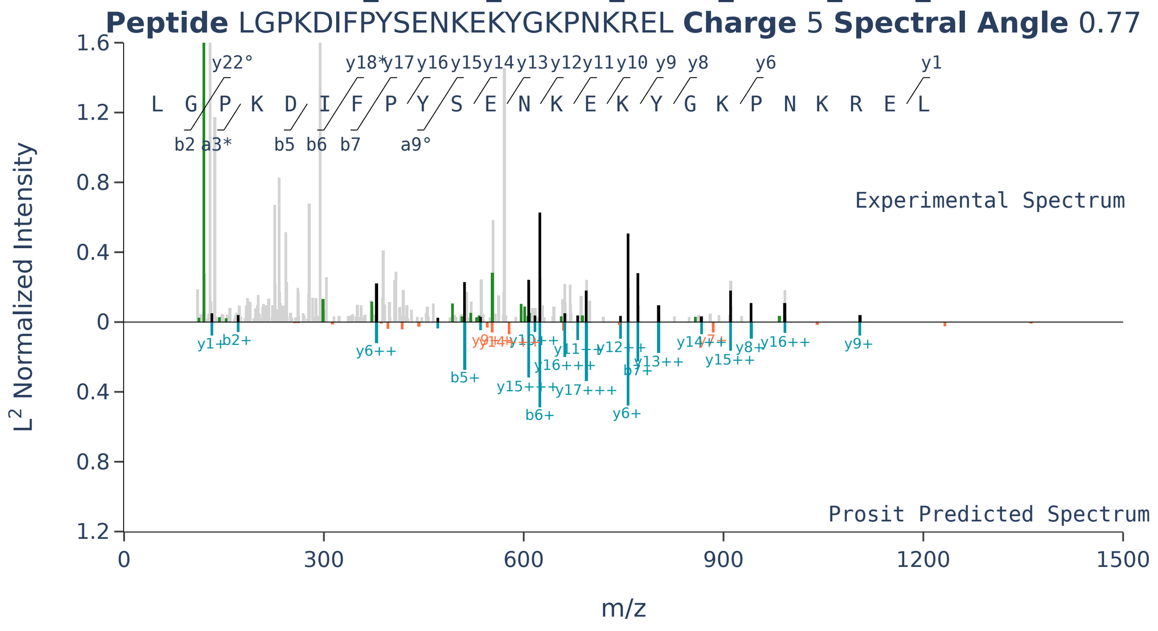

Source HRoetschke_271222_090123_HFGoe_LEDGF_b1R2_24h **Scan** 16275
Peptide KFKNMFLVGKETSVSKE **Charge** 3 **Spectral Angle** 0.88

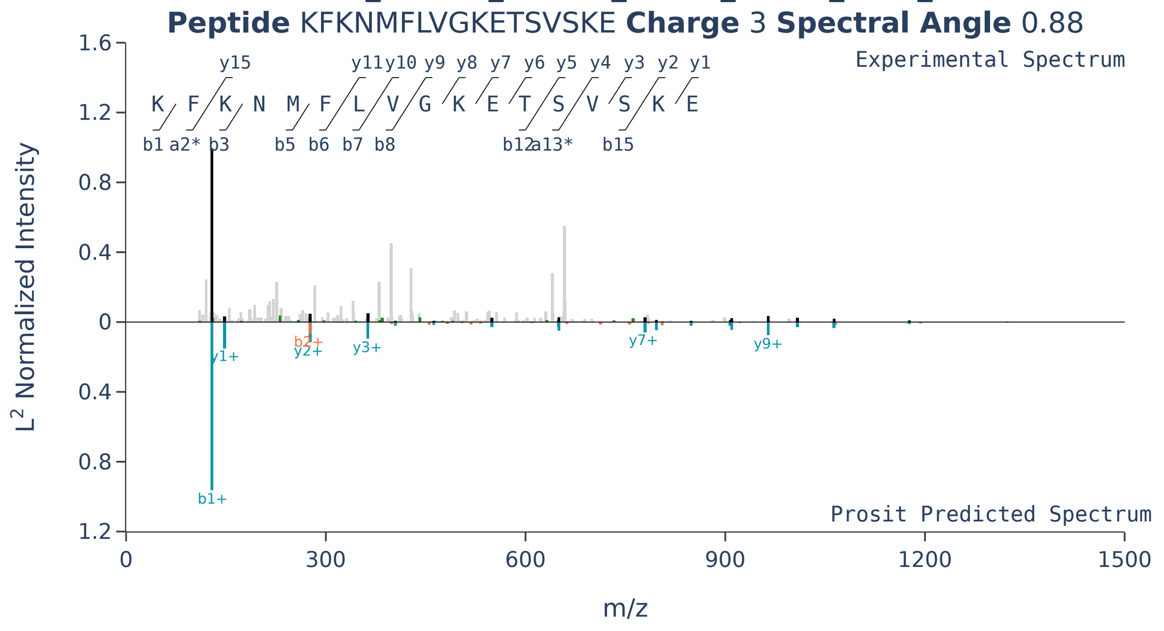

Source HRoetschke_271222_090123_HFGoe_LEDGF_b1R2_24h **Scan** 6939
Peptide SVNLKVSPKRGRTA **Charge** 3 **Spectral Angle** 0.85

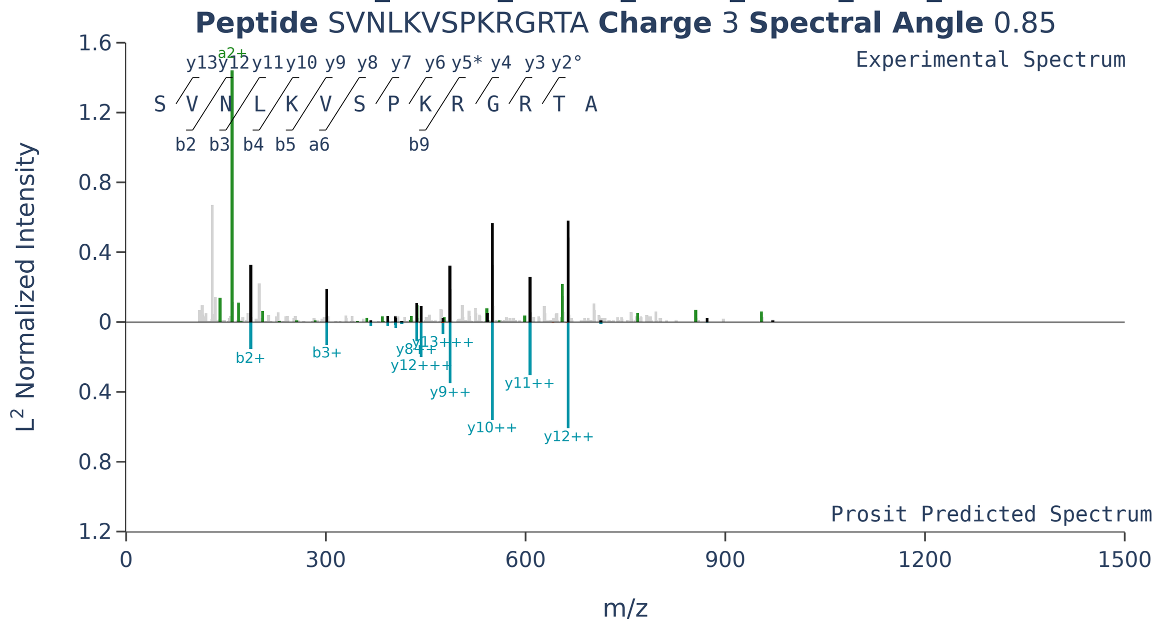

Source HRoetschke_271222_090123_HFGoe_LEDGF_b2R1_4h **Scan** 13807
Peptide FLVGEGDKETSVSKE **Charge** 3 **Spectral Angle** 0.72

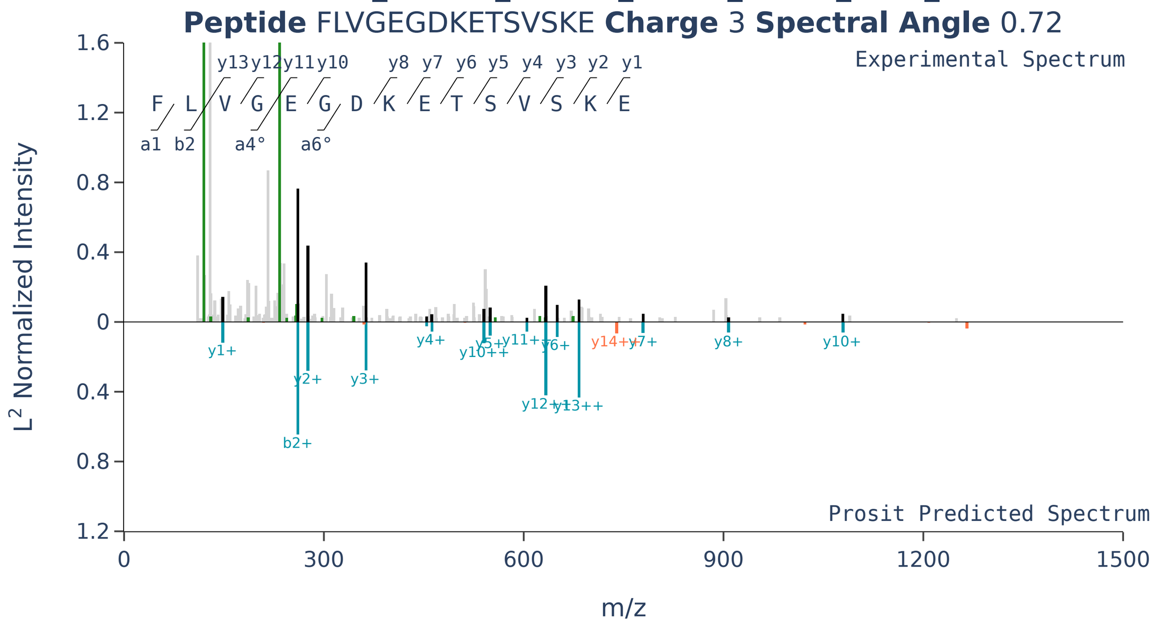

Source HRoetschke_271222_090123_HFGoe_LEDGF_b1R1_4h **Scan** 20007
Peptide DNNPKVKFSTAF **Charge** 2 **Spectral Angle** 0.84

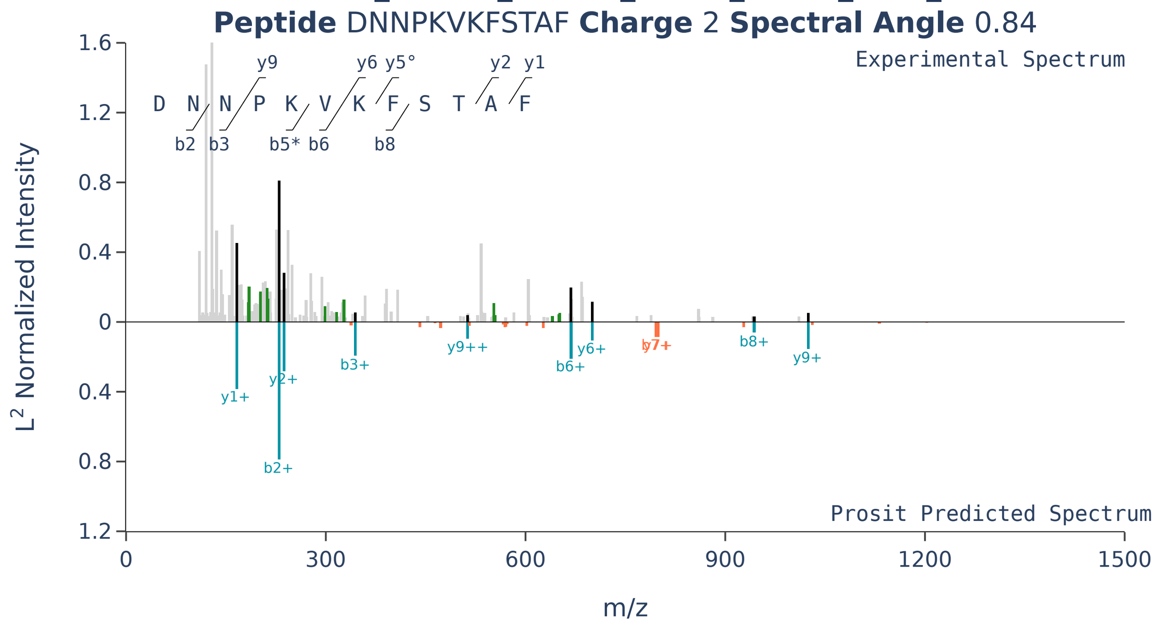

Source HRoetschke_271222_090123_HFGoe_LEDGF_b2R1_4h **Scan** 17002
Peptide ASHETAFLGPKDIFPYSENKEKYGKPNKR **Charge** 4 **Spectral Angle** 0.79

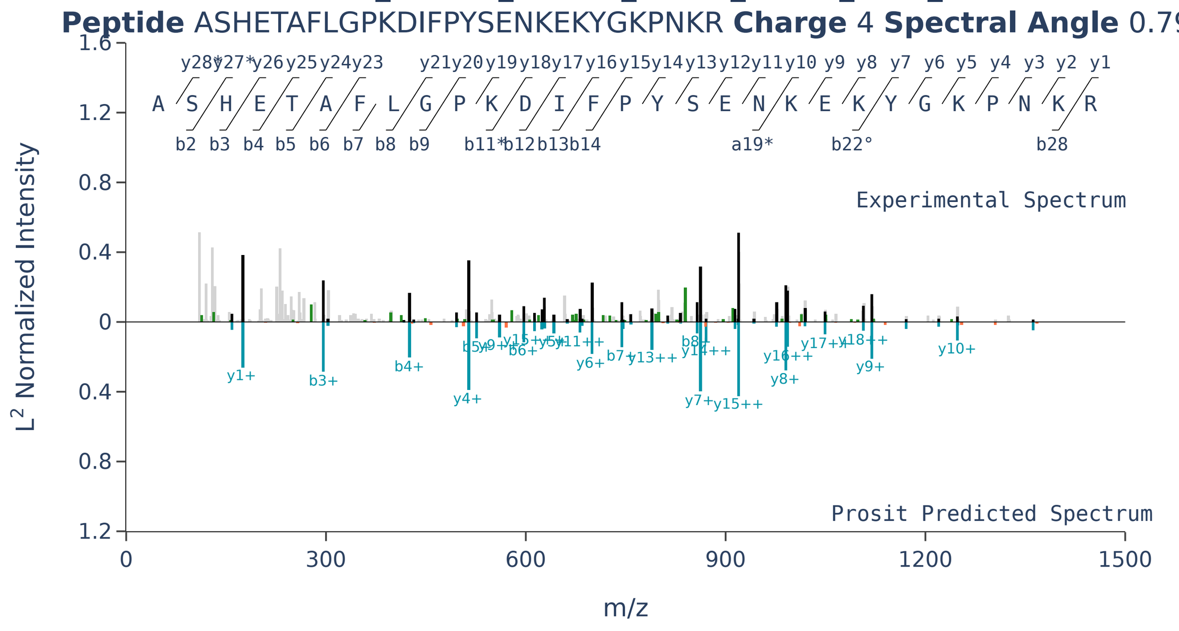

Source HRoetschke_271222_090123_HFGoe_LEDGF_b1R1_4h **Scan** 21483
Peptide NPMTRDFKPGDLIFAKMK **Charge** 3 **Spectral Angle** 0.74

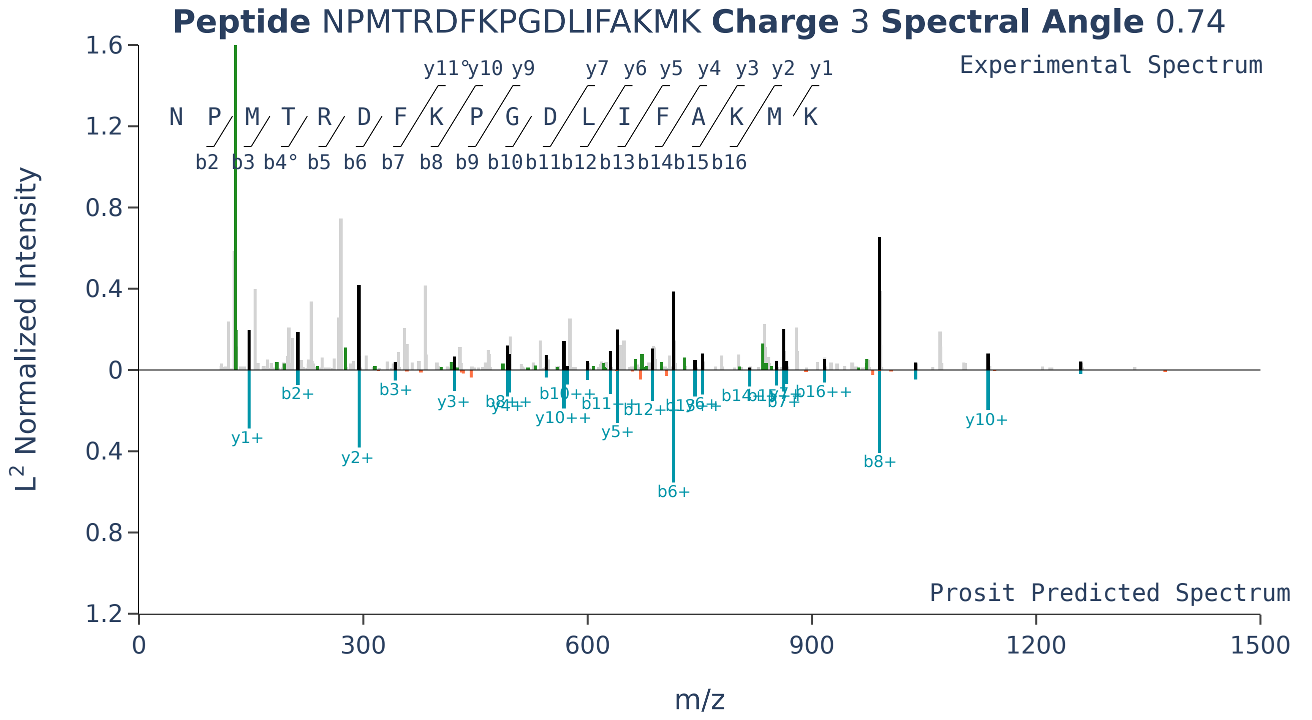

Source HRoetschke_271222_090123_HFGoe_LEDGF_b1R1_4h **Scan** 17051
Peptide FLGPKDIFPYSENKEKYGKPNKAR **Charge** 4 **Spectral Angle** 0.81

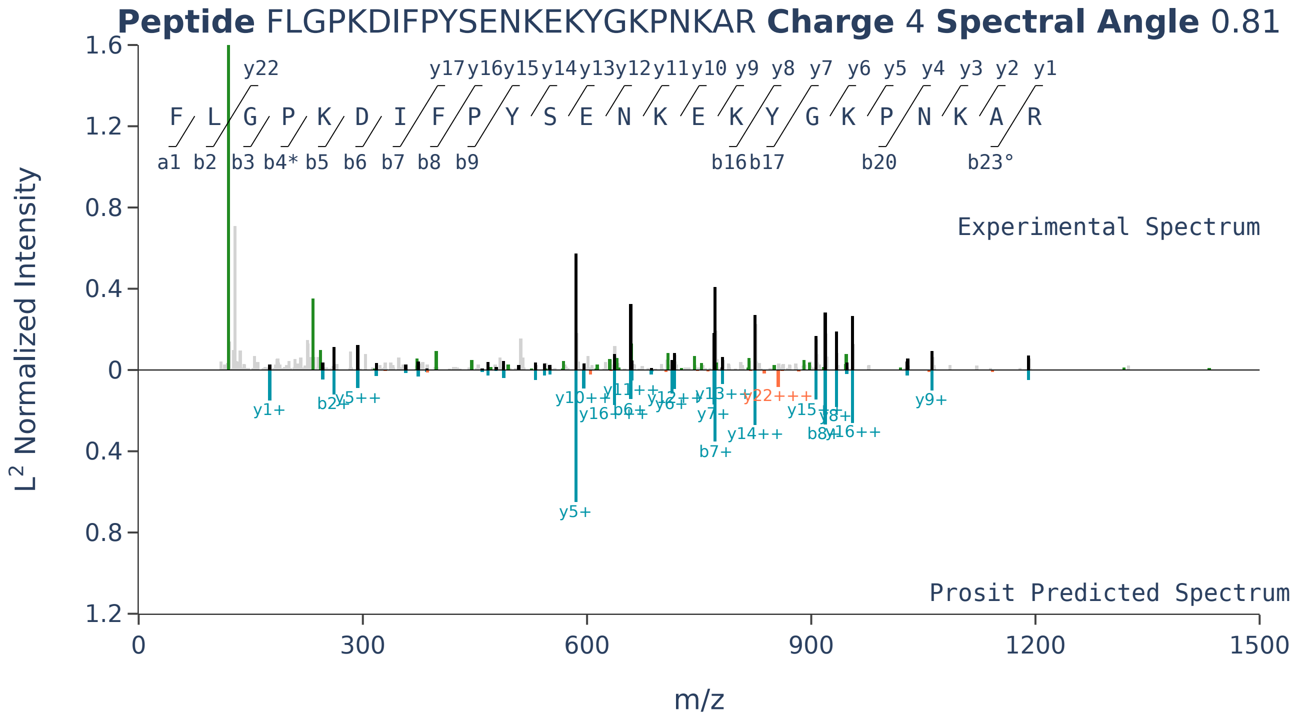

Source HRoetschke_271222_090123_HFGoe_LEDGF_b1R2_24h **Scan** 17757
Peptide GVDEVPDGAVKPPTNKL **Charge** 3 **Spectral Angle** 0.77

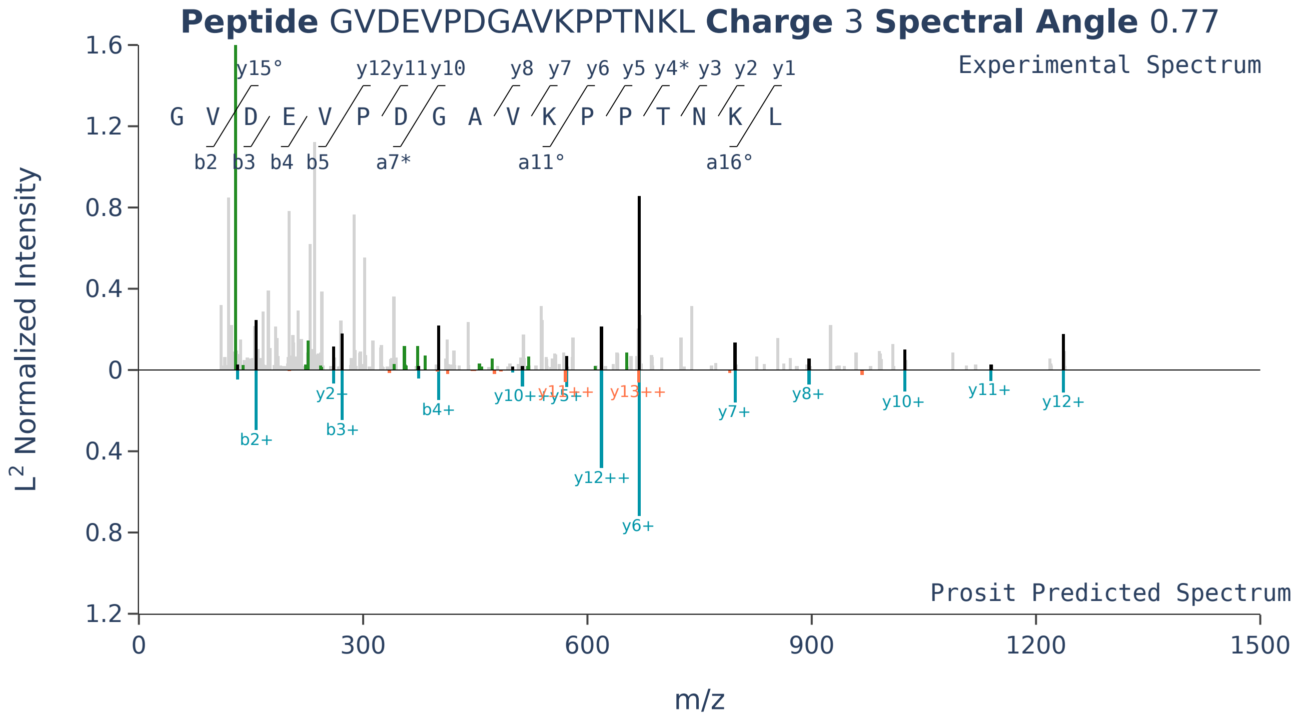

Source HRoetschke_271222_090123_HFGoe_LEDGF_b1R1_24h **Scan** 16040
Peptide FLGPKDIFPYSENKEKYGKPNKRKA **Charge** 5 **Spectral Angle** 0.79

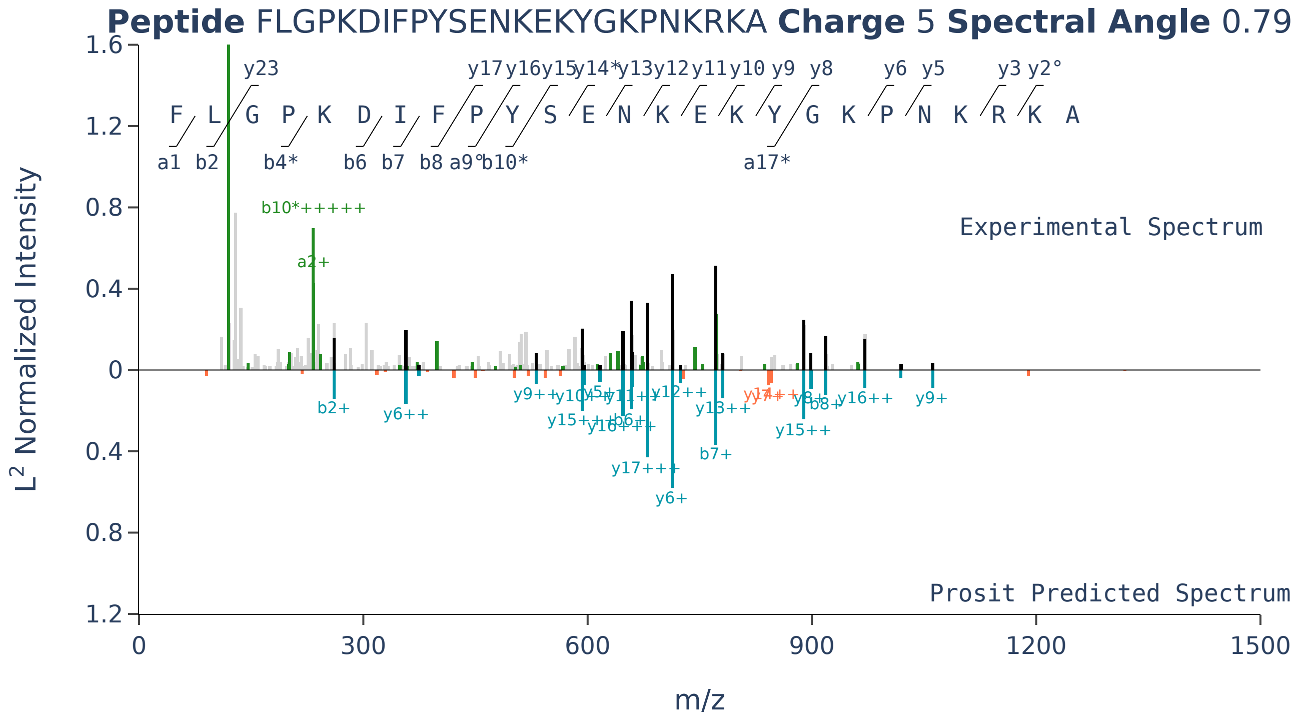

Source HRoetschke_271222_090123_HFGoe_LEDGF_b2R1_4h **Scan** 13443
Peptide ASLMDSRLQR **Charge** 3 **Spectral Angle** 0.82

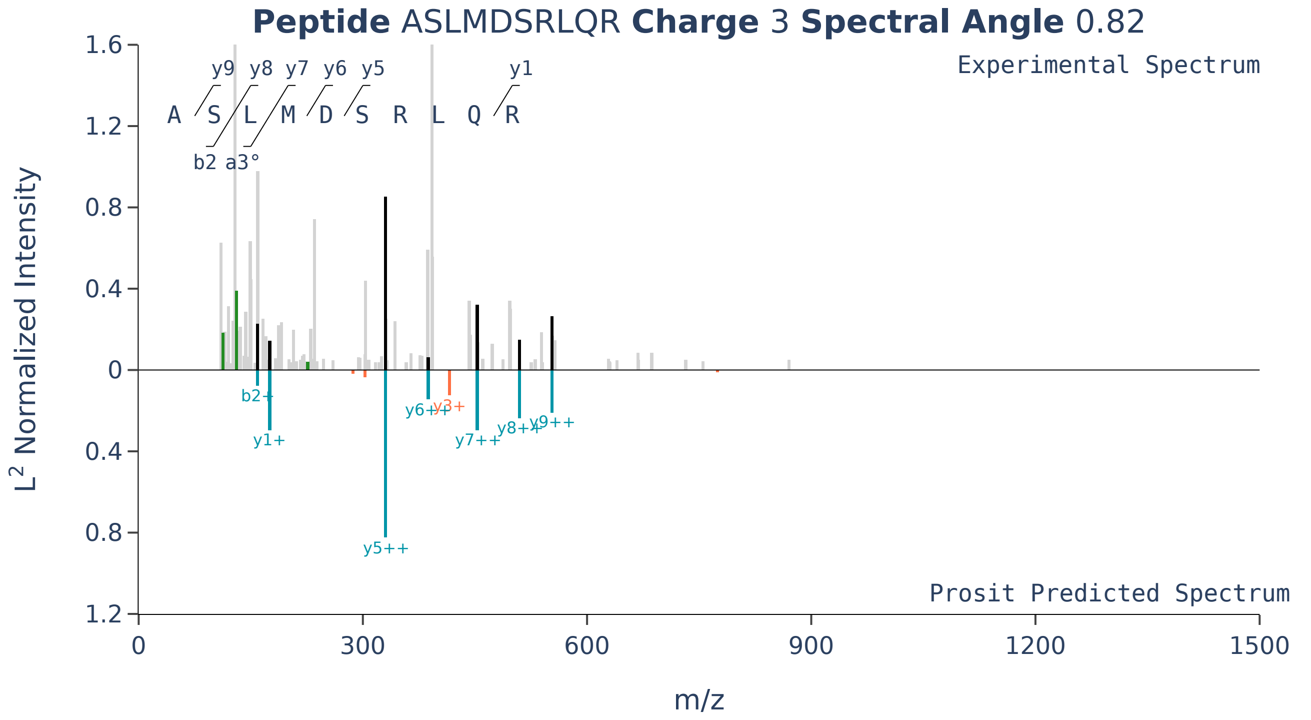

Source HRoetschke_271222_090123_HFGoe_LEDGF_b1R2_4h **Scan** 19115
Peptide ATASFLGPKDIFPYSENKEKYGKPNKR **Charge** 5 **Spectral Angle** 0.78

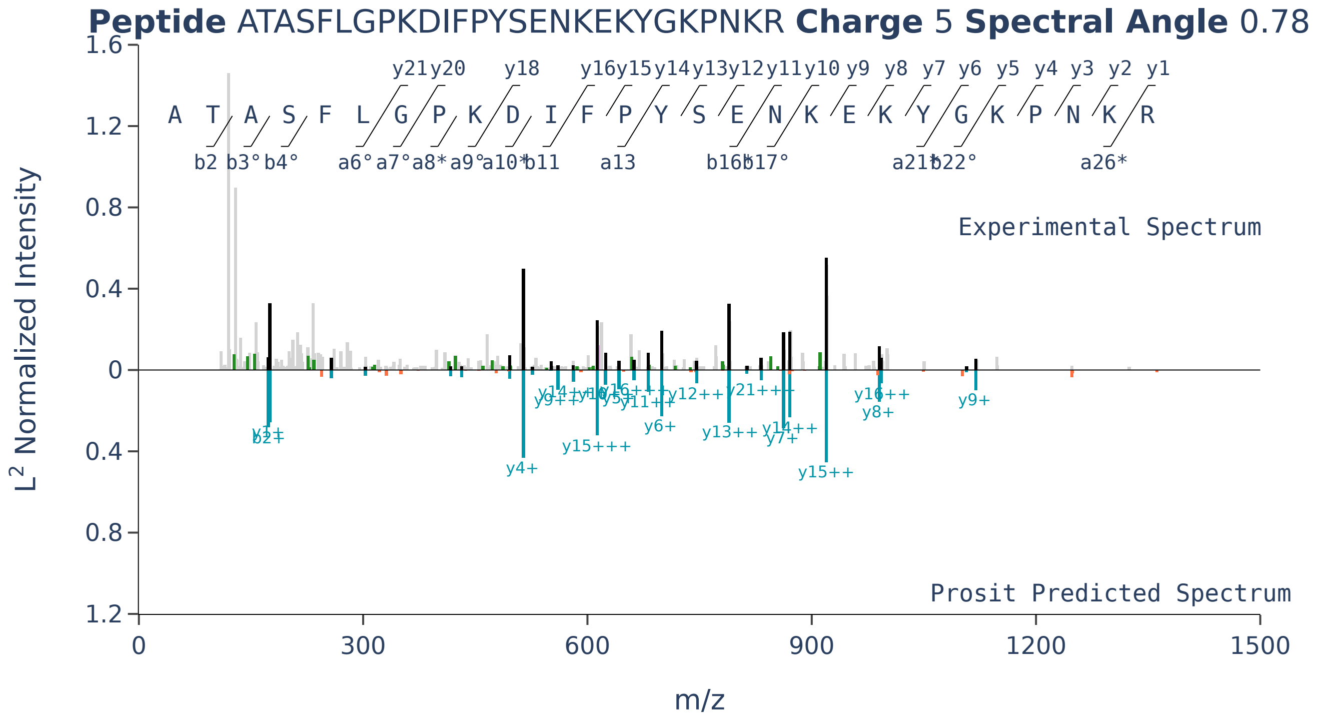

Source HRoetschke_271222_090123_HFGoe_LEDGF_b1R1_4h **Scan** 20743
Peptide EVPDGHWPARVDEVPDGAVKPPTN **Charge** 3 **Spectral Angle** 0.95

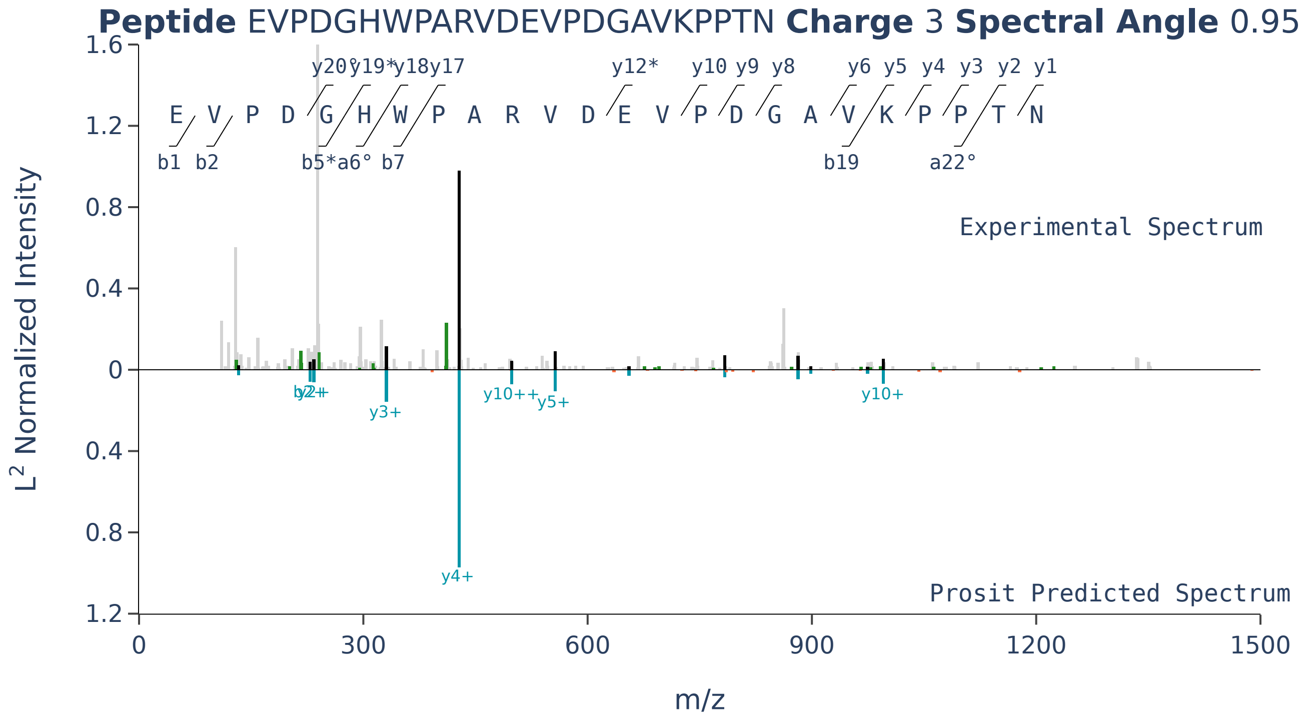

Source HRoetschke_271222_090123_HFGoe_LEDGF_b2R1_24h **Scan** 19506
Peptide GMTDSRLQRIHAEIKNSL **Charge** 3 **Spectral Angle** 0.83

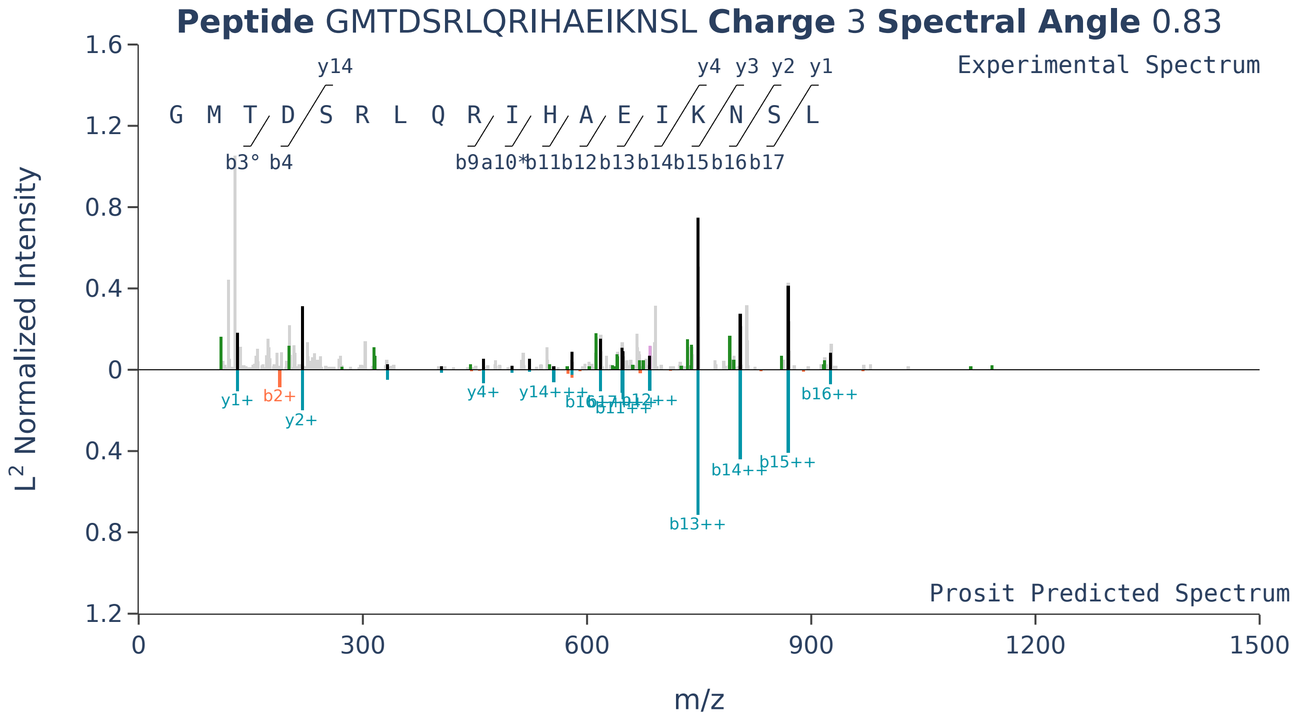

Source HRoetschke_271222_090123_HFGoe_LEDGF_b2R2_2h **Scan** 11709
Peptide GPNGMTRDFKPGD **Charge** 3 **Spectral Angle** 0.8

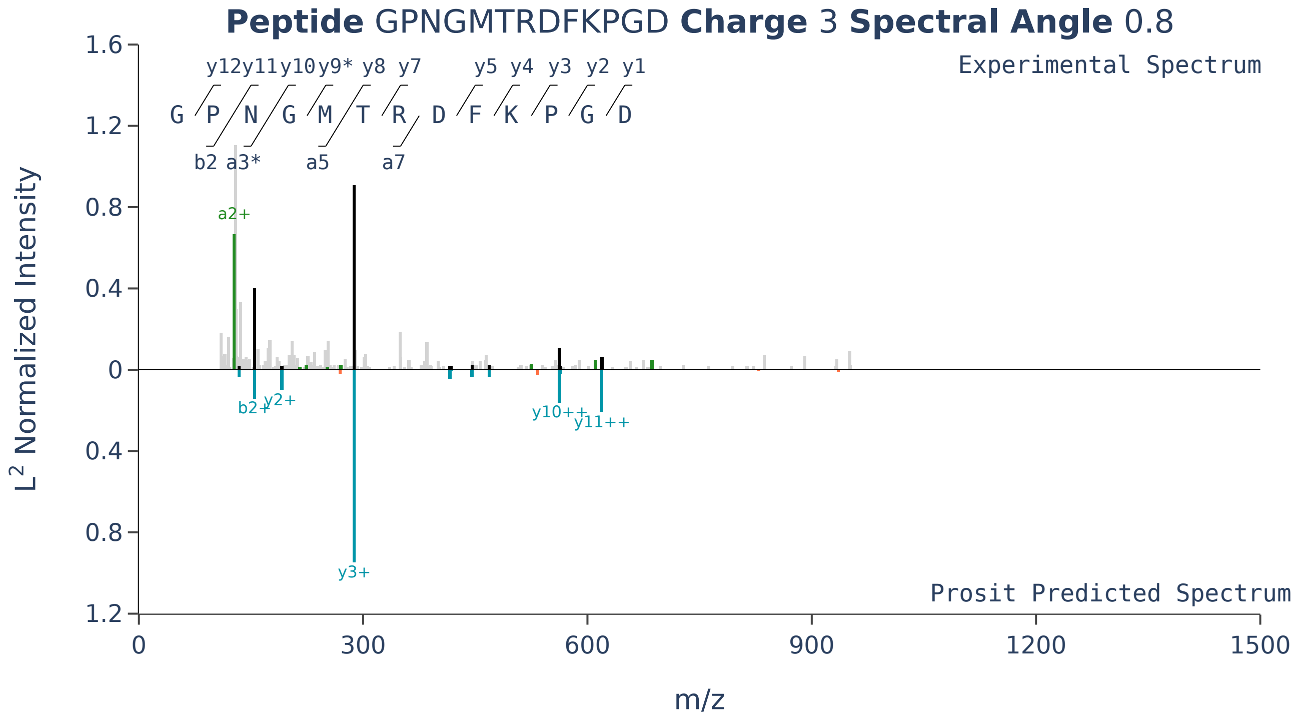

Source HRoetschke_271222_090123_HFGoe_LEDGF_b2R1_4h **Scan** 11638
Peptide DNNPKVKFSA **Charge** 2 **Spectral Angle** 0.8

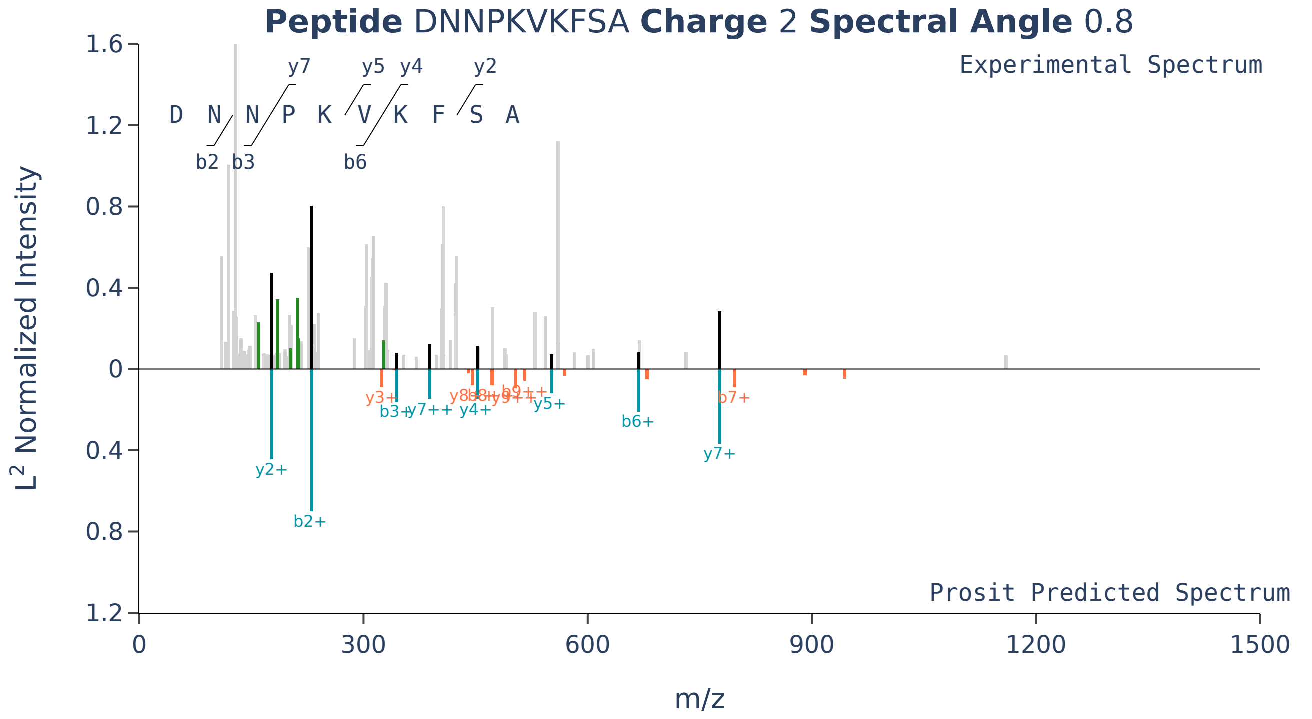

Source HRoetschke_221222_180123_HFGoe_RF1_b1R1_4h **Scan** 11183
Peptide DRSDIIQ **Charge** 2 **Spectral Angle** 0.94

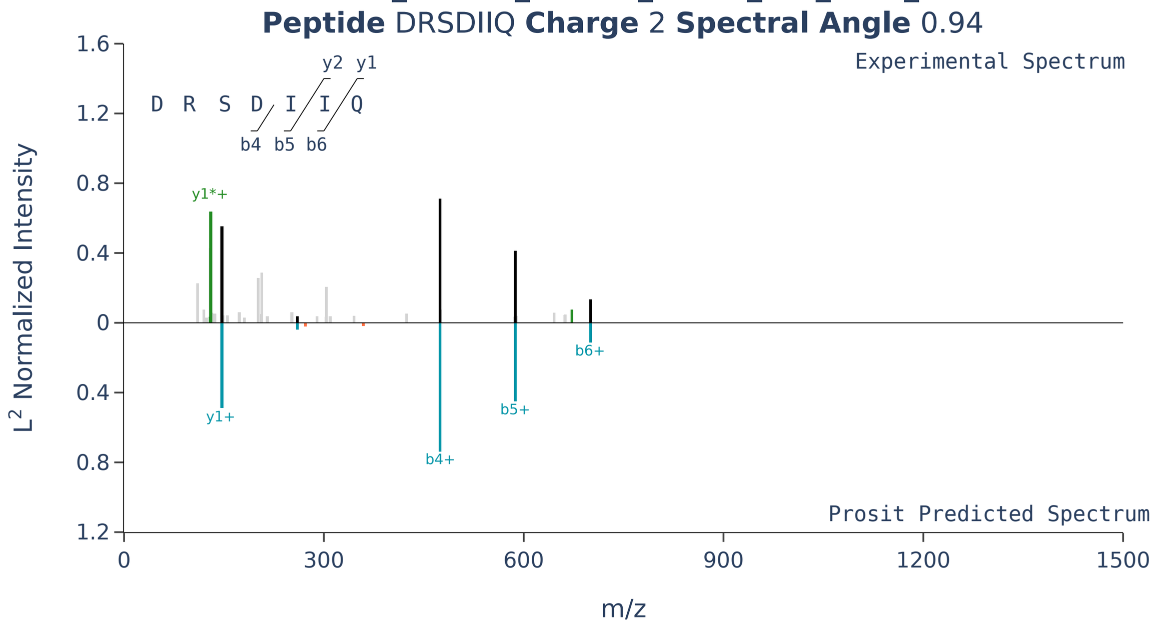

Source HRoetschke_221222_180123_HFGoe_RF1_b1R1_24h **Scan** 17486
Peptide AALFADG **Charge** 1 **Spectral Angle** 0.96

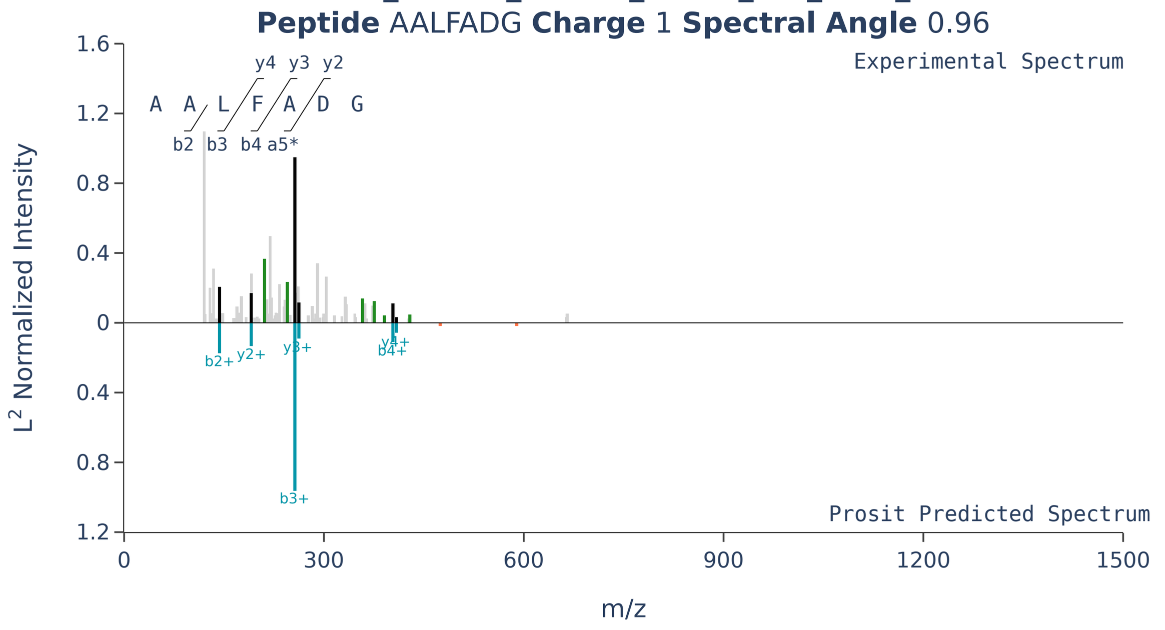

Source HRoetschke_221222_180123_HFGoe_RF1_b2R1_24h **Scan** 19051
Peptide ELLLPKD **Charge** 2 **Spectral Angle** 0.97

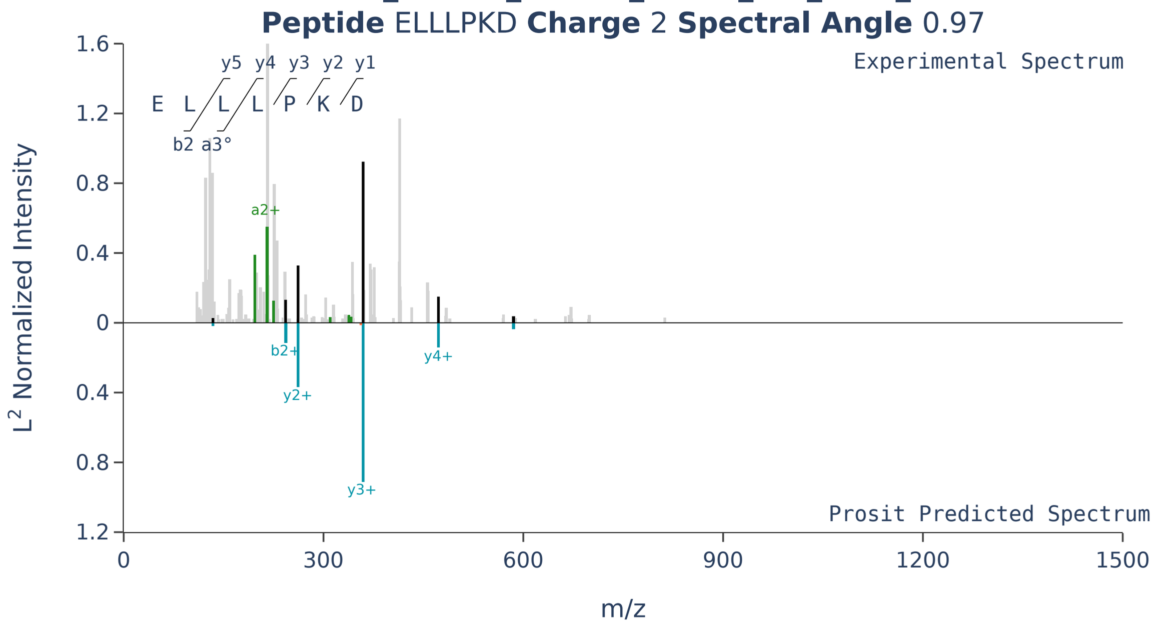

Source HRoetschke_221222_180123_HFGoe_RF1_b2R2_2h **Scan** 18682
Peptide SLLLPKD **Charge** 2 **Spectral Angle** 0.92

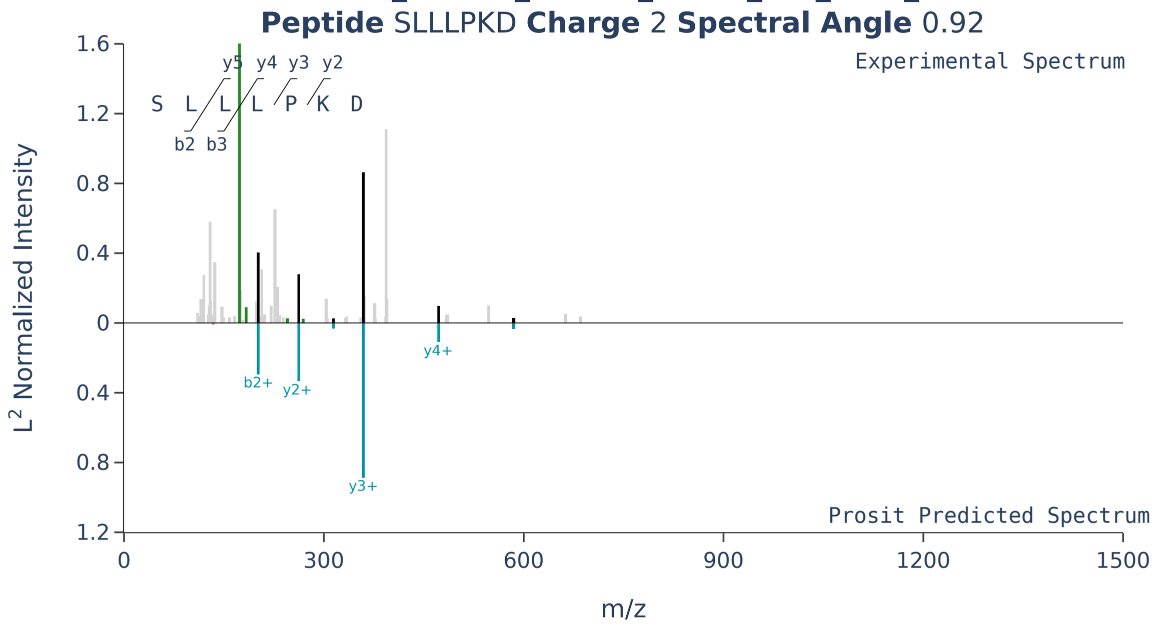

Source HRoetschke_221222_180123_HFGoe_RF1_b1R1_2h **Scan** 19073
Peptide PTGIVVECAD **Charge** 1 **Spectral Angle** 0.91

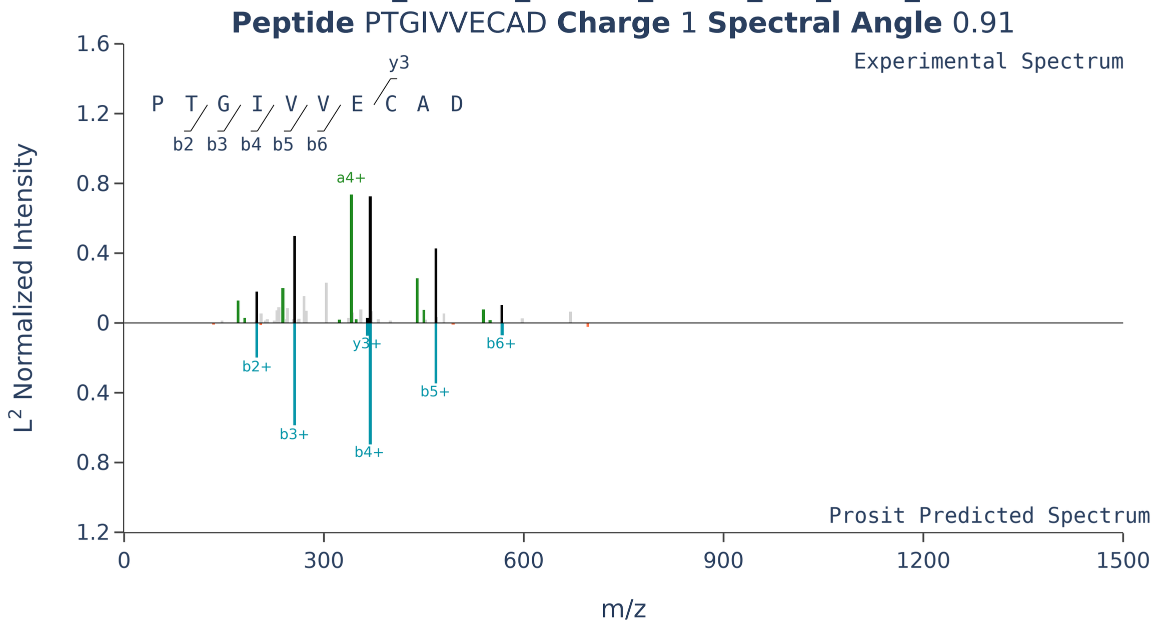

Source HRoetschke_221222_180123_HFGoe_RF1_b2R1_4h **Scan** 19618
Peptide DLLLPKD **Charge** 2 **Spectral Angle** 0.96

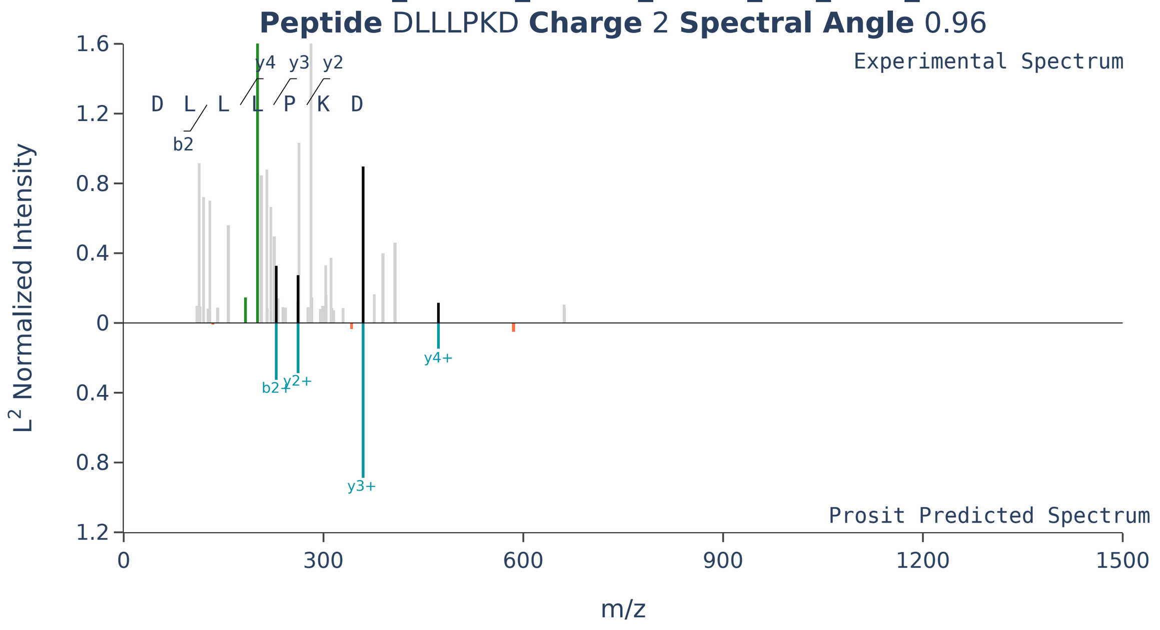

Source HRoetschke_221222_180123_HFGoe_RF1_b1R1_2h **Scan** 18991
Peptide TLLLPKD **Charge** 2 **Spectral Angle** 0.94

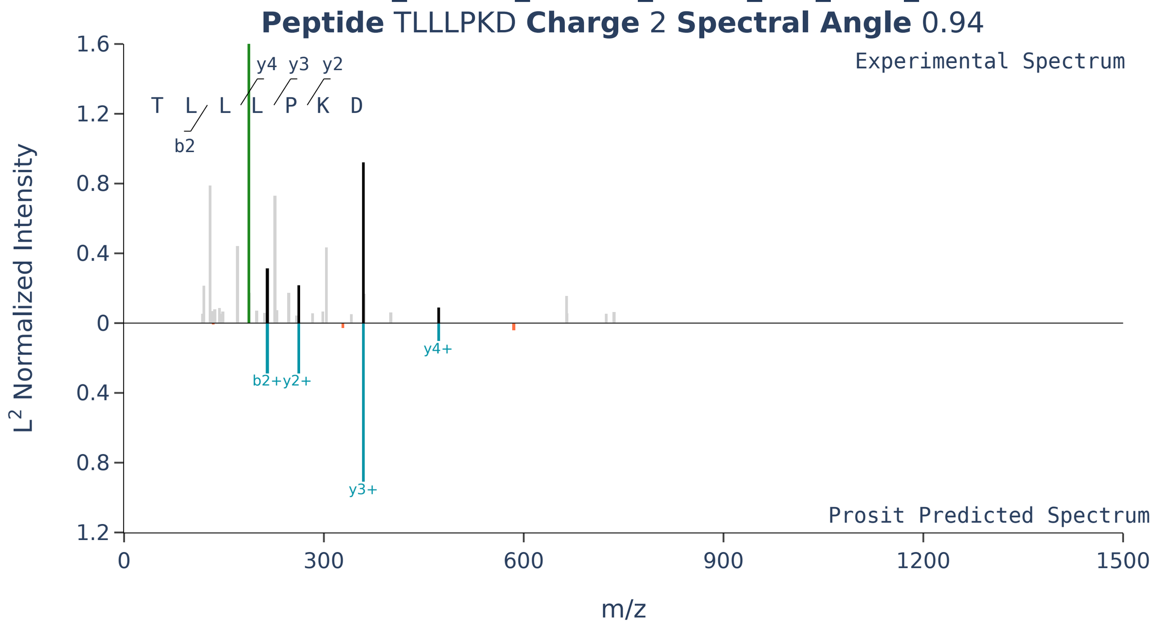

Source HRoetschke_221222_180123_HFGoe_RF1_b1R1_4h **Scan** 9575
Peptide CGDGVYGRLK **Charge** 3 **Spectral Angle** 0.91

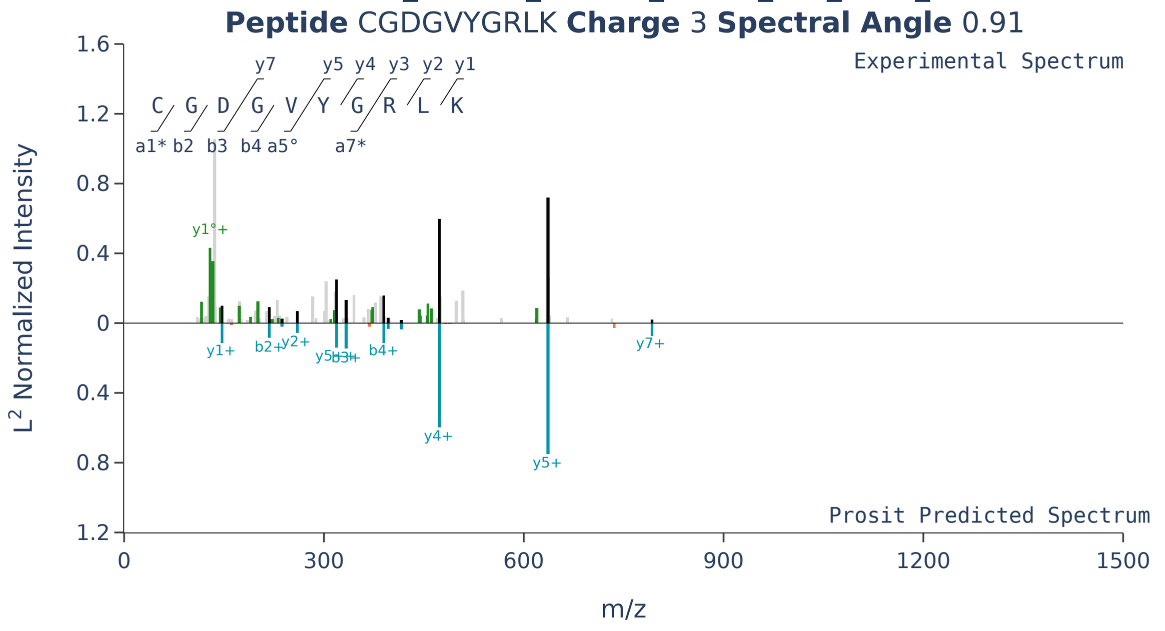

Source HRoetschke_221222_180123_HFGoe_RF1_b1R2_4h **Scan** 7883
Peptide RALSITH **Charge** 2 **Spectral Angle** 0.92

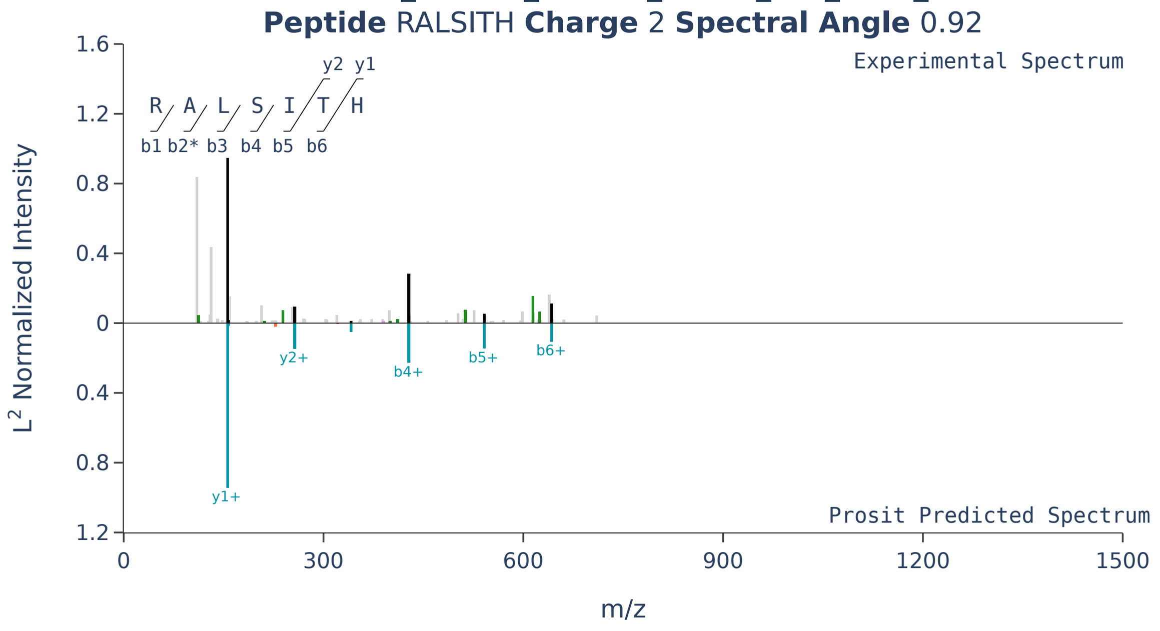

Source HRoetschke_221222_180123_HFGoe_RF1_b1R2_2h **Scan** 21736
Peptide SVLLLPKD **Charge** 2 **Spectral Angle** 0.88

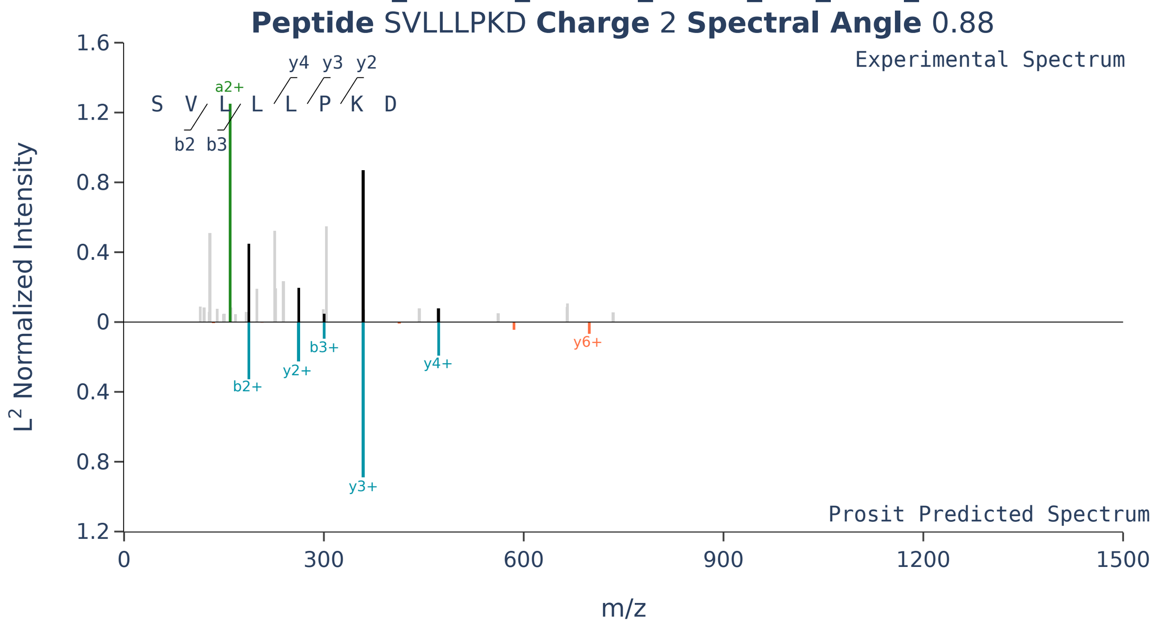

Source HRoetschke_221222_180123_HFGoe_RF1_b2R1_2h **Scan** 18521
Peptide PTGIVVECEG **Charge** 1 **Spectral Angle** 0.89

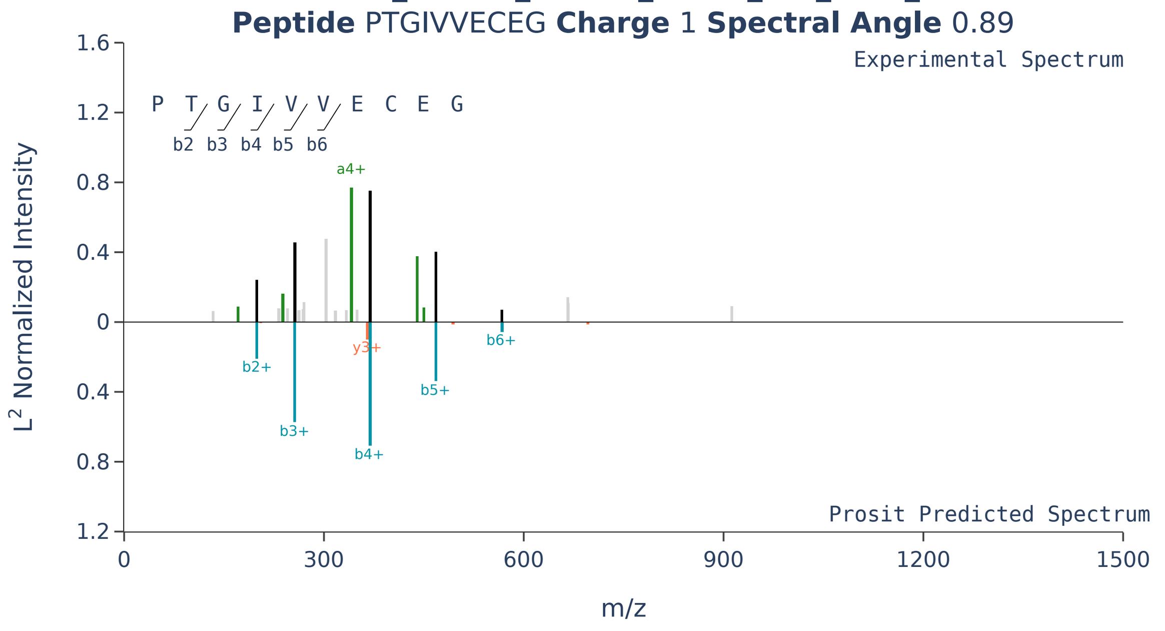

Source HRoetschke_221222_180123_HFGoe_RF1_b2R2_1h **Scan** 18593
Peptide PTGIVVECDA **Charge** 1 **Spectral Angle** 0.88

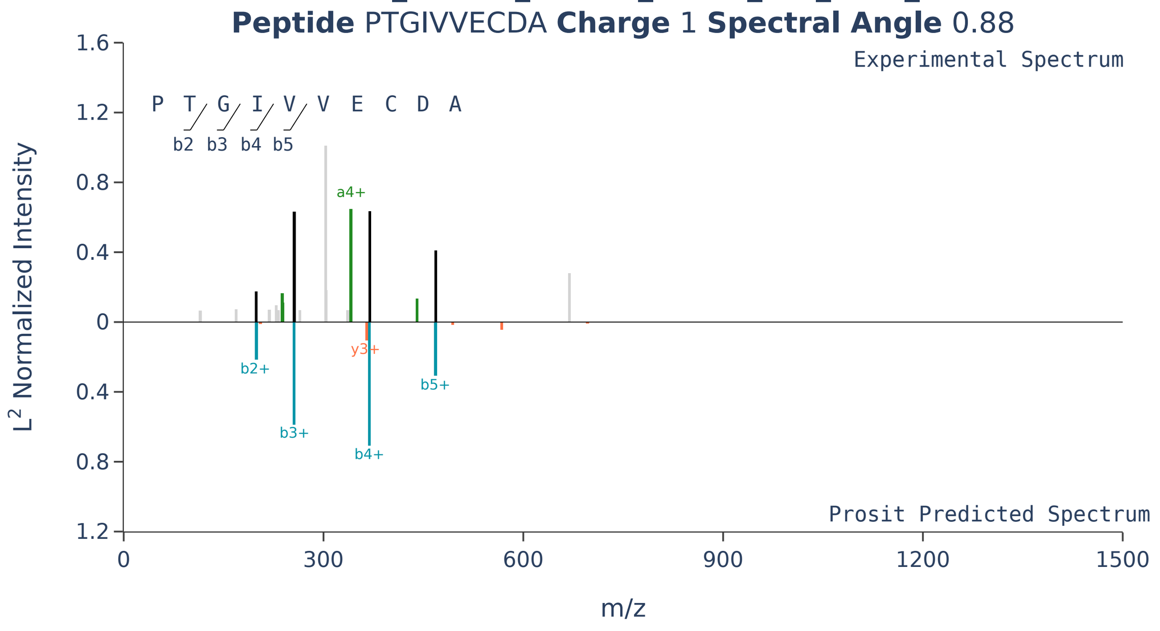

Source HRoetschke_221222_180123_HFGoe_RF1_b2R2_24h **Scan** 15815
Peptide SLYRLDE **Charge** 2 **Spectral Angle** 0.92

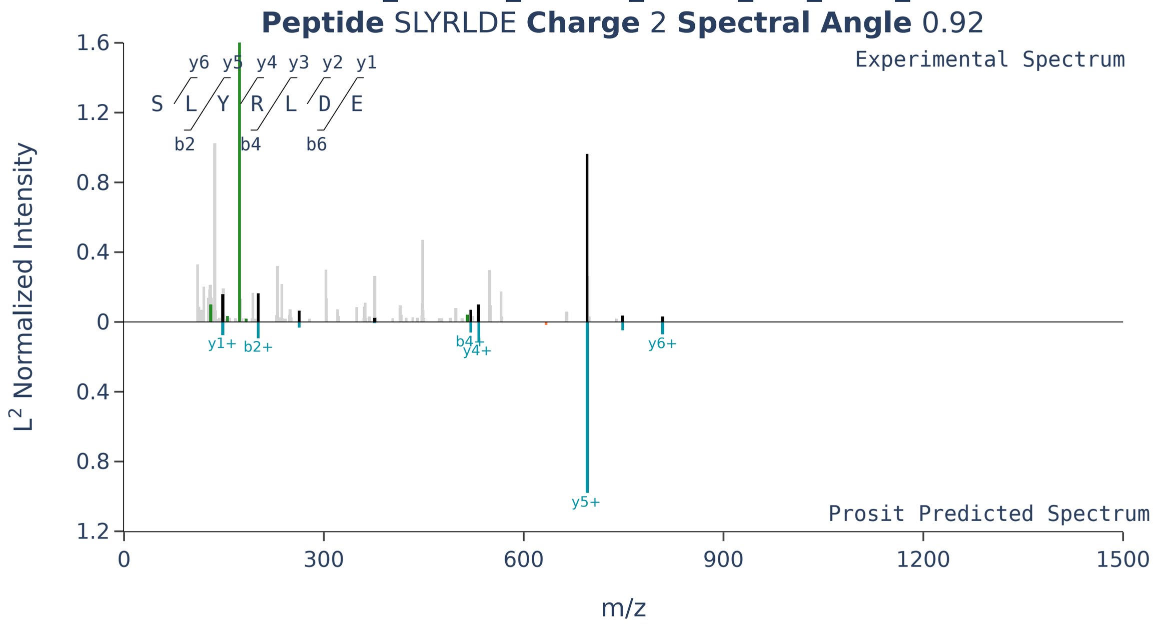

Source HRoetschke_221222_180123_HFGoe_RF1_b2R1_1h **Scan** 15207
Peptide GAQHVNTTDSAIRI **Charge** 3 **Spectral Angle** 0.88

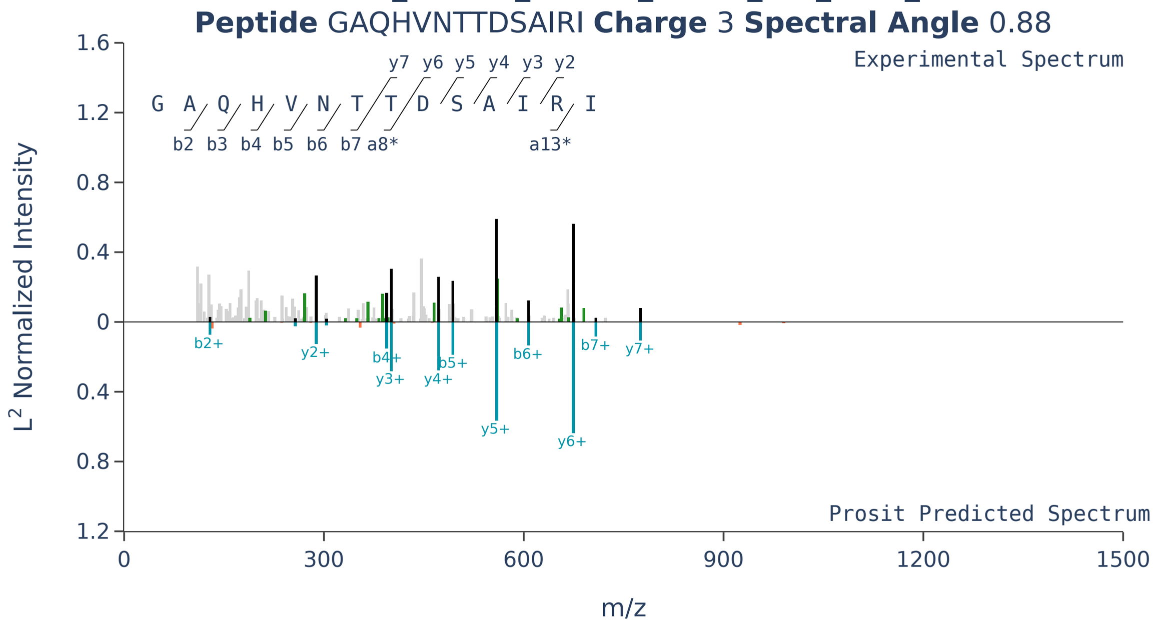

Source HRoetschke_221222_180123_HFGoe_RF1_b2R1_24h **Scan** 13838
Peptide DDWQQVQ **Charge** 2 **Spectral Angle** 0.92

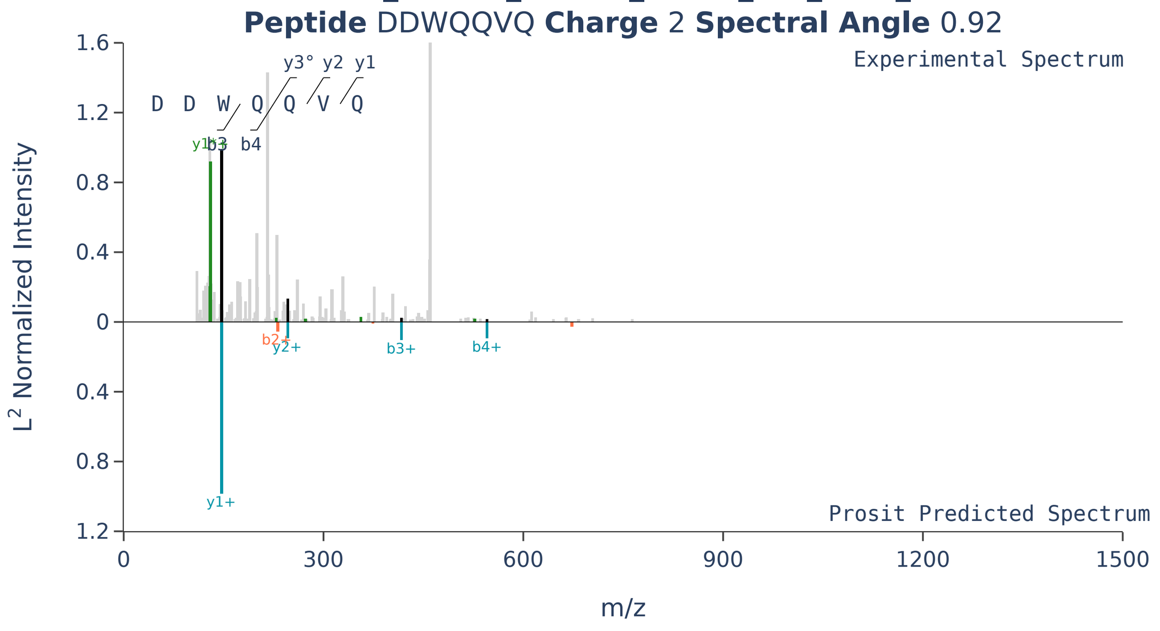

Source HRoetschke_221222_180123_HFGoe_RF1_b1R2_2h **Scan** 19542
Peptide CGDGVYGRLKF **Charge** 2 **Spectral Angle** 0.86

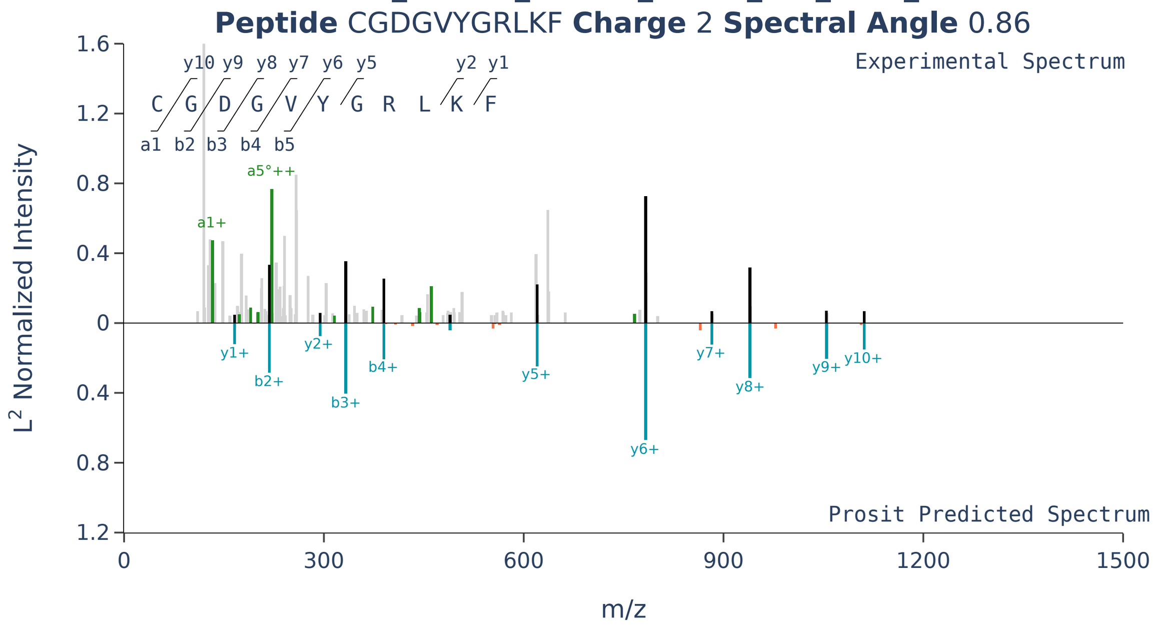

Source HRoetschke_221222_180123_HFGoe_RF1_b2R1_2h **Scan** 10535
Peptide AQLSDVSRCA **Charge** 2 **Spectral Angle** 0.75

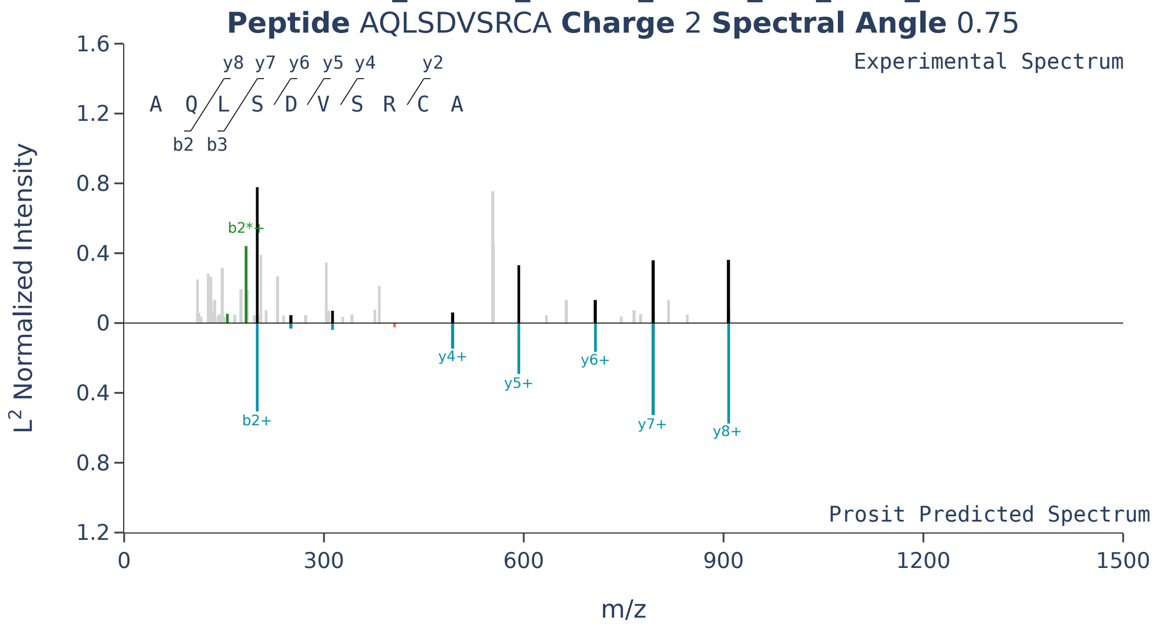

Source HRoetschke_221222_180123_HFGoe_RF1_b1R1_24h **Scan** 7869
Peptide GTIRITH **Charge** 2 **Spectral Angle** 0.91

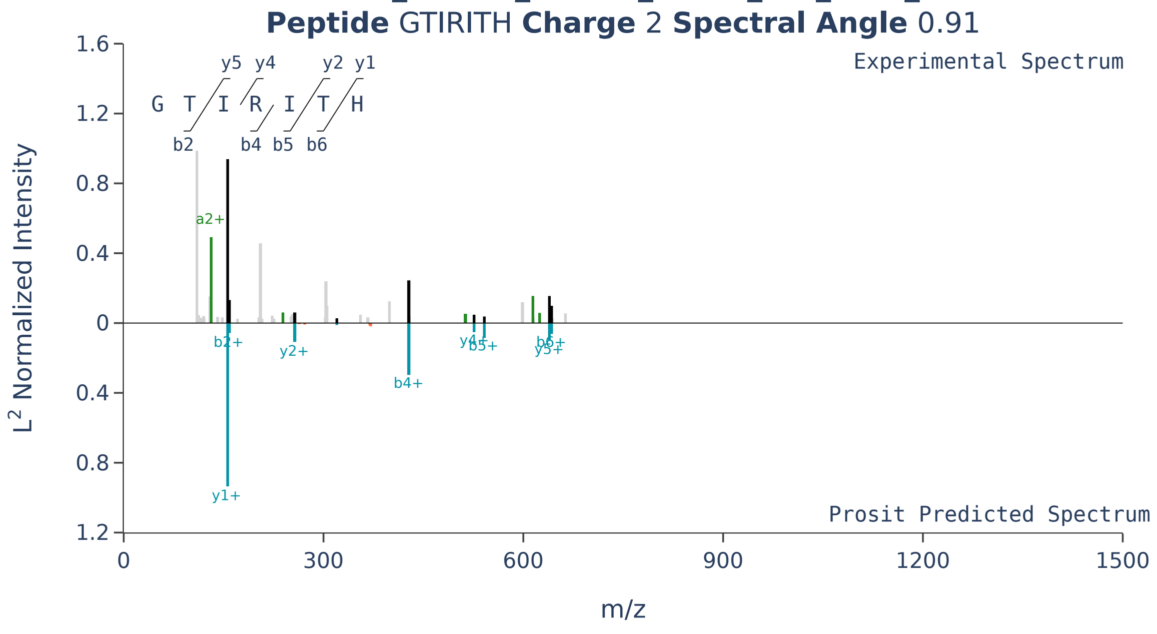

Source HRoetschke_221222_180123_HFGoe_RF1_b1R2_24h **Scan** 12462
Peptide EMNFPQGRVTDH **Charge** 3 **Spectral Angle** 0.8

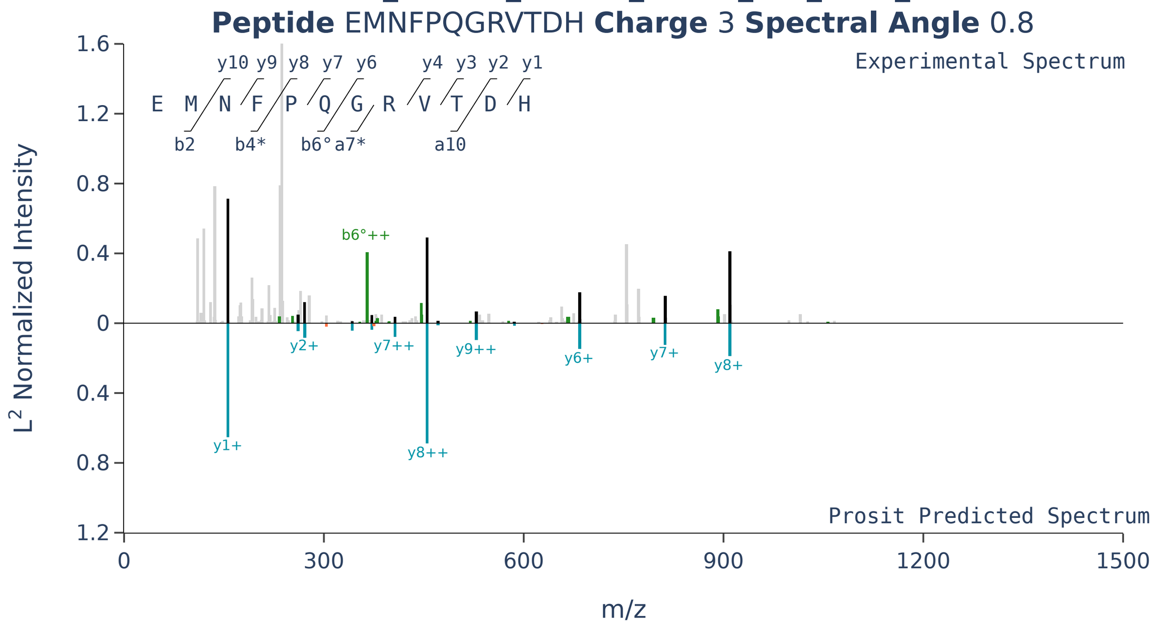

Source HRoetschke_221222_180123_HFGoe_RF1_b1R2_2h **Scan** 17248
Peptide QDERSQLLLPKD **Charge** 2 **Spectral Angle** 0.91

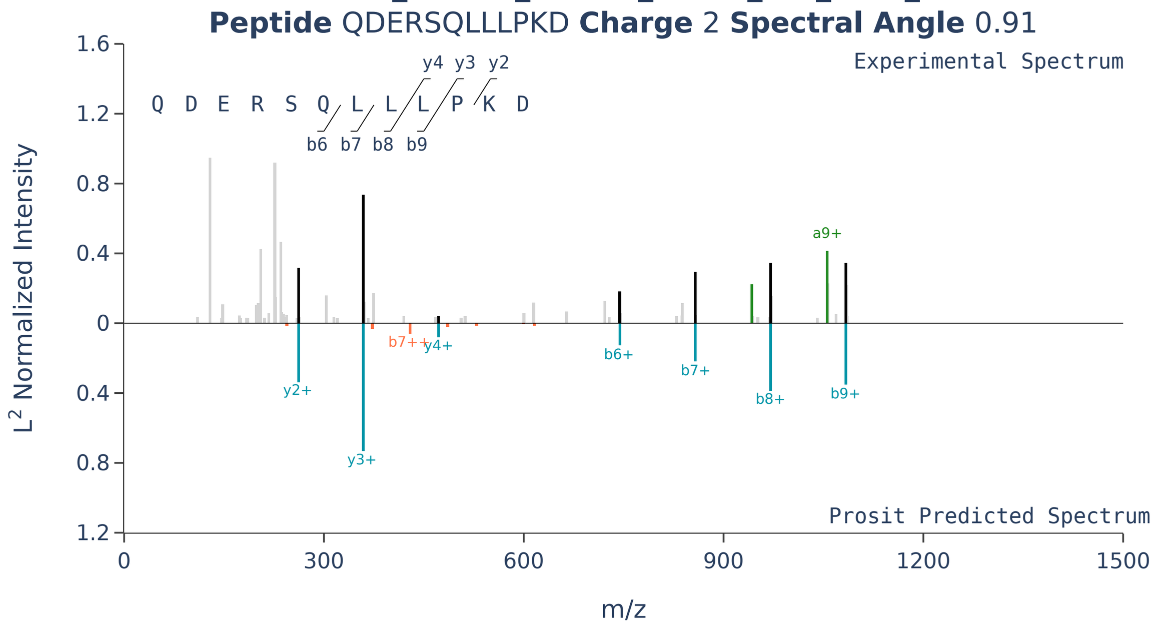

Source HRoetschke_221222_180123_HFGoe_RF1_b2R2_4h **Scan** 16780
Peptide RAGTGGDELLLPKD **Charge** 2 **Spectral Angle** 0.87

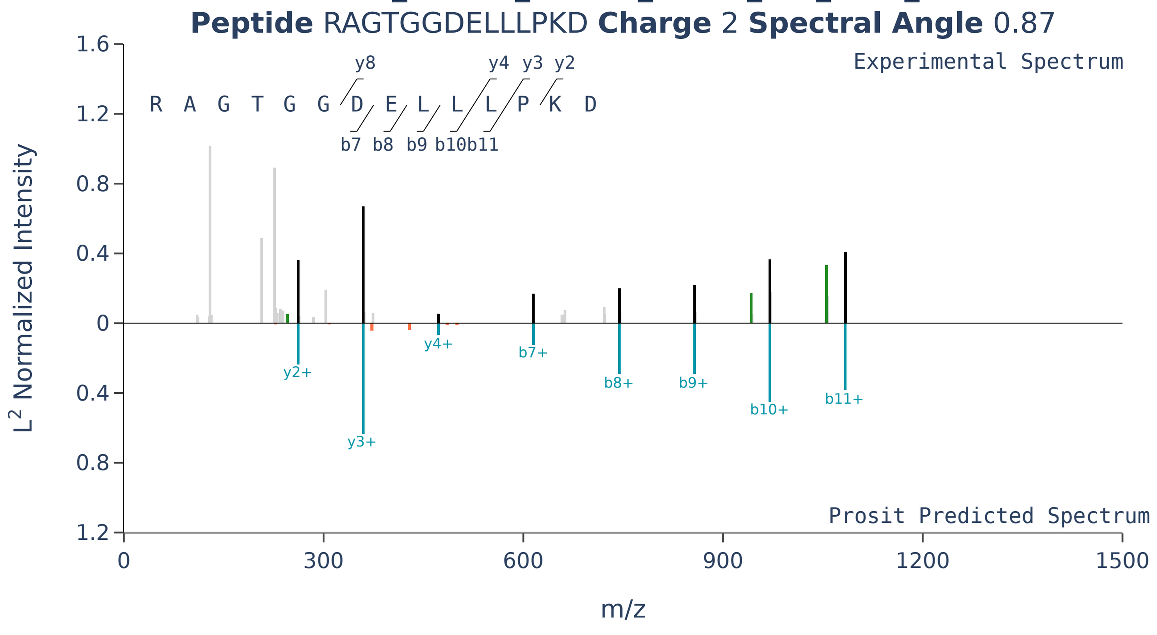

Source HRoetschke_221222_180123_HFGoe_RF1_b1R2_24h **Scan** 15104
Peptide RAGTGGDLLLPKD **Charge** 3 **Spectral Angle** 0.92

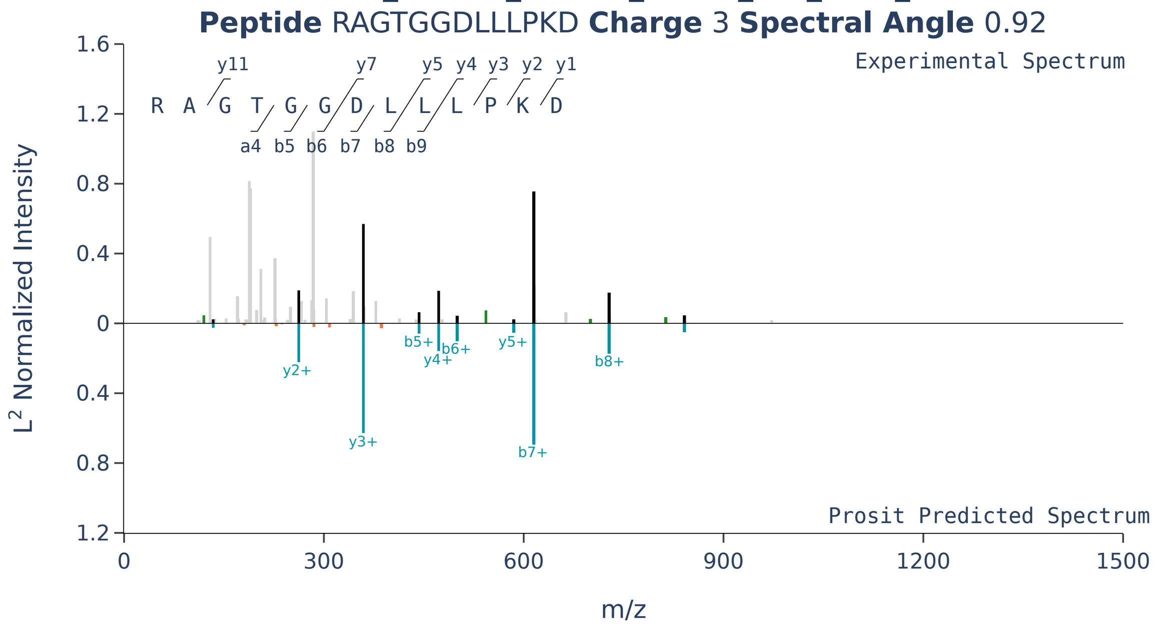

Source HRoetschke_221222_180123_HFGoe_RF1_b1R1_2h **Scan** 19911
Peptide RAGTGGDEQVLLLPKD **Charge** 3 **Spectral Angle** 0.85

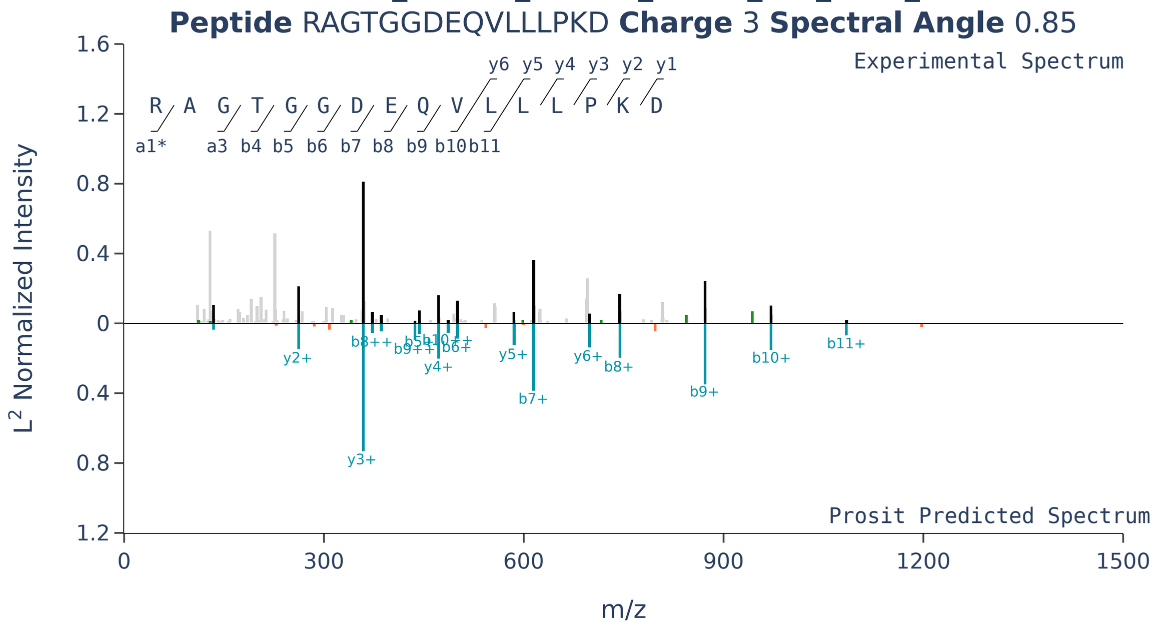

Source HRoetschke_221222_180123_HFGoe_RF1_b2R1_1h **Scan** 15287
Peptide AQQHVNTTDSAIRI **Charge** 3 **Spectral Angle** 0.88

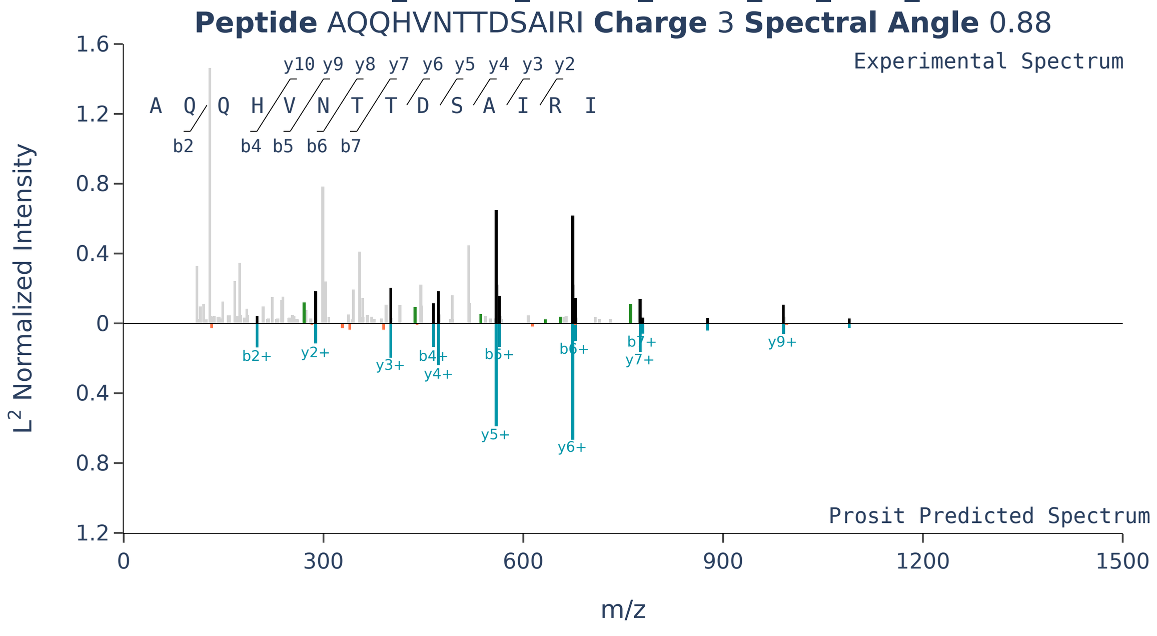

Source HRoetschke_221222_180123_HFGoe_RF1_b2R1_24h **Scan** 19187
Peptide GRVTDQVLL **Charge** 2 **Spectral Angle** 0.86

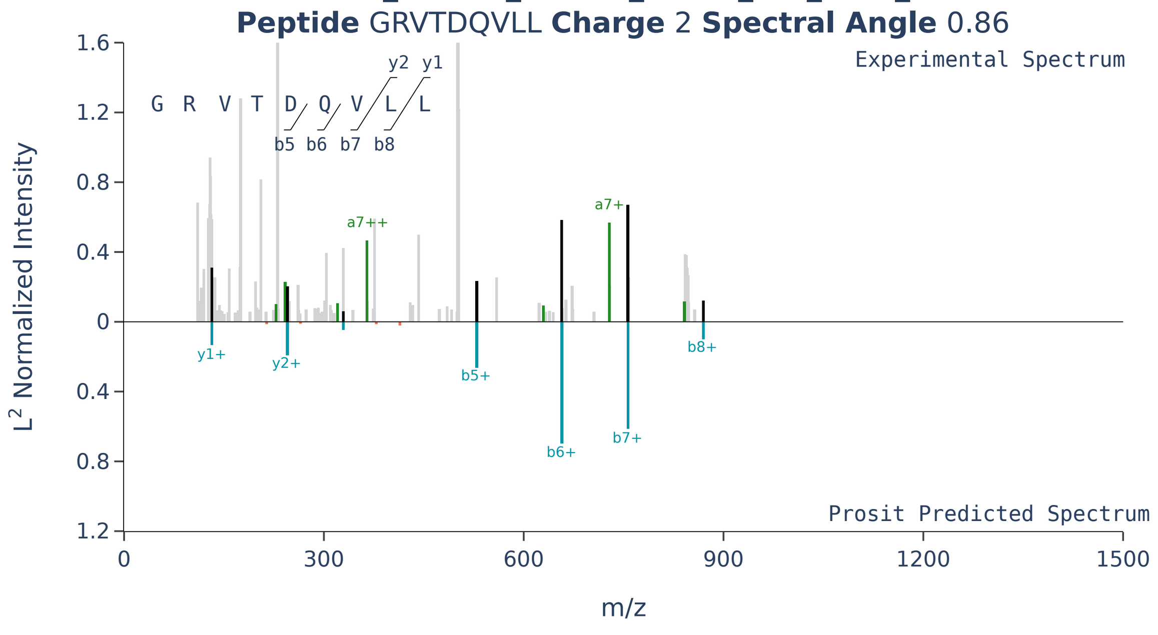

Source HRoetschke_221222_180123_HFGoe_RF1_b1R2_2h **Scan** 8499
Peptide GTGGDEASRYAEA **Charge** 2 **Spectral Angle** 0.84

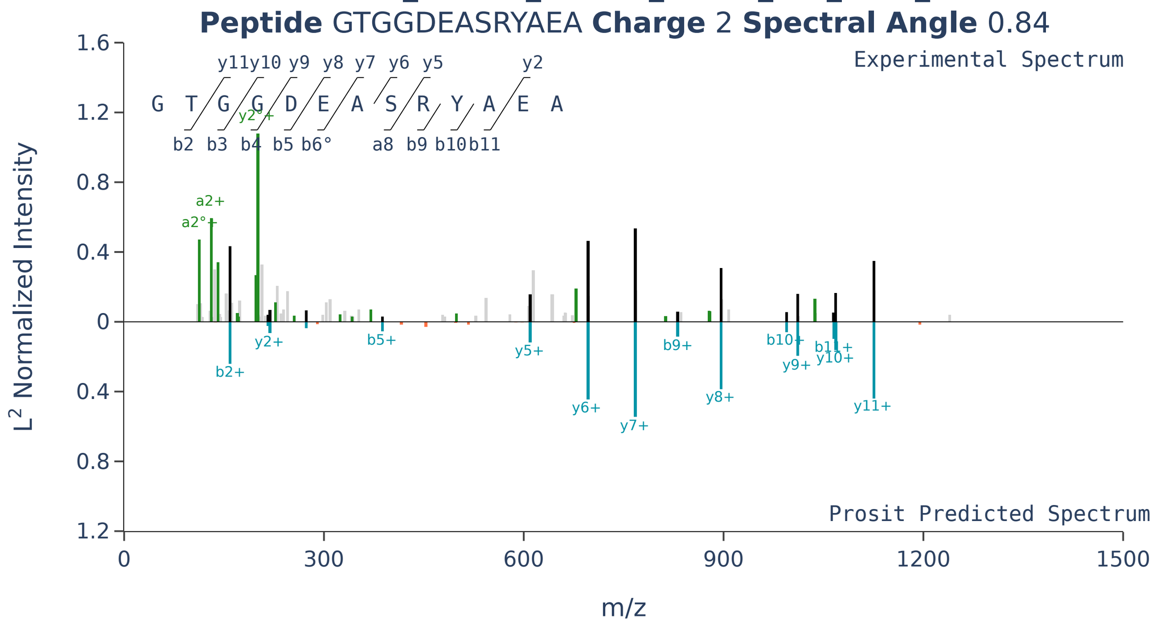

Source HRoetschke_221222_180123_HFGoe_RF1_b1R2_0h **Scan** 12573
Peptide AADQERFRAL **Charge** 3 **Spectral Angle** 0.79

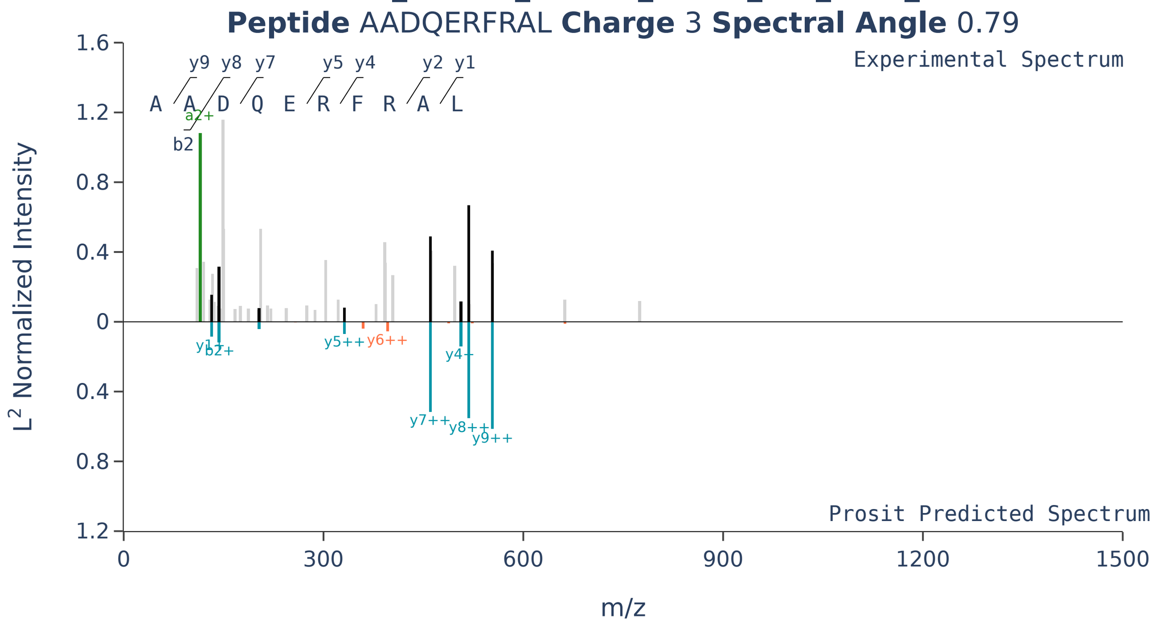

Source HRoetschke_221222_180123_HFGoe_RF1_b1R1_4h **Scan** 15023
Peptide STLYRLDE **Charge** 2 **Spectral Angle** 0.87

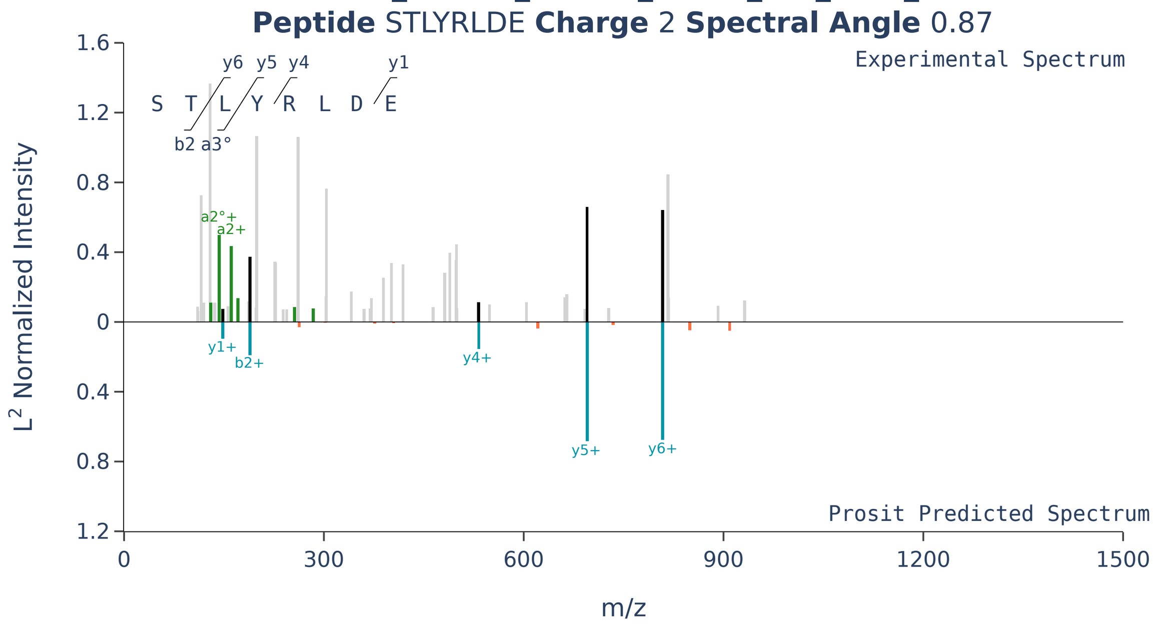

Source HRoetschke_221222_180123_HFGoe_RF1_b1R2_2h **Scan** 7983
Peptide DQQTIAD **Charge** 2 **Spectral Angle** 0.69

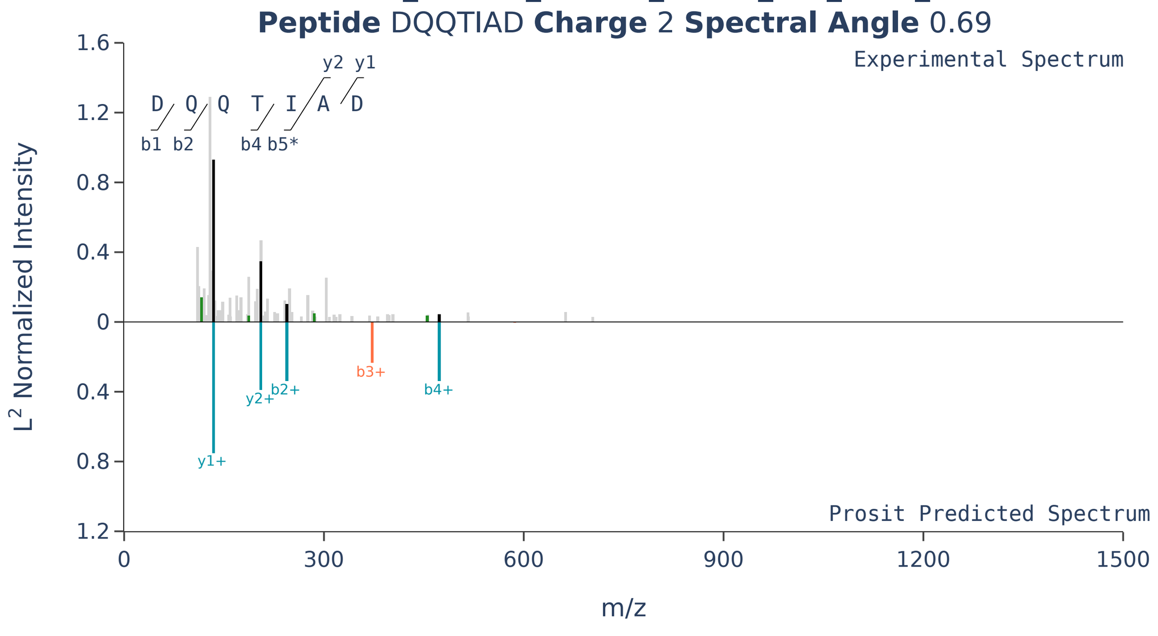

Source HRoetschke_221222_180123_HFGoe_RF1_b1R2_2h **Scan** 12062
Peptide RHELLLPKD **Charge** 3 **Spectral Angle** 0.88

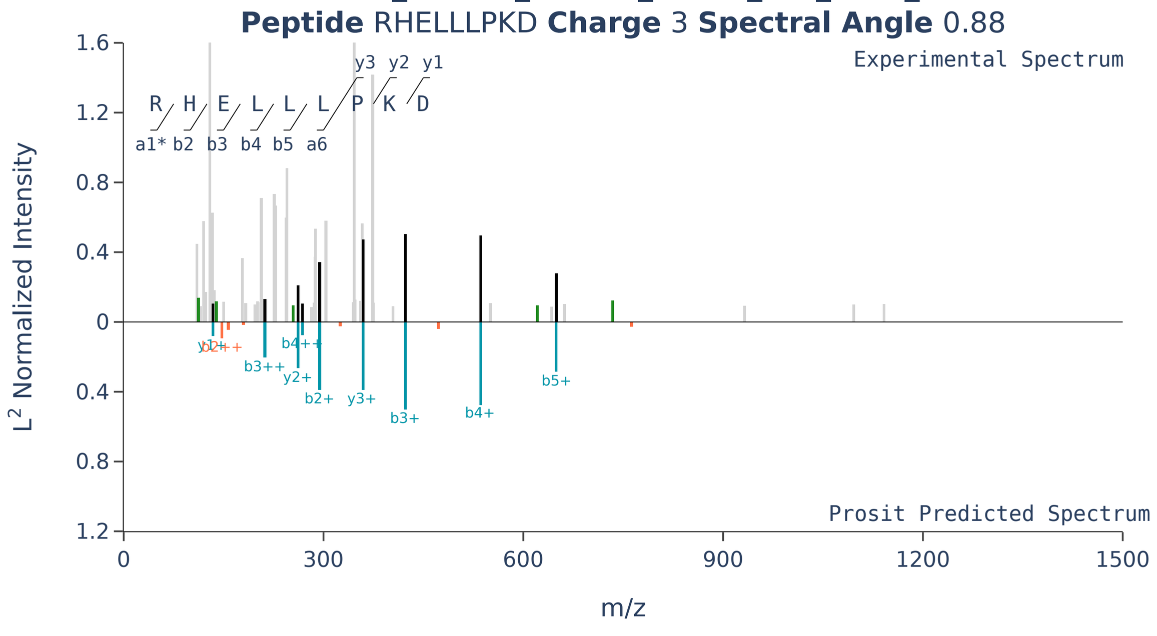

Source HRoetschke_221222_180123_HFGoe_RF1_b1R1_24h **Scan** 14815
Peptide GTLYRLDE **Charge** 2 **Spectral Angle** 0.86

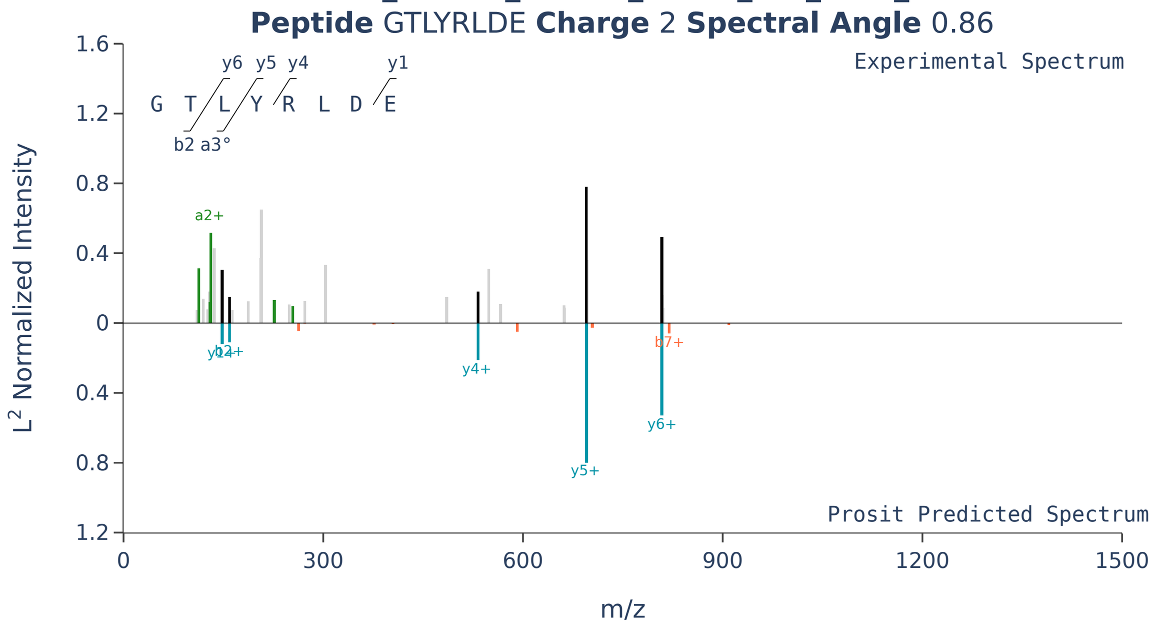

Source HRoetschke_221222_180123_HFGoe_RF1_b2R1_4h **Scan** 20495
Peptide PTGIVAEM **Charge** 1 **Spectral Angle** 0.88

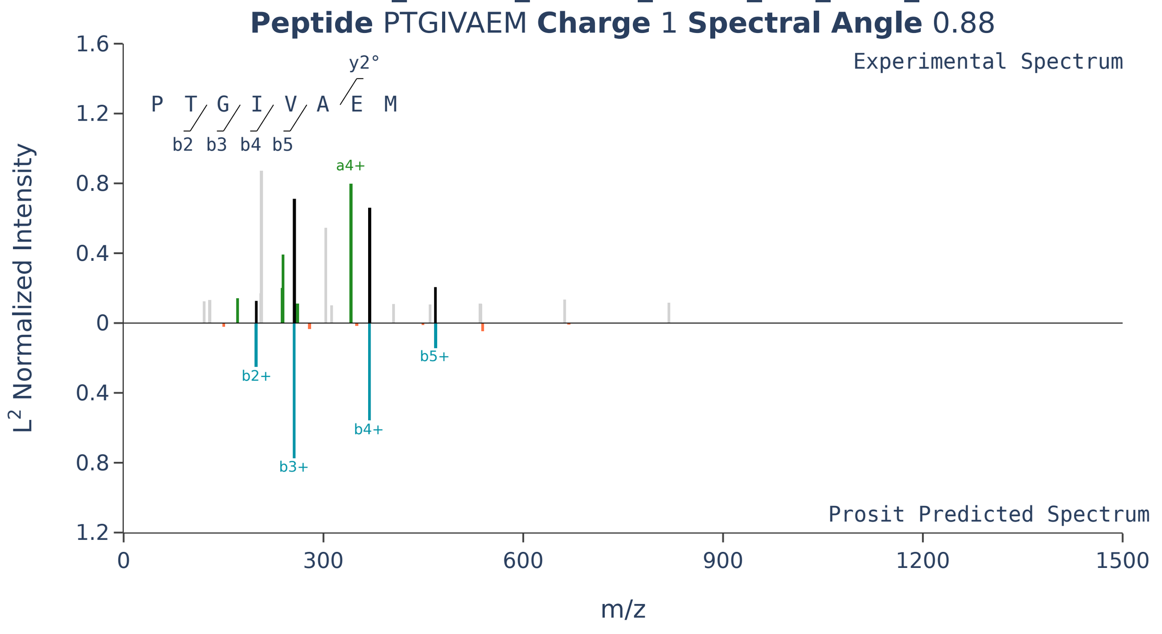

Source HRoetschke_221222_180123_HFGoe_RF1_b1R1_2h **Scan** 18546
Peptide CGDGVYGRLKFE **Charge** 2 **Spectral Angle** 0.83

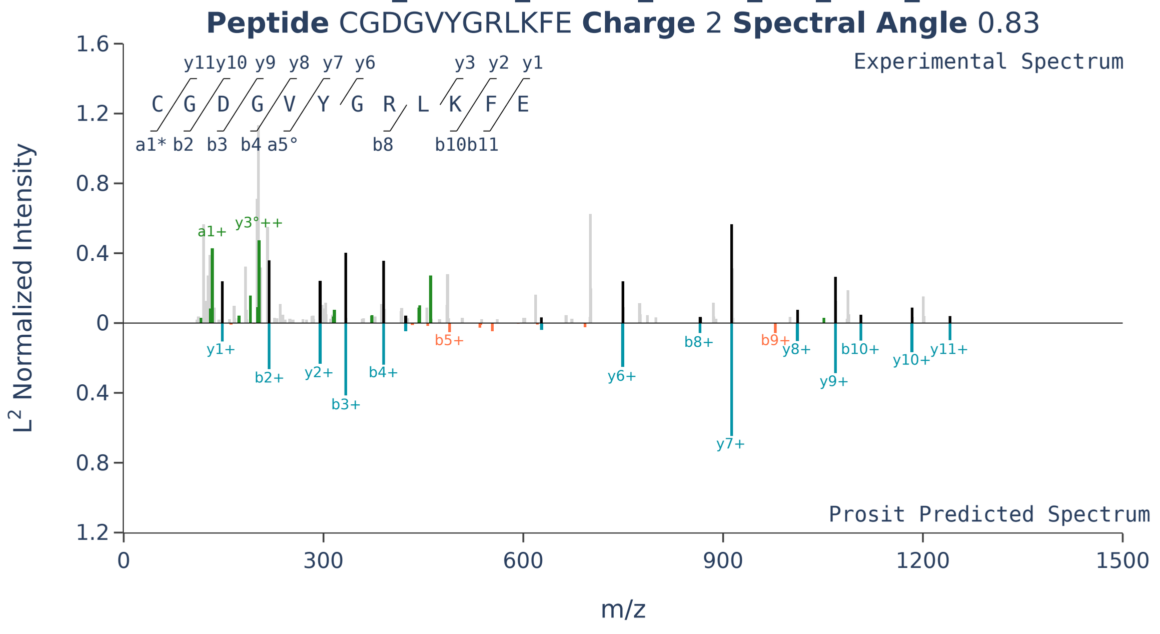

Source HRoetschke_221222_180123_HFGoe_RF1_b1R1_4h **Scan** 10347
Peptide RSSQQLQV **Charge** 2 **Spectral Angle** 0.9

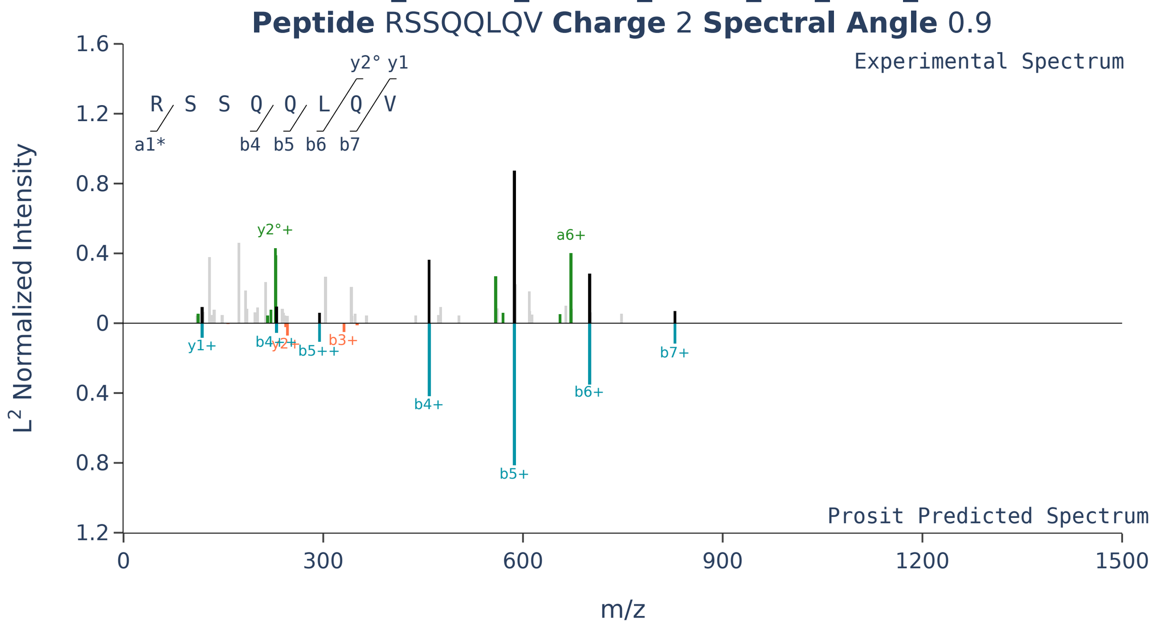

Source HRoetschke_221222_180123_HFGoe_RF1_b2R2_24h **Scan** 17134
Peptide GEHGGYKEIIAKIC **Charge** 4 **Spectral Angle** 0.8

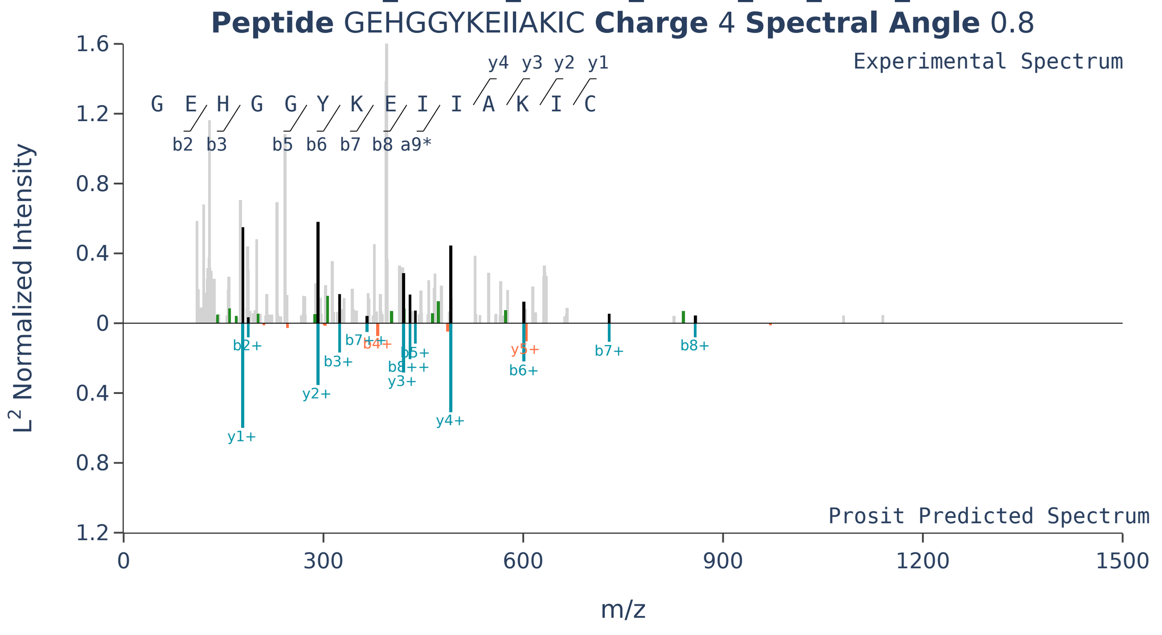

Source HRoetschke_221222_180123_HFGoe_RF1_b1R1_2h **Scan** 19138
Peptide LGLLPKD **Charge** 2 **Spectral Angle** 0.84

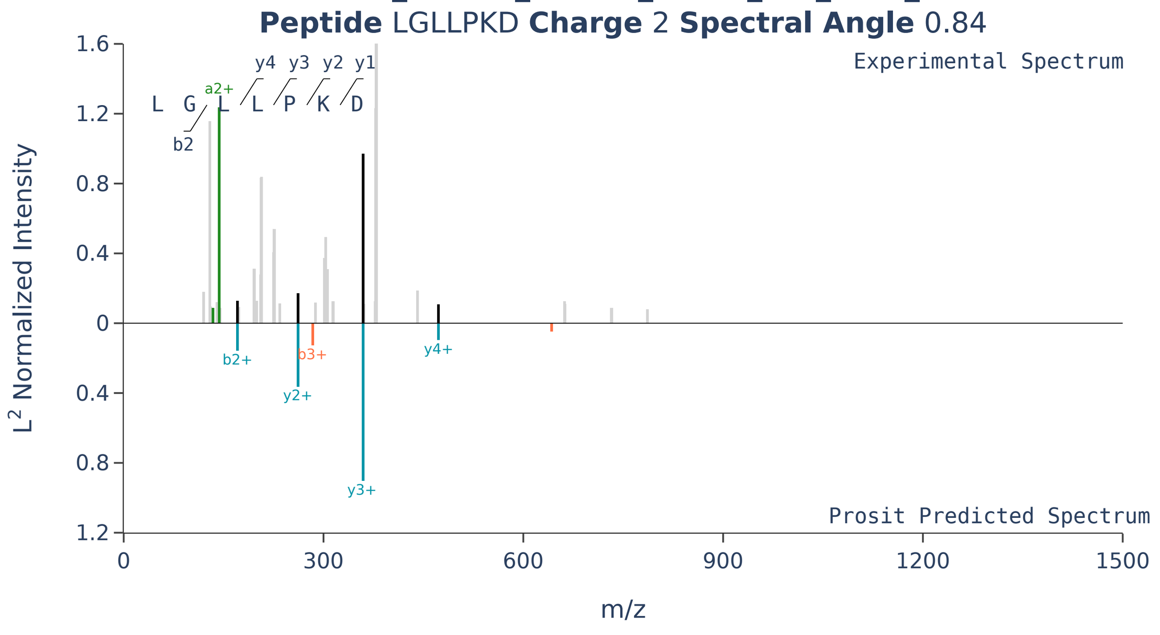

Source HRoetschke_221222_180123_HFGoe_RF1_b2R1_24h **Scan** 17570
Peptide SEGEHGGYKEIIAKIC **Charge** 3 **Spectral Angle** 0.85

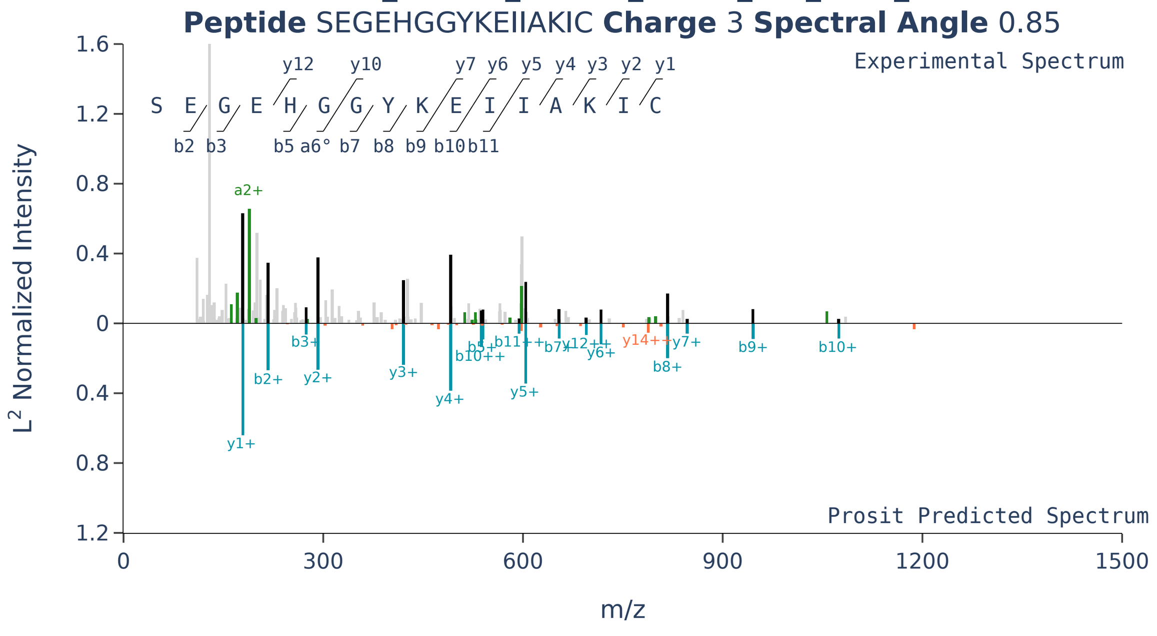

Source HRoetschke_221222_180123_HFGoe_RF1_b2R1_24h **Scan** 18133
Peptide TYNFPQGRNL **Charge** 2 **Spectral Angle** 0.86

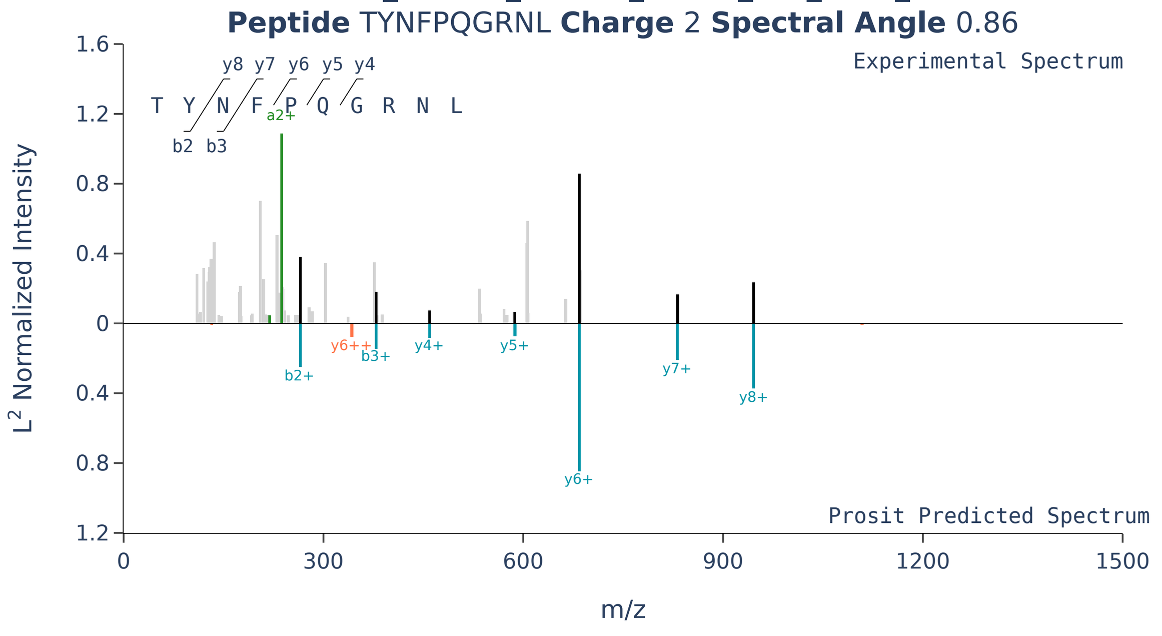

Source HRoetschke_221222_180123_HFGoe_RF1_b1R2_2h **Scan** 21969
Peptide TVLLLPKD **Charge** 2 **Spectral Angle** 0.81

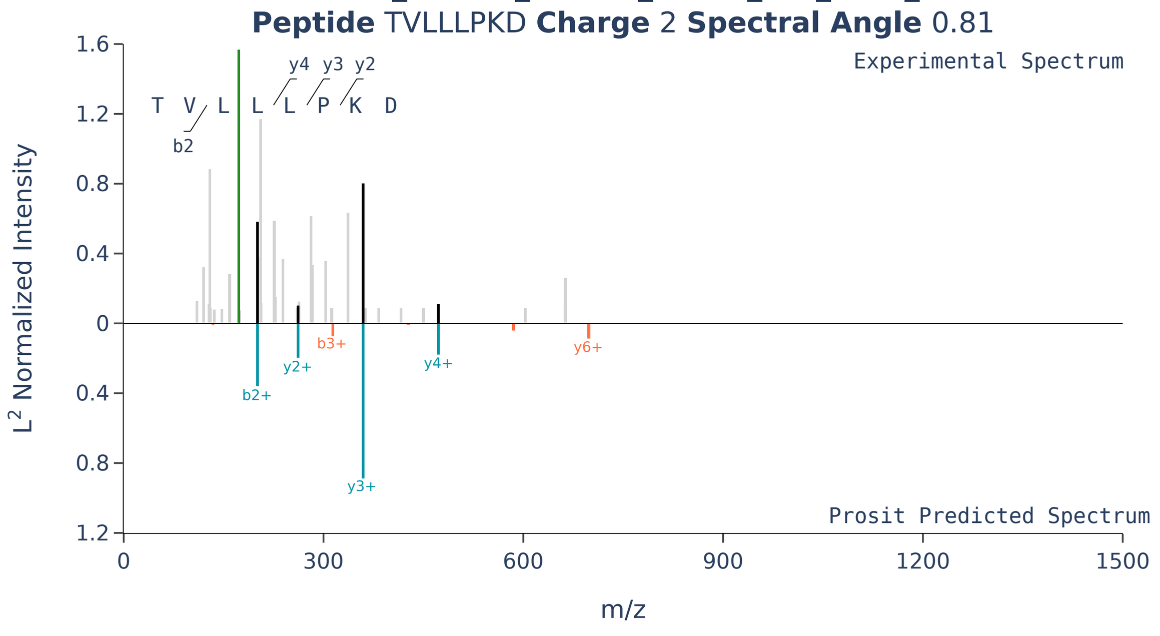

Source HRoetschke_221222_180123_HFGoe_RF1_b2R2_2h **Scan** 11142
Peptide GGDEAQTI **Charge** 1 **Spectral Angle** 0.79

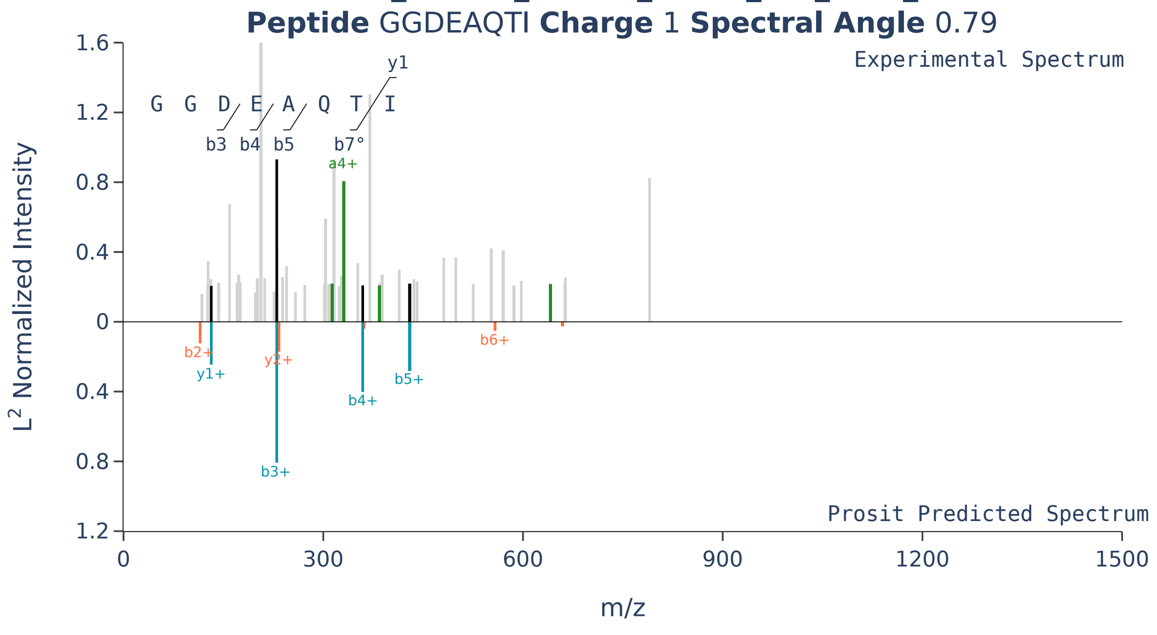

Source HRoetschke_221222_180123_HFGoe_RF1_b1R1_4h **Scan** 12201
Peptide FRSSEAAL **Charge** 2 **Spectral Angle** 0.82

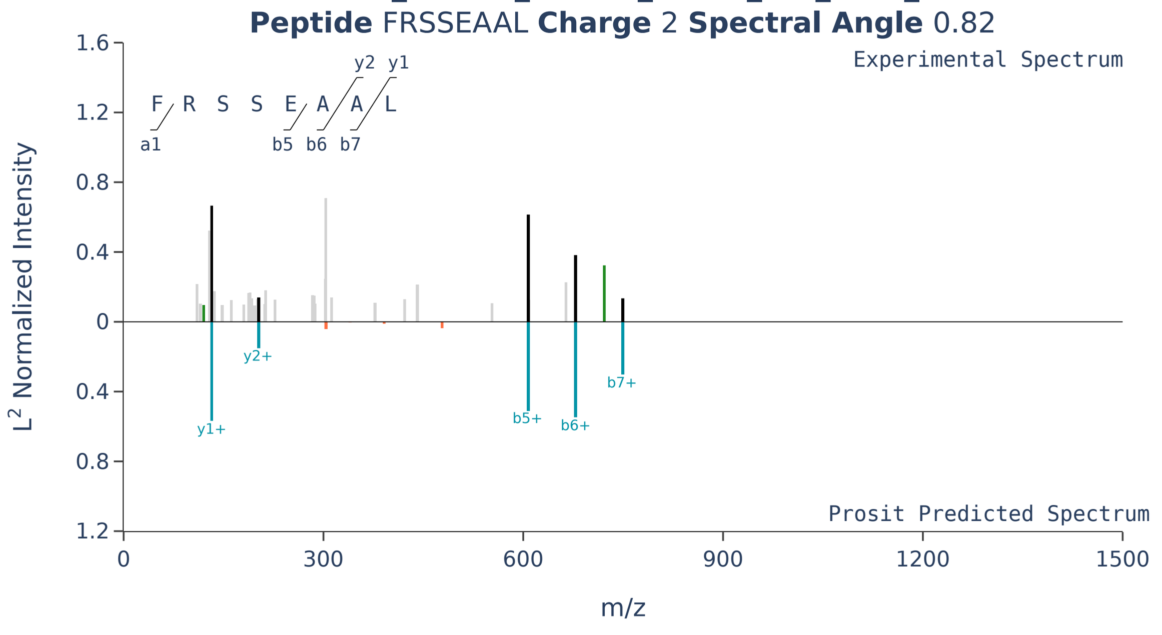

Source HRoetschke_221222_180123_HFGoe_RF1_b1R1_2h **Scan** 11761
Peptide SREYAQLSA **Charge** 2 **Spectral Angle** 0.83

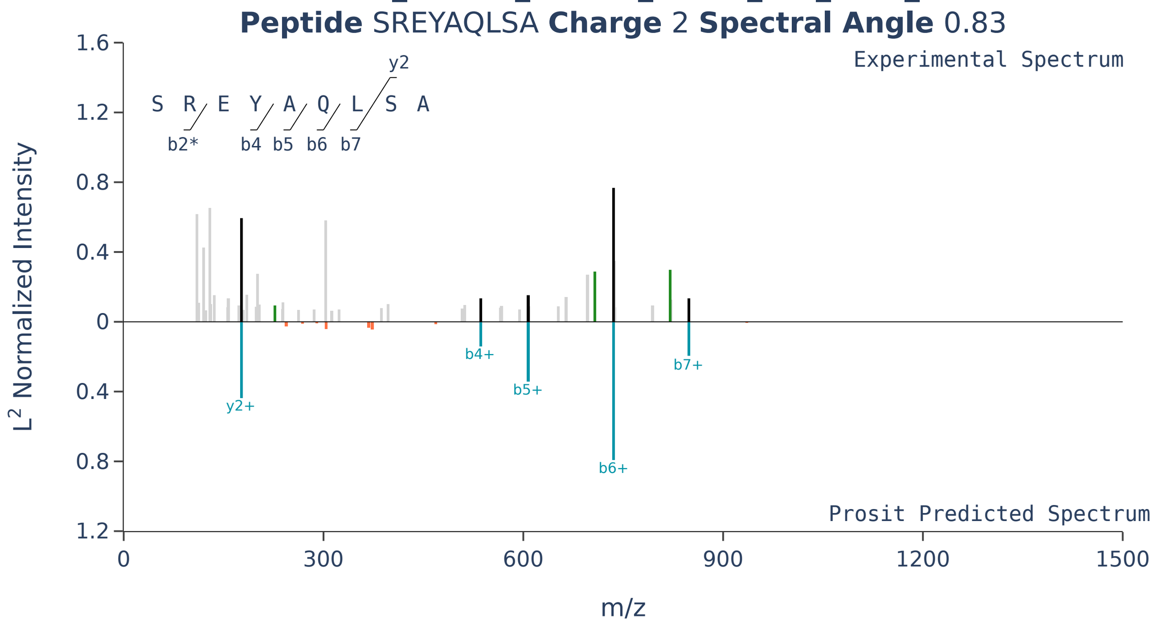

Source HRoetschke_221222_180123_HFGoe_RF1_b2R1_24h **Scan** 11325
Peptide RAGTGGDEQVL **Charge** 2 **Spectral Angle** 0.8

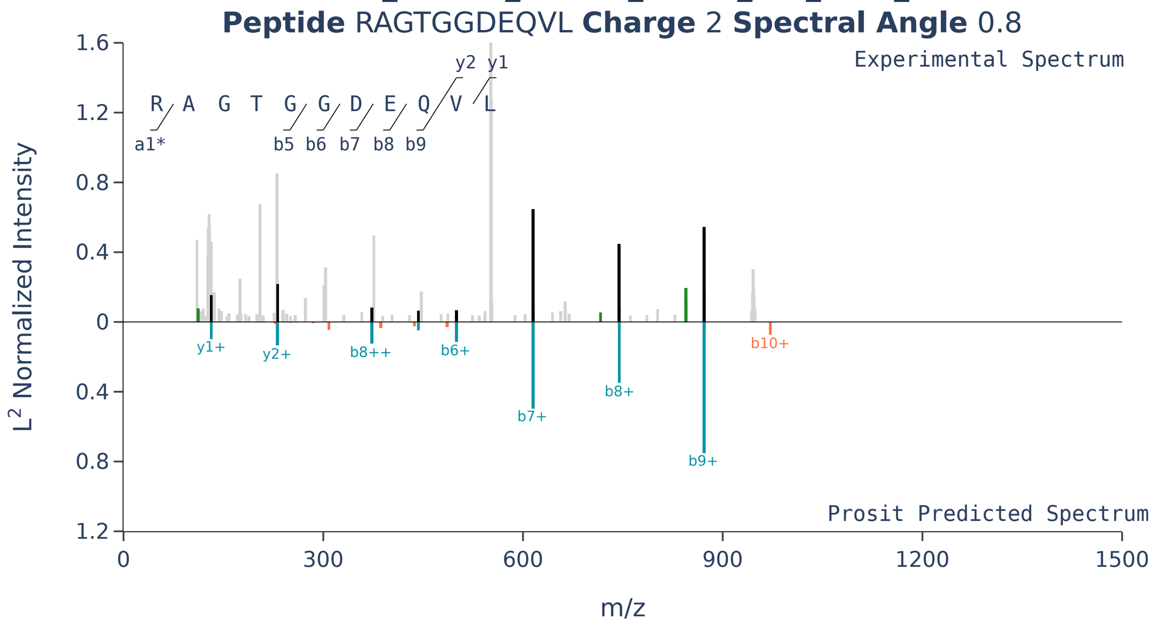

Source HRoetschke_221222_180123_HFGoe_RF1_b1R2_0h **Scan** 20883
Peptide PTGIIVVE **Charge** 1 **Spectral Angle** 0.8

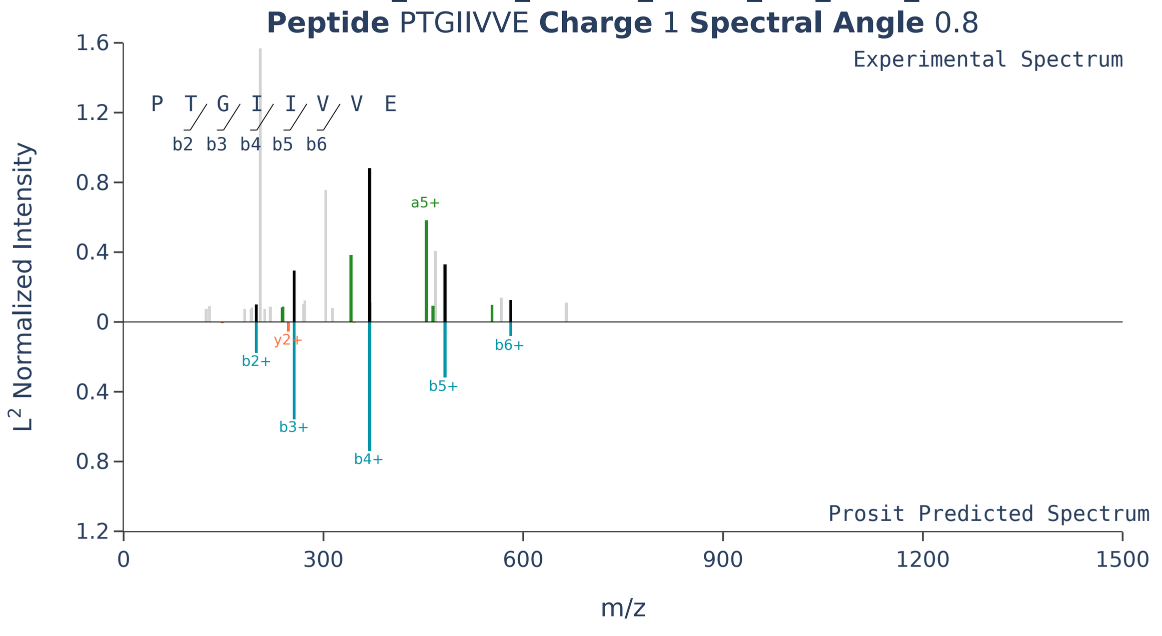

Source HRoetschke_221222_180123_HFGoe_RF1_b2R1_24h **Scan** 14272
Peptide EYAQLSDVSRCDG **Charge** 2 **Spectral Angle** 0.81

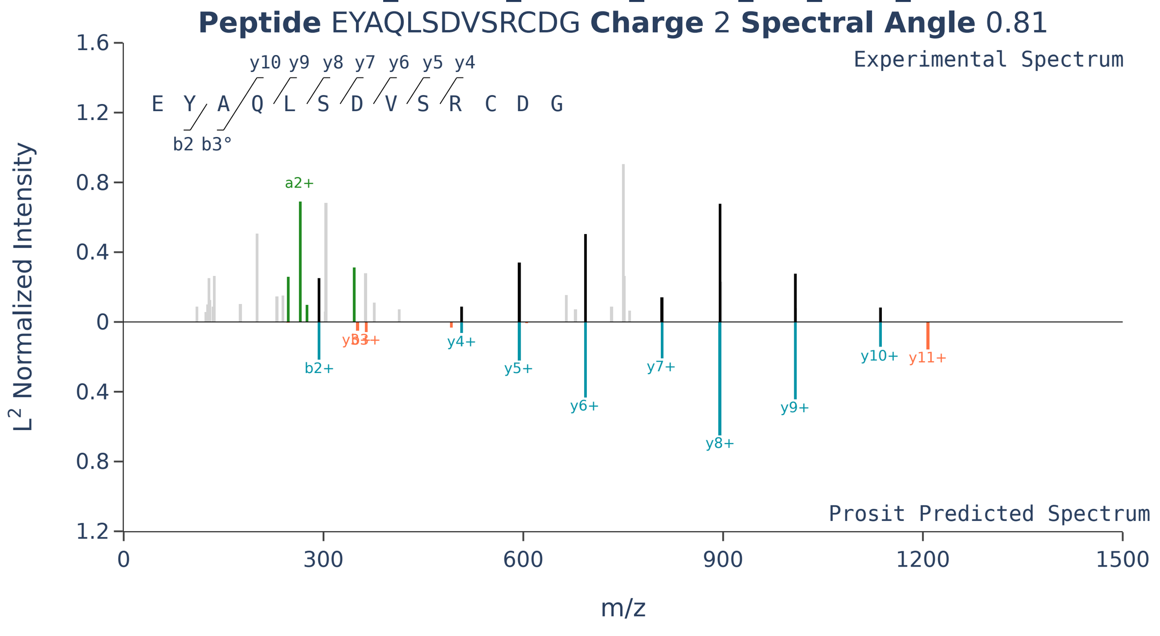

Source HRoetschke_221222_180123_HFGoe_RF1_b2R2_24h **Scan** 6608
Peptide SRYAEATGGDEA **Charge** 2 **Spectral Angle** 0.8

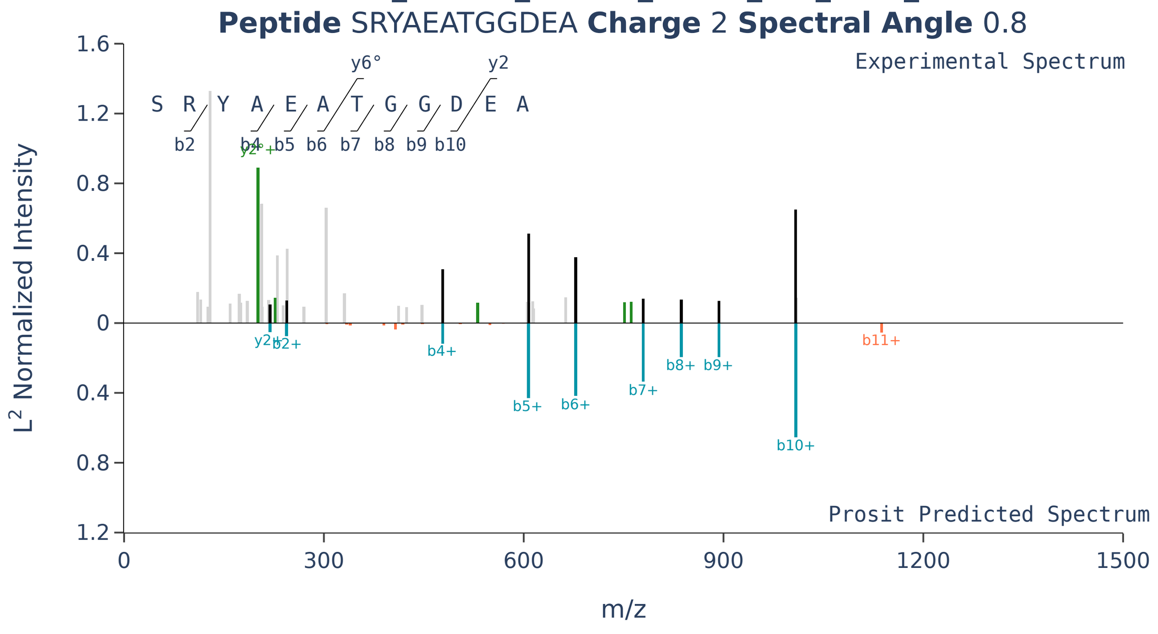

Source HRoetschke_221222_180123_HFGoe_RF1_b2R2_1h **Scan** 11379
Peptide NFPQGRVSE **Charge** 2 **Spectral Angle** 0.9

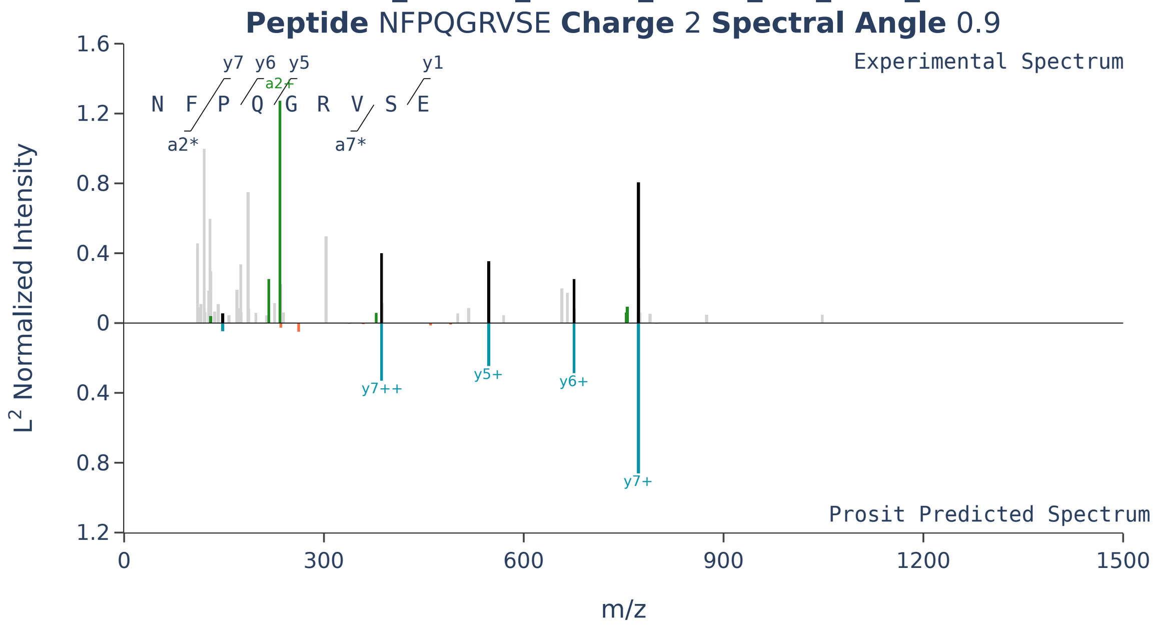

Source HRoetschke_221222_180123_HFGoe_RF1_b2R2_24h **Scan** 9587
Peptide KLEALSD **Charge** 2 **Spectral Angle** 0.9

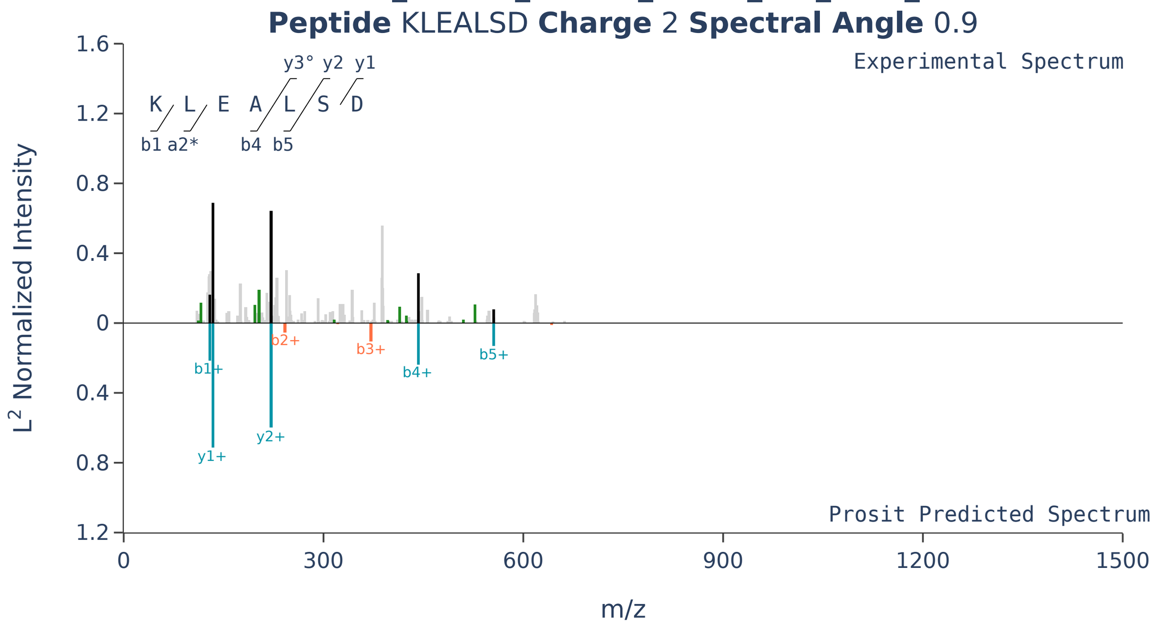

Source HRoetschke_221222_180123_HFGoe_RF1_b2R1_24h **Scan** 19603
Peptide TGGDLLLPKD **Charge** 2 **Spectral Angle** 0.88

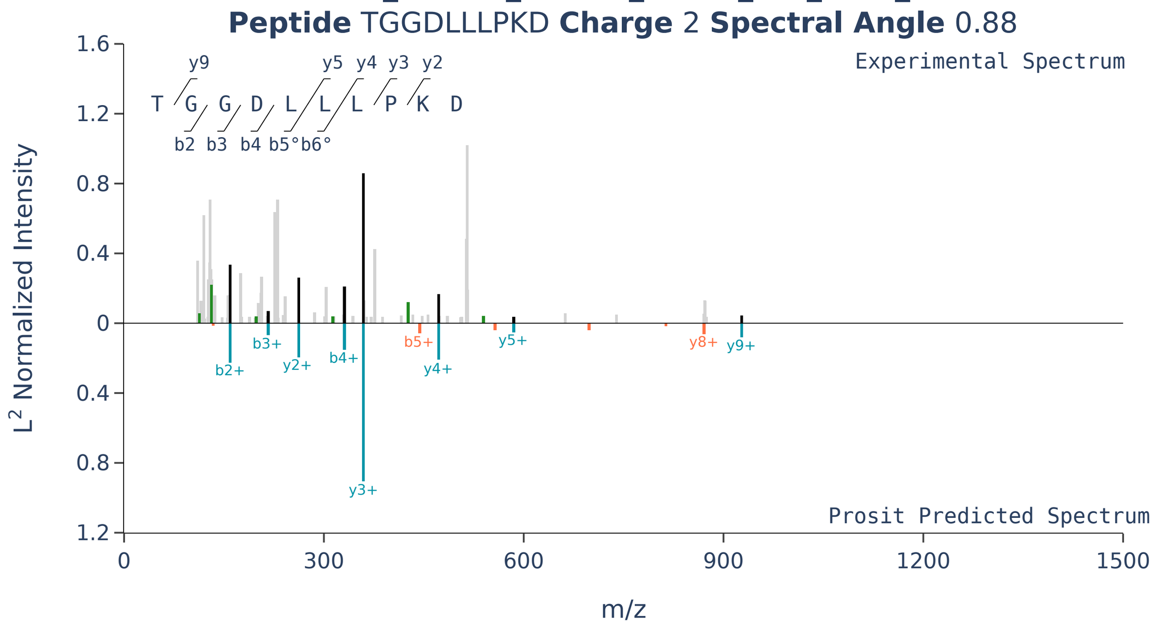

Source HRoetschke_221222_180123_HFGoe_RF1_b1R1_2h **Scan** 8582
Peptide SRYAEAKL **Charge** 2 **Spectral Angle** 0.88

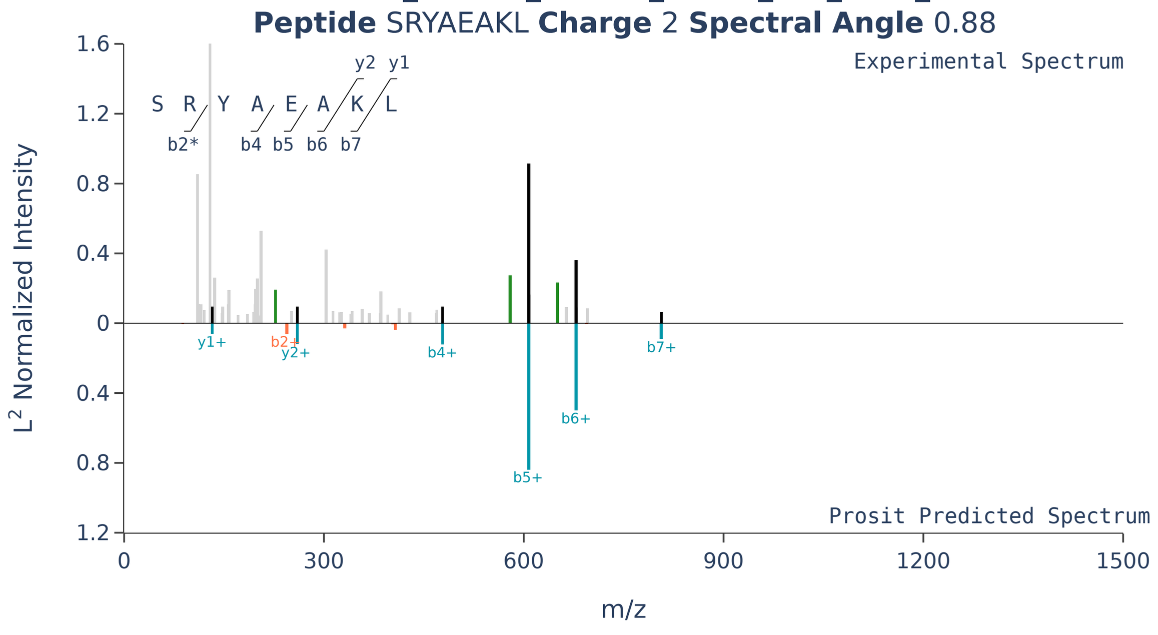

Source HRoetschke_221222_180123_HFGoe_RF1_b1R2_24h **Scan** 14039
Peptide CGDGVYGRL **Charge** 2 **Spectral Angle** 0.87

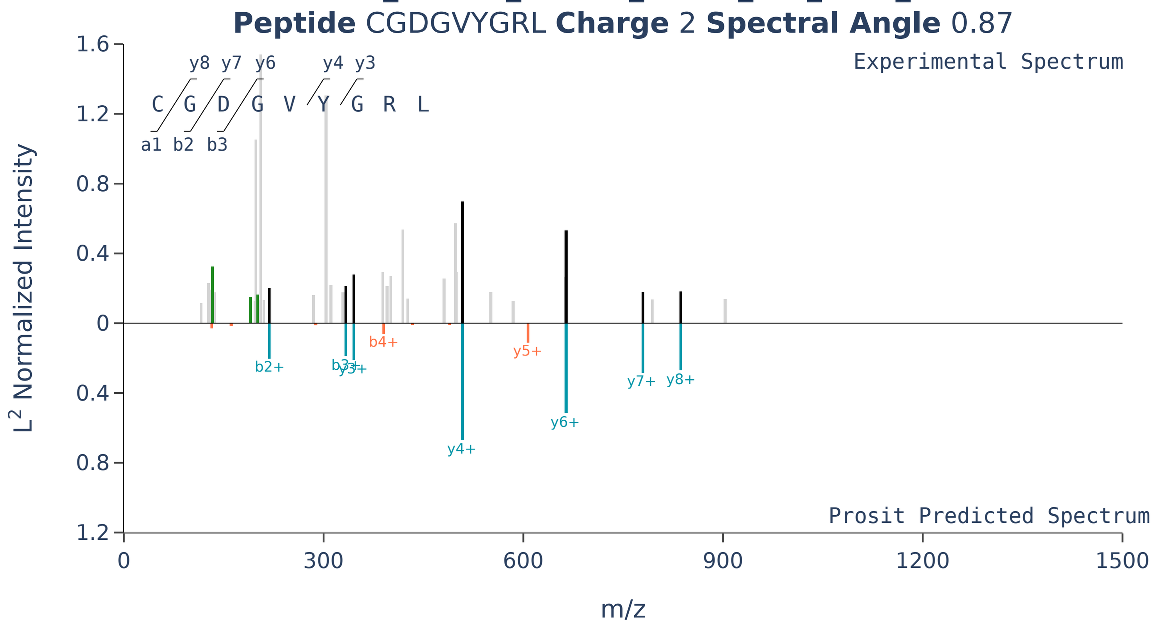

Source HRoetschke_221222_180123_HFGoe_RF1_b2R2_4h **Scan** 8058
Peptide VTDHRINLTR **Charge** 3 **Spectral Angle** 0.77

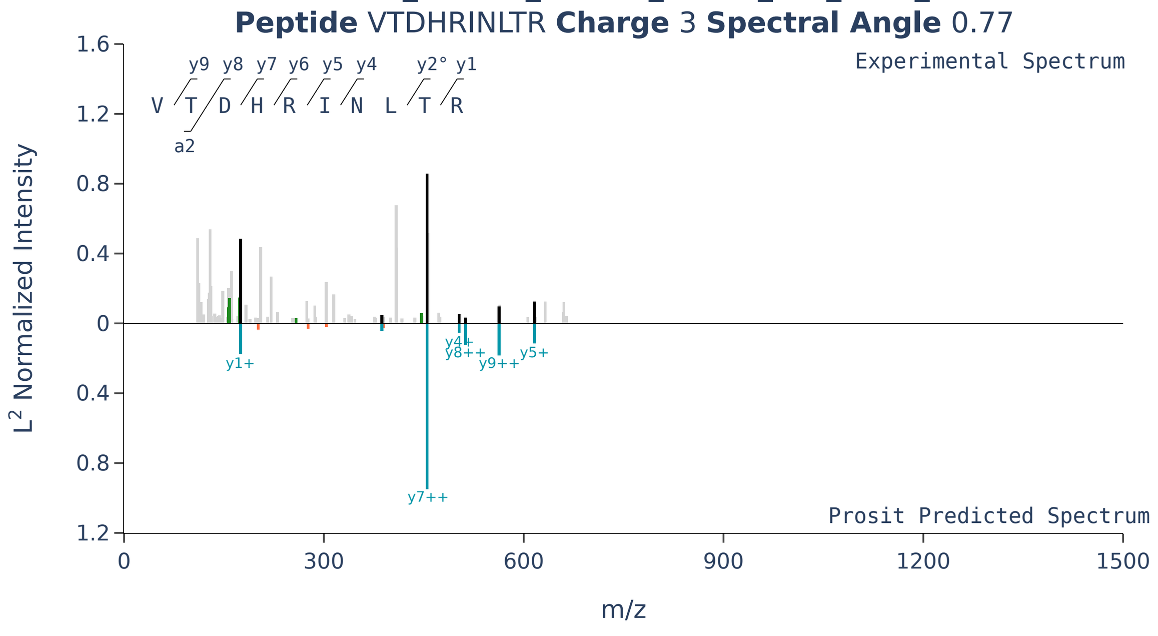

Source HRoetschke_221222_180123_HFGoe_RF1_b2R1_4h **Scan** 9888
Peptide AQLSDVSRCT **Charge** 2 **Spectral Angle** 0.73

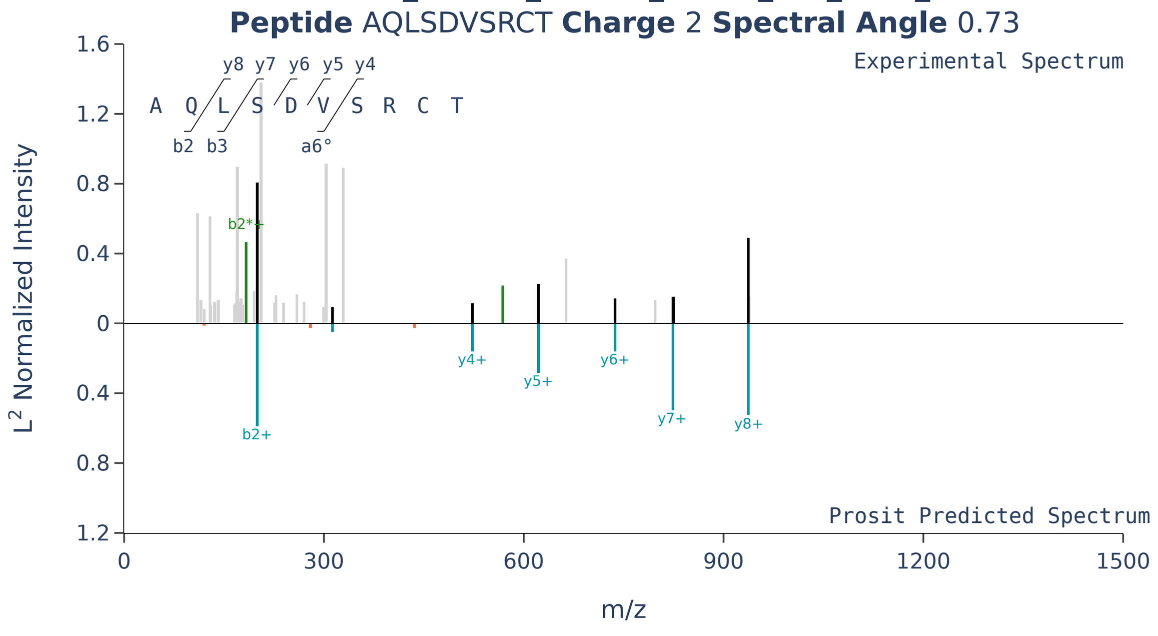

Source HRoetschke_221222_180123_HFGoe_RF1_b2R2_1h **Scan** 11344
Peptide MKEYAQLSDVSR **Charge** 3 **Spectral Angle** 0.83

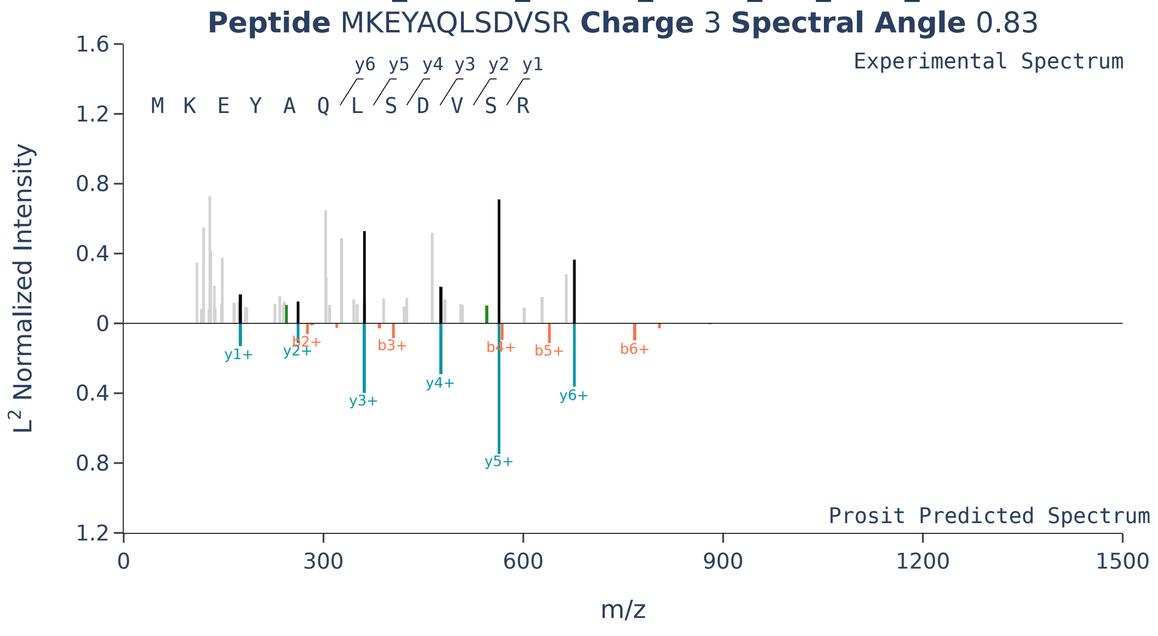

Source HRoetschke_221222_180123_HFGoe_RF1_b2R1_24h **Scan** 8910
Peptide AQLSDVSRCGD **Charge** 2 **Spectral Angle** 0.77

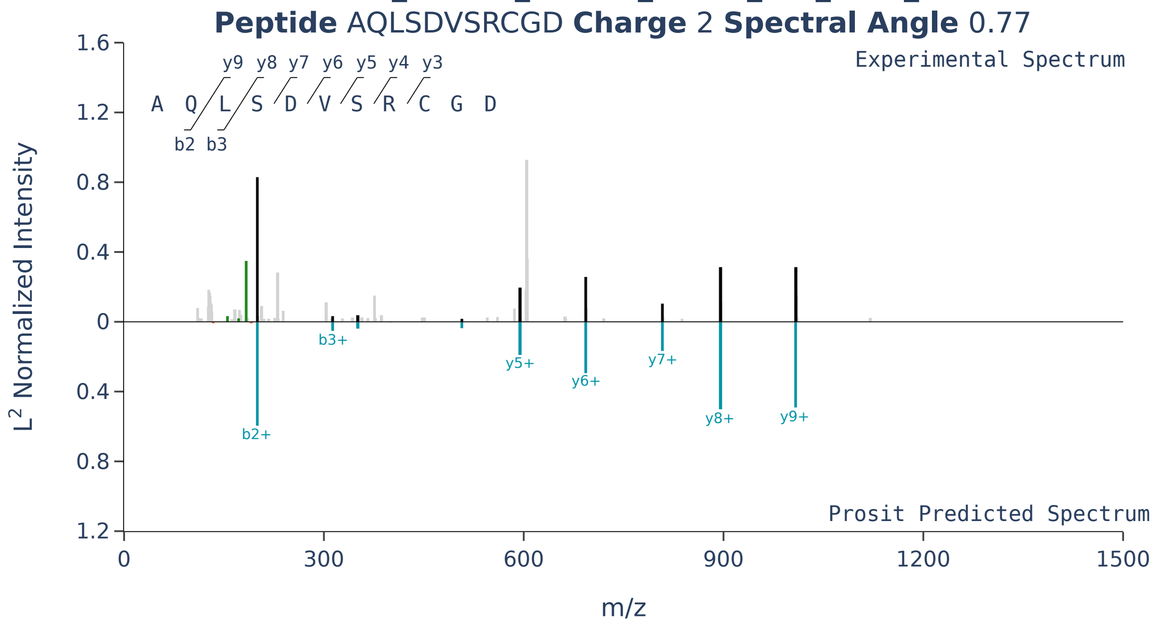

Source HRoetschke_221222_180123_HFGoe_RF1_b1R2_2h **Scan** 19892
Peptide FALEVRA **Charge** 1 **Spectral Angle** 0.83

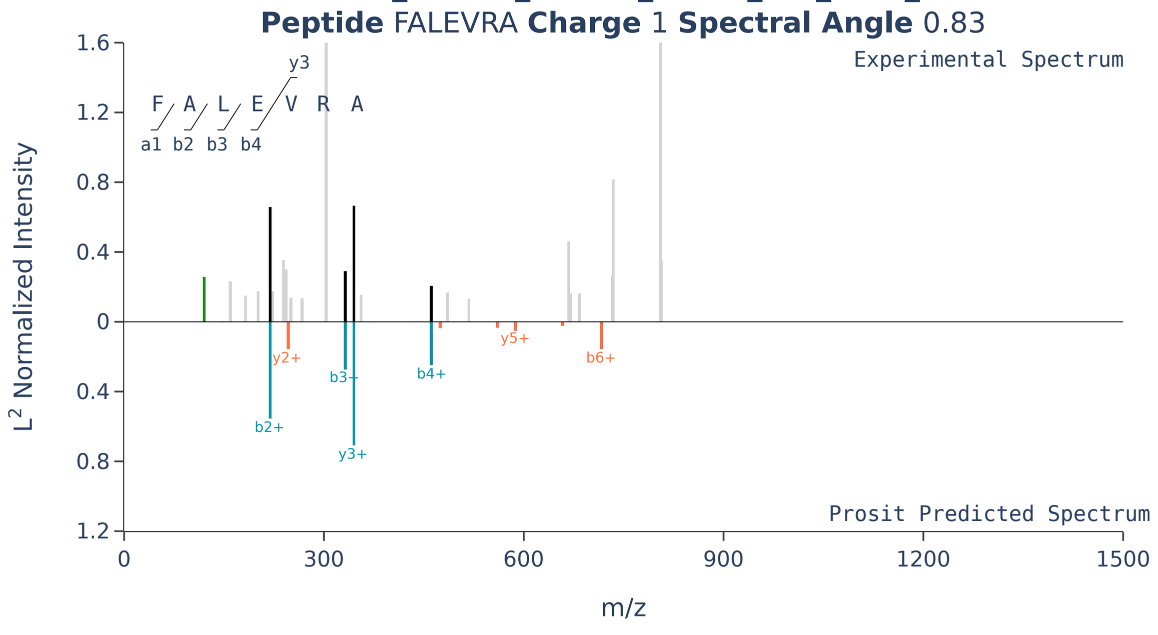

Source HRoetschke_221222_180123_HFGoe_RF1_b1R2_2h **Scan** 8651
Peptide LEQSQGRI **Charge** 2 **Spectral Angle** 0.8

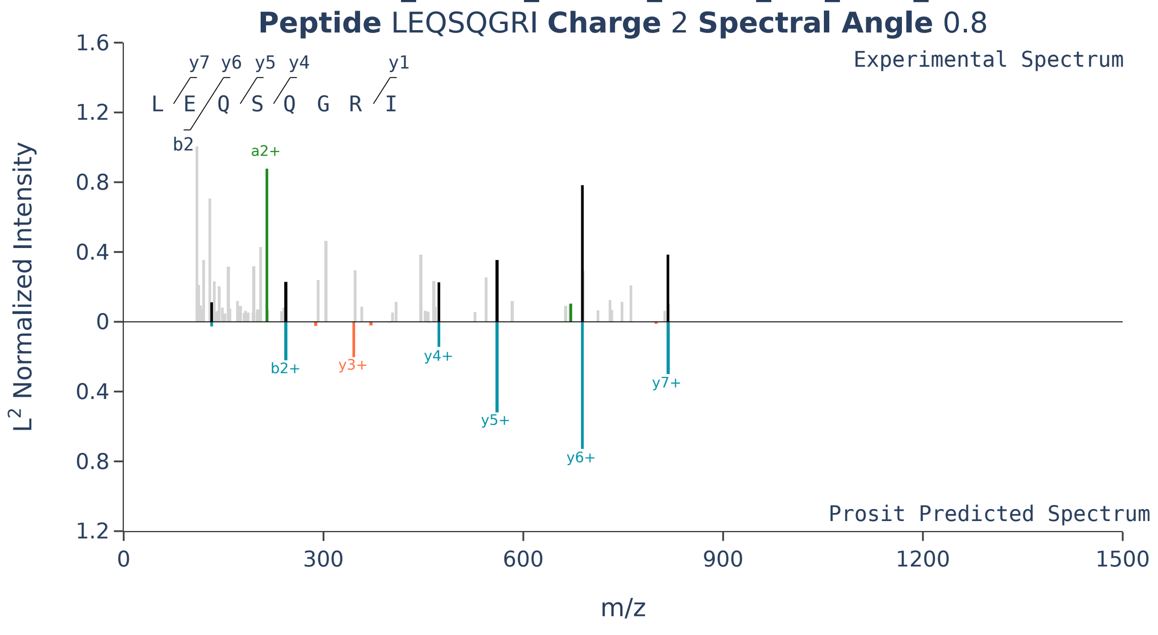

Source HRoetschke_221222_180123_HFGoe_RF1_b2R1_2h **Scan** 16968
Peptide EVMLLPKD **Charge** 2 **Spectral Angle** 0.92

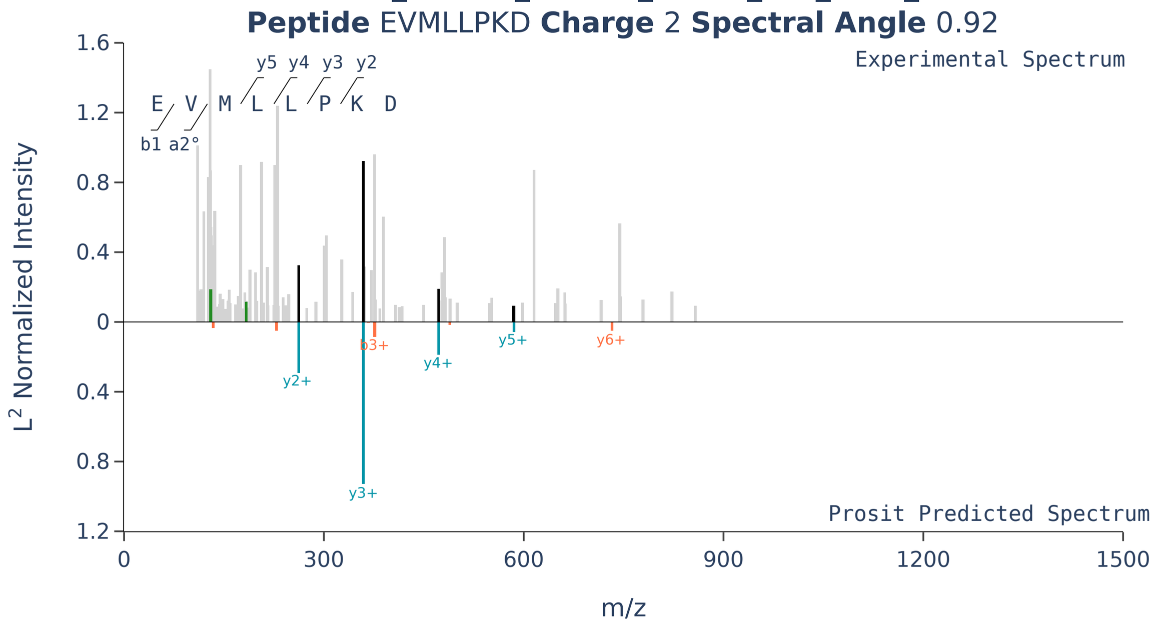

Source HRoetschke_221222_180123_HFGoe_RF1_b2R1_24h **Scan** 11901
Peptide DHRINLTA **Charge** 2 **Spectral Angle** 0.84

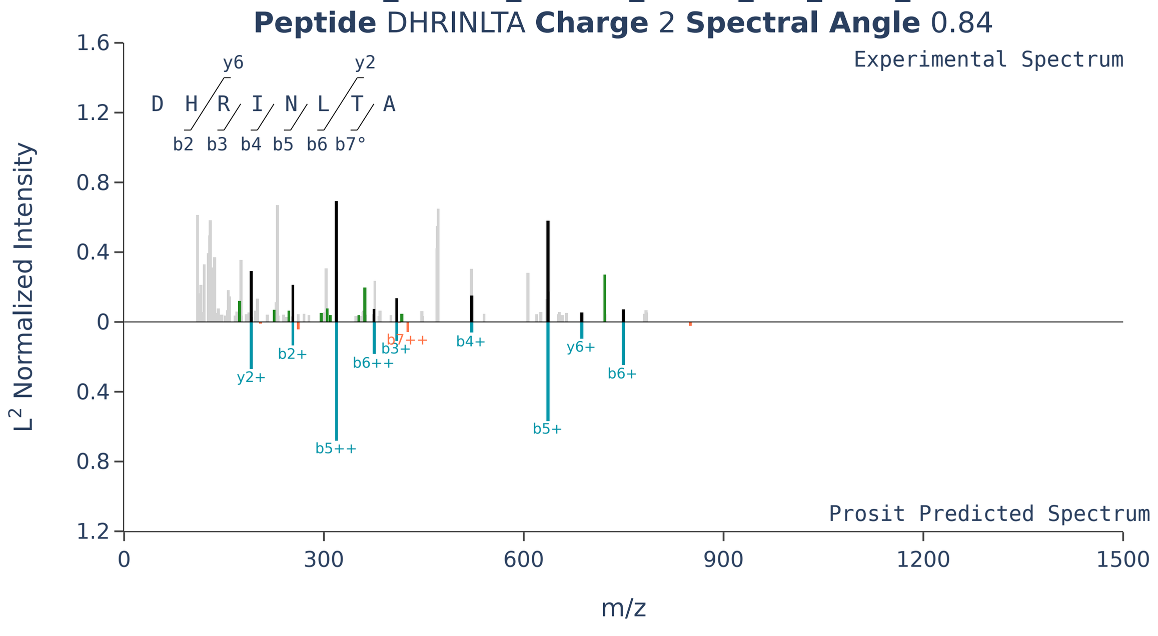

Source HRoetschke_221222_180123_HFGoe_RF1_b1R1_2h **Scan** 12826
Peptide AQMDGVYGRLK **Charge** 3 **Spectral Angle** 0.86

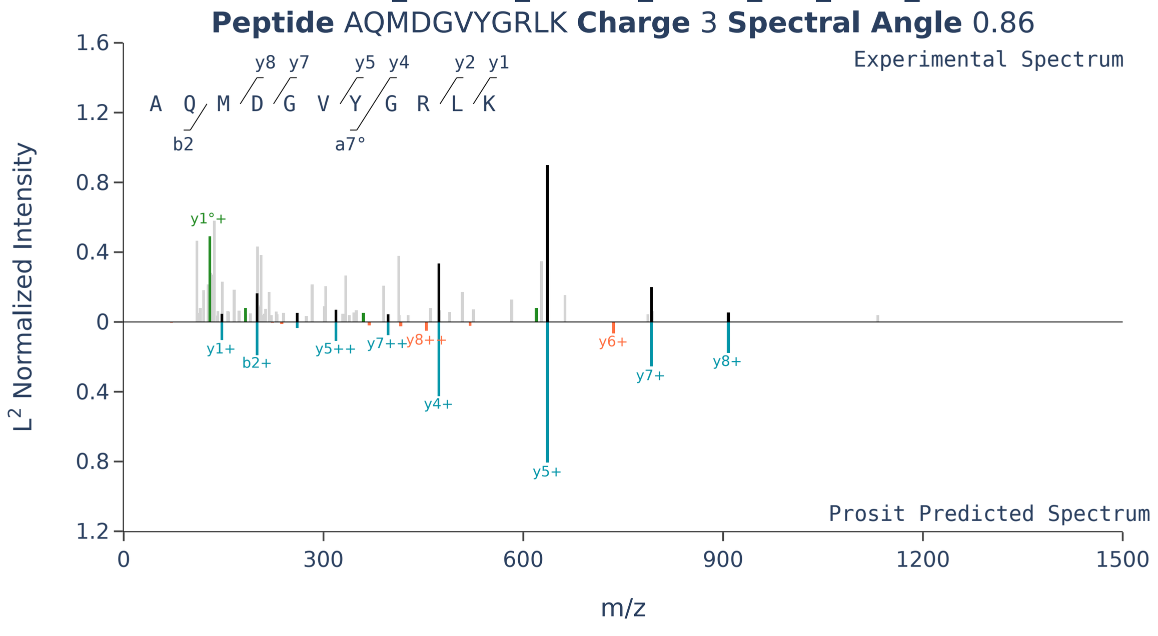

Source HRoetschke_221222_180123_HFGoe_RF1_b1R2_2h **Scan** 12898
Peptide DTFRSSQQL **Charge** 2 **Spectral Angle** 0.73

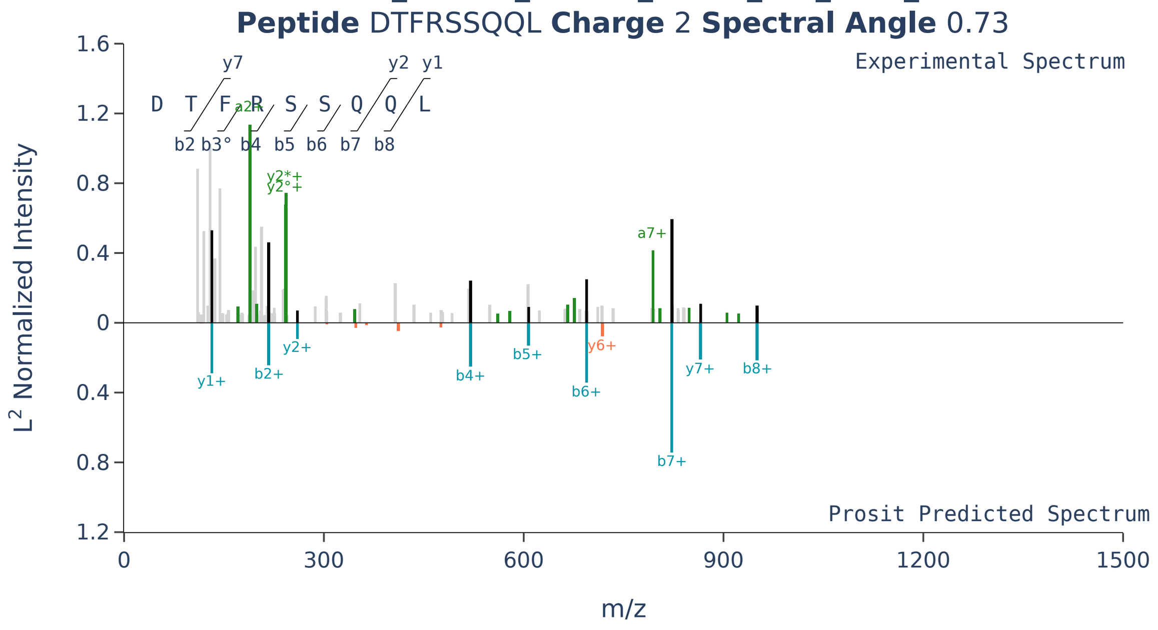

Source HRoetschke_221222_180123_HFGoe_RF1_b2R2_1h **Scan** 17088
Peptide ALSREQALLGD **Charge** 2 **Spectral Angle** 0.82

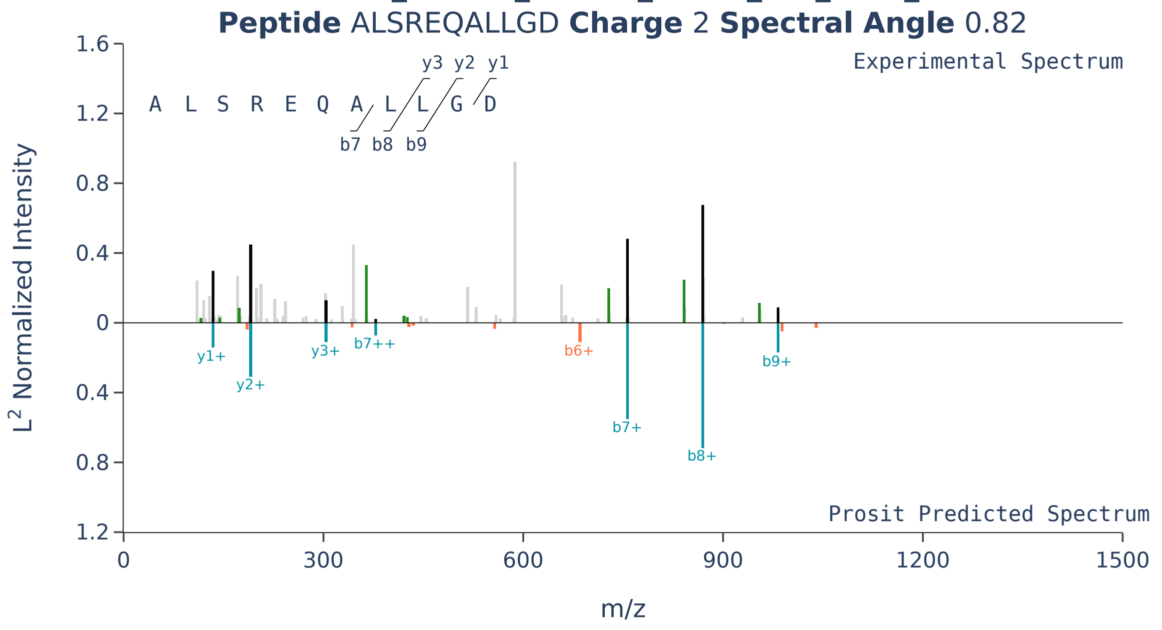

Source HRoetschke_221222_180123_HFGoe_RF1_b2R2_4h **Scan** 8831
Peptide AQLSDVSRCDG **Charge** 2 **Spectral Angle** 0.73

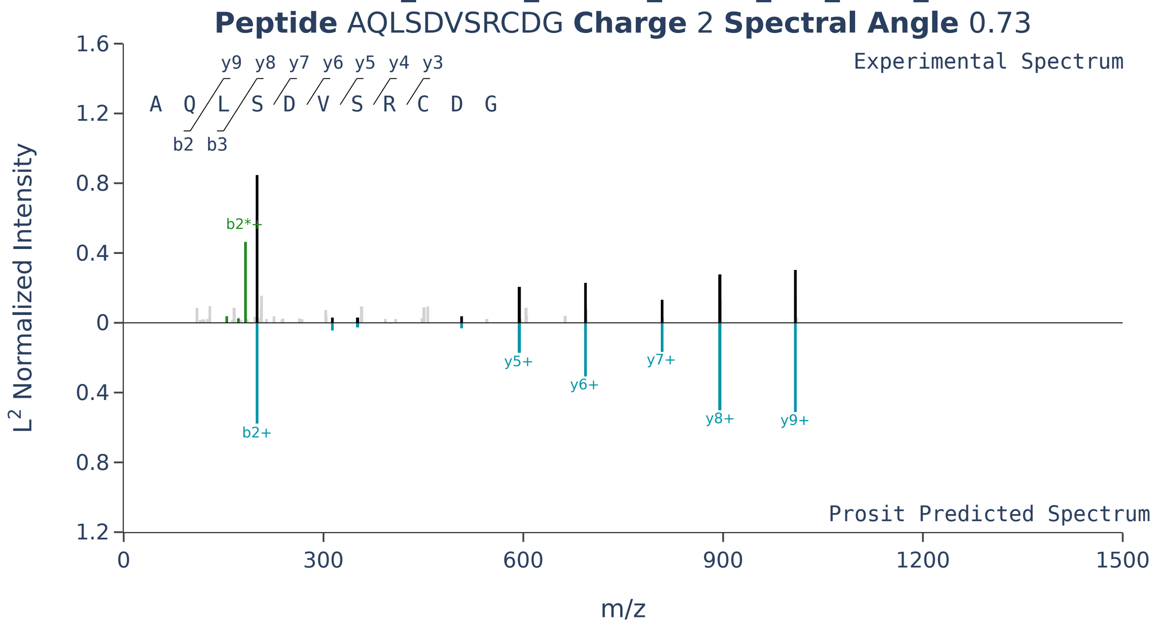

Source HRoetschke_221222_180123_HFGoe_RF1_b2R2_2h **Scan** 19458
Peptide EQQLQVLQ **Charge** 1 **Spectral Angle** 0.73

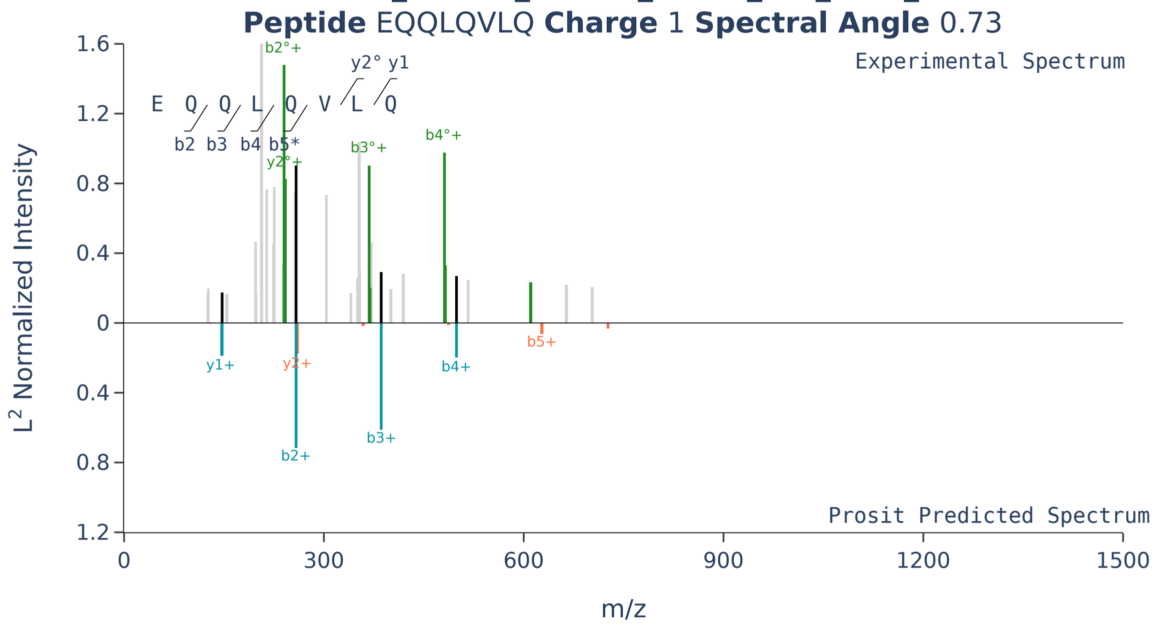

Source HRoetschke_221222_180123_HFGoe_RF1_b2R2_1h **Scan** 13191
Peptide REYAEAL **Charge** 2 **Spectral Angle** 0.83

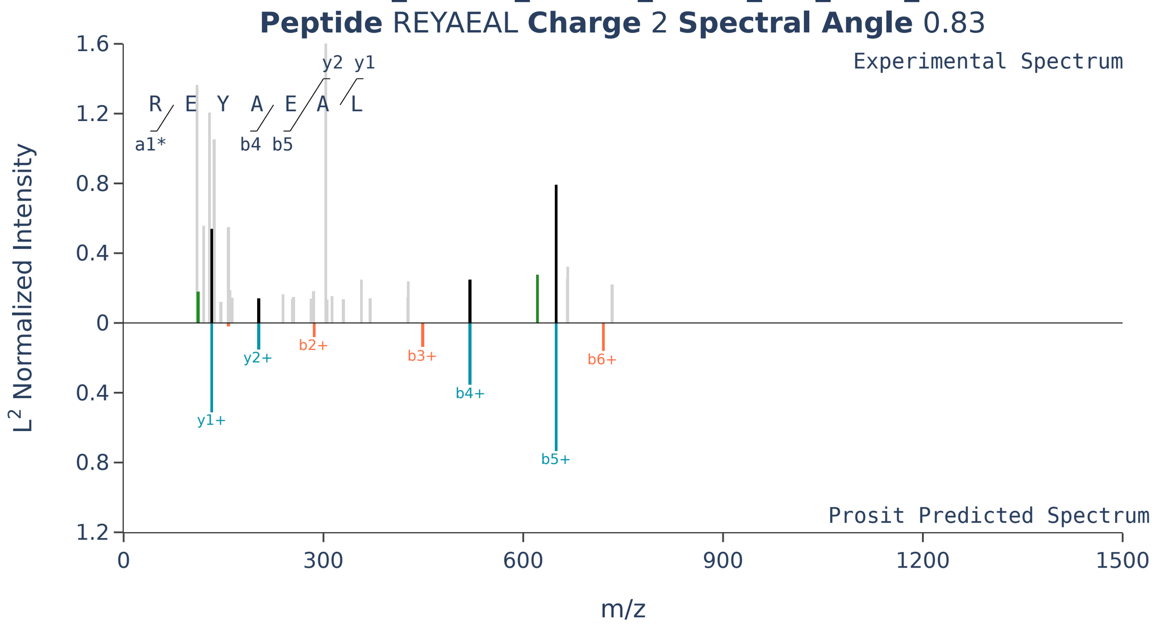

Source HRoetschke_221222_180123_HFGoe_RF1_b1R1_4h **Scan** 17882
Peptide LLGDASRCF **Charge** 2 **Spectral Angle** 0.84

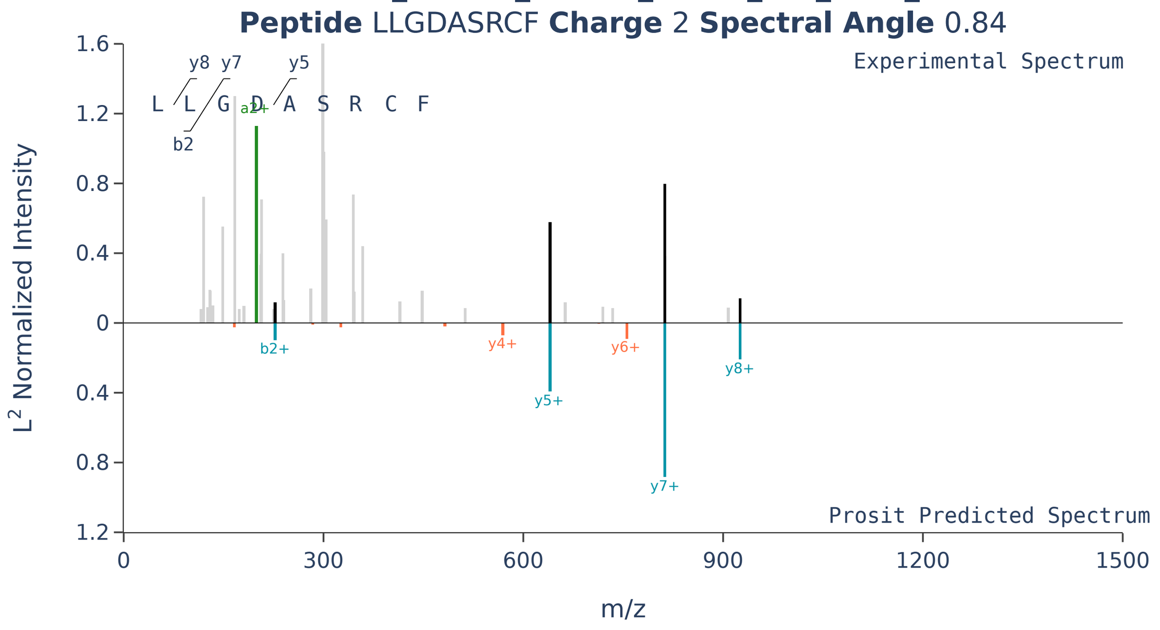

Source HRoetschke_221222_180123_HFGoe_RF1_b2R1_2h **Scan** 19332
Peptide TDWQQVAG **Charge** 1 **Spectral Angle** 0.93

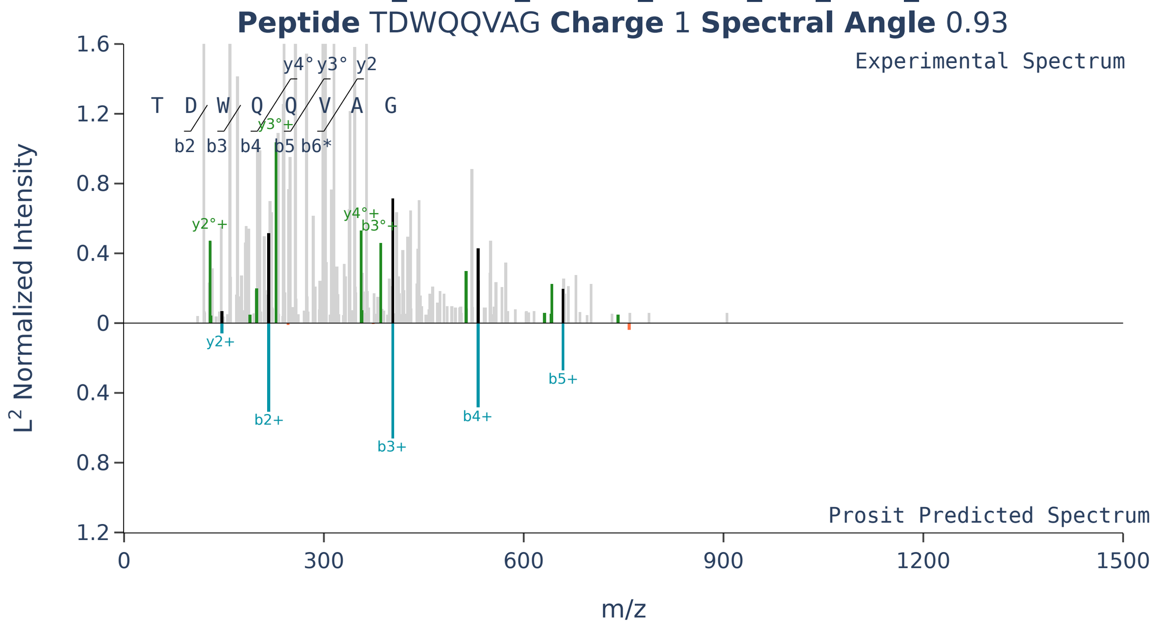

Source HRoetschke_221222_180123_HFGoe_RF1_b2R2_24h **Scan** 15536
Peptide RAGLLLPKD **Charge** 2 **Spectral Angle** 0.86

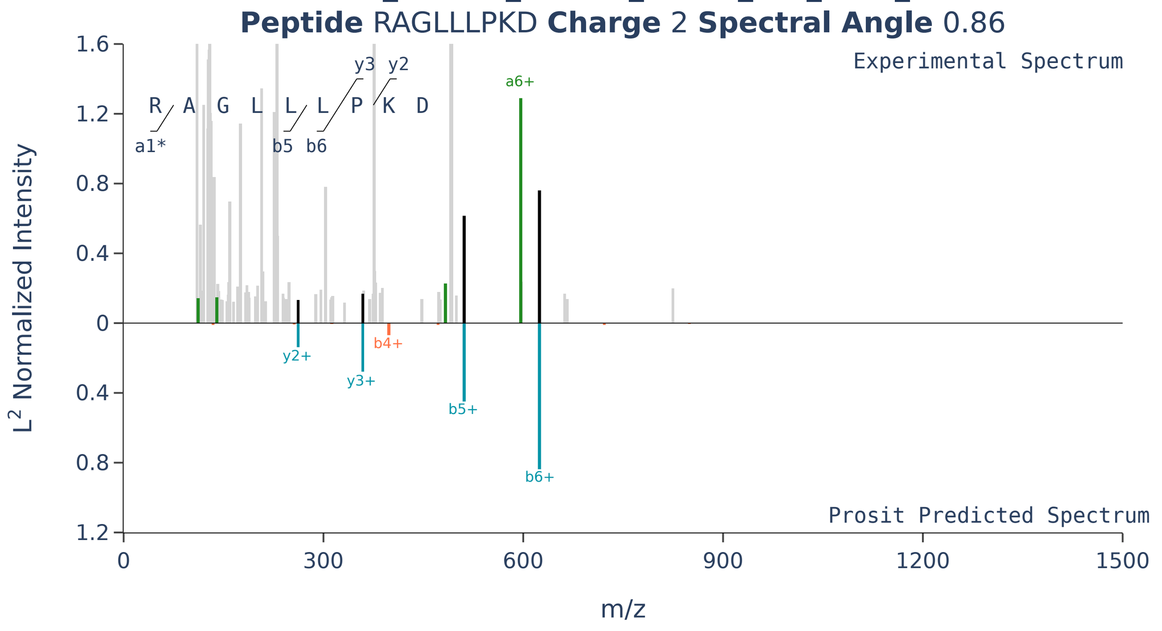

Source HRoetschke_221222_180123_HFGoe_RF1_b1R2_4h **Scan** 10508
Peptide SRYAEAQQL **Charge** 2 **Spectral Angle** 0.81

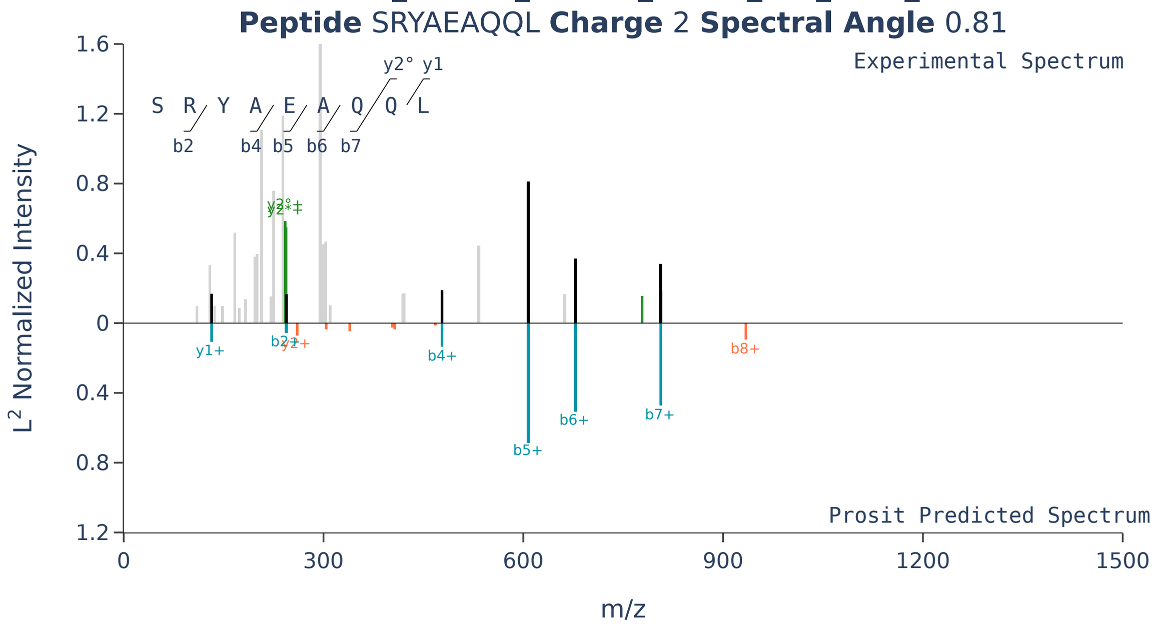

Source HRoetschke_221222_180123_HFGoe_RF1_b1R1_2h **Scan** 13369
Peptide STRGAGGQHVNTTDSAIRI **Charge** 4 **Spectral Angle** 0.81

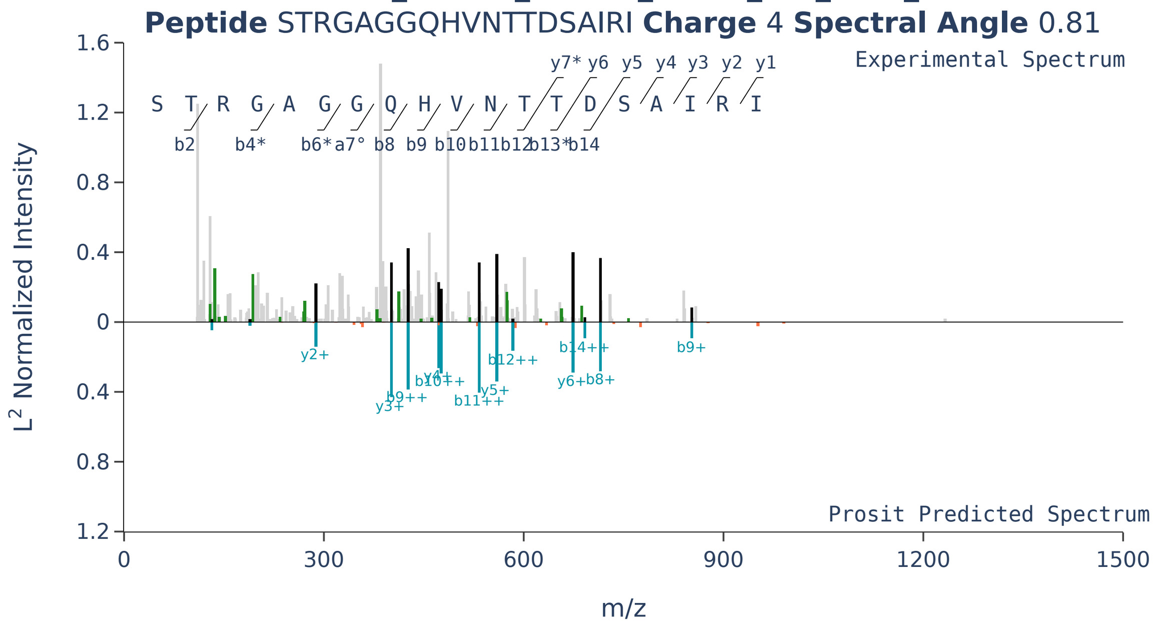

Source HRoetschke_221222_180123_HFGoe_RF1_b1R1_4h **Scan** 16446
Peptide QADQLAMLD **Charge** 1 **Spectral Angle** 0.85

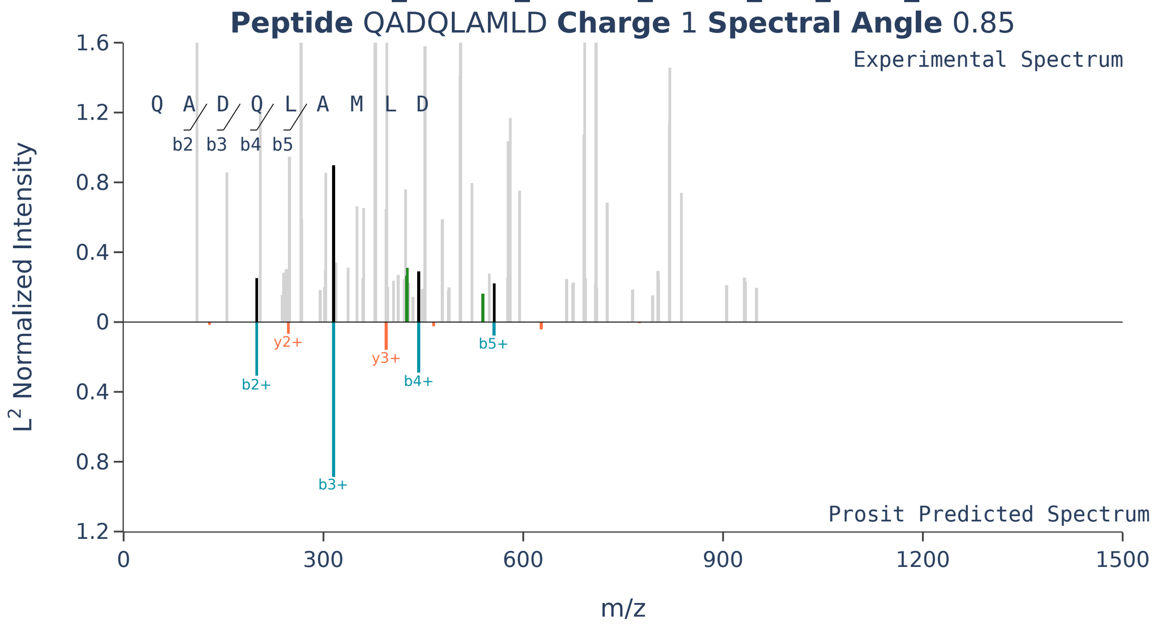

Source HRoetschke_221222_180123_HFGoe_RF1_b2R2_4h **Scan** 13589
Peptide DQLAAAIR **Charge** 2 **Spectral Angle** 0.74

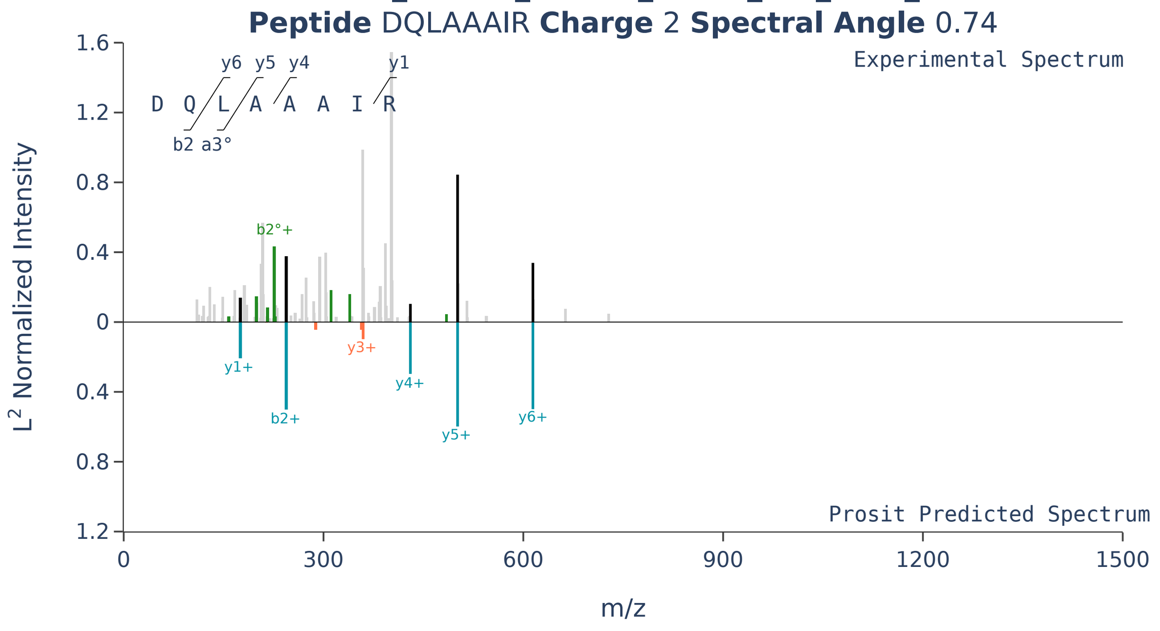

Source HRoetschke_221222_180123_HFGoe_RF1_b2R2_24h **Scan** 11452
Peptide DRSNRTYNFPQ **Charge** 3 **Spectral Angle** 0.81

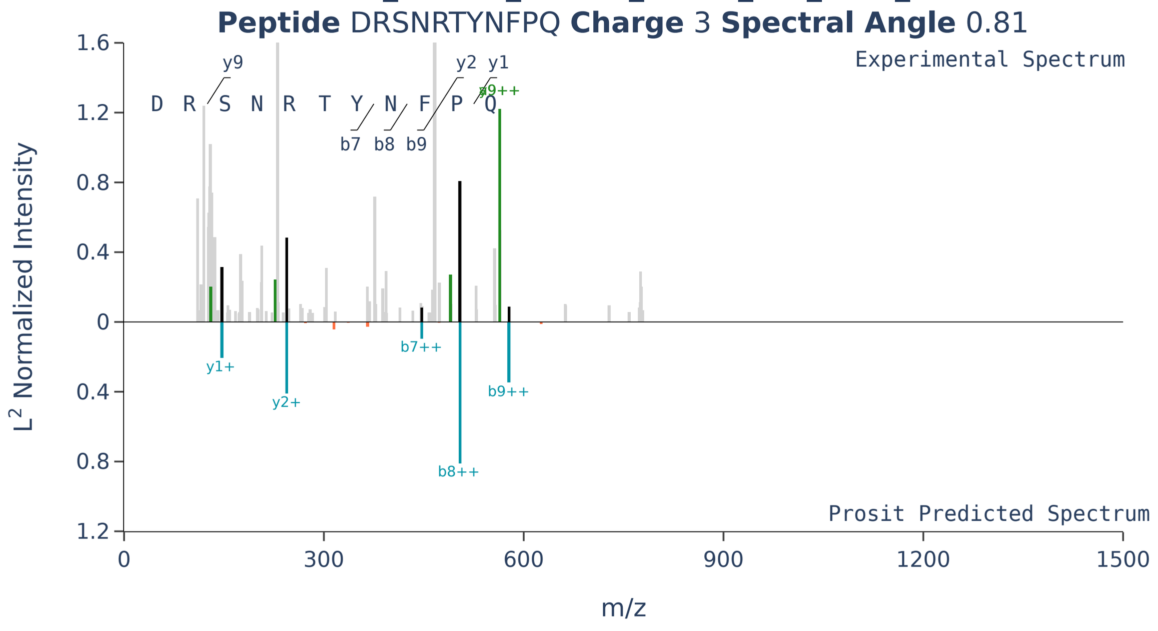

Source HRoetschke_221222_180123_HFGoe_RF1_b2R2_4h **Scan** 12514
Peptide DTFRSSGQQL **Charge** 2 **Spectral Angle** 0.76

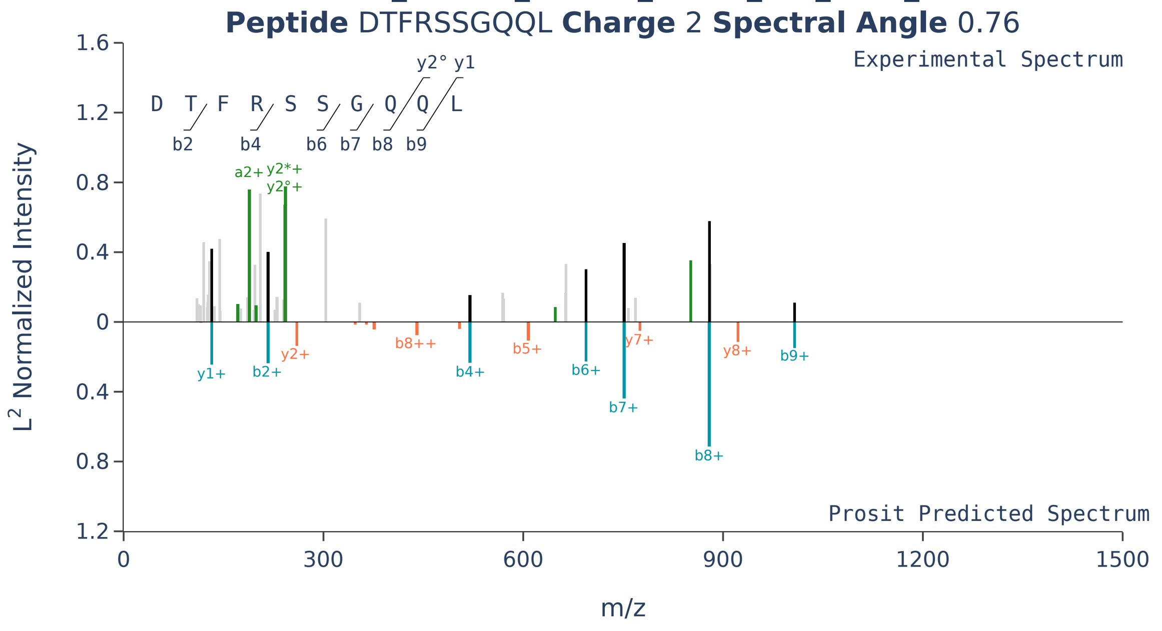

Source HRoetschke_221222_180123_HFGoe_RF1_b2R2_2h **Scan** 11407
Peptide ALSRREYAQLSD **Charge** 3 **Spectral Angle** 0.8

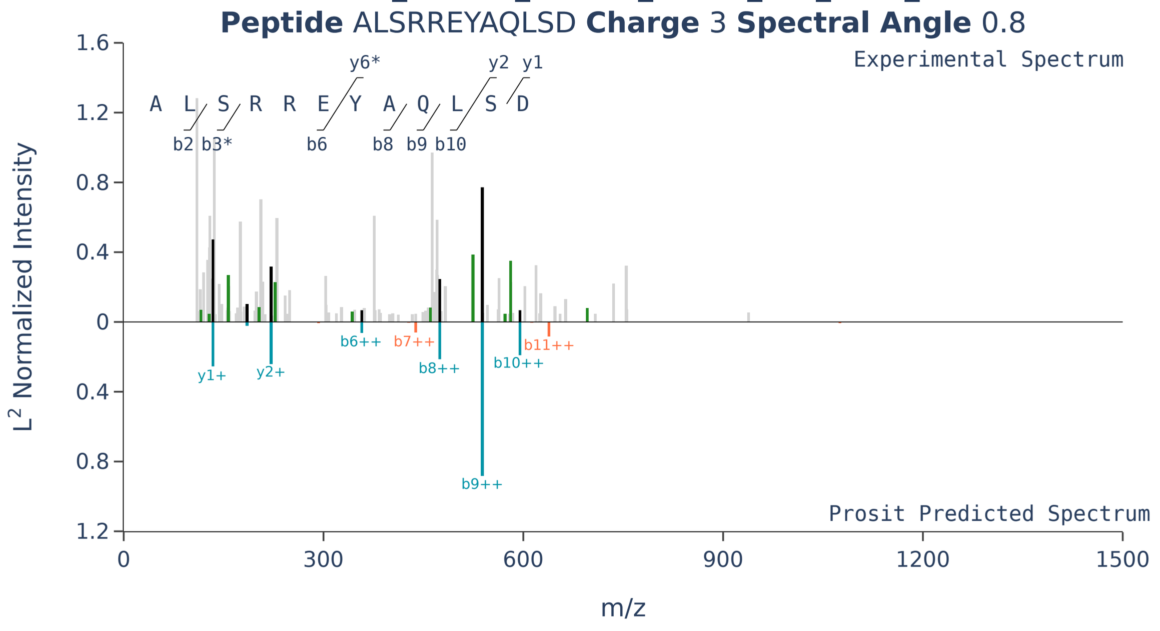

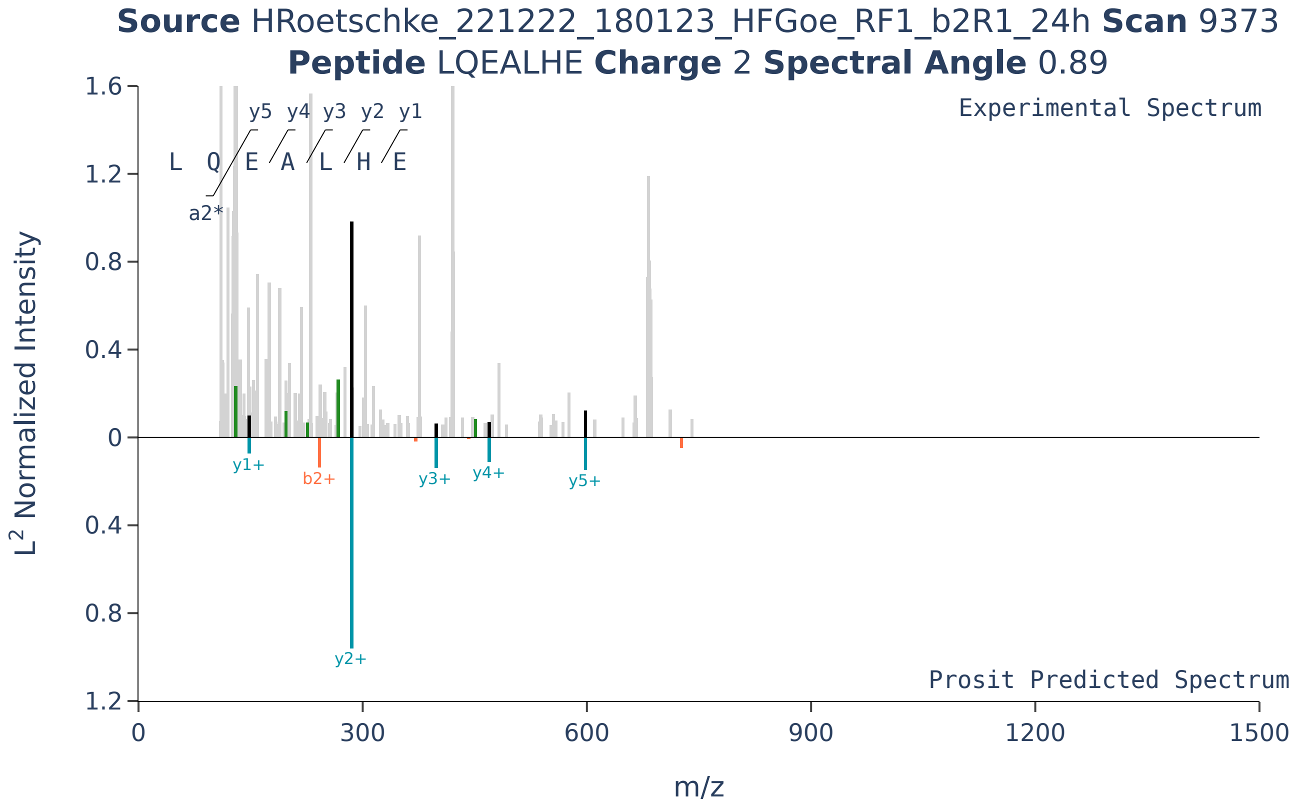

Source HRoetschke_221222_180123_HFGoe_RF1_b2R1_24h Scan 9373
Peptide LQEALHE Charge 2 Spectral Angle 0.89
Experimental Spectrum
Prosit Predicted Spectrum
L² Normalized Intensity
m/z

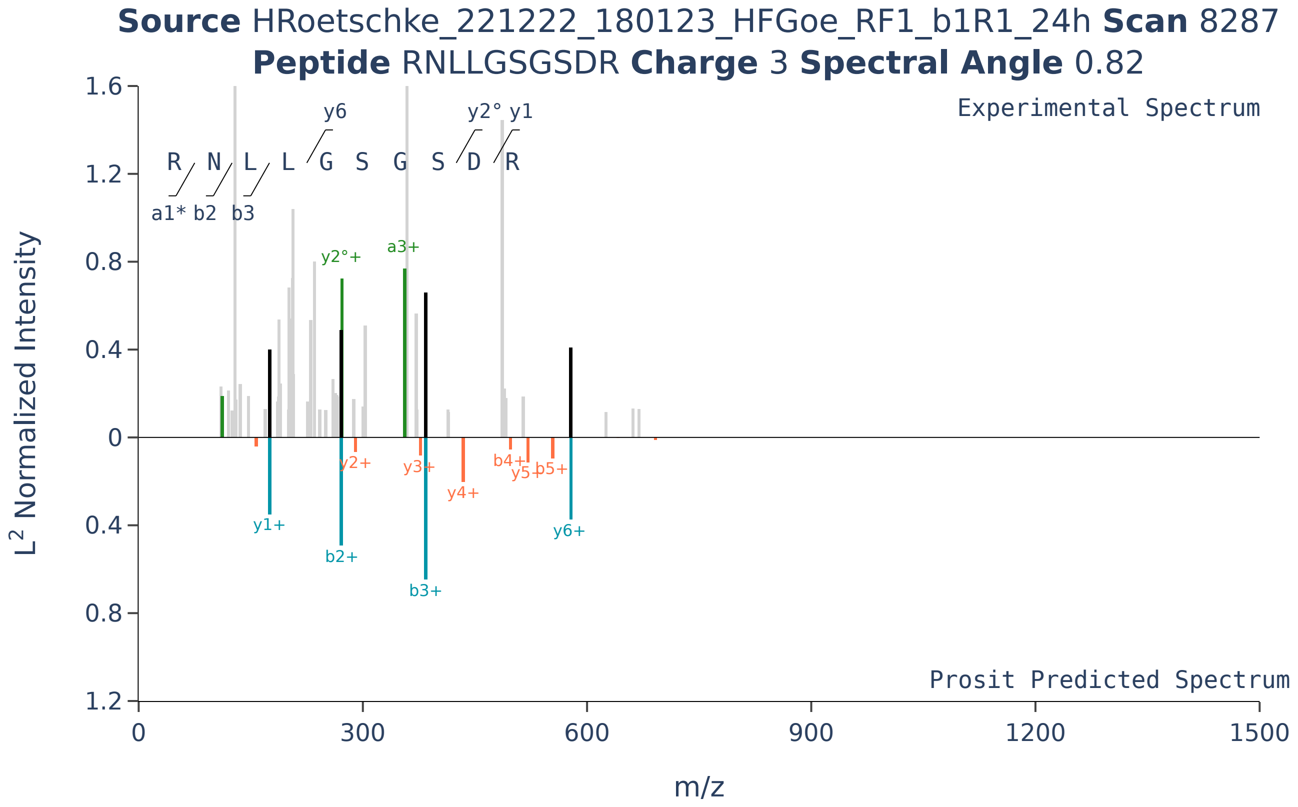

Source HRoetschke_221222_180123_HFGoe_RF1_b1R1_24h Scan 8287
Peptide RNLLGSGSDR Charge 3 Spectral Angle 0.82
Experimental Spectrum
Prosit Predicted Spectrum
L² Normalized Intensity
m/z

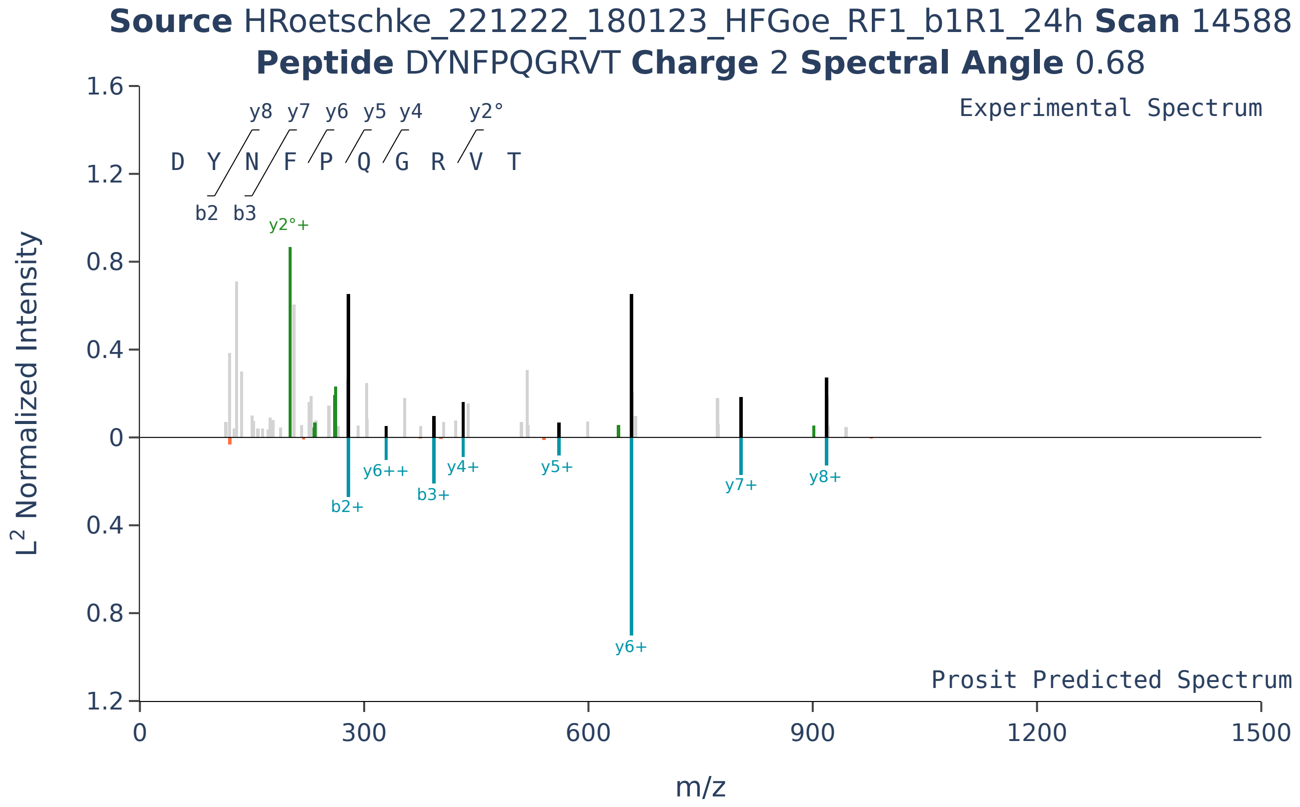

Source HRoetschke_221222_180123_HFGoe_RF1_b1R1_24h Scan 14588
Peptide DYNFPQGRVT Charge 2 Spectral Angle 0.68
Experimental Spectrum
Prosit Predicted Spectrum
L² Normalized Intensity
m/z

Source HRoetschke_271222_100123_HFGoe_UBE2K_b2R2_24h **Scan** 9914
Peptide HMANIAV **Charge** 2 **Spectral Angle** 0.95

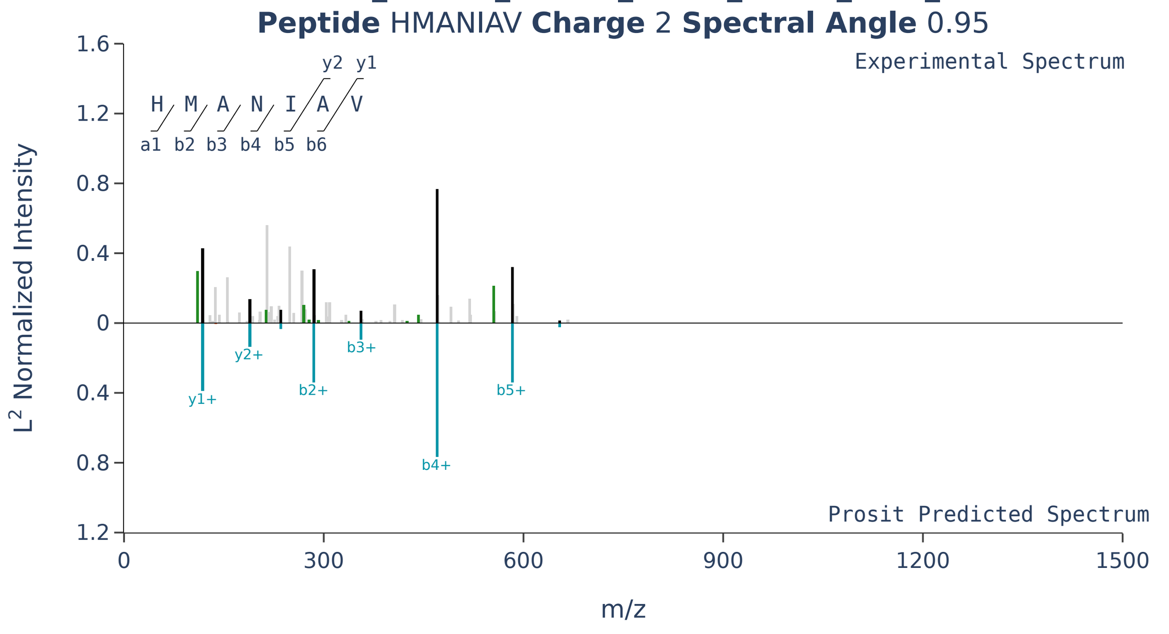

Source HRoetschke_271222_100123_HFGoe_UBE2K_b1R1_24h **Scan** 6398
Peptide HMANIAVQR **Charge** 3 **Spectral Angle** 0.94

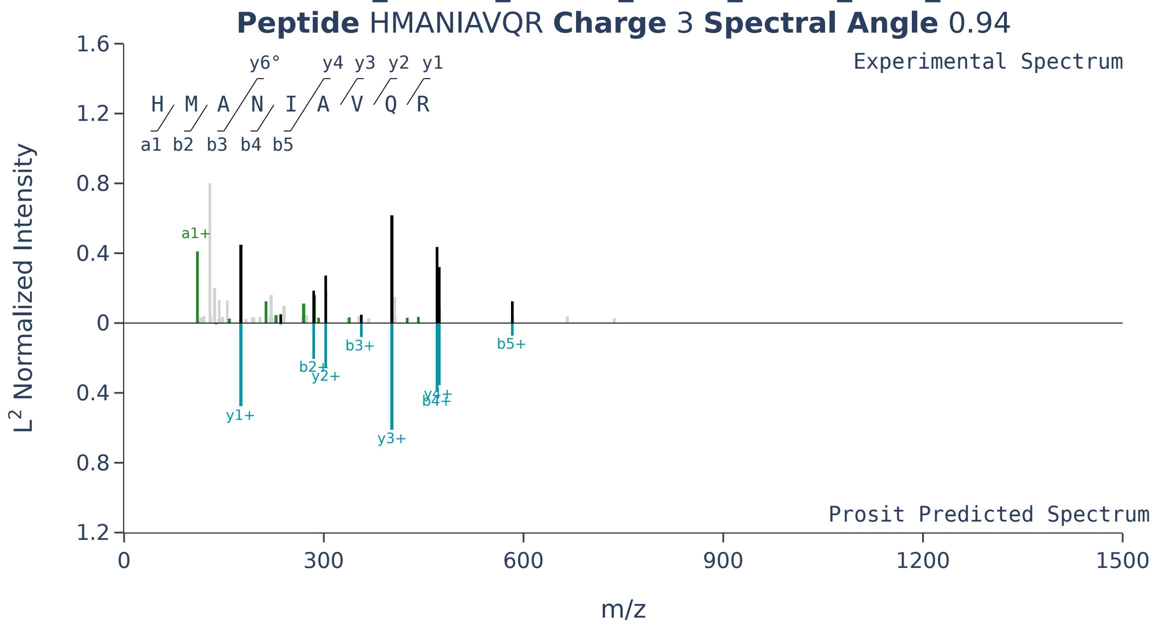

Source HRoetschke_271222_100123_HFGoe_UBE2K_b1R2_24h **Scan** 14481
Peptide SSNIAVQRI **Charge** 2 **Spectral Angle** 0.95

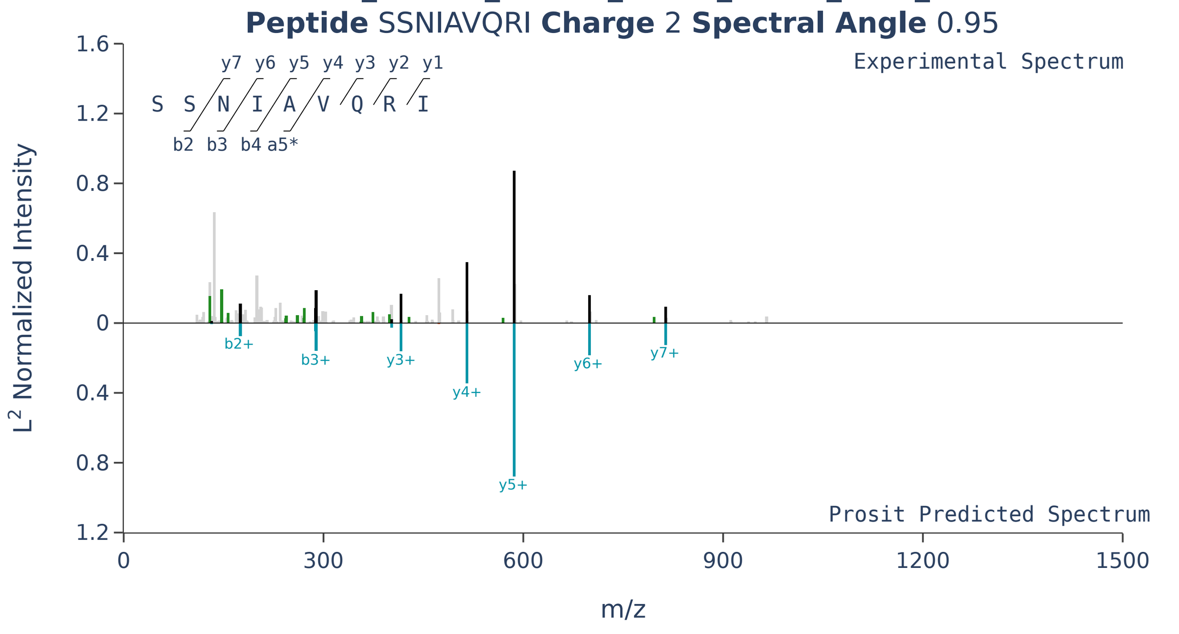

Source HRoetschke_271222_100123_HFGoe_UBE2K_b1R1_24h **Scan** 13609
Peptide SNIAVQRI **Charge** 2 **Spectral Angle** 0.89

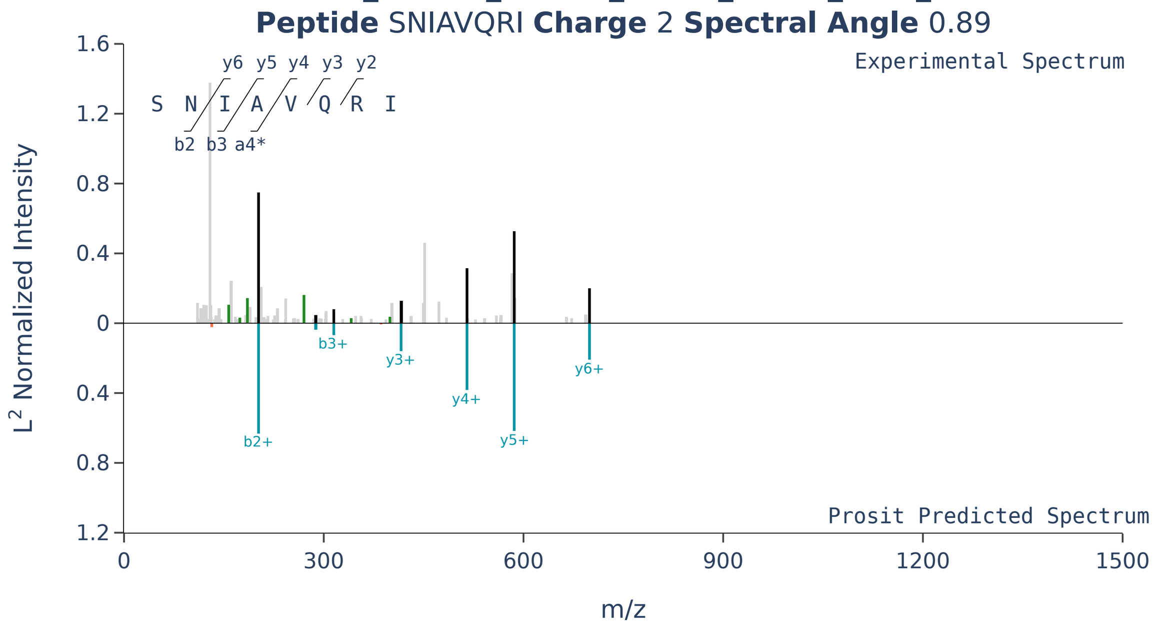

Source HRoetschke_271222_100123_HFGoe_UBE2K_b1R2_24h **Scan** 14059
Peptide PNISSVTDA **Charge** 1 **Spectral Angle** 0.95

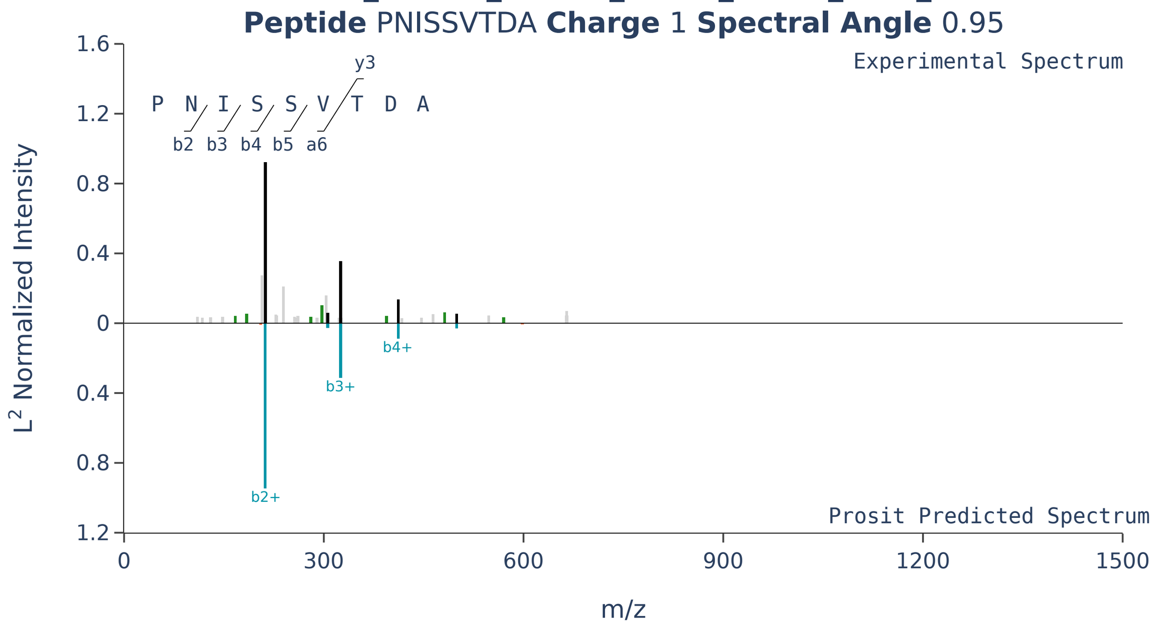

Source HRoetschke_271222_100123_HFGoe_UBE2K_b1R1_24h **Scan** 6520
Peptide HMANIAVQRIKR **Charge** 4 **Spectral Angle** 0.9

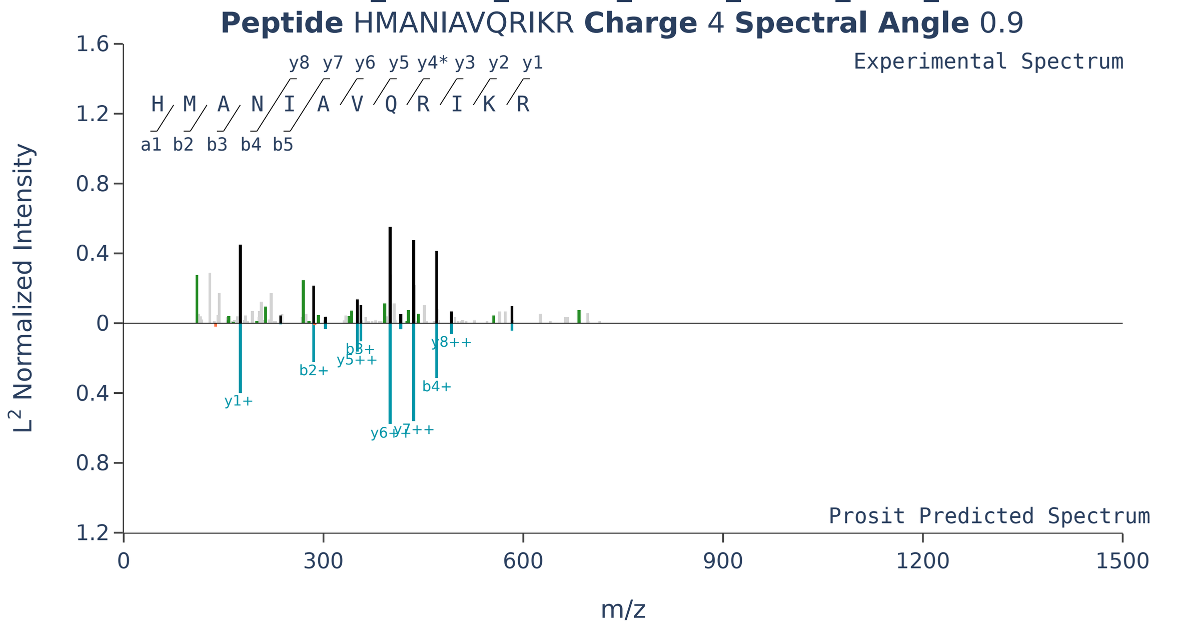

Source HRoetschke_271222_100123_HFGoe_UBE2K_b2R2_4h **Scan** 11374
Peptide HMANIAVQRI **Charge** 3 **Spectral Angle** 0.91

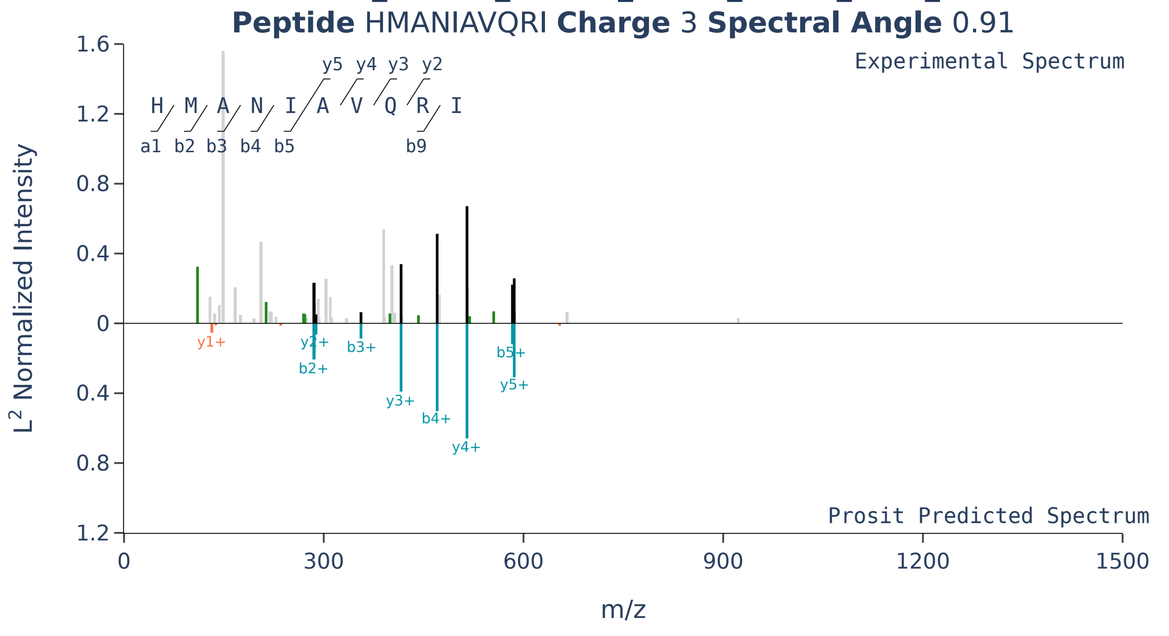

Source HRoetschke_271222_100123_HFGoe_UBE2K_b1R1_24h **Scan** 11109
Peptide RGENFTELRG **Charge** 3 **Spectral Angle** 0.9

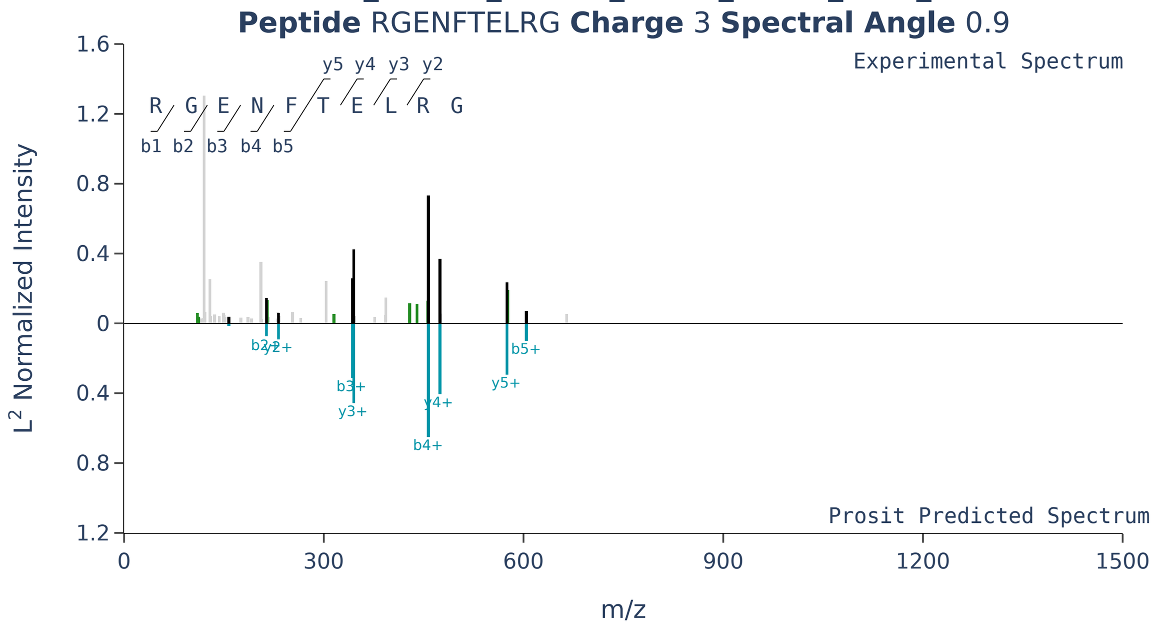

Source HRoetschke_271222_100123_HFGoe_UBE2K_b2R1_24h **Scan** 8147
Peptide MANIATAT **Charge** 1 **Spectral Angle** 0.88

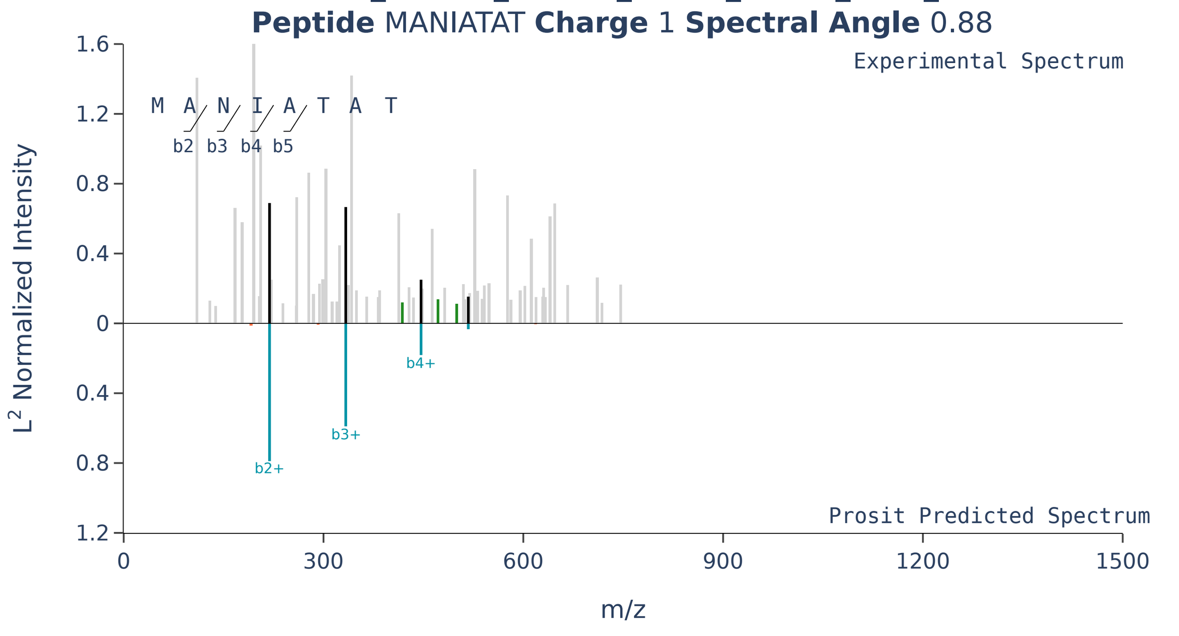

Source HRoetschke_271222_100123_HFGoe_UBE2K_b1R2_24h **Scan** 16228
Peptide RGTGAICL **Charge** 2 **Spectral Angle** 0.89

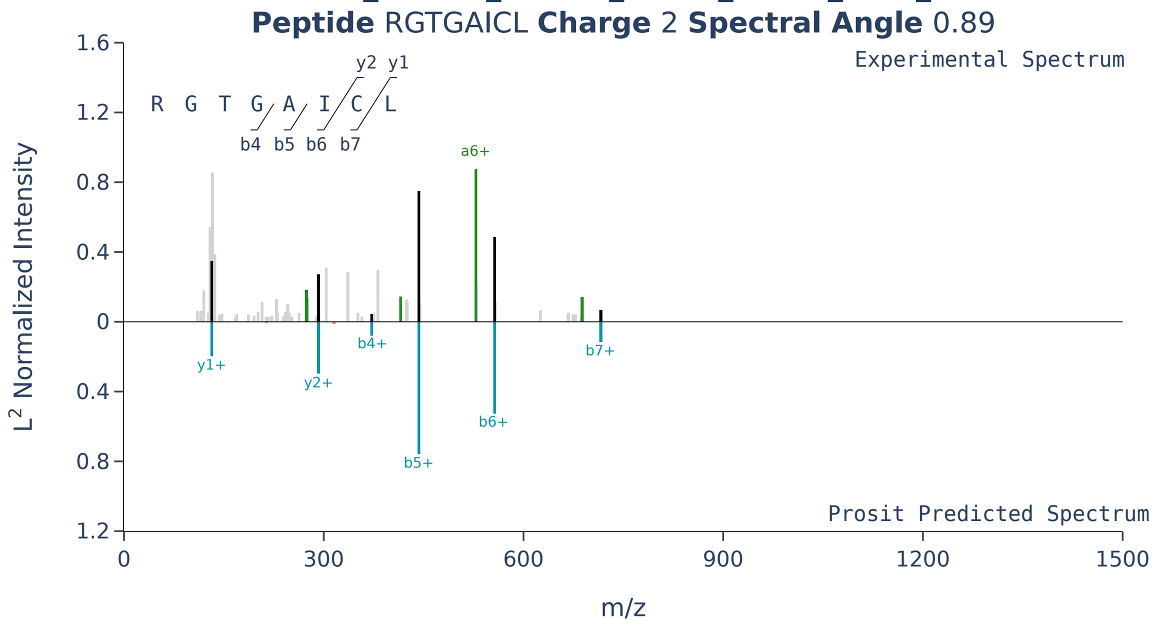

Source HRoetschke_271222_100123_HFGoe_UBE2K_b1R2_24h **Scan** 14922
Peptide TGNIAVQRI **Charge** 2 **Spectral Angle** 0.87

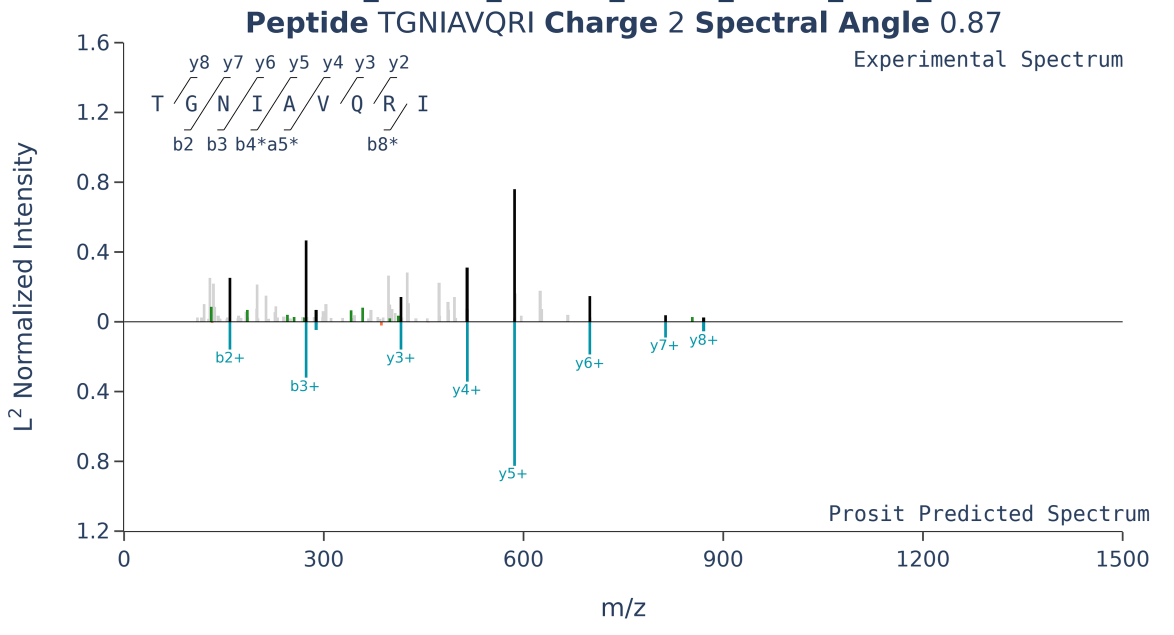

Source HRoetschke_271222_100123_HFGoe_UBE2K_b1R1_24h **Scan** 12864
Peptide KSEETNIAVQRI **Charge** 3 **Spectral Angle** 0.91

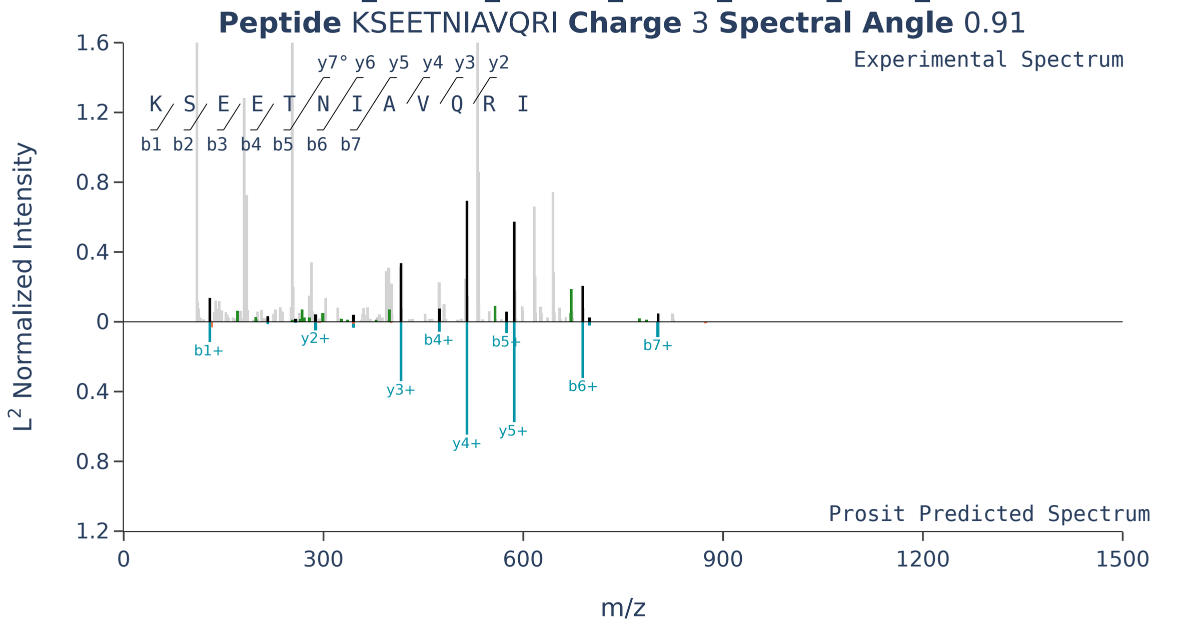

Source HRoetschke_271222_100123_HFGoe_UBE2K_b1R2_4h **Scan** 12606
Peptide ENFTELRGD **Charge** 2 **Spectral Angle** 0.91

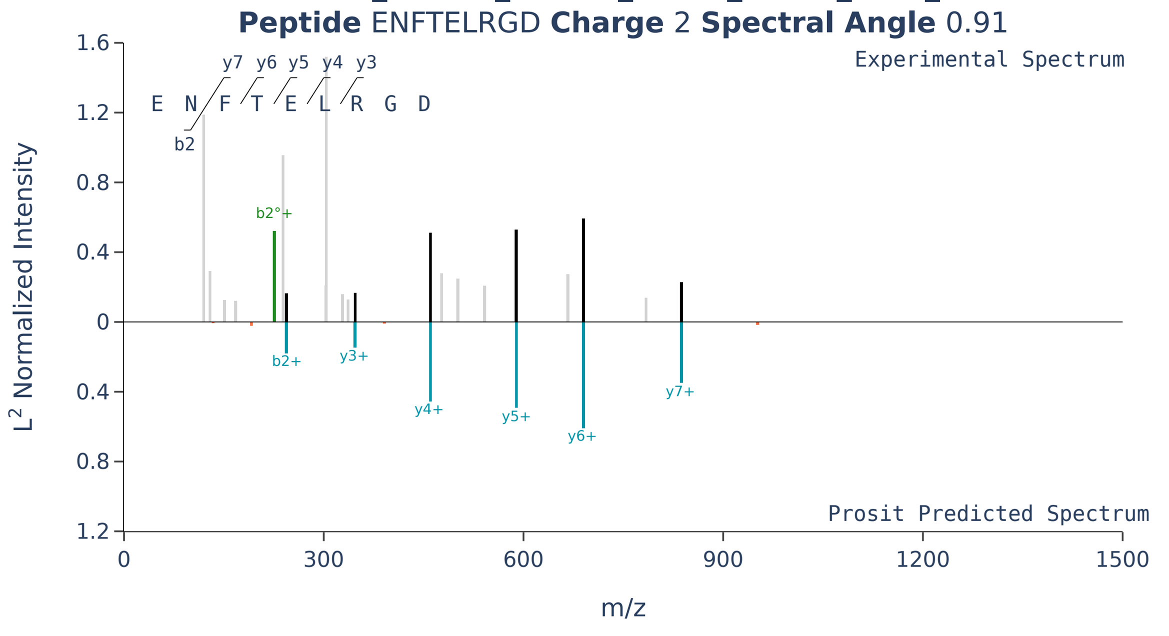

Source HRoetschke_271222_100123_HFGoe_UBE2K_b2R1_24h **Scan** 9817
Peptide LMGFDRN **Charge** 2 **Spectral Angle** 0.93

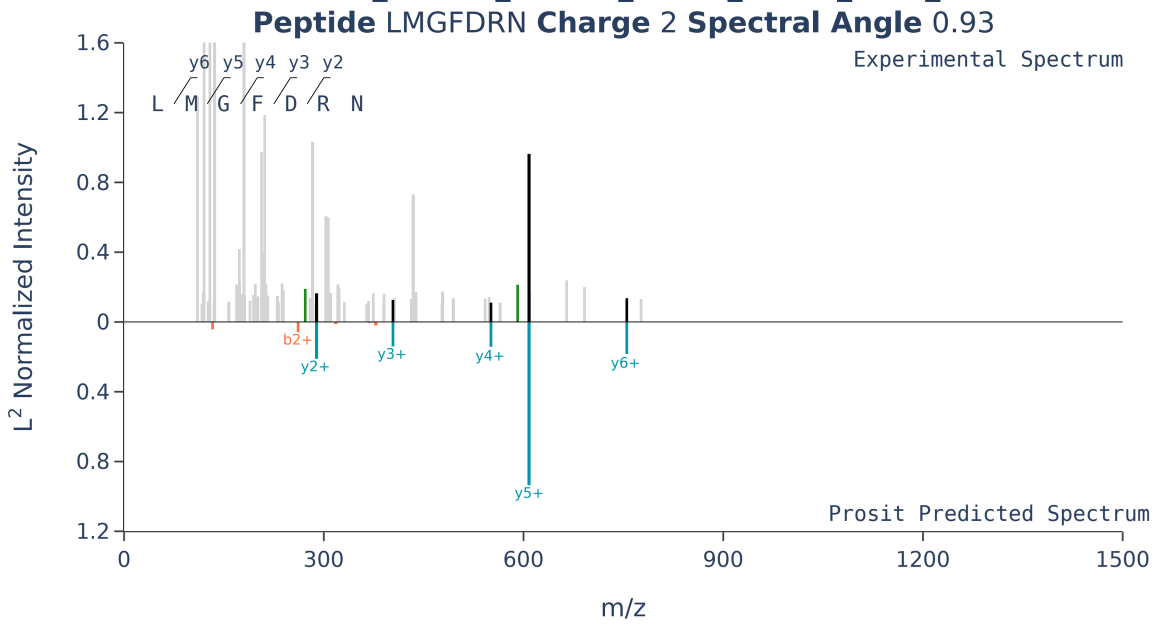

Source HRoetschke_271222_100123_HFGoe_UBE2K_b1R2_24h **Scan** 12430
Peptide RGEIAGEIAG **Charge** 2 **Spectral Angle** 0.91

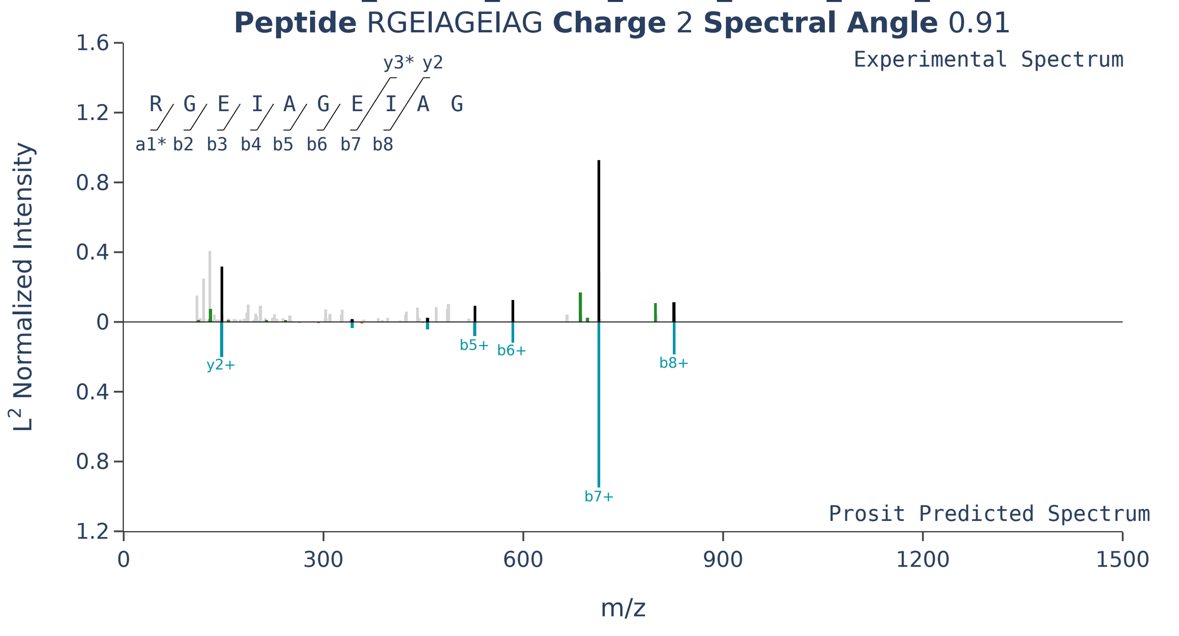

Source HRoetschke_271222_100123_HFGoe_UBE2K_b1R2_24h **Scan** 14939
Peptide ENFTELRE **Charge** 2 **Spectral Angle** 0.81

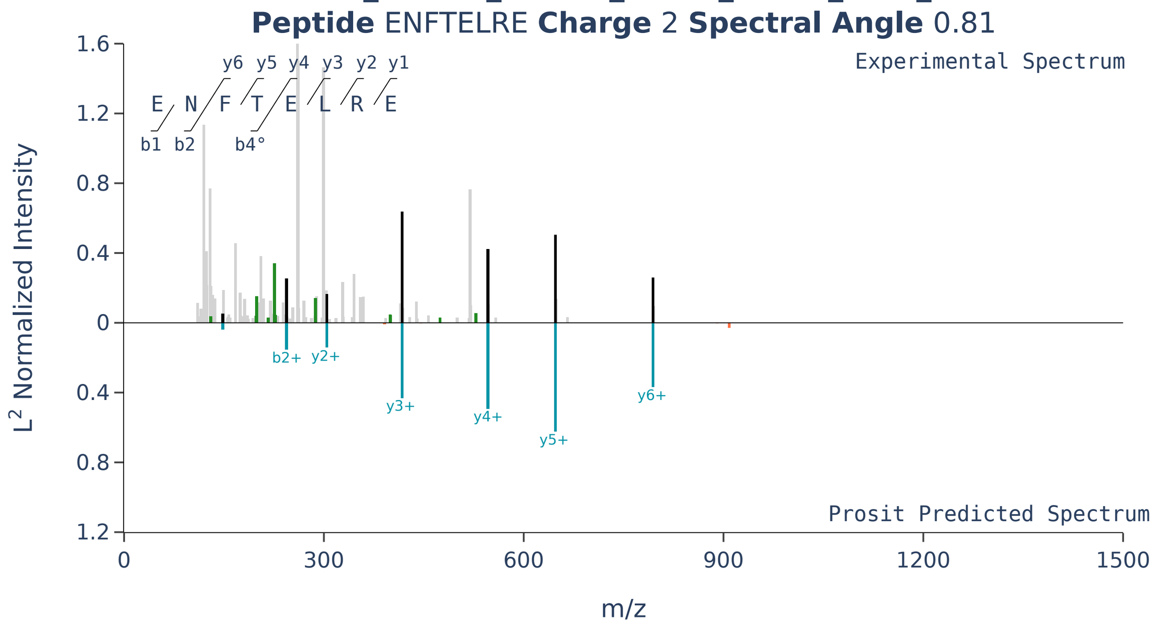

Source HRoetschke_271222_100123_HFGoe_UBE2K_b2R1_24h **Scan** 11277
Peptide KQTAEIKI **Charge** 2 **Spectral Angle** 0.94

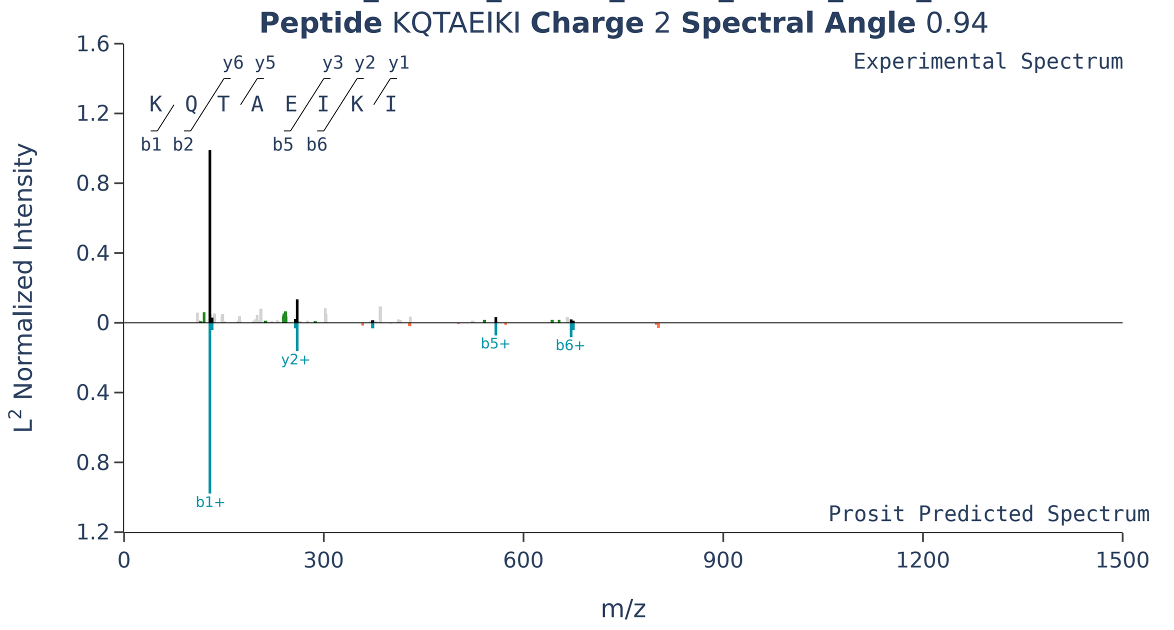

Source HRoetschke_271222_100123_HFGoe_UBE2K_b1R2_24h **Scan** 12681
Peptide KQTANIAVQRI **Charge** 3 **Spectral Angle** 0.88

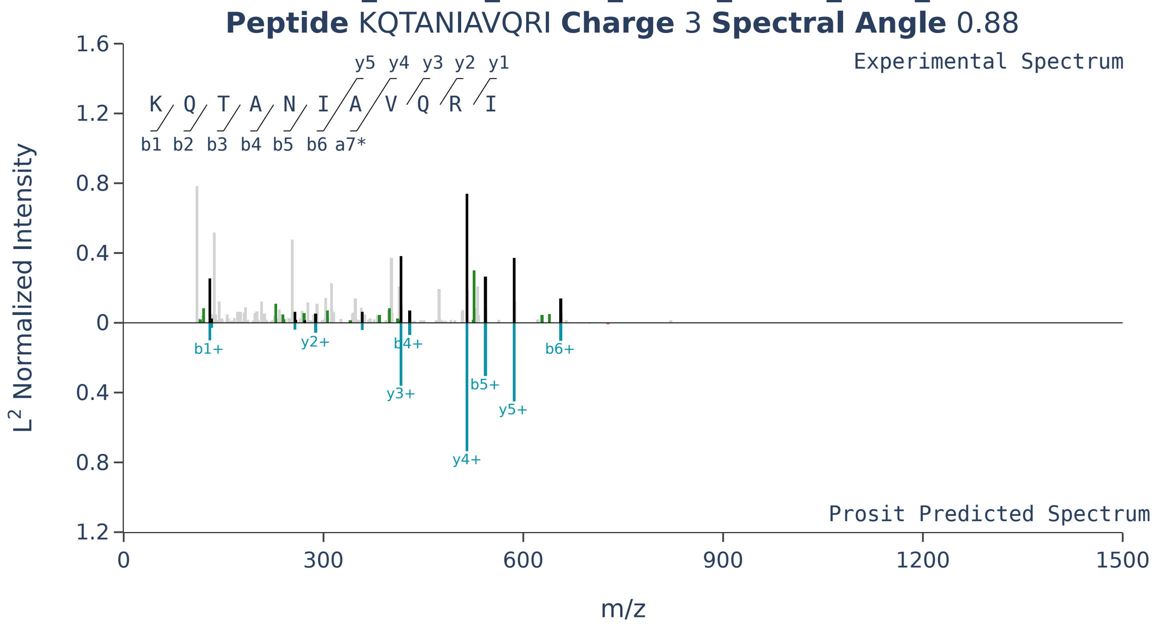

Source HRoetschke_271222_100123_HFGoe_UBE2K_b1R2_24h **Scan** 11086
Peptide MAGFDRNA **Charge** 1 **Spectral Angle** 0.9

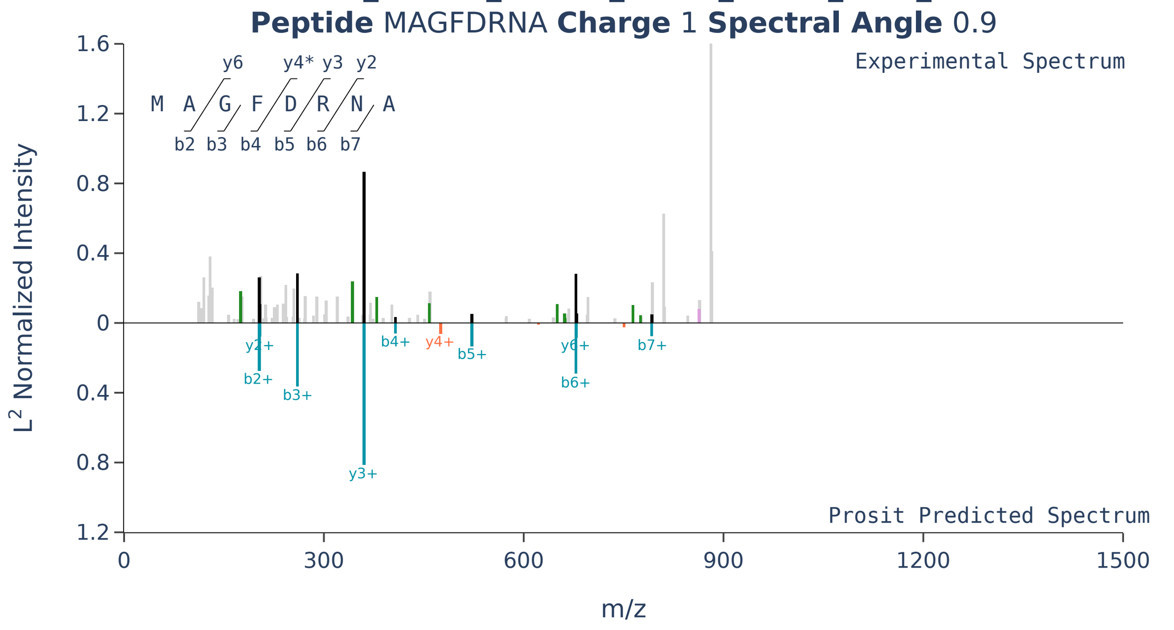

Source HRoetschke_271222_100123_HFGoe_UBE2K_b2R2_4h **Scan** 13356
Peptide SSENFTELRG **Charge** 2 **Spectral Angle** 0.89

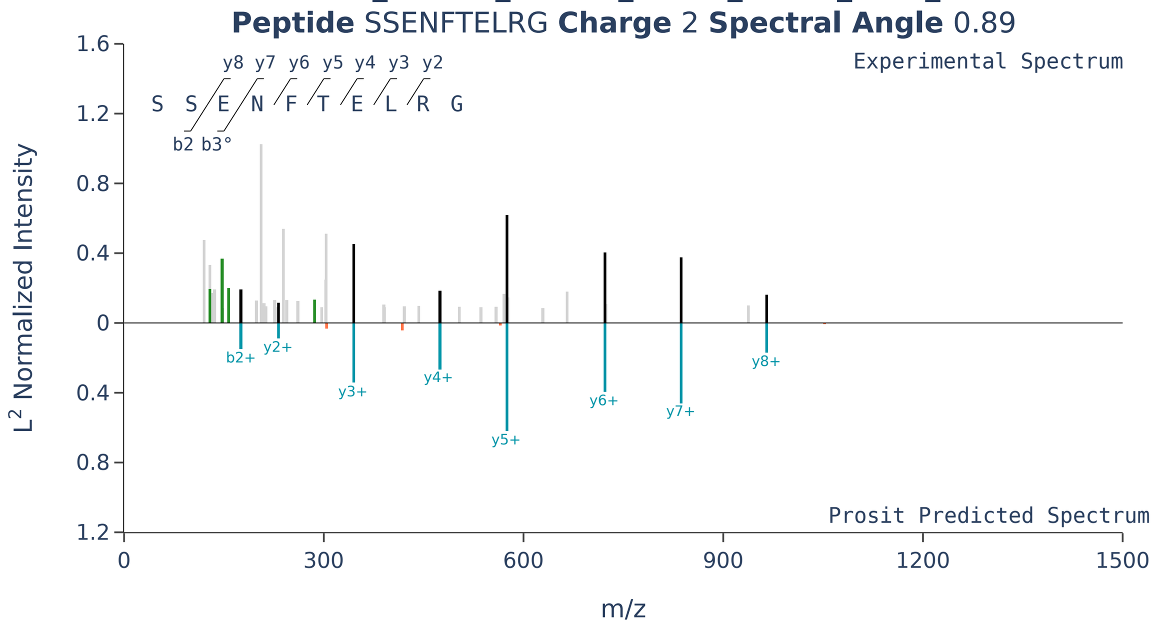

Source HRoetschke_271222_100123_HFGoe_UBE2K_b1R2_24h **Scan** 14436
Peptide EMAGPPDTPYEGGRY **Charge** 2 **Spectral Angle** 0.83

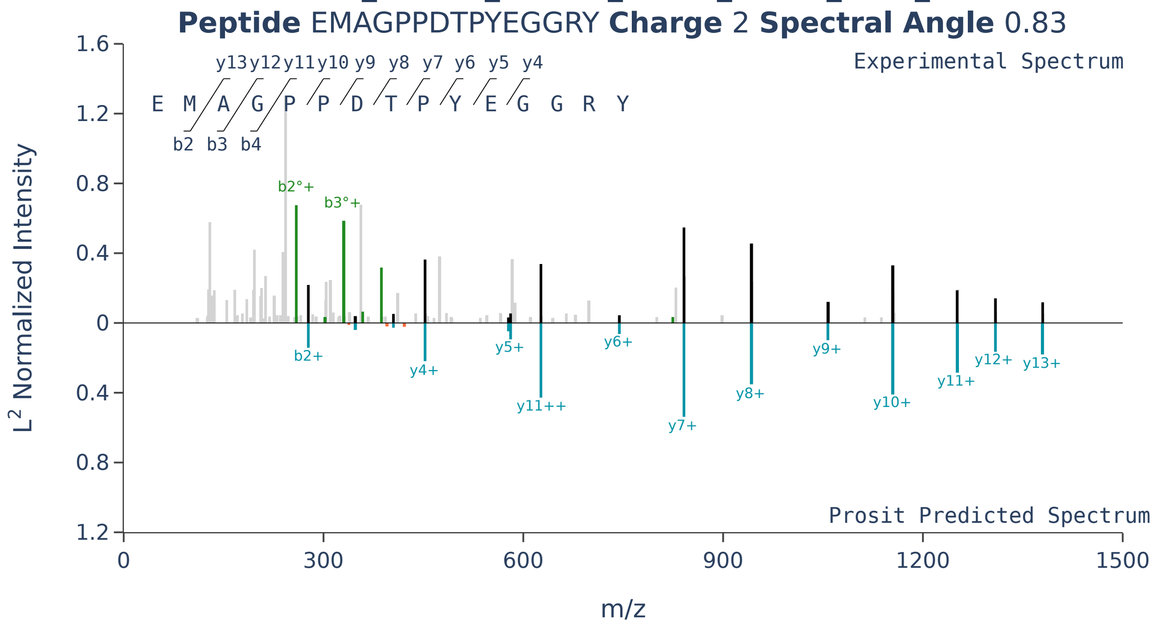

Source HRoetschke_271222_100123_HFGoe_UBE2K_b1R2_24h **Scan** 11313
Peptide TKENFTELRG **Charge** 2 **Spectral Angle** 0.85

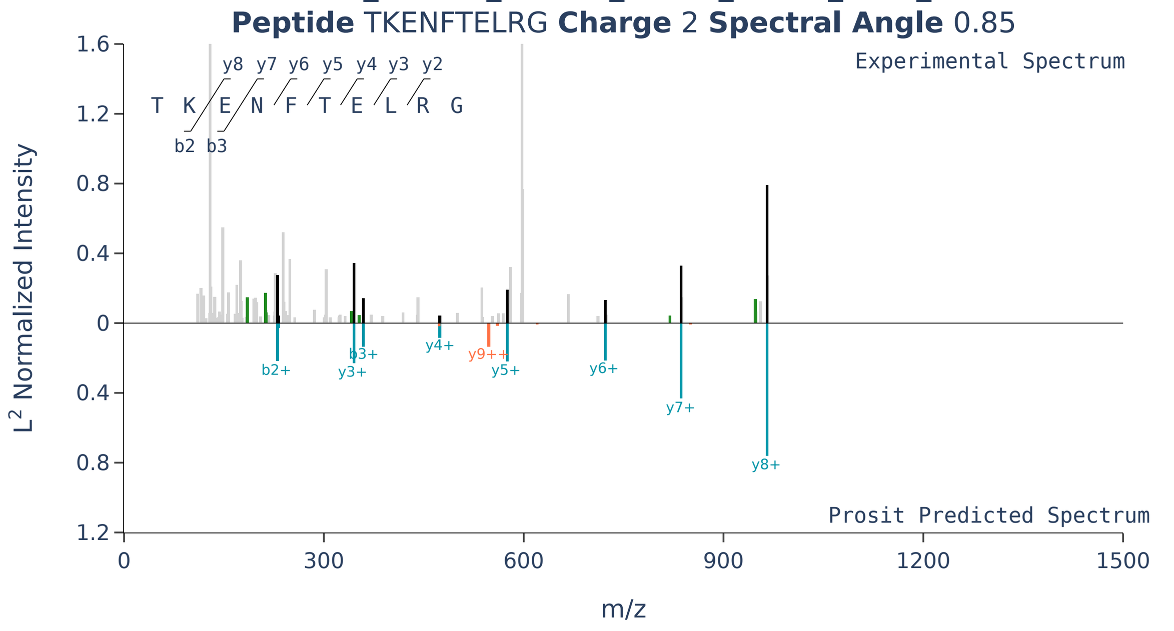

Source HRoetschke_271222_100123_HFGoe_UBE2K_b2R2_4h **Scan** 8251
Peptide VETARGEI **Charge** 2 **Spectral Angle** 0.89

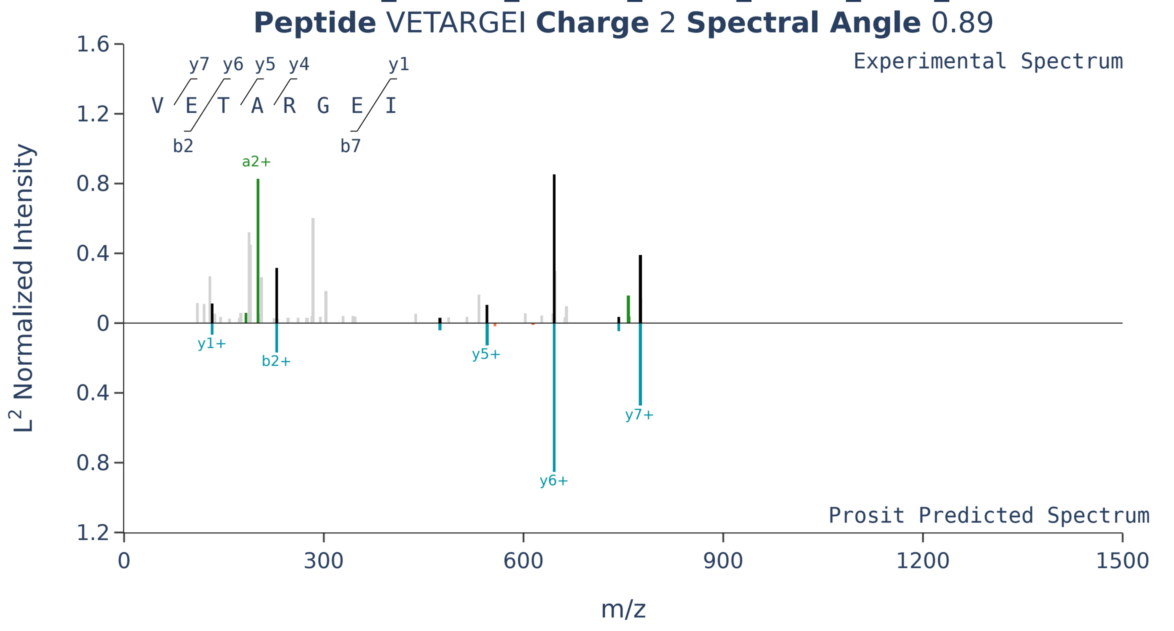

Source HRoetschke_271222_100123_HFGoe_UBE2K_b1R1_24h **Scan** 11219
Peptide SSKVDLVD **Charge** 2 **Spectral Angle** 0.83

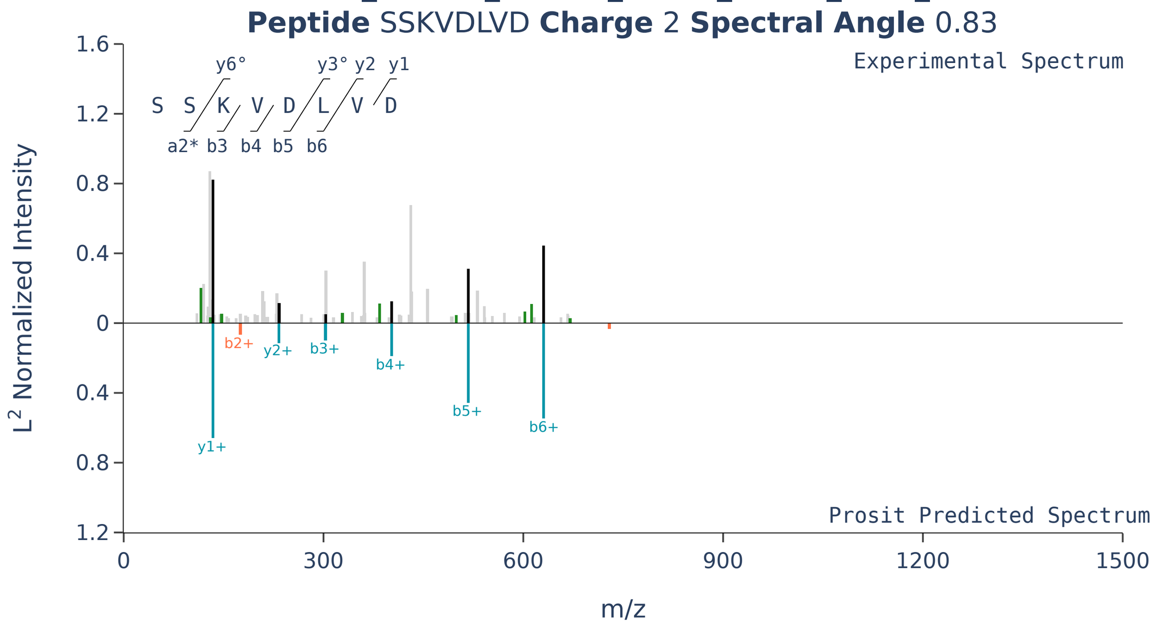

Source HRoetschke_271222_100123_HFGoe_UBE2K_b1R2_24h **Scan** 5853
Peptide RGAAAEPDDPQD **Charge** 2 **Spectral Angle** 0.95

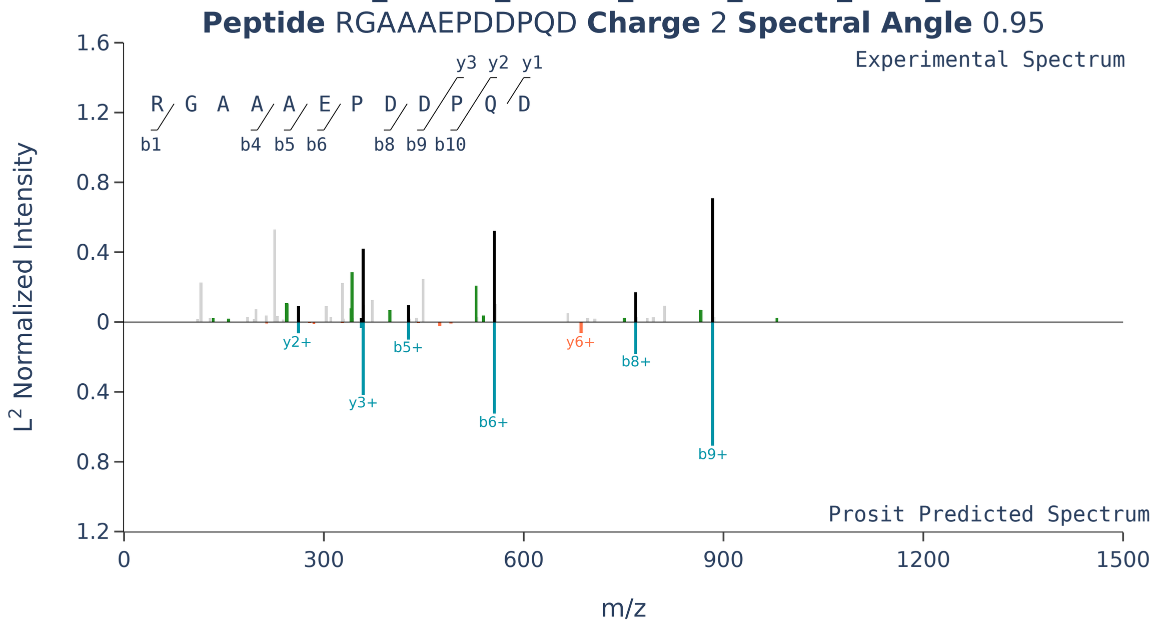

Source HRoetschke_271222_100123_HFGoe_UBE2K_b1R2_24h **Scan** 13376
Peptide DQYKQNPEMFKQTA **Charge** 2 **Spectral Angle** 0.92

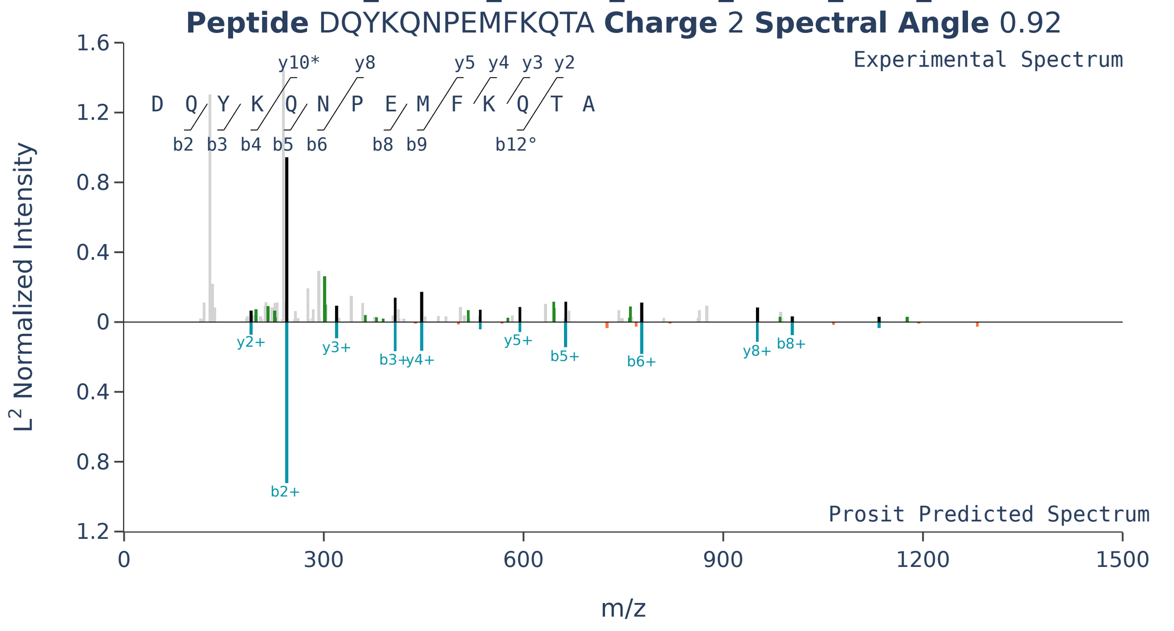

Source HRoetschke_271222_100123_HFGoe_UBE2K_b2R1_24h **Scan** 13771
Peptide ENFTELRQD **Charge** 2 **Spectral Angle** 0.83

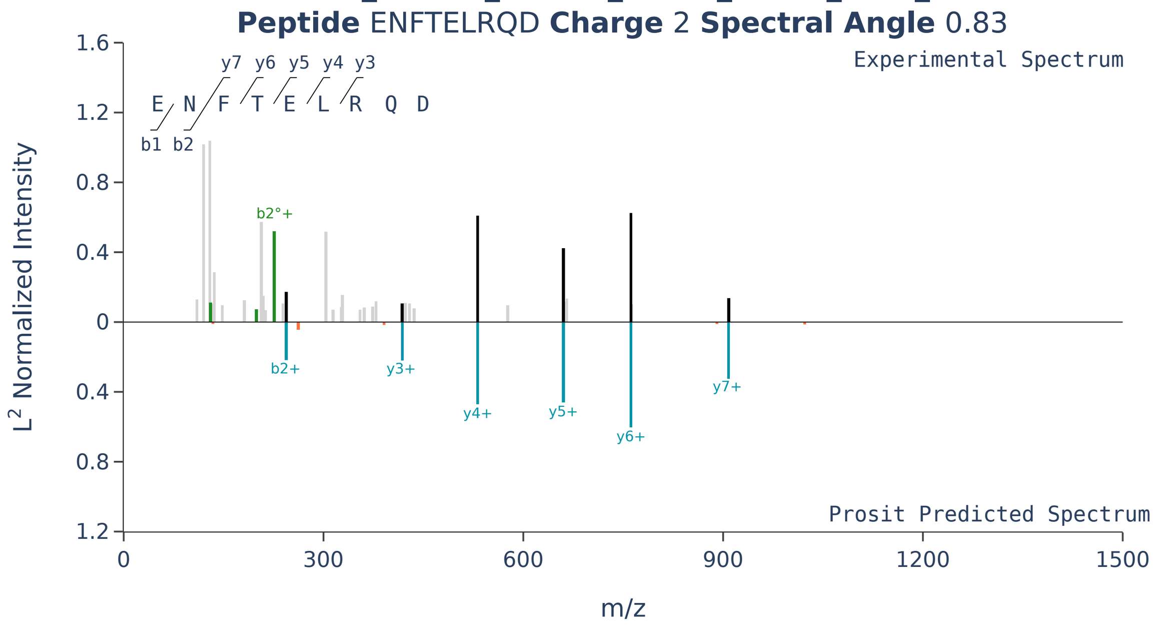

Source HRoetschke_271222_100123_HFGoe_UBE2K_b1R2_24h **Scan** 16577
Peptide SSEIKIPET **Charge** 2 **Spectral Angle** 0.82

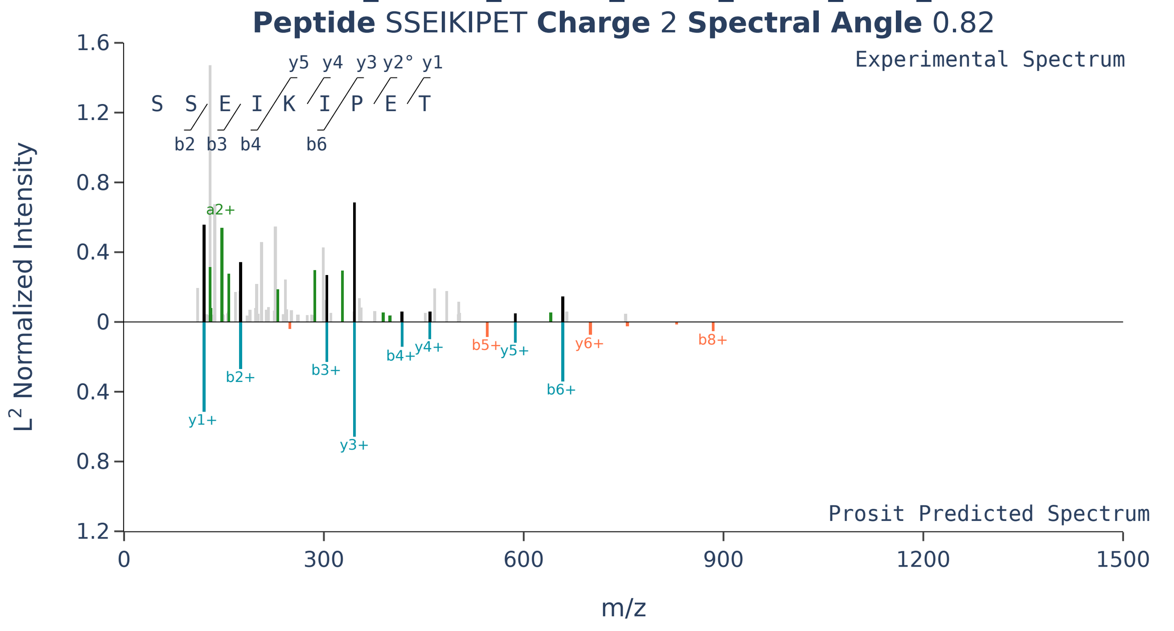

Source HRoetschke_271222_100123_HFGoe_UBE2K_b1R2_24h **Scan** 9235
Peptide SSKSWDVETRG **Charge** 3 **Spectral Angle** 0.92

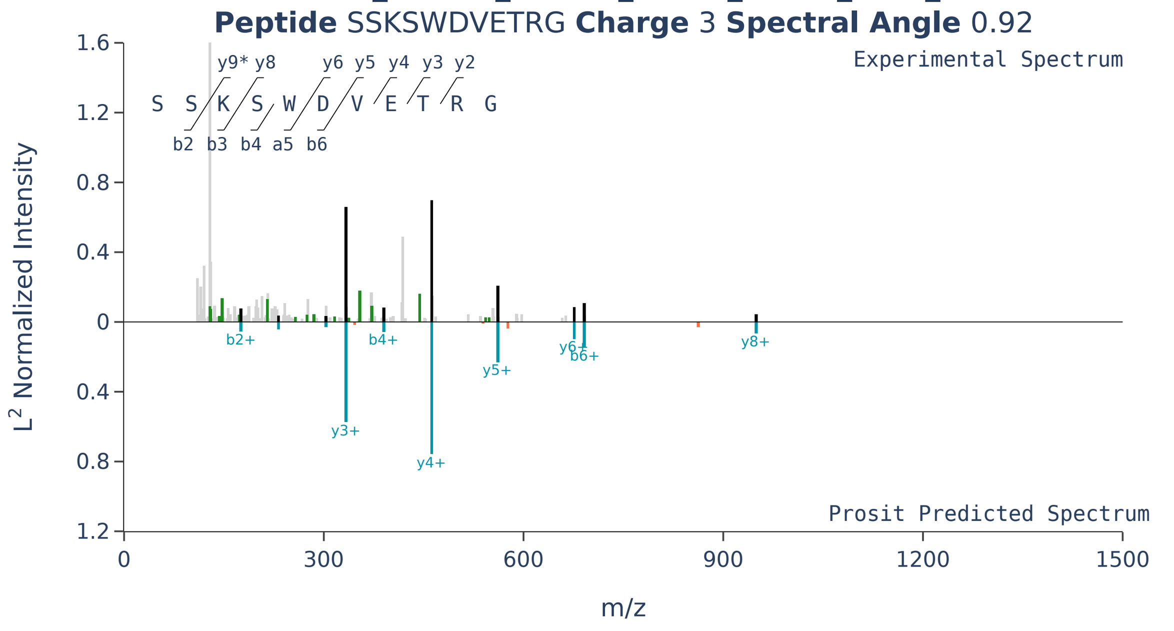

Source HRoetschke_271222_100123_HFGoe_UBE2K_b1R2_24h **Scan** 14938
Peptide ENFTELRGTE **Charge** 2 **Spectral Angle** 0.81

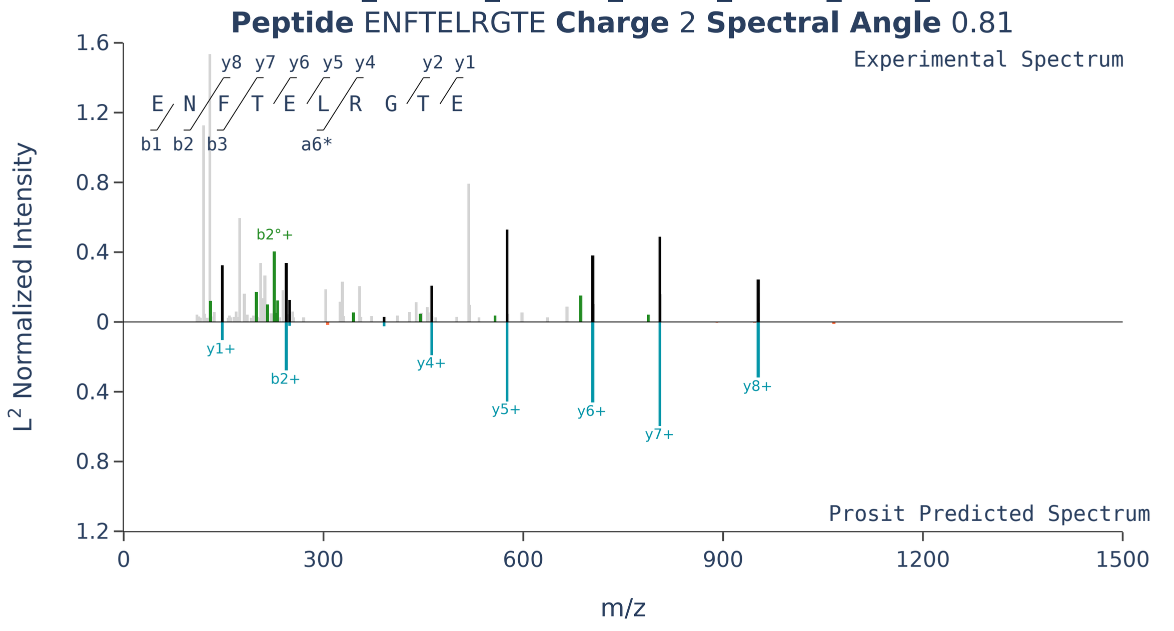

Source HRoetschke_271222_100123_HFGoe_UBE2K_b1R1_24h **Scan** 10483
Peptide ENFTELRGH **Charge** 3 **Spectral Angle** 0.86

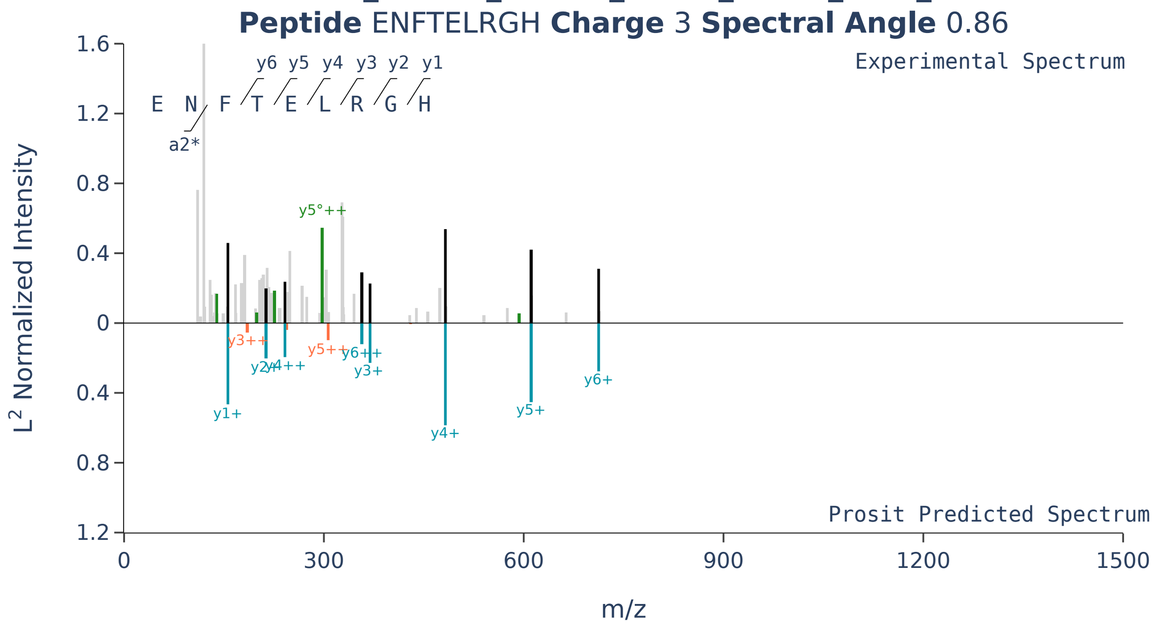

Source HRoetschke_271222_100123_HFGoe_UBE2K_b2R1_24h **Scan** 9964
Peptide SSAMGFDRN **Charge** 2 **Spectral Angle** 0.9

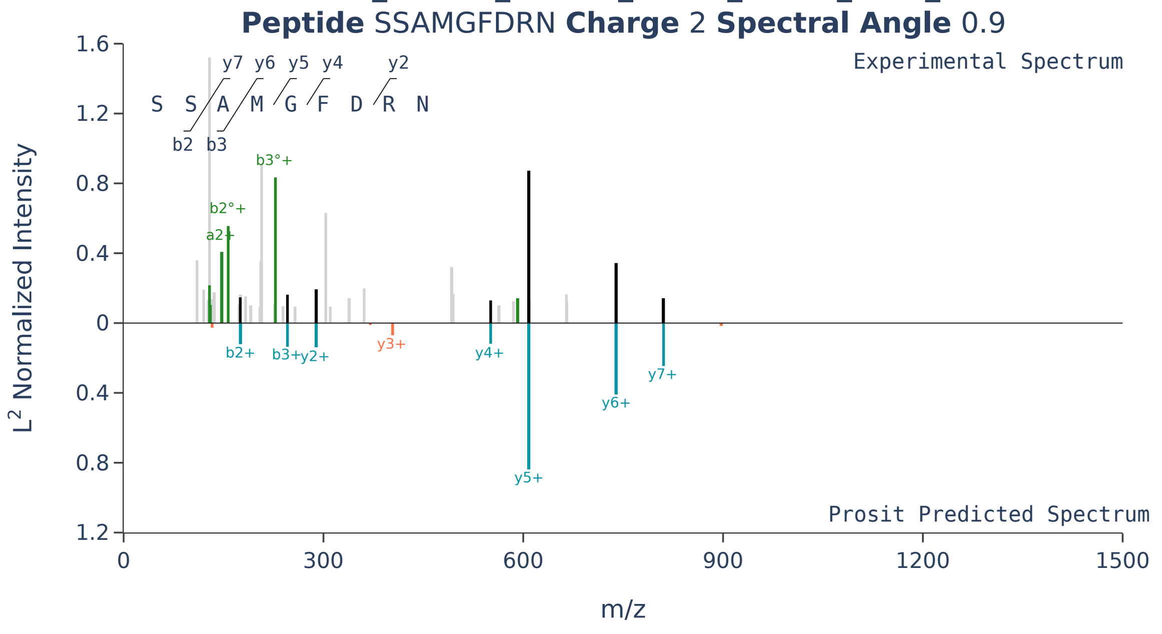

Source HRoetschke_271222_100123_HFGoe_UBE2K_b1R2_24h **Scan** 9250
Peptide VYAGAPVSR **Charge** 2 **Spectral Angle** 0.82

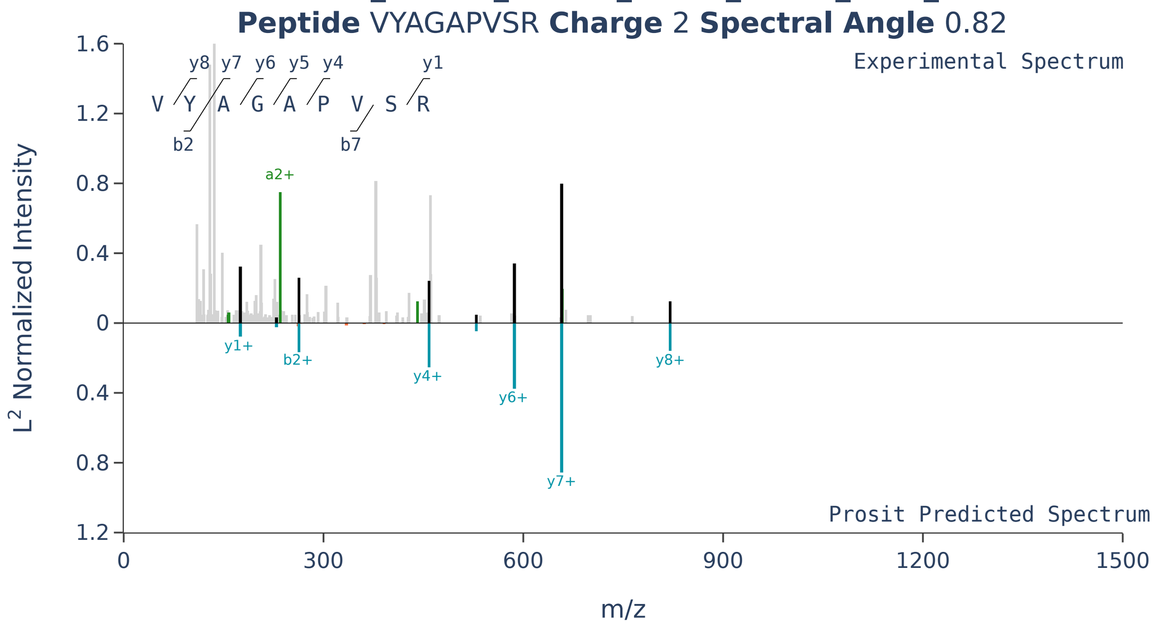

Source HRoetschke_271222_100123_HFGoe_UBE2K_b1R1_24h **Scan** 15353
Peptide EETSNIAVQRI **Charge** 2 **Spectral Angle** 0.9

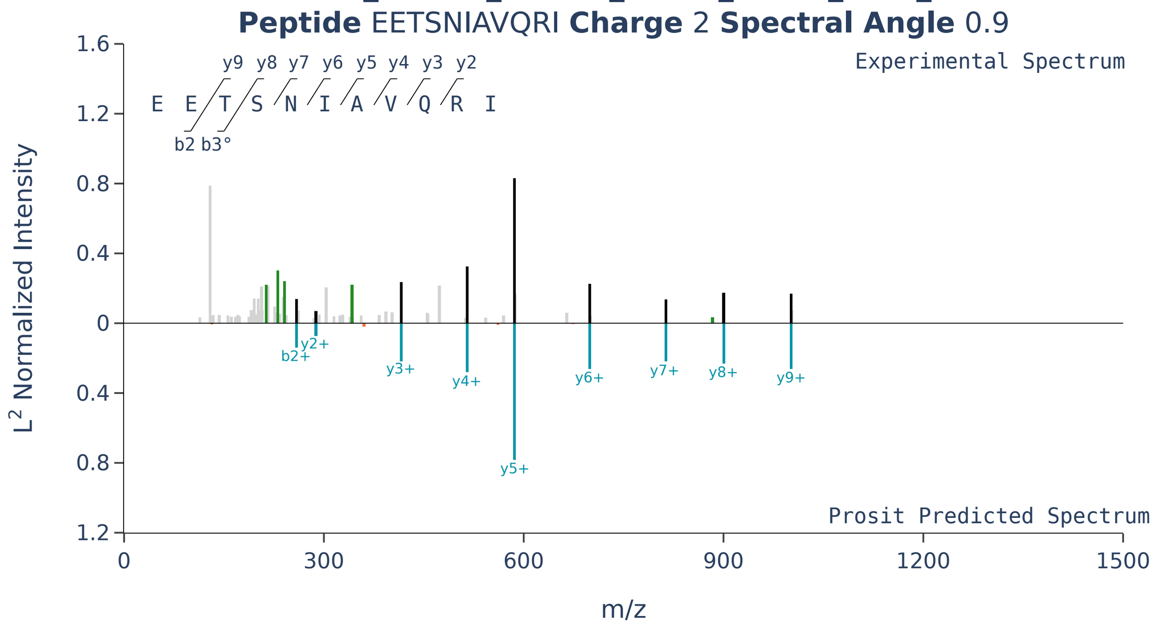

Source HRoetschke_271222_100123_HFGoe_UBE2K_b1R1_2h **Scan** 13470
Peptide ENFTELREGG **Charge** 2 **Spectral Angle** 0.85

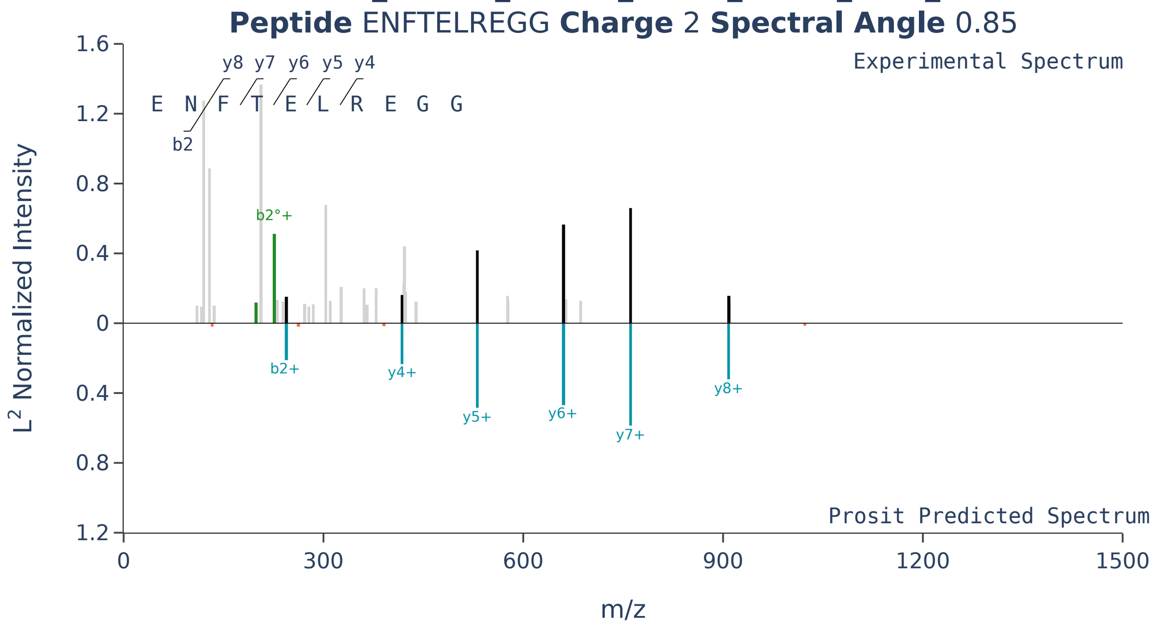

Source HRoetschke_271222_100123_HFGoe_UBE2K_b1R2_24h **Scan** 8342
Peptide HMANIAVQ **Charge** 1 **Spectral Angle** 0.85

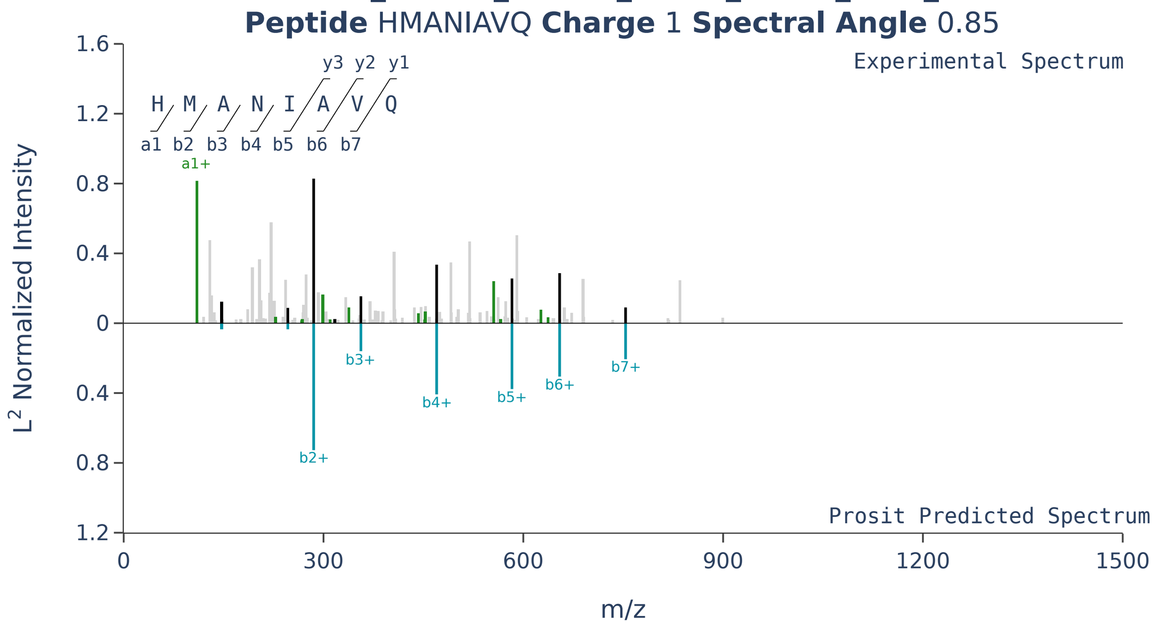

Source HRoetschke_271222_100123_HFGoe_UBE2K_b1R1_24h **Scan** 15743
Peptide EETNIAVQRI **Charge** 2 **Spectral Angle** 0.87

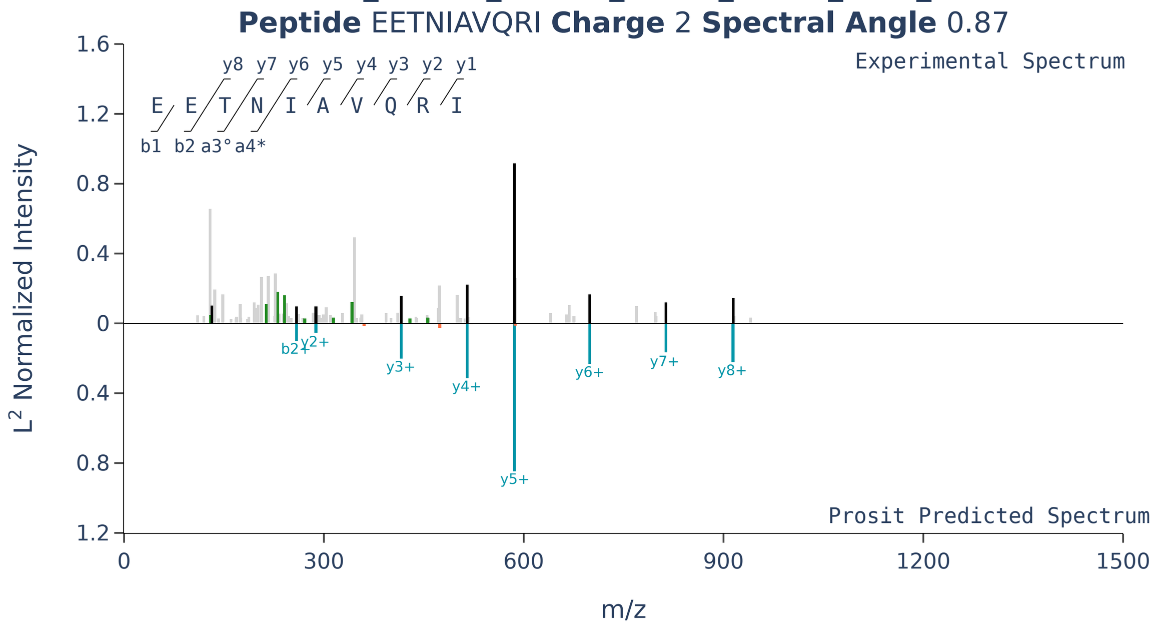

Source HRoetschke_271222_100123_HFGoe_UBE2K_b2R1_24h **Scan** 7872
Peptide HMANIAVQRIK **Charge** 3 **Spectral Angle** 0.89

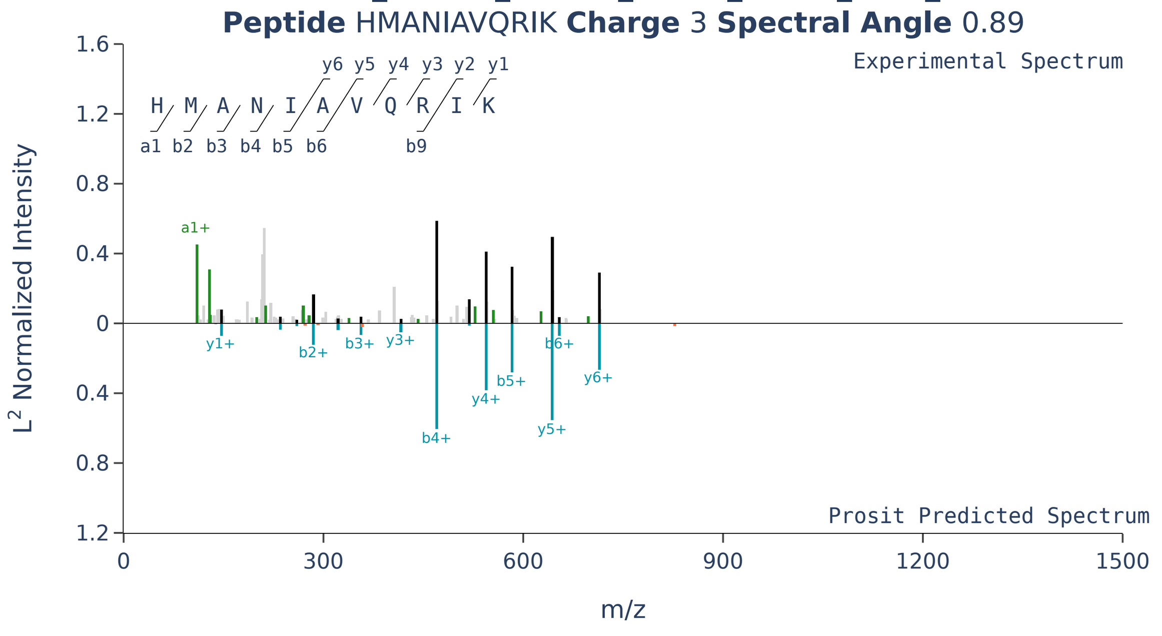

Source HRoetschke_271222_100123_HFGoe_UBE2K_b2R2_4h **Scan** 6717
Peptide HMANIAVQRIKRE **Charge** 4 **Spectral Angle** 0.84

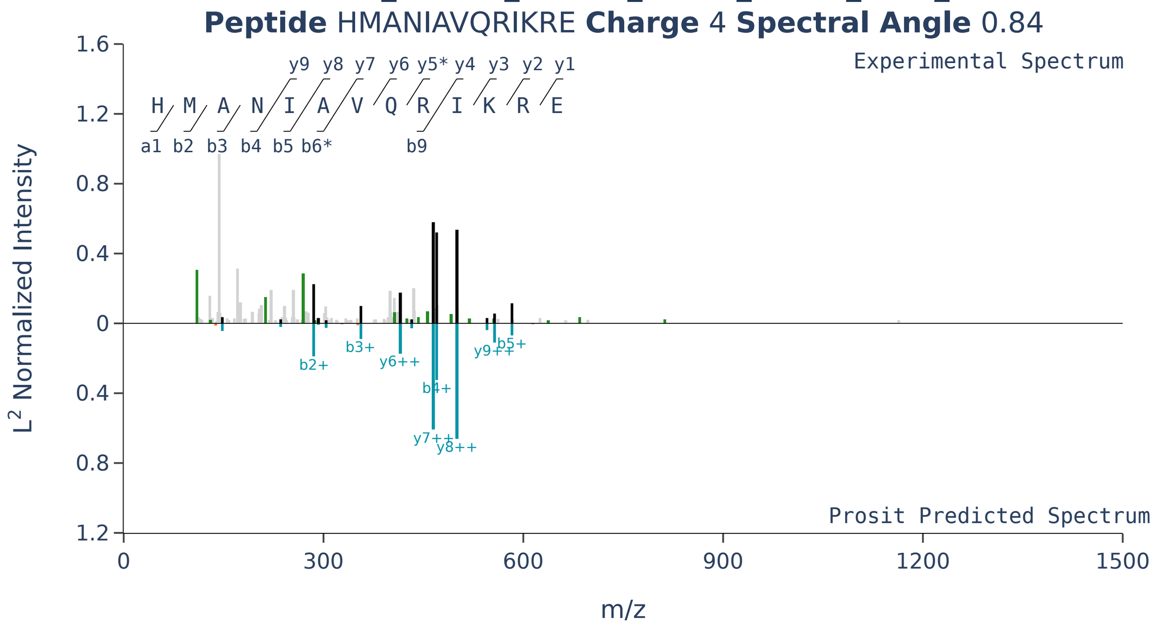

Source HRoetschke_271222_100123_HFGoe_UBE2K_b1R2_24h **Scan** 8202
Peptide VETLRGE **Charge** 2 **Spectral Angle** 0.88

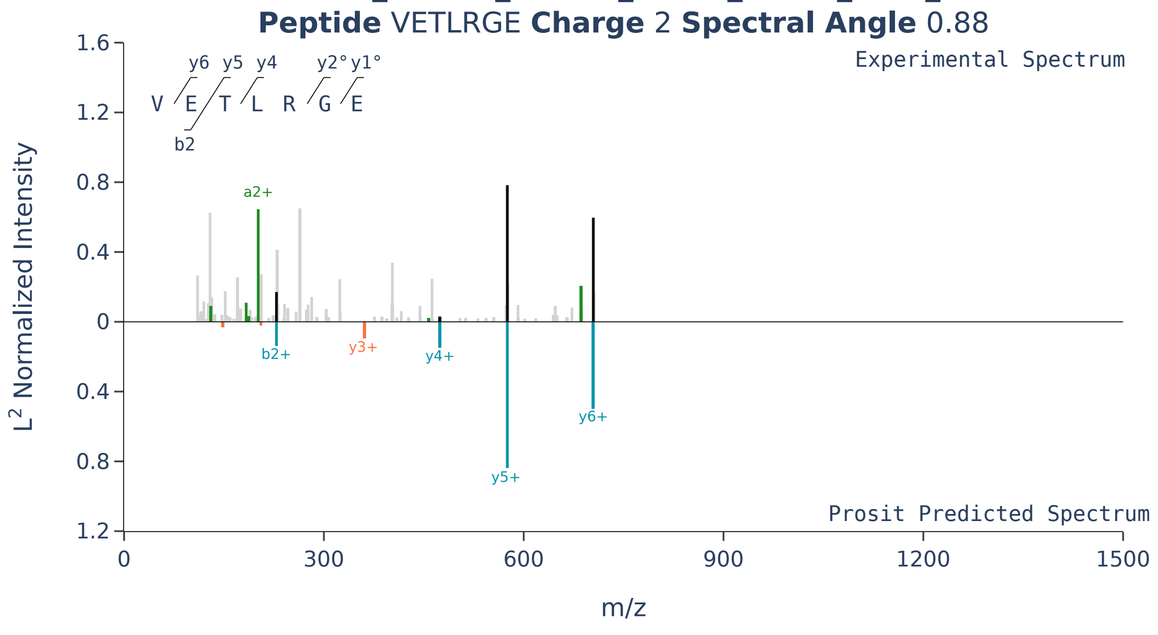

Source HRoetschke_271222_100123_HFGoe_UBE2K_b1R2_24h **Scan** 7215
Peptide AGAPVDT **Charge** 1 **Spectral Angle** 0.75

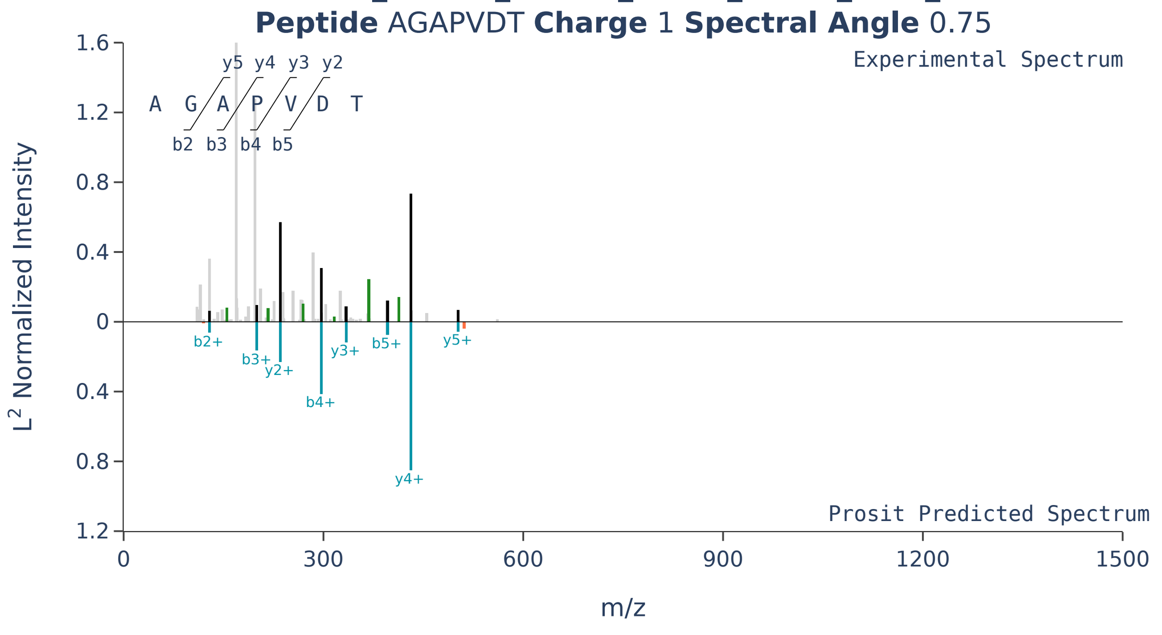

Source HRoetschke_271222_100123_HFGoe_UBE2K_b1R2_24h **Scan** 16287
Peptide LSSKSWDVETAE **Charge** 2 **Spectral Angle** 0.85

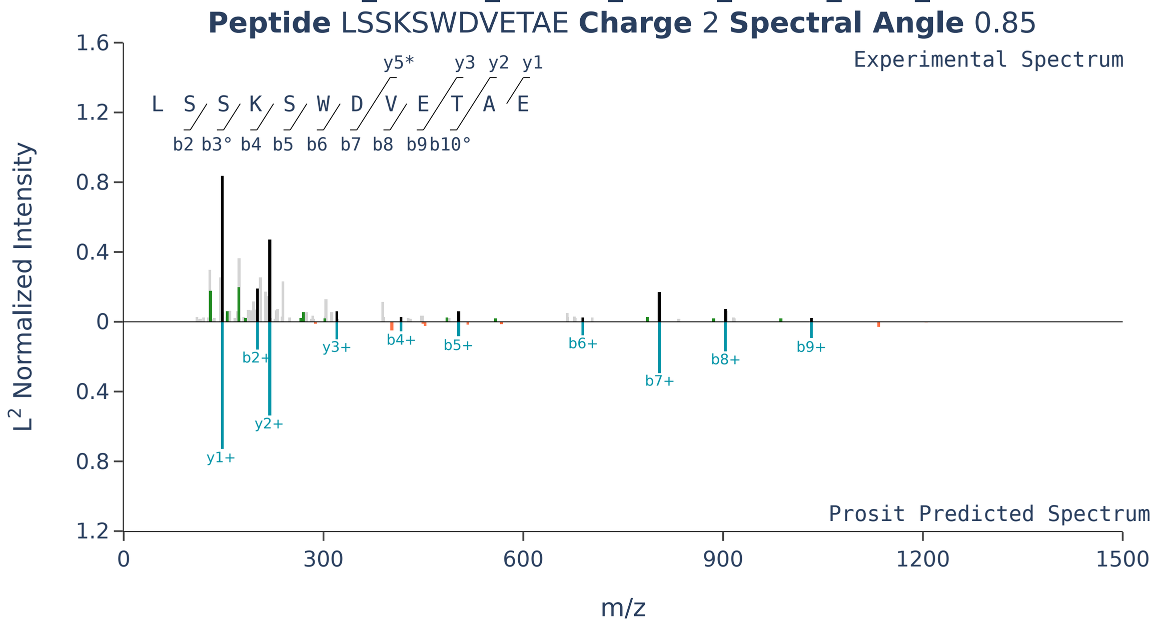

Source HRoetschke_271222_100123_HFGoe_UBE2K_b2R1_2h **Scan** 19309
Peptide ETALLLSN **Charge** 1 **Spectral Angle** 0.81

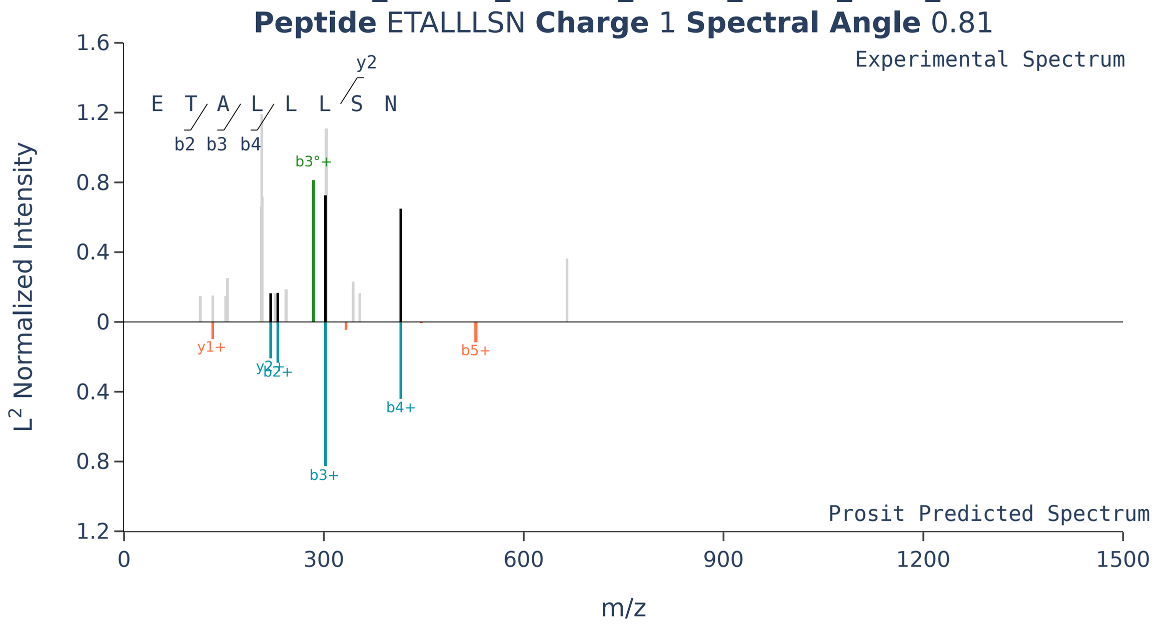

Source HRoetschke_271222_100123_HFGoe_UBE2K_b1R2_24h **Scan** 19735
Peptide SSEIKIPETY **Charge** 2 **Spectral Angle** 0.85

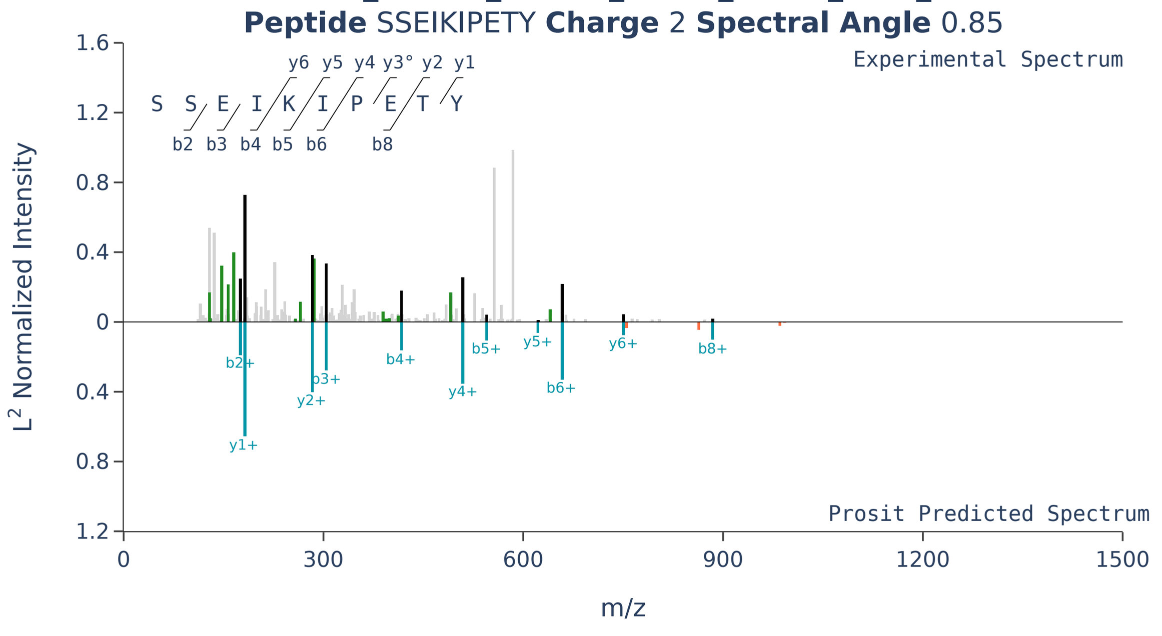

Source HRoetschke_271222_100123_HFGoe_UBE2K_b1R2_4h **Scan** 20005
Peptide TELLLTARL **Charge** 2 **Spectral Angle** 0.78

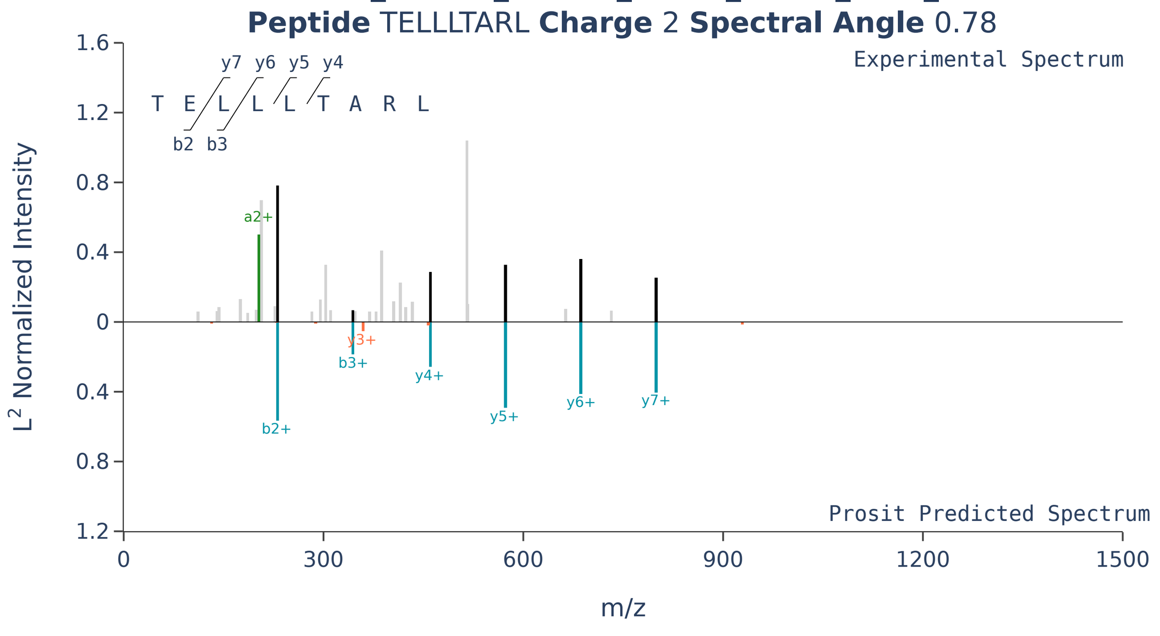

Source HRoetschke_271222_100123_HFGoe_UBE2K_b1R2_24h **Scan** 10521
Peptide TGDILKD **Charge** 2 **Spectral Angle** 0.85

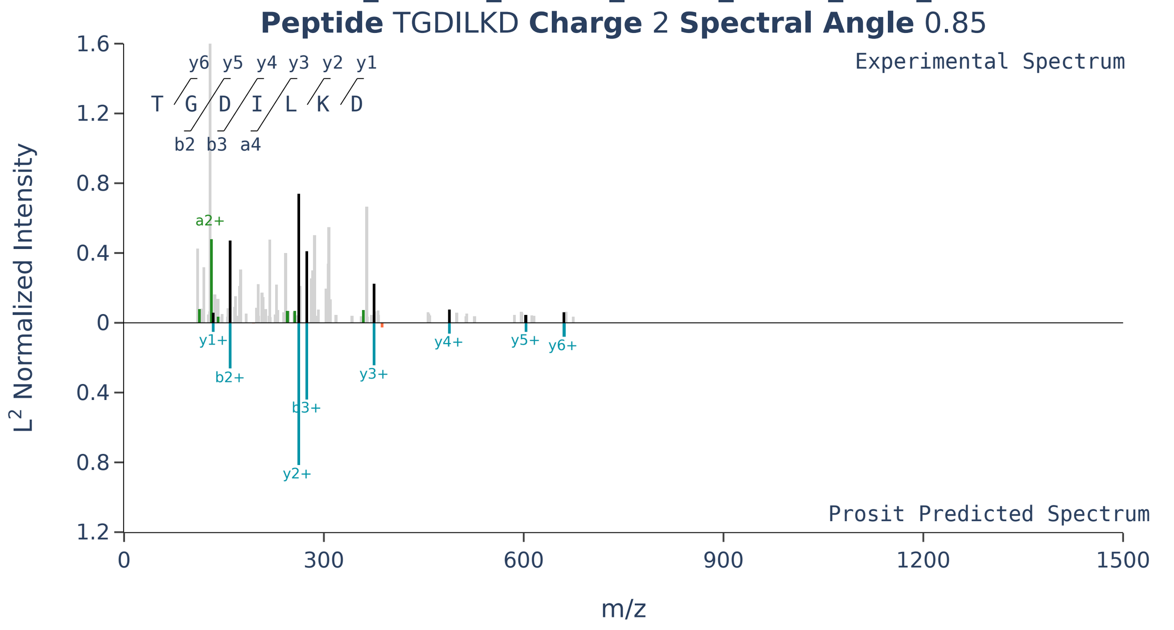

Source HRoetschke_271222_100123_HFGoe_UBE2K_b1R2_24h **Scan** 13896
Peptide AAGPPDTPYEGGRY **Charge** 2 **Spectral Angle** 0.86

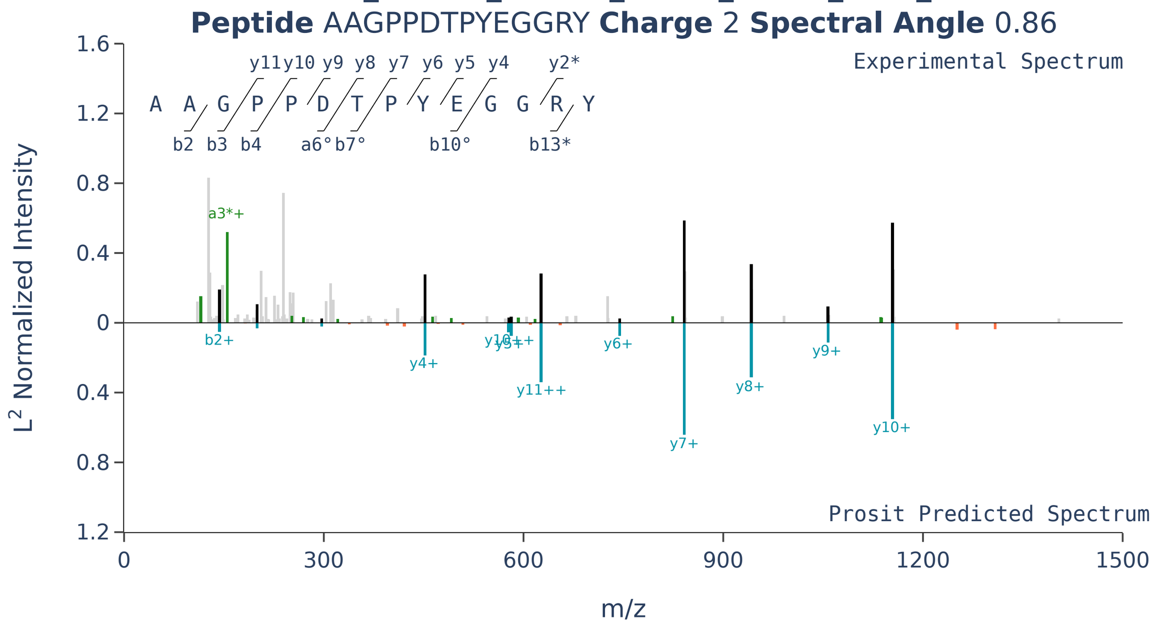

Source HRoetschke_271222_100123_HFGoe_UBE2K_b1R2_24h **Scan** 16628
Peptide ENFTELRDA **Charge** 2 **Spectral Angle** 0.85

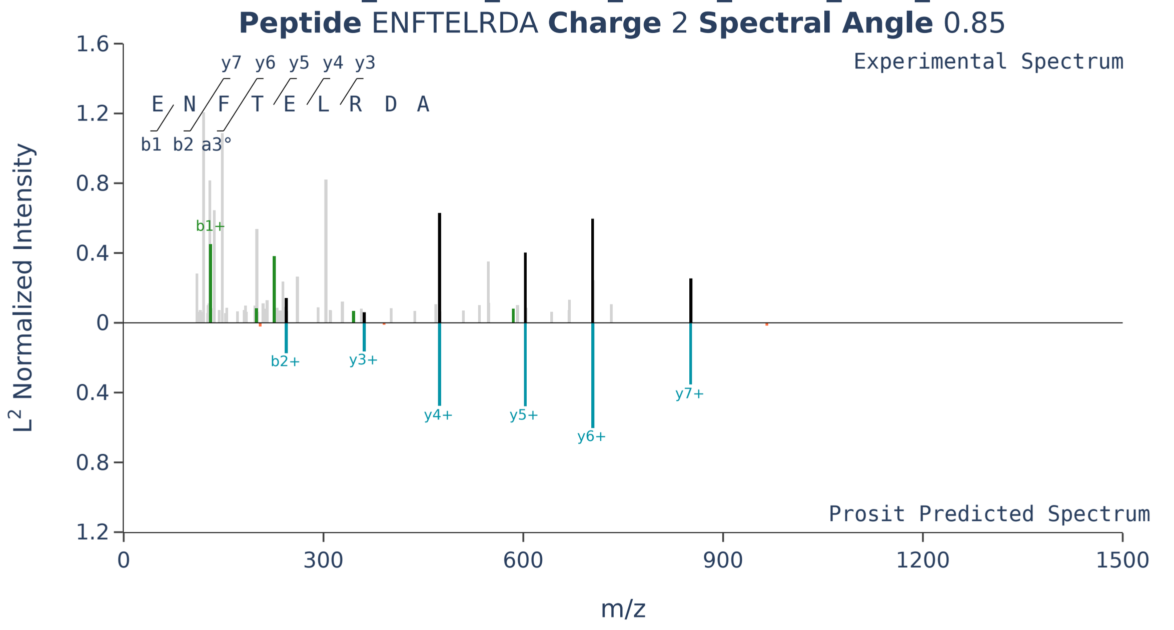

Source HRoetschke_271222_100123_HFGoe_UBE2K_b2R1_4h **Scan** 14402
Peptide ENFTETELRG **Charge** 2 **Spectral Angle** 0.8

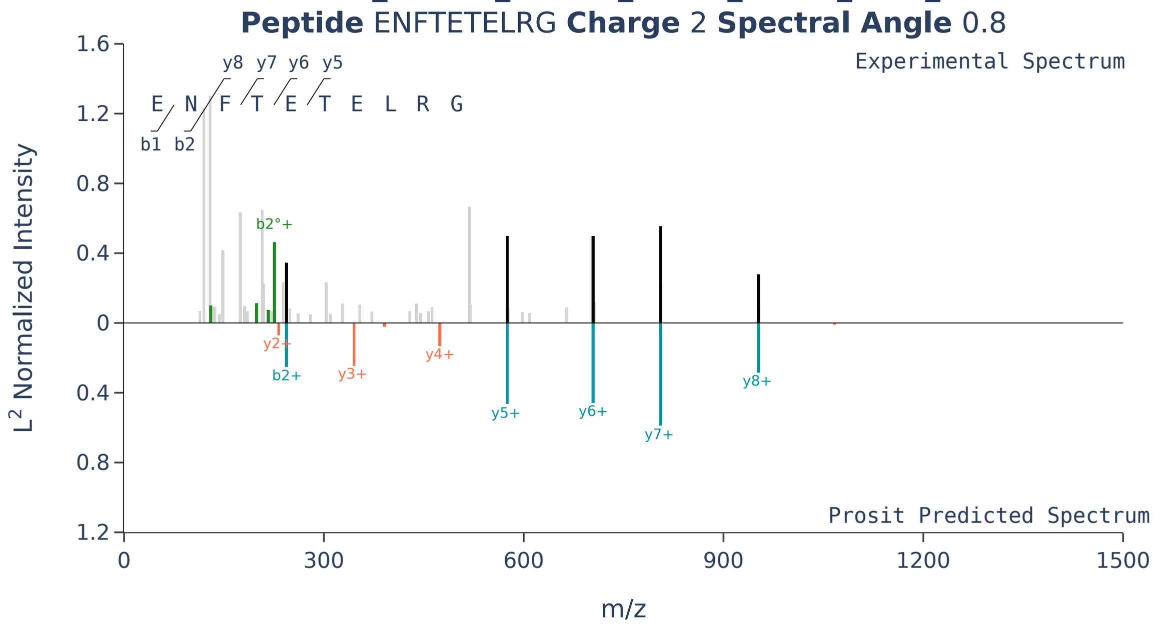

Source HRoetschke_271222_100123_HFGoe_UBE2K_b1R2_24h **Scan** 15564
Peptide QANIAVQRI **Charge** 2 **Spectral Angle** 0.83

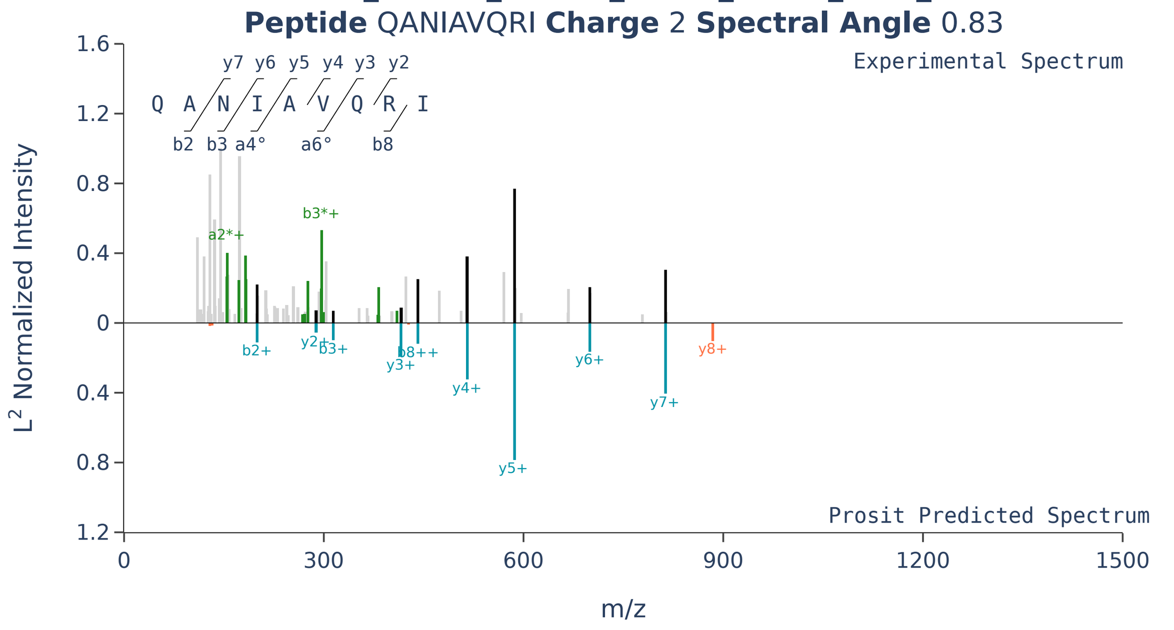

Source HRoetschke_271222_100123_HFGoe_UBE2K_b1R2_24h **Scan** 10731
Peptide DQYKQNPEMFKQTAR **Charge** 3 **Spectral Angle** 0.82

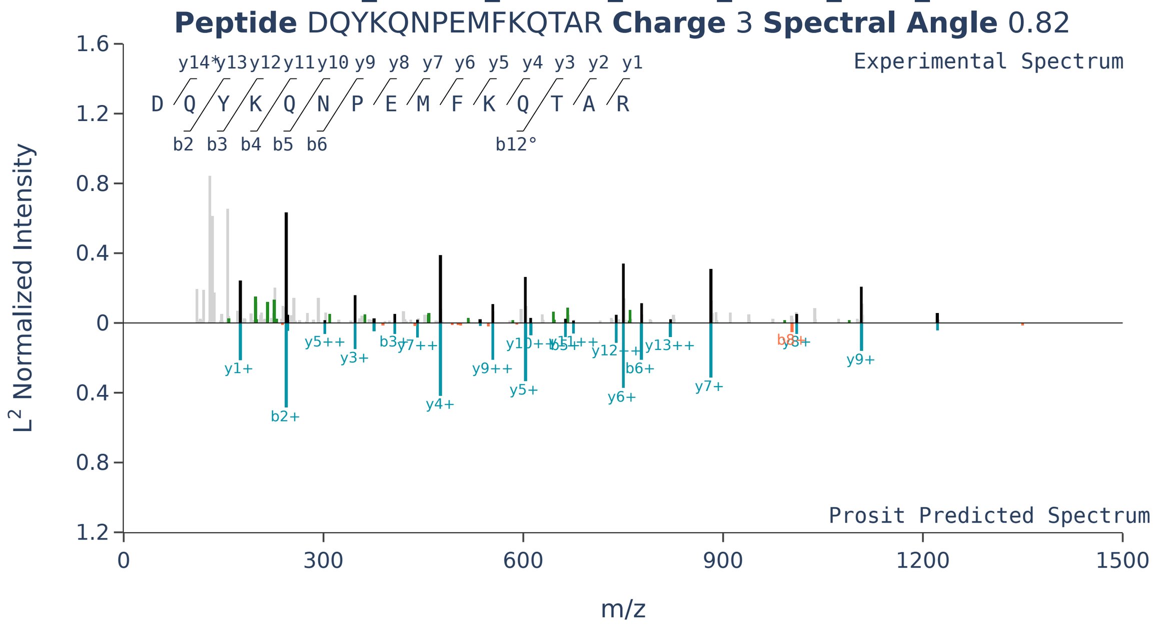

Source HRoetschke_271222_100123_HFGoe_UBE2K_b2R1_4h **Scan** 14950
Peptide KIENFTELRG **Charge** 2 **Spectral Angle** 0.75

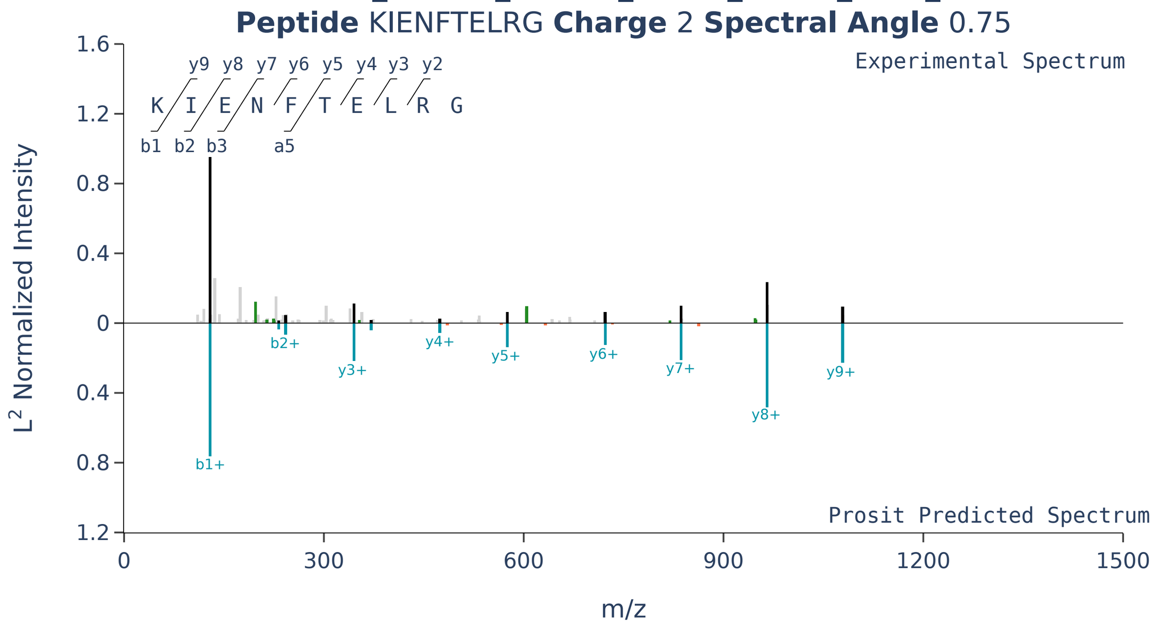

Source HRoetschke_271222_100123_HFGoe_UBE2K_b1R2_24h **Scan** 16463
Peptide RGEIKIPETY **Charge** 3 **Spectral Angle** 0.78

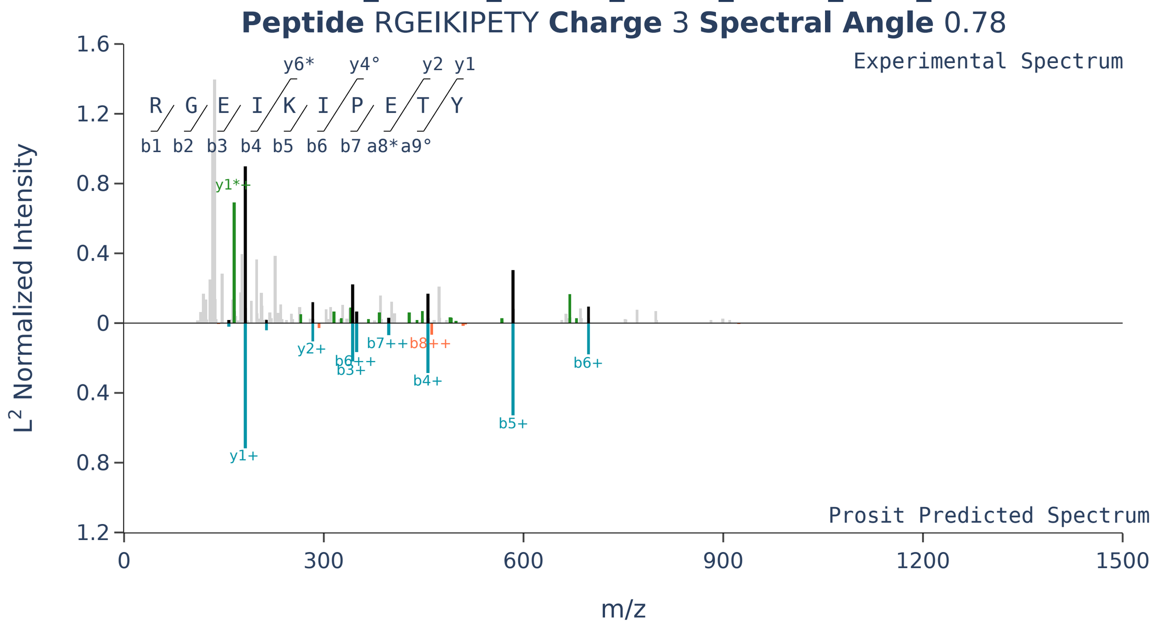

Source HRoetschke_271222_100123_HFGoe_UBE2K_b2R2_2h **Scan** 12689
Peptide ENFTELRGEE **Charge** 2 **Spectral Angle** 0.86

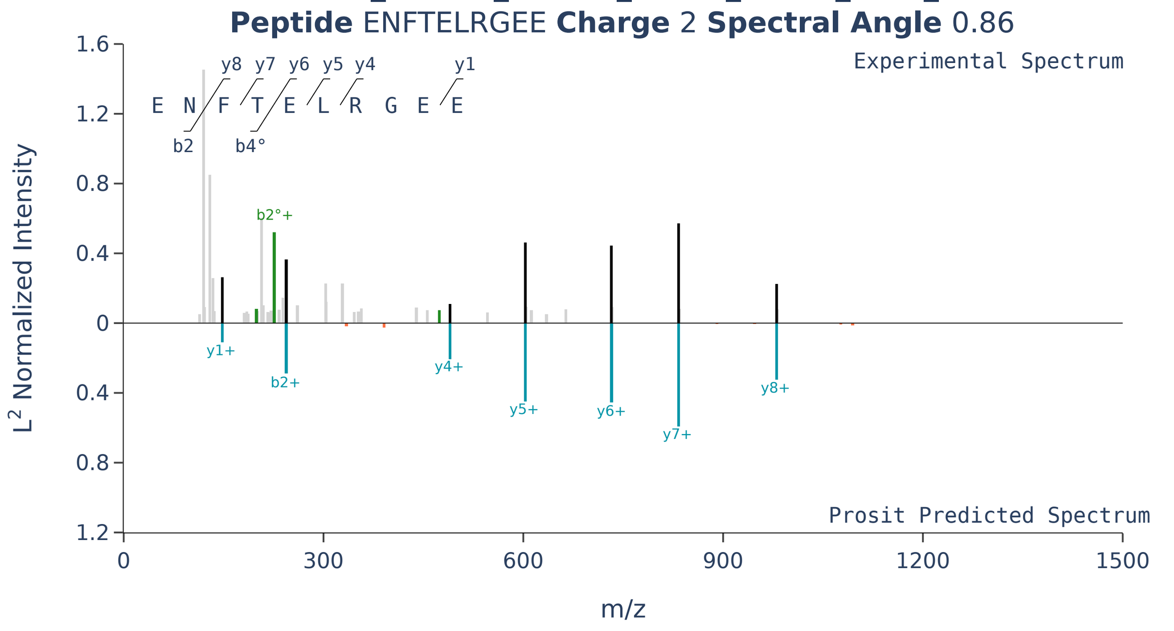

Source HRoetschke_271222_100123_HFGoe_UBE2K_b1R1_2h **Scan** 13581
Peptide ENFTELRGTG **Charge** 2 **Spectral Angle** 0.85

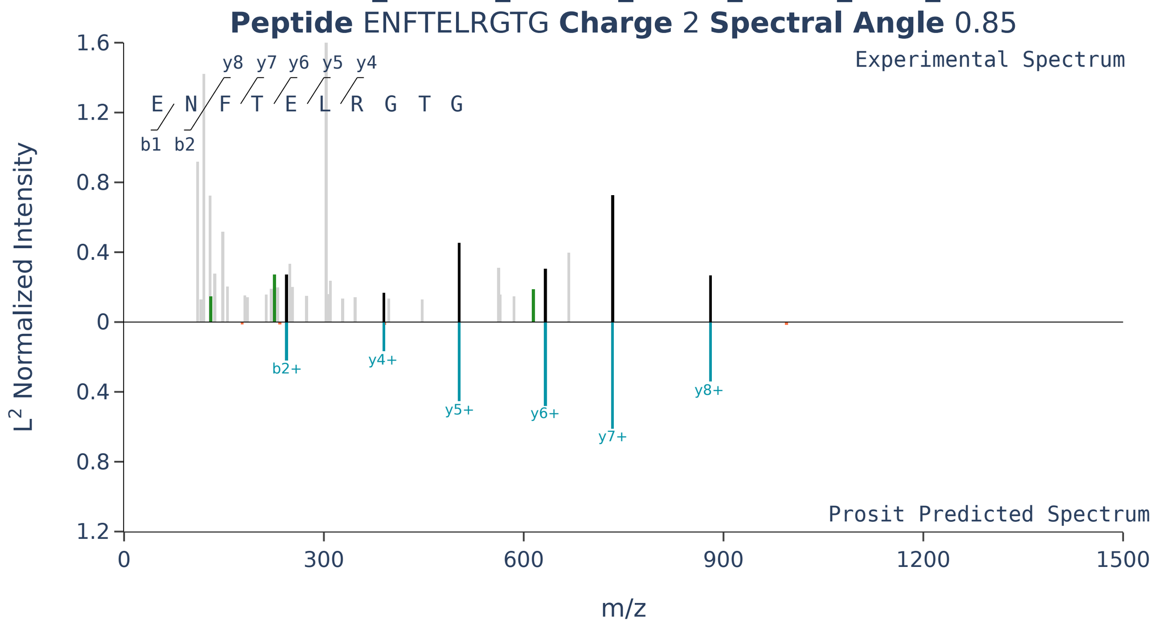

Source HRoetschke_271222_100123_HFGoe_UBE2K_b1R2_24h **Scan** 16442
Peptide TKENFTEL **Charge** 2 **Spectral Angle** 0.84

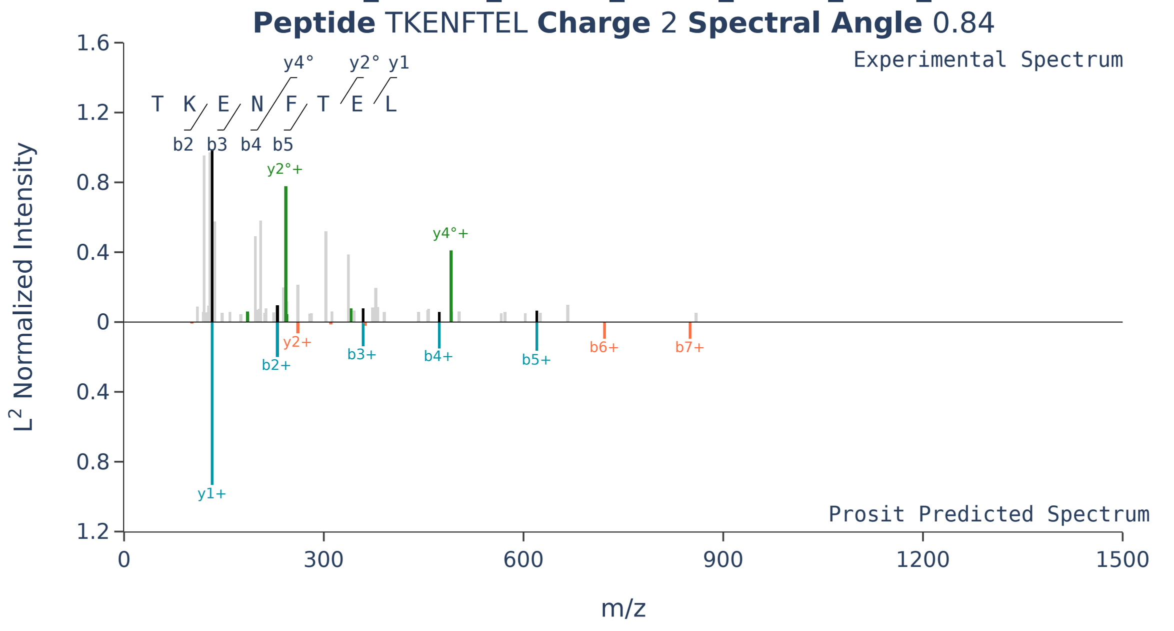

Source HRoetschke_271222_100123_HFGoe_UBE2K_b1R2_24h **Scan** 15696
Peptide RGEIAAGPPDTPYE **Charge** 2 **Spectral Angle** 0.78

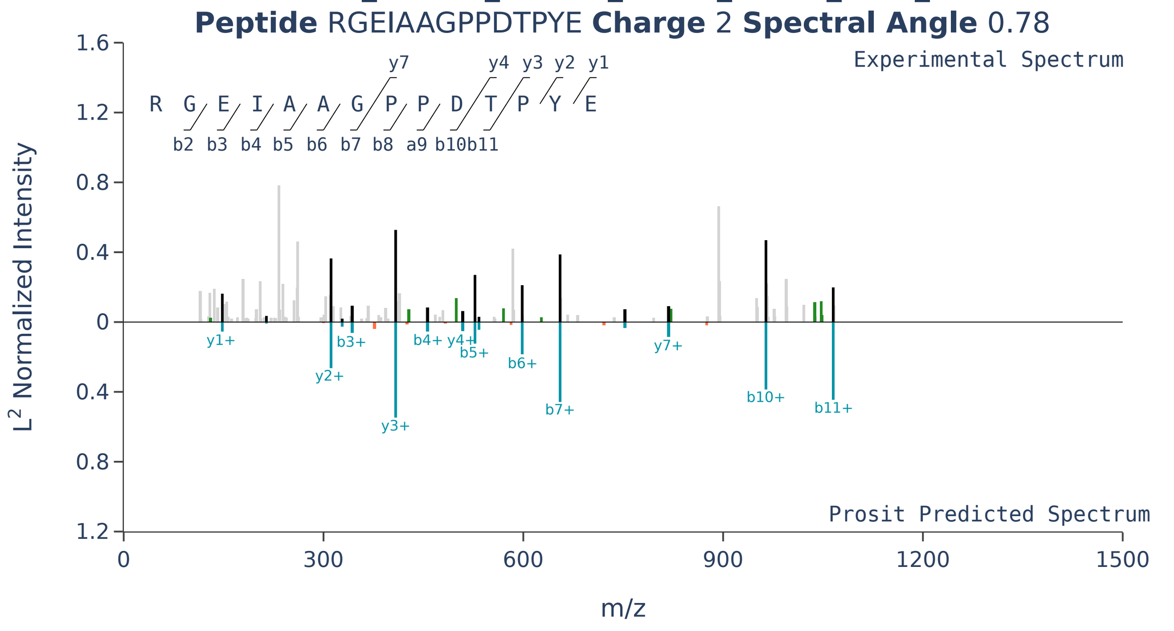

Source HRoetschke_271222_100123_HFGoe_UBE2K_b1R2_24h **Scan** 9161
Peptide GRKVDLVD **Charge** 2 **Spectral Angle** 0.87

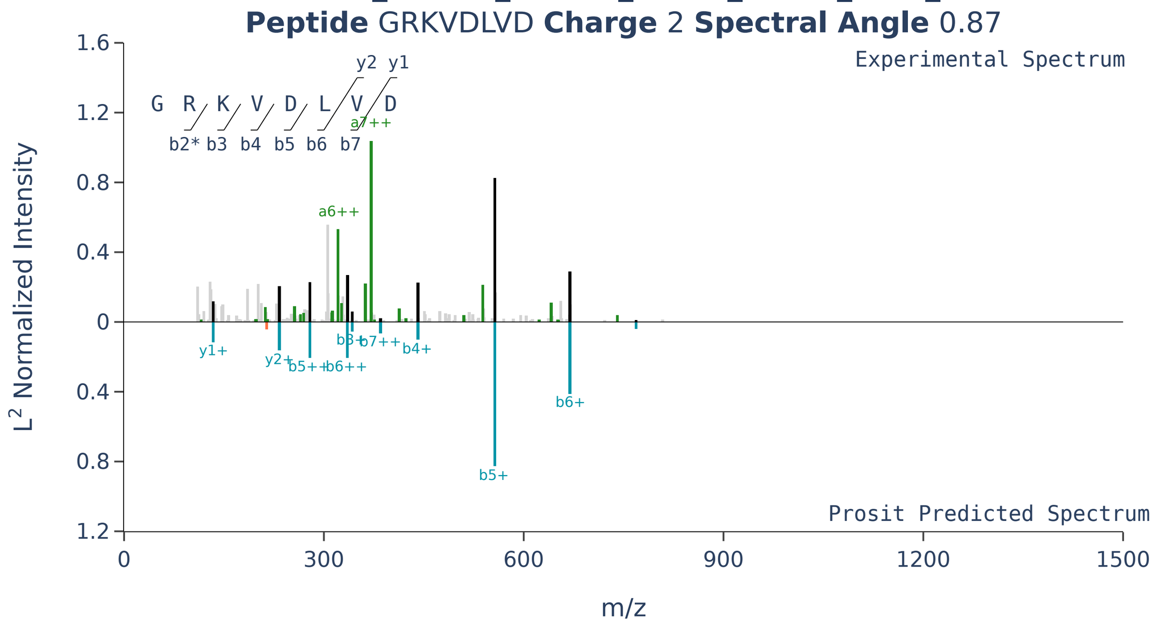

Source HRoetschke_271222_100123_HFGoe_UBE2K_b1R1_24h **Scan** 13605
Peptide GGANIAVQRI **Charge** 2 **Spectral Angle** 0.89

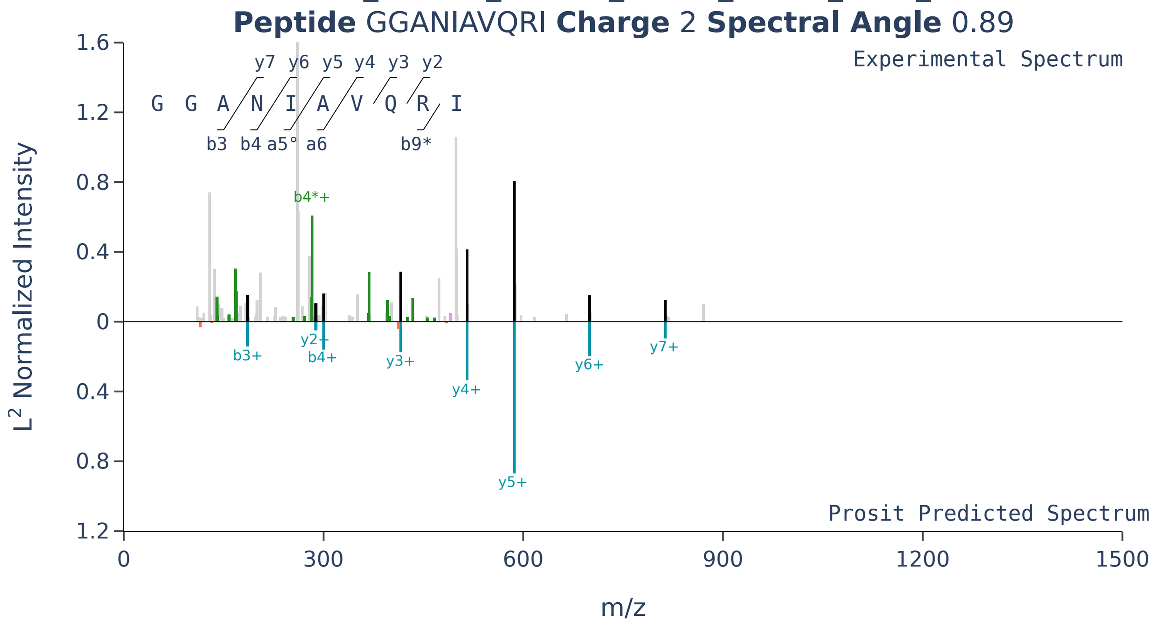

Source HRoetschke_271222_100123_HFGoe_UBE2K_b1R2_24h **Scan** 9193
Peptide SSKSWDVETR **Charge** 3 **Spectral Angle** 0.87

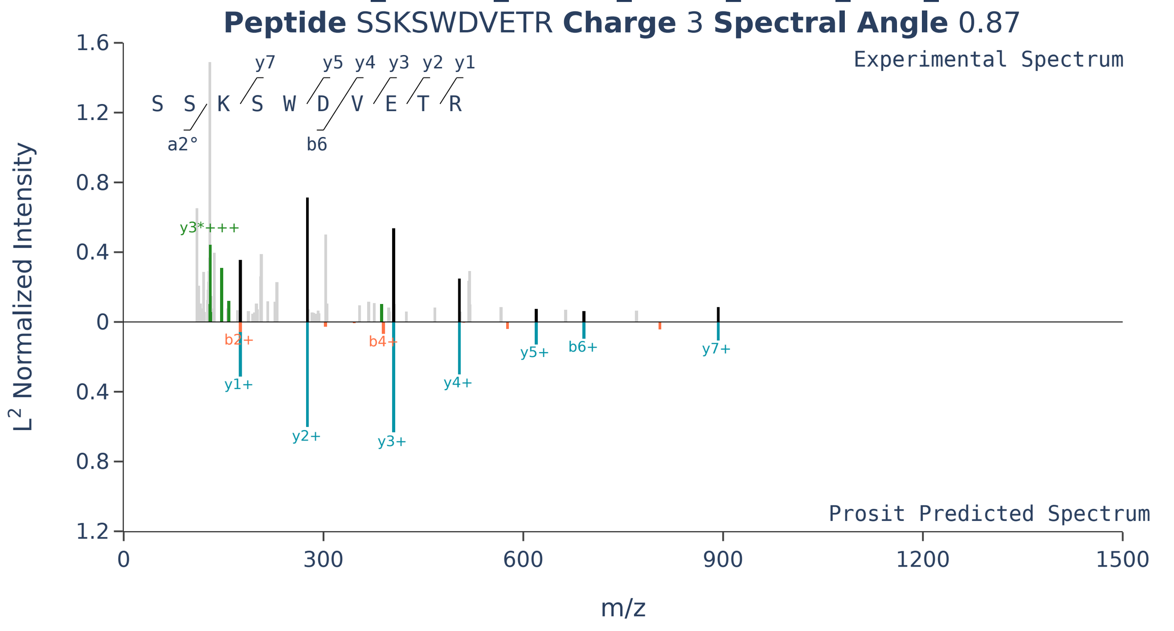

Source HRoetschke_271222_100123_HFGoe_UBE2K_b1R1_24h **Scan** 10875
Peptide TGKIPETY **Charge** 2 **Spectral Angle** 0.83

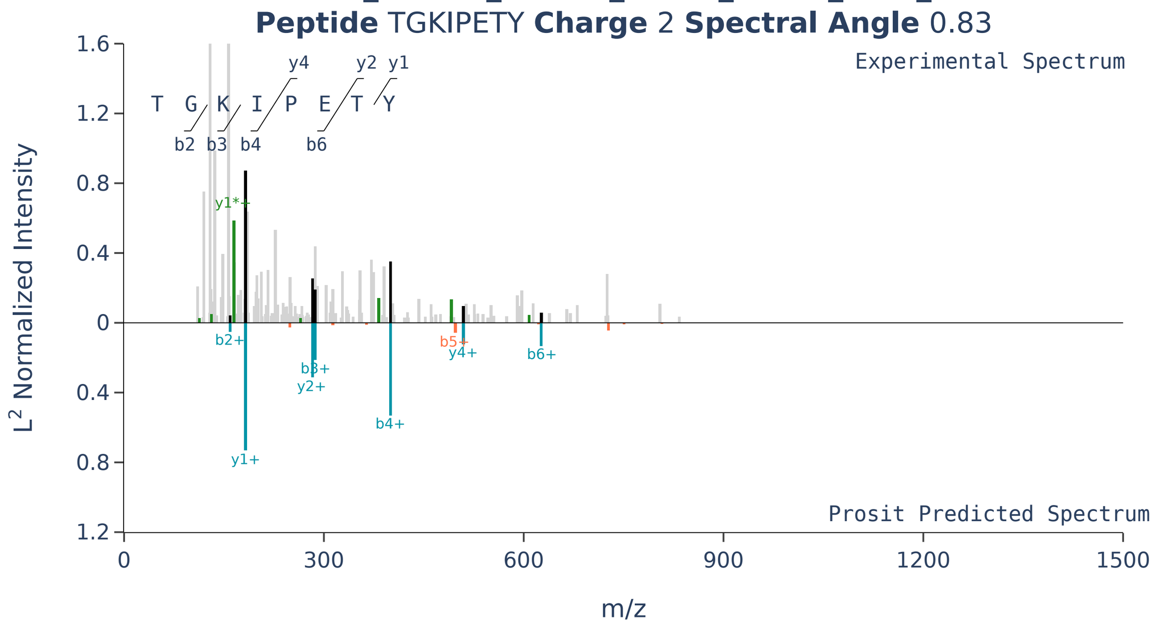

Source HRoetschke_271222_100123_HFGoe_UBE2K_b2R1_24h **Scan** 8891
Peptide RGAVVANQY **Charge** 2 **Spectral Angle** 0.81

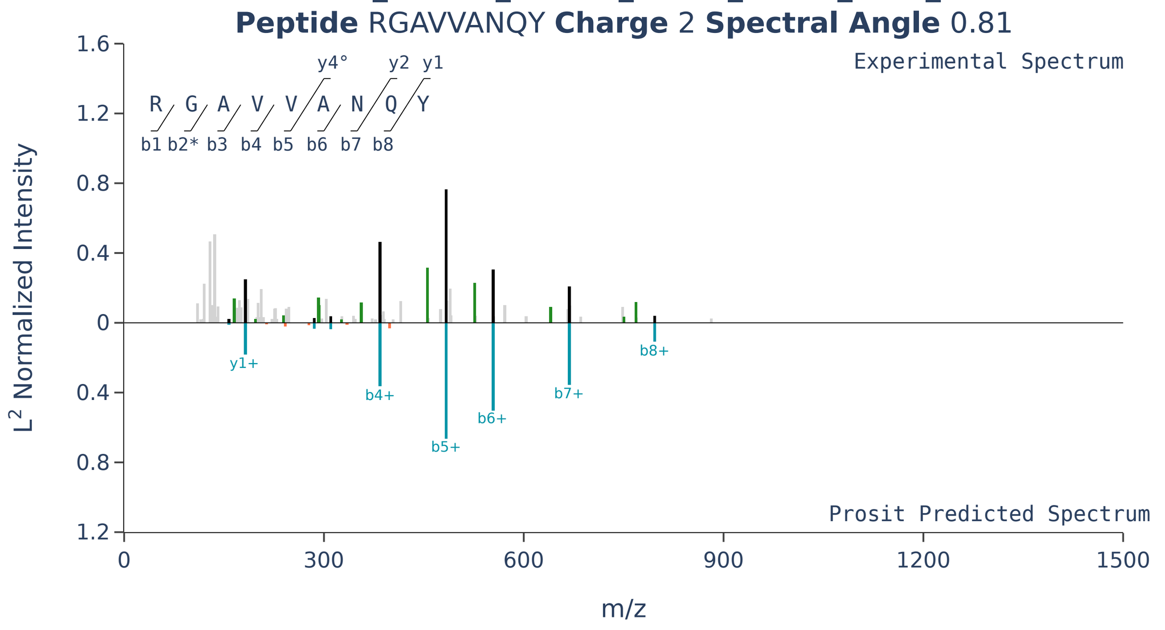

Source HRoetschke_271222_100123_HFGoe_UBE2K_b1R2_4h **Scan** 10312
Peptide AHVYAGAPVSE **Charge** 2 **Spectral Angle** 0.81

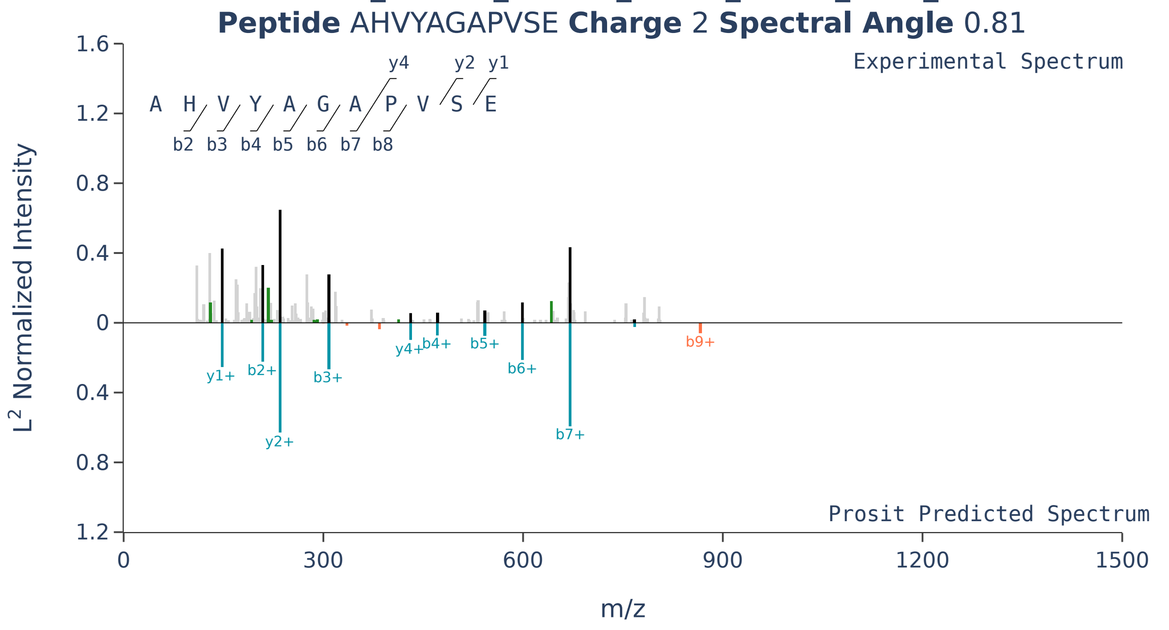

Source HRoetschke_271222_100123_HFGoe_UBE2K_b2R2_24h **Scan** 7658
Peptide VETRGEI **Charge** 2 **Spectral Angle** 0.94

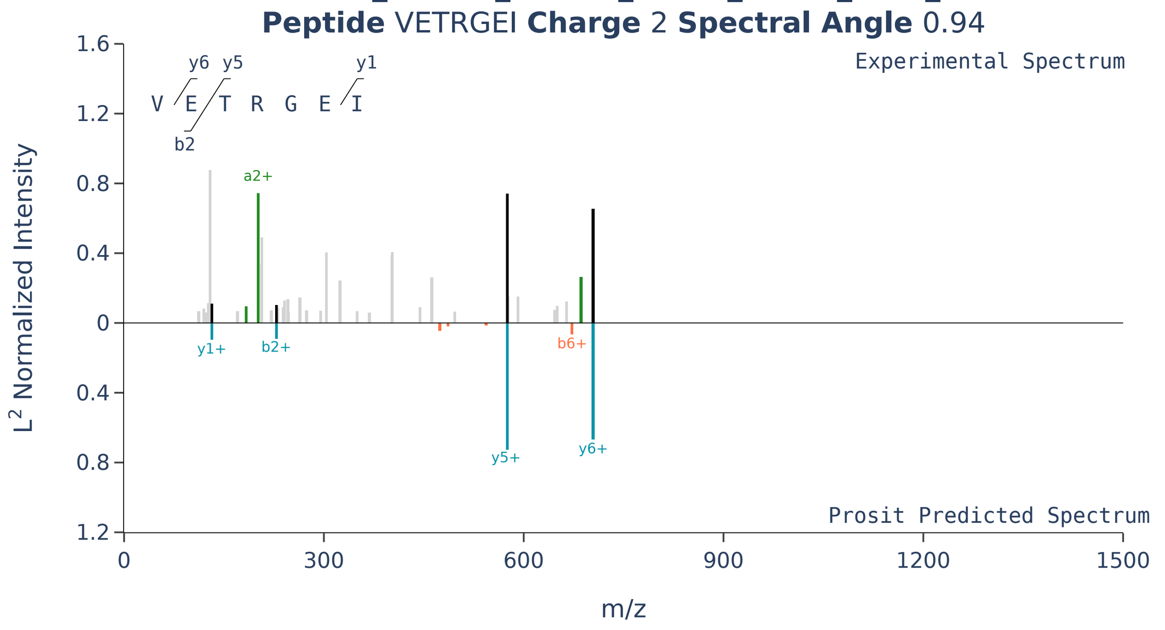

Source HRoetschke_271222_100123_HFGoe_UBE2K_b1R2_24h **Scan** 9218
Peptide AHAVVANQY **Charge** 2 **Spectral Angle** 0.86

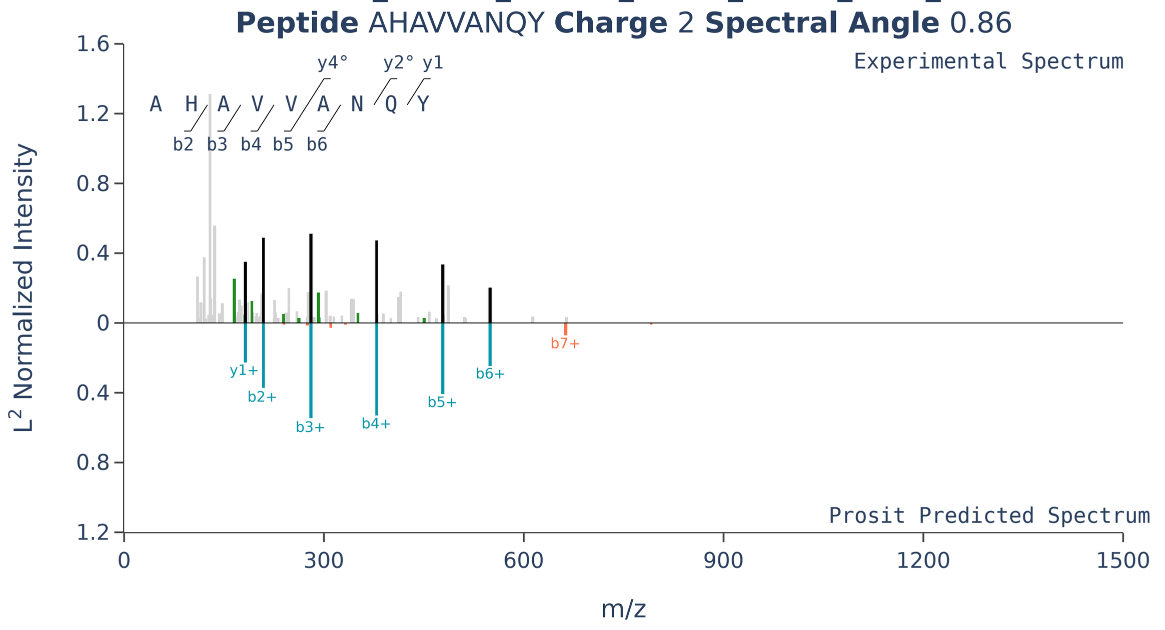

Source HRoetschke_271222_100123_HFGoe_UBE2K_b1R2_24h **Scan** 13772
Peptide AMGFDRNEL **Charge** 2 **Spectral Angle** 0.9

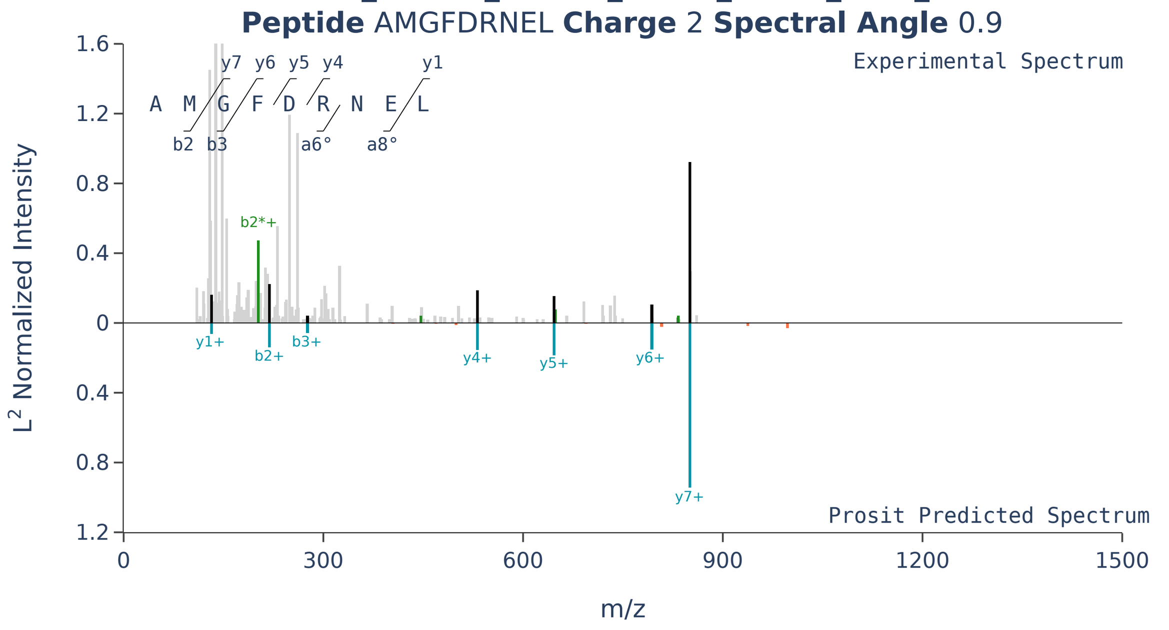

Source HRoetschke_271222_100123_HFGoe_UBE2K_b2R1_4h **Scan** 17721
Peptide LDKVRFI **Charge** 2 **Spectral Angle** 0.9

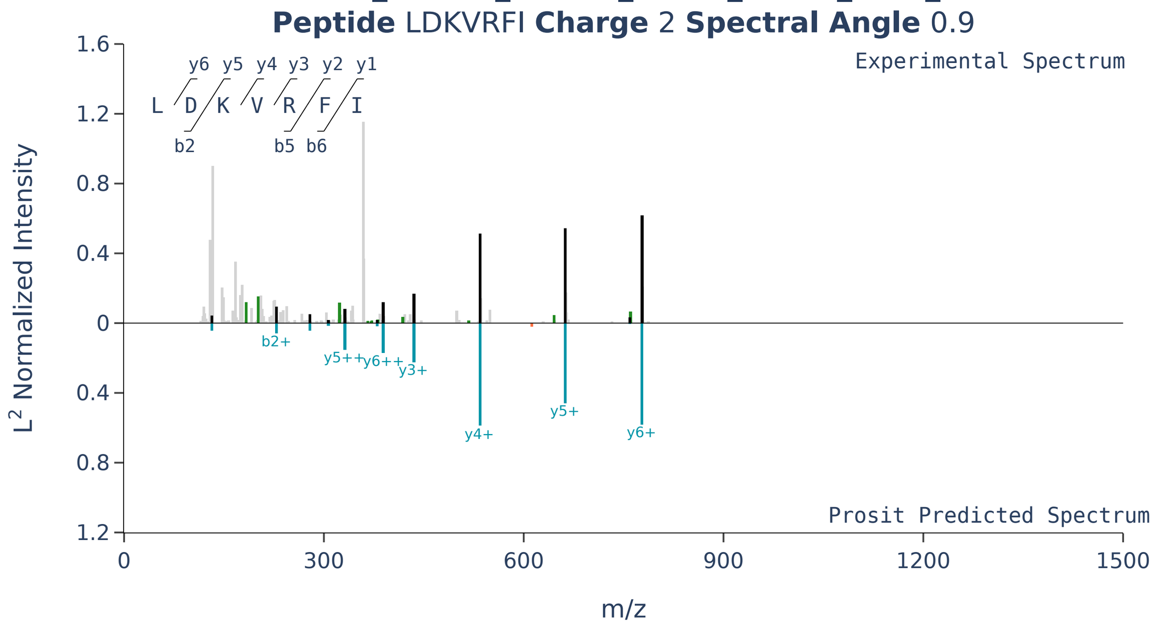

Source HRoetschke_271222_100123_HFGoe_UBE2K_b1R2_24h **Scan** 14007
Peptide TGAGPPDTPYEGGRY **Charge** 2 **Spectral Angle** 0.86

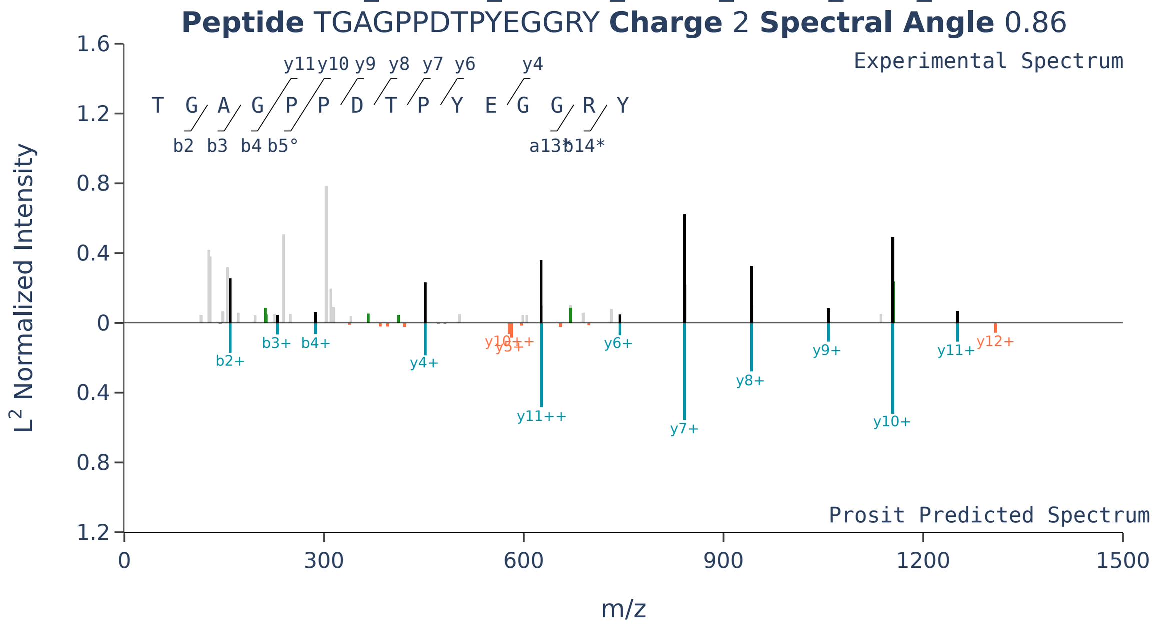

Source HRoetschke_271222_100123_HFGoe_UBE2K_b1R2_24h **Scan** 10524
Peptide ELRGEIAGE **Charge** 2 **Spectral Angle** 0.84

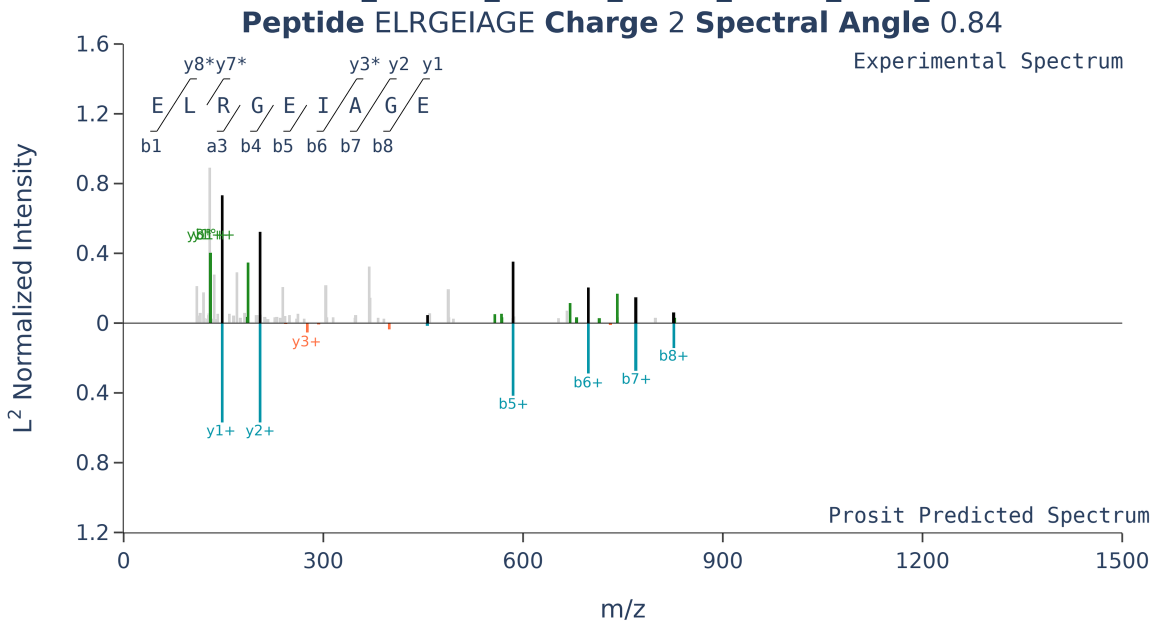

Source HRoetschke_271222_100123_HFGoe_UBE2K_b2R2_4h **Scan** 21056
Peptide ALLAAAGEIA **Charge** 1 **Spectral Angle** 0.86

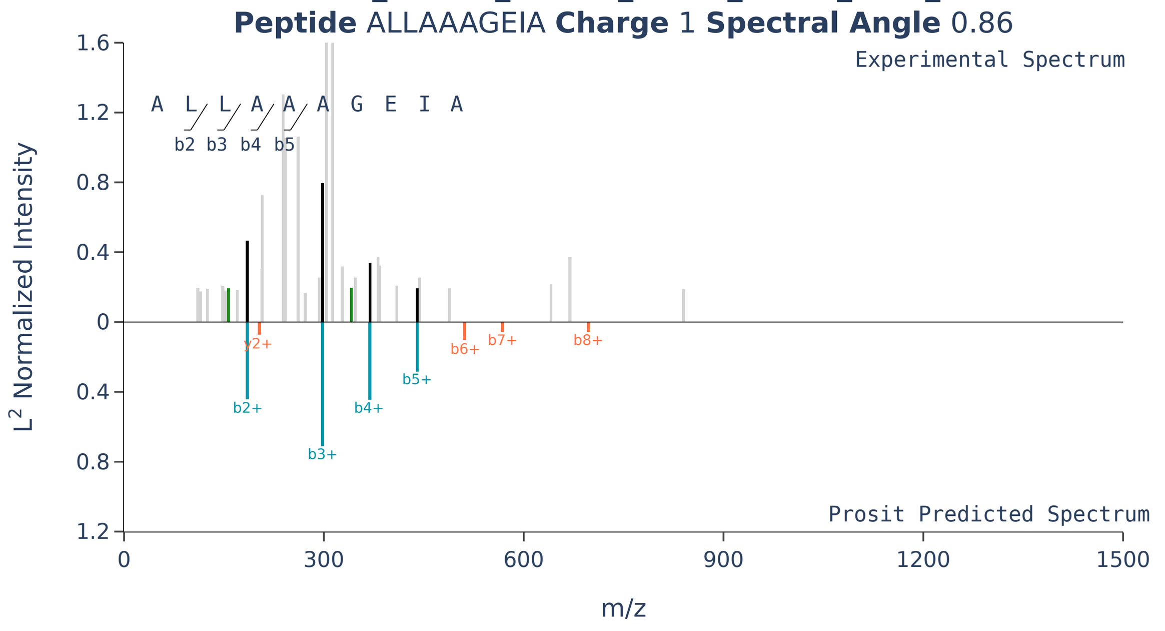

Source HRoetschke_271222_100123_HFGoe_UBE2K_b1R2_24h **Scan** 8765
Peptide RGEIAGRGEI **Charge** 3 **Spectral Angle** 0.91

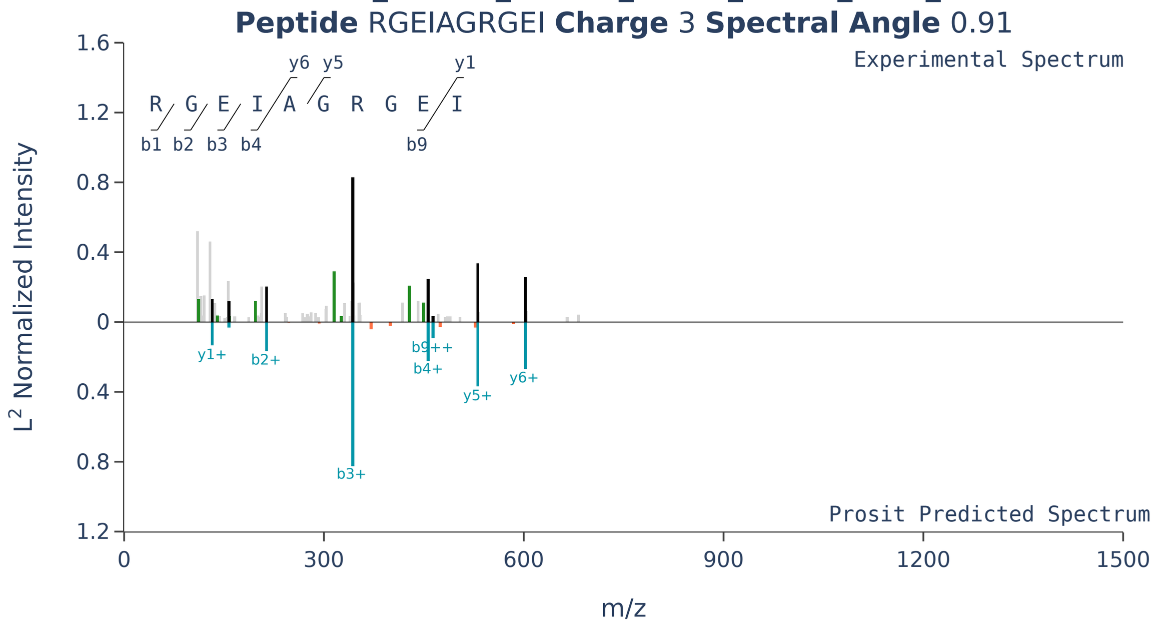

Source HRoetschke_271222_100123_HFGoe_UBE2K_b1R2_24h **Scan** 9344
Peptide GRAVVANQY **Charge** 2 **Spectral Angle** 0.8

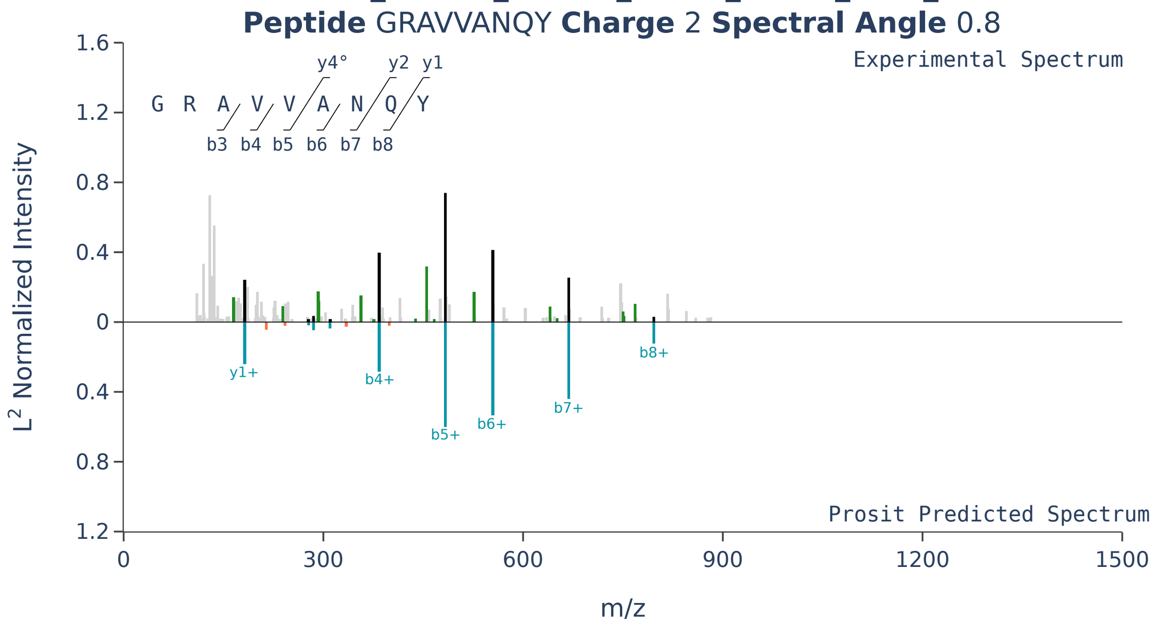

Source HRoetschke_271222_100123_HFGoe_UBE2K_b1R2_24h **Scan** 18501
Peptide ENFTELRGEV **Charge** 2 **Spectral Angle** 0.85

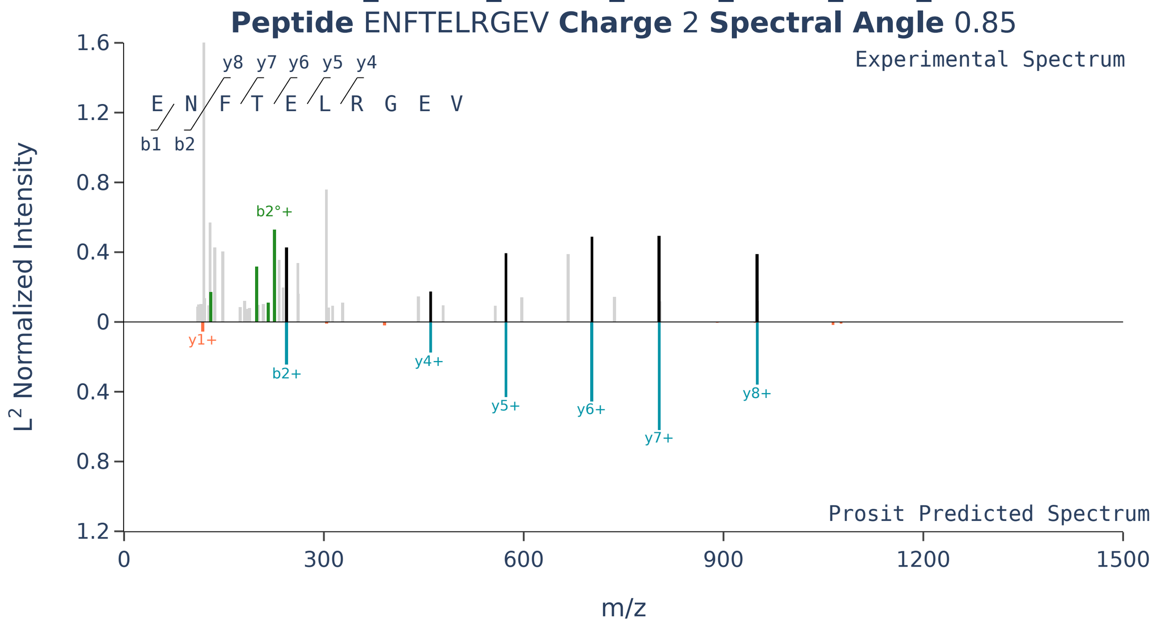

Source HRoetschke_271222_100123_HFGoe_UBE2K_b2R2_24h **Scan** 13526
Peptide SANIAVQRI **Charge** 2 **Spectral Angle** 0.78

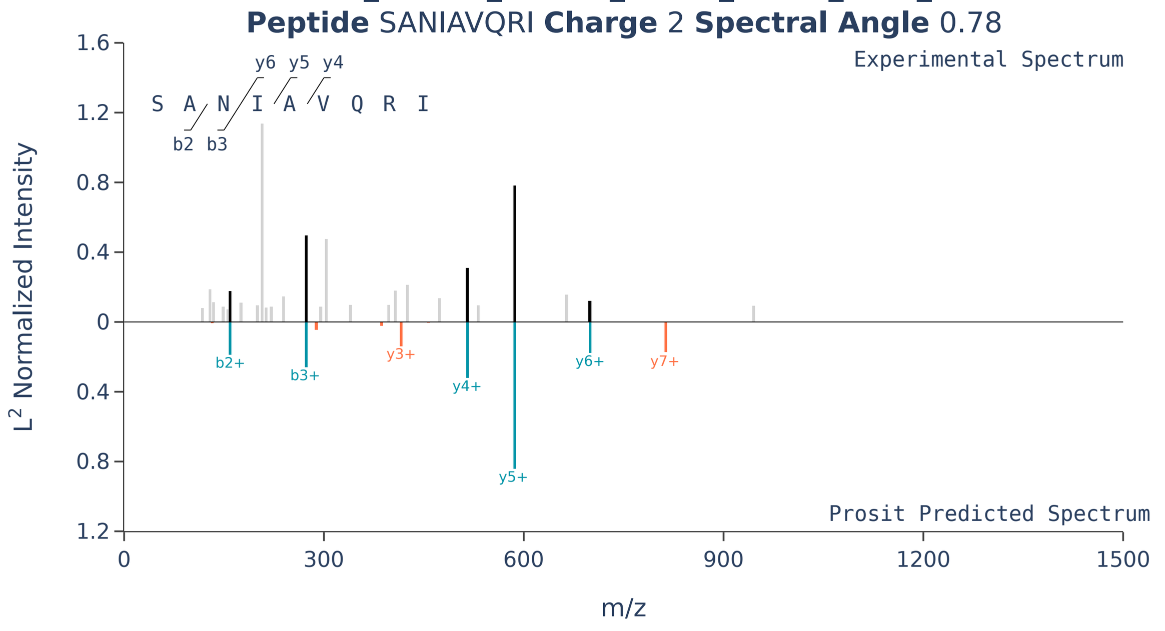

Source HRoetschke_271222_100123_HFGoe_UBE2K_b1R2_24h **Scan** 14886
Peptide ENFTELRGETG **Charge** 2 **Spectral Angle** 0.84

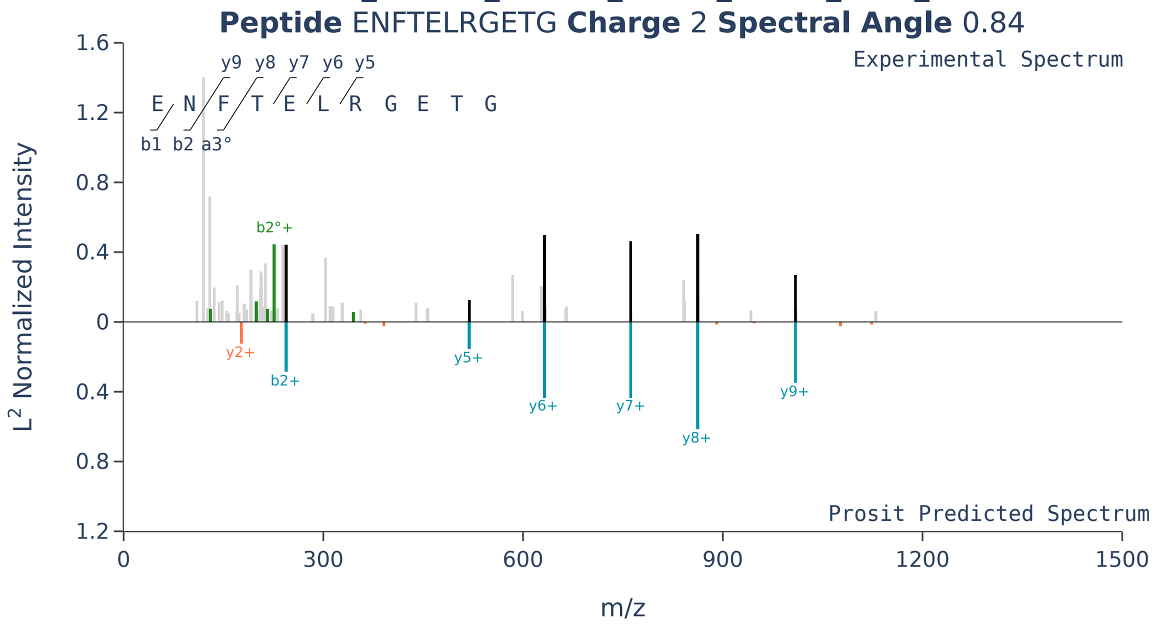

Source HRoetschke_271222_100123_HFGoe_UBE2K_b1R2_2h **Scan** 12558
Peptide ANNIAVQRI **Charge** 2 **Spectral Angle** 0.75

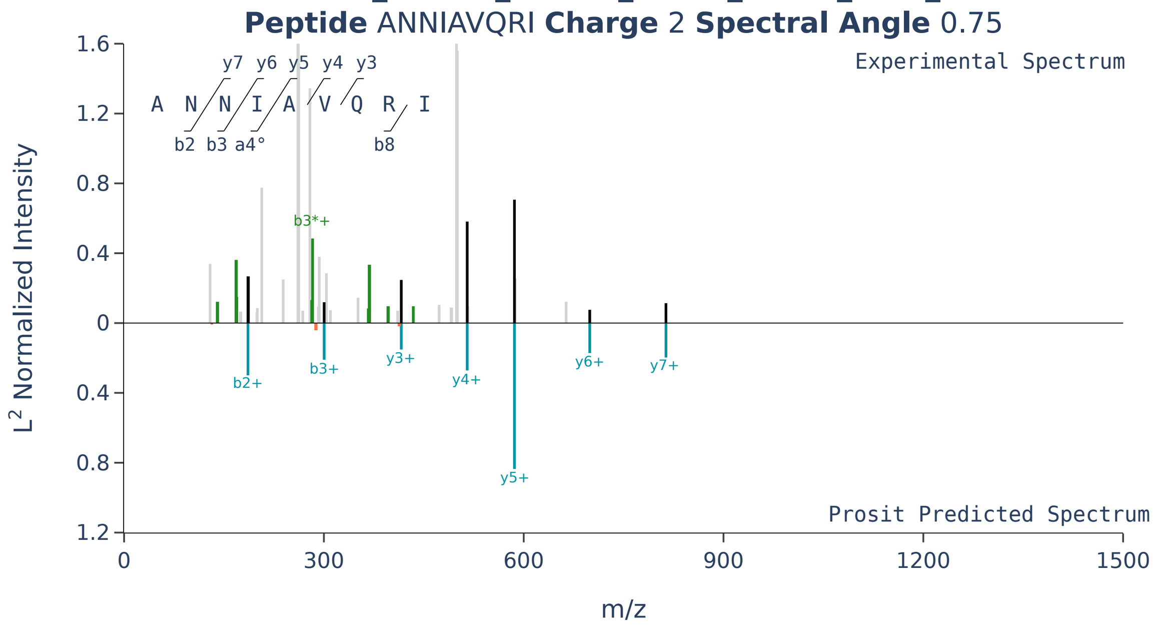

Source HRoetschke_271222_100123_HFGoe_UBE2K_b1R2_24h **Scan** 15605
Peptide GARGEIAGPPDTPYE **Charge** 2 **Spectral Angle** 0.81

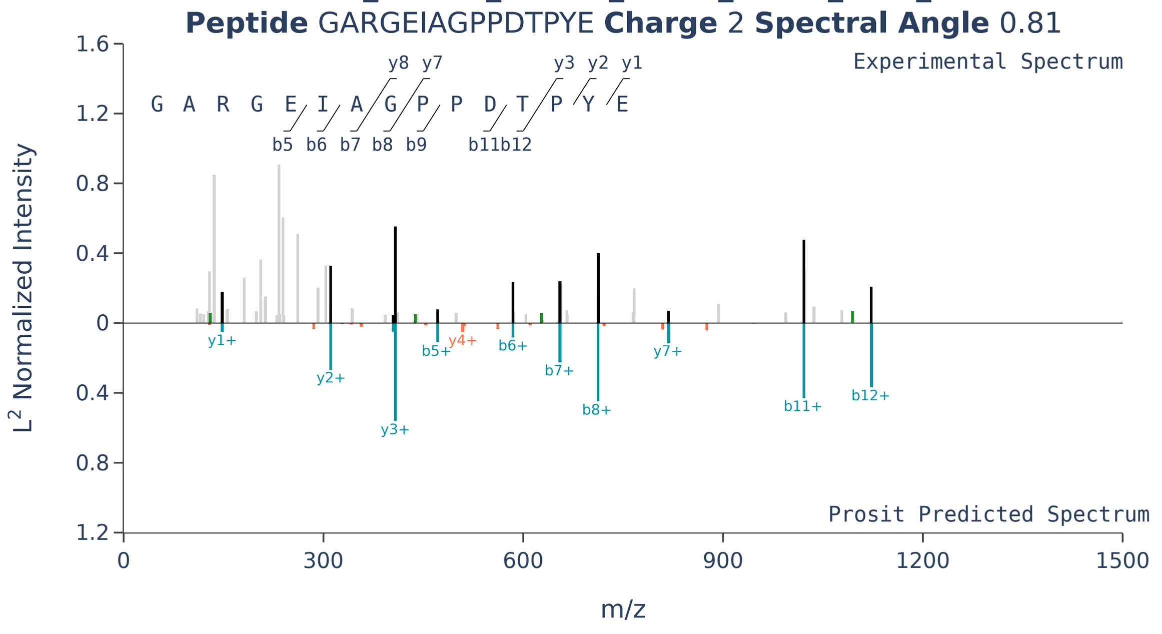

Source HRoetschke_271222_100123_HFGoe_UBE2K_b1R1_2h **Scan** 8934
Peptide VETATLRGE **Charge** 2 **Spectral Angle** 0.83

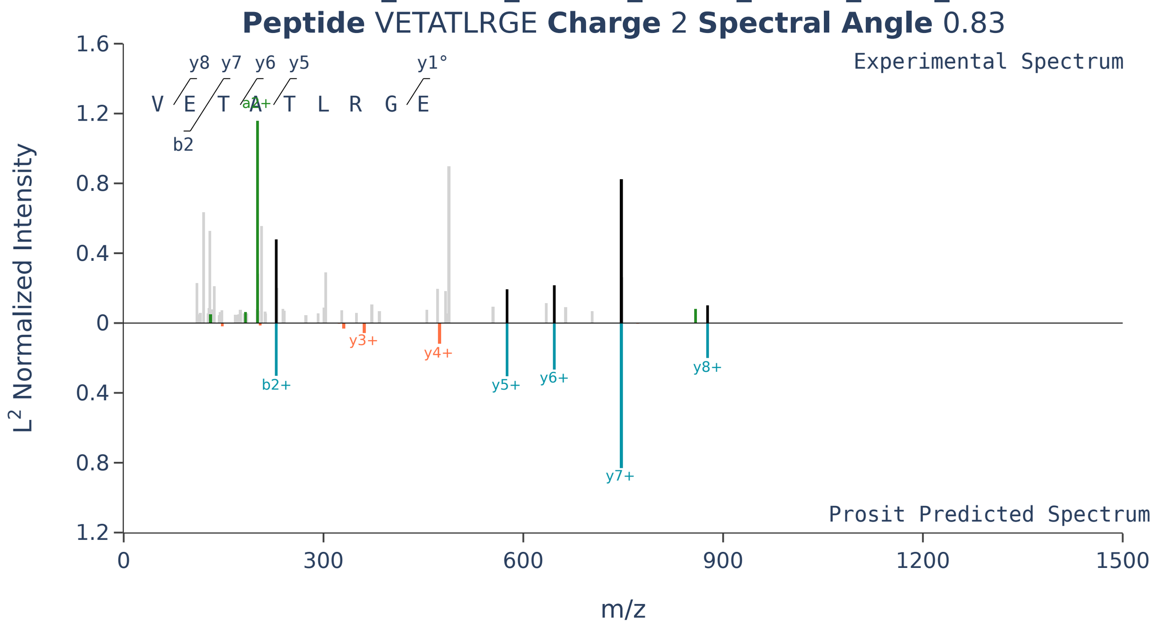

Source HRoetschke_271222_100123_HFGoe_UBE2K_b1R2_24h **Scan** 9258
Peptide SSKSWDVETGR **Charge** 2 **Spectral Angle** 0.83

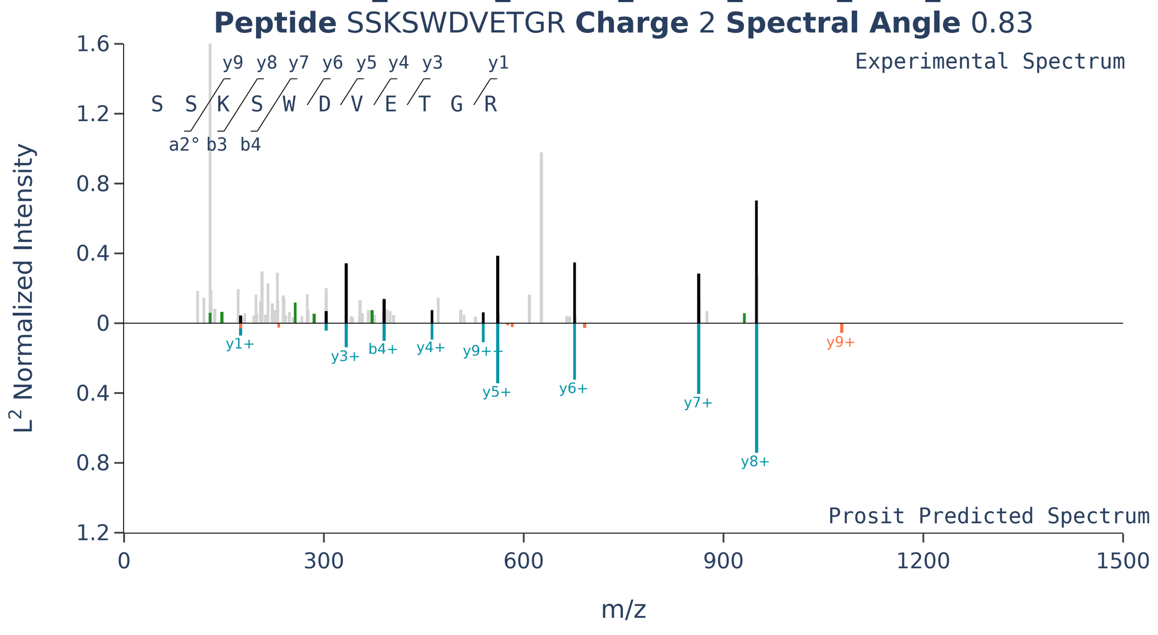

Source HRoetschke_271222_100123_HFGoe_UBE2K_b1R1_2h **Scan** 16919
Peptide ENFTELRDAV **Charge** 2 **Spectral Angle** 0.85

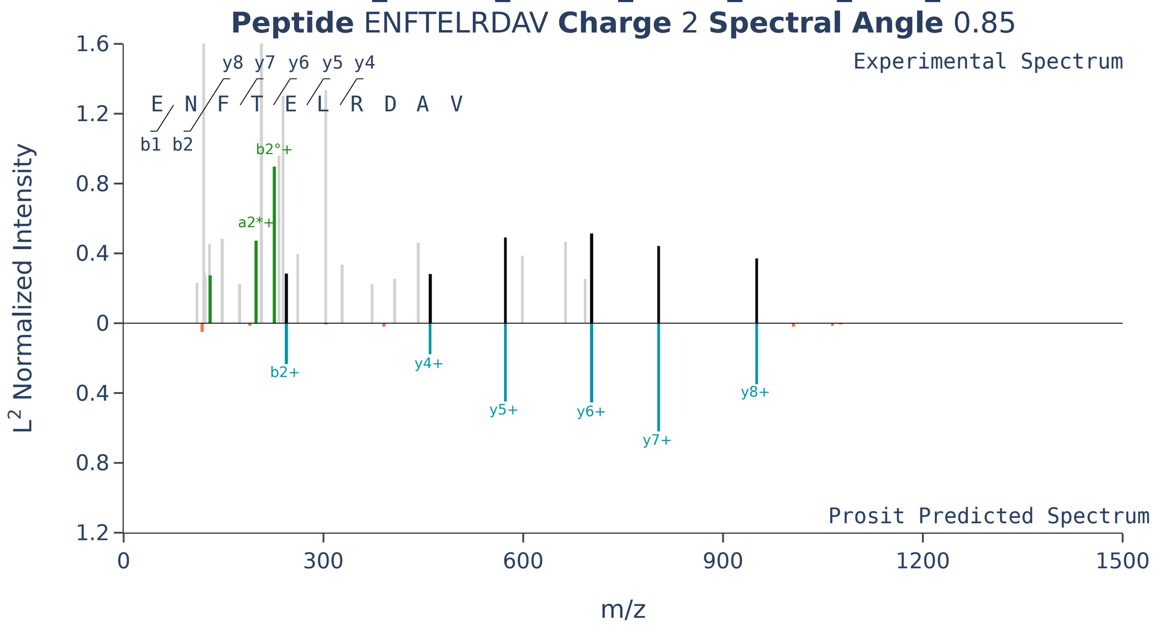

Source HRoetschke_271222_100123_HFGoe_UBE2K_b2R1_4h **Scan** 13448
Peptide VDENFTELRGRGE **Charge** 3 **Spectral Angle** 0.8

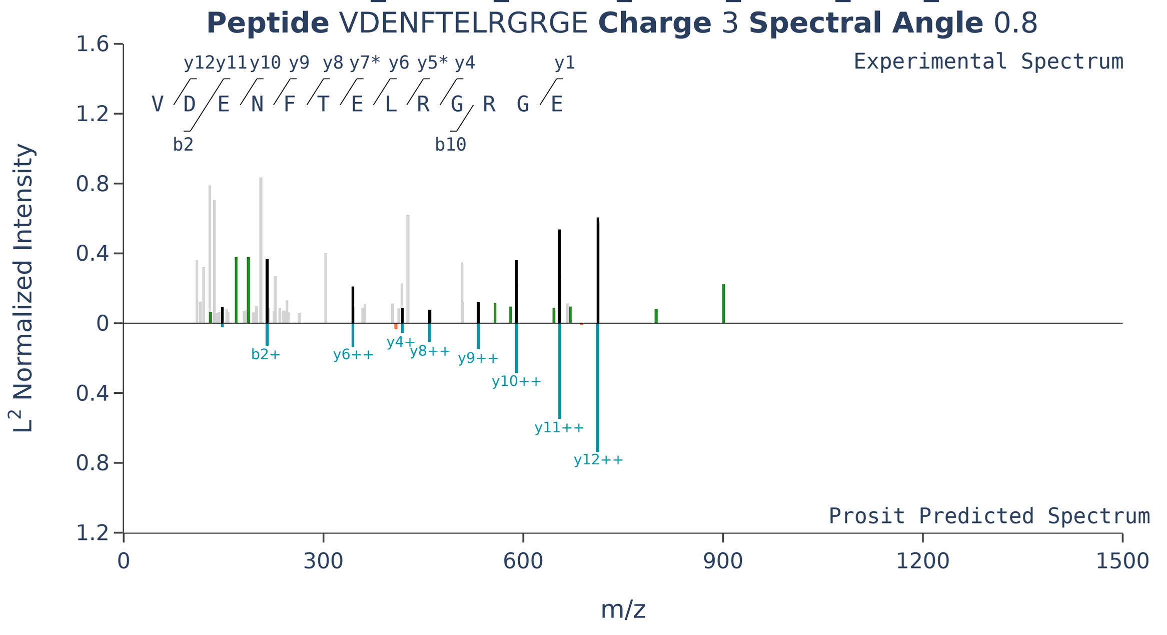

Source HRoetschke_271222_100123_HFGoe_UBE2K_b1R2_24h **Scan** 20057
Peptide TGEIKIPETY **Charge** 2 **Spectral Angle** 0.8

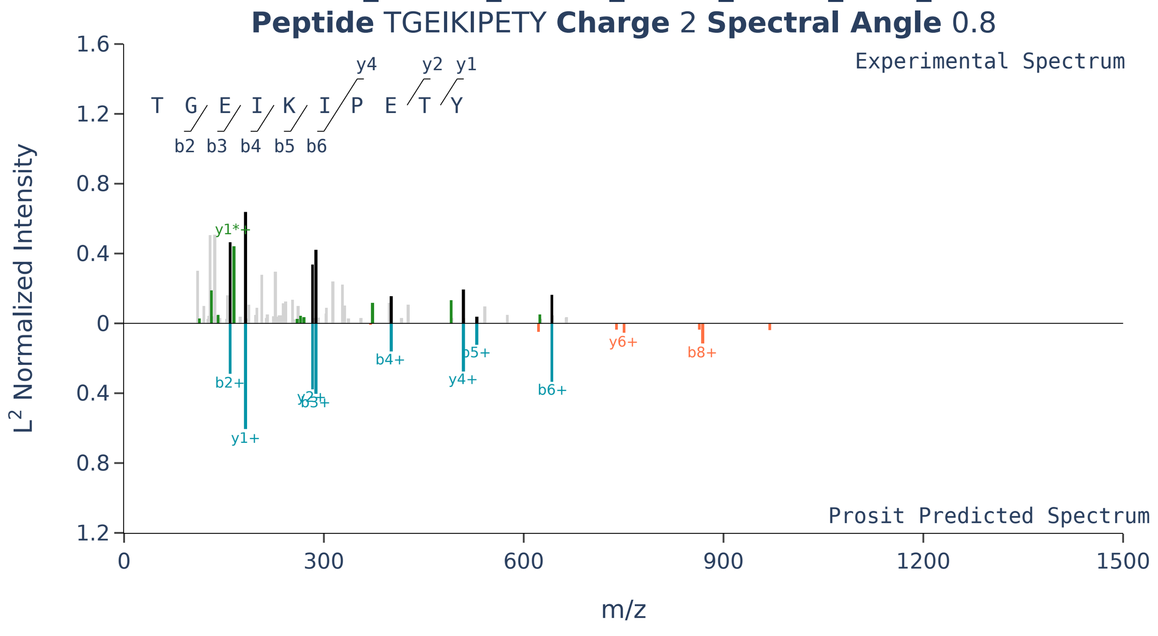

Source HRoetschke_271222_100123_HFGoe_UBE2K_b2R1_4h **Scan** 7891
Peptide AHVYAGAPVSR **Charge** 2 **Spectral Angle** 0.83

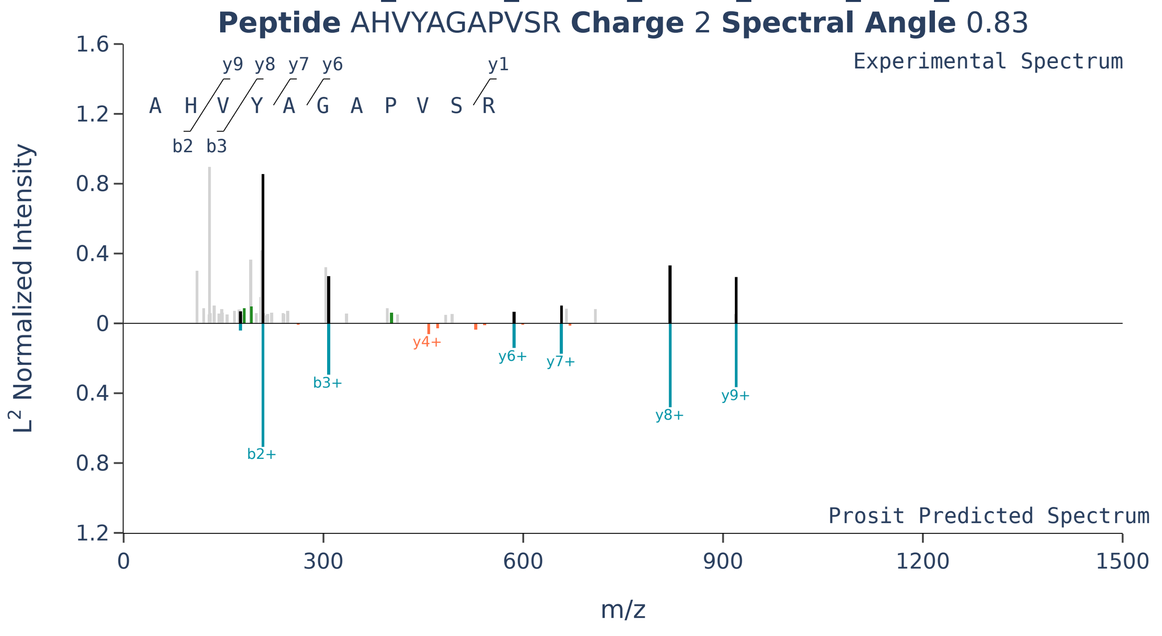

Source HRoetschke_271222_100123_HFGoe_UBE2K_b1R2_24h **Scan** 15585
Peptide TGRGEIAGPPDTPYE **Charge** 2 **Spectral Angle** 0.79

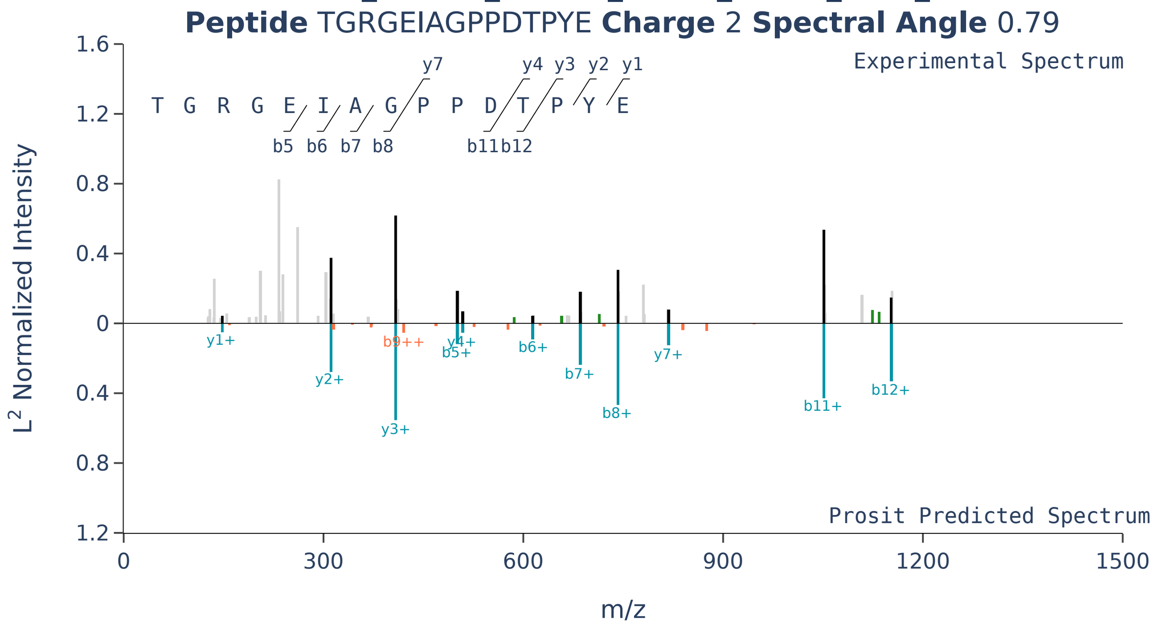

Source HRoetschke_271222_100123_HFGoe_UBE2K_b1R2_24h **Scan** 15555
Peptide RGEIAGKVDLVD **Charge** 3 **Spectral Angle** 0.73

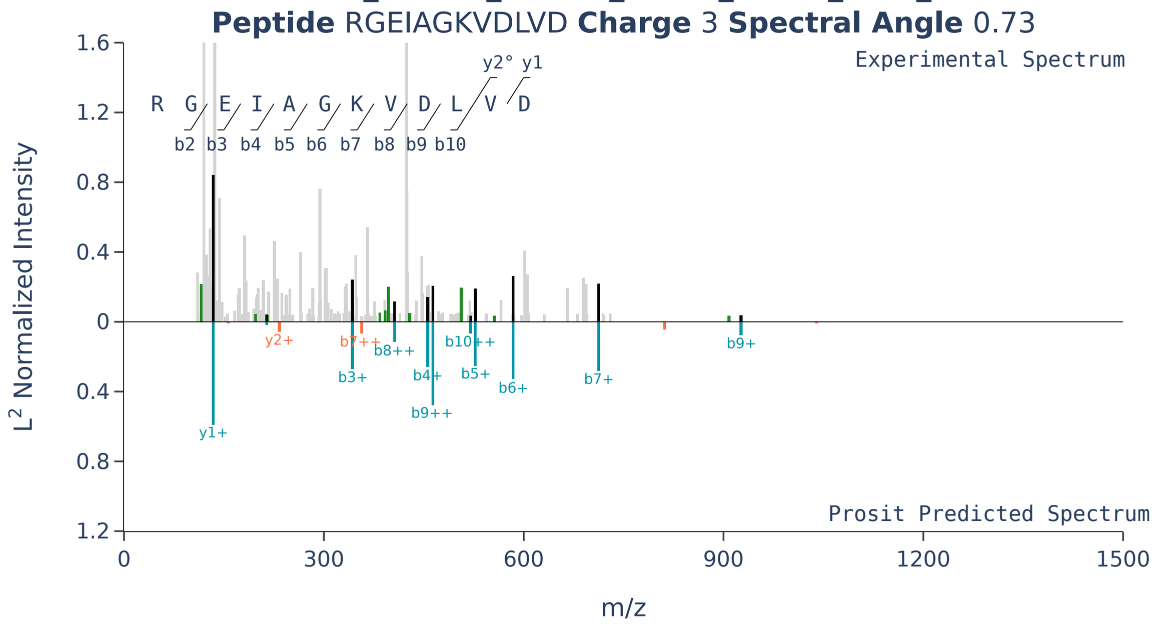

Source HRoetschke_271222_100123_HFGoe_UBE2K_b1R1_24h **Scan** 9490
Peptide KEVLKVET **Charge** 2 **Spectral Angle** 0.83

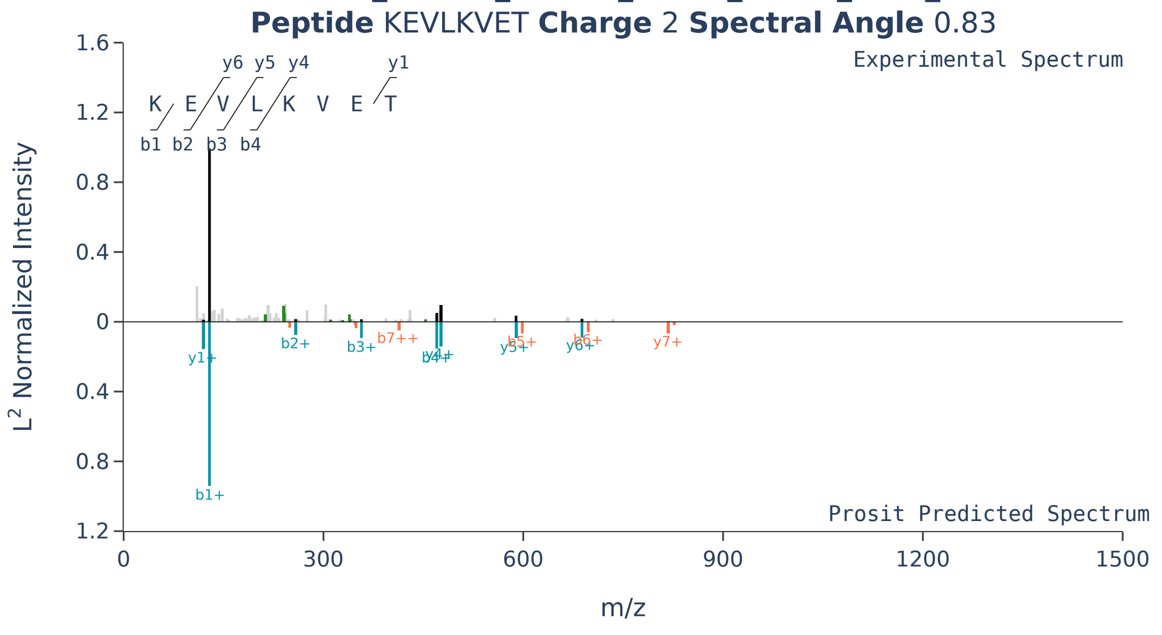

Source HRoetschke_271222_100123_HFGoe_UBE2K_b1R1_24h **Scan** 11719
Peptide KQNPEMFKQTAY **Charge** 3 **Spectral Angle** 0.86

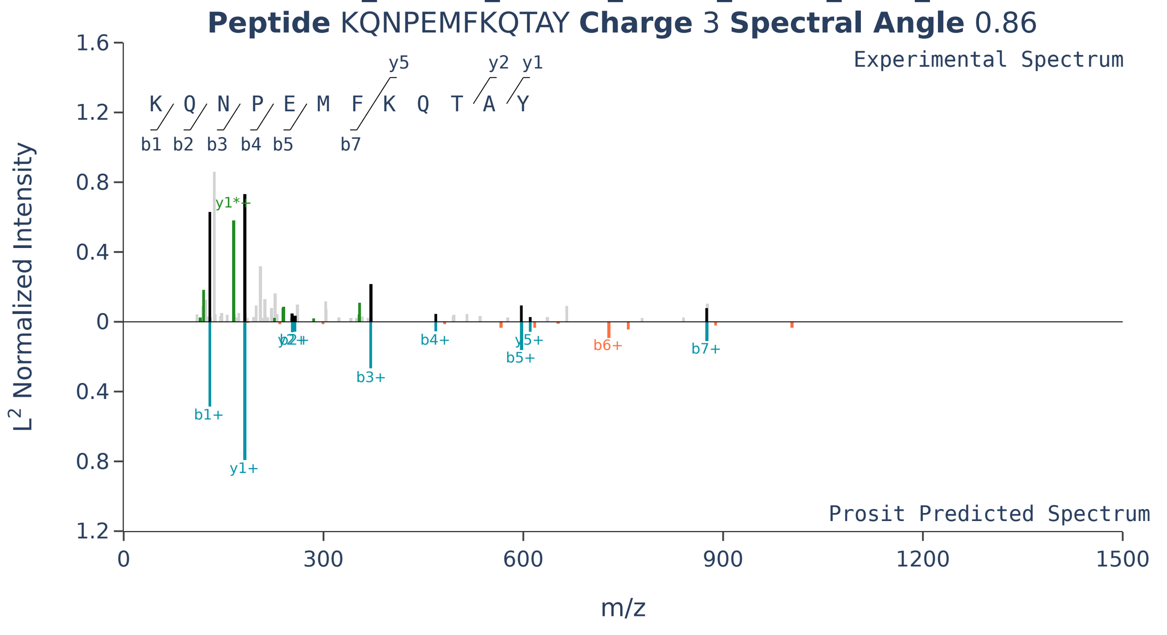

Source HRoetschke_271222_100123_HFGoe_UBE2K_b2R1_4h **Scan** 15091
Peptide ARGEIAGPPDTPYE **Charge** 2 **Spectral Angle** 0.76

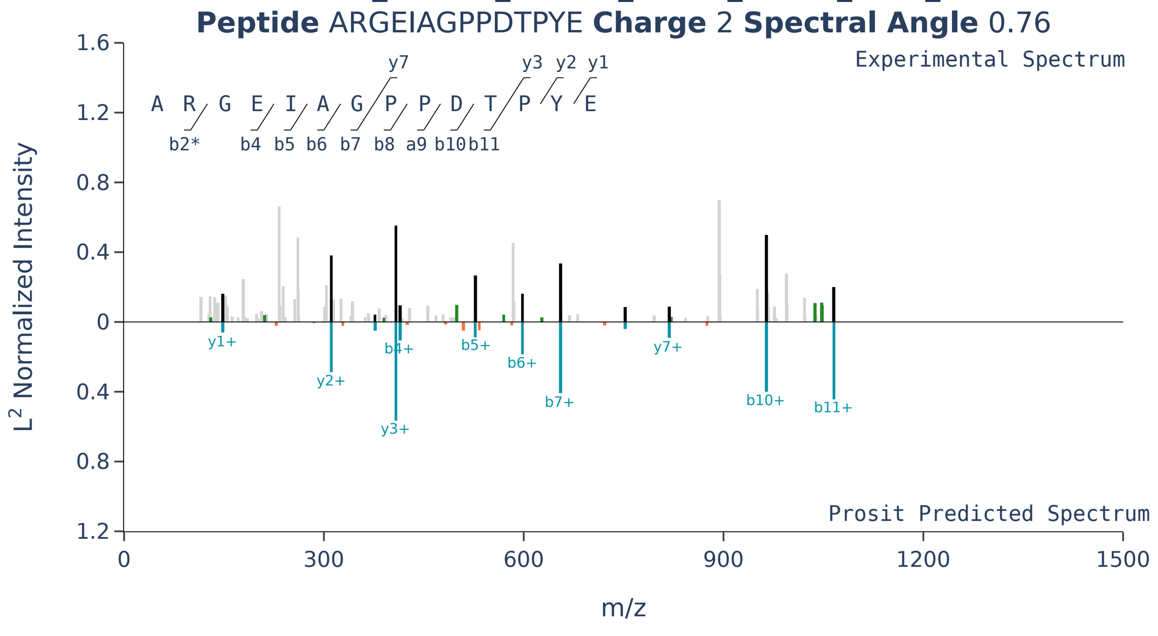

Source HRoetschke_271222_100123_HFGoe_UBE2K_b1R2_24h **Scan** 20041
Peptide GAEIKIPETY **Charge** 2 **Spectral Angle** 0.77

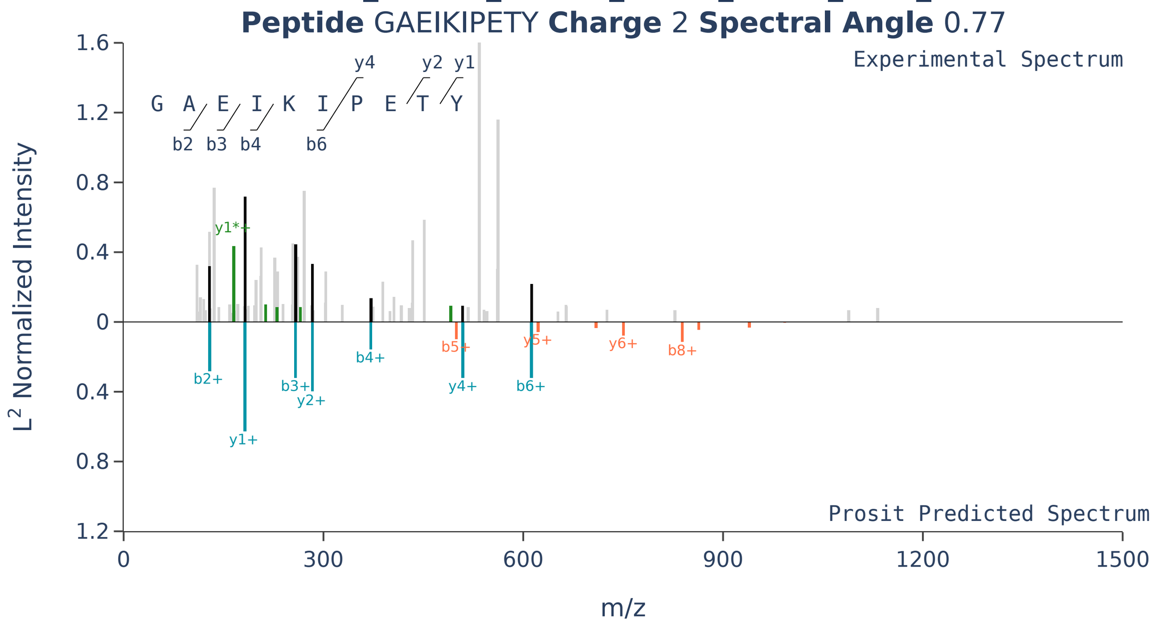

Source HRoetschke_271222_100123_HFGoe_UBE2K_b2R2_4h **Scan** 13557
Peptide ENFTELRGET **Charge** 2 **Spectral Angle** 0.8

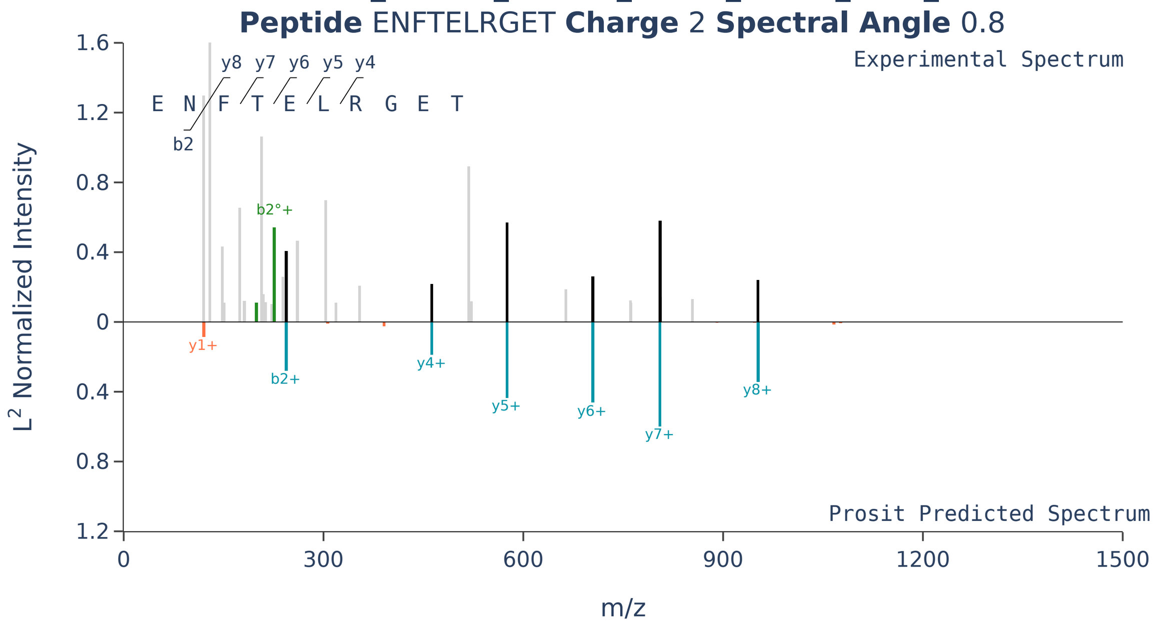

Source HRoetschke_271222_100123_HFGoe_UBE2K_b1R1_24h **Scan** 9489
Peptide KQTAKIPETY **Charge** 3 **Spectral Angle** 0.78

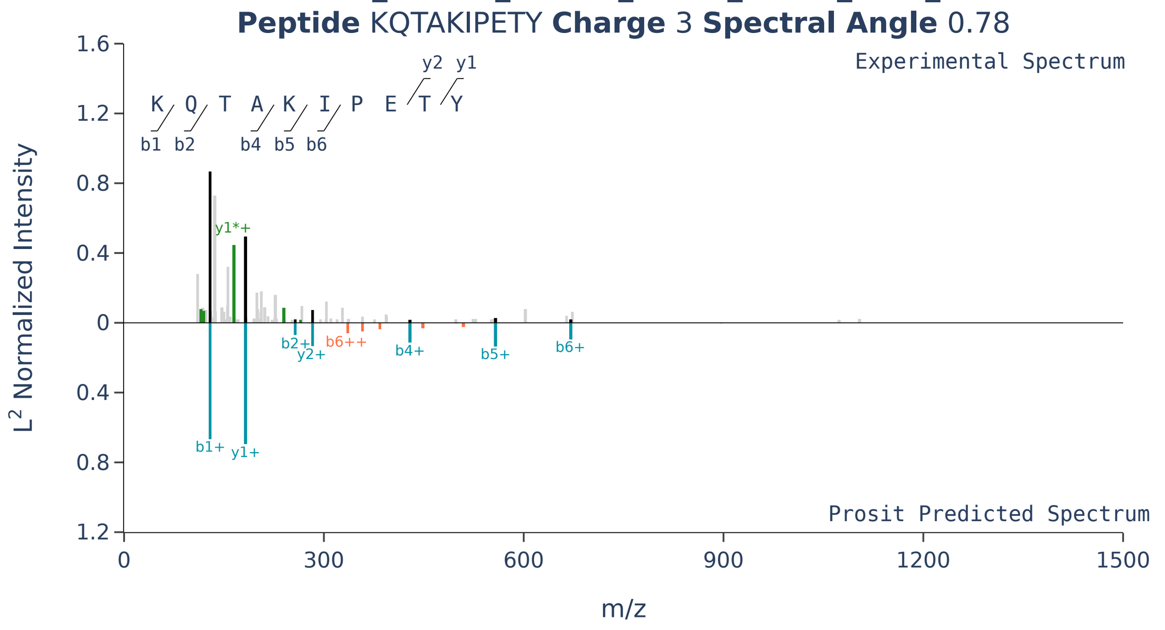

Source HRoetschke_271222_100123_HFGoe_UBE2K_b1R2_24h **Scan** 14067
Peptide RTEIKIPET **Charge** 2 **Spectral Angle** 0.81

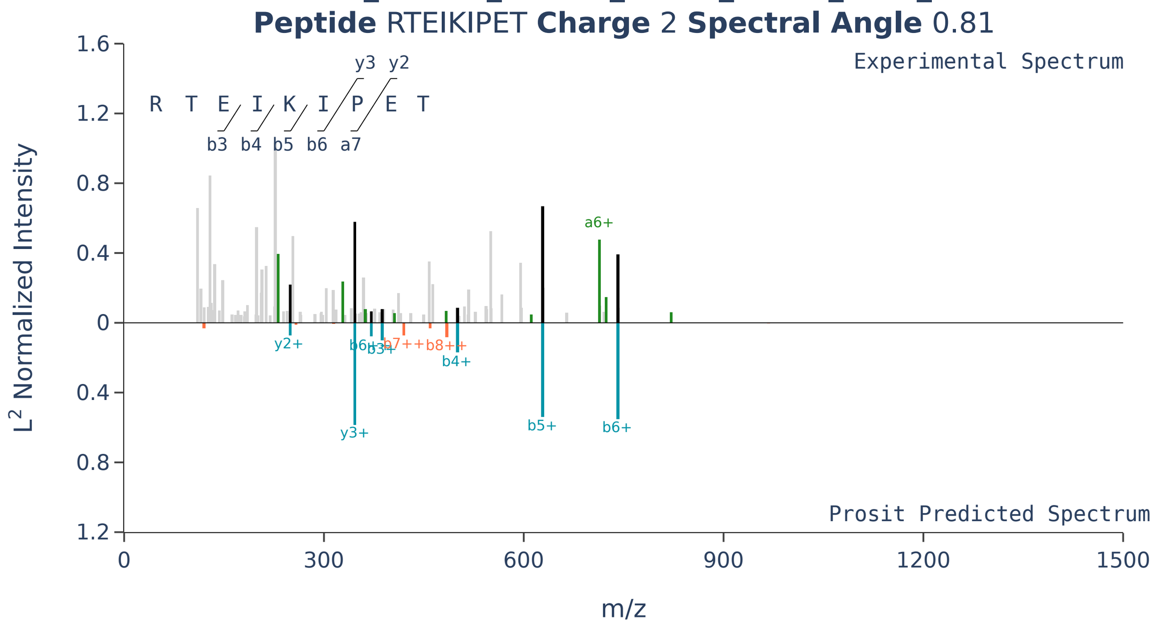

Source HRoetschke_271222_100123_HFGoe_UBE2K_b1R1_24h **Scan** 12324
Peptide RGRGEIAGPPDTPYE **Charge** 3 **Spectral Angle** 0.76

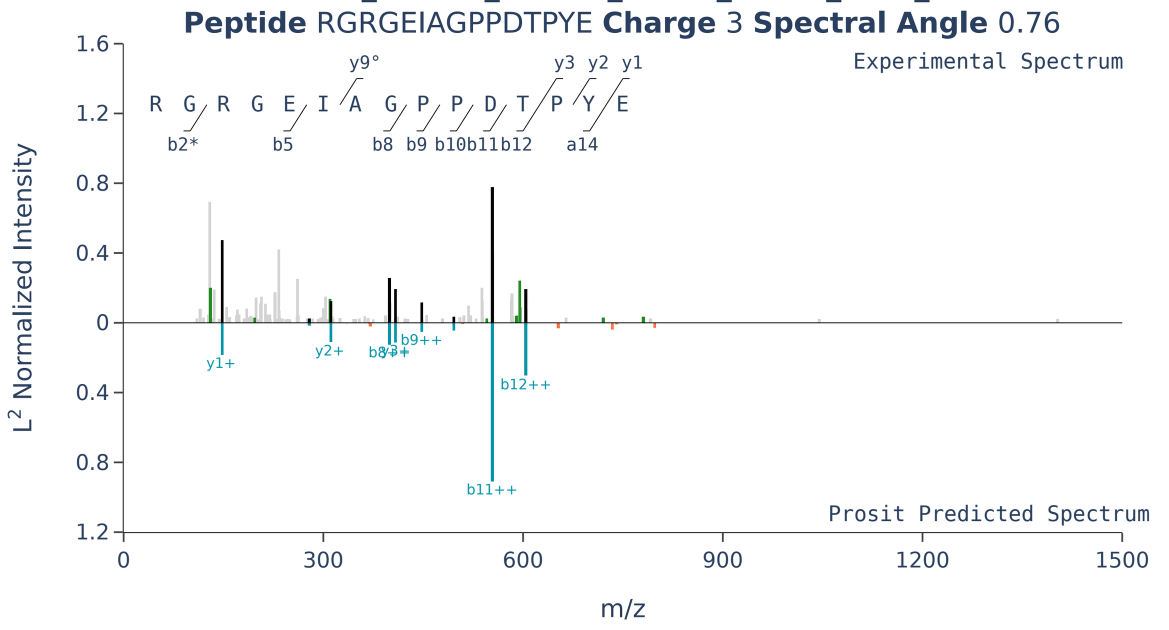

Source HRoetschke_271222_100123_HFGoe_UBE2K_b2R1_4h **Scan** 12529
Peptide VYAGAPVDT **Charge** 1 **Spectral Angle** 0.77

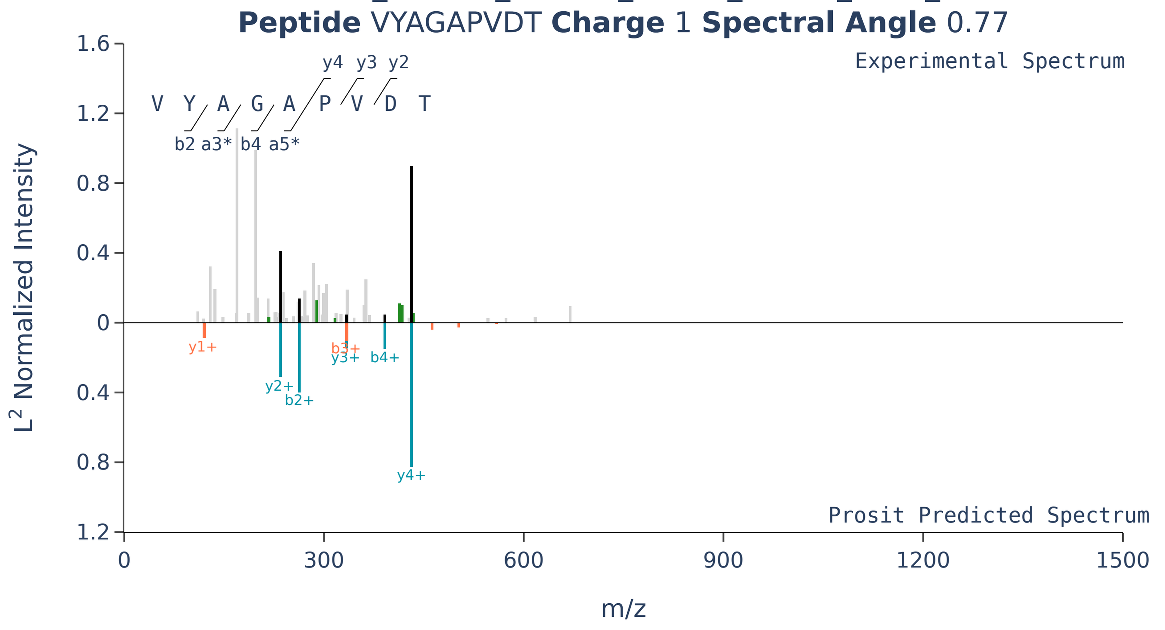

Source HRoetschke_271222_100123_HFGoe_UBE2K_b1R2_24h **Scan** 7715
Peptide SSNIAVQRIKRE **Charge** 3 **Spectral Angle** 0.85

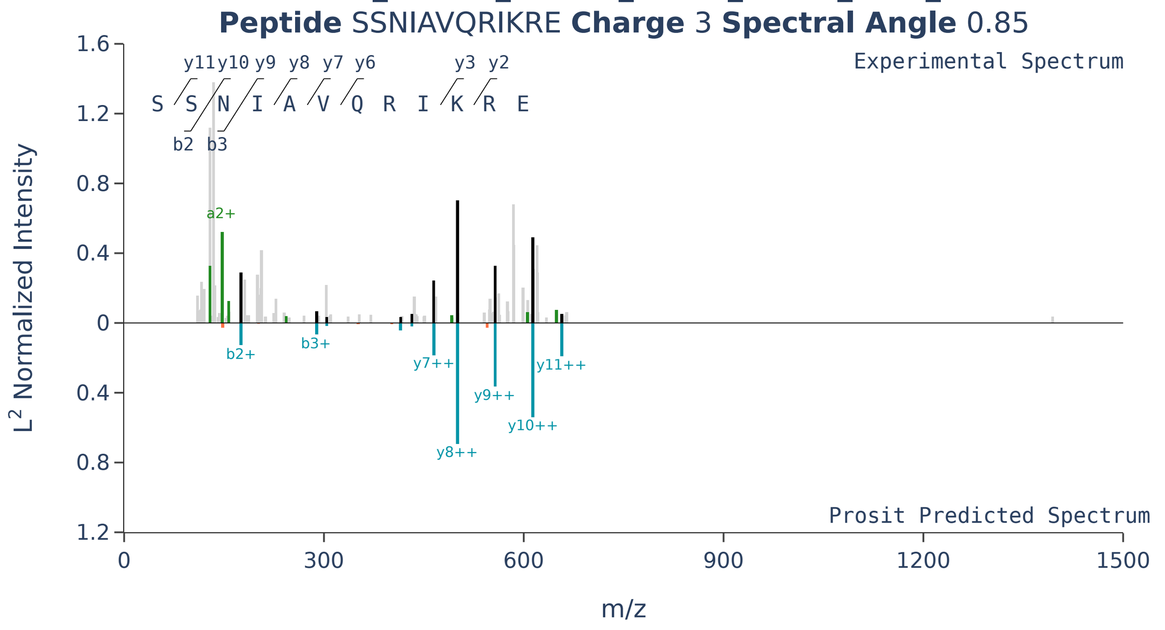

Source HRoetschke_271222_100123_HFGoe_UBE2K_b1R2_24h **Scan** 7737
Peptide NIAVQRIKREE **Charge** 3 **Spectral Angle** 0.89

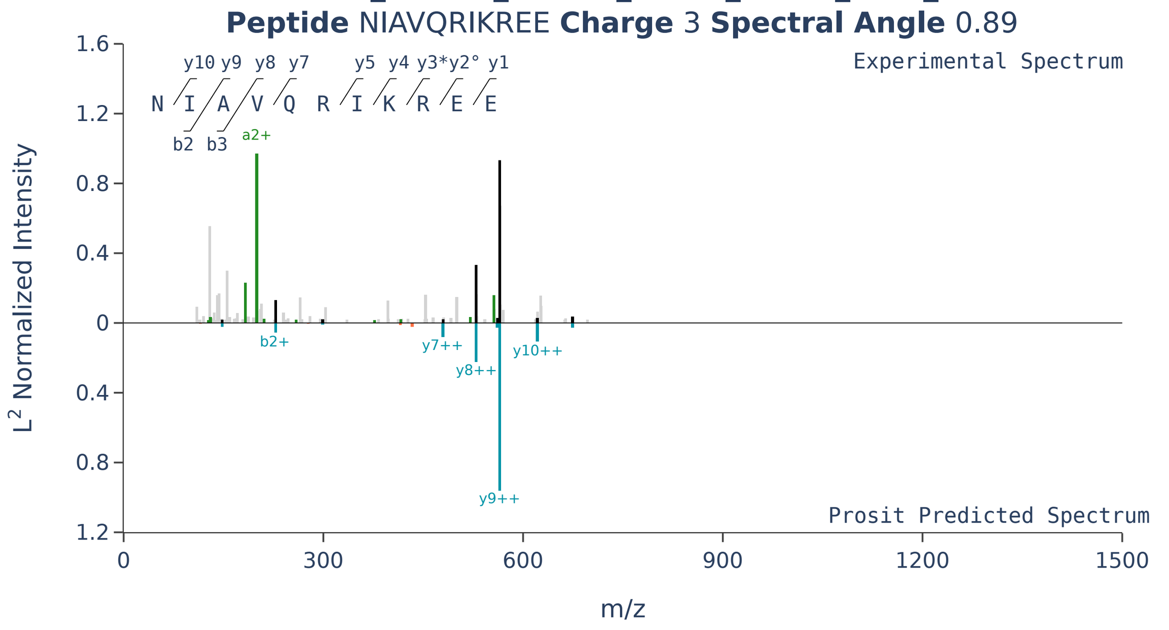

Source HRoetschke_271222_100123_HFGoe_UBE2K_b1R2_24h **Scan** 10156
Peptide KEVLKSEL **Charge** 2 **Spectral Angle** 0.83

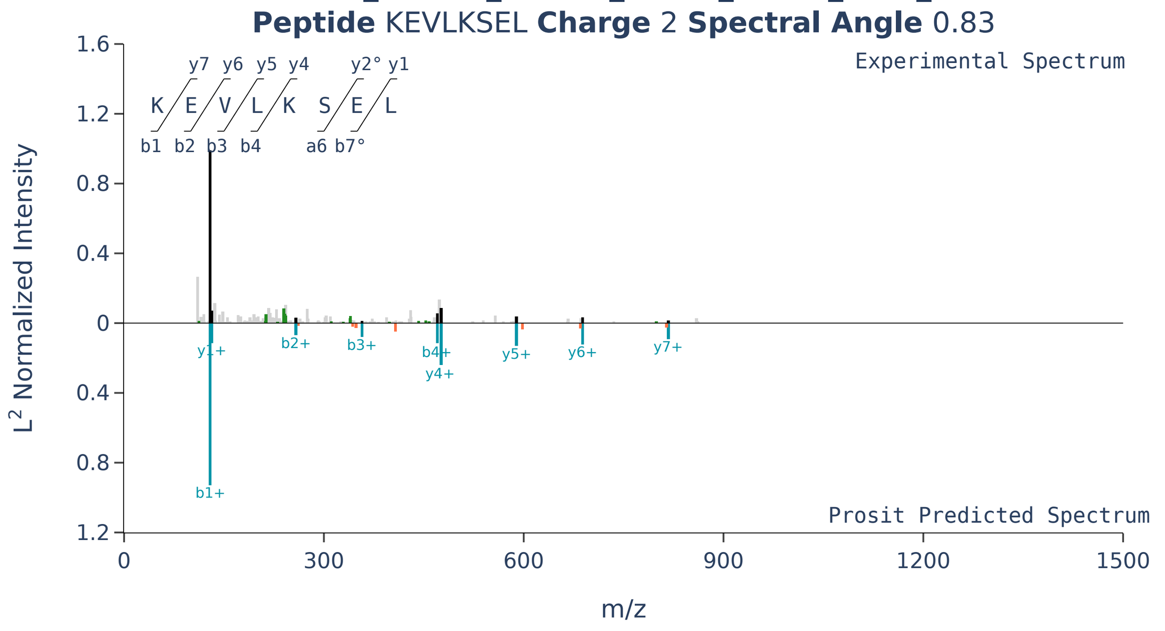

Source HRoetschke_271222_100123_HFGoe_UBE2K_b1R2_2h **Scan** 10677
Peptide KSWDVETE **Charge** 2 **Spectral Angle** 0.8

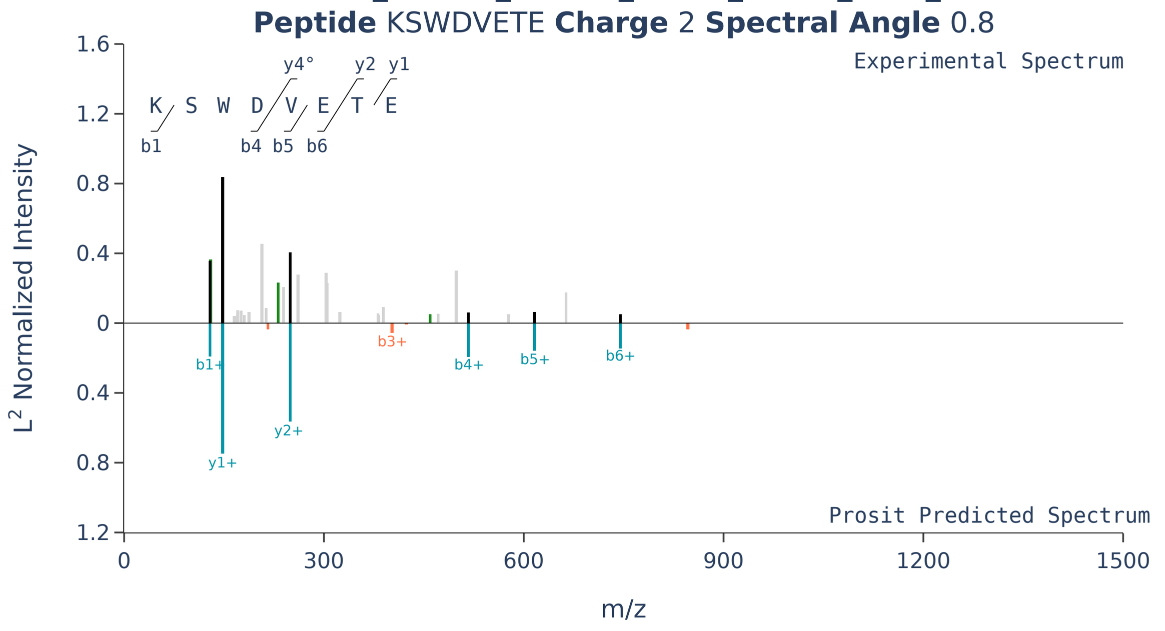

Source HRoetschke_271222_100123_HFGoe_UBE2K_b2R2_4h **Scan** 6402
Peptide ENLCKQTAR **Charge** 3 **Spectral Angle** 0.85

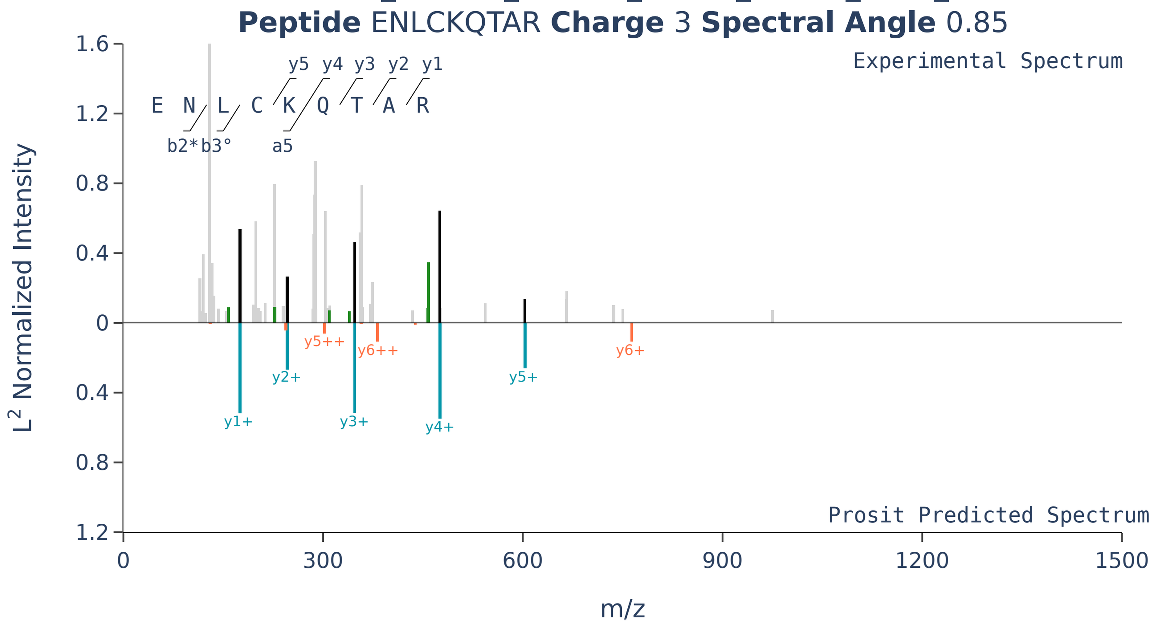

Source HRoetschke_271222_100123_HFGoe_UBE2K_b1R1_24h **Scan** 12264
Peptide KSWDVETAE **Charge** 2 **Spectral Angle** 0.85

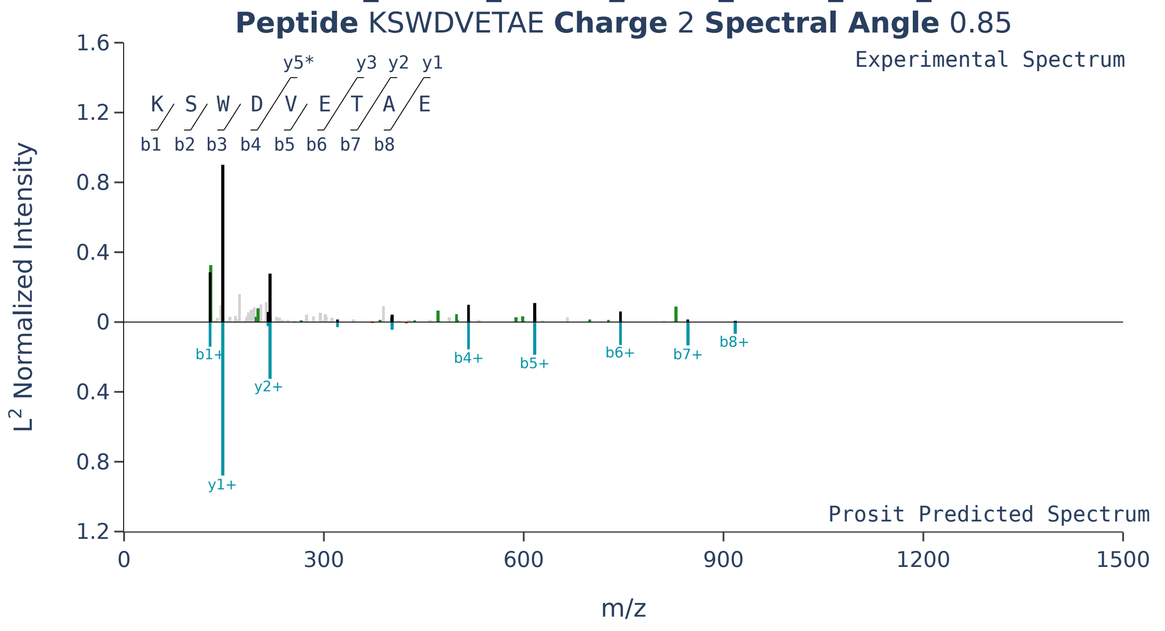

Source HRoetschke_271222_100123_HFGoe_UBE2K_b1R1_2h **Scan** 12473
Peptide ENLTELRG **Charge** 2 **Spectral Angle** 0.79

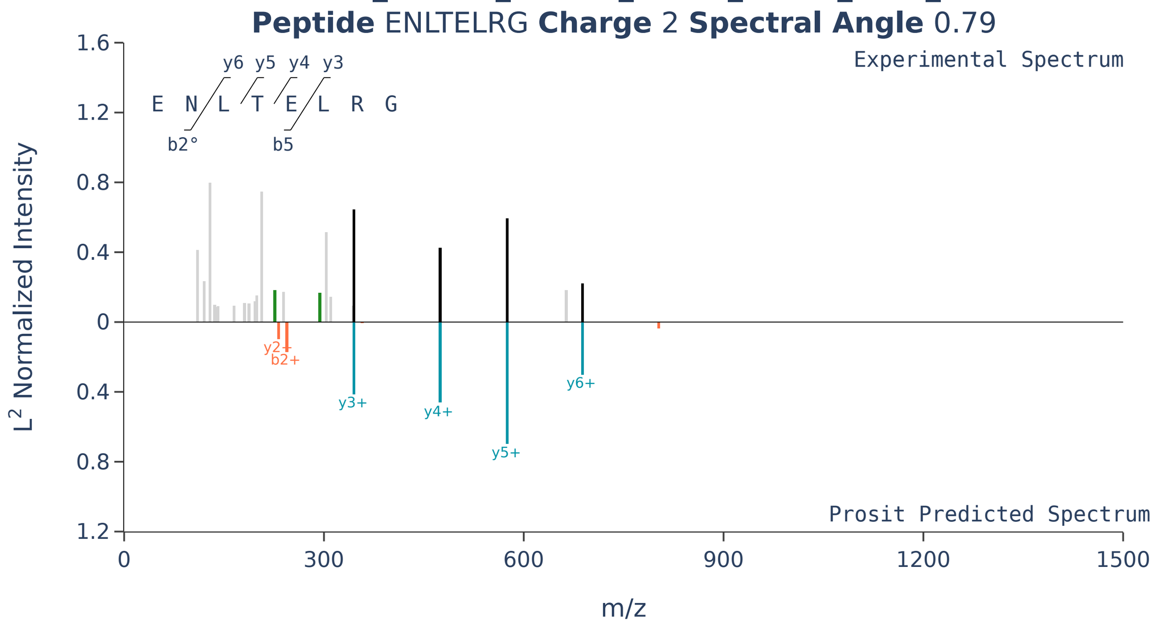

Source HRoetschke_271222_100123_HFGoe_UBE2K_b2R2_24h **Scan** 12185
Peptide DVETATVETA **Charge** 2 **Spectral Angle** 0.8

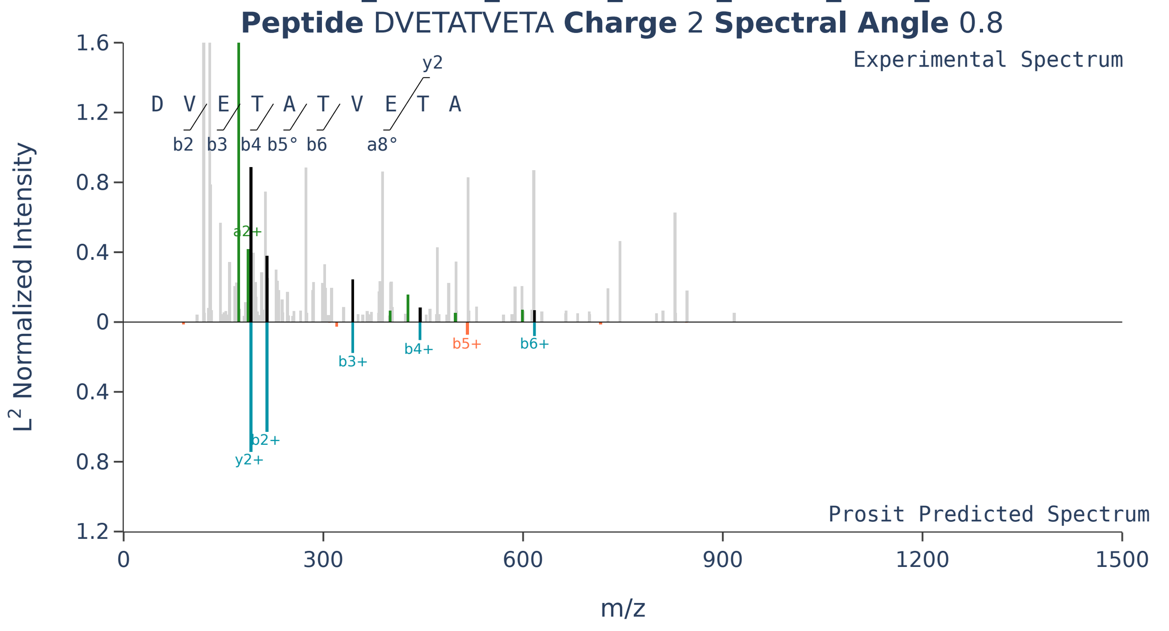

Source HRoetschke_271222_100123_HFGoe_UBE2K_b2R2_4h **Scan** 11988
Peptide SSKSWDVETAE **Charge** 2 **Spectral Angle** 0.86

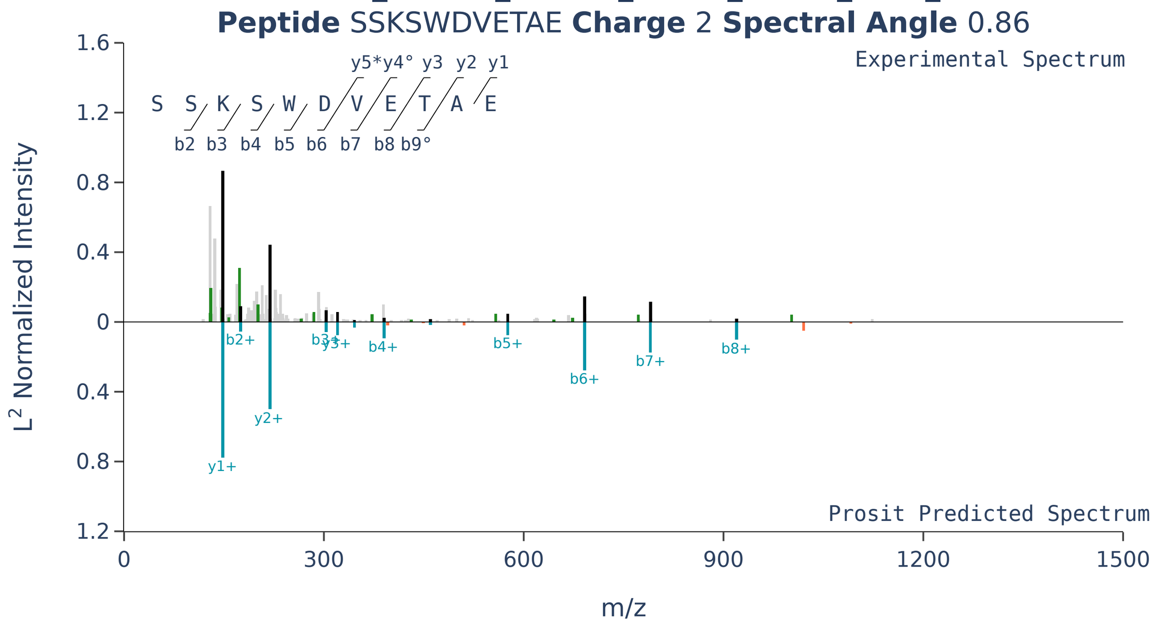

Source HRoetschke_271222_100123_HFGoe_UBE2K_b1R1_2h **Scan** 7890
Peptide VETARGEIAG **Charge** 2 **Spectral Angle** 0.78

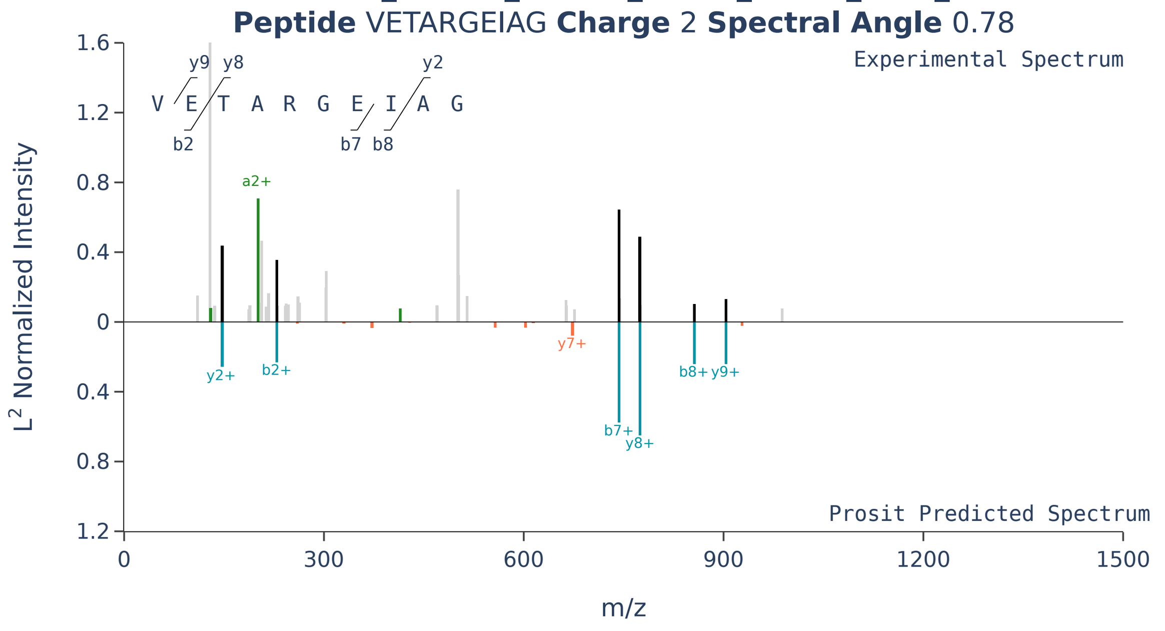

Source HRoetschke_271222_100123_HFGoe_UBE2K_b1R2_24h **Scan** 15440
Peptide KGEIAGPPDTPYE **Charge** 2 **Spectral Angle** 0.72

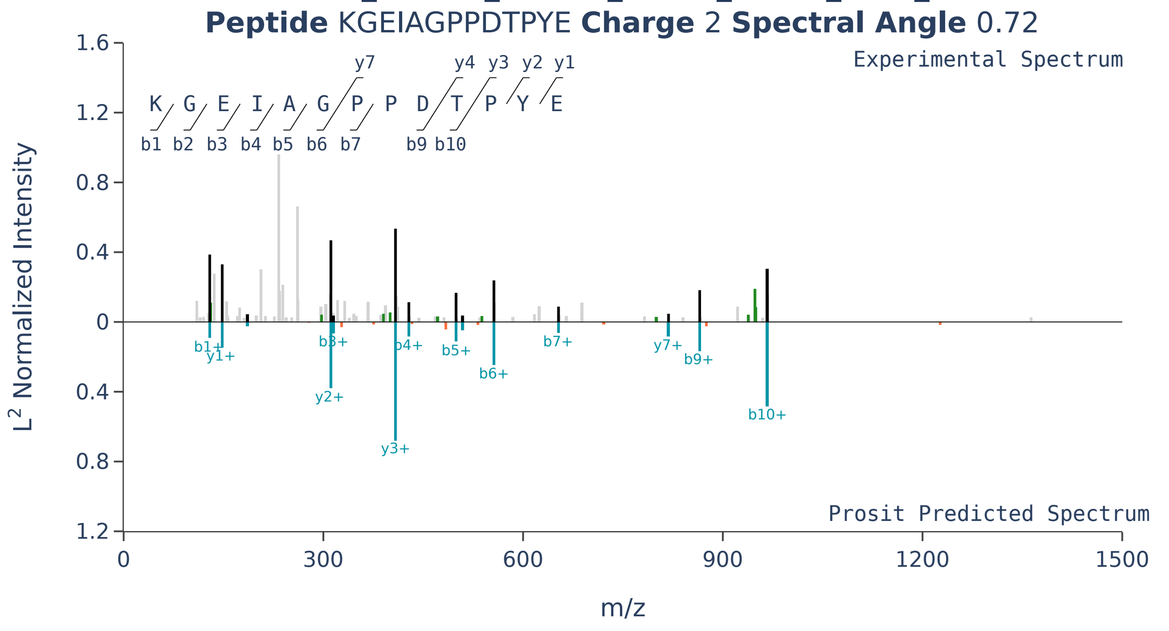

Source HRoetschke_271222_100123_HFGoe_UBE2K_b1R2_24h **Scan** 13382
Peptide AAGPPDTPYE **Charge** 1 **Spectral Angle** 0.79

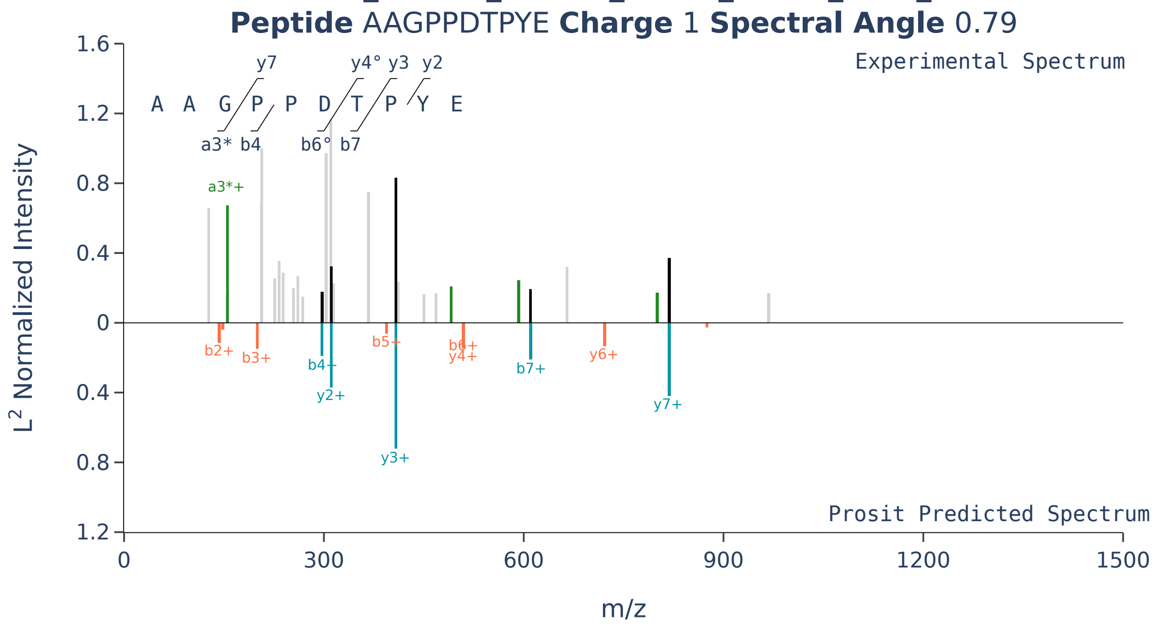

Source HRoetschke_271222_100123_HFGoe_UBE2K_b1R2_24h **Scan** 10910
Peptide ENFTELRGGR **Charge** 3 **Spectral Angle** 0.87

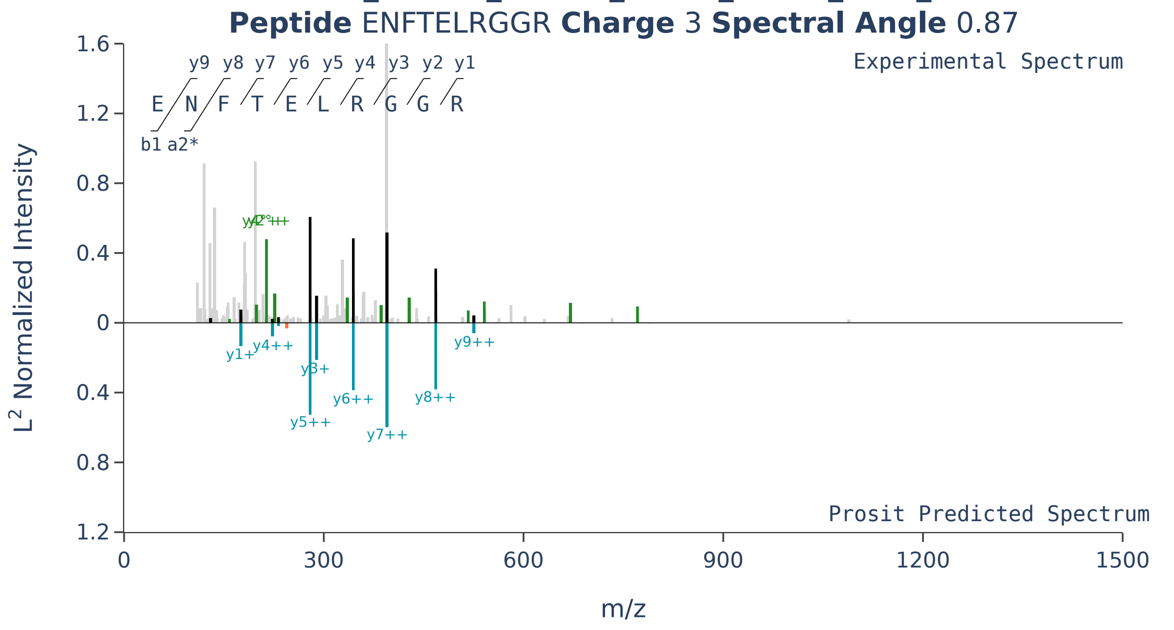

Source HRoetschke_271222_100123_HFGoe_UBE2K_b1R2_24h **Scan** 15103
Peptide RGIAGPPDTPYEGGRY **Charge** 3 **Spectral Angle** 0.81

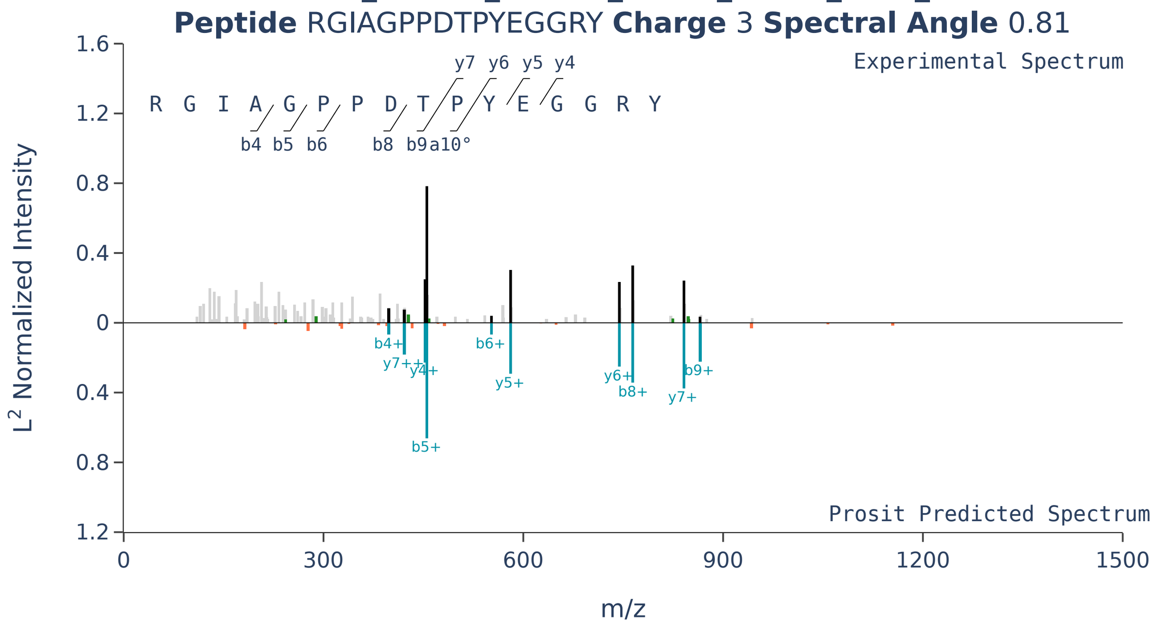

Source HRoetschke_271222_100123_HFGoe_UBE2K_b2R2_24h **Scan** 11895
Peptide VYAGAPVSE **Charge** 1 **Spectral Angle** 0.81

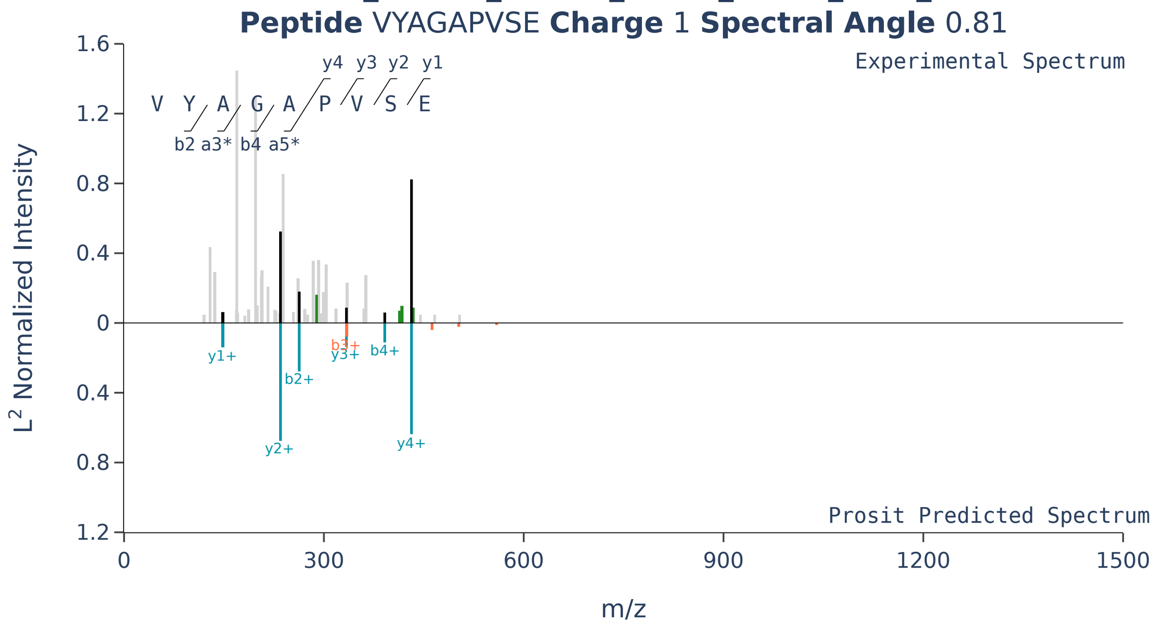

Source HRoetschke_271222_100123_HFGoe_UBE2K_b1R1_1h **Scan** 13584
Peptide SPEYTLE **Charge** 2 **Spectral Angle** 0.68

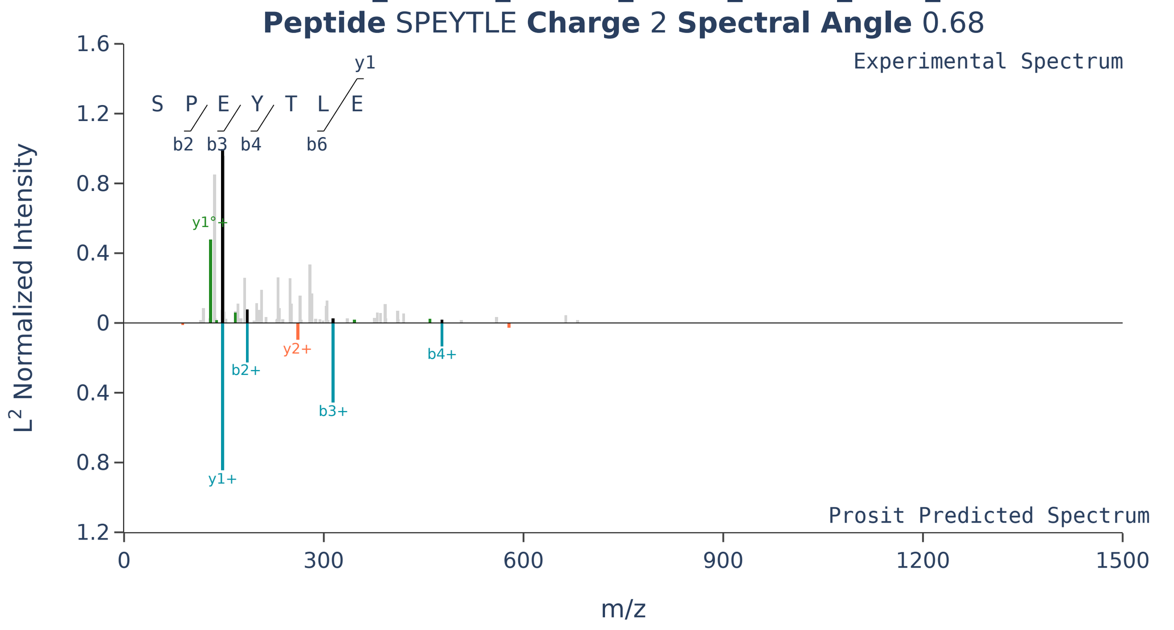

Source HRoetschke_271222_100123_HFGoe_UBE2K_b2R1_4h **Scan** 8998
Peptide AGAPVSVS **Charge** 1 **Spectral Angle** 0.83

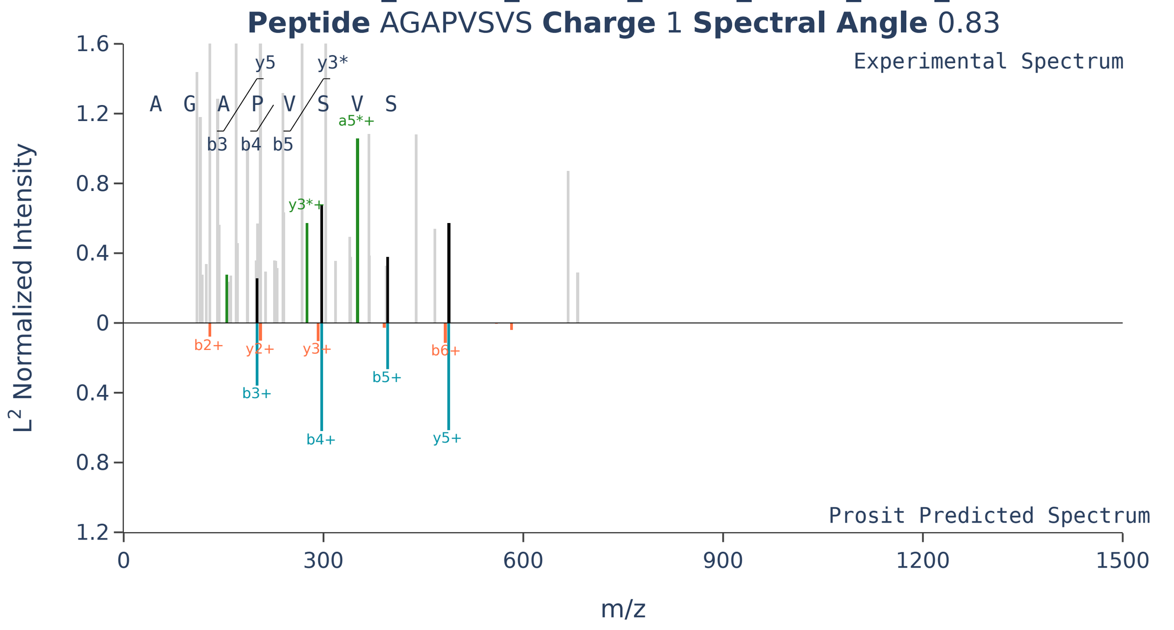

Source HRoetschke_271222_100123_HFGoe_UBE2K_b1R1_24h **Scan** 6915
Peptide PEYTKKIEE **Charge** 3 **Spectral Angle** 0.83

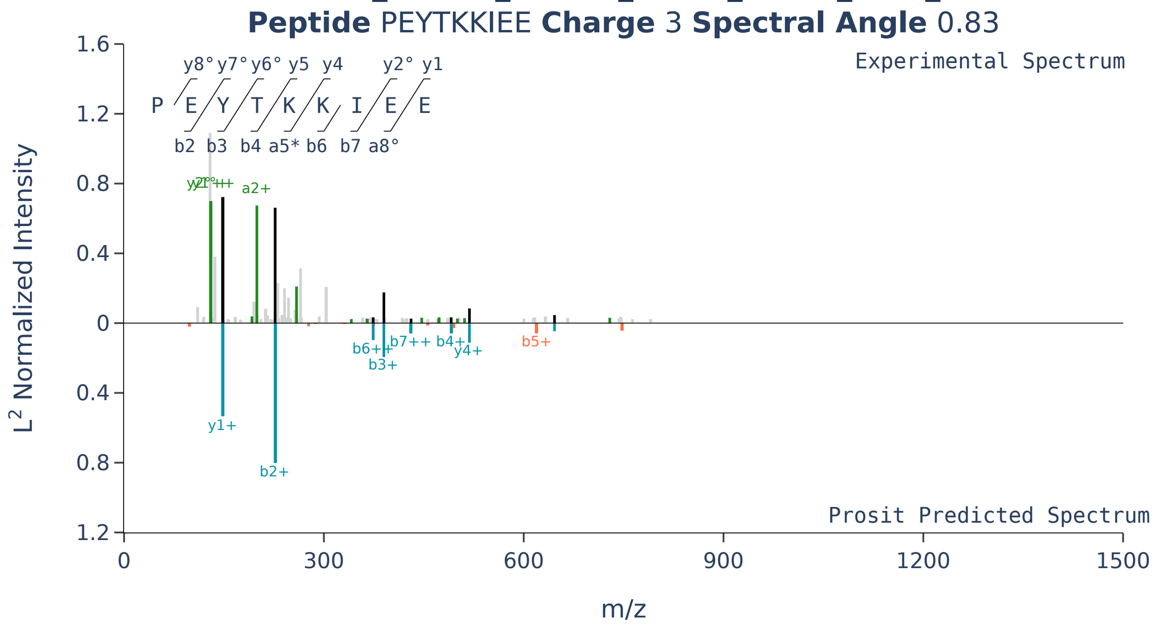

Source HRoetschke_271222_100123_HFGoe_UBE2K_b1R2_24h **Scan** 17294
Peptide RTEIKIPETY **Charge** 2 **Spectral Angle** 0.77

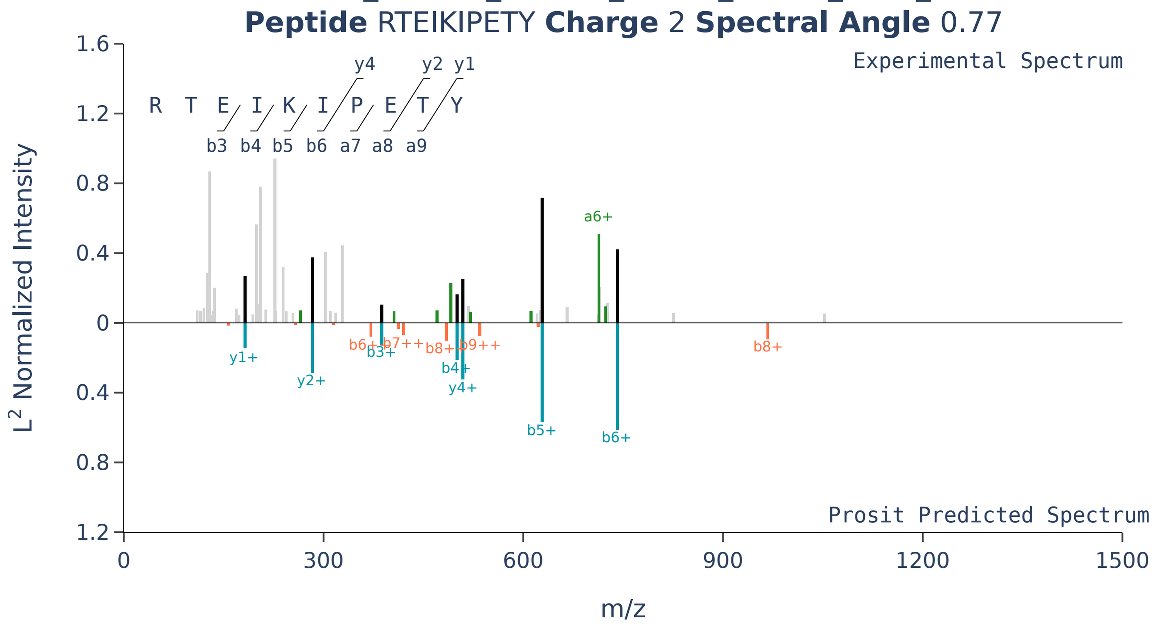

Source HRoetschke_271222_100123_HFGoe_UBE2K_b1R2_2h **Scan** 9582
Peptide RGAGPPDTPYE **Charge** 2 **Spectral Angle** 0.9

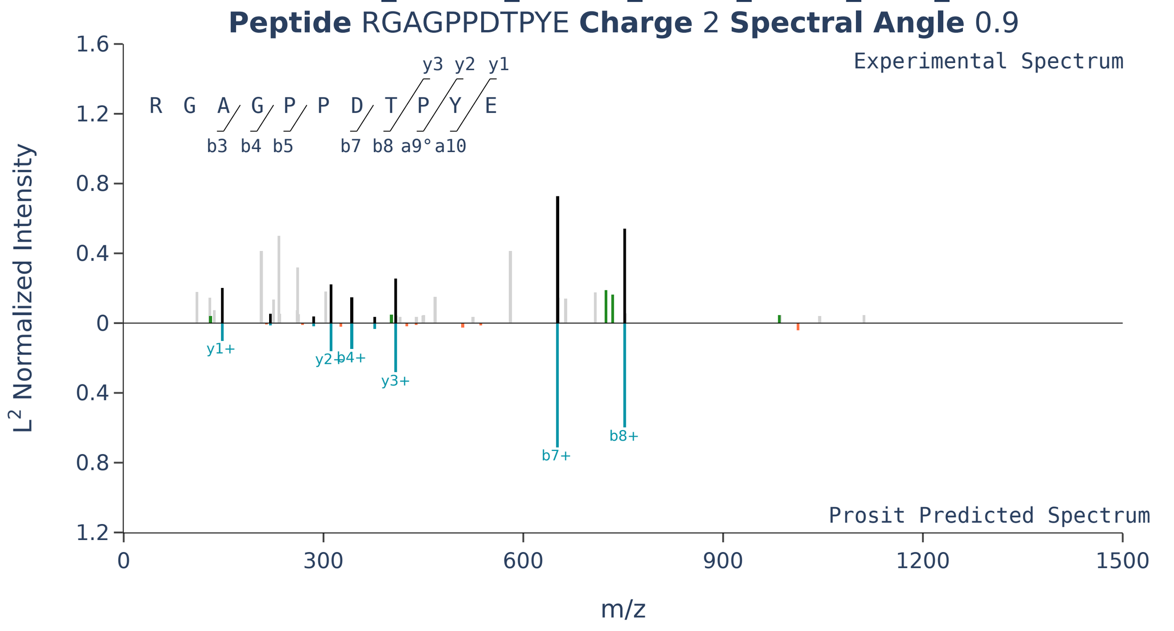

Source HRoetschke_271222_100123_HFGoe_UBE2K_b1R2_24h **Scan** 17094
Peptide PNISSVTGRGEI **Charge** 2 **Spectral Angle** 0.76

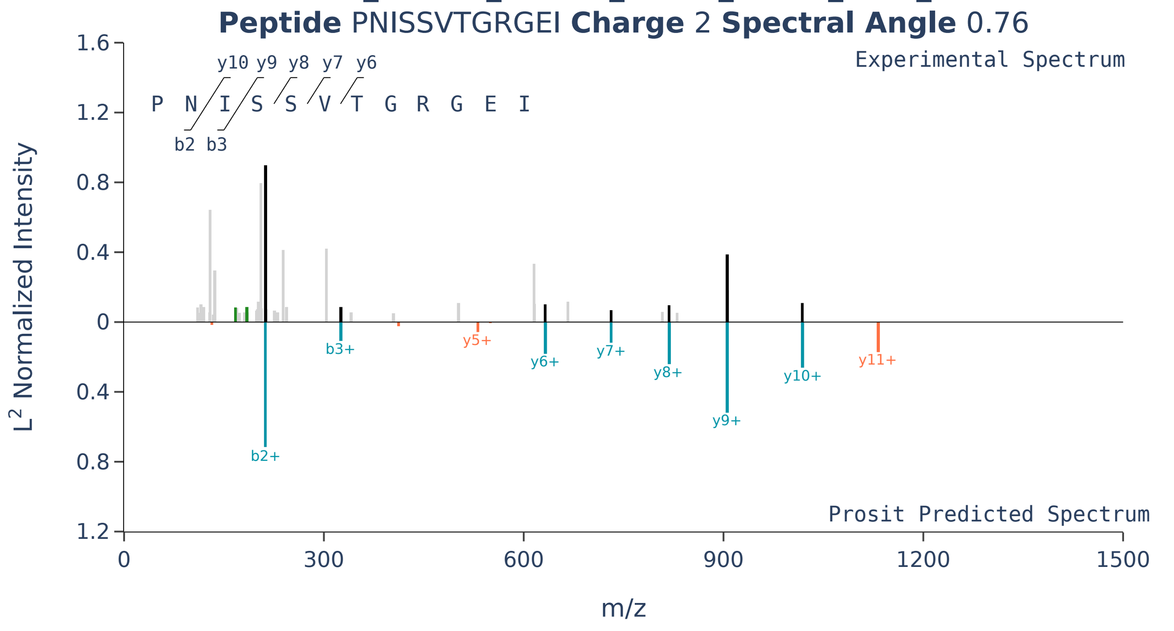

Source HRoetschke_271222_100123_HFGoe_UBE2K_b2R2_24h **Scan** 5796
Peptide GRAAAEPDDPQD **Charge** 2 **Spectral Angle** 0.88

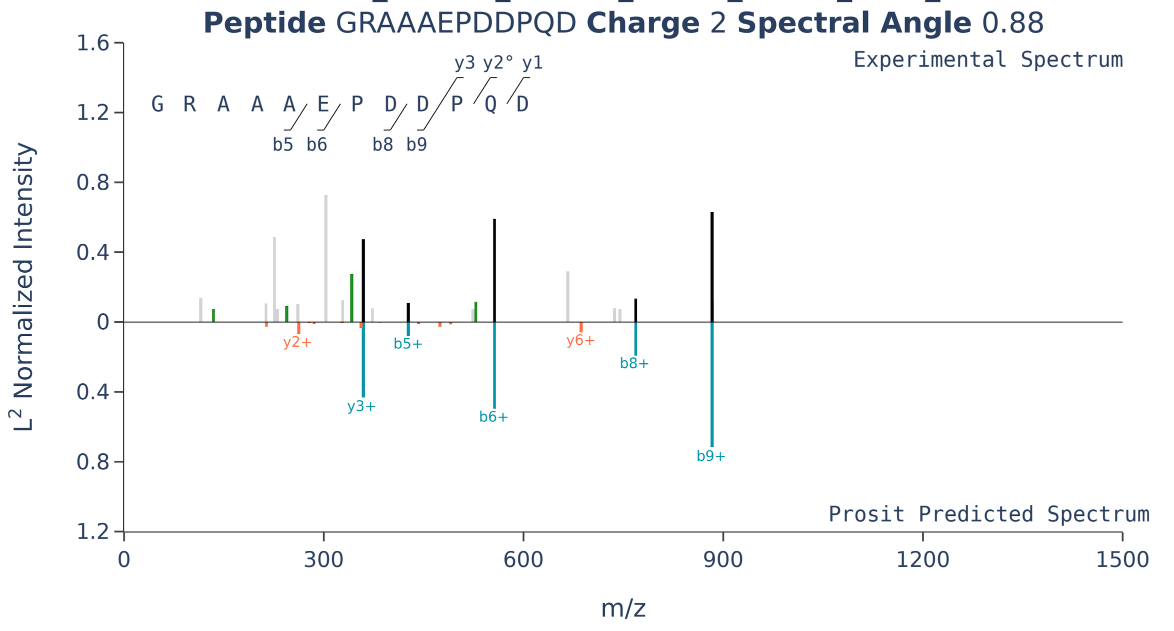

Source HRoetschke_271222_100123_HFGoe_UBE2K_b1R2_24h **Scan** 11869
Peptide SSKSWDVETG **Charge** 2 **Spectral Angle** 0.81

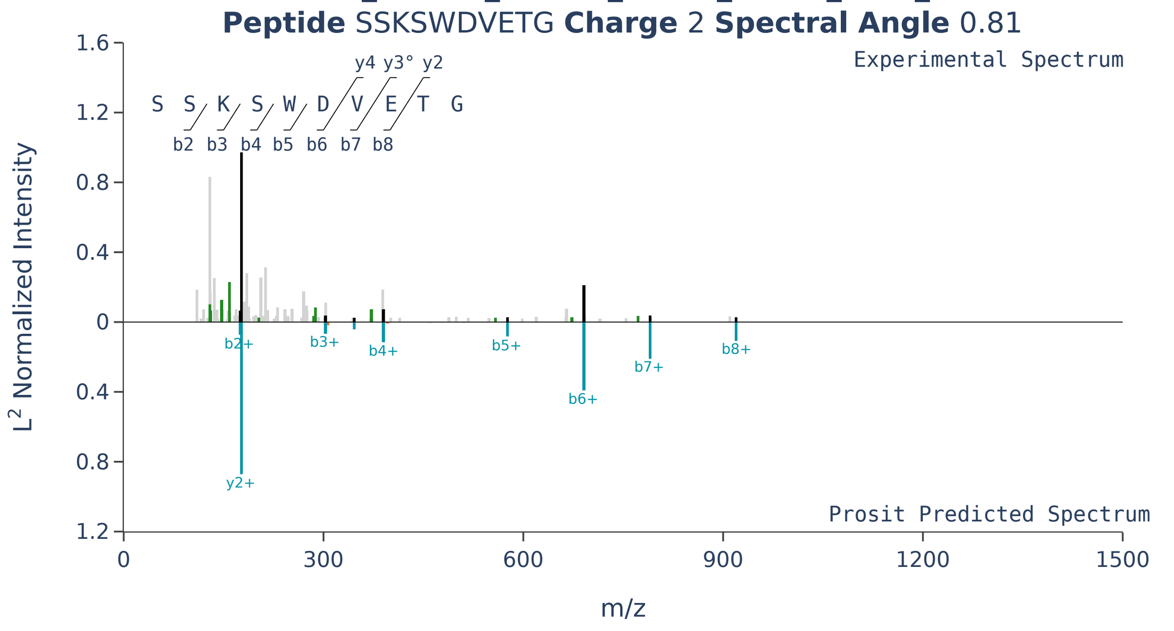

Source HRoetschke_271222_100123_HFGoe_UBE2K_b1R2_24h **Scan** 14523
Peptide ENFTELRGEETS **Charge** 2 **Spectral Angle** 0.72

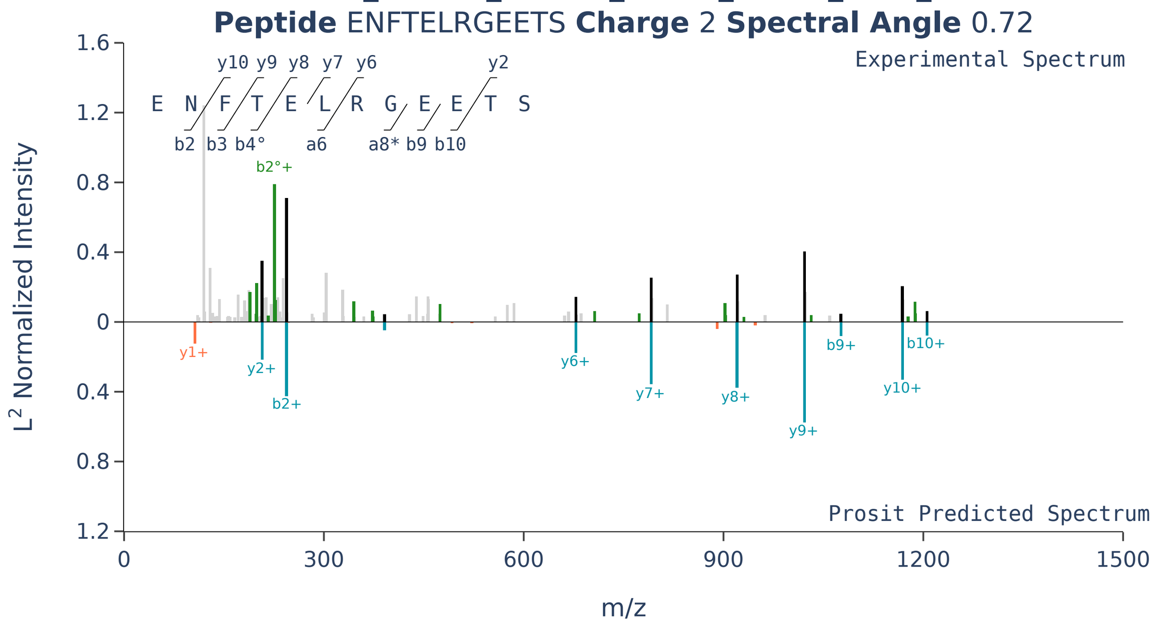

Source HRoetschke_271222_100123_HFGoe_UBE2K_b2R2_1h **Scan** 10093
Peptide AHVYAGAPVSG **Charge** 2 **Spectral Angle** 0.82

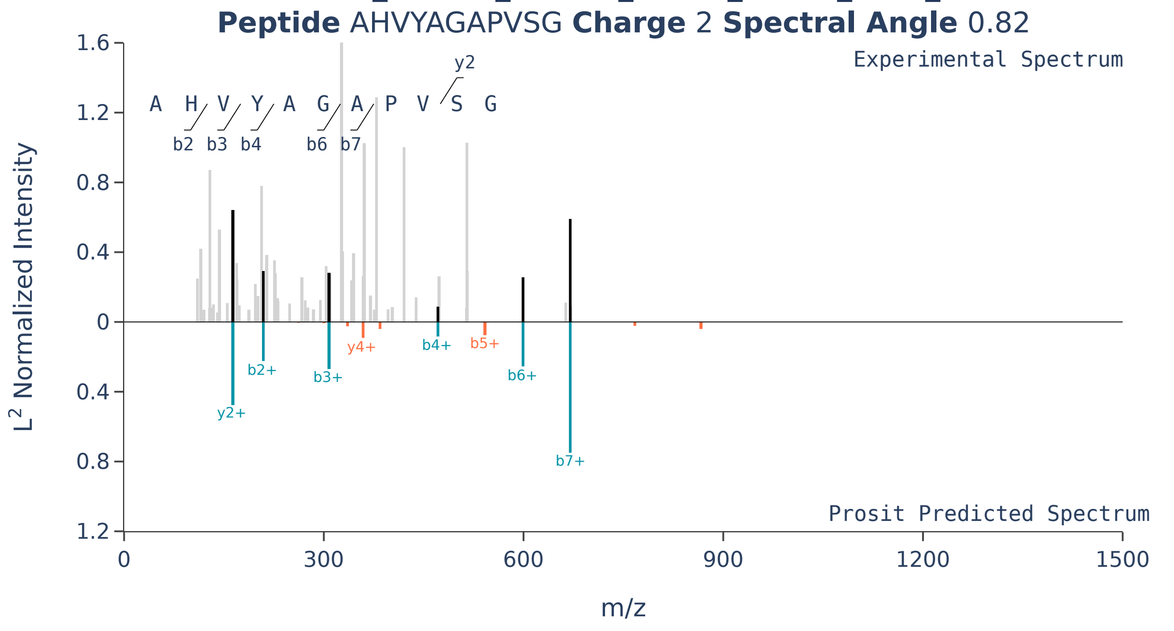

Source HRoetschke_271222_100123_HFGoe_UBE2K_b1R2_24h **Scan** 11507
Peptide RNAPPDTPYEGGRY **Charge** 3 **Spectral Angle** 0.74

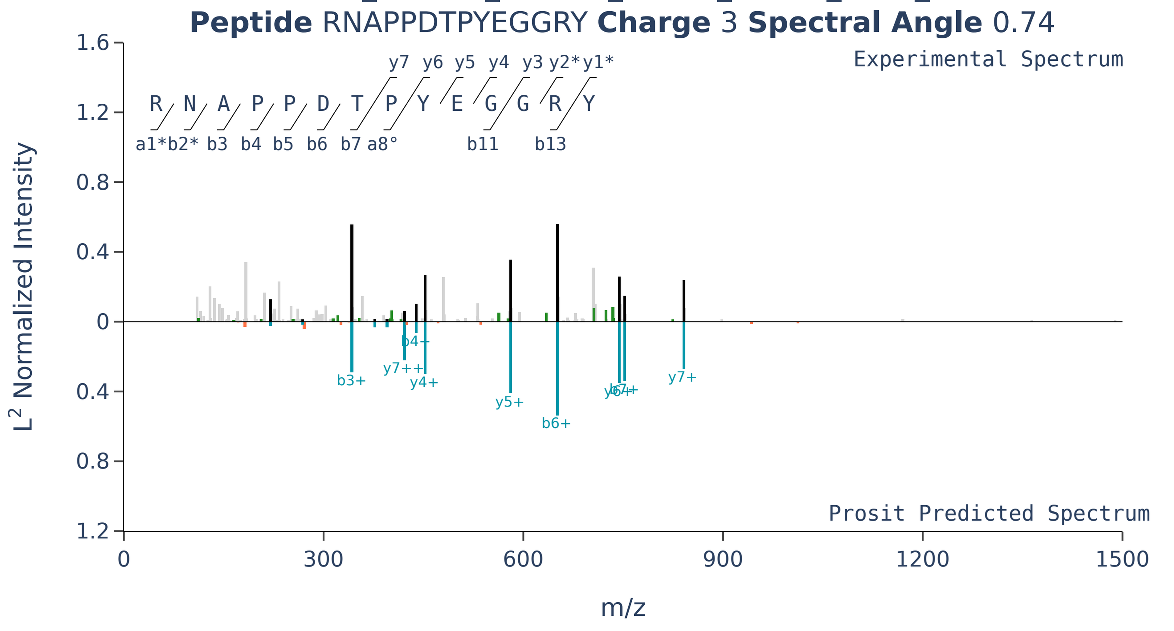

Source HRoetschke_271222_100123_HFGoe_UBE2K_b1R1_24h **Scan** 9655
Peptide EMGFDRN **Charge** 2 **Spectral Angle** 0.74

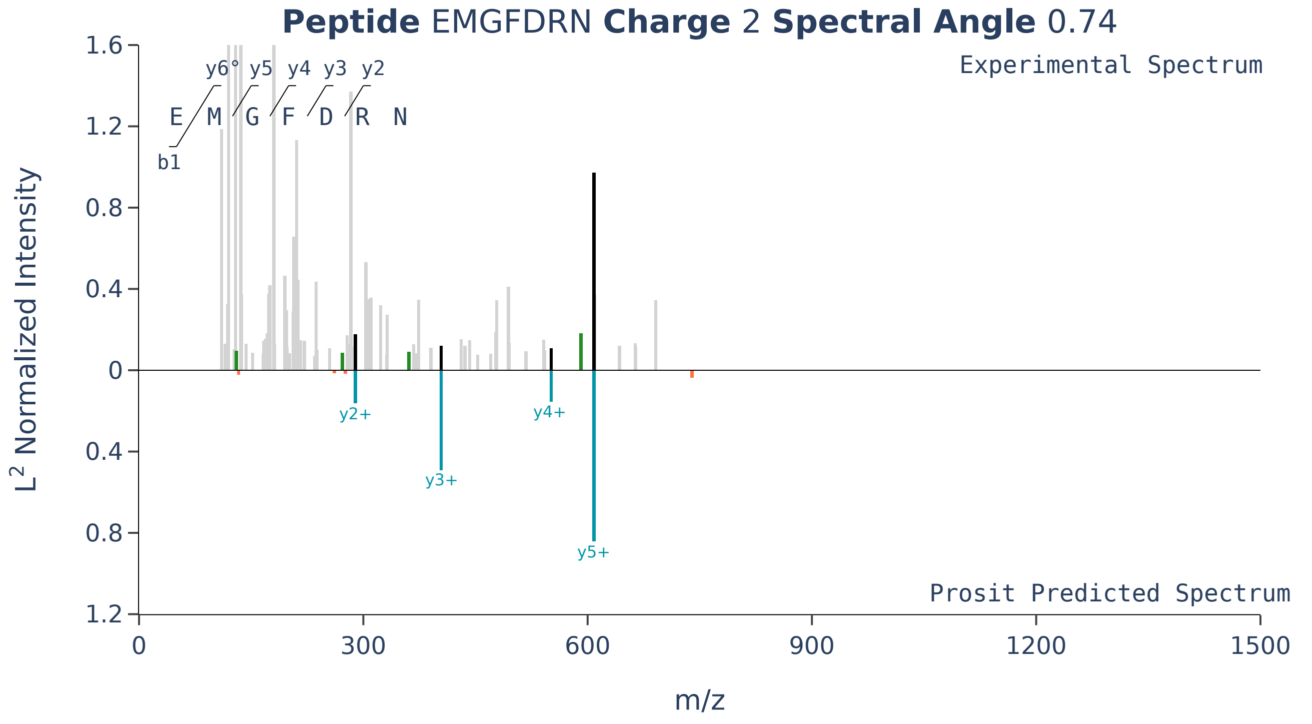

Source HRoetschke_271222_100123_HFGoe_UBE2K_b1R2_24h **Scan** 13695
Peptide SSAGPPDTPYEGGRY **Charge** 2 **Spectral Angle** 0.84

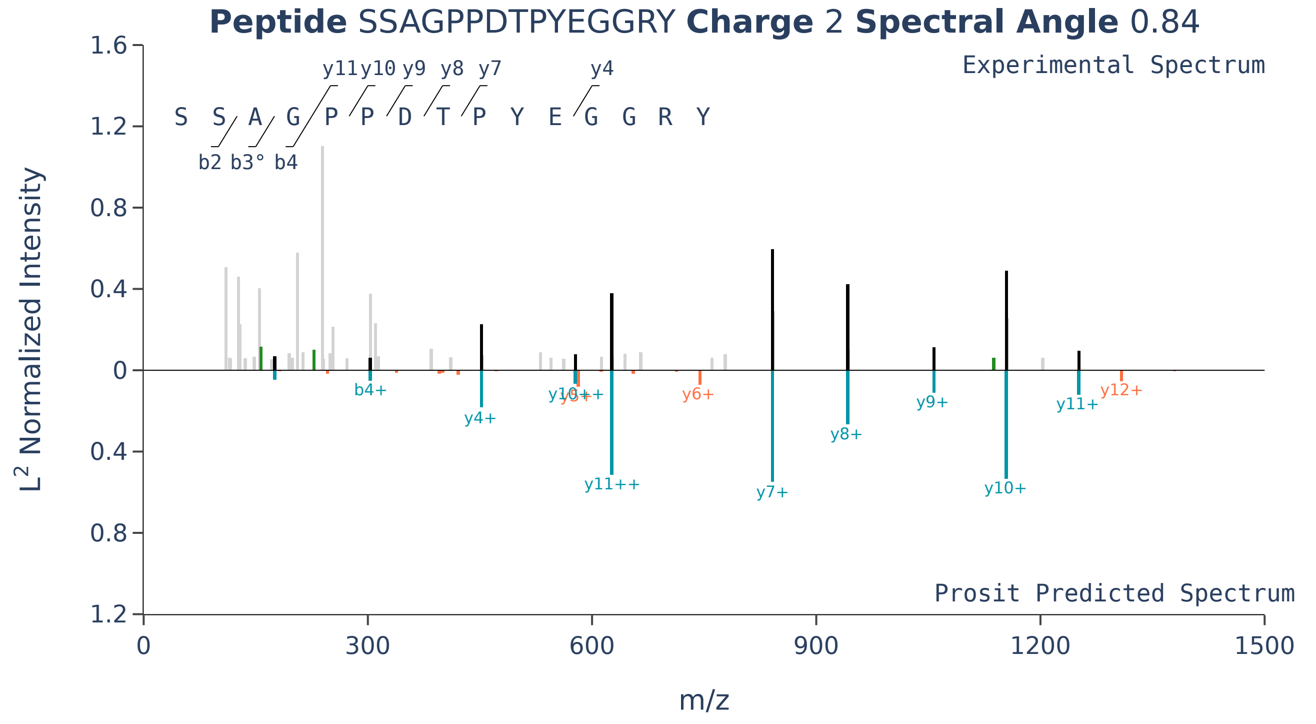

Source HRoetschke_271222_100123_HFGoe_UBE2K_b2R2_24h **Scan** 8559
Peptide RGKVDLVD **Charge** 2 **Spectral Angle** 0.82

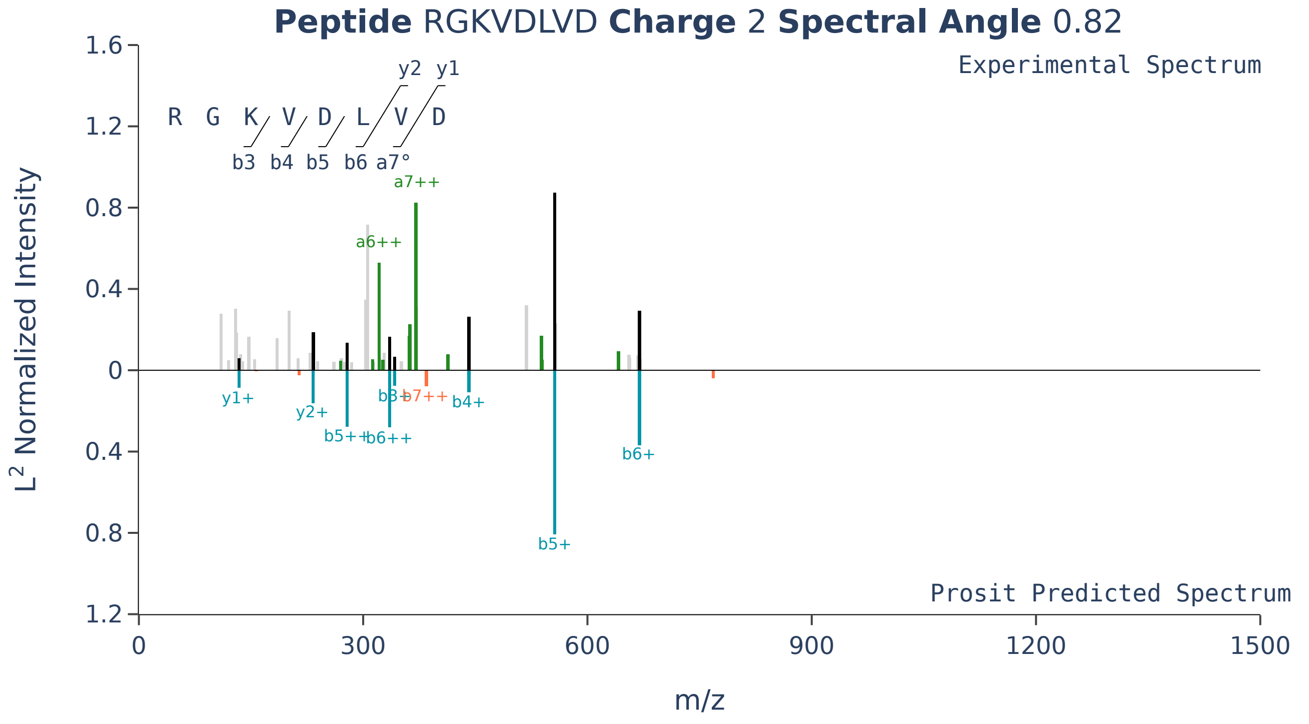

Source HRoetschke_271222_100123_HFGoe_UBE2K_b2R1_24h **Scan** 13481
Peptide MAGPPDTPYEGGRY **Charge** 2 **Spectral Angle** 0.72

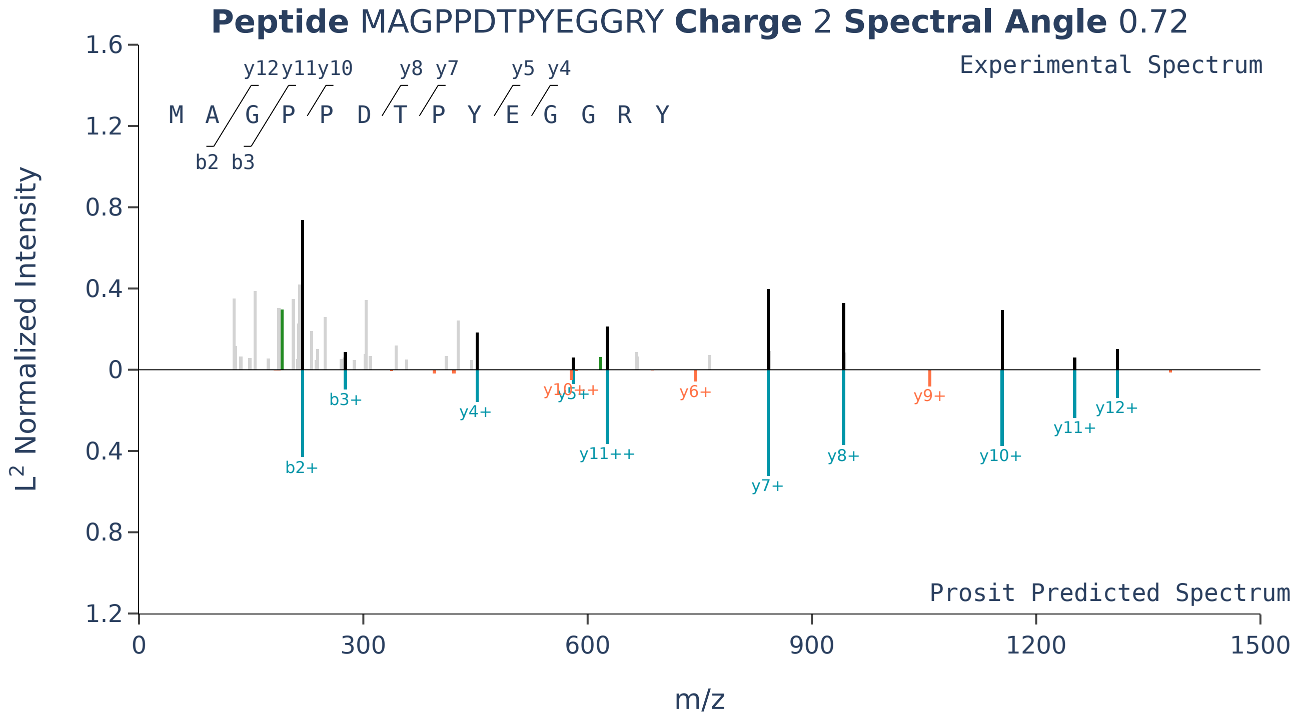

Source HRoetschke_271222_100123_HFGoe_UBE2K_b1R2_24h **Scan** 15450
Peptide KQNPEMFKQTAL **Charge** 3 **Spectral Angle** 0.83

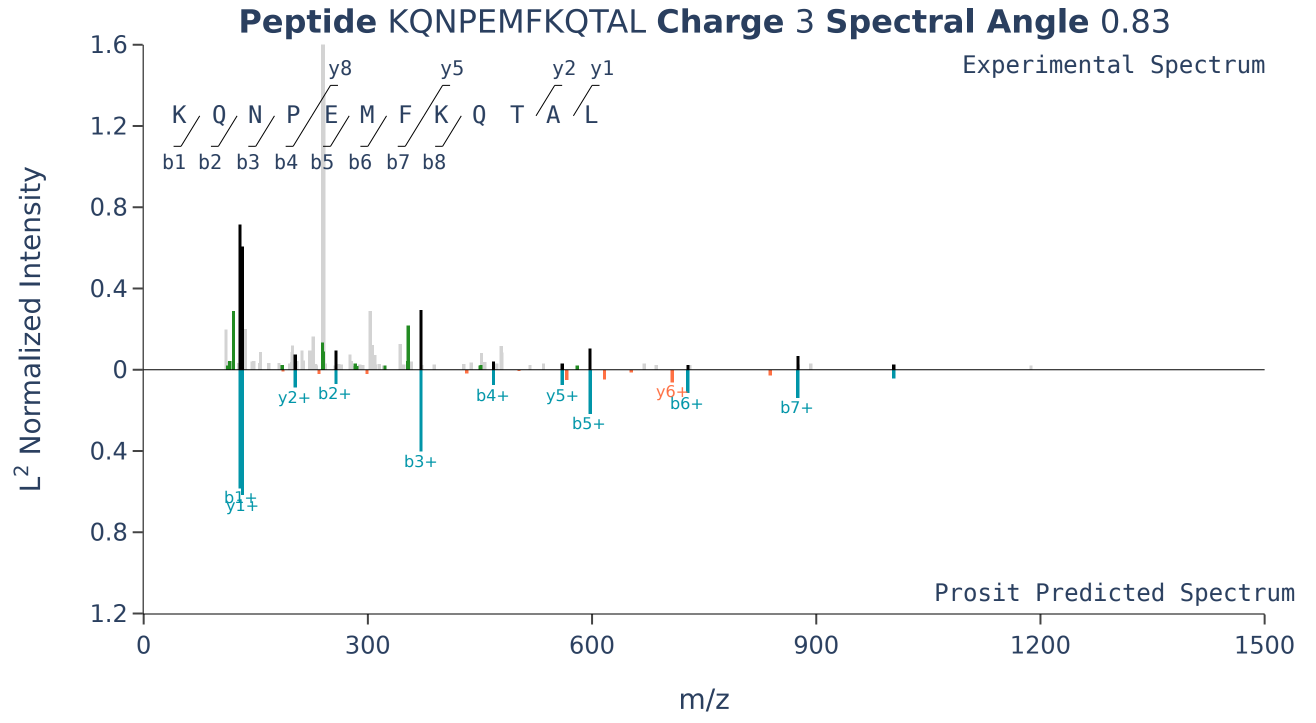

Source HRoetschke_271222_100123_HFGoe_UBE2K_b1R1_24h **Scan** 8246
Peptide LRGEAAAEPDDPQD **Charge** 2 **Spectral Angle** 0.83

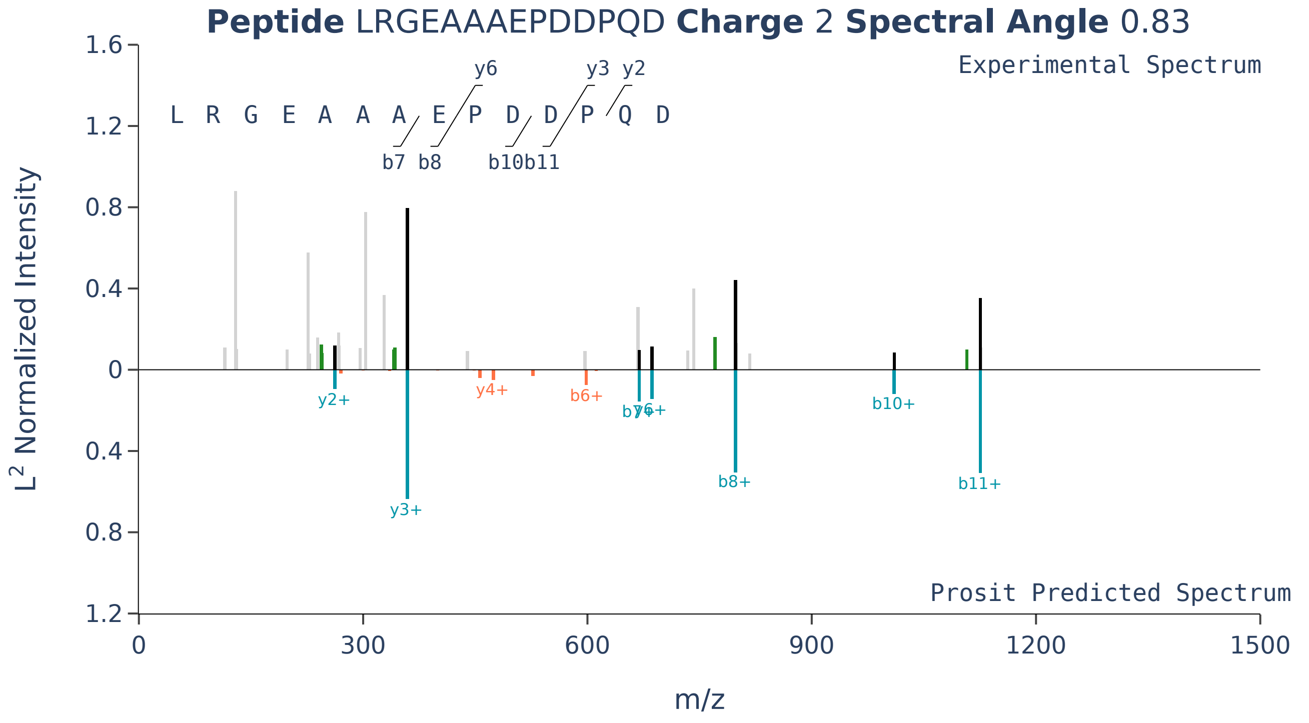

Source HRoetschke_271222_100123_HFGoe_UBE2K_b1R2_24h **Scan** 14693
Peptide DQNFTELRG **Charge** 2 **Spectral Angle** 0.78

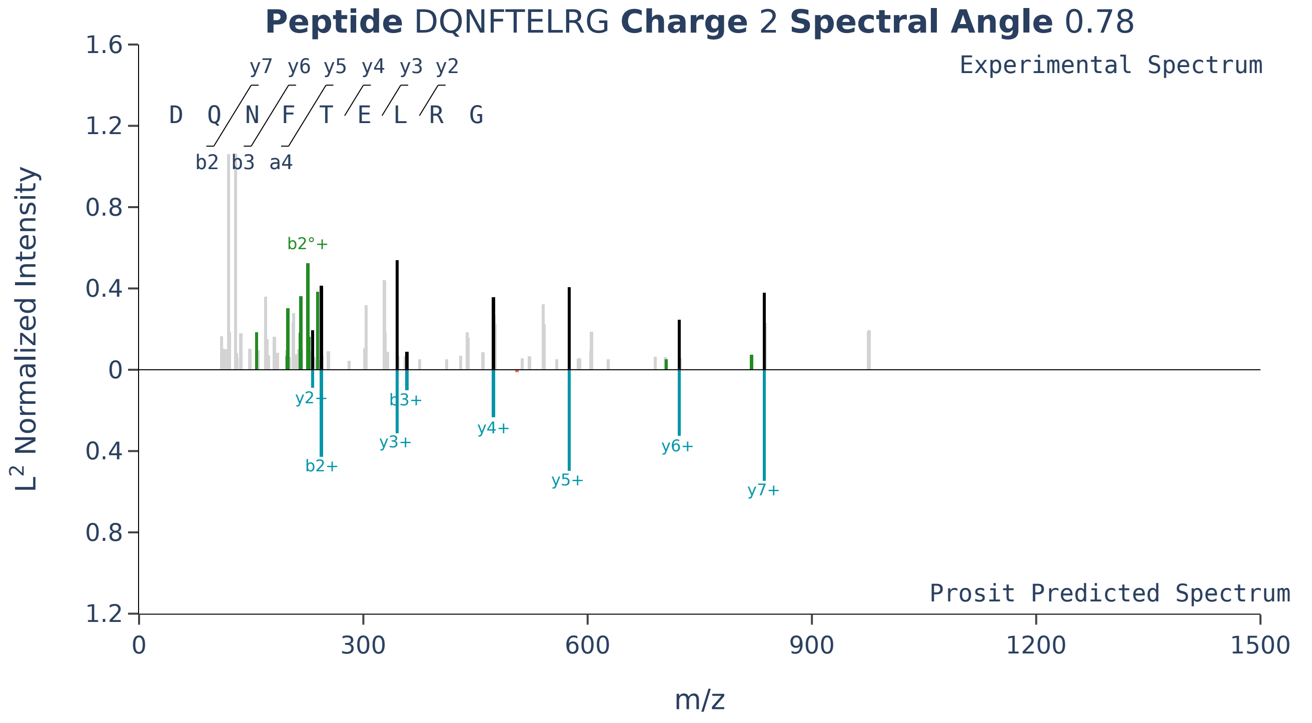

Source HRoetschke_271222_100123_HFGoe_UBE2K_b1R2_24h **Scan** 11200
Peptide ENFTELRGRGE **Charge** 2 **Spectral Angle** 0.77

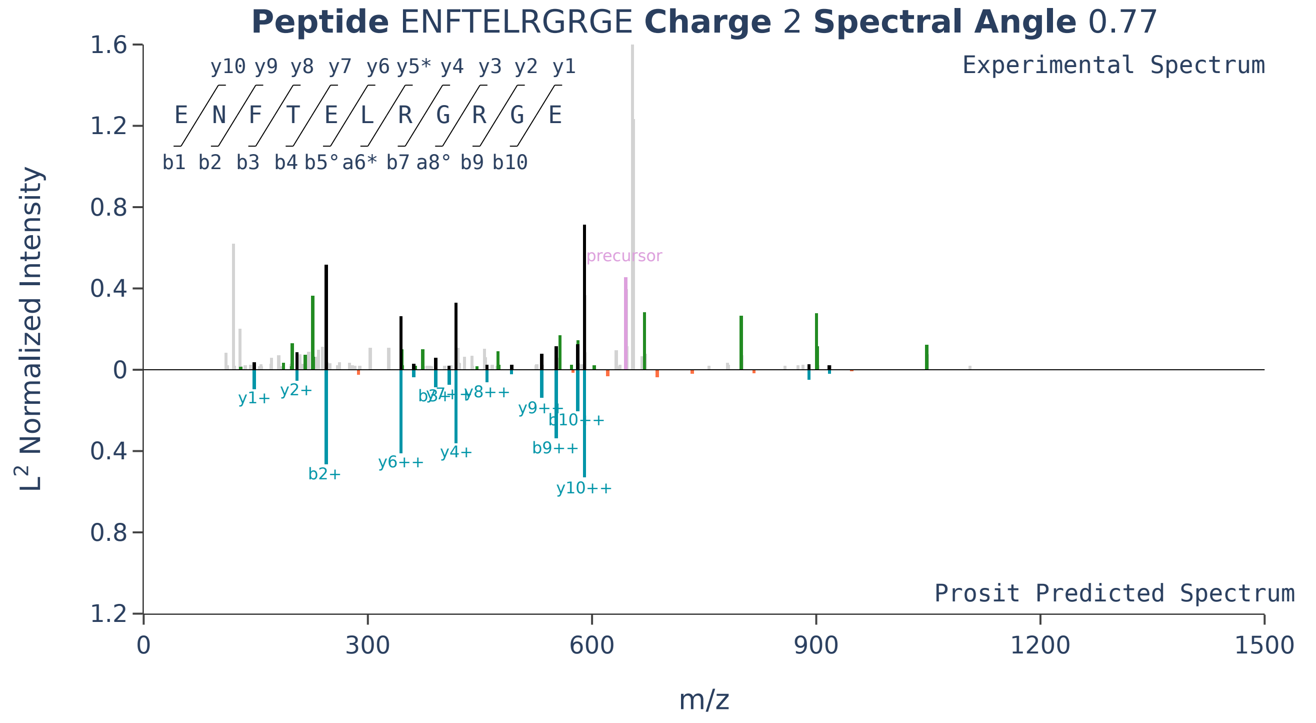

Source HRoetschke_271222_100123_HFGoe_UBE2K_b1R1_24h **Scan** 12561
Peptide RGEIAGEL **Charge** 2 **Spectral Angle** 0.77

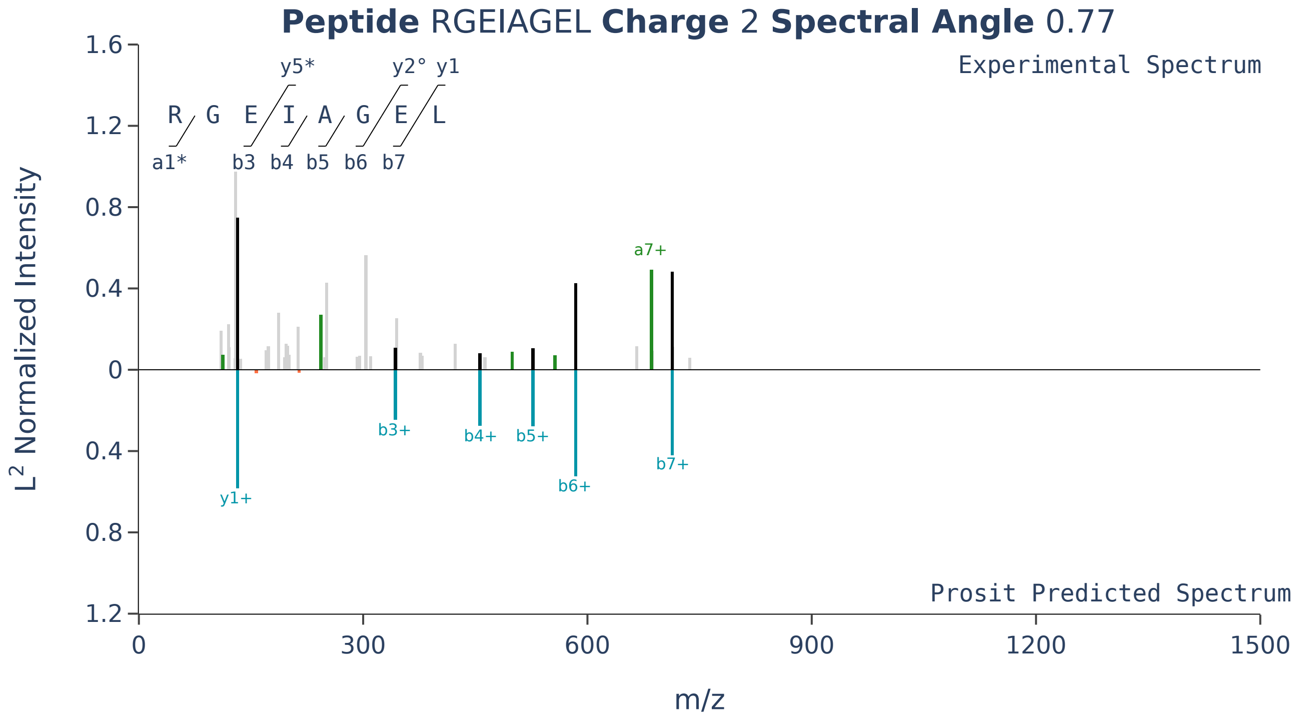

Source HRoetschke_271222_100123_HFGoe_UBE2K_b2R1_4h **Scan** 18761
Peptide ENFTELRGEM **Charge** 2 **Spectral Angle** 0.8

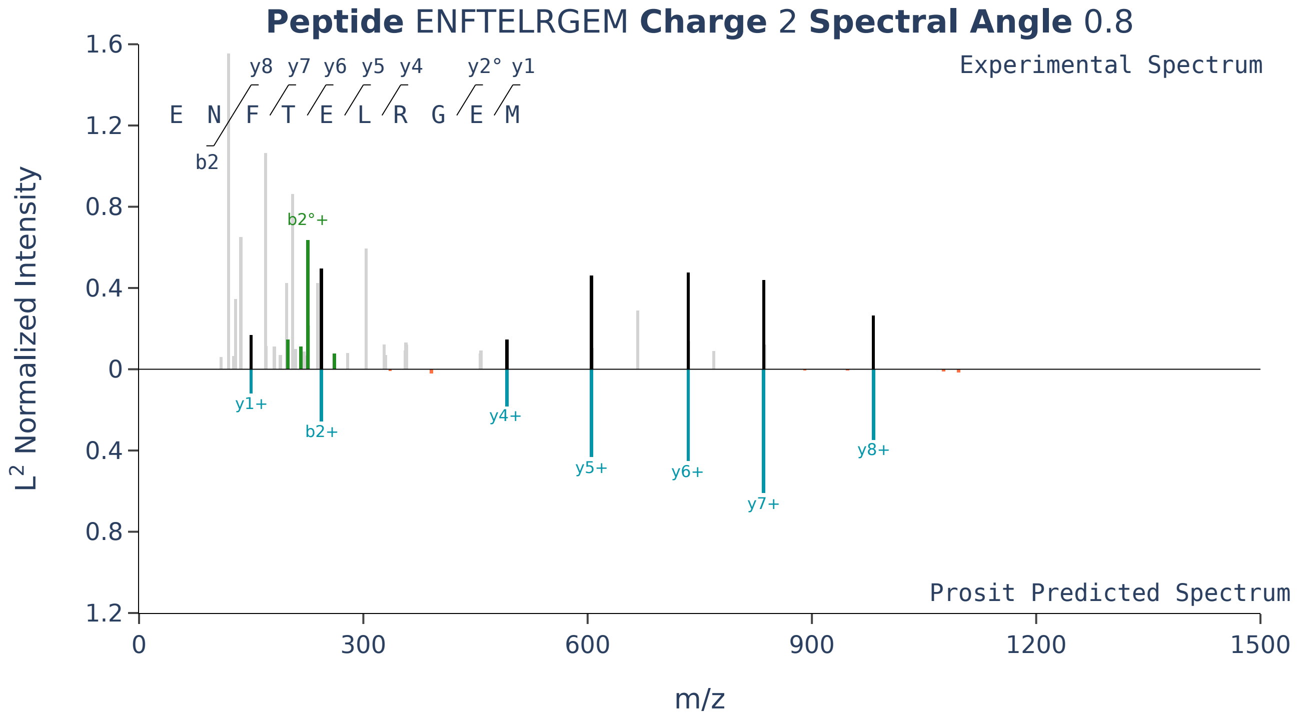

Source HRoetschke_231222_070123_HFGoe_UBE2S_b1R1_24h **Scan** 10900
Peptide PESALNEEASST **Charge** 1 **Spectral Angle** 0.89

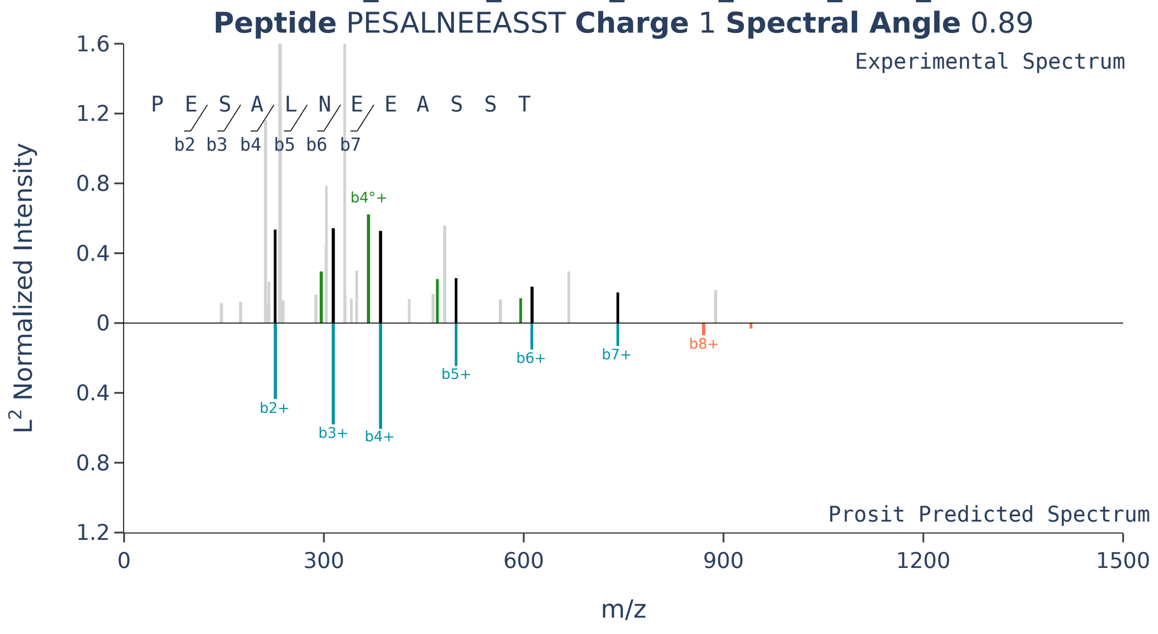

Source HRoetschke_231222_070123_HFGoe_UBE2S_b1R1_2h **Scan** 6743
Peptide RAEAGALNE **Charge** 2 **Spectral Angle** 0.9

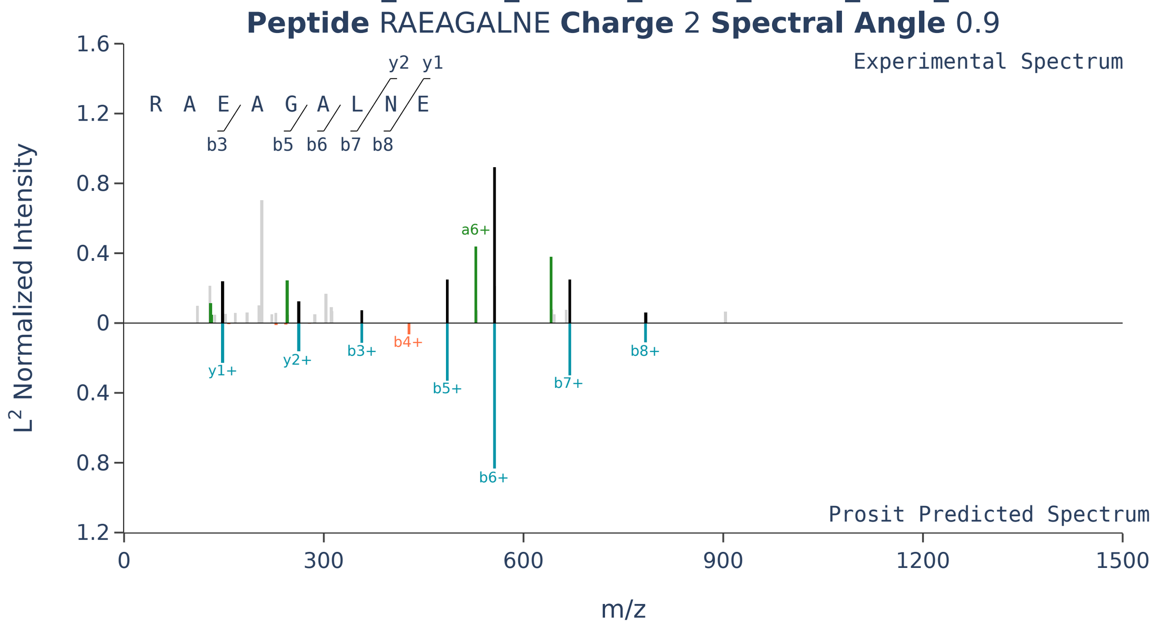

Source HRoetschke_231222_070123_HFGoe_UBE2S_b1R2_2h **Scan** 8050
Peptide HGGAGGPSGL **Charge** 2 **Spectral Angle** 0.93

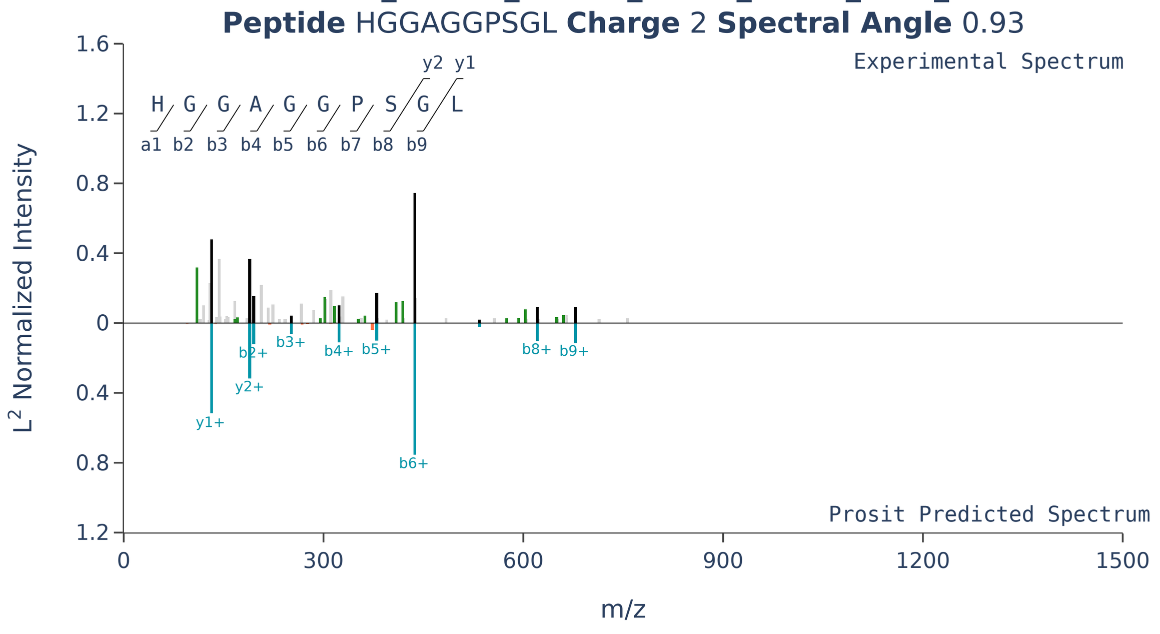

Source HRoetschke_231222_070123_HFGoe_UBE2S_b1R1_24h **Scan** 6815
Peptide HGGAGGPSGAGRL **Charge** 3 **Spectral Angle** 0.84

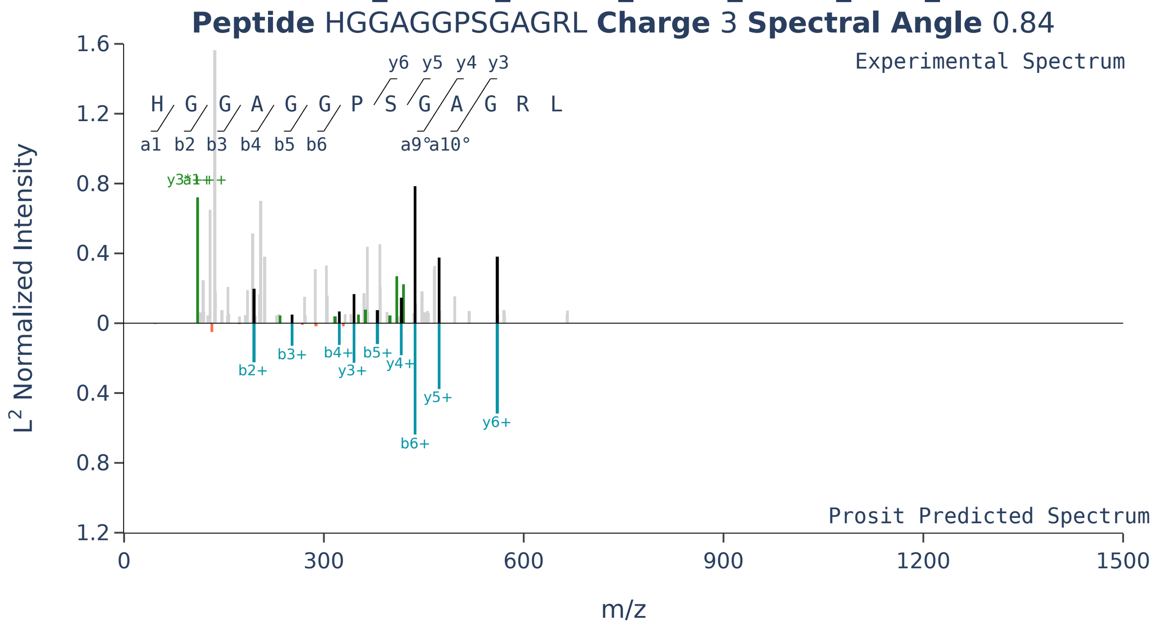

Source HRoetschke_231222_070123_HFGoe_UBE2S_b1R2_24h **Scan** 10169
Peptide LIHPNPESE **Charge** 2 **Spectral Angle** 0.87

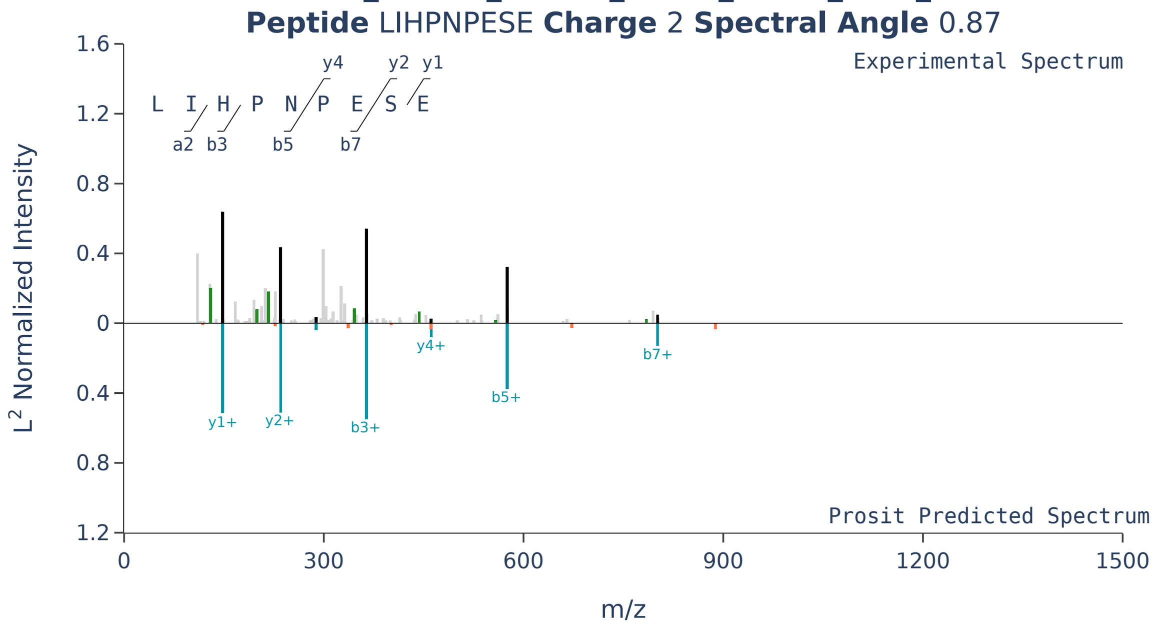

Source HRoetschke_231222_070123_HFGoe_UBE2S_b1R2_24h **Scan** 7302
Peptide HGGAGGPSGEAGRL **Charge** 3 **Spectral Angle** 0.91

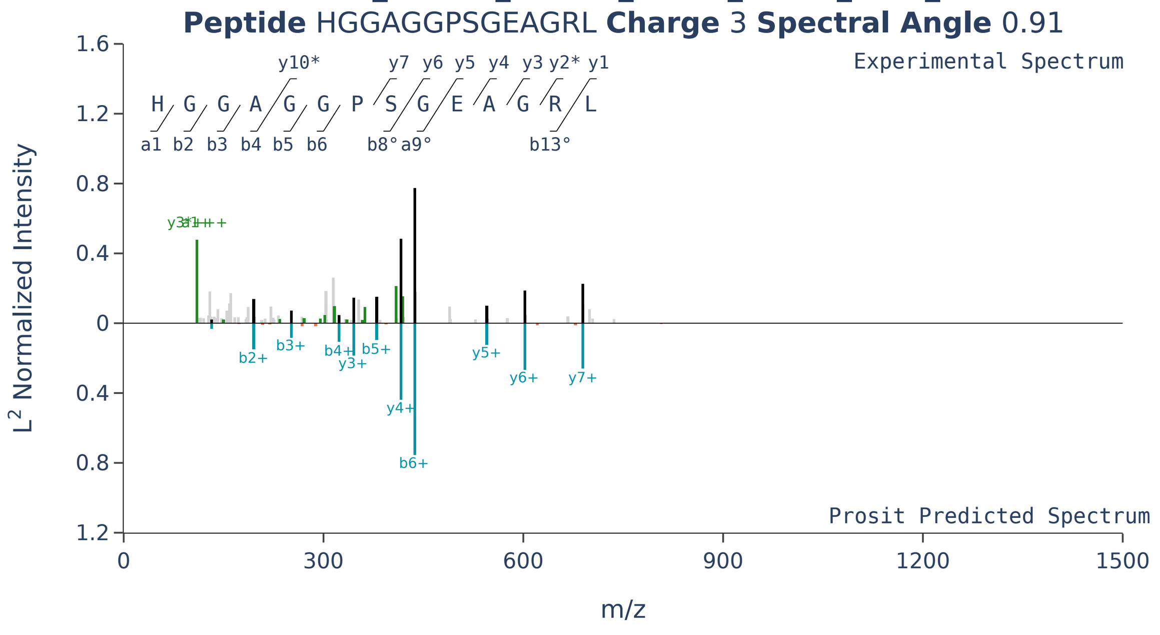

Source HRoetschke_231222_070123_HFGoe_UBE2S_b1R1_24h **Scan** 9233
Peptide LIHPNPESN **Charge** 2 **Spectral Angle** 0.89

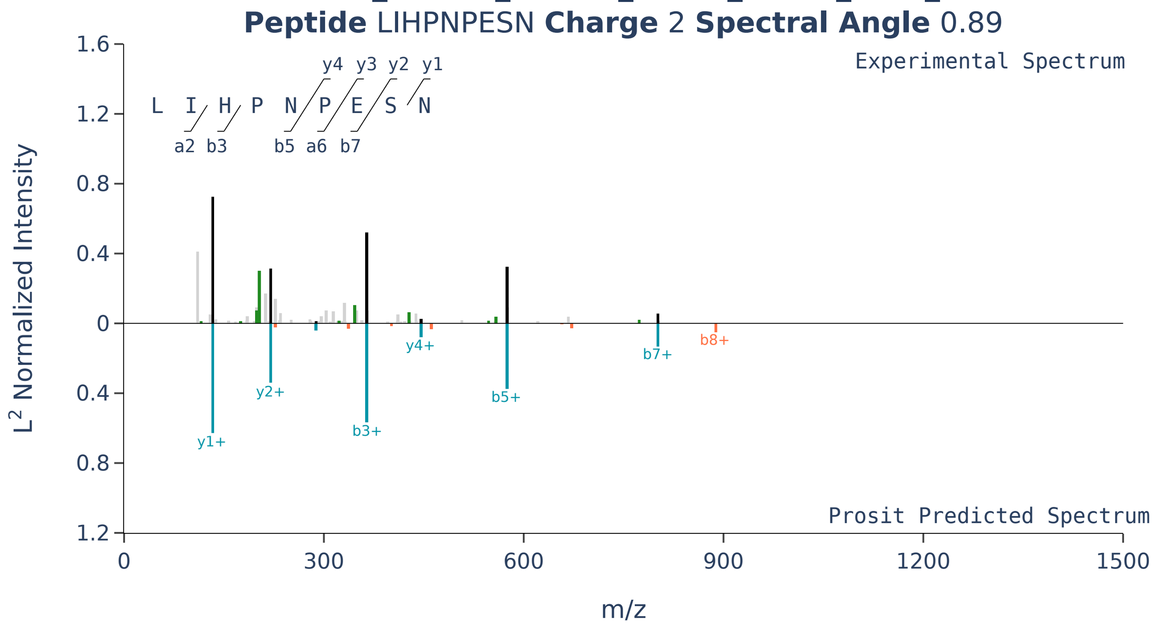

Source HRoetschke_231222_070123_HFGoe_UBE2S_b1R1_24h **Scan** 6272
Peptide ASGRAEAGRAL **Charge** 3 **Spectral Angle** 0.91

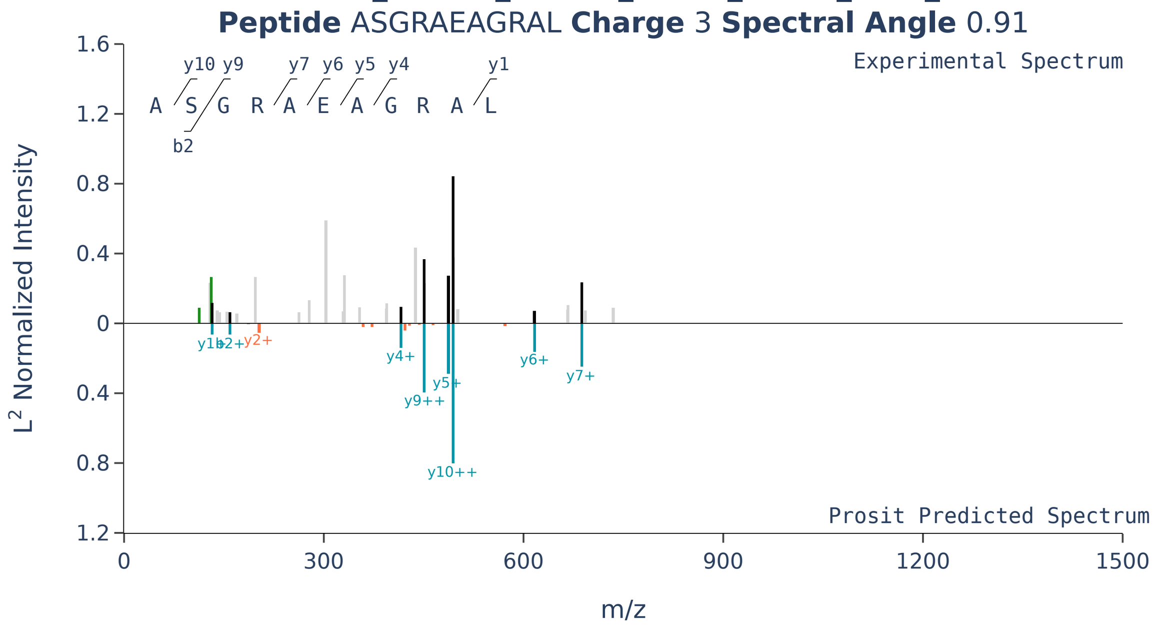

Source HRoetschke_231222_070123_HFGoe_UBE2S_b2R2_24h **Scan** 8293
Peptide ASSTDPGAPGGPGGTE **Charge** 2 **Spectral Angle** 0.91

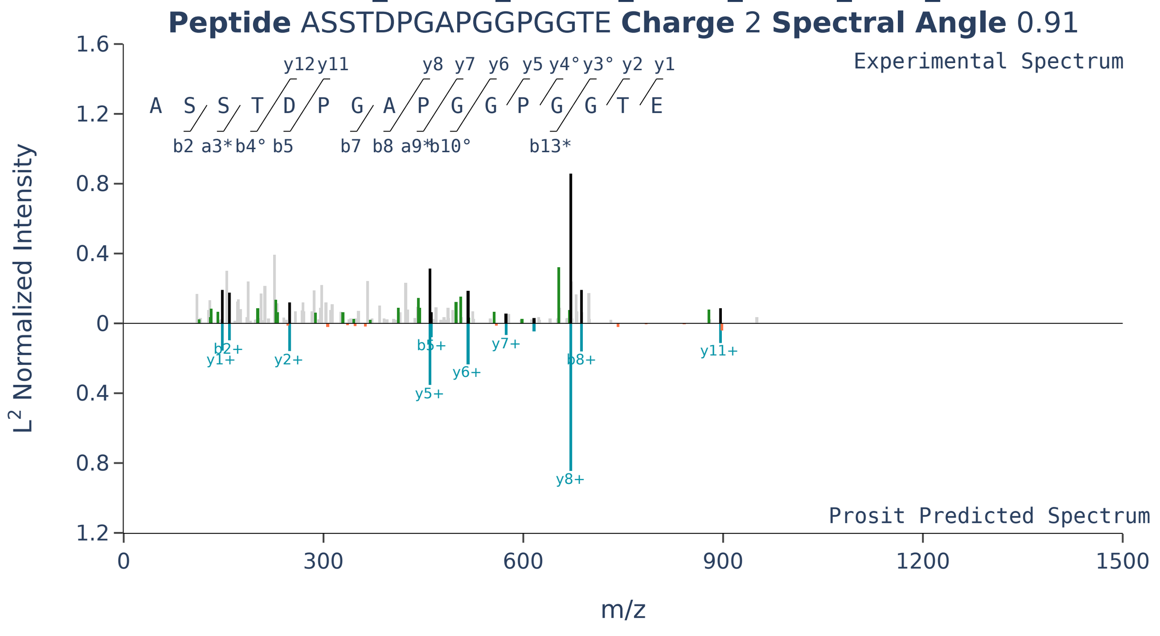

Source HRoetschke_231222_070123_HFGoe_UBE2S_b1R1_24h **Scan** 6091
Peptide SSTDPGAPGGPGGKD **Charge** 2 **Spectral Angle** 0.85

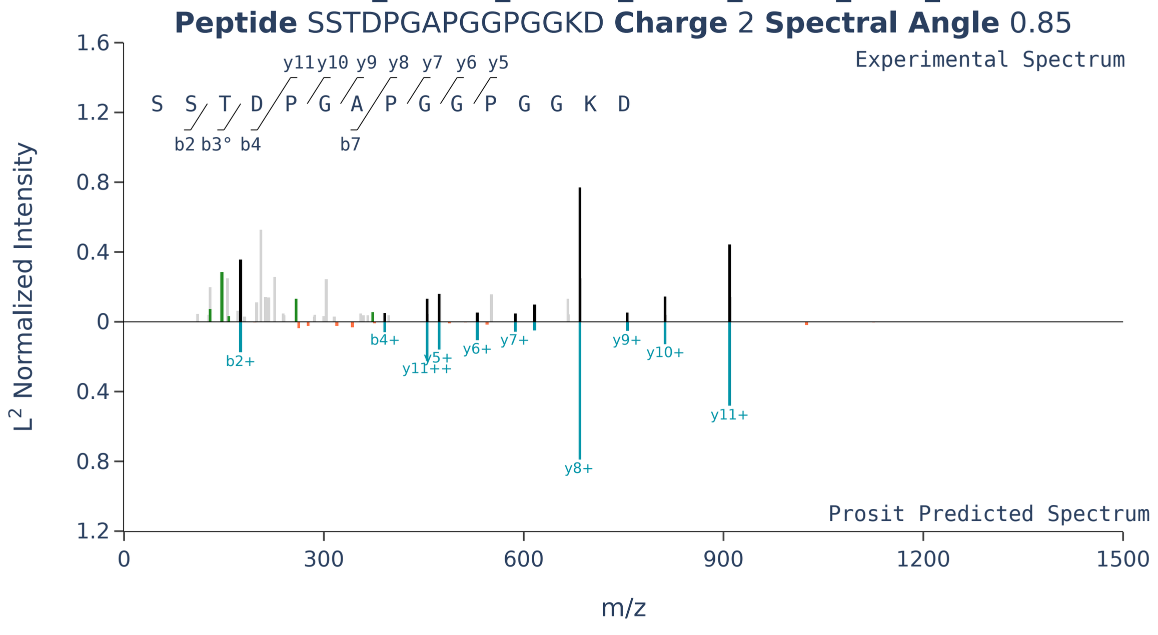

Source HRoetschke_231222_070123_HFGoe_UBE2S_b1R2_24h **Scan** 8830
Peptide AGASSTDPGAPGGPGGAE **Charge** 2 **Spectral Angle** 0.89

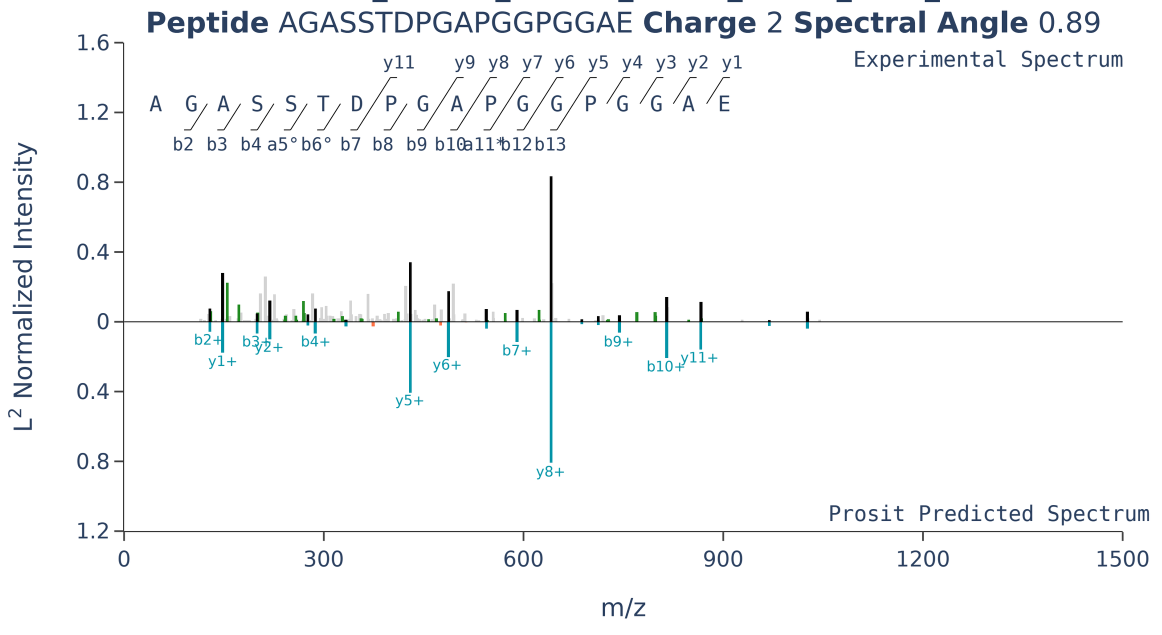

Source HRoetschke_231222_070123_HFGoe_UBE2S_b1R1_24h **Scan** 8933
Peptide GAASSTDPGAPGGPGGAE **Charge** 2 **Spectral Angle** 0.9

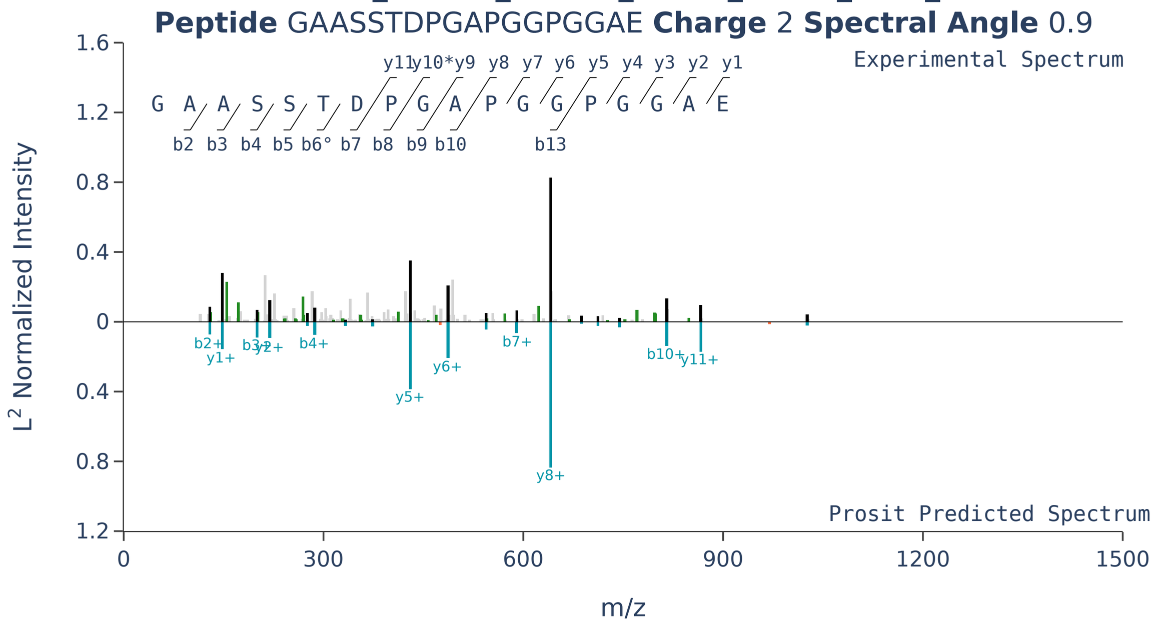

Source HRoetschke_231222_070123_HFGoe_UBE2S_b2R1_24h **Scan** 7410
Peptide SSTDPGAPGGPGGRAEA **Charge** 2 **Spectral Angle** 0.88

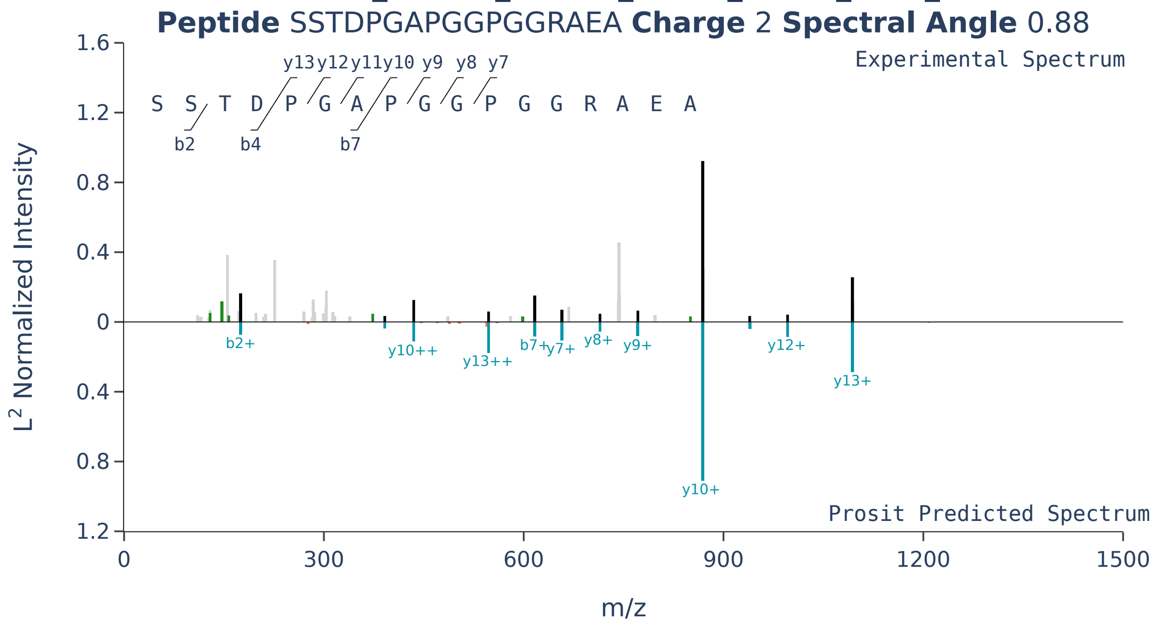

Source HRoetschke_231222_070123_HFGoe_UBE2S_b2R2_24h **Scan** 8074
Peptide HGGAGGPSGLTD **Charge** 2 **Spectral Angle** 0.85

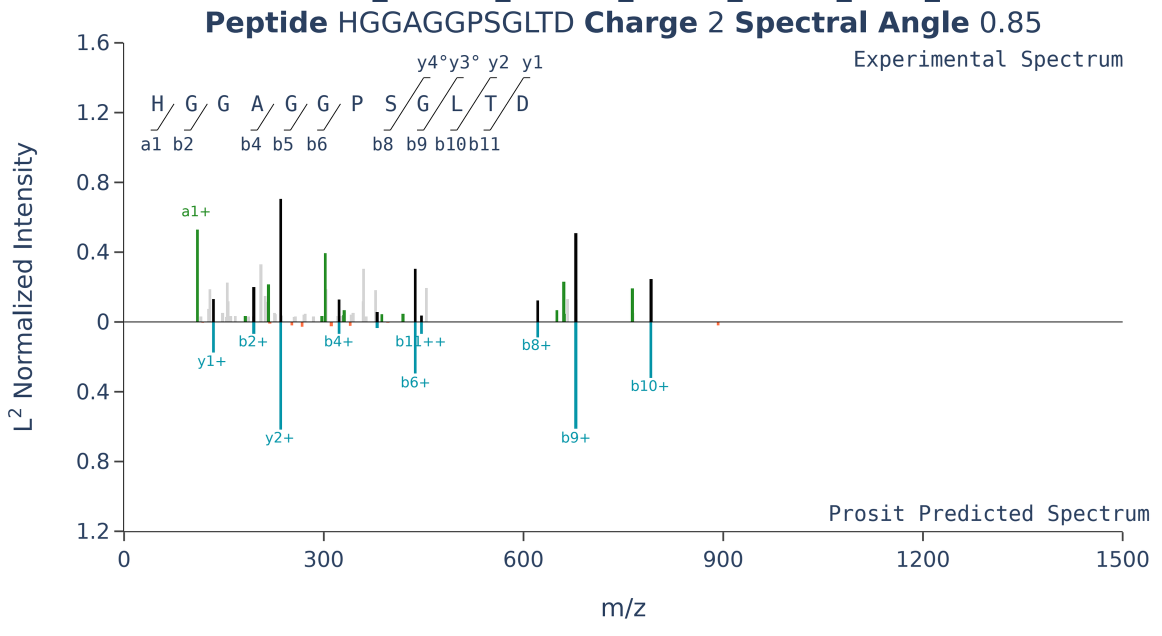

Source HRoetschke_231222_070123_HFGoe_UBE2S_b1R2_24h **Scan** 11892
Peptide NYEEYAAARA **Charge** 2 **Spectral Angle** 0.85

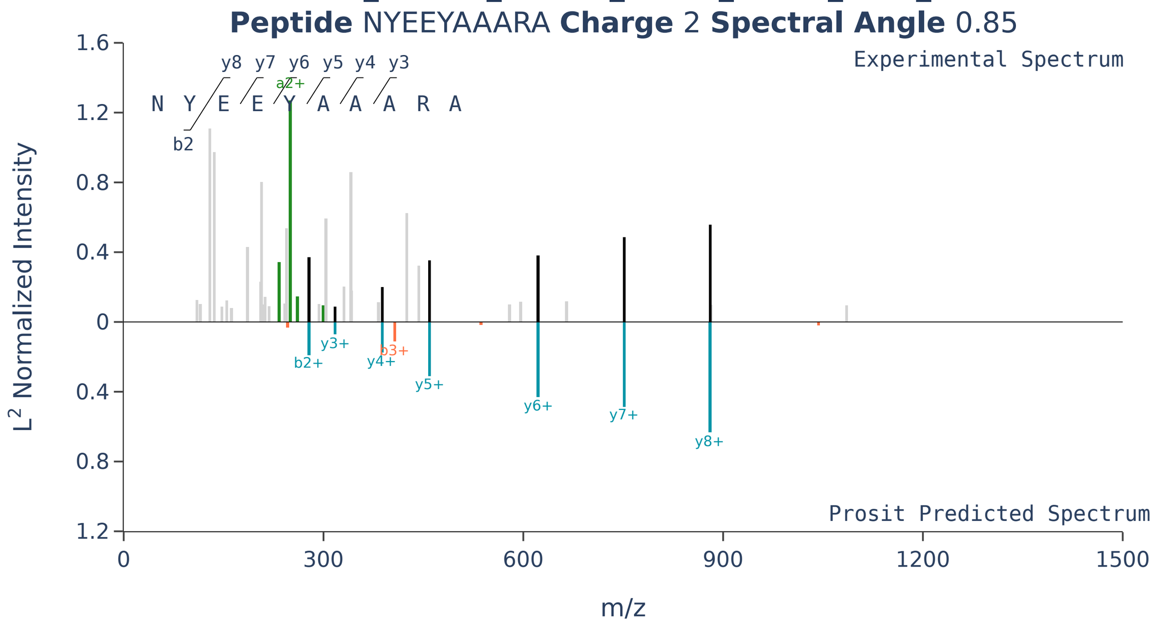

Source HRoetschke_231222_070123_HFGoe_UBE2S_b1R1_24h **Scan** 8003
Peptide SSTDPGAPGGPNE **Charge** 2 **Spectral Angle** 0.89

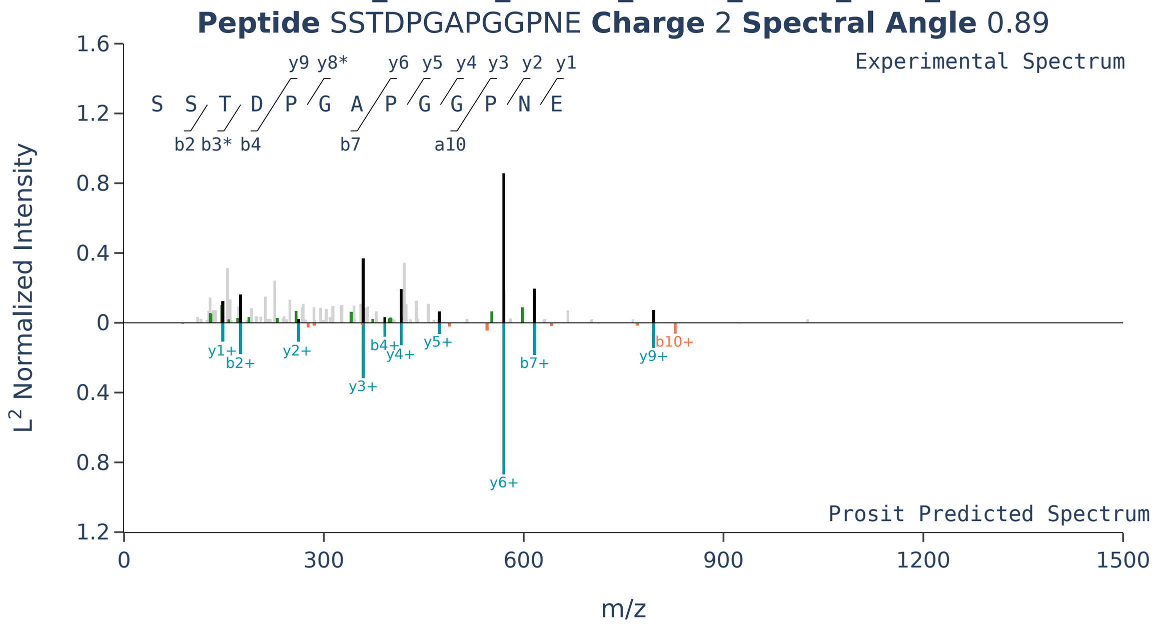

Source HRoetschke_231222_070123_HFGoe_UBE2S_b2R1_24h **Scan** 8469
Peptide RAEAGRALM **Charge** 2 **Spectral Angle** 0.82

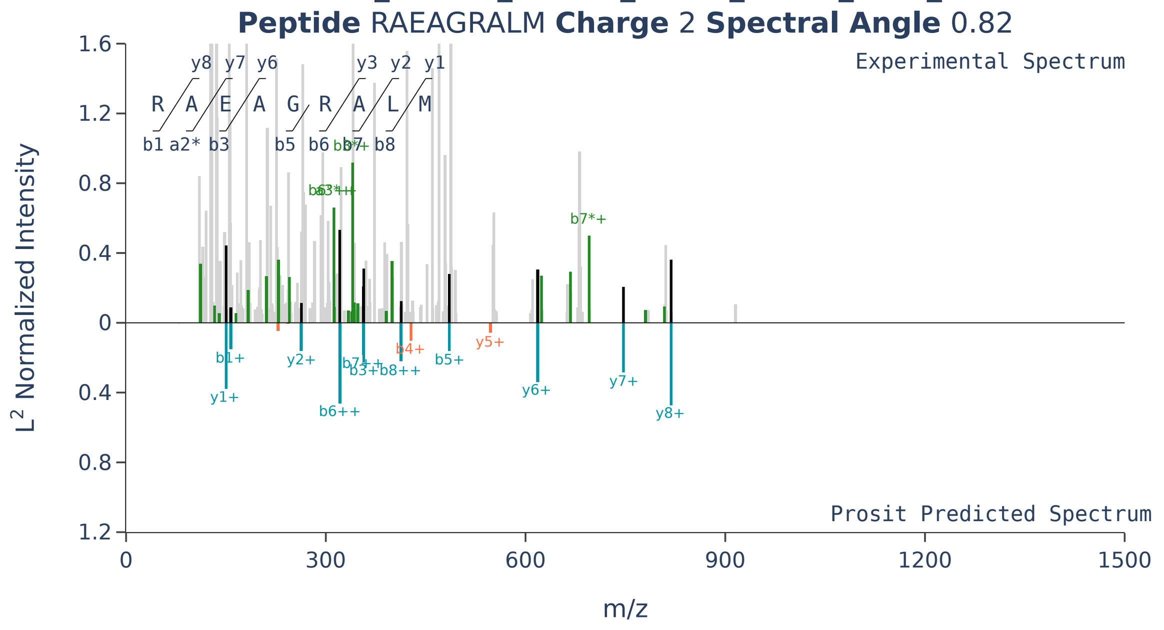

Source HRoetschke_231222_070123_HFGoe_UBE2S_b2R1_24h **Scan** 7276
Peptide RAEAGRALMN **Charge** 2 **Spectral Angle** 0.84

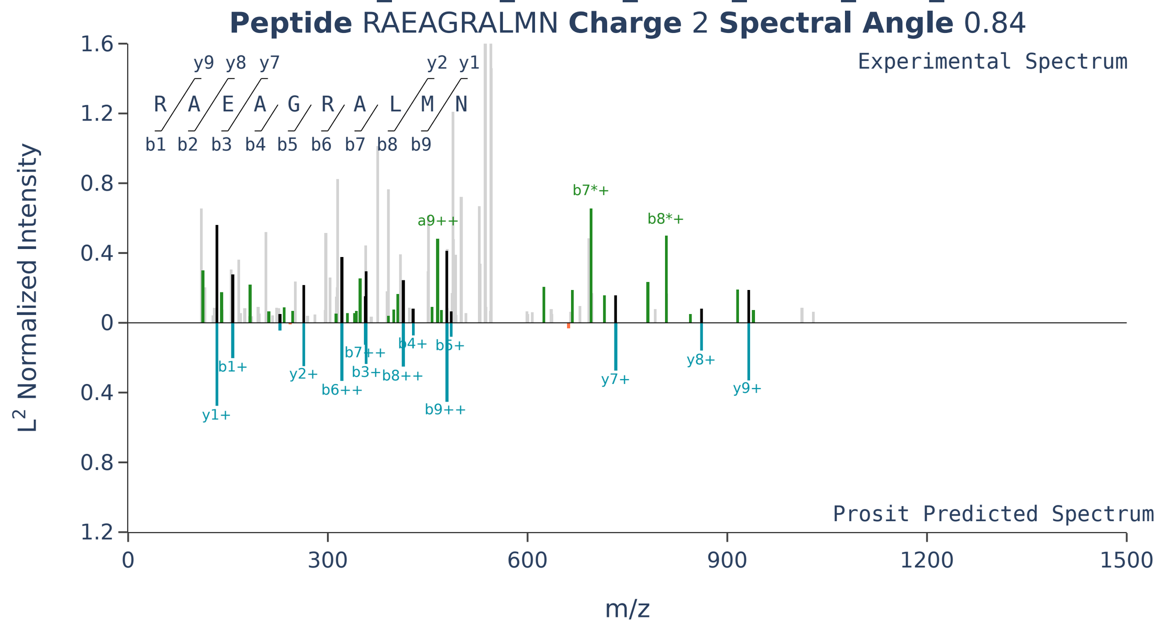

Source HRoetschke_231222_070123_HFGoe_UBE2S_b1R1_2h **Scan** 6627
Peptide RAEAGMNSNVE **Charge** 2 **Spectral Angle** 0.81

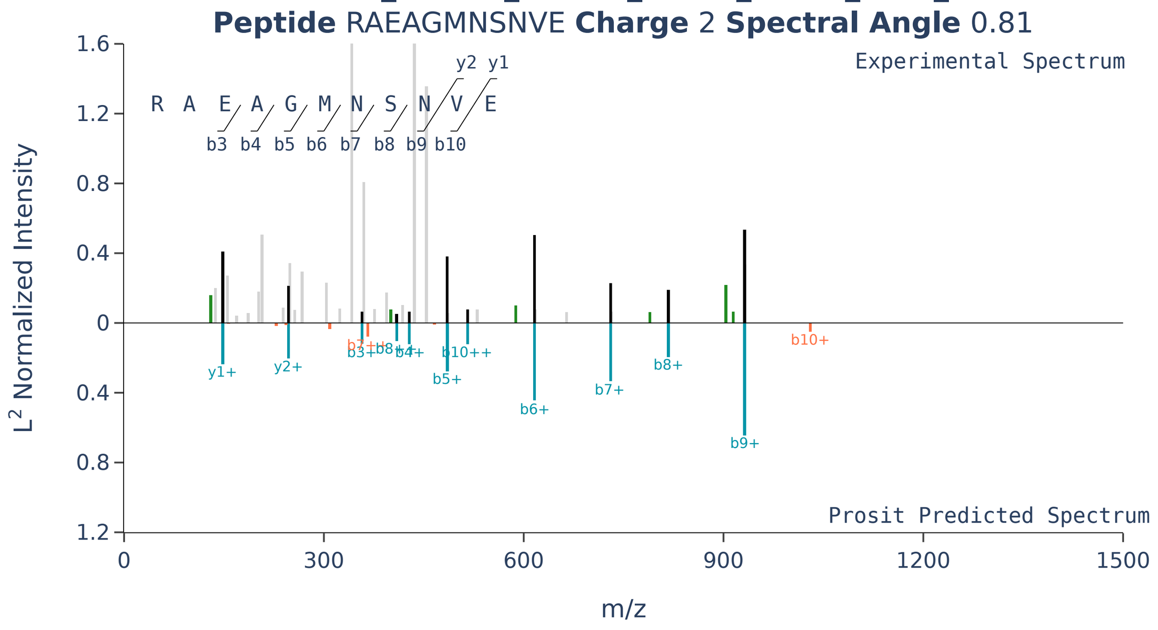

Source HRoetschke_231222_070123_HFGoe_UBE2S_b1R1_24h **Scan** 8228
Peptide HGGAGGPSGMNSNV **Charge** 2 **Spectral Angle** 0.86

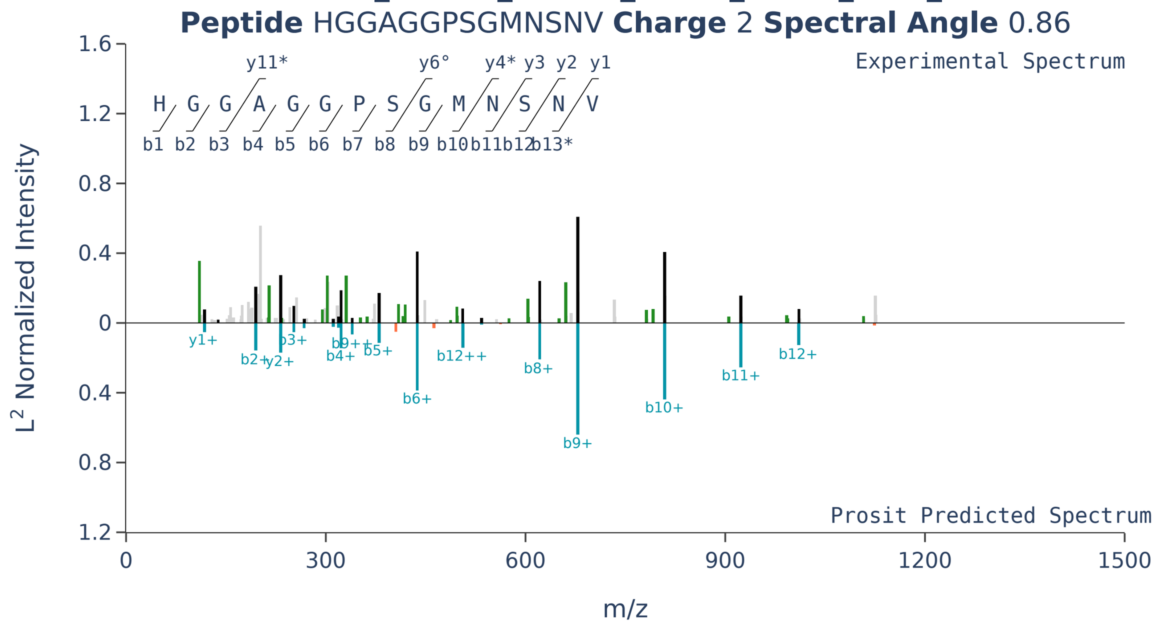

Source HRoetschke_231222_070123_HFGoe_UBE2S_b1R2_24h **Scan** 13891
Peptide HGGAGGPSGTDLQV **Charge** 2 **Spectral Angle** 0.85

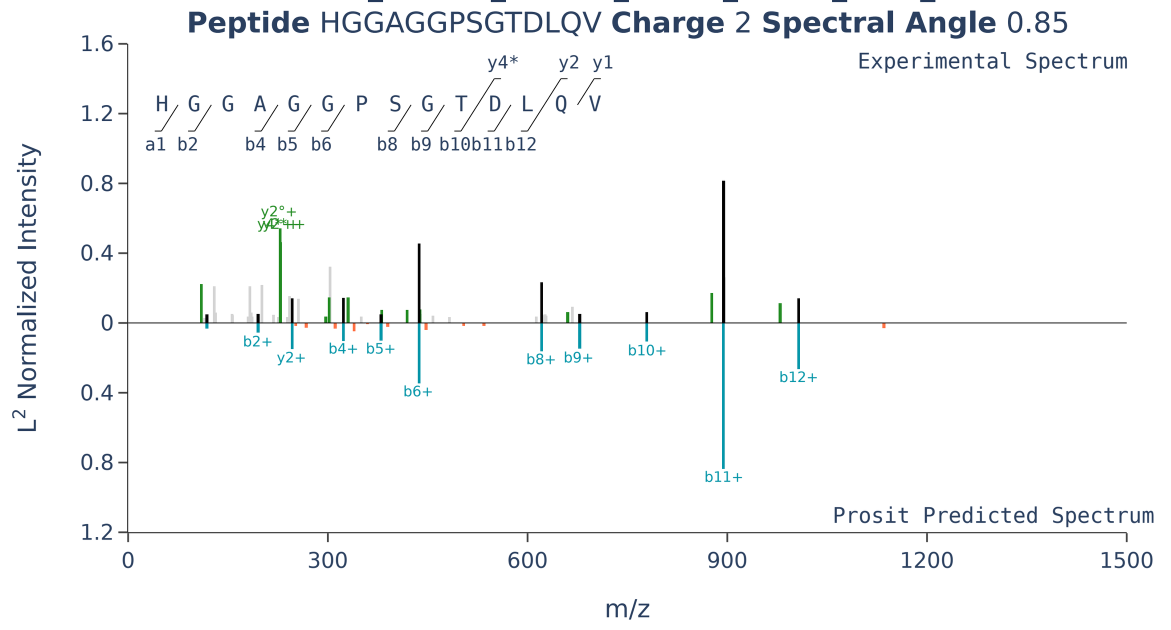

Source HRoetschke_231222_070123_HFGoe_UBE2S_b1R2_24h **Scan** 12821
Peptide ASGTEASSTDPGAPGGPGGRAL **Charge** 2 **Spectral Angle** 0.92

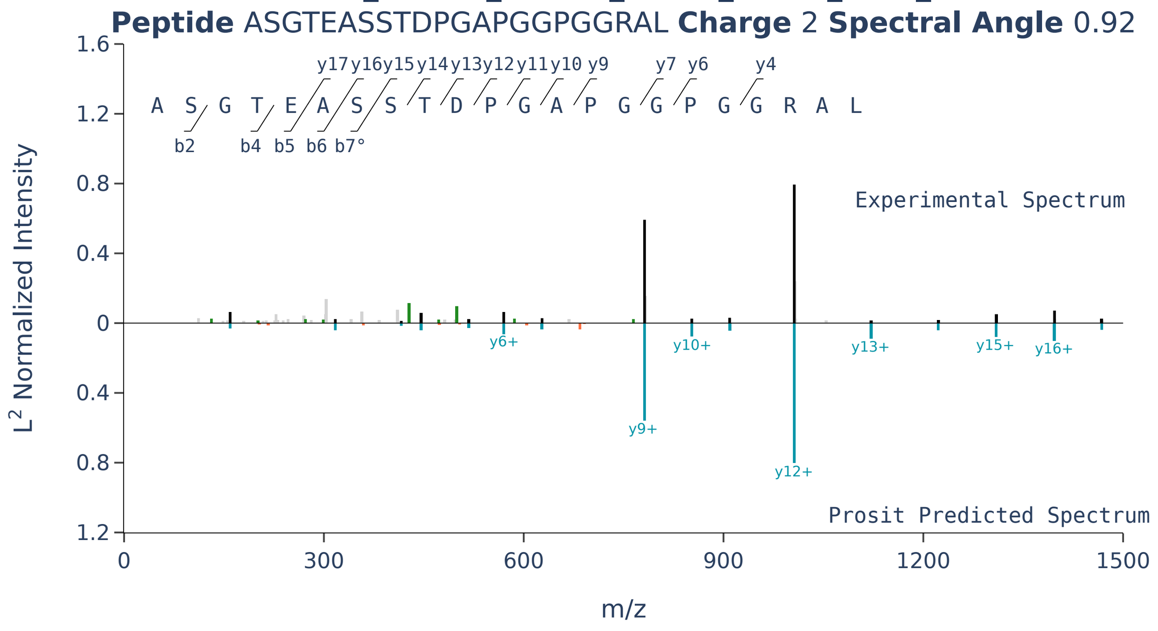

Source HRoetschke_231222_070123_HFGoe_UBE2S_b2R1_24h **Scan** 6668
Peptide RAEAGEAGRL **Charge** 2 **Spectral Angle** 0.83

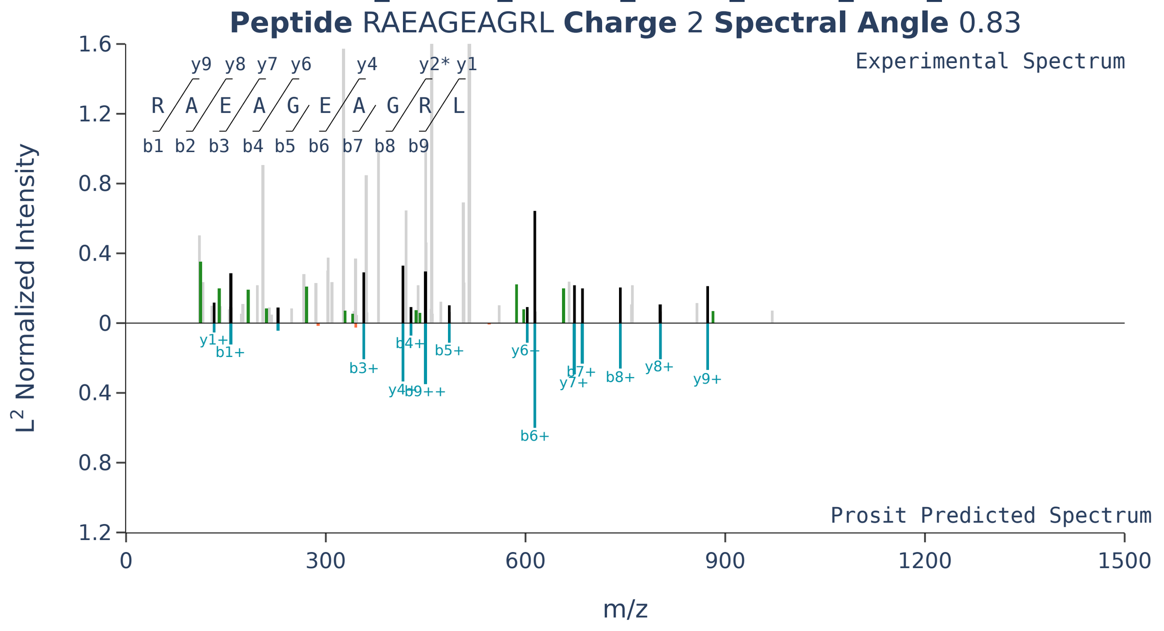

Source HRoetschke_231222_070123_HFGoe_UBE2S_b1R2_4h **Scan** 12752
Peptide GIRLTIK **Charge** 2 **Spectral Angle** 0.79

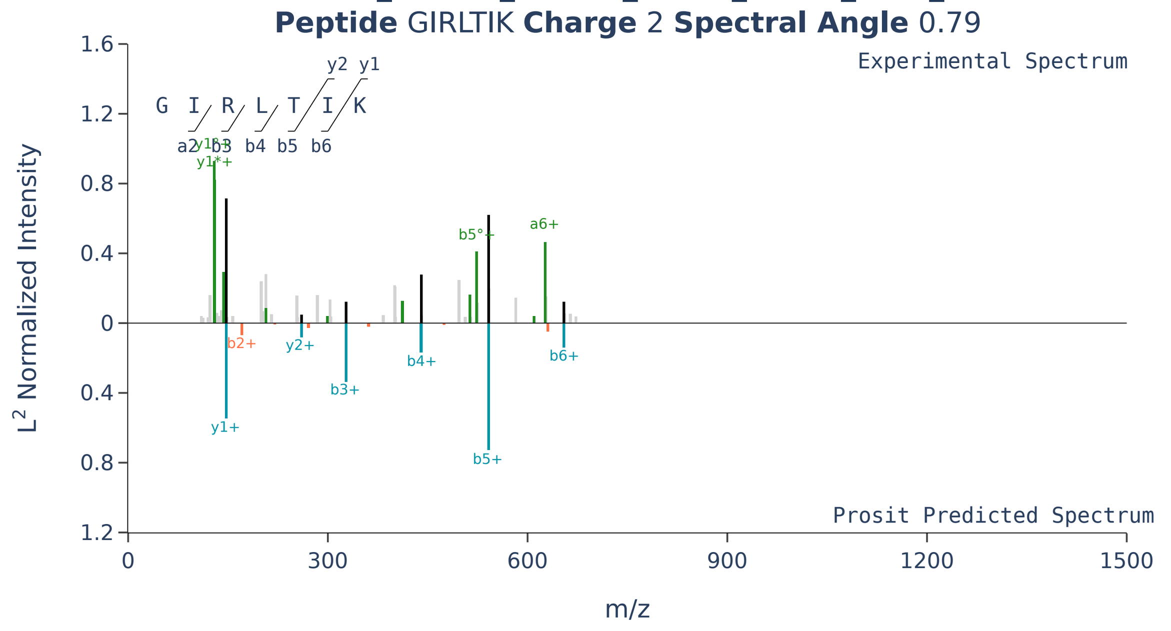

Source HRoetschke_231222_070123_HFGoe_UBE2S_b2R1_24h **Scan** 8154
Peptide ASGTEARL **Charge** 2 **Spectral Angle** 0.8

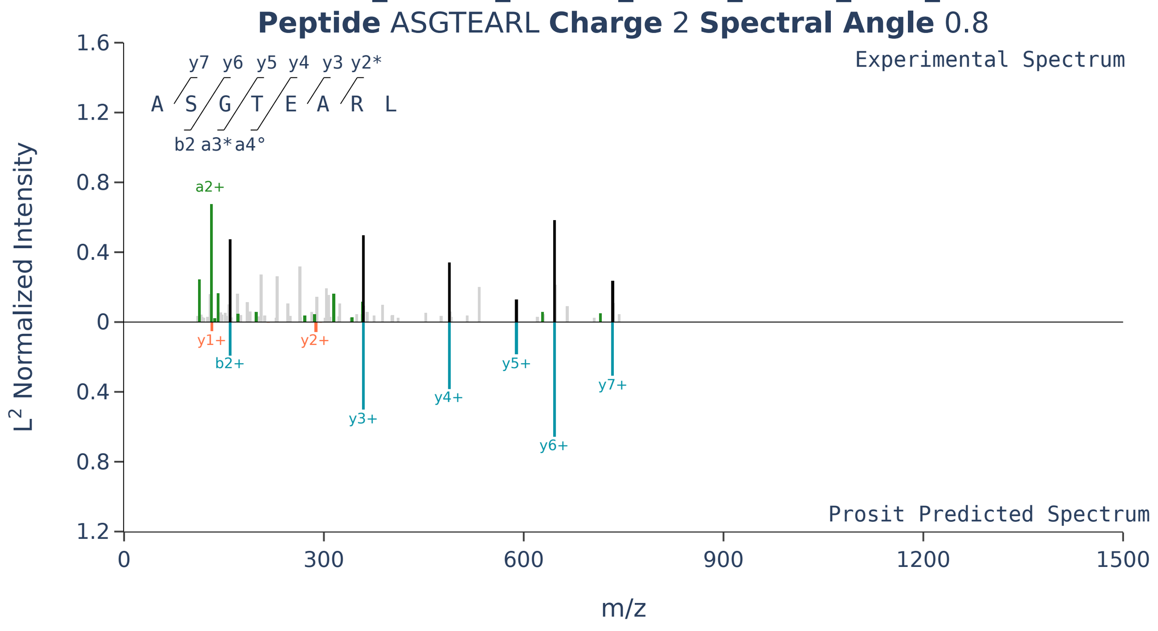

Source HRoetschke_231222_070123_HFGoe_UBE2S_b1R2_24h **Scan** 10156
Peptide PESALNTEASS **Charge** 1 **Spectral Angle** 0.84

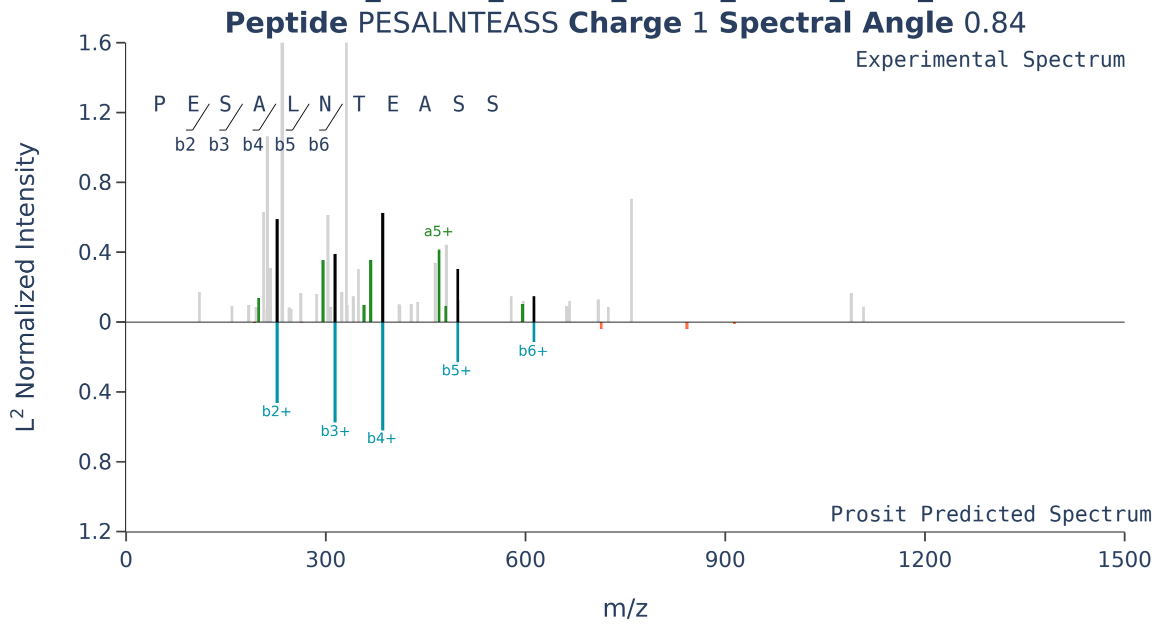

Source HRoetschke_231222_070123_HFGoe_UBE2S_b1R1_24h **Scan** 20066
Peptide EVDPPDGIKVFPNE **Charge** 2 **Spectral Angle** 0.83

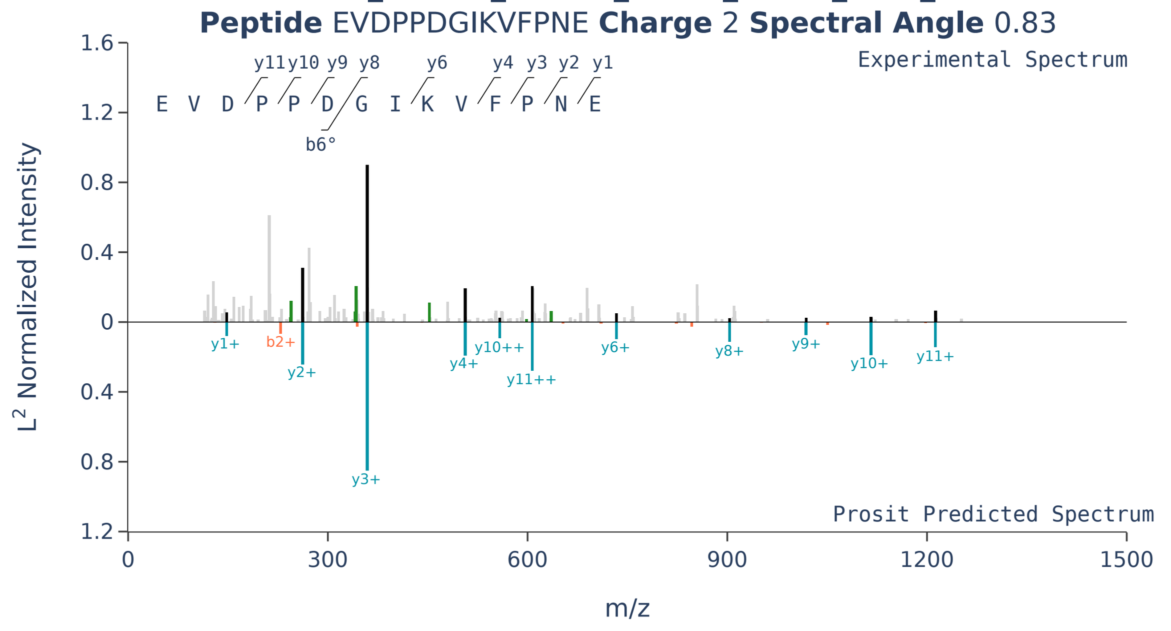

Source HRoetschke_231222_070123_HFGoe_UBE2S_b1R1_24h **Scan** 9263
Peptide AGGPSGEAGRL **Charge** 2 **Spectral Angle** 0.86

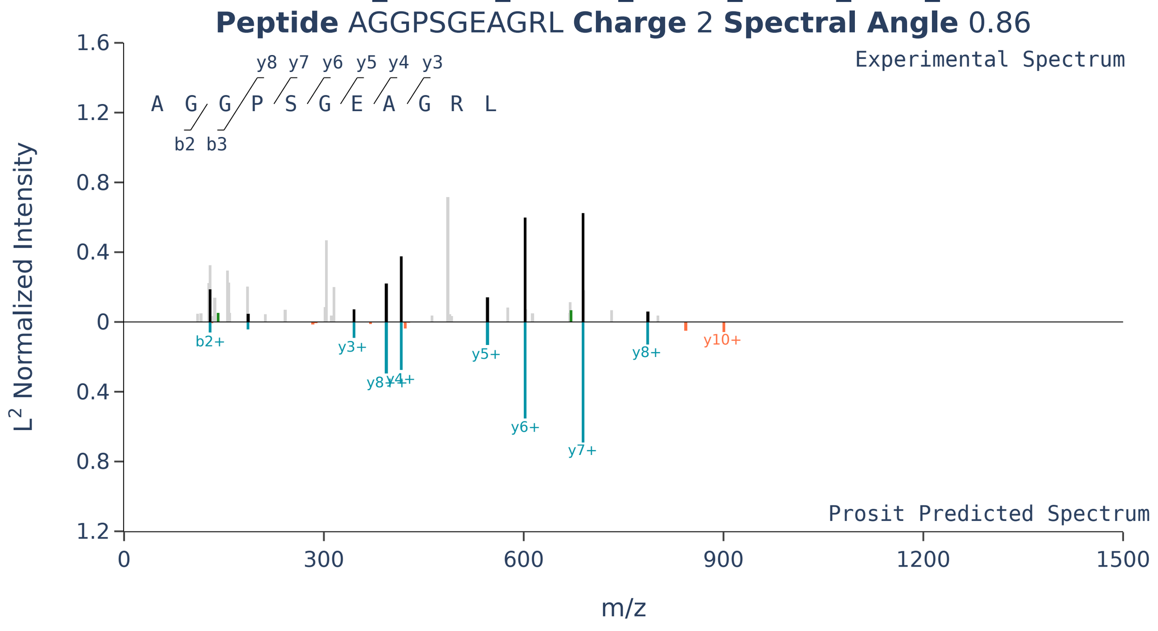

Source HRoetschke_231222_070123_HFGoe_UBE2S_b1R1_24h **Scan** 11568
Peptide GRALMNSNV **Charge** 2 **Spectral Angle** 0.87

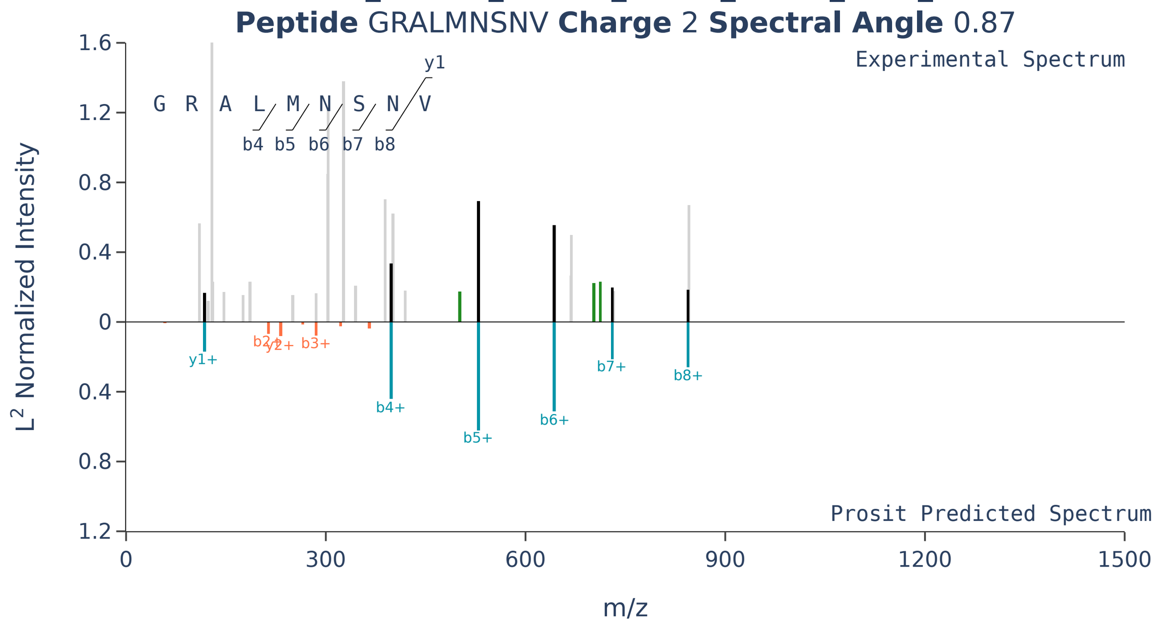

Source HRoetschke_231222_070123_HFGoe_UBE2S_b1R1_24h **Scan** 9258
Peptide TEIHGGAGGPSGRLA **Charge** 3 **Spectral Angle** 0.9

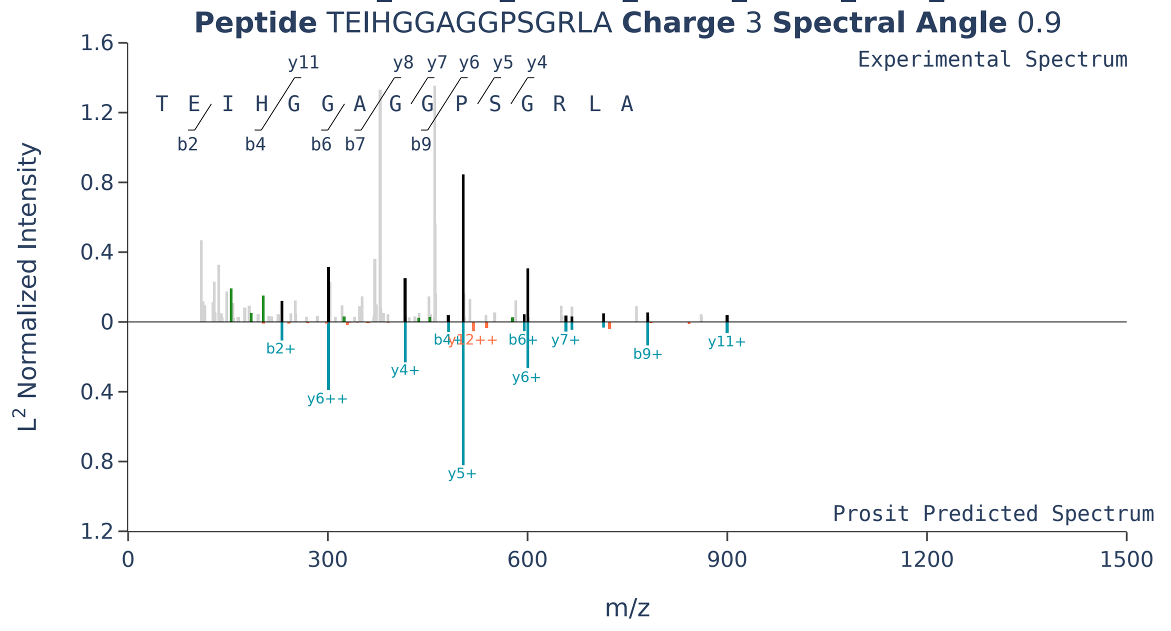

Source HRoetschke_231222_070123_HFGoe_UBE2S_b2R1_1h **Scan** 7030
Peptide HGGAGGPSGTIE **Charge** 2 **Spectral Angle** 0.83

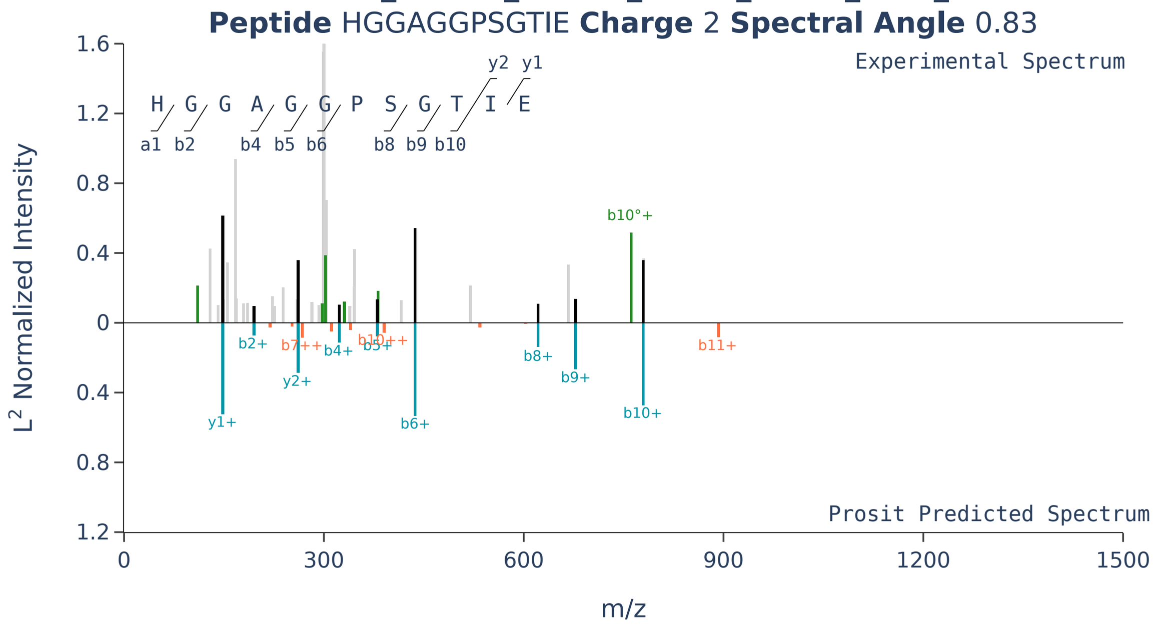

Source HRoetschke_231222_070123_HFGoe_UBE2S_b2R2_2h **Scan** 9284
Peptide KDFPASPPRA **Charge** 3 **Spectral Angle** 0.85

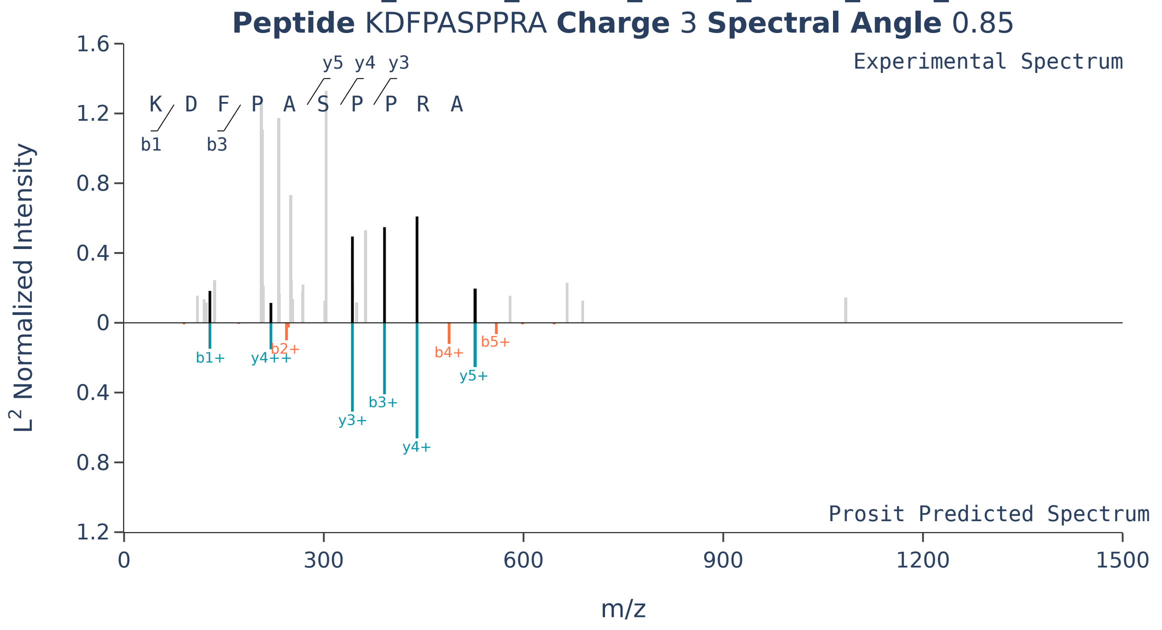

Source HRoetschke_231222_070123_HFGoe_UBE2S_b1R1_24h **Scan** 7144
Peptide HPNPESM **Charge** 2 **Spectral Angle** 0.81

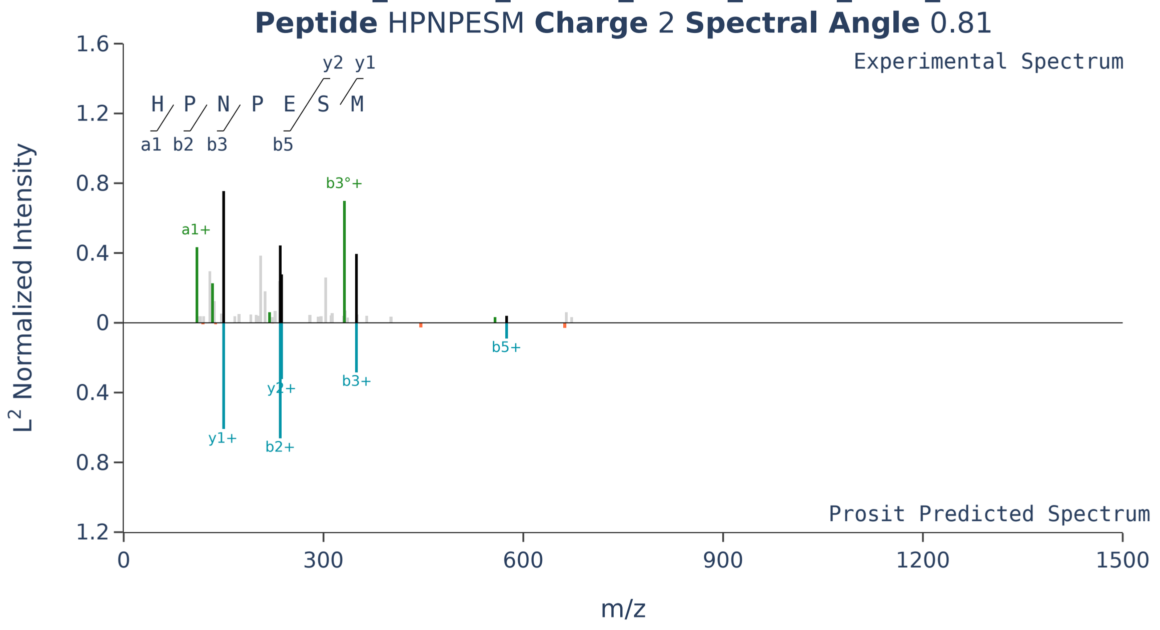

Source HRoetschke_231222_070123_HFGoe_UBE2S_b1R1_24h **Scan** 13862
Peptide PNVGEDLTD **Charge** 2 **Spectral Angle** 0.84

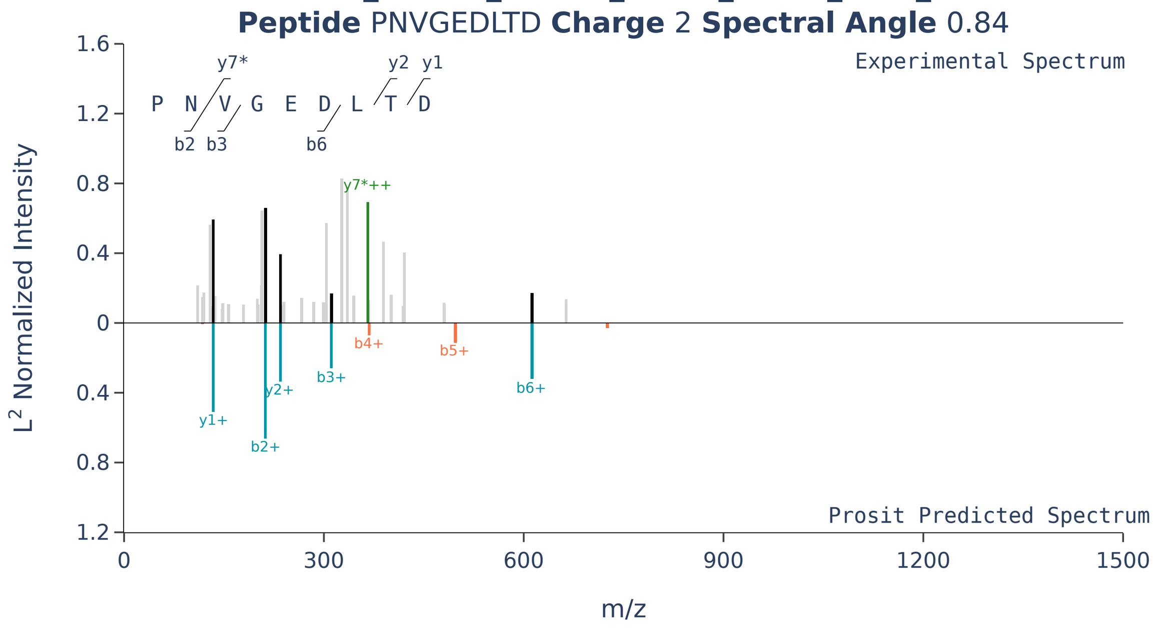

Source HRoetschke_231222_070123_HFGoe_UBE2S_b1R1_24h **Scan** 8649
Peptide AASSTDPGAPGGPGGAE **Charge** 2 **Spectral Angle** 0.88

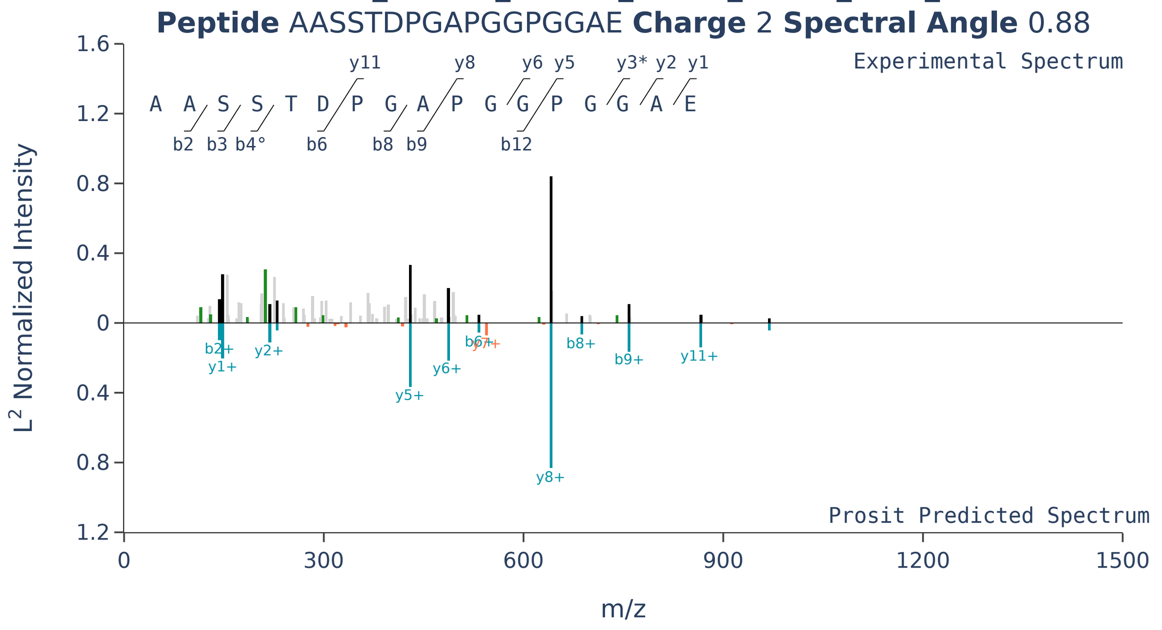

Source HRoetschke_231222_070123_HFGoe_UBE2S_b2R2_4h **Scan** 7634
Peptide SSTDPGAPGGPGGE **Charge** 2 **Spectral Angle** 0.9

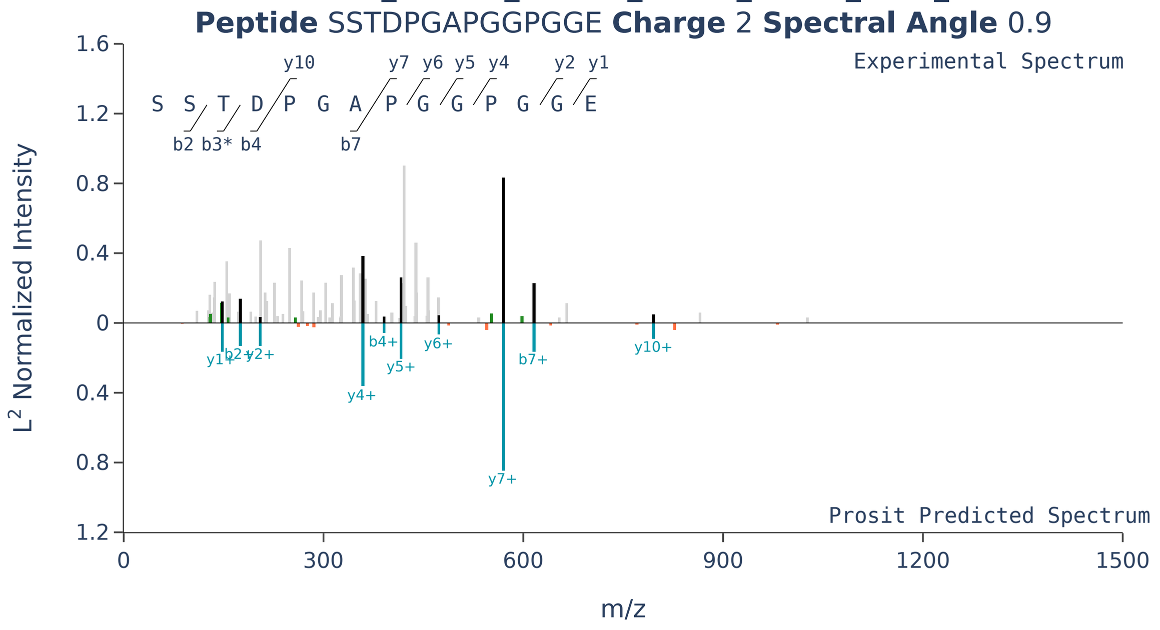

Source HRoetschke_231222_070123_HFGoe_UBE2S_b1R1_24h **Scan** 8960
Peptide ANGEICVKD **Charge** 2 **Spectral Angle** 0.75

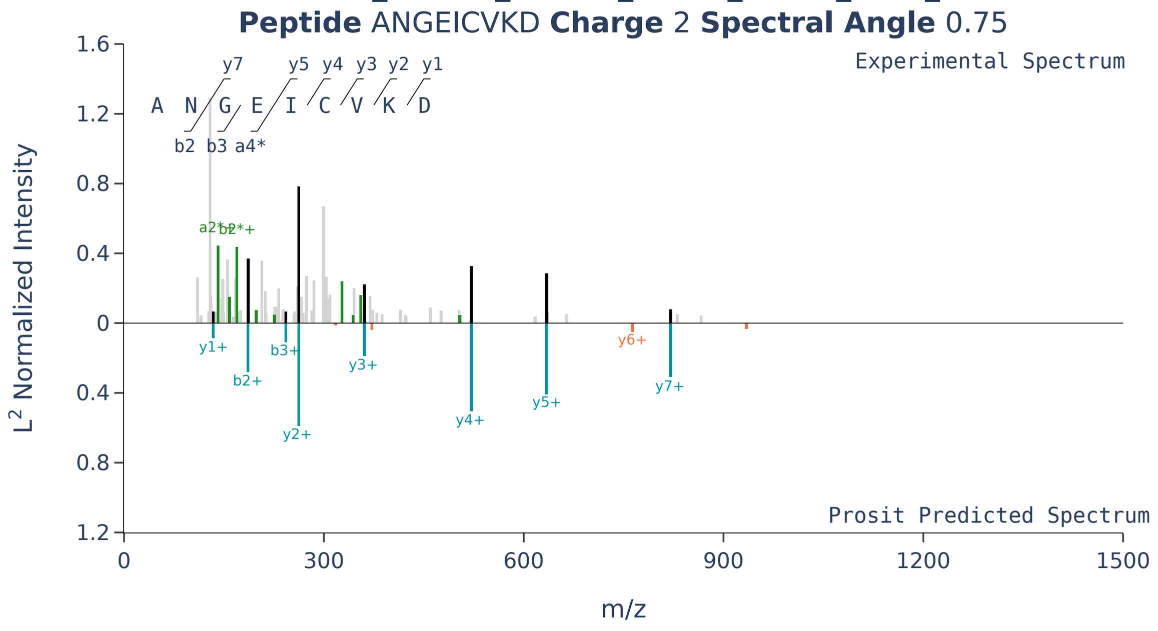

Source HRoetschke_231222_070123_HFGoe_UBE2S_b1R2_24h **Scan** 10229
Peptide LIHPNPESTD **Charge** 2 **Spectral Angle** 0.87

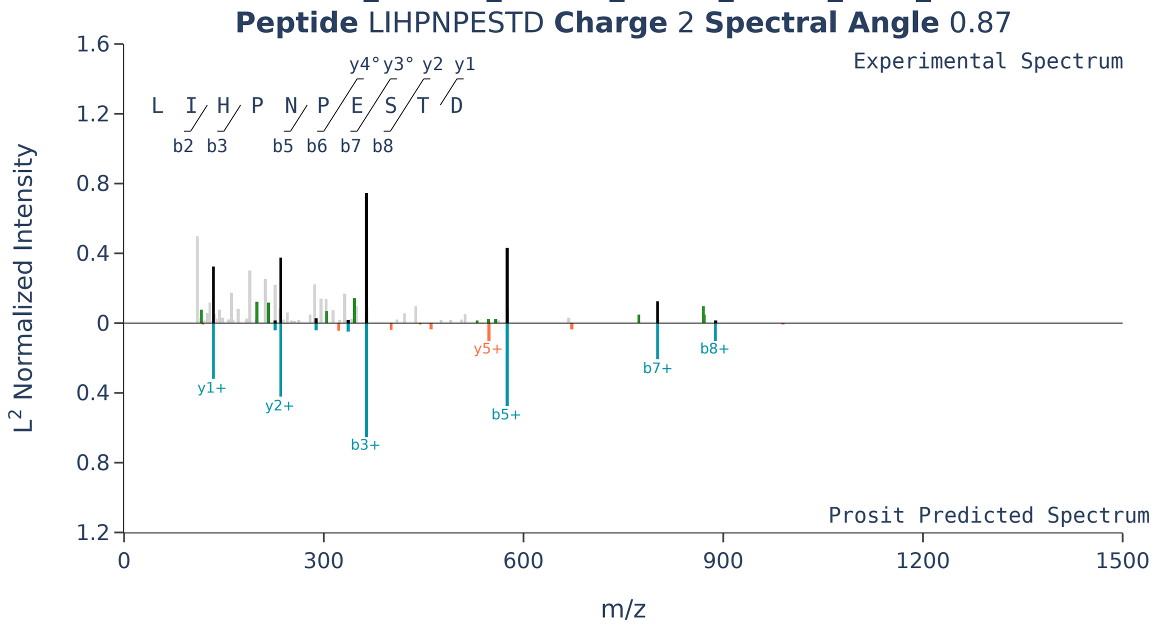

Source HRoetschke_231222_070123_HFGoe_UBE2S_b2R1_24h **Scan** 17285
Peptide DGEICVNVLKR **Charge** 3 **Spectral Angle** 0.82

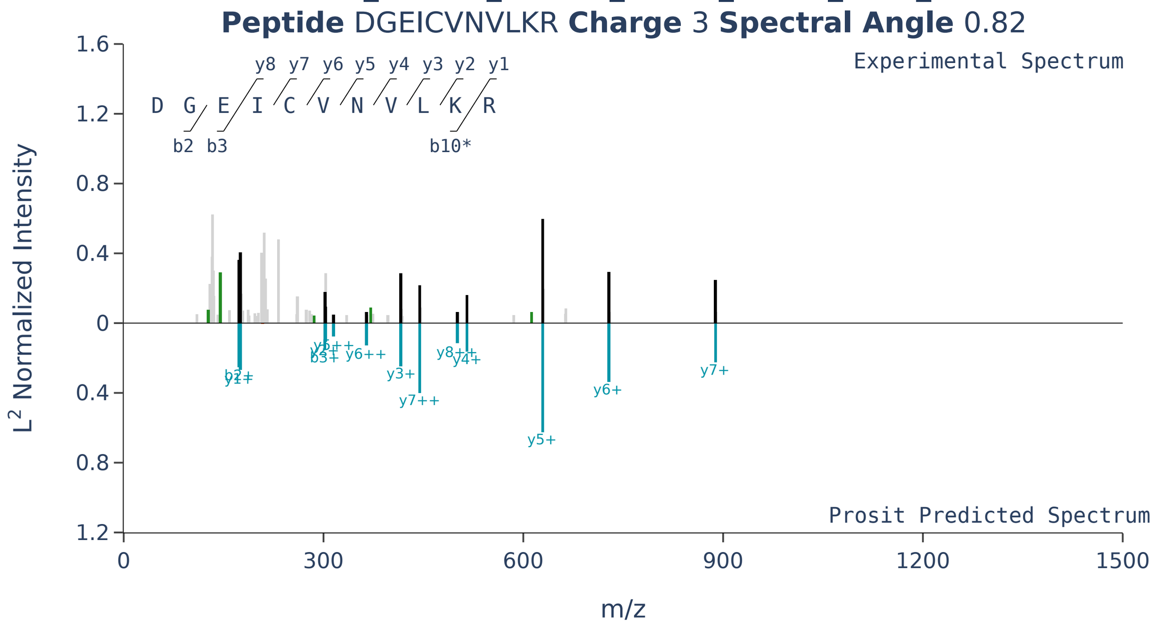

Source HRoetschke_231222_070123_HFGoe_UBE2S_b1R2_24h **Scan** 8503
Peptide YKEVTMN **Charge** 2 **Spectral Angle** 0.9

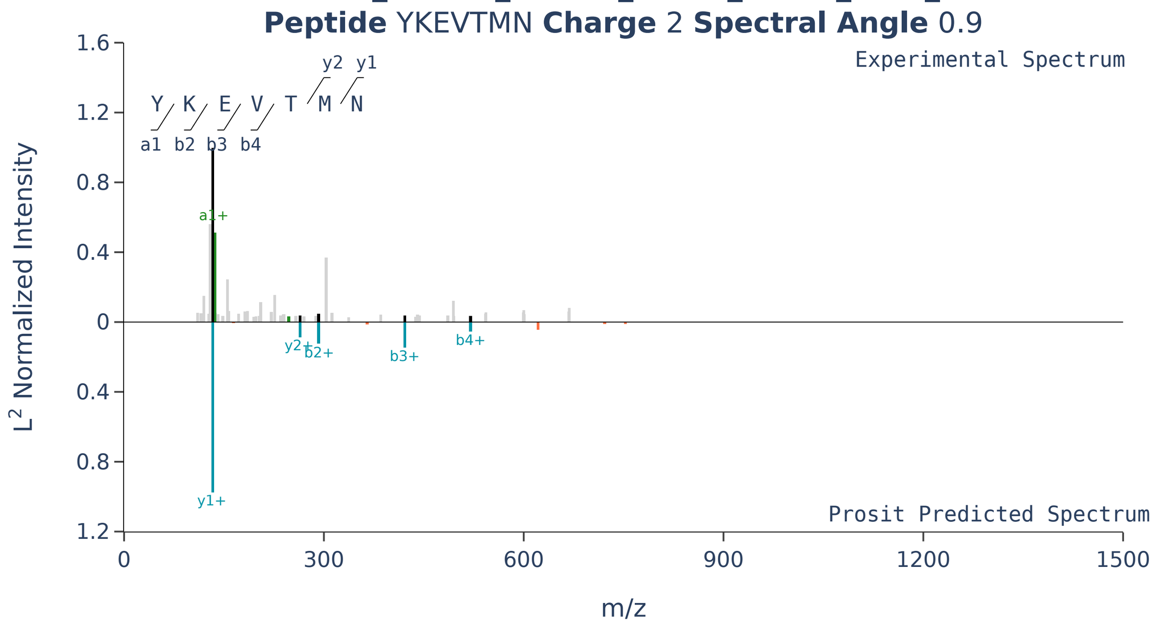

Source HRoetschke_231222_070123_HFGoe_UBE2S_b1R1_24h **Scan** 12207
Peptide ASSTDPGAPGGPGGRAL **Charge** 2 **Spectral Angle** 0.89

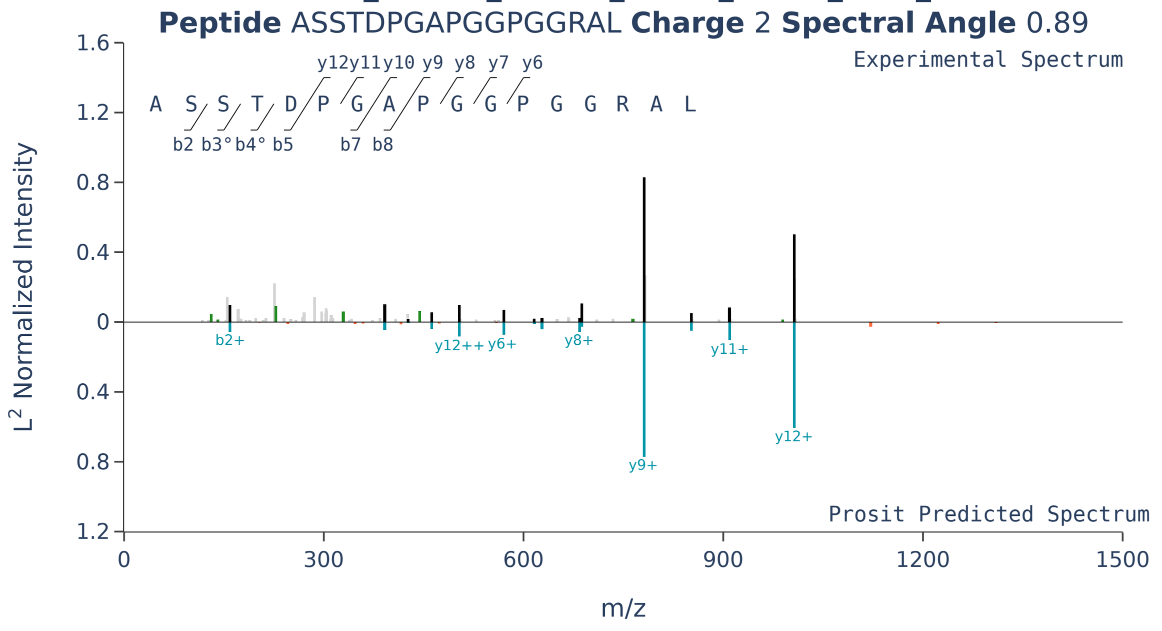

Source HRoetschke_231222_070123_HFGoe_UBE2S_b1R2_24h **Scan** 15465
Peptide ASADPPDGIKV **Charge** 2 **Spectral Angle** 0.88

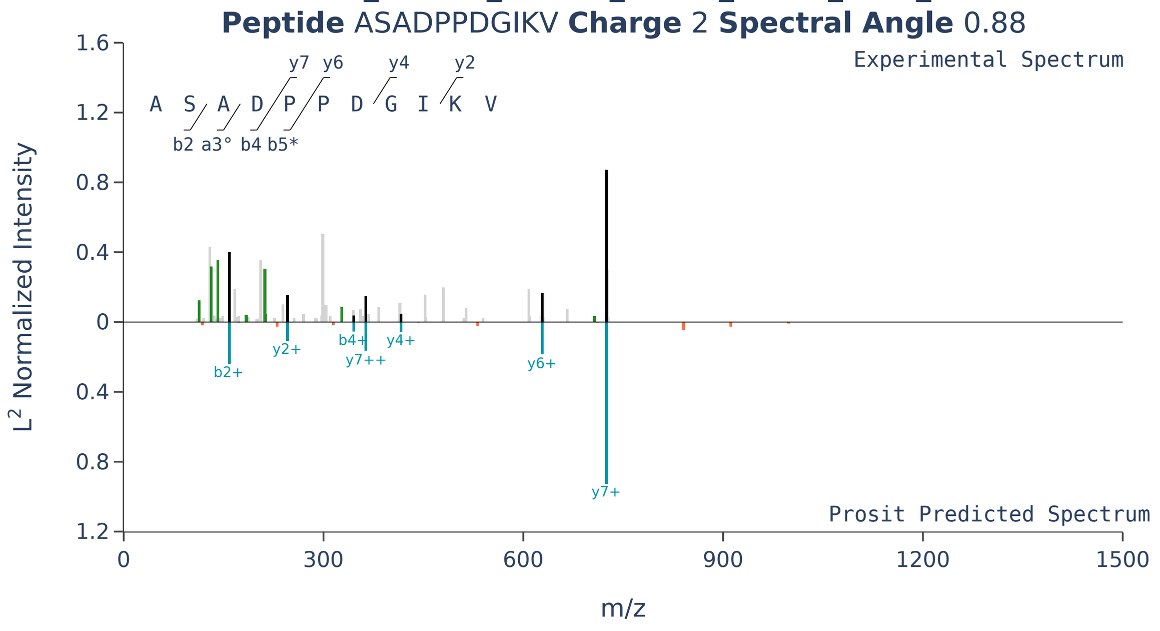

Source HRoetschke_231222_070123_HFGoe_UBE2S_b1R1_24h **Scan** 9322
Peptide HGGAGGPSGTTL **Charge** 2 **Spectral Angle** 0.82

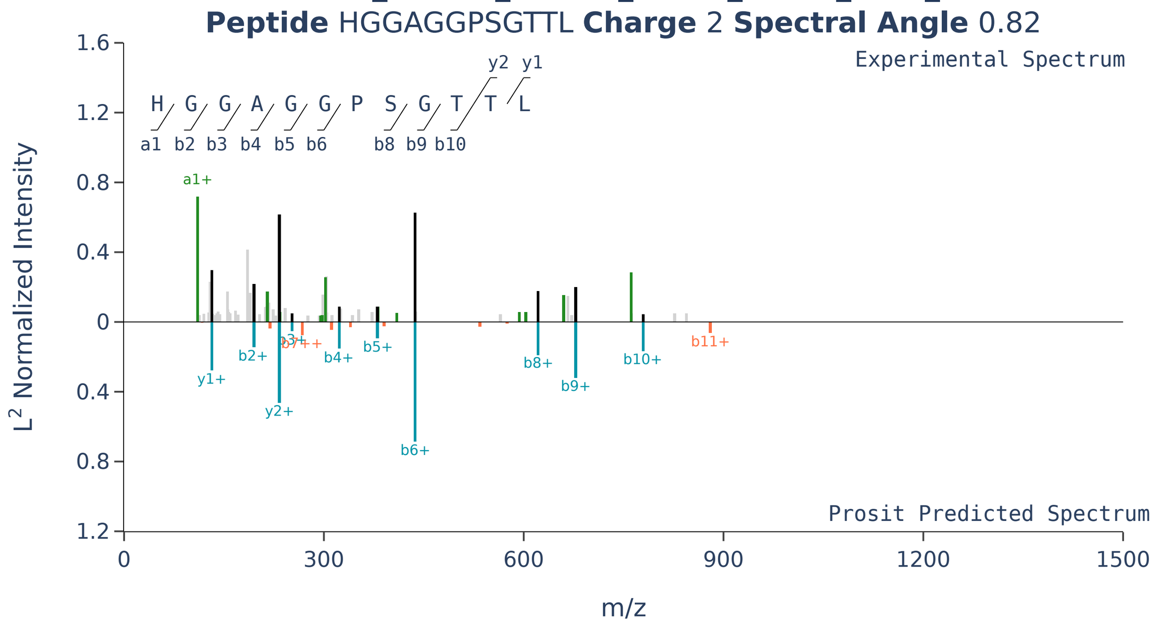

Source HRoetschke_231222_070123_HFGoe_UBE2S_b2R1_4h **Scan** 19212
Peptide QTADPPDGIKVFPNE **Charge** 2 **Spectral Angle** 0.83

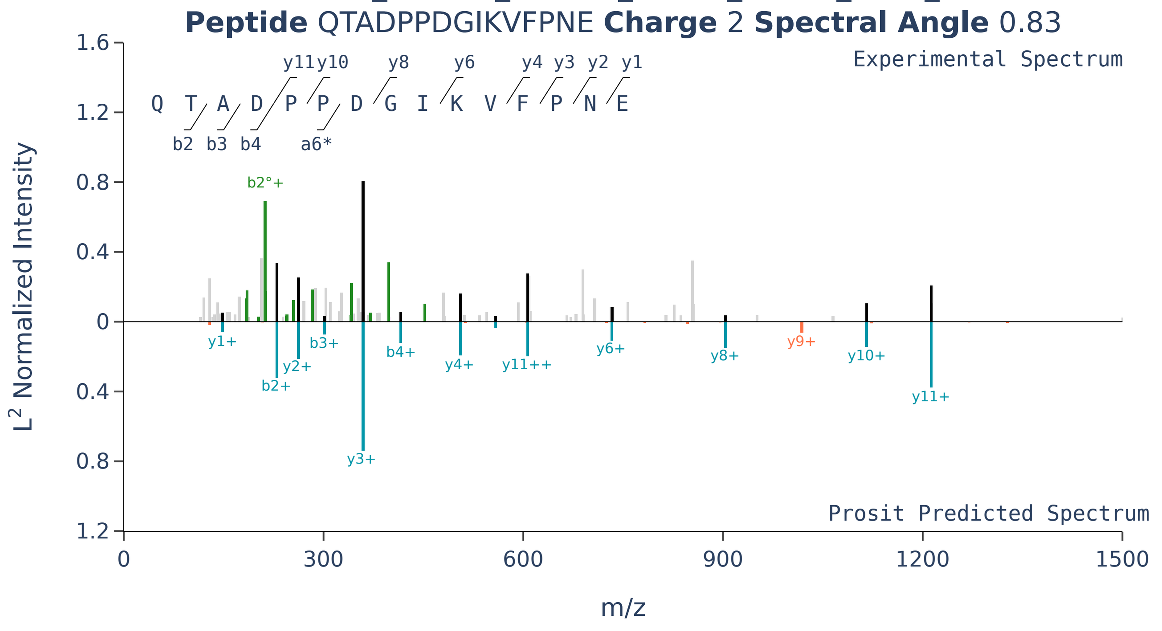

Source HRoetschke_231222_070123_HFGoe_UBE2S_b1R2_2h **Scan** 6698
Peptide GPEGTPGGPGG **Charge** 1 **Spectral Angle** 0.91

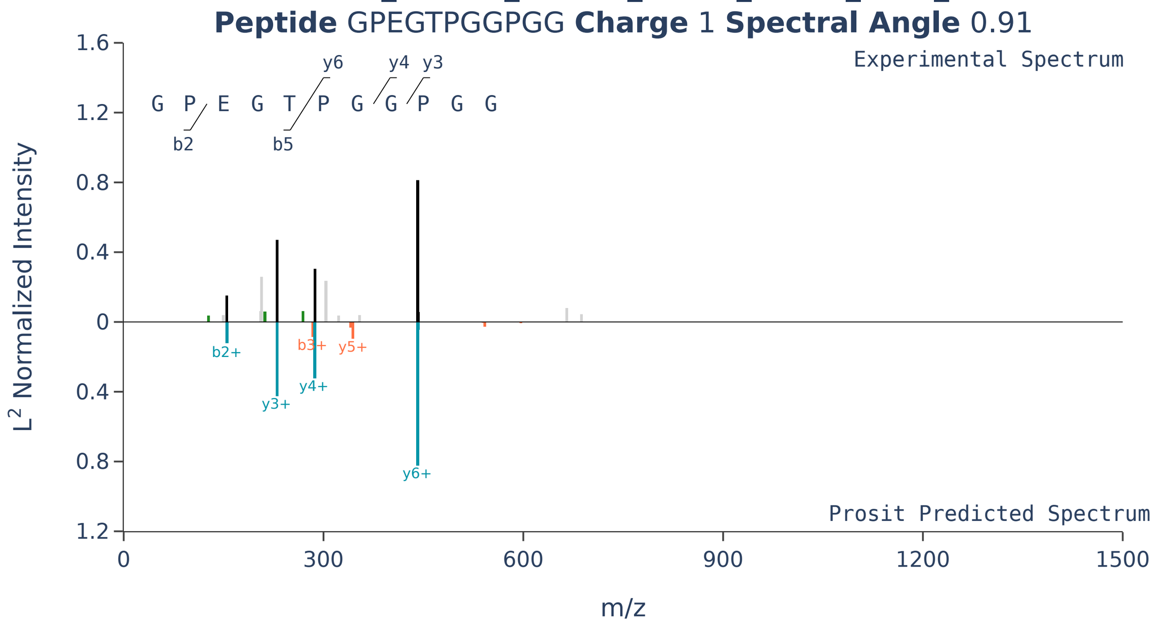

Source HRoetschke_231222_070123_HFGoe_UBE2S_b1R2_4h **Scan** 7280
Peptide HGGAGGPSGMNSNVE **Charge** 2 **Spectral Angle** 0.78

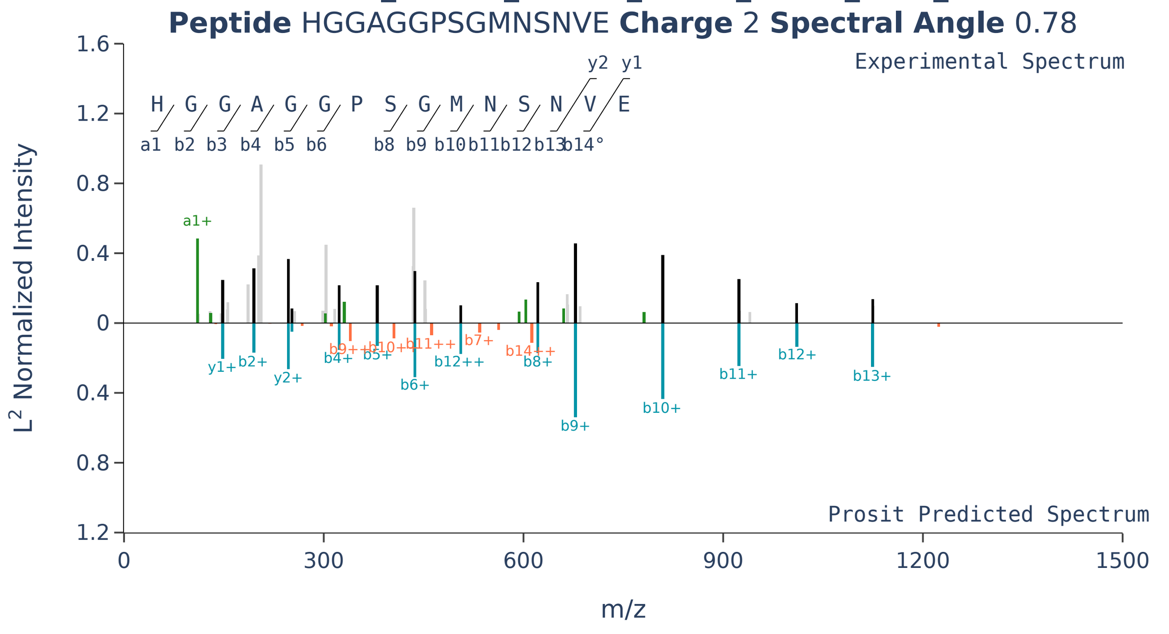

Source HRoetschke_231222_070123_HFGoe_UBE2S_b1R2_24h **Scan** 6987
Peptide VYKEVTE **Charge** 2 **Spectral Angle** 0.78

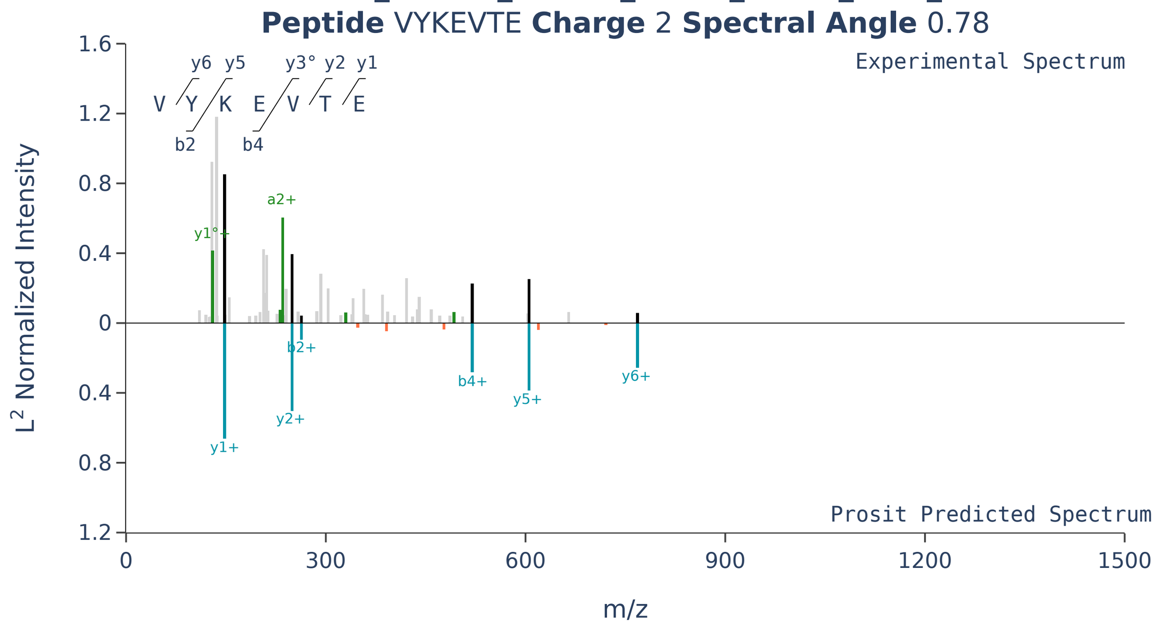

Source HRoetschke_231222_070123_HFGoe_UBE2S_b2R1_24h **Scan** 11975
Peptide SSTDPGAPGGPGGRAL **Charge** 2 **Spectral Angle** 0.86

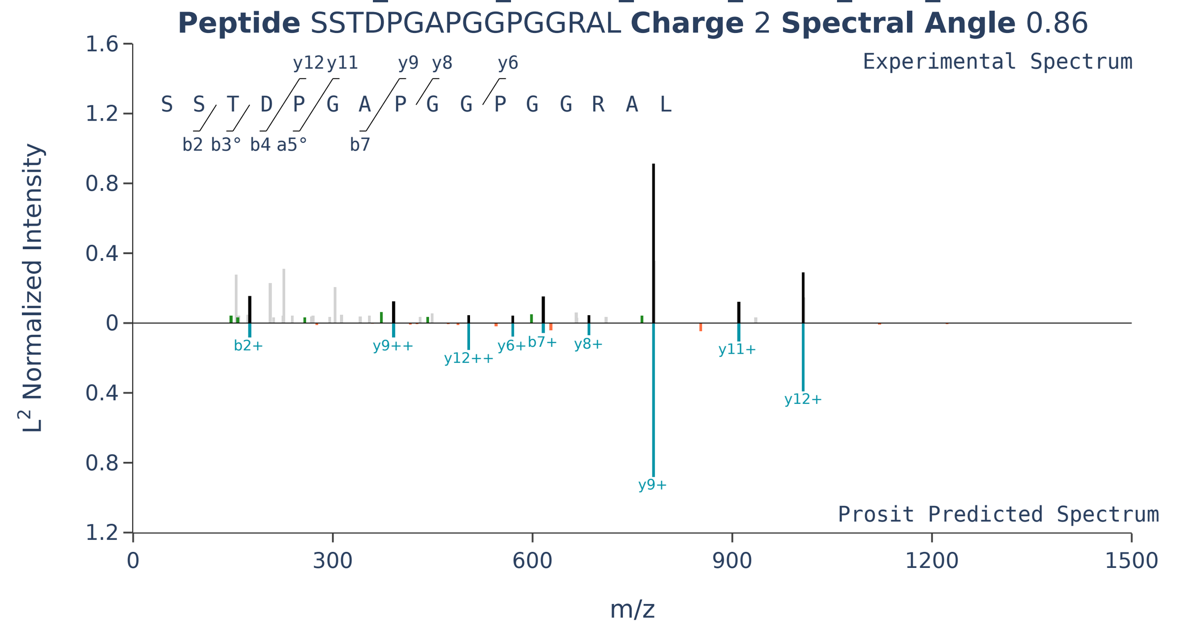

Source HRoetschke_231222_070123_HFGoe_UBE2S_b2R1_4h **Scan** 6747
Peptide GRAEAMNSNVE **Charge** 2 **Spectral Angle** 0.8

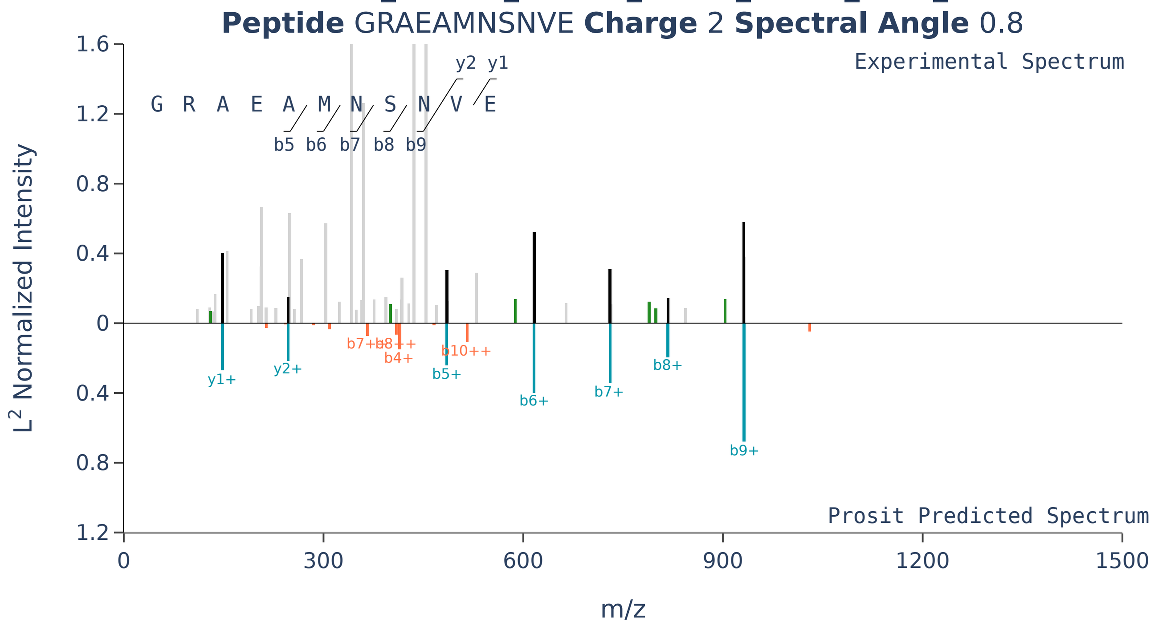

Source HRoetschke_231222_070123_HFGoe_UBE2S_b1R1_24h **Scan** 10941
Peptide RAEAGRALMNSNV **Charge** 3 **Spectral Angle** 0.82

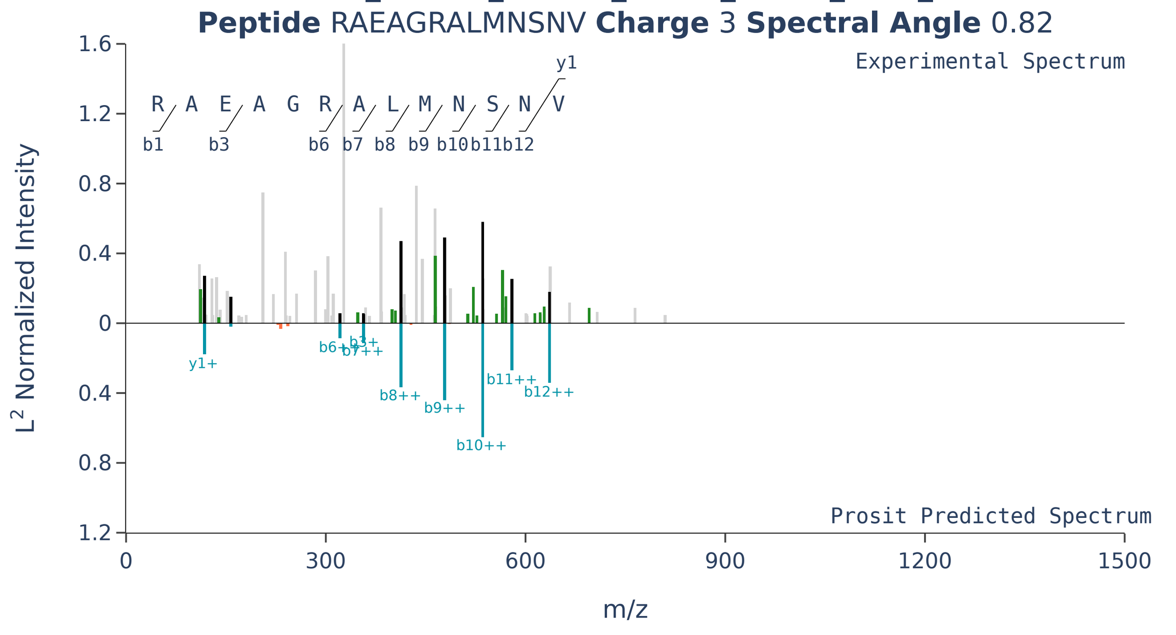

Source HRoetschke_231222_070123_HFGoe_UBE2S_b1R2_24h **Scan** 6106
Peptide ASSTDPGAPGGPGGKD **Charge** 2 **Spectral Angle** 0.88

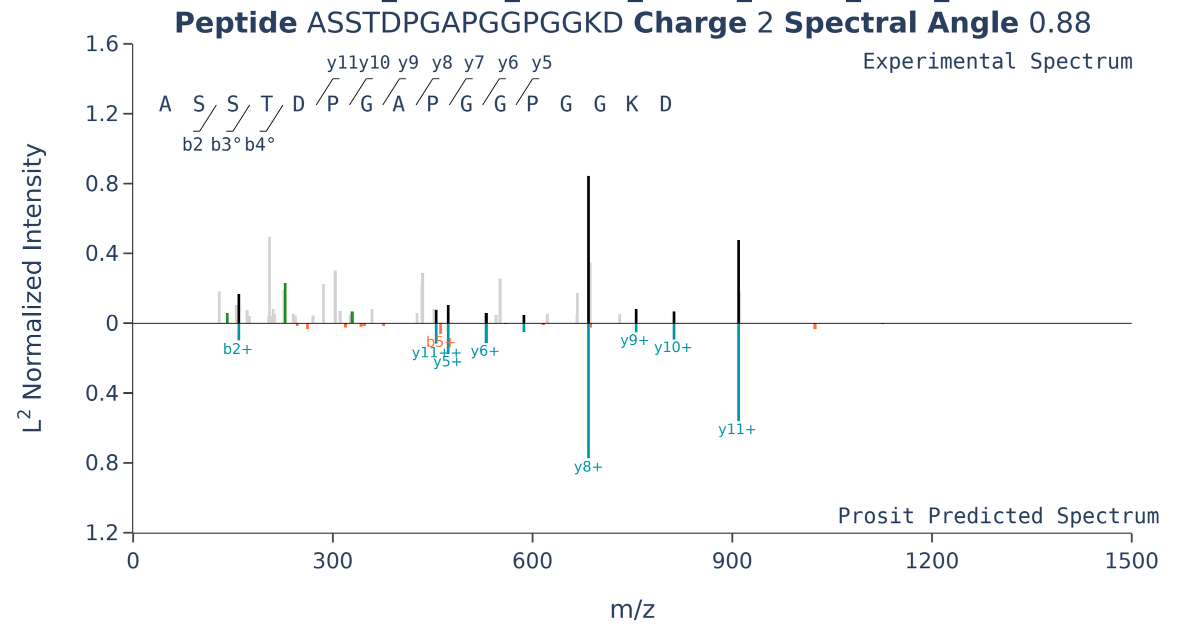

Source HRoetschke_231222_070123_HFGoe_UBE2S_b1R1_24h **Scan** 7726
Peptide LIHPNPESGKD **Charge** 3 **Spectral Angle** 0.81

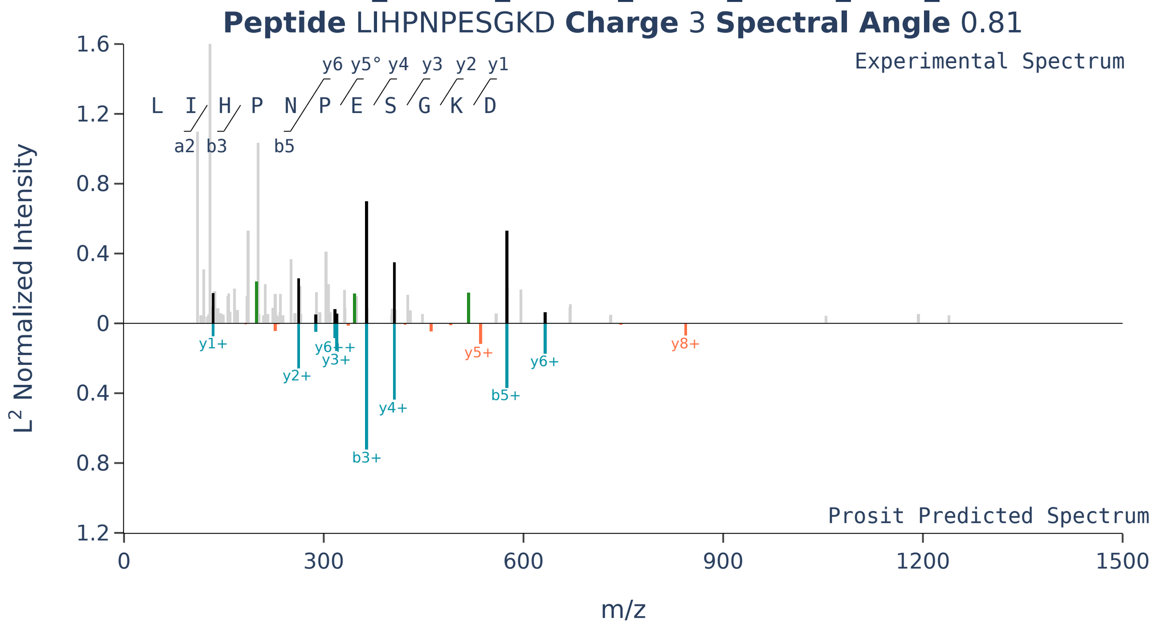

Source HRoetschke_231222_070123_HFGoe_UBE2S_b2R1_24h **Scan** 13059
Peptide GRAEAANGEICV **Charge** 2 **Spectral Angle** 0.87

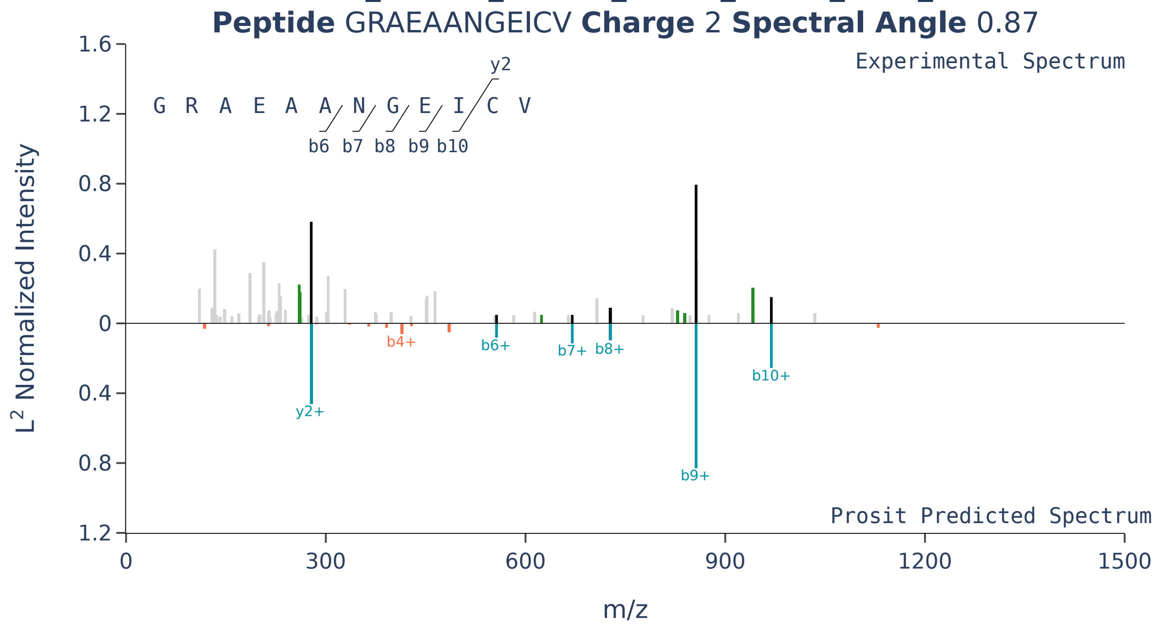

Source HRoetschke_231222_070123_HFGoe_UBE2S_b1R1_2h **Scan** 12219
Peptide HGGAGGPSGLQV **Charge** 2 **Spectral Angle** 0.85

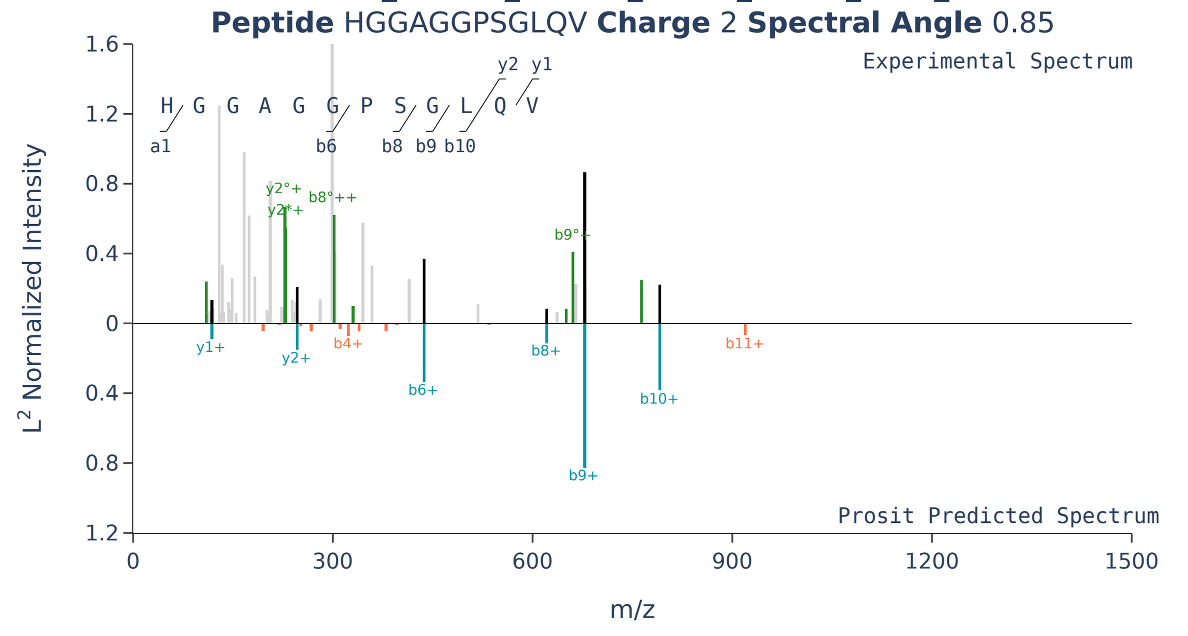

Source HRoetschke_231222_070123_HFGoe_UBE2S_b1R2_4h **Scan** 5711
Peptide DPGAPGGPGGR **Charge** 2 **Spectral Angle** 0.85

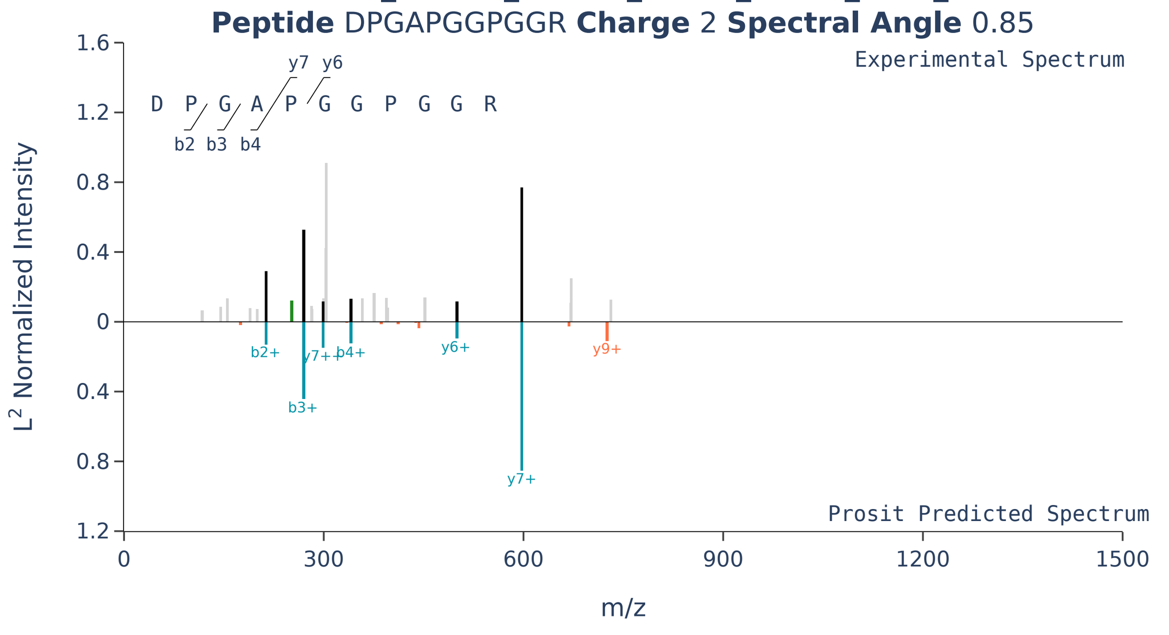

Source HRoetschke_231222_070123_HFGoe_UBE2S_b1R1_24h **Scan** 7414
Peptide DPGAPGGPGGE **Charge** 2 **Spectral Angle** 0.79

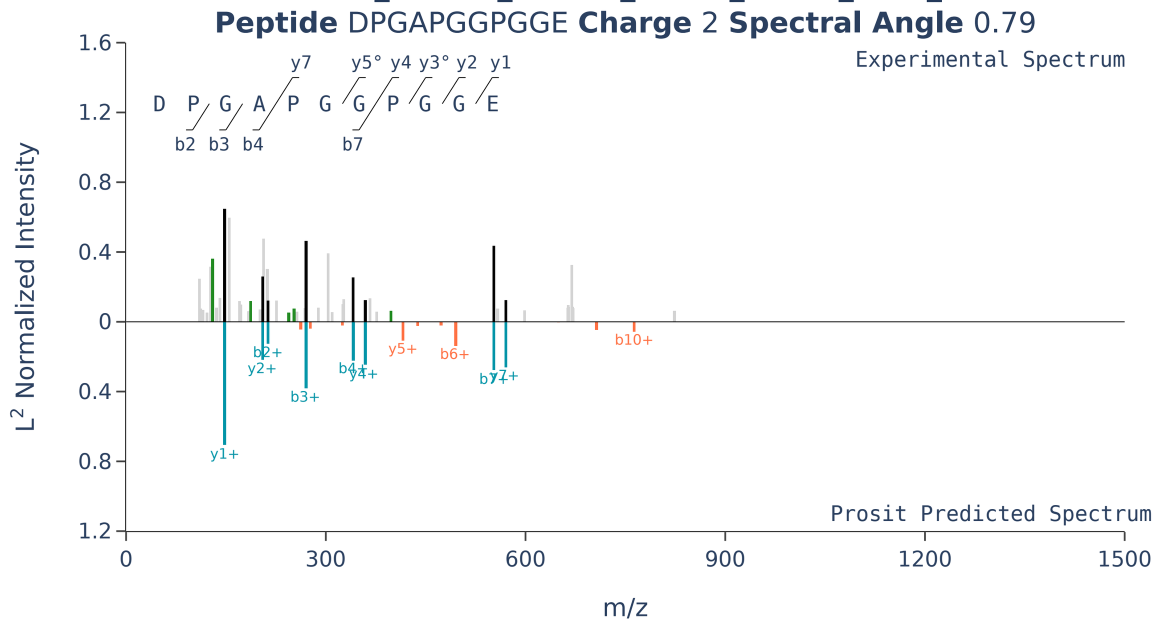

Source HRoetschke_231222_070123_HFGoe_UBE2S_b1R2_2h **Scan** 8265
Peptide SSTDPGAPGGPGGLRR **Charge** 3 **Spectral Angle** 0.88

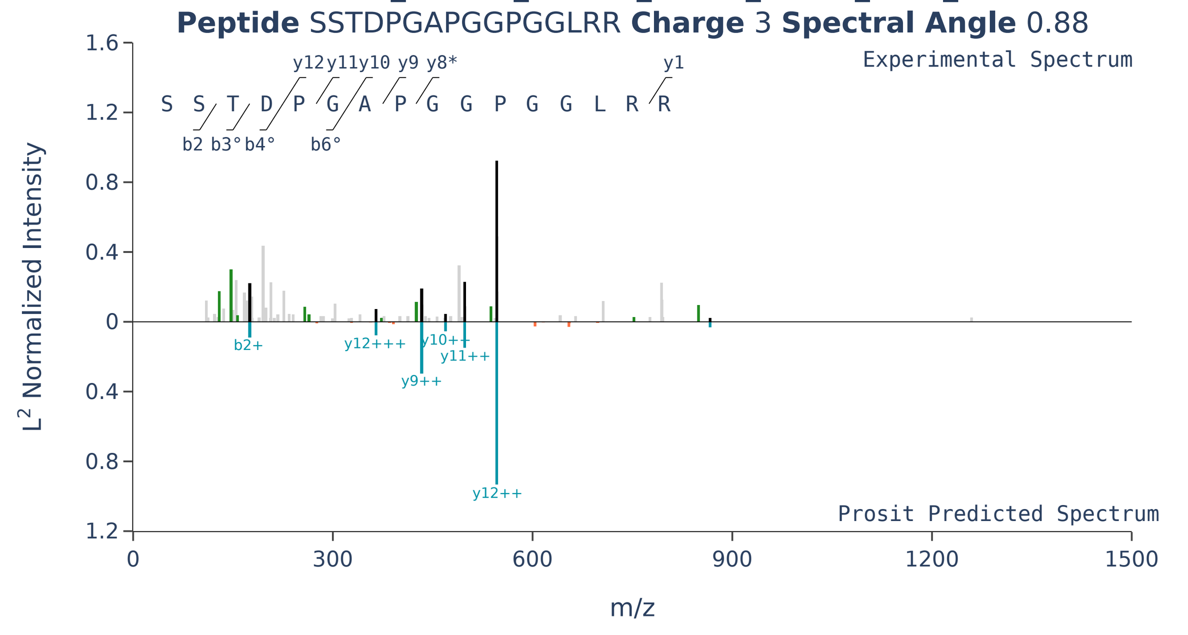

Source HRoetschke_231222_070123_HFGoe_UBE2S_b1R1_24h **Scan** 9350
Peptide ASSTDPGAPGGPGGTEA **Charge** 2 **Spectral Angle** 0.84

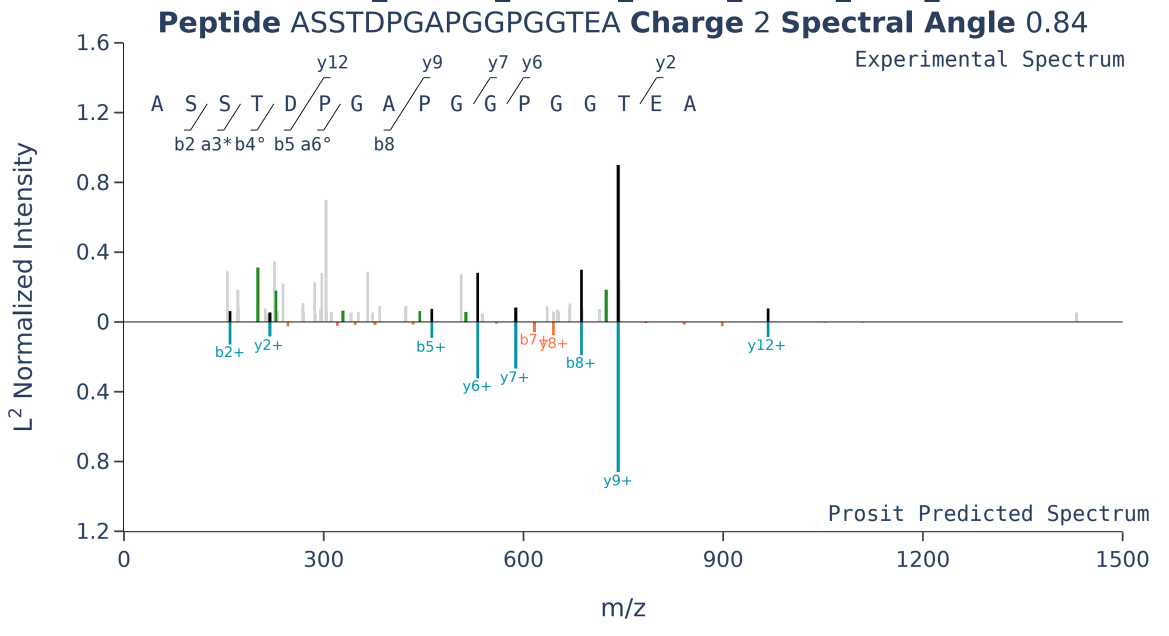

Source HRoetschke_231222_070123_HFGoe_UBE2S_b1R1_24h **Scan** 11143
Peptide HGGAGGPSGRLL **Charge** 3 **Spectral Angle** 0.81

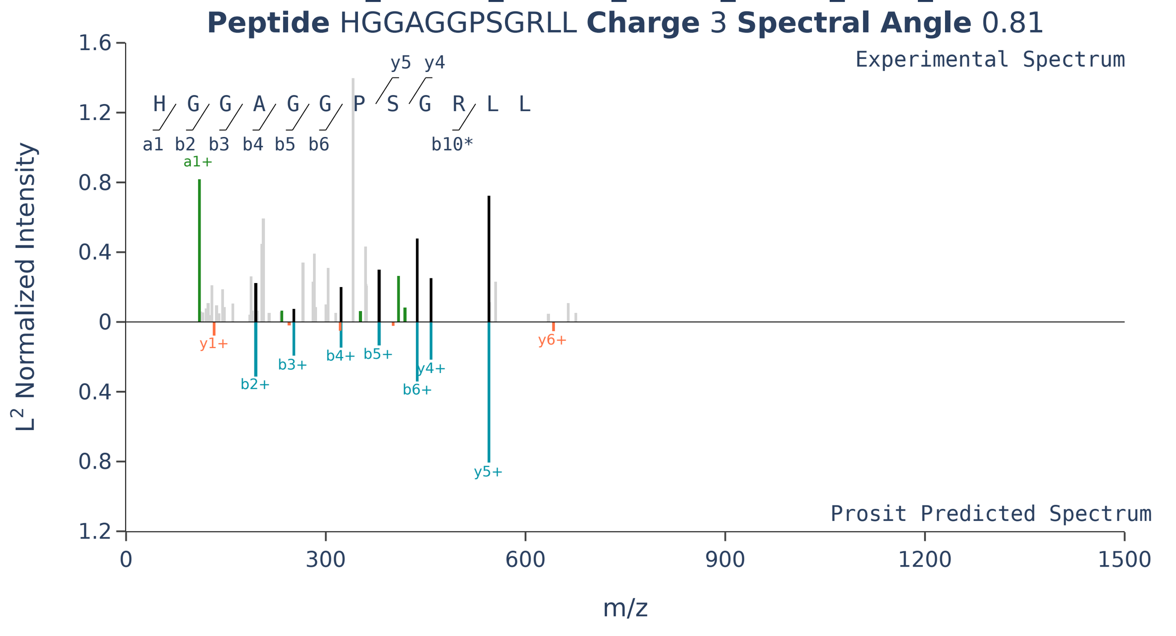

Source HRoetschke_231222_070123_HFGoe_UBE2S_b1R1_24h **Scan** 7098
Peptide TEARAEAGRAL **Charge** 3 **Spectral Angle** 0.87

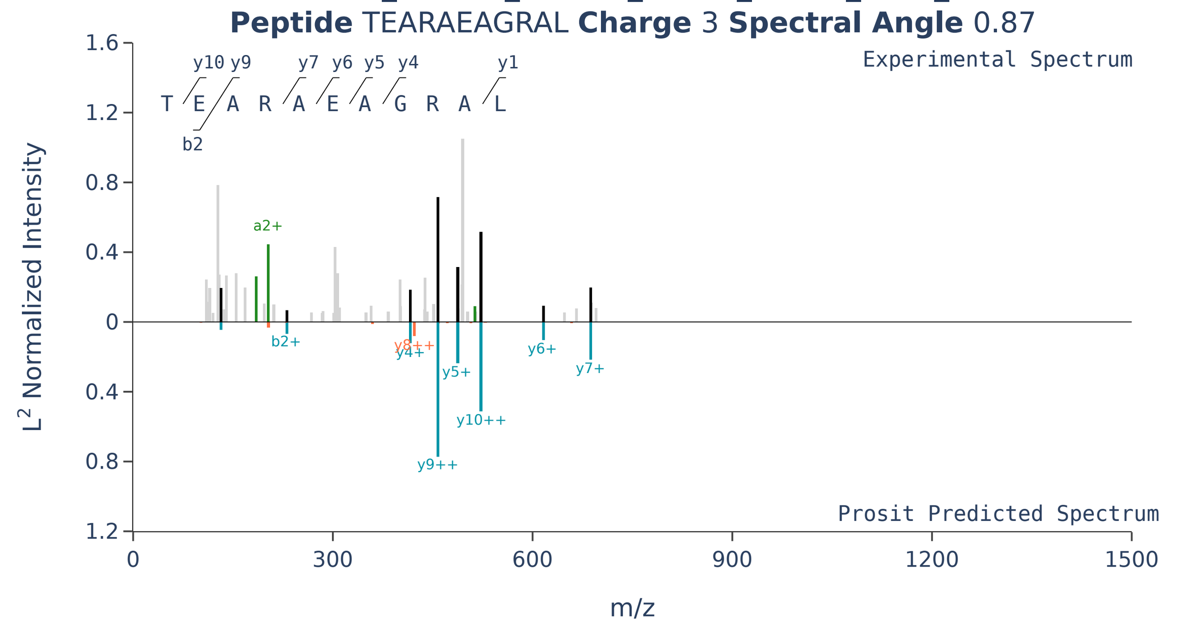

Source HRoetschke_231222_070123_HFGoe_UBE2S_b1R1_24h **Scan** 6201
Peptide RAEAGRALATD **Charge** 3 **Spectral Angle** 0.75

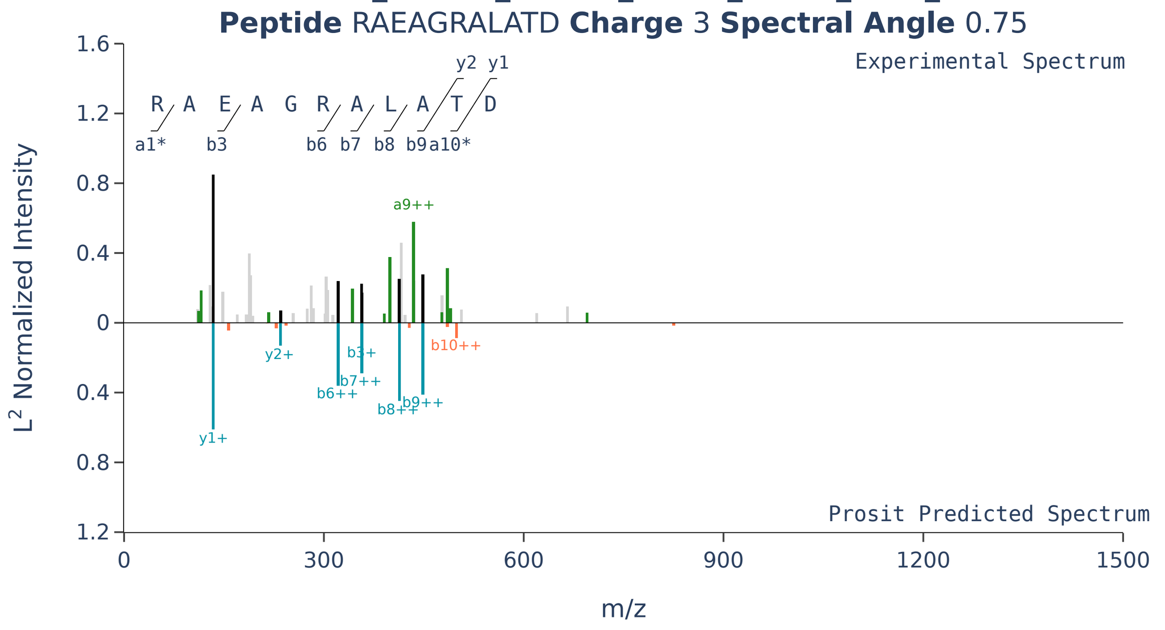

Source HRoetschke_231222_070123_HFGoe_UBE2S_b1R1_24h **Scan** 6591
Peptide TEIHGGAGGPSGTAD **Charge** 2 **Spectral Angle** 0.82

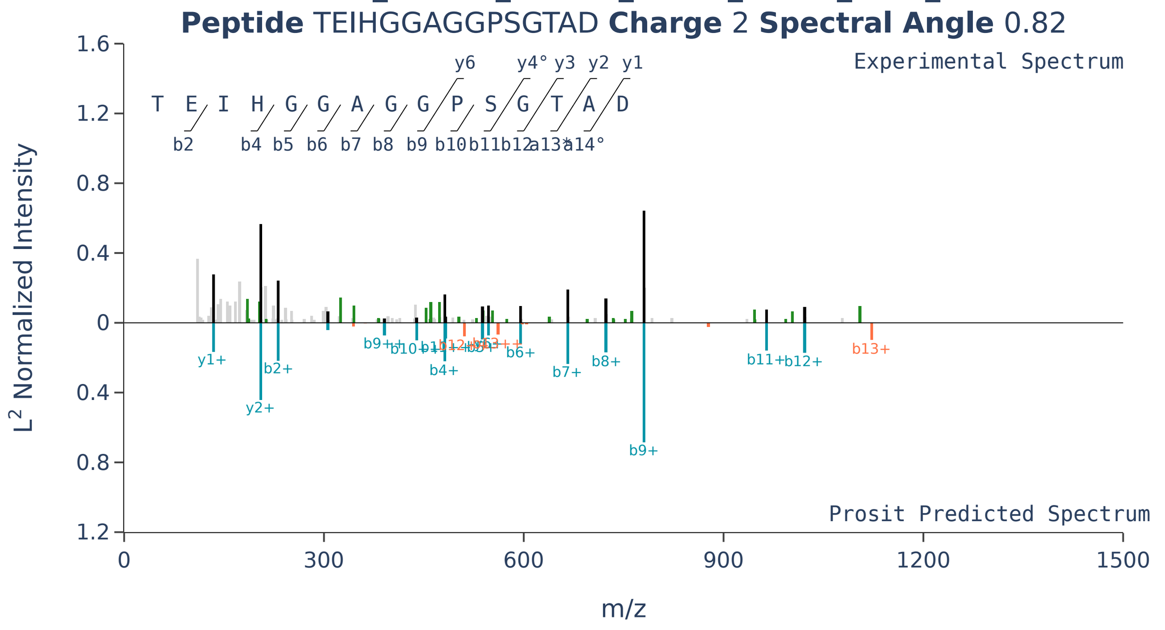

Source HRoetschke_231222_070123_HFGoe_UBE2S_b1R1_24h **Scan** 10689
Peptide LIHPNPESTE **Charge** 2 **Spectral Angle** 0.88

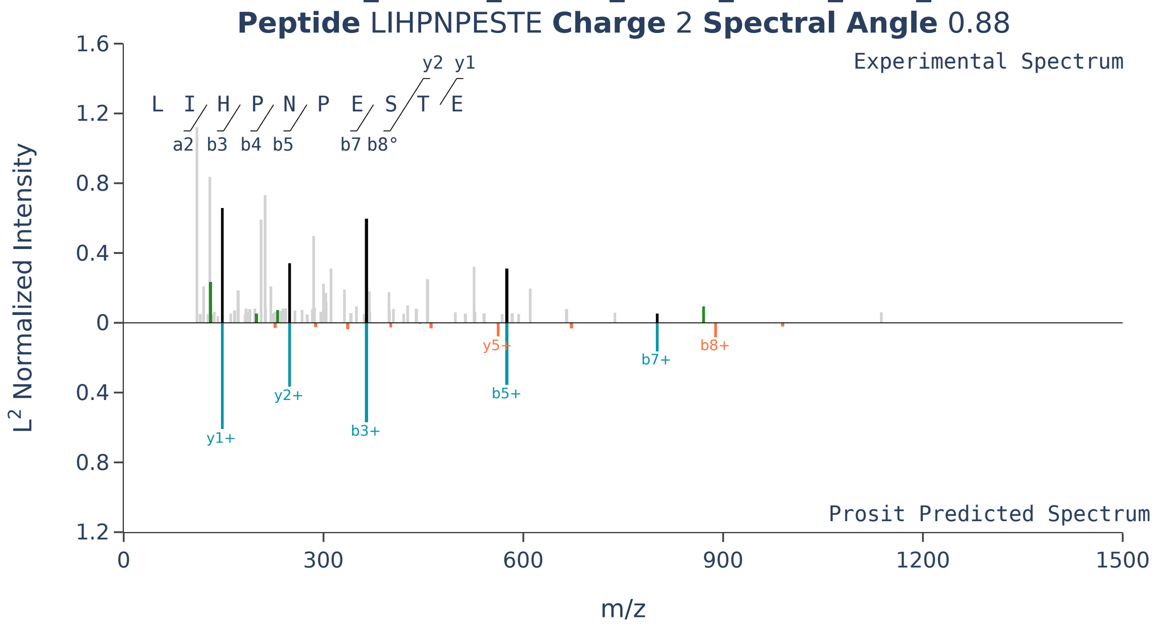

Source HRoetschke_231222_070123_HFGoe_UBE2S_b1R2_24h **Scan** 9682
Peptide TEIHGGAGGPSGM **Charge** 2 **Spectral Angle** 0.83

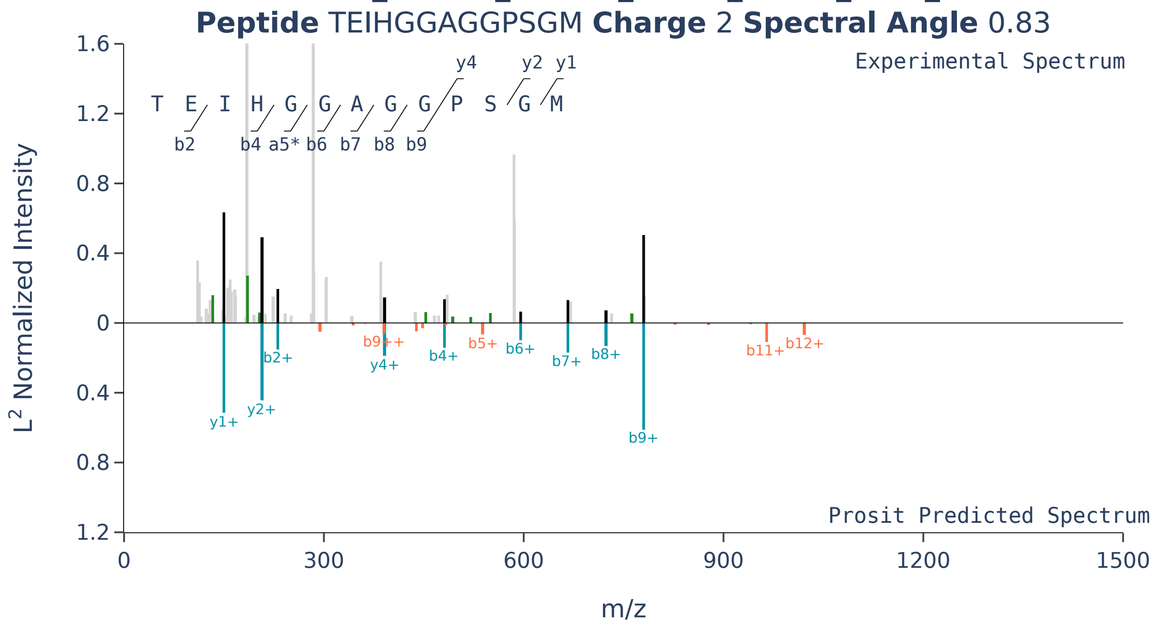

Source HRoetschke_231222_070123_HFGoe_UBE2S_b1R2_4h **Scan** 7011
Peptide DPGAPGGPNE **Charge** 2 **Spectral Angle** 0.79

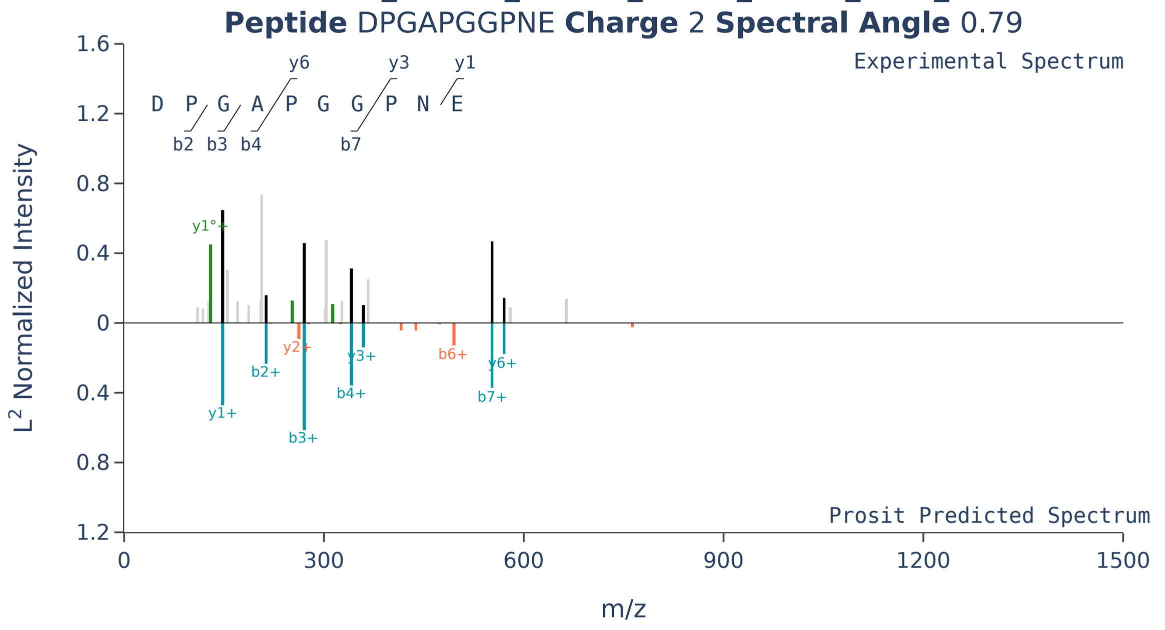

Source HRoetschke_231222_070123_HFGoe_UBE2S_b2R1_24h **Scan** 12546
Peptide RLLTEIHGGAGGPSGNE **Charge** 3 **Spectral Angle** 0.86

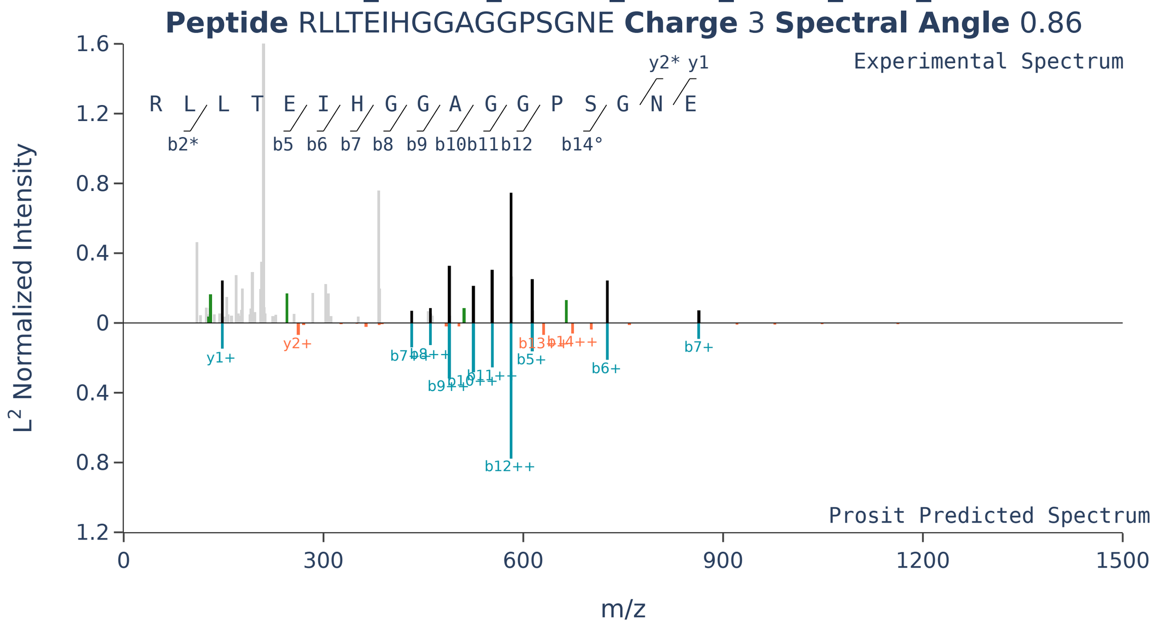

Source HRoetschke_231222_070123_HFGoe_UBE2S_b1R2_4h **Scan** 6025
Peptide RAEAGRALAE **Charge** 2 **Spectral Angle** 0.75

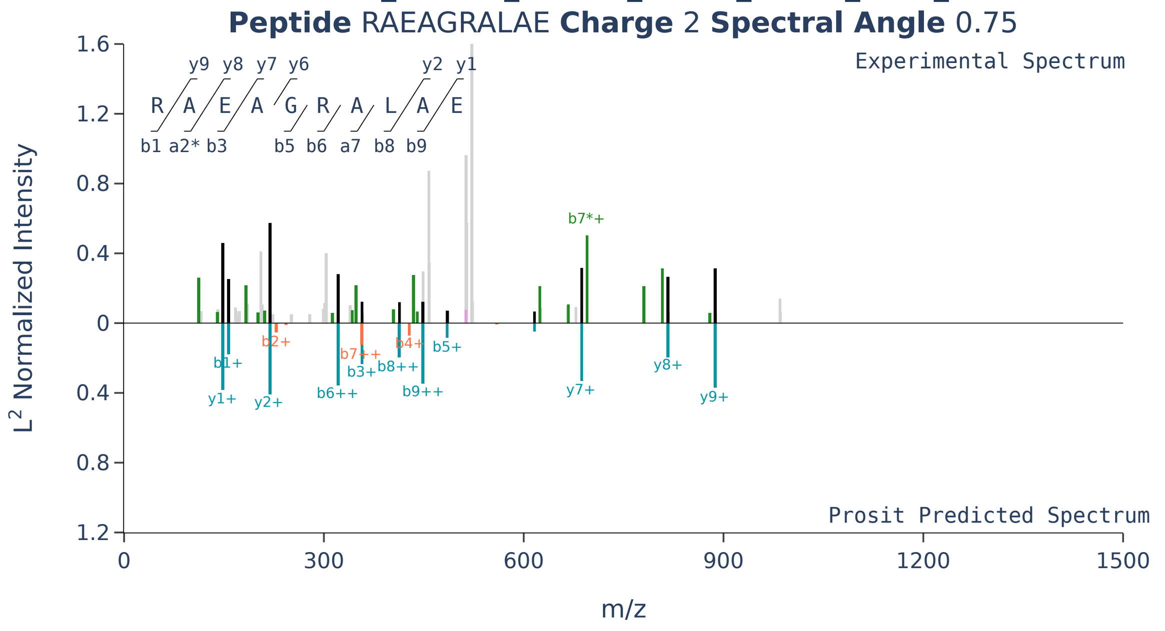

Source HRoetschke_231222_070123_HFGoe_UBE2S_b2R1_24h **Scan** 10137
Peptide PESALNEASST **Charge** 1 **Spectral Angle** 0.77

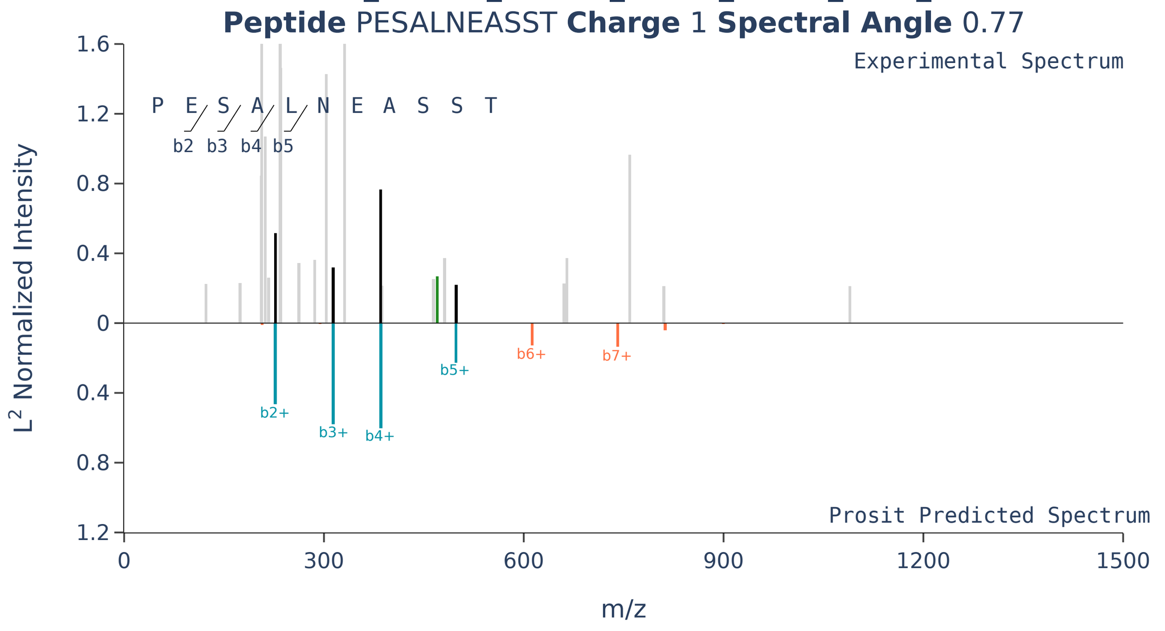

Source HRoetschke_231222_070123_HFGoe_UBE2S_b2R1_24h **Scan** 8580
Peptide ASSTDPGAAPGGPGGAE **Charge** 2 **Spectral Angle** 0.83

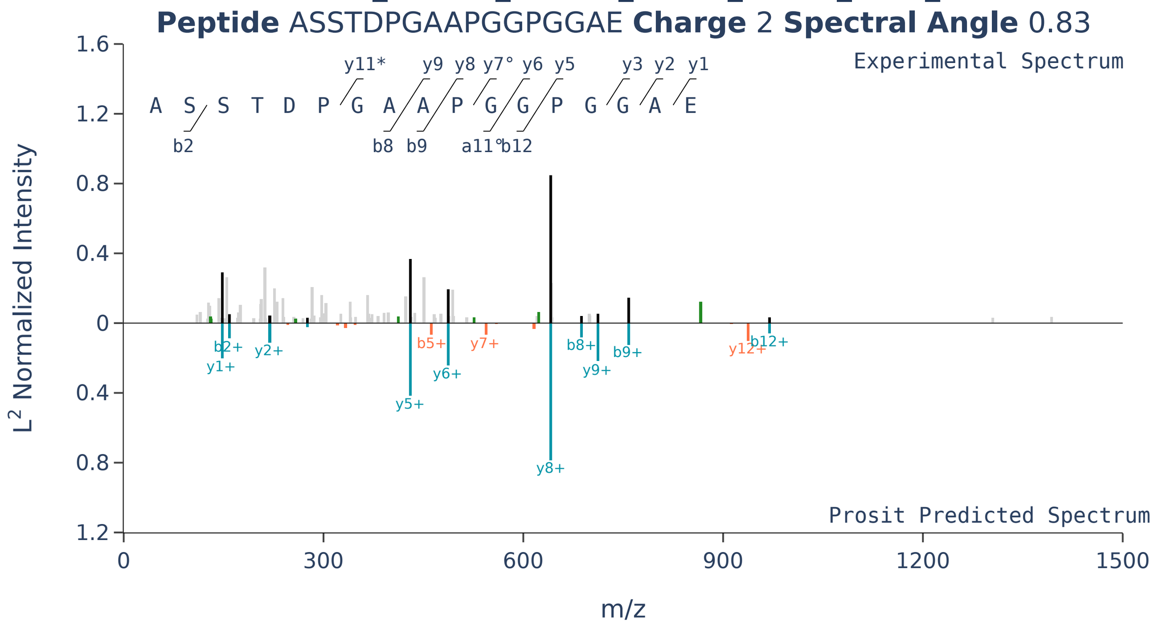

Source HRoetschke_231222_070123_HFGoe_UBE2S_b1R1_24h **Scan** 7818
Peptide VYKEVTA **Charge** 2 **Spectral Angle** 0.84

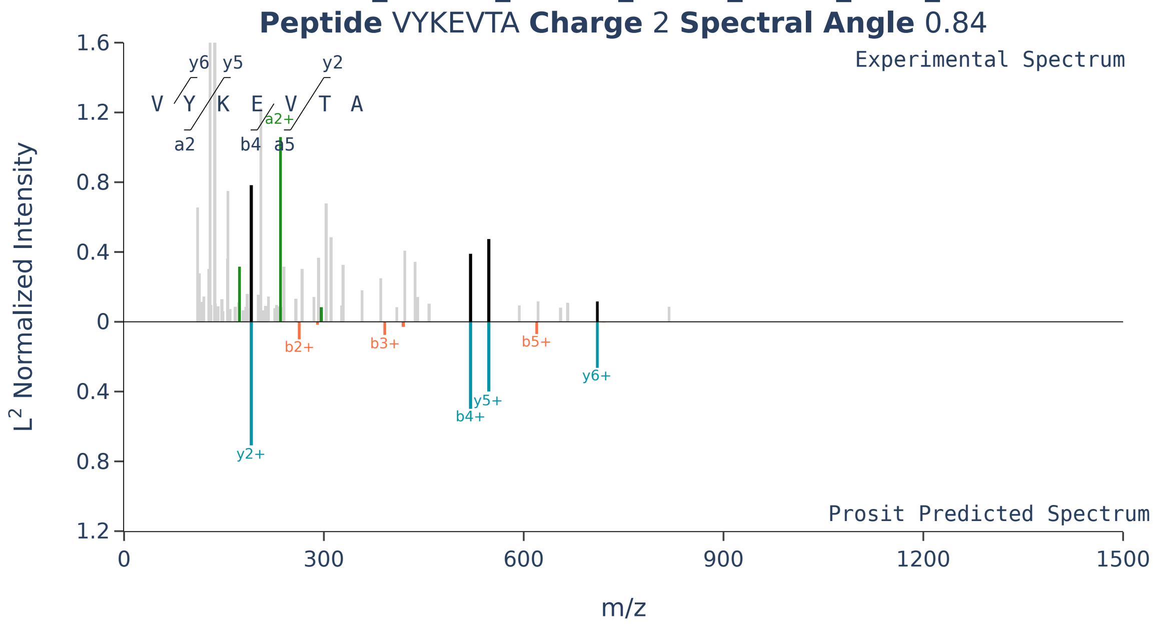

Source HRoetschke_231222_070123_HFGoe_UBE2S_b1R1_24h **Scan** 6043
Peptide TEIHGGAGGPSDG **Charge** 2 **Spectral Angle** 0.82

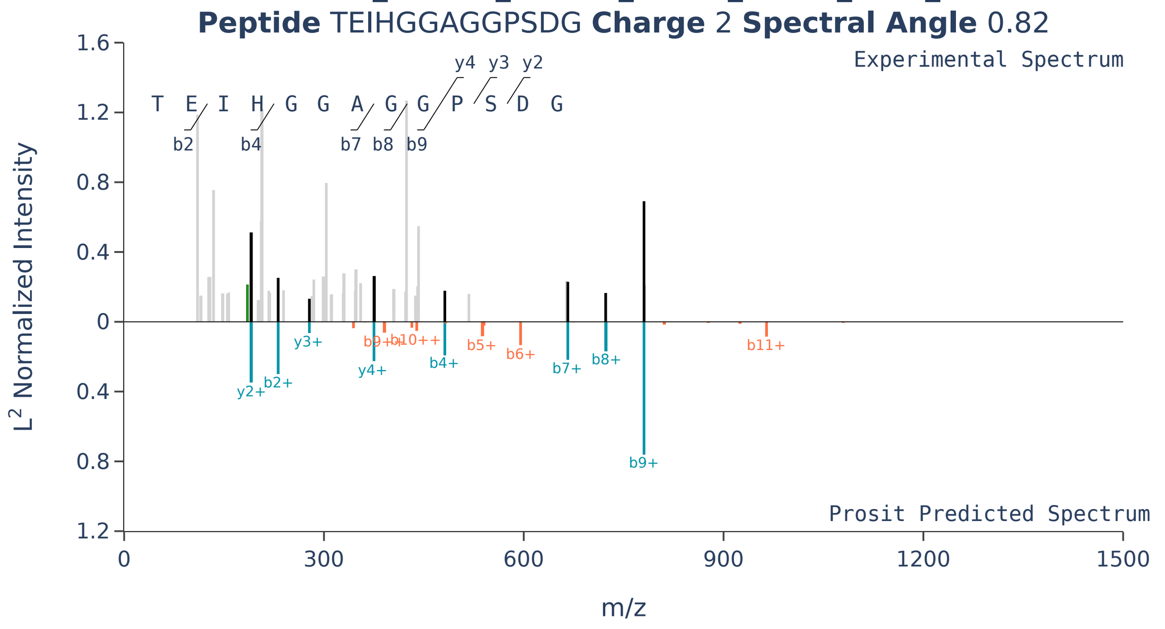

Source HRoetschke_231222_070123_HFGoe_UBE2S_b2R1_2h **Scan** 8075
Peptide GASSTDPGAPGGPGGAE **Charge** 2 **Spectral Angle** 0.86

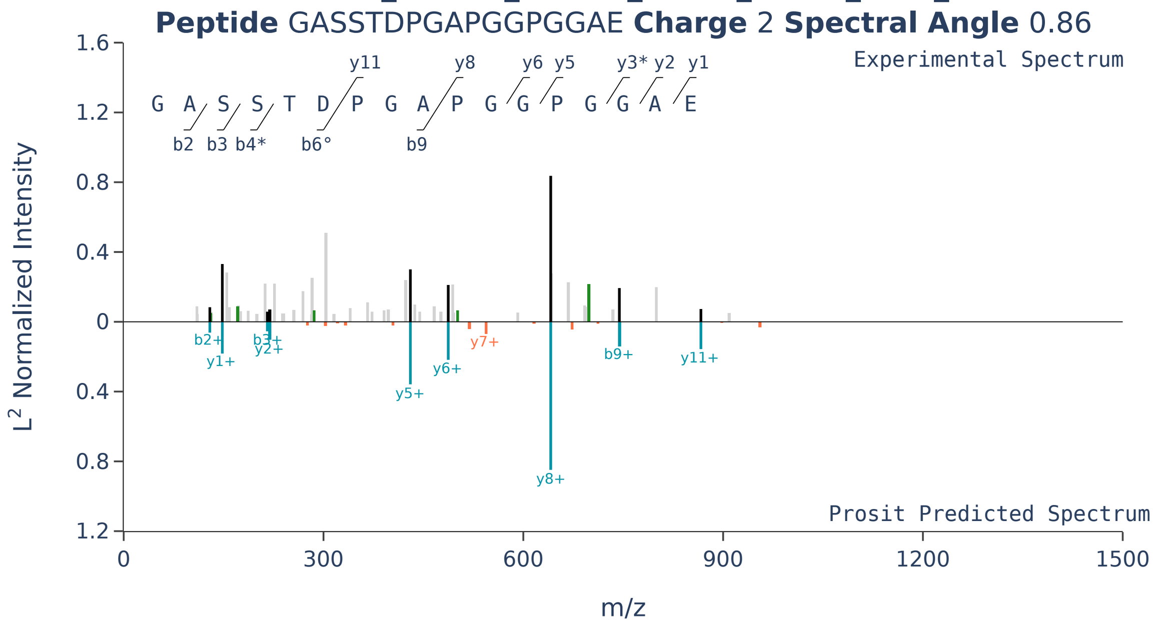

Source HRoetschke_231222_070123_HFGoe_UBE2S_b1R2_24h **Scan** 15259
Peptide HGGAGGPSGLLE **Charge** 2 **Spectral Angle** 0.86

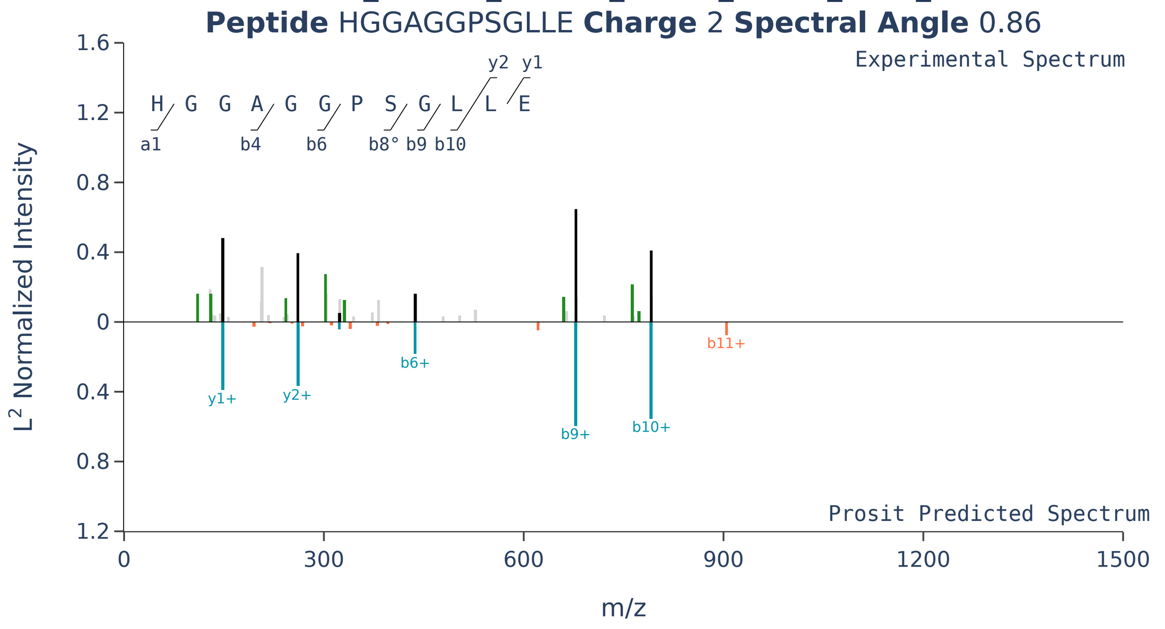

Source HRoetschke_231222_070123_HFGoe_UBE2S_b1R2_2h **Scan** 6974
Peptide DPGAAEAG **Charge** 1 **Spectral Angle** 0.82

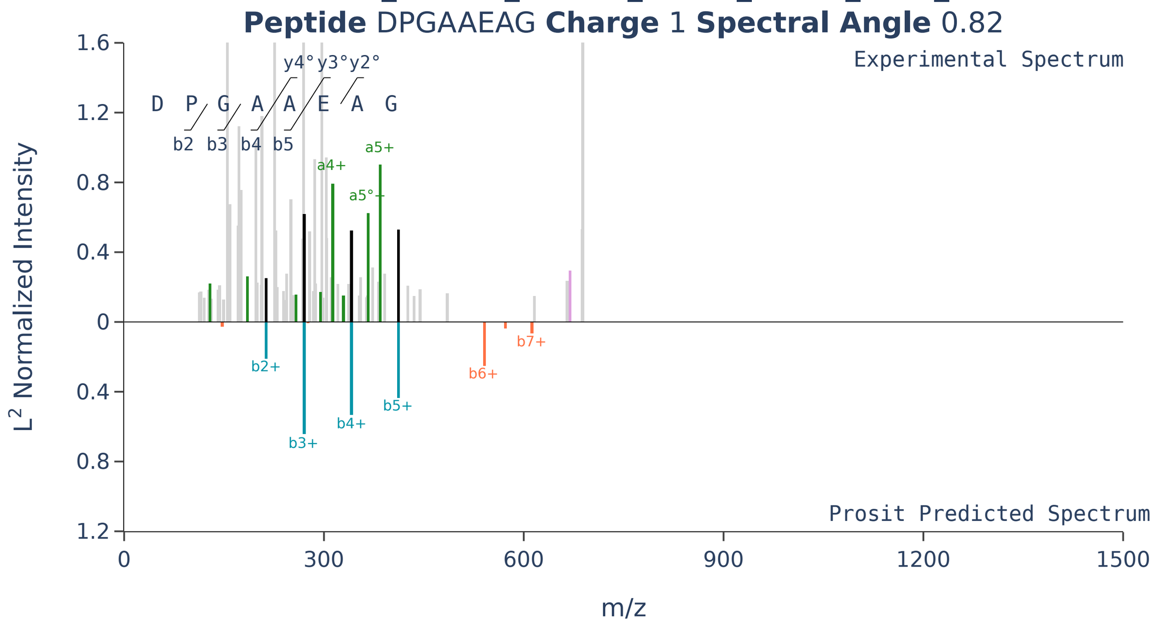

Source HRoetschke_231222_070123_HFGoe_UBE2S_b1R2_24h **Scan** 10040
Peptide IHGGAGGPSGEDLTD **Charge** 2 **Spectral Angle** 0.82

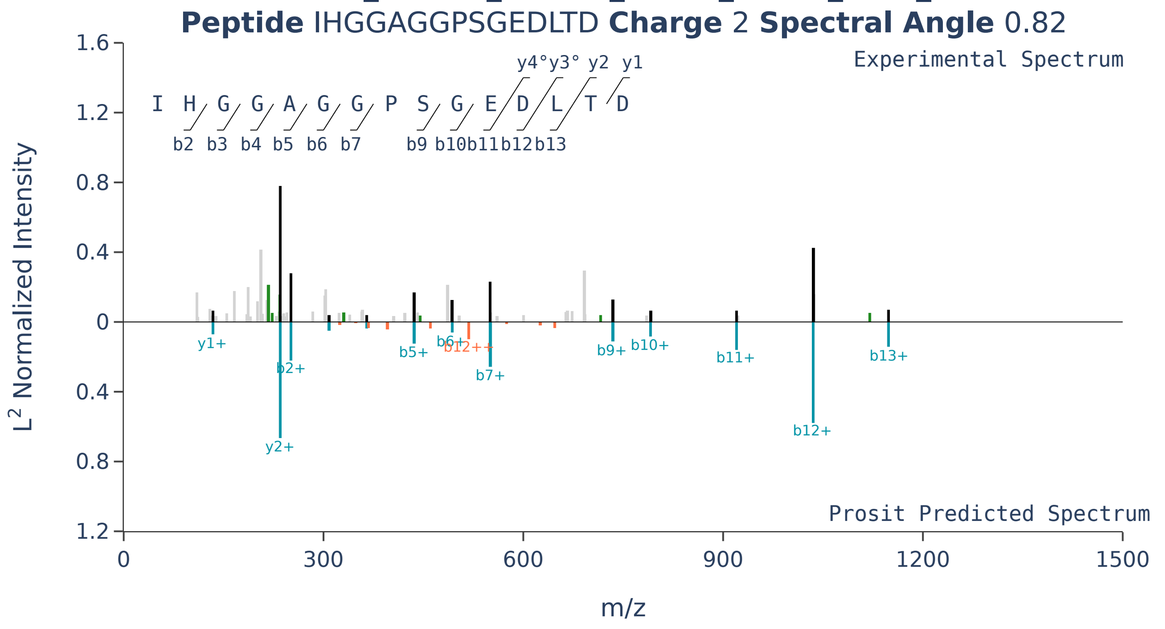

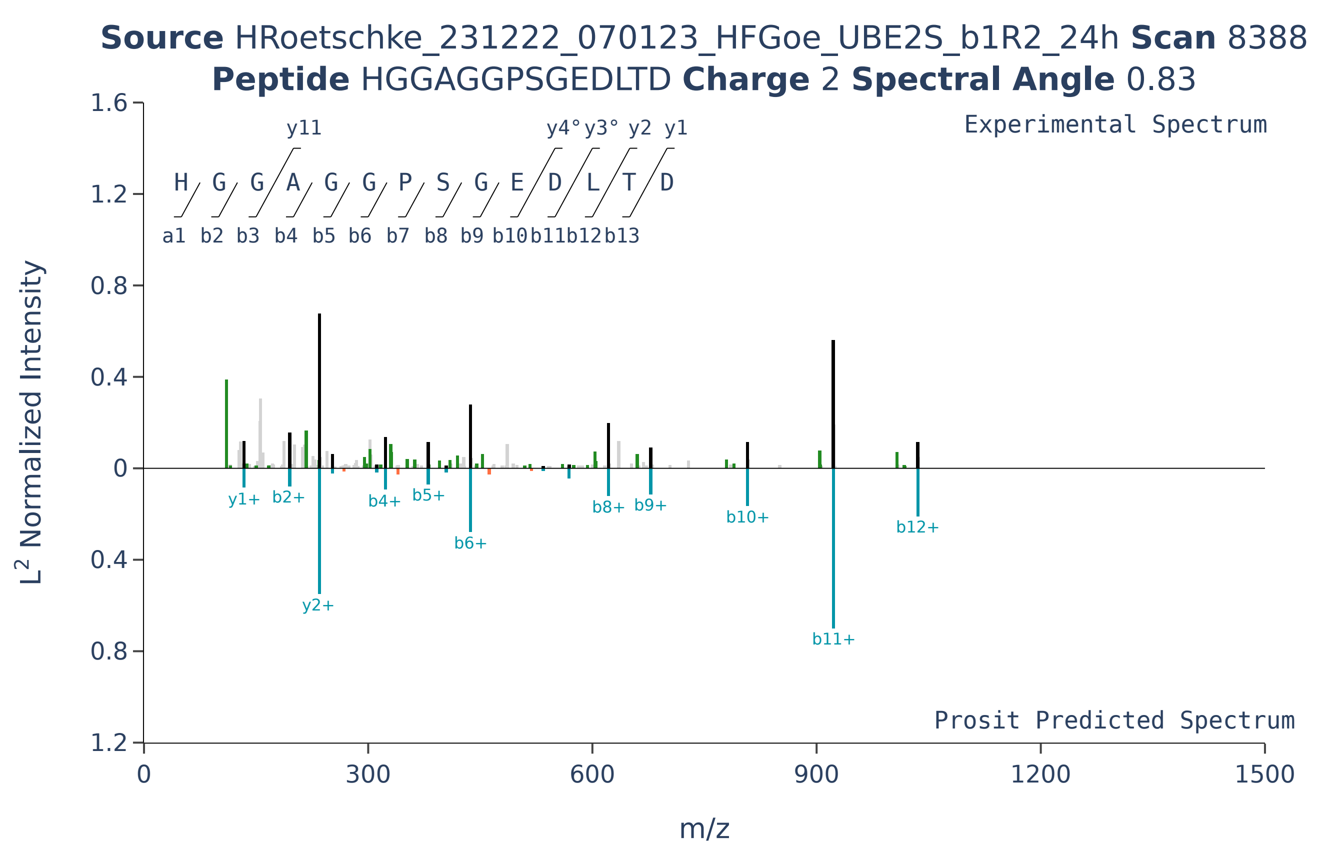

Source HRoetschke_231222_070123_HFGoe_UBE2S_b1R2_24h Scan 8388
Peptide HGGAGGPSGEDLTD Charge 2 Spectral Angle 0.83
Experimental Spectrum
Prosit Predicted Spectrum
H G G A G G P S G E D L T D
y11 y4° y3° y2 y1
a1 b2 b3 b4 b5 b6 b7 b8 b9 b10 b11 b12 b13
y1+ b2+ b4+ b5+ b6+ y2+ b8+ b9+ b10+ b11+ b12+
L² Normalized Intensity
m/z
0 300 600 900 1200 1500
1.6 1.2 0.8 0.4 0 0.4 0.8 1.2
