# Supplementary material for: Protein degradation by human 20S proteasomes elucidates the interplay between peptide hydrolysis and splicing
Source: Nat Commun. 2024 Feb 7;15:1147. doi: 10.1038/s41467-024-45339-3 (PMC10850103; doi:10.1038/s41467-024-45339-3)

Supplementary Data 5. MS2 spectra of a selection of non-spliced peptides identified in the protein digestions compared to their cognate synthetic peptides. Plots comparing (i) the experimental spectrum for an identified peptide on the positive y-axis against the spectrum for the cognate synthetic peptide on the negative y-axis (left), (ii) the experimental spectrum for an identified peptide on the positive y-axis against the Prosit predicted spectrum for that peptide on the negative y-axis (middle), and (iii) the spectrum for the cognate synthetic peptide on the positive y-axis against the Prosit-predicted spectrum for that peptide on the negative y-axis (right). Comparisons are shown for a random selection of 10 non-spliced peptides.

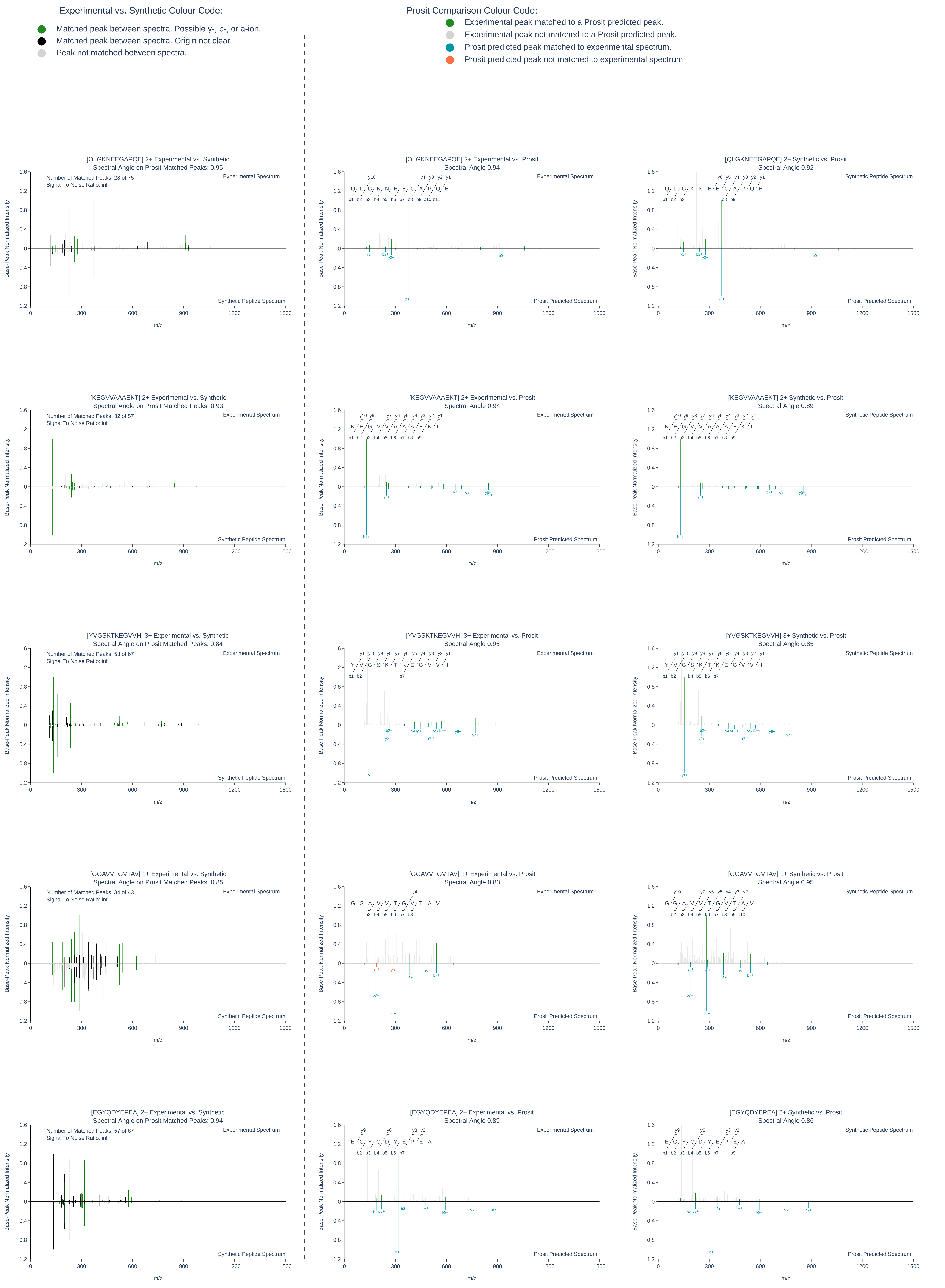
Experimental vs. Synthetic Colour Code:
Matched peak between spectra. Possible y-, b-, or a-ion.
Matched peak between spectra. Origin not clear.
Peak not matched between spectra.
Prosit Comparison Colour Code:
Experimental peak matched to a Prosit predicted peak.
Experimental peak not matched to a Prosit predicted peak.
Prosit predicted peak matched to experimental spectrum.
Prosit predicted peak not matched to experimental spectrum.
[QLGKNEEGAPQE] 2+ Experimental vs. Synthetic
Spectral Angle on Prosit Matched Peaks: 0.95
Number of Matched Peaks: 28 of 75
Signal To Noise Ratio: inf
[QLGKNEEGAPQE] 2+ Experimental vs. Prosit
Spectral Angle 0.94
[QLGKNEEGAPQE] 2+ Synthetic vs. Prosit
Spectral Angle 0.92
[KEGVVAAAEKT] 2+ Experimental vs. Synthetic
Spectral Angle on Prosit Matched Peaks: 0.93
Number of Matched Peaks: 32 of 57
Signal To Noise Ratio: inf
[KEGVVAAAEKT] 2+ Experimental vs. Prosit
Spectral Angle 0.94
[KEGVVAAAEKT] 2+ Synthetic vs. Prosit
Spectral Angle 0.89
[YVGSKTKEGVVH] 3+ Experimental vs. Synthetic
Spectral Angle on Prosit Matched Peaks: 0.84
Number of Matched Peaks: 53 of 67
Signal To Noise Ratio: inf
[YVGSKTKEGVVH] 3+ Experimental vs. Prosit
Spectral Angle 0.95
[YVGSKTKEGVVH] 3+ Synthetic vs. Prosit
Spectral Angle 0.85
[GGAVVTGVTAV] 1+ Experimental vs. Synthetic
Spectral Angle on Prosit Matched Peaks: 0.85
Number of Matched Peaks: 34 of 43
Signal To Noise Ratio: inf
[GGAVVTGVTAV] 1+ Experimental vs. Prosit
Spectral Angle 0.83
[GGAVVTGVTAV] 1+ Synthetic vs. Prosit
Spectral Angle 0.95
[EGYQDYEPEA] 2+ Experimental vs. Synthetic
Spectral Angle on Prosit Matched Peaks: 0.94
Number of Matched Peaks: 57 of 67
Signal To Noise Ratio: inf
[EGYQDYEPEA] 2+ Experimental vs. Prosit
Spectral Angle 0.89
[EGYQDYEPEA] 2+ Synthetic vs. Prosit
Spectral Angle 0.86
Experimental Spectrum
Synthetic Peptide Spectrum
Prosit Predicted Spectrum
Base-Peak Normalized Intensity
m/z

Experimental vs. Synthetic Colour Code:

- Matched peak between spectra. Possible y-, b-, or a-ion.
- Matched peak between spectra. Origin not clear.
- Peak not matched between spectra.

Prosit Comparison Colour Code:

- Experimental peak matched to a Prosit predicted peak.
- Experimental peak not matched to a Prosit predicted peak.
- Prosit predicted peak matched to experimental spectrum.
- Prosit predicted peak not matched to experimental spectrum.

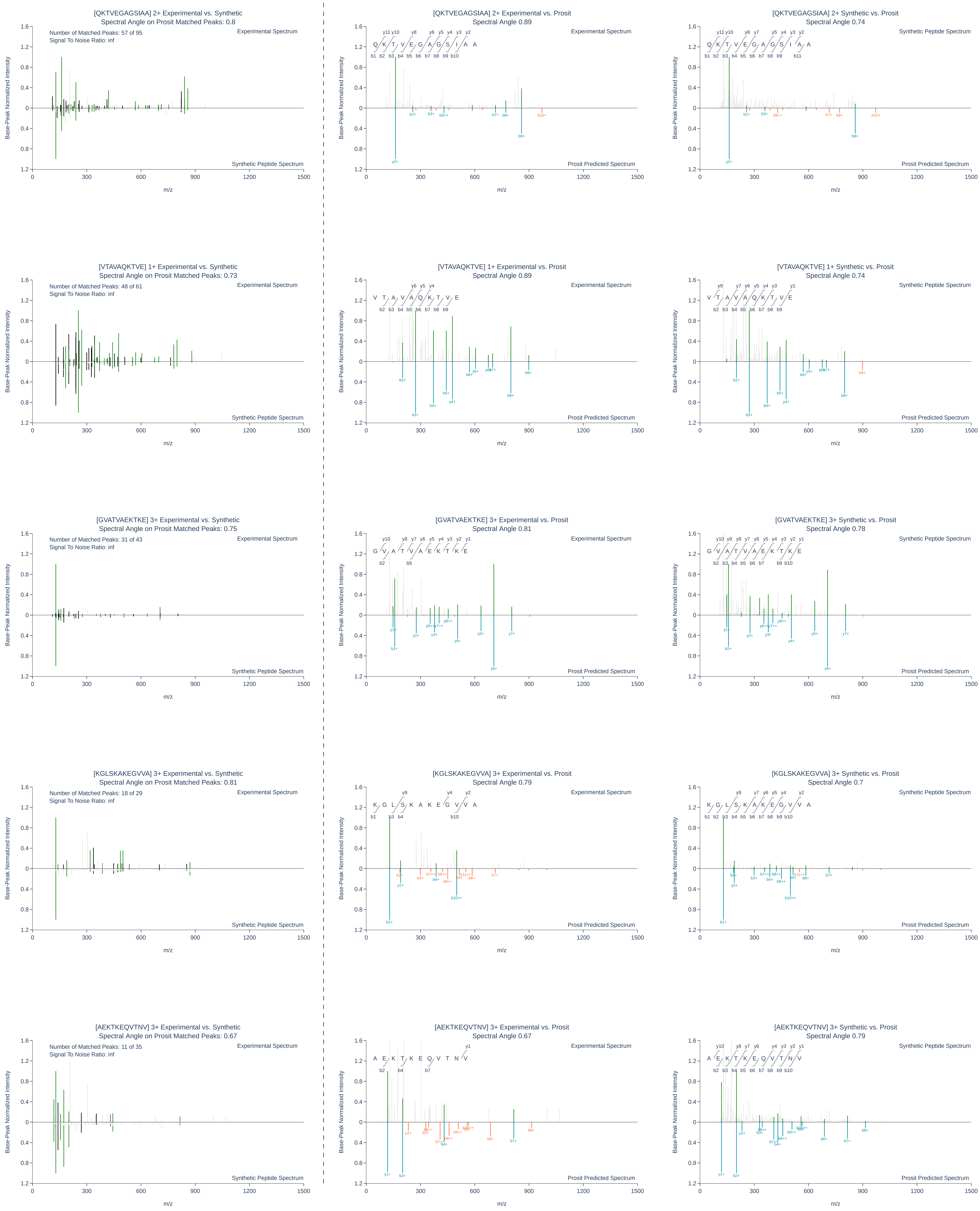

Supplement: Supplementary file 8 — Supplementary Data 5 [file 41467_2024_45339_MOESM8_ESM.pdf]
