# Supplementary material for: Protein degradation by human 20S proteasomes elucidates the interplay between peptide hydrolysis and splicing
Source: Nat Commun. 2024 Feb 7;15:1147. doi: 10.1038/s41467-024-45339-3 (PMC10850103; doi:10.1038/s41467-024-45339-3)
Supplement: Supplementary file 9 — Supplementary Data 6 [file 41467_2024_45339_MOESM9_ESM.pdf]

Supplementary Data 6. MS2 spectra of a selection of *cis*-spliced peptides identified in the protein digestions compared to their cognate synthetic peptides. Plots comparing (i) the experimental spectrum for an identified peptide on the positive y-axis against the spectrum for the cognate synthetic peptide on the negative y-axis (left), (ii) the experimental spectrum for an identified peptide on the positive y-axis against the Prosit-predicted spectrum for that peptide on the negative y-axis (middle), and (iii) the spectrum for the cognate synthetic peptide on the positive y-axis against the Prosit-predicted spectrum for that peptide on the negative y-axis (right). Comparisons are shown for a random selection of 22 spliced peptides.

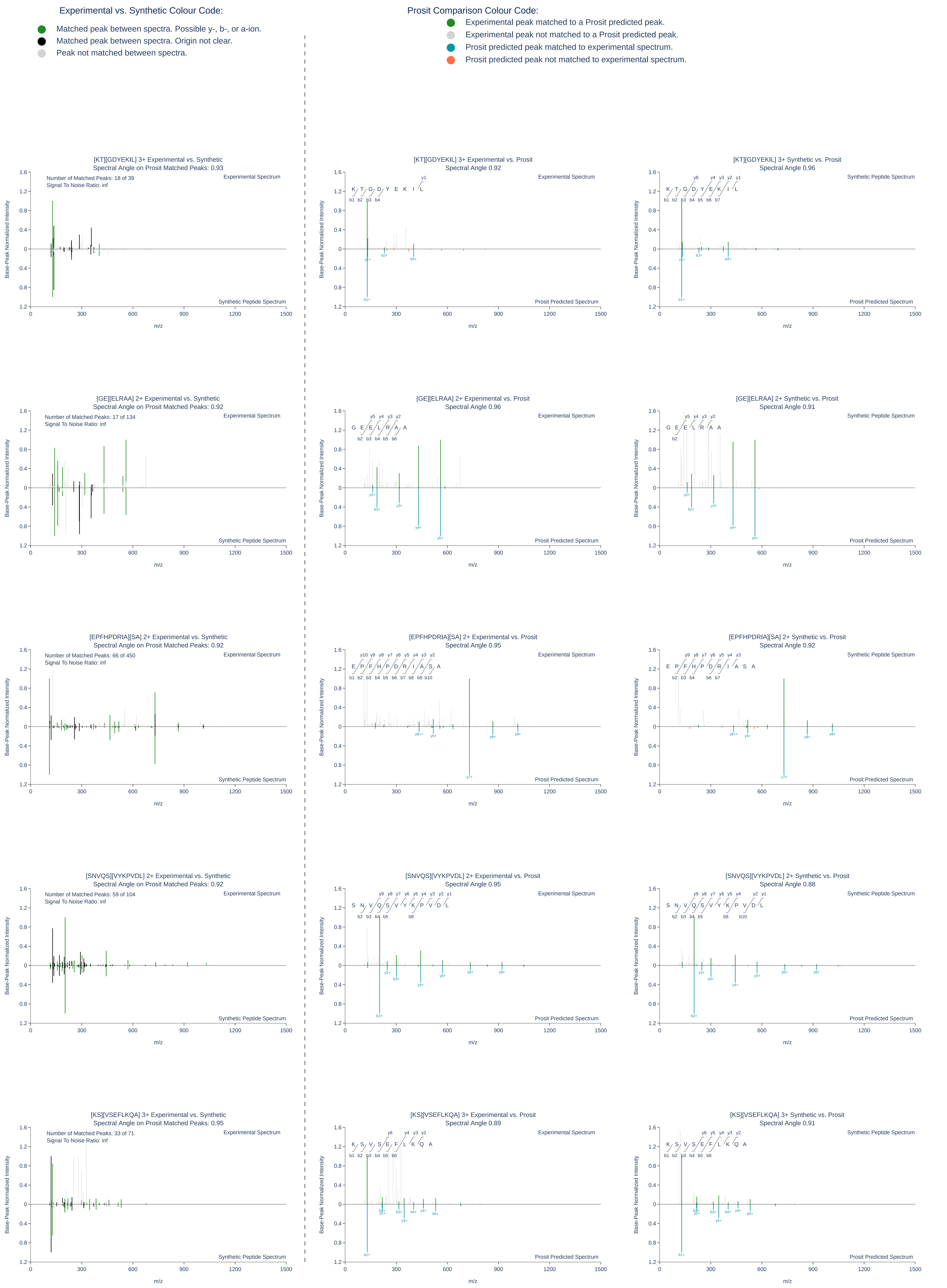

Experimental vs. Synthetic Colour Code:
Matched peak between spectra. Possible y-, b-, or a-ion.
Matched peak between spectra. Origin not clear.
Peak not matched between spectra.
Prosit Comparison Colour Code:
Experimental peak matched to a Prosit predicted peak.
Experimental peak not matched to a Prosit predicted peak.
Prosit predicted peak matched to experimental spectrum.
Prosit predicted peak not matched to experimental spectrum.
[KT][GDYEKIL] 3+ Experimental vs. Synthetic
Spectral Angle on Prosit Matched Peaks: 0.93
Number of Matched Peaks: 18 of 39
Signal To Noise Ratio: inf
[KT][GDYEKIL] 3+ Experimental vs. Prosit
Spectral Angle 0.92
[KT][GDYEKIL] 3+ Synthetic vs. Prosit
Spectral Angle 0.96
[GE][ELRAA] 2+ Experimental vs. Synthetic
Spectral Angle on Prosit Matched Peaks: 0.92
Number of Matched Peaks: 17 of 134
Signal To Noise Ratio: inf
[GE][ELRAA] 2+ Experimental vs. Prosit
Spectral Angle 0.96
[GE][ELRAA] 2+ Synthetic vs. Prosit
Spectral Angle 0.91
[EPFHPDRIA][SA] 2+ Experimental vs. Synthetic
Spectral Angle on Prosit Matched Peaks: 0.92
Number of Matched Peaks: 66 of 450
Signal To Noise Ratio: inf
[EPFHPDRIA][SA] 2+ Experimental vs. Prosit
Spectral Angle 0.95
[EPFHPDRIA][SA] 2+ Synthetic vs. Prosit
Spectral Angle 0.92
[SNVQS][VYKPVDL] 2+ Experimental vs. Synthetic
Spectral Angle on Prosit Matched Peaks: 0.92
Number of Matched Peaks: 59 of 104
Signal To Noise Ratio: inf
[SNVQS][VYKPVDL] 2+ Experimental vs. Prosit
Spectral Angle 0.95
[SNVQS][VYKPVDL] 2+ Synthetic vs. Prosit
Spectral Angle 0.88
[KS][VSEFLKQA] 3+ Experimental vs. Synthetic
Spectral Angle on Prosit Matched Peaks: 0.95
Number of Matched Peaks: 33 of 71
Signal To Noise Ratio: inf
[KS][VSEFLKQA] 3+ Experimental vs. Prosit
Spectral Angle 0.89
[KS][VSEFLKQA] 3+ Synthetic vs. Prosit
Spectral Angle 0.91
Experimental Spectrum
Synthetic Peptide Spectrum
Prosit Predicted Spectrum
Base-Peak Normalized Intensity
m/z

Experimental vs. Synthetic Colour Code:

- Matched peak between spectra. Possible y-, b-, or a-ion.
- Matched peak between spectra. Origin not clear.
- Peak not matched between spectra.

Prosit Comparison Colour Code:

- Experimental peak matched to a Prosit predicted peak.
- Experimental peak not matched to a Prosit predicted peak.
- Prosit predicted peak matched to experimental spectrum.
- Prosit predicted peak not matched to experimental spectrum.

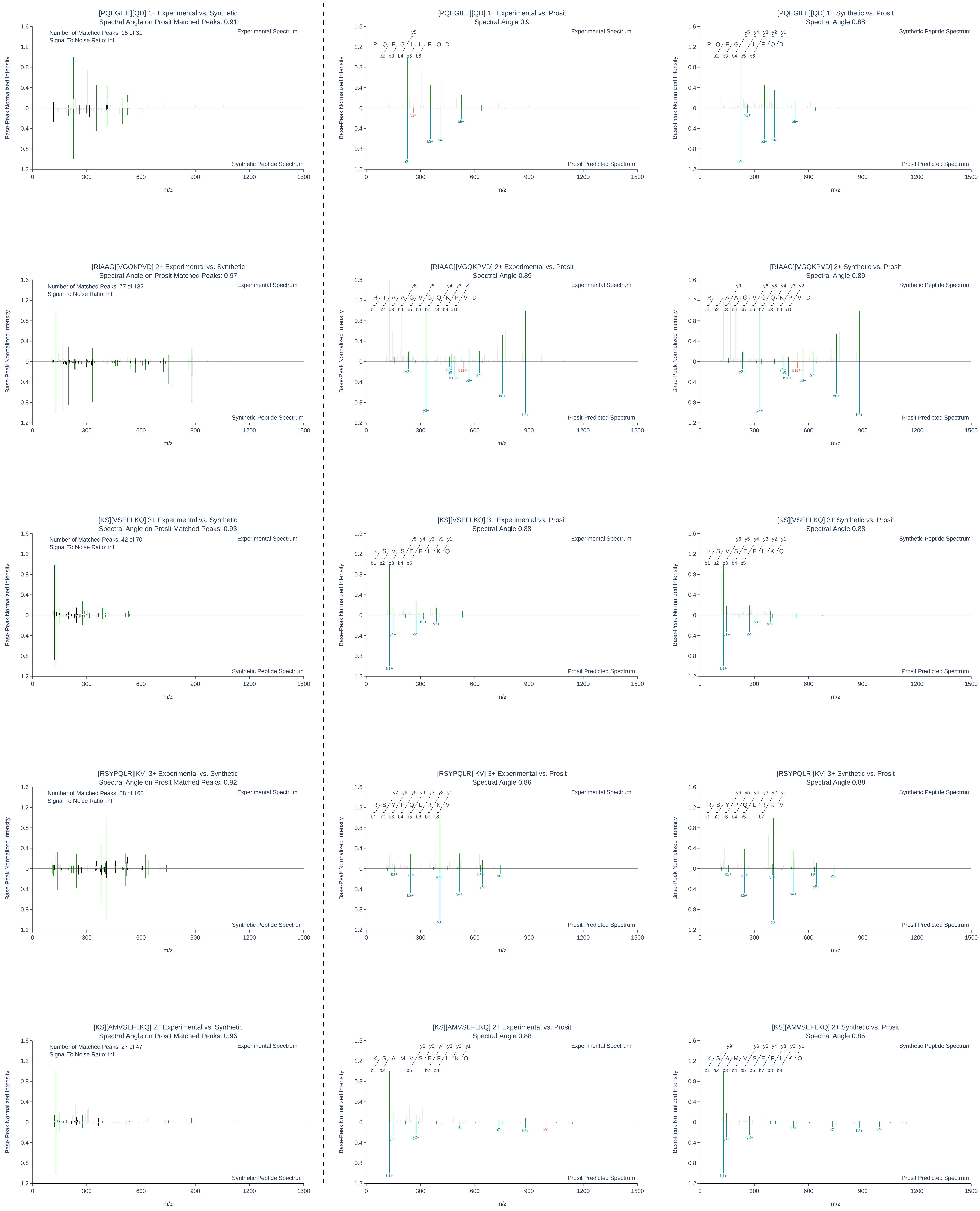

Experimental vs. Synthetic Colour Code:

- Matched peak between spectra. Possible y-, b-, or a-ion.
- Matched peak between spectra. Origin not clear.
- Peak not matched between spectra.

Prosit Comparison Colour Code:

- Experimental peak matched to a Prosit predicted peak.
- Experimental peak not matched to a Prosit predicted peak.
- Prosit predicted peak matched to experimental spectrum.
- Prosit predicted peak not matched to experimental spectrum.

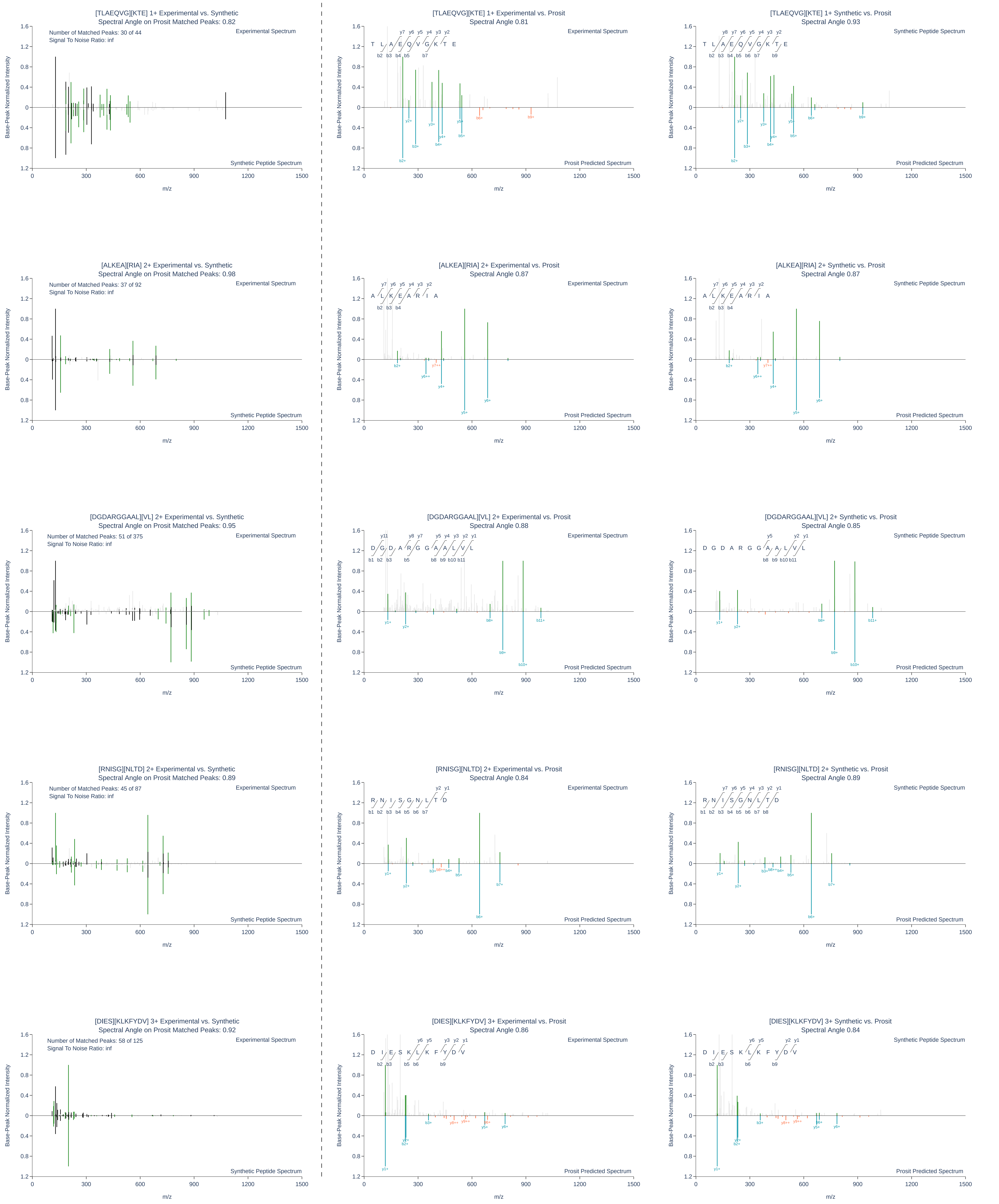

Experimental vs. Synthetic Colour Code:

- Matched peak between spectra. Possible y-, b-, or a-ion.
- Matched peak between spectra. Origin not clear.
- Peak not matched between spectra.

Prosit Comparison Colour Code:

- Experimental peak matched to a Prosit predicted peak.
- Experimental peak not matched to a Prosit predicted peak.
- Prosit predicted peak matched to experimental spectrum.
- Prosit predicted peak not matched to experimental spectrum.

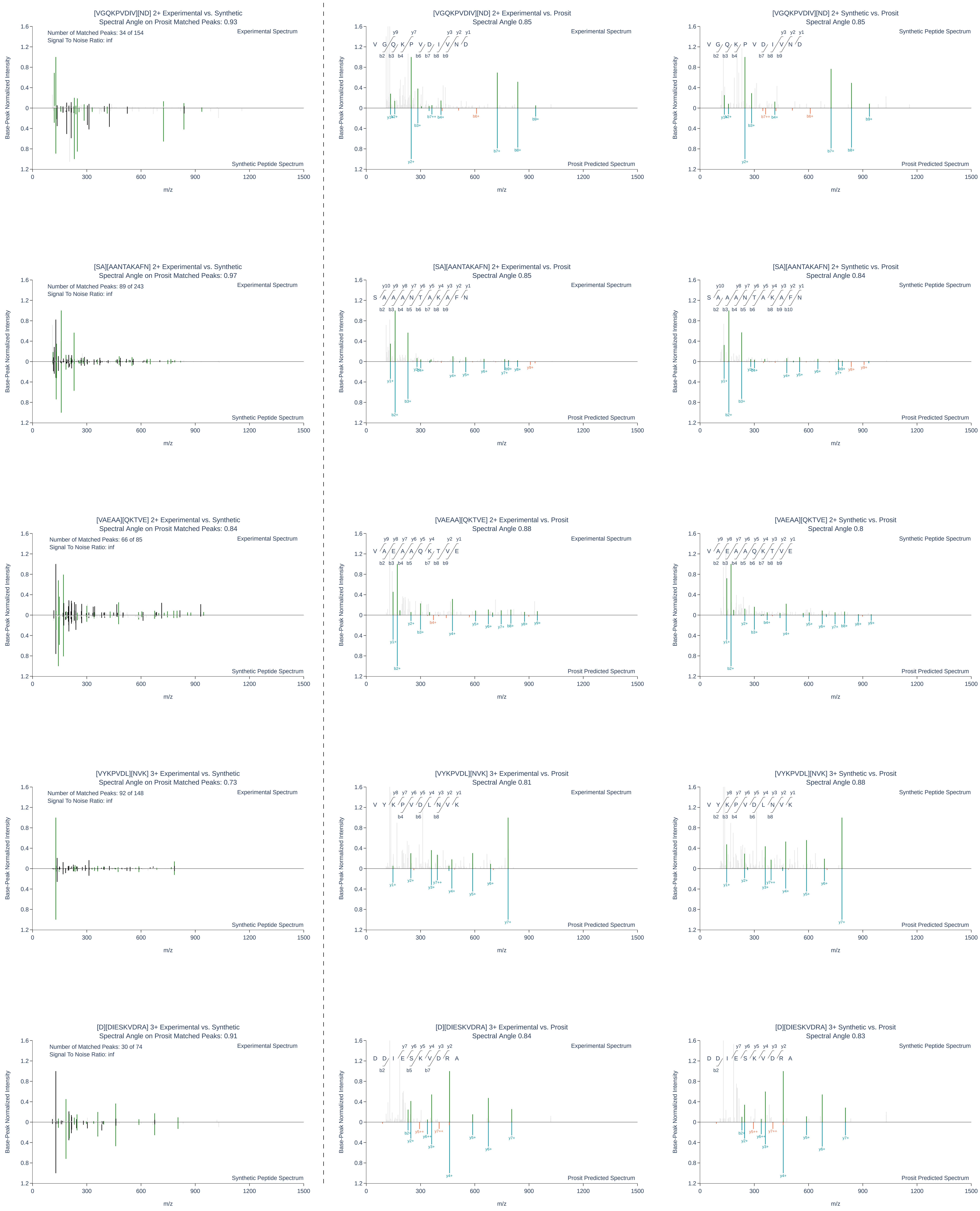

Experimental vs. Synthetic Colour Code:

- Matched peak between spectra. Possible y-, b-, or a-ion.
- Matched peak between spectra. Origin not clear.
- Peak not matched between spectra.

Prosit Comparison Colour Code:

- Experimental peak matched to a Prosit predicted peak.
- Experimental peak not matched to a Prosit predicted peak.
- Prosit predicted peak matched to experimental spectrum.
- Prosit predicted peak not matched to experimental spectrum.

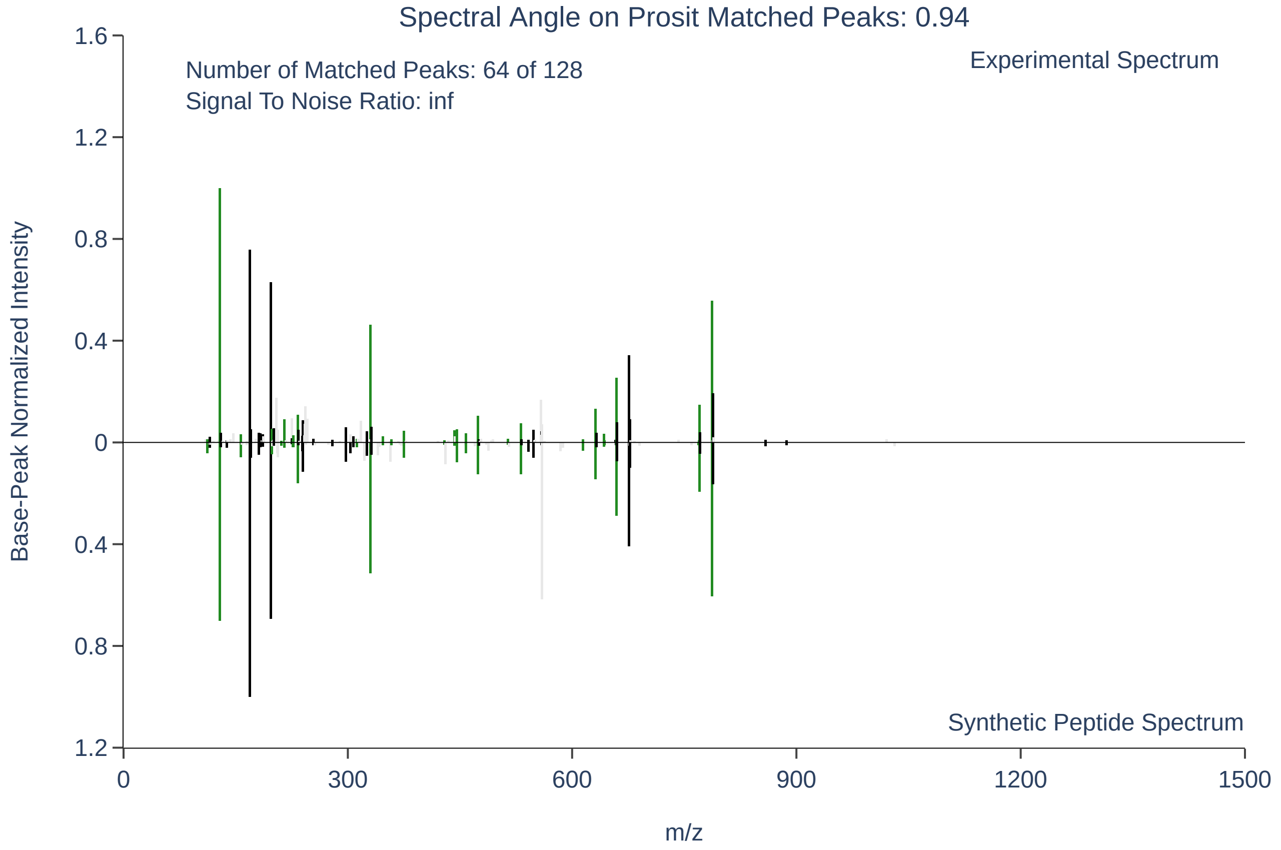

[RSM][VGQKPVD] 2+ Experimental vs. Synthetic
Spectral Angle on Prosit Matched Peaks: 0.94

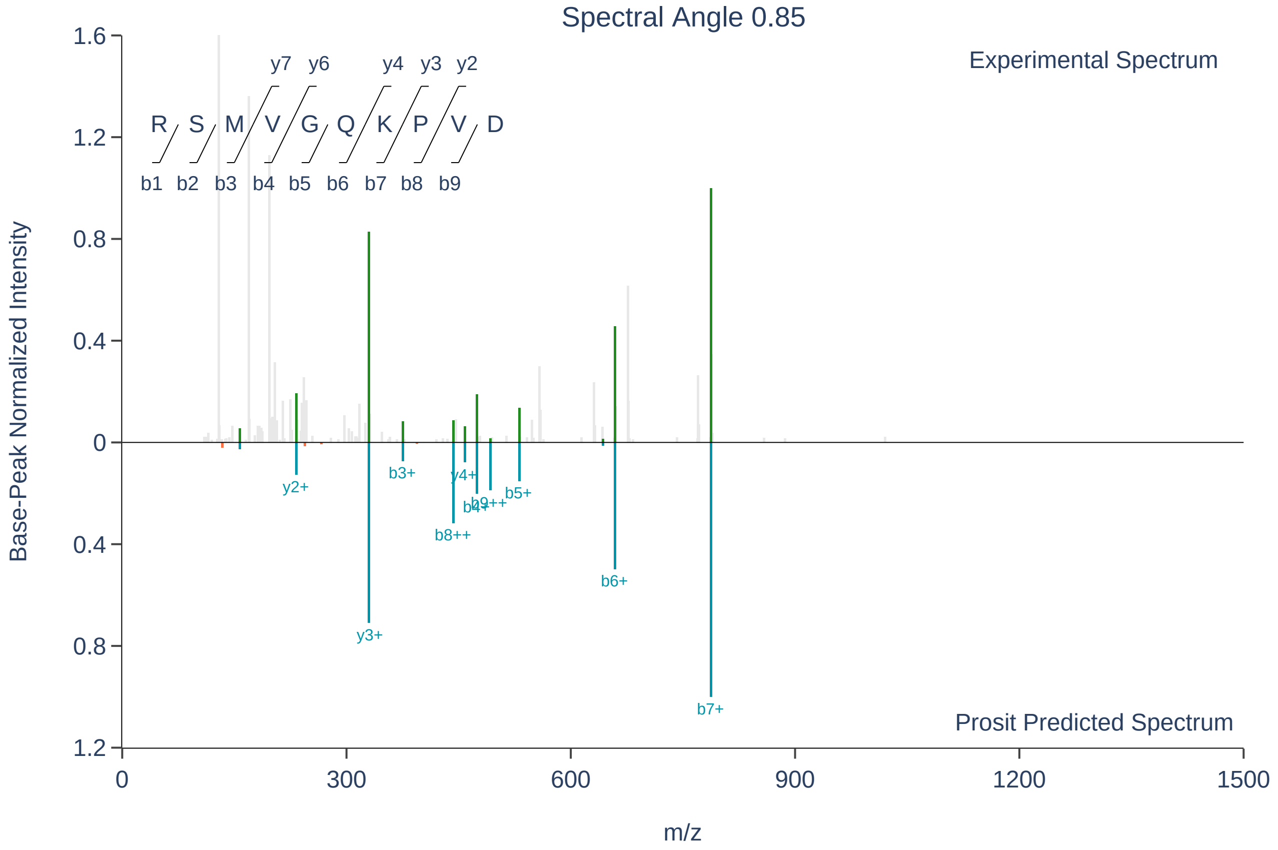

[RSM][VGQKPVD] 2+ Experimental vs. Prosit
Spectral Angle 0.85

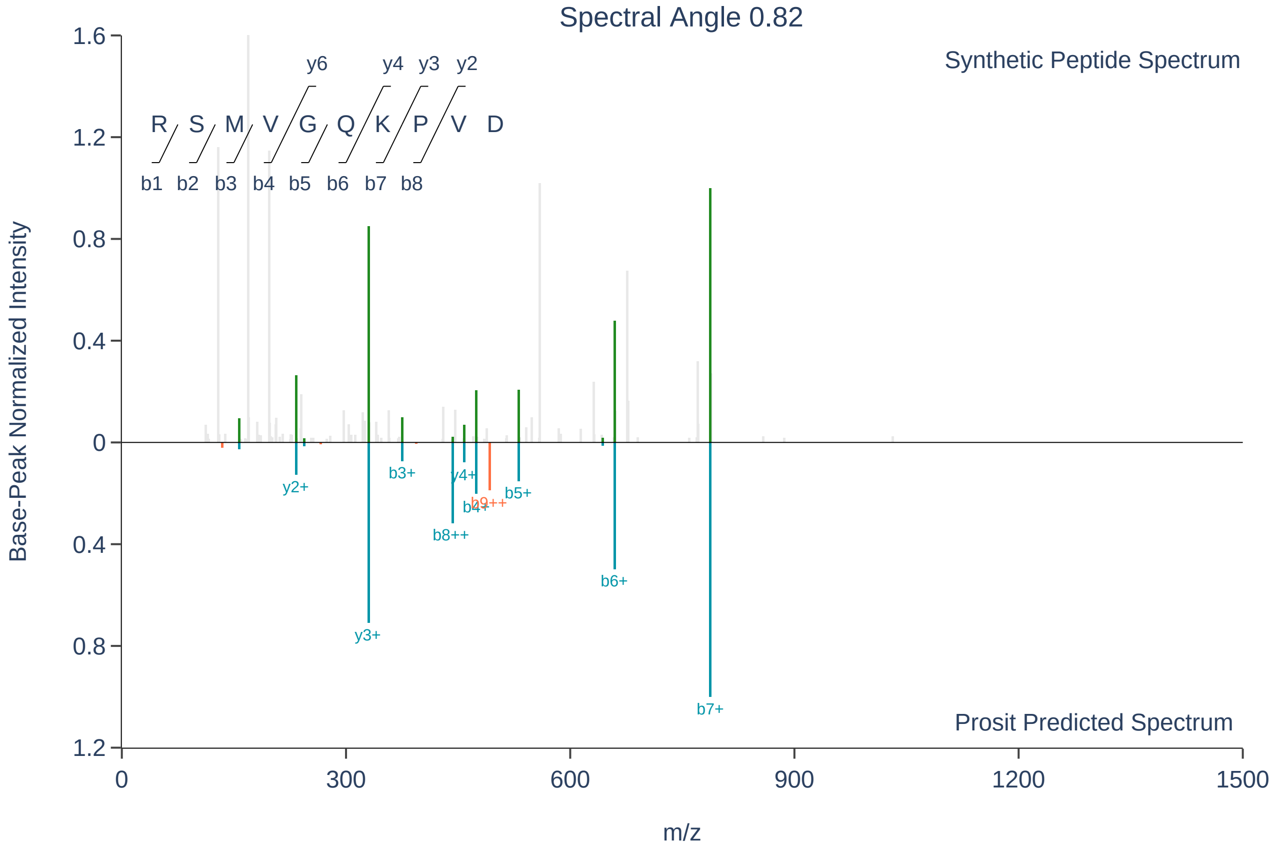

[RSM][VGQKPVD] 2+ Synthetic vs. Prosit
Spectral Angle 0.82

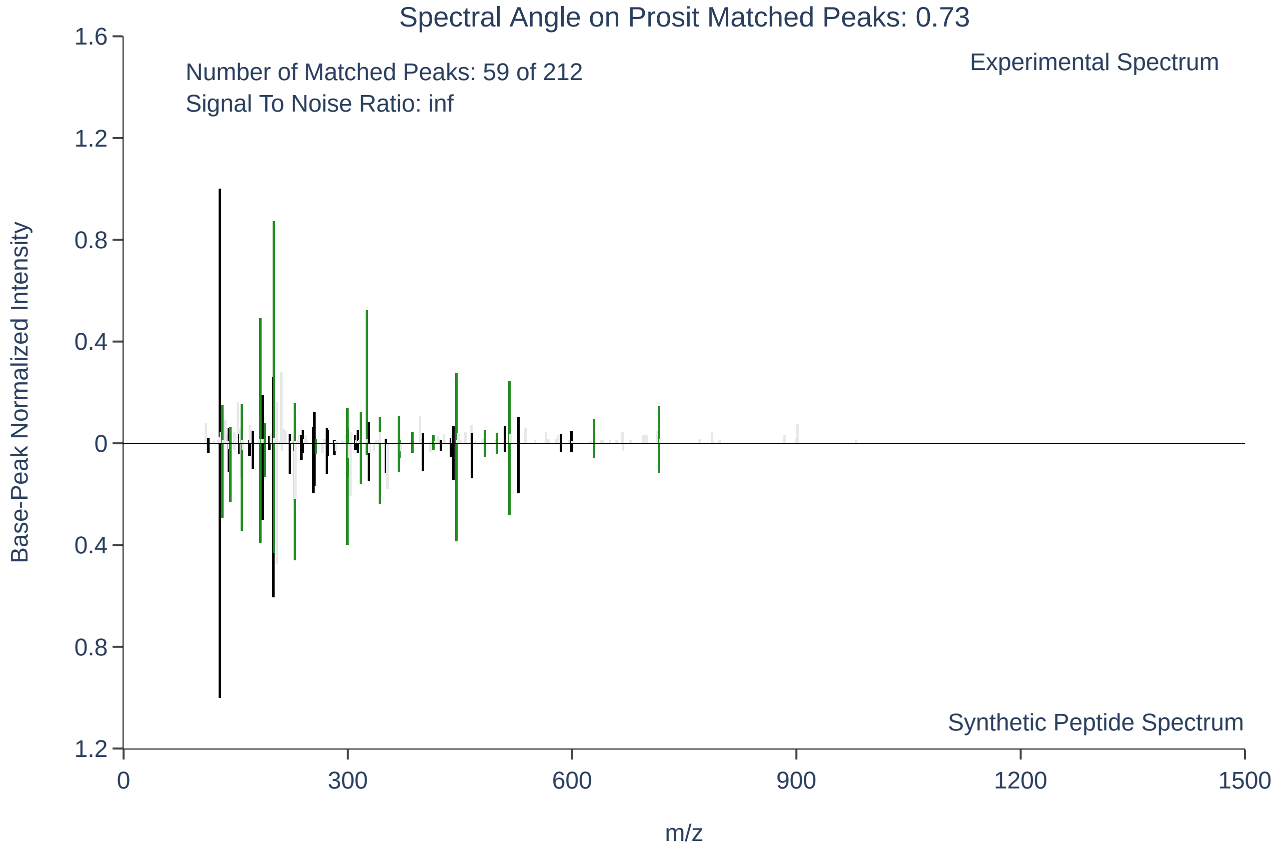

[LDN][ASLAKQGL] 2+ Experimental vs. Synthetic
Spectral Angle on Prosit Matched Peaks: 0.73

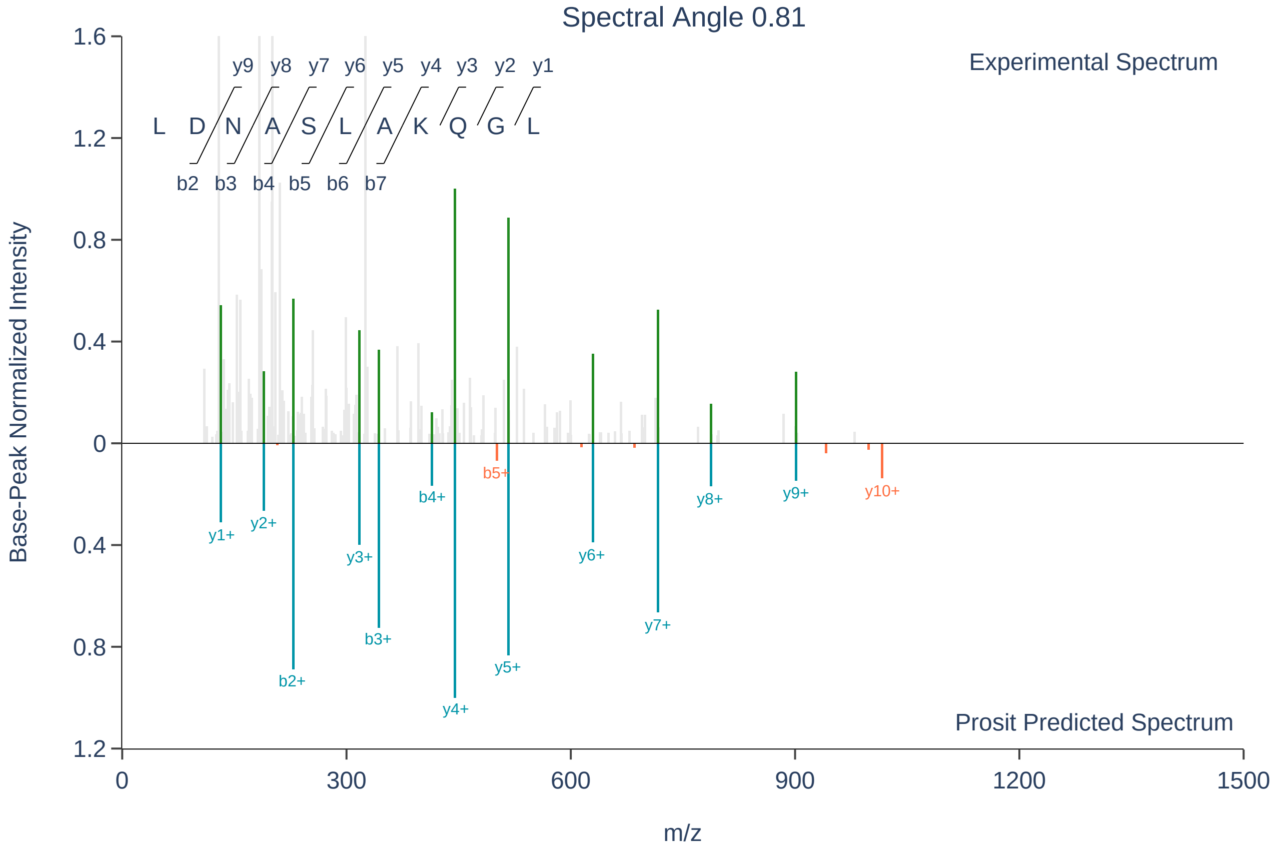

[LDN][ASLAKQGL] 2+ Experimental vs. Prosit
Spectral Angle 0.81

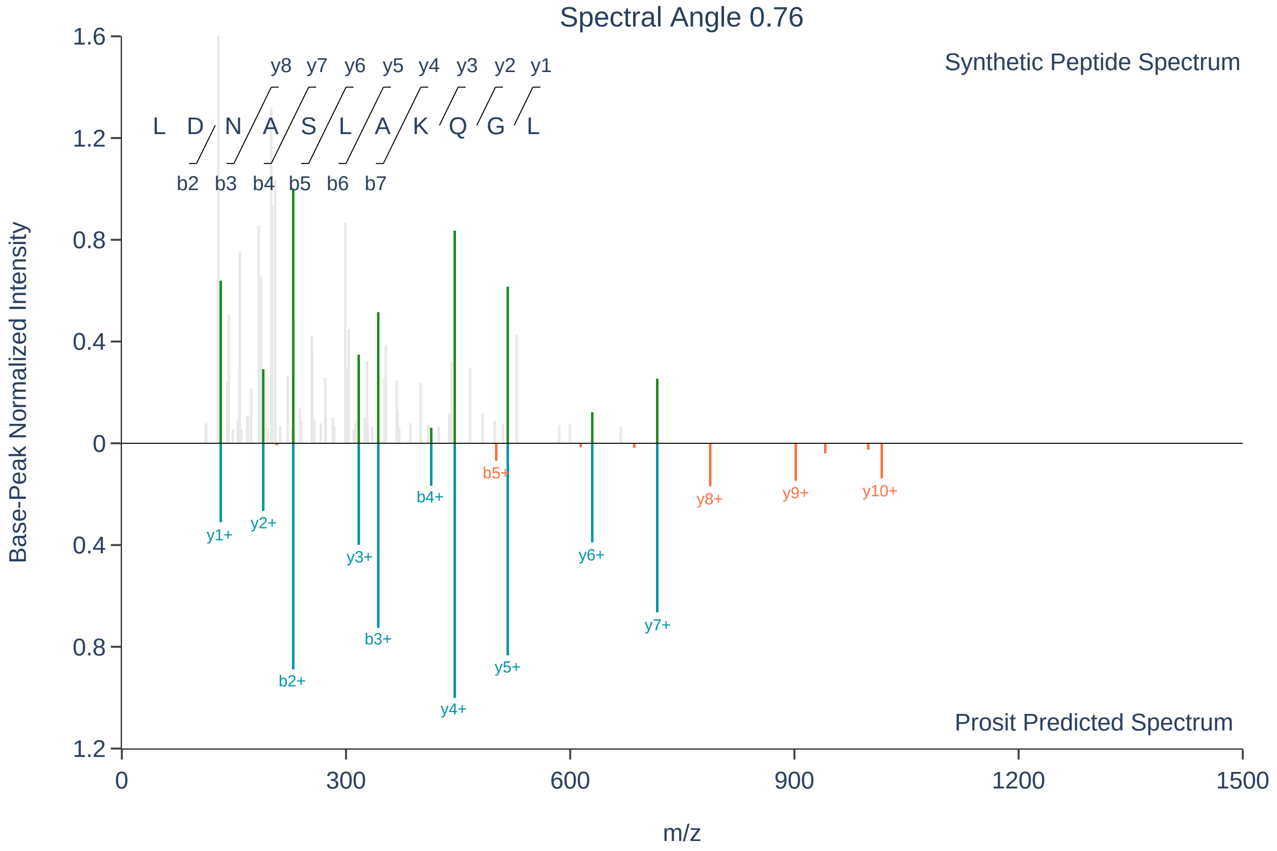

[LDN][ASLAKQGL] 2+ Synthetic vs. Prosit
Spectral Angle 0.76
