# Supplementary material for: Protein degradation by human 20S proteasomes elucidates the interplay between peptide hydrolysis and splicing
Source: Nat Commun. 2024 Feb 7;15:1147. doi: 10.1038/s41467-024-45339-3 (PMC10850103; doi:10.1038/s41467-024-45339-3)

Supplementary Data 7. MS2 spectra of two representative spliced peptides identified in the protein digestions compared to isobaric non-spliced peptide competing for the same MS2 spectrum, and their cognate synthetic peptides. Plots comparing (i) the experimental spectrum for an identified spliced peptide (top) or an isobaric non-spliced peptide (bottom) on the positive y-axis against the spectrum for the cognate synthetic peptide on the negative y-axis (left), (ii) the experimental spectrum for an identified peptide on the positive y-axis against the Prosit-predicted spectrum for that peptide on the negative y-axis (middle), and (iii) the spectrum for the cognate synthetic peptide on the positive y-axis against the Prosit-predicted spectrum for that peptide on the negative y-axis (right).

Comparison of Spliced Peptide GQLGKNEEGAPQE against Canonical Peptide QLGKNEEGAPQEG

Experimental vs. Synthetic Colour Code:

- Matched peak between spectra. Possible y-, b-, or a-ion.
- Matched peak between spectra. Origin not clear.
- Peak not matched between spectra.

Prosit Comparison Colour Code:

- Experimental peak matched to a Prosit predicted peak.
- Experimental peak not matched to a Prosit predicted peak.
- Prosit predicted peak matched to experimental spectrum.
- Prosit predicted peak not matched to experimental spectrum.

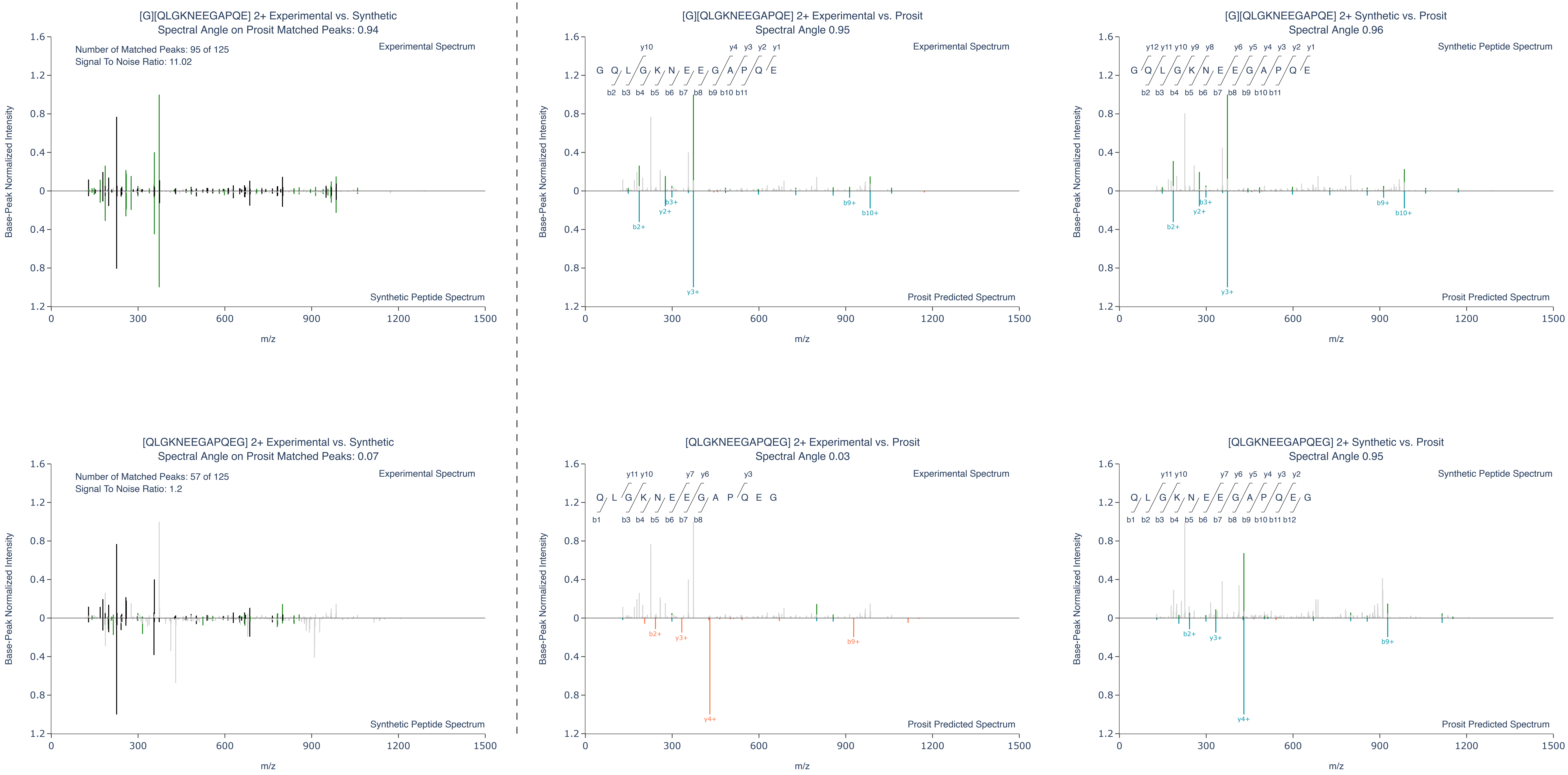

Comparison of Spliced Peptide KSEKLDFKDRVQSL against Canonical Peptide SEKLDFKDRVQSKL

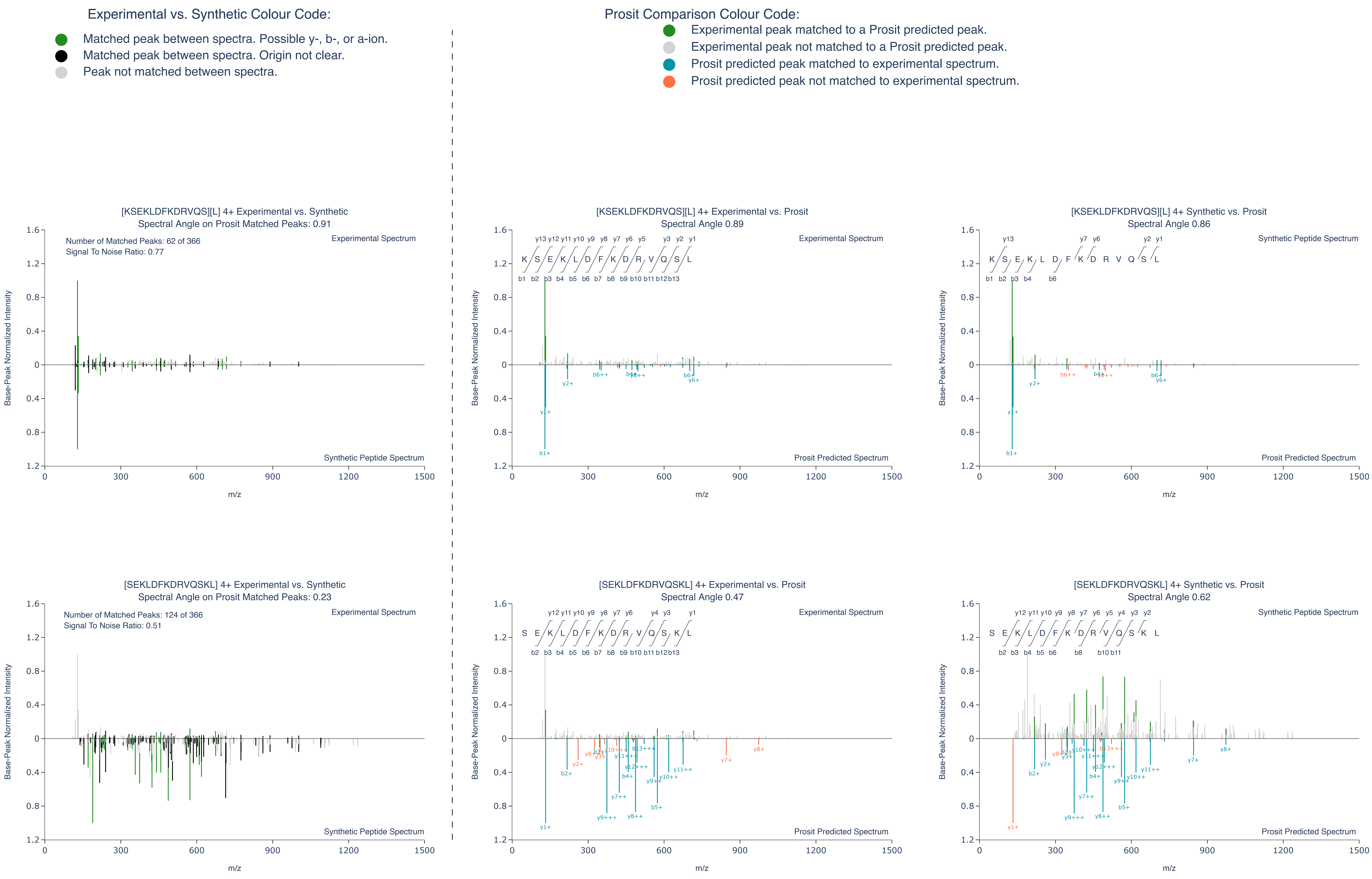

Supplement: Supplementary file 10 — Supplementary Data 7 [file 41467_2024_45339_MOESM10_ESM.pdf]
