# Supplementary material for: Protein degradation by human 20S proteasomes elucidates the interplay between peptide hydrolysis and splicing
Source: Nat Commun. 2024 Feb 7;15:1147. doi: 10.1038/s41467-024-45339-3 (PMC10850103; doi:10.1038/s41467-024-45339-3)

Supplementary Data 8. MS2 spectra of spliced peptides identified in the protein digestions compared to isobaric non-spliced peptide competing for the same MS2 spectrum. Plots comparing the experimental spectrum for an identified peptide on the positive y-axis against the Prosit-predicted spectrum for that peptide on the negative y-axis. Plots for the identified spliced peptides are shown on the left and the corresponding isobaric non-spliced peptides on the right. Comparisons are shown for the best scoring spectrum of all spliced peptides identified at 1% FDR (both *cis*- and homologous *trans*- identifications are shown) and the non-spliced competitor with highest spectral angle is shown. PSMs with iRT prediction error outside of 4.25 (the 95% confidence interval in the Prosit original identifications) are highlighted in red.

inSPIRE isobaric peptide comparisons for spliced assignments

Experimental Spectrum Colour Code:

- Experimental peak matched to a Prosit predicted peak.
- Possible ion unknown to Prosit.
- Precursor matched peak.
- Experimental peak not matched to any potential ion.

Prosit Spectrum Colour Code:

- Prosit predicted peak matched to experimental spectrum.
- Prosit predicted peak not matched to experimental spectrum.

Additional Notes:

° indicates an ion with loss of H_2O.

* indicates an ion with loss of NH_3.

Loading [MathJax]/extensions/MathMenu.js

Source WSoh_301121_021221_HFGoe_M1_AnnexinA1_24h_R2 **Scan** 10880
Peptide SKGGPGSEQEYVQTVKS **Charge** 3 **Spectral Angle** 0.86
Spearman Correlation 0.89 **iRT Error:** 0.01

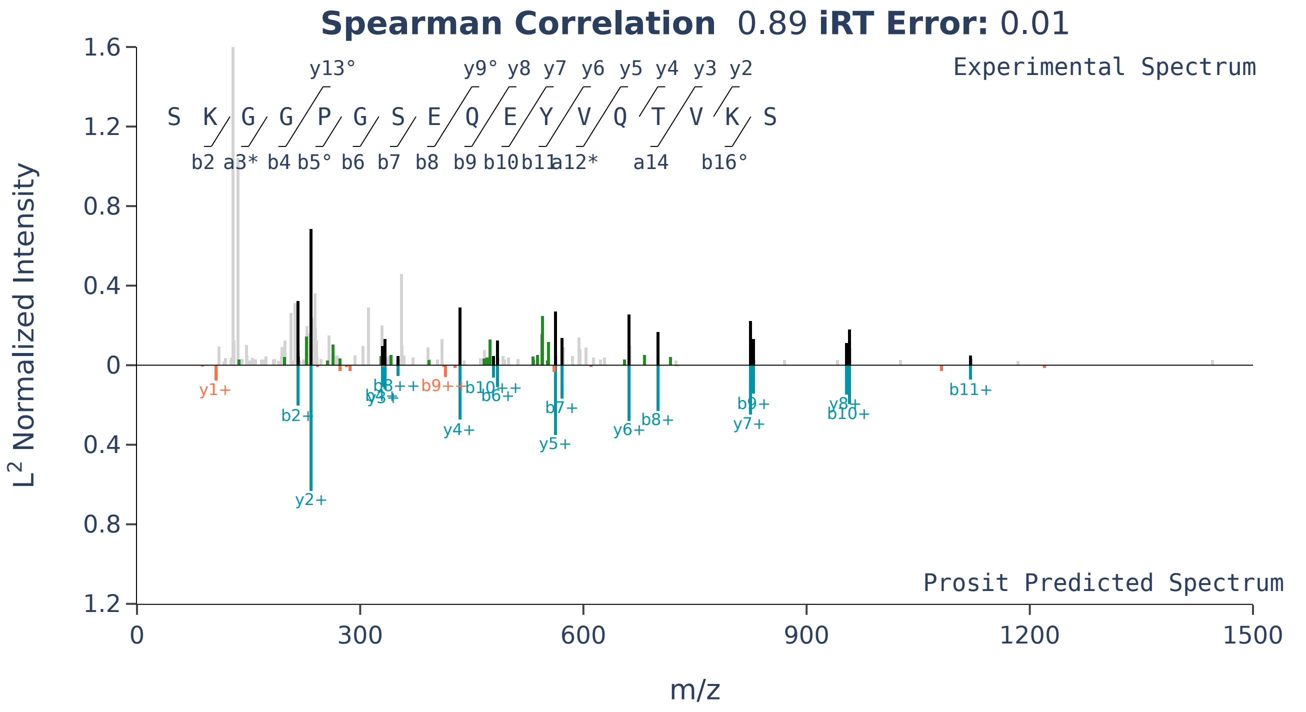

Source WSoh_301121_021221_HFGoe_M1_AnnexinA1_24h_R2 **Scan** 10880
Peptide EQEYVQTVKSSKGGPGS **Charge** 3 **Spectral Angle** 0.09
Spearman Correlation -0.11 **iRT Error:** 12.5

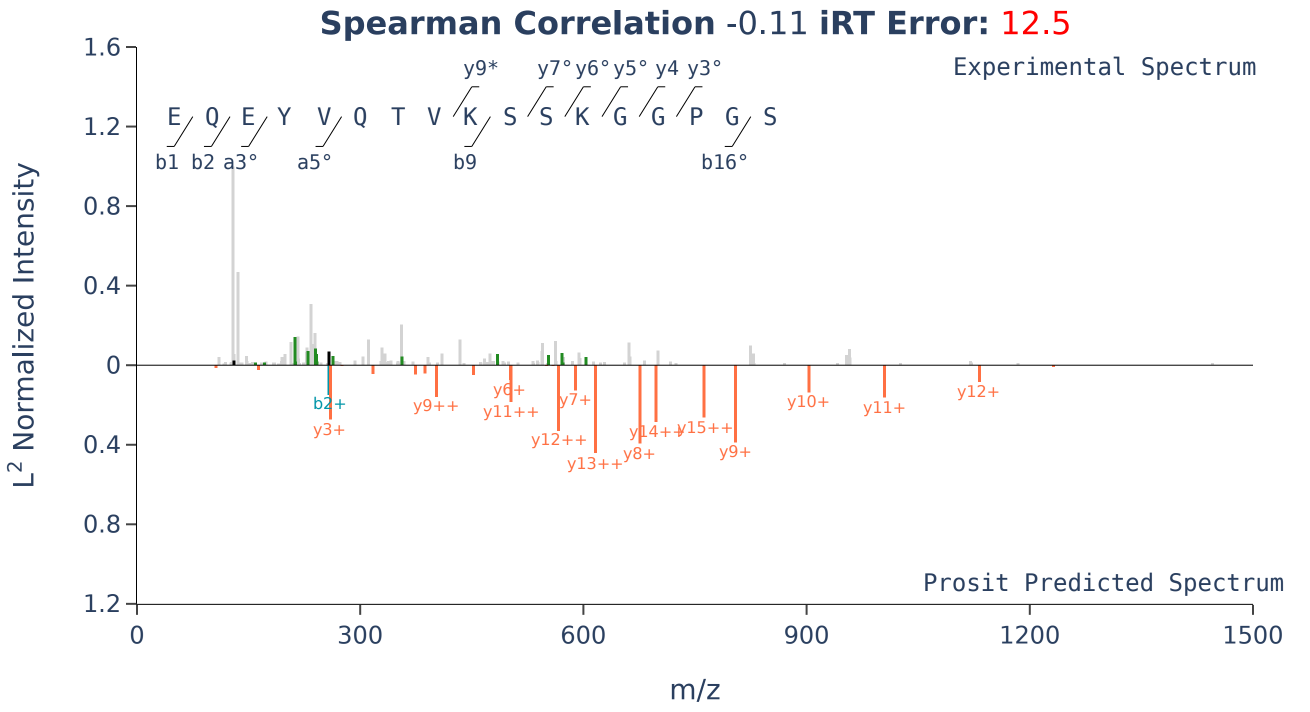

Source WSoh_301121_021221_HFGoe_M2_AnnexinA1_2h_R2 **Scan** 7974
Peptide GEELRAA **Charge** 2 **Spectral Angle** 0.96
Spearman Correlation 1.0 **iRT Error:** 0.07

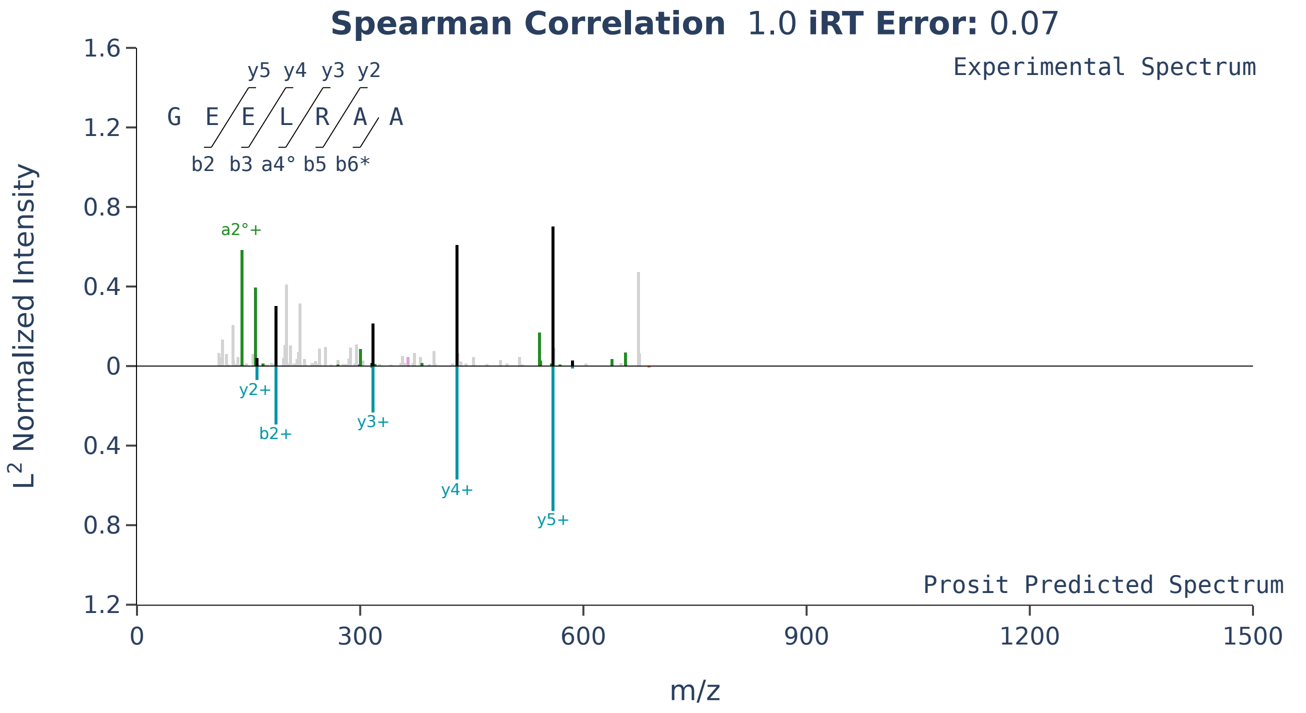

Source WSoh_301121_021221_HFGoe_M2_AnnexinA1_2h_R2 **Scan** 7974
Peptide ADELRAA **Charge** 2 **Spectral Angle** 0.95
Spearman Correlation 0.98 **iRT Error:** 44.11

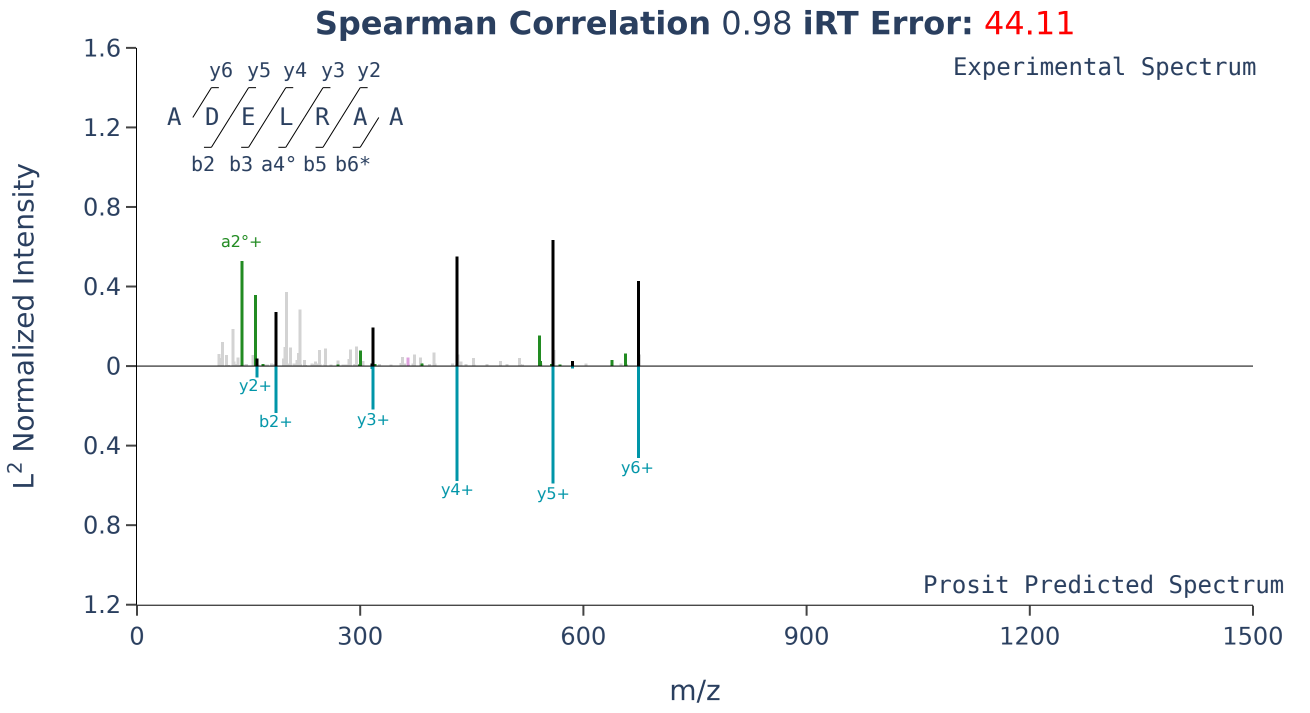

Source WSoh_301121_021221_HFGoe_M2_AnnexinA1_24h_R1 **Scan** 9665
Peptide SKGGPGSQEYVQTVKS **Charge** 3 **Spectral Angle** 0.86
Spearman Correlation 0.86 **iRT Error:** 0.09

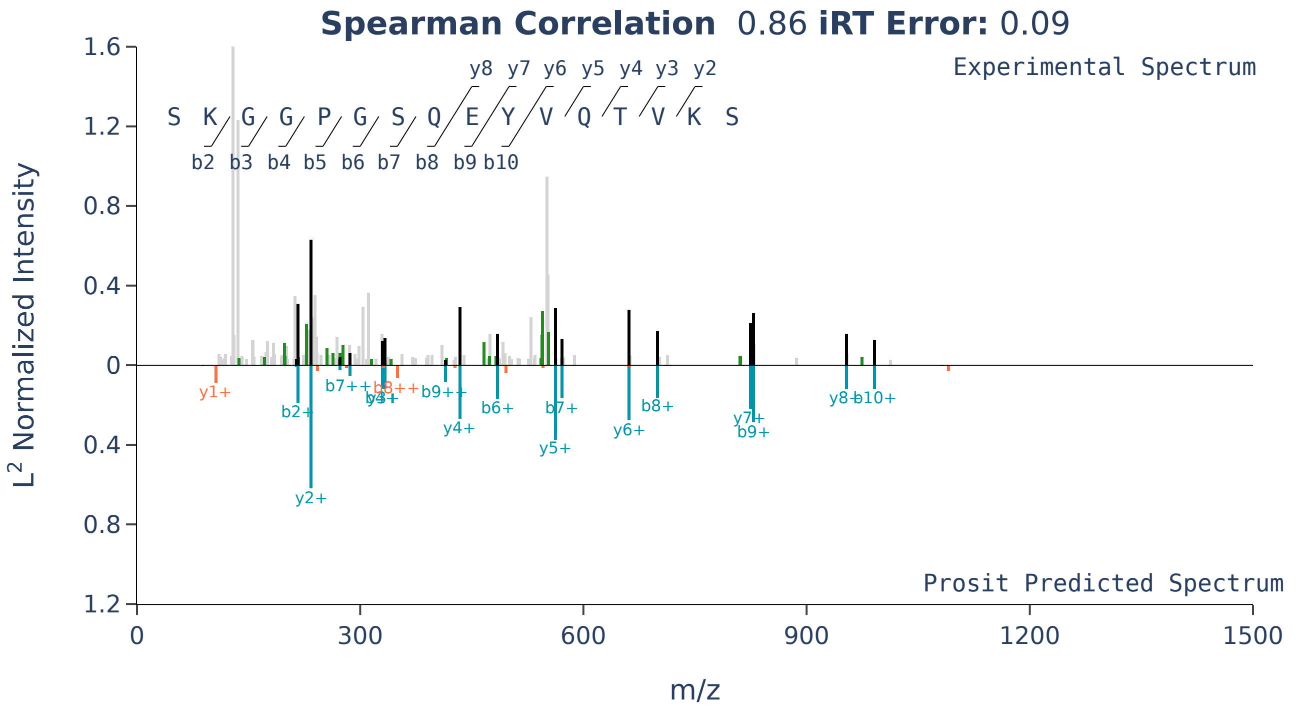

Source WSoh_301121_021221_HFGoe_M2_AnnexinA1_24h_R1 **Scan** 9665
Peptide QEYVQTVKSSKGGPGS **Charge** 3 **Spectral Angle** 0.08
Spearman Correlation 0.06 **iRT Error:** 10.51

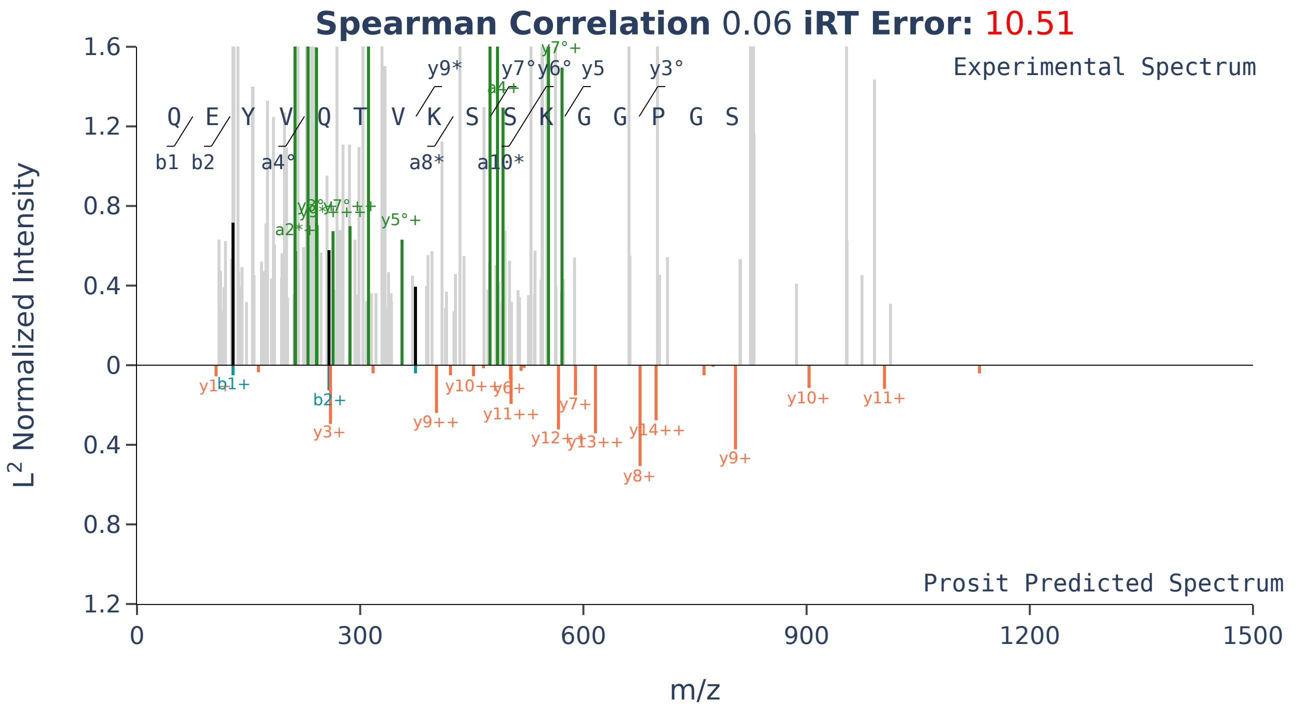

Source WSoh_301121_021221_HFGoe_M1_AnnexinA1_24h_R1 **Scan** 12286
Peptide KDITSDTSGDFRNAA **Charge** 3 **Spectral Angle** 0.75
Spearman Correlation 0.92 **iRT Error:** 0.14

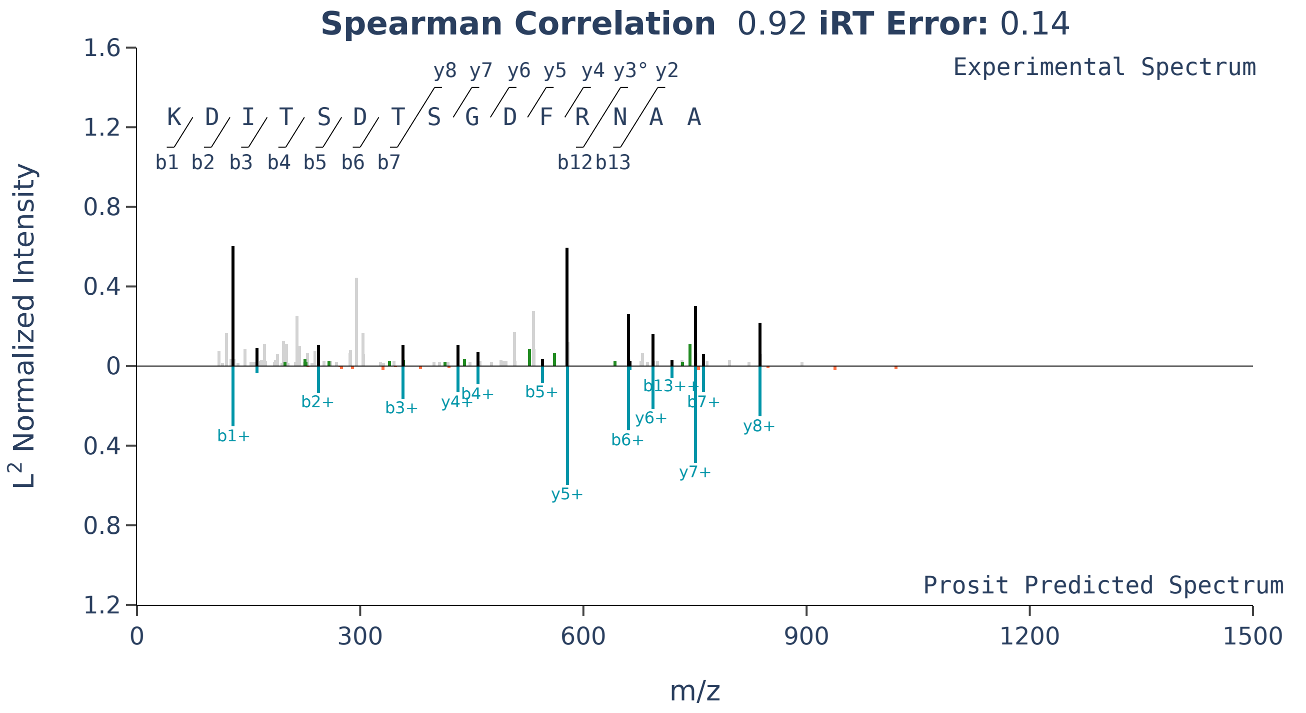

Source WSoh_301121_021221_HFGoe_M1_AnnexinA1_24h_R1 **Scan** 12286
Peptide AKDITSDTSGDFRNA **Charge** 3 **Spectral Angle** 0.7
Spearman Correlation 0.61 **iRT Error:** 3.28

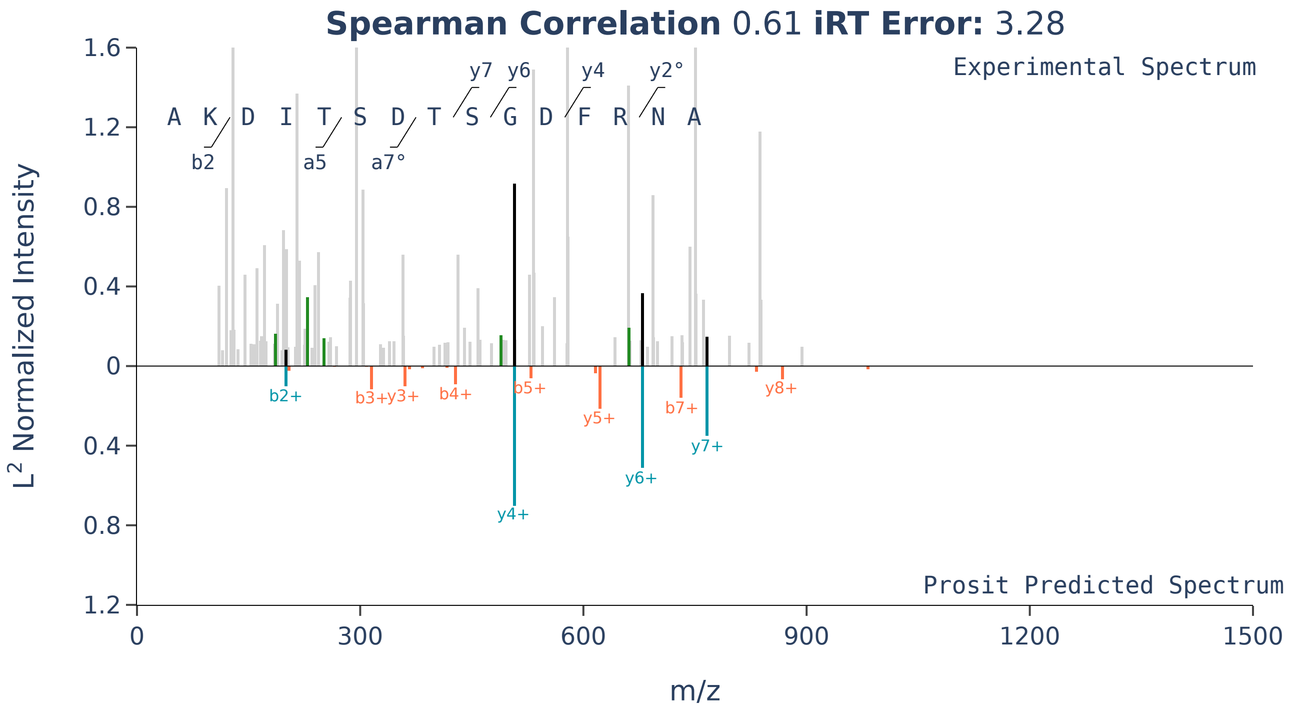

Source WSoh_301121_021221_HFGoe_M2_AnnexinA1_24h_R1 **Scan** 17744
Peptide LELKGDLAD **Charge** 2 **Spectral Angle** 0.76
Spearman Correlation 0.88 **iRT Error:** 0.34

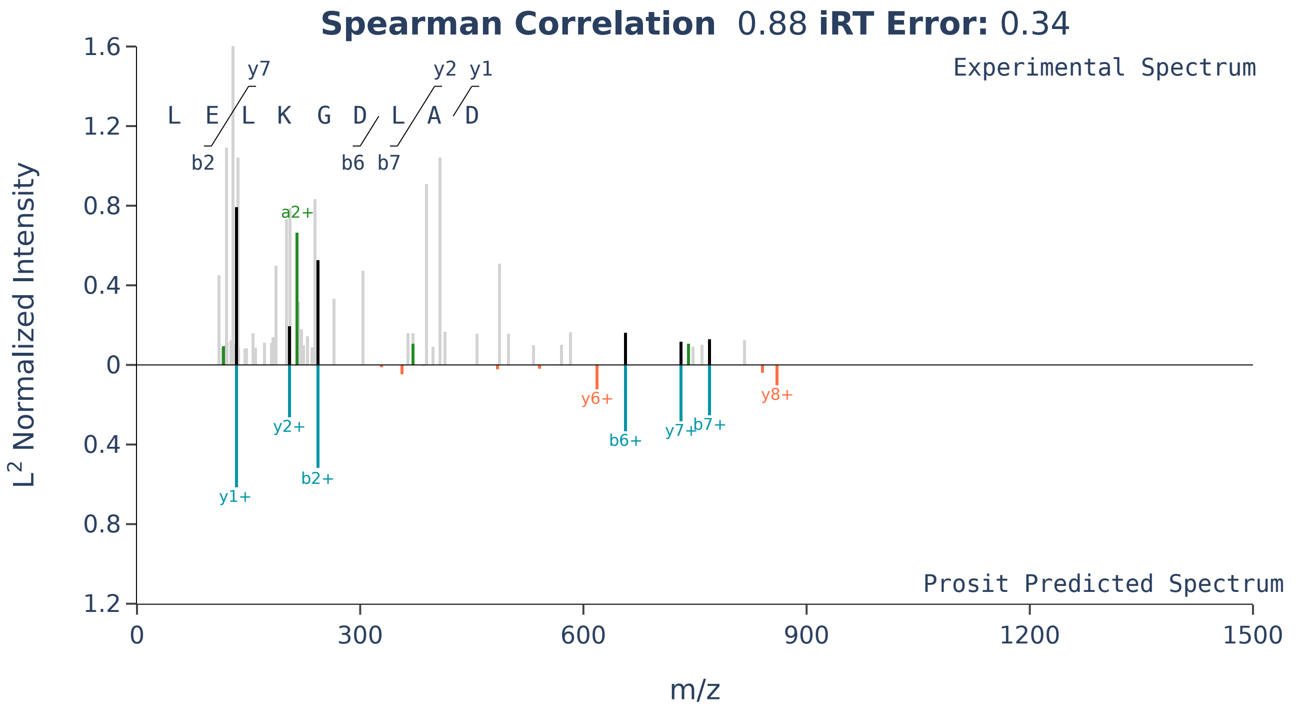

Source WSoh_301121_021221_HFGoe_M2_AnnexinA1_24h_R1 **Scan** 17744
Peptide TGKPLDETL **Charge** 2 **Spectral Angle** 0.54
Spearman Correlation 0.42 **iRT Error:** 6.4

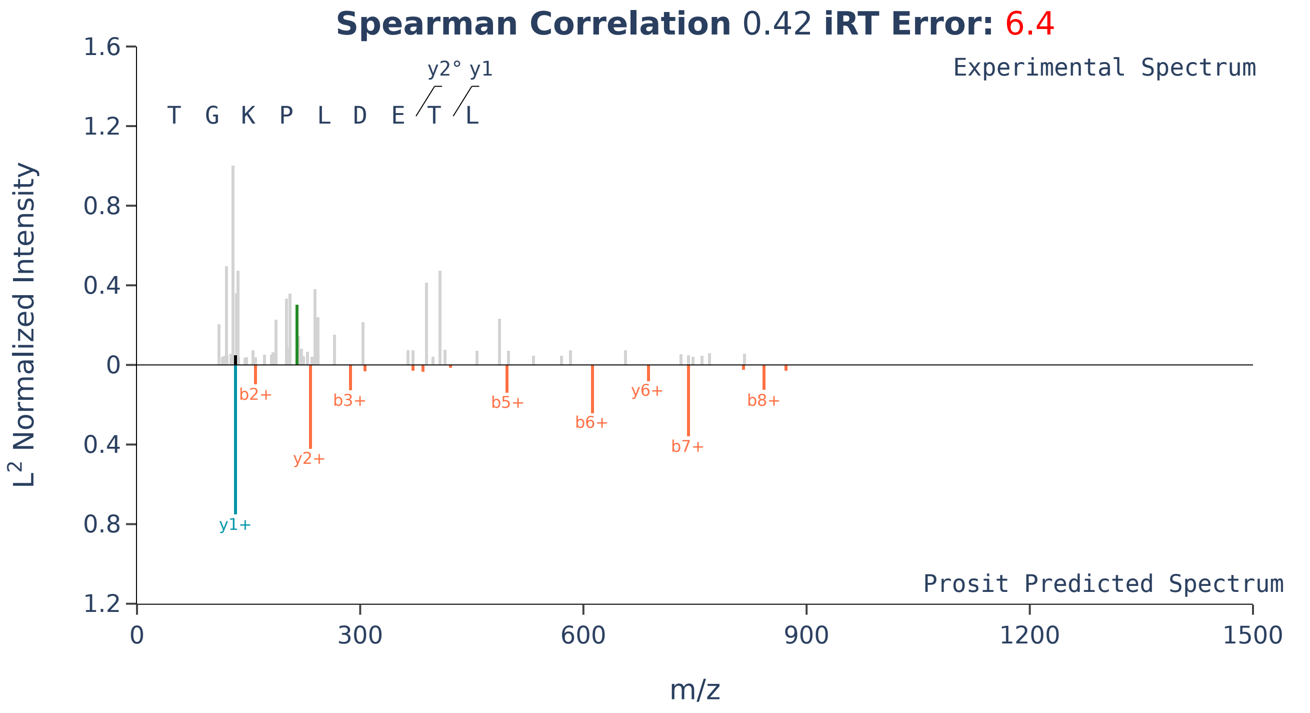

Source WSoh_301121_021221_HFGoe_M1_AnnexinA1_24h_R1 **Scan** 14676
Peptide SKGGPGSEQEYVQTV **Charge** 2 **Spectral Angle** 0.84
Spearman Correlation 0.94 **iRT Error:** 0.34

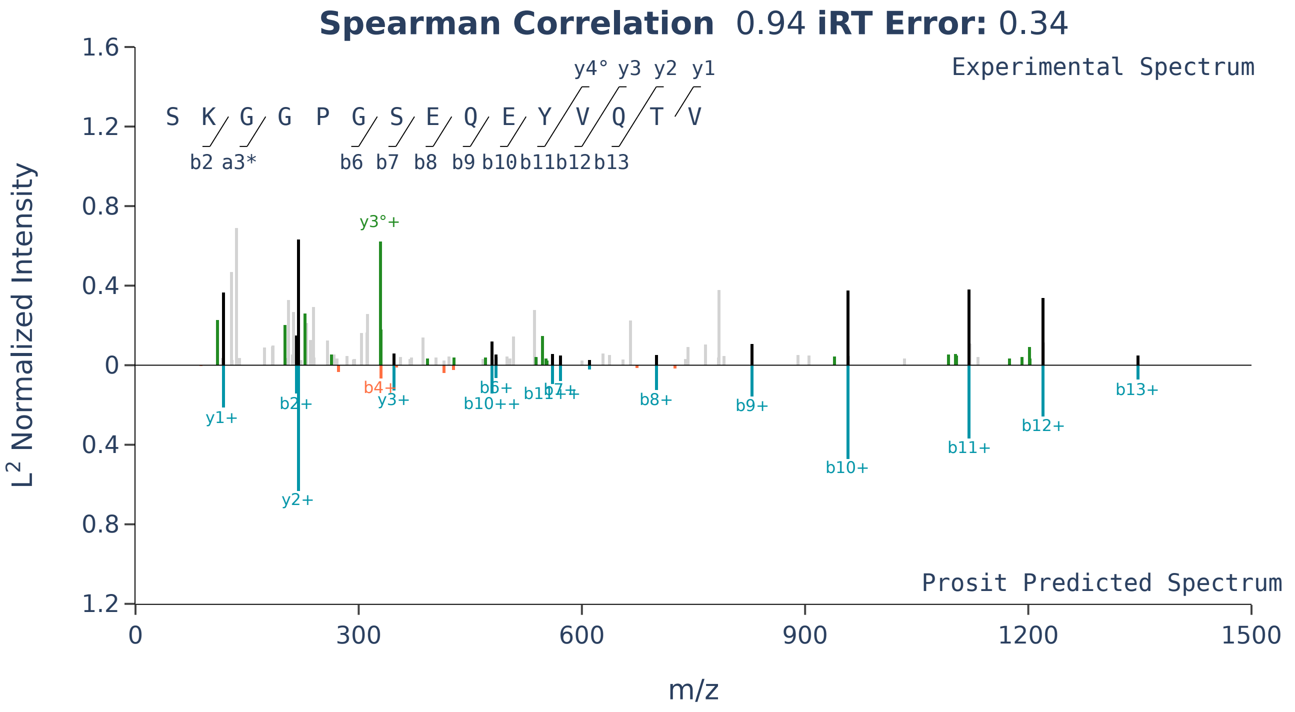

Source WSoh_301121_021221_HFGoe_M1_AnnexinA1_24h_R1 **Scan** 14676
Peptide SPYPTFNPSSDVAAL **Charge** 2 **Spectral Angle** 0.1
Spearman Correlation 0.19 **iRT Error:** 78.42

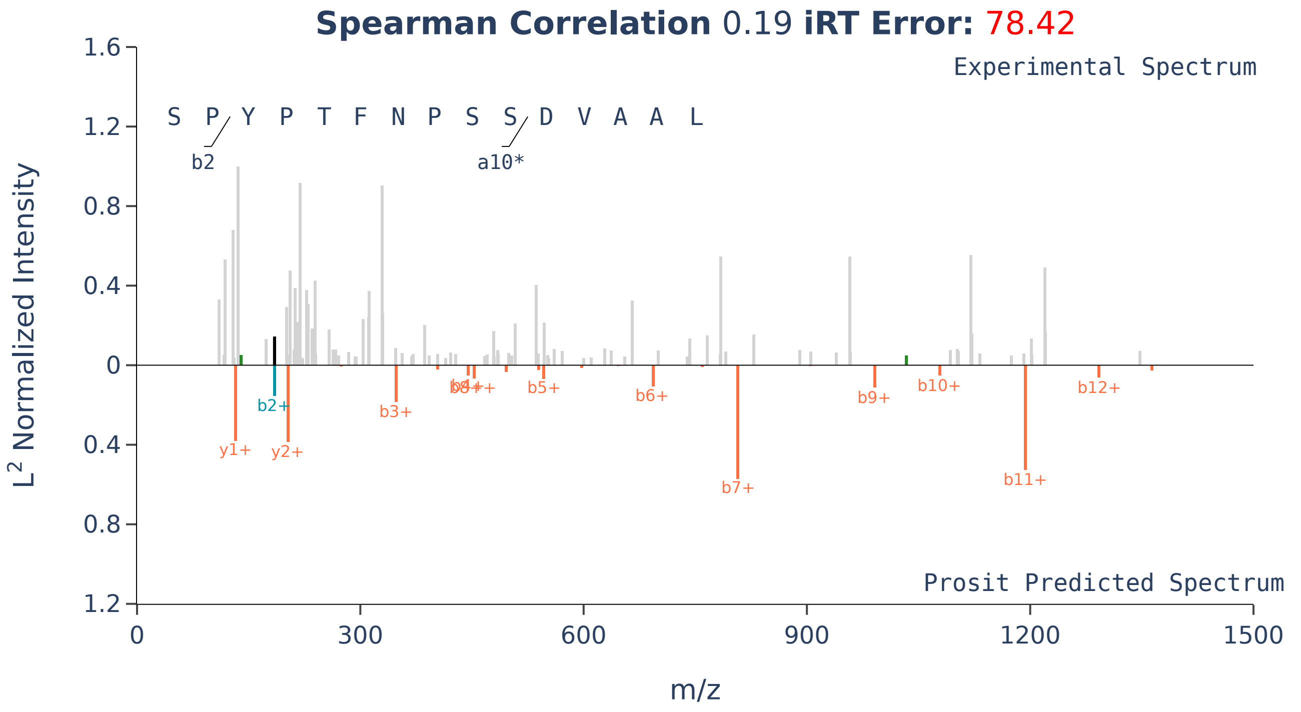

Source WSoh_301121_021221_HFGoe_M2_AnnexinA1_24h_R1 **Scan** 13674
Peptide EKILVGD **Charge** 1 **Spectral Angle** 0.87
Spearman Correlation 0.98 **iRT Error:** 0.37

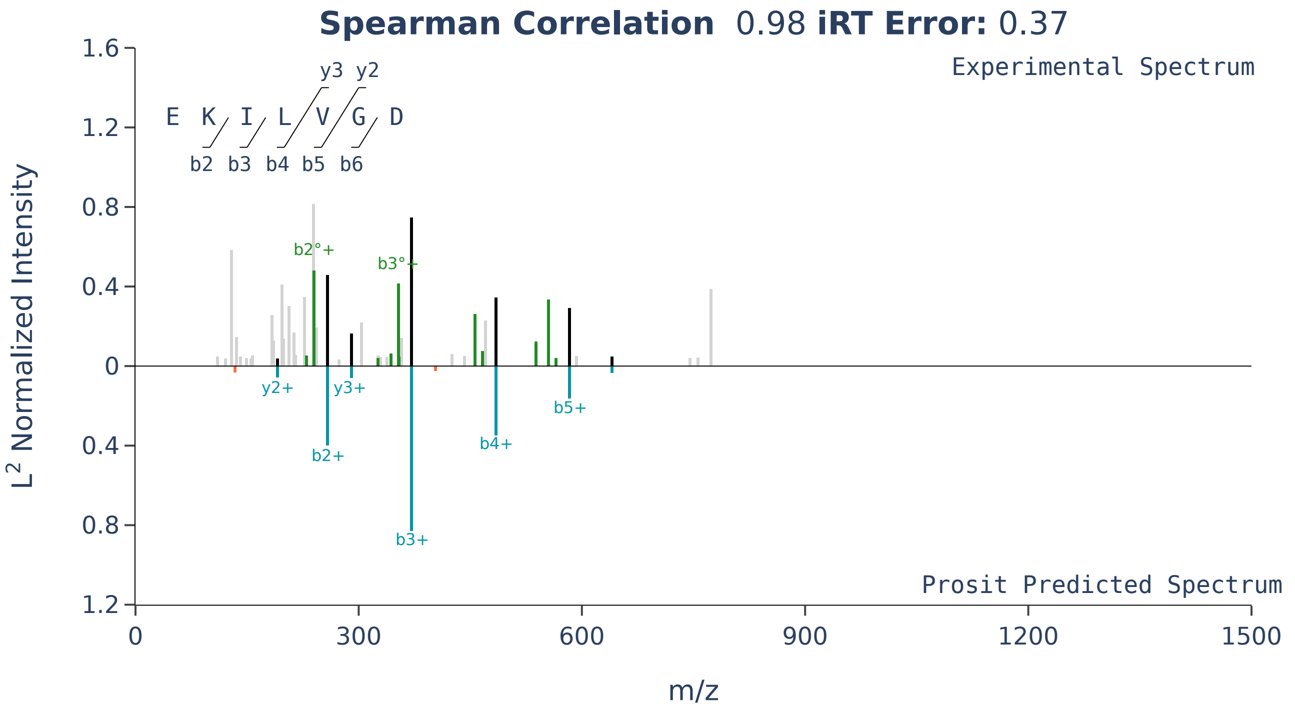

Source WSoh_301121_021221_HFGoe_M2_AnnexinA1_24h_R1 **Scan** 13674
Peptide AGERRKG **Charge** 1 **Spectral Angle** 0.0
Spearman Correlation 0.0 **iRT Error:** 35.94

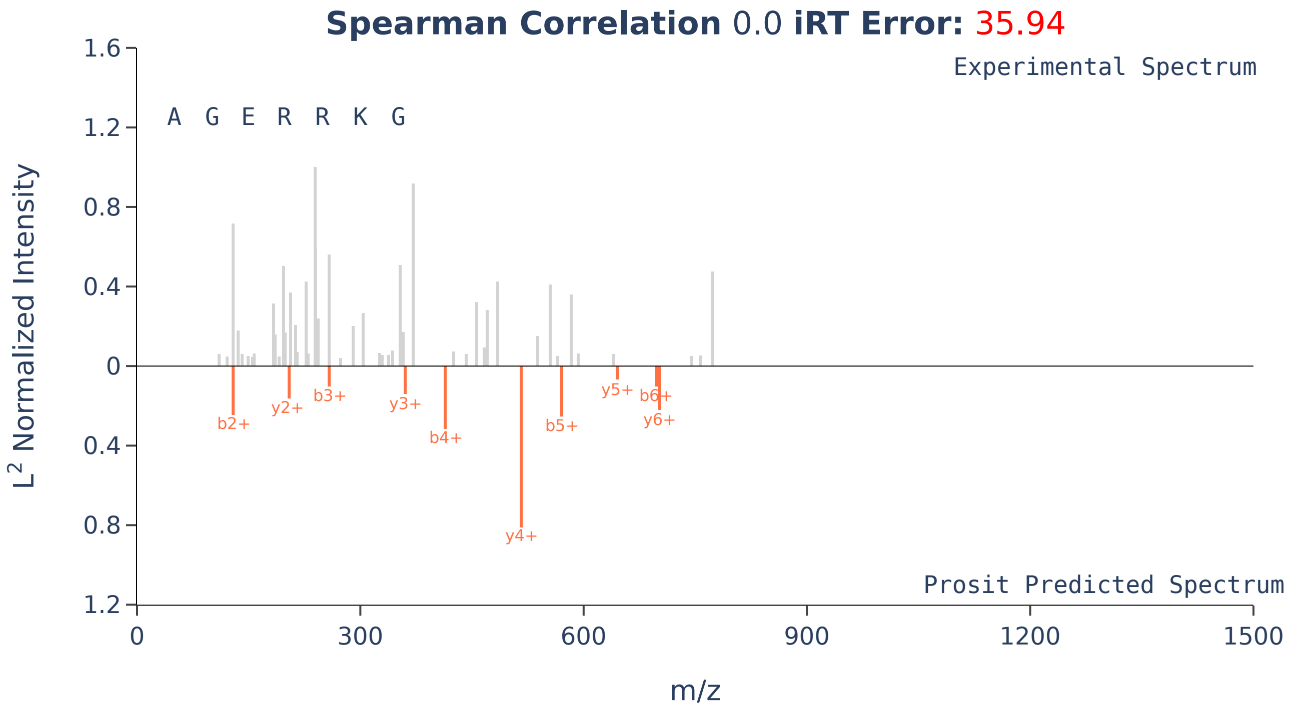

Source WSoh_301121_021221_HFGoe_M2_AnnexinA1_4h_R1 **Scan** 6537
Peptide RDINRVYKE **Charge** 3 **Spectral Angle** 0.82
Spearman Correlation 0.93 **iRT Error:** 0.6

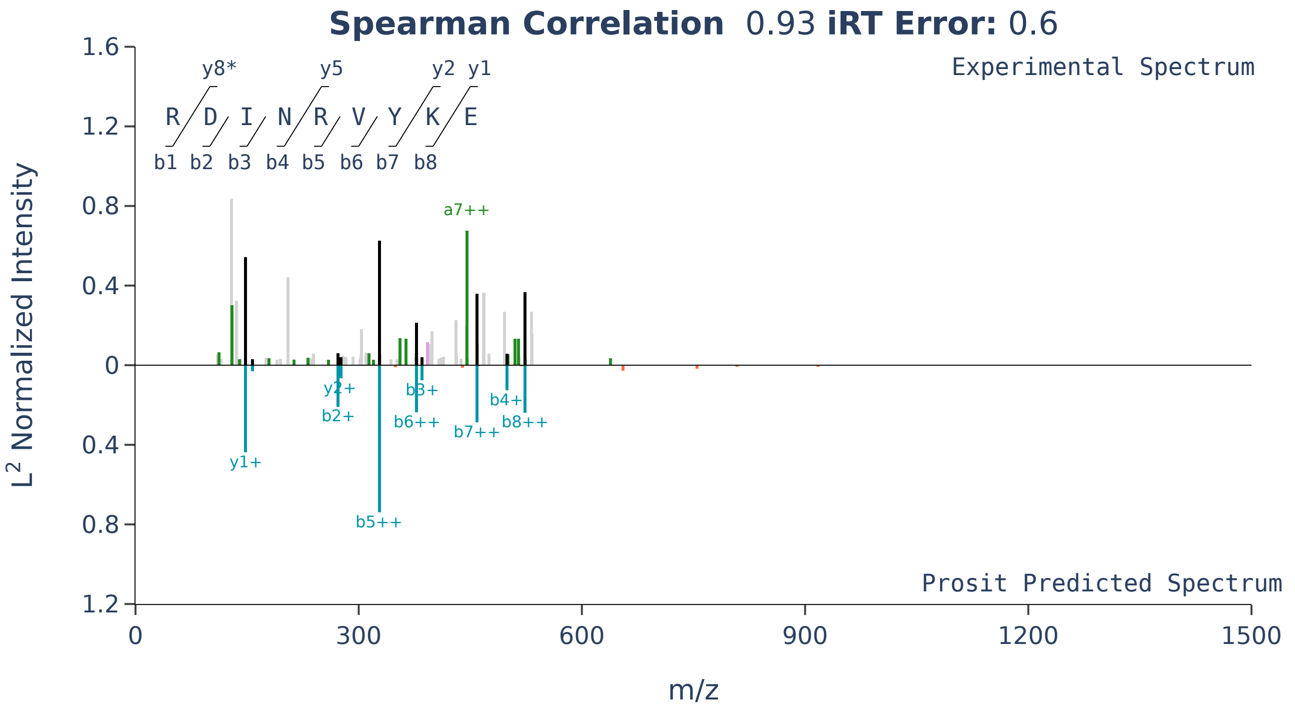

Source WSoh_301121_021221_HFGoe_M2_AnnexinA1_4h_R1 **Scan** 6537
Peptide ALYEAGERRK **Charge** 3 **Spectral Angle** 0.0
Spearman Correlation 0.0 **iRT Error:** 7.53

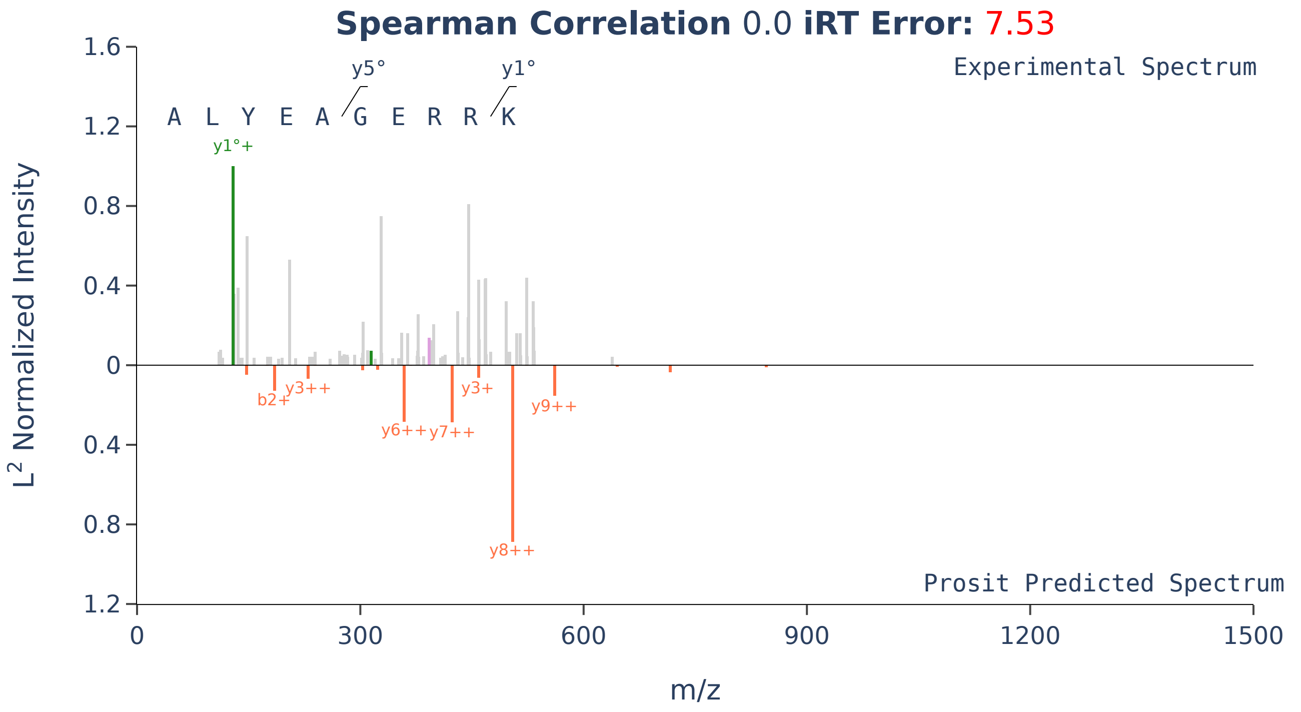

Source WSoh_301121_021221_HFGoe_M2_AnnexinA1_2h_R2 **Scan** 11583
Peptide ASDARALYE **Charge** 2 **Spectral Angle** 0.83
Spearman Correlation 0.93 **iRT Error:** 0.65

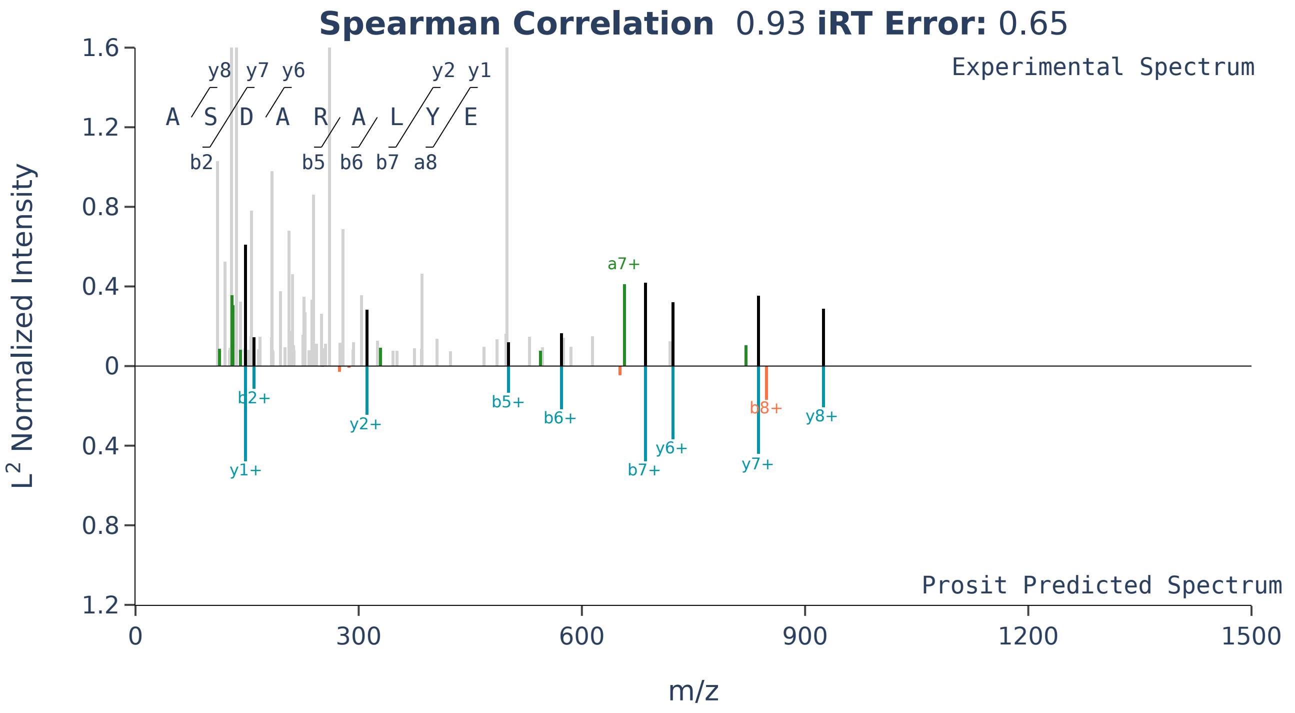

Source WSoh_301121_021221_HFGoe_M2_AnnexinA1_2h_R2 **Scan** 11583
Peptide SDARALYEA **Charge** 2 **Spectral Angle** 0.23
Spearman Correlation 0.18 **iRT Error:** 7.5

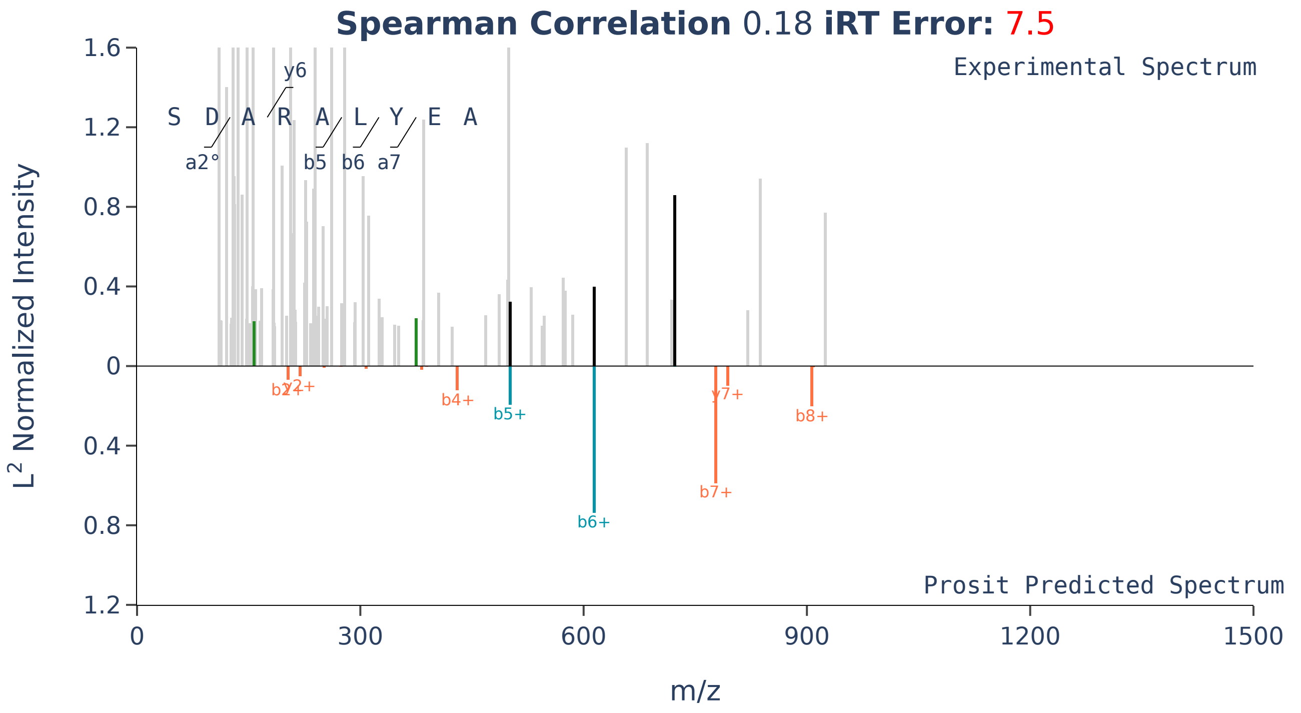

Source WSoh_301121_021221_HFGoe_M2_AnnexinA1_1h_R1 **Scan** 10913
Peptide AGEEYVQT **Charge** 1 **Spectral Angle** 0.82
Spearman Correlation 0.88 **iRT Error:** 0.72

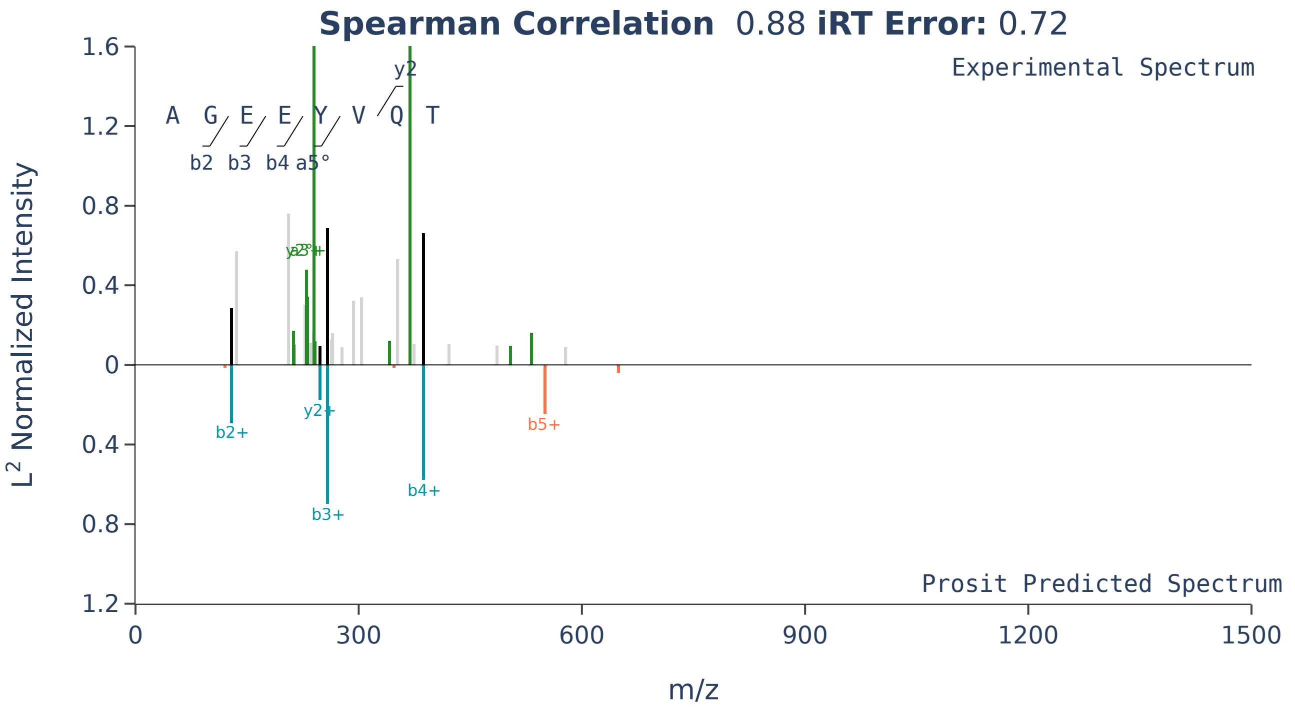

Source WSoh_301121_021221_HFGoe_M2_AnnexinA1_1h_R1 **Scan** 10913
Peptide EQEYVQT **Charge** 1 **Spectral Angle** 0.88
Spearman Correlation 0.79 **iRT Error:** 6.73

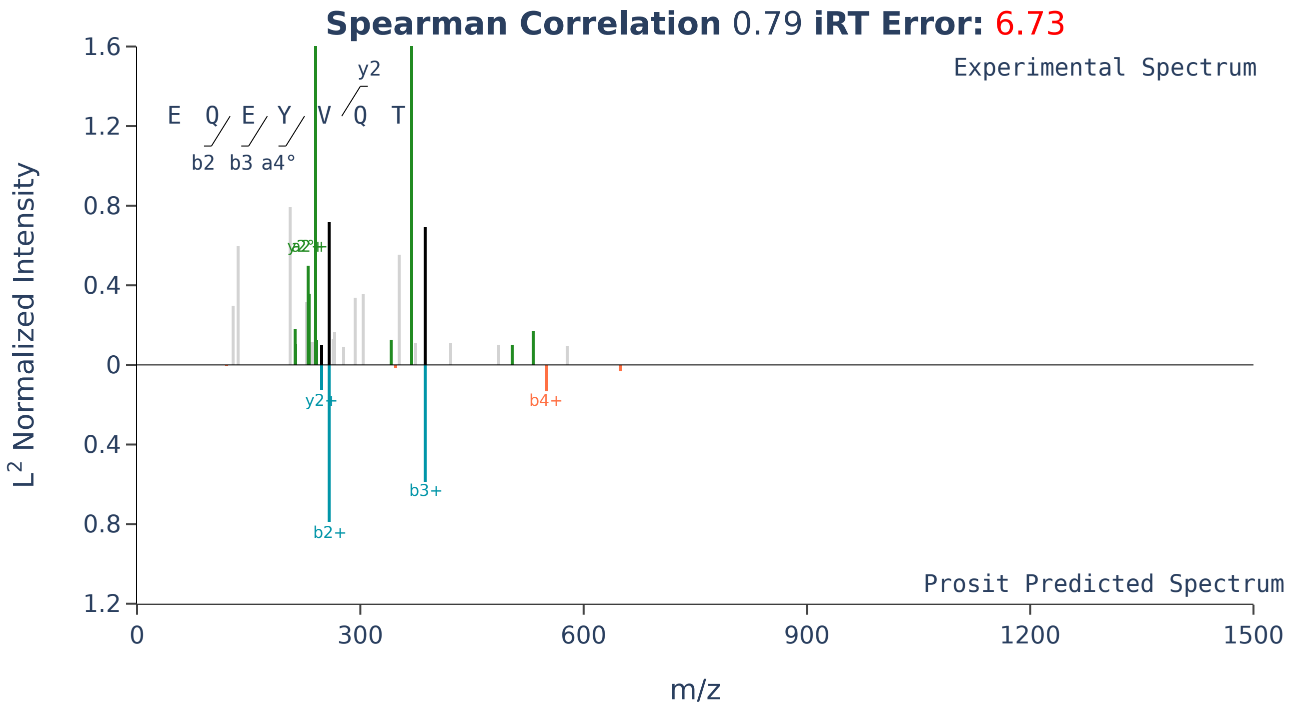

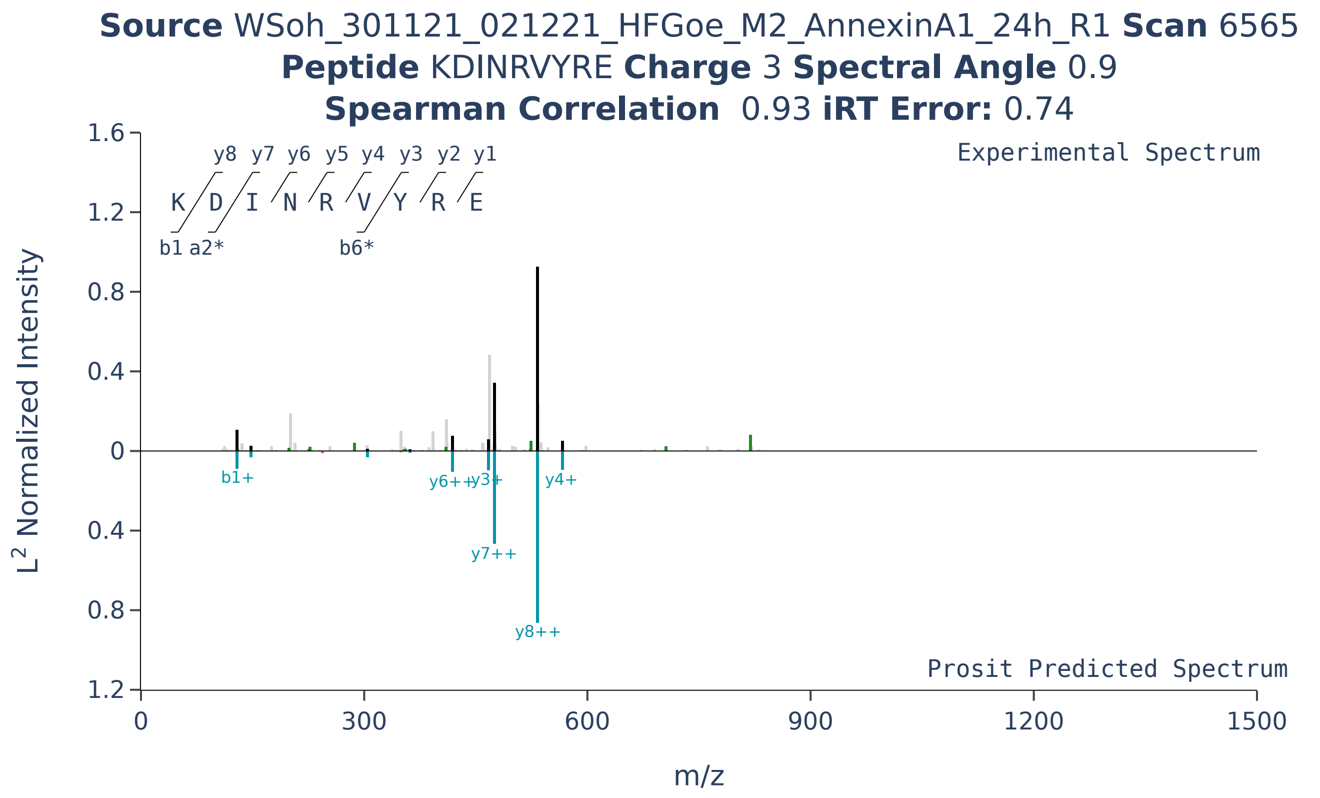
Source WSoh_301121_021221_HFGoe_M2_AnnexinA1_24h_R1 Scan 6565
Peptide KDINRVYRE Charge 3 Spectral Angle 0.9
Spearman Correlation 0.93 iRT Error: 0.74
Experimental Spectrum
Prosit Predicted Spectrum
L² Normalized Intensity
m/z

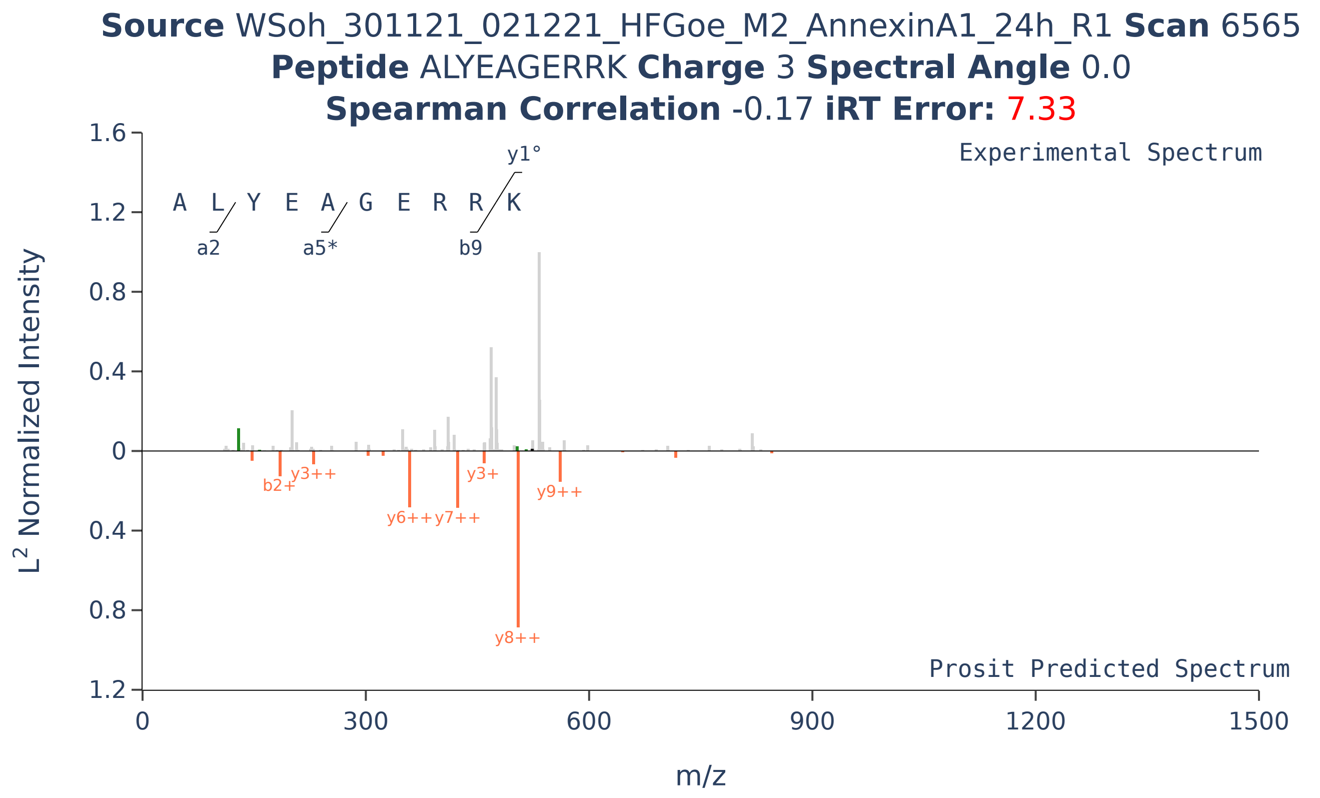
Source WSoh_301121_021221_HFGoe_M2_AnnexinA1_24h_R1 Scan 6565
Peptide ALYEAGERRK Charge 3 Spectral Angle 0.0
Spearman Correlation -0.17 iRT Error: 7.33
Experimental Spectrum
Prosit Predicted Spectrum
L² Normalized Intensity
m/z

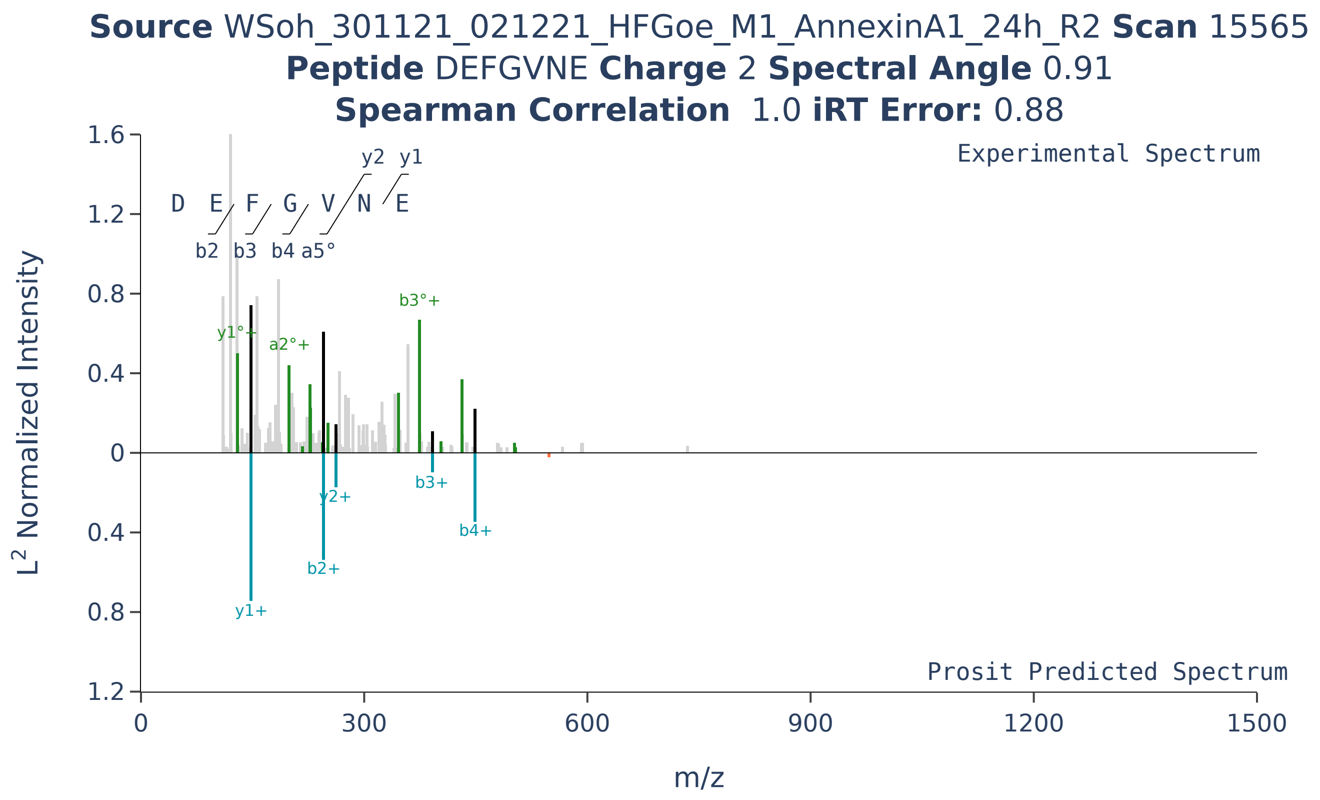
Source WSoh_301121_021221_HFGoe_M1_AnnexinA1_24h_R2 Scan 15565
Peptide DEFGVNE Charge 2 Spectral Angle 0.91
Spearman Correlation 1.0 iRT Error: 0.88
Experimental Spectrum
Prosit Predicted Spectrum
L² Normalized Intensity
m/z

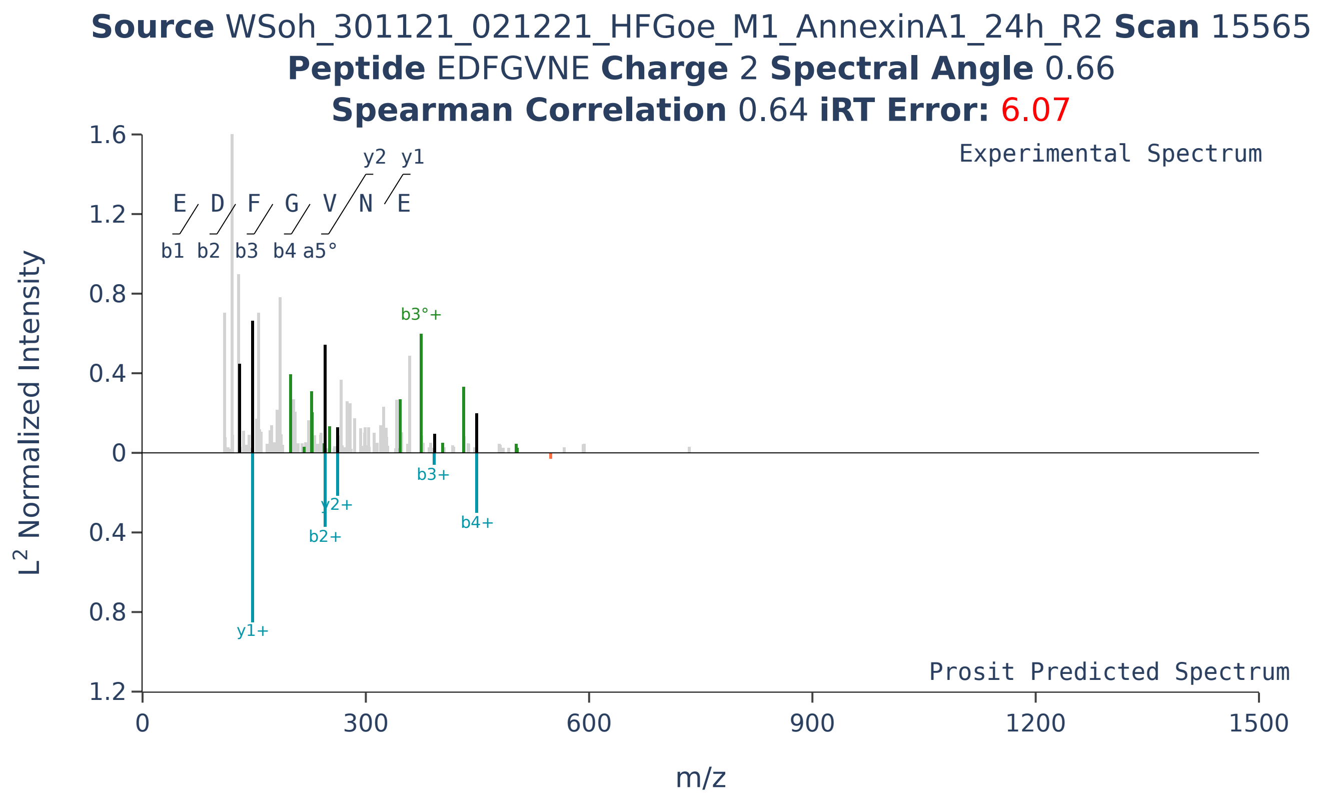
Source WSoh_301121_021221_HFGoe_M1_AnnexinA1_24h_R2 Scan 15565
Peptide EDFGVNE Charge 2 Spectral Angle 0.66
Spearman Correlation 0.64 iRT Error: 6.07
Experimental Spectrum
Prosit Predicted Spectrum
L² Normalized Intensity
m/z

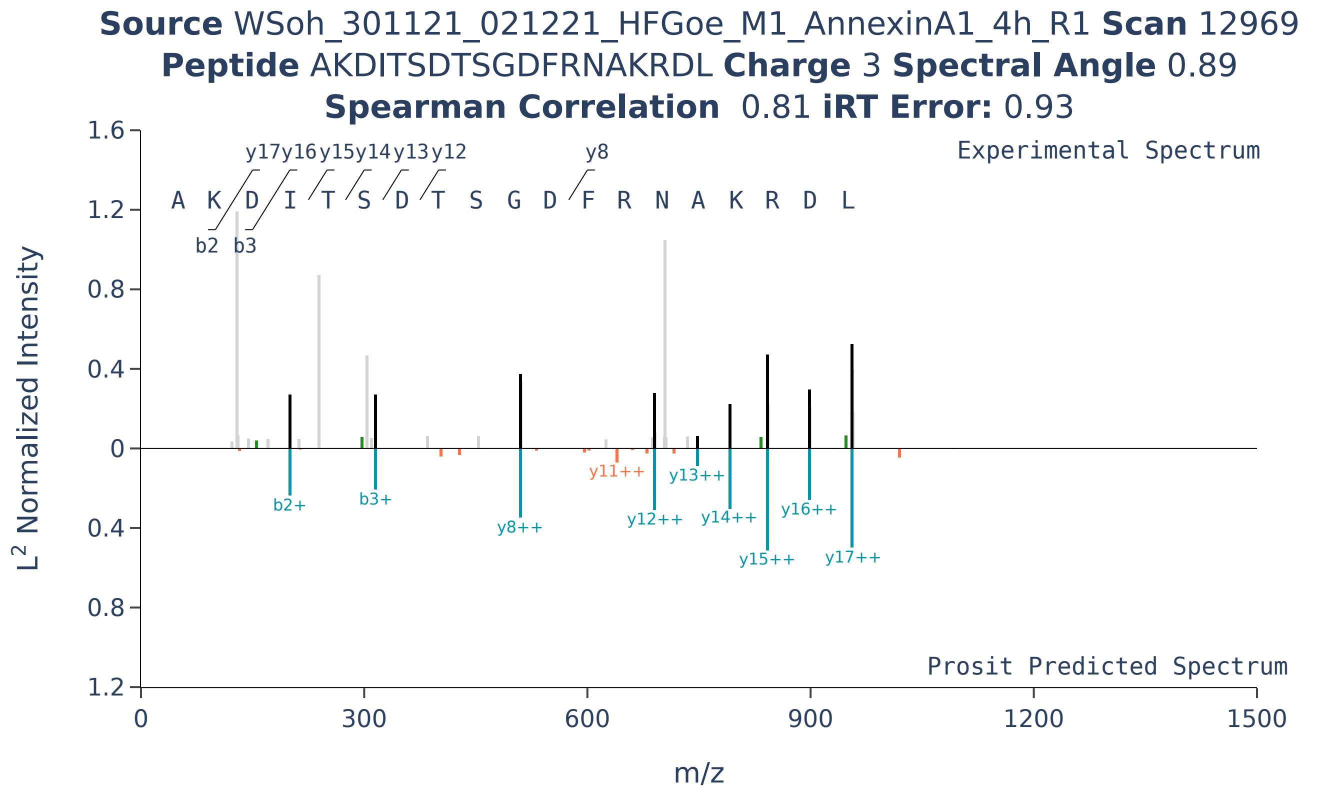
Source WSoh_301121_021221_HFGoe_M1_AnnexinA1_4h_R1 Scan 12969
Peptide AKDITSDTSGDFRNAKRDL Charge 3 Spectral Angle 0.89
Spearman Correlation 0.81 iRT Error: 0.93
Experimental Spectrum
Prosit Predicted Spectrum
L² Normalized Intensity
m/z

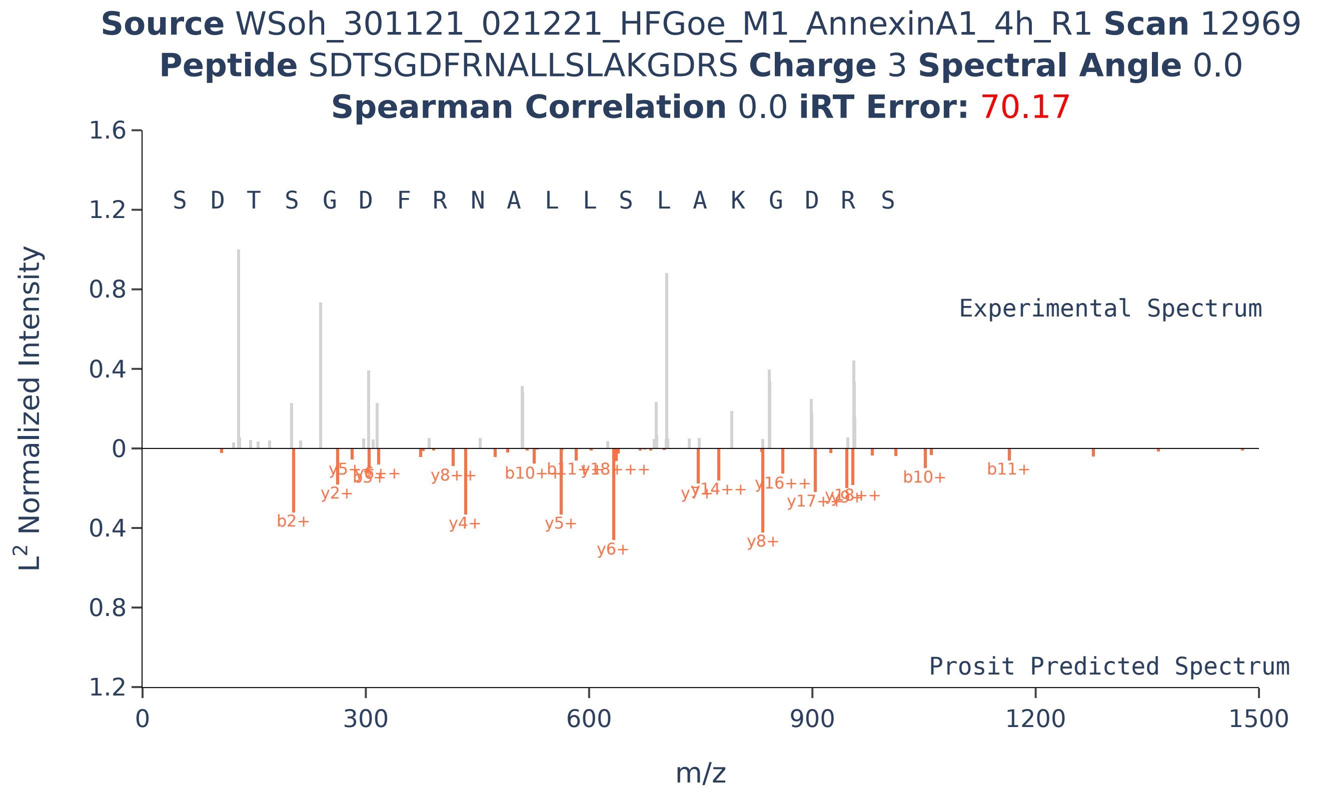
Source WSoh_301121_021221_HFGoe_M1_AnnexinA1_4h_R1 Scan 12969
Peptide SDTSGDFRNALLSLAKGDRS Charge 3 Spectral Angle 0.0
Spearman Correlation 0.0 iRT Error: 70.17
Experimental Spectrum
Prosit Predicted Spectrum
L² Normalized Intensity
m/z

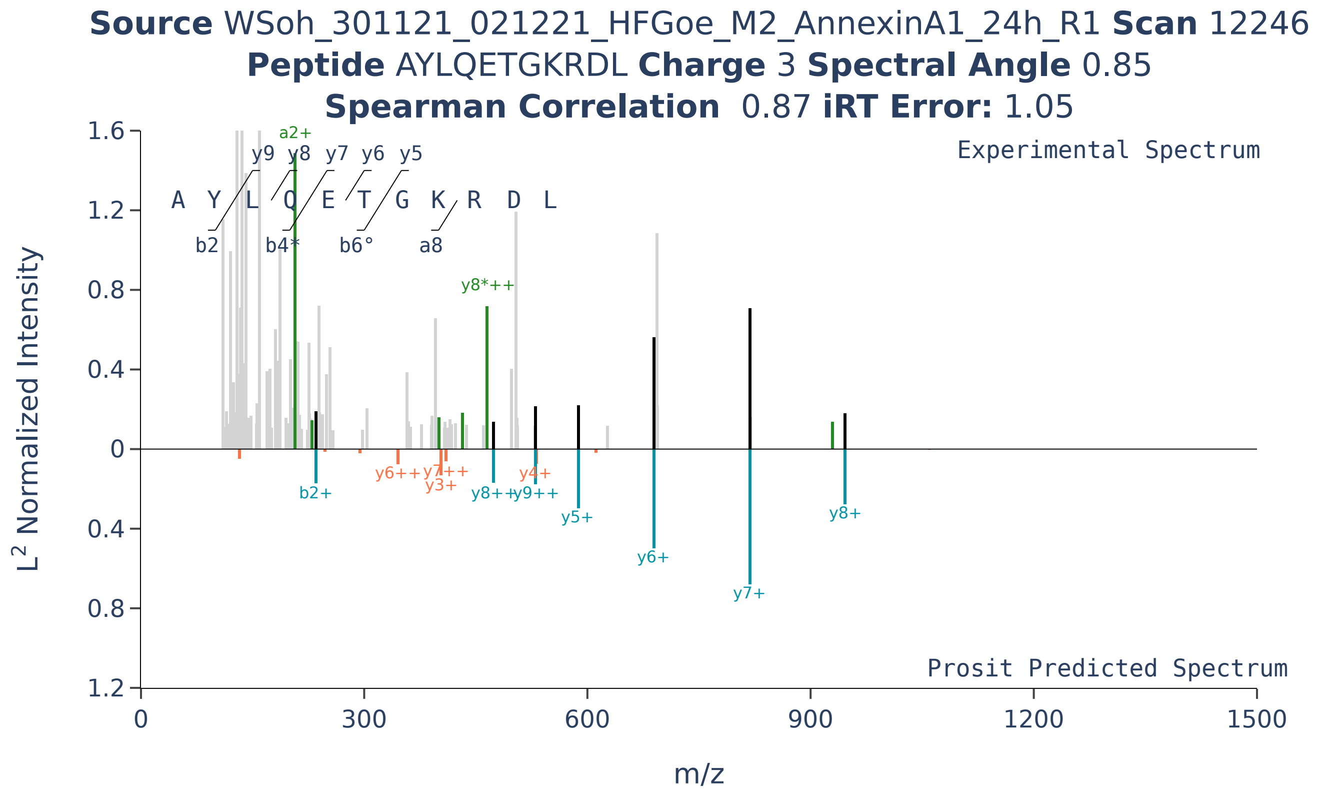
Source WSoh_301121_021221_HFGoe_M2_AnnexinA1_24h_R1 Scan 12246
Peptide AYLQETGKRDL Charge 3 Spectral Angle 0.85
Spearman Correlation 0.87 iRT Error: 1.05
Experimental Spectrum
Prosit Predicted Spectrum
L² Normalized Intensity
m/z

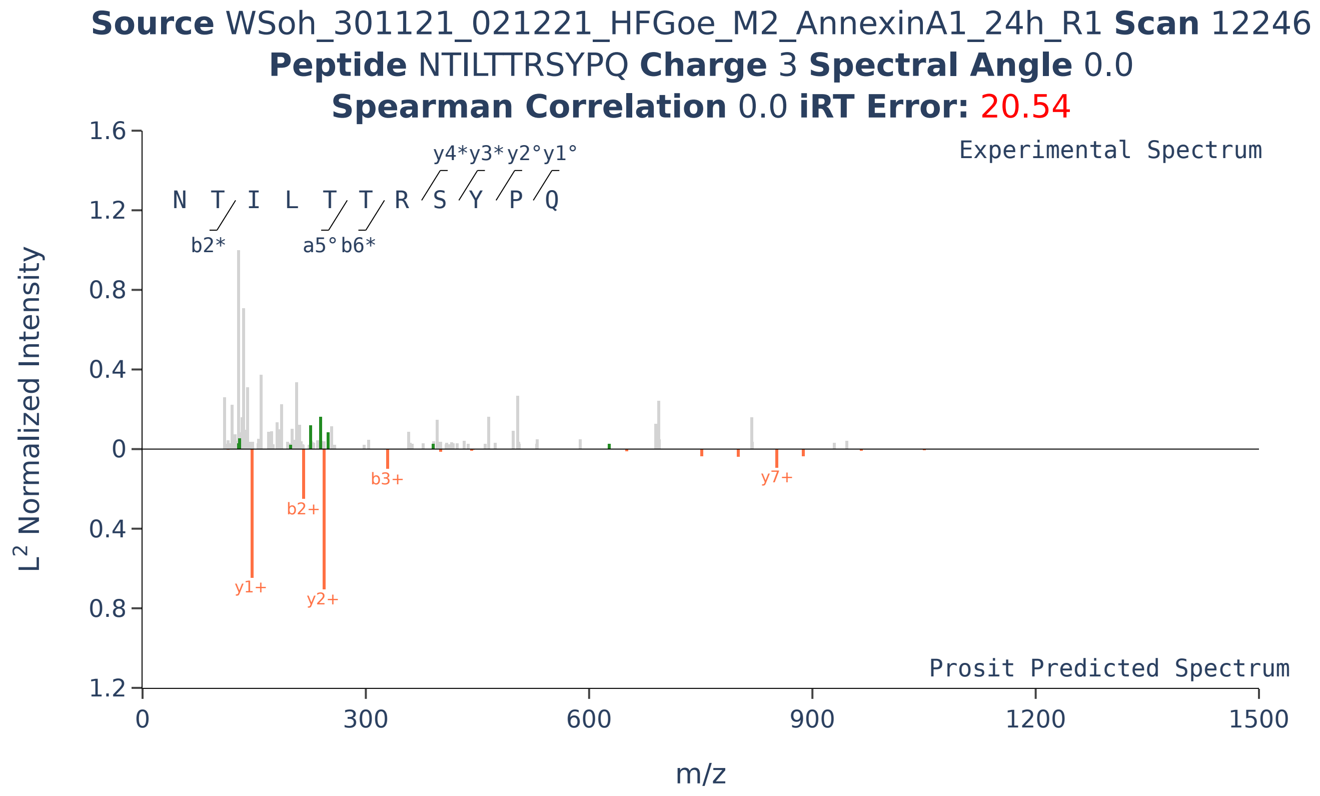
Source WSoh_301121_021221_HFGoe_M2_AnnexinA1_24h_R1 Scan 12246
Peptide NTILTTRSYPQ Charge 3 Spectral Angle 0.0
Spearman Correlation 0.0 iRT Error: 20.54
Experimental Spectrum
Prosit Predicted Spectrum
L² Normalized Intensity
m/z

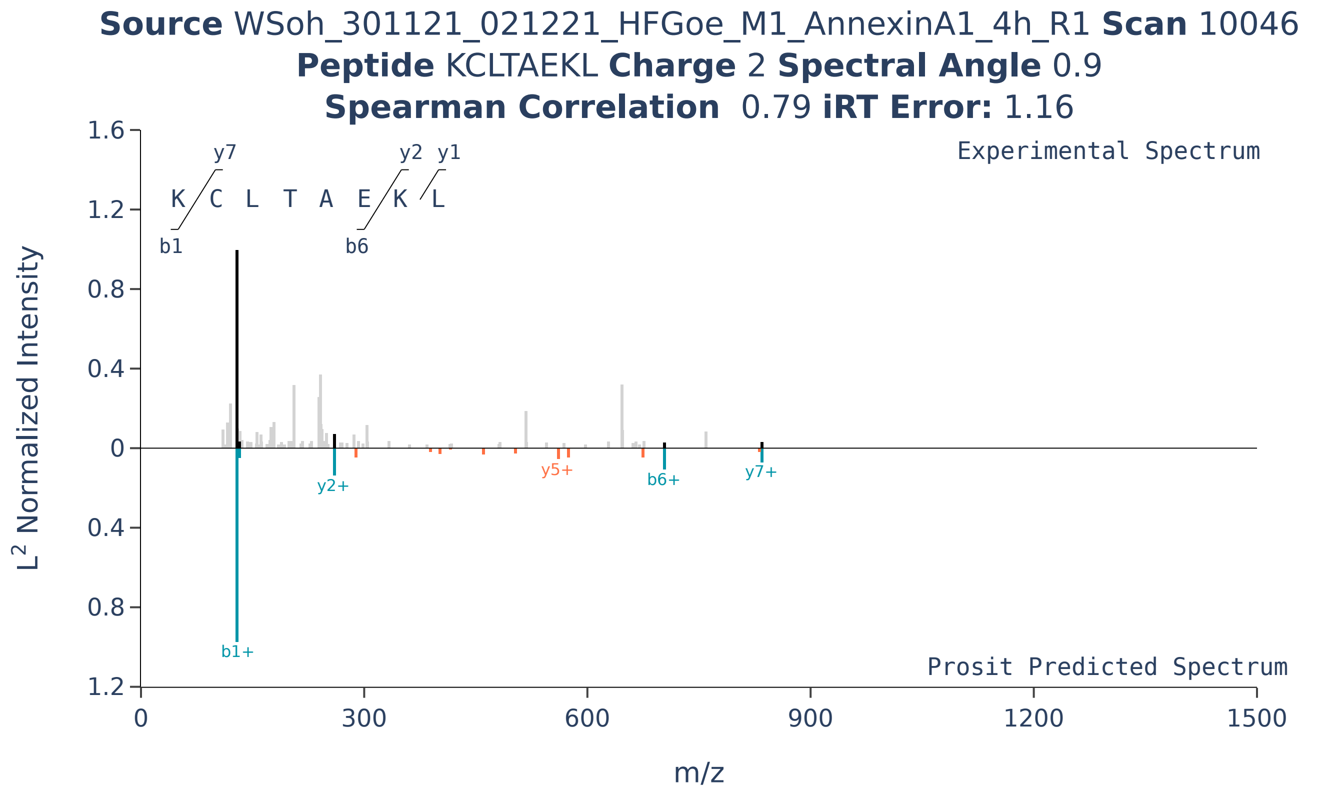
Source WSoh_301121_021221_HFGoe_M1_AnnexinA1_4h_R1 Scan 10046
Peptide KCLTAEKL Charge 2 Spectral Angle 0.9
Spearman Correlation 0.79 iRT Error: 1.16
Experimental Spectrum
Prosit Predicted Spectrum
L² Normalized Intensity
m/z

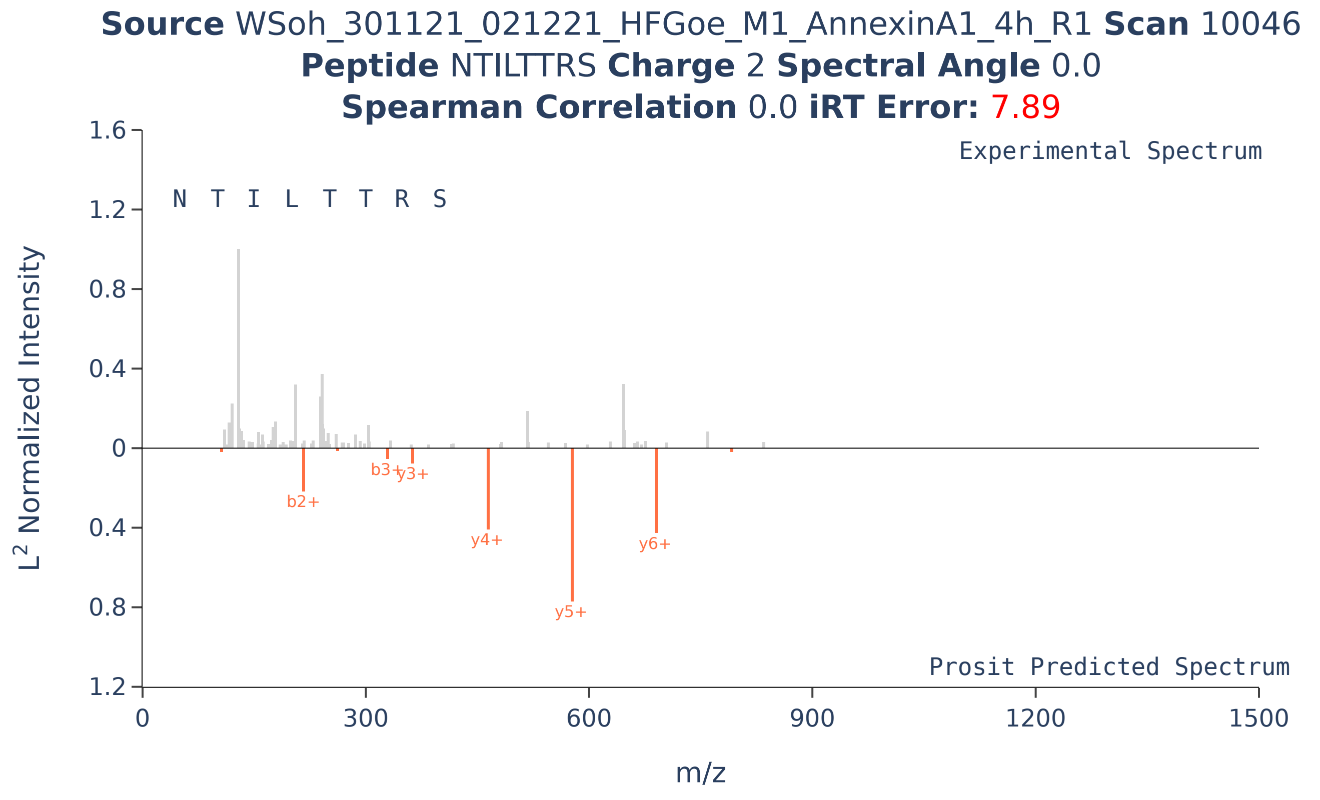
Source WSoh_301121_021221_HFGoe_M1_AnnexinA1_4h_R1 Scan 10046
Peptide NTILTTRS Charge 2 Spectral Angle 0.0
Spearman Correlation 0.0 iRT Error: 7.89
Experimental Spectrum
Prosit Predicted Spectrum
L² Normalized Intensity
m/z

Source WSoh_301121_021221_HFGoe_M2_AnnexinA1_4h_R2 **Scan** 20269
Peptide KSPAFFAEKL **Charge** 3 **Spectral Angle** 0.84
Spearman Correlation 0.88 **iRT Error:** 1.43

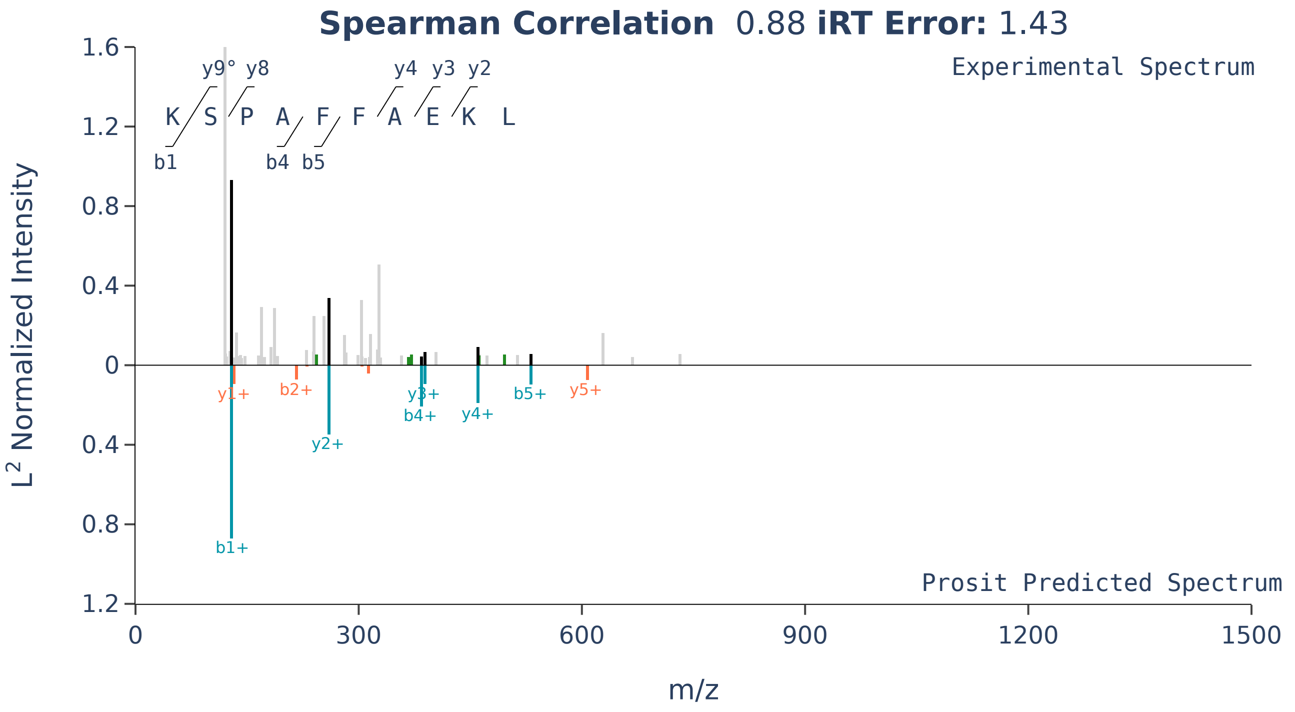

Source WSoh_301121_021221_HFGoe_M2_AnnexinA1_4h_R2 **Scan** 20269
Peptide SKPAFFAEKL **Charge** 3 **Spectral Angle** 0.71
Spearman Correlation 0.54 **iRT Error:** 4.14

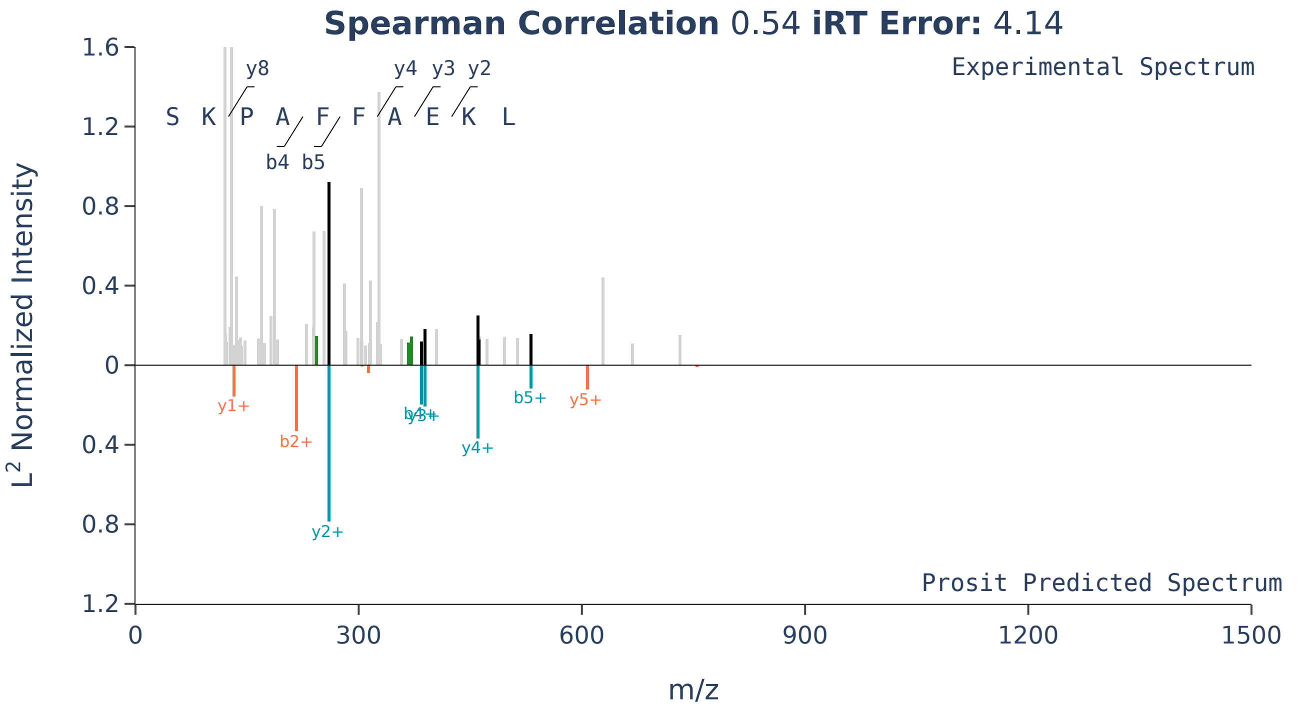

Source WSoh_301121_021221_HFGoe_M1_AnnexinA1_24h_R2 **Scan** 9887
Peptide SKGGPGQEYVQT **Charge** 2 **Spectral Angle** 0.81
Spearman Correlation 0.86 **iRT Error:** 2.66

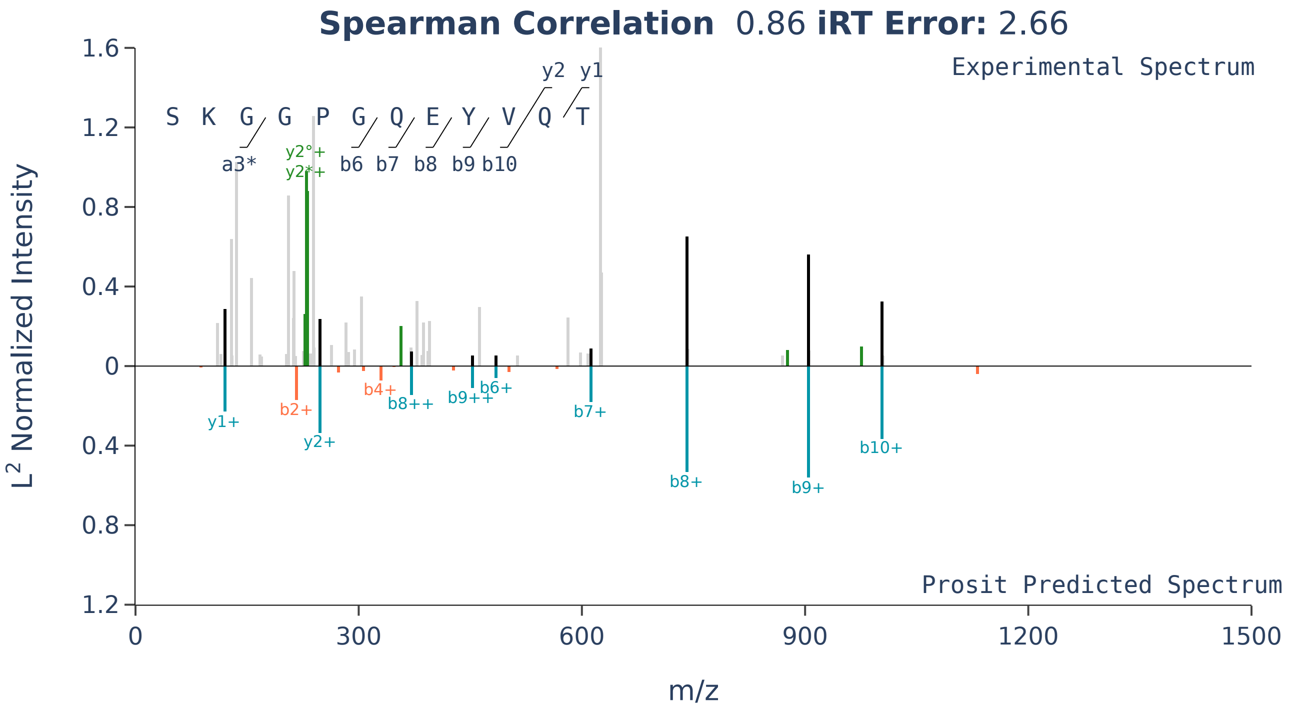

Source WSoh_301121_021221_HFGoe_M1_AnnexinA1_24h_R2 **Scan** 9887
Peptide AMKGLGTDEDTL **Charge** 2 **Spectral Angle** 0.0
Spearman Correlation 0.0 **iRT Error:** 46.43

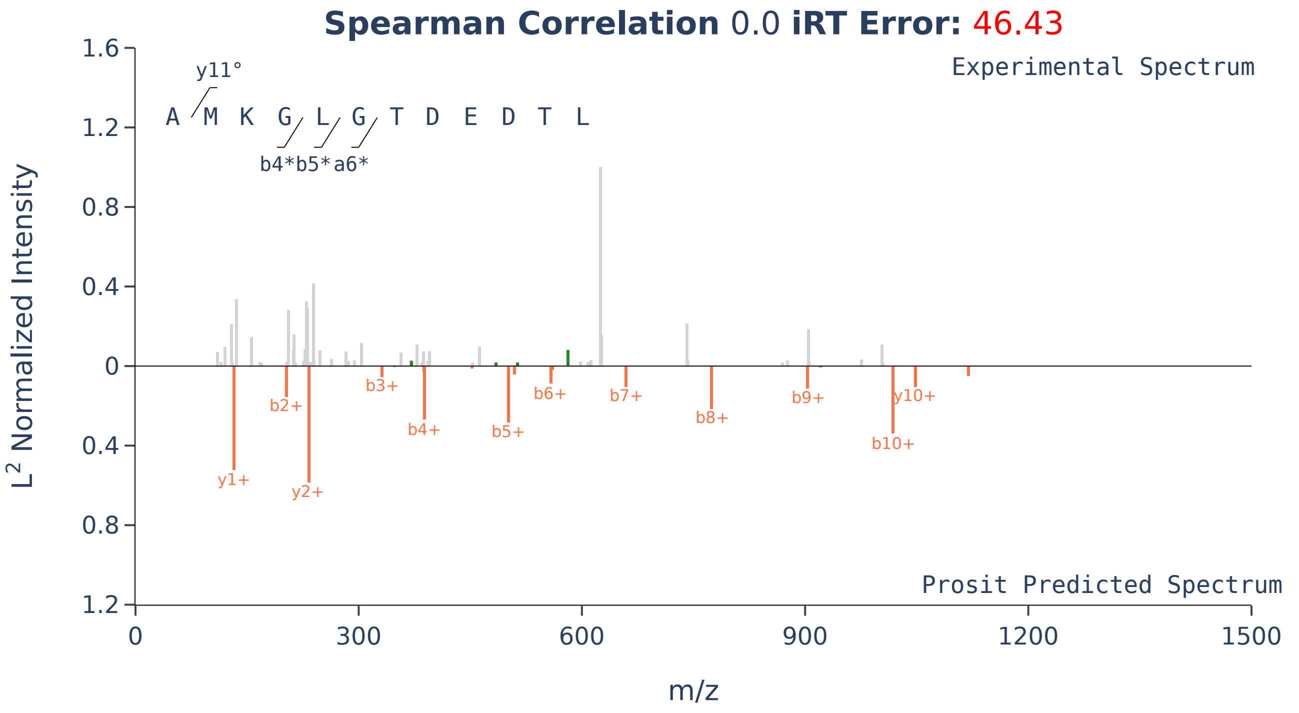

Source WSoh_301121_021221_HFGoe_M2_AnnexinA1_24h_R1 **Scan** 18378
Peptide EQEYVQTVKSETL **Charge** 2 **Spectral Angle** 0.81
Spearman Correlation 0.87 **iRT Error:** 2.67

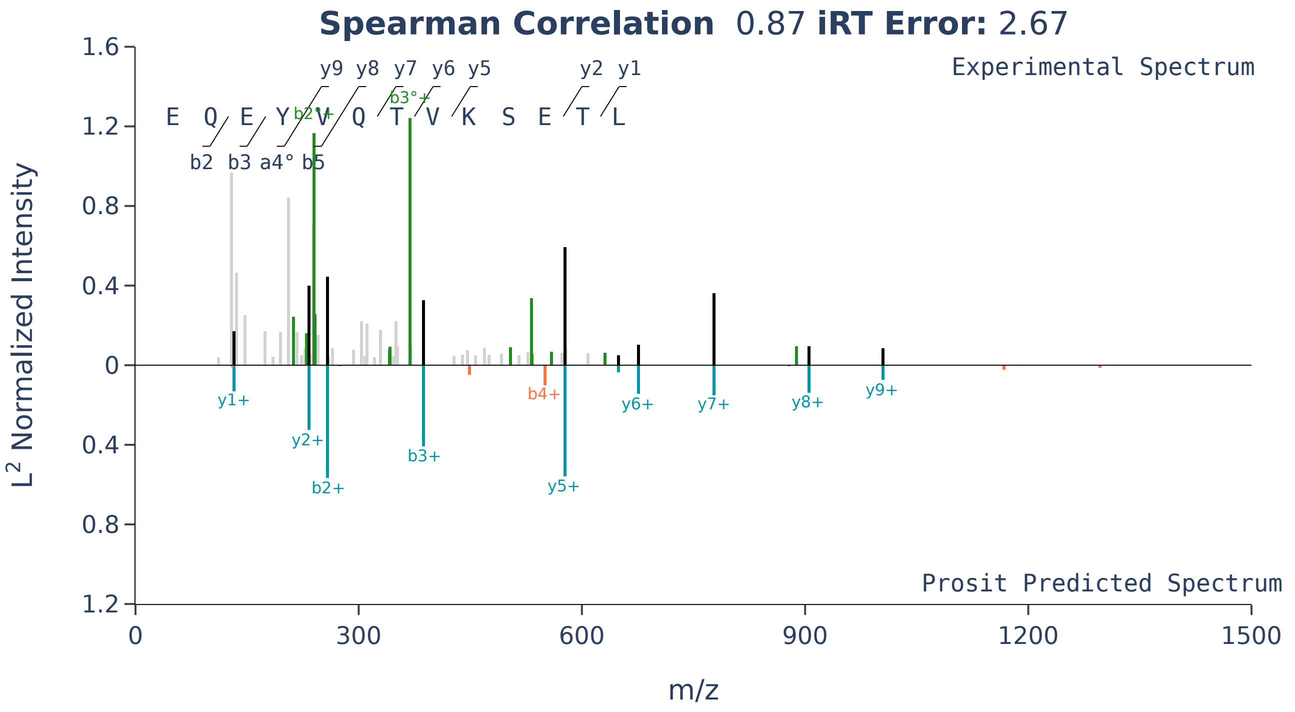

Source WSoh_301121_021221_HFGoe_M2_AnnexinA1_24h_R1 **Scan** 18378
Peptide FLKQAWFIENEE **Charge** 2 **Spectral Angle** 0.0
Spearman Correlation 0.0 **iRT Error:** 44.4

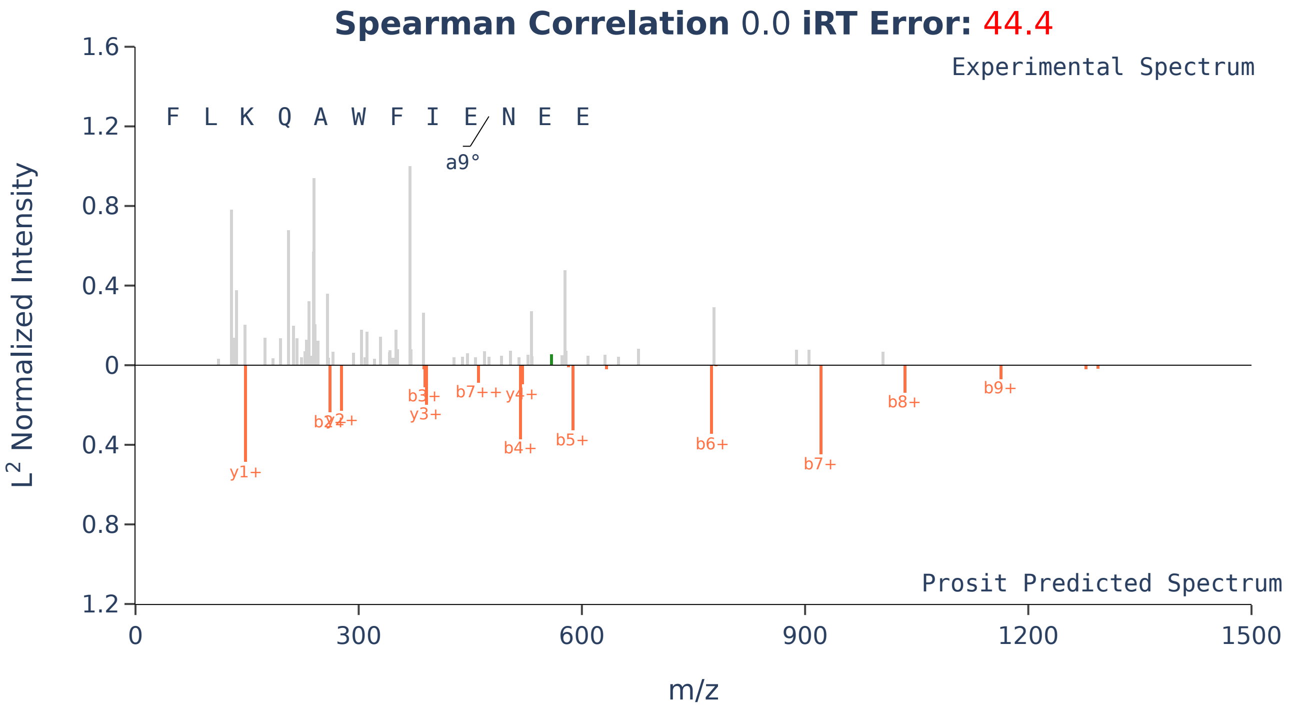

Source WSoh_301121_021221_HFGoe_M2_AnnexinA1_24h_R2 **Scan** 16243
Peptide DRSYPQL **Charge** 2 **Spectral Angle** 0.89
Spearman Correlation 0.85 **iRT Error:** 2.78

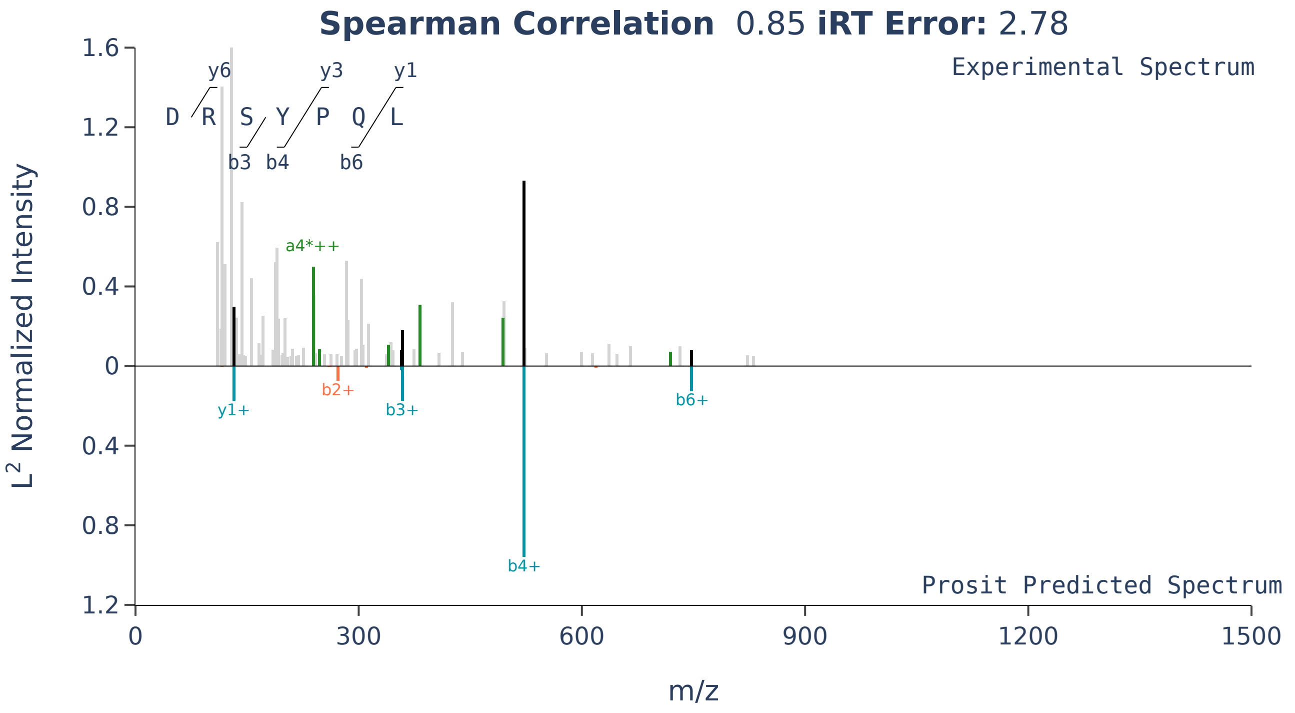

Source WSoh_301121_021221_HFGoe_M2_AnnexinA1_24h_R2 **Scan** 16243
Peptide TKYSKHD **Charge** 2 **Spectral Angle** 0.0
Spearman Correlation 0.0 **iRT Error:** 47.31

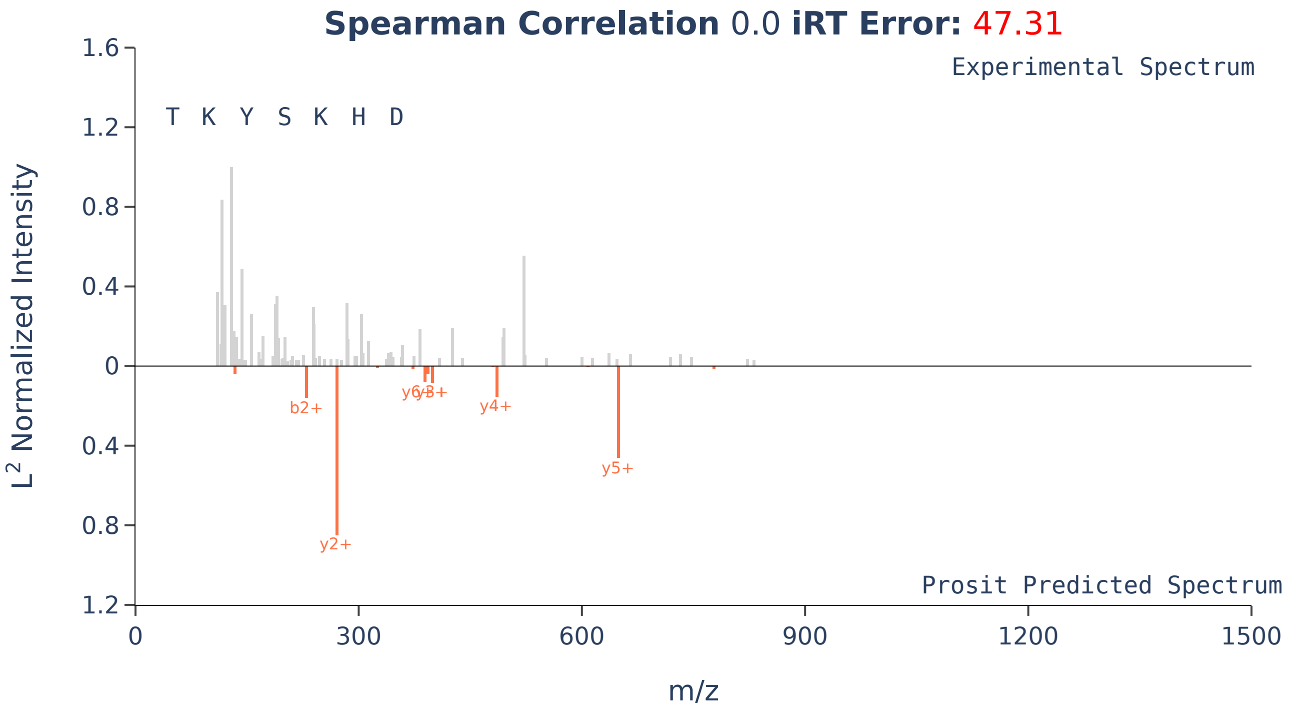

Source WSoh_301121_021221_HFGoe_M1_AnnexinA1_2h_R2 **Scan** 12601
Peptide RSYPQLRVS **Charge** 2 **Spectral Angle** 0.82
Spearman Correlation 0.87 **iRT Error:** 3.11

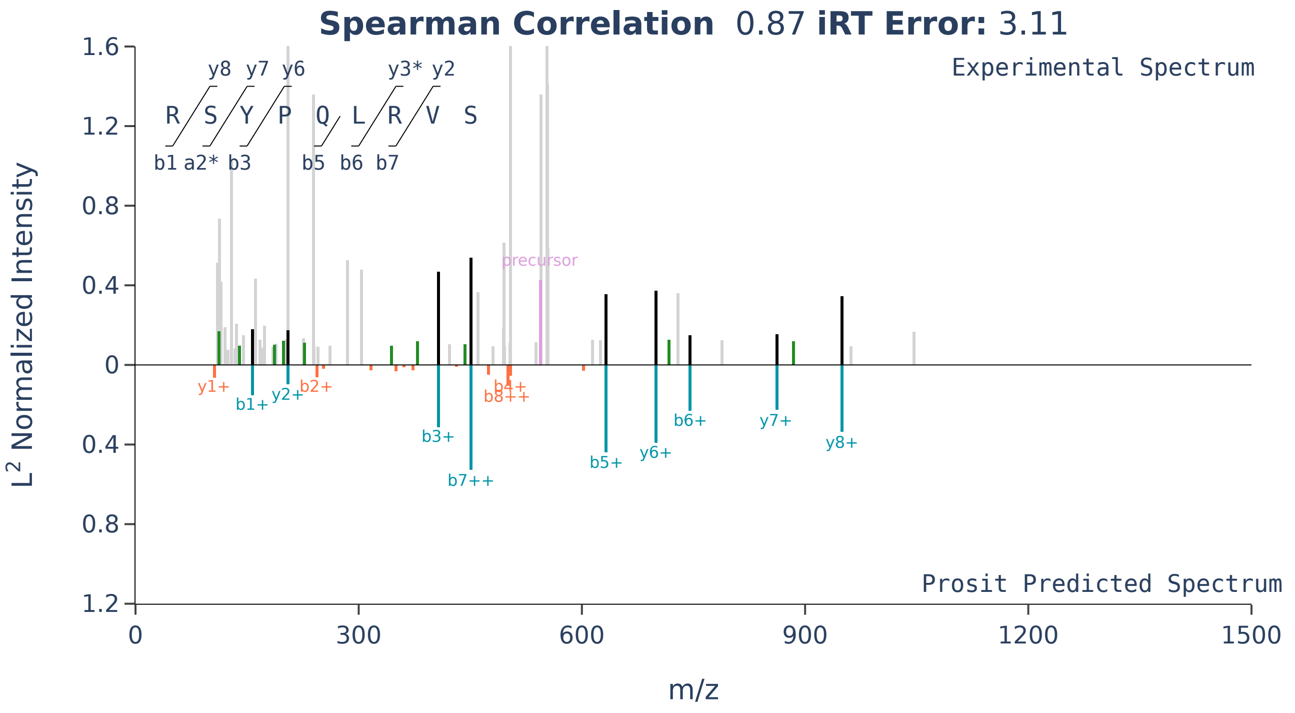

Source WSoh_301121_021221_HFGoe_M1_AnnexinA1_2h_R2 **Scan** 12601
Peptide QRQQIKAAY **Charge** 2 **Spectral Angle** 0.0
Spearman Correlation 0.0 **iRT Error:** 31.28

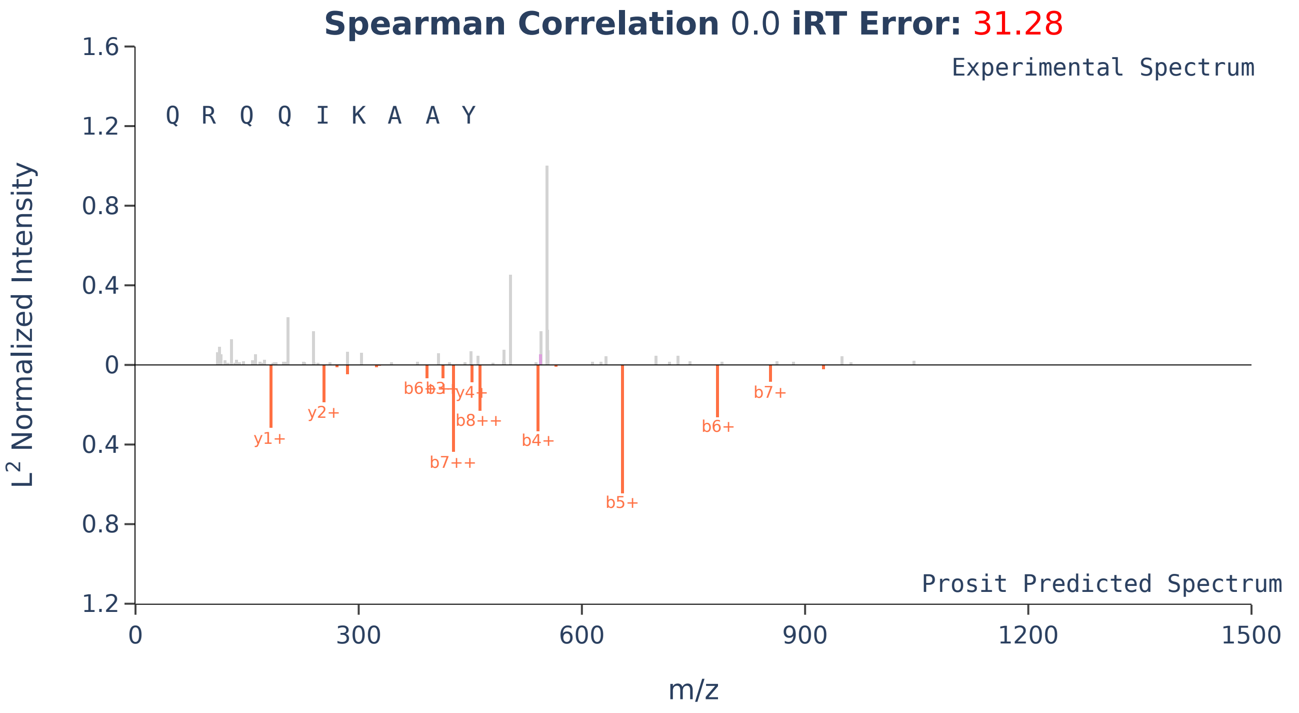

Source WSoh_301121_021221_HFGoe_M2_AnnexinA1_24h_R2 **Scan** 10534
Peptide SKGGPGSEQEYVQT **Charge** 2 **Spectral Angle** 0.86
Spearman Correlation 0.89 **iRT Error:** 3.32

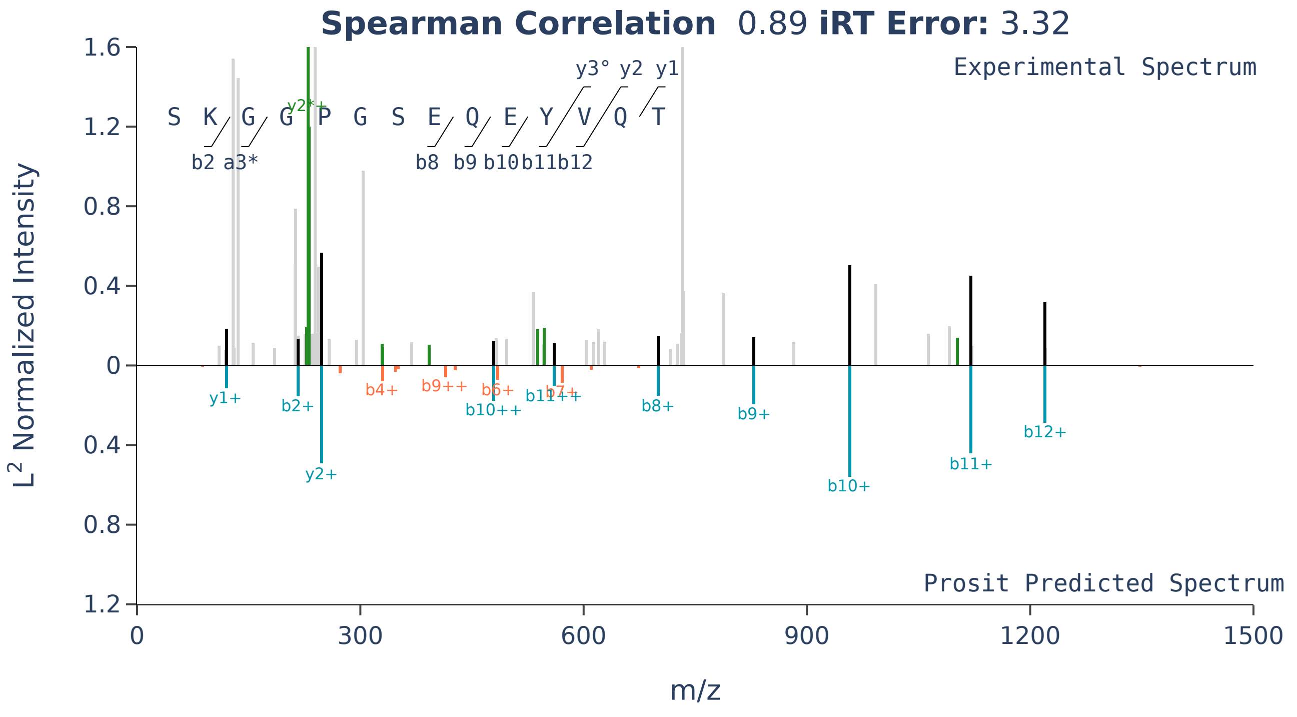

Source WSoh_301121_021221_HFGoe_M2_AnnexinA1_24h_R2 **Scan** 10534
Peptide DLADSDARALYEAG **Charge** 2 **Spectral Angle** 0.0
Spearman Correlation 0.0 **iRT Error:** 58.1

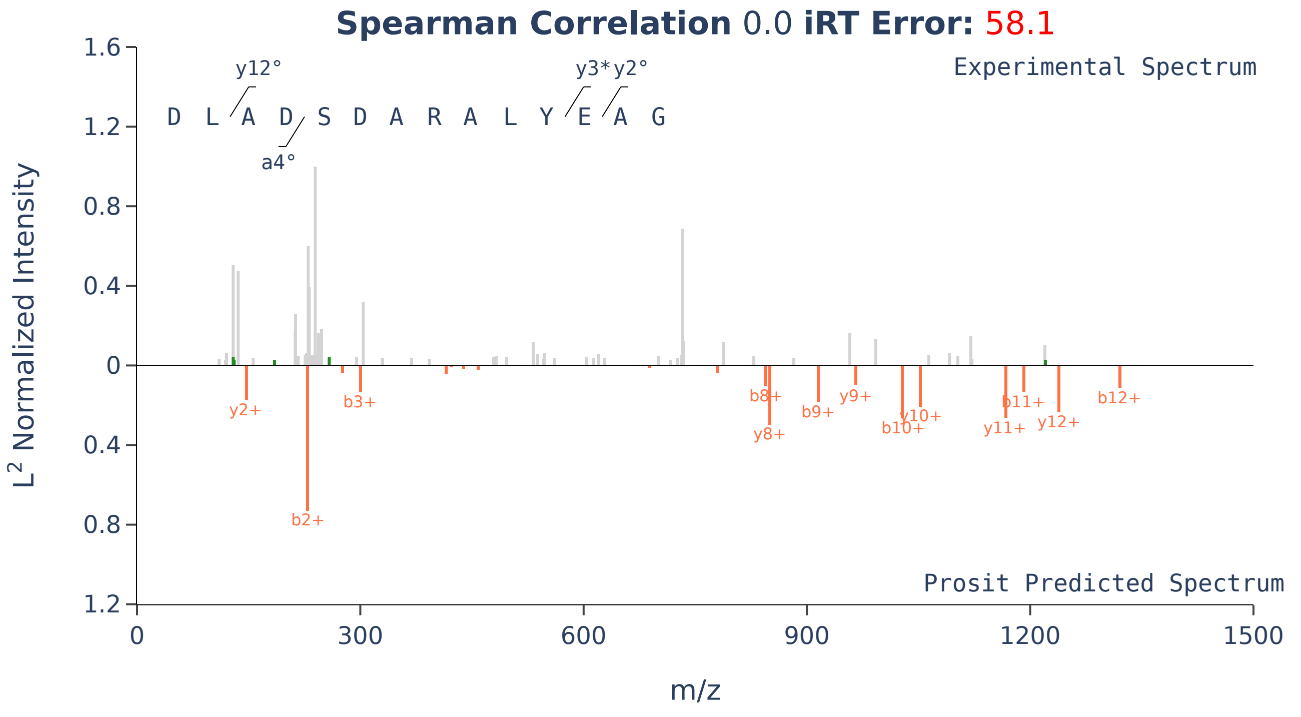

Source WSoh_301121_021221_HFGoe_M2_AnnexinA1_24h_R2 **Scan** 10911
Peptide AKDITSDTSGDFRNKRDL **Charge** 4 **Spectral Angle** 0.93
Spearman Correlation 0.88 **iRT Error:** 3.71

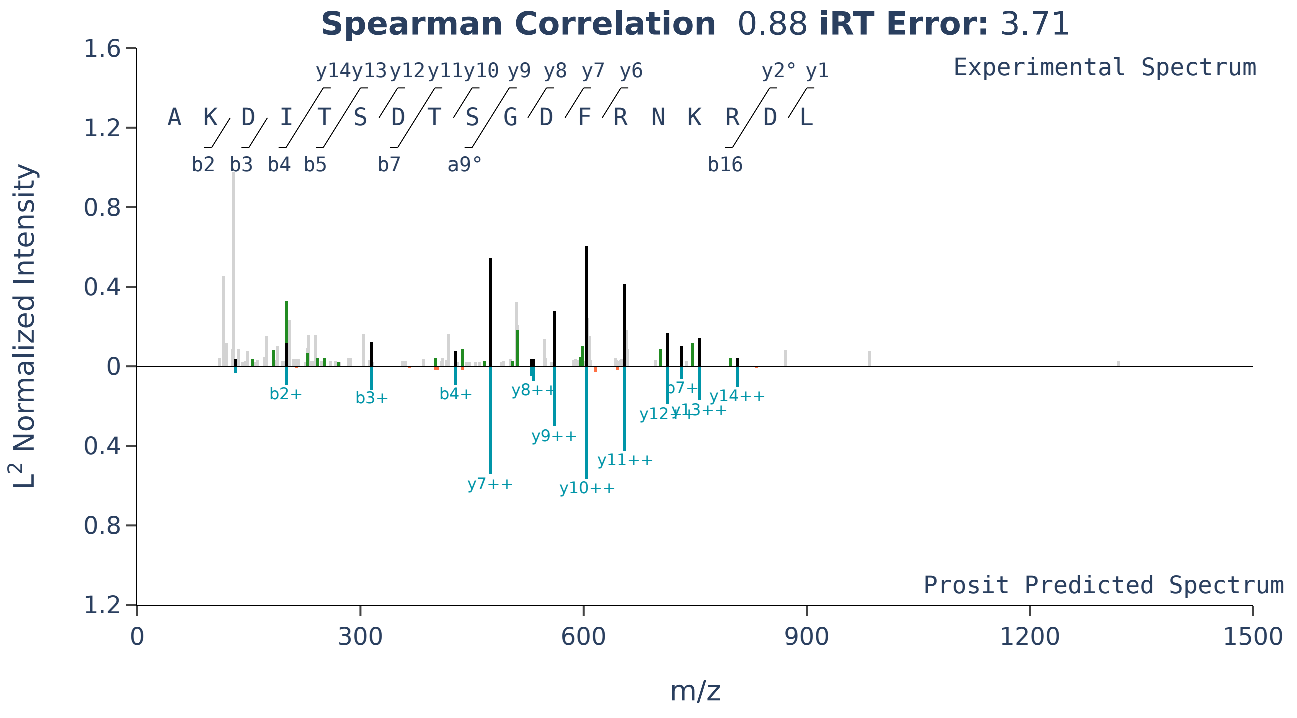

Source WSoh_301121_021221_HFGoe_M2_AnnexinA1_24h_R2 **Scan** 10911
Peptide KRDLAKDITSDTSGDFRN **Charge** 4 **Spectral Angle** 0.2
Spearman Correlation 0.23 **iRT Error:** 14.89

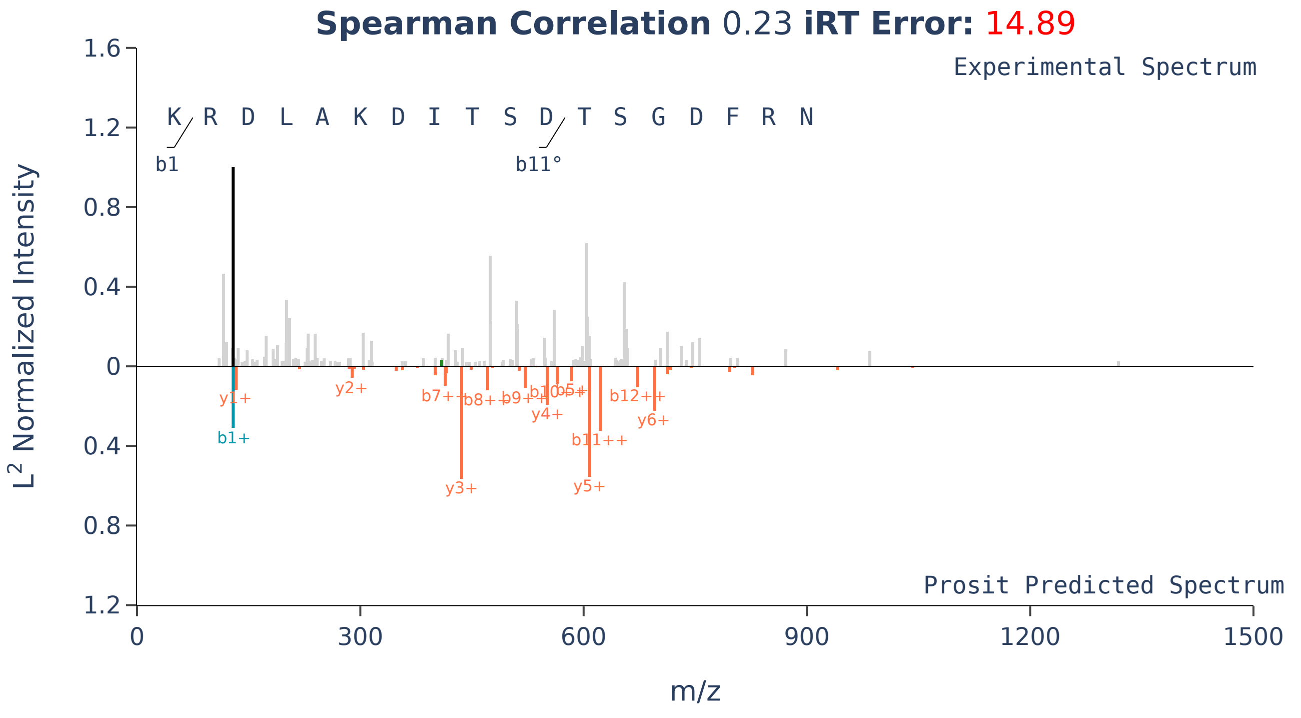

Source WSoh_301121_021221_HFGoe_M2_AnnexinA1_24h_R2 **Scan** 12750
Peptide AGERRKGTDDVNVF **Charge** 3 **Spectral Angle** 0.89
Spearman Correlation 0.88 **iRT Error:** 3.92

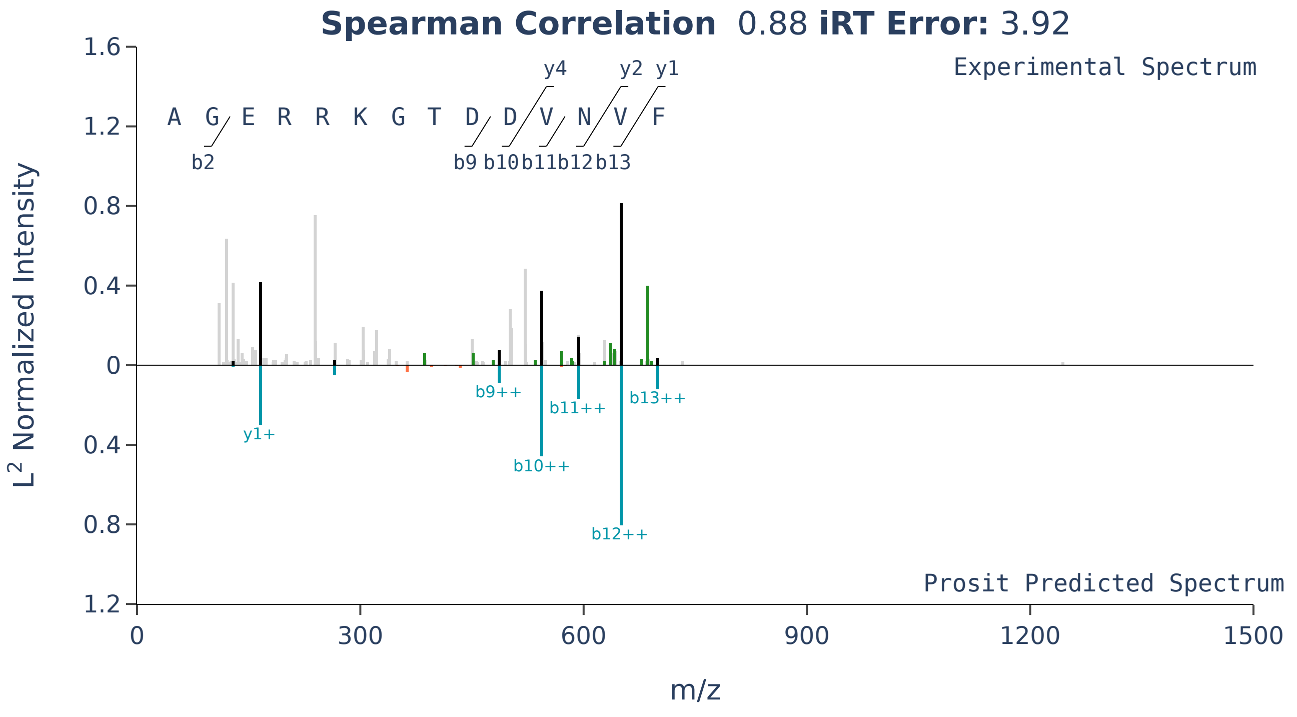

Source WSoh_301121_021221_HFGoe_M2_AnnexinA1_24h_R2 **Scan** 12750
Peptide DIKAFYQKMYGIS **Charge** 3 **Spectral Angle** 0.0
Spearman Correlation 0.0 **iRT Error:** 73.74

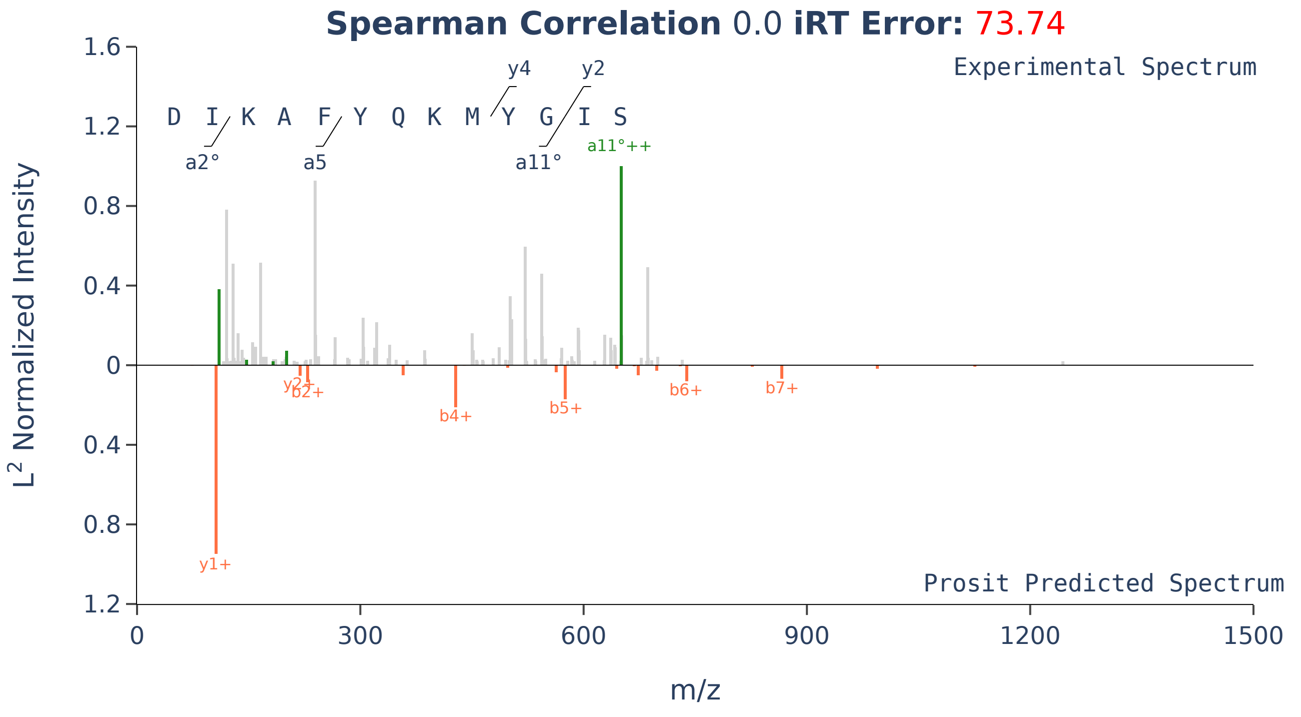

Source WSoh_301121_021221_HFGoe_M1_AnnexinA1_4h_R2 **Scan** 14611
Peptide KTGDYEKIL **Charge** 3 **Spectral Angle** 0.92
Spearman Correlation 0.82 **iRT Error:** 4.03

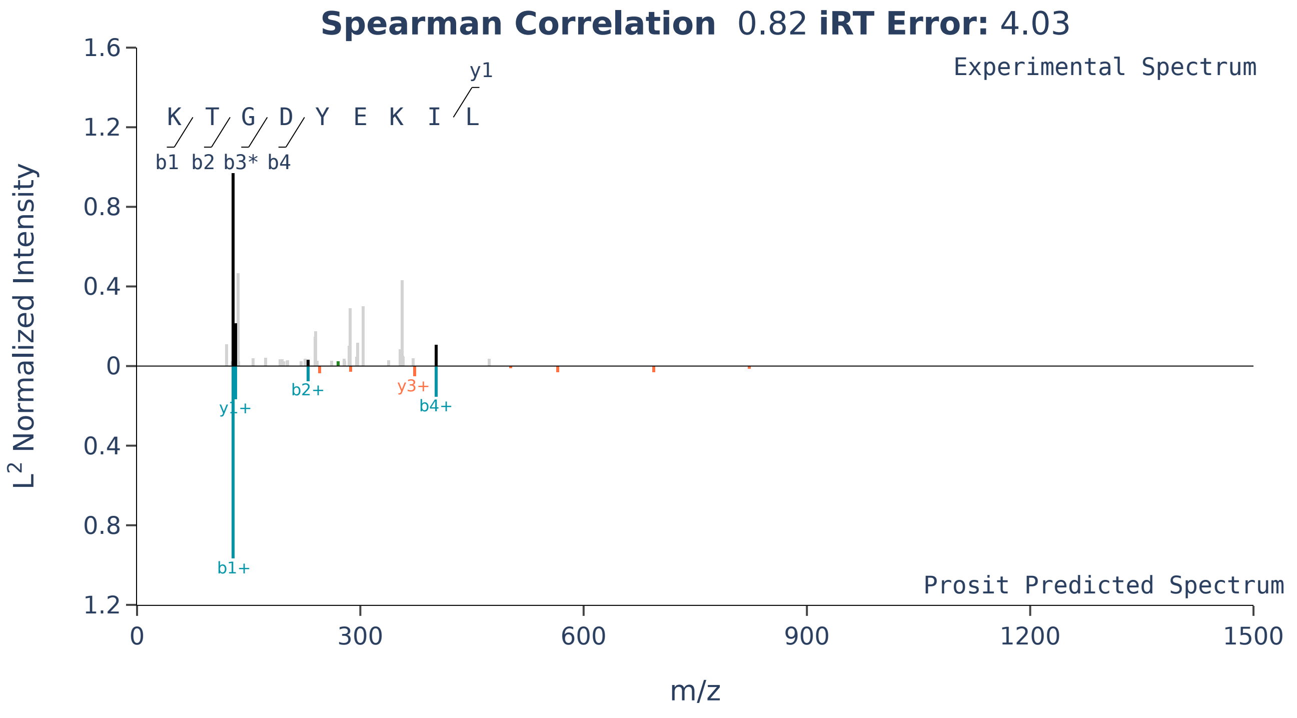

Source WSoh_301121_021221_HFGoe_M1_AnnexinA1_4h_R2 **Scan** 14611
Peptide TKGDYEKIL **Charge** 3 **Spectral Angle** 0.6
Spearman Correlation 0.7 **iRT Error:** 2.39

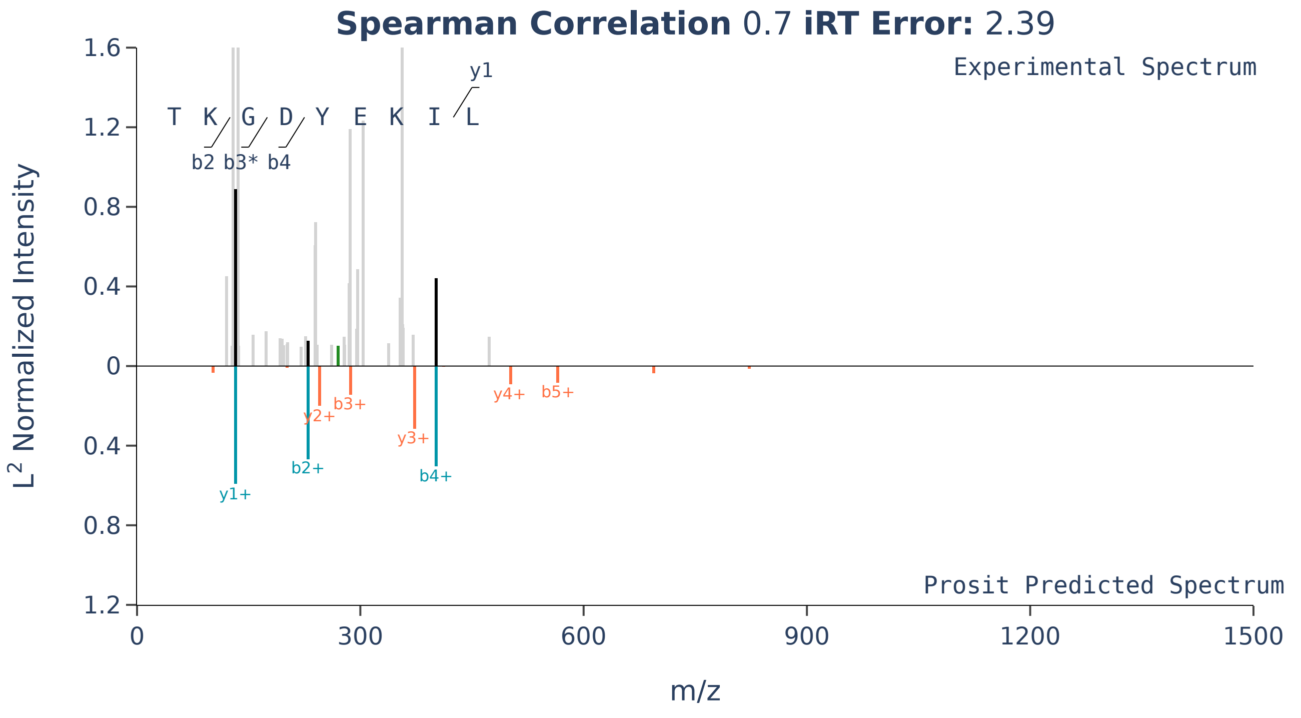

Source WSoh_301121_021221_HFGoe_M1_AnnexinA1_24h_R1 **Scan** 12906
Peptide DAGERRKGTDVNVF **Charge** 3 **Spectral Angle** 0.91
Spearman Correlation 0.83 **iRT Error:** 4.34

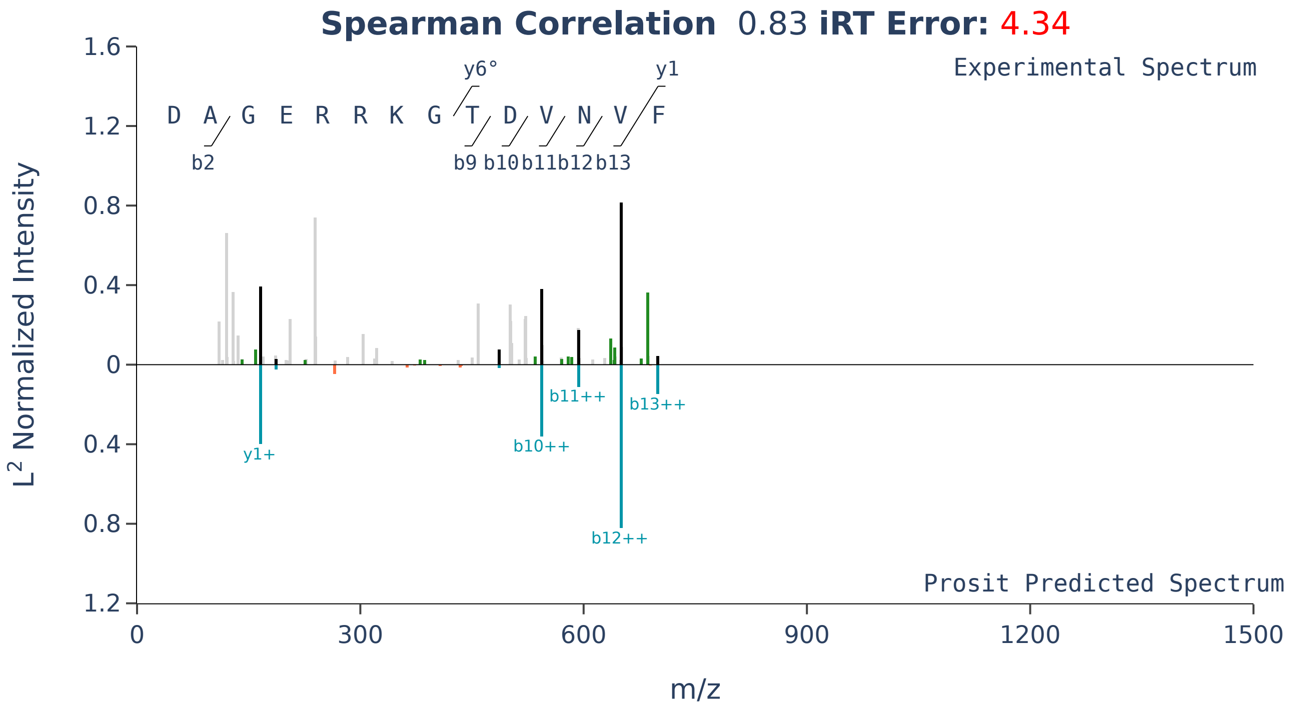

Source WSoh_301121_021221_HFGoe_M1_AnnexinA1_24h_R1 **Scan** 12906
Peptide DIKAFYQKMYGIS **Charge** 3 **Spectral Angle** 0.0
Spearman Correlation 0.0 **iRT Error:** 73.88

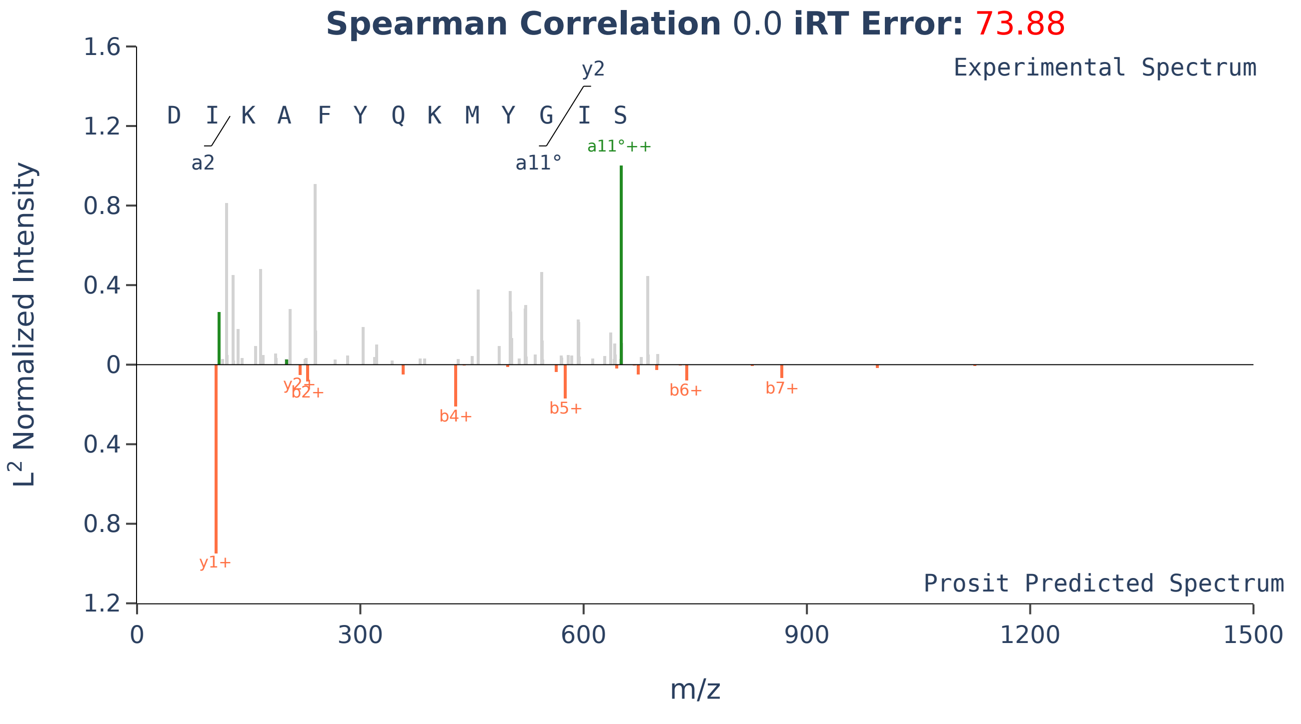

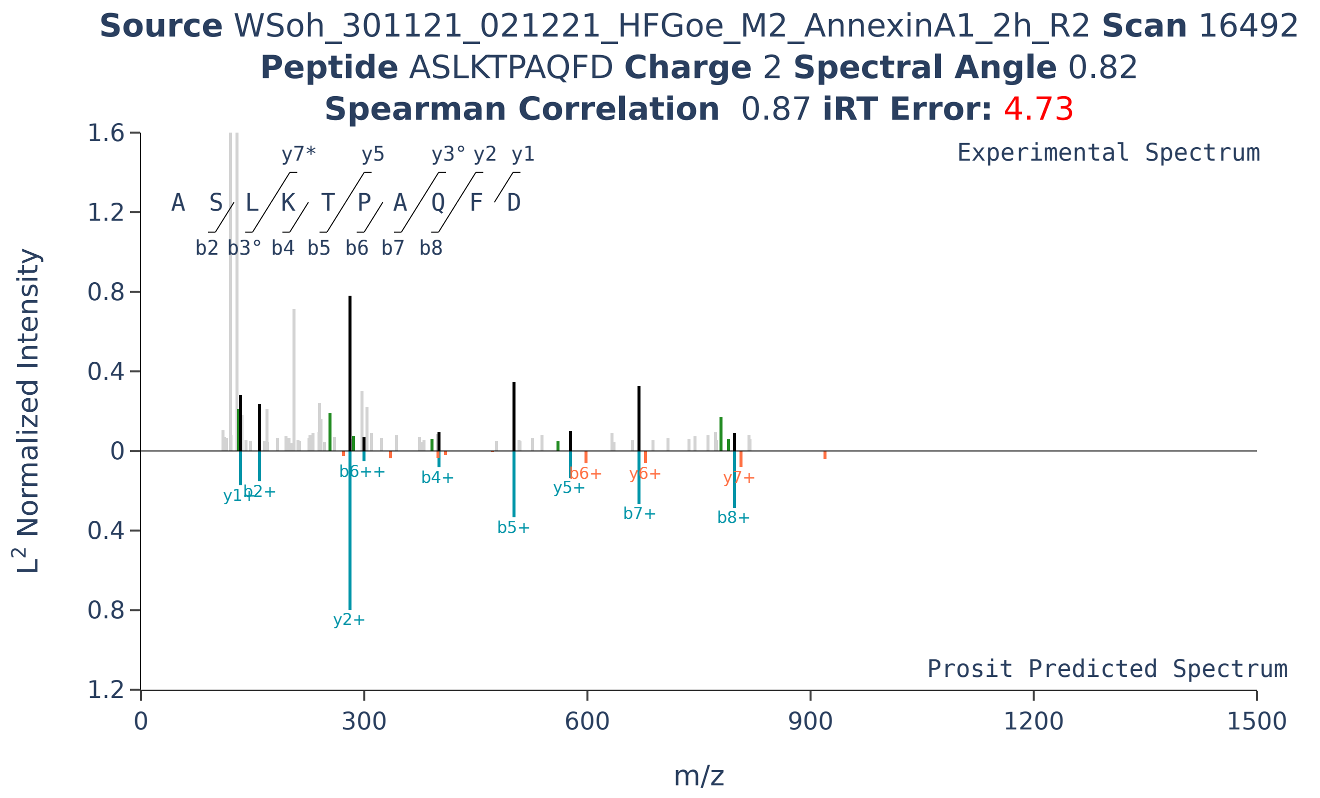
Source WSoh_301121_021221_HFGoe_M2_AnnexinA1_2h_R2 Scan 16492
Peptide ASLKTPAQFD Charge 2 Spectral Angle 0.82
Spearman Correlation 0.87 iRT Error: 4.73
Experimental Spectrum
Prosit Predicted Spectrum
L2 Normalized Intensity
m/z

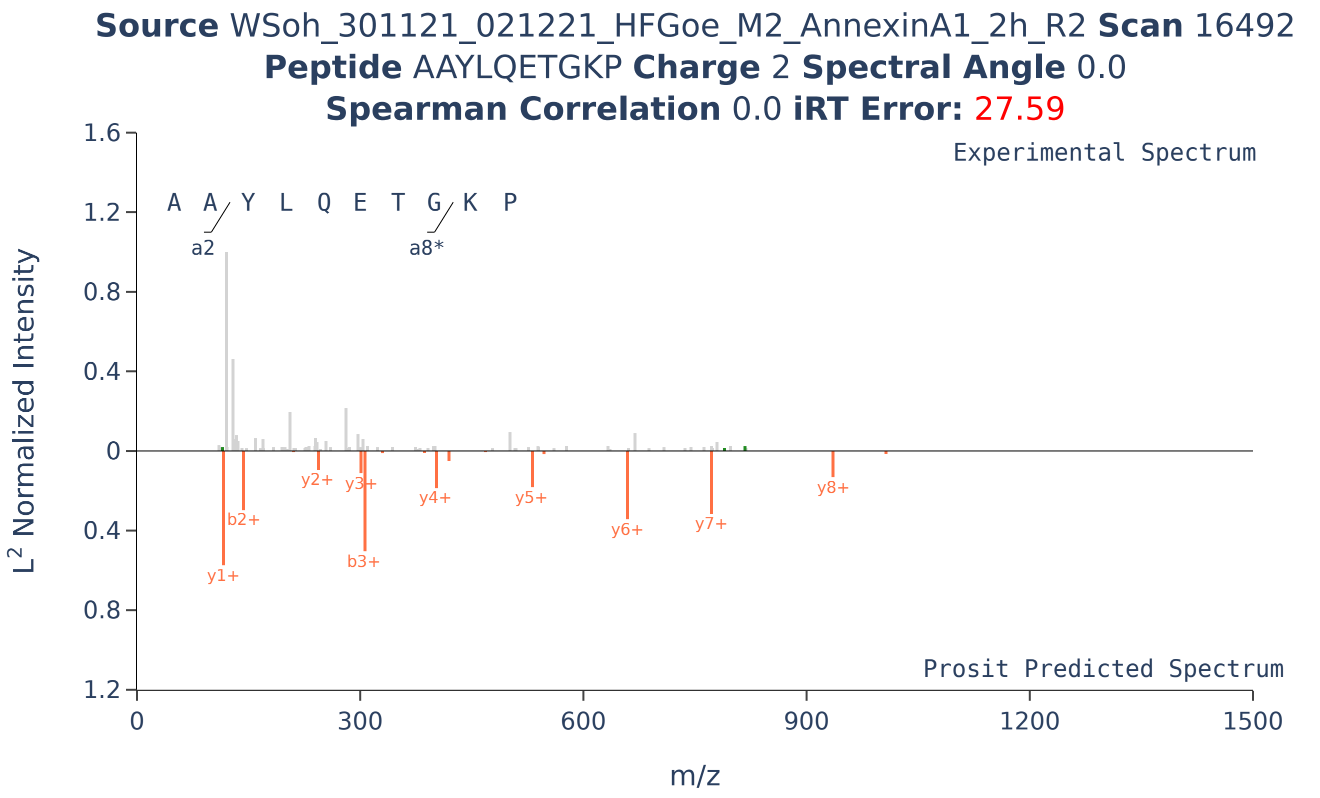
Source WSoh_301121_021221_HFGoe_M2_AnnexinA1_2h_R2 Scan 16492
Peptide AAYLQETGKP Charge 2 Spectral Angle 0.0
Spearman Correlation 0.0 iRT Error: 27.59
Experimental Spectrum
Prosit Predicted Spectrum
L2 Normalized Intensity
m/z

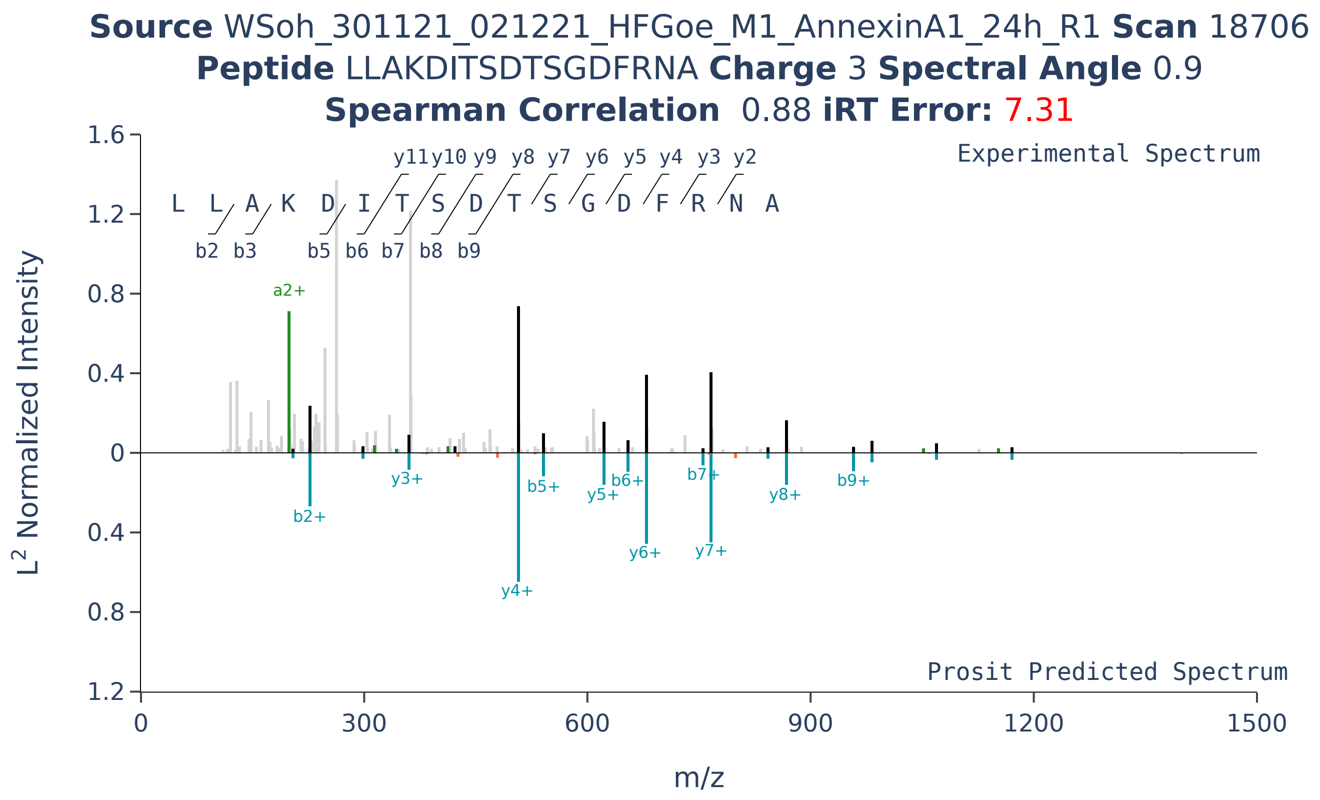
Source WSoh_301121_021221_HFGoe_M1_AnnexinA1_24h_R1 Scan 18706
Peptide LLAKDITSDTSGDFRNA Charge 3 Spectral Angle 0.9
Spearman Correlation 0.88 iRT Error: 7.31
Experimental Spectrum
Prosit Predicted Spectrum
L2 Normalized Intensity
m/z

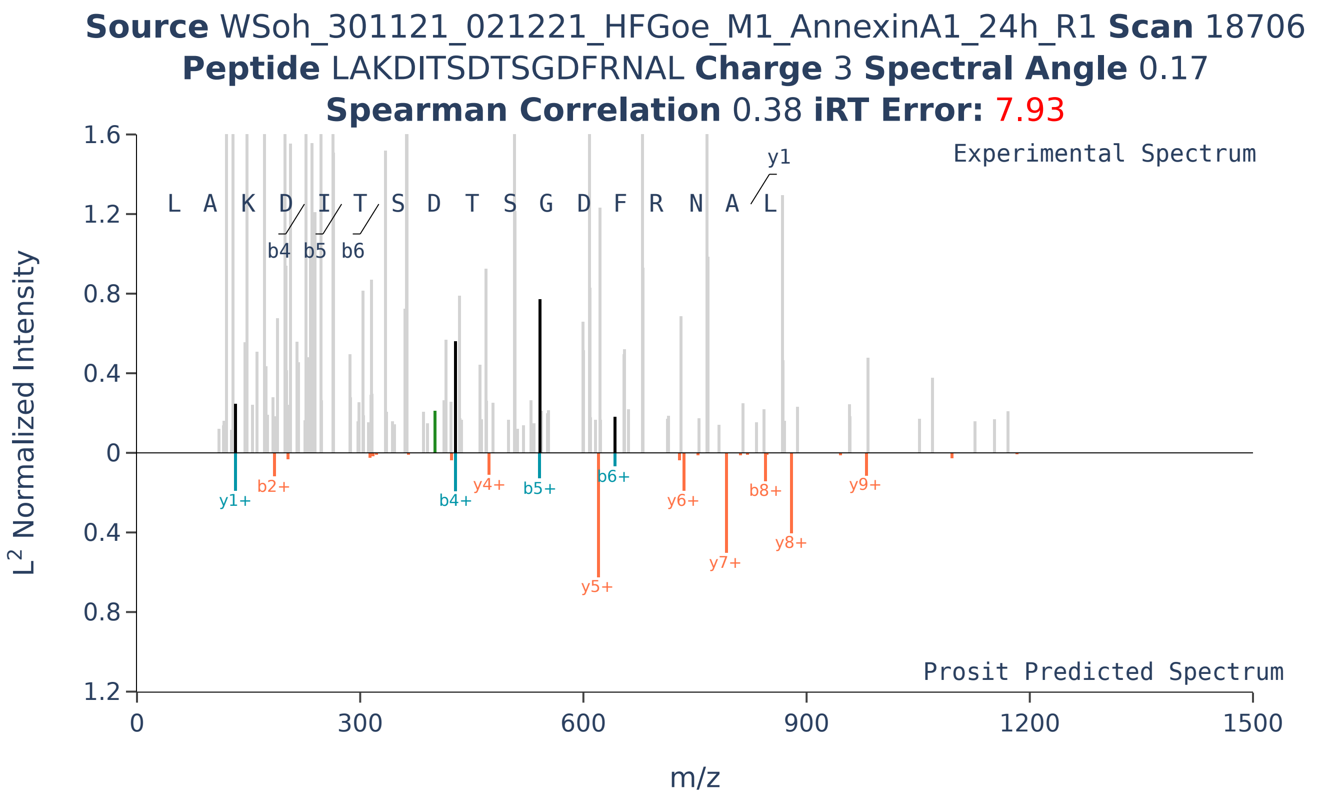
Source WSoh_301121_021221_HFGoe_M1_AnnexinA1_24h_R1 Scan 18706
Peptide LAKDITSDTSGDFRNAL Charge 3 Spectral Angle 0.17
Spearman Correlation 0.38 iRT Error: 7.93
Experimental Spectrum
Prosit Predicted Spectrum
L2 Normalized Intensity
m/z

Source W_Soh_160821_170921_Goe_aSyn_A1_1h_R2 **Scan** 14829
Peptide PQEGILEDQE **Charge** 1 **Spectral Angle** 0.89
Spearman Correlation 0.86 **iRT Error:** 0.31

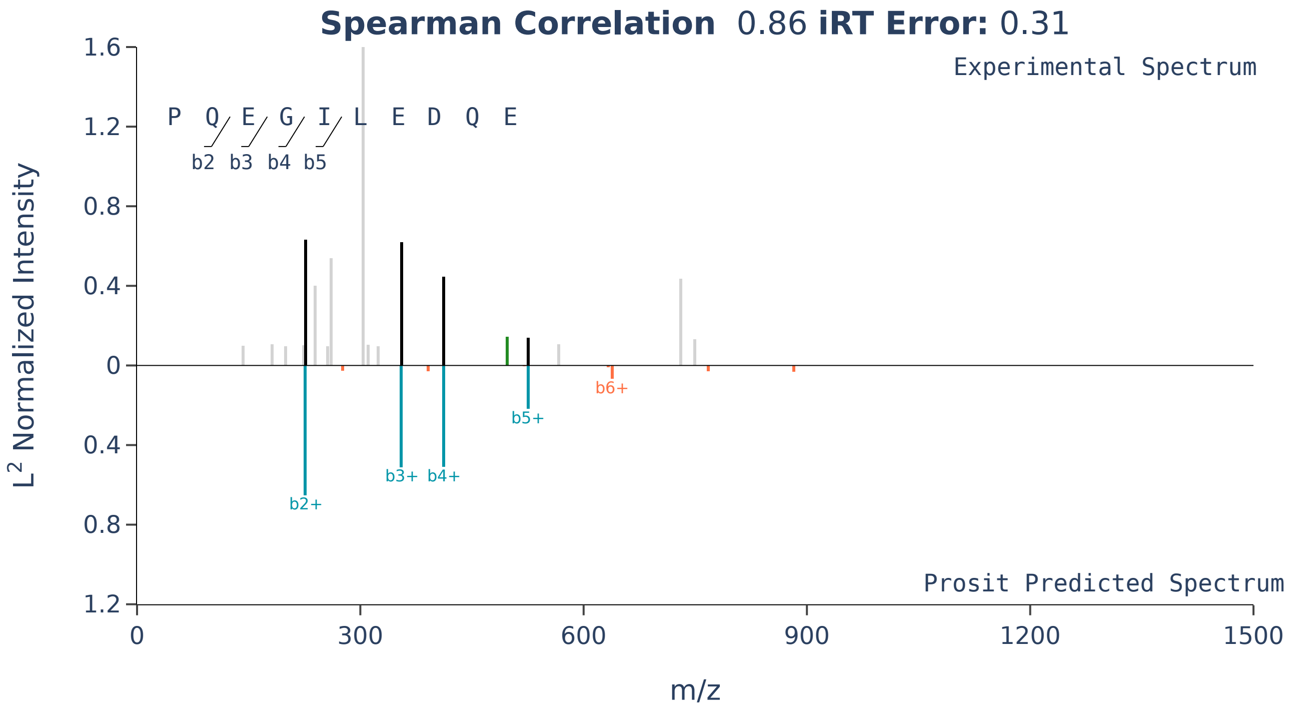

Source W_Soh_160821_170921_Goe_aSyn_A1_1h_R2 **Scan** 14829
Peptide EGAPQEGILED **Charge** 1 **Spectral Angle** 0.15
Spearman Correlation 0.13 **iRT Error:** 11.71

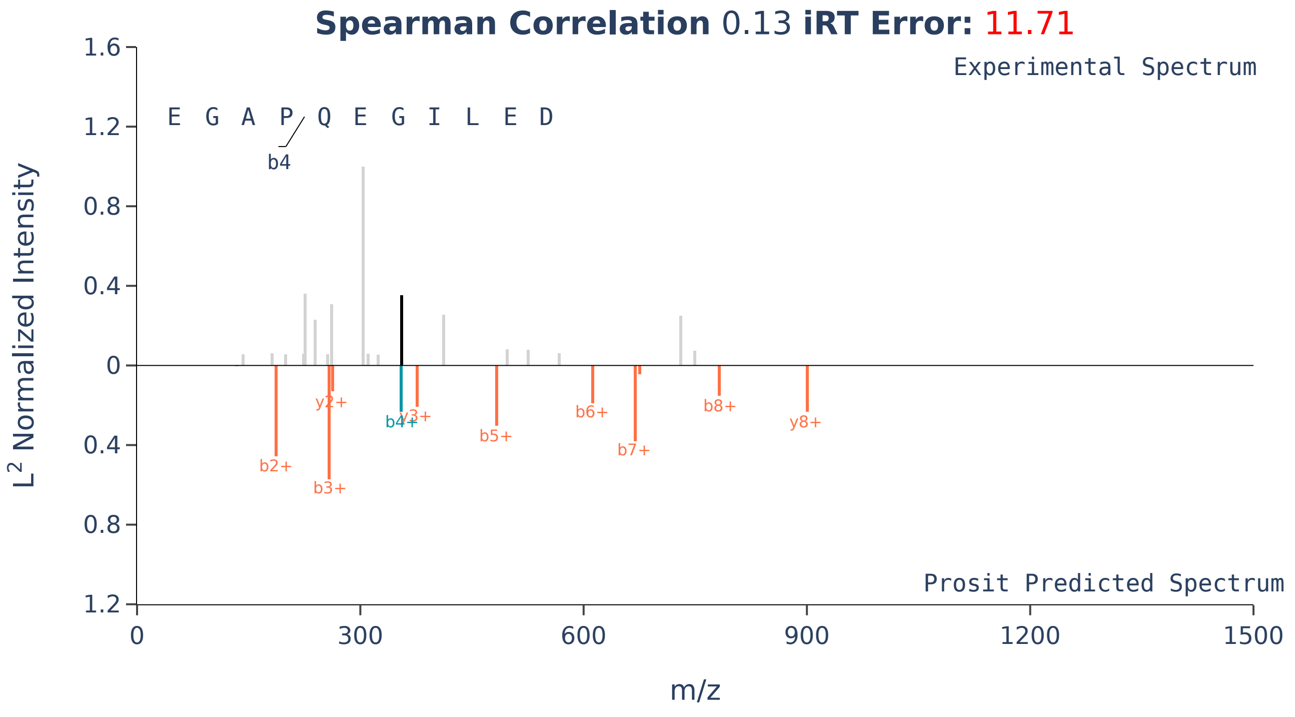

Source W_Soh_160821_170921_Goe_aSyn_A2_24h_R2 **Scan** 16725
Peptide PQEGILEDEGA **Charge** 1 **Spectral Angle** 0.91
Spearman Correlation 0.92 **iRT Error:** 0.67

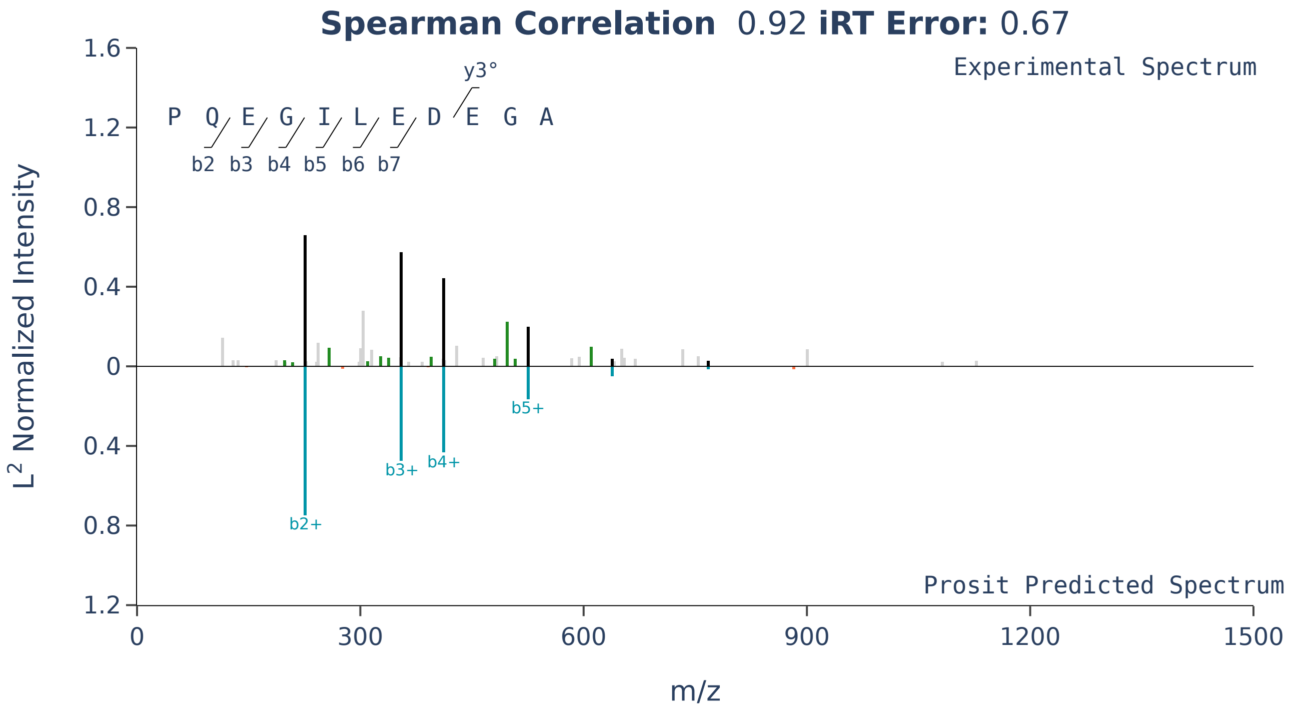

Source W_Soh_160821_170921_Goe_aSyn_A2_24h_R2 **Scan** 16725
Peptide EGAPQEGILED **Charge** 1 **Spectral Angle** 0.28
Spearman Correlation 0.77 **iRT Error:** 3.88

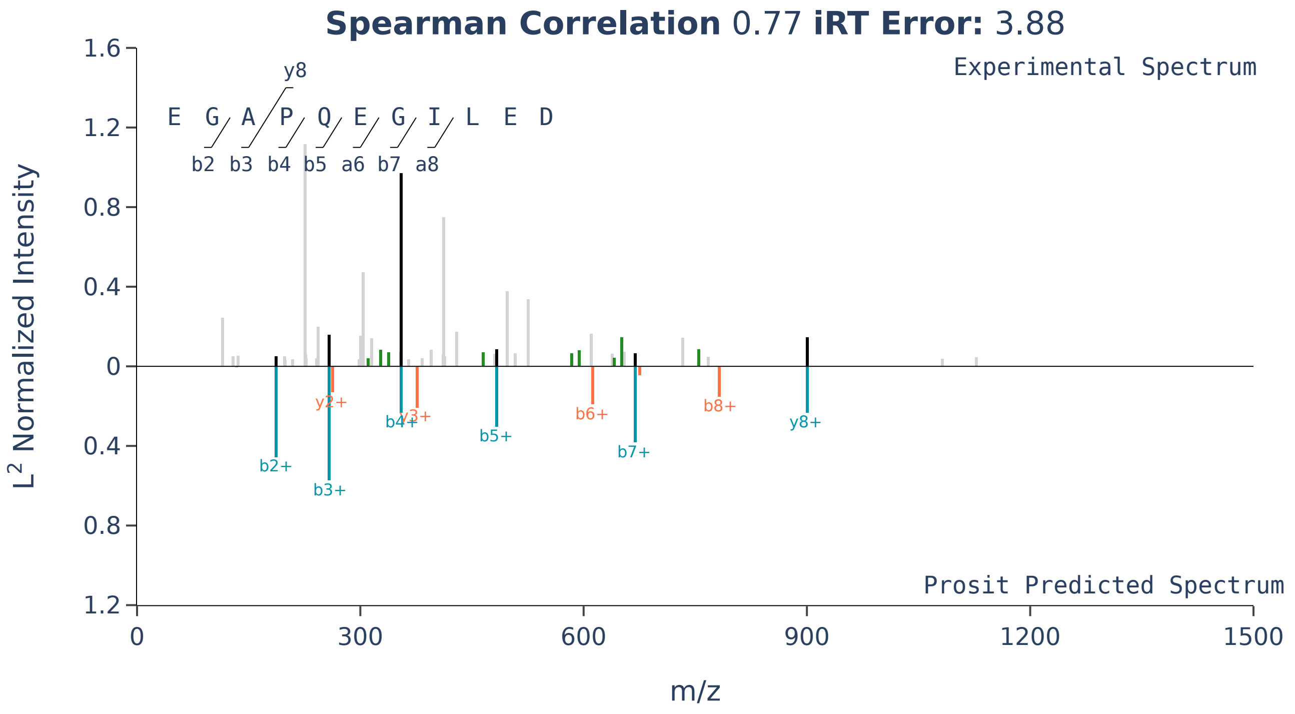

Source W_Soh_160821_170921_Goe_aSyn_A1_24h_R2 **Scan** 10619
Peptide DPNEAYEMPSE **Charge** 1 **Spectral Angle** 0.76
Spearman Correlation 0.91 **iRT Error:** 0.69

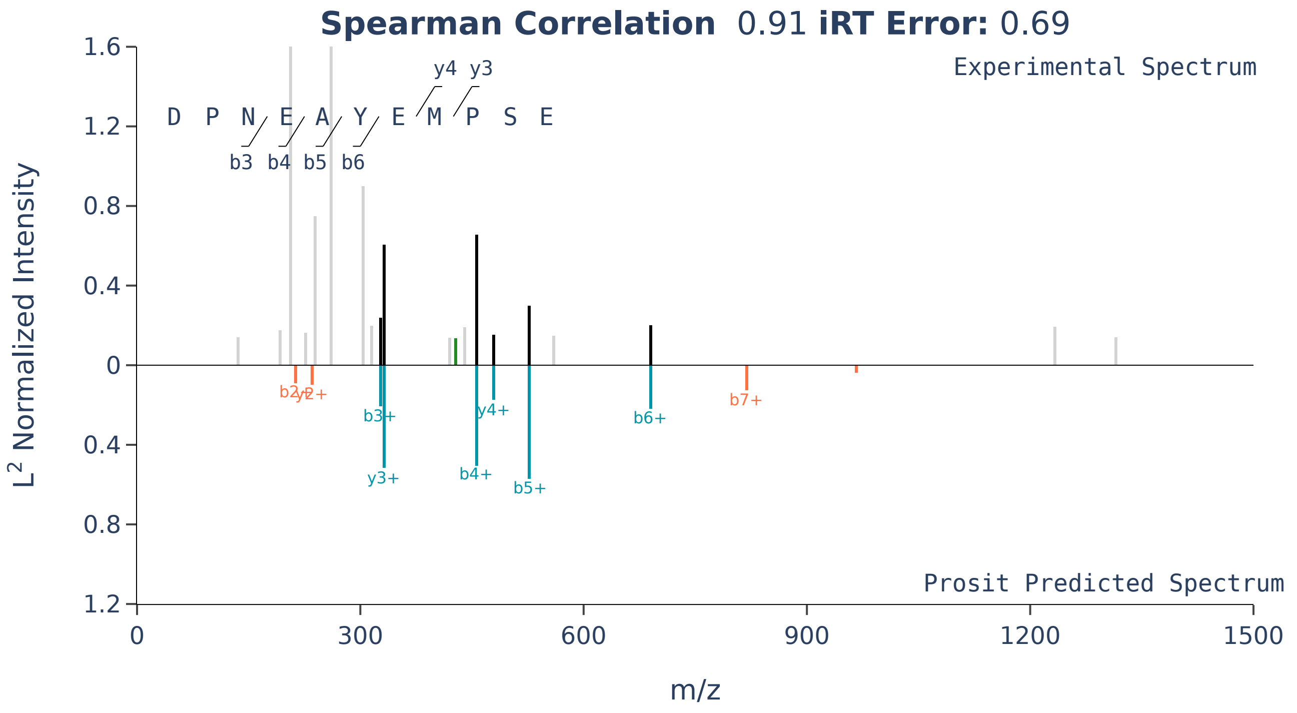

Source W_Soh_160821_170921_Goe_aSyn_A1_24h_R2 **Scan** 10619
Peptide PDNEAYEMPSE **Charge** 1 **Spectral Angle** 0.59
Spearman Correlation 0.58 **iRT Error:** 5.8

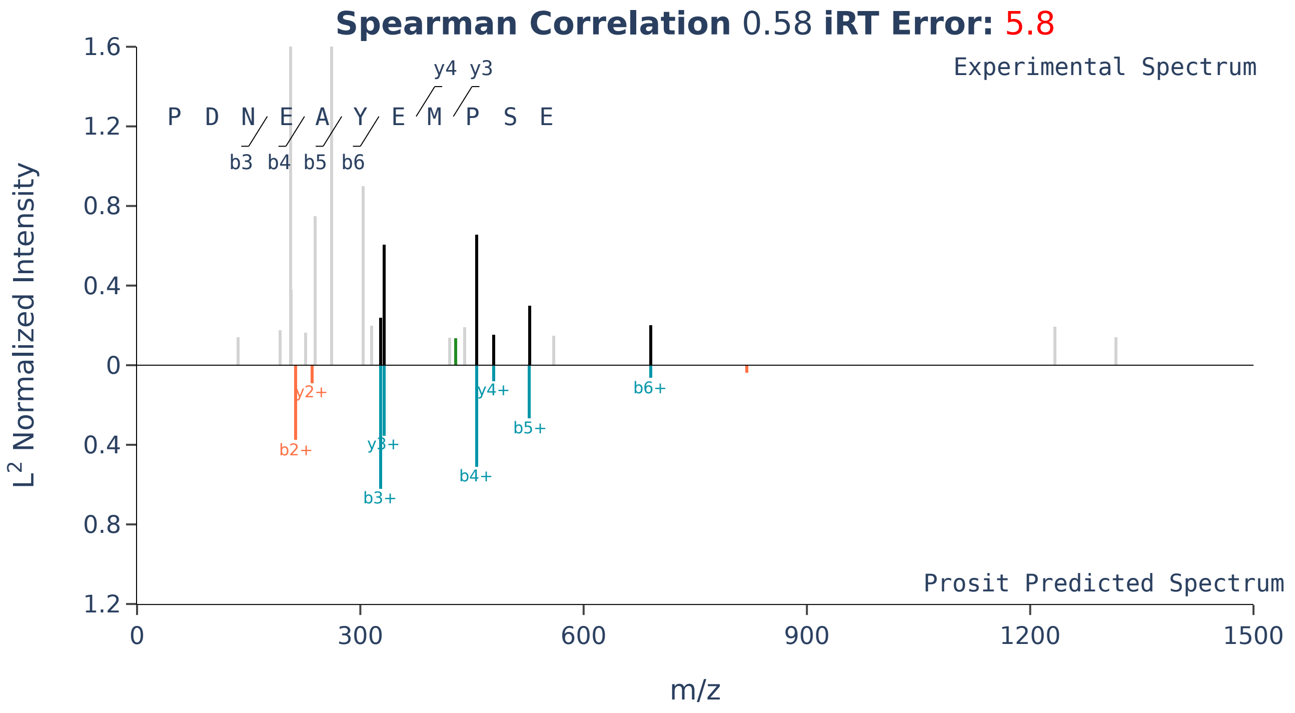

Source W_Soh_160821_170921_Goe_aSyn_A2_30'_R1 **Scan** 13589
Peptide PQEGILEQD **Charge** 1 **Spectral Angle** 0.9
Spearman Correlation 0.91 **iRT Error:** 0.92

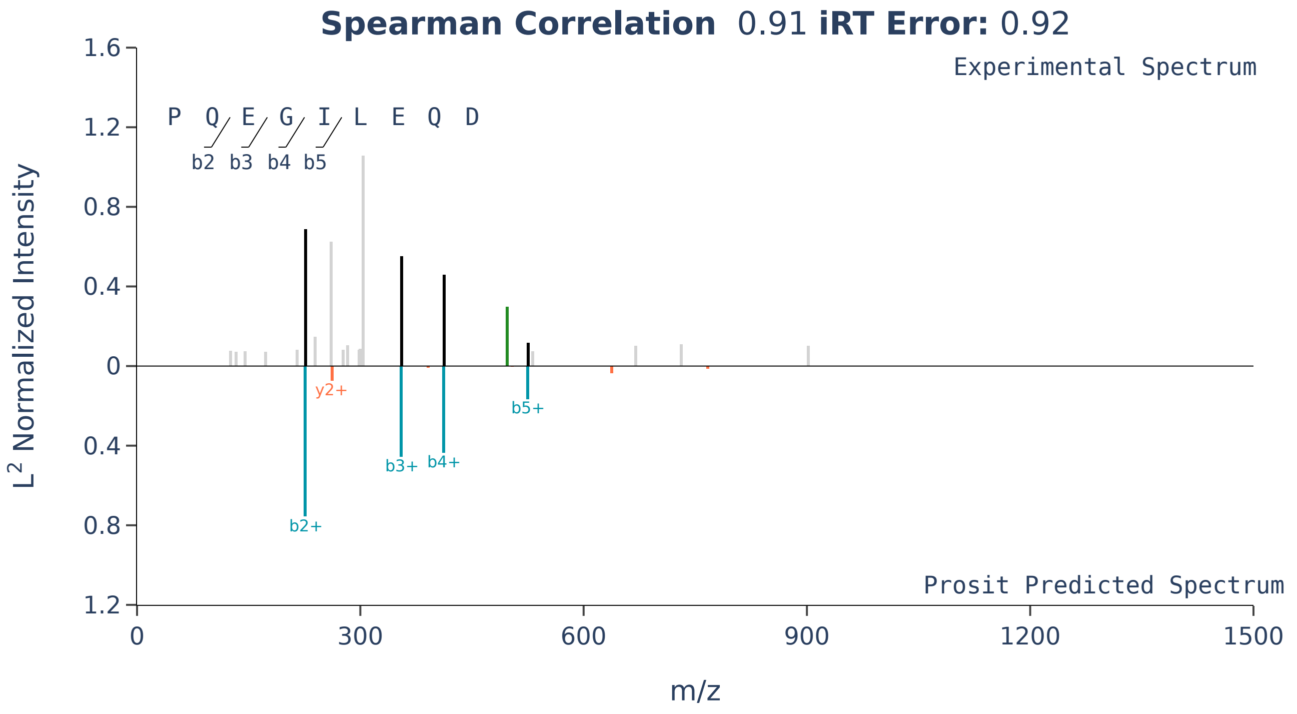

Source W_Soh_160821_170921_Goe_aSyn_A2_30'_R1 **Scan** 13589
Peptide GAPQEGILED **Charge** 1 **Spectral Angle** 0.36
Spearman Correlation 0.46 **iRT Error:** 8.01

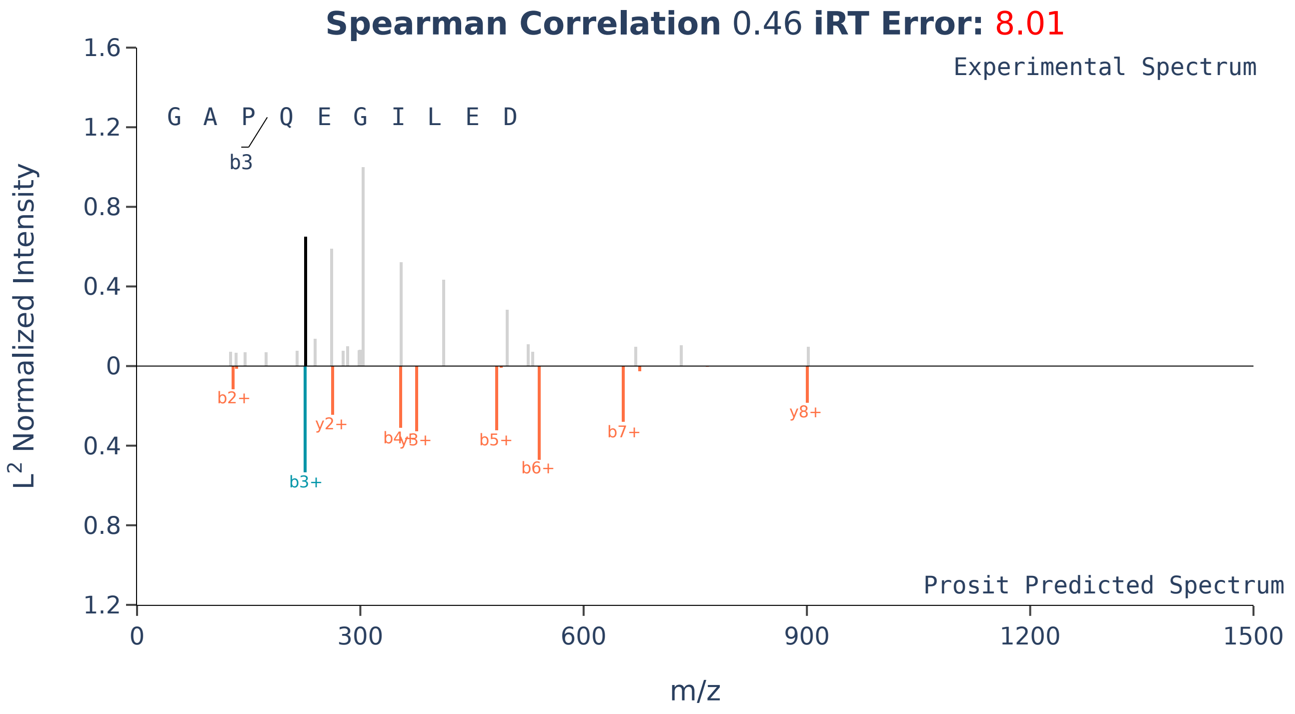

Source W_Soh_160821_170921_Goe_aSyn_A1_24h_R2 **Scan** 16566
Peptide PQEGILEDGA **Charge** 1 **Spectral Angle** 0.92
Spearman Correlation 0.89 **iRT Error:** 1.14

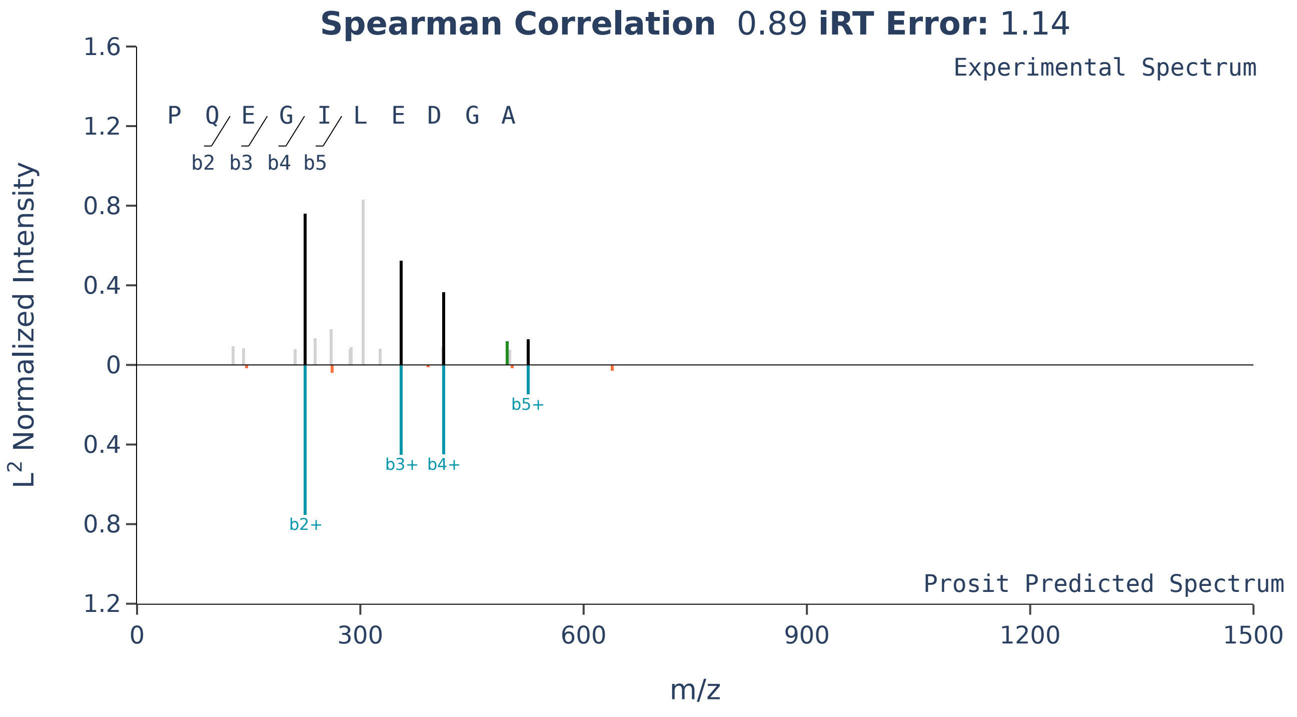

Source W_Soh_160821_170921_Goe_aSyn_A1_24h_R2 **Scan** 16566
Peptide GAPQEGILED **Charge** 1 **Spectral Angle** 0.36
Spearman Correlation 0.46 **iRT Error:** 3.14

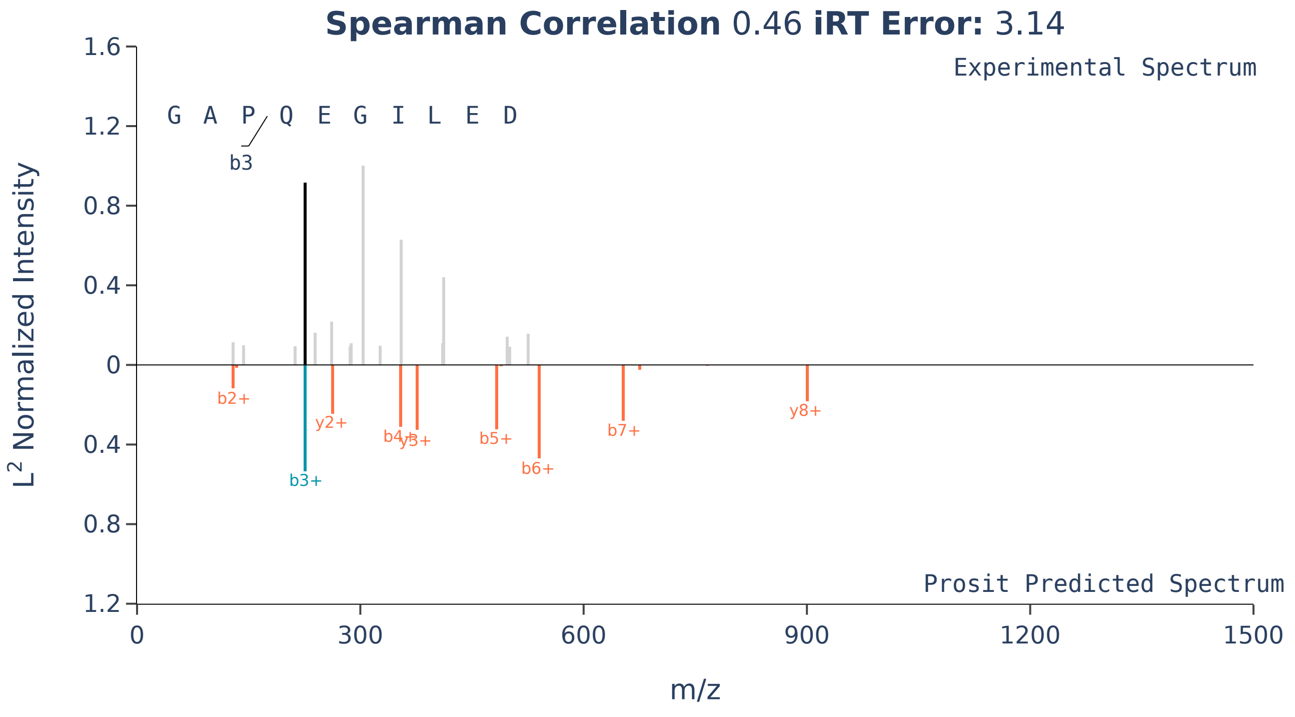

Source W_Soh_160821_170921_Goe_aSyn_A1_24h_R2 **Scan** 17951
Peptide PDNEAYEMVDPD **Charge** 1 **Spectral Angle** 0.85
Spearman Correlation 0.92 **iRT Error:** 1.16

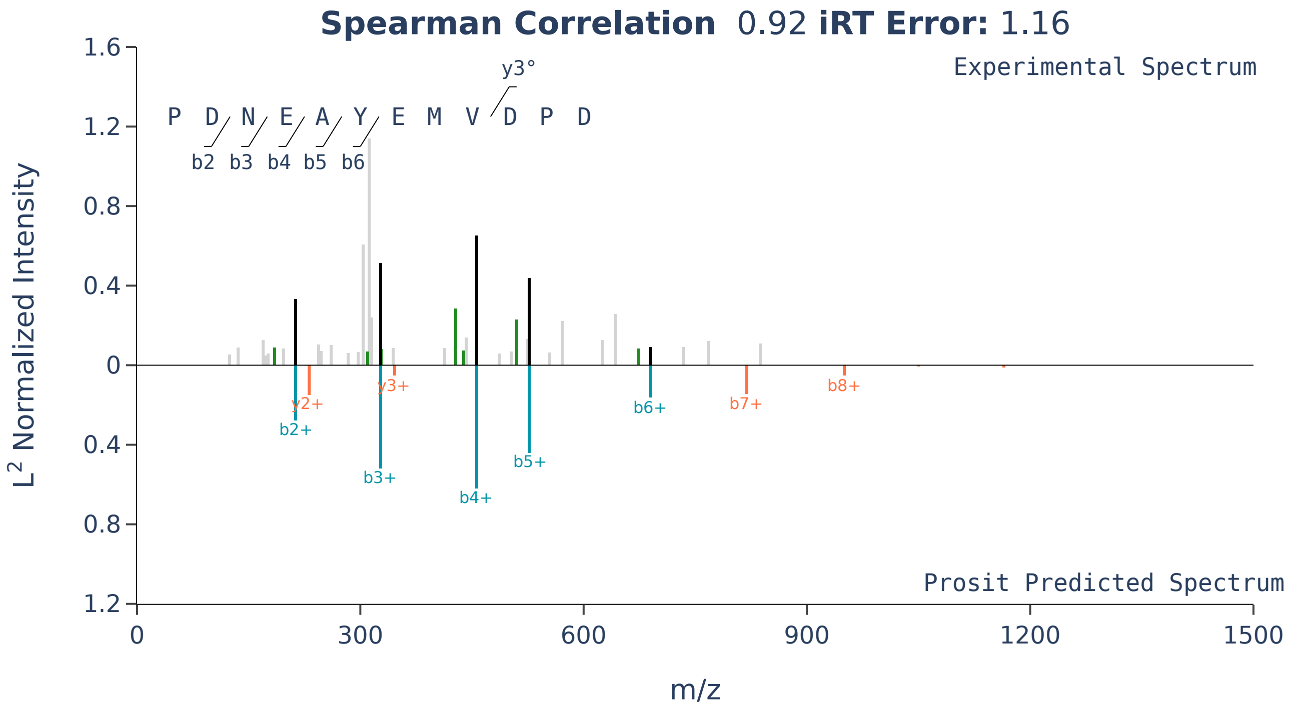

Source W_Soh_160821_170921_Goe_aSyn_A1_24h_R2 **Scan** 17951
Peptide EDMPVDPDNEAY **Charge** 1 **Spectral Angle** 0.0
Spearman Correlation 0.0 **iRT Error:** 0.61

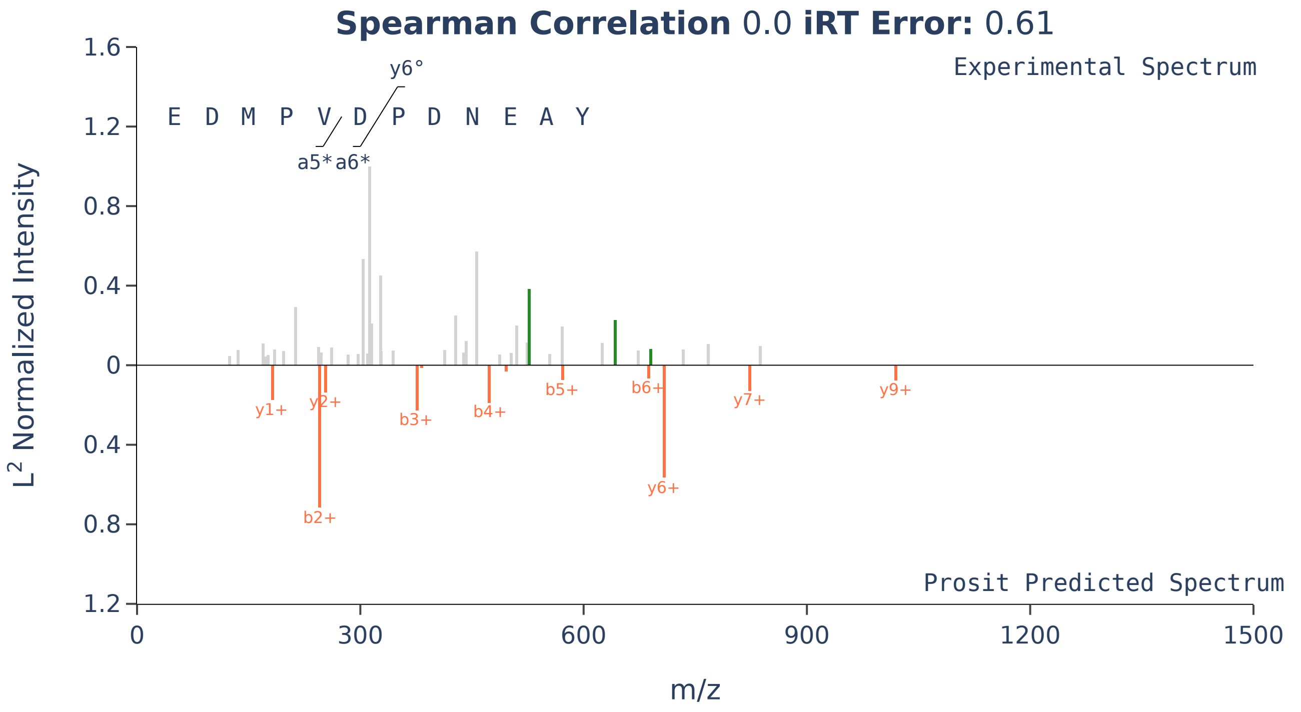

Source W_Soh_160821_170921_Goe_aSyn_A1_24h_R1 **Scan** 13983
Peptide MDVFMKKGLS **Charge** 2 **Spectral Angle** 0.8
Spearman Correlation 0.85 **iRT Error:** 1.19

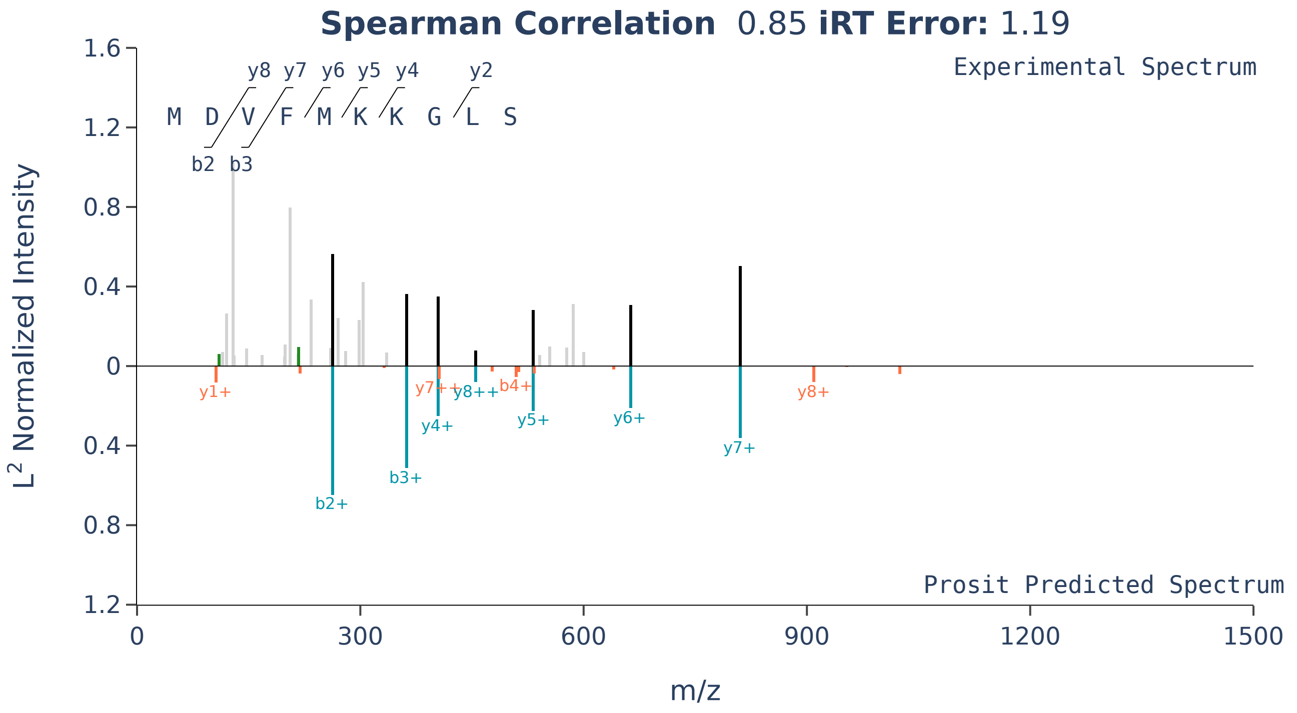

Source W_Soh_160821_170921_Goe_aSyn_A1_24h_R1 **Scan** 13983
Peptide MDVFMKGLSK **Charge** 2 **Spectral Angle** 0.27
Spearman Correlation 0.49 **iRT Error:** 3.84

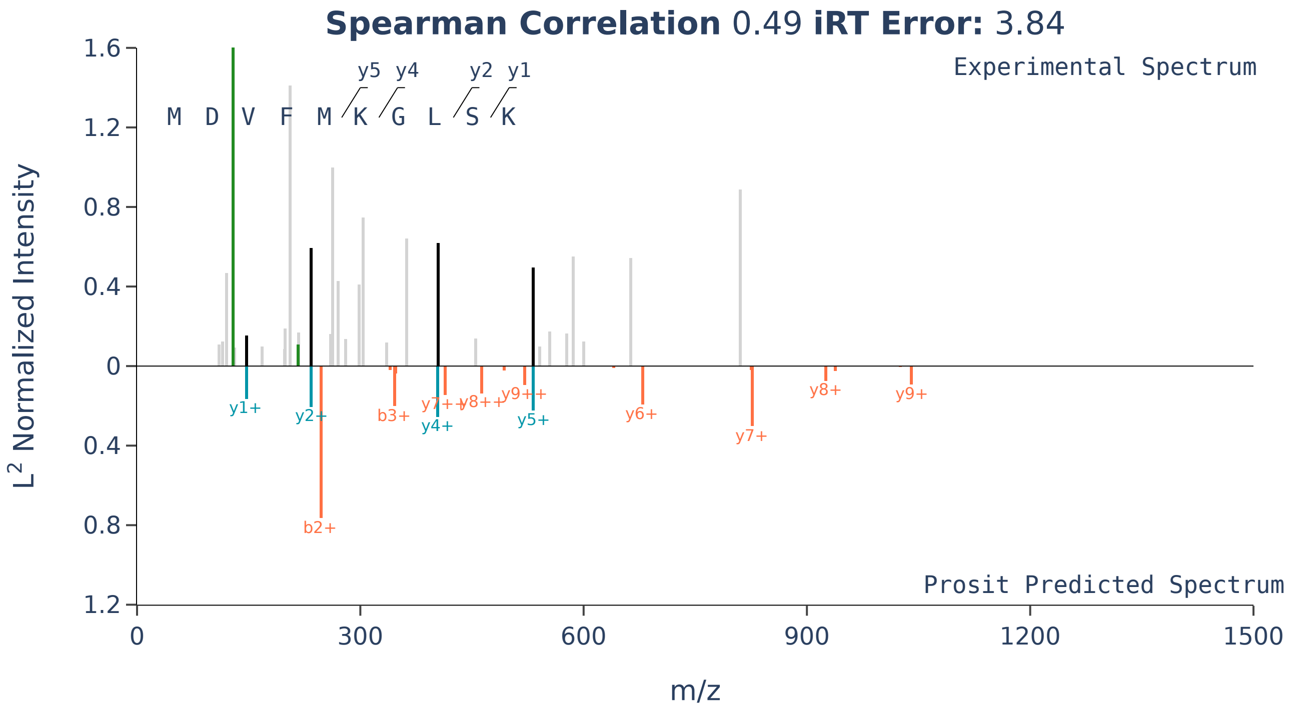

Source W_Soh_160821_170921_Goe_aSyn_A1_6h_R2 **Scan** 17655
Peptide PDNEAYEDMPVD **Charge** 1 **Spectral Angle** 0.78
Spearman Correlation 0.86 **iRT Error:** 1.41

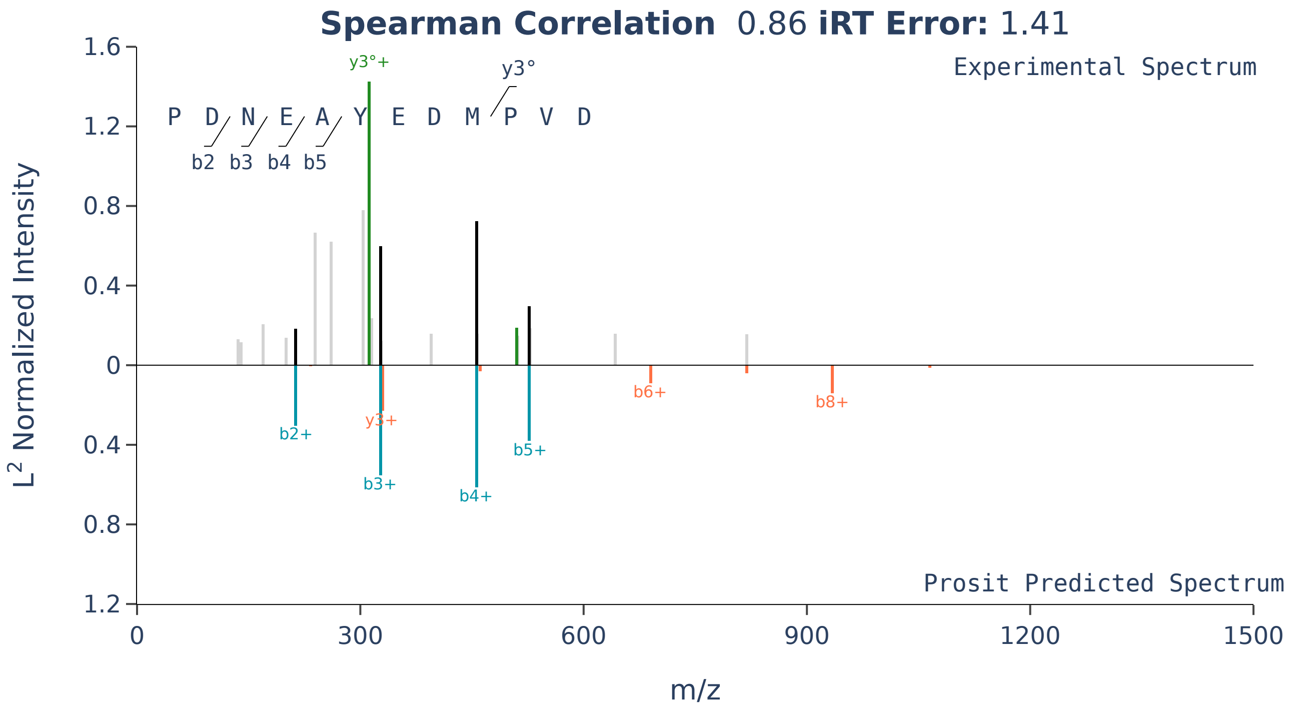

Source W_Soh_160821_170921_Goe_aSyn_A1_6h_R2 **Scan** 17655
Peptide EDMPVDPDNEAY **Charge** 1 **Spectral Angle** 0.0
Spearman Correlation 0.0 **iRT Error:** 2.95

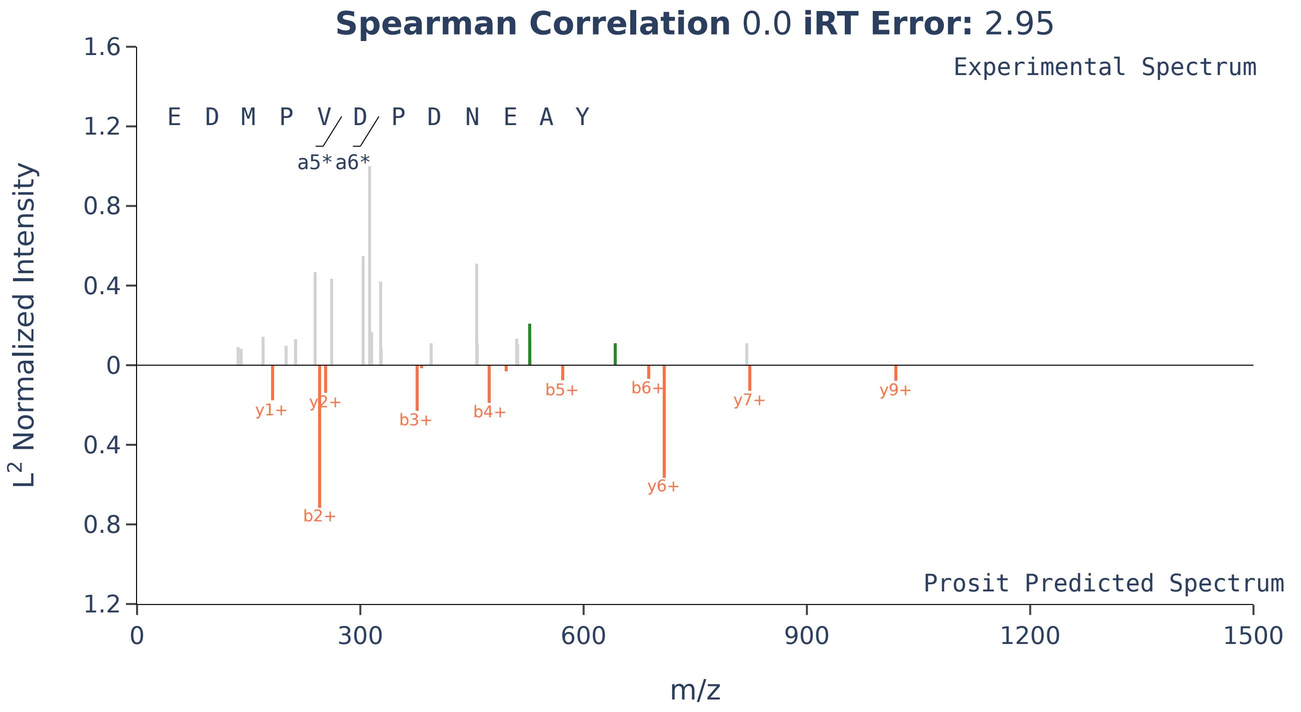

Source W_Soh_160821_170921_Goe_aSyn_A1_3h_R2 **Scan** 6430
Peptide GQLGKNEEGAPQE **Charge** 2 **Spectral Angle** 0.95
Spearman Correlation 0.91 **iRT Error:** 1.43

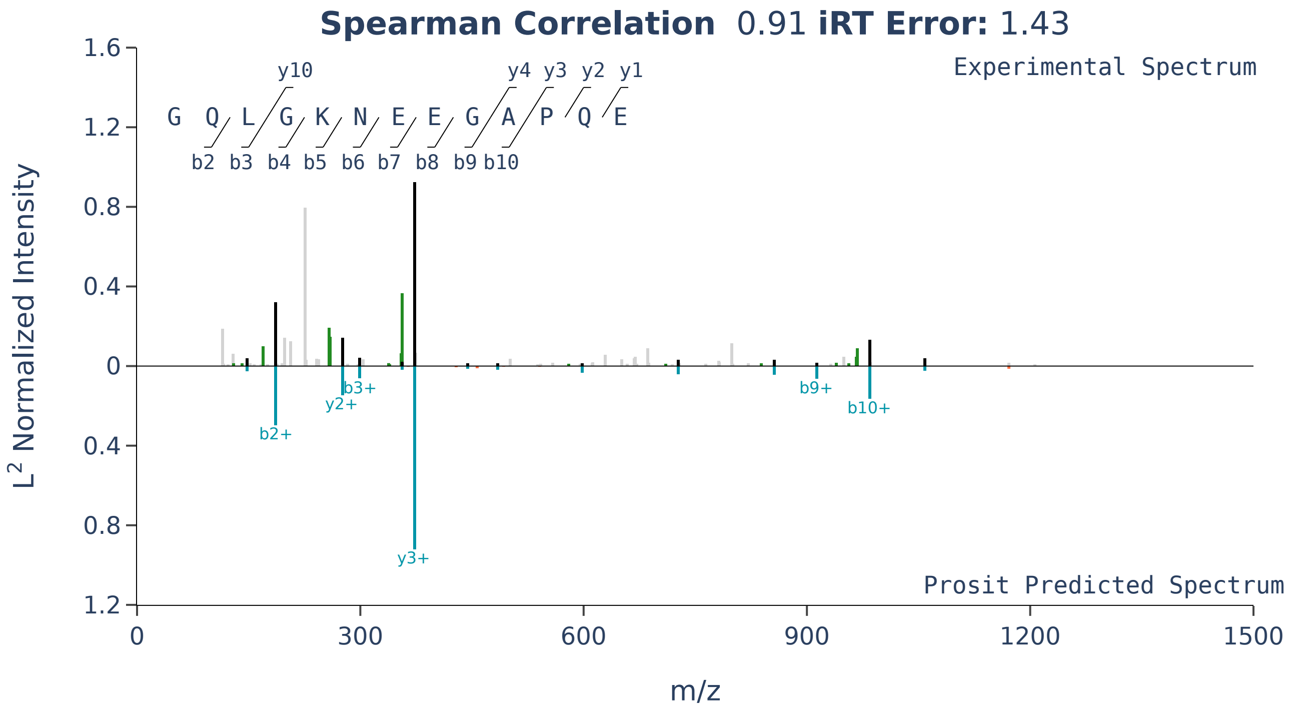

Source W_Soh_160821_170921_Goe_aSyn_A1_3h_R2 **Scan** 6430
Peptide QLGKNEEGAPQEG **Charge** 2 **Spectral Angle** 0.03
Spearman Correlation 0.06 **iRT Error:** 0.87

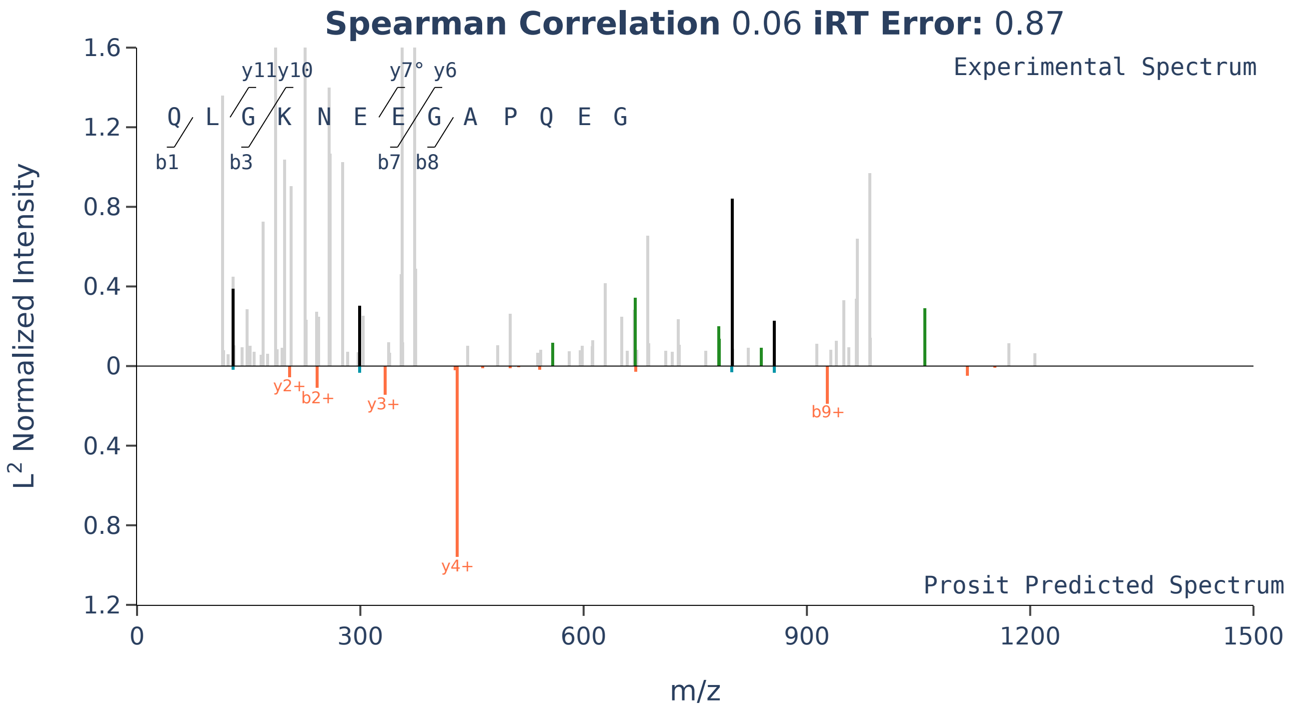

Source W_Soh_160821_170921_Goe_aSyn_A1_24h_R1 **Scan** 16985
Peptide PQEGILEEGA **Charge** 1 **Spectral Angle** 0.96
Spearman Correlation 0.92 **iRT Error:** 1.51

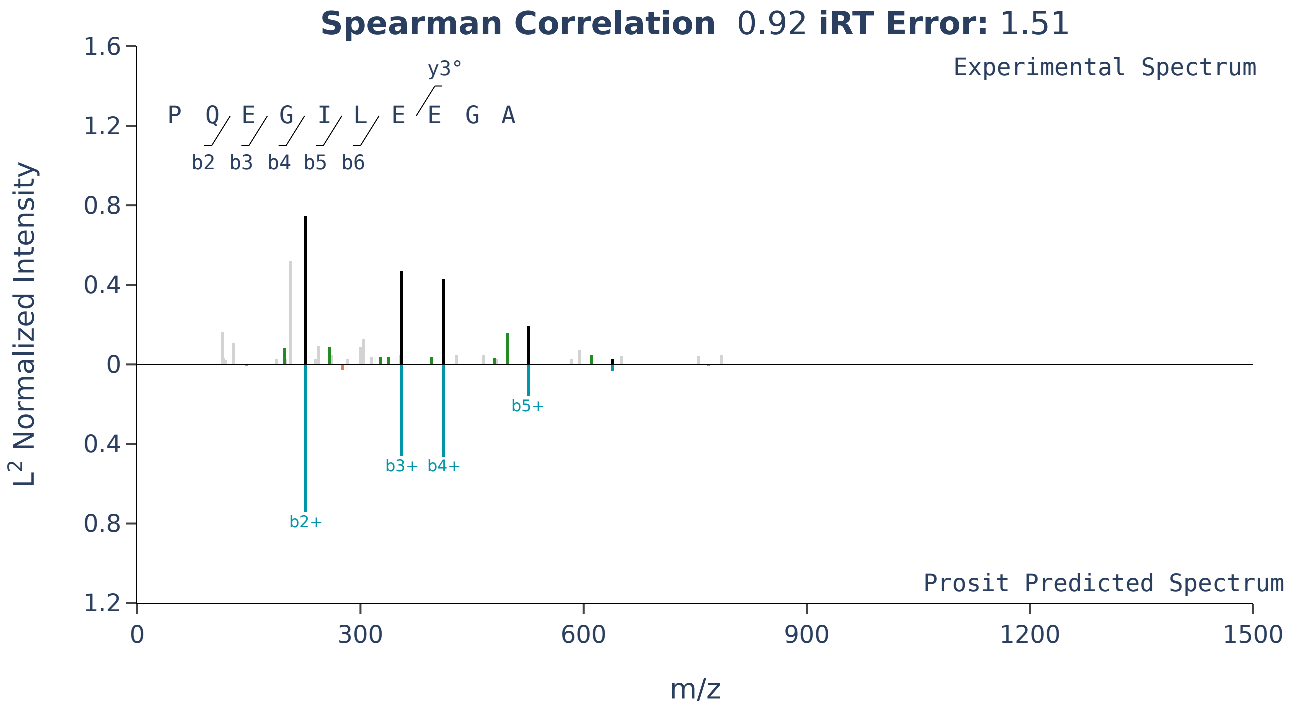

Source W_Soh_160821_170921_Goe_aSyn_A1_24h_R1 **Scan** 16985
Peptide EEGAPQEGIL **Charge** 1 **Spectral Angle** 0.0
Spearman Correlation 0.0 **iRT Error:** 15.84

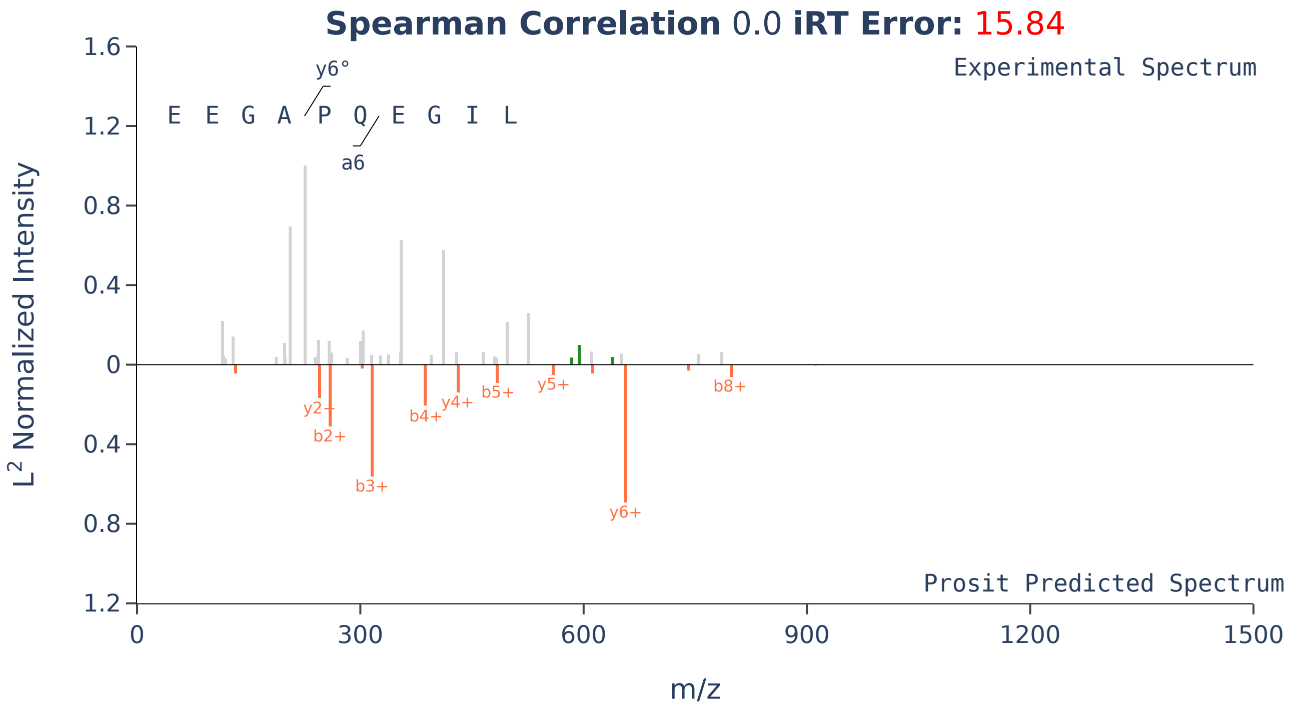

Source W_Soh_160821_170921_Goe_aSyn_A1_3h_R2 **Scan** 15357
Peptide DMVFMKGLSKAKEGVVA **Charge** 3 **Spectral Angle** 0.87
Spearman Correlation 0.87 **iRT Error:** 3.75

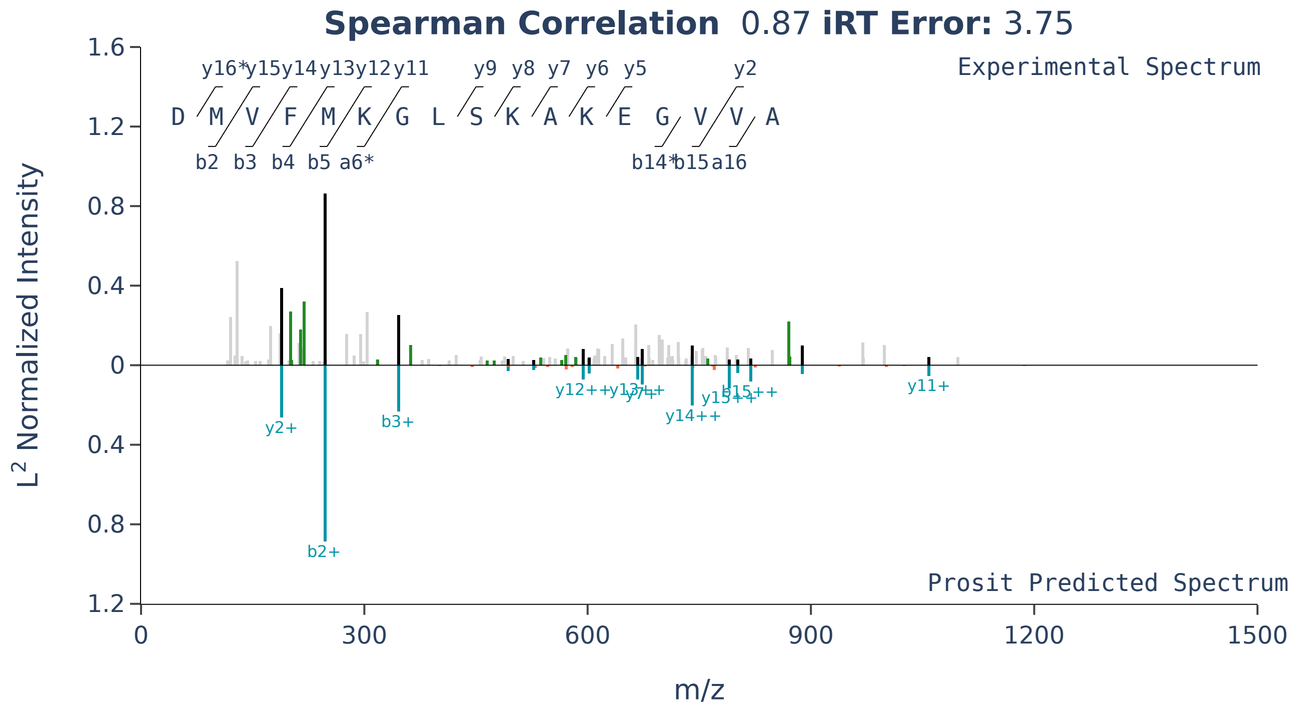

Source W_Soh_160821_170921_Goe_aSyn_A1_3h_R2 **Scan** 15357
Peptide MDVFMKGLSKAKEGVVA **Charge** 3 **Spectral Angle** 0.32
Spearman Correlation 0.51 **iRT Error:** 10.45

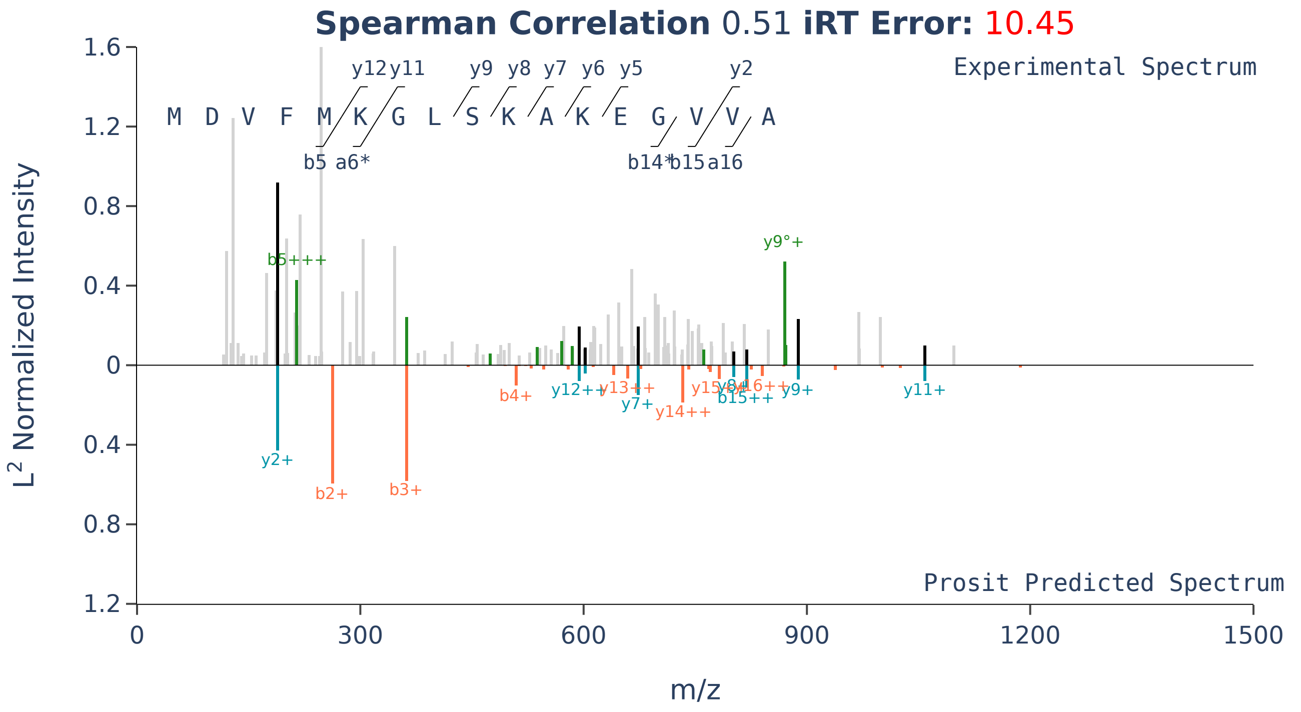

Source W_Soh_160821_170921_Goe_aSyn_A1_6h_R1 **Scan** 16528
Peptide PQEGILENEE **Charge** 1 **Spectral Angle** 0.94
Spearman Correlation 0.92 **iRT Error:** 3.97

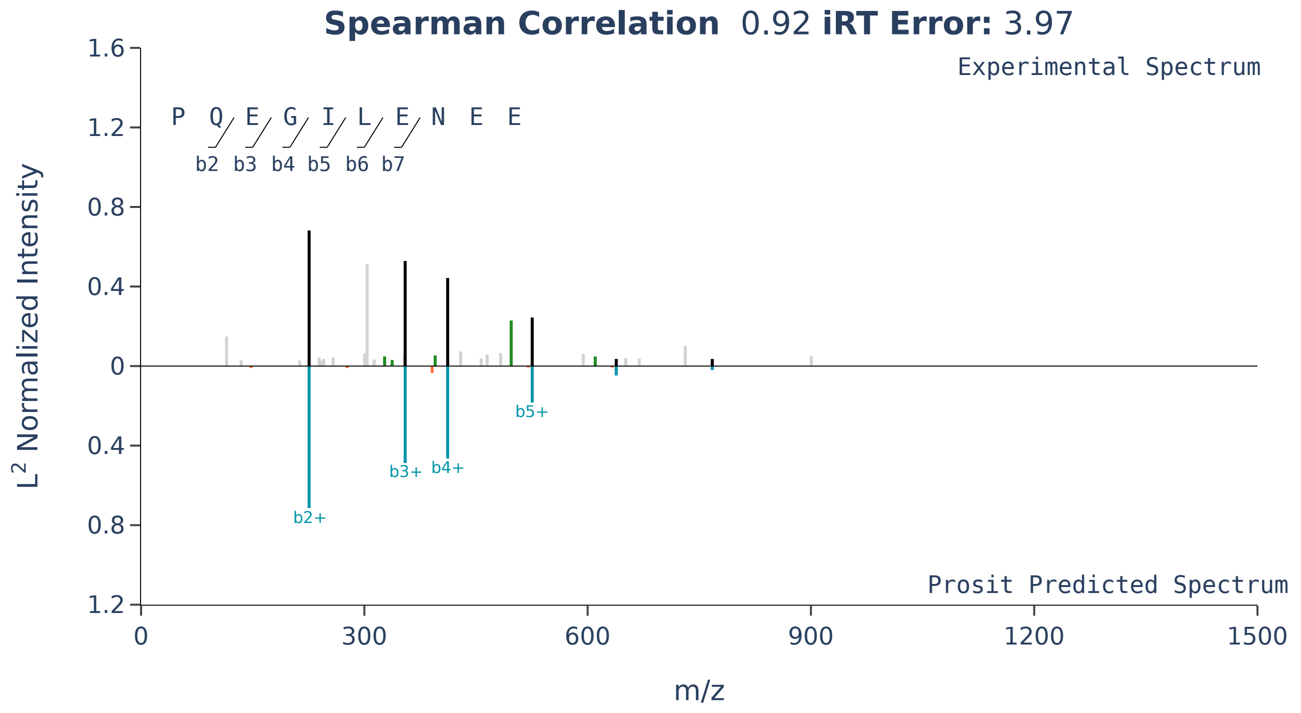

Source W_Soh_160821_170921_Goe_aSyn_A1_6h_R1 **Scan** 16528
Peptide EGAPQEGILED **Charge** 1 **Spectral Angle** 0.23
Spearman Correlation 0.58 **iRT Error:** 7.97

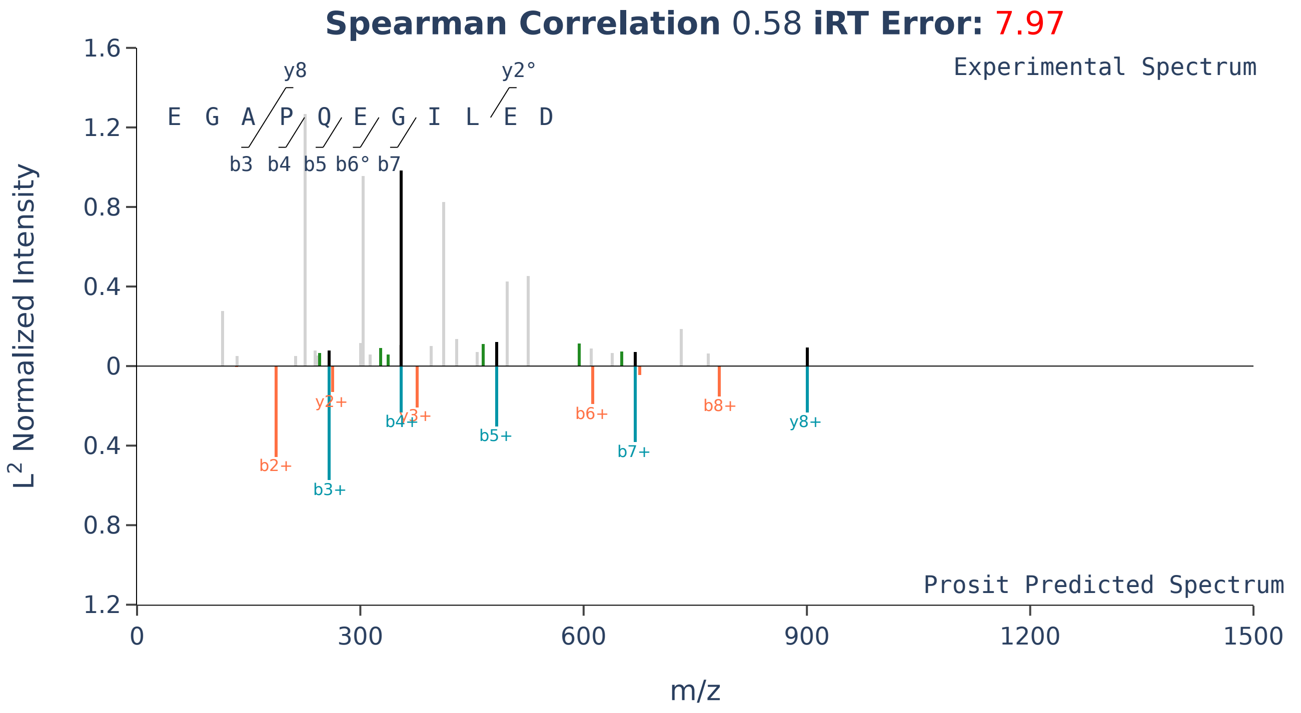

Source W_Soh_160821_170921_Goe_aSyn_A1_3h_R2 **Scan** 19625
Peptide AMDVFMKGLSKAKEGVVA **Charge** 3 **Spectral Angle** 0.83
Spearman Correlation 0.9 **iRT Error:** 6.7

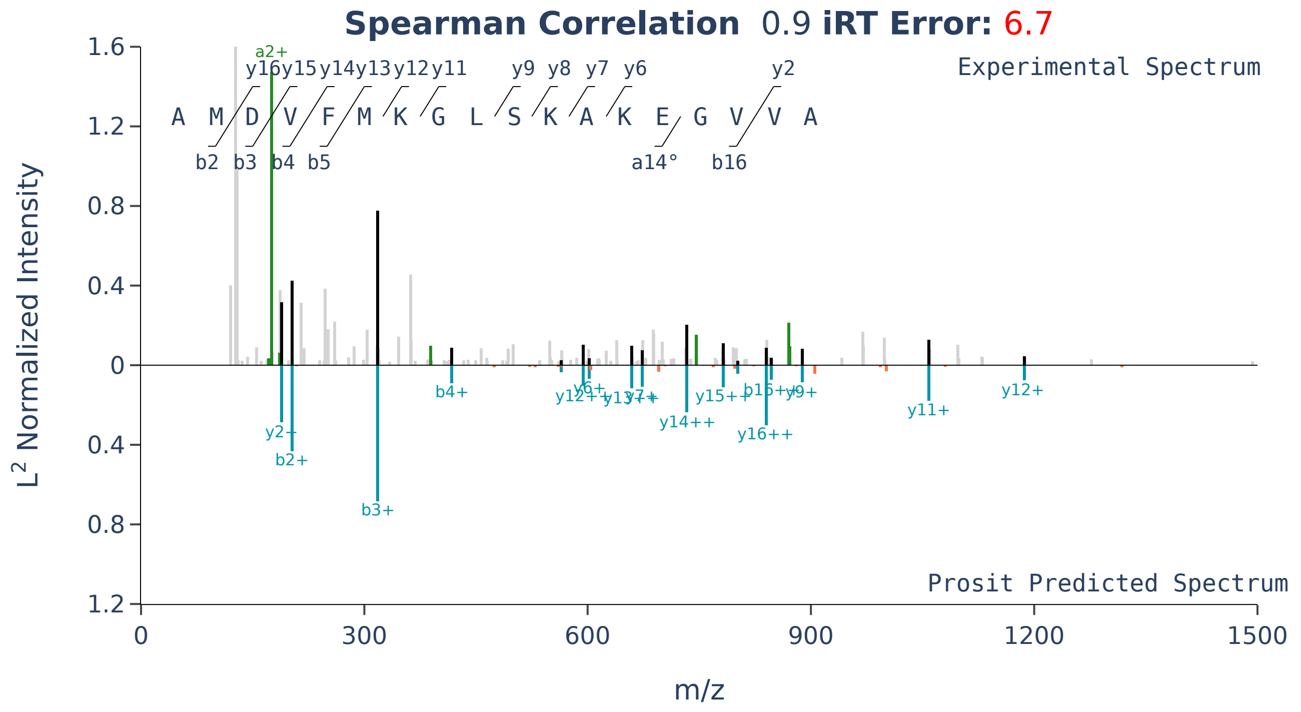

Source W_Soh_160821_170921_Goe_aSyn_A1_3h_R2 **Scan** 19625
Peptide MDVFMKGLSKAKEGVVAA **Charge** 3 **Spectral Angle** 0.63
Spearman Correlation 0.51 **iRT Error:** 0.76

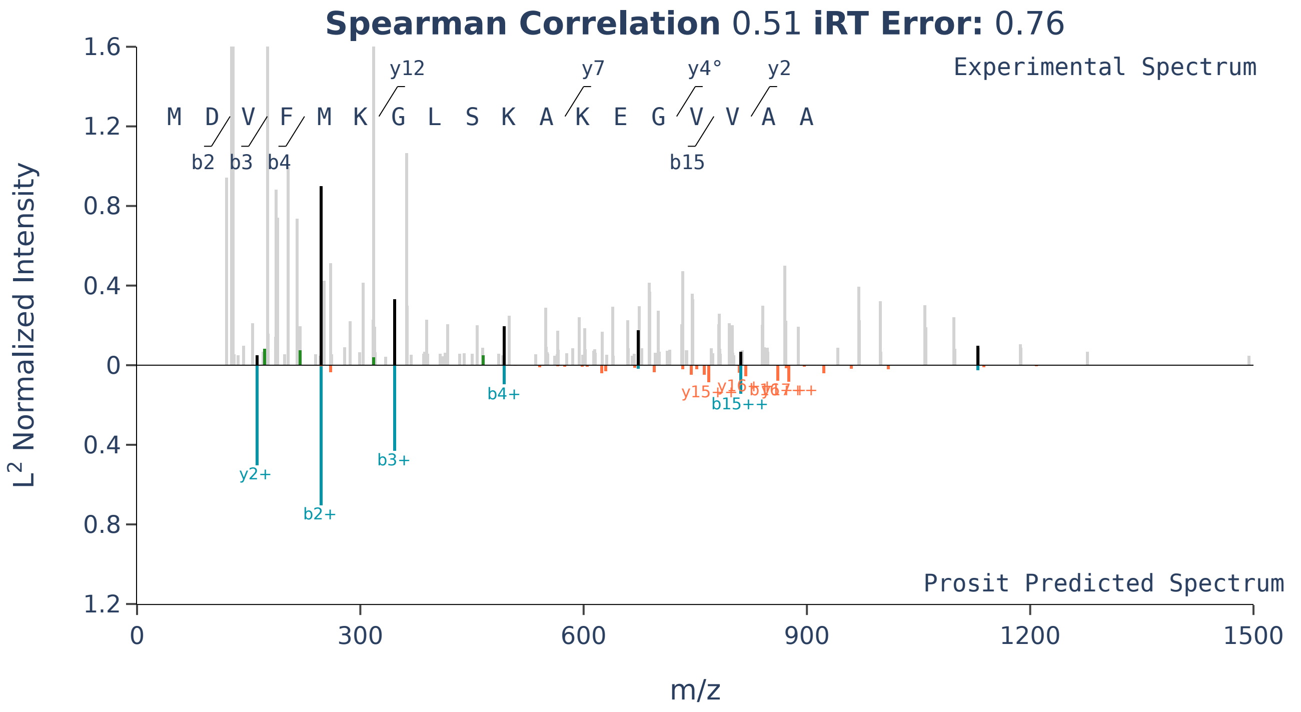

Source W_Soh_160821_170921_Goe_aSyn_A2_30'_R2 **Scan** 10640
Peptide AGKTKEGVLYVGA **Charge** 3 **Spectral Angle** 0.85
Spearman Correlation 0.89 **iRT Error:** 8.37

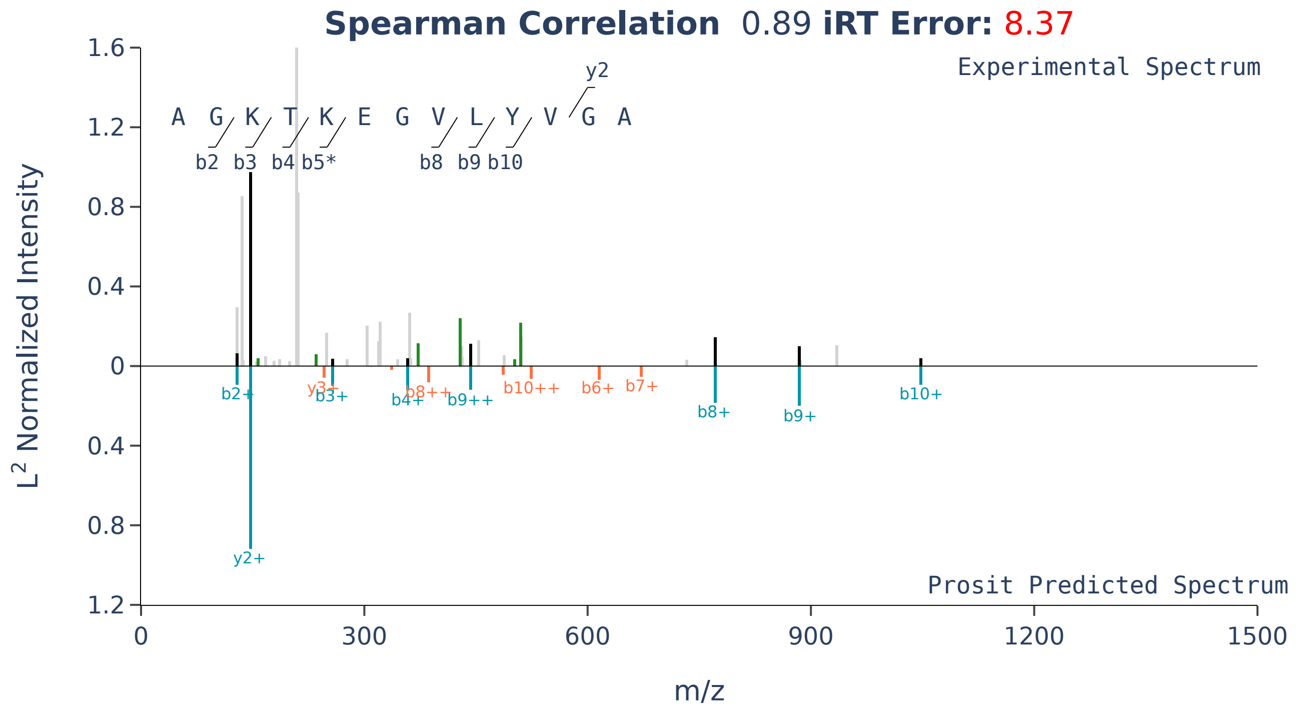

Source W_Soh_160821_170921_Goe_aSyn_A2_30'_R2 **Scan** 10640
Peptide AAGKTKEGVLYVG **Charge** 3 **Spectral Angle** 0.0
Spearman Correlation 0.0 **iRT Error:** 6.8

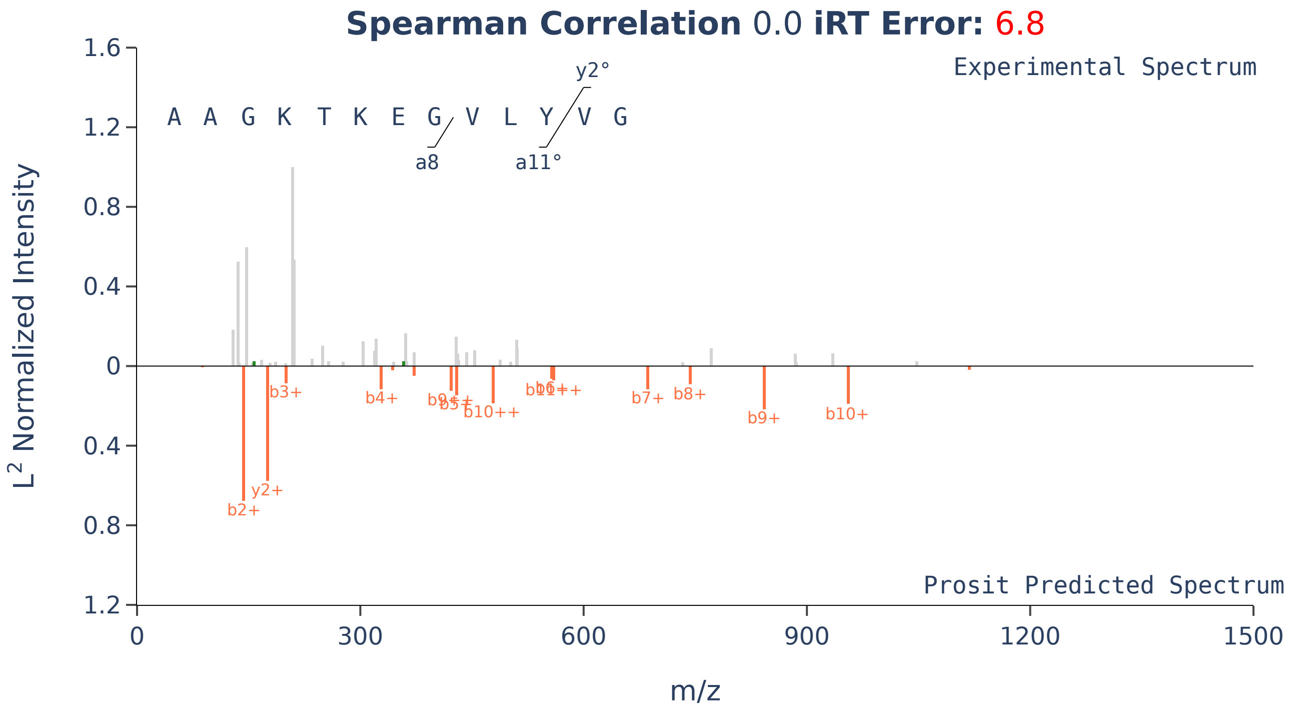

Source W_Soh_160821_170921_Goe_aSyn_A1_6h_R1 **Scan** 21197
Peptide NEAYEMPMPSEEGYQDYEPEA **Charge** 2 **Spectral Angle** 0.81
Spearman Correlation 0.91 **iRT Error:** 8.54

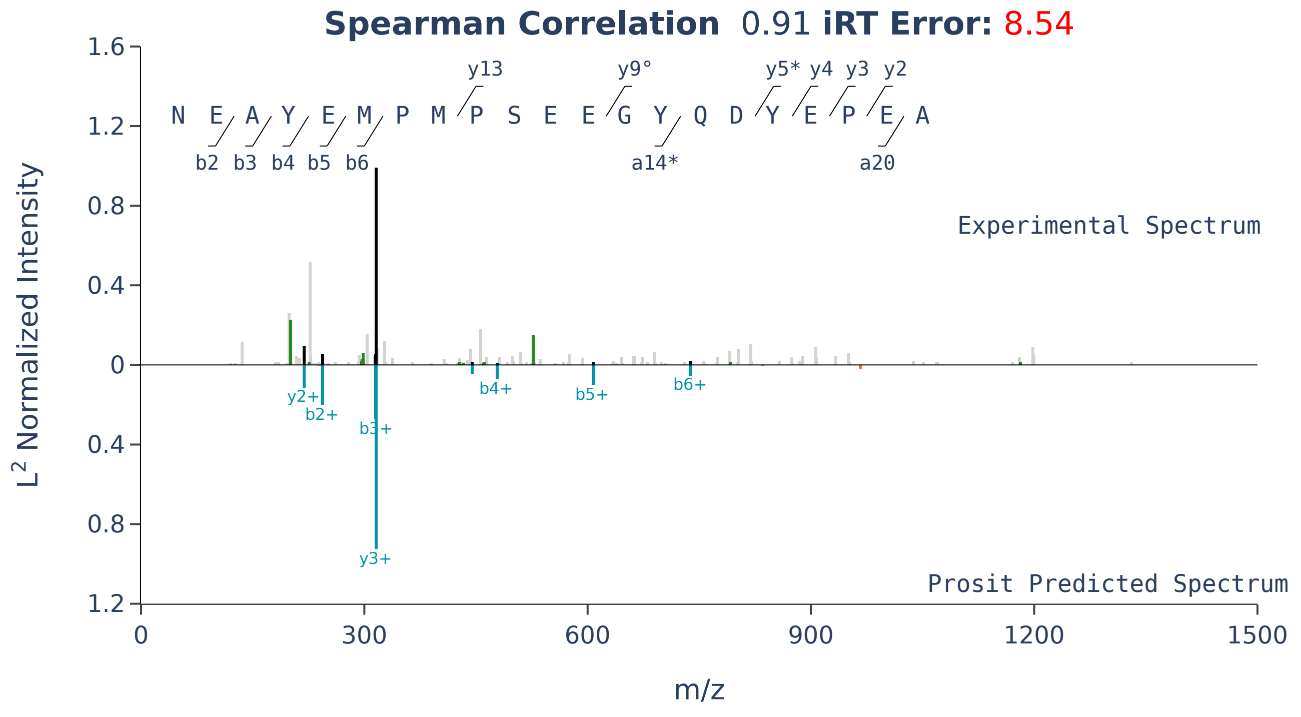

Source W_Soh_160821_170921_Goe_aSyn_A1_6h_R1 **Scan** 21197
Peptide MPVDPDNEAYEMPSEEGYQDY **Charge** 2 **Spectral Angle** 0.05
Spearman Correlation -0.11 **iRT Error:** 7.48

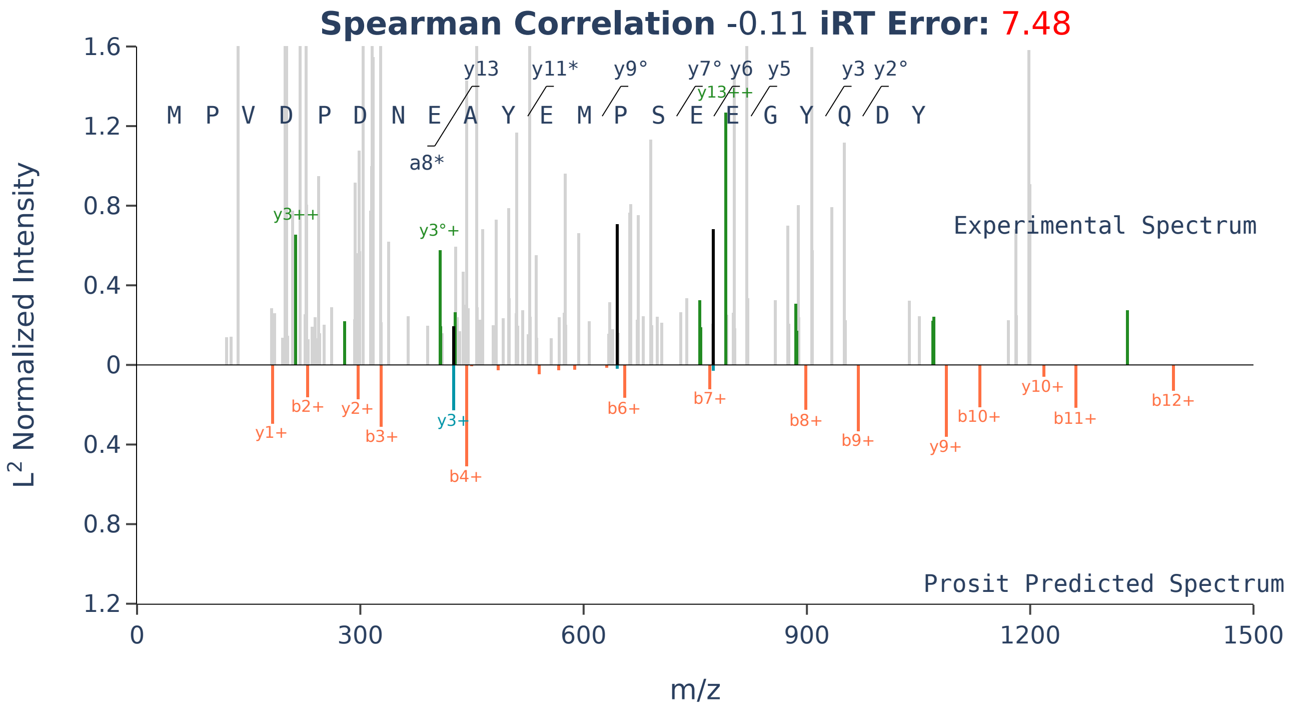

Source HRoetschke_291222_040123_Fu_CaM_b1R2_2h **Scan** 7533
Peptide ADQLLTEEQ **Charge** 1 **Spectral Angle** 0.87
Spearman Correlation 0.89 **iRT Error:** 0.14

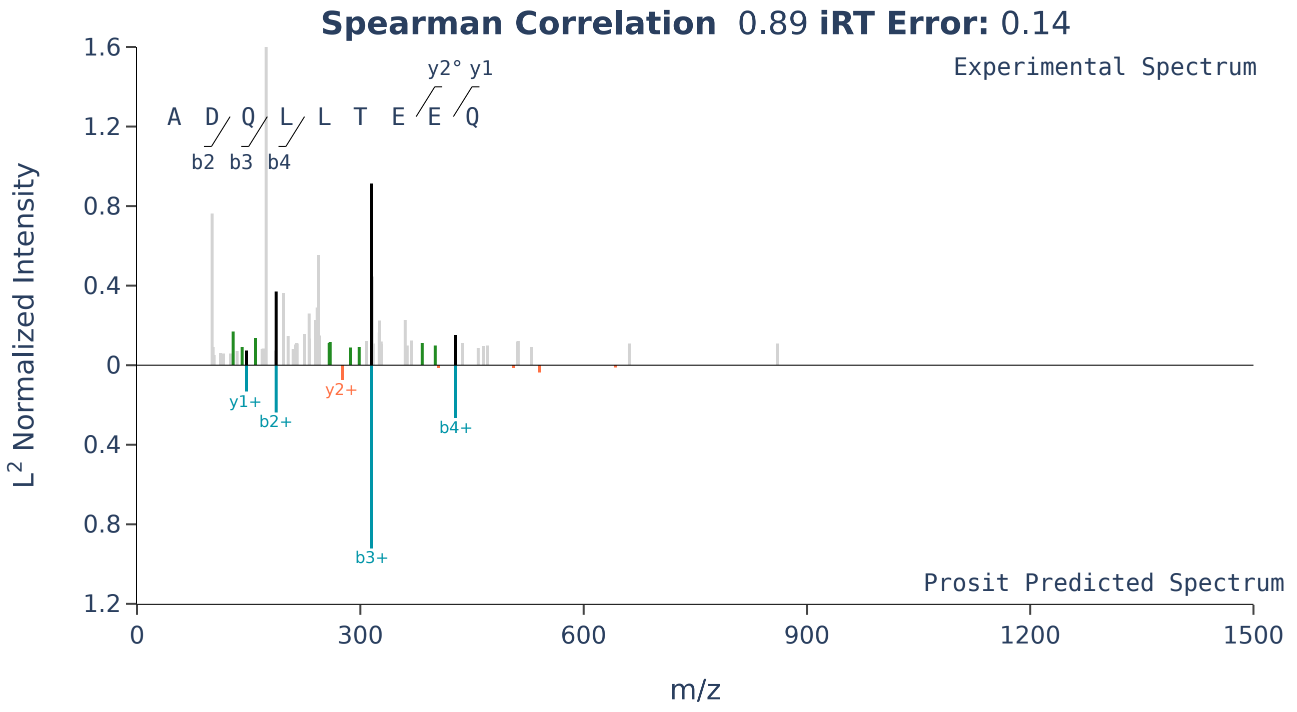

Source HRoetschke_291222_040123_Fu_CaM_b1R2_2h **Scan** 7533
Peptide DQLTEEQIA **Charge** 1 **Spectral Angle** 0.79
Spearman Correlation 0.65 **iRT Error:** 4.9

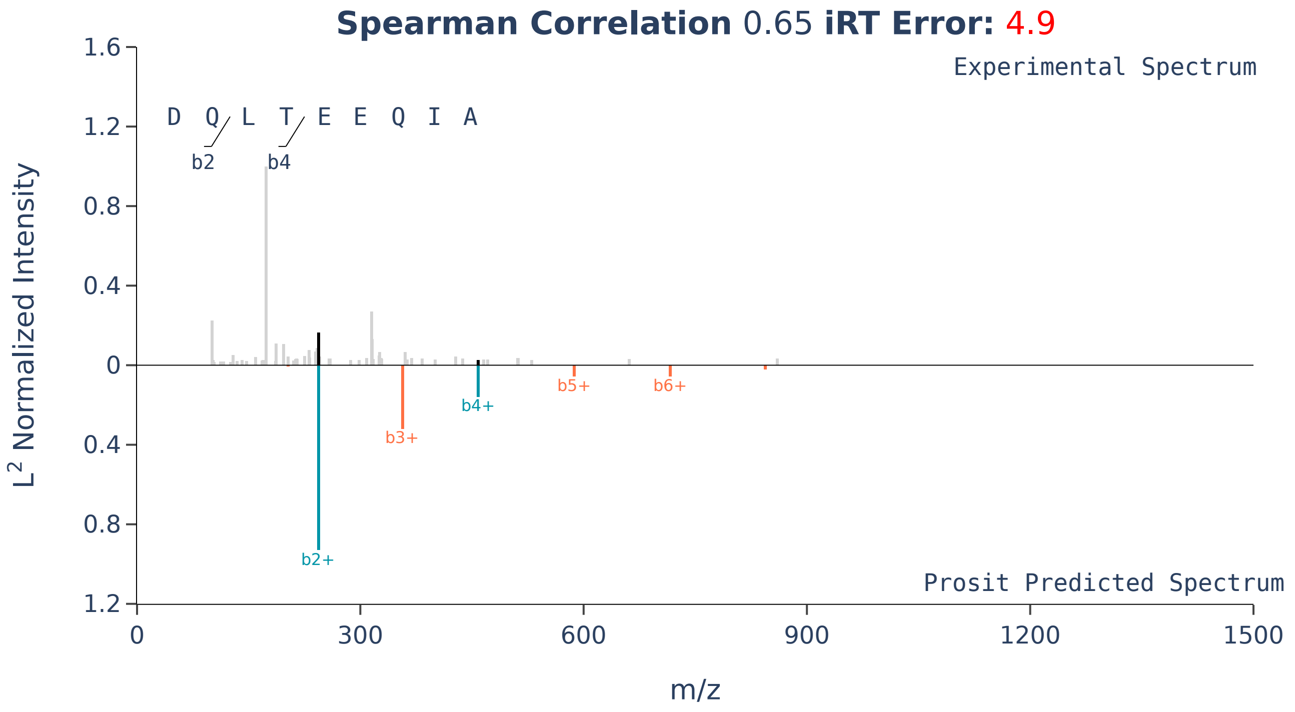

Source HRoetschke_291222_040123_Fu_CaM_b2R1_1h **Scan** 2364
Peptide EKLGTVMR **Charge** 2 **Spectral Angle** 0.76
Spearman Correlation 0.89 **iRT Error:** 0.61

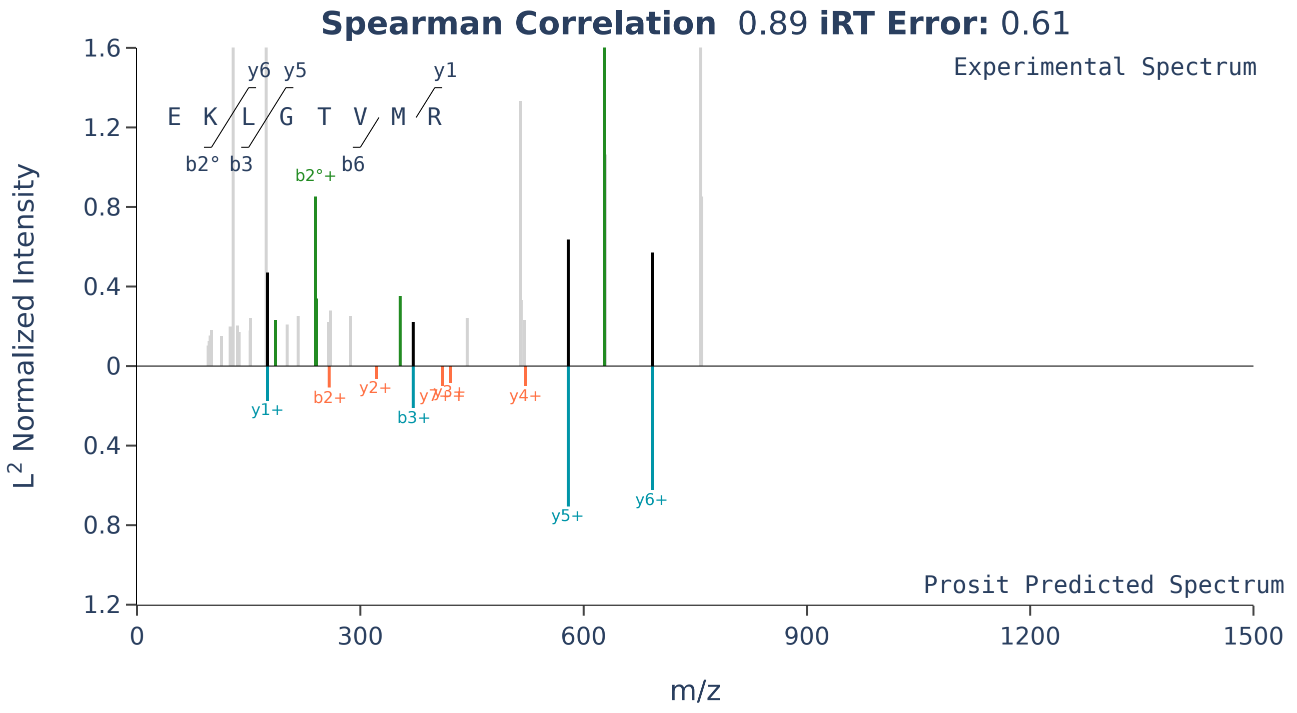

Source HRoetschke_291222_040123_Fu_CaM_b2R1_1h **Scan** 2364
Peptide LGTVMRSLG **Charge** 2 **Spectral Angle** 0.41
Spearman Correlation 0.45 **iRT Error:** 33.54

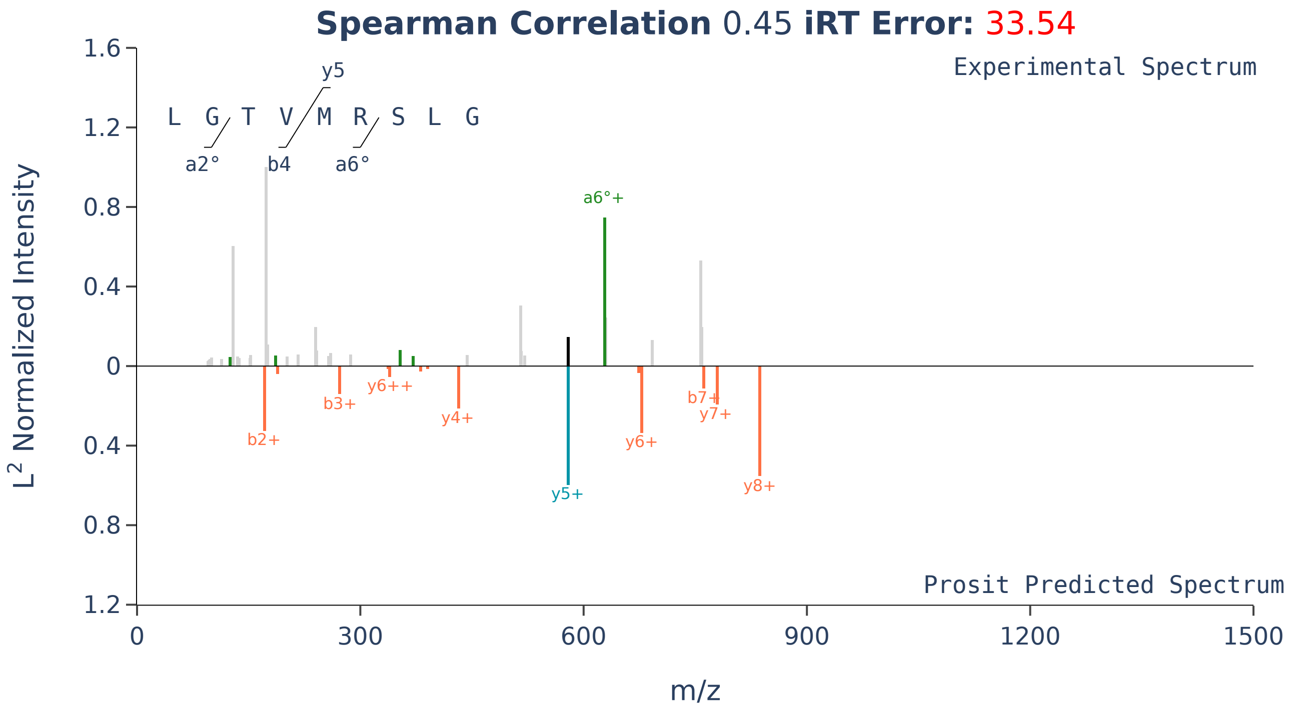

Source HRoetschke_291222_040123_Fu_CaM_b1R1_24h **Scan** 2719
Peptide ARSLGQNPTE **Charge** 2 **Spectral Angle** 0.89
Spearman Correlation 0.85 **iRT Error:** 0.94

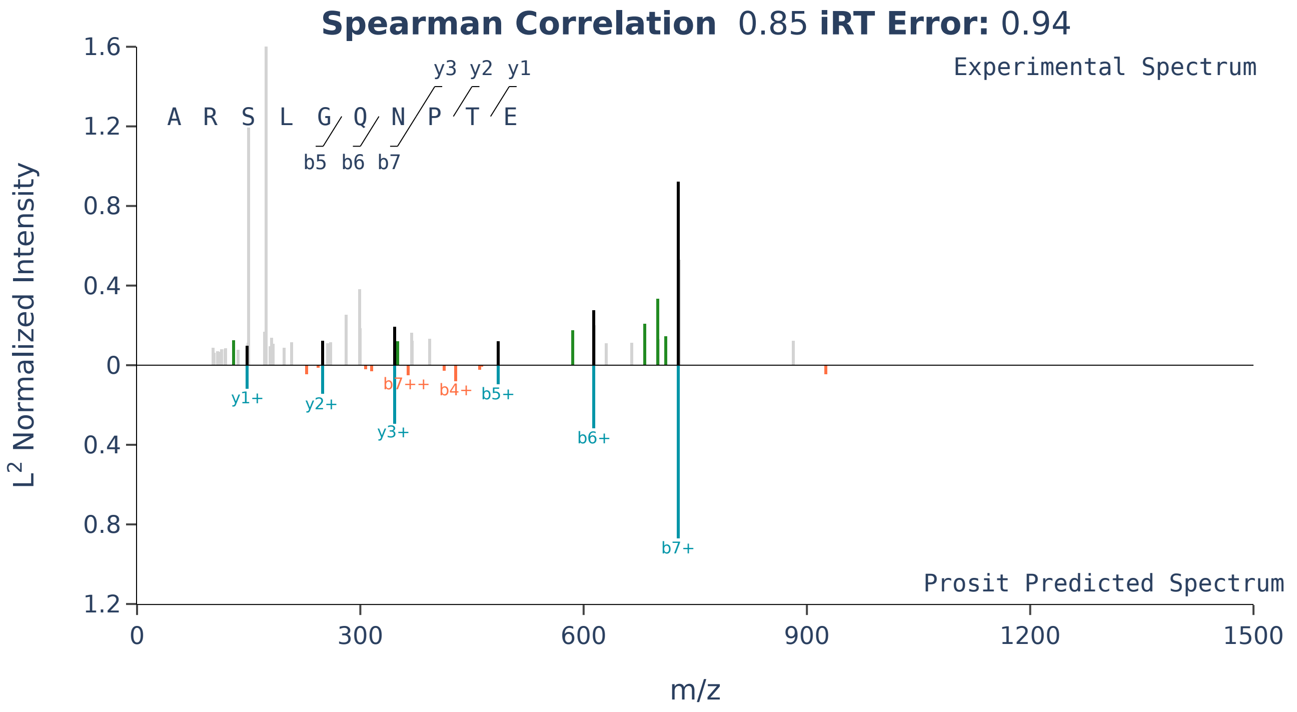

Source HRoetschke_291222_040123_Fu_CaM_b1R1_24h **Scan** 2719
Peptide RSLGQNPTEA **Charge** 2 **Spectral Angle** 0.0
Spearman Correlation 0.0 **iRT Error:** 5.47

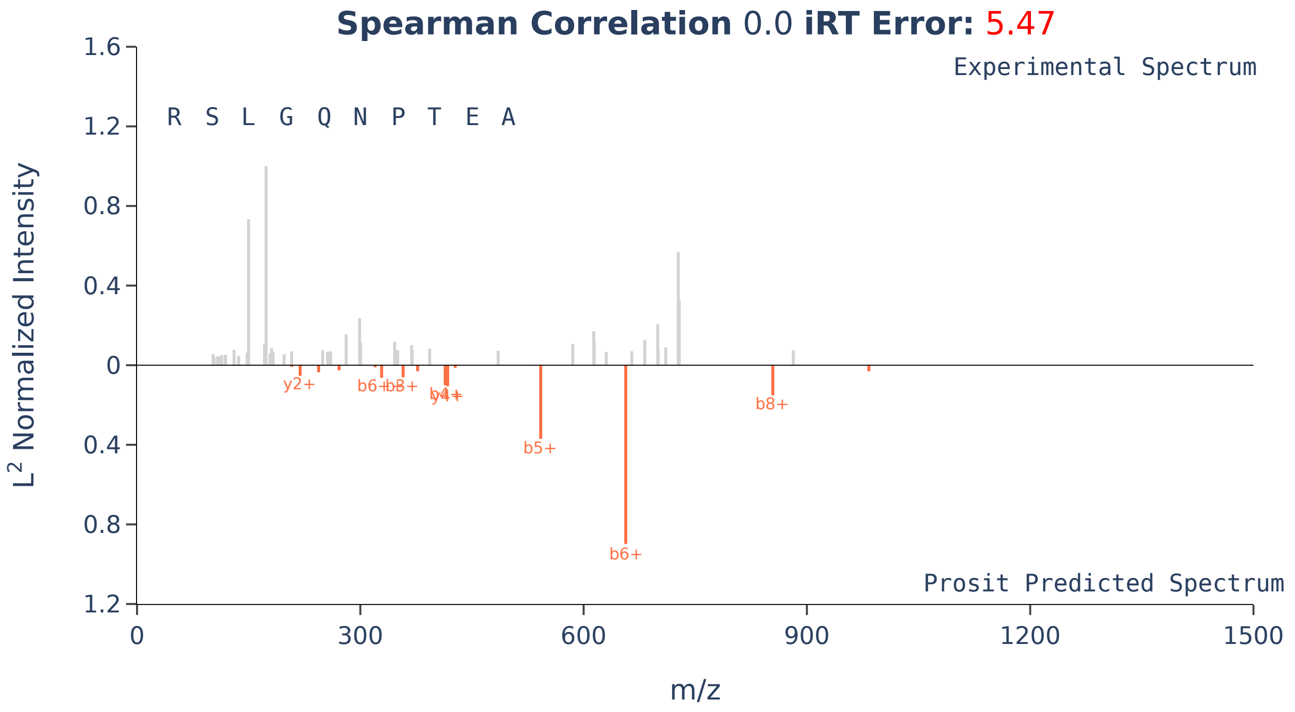

Source HRoetschke_291222_040123_Fu_CaM_b2R2_2h **Scan** 11217
Peptide PEFFLTM **Charge** 2 **Spectral Angle** 0.89
Spearman Correlation 0.91 **iRT Error:** 1.88

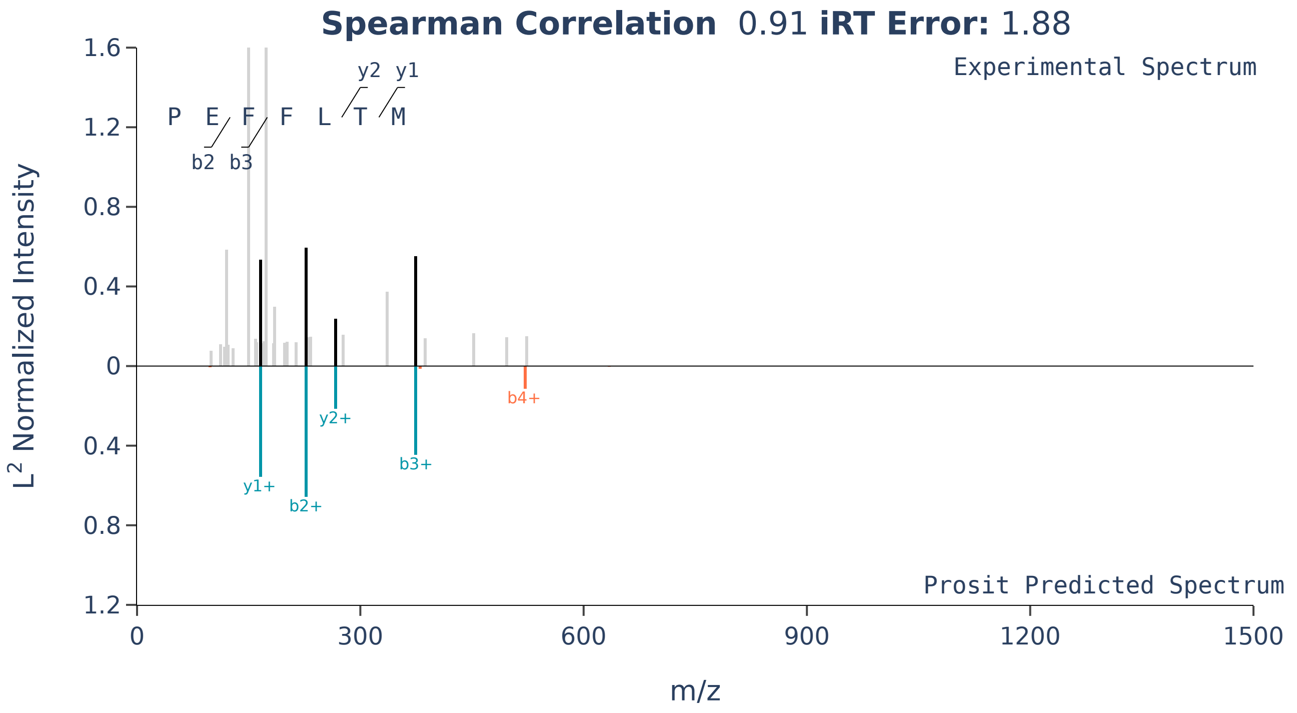

Source HRoetschke_291222_040123_Fu_CaM_b2R2_2h **Scan** 11217
Peptide FPEFLTM **Charge** 2 **Spectral Angle** 0.68
Spearman Correlation 0.85 **iRT Error:** 0.7

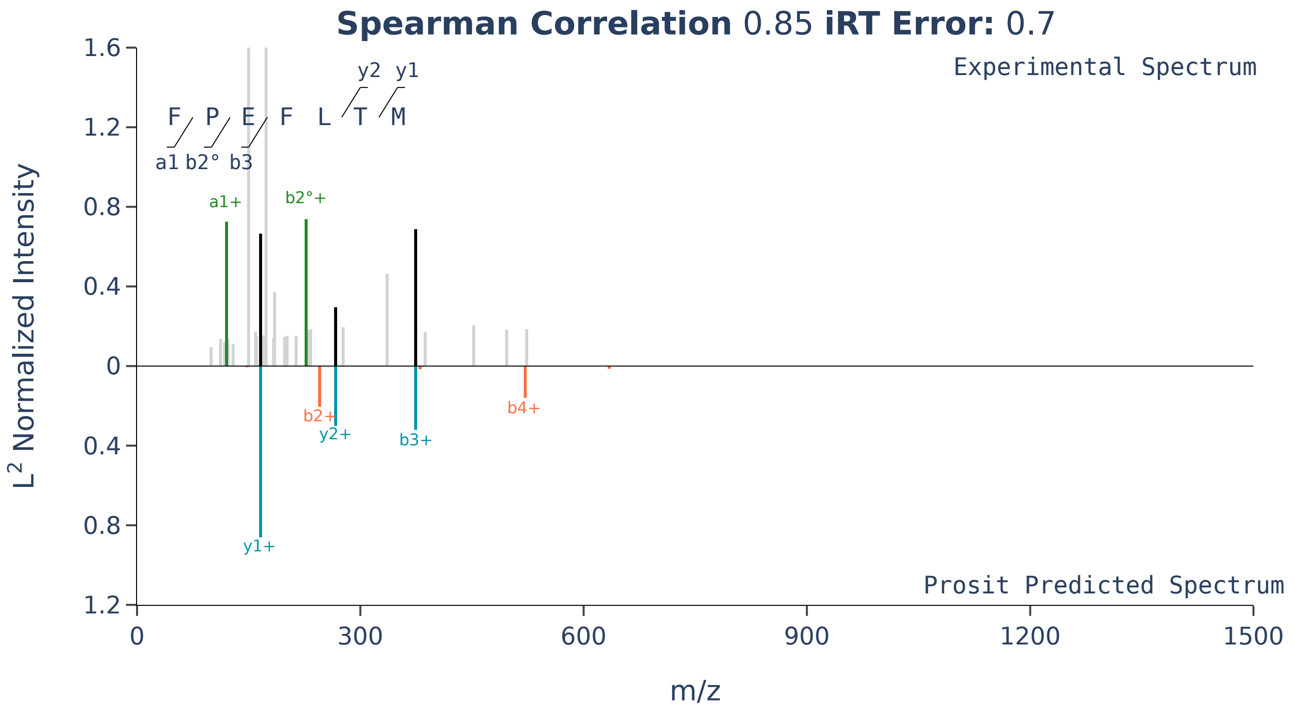

Source Ncheng_210623_230623_HFGoe_FFH_20S_25_1_A1_4h_R2 **Scan** 22455
Peptide NVKSLTPGQEFVKIVRNE **Charge** 3 **Spectral Angle** 0.86
Spearman Correlation 0.94 **iRT Error:** 0.0

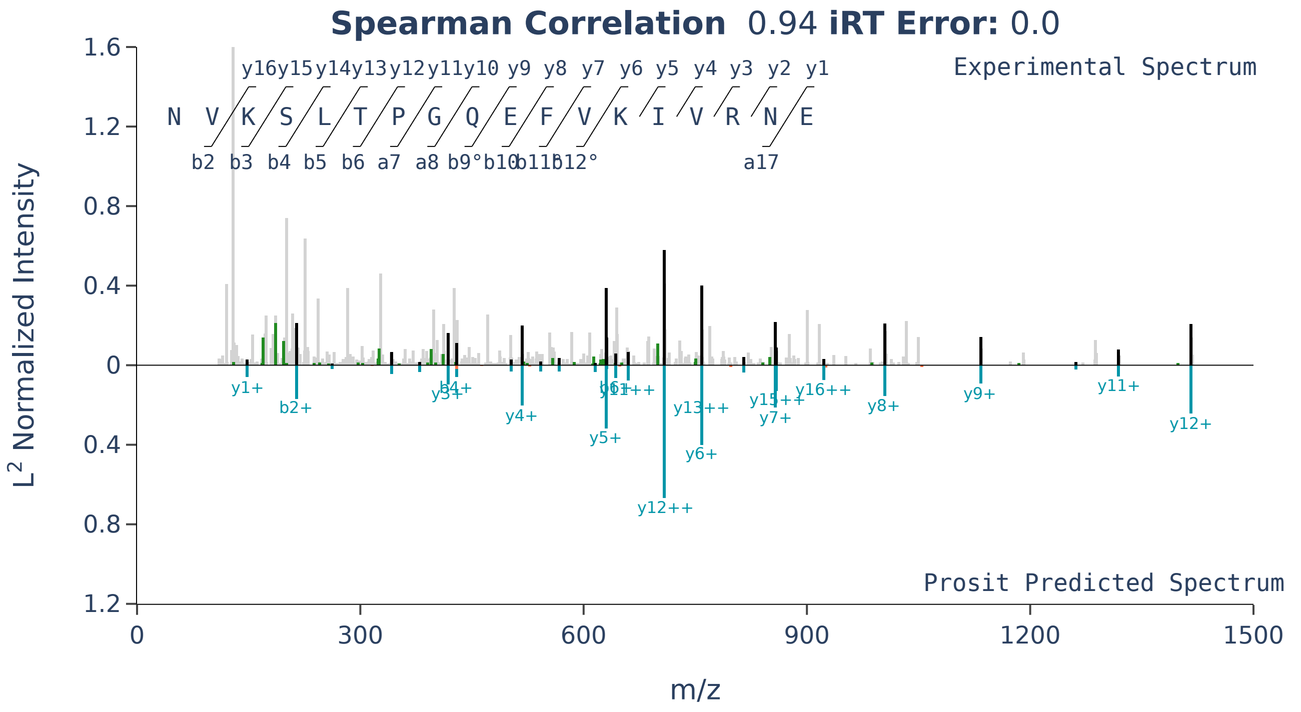

Source Ncheng_210623_230623_HFGoe_FFH_20S_25_1_A1_4h_R2 **Scan** 22455
Peptide EVNKSLTPGQEFVKIVRN **Charge** 3 **Spectral Angle** 0.37
Spearman Correlation 0.64 **iRT Error:** 7.42

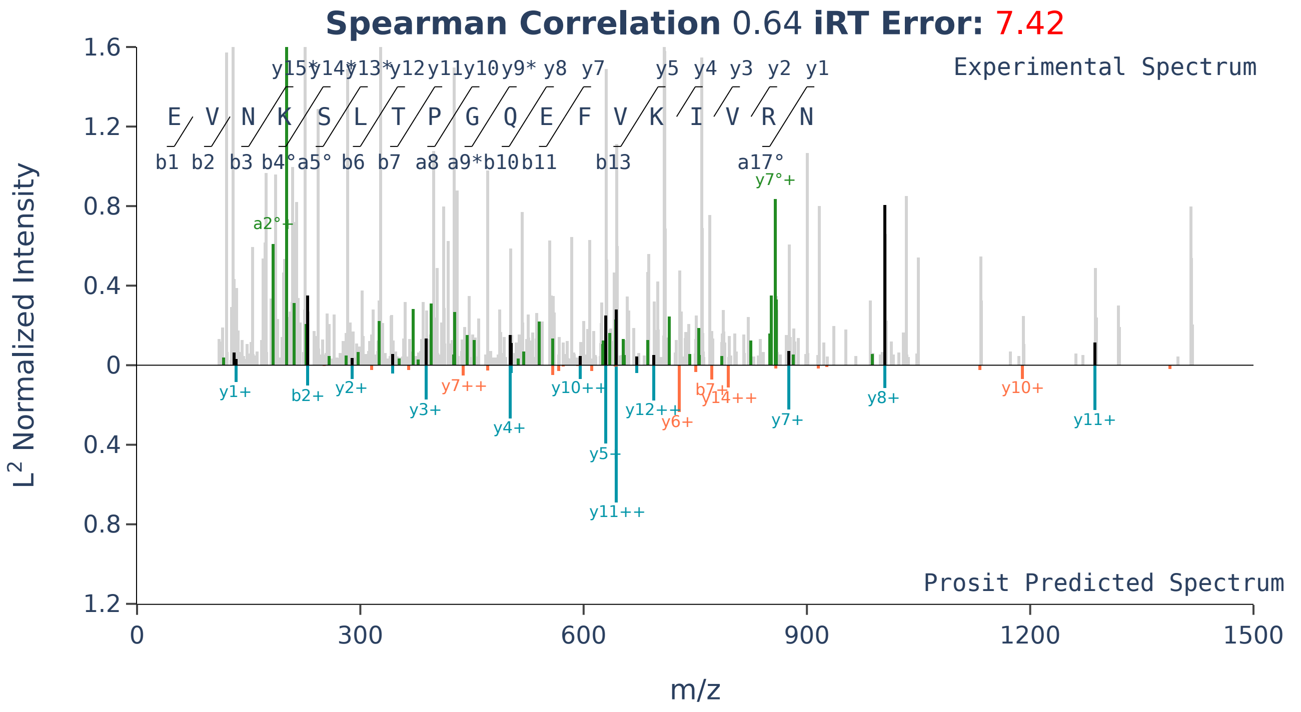

Source Ncheng_210623_230623_HFGoe_FFH_20S_25_1_A1_24h_R2 **Scan** 16060
Peptide MGKLPGMGQIPDVNKS **Charge** 2 **Spectral Angle** 0.75
Spearman Correlation 0.85 **iRT Error:** 0.01

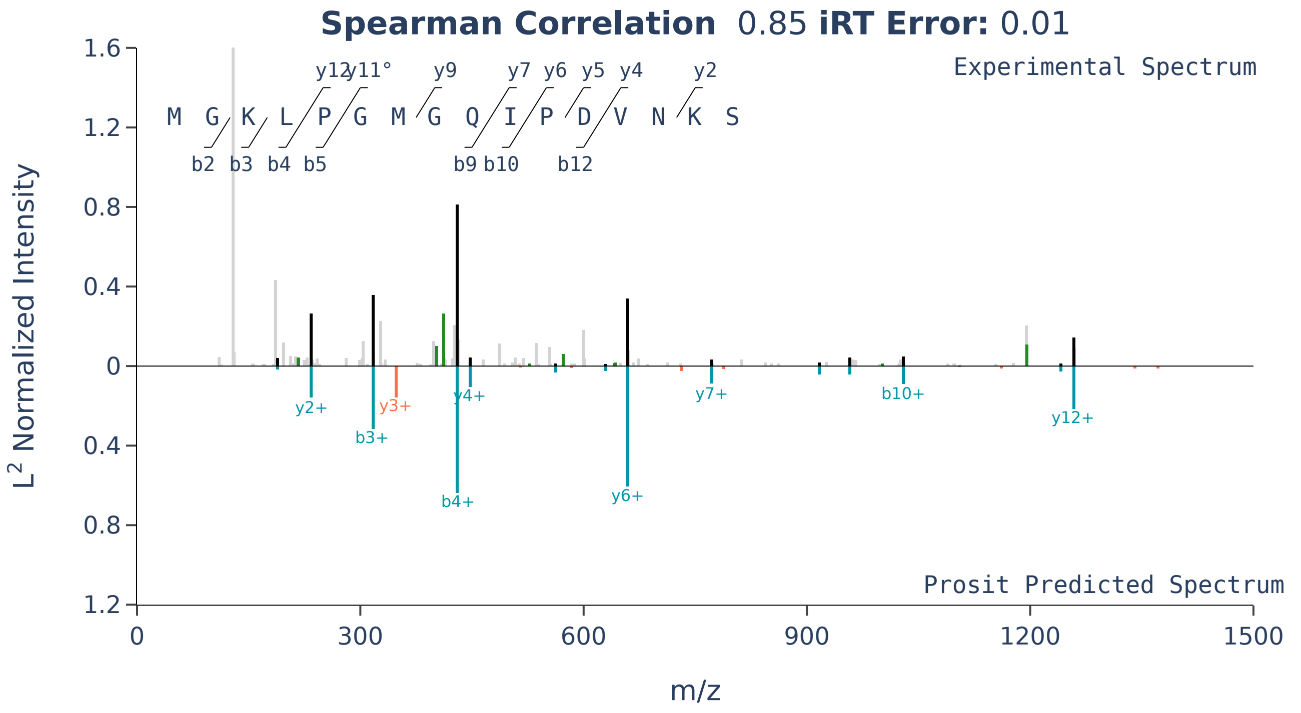

Source Ncheng_210623_230623_HFGoe_FFH_20S_25_1_A1_24h_R2 **Scan** 16060
Peptide MGKLPGMGQIPDNVKS **Charge** 2 **Spectral Angle** 0.47
Spearman Correlation 0.52 **iRT Error:** 9.72

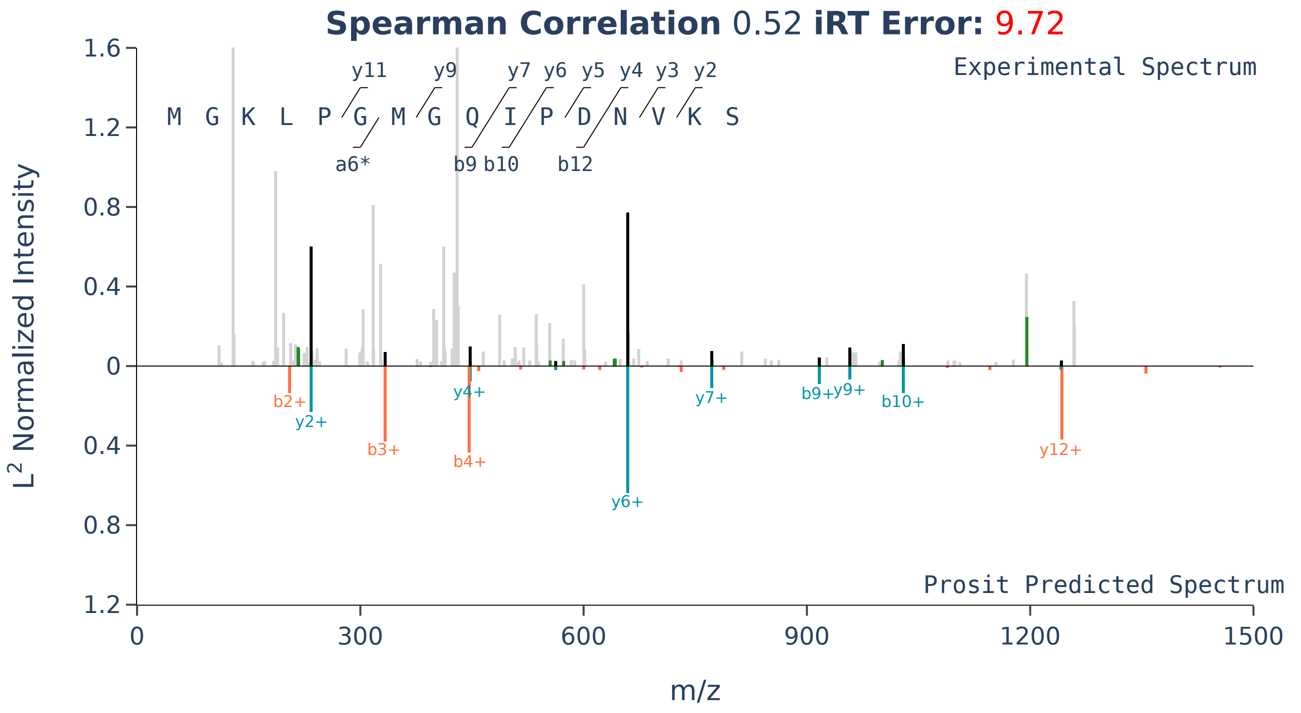

Source Ncheng_210623_230623_HFGoe_FFH_20S_25_1_A1_4h_R1 **Scan** 20842
Peptide SAALEPFHPDRI **Charge** 3 **Spectral Angle** 0.82
Spearman Correlation 0.91 **iRT Error:** 0.05

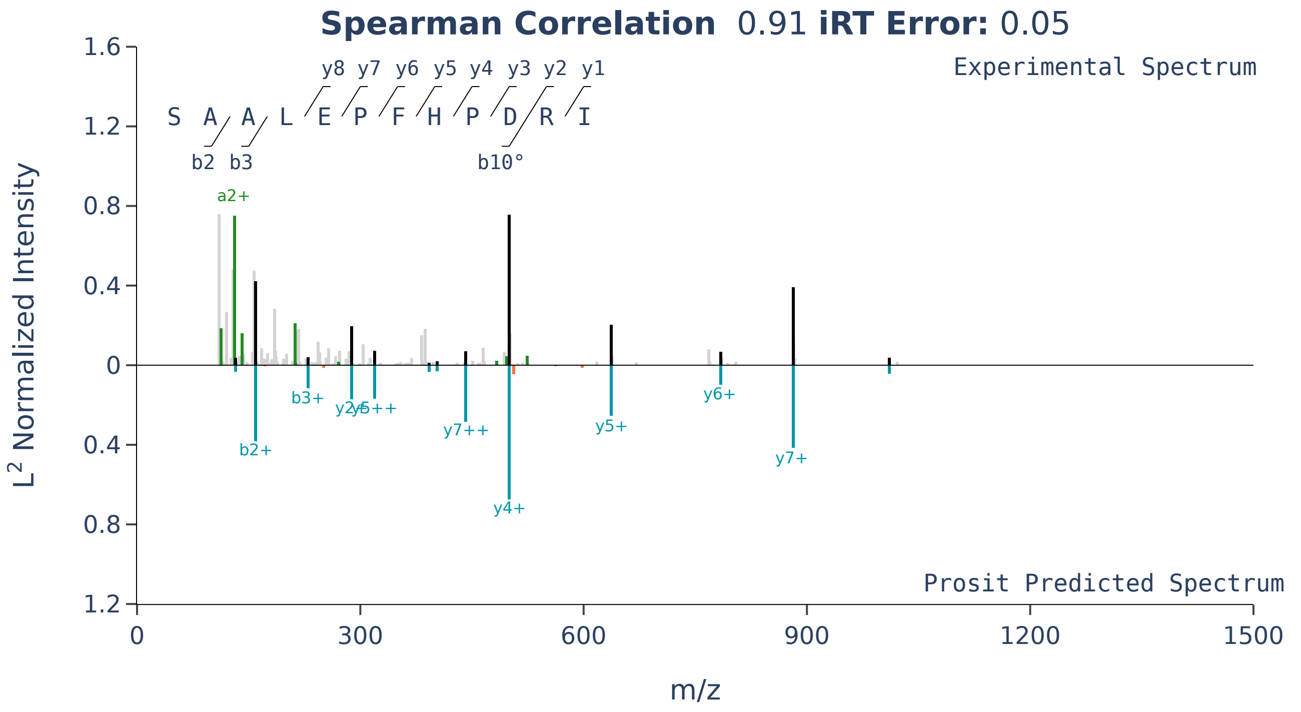

Source Ncheng_210623_230623_HFGoe_FFH_20S_25_1_A1_4h_R1 **Scan** 20842
Peptide ALEPFHPDRIAS **Charge** 3 **Spectral Angle** 0.16
Spearman Correlation 0.12 **iRT Error:** 13.18

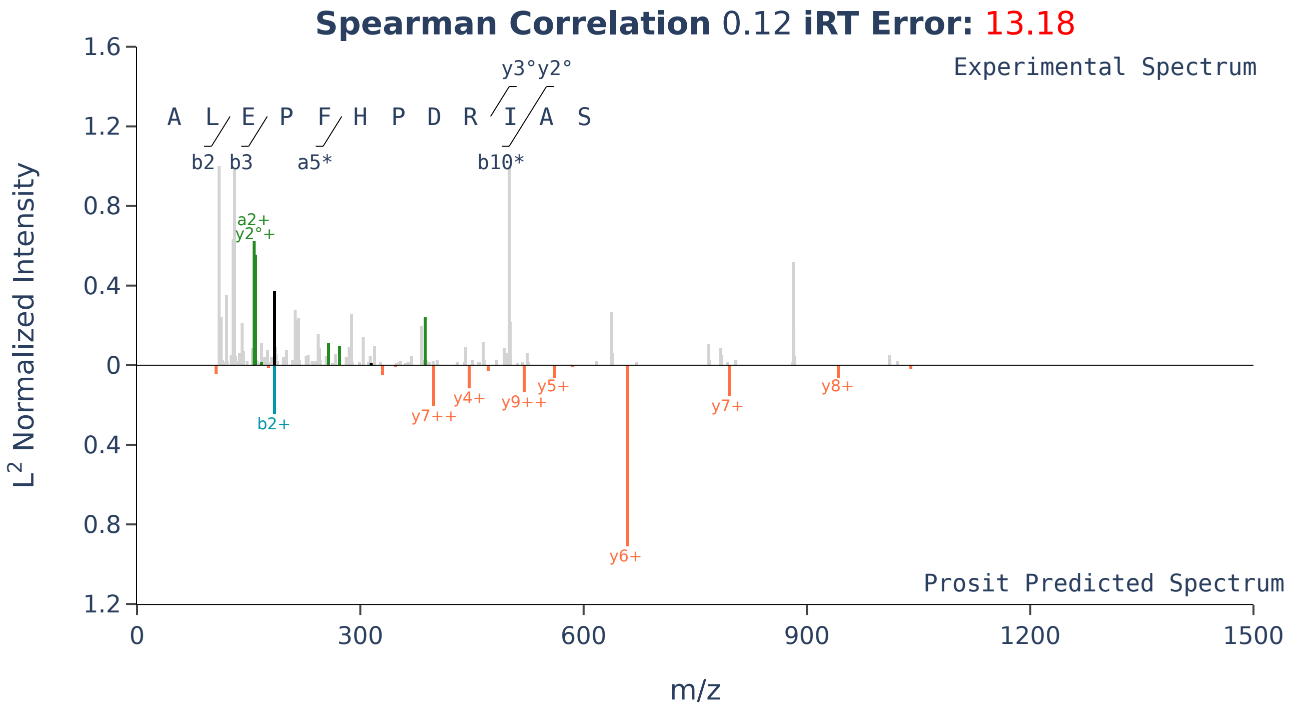

Source Ncheng_210623_230623_HFGoe_FFH_20S_25_1_A2_24h_R1 **Scan** 10015
Peptide GGMAVGQKPVD **Charge** 2 **Spectral Angle** 0.76
Spearman Correlation 0.91 **iRT Error:** 0.06

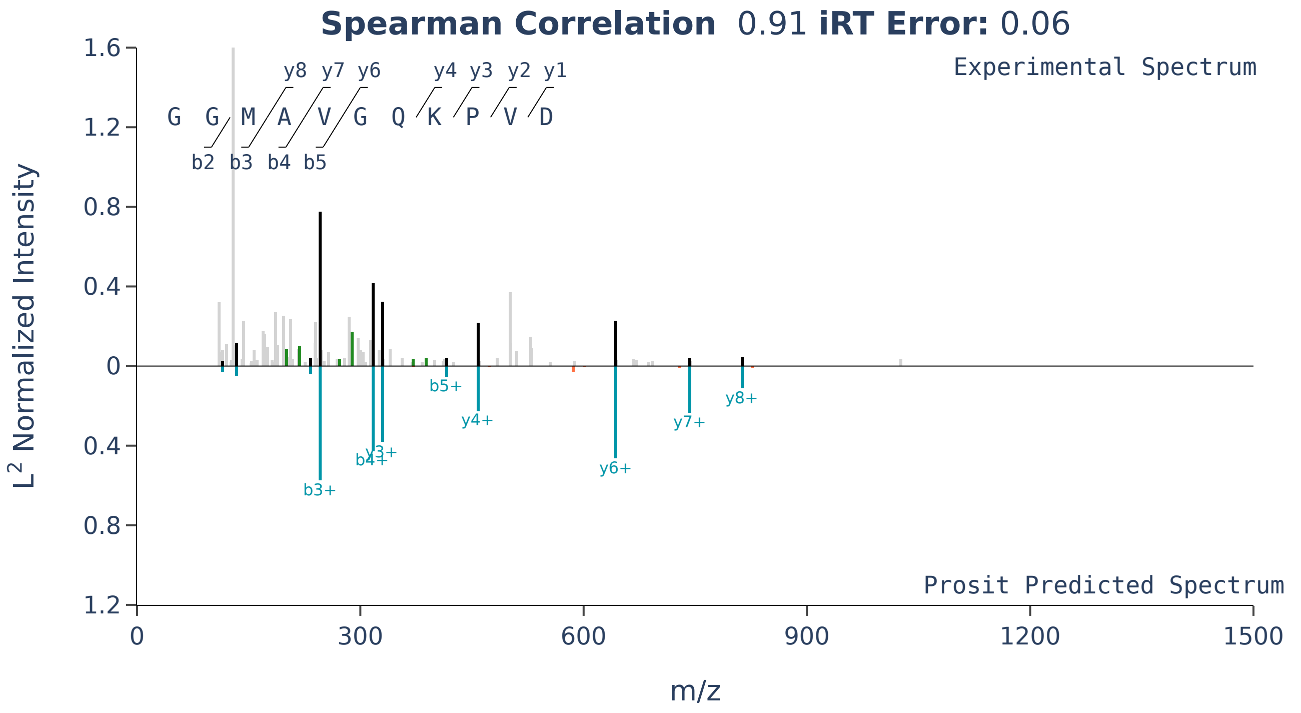

Source Ncheng_210623_230623_HFGoe_FFH_20S_25_1_A2_24h_R1 **Scan** 10015
Peptide GMGQIPDNVK **Charge** 2 **Spectral Angle** 0.04
Spearman Correlation 0.02 **iRT Error:** 21.78

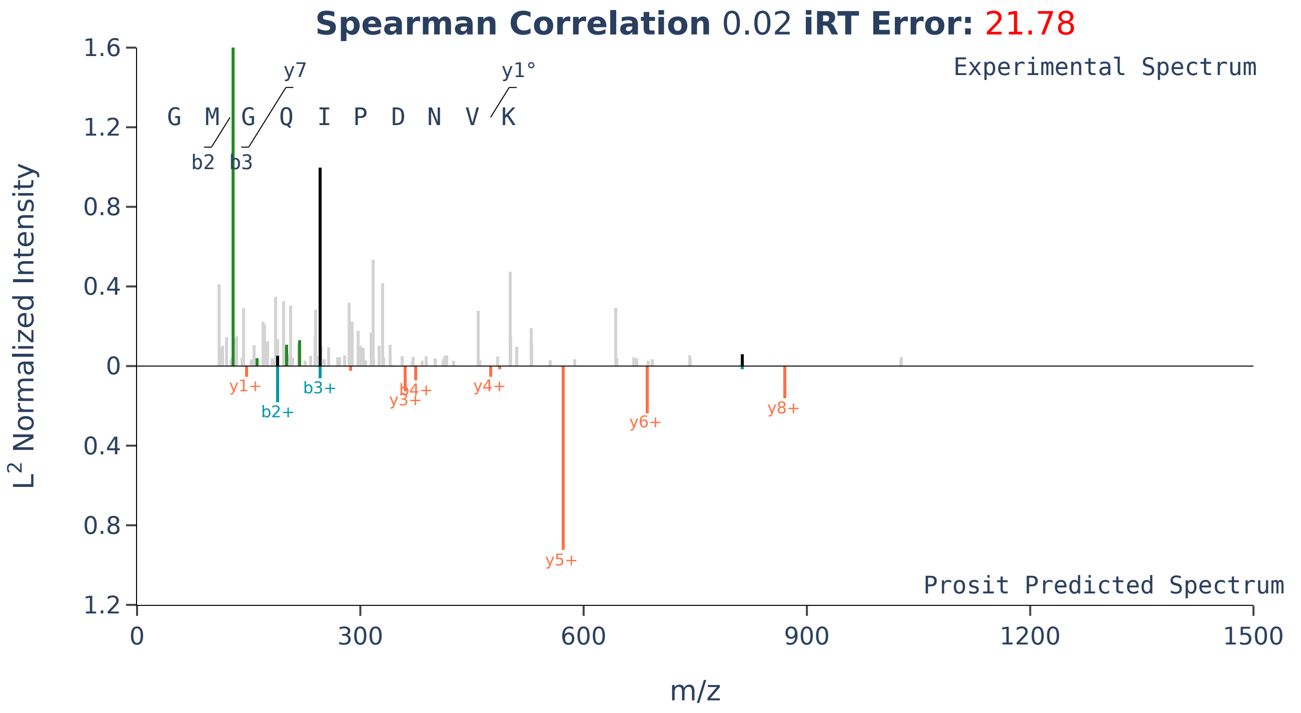

Source Ncheng_210623_230623_HFGoe_FFH_20S_25_1_A1_4h_R1 **Scan** 15069
Peptide RNIAMTGQDAANTAKAFN **Charge** 3 **Spectral Angle** 0.81
Spearman Correlation 0.87 **iRT Error:** 0.07

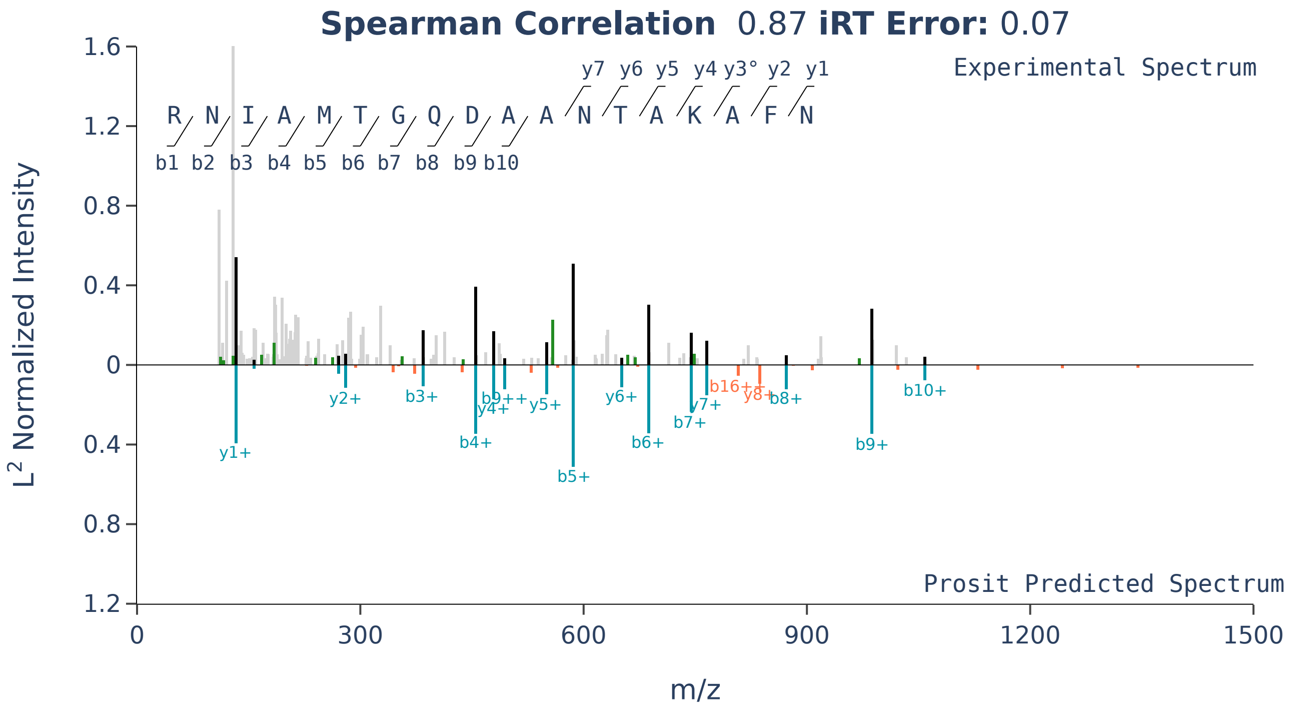

Source Ncheng_210623_230623_HFGoe_FFH_20S_25_1_A1_4h_R1 **Scan** 15069
Peptide LNDFLEQLRQMKNMGG **Charge** 3 **Spectral Angle** 0.11
Spearman Correlation 0.26 **iRT Error:** 76.69

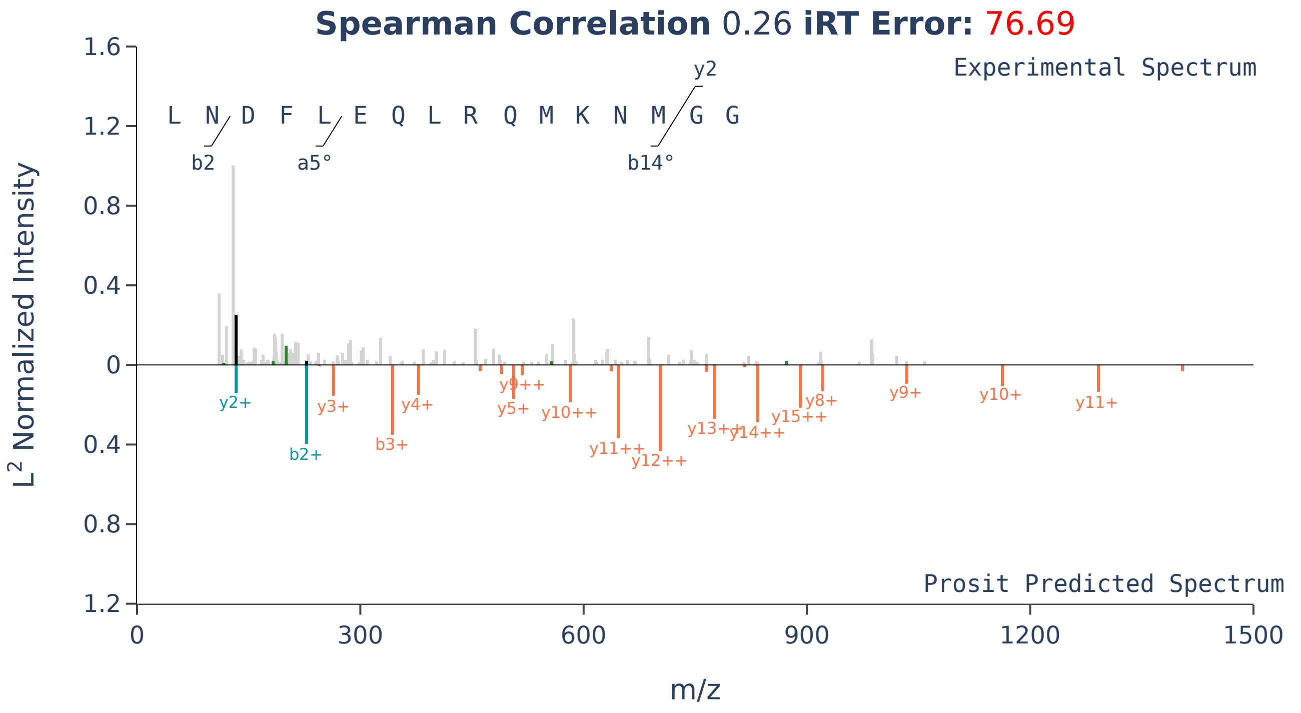

Source Ncheng_210623_230623_HFGoe_FFH_20S_25_1_A1_2h_R1 **Scan** 17367
Peptide ANIPDNVKSQMDDKVLVRME **Charge** 3 **Spectral Angle** 0.81
Spearman Correlation 0.86 **iRT Error:** 0.1

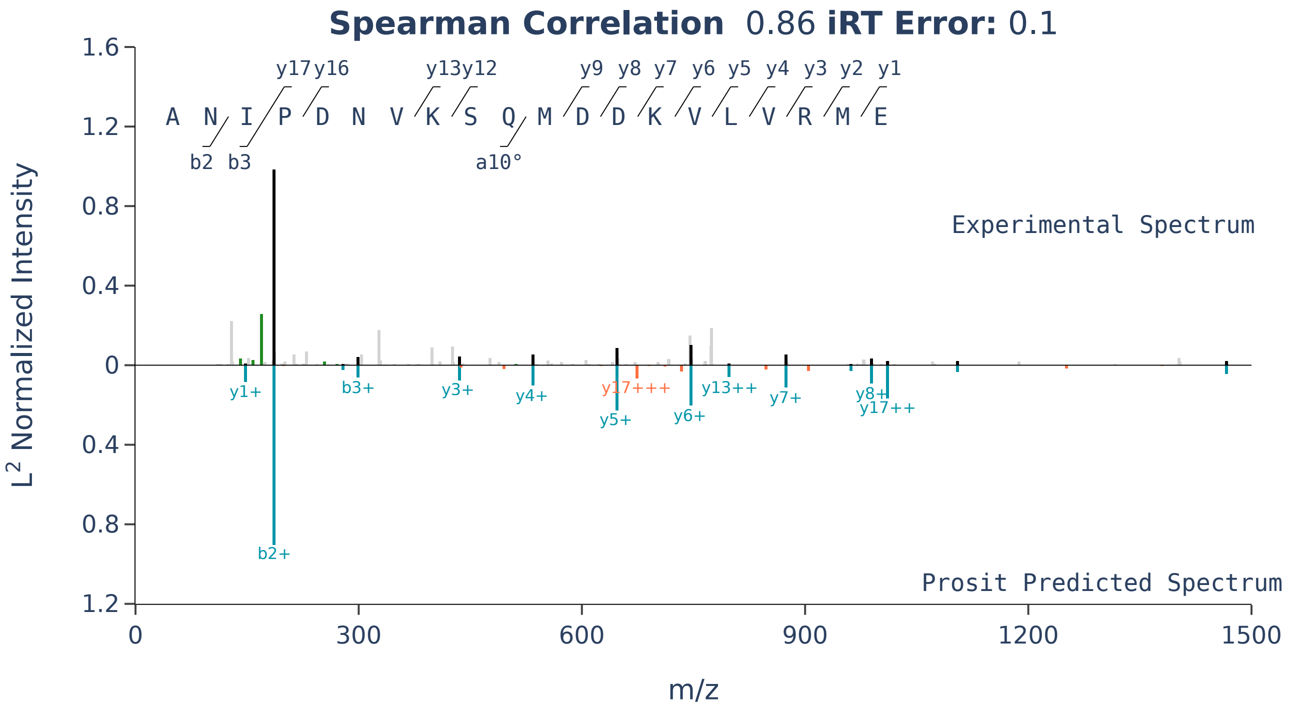

Source Ncheng_210623_230623_HFGoe_FFH_20S_25_1_A1_2h_R1 **Scan** 17367
Peptide GQIPDNVKSQMDDKVLVRME **Charge** 3 **Spectral Angle** 0.85
Spearman Correlation 0.82 **iRT Error:** 2.2

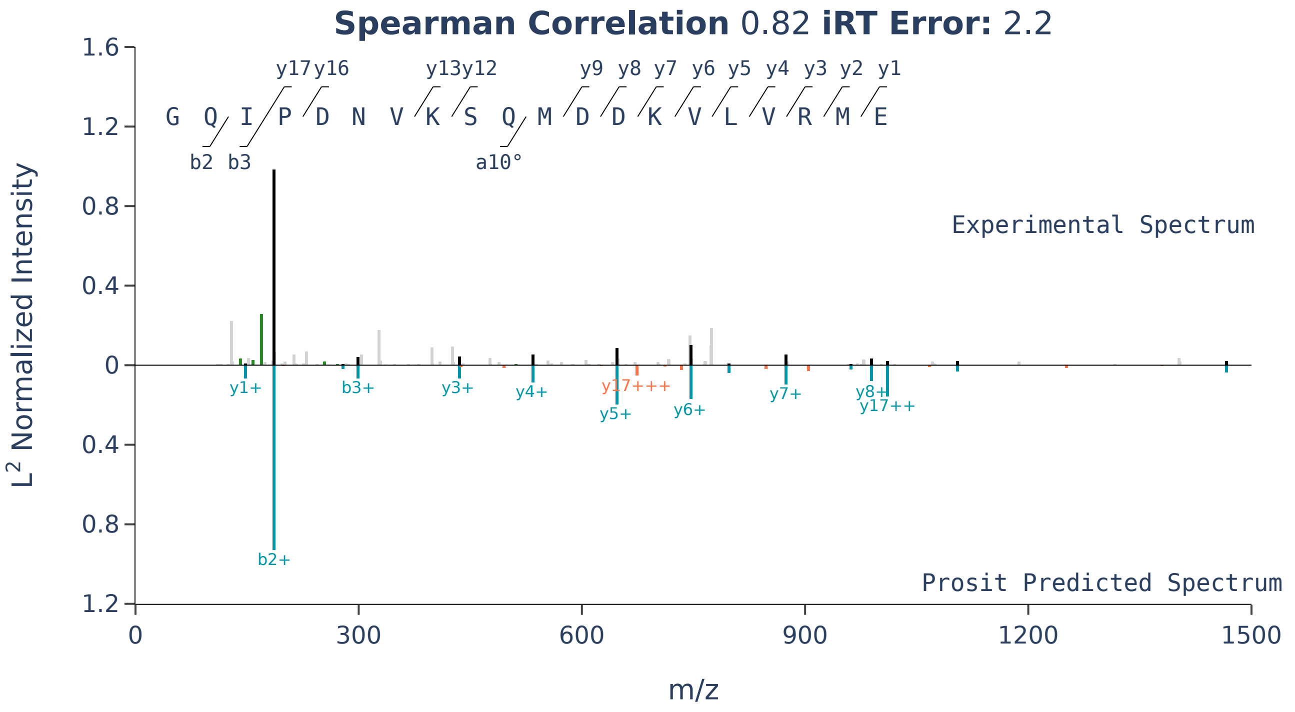

Source Ncheng_210623_230623_HFGoe_FFH_20S_25_1_A1_4h_R1 **Scan** 15574
Peptide ALEPFHPDRIASKTE **Charge** 3 **Spectral Angle** 0.71
Spearman Correlation 0.88 **iRT Error:** 0.11

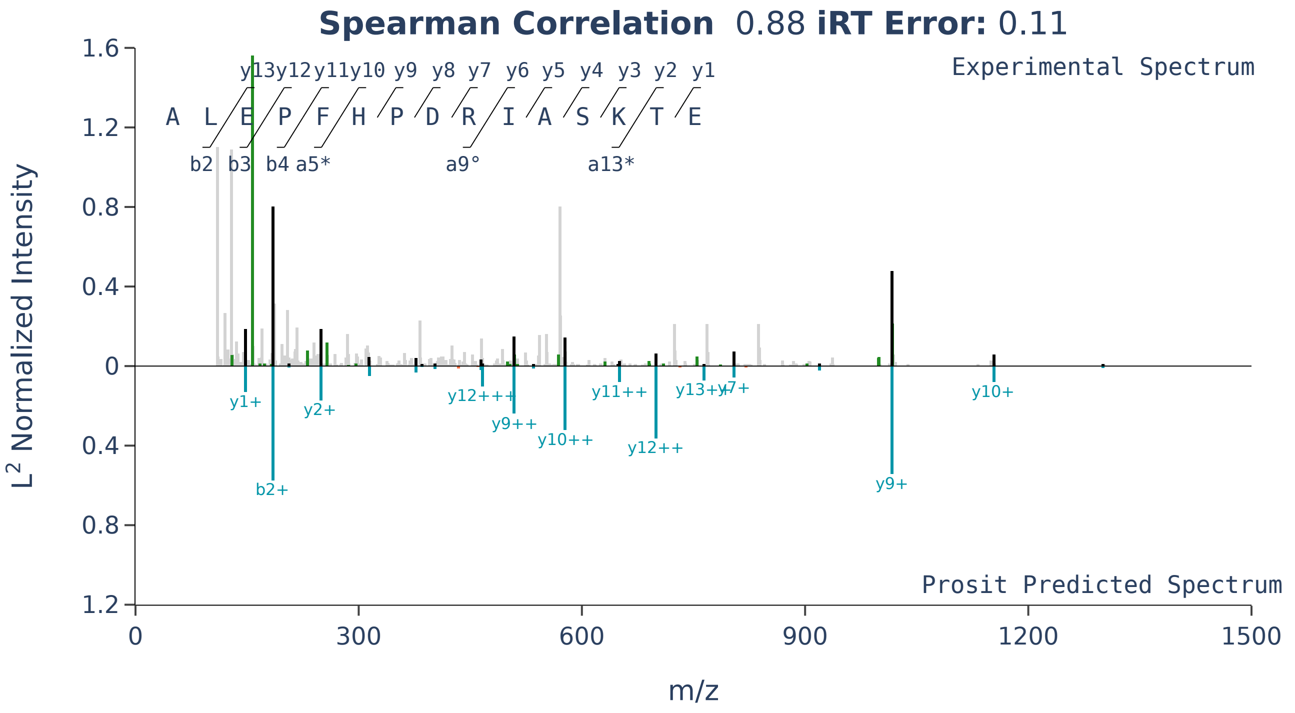

Source Ncheng_210623_230623_HFGoe_FFH_20S_25_1_A1_4h_R1 **Scan** 15574
Peptide KTEALEPFHPDRIAS **Charge** 3 **Spectral Angle** 0.39
Spearman Correlation 0.16 **iRT Error:** 5.89

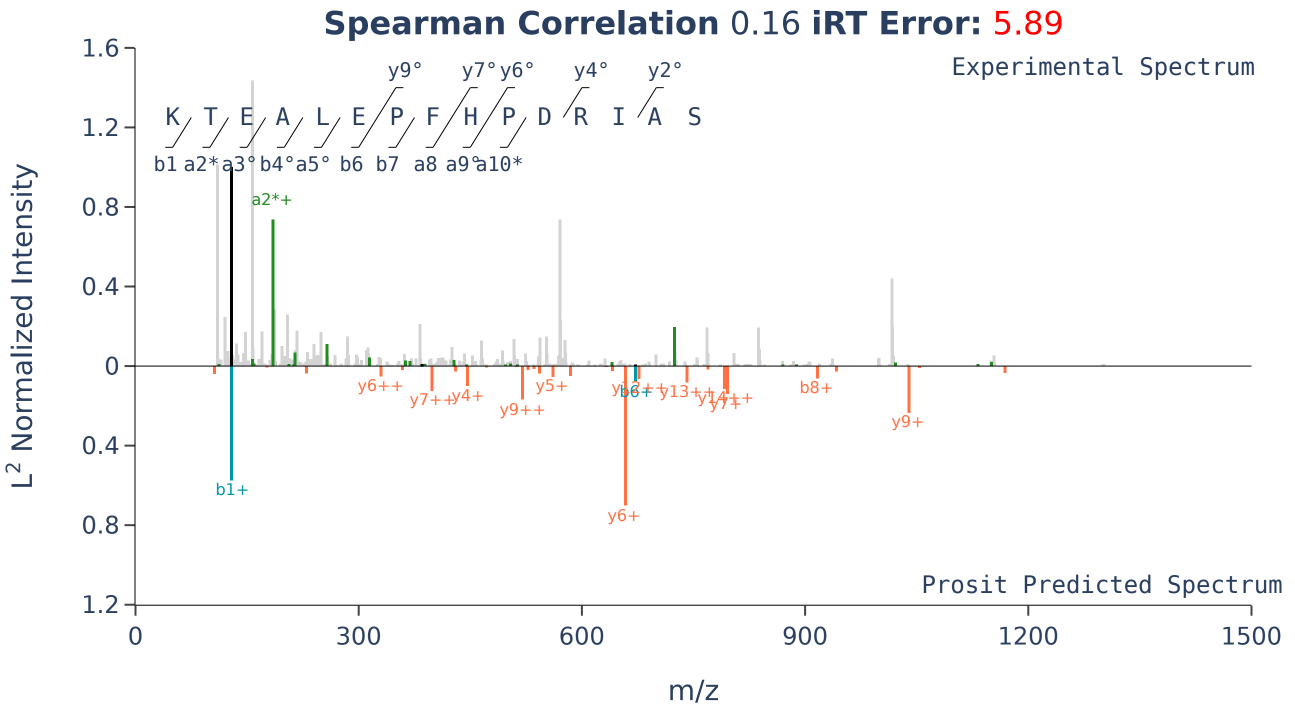

Source Ncheng_210623_230623_HFGoe_FFH_20S_25_1_A2_24h_R1 **Scan** 20487
Peptide PGMGQIGDV **Charge** 1 **Spectral Angle** 0.76
Spearman Correlation 0.89 **iRT Error:** 0.11

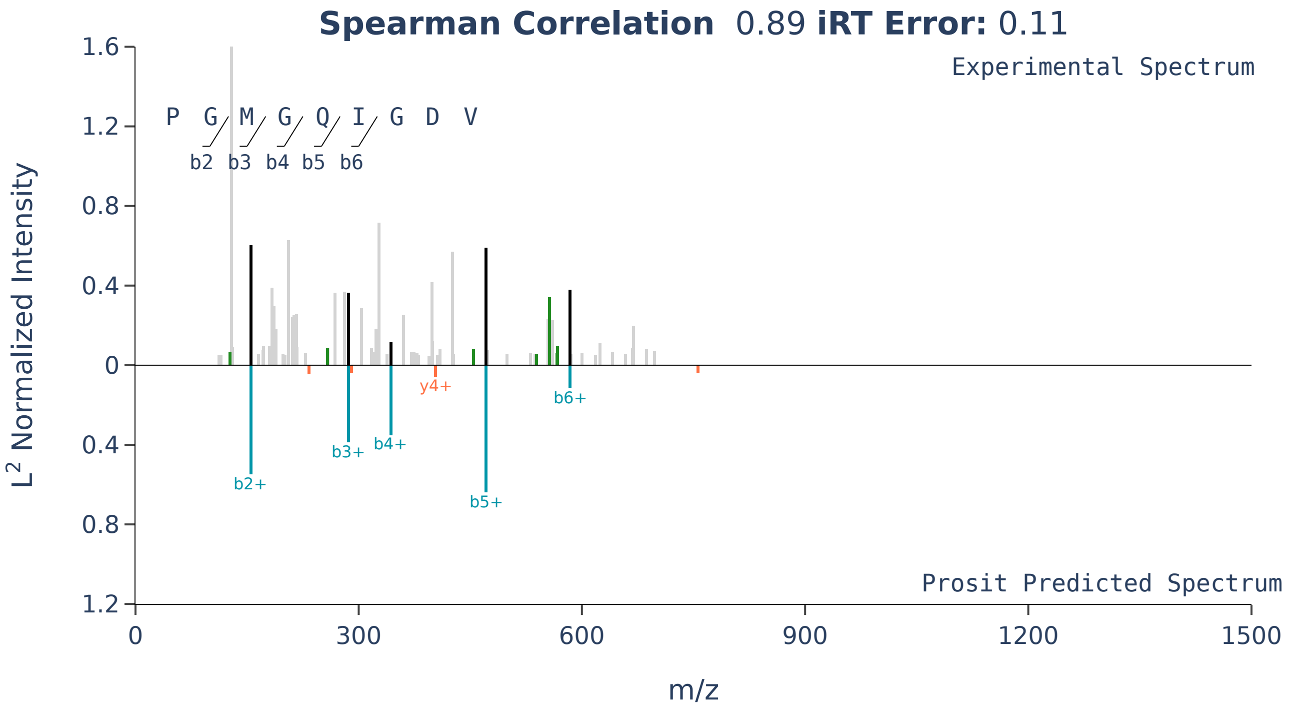

Source Ncheng_210623_230623_HFGoe_FFH_20S_25_1_A2_24h_R1 **Scan** 20487
Peptide MGQIPDNV **Charge** 1 **Spectral Angle** 0.38
Spearman Correlation 0.45 **iRT Error:** 16.89

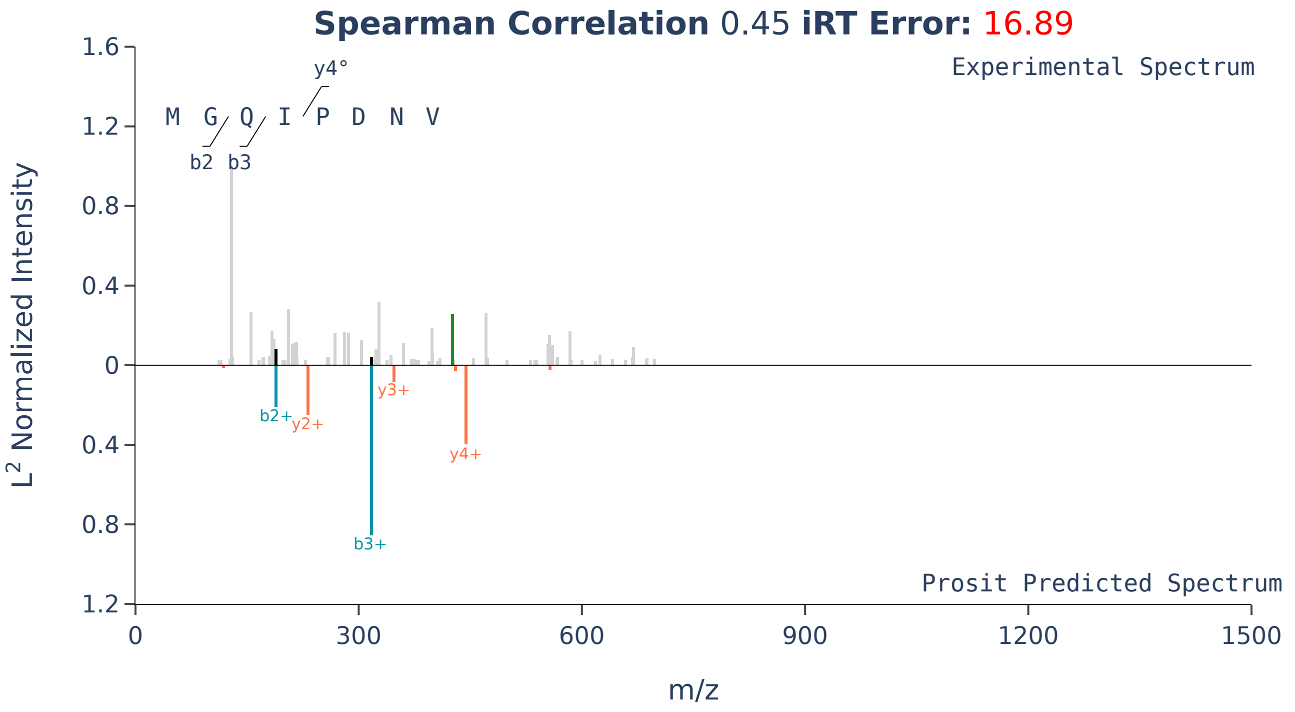

Source Ncheng_210623_230623_HFGoe_FFH_20S_25_1_A2_24h_R2 **Scan** 12142
Peptide DVYRPAAIKQA **Charge** 2 **Spectral Angle** 0.78
Spearman Correlation 0.89 **iRT Error:** 0.14

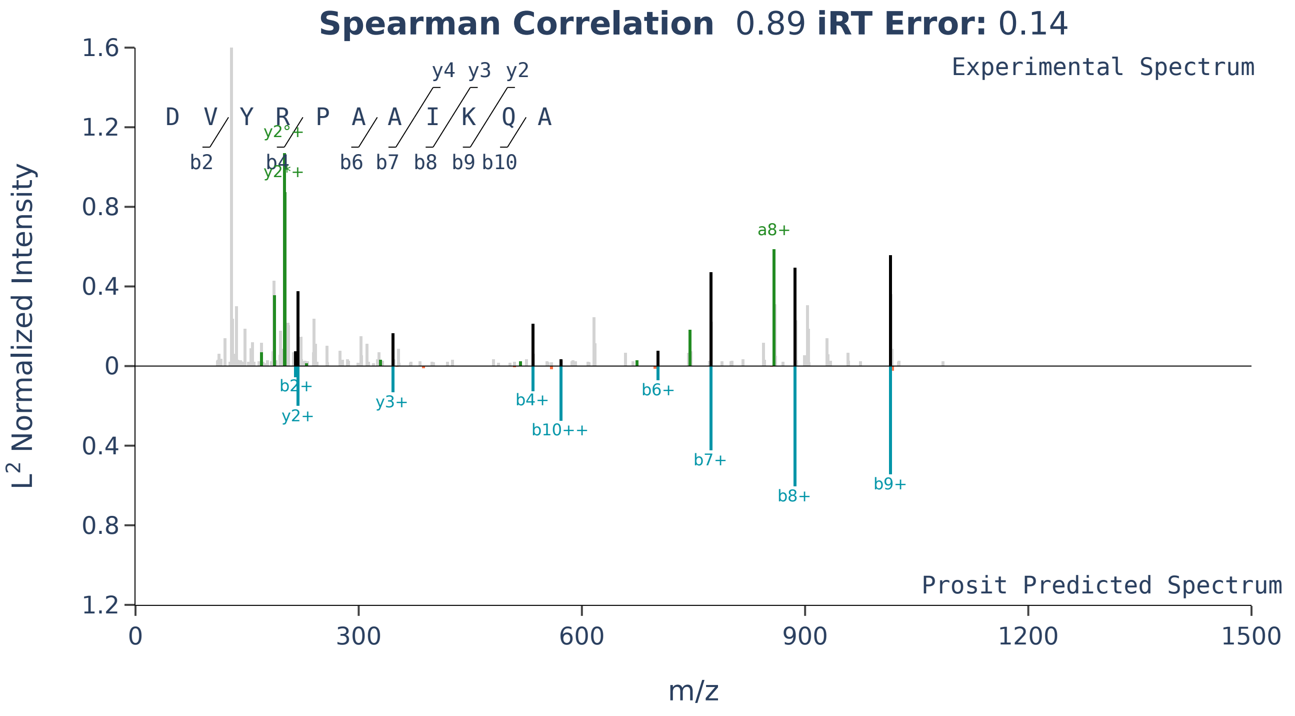

Source Ncheng_210623_230623_HFGoe_FFH_20S_25_1_A2_24h_R2 **Scan** 12142
Peptide GSRKRRIAAGCG **Charge** 2 **Spectral Angle** 0.03
Spearman Correlation -0.19 **iRT Error:** 52.73

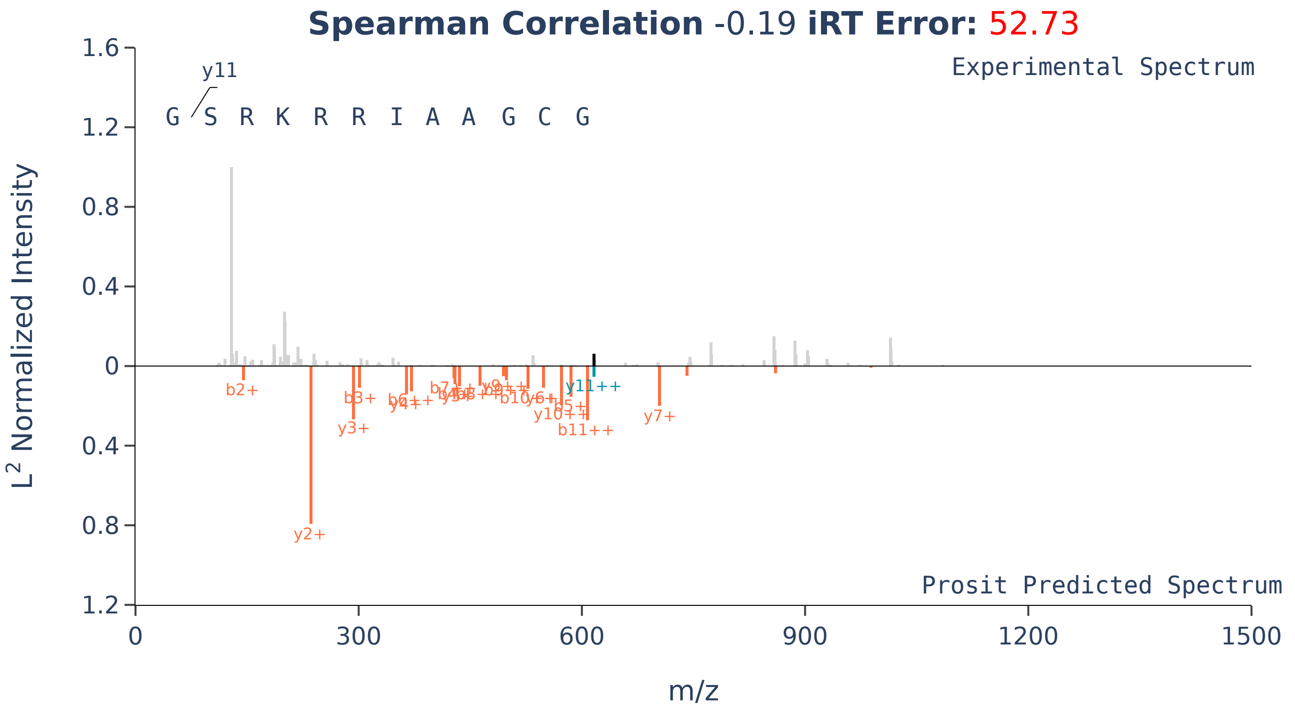

Source Ncheng_210623_230623_HFGoe_FFH_20S_25_1_A2_2h_R2 **Scan** 32174
Peptide NMMASLMGKLPGMGQIPDNVKSQMDD **Charge** 3 **Spectral Angle** 0.75
Spearman Correlation 0.93 **iRT Error:** 0.15

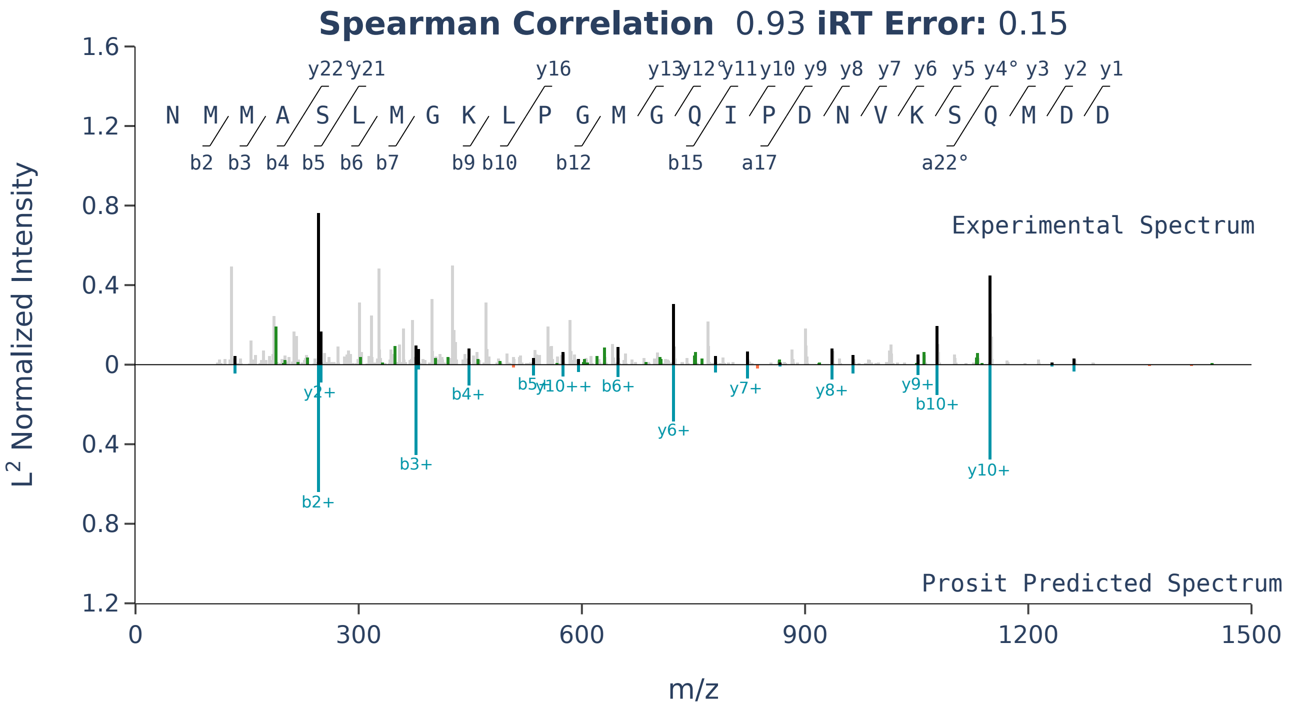

Source Ncheng_210623_230623_HFGoe_FFH_20S_25_1_A2_2h_R2 **Scan** 32174
Peptide MGGMASLMGKLPGMGQIPDNVKSQMDD **Charge** 3 **Spectral Angle** 0.82
Spearman Correlation 0.93 **iRT Error:** 11.56

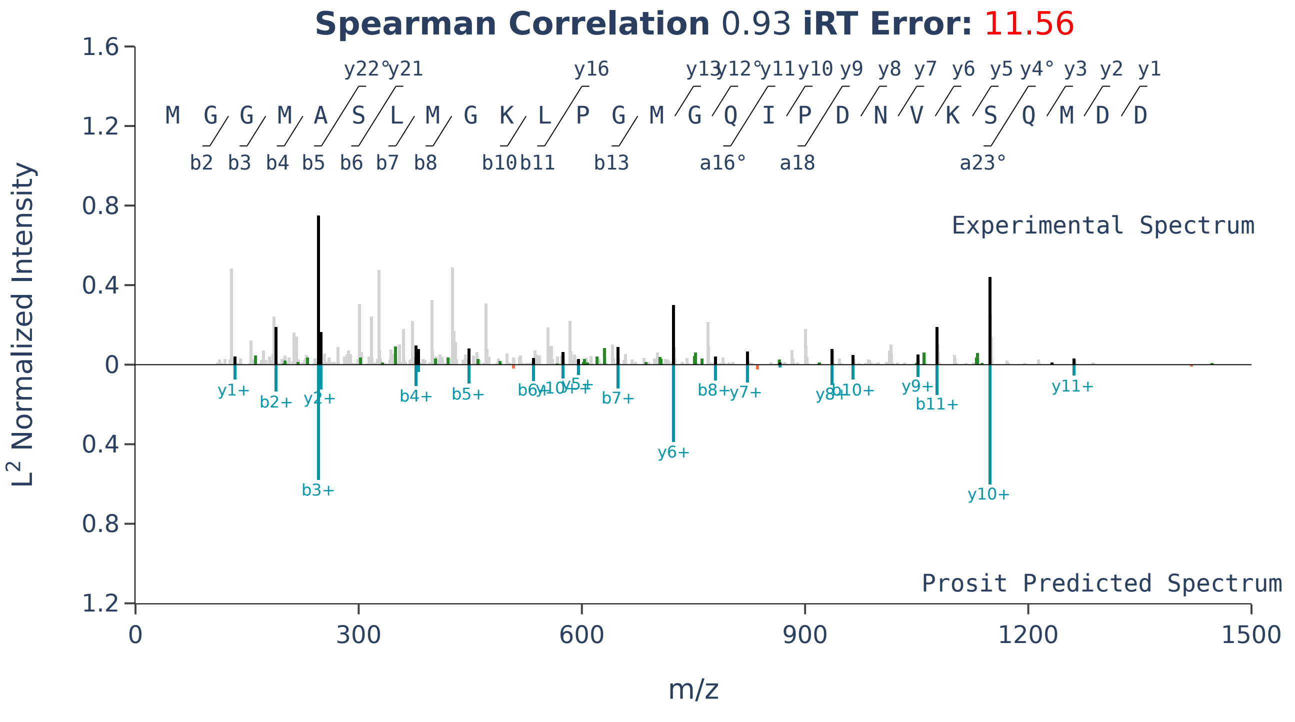

Source Ncheng_210623_230623_HFGoe_FFH_20S_25_1_A1_24h_R1 **Scan** 7039
Peptide KNSLTPGQE **Charge** 1 **Spectral Angle** 0.84
Spearman Correlation 0.89 **iRT Error:** 0.17

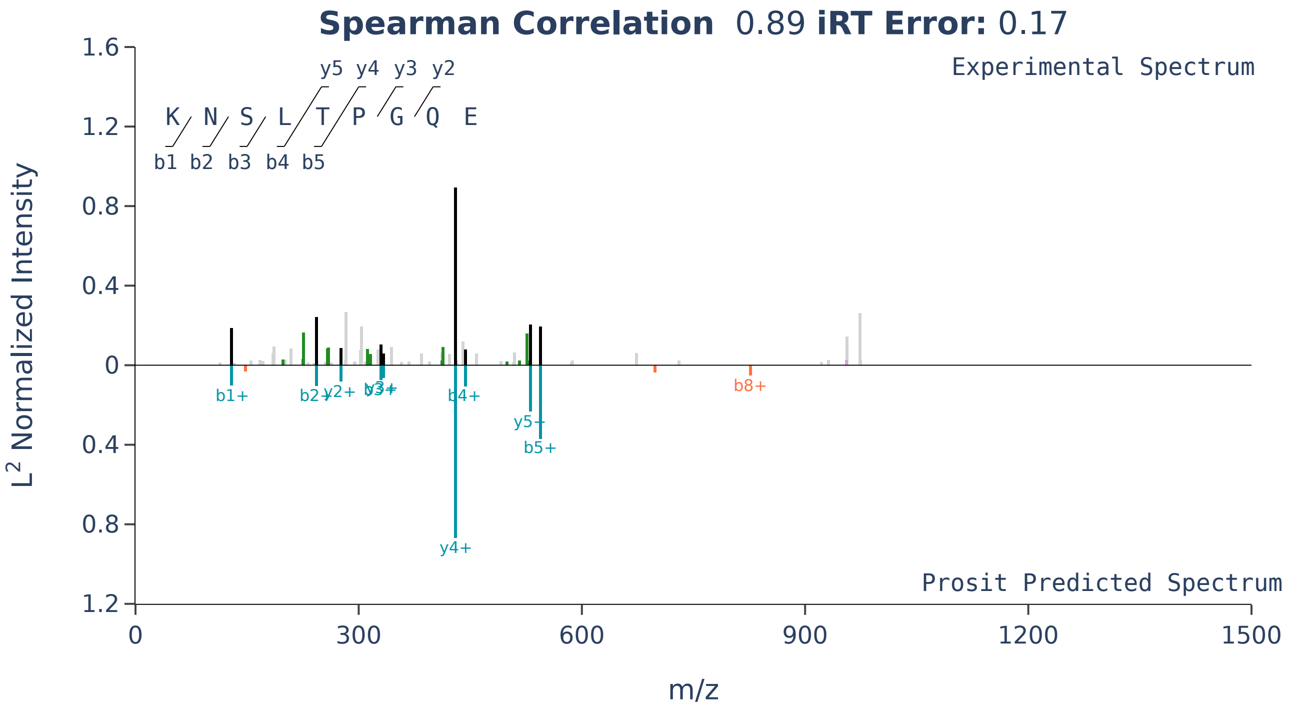

Source Ncheng_210623_230623_HFGoe_FFH_20S_25_1_A1_24h_R1 **Scan** 7039
Peptide ENQTLNLAA **Charge** 1 **Spectral Angle** 0.0
Spearman Correlation 0.0 **iRT Error:** 56.08

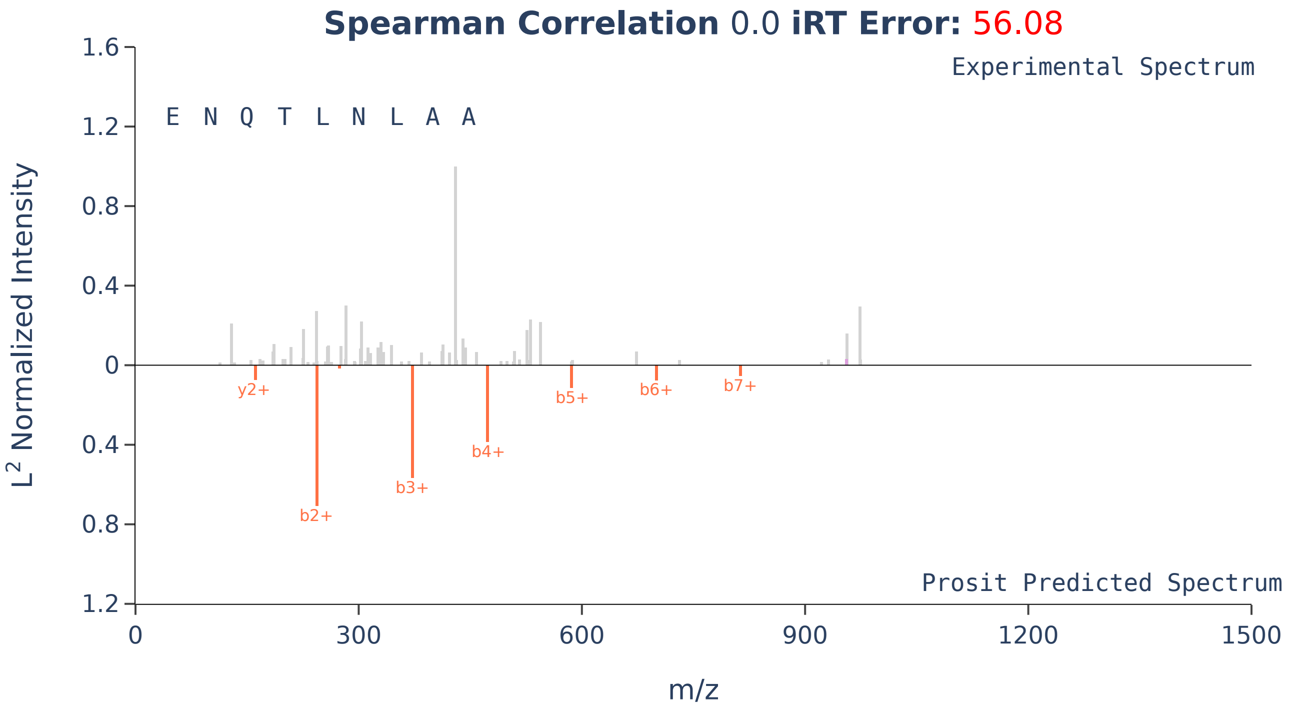

Source Ncheng_210623_230623_HFGoe_FFH_20S_25_1_A2_24h_R1 **Scan** 15017
Peptide DKVLVRMEAA **Charge** 2 **Spectral Angle** 0.9
Spearman Correlation 0.89 **iRT Error:** 0.19

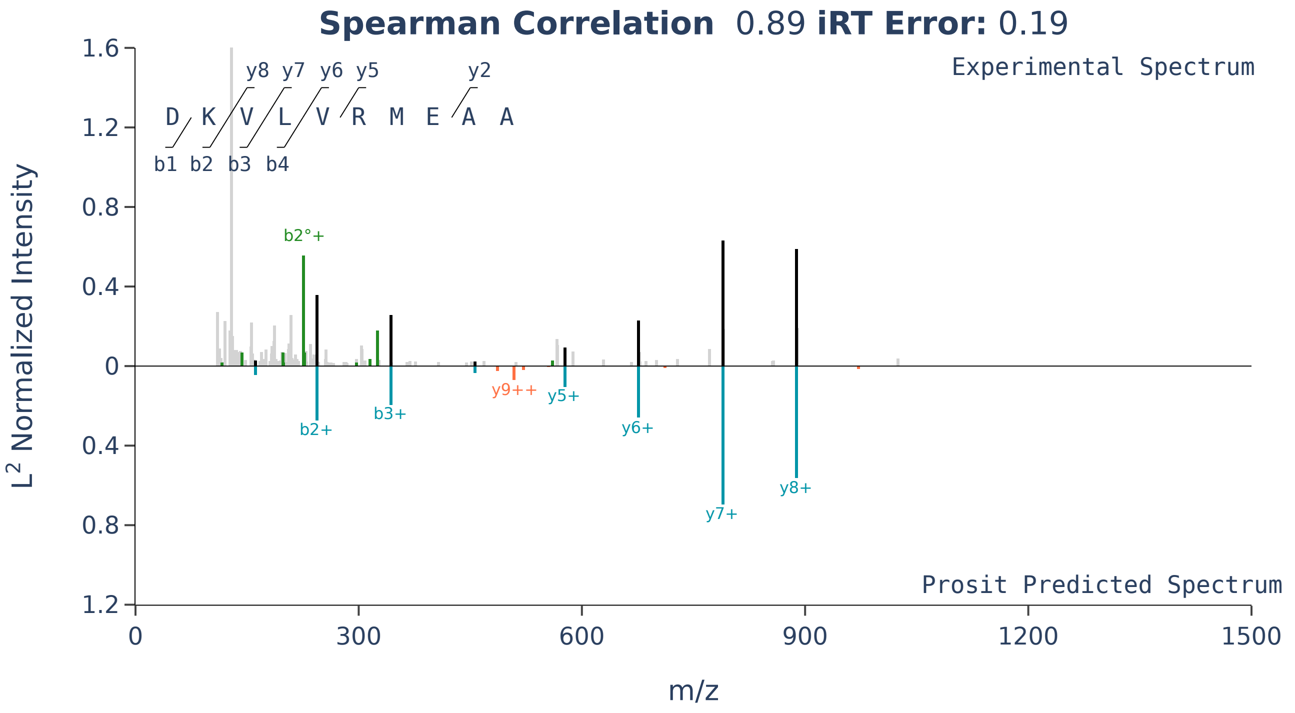

Source Ncheng_210623_230623_HFGoe_FFH_20S_25_1_A2_24h_R1 **Scan** 15017
Peptide ASRILGMGDVL **Charge** 2 **Spectral Angle** 0.04
Spearman Correlation 0.06 **iRT Error:** 71.72

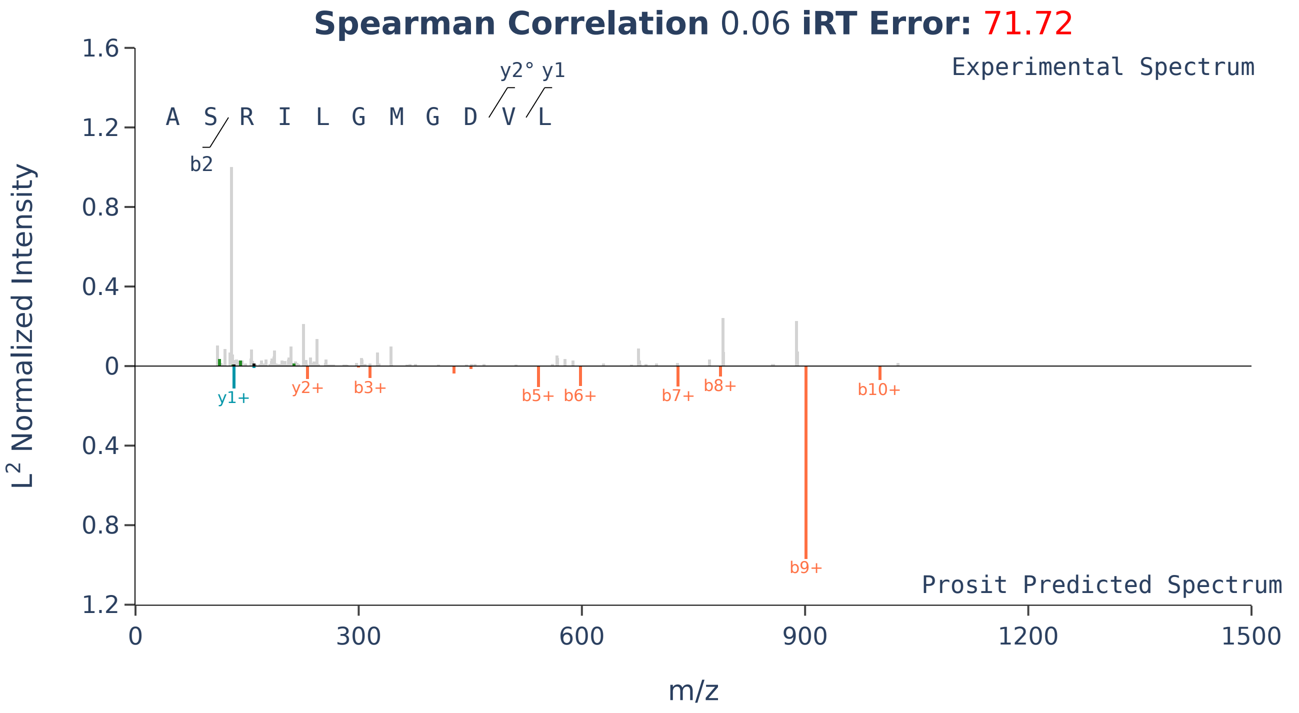

Source Ncheng_210623_230623_HFGoe_FFH_20S_25_1_A2_24h_R1 **Scan** 15438
Peptide TGAMTGQDAANTAKAFNE **Charge** 2 **Spectral Angle** 0.93
Spearman Correlation 0.87 **iRT Error:** 0.2

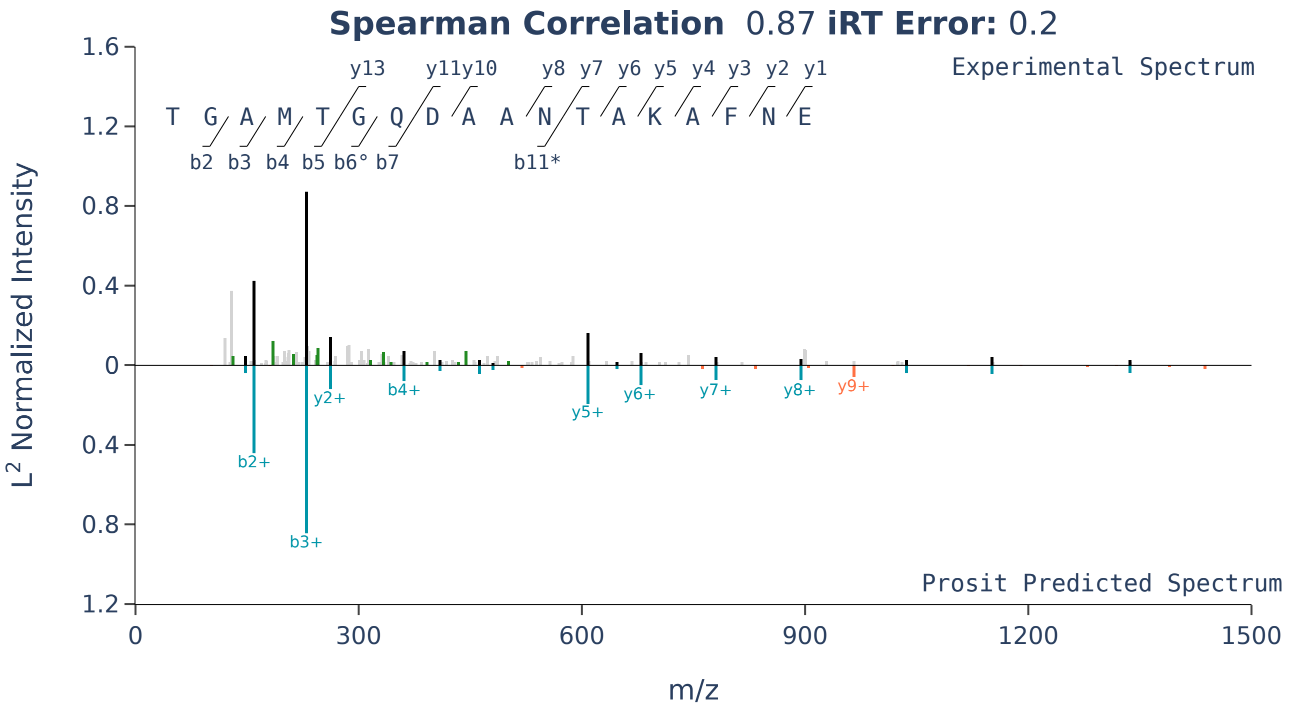

Source Ncheng_210623_230623_HFGoe_FFH_20S_25_1_A2_24h_R1 **Scan** 15438
Peptide GGMAKMMRSMKGMMPPG **Charge** 2 **Spectral Angle** 0.0
Spearman Correlation 0.0 **iRT Error:** 22.08

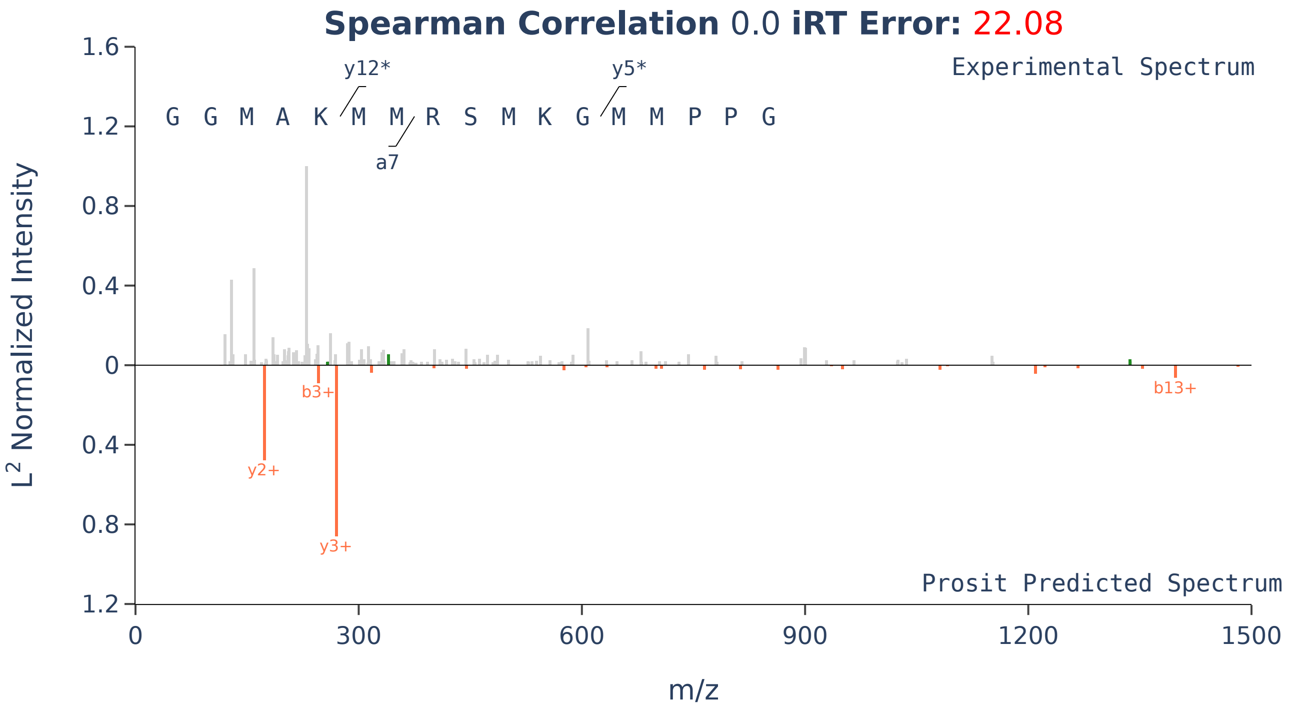

Source Ncheng_210623_230623_HFGoe_FFH_20S_25_1_A1_2h_R2 **Scan** 19329
Peptide GMKLPGMGQIPDNVKSQMD **Charge** 3 **Spectral Angle** 0.75
Spearman Correlation 0.9 **iRT Error:** 0.26

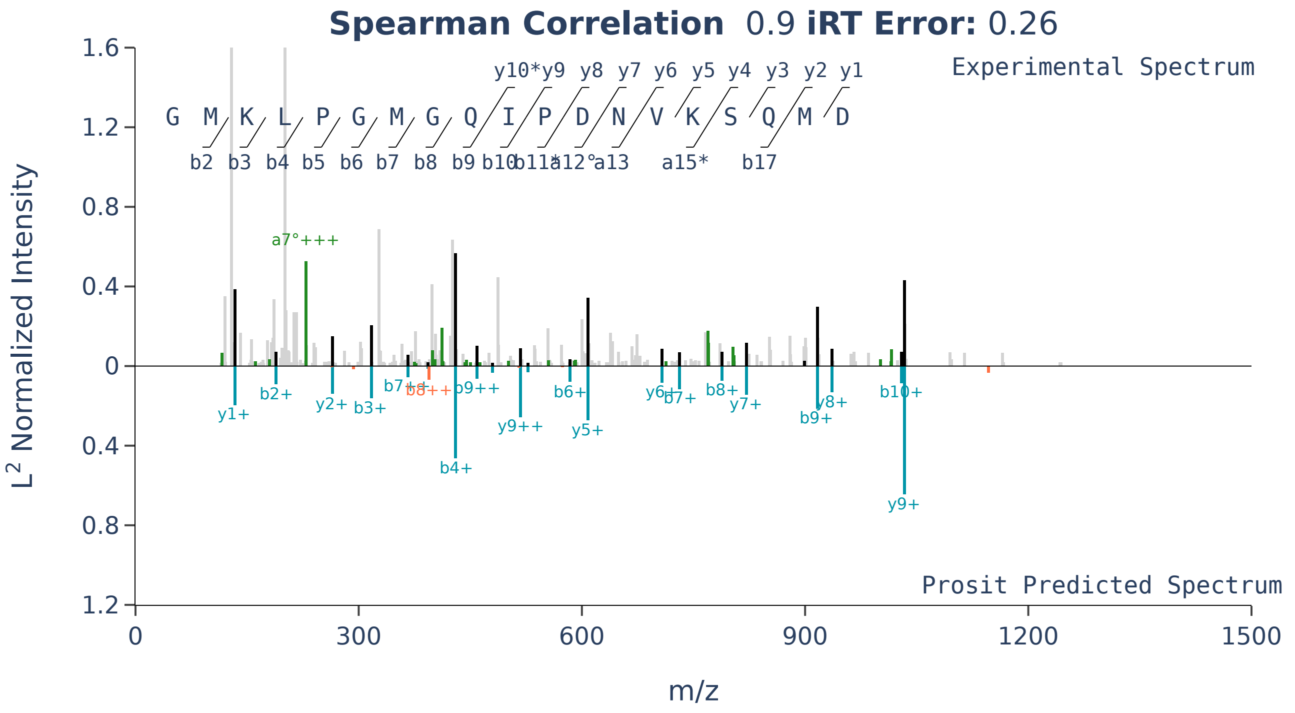

Source Ncheng_210623_230623_HFGoe_FFH_20S_25_1_A1_2h_R2 **Scan** 19329
Peptide MGKLPGMGQIPDNVKSQMD **Charge** 3 **Spectral Angle** 0.33
Spearman Correlation 0.3 **iRT Error:** 2.54

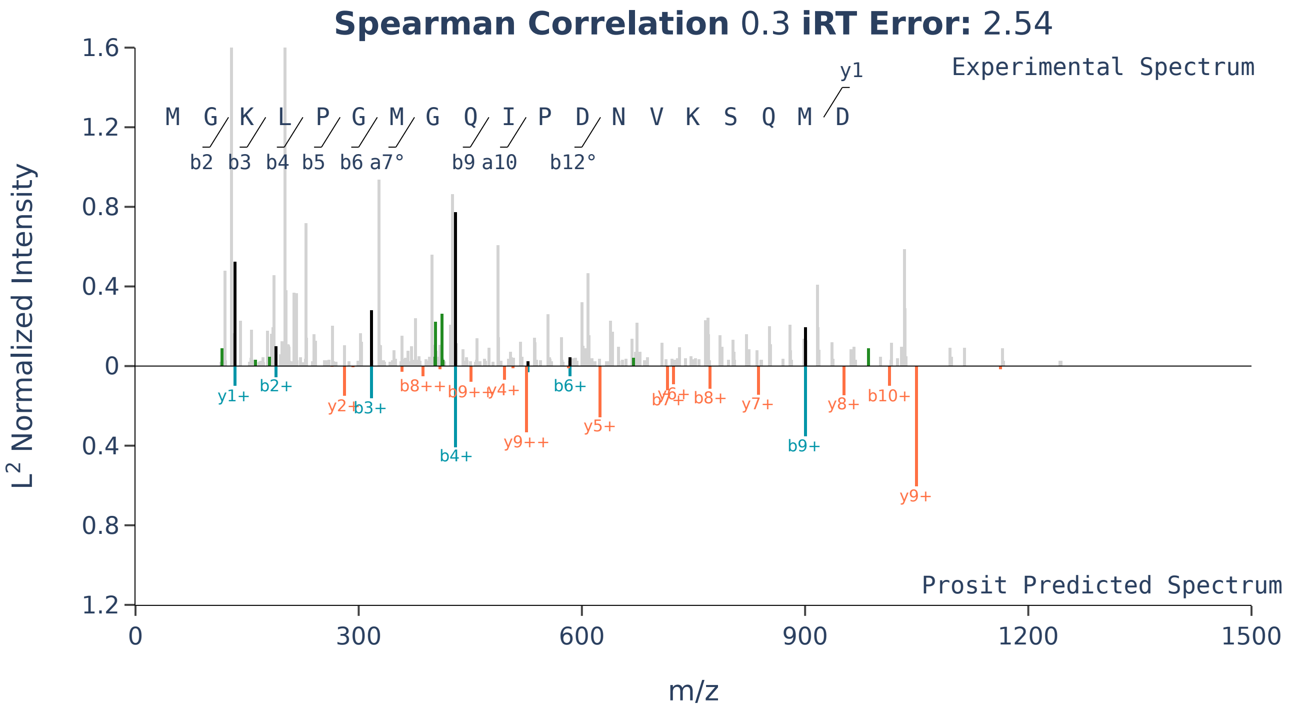

Source Ncheng_210623_230623_HFGoe_FFH_20S_25_1_A1_24h_R1 **Scan** 19240
Peptide ALEPFHPDRIDTA **Charge** 2 **Spectral Angle** 0.79
Spearman Correlation 0.85 **iRT Error:** 0.29

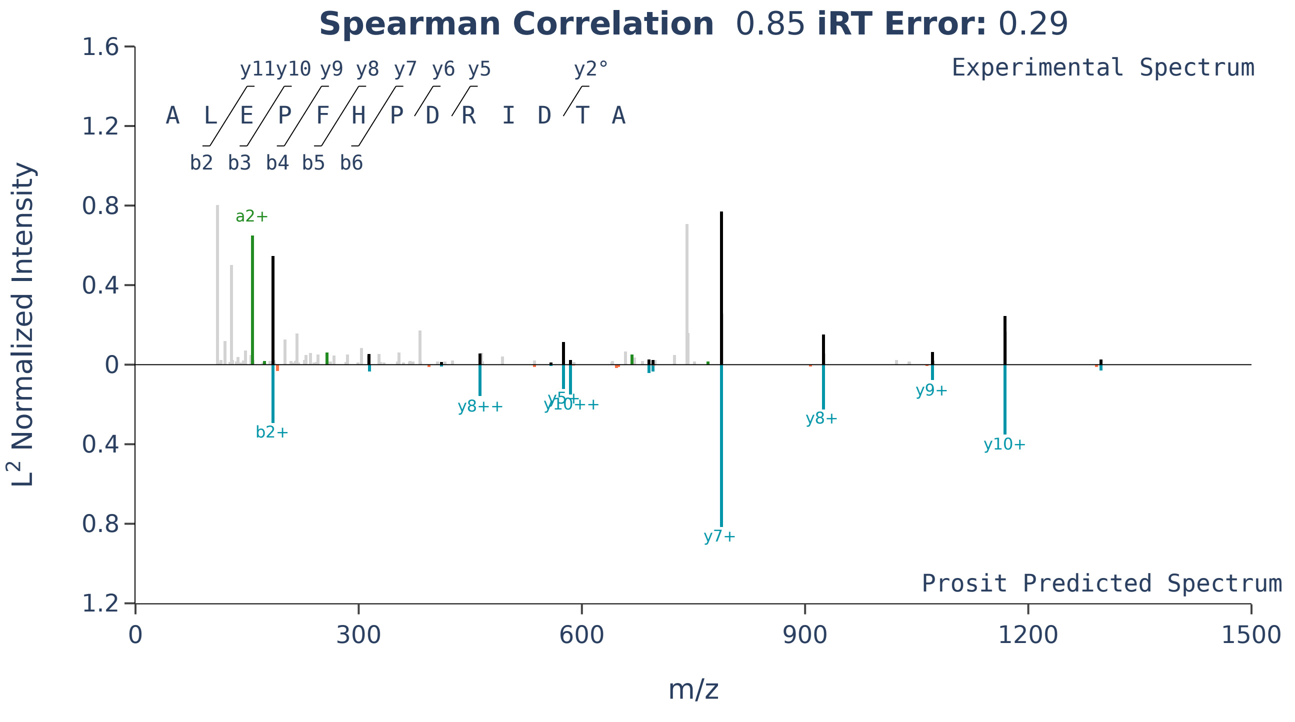

Source Ncheng_210623_230623_HFGoe_FFH_20S_25_1_A1_24h_R1 **Scan** 19240
Peptide MDEIKQVHASINP **Charge** 2 **Spectral Angle** 0.0
Spearman Correlation 0.0 **iRT Error:** 19.71

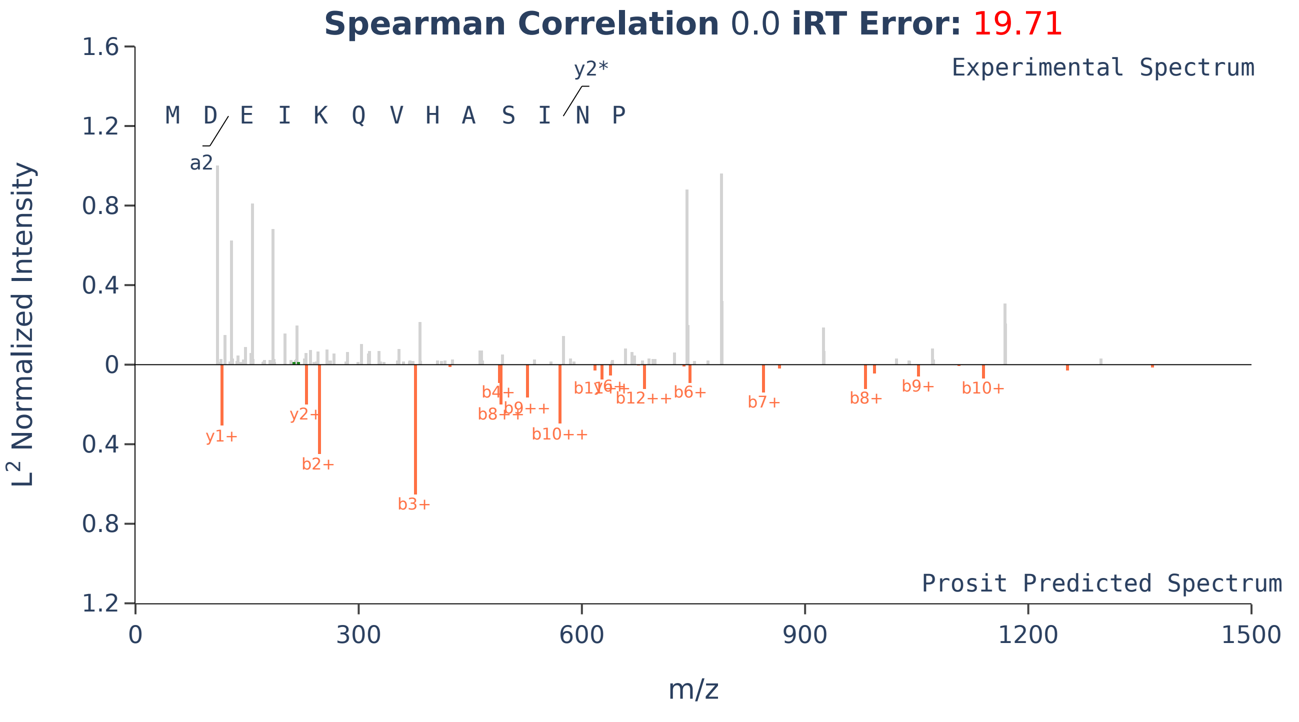

Source Ncheng_210623_230623_HFGoe_FFH_20S_25_1_A1_2h_R2 **Scan** 11881
Peptide KTEAMTGQDAANTAKAFN **Charge** 3 **Spectral Angle** 0.72
Spectral Correlation 0.91 **iRT Error:** 0.31

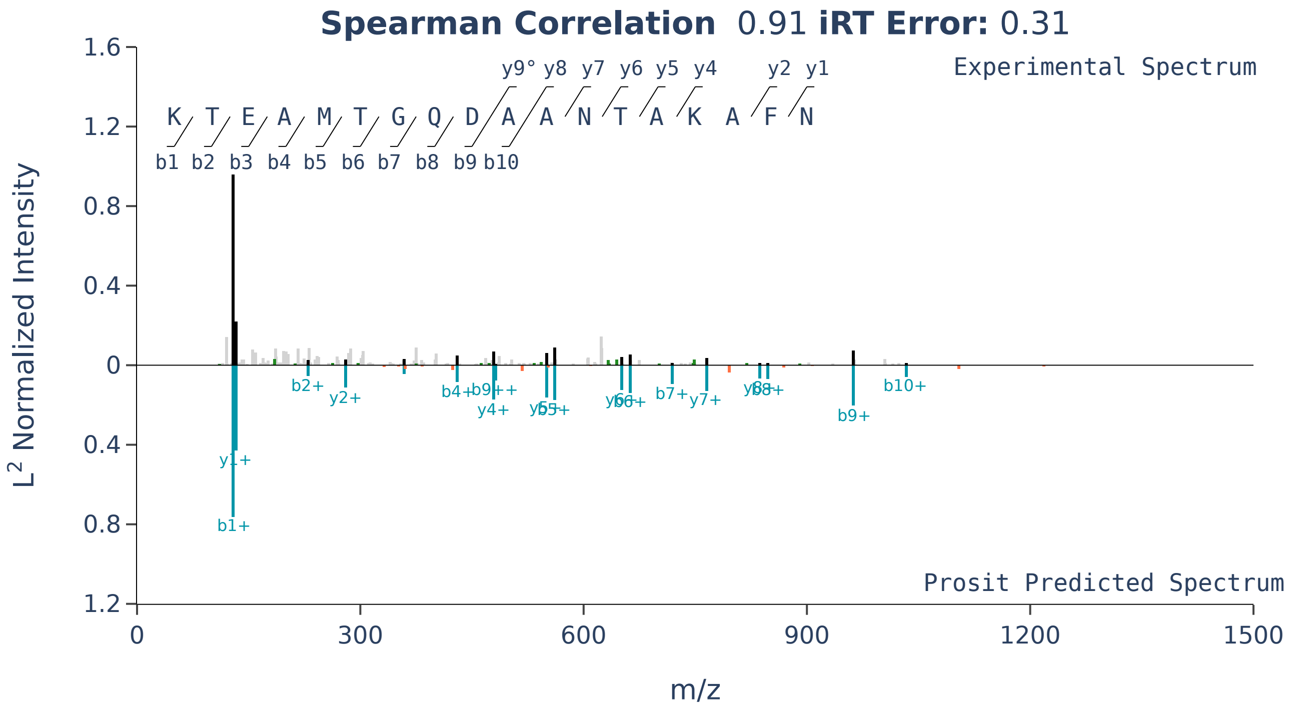

Source Ncheng_210623_230623_HFGoe_FFH_20S_25_1_A1_2h_R2 **Scan** 11881
Peptide DFLEQLRQMKNMGGMA **Charge** 3 **Spectral Angle** 0.17
Spearman Correlation 0.28 **iRT Error:** 105.73

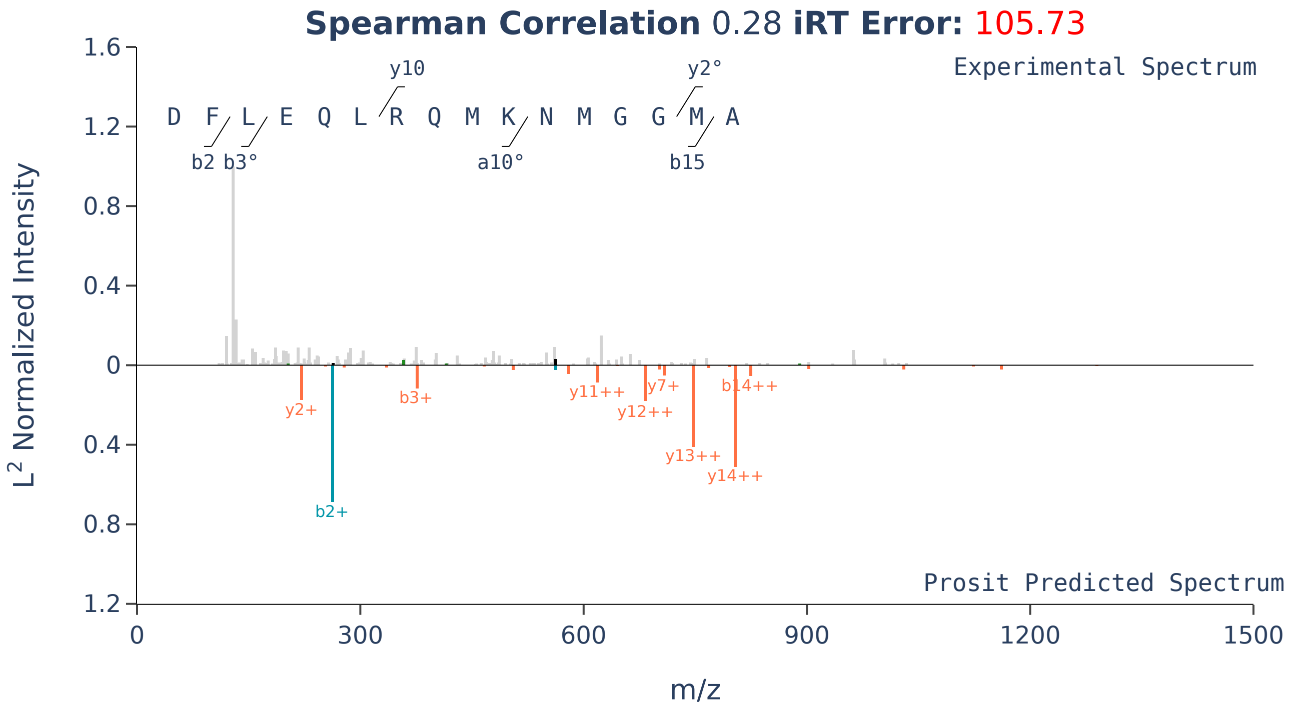

Source Ncheng_210623_230623_HFGoe_FFH_20S_25_1_A2_4h_R2 **Scan** 17219
Peptide MREAIINSMTMKE **Charge** 3 **Spectral Angle** 0.81
Spearman Correlation 0.91 **iRT Error:** 0.33

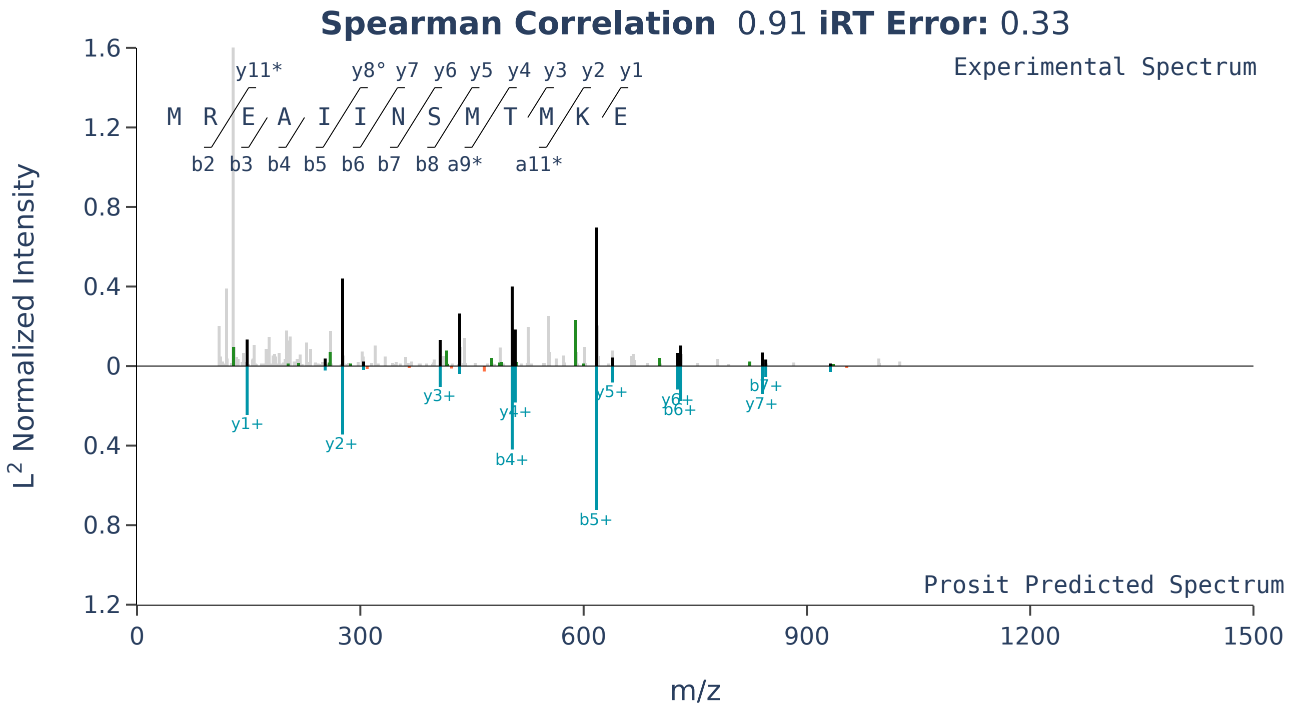

Source Ncheng_210623_230623_HFGoe_FFH_20S_25_1_A2_4h_R2 **Scan** 17219
Peptide MEAIINSMTMKER **Charge** 3 **Spectral Angle** 0.23
Spearman Correlation 0.26 **iRT Error:** 2.18

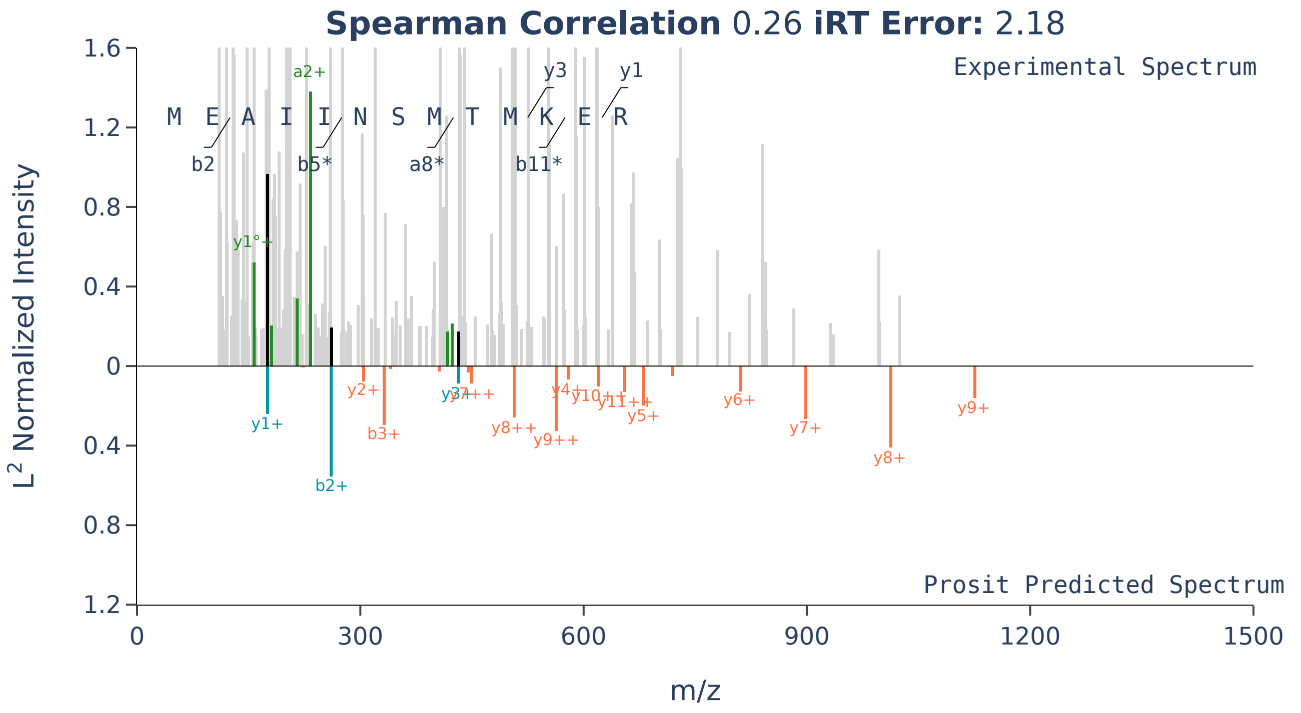

Source Ncheng_210623_230623_HFGoe_FFH_20S_25_1_A1_4h_R2 **Scan** 18893
Peptide GKLPGMMGQIPDNVKSQM **Charge** 3 **Spectral Angle** 0.74
Spearman Correlation 0.87 **iRT Error:** 0.33

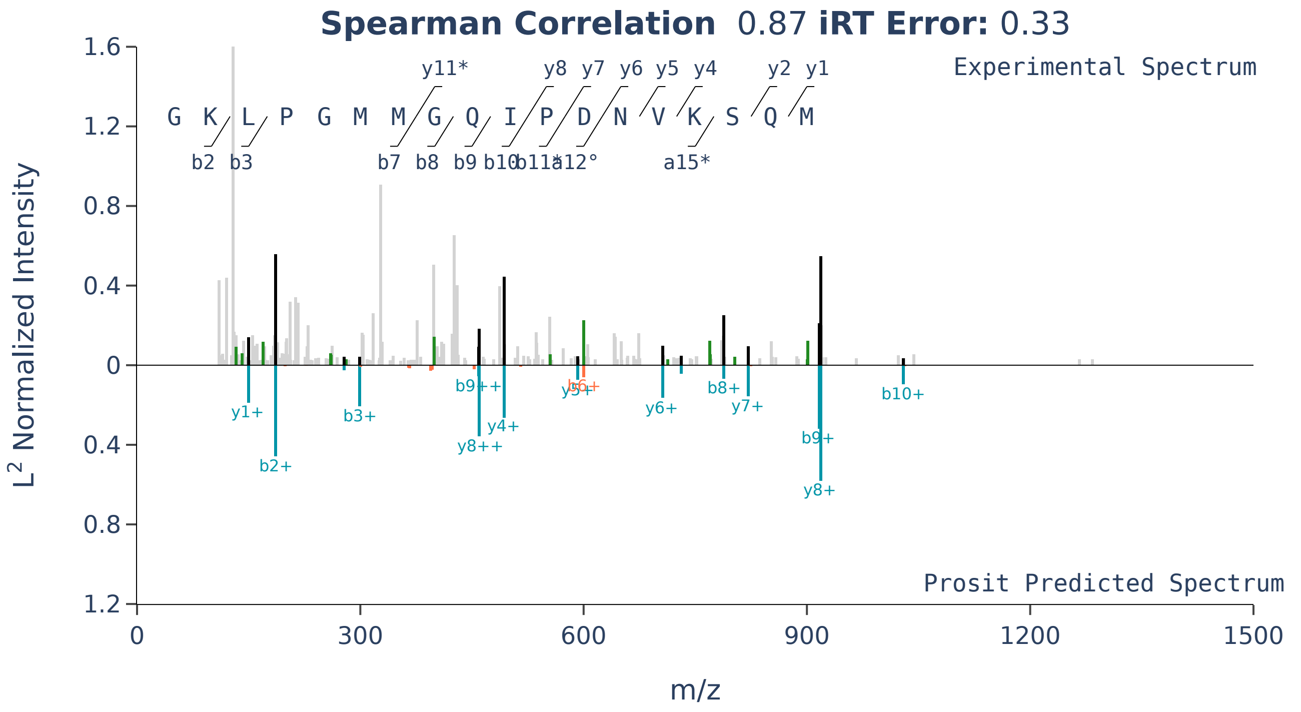

Source Ncheng_210623_230623_HFGoe_FFH_20S_25_1_A1_4h_R2 **Scan** 18893
Peptide MGKLPGMGQIPDNVKSQM **Charge** 3 **Spectral Angle** 0.22
Spearman Correlation 0.2 **iRT Error:** 4.9

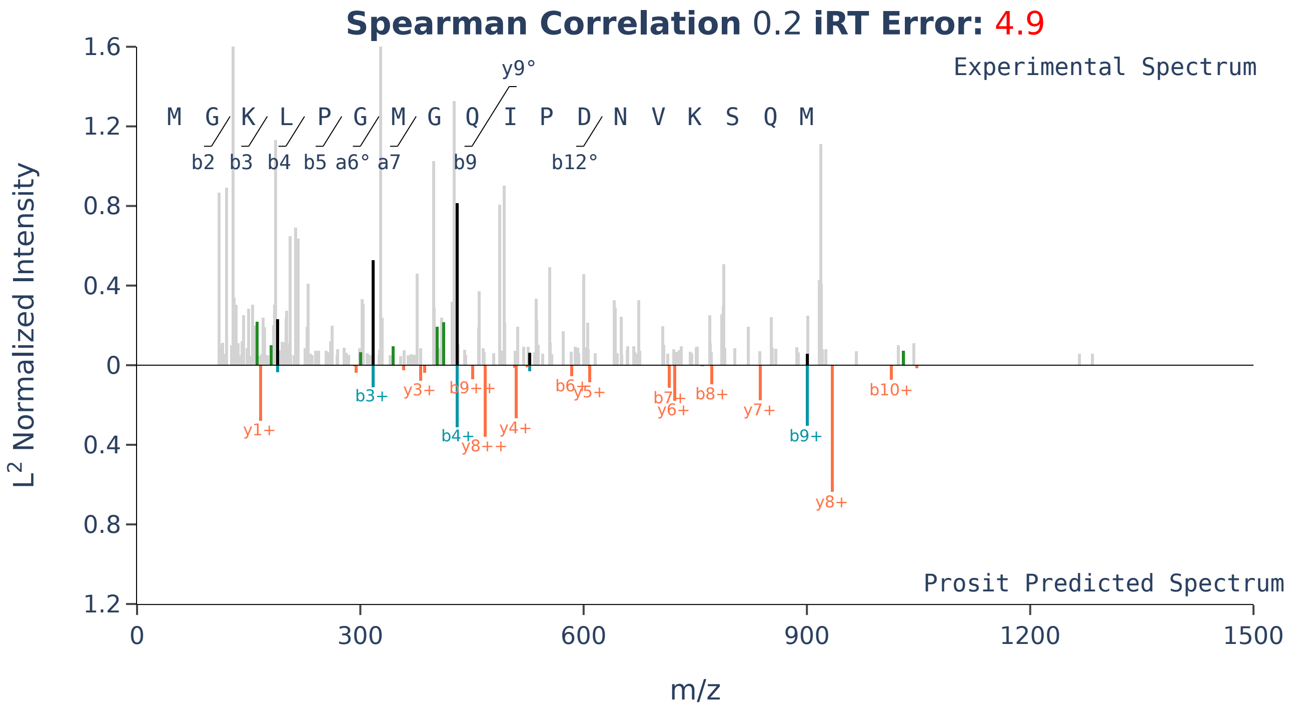

Source Ncheng_210623_230623_HFGoe_FFH_20S_25_1_A1_24h_R1 **Scan** 19244
Peptide GMKLPGMGQIPDNVKSQM **Charge** 2 **Spectral Angle** 0.83
Spearman Correlation 0.82 **iRT Error:** 0.34

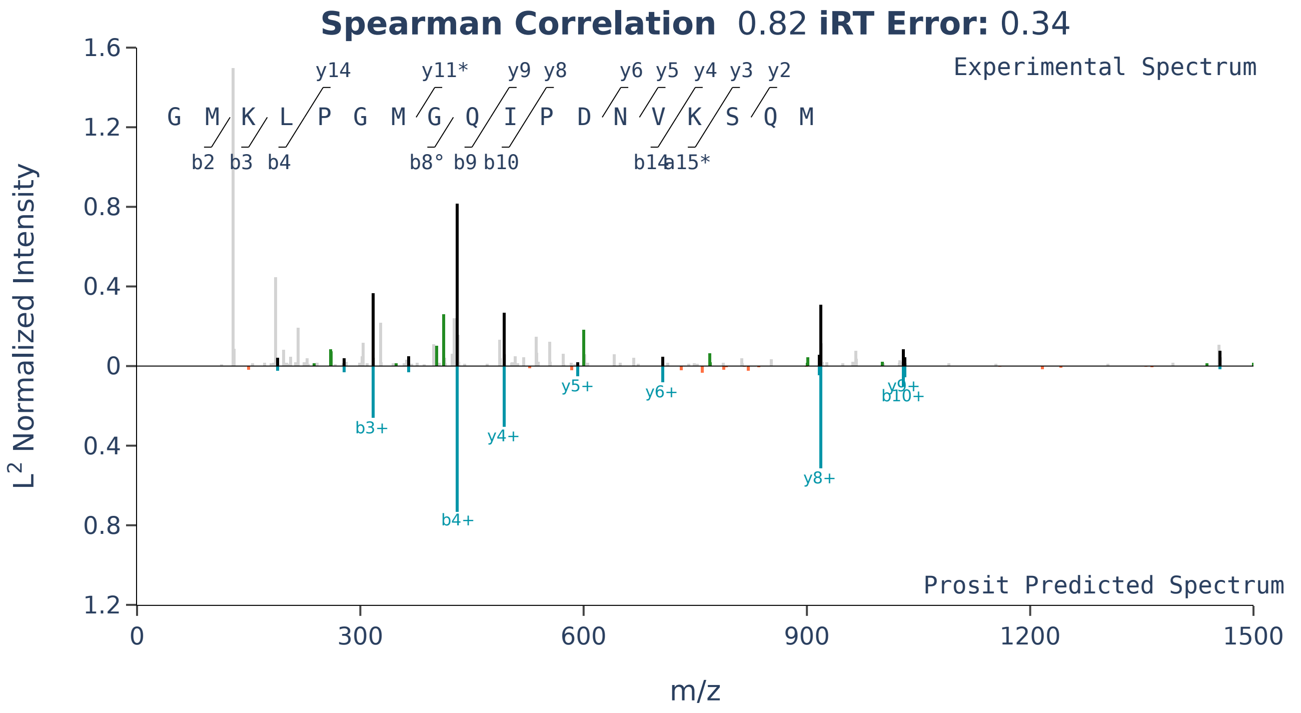

Source Ncheng_210623_230623_HFGoe_FFH_20S_25_1_A1_24h_R1 **Scan** 19244
Peptide MGKLPGMGQIPDNVKSQM **Charge** 2 **Spectral Angle** 0.46
Spearman Correlation 0.56 **iRT Error:** 5.01

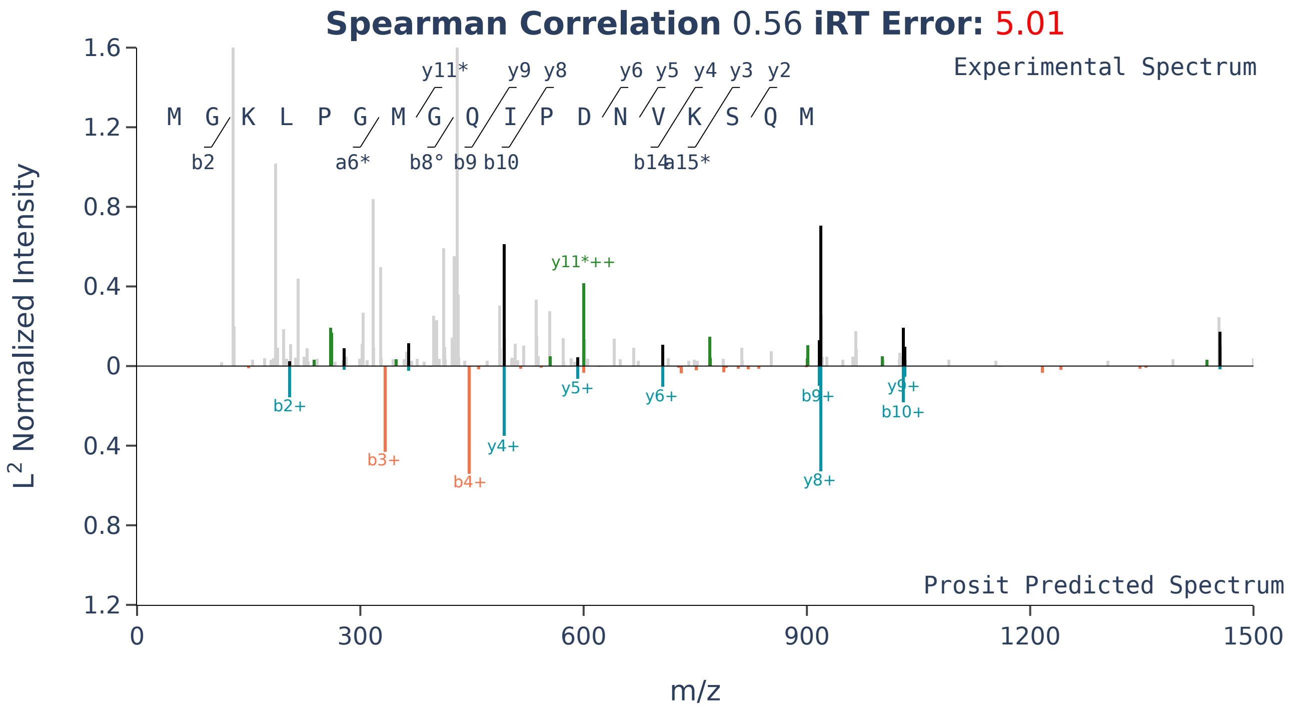

Source Ncheng_210623_230623_HFGoe_FFH_20S_25_1_A2_24h_R2 **Scan** 12384
Peptide FNAMTGQDAANTAKA **Charge** 2 **Spectral Angle** 0.87
Spearman Correlation 0.84 **iRT Error:** 0.34

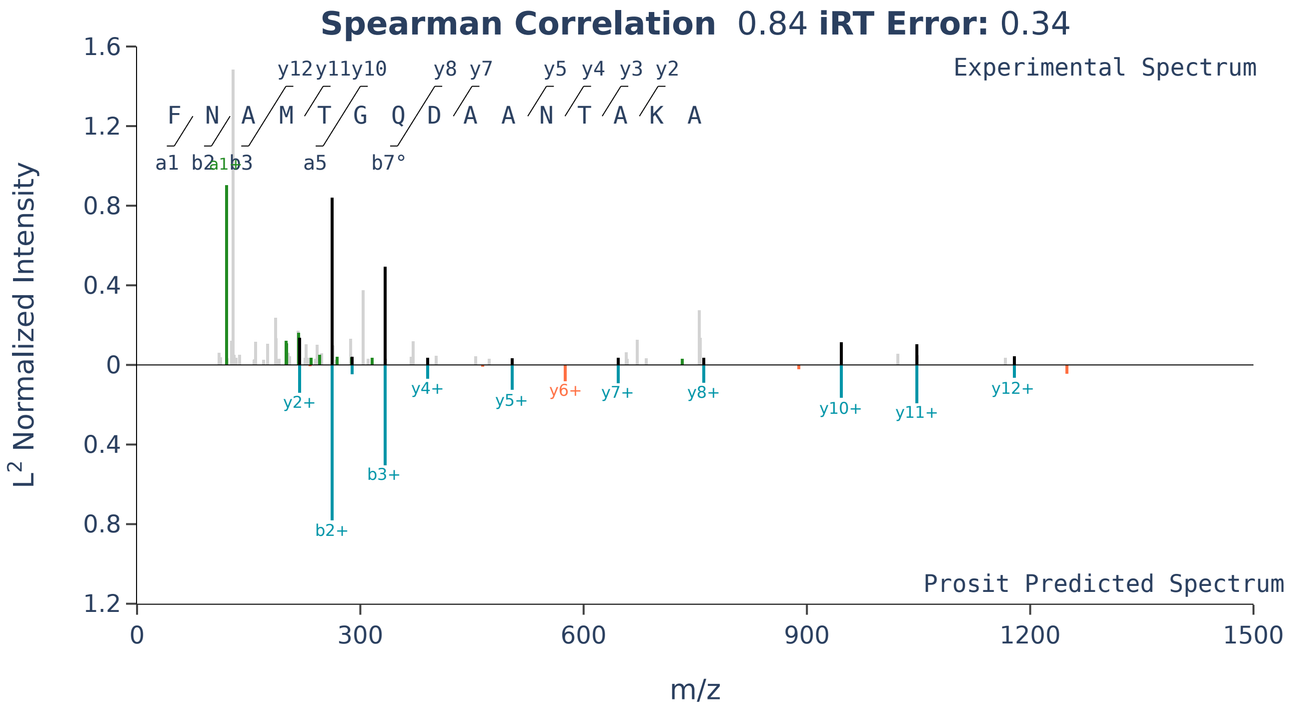

Source Ncheng_210623_230623_HFGoe_FFH_20S_25_1_A2_24h_R2 **Scan** 12384
Peptide AMTGQDAANTAKAFN **Charge** 2 **Spectral Angle** 0.49
Spearman Correlation 0.42 **iRT Error:** 6.51

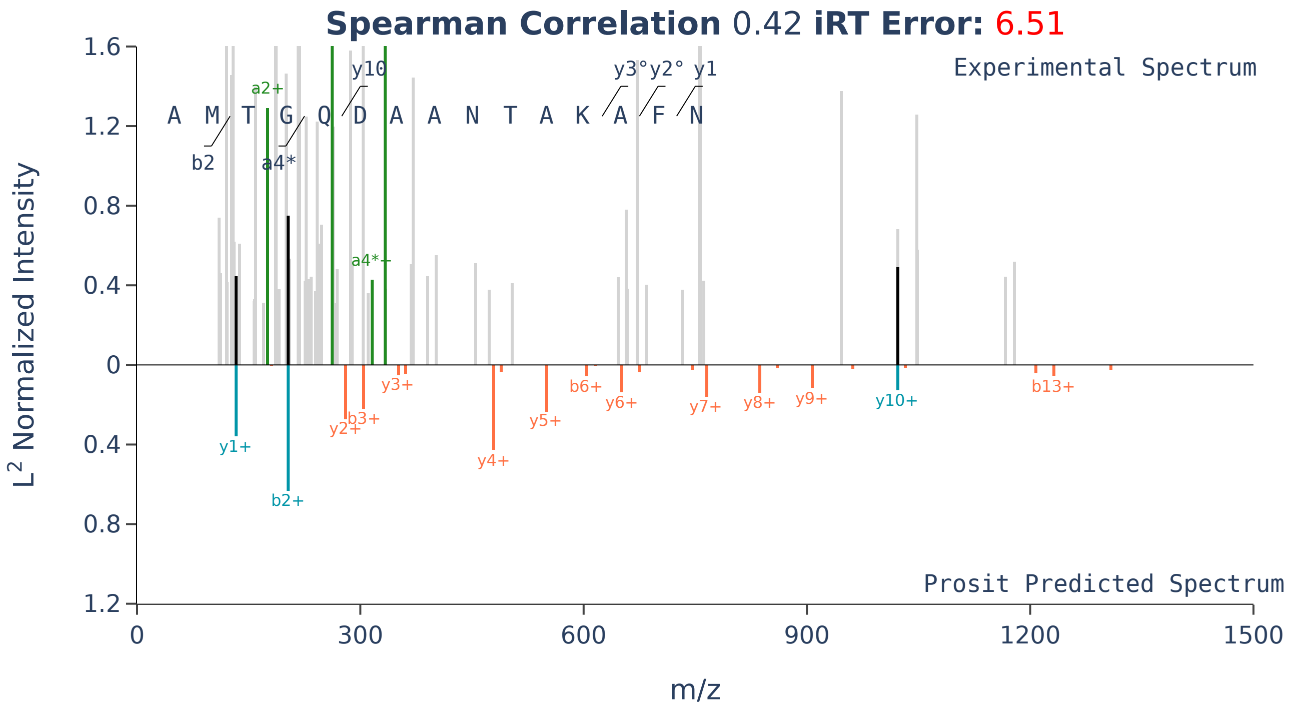

Source Ncheng_210623_230623_HFGoe_FFH_20S_25_1_A1_2h_R1 **Scan** 6805
Peptide GGKSLTPGQE **Charge** 2 **Spectral Angle** 0.75
Spearman Correlation 0.9 **iRT Error:** 0.34

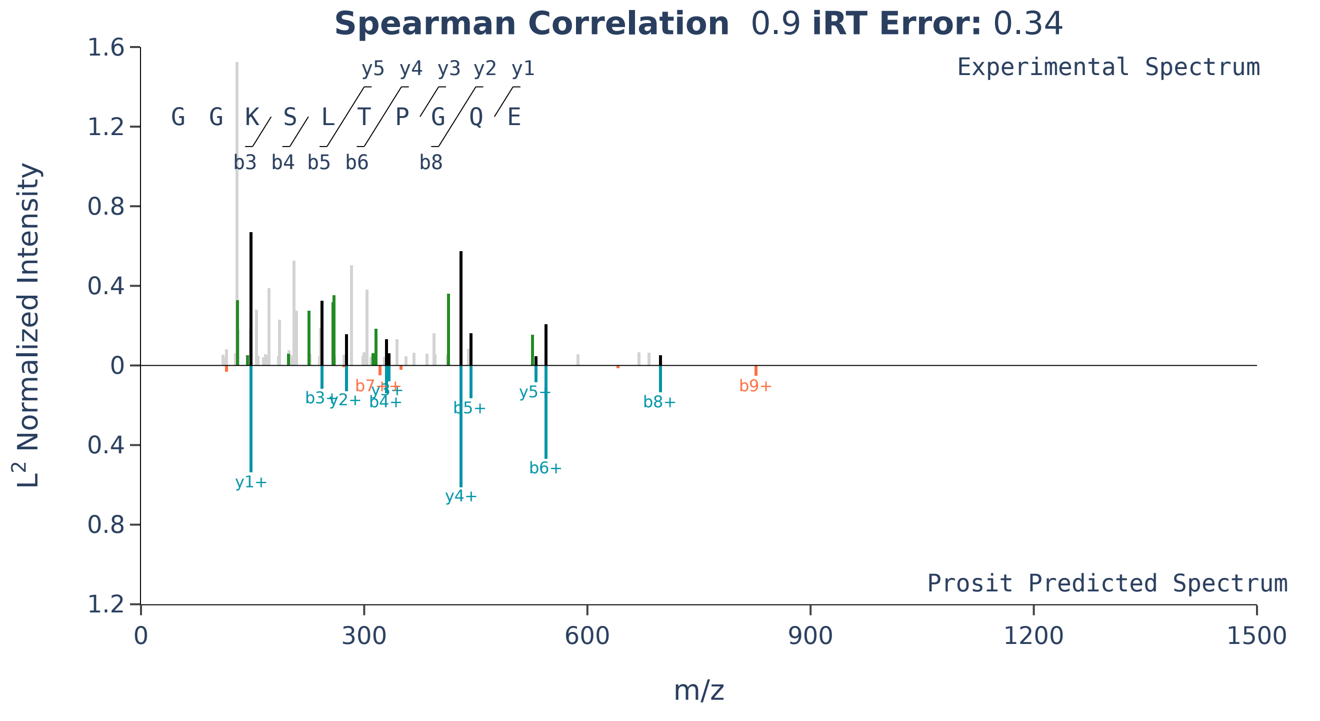

Source Ncheng_210623_230623_HFGoe_FFH_20S_25_1_A1_2h_R1 **Scan** 6805
Peptide ENQTLNLAA **Charge** 2 **Spectral Angle** 0.01
Spearman Correlation -0.23 **iRT Error:** 52.96

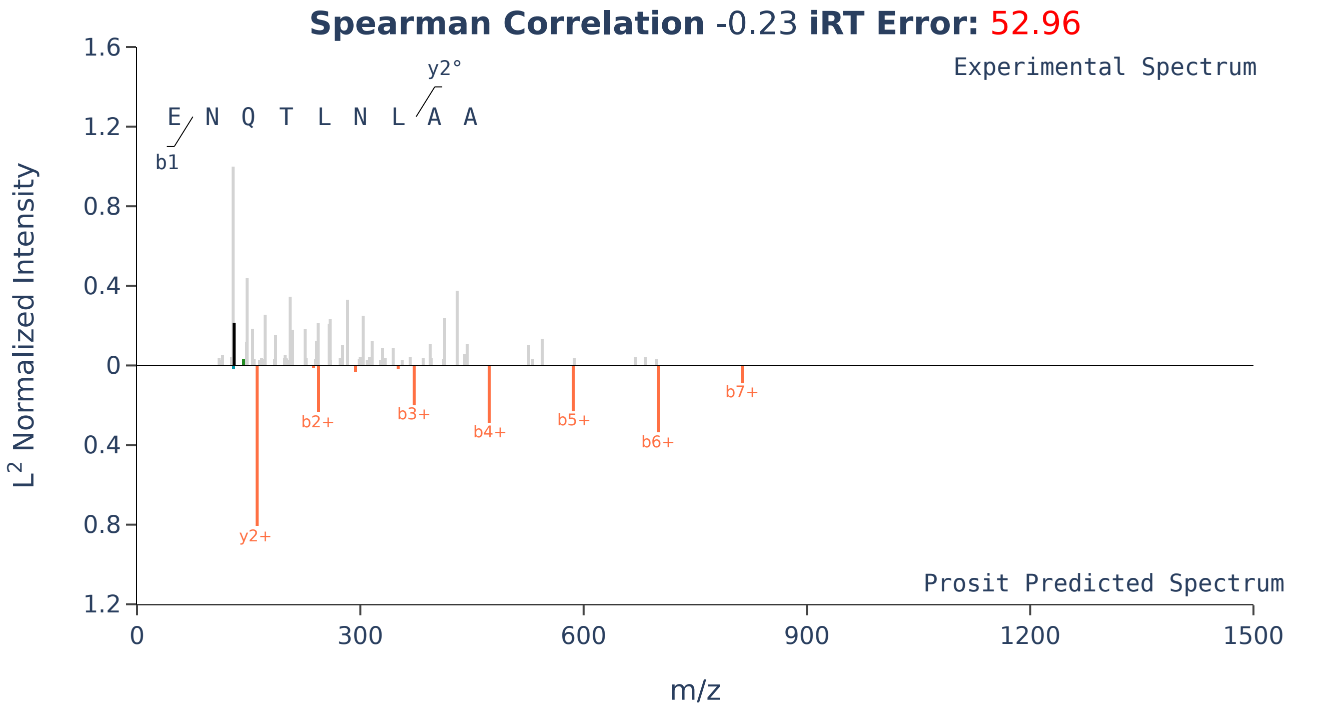

Source Ncheng_210623_230623_HFGoe_FFH_20S_25_1_A2_2h_R2 **Scan** 18045
Peptide KLPGMGMGQIPDNVKSQMDD **Charge** 3 **Spectral Angle** 0.84
Spearman Correlation 0.86 **iRT Error:** 0.34

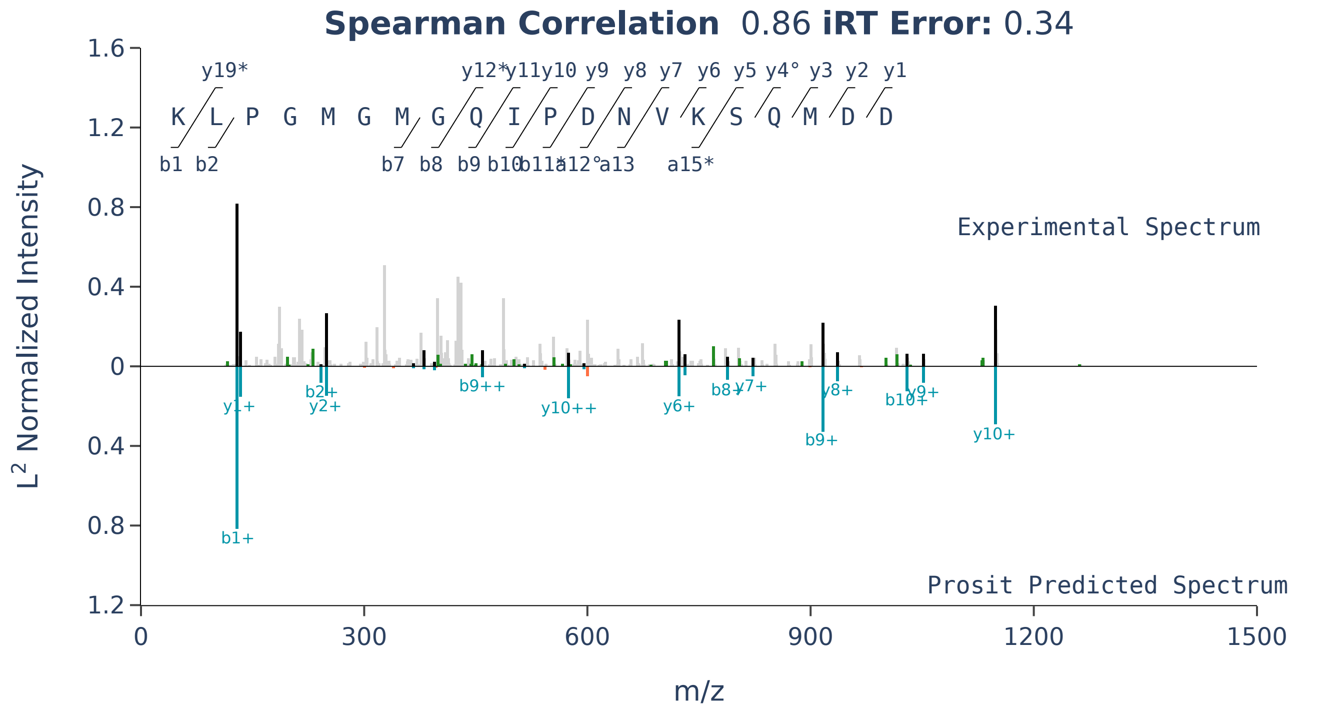

Source Ncheng_210623_230623_HFGoe_FFH_20S_25_1_A2_2h_R2 **Scan** 18045
Peptide MGKLPGMGMGQIPDNVKSQMDD **Charge** 3 **Spectral Angle** 0.35
Spearman Correlation 0.21 **iRT Error:** 9.48

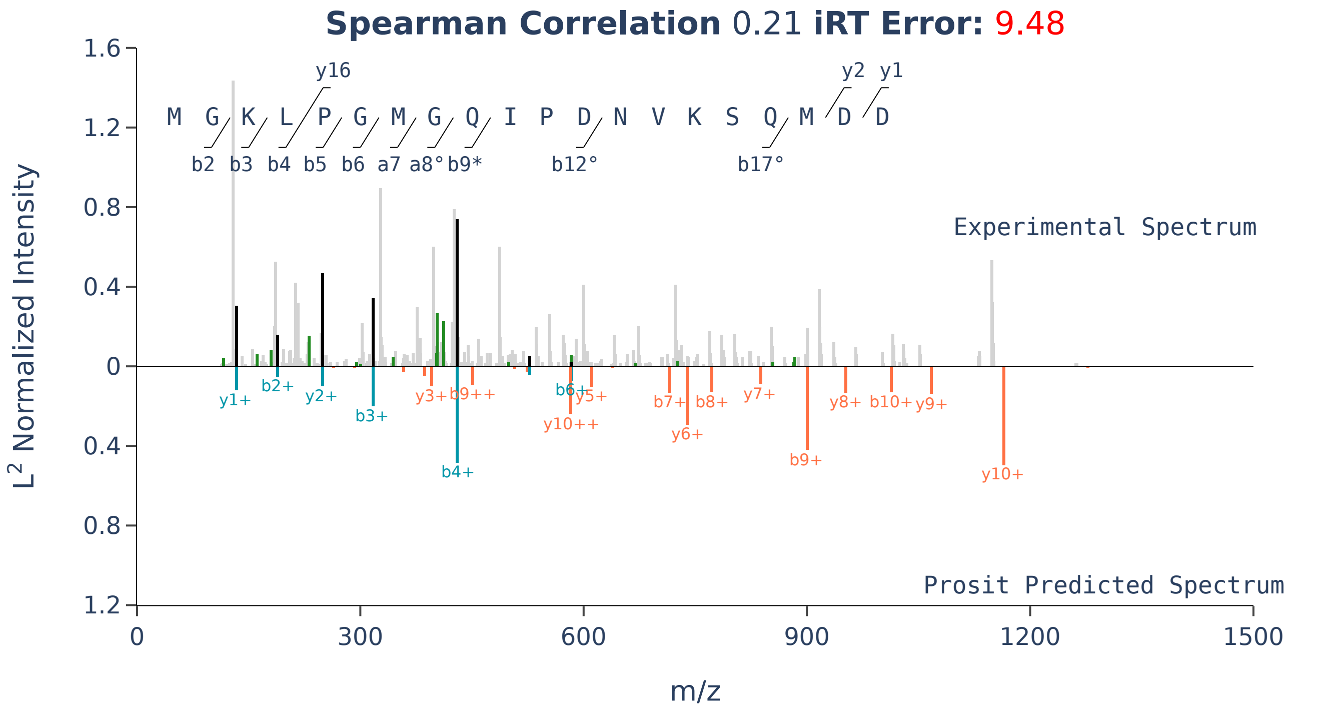

Source Ncheng_210623_230623_HFGoe_FFH_20S_25_1_A1_2h_R2 **Scan** 17593
Peptide KLPGMGQIPDNVKSQDM **Charge** 2 **Spectral Angle** 0.84
Spearman Correlation 0.87 **iRT Error:** 0.38

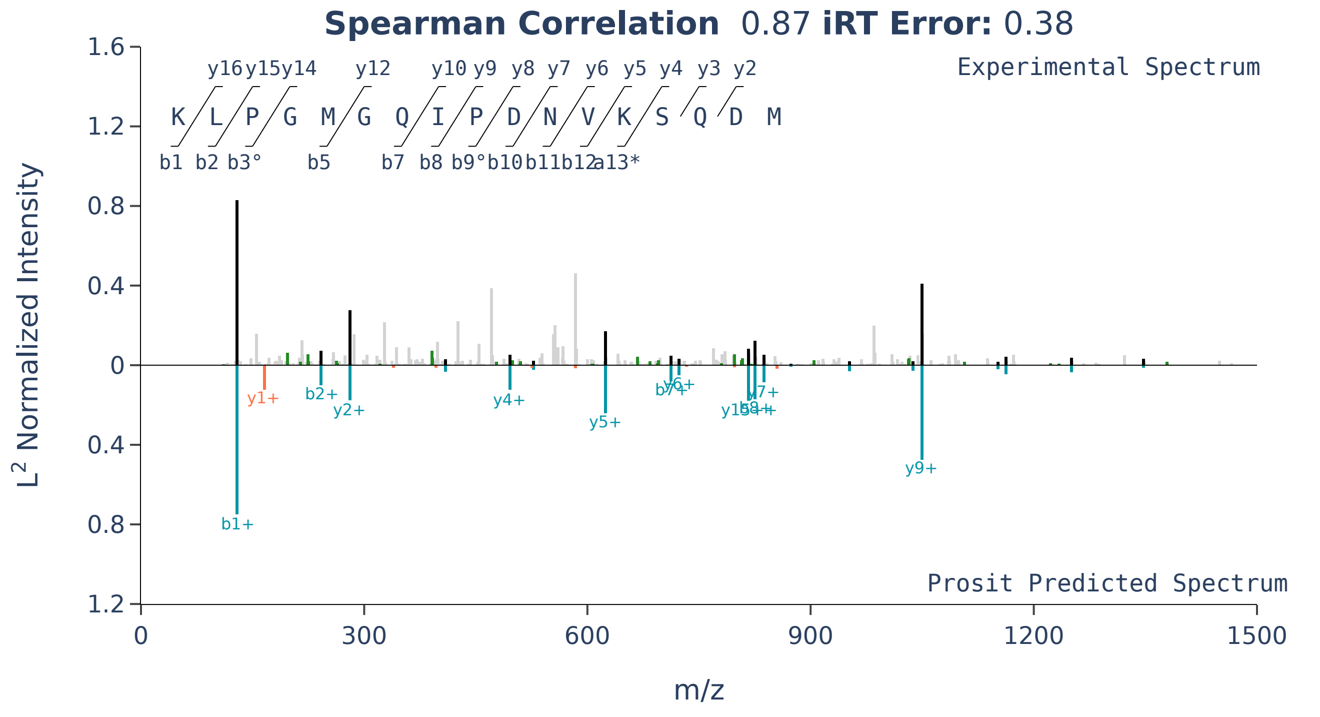

Source Ncheng_210623_230623_HFGoe_FFH_20S_25_1_A1_2h_R2 **Scan** 17593
Peptide KLPGMGQIPDNVKSQMD **Charge** 2 **Spectral Angle** 0.54
Spearman Correlation 0.47 **iRT Error:** 12.63

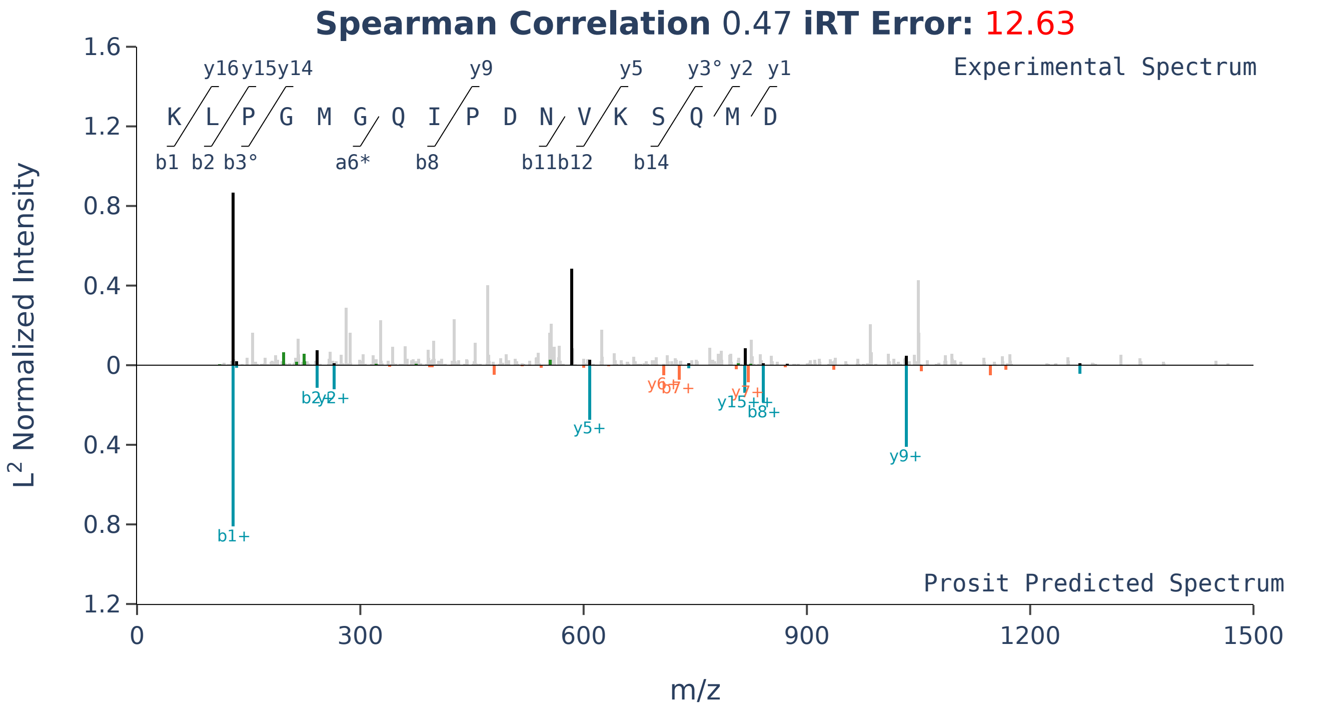

Source Ncheng_210623_230623_HFGoe_FFH_20S_25_1_A2_24h_R2 **Scan** 9932
Peptide GQIPDNVKTG **Charge** 1 **Spectral Angle** 0.85
Spearman Correlation 0.9 **iRT Error:** 0.38

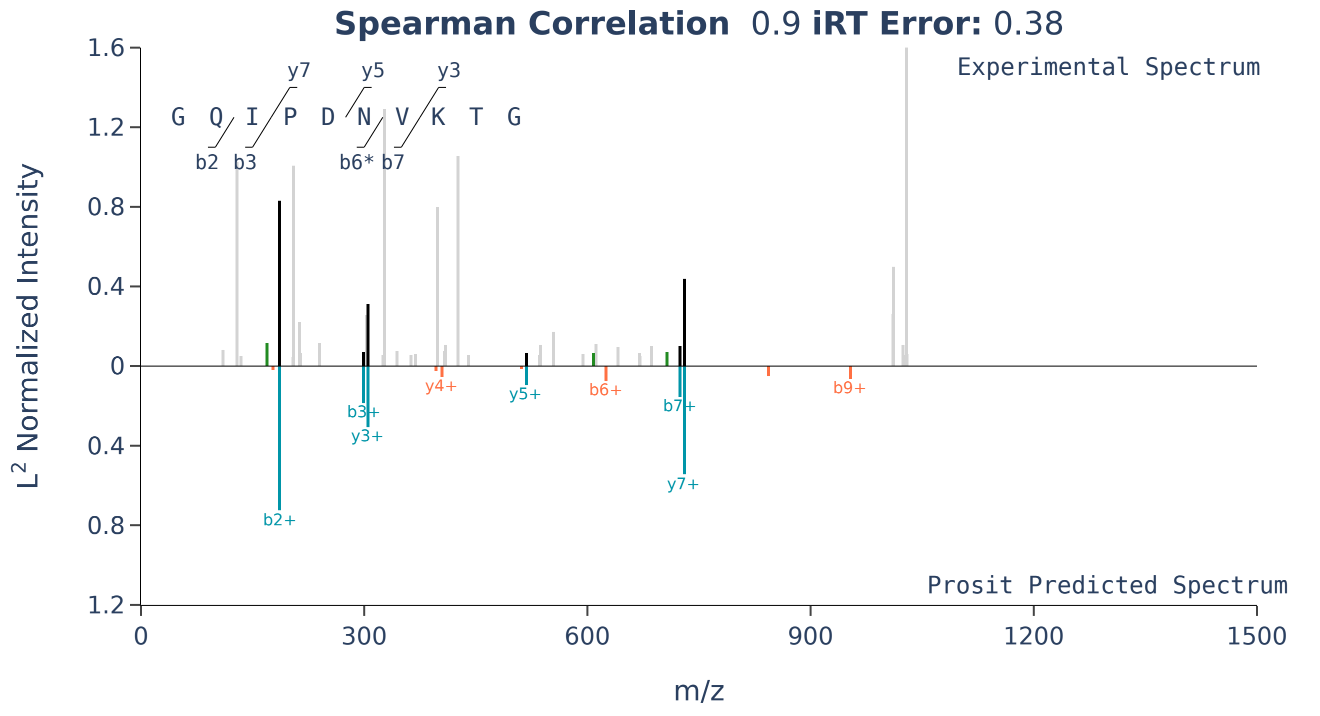

Source Ncheng_210623_230623_HFGoe_FFH_20S_25_1_A2_24h_R2 **Scan** 9932
Peptide QIPDNVKSQ **Charge** 1 **Spectral Angle** 0.0
Spearman Correlation 0.0 **iRT Error:** 10.6

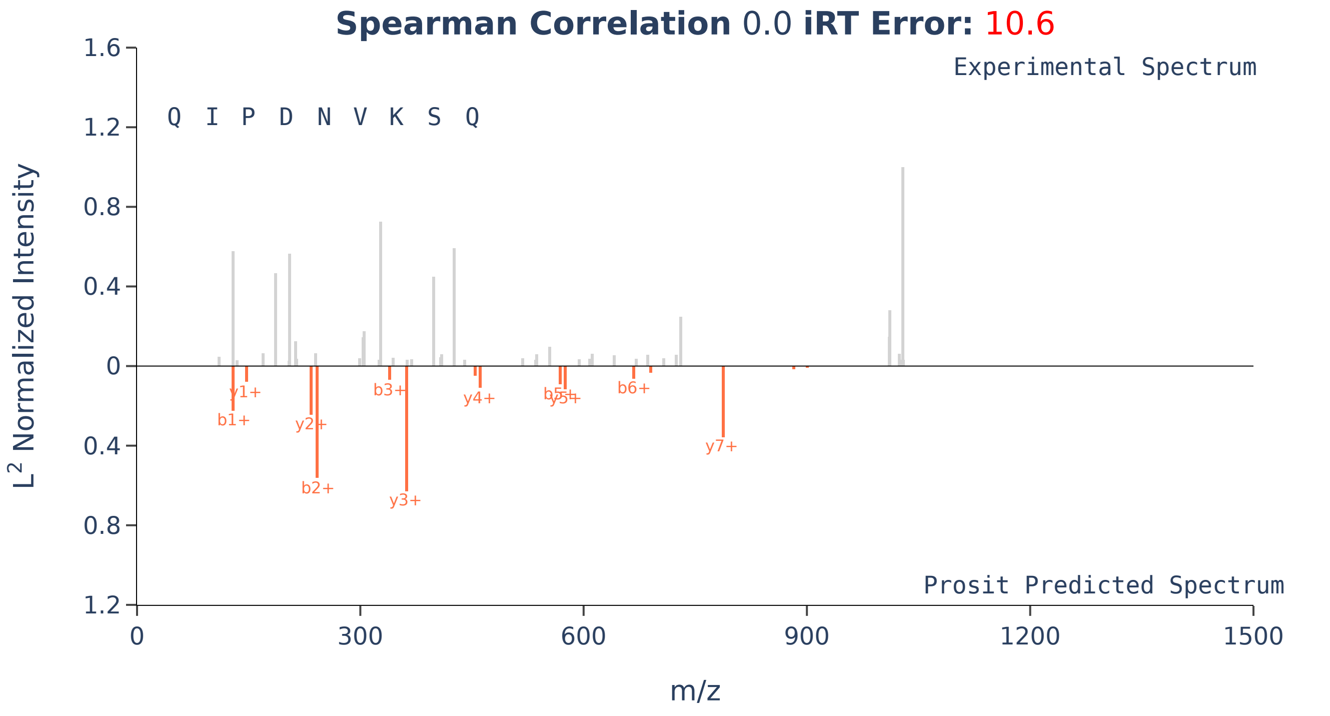

Source Ncheng_210623_230623_HFGoe_FFH_20S_25_1_A2_4h_R2 **Scan** 30387
Peptide LGGHEVNKSLTPGQEFVKIVRNELVA **Charge** 2 **Spectral Angle** 0.72
Spearman Correlation 0.87 **iRT Error:** 0.42

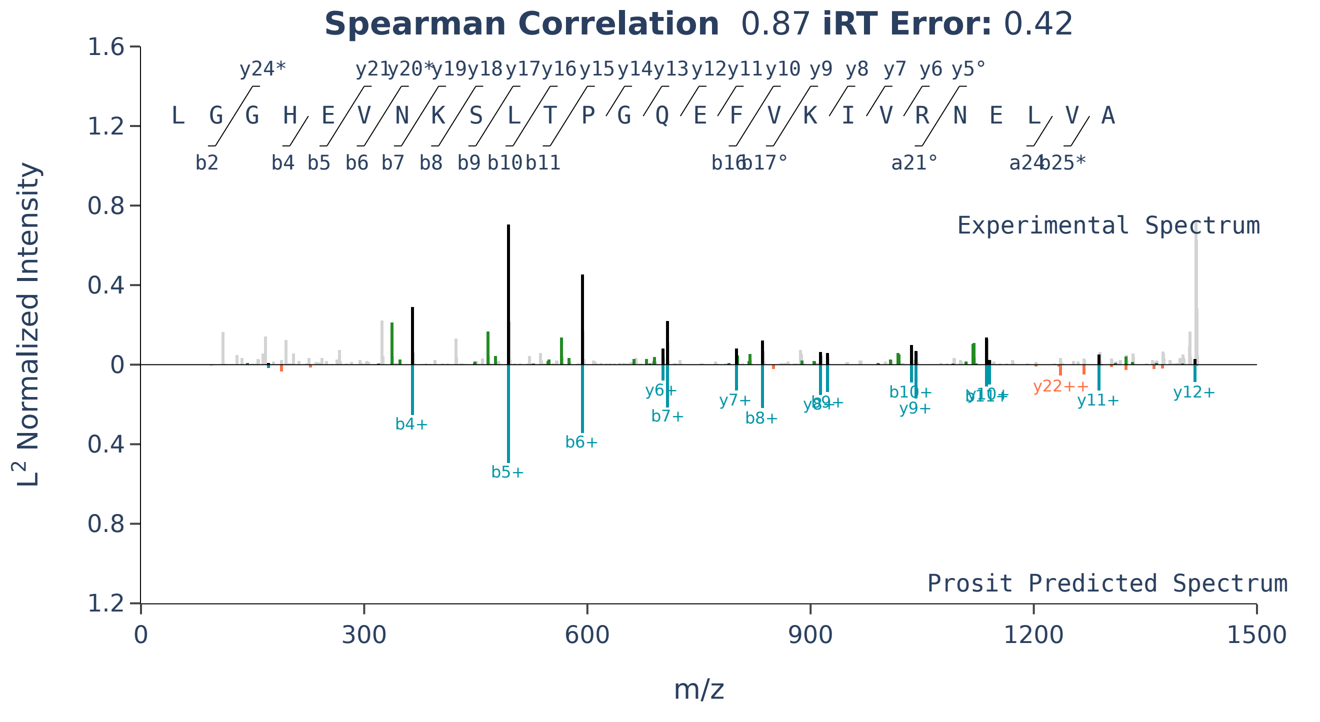

Source Ncheng_210623_230623_HFGoe_FFH_20S_25_1_A2_4h_R2 **Scan** 30387
Peptide VGHEVNKSLTPGQEFVKIVRNELVAA **Charge** 2 **Spectral Angle** 0.44
Spearman Correlation 0.45 **iRT Error:** 7.05

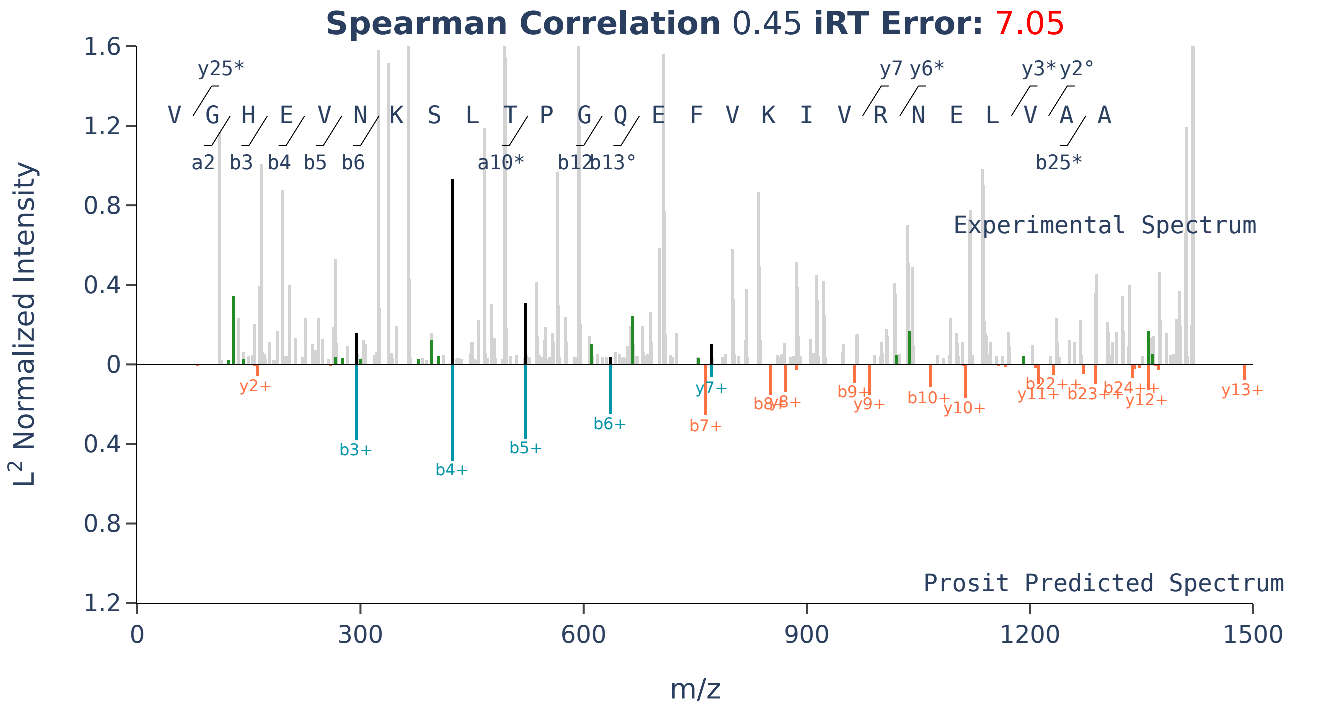

Source Ncheng_210623_230623_HFGoe_FFH_20S_25_1_A1_2h_R2 **Scan** 10534
Peptide KVLVRMED **Charge** 2 **Spectral Angle** 0.69
Spearman Correlation 0.95 **iRT Error:** 0.42

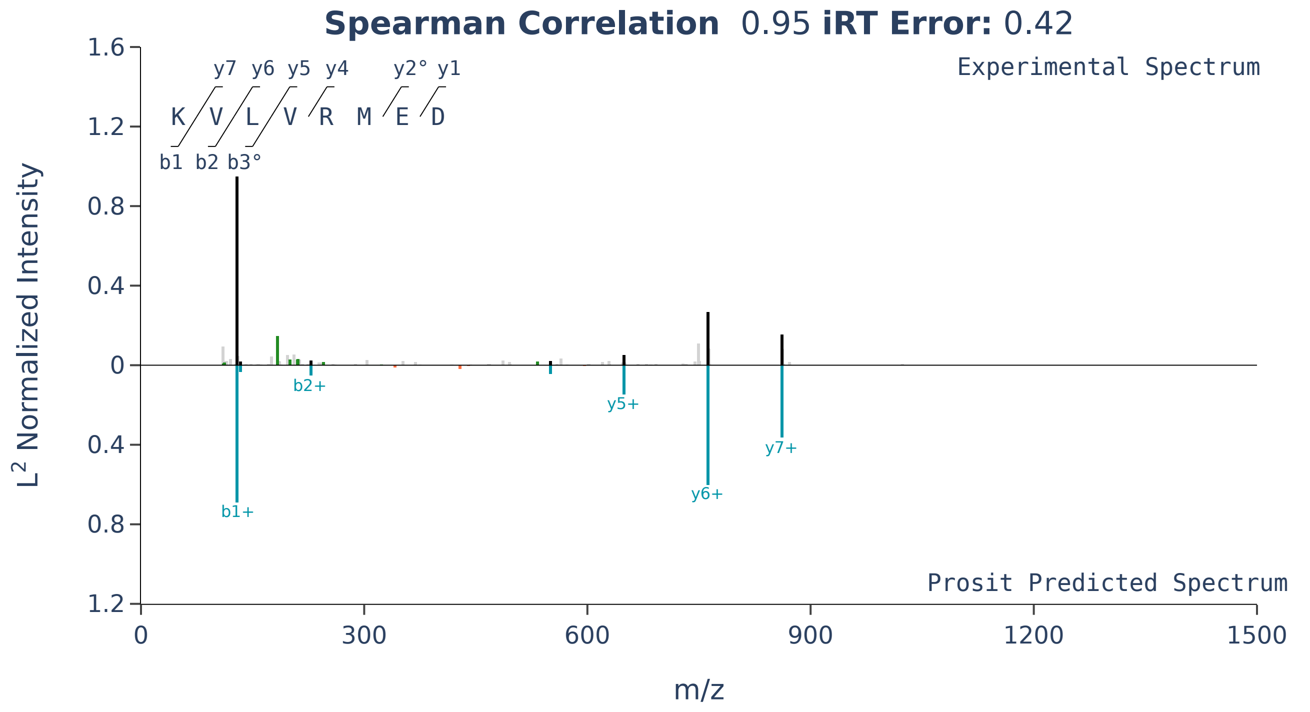

Source Ncheng_210623_230623_HFGoe_FFH_20S_25_1_A1_2h_R2 **Scan** 10534
Peptide DKVLVRME **Charge** 2 **Spectral Angle** 0.47
Spearman Correlation 0.33 **iRT Error:** 13.87

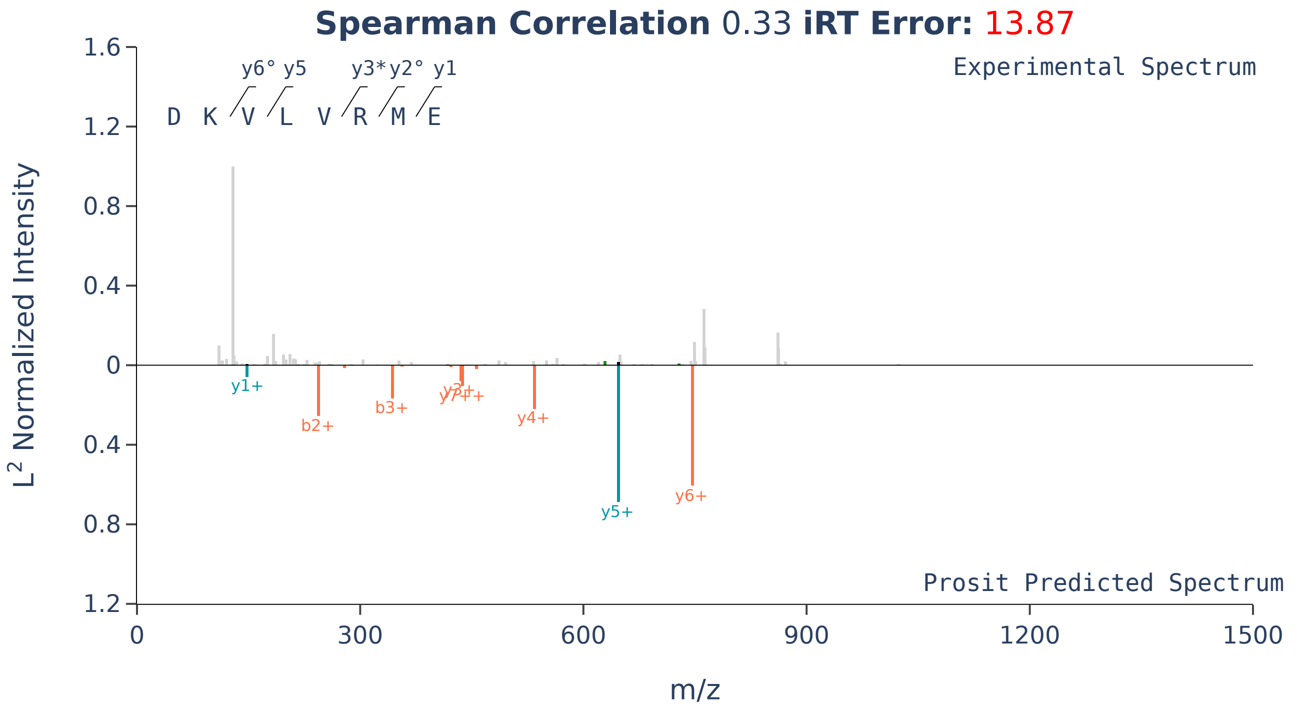

Source Ncheng_210623_230623_HFGoe_FFH_20S_25_1_A1_4h_R1 **Scan** 12044
Peptide PDNVKSQMDADVA **Charge** 1 **Spectral Angle** 0.89
Spearman Correlation 0.93 **iRT Error:** 0.42

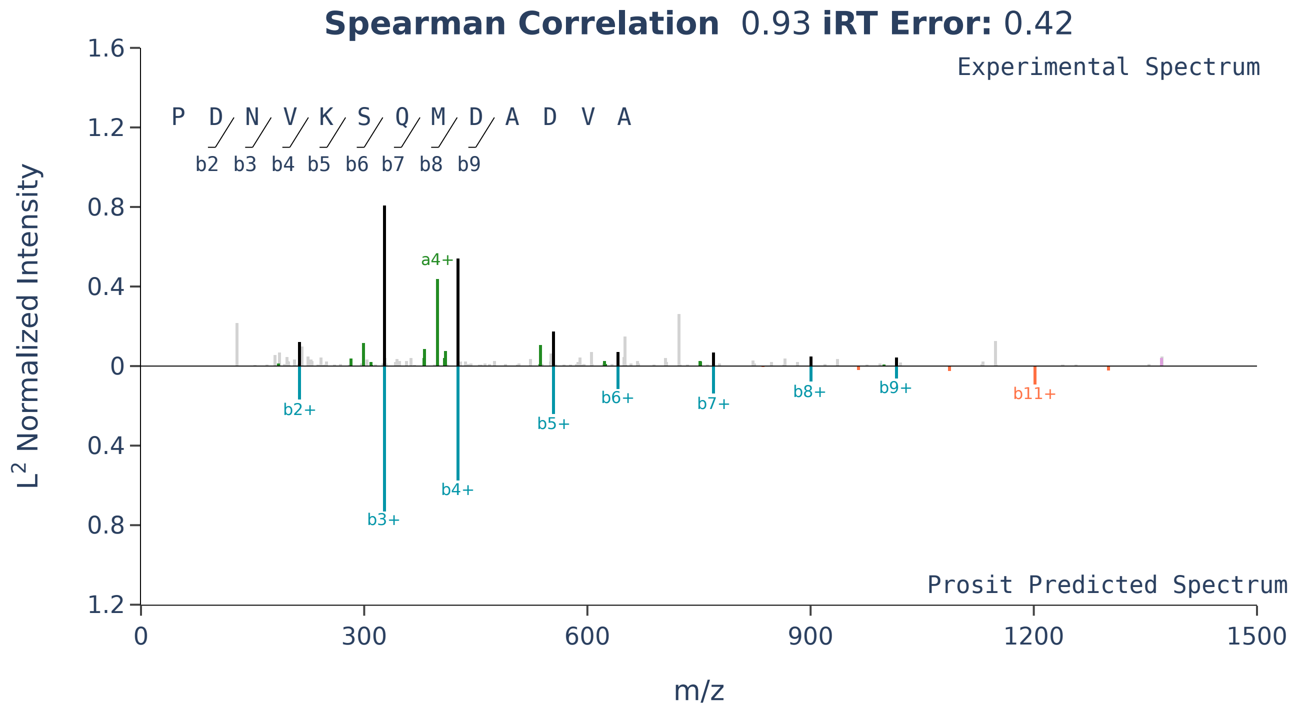

Source Ncheng_210623_230623_HFGoe_FFH_20S_25_1_A1_4h_R1 **Scan** 12044
Peptide QIPDNVKSQMDD **Charge** 1 **Spectral Angle** 0.8
Spearman Correlation 0.62 **iRT Error:** 4.09

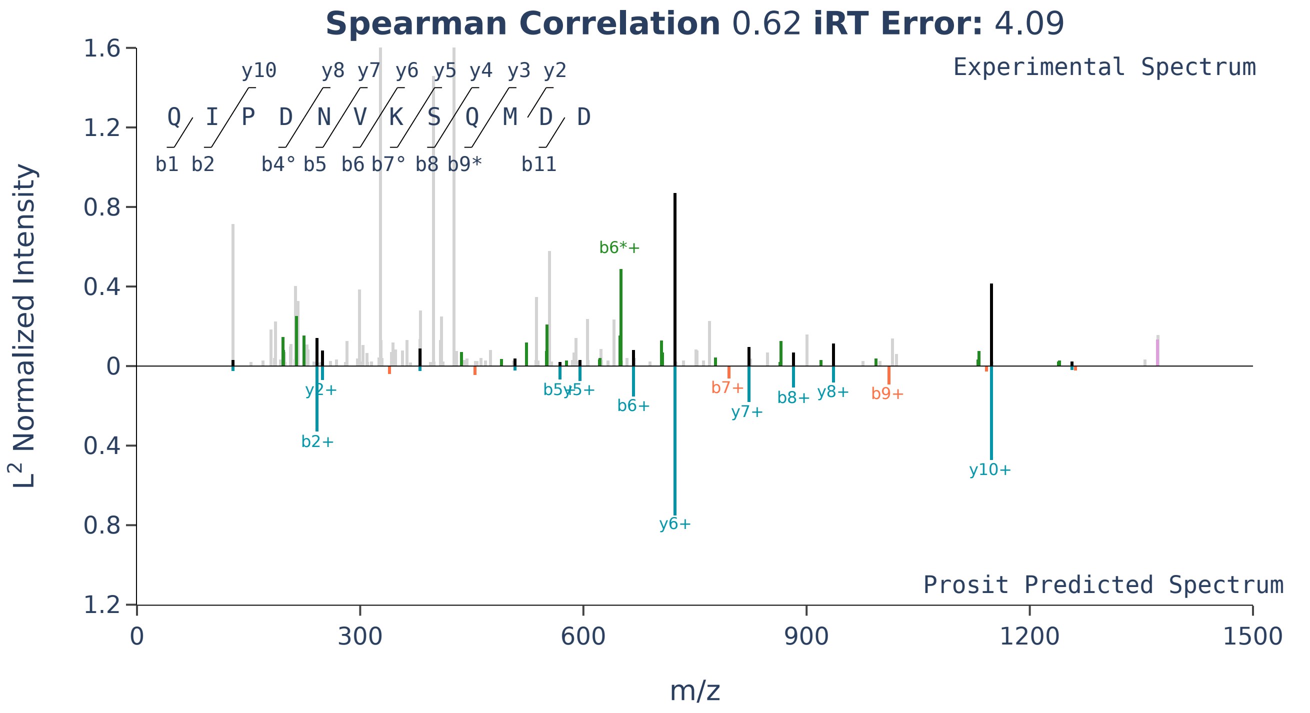

Source Ncheng_210623_230623_HFGoe_FFH_20S_25_1_A2_2h_R1 **Scan** 19140
Peptide GMKLPGMGQIPDNVKSQMDD **Charge** 3 **Spectral Angle** 0.76
Spearman Correlation 0.87 **iRT Error:** 0.43

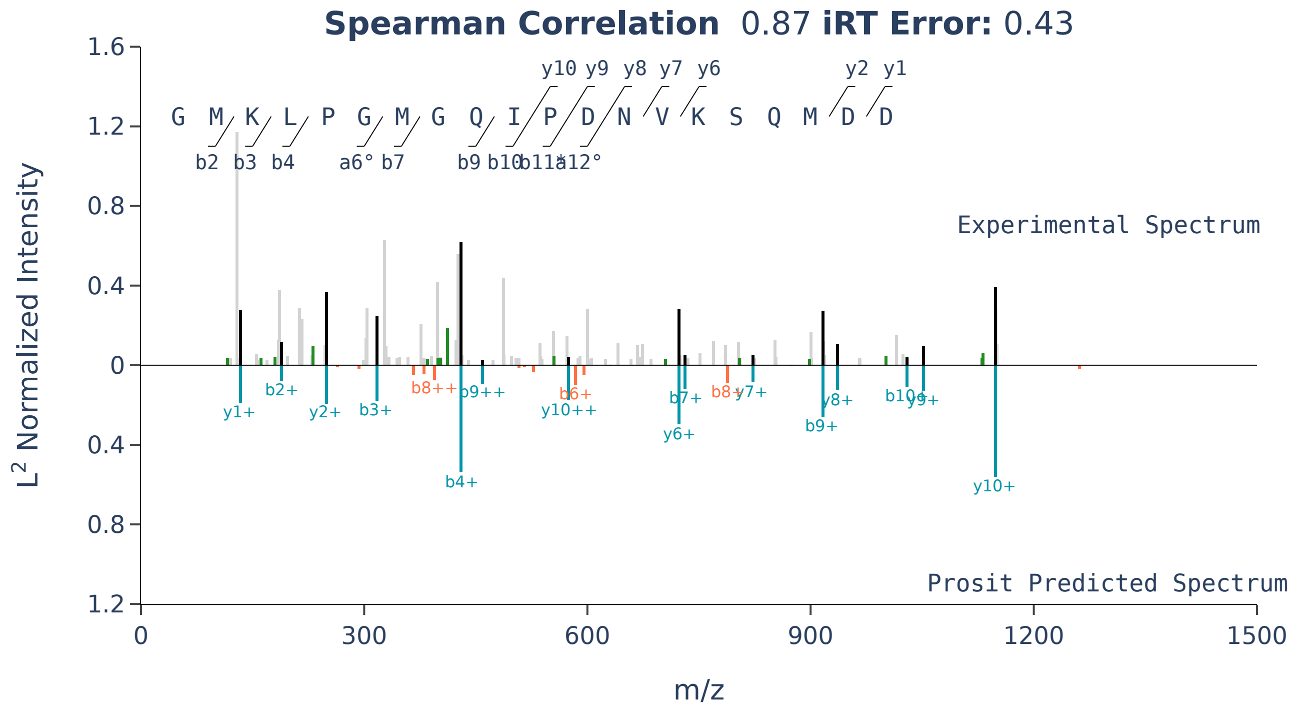

Source Ncheng_210623_230623_HFGoe_FFH_20S_25_1_A2_2h_R1 **Scan** 19140
Peptide MGKLPGMGQIPDNVKSQMDD **Charge** 3 **Spectral Angle** 0.35
Spearman Correlation 0.28 **iRT Error:** 3.89

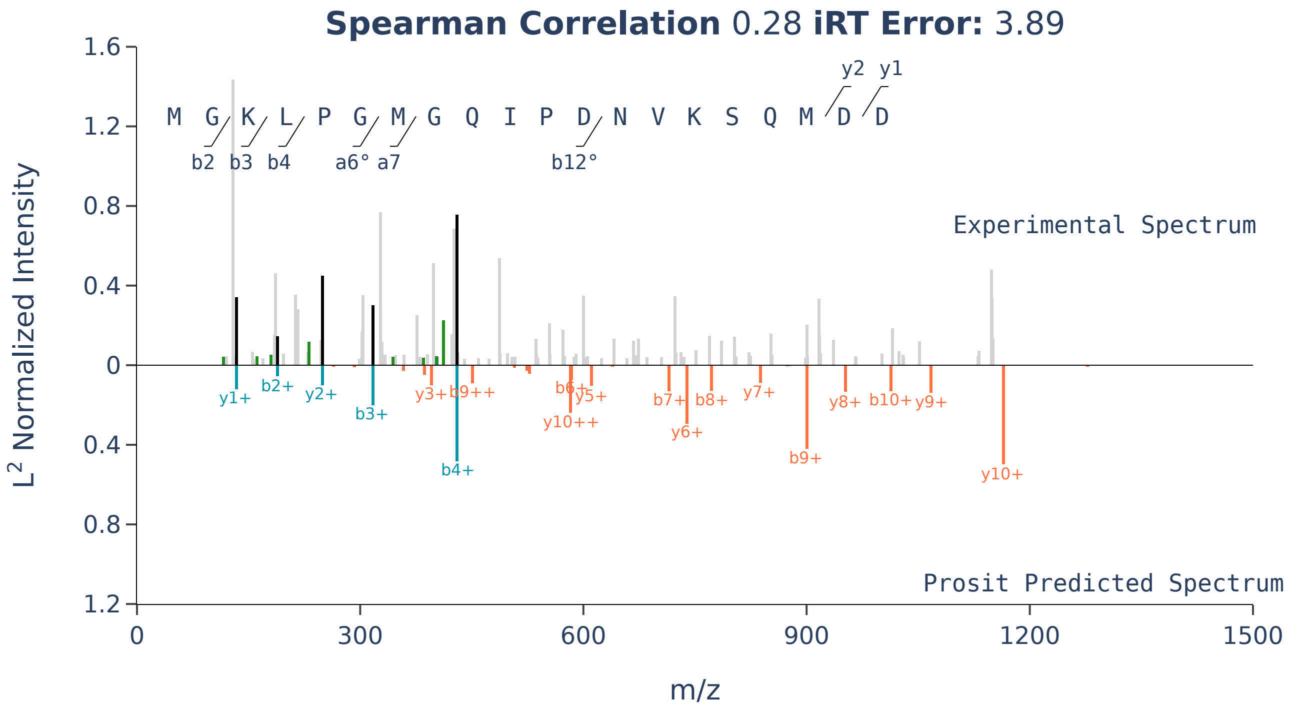

Source Ncheng_210623_230623_HFGoe_FFH_20S_25_1_A1_24h_R1 **Scan** 14727
Peptide LGVGEVGQKPVD **Charge** 2 **Spectral Angle** 0.72
Spearman Correlation 0.9 **iRT Error:** 0.46

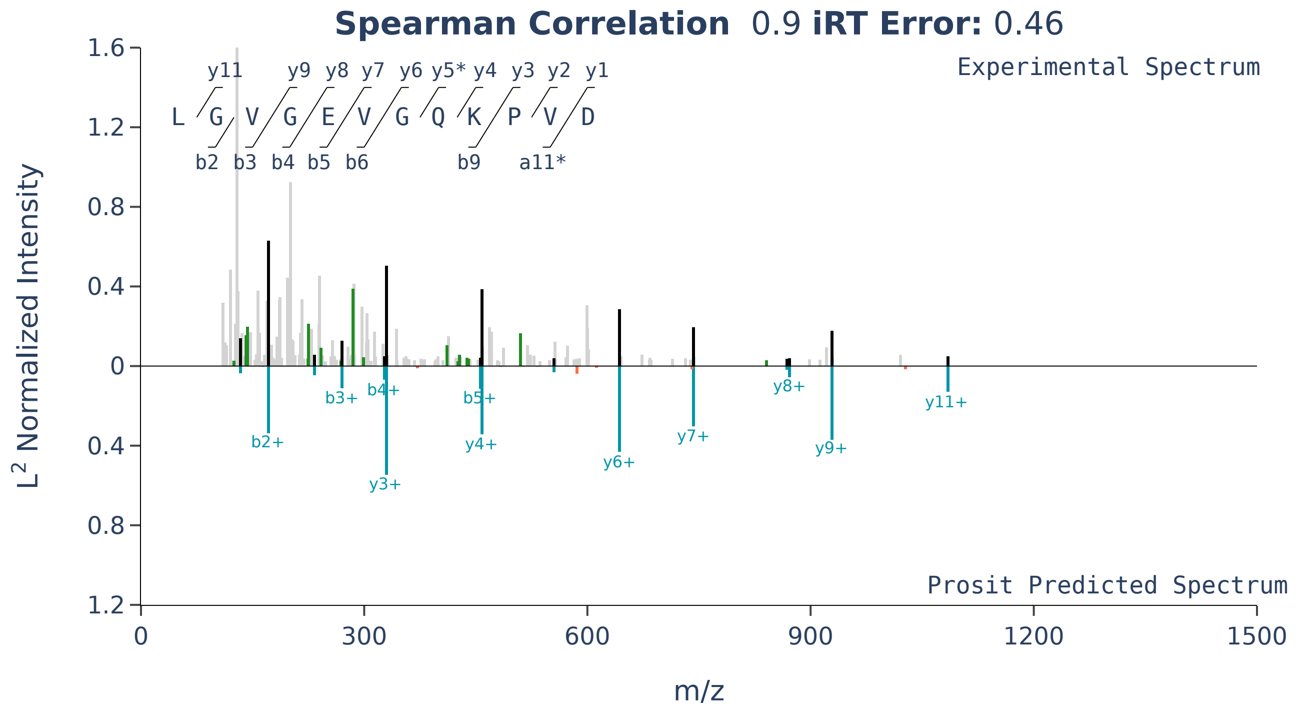

Source Ncheng_210623_230623_HFGoe_FFH_20S_25_1_A1_24h_R1 **Scan** 14727
Peptide EADVALPVVRE **Charge** 2 **Spectral Angle** 0.59
Spearman Correlation 0.39 **iRT Error:** 38.6

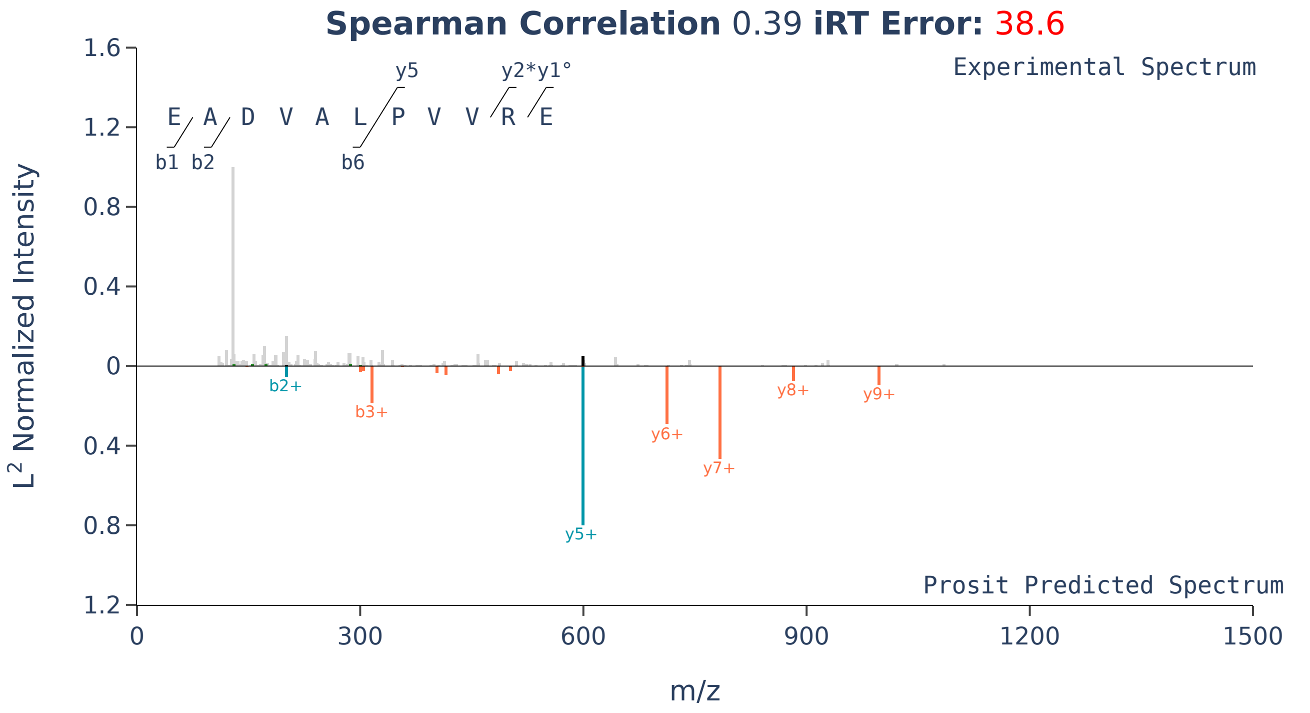

Source Ncheng_210623_230623_HFGoe_FFH_20S_25_1_A2_2h_R2 **Scan** 17909
Peptide KLPGMGMGQIPDNVKSQMD **Charge** 3 **Spectral Angle** 0.78
Spearman Correlation 0.86 **iRT Error:** 0.47

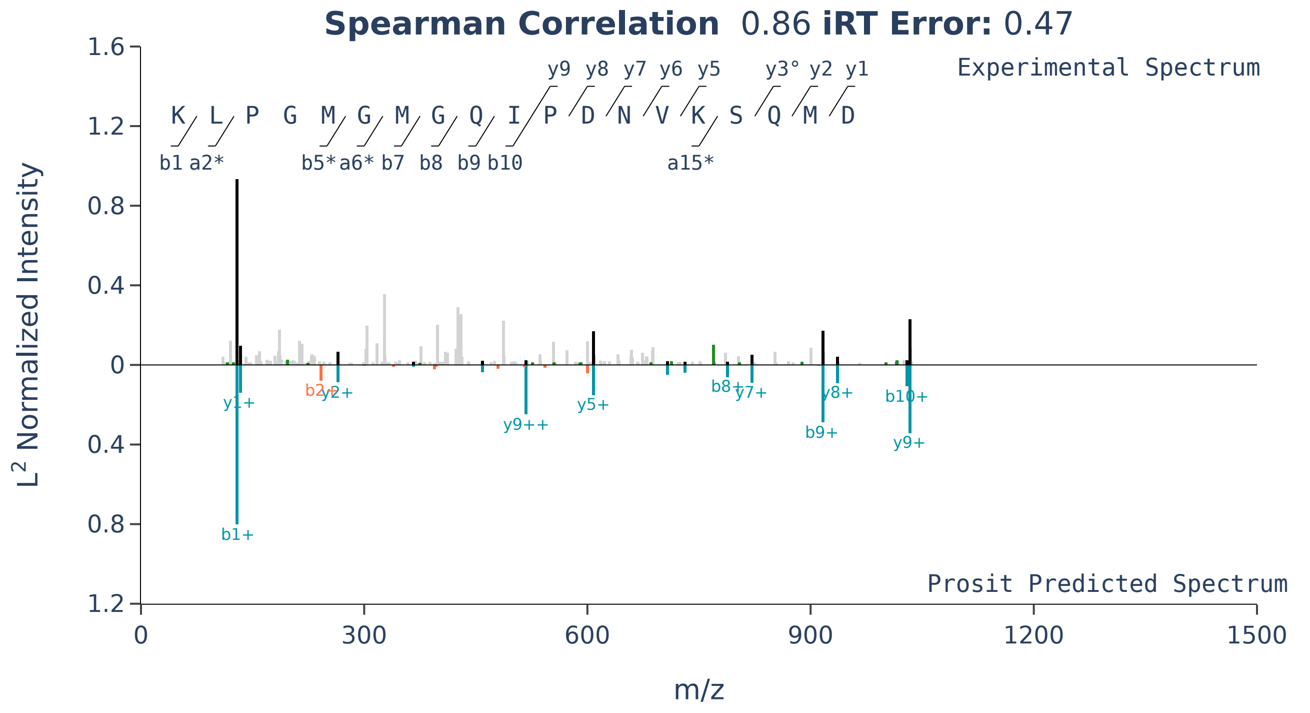

Source Ncheng_210623_230623_HFGoe_FFH_20S_25_1_A2_2h_R2 **Scan** 17909
Peptide MGKLPGMGQIPDNVKSQMD **Charge** 3 **Spectral Angle** 0.3
Spearman Correlation 0.24 **iRT Error:** 9.67

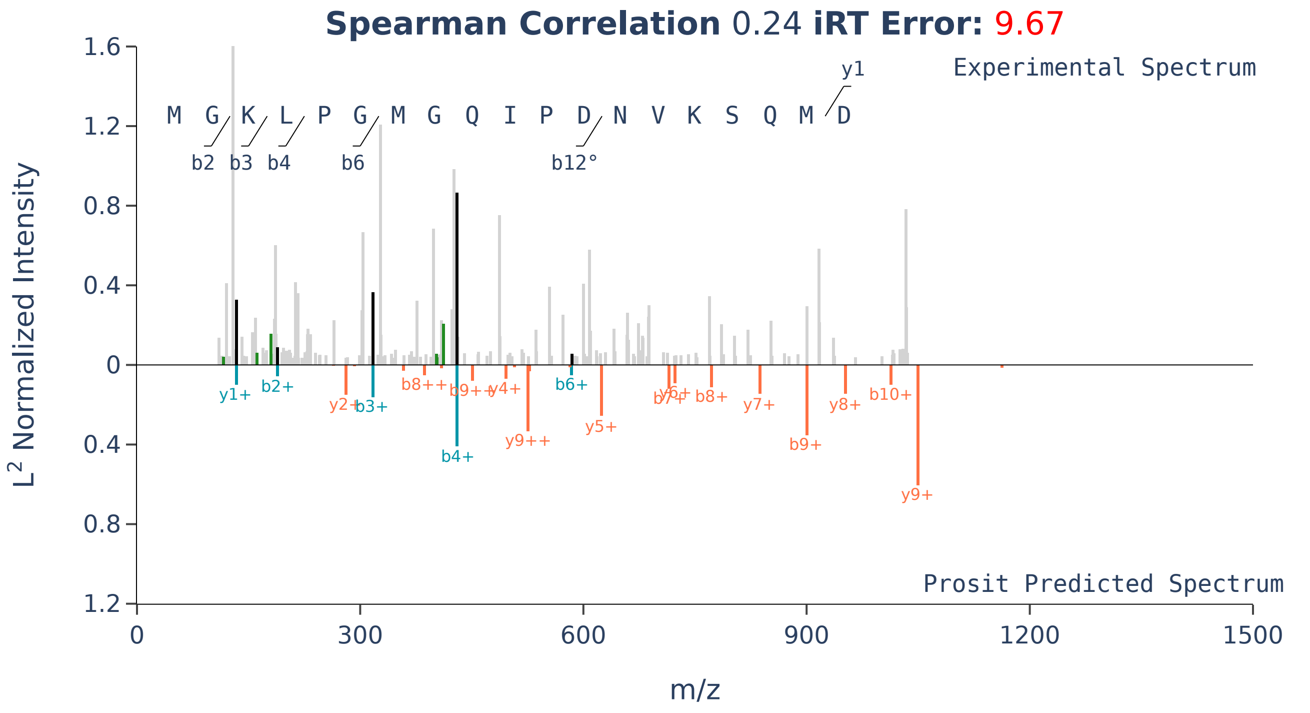

Source Ncheng_210623_230623_HFGoe_FFH_20S_25_1_A2_4h_R2 **Scan** 20370
Peptide MGKLPGMGQIPDNVKSMAG **Charge** 2 **Spectral Angle** 0.84
Spearman Correlation 0.87 **iRT Error:** 0.48

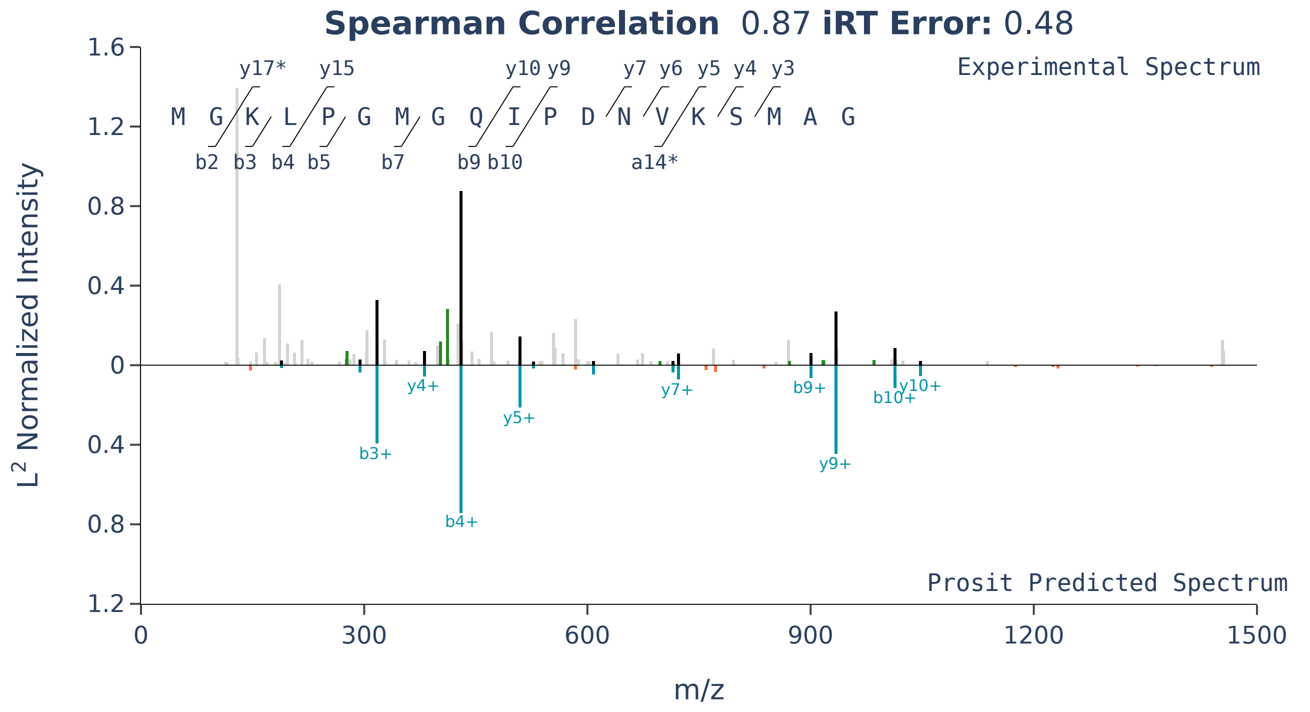

Source Ncheng_210623_230623_HFGoe_FFH_20S_25_1_A2_4h_R2 **Scan** 20370
Peptide MGKLPGMGQIPDNVKSQM **Charge** 2 **Spectral Angle** 0.16
Spearman Correlation 0.28 **iRT Error:** 3.28

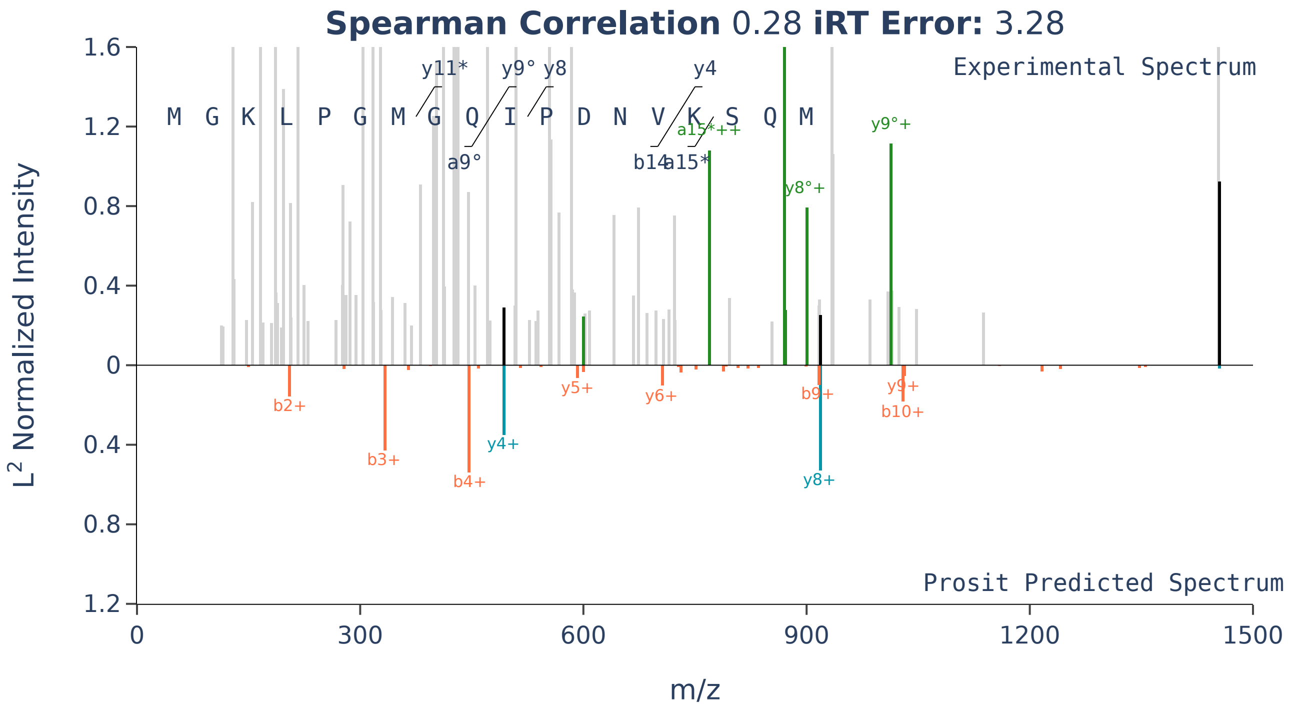

Source Ncheng_210623_230623_HFGoe_FFH_20S_25_1_A2_24h_R2 **Scan** 15240
Peptide TAMTGQDAANTAKAFNE **Charge** 2 **Spectral Angle** 0.84
Spearman Correlation 0.83 **iRT Error:** 0.49

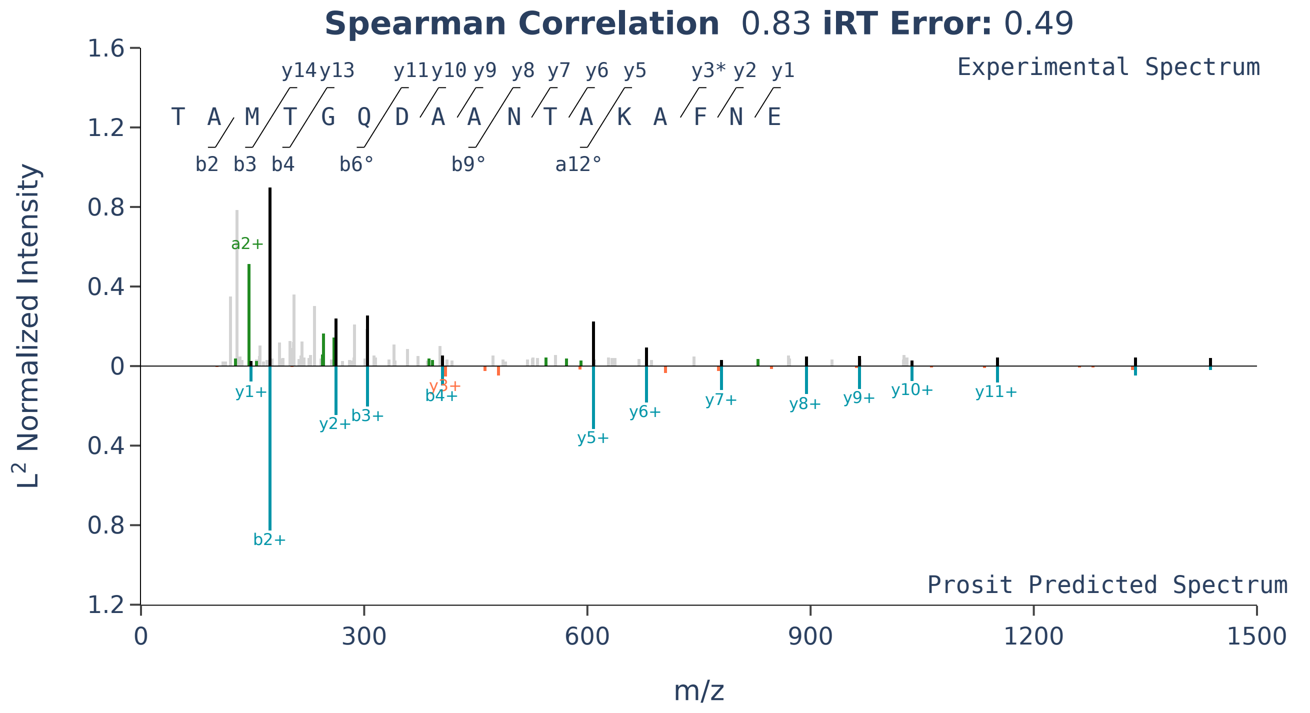

Source Ncheng_210623_230623_HFGoe_FFH_20S_25_1_A2_24h_R2 **Scan** 15240
Peptide GGMAKMMRSMKGMMPP **Charge** 2 **Spectral Angle** 0.0
Spearman Correlation 0.0 **iRT Error:** 26.26

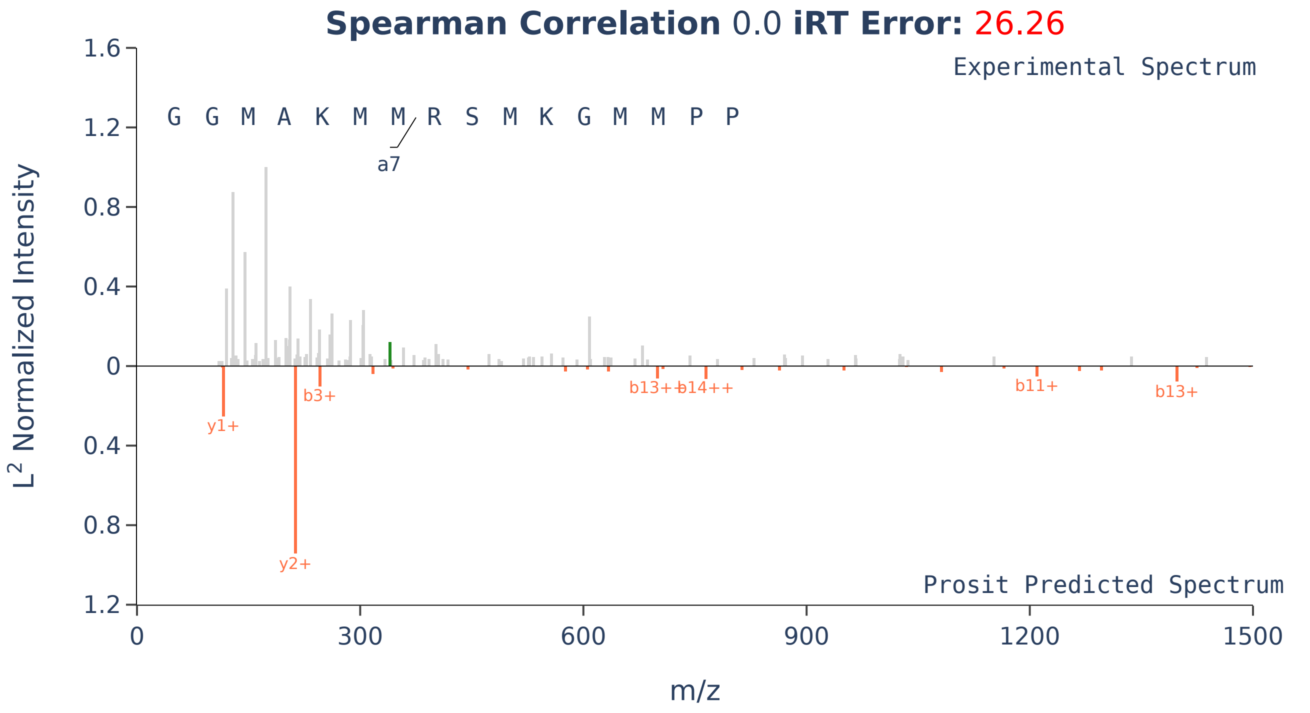

Source Ncheng_210623_230623_HFGoe_FFH_20S_25_1_A1_2h_R2 **Scan** 27413
Peptide AMTGQDAANTAKAFNEALPLTDG **Charge** 2 **Spectral Angle** 0.88
Spearman Correlation 0.87 **iRT Error:** 0.53

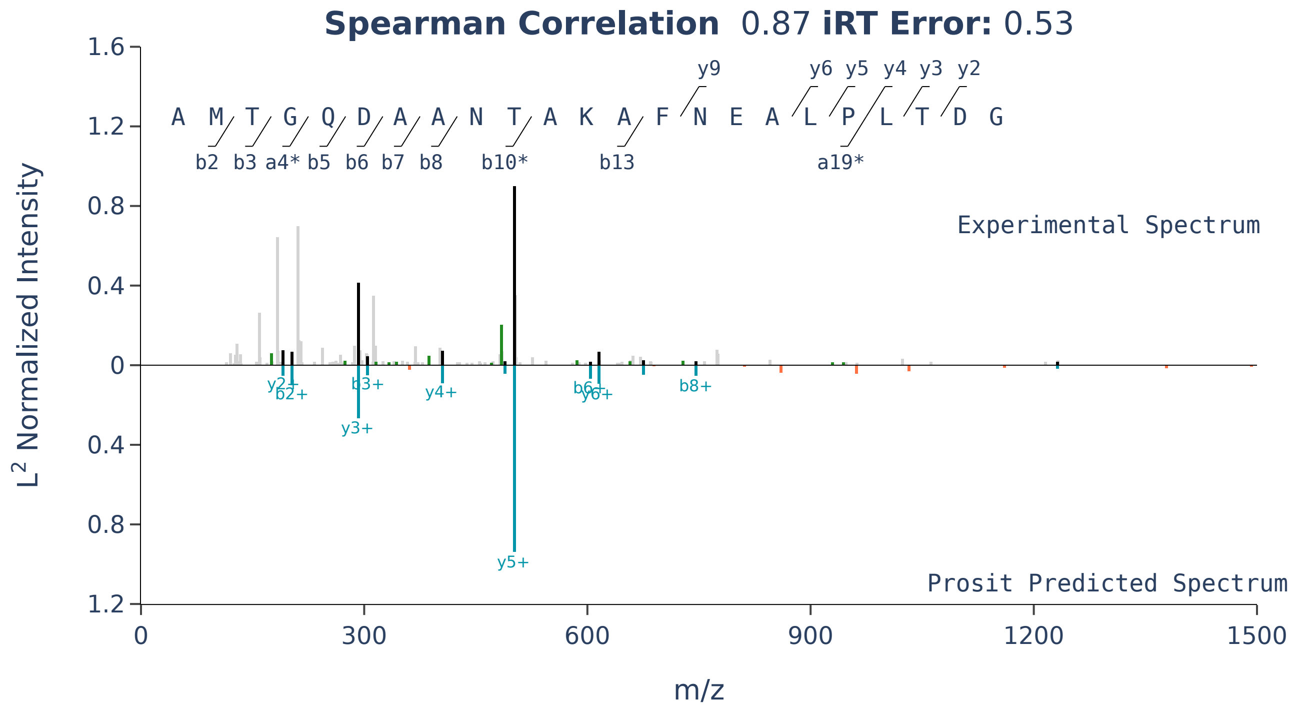

Source Ncheng_210623_230623_HFGoe_FFH_20S_25_1_A1_2h_R2 **Scan** 27413
Peptide DAMTGQDAANTAKAFNEALPLTG **Charge** 2 **Spectral Angle** 0.04
Spearman Correlation 0.07 **iRT Error:** 7.45

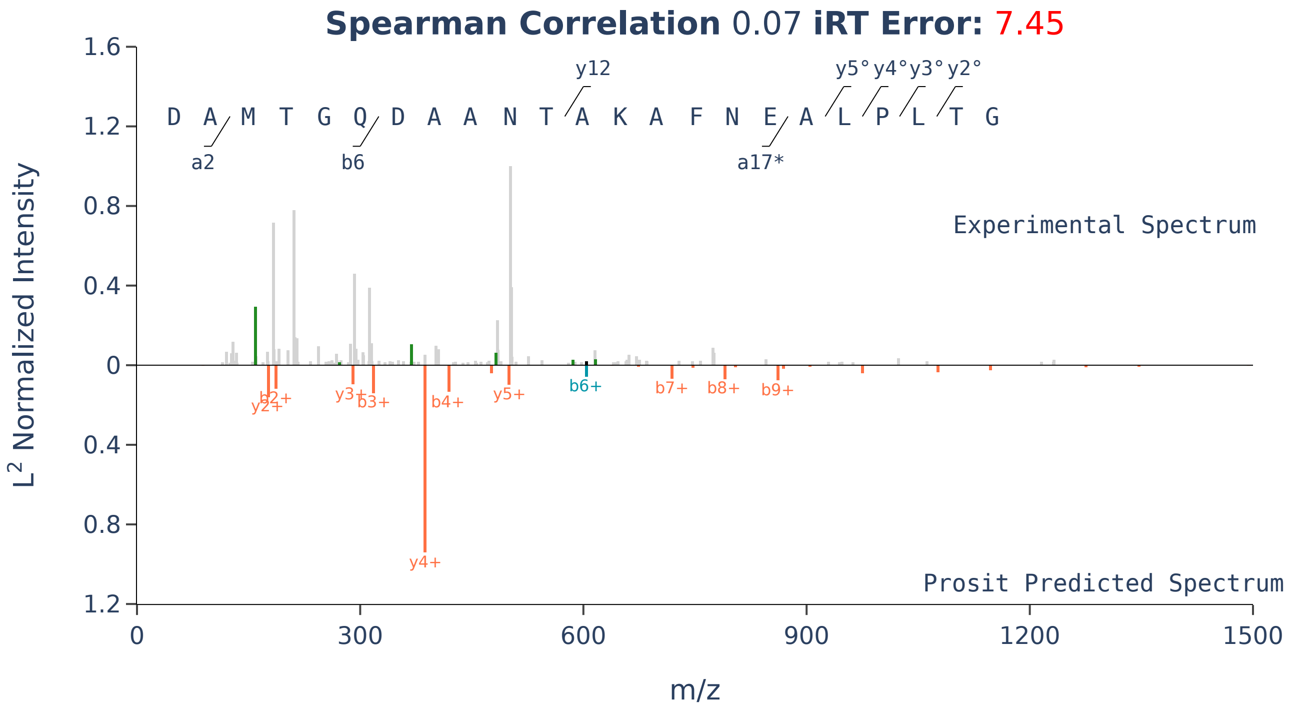

Source Ncheng_210623_230623_HFGoe_FFH_20S_25_1_A2_2h_R2 **Scan** 18366
Peptide GKLPGMMGQIPDNVKSQMDD **Charge** 3 **Spectral Angle** 0.74
Spearman Correlation 0.86 **iRT Error:** 0.57

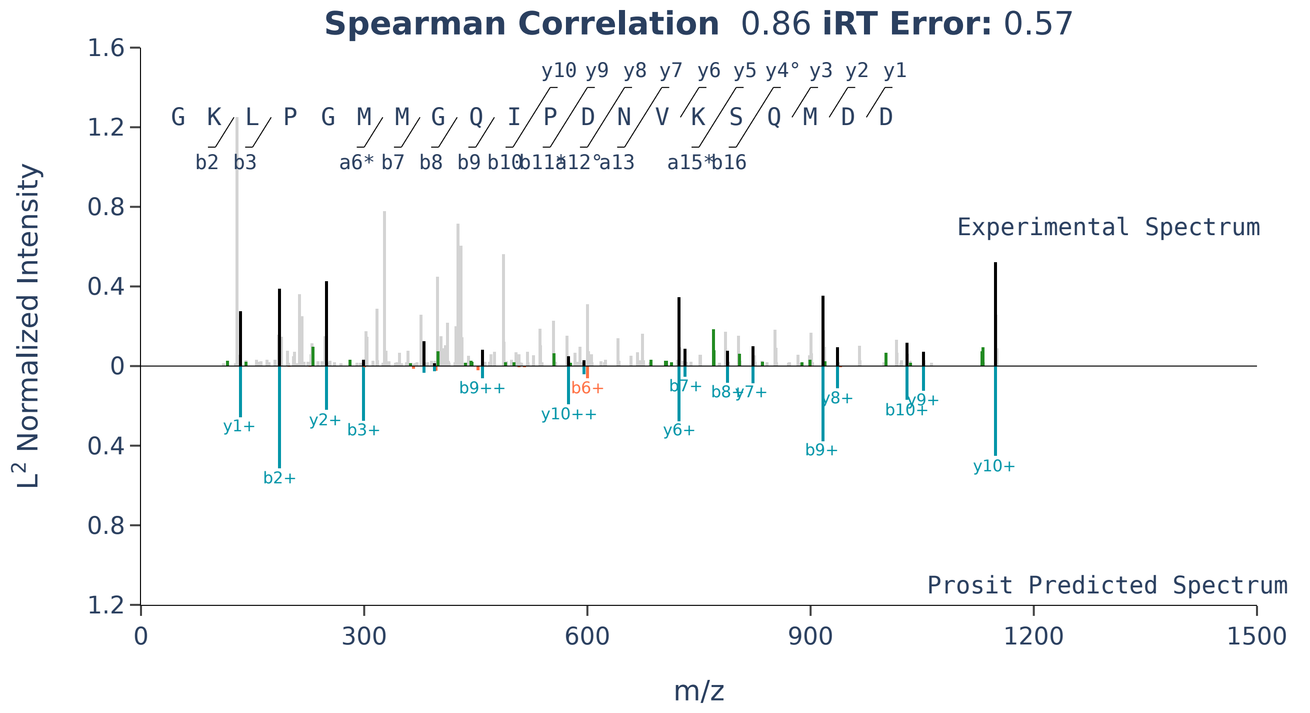

Source Ncheng_210623_230623_HFGoe_FFH_20S_25_1_A2_2h_R2 **Scan** 18366
Peptide MGKLPGMGQIPDNVKSQMDD **Charge** 3 **Spectral Angle** 0.34
Spearman Correlation 0.21 **iRT Error:** 7.85

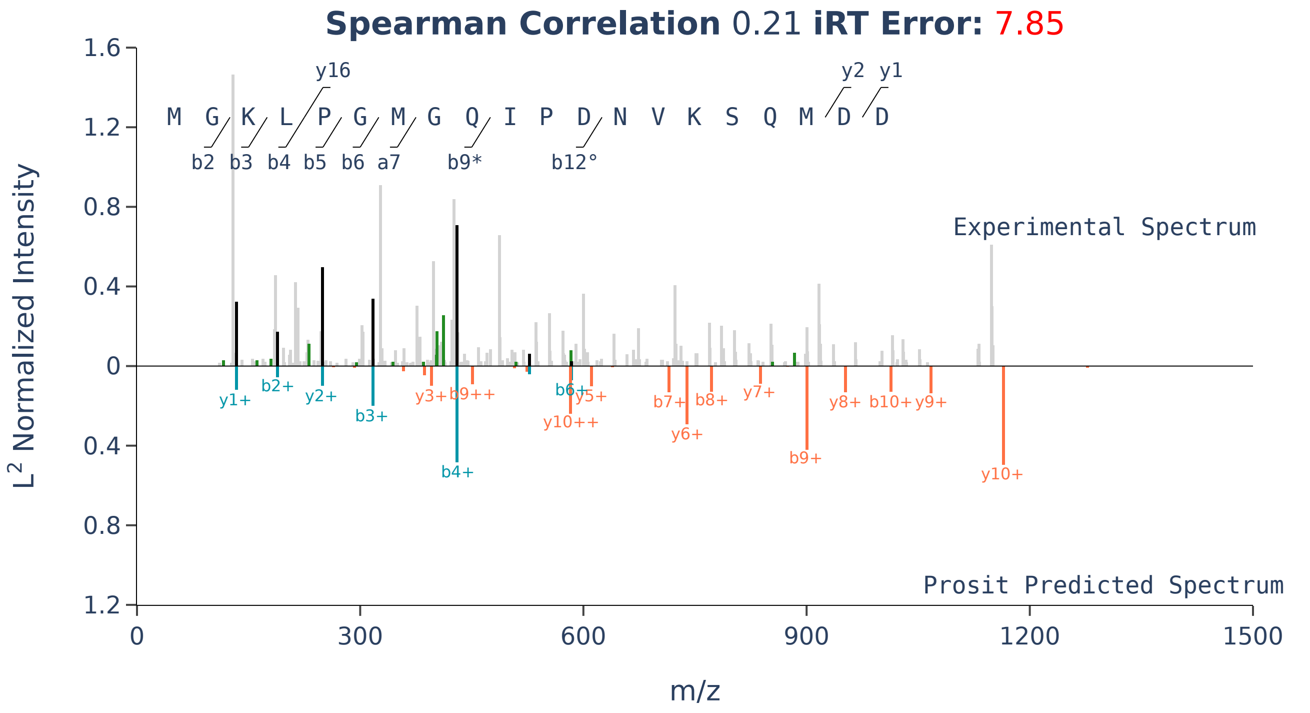

Source Ncheng_210623_230623_HFGoe_FFH_20S_25_1_A2_1h_R1 **Scan** 30536
Peptide NLHEVNKSLTPGQEFVKIVRNELVAAM **Charge** 5 **Spectral Angle** 0.89
Spearman Correlation 0.82 **iRT Error:** 0.57

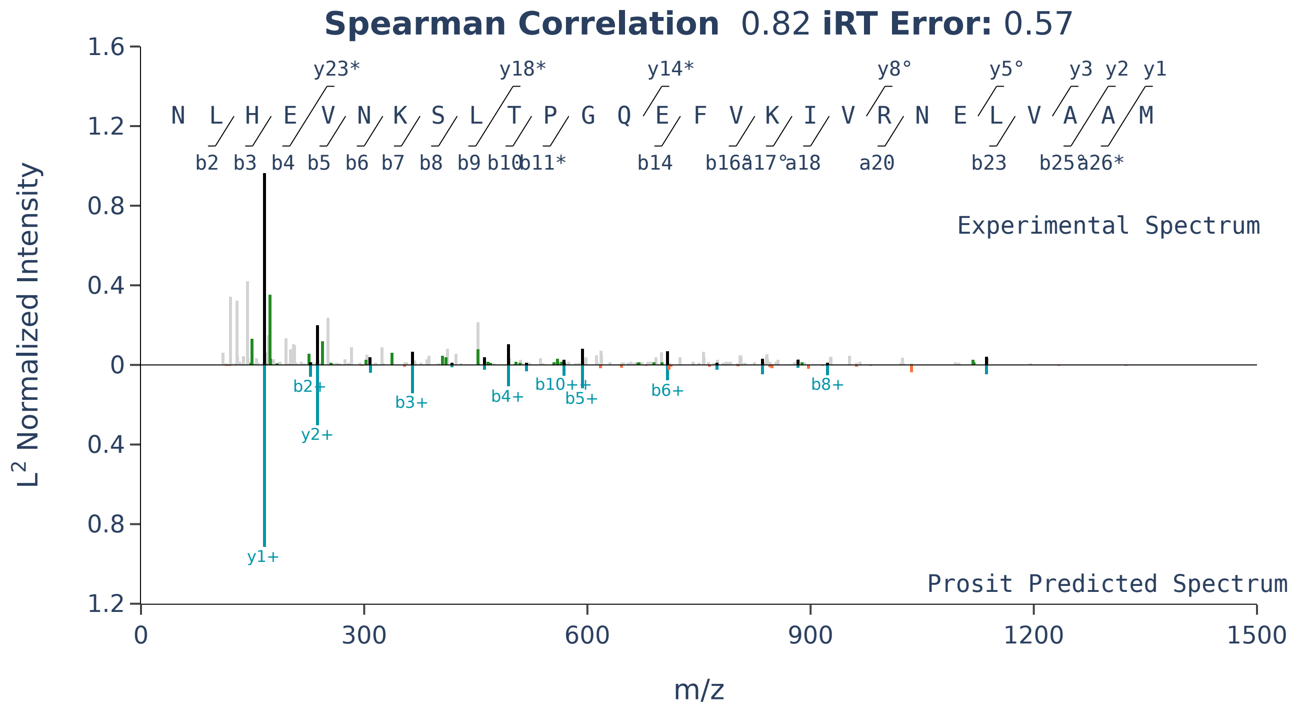

Source Ncheng_210623_230623_HFGoe_FFH_20S_25_1_A2_1h_R1 **Scan** 30536
Peptide KLASKLKKGDGFDLNDFLEQLRQMKN **Charge** 5 **Spectral Angle** 0.55
Spearman Correlation 0.23 **iRT Error:** 26.83

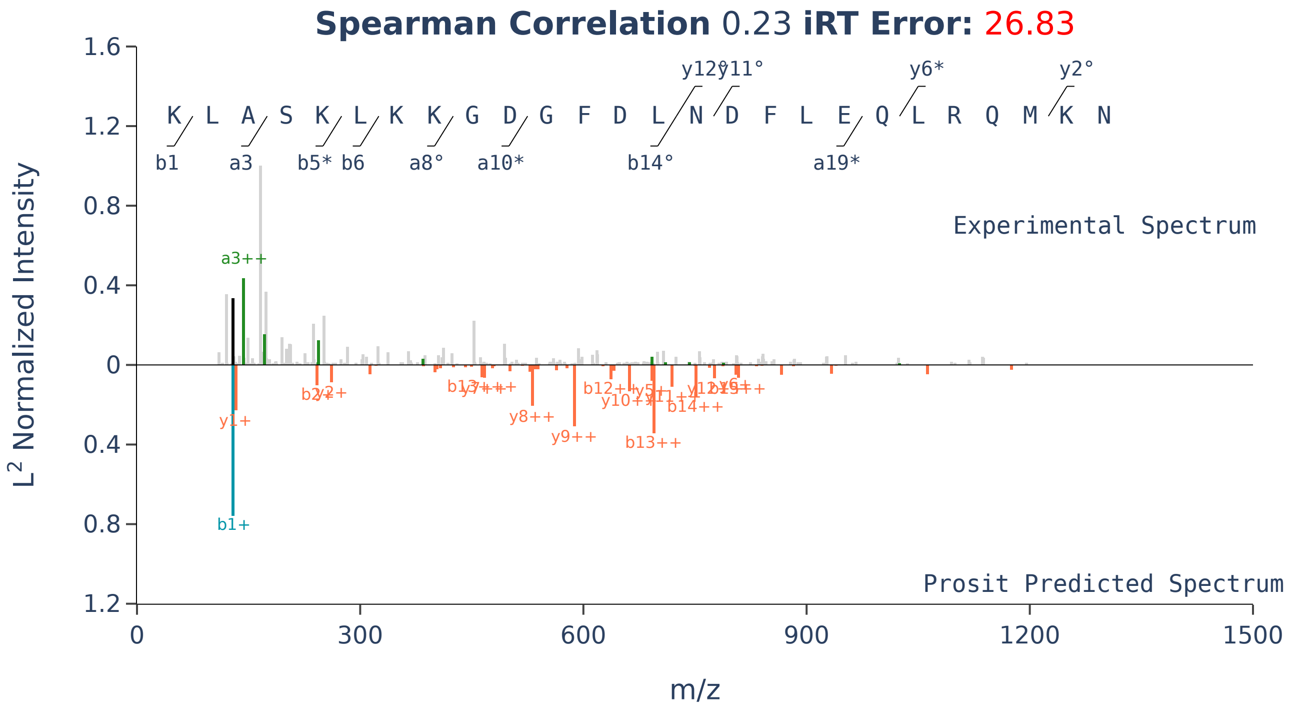

Source Ncheng_210623_230623_HFGoe_FFH_20S_25_1_A2_24h_R1 **Scan** 14319
Peptide EAMTGQDAANTAKAFN **Charge** 2 **Spectral Angle** 0.8
Spearman Correlation 0.89 **iRT Error:** 0.58

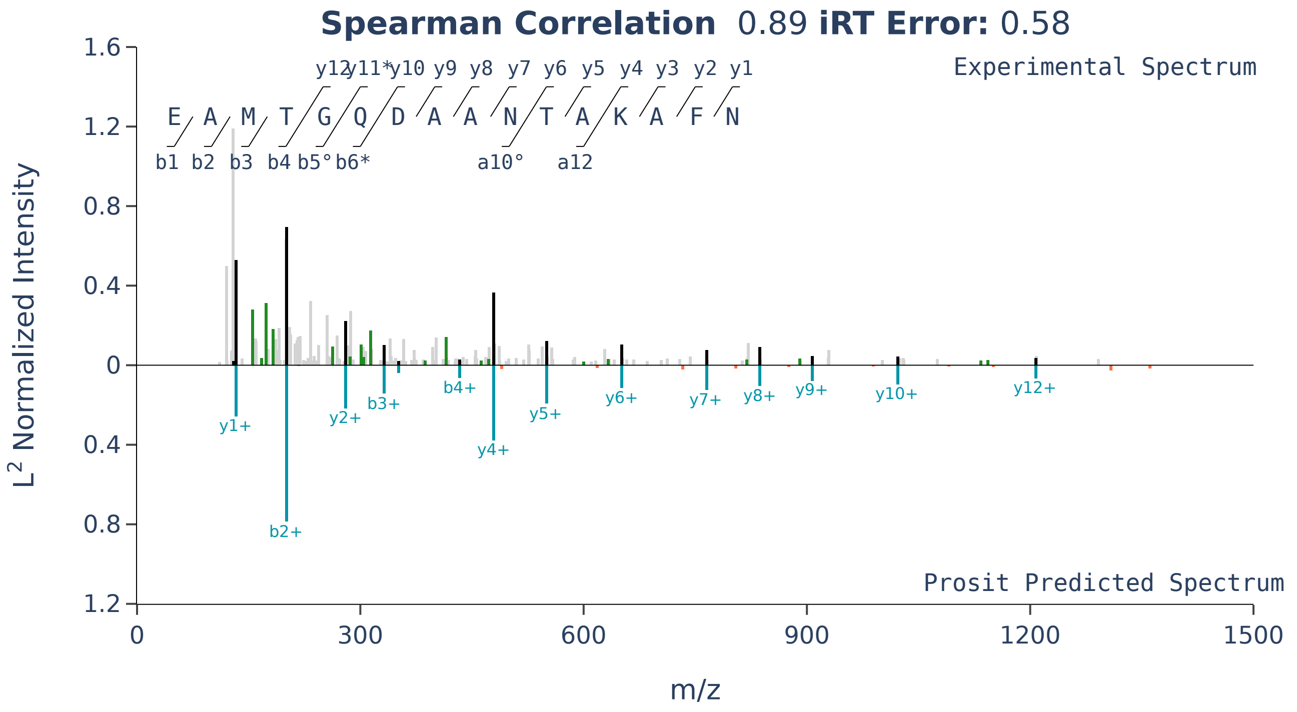

Source Ncheng_210623_230623_HFGoe_FFH_20S_25_1_A2_24h_R1 **Scan** 14319
Peptide AMTGQDAANTAKAFNE **Charge** 2 **Spectral Angle** 0.49
Spearman Correlation 0.34 **iRT Error:** 2.46

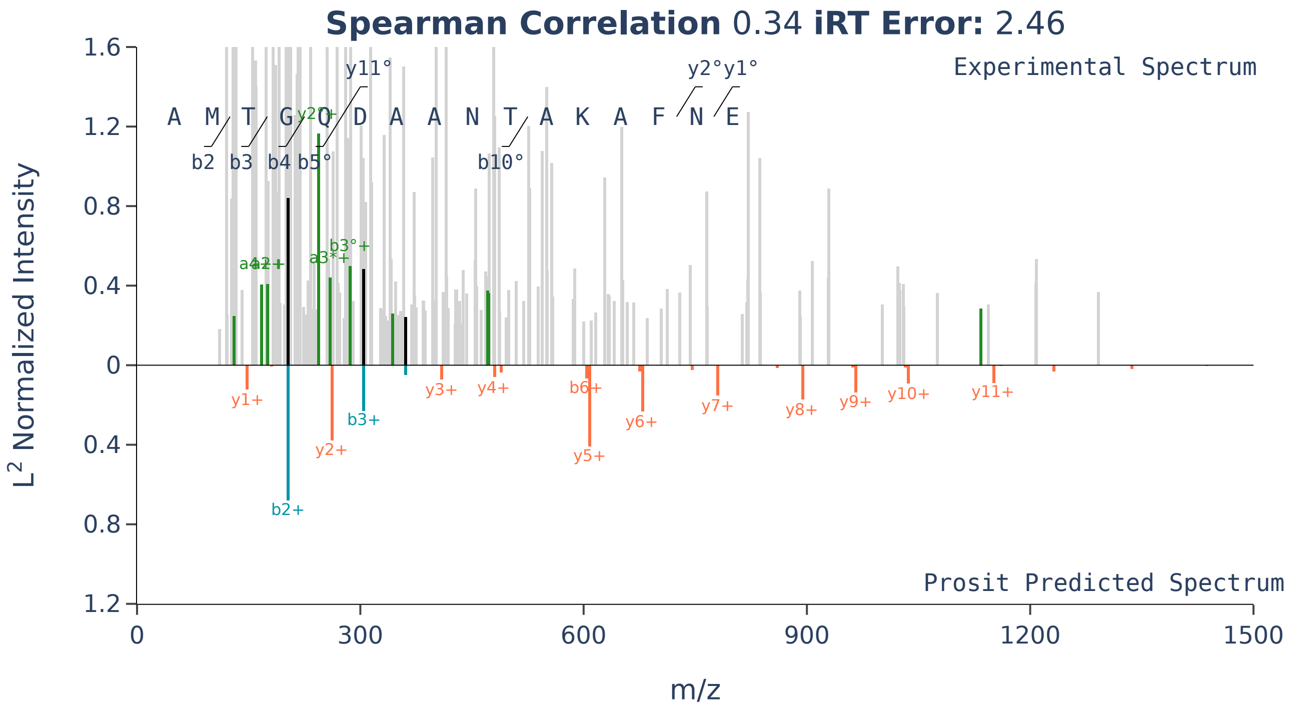

Source Ncheng_210623_230623_HFGoe_FFH_20S_25_1_A1_24h_R1 **Scan** 9747
Peptide KAFNEVGQKPVD **Charge** 2 **Spectral Angle** 0.74
Spearman Correlation 0.88 **iRT Error:** 0.61

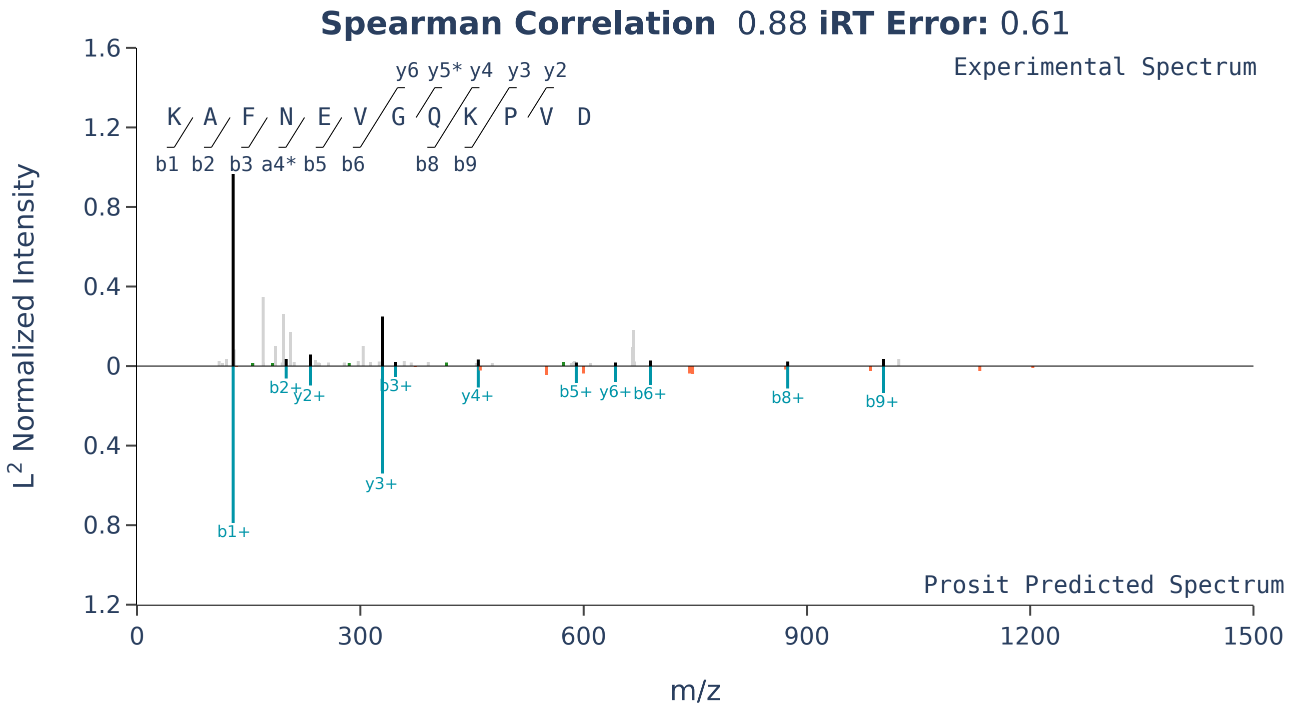

Source Ncheng_210623_230623_HFGoe_FFH_20S_25_1_A1_24h_R1 **Scan** 9747
Peptide SGRGRLTEDNVK **Charge** 2 **Spectral Angle** 0.0
Spearman Correlation 0.0 **iRT Error:** 27.42

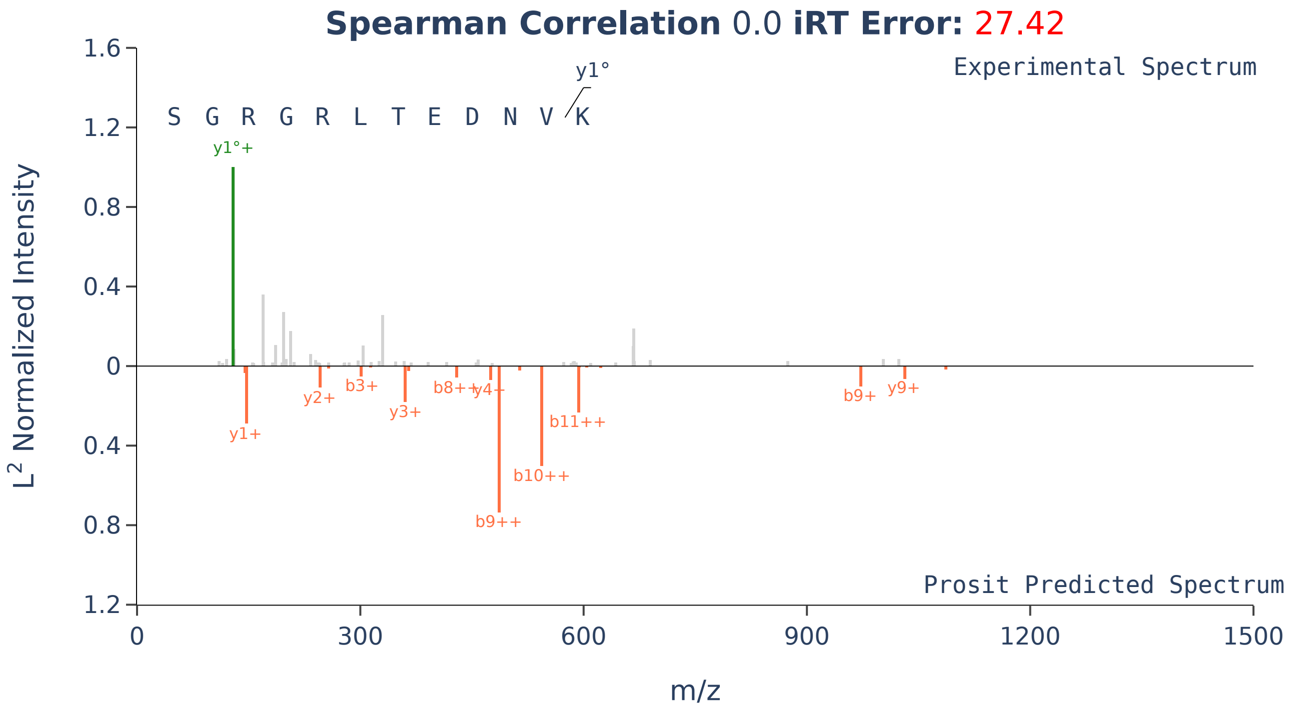

Source Ncheng_210623_230623_HFGoe_FFH_20S_25_1_A1_4h_R1 **Scan** 9498
Peptide QAEKLASSRTL **Charge** 2 **Spectral Angle** 0.85
Spearman Correlation 0.83 **iRT Error:** 0.61

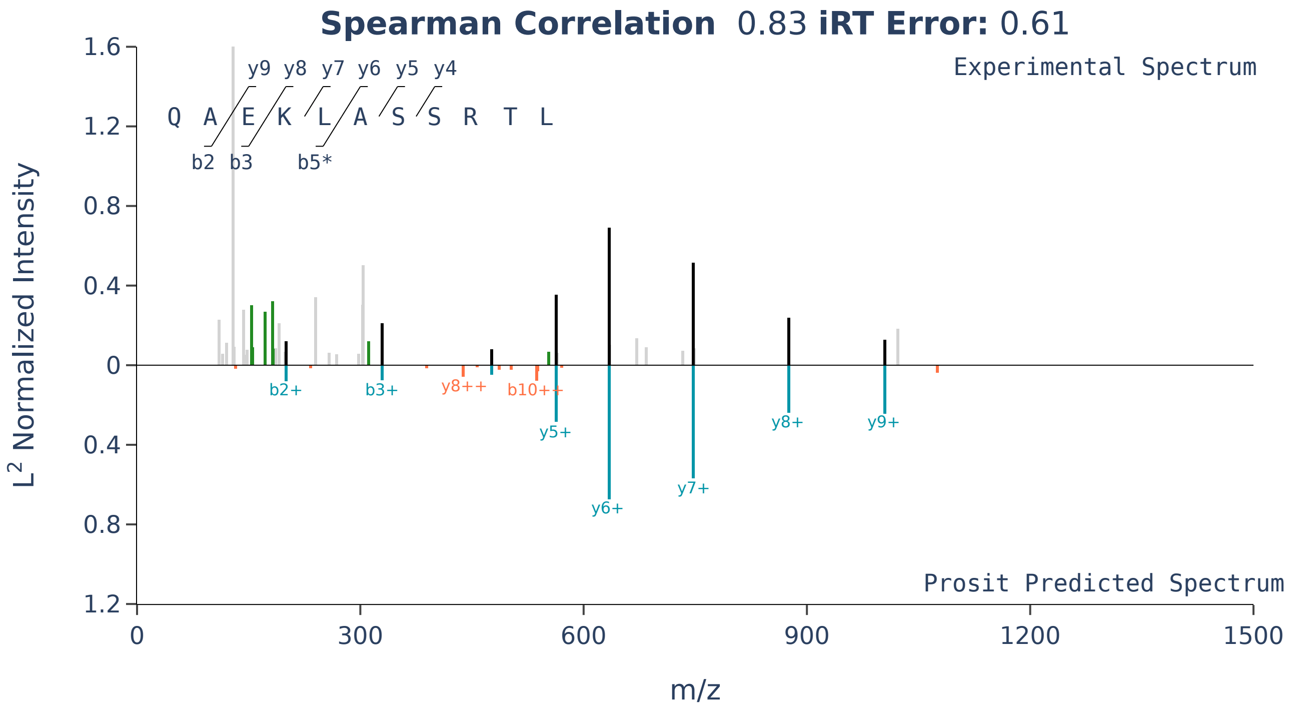

Source Ncheng_210623_230623_HFGoe_FFH_20S_25_1_A1_4h_R1 **Scan** 9498
Peptide GLQGAGKTTSVGK **Charge** 2 **Spectral Angle** 0.0
Spearman Correlation 0.0 **iRT Error:** 26.12

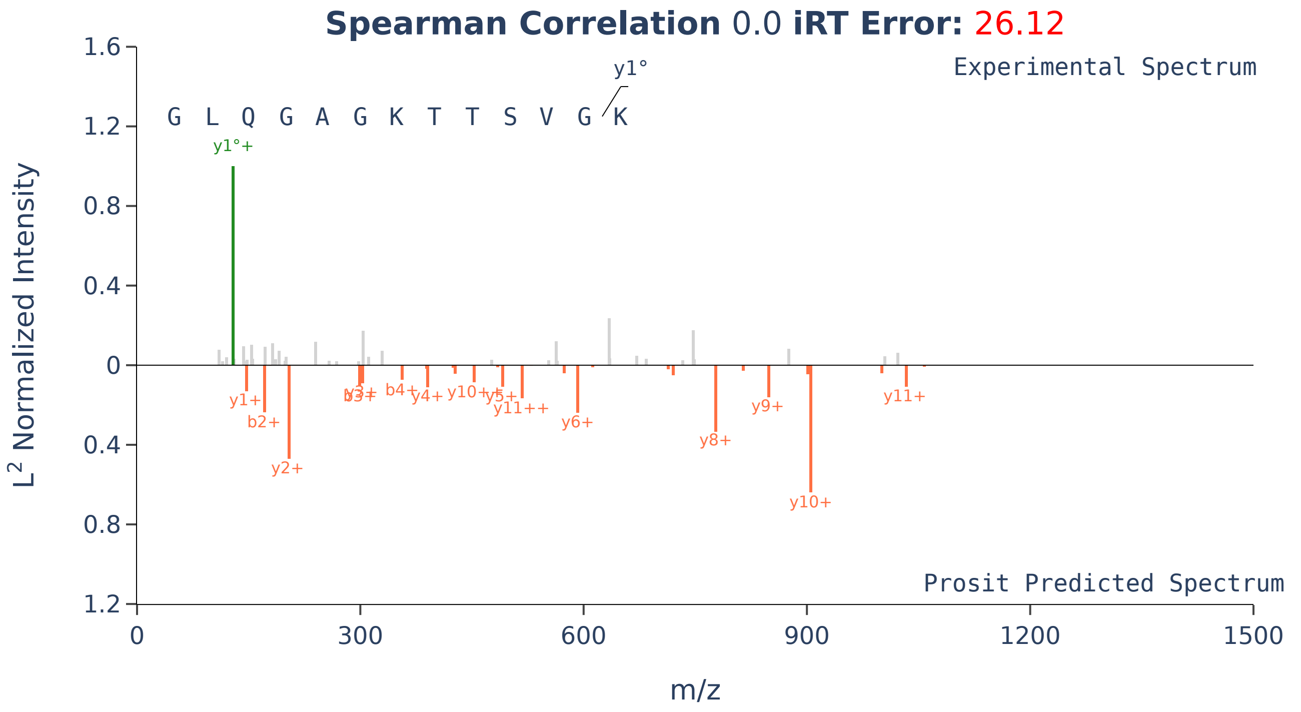

Source Ncheng_210623_230623_HFGoe_FFH_20S_25_1_A2_4h_R2 **Scan** 19091
Peptide KLPGMGMGQIPDNVKSQM **Charge** 3 **Spectral Angle** 0.79
Spearman Correlation 0.85 **iRT Error:** 0.71

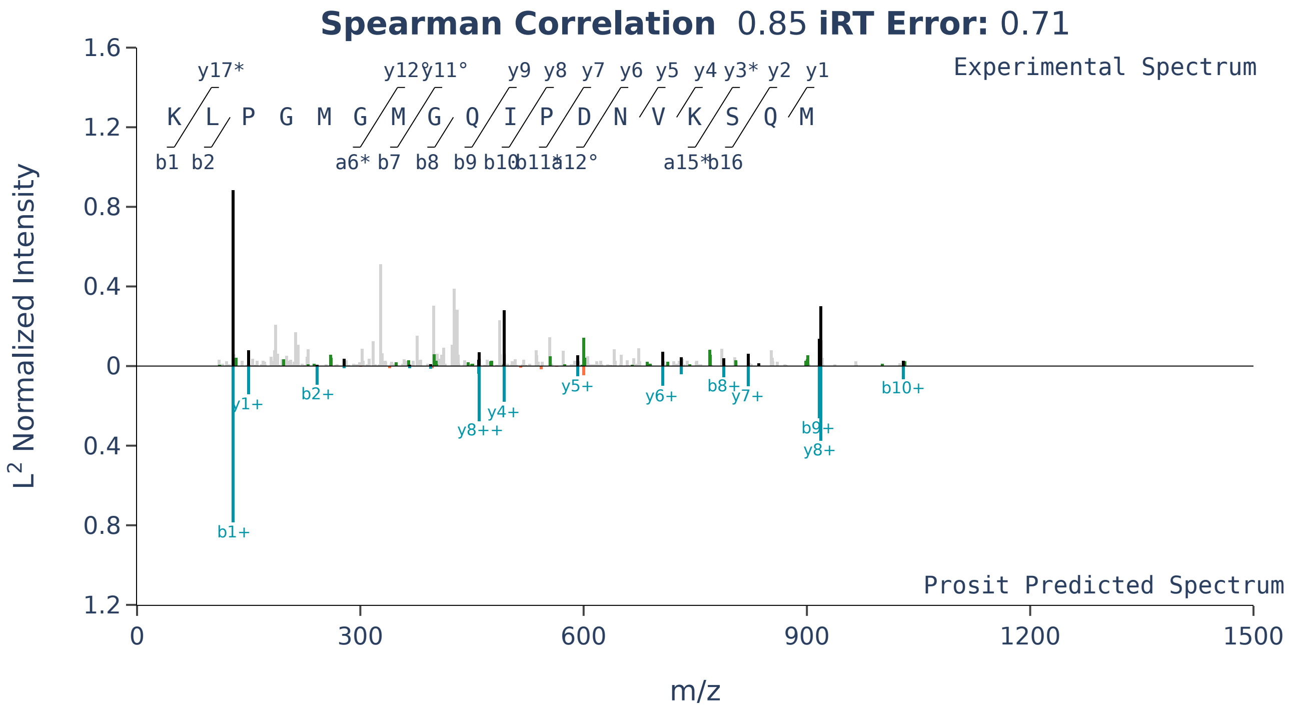

Source Ncheng_210623_230623_HFGoe_FFH_20S_25_1_A2_4h_R2 **Scan** 19091
Peptide MGKLPGMGQIPDNVKSQM **Charge** 3 **Spectral Angle** 0.22
Spearman Correlation 0.08 **iRT Error:** 5.38

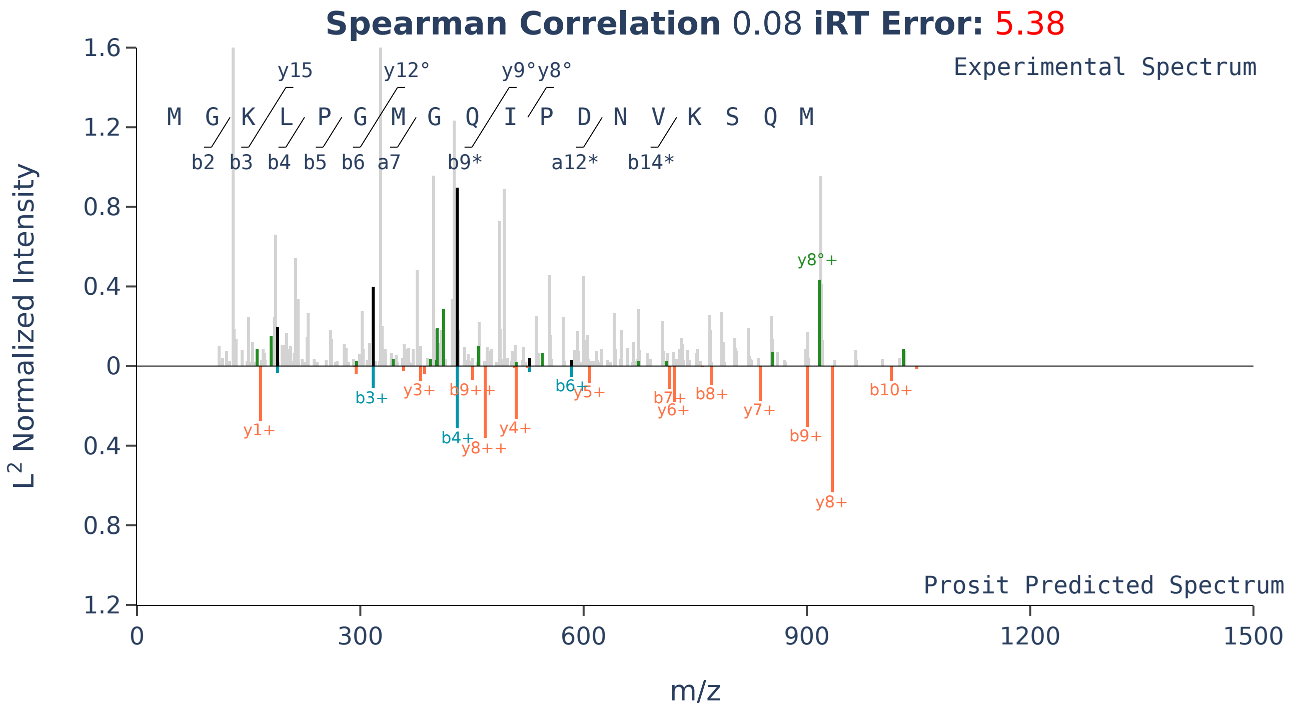

Source Ncheng_210623_230623_HFGoe_FFH_20S_25_1_A1_4h_R2 **Scan** 17070
Peptide ESKPIKFLGVGEKTE **Charge** 2 **Spectral Angle** 0.79
Spearman Correlation 0.85 **iRT Error:** 0.71

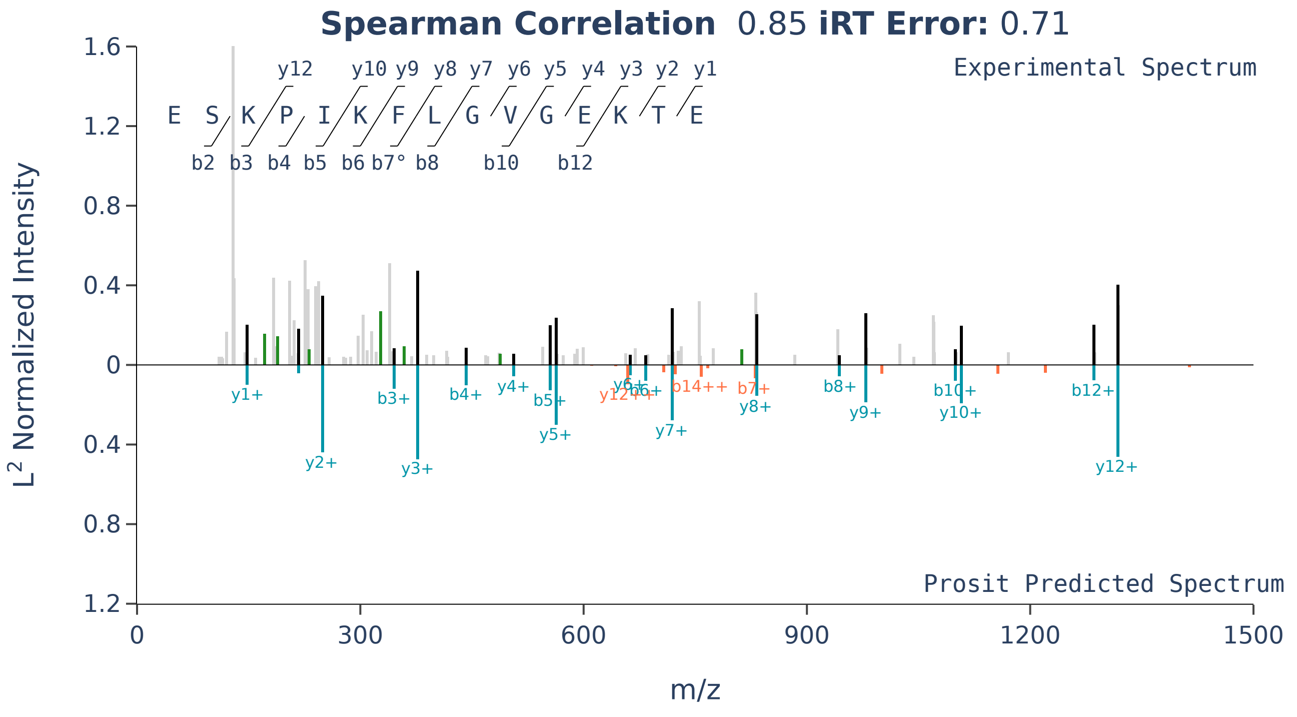

Source Ncheng_210623_230623_HFGoe_FFH_20S_25_1_A1_4h_R2 **Scan** 17070
Peptide SMTMKERAKPEIIK **Charge** 2 **Spectral Angle** 0.11
Spearman Correlation 0.12 **iRT Error:** 43.31

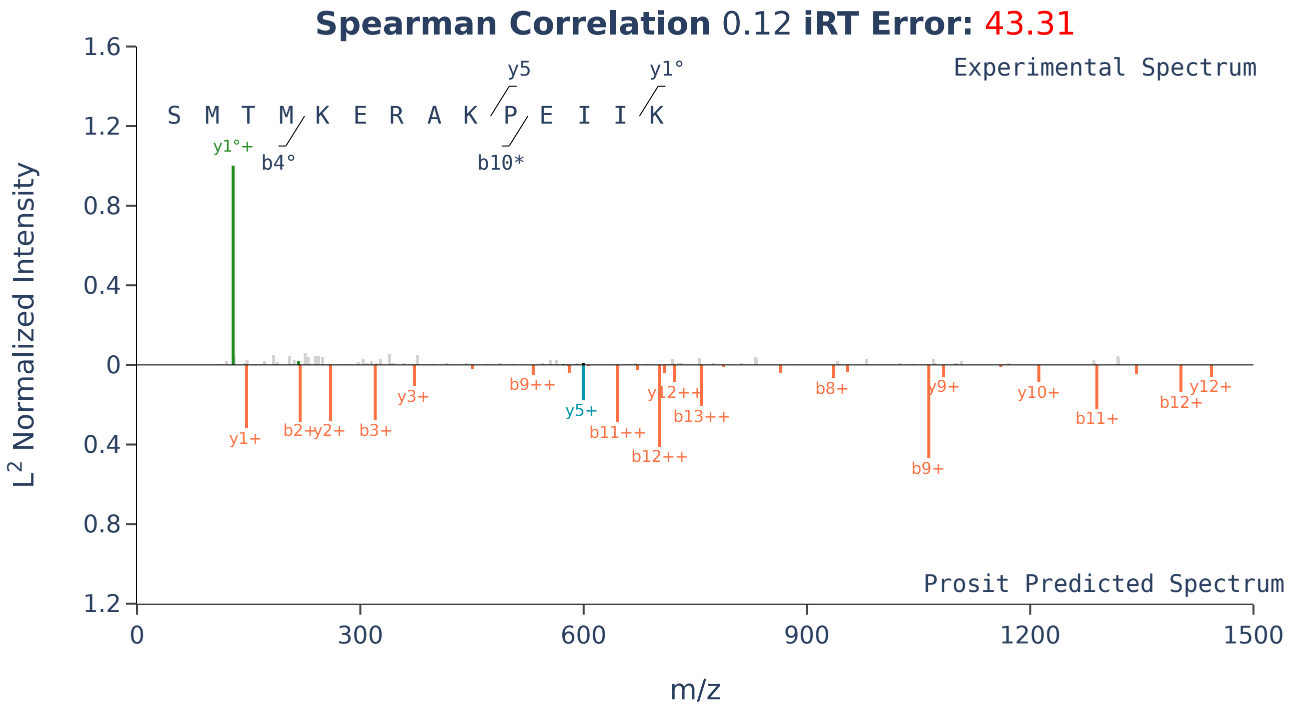

Source Ncheng_210623_230623_HFGoe_FFH_20S_25_1_A1_24h_R2 **Scan** 6468
Peptide VKIVRGDA **Charge** 2 **Spectral Angle** 0.89
Spearman Correlation 0.81 **iRT Error:** 0.73

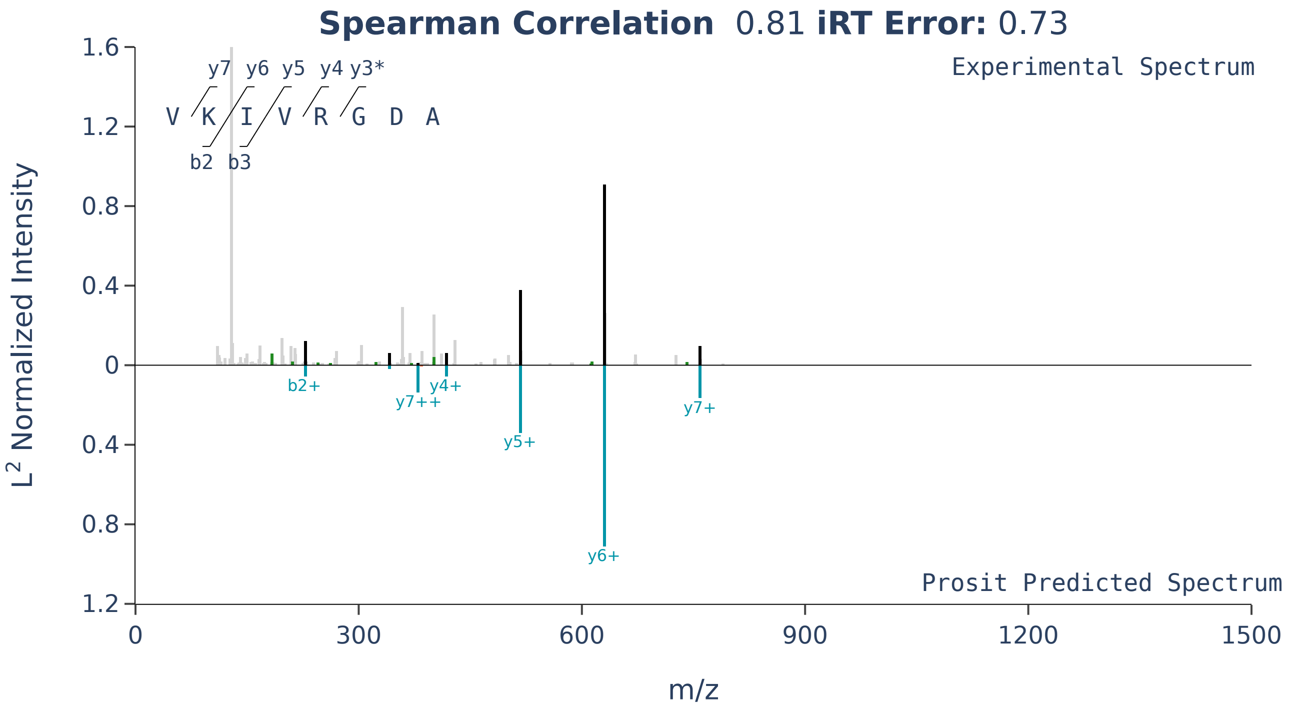

Source Ncheng_210623_230623_HFGoe_FFH_20S_25_1_A1_24h_R2 **Scan** 6468
Peptide DVNRLLK **Charge** 2 **Spectral Angle** 0.27
Spearman Correlation 0.31 **iRT Error:** 35.28

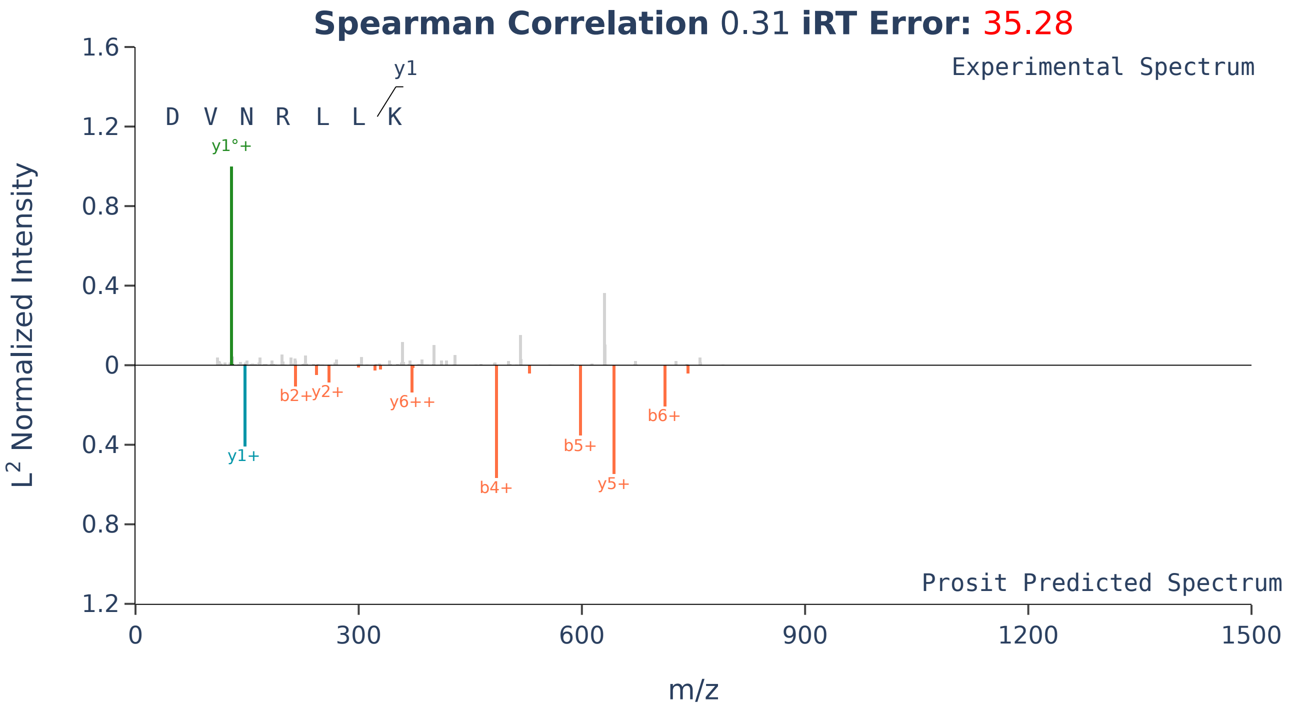

Source Ncheng_210623_230623_HFGoe_FFH_20S_25_1_A1_4h_R2 **Scan** 18824
Peptide ALEPFHPDRIASE **Charge** 3 **Spectral Angle** 0.84
Spearman Correlation 0.87 **iRT Error:** 0.74

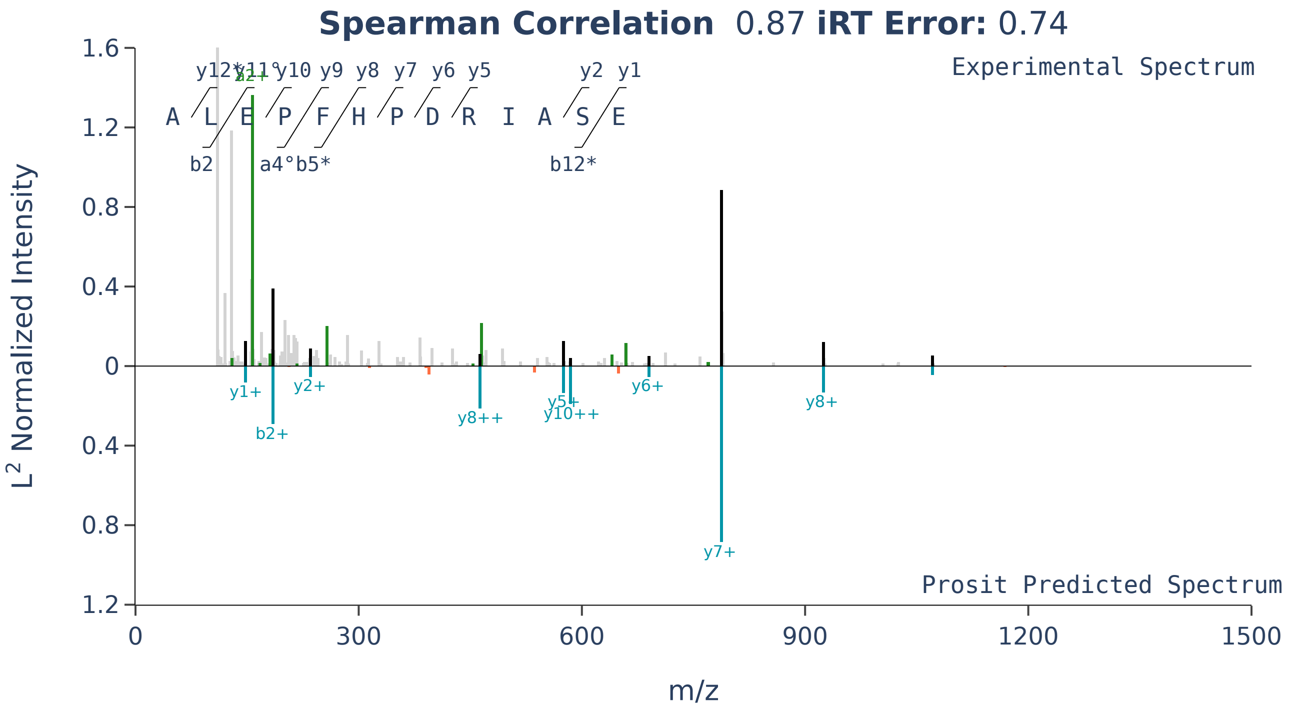

Source Ncheng_210623_230623_HFGoe_FFH_20S_25_1_A1_4h_R2 **Scan** 18824
Peptide MDEIKQVHASINP **Charge** 3 **Spectral Angle** 0.0
Spearman Correlation 0.0 **iRT Error:** 14.95

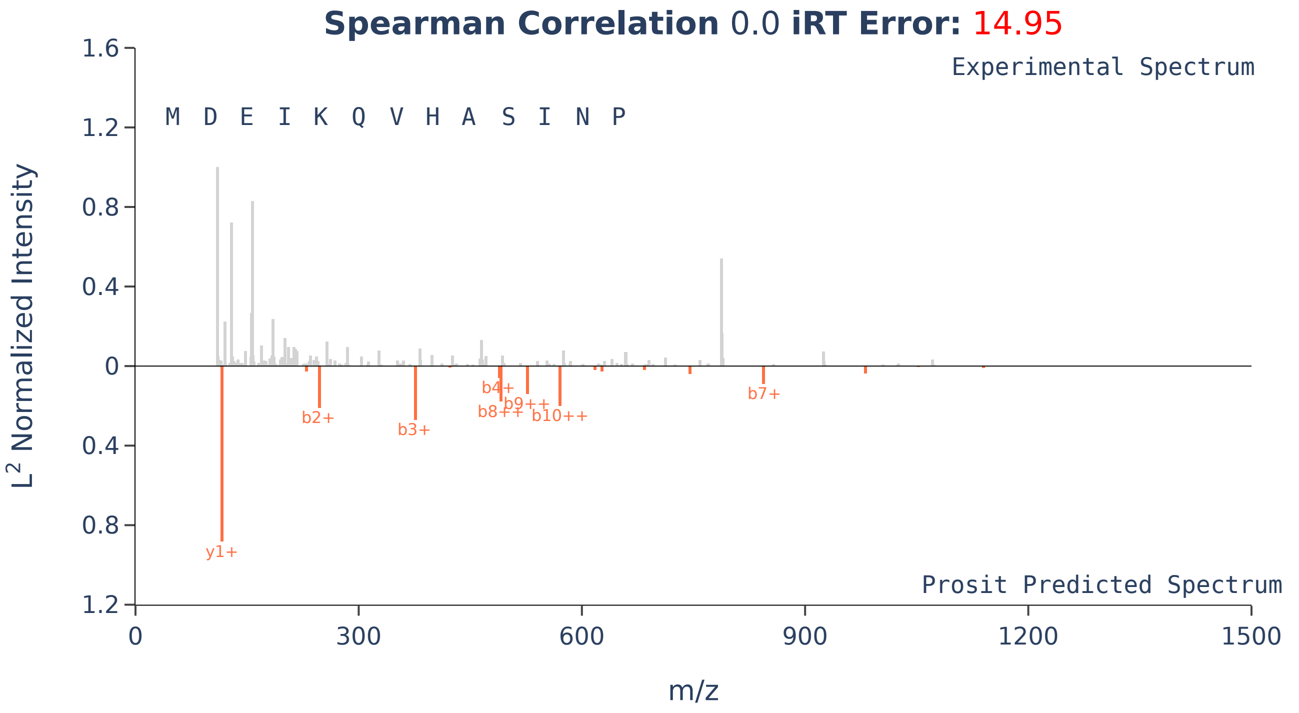

Source Ncheng_210623_230623_HFGoe_FFH_20S_25_1_A1_4h_R1 **Scan** 22260
Peptide DMGKLPGMGQIPDNVKSQ **Charge** 3 **Spectral Angle** 0.76
Spearman Correlation 0.91 **iRT Error:** 0.77

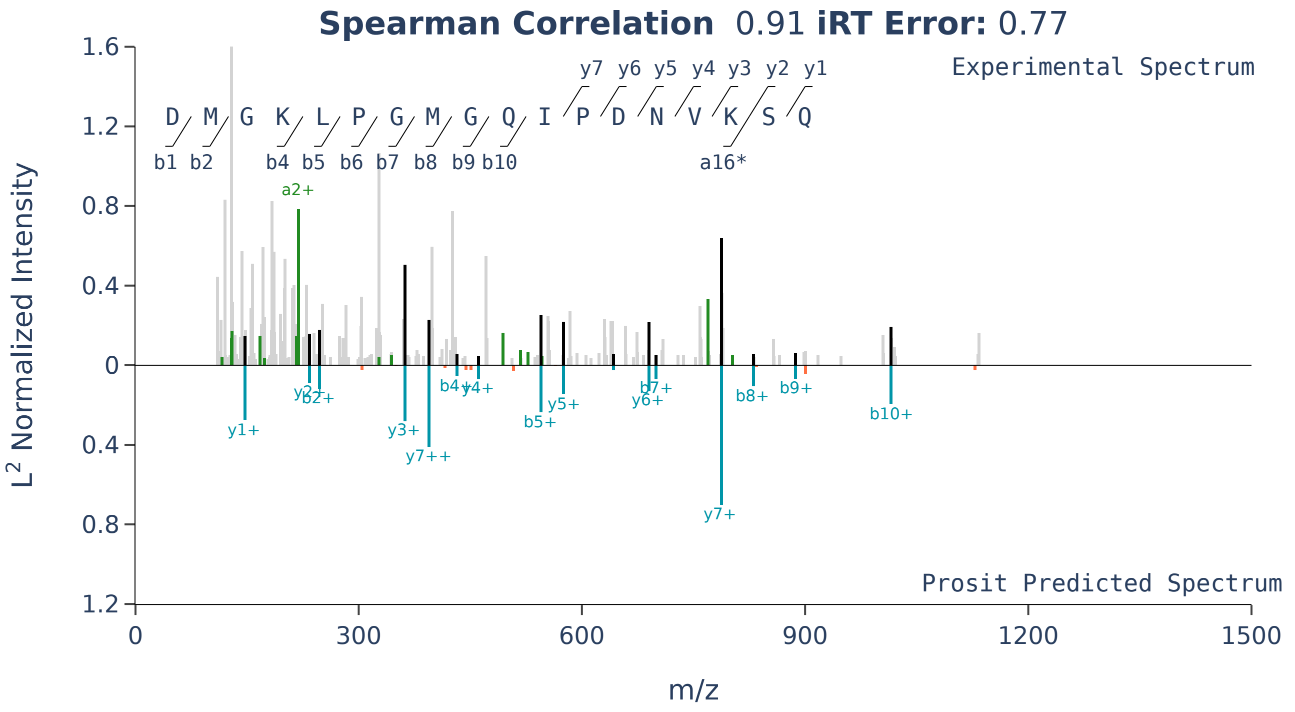

Source Ncheng_210623_230623_HFGoe_FFH_20S_25_1_A1_4h_R1 **Scan** 22260
Peptide GKLPGMGQIPDNVKSQMD **Charge** 3 **Spectral Angle** 0.44
Spearman Correlation 0.47 **iRT Error:** 12.43

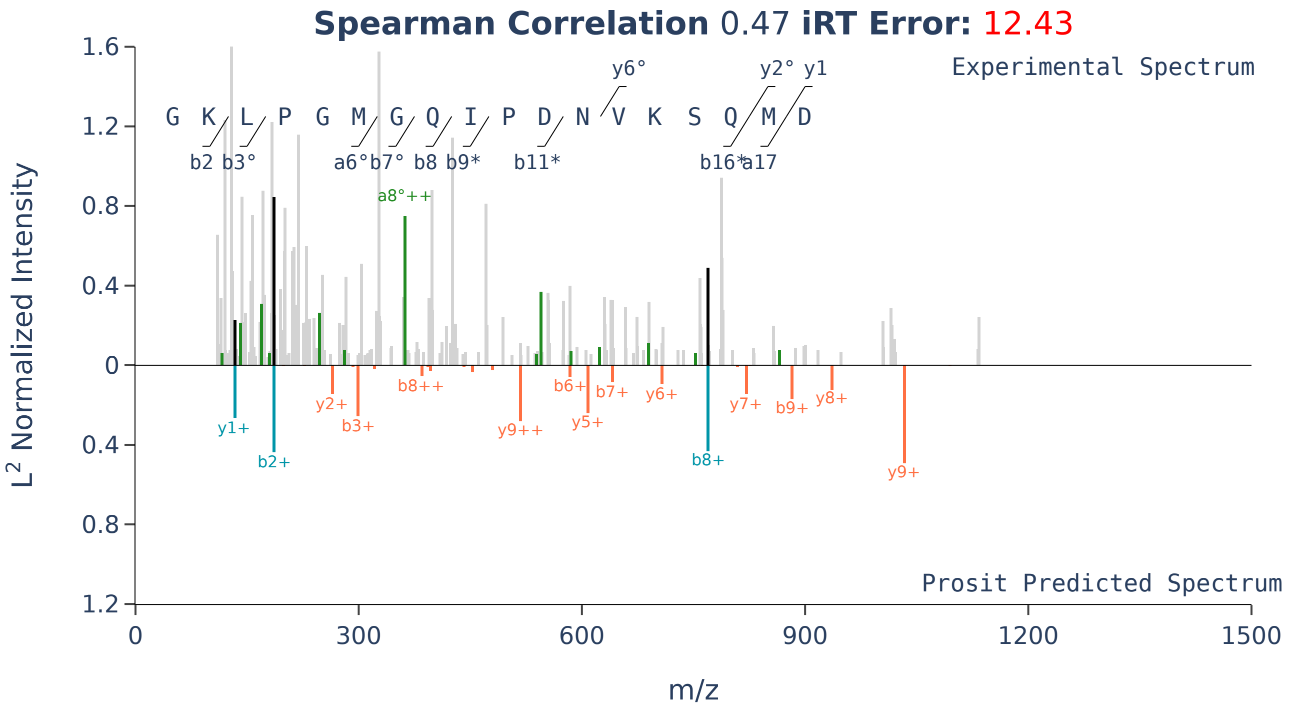

Source Ncheng_210623_230623_HFGoe_FFH_20S_25_1_A1_4h_R1 **Scan** 20545
Peptide AALEPFHPDRI **Charge** 3 **Spectral Angle** 0.76
Spearman Correlation 0.87 **iRT Error:** 0.79

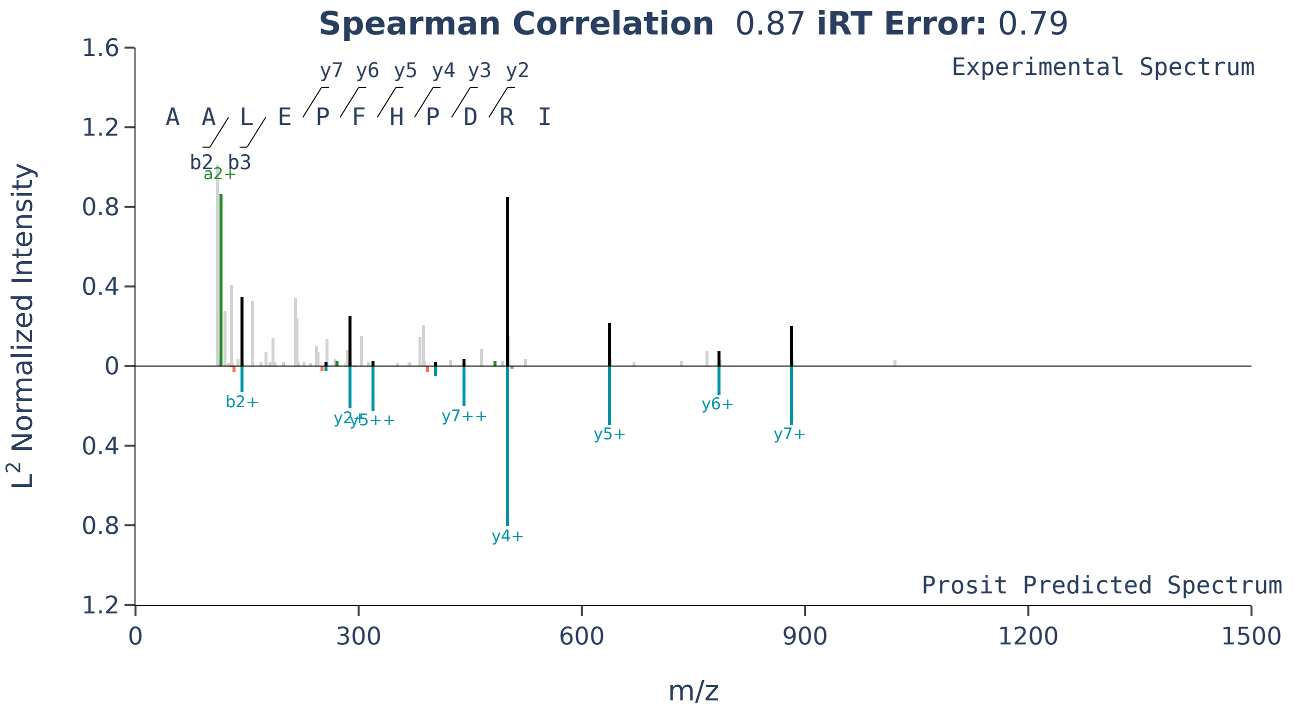

Source Ncheng_210623_230623_HFGoe_FFH_20S_25_1_A1_4h_R1 **Scan** 20545
Peptide ALEPFHPDRIA **Charge** 3 **Spectral Angle** 0.1
Spearman Correlation 0.22 **iRT Error:** 6.46

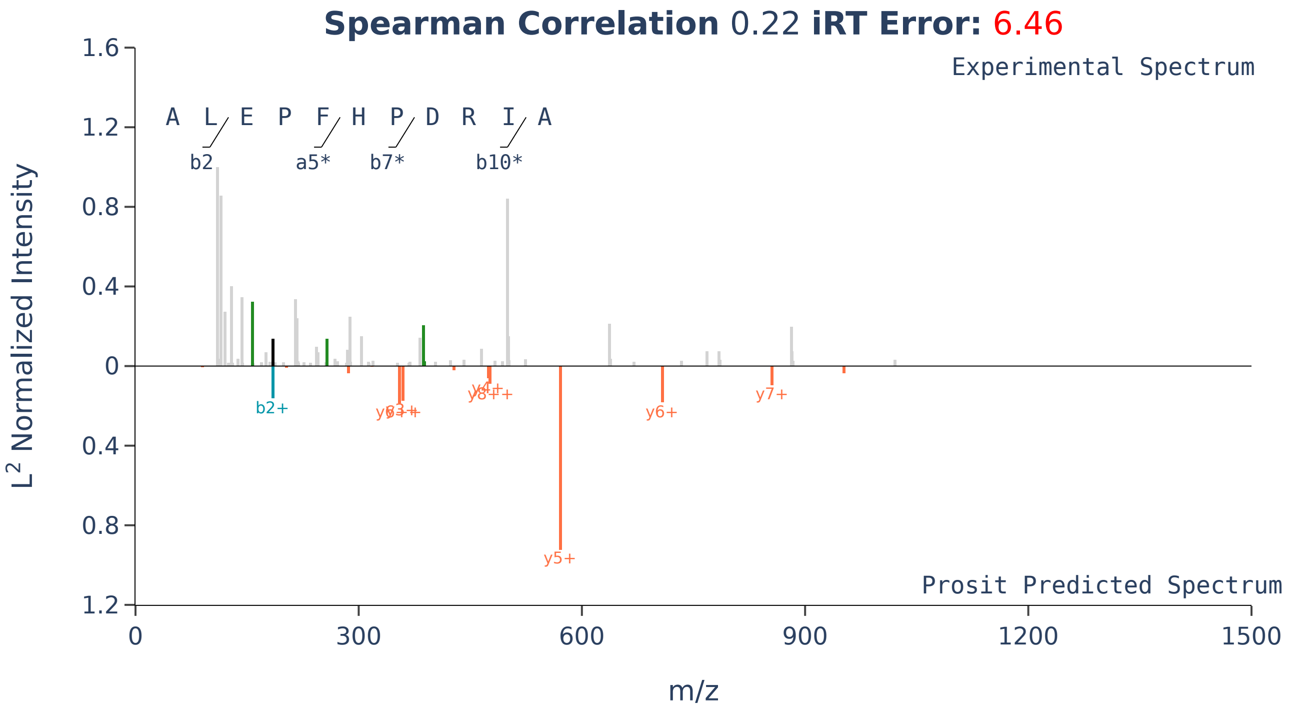

Source Ncheng_210623_230623_HFGoe_FFH_20S_25_1_A1_4h_R2 **Scan** 9528
Peptide SRTLAEQVG **Charge** 2 **Spectral Angle** 0.85
Spearman Correlation 0.89 **iRT Error:** 0.82

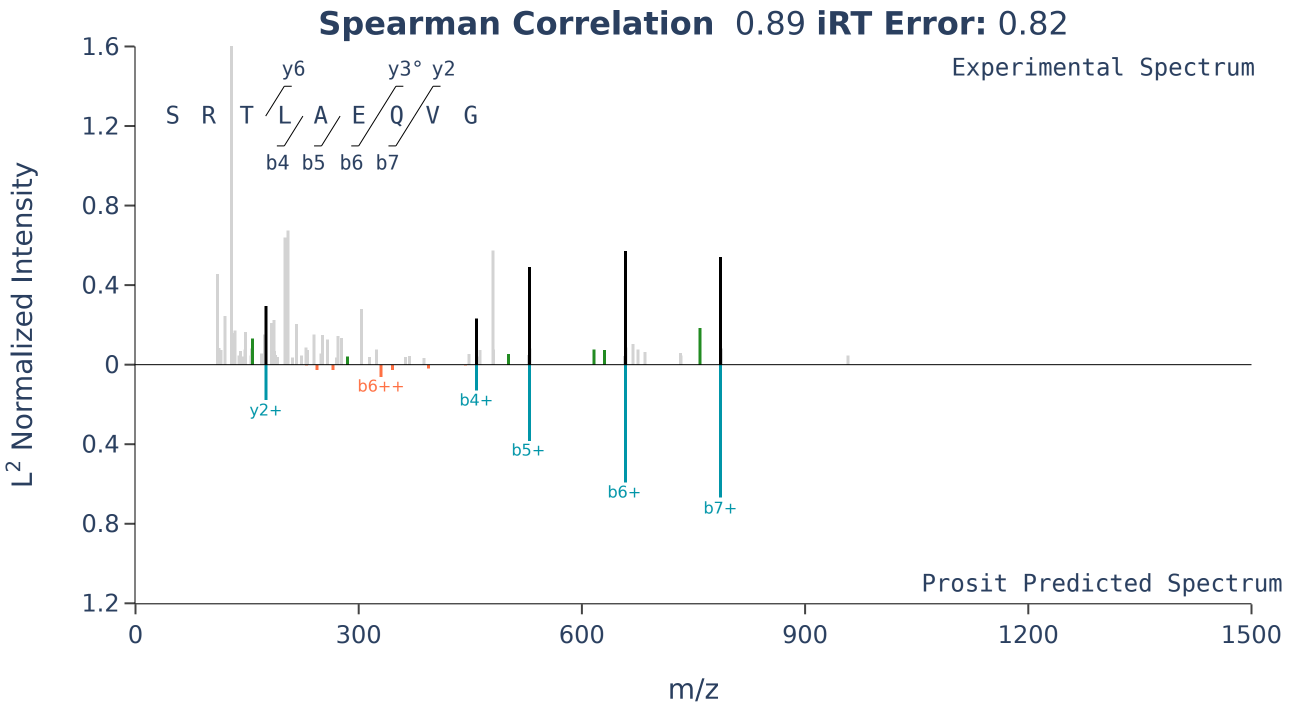

Source Ncheng_210623_230623_HFGoe_FFH_20S_25_1_A1_4h_R2 **Scan** 9528
Peptide DNVKDTLR **Charge** 2 **Spectral Angle** 0.0
Spearman Correlation 0.0 **iRT Error:** 12.17

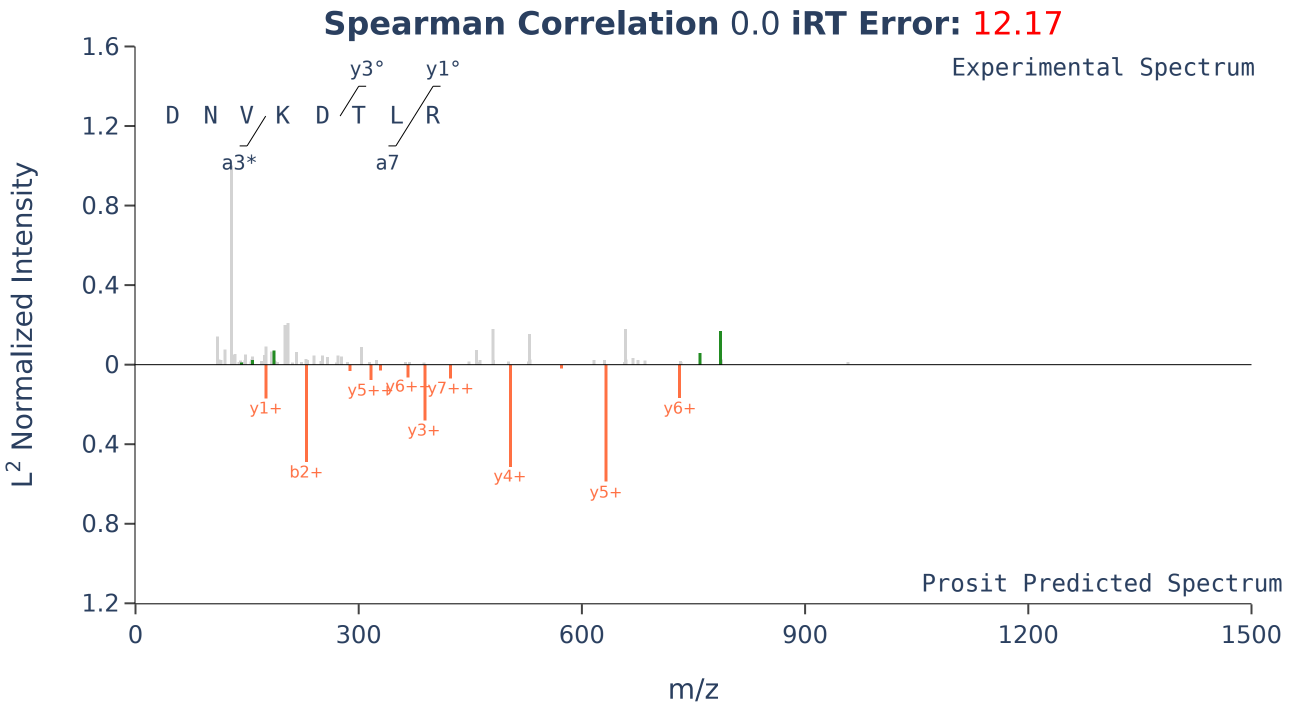

Source Ncheng_210623_230623_HFGoe_FFH_20S_25_1_A2_24h_R2 **Scan** 9799
Peptide GMQVGQKPVD **Charge** 2 **Spectral Angle** 0.78
Spearman Correlation 0.91 **iRT Error:** 0.85

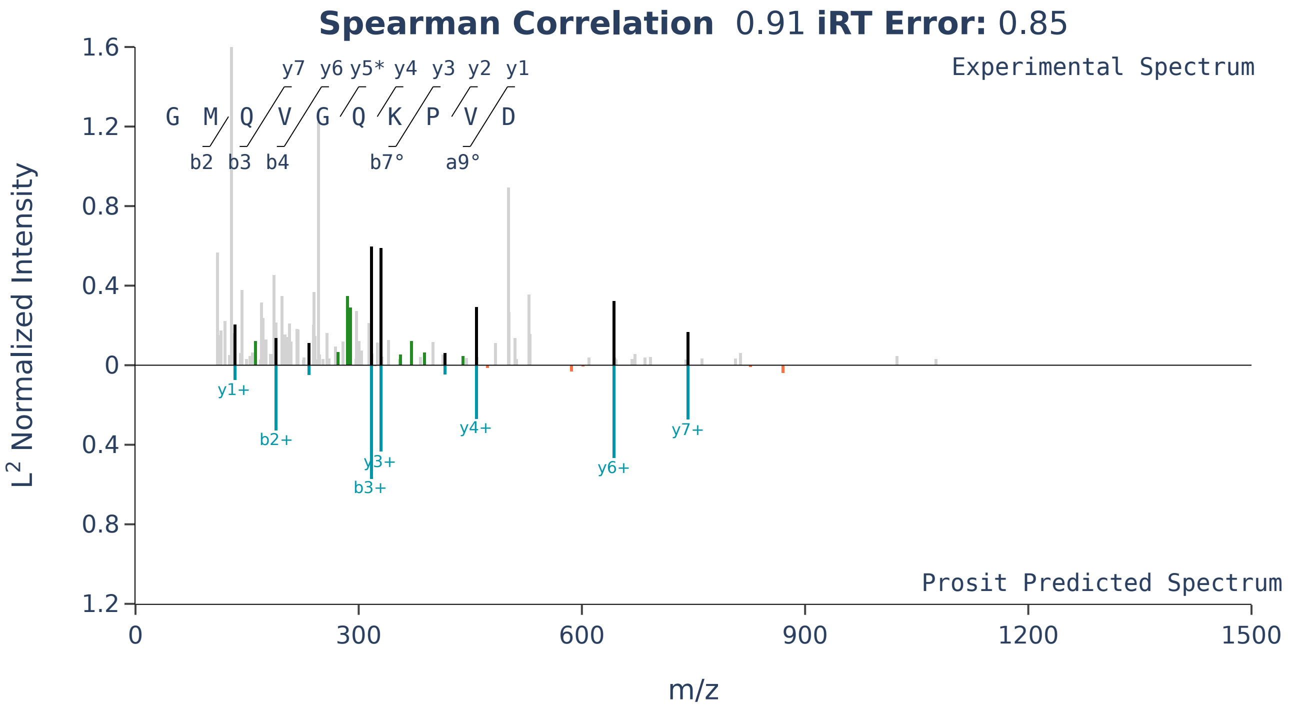

Source Ncheng_210623_230623_HFGoe_FFH_20S_25_1_A2_24h_R2 **Scan** 9799
Peptide GMGQIPDNVK **Charge** 2 **Spectral Angle** 0.05
Spearman Correlation 0.07 **iRT Error:** 22.18

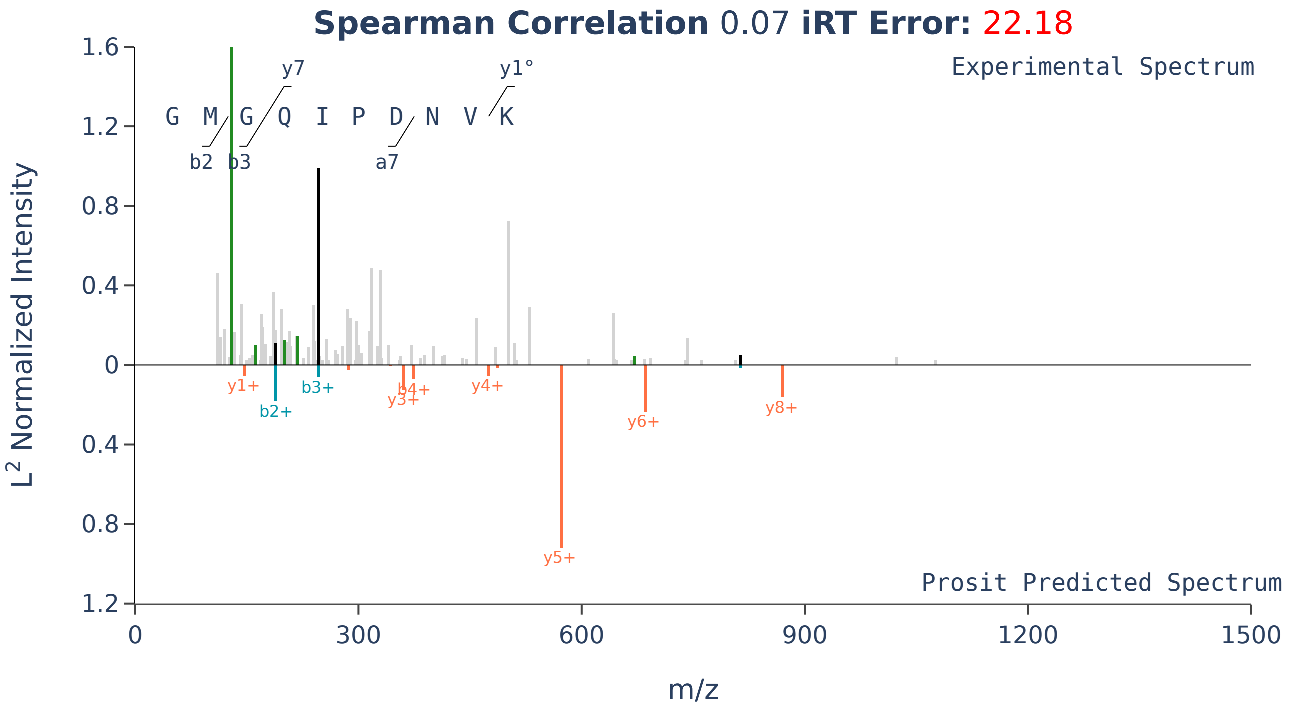

Source Ncheng_210623_230623_HFGoe_FFH_20S_25_1_A1_24h_R1 **Scan** 14870
Peptide GVGEKTEAMTGQDAANTAKAFN **Charge** 3 **Spectral Angle** 0.8
Spearman Correlation 0.91 **iRT Error:** 0.85

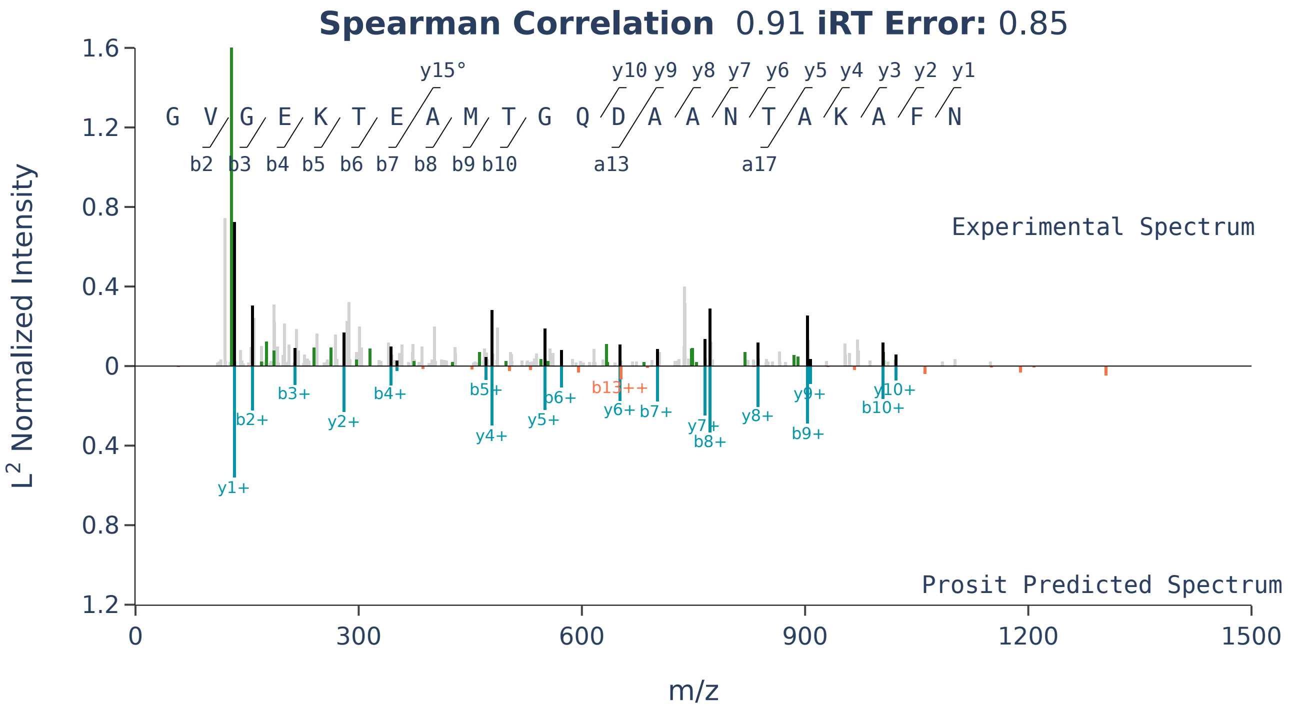

Source Ncheng_210623_230623_HFGoe_FFH_20S_25_1_A1_24h_R1 **Scan** 14870
Peptide DLNDFLEQLRQMKNMGGMA **Charge** 3 **Spectral Angle** 0.2
Spearman Correlation 0.15 **iRT Error:** 119.64

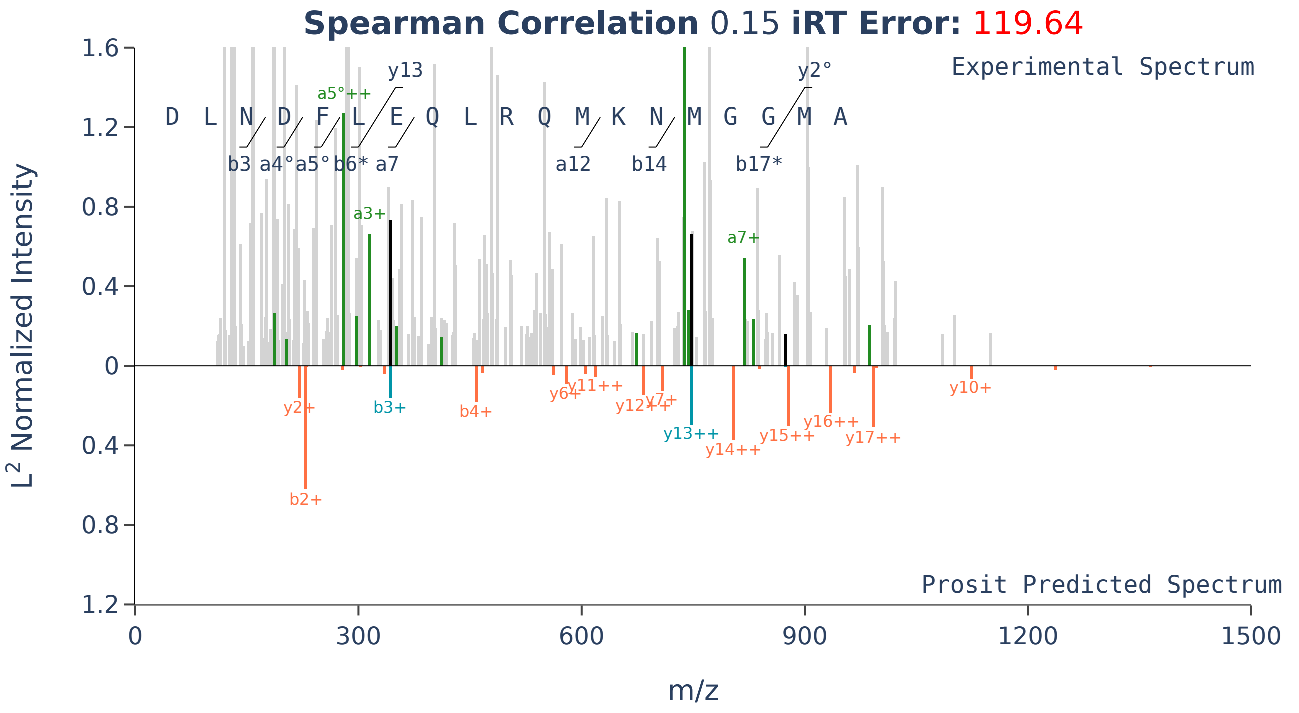

Source Ncheng_210623_230623_HFGoe_FFH_20S_25_1_A2_24h_R2 **Scan** 11464
Peptide DVYRPAAIKQT **Charge** 2 **Spectral Angle** 0.73
Spearman Correlation 0.88 **iRT Error:** 0.87

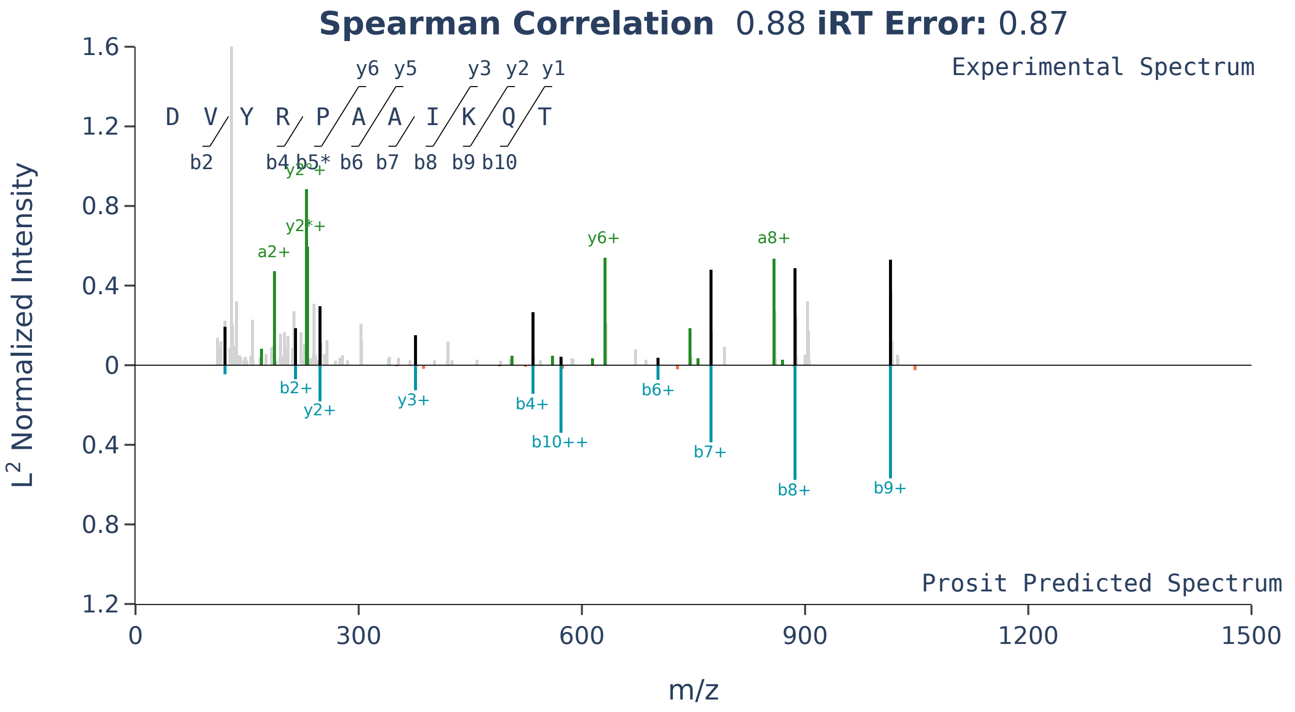

Source Ncheng_210623_230623_HFGoe_FFH_20S_25_1_A2_24h_R2 **Scan** 11464
Peptide QEFVKIVRNE **Charge** 2 **Spectral Angle** 0.02
Spearman Correlation -0.3 **iRT Error:** 18.04

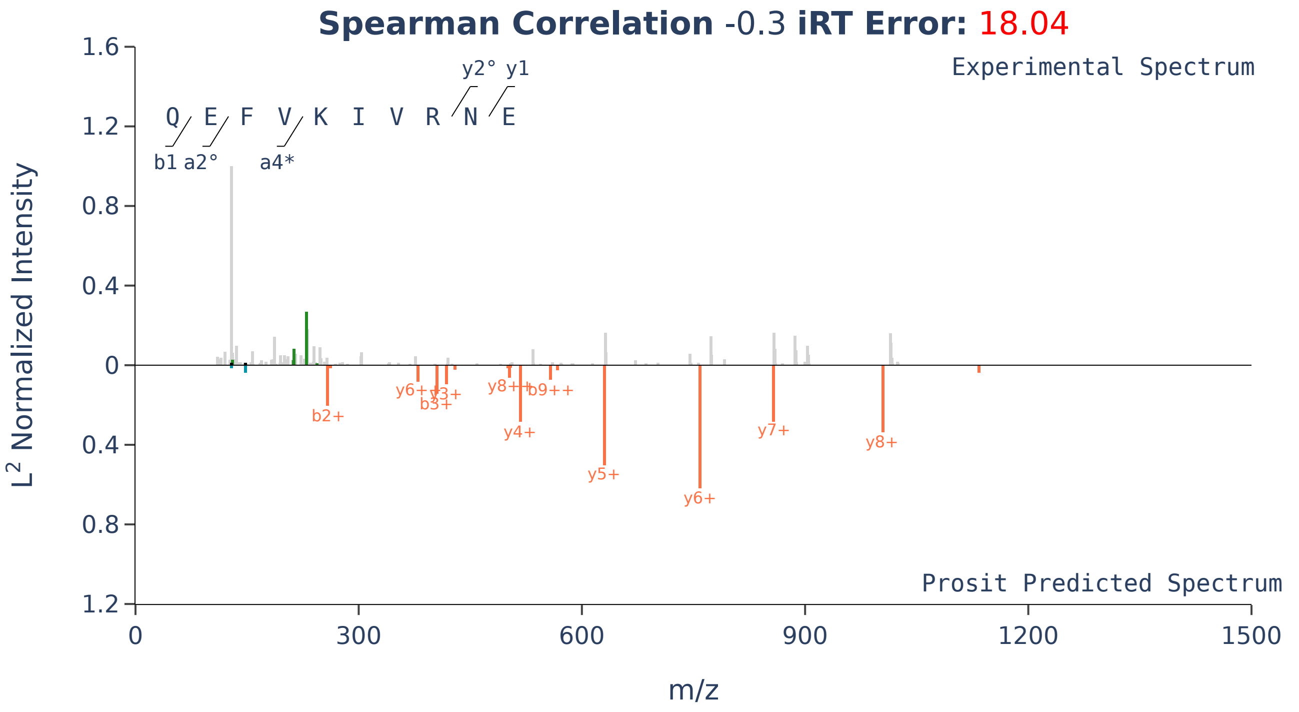

Source Ncheng_210623_230623_HFGoe_FFH_20S_25_1_A2_24h_R2 **Scan** 10004
Peptide GKSINPVET **Charge** 2 **Spectral Angle** 0.84
Spearman Correlation 0.89 **iRT Error:** 0.98

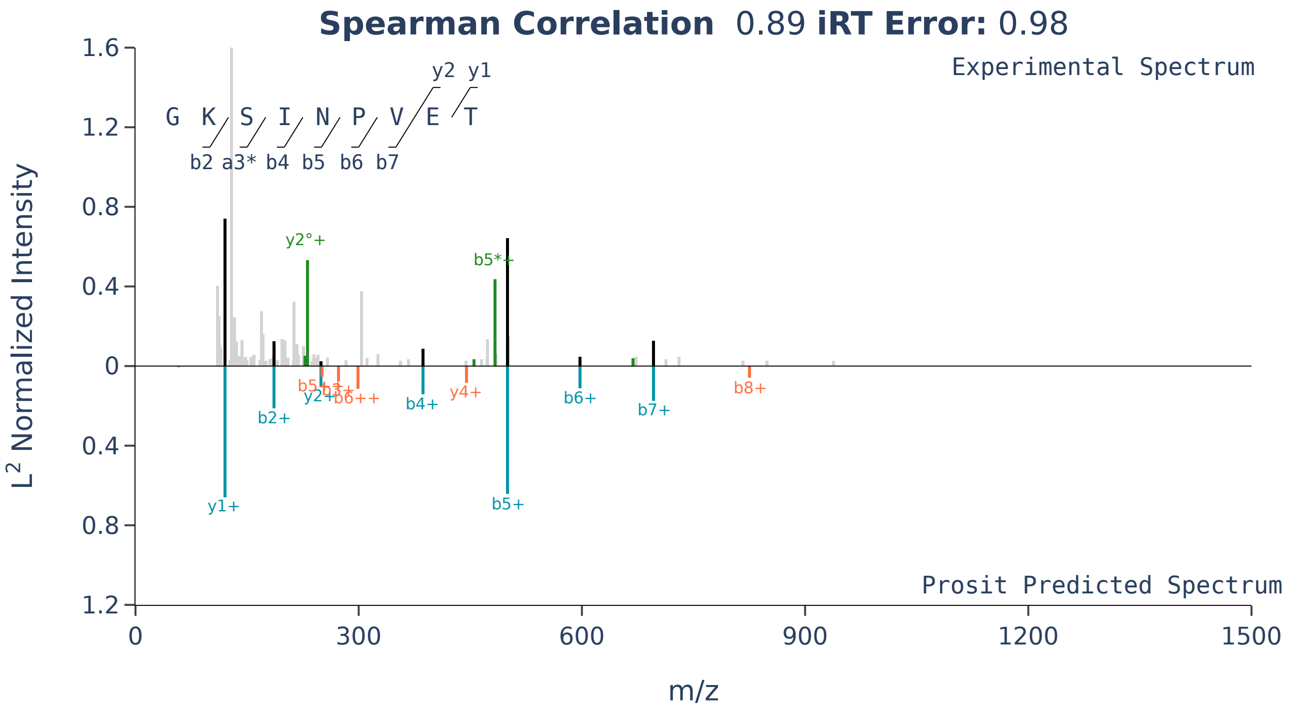

Source Ncheng_210623_230623_HFGoe_FFH_20S_25_1_A2_24h_R2 **Scan** 10004
Peptide EVNKSLTPG **Charge** 2 **Spectral Angle** 0.01
Spearman Correlation -0.24 **iRT Error:** 8.32

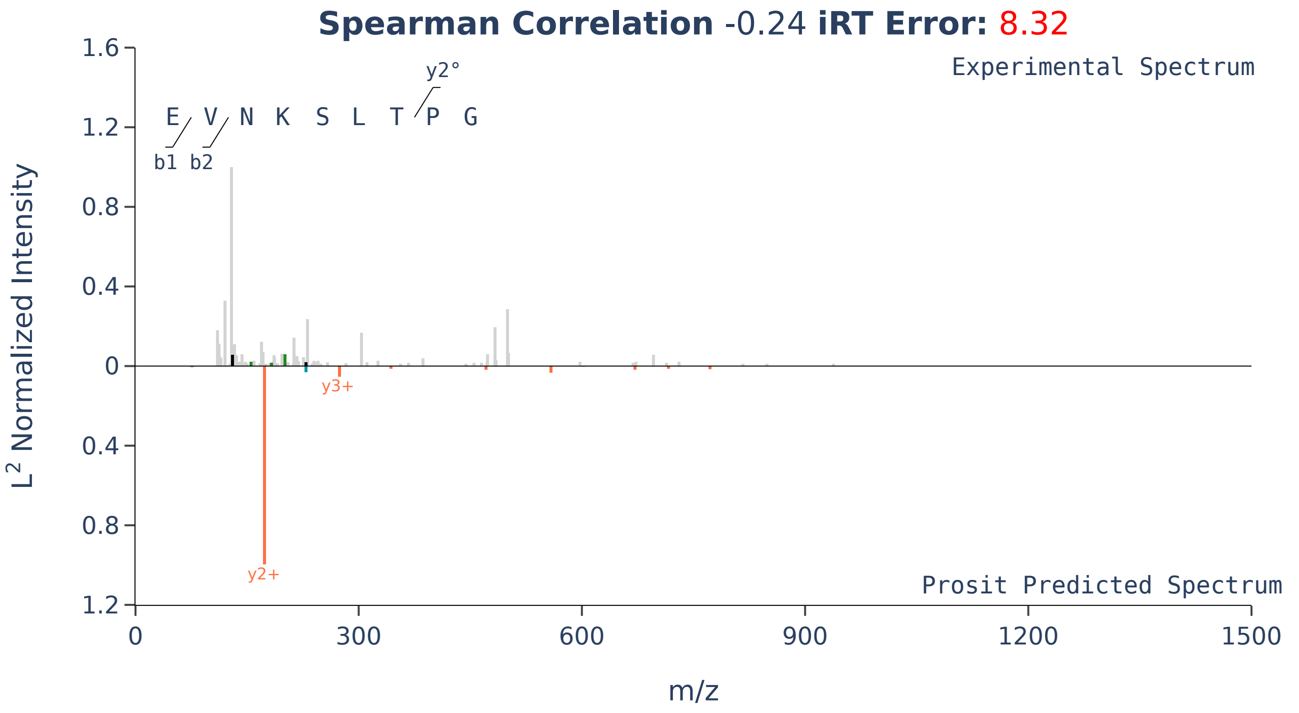

Source Ncheng_210623_230623_HFGoe_FFH_20S_25_1_A2_24h_R2 **Scan** 10292
Peptide NKMGGMASL **Charge** 2 **Spectral Angle** 0.74
Spearman Correlation 0.88 **iRT Error:** 0.98

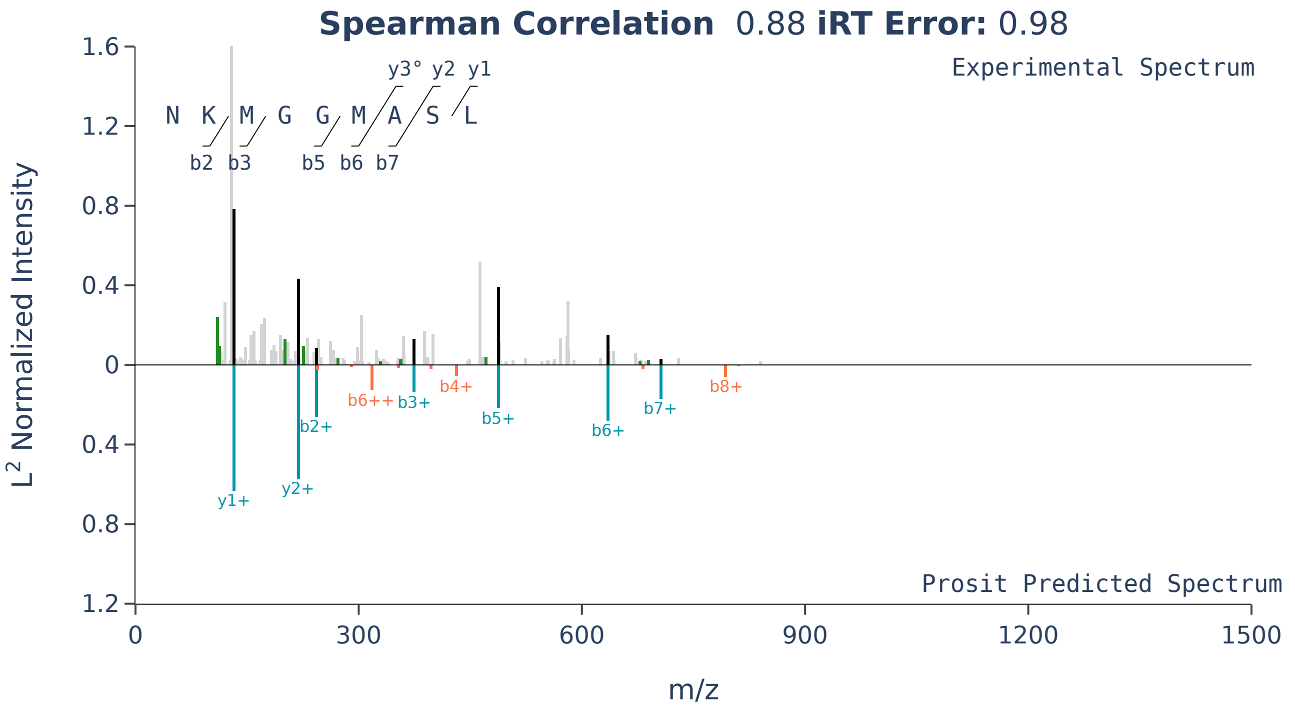

Source Ncheng_210623_230623_HFGoe_FFH_20S_25_1_A2_24h_R2 **Scan** 10292
Peptide KNMGGMASL **Charge** 2 **Spectral Angle** 0.41
Spearman Correlation 0.79 **iRT Error:** 16.39

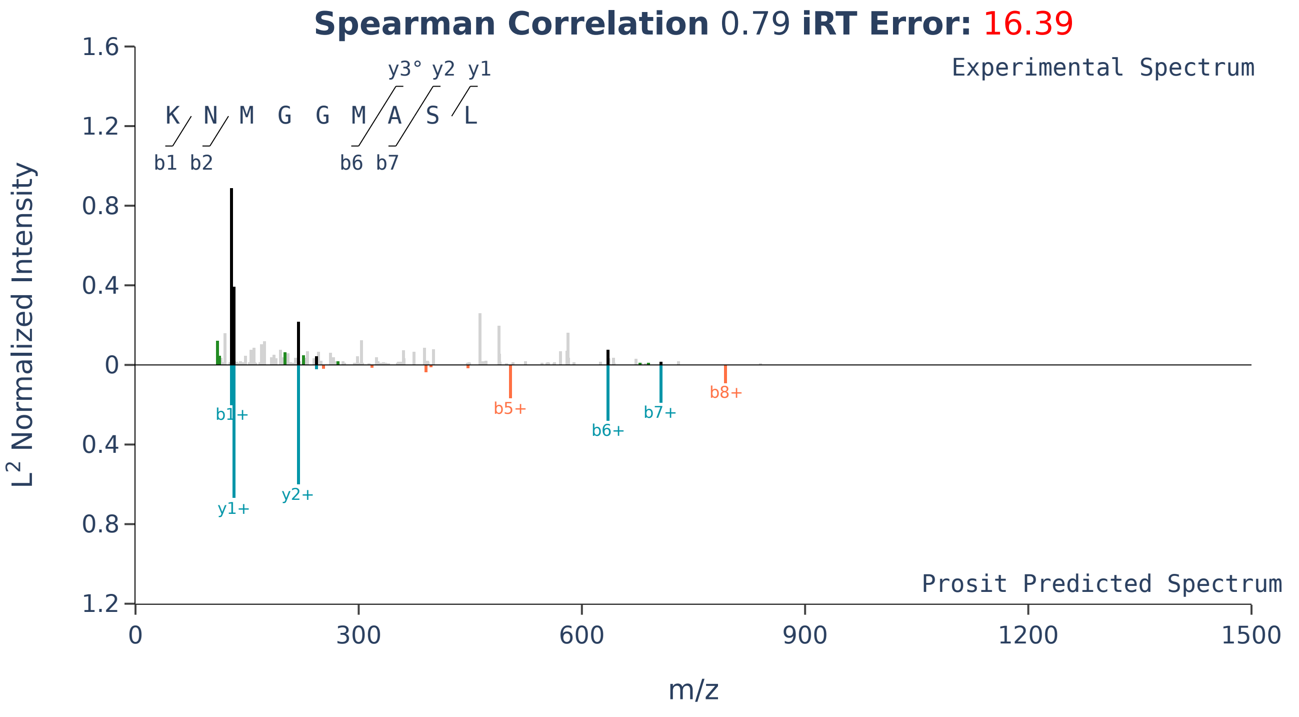

Source Ncheng_210623_230623_HFGoe_FFH_20S_25_1_A1_24h_R1 **Scan** 10843
Peptide NISTLRE **Charge** 2 **Spectral Angle** 0.78
Spearman Correlation 0.85 **iRT Error:** 1.0

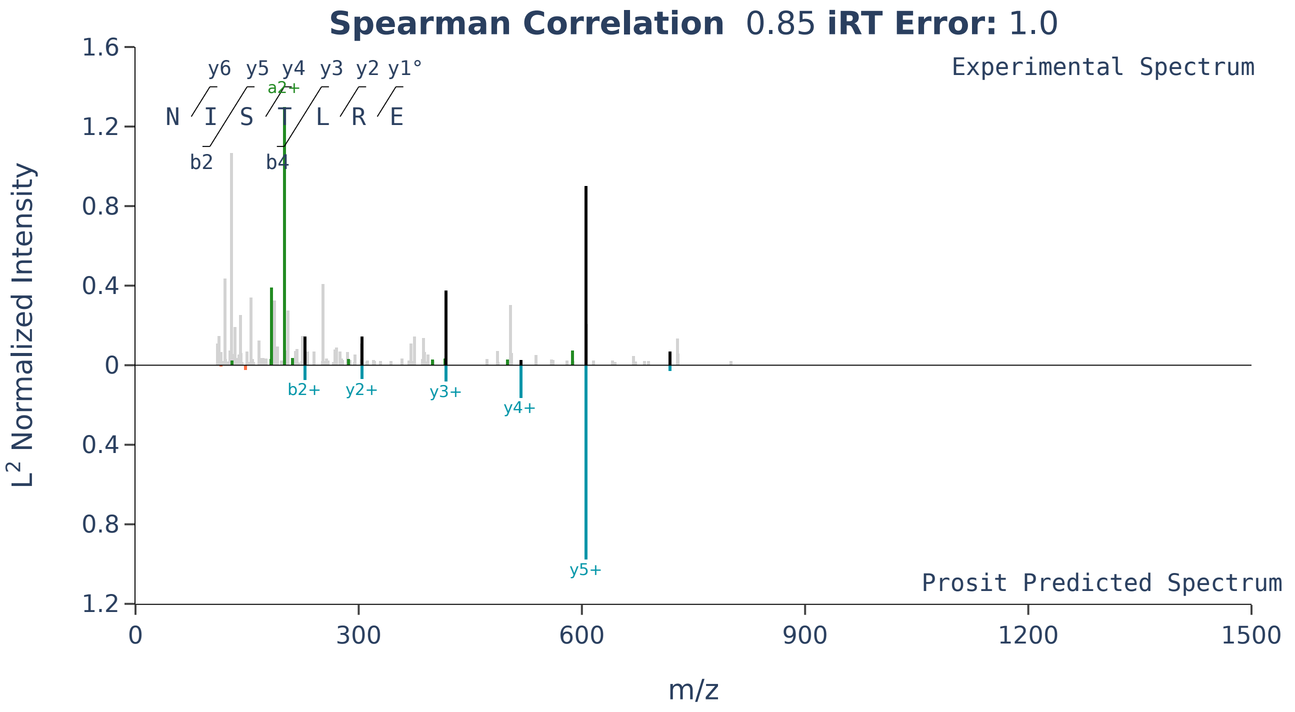

Source Ncheng_210623_230623_HFGoe_FFH_20S_25_1_A1_24h_R1 **Scan** 10843
Peptide GLQGAGKTT **Charge** 2 **Spectral Angle** 0.39
Spearman Correlation 0.4 **iRT Error:** 16.89

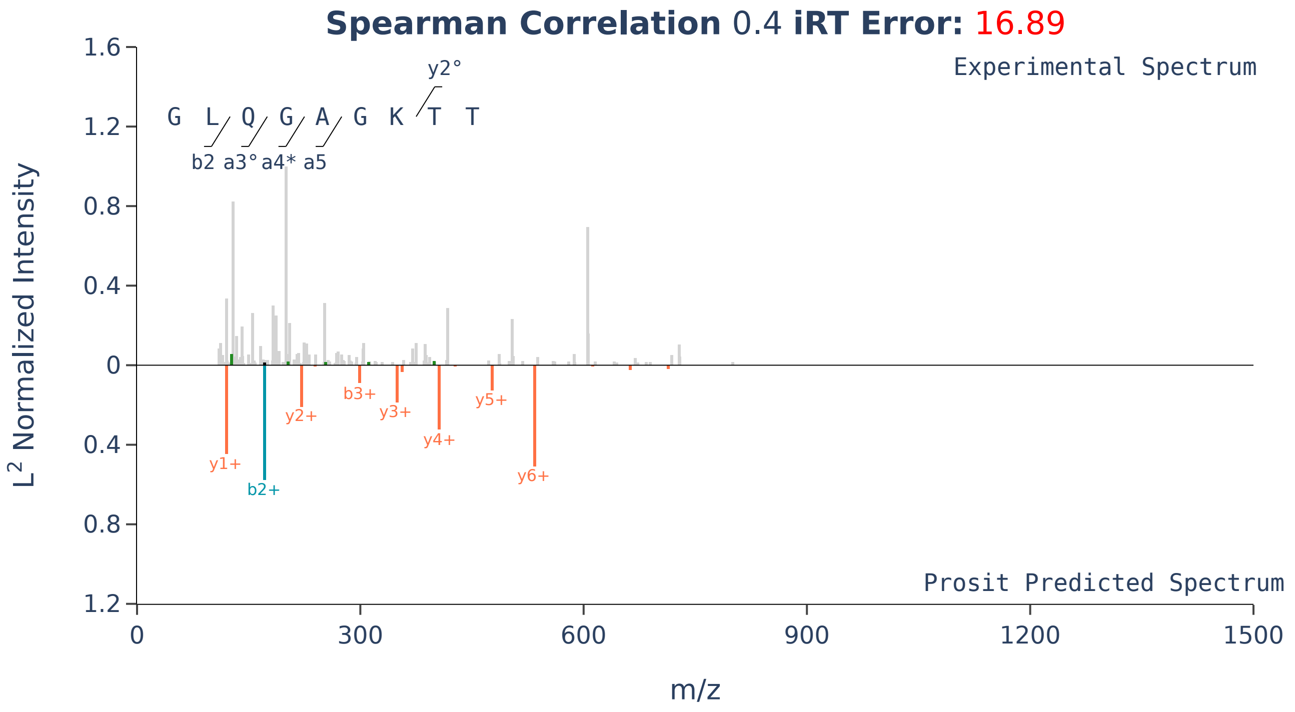

Source Ncheng_210623_230623_HFGoe_FFH_20S_25_1_A2_4h_R2 **Scan** 21666
Peptide MGKLPGMGQIPDNVKGMAS **Charge** 2 **Spectral Angle** 0.88
Spearman Correlation 0.87 **iRT Error:** 1.01

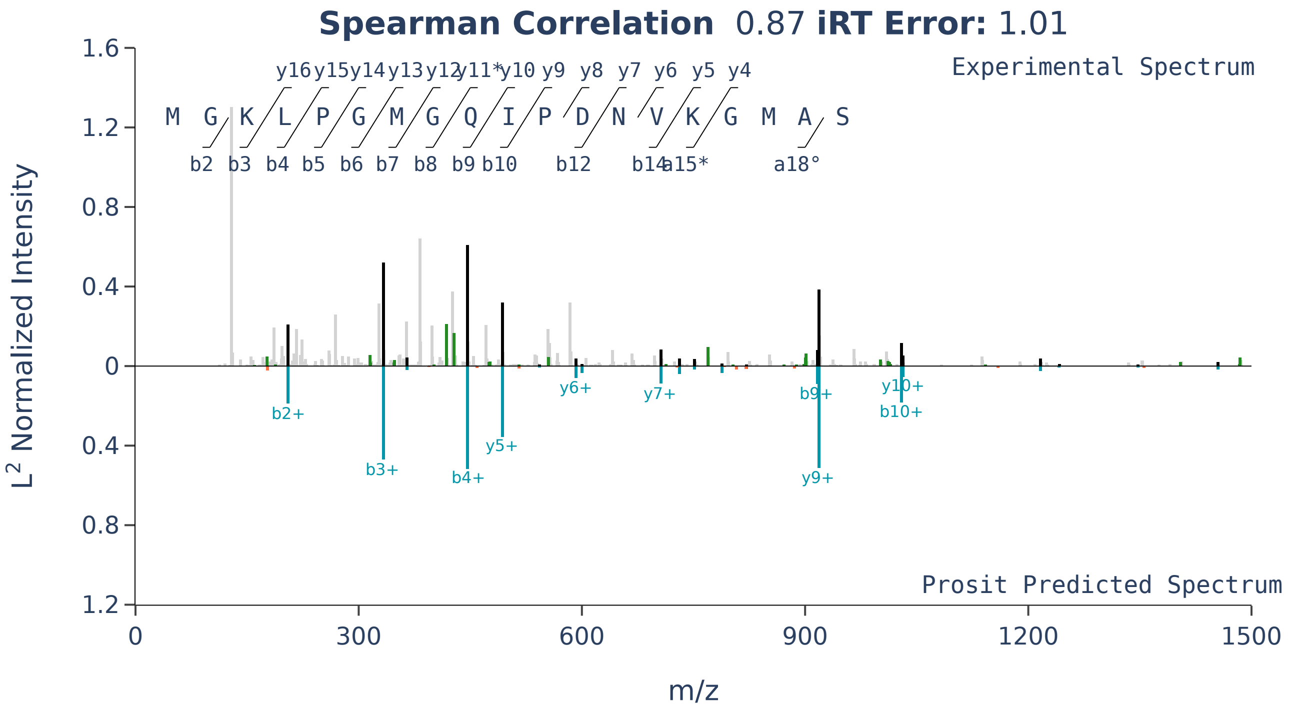

Source Ncheng_210623_230623_HFGoe_FFH_20S_25_1_A2_4h_R2 **Scan** 21666
Peptide MGKLPGMGQIPDNVKSQM **Charge** 2 **Spectral Angle** 0.48
Spearman Correlation 0.79 **iRT Error:** 18.1

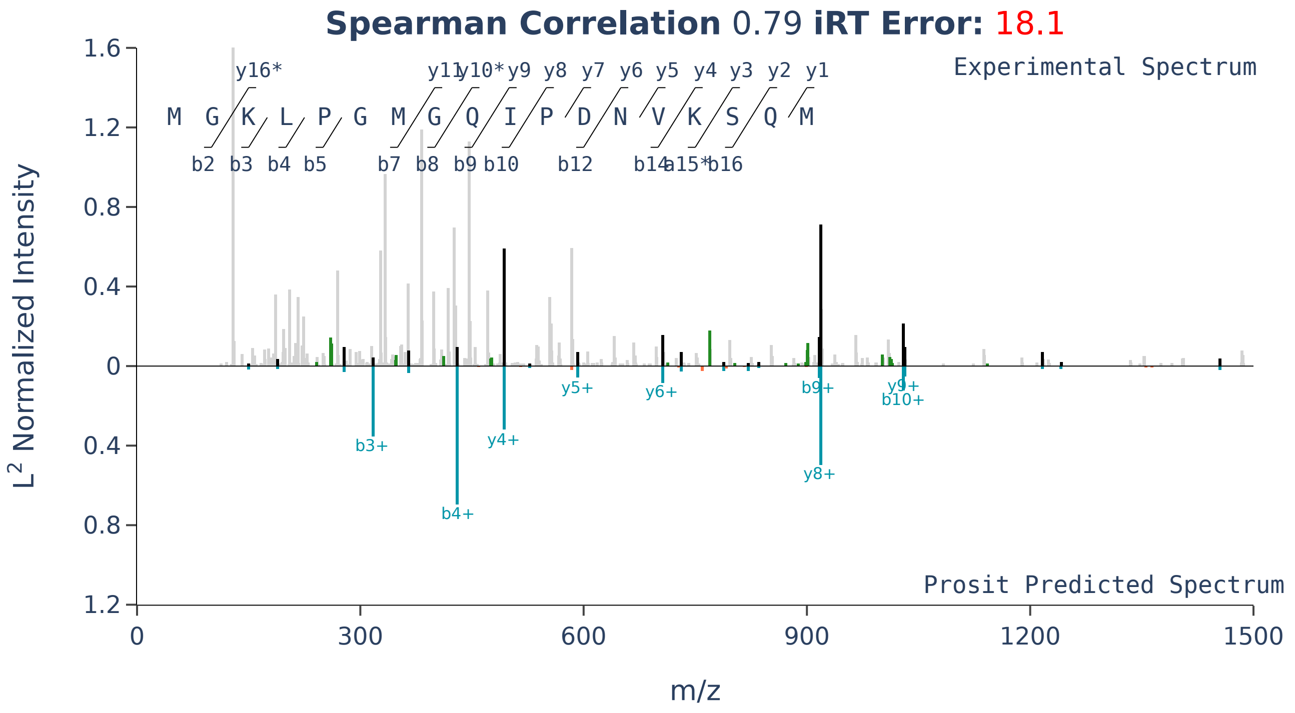

Source Ncheng_210623_230623_HFGoe_FFH_20S_25_1_A1_24h_R2 **Scan** 17836
Peptide LGKGHEVNKSLTPGQEFVKIVRN **Charge** 2 **Spectral Angle** 0.8
Spearman Correlation 0.87 **iRT Error:** 1.02

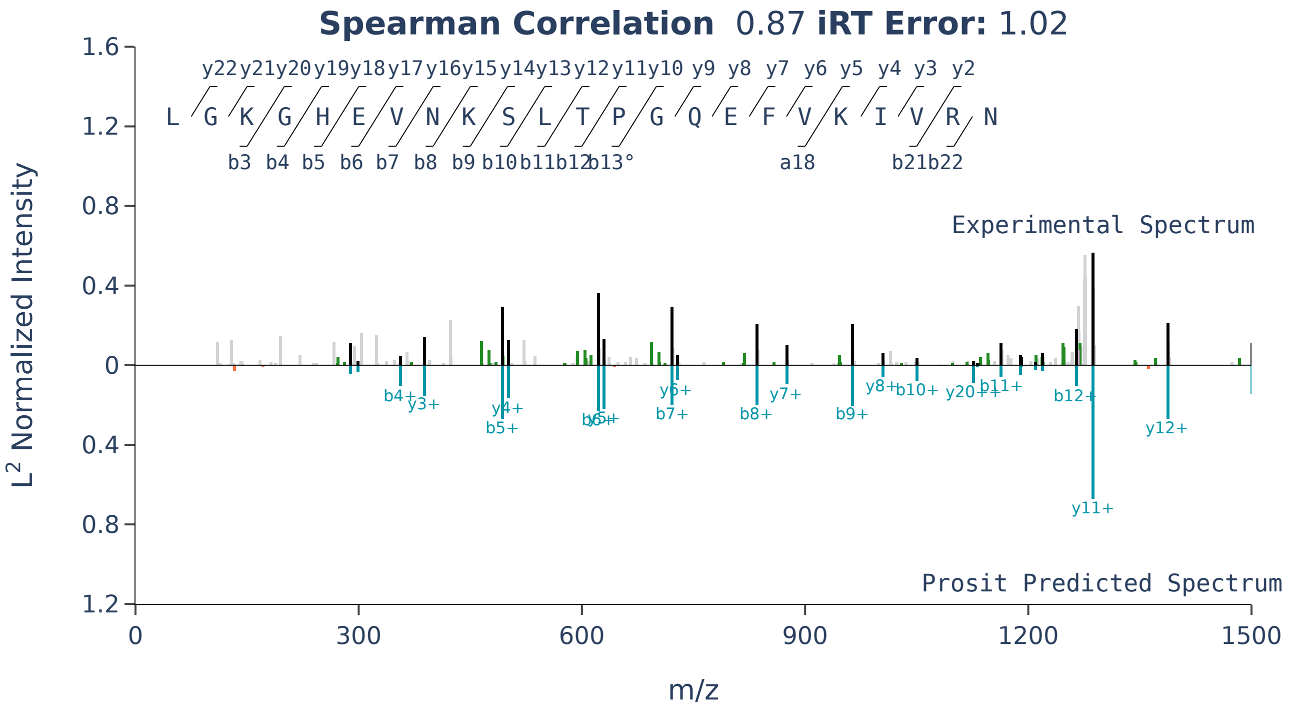

Source Ncheng_210623_230623_HFGoe_FFH_20S_25_1_A1_24h_R2 **Scan** 17836
Peptide KAVGHEVNKSLTPGQEFVKIVRN **Charge** 2 **Spectral Angle** 0.76
Spearman Correlation 0.73 **iRT Error:** 1.67

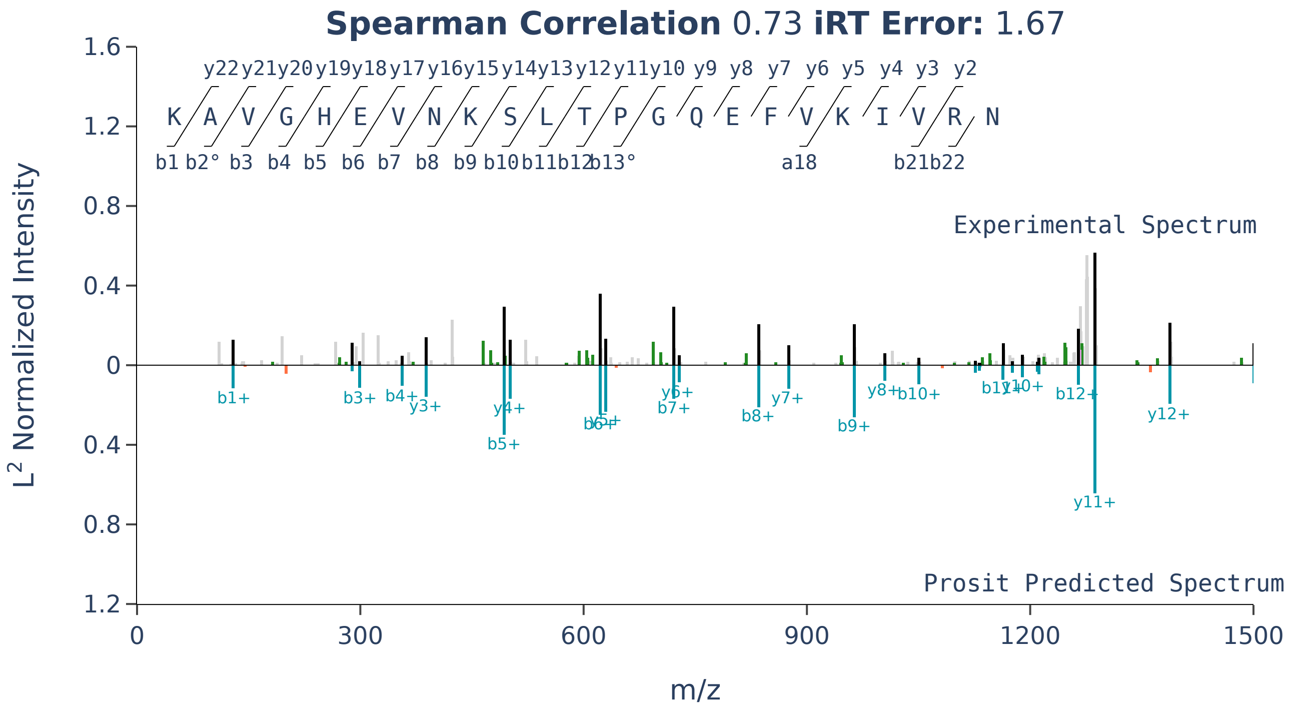

Source Ncheng_210623_230623_HFGoe_FFH_20S_25_1_A2_24h_R2 **Scan** 20718
Peptide TGLEPFHPDRI **Charge** 3 **Spectral Angle** 0.75
Spearman Correlation 0.87 **iRT Error:** 1.1

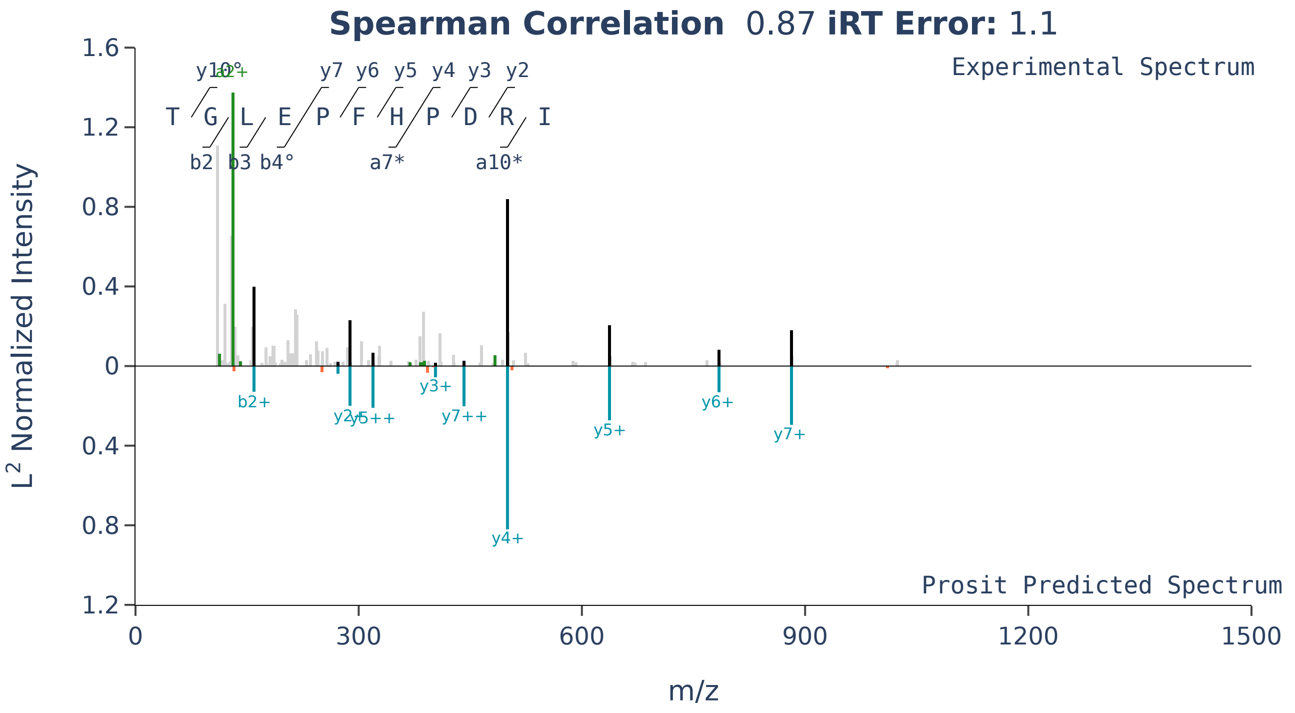

Source Ncheng_210623_230623_HFGoe_FFH_20S_25_1_A2_24h_R2 **Scan** 20718
Peptide LEPFHPDRIAS **Charge** 3 **Spectral Angle** 0.1
Spearman Correlation -0.01 **iRT Error:** 21.39

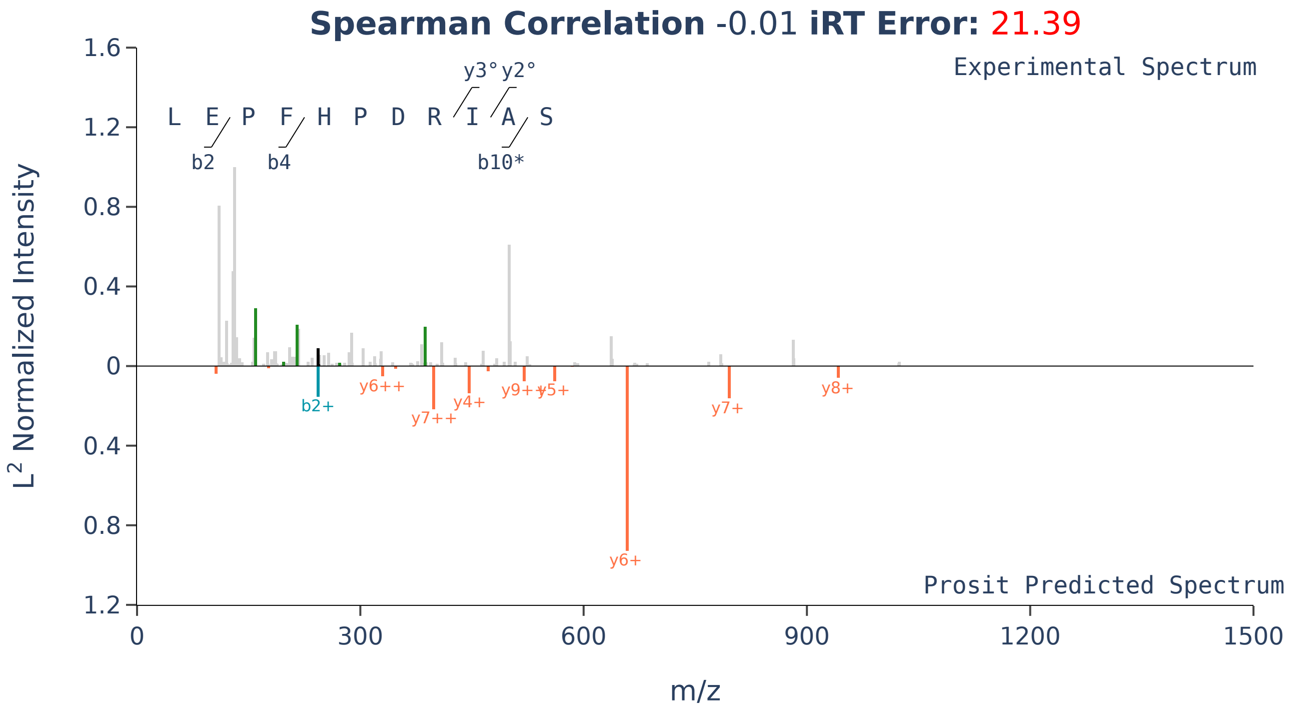

Source Ncheng_210623_230623_HFGoe_FFH_20S_25_1_A2_1h_R1 **Scan** 23336
Peptide DMGKLPGMGQIPDNVKSQMD **Charge** 3 **Spectral Angle** 0.73
Spearman Correlation 0.89 **iRT Error:** 1.12

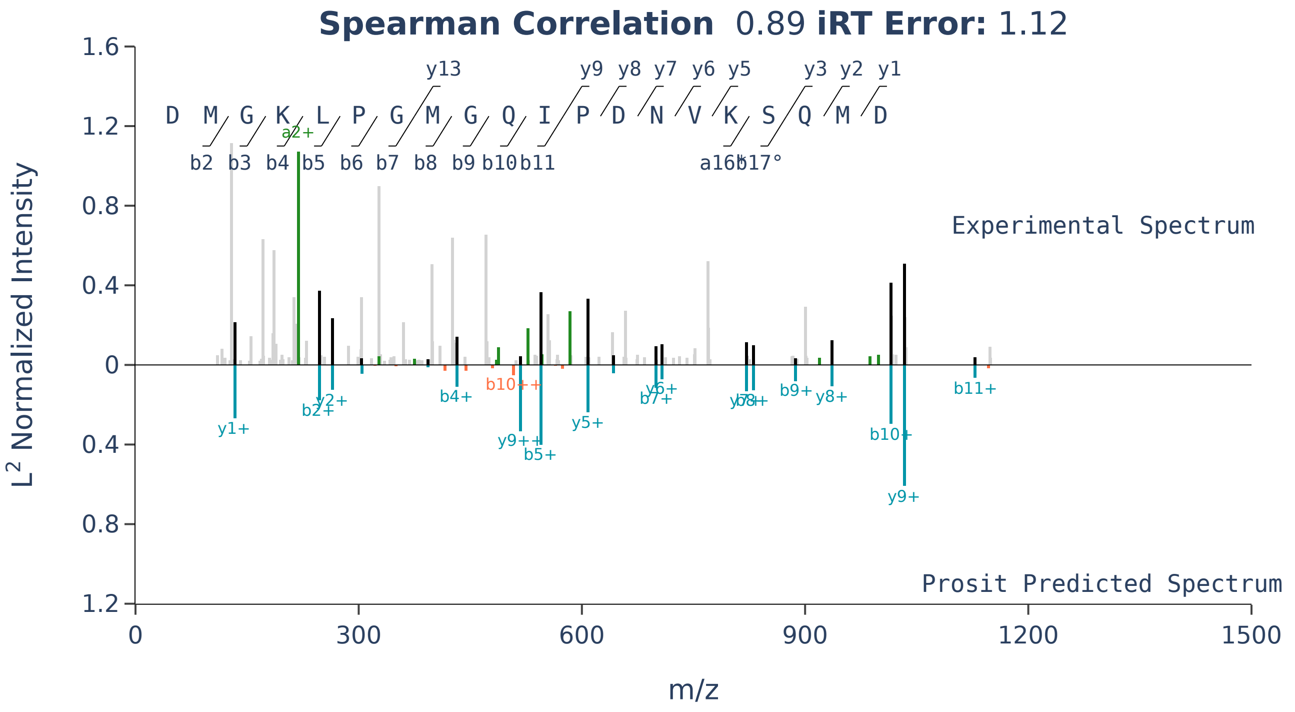

Source Ncheng_210623_230623_HFGoe_FFH_20S_25_1_A2_1h_R1 **Scan** 23336
Peptide MGKLPGMGQIPDNVKSQMDD **Charge** 3 **Spectral Angle** 0.5
Spearman Correlation 0.62 **iRT Error:** 12.87

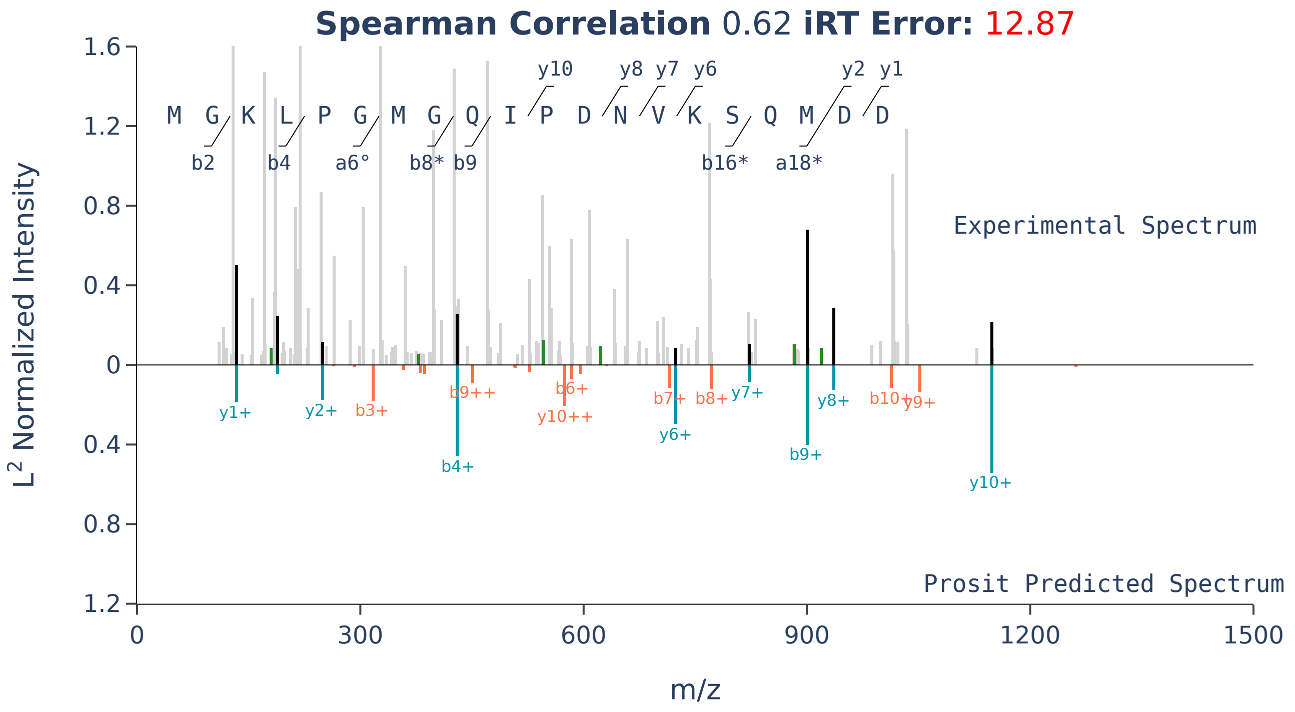

Source Ncheng_210623_230623_HFGoe_FFH_20S_25_1_A1_2h_R1 **Scan** 6134
Peptide EKLQVHA **Charge** 1 **Spectral Angle** 0.76
Spearman Correlation 0.9 **iRT Error:** 1.14

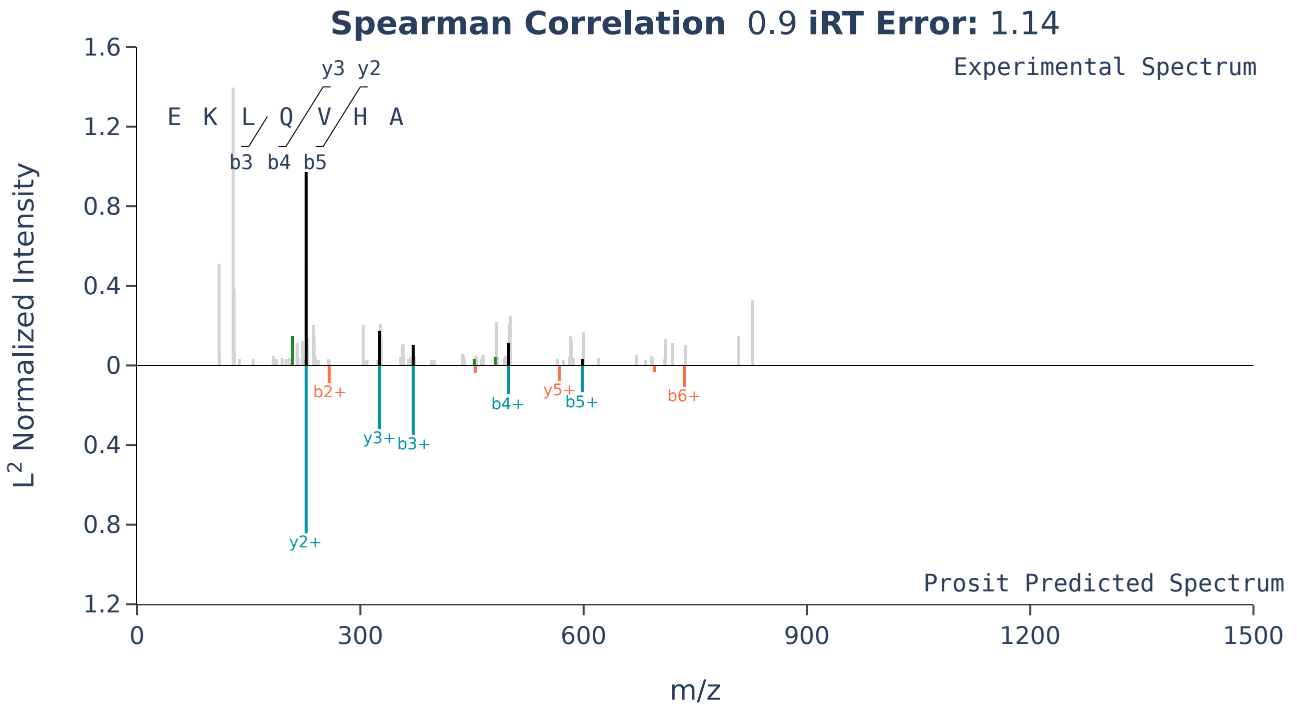

Source Ncheng_210623_230623_HFGoe_FFH_20S_25_1_A1_2h_R1 **Scan** 6134
Peptide EIKQVHA **Charge** 1 **Spectral Angle** 0.8
Spearman Correlation 0.84 **iRT Error:** 85.44

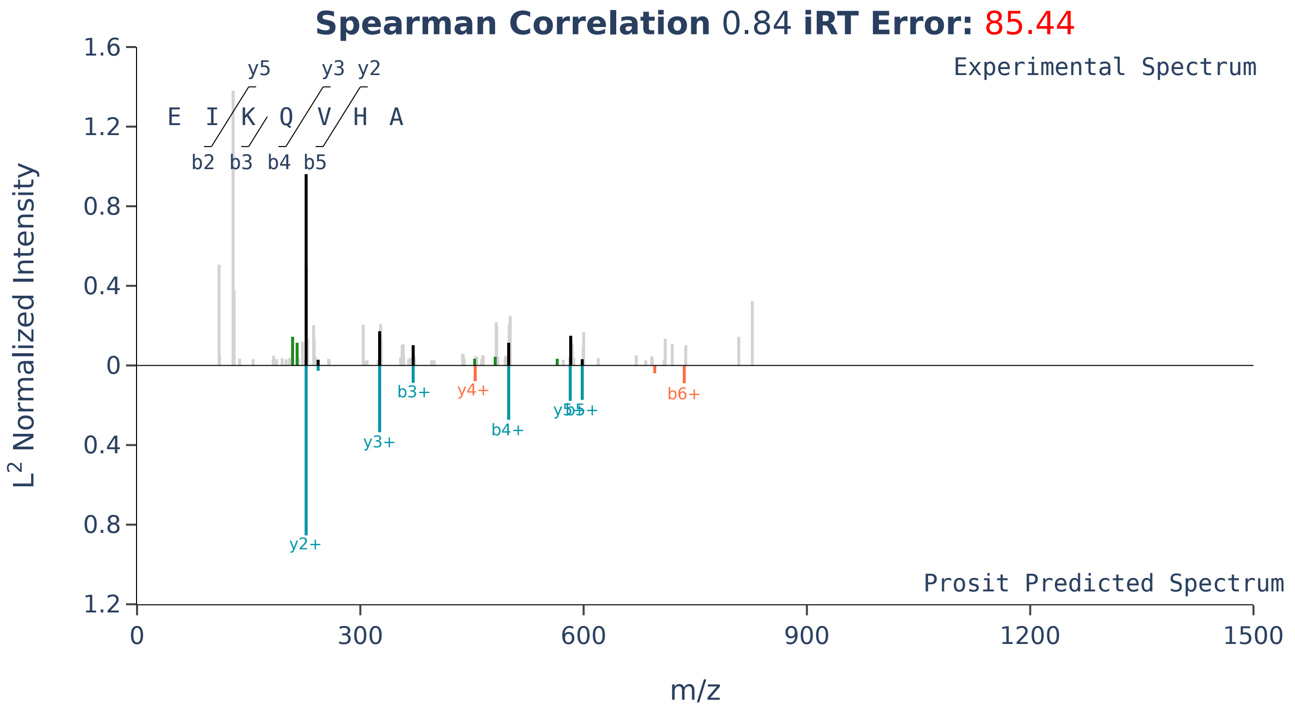

Source Ncheng_210623_230623_HFGoe_FFH_20S_25_1_A2_24h_R2 **Scan** 28381
Peptide PPAVVLAAMG **Charge** 1 **Spectral Angle** 0.86
Spearman Correlation 0.94 **iRT Error:** 1.14

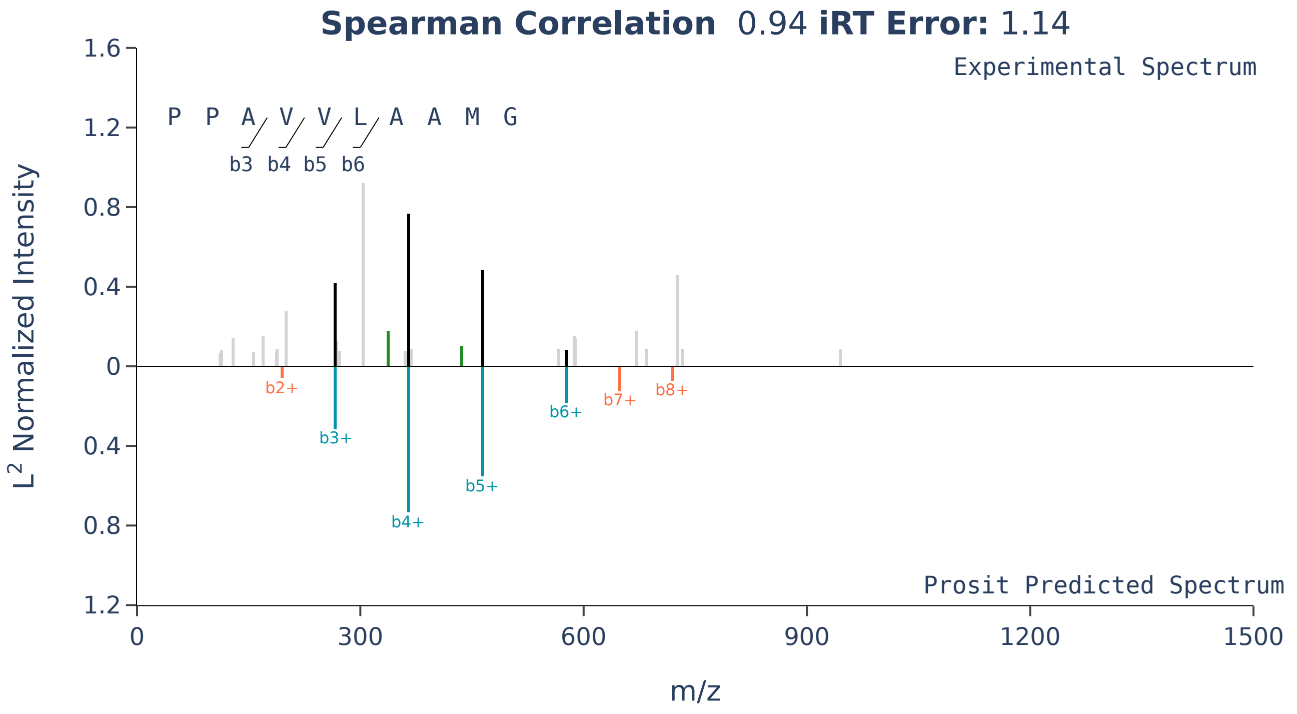

Source Ncheng_210623_230623_HFGoe_FFH_20S_25_1_A2_24h_R2 **Scan** 28381
Peptide QPPAVVLMA **Charge** 1 **Spectral Angle** 0.0
Spearman Correlation 0.0 **iRT Error:** 8.35

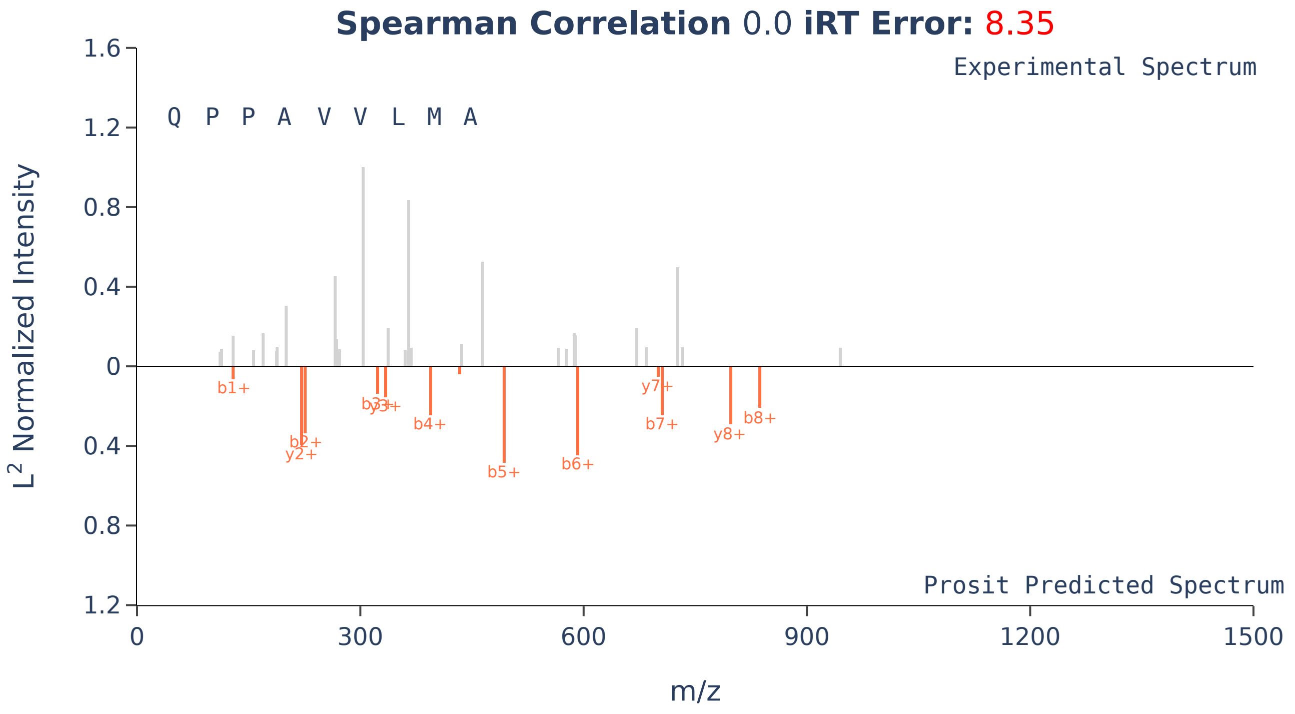

Source Ncheng_210623_230623_HFGoe_FFH_20S_25_1_A2_24h_R1 **Scan** 13892
Peptide AMTGQDAANTAKAFND **Charge** 2 **Spectral Angle** 0.79
Spearman Correlation 0.88 **iRT Error:** 1.15

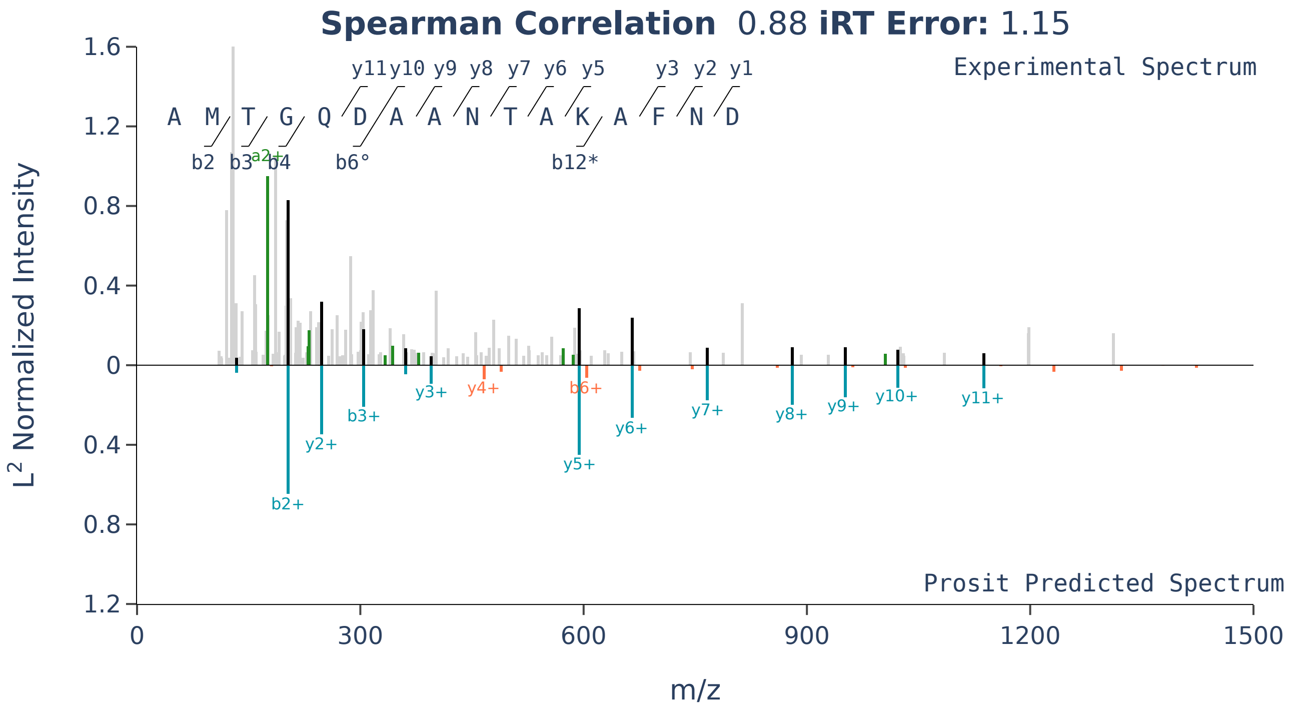

Source Ncheng_210623_230623_HFGoe_FFH_20S_25_1_A2_24h_R1 **Scan** 13892
Peptide DAMTGQDAANTAKAFN **Charge** 2 **Spectral Angle** 0.51
Spearman Correlation 0.63 **iRT Error:** 7.59

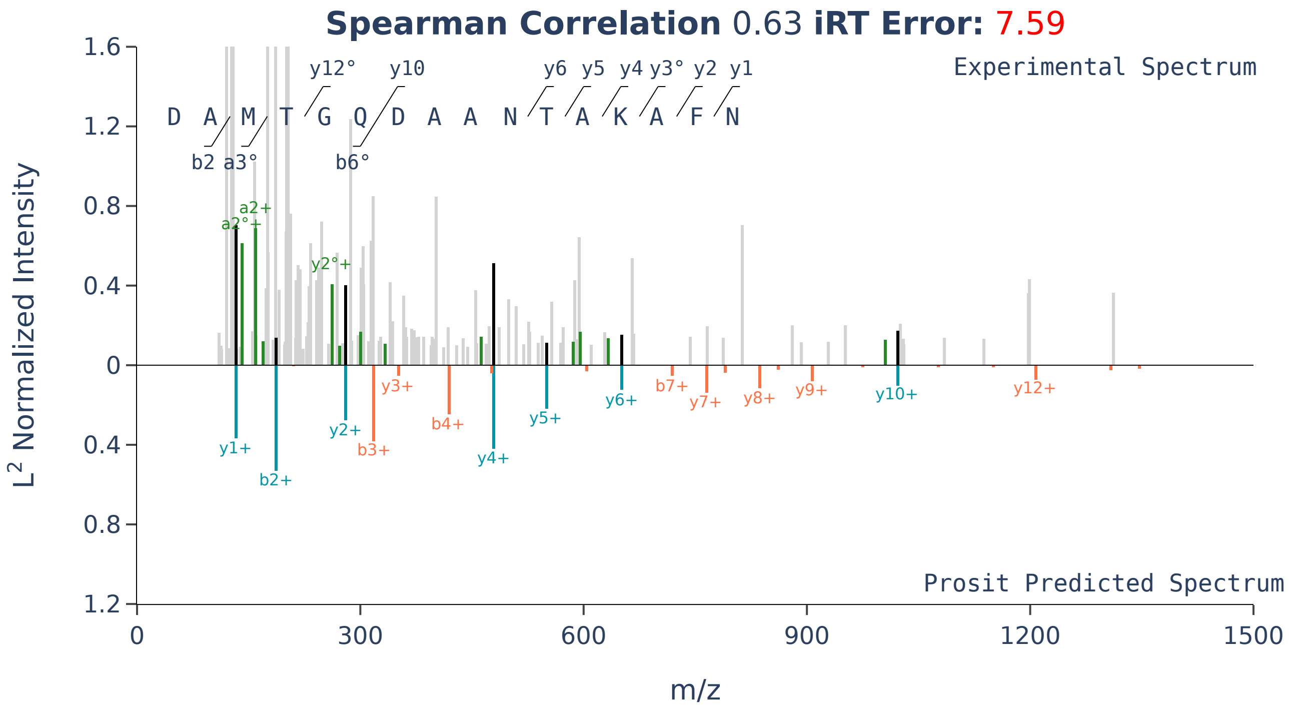

Source Ncheng_210623_230623_HFGoe_FFH_20S_25_1_A2_24h_R2 **Scan** 8078
Peptide GEAANTAKAFN **Charge** 2 **Spectral Angle** 0.82
Spearman Correlation 0.87 **iRT Error:** 1.21

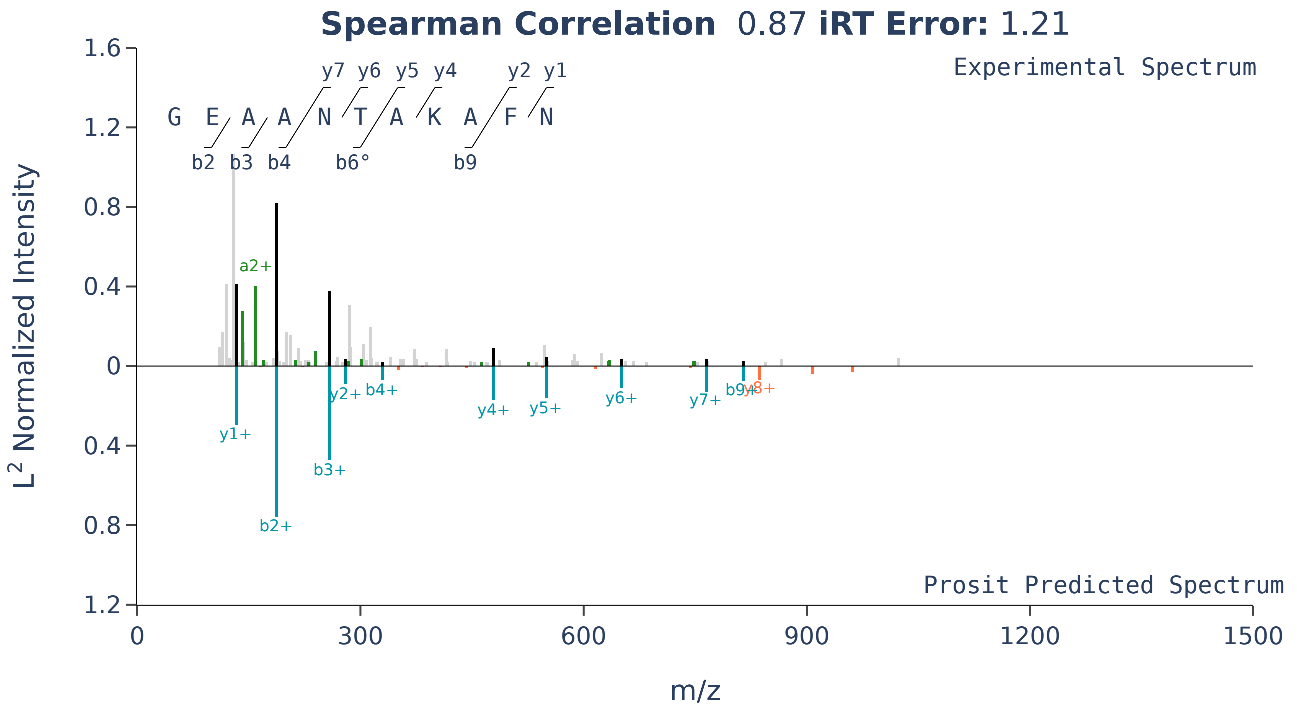

Source Ncheng_210623_230623_HFGoe_FFH_20S_25_1_A2_24h_R2 **Scan** 8078
Peptide GQDAANTAKAF **Charge** 2 **Spectral Angle** 0.37
Spearman Correlation 0.33 **iRT Error:** 14.33

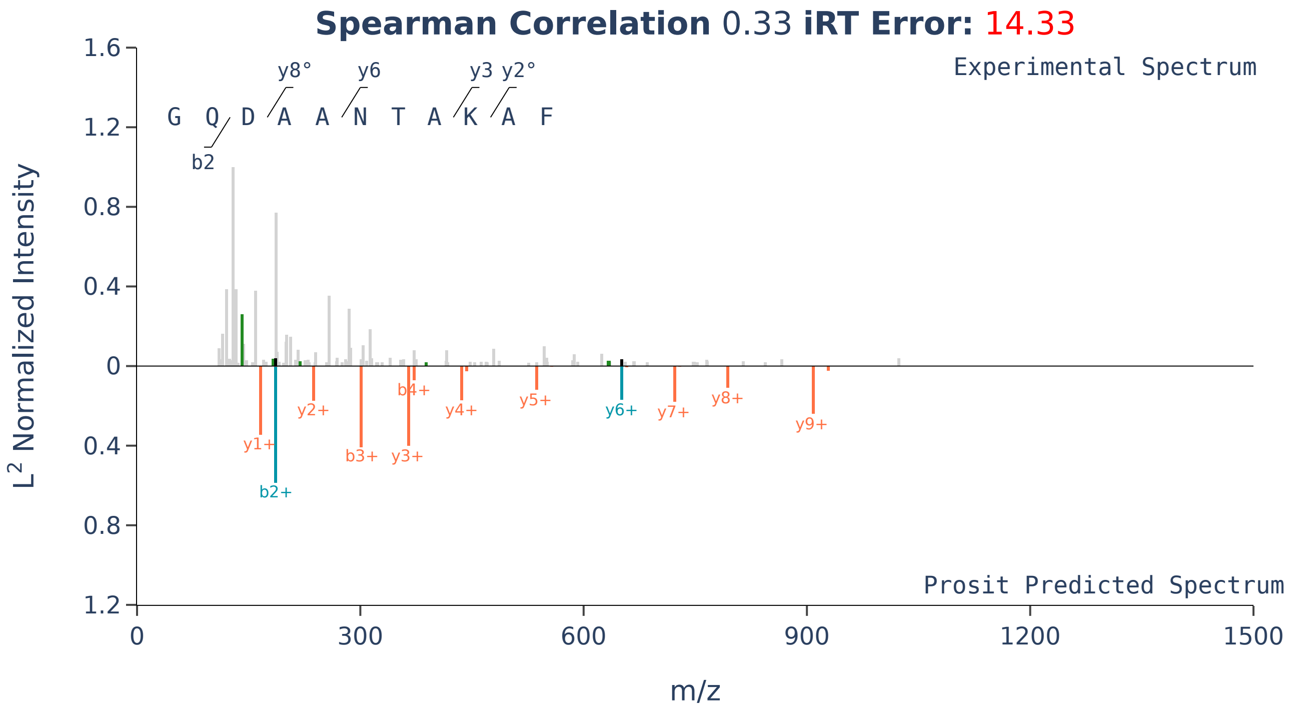

Source Ncheng_210623_230623_HFGoe_FFH_20S_25_1_A2_4h_R1 **Scan** 20918
Peptide GHEVNKSLTPGQEFVKIVRQD **Charge** 2 **Spectral Angle** 0.8
Spearman Correlation 0.88 **iRT Error:** 1.29

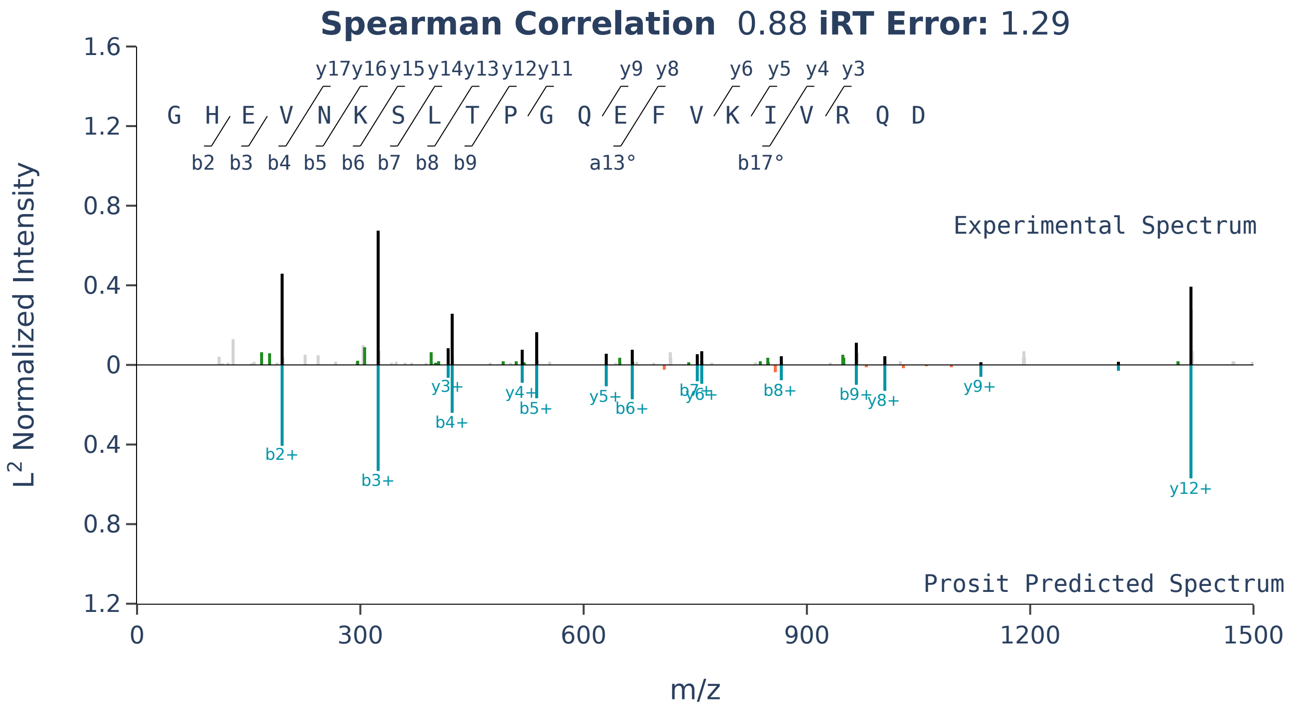

Source Ncheng_210623_230623_HFGoe_FFH_20S_25_1_A2_4h_R1 **Scan** 20918
Peptide GHEVNKSLTPGQEFVKIVRNE **Charge** 2 **Spectral Angle** 0.81
Spearman Correlation 0.88 **iRT Error:** 2.09

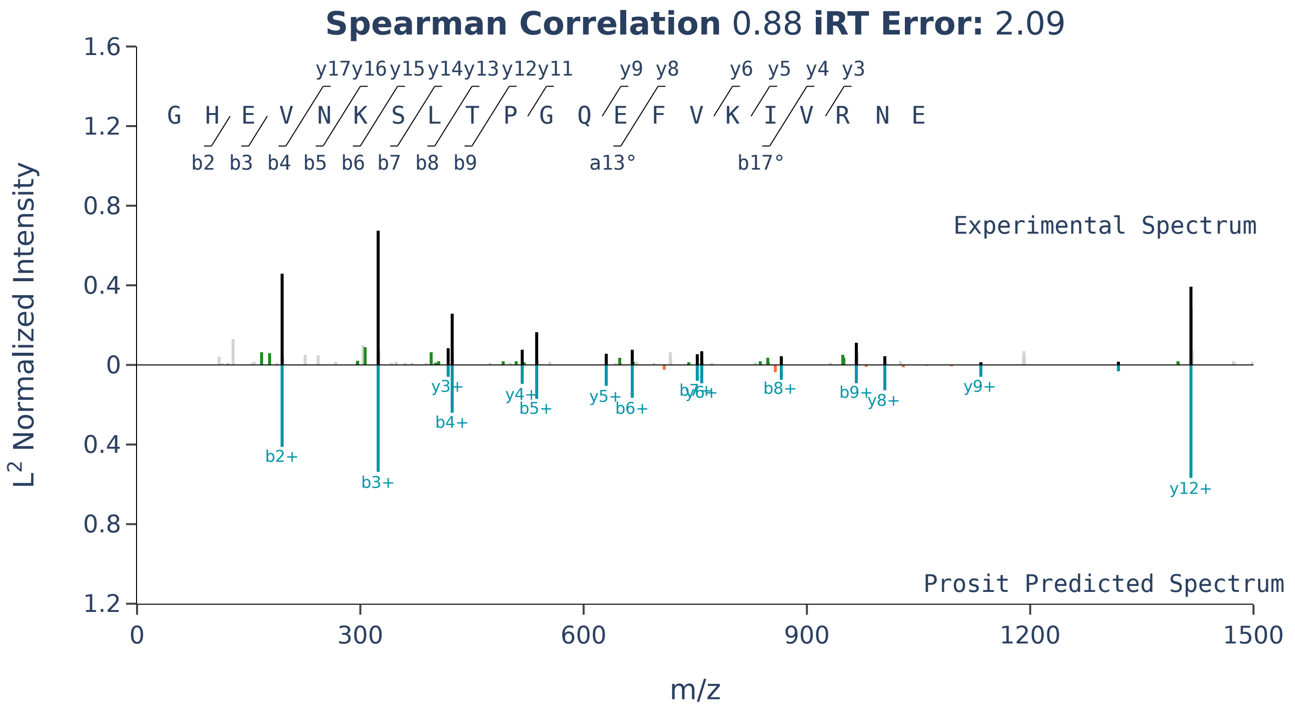

Source Ncheng_210623_230623_HFGoe_FFH_20S_25_1_A2_2h_R2 **Scan** 18096
Peptide GMKLPGMGQIPDNVKSQ **Charge** 2 **Spectral Angle** 0.8
Spearman Correlation 0.89 **iRT Error:** 1.3

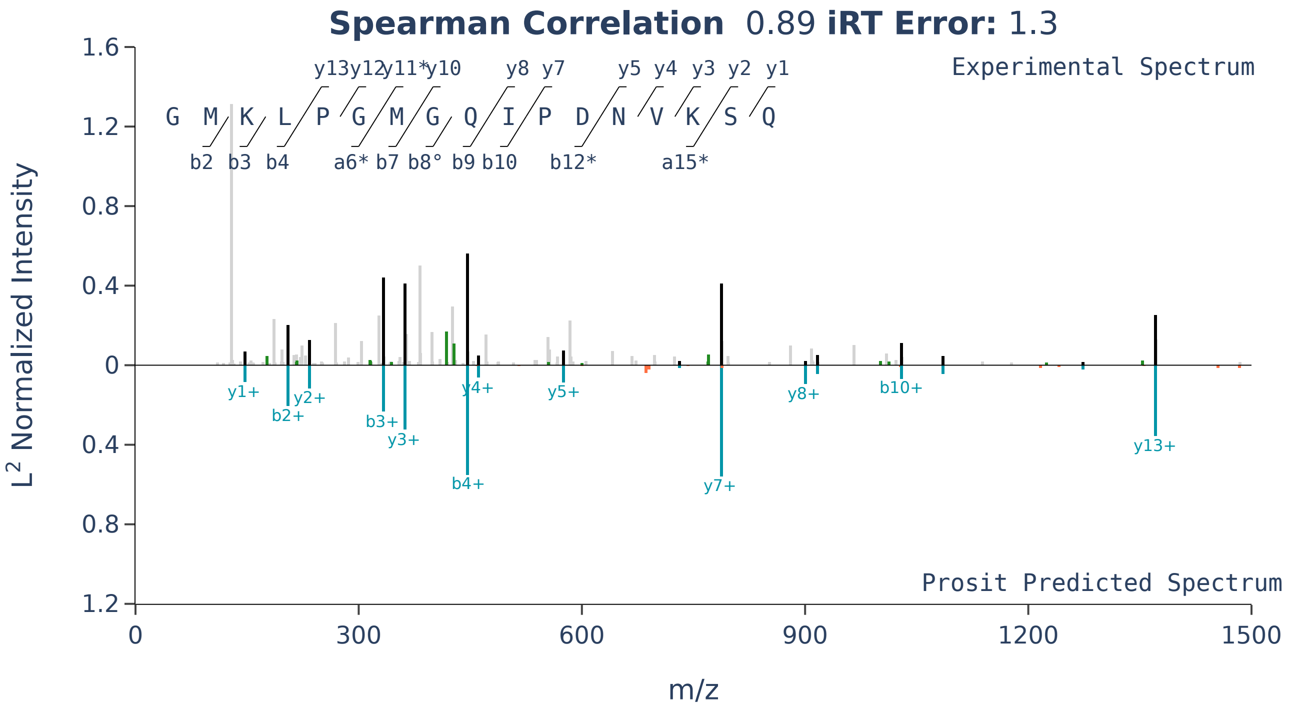

Source Ncheng_210623_230623_HFGoe_FFH_20S_25_1_A2_2h_R2 **Scan** 18096
Peptide GKLPGMGQIPDNVKSQM **Charge** 2 **Spectral Angle** 0.32
Spearman Correlation 0.28 **iRT Error:** 4.31

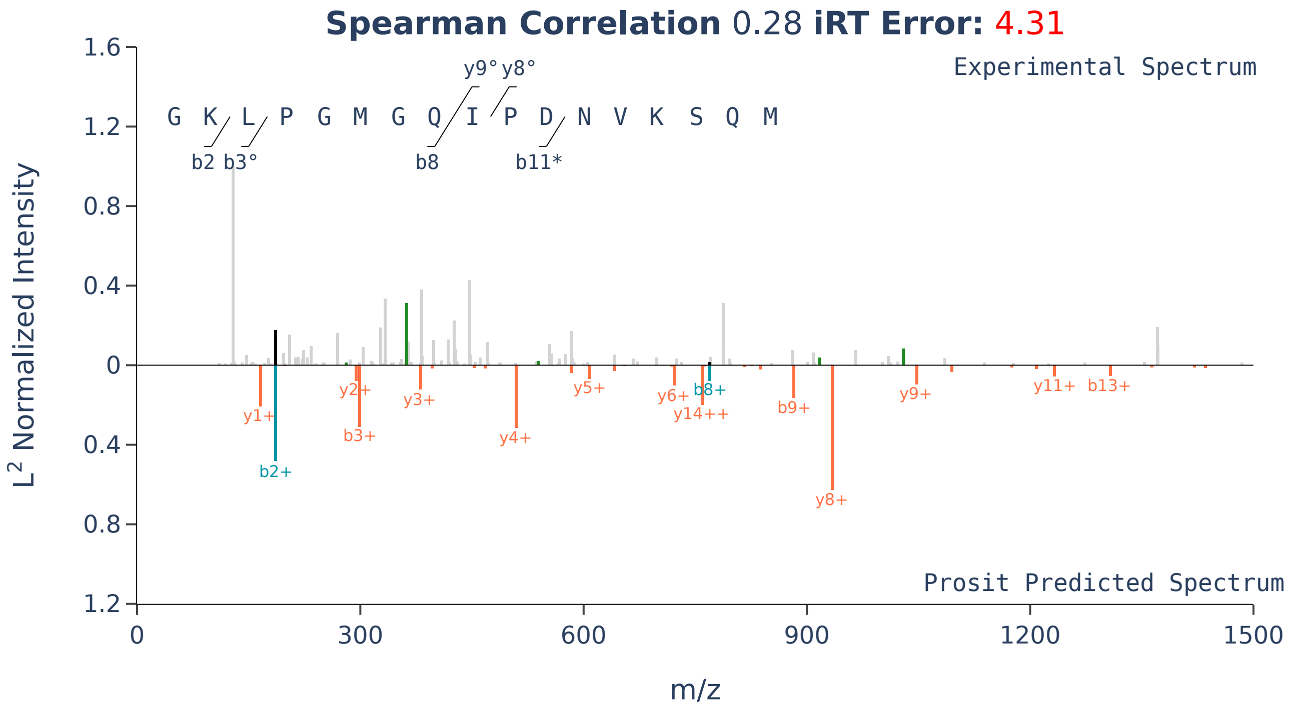

Source Ncheng_210623_230623_HFGoe_FFH_20S_25_1_A1_24h_R2 **Scan** 22068
Peptide AGKGVDFFPSD **Charge** 2 **Spectral Angle** 0.86
Spearman Correlation 0.86 **iRT Error:** 1.33

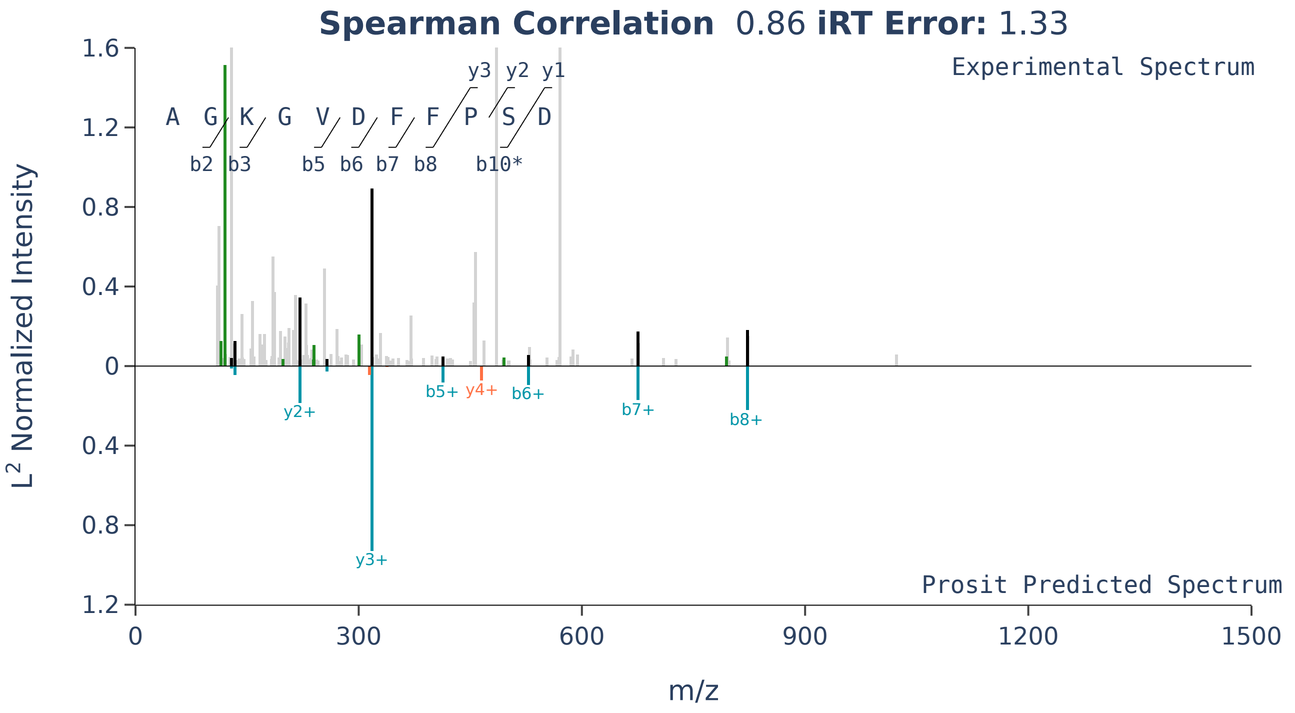

Source Ncheng_210623_230623_HFGoe_FFH_20S_25_1_A1_24h_R2 **Scan** 22068
Peptide DFFPSDVGQK **Charge** 2 **Spectral Angle** 0.27
Spearman Correlation 0.39 **iRT Error:** 7.41

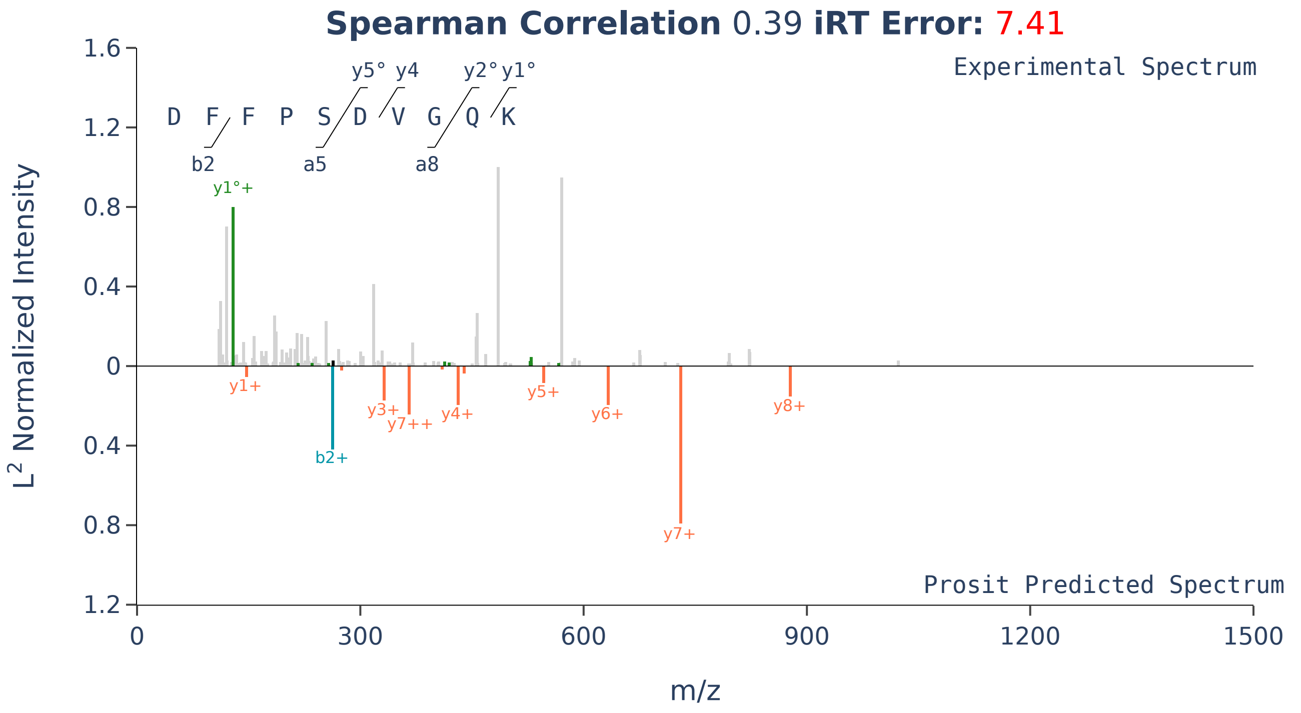

Source Ncheng_210623_230623_HFGoe_FFH_20S_25_1_A2_4h_R1 **Scan** 13525
Peptide KNMGGIIN **Charge** 1 **Spectral Angle** 0.78
Spearman Correlation 0.92 **iRT Error:** 1.37

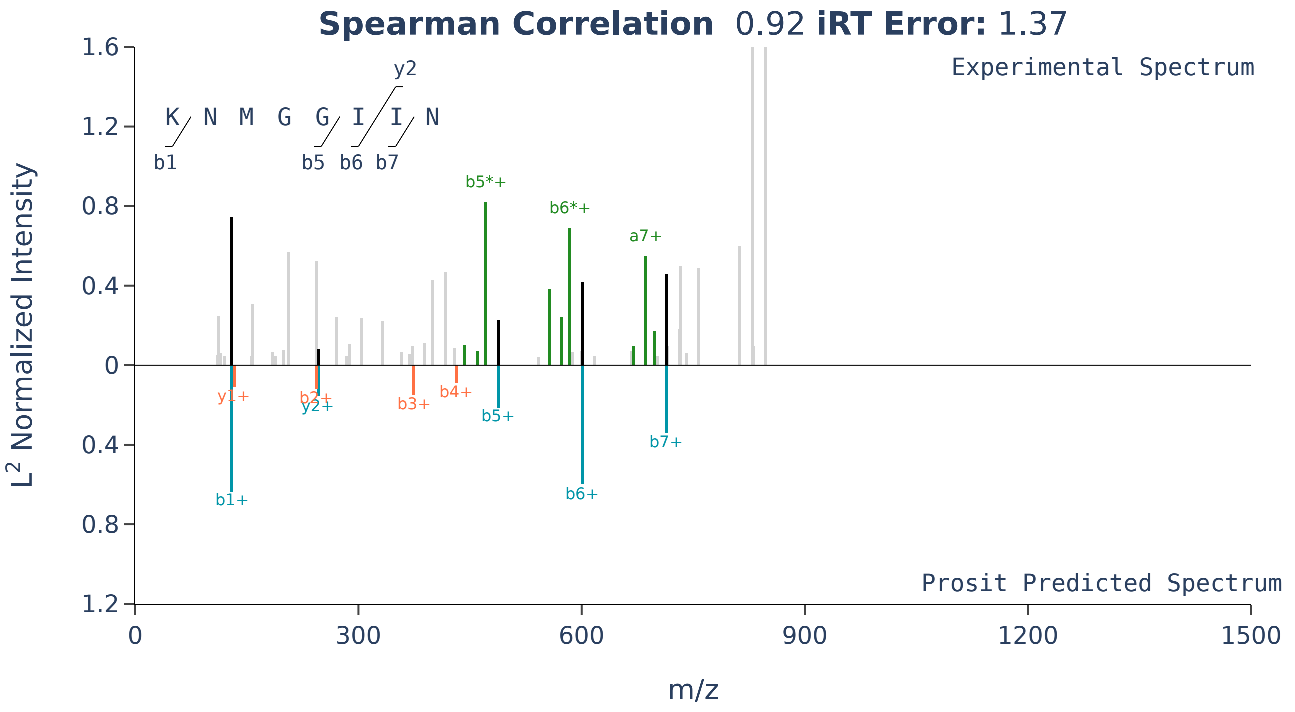

Source Ncheng_210623_230623_HFGoe_FFH_20S_25_1_A2_4h_R1 **Scan** 13525
Peptide RMEAIIN **Charge** 1 **Spectral Angle** 0.72
Spearman Correlation 0.63 **iRT Error:** 12.11

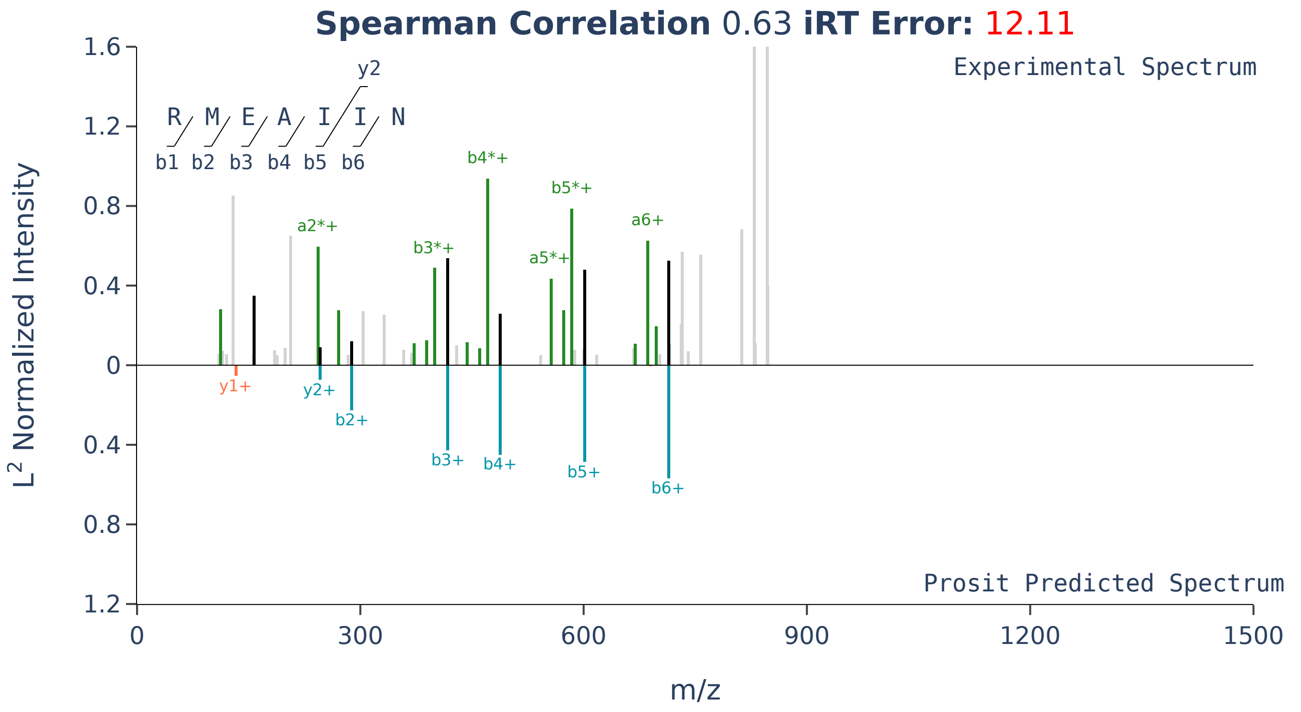

Source Ncheng_210623_230623_HFGoe_FFH_20S_25_1_A2_24h_R2 **Scan** 12714
Peptide PDNVKSQMDDGLQ **Charge** 1 **Spectral Angle** 0.86
Spearman Correlation 0.92 **iRT Error:** 1.39

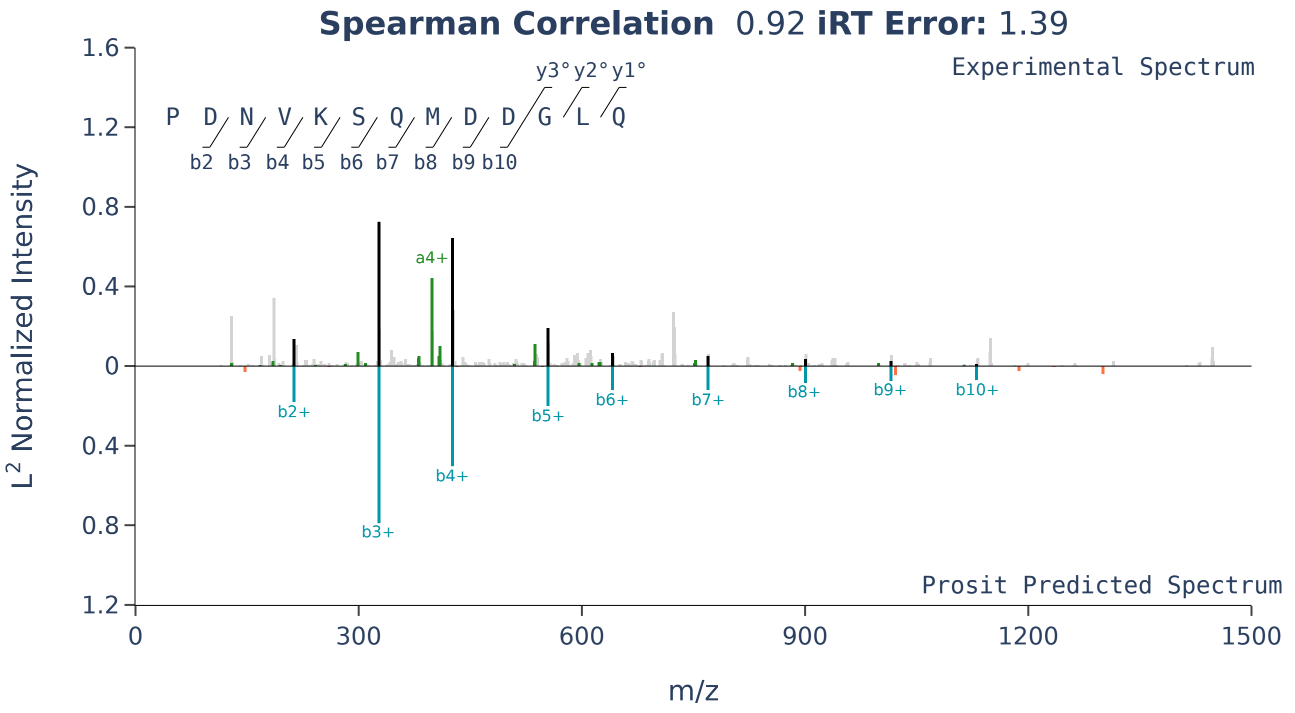

Source Ncheng_210623_230623_HFGoe_FFH_20S_25_1_A2_24h_R2 **Scan** 12714
Peptide GQIPDNVKSQMDD **Charge** 1 **Spectral Angle** 0.77
Spearman Correlation 0.54 **iRT Error:** 3.58

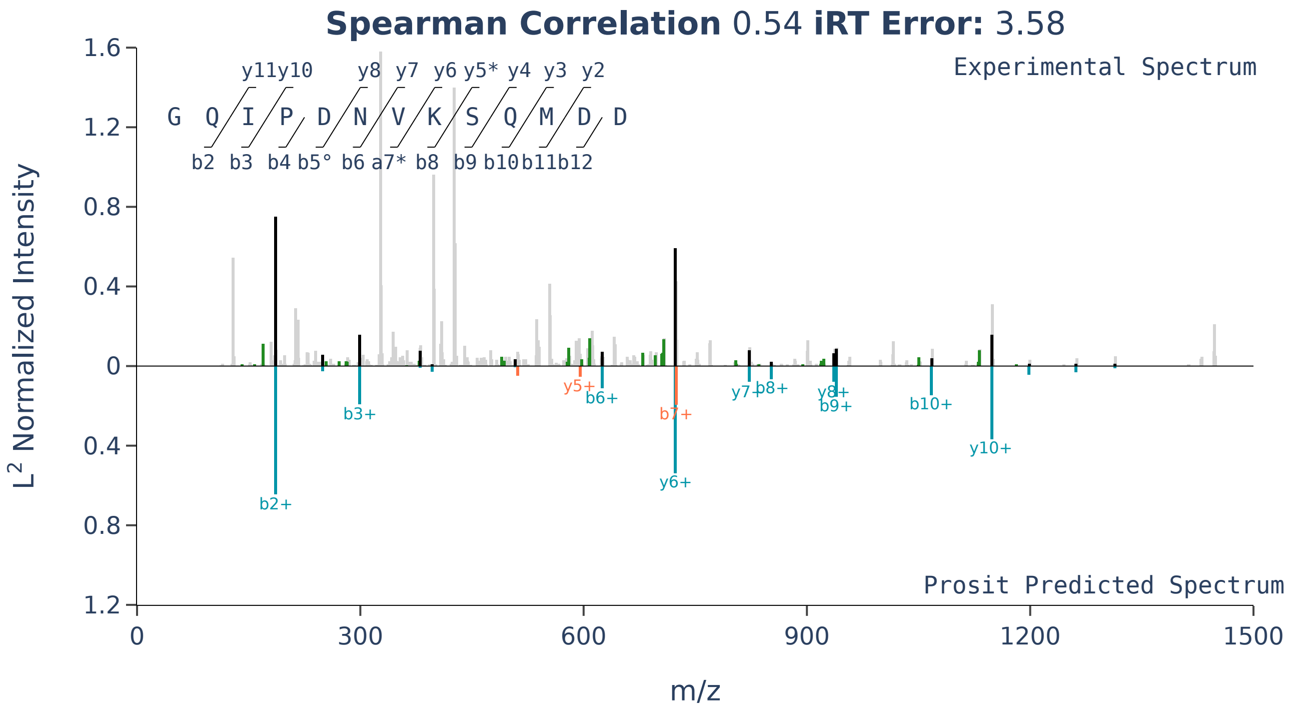

Source Ncheng_210623_230623_HFGoe_FFH_20S_25_1_A1_24h_R2 **Scan** 19150
Peptide ALEPFHPGRLTED **Charge** 2 **Spectral Angle** 0.82
Spearman Correlation 0.88 **iRT Error:** 1.45

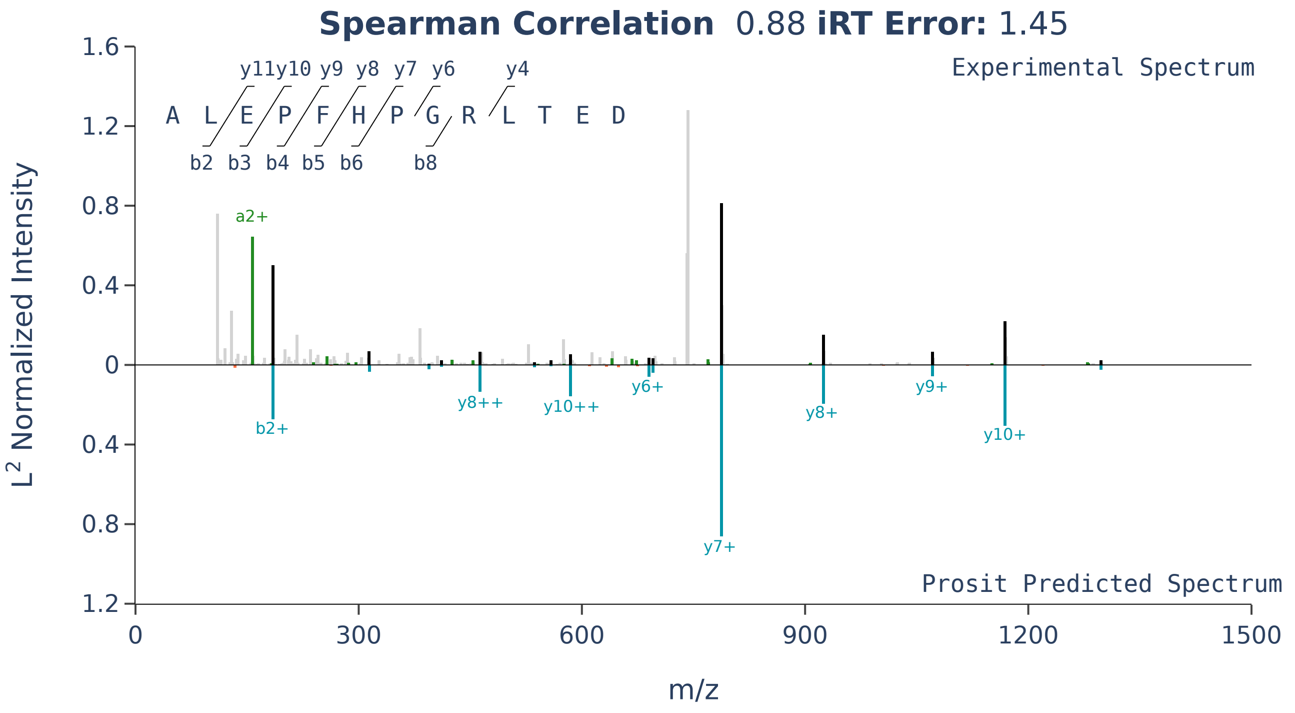

Source Ncheng_210623_230623_HFGoe_FFH_20S_25_1_A1_24h_R2 **Scan** 19150
Peptide MDEIKQVHASINP **Charge** 2 **Spectral Angle** 0.13
Spearman Correlation 0.2 **iRT Error:** 18.74

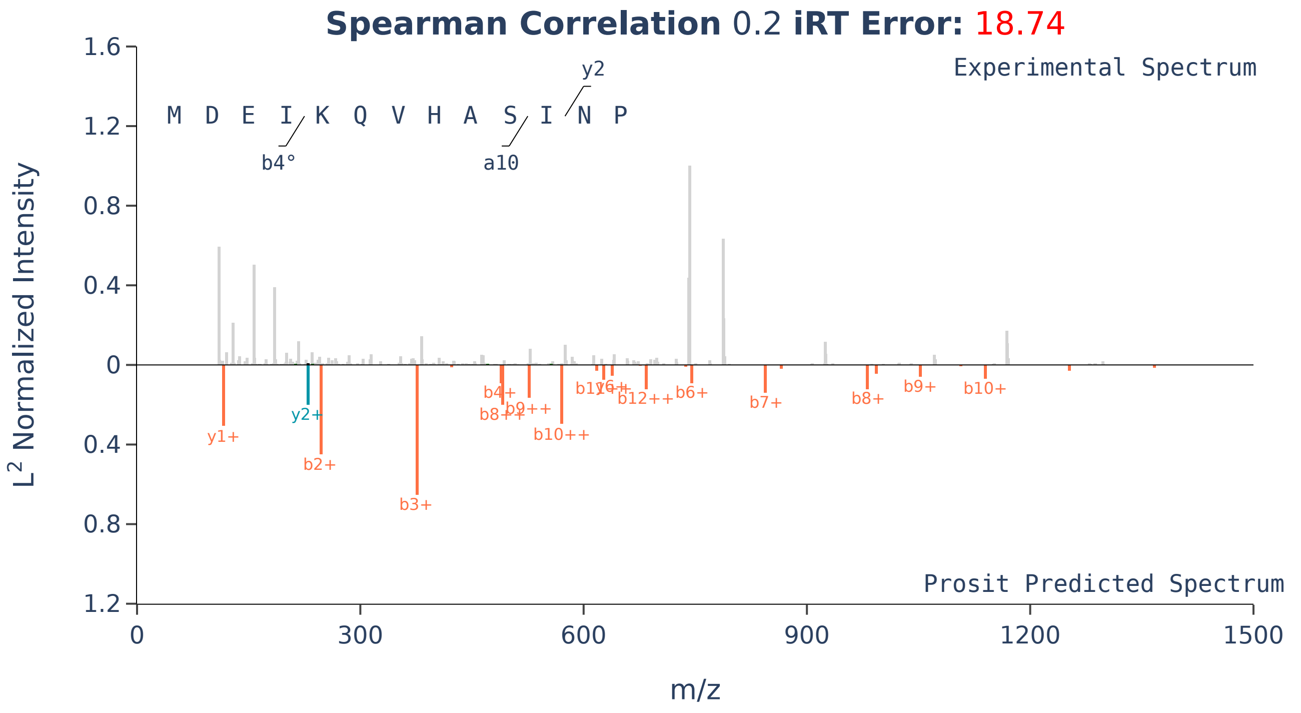

Source Ncheng_210623_230623_HFGoe_FFH_20S_25_1_A1_2h_R2 **Scan** 6546
Peptide KVLVKTE **Charge** 2 **Spectral Angle** 0.87
Spearman Correlation 0.88 **iRT Error:** 1.46

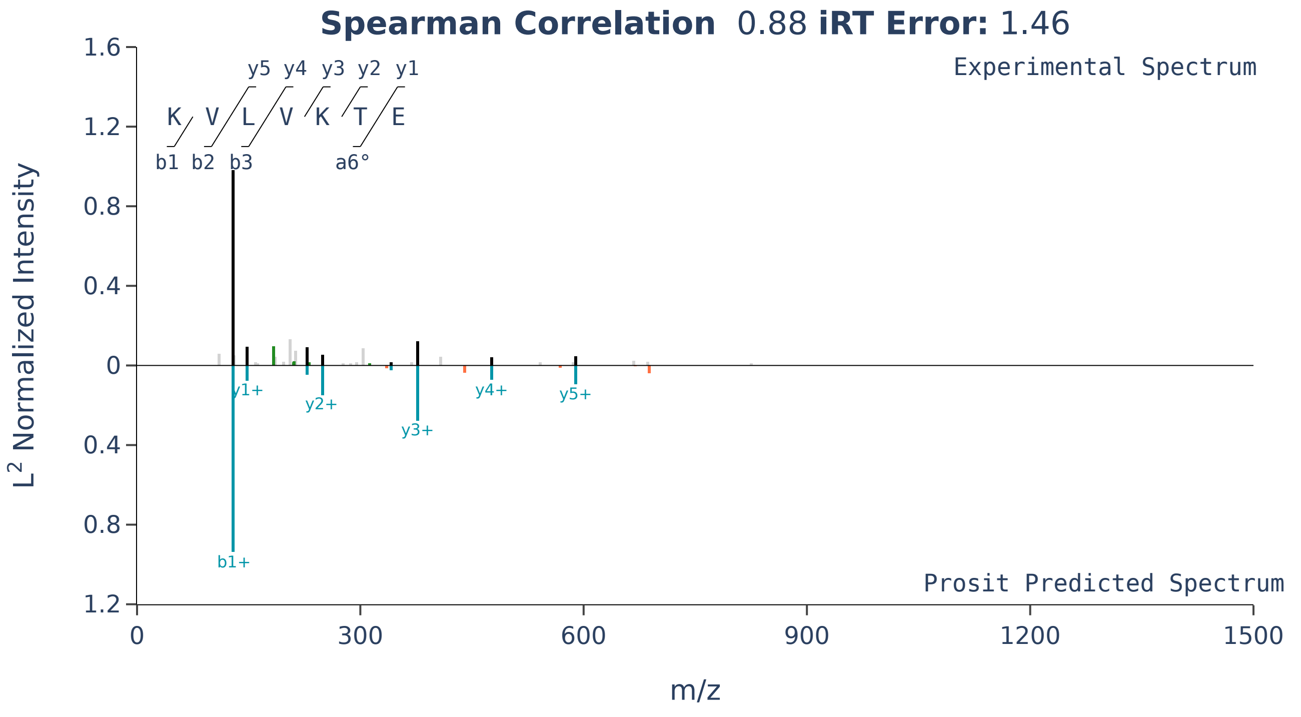

Source Ncheng_210623_230623_HFGoe_FFH_20S_25_1_A1_2h_R2 **Scan** 6546
Peptide TGVVLTKV **Charge** 2 **Spectral Angle** 0.0
Spearman Correlation 0.0 **iRT Error:** 52.27

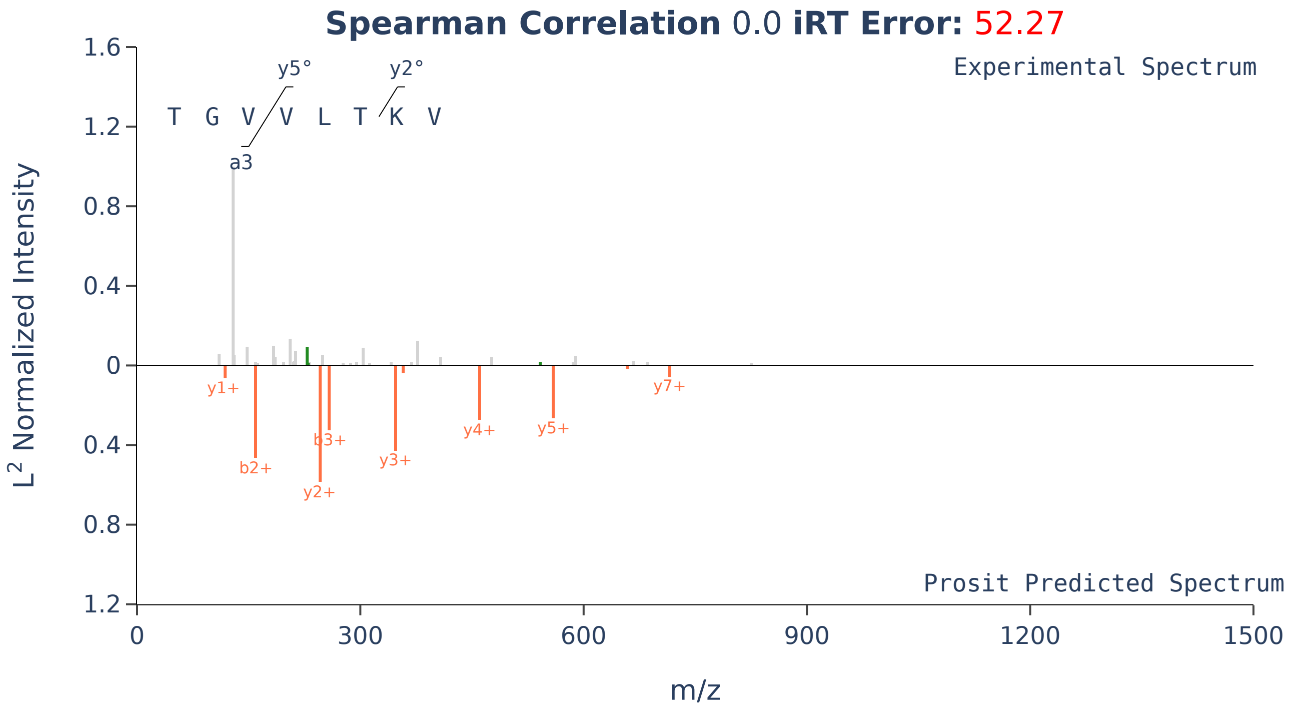

Source Ncheng_210623_230623_HFGoe_FFH_20S_25_1_A2_24h_R1 **Scan** 6554
Peptide GAGLQGAGKTT **Charge** 2 **Spectral Angle** 0.76
Spearman Correlation 0.92 **iRT Error:** 1.48

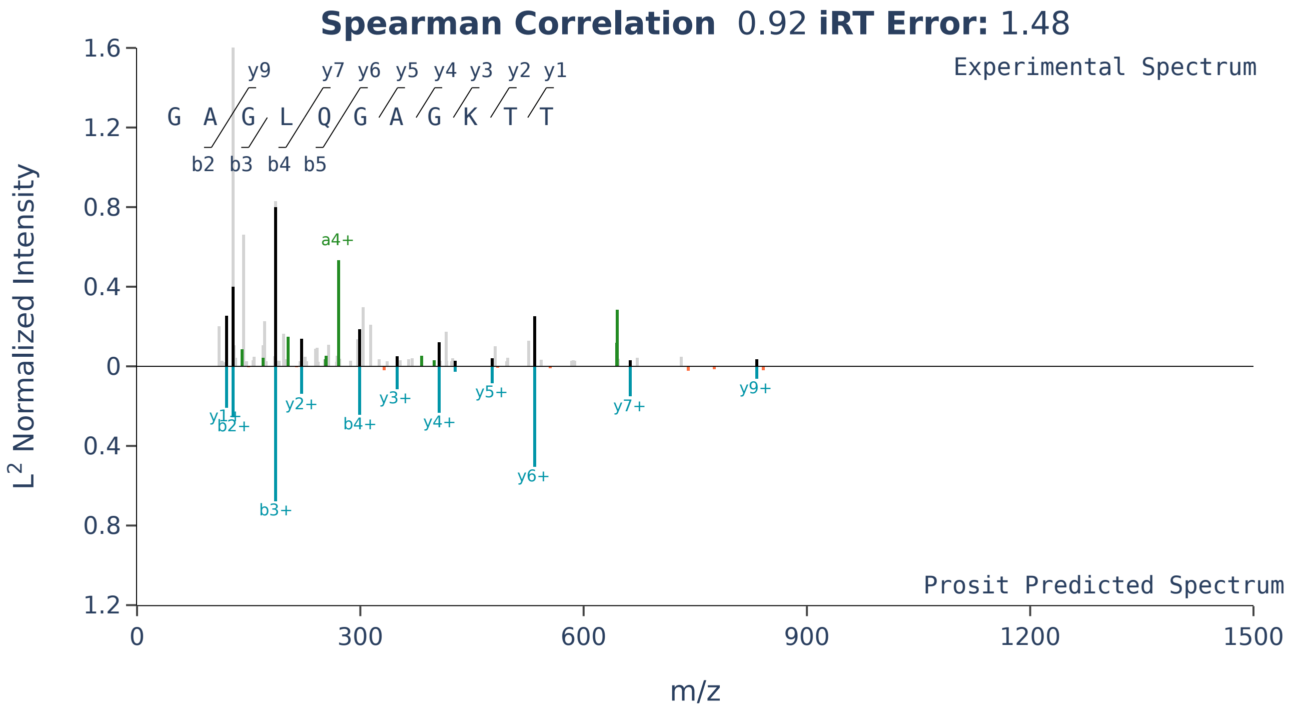

Source Ncheng_210623_230623_HFGoe_FFH_20S_25_1_A2_24h_R1 **Scan** 6554
Peptide DNVKDTLR **Charge** 2 **Spectral Angle** 0.0
Spearman Correlation 0.0 **iRT Error:** 6.6

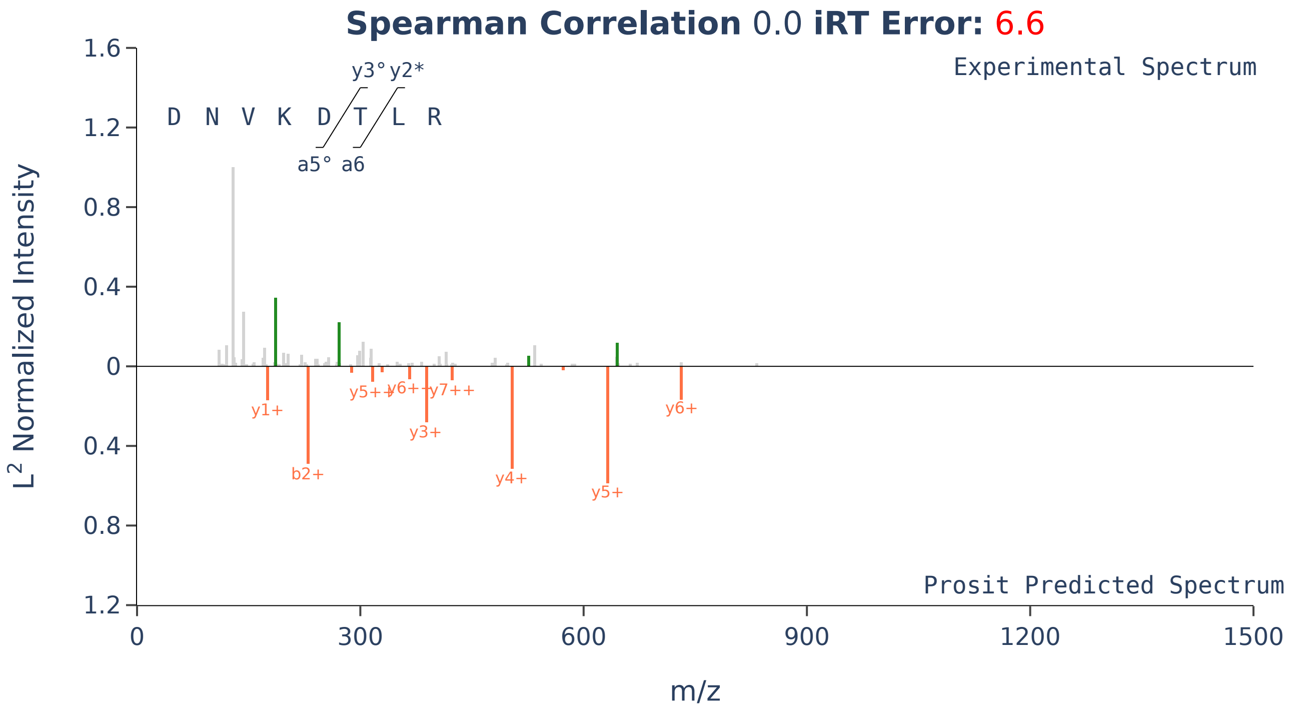

Source Ncheng_210623_230623_HFGoe_FFH_20S_25_1_A1_4h_R1 **Scan** 15330
Peptide KLKFYDVGQKPVD **Charge** 2 **Spectral Angle** 0.82
Spearman Correlation 0.85 **iRT Error:** 1.49

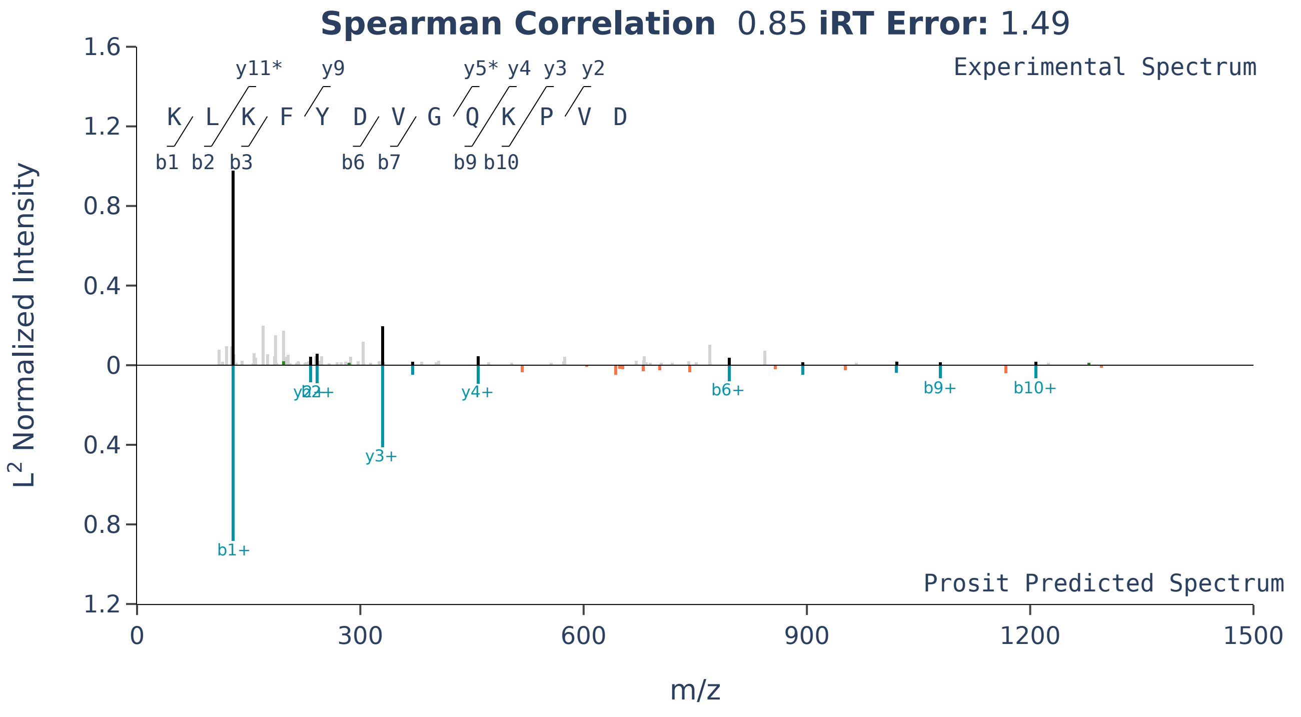

Source Ncheng_210623_230623_HFGoe_FFH_20S_25_1_A1_4h_R1 **Scan** 15330
Peptide VLLVDTAGRLHVDE **Charge** 2 **Spectral Angle** 0.0
Spearman Correlation 0.0 **iRT Error:** 37.82

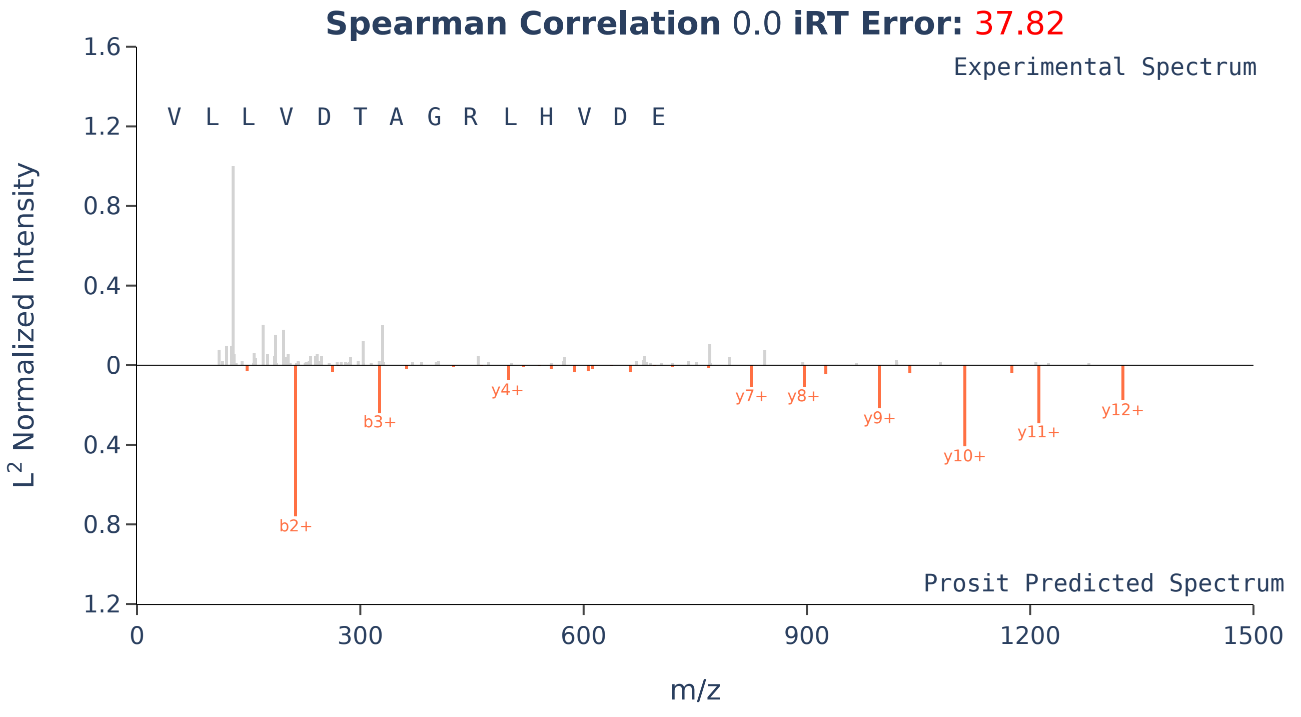

Source Ncheng_210623_230623_HFGoe_FFH_20S_25_1_A1_24h_R2 **Scan** 12401
Peptide TKETLAEQV **Charge** 2 **Spectral Angle** 0.68
Spearman Correlation 0.92 **iRT Error:** 1.5

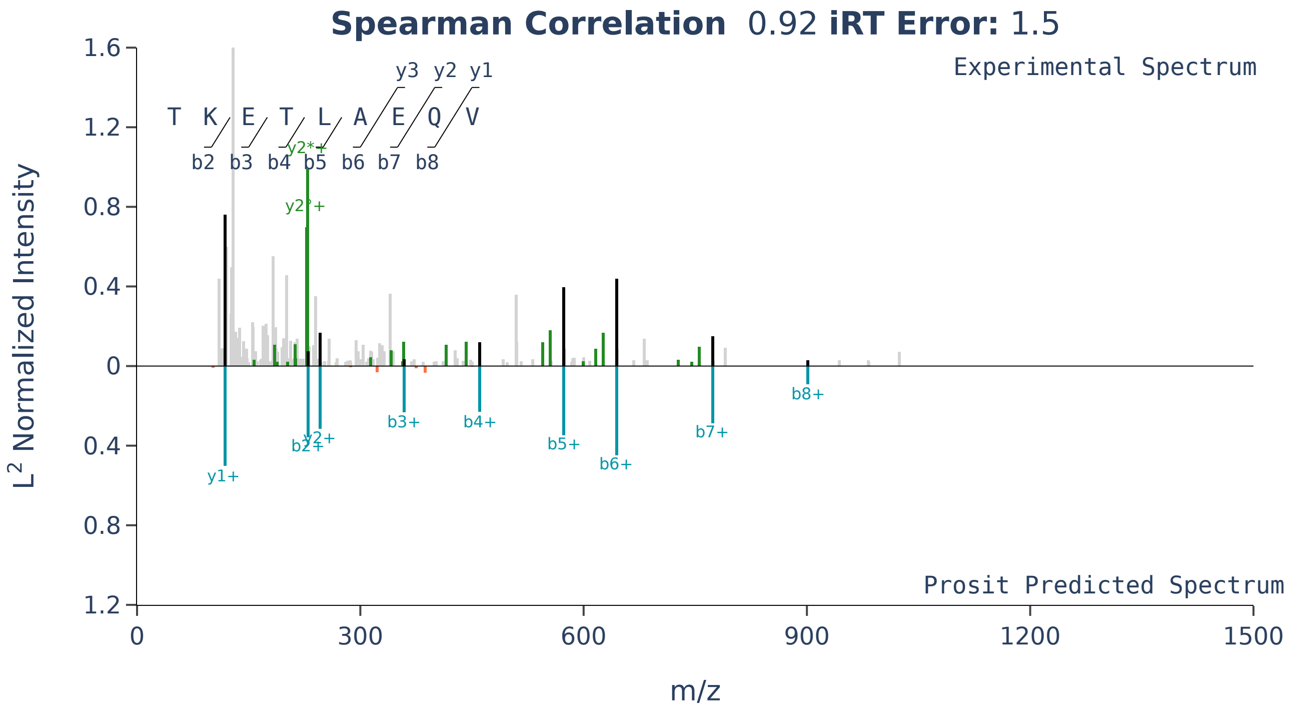

Source Ncheng_210623_230623_HFGoe_FFH_20S_25_1_A1_24h_R2 **Scan** 12401
Peptide ASRILGMGDV **Charge** 2 **Spectral Angle** 0.33
Spearman Correlation 0.36 **iRT Error:** 50.93

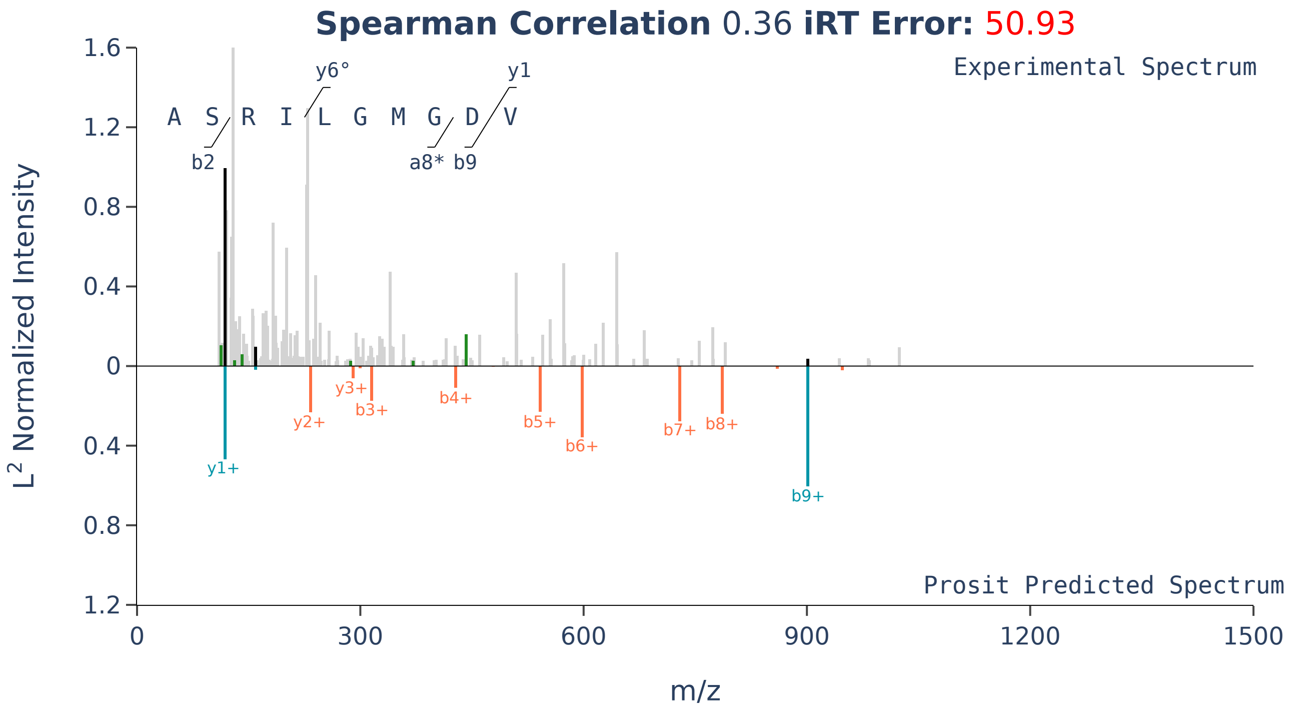

Source Ncheng_210623_230623_HFGoe_FFH_20S_25_1_A1_24h_R2 **Scan** 16240
Peptide QVGAMTGQDAANTAKAFN **Charge** 2 **Spectral Angle** 0.81
Spearman Correlation 0.96 **iRT Error:** 1.51

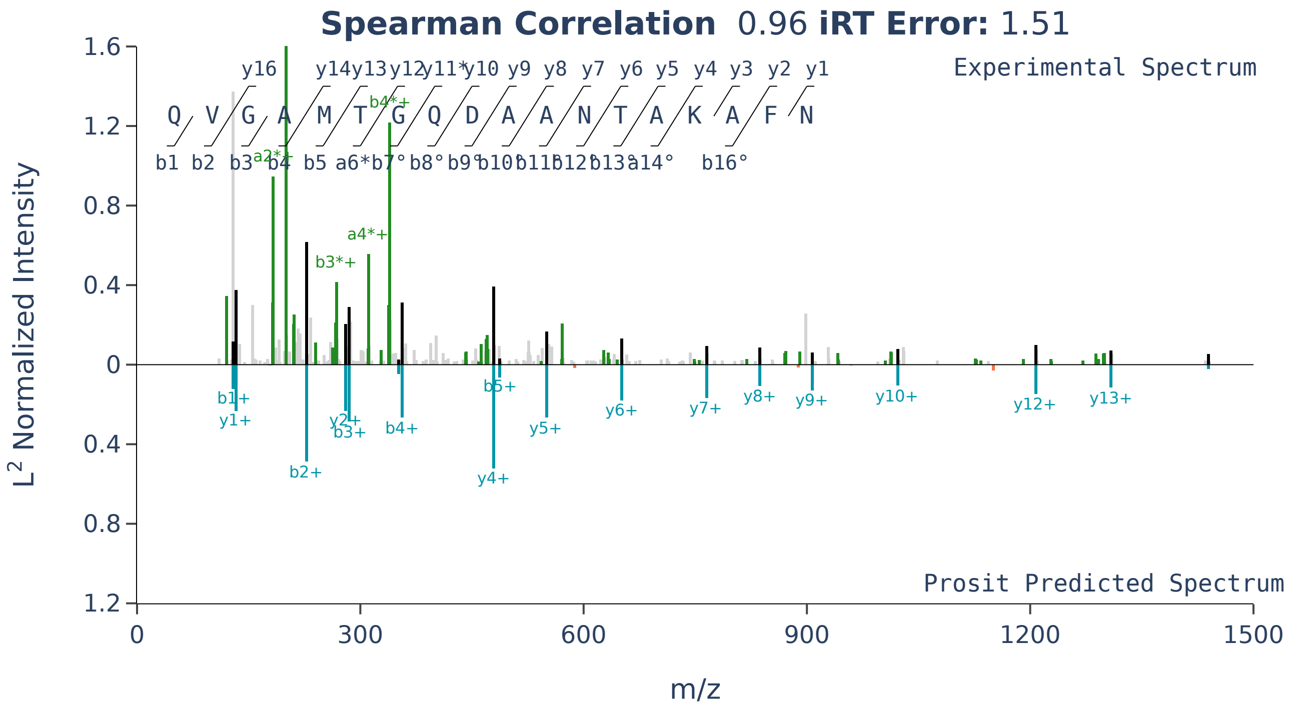

Source Ncheng_210623_230623_HFGoe_FFH_20S_25_1_A1_24h_R2 **Scan** 16240
Peptide LHVDEAMMDEIKQVH **Charge** 2 **Spectral Angle** 0.6
Spearman Correlation 0.32 **iRT Error:** 33.16

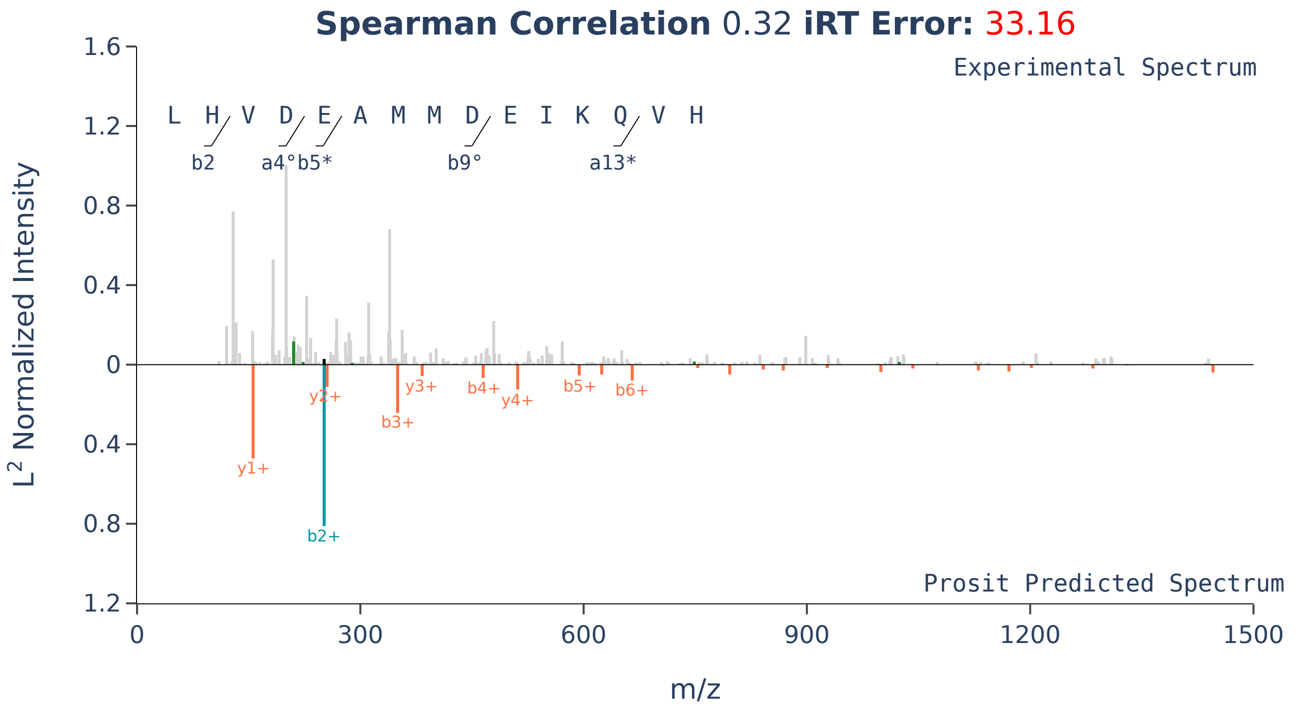

Source Ncheng_210623_230623_HFGoe_FFH_20S_25_1_A1_24h_R1 **Scan** 12217
Peptide TDKPIKF **Charge** 2 **Spectral Angle** 0.75
Spearman Correlation 0.95 **iRT Error:** 1.52

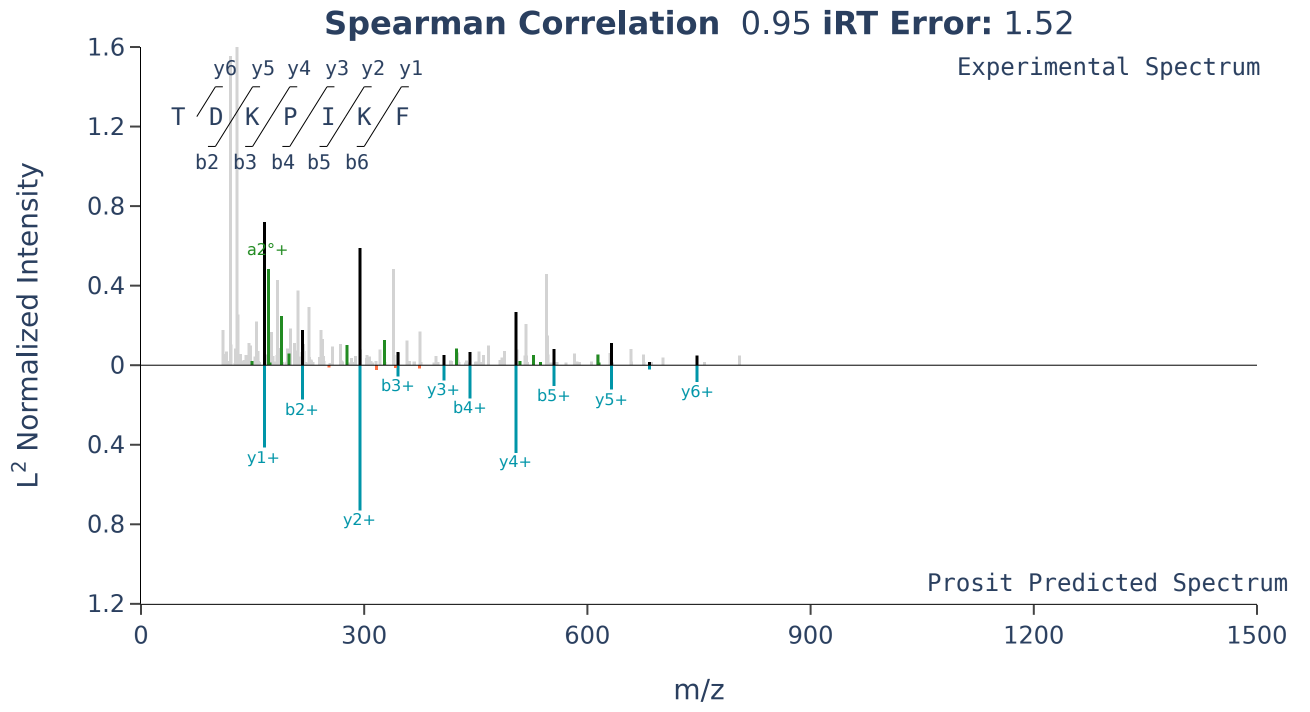

Source Ncheng_210623_230623_HFGoe_FFH_20S_25_1_A1_24h_R1 **Scan** 12217
Peptide AGKTTSVGK **Charge** 2 **Spectral Angle** 0.15
Spearman Correlation 0.09 **iRT Error:** 46.3

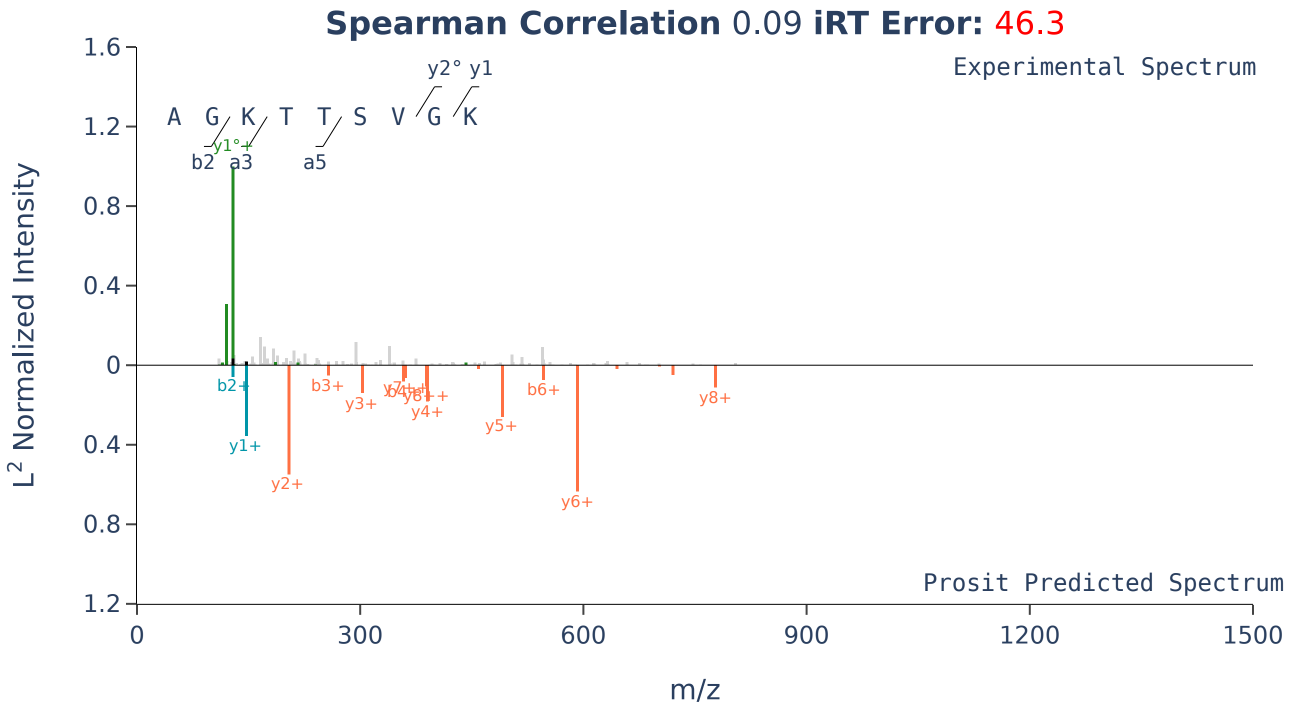

Source Ncheng_210623_230623_HFGoe_FFH_20S_25_1_A2_4h_R2 **Scan** 12047
Peptide TLAEVGQKPVD **Charge** 2 **Spectral Angle** 0.81
Spearman Correlation 0.9 **iRT Error:** 1.55

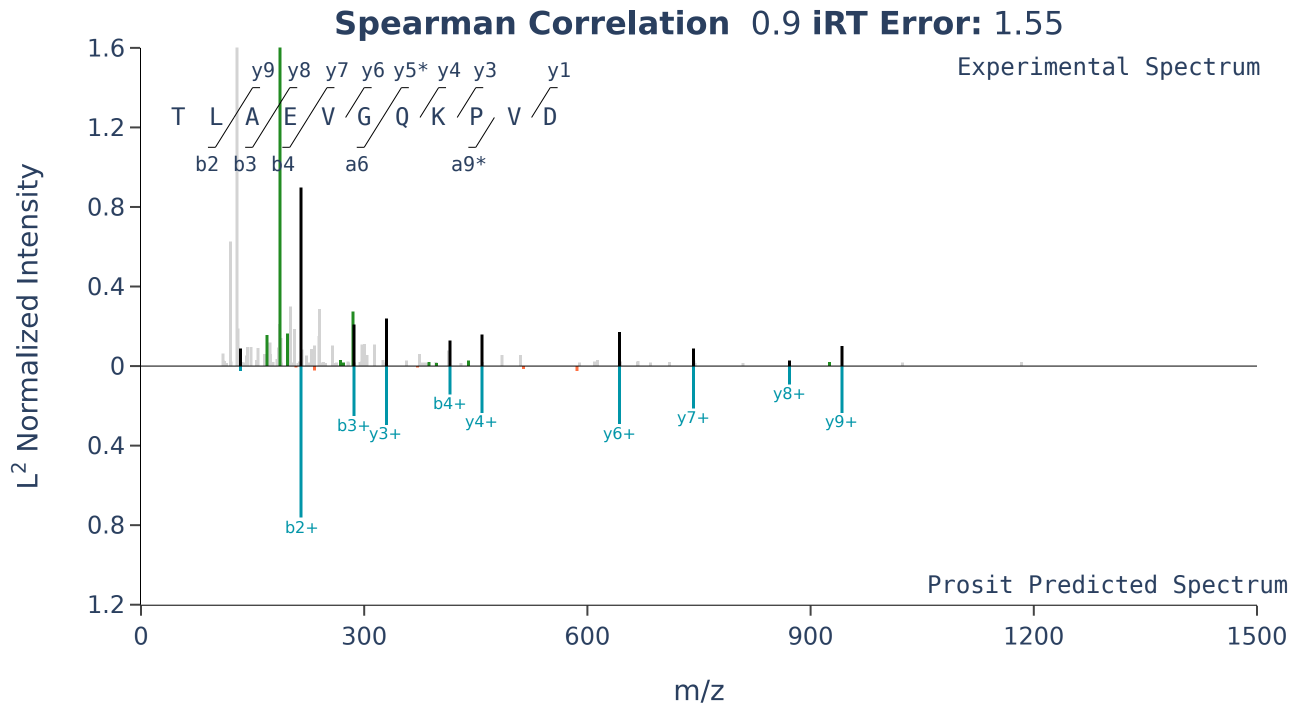

Source Ncheng_210623_230623_HFGoe_FFH_20S_25_1_A2_4h_R2 **Scan** 12047
Peptide SDVGQKPVDIV **Charge** 2 **Spectral Angle** 0.28
Spearman Correlation 0.34 **iRT Error:** 34.25

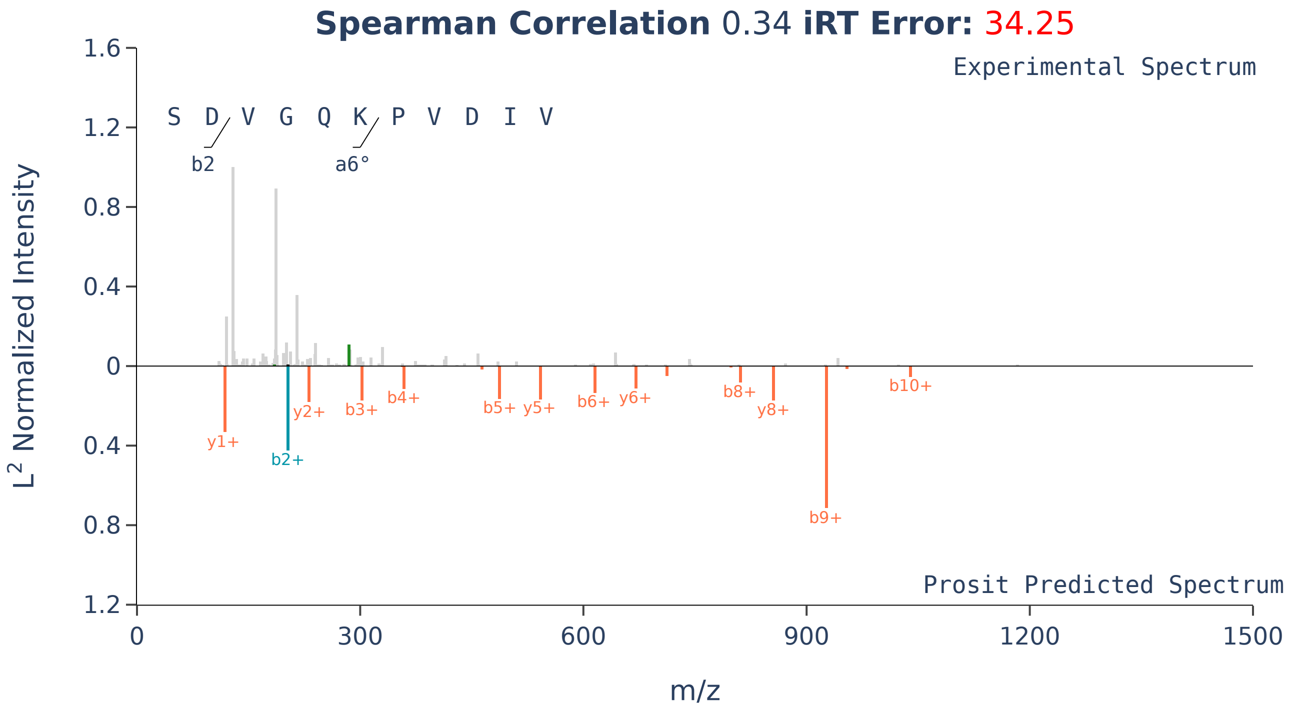

Source Ncheng_210623_230623_HFGoe_FFH_20S_25_1_A1_2h_R2 **Scan** 9432
Peptide RSTLAEQVG **Charge** 2 **Spectral Angle** 0.89
Spearman Correlation 0.9 **iRT Error:** 1.58

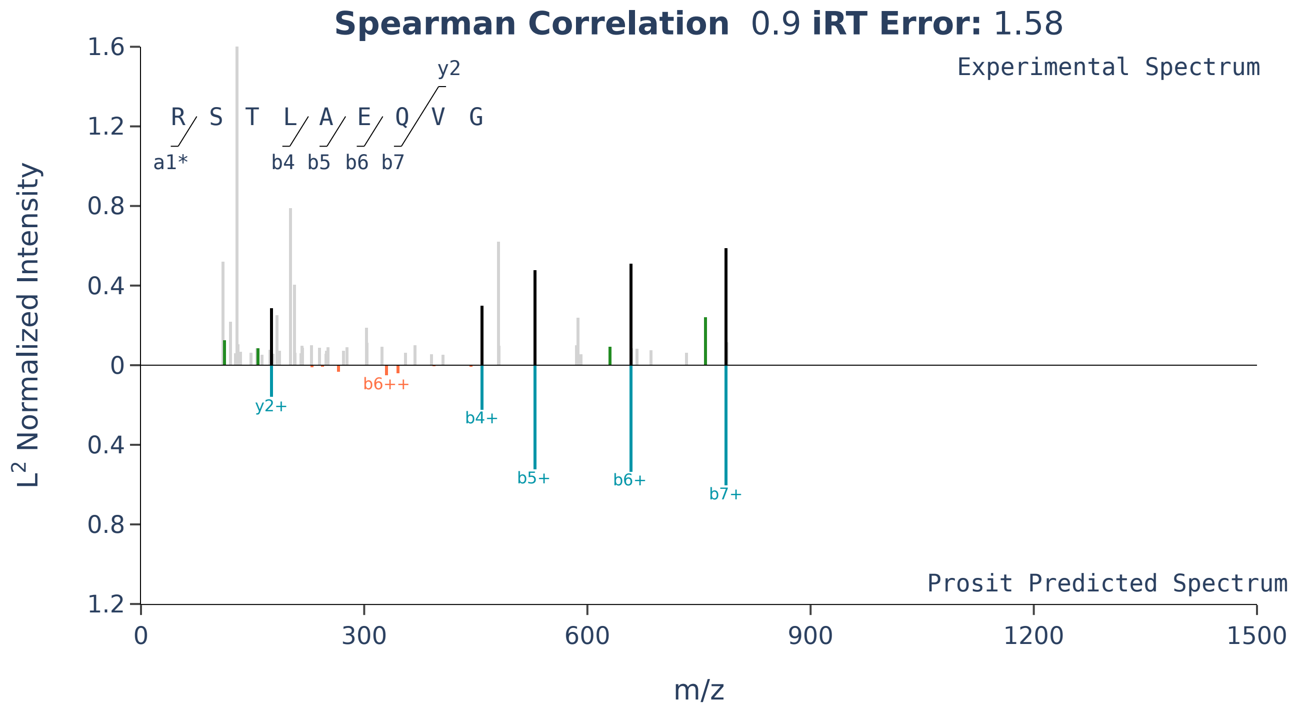

Source Ncheng_210623_230623_HFGoe_FFH_20S_25_1_A1_2h_R2 **Scan** 9432
Peptide DNVKDTLR **Charge** 2 **Spectral Angle** 0.0
Spearman Correlation 0.0 **iRT Error:** 13.17

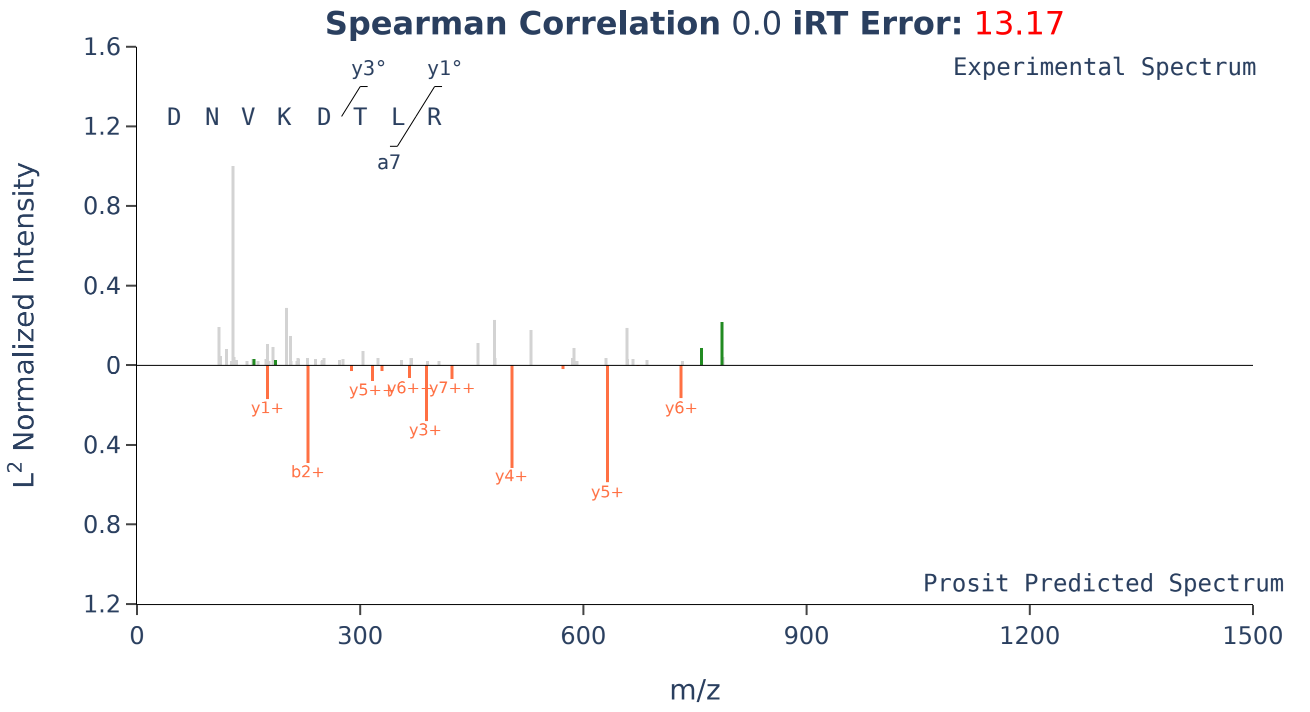

Source Ncheng_210623_230623_HFGoe_FFH_20S_25_1_A1_4h_R1 **Scan** 15645
Peptide MGKLPGMGQIPDNVKNT **Charge** 2 **Spectral Angle** 0.88
Spearman Correlation 0.89 **iRT Error:** 1.6

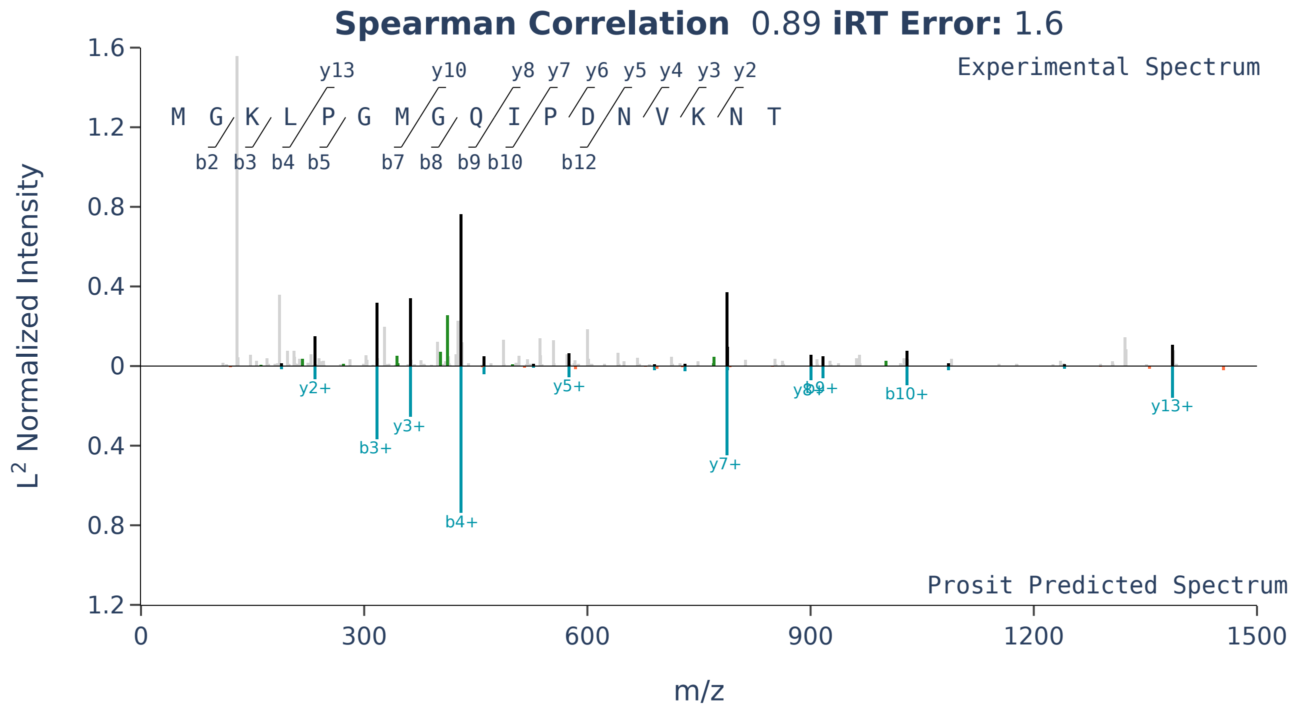

Source Ncheng_210623_230623_HFGoe_FFH_20S_25_1_A1_4h_R1 **Scan** 15645
Peptide GKLPGMGQIPDNVKSQM **Charge** 2 **Spectral Angle** 0.33
Spearman Correlation 0.35 **iRT Error:** 9.45

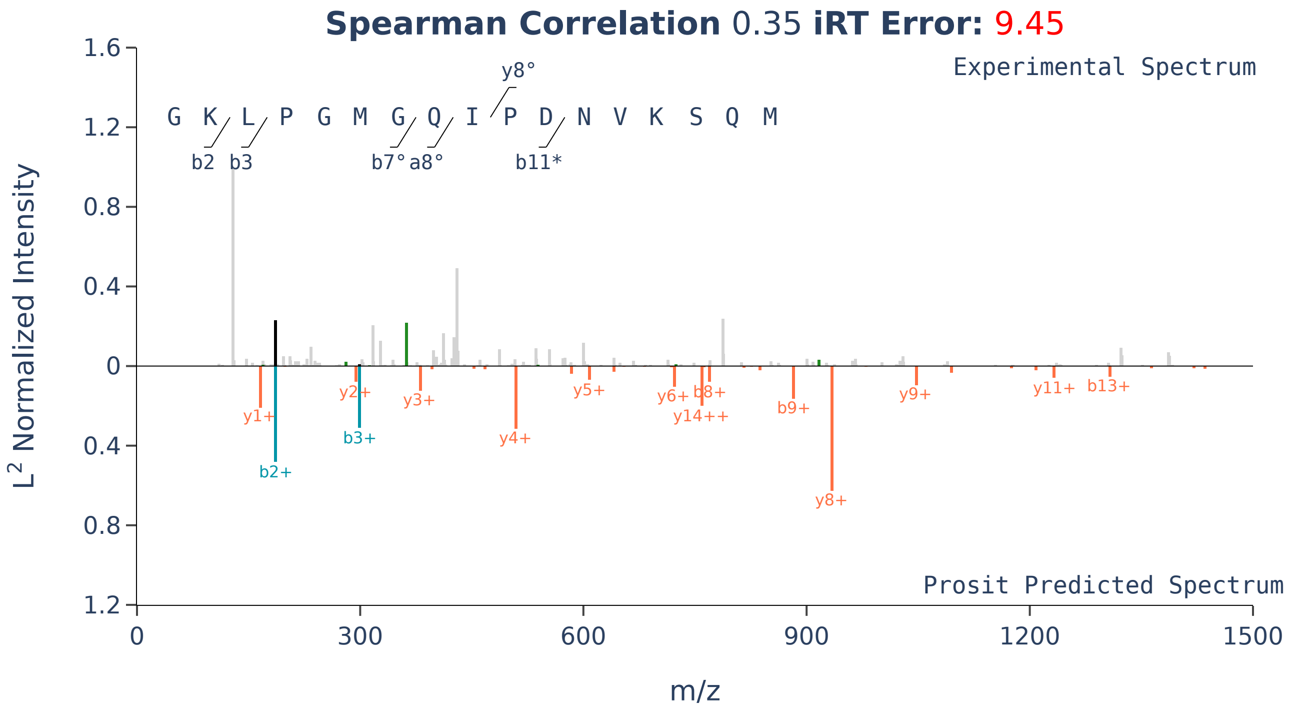

Source Ncheng_210623_230623_HFGoe_FFH_20S_25_1_A1_24h_R2 **Scan** 9873
Peptide GQIPDNVKAS **Charge** 2 **Spectral Angle** 0.85
Spearman Correlation 0.84 **iRT Error:** 1.61

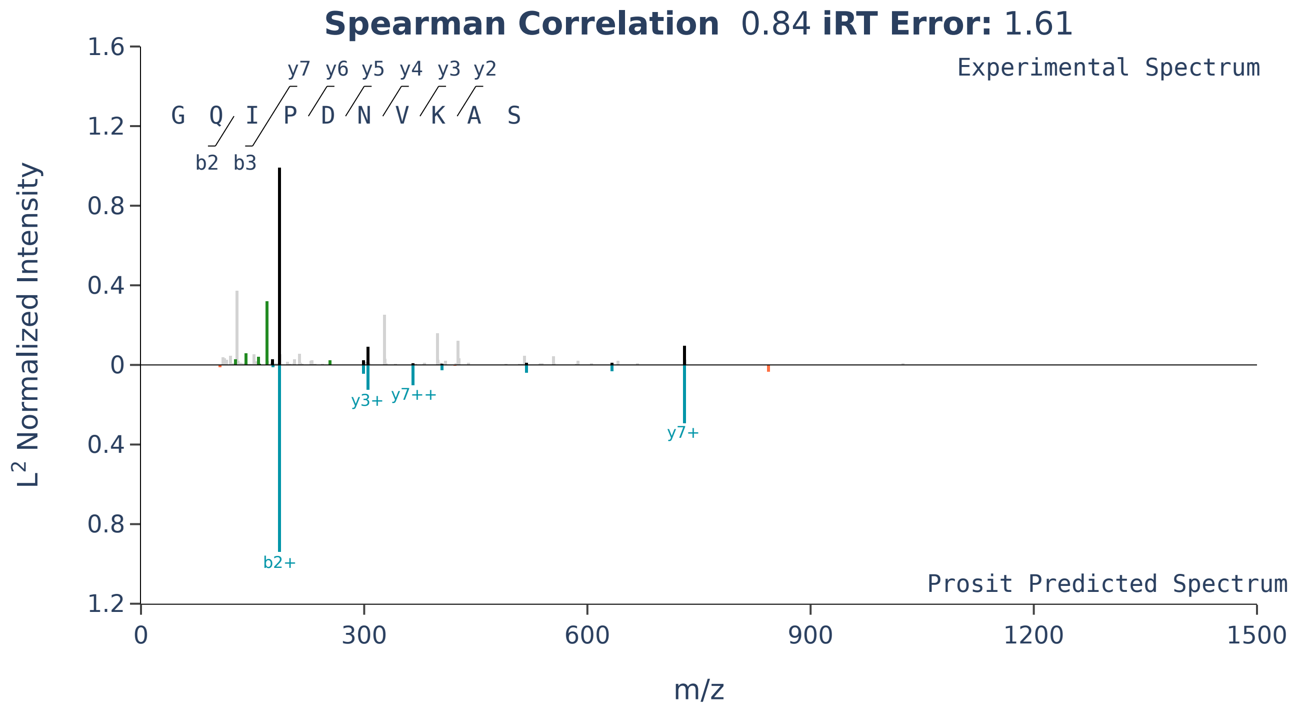

Source Ncheng_210623_230623_HFGoe_FFH_20S_25_1_A1_24h_R2 **Scan** 9873
Peptide QIPDNVKSQ **Charge** 2 **Spectral Angle** 0.24
Spearman Correlation 0.3 **iRT Error:** 10.58

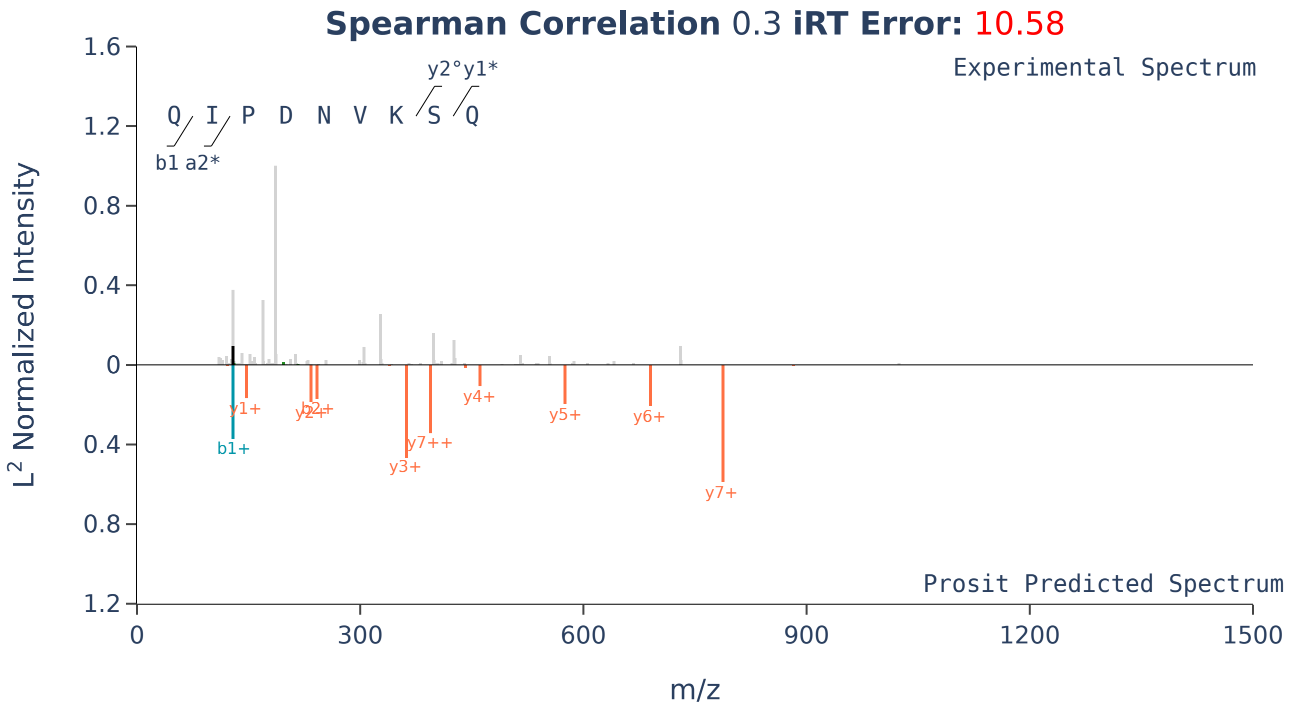

Source Ncheng_210623_230623_HFGoe_FFH_20S_25_1_A1_24h_R2 **Scan** 8495
Peptide MGAKMMRSM **Charge** 3 **Spectral Angle** 0.87
Spearman Correlation 0.82 **iRT Error:** 1.66

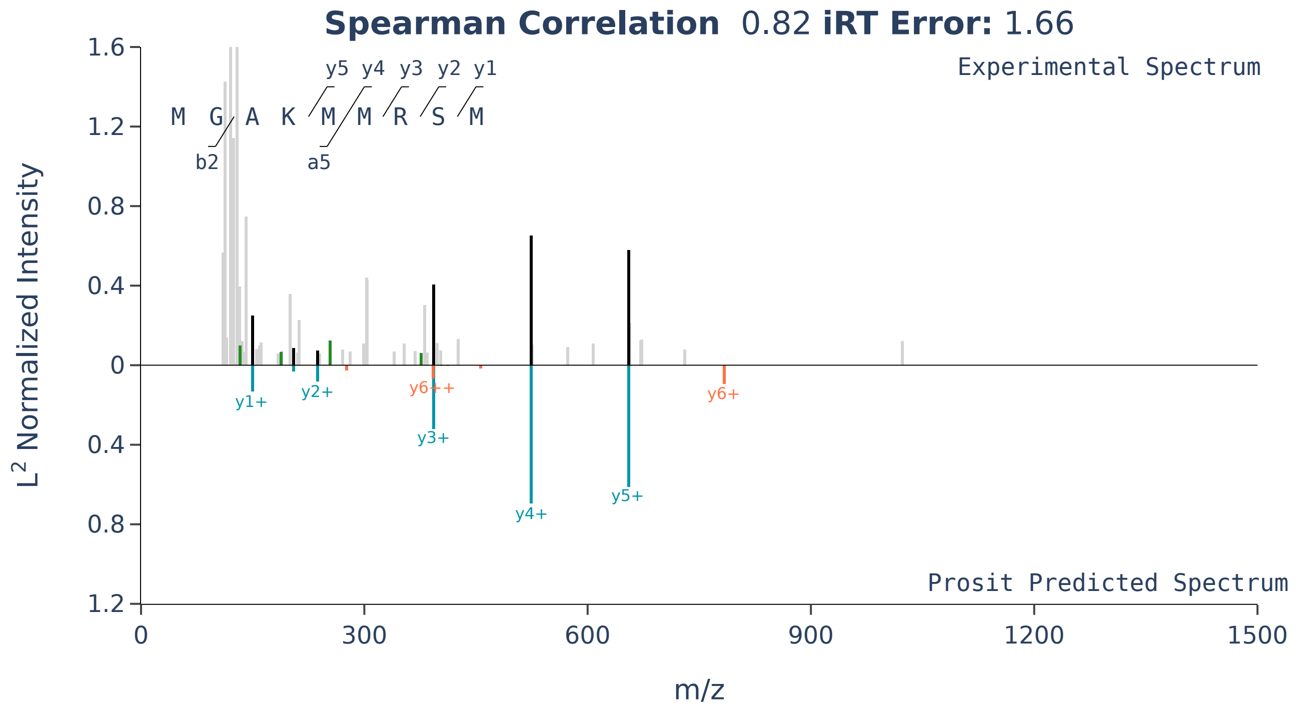

Source Ncheng_210623_230623_HFGoe_FFH_20S_25_1_A1_24h_R2 **Scan** 8495
Peptide GMAKMMRSM **Charge** 3 **Spectral Angle** 0.34
Spearman Correlation 0.41 **iRT Error:** 4.81

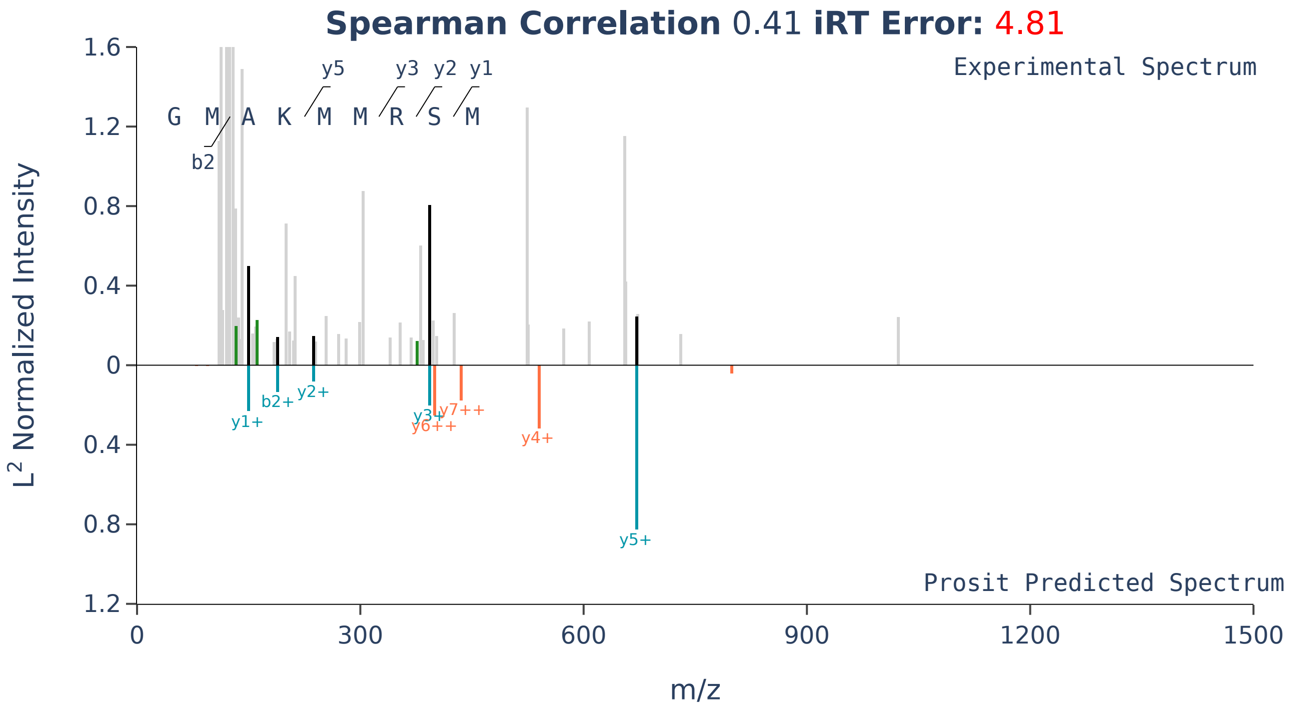

Source Ncheng_210623_230623_HFGoe_FFH_20S_25_1_A2_4h_R1 **Scan** 14560
Peptide HASINPVEVGQKPVD **Charge** 3 **Spectral Angle** 0.85
Spearman Correlation 0.86 **iRT Error:** 1.66

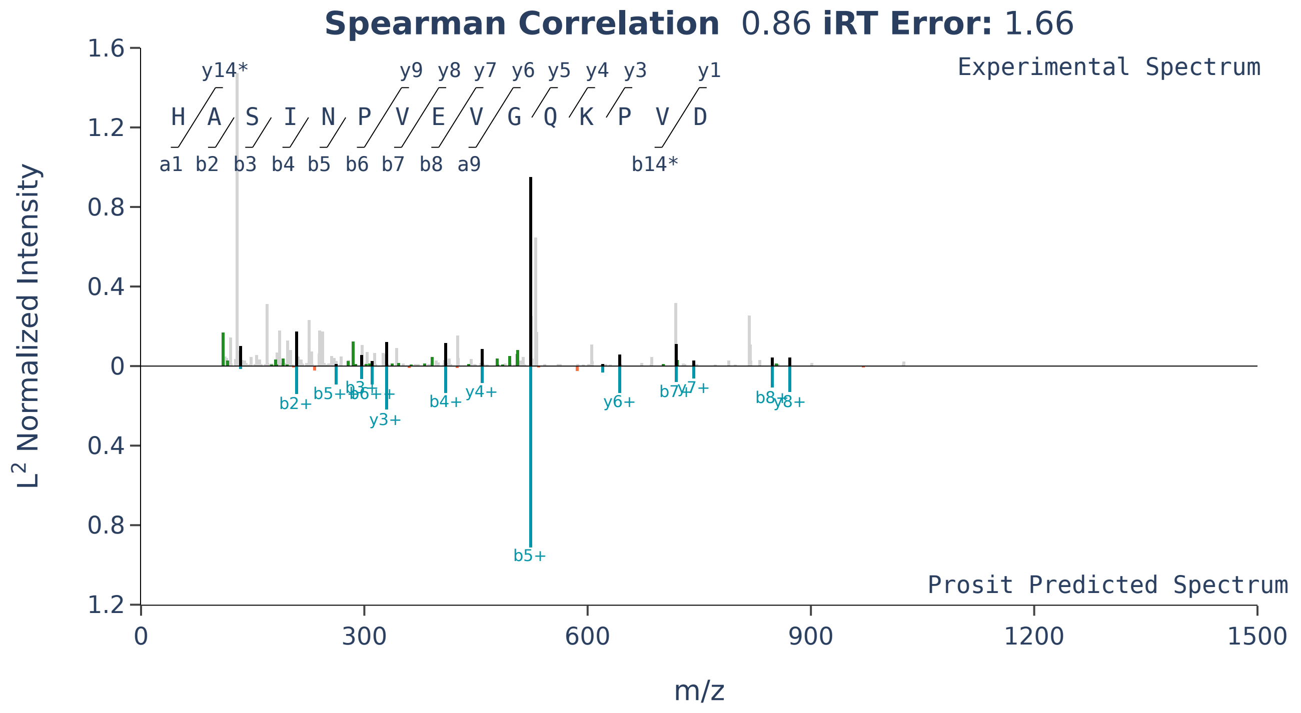

Source Ncheng_210623_230623_HFGoe_FFH_20S_25_1_A2_4h_R1 **Scan** 14560
Peptide VQDVNRLLKQFDD **Charge** 3 **Spectral Angle** 0.14
Spearman Correlation 0.32 **iRT Error:** 51.57

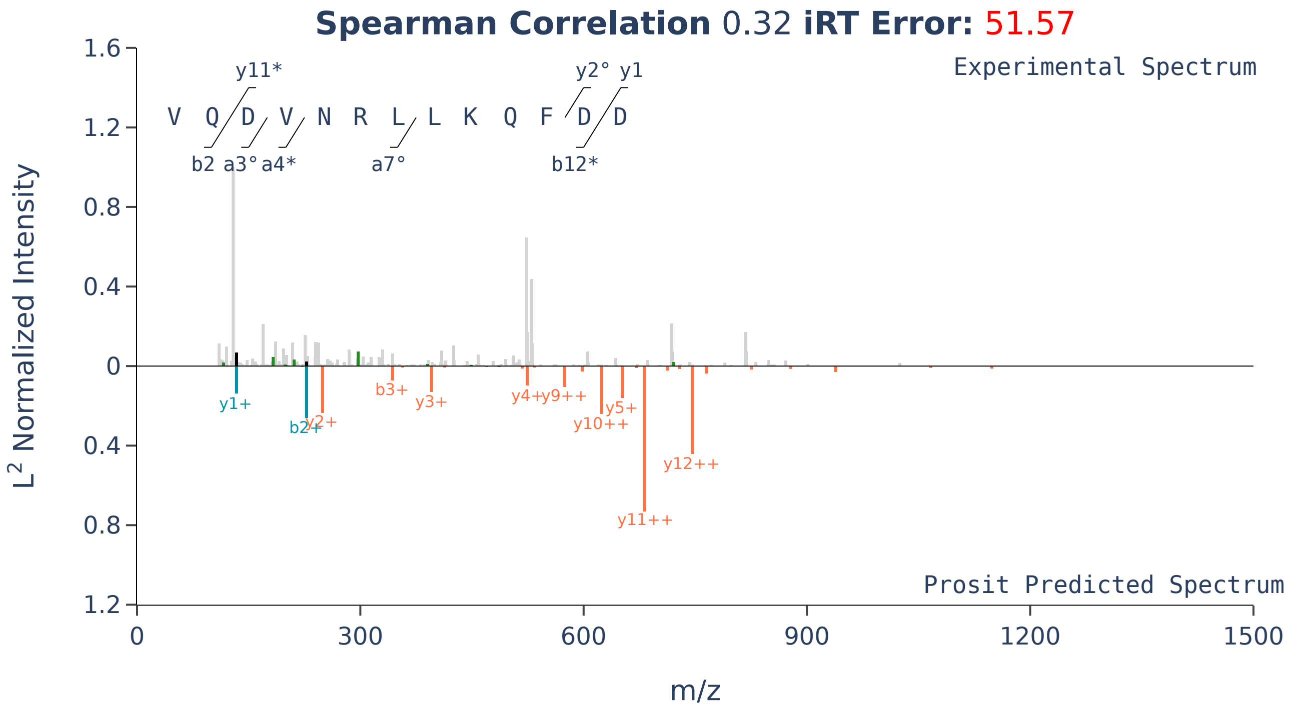

Source Ncheng_210623_230623_HFGoe_FFH_20S_25_1_A1_4h_R1 **Scan** 22422
Peptide GKVDFFPSD **Charge** 2 **Spectral Angle** 0.77
Spearman Correlation 0.97 **iRT Error:** 1.68

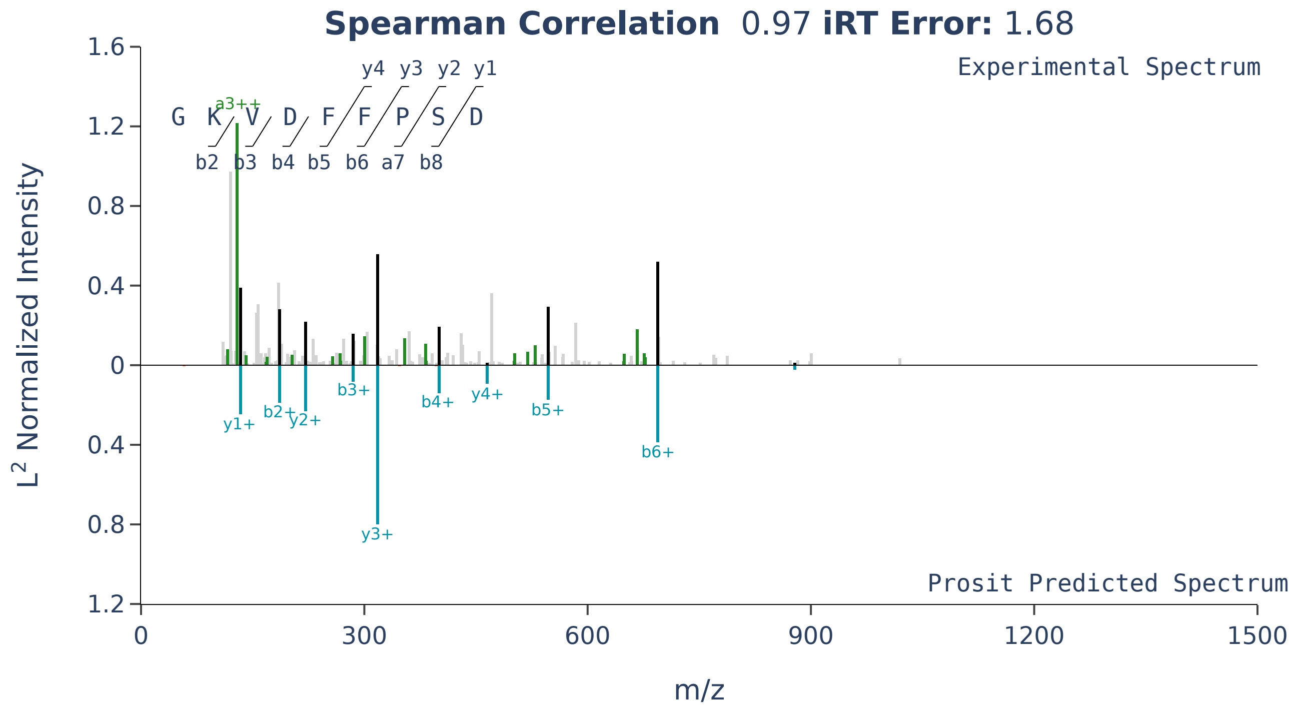

Source Ncheng_210623_230623_HFGoe_FFH_20S_25_1_A1_4h_R1 **Scan** 22422
Peptide AEQVGVDFF **Charge** 2 **Spectral Angle** 0.05
Spearman Correlation 0.0 **iRT Error:** 51.84

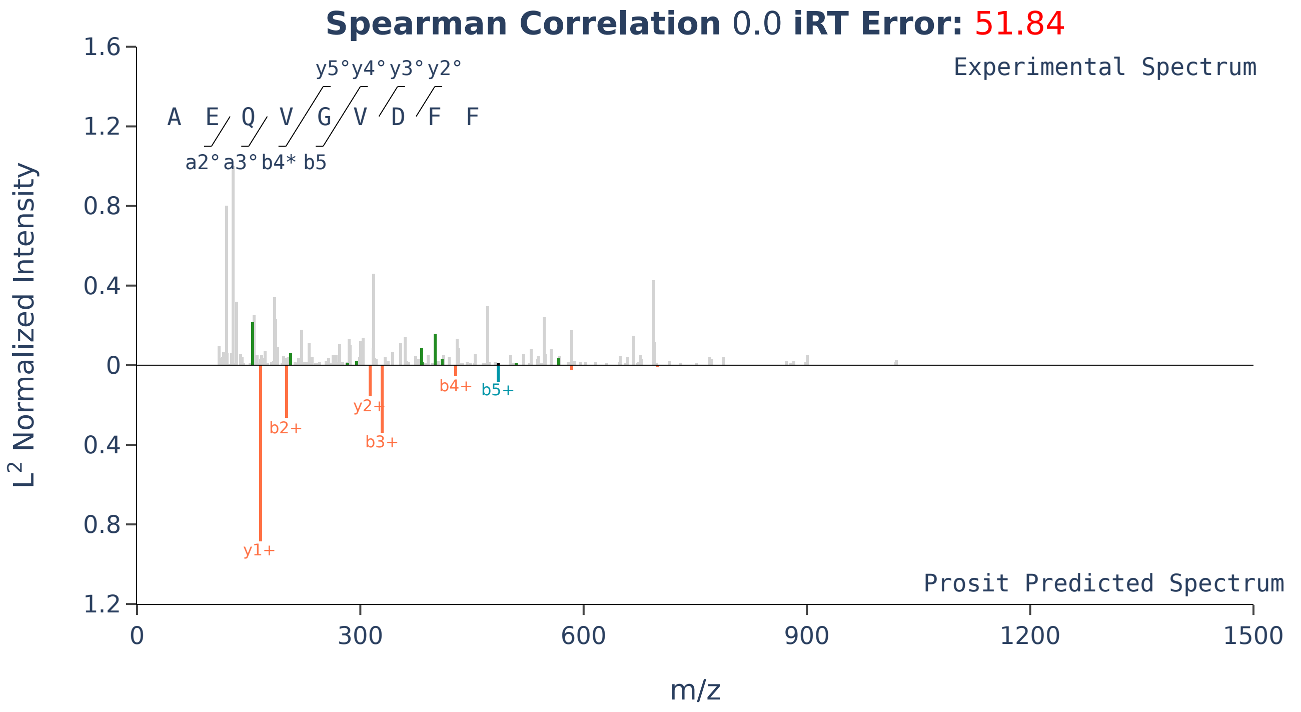

Source Ncheng_210623_230623_HFGoe_FFH_20S_25_1_A2_4h_R1 **Scan** 15998
Peptide KLPGMGMGQIPDNVKSQ **Charge** 2 **Spectral Angle** 0.84
Spearman Correlation 0.83 **iRT Error:** 1.68

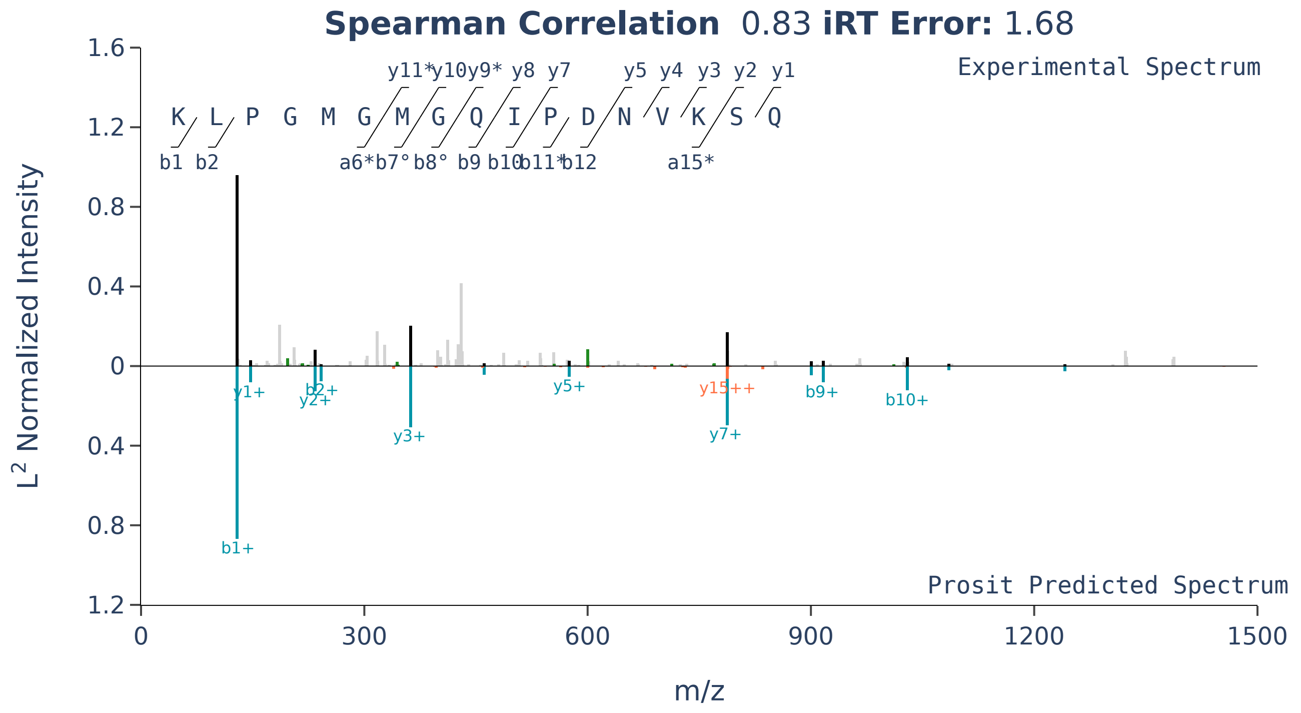

Source Ncheng_210623_230623_HFGoe_FFH_20S_25_1_A2_4h_R1 **Scan** 15998
Peptide GKLPGMGMGQIPDNVKSQM **Charge** 2 **Spectral Angle** 0.32
Spearman Correlation 0.28 **iRT Error:** 7.28

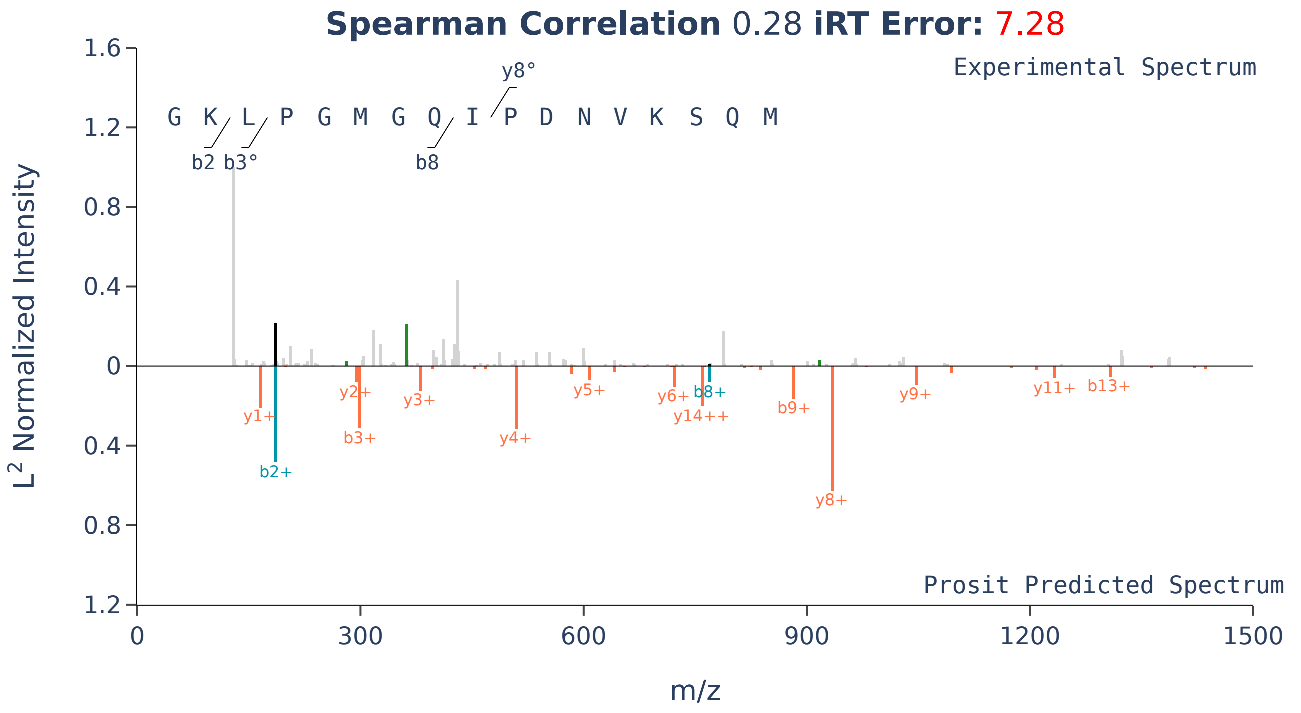

Source Ncheng_210623_230623_HFGoe_FFH_20S_25_1_A1_4h_R1 **Scan** 30183
Peptide AVGHEVNKSLTPGQEFVKIVRNELAV **Charge** 2 **Spectral Angle** 0.78
Spearman Correlation 0.87 **iRT Error:** 1.69

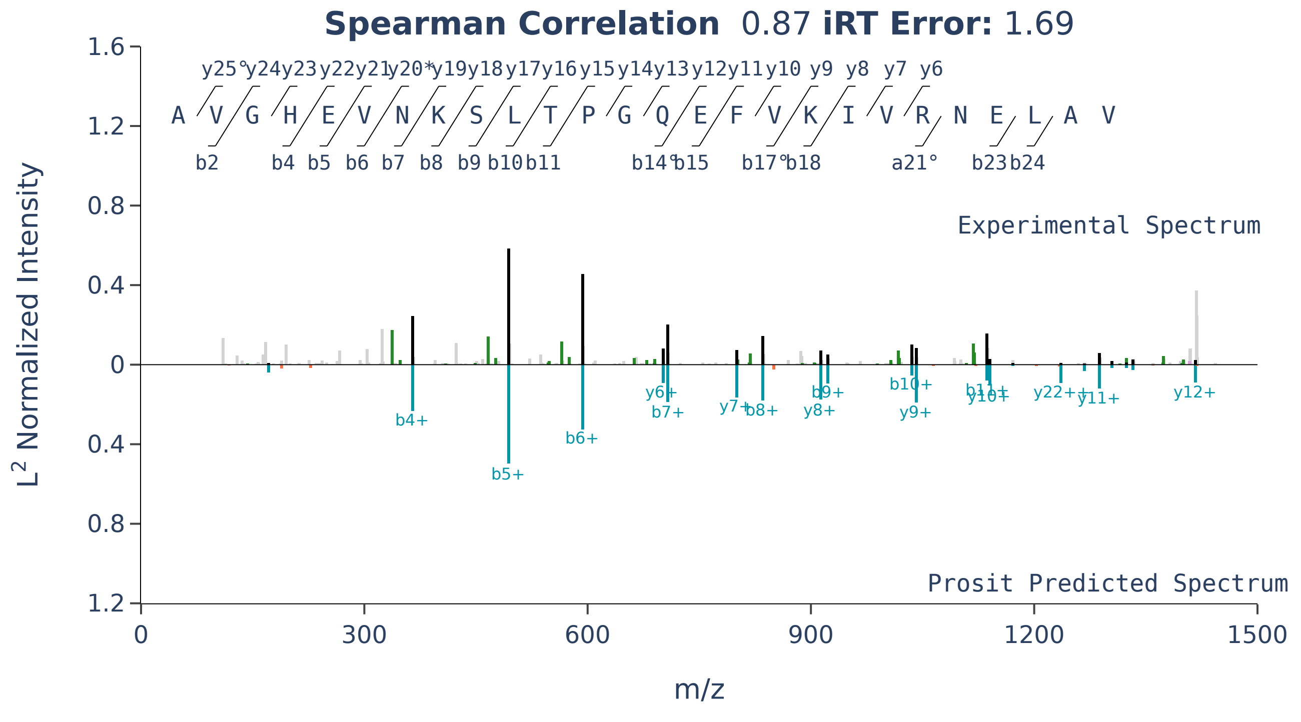

Source Ncheng_210623_230623_HFGoe_FFH_20S_25_1_A1_4h_R1 **Scan** 30183
Peptide VGHEVNKSLTPGQEFVKIVRNELVAA **Charge** 2 **Spectral Angle** 0.45
Spearman Correlation 0.39 **iRT Error:** 3.96

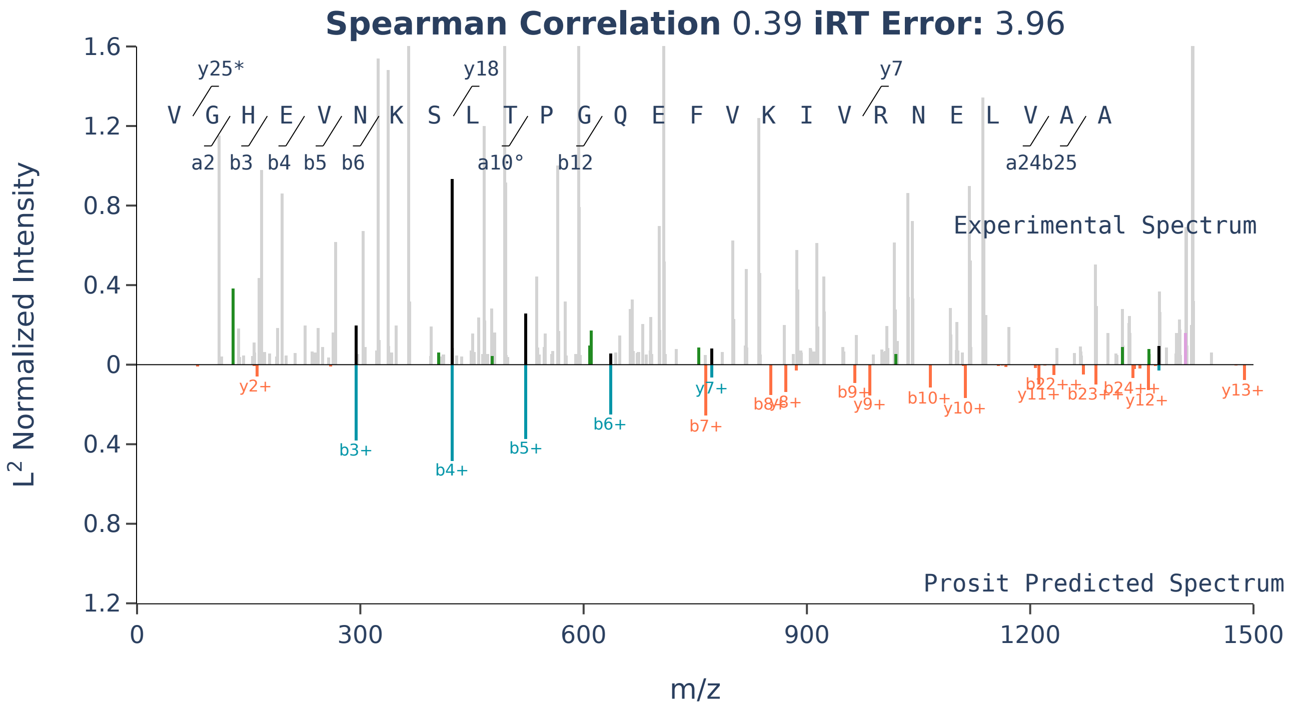

Source Ncheng_210623_230623_HFGoe_FFH_20S_25_1_A1_24h_R2 **Scan** 25388
Peptide ARGVGVDFFPSD **Charge** 2 **Spectral Angle** 0.88
Spearman Correlation 0.85 **iRT Error:** 1.71

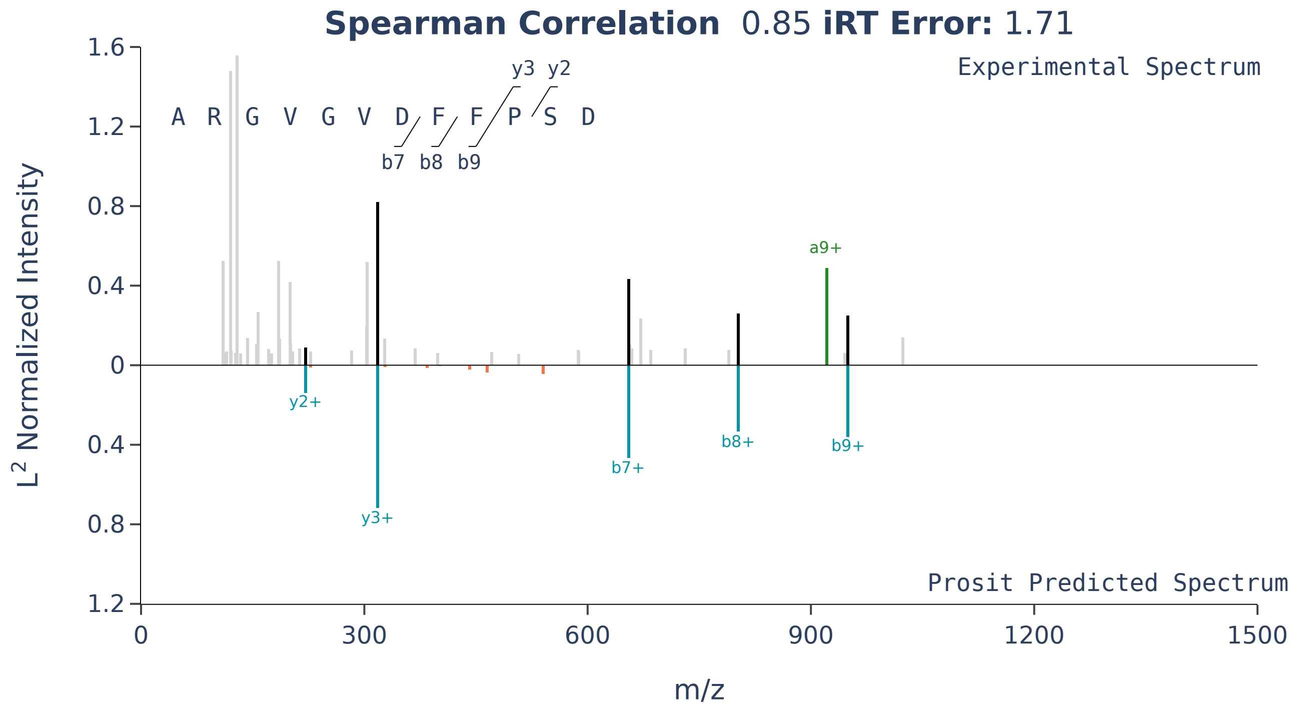

Source Ncheng_210623_230623_HFGoe_FFH_20S_25_1_A1_24h_R2 **Scan** 25388
Peptide EAIINSMTMKE **Charge** 2 **Spectral Angle** 0.0
Spearman Correlation 0.0 **iRT Error:** 31.29

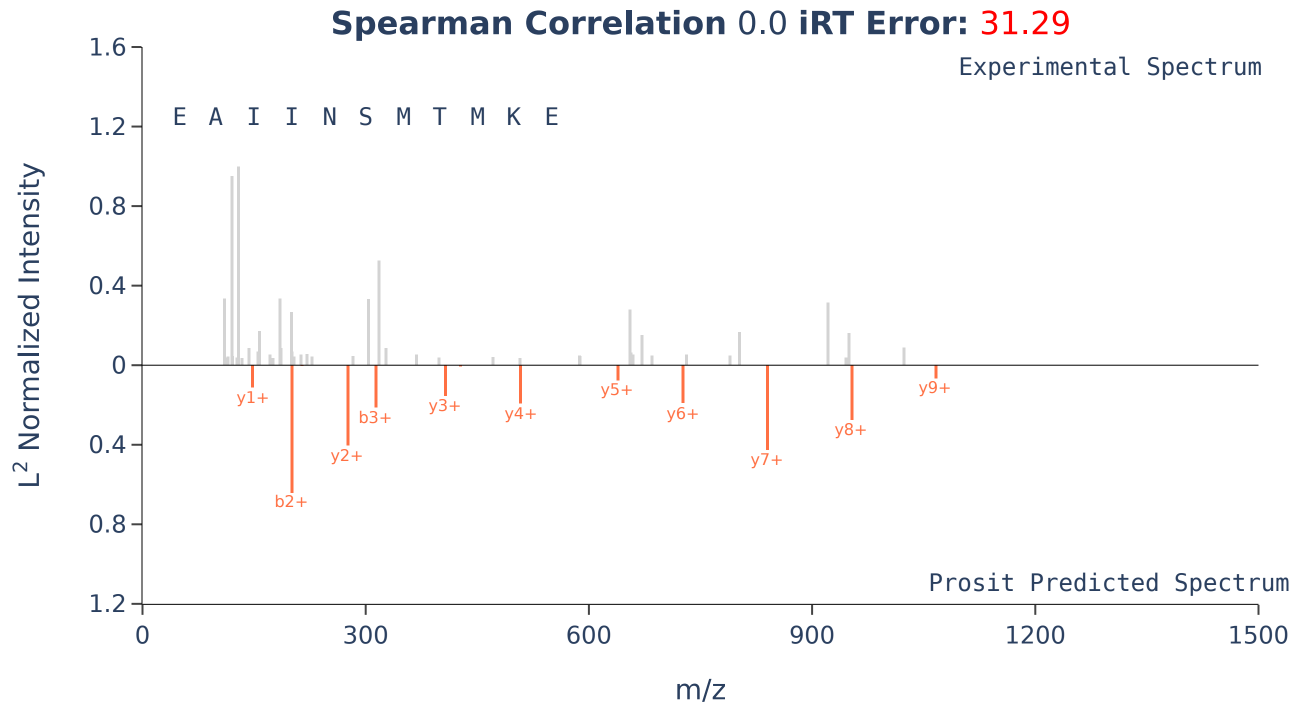

Source Ncheng_210623_230623_HFGoe_FFH_20S_25_1_A2_24h_R1 **Scan** 16894
Peptide MREAIINSM **Charge** 2 **Spectral Angle** 0.75
Spearman Correlation 0.88 **iRT Error:** 1.73

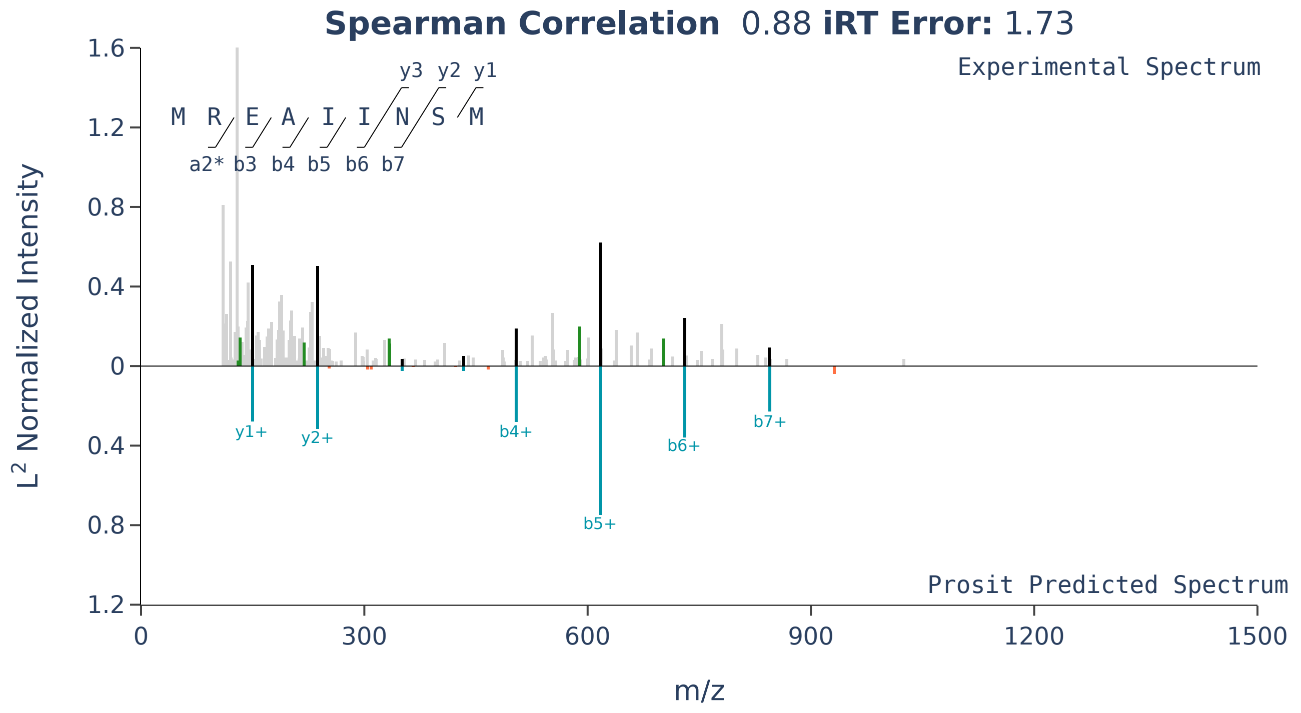

Source Ncheng_210623_230623_HFGoe_FFH_20S_25_1_A2_24h_R1 **Scan** 16894
Peptide RMEAIINSM **Charge** 2 **Spectral Angle** 0.6
Spearman Correlation 0.57 **iRT Error:** 12.05

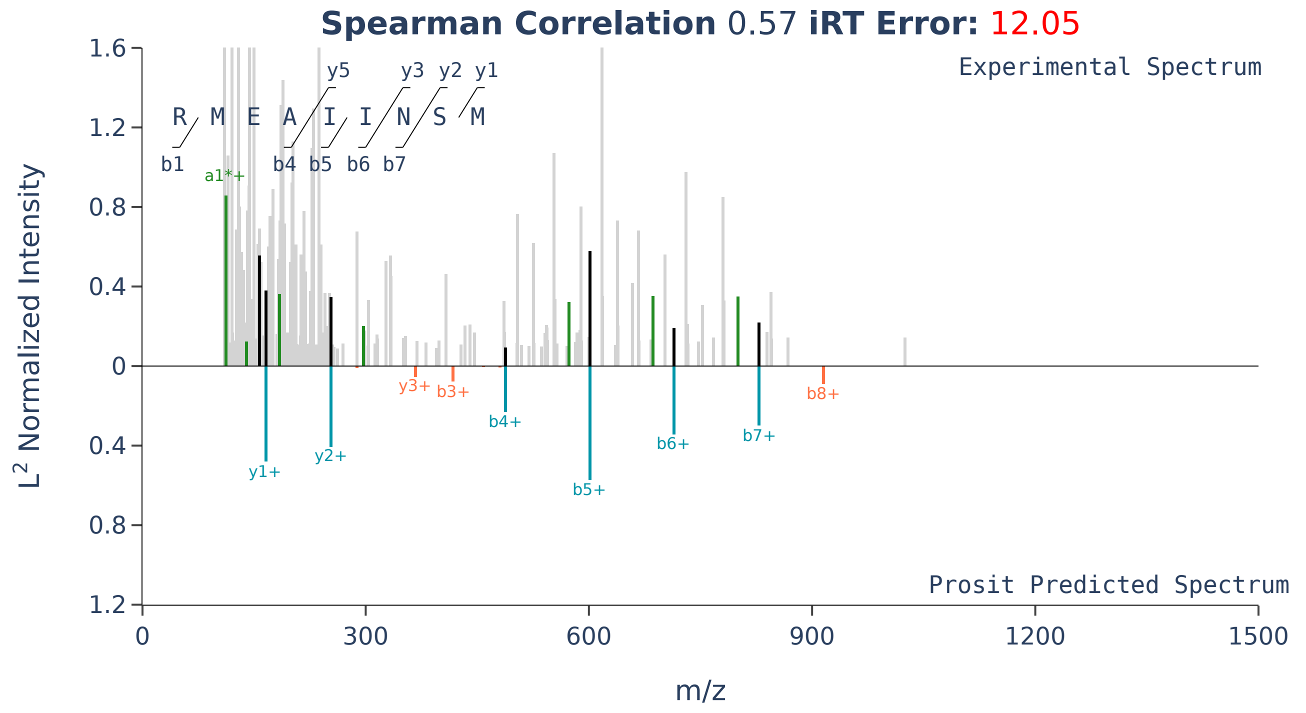

Source Ncheng_210623_230623_HFGoe_FFH_20S_25_1_A2_24h_R1 **Scan** 12769
Peptide AMTGQDAANTAKL **Charge** 2 **Spectral Angle** 0.67
Spearman Correlation 0.94 **iRT Error:** 1.73

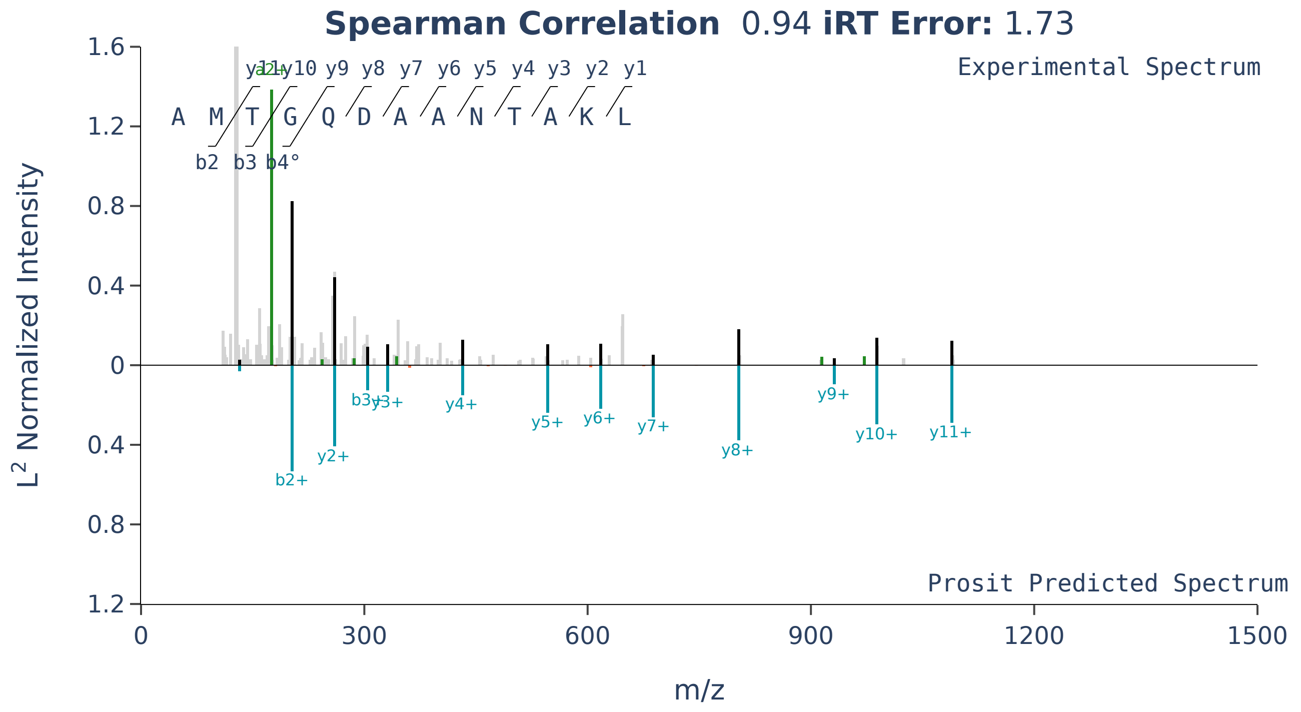

Source Ncheng_210623_230623_HFGoe_FFH_20S_25_1_A2_24h_R1 **Scan** 12769
Peptide EQLRQMKNMGG **Charge** 2 **Spectral Angle** 0.11
Spearman Correlation 0.27 **iRT Error:** 20.1

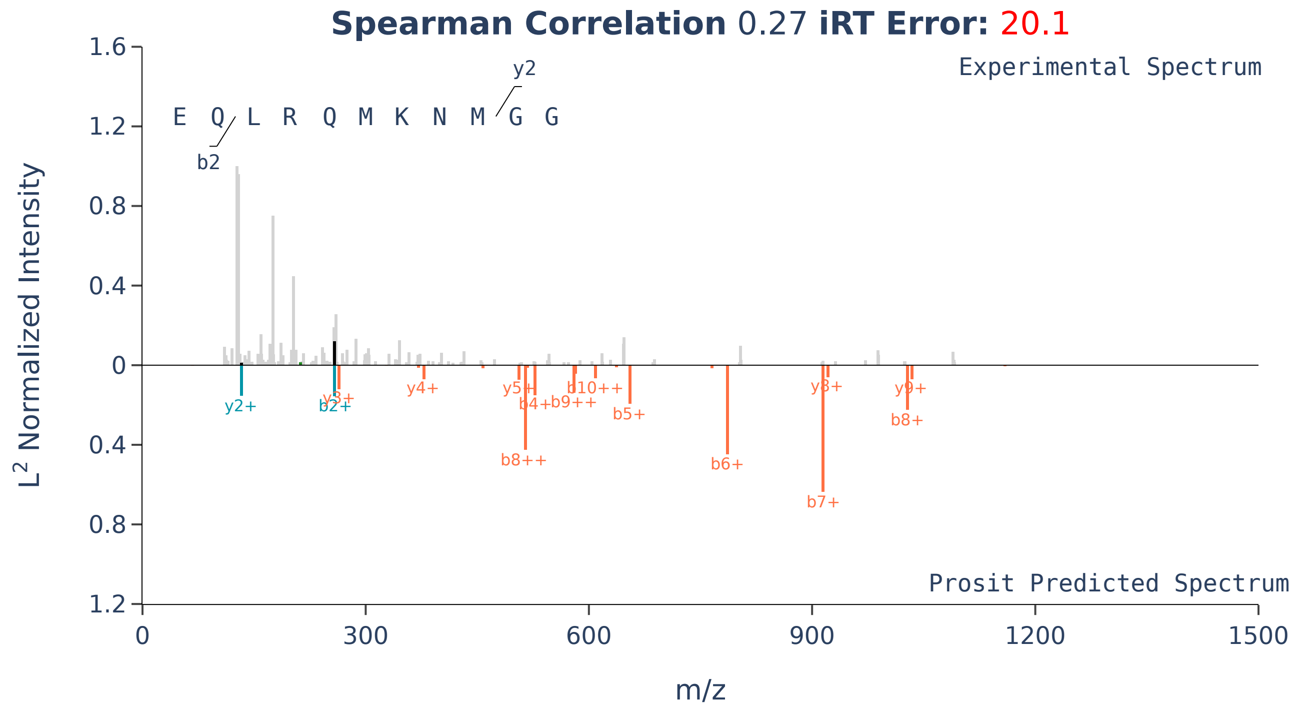

Source Ncheng_210623_230623_HFGoe_FFH_20S_25_1_A1_4h_R2 **Scan** 15031
Peptide SAAMTGQDAANTAKAFNE **Charge** 2 **Spectral Angle** 0.93
Spearman Correlation 0.9 **iRT Error:** 1.74

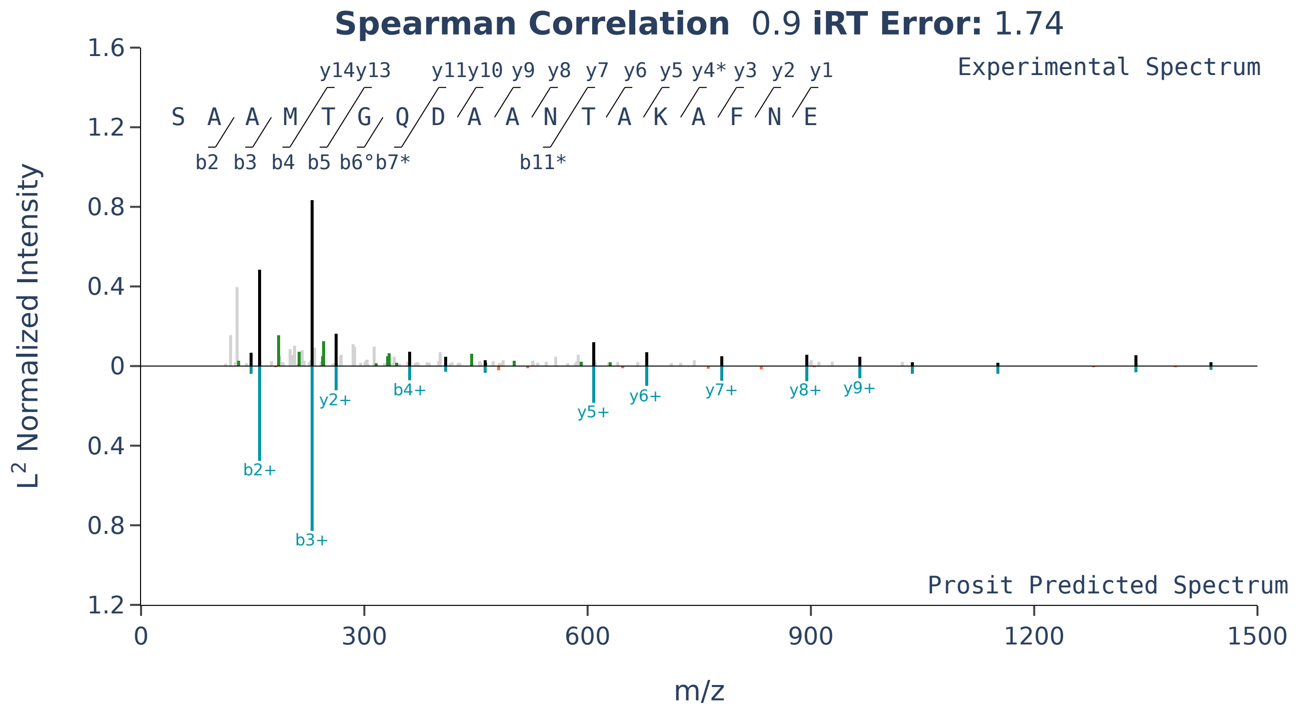

Source Ncheng_210623_230623_HFGoe_FFH_20S_25_1_A1_4h_R2 **Scan** 15031
Peptide GGMAKMMRSMKGMMPPG **Charge** 2 **Spectral Angle** 0.0
Spearman Correlation 0.0 **iRT Error:** 24.19

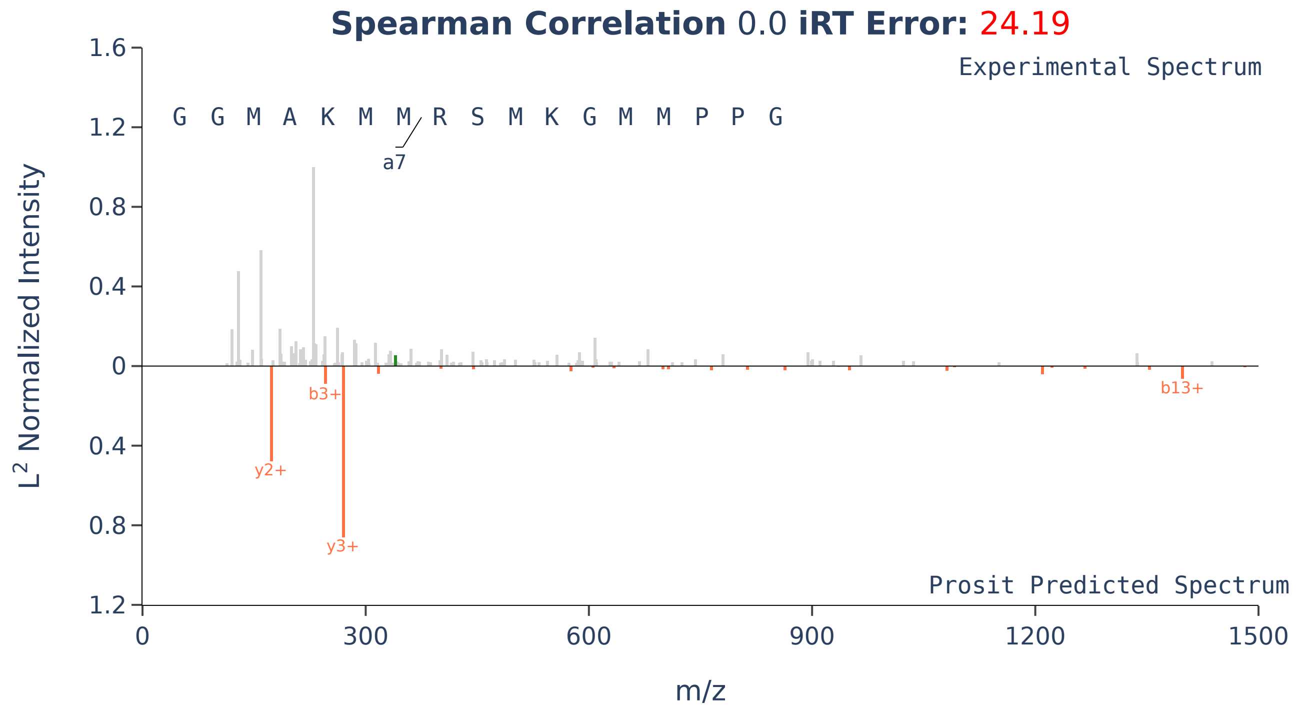

Source Ncheng_210623_230623_HFGoe_FFH_20S_25_1_A1_4h_R1 **Scan** 29016
Peptide QVGGVDFFPSD **Charge** 2 **Spectral Angle** 0.92
Spearman Correlation 0.83 **iRT Error:** 1.75

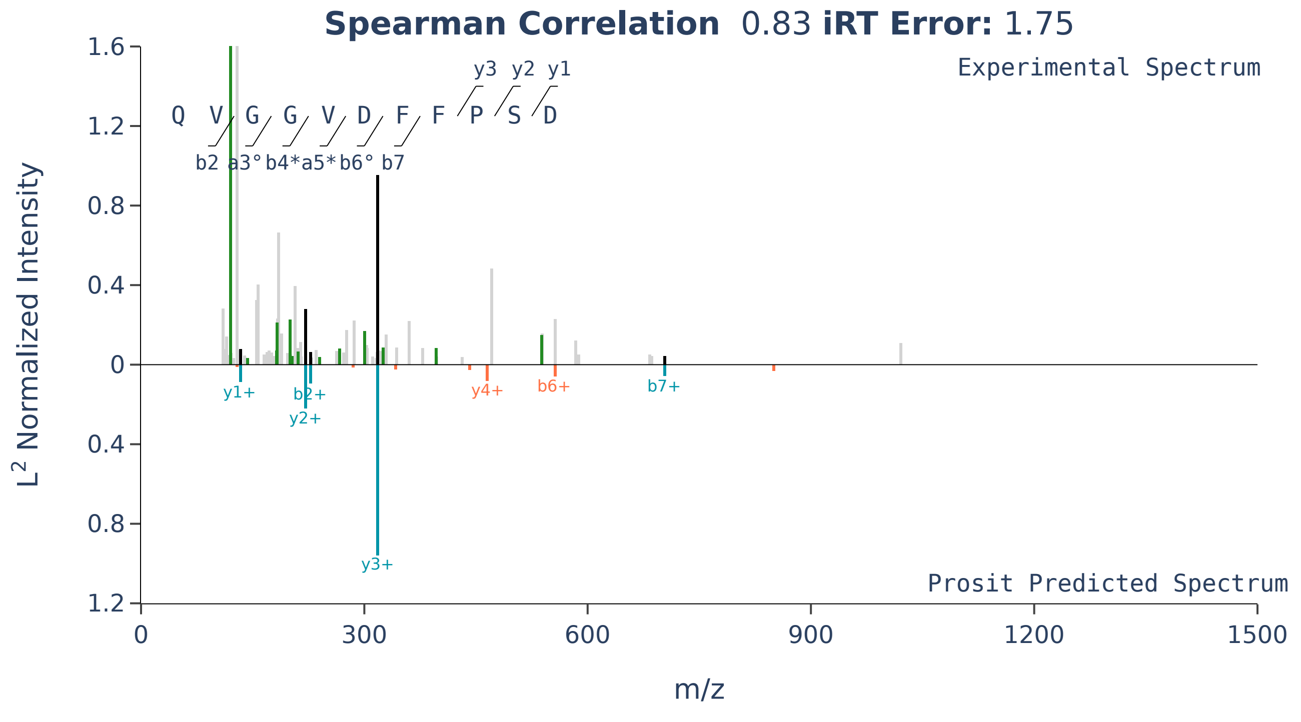

Source Ncheng_210623_230623_HFGoe_FFH_20S_25_1_A1_4h_R1 **Scan** 29016
Peptide QMKNMGGMASL **Charge** 2 **Spectral Angle** 0.0
Spearman Correlation 0.0 **iRT Error:** 50.51

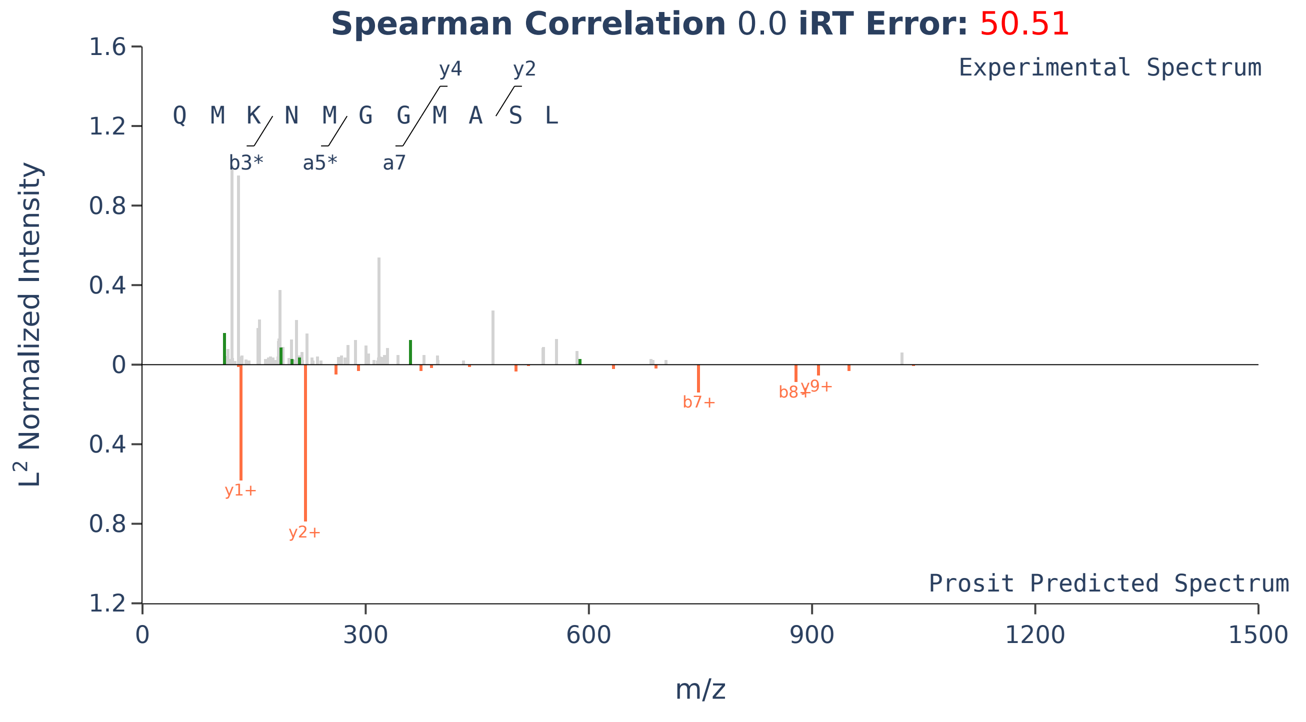

Source Ncheng_210623_230623_HFGoe_FFH_20S_25_1_A1_4h_R1 **Scan** 12244
Peptide RMEAIINSMTMKERKA **Charge** 3 **Spectral Angle** 0.85
Spearman Correlation 0.84 **iRT Error:** 1.78

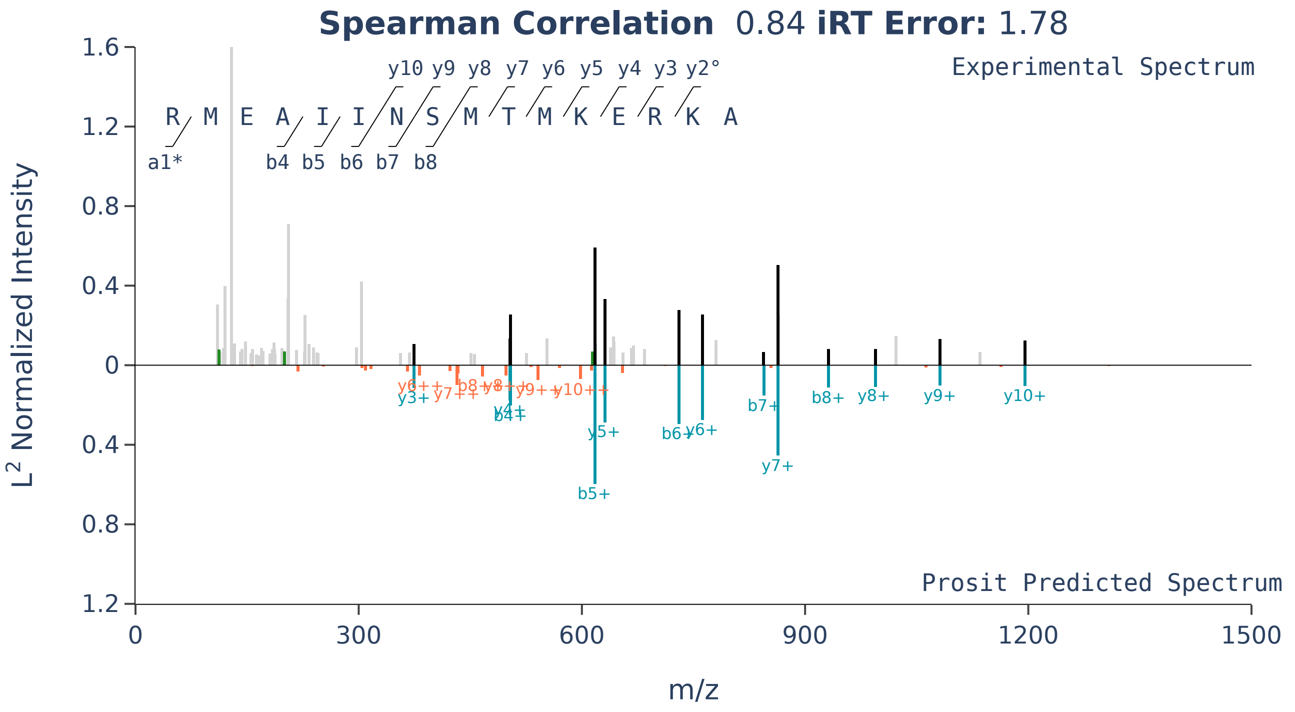

Source Ncheng_210623_230623_HFGoe_FFH_20S_25_1_A1_4h_R1 **Scan** 12244
Peptide RMEAIINSMTMKERAK **Charge** 3 **Spectral Angle** 0.16
Spearman Correlation 0.29 **iRT Error:** 7.68

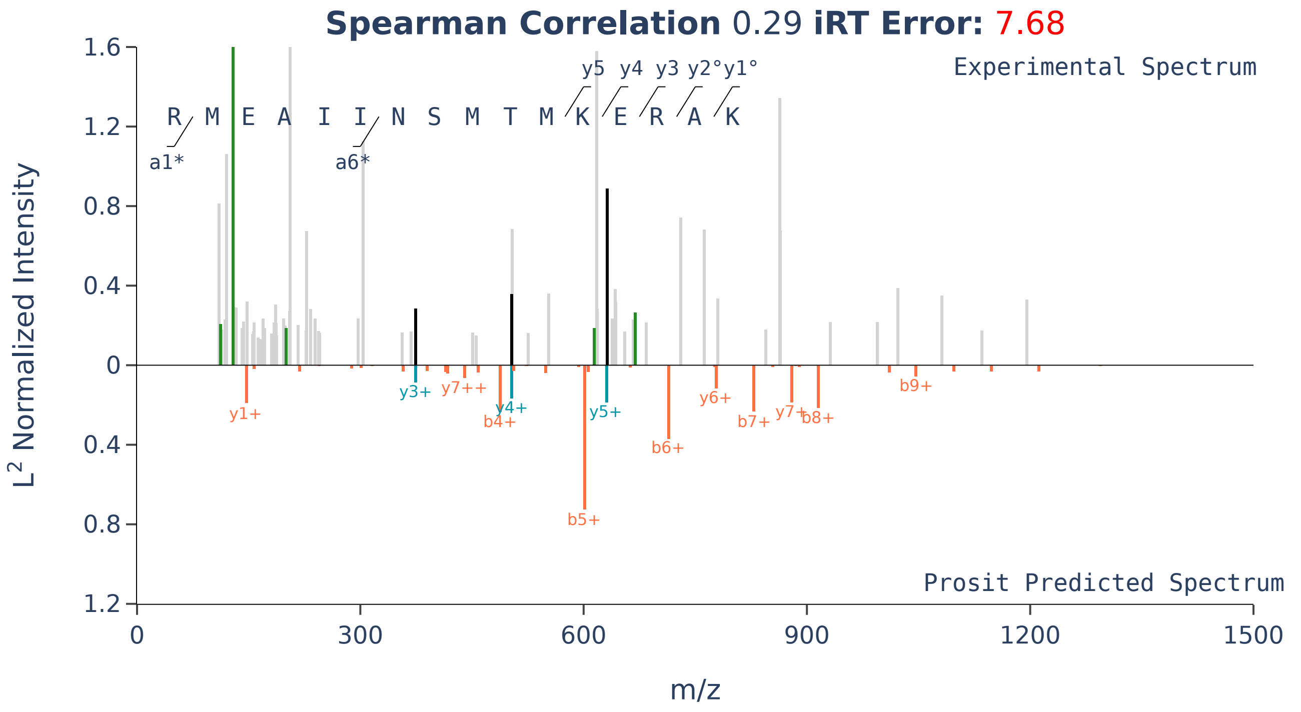

Source Ncheng_210623_230623_HFGoe_FFH_20S_25_1_A1_2h_R1 **Scan** 10496
Peptide KVLVRMDE **Charge** 2 **Spectral Angle** 0.69
Spearman Correlation 0.93 **iRT Error:** 1.92

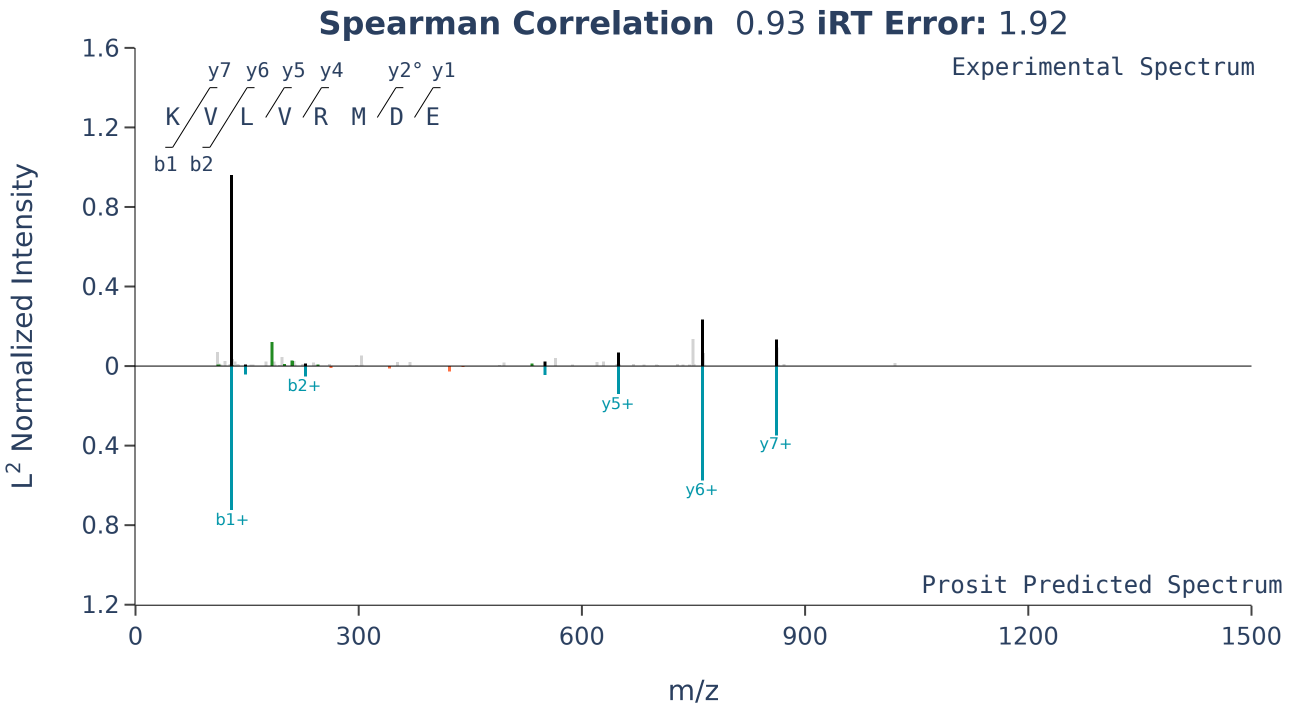

Source Ncheng_210623_230623_HFGoe_FFH_20S_25_1_A1_2h_R1 **Scan** 10496
Peptide DKVLVRME **Charge** 2 **Spectral Angle** 0.41
Spearman Correlation 0.33 **iRT Error:** 14.42

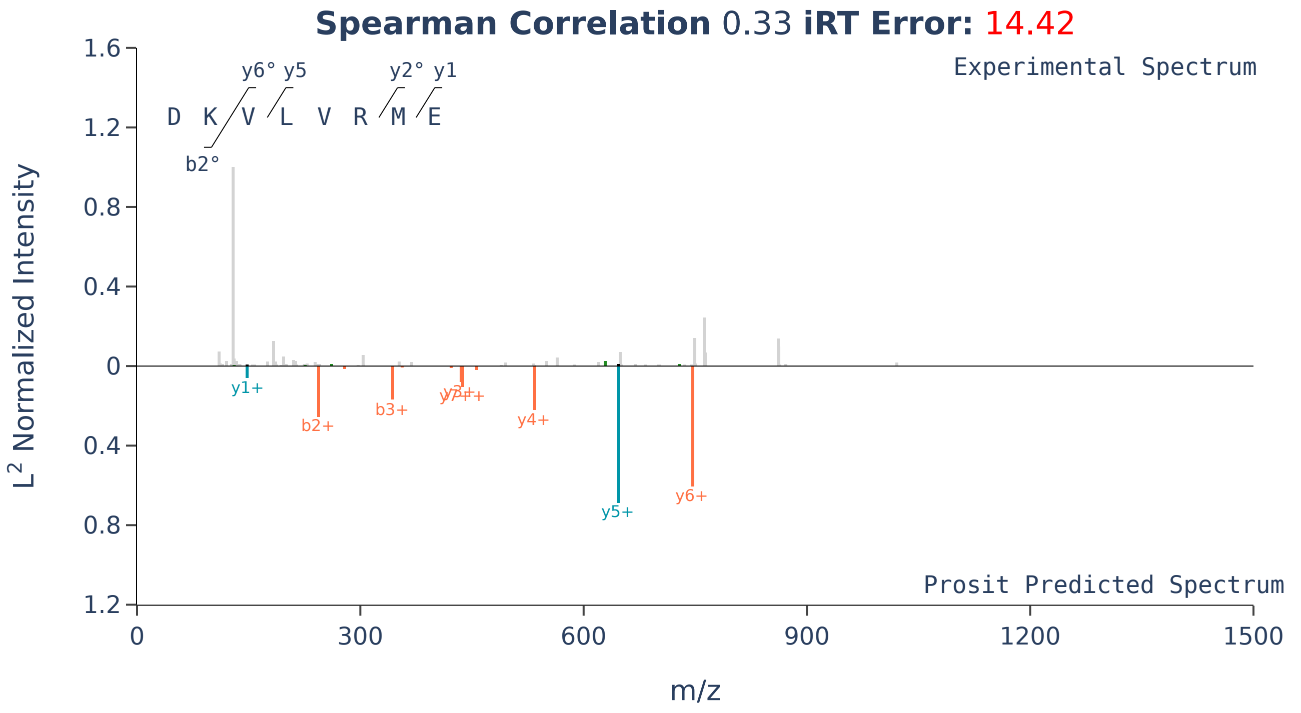

Source Ncheng_210623_230623_HFGoe_FFH_20S_25_1_A1_24h_R1 **Scan** 17734
Peptide TDKPIKFLGVGEKTE **Charge** 2 **Spectral Angle** 0.81
Spearman Correlation 0.88 **iRT Error:** 1.98

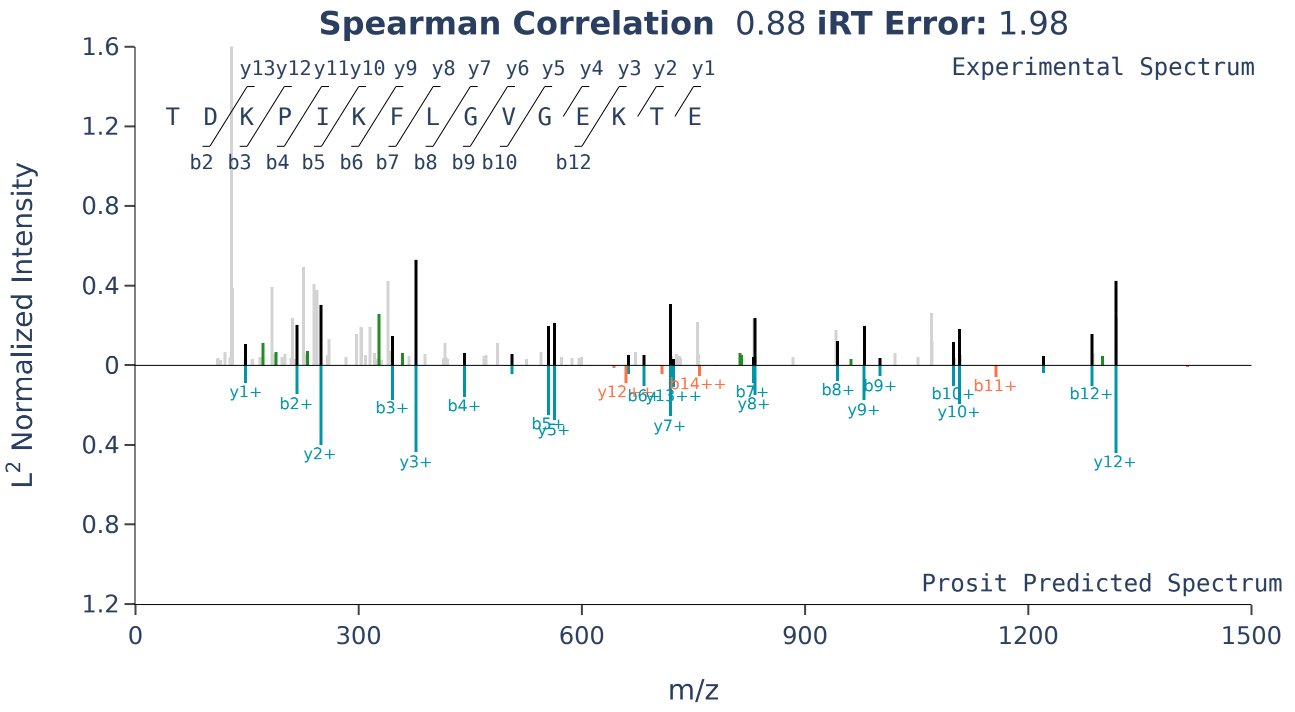

Source Ncheng_210623_230623_HFGoe_FFH_20S_25_1_A1_24h_R1 **Scan** 17734
Peptide SMTMKERAKPEIIK **Charge** 2 **Spectral Angle** 0.23
Spearman Correlation 0.34 **iRT Error:** 48.58

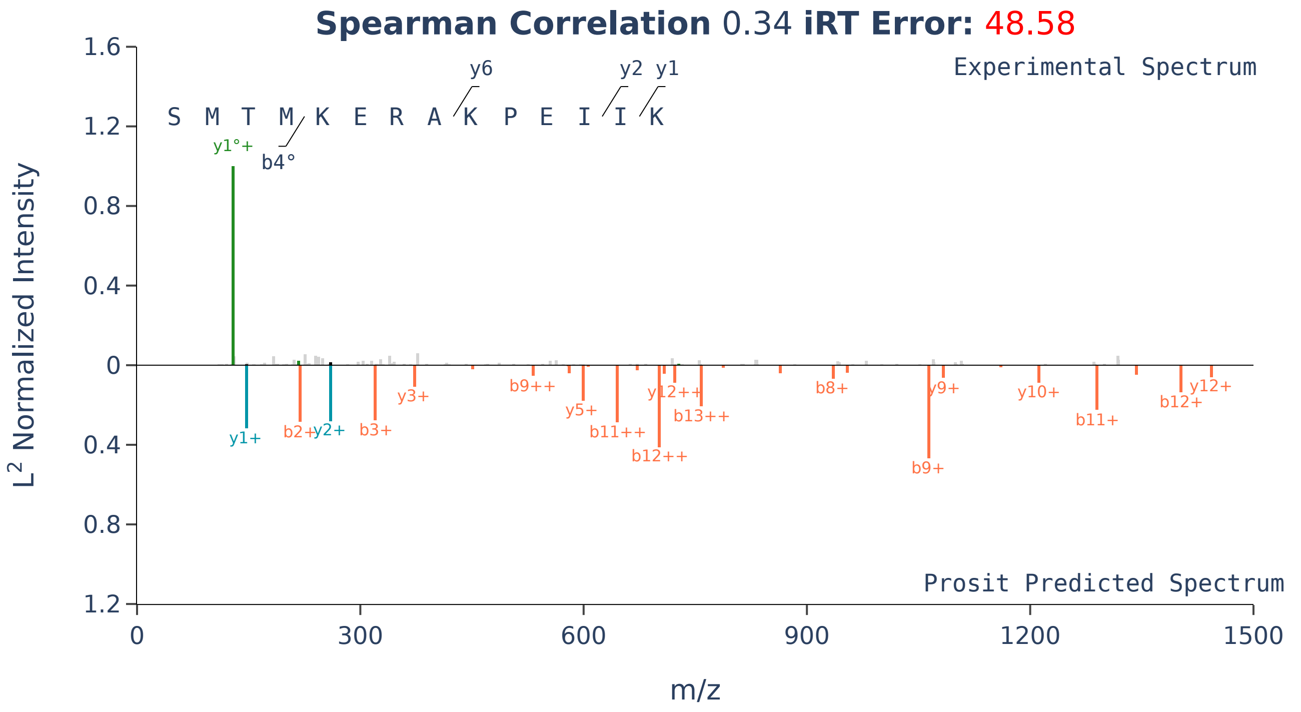

Source Ncheng_210623_230623_HFGoe_FFH_20S_25_1_A2_4h_R1 **Scan** 36572
Peptide PLTGVVEAII **Charge** 1 **Spectral Angle** 0.84
Spearman Correlation 0.88 **iRT Error:** 2.01

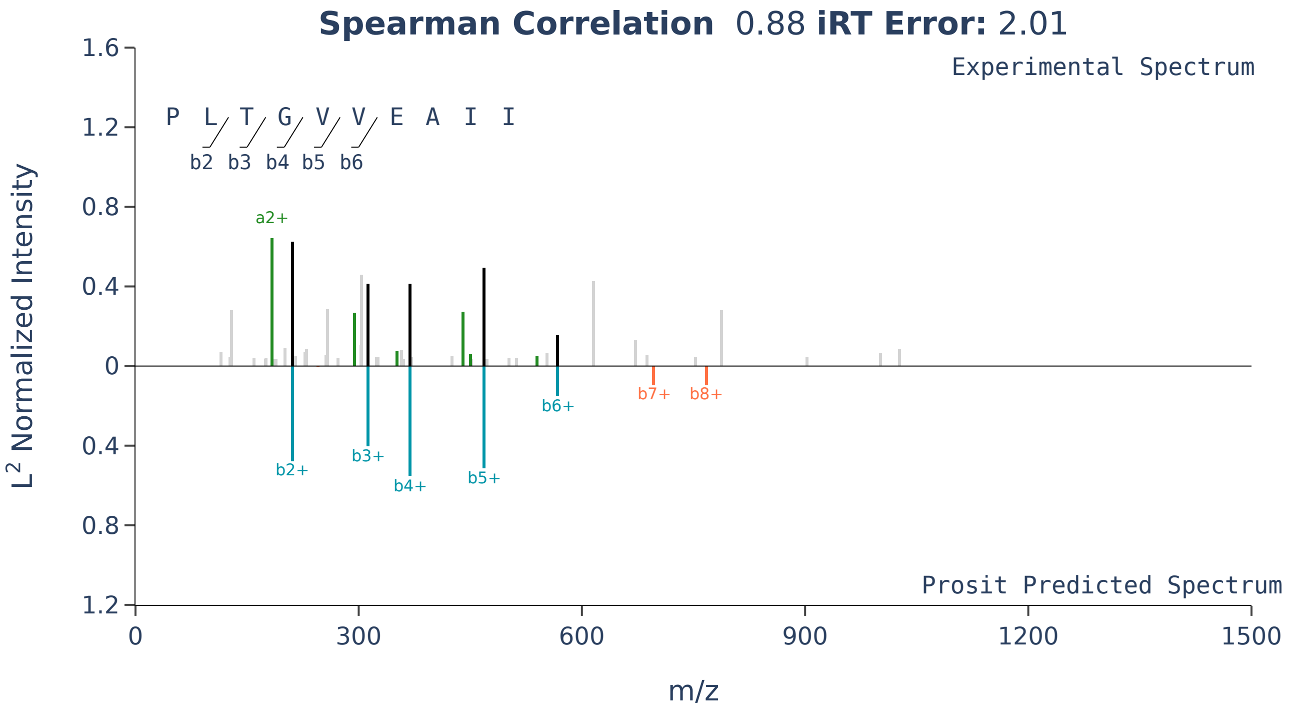

Source Ncheng_210623_230623_HFGoe_FFH_20S_25_1_A2_4h_R1 **Scan** 36572
Peptide EALPLTGVVL **Charge** 1 **Spectral Angle** 0.62
Spearman Correlation 0.5 **iRT Error:** 3.5

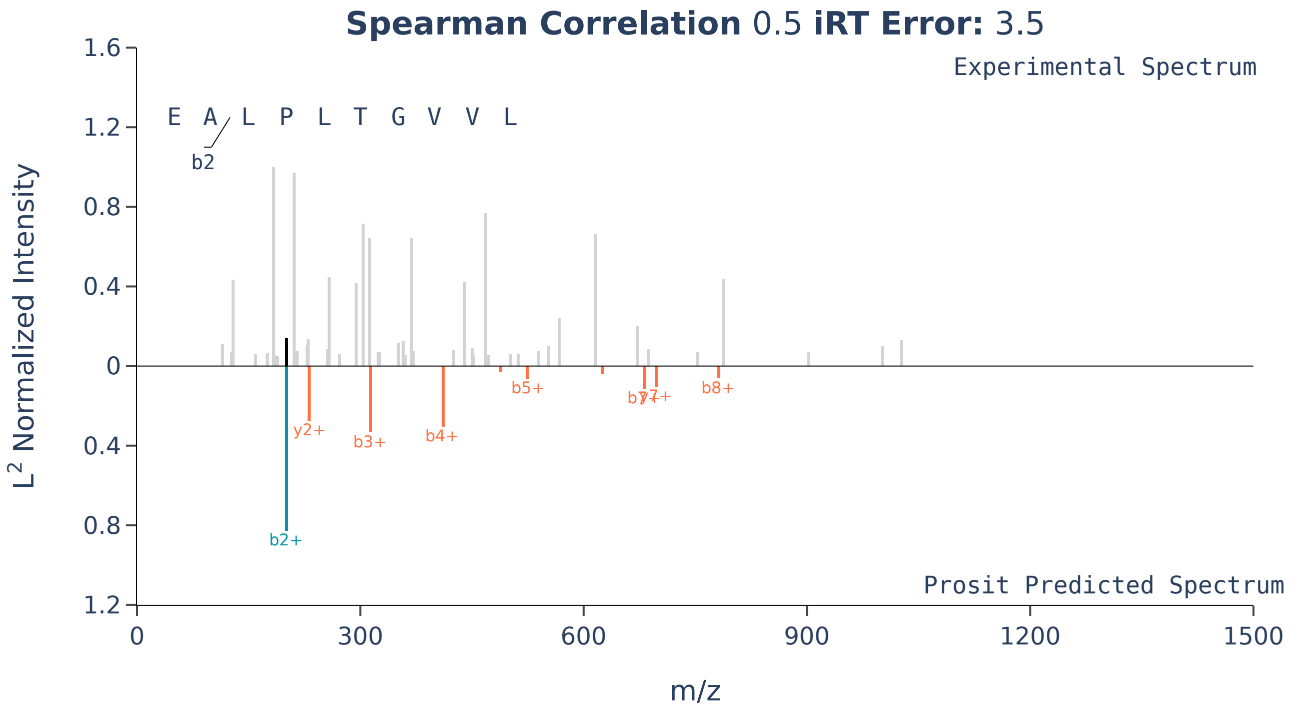

Source Ncheng_210623_230623_HFGoe_FFH_20S_25_1_A1_4h_R2 **Scan** 15446
Peptide RIAAGAMTGQDAANTAKAFN **Charge** 3 **Spectral Angle** 0.82
Spearman Correlation 0.88 **iRT Error:** 2.02

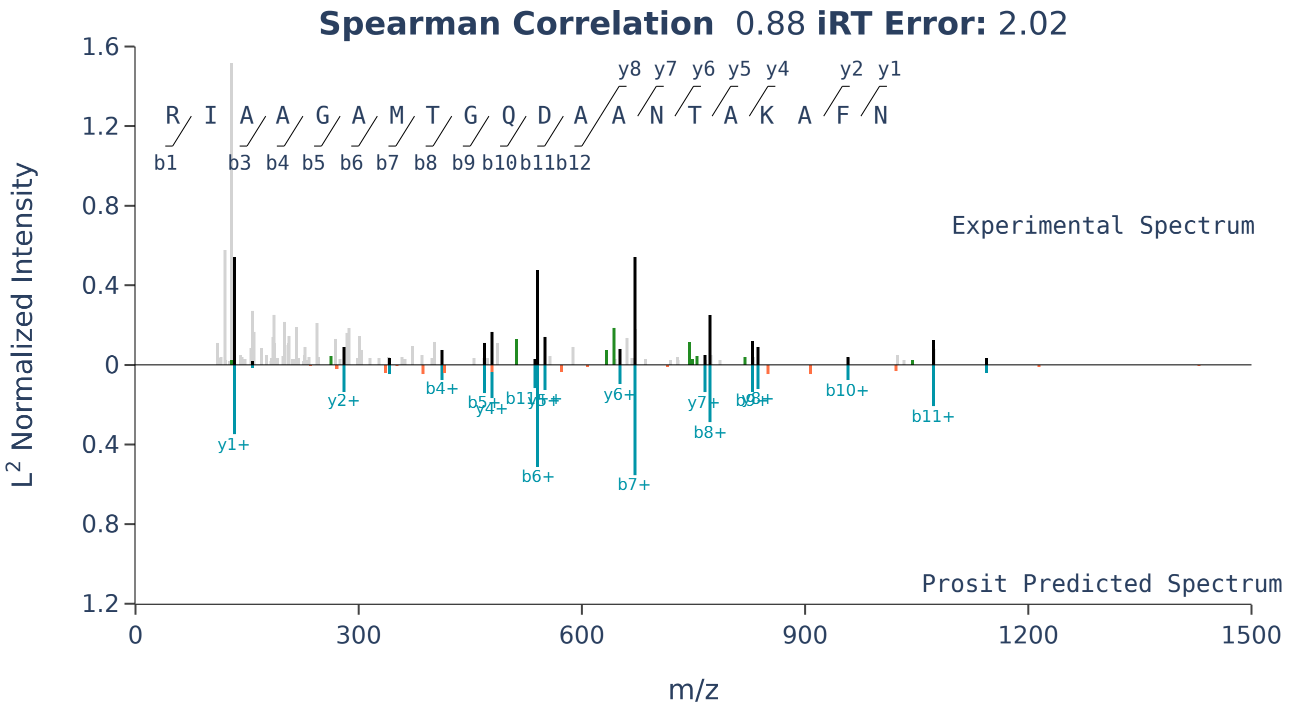

Source Ncheng_210623_230623_HFGoe_FFH_20S_25_1_A1_4h_R2 **Scan** 15446
Peptide MEAIINSMTMKERAKPE **Charge** 3 **Spectral Angle** 0.04
Spearman Correlation 0.08 **iRT Error:** 11.34

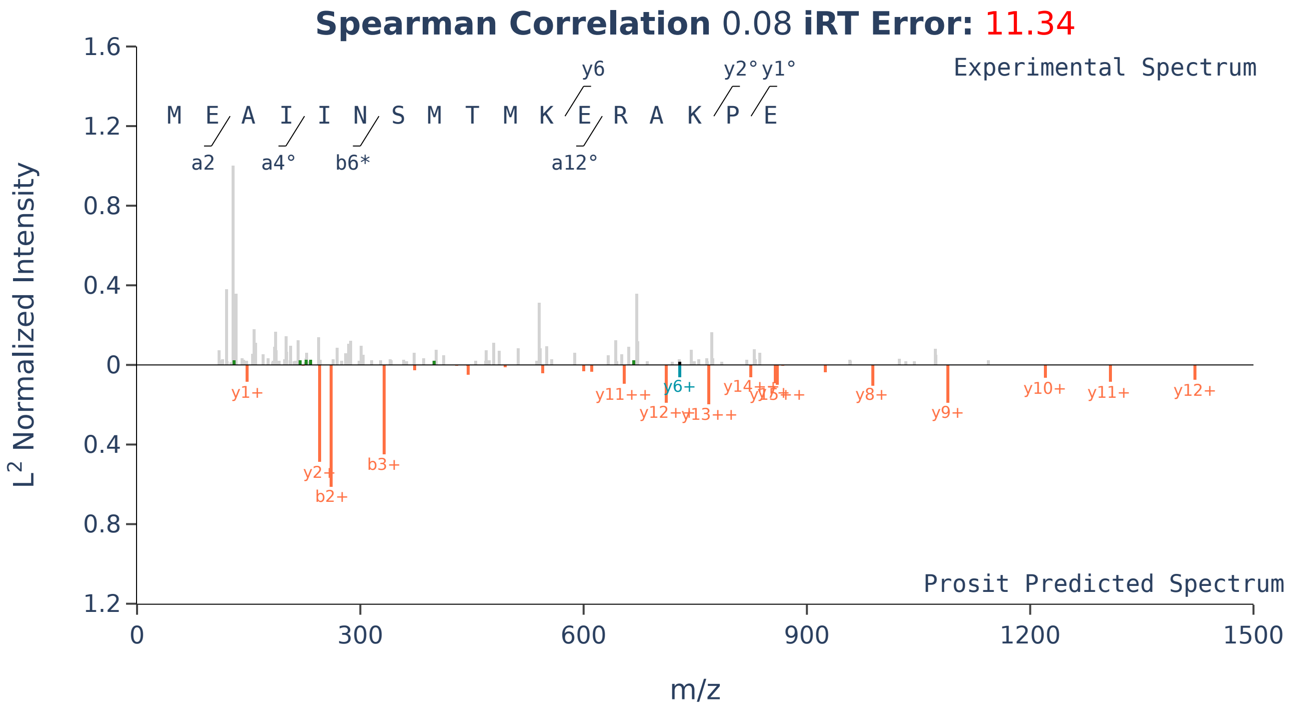

Source Ncheng_210623_230623_HFGoe_FFH_20S_25_1_A2_24h_R1 **Scan** 15564
Peptide EALPLTGKTE **Charge** 2 **Spectral Angle** 0.7
Spearman Correlation 0.91 **iRT Error:** 2.03

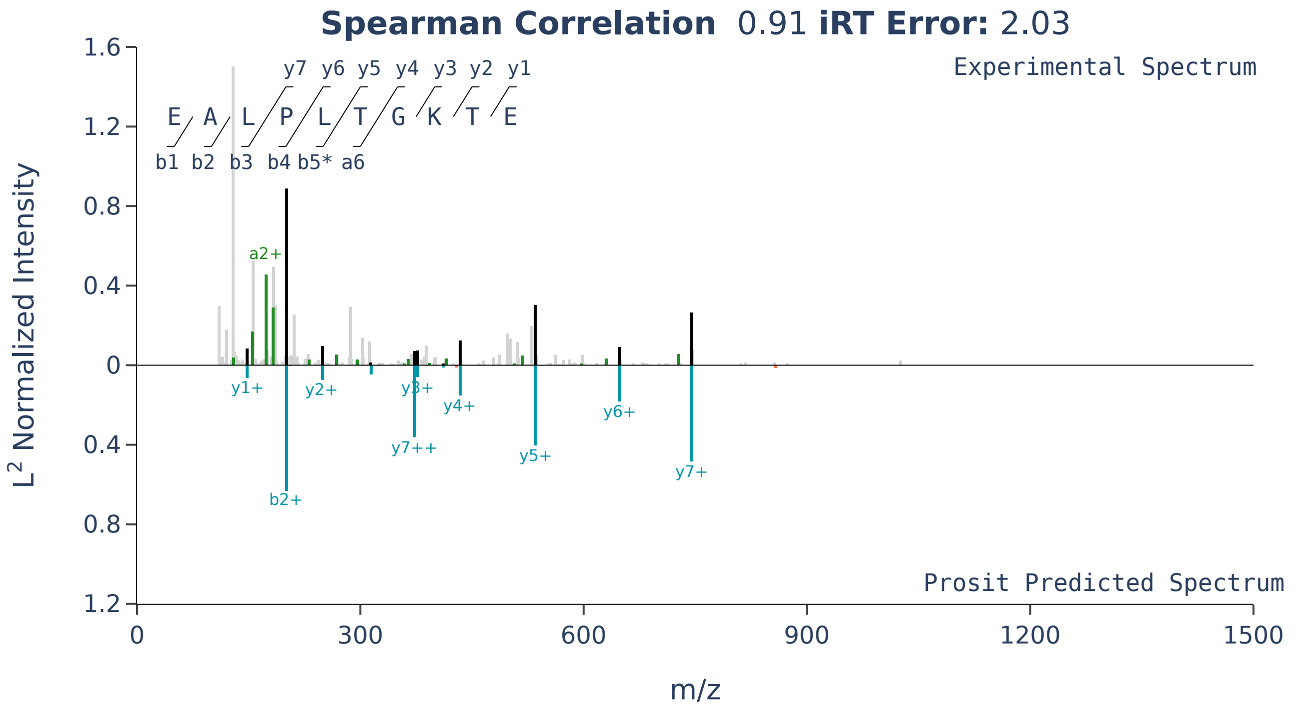

Source Ncheng_210623_230623_HFGoe_FFH_20S_25_1_A2_24h_R1 **Scan** 15564
Peptide LETLAEQVGV **Charge** 2 **Spectral Angle** 0.27
Spearman Correlation 0.37 **iRT Error:** 42.49

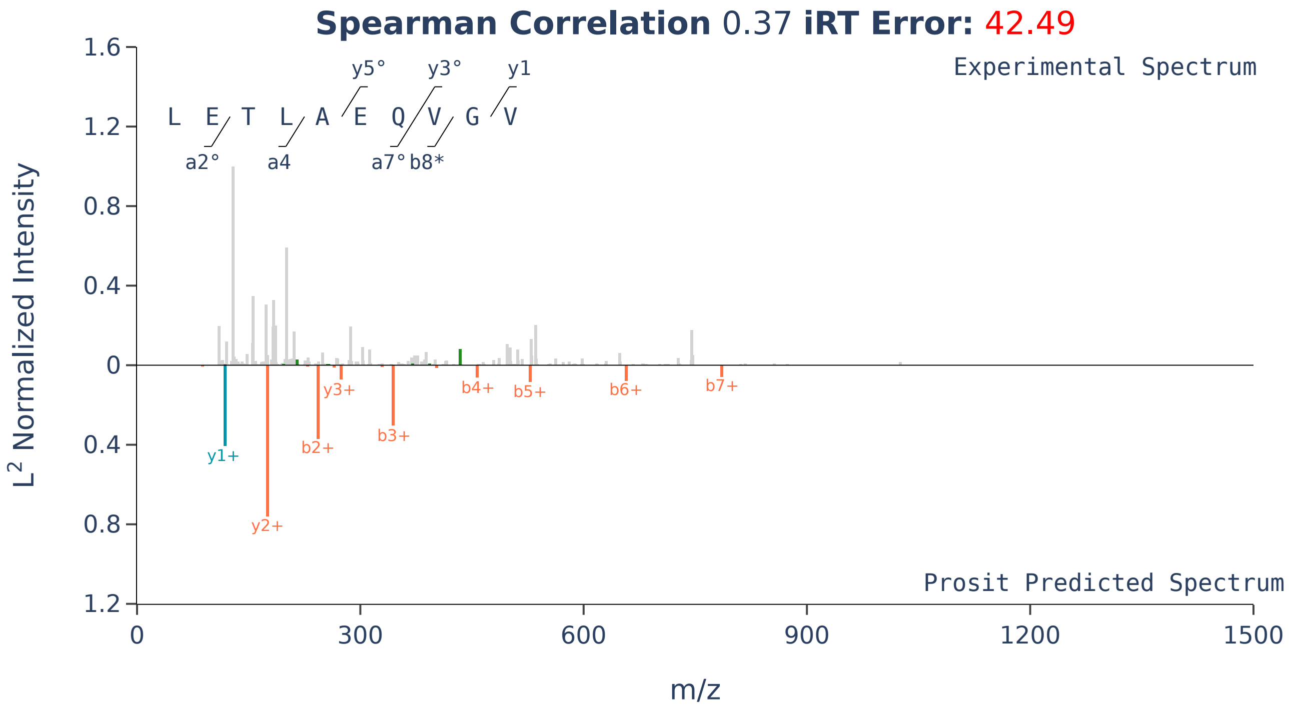

Source Ncheng_210623_230623_HFGoe_FFH_20S_25_1_A1_4h_R1 **Scan** 16055
Peptide QVQMTGQDAANTAKAFN **Charge** 3 **Spectral Angle** 0.82
Spearman Correlation 0.91 **iRT Error:** 2.27

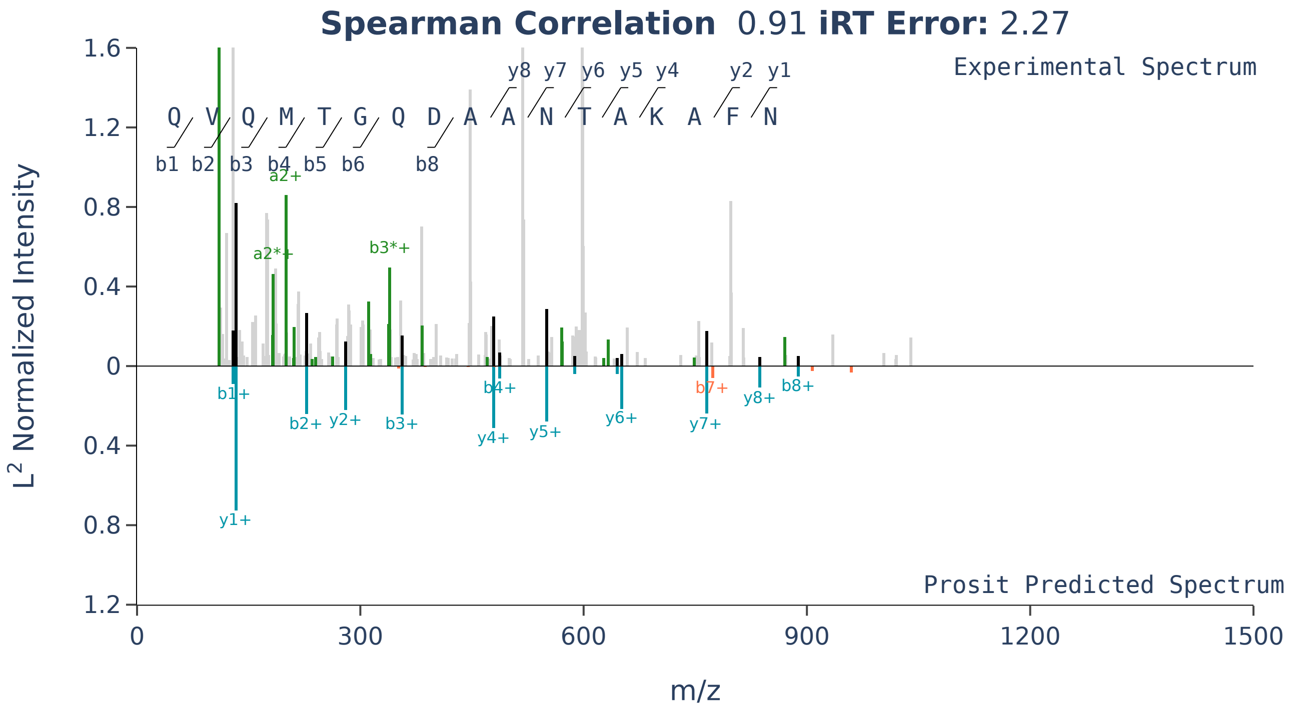

Source Ncheng_210623_230623_HFGoe_FFH_20S_25_1_A1_4h_R1 **Scan** 16055
Peptide LHVDEAMMDEIKQVH **Charge** 3 **Spectral Angle** 0.0
Spearman Correlation 0.0 **iRT Error:** 35.07

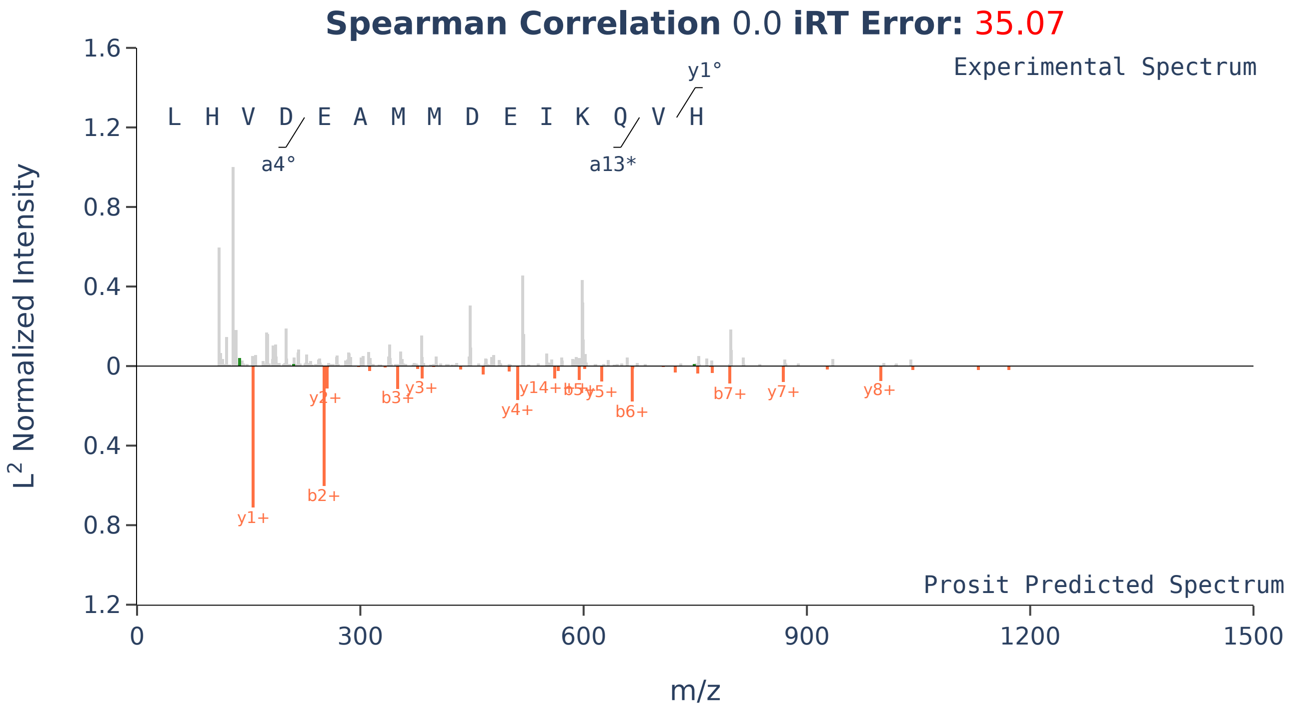

Source Ncheng_210623_230623_HFGoe_FFH_20S_25_1_A2_24h_R2 **Scan** 24170
Peptide PPAVVGAAL **Charge** 1 **Spectral Angle** 0.87
Spearman Correlation 0.89 **iRT Error:** 2.32

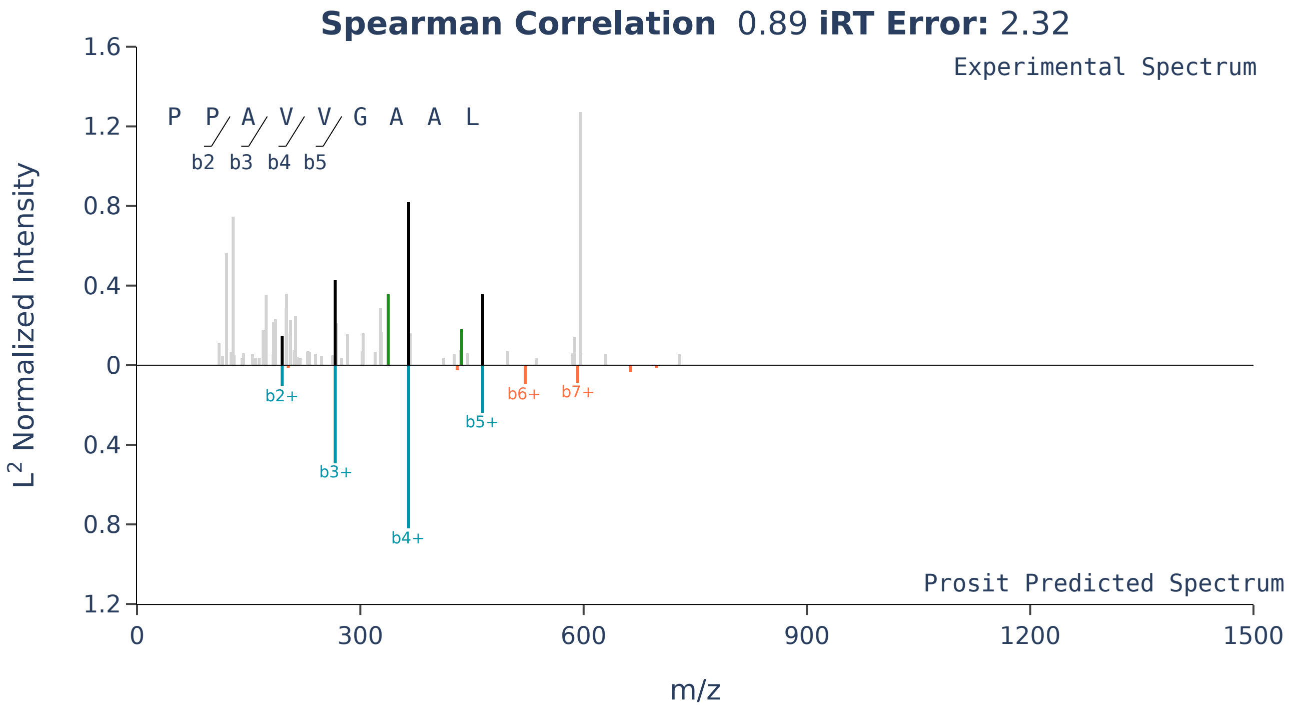

Source Ncheng_210623_230623_HFGoe_FFH_20S_25_1_A2_24h_R2 **Scan** 24170
Peptide AQPPAVVL **Charge** 1 **Spectral Angle** 0.91
Spearman Correlation 0.76 **iRT Error:** 1.95

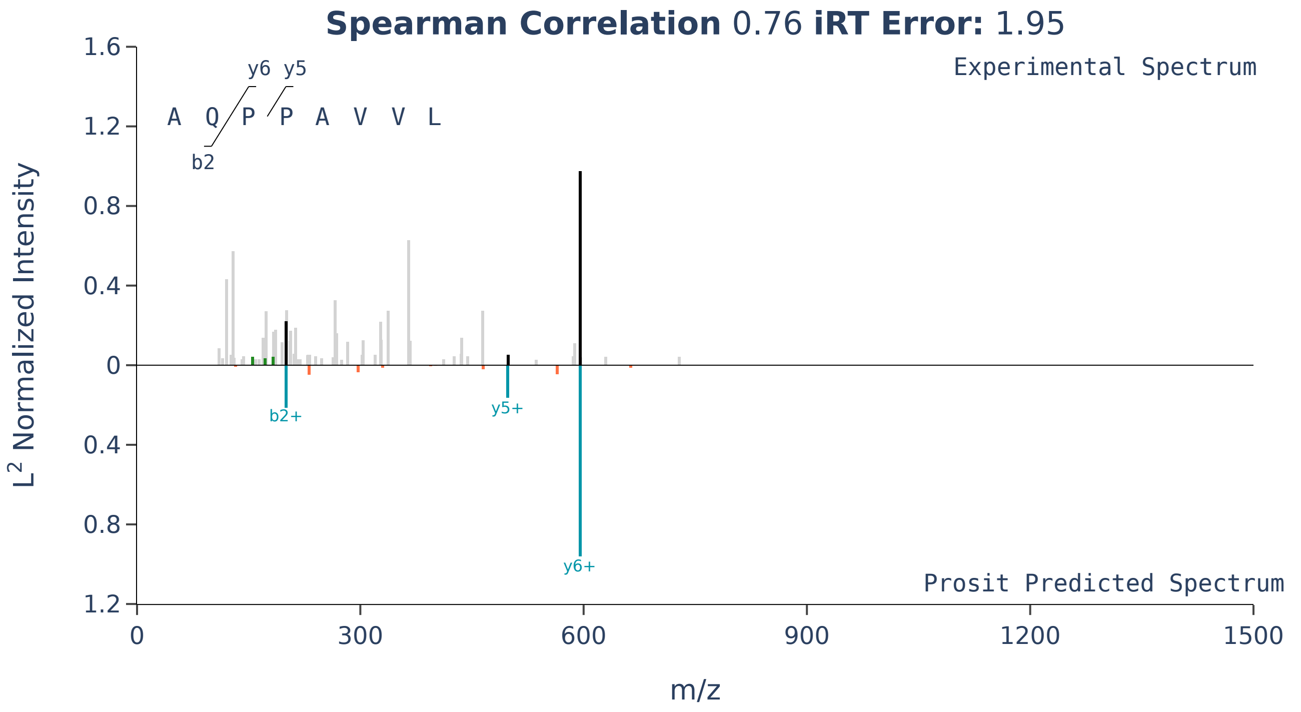

Source Ncheng_210623_230623_HFGoe_FFH_20S_25_1_A1_2h_R1 **Scan** 15407
Peptide EIKQVHASINPEV **Charge** 2 **Spectral Angle** 0.85
Spearman Correlation 0.87 **iRT Error:** 2.33

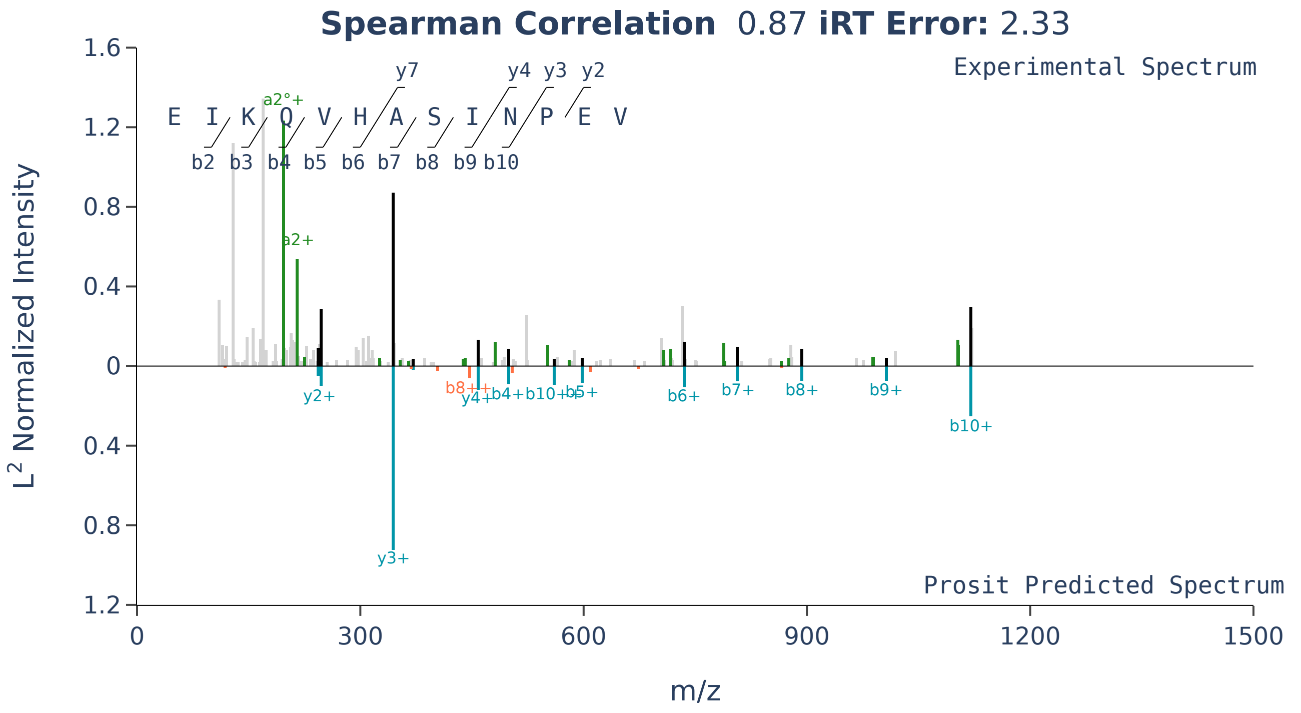

Source Ncheng_210623_230623_HFGoe_FFH_20S_25_1_A1_2h_R1 **Scan** 15407
Peptide EIKQVHASINPVE **Charge** 2 **Spectral Angle** 0.68
Spearman Correlation 0.59 **iRT Error:** 8.08

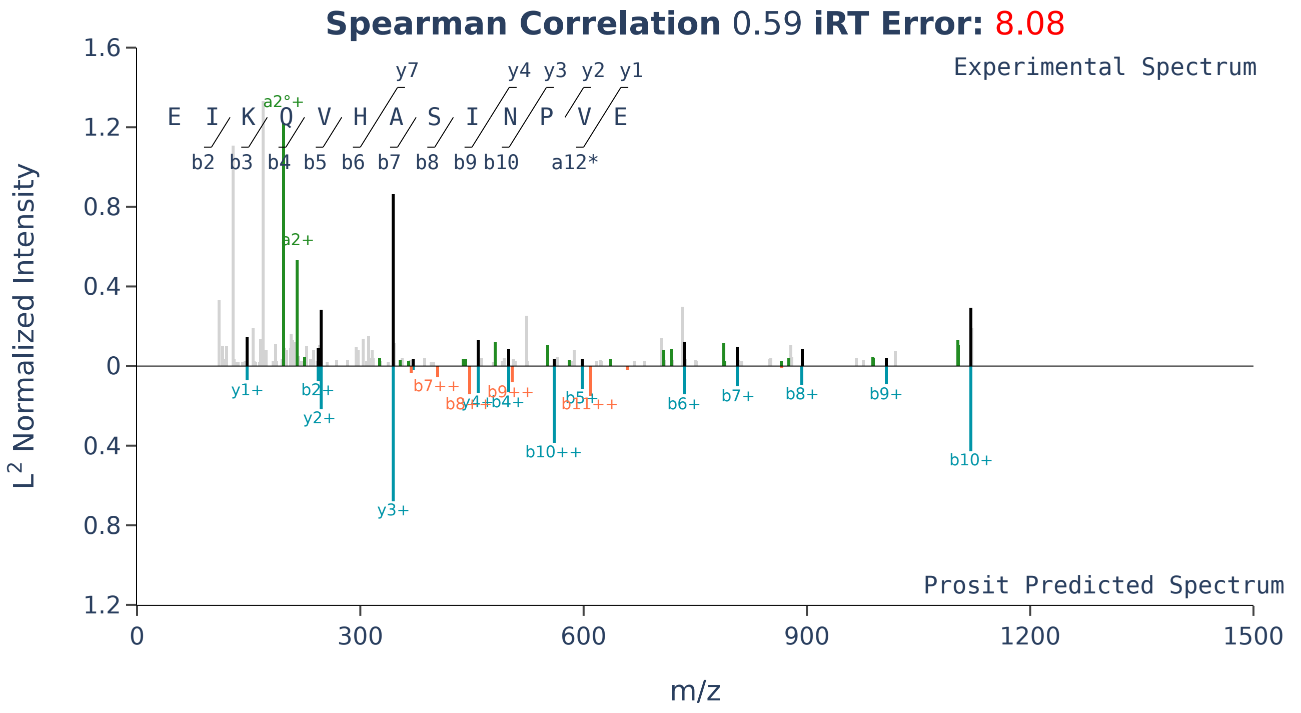

Source Ncheng_210623_230623_HFGoe_FFH_20S_25_1_A2_4h_R1 **Scan** 20857
Peptide QTLEPFHPDRI **Charge** 3 **Spectral Angle** 0.81
Spearman Correlation 0.85 **iRT Error:** 2.34

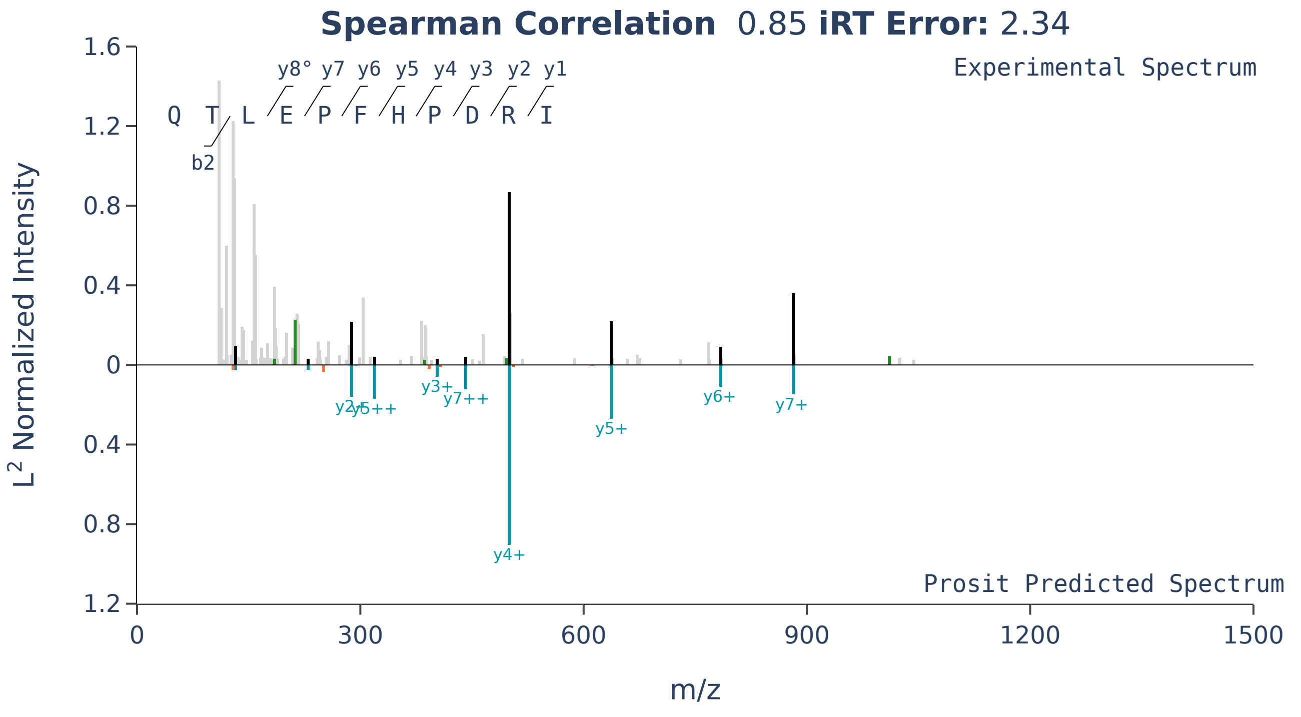

Source Ncheng_210623_230623_HFGoe_FFH_20S_25_1_A2_4h_R1 **Scan** 20857
Peptide ALEPFHPDRIAS **Charge** 3 **Spectral Angle** 0.2
Spearman Correlation 0.51 **iRT Error:** 12.18

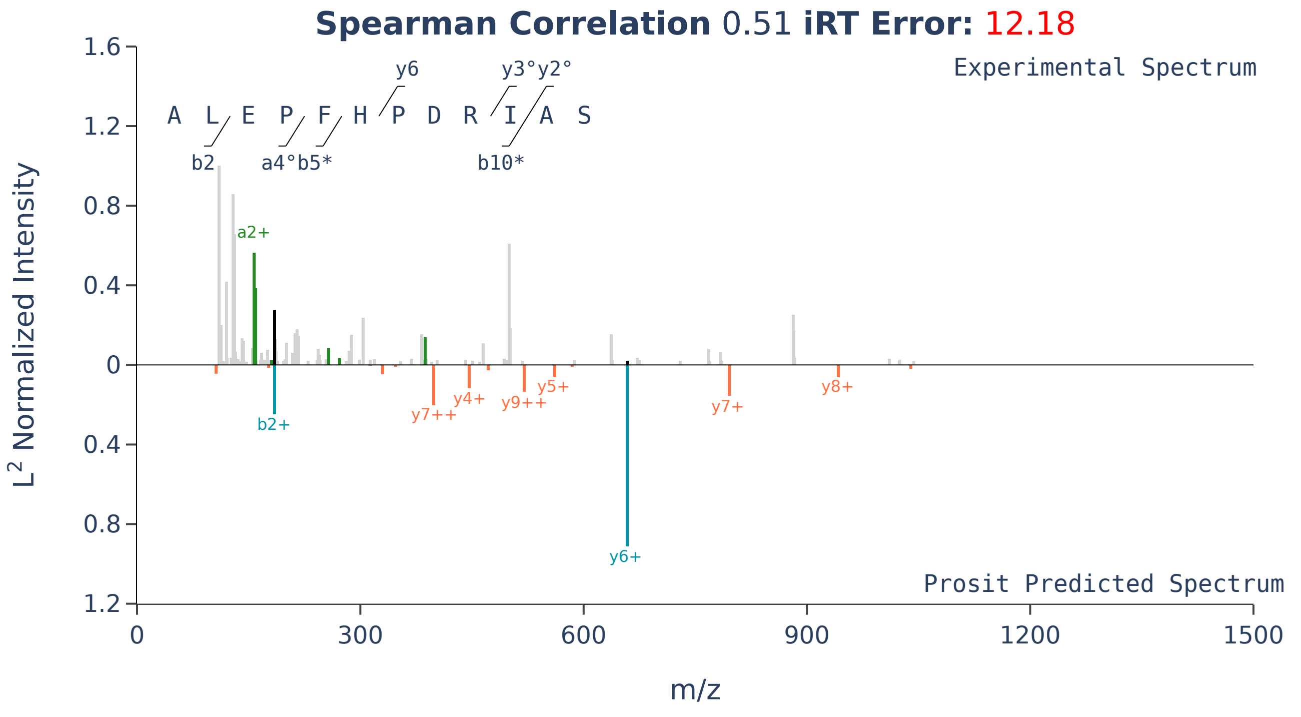

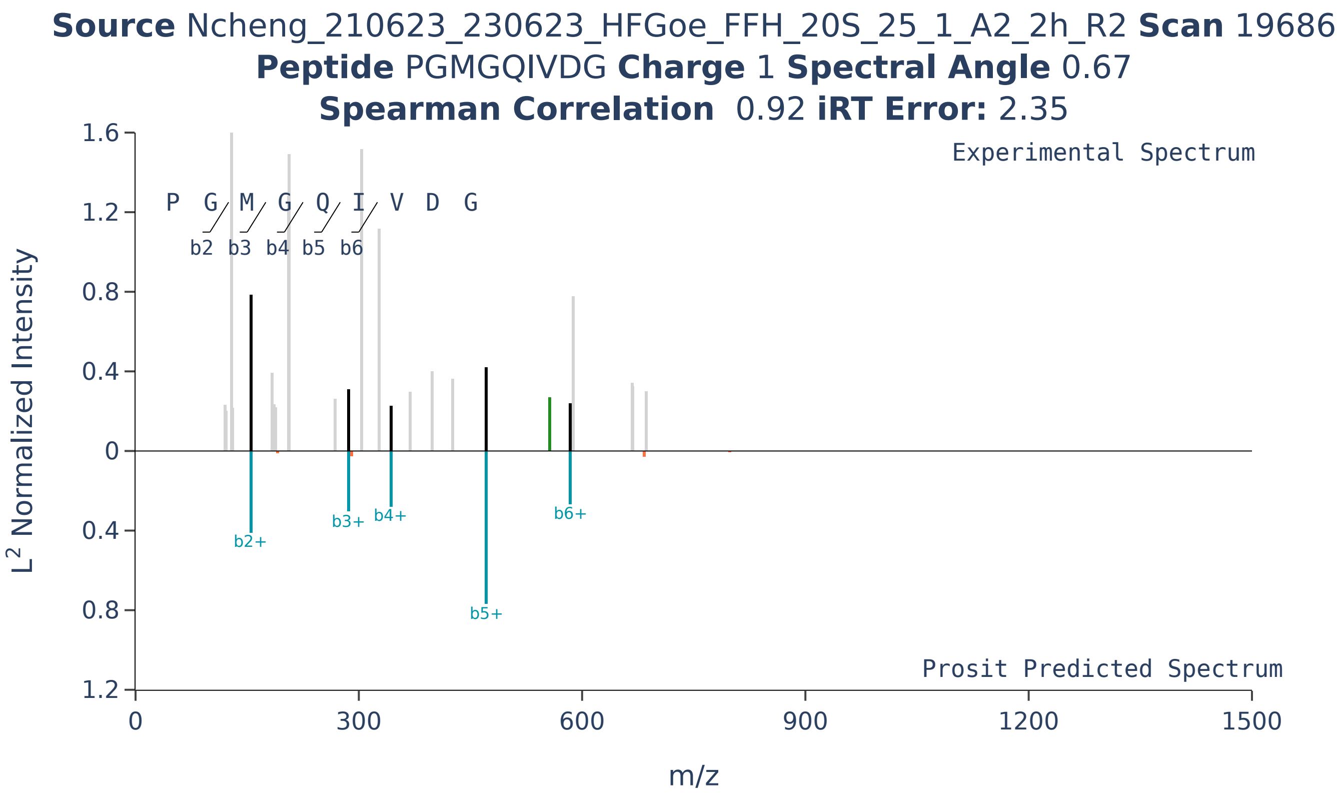
Source Ncheng_210623_230623_HFGoe_FFH_20S_25_1_A2_2h_R2 Scan 19686
Peptide PGMGQIVDG Charge 1 Spectral Angle 0.67
Spearman Correlation 0.92 iRT Error: 2.35
Experimental Spectrum
Prosit Predicted Spectrum
L² Normalized Intensity
m/z

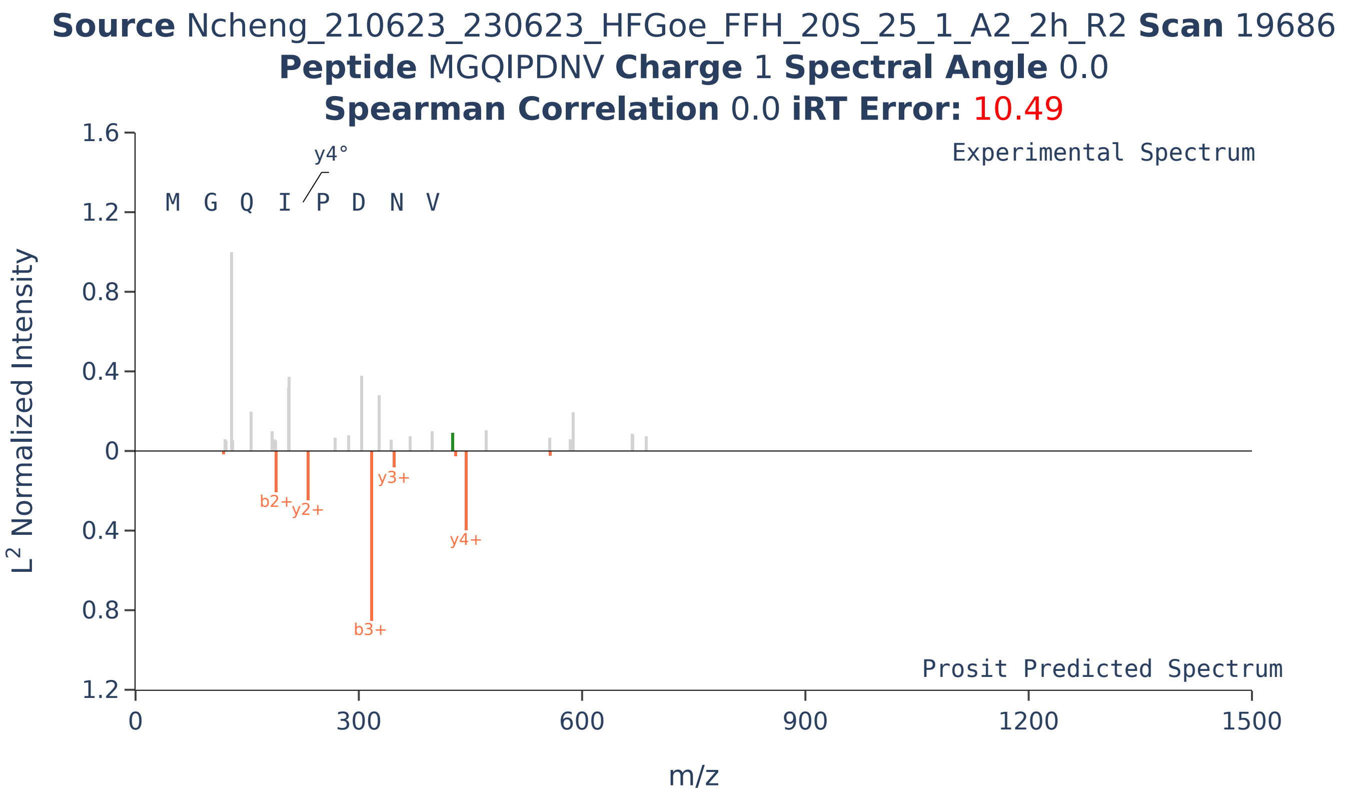
Source Ncheng_210623_230623_HFGoe_FFH_20S_25_1_A2_2h_R2 Scan 19686
Peptide MGQIPDNV Charge 1 Spectral Angle 0.0
Spearman Correlation 0.0 iRT Error: 10.49
Experimental Spectrum
Prosit Predicted Spectrum
L² Normalized Intensity
m/z

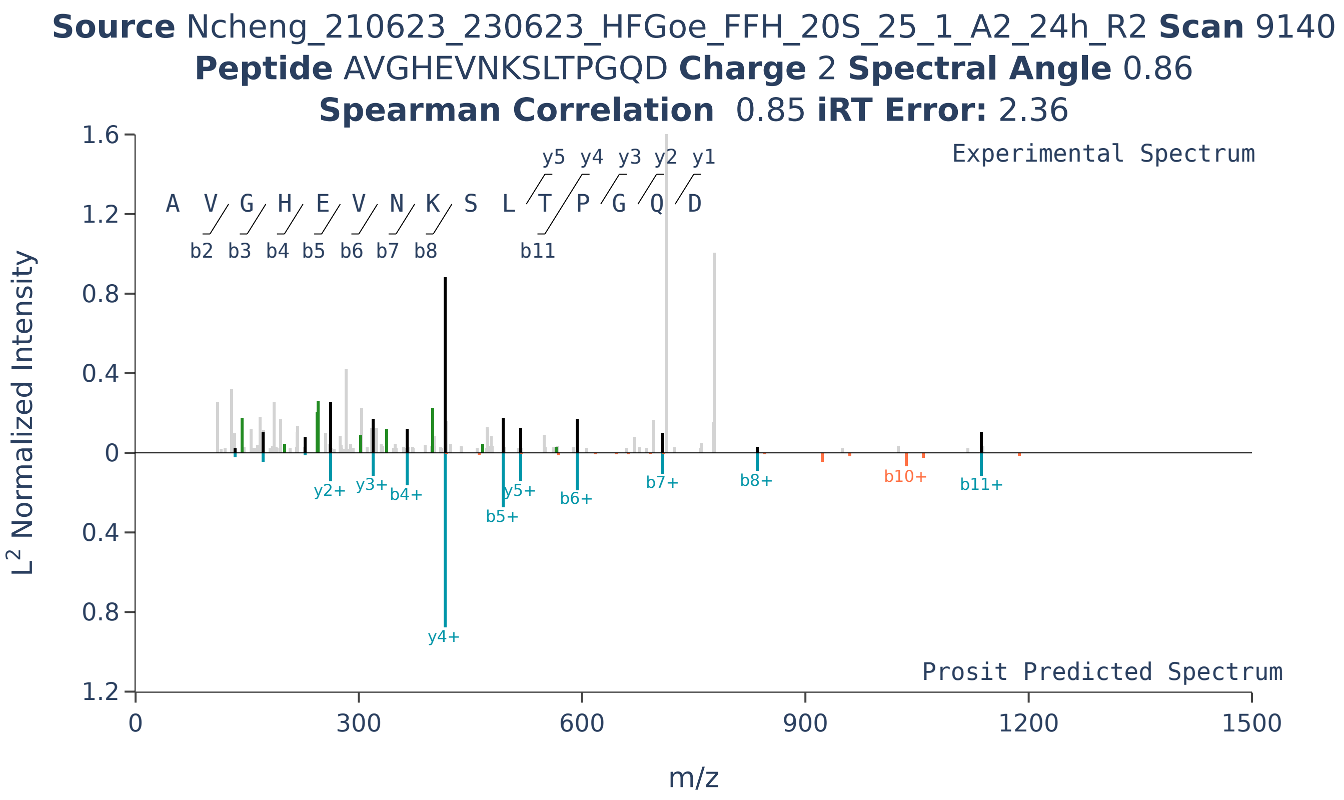
Source Ncheng_210623_230623_HFGoe_FFH_20S_25_1_A2_24h_R2 Scan 9140
Peptide AVGHEVNKSLTPGQD Charge 2 Spectral Angle 0.86
Spearman Correlation 0.85 iRT Error: 2.36
Experimental Spectrum
Prosit Predicted Spectrum
L² Normalized Intensity
m/z

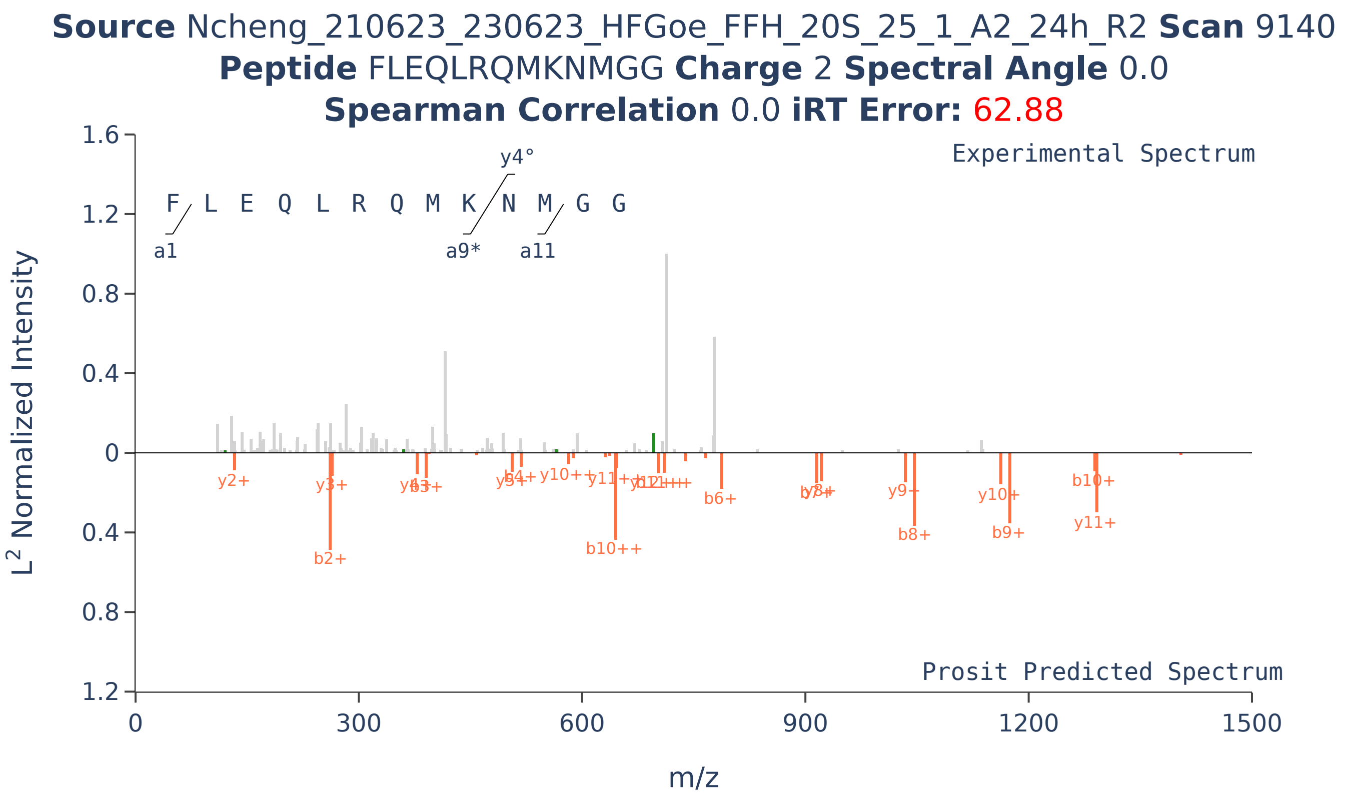
Source Ncheng_210623_230623_HFGoe_FFH_20S_25_1_A2_24h_R2 Scan 9140
Peptide FLEQLRQMKNMGG Charge 2 Spectral Angle 0.0
Spearman Correlation 0.0 iRT Error: 62.88
Experimental Spectrum
Prosit Predicted Spectrum
L² Normalized Intensity
m/z

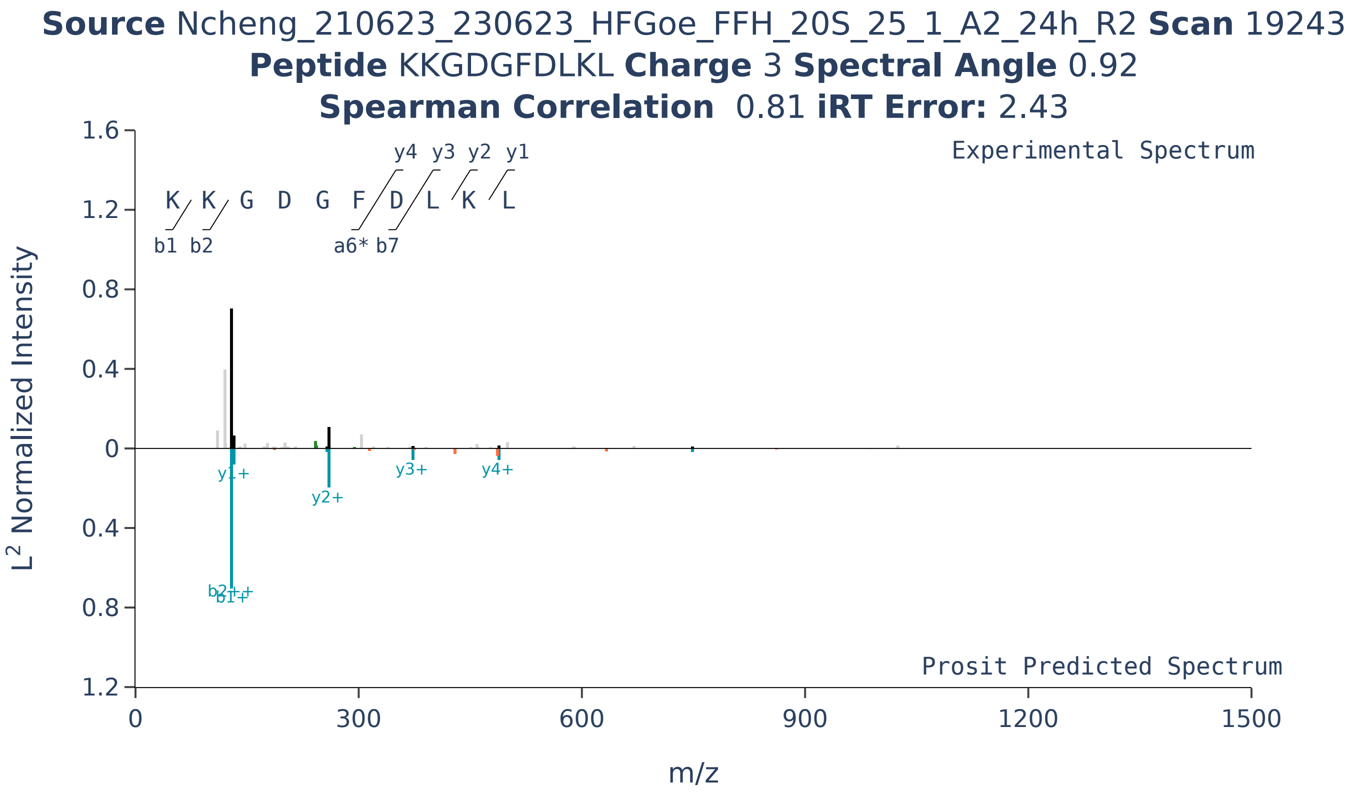
Source Ncheng_210623_230623_HFGoe_FFH_20S_25_1_A2_24h_R2 Scan 19243
Peptide KKGDGFDLKL Charge 3 Spectral Angle 0.92
Spearman Correlation 0.81 iRT Error: 2.43
Experimental Spectrum
Prosit Predicted Spectrum
L² Normalized Intensity
m/z

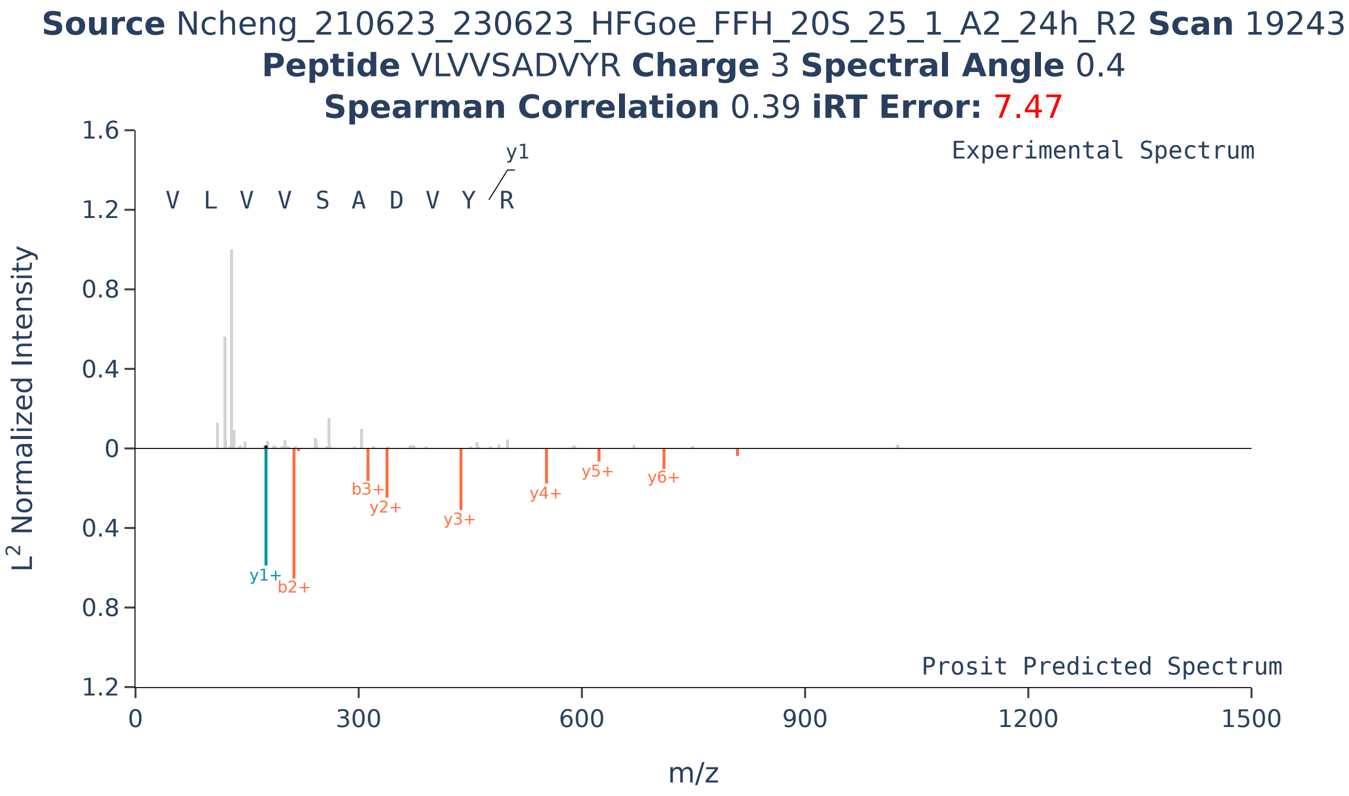
Source Ncheng_210623_230623_HFGoe_FFH_20S_25_1_A2_24h_R2 Scan 19243
Peptide VLVVSADVYR Charge 3 Spectral Angle 0.4
Spearman Correlation 0.39 iRT Error: 7.47
Experimental Spectrum
Prosit Predicted Spectrum
L² Normalized Intensity
m/z

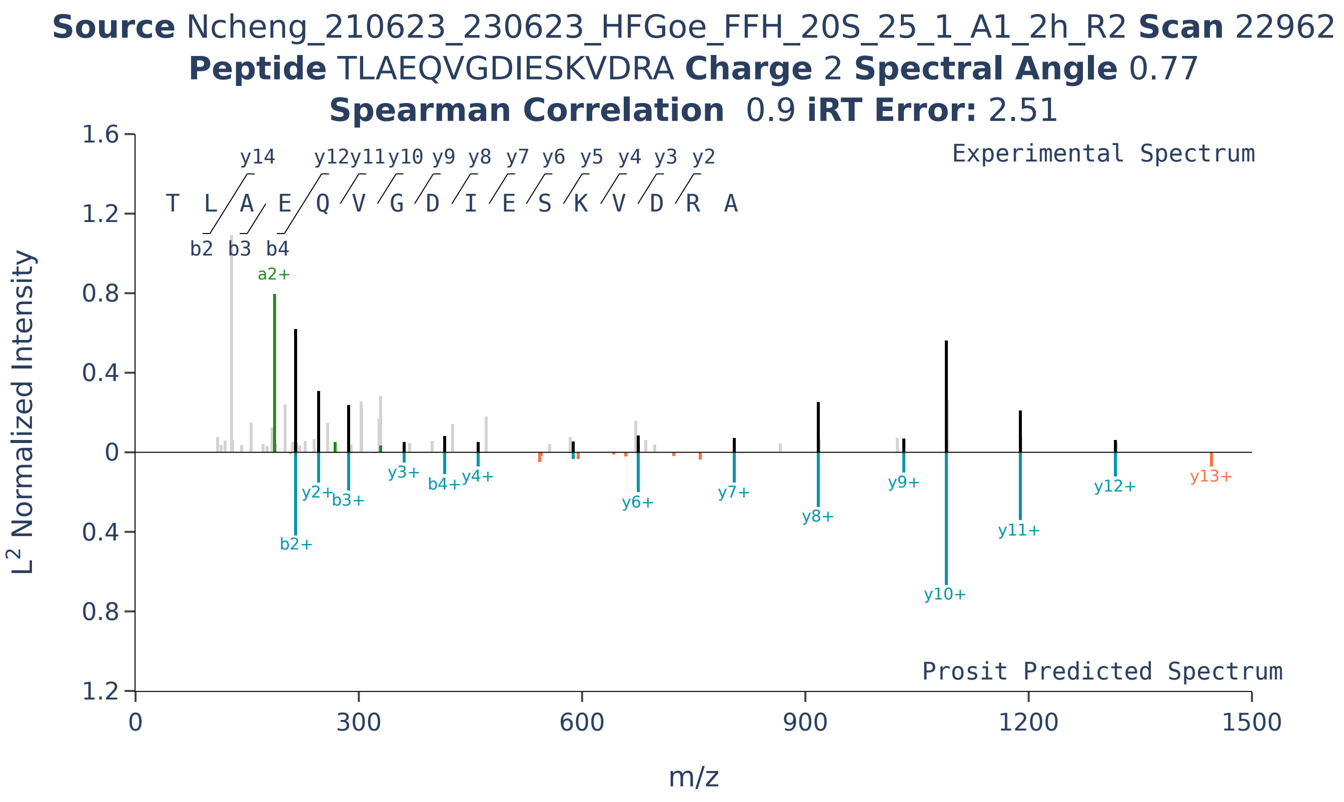
Source Ncheng_210623_230623_HFGoe_FFH_20S_25_1_A1_2h_R2 Scan 22962
Peptide TLAEQVGDIESKVDRA Charge 2 Spectral Angle 0.77
Spearman Correlation 0.9 iRT Error: 2.51
Experimental Spectrum
Prosit Predicted Spectrum
L² Normalized Intensity
m/z

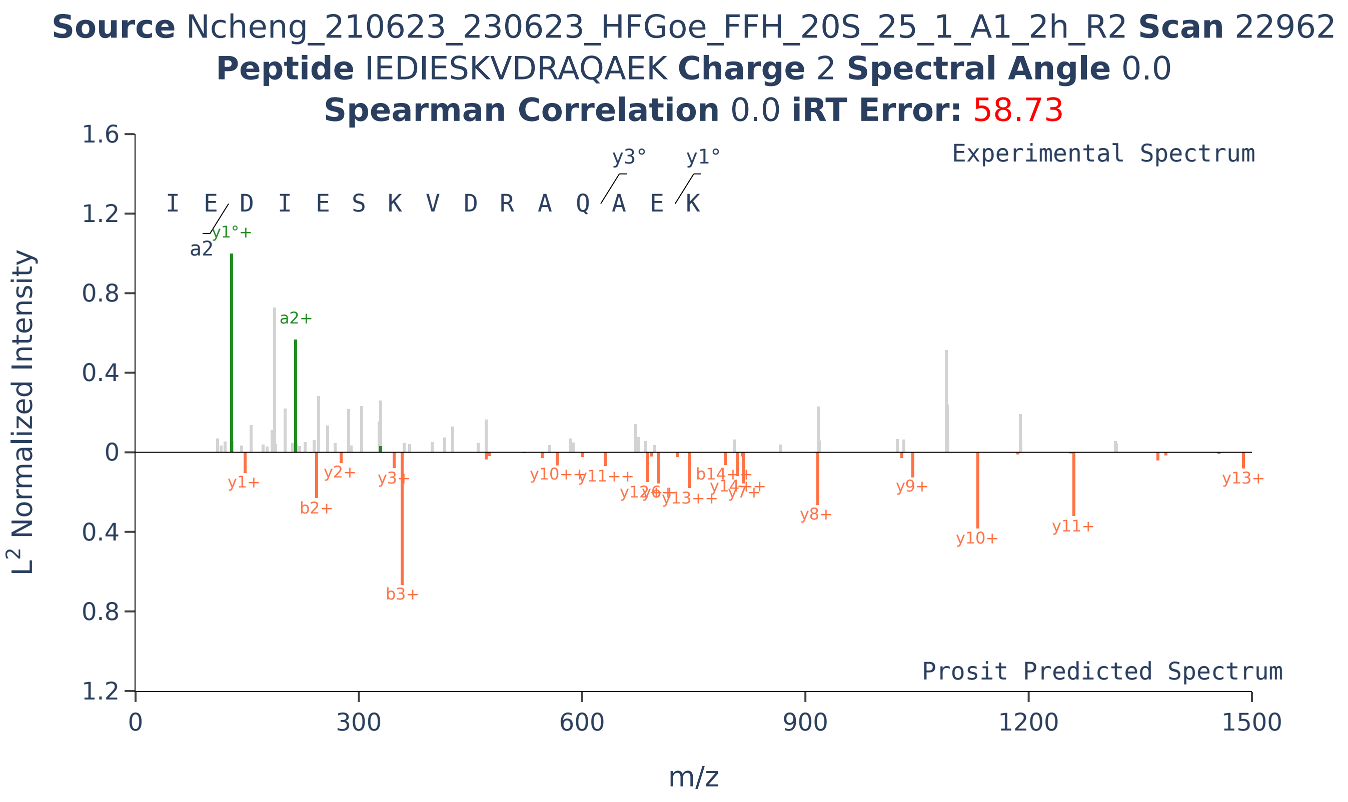
Source Ncheng_210623_230623_HFGoe_FFH_20S_25_1_A1_2h_R2 Scan 22962
Peptide IEDIESKVDRAQAEK Charge 2 Spectral Angle 0.0
Spearman Correlation 0.0 iRT Error: 58.73
Experimental Spectrum
Prosit Predicted Spectrum
L² Normalized Intensity
m/z

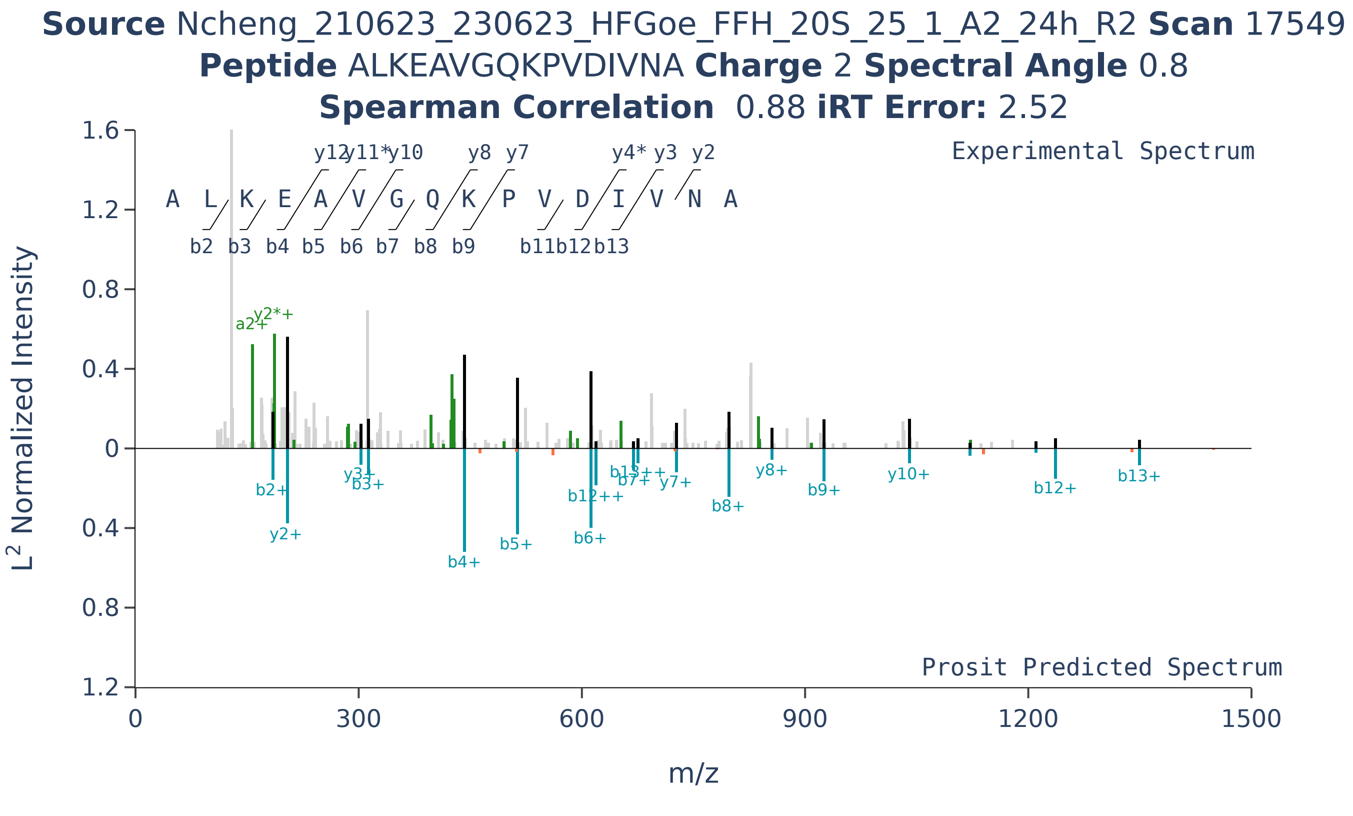
Source Ncheng_210623_230623_HFGoe_FFH_20S_25_1_A2_24h_R2 Scan 17549
Peptide ALKEAVGQKPVDIVNA Charge 2 Spectral Angle 0.8
Spearman Correlation 0.88 iRT Error: 2.52
Experimental Spectrum
Prosit Predicted Spectrum
L² Normalized Intensity
m/z

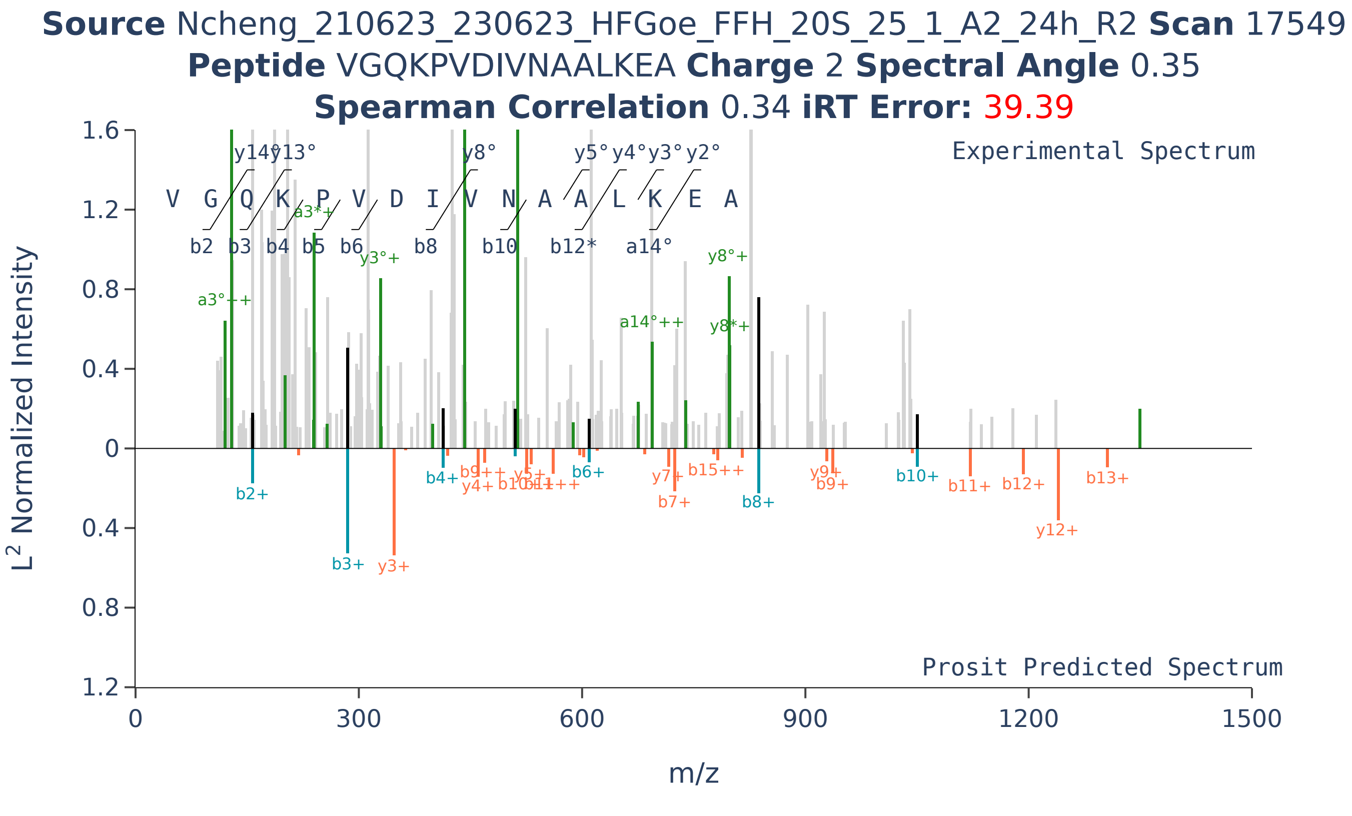
Source Ncheng_210623_230623_HFGoe_FFH_20S_25_1_A2_24h_R2 Scan 17549
Peptide VGQKPVDIVNAALKEA Charge 2 Spectral Angle 0.35
Spearman Correlation 0.34 iRT Error: 39.39
Experimental Spectrum
Prosit Predicted Spectrum
L² Normalized Intensity
m/z

Source Ncheng_210623_230623_HFGoe_FFH_20S_25_1_A2_24h_R2 **Scan** 9209
Peptide MKNGGMASL **Charge** 2 **Spectral Angle** 0.76
Spearman Correlation 0.87 **iRT Error:** 2.54

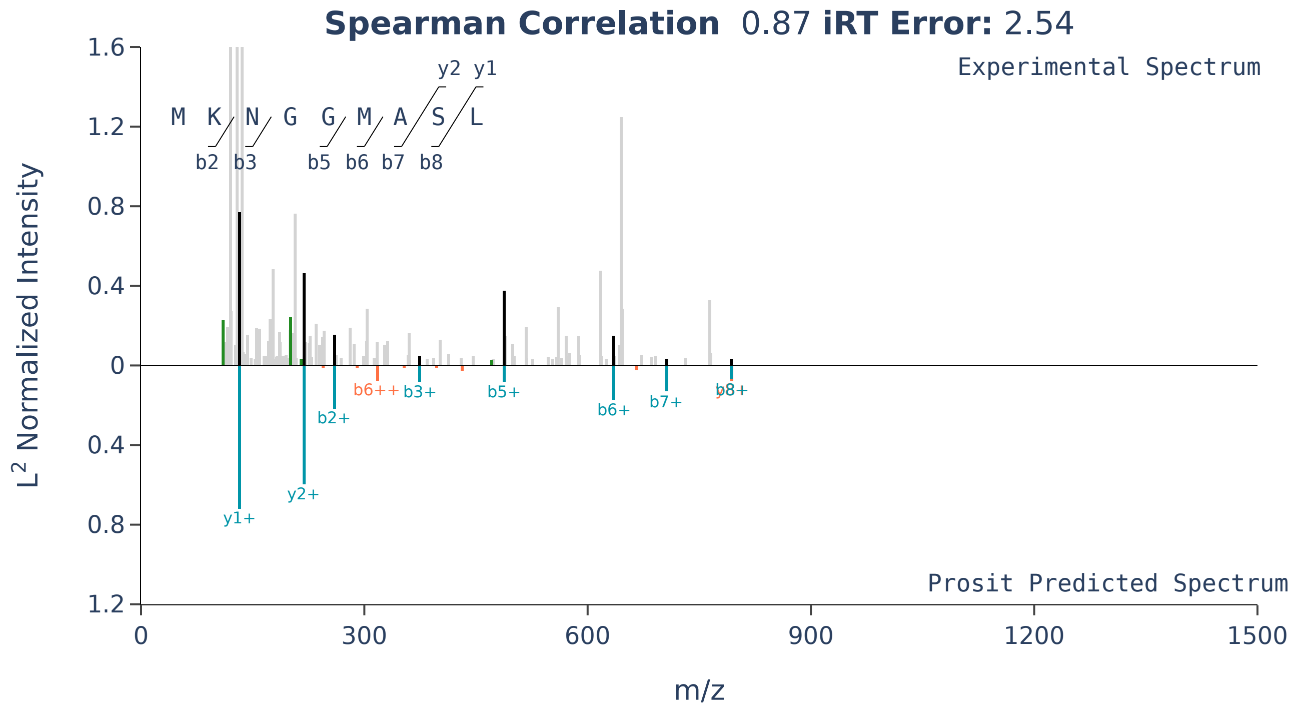

Source Ncheng_210623_230623_HFGoe_FFH_20S_25_1_A2_24h_R2 **Scan** 9209
Peptide KNMGGMASL **Charge** 2 **Spectral Angle** 0.28
Spearman Correlation 0.82 **iRT Error:** 21.92

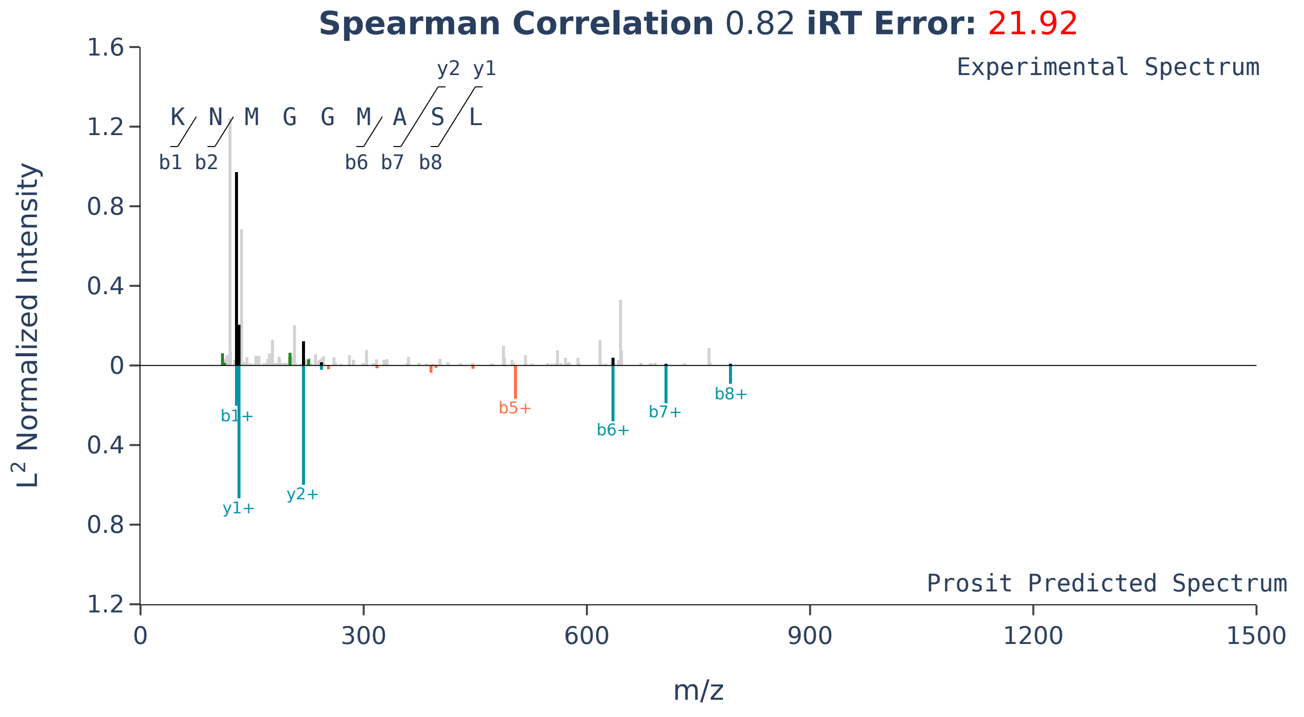

Source Ncheng_210623_230623_HFGoe_FFH_20S_25_1_A1_24h_R1 **Scan** 5629
Peptide KNMGGMAR **Charge** 2 **Spectral Angle** 0.8
Spearman Correlation 0.96 **iRT Error:** 2.57

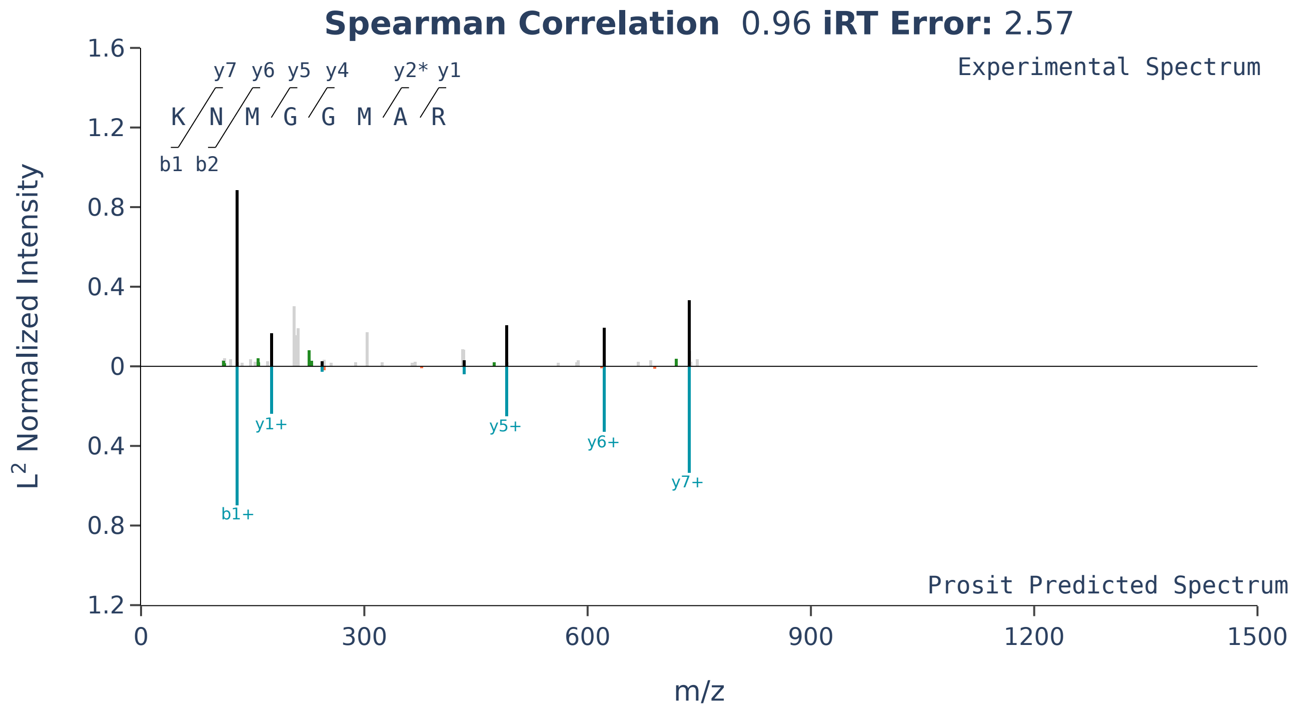

Source Ncheng_210623_230623_HFGoe_FFH_20S_25_1_A1_24h_R1 **Scan** 5629
Peptide RQMKNMG **Charge** 2 **Spectral Angle** 0.01
Spearman Correlation -0.41 **iRT Error:** 105.69

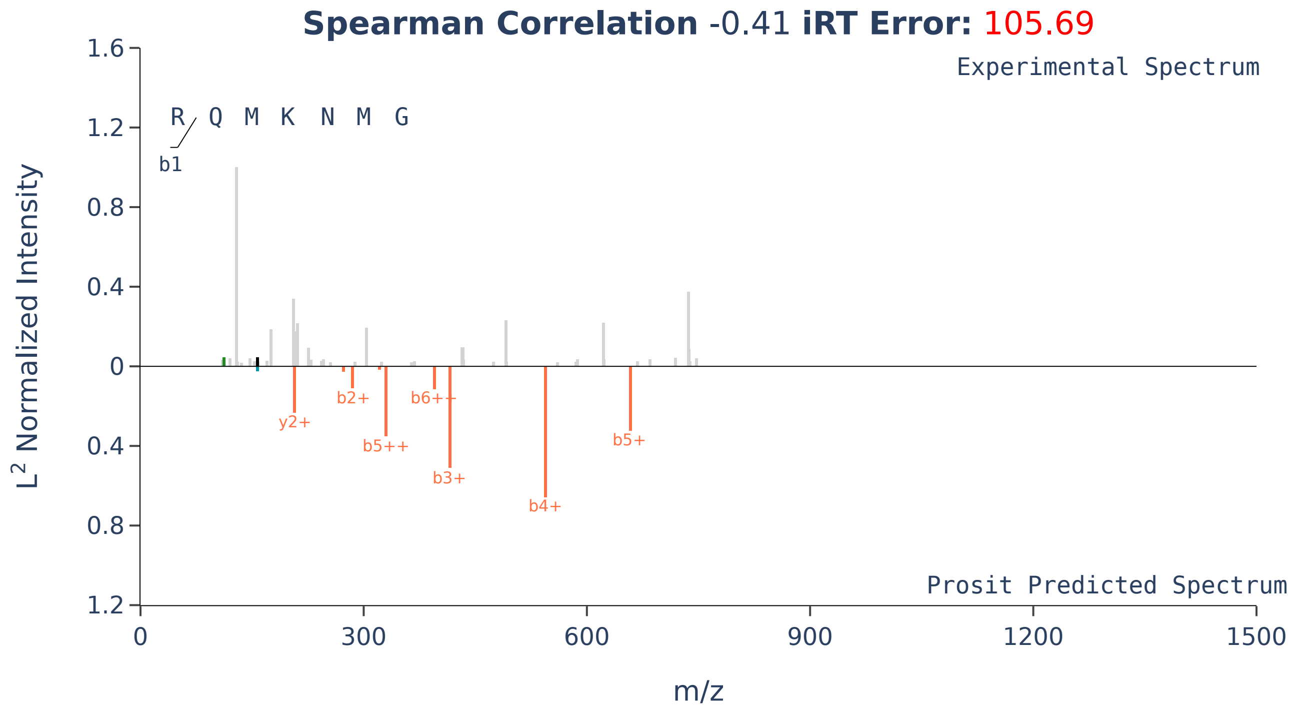

Source Ncheng_210623_230623_HFGoe_FFH_20S_25_1_A2_2h_R1 **Scan** 18566
Peptide MKGLPGMGQIPDNVKSQ **Charge** 3 **Spectral Angle** 0.78
Spearman Correlation 0.91 **iRT Error:** 2.6

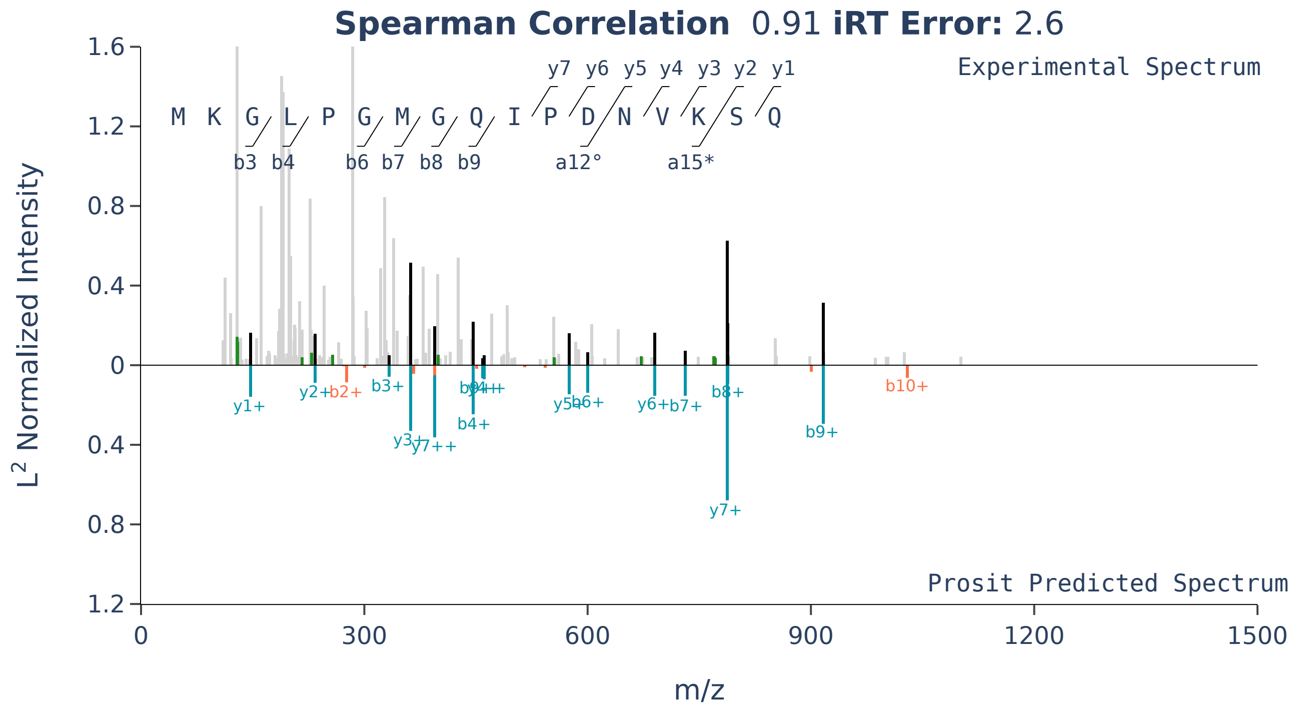

Source Ncheng_210623_230623_HFGoe_FFH_20S_25_1_A2_2h_R1 **Scan** 18566
Peptide GKLPGMGQIPDNVKSQM **Charge** 3 **Spectral Angle** 0.22
Spearman Correlation 0.34 **iRT Error:** 6.56

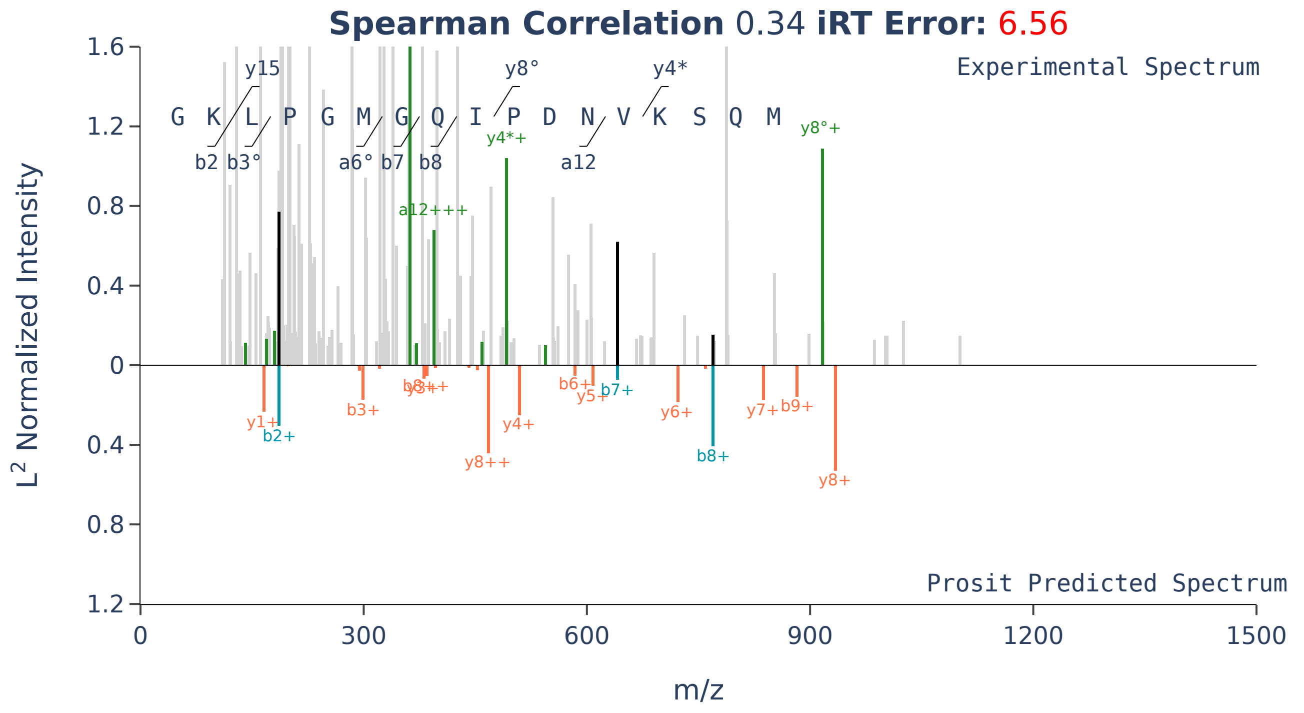

Source Ncheng_210623_230623_HFGoe_FFH_20S_25_1_A2_24h_R1 **Scan** 12586
Peptide MPPGFPGRSR **Charge** 2 **Spectral Angle** 0.79
Spearman Correlation 0.91 **iRT Error:** 2.63

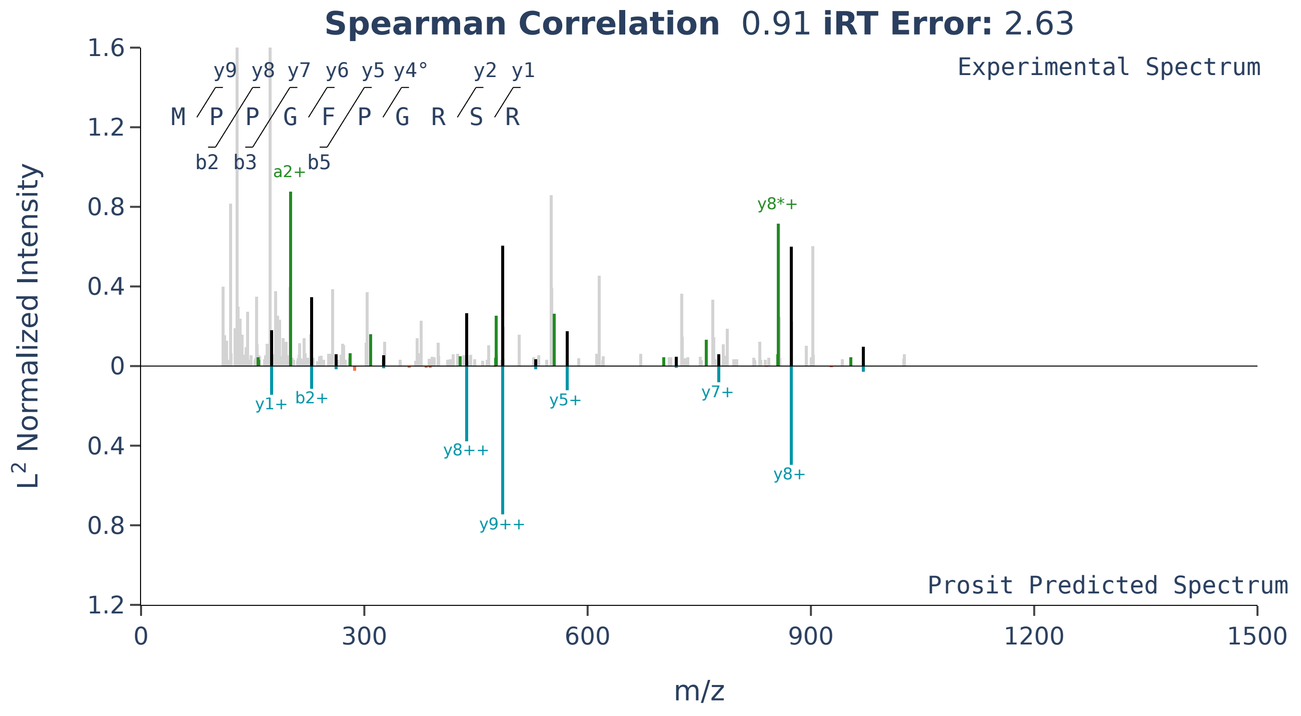

Source Ncheng_210623_230623_HFGoe_FFH_20S_25_1_A2_24h_R1 **Scan** 12586
Peptide ENQTLNLAAQ **Charge** 2 **Spectral Angle** 0.0
Spearman Correlation 0.0 **iRT Error:** 23.55

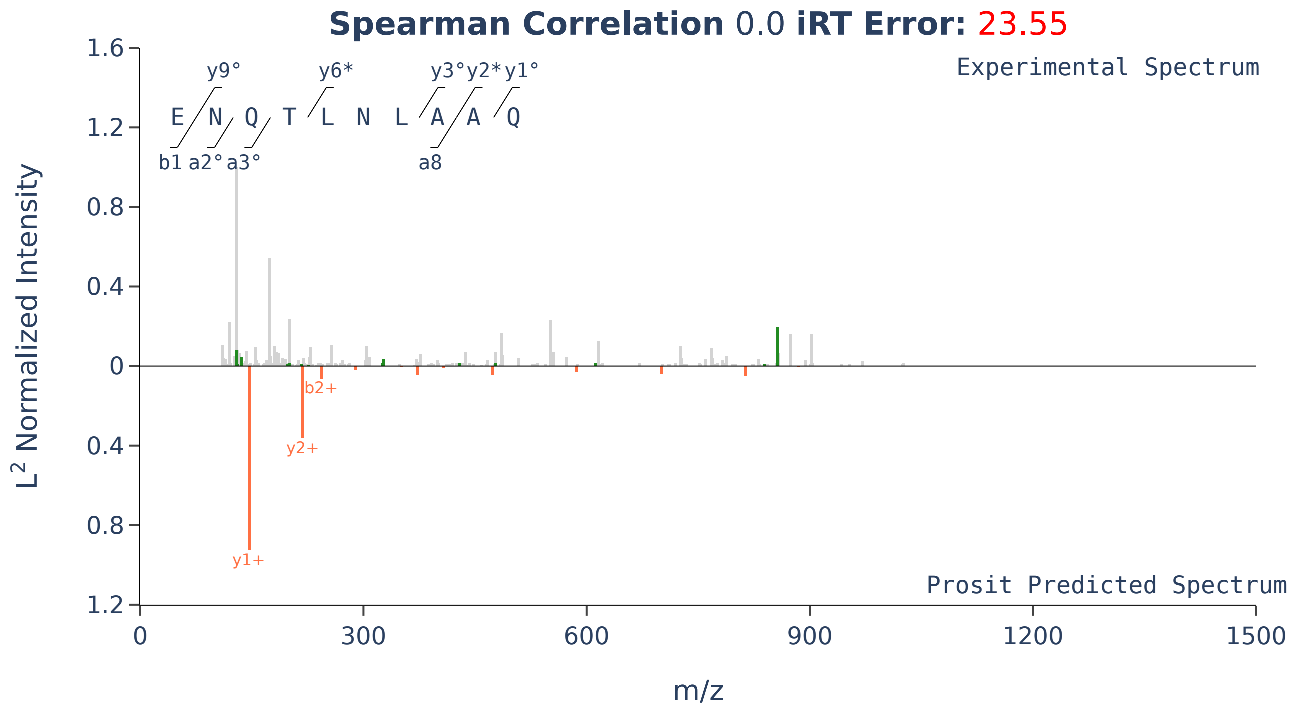

Source Ncheng_210623_230623_HFGoe_FFH_20S_25_1_A2_4h_R2 **Scan** 34440
Peptide PLTGVVLTLEA **Charge** 1 **Spectral Angle** 0.79
Spearman Correlation 0.91 **iRT Error:** 2.82

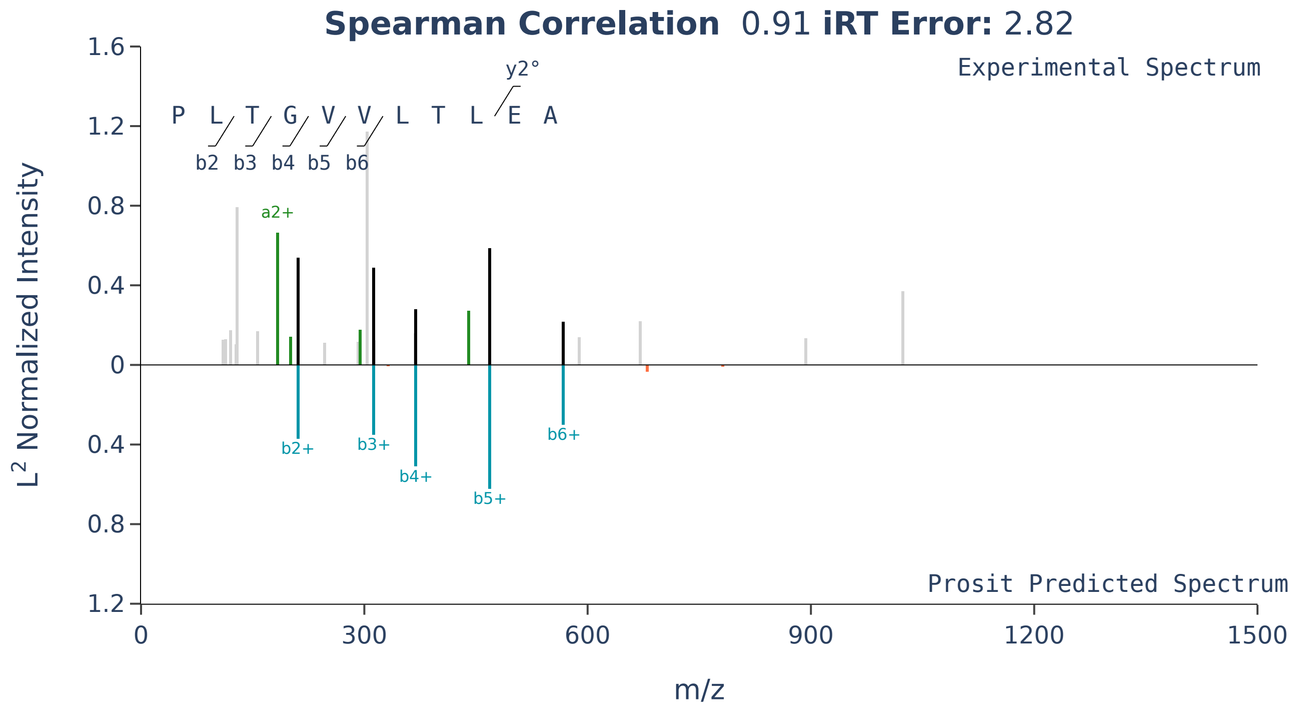

Source Ncheng_210623_230623_HFGoe_FFH_20S_25_1_A2_4h_R2 **Scan** 34440
Peptide EALPLTGVVLT **Charge** 1 **Spectral Angle** 0.6
Spearman Correlation 0.48 **iRT Error:** 4.31

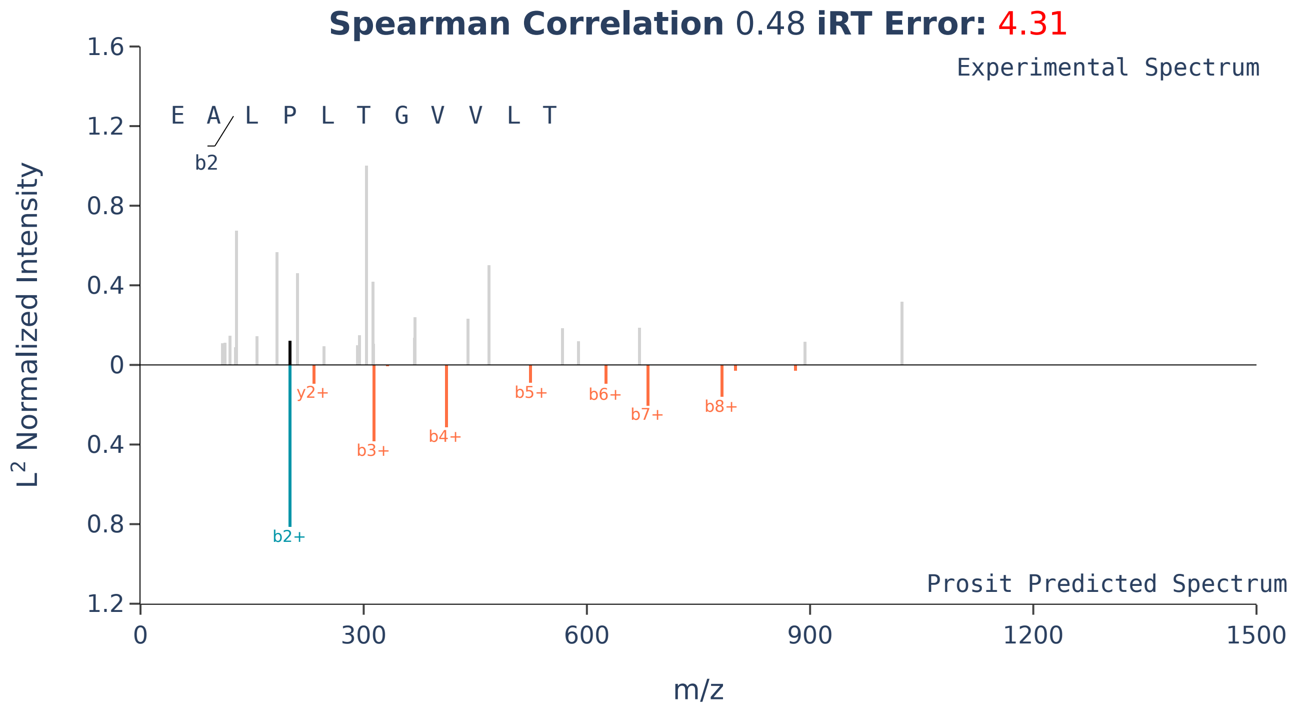

Source Ncheng_210623_230623_HFGoe_FFH_20S_25_1_A2_2h_R1 **Scan** 26158
Peptide EALPLTGAMTGQDAANTAKAFN **Charge** 3 **Spectral Angle** 0.91
Spearman Correlation 0.87 **iRT Error:** 2.85

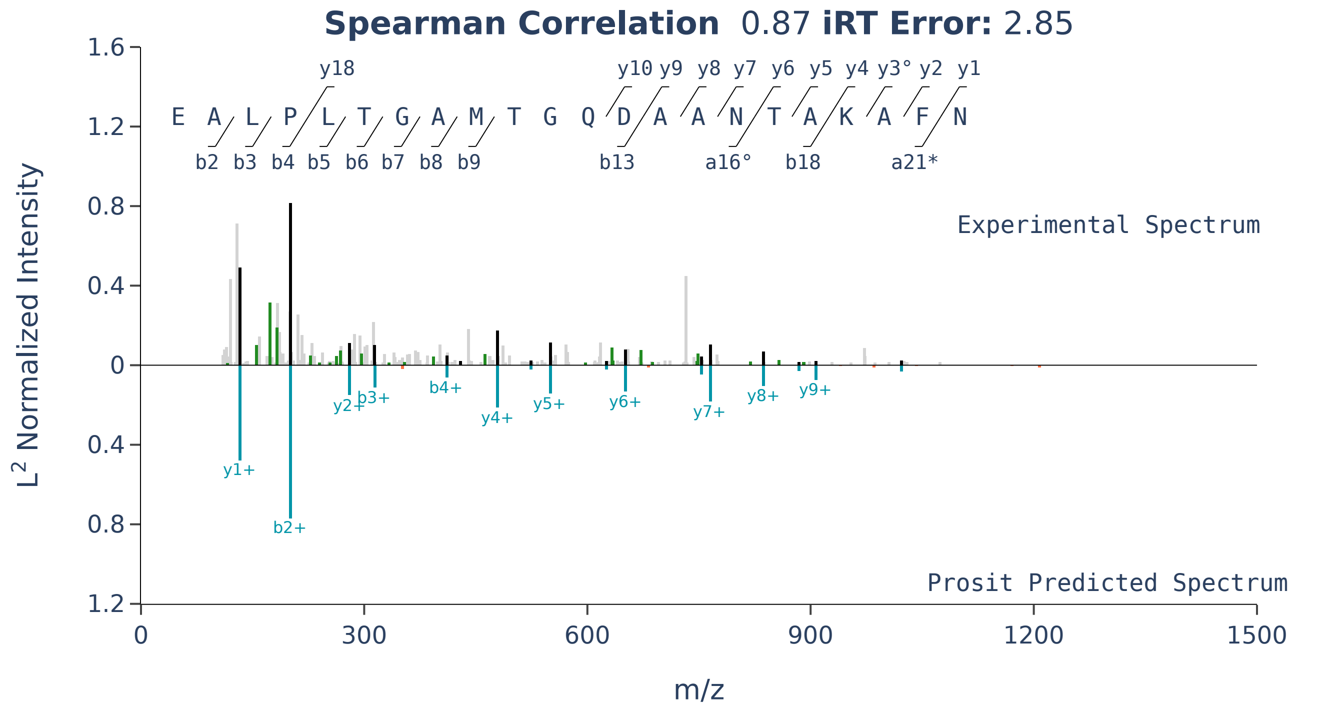

Source Ncheng_210623_230623_HFGoe_FFH_20S_25_1_A2_2h_R1 **Scan** 26158
Peptide AMTGQDAANTAKAFNEALPLTG **Charge** 3 **Spectral Angle** 0.02
Spearman Correlation 0.06 **iRT Error:** 5.7

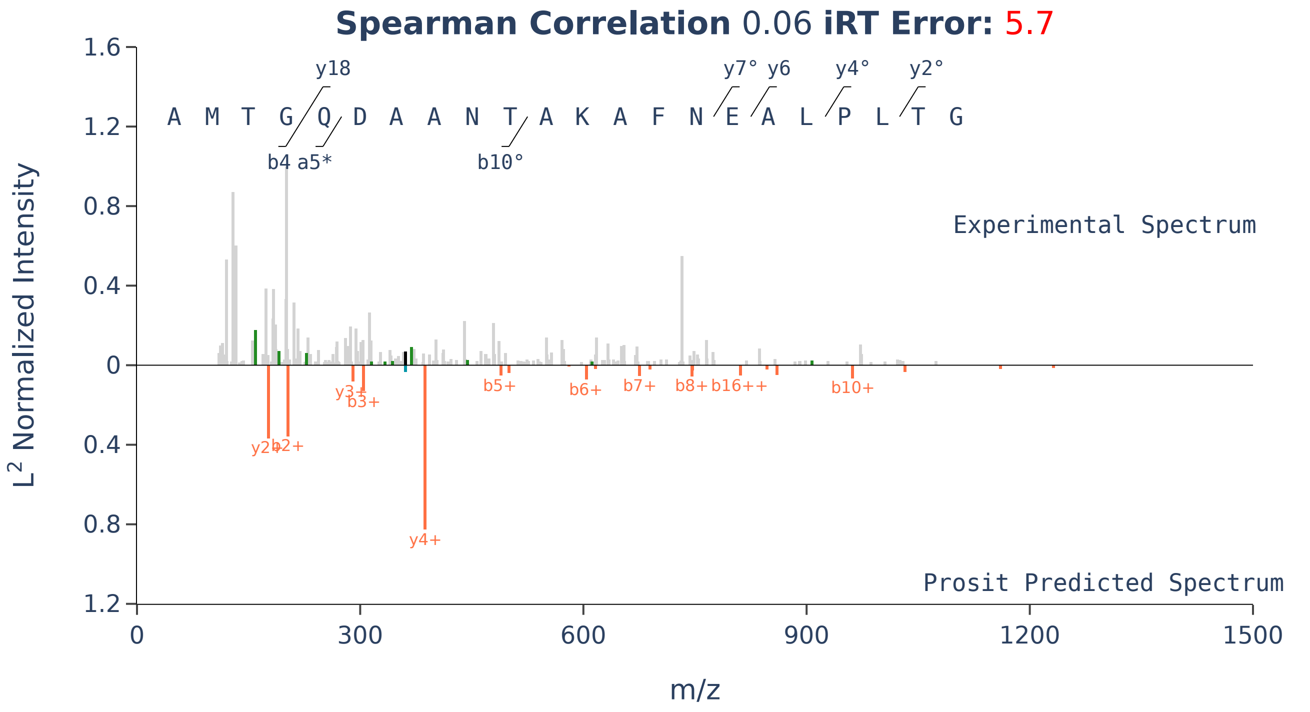

Source Ncheng_210623_230623_HFGoe_FFH_20S_25_1_A2_4h_R1 **Scan** 28493
Peptide DLMGKLPGMGQIPDNVKSQMD **Charge** 3 **Spectral Angle** 0.71
Spearman Correlation 0.91 **iRT Error:** 2.88

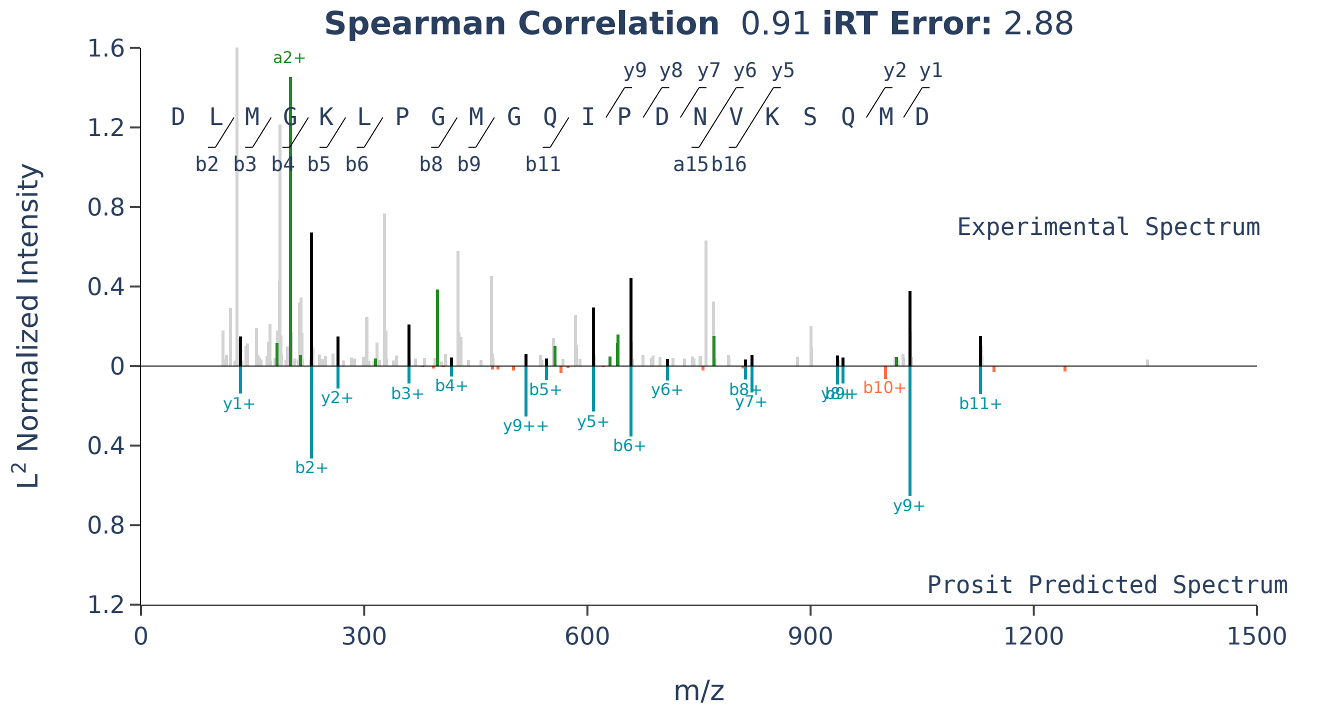

Source Ncheng_210623_230623_HFGoe_FFH_20S_25_1_A2_4h_R1 **Scan** 28493
Peptide LMGKLPGMGQIPDNVKSQMDD **Charge** 3 **Spectral Angle** 0.21
Spearman Correlation 0.38 **iRT Error:** 22.16

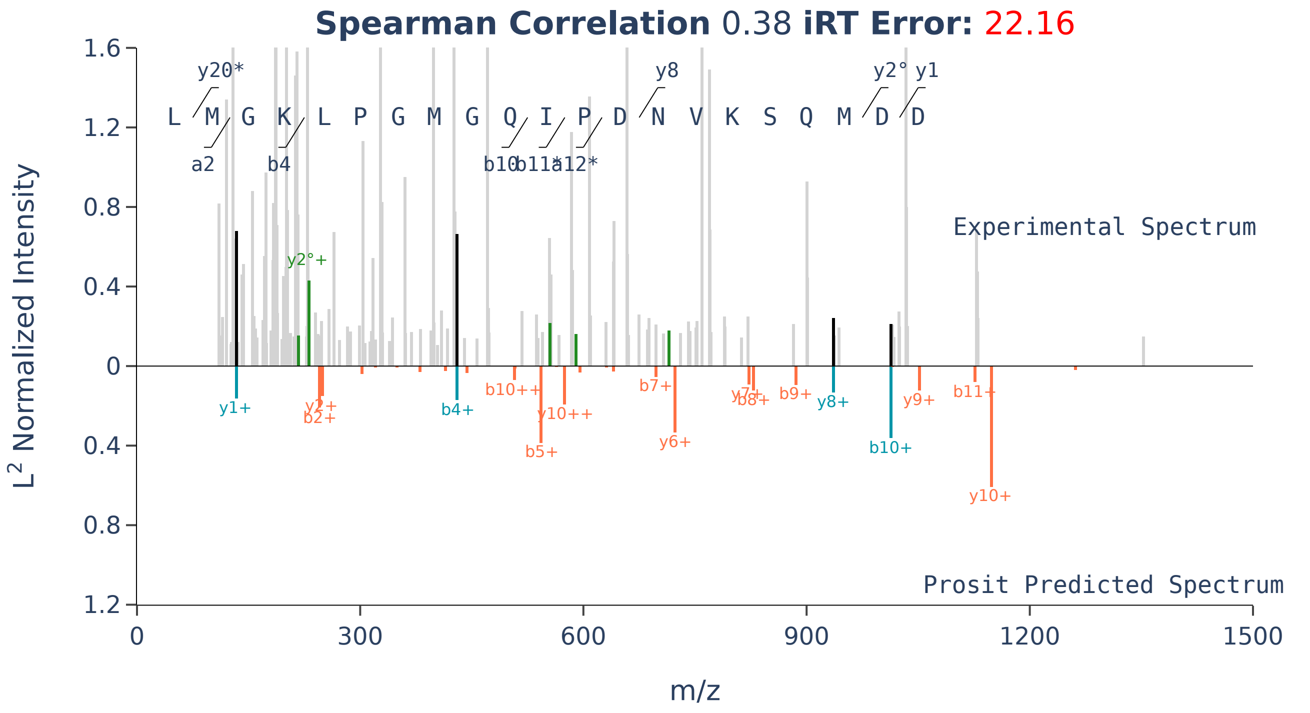

Source Ncheng_210623_230623_HFGoe_FFH_20S_25_1_A1_24h_R1 **Scan** 12625
Peptide EPFHPDGRLT **Charge** 1 **Spectral Angle** 0.89
Spearman Correlation 0.85 **iRT Error:** 2.9

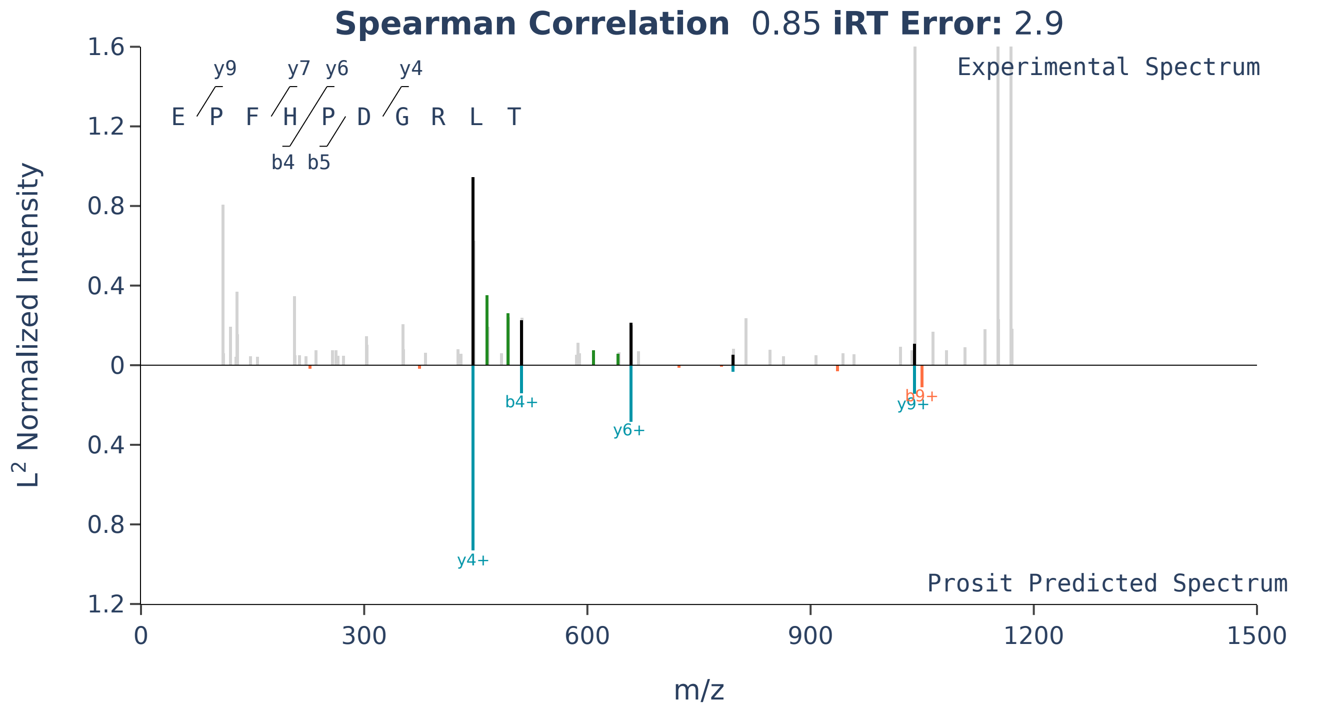

Source Ncheng_210623_230623_HFGoe_FFH_20S_25_1_A1_24h_R1 **Scan** 12625
Peptide EPFHPDRIAS **Charge** 1 **Spectral Angle** 0.48
Spearman Correlation 0.59 **iRT Error:** 2.9

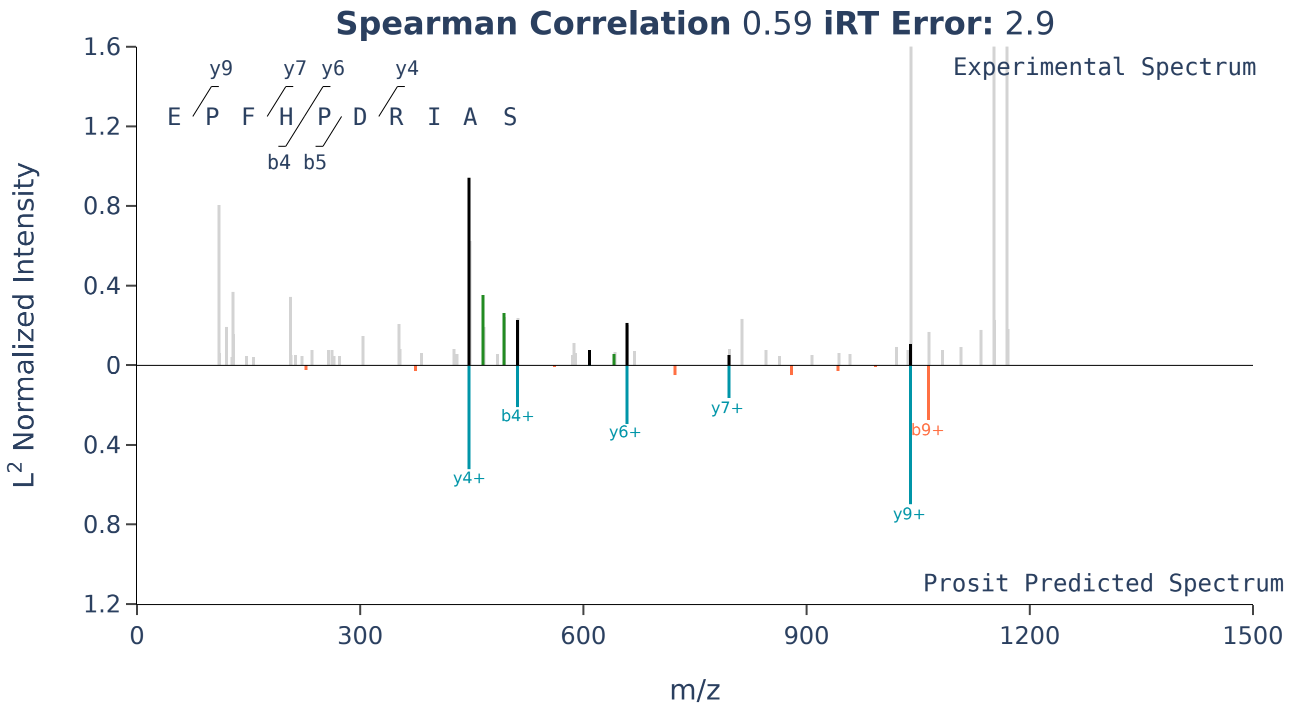

Source Ncheng_210623_230623_HFGoe_FFH_20S_25_1_A2_24h_R2 **Scan** 6831
Peptide ALKEARME **Charge** 2 **Spectral Angle** 0.84
Spearman Correlation 0.86 **iRT Error:** 2.93

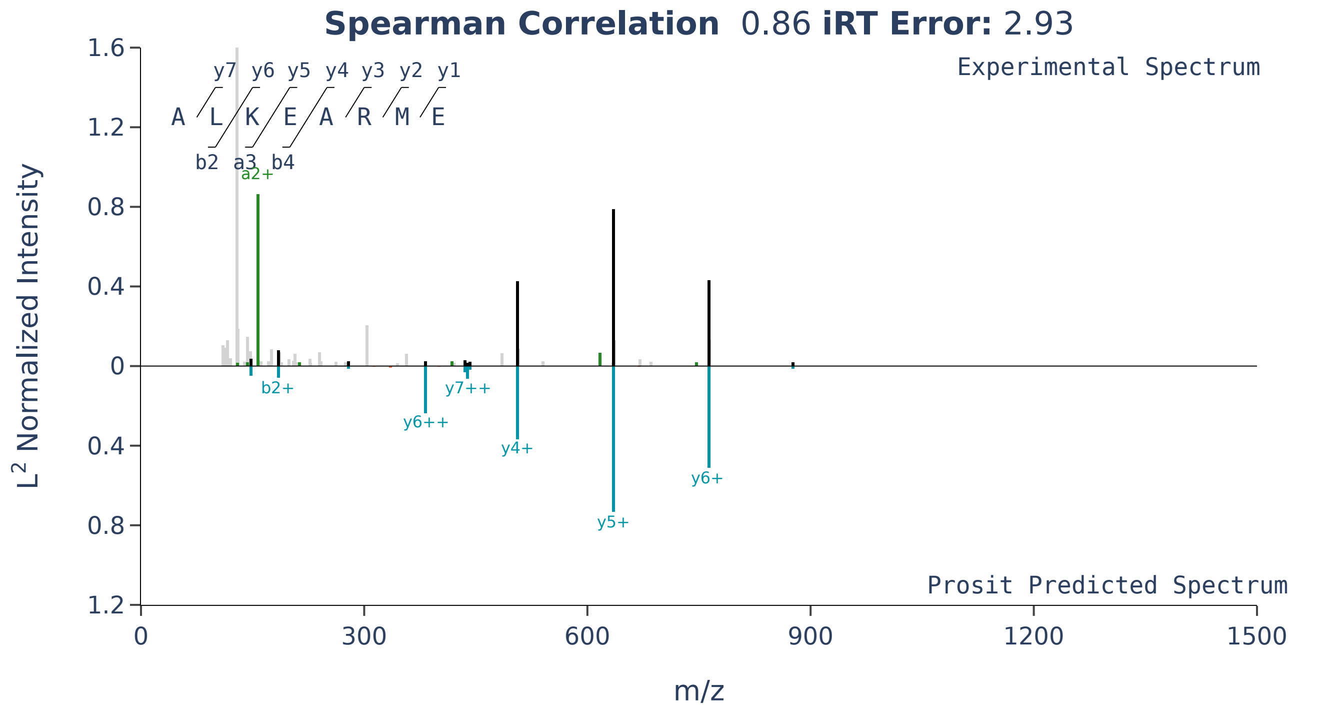

Source Ncheng_210623_230623_HFGoe_FFH_20S_25_1_A2_24h_R2 **Scan** 6831
Peptide SRILGMGDV **Charge** 2 **Spectral Angle** 0.0
Spearman Correlation 0.0 **iRT Error:** 80.77

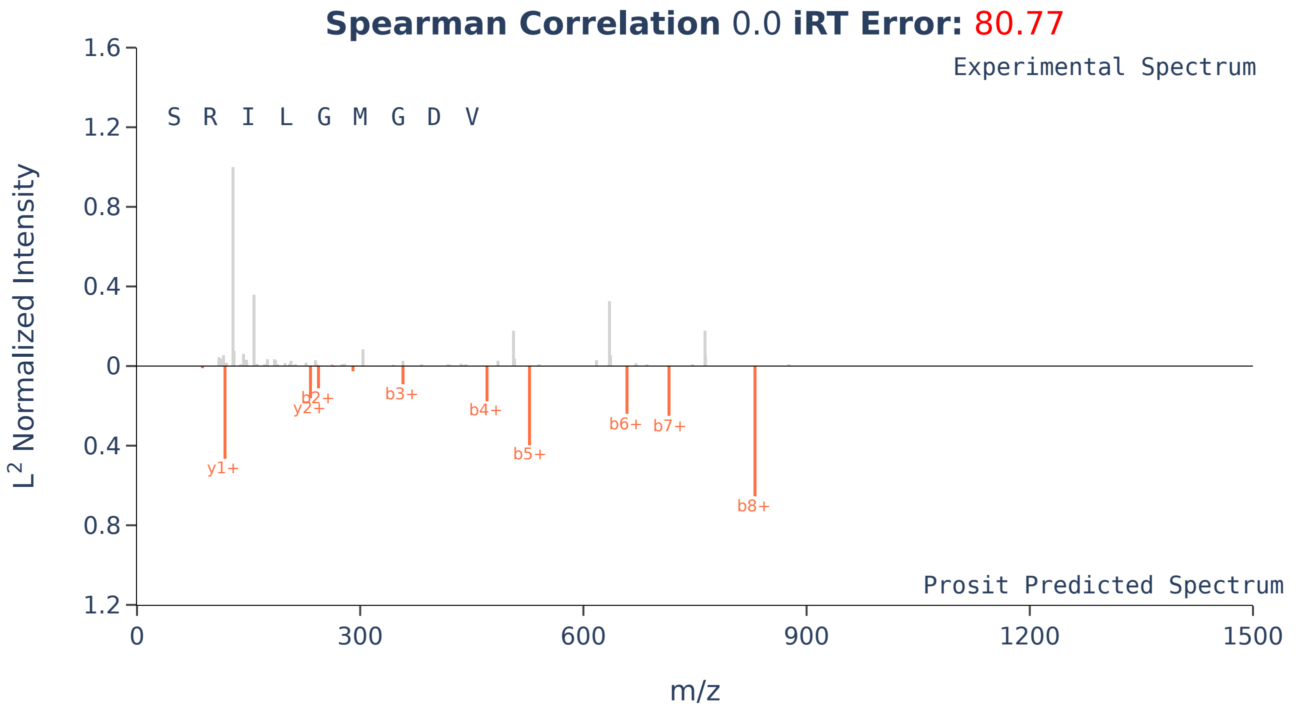

Source Ncheng_210623_230623_HFGoe_FFH_20S_25_1_A2_24h_R1 **Scan** 20786
Peptide SALEPFHPDRI **Charge** 3 **Spectral Angle** 0.76
Spearman Correlation 0.88 **iRT Error:** 3.05

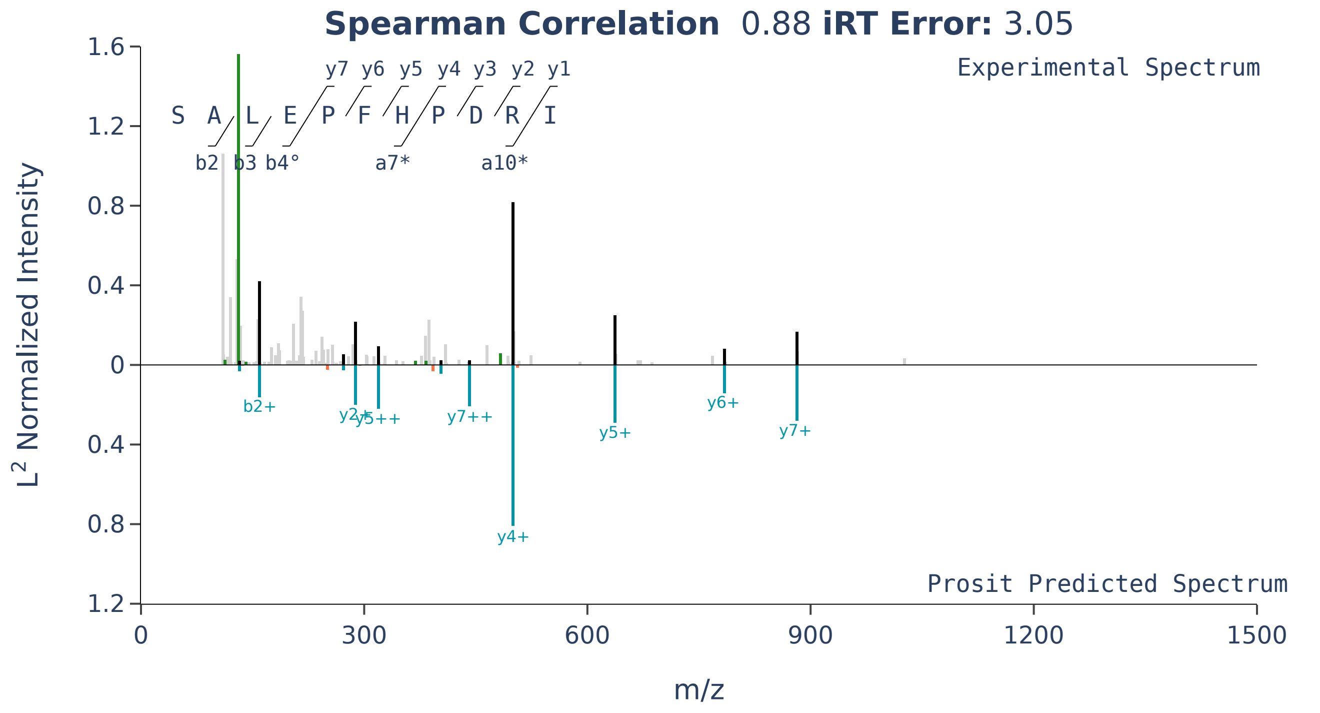

Source Ncheng_210623_230623_HFGoe_FFH_20S_25_1_A2_24h_R1 **Scan** 20786
Peptide LEPFHPDRIAS **Charge** 3 **Spectral Angle** 0.1
Spearman Correlation 0.26 **iRT Error:** 22.99

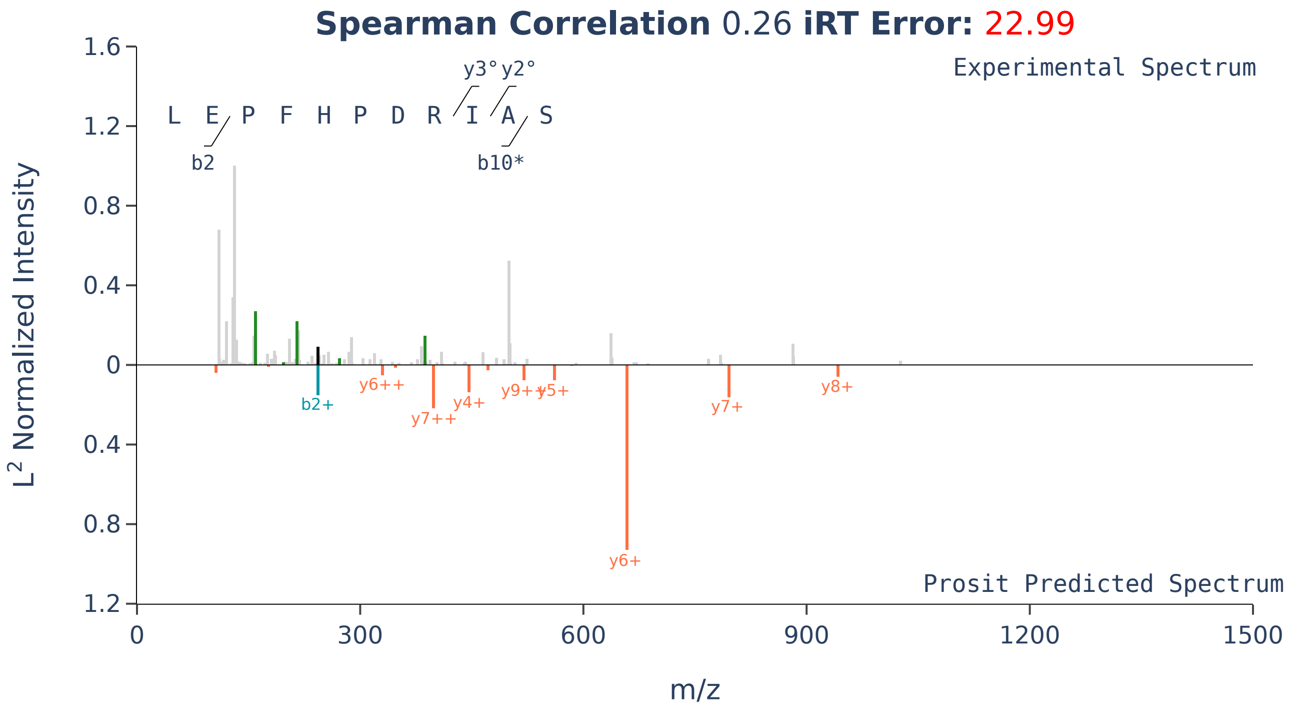

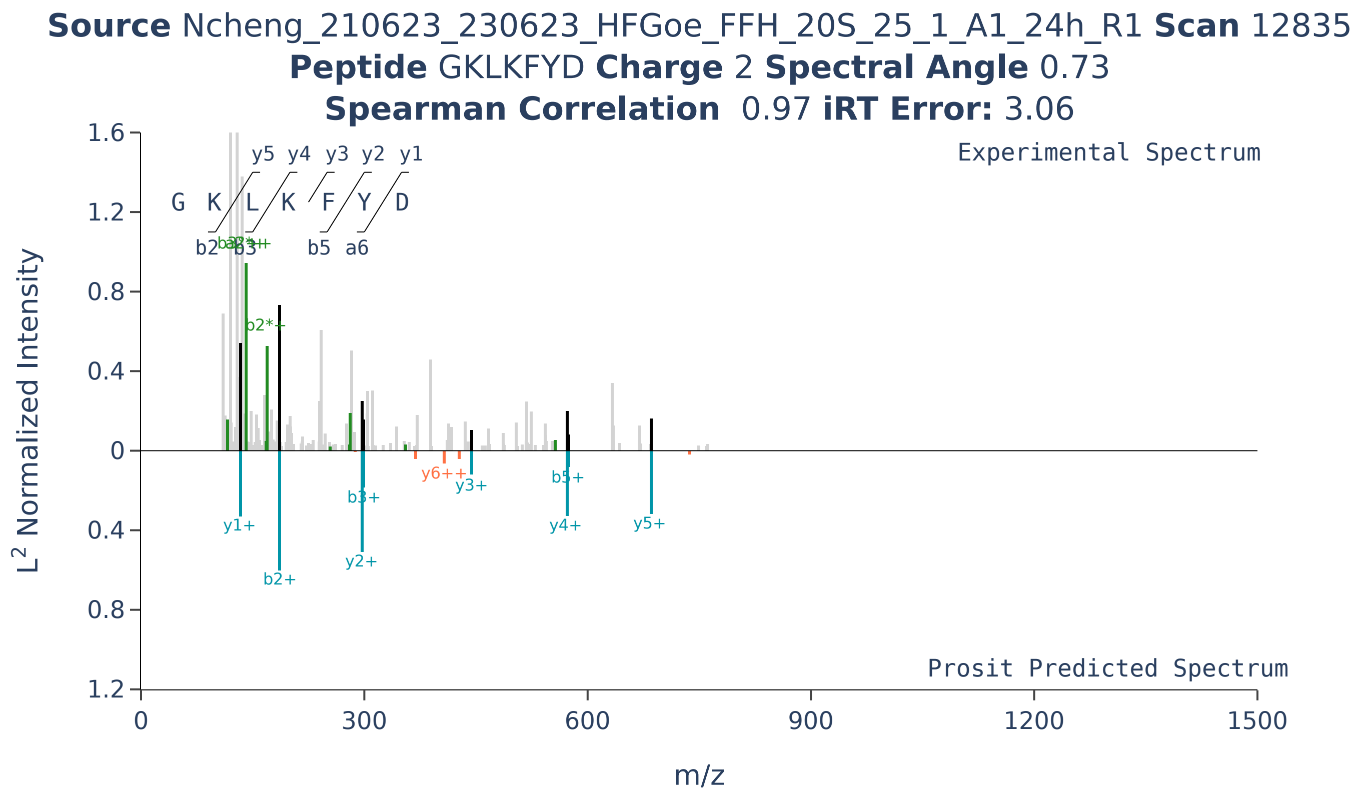
Source Ncheng_210623_230623_HFGoe_FFH_20S_25_1_A1_24h_R1 **Scan** 12835
Peptide GKLKFYD **Charge** 2 **Spectral Angle** 0.73
Spearman Correlation 0.97 **iRT Error:** 3.06
Experimental Spectrum
Prosit Predicted Spectrum
L^2 Normalized Intensity
m/z

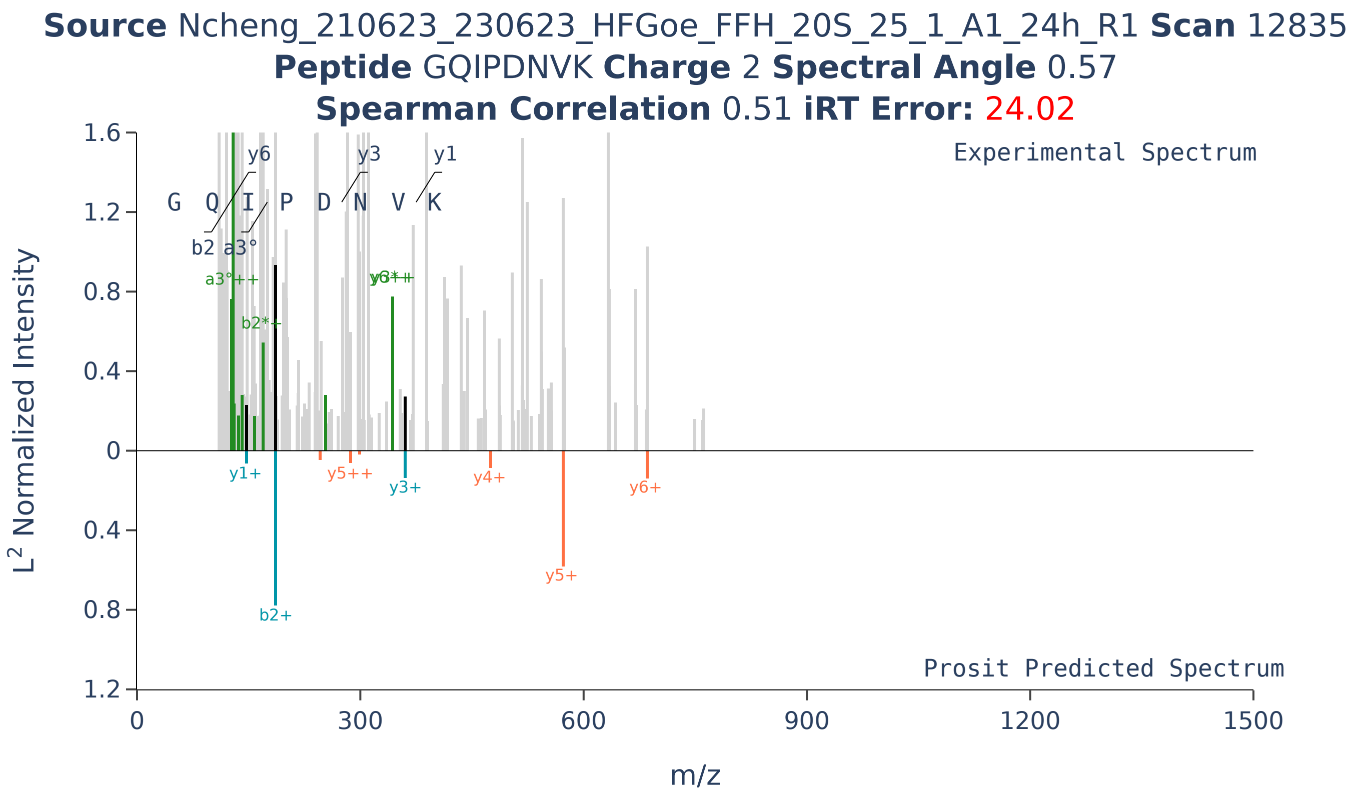
Source Ncheng_210623_230623_HFGoe_FFH_20S_25_1_A1_24h_R1 **Scan** 12835
Peptide GQIPDNVK **Charge** 2 **Spectral Angle** 0.57
Spearman Correlation 0.51 **iRT Error:** 24.02
Experimental Spectrum
Prosit Predicted Spectrum
L^2 Normalized Intensity
m/z

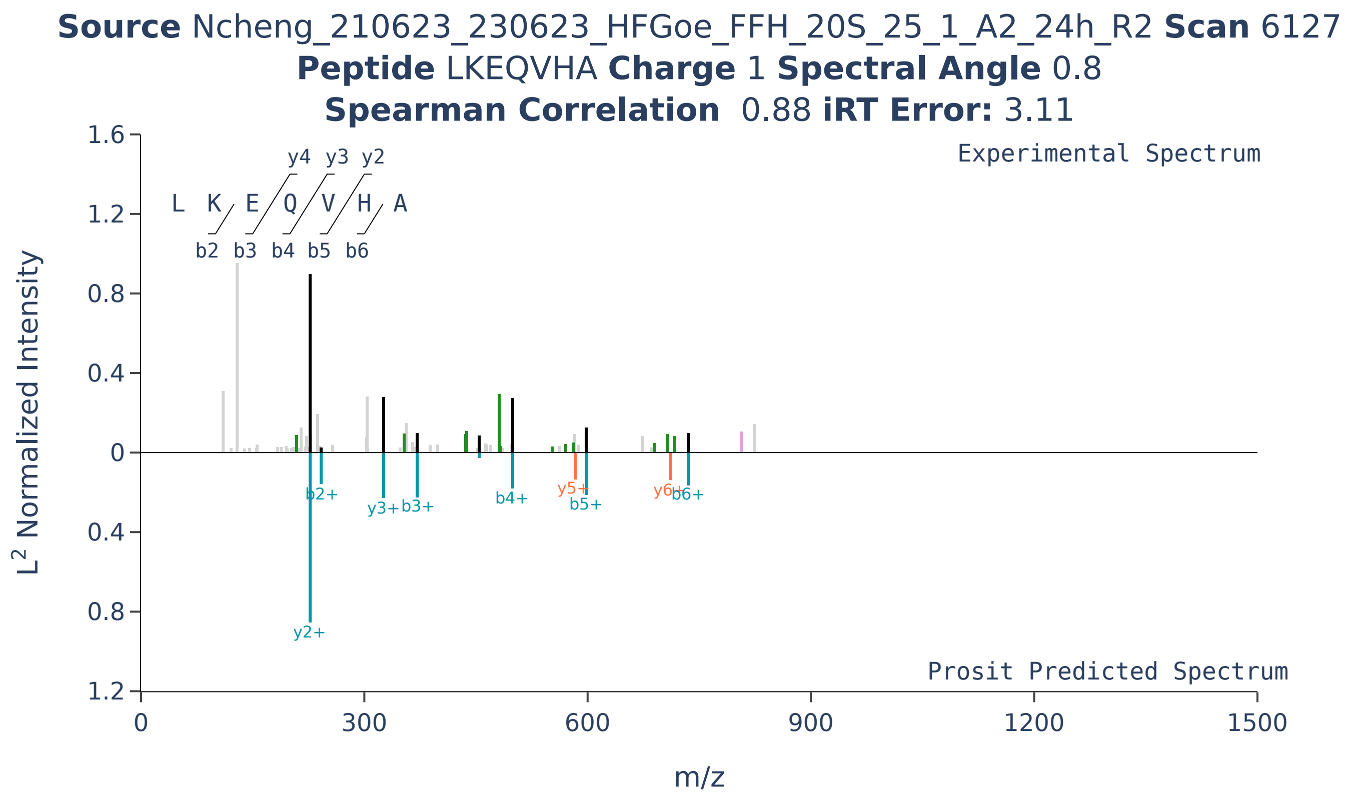
Source Ncheng_210623_230623_HFGoe_FFH_20S_25_1_A2_24h_R2 **Scan** 6127
Peptide LKEQVHA **Charge** 1 **Spectral Angle** 0.8
Spearman Correlation 0.88 **iRT Error:** 3.11
Experimental Spectrum
Prosit Predicted Spectrum
L^2 Normalized Intensity
m/z

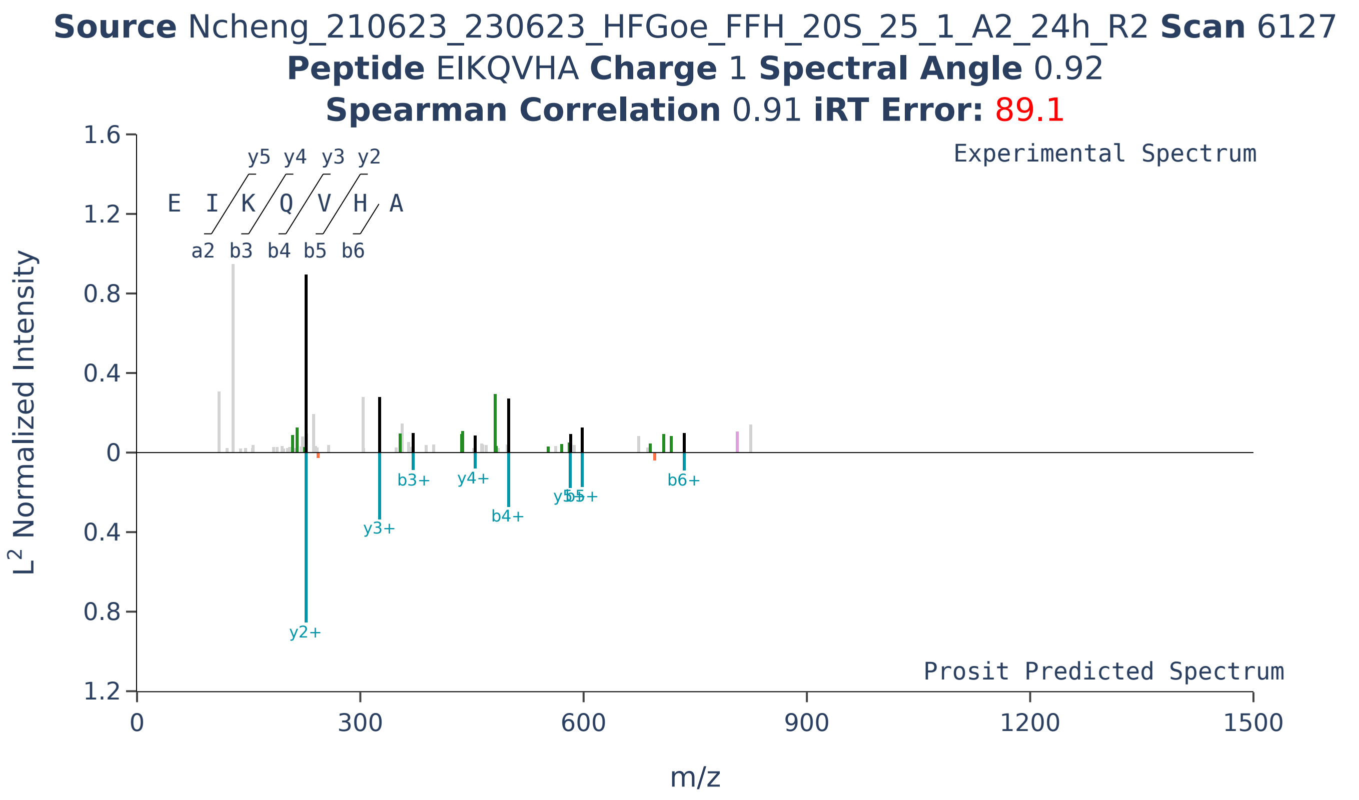
Source Ncheng_210623_230623_HFGoe_FFH_20S_25_1_A2_24h_R2 **Scan** 6127
Peptide EIKQVHA **Charge** 1 **Spectral Angle** 0.92
Spearman Correlation 0.91 **iRT Error:** 89.1
Experimental Spectrum
Prosit Predicted Spectrum
L^2 Normalized Intensity
m/z

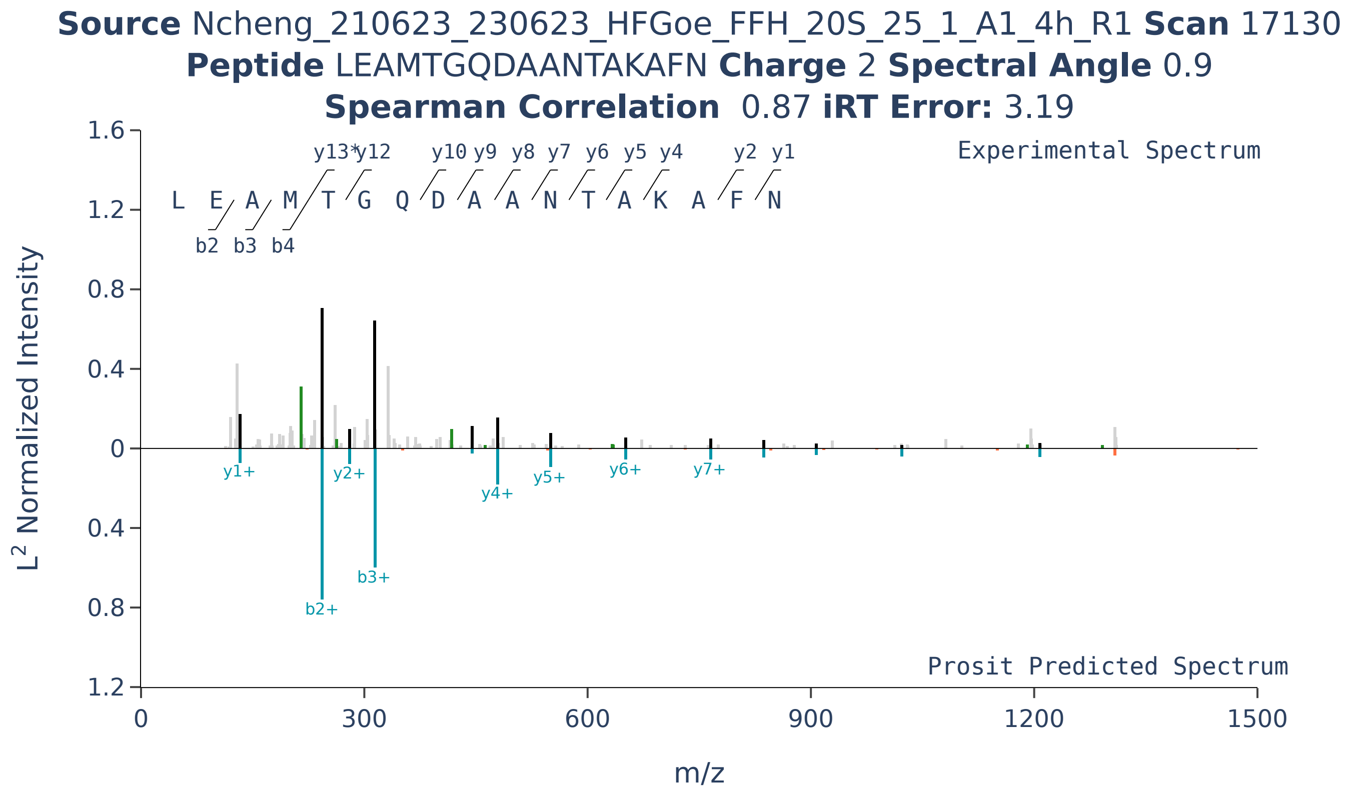
Source Ncheng_210623_230623_HFGoe_FFH_20S_25_1_A1_4h_R1 **Scan** 17130
Peptide LEAMTGQDAANTAKAFN **Charge** 2 **Spectral Angle** 0.9
Spearman Correlation 0.87 **iRT Error:** 3.19
Experimental Spectrum
Prosit Predicted Spectrum
L^2 Normalized Intensity
m/z

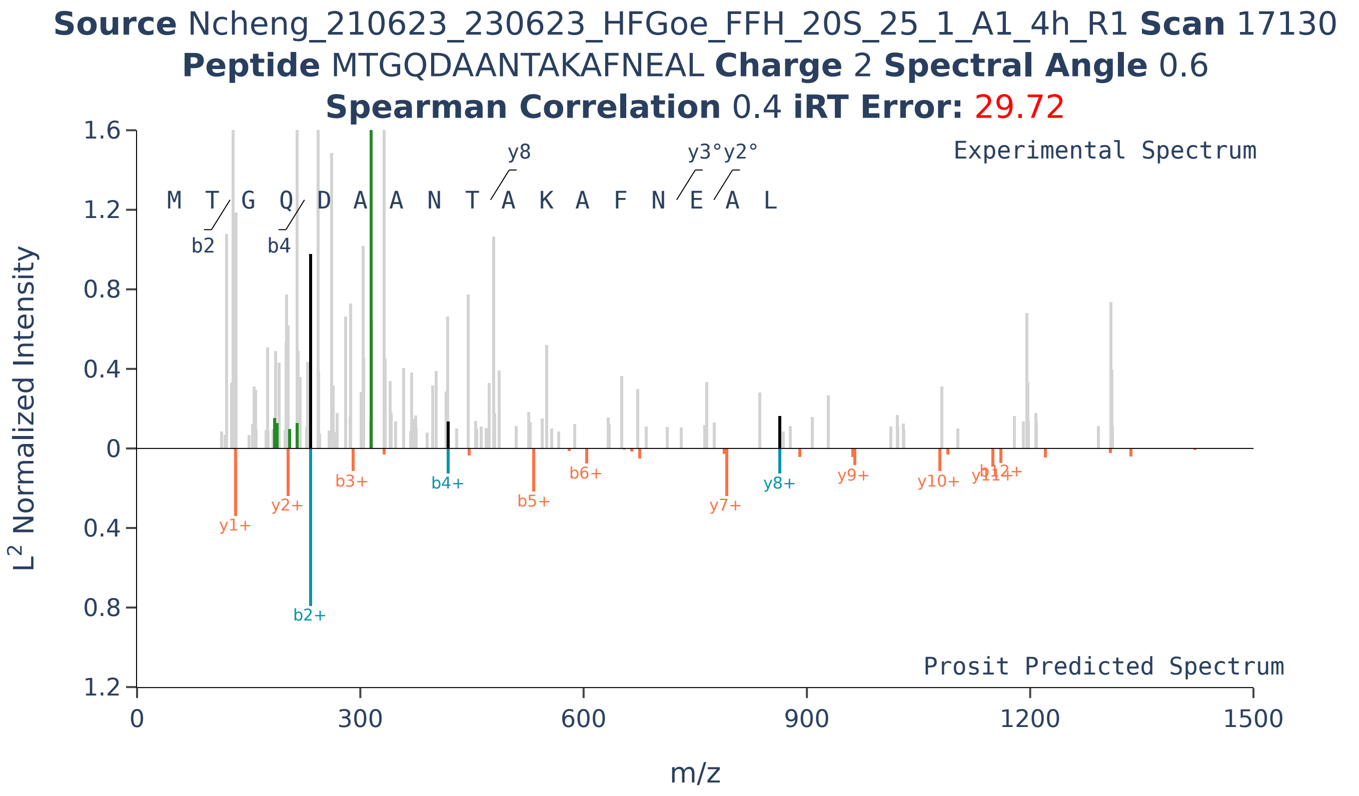
Source Ncheng_210623_230623_HFGoe_FFH_20S_25_1_A1_4h_R1 **Scan** 17130
Peptide MTGQDAANTAKAFNEAL **Charge** 2 **Spectral Angle** 0.6
Spearman Correlation 0.4 **iRT Error:** 29.72
Experimental Spectrum
Prosit Predicted Spectrum
L^2 Normalized Intensity
m/z

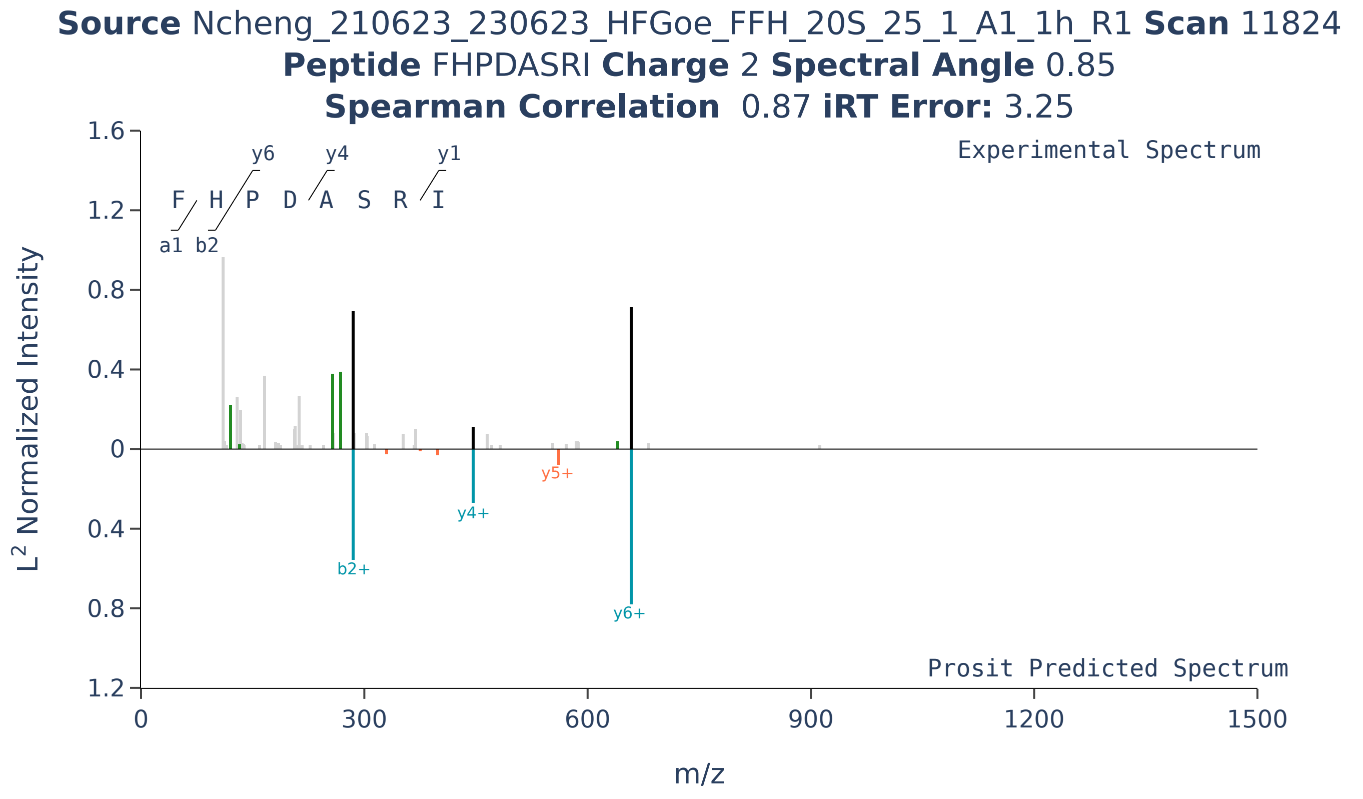
Source Ncheng_210623_230623_HFGoe_FFH_20S_25_1_A1_1h_R1 **Scan** 11824
Peptide FHPDASRI **Charge** 2 **Spectral Angle** 0.85
Spearman Correlation 0.87 **iRT Error:** 3.25
Experimental Spectrum
Prosit Predicted Spectrum
L^2 Normalized Intensity
m/z

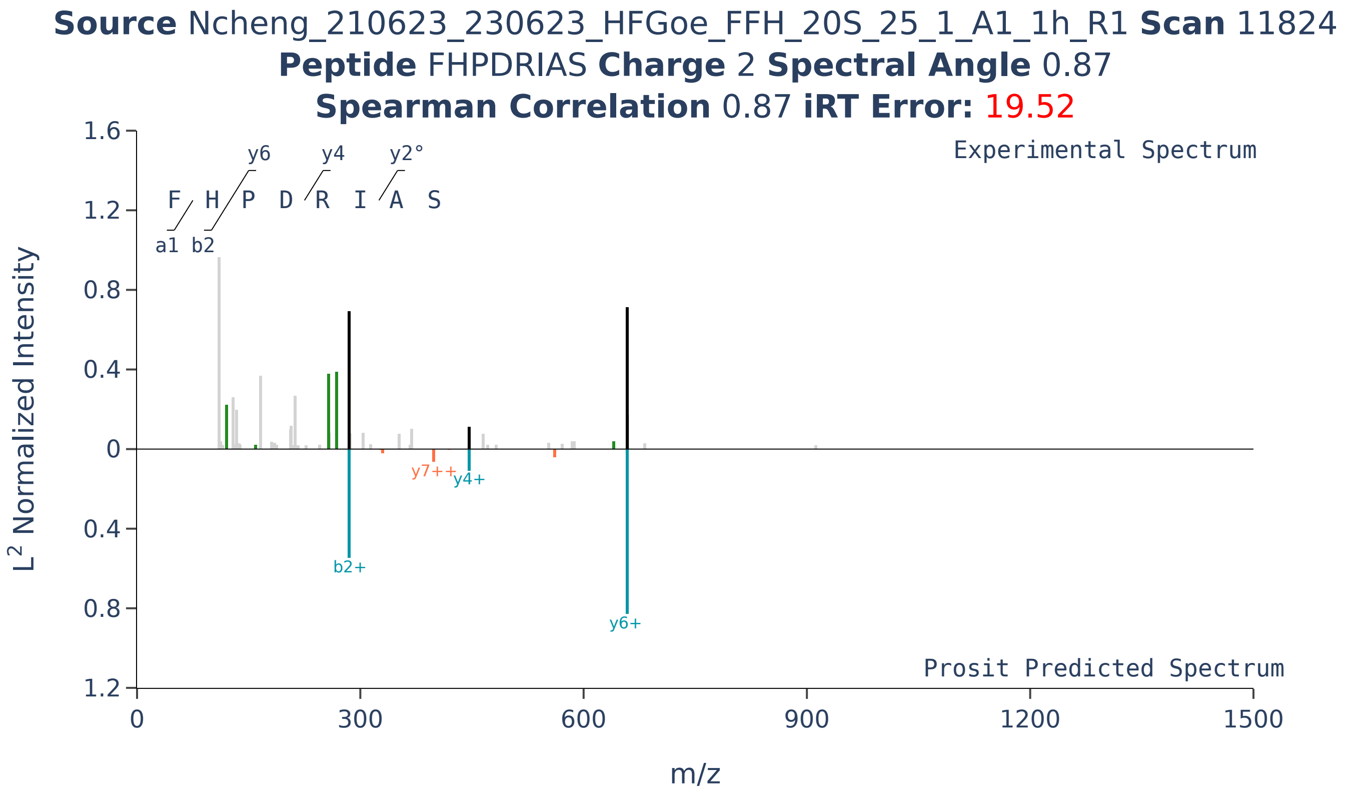
Source Ncheng_210623_230623_HFGoe_FFH_20S_25_1_A1_1h_R1 **Scan** 11824
Peptide FHPDRIAS **Charge** 2 **Spectral Angle** 0.87
Spearman Correlation 0.87 **iRT Error:** 19.52
Experimental Spectrum
Prosit Predicted Spectrum
L^2 Normalized Intensity
m/z

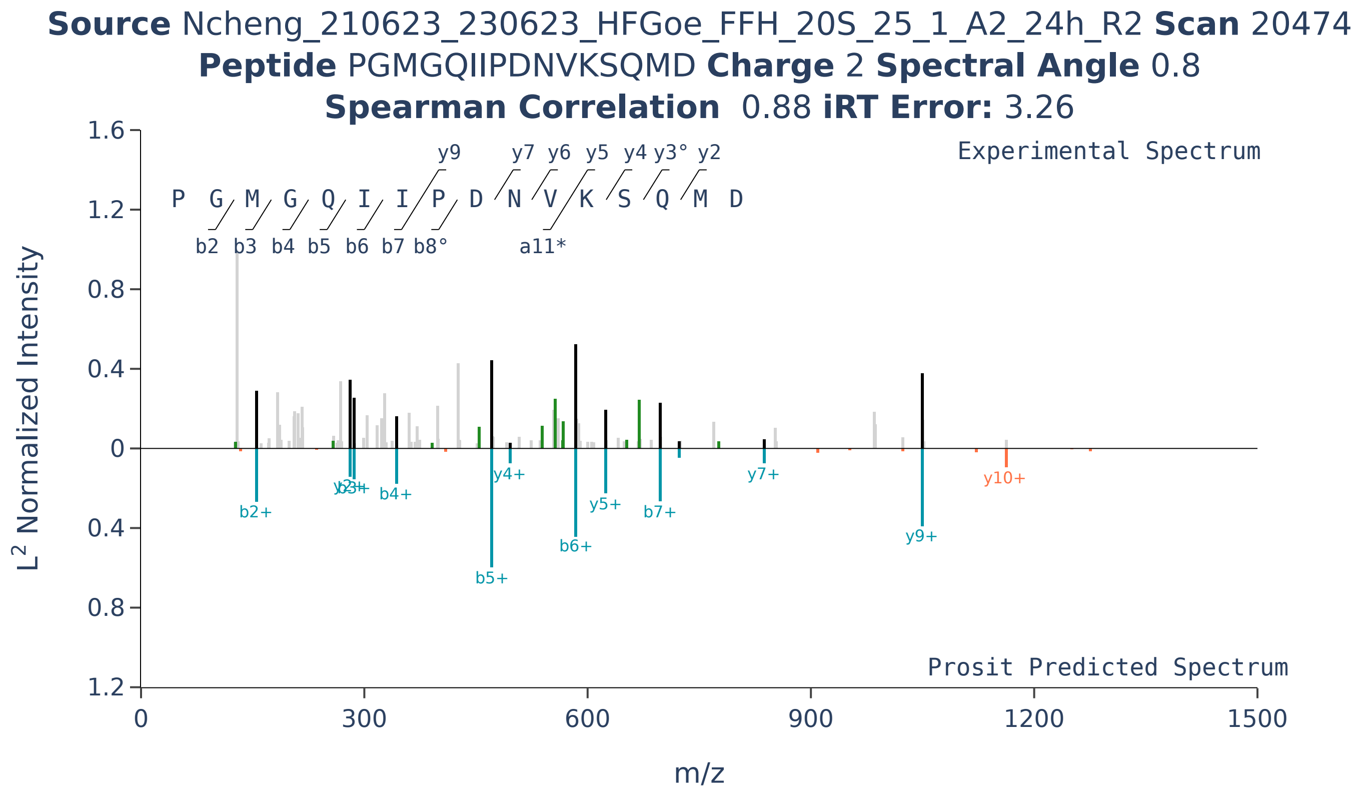
Source Ncheng_210623_230623_HFGoe_FFH_20S_25_1_A2_24h_R2 **Scan** 20474
Peptide PGMGQIIPDNVKSQMD **Charge** 2 **Spectral Angle** 0.8
Spearman Correlation 0.88 **iRT Error:** 3.26
Experimental Spectrum
Prosit Predicted Spectrum
L^2 Normalized Intensity
m/z

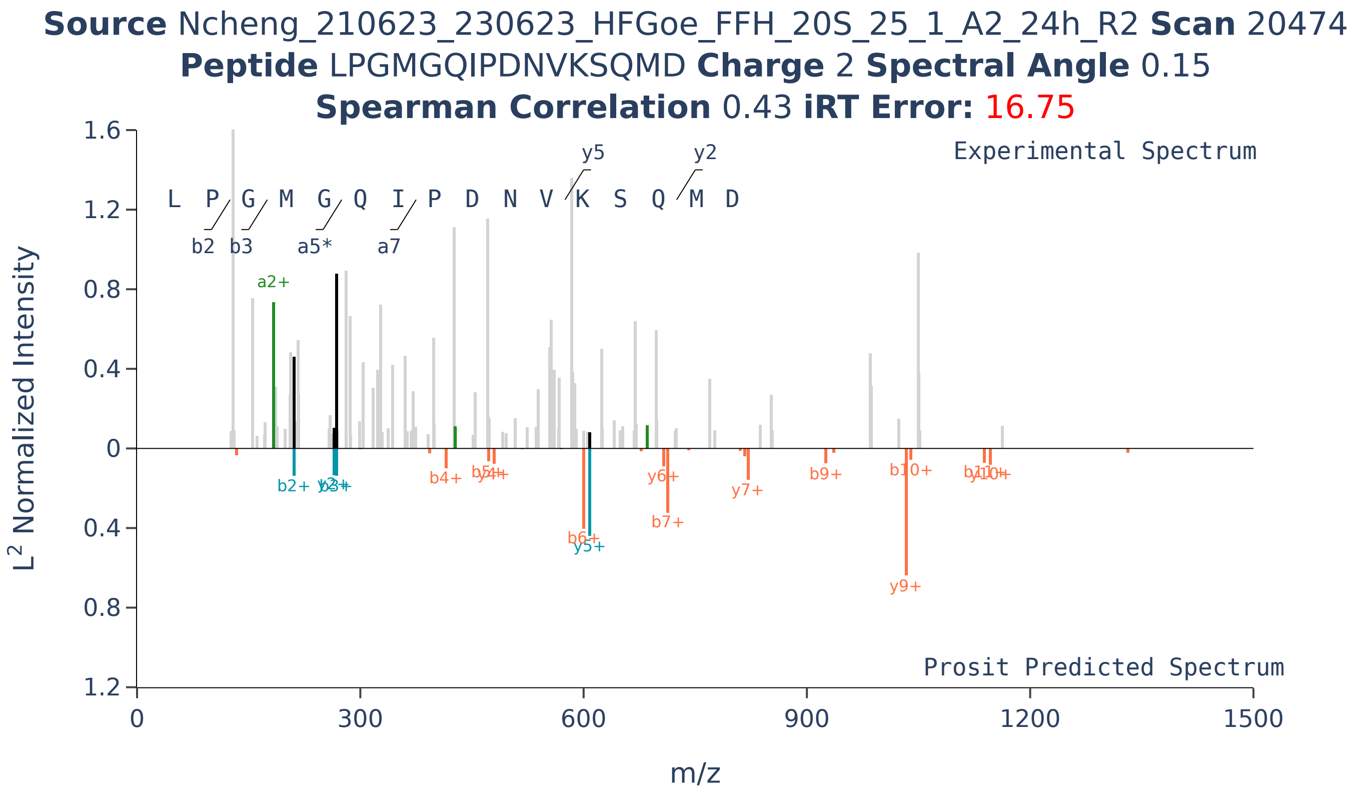
Source Ncheng_210623_230623_HFGoe_FFH_20S_25_1_A2_24h_R2 **Scan** 20474
Peptide LPGMGQIPDNVKSQMD **Charge** 2 **Spectral Angle** 0.15
Spearman Correlation 0.43 **iRT Error:** 16.75
Experimental Spectrum
Prosit Predicted Spectrum
L^2 Normalized Intensity
m/z

Source Ncheng_210623_230623_HFGoe_FFH_20S_25_1_A2_2h_R2 **Scan** 9431
Peptide VHDEAMMD **Charge** 1 **Spectral Angle** 0.75
Spearman Correlation 0.94 **iRT Error:** 3.44

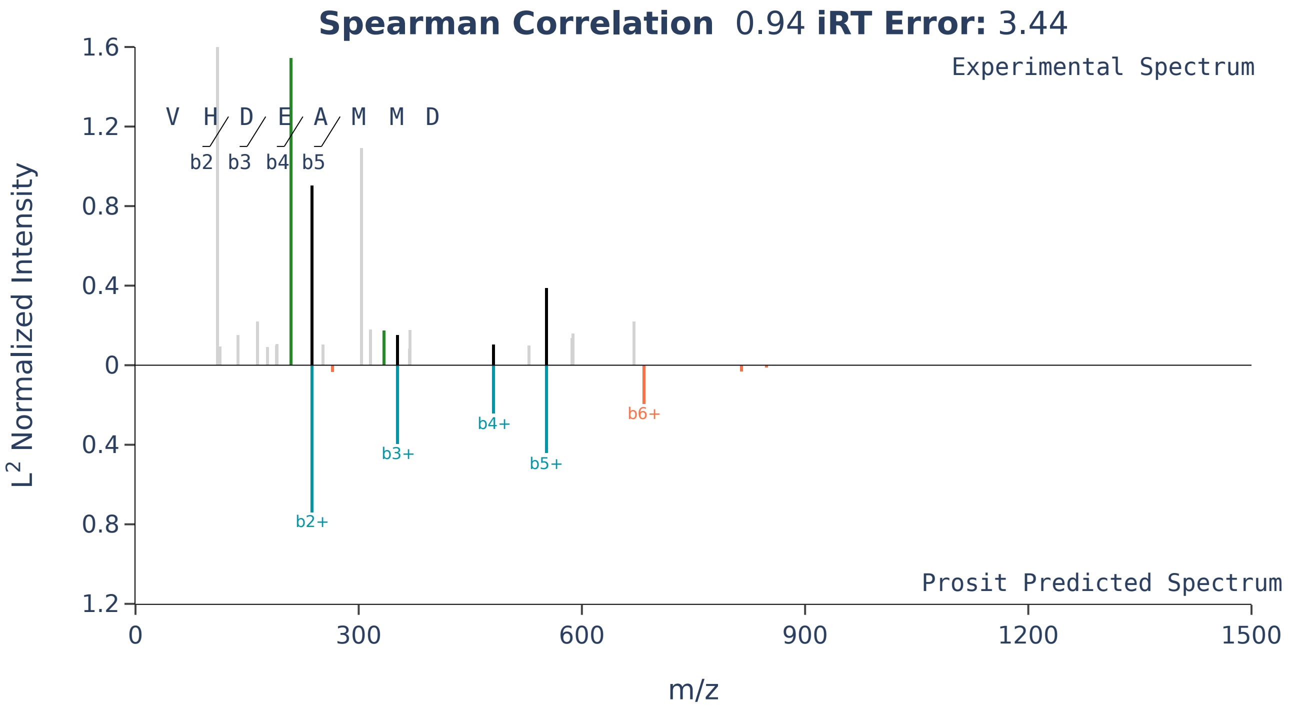

Source Ncheng_210623_230623_HFGoe_FFH_20S_25_1_A2_2h_R2 **Scan** 9431
Peptide HVDEAMMD **Charge** 1 **Spectral Angle** 0.75
Spearman Correlation 0.59 **iRT Error:** 12.65

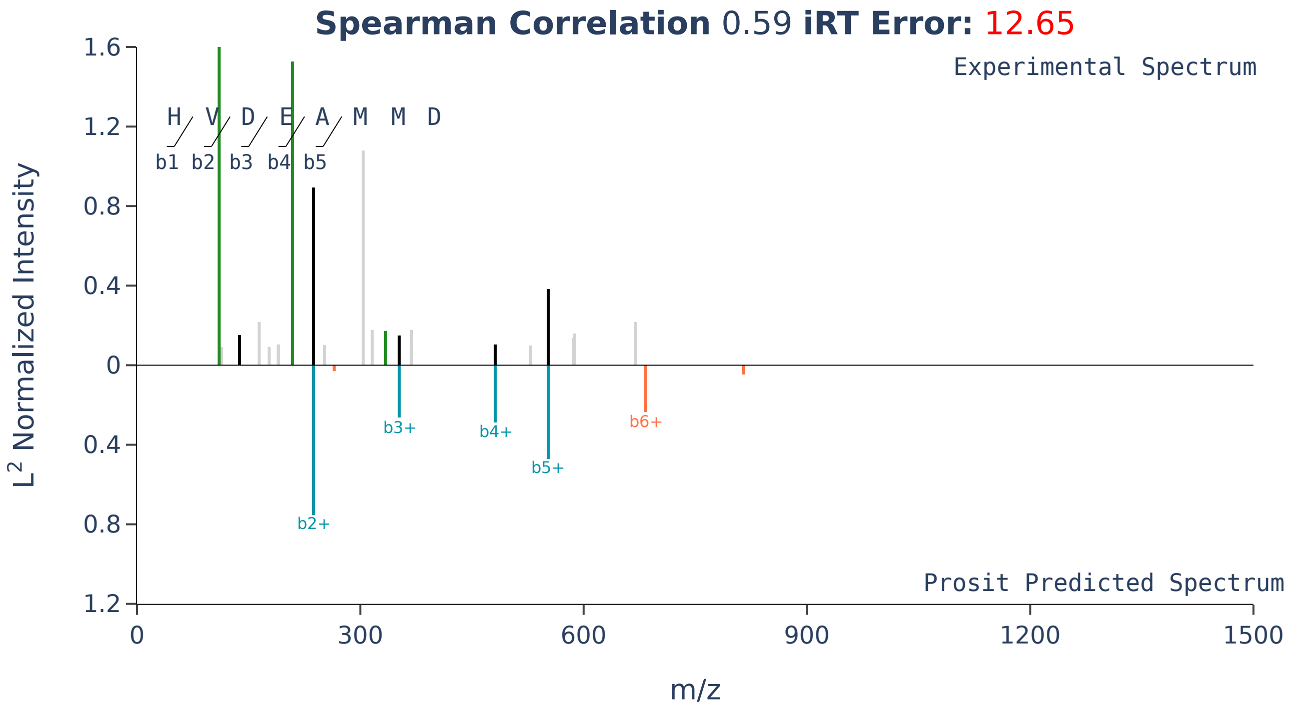

Source Ncheng_210623_230623_HFGoe_FFH_20S_25_1_A1_4h_R1 **Scan** 8519
Peptide ALKEAVGQKPVD **Charge** 2 **Spectral Angle** 0.81
Spearman Correlation 0.86 **iRT Error:** 3.47

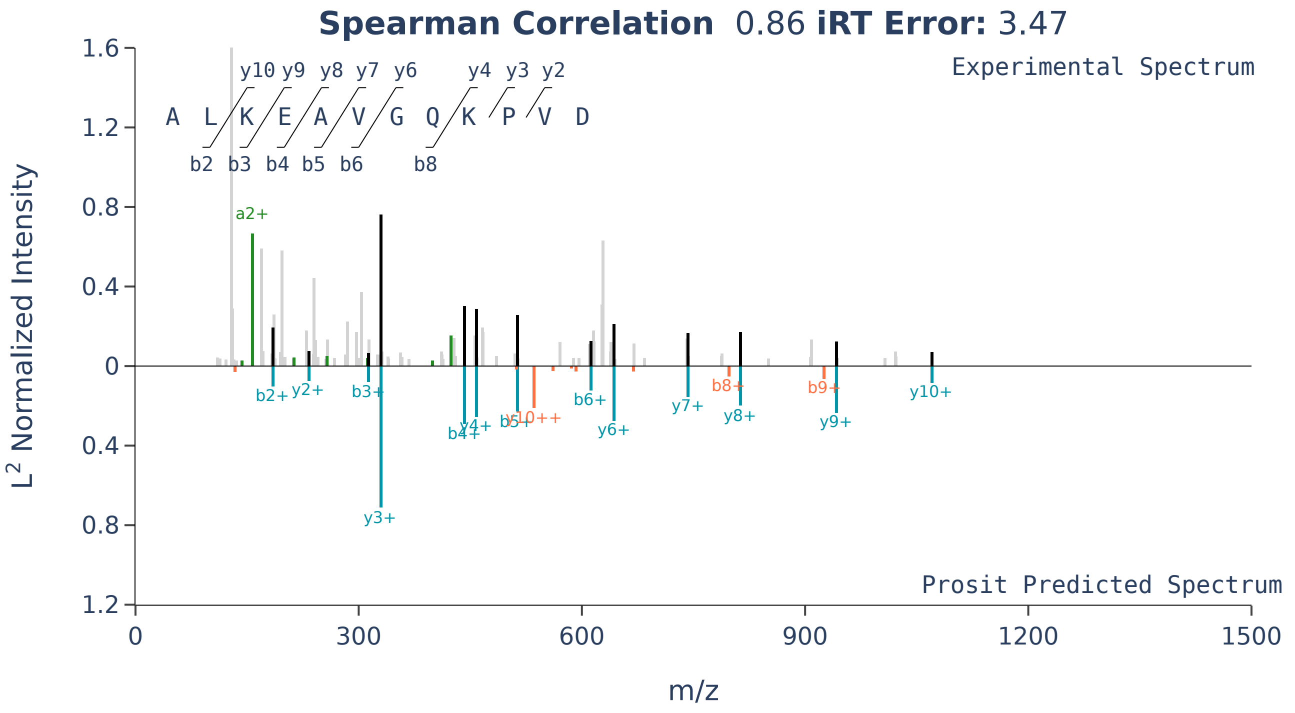

Source Ncheng_210623_230623_HFGoe_FFH_20S_25_1_A1_4h_R1 **Scan** 8519
Peptide LKEAKLKFYD **Charge** 2 **Spectral Angle** 0.17
Spearman Correlation 0.11 **iRT Error:** 28.1

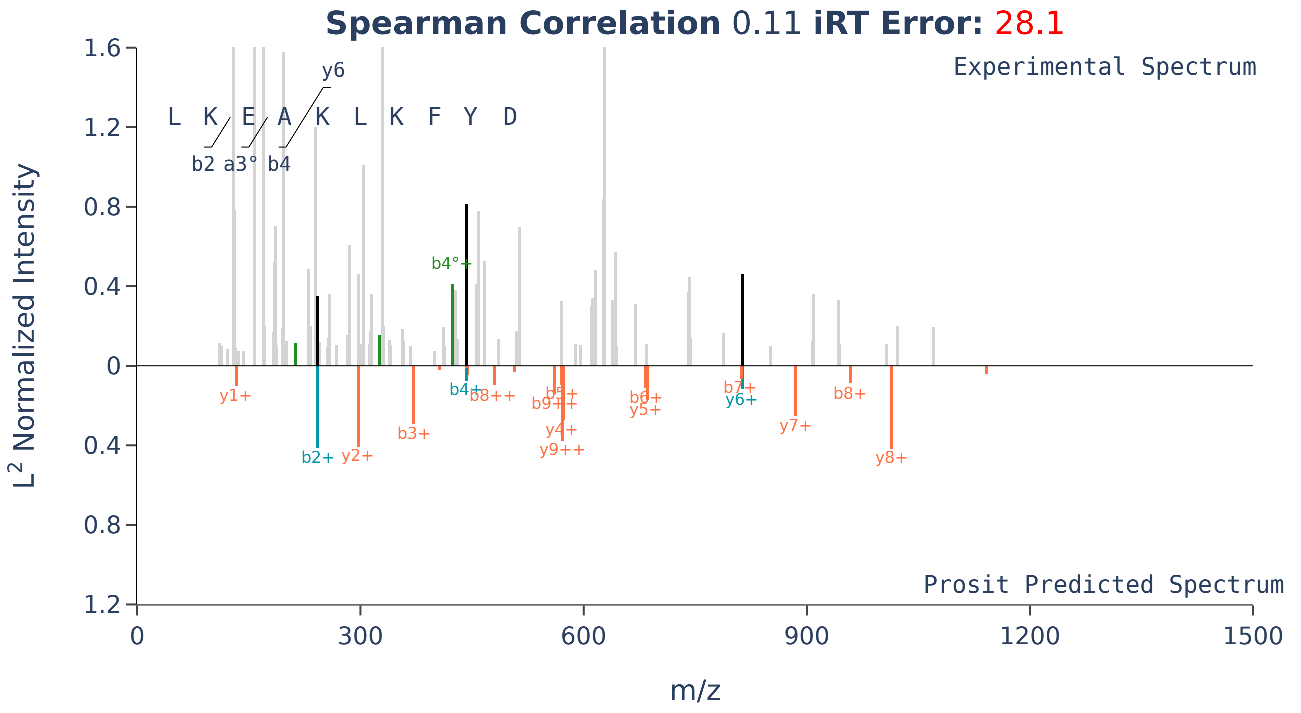

Source Ncheng_210623_230623_HFGoe_FFH_20S_25_1_A1_2h_R1 **Scan** 28025
Peptide AANTAKAFNEALPLTGAMTGQD **Charge** 2 **Spectral Angle** 0.81
Spearman Correlation 0.93 **iRT Error:** 3.49

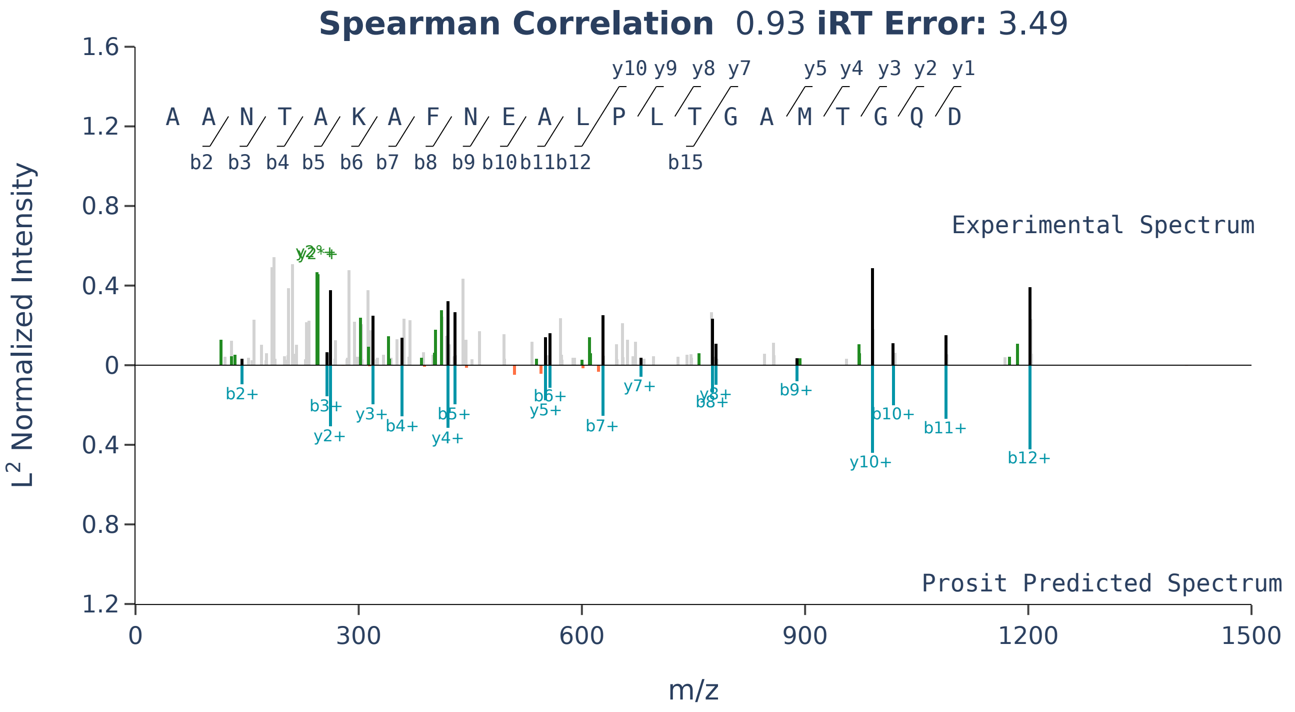

Source Ncheng_210623_230623_HFGoe_FFH_20S_25_1_A1_2h_R1 **Scan** 28025
Peptide AMTGQDAANTAKAFNEALPLTG **Charge** 2 **Spectral Angle** 0.2
Spearman Correlation 0.41 **iRT Error:** 2.82

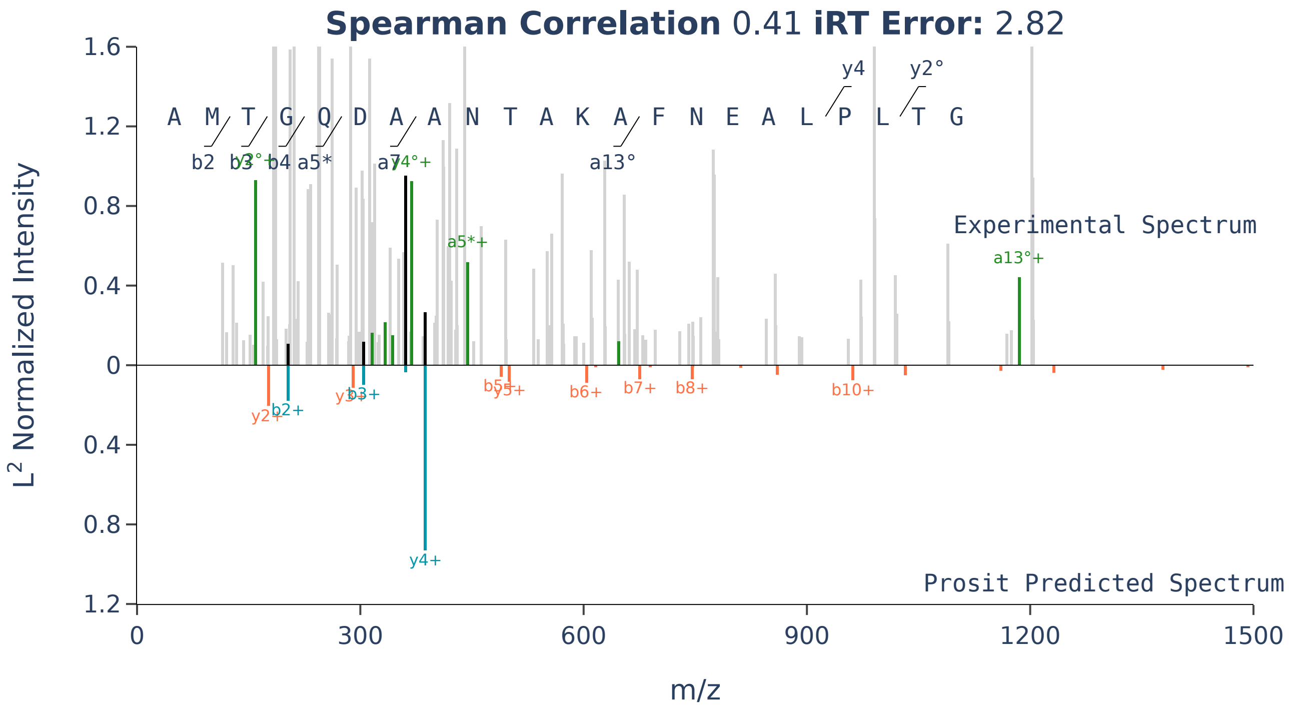

Source Ncheng_210623_230623_HFGoe_FFH_20S_25_1_A1_4h_R1 **Scan** 10874
Peptide ASKKGDGFDLND **Charge** 1 **Spectral Angle** 0.77
Spearman Correlation 0.91 **iRT Error:** 3.75

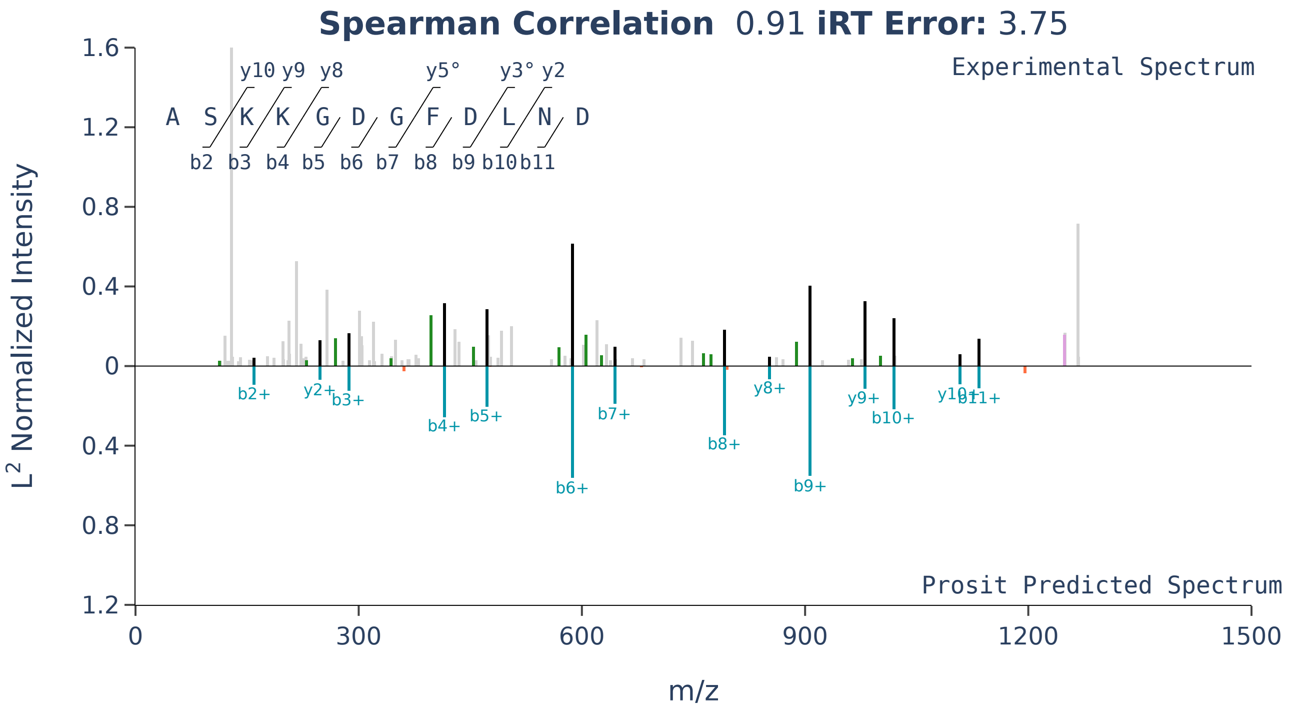

Source Ncheng_210623_230623_HFGoe_FFH_20S_25_1_A1_4h_R1 **Scan** 10874
Peptide VGVDFFPSDVGQ **Charge** 1 **Spectral Angle** 0.0
Spearman Correlation 0.0 **iRT Error:** 104.44

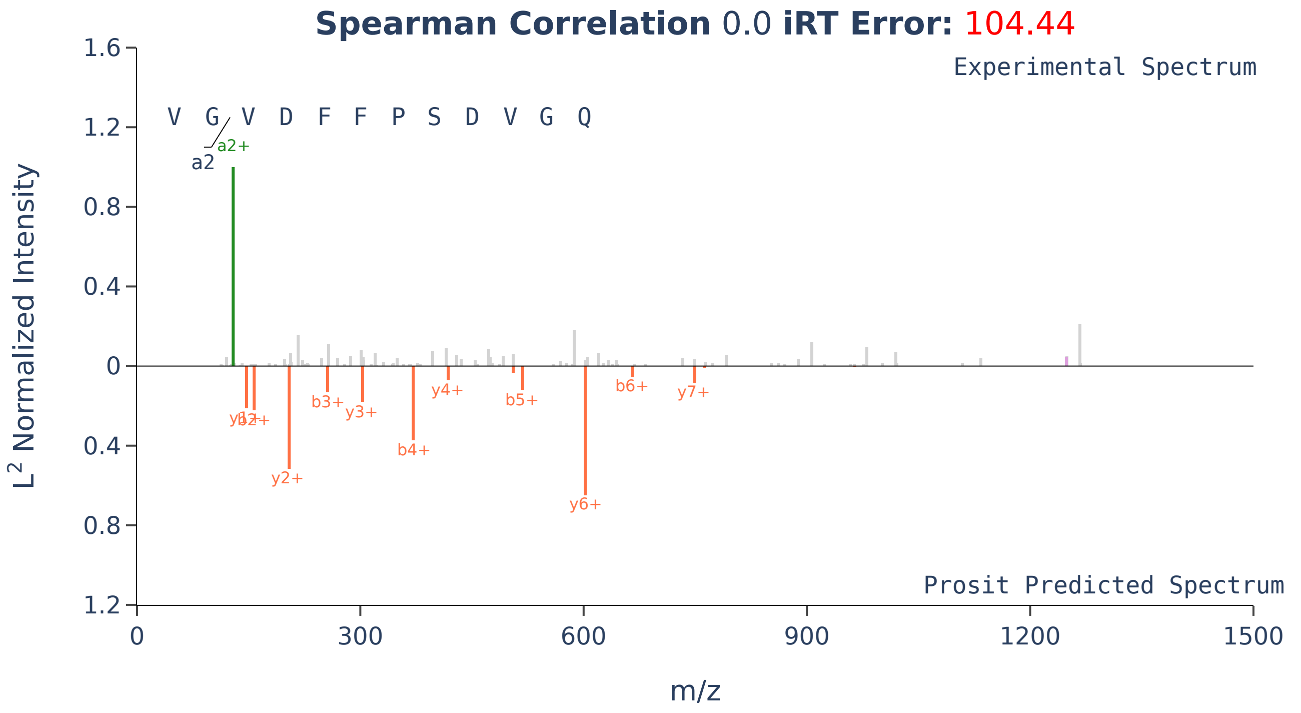

Source Ncheng_210623_230623_HFGoe_FFH_20S_25_1_A1_24h_R1 **Scan** 13153
Peptide DEIKQVHLA **Charge** 2 **Spectral Angle** 0.77
Spearman Correlation 0.88 **iRT Error:** 3.76

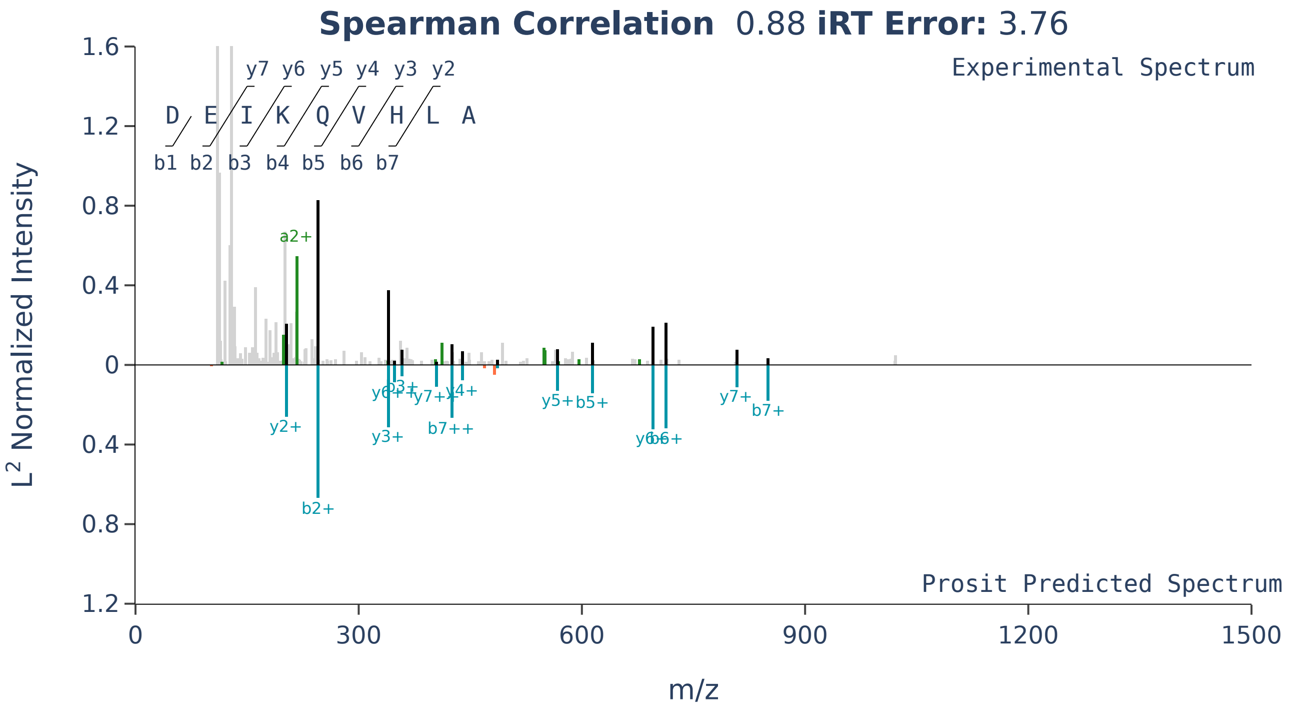

Source Ncheng_210623_230623_HFGoe_FFH_20S_25_1_A1_24h_R1 **Scan** 13153
Peptide QTLNLAAQPP **Charge** 2 **Spectral Angle** 0.0
Spearman Correlation -0.17 **iRT Error:** 34.87

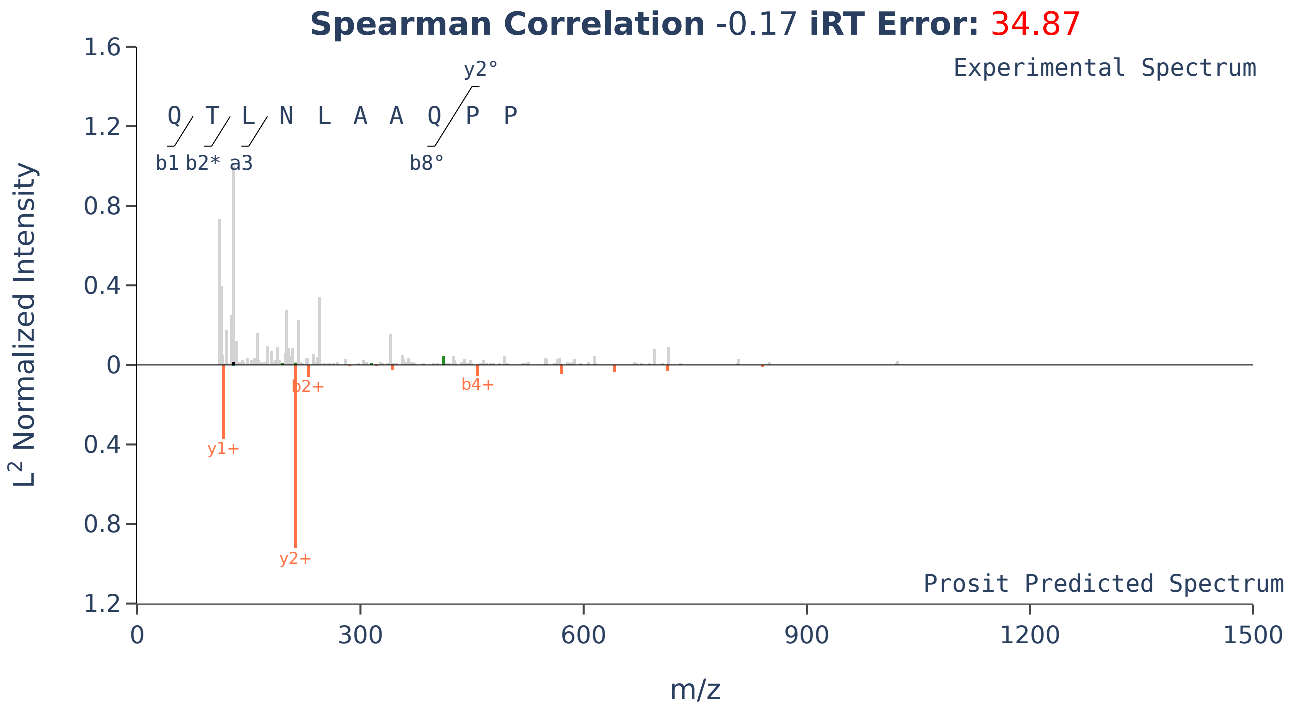

Source Ncheng_210623_230623_HFGoe_FFH_20S_25_1_A2_24h_R2 **Scan** 23499
Peptide PGQEFVSLT **Charge** 1 **Spectral Angle** 0.88
Spearman Correlation 0.91 **iRT Error:** 3.77

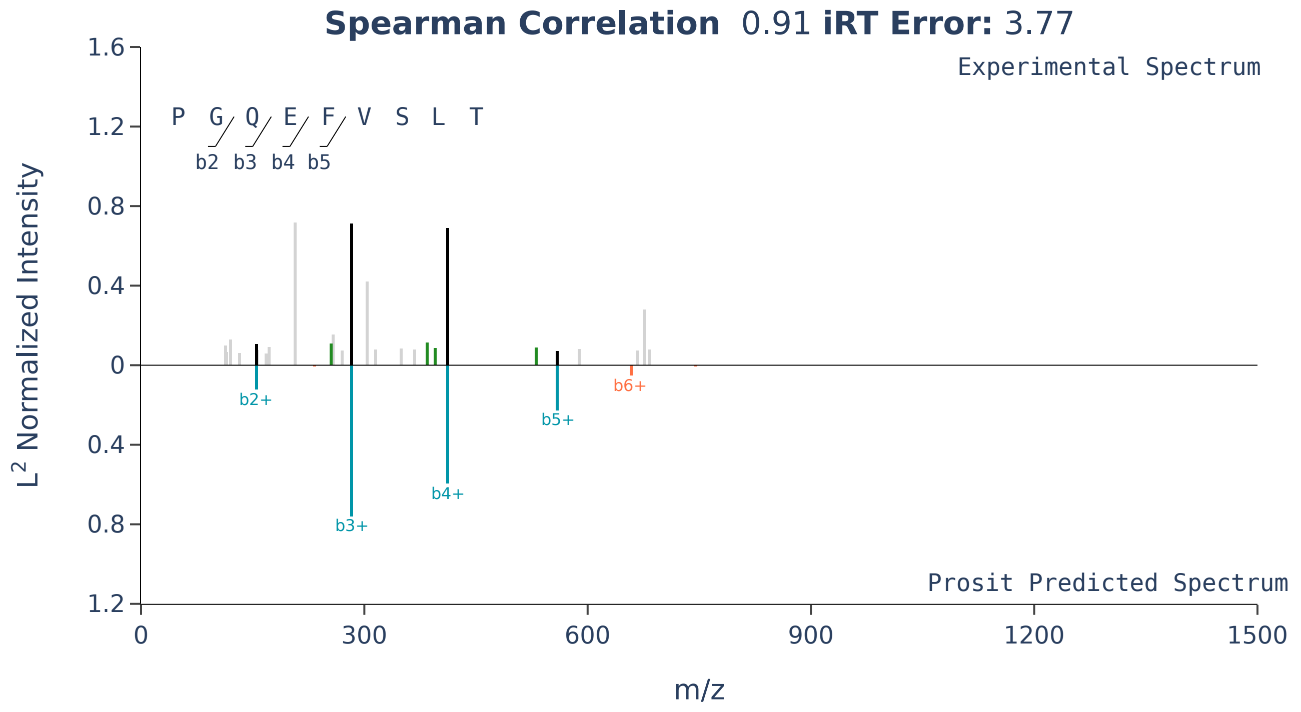

Source Ncheng_210623_230623_HFGoe_FFH_20S_25_1_A2_24h_R2 **Scan** 23499
Peptide LAEQVGVDF **Charge** 1 **Spectral Angle** 0.0
Spearman Correlation 0.0 **iRT Error:** 12.38

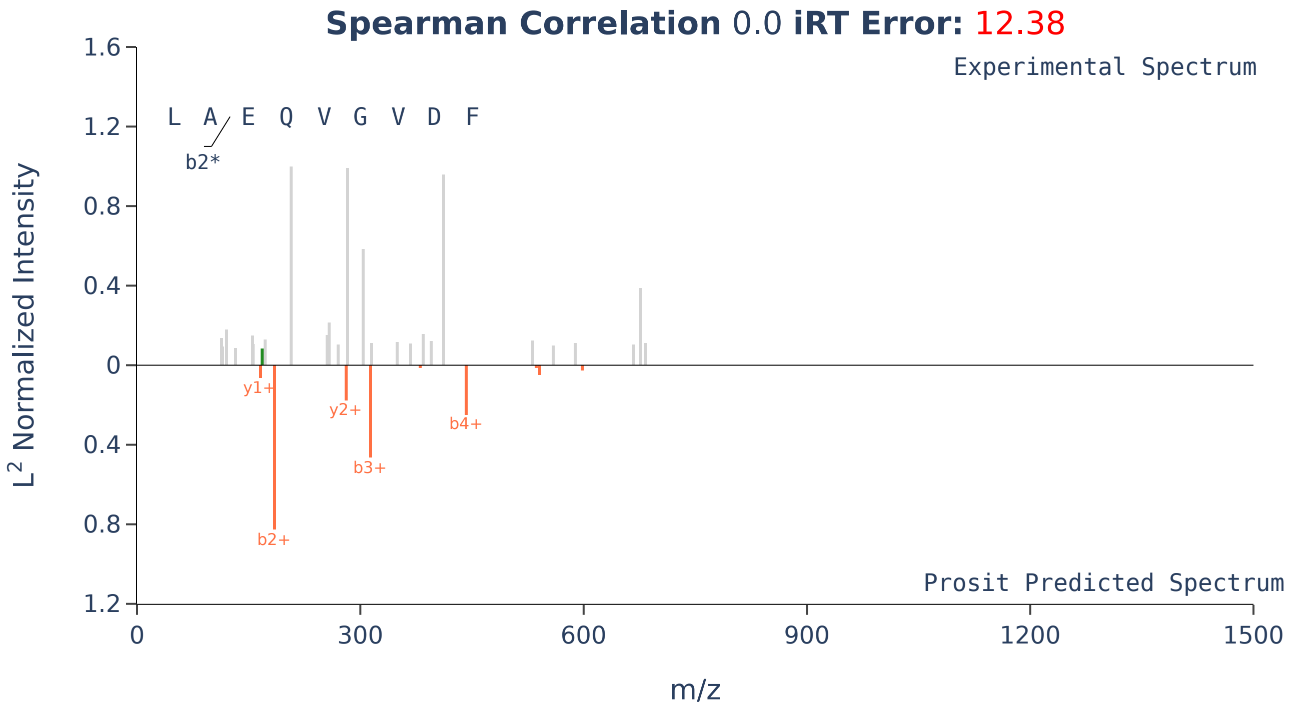

Source Ncheng_210623_230623_HFGoe_FFH_20S_25_1_A1_4h_R2 **Scan** 26654
Peptide SAMGKLPGMGQIPDNV **Charge** 2 **Spectral Angle** 0.89
Spearman Correlation 0.85 **iRT Error:** 3.89

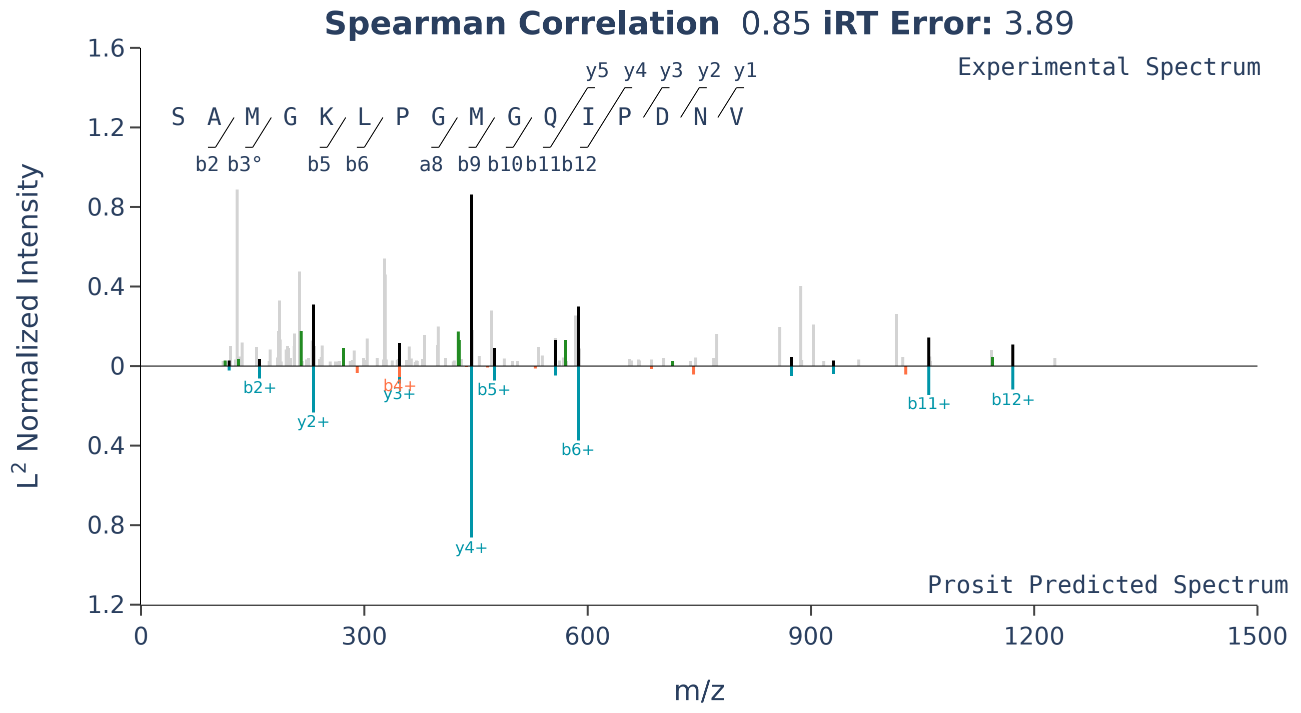

Source Ncheng_210623_230623_HFGoe_FFH_20S_25_1_A1_4h_R2 **Scan** 26654
Peptide LPGMGQIPDNVKSQM **Charge** 2 **Spectral Angle** 0.28
Spearman Correlation 0.29 **iRT Error:** 15.74

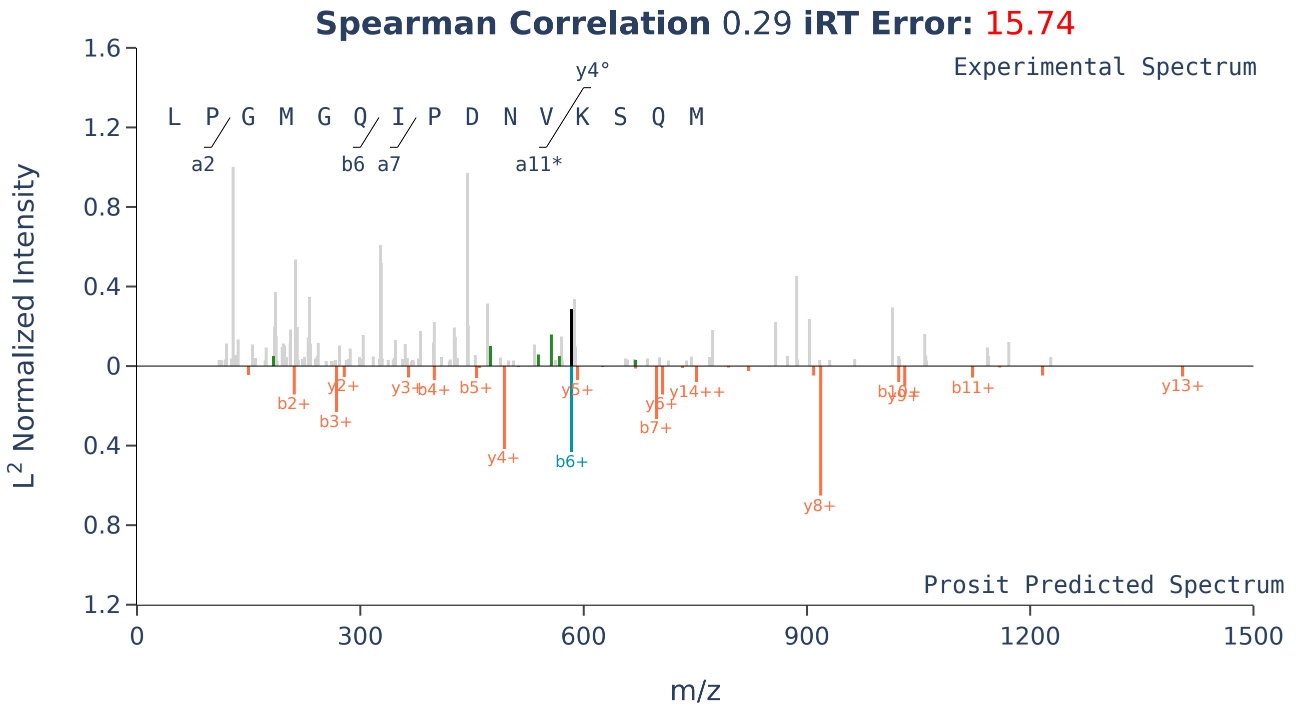

Source Ncheng_210623_230623_HFGoe_FFH_20S_25_1_A1_4h_R1 **Scan** 28201
Peptide PPAVVLMAAAG **Charge** 1 **Spectral Angle** 0.87
Spearman Correlation 0.95 **iRT Error:** 3.99

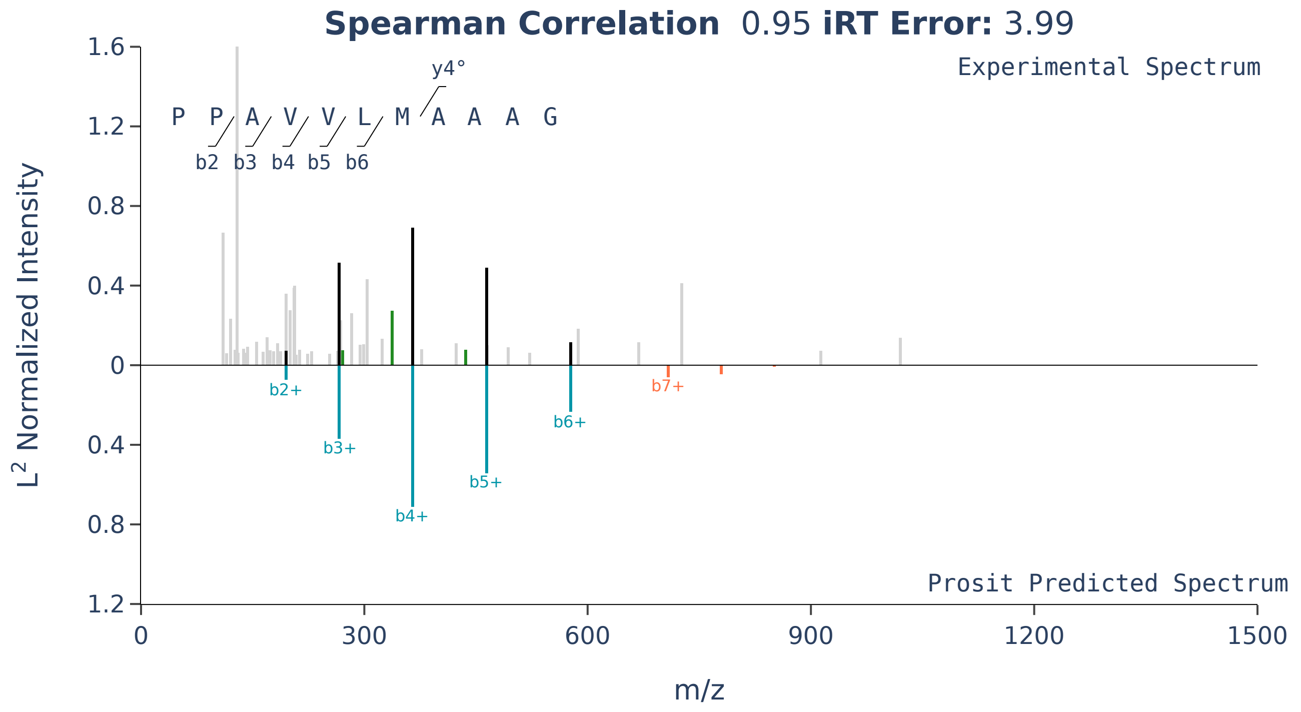

Source Ncheng_210623_230623_HFGoe_FFH_20S_25_1_A1_4h_R1 **Scan** 28201
Peptide AQPPAVVLMA **Charge** 1 **Spectral Angle** 0.0
Spearman Correlation 0.0 **iRT Error:** 1.86

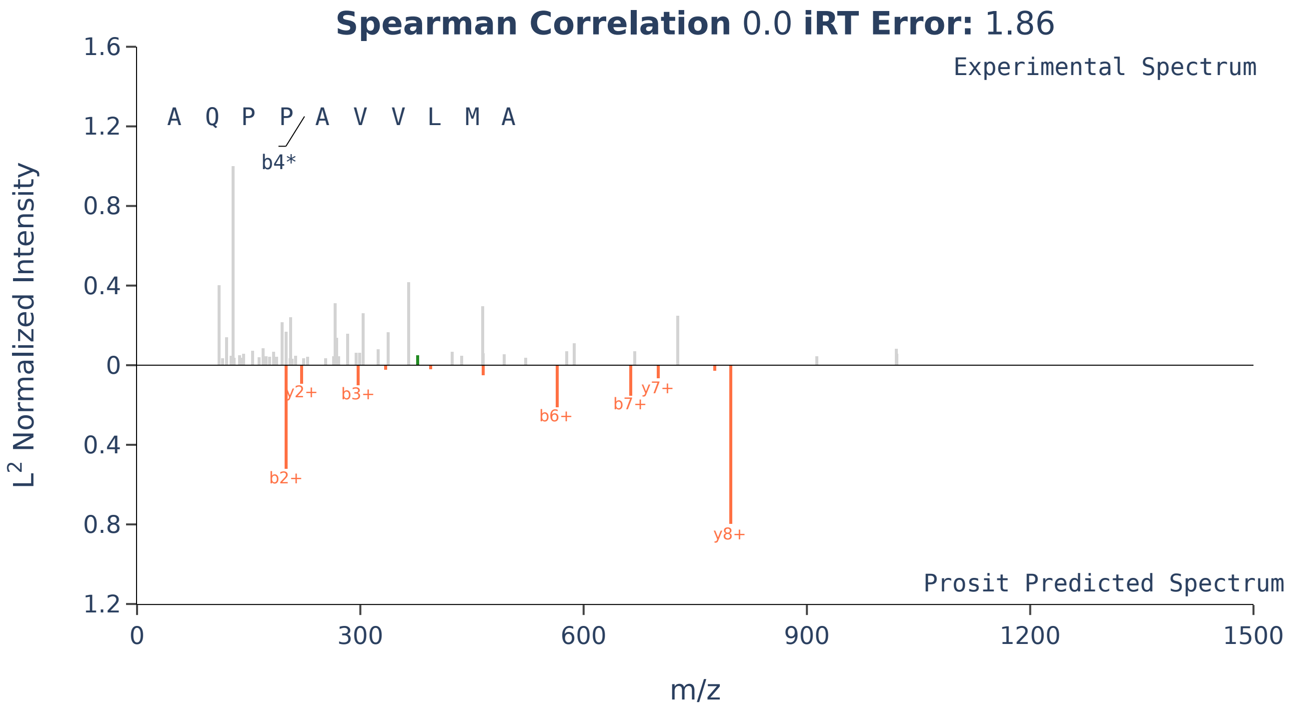

Source Ncheng_210623_230623_HFGoe_FFH_20S_25_1_A1_2h_R2 **Scan** 12931
Peptide PDNVKSQMNLA **Charge** 1 **Spectral Angle** 0.87
Spearman Correlation 0.89 **iRT Error:** 4.24

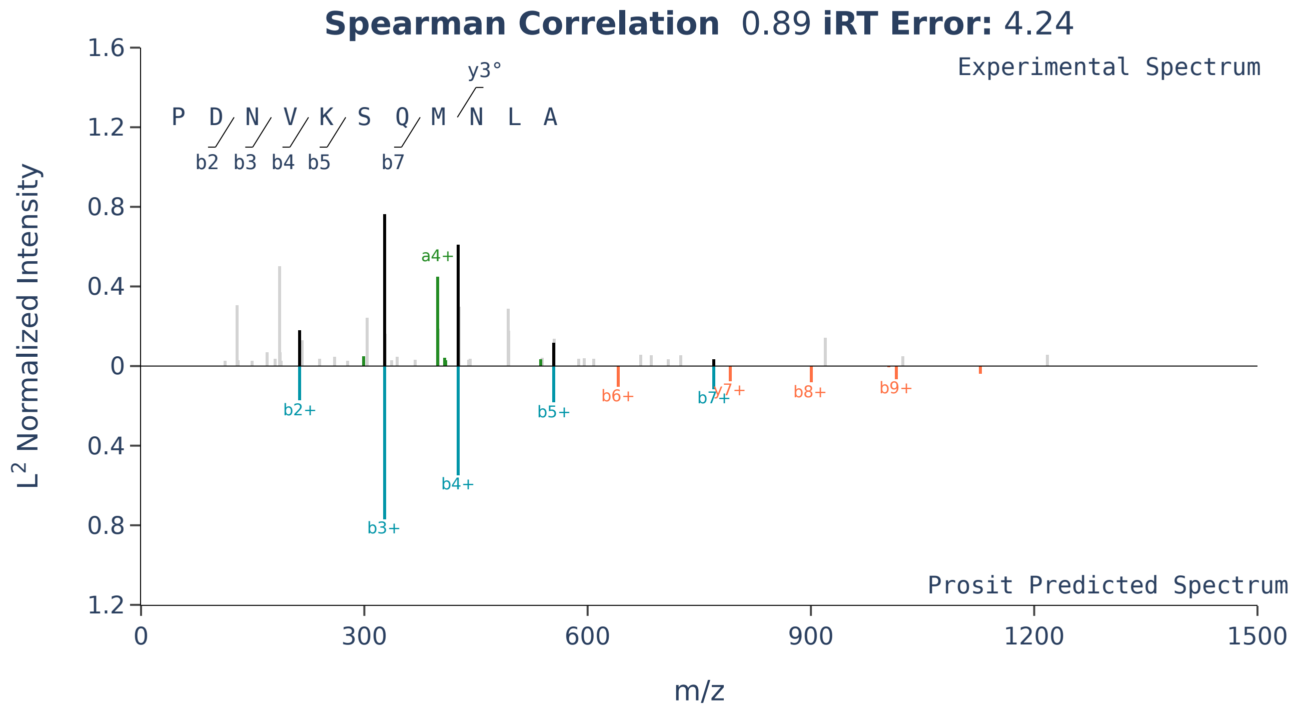

Source Ncheng_210623_230623_HFGoe_FFH_20S_25_1_A1_2h_R2 **Scan** 12931
Peptide MGQIPDNVKSQ **Charge** 1 **Spectral Angle** 0.0
Spearman Correlation 0.0 **iRT Error:** 5.26

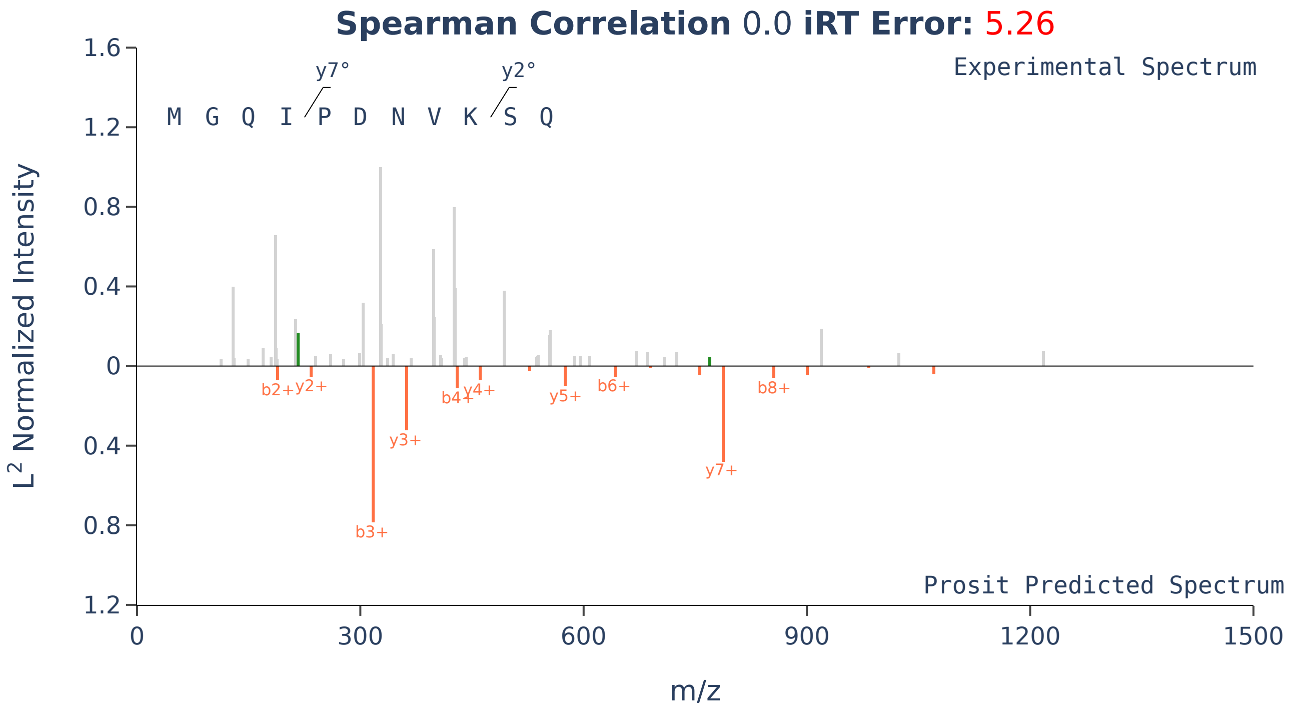

Source Ncheng_210623_230623_HFGoe_FFH_20S_25_1_A1_2h_R2 **Scan** 17535
Peptide ETLAEQVGVGQKPVD **Charge** 2 **Spectral Angle** 0.88
Spearman Correlation 0.9 **iRT Error:** 4.55

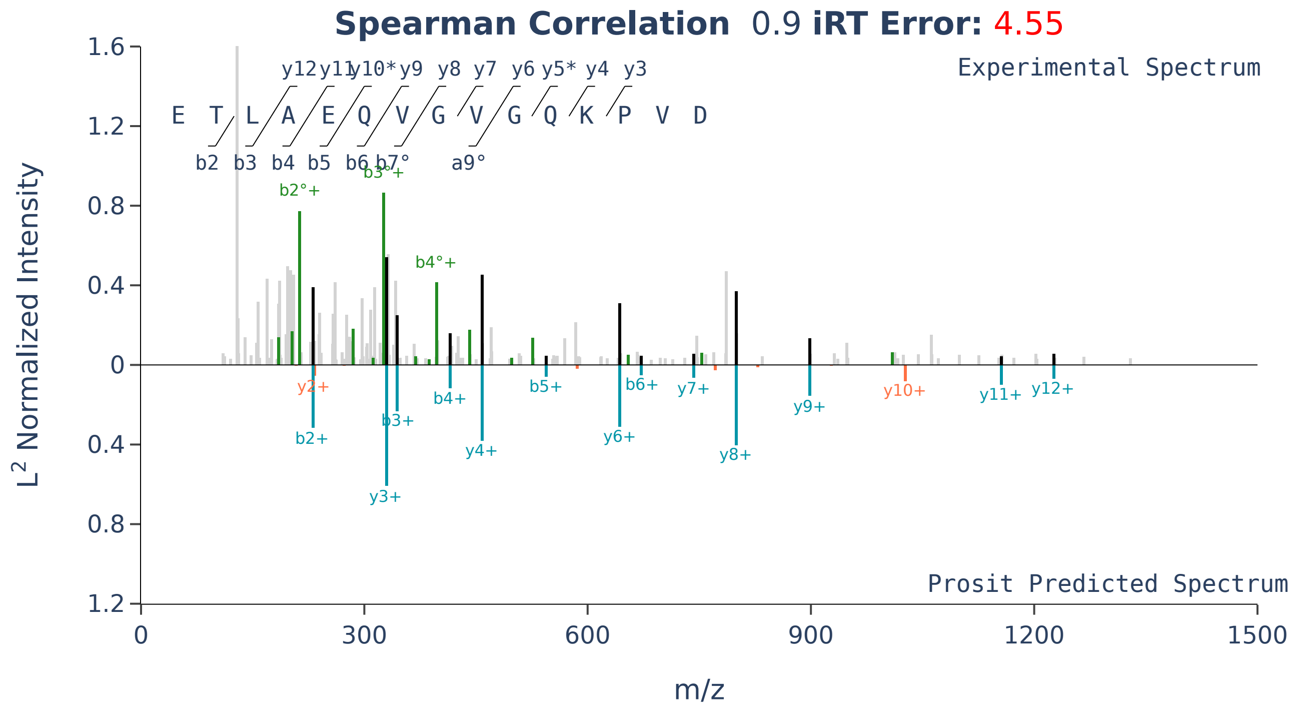

Source Ncheng_210623_230623_HFGoe_FFH_20S_25_1_A1_2h_R2 **Scan** 17535
Peptide LMGKLPGMGQIPDNV **Charge** 2 **Spectral Angle** 0.01
Spearman Correlation -0.12 **iRT Error:** 49.39

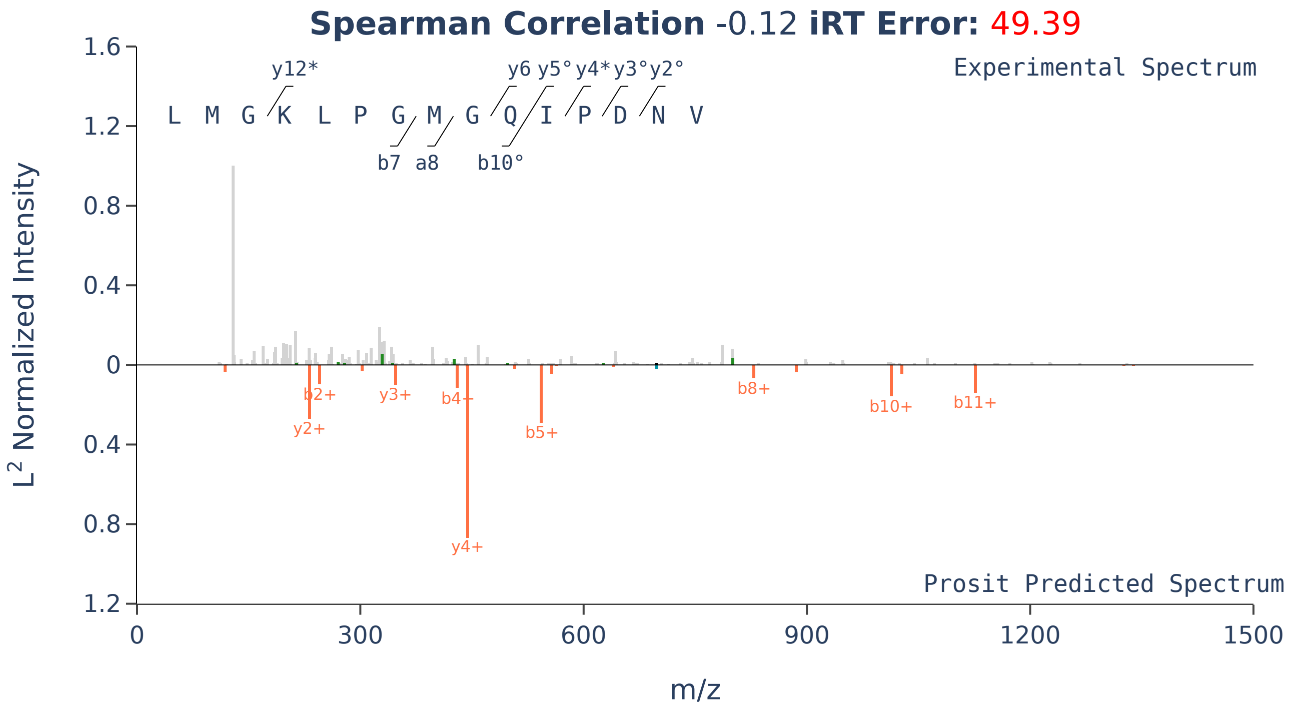

Source Ncheng_210623_230623_HFGoe_FFH_20S_25_1_A2_4h_R1 **Scan** 8342
Peptide ALKEARIA **Charge** 2 **Spectral Angle** 0.87
Spearman Correlation 0.88 **iRT Error:** 4.56

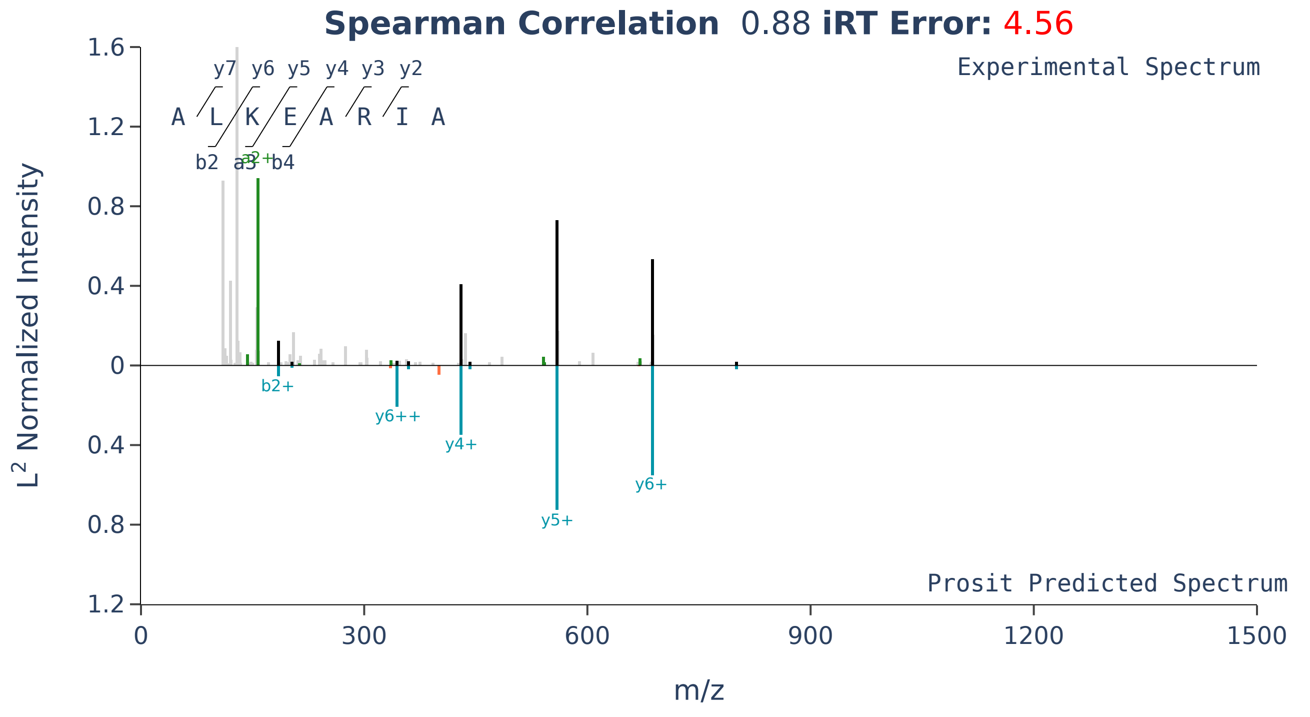

Source Ncheng_210623_230623_HFGoe_FFH_20S_25_1_A2_4h_R1 **Scan** 8342
Peptide KIVRNEL **Charge** 2 **Spectral Angle** 0.37
Spearman Correlation 0.03 **iRT Error:** 2.48

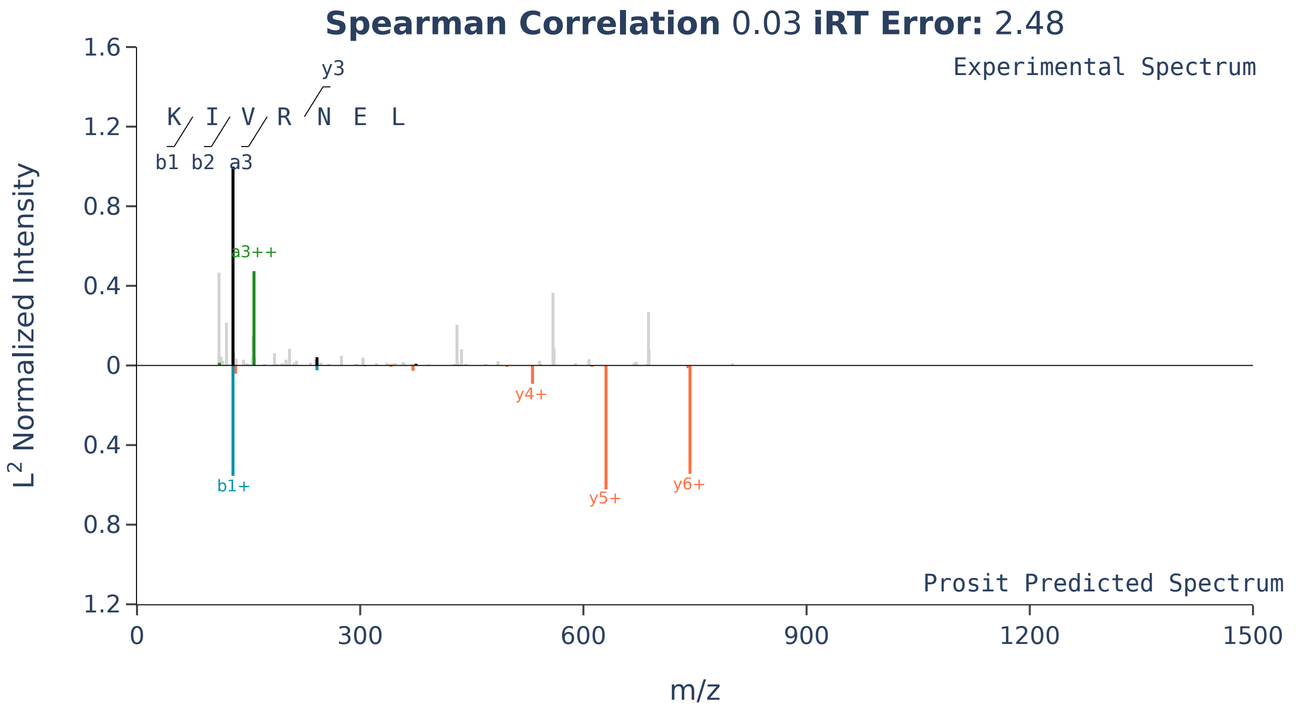

Source Ncheng_210623_230623_HFGoe_FFH_20S_25_1_A2_2h_R2 **Scan** 9209
Peptide ESKVDNL **Charge** 2 **Spectral Angle** 0.91
Spearman Correlation 0.85 **iRT Error:** 4.62

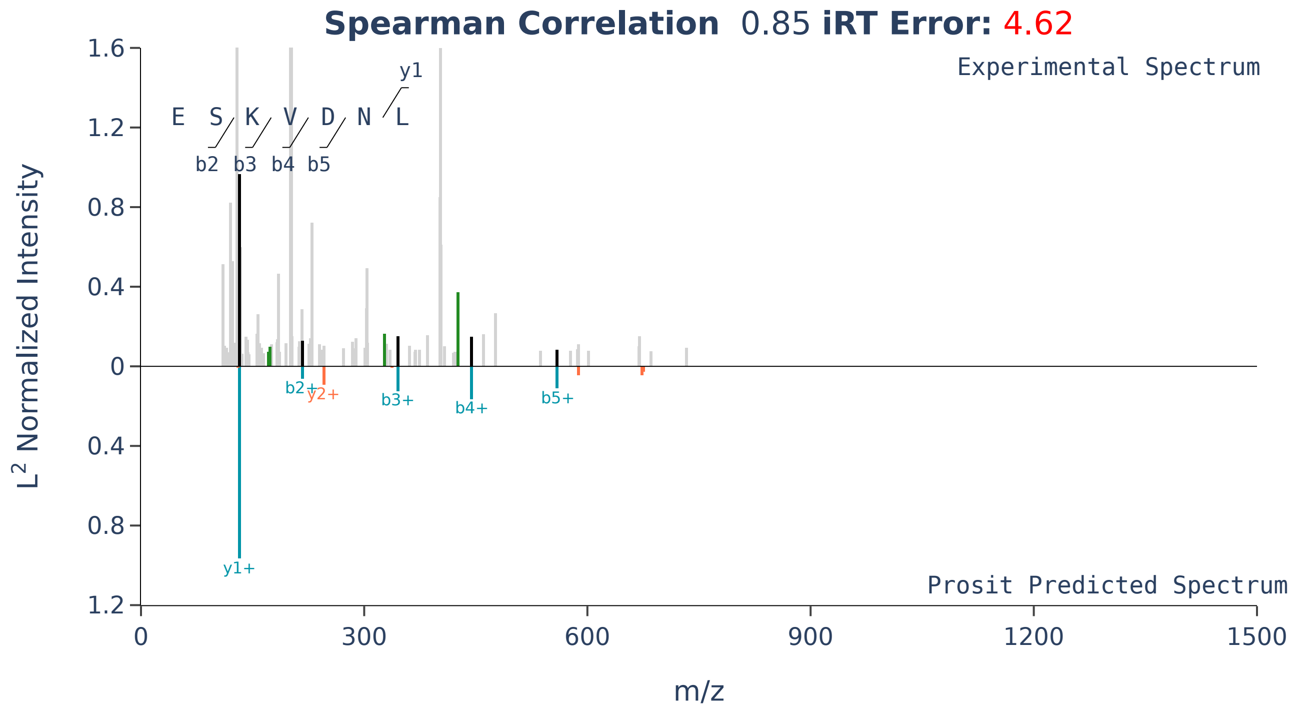

Source Ncheng_210623_230623_HFGoe_FFH_20S_25_1_A2_2h_R2 **Scan** 9209
Peptide DNVKDTL **Charge** 2 **Spectral Angle** 0.81
Spearman Correlation 0.8 **iRT Error:** 10.62

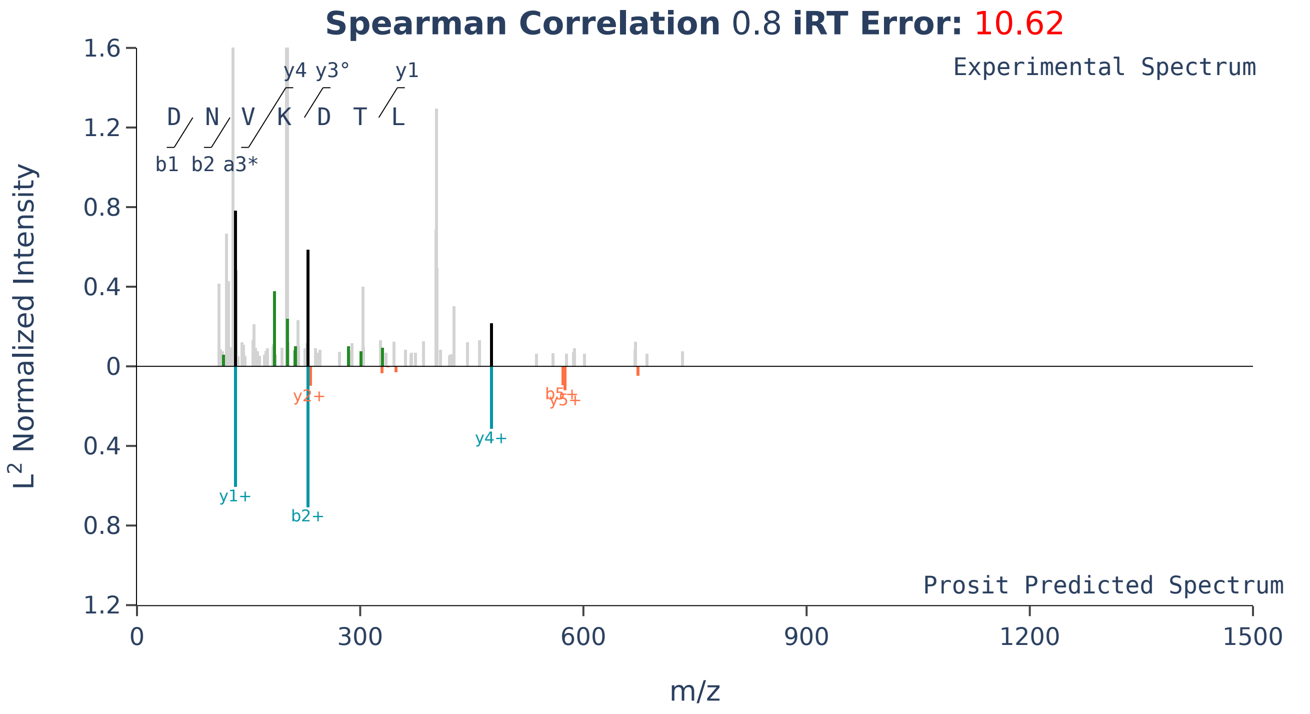

Source Ncheng_210623_230623_HFGoe_FFH_20S_25_1_A2_2h_R1 **Scan** 14832
Peptide MRSTLAEQV **Charge** 2 **Spectral Angle** 0.83
Spearman Correlation 0.91 **iRT Error:** 4.65

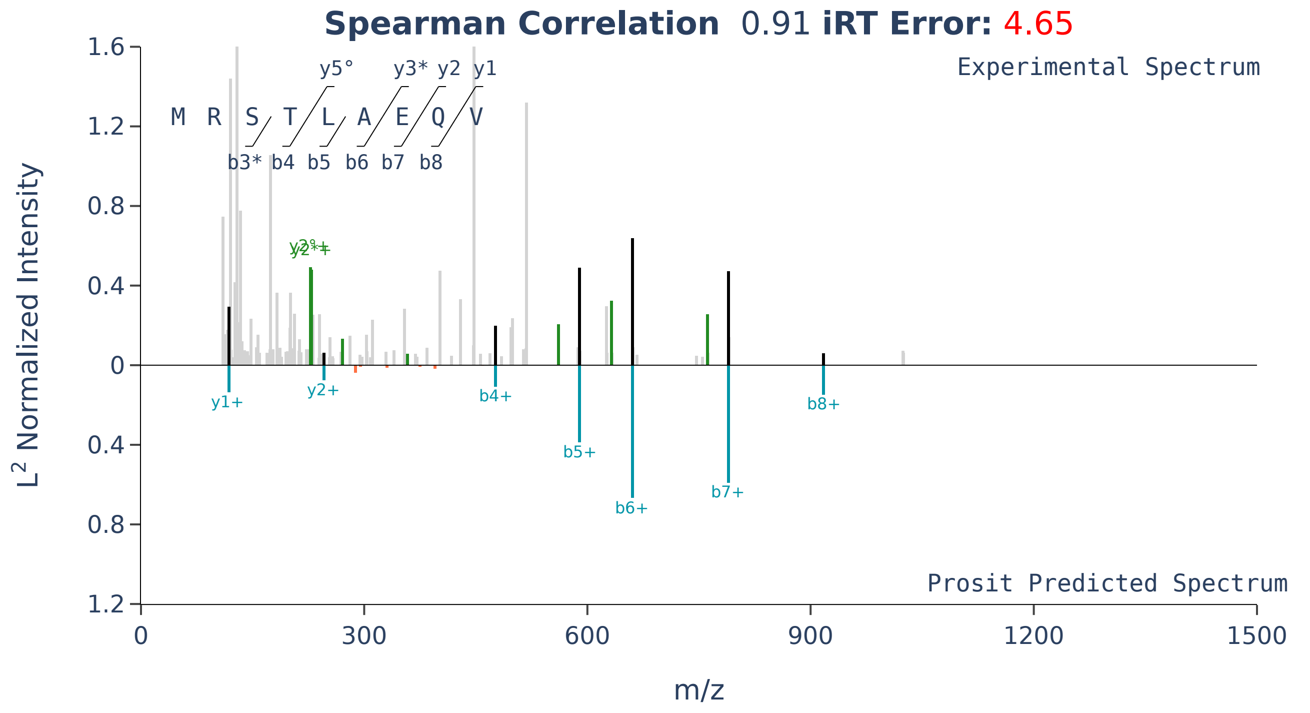

Source Ncheng_210623_230623_HFGoe_FFH_20S_25_1_A2_2h_R1 **Scan** 14832
Peptide LRQMKNMGG **Charge** 2 **Spectral Angle** 0.0
Spearman Correlation 0.0 **iRT Error:** 50.54

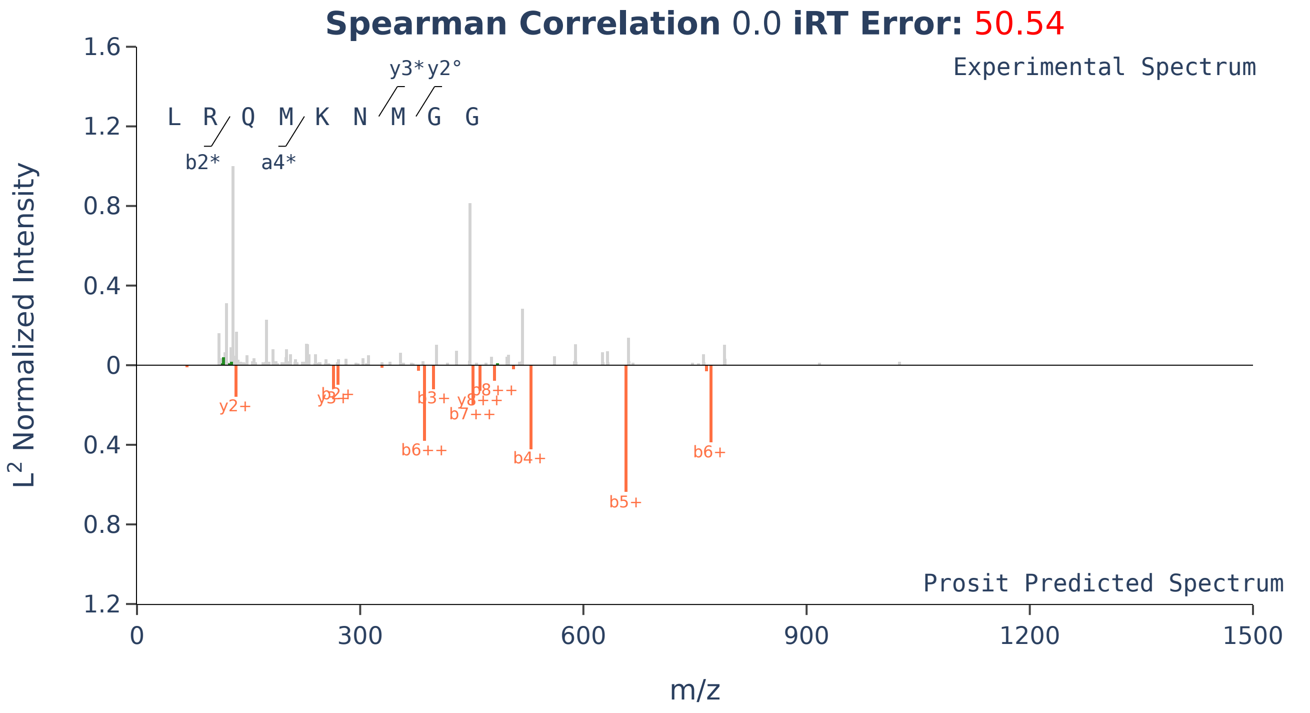

Source Ncheng_210623_230623_HFGoe_FFH_20S_25_1_A1_24h_R2 **Scan** 17102
Peptide ASQDVNRLL **Charge** 2 **Spectral Angle** 0.79
Spearman Correlation 0.9 **iRT Error:** 4.66

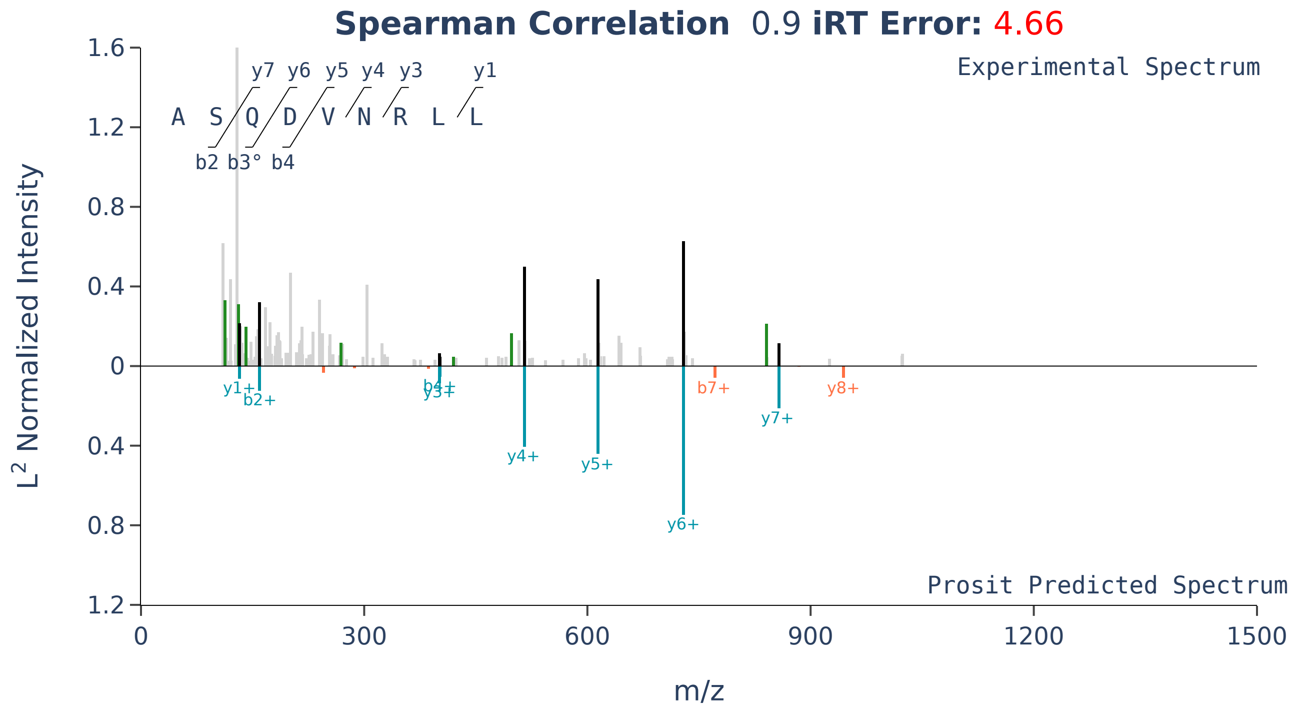

Source Ncheng_210623_230623_HFGoe_FFH_20S_25_1_A1_24h_R2 **Scan** 17102
Peptide VVLMAGLQGAG **Charge** 2 **Spectral Angle** 0.0
Spearman Correlation 0.0 **iRT Error:** 48.64

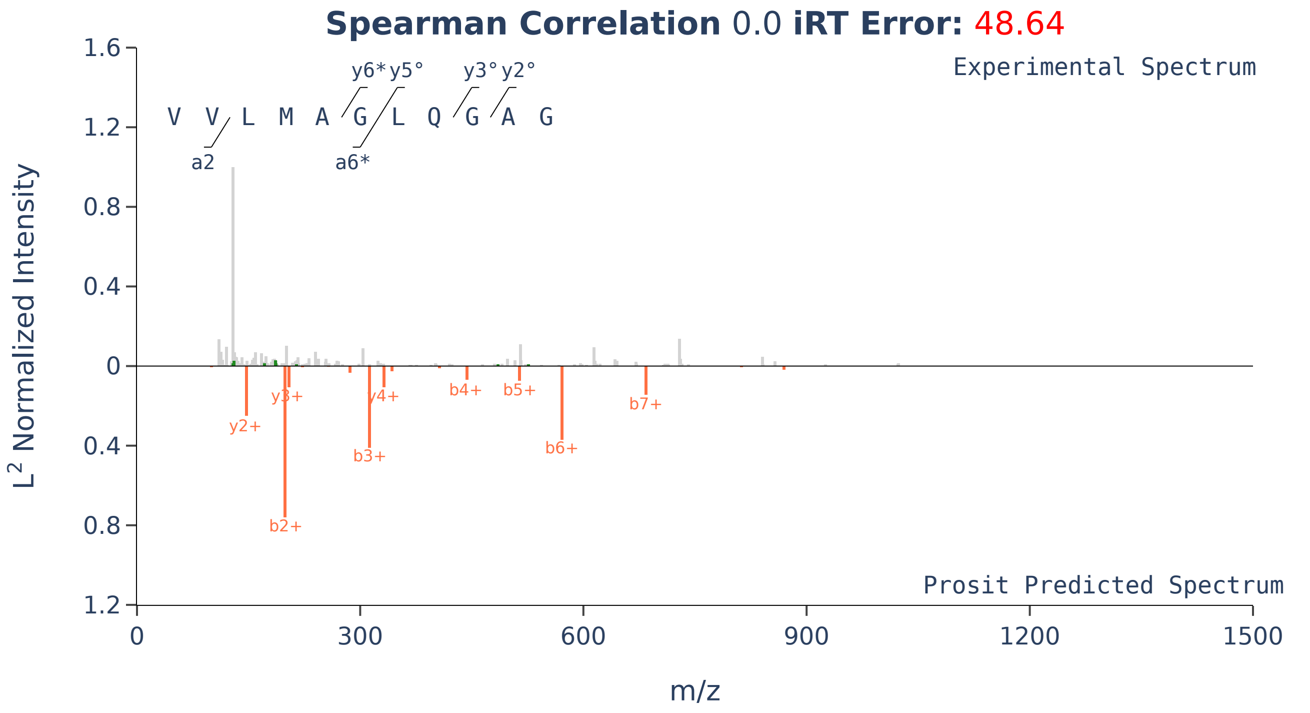

Source Ncheng_210623_230623_HFGoe_FFH_20S_25_1_A2_2h_R2 **Scan** 15329
Peptide TGKPIKFLGVGEKTED **Charge** 2 **Spectral Angle** 0.85
Spearman Correlation 0.92 **iRT Error:** 4.68

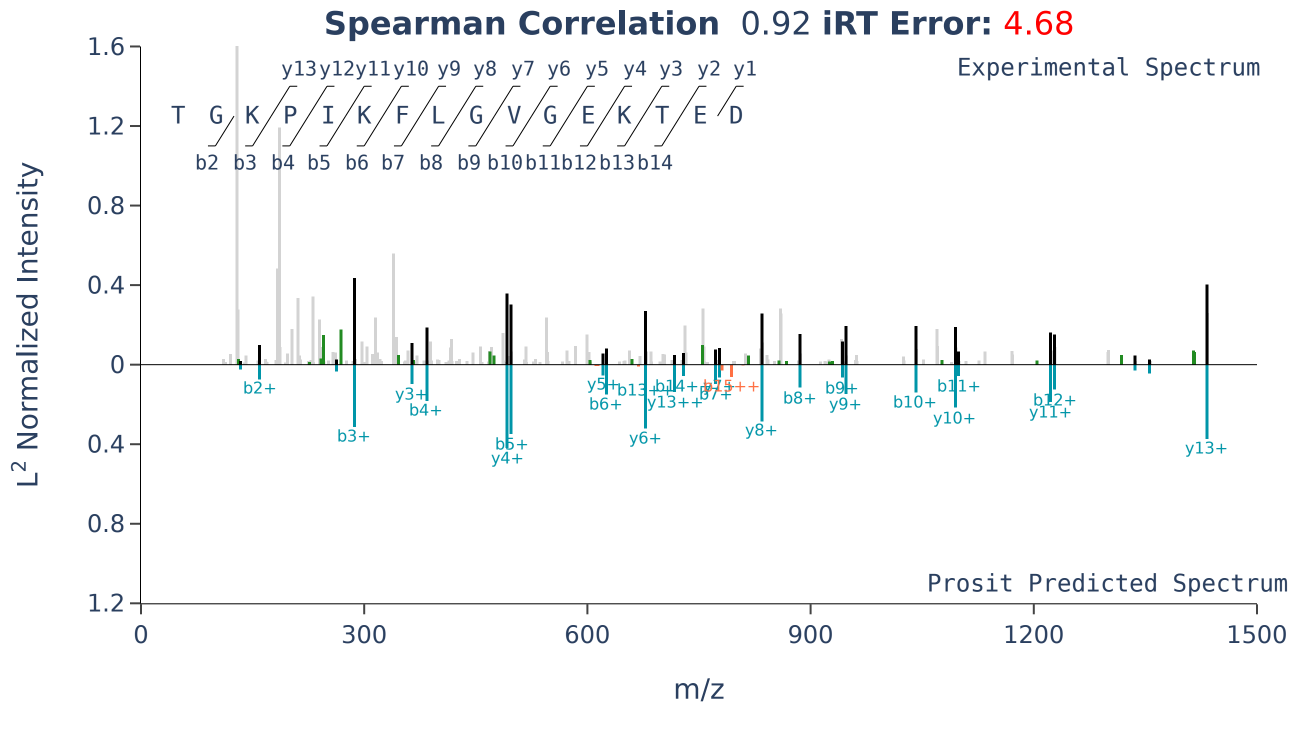

Source Ncheng_210623_230623_HFGoe_FFH_20S_25_1_A2_2h_R2 **Scan** 15329
Peptide MTMKERAKPEIIKGS **Charge** 2 **Spectral Angle** 0.03
Spearman Correlation -0.37 **iRT Error:** 38.42

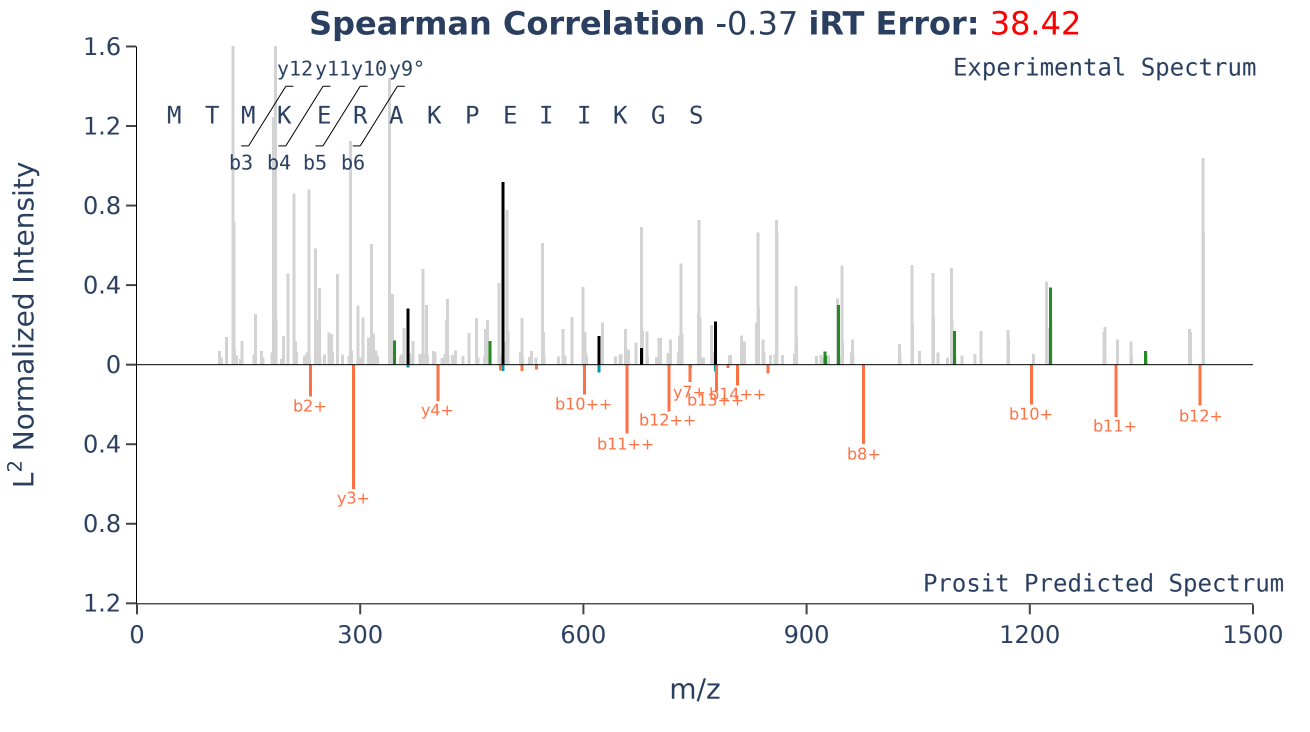

Source Ncheng_210623_230623_HFGoe_FFH_20S_25_1_A2_24h_R2 **Scan** 17668
Peptide GKLKFYDV **Charge** 2 **Spectral Angle** 0.74
Spearman Correlation 0.92 **iRT Error:** 4.84

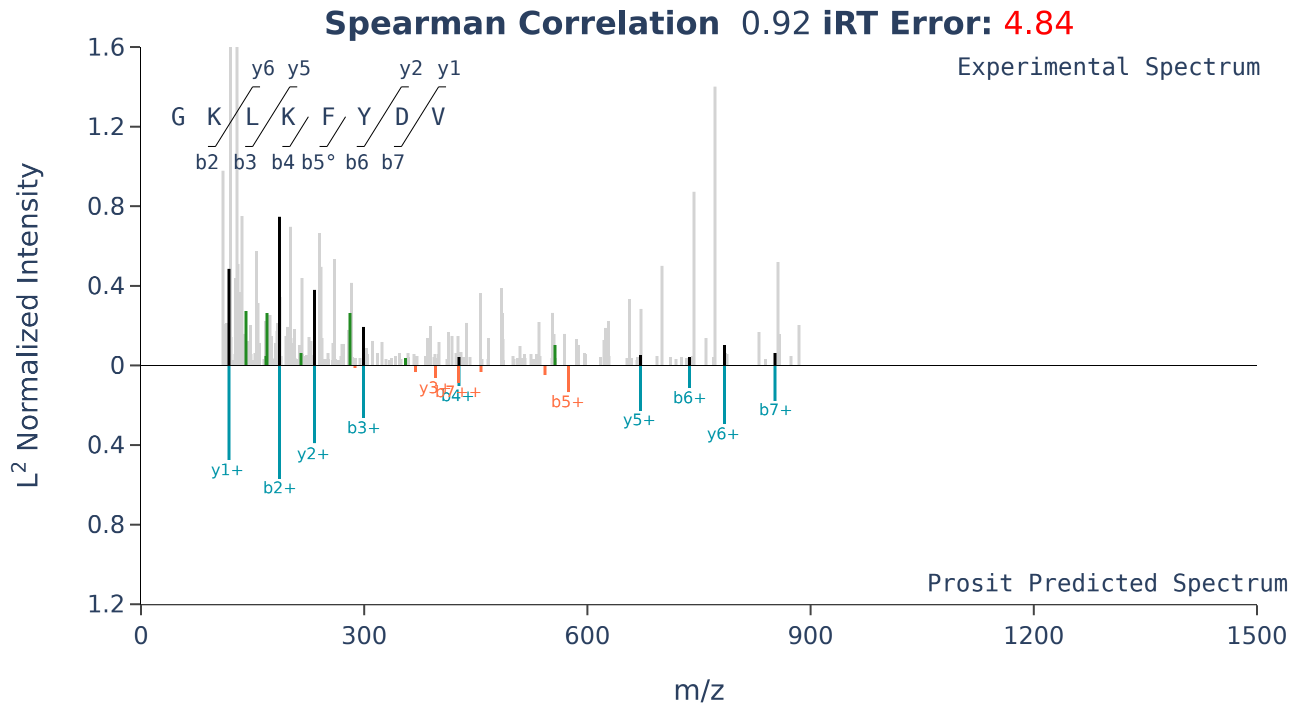

Source Ncheng_210623_230623_HFGoe_FFH_20S_25_1_A2_24h_R2 **Scan** 17668
Peptide GQKPVDIVN **Charge** 2 **Spectral Angle** 0.05
Spearman Correlation -0.16 **iRT Error:** 24.7

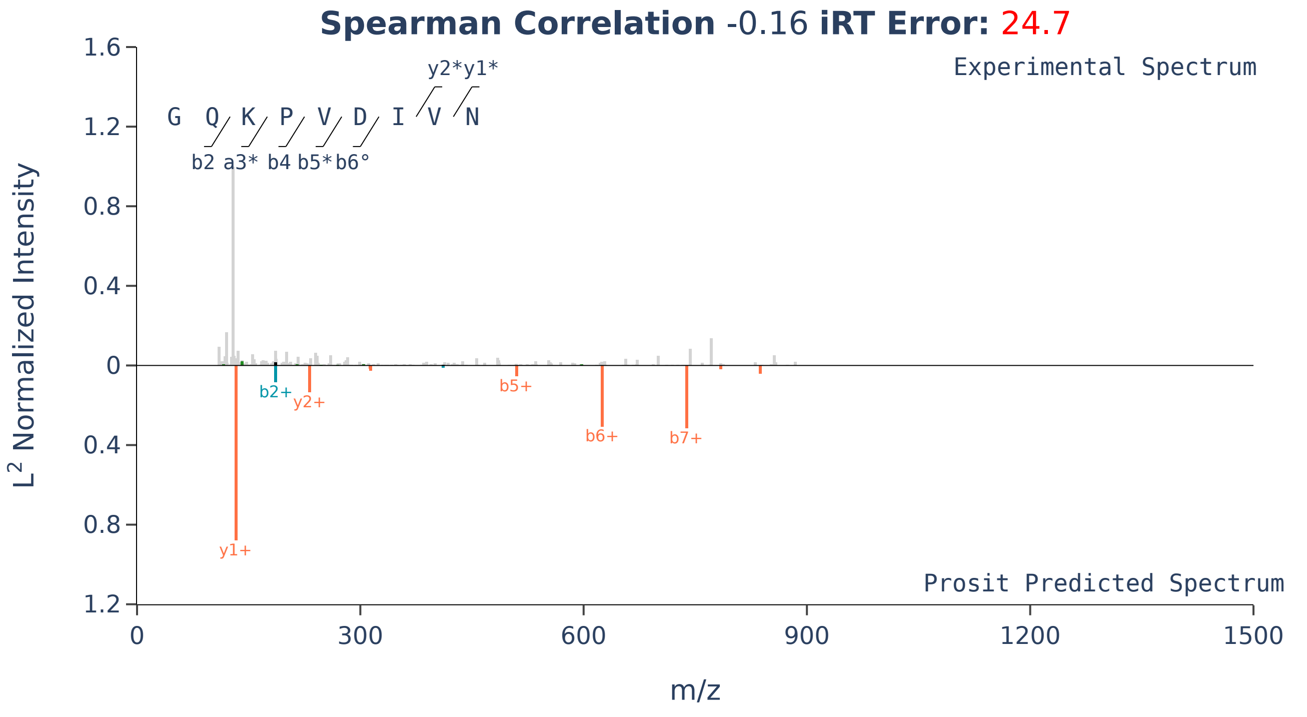

Source Ncheng_210623_230623_HFGoe_FFH_20S_25_1_A1_4h_R2 **Scan** 32197
Peptide DVGLSLIE **Charge** 2 **Spectral Angle** 0.88
Spearman Correlation 0.93 **iRT Error:** 5.04

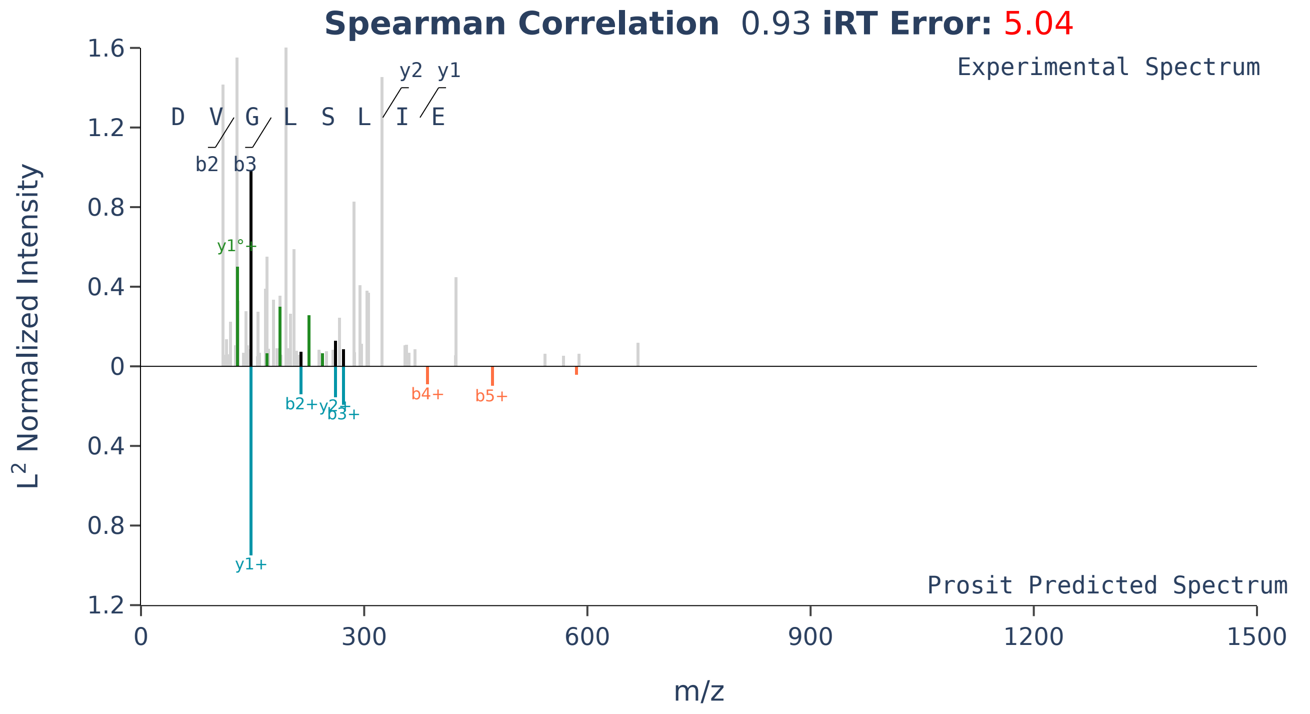

Source Ncheng_210623_230623_HFGoe_FFH_20S_25_1_A1_4h_R2 **Scan** 32197
Peptide DVLLVDTA **Charge** 2 **Spectral Angle** 0.52
Spearman Correlation 0.65 **iRT Error:** 36.11

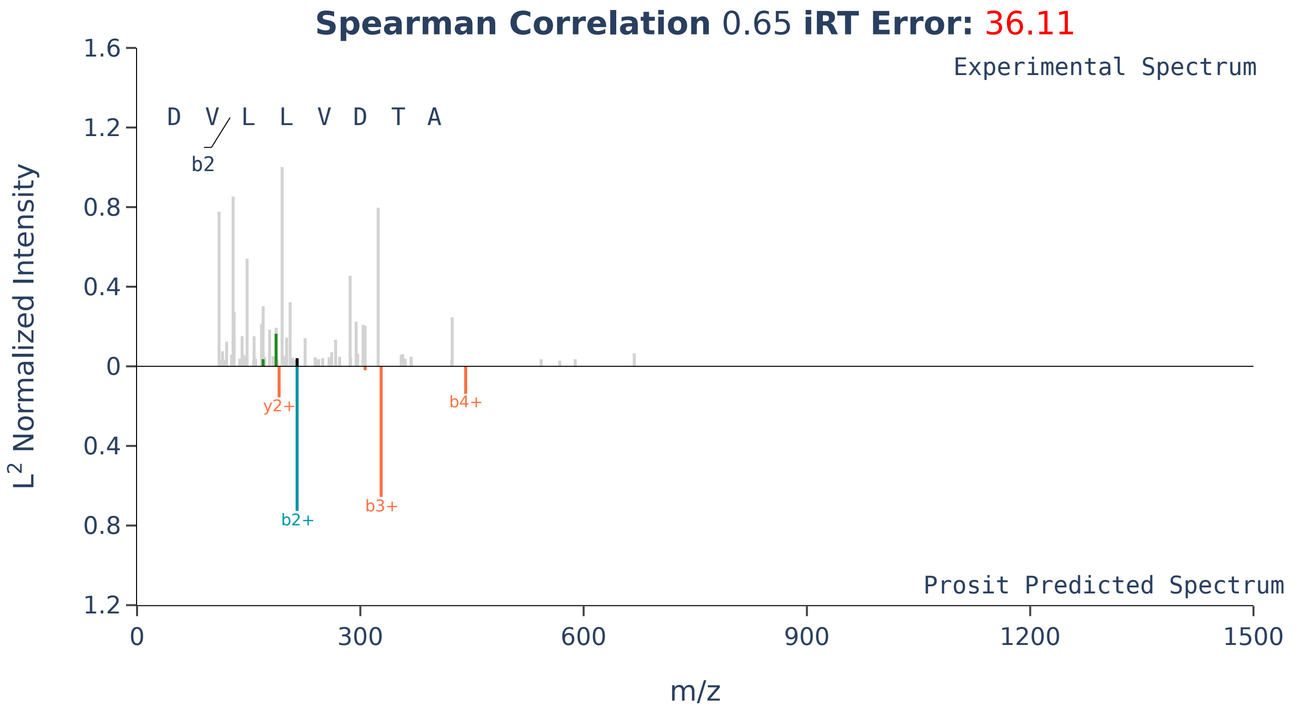

Source Ncheng_210623_230623_HFGoe_FFH_20S_25_1_A2_4h_R1 **Scan** 18978
Peptide QVQLEPFHPDRIASR **Charge** 4 **Spectral Angle** 0.82
Spearman Correlation 0.91 **iRT Error:** 5.09

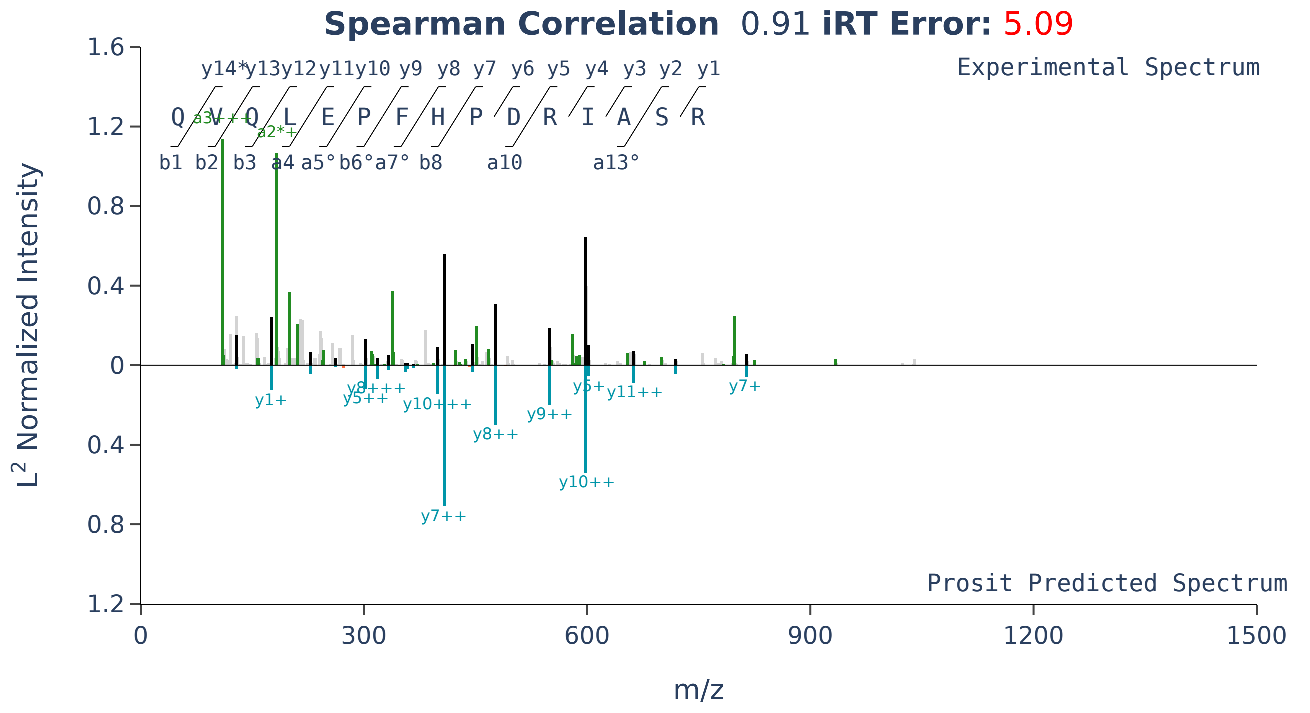

Source Ncheng_210623_230623_HFGoe_FFH_20S_25_1_A2_4h_R1 **Scan** 18978
Peptide DEIKQVHASINPVETL **Charge** 4 **Spectral Angle** 0.38
Spearman Correlation 0.29 **iRT Error:** 24.16

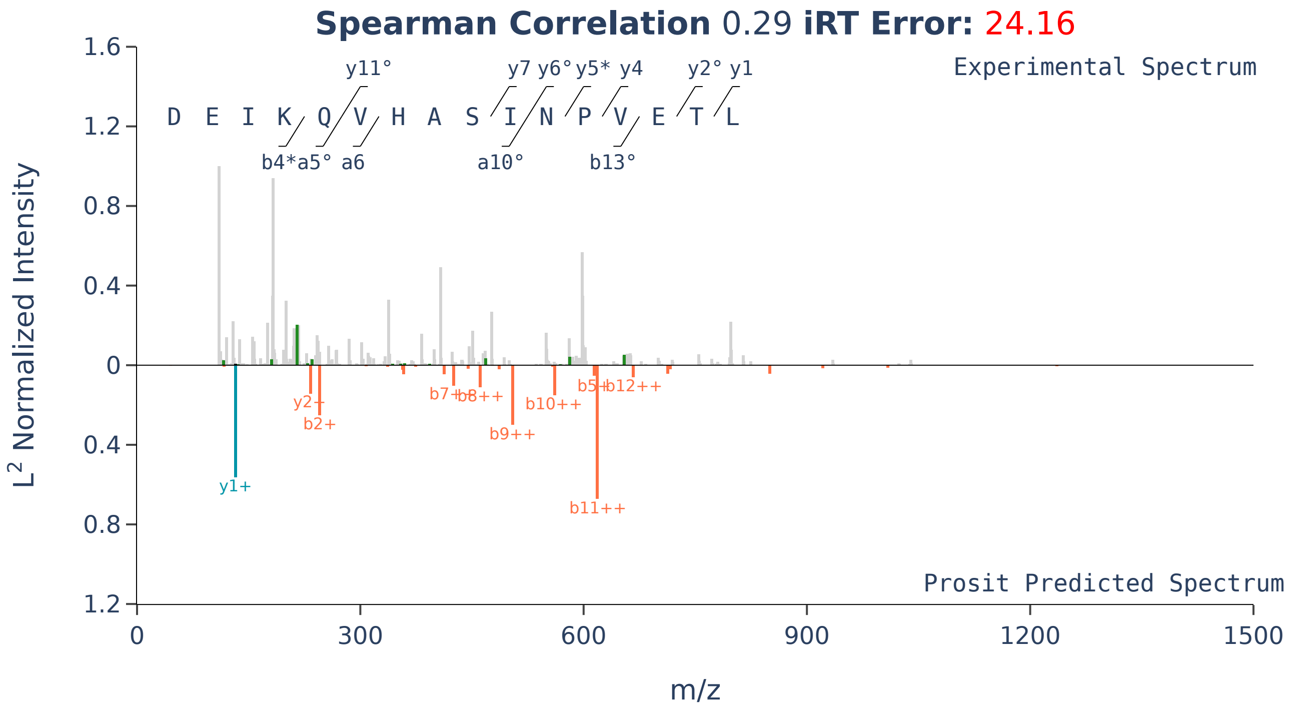

Source Ncheng_210623_230623_HFGoe_FFH_20S_25_1_A1_2h_R2 **Scan** 11160
Peptide MDIESKVDRA **Charge** 3 **Spectral Angle** 0.79
Spearman Correlation 0.91 **iRT Error:** 5.18

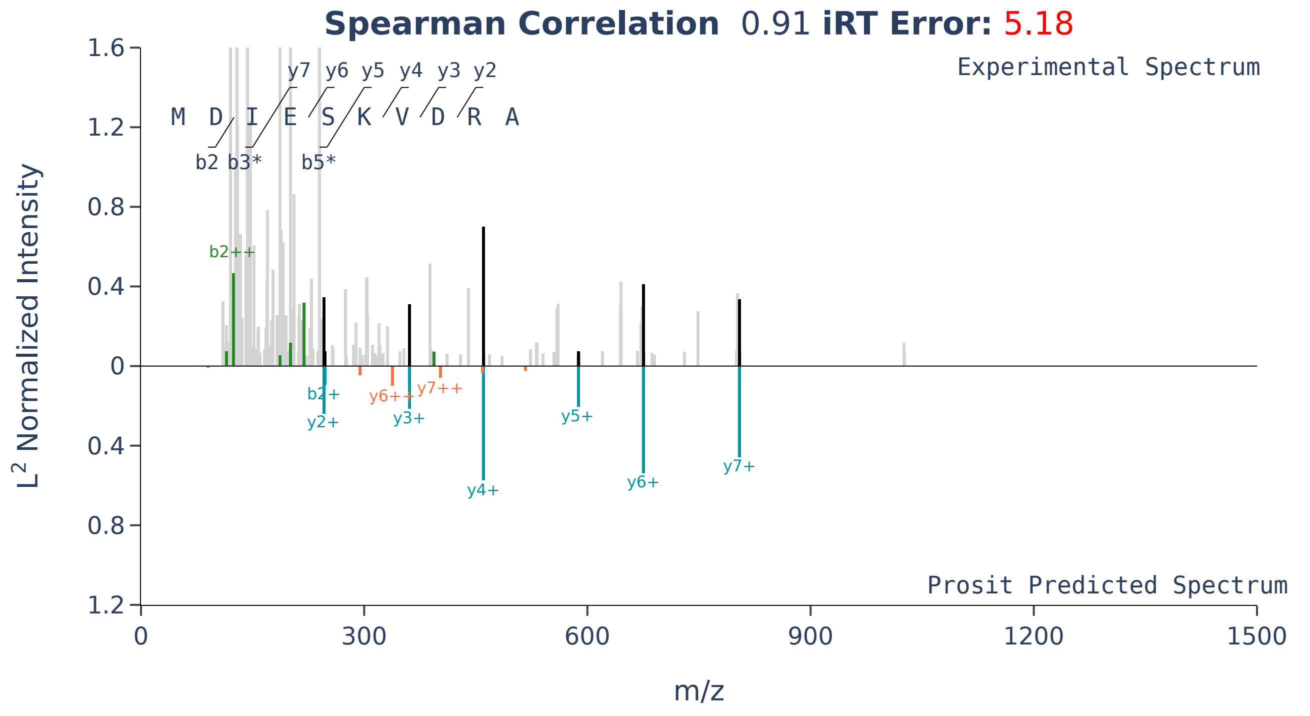

Source Ncheng_210623_230623_HFGoe_FFH_20S_25_1_A1_2h_R2 **Scan** 11160
Peptide NVKSQMDDKV **Charge** 3 **Spectral Angle** 0.02
Spearman Correlation -0.41 **iRT Error:** 7.97

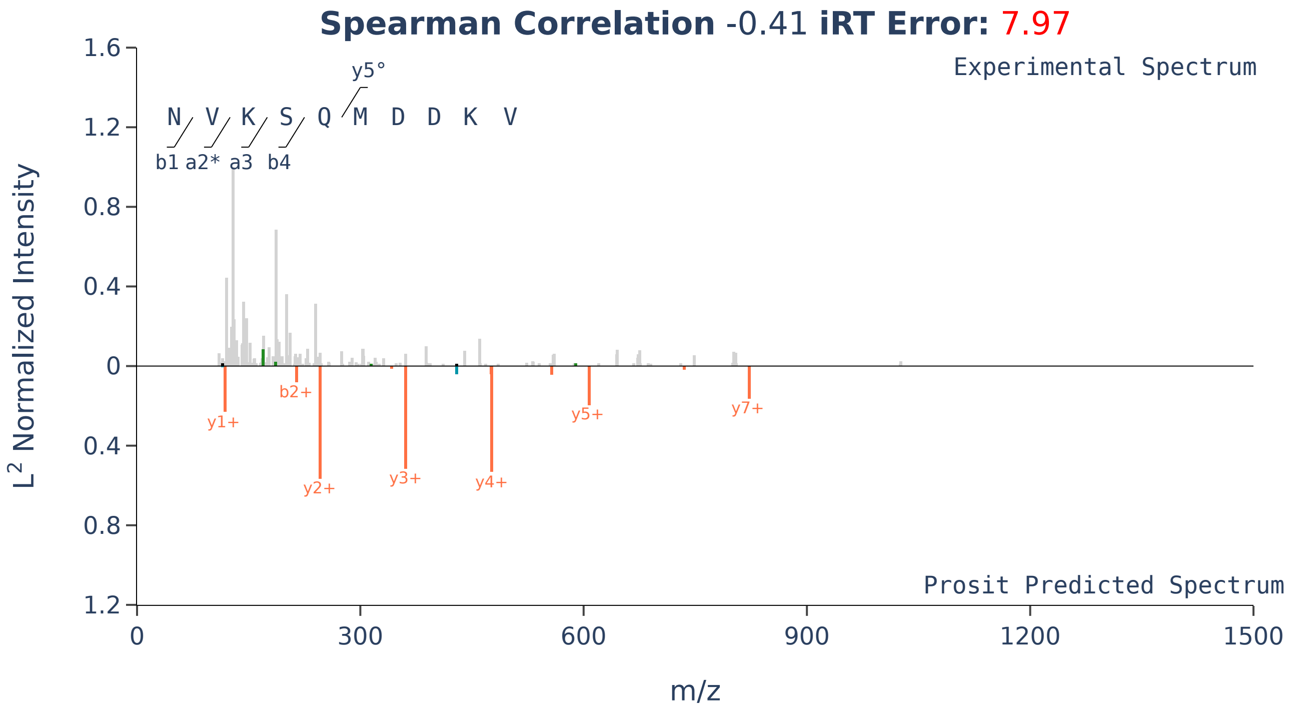

Source Ncheng_210623_230623_HFGoe_FFH_20S_25_1_A2_4h_R2 **Scan** 36873
Peptide PLTGVVLEAL **Charge** 1 **Spectral Angle** 0.82
Spearman Correlation 0.87 **iRT Error:** 5.42

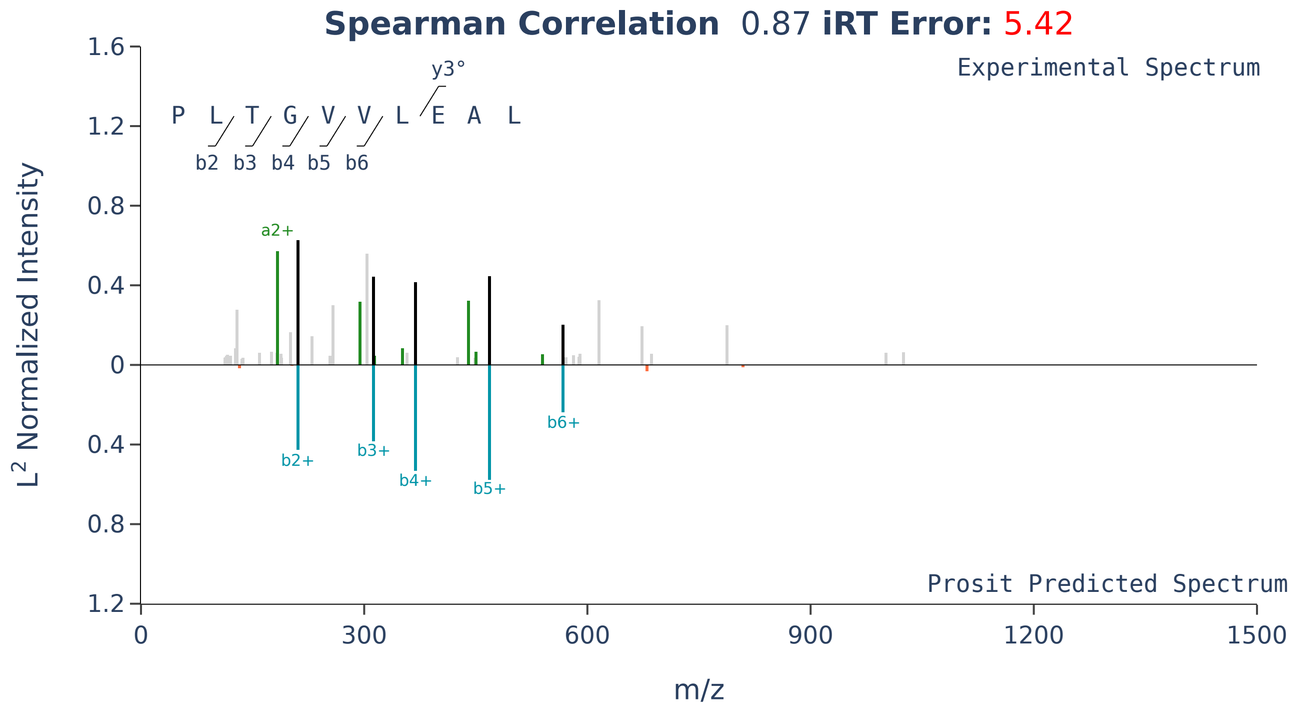

Source Ncheng_210623_230623_HFGoe_FFH_20S_25_1_A2_4h_R2 **Scan** 36873
Peptide EALPLTGVVL **Charge** 1 **Spectral Angle** 0.69
Spearman Correlation 0.67 **iRT Error:** 3.12

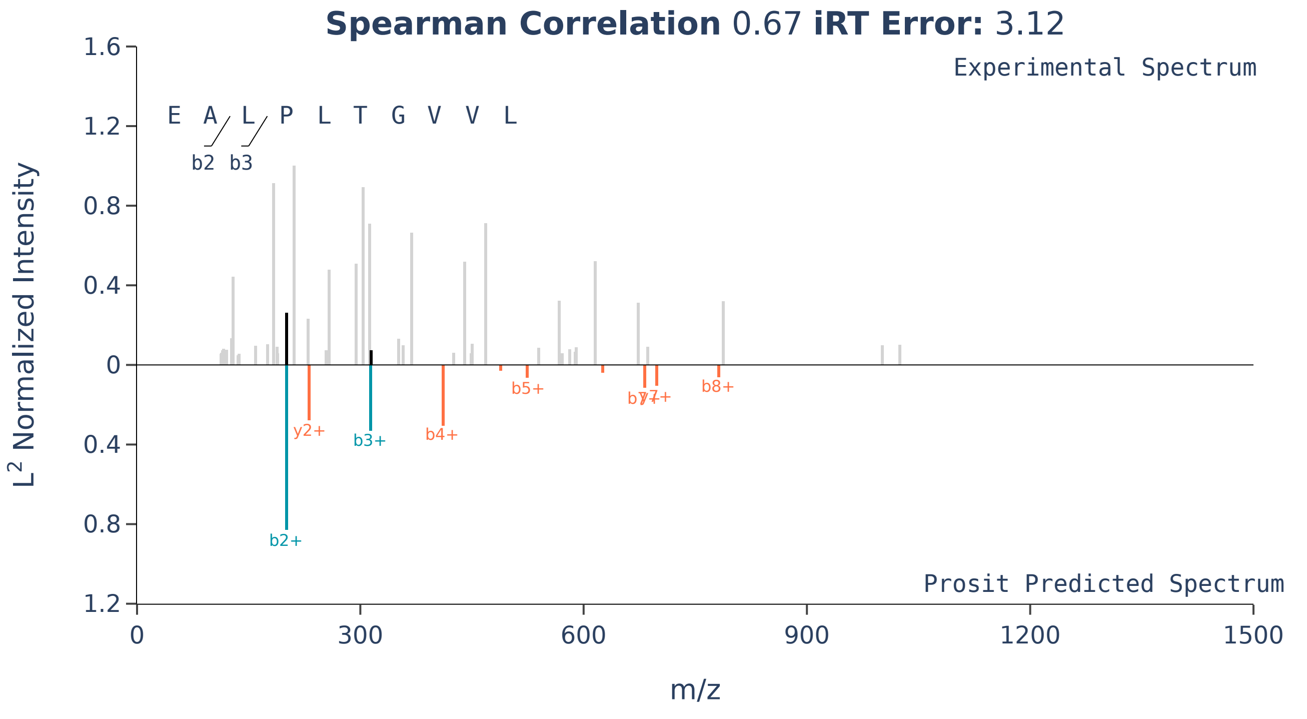

Source Ncheng_210623_230623_HFGoe_FFH_20S_25_1_A2_1h_R1 **Scan** 11614
Peptide PDNVKSQMEQVG **Charge** 1 **Spectral Angle** 0.85
Spearman Correlation 0.95 **iRT Error:** 5.5

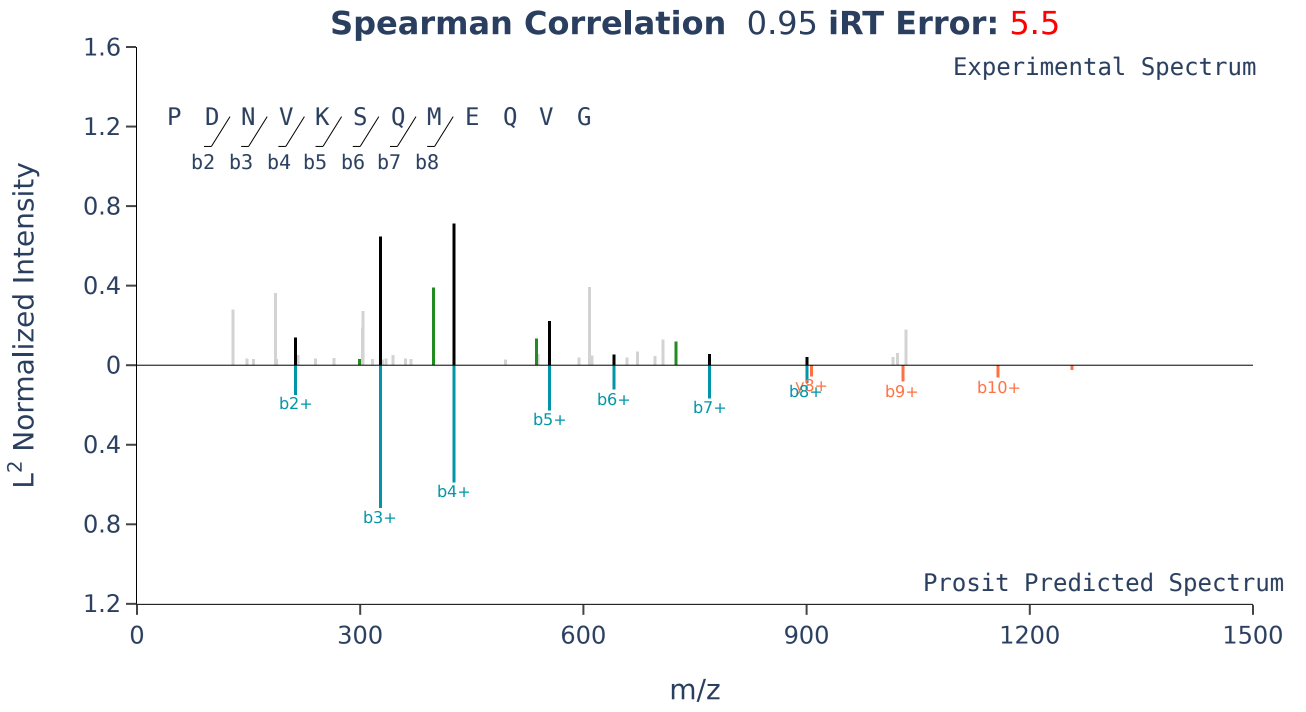

Source Ncheng_210623_230623_HFGoe_FFH_20S_25_1_A2_1h_R1 **Scan** 11614
Peptide GQIPDNVKSQMD **Charge** 1 **Spectral Angle** 0.77
Spearman Correlation 0.54 **iRT Error:** 3.38

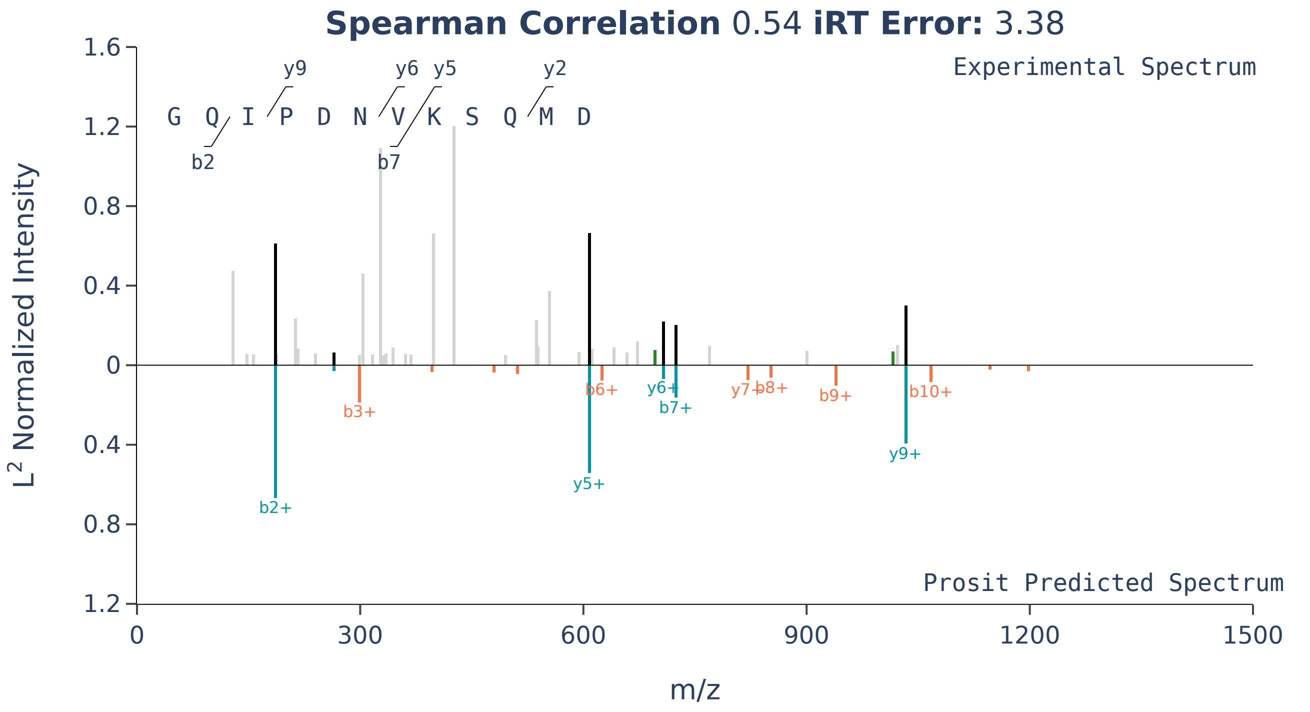

Source Ncheng_210623_230623_HFGoe_FFH_20S_25_1_A1_1h_R1 **Scan** 11245
Peptide PDNVKSQMADVA **Charge** 1 **Spectral Angle** 0.84
Spearman Correlation 0.91 **iRT Error:** 5.52

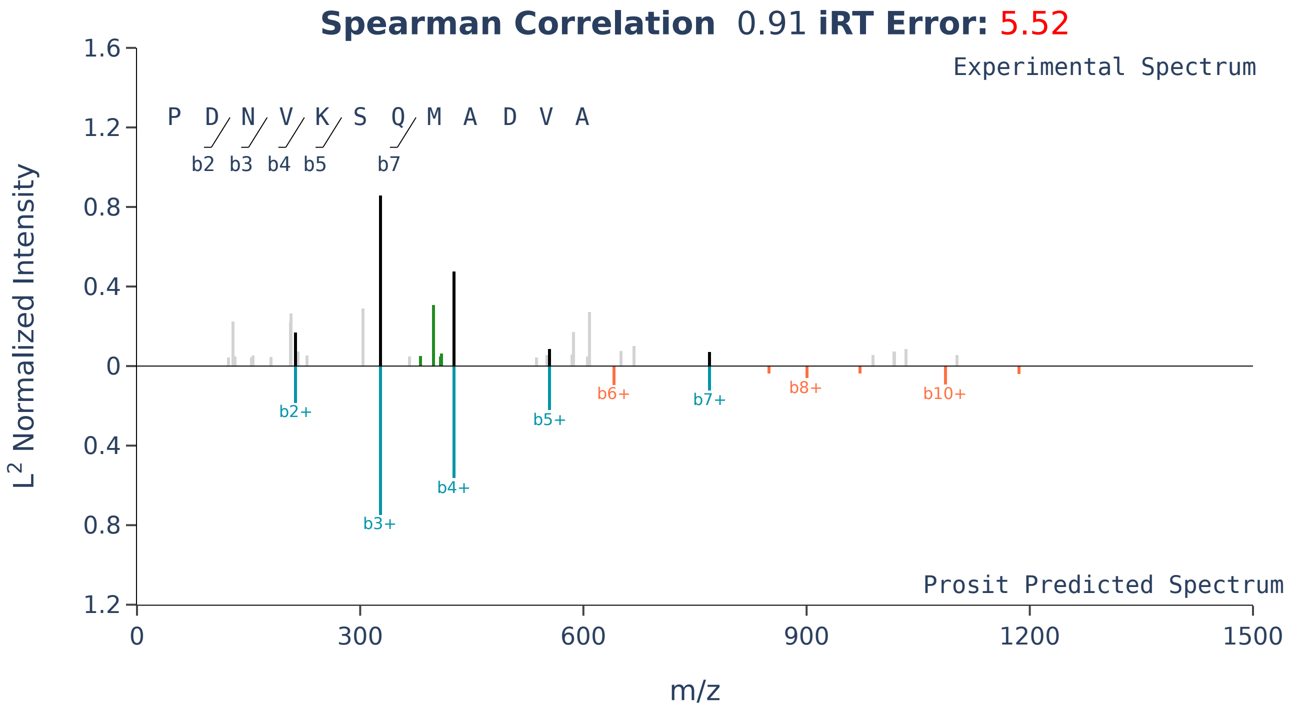

Source Ncheng_210623_230623_HFGoe_FFH_20S_25_1_A1_1h_R1 **Scan** 11245
Peptide QIPDNVKSQMD **Charge** 1 **Spectral Angle** 0.57
Spearman Correlation 0.55 **iRT Error:** 3.39

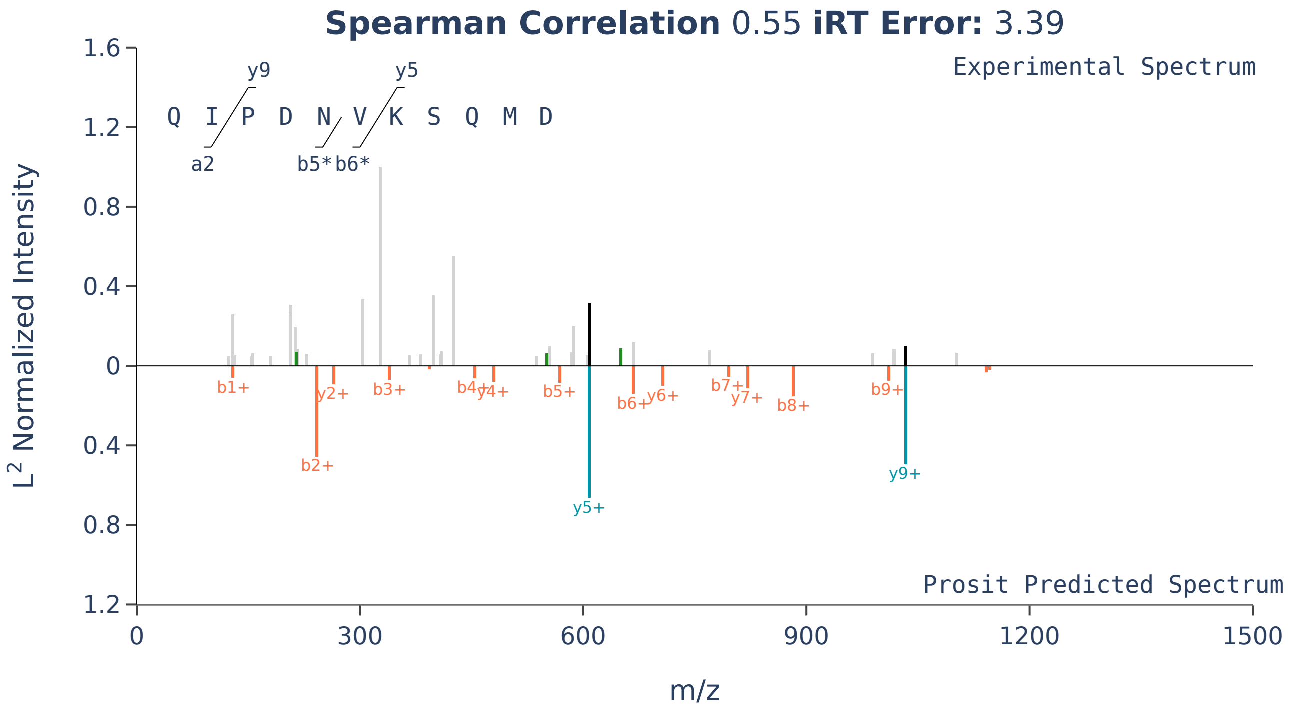

Source Ncheng_210623_230623_HFGoe_FFH_20S_25_1_A2_4h_R1 **Scan** 6886
Peptide NKSLTPGDAA **Charge** 1 **Spectral Angle** 0.8
Spearman Correlation 0.9 **iRT Error:** 5.54

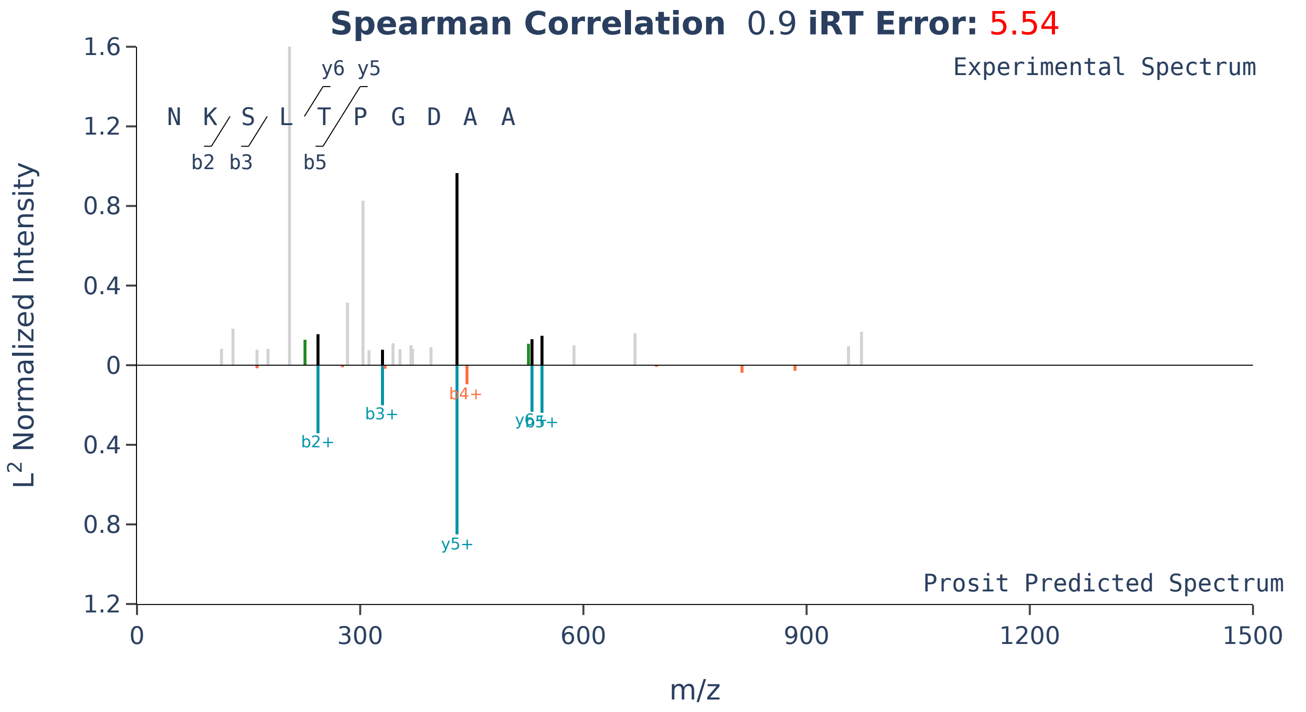

Source Ncheng_210623_230623_HFGoe_FFH_20S_25_1_A2_4h_R1 **Scan** 6886
Peptide ENQTLNLAA **Charge** 1 **Spectral Angle** 0.0
Spearman Correlation 0.0 **iRT Error:** 52.63

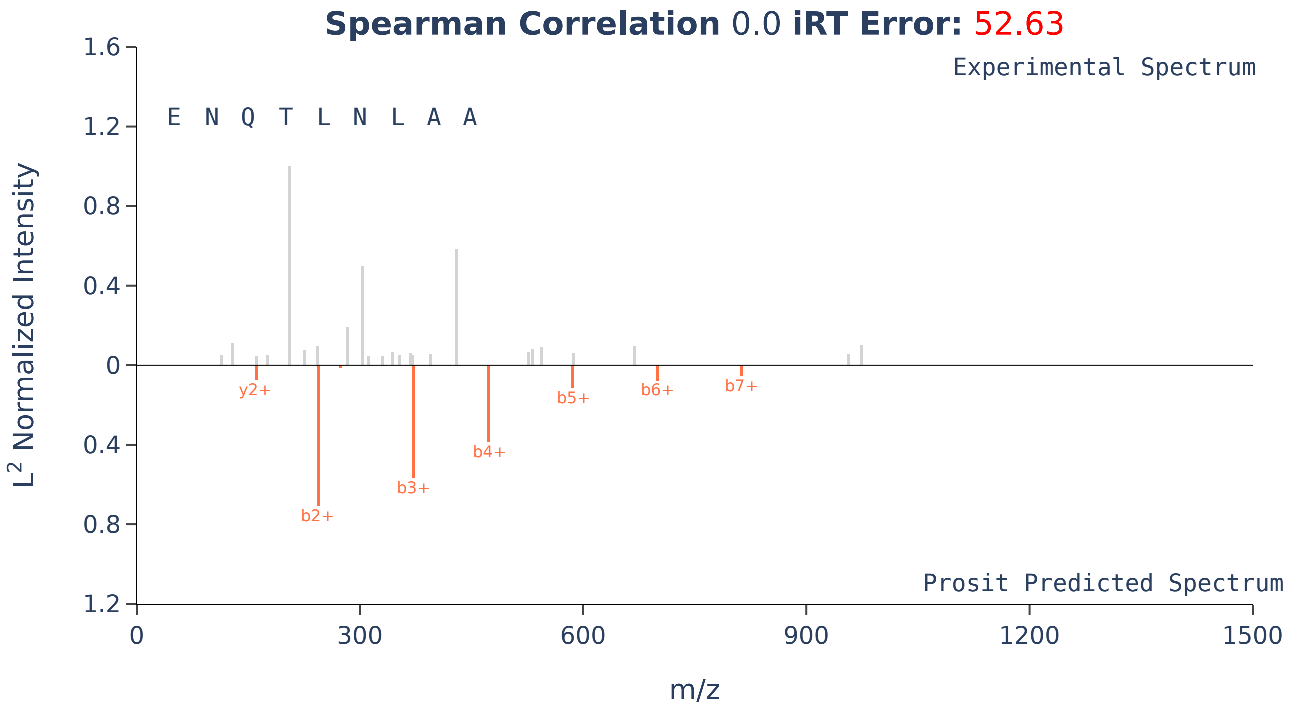

Source Ncheng_210623_230623_HFGoe_FFH_20S_25_1_A2_2h_R2 **Scan** 28231
Peptide DSLMGKLPGMGQIPDNVKSQMD **Charge** 3 **Spectral Angle** 0.74
Spearman Correlation 0.93 **iRT Error:** 5.59

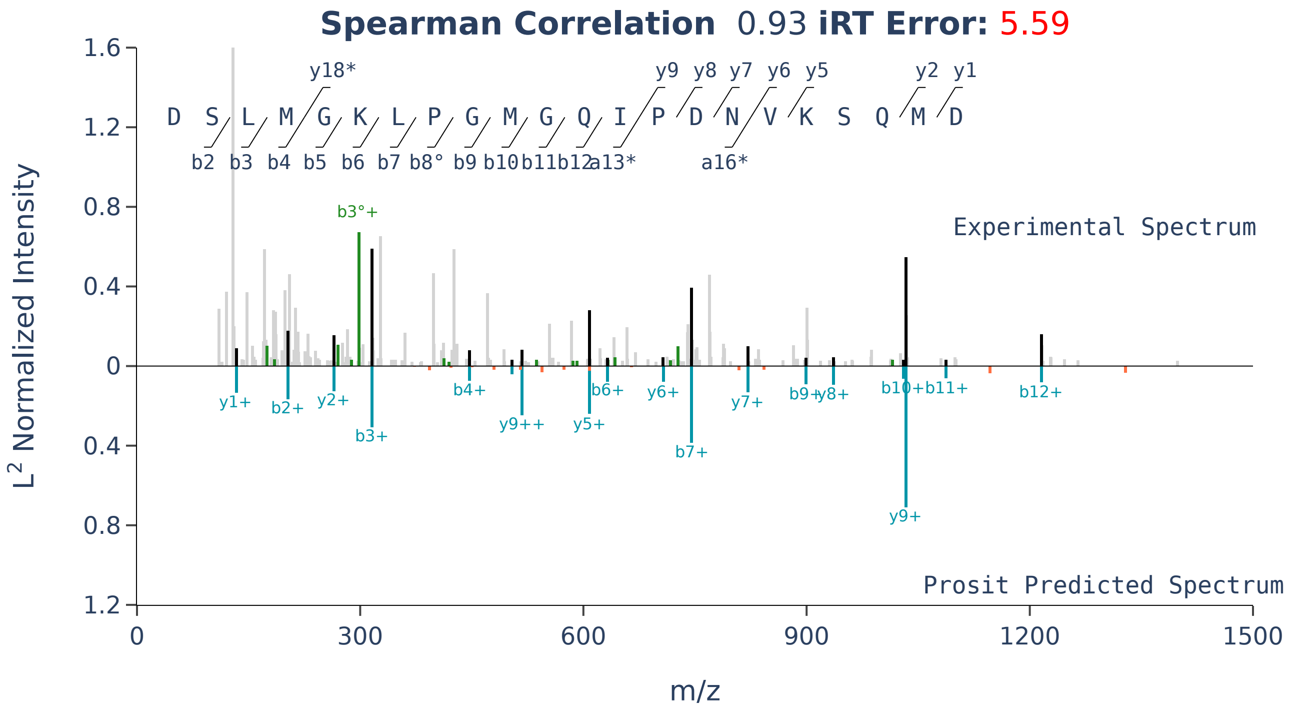

Source Ncheng_210623_230623_HFGoe_FFH_20S_25_1_A2_2h_R2 **Scan** 28231
Peptide SLMGKLPGMGQIPDNVKSQMDD **Charge** 3 **Spectral Angle** 0.31
Spearman Correlation 0.32 **iRT Error:** 14.46

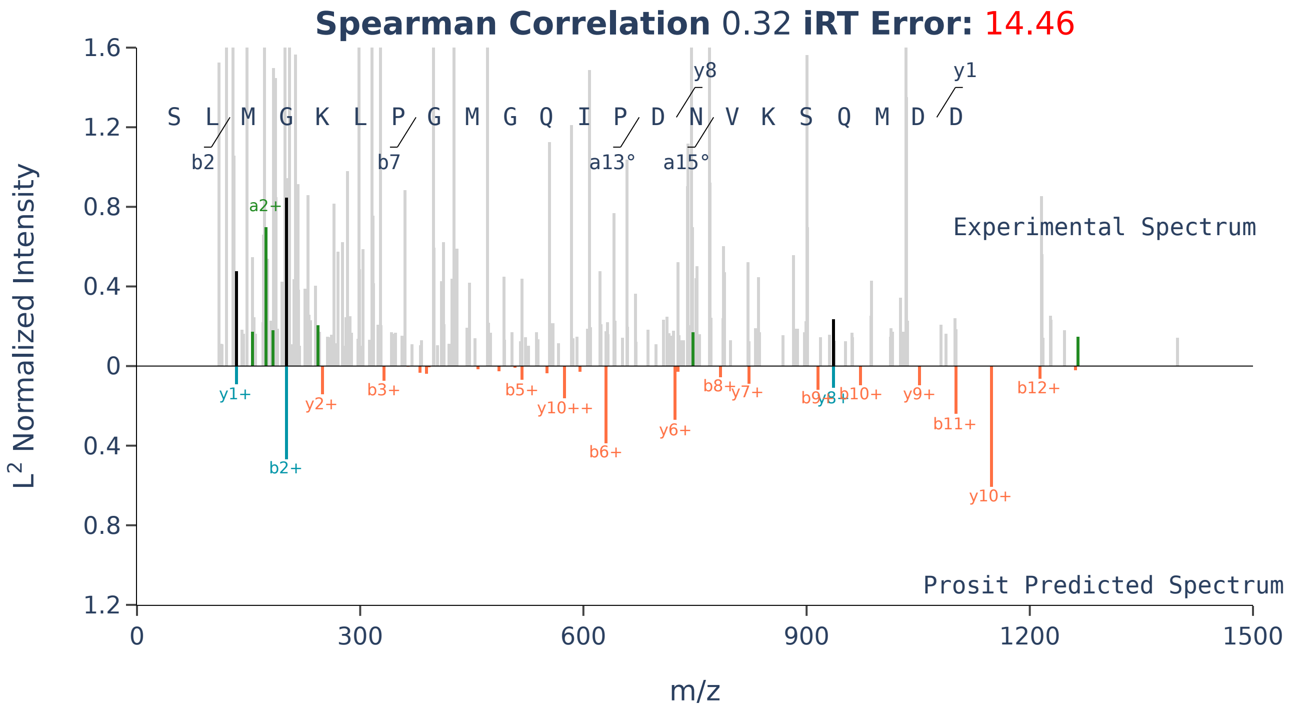

Source Ncheng_210623_230623_HFGoe_FFH_20S_25_1_A2_4h_R2 **Scan** 17017
Peptide MKGMMPPGFPGRSR **Charge** 3 **Spectral Angle** 0.88
Spearman Correlation 0.86 **iRT Error:** 5.66

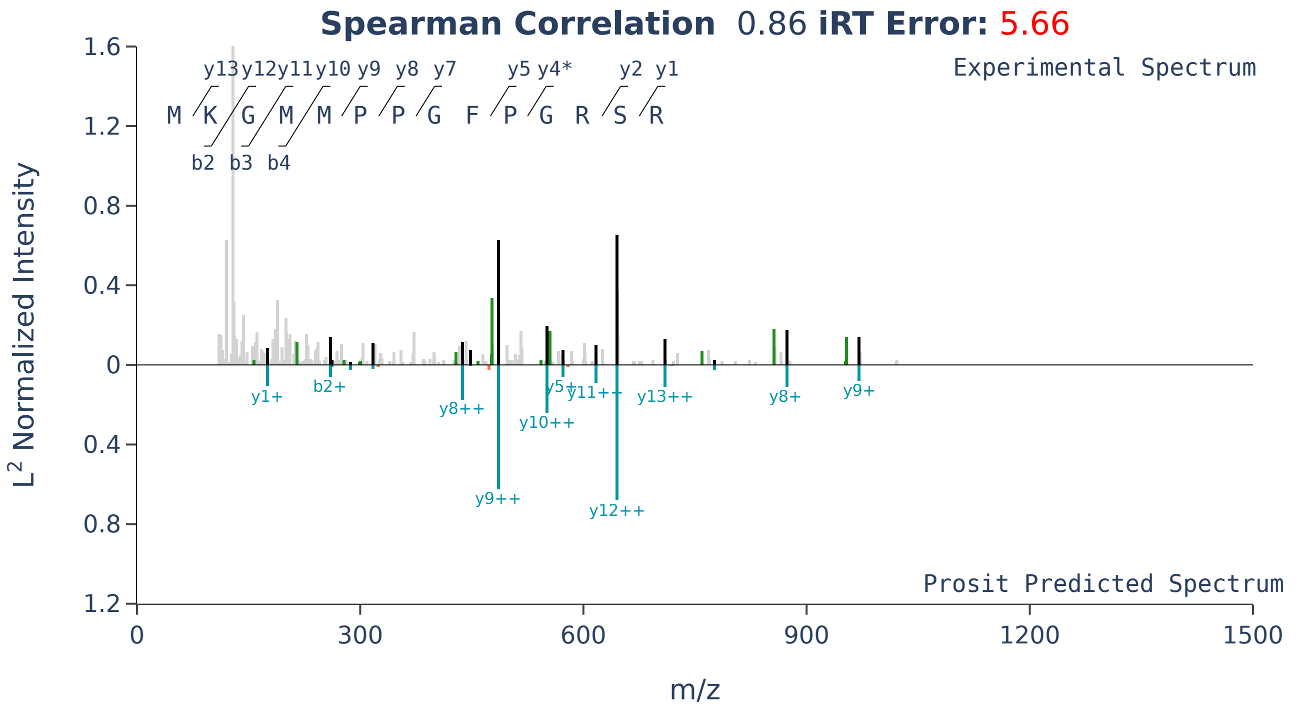

Source Ncheng_210623_230623_HFGoe_FFH_20S_25_1_A2_4h_R2 **Scan** 17017
Peptide RSMKGMMPPGFPGR **Charge** 3 **Spectral Angle** 0.06
Spearman Correlation -0.2 **iRT Error:** 2.56

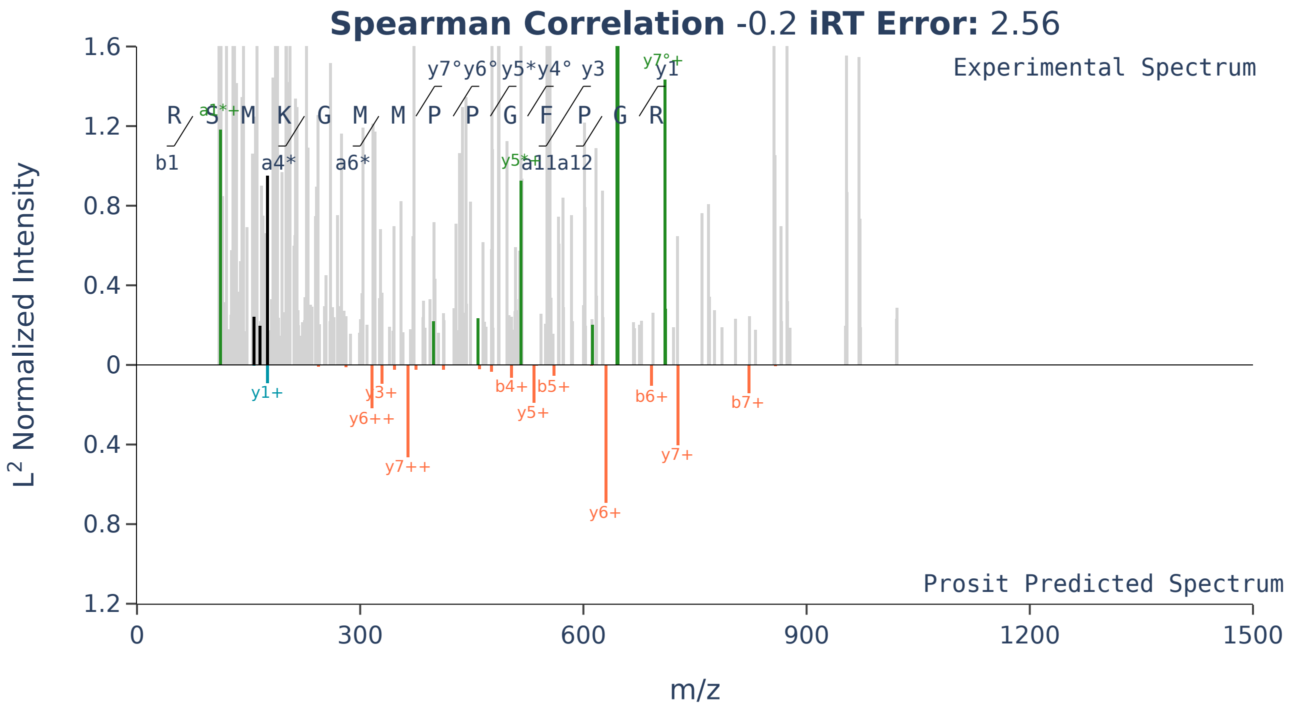

Source Ncheng_210623_230623_HFGoe_FFH_20S_25_1_A2_24h_R2 **Scan** 21558
Peptide SAMGKLPGMGQIPDNVKSQ **Charge** 2 **Spectral Angle** 0.87
Spearman Correlation 0.87 **iRT Error:** 5.86

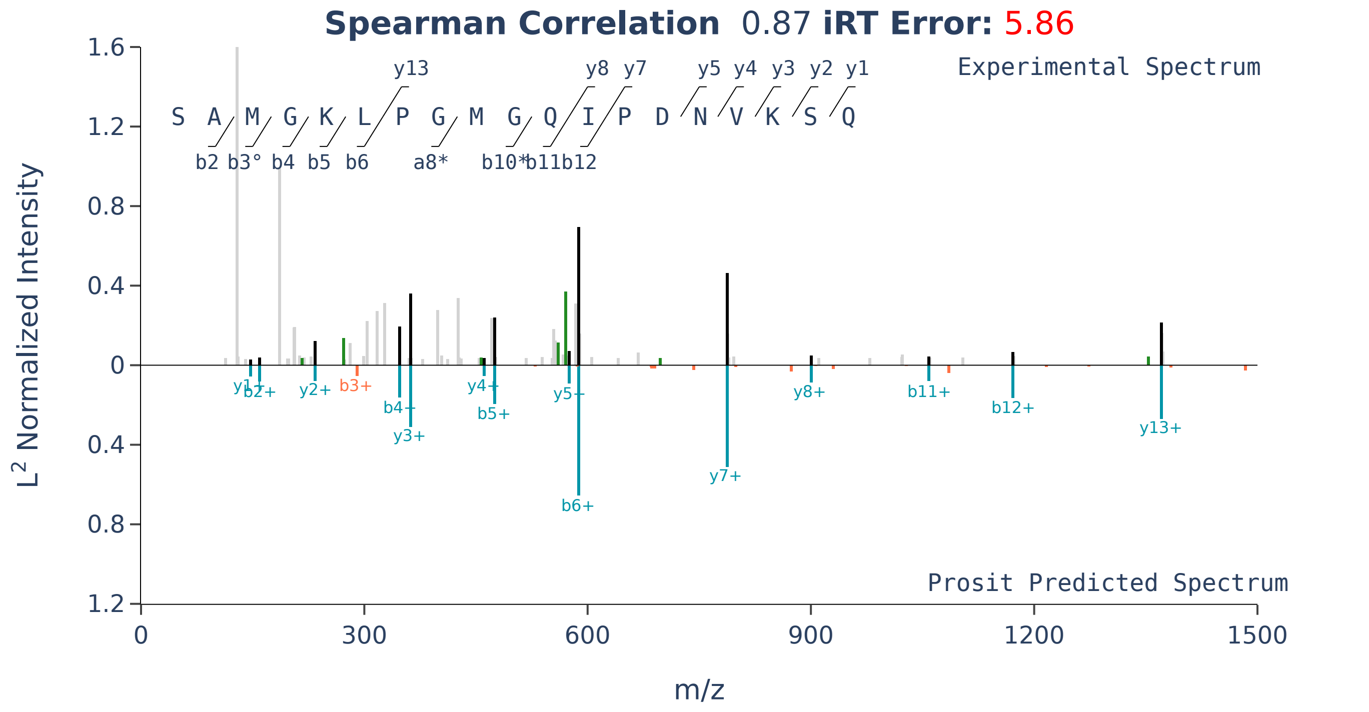

Source Ncheng_210623_230623_HFGoe_FFH_20S_25_1_A2_24h_R2 **Scan** 21558
Peptide LKQFDDMQRMMKKMK **Charge** 2 **Spectral Angle** 0.0
Spearman Correlation 0.0 **iRT Error:** 23.49

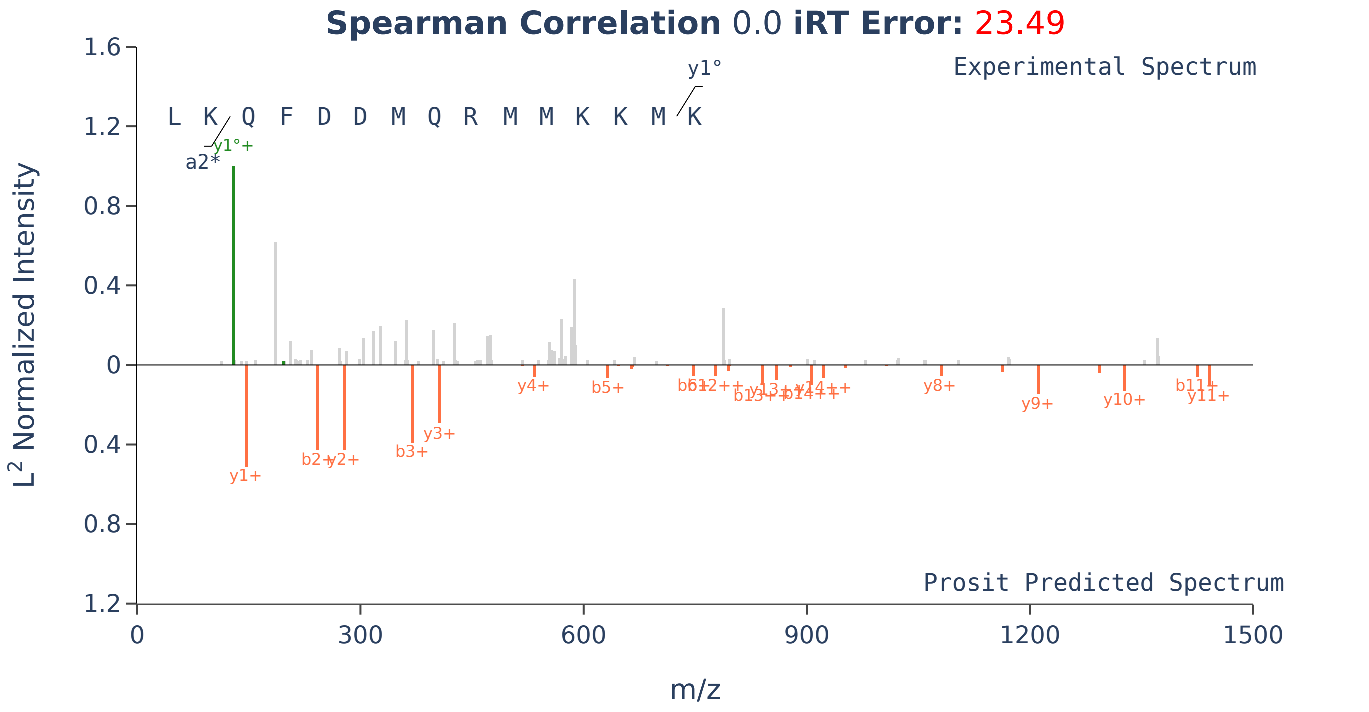

Source Ncheng_210623_230623_HFGoe_FFH_20S_25_1_A1_4h_R2 **Scan** 11184
Peptide TLAEQVGE **Charge** 1 **Spectral Angle** 0.91
Spearman Correlation 1.0 **iRT Error:** 5.93

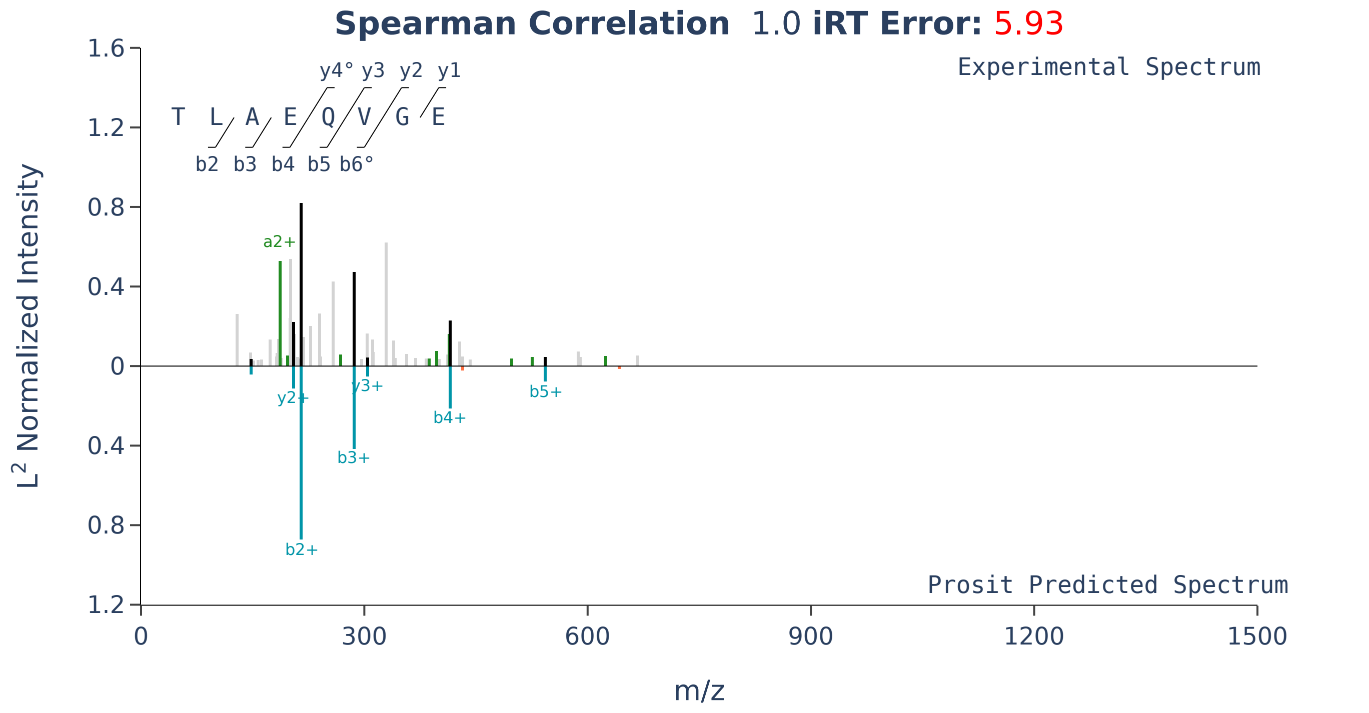

Source Ncheng_210623_230623_HFGoe_FFH_20S_25_1_A1_4h_R2 **Scan** 11184
Peptide ETLAEQVG **Charge** 1 **Spectral Angle** 0.18
Spearman Correlation 0.2 **iRT Error:** 12.84

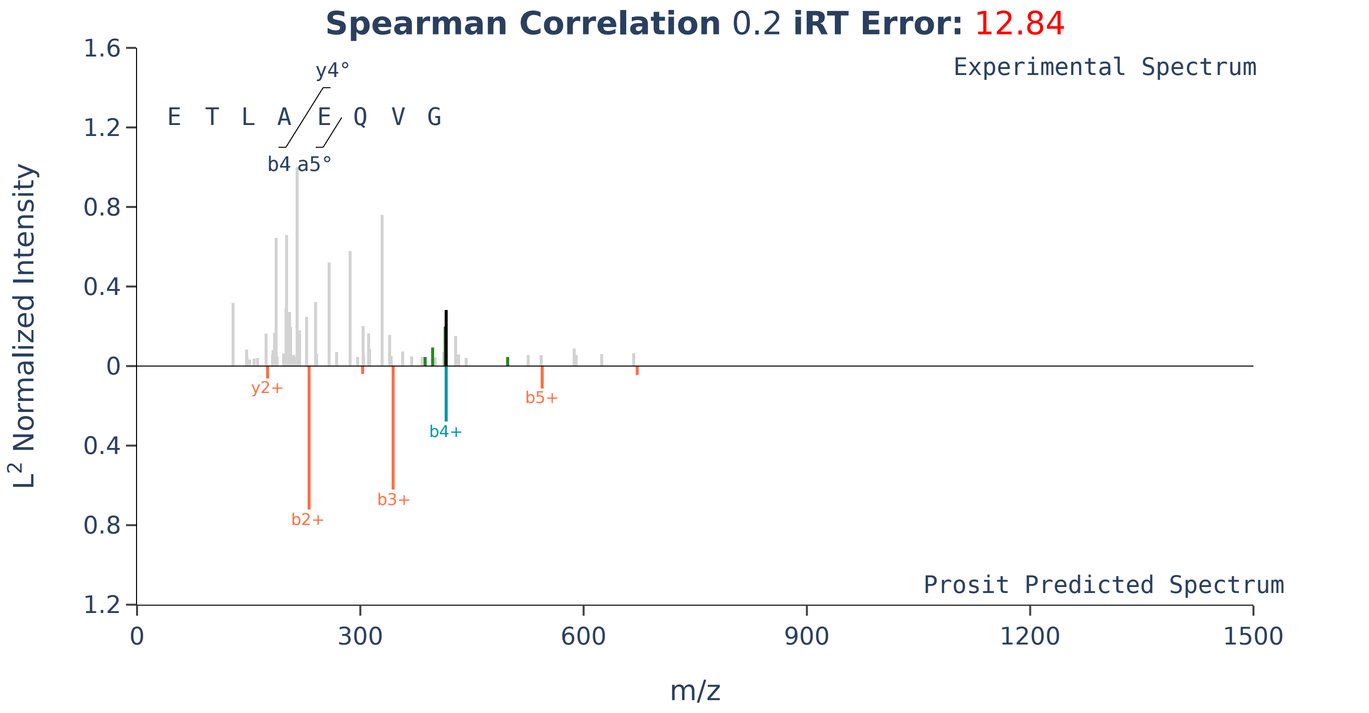

Source Ncheng_210623_230623_HFGoe_FFH_20S_25_1_A2_4h_R2 **Scan** 21749
Peptide GMQKLPGMGQIPDNVKS **Charge** 3 **Spectral Angle** 0.87
Spearman Correlation 0.91 **iRT Error:** 6.7

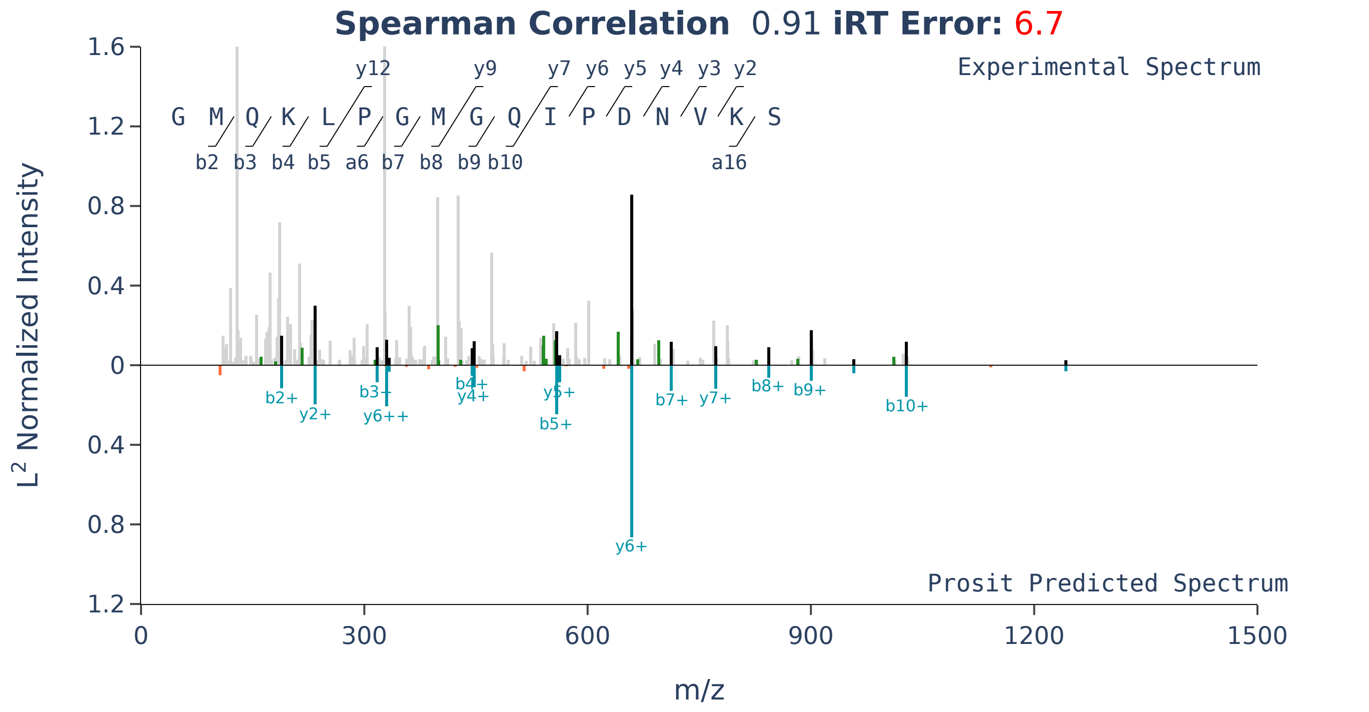

Source Ncheng_210623_230623_HFGoe_FFH_20S_25_1_A2_4h_R2 **Scan** 21749
Peptide GKLPGMGQIPDNVKSQM **Charge** 3 **Spectral Angle** 0.33
Spearman Correlation 0.63 **iRT Error:** 4.74

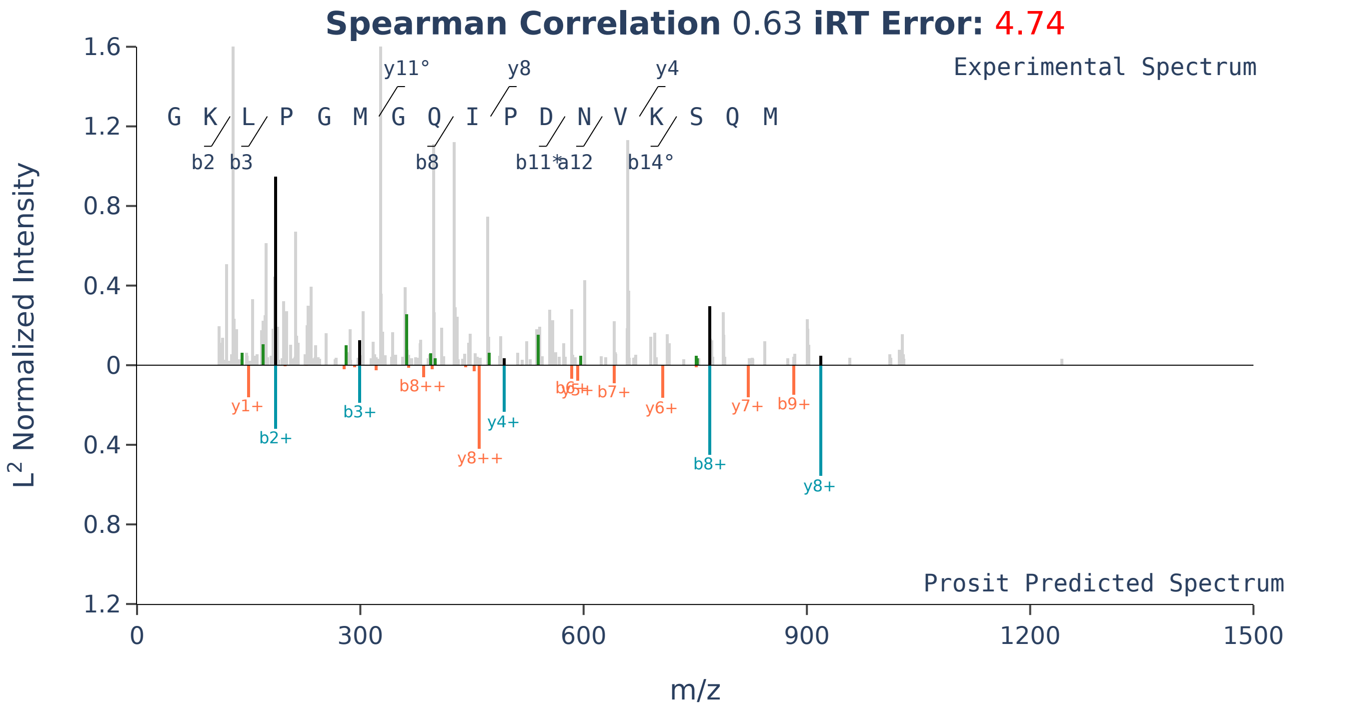

Source Ncheng_210623_230623_HFGoe_FFH_20S_25_1_A2_4h_R1 **Scan** 27735
Peptide PGMGQIIPDNVKSQMDDKVLVRME **Charge** 3 **Spectral Angle** 0.78
Spearman Correlation 0.93 **iRT Error:** 6.8

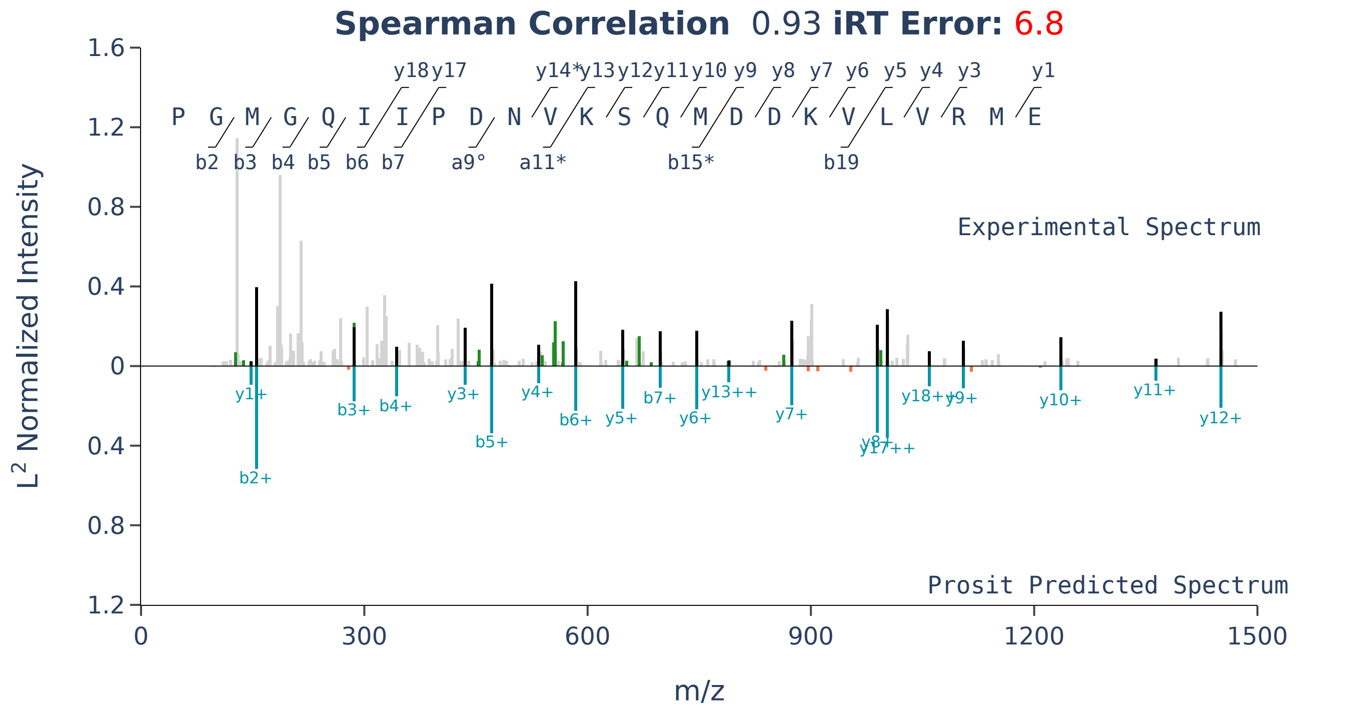

Source Ncheng_210623_230623_HFGoe_FFH_20S_25_1_A2_4h_R1 **Scan** 27735
Peptide LPGMGQIPDNVKSQMDDKVLVRME **Charge** 3 **Spectral Angle** 0.69
Spearman Correlation 0.91 **iRT Error:** 4.48

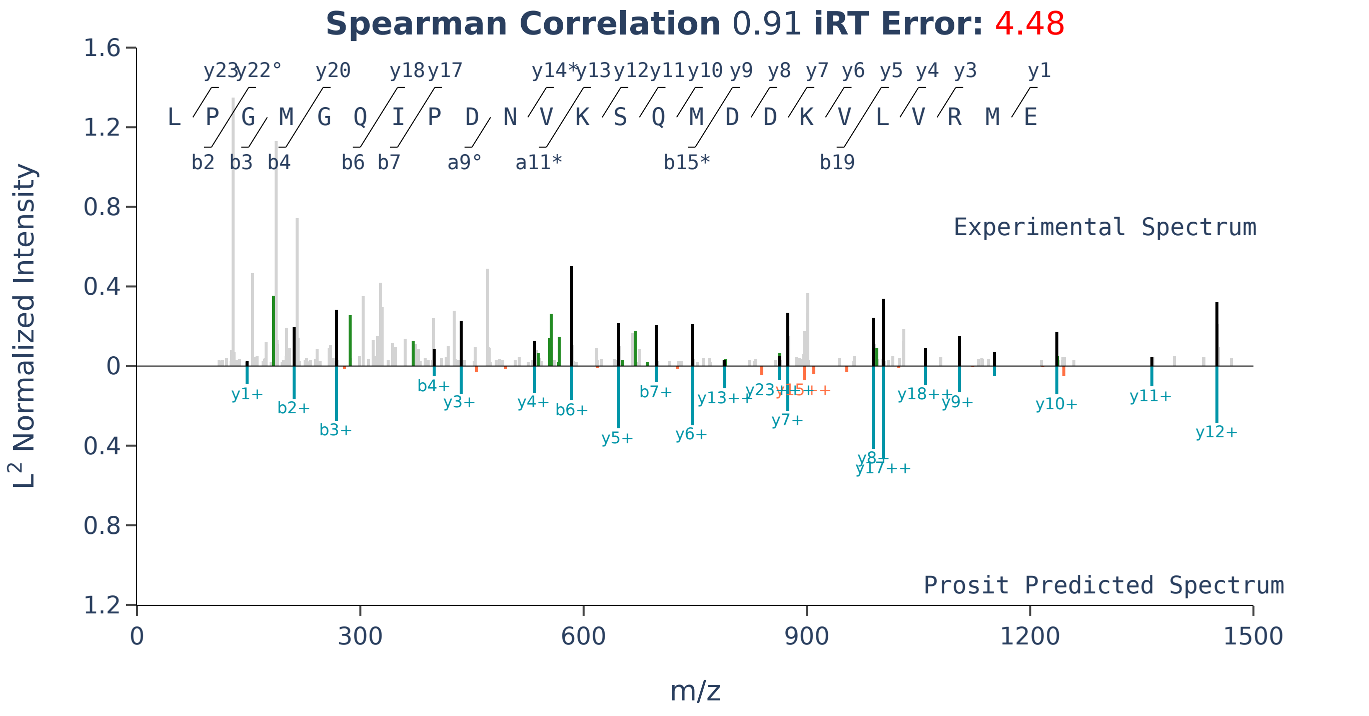

Source Ncheng_210623_230623_HFGoe_FFH_20S_25_1_A1_24h_R1 **Scan** 9986
Peptide AEQVGAMTGQD **Charge** 2 **Spectral Angle** 0.91
Spearman Correlation 0.91 **iRT Error:** 7.02

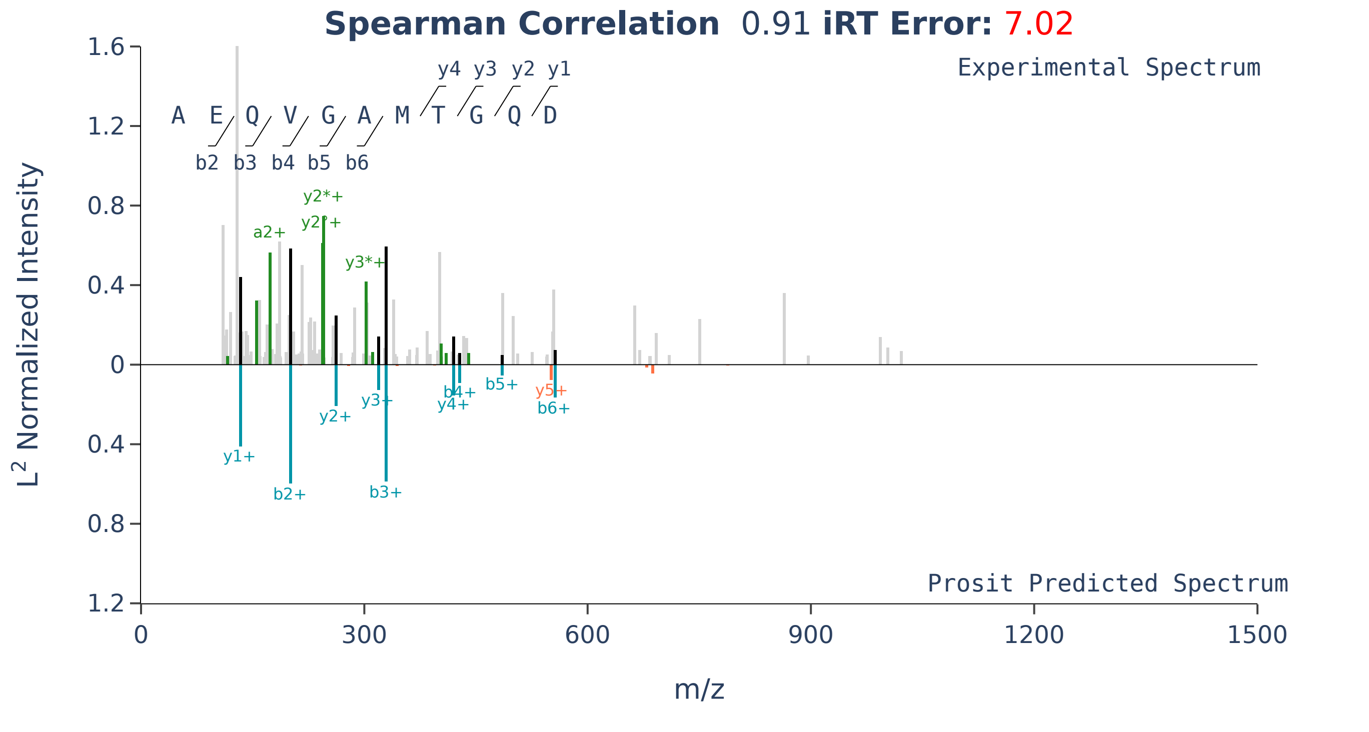

Source Ncheng_210623_230623_HFGoe_FFH_20S_25_1_A1_24h_R1 **Scan** 9986
Peptide AMGEENQTLN **Charge** 2 **Spectral Angle** 0.06
Spearman Correlation 0.29 **iRT Error:** 6.26

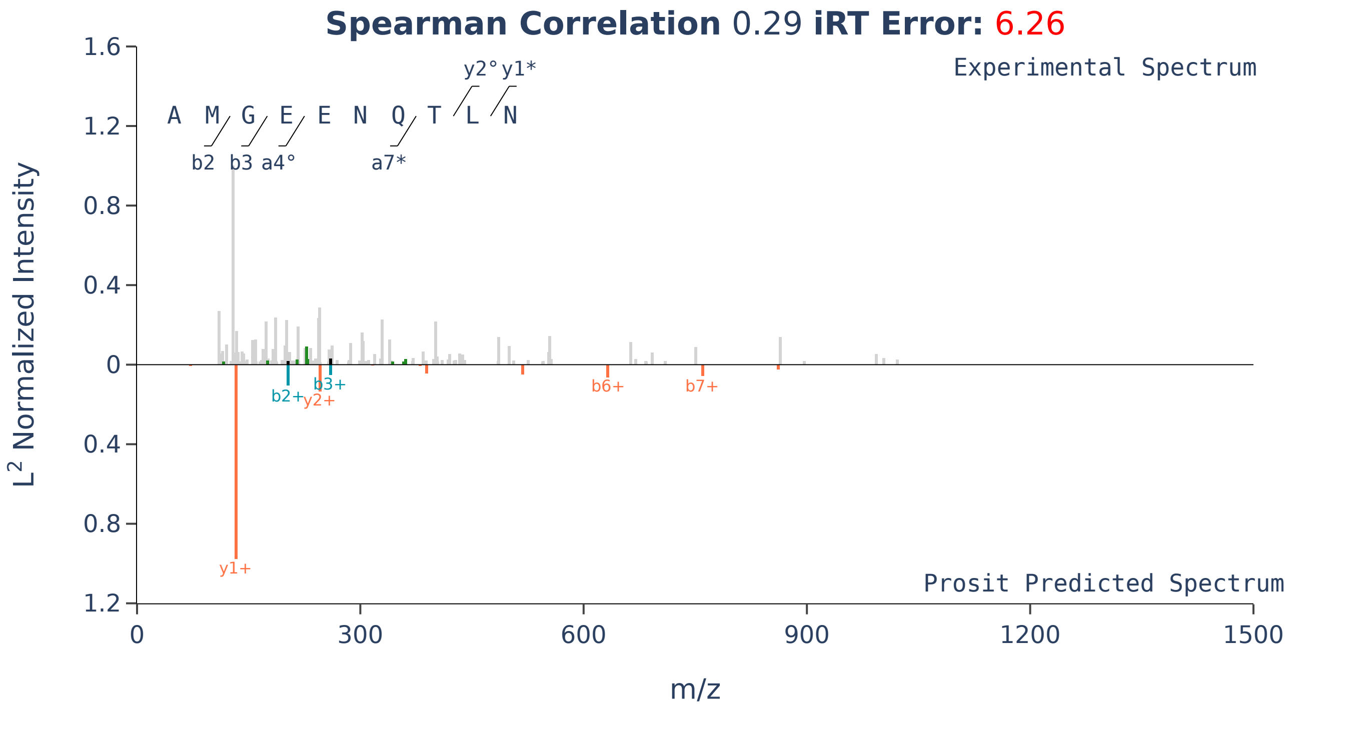

Source Ncheng_210623_230623_HFGoe_FFH_20S_25_1_A1_4h_R2 **Scan** 12085
Peptide KDTPIKF **Charge** 2 **Spectral Angle** 0.9
Spearman Correlation 0.93 **iRT Error:** 7.78

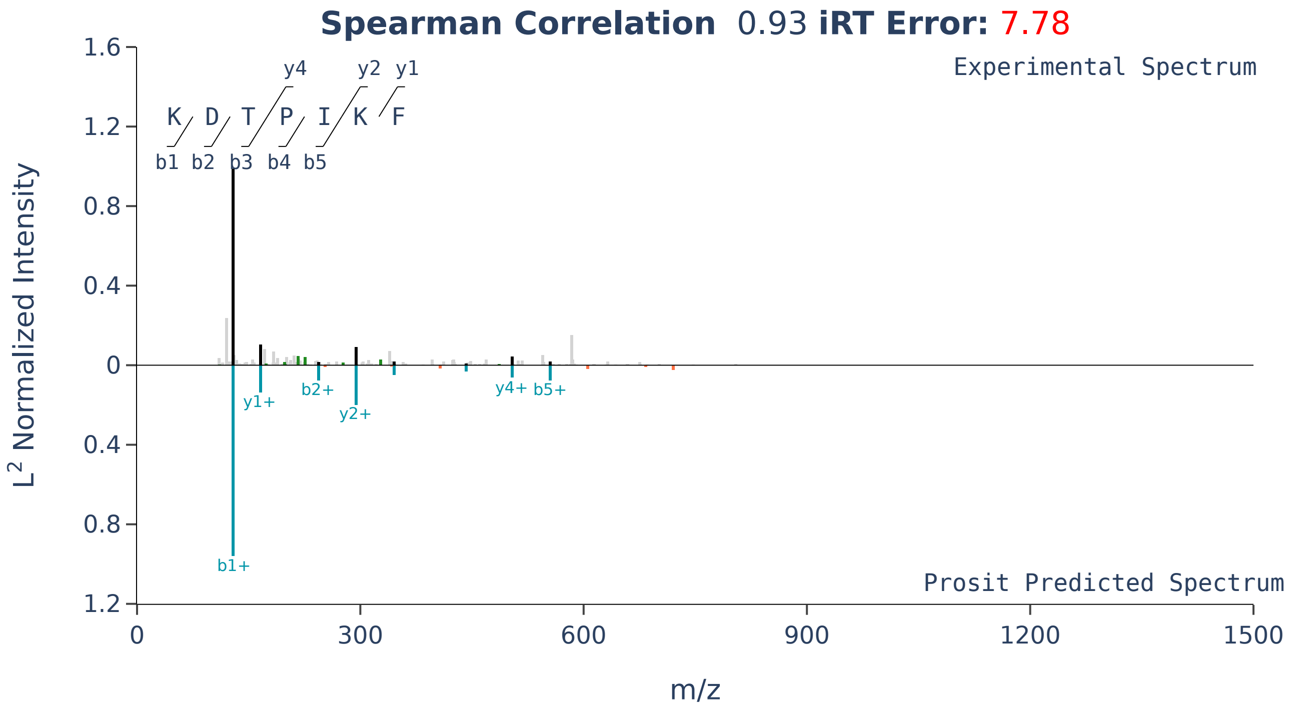

Source Ncheng_210623_230623_HFGoe_FFH_20S_25_1_A1_4h_R2 **Scan** 12085
Peptide AGKTTSVGK **Charge** 2 **Spectral Angle** 0.14
Spearman Correlation 0.09 **iRT Error:** 46.62

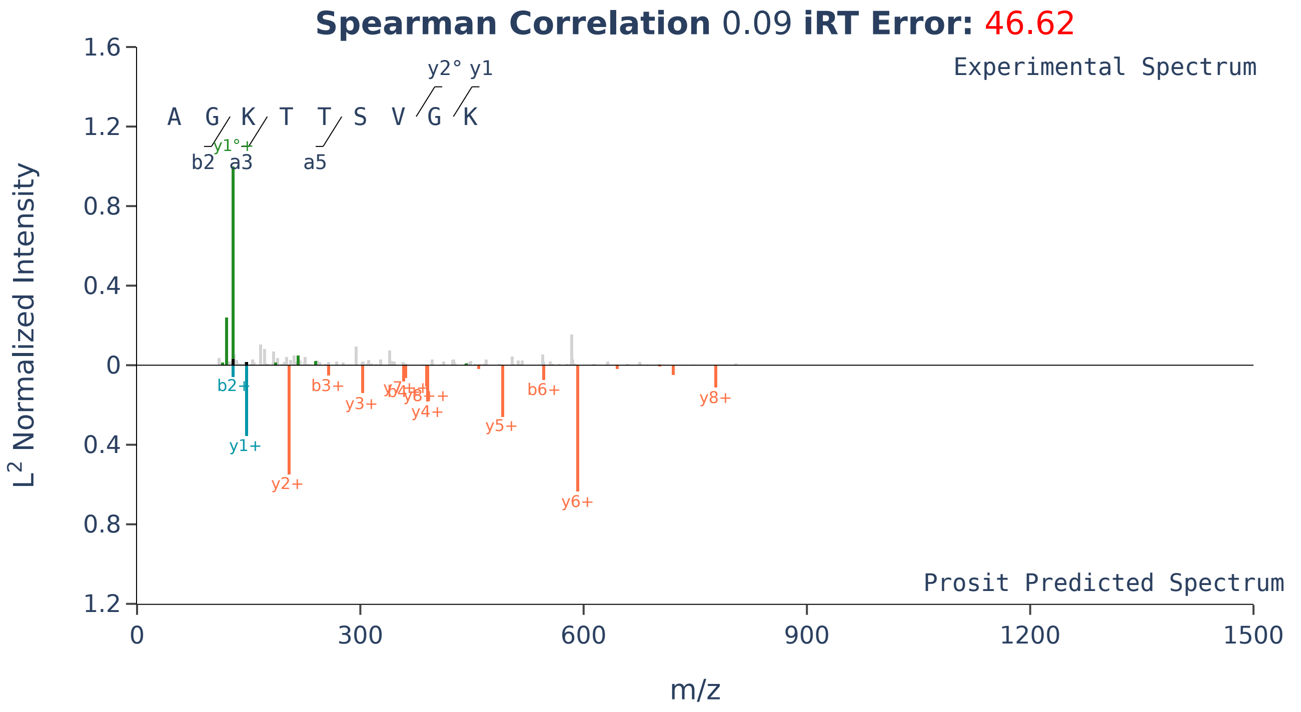

Source Ncheng_210623_230623_HFGoe_FFH_20S_25_1_A1_2h_R1 **Scan** 11036
Peptide TLAEQVDA **Charge** 1 **Spectral Angle** 0.89
Spearman Correlation 0.99 **iRT Error:** 8.09

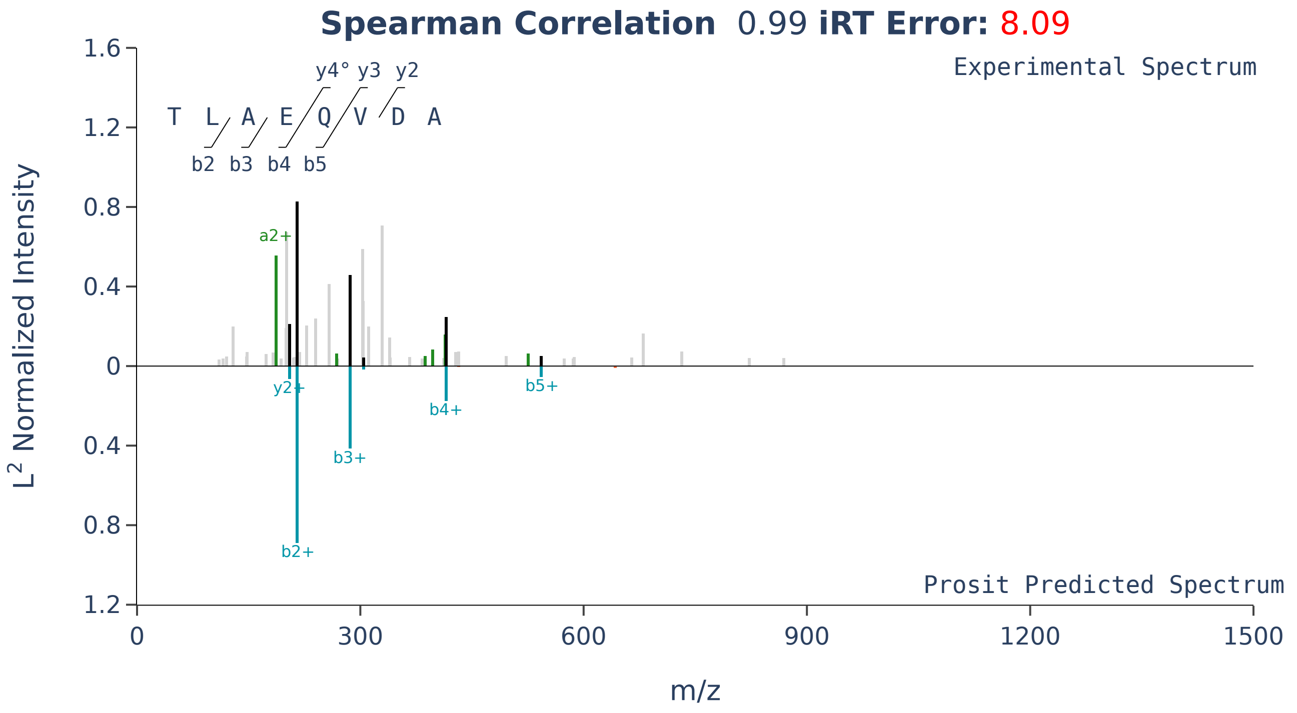

Source Ncheng_210623_230623_HFGoe_FFH_20S_25_1_A1_2h_R1 **Scan** 11036
Peptide ETLAEQVG **Charge** 1 **Spectral Angle** 0.18
Spearman Correlation 0.2 **iRT Error:** 12.72

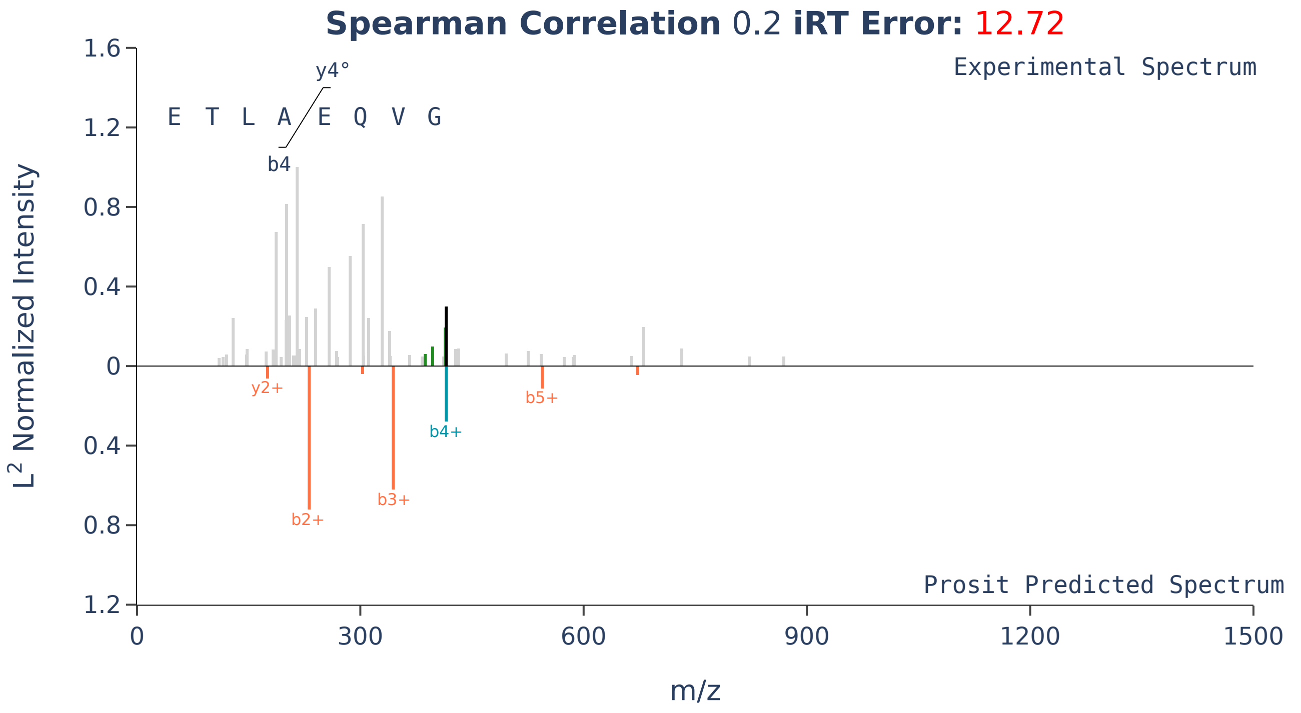

Source Ncheng_210623_230623_HFGoe_FFH_20S_25_1_A2_24h_R2 **Scan** 14771
Peptide VGQKPVDIVNDA **Charge** 2 **Spectral Angle** 0.84
Spearman Correlation 0.91 **iRT Error:** 8.39

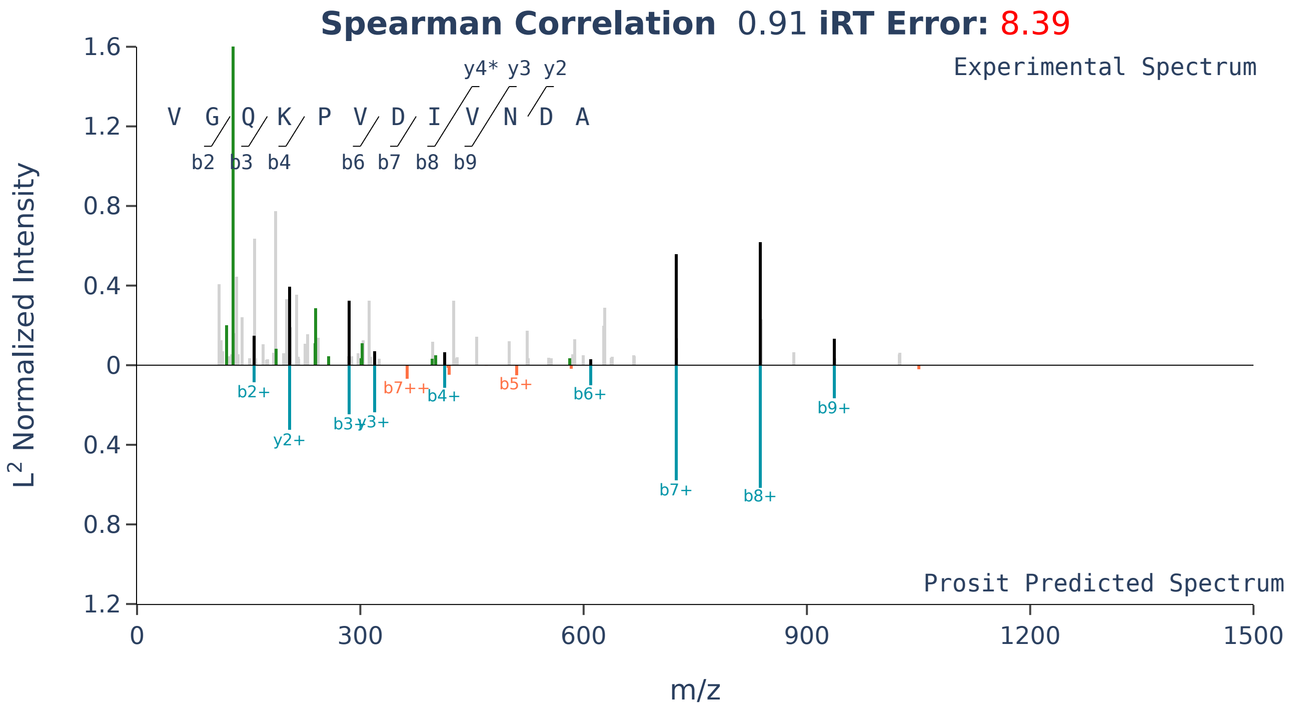

Source Ncheng_210623_230623_HFGoe_FFH_20S_25_1_A2_24h_R2 **Scan** 14771
Peptide DVGQKPVDIVNA **Charge** 2 **Spectral Angle** 0.06
Spearman Correlation 0.12 **iRT Error:** 17.87

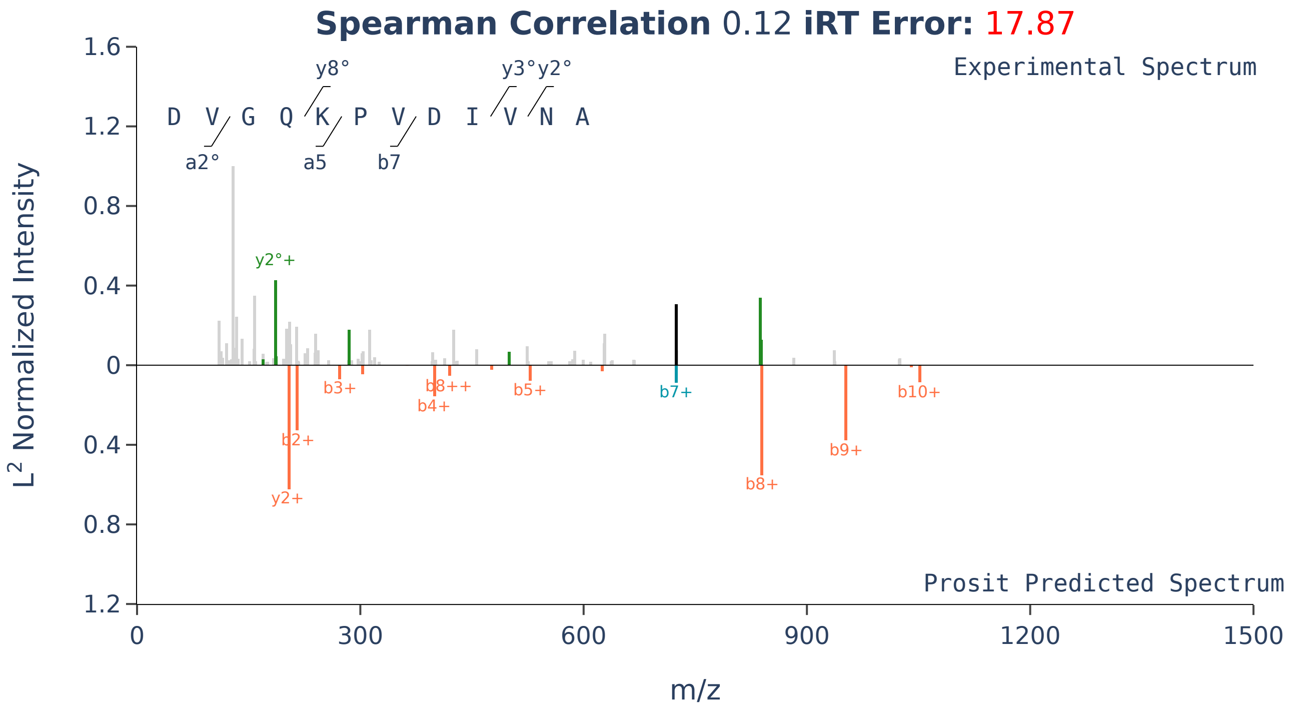

Source Ncheng_210623_230623_HFGoe_FFH_20S_25_1_A2_24h_R2 **Scan** 7640
Peptide DAIESKVD **Charge** 2 **Spectral Angle** 0.85
Spearman Correlation 0.95 **iRT Error:** 8.93

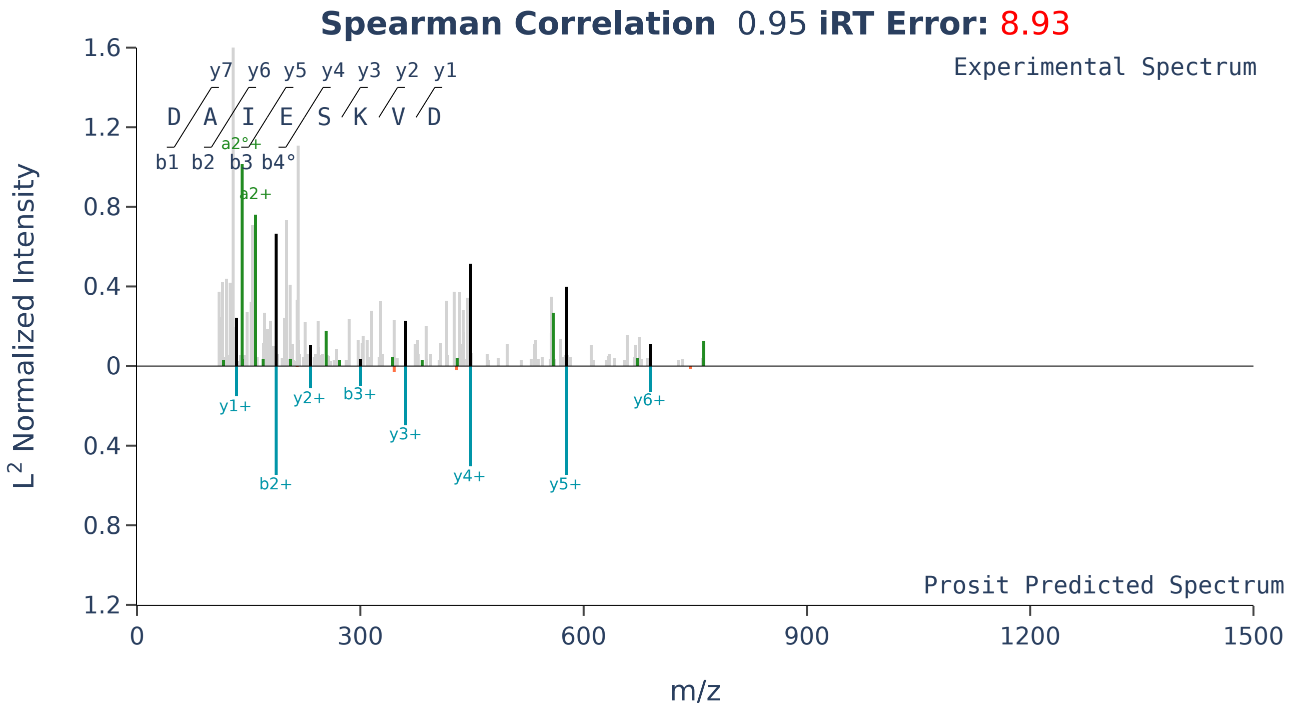

Source Ncheng_210623_230623_HFGoe_FFH_20S_25_1_A2_24h_R2 **Scan** 7640
Peptide GEKTEALE **Charge** 2 **Spectral Angle** 0.27
Spearman Correlation -0.03 **iRT Error:** 1.42

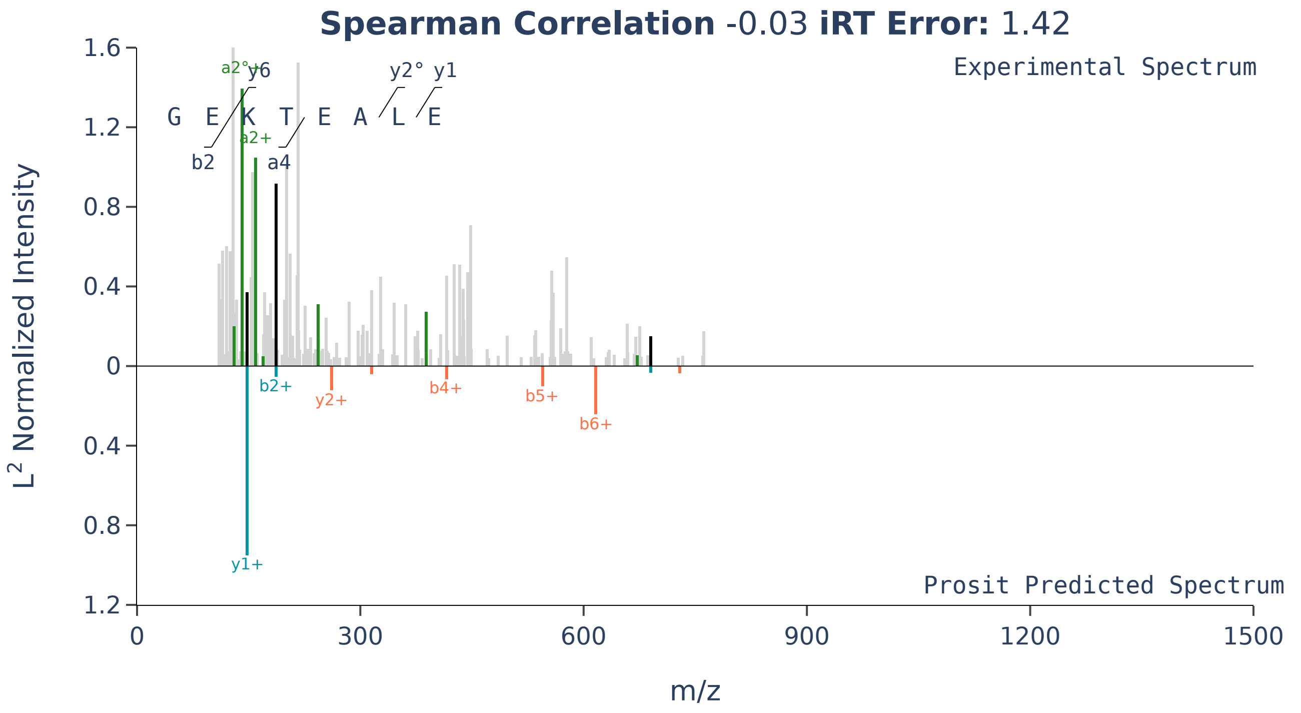

Source Ncheng_210623_230623_HFGoe_FFH_20S_25_1_A1_24h_R1 **Scan** 28310
Peptide PPAVVLMAAG **Charge** 1 **Spectral Angle** 0.96
Spearman Correlation 0.98 **iRT Error:** 9.66

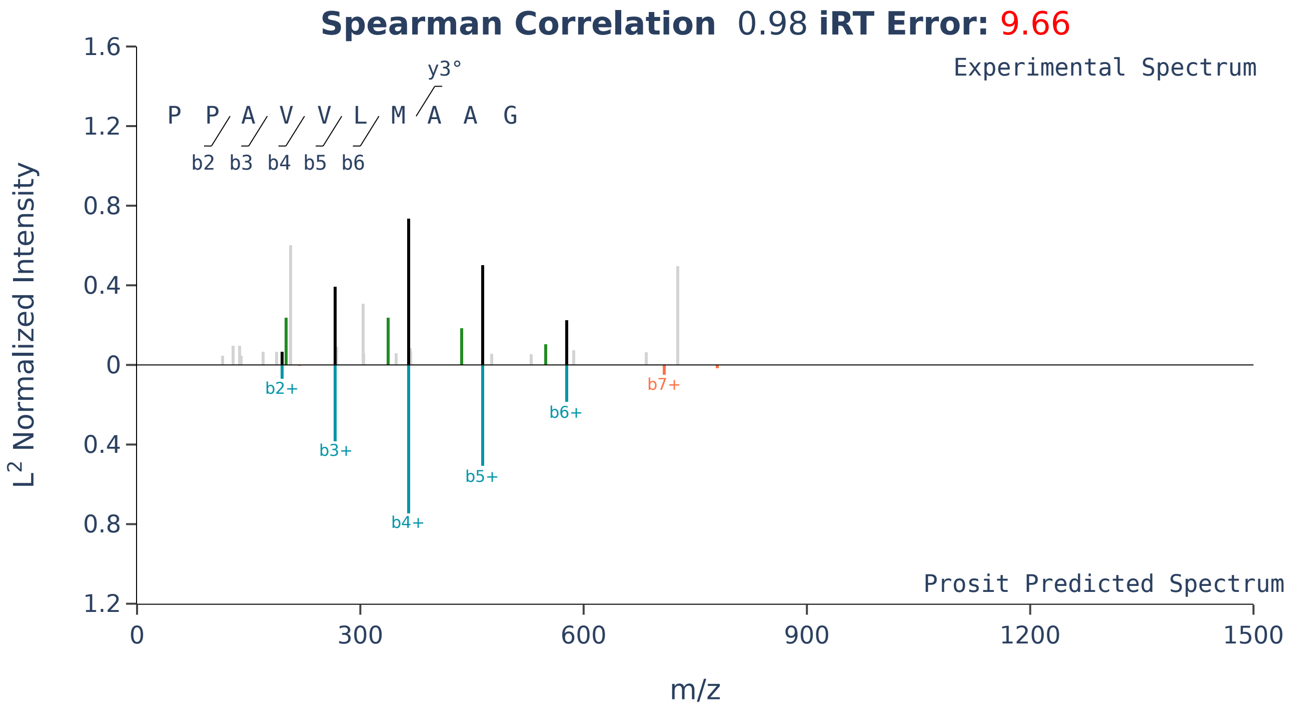

Source Ncheng_210623_230623_HFGoe_FFH_20S_25_1_A1_24h_R1 **Scan** 28310
Peptide QPPAVVLMA **Charge** 1 **Spectral Angle** 0.0
Spearman Correlation 0.0 **iRT Error:** 10.78

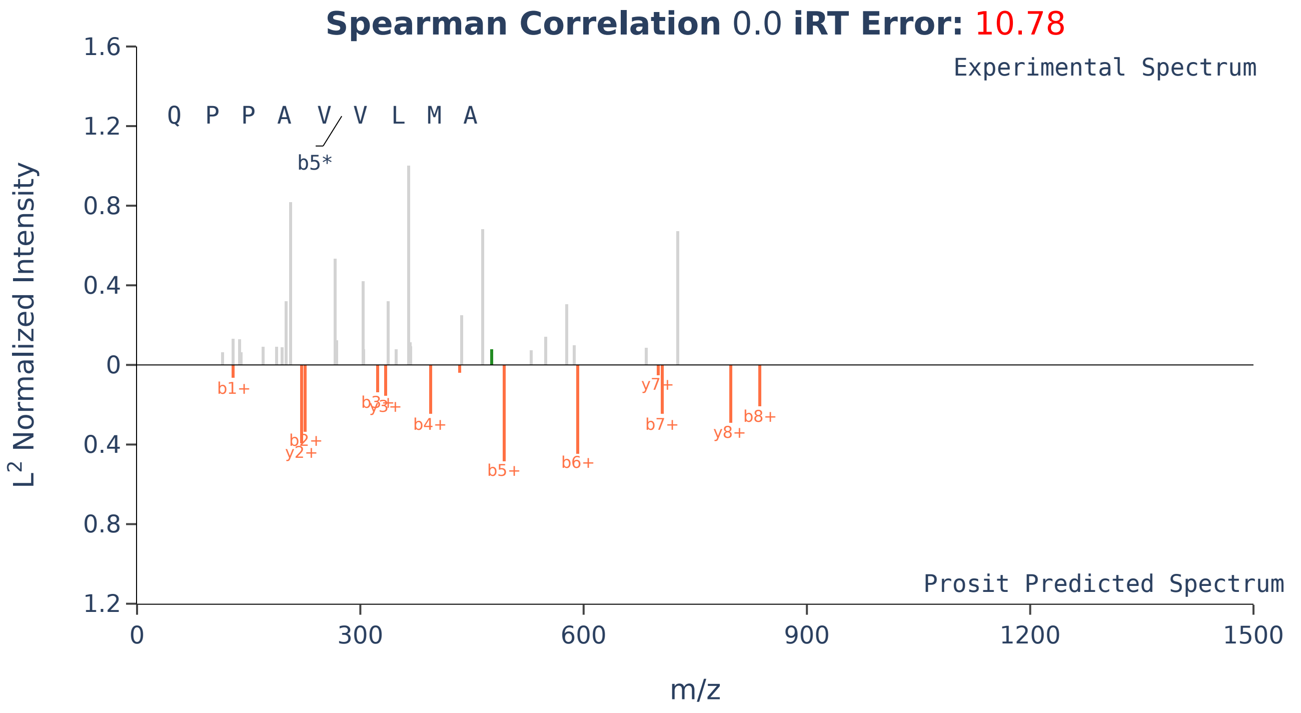

Source Ncheng_210623_230623_HFGoe_FFH_20S_25_1_A2_24h_R1 **Scan** 25916
Peptide GAGKVGVDFFPSD **Charge** 2 **Spectral Angle** 0.93
Spearman Correlation 0.91 **iRT Error:** 10.1

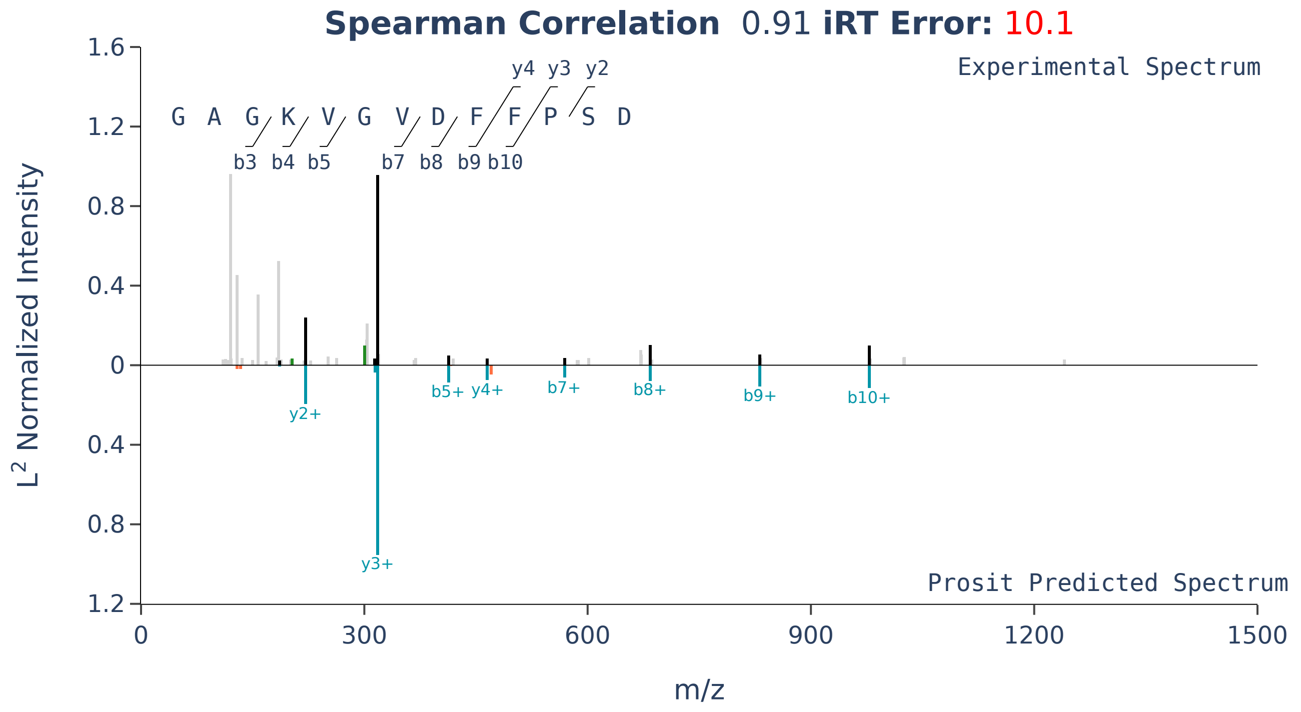

Source Ncheng_210623_230623_HFGoe_FFH_20S_25_1_A2_24h_R1 **Scan** 25916
Peptide GVDFFPSDVGQK **Charge** 2 **Spectral Angle** 0.14
Spearman Correlation 0.13 **iRT Error:** 13.97

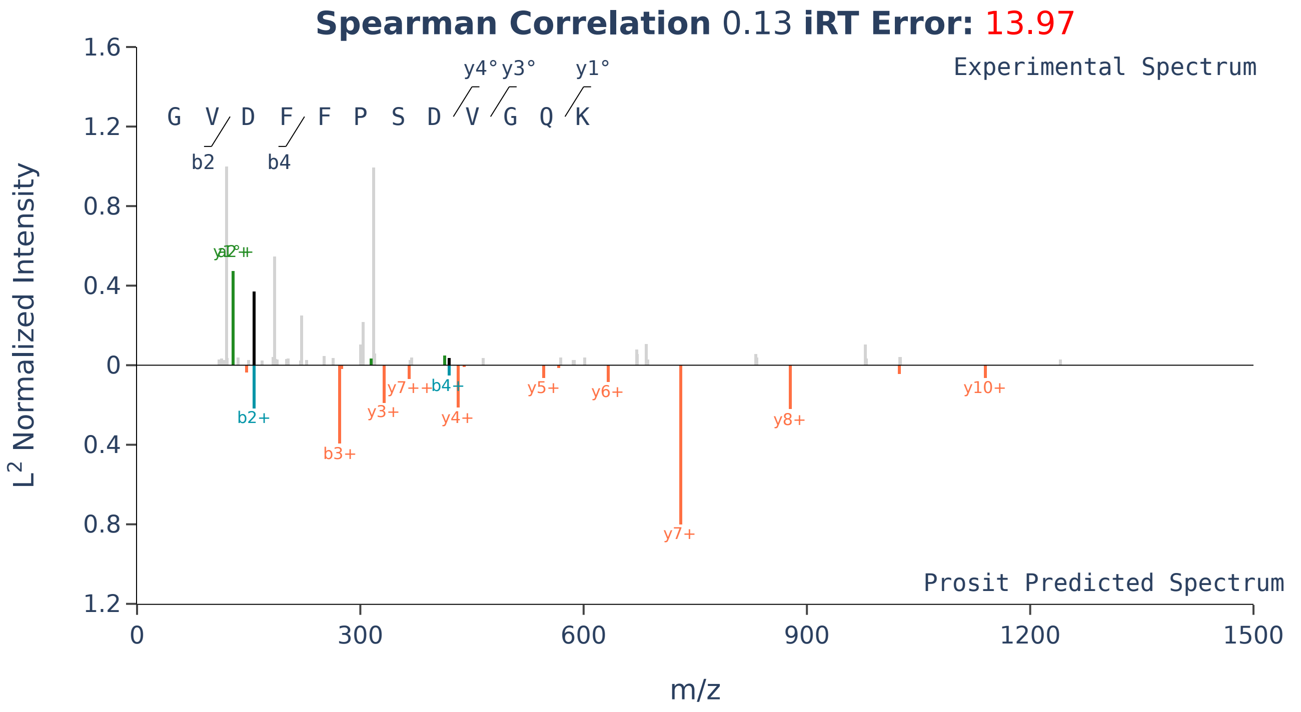

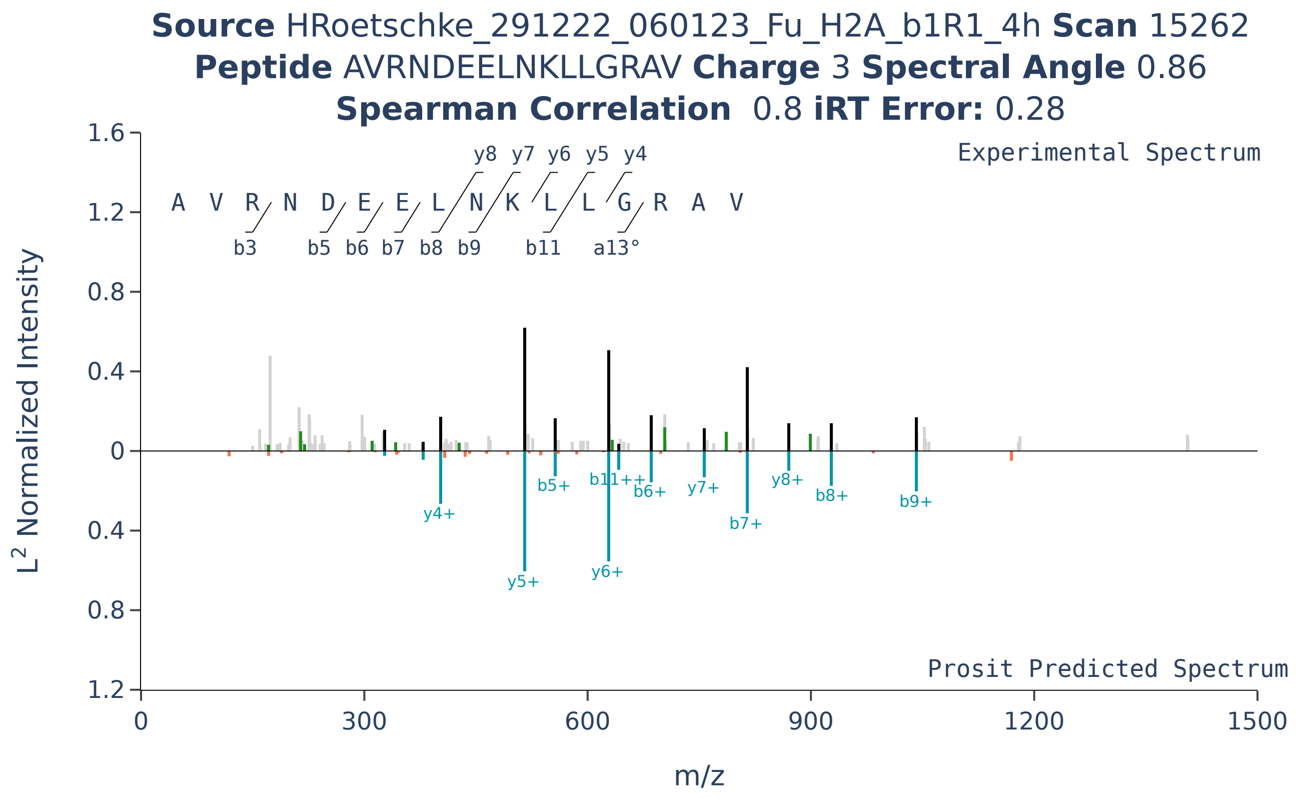
Source HRoetschke_291222_060123_Fu_H2A_b1R1_4h Scan 15262
Peptide AVRNDEELNKLLGRAV Charge 3 Spectral Angle 0.86
Spearman Correlation 0.8 iRT Error: 0.28
Experimental Spectrum
Prosit Predicted Spectrum
L² Normalized Intensity
m/z

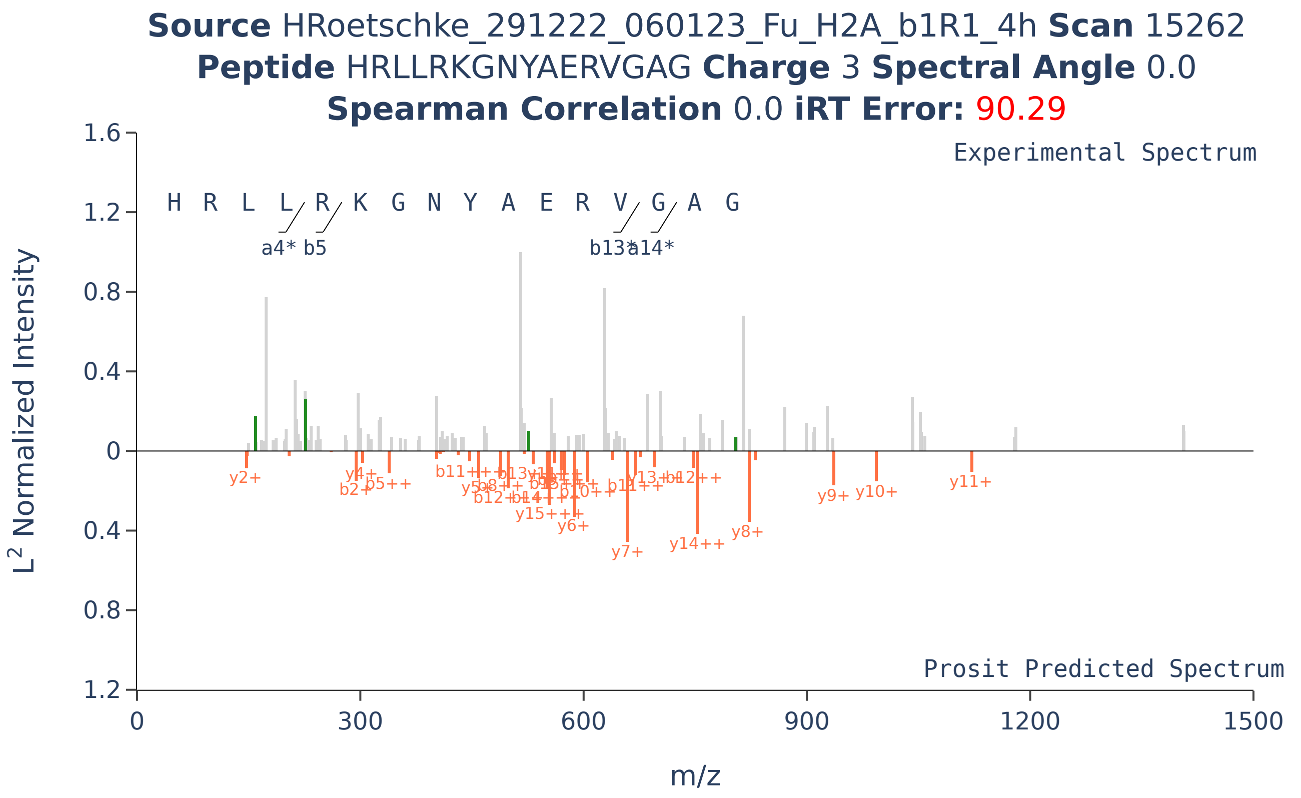
Source HRoetschke_291222_060123_Fu_H2A_b1R1_4h Scan 15262
Peptide HRLLRKGNYAERVGAG Charge 3 Spectral Angle 0.0
Spearman Correlation 0.0 iRT Error: 90.29
Experimental Spectrum
Prosit Predicted Spectrum
L² Normalized Intensity
m/z

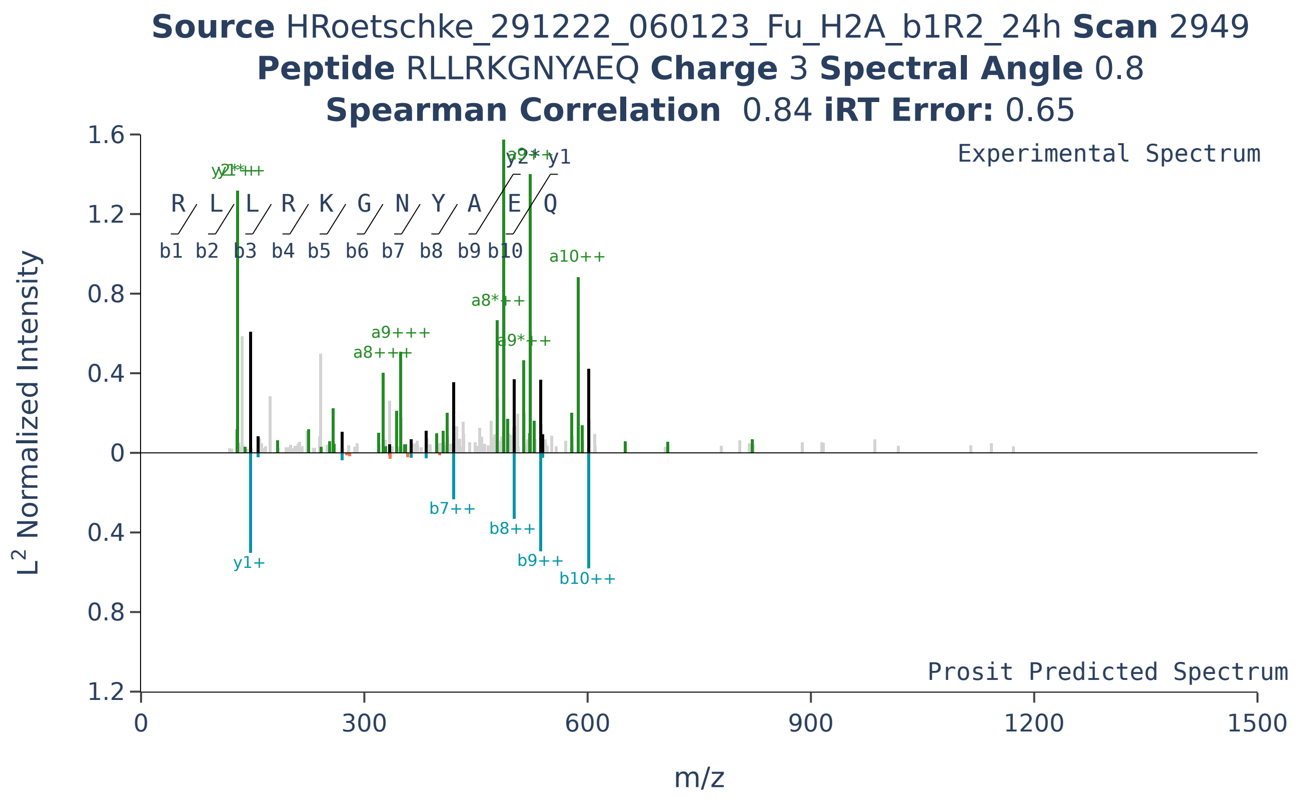
Source HRoetschke_291222_060123_Fu_H2A_b1R2_24h Scan 2949
Peptide RLLRKGNYAEQ Charge 3 Spectral Angle 0.8
Spearman Correlation 0.84 iRT Error: 0.65
Experimental Spectrum
Prosit Predicted Spectrum
L² Normalized Intensity
m/z

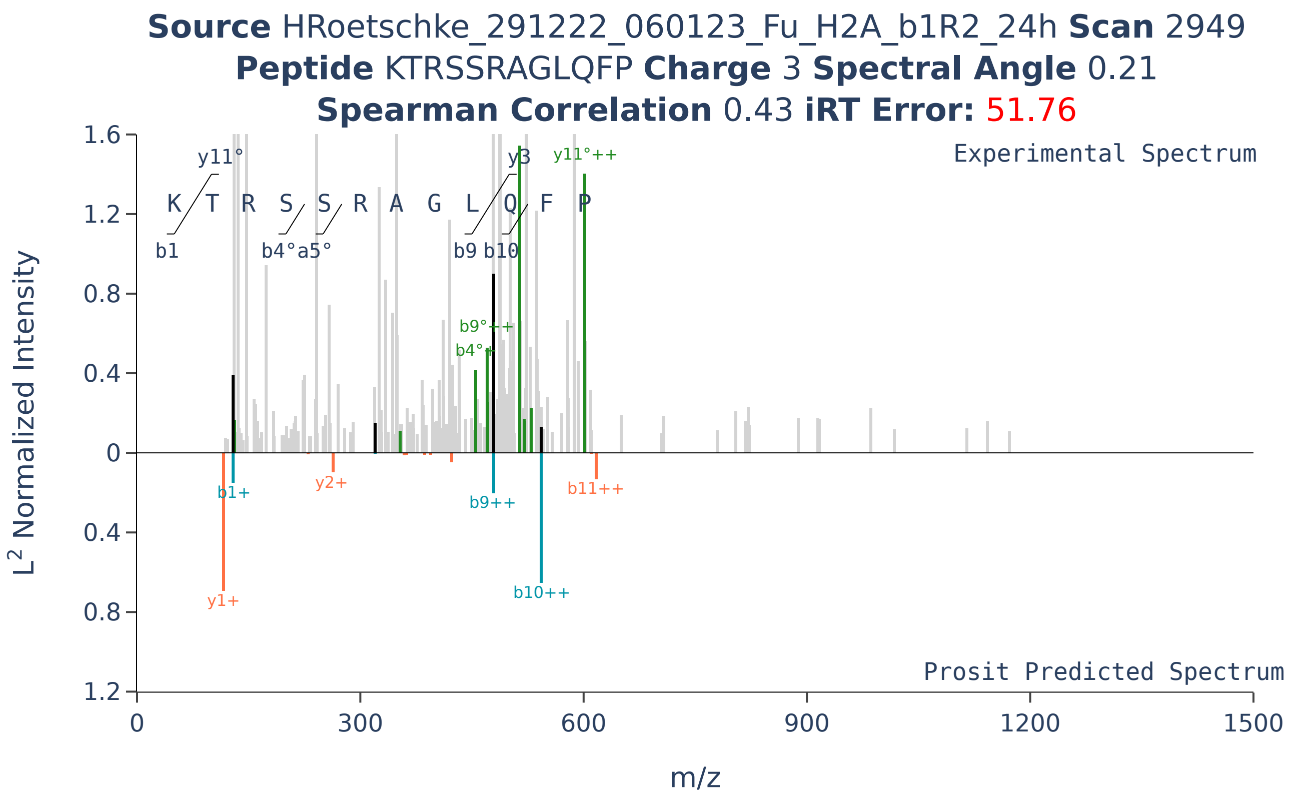
Source HRoetschke_291222_060123_Fu_H2A_b1R2_24h Scan 2949
Peptide KTRSSRAGLQFP Charge 3 Spectral Angle 0.21
Spearman Correlation 0.43 iRT Error: 51.76
Experimental Spectrum
Prosit Predicted Spectrum
L² Normalized Intensity
m/z

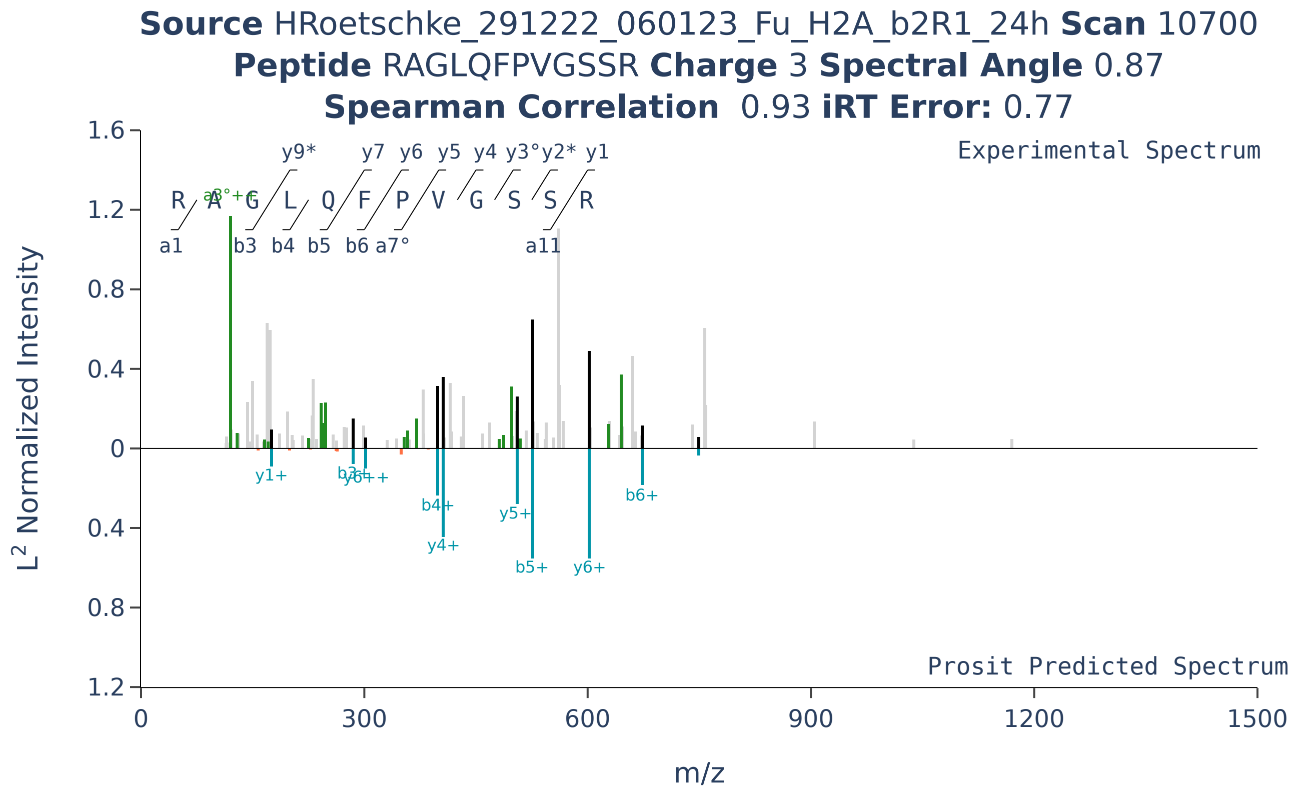
Source HRoetschke_291222_060123_Fu_H2A_b2R1_24h Scan 10700
Peptide RAGLQFPVGSSR Charge 3 Spectral Angle 0.87
Spearman Correlation 0.93 iRT Error: 0.77
Experimental Spectrum
Prosit Predicted Spectrum
L² Normalized Intensity
m/z

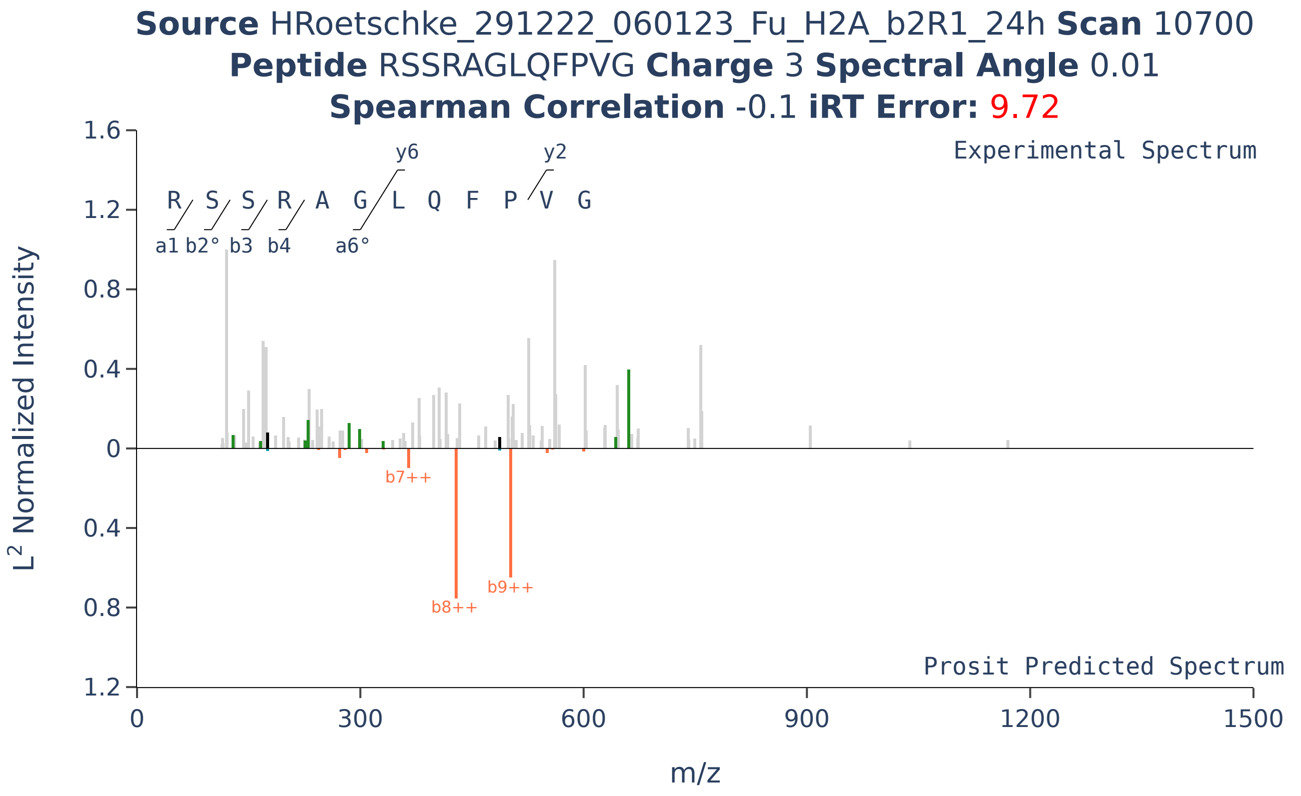
Source HRoetschke_291222_060123_Fu_H2A_b2R1_24h Scan 10700
Peptide RSSRAGLQFPVG Charge 3 Spectral Angle 0.01
Spearman Correlation -0.1 iRT Error: 9.72
Experimental Spectrum
Prosit Predicted Spectrum
L² Normalized Intensity
m/z

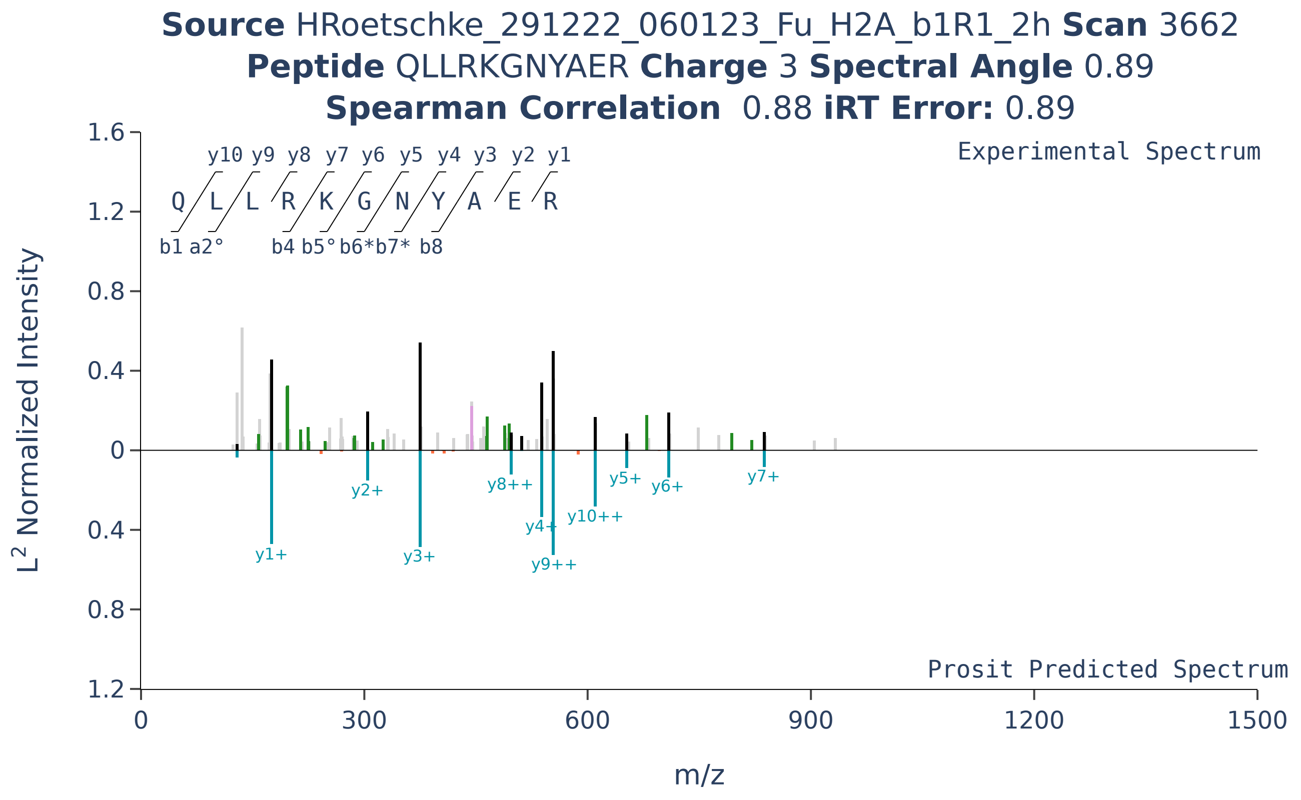
Source HRoetschke_291222_060123_Fu_H2A_b1R1_2h Scan 3662
Peptide QLLRKGNYAER Charge 3 Spectral Angle 0.89
Spearman Correlation 0.88 iRT Error: 0.89
Experimental Spectrum
Prosit Predicted Spectrum
L² Normalized Intensity
m/z

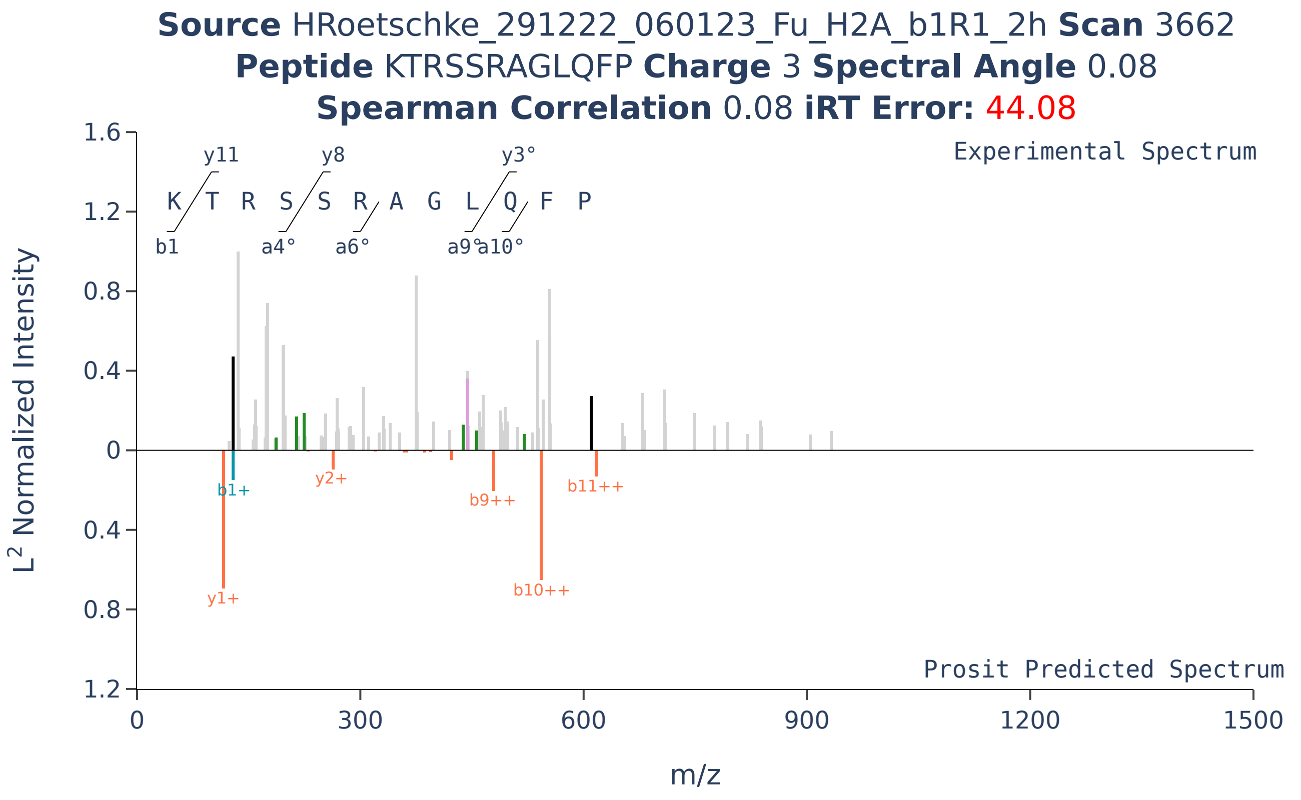
Source HRoetschke_291222_060123_Fu_H2A_b1R1_2h Scan 3662
Peptide KTRSSRAGLQFP Charge 3 Spectral Angle 0.08
Spearman Correlation 0.08 iRT Error: 44.08
Experimental Spectrum
Prosit Predicted Spectrum
L² Normalized Intensity
m/z

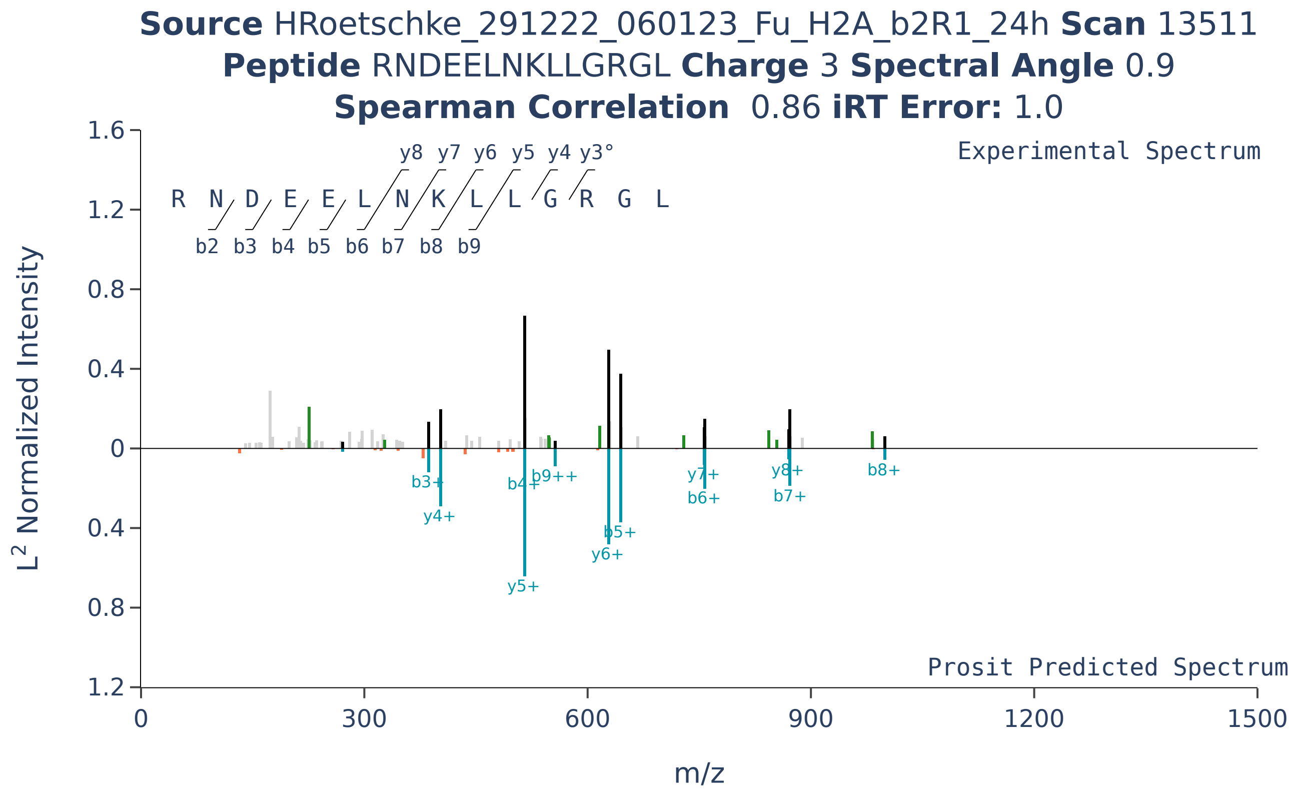
Source HRoetschke_291222_060123_Fu_H2A_b2R1_24h Scan 13511
Peptide RNDEELNKLLGRGL Charge 3 Spectral Angle 0.9
Spearman Correlation 0.86 iRT Error: 1.0
Experimental Spectrum
Prosit Predicted Spectrum
L² Normalized Intensity
m/z

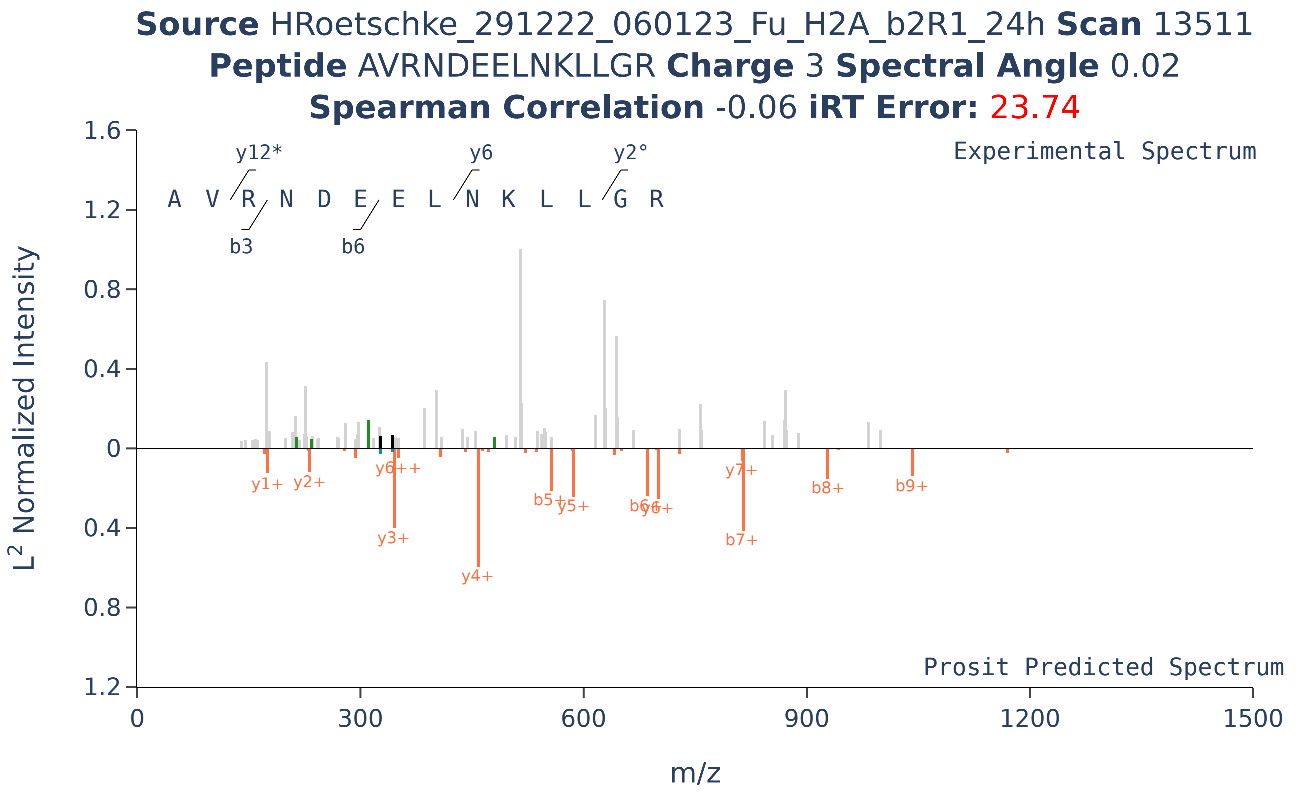
Source HRoetschke_291222_060123_Fu_H2A_b2R1_24h Scan 13511
Peptide AVRNDEELNKLLGR Charge 3 Spectral Angle 0.02
Spearman Correlation -0.06 iRT Error: 23.74
Experimental Spectrum
Prosit Predicted Spectrum
L² Normalized Intensity
m/z

Source HRoetschke_291222_060123_Fu_H2A_b1R1_2h **Scan** 12447
Peptide VRNDEELNKLLRAG **Charge** 4 **Spectral Angle** 0.84
Spearman Correlation 0.82 **iRT Error:** 1.2

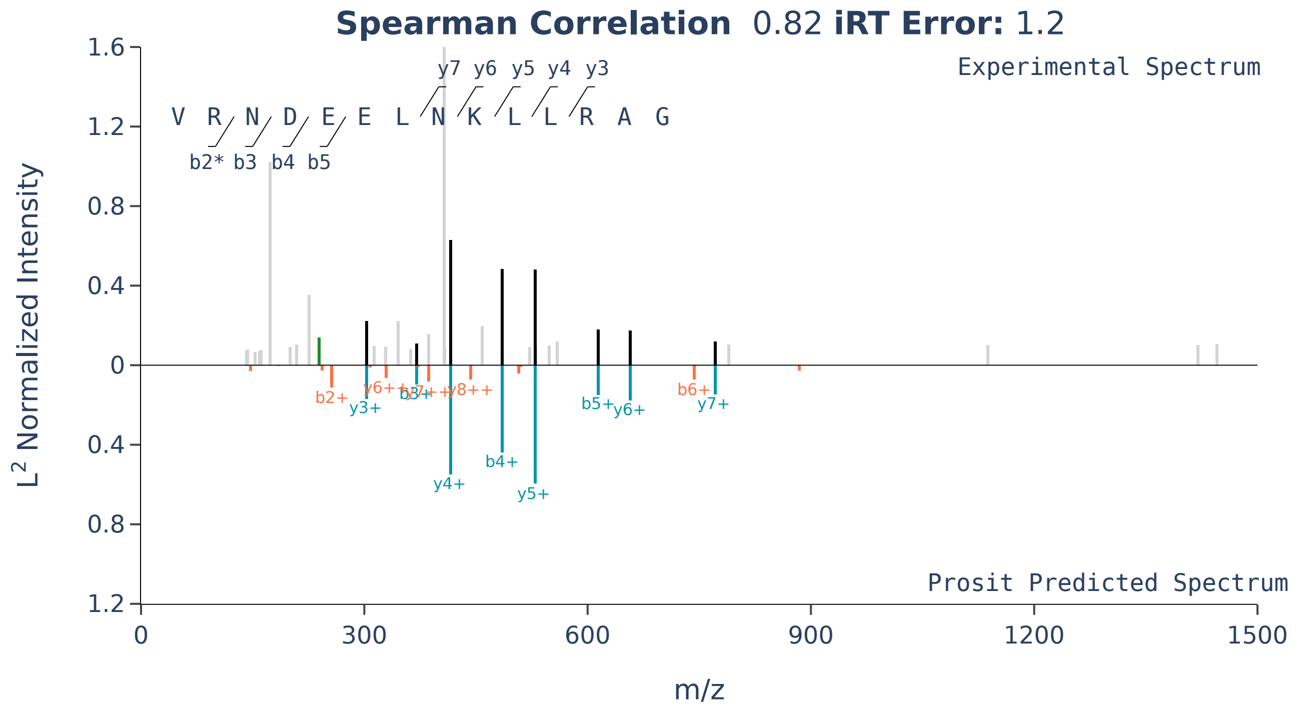

Source HRoetschke_291222_060123_Fu_H2A_b1R1_2h **Scan** 12447
Peptide AVRNDEELNKLLGR **Charge** 4 **Spectral Angle** 0.67
Spearman Correlation 0.42 **iRT Error:** 6.79

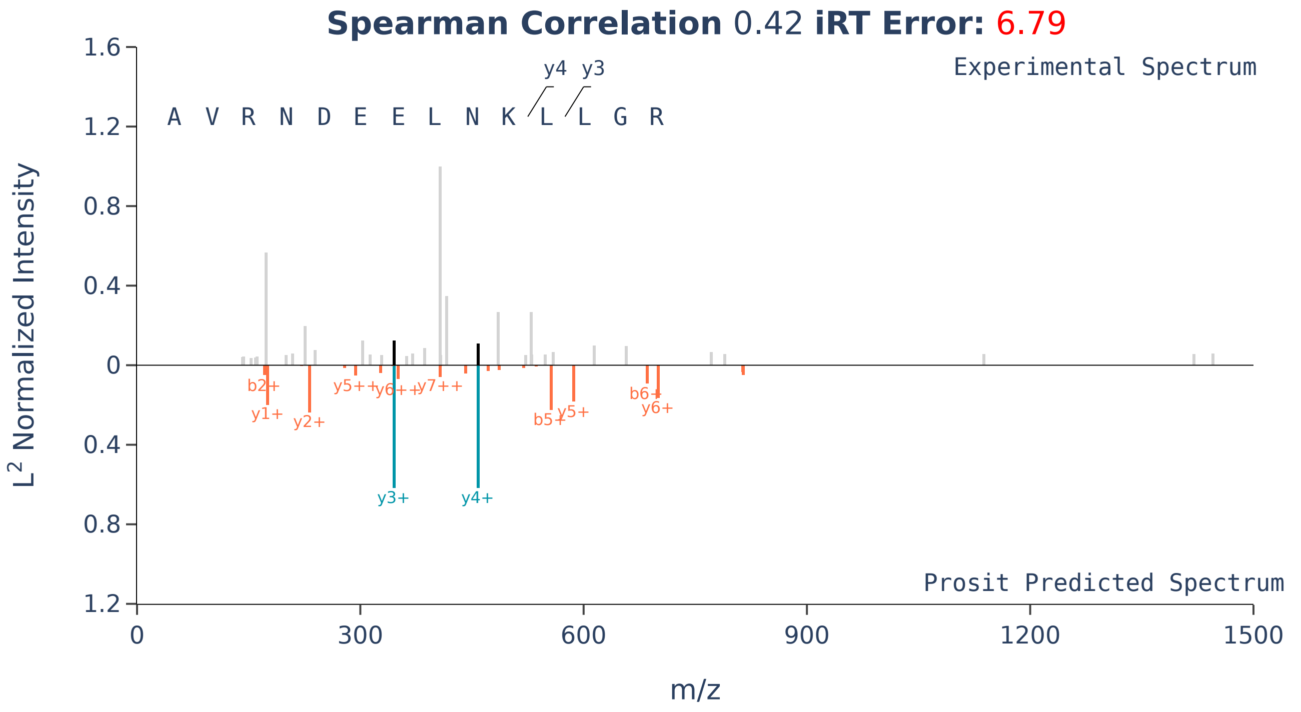

Source HRoetschke_291222_060123_Fu_H2A_b1R1_4h **Scan** 14303
Peptide VRNDEELNKLLGA **Charge** 2 **Spectral Angle** 0.86
Spearman Correlation 0.82 **iRT Error:** 1.3

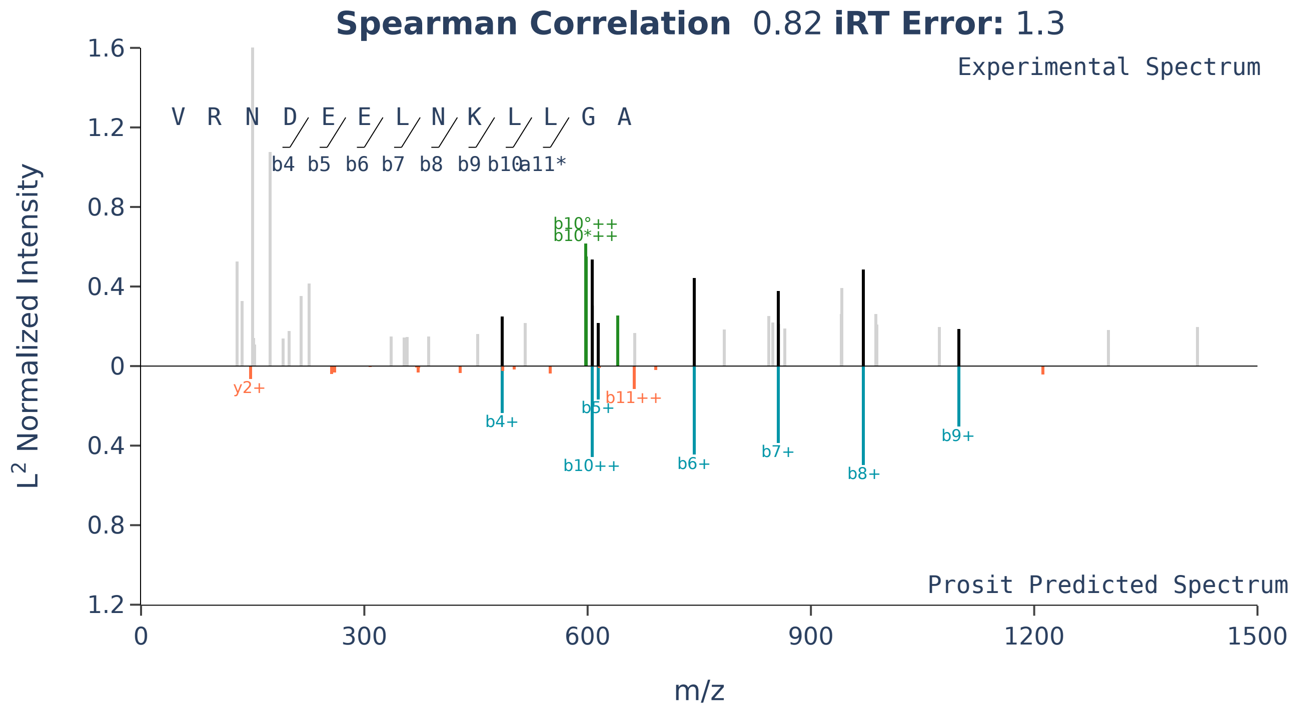

Source HRoetschke_291222_060123_Fu_H2A_b1R1_4h **Scan** 14303
Peptide AVRNDEELNKLLG **Charge** 2 **Spectral Angle** 0.0
Spearman Correlation 0.0 **iRT Error:** 5.52

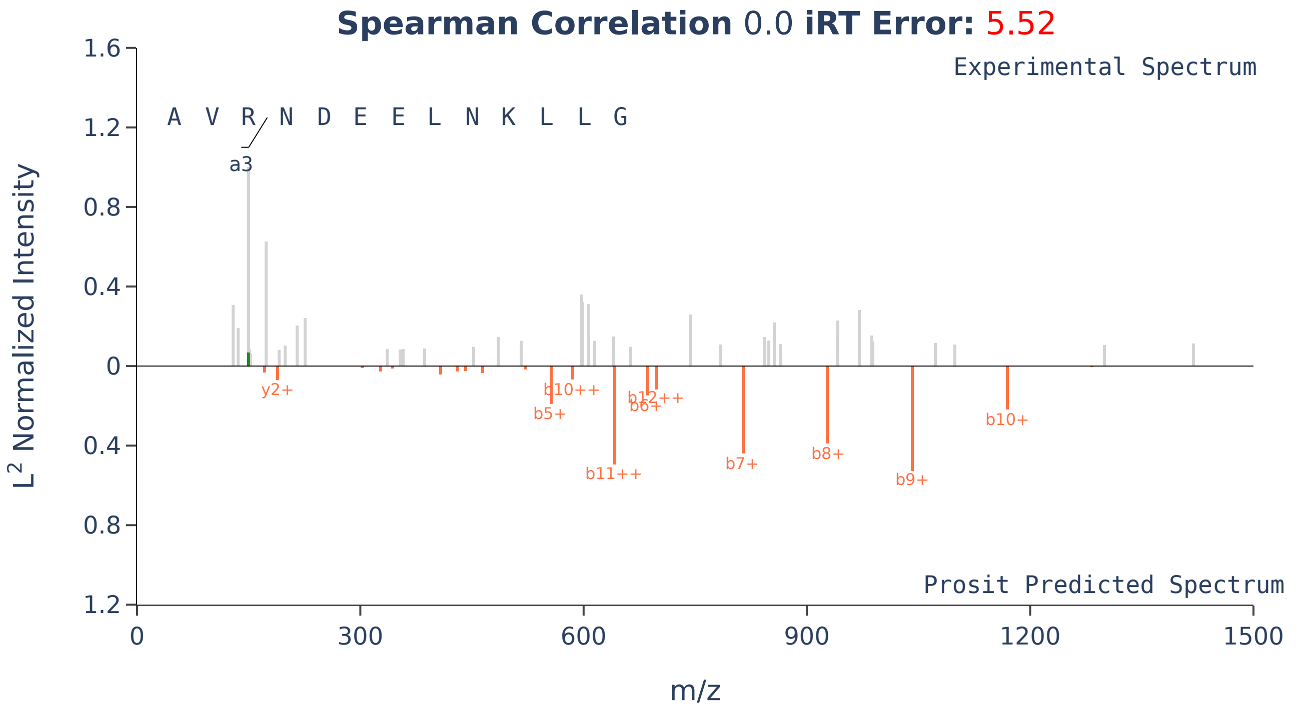

Source HRoetschke_291222_060123_Fu_H2A_b1R2_4h **Scan** 12857
Peptide VRNDEELNKLLGRA **Charge** 4 **Spectral Angle** 0.86
Spearman Correlation 0.83 **iRT Error:** 1.73

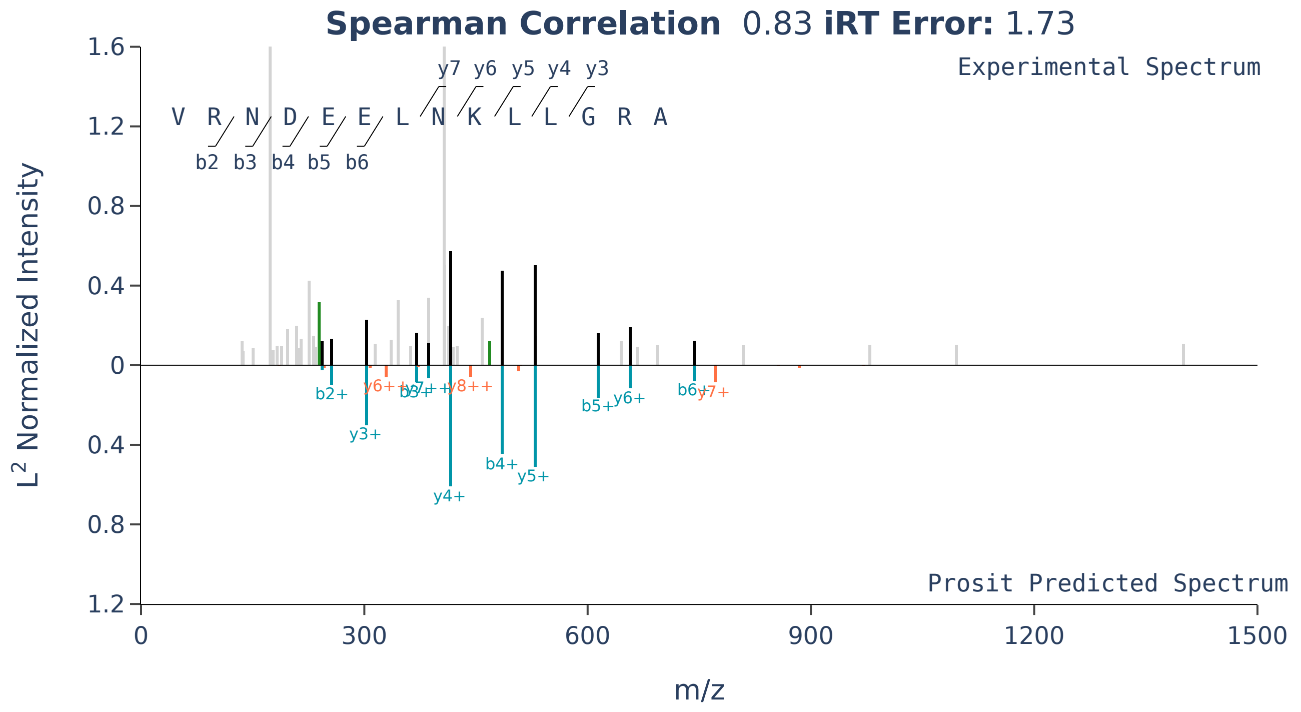

Source HRoetschke_291222_060123_Fu_H2A_b1R2_4h **Scan** 12857
Peptide AVRNDEELNKLLGR **Charge** 4 **Spectral Angle** 0.7
Spearman Correlation 0.51 **iRT Error:** 5.72

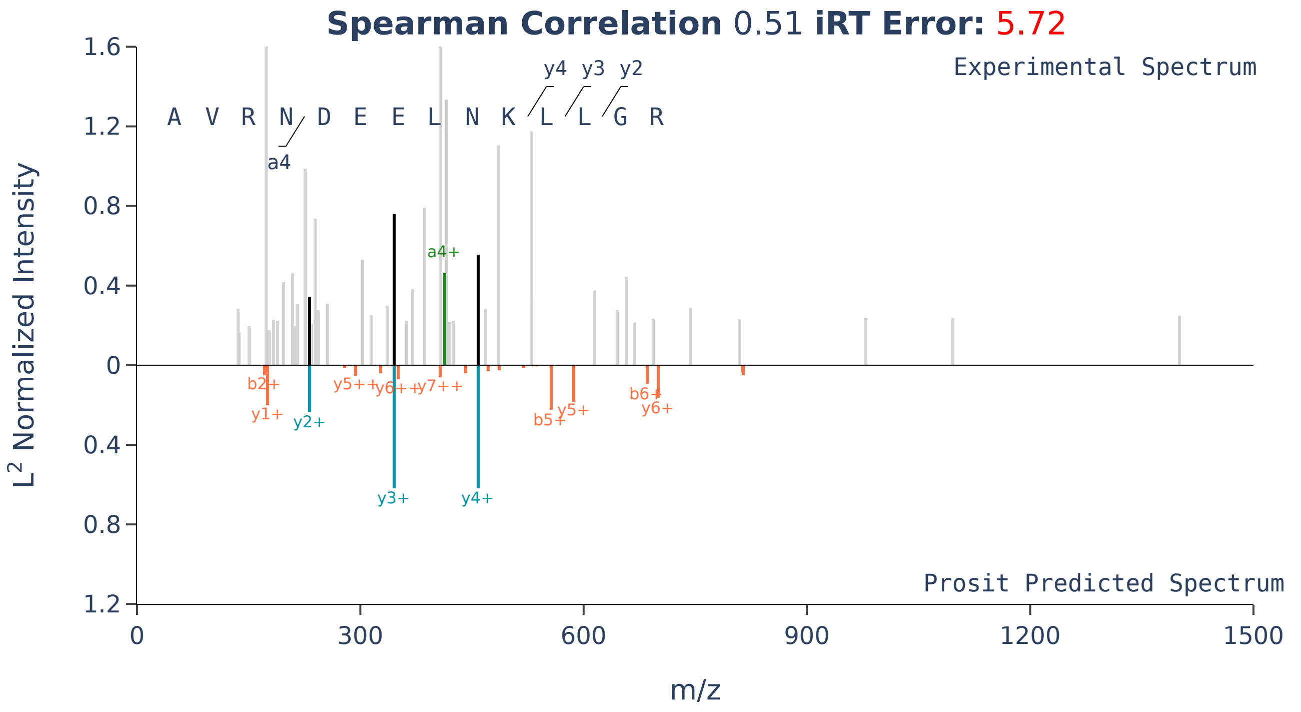

Source HRoetschke_291222_060123_Fu_H2A_b1R1_4h **Scan** 14644
Peptide ARNDEELNKLLGRV **Charge** 4 **Spectral Angle** 0.87
Spearman Correlation 0.83 **iRT Error:** 2.16

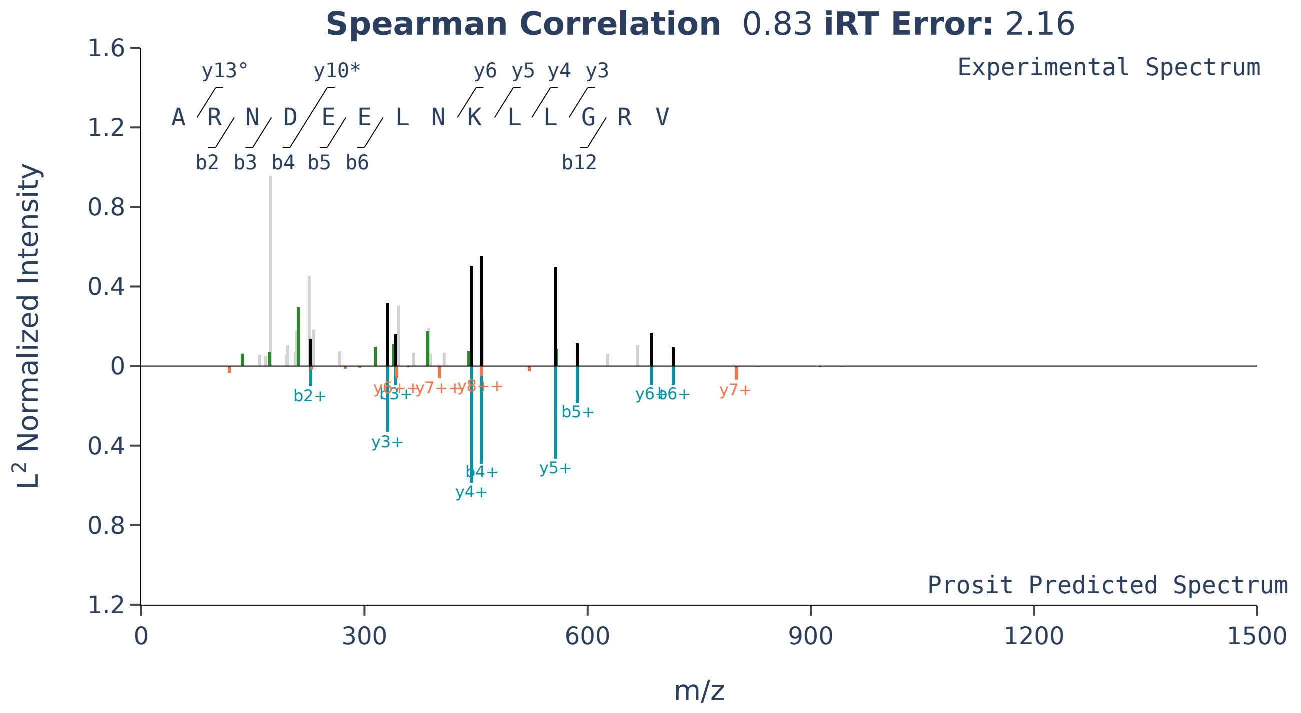

Source HRoetschke_291222_060123_Fu_H2A_b1R1_4h **Scan** 14644
Peptide AVRNDEELNKLLGR **Charge** 4 **Spectral Angle** 0.69
Spearman Correlation 0.51 **iRT Error:** 18.49

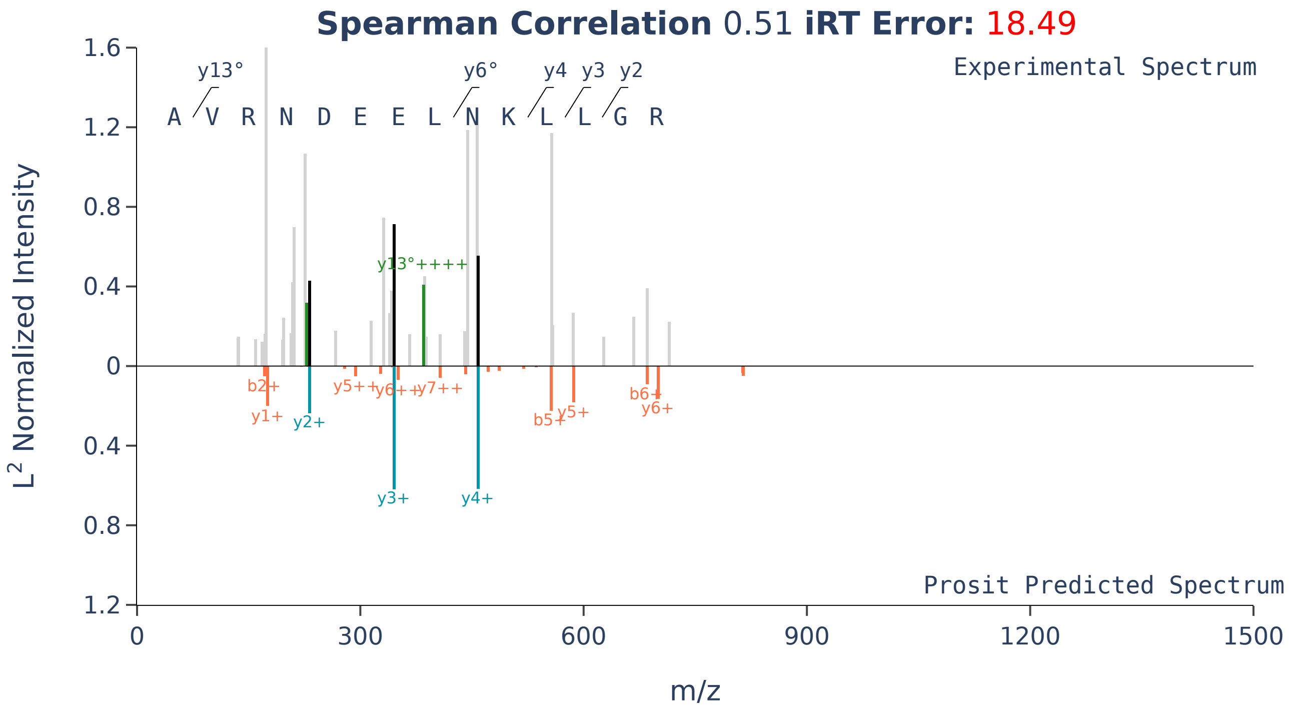

Source HRoetschke_291222_060123_Fu_H2A_b2R1_24h **Scan** 12640
Peptide QAPVYLA **Charge** 2 **Spectral Angle** 0.73
Spearman Correlation 0.93 **iRT Error:** 3.83

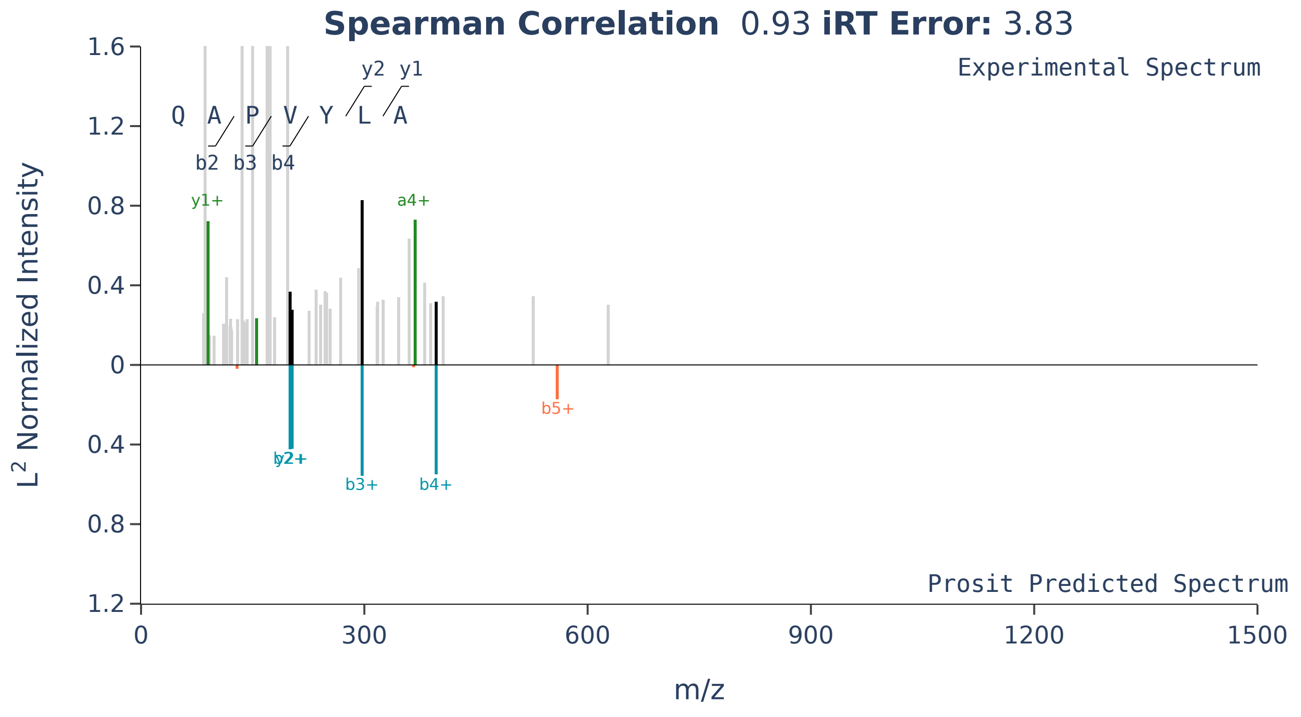

Source HRoetschke_291222_060123_Fu_H2A_b2R1_24h **Scan** 12640
Peptide AGAPVYLA **Charge** 2 **Spectral Angle** 0.47
Spearman Correlation 0.52 **iRT Error:** 1.51

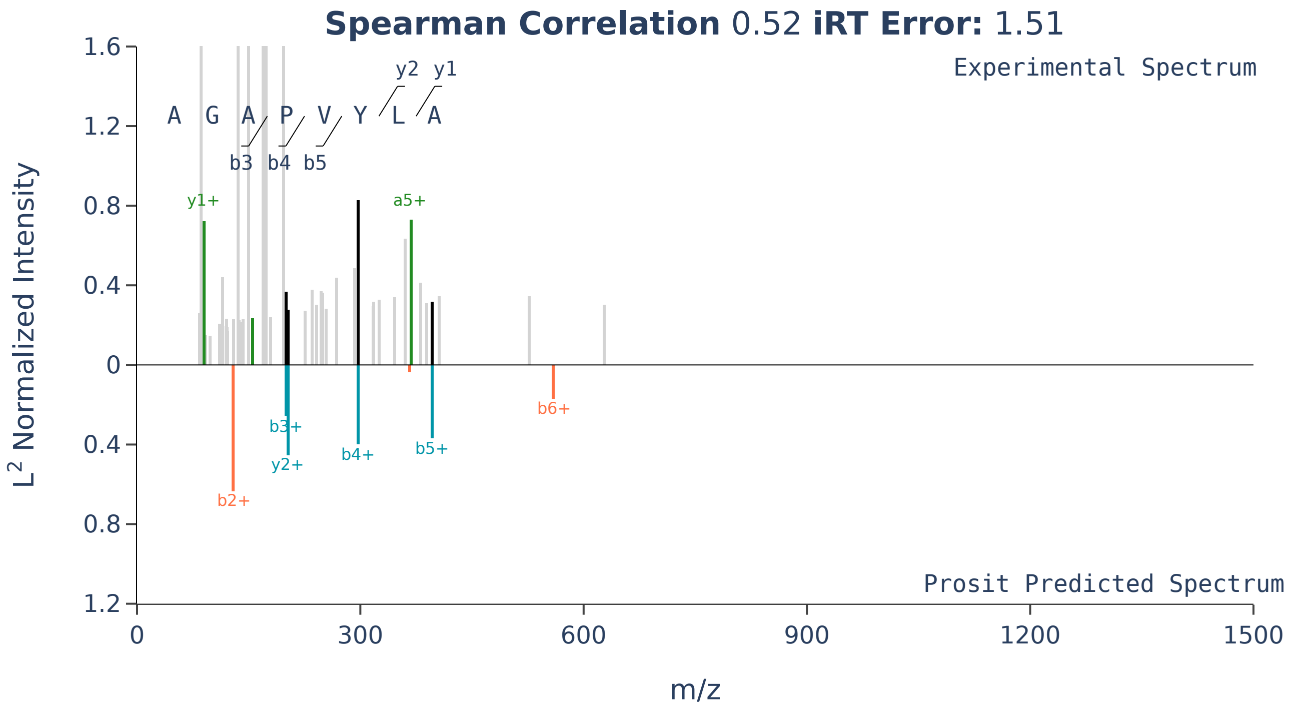

Source HRoetschke_291222_060123_Fu_H2A_b1R1_1h **Scan** 9889
Peptide AVRNDLQF **Charge** 2 **Spectral Angle** 0.92
Spearman Correlation 0.85 **iRT Error:** 5.48

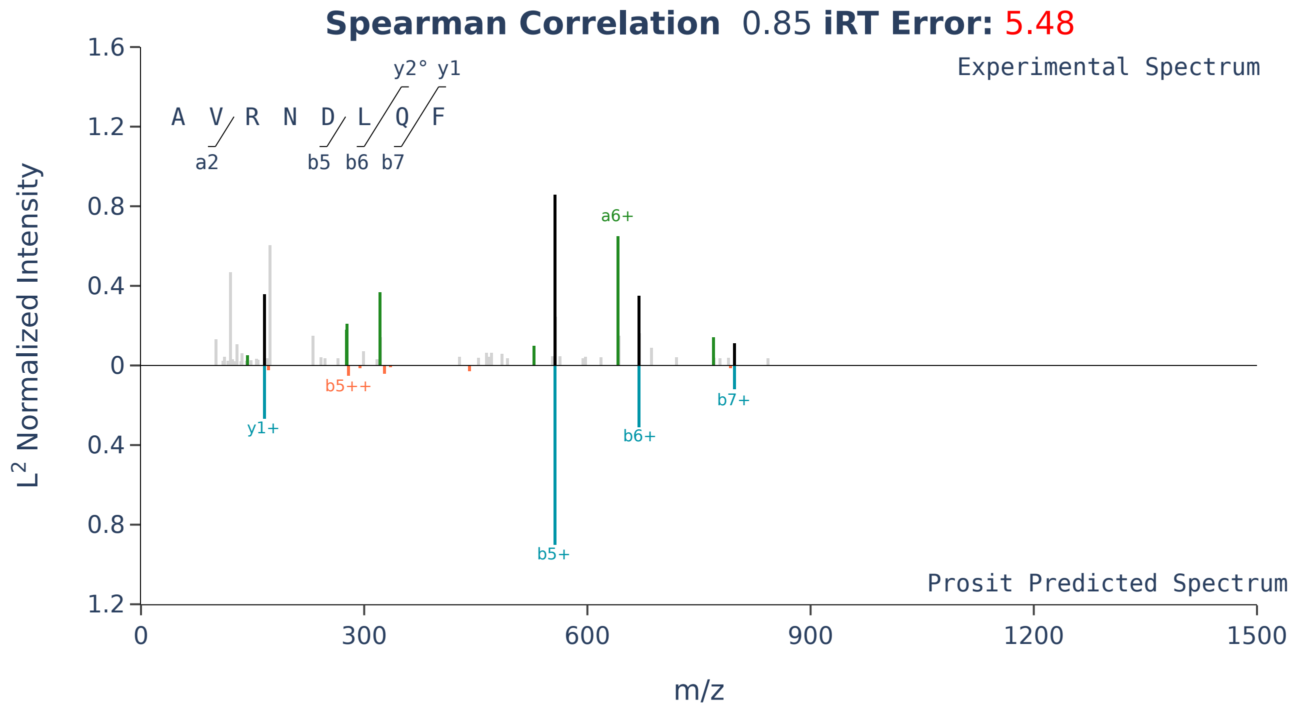

Source HRoetschke_291222_060123_Fu_H2A_b1R1_1h **Scan** 9889
Peptide SSRAGLQFP **Charge** 2 **Spectral Angle** 0.0
Spearman Correlation 0.0 **iRT Error:** 14.07

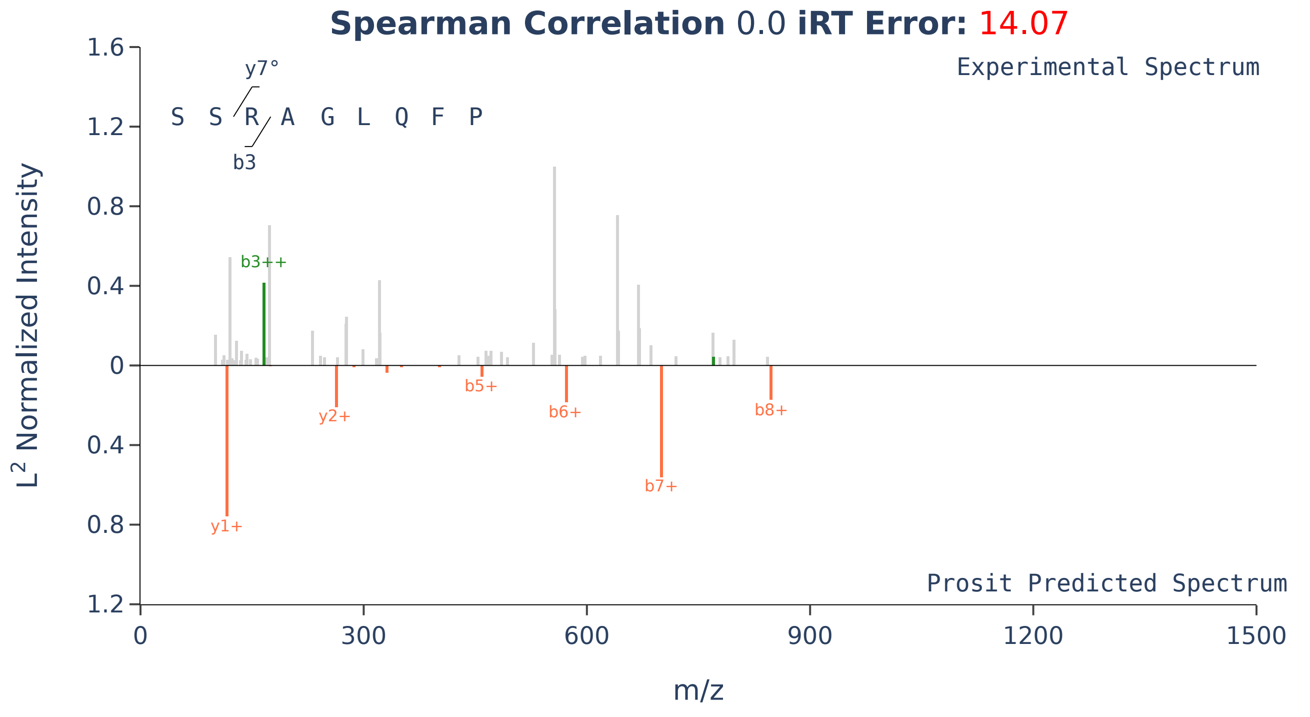

Source HRoetschke_291222_060123_Fu_H2A_b1R2_24h **Scan** 9665
Peptide SSRAGLQFA **Charge** 2 **Spectral Angle** 0.87
Spearman Correlation 0.88 **iRT Error:** 6.46

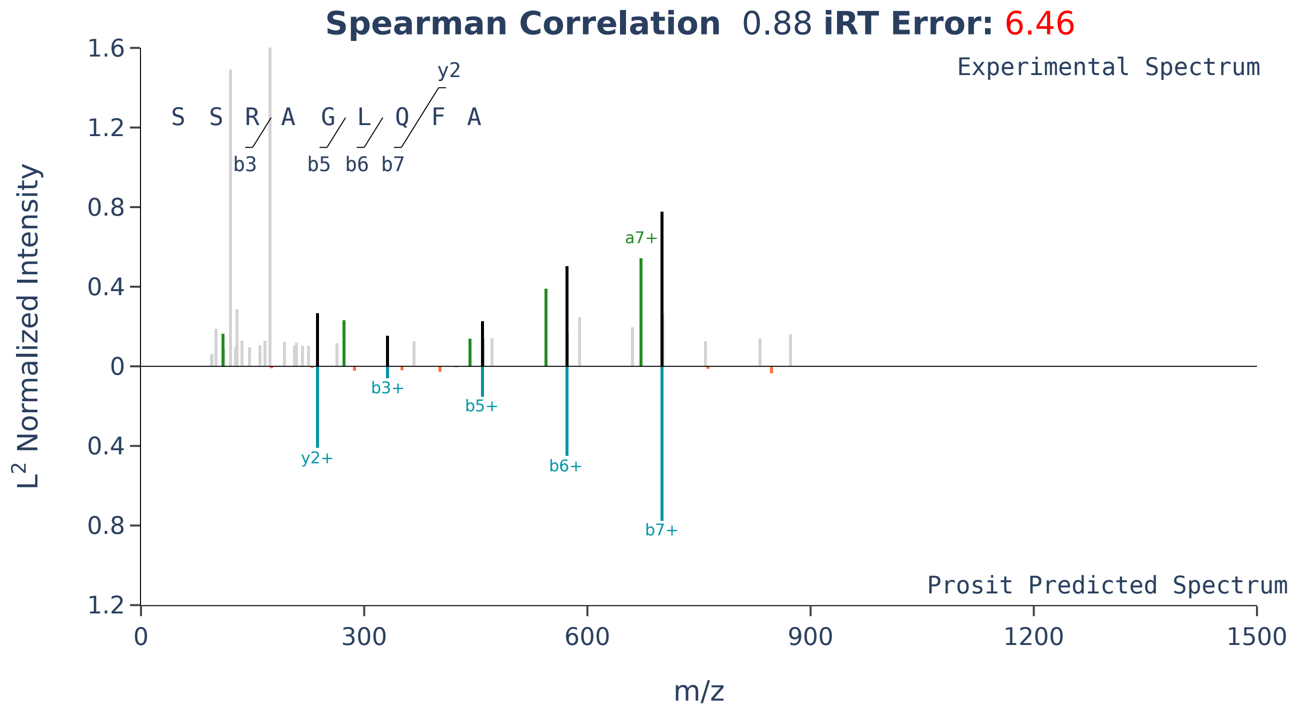

Source HRoetschke_291222_060123_Fu_H2A_b1R2_24h **Scan** 9665
Peptide KGNYAERV **Charge** 2 **Spectral Angle** 0.37
Spearman Correlation 0.37 **iRT Error:** 44.5

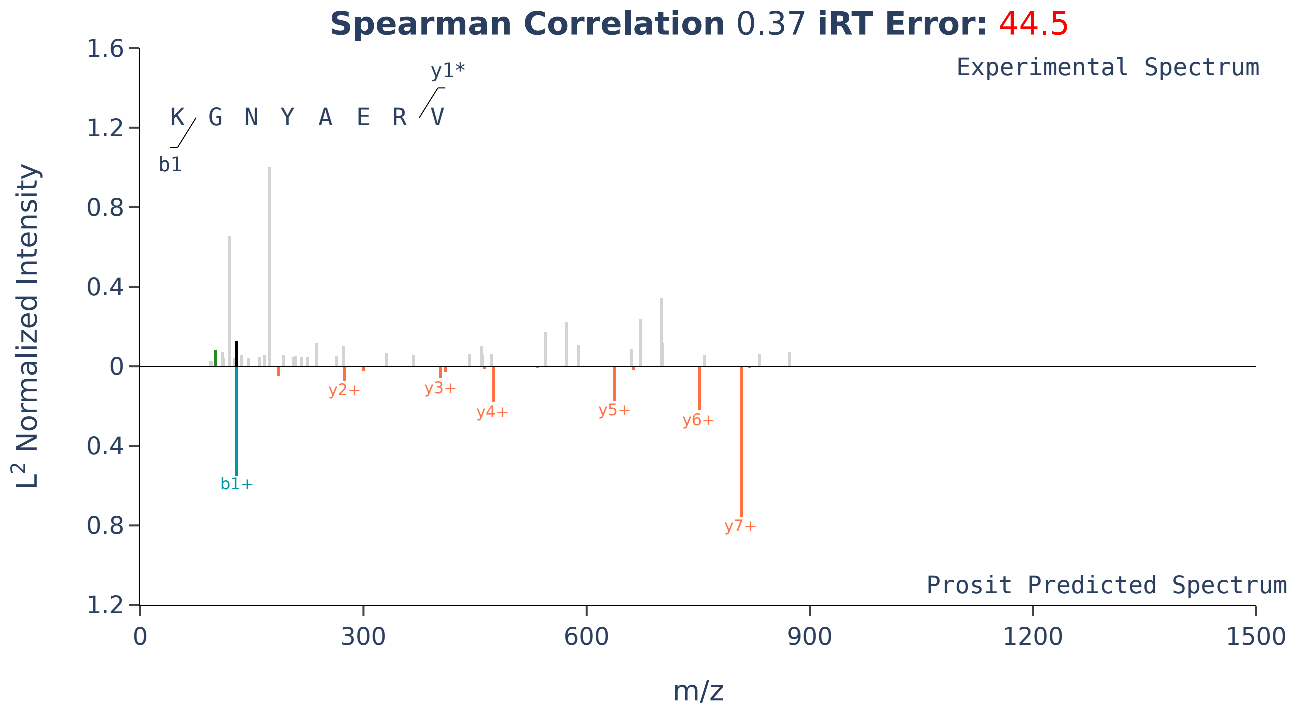

Source HRoetschke_291222_060123_Fu_H2A_b1R1_24h **Scan** 8414
Peptide QFPVGRVHLR **Charge** 3 **Spectral Angle** 0.88
Spearman Correlation 0.92 **iRT Error:** 6.63

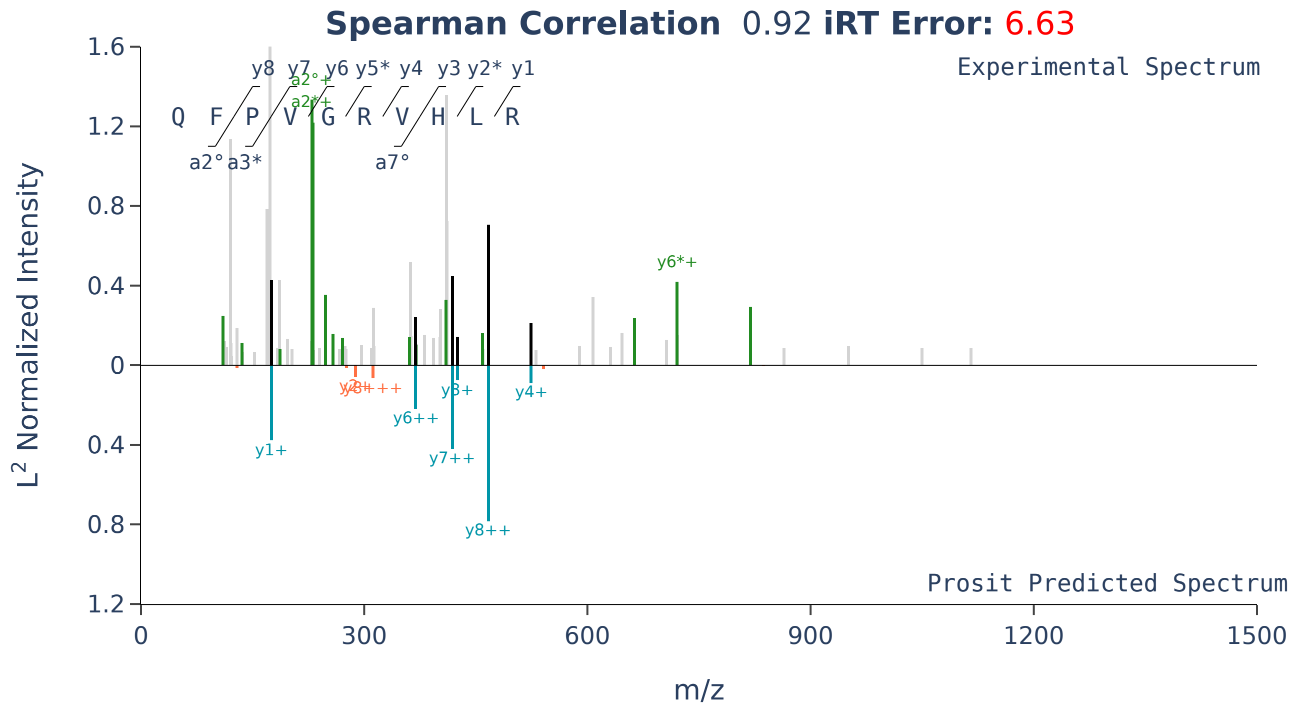

Source HRoetschke_291222_060123_Fu_H2A_b1R1_24h **Scan** 8414
Peptide LQFPVGRVHR **Charge** 3 **Spectral Angle** 0.41
Spearman Correlation 0.39 **iRT Error:** 10.02

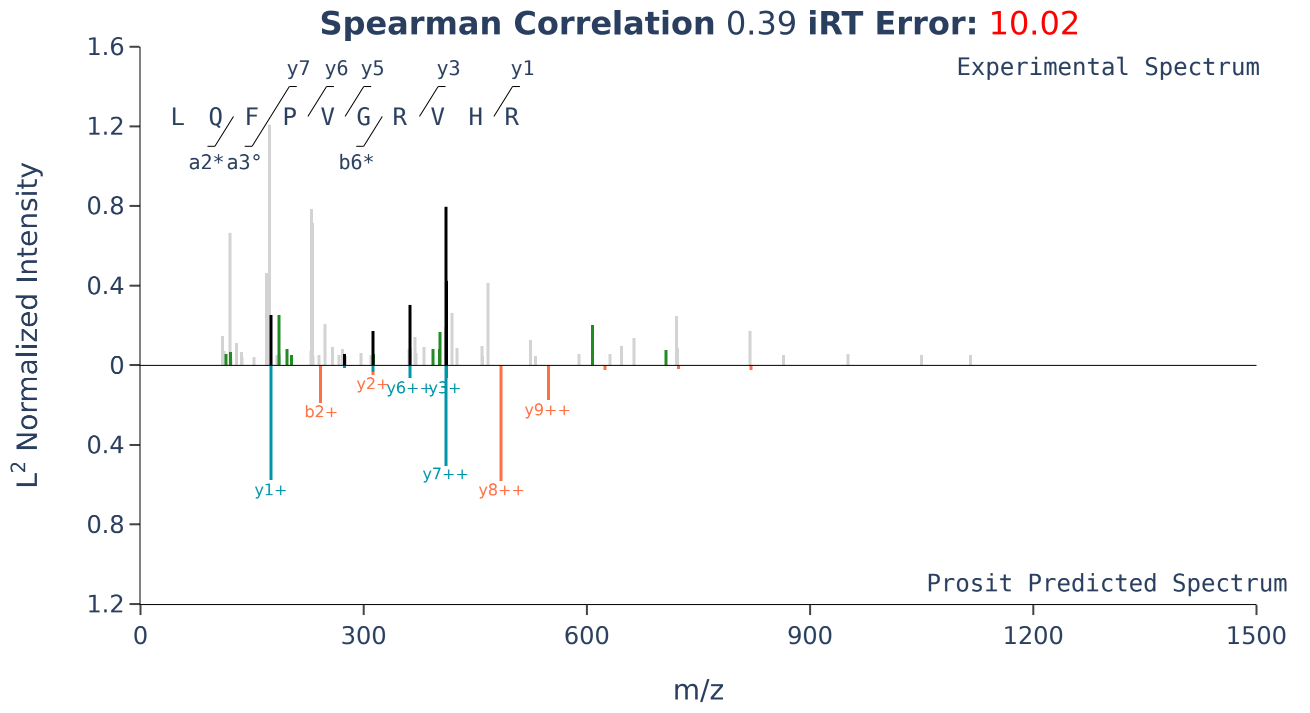

Source HRoetschke_291222_060123_Fu_H2A_b1R2_24h **Scan** 10084
Peptide ASSRAGLQF **Charge** 2 **Spectral Angle** 0.83
Spearman Correlation 0.9 **iRT Error:** 6.98

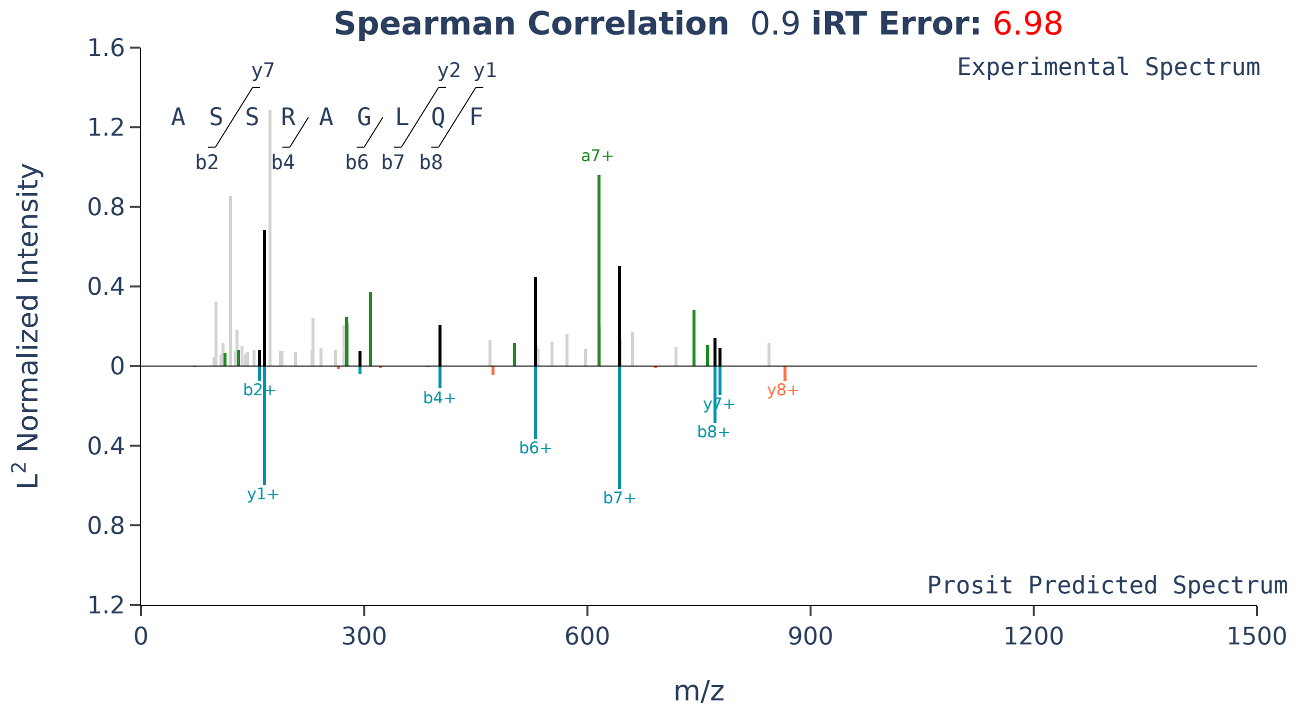

Source HRoetschke_291222_060123_Fu_H2A_b1R2_24h **Scan** 10084
Peptide KGNYAERV **Charge** 2 **Spectral Angle** 0.37
Spearman Correlation 0.37 **iRT Error:** 49.24

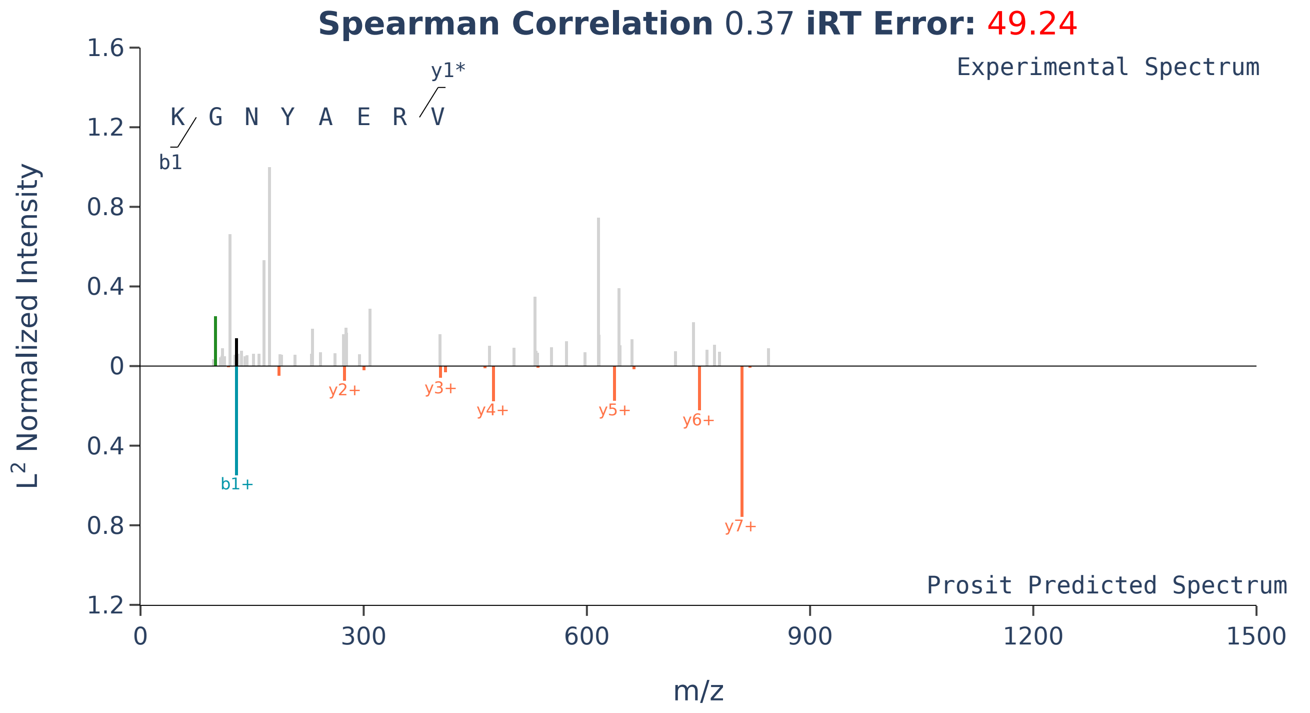

Source HRoetschke_291222_080123_Fu_H2B_b1R1_4h **Scan** 3288
Peptide KTQVTKYTSA **Charge** 3 **Spectral Angle** 0.92
Spearman Correlation 0.82 **iRT Error:** 0.0

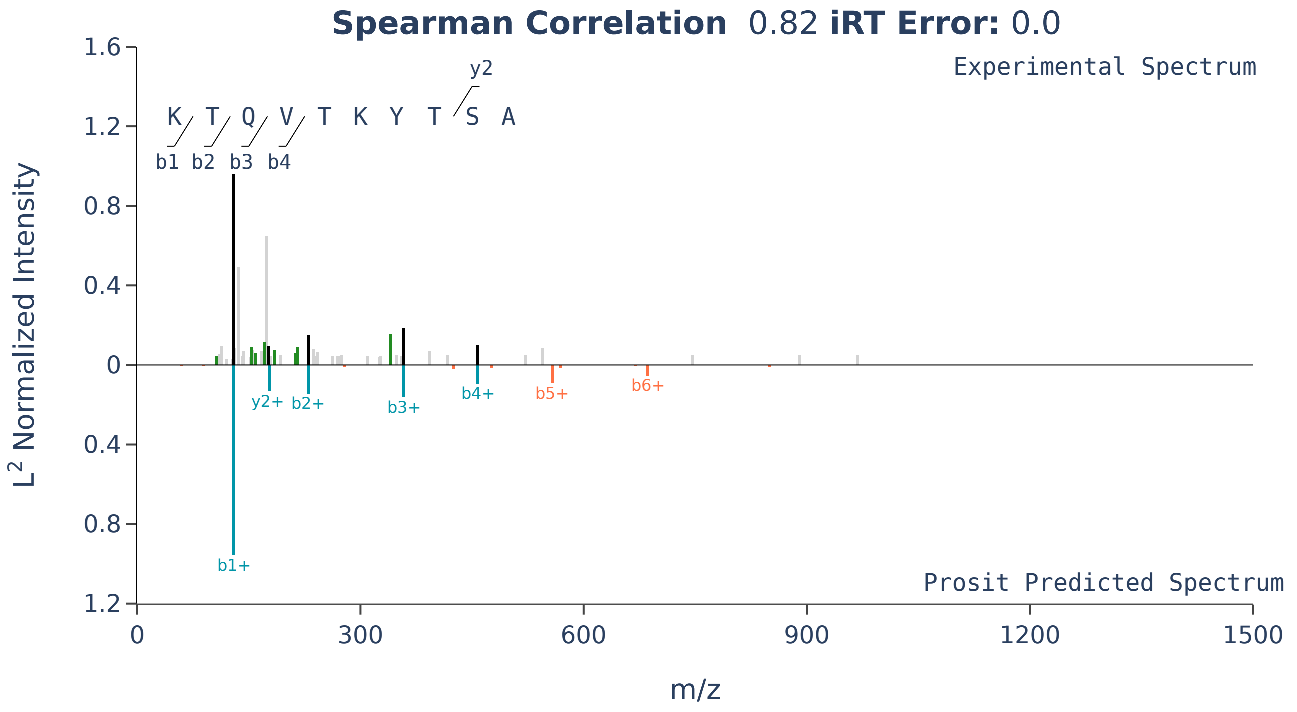

Source HRoetschke_291222_080123_Fu_H2B_b1R1_4h **Scan** 3288
Peptide GTKAVTKYTSA **Charge** 3 **Spectral Angle** 0.81
Spearman Correlation 0.73 **iRT Error:** 0.69

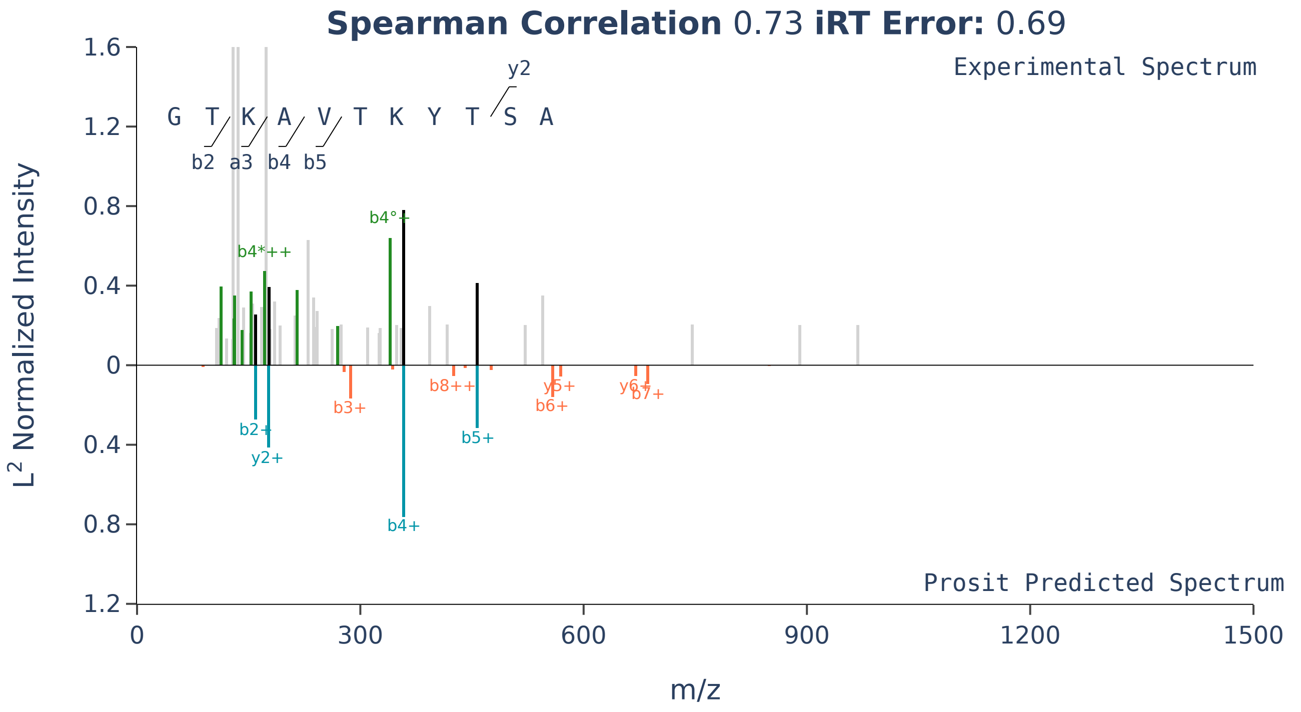

Source HRoetschke_291222_080123_Fu_H2B_b2R1_2h **Scan** 12766
Peptide MAKSIMNSF **Charge** 2 **Spectral Angle** 0.81
Spearman Correlation 0.92 **iRT Error:** 0.32

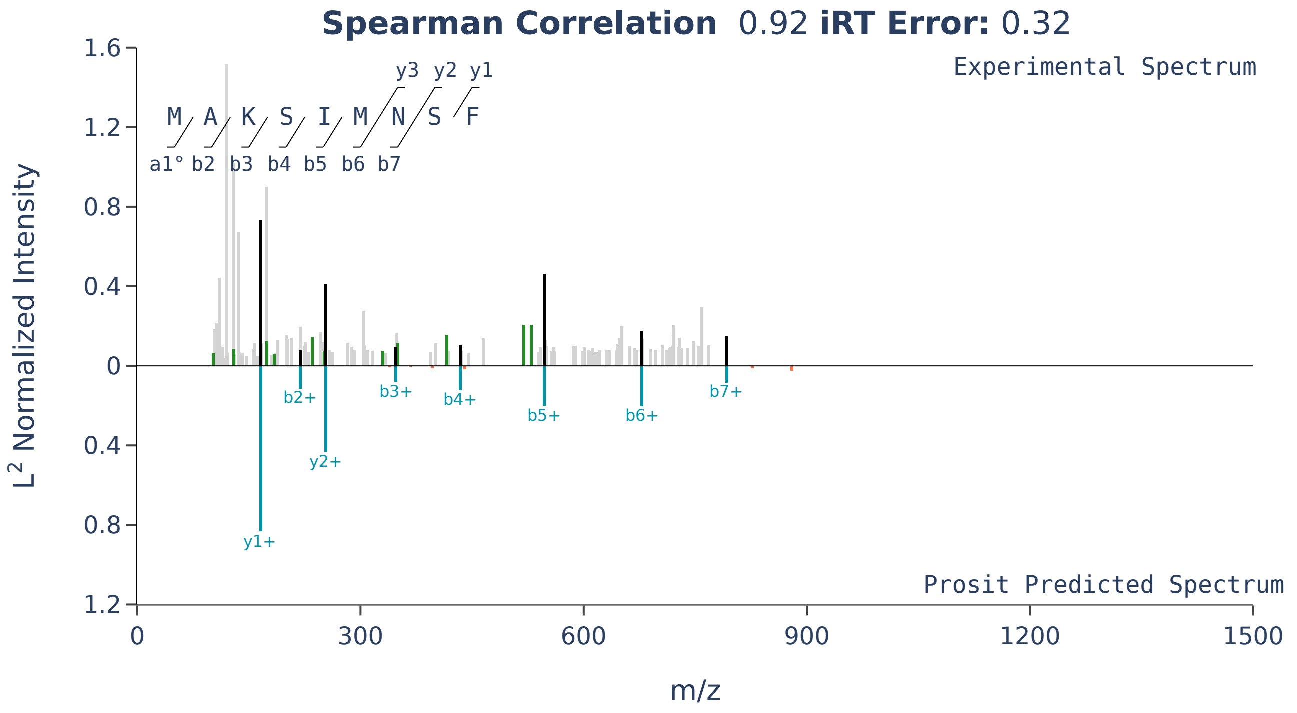

Source HRoetschke_291222_080123_Fu_H2B_b2R1_2h **Scan** 12766
Peptide KAMSIMNSF **Charge** 2 **Spectral Angle** 0.62
Spearman Correlation 0.68 **iRT Error:** 8.42

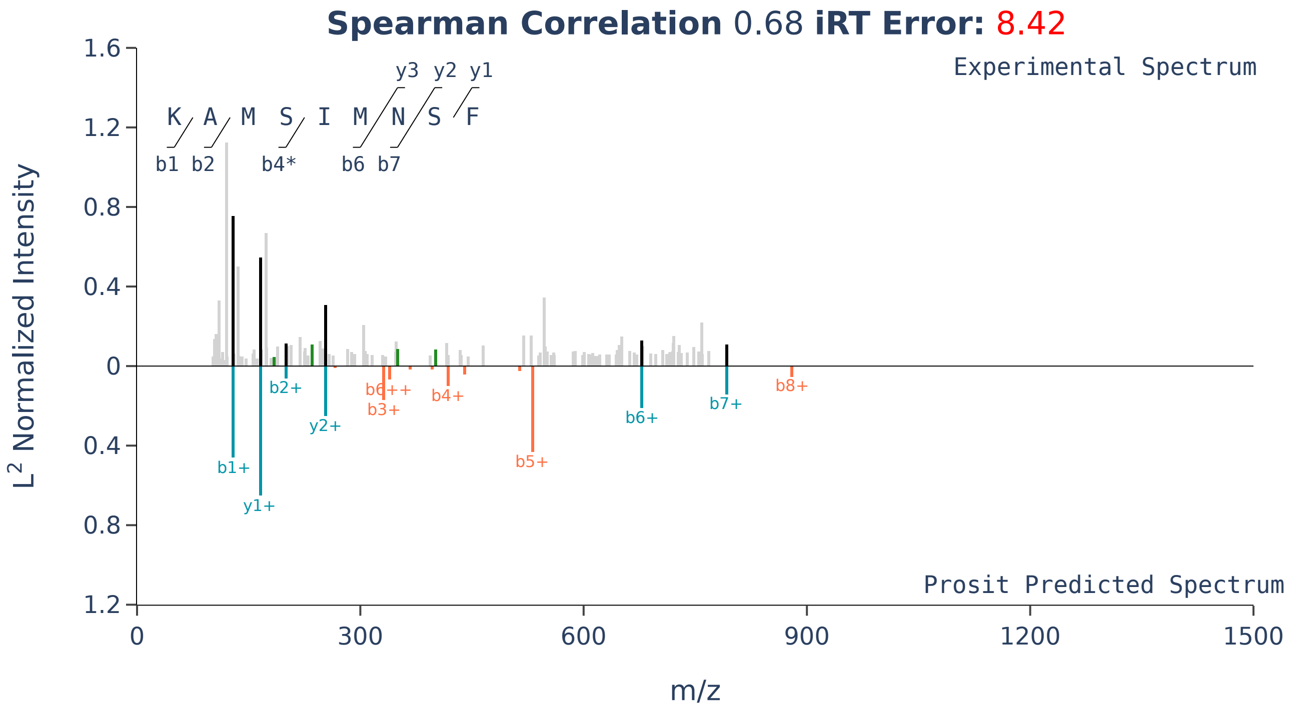

Source HRoetschke_291222_080123_Fu_H2B_b1R2_24h **Scan** 4252
Peptide HPDTGISSKQV **Charge** 3 **Spectral Angle** 0.88
Spearman Correlation 0.84 **iRT Error:** 0.97

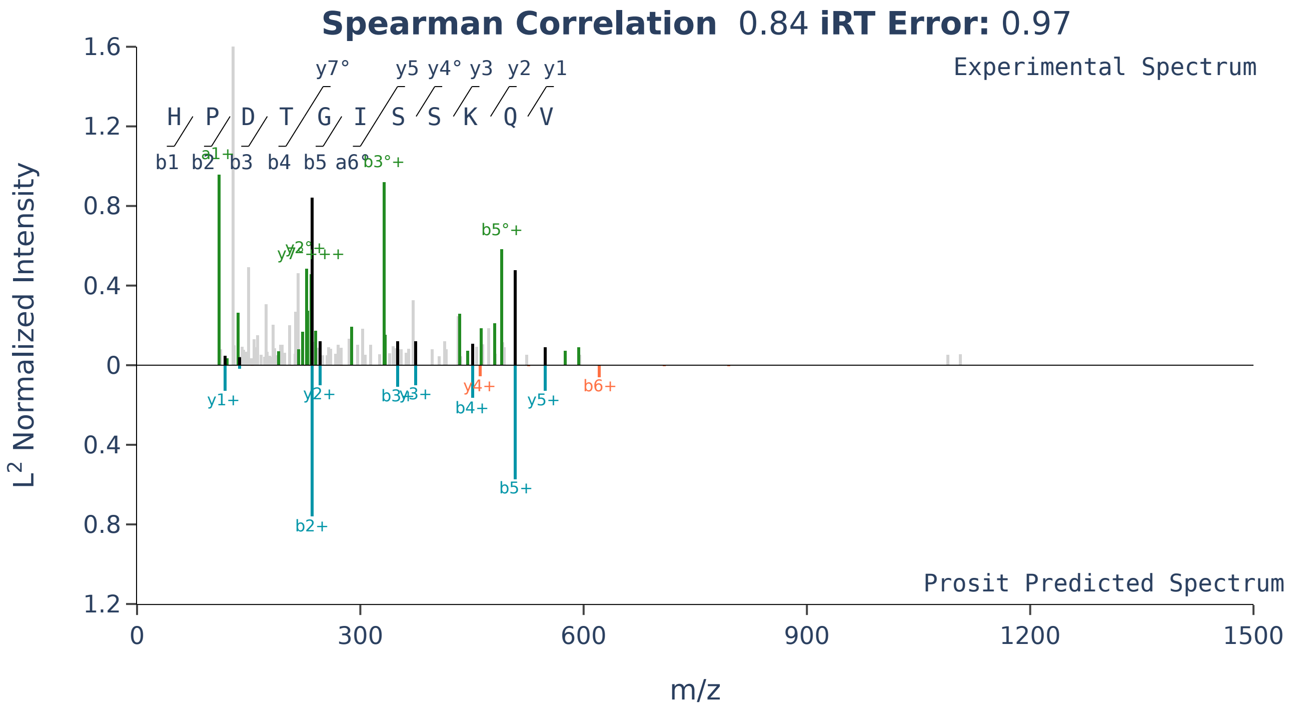

Source HRoetschke_291222_080123_Fu_H2B_b1R2_24h **Scan** 4252
Peptide QVHPDTGISSK **Charge** 3 **Spectral Angle** 0.19
Spearman Correlation -0.01 **iRT Error:** 6.08

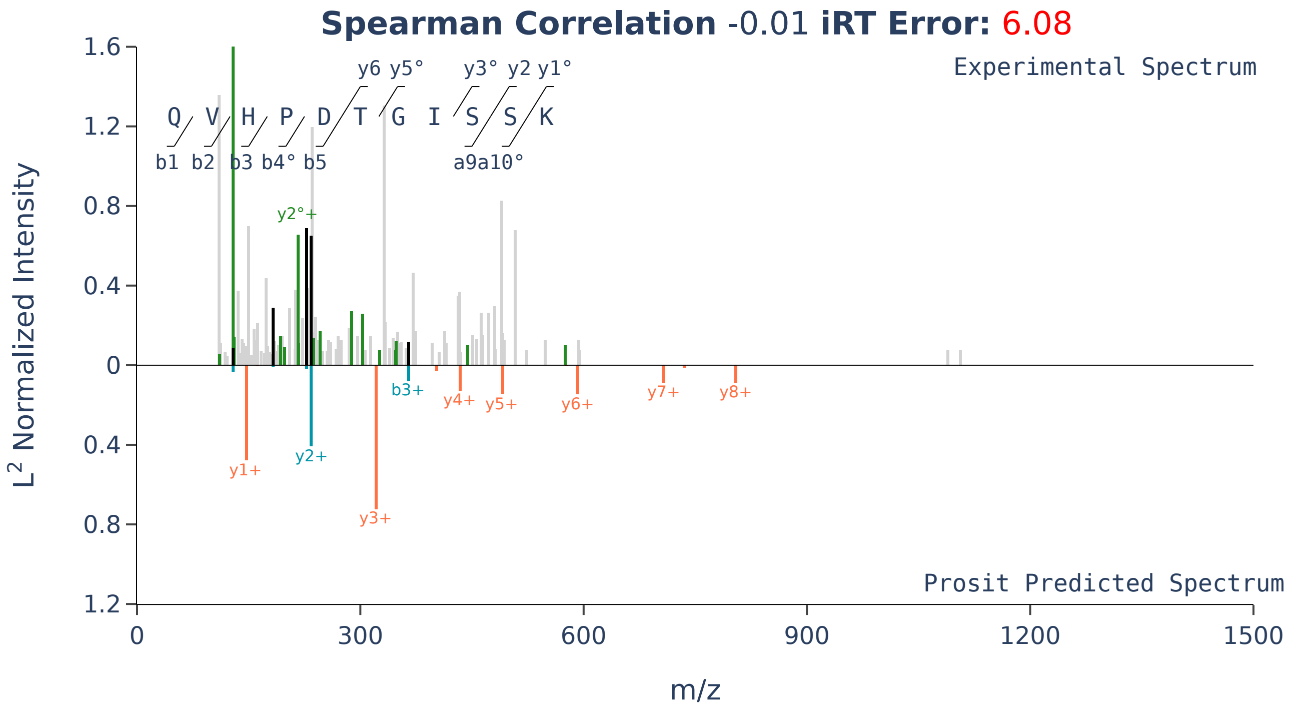

Source HRoetschke_291222_080123_Fu_H2B_b2R1_24h **Scan** 3626
Peptide KQVHPDTGISSA **Charge** 2 **Spectral Angle** 0.82
Spearman Correlation 0.93 **iRT Error:** 1.36

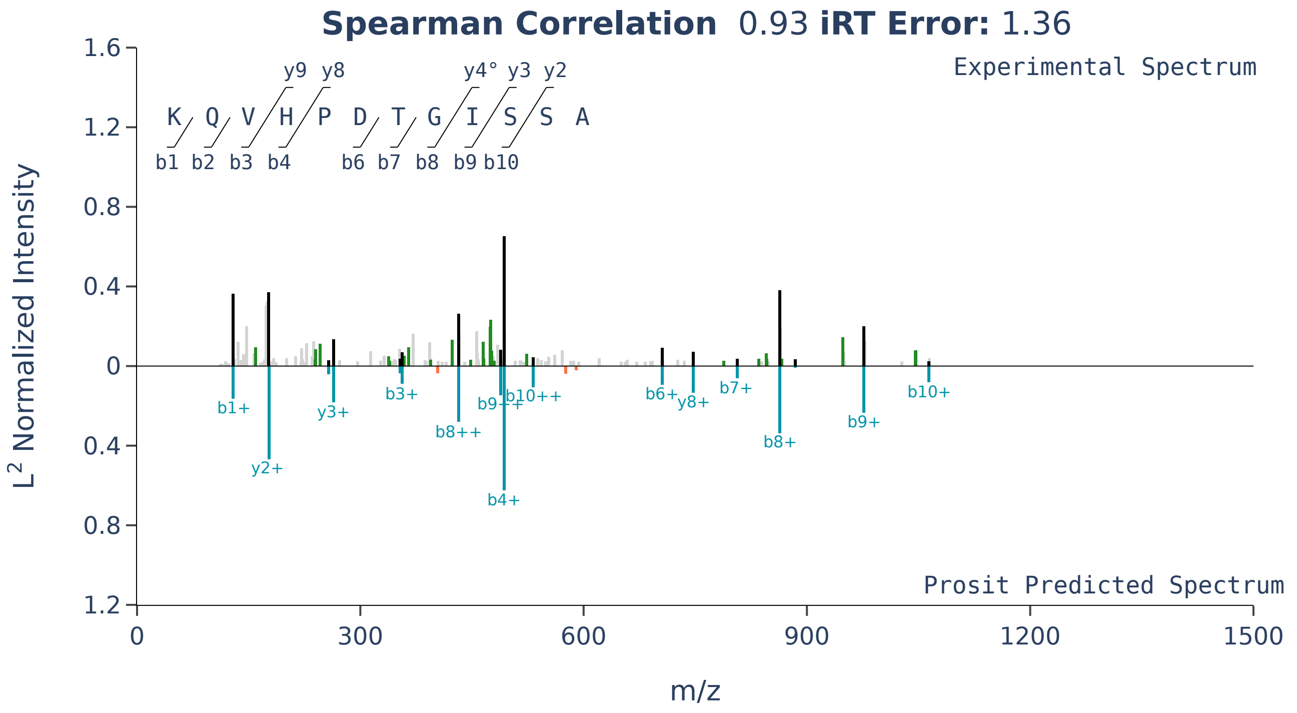

Source HRoetschke_291222_080123_Fu_H2B_b2R1_24h **Scan** 3626
Peptide QVHPDTGISSKA **Charge** 2 **Spectral Angle** 0.25
Spearman Correlation 0.1 **iRT Error:** 2.76

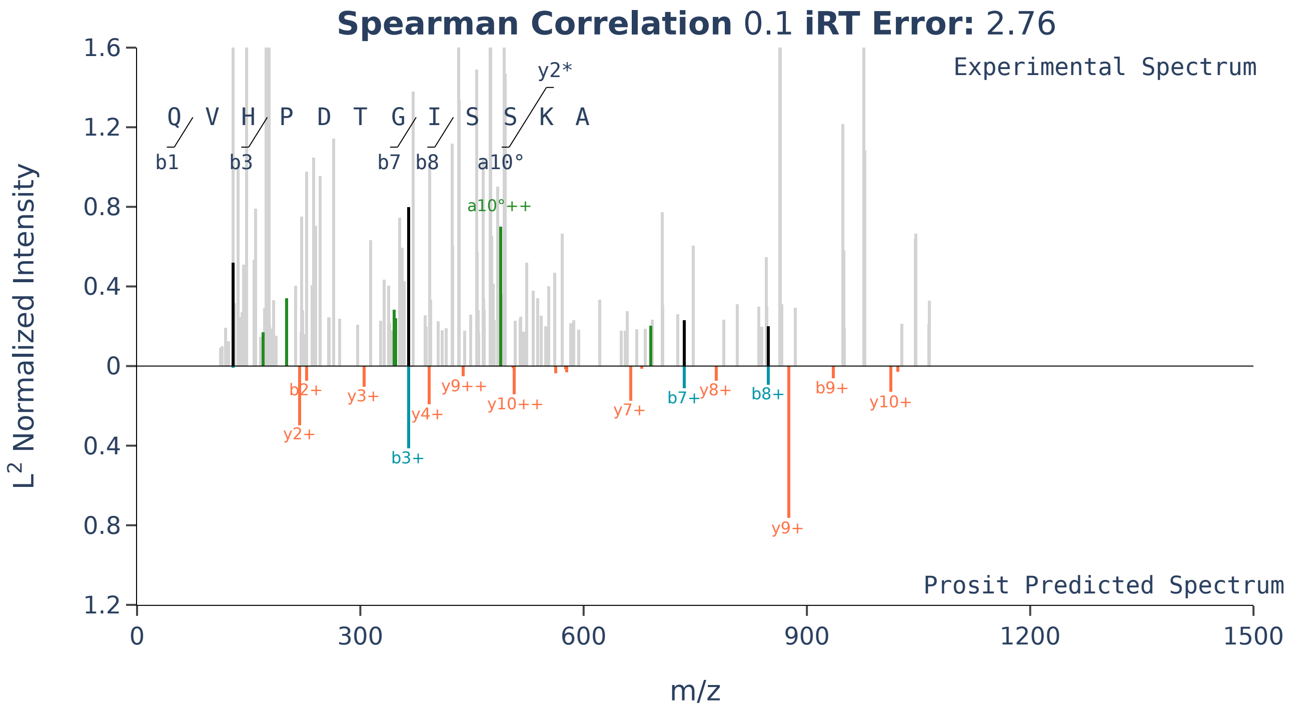

Source HRoetschke_291222_080123_Fu_H2B_b2R2_24h **Scan** 3764
Peptide KQVHPDTGISTG **Charge** 2 **Spectral Angle** 0.87
Spearman Correlation 0.92 **iRT Error:** 1.6

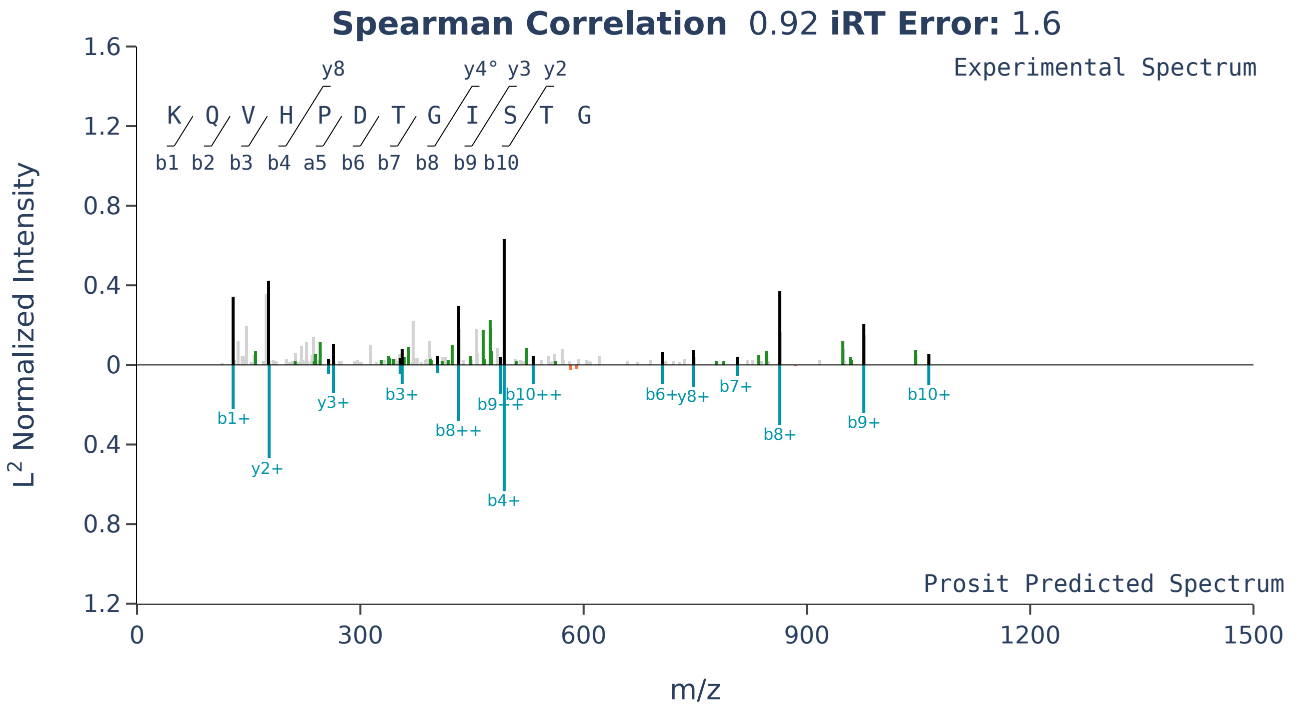

Source HRoetschke_291222_080123_Fu_H2B_b2R2_24h **Scan** 3764
Peptide QVHPDTGISSKA **Charge** 2 **Spectral Angle** 0.24
Spearman Correlation 0.06 **iRT Error:** 2.33

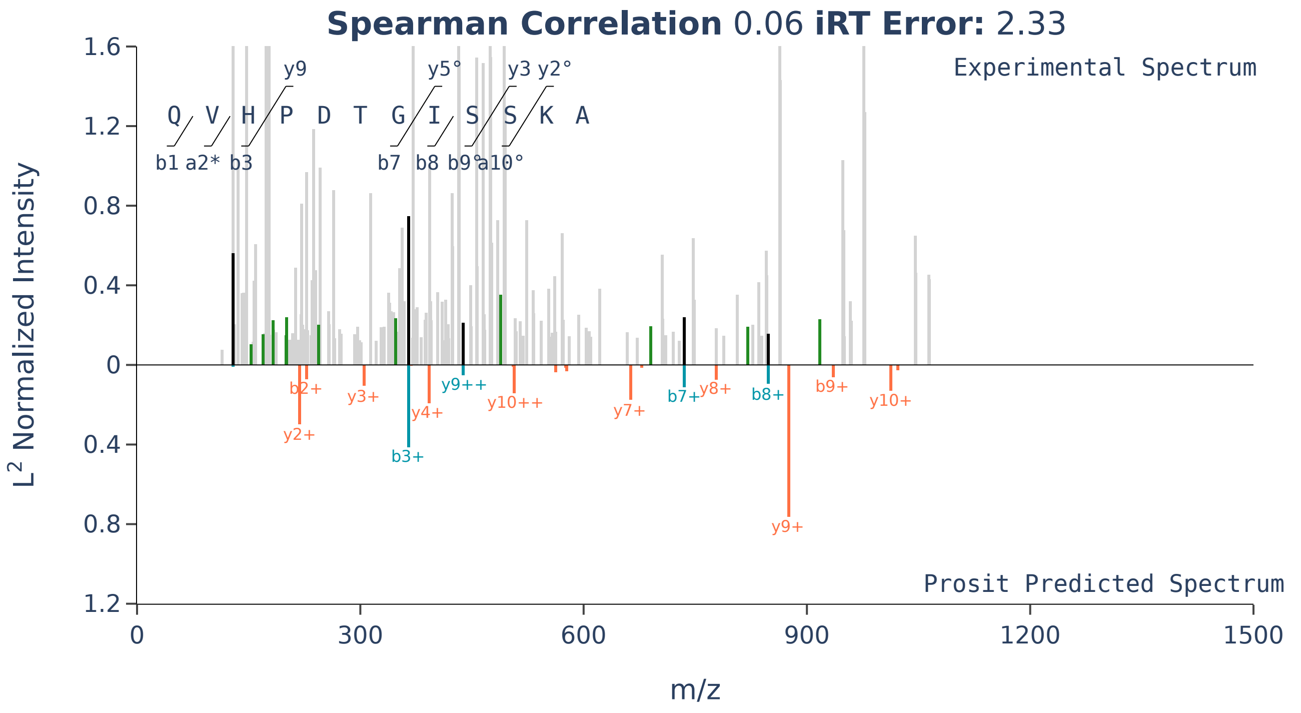

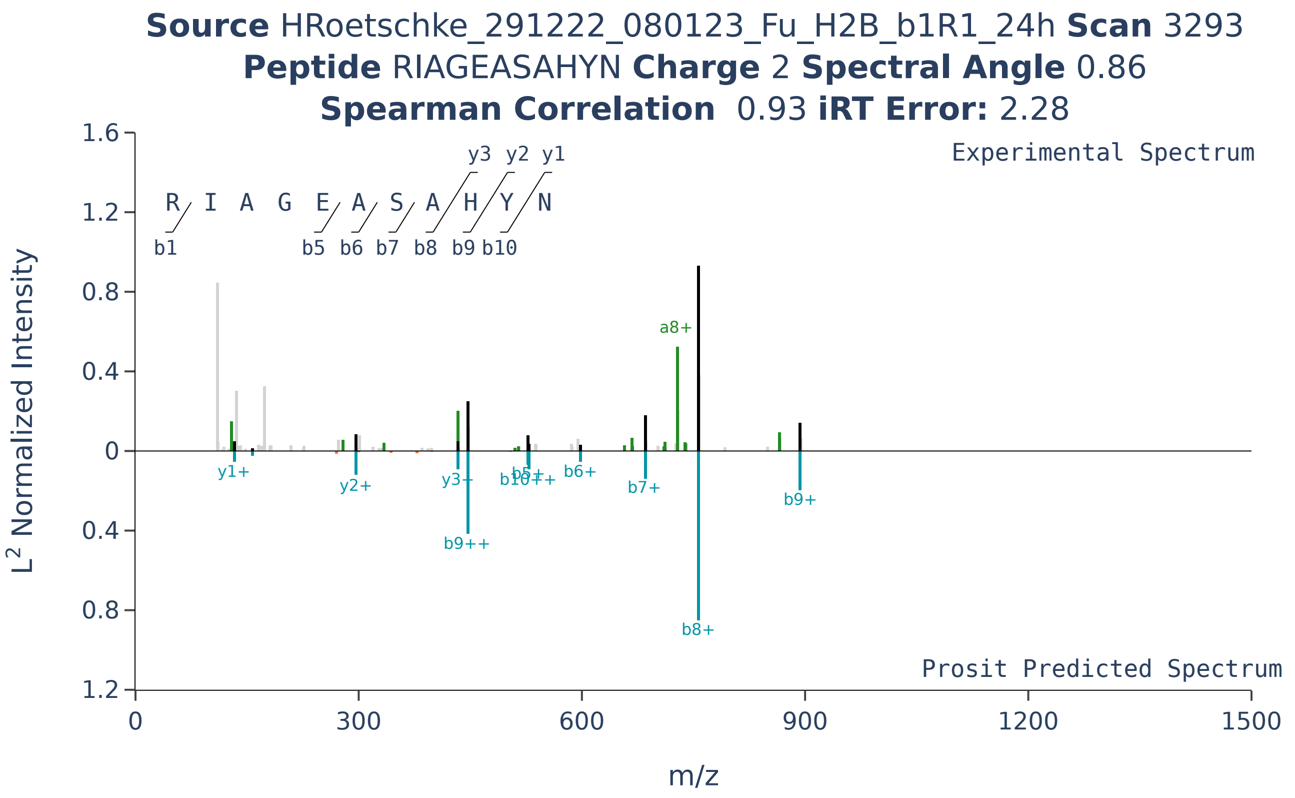
Source HRoetschke_291222_080123_Fu_H2B_b1R1_24h Scan 3293
Peptide RIAGEASAHYN Charge 2 Spectral Angle 0.86
Spearman Correlation 0.93 iRT Error: 2.28
Experimental Spectrum
Prosit Predicted Spectrum
L² Normalized Intensity
m/z

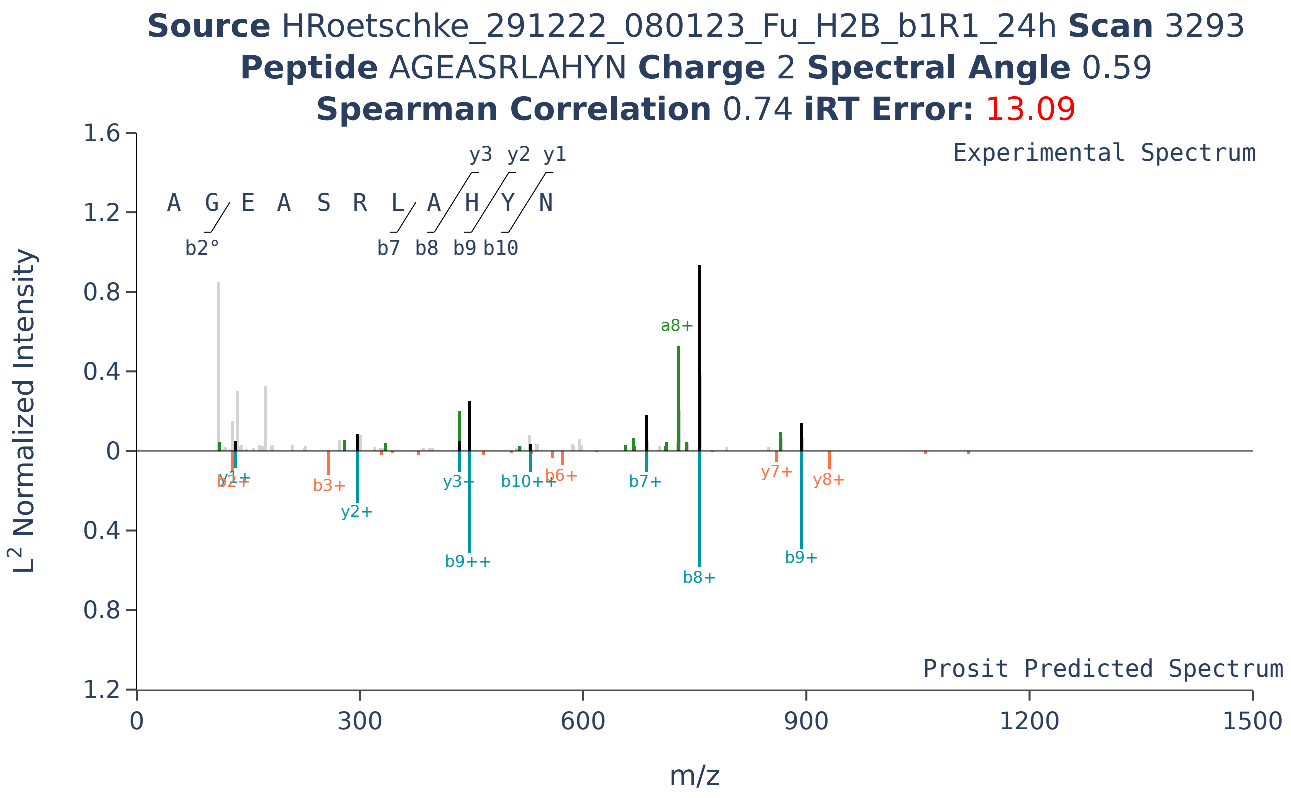
Source HRoetschke_291222_080123_Fu_H2B_b1R1_24h Scan 3293
Peptide AGEASRLAHYN Charge 2 Spectral Angle 0.59
Spearman Correlation 0.74 iRT Error: 13.09
Experimental Spectrum
Prosit Predicted Spectrum
L² Normalized Intensity
m/z

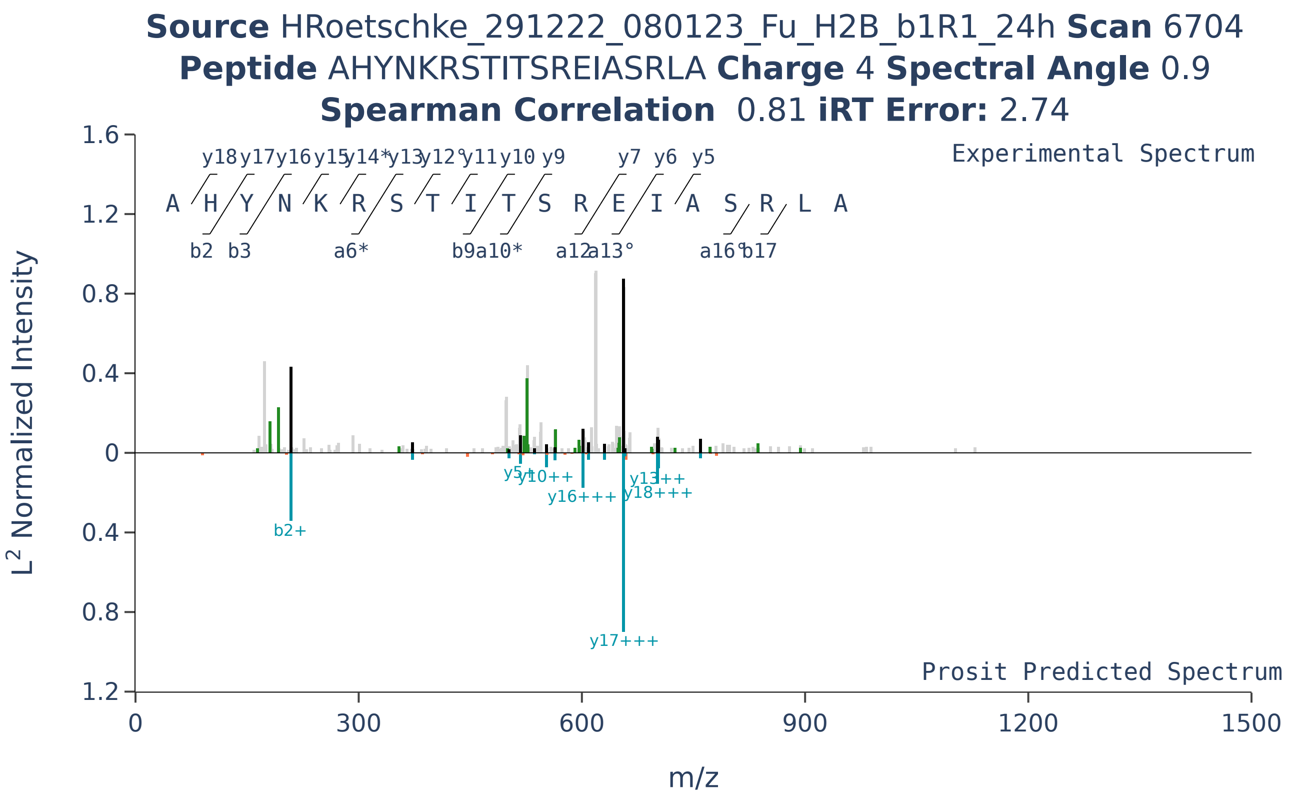
Source HRoetschke_291222_080123_Fu_H2B_b1R1_24h Scan 6704
Peptide AHYNKRSTITSREIASRLA Charge 4 Spectral Angle 0.9
Spearman Correlation 0.81 iRT Error: 2.74
Experimental Spectrum
Prosit Predicted Spectrum
L² Normalized Intensity
m/z

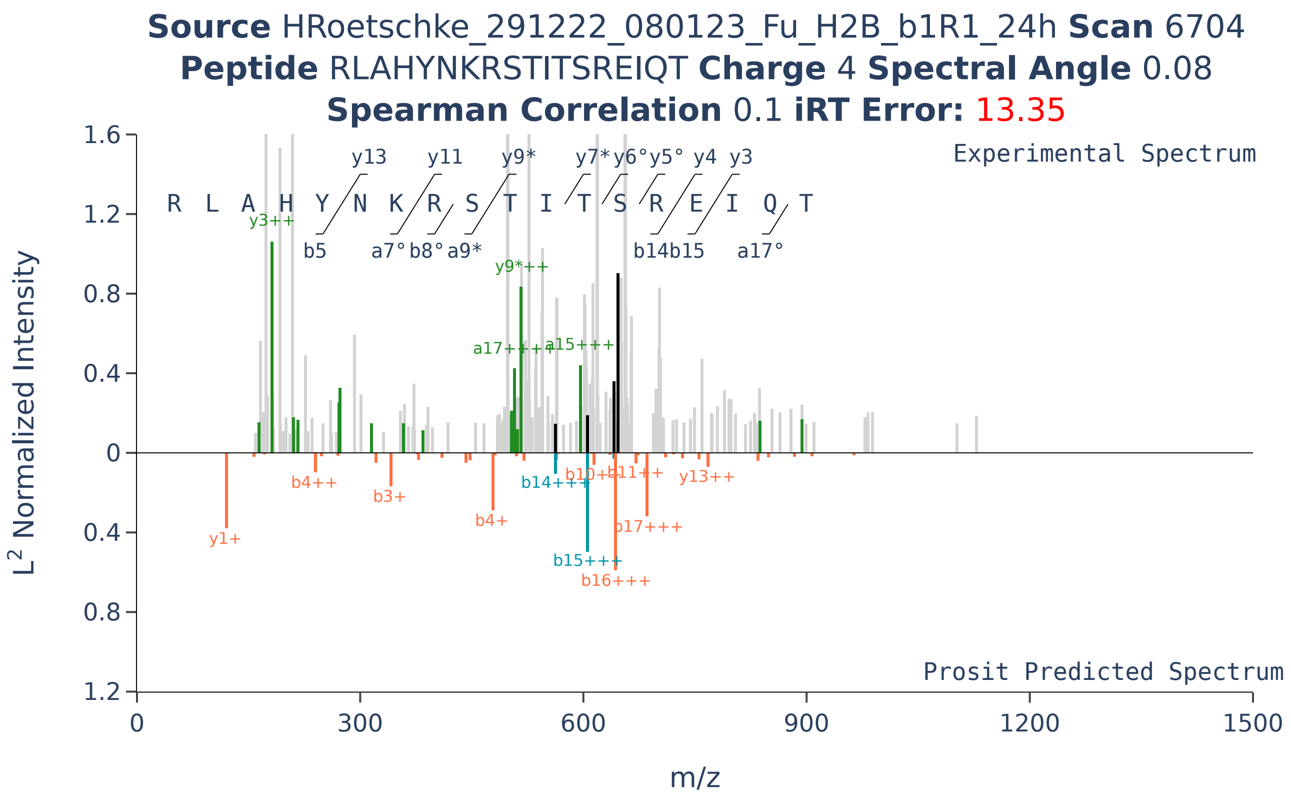
Source HRoetschke_291222_080123_Fu_H2B_b1R1_24h Scan 6704
Peptide RLAHYNKRSTITSREIQT Charge 4 Spectral Angle 0.08
Spearman Correlation 0.1 iRT Error: 13.35
Experimental Spectrum
Prosit Predicted Spectrum
L² Normalized Intensity
m/z

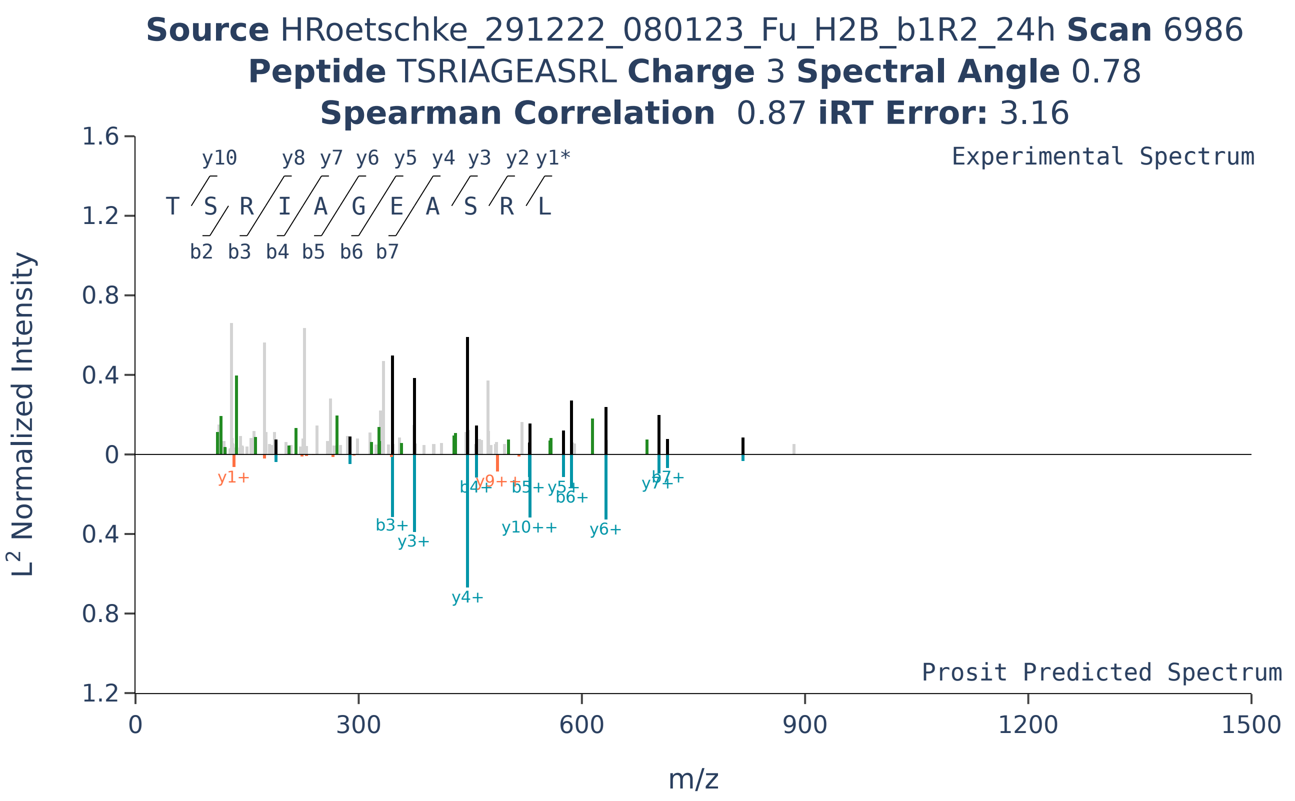
Source HRoetschke_291222_080123_Fu_H2B_b1R2_24h Scan 6986
Peptide TSRIAGEASRL Charge 3 Spectral Angle 0.78
Spearman Correlation 0.87 iRT Error: 3.16
Experimental Spectrum
Prosit Predicted Spectrum
L² Normalized Intensity
m/z

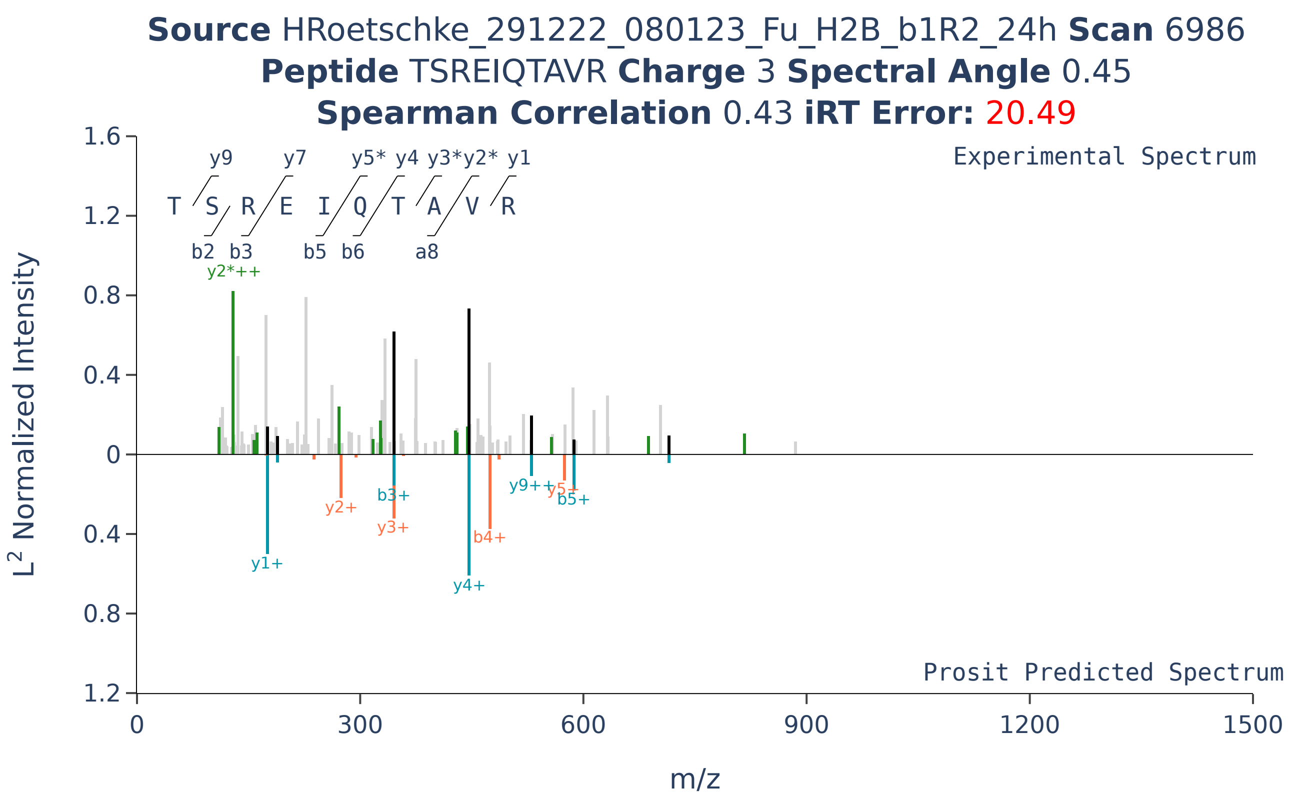
Source HRoetschke_291222_080123_Fu_H2B_b1R2_24h Scan 6986
Peptide TSREIQTAVR Charge 3 Spectral Angle 0.45
Spearman Correlation 0.43 iRT Error: 20.49
Experimental Spectrum
Prosit Predicted Spectrum
L² Normalized Intensity
m/z

Source HRoetschke_271222_120123_HFGoe_H3_b2R2_24h **Scan** 18082
Peptide ALQDFKTDL **Charge** 2 **Spectral Angle** 0.72
Spearman Correlation 0.91 **iRT Error:** 0.23

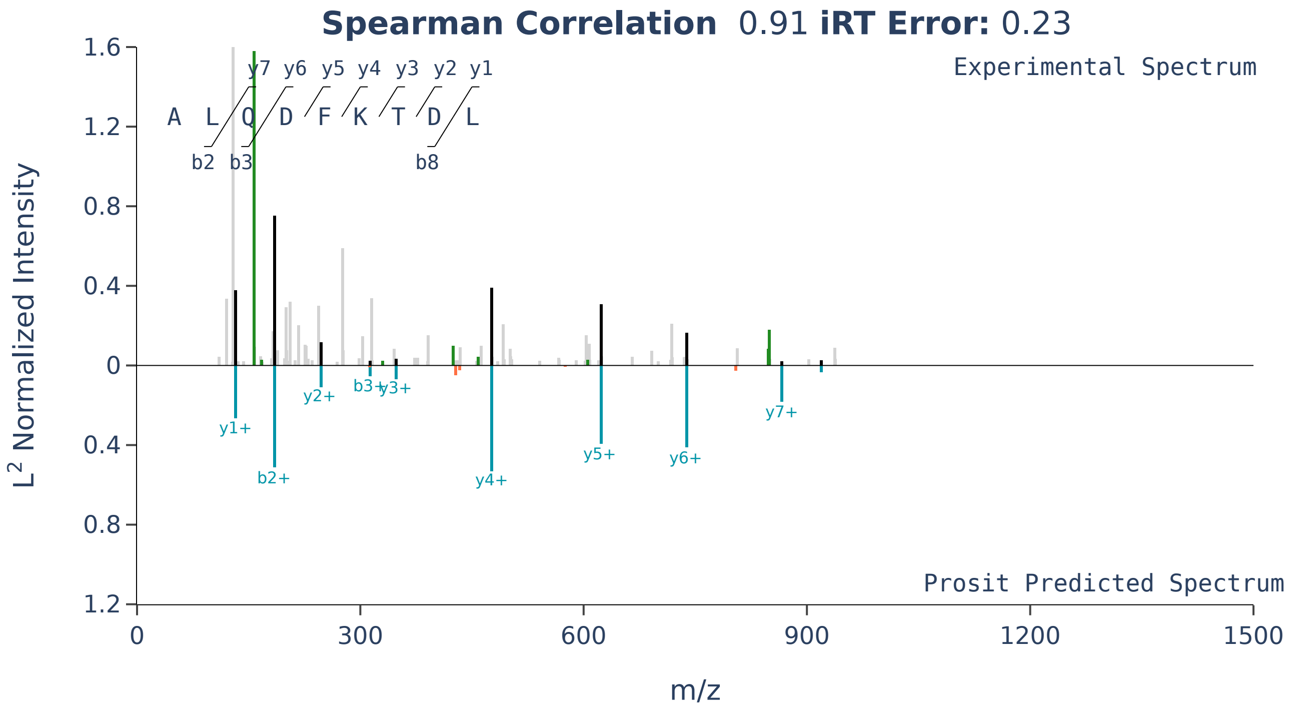

Source HRoetschke_271222_120123_HFGoe_H3_b2R2_24h **Scan** 18082
Peptide IAQDFKTDL **Charge** 2 **Spectral Angle** 0.68
Spearman Correlation 0.89 **iRT Error:** 35.35

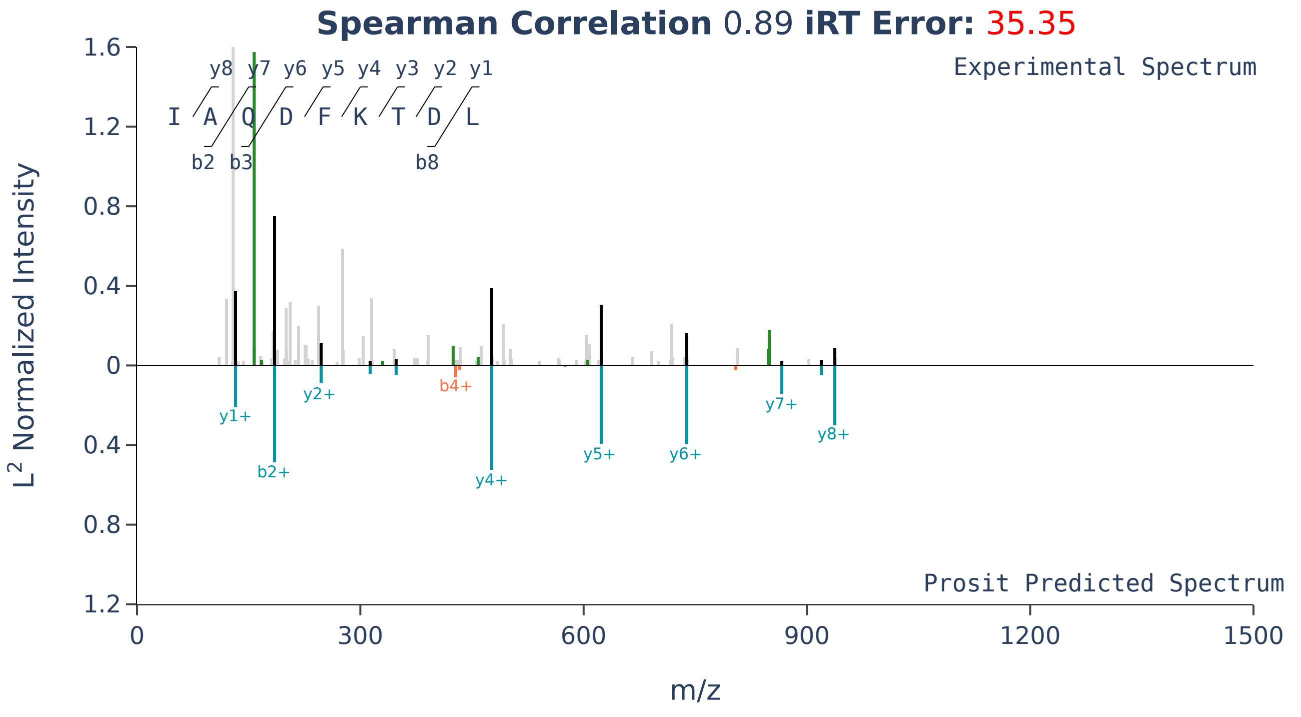

Source HRoetschke_271222_120123_HFGoe_H3_b2R2_2h **Scan** 8971
Peptide TGGSAVMA **Charge** 1 **Spectral Angle** 0.65
Spearman Correlation 0.93 **iRT Error:** 0.3

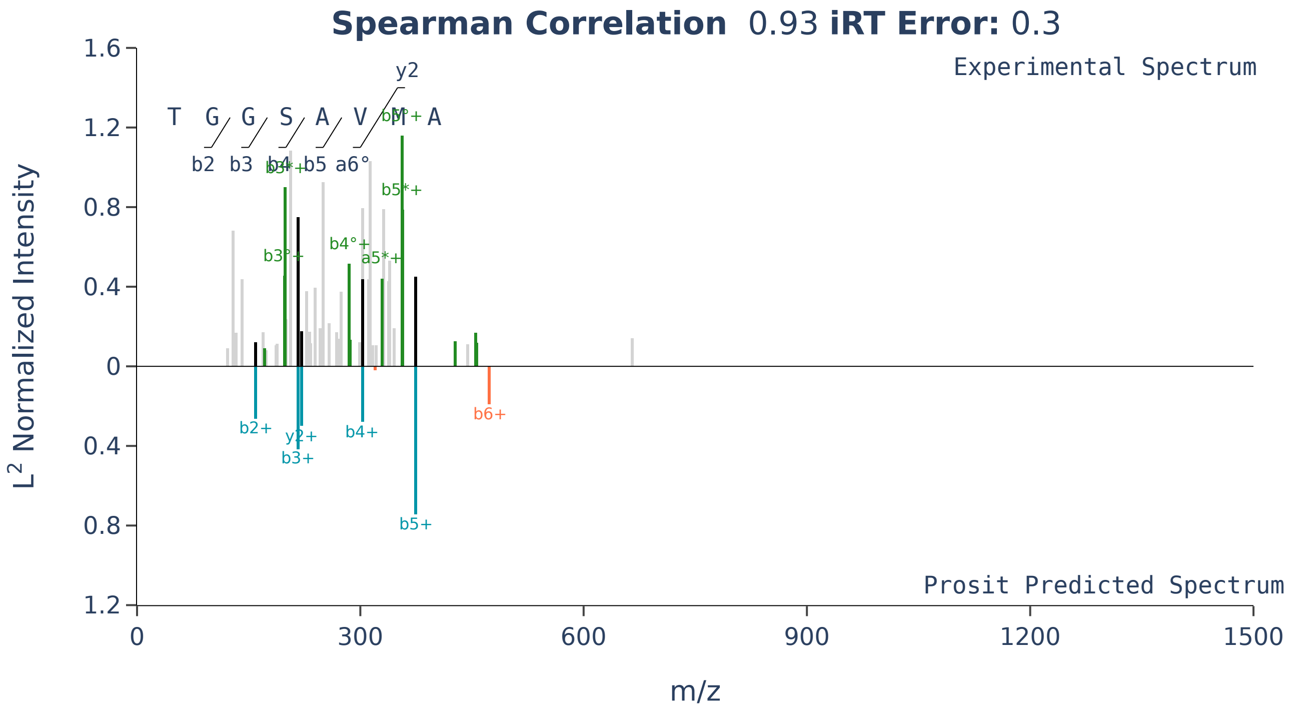

Source HRoetschke_271222_120123_HFGoe_H3_b2R2_2h **Scan** 8971
Peptide QSSAVMA **Charge** 1 **Spectral Angle** 0.67
Spearman Correlation 0.89 **iRT Error:** 10.57

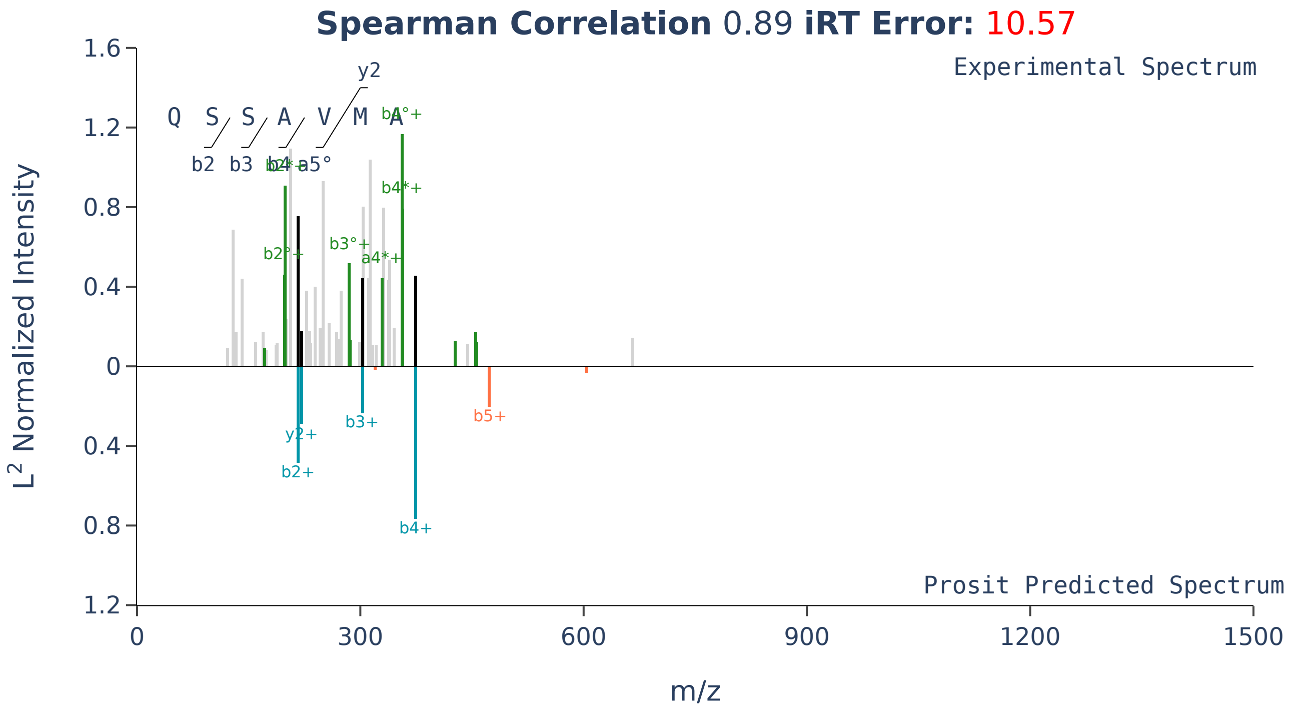

Source HRoetschke_271222_120123_HFGoe_H3_b1R1_24h **Scan** 13802
Peptide AQDFKTDLRY **Charge** 3 **Spectral Angle** 0.87
Spearman Correlation 0.91 **iRT Error:** 0.41

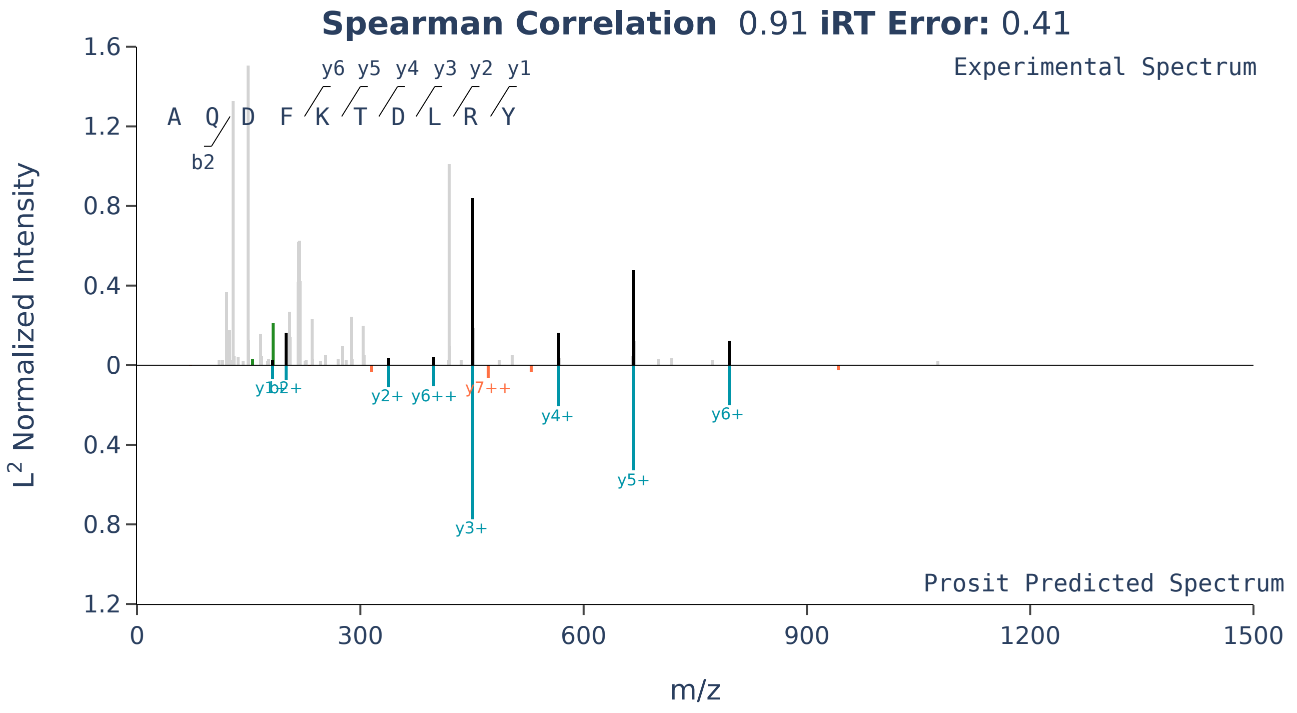

Source HRoetschke_271222_120123_HFGoe_H3_b1R1_24h **Scan** 13802
Peptide DFKTDLRFQS **Charge** 3 **Spectral Angle** 0.0
Spearman Correlation 0.0 **iRT Error:** 24.75

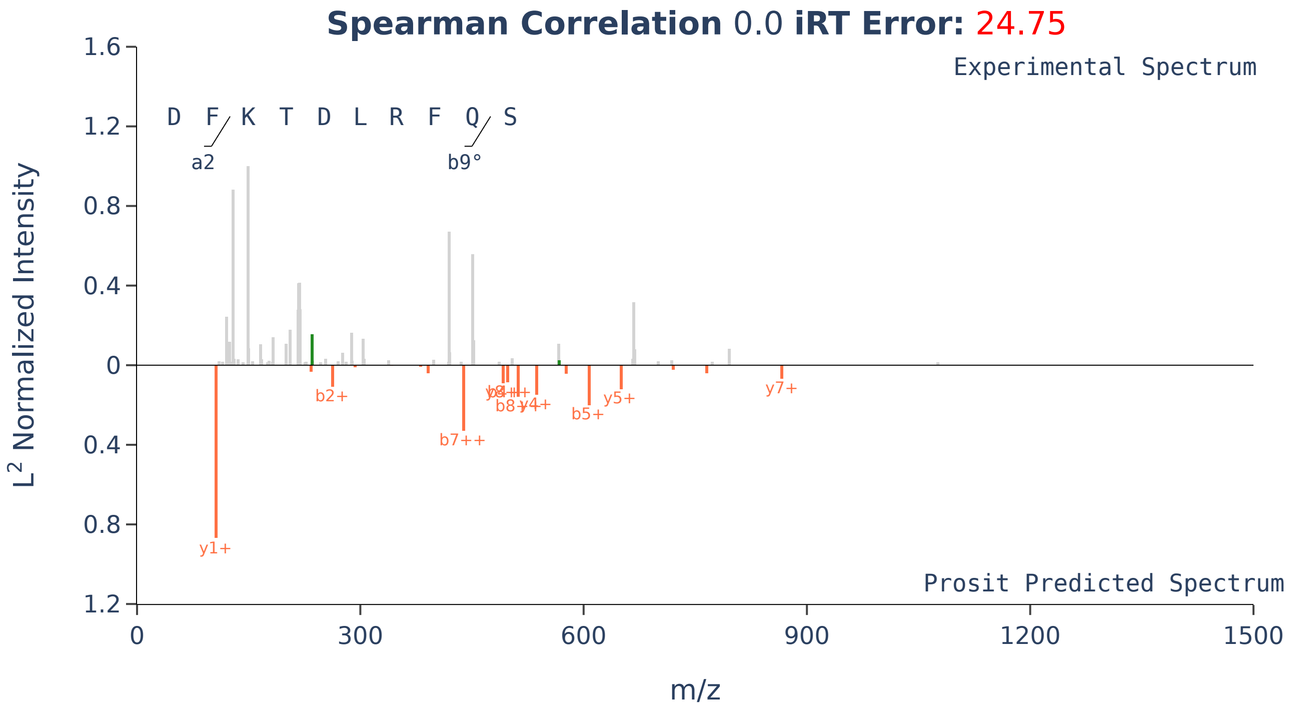

Source HRoetschke_271222_120123_HFGoe_H3_b2R2_4h **Scan** 18845
Peptide DTNFKTDLRF **Charge** 3 **Spectral Angle** 0.87
Spearman Correlation 0.91 **iRT Error:** 0.83

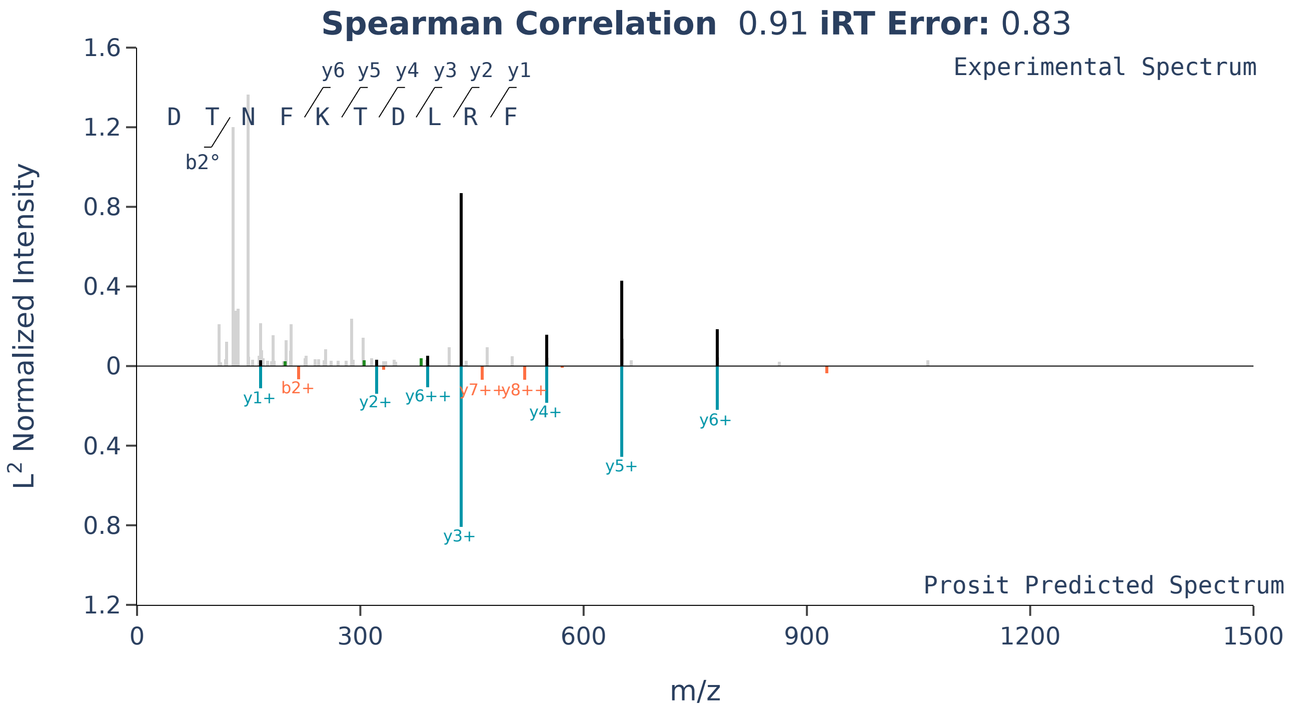

Source HRoetschke_271222_120123_HFGoe_H3_b2R2_4h **Scan** 18845
Peptide DFKTDLRFQS **Charge** 3 **Spectral Angle** 0.0
Spearman Correlation 0.0 **iRT Error:** 10.02

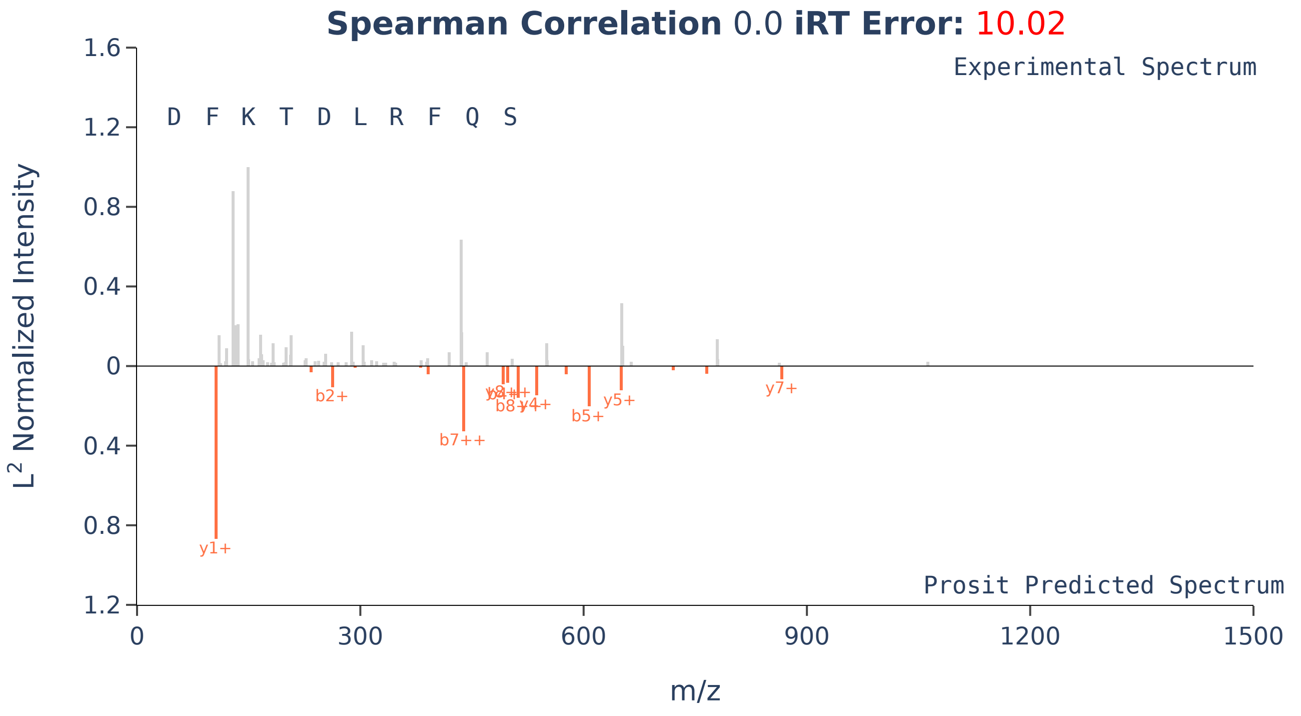

Source HRoetschke_271222_120123_HFGoe_H3_b2R1_2h **Scan** 17832
Peptide FEDTNLLC **Charge** 2 **Spectral Angle** 0.93
Spearman Correlation 0.94 **iRT Error:** 1.19

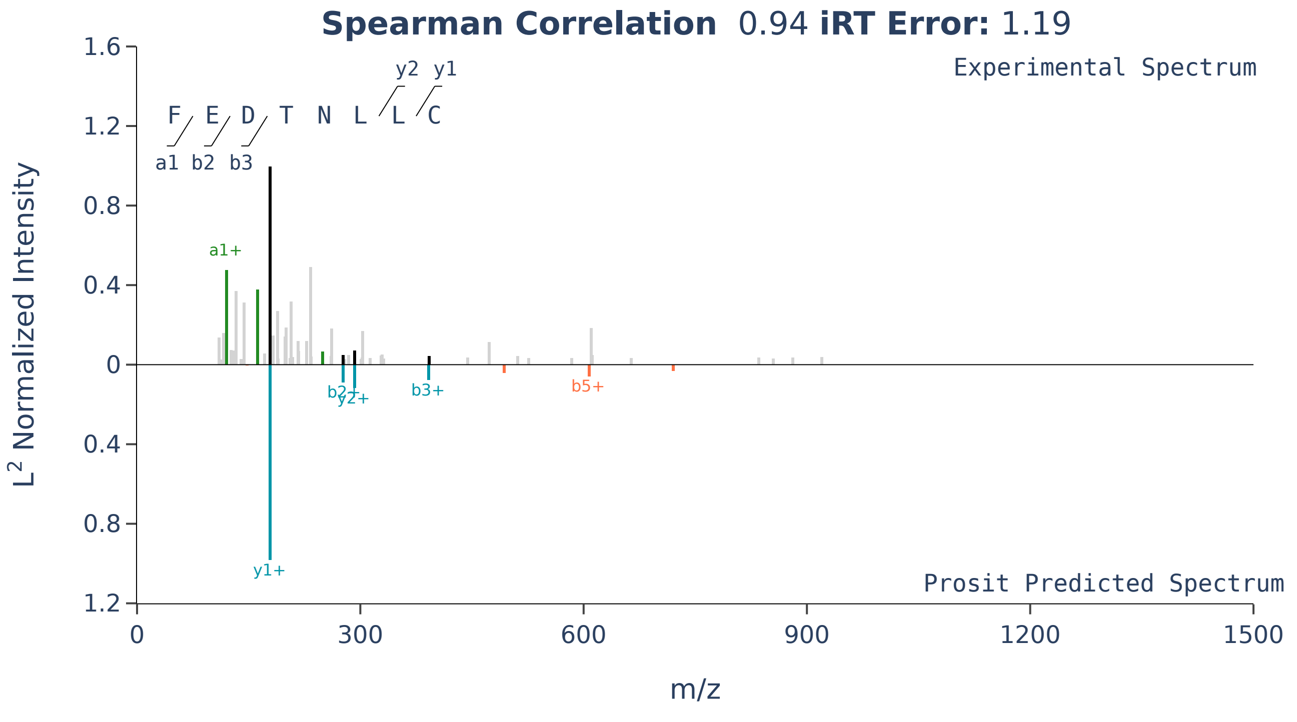

Source HRoetschke_271222_120123_HFGoe_H3_b2R1_2h **Scan** 17832
Peptide LFEDTNLC **Charge** 2 **Spectral Angle** 0.95
Spearman Correlation 0.81 **iRT Error:** 5.69

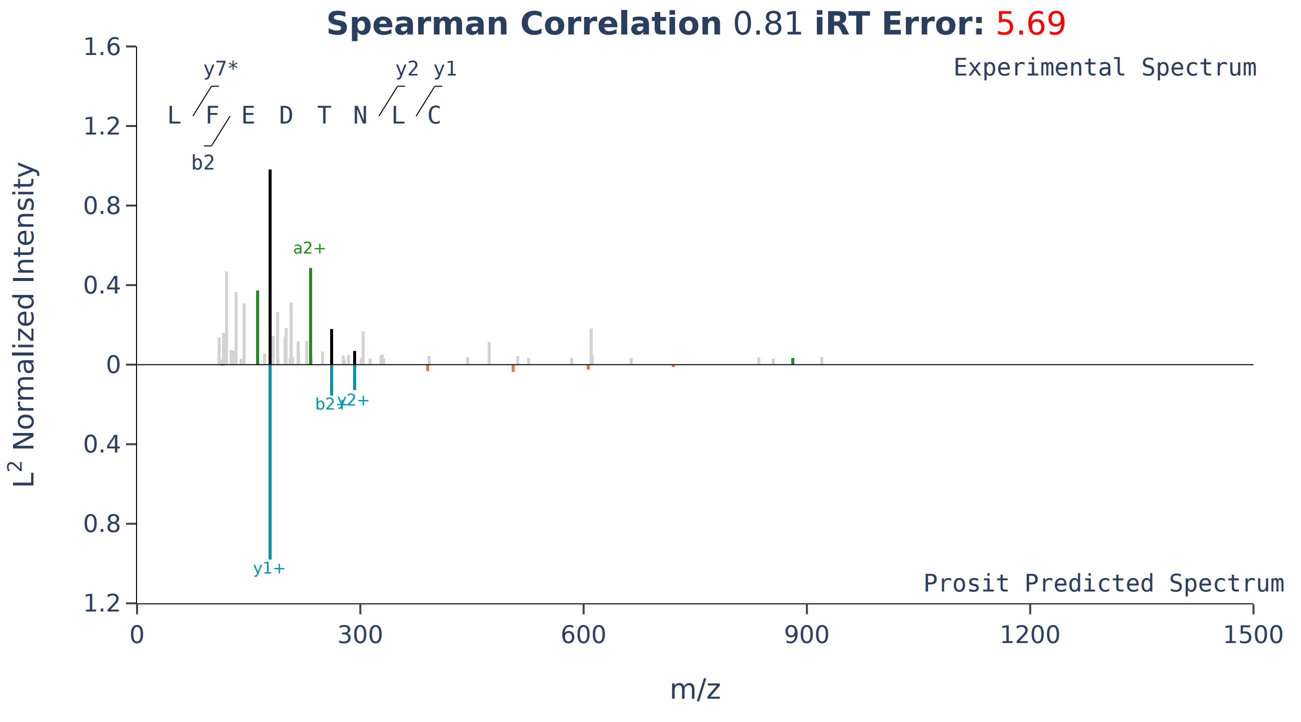

Source HRoetschke_271222_120123_HFGoe_H3_b2R1_4h **Scan** 13959
Peptide AQDFKTLVRE **Charge** 2 **Spectral Angle** 0.8
Spearman Correlation 0.87 **iRT Error:** 1.6

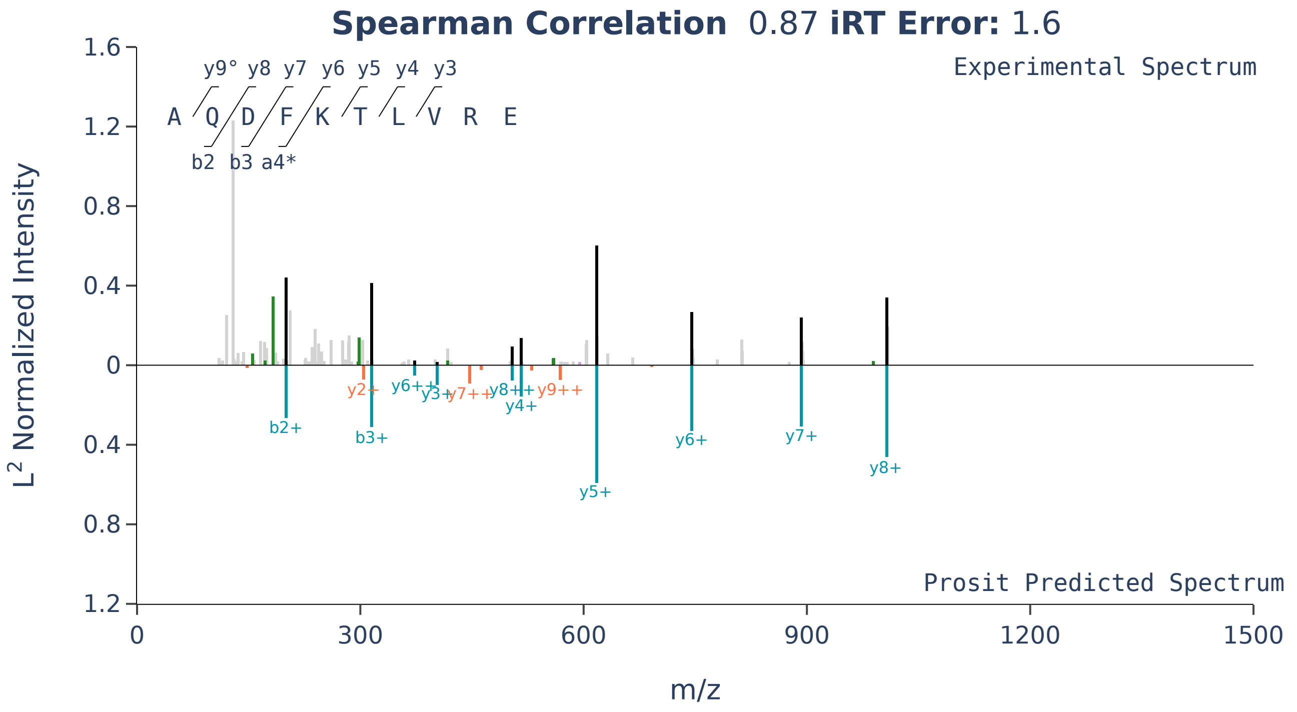

Source HRoetschke_271222_120123_HFGoe_H3_b2R1_4h **Scan** 13959
Peptide IAQDFKTDLR **Charge** 2 **Spectral Angle** 0.32
Spearman Correlation 0.62 **iRT Error:** 8.18

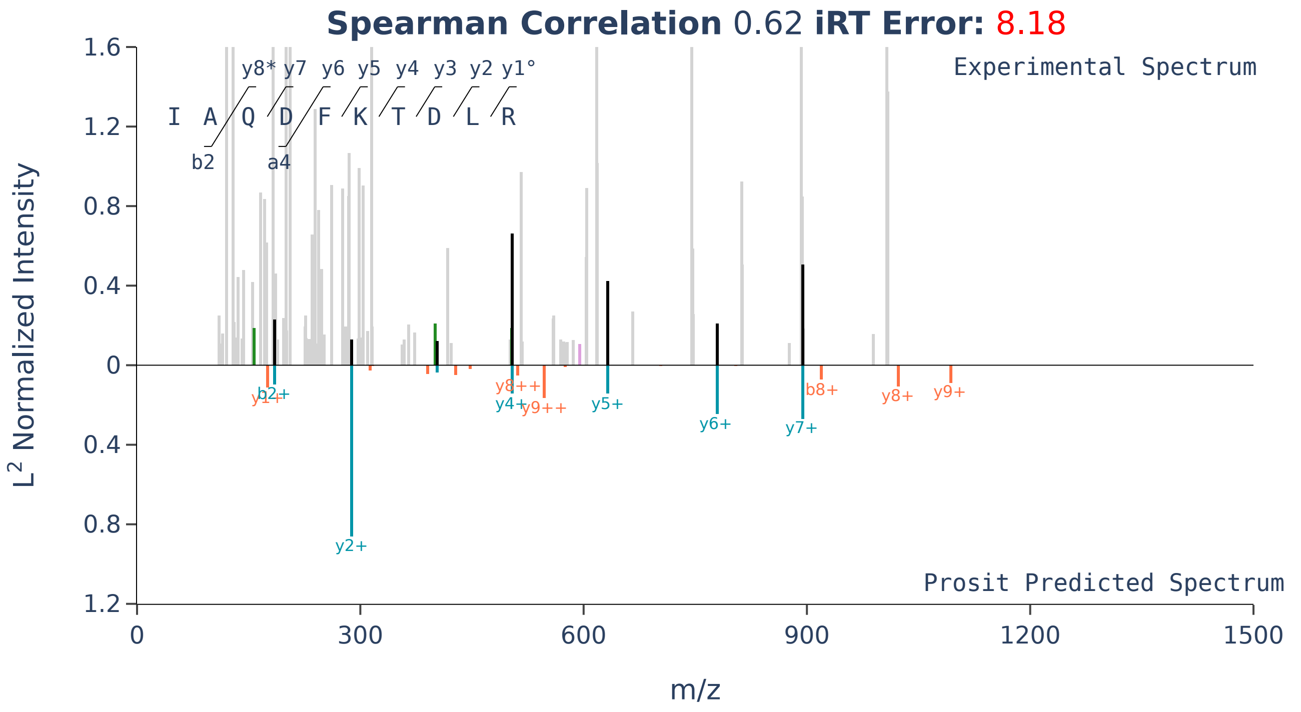

Source HRoetschke_271222_120123_HFGoe_H3_b1R1_4h **Scan** 17738
Peptide SQDFKTDLRF **Charge** 3 **Spectral Angle** 0.86
Spearman Correlation 0.9 **iRT Error:** 1.78

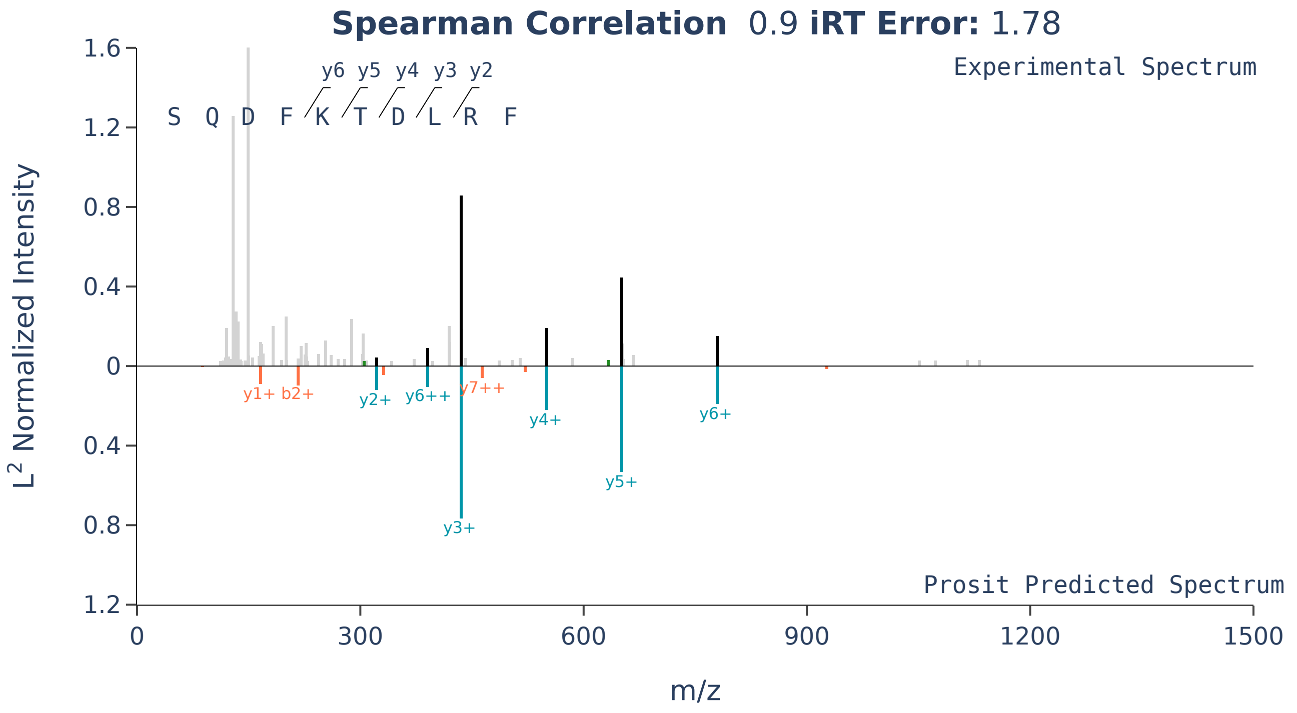

Source HRoetschke_271222_120123_HFGoe_H3_b1R1_4h **Scan** 17738
Peptide DFKTDLRFQS **Charge** 3 **Spectral Angle** 0.0
Spearman Correlation 0.0 **iRT Error:** 2.99

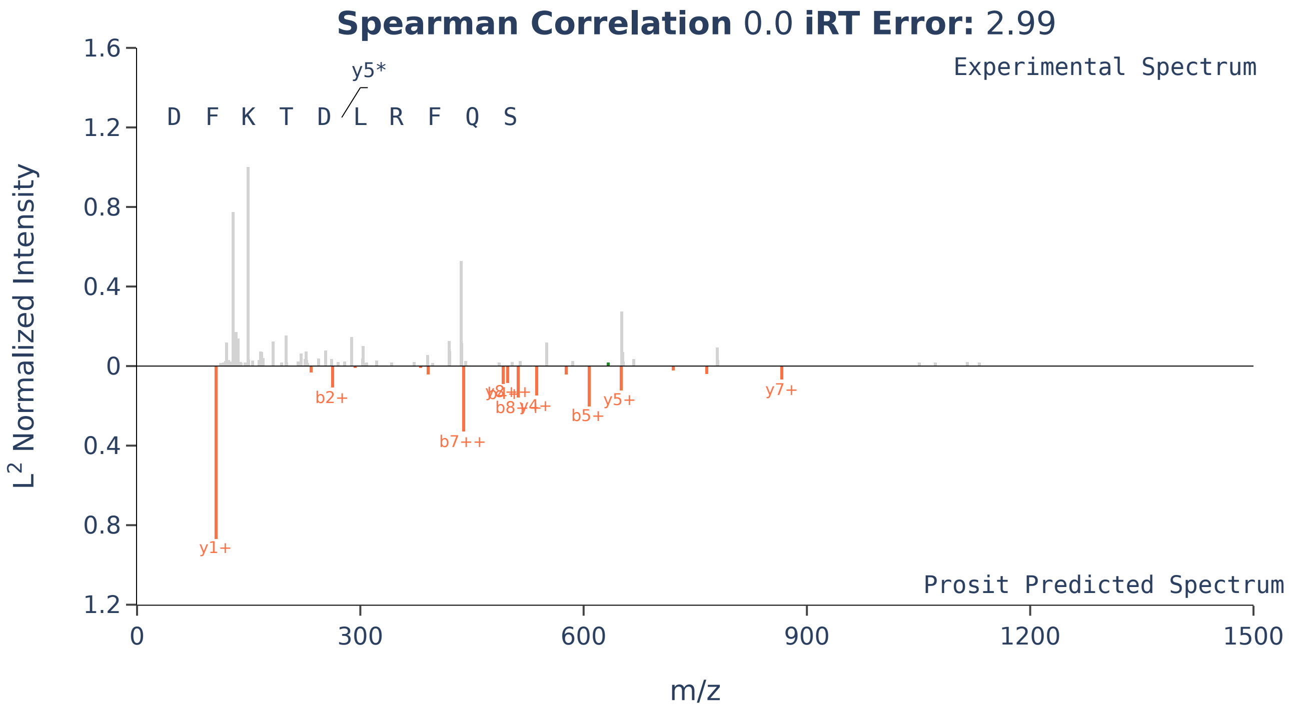

Source HRoetschke_271222_120123_HFGoe_H3_b1R2_2h **Scan** 16251
Peptide SEAAYLVA **Charge** 2 **Spectral Angle** 0.78
Spearman Correlation 0.96 **iRT Error:** 1.92

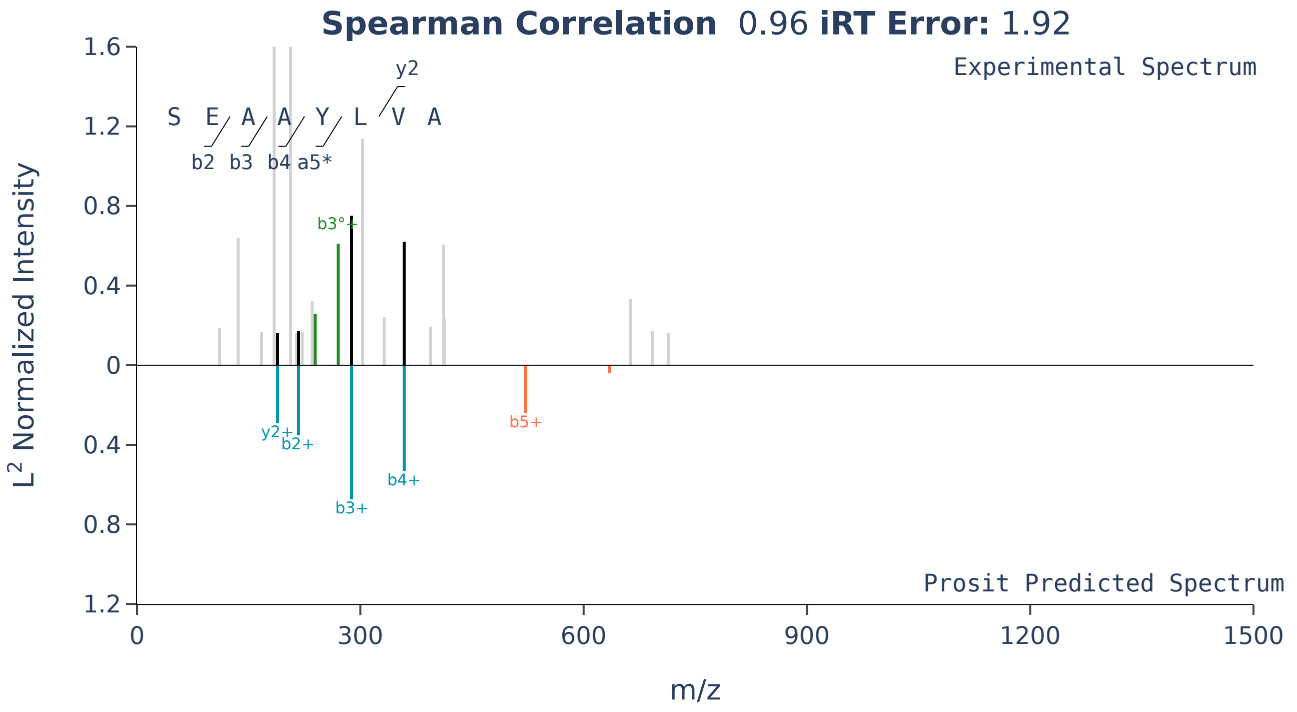

Source HRoetschke_271222_120123_HFGoe_H3_b1R2_2h **Scan** 16251
Peptide ASEAYLVA **Charge** 2 **Spectral Angle** 0.68
Spearman Correlation 0.85 **iRT Error:** 3.63

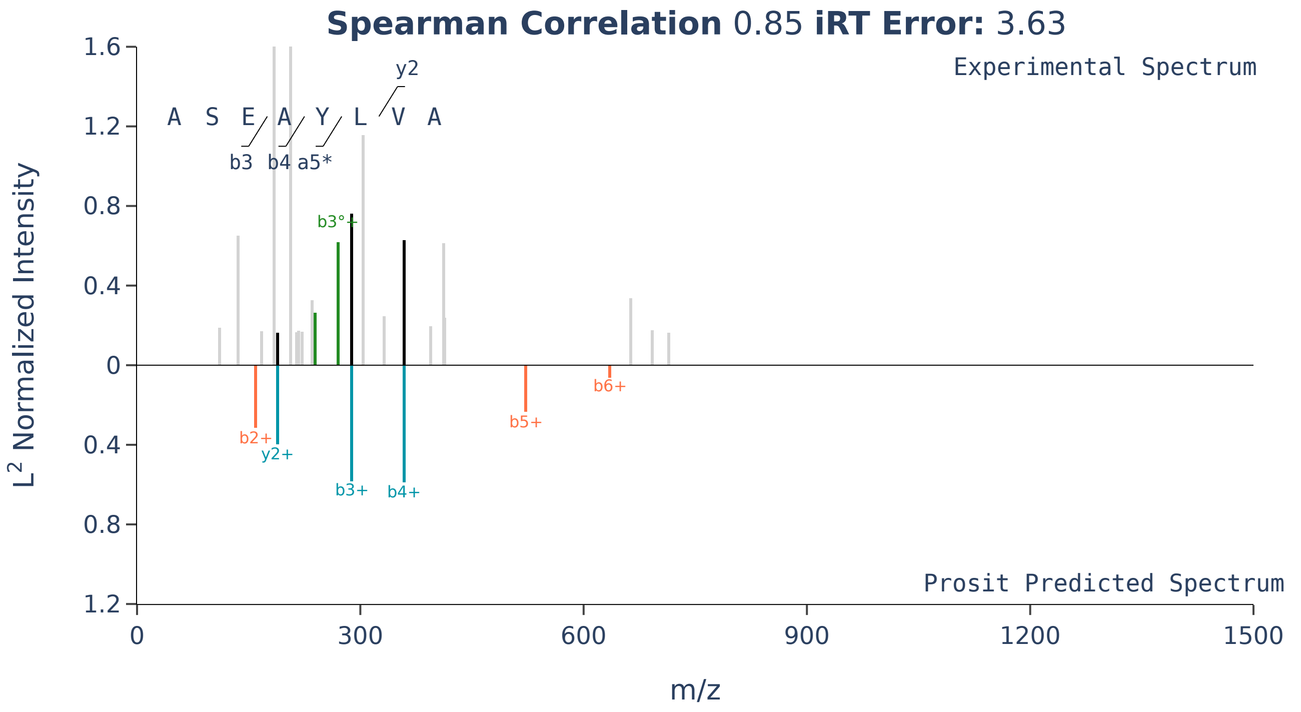

Source HRoetschke_271222_120123_HFGoe_H3_b2R2_2h **Scan** 8494
Peptide IAQDDLR **Charge** 2 **Spectral Angle** 0.8
Spearman Correlation 0.94 **iRT Error:** 2.48

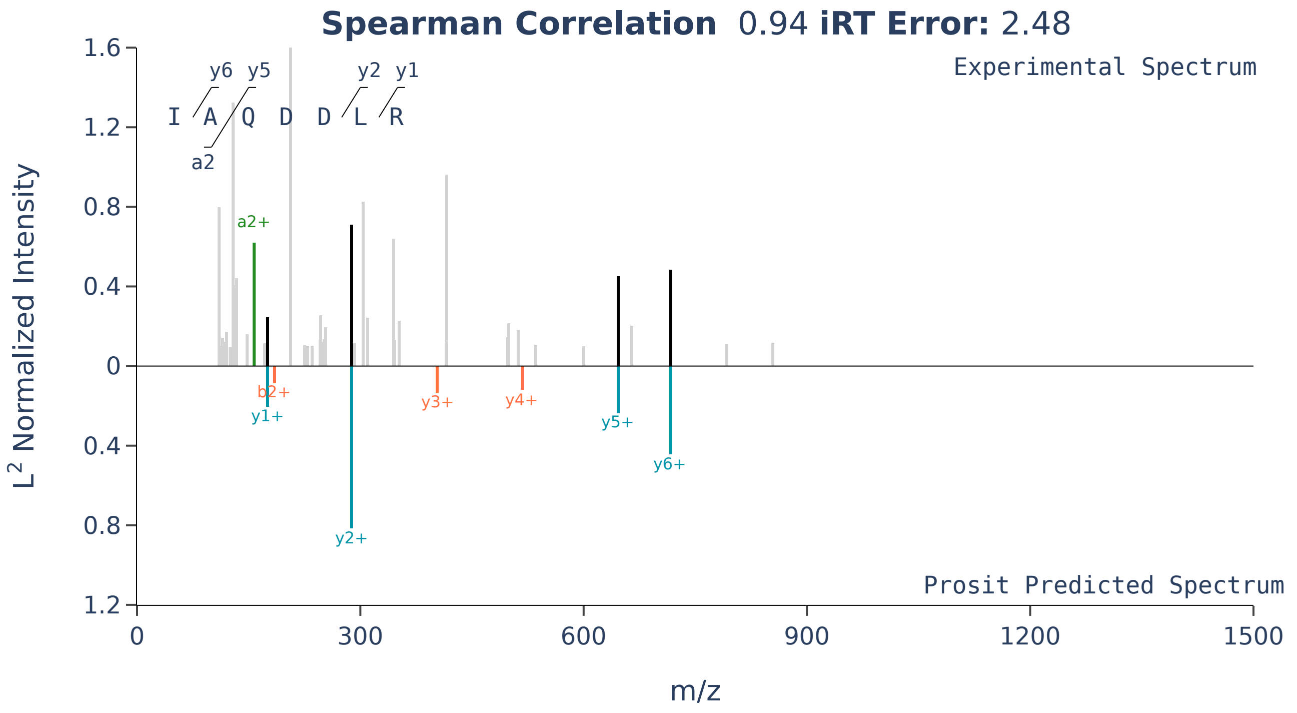

Source HRoetschke_271222_120123_HFGoe_H3_b2R2_2h **Scan** 8494
Peptide VREIAQD **Charge** 2 **Spectral Angle** 0.29
Spearman Correlation 0.5 **iRT Error:** 5.3

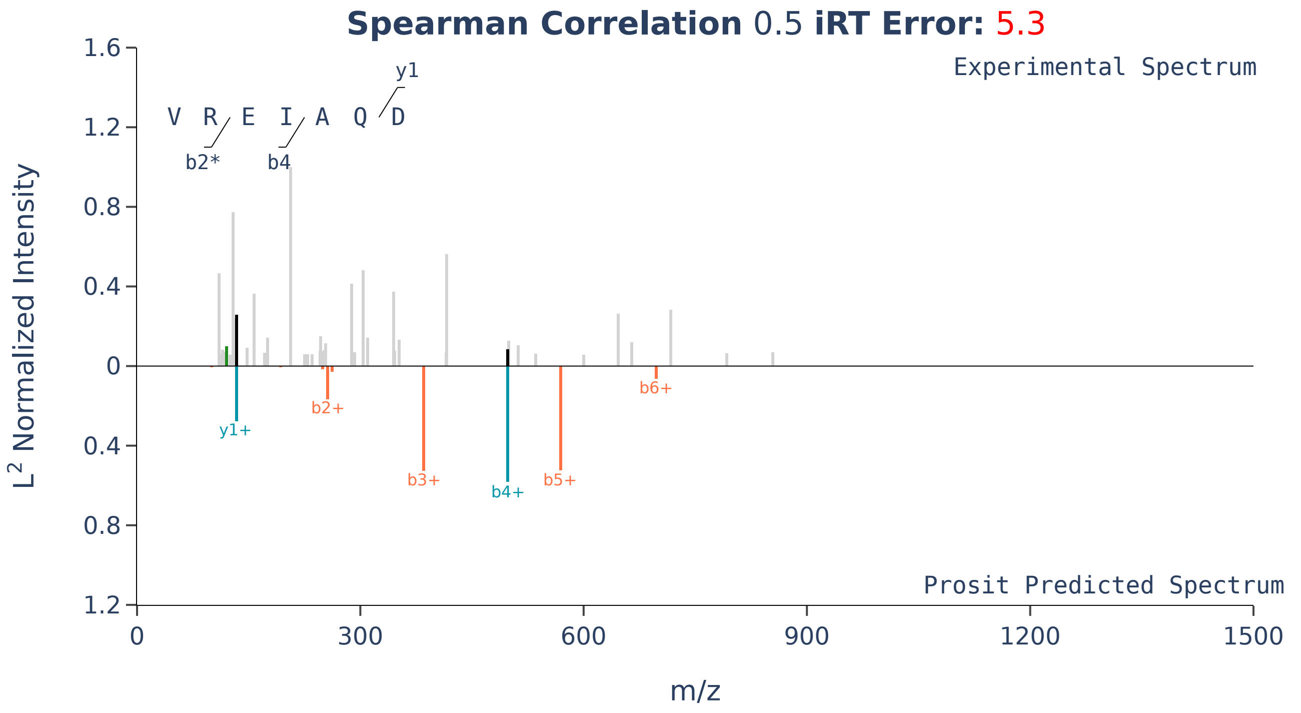

Source HRoetschke_271222_120123_HFGoe_H3_b2R1_4h **Scan** 18080
Peptide QSSAVMTDLRF **Charge** 3 **Spectral Angle** 0.83
Spearman Correlation 0.87 **iRT Error:** 4.13

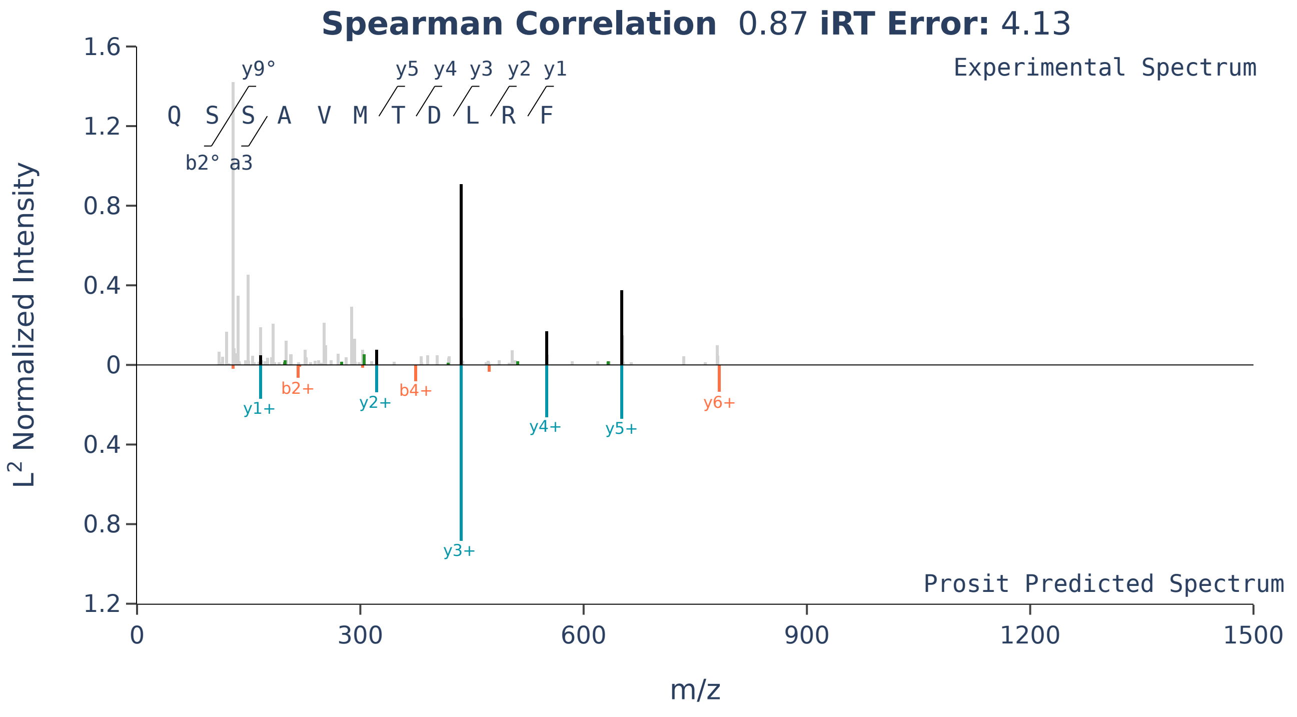

Source HRoetschke_271222_120123_HFGoe_H3_b2R1_4h **Scan** 18080
Peptide TDLRFQSSAVM **Charge** 3 **Spectral Angle** 0.15
Spearman Correlation 0.45 **iRT Error:** 12.78

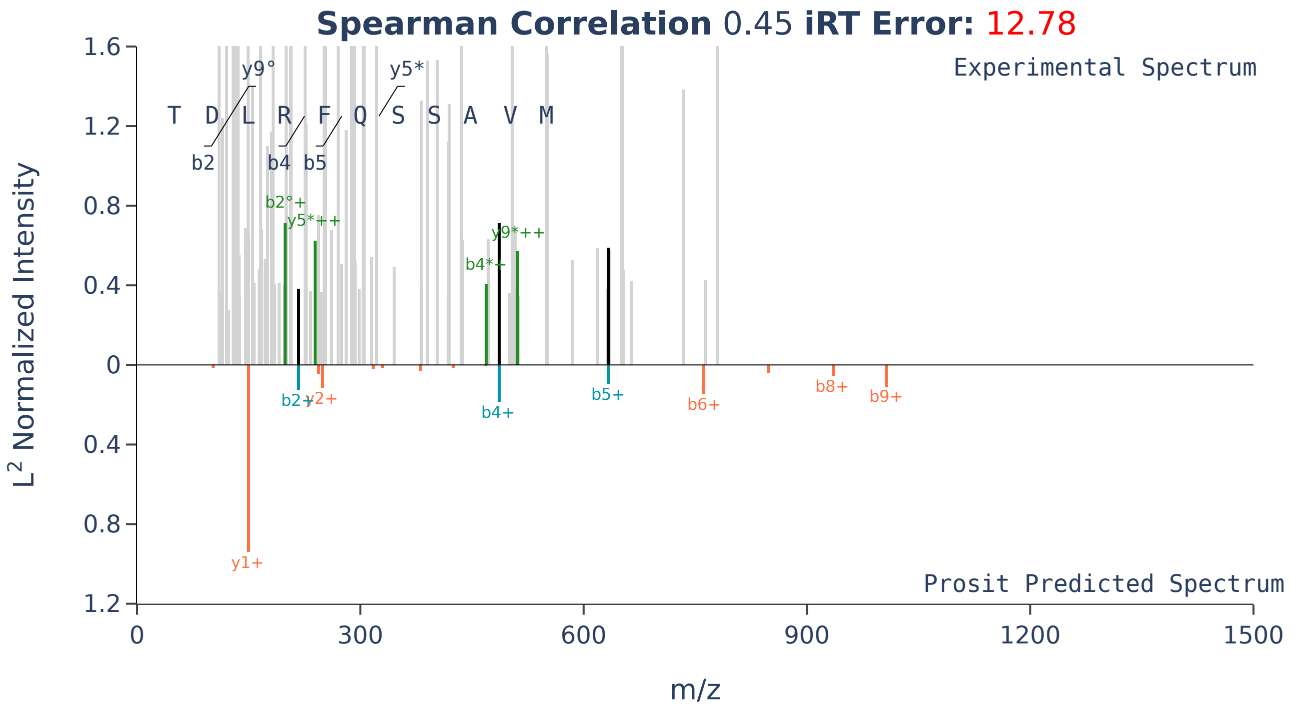

Source HRoetschke_271222_120123_HFGoe_H3_b2R2_2h **Scan** 10913
Peptide RFQSSDL **Charge** 2 **Spectral Angle** 0.83
Spearman Correlation 0.98 **iRT Error:** 4.96

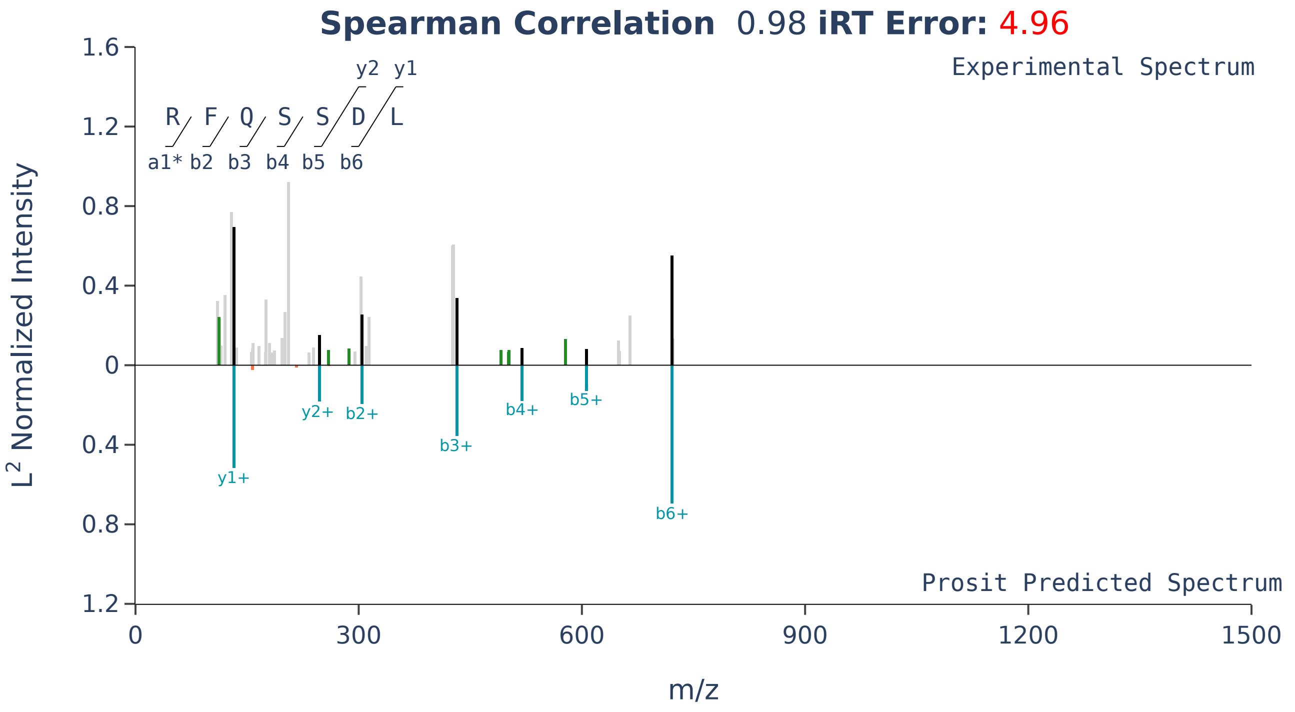

Source HRoetschke_271222_120123_HFGoe_H3_b2R2_2h **Scan** 10913
Peptide DLRFQSS **Charge** 2 **Spectral Angle** 0.0
Spearman Correlation 0.0 **iRT Error:** 12.23

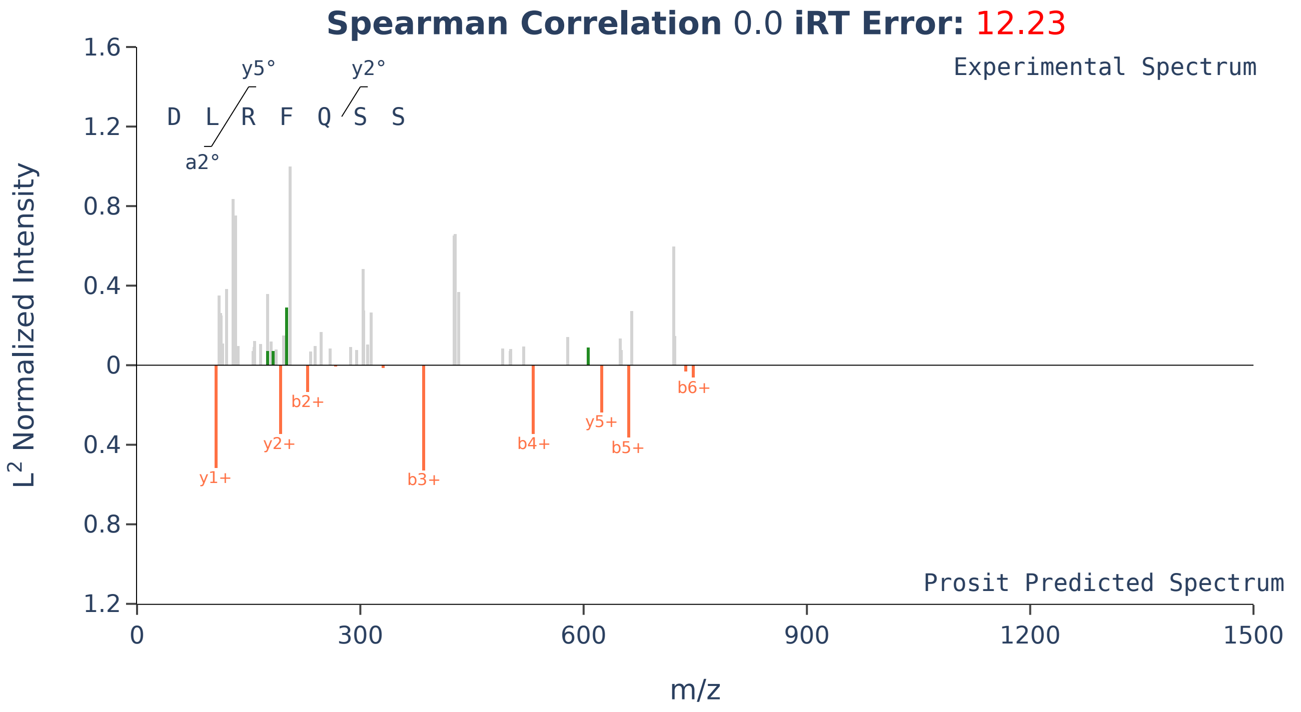

Source HRoetschke_271222_120123_HFGoe_H3_b1R1_24h **Scan** 13971
Peptide AQDFKTEL **Charge** 2 **Spectral Angle** 0.82
Spearman Correlation 0.88 **iRT Error:** 5.58

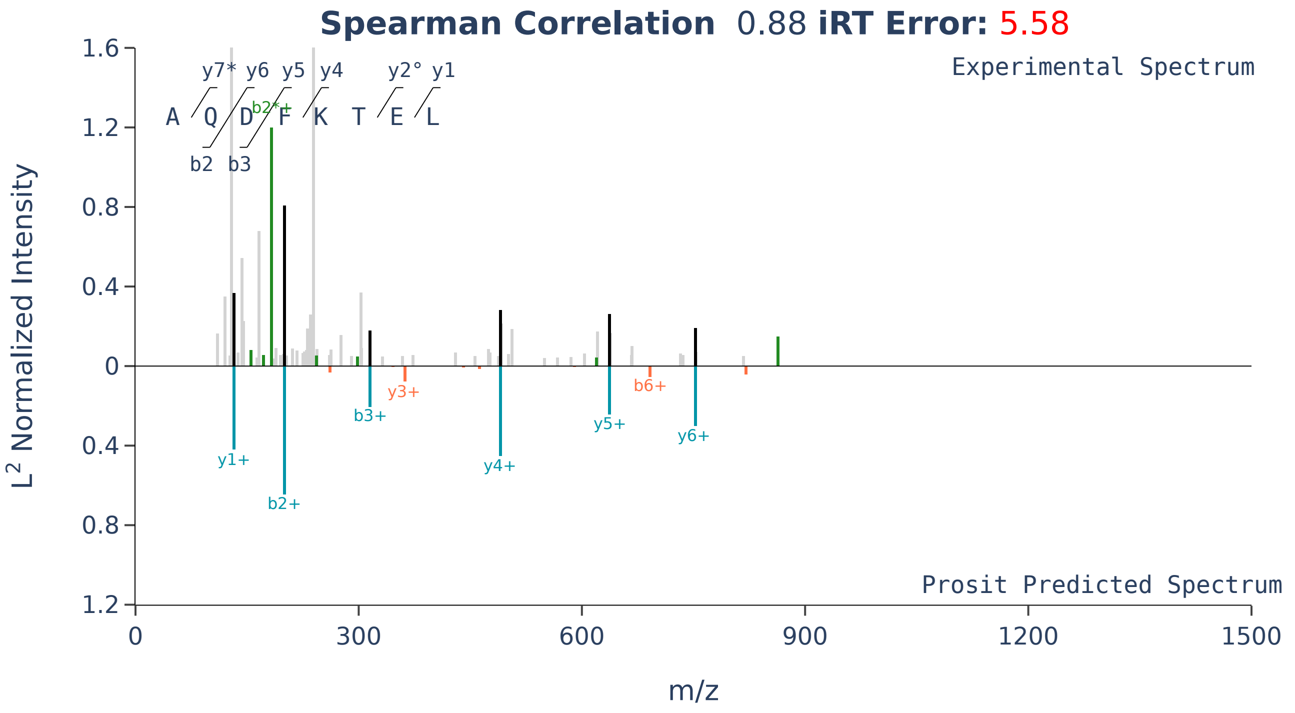

Source HRoetschke_271222_120123_HFGoe_H3_b1R1_24h **Scan** 13971
Peptide EIAQDFKT **Charge** 2 **Spectral Angle** 0.12
Spearman Correlation 0.15 **iRT Error:** 14.37

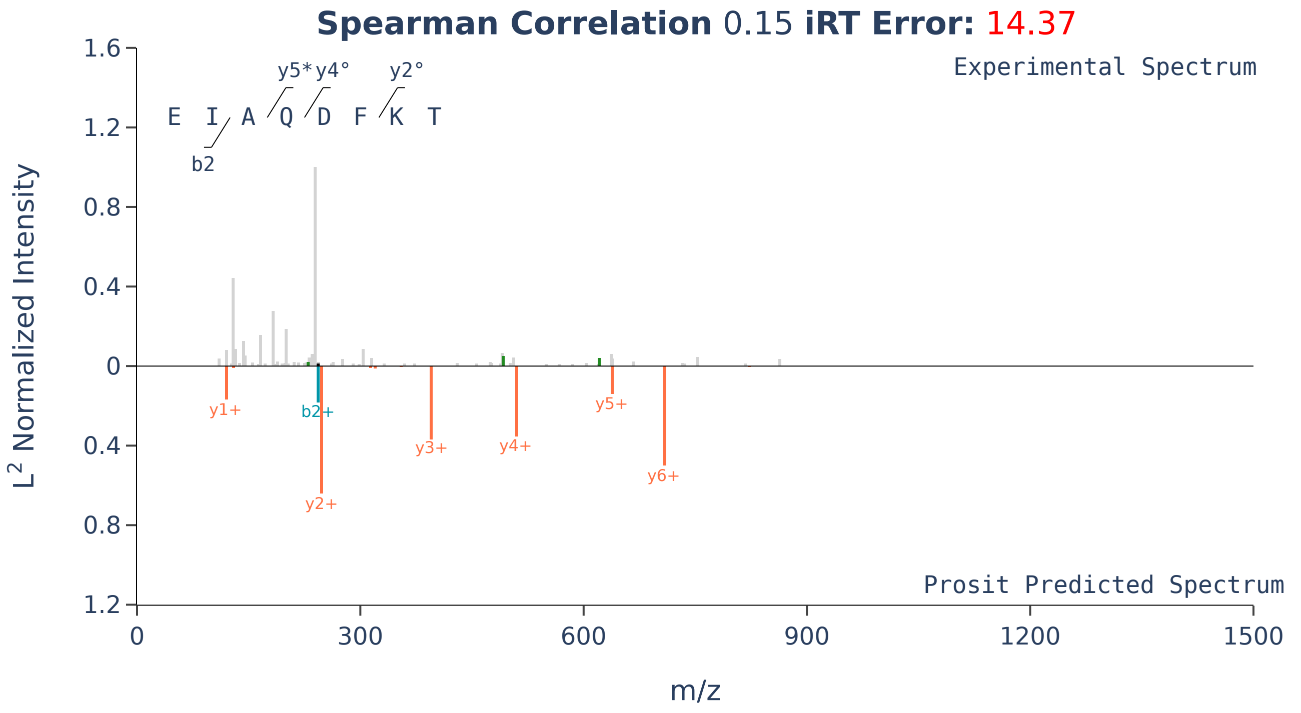

Source HRoetschke_271222_120123_HFGoe_H3_b2R1_24h **Scan** 11142
Peptide RFQSSEL **Charge** 2 **Spectral Angle** 0.79
Spearman Correlation 0.95 **iRT Error:** 6.91

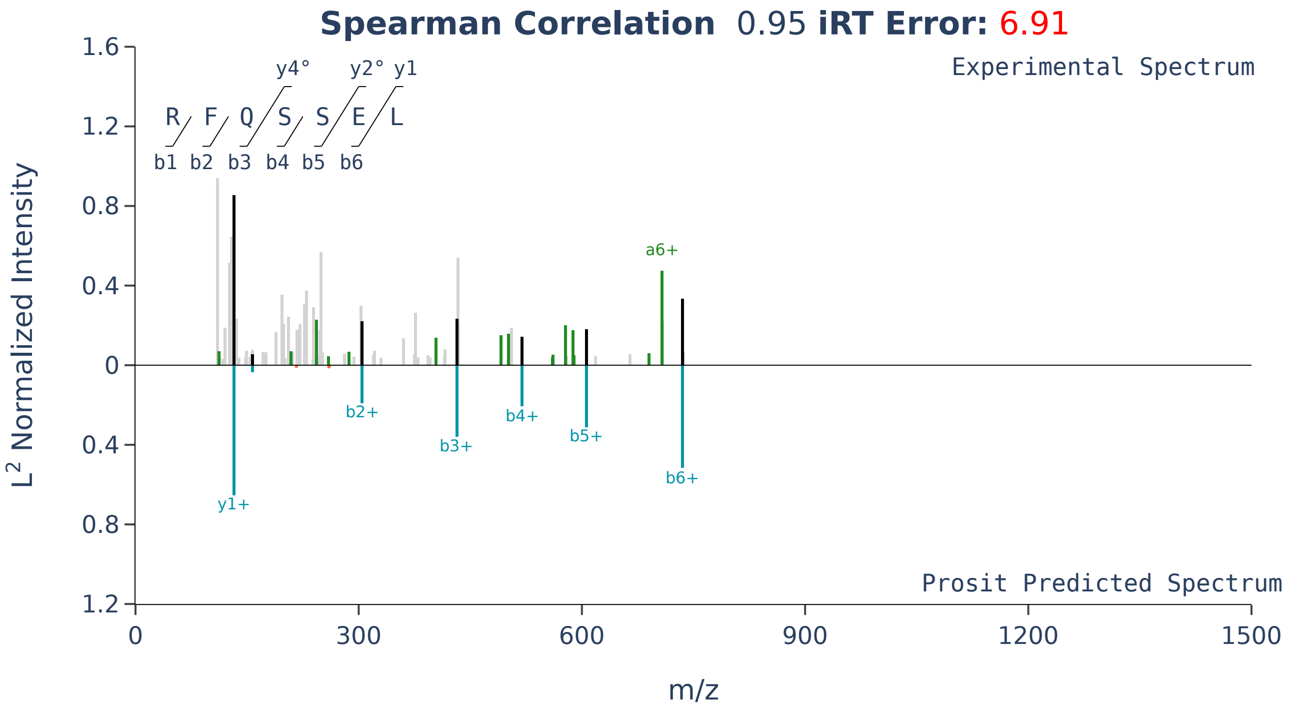

Source HRoetschke_271222_120123_HFGoe_H3_b2R1_24h **Scan** 11142
Peptide TDLRFQS **Charge** 2 **Spectral Angle** 0.18
Spearman Correlation 0.31 **iRT Error:** 16.89

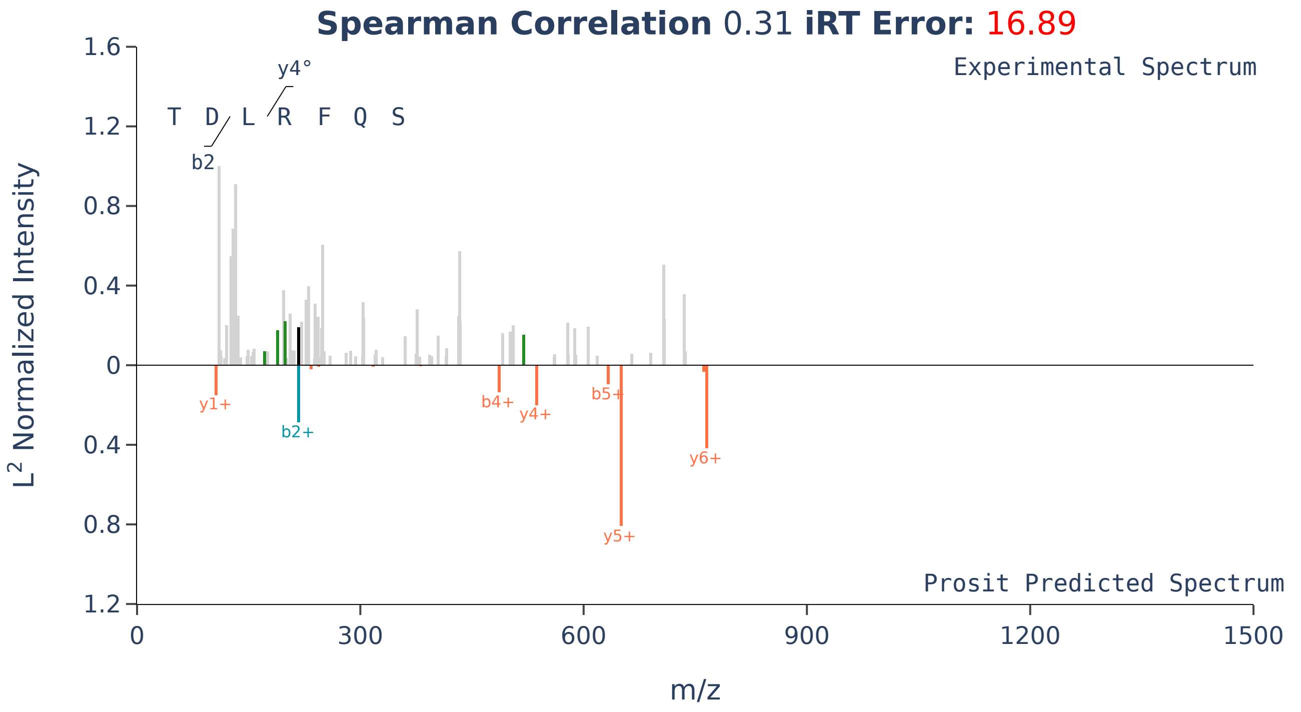

Source HRoetschke_271222_120123_HFGoe_H3_b2R1_24h **Scan** 17051
Peptide STELLRL **Charge** 2 **Spectral Angle** 0.86
Spearman Correlation 0.94 **iRT Error:** 7.56

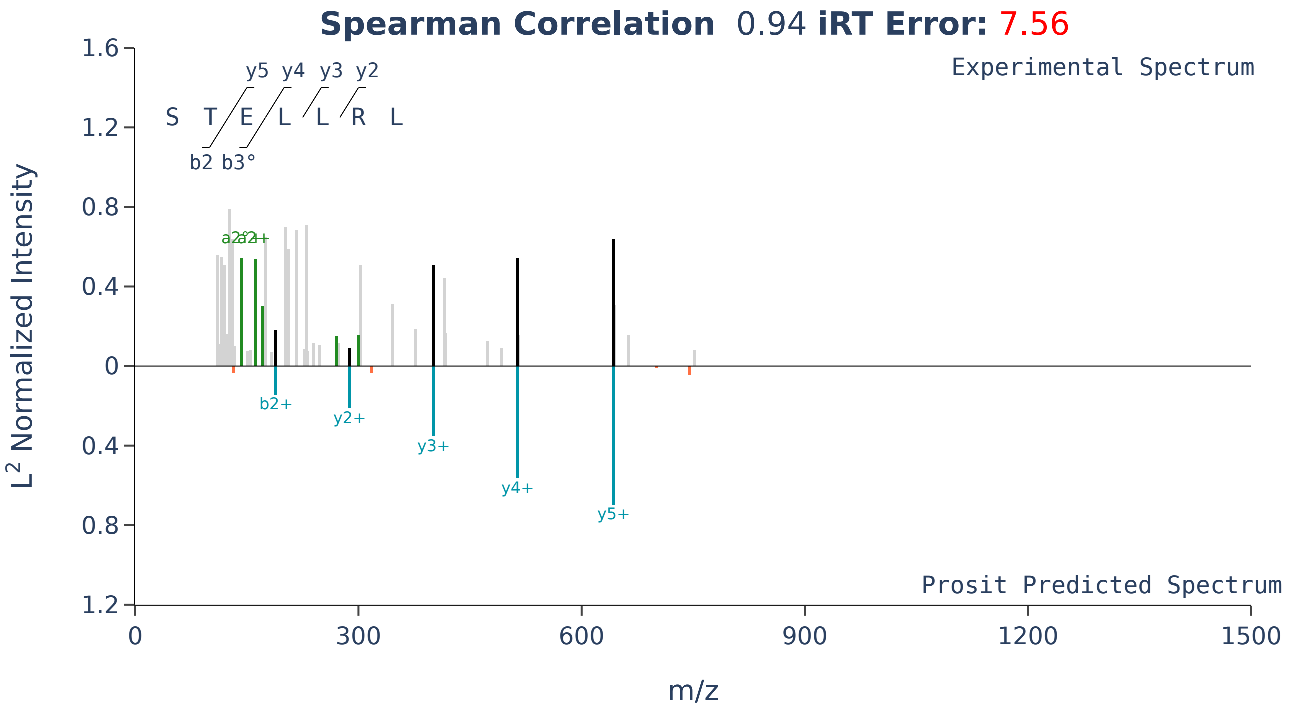

Source HRoetschke_271222_120123_HFGoe_H3_b2R1_24h **Scan** 17051
Peptide STELLIR **Charge** 2 **Spectral Angle** 0.82
Spearman Correlation 0.88 **iRT Error:** 33.58

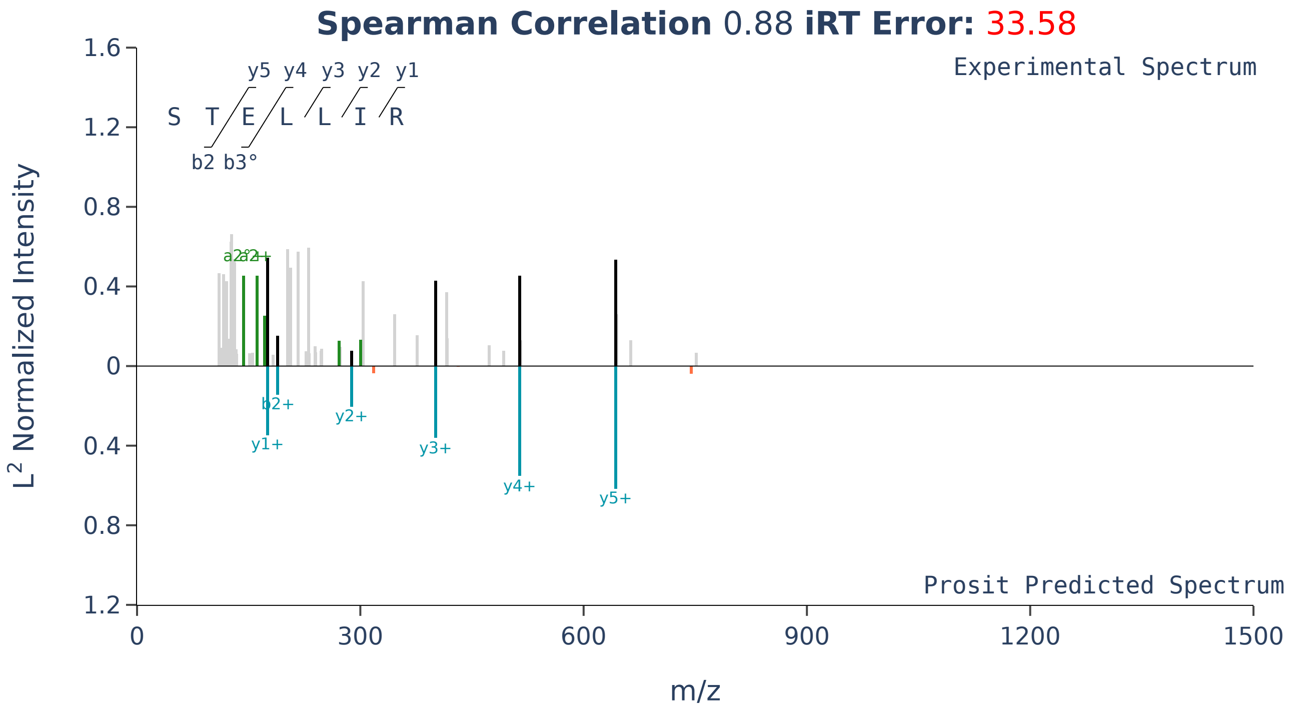

Source HRoetschke_271222_120123_HFGoe_H3_b1R1_1h **Scan** 11387
Peptide EDNLCAIHAKRV **Charge** 3 **Spectral Angle** 0.77
Spearman Correlation 0.94 **iRT Error:** 7.65

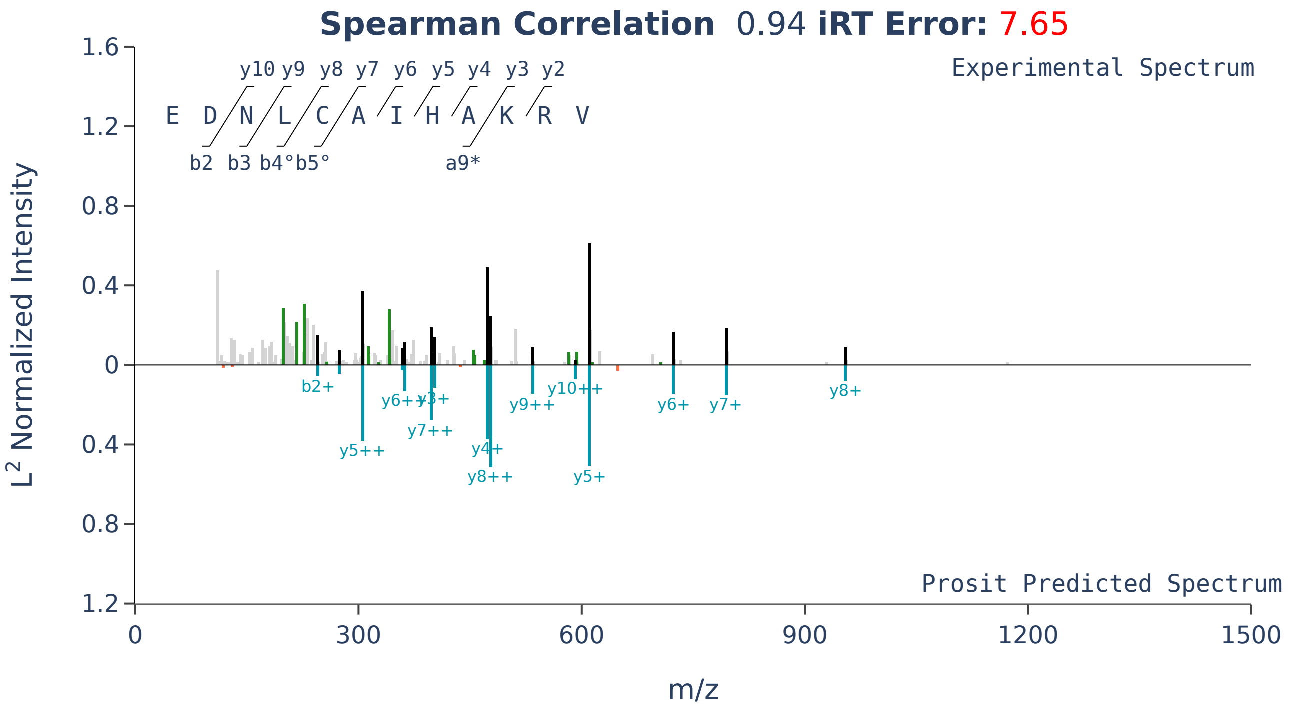

Source HRoetschke_271222_120123_HFGoe_H3_b1R1_1h **Scan** 11387
Peptide AYLVALFEDTNL **Charge** 3 **Spectral Angle** 0.0
Spearman Correlation 0.0 **iRT Error:** 135.72

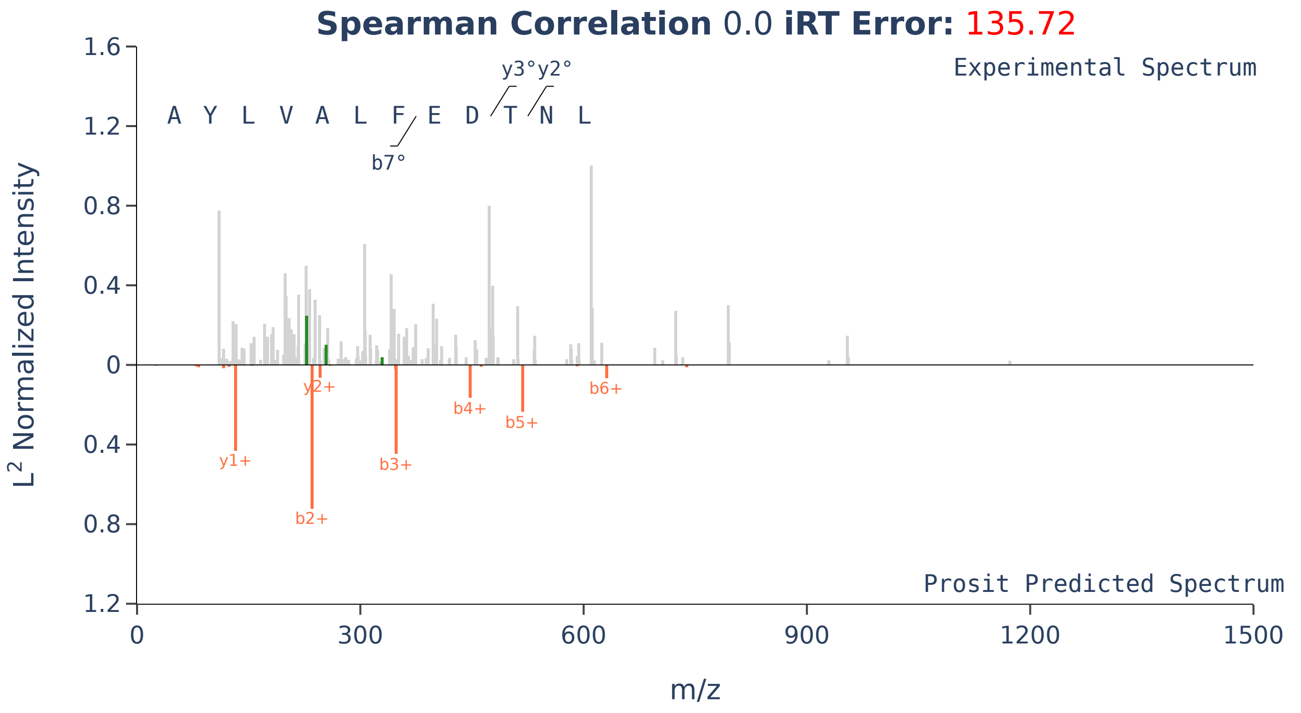

Source HRoetschke_291222_140123_HFGoe_H4_b1R1_0h **Scan** 13964
Peptide TRGVLIYEETR **Charge** 3 **Spectral Angle** 0.86
Spearman Correlation 0.95 **iRT Error:** 0.0

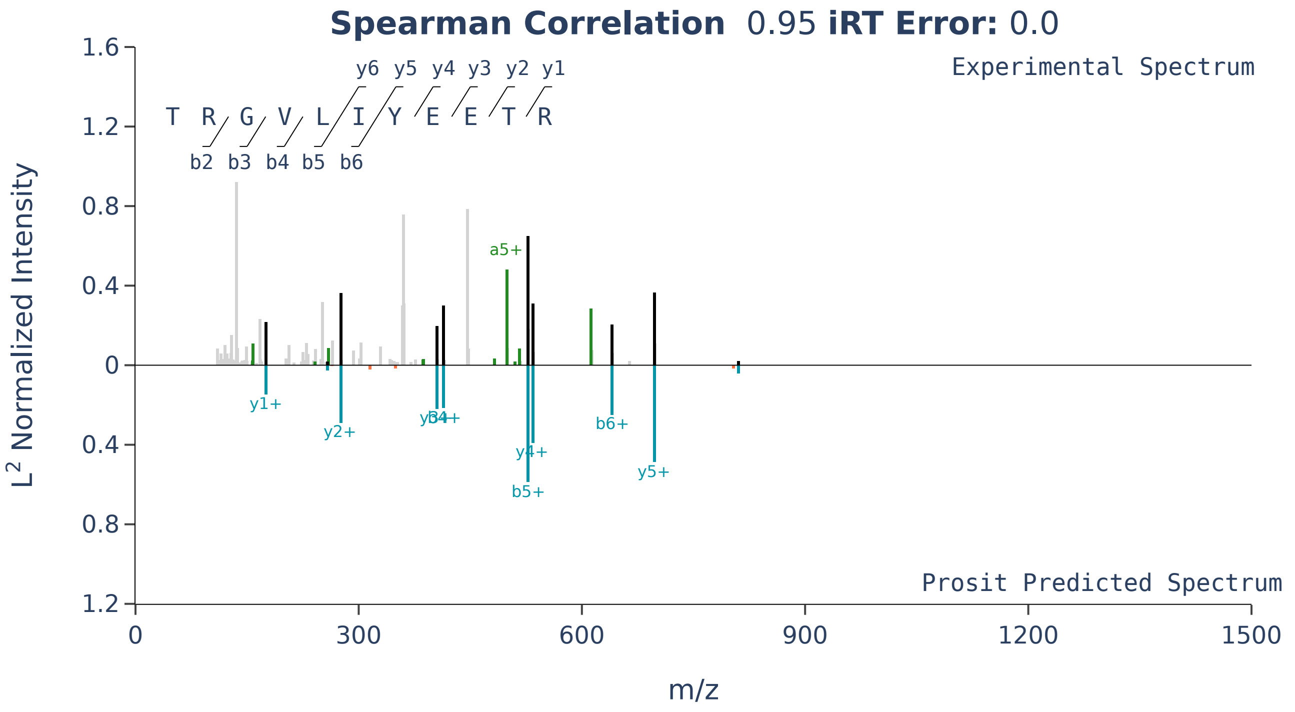

Source HRoetschke_291222_140123_HFGoe_H4_b1R1_0h **Scan** 13964
Peptide RISGLIYEETR **Charge** 3 **Spectral Angle** 0.86
Spearman Correlation 0.89 **iRT Error:** 61.18

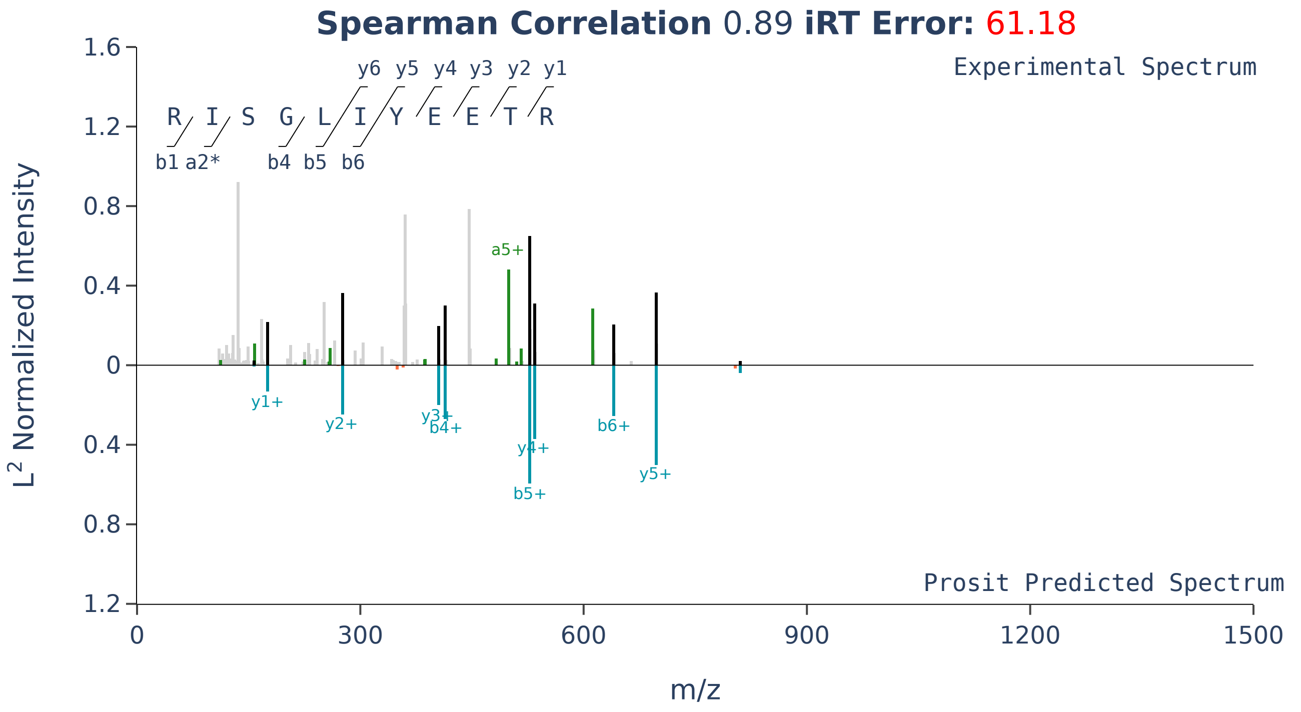

Source HRoetschke_291222_140123_HFGoe_H4_b1R1_2h **Scan** 15108
Peptide SGRGLIYEET **Charge** 1 **Spectral Angle** 0.64
Spearman Correlation 0.94 **iRT Error:** 0.06

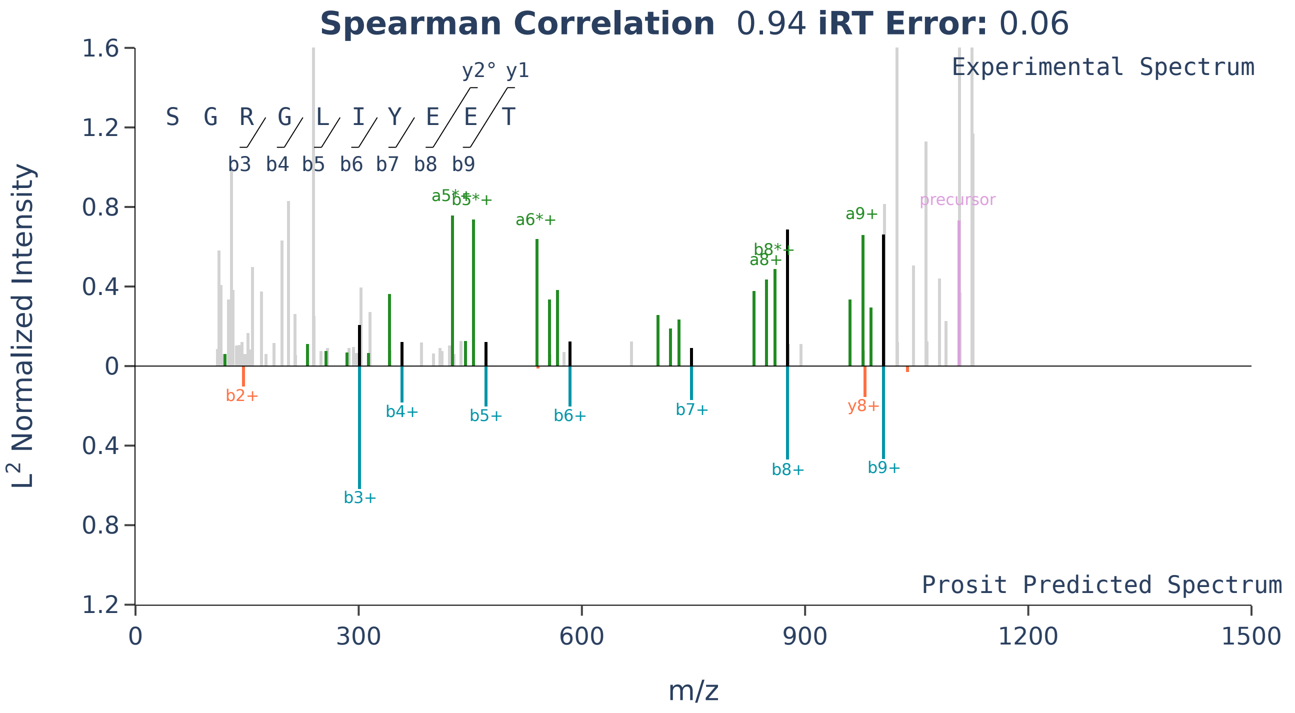

Source HRoetschke_291222_140123_HFGoe_H4_b1R1_2h **Scan** 15108
Peptide SGLIYEETRG **Charge** 1 **Spectral Angle** 0.59
Spearman Correlation 0.43 **iRT Error:** 1.01

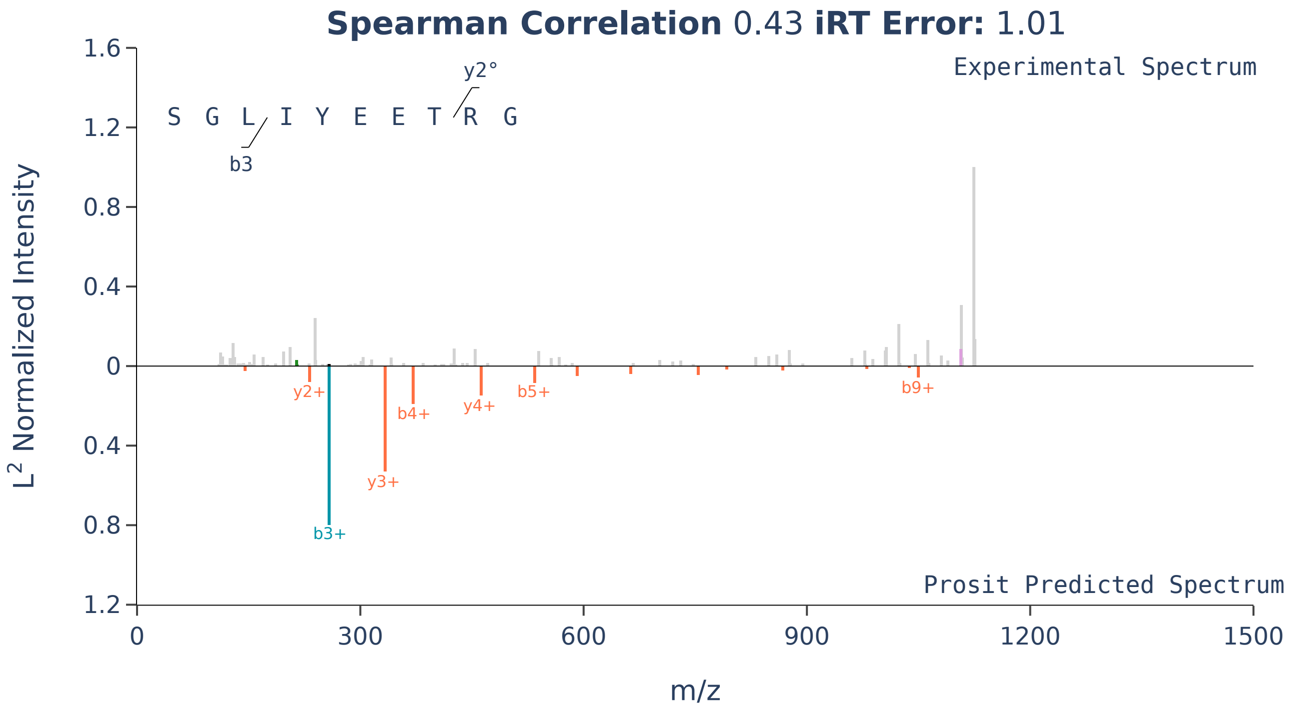

Source HRoetschke_291222_140123_HFGoe_H4_b1R1_24h **Scan** 16355
Peptide TAMDVVVY **Charge** 1 **Spectral Angle** 0.77
Spearman Correlation 0.96 **iRT Error:** 0.11

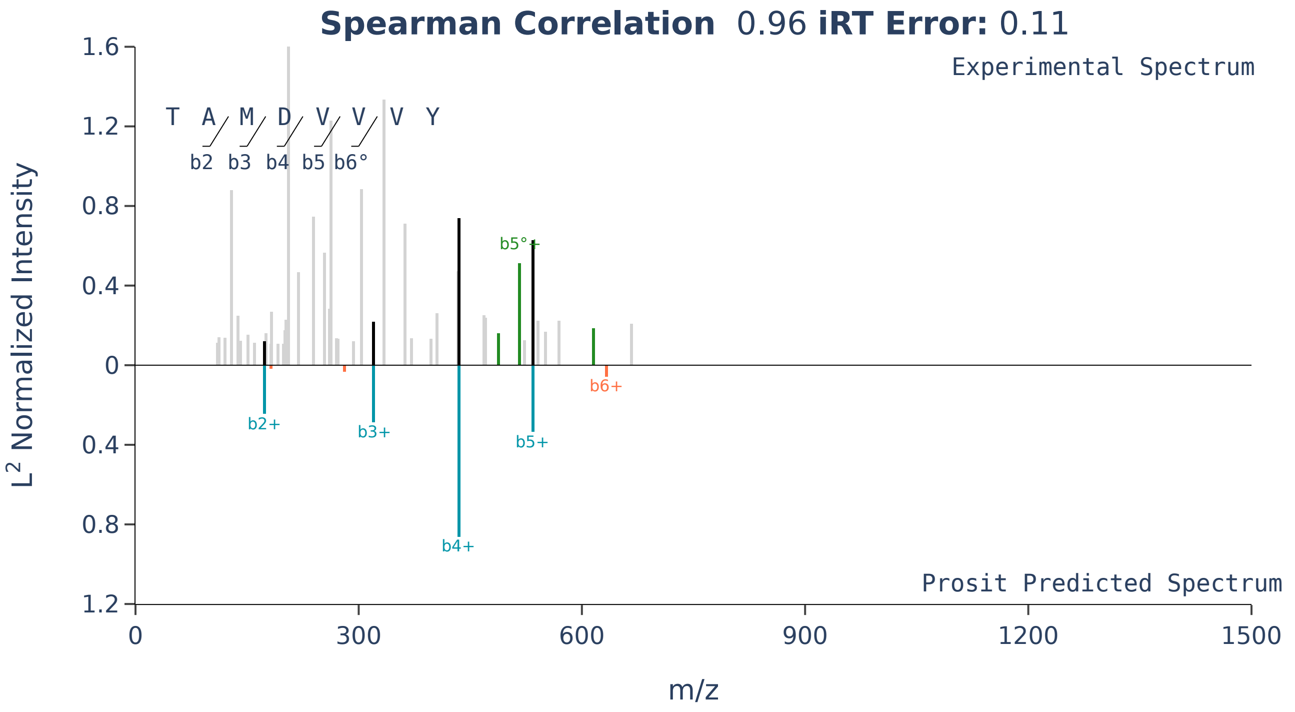

Source HRoetschke_291222_140123_HFGoe_H4_b1R1_24h **Scan** 16355
Peptide VTAMDVVY **Charge** 1 **Spectral Angle** 0.73
Spearman Correlation 0.67 **iRT Error:** 5.19

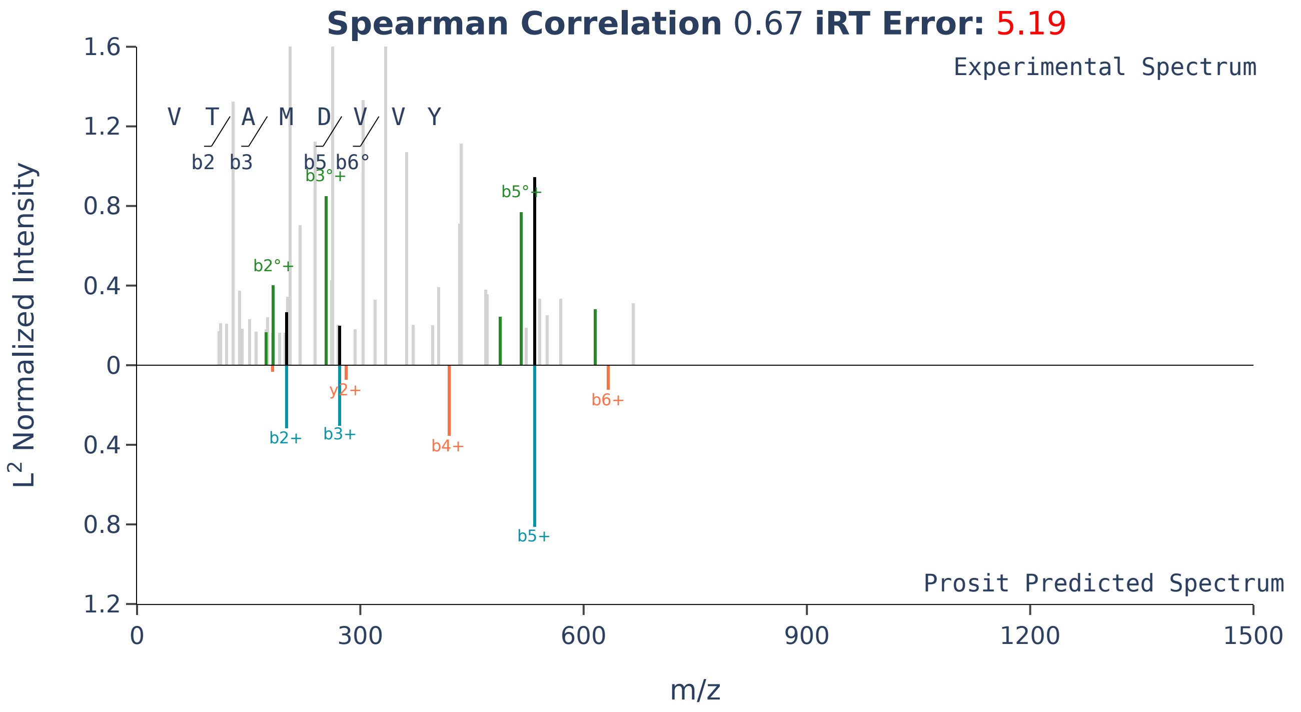

Source HRoetschke_291222_140123_HFGoe_H4_b1R1_24h **Scan** 9861
Peptide RDNIQGITKPAIRRAIRR **Charge** 5 **Spectral Angle** 0.77
Spearman Correlation 0.84 **iRT Error:** 0.11

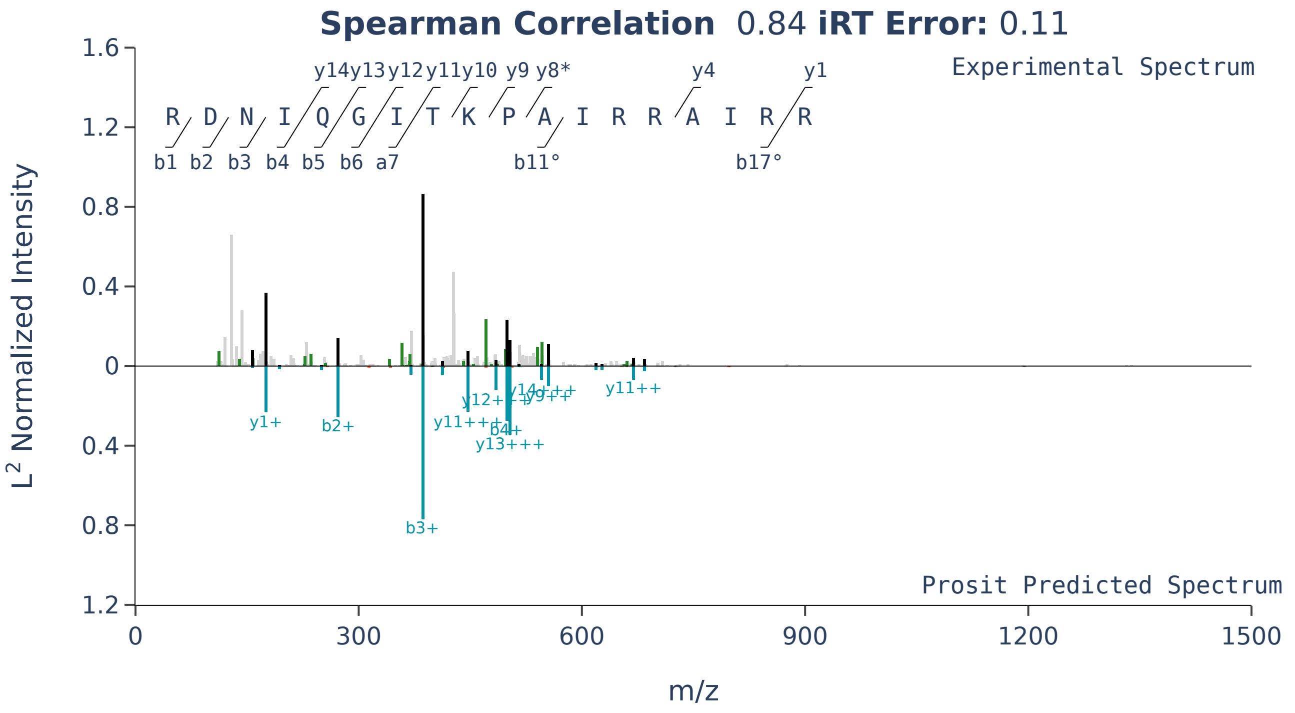

Source HRoetschke_291222_140123_HFGoe_H4_b1R1_24h **Scan** 9861
Peptide RDNIQGITKPAIRRLARR **Charge** 5 **Spectral Angle** 0.77
Spearman Correlation 0.84 **iRT Error:** 9.36

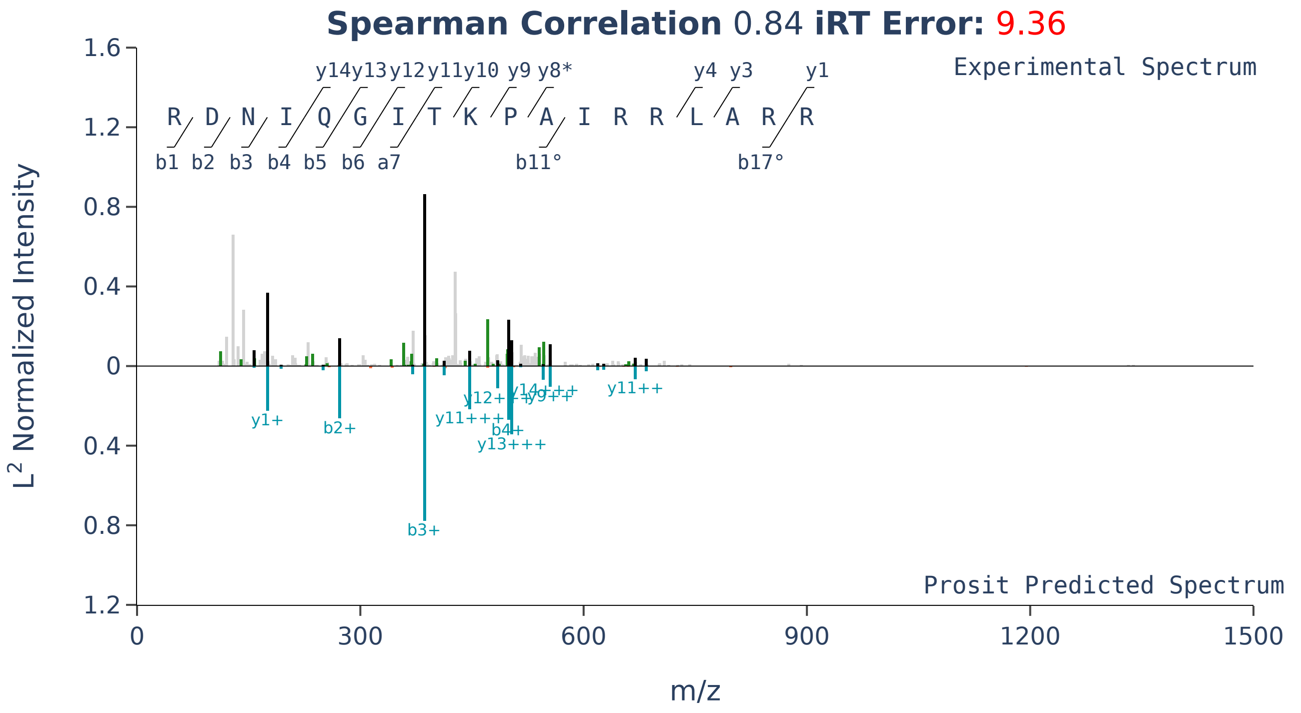

Source HRoetschke_291222_140123_HFGoe_H4_b2R1_24h **Scan** 13166
Peptide GKGGSGLIYE **Charge** 2 **Spectral Angle** 0.78
Spearman Correlation 0.93 **iRT Error:** 0.36

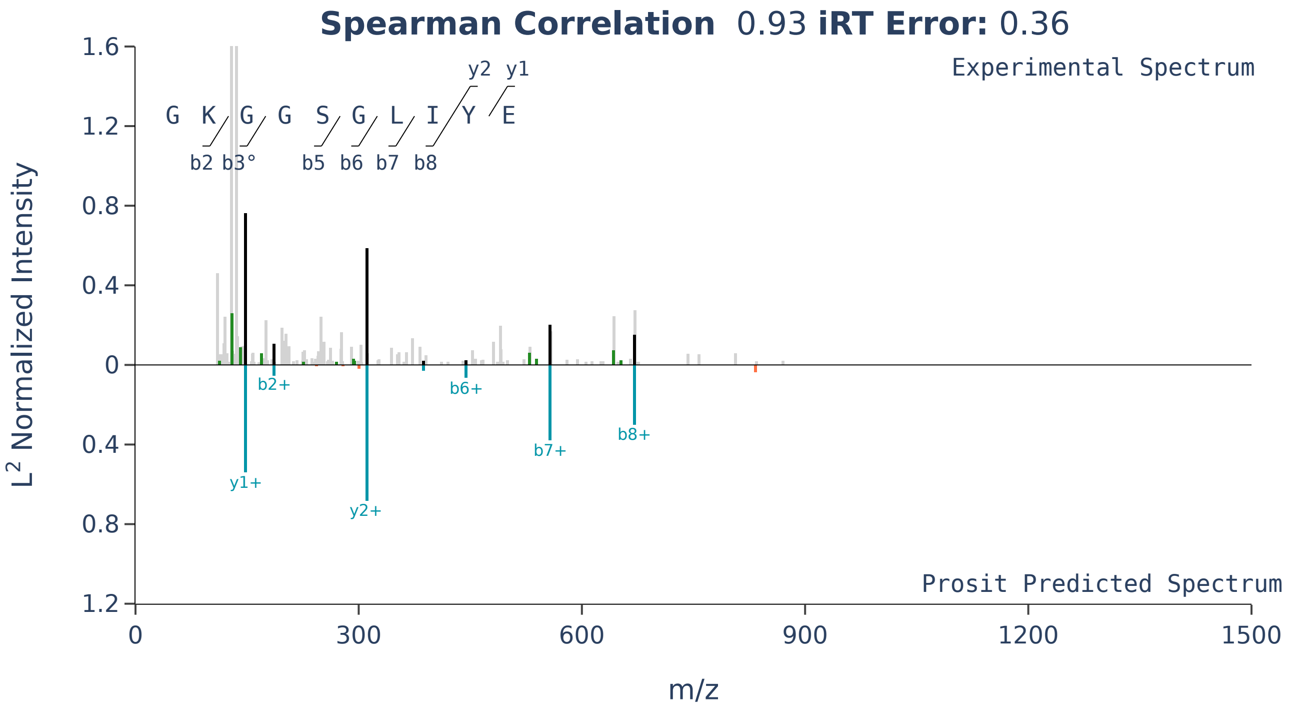

Source HRoetschke_291222_140123_HFGoe_H4_b2R1_24h **Scan** 13166
Peptide GLIYEETR **Charge** 2 **Spectral Angle** 0.07
Spearman Correlation -0.23 **iRT Error:** 0.72

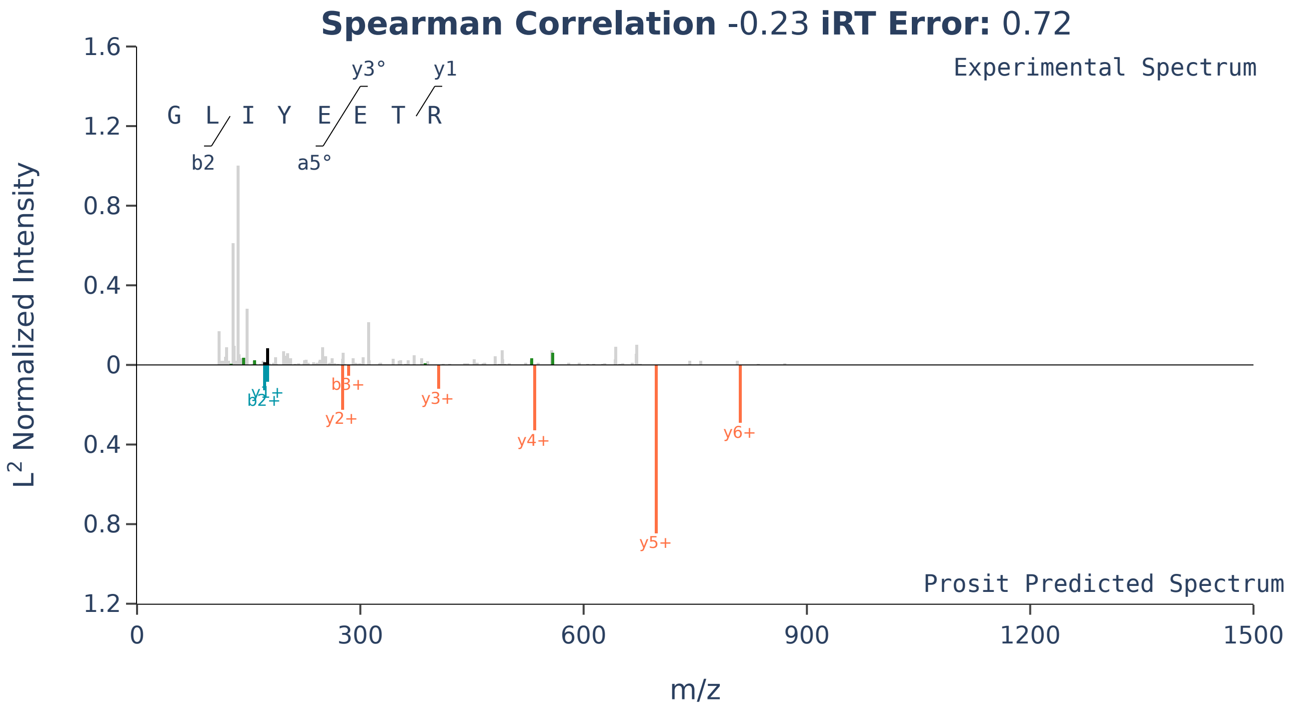

Source HRoetschke_291222_140123_HFGoe_H4_b1R1_2h **Scan** 15190
Peptide ISGLIYEETARRGGVKRI **Charge** 5 **Spectral Angle** 0.7
Spearman Correlation 0.88 **iRT Error:** 0.39

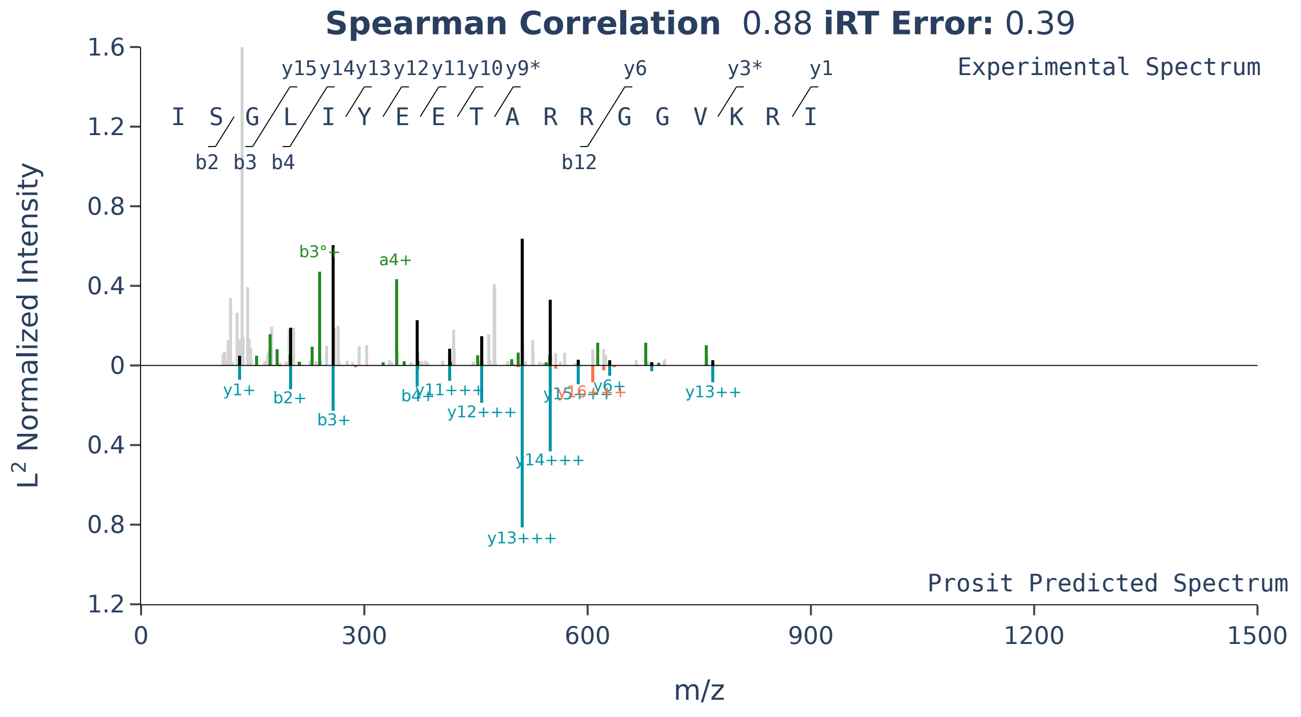

Source HRoetschke_291222_140123_HFGoe_H4_b1R1_2h **Scan** 15190
Peptide LARRGGVKRISGLIYEET **Charge** 5 **Spectral Angle** 0.34
Spearman Correlation 0.28 **iRT Error:** 1.72

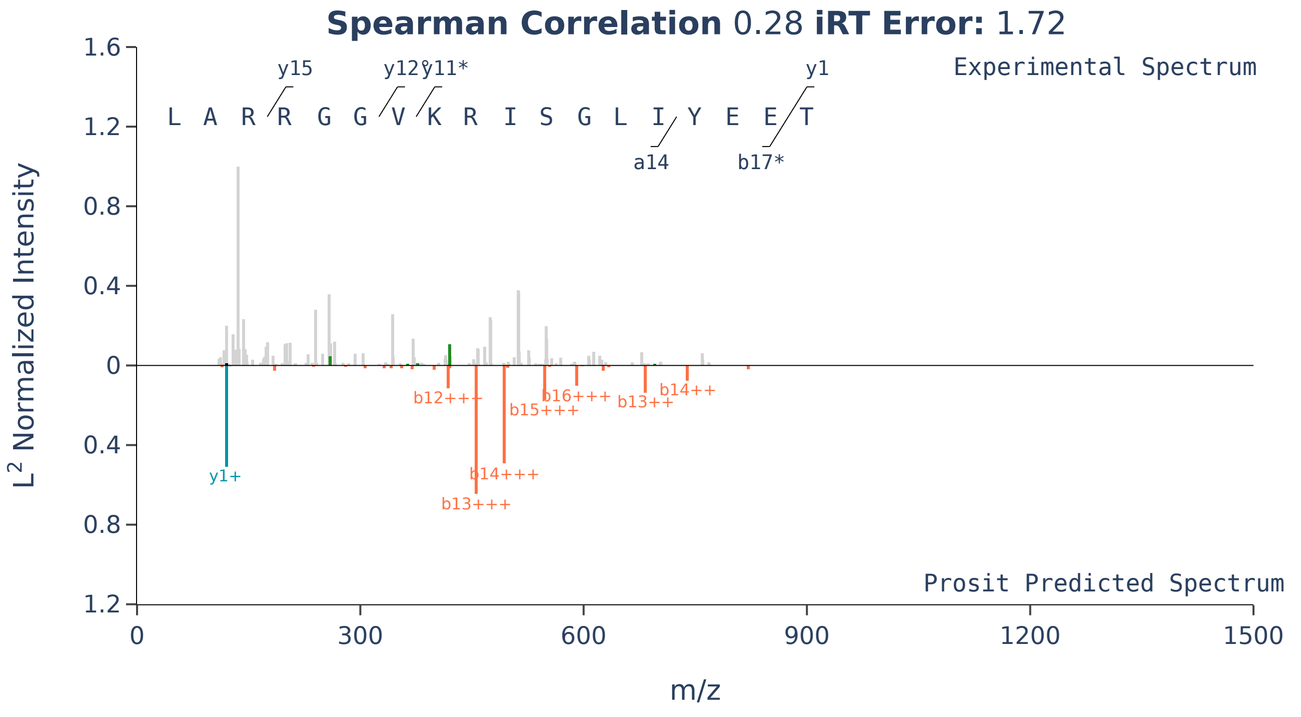

Source HRoetschke_291222_140123_HFGoe_H4_b2R1_4h **Scan** 9874
Peptide TKPAIRLR **Charge** 2 **Spectral Angle** 0.86
Spearman Correlation 0.89 **iRT Error:** 0.4

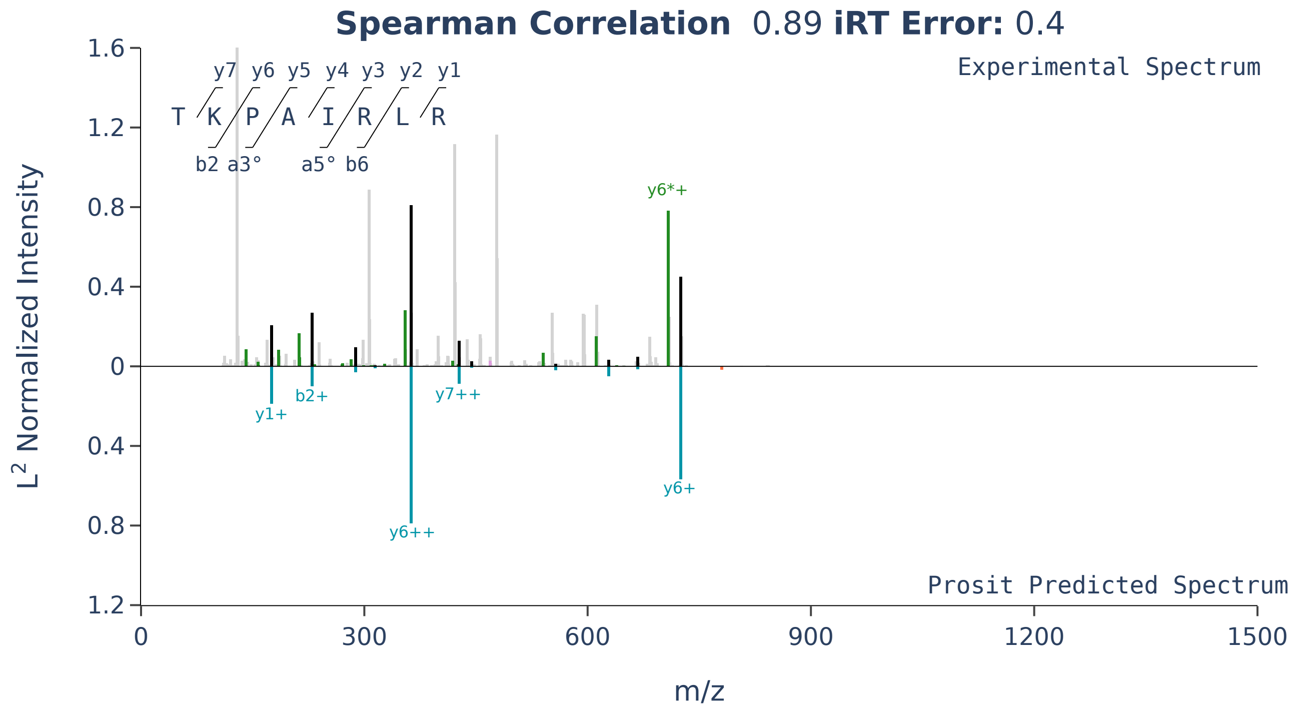

Source HRoetschke_291222_140123_HFGoe_H4_b2R1_4h **Scan** 9874
Peptide ITKPAIRR **Charge** 2 **Spectral Angle** 0.47
Spearman Correlation 0.72 **iRT Error:** 98.24

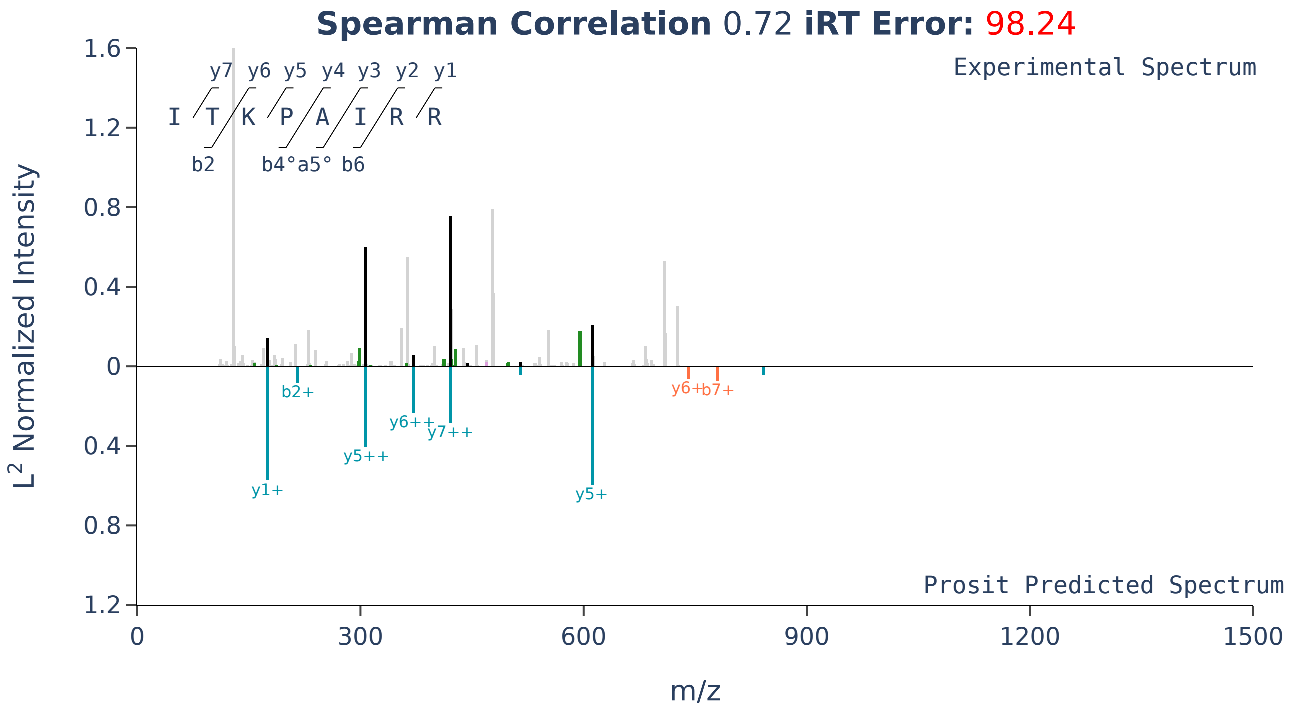

Source HRoetschke_291222_140123_HFGoe_H4_b1R2_1h **Scan** 8743
Peptide VYALKRGA **Charge** 2 **Spectral Angle** 0.88
Spearman Correlation 0.93 **iRT Error:** 0.58

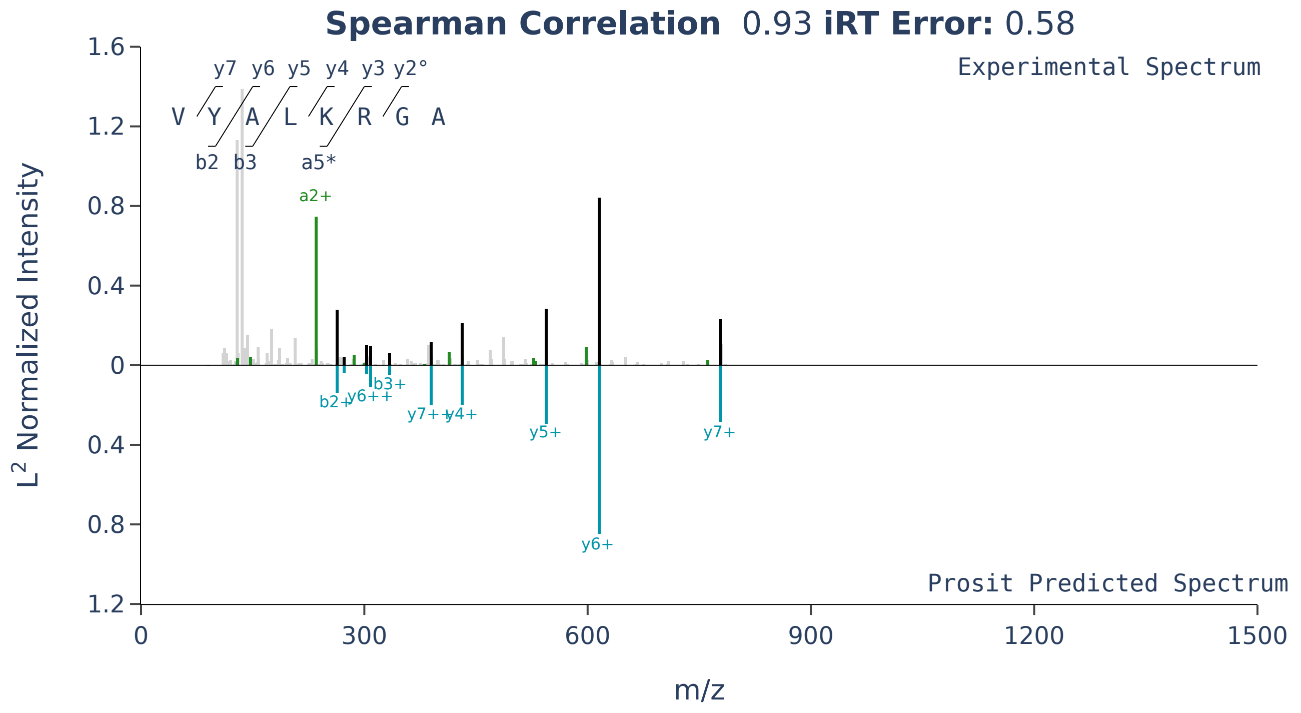

Source HRoetschke_291222_140123_HFGoe_H4_b1R2_1h **Scan** 8743
Peptide VYALKRQ **Charge** 2 **Spectral Angle** 0.89
Spearman Correlation 0.95 **iRT Error:** 131.44

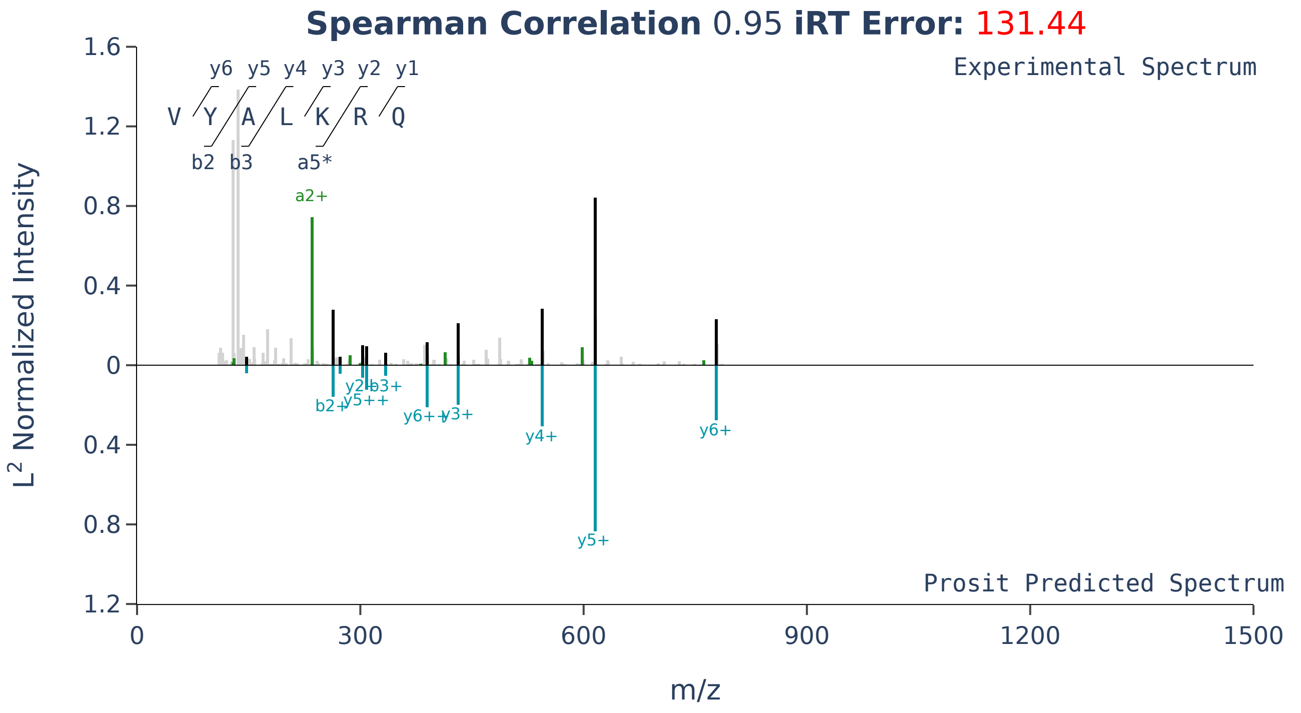

Source HRoetschke_291222_140123_HFGoe_H4_b1R1_2h **Scan** 22550
Peptide AMDVVYALLKRQGRT **Charge** 3 **Spectral Angle** 0.76
Spearman Correlation 0.89 **iRT Error:** 0.78

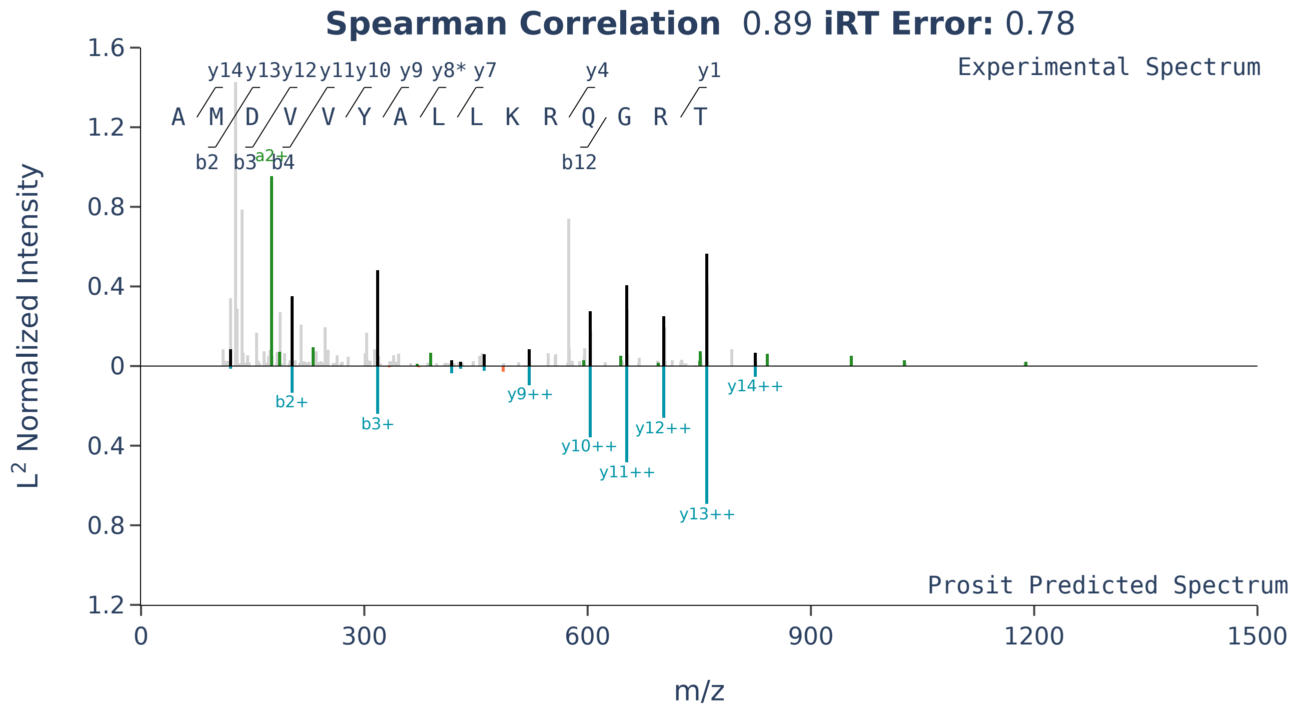

Source HRoetschke_291222_140123_HFGoe_H4_b1R1_2h **Scan** 22550
Peptide AMDVVYALKRQGRTL **Charge** 3 **Spectral Angle** 0.54
Spearman Correlation 0.84 **iRT Error:** 25.75

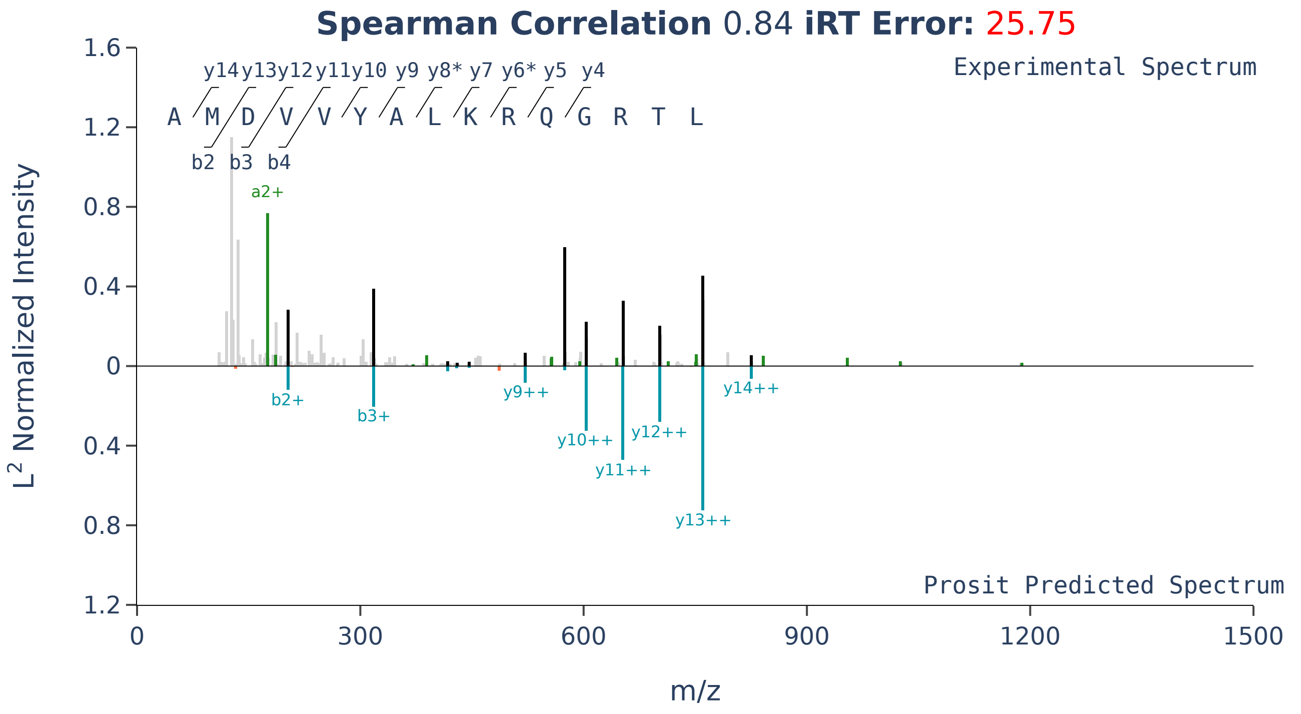

Source HRoetschke_291222_140123_HFGoe_H4_b2R1_1h **Scan** 8501
Peptide VYALGAKR **Charge** 2 **Spectral Angle** 0.87
Spearman Correlation 0.92 **iRT Error:** 0.8

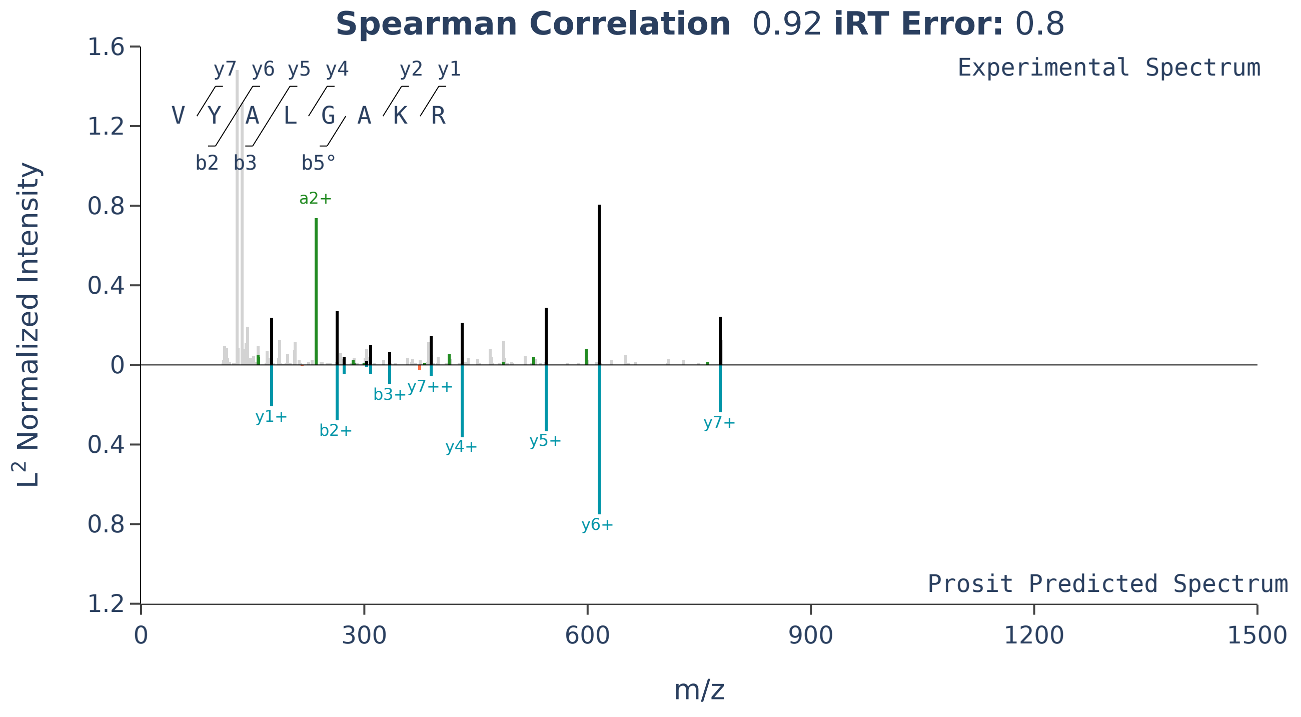

Source HRoetschke_291222_140123_HFGoe_H4_b2R1_1h **Scan** 8501
Peptide VYALKRQ **Charge** 2 **Spectral Angle** 0.91
Spearman Correlation 0.96 **iRT Error:** 125.13

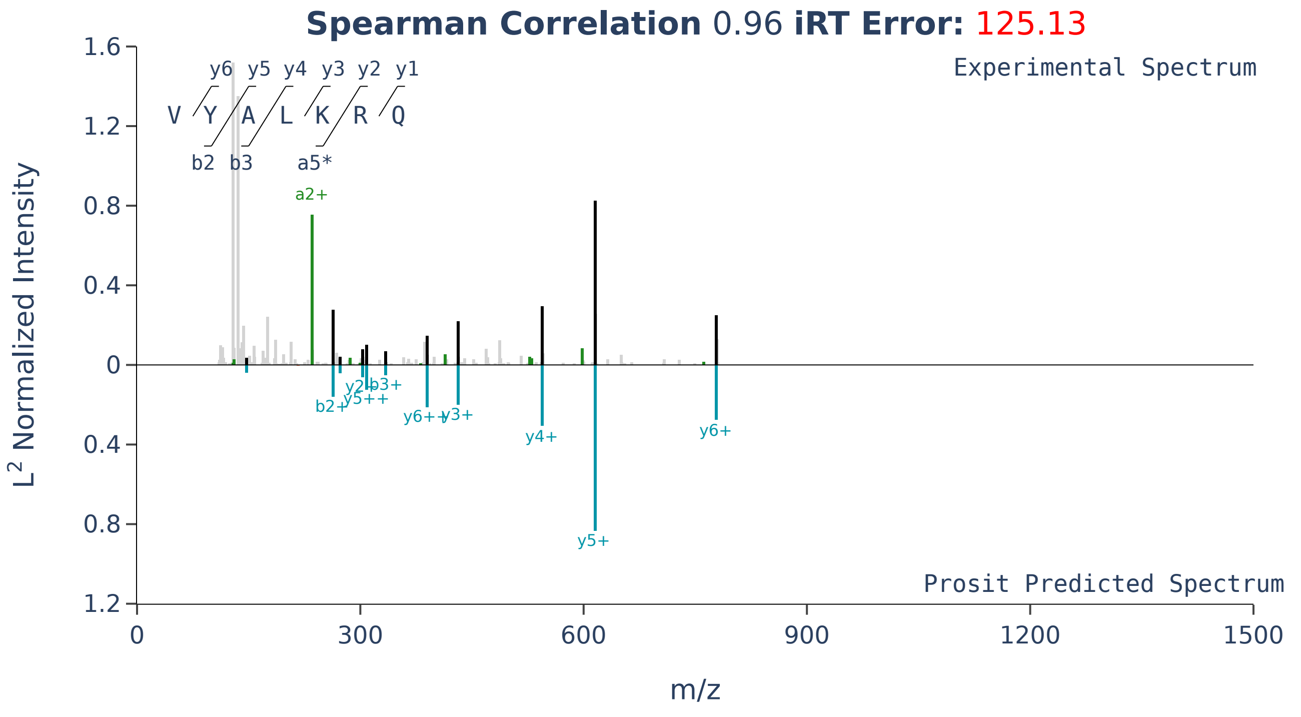

Source HRoetschke_291222_140123_HFGoe_H4_b1R2_2h **Scan** 20854
Peptide DNRISGLIYE **Charge** 2 **Spectral Angle** 0.86
Spearman Correlation 0.87 **iRT Error:** 0.88

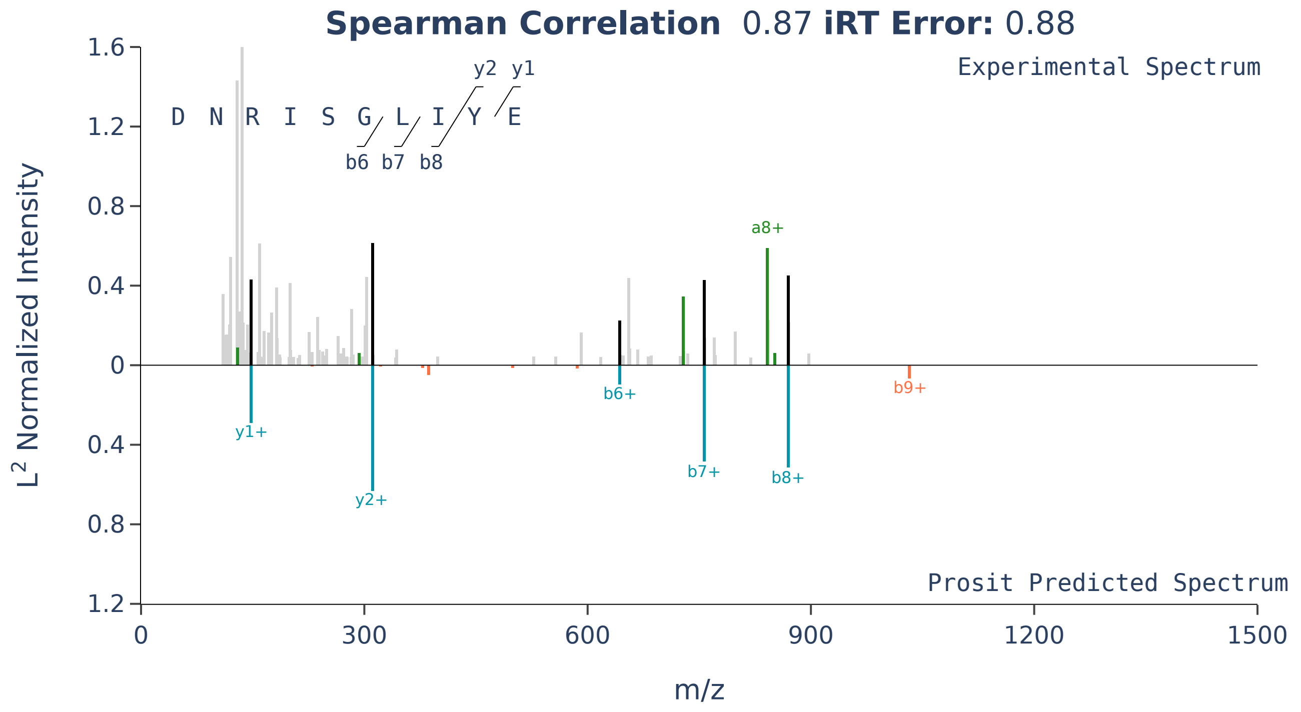

Source HRoetschke_291222_140123_HFGoe_H4_b1R2_2h **Scan** 20854
Peptide ENVIRDAVTY **Charge** 2 **Spectral Angle** 0.7
Spearman Correlation 0.69 **iRT Error:** 23.8

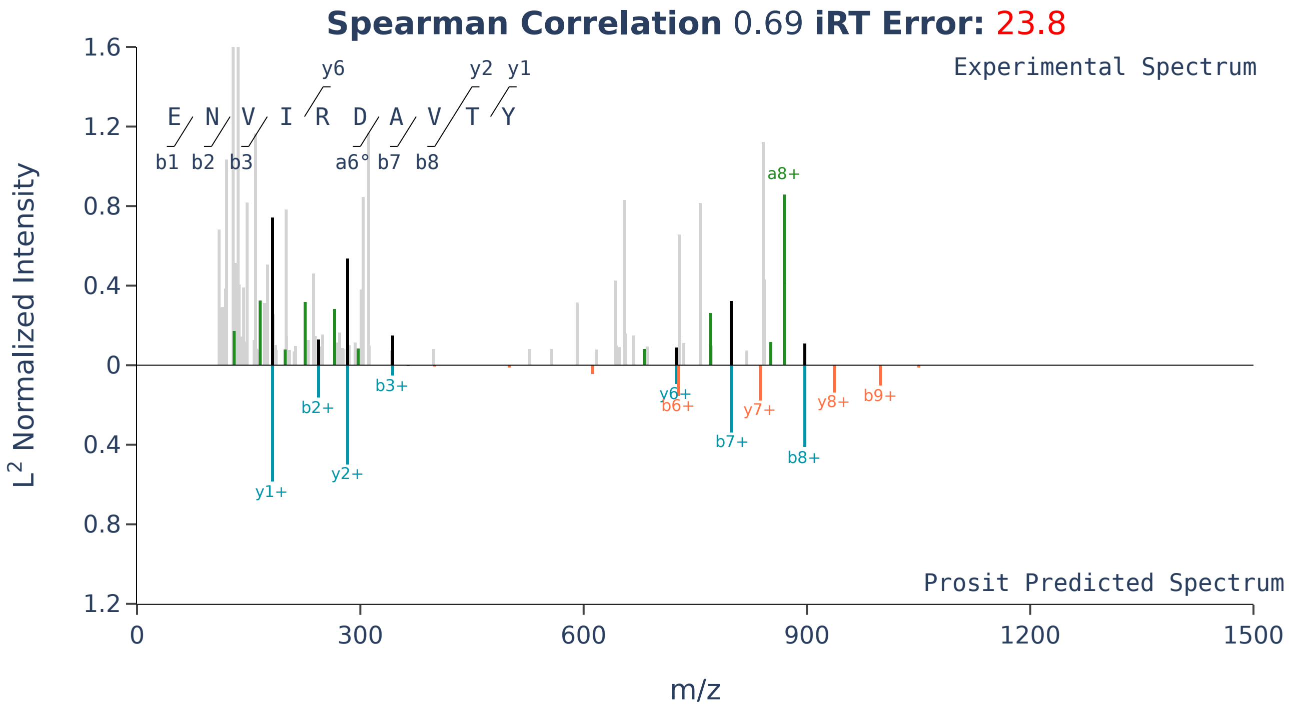

Source HRoetschke_291222_140123_HFGoe_H4_b1R1_24h **Scan** 13322
Peptide RISGLYE **Charge** 2 **Spectral Angle** 0.87
Spearman Correlation 0.92 **iRT Error:** 1.36

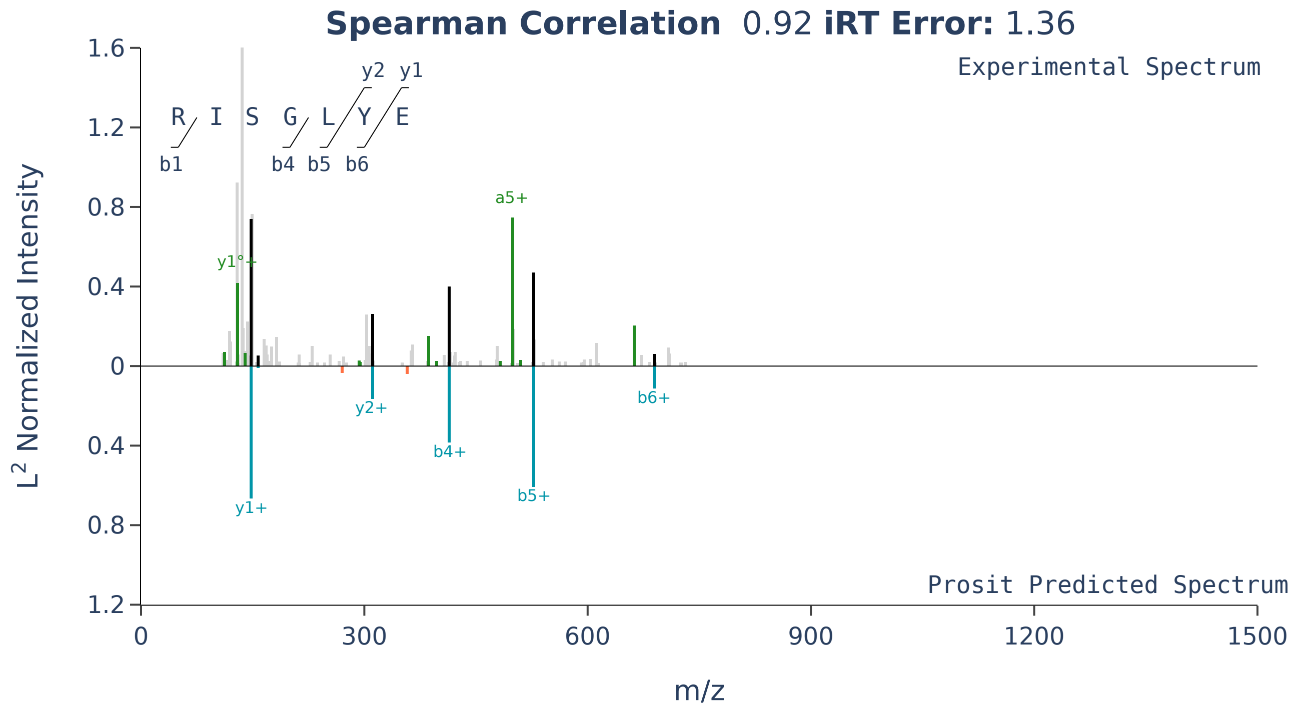

Source HRoetschke_291222_140123_HFGoe_H4_b1R1_24h **Scan** 13322
Peptide IRDAVTY **Charge** 2 **Spectral Angle** 0.55
Spearman Correlation 0.54 **iRT Error:** 14.81

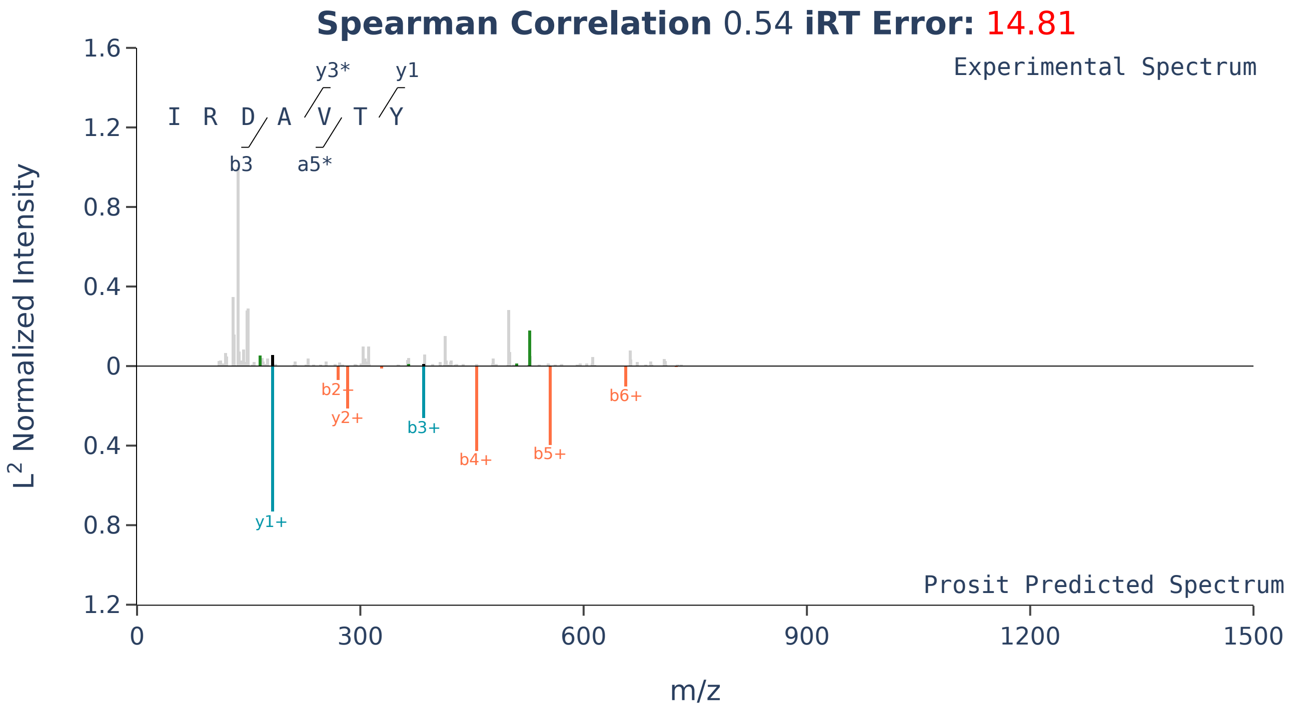

Source HRoetschke_291222_140123_HFGoe_H4_b2R1_4h **Scan** 5355
Peptide HAKRKTVTET **Charge** 3 **Spectral Angle** 0.91
Spearman Correlation 0.82 **iRT Error:** 1.75

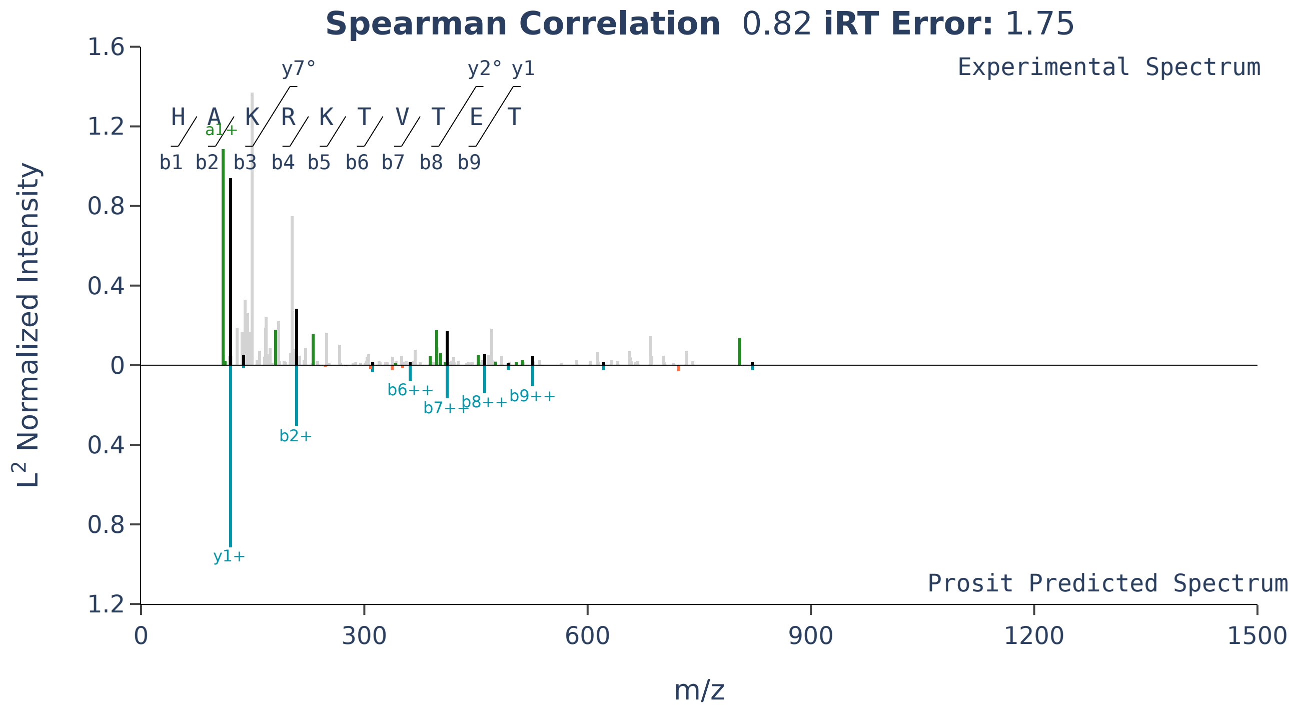

Source HRoetschke_291222_140123_HFGoe_H4_b2R1_4h **Scan** 5355
Peptide TEHAKRKTVT **Charge** 3 **Spectral Angle** 0.5
Spearman Correlation 0.86 **iRT Error:** 90.65

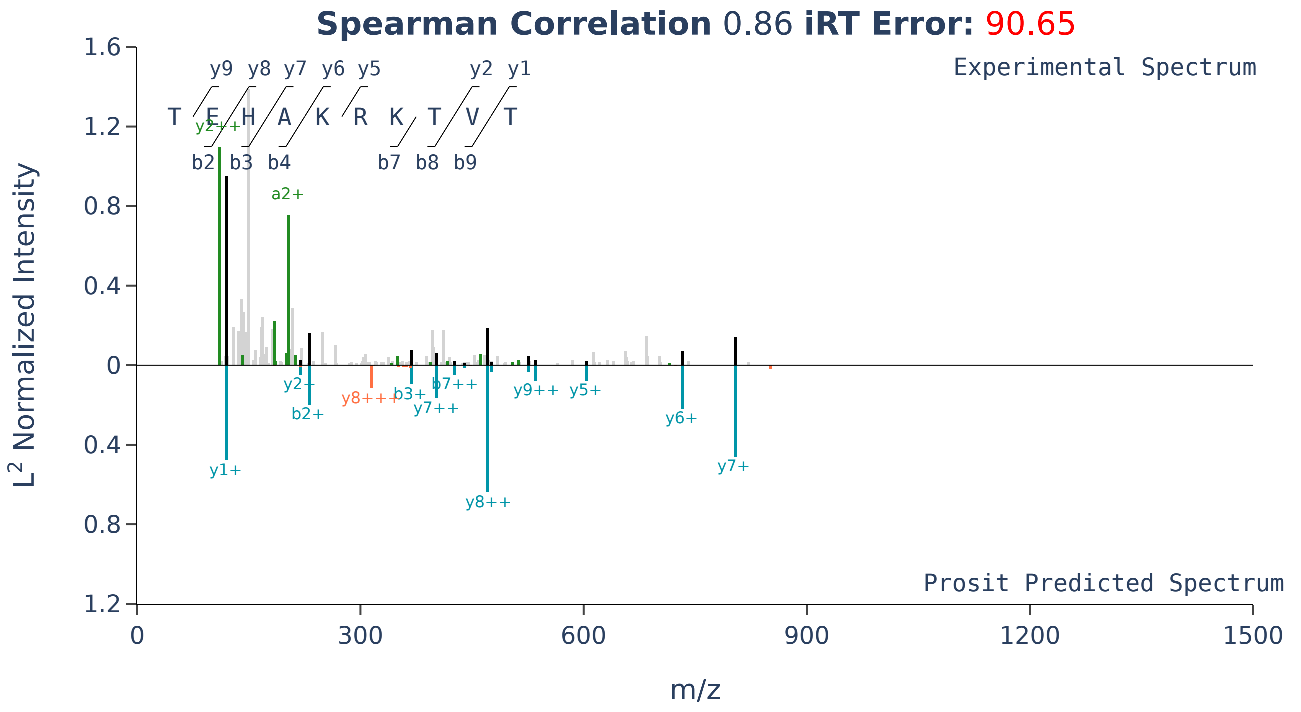

Source HRoetschke_291222_140123_HFGoe_H4_b1R1_2h **Scan** 15117
Peptide MDVVYALKRQGRTA **Charge** 4 **Spectral Angle** 0.89
Spearman Correlation 0.86 **iRT Error:** 1.78

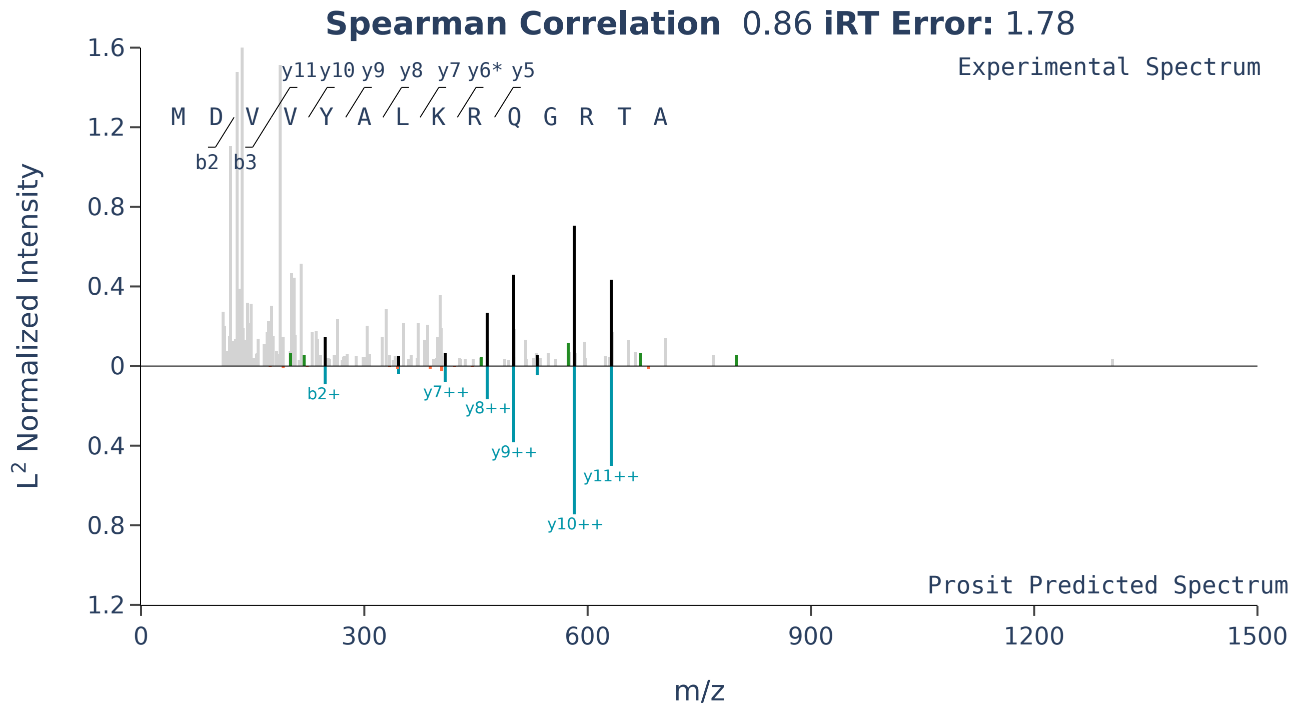

Source HRoetschke_291222_140123_HFGoe_H4_b1R1_2h **Scan** 15117
Peptide TAMDVVYALKRQGR **Charge** 4 **Spectral Angle** 0.08
Spearman Correlation 0.18 **iRT Error:** 20.72

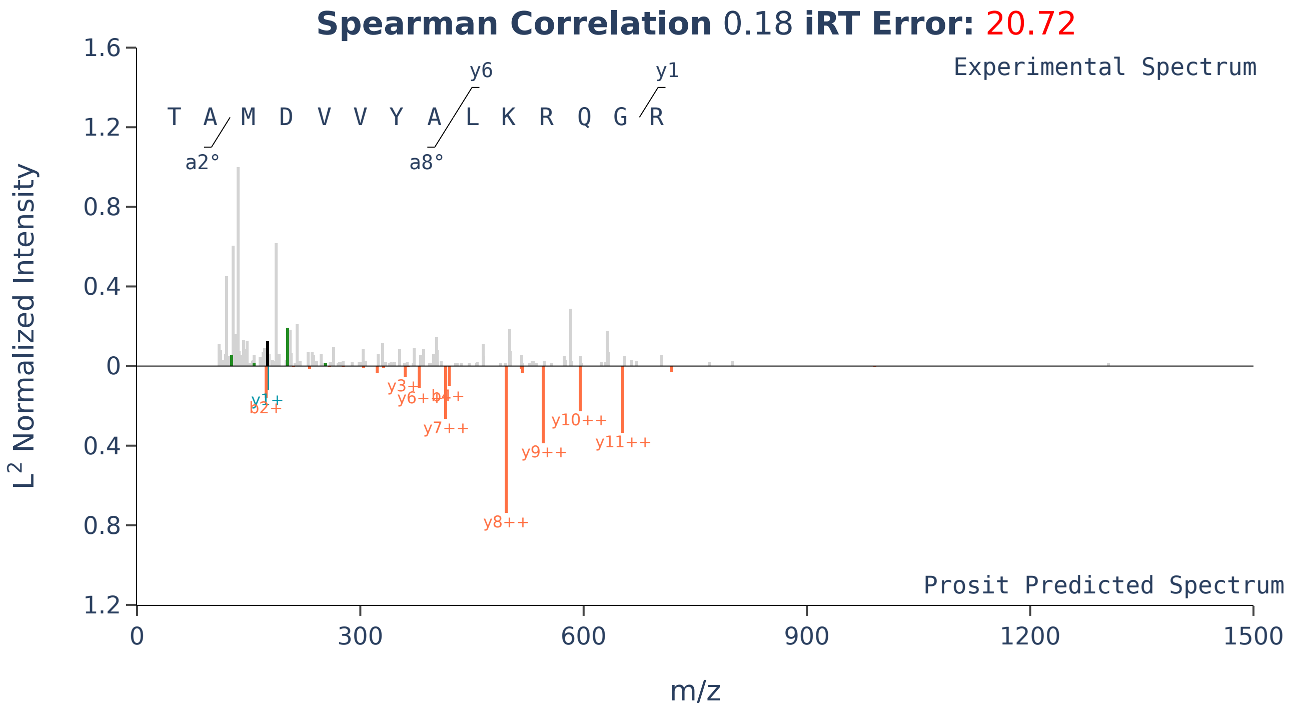

Source HRoetschke_291222_140123_HFGoe_H4_b1R1_1h **Scan** 21287
Peptide DNRISGLIYEET **Charge** 2 **Spectral Angle** 0.8
Spearman Correlation 0.91 **iRT Error:** 2.69

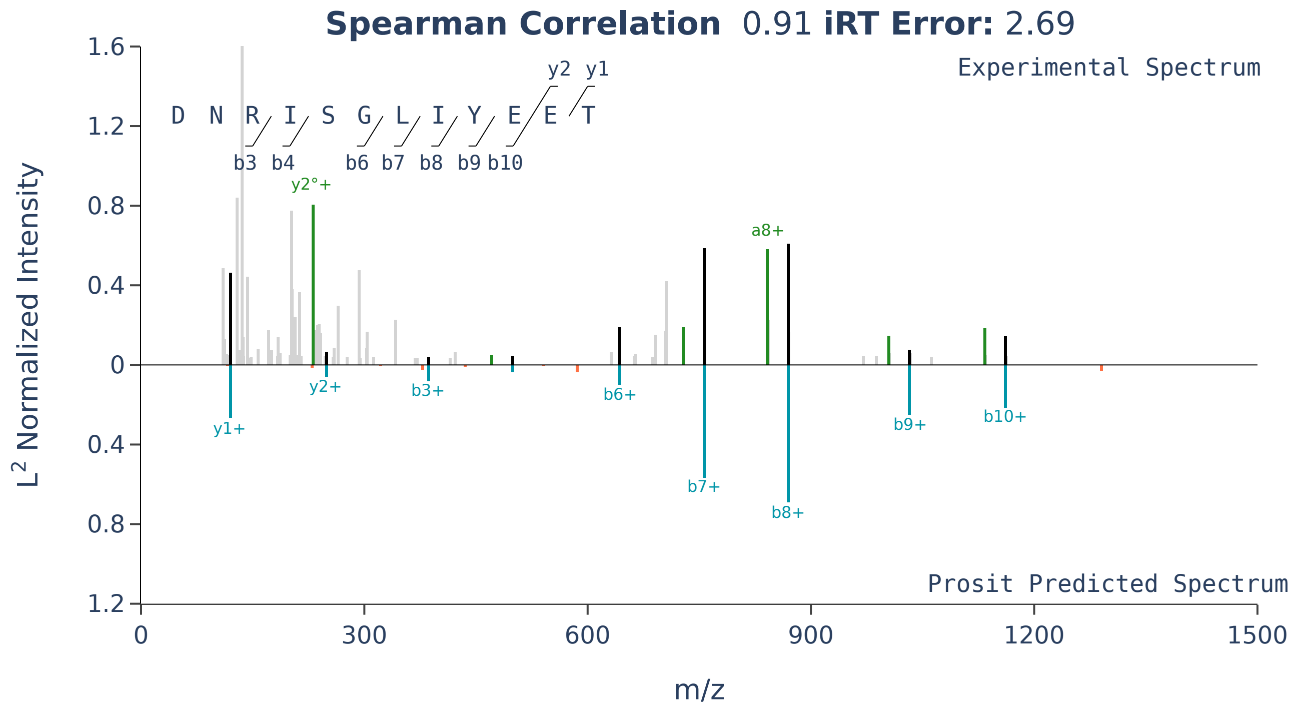

Source HRoetschke_291222_140123_HFGoe_H4_b1R1_1h **Scan** 21287
Peptide ENVIRDAVTYTE **Charge** 2 **Spectral Angle** 0.37
Spearman Correlation 0.55 **iRT Error:** 18.63

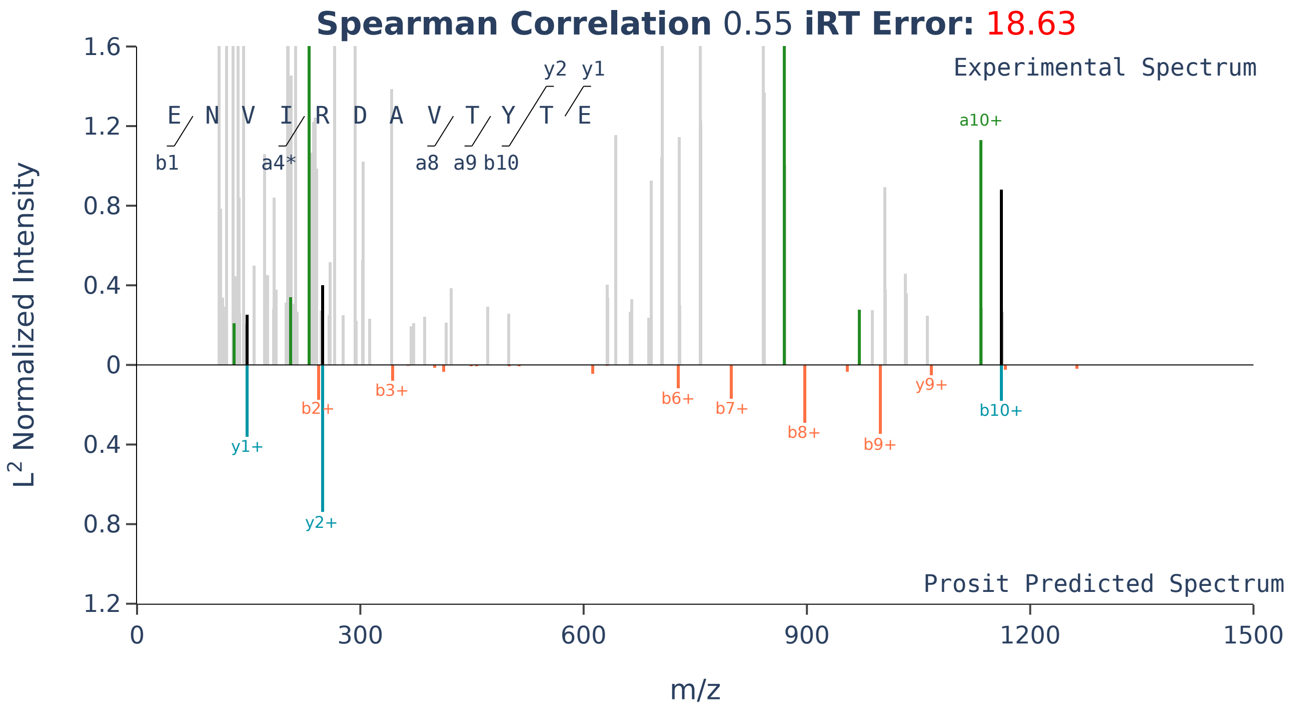

Source HRoetschke_291222_140123_HFGoe_H4_b1R1_2h **Scan** 14573
Peptide SGLIYEETRGGVKRI **Charge** 3 **Spectral Angle** 0.86
Spearman Correlation 0.93 **iRT Error:** 2.99

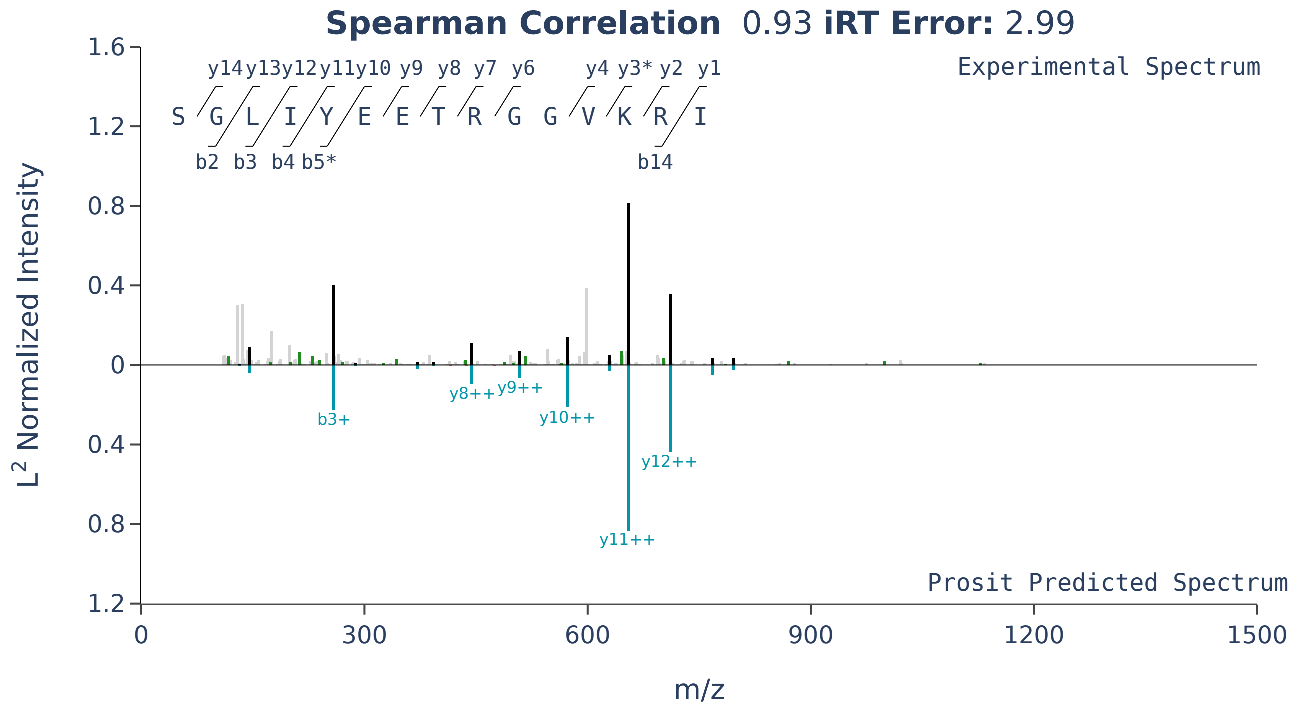

Source HRoetschke_291222_140123_HFGoe_H4_b1R1_2h **Scan** 14573
Peptide RGGVKRISGLIYEET **Charge** 3 **Spectral Angle** 0.01
Spearman Correlation 0.1 **iRT Error:** 12.84

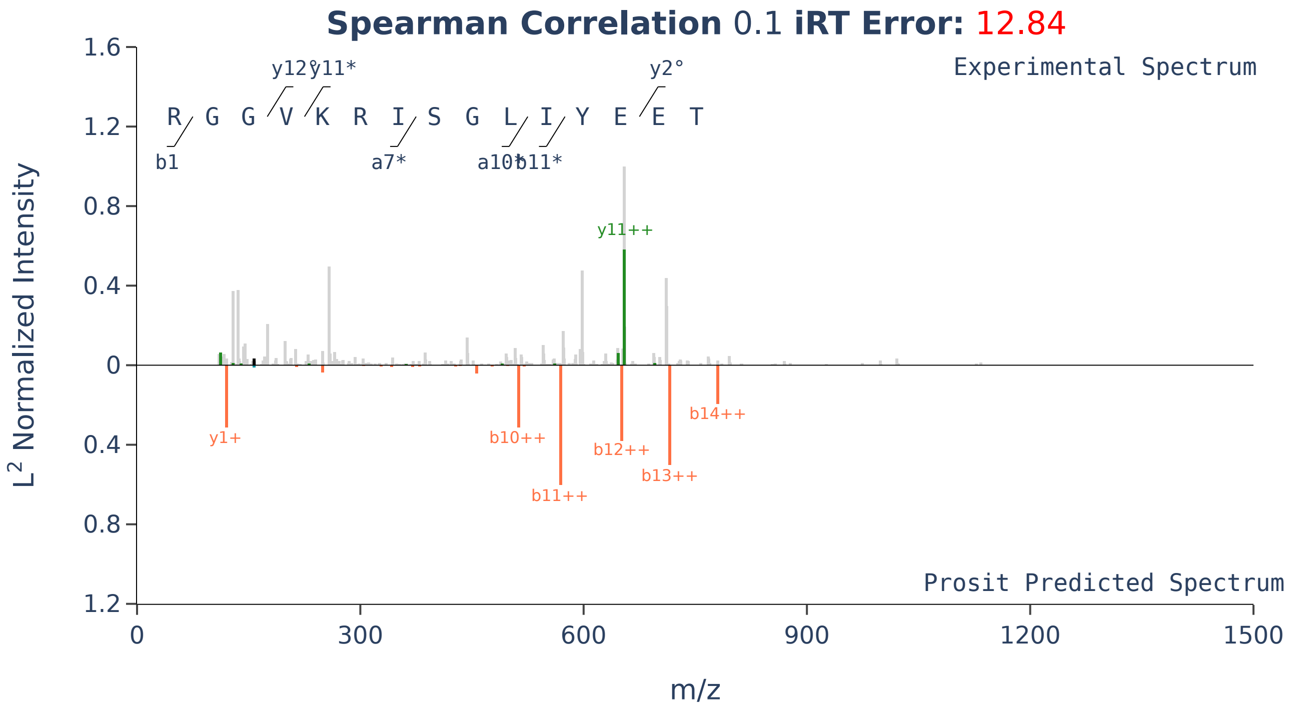

Source HRoetschke_291222_140123_HFGoe_H4_b1R2_24h **Scan** 18414
Peptide AMDVLYG **Charge** 1 **Spectral Angle** 0.92
Spearman Correlation 0.93 **iRT Error:** 3.1

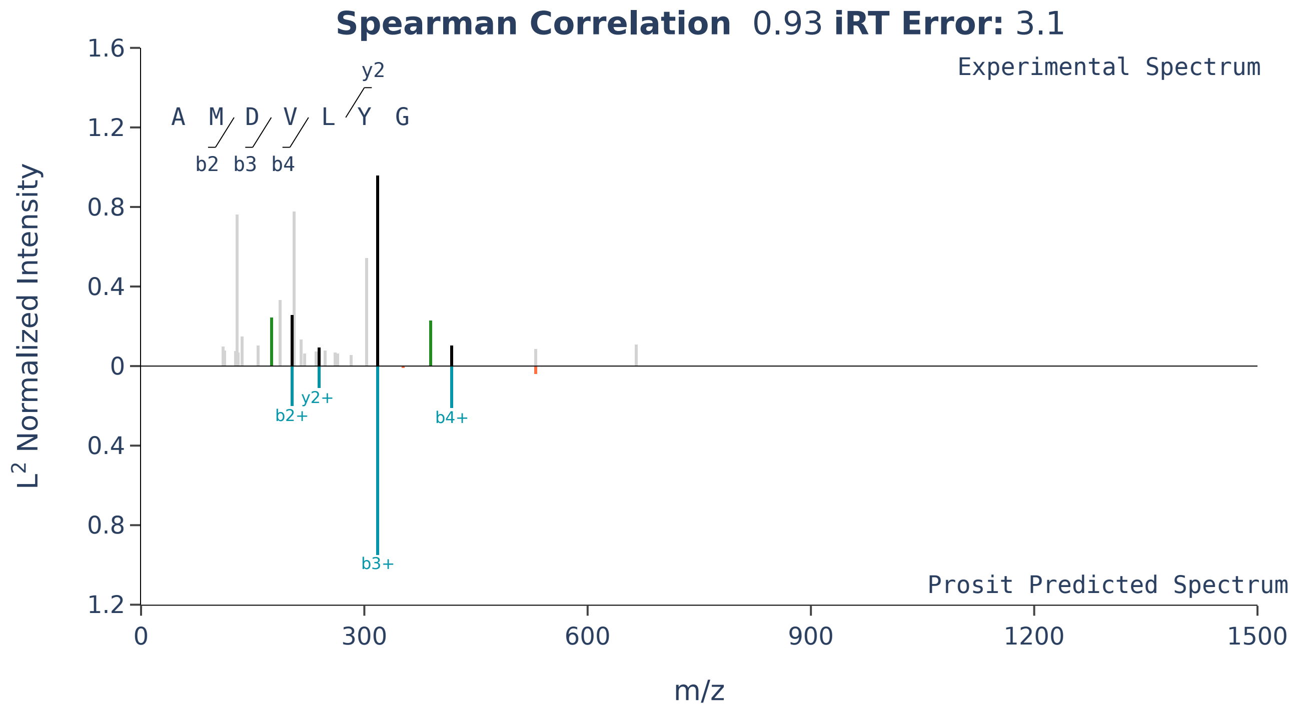

Source HRoetschke_291222_140123_HFGoe_H4_b1R2_24h **Scan** 18414
Peptide AMDVVYA **Charge** 1 **Spectral Angle** 0.86
Spearman Correlation 0.87 **iRT Error:** 16.1

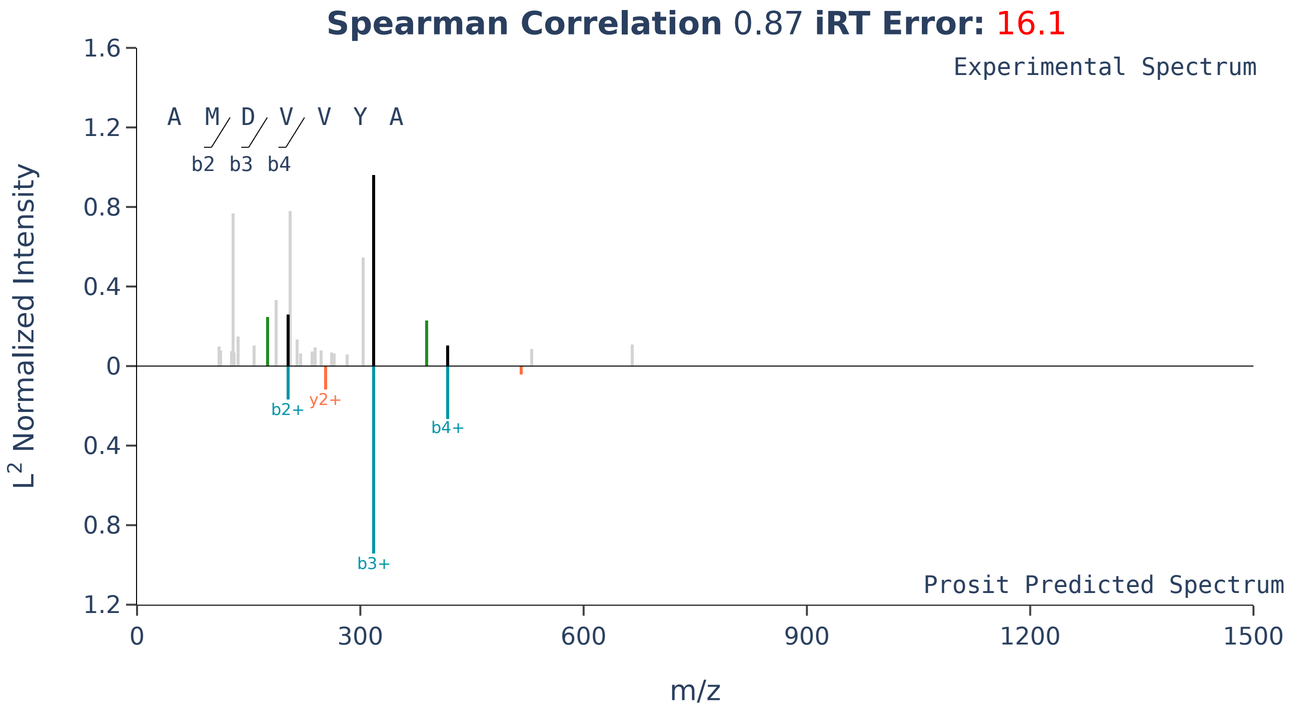

Source HRoetschke_291222_140123_HFGoe_H4_b1R2_1h **Scan** 14764
Peptide ENNIQGITKPAIRRL **Charge** 3 **Spectral Angle** 0.83
Spearman Correlation 0.89 **iRT Error:** 3.23

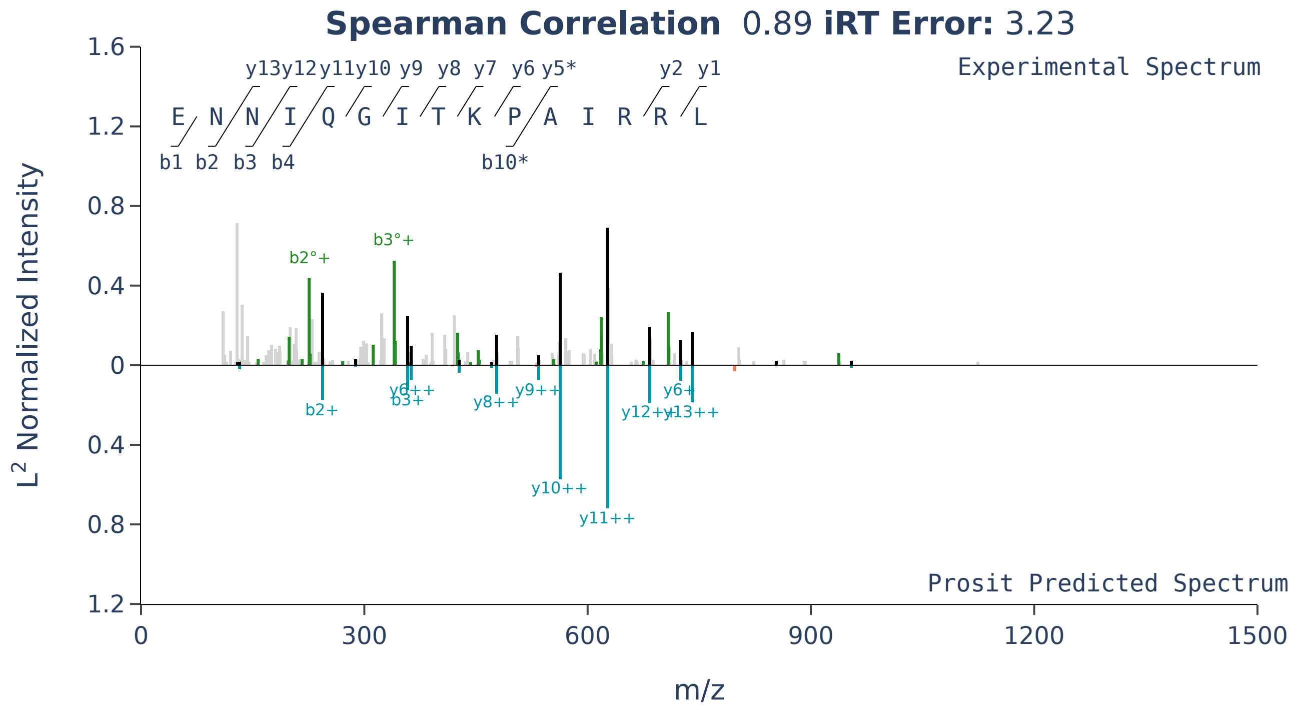

Source HRoetschke_291222_140123_HFGoe_H4_b1R2_1h **Scan** 14764
Peptide KRKTVTAMDVVYALK **Charge** 3 **Spectral Angle** 0.21
Spearman Correlation 0.24 **iRT Error:** 12.8

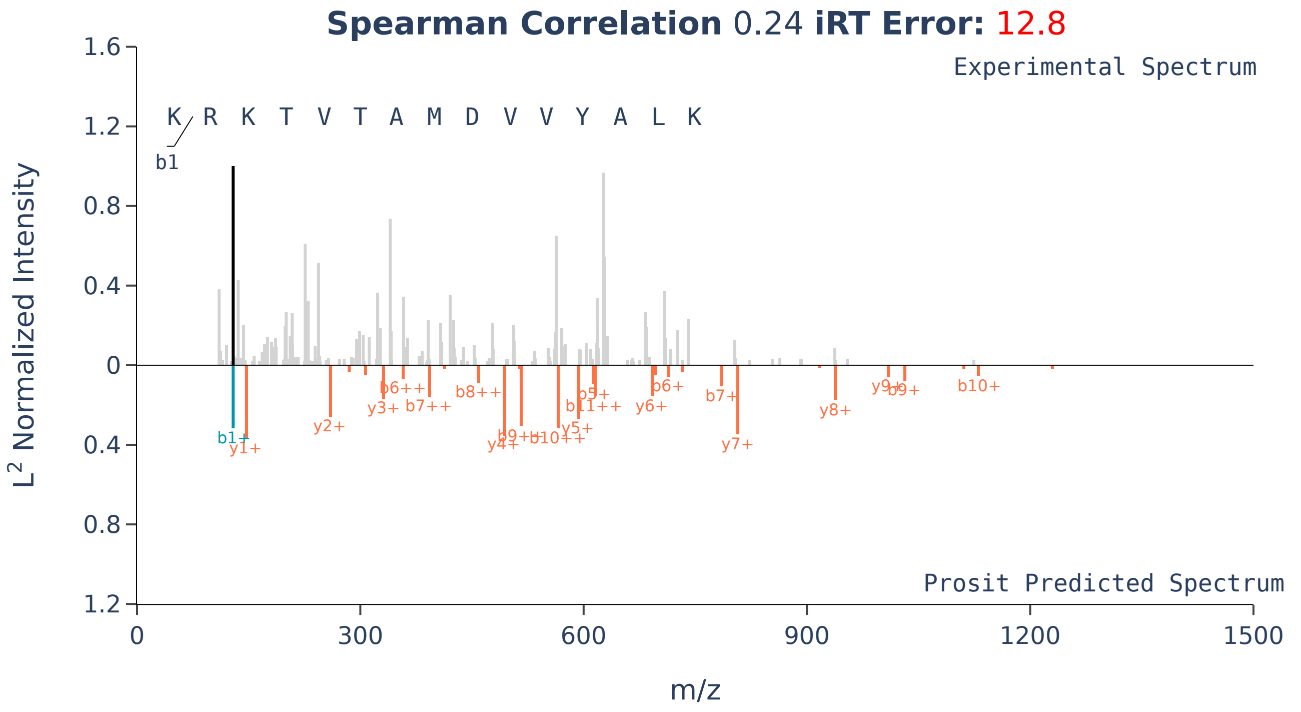

Source HRoetschke_291222_140123_HFGoe_H4_b1R2_2h **Scan** 7560
Peptide VYALKARRGG **Charge** 3 **Spectral Angle** 0.95
Spearman Correlation 0.82 **iRT Error:** 4.3

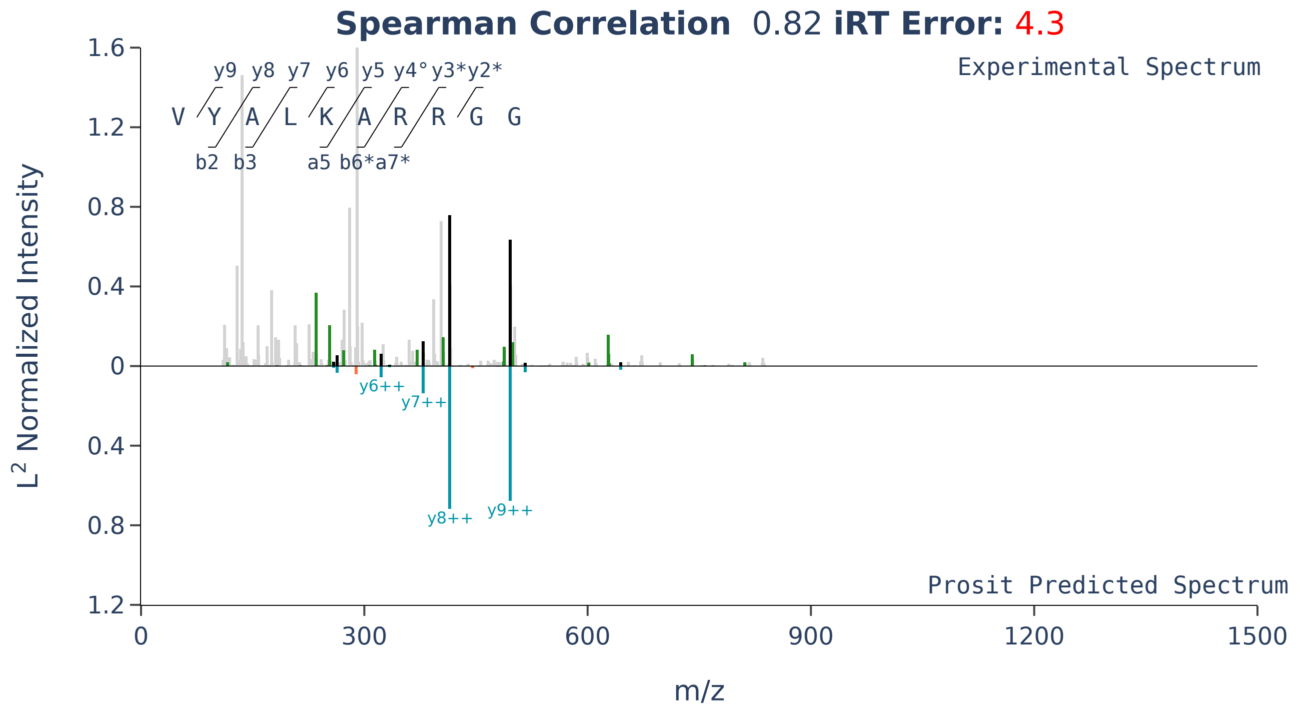

Source HRoetschke_291222_140123_HFGoe_H4_b1R2_2h **Scan** 7560
Peptide VYALKRQGR **Charge** 3 **Spectral Angle** 0.9
Spearman Correlation 0.99 **iRT Error:** 111.04

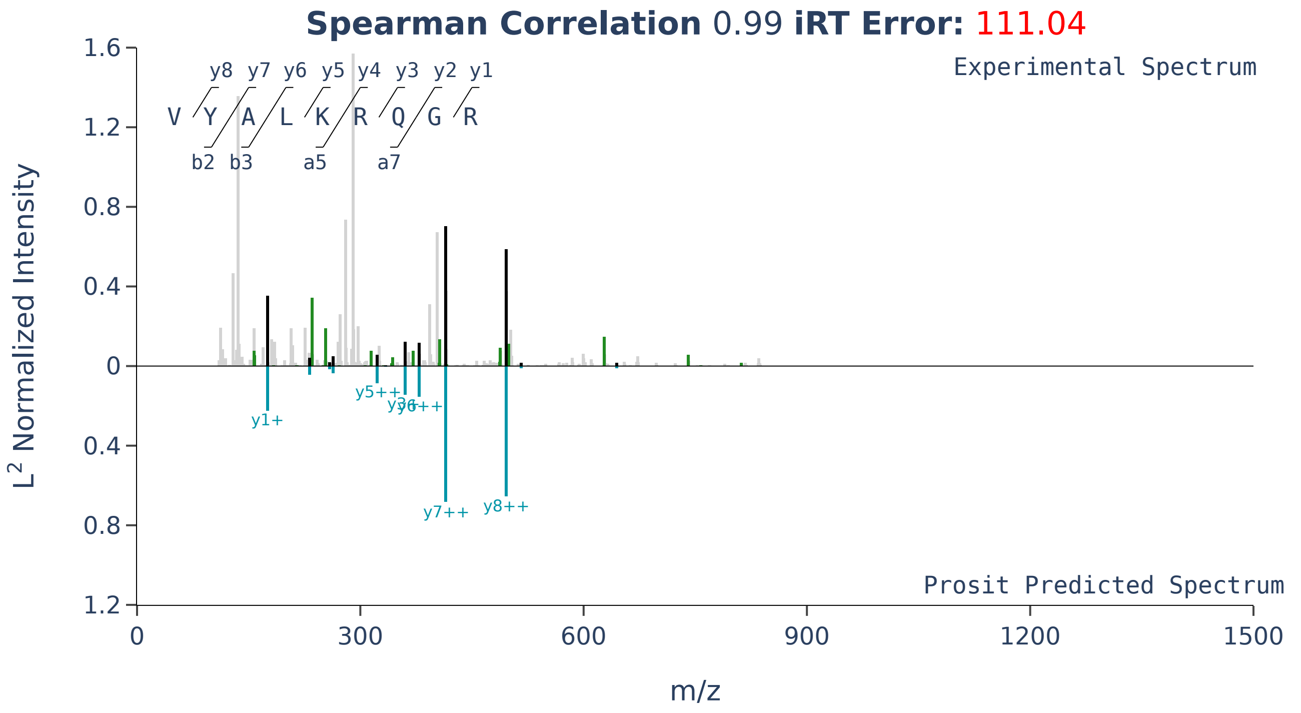

Source HRoetschke_291222_140123_HFGoe_H4_b1R1_4h **Scan** 10180
Peptide TERGVLK **Charge** 2 **Spectral Angle** 0.79
Spearman Correlation 0.9 **iRT Error:** 4.37

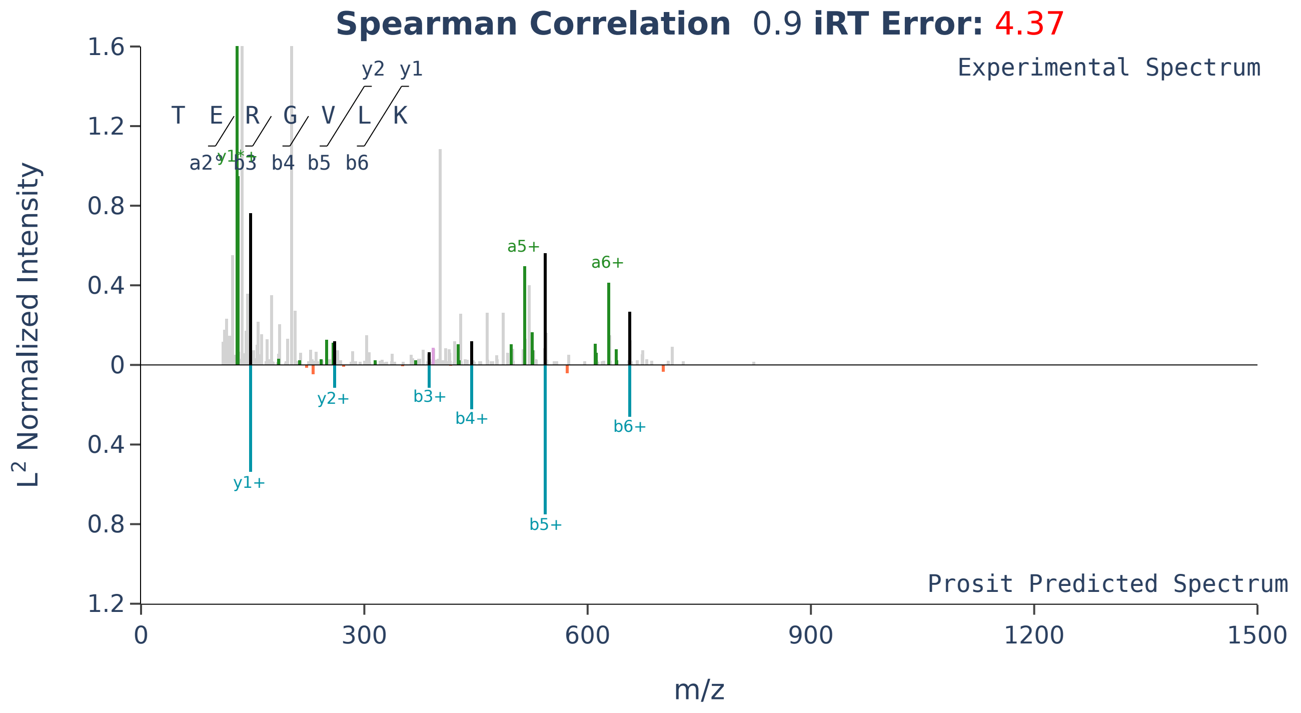

Source HRoetschke_291222_140123_HFGoe_H4_b1R1_4h **Scan** 10180
Peptide ETRGVLK **Charge** 2 **Spectral Angle** 0.86
Spearman Correlation 0.96 **iRT Error:** 106.3

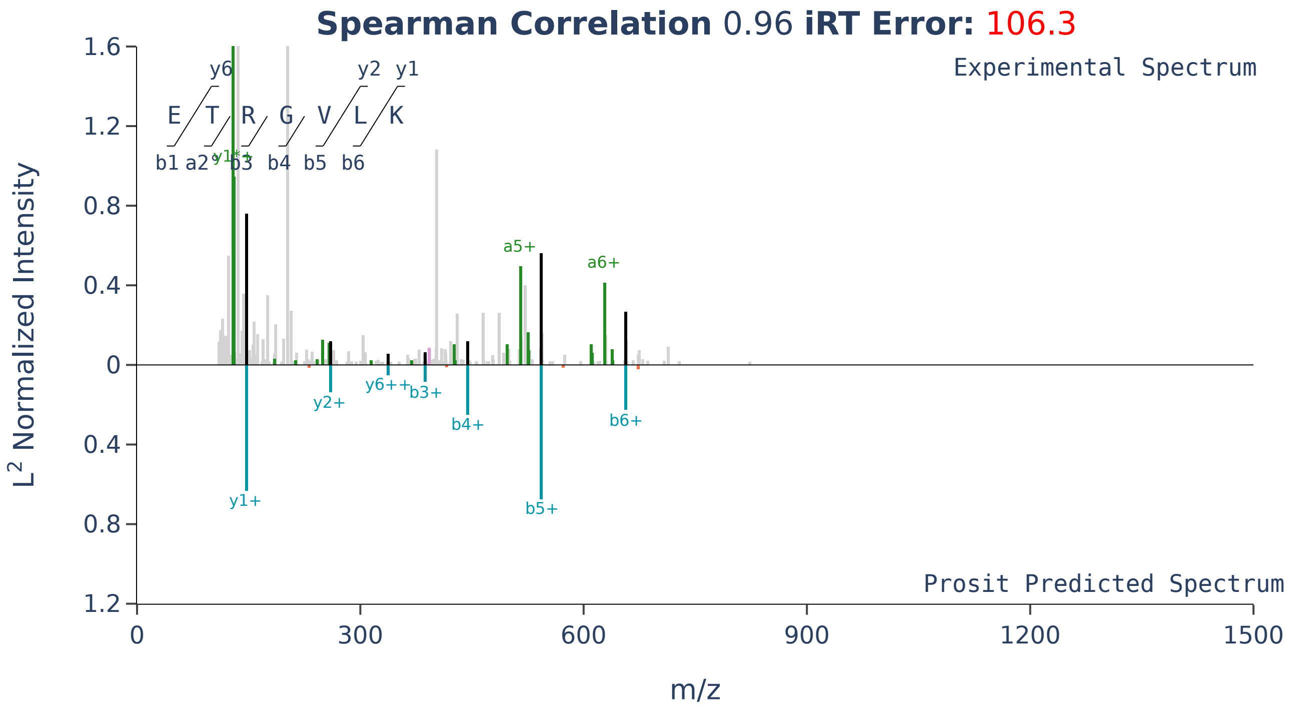

Source HRoetschke_291222_140123_HFGoe_H4_b1R1_24h **Scan** 13962
Peptide RSGLIYEET **Charge** 2 **Spectral Angle** 0.75
Spearman Correlation 0.96 **iRT Error:** 5.49

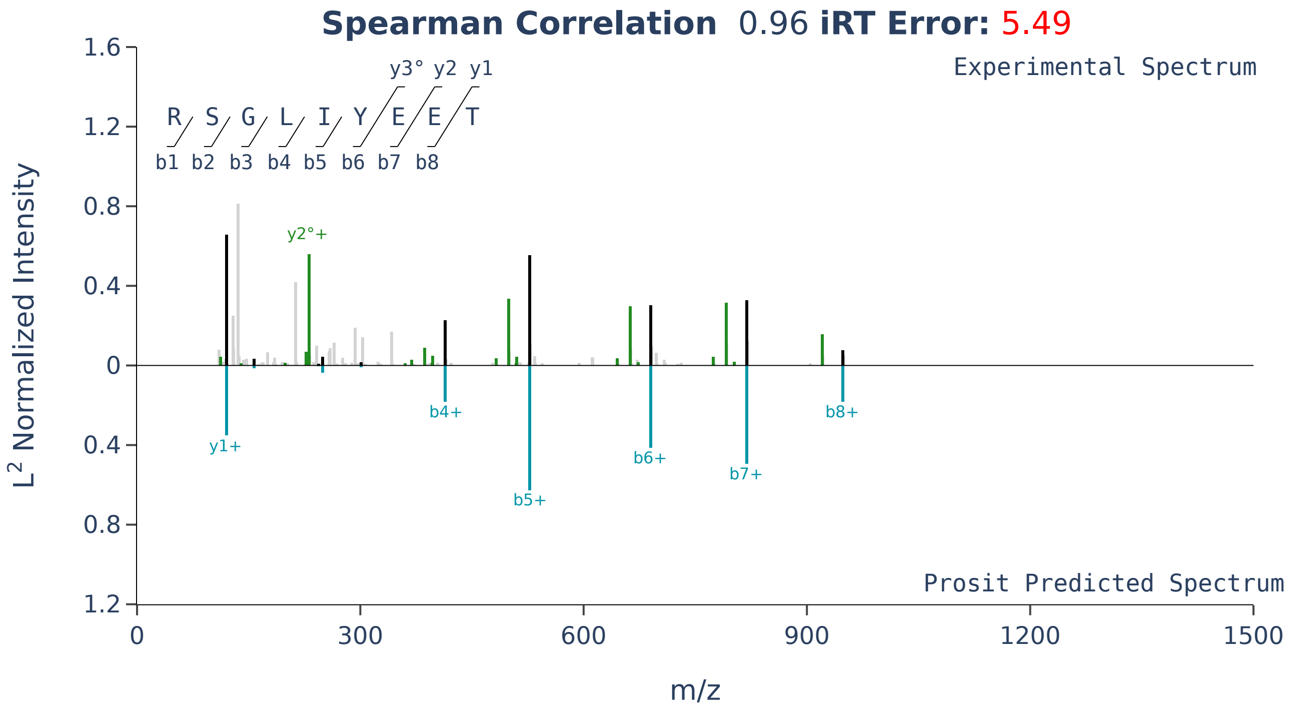

Source HRoetschke_291222_140123_HFGoe_H4_b1R1_24h **Scan** 13962
Peptide IRDAVTYTE **Charge** 2 **Spectral Angle** 0.29
Spearman Correlation 0.39 **iRT Error:** 21.32

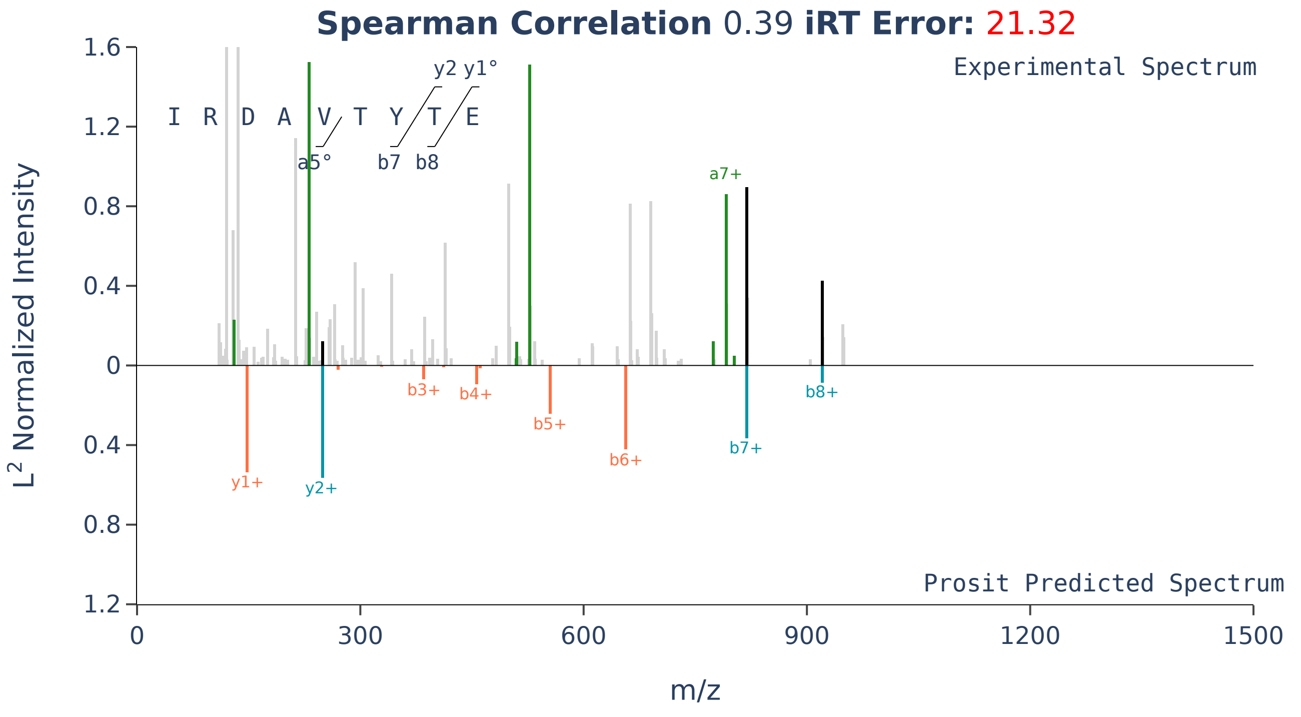

Source HRoetschke_291222_140123_HFGoe_H4_b1R1_2h **Scan** 19779
Peptide TKVTAMDVVY **Charge** 2 **Spectral Angle** 0.8
Spearman Correlation 0.96 **iRT Error:** 6.87

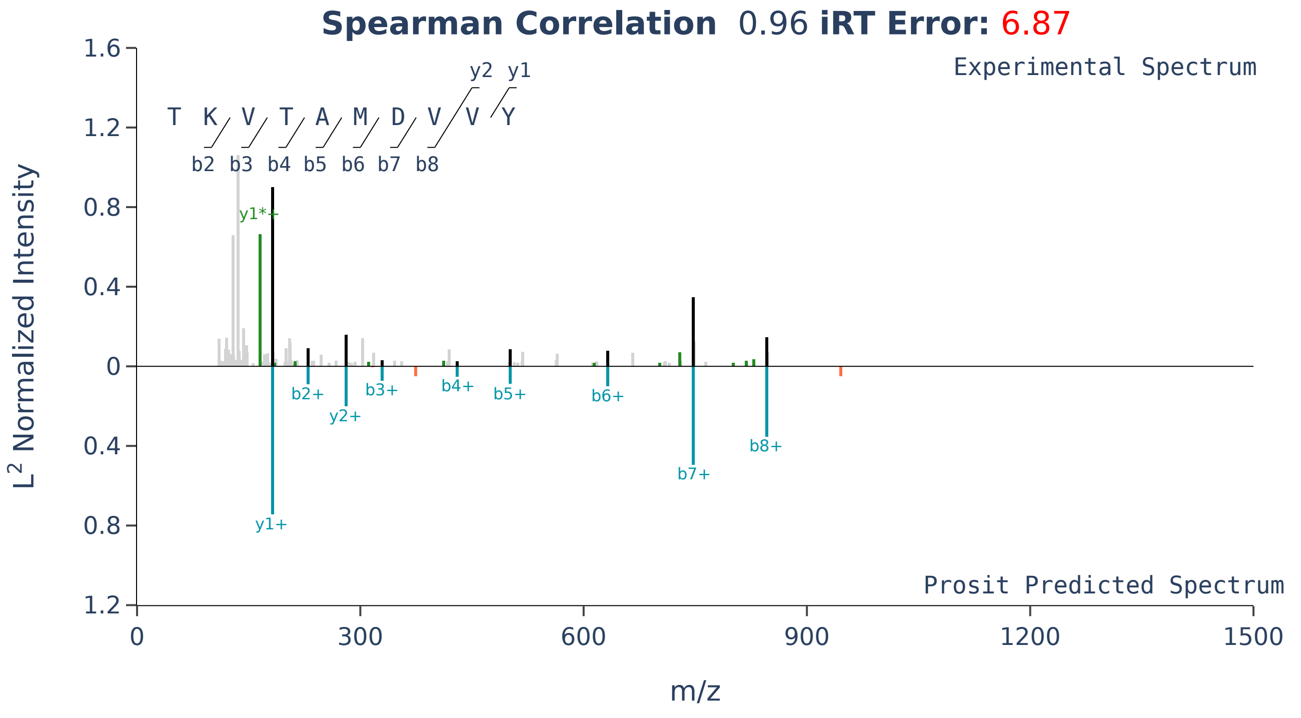

Source HRoetschke_291222_140123_HFGoe_H4_b1R1_2h **Scan** 19779
Peptide KTVTAMDVVY **Charge** 2 **Spectral Angle** 0.58
Spearman Correlation 0.63 **iRT Error:** 7.84

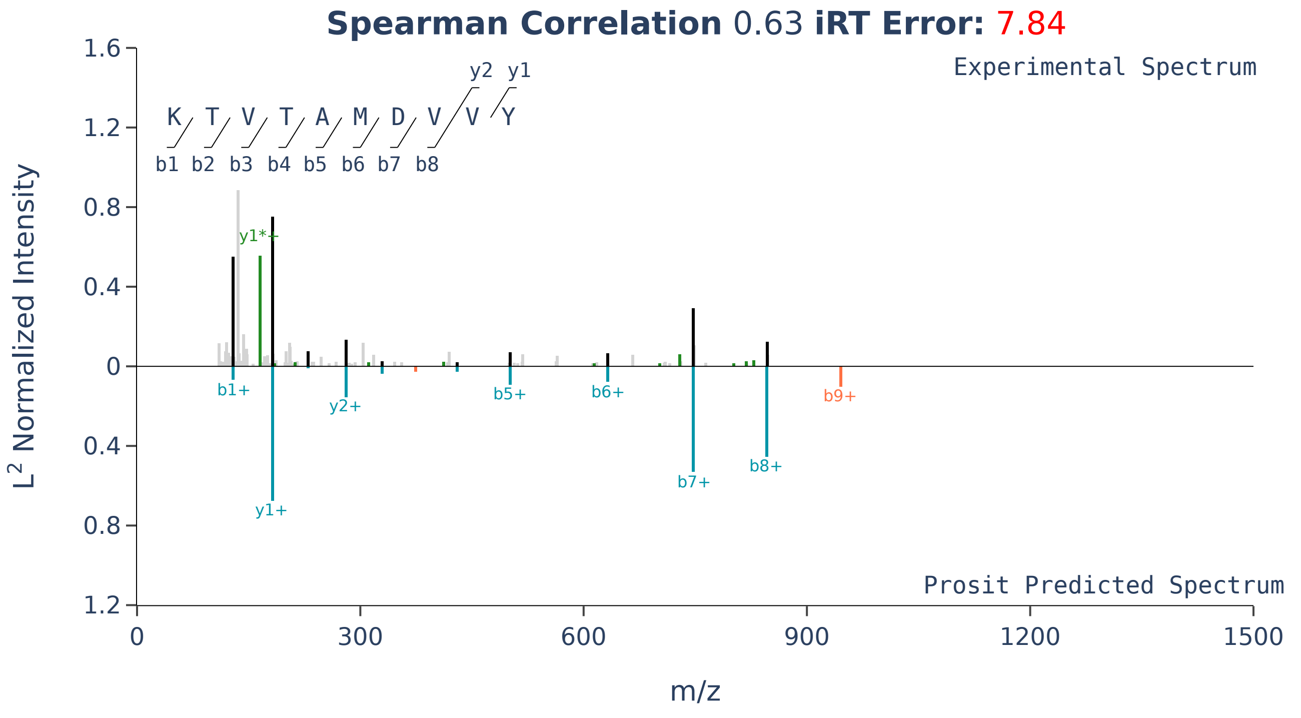

Source HRoetschke_291222_140123_HFGoe_H4_b1R2_2h **Scan** 10829
Peptide GITKPAIAIRR **Charge** 3 **Spectral Angle** 0.87
Spearman Correlation 0.93 **iRT Error:** 8.99

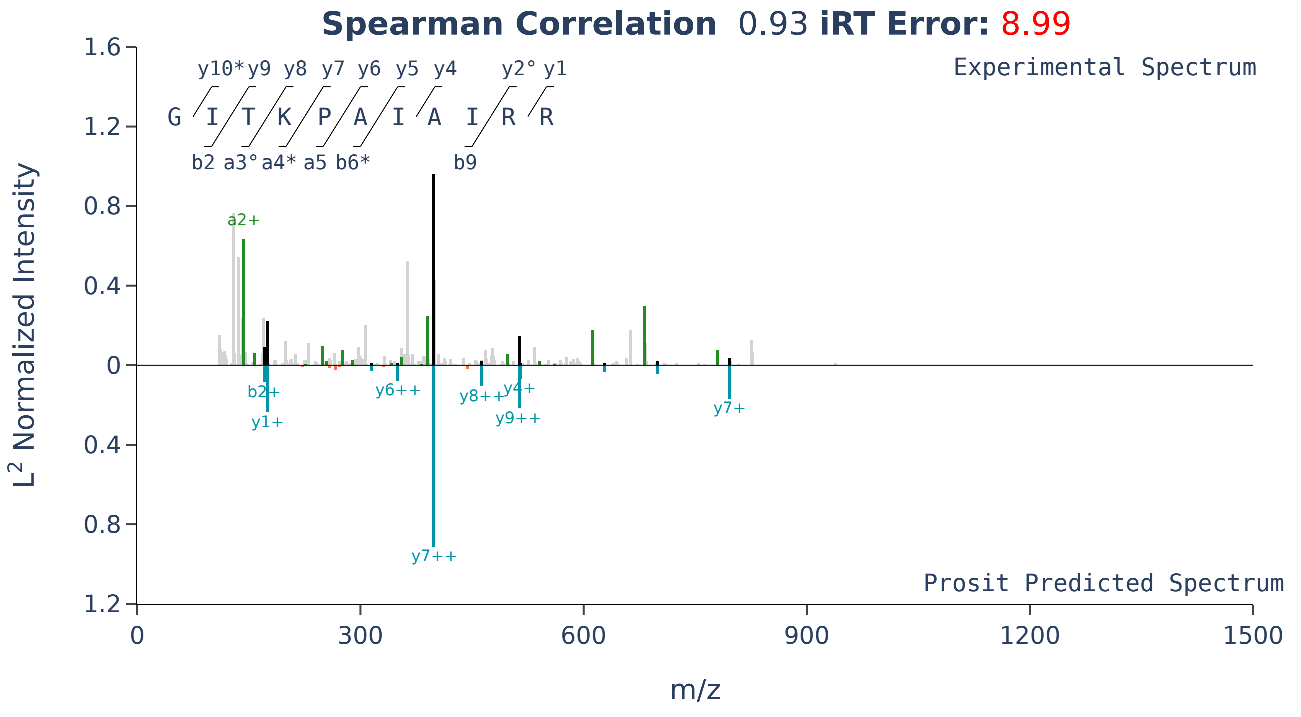

Source HRoetschke_291222_140123_HFGoe_H4_b1R2_2h **Scan** 10829
Peptide GITKPAIRRLA **Charge** 3 **Spectral Angle** 0.46
Spearman Correlation 0.68 **iRT Error:** 8.57

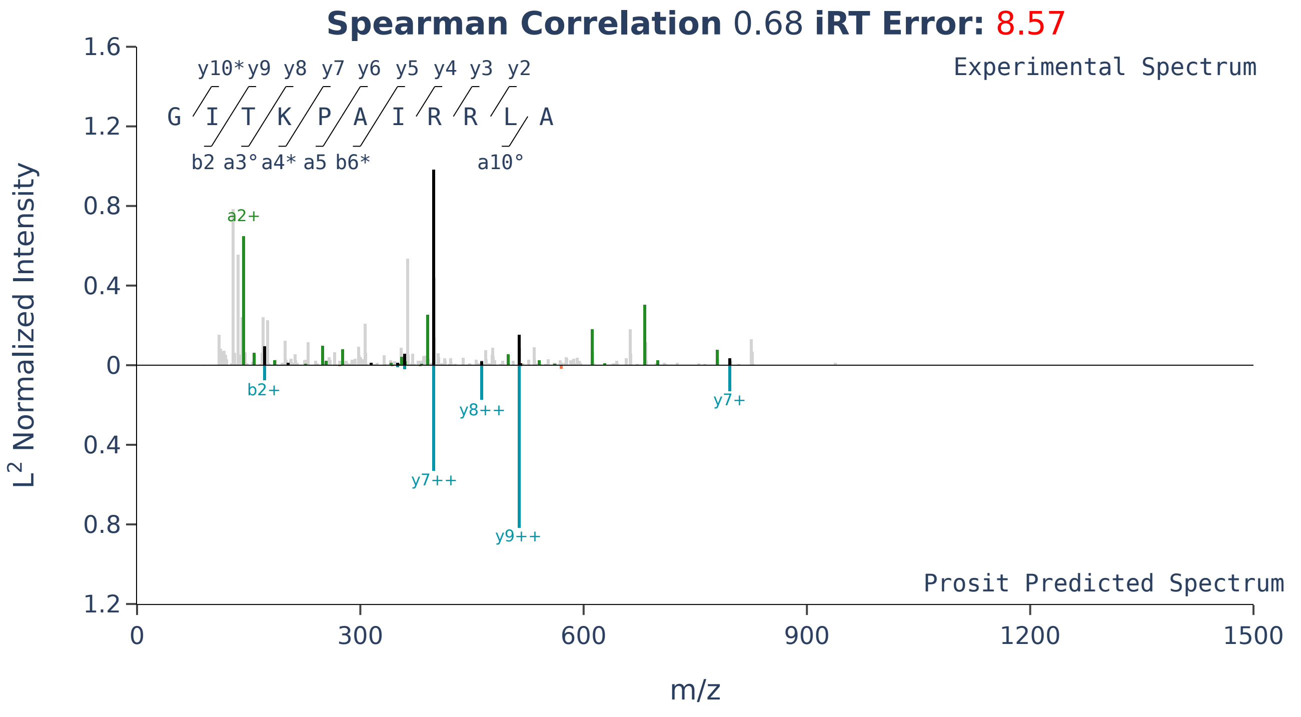

Source HRoetschke_291222_140123_HFGoe_H4_b2R2_24h **Scan** 10371
Peptide LAKRQGRTL **Charge** 2 **Spectral Angle** 0.96
Spearman Correlation 0.92 **iRT Error:** 9.26

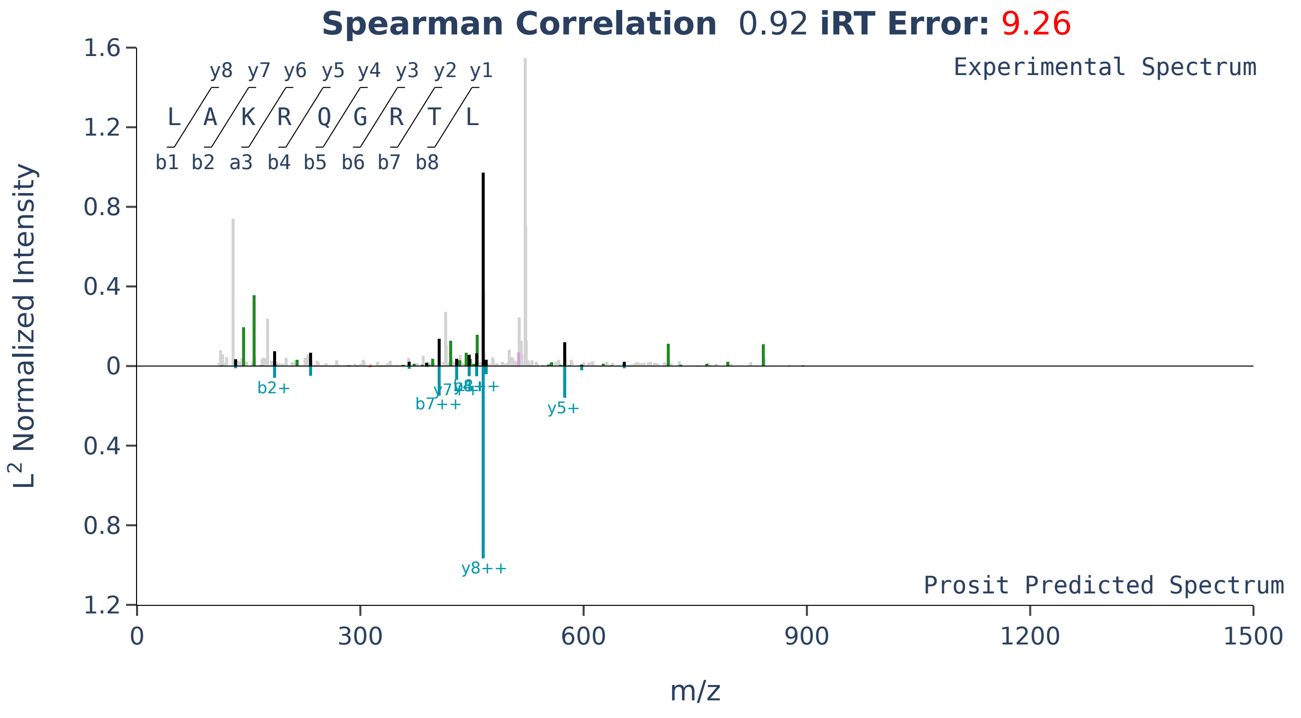

Source HRoetschke_291222_140123_HFGoe_H4_b2R2_24h **Scan** 10371
Peptide RGGVKRISGL **Charge** 2 **Spectral Angle** 0.28
Spearman Correlation 0.06 **iRT Error:** 12.92

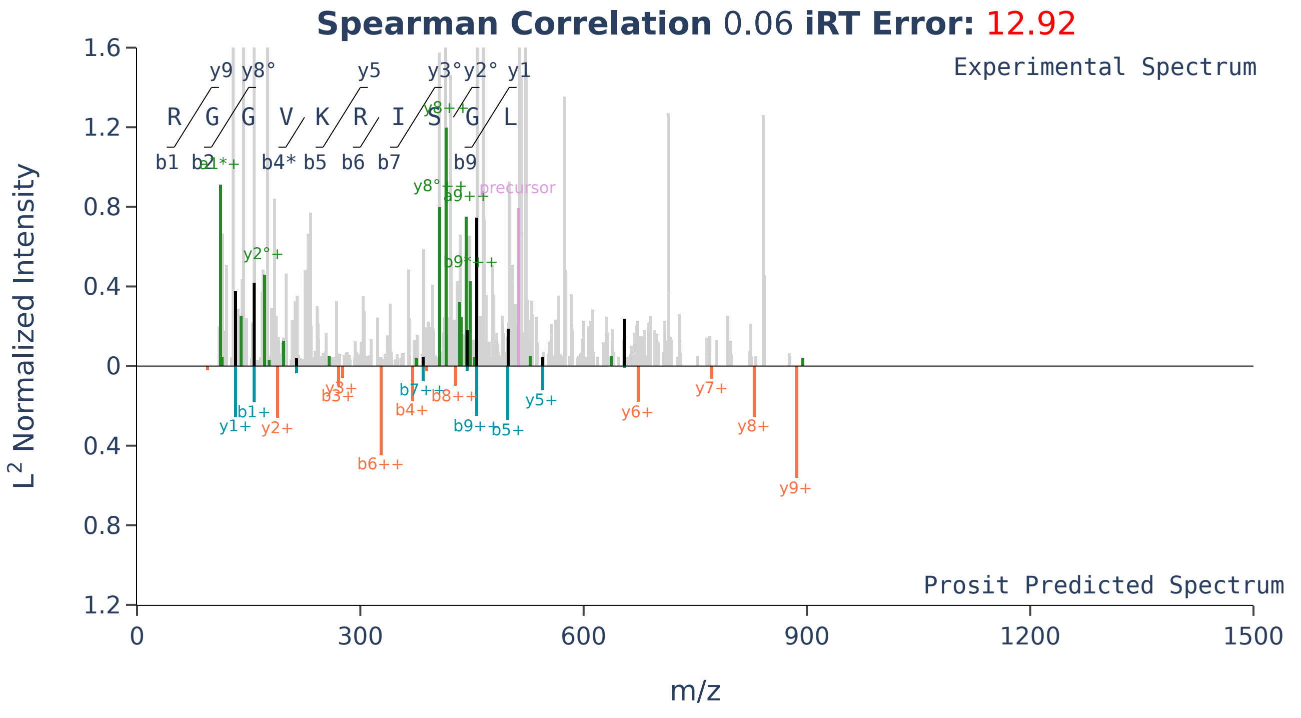

Source HRoetschke_291222_140123_HFGoe_H4_b1R1_24h **Scan** 6651
Peptide GRNIQGITK **Charge** 2 **Spectral Angle** 0.87
Spearman Correlation 0.95 **iRT Error:** 10.85

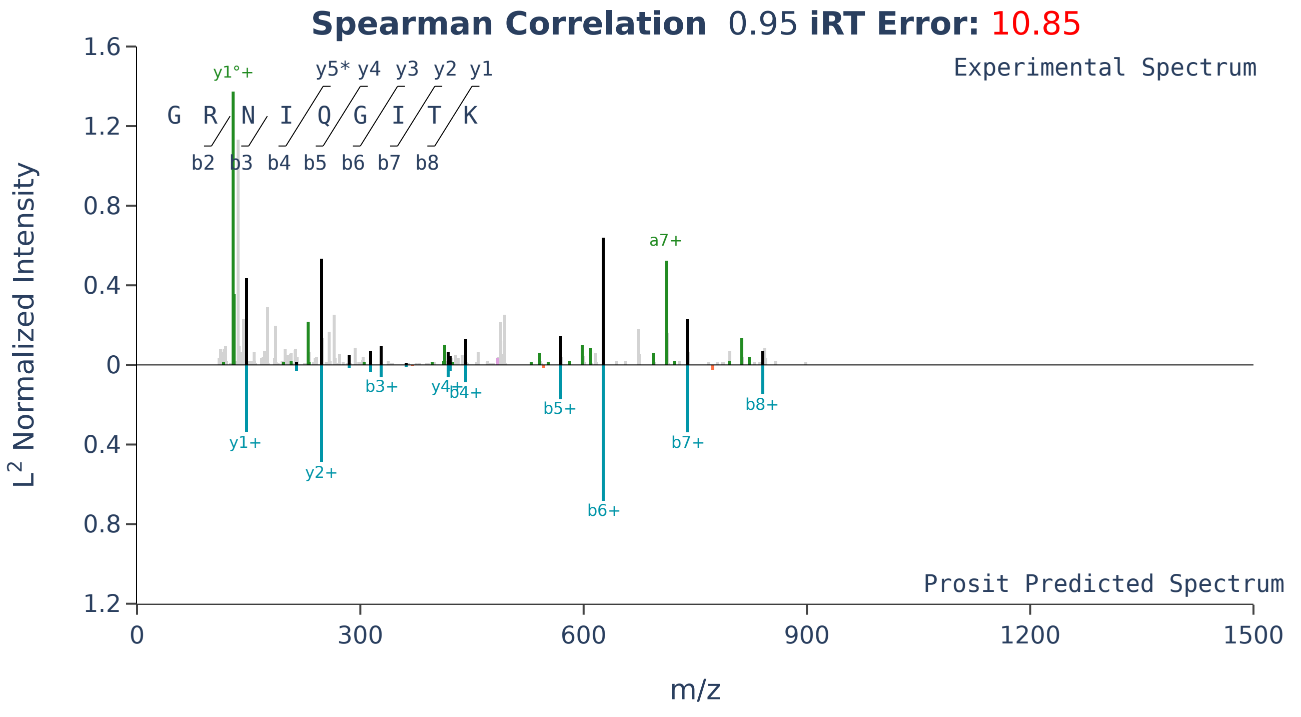

Source HRoetschke_291222_140123_HFGoe_H4_b1R1_24h **Scan** 6651
Peptide GKGGKGLGKGGA **Charge** 2 **Spectral Angle** 0.27
Spearman Correlation 0.03 **iRT Error:** 4.08

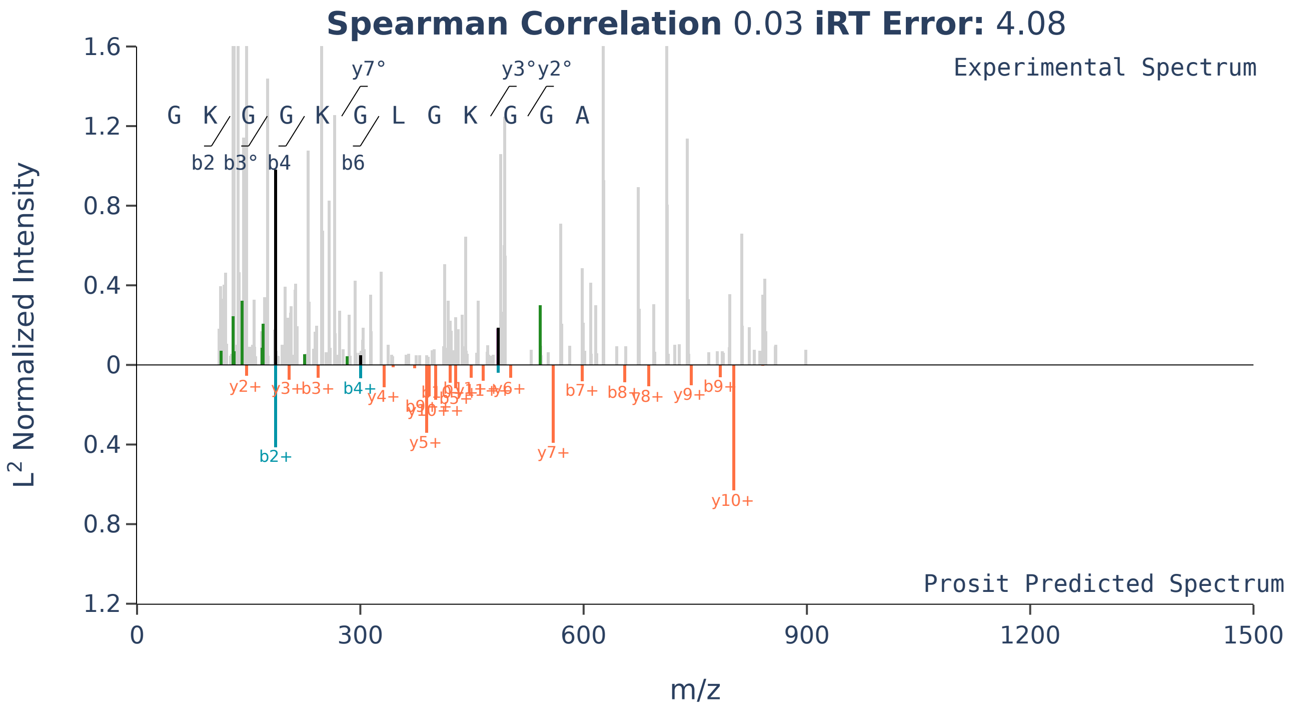

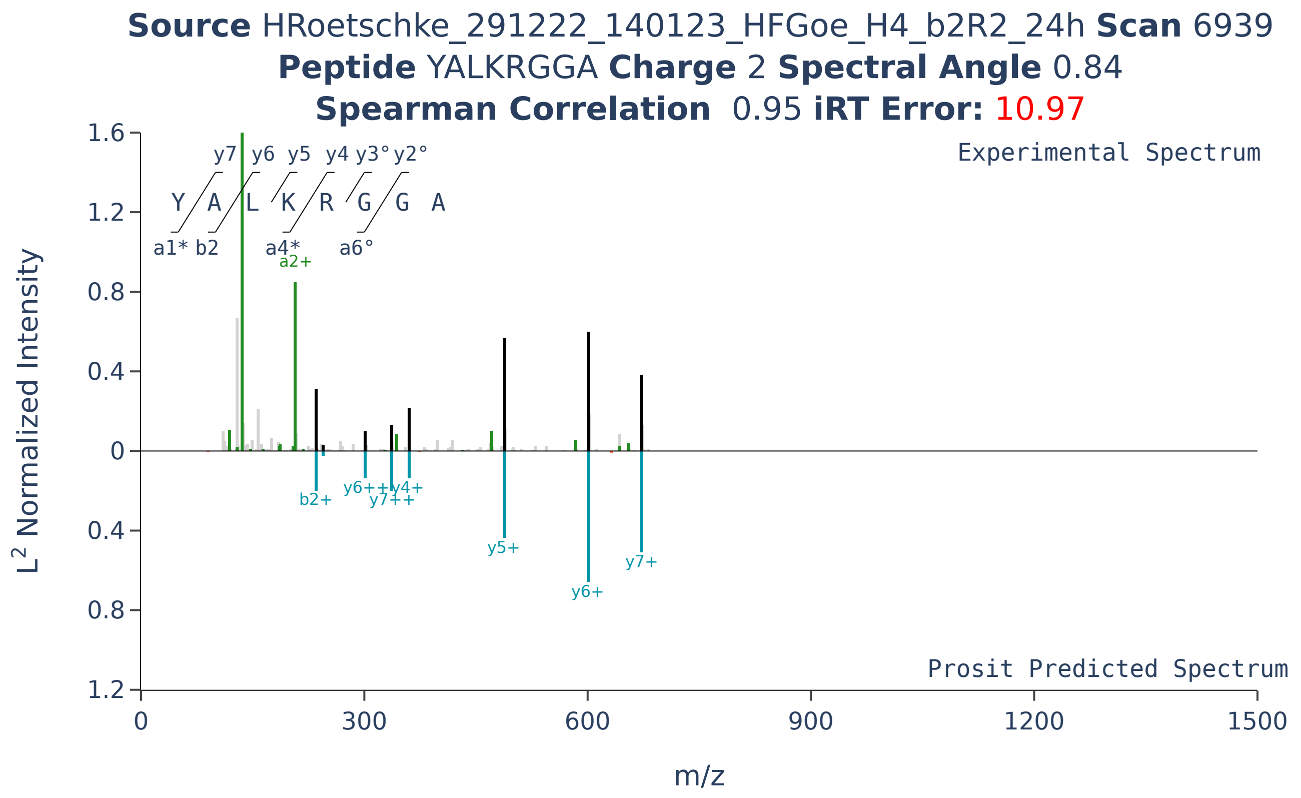
Source HRoetschke_291222_140123_HFGoe_H4_b2R2_24h **Scan** 6939
Peptide YALKRGGA **Charge** 2 **Spectral Angle** 0.84
Spearman Correlation 0.95 **iRT Error:** 10.97
Experimental Spectrum
Prosit Predicted Spectrum
L² Normalized Intensity
m/z

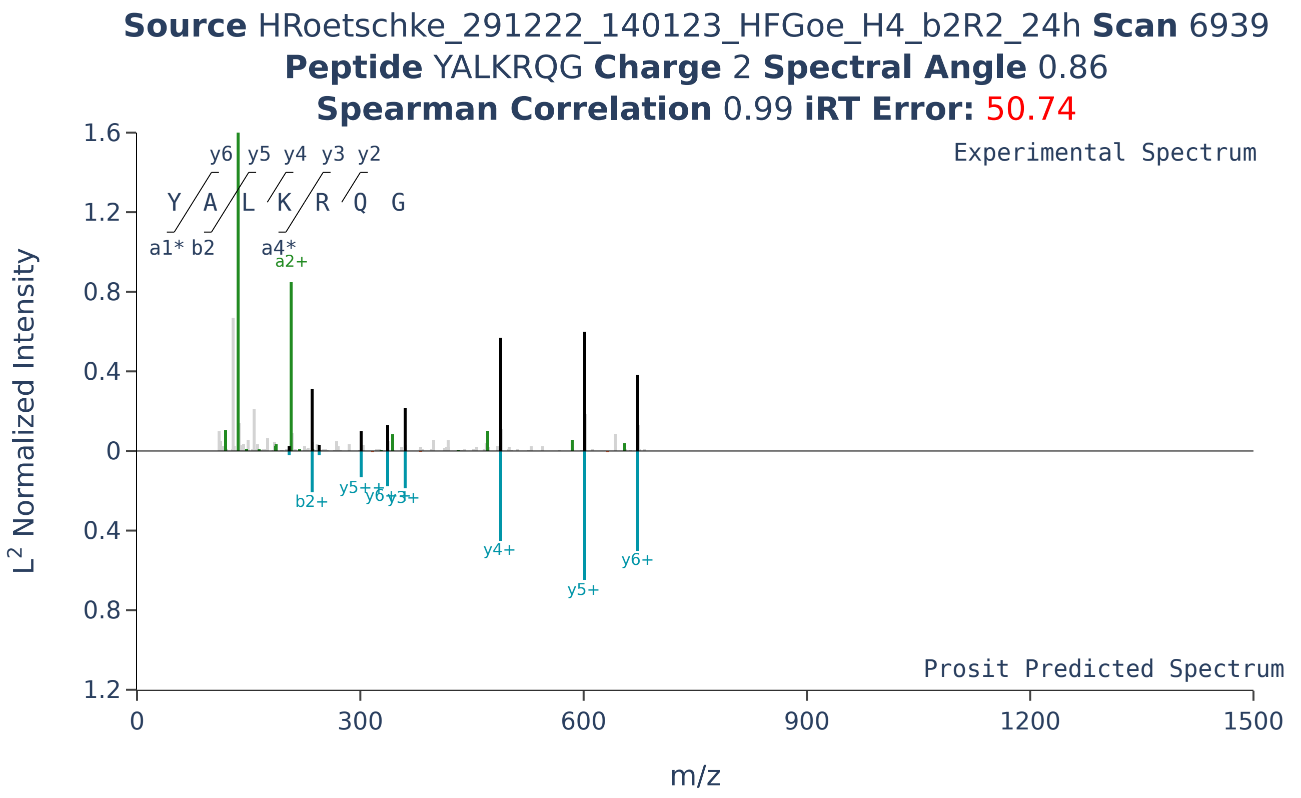
Source HRoetschke_291222_140123_HFGoe_H4_b2R2_24h **Scan** 6939
Peptide YALKRQG **Charge** 2 **Spectral Angle** 0.86
Spearman Correlation 0.99 **iRT Error:** 50.74
Experimental Spectrum
Prosit Predicted Spectrum
L² Normalized Intensity
m/z

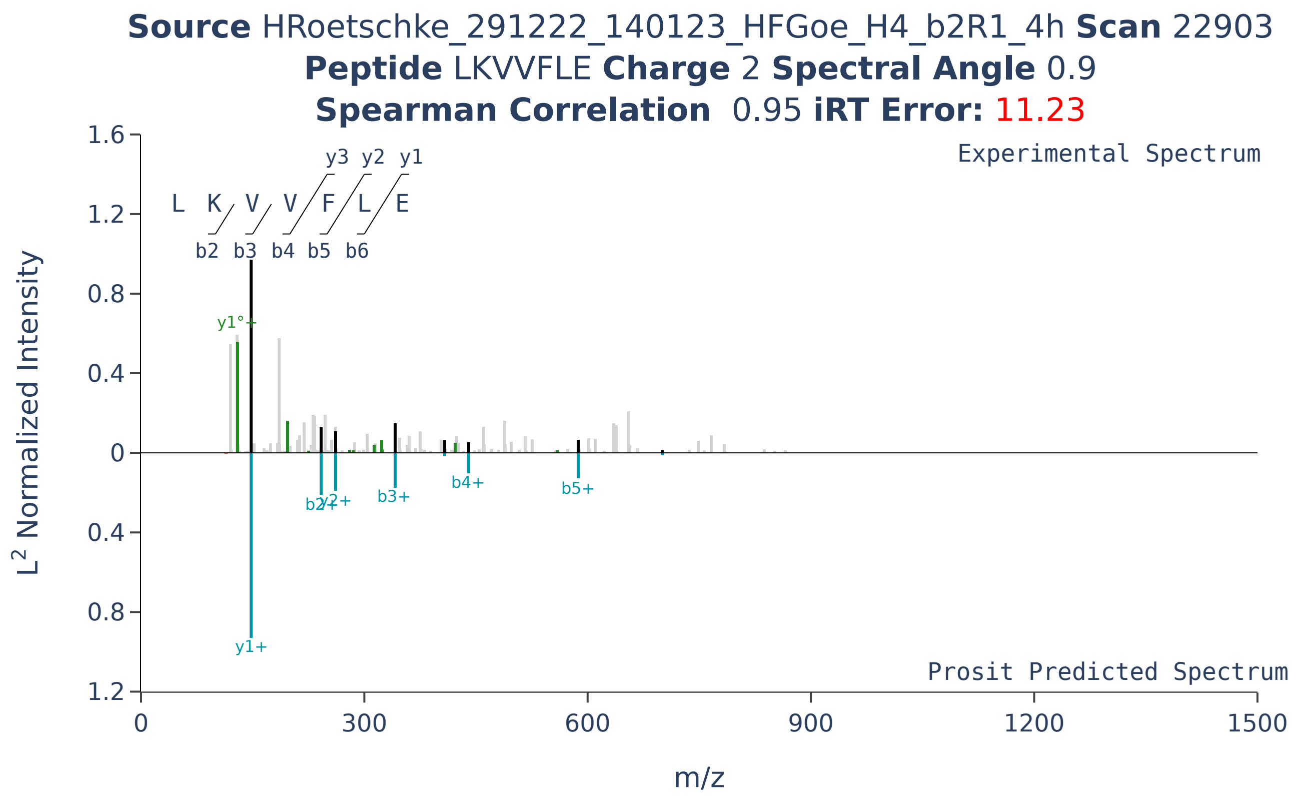
Source HRoetschke_291222_140123_HFGoe_H4_b2R1_4h **Scan** 22903
Peptide LKVVFLE **Charge** 2 **Spectral Angle** 0.9
Spearman Correlation 0.95 **iRT Error:** 11.23
Experimental Spectrum
Prosit Predicted Spectrum
L² Normalized Intensity
m/z

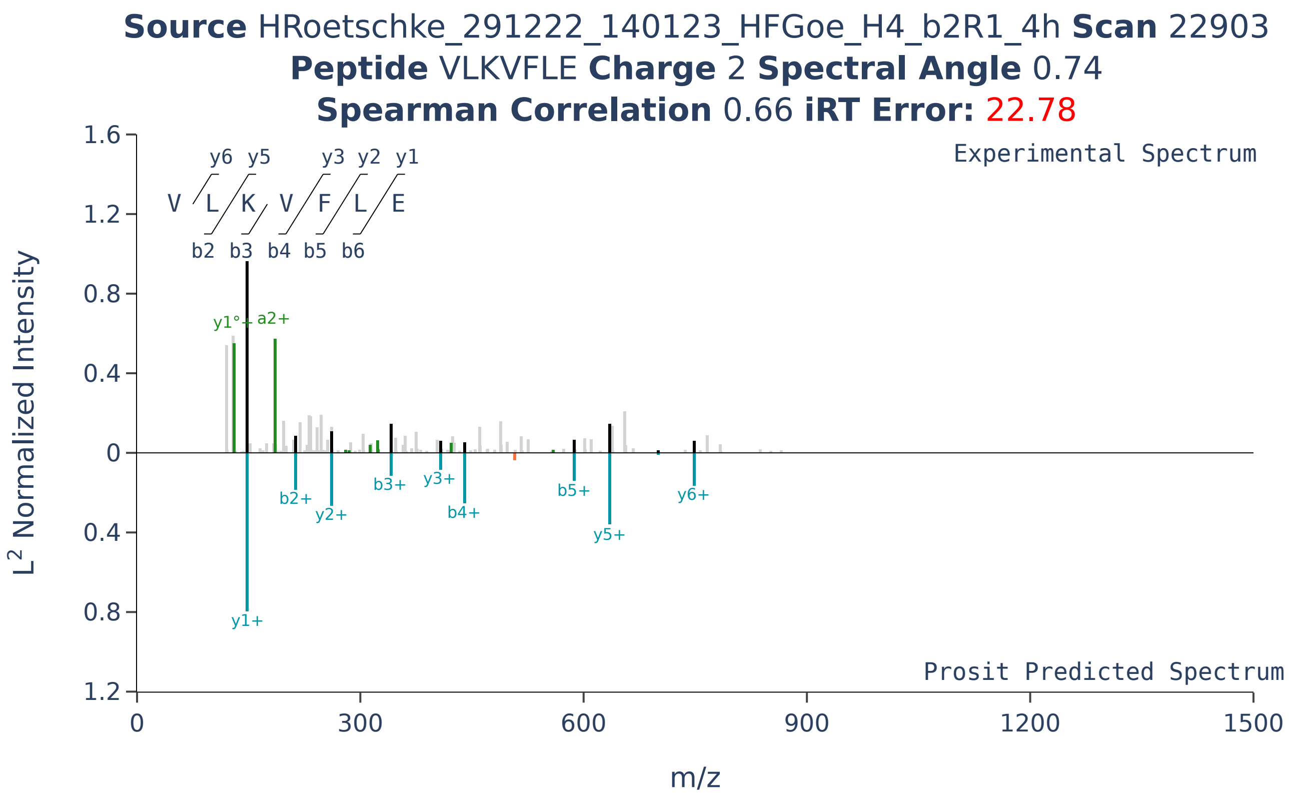
Source HRoetschke_291222_140123_HFGoe_H4_b2R1_4h **Scan** 22903
Peptide VLKVFLE **Charge** 2 **Spectral Angle** 0.74
Spearman Correlation 0.66 **iRT Error:** 22.78
Experimental Spectrum
Prosit Predicted Spectrum
L² Normalized Intensity
m/z

Source WSoh_221121_011221_HFGoe_L1_hTau_1h_R2 **Scan** 12661
Peptide QSKLDFKDRV **Charge** 3 **Spectral Angle** 0.85
Spearman Correlation 0.85 **iRT Error:** 0.0

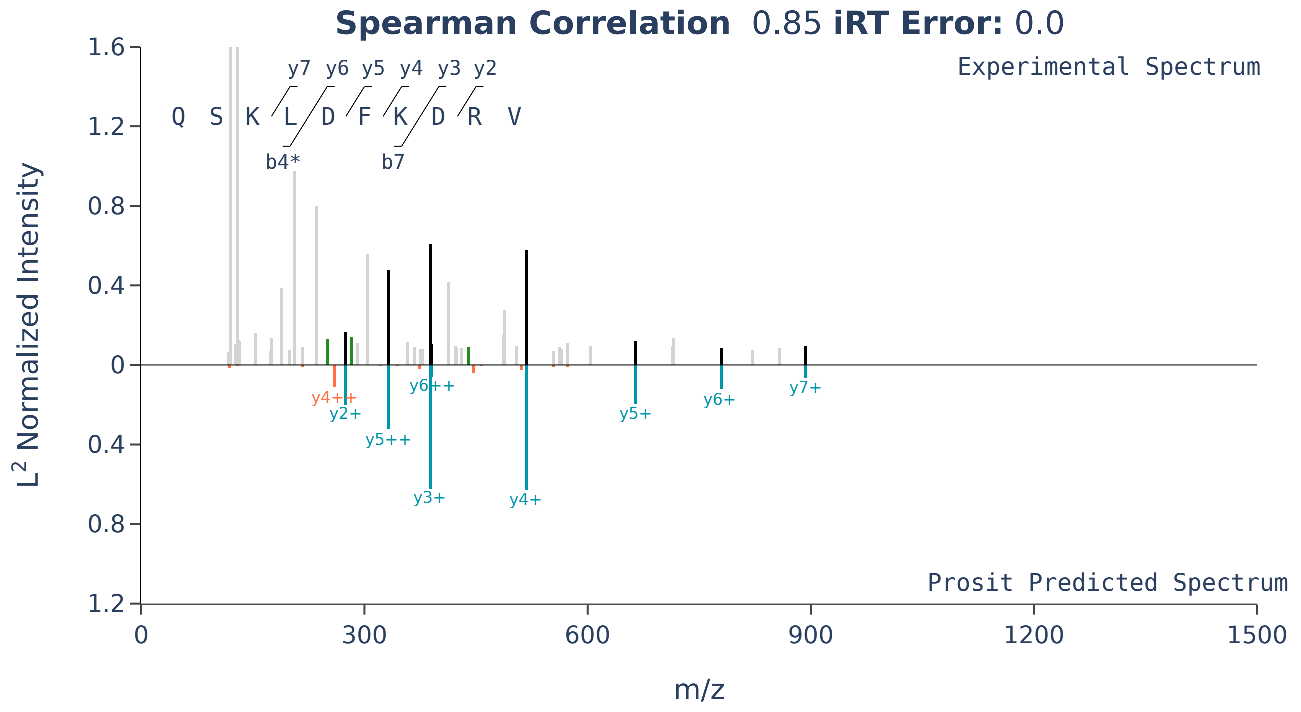

Source WSoh_221121_011221_HFGoe_L1_hTau_1h_R2 **Scan** 12661
Peptide LDFKDRVQSK **Charge** 3 **Spectral Angle** 0.46
Spearman Correlation 0.48 **iRT Error:** 15.12

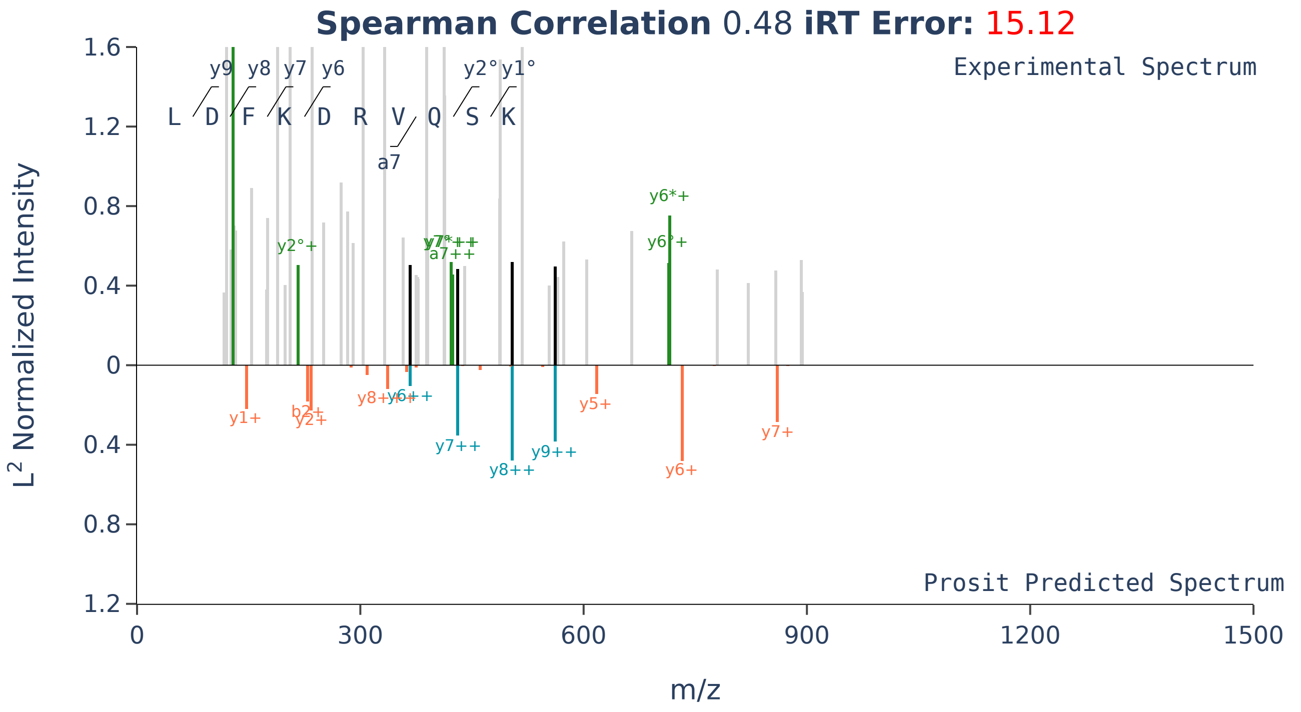

Source WSoh_221121_011221_HFGoe_L1_hTau_24h_R2 **Scan** 16837
Peptide APVPMPDLKNVKGGK **Charge** 3 **Spectral Angle** 0.75
Spearman Correlation 0.9 **iRT Error:** 0.03

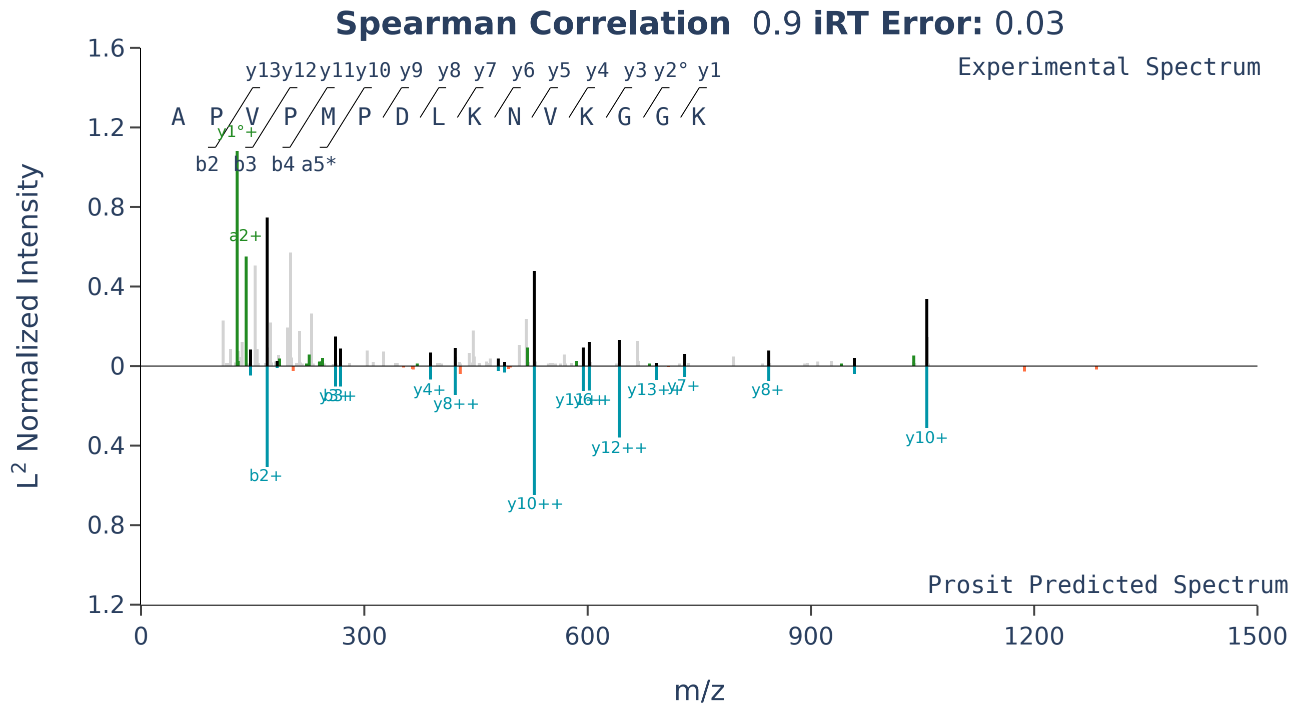

Source WSoh_221121_011221_HFGoe_L1_hTau_24h_R2 **Scan** 16837
Peptide ADGKTKIATPRGAAPP **Charge** 3 **Spectral Angle** 0.01
Spearman Correlation -0.07 **iRT Error:** 43.03

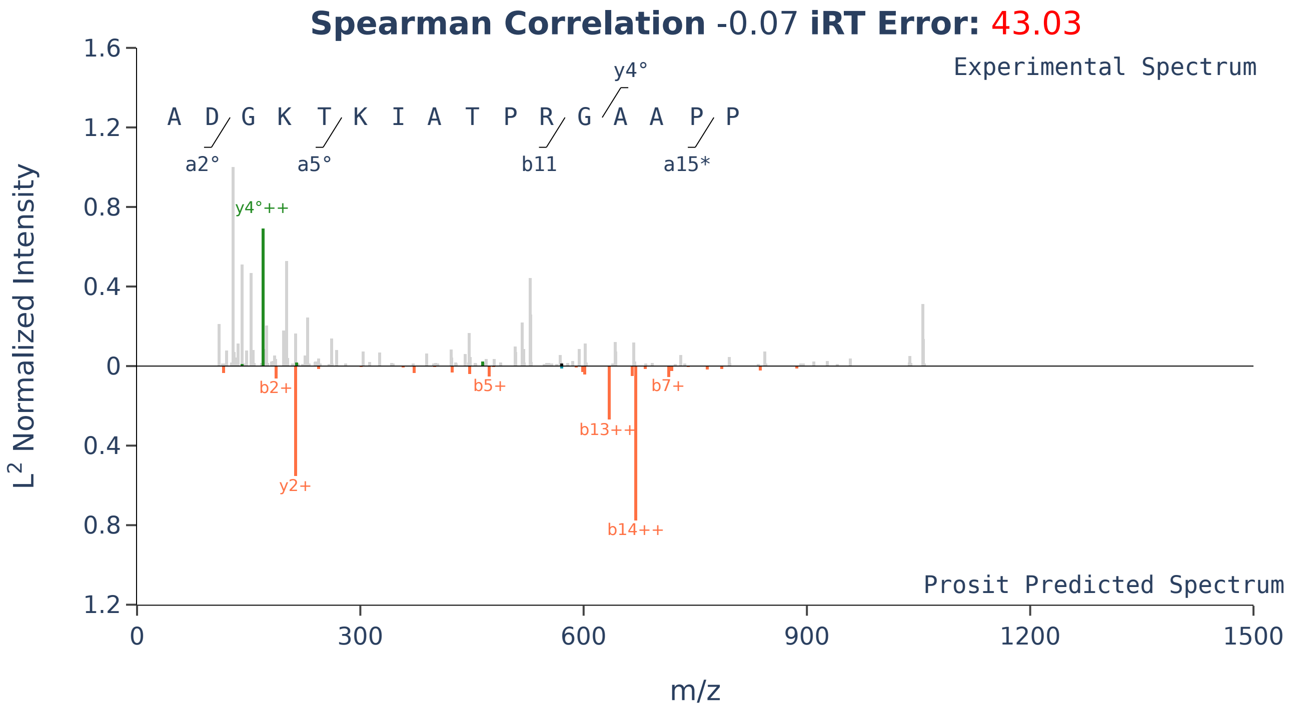

Source WSoh_221121_011221_HFGoe_L2_hTau_1h_R2 **Scan** 9418
Peptide DFKDRVQSA **Charge** 2 **Spectral Angle** 0.89
Spearman Correlation 0.95 **iRT Error:** 0.03

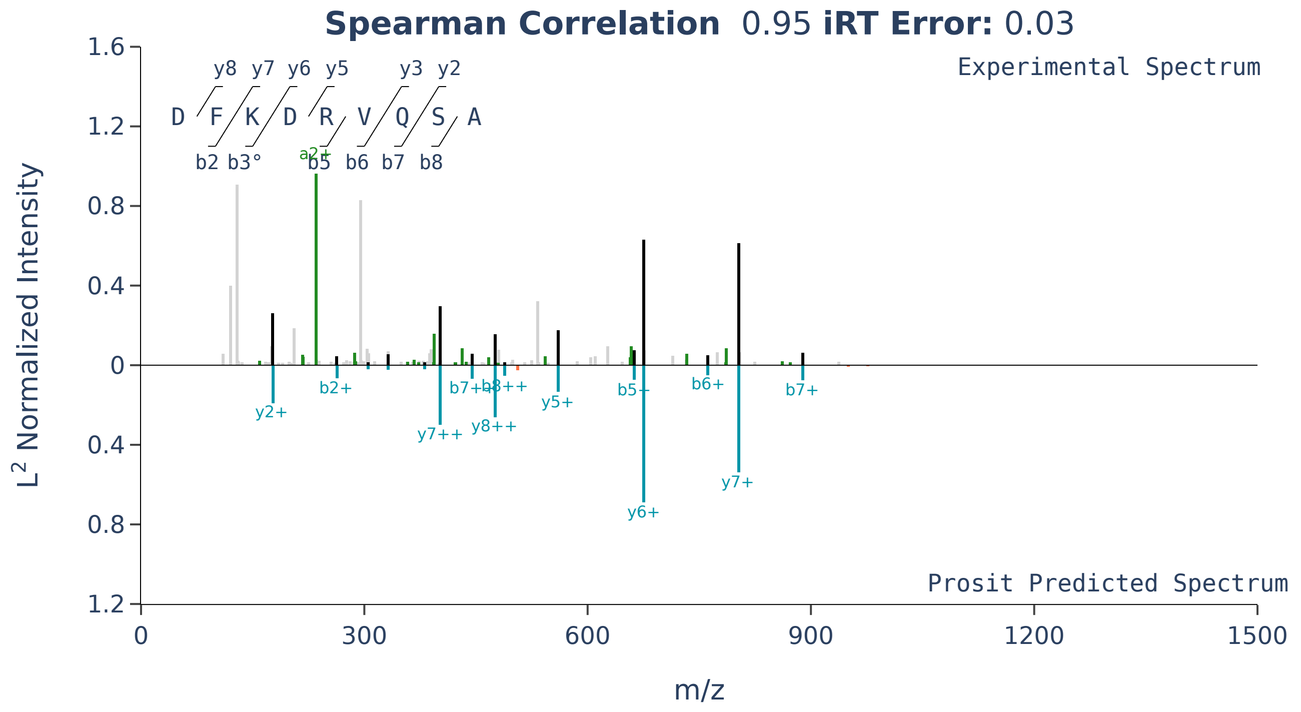

Source WSoh_221121_011221_HFGoe_L2_hTau_1h_R2 **Scan** 9418
Peptide CGSLGNIHHK **Charge** 2 **Spectral Angle** 0.0
Spearman Correlation 0.0 **iRT Error:** 21.37

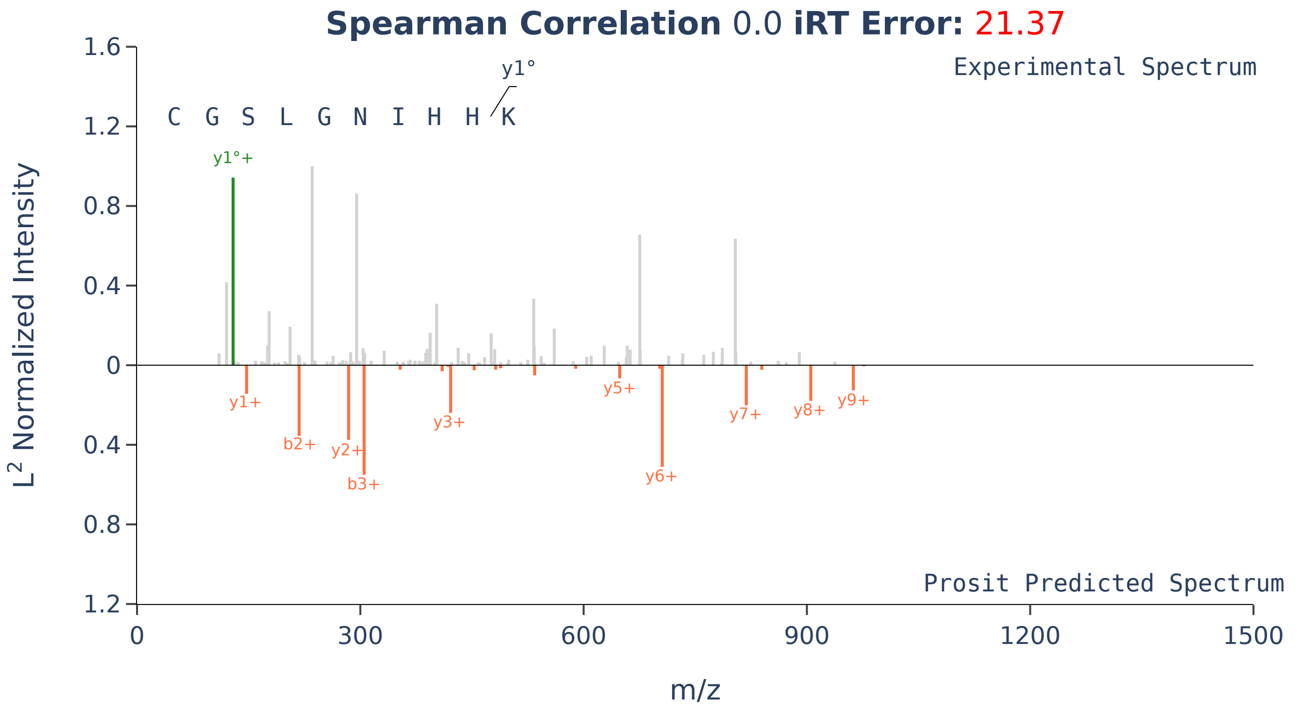

Source WSoh_221121_011221_HFGoe_L2_hTau_24h_R1 **Scan** 5702
Peptide RQEKIETH **Charge** 2 **Spectral Angle** 0.86
Spearman Correlation 0.81 **iRT Error:** 0.04

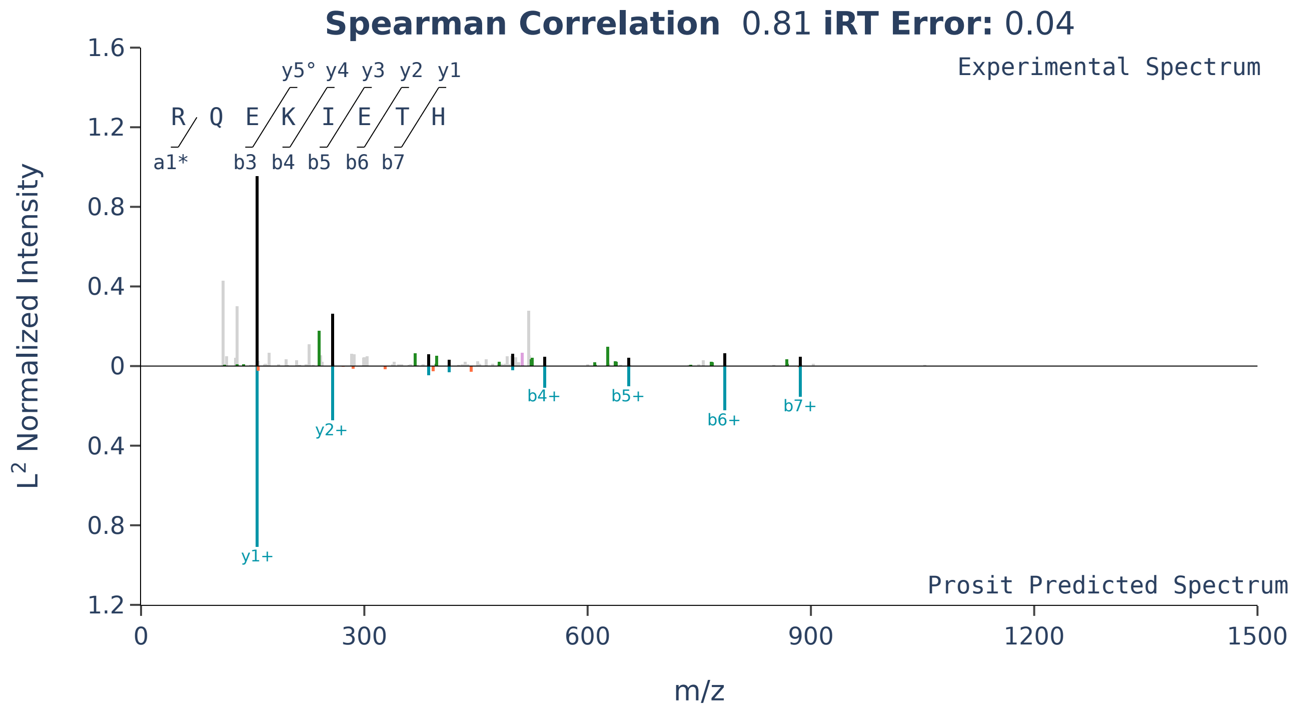

Source WSoh_221121_011221_HFGoe_L2_hTau_24h_R1 **Scan** 5702
Peptide GGGNKKIETH **Charge** 2 **Spectral Angle** 0.93
Spearman Correlation 0.81 **iRT Error:** 58.44

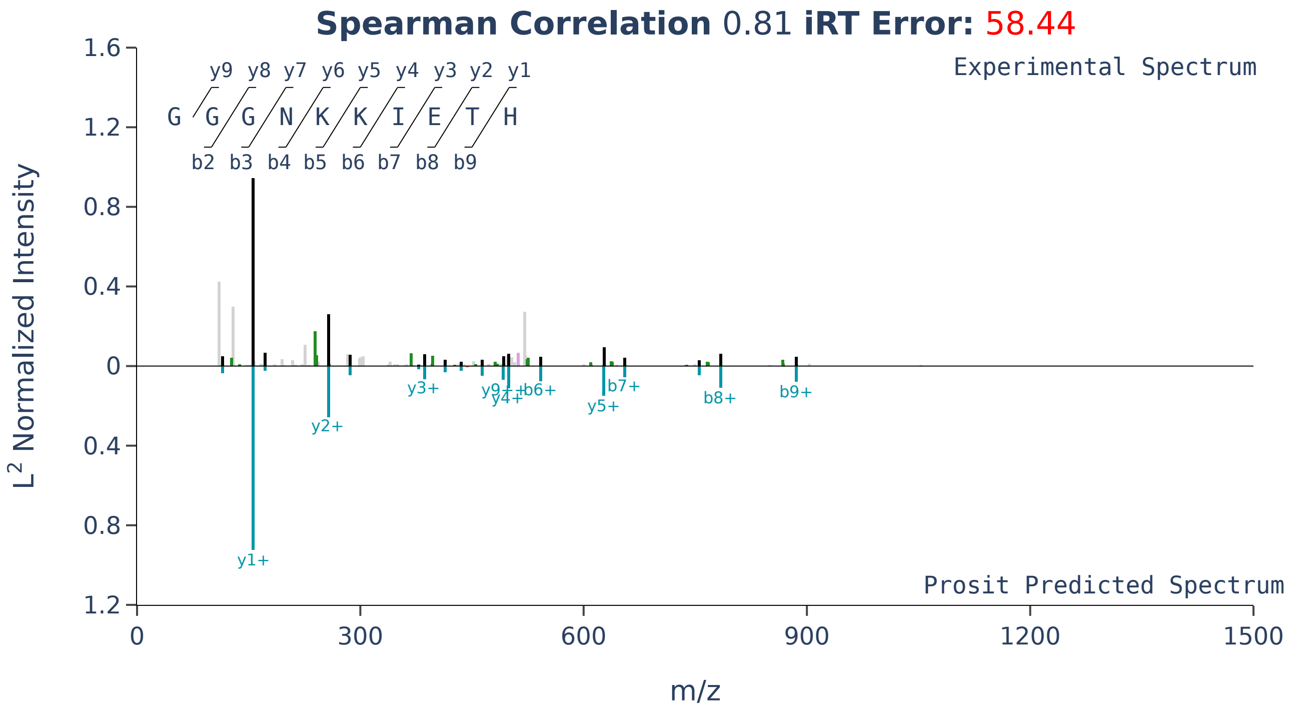

Source WSoh_221121_011221_HFGoe_L2_hTau_24h_R2 **Scan** 12911
Peptide SPGSPGKQAAAQPHTEIPEGTTAEE **Charge** 3 **Spectral Angle** 0.82
Spearman Correlation 0.82 **iRT Error:** 0.04

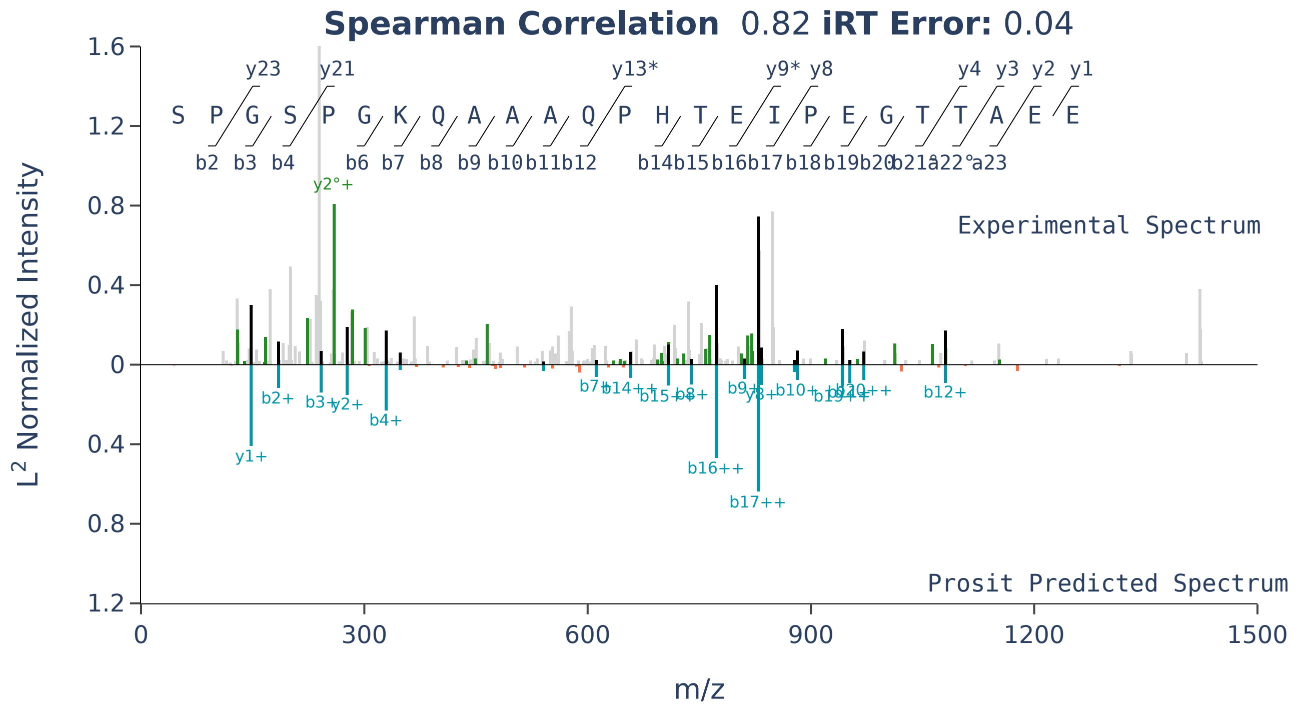

Source WSoh_221121_011221_HFGoe_L2_hTau_24h_R2 **Scan** 12911
Peptide EGAPGKQAAAQPHTEIPEGTTAEEA **Charge** 3 **Spectral Angle** 0.12
Spearman Correlation 0.28 **iRT Error:** 6.08

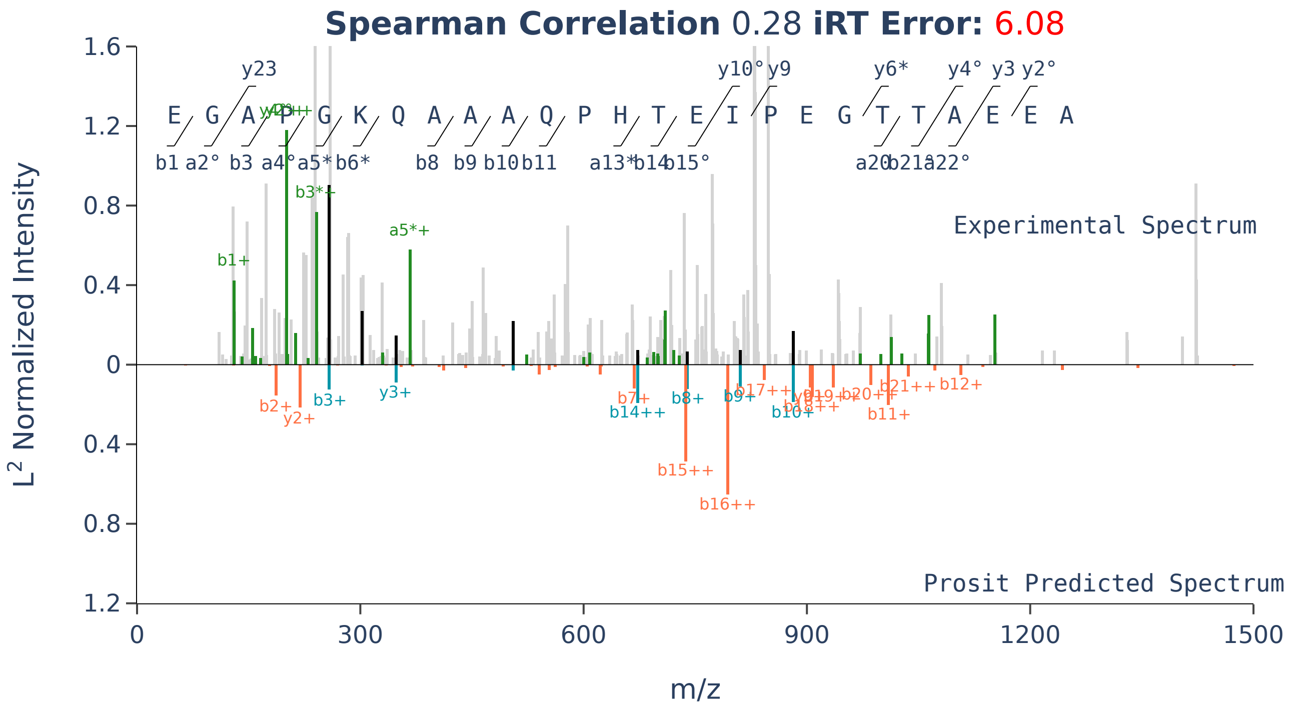

Source WSoh_221121_011221_HFGoe_L1_hTau_4h_R1 **Scan** 12430
Peptide QEAPGKQAAAQPHTEIPEGTTAEE **Charge** 3 **Spectral Angle** 0.67
Spearman Correlation 0.9 **iRT Error:** 0.05

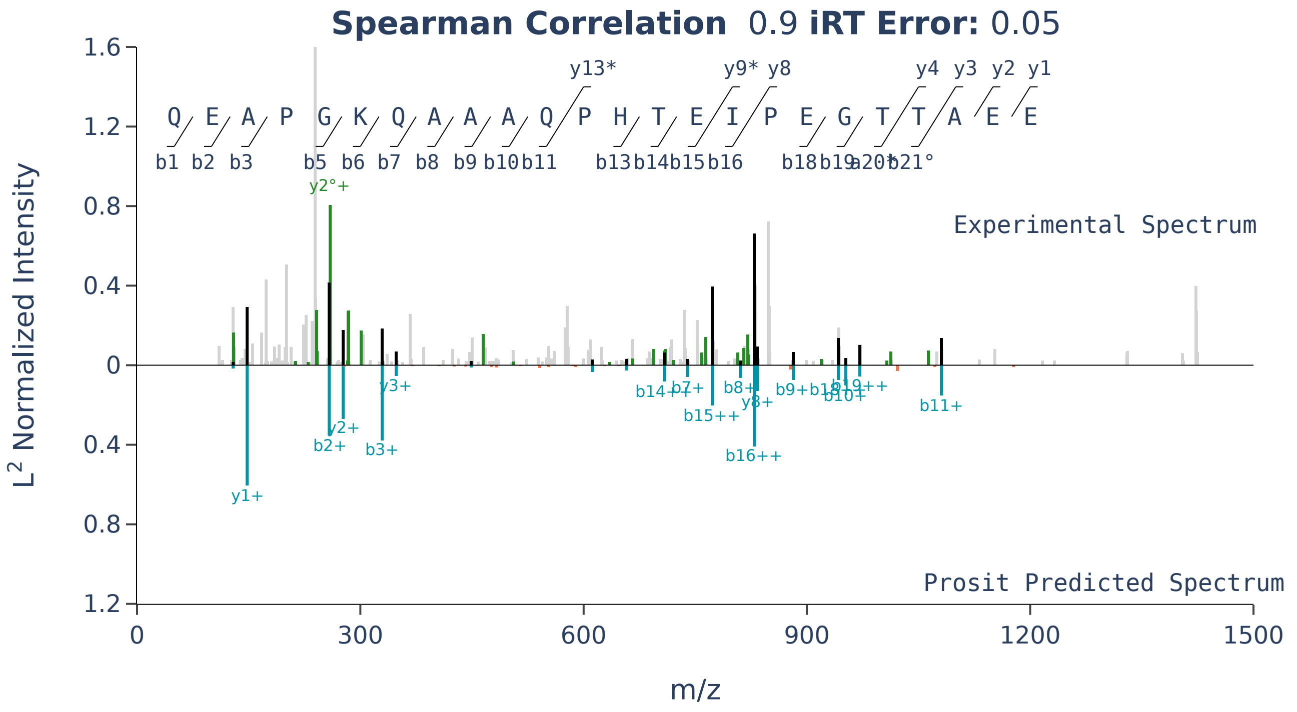

Source WSoh_221121_011221_HFGoe_L1_hTau_4h_R1 **Scan** 12430
Peptide EGAPGKQAAAQPHTEIPEGTTAEEA **Charge** 3 **Spectral Angle** 0.12
Spearman Correlation 0.26 **iRT Error:** 5.05

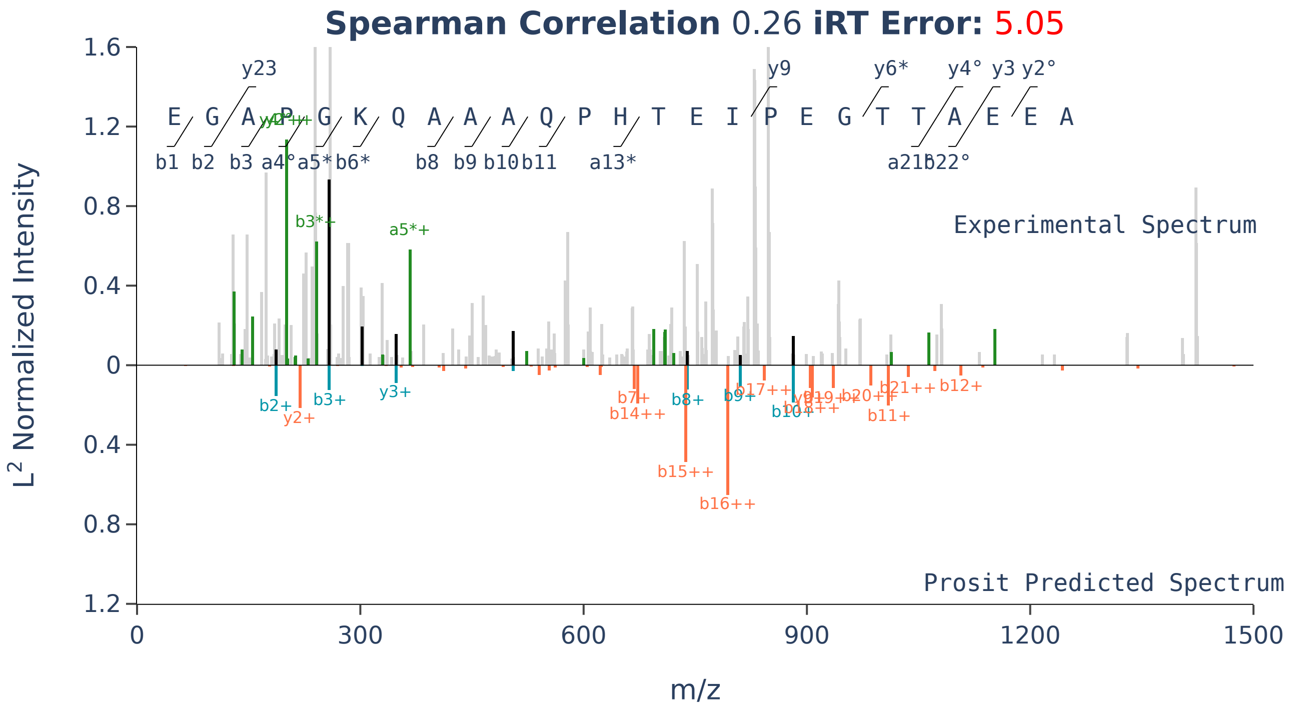

Source WSoh_221121_011221_HFGoe_L2_hTau_24h_R1 **Scan** 11432
Peptide NITHVPGGGNKKIETHKLTFREGG **Charge** 3 **Spectral Angle** 0.79
Spearman Correlation 0.89 **iRT Error:** 0.06

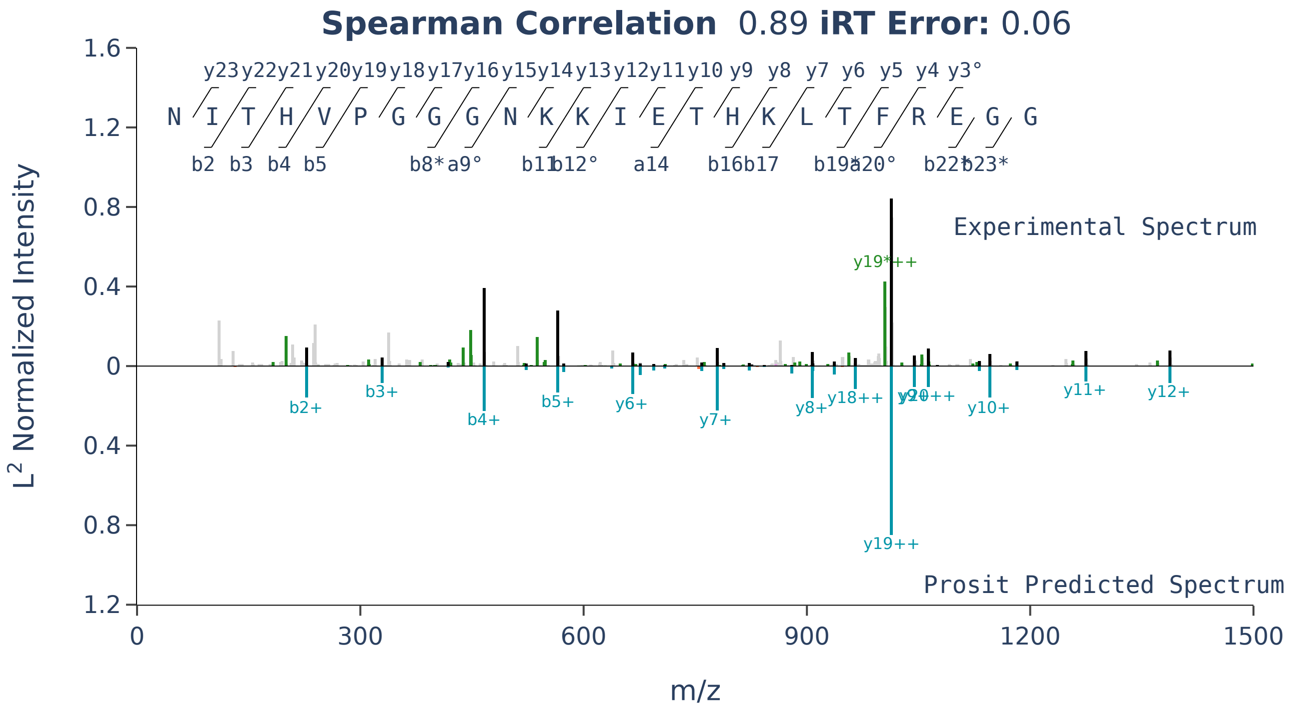

Source WSoh_221121_011221_HFGoe_L2_hTau_24h_R1 **Scan** 11432
Peptide NITHVPGGGNKKIETHKLTFREN **Charge** 3 **Spectral Angle** 0.84
Spearman Correlation 0.83 **iRT Error:** 1.41

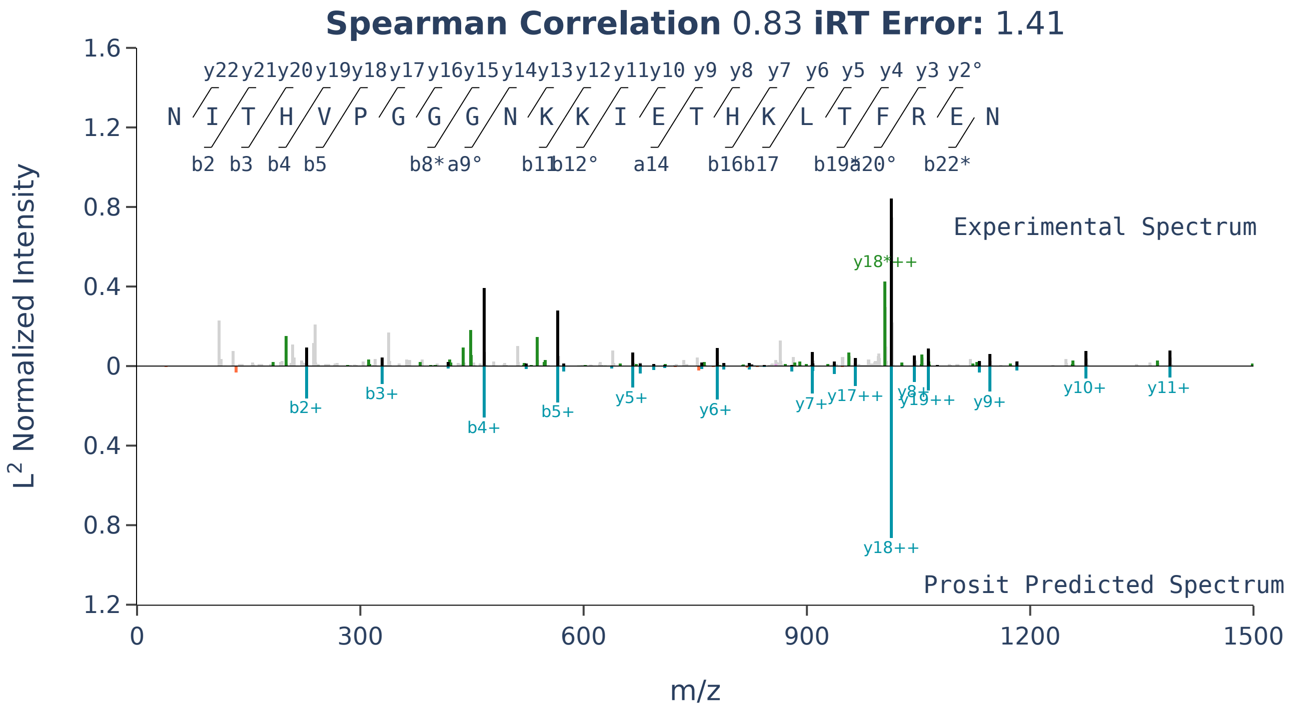

Source WSoh_221121_011221_HFGoe_L1_hTau_24h_R1 **Scan** 13863
Peptide KHLTFRENAKAKTDHGAEIVYKSPVV **Charge** 4 **Spectral Angle** 0.83
Spearman Correlation 0.82 **iRT Error:** 0.07

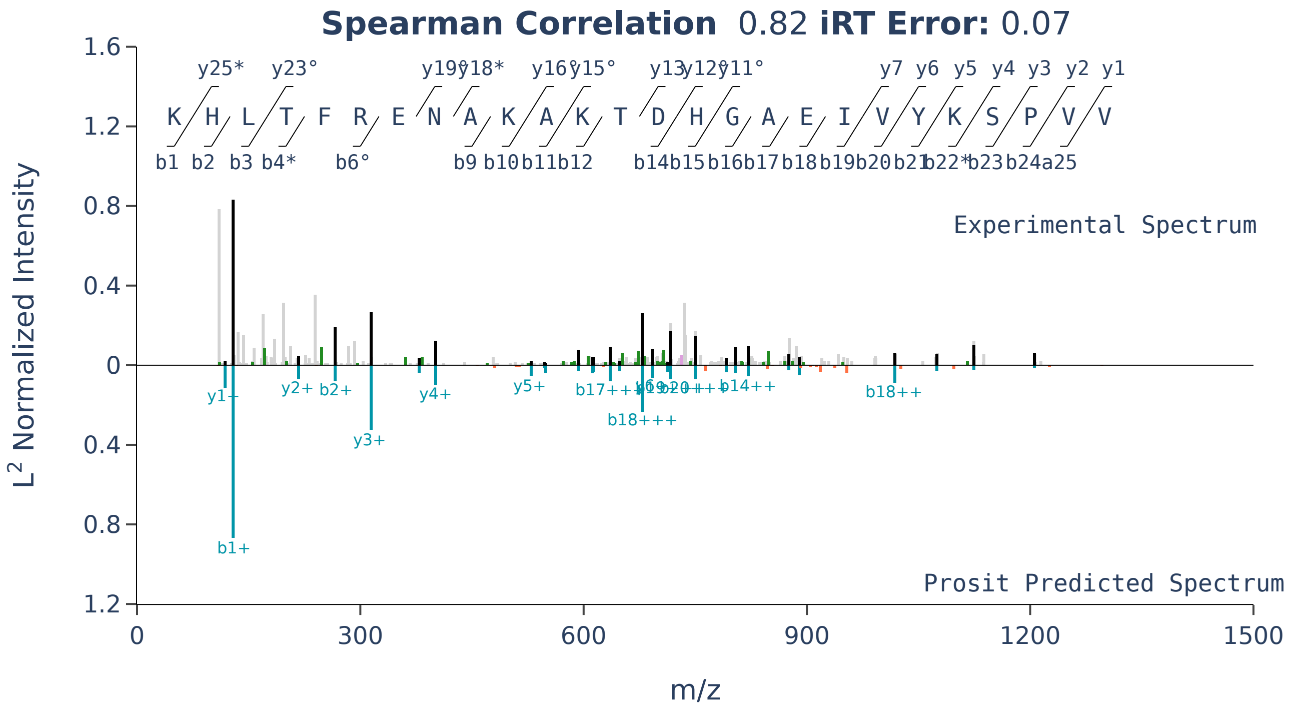

Source WSoh_221121_011221_HFGoe_L1_hTau_24h_R1 **Scan** 13863
Peptide HKLTFRENAKAKTDHGAEIVYKSPVV **Charge** 4 **Spectral Angle** 0.77
Spearman Correlation 0.86 **iRT Error:** 2.0

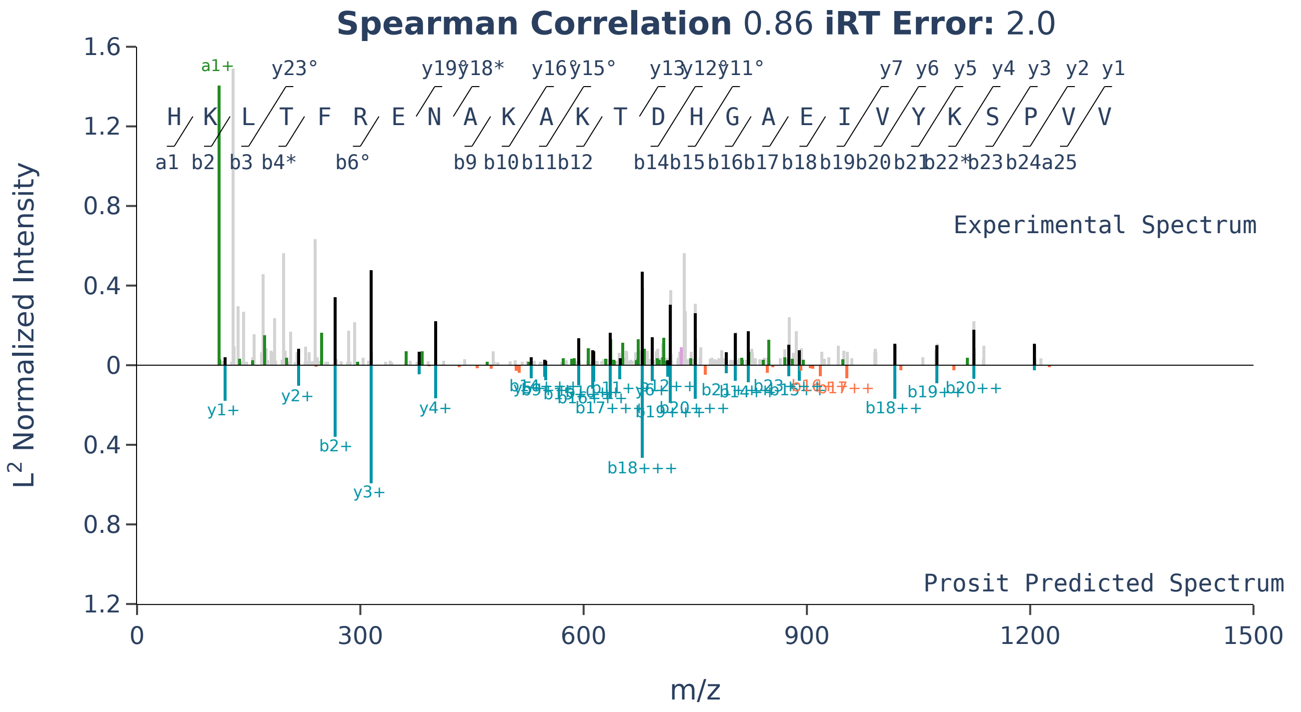

Source WSoh_221121_011221_HFGoe_L1_hTau_24h_R2 **Scan** 13760
Peptide AAQPHTEIPEGTTAEEAGEGAPGKQA **Charge** 3 **Spectral Angle** 0.91
Spearman Correlation 0.88 **iRT Error:** 0.07

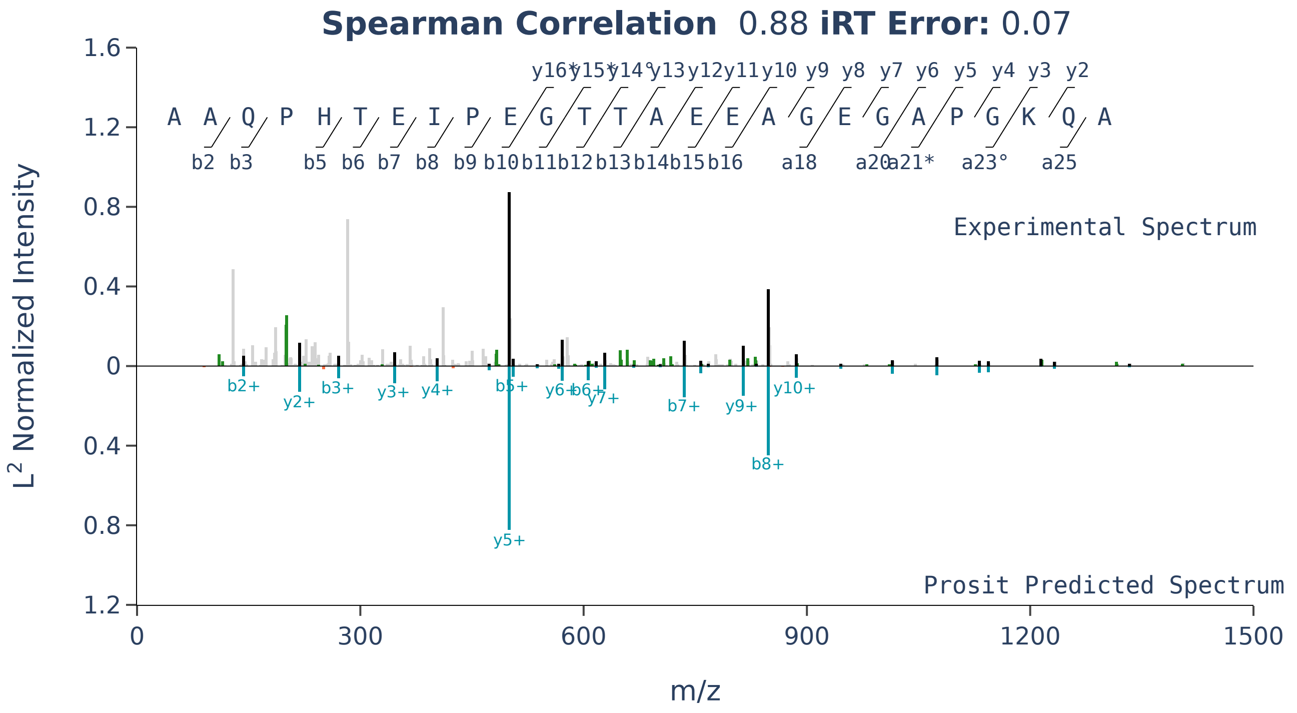

Source WSoh_221121_011221_HFGoe_L1_hTau_24h_R2 **Scan** 13760
Peptide VVSGDTSPRHLSNVSSTGSIDMVDS **Charge** 3 **Spectral Angle** 0.0
Spearman Correlation 0.0 **iRT Error:** 28.39

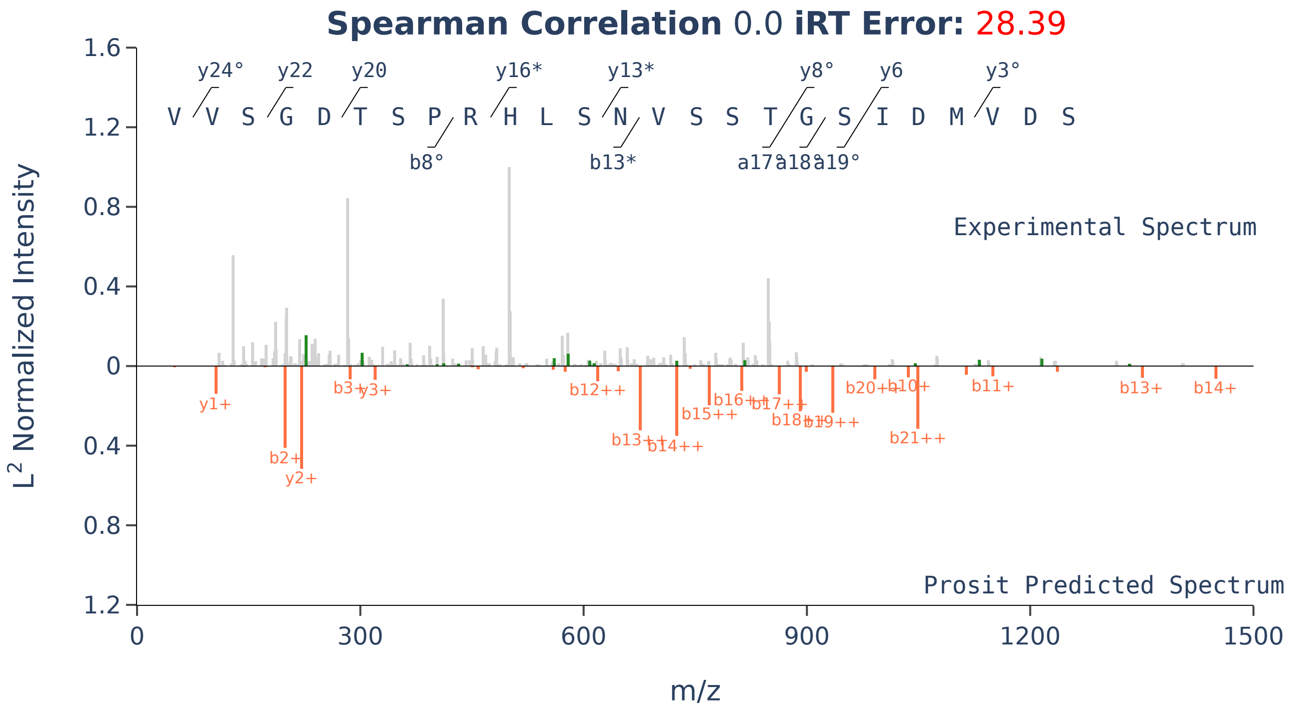

Source WSoh_221121_011221_HFGoe_L2_hTau_24h_R2 **Scan** 12350
Peptide PGKQAAAQQPHTEIPEGTTAEE **Charge** 3 **Spectral Angle** 0.82
Spearman Correlation 0.87 **iRT Error:** 0.08

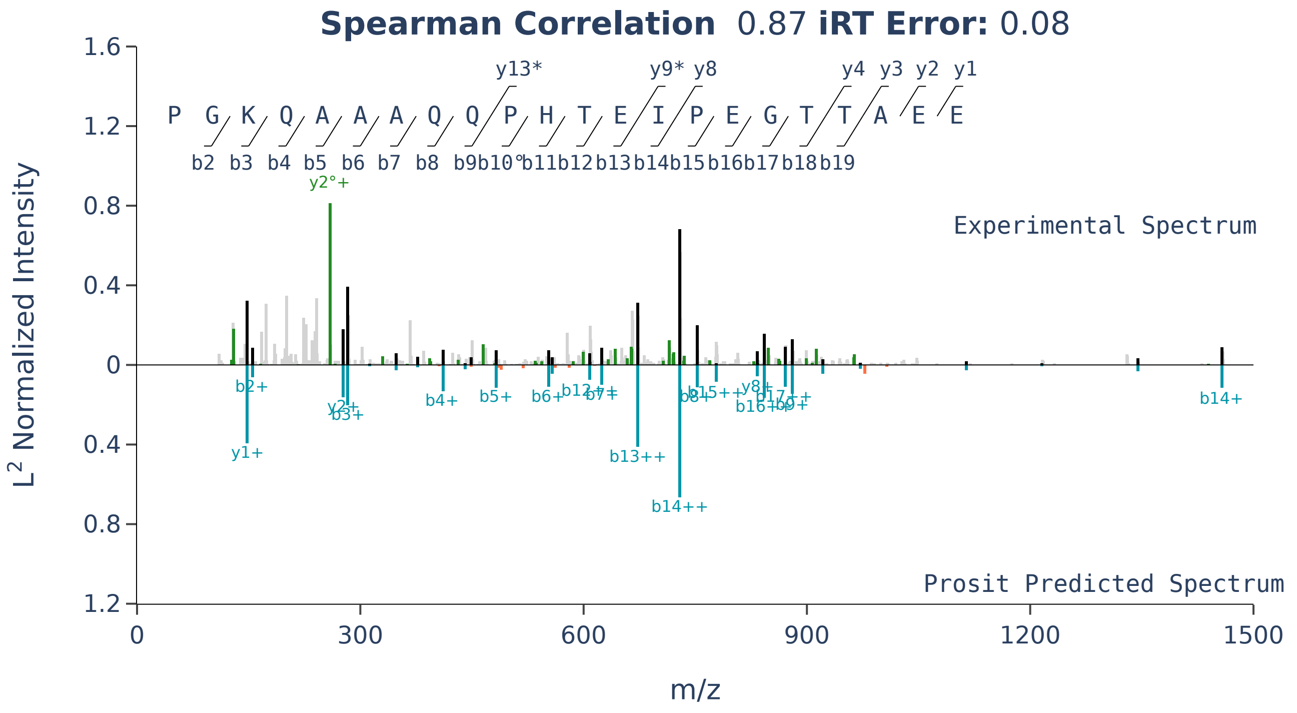

Source WSoh_221121_011221_HFGoe_L2_hTau_24h_R2 **Scan** 12350
Peptide EGAPGKQAAAQPHTEIPEGTTAE **Charge** 3 **Spectral Angle** 0.24
Spearman Correlation 0.25 **iRT Error:** 3.18

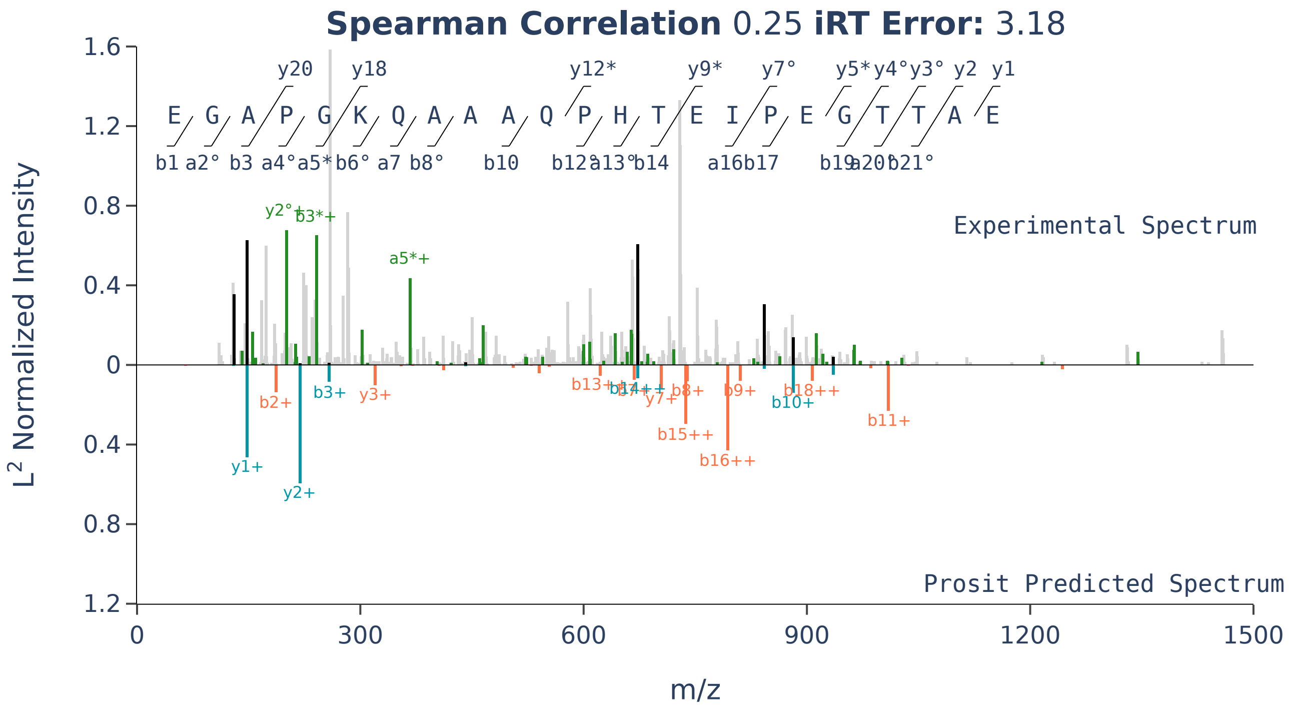

Source WSoh_221121_011221_HFGoe_L1_hTau_24h_R2 **Scan** 8310
Peptide KSEKLDFKDRVQSR **Charge** 3 **Spectral Angle** 0.86
Spearman Correlation 0.86 **iRT Error:** 0.11

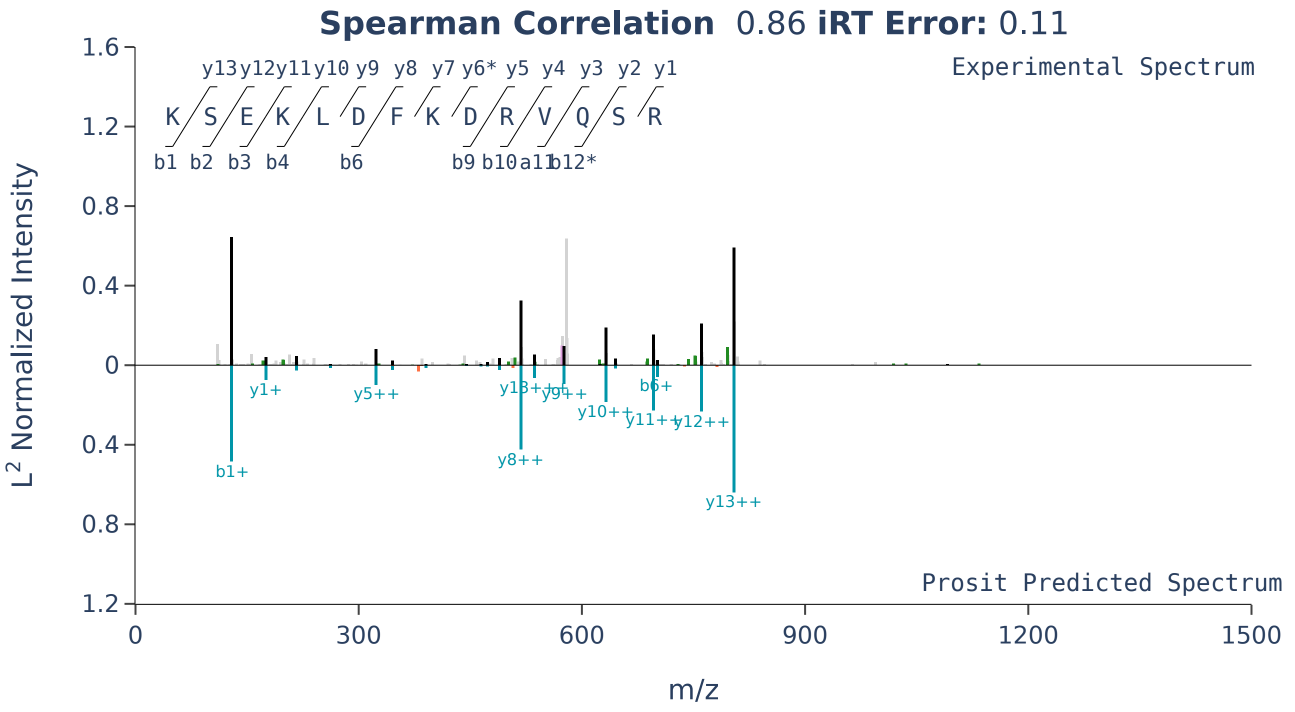

Source WSoh_221121_011221_HFGoe_L1_hTau_24h_R2 **Scan** 8310
Peptide ADGKTKIATPRGAAPPGQ **Charge** 3 **Spectral Angle** 0.03
Spearman Correlation 0.09 **iRT Error:** 1.57

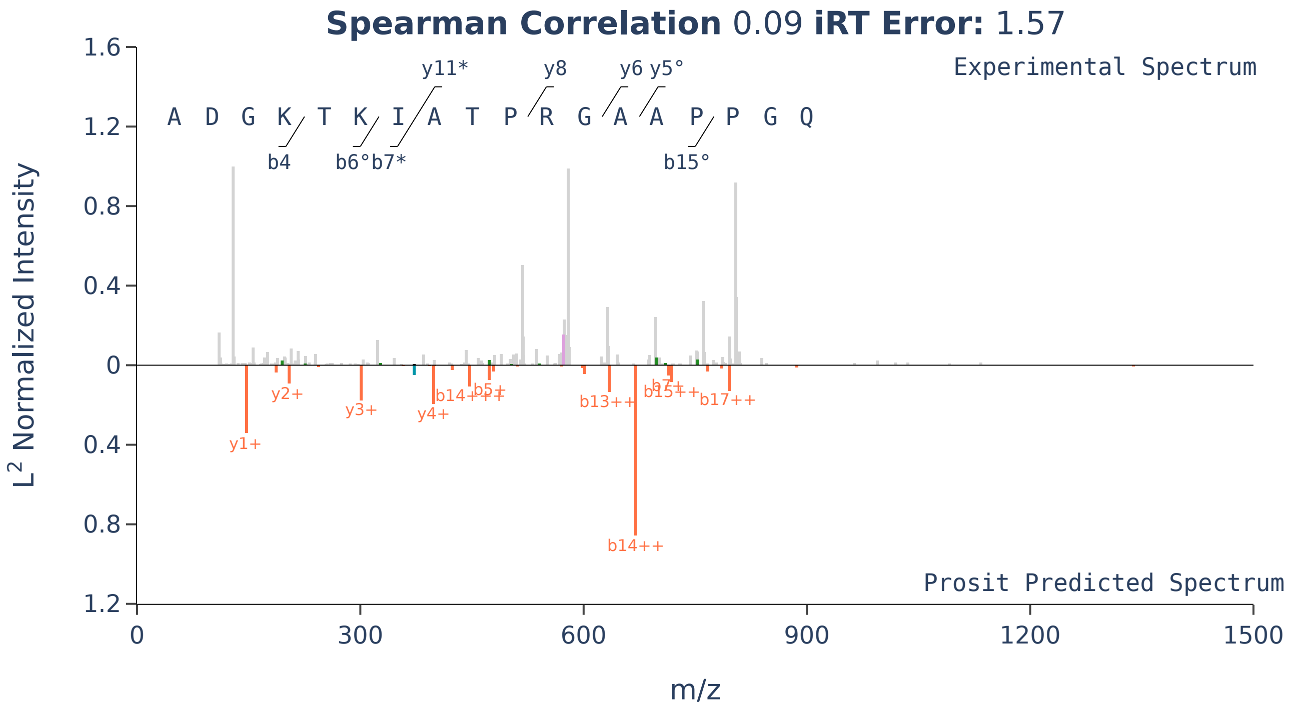

Source WSoh_221121_011221_HFGoe_L1_hTau_24h_R1 **Scan** 18222
Peptide GAIGDTPSLEDEAAGHVTQARMVSKSKD **Charge** 4 **Spectral Angle** 0.77
Spearman Correlation 0.87 **iRT Error:** 0.13

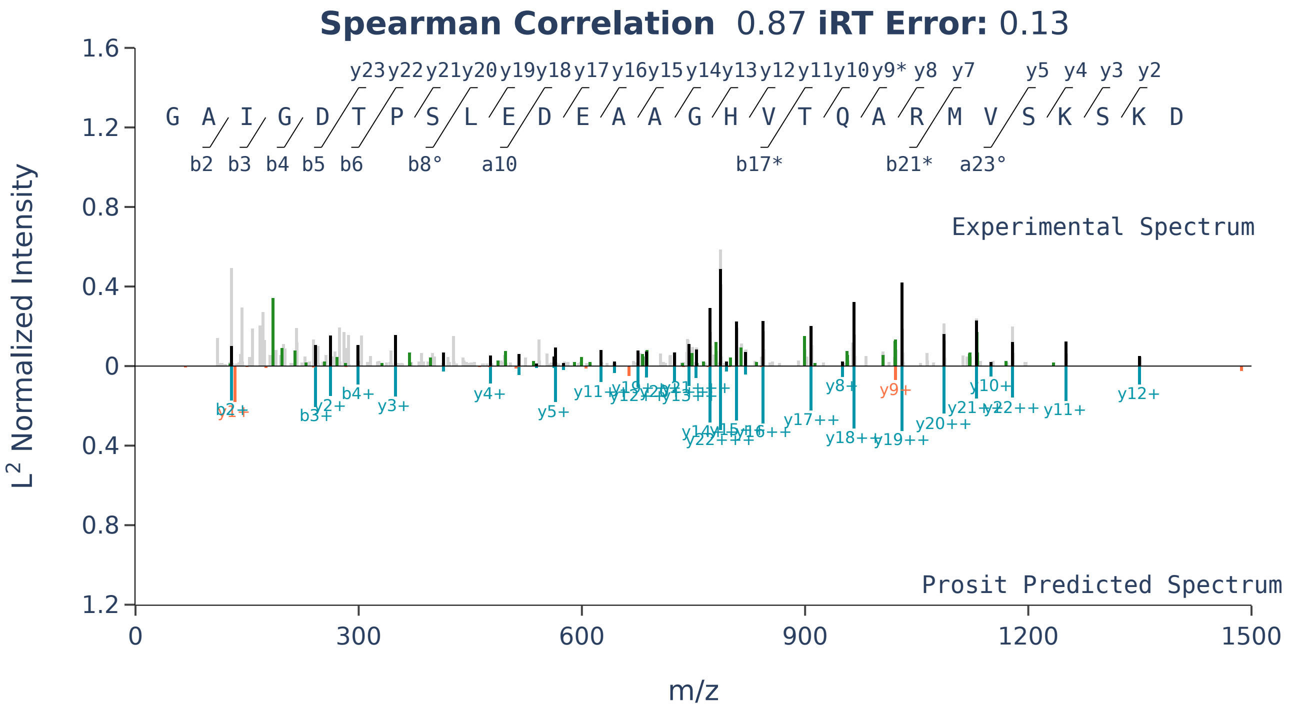

Source WSoh_221121_011221_HFGoe_L1_hTau_24h_R1 **Scan** 18222
Peptide AGIGDTPSLEDEAAGHVTQARMVSKSKD **Charge** 4 **Spectral Angle** 0.77
Spearman Correlation 0.87 **iRT Error:** 1.09

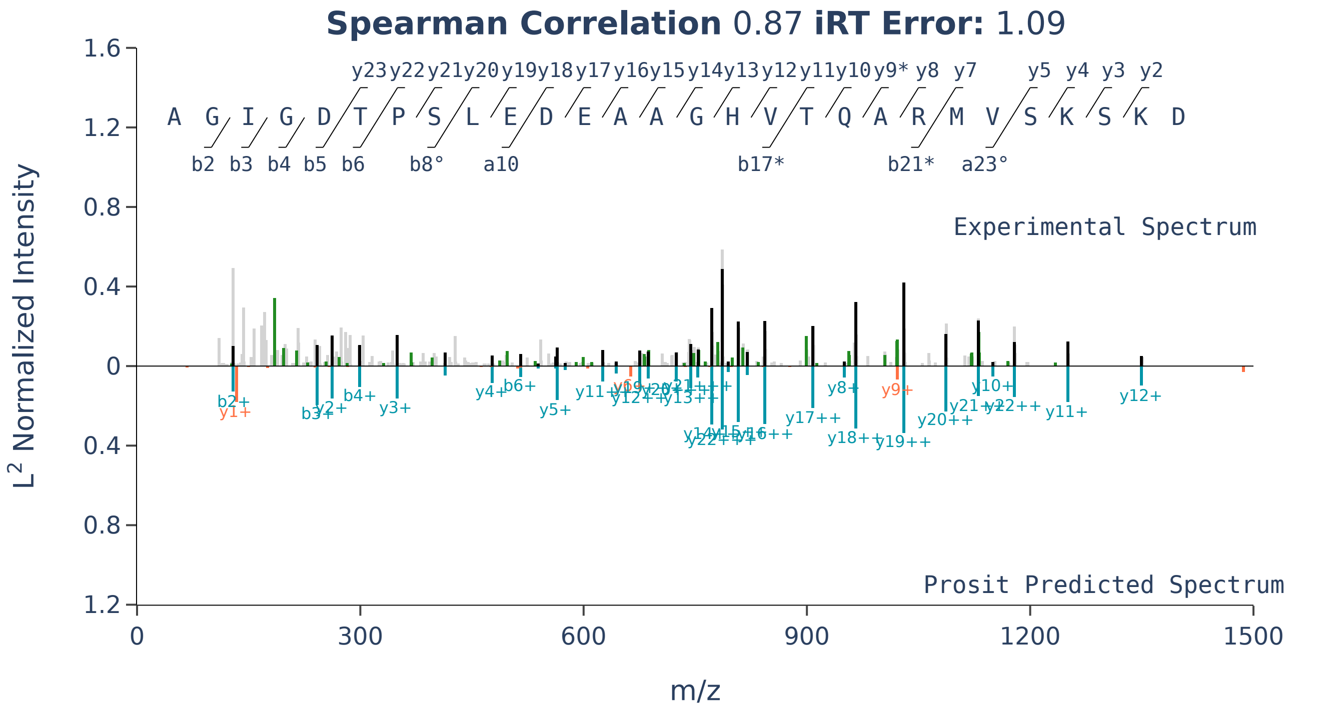

Source WSoh_221121_011221_HFGoe_L1_hTau_24h_R2 **Scan** 7750
Peptide PPAPKTPPSSD **Charge** 2 **Spectral Angle** 0.81
Spearman Correlation 0.89 **iRT Error:** 0.14

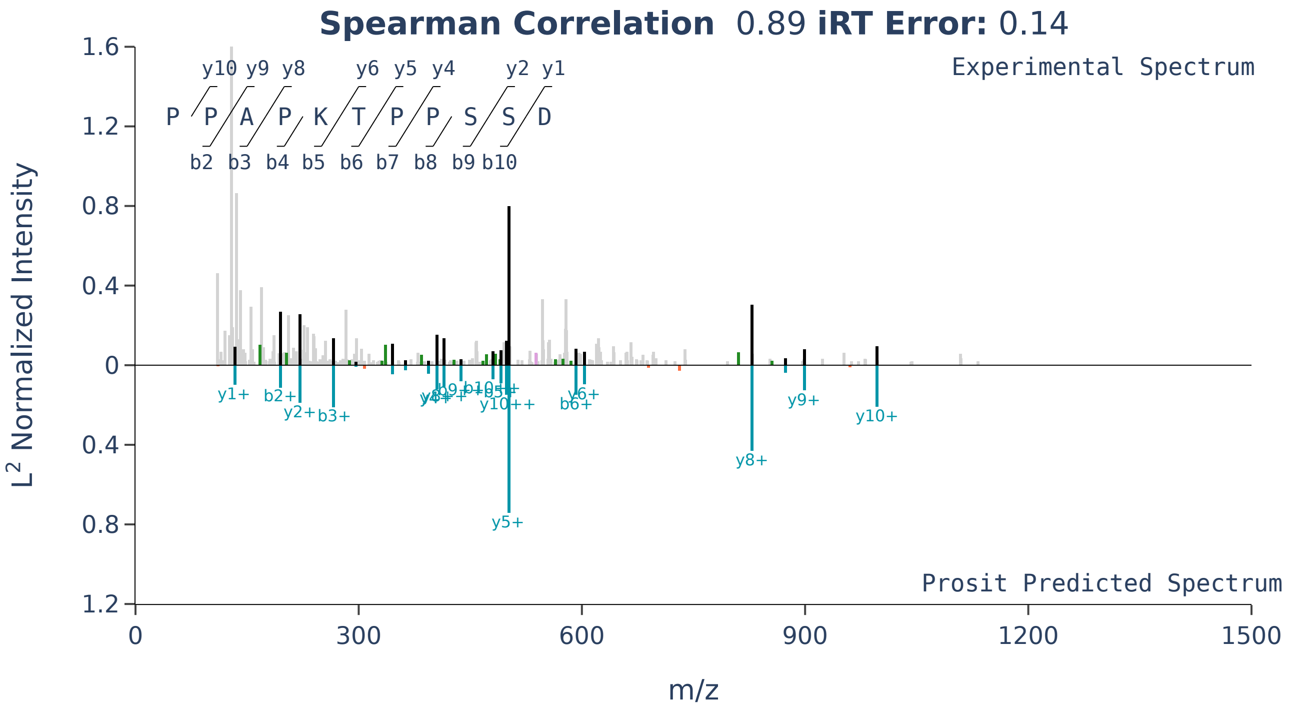

Source WSoh_221121_011221_HFGoe_L1_hTau_24h_R2 **Scan** 7750
Peptide PKTPPSSGEPP **Charge** 2 **Spectral Angle** 0.07
Spearman Correlation 0.26 **iRT Error:** 0.34

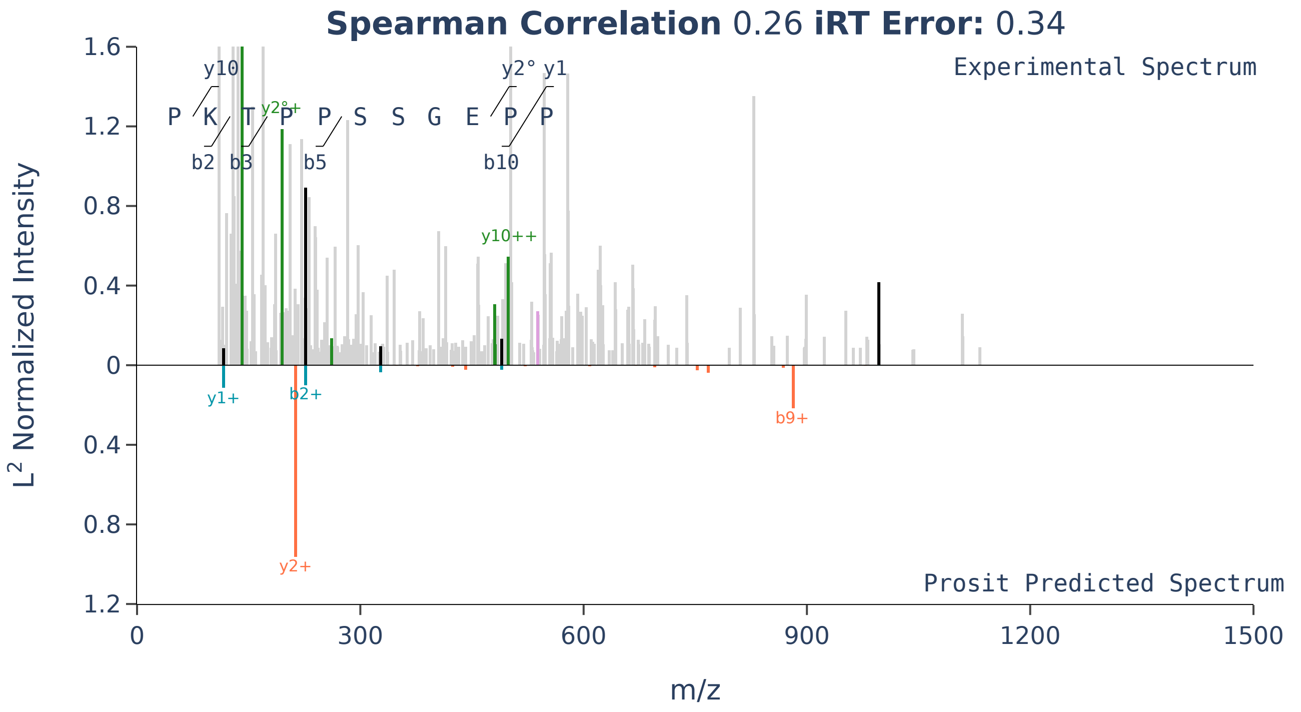

Source WSoh_221121_011221_HFGoe_L1_hTau_4h_R1 **Scan** 7460
Peptide DFKDRVQSKA **Charge** 2 **Spectral Angle** 0.86
Spearman Correlation 0.94 **iRT Error:** 0.15

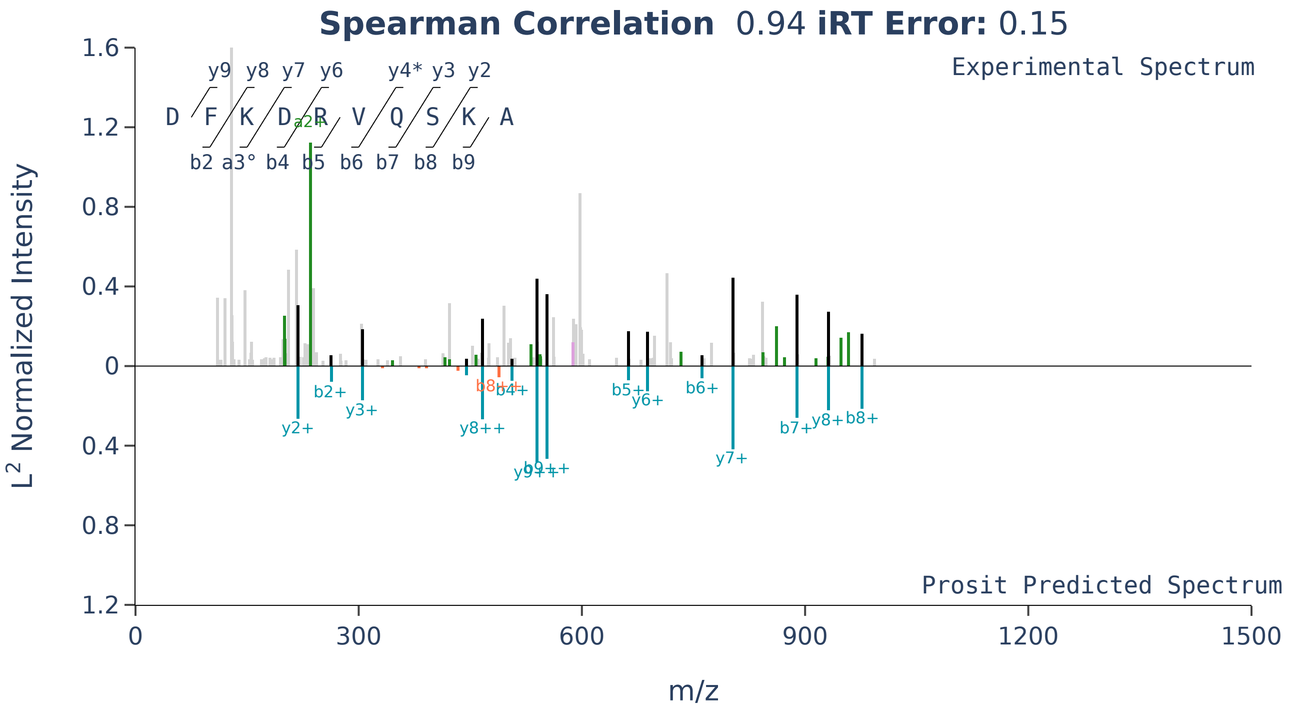

Source WSoh_221121_011221_HFGoe_L1_hTau_4h_R1 **Scan** 7460
Peptide KQAAAQPHTEI **Charge** 2 **Spectral Angle** 0.55
Spearman Correlation 0.25 **iRT Error:** 6.91

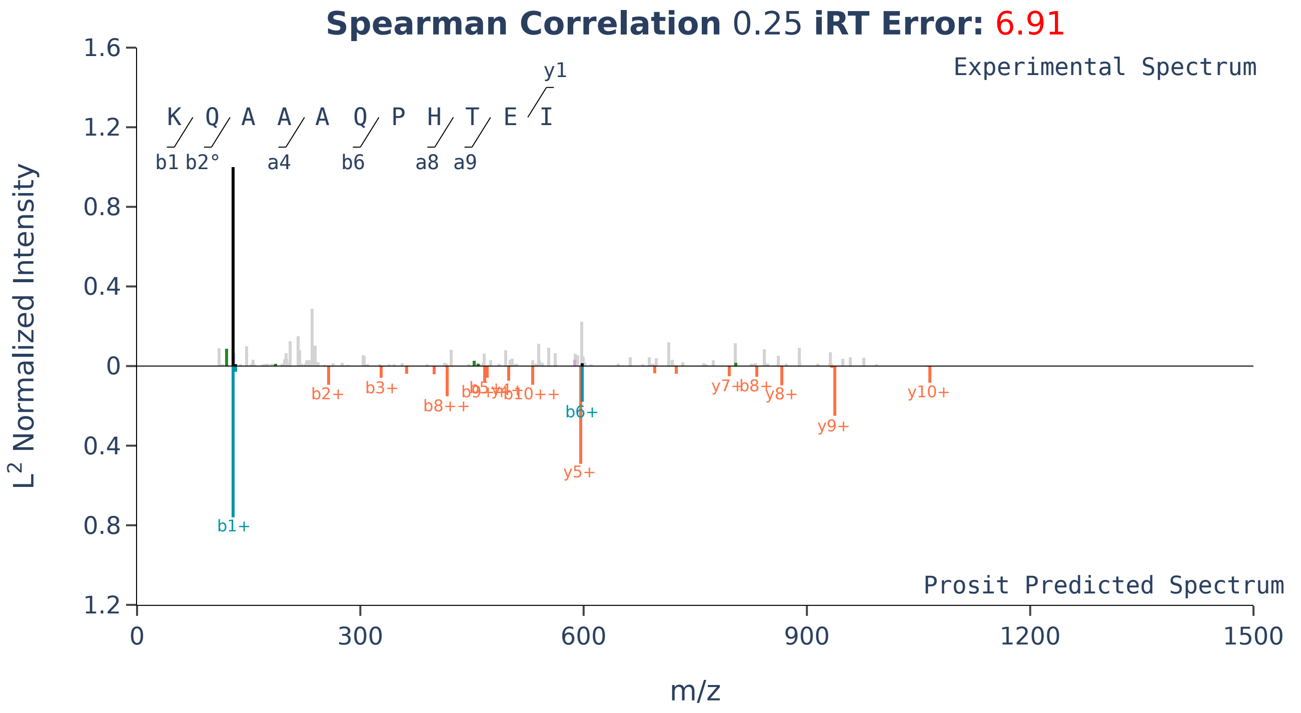

Source WSoh_221121_011221_HFGoe_L2_hTau_24h_R1 **Scan** 19420
Peptide NKKLDLSNVQSL **Charge** 2 **Spectral Angle** 0.84
Spearman Correlation 0.92 **iRT Error:** 0.18

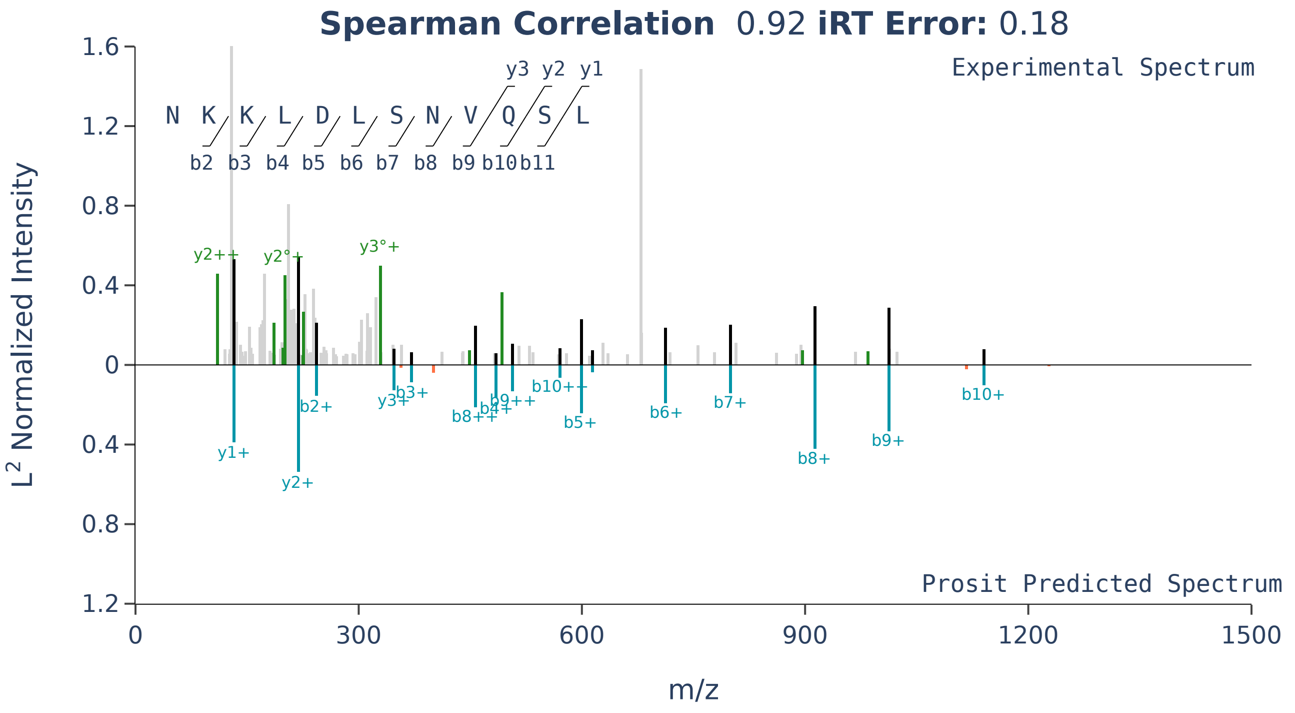

Source WSoh_221121_011221_HFGoe_L2_hTau_24h_R1 **Scan** 19420
Peptide INKKLDLSNVQS **Charge** 2 **Spectral Angle** 0.25
Spearman Correlation 0.31 **iRT Error:** 33.2

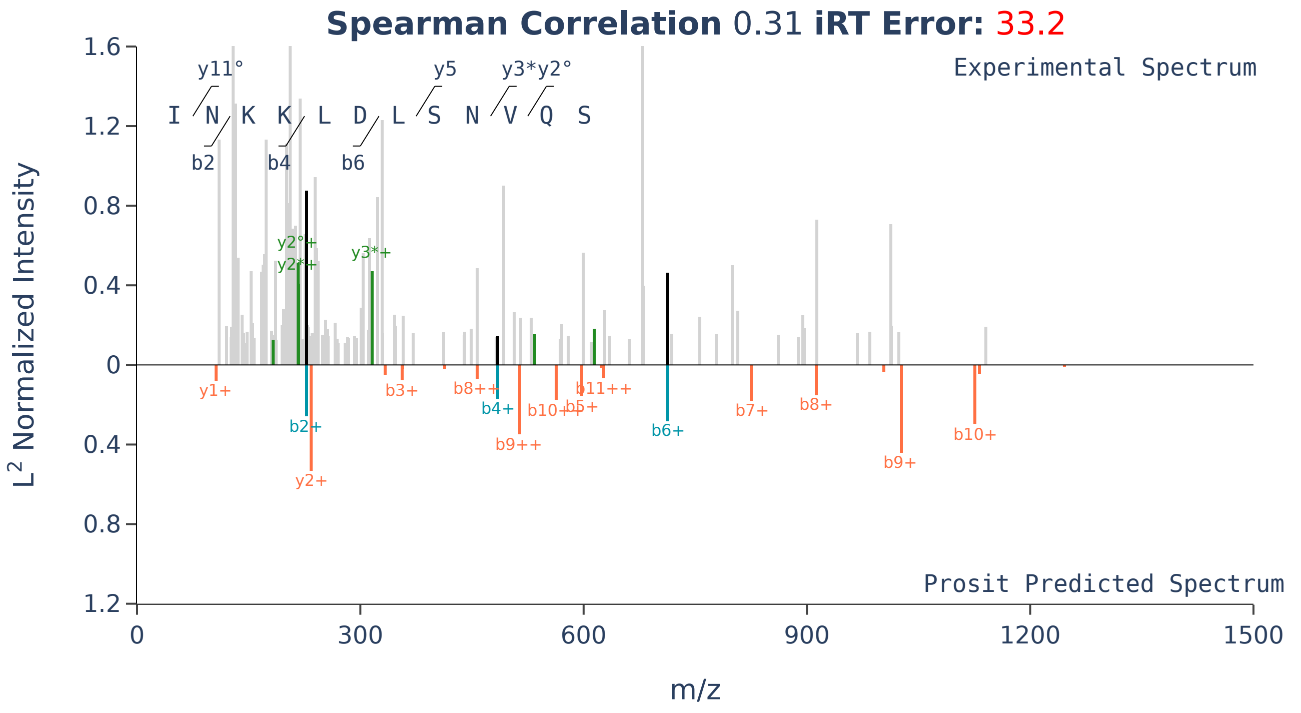

Source WSoh_221121_011221_HFGoe_L1_hTau_24h_R2 **Scan** 18968
Peptide EGAPGKQAVTAPLVD **Charge** 2 **Spectral Angle** 0.82
Spearman Correlation 0.94 **iRT Error:** 0.23

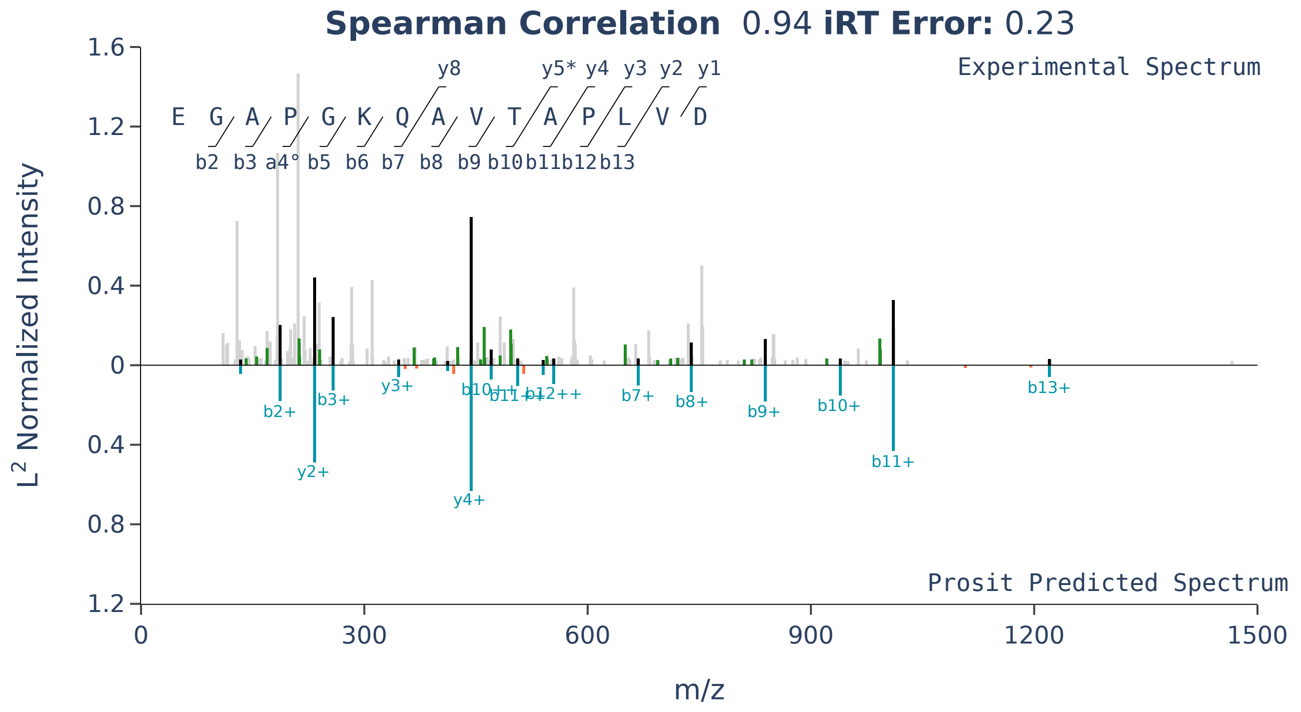

Source WSoh_221121_011221_HFGoe_L1_hTau_24h_R2 **Scan** 18968
Peptide VTAPLVDEGAPGKQA **Charge** 2 **Spectral Angle** 0.46
Spearman Correlation 0.48 **iRT Error:** 10.13

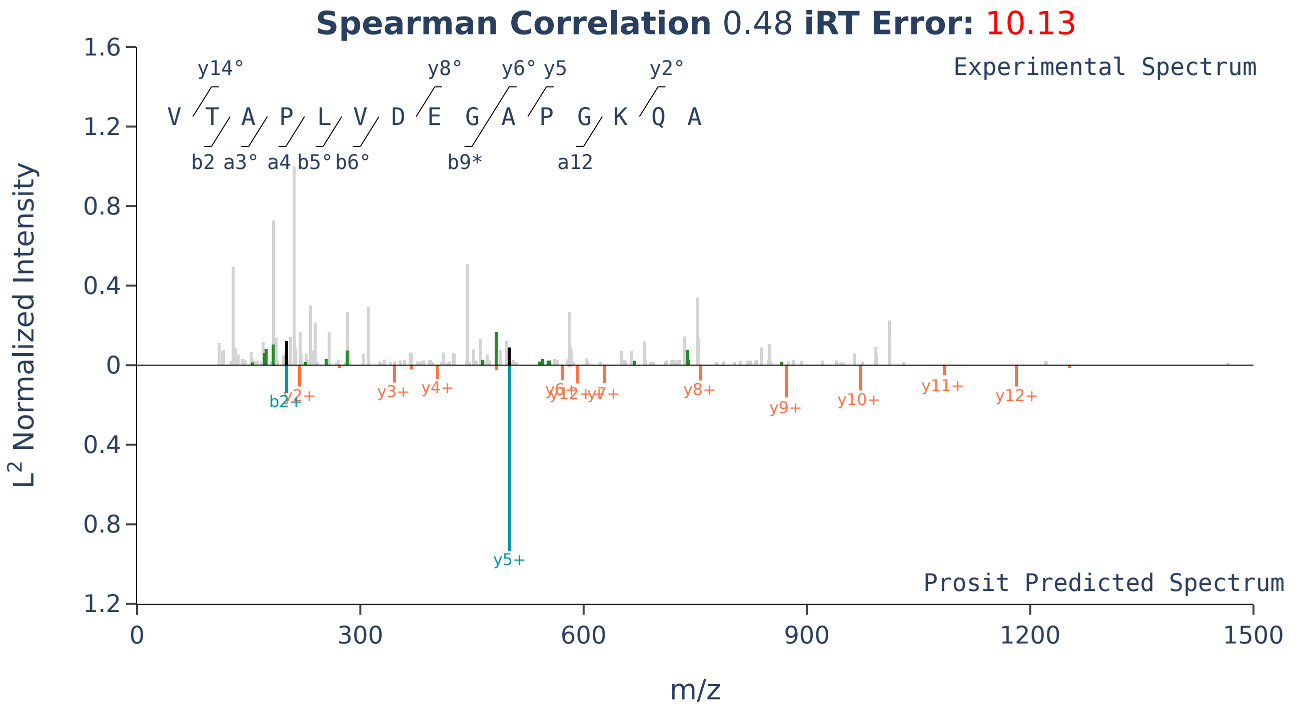

Source WSoh_221121_011221_HFGoe_L2_hTau_24h_R2 **Scan** 21832
Peptide DEAPRQEFEVME **Charge** 2 **Spectral Angle** 0.89
Spearman Correlation 0.98 **iRT Error:** 0.24

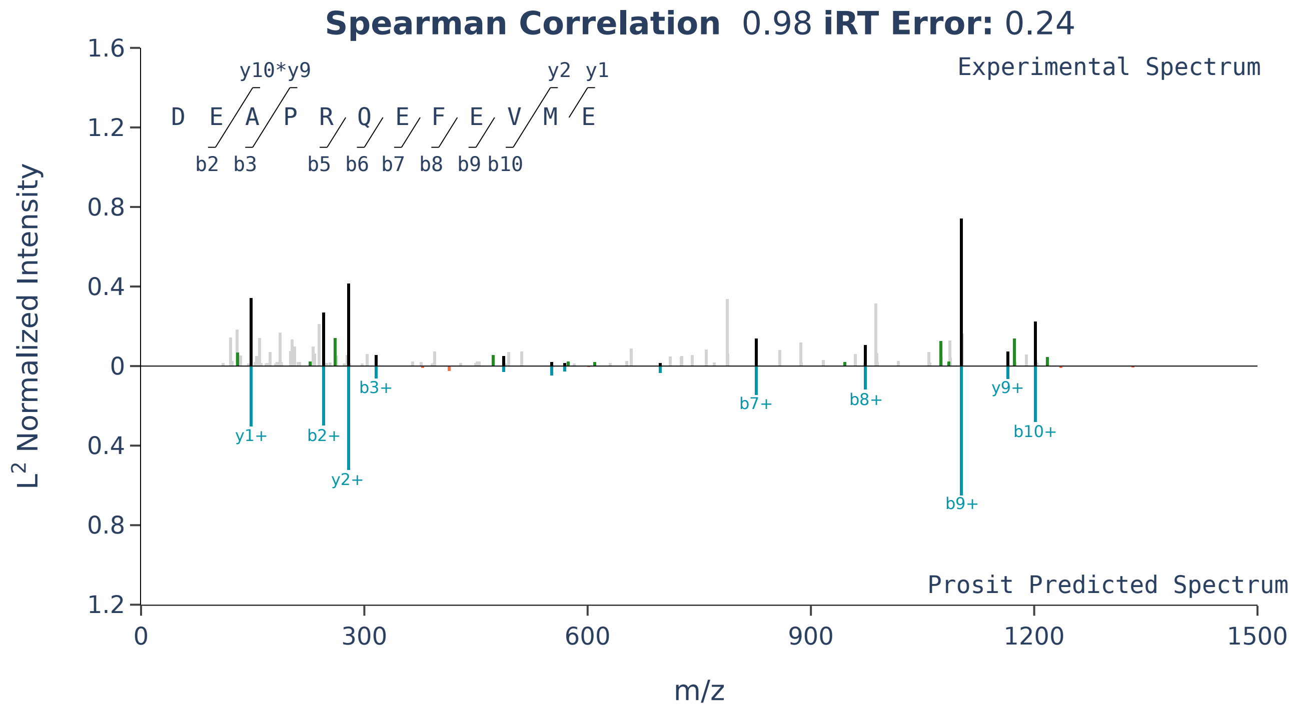

Source WSoh_221121_011221_HFGoe_L2_hTau_24h_R2 **Scan** 21832
Peptide AEPRQEFEVMED **Charge** 2 **Spectral Angle** 0.84
Spearman Correlation 0.7 **iRT Error:** 9.57

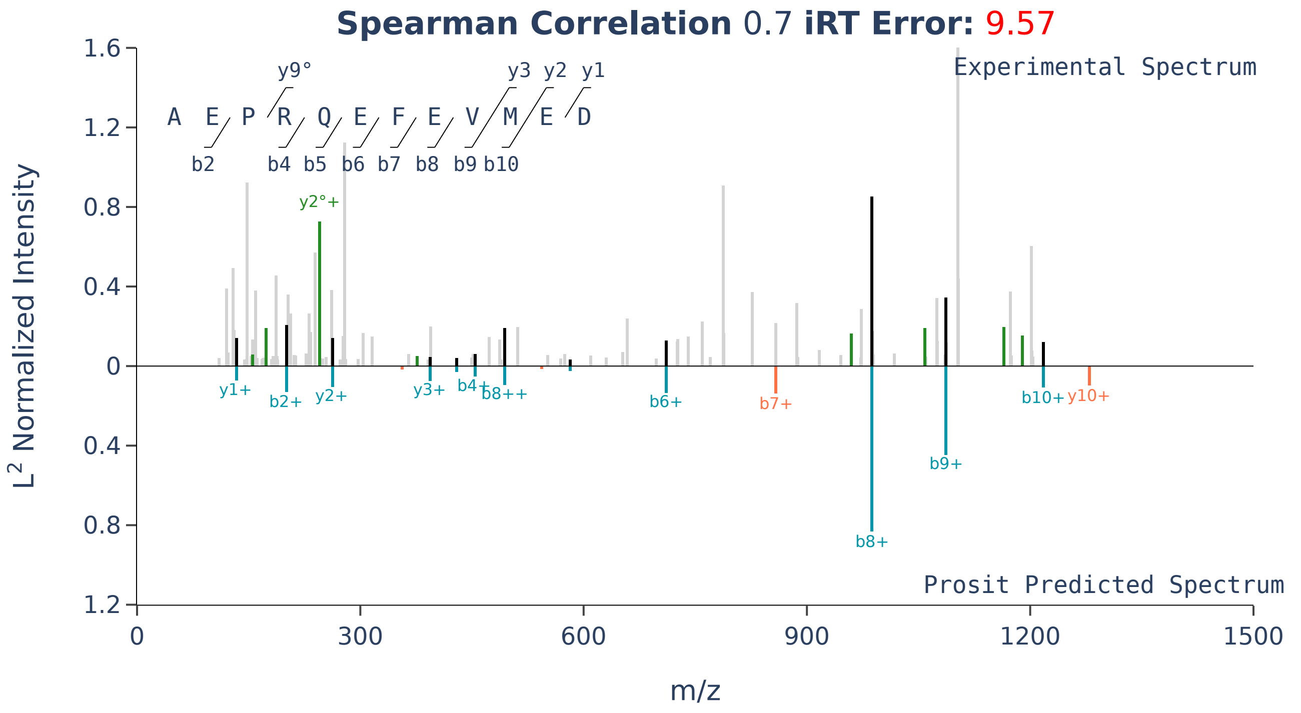

Source WSoh_221121_011221_HFGoe_L2_hTau_24h_R2 **Scan** 13706
Peptide AQQPHTEIPEGTTAEE **Charge** 2 **Spectral Angle** 0.85
Spearman Correlation 0.92 **iRT Error:** 0.26

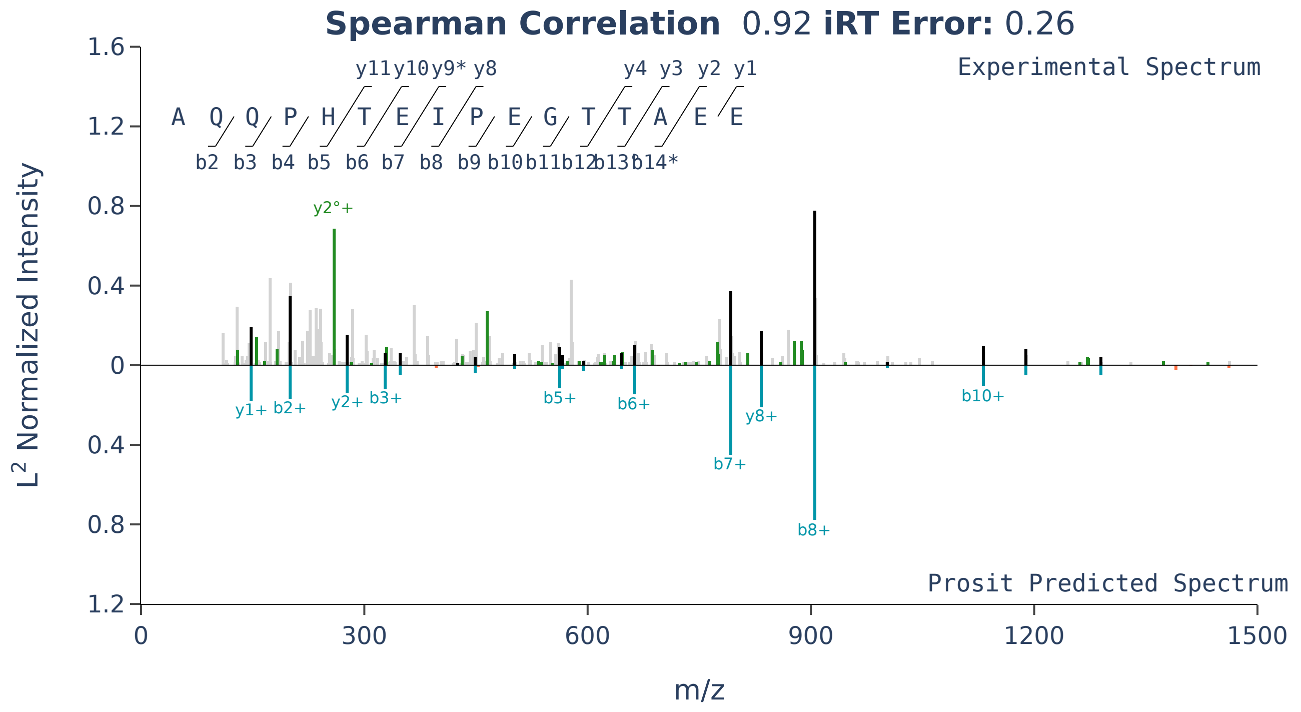

Source WSoh_221121_011221_HFGoe_L2_hTau_24h_R2 **Scan** 13706
Peptide AQPHTEIPEGTTAEEAG **Charge** 2 **Spectral Angle** 0.54
Spearman Correlation 0.45 **iRT Error:** 2.46

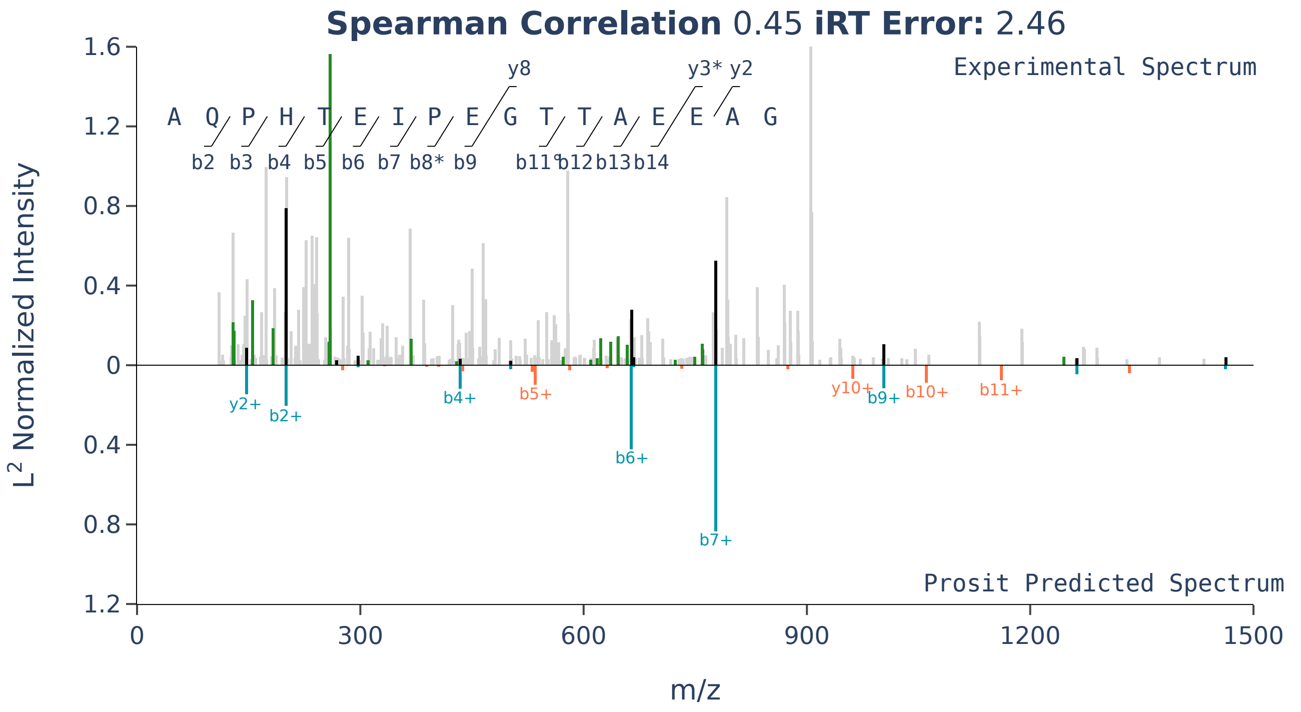

Source WSoh_221121_011221_HFGoe_L1_hTau_1h_R1 **Scan** 9077
Peptide DFKDRVQTG **Charge** 2 **Spectral Angle** 0.88
Spearman Correlation 0.92 **iRT Error:** 0.28

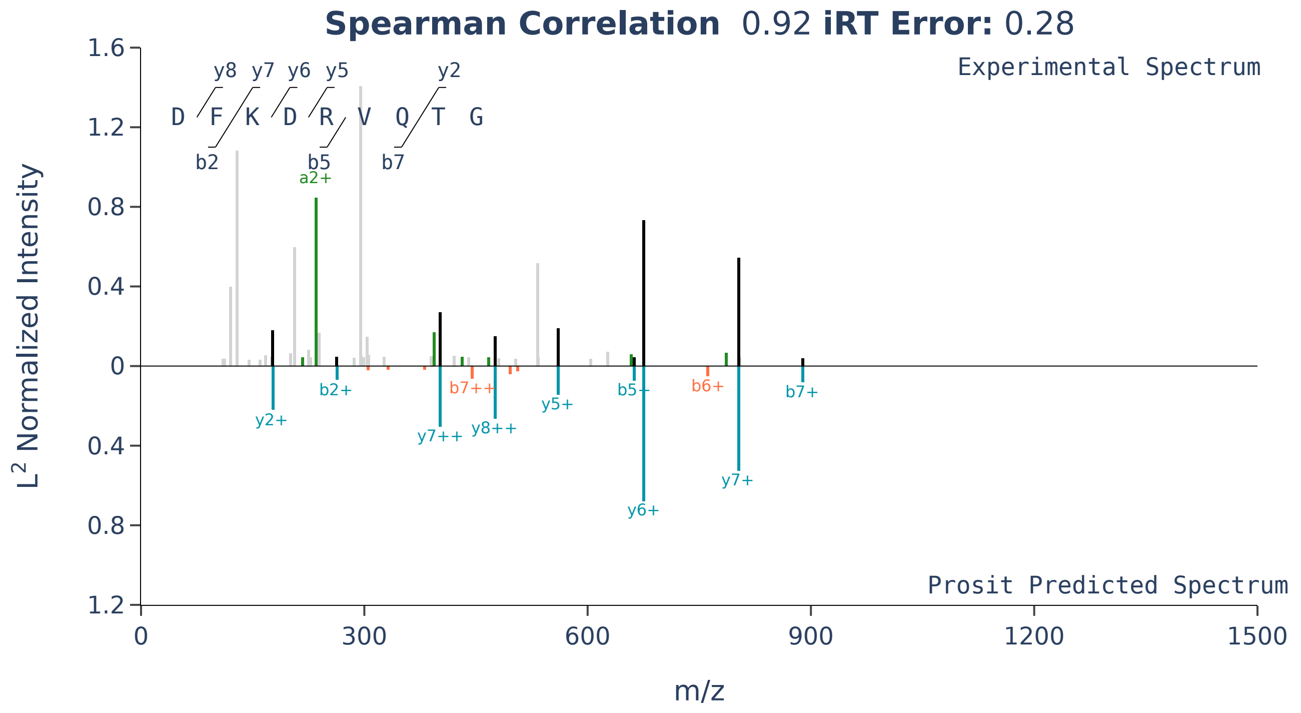

Source WSoh_221121_011221_HFGoe_L1_hTau_1h_R1 **Scan** 9077
Peptide QAAAQPHTEI **Charge** 2 **Spectral Angle** 0.0
Spearman Correlation 0.0 **iRT Error:** 10.59

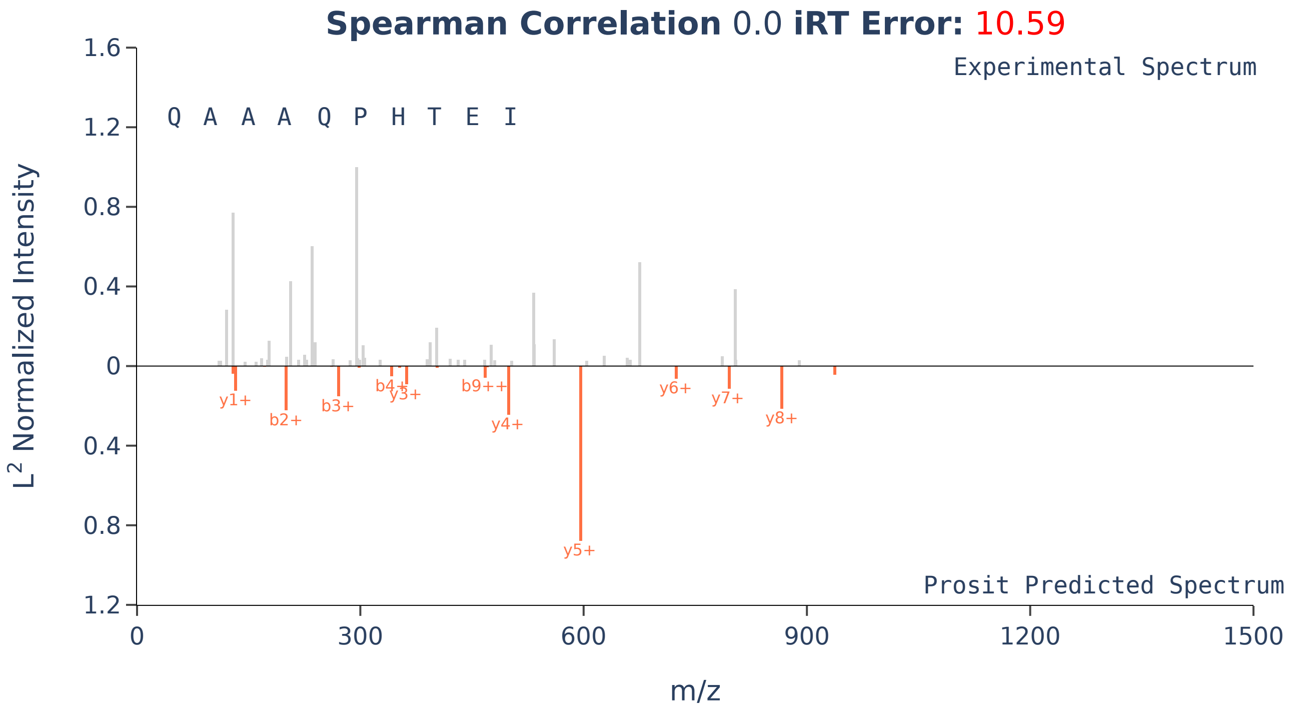

Source WSoh_221121_011221_HFGoe_L2_hTau_24h_R1 **Scan** 16582
Peptide DGSEEPGSETSDAKSTSPLQTPTE **Charge** 3 **Spectral Angle** 0.93
Spearman Correlation 0.88 **iRT Error:** 0.29

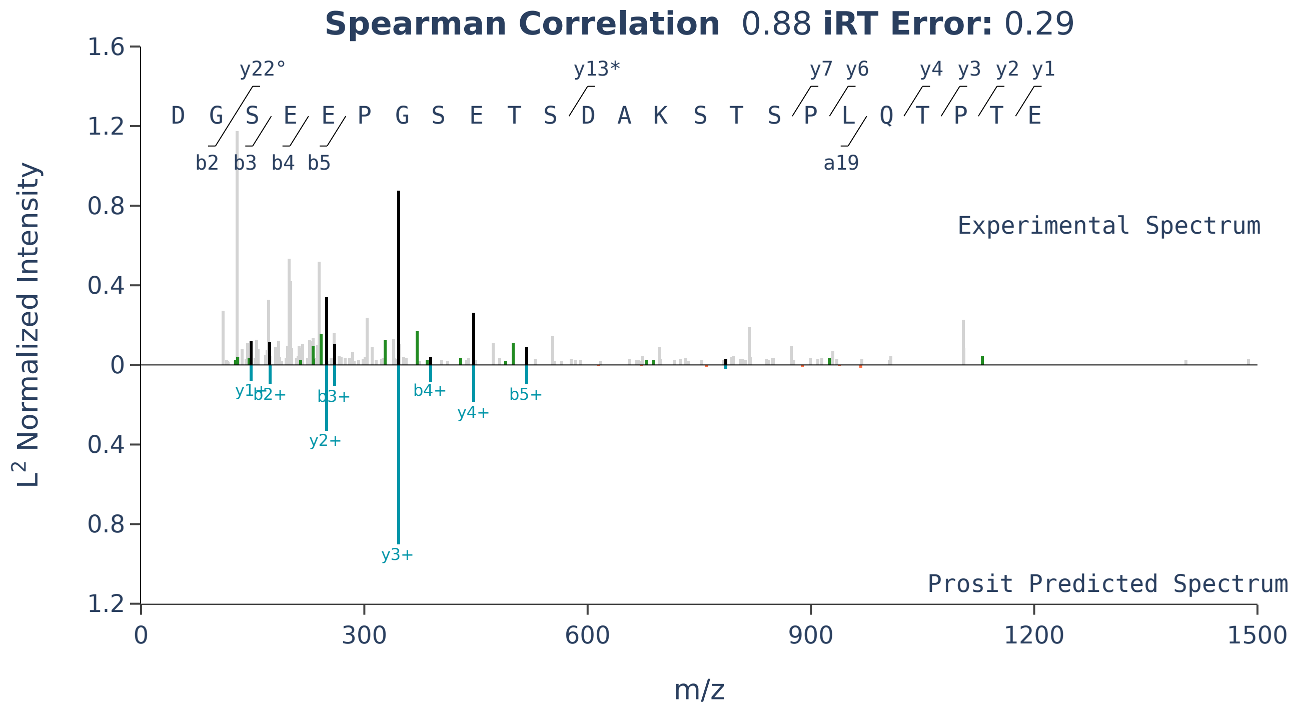

Source WSoh_221121_011221_HFGoe_L2_hTau_24h_R1 **Scan** 16582
Peptide SPLQTPTEDGSEEPGSETSDAKST **Charge** 3 **Spectral Angle** 0.24
Spearman Correlation 0.32 **iRT Error:** 12.53

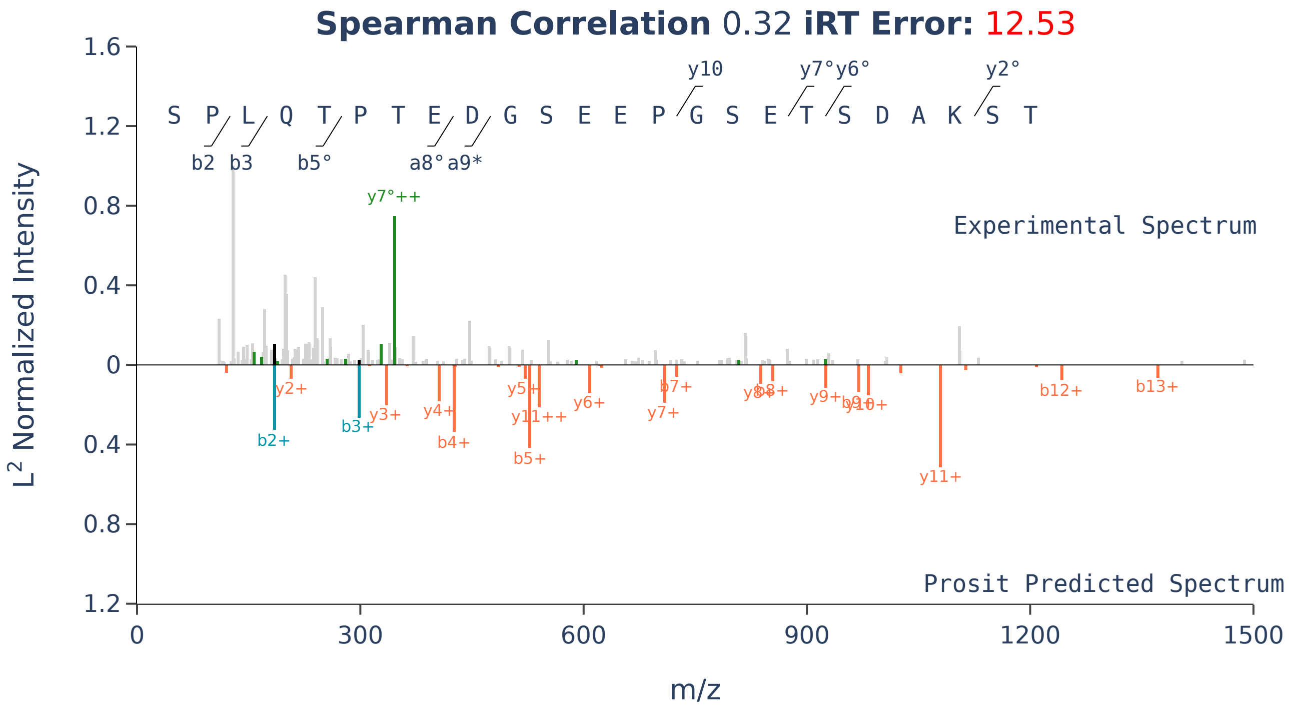

Source WSoh_221121_011221_HFGoe_L2_hTau_2h_R1 **Scan** 12718
Peptide KSEKLDFKDRVQSV **Charge** 4 **Spectral Angle** 0.87
Spearman Correlation 0.84 **iRT Error:** 0.34

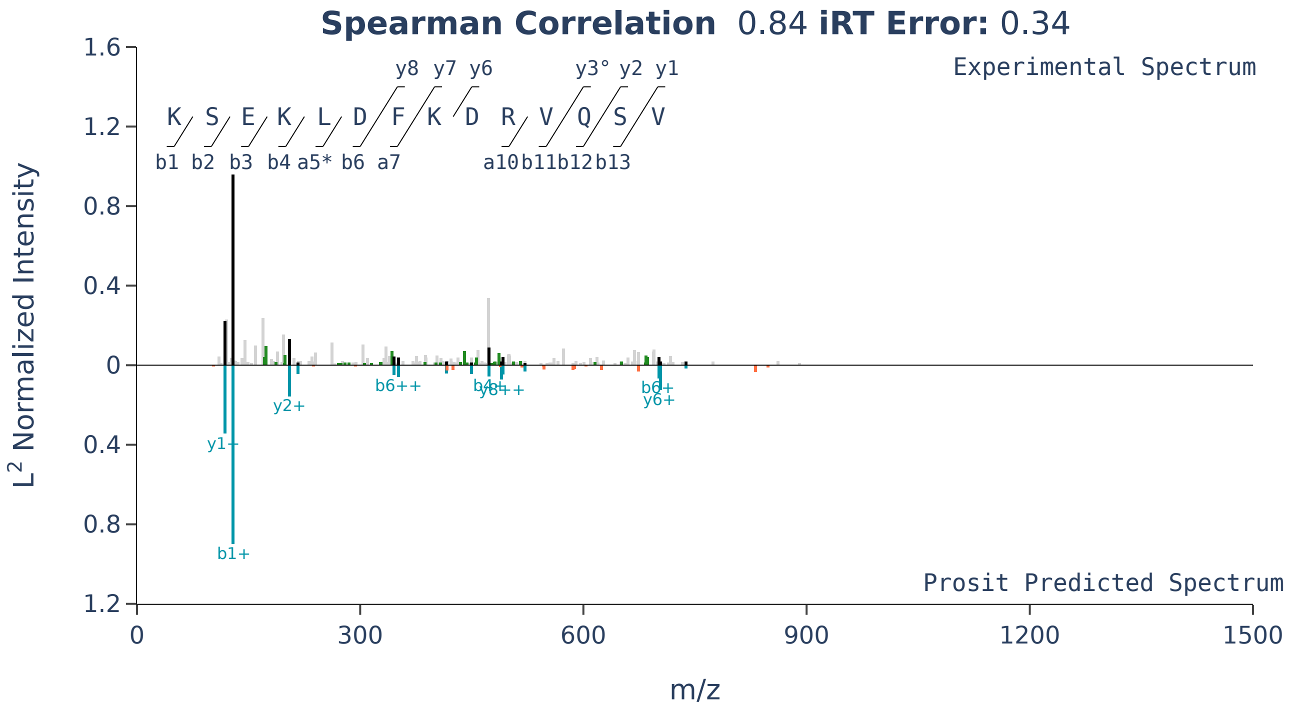

Source WSoh_221121_011221_HFGoe_L2_hTau_2h_R1 **Scan** 12718
Peptide VKSEKLDFKDRVQS **Charge** 4 **Spectral Angle** 0.09
Spearman Correlation -0.02 **iRT Error:** 14.63

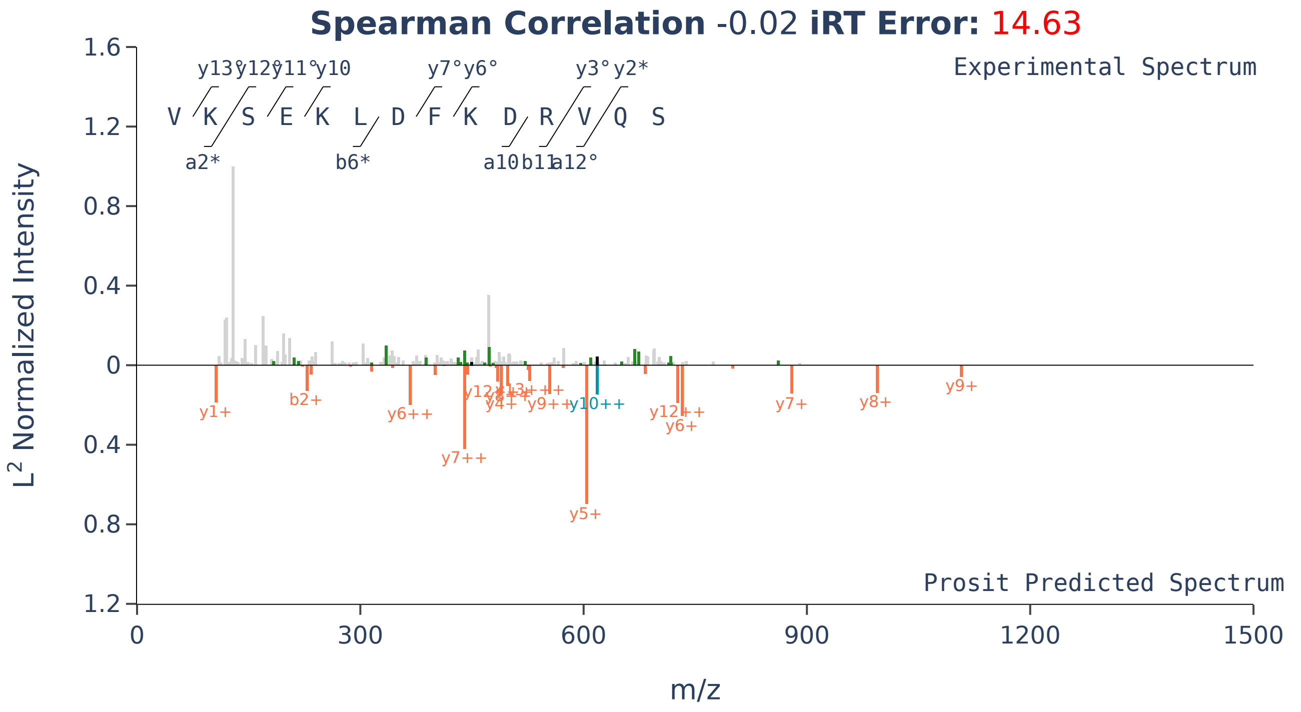

Source WSoh_221121_011221_HFGoe_L1_hTau_4h_R2 **Scan** 11667
Peptide NLDRKDQGGYTMHQDQEGDTDAGLKE **Charge** 5 **Spectral Angle** 0.78
Spearman Correlation 0.89 **iRT Error:** 0.35

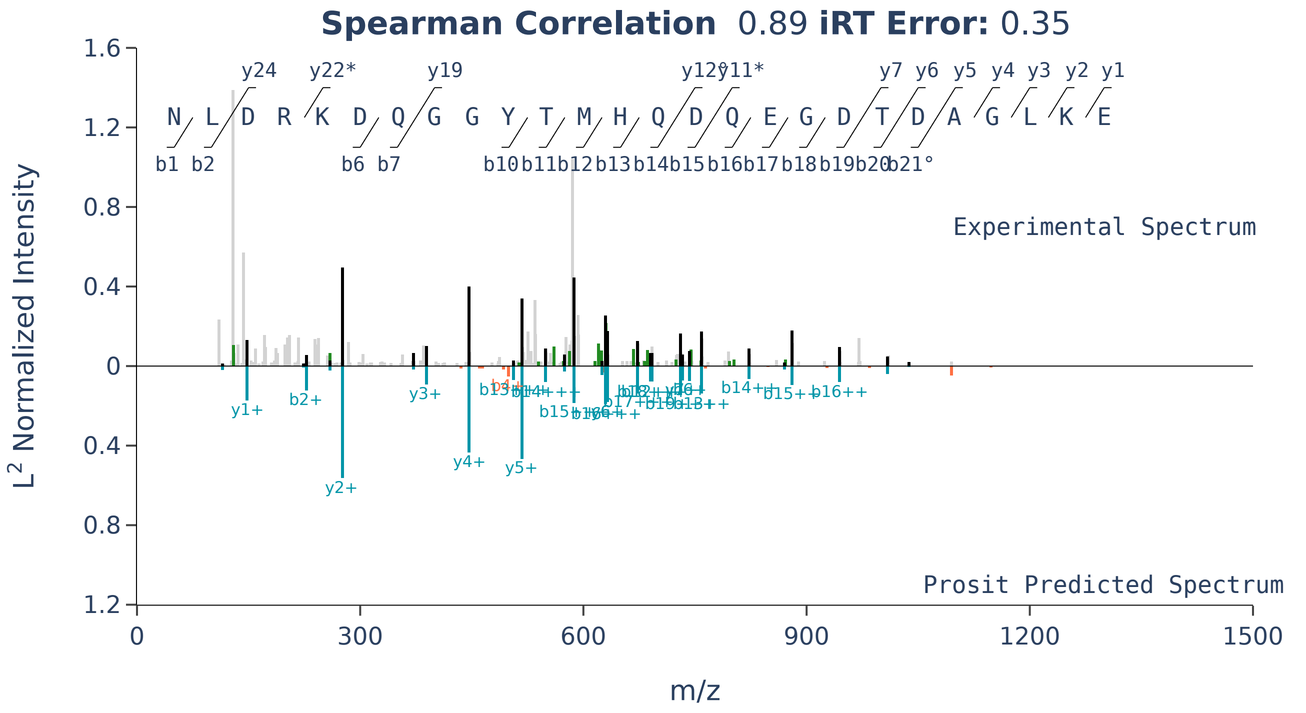

Source WSoh_221121_011221_HFGoe_L1_hTau_4h_R2 **Scan** 11667
Peptide GLGDRKDQGGYTMHQDQEGDTDAGLKE **Charge** 5 **Spectral Angle** 0.77
Spearman Correlation 0.85 **iRT Error:** 0.62

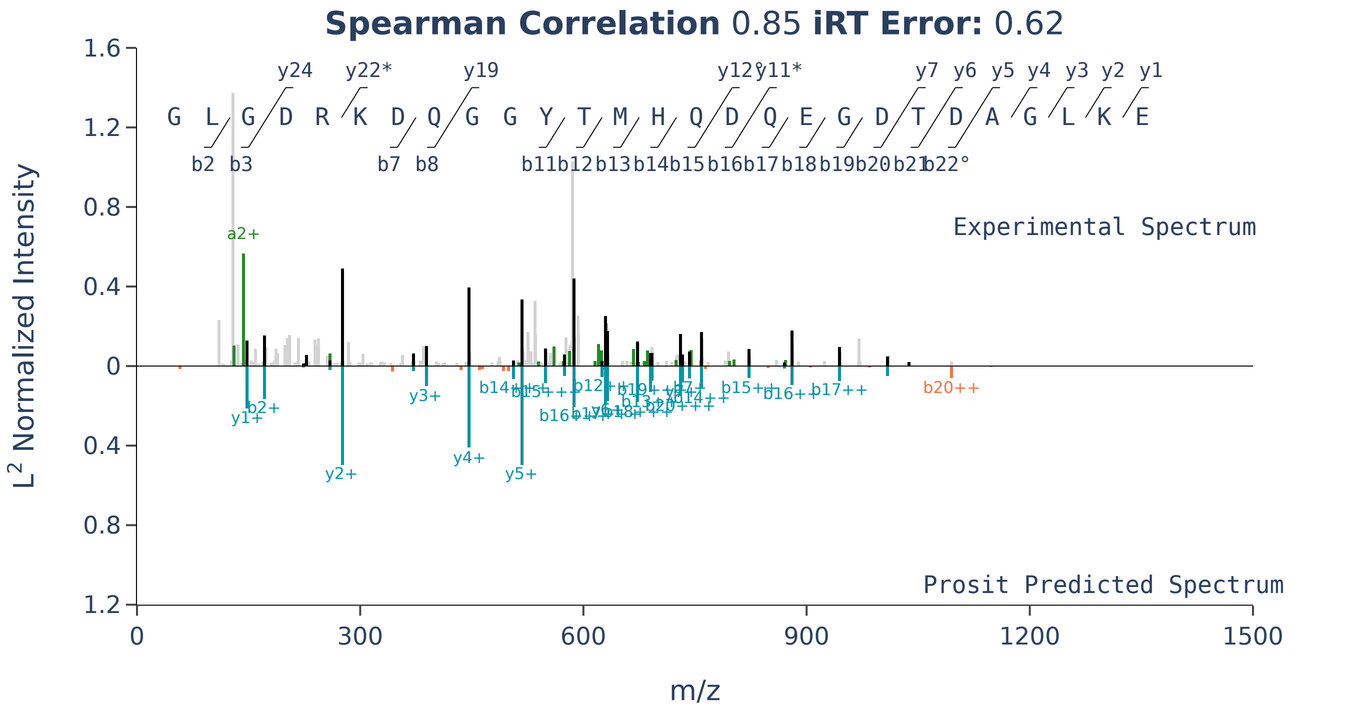

Source WSoh_221121_011221_HFGoe_L1_hTau_24h_R2 **Scan** 14450
Peptide QEGKAKTDHGAEIVYKSPVV **Charge** 2 **Spectral Angle** 0.82
Spearman Correlation 0.85 **iRT Error:** 0.46

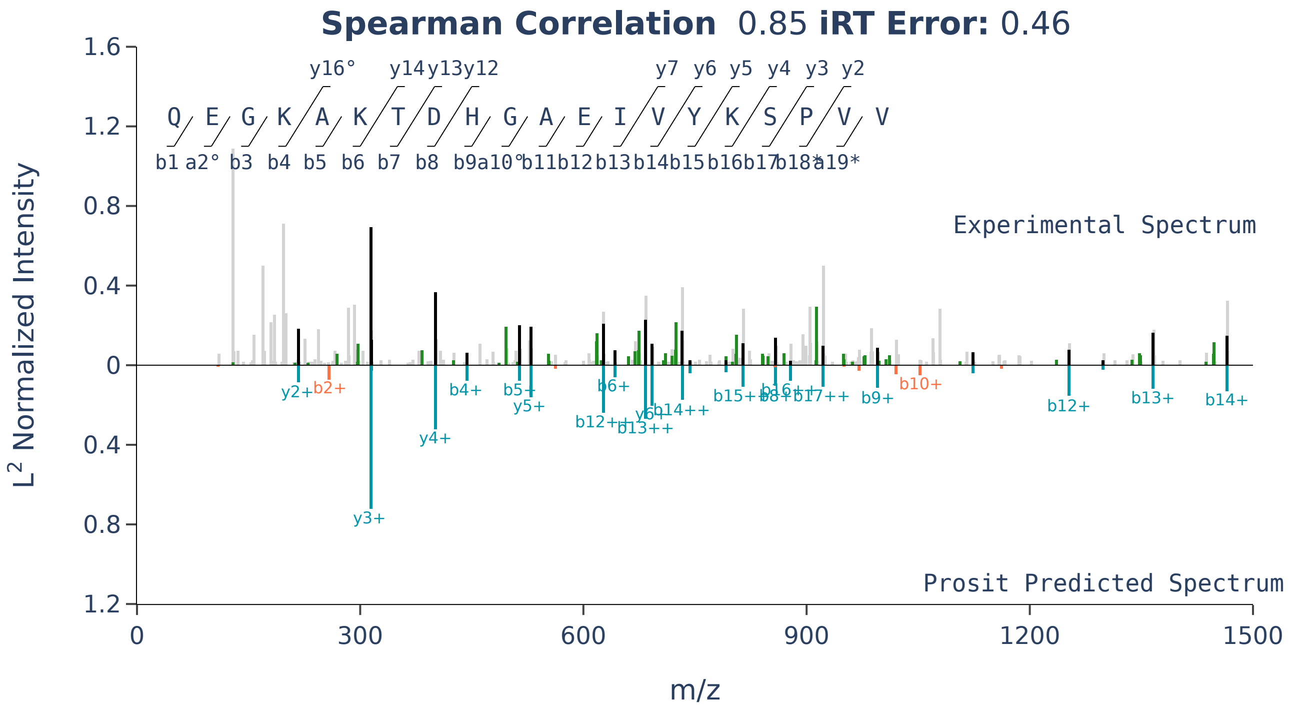

Source WSoh_221121_011221_HFGoe_L1_hTau_24h_R2 **Scan** 14450
Peptide ENAKAKTDHGAEIVYKSPVV **Charge** 2 **Spectral Angle** 0.77
Spearman Correlation 0.84 **iRT Error:** 1.99

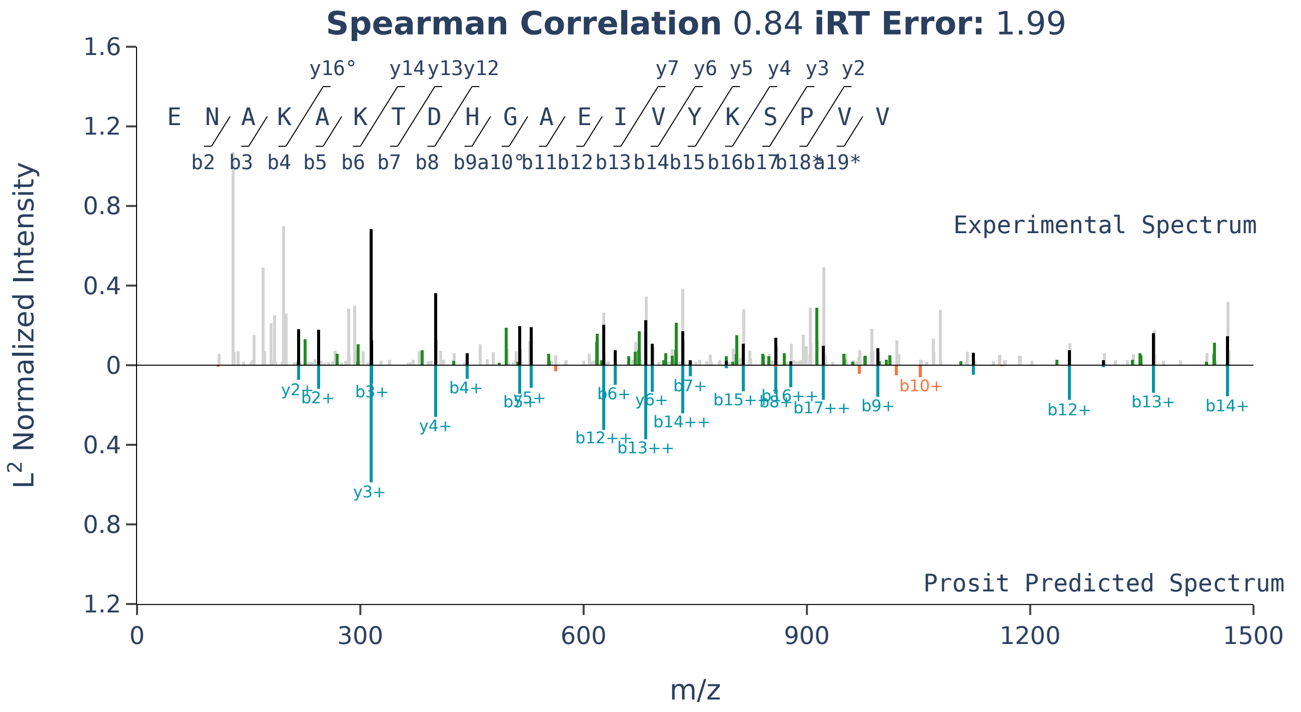

Source WSoh_221121_011221_HFGoe_L2_hTau_24h_R2 **Scan** 12343
Peptide KSEKLDFKDRVQNLK **Charge** 2 **Spectral Angle** 0.77
Spearman Correlation 0.86 **iRT Error:** 0.48

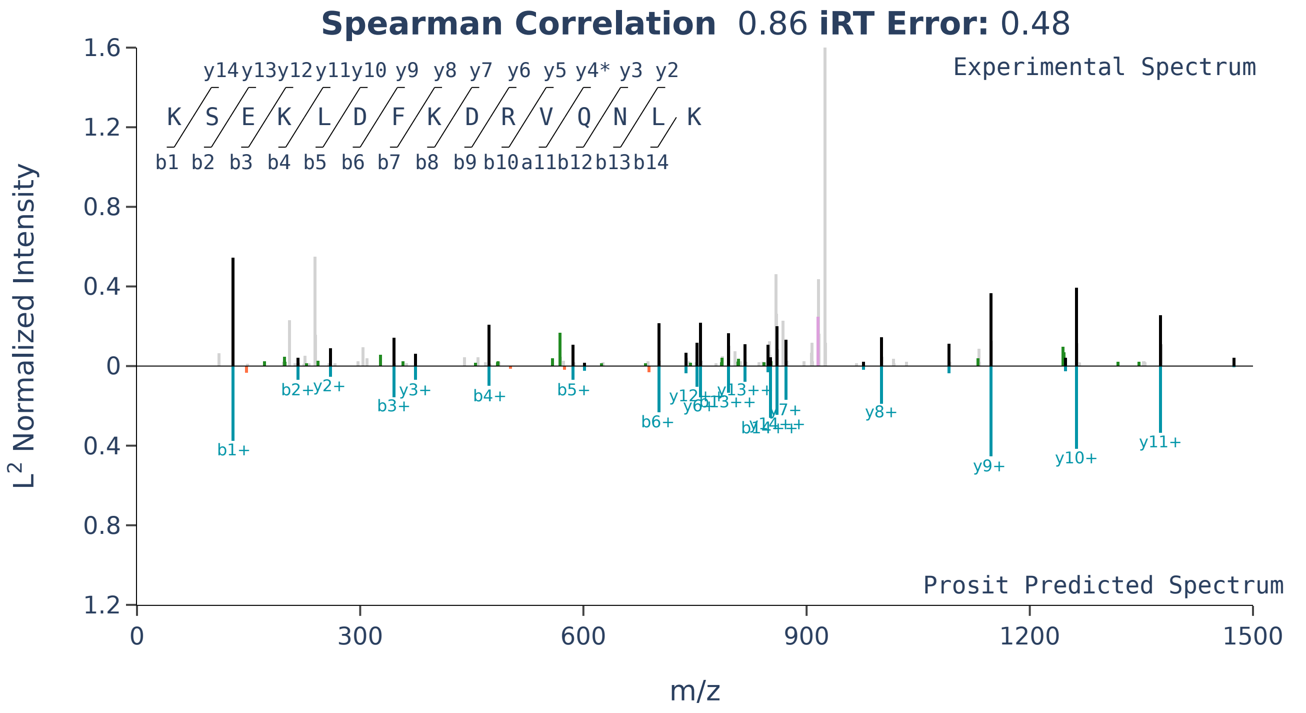

Source WSoh_221121_011221_HFGoe_L2_hTau_24h_R2 **Scan** 12343
Peptide PPKSPSSAKSRLQTAPVP **Charge** 2 **Spectral Angle** 0.09
Spearman Correlation 0.12 **iRT Error:** 3.97

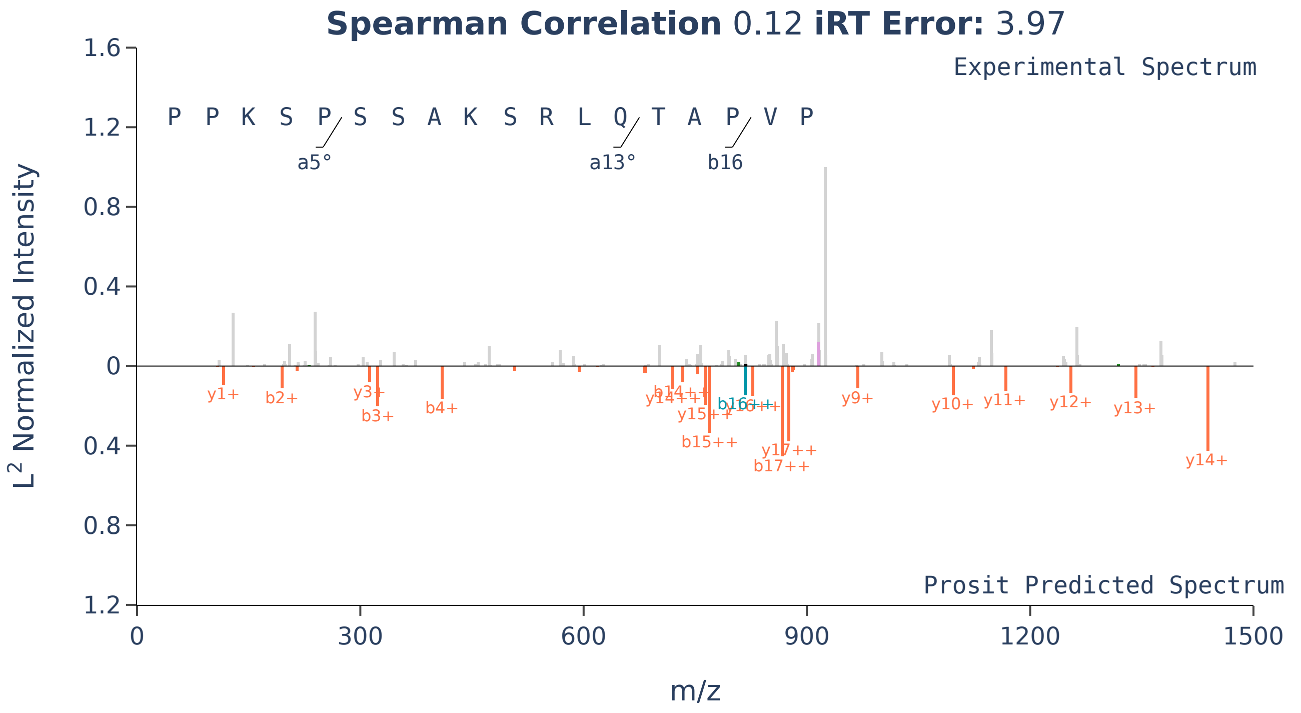

Source WSoh_221121_011221_HFGoe_L2_hTau_24h_R1 **Scan** 11766
Peptide LGGDRKDQGGYTMHQDQEGDTDAGLKE **Charge** 5 **Spectral Angle** 0.78
Spearman Correlation 0.92 **iRT Error:** 0.55

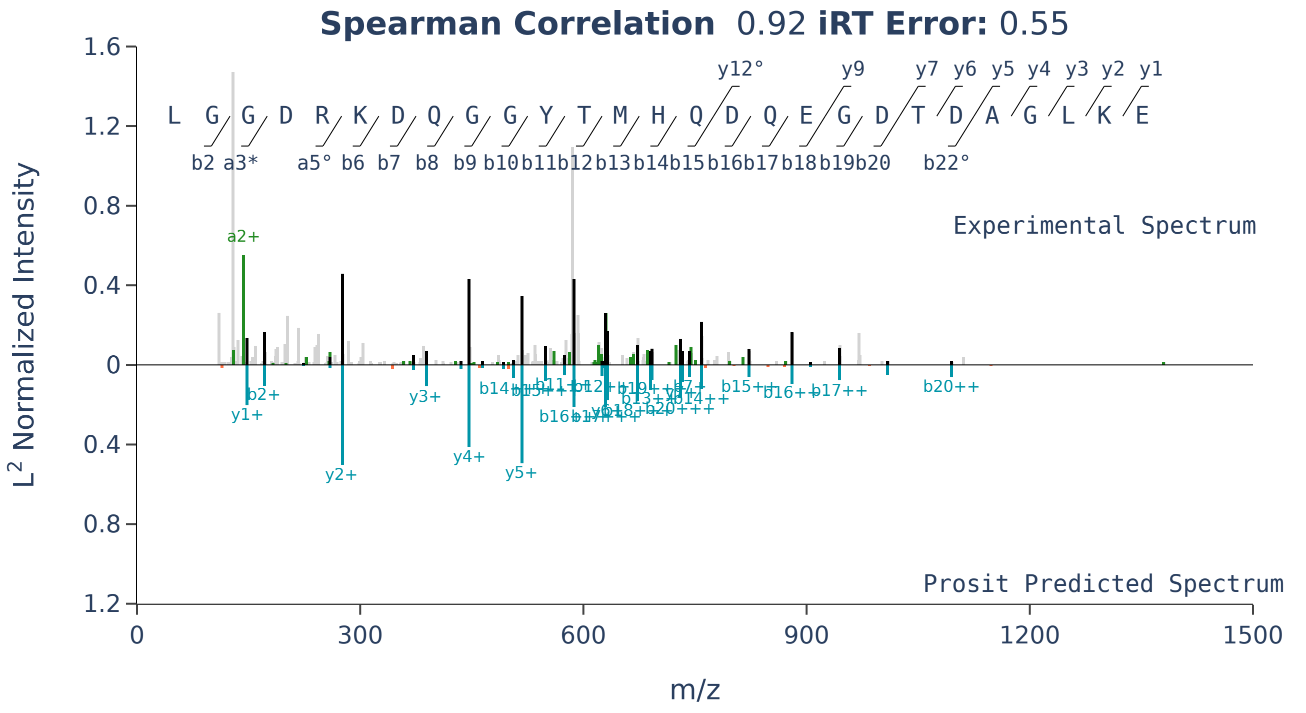

Source WSoh_221121_011221_HFGoe_L2_hTau_24h_R1 **Scan** 11766
Peptide GLGDRKDQGGYTMHQDQEGDTDAGLKE **Charge** 5 **Spectral Angle** 0.77
Spearman Correlation 0.9 **iRT Error:** 1.77

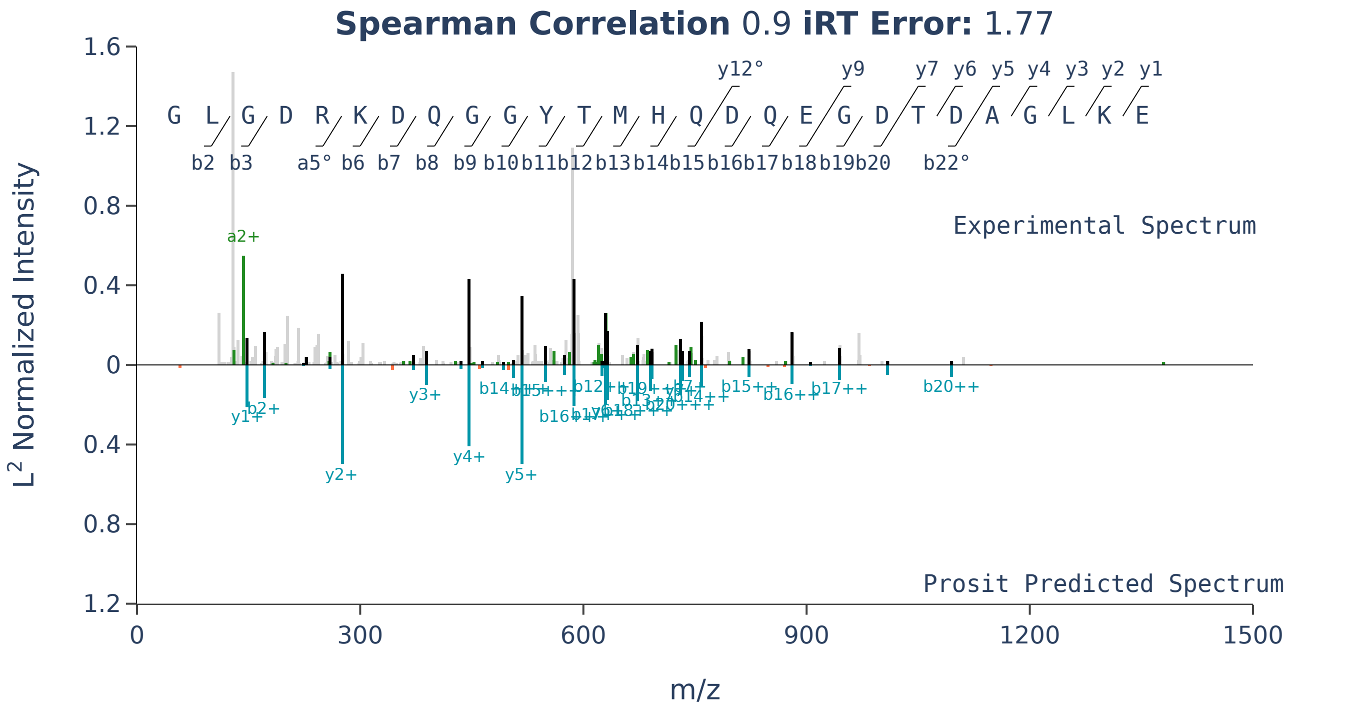

Source WSoh_221121_011221_HFGoe_L2_hTau_24h_R2 **Scan** 19113
Peptide DHAGTYGLGDRKDAEPRQEFEVME **Charge** 3 **Spectral Angle** 0.85
Spearman Correlation 0.83 **iRT Error:** 0.59

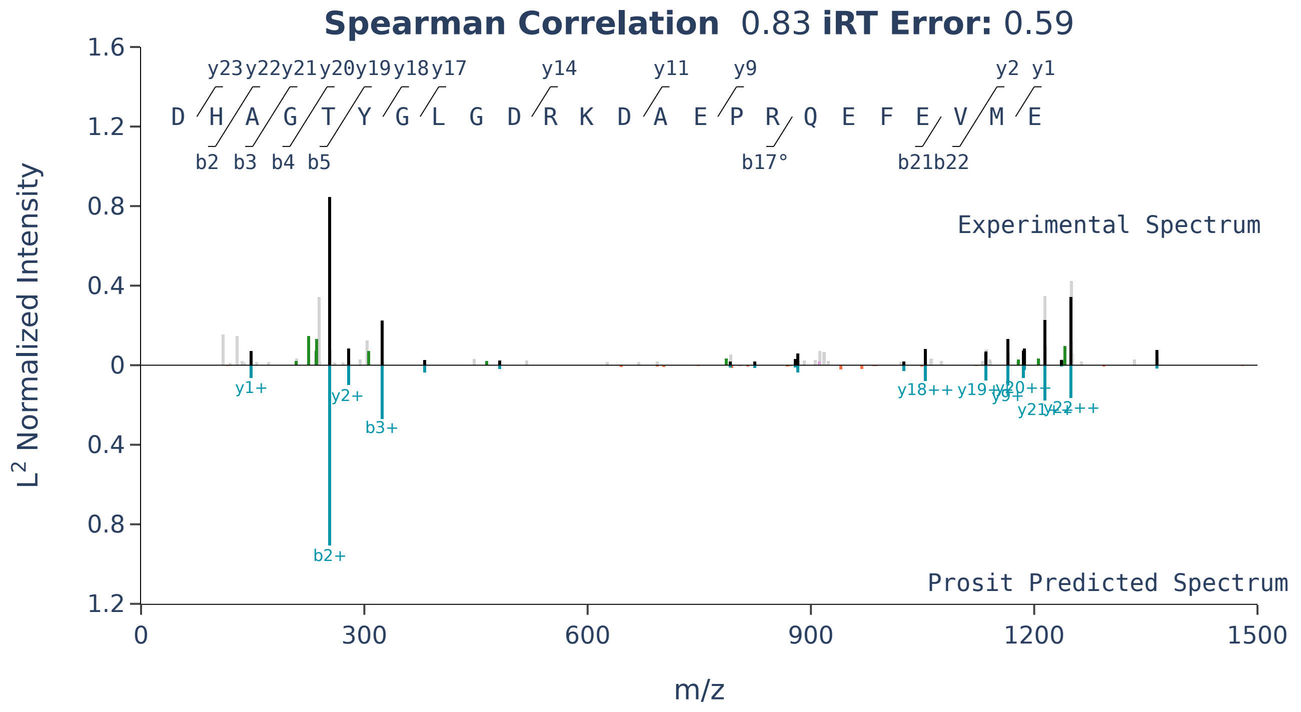

Source WSoh_221121_011221_HFGoe_L2_hTau_24h_R2 **Scan** 19113
Peptide AEPRQEFEVMEDHAGTYGLGDRKD **Charge** 3 **Spectral Angle** 0.05
Spearman Correlation 0.04 **iRT Error:** 4.71

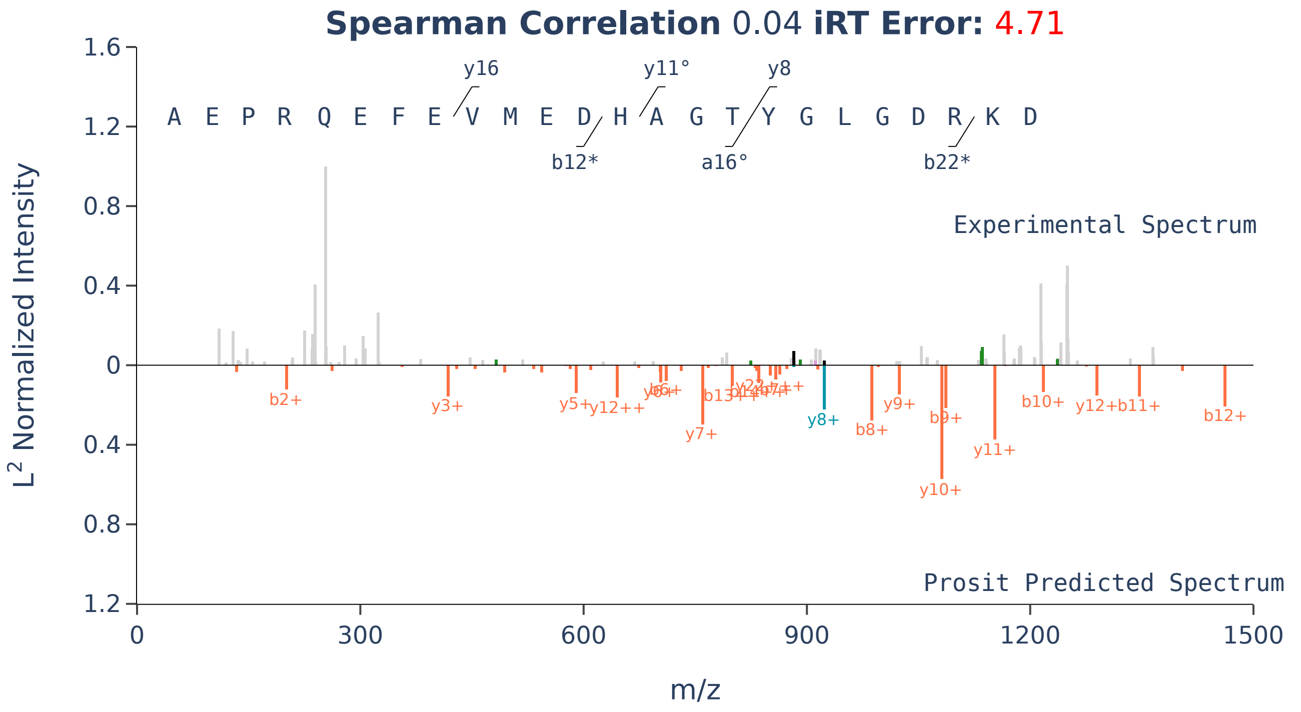

Source WSoh_221121_011221_HFGoe_L1_hTau_1h_R2 **Scan** 10728
Peptide PGSPGTPGSRSRTSLP **Charge** 3 **Spectral Angle** 0.87
Spearman Correlation 0.89 **iRT Error:** 0.59

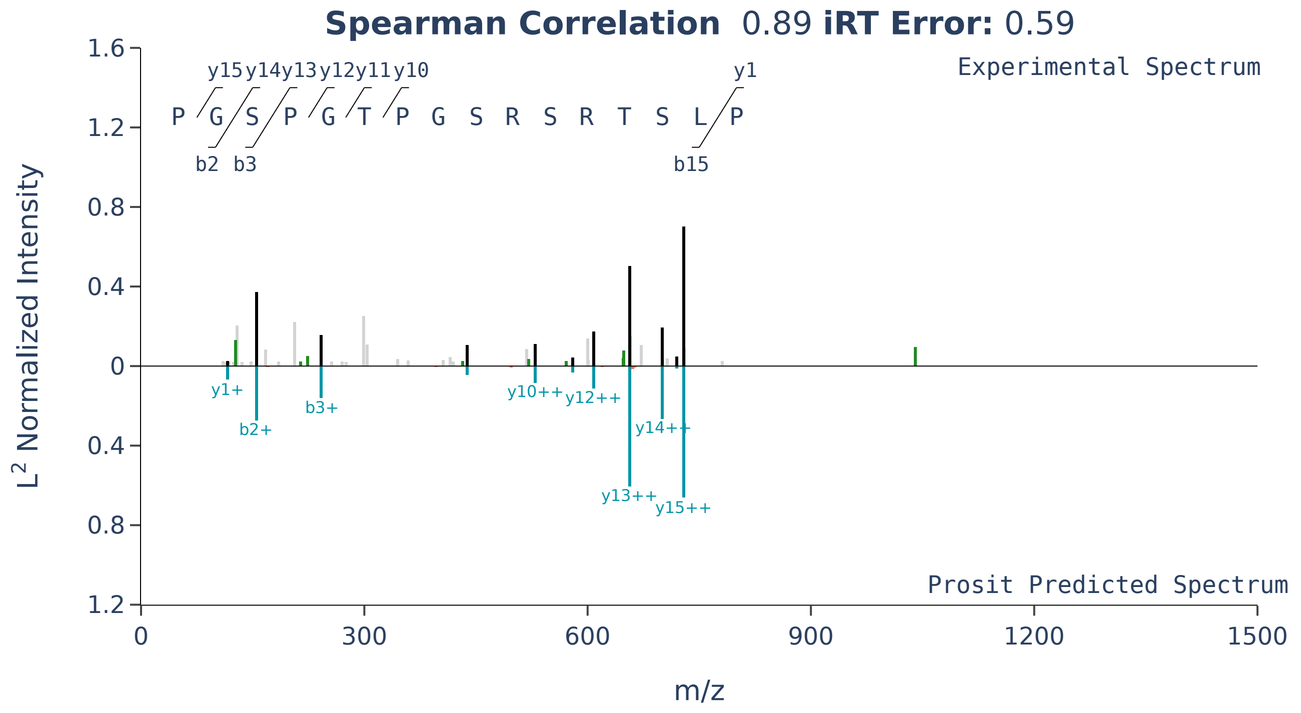

Source WSoh_221121_011221_HFGoe_L1_hTau_1h_R2 **Scan** 10728
Peptide GSPGTPGSRSRTPSLP **Charge** 3 **Spectral Angle** 0.45
Spearman Correlation 0.32 **iRT Error:** 10.84

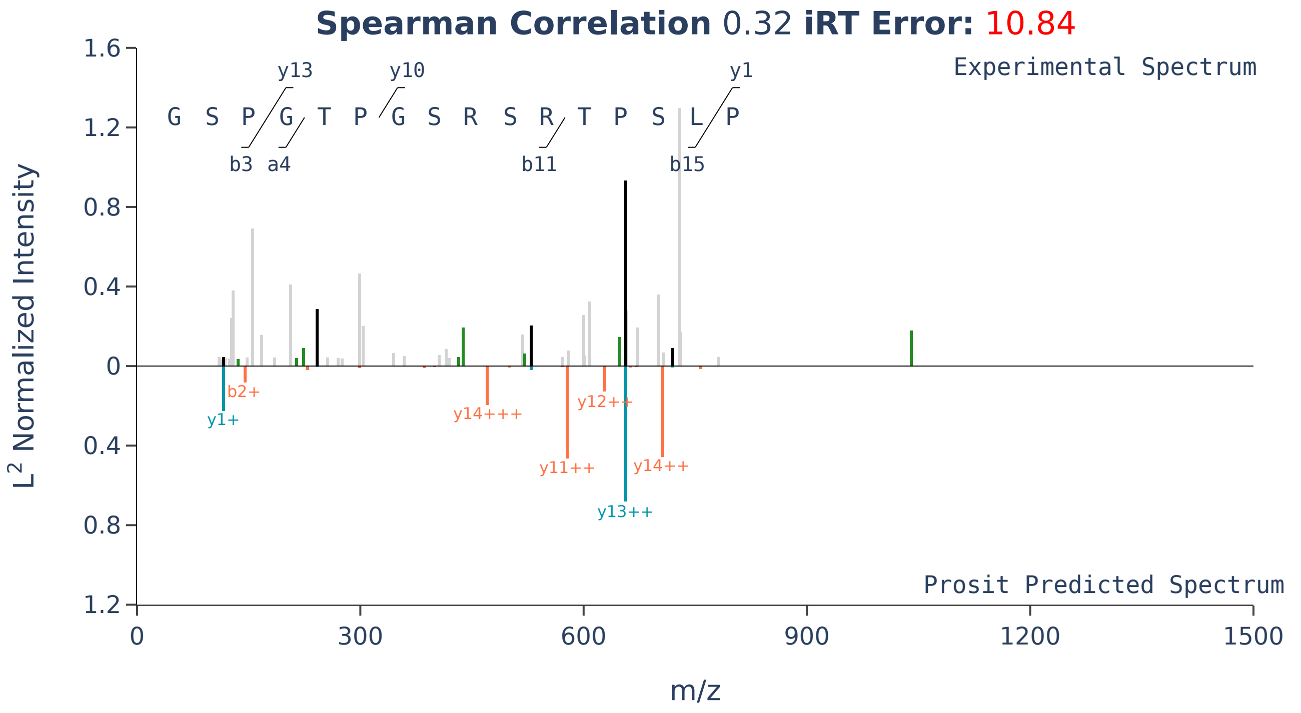

Source WSoh_221121_011221_HFGoe_L2_hTau_24h_R2 **Scan** 12721
Peptide GEAPGKQAAAQPHTEIPEGTTAE **Charge** 3 **Spectral Angle** 0.79
Spearman Correlation 0.86 **iRT Error:** 0.6

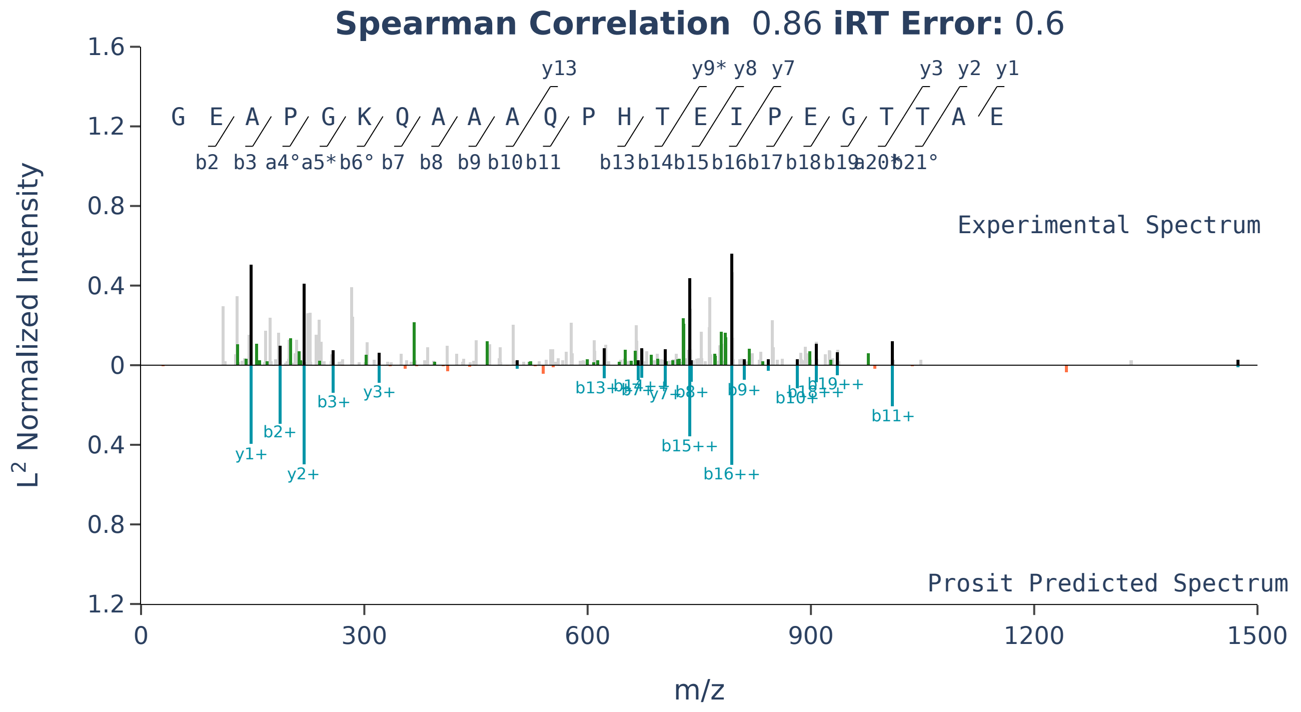

Source WSoh_221121_011221_HFGoe_L2_hTau_24h_R2 **Scan** 12721
Peptide GAPGKQAAAQPHTEIPEGTTAEE **Charge** 3 **Spectral Angle** 0.37
Spearman Correlation 0.44 **iRT Error:** 0.16

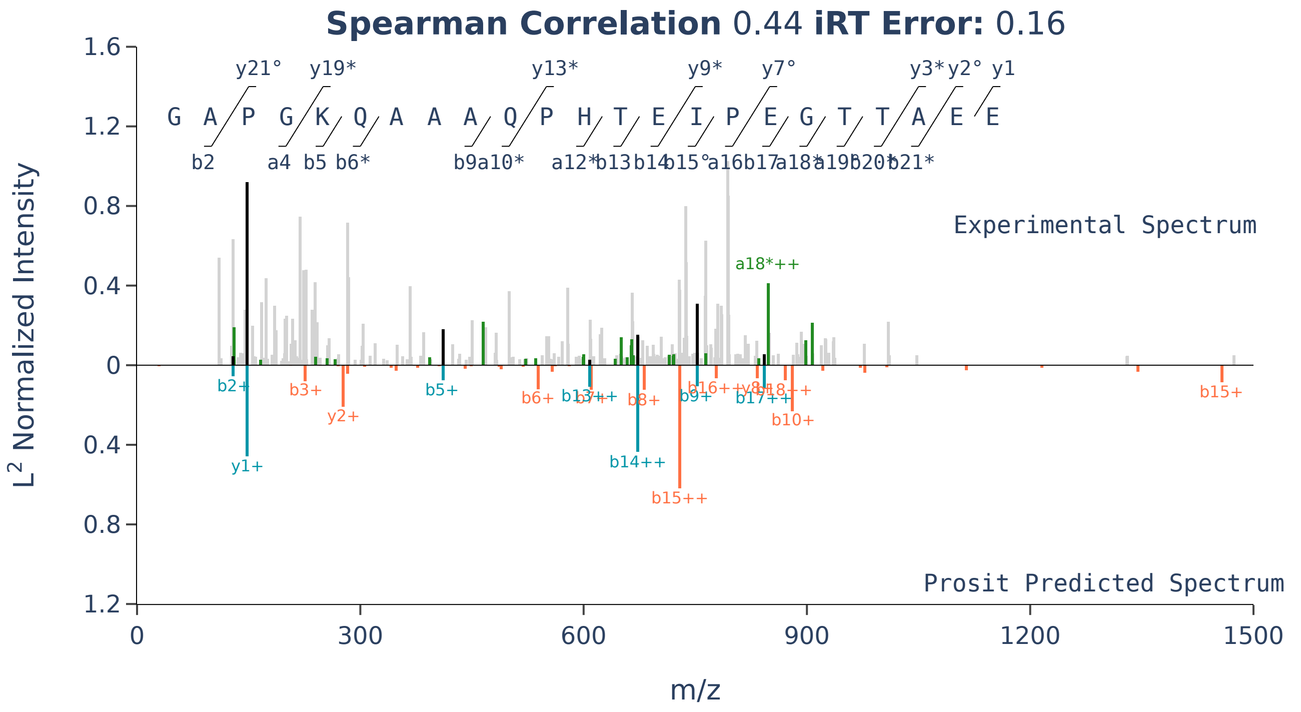

Source WSoh_221121_011221_HFGoe_L2_hTau_2h_R2 **Scan** 6237
Peptide TRIPAKTA **Charge** 2 **Spectral Angle** 0.87
Spearman Correlation 0.9 **iRT Error:** 0.61

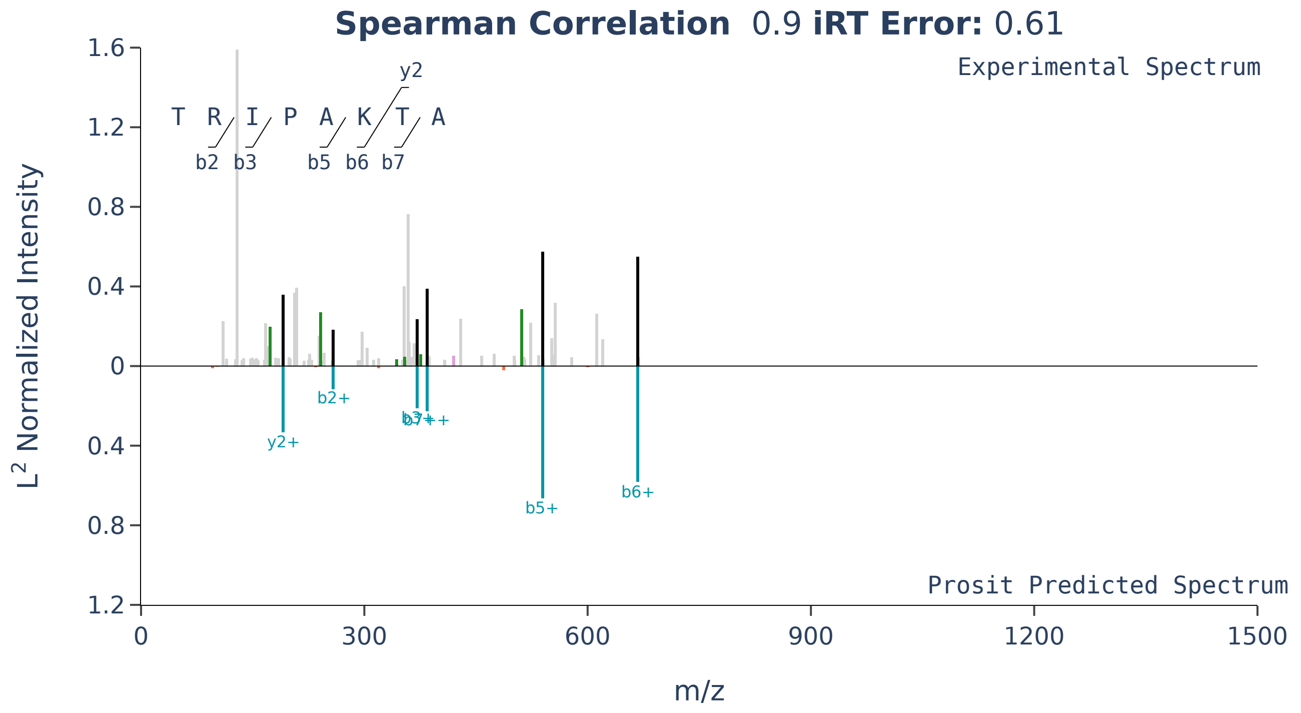

Source WSoh_221121_011221_HFGoe_L2_hTau_2h_R2 **Scan** 6237
Peptide ATRIPAKT **Charge** 2 **Spectral Angle** 0.04
Spearman Correlation -0.08 **iRT Error:** 4.26

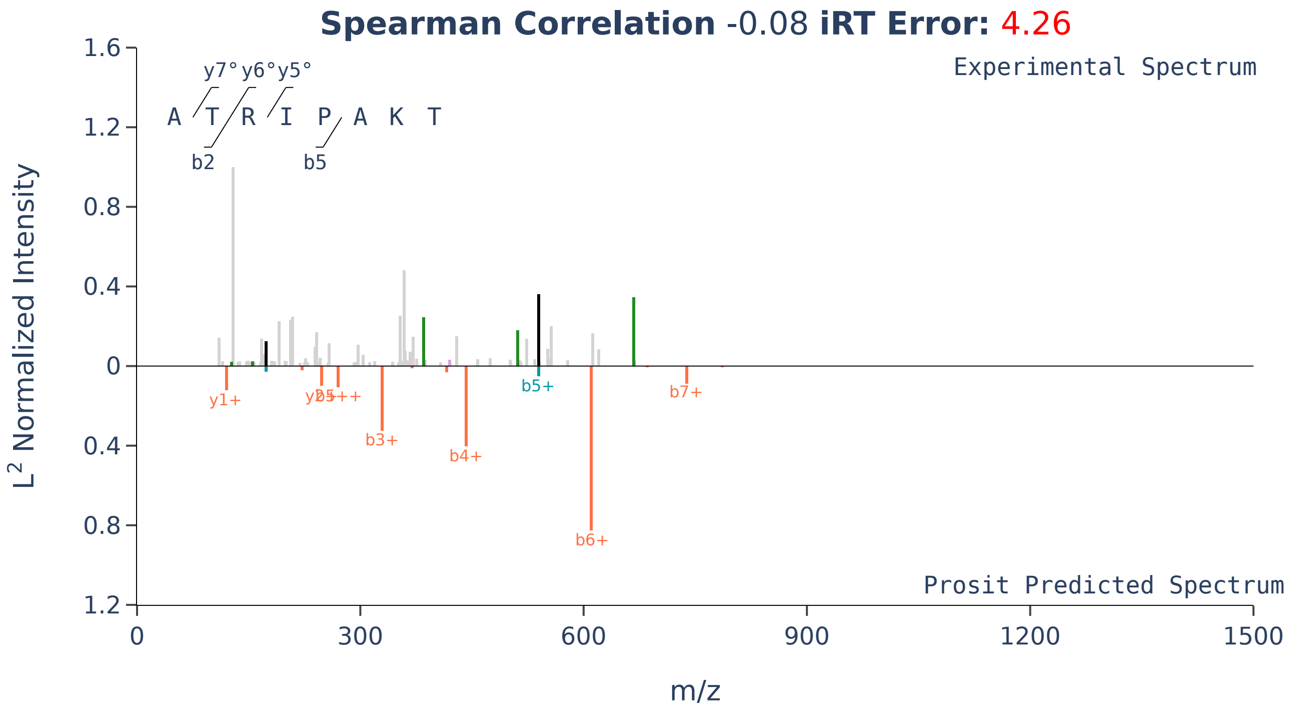

Source WSoh_221121_011221_HFGoe_L1_hTau_24h_R2 **Scan** 21018
Peptide SNVSSAEPRQEFEVME **Charge** 2 **Spectral Angle** 0.9
Spearman Correlation 0.91 **iRT Error:** 0.66

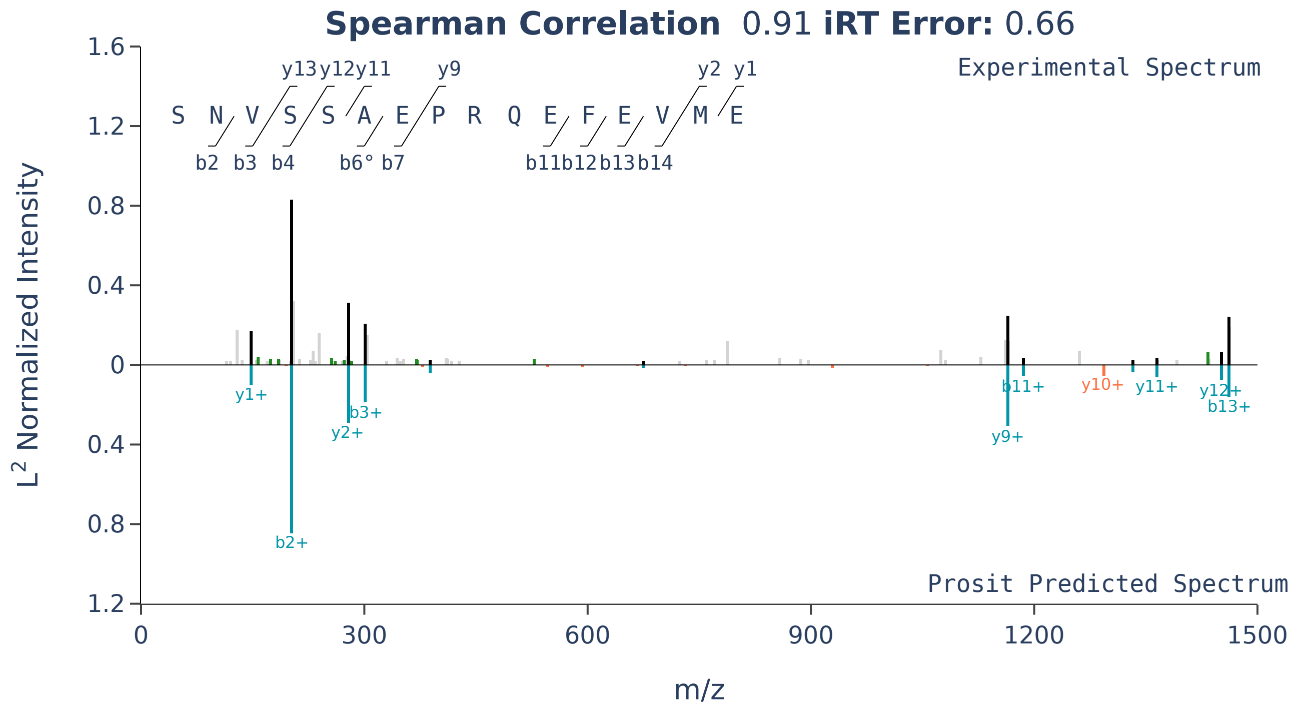

Source WSoh_221121_011221_HFGoe_L1_hTau_24h_R2 **Scan** 21018
Peptide SSGEPPKSGDRSGYSSPGS **Charge** 2 **Spectral Angle** 0.0
Spearman Correlation -0.24 **iRT Error:** 84.88

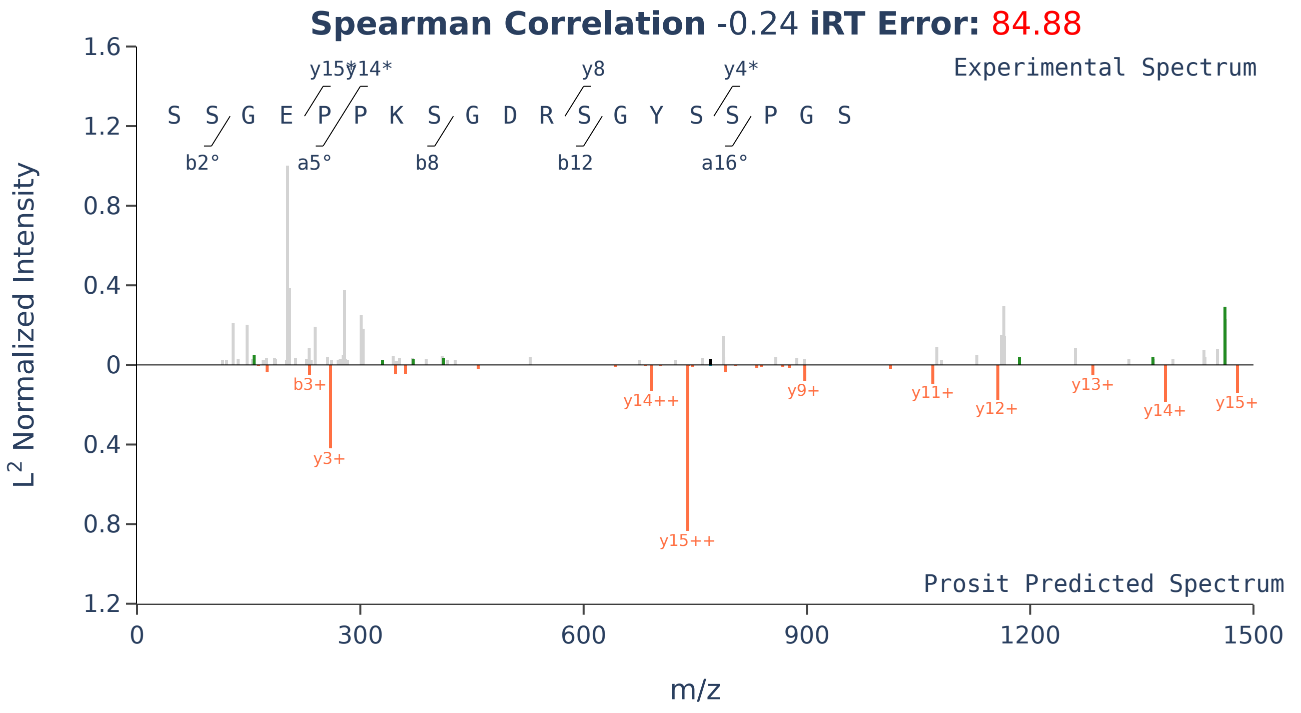

Source WSoh_221121_011221_HFGoe_L1_hTau_4h_R2 **Scan** 11381
Peptide NITHVPGGGNKKIETHKLTFRDAG **Charge** 3 **Spectral Angle** 0.8
Spearman Correlation 0.89 **iRT Error:** 0.72

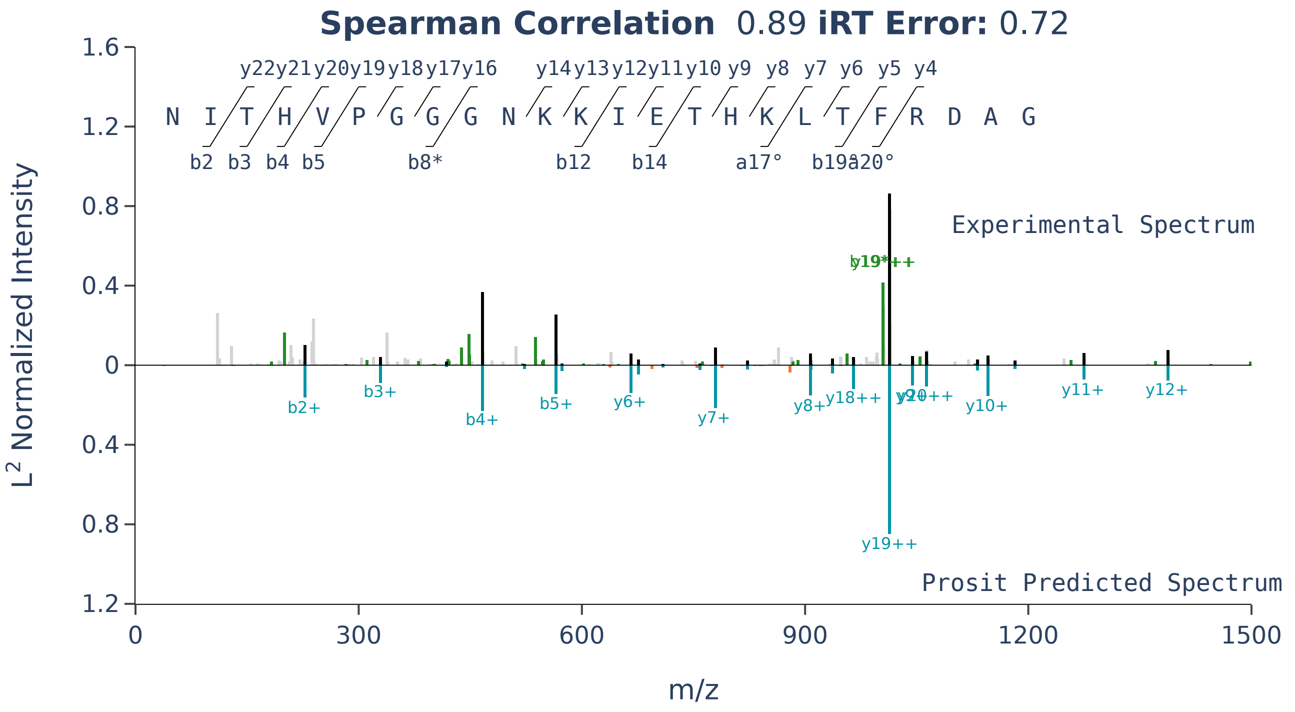

Source WSoh_221121_011221_HFGoe_L1_hTau_4h_R2 **Scan** 11381
Peptide NITHVPGGGNKKIETHKLTFREN **Charge** 3 **Spectral Angle** 0.85
Spearman Correlation 0.85 **iRT Error:** 2.76

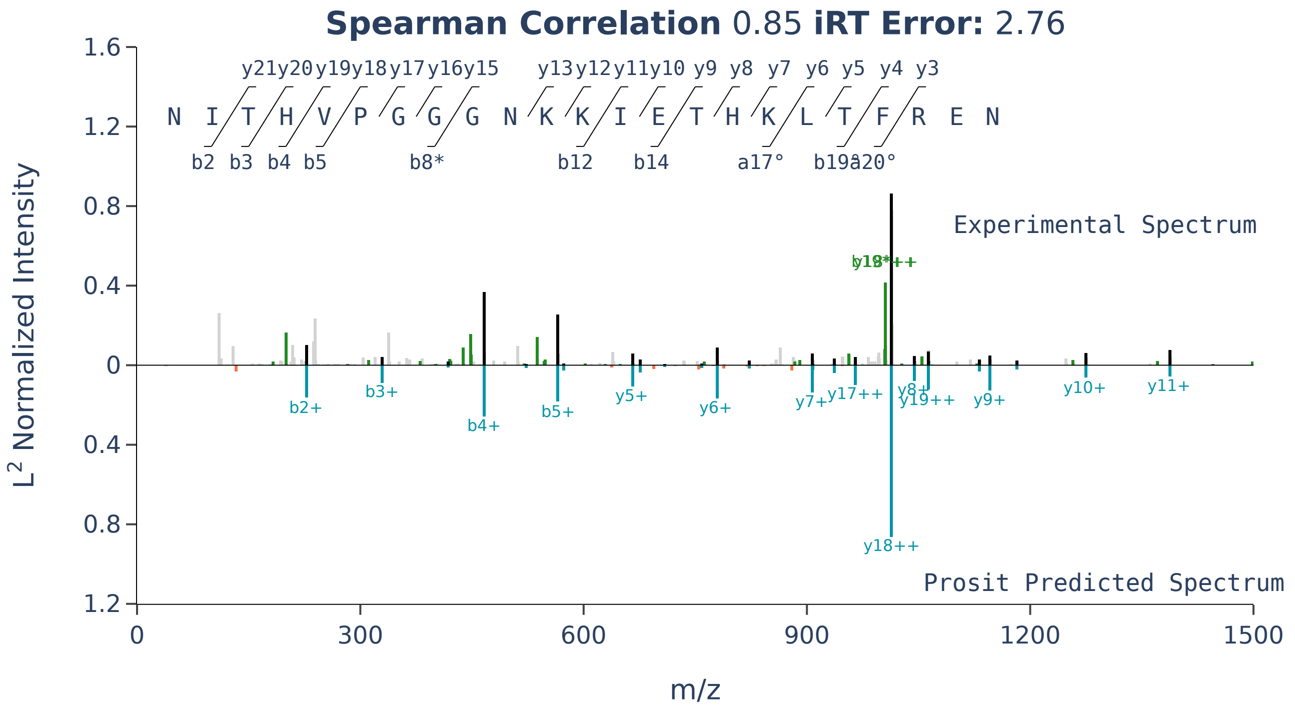

Source WSoh_221121_011221_HFGoe_L2_hTau_24h_R2 **Scan** 7824
Peptide THVPGGGNKKIETHGLG **Charge** 2 **Spectral Angle** 0.92
Spearman Correlation 0.78 **iRT Error:** 0.72

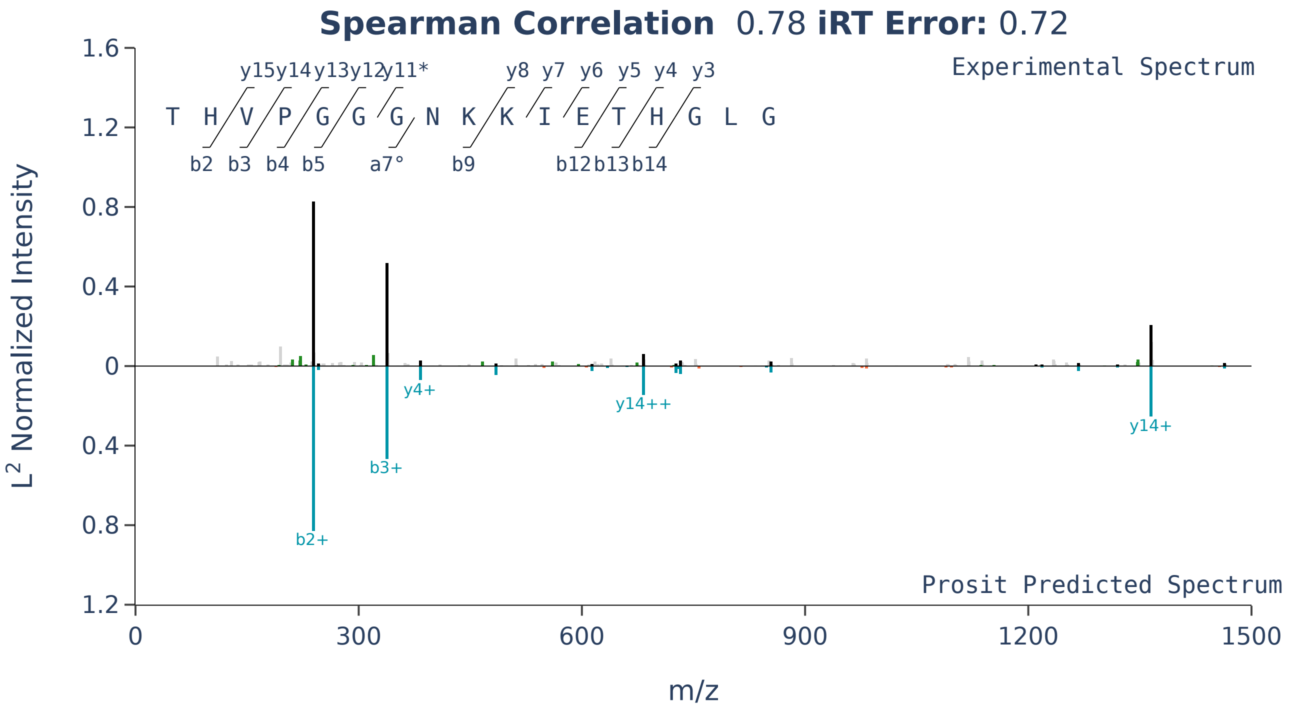

Source WSoh_221121_011221_HFGoe_L2_hTau_24h_R2 **Scan** 7824
Peptide NITHVPGGGNKKIETH **Charge** 2 **Spectral Angle** 0.7
Spearman Correlation 0.51 **iRT Error:** 5.12

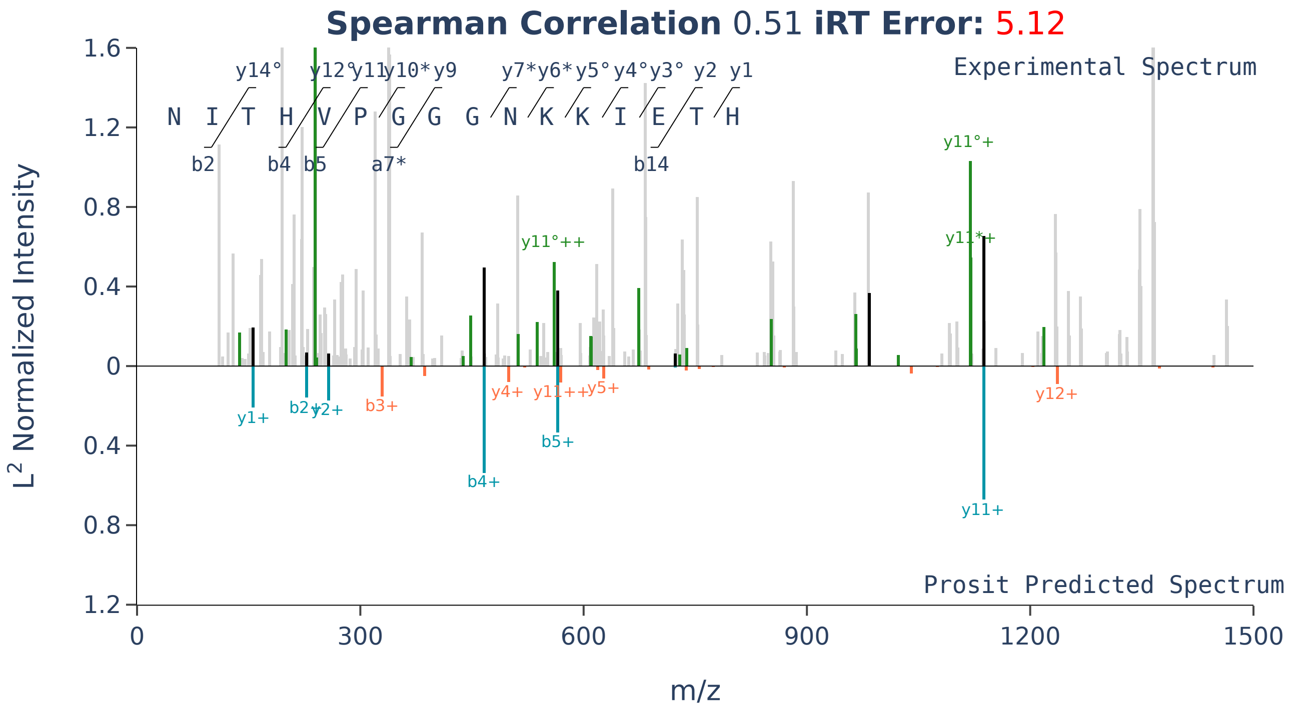

Source WSoh_221121_011221_HFGoe_L1_hTau_2h_R1 **Scan** 12663
Peptide DFKDRVQSV **Charge** 2 **Spectral Angle** 0.92
Spearman Correlation 0.95 **iRT Error:** 0.81

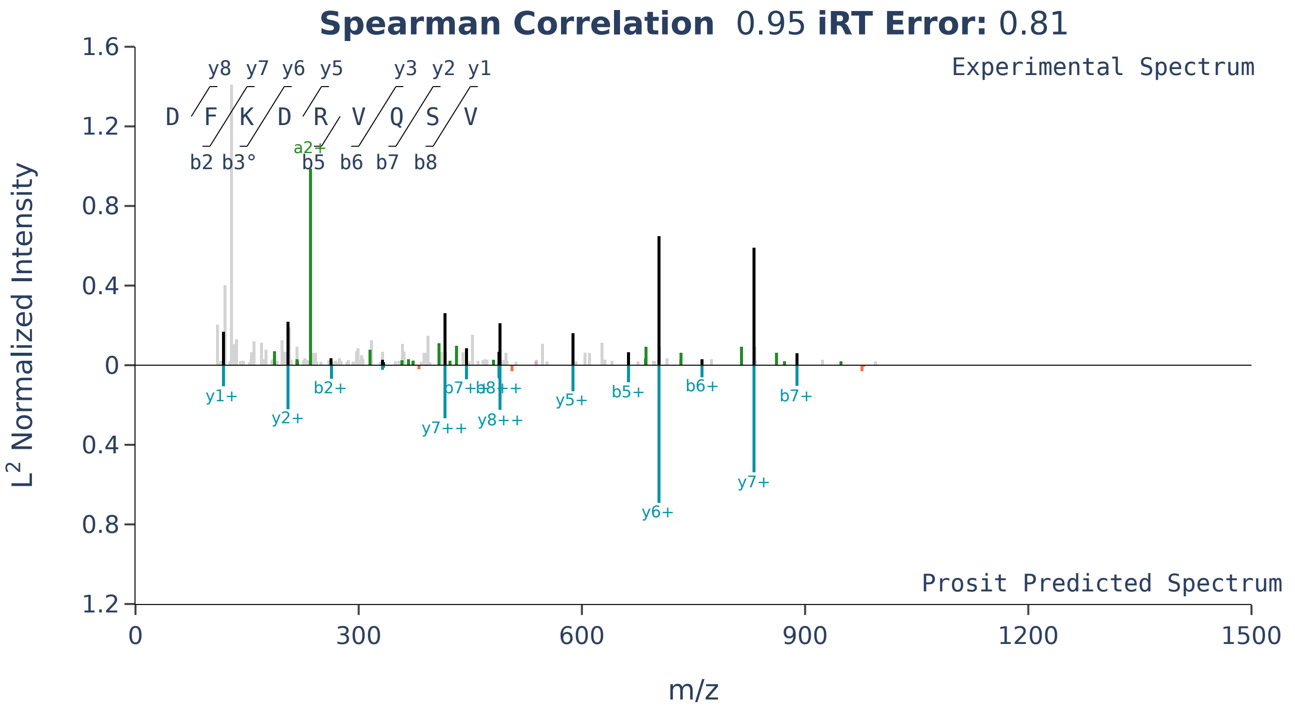

Source WSoh_221121_011221_HFGoe_L1_hTau_2h_R1 **Scan** 12663
Peptide KVTSKCGSLGN **Charge** 2 **Spectral Angle** 0.75
Spearman Correlation -0.04 **iRT Error:** 37.78

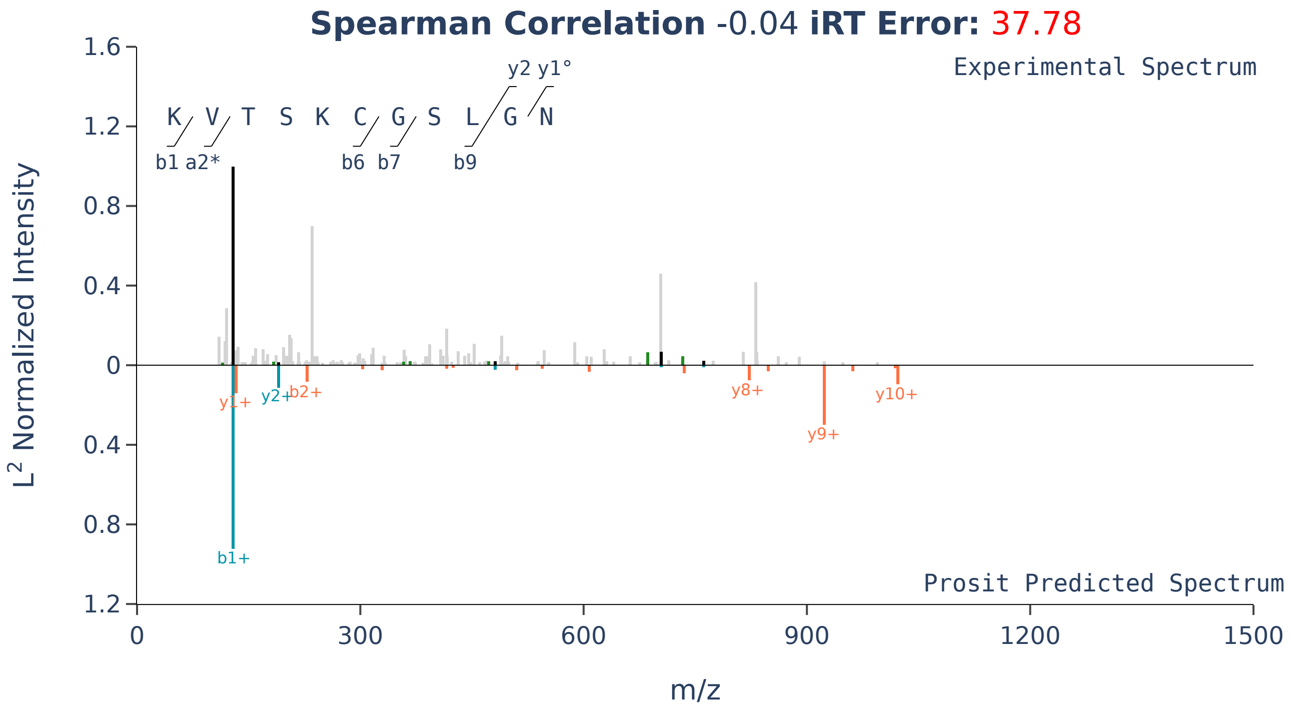

Source WSoh_221121_011221_HFGoe_L1_hTau_4h_R2 **Scan** 10576
Peptide KLDFKDRVQSG **Charge** 3 **Spectral Angle** 0.76
Spearman Correlation 0.9 **iRT Error:** 0.83

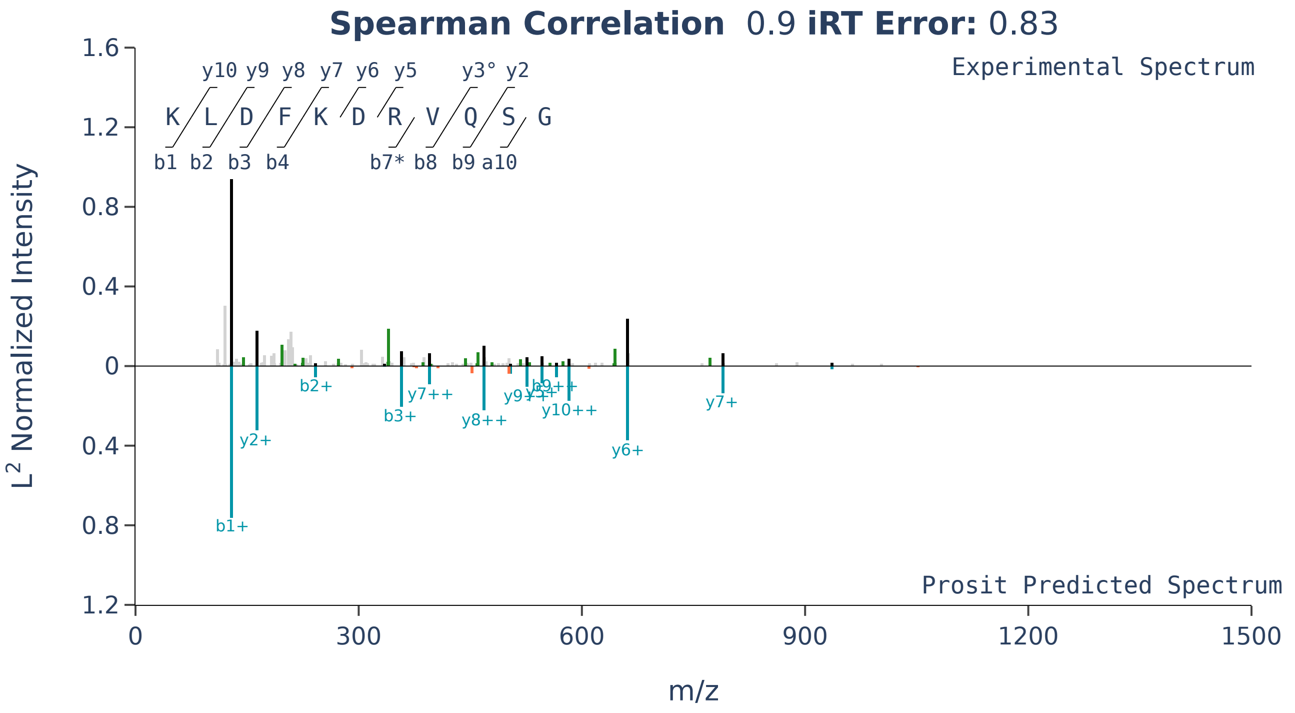

Source WSoh_221121_011221_HFGoe_L1_hTau_4h_R2 **Scan** 10576
Peptide DFKDRVQSKIG **Charge** 3 **Spectral Angle** 0.05
Spearman Correlation -0.07 **iRT Error:** 10.16

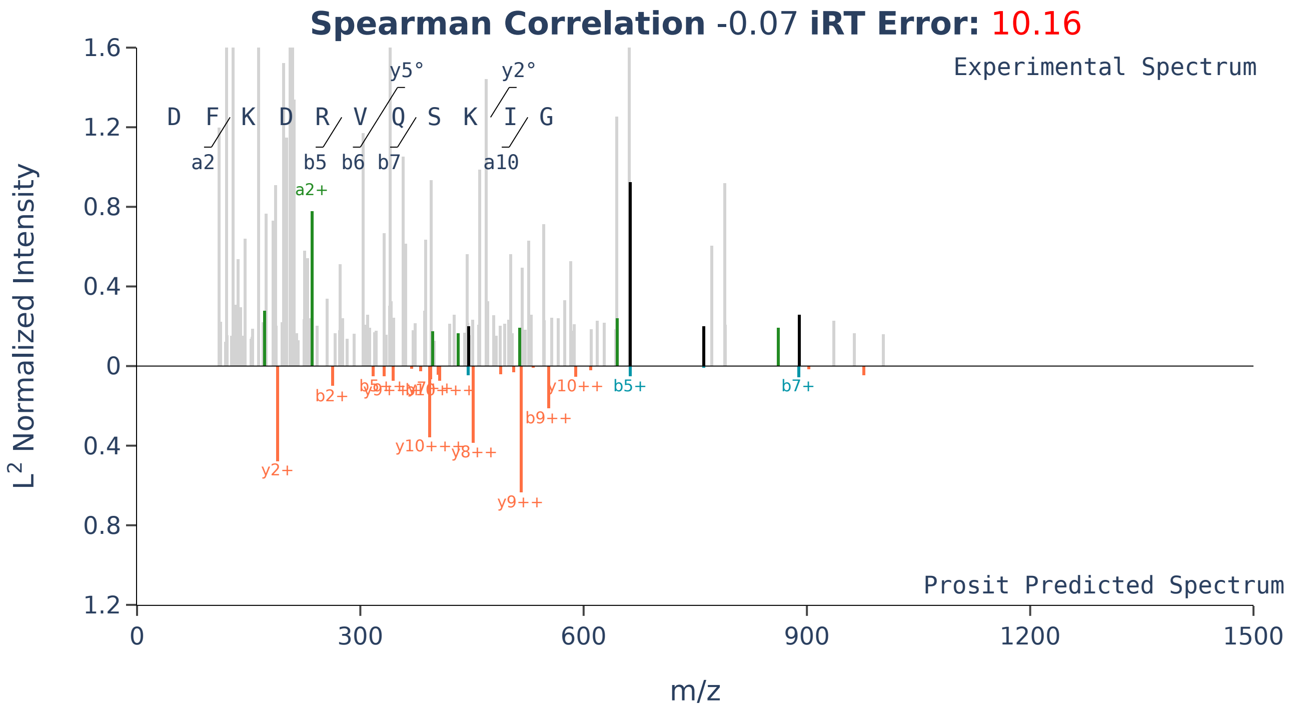

Source WSoh_221121_011221_HFGoe_L1_hTau_4h_R1 **Scan** 13970
Peptide DAAQPHTEIPEGTTAE **Charge** 2 **Spectral Angle** 0.89
Spearman Correlation 0.92 **iRT Error:** 0.84

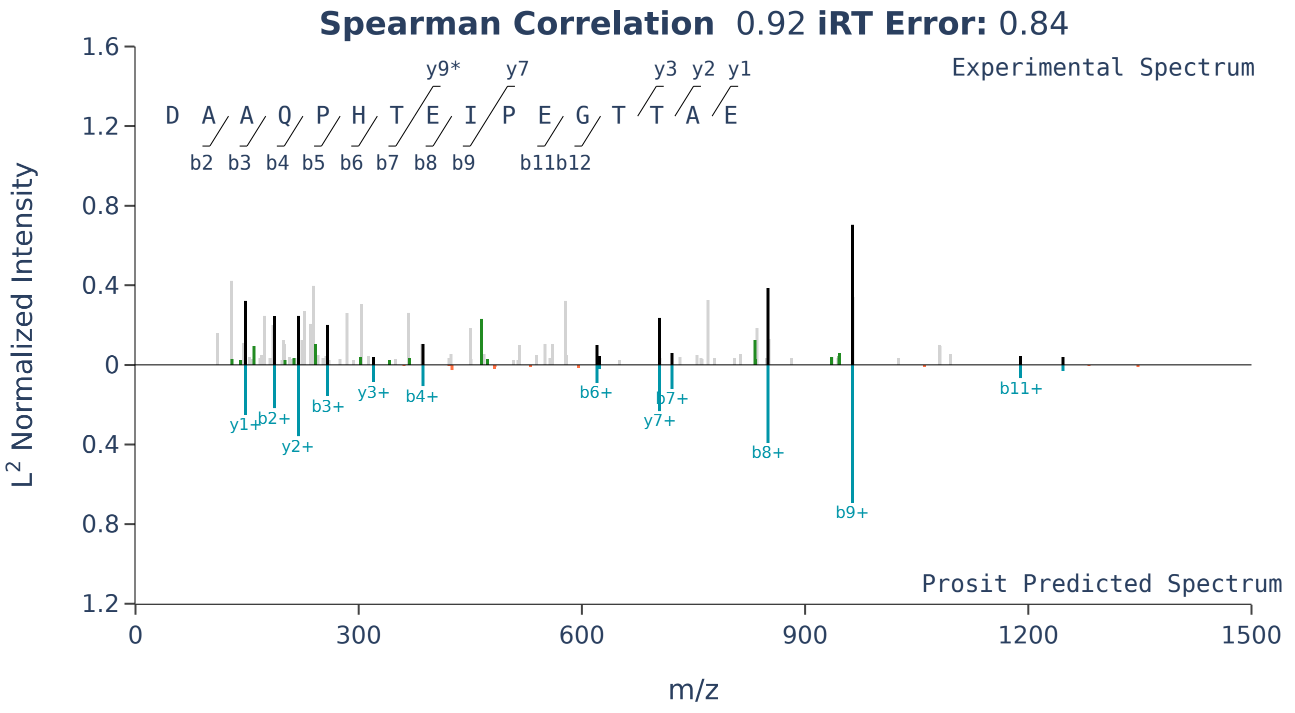

Source WSoh_221121_011221_HFGoe_L1_hTau_4h_R1 **Scan** 13970
Peptide QPHTEIPEGTTAEEAG **Charge** 2 **Spectral Angle** 0.0
Spearman Correlation 0.0 **iRT Error:** 4.65

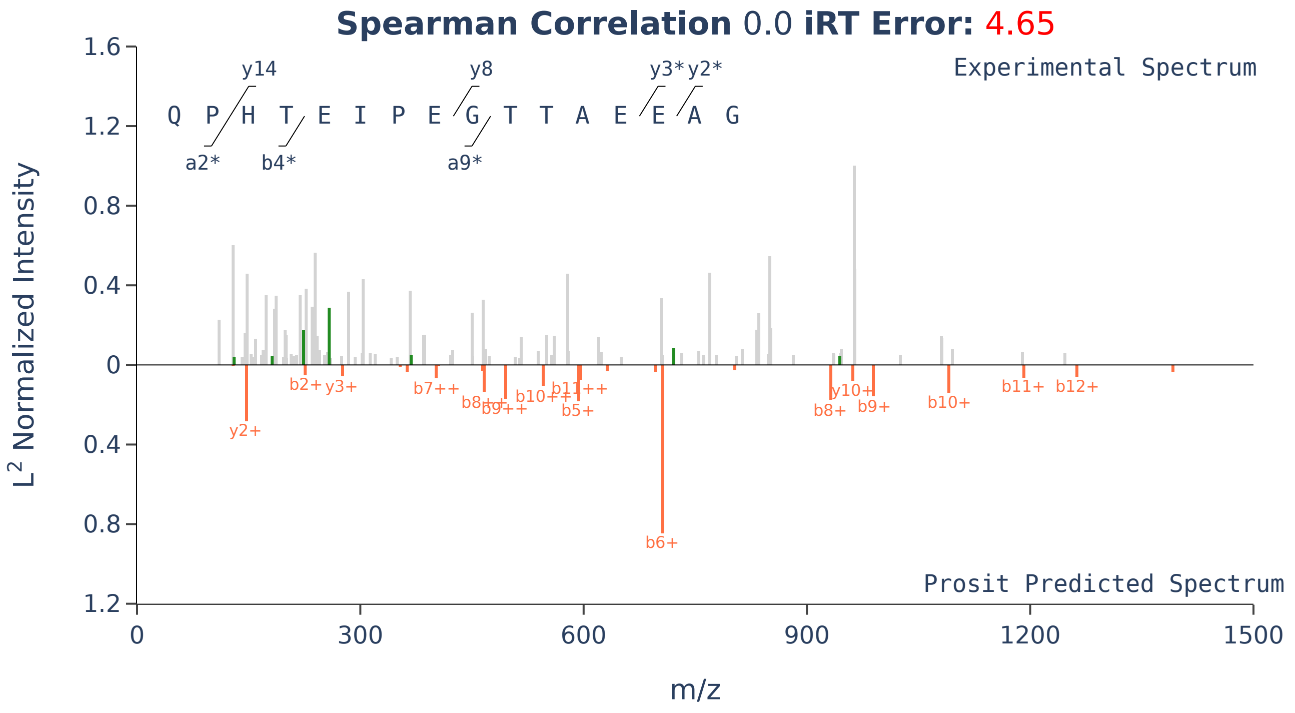

Source WSoh_221121_011221_HFGoe_L2_hTau_24h_R2 **Scan** 7527
Peptide EHAGTYGLGDRKD **Charge** 3 **Spectral Angle** 0.9
Spearman Correlation 0.89 **iRT Error:** 0.85

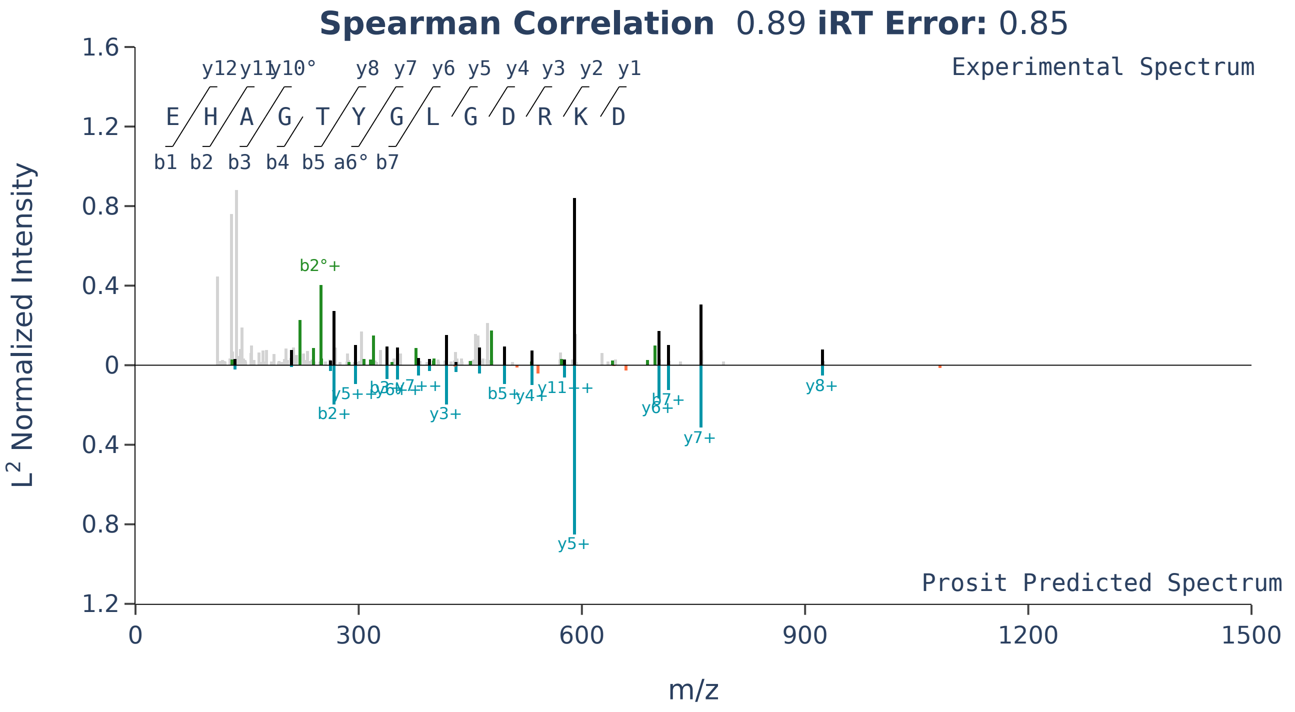

Source WSoh_221121_011221_HFGoe_L2_hTau_24h_R2 **Scan** 7527
Peptide GTTAEEAGIGDTPSL **Charge** 3 **Spectral Angle** 0.02
Spearman Correlation 0.3 **iRT Error:** 82.8

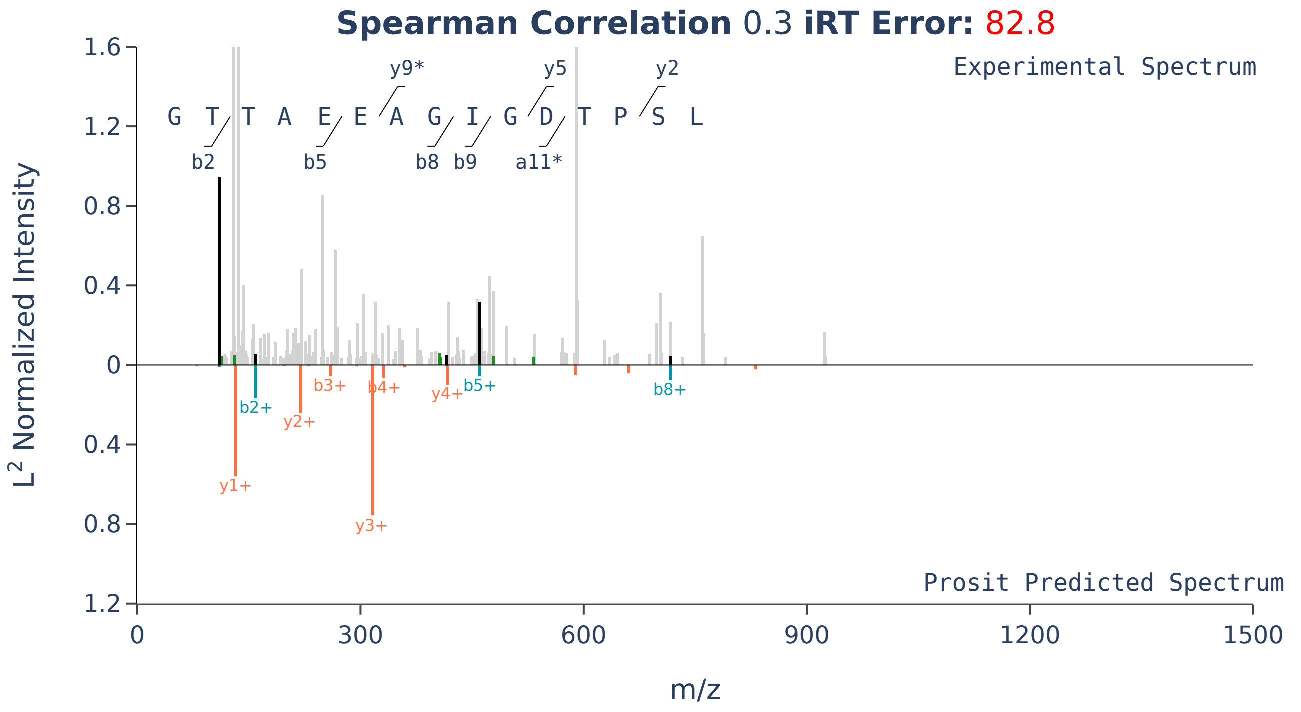

Source WSoh_221121_011221_HFGoe_L2_hTau_24h_R1 **Scan** 18118
Peptide DLSNVQSKL **Charge** 2 **Spectral Angle** 0.87
Spearman Correlation 0.98 **iRT Error:** 0.92

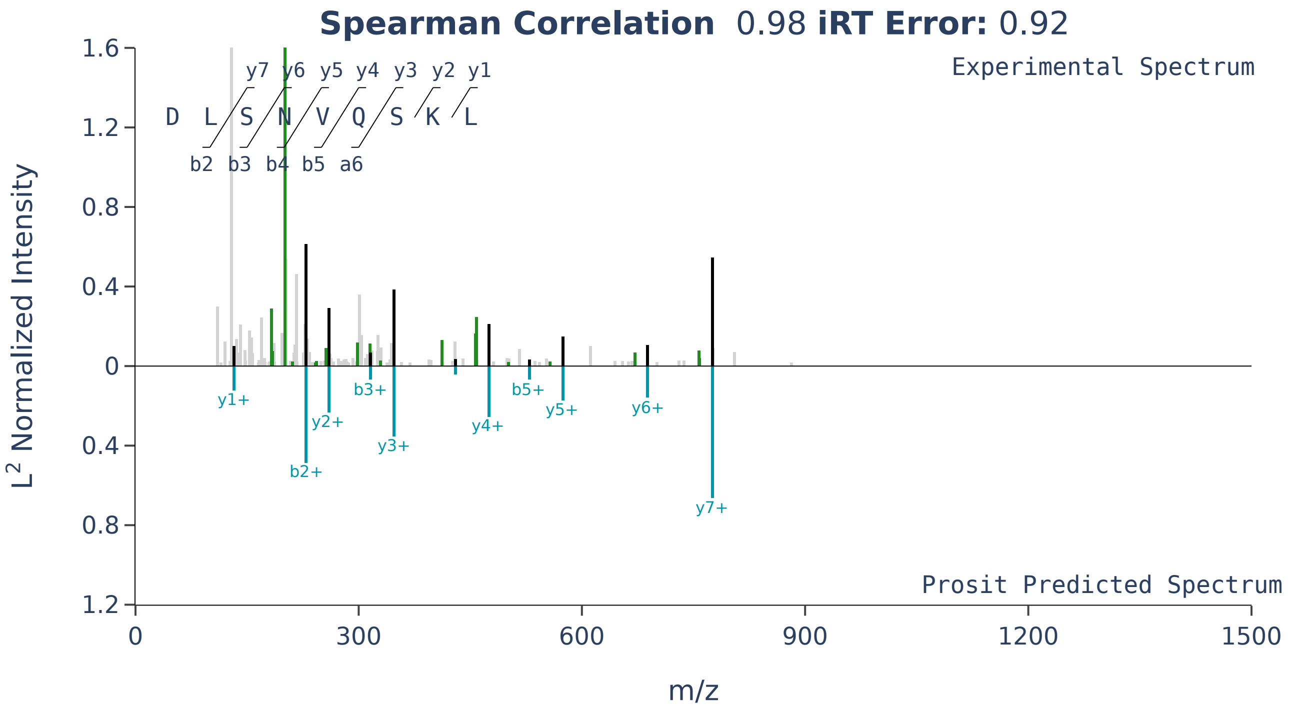

Source WSoh_221121_011221_HFGoe_L2_hTau_24h_R1 **Scan** 18118
Peptide GQVEVKSEK **Charge** 2 **Spectral Angle** 0.09
Spearman Correlation 0.14 **iRT Error:** 31.94

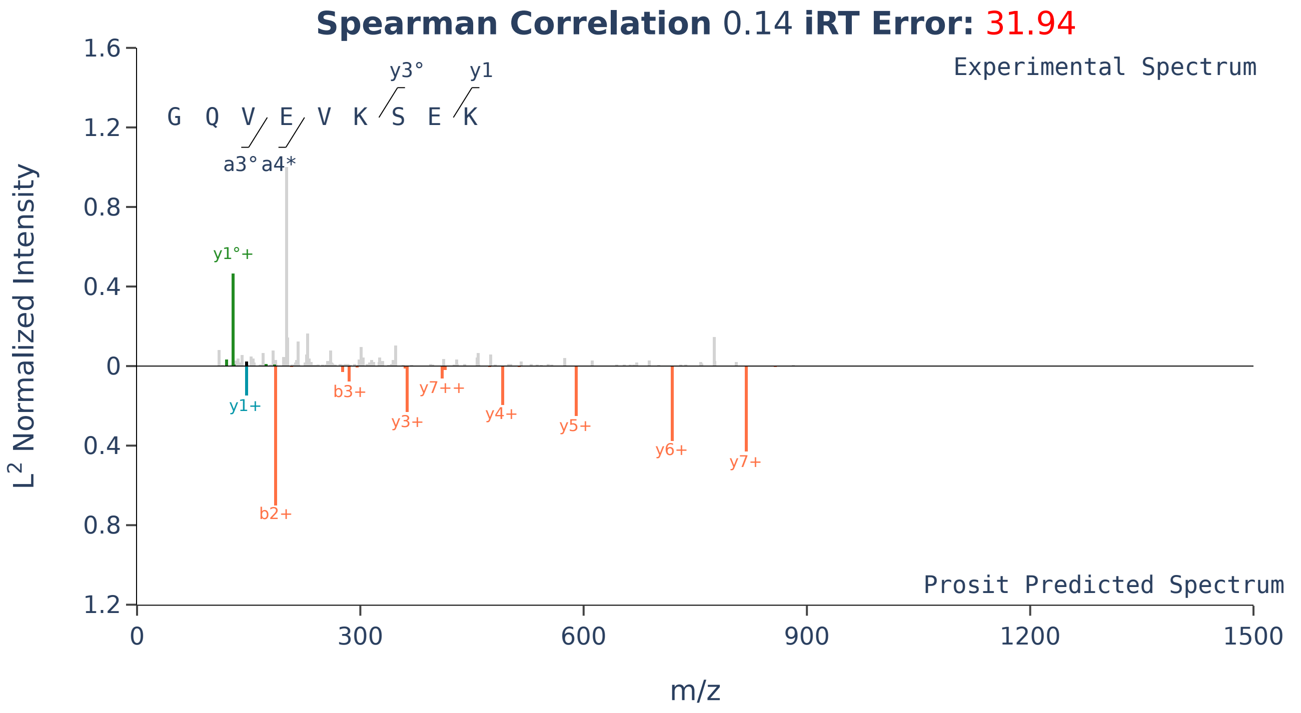

Source WSoh_221121_011221_HFGoe_L2_hTau_24h_R1 **Scan** 20274
Peptide EGAIGDTPSLEDEAAGHVTQARMVSK **Charge** 4 **Spectral Angle** 0.81
Spearman Correlation 0.85 **iRT Error:** 1.03

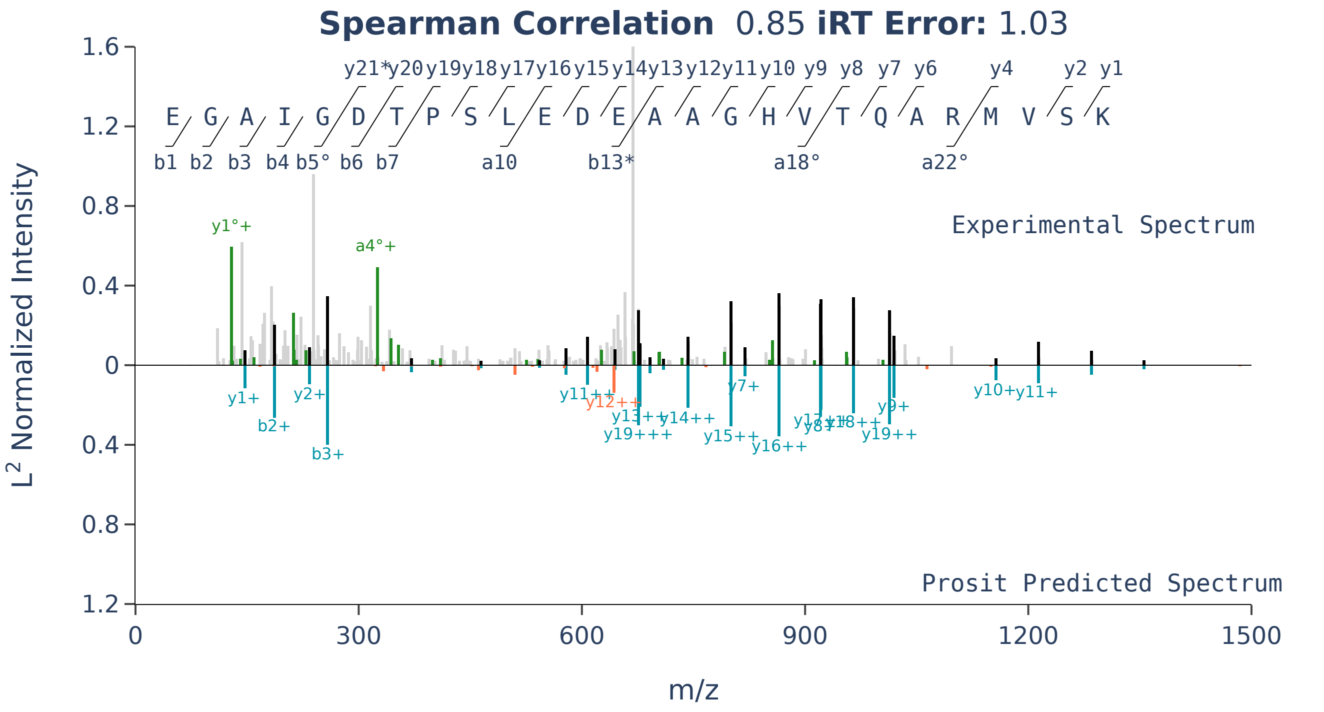

Source WSoh_221121_011221_HFGoe_L2_hTau_24h_R1 **Scan** 20274
Peptide EAGIGDTPSLEDEAAGHVTQARMVSK **Charge** 4 **Spectral Angle** 0.79
Spearman Correlation 0.84 **iRT Error:** 0.65

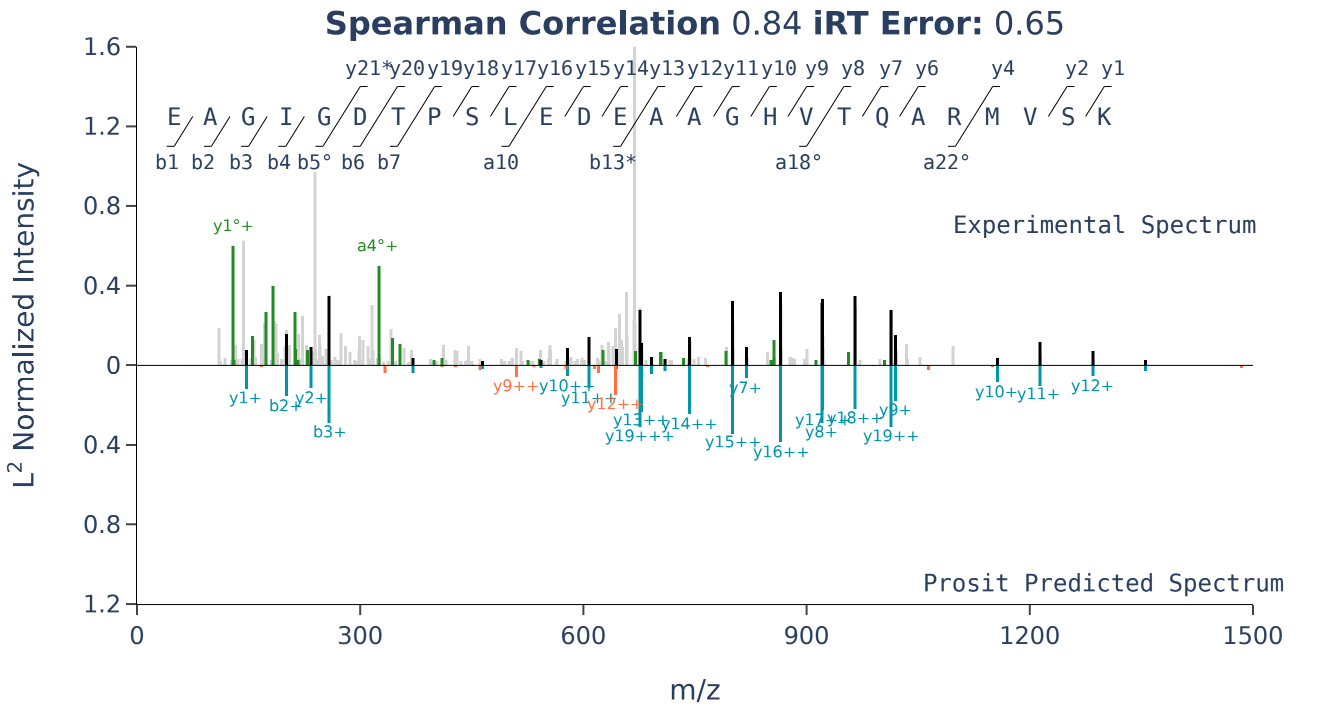

Source WSoh_221121_011221_HFGoe_L2_hTau_4h_R2 **Scan** 13927
Peptide SKHQPGGGKVQIINKKLDL **Charge** 3 **Spectral Angle** 0.81
Spearman Correlation 0.84 **iRT Error:** 1.07

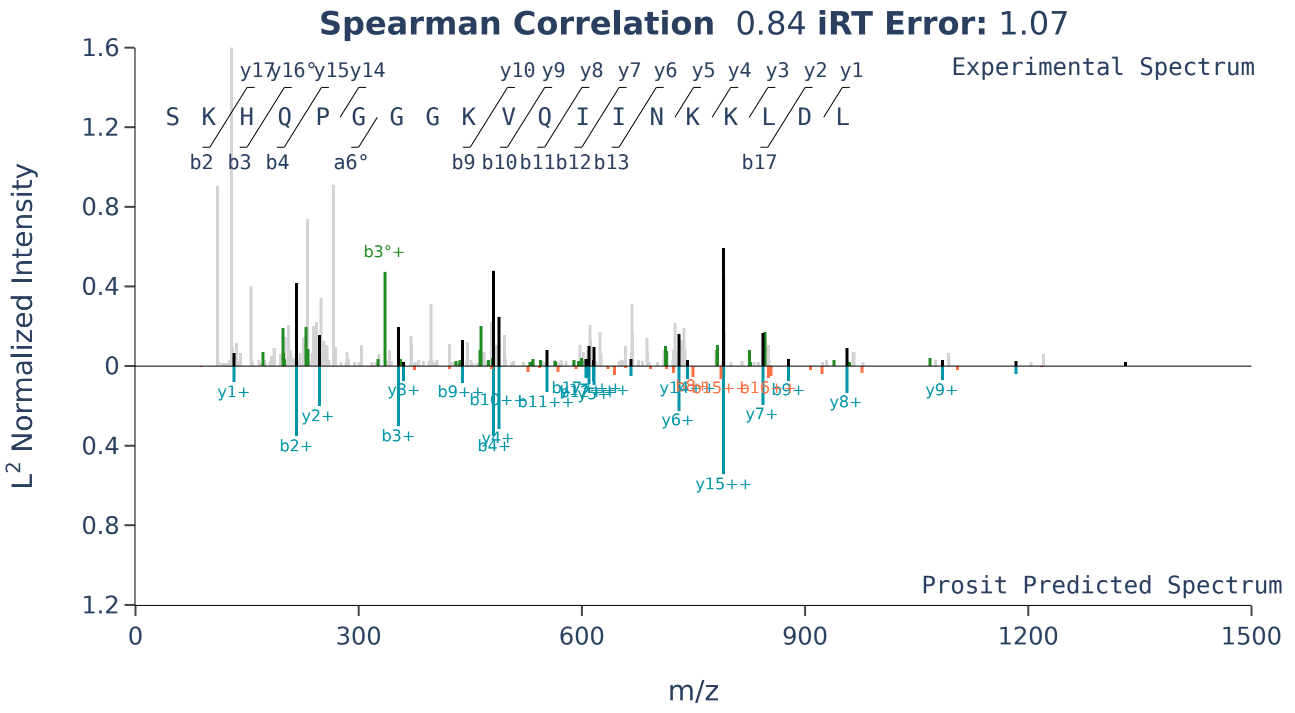

Source WSoh_221121_011221_HFGoe_L2_hTau_4h_R2 **Scan** 13927
Peptide KHQPGGGKVQIINKKLDLS **Charge** 3 **Spectral Angle** 0.66
Spearman Correlation 0.23 **iRT Error:** 7.45

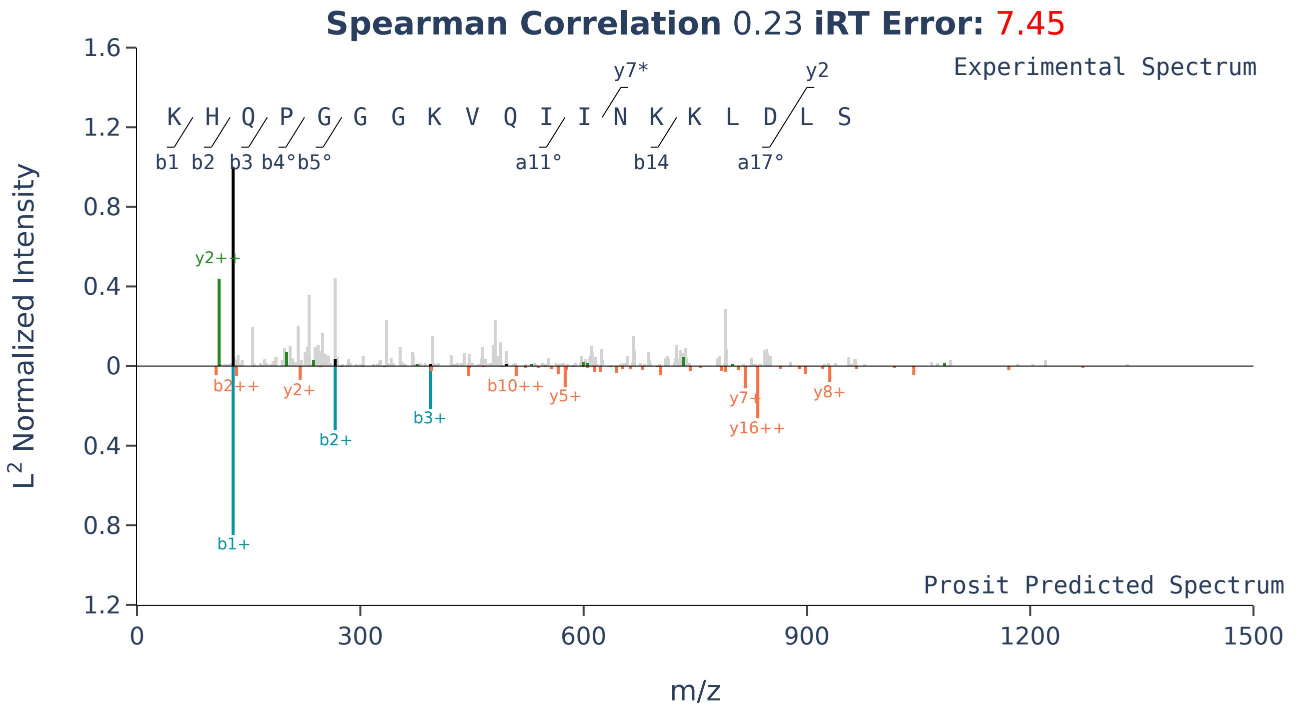

Source WSoh_221121_011221_HFGoe_L1_hTau_24h_R2 **Scan** 11519
Peptide AAQPHTEIPEGTEGAPGKQA **Charge** 2 **Spectral Angle** 0.85
Spearman Correlation 0.89 **iRT Error:** 1.08

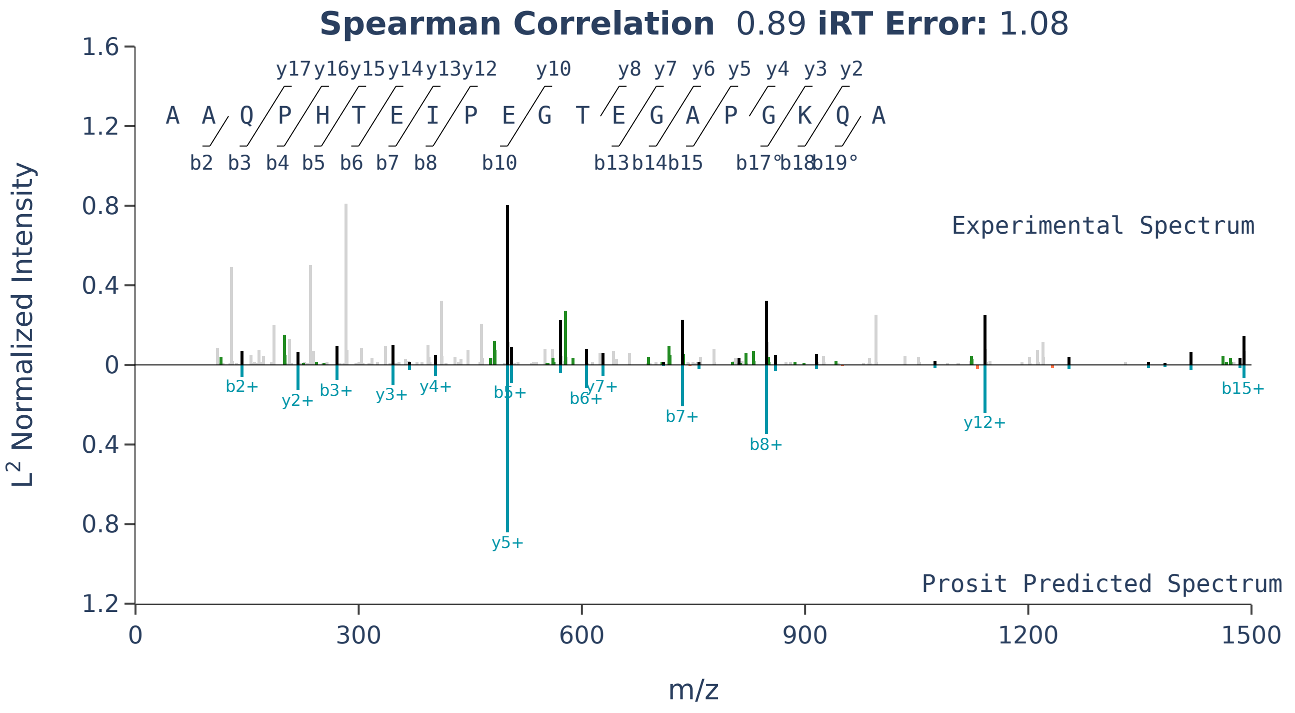

Source WSoh_221121_011221_HFGoe_L1_hTau_24h_R2 **Scan** 11519
Peptide EGAPGKQAAAQPHTEIPEGT **Charge** 2 **Spectral Angle** 0.01
Spearman Correlation -0.13 **iRT Error:** 2.92

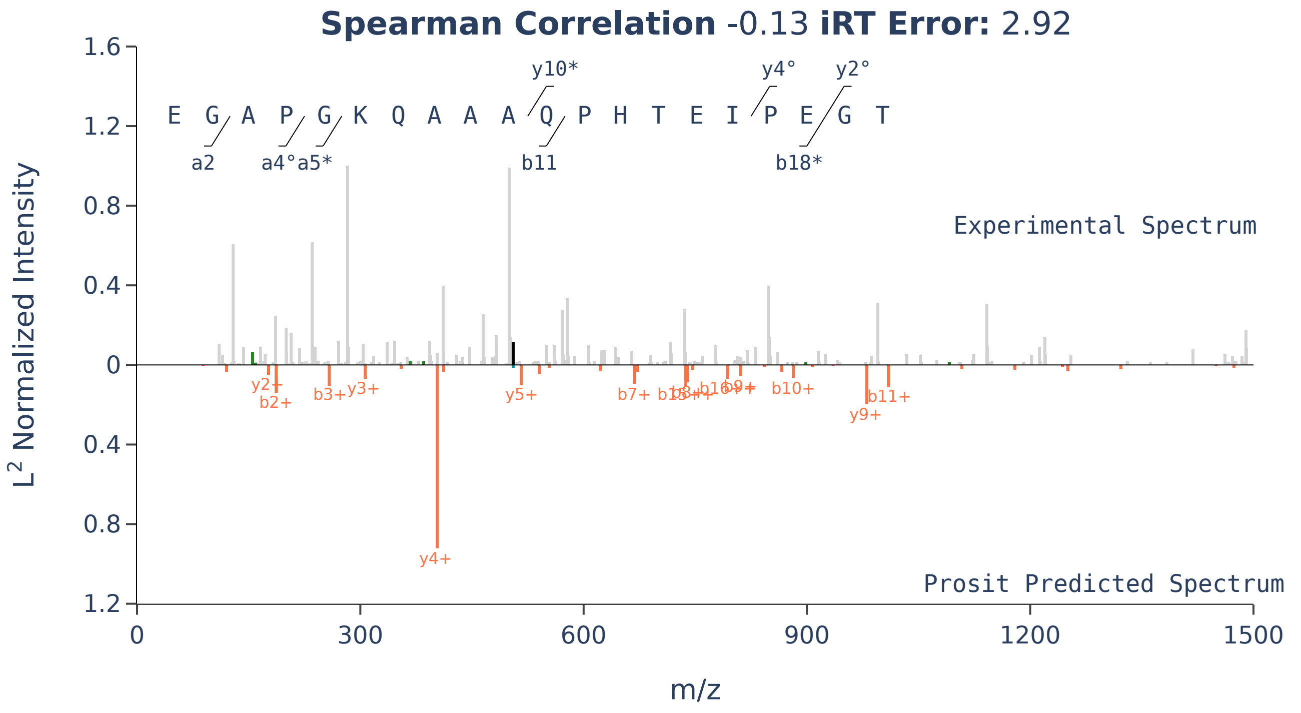

Source WSoh_221121_011221_HFGoe_L1_hTau_24h_R1 **Scan** 7612
Peptide PGGGNKKIETHVY **Charge** 3 **Spectral Angle** 0.83
Spearman Correlation 0.88 **iRT Error:** 1.13

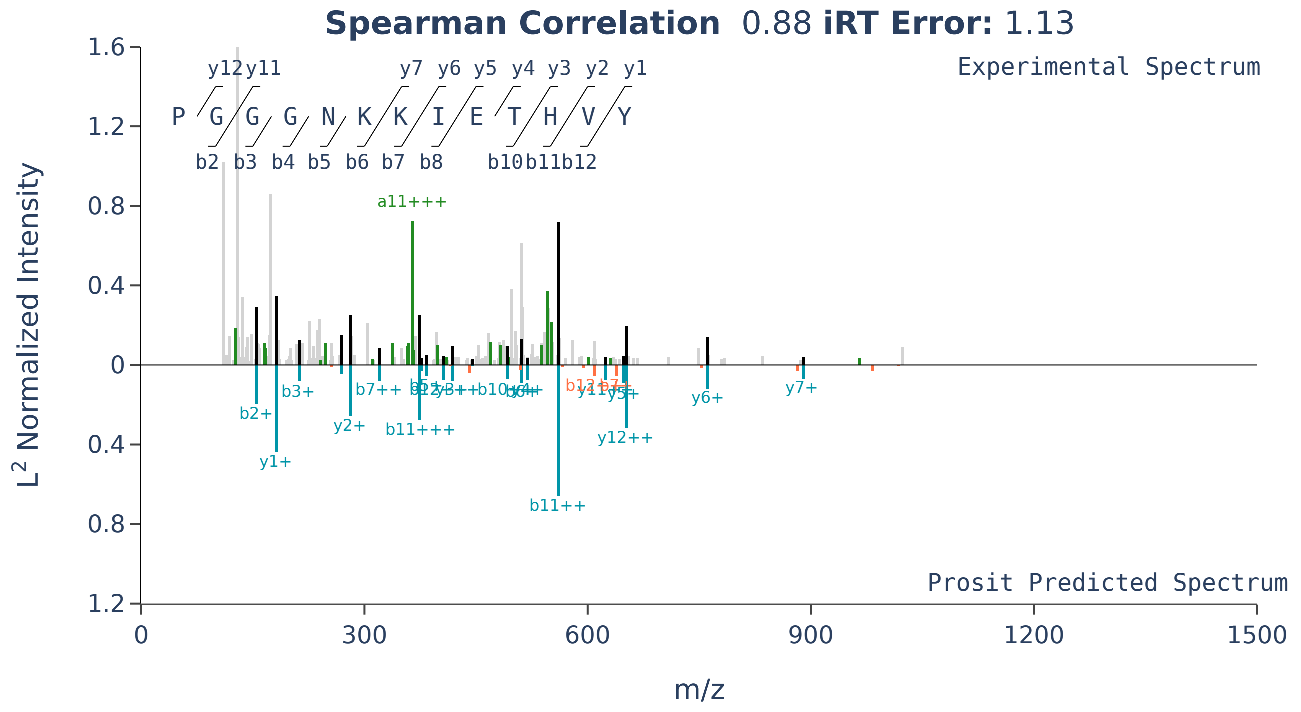

Source WSoh_221121_011221_HFGoe_L1_hTau_24h_R1 **Scan** 7612
Peptide SPGTPGSRSRTPSL **Charge** 3 **Spectral Angle** 0.01
Spearman Correlation -0.14 **iRT Error:** 16.37

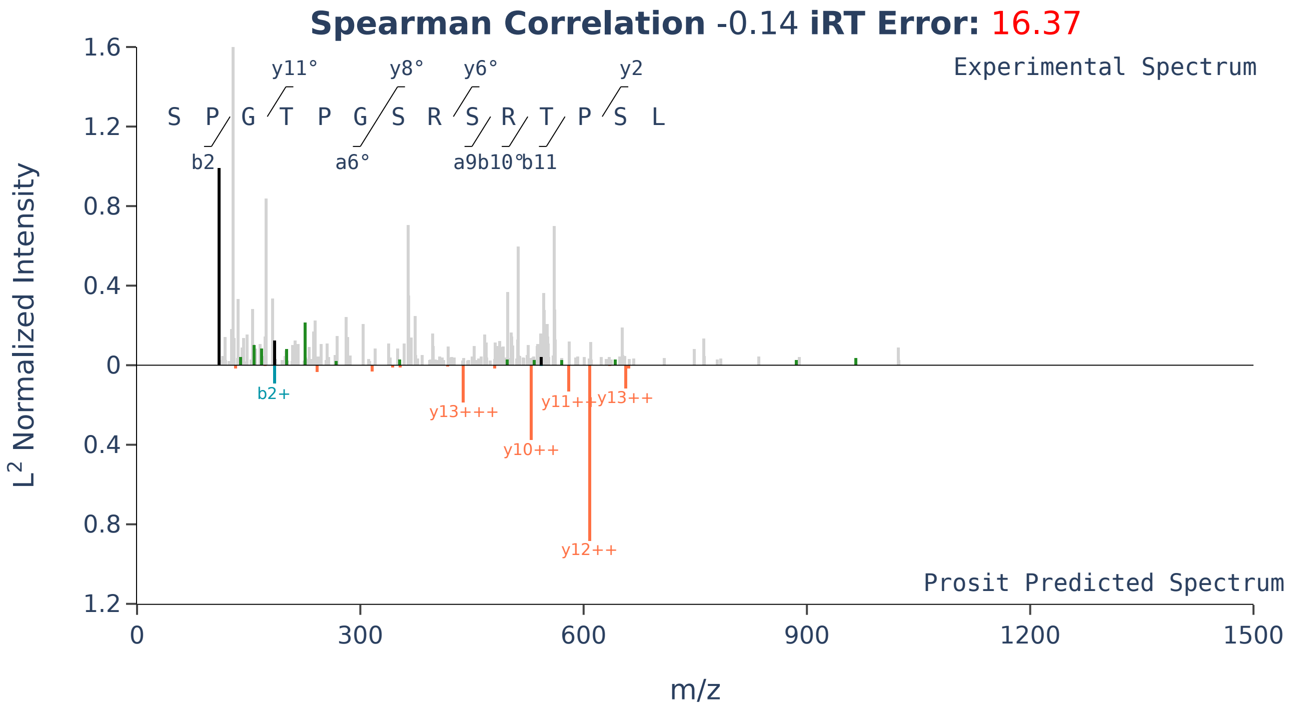

Source WSoh_221121_011221_HFGoe_L1_hTau_24h_R2 **Scan** 8783
Peptide KGSLGNIHHKPGGGQVEV **Charge** 2 **Spectral Angle** 0.85
Spearman Correlation 0.92 **iRT Error:** 1.15

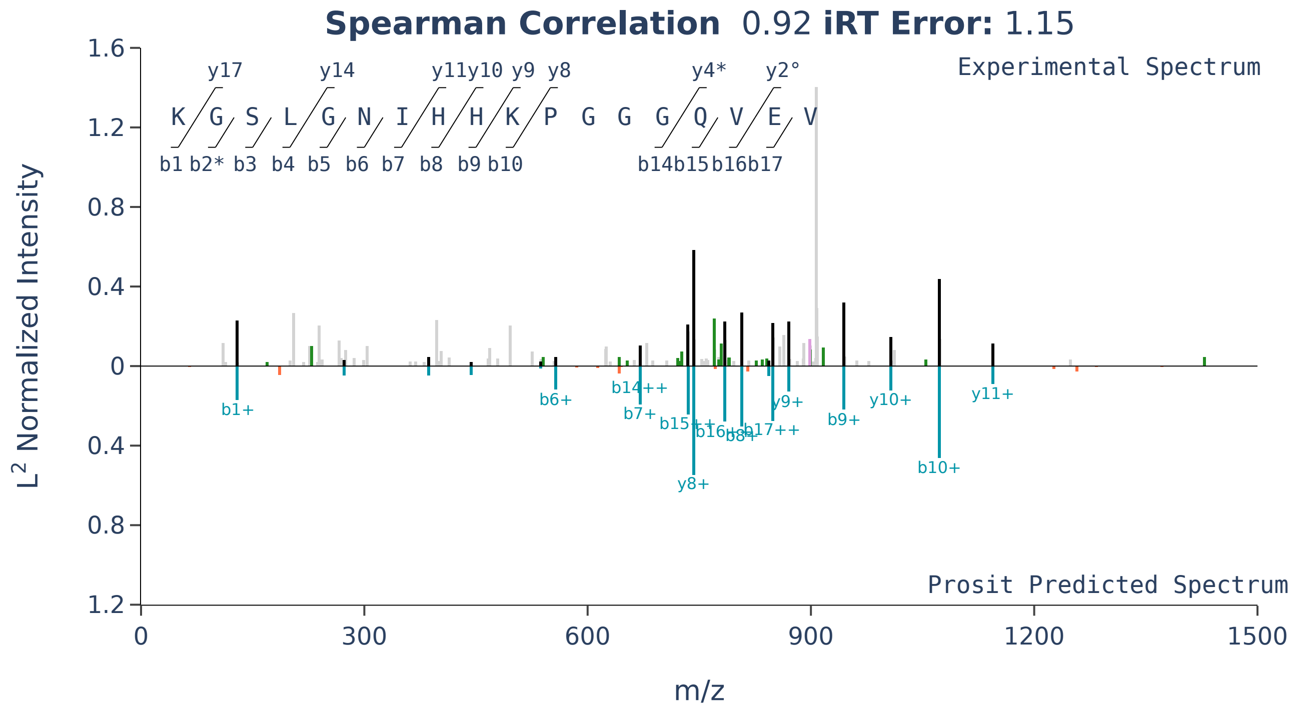

Source WSoh_221121_011221_HFGoe_L1_hTau_24h_R2 **Scan** 8783
Peptide GSLGNIHHKPGGGQVEVK **Charge** 2 **Spectral Angle** 0.52
Spearman Correlation 0.45 **iRT Error:** 5.05

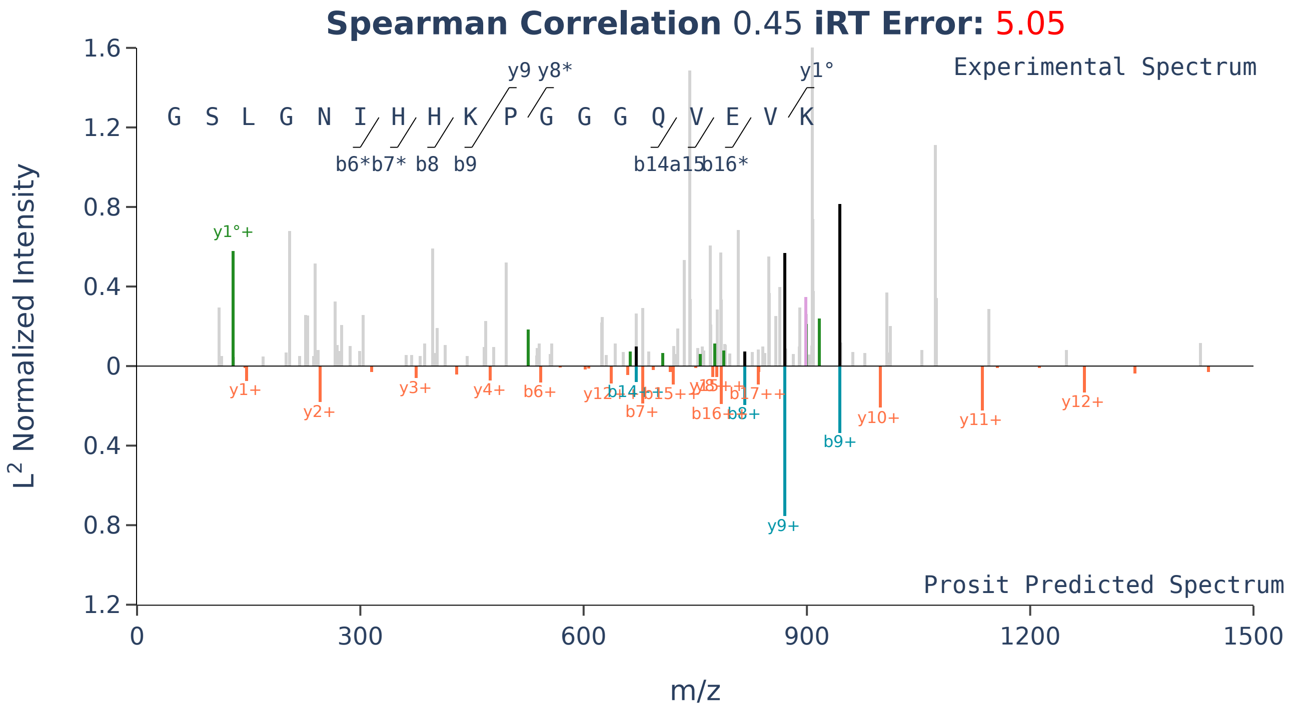

Source WSoh_221121_011221_HFGoe_L1_hTau_24h_R1 **Scan** 17679
Peptide KLDFKDRVQSL **Charge** 2 **Spectral Angle** 0.8
Spearman Correlation 0.91 **iRT Error:** 1.32

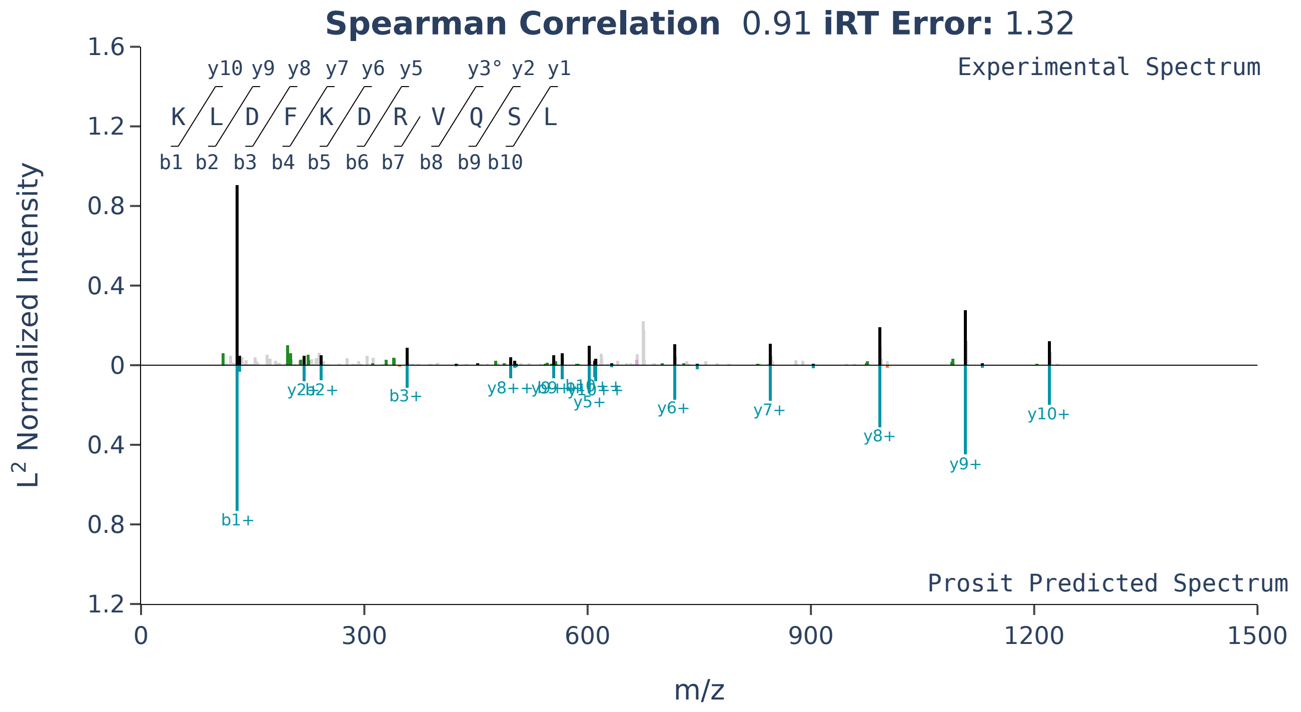

Source WSoh_221121_011221_HFGoe_L1_hTau_24h_R1 **Scan** 17679
Peptide LDFKDRVQSKI **Charge** 2 **Spectral Angle** 0.41
Spearman Correlation 0.43 **iRT Error:** 4.81

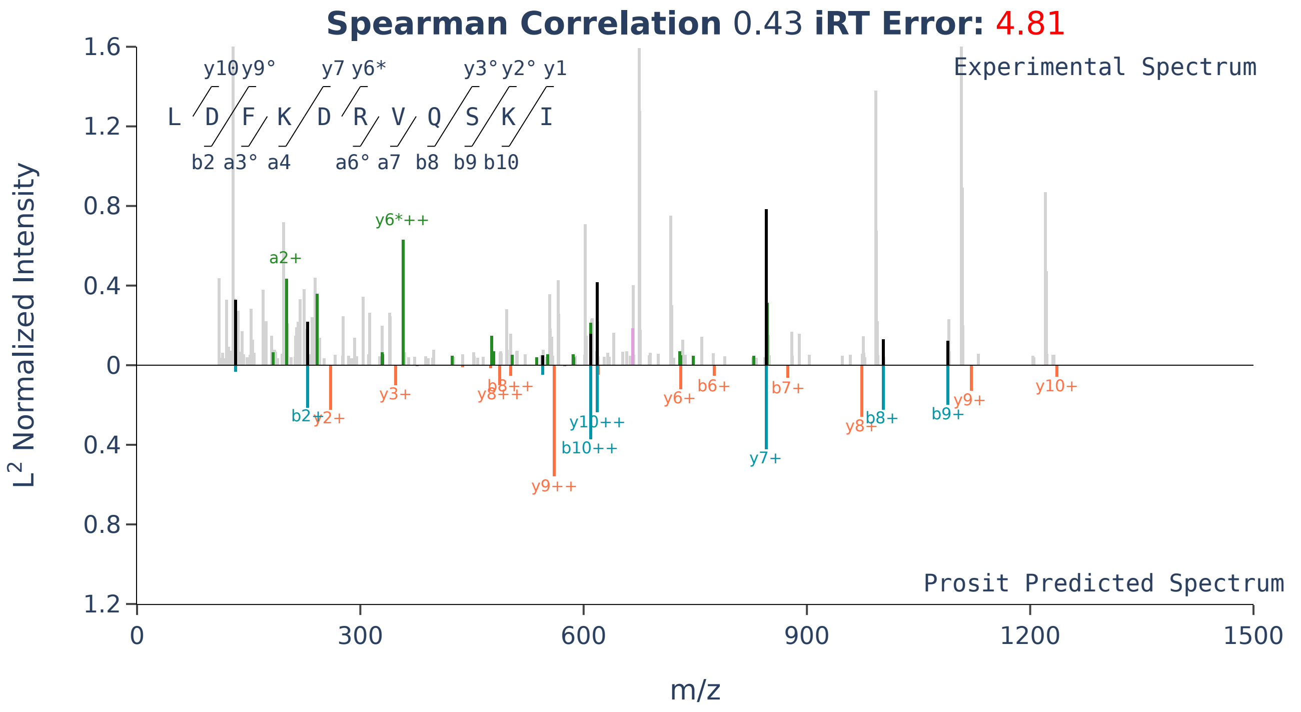

Source WSoh_221121_011221_HFGoe_L1_hTau_2h_R1 **Scan** 6117
Peptide SKHVPGGGSV **Charge** 2 **Spectral Angle** 0.86
Spearman Correlation 0.81 **iRT Error:** 1.37

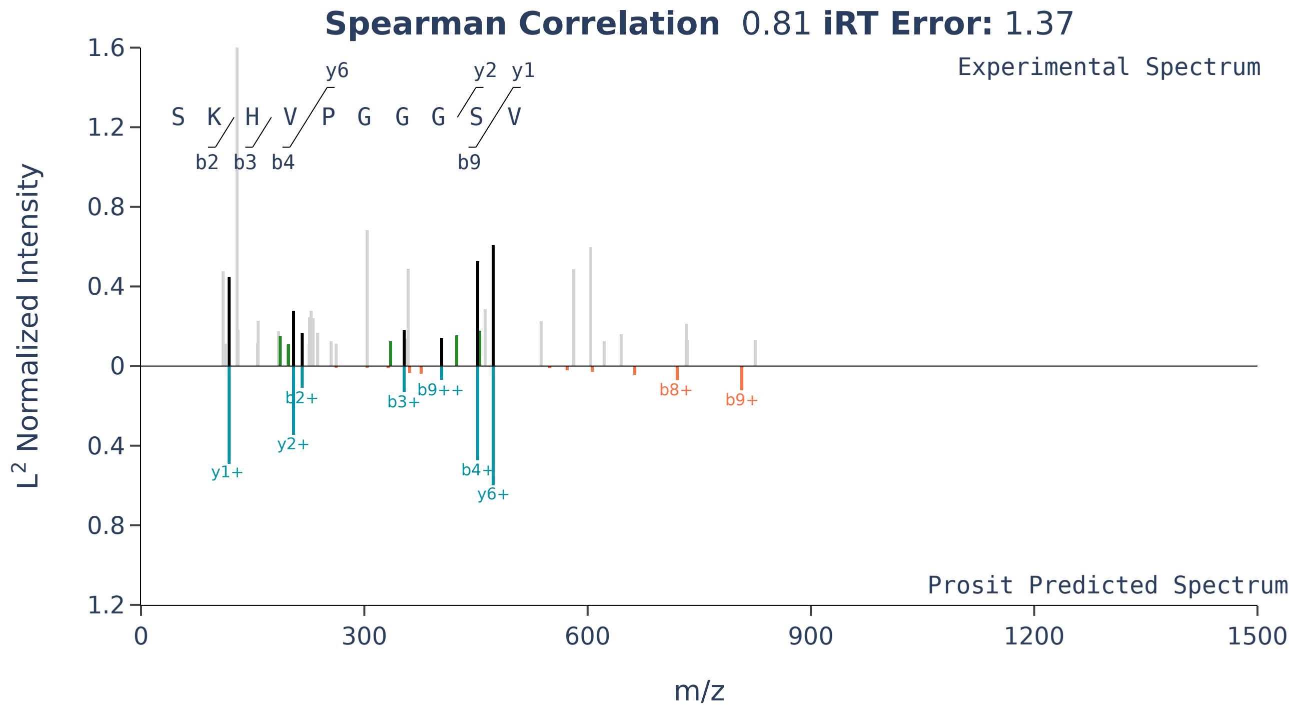

Source WSoh_221121_011221_HFGoe_L1_hTau_2h_R1 **Scan** 6117
Peptide AAPPGQKGQA **Charge** 2 **Spectral Angle** 0.0
Spearman Correlation 0.0 **iRT Error:** 8.57

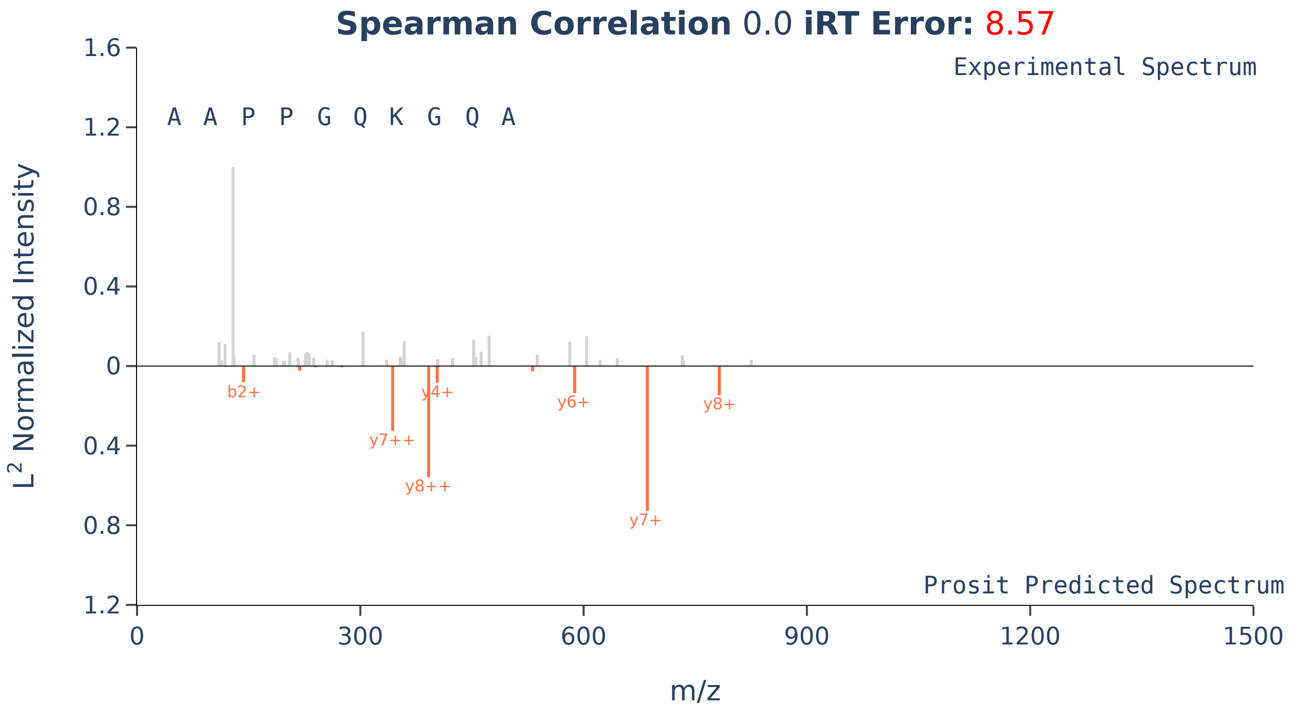

Source WSoh_221121_011221_HFGoe_L1_hTau_24h_R1 **Scan** 10847
Peptide AAQPHTEIPEGEGAPGKQA **Charge** 2 **Spectral Angle** 0.83
Spearman Correlation 0.9 **iRT Error:** 1.41

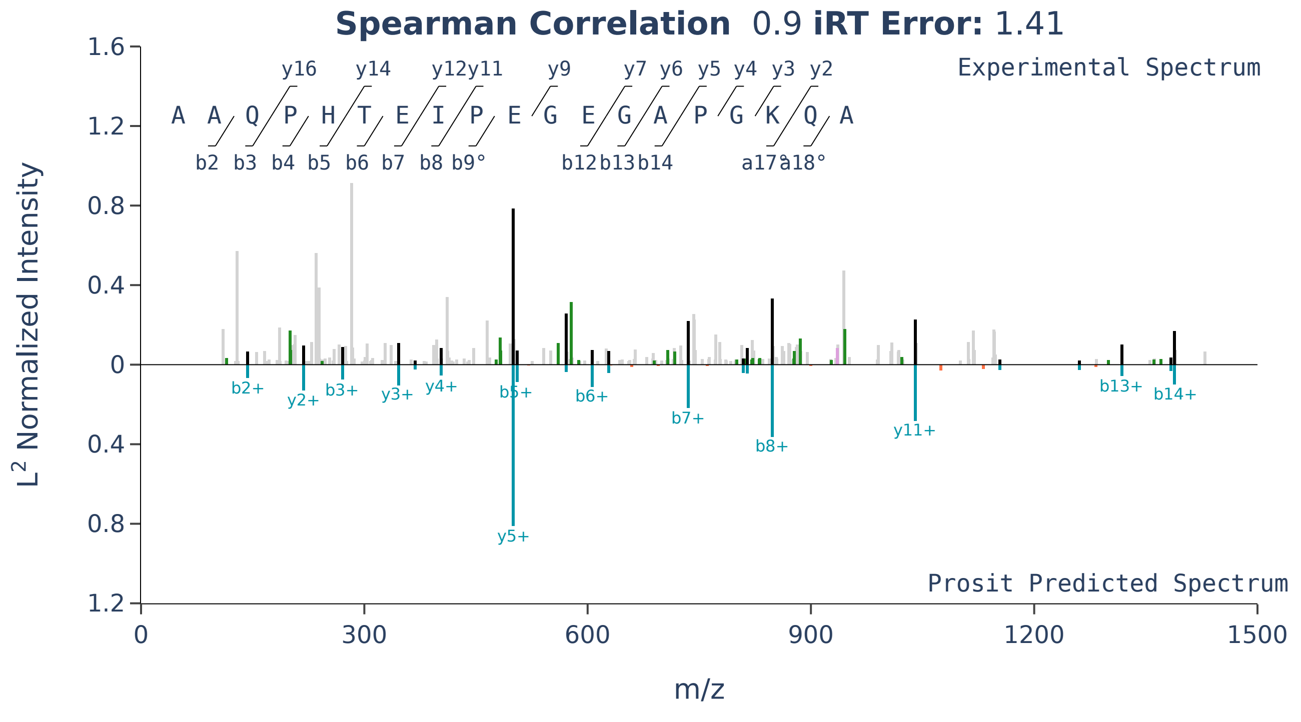

Source WSoh_221121_011221_HFGoe_L1_hTau_24h_R1 **Scan** 10847
Peptide EGAPGKQAAAQPHTEIPEG **Charge** 2 **Spectral Angle** 0.04
Spearman Correlation 0.18 **iRT Error:** 4.94

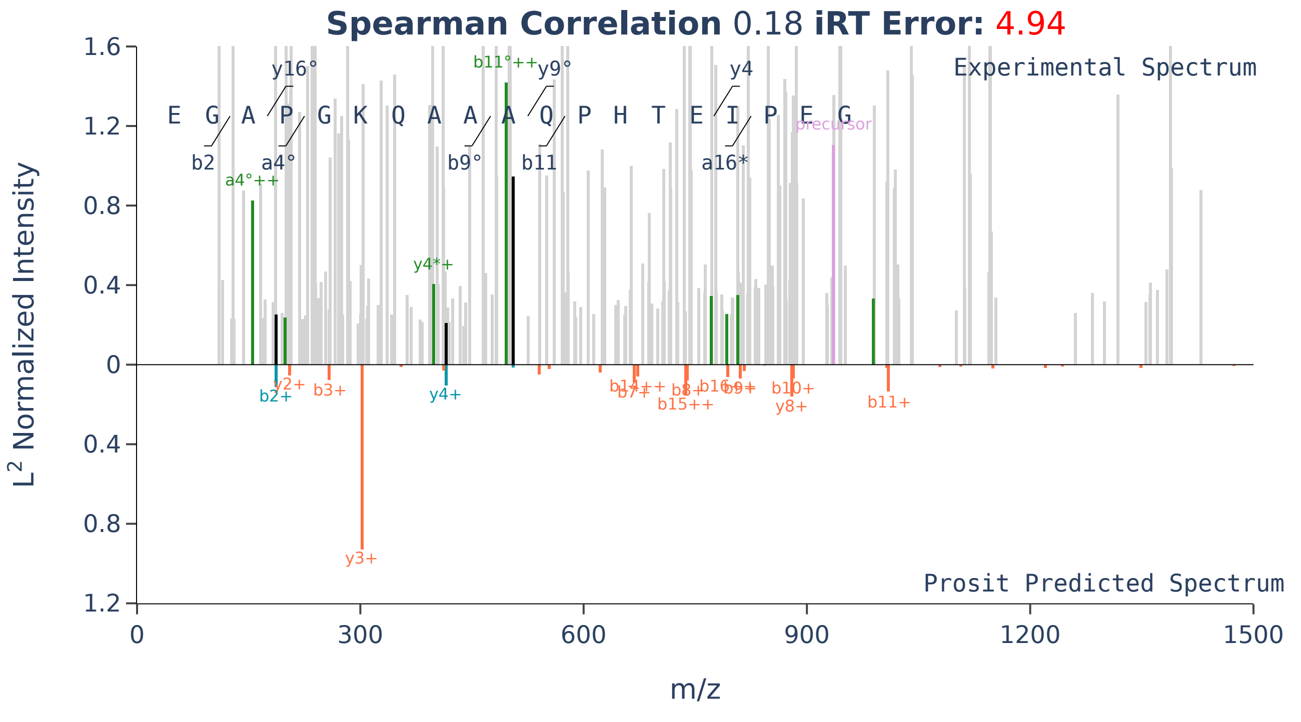

Source WSoh_221121_011221_HFGoe_L2_hTau_24h_R1 **Scan** 9382
Peptide KNEKLDFKDRVQS **Charge** 2 **Spectral Angle** 0.77
Spearman Correlation 0.95 **iRT Error:** 1.42

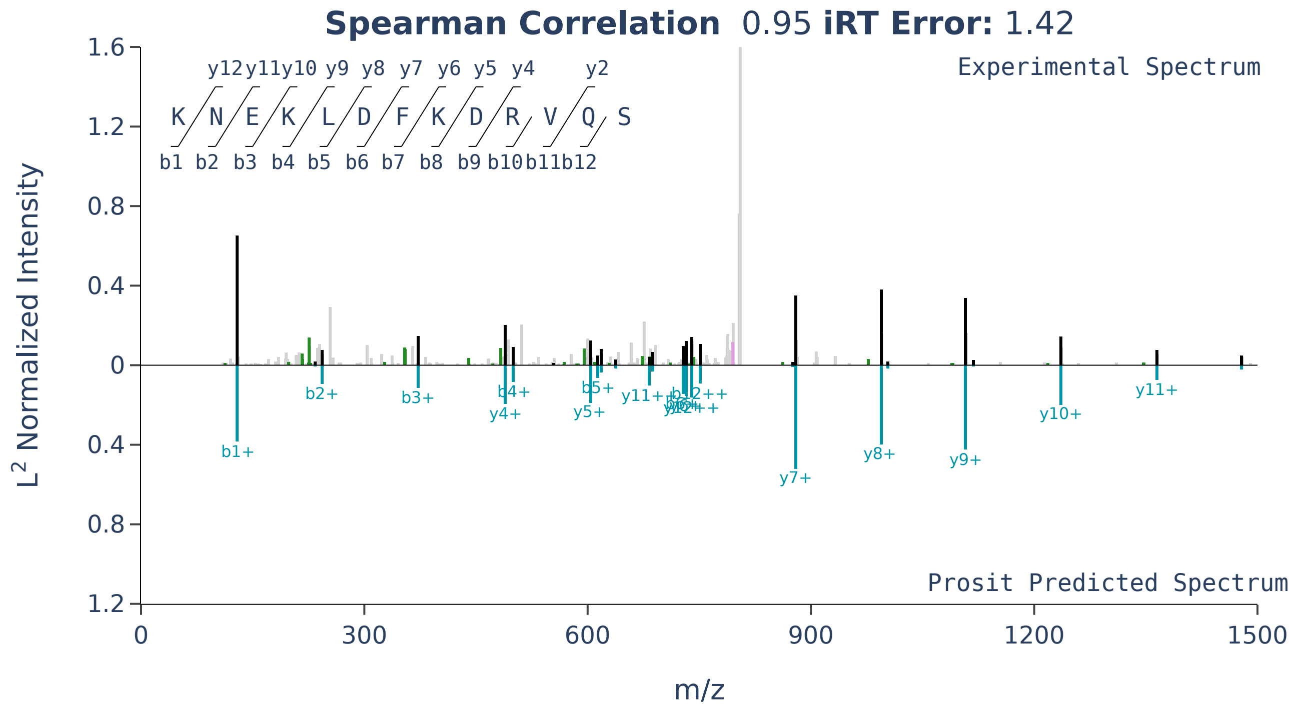

Source WSoh_221121_011221_HFGoe_L2_hTau_24h_R1 **Scan** 9382
Peptide FKDRVQSKIGSLDN **Charge** 2 **Spectral Angle** 0.3
Spearman Correlation 0.39 **iRT Error:** 21.53

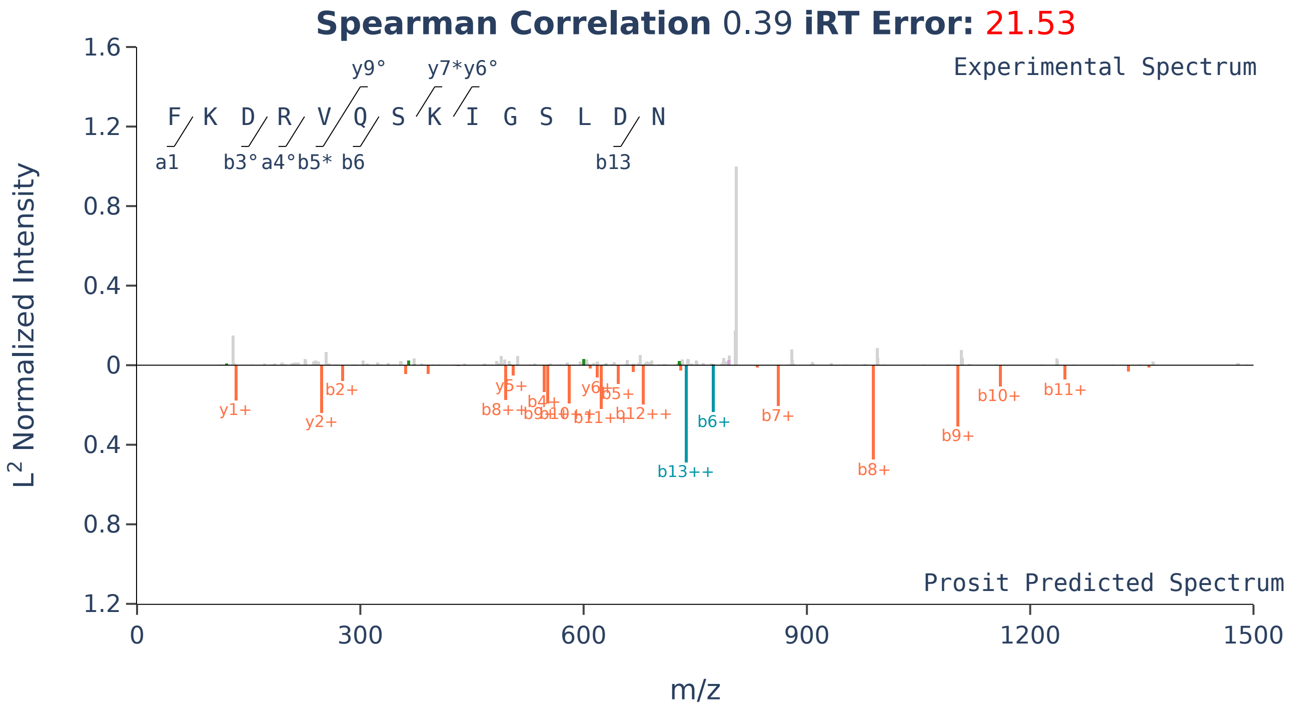

Source WSoh_221121_011221_HFGoe_L1_hTau_4h_R1 **Scan** 18412
Peptide GSDTSPRHLSNVSSTGSIDMV **Charge** 3 **Spectral Angle** 0.88
Spearman Correlation 0.95 **iRT Error:** 1.49

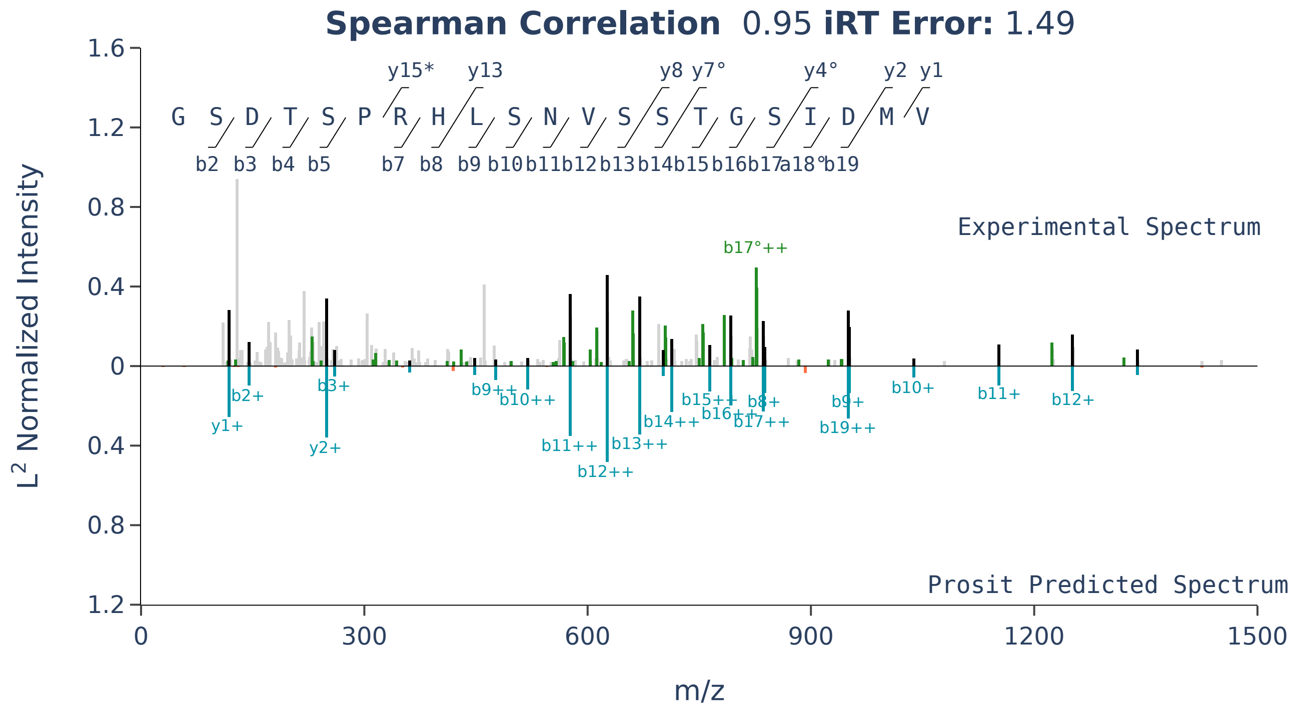

Source WSoh_221121_011221_HFGoe_L1_hTau_4h_R1 **Scan** 18412
Peptide VSGDTSPRHLSNVSSTGSIDM **Charge** 3 **Spectral Angle** 0.21
Spearman Correlation 0.24 **iRT Error:** 11.73

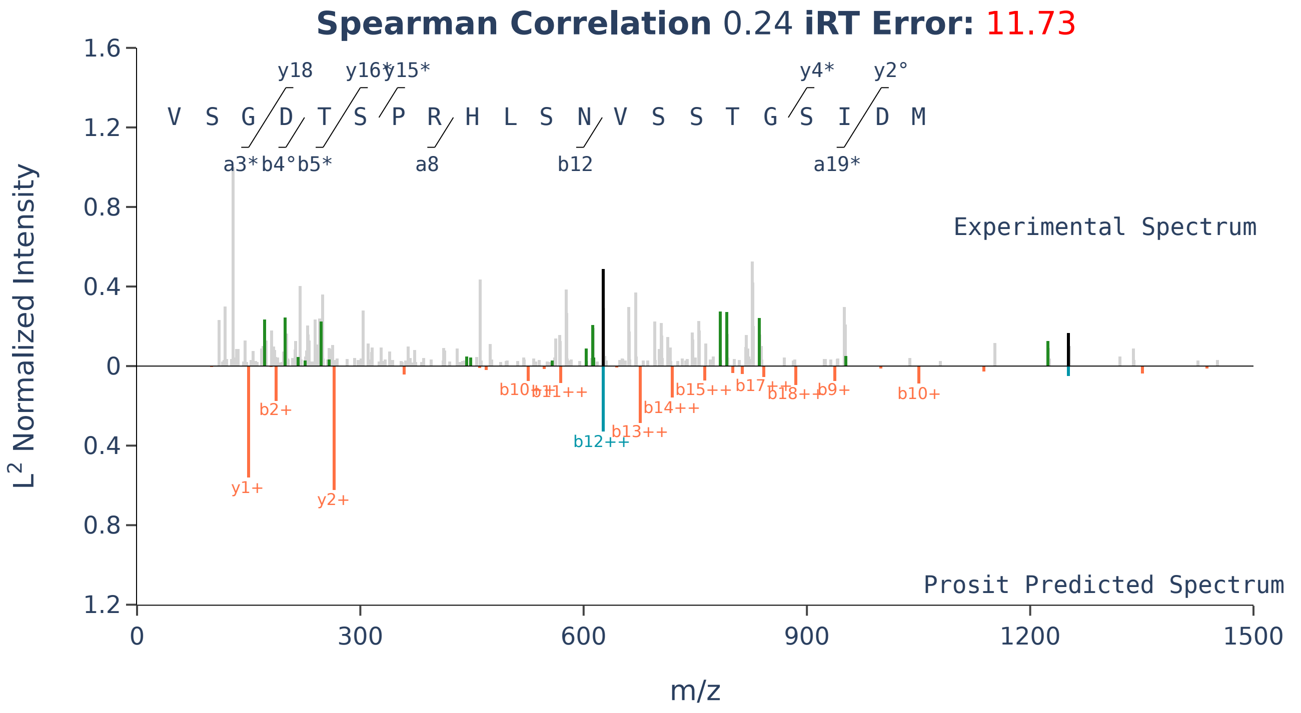

Source WSoh_221121_011221_HFGoe_L2_hTau_4h_R2 **Scan** 19485
Peptide AGIGDTPSLEDEAAGHVTQARMVKS **Charge** 4 **Spectral Angle** 0.85
Spearman Correlation 0.86 **iRT Error:** 1.5

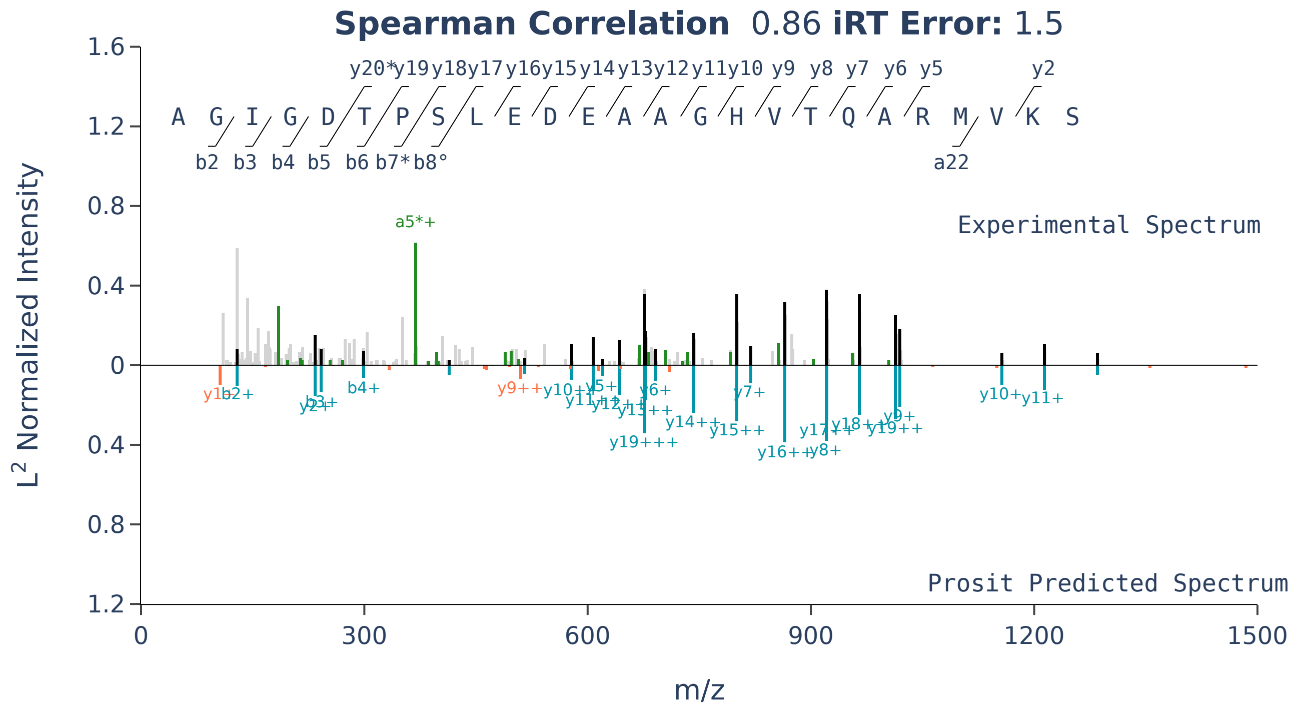

Source WSoh_221121_011221_HFGoe_L2_hTau_4h_R2 **Scan** 19485
Peptide AGIGDTPSLEDEAAGHVTQARMVSK **Charge** 4 **Spectral Angle** 0.84
Spearman Correlation 0.85 **iRT Error:** 2.96

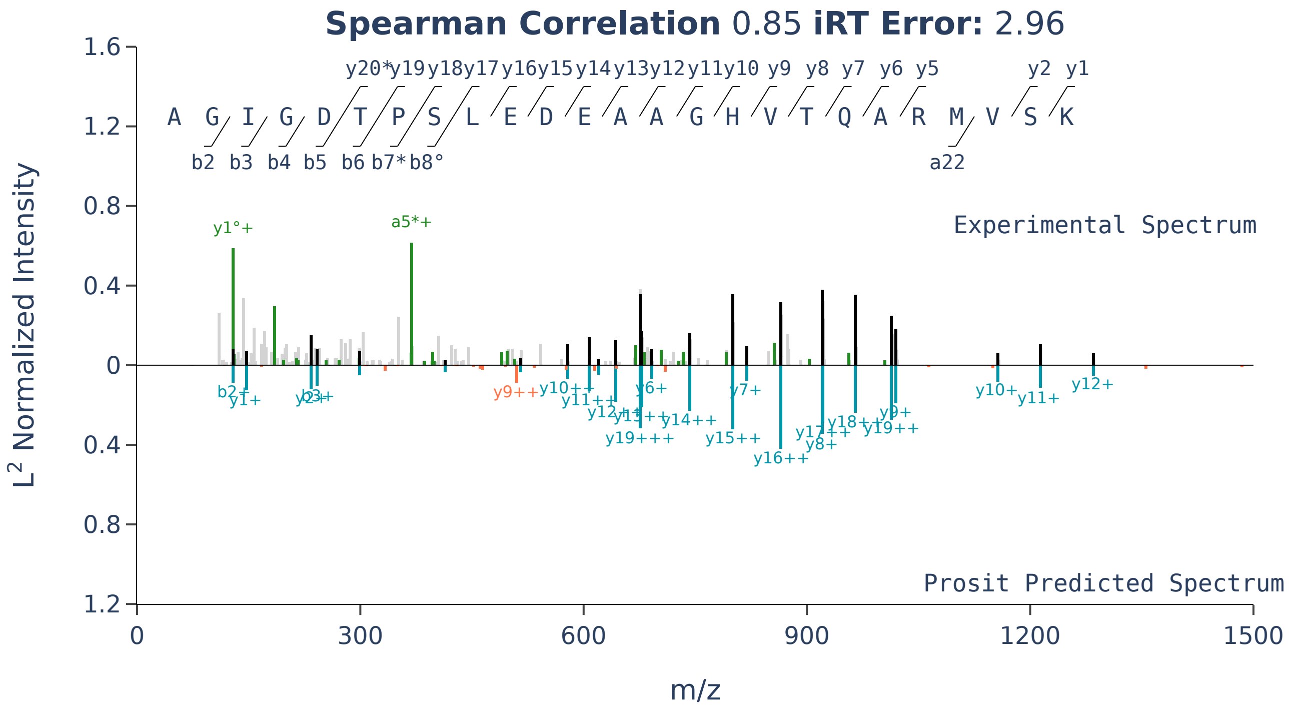

Source WSoh_221121_011221_HFGoe_L1_hTau_4h_R2 **Scan** 14502
Peptide VRQSKIGSLDNITHVPGGGNKKIETHKL **Charge** 5 **Spectral Angle** 0.82
Spearman Correlation 0.83 **iRT Error:** 1.51

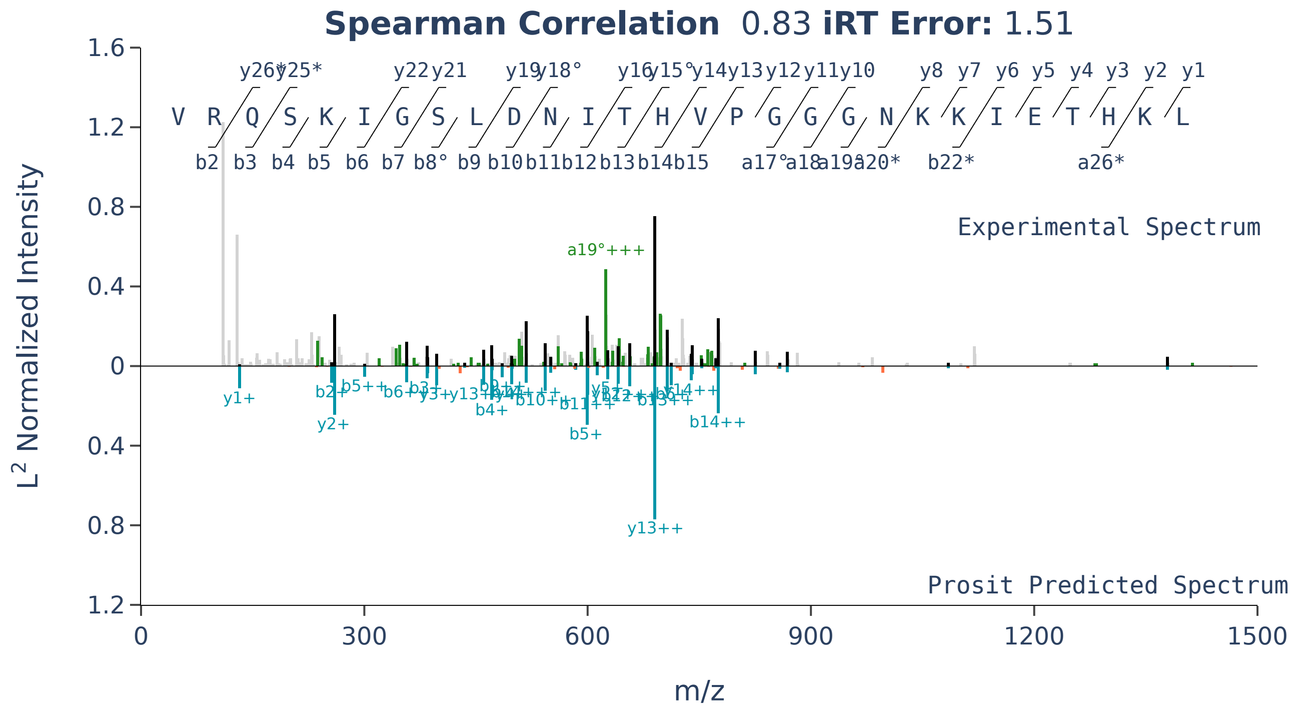

Source WSoh_221121_011221_HFGoe_L1_hTau_4h_R2 **Scan** 14502
Peptide RVQSKIGSLDNITHVPGGGNKKIETHKL **Charge** 5 **Spectral Angle** 0.81
Spearman Correlation 0.83 **iRT Error:** 5.17

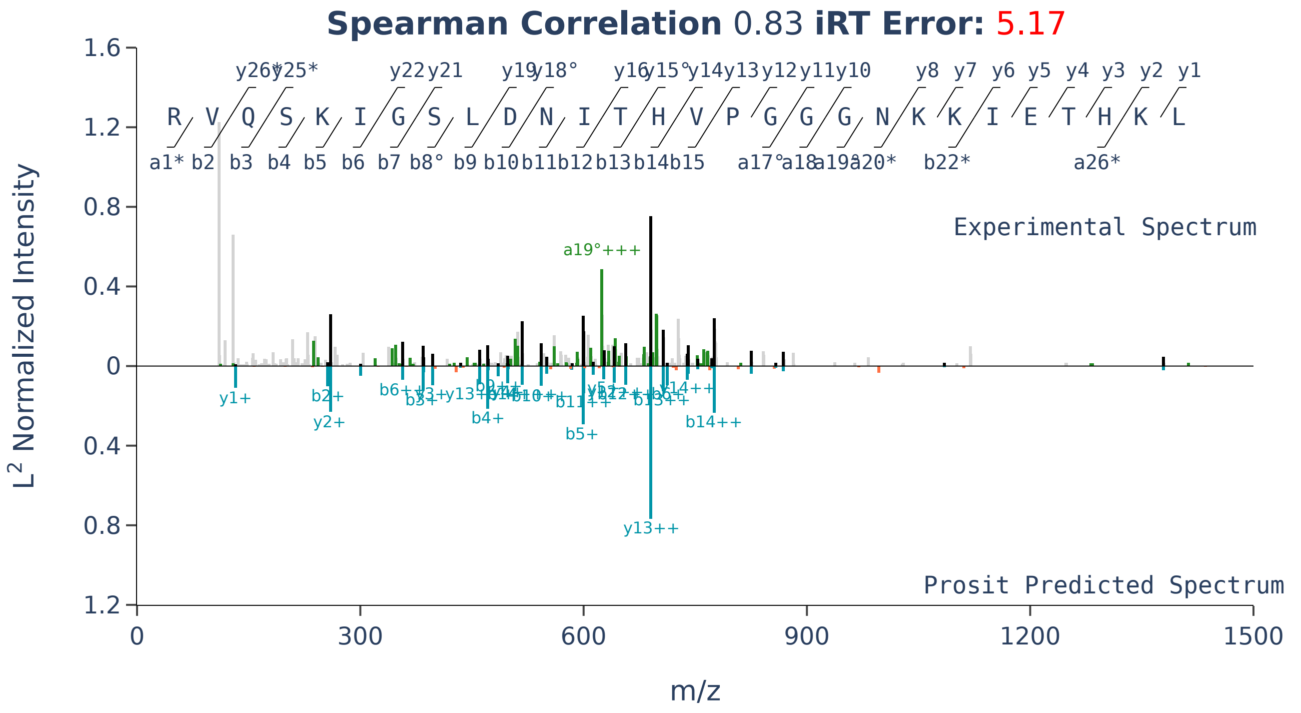

Source WSoh_221121_011221_HFGoe_L1_hTau_24h_R2 **Scan** 19839
Peptide EAAGHVTQAAGIGDTPSLED **Charge** 2 **Spectral Angle** 0.87
Spearman Correlation 0.91 **iRT Error:** 1.51

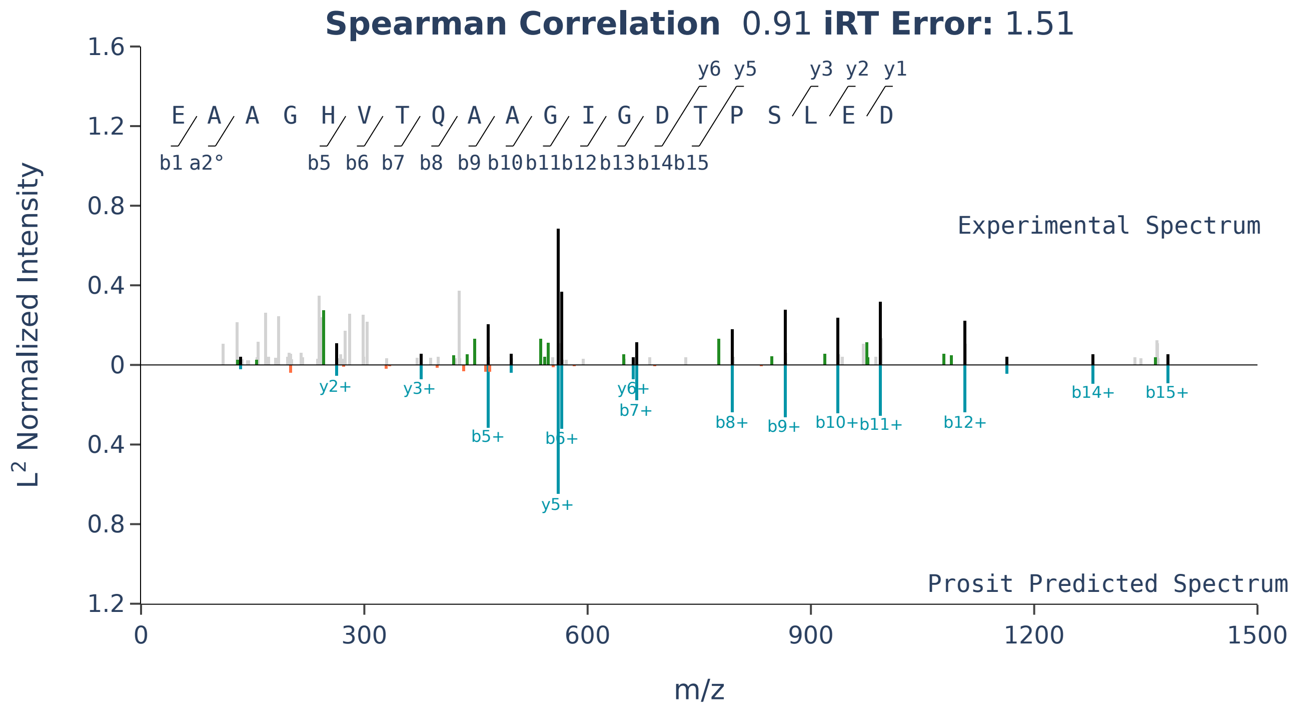

Source WSoh_221121_011221_HFGoe_L1_hTau_24h_R2 **Scan** 19839
Peptide AGIGDTPSLEDEAAGHVTQA **Charge** 2 **Spectral Angle** 0.21
Spearman Correlation 0.26 **iRT Error:** 8.64

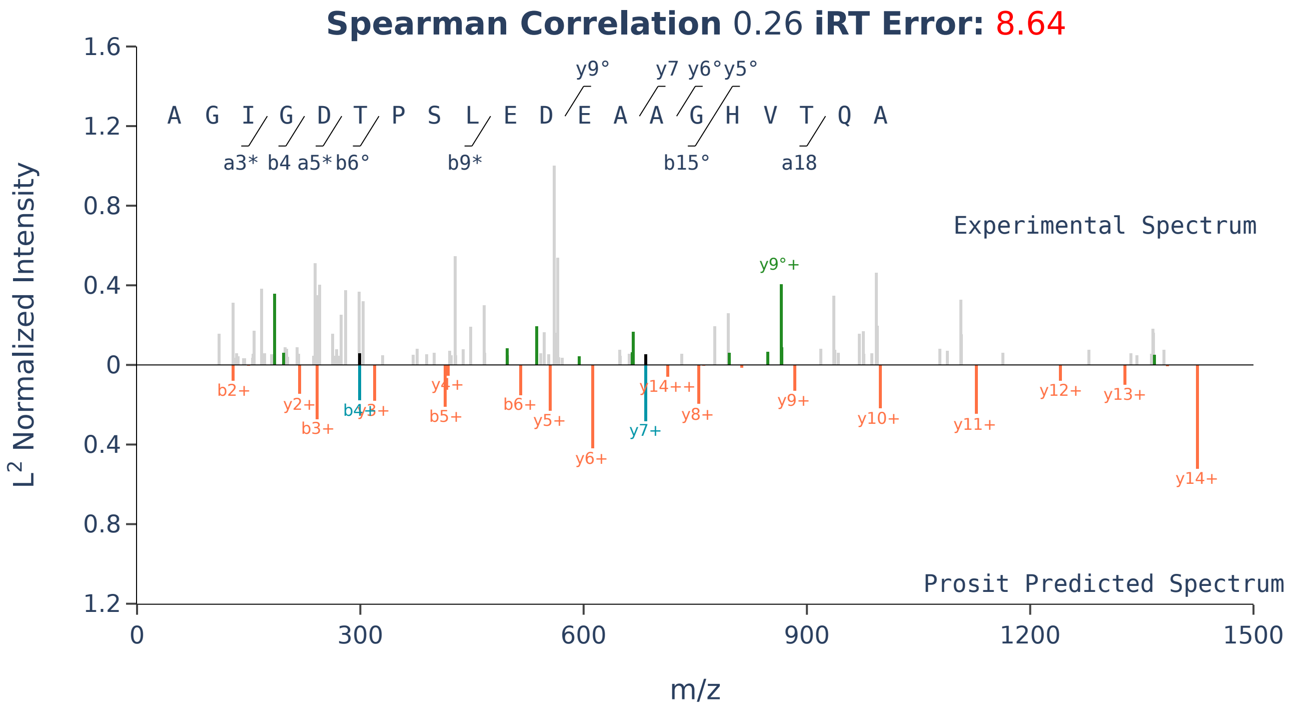

Source WSoh_221121_011221_HFGoe_L2_hTau_2h_R1 **Scan** 11913
Peptide DNITHVPGGGNKKIETHLS **Charge** 3 **Spectral Angle** 0.83
Spearman Correlation 0.84 **iRT Error:** 1.51

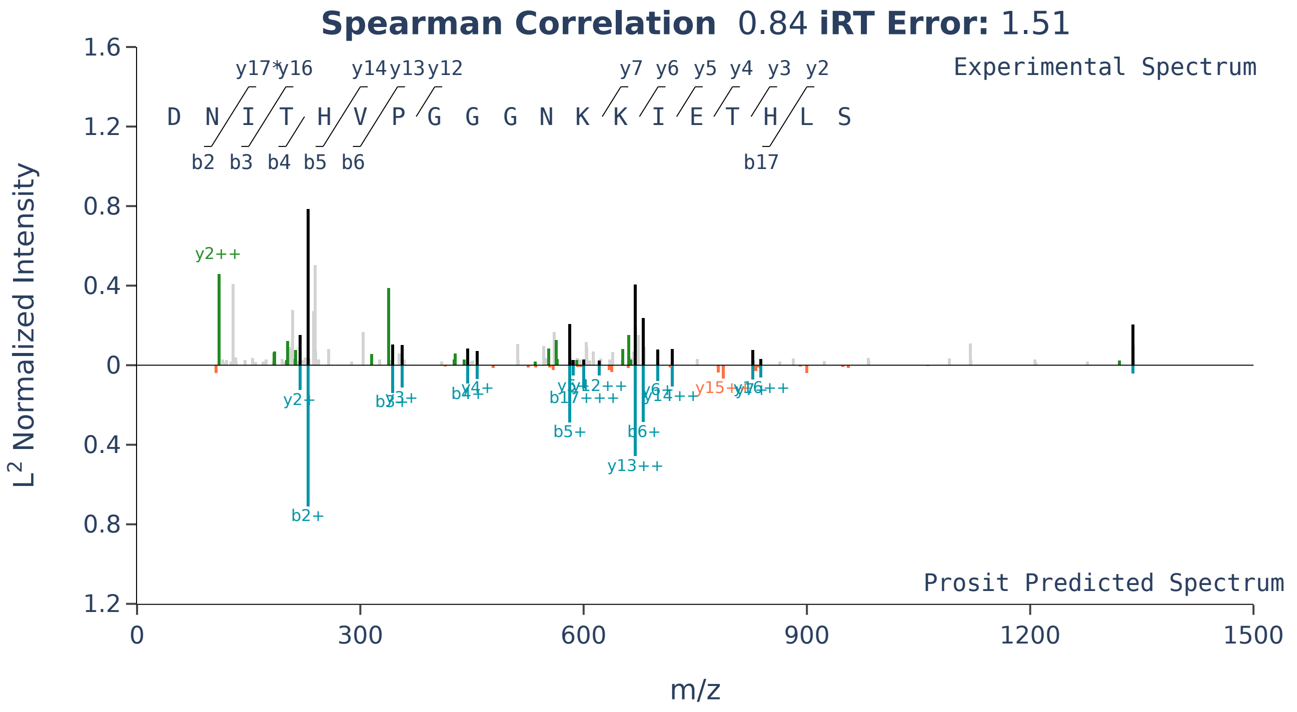

Source WSoh_221121_011221_HFGoe_L2_hTau_2h_R1 **Scan** 11913
Peptide RENAKAKTDHGAEIVYKS **Charge** 3 **Spectral Angle** 0.03
Spearman Correlation 0.04 **iRT Error:** 32.94

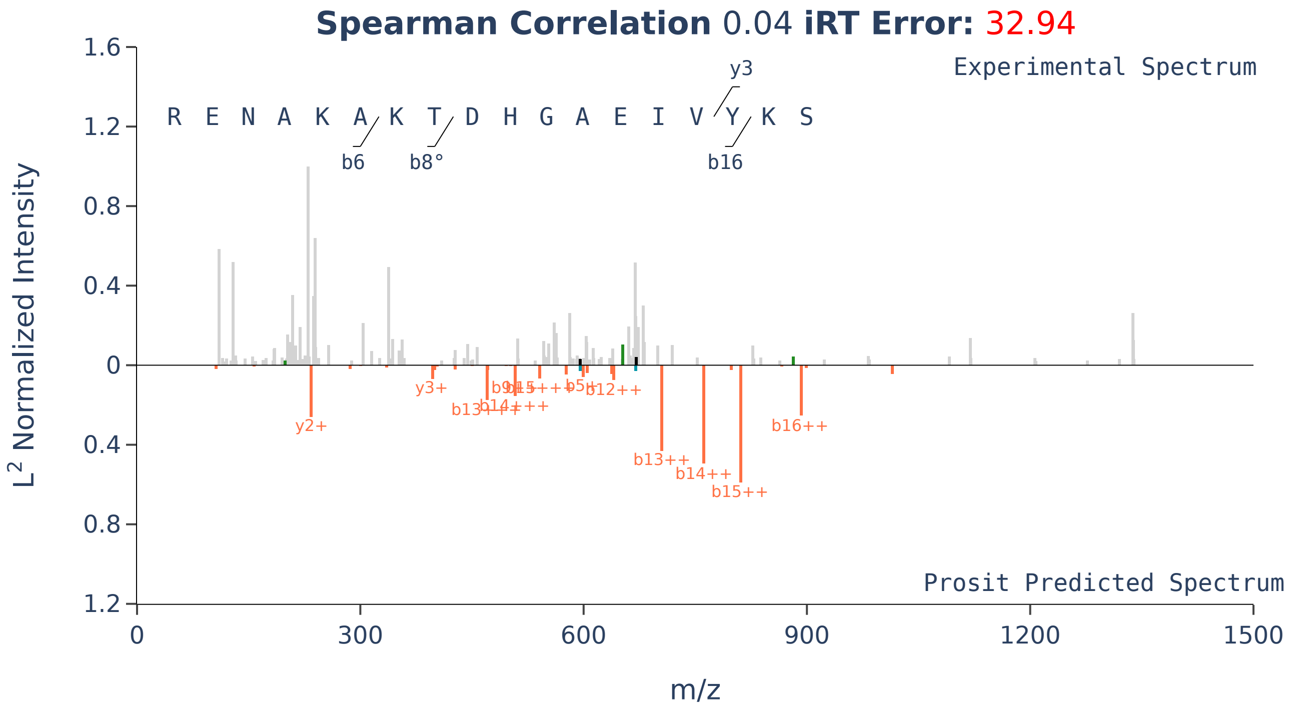

Source WSoh_221121_011221_HFGoe_L1_hTau_24h_R1 **Scan** 8682
Peptide KSSLGNIHHKPGGGQVEV **Charge** 2 **Spectral Angle** 0.84
Spearman Correlation 0.91 **iRT Error:** 1.52

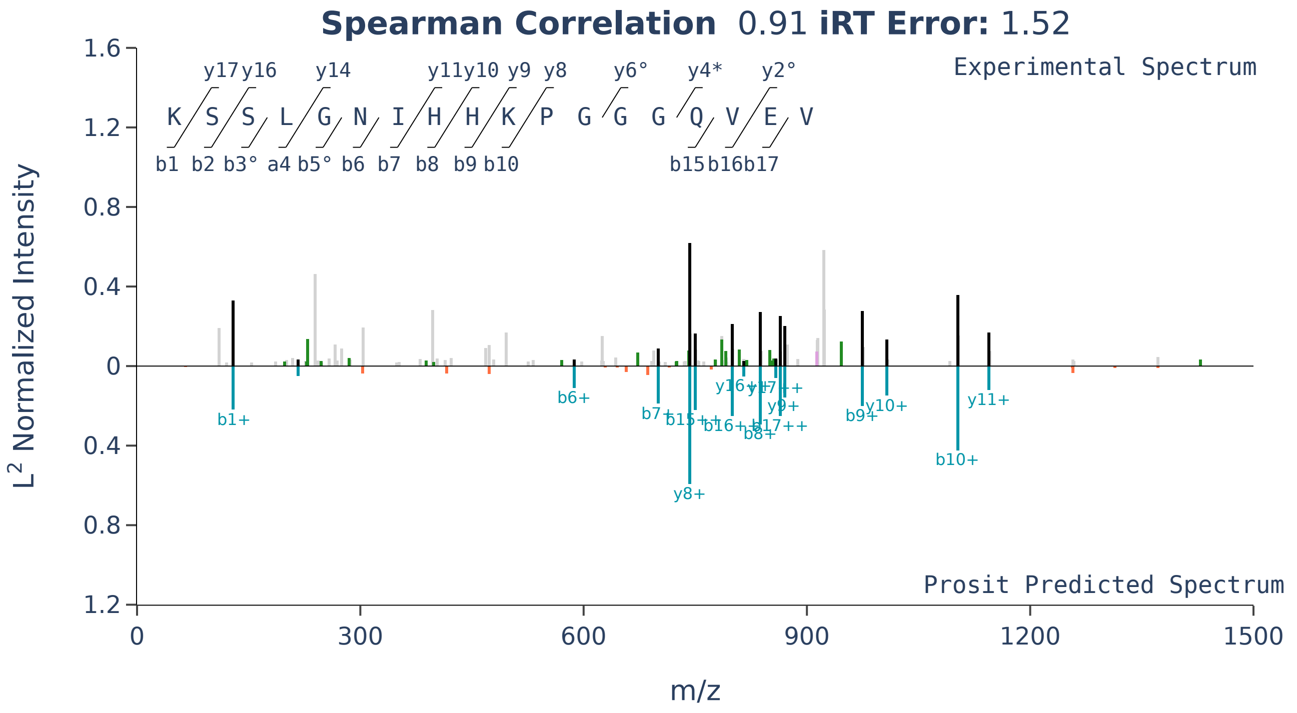

Source WSoh_221121_011221_HFGoe_L1_hTau_24h_R1 **Scan** 8682
Peptide NIHHKPGGGQVEVKSEK **Charge** 2 **Spectral Angle** 0.0
Spearman Correlation -0.18 **iRT Error:** 28.92

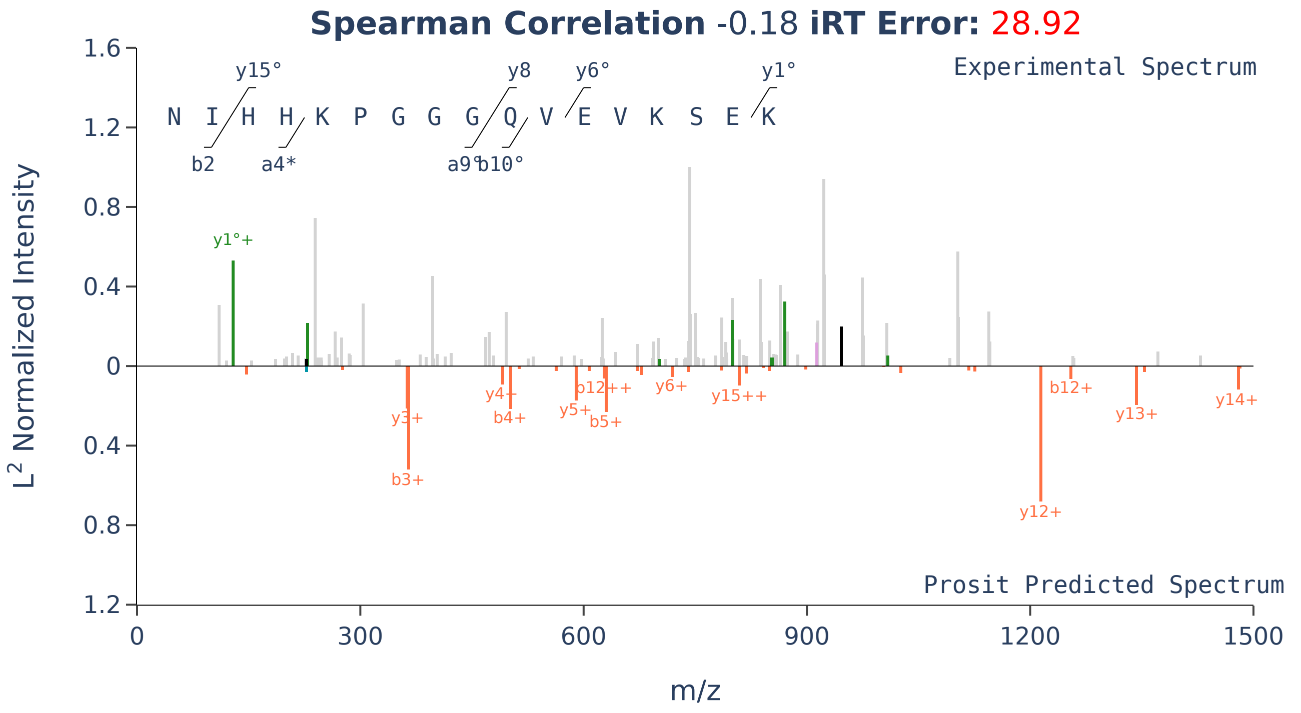

Source WSoh_221121_011221_HFGoe_L1_hTau_24h_R2 **Scan** 7911
Peptide DFKDRVQSN **Charge** 2 **Spectral Angle** 0.78
Spearman Correlation 0.95 **iRT Error:** 1.53

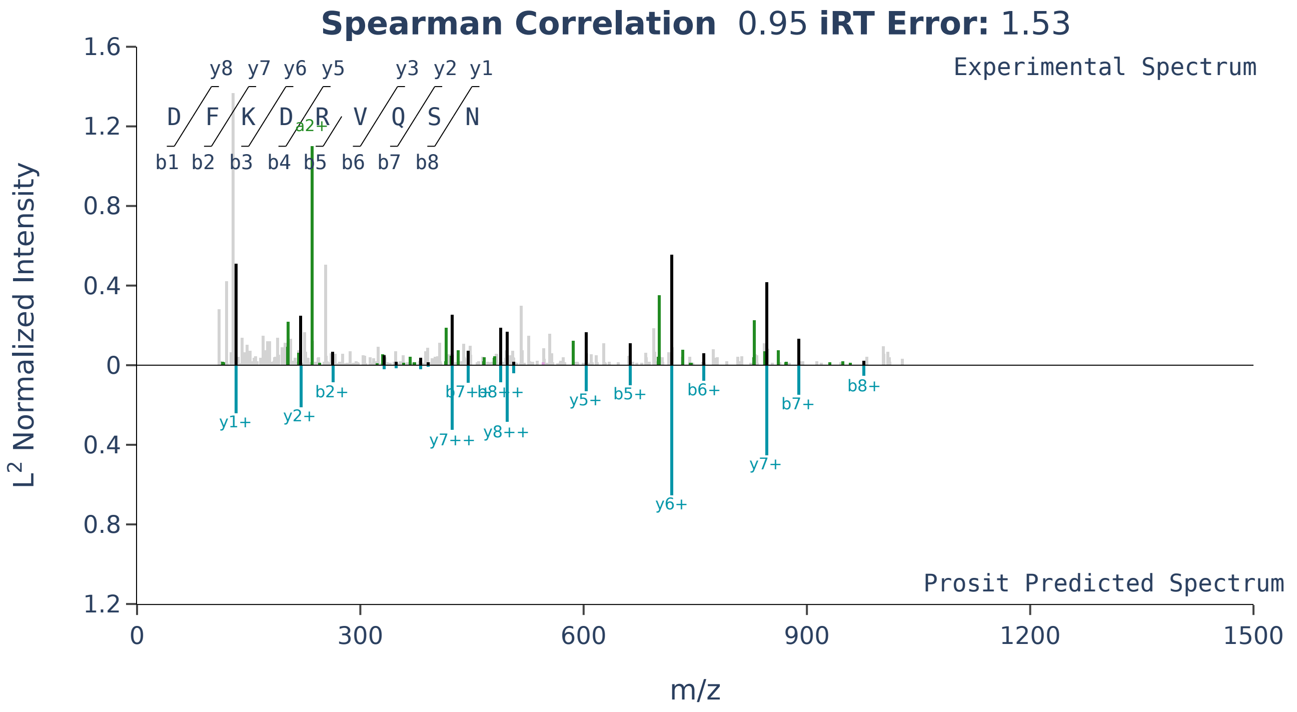

Source WSoh_221121_011221_HFGoe_L1_hTau_24h_R2 **Scan** 7911
Peptide LGDRKDQGGY **Charge** 2 **Spectral Angle** 0.09
Spearman Correlation 0.15 **iRT Error:** 8.41

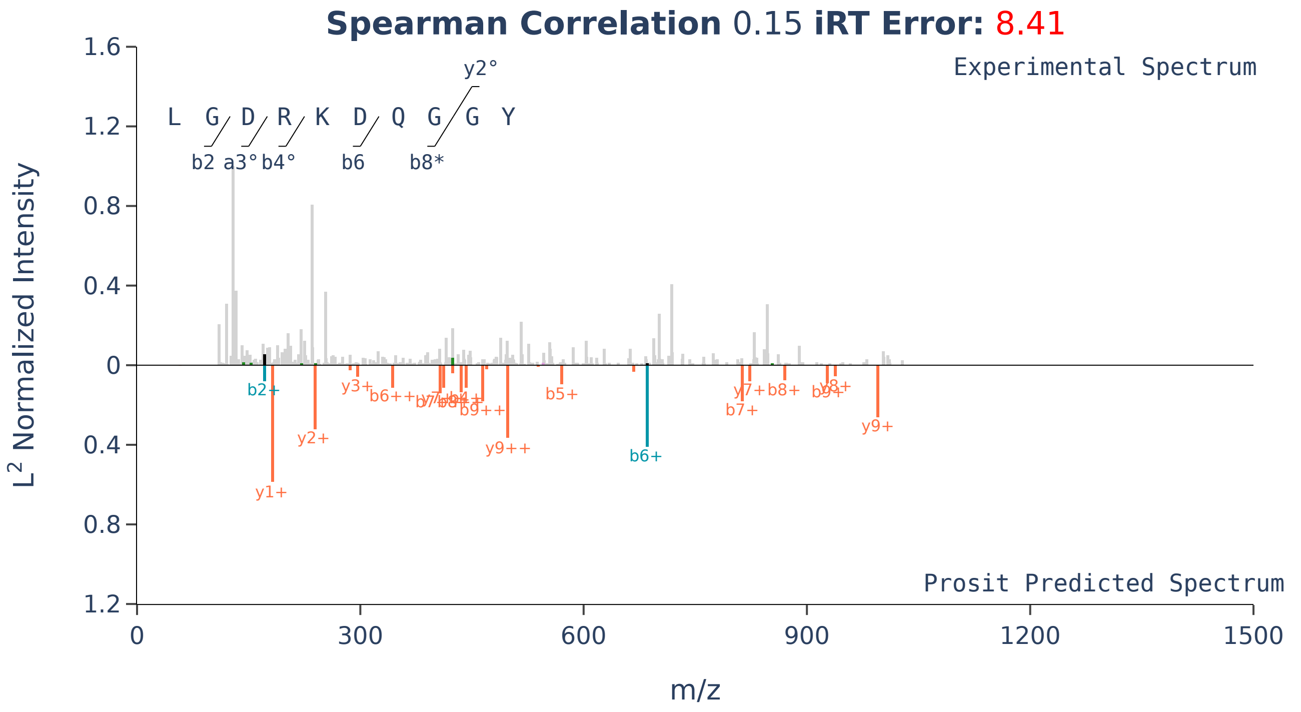

Source WSoh_221121_011221_HFGoe_L1_hTau_24h_R1 **Scan** 18487
Peptide PVPMPDLKNVKN **Charge** 3 **Spectral Angle** 0.81
Spearman Correlation 0.9 **iRT Error:** 1.57

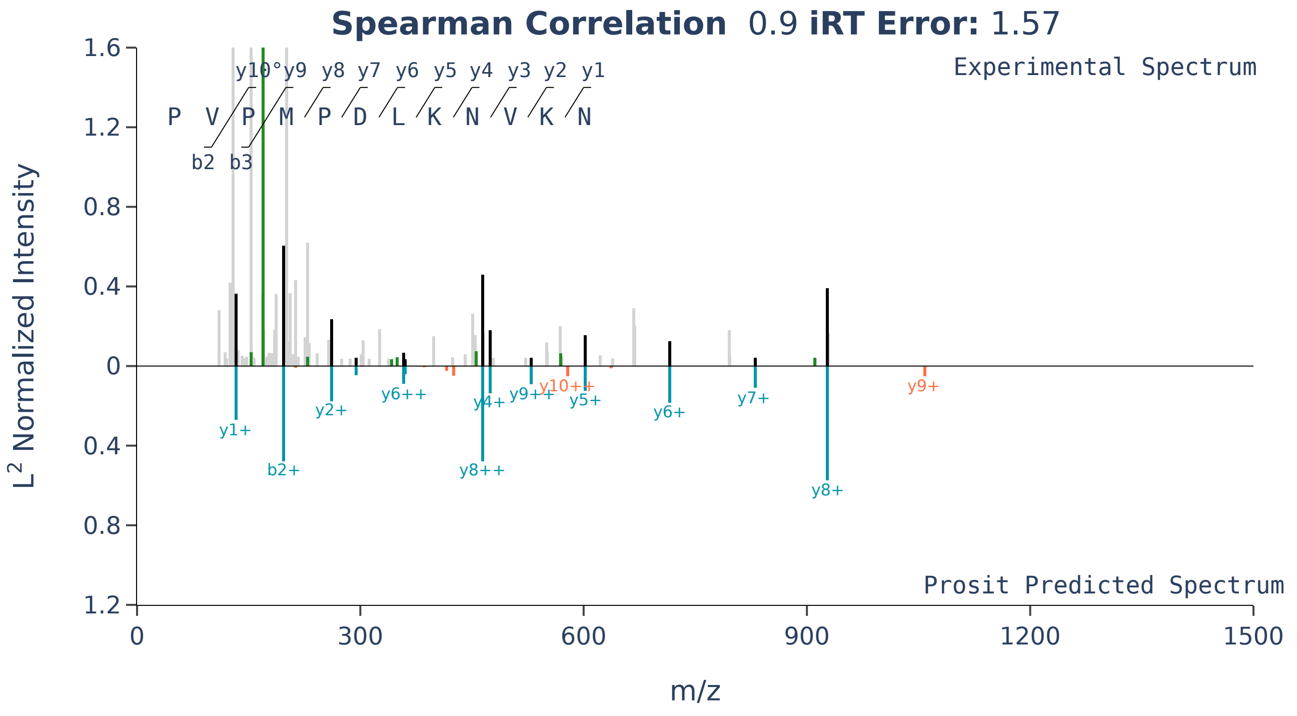

Source WSoh_221121_011221_HFGoe_L1_hTau_24h_R1 **Scan** 18487
Peptide RTPSLPTPPTRE **Charge** 3 **Spectral Angle** 0.0
Spearman Correlation 0.0 **iRT Error:** 20.61

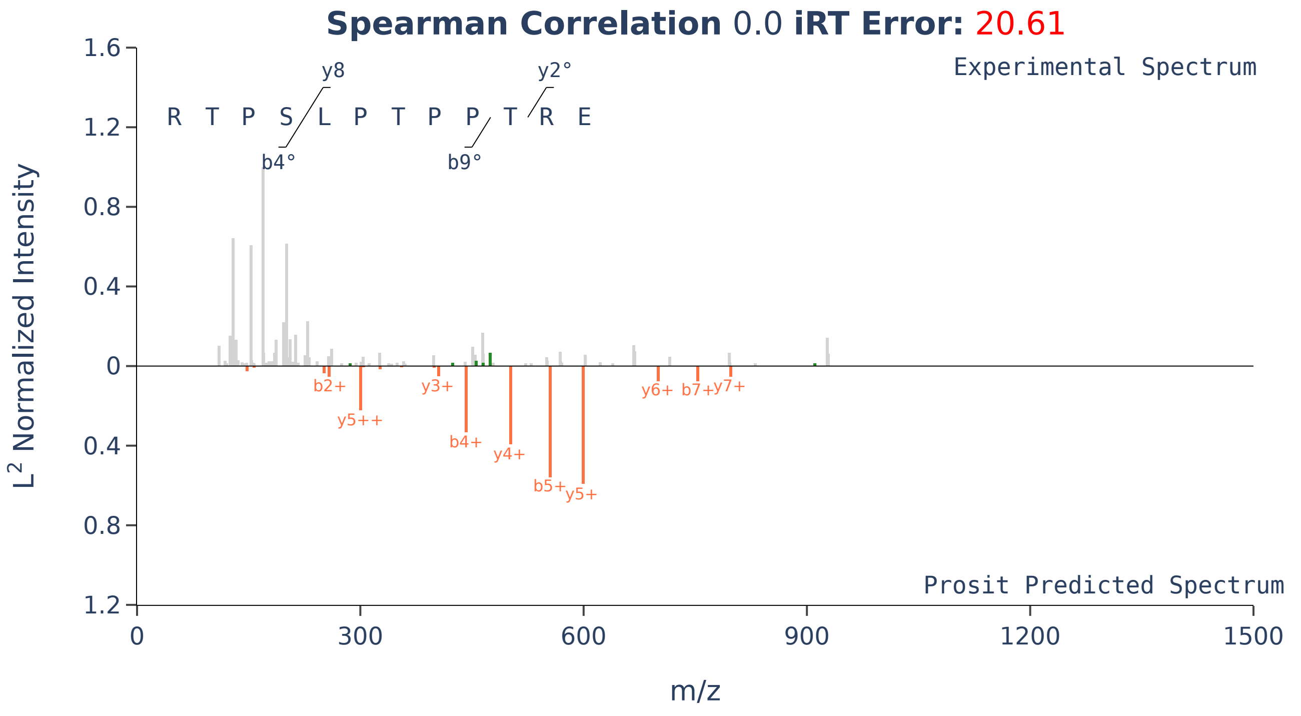

Source WSoh_221121_011221_HFGoe_L2_hTau_24h_R2 **Scan** 13236
Peptide DLSNVQSKCGSKDLG **Charge** 3 **Spectral Angle** 0.79
Spearman Correlation 0.87 **iRT Error:** 1.6

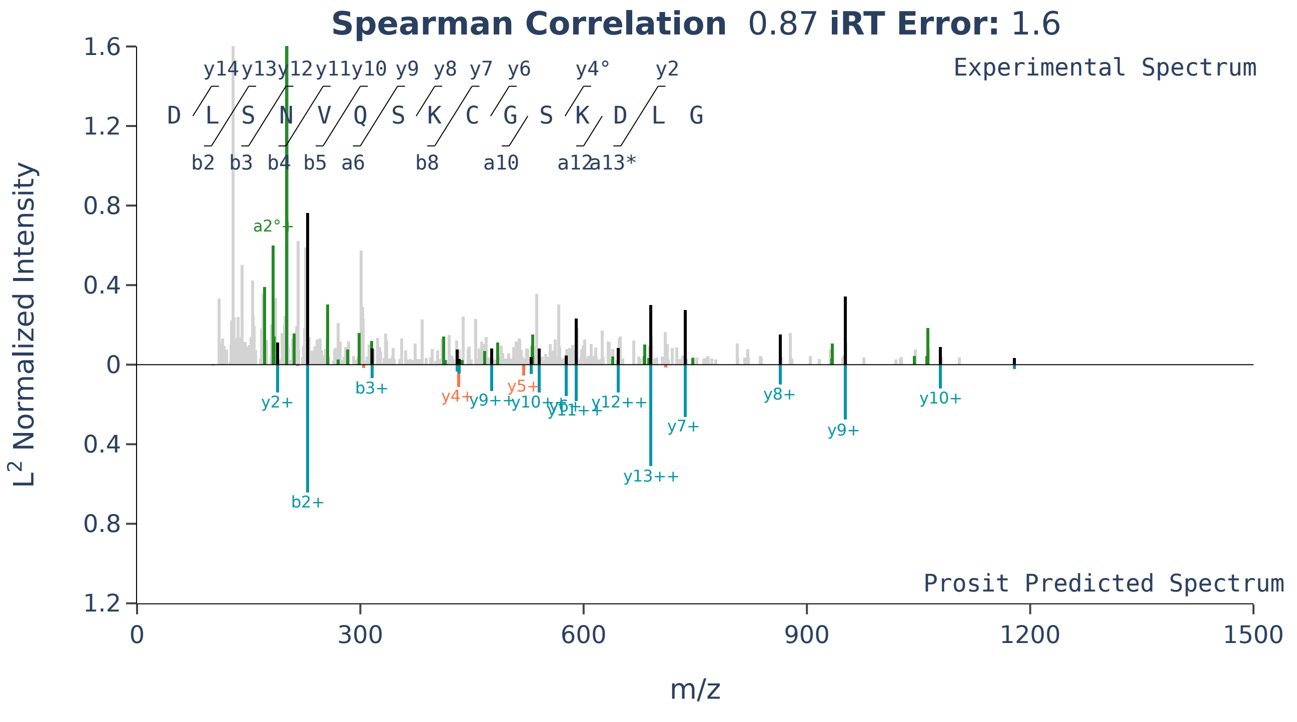

Source WSoh_221121_011221_HFGoe_L2_hTau_24h_R2 **Scan** 13236
Peptide QAAAQPHTEIPEGTT **Charge** 3 **Spectral Angle** 0.08
Spearman Correlation 0.04 **iRT Error:** 0.66

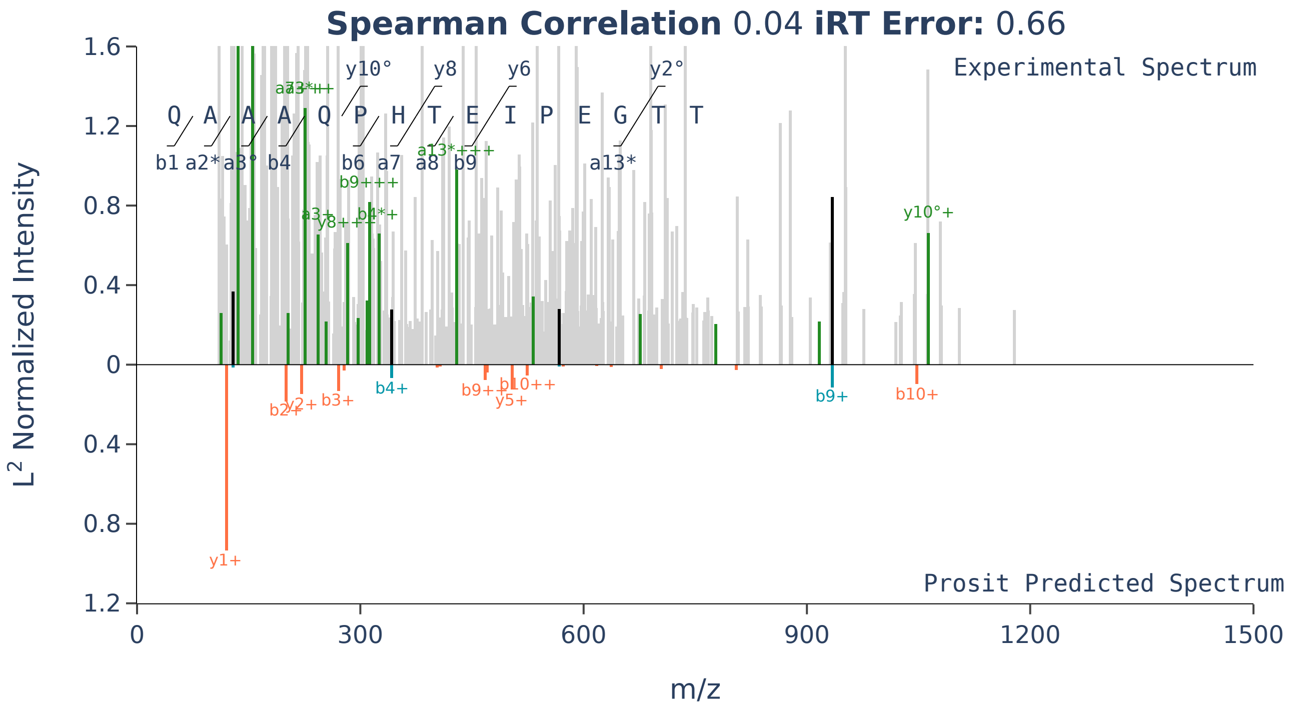

Source WSoh_221121_011221_HFGoe_L2_hTau_24h_R2 **Scan** 7404
Peptide GDTSPRHLSTQA **Charge** 3 **Spectral Angle** 0.87
Spearman Correlation 0.88 **iRT Error:** 1.6

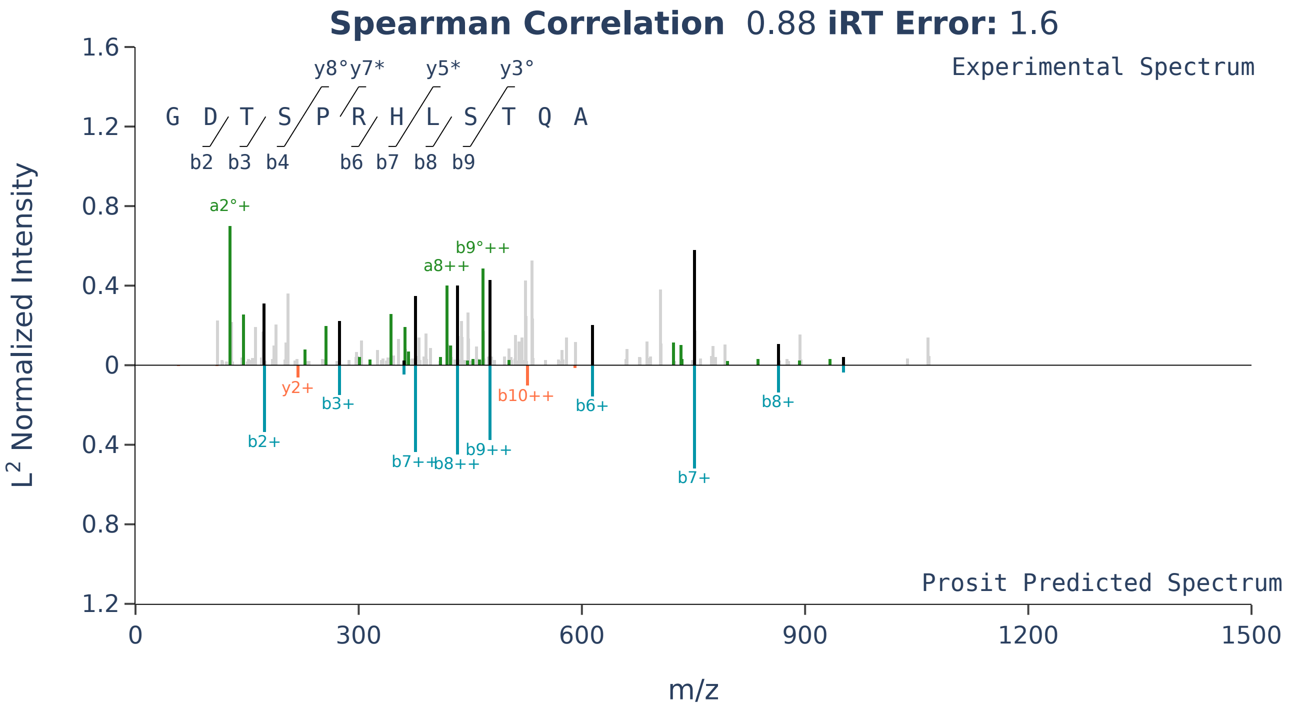

Source WSoh_221121_011221_HFGoe_L2_hTau_24h_R2 **Scan** 7404
Peptide VSGDTSPRHLSN **Charge** 3 **Spectral Angle** 0.16
Spearman Correlation 0.23 **iRT Error:** 7.29

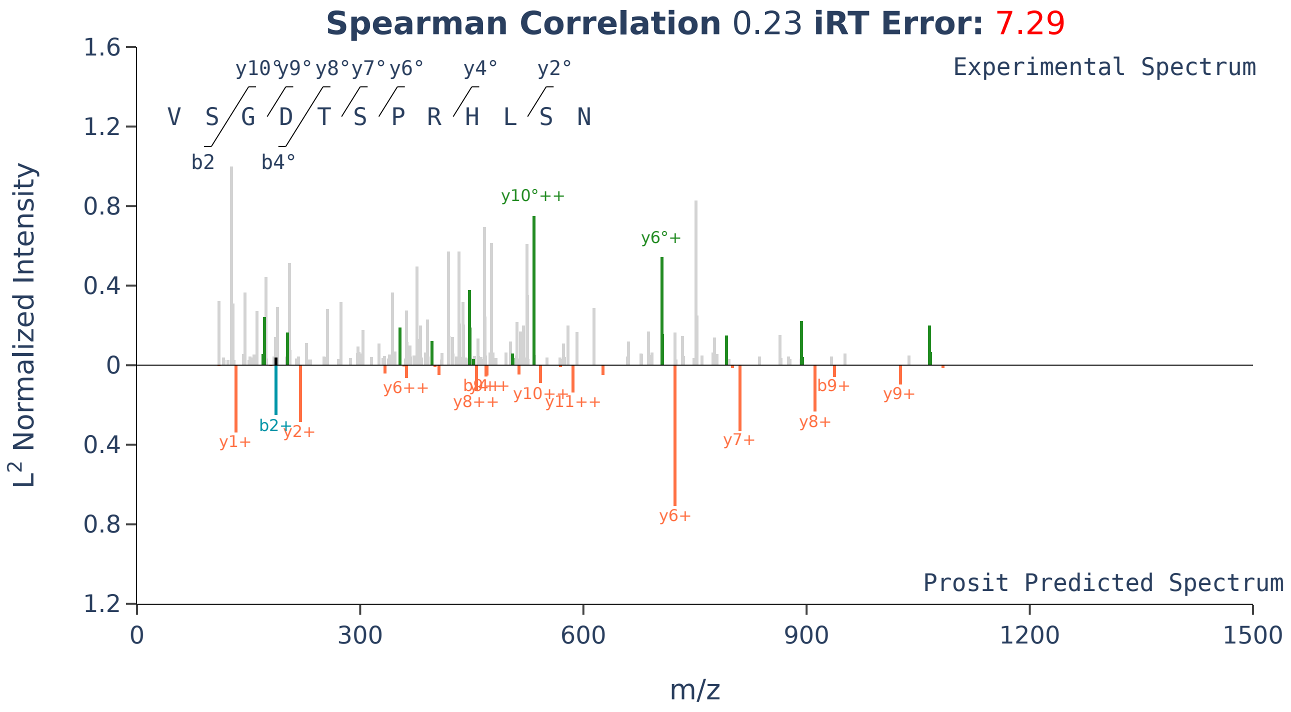

Source WSoh_221121_011221_HFGoe_L2_hTau_4h_R2 **Scan** 11362
Peptide NITHVPGGGNKKIETHKLTFRADG **Charge** 3 **Spectral Angle** 0.82
Spearman Correlation 0.87 **iRT Error:** 1.62

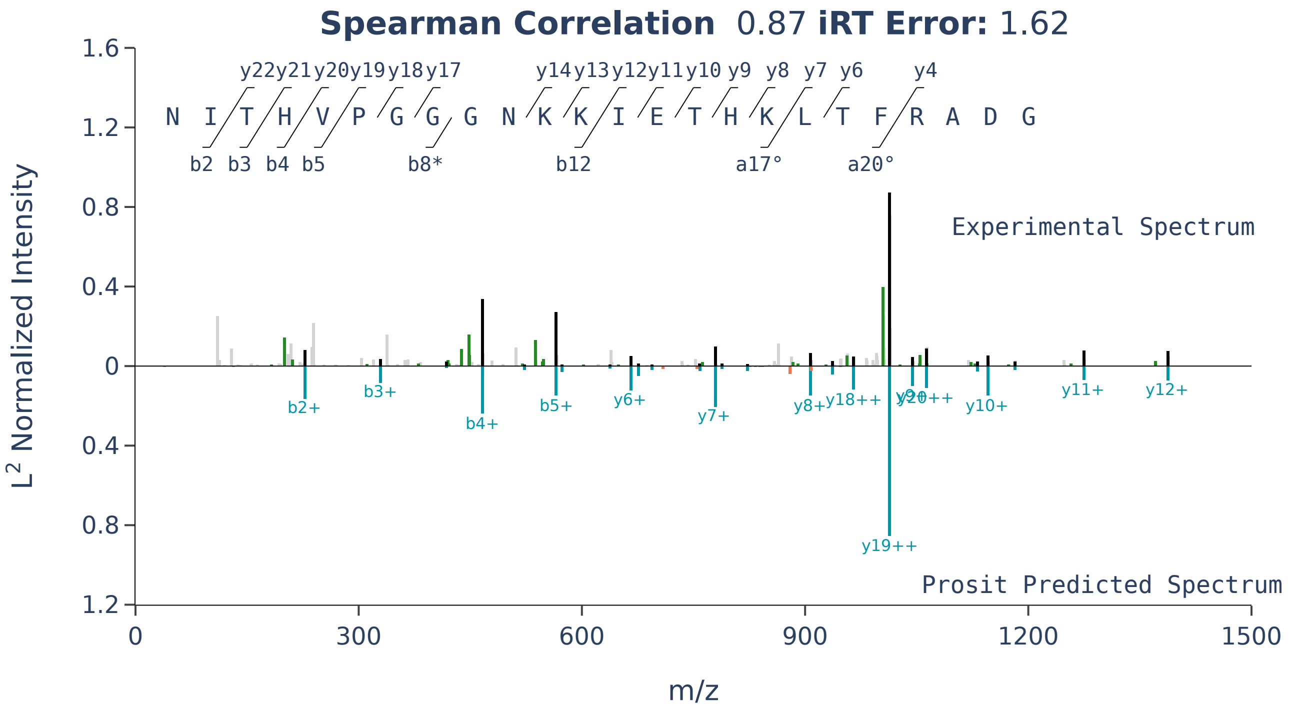

Source WSoh_221121_011221_HFGoe_L2_hTau_4h_R2 **Scan** 11362
Peptide NITHVPGGGNKKIETHKLTFREN **Charge** 3 **Spectral Angle** 0.85
Spearman Correlation 0.85 **iRT Error:** 2.71

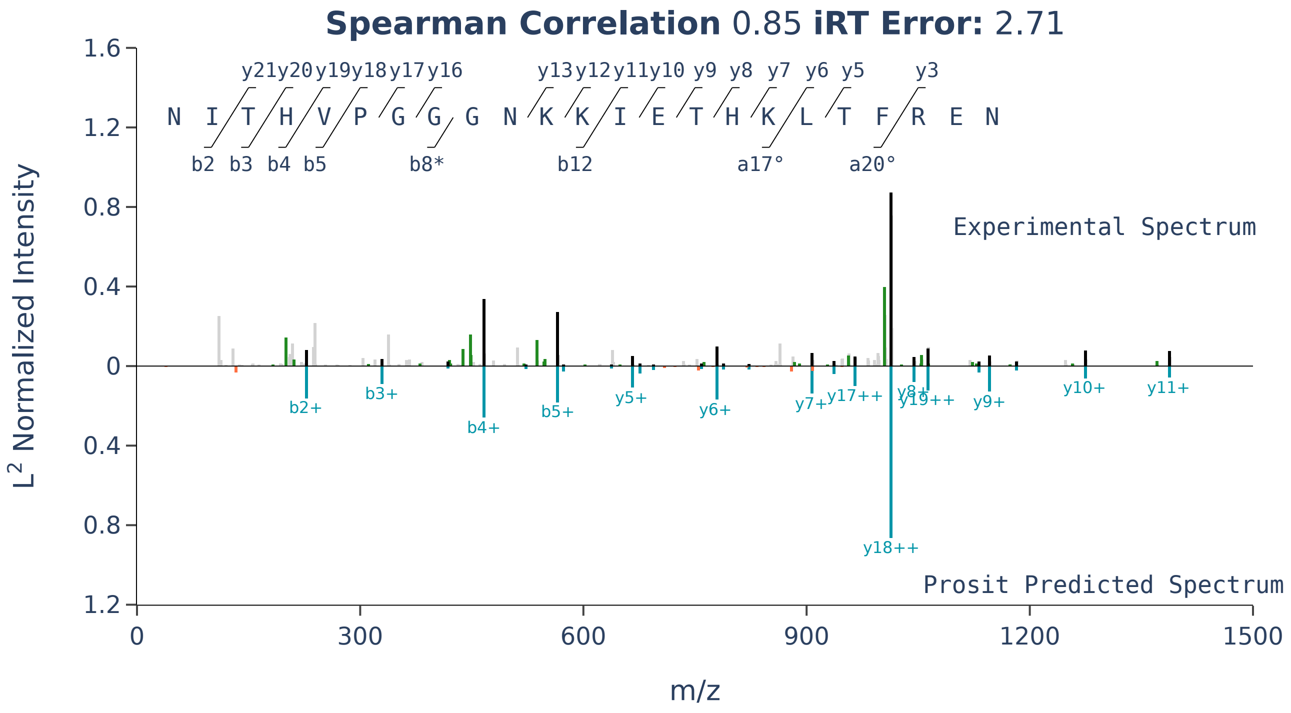

Source WSoh_221121_011221_HFGoe_L2_hTau_24h_R1 **Scan** 8928
Peptide SNVQSRLQT **Charge** 2 **Spectral Angle** 0.85
Spearman Correlation 0.94 **iRT Error:** 1.63

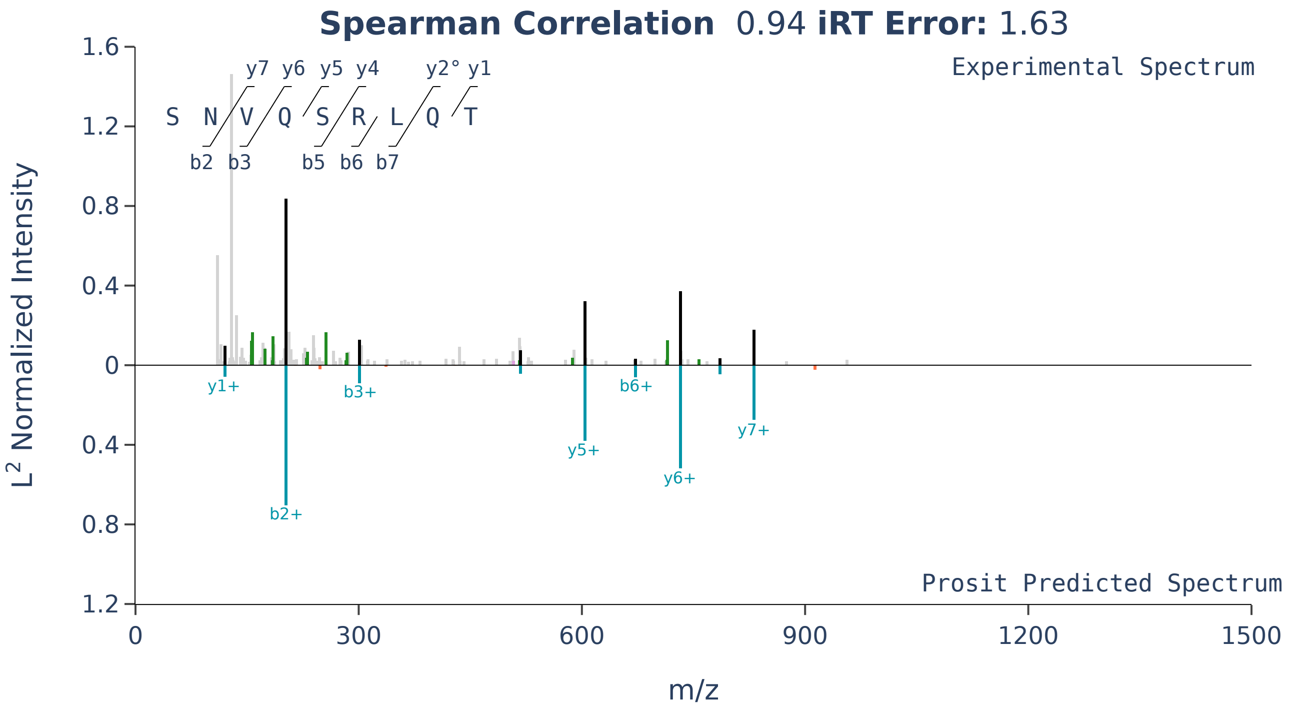

Source WSoh_221121_011221_HFGoe_L2_hTau_24h_R1 **Scan** 8928
Peptide RENAKAKTD **Charge** 2 **Spectral Angle** 0.02
Spearman Correlation -0.29 **iRT Error:** 55.58

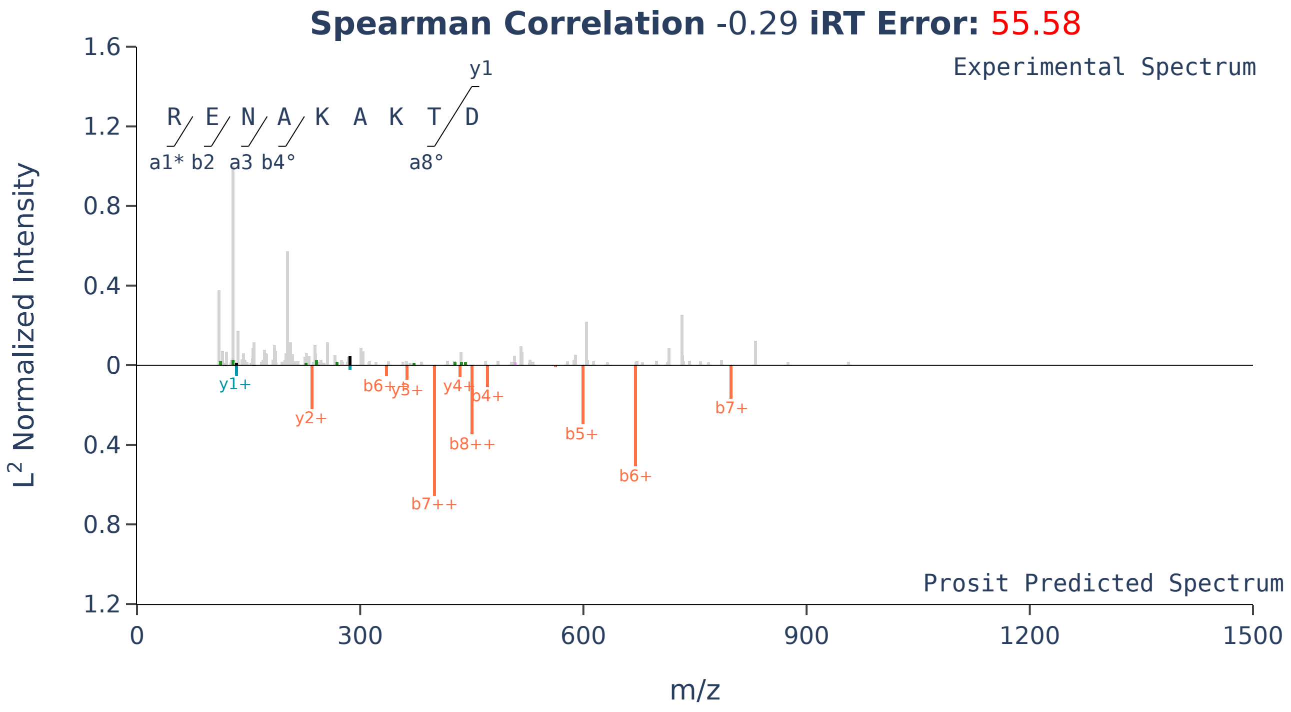

Source WSoh_221121_011221_HFGoe_L1_hTau_0h_R1 **Scan** 5795
Peptide FREPRGA **Charge** 2 **Spectral Angle** 0.85
Spearman Correlation 0.82 **iRT Error:** 1.64

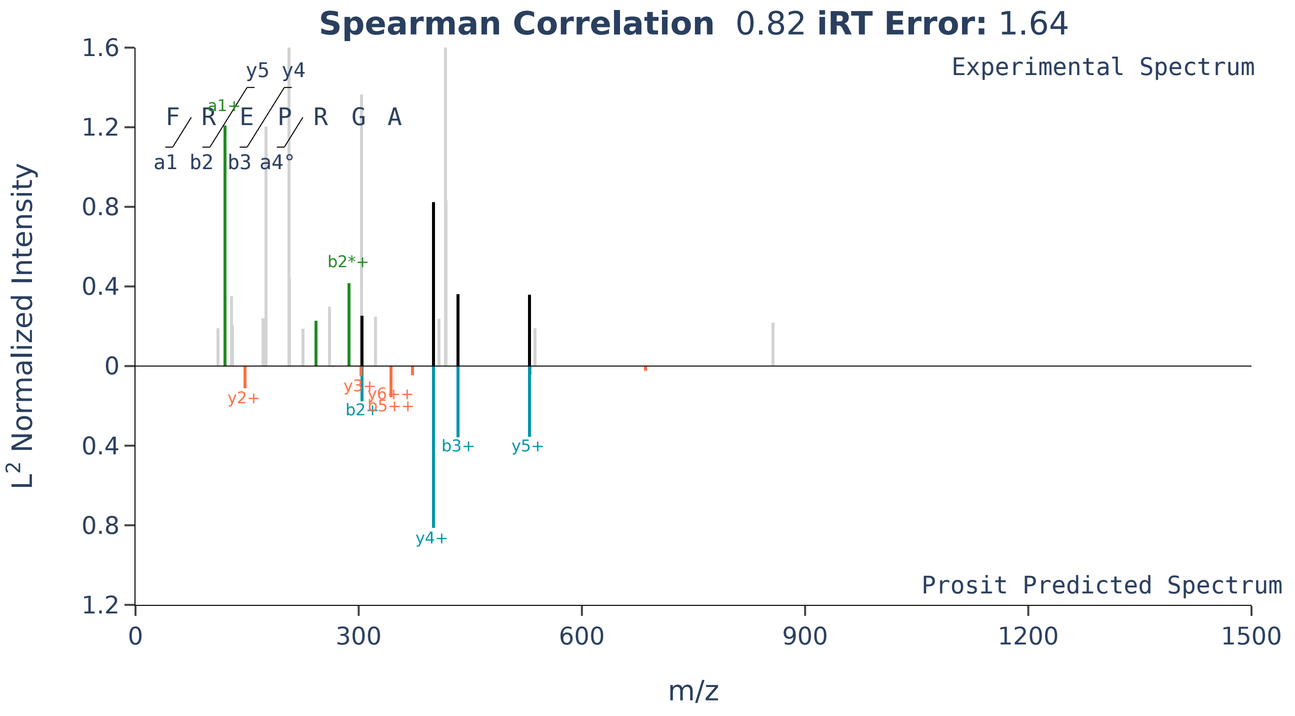

Source WSoh_221121_011221_HFGoe_L1_hTau_0h_R1 **Scan** 5795
Peptide IGSLDNIT **Charge** 2 **Spectral Angle** 0.3
Spearman Correlation 0.39 **iRT Error:** 76.05

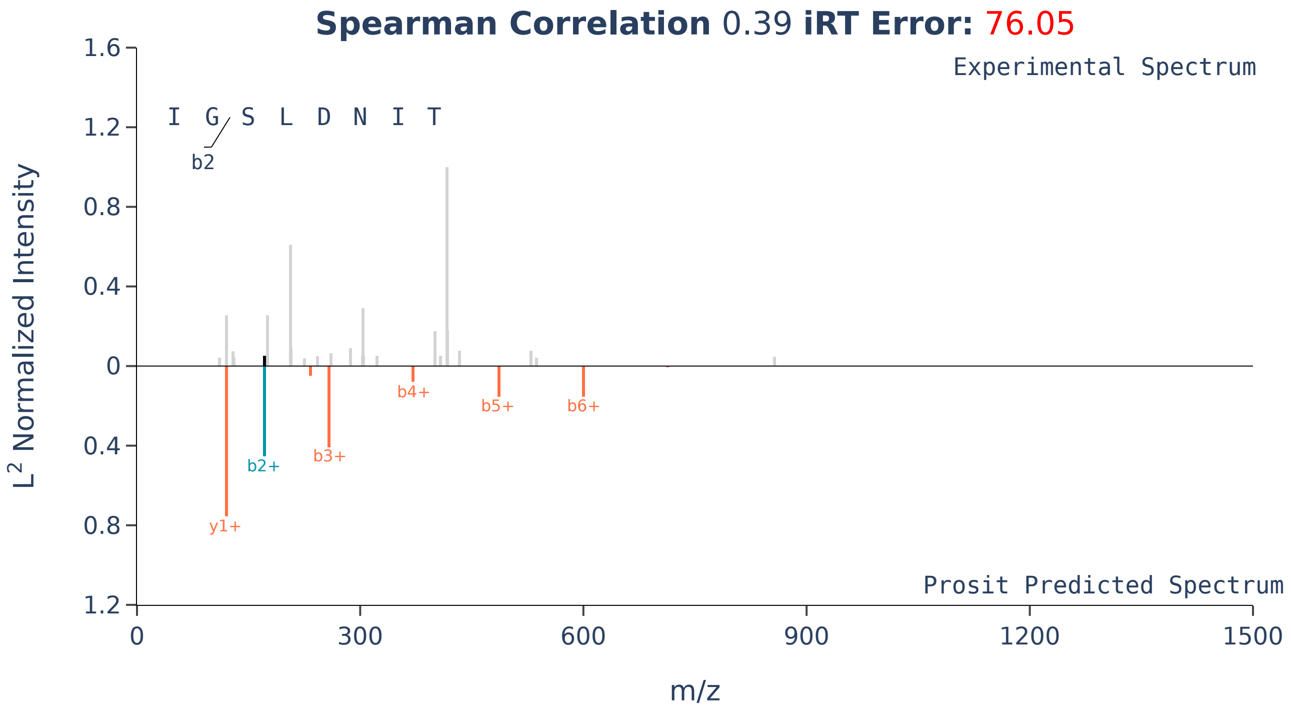

Source WSoh_221121_011221_HFGoe_L1_hTau_24h_R1 **Scan** 21914
Peptide SNVQSVYKPVDL **Charge** 2 **Spectral Angle** 0.95
Spearman Correlation 0.94 **iRT Error:** 1.69

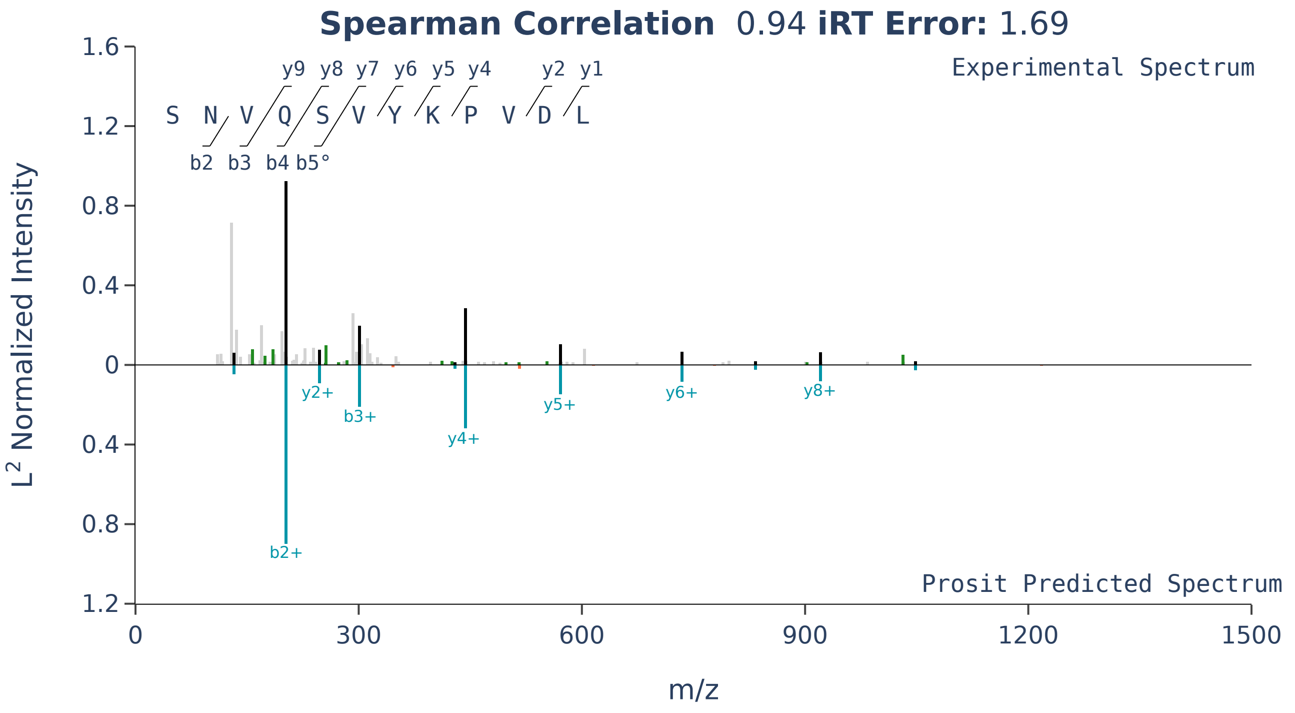

Source WSoh_221121_011221_HFGoe_L1_hTau_24h_R1 **Scan** 21914
Peptide PRGAAPPGQKGQAN **Charge** 2 **Spectral Angle** 0.0
Spearman Correlation 0.0 **iRT Error:** 100.03

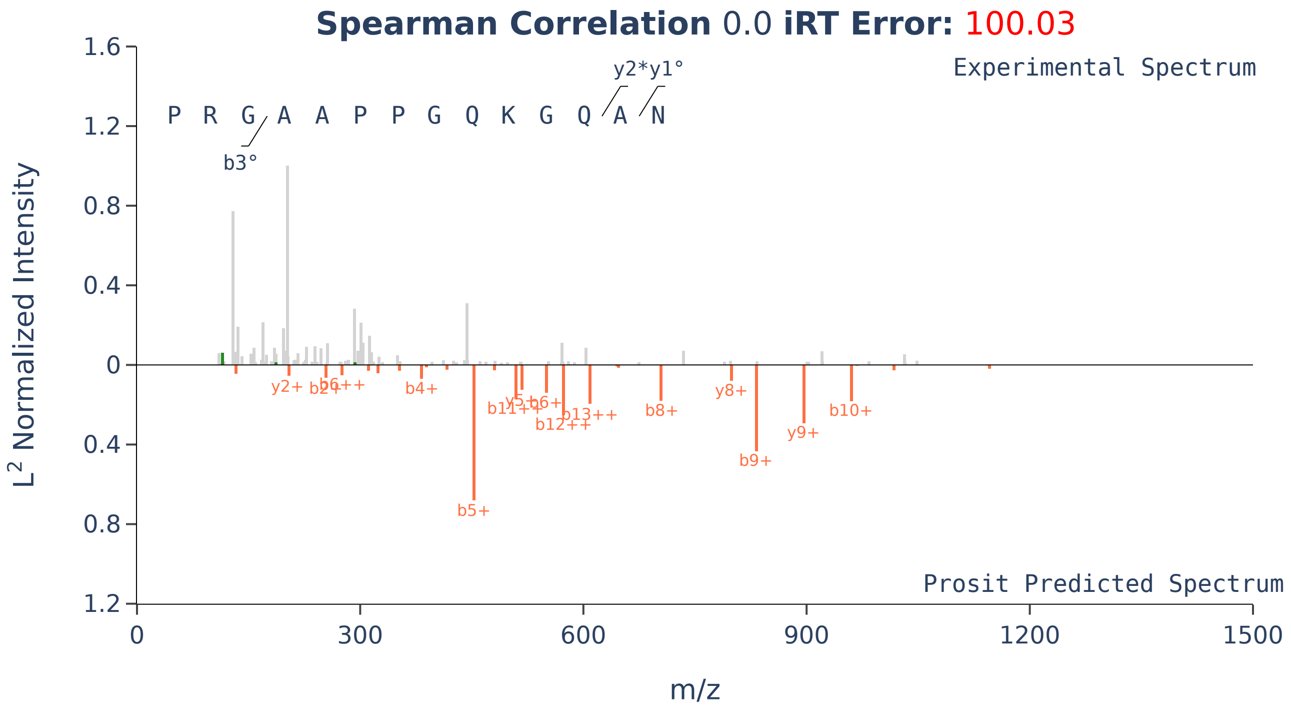

Source WSoh_221121_011221_HFGoe_L1_hTau_4h_R1 **Scan** 6430
Peptide SKVTSKHQPGGGKVQIINK **Charge** 3 **Spectral Angle** 0.83
Spearman Correlation 0.86 **iRT Error:** 1.82

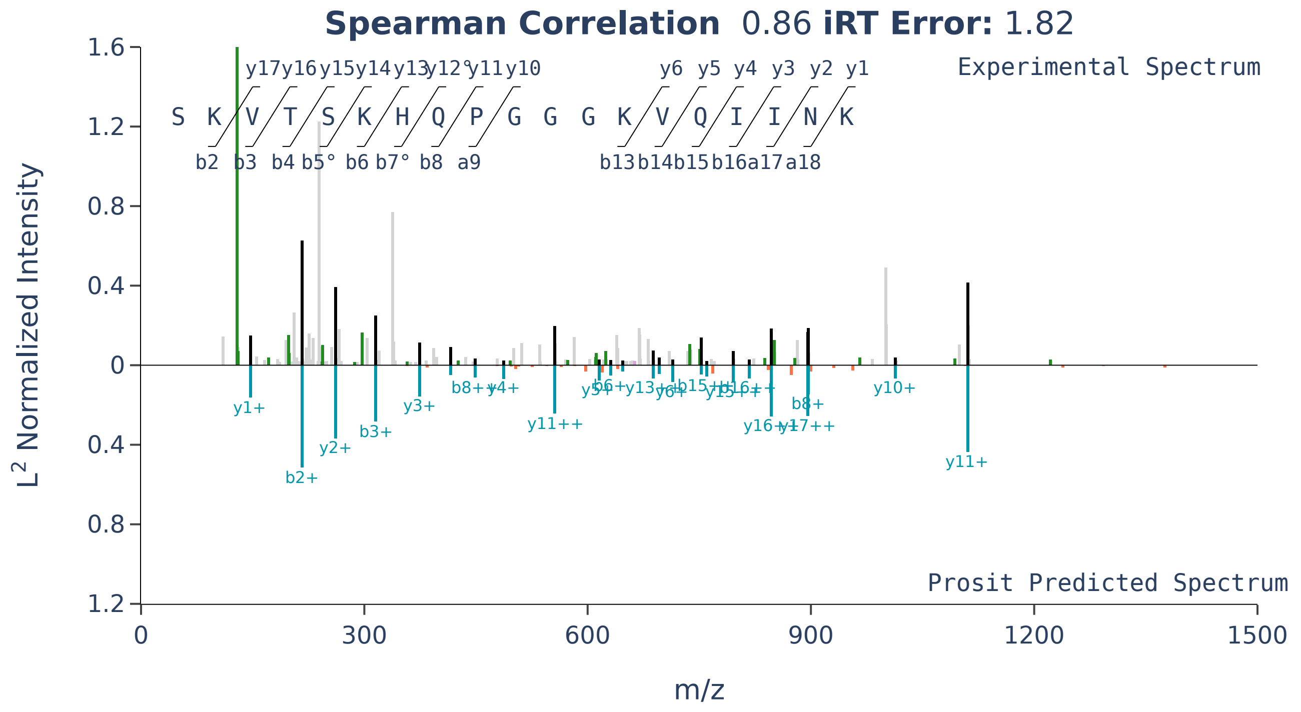

Source WSoh_221121_011221_HFGoe_L1_hTau_4h_R1 **Scan** 6430
Peptide IKHVPGGGSVQIVYKPVDL **Charge** 3 **Spectral Angle** 0.0
Spearman Correlation 0.0 **iRT Error:** 83.81

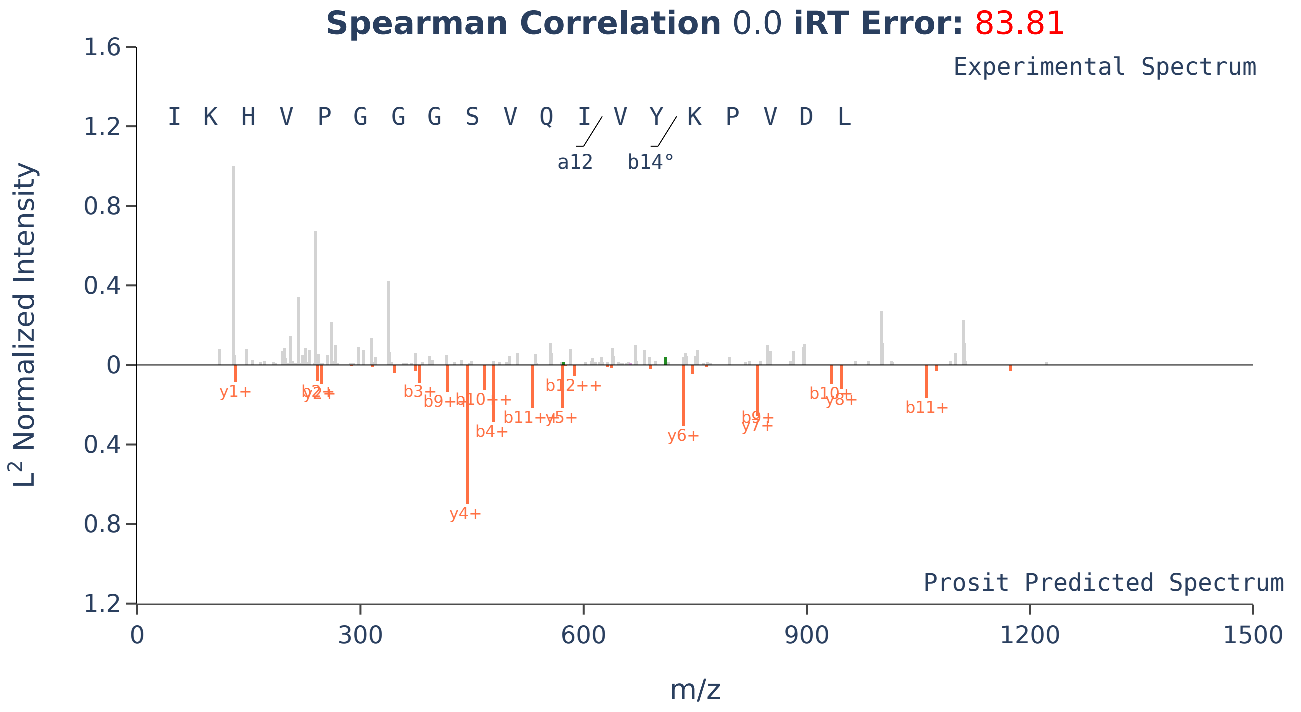

Source WSoh_221121_011221_HFGoe_L1_hTau_24h_R2 **Scan** 12601
Peptide VEVSLGNIHHKPGGGQ **Charge** 3 **Spectral Angle** 0.8
Spearman Correlation 0.93 **iRT Error:** 1.94

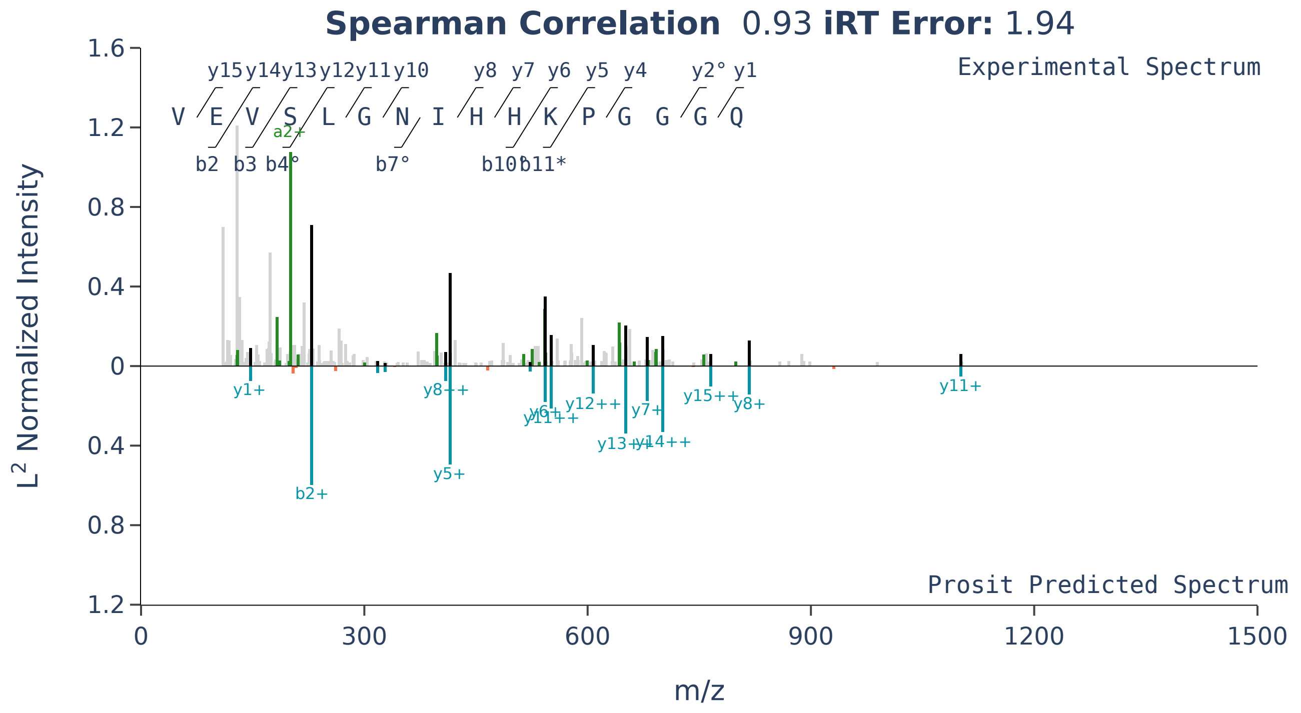

Source WSoh_221121_011221_HFGoe_L1_hTau_24h_R2 **Scan** 12601
Peptide SLGNIHHKPGGGQVEV **Charge** 3 **Spectral Angle** 0.37
Spearman Correlation 0.77 **iRT Error:** 8.66

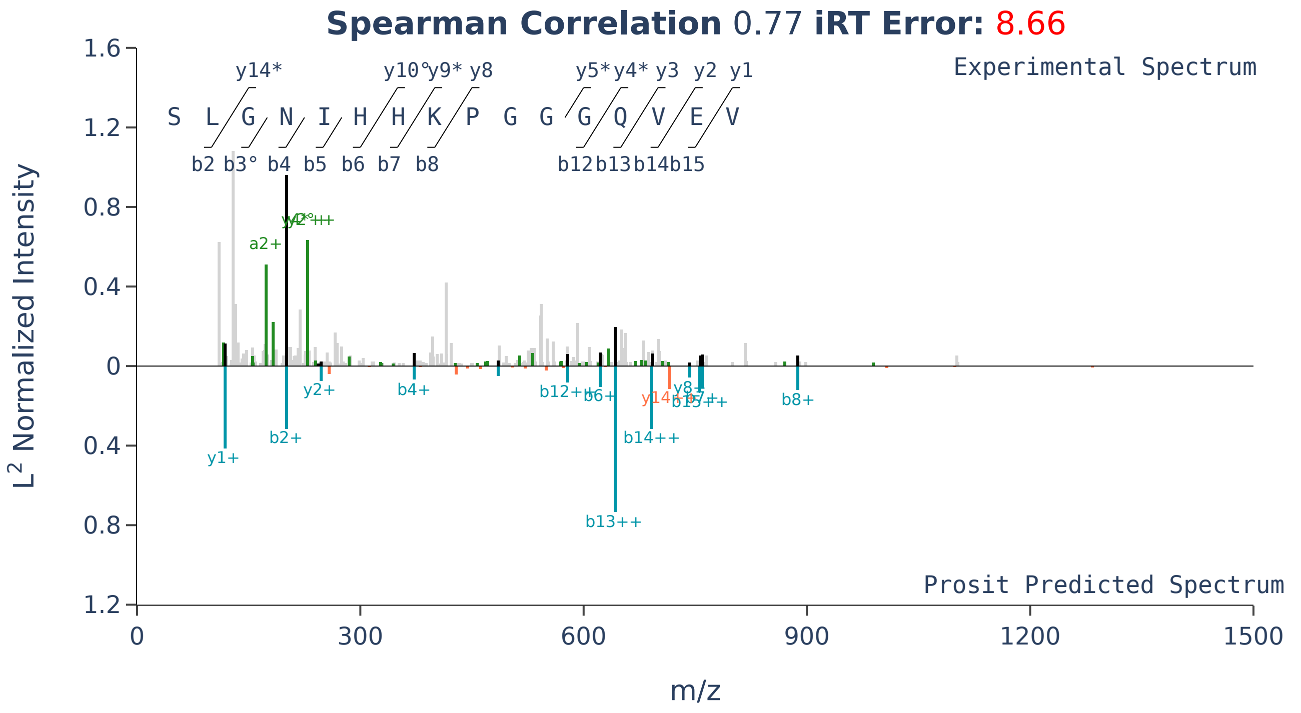

Source WSoh_221121_011221_HFGoe_L2_hTau_24h_R2 **Scan** 8194
Peptide VTKVTSKCGSLGNIHHKPGGGQVE **Charge** 3 **Spectral Angle** 0.84
Spearman Correlation 0.85 **iRT Error:** 1.98

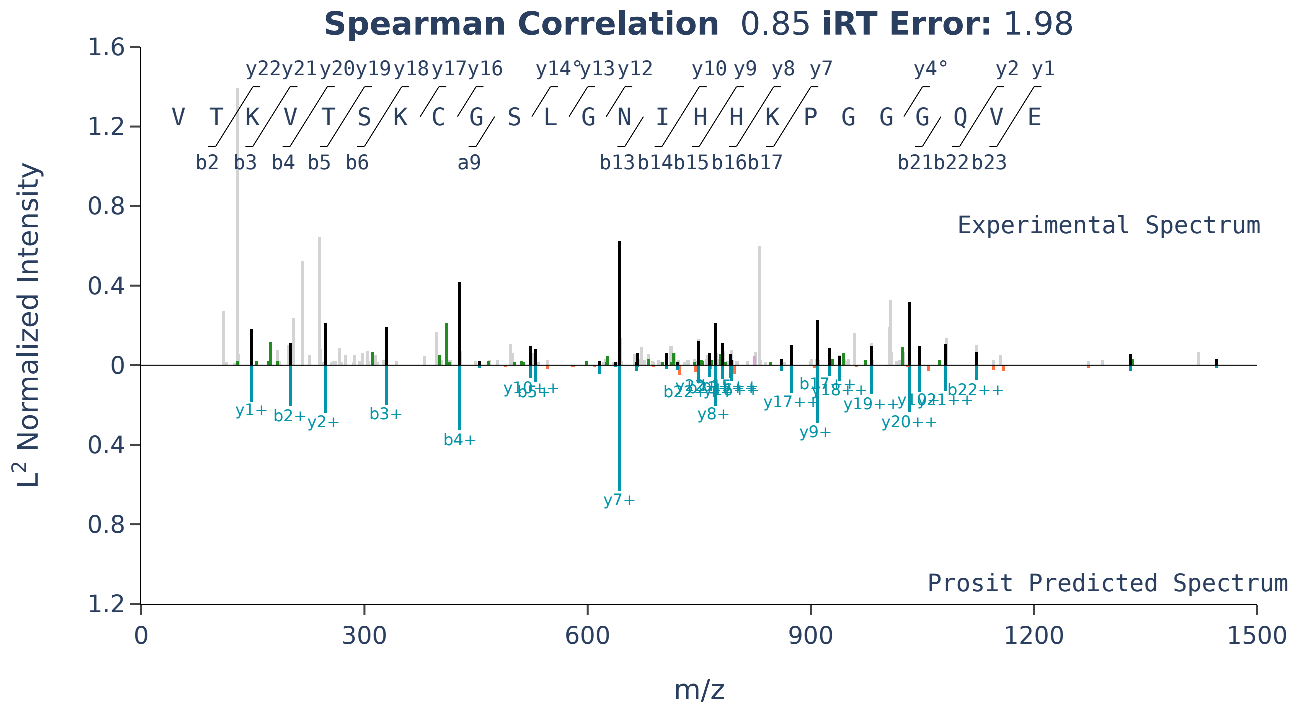

Source WSoh_221121_011221_HFGoe_L2_hTau_24h_R2 **Scan** 8194
Peptide LSKVTSKCGSLGNIHHKPGGGQVE **Charge** 3 **Spectral Angle** 0.86
Spearman Correlation 0.85 **iRT Error:** 5.99

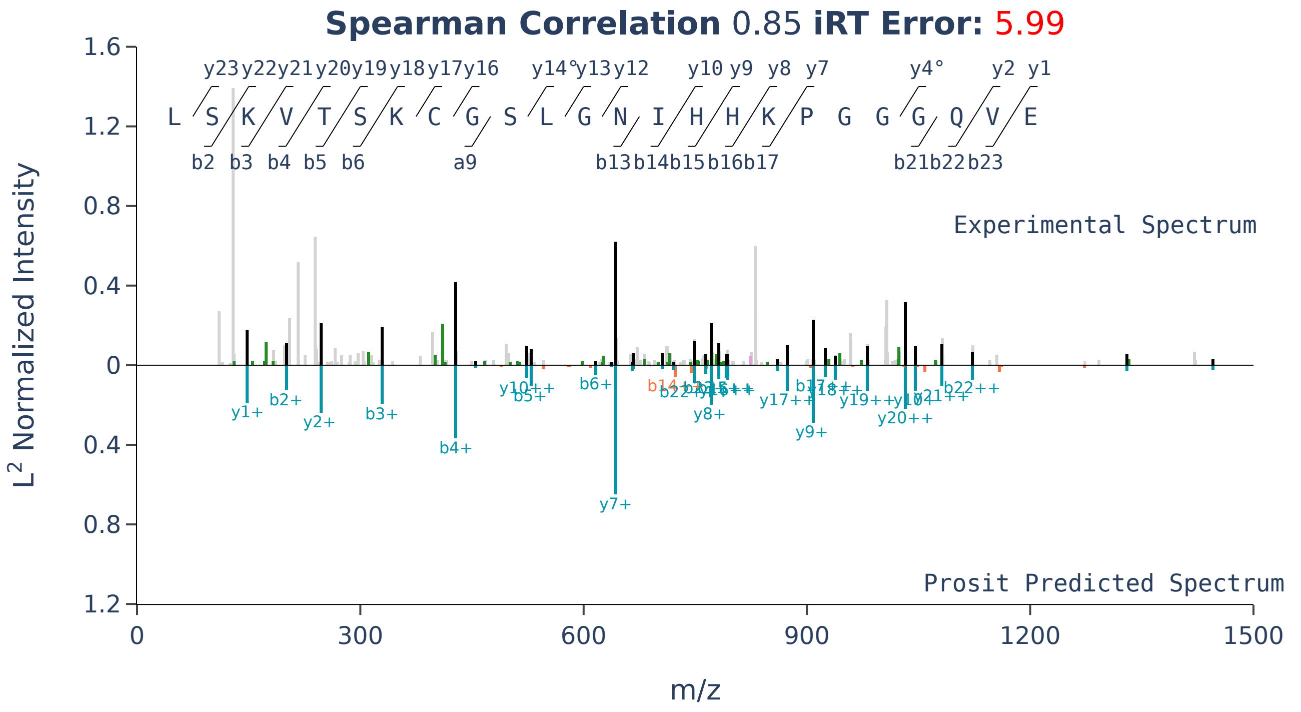

Source WSoh_221121_011221_HFGoe_L2_hTau_24h_R1 **Scan** 21175
Peptide RLQTAPVPMPDLKNSKIG **Charge** 3 **Spectral Angle** 0.86
Spearman Correlation 0.82 **iRT Error:** 1.99

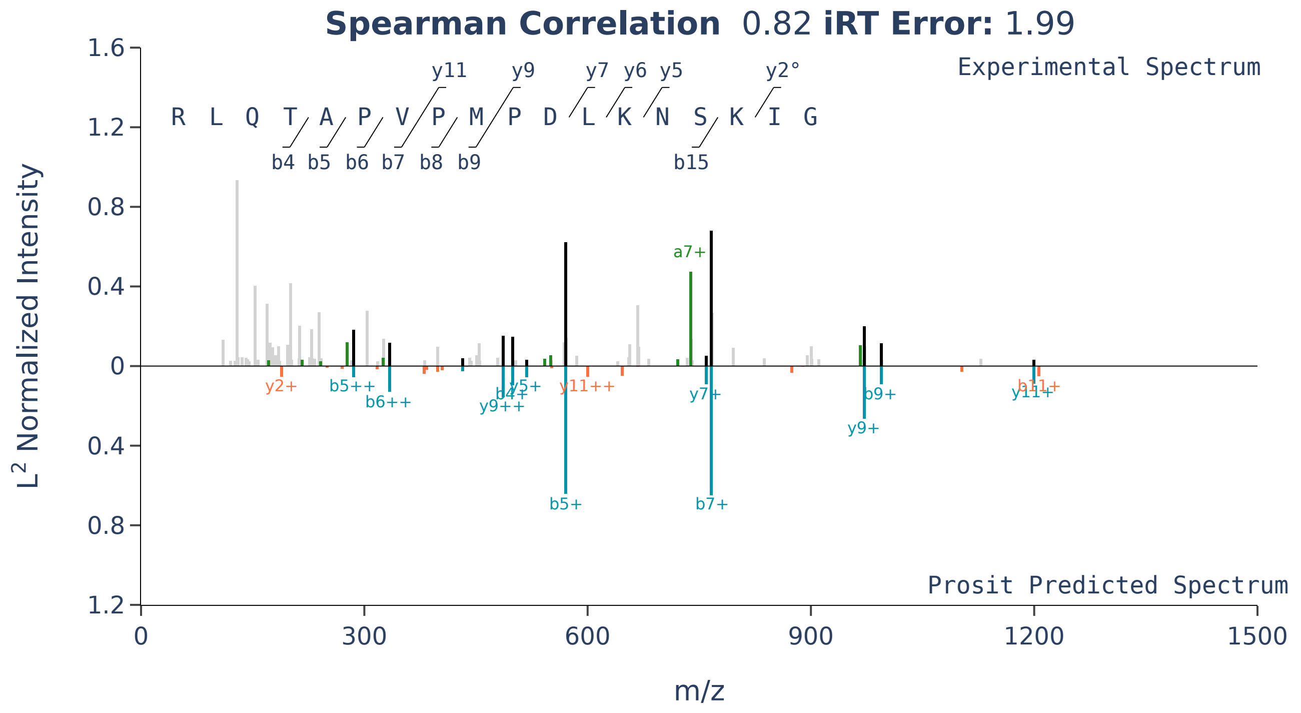

Source WSoh_221121_011221_HFGoe_L2_hTau_24h_R1 **Scan** 21175
Peptide AKSRLQTAPVPMPDLKNV **Charge** 3 **Spectral Angle** 0.0
Spearman Correlation 0.0 **iRT Error:** 2.12

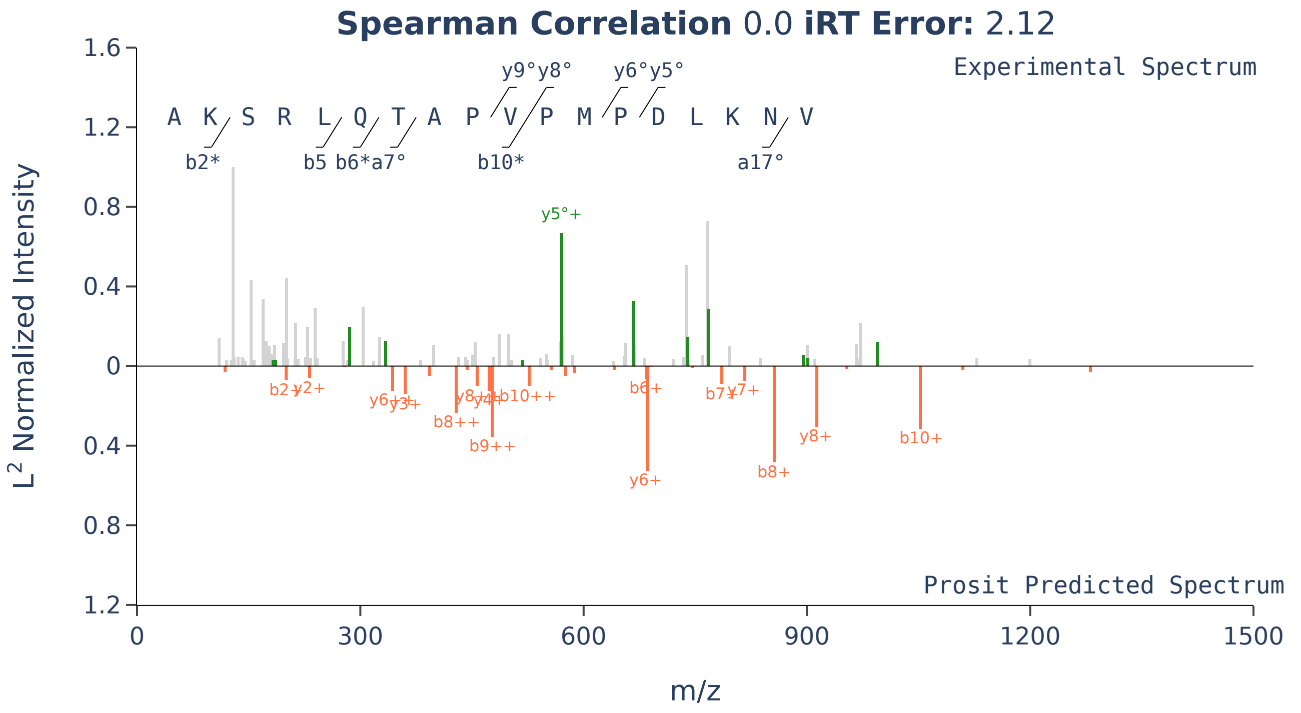

Source WSoh_221121_011221_HFGoe_L2_hTau_4h_R2 **Scan** 11529
Peptide KHLTFRENAKAKTDHGAEIVY **Charge** 4 **Spectral Angle** 0.88
Spearman Correlation 0.83 **iRT Error:** 2.07

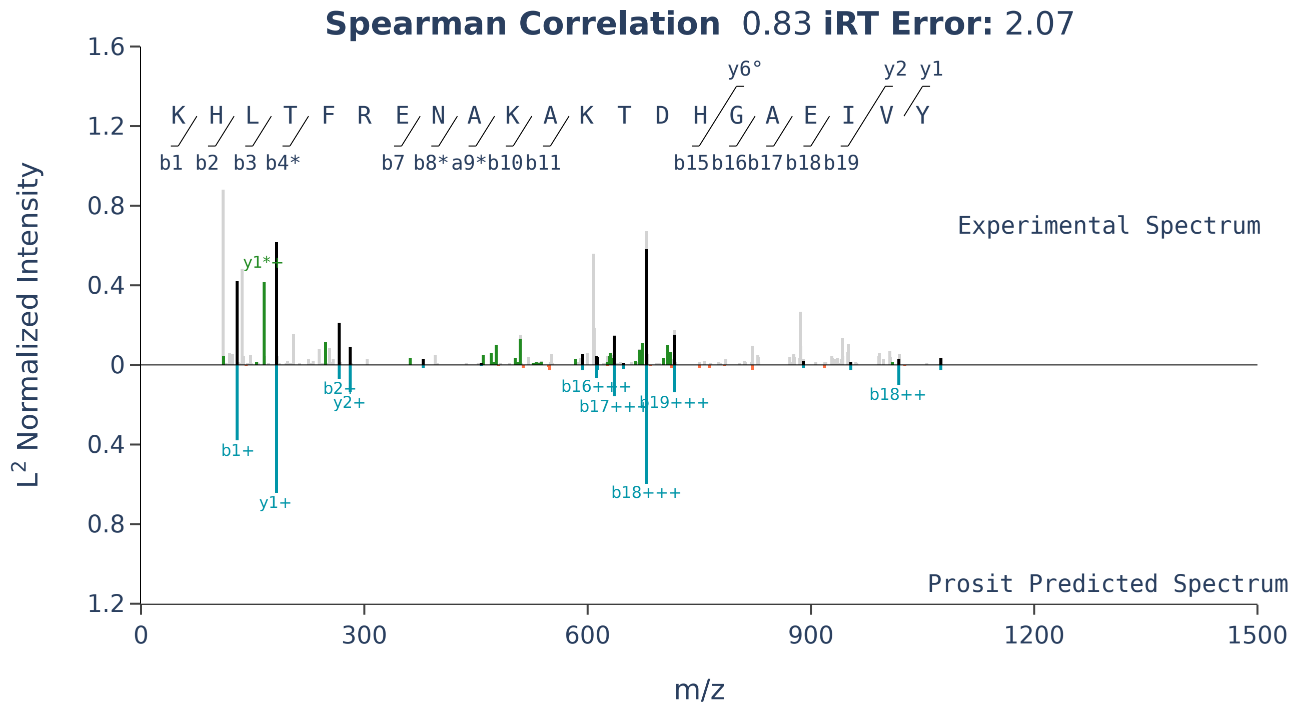

Source WSoh_221121_011221_HFGoe_L2_hTau_4h_R2 **Scan** 11529
Peptide HKLTFRENAKAKTDHGAEIVY **Charge** 4 **Spectral Angle** 0.88
Spearman Correlation 0.8 **iRT Error:** 0.31

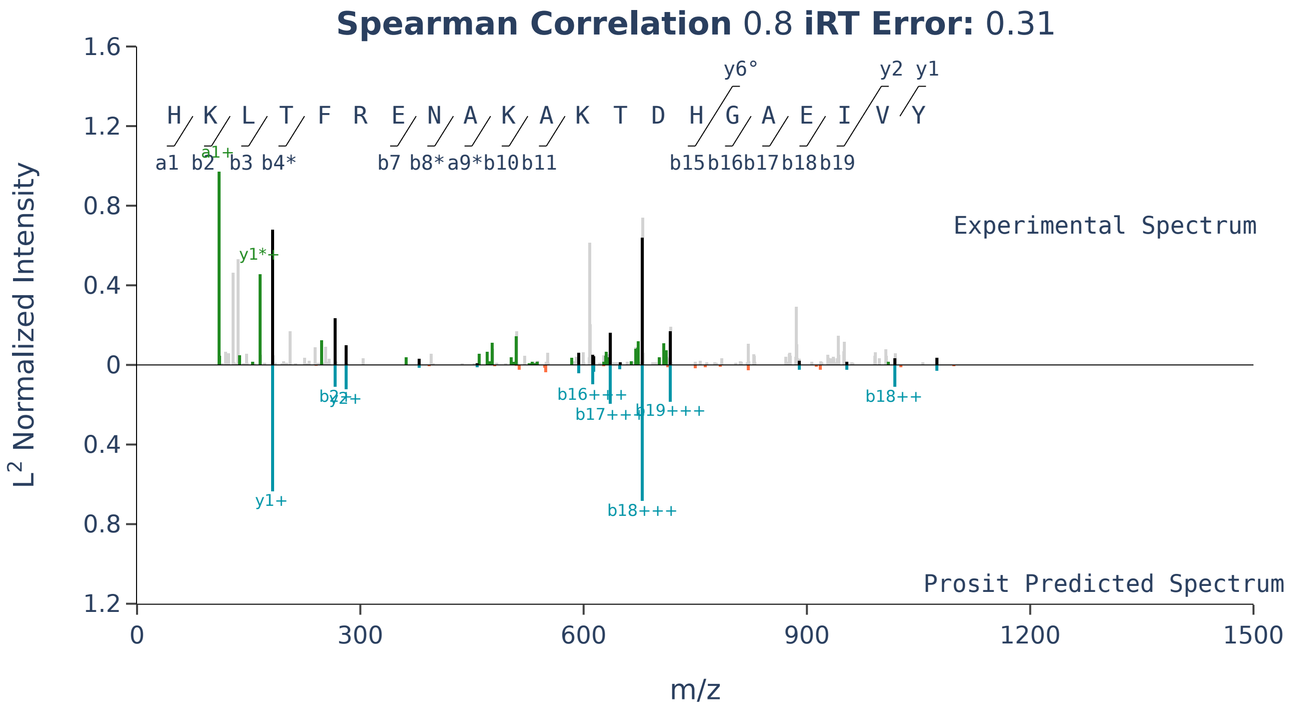

Source WSoh_221121_011221_HFGoe_L1_hTau_24h_R1 **Scan** 14255
Peptide SNVQAPV **Charge** 1 **Spectral Angle** 0.84
Spearman Correlation 0.88 **iRT Error:** 2.14

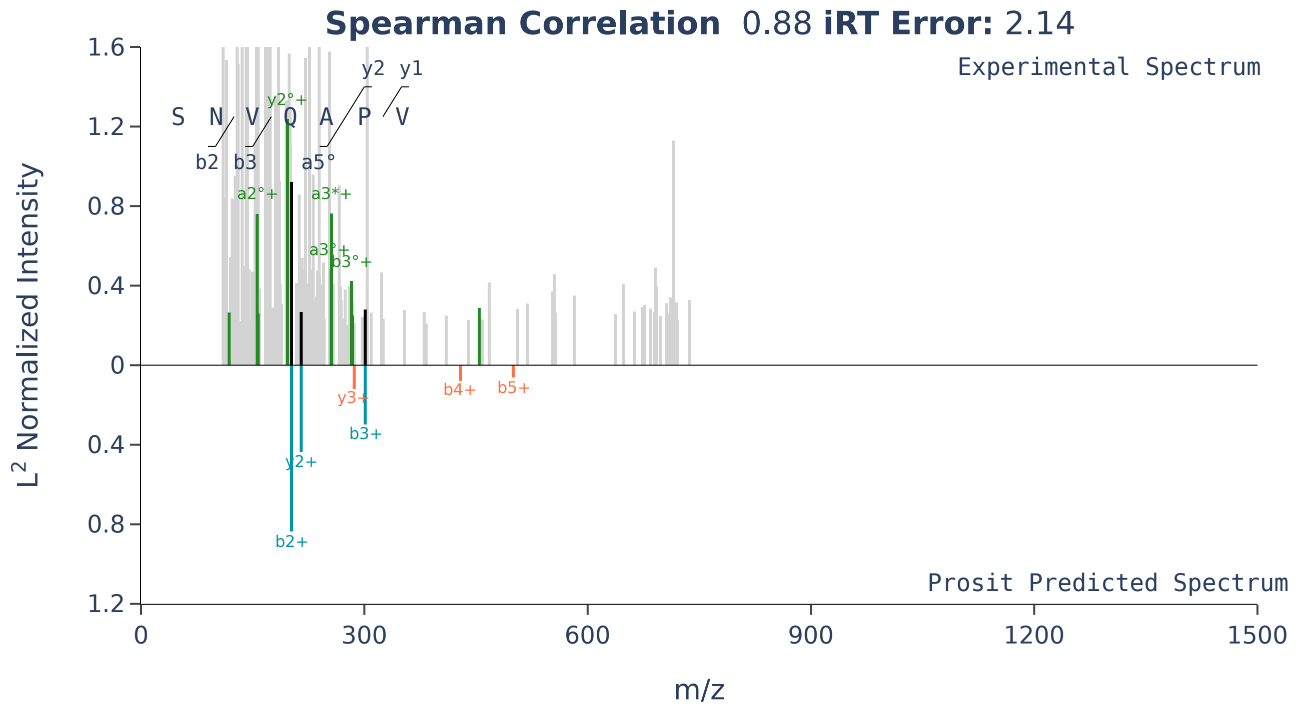

Source WSoh_221121_011221_HFGoe_L1_hTau_24h_R1 **Scan** 14255
Peptide PGGGSVQI **Charge** 1 **Spectral Angle** 0.15
Spearman Correlation 0.08 **iRT Error:** 9.69

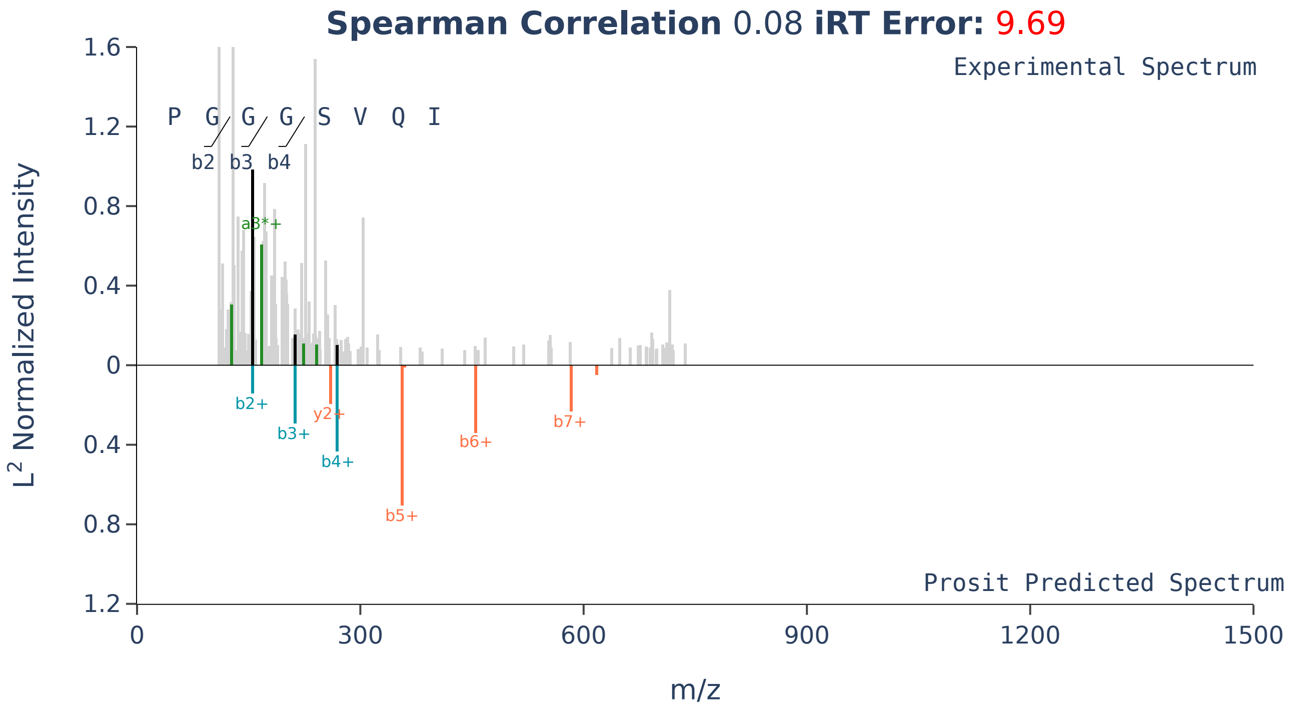

Source WSoh_221121_011221_HFGoe_L2_hTau_24h_R1 **Scan** 14761
Peptide QSTPSLPTPPTREPKKV **Charge** 4 **Spectral Angle** 0.88
Spearman Correlation 0.89 **iRT Error:** 2.14

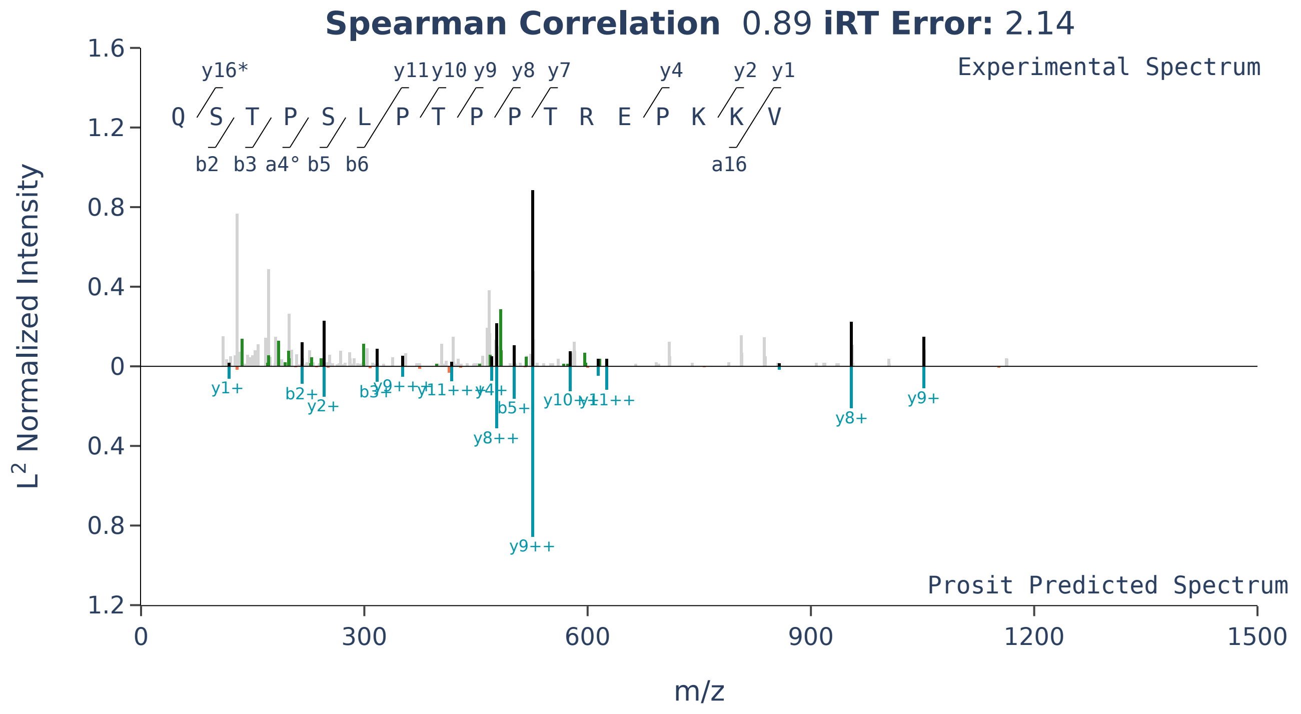

Source WSoh_221121_011221_HFGoe_L2_hTau_24h_R1 **Scan** 14761
Peptide EKLDFKDRVQSKIGSL **Charge** 4 **Spectral Angle** 0.29
Spearman Correlation 0.26 **iRT Error:** 25.68

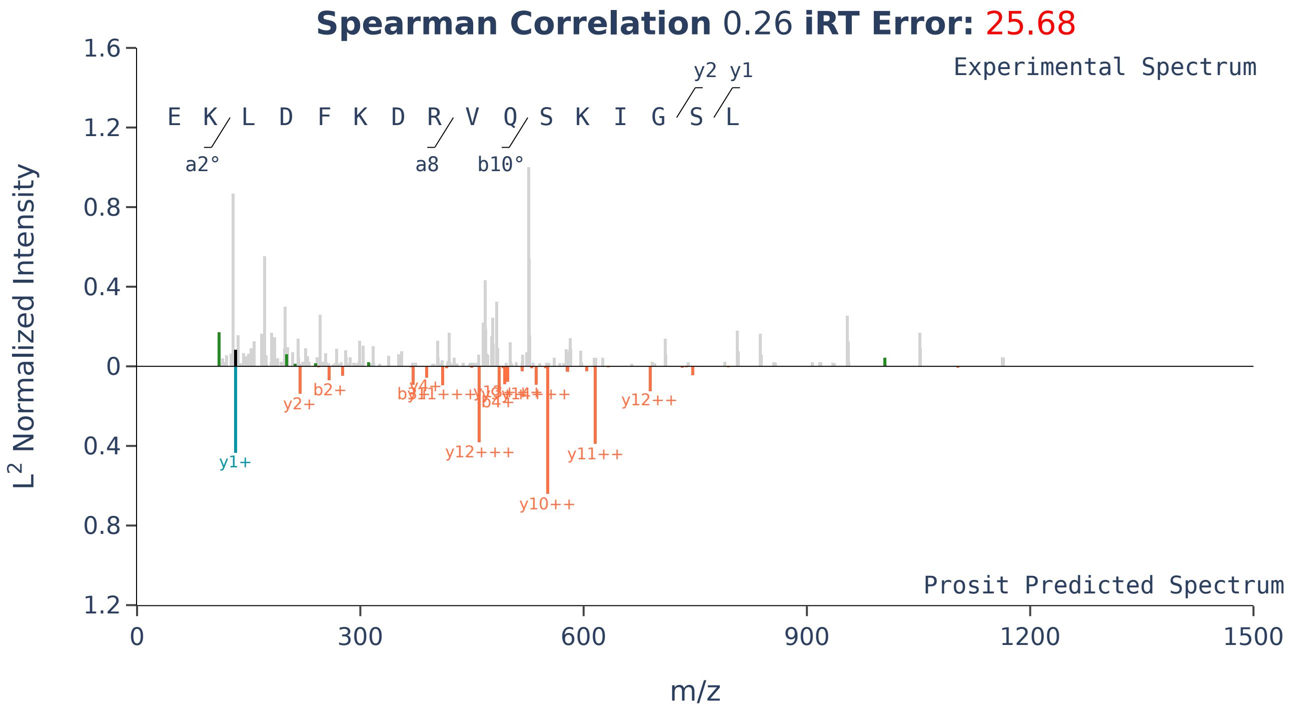

Source WSoh_221121_011221_HFGoe_L2_hTau_1h_R1 **Scan** 12361
Peptide ADEVSAKL **Charge** 2 **Spectral Angle** 0.83
Spearman Correlation 0.87 **iRT Error:** 2.22

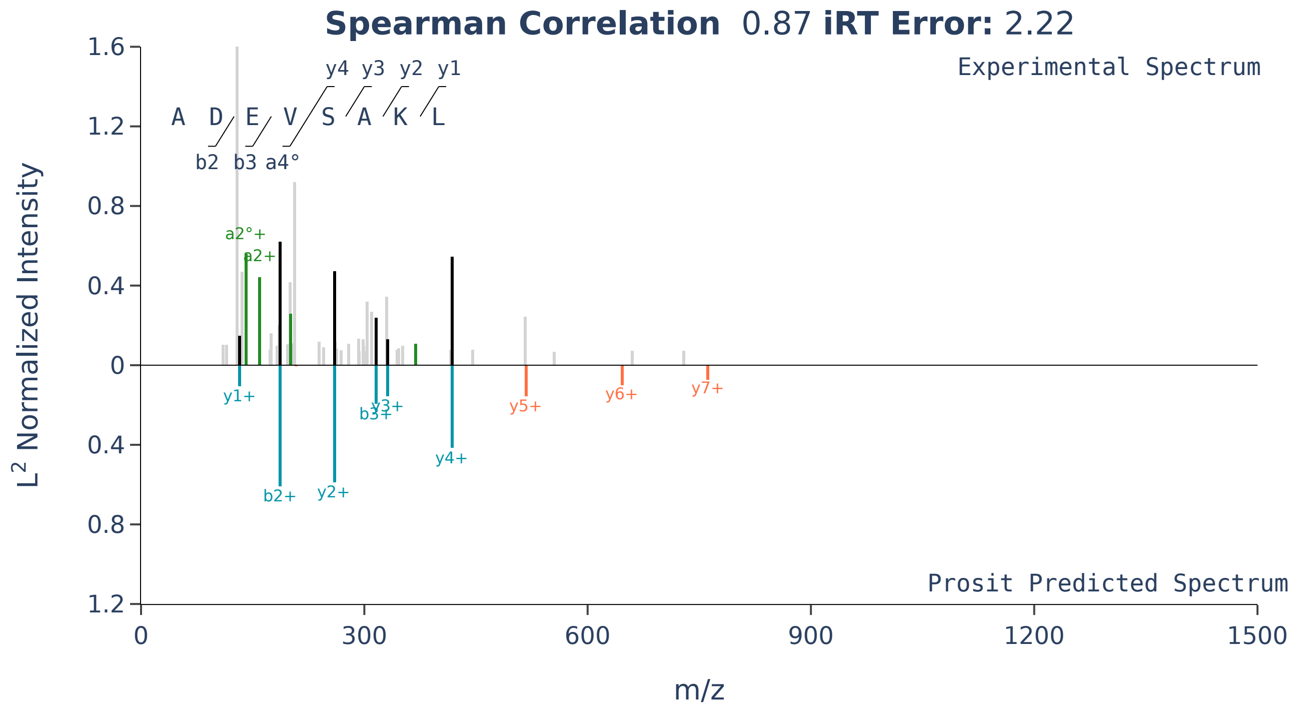

Source WSoh_221121_011221_HFGoe_L2_hTau_1h_R1 **Scan** 12361
Peptide IGSLDNIT **Charge** 2 **Spectral Angle** 0.0
Spearman Correlation 0.0 **iRT Error:** 41.02

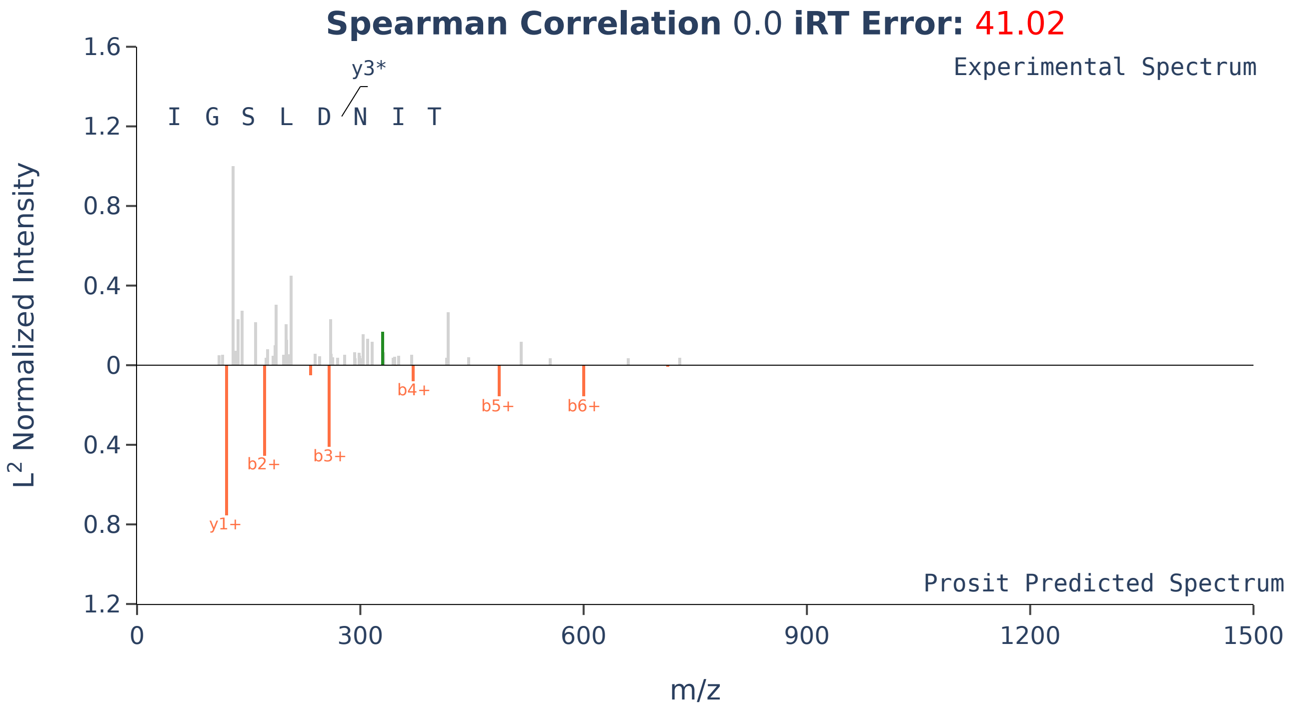

Source WSoh_221121_011221_HFGoe_L2_hTau_2h_R1 **Scan** 7861
Peptide DFKDRVQSS **Charge** 2 **Spectral Angle** 0.88
Spearman Correlation 0.87 **iRT Error:** 2.23

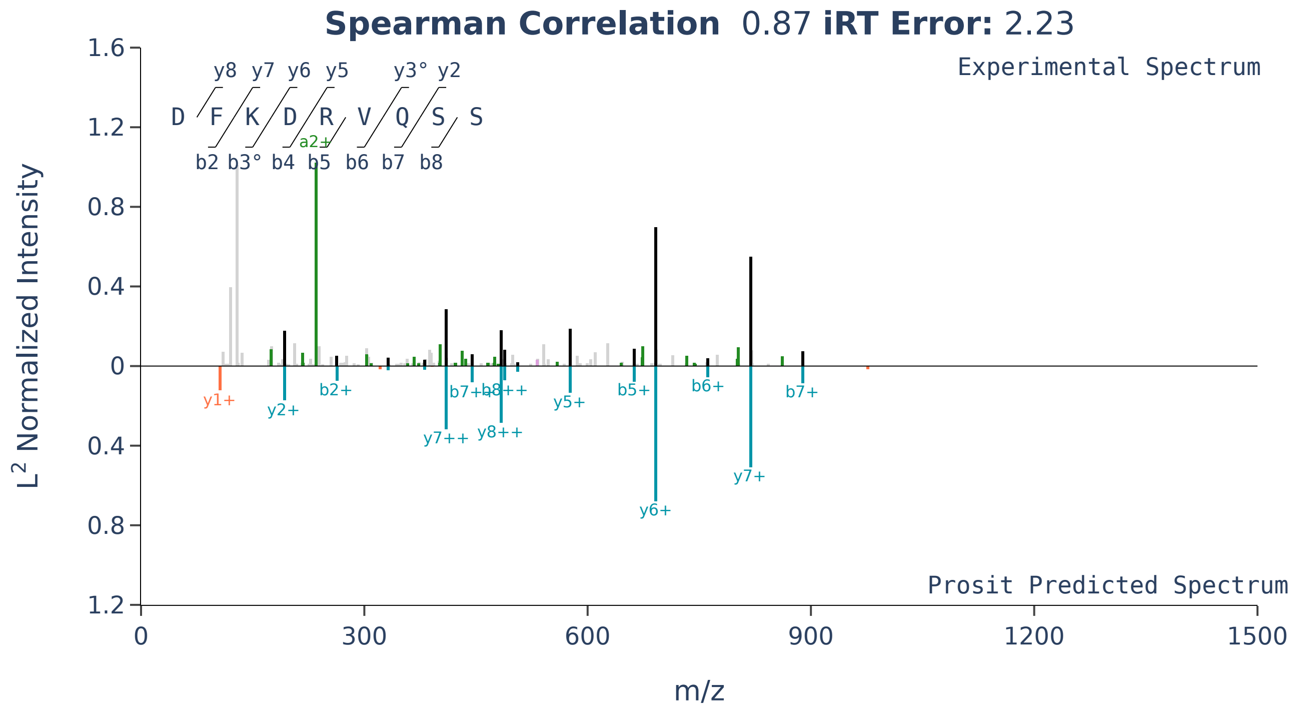

Source WSoh_221121_011221_HFGoe_L2_hTau_2h_R1 **Scan** 7861
Peptide GTYGLGDRKD **Charge** 2 **Spectral Angle** 0.32
Spearman Correlation 0.11 **iRT Error:** 3.97

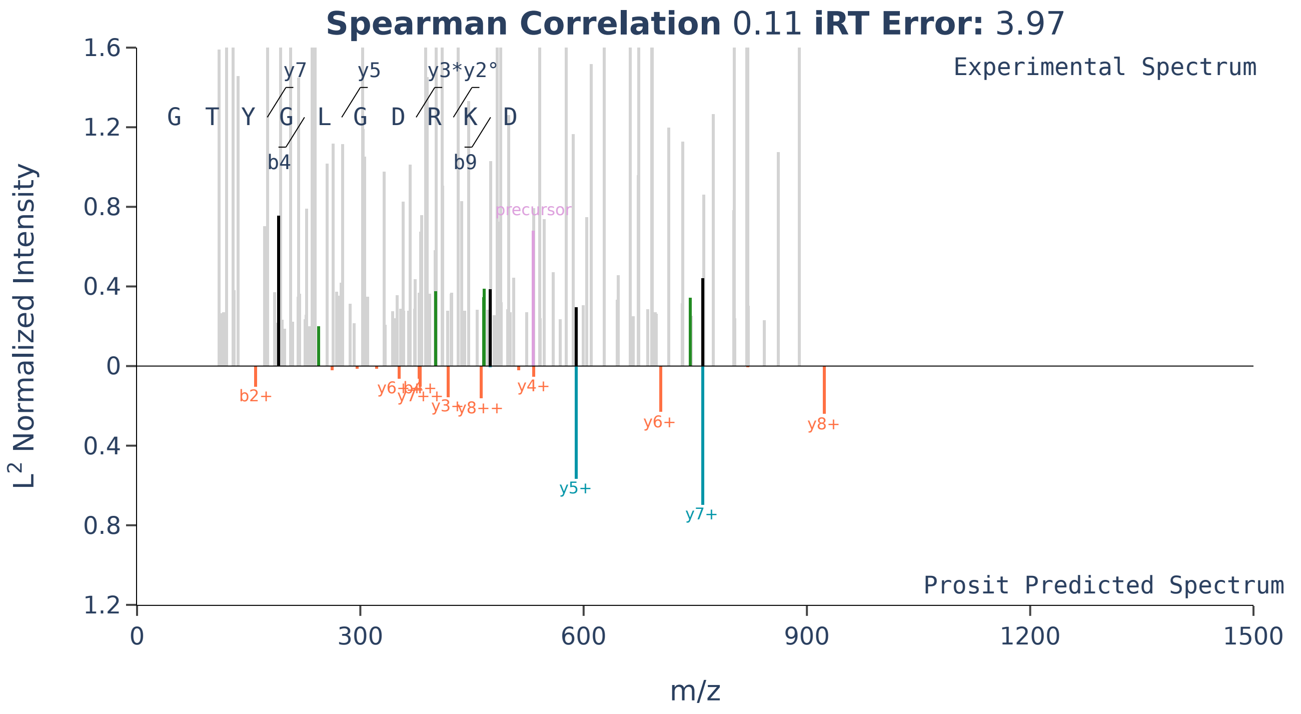

Source WSoh_221121_011221_HFGoe_L1_hTau_4h_R1 **Scan** 8274
Peptide RVQSKPGGGQVEV **Charge** 3 **Spectral Angle** 0.82
Spearman Correlation 0.87 **iRT Error:** 2.24

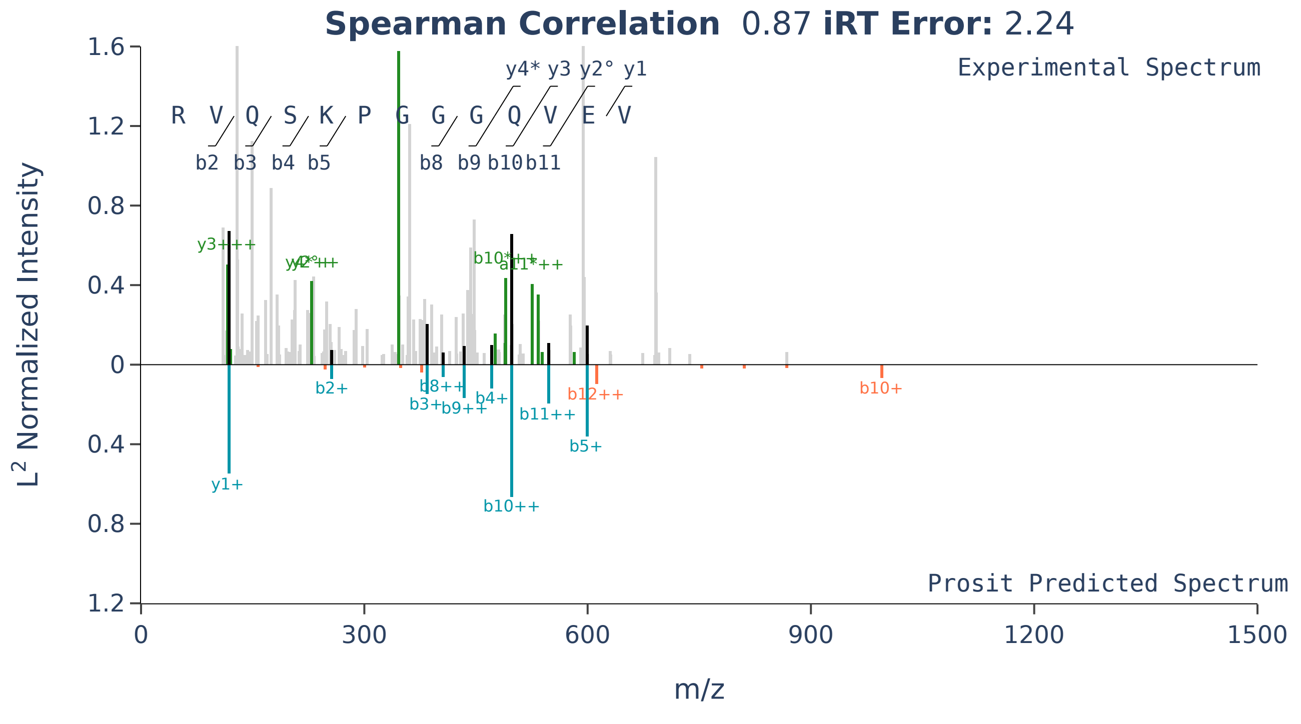

Source WSoh_221121_011221_HFGoe_L1_hTau_4h_R1 **Scan** 8274
Peptide HVPGGGSVQIVYK **Charge** 3 **Spectral Angle** 0.0
Spearman Correlation 0.0 **iRT Error:** 37.75

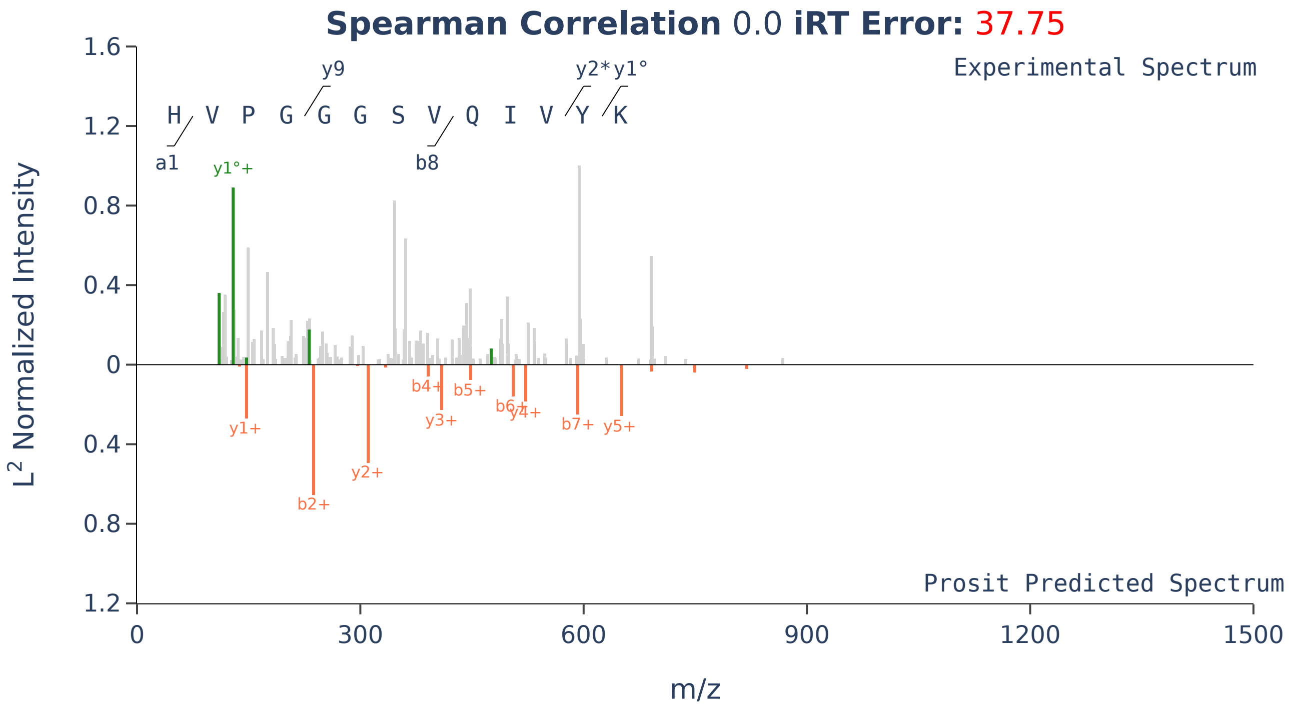

Source WSoh_221121_011221_HFGoe_L2_hTau_2h_R1 **Scan** 17801
Peptide DLSNVNIT **Charge** 1 **Spectral Angle** 0.84
Spearman Correlation 0.89 **iRT Error:** 2.25

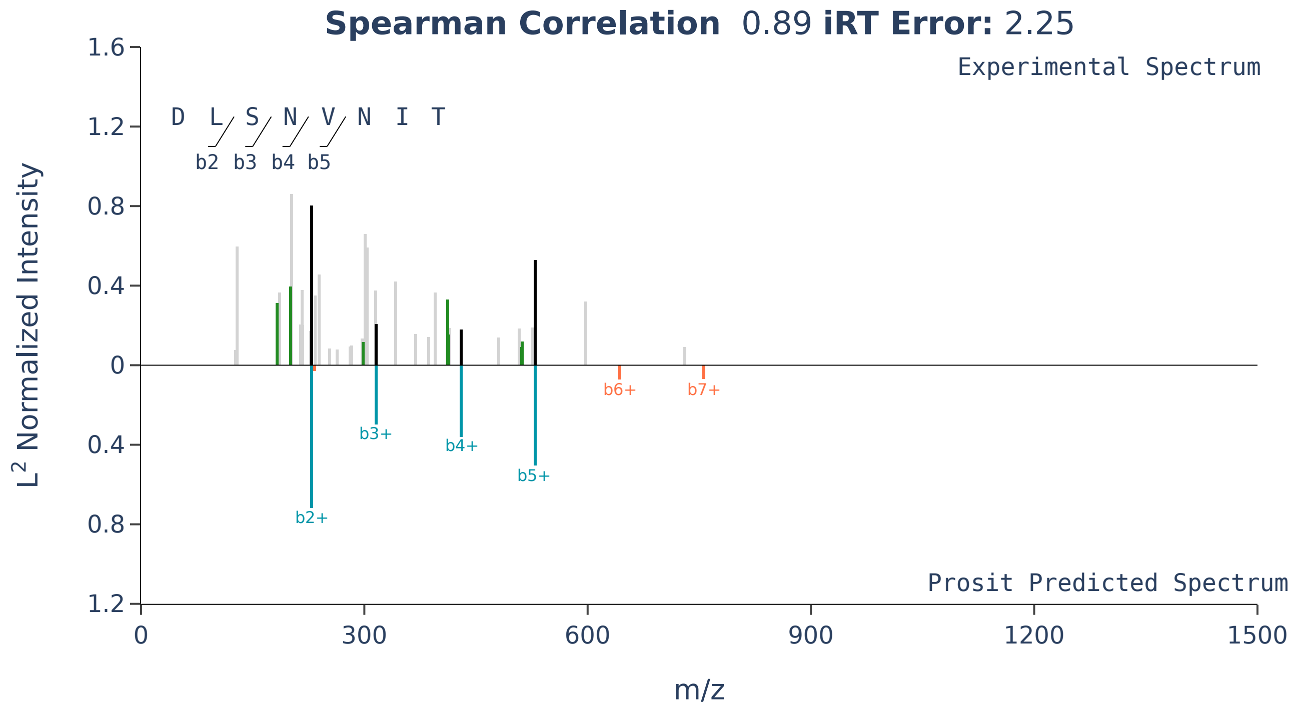

Source WSoh_221121_011221_HFGoe_L2_hTau_2h_R1 **Scan** 17801
Peptide GQVEVKSE **Charge** 1 **Spectral Angle** 0.0
Spearman Correlation 0.0 **iRT Error:** 0.83

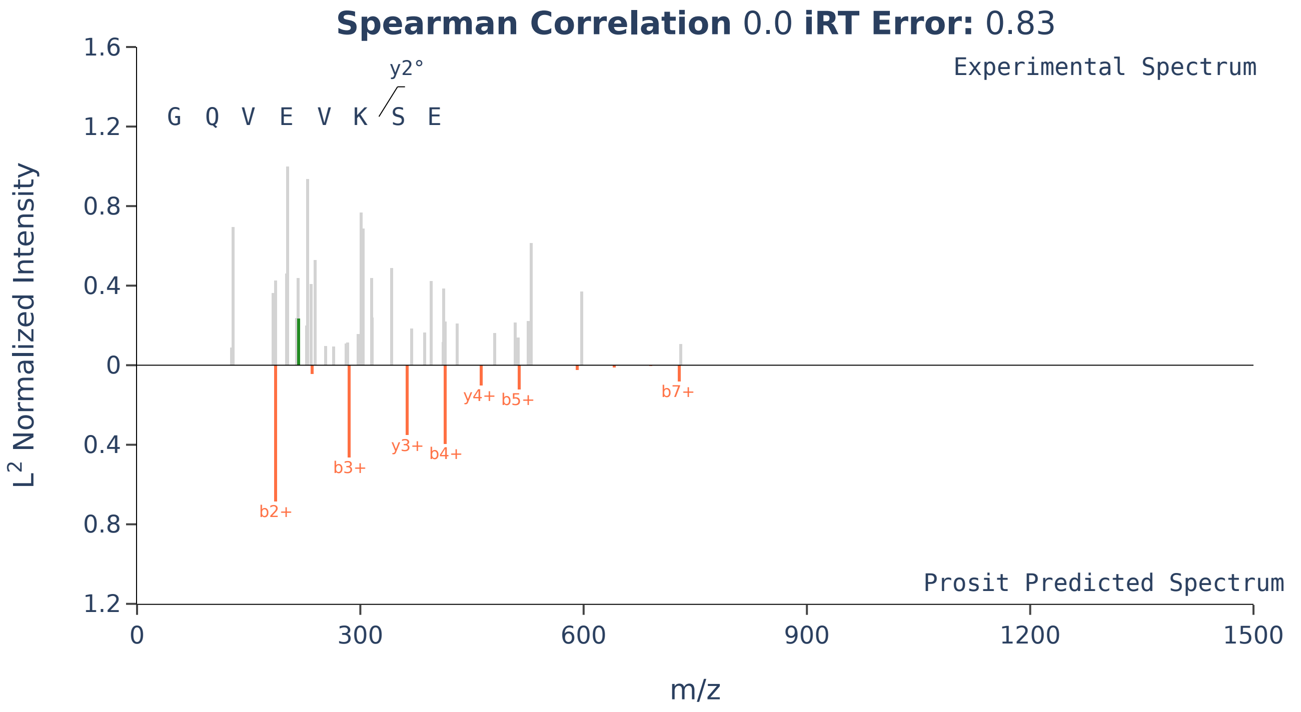

Source WSoh_221121_011221_HFGoe_L1_hTau_24h_R1 **Scan** 19421
Peptide GADQEGDTDAGLKESPLQTPTE **Charge** 3 **Spectral Angle** 0.92
Spearman Correlation 0.8 **iRT Error:** 2.25

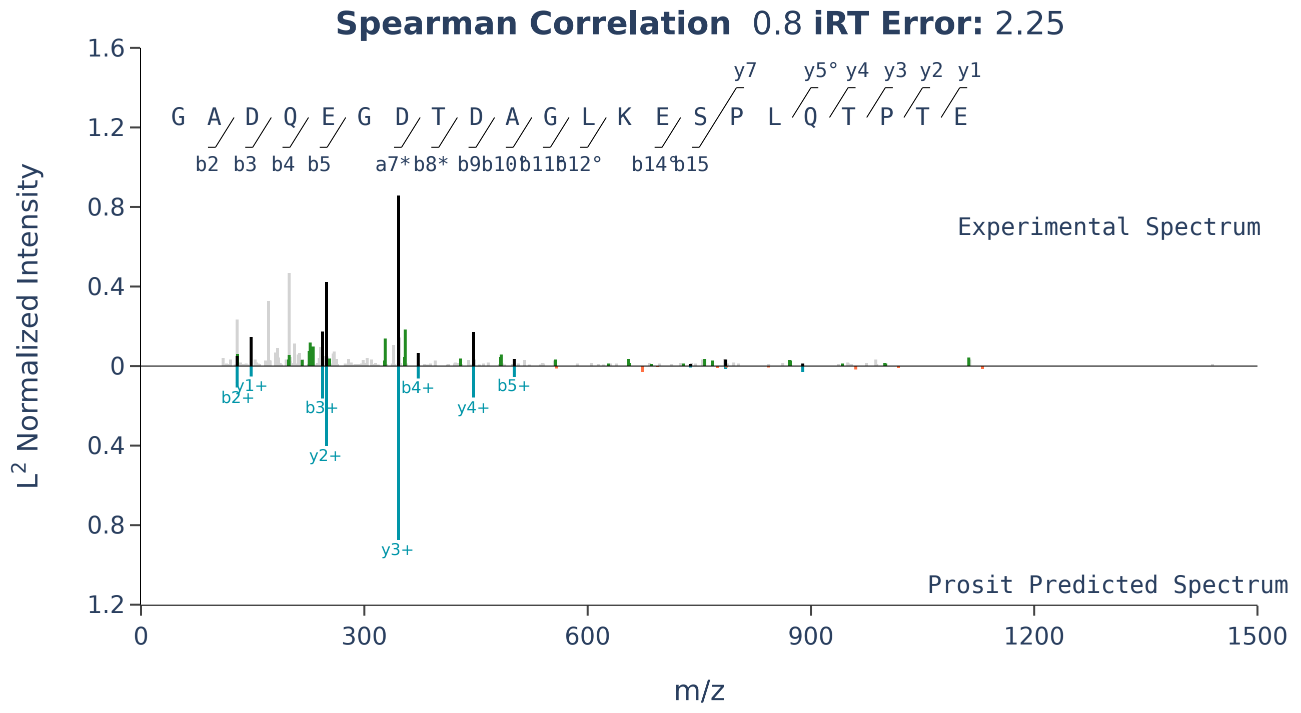

Source WSoh_221121_011221_HFGoe_L1_hTau_24h_R1 **Scan** 19421
Peptide QDQEGDTDAGLKESPLQTPTE **Charge** 3 **Spectral Angle** 0.93
Spearman Correlation 0.72 **iRT Error:** 2.78

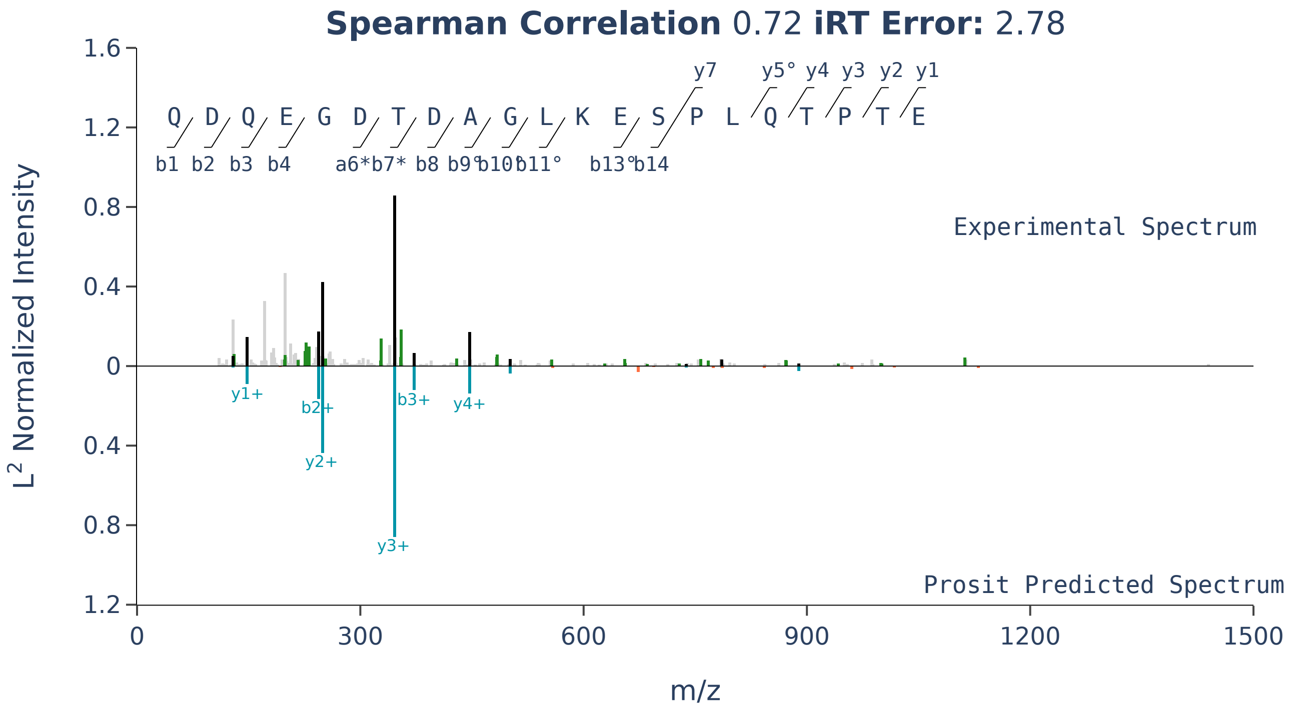

Source WSoh_221121_011221_HFGoe_L2_hTau_24h_R2 **Scan** 21509
Peptide PSLEDEAADNIT **Charge** 1 **Spectral Angle** 0.81
Spearman Correlation 0.92 **iRT Error:** 2.31

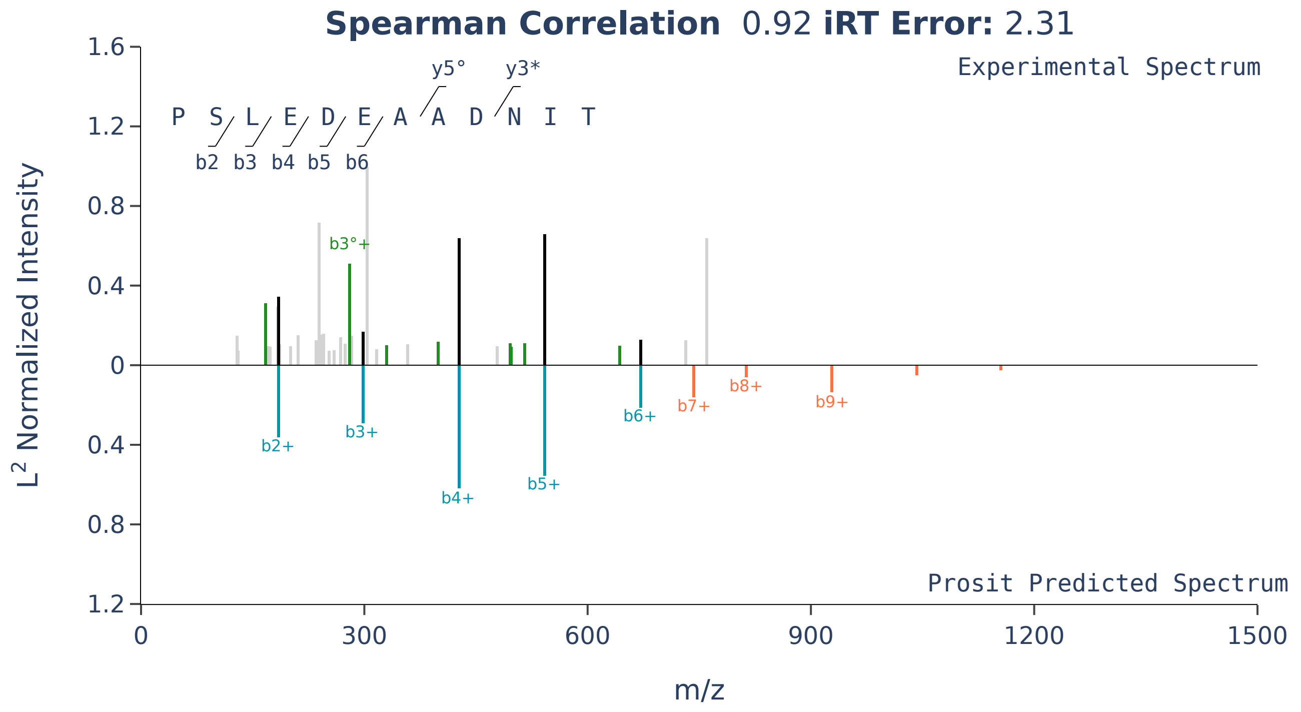

Source WSoh_221121_011221_HFGoe_L2_hTau_24h_R2 **Scan** 21509
Peptide IGDTPSLEDEAAG **Charge** 1 **Spectral Angle** 0.18
Spearman Correlation 0.31 **iRT Error:** 14.6

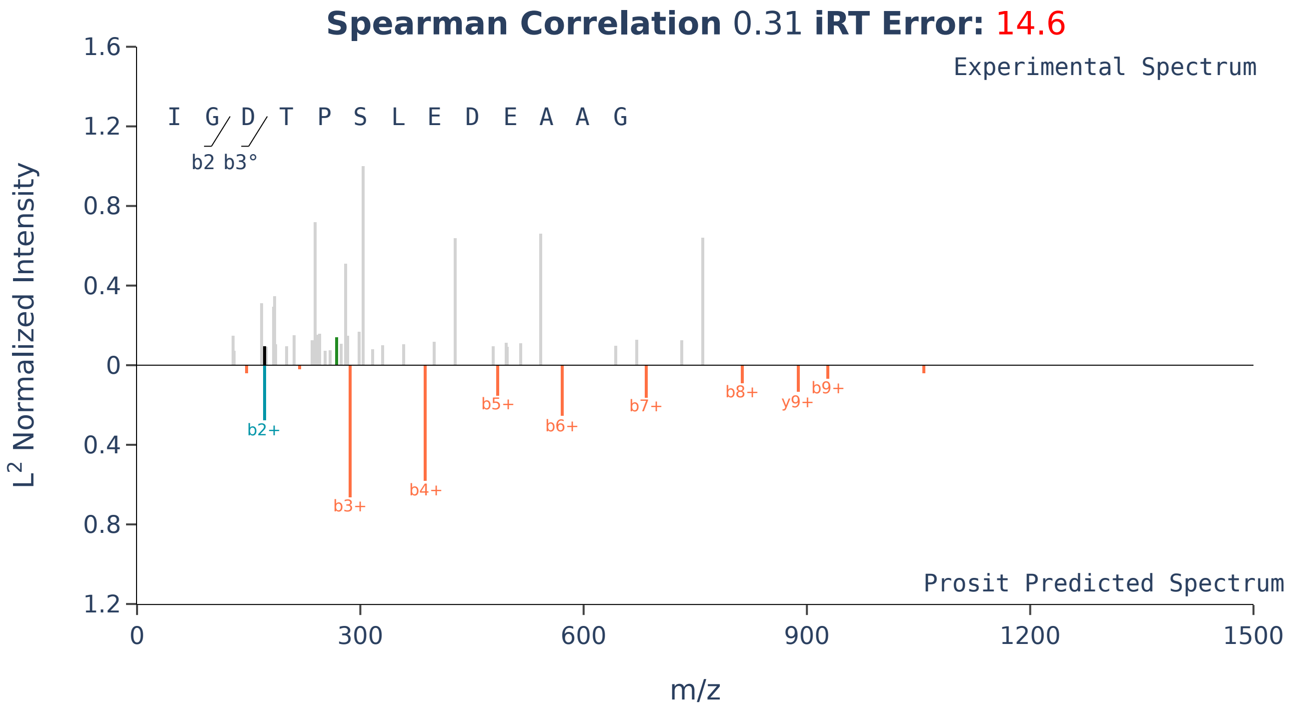

Source WSoh_221121_011221_HFGoe_L2_hTau_4h_R2 **Scan** 15310
Peptide AAQPHTEIPEGRMV **Charge** 3 **Spectral Angle** 0.89
Spearman Correlation 0.94 **iRT Error:** 2.31

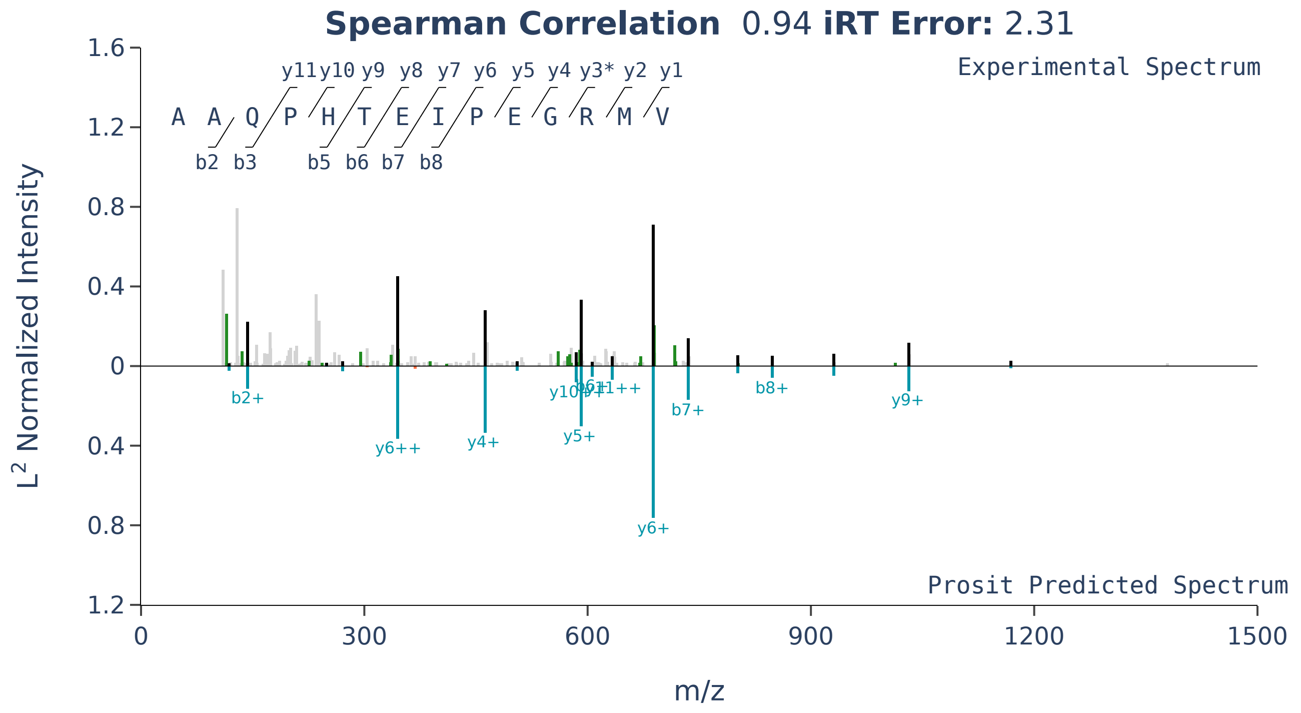

Source WSoh_221121_011221_HFGoe_L2_hTau_4h_R2 **Scan** 15310
Peptide GTGSDDKKAKGADGKT **Charge** 3 **Spectral Angle** 0.44
Spearman Correlation 0.25 **iRT Error:** 76.85

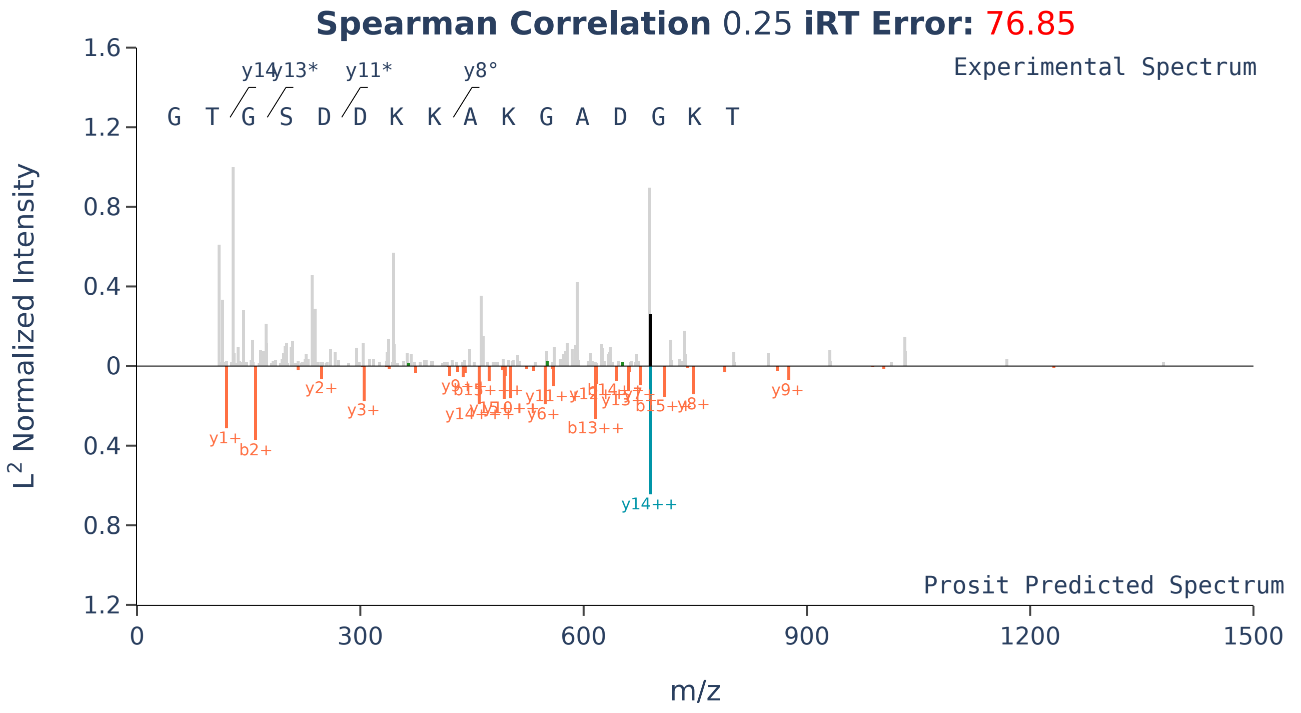

Source WSoh_221121_011221_HFGoe_L2_hTau_2h_R1 **Scan** 9629
Peptide KSEKLDFKDRVQST **Charge** 2 **Spectral Angle** 0.83
Spearman Correlation 0.93 **iRT Error:** 2.35

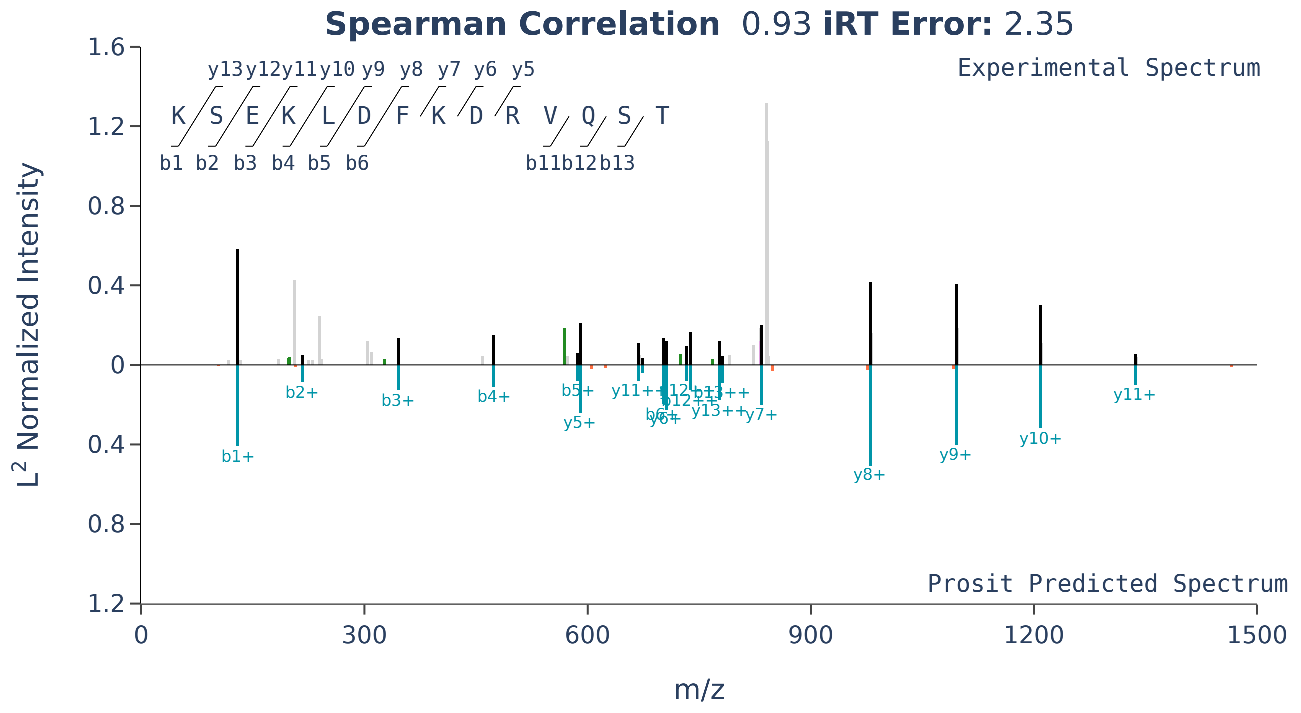

Source WSoh_221121_011221_HFGoe_L2_hTau_2h_R1 **Scan** 9629
Peptide LSKVTSKCGSLGNIHH **Charge** 2 **Spectral Angle** 0.02
Spearman Correlation -0.26 **iRT Error:** 3.08

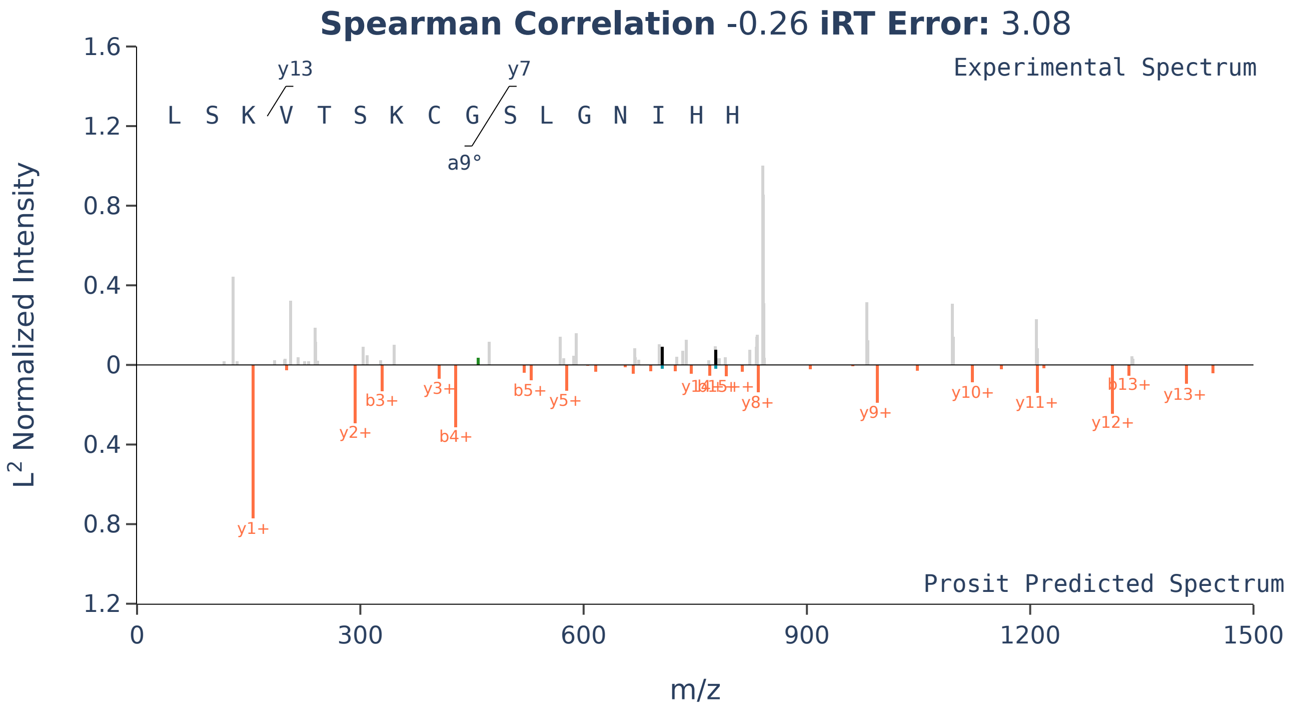

Source WSoh_221121_011221_HFGoe_L1_hTau_1h_R1 **Scan** 7833
Peptide DFKDRVQSG **Charge** 2 **Spectral Angle** 0.9
Spearman Correlation 0.93 **iRT Error:** 2.39

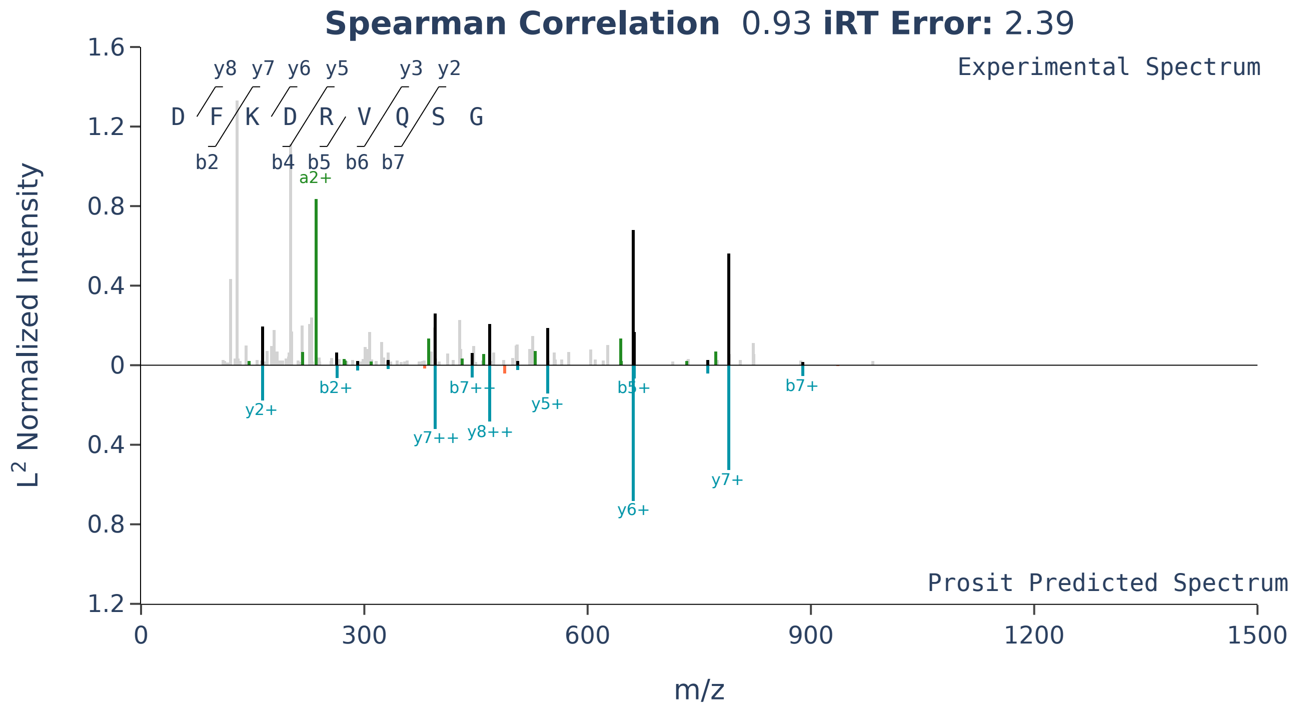

Source WSoh_221121_011221_HFGoe_L1_hTau_1h_R1 **Scan** 7833
Peptide YGLGDRKDQ **Charge** 2 **Spectral Angle** 0.07
Spearman Correlation -0.12 **iRT Error:** 2.95

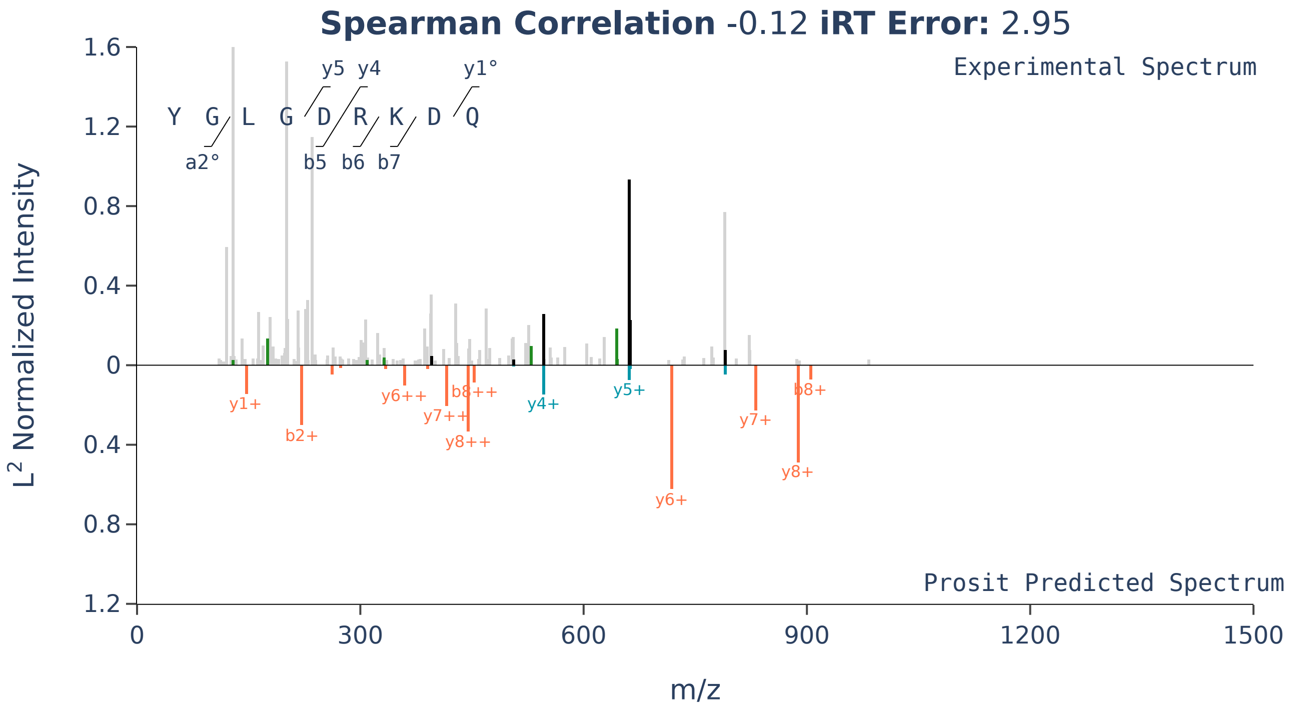

Source WSoh_221121_011221_HFGoe_L2_hTau_4h_R1 **Scan** 11250
Peptide NITHVPGGGNKKIETHKLTFRQD **Charge** 3 **Spectral Angle** 0.81
Spearman Correlation 0.85 **iRT Error:** 2.4

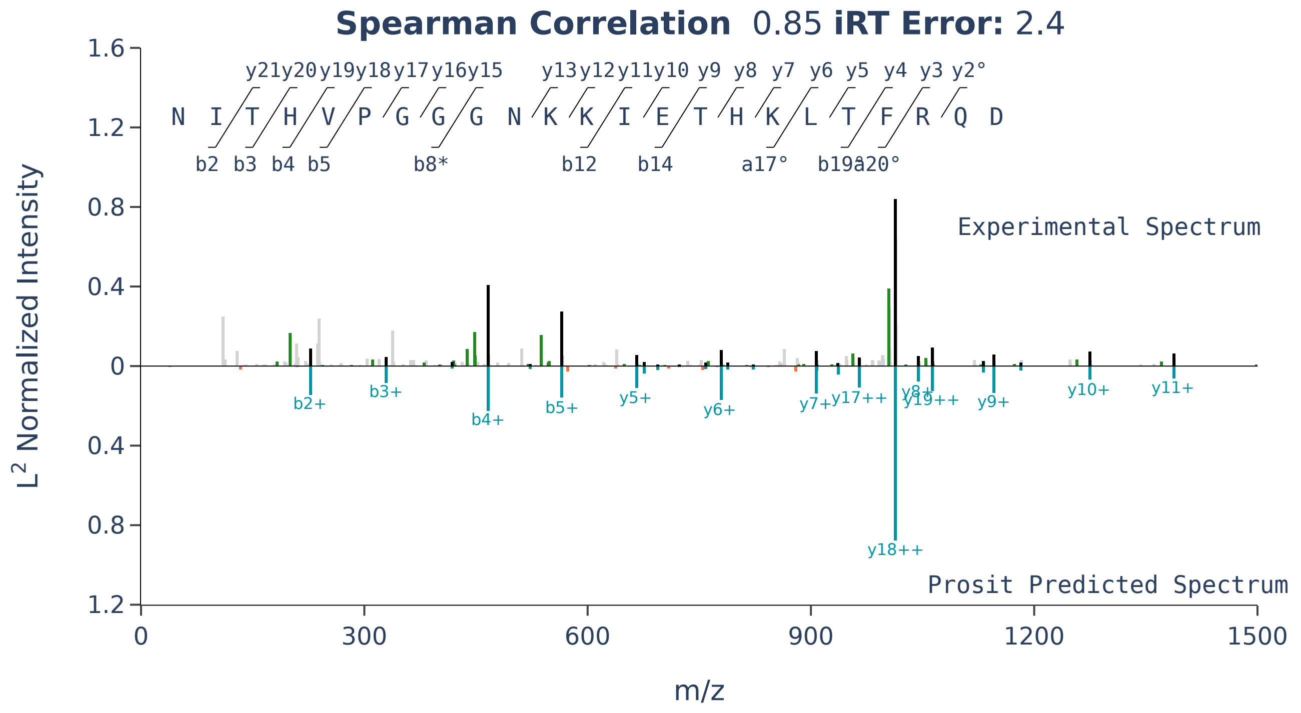

Source WSoh_221121_011221_HFGoe_L2_hTau_4h_R1 **Scan** 11250
Peptide NITHVPGGGNKKIETHKLTFREN **Charge** 3 **Spectral Angle** 0.83
Spearman Correlation 0.84 **iRT Error:** 2.87

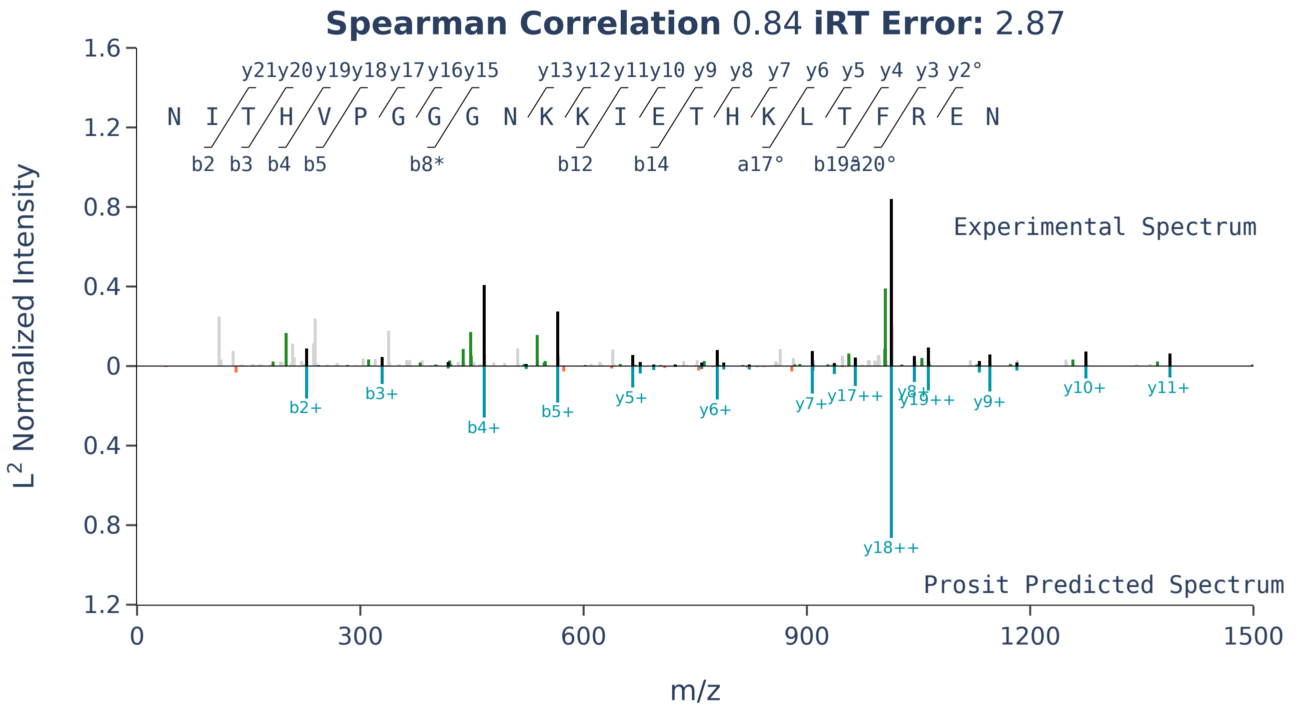

Source WSoh_221121_011221_HFGoe_L1_hTau_24h_R1 **Scan** 20907
Peptide QPGPVPMPDLKNVKS **Charge** 2 **Spectral Angle** 0.88
Spearman Correlation 0.85 **iRT Error:** 2.53

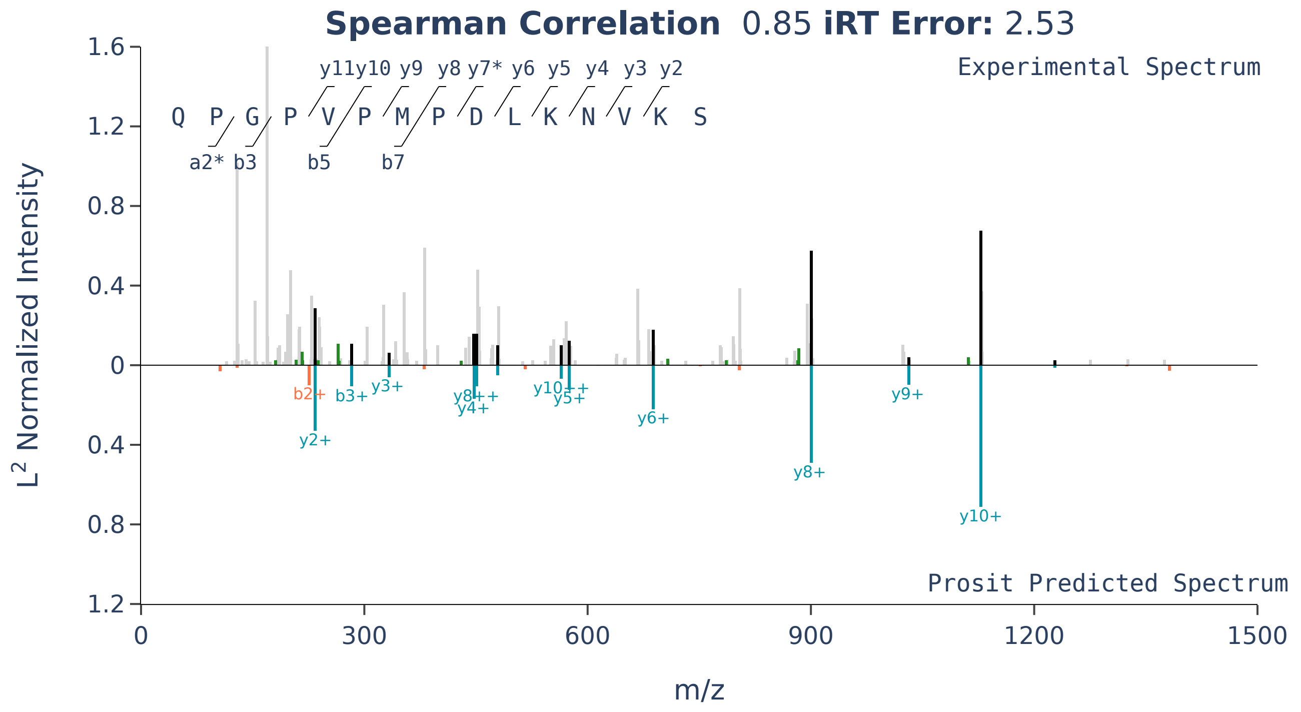

Source WSoh_221121_011221_HFGoe_L1_hTau_24h_R1 **Scan** 20907
Peptide FKDRVQSKIGSLDN **Charge** 2 **Spectral Angle** 0.0
Spearman Correlation 0.0 **iRT Error:** 43.21

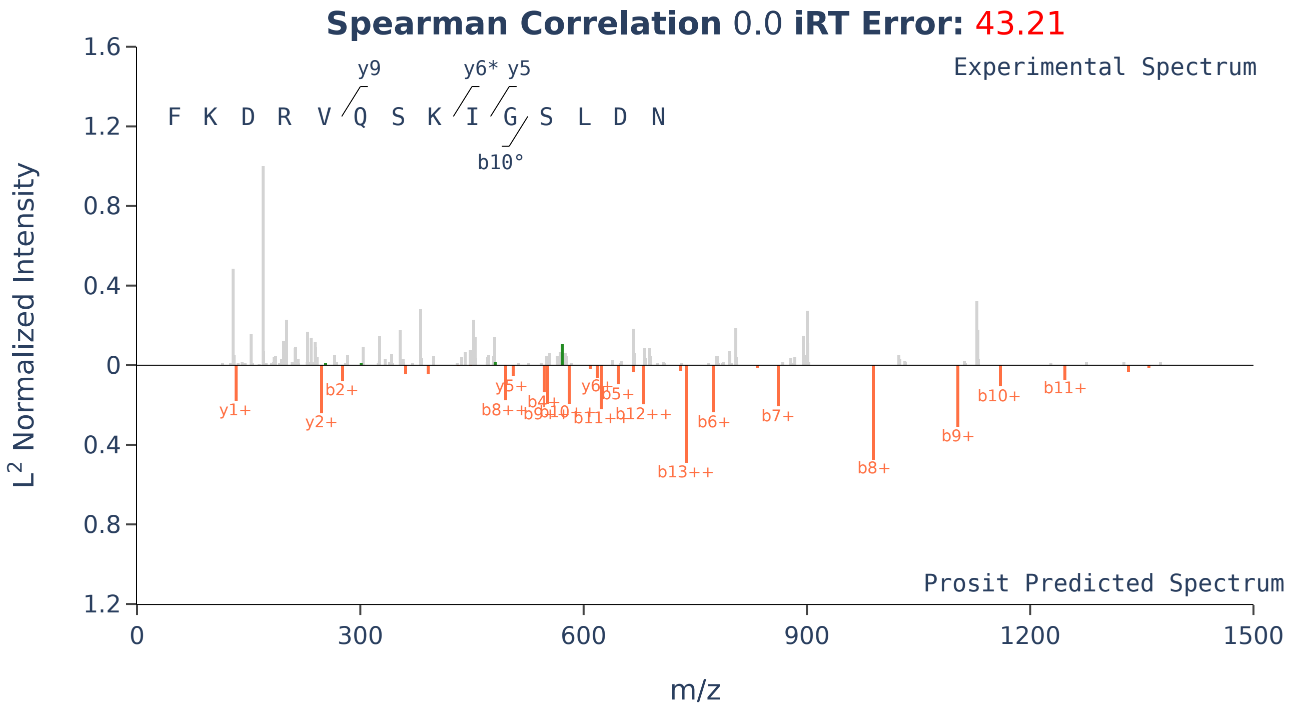

Source WSoh_221121_011221_HFGoe_L1_hTau_4h_R2 **Scan** 15619
Peptide KSEKLDFKDRSVQI **Charge** 2 **Spectral Angle** 0.67
Spearman Correlation 0.94 **iRT Error:** 2.53

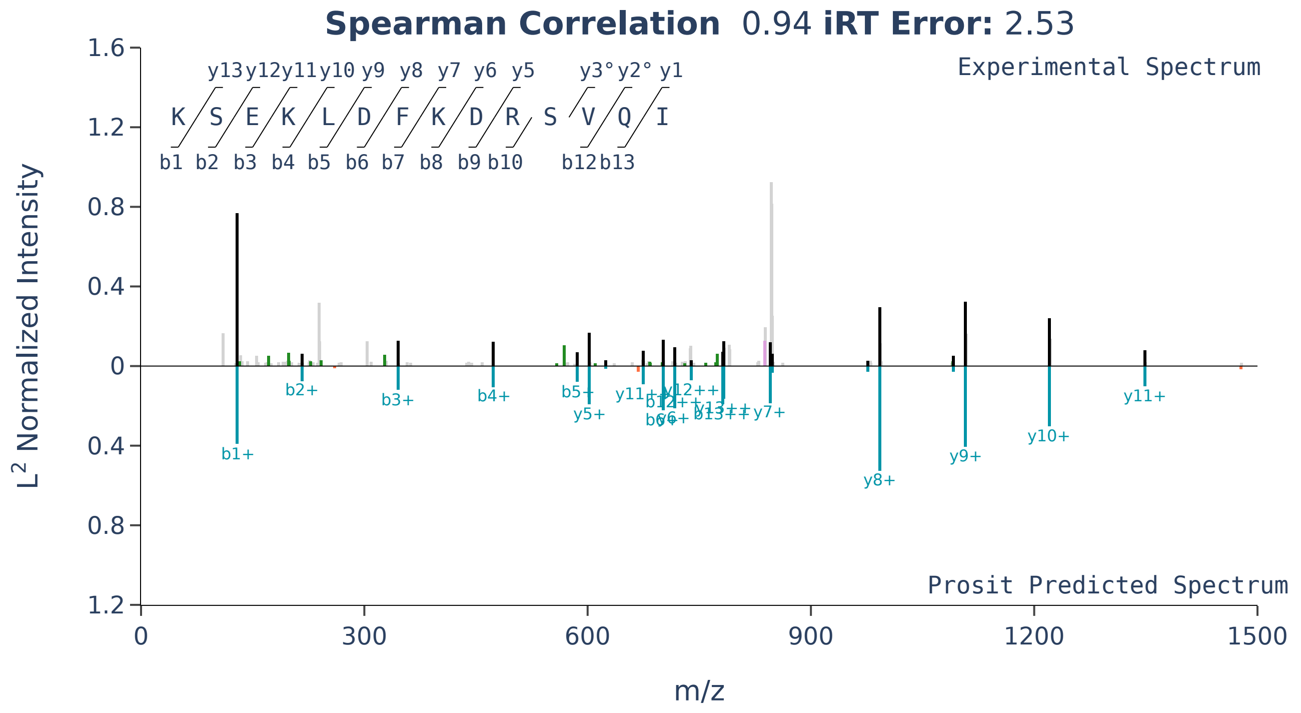

Source WSoh_221121_011221_HFGoe_L1_hTau_4h_R2 **Scan** 15619
Peptide RGAAPPGQKGQANATRI **Charge** 2 **Spectral Angle** 0.23
Spearman Correlation 0.22 **iRT Error:** 51.21

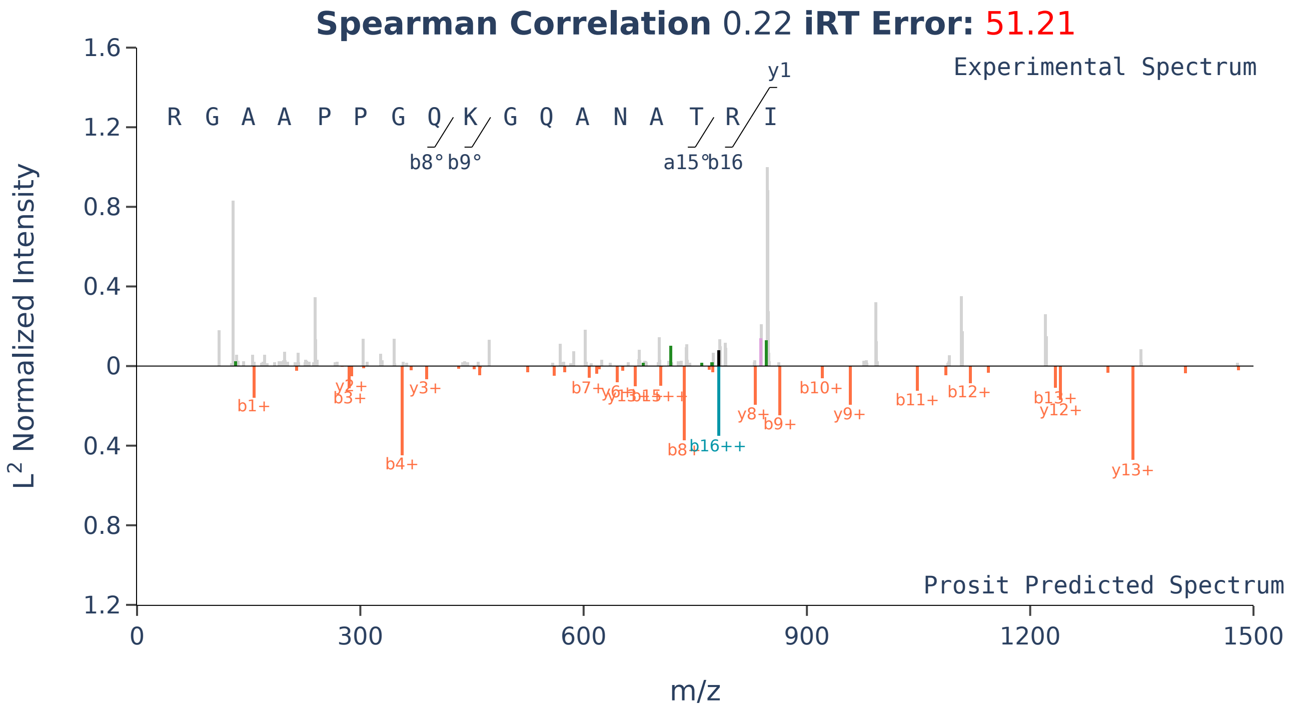

Source WSoh_221121_011221_HFGoe_L1_hTau_24h_R1 **Scan** 8667
Peptide VGSLGNIHHKPGGGQVE **Charge** 2 **Spectral Angle** 0.83
Spearman Correlation 0.88 **iRT Error:** 2.57

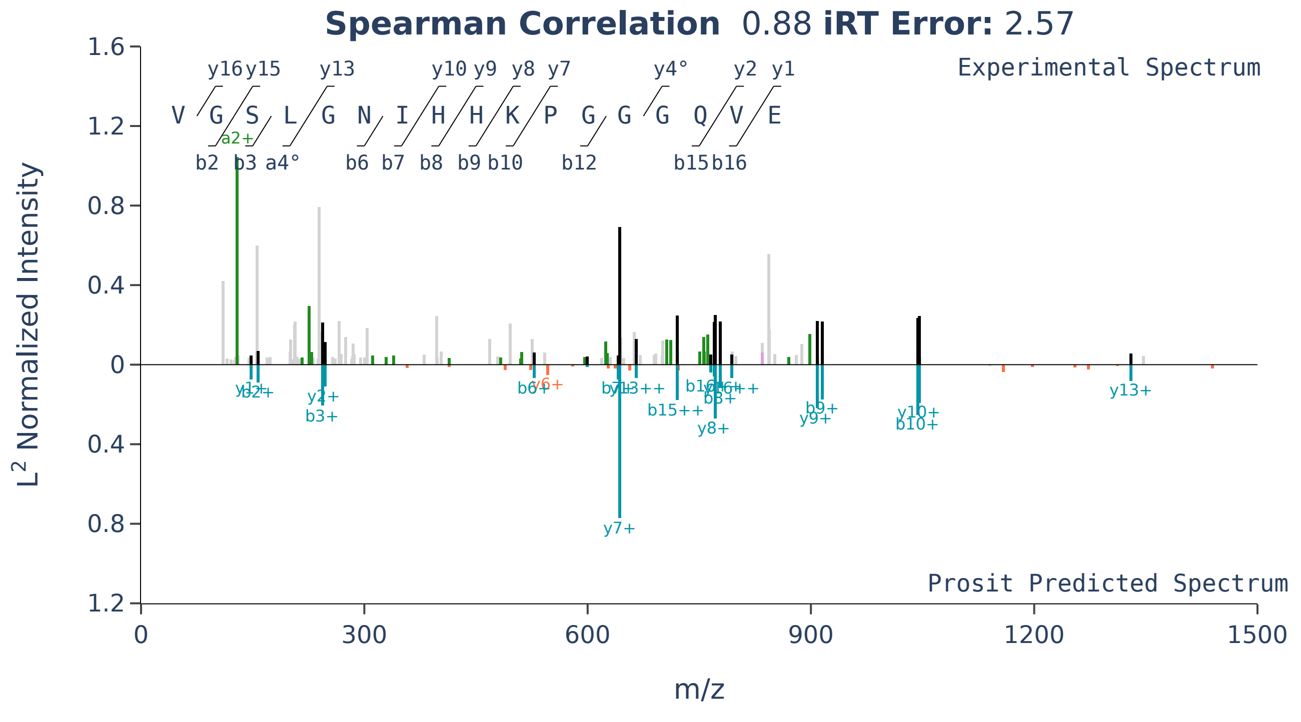

Source WSoh_221121_011221_HFGoe_L1_hTau_24h_R1 **Scan** 8667
Peptide GSLGNIHHKPGGGQVEV **Charge** 2 **Spectral Angle** 0.13
Spearman Correlation 0.26 **iRT Error:** 13.04

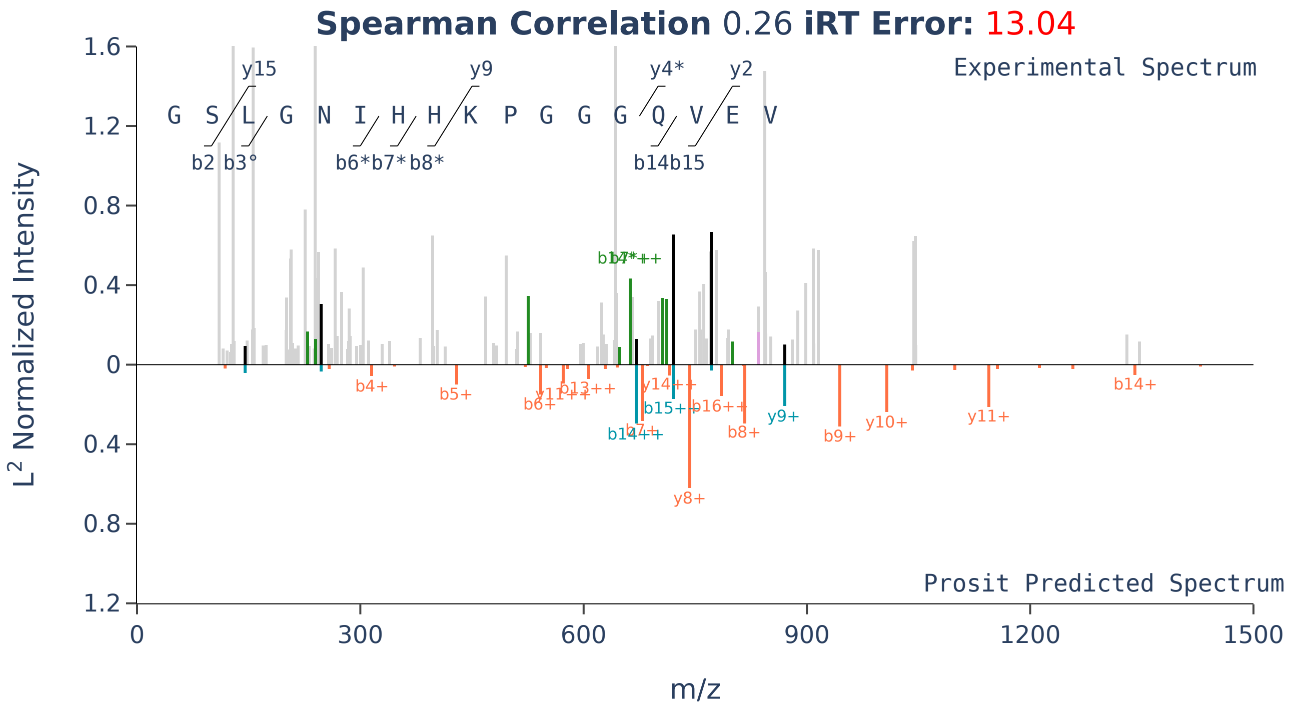

Source WSoh_221121_011221_HFGoe_L1_hTau_24h_R1 **Scan** 17621
Peptide APVPMPDLKNVKSH **Charge** 3 **Spectral Angle** 0.85
Spearman Correlation 0.89 **iRT Error:** 2.59

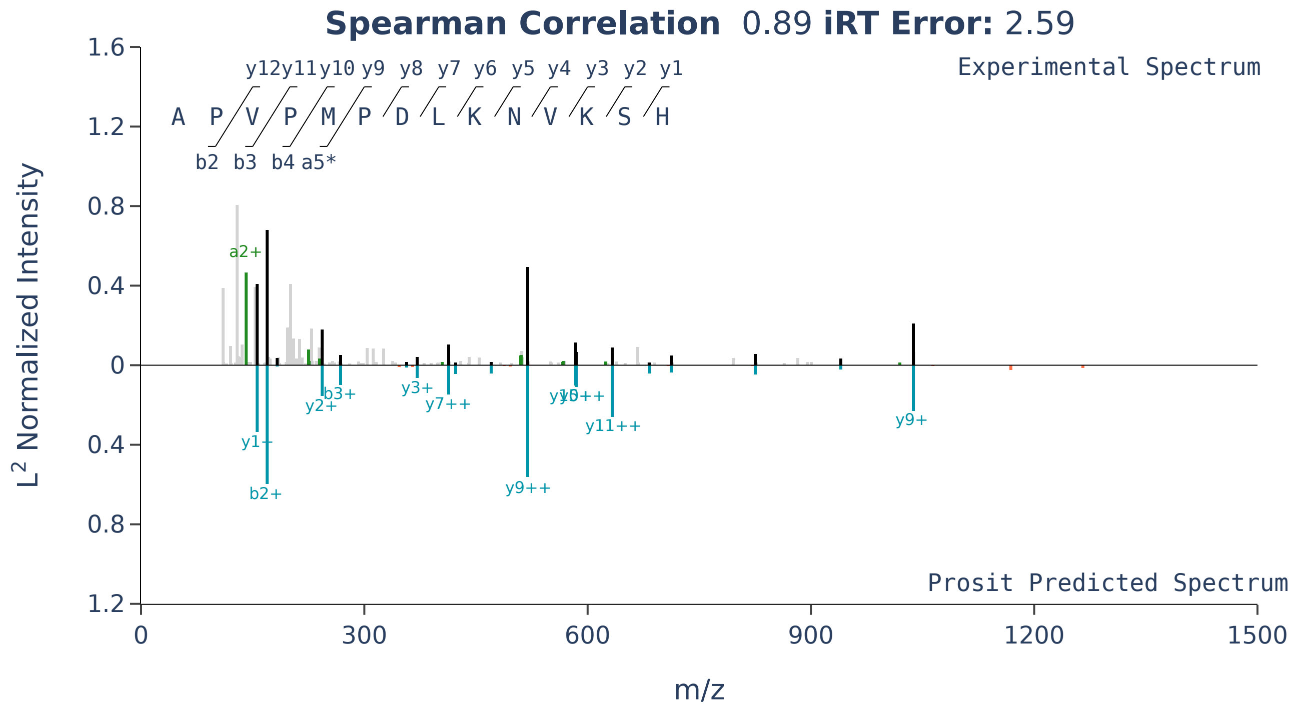

Source WSoh_221121_011221_HFGoe_L1_hTau_24h_R1 **Scan** 17621
Peptide DLKNVKSKIGSTEN **Charge** 3 **Spectral Angle** 0.0
Spearman Correlation 0.0 **iRT Error:** 38.6

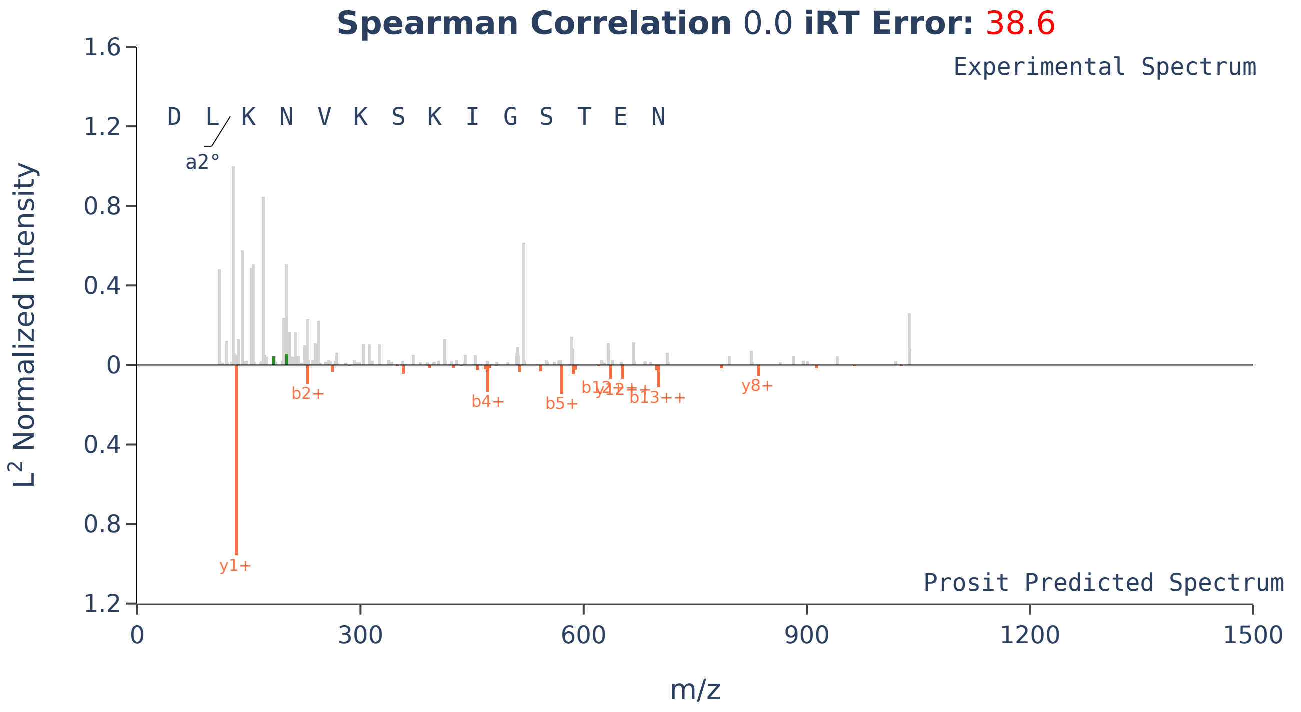

Source WSoh_221121_011221_HFGoe_L2_hTau_24h_R1 **Scan** 11859
Peptide AAQPHTEIPEGTQE **Charge** 2 **Spectral Angle** 0.9
Spearman Correlation 0.91 **iRT Error:** 2.59

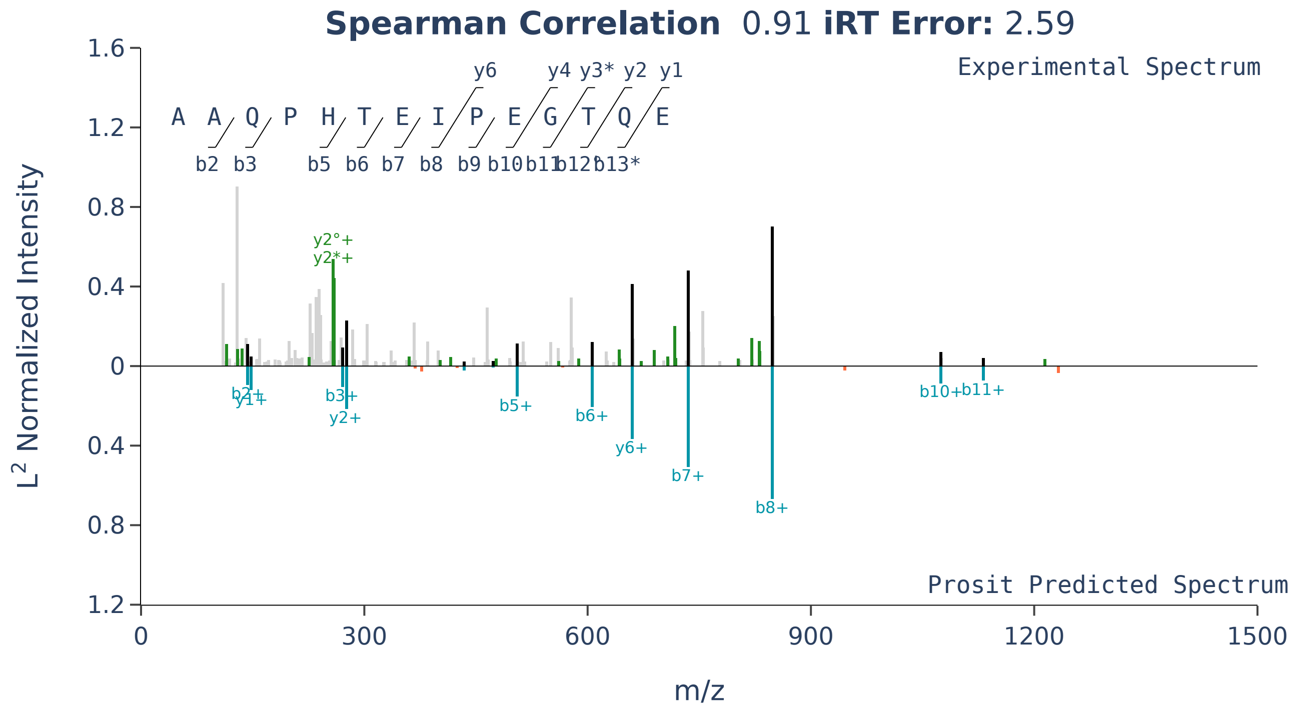

Source WSoh_221121_011221_HFGoe_L2_hTau_24h_R1 **Scan** 11859
Peptide SSTGSIDMVDSPQLA **Charge** 2 **Spectral Angle** 0.03
Spearman Correlation -0.11 **iRT Error:** 72.75

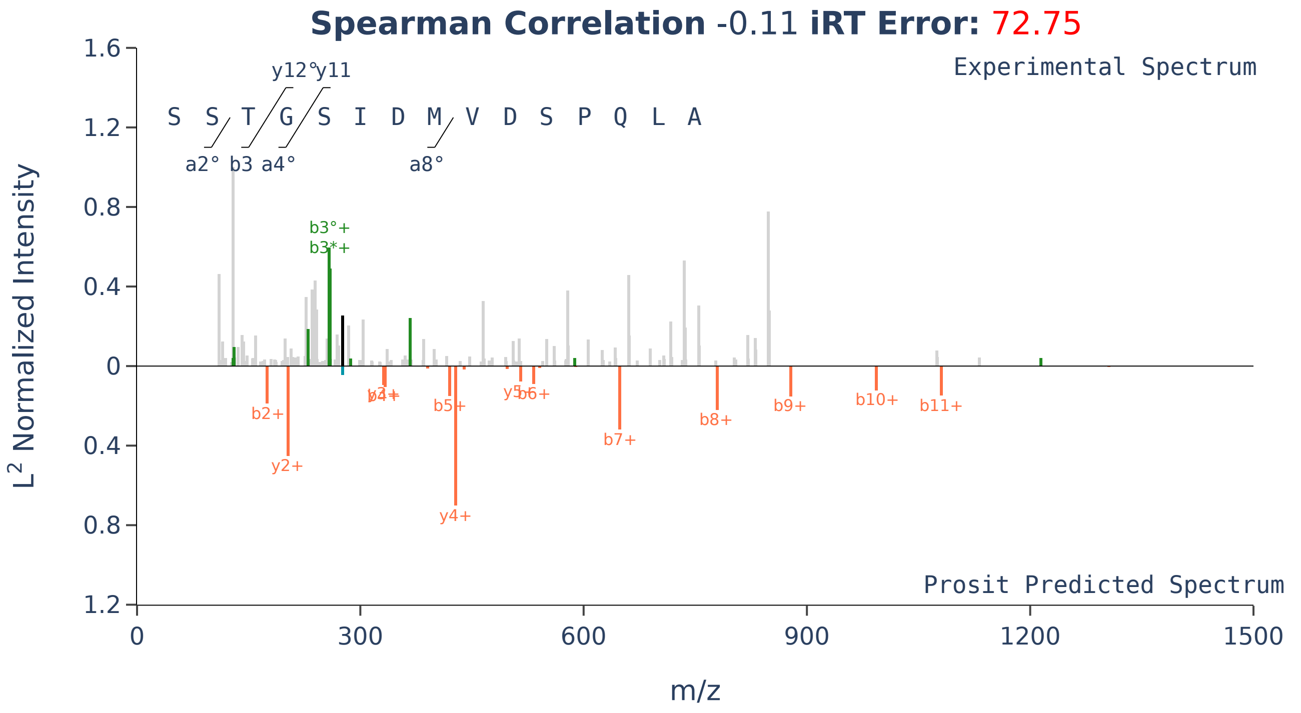

Source WSoh_221121_011221_HFGoe_L2_hTau_24h_R1 **Scan** 21500
Peptide PSLEDEAAGIGDT **Charge** 1 **Spectral Angle** 0.81
Spearman Correlation 0.9 **iRT Error:** 2.63

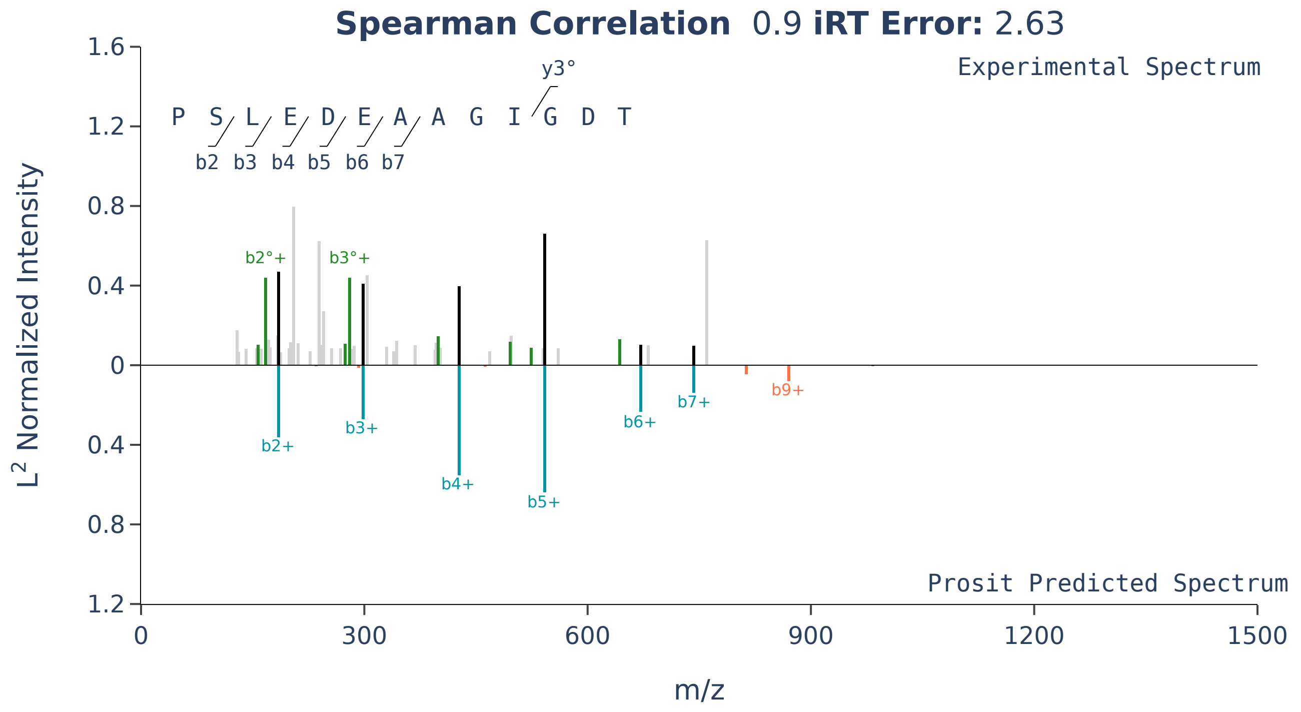

Source WSoh_221121_011221_HFGoe_L2_hTau_24h_R1 **Scan** 21500
Peptide IGDTPSLEDEAAG **Charge** 1 **Spectral Angle** 0.43
Spearman Correlation 0.57 **iRT Error:** 14.32

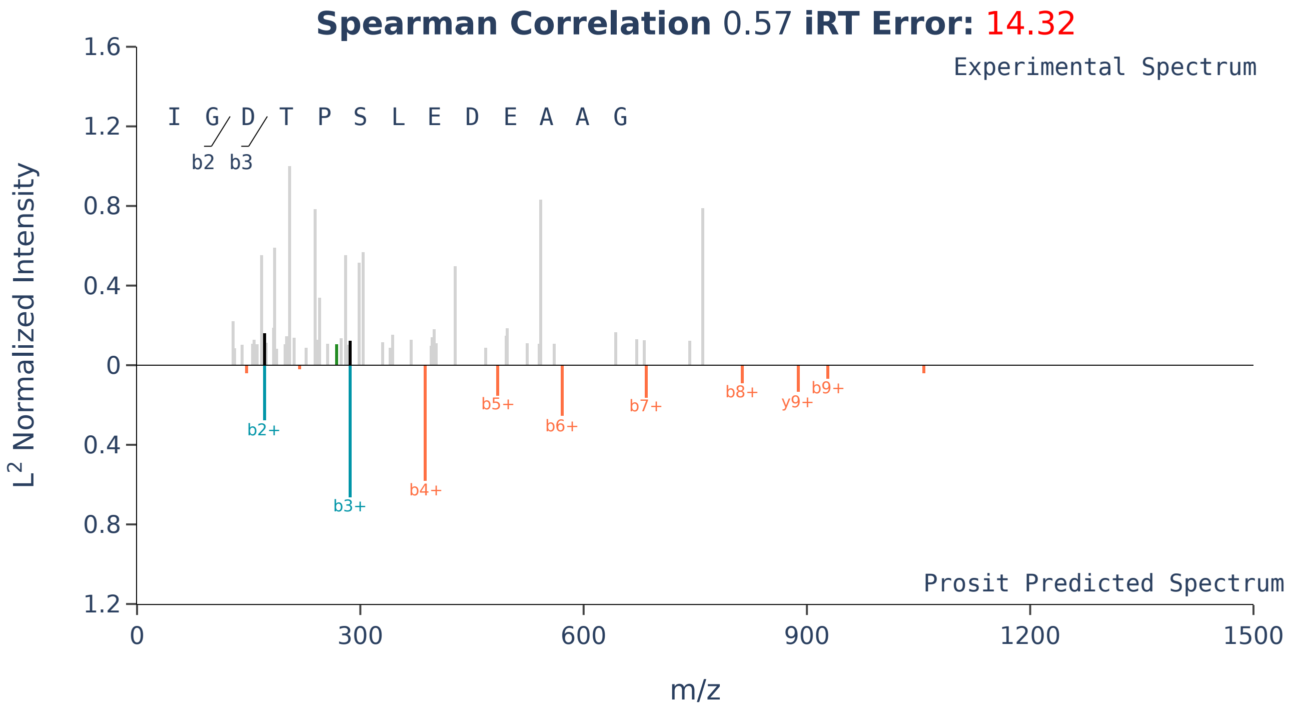

Source WSoh_221121_011221_HFGoe_L2_hTau_24h_R2 **Scan** 10988
Peptide SGSLGNIHHKPGGGQVEV **Charge** 2 **Spectral Angle** 0.78
Spearman Correlation 0.9 **iRT Error:** 2.67

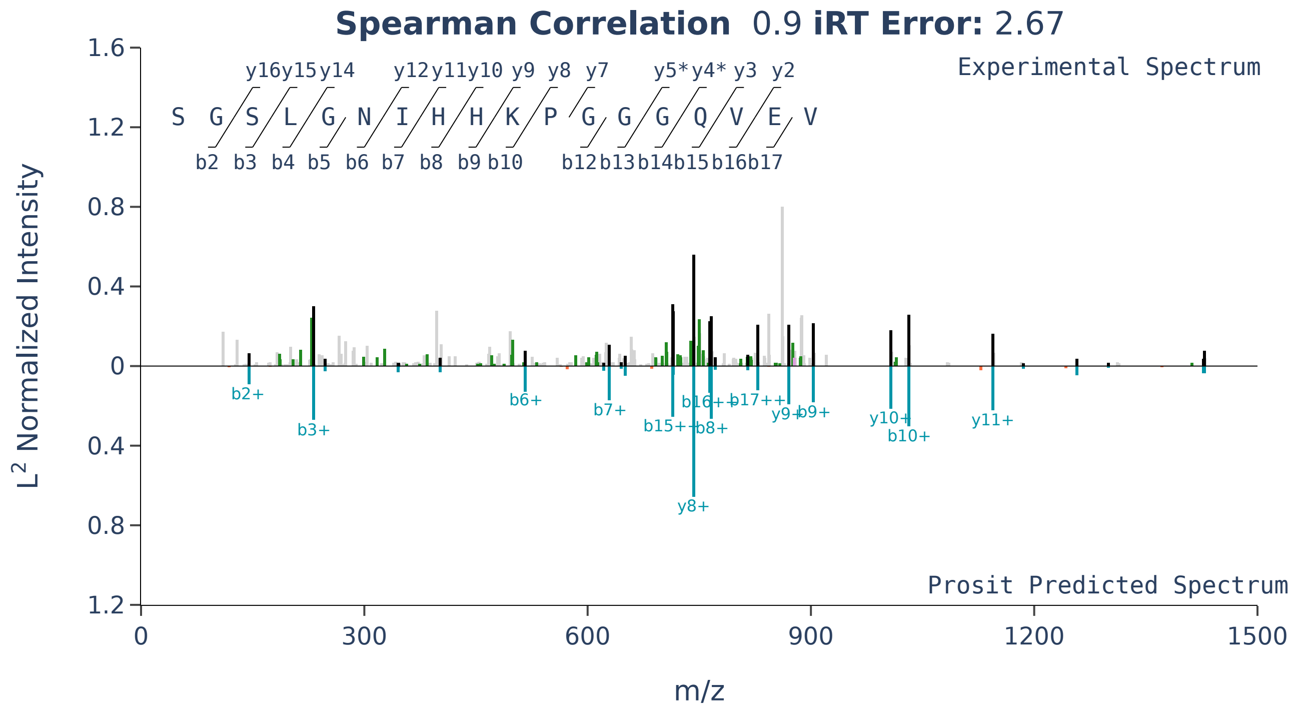

Source WSoh_221121_011221_HFGoe_L2_hTau_24h_R2 **Scan** 10988
Peptide AGHVTQARMVSKSKDGT **Charge** 2 **Spectral Angle** 0.05
Spearman Correlation -0.03 **iRT Error:** 38.19

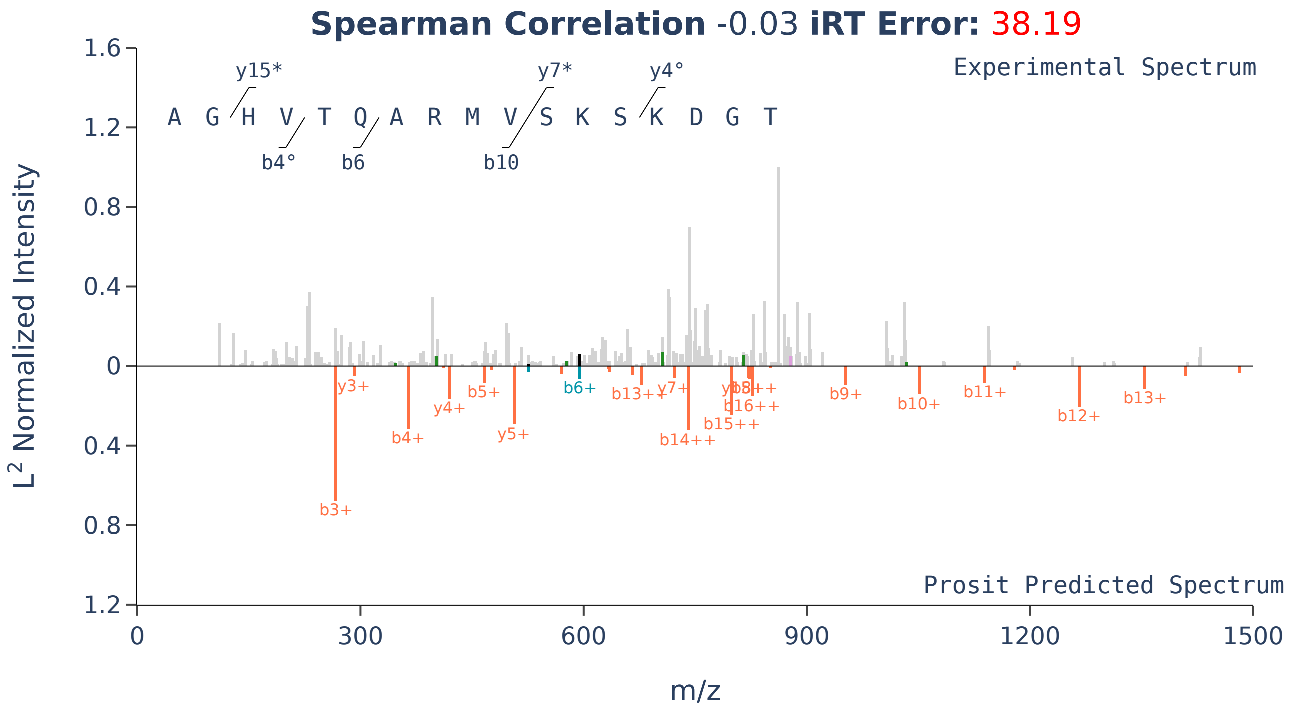

Source WSoh_221121_011221_HFGoe_L1_hTau_24h_R1 **Scan** 12489
Peptide DSLGNIHHKPGGGQVEV **Charge** 2 **Spectral Angle** 0.83
Spearman Correlation 0.86 **iRT Error:** 2.72

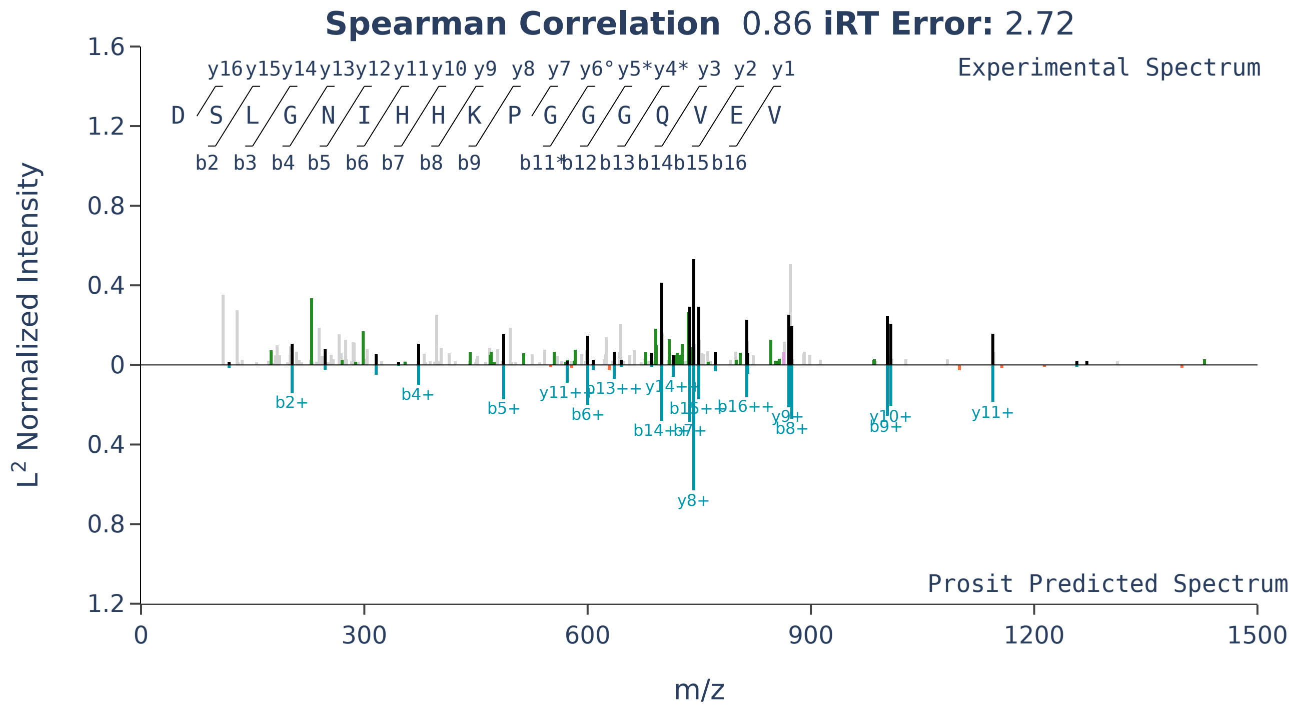

Source WSoh_221121_011221_HFGoe_L1_hTau_24h_R1 **Scan** 12489
Peptide SNVQSKCGSKDNIKHV **Charge** 2 **Spectral Angle** 0.04
Spearman Correlation -0.07 **iRT Error:** 38.15

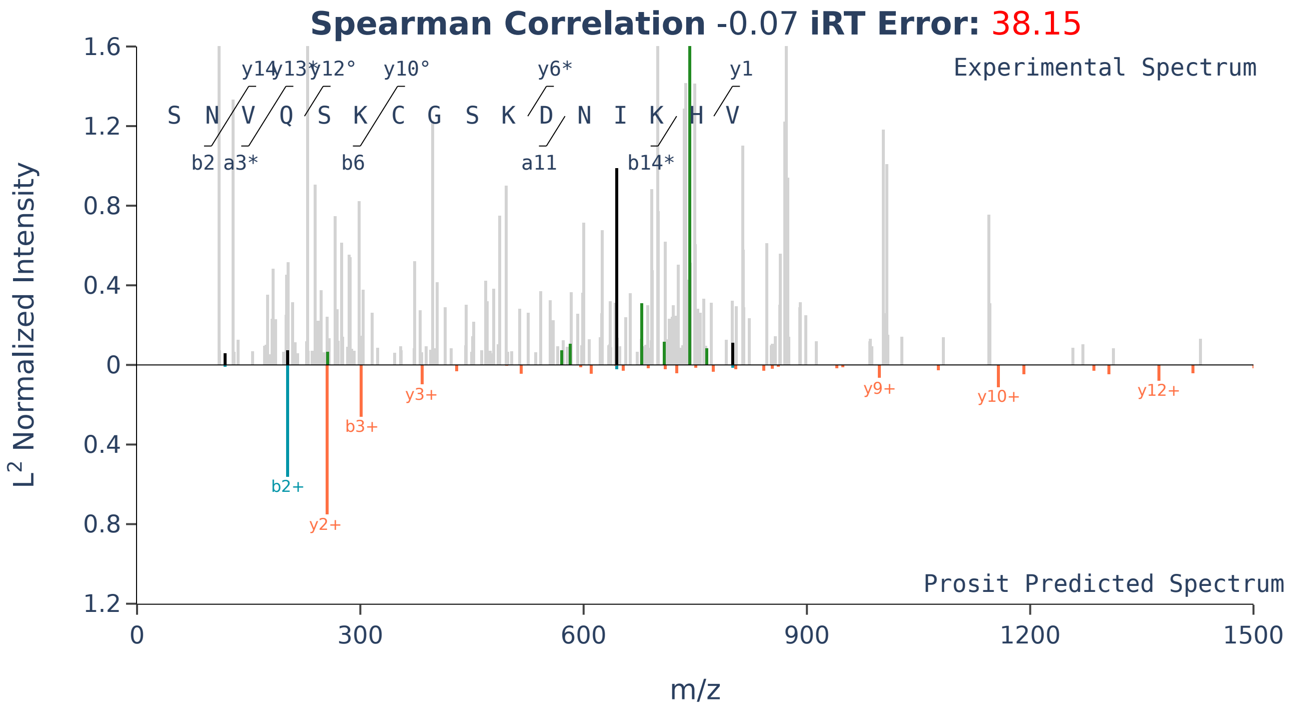

Source WSoh_221121_011221_HFGoe_L1_hTau_4h_R2 **Scan** 11686
Peptide SKHVPGGGSVQI **Charge** 3 **Spectral Angle** 0.89
Spearman Correlation 0.89 **iRT Error:** 2.74

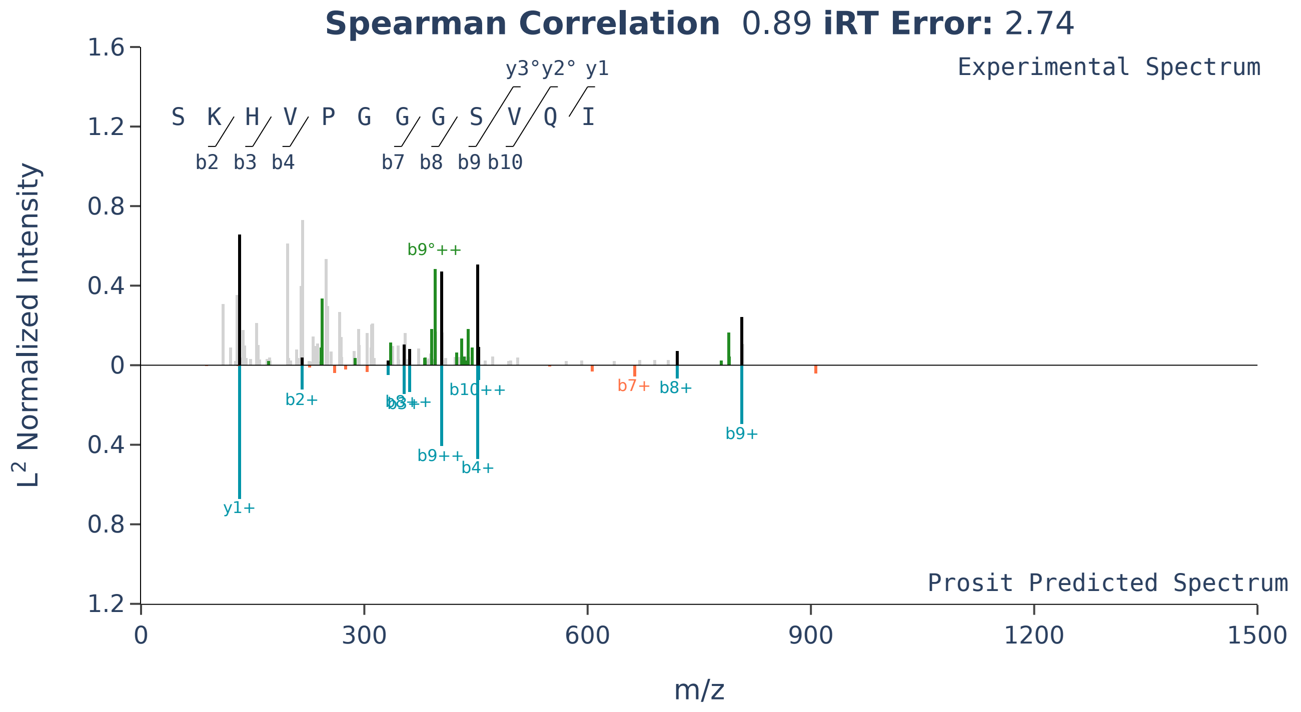

Source WSoh_221121_011221_HFGoe_L1_hTau_4h_R2 **Scan** 11686
Peptide TFRENAKAKT **Charge** 3 **Spectral Angle** 0.0
Spearman Correlation 0.0 **iRT Error:** 38.33

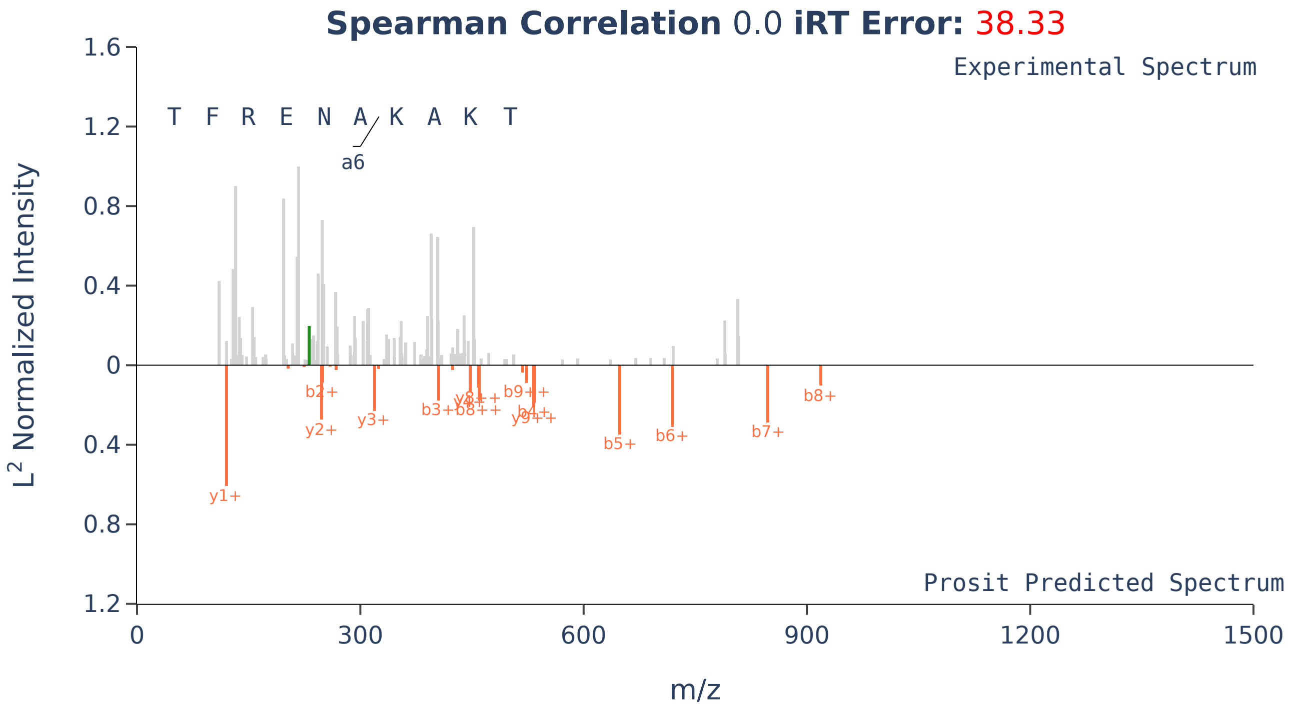

Source WSoh_221121_011221_HFGoe_L2_hTau_24h_R1 **Scan** 22749
Peptide LQTAPVPMPDLKNVKGG **Charge** 2 **Spectral Angle** 0.88
Spearman Correlation 0.86 **iRT Error:** 2.78

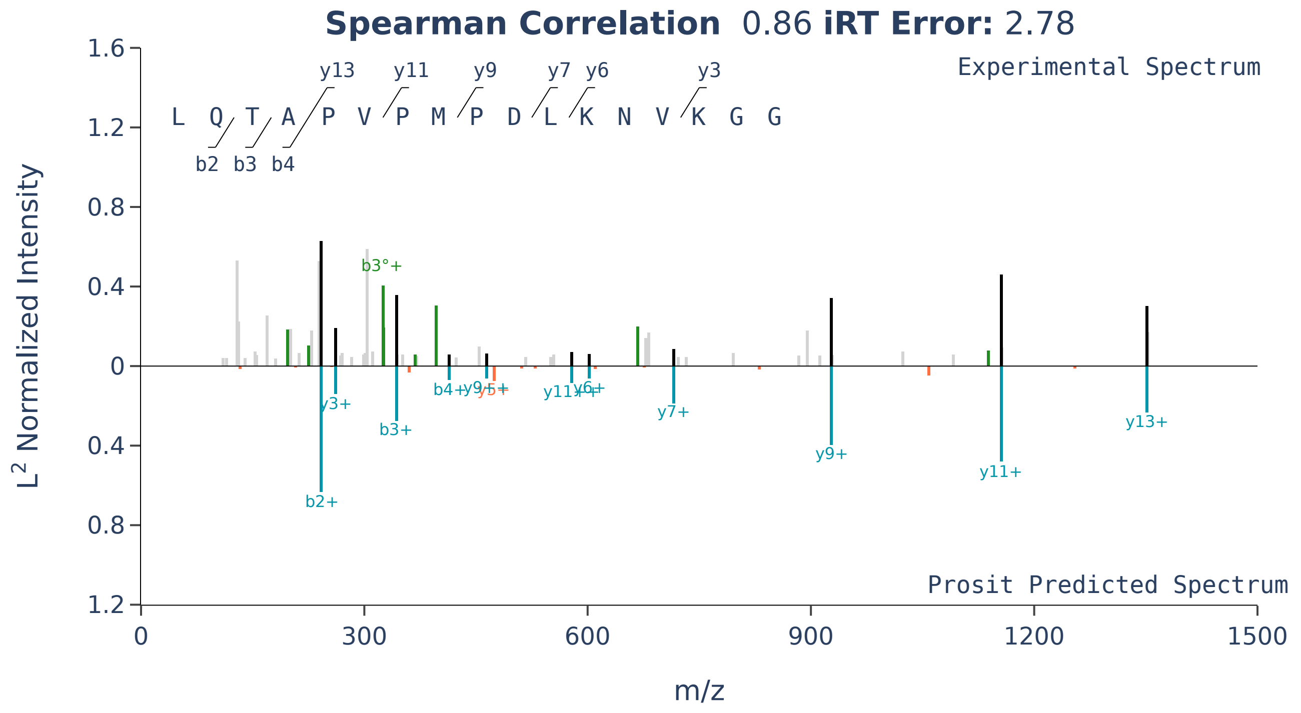

Source WSoh_221121_011221_HFGoe_L2_hTau_24h_R1 **Scan** 22749
Peptide HVPGGGSVQIVYKPVDL **Charge** 2 **Spectral Angle** 0.0
Spearman Correlation 0.0 **iRT Error:** 5.41

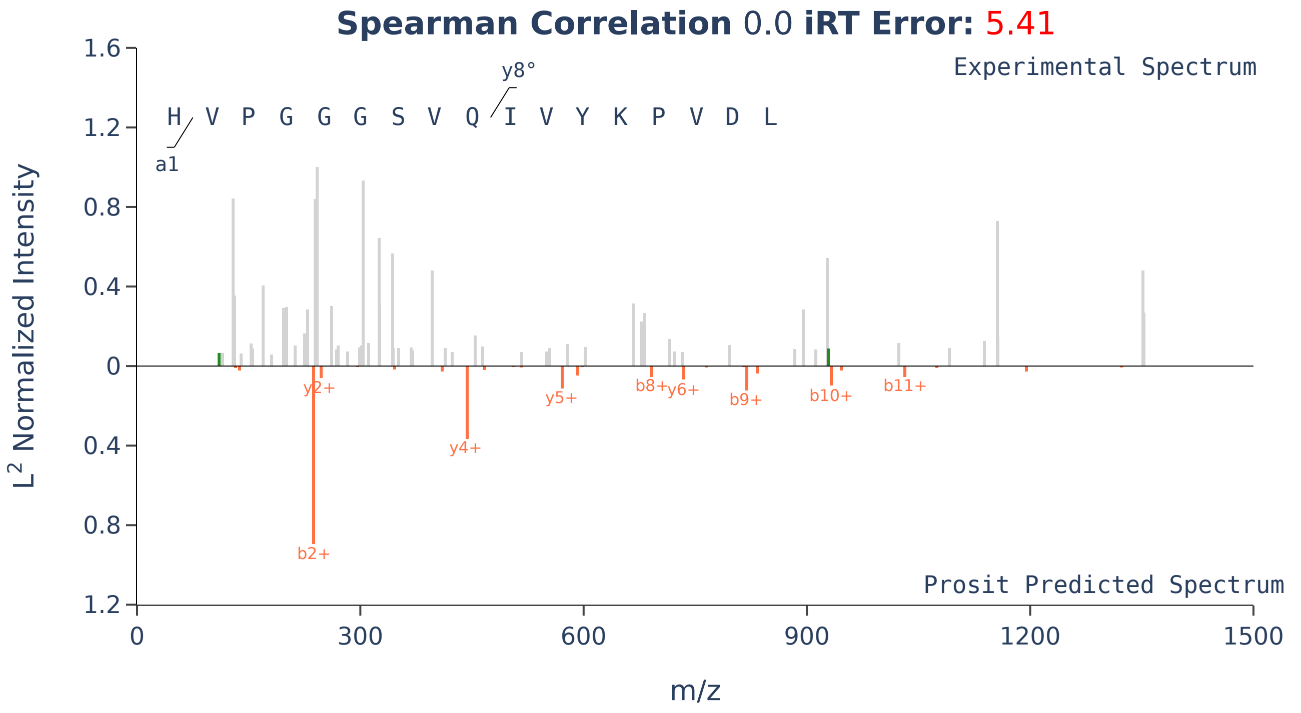

Source WSoh_221121_011221_HFGoe_L1_hTau_4h_R2 **Scan** 13029
Peptide DLSNVQSKCSKDNI **Charge** 2 **Spectral Angle** 0.84
Spearman Correlation 0.88 **iRT Error:** 2.99

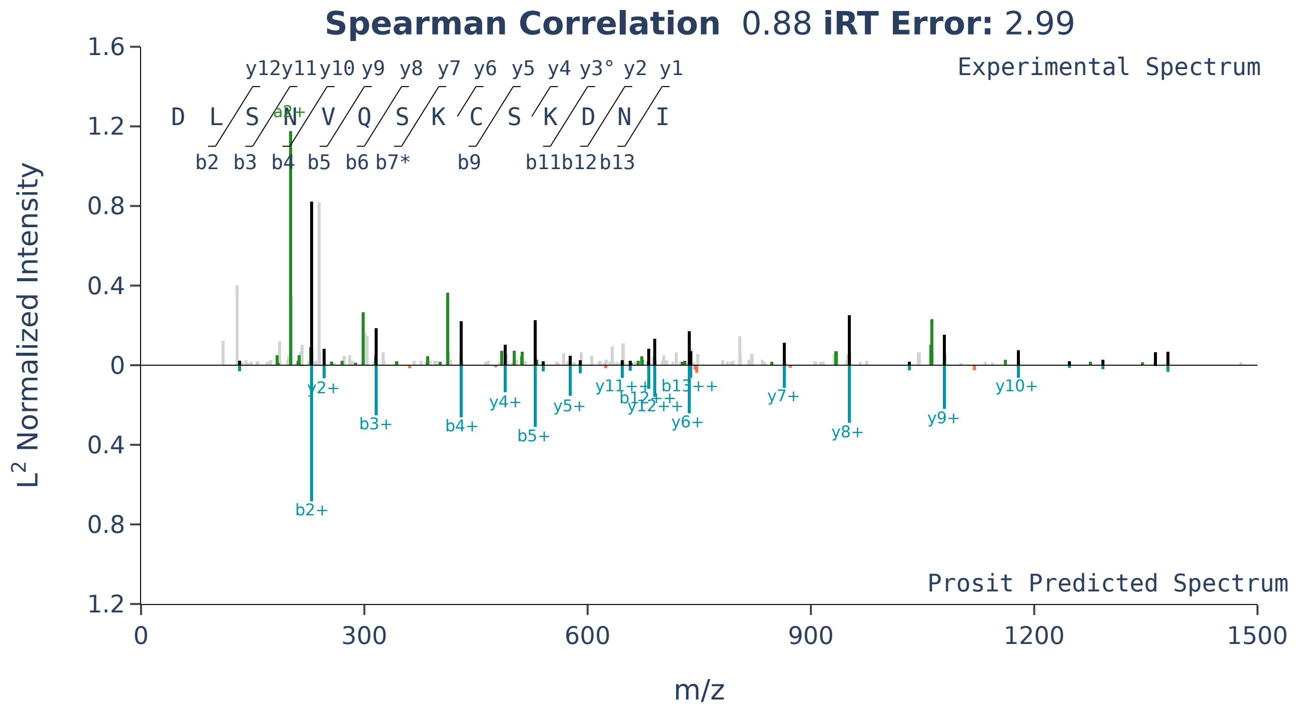

Source WSoh_221121_011221_HFGoe_L1_hTau_4h_R2 **Scan** 13029
Peptide QAAAQPHTEIPEGTT **Charge** 2 **Spectral Angle** 0.14
Spearman Correlation 0.22 **iRT Error:** 1.88

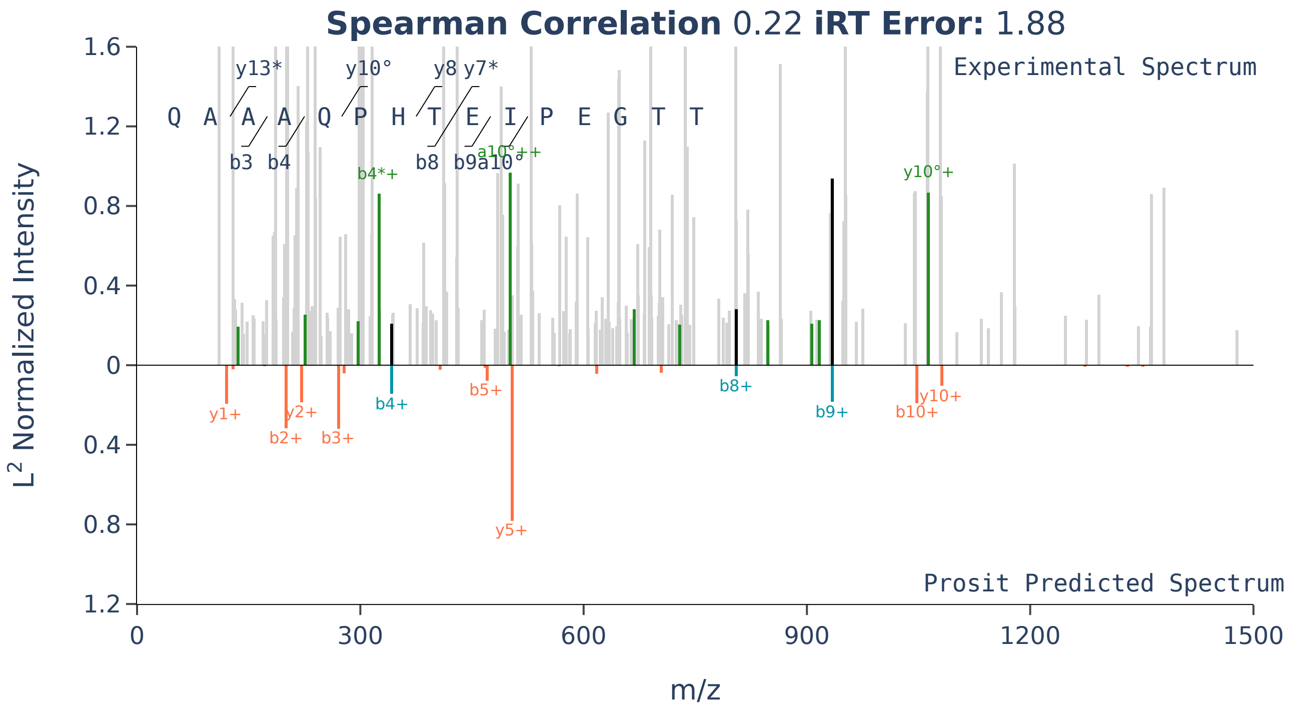

Source WSoh_221121_011221_HFGoe_L1_hTau_24h_R1 **Scan** 5877
Peptide SNVQSKCSKDNIKHV **Charge** 2 **Spectral Angle** 0.95
Spearman Correlation 0.88 **iRT Error:** 3.13

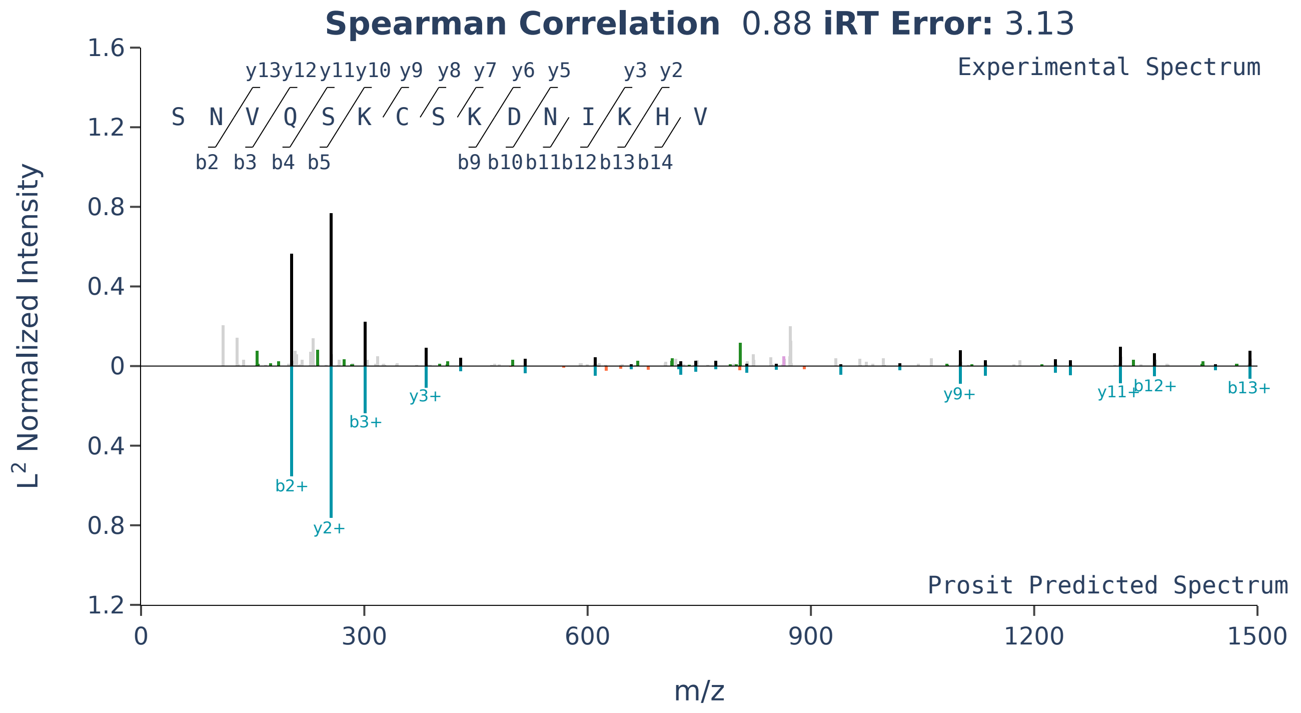

Source WSoh_221121_011221_HFGoe_L1_hTau_24h_R1 **Scan** 5877
Peptide FRENAKAKTDHGAEI **Charge** 2 **Spectral Angle** 0.09
Spearman Correlation 0.08 **iRT Error:** 4.92

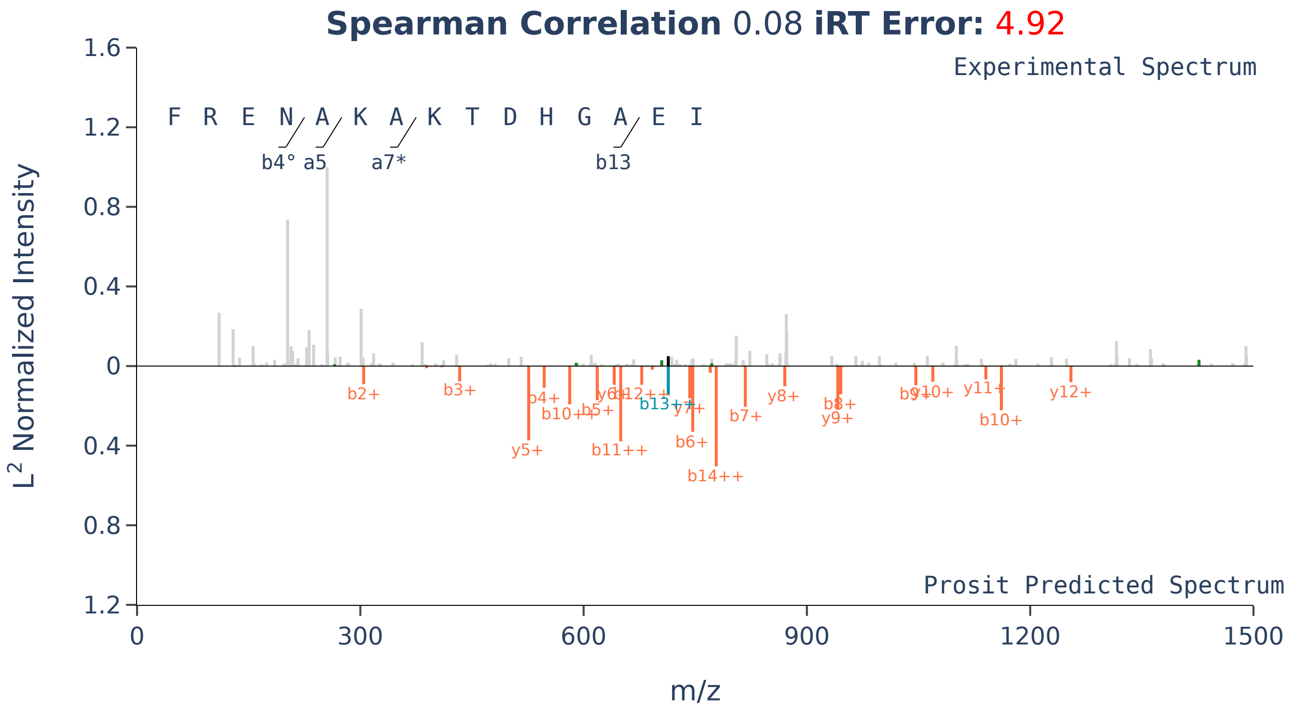

Source WSoh_221121_011221_HFGoe_L2_hTau_24h_R1 **Scan** 20816
Peptide QTAPVPMPDLKNVKSG **Charge** 3 **Spectral Angle** 0.76
Spearman Correlation 0.9 **iRT Error:** 3.14

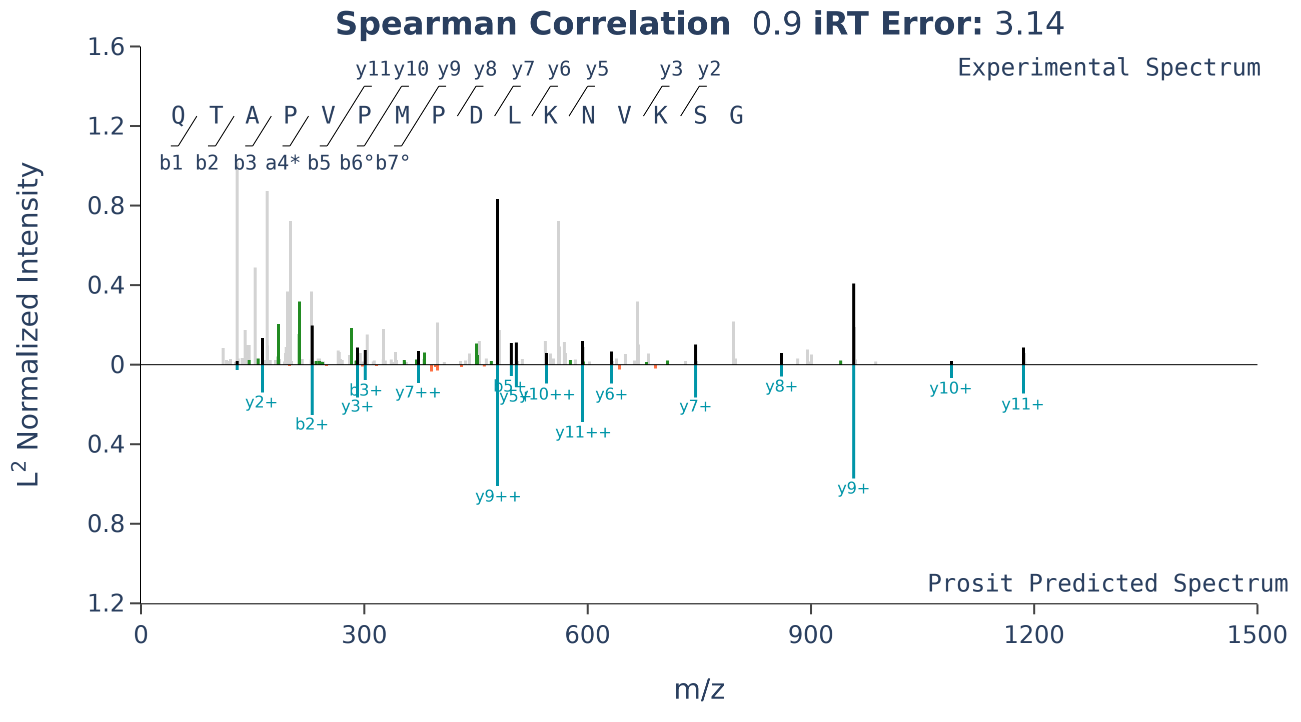

Source WSoh_221121_011221_HFGoe_L2_hTau_24h_R1 **Scan** 20816
Peptide SRSRTPSLPTPPTRE **Charge** 3 **Spectral Angle** 0.06
Spearman Correlation 0.13 **iRT Error:** 43.88

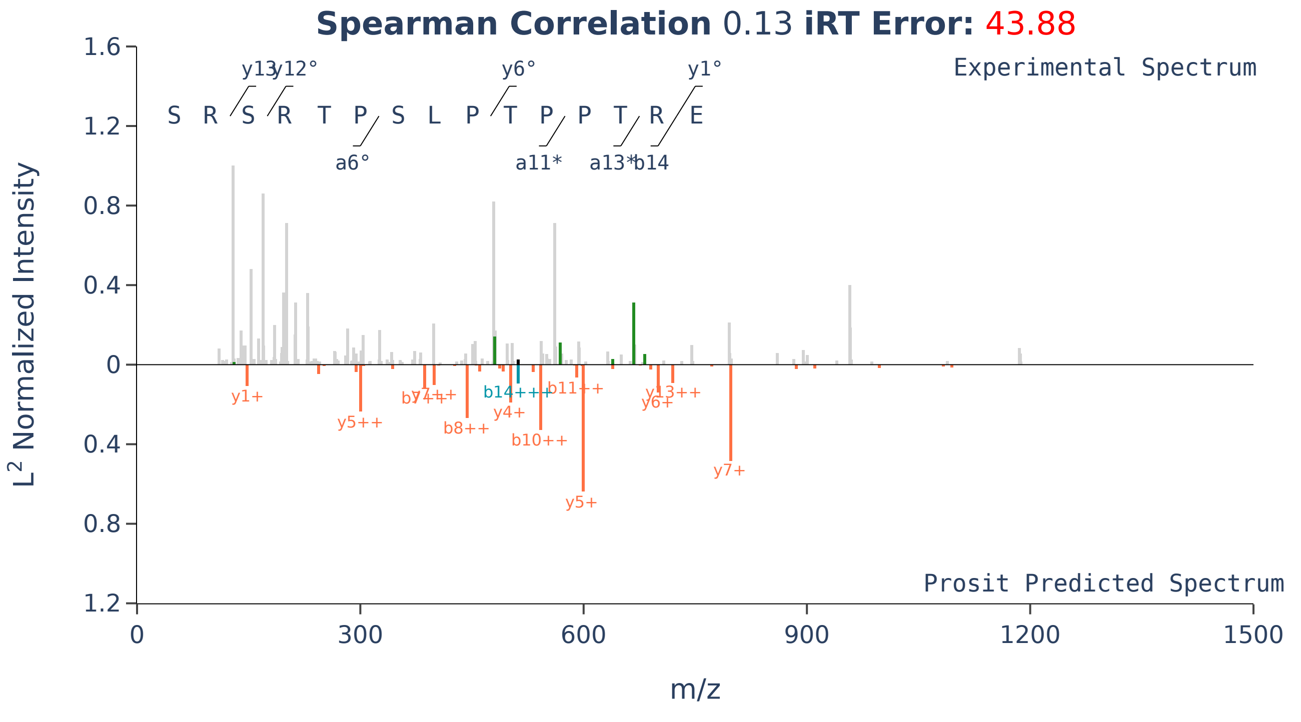

Source WSoh_221121_011221_HFGoe_L1_hTau_24h_R2 **Scan** 22709
Peptide EDEAAGHVTQAAGIGDTPSL **Charge** 2 **Spectral Angle** 0.92
Spearman Correlation 0.89 **iRT Error:** 3.19

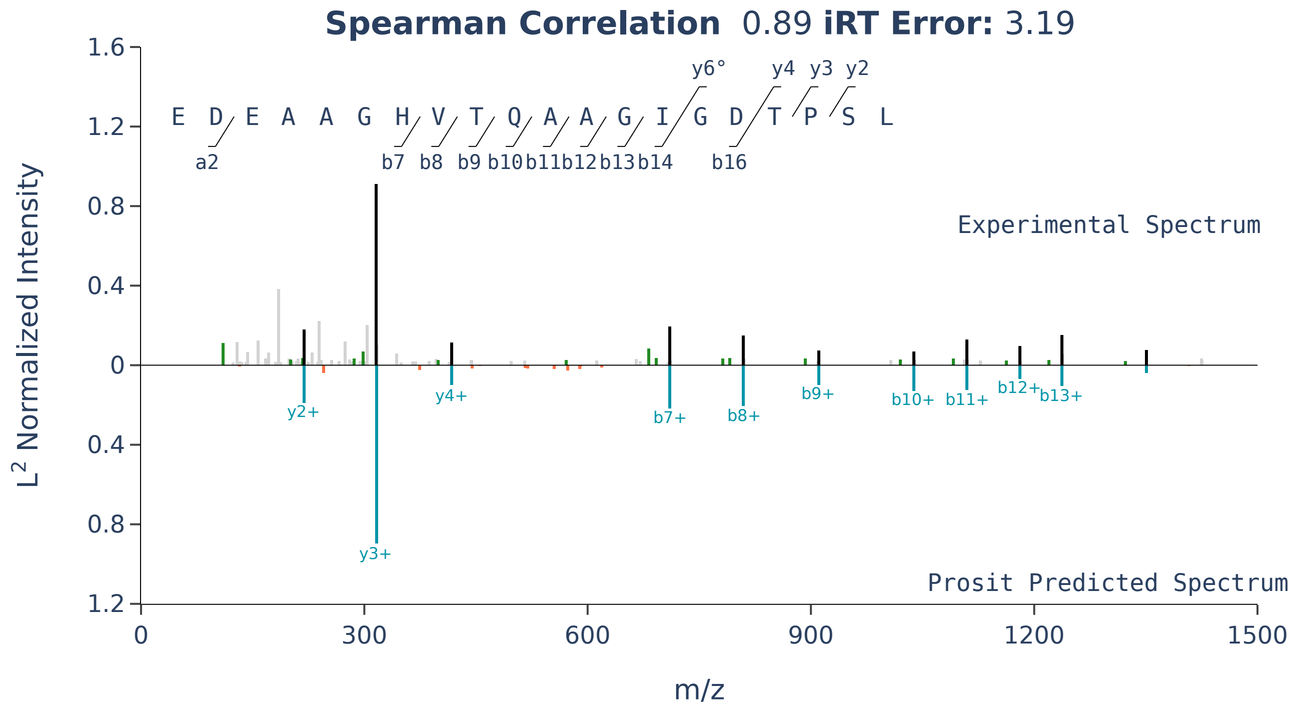

Source WSoh_221121_011221_HFGoe_L1_hTau_24h_R2 **Scan** 22709
Peptide AGIGDTPSLEDEAAGHVTQA **Charge** 2 **Spectral Angle** 0.31
Spearman Correlation 0.4 **iRT Error:** 8.64

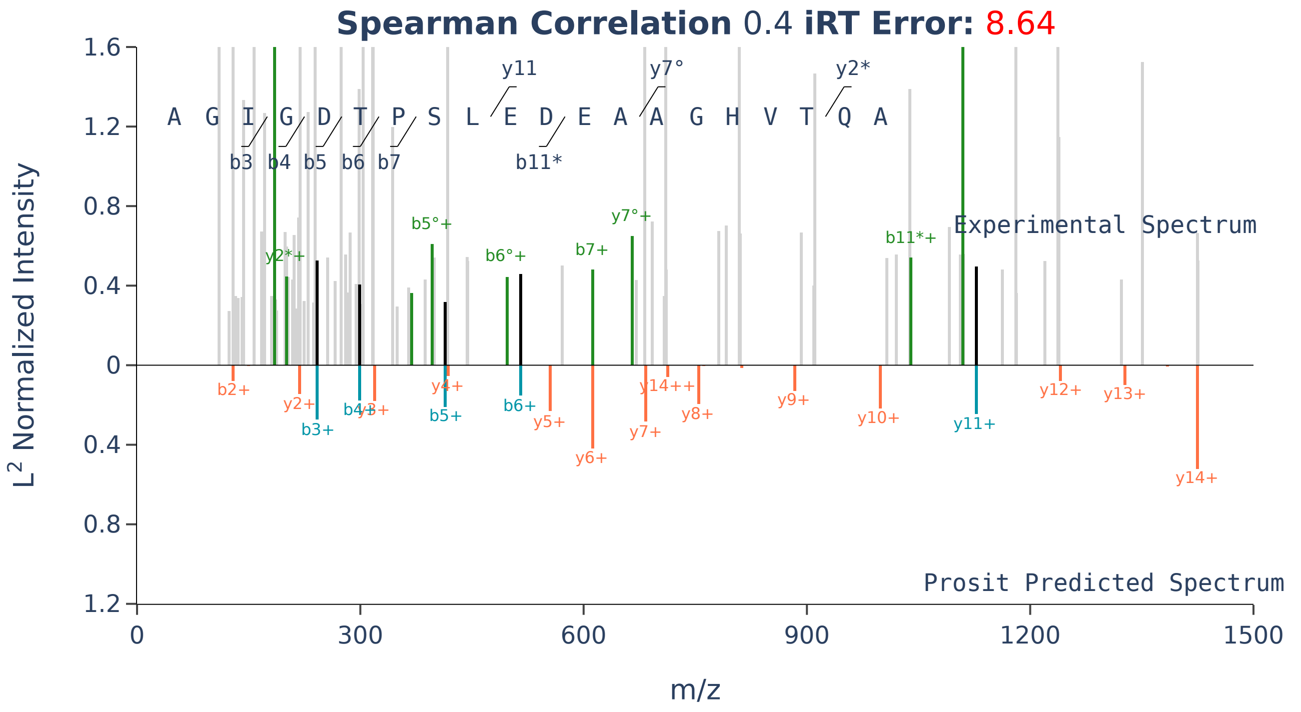

Source WSoh_221121_011221_HFGoe_L1_hTau_24h_R1 **Scan** 11151
Peptide GTLGNIHHKPGGGQVEV **Charge** 2 **Spectral Angle** 0.84
Spearman Correlation 0.89 **iRT Error:** 3.21

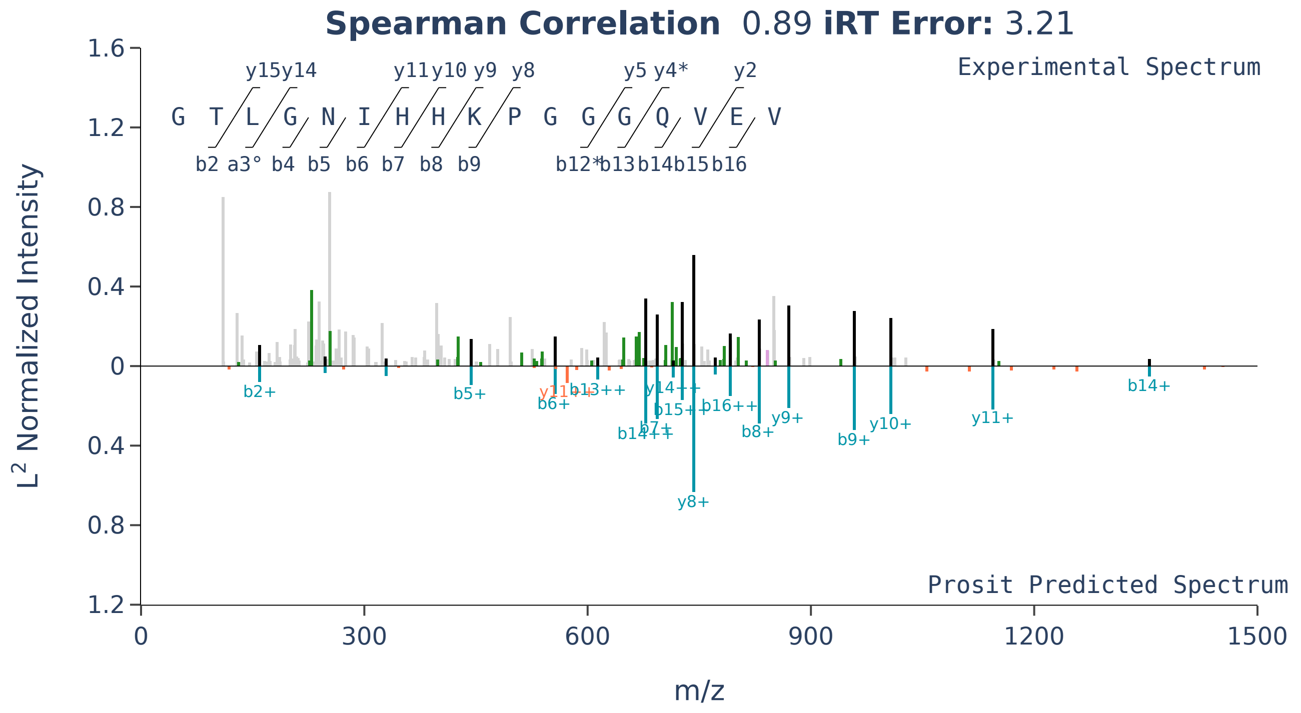

Source WSoh_221121_011221_HFGoe_L1_hTau_24h_R1 **Scan** 11151
Peptide EAAGHVTQARMVSKSK **Charge** 2 **Spectral Angle** 0.05
Spearman Correlation 0.03 **iRT Error:** 35.28

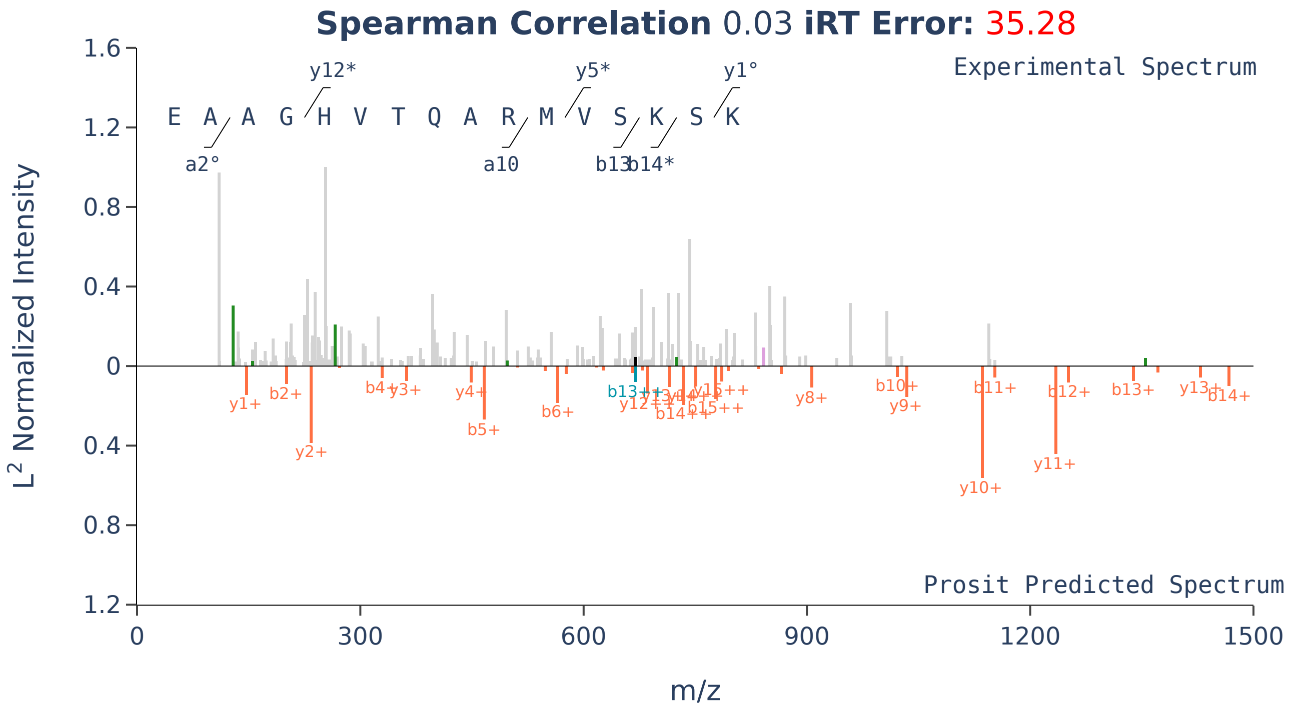

Source WSoh_221121_011221_HFGoe_L2_hTau_4h_R1 **Scan** 8799
Peptide KSEKLDFKDRVQN **Charge** 2 **Spectral Angle** 0.77
Spearman Correlation 0.95 **iRT Error:** 3.24

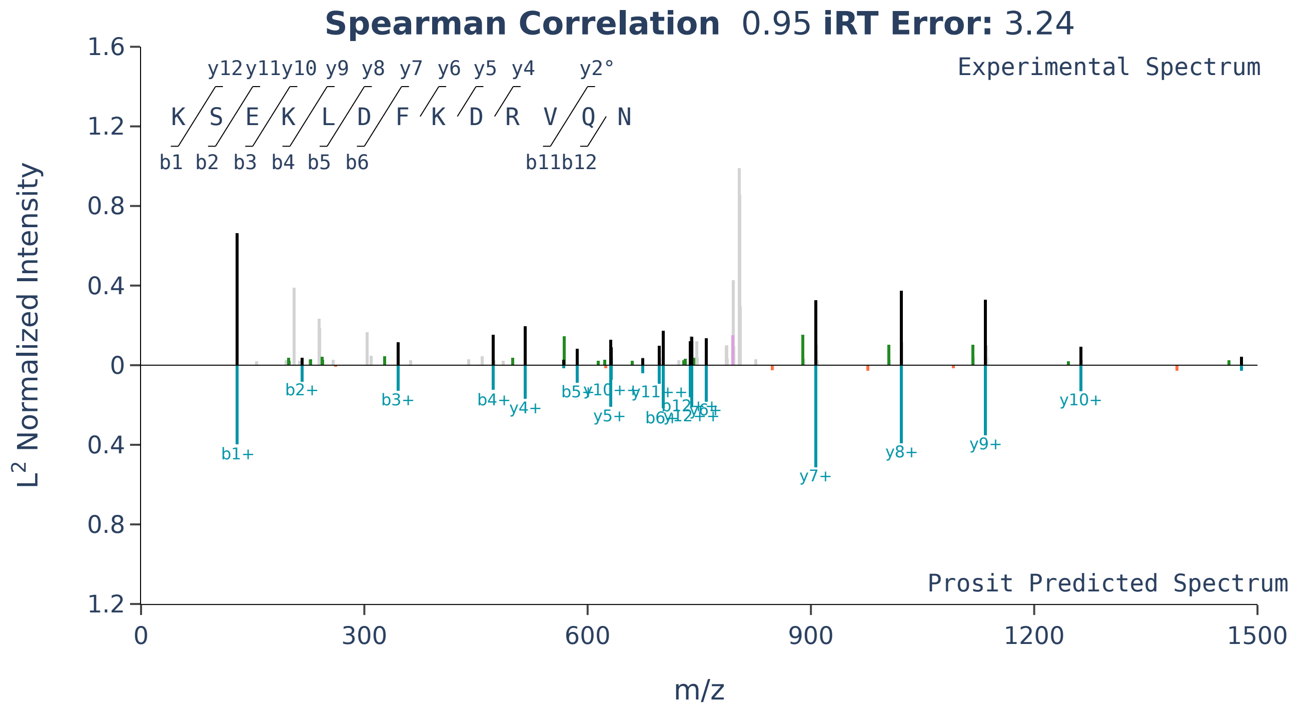

Source WSoh_221121_011221_HFGoe_L2_hTau_4h_R1 **Scan** 8799
Peptide FKDRVQSKIGSLDN **Charge** 2 **Spectral Angle** 0.32
Spearman Correlation 0.32 **iRT Error:** 23.24

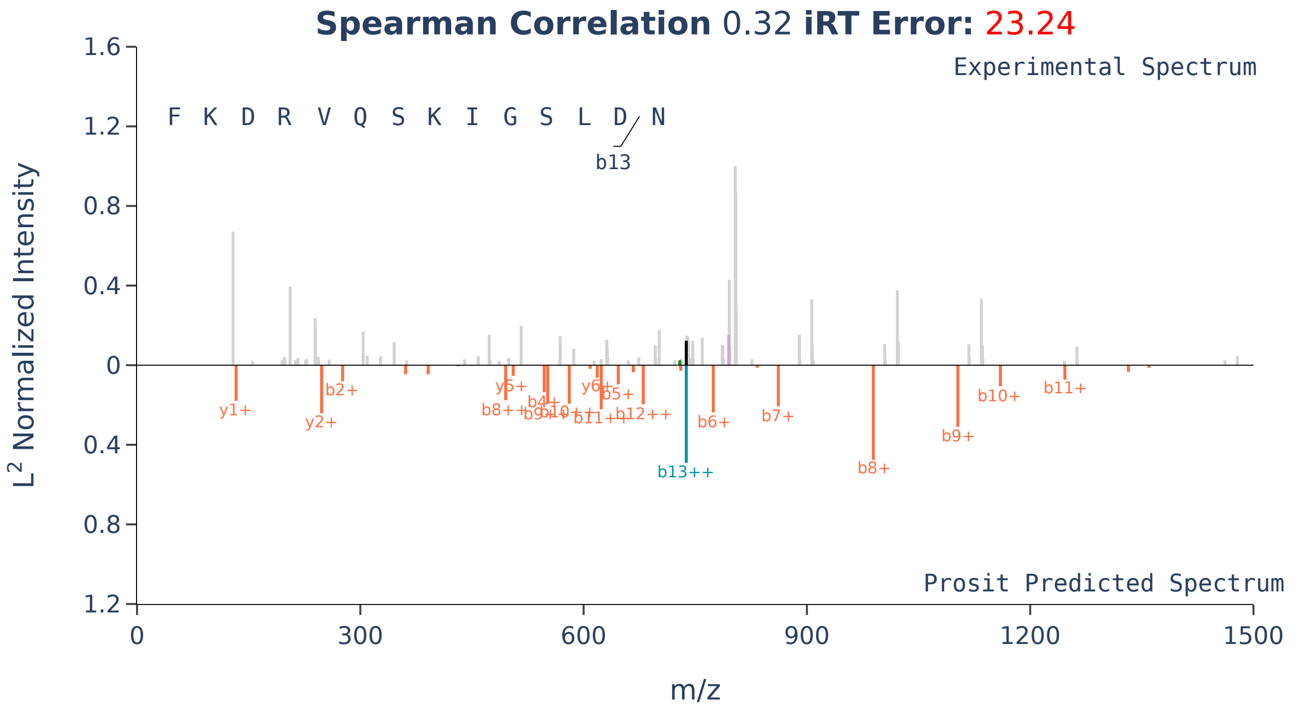

Source WSoh_221121_011221_HFGoe_L2_hTau_24h_R1 **Scan** 10872
Peptide SSLGNIHHKPGGGQVEV **Charge** 2 **Spectral Angle** 0.84
Spearman Correlation 0.84 **iRT Error:** 3.28

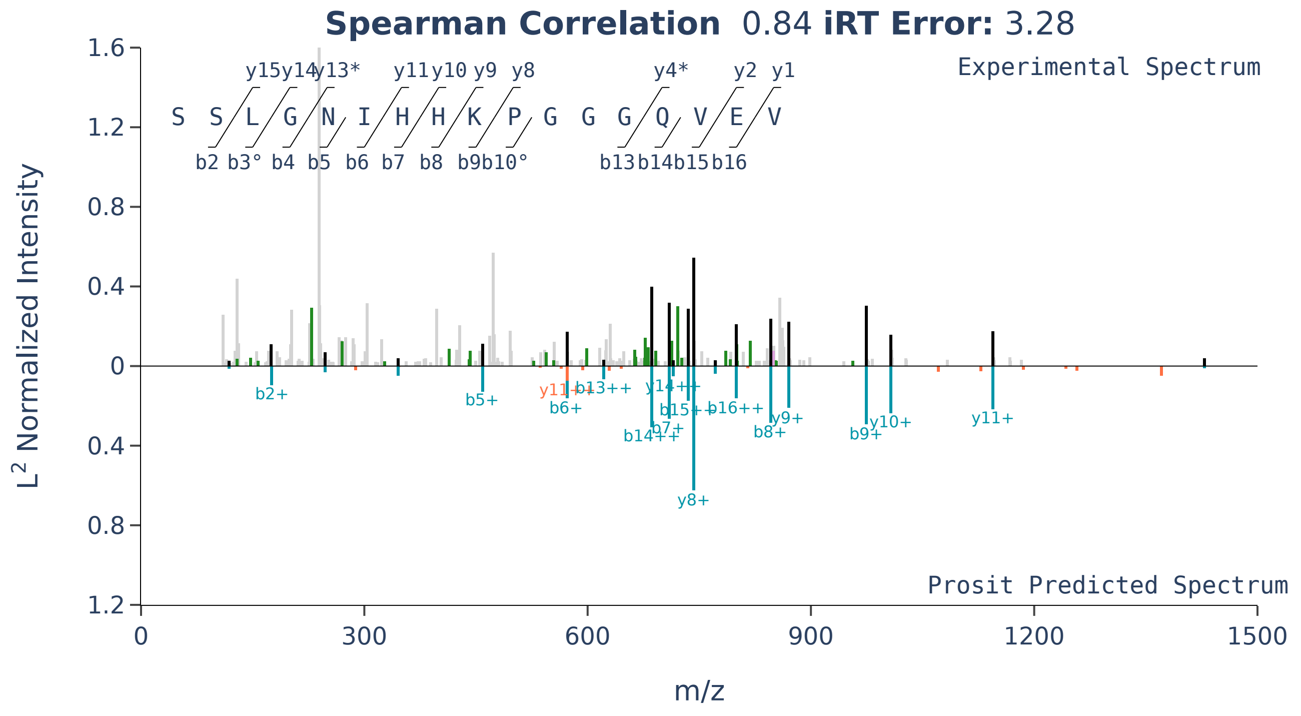

Source WSoh_221121_011221_HFGoe_L2_hTau_24h_R1 **Scan** 10872
Peptide NIHHKPGGGQVEVKSE **Charge** 2 **Spectral Angle** 0.01
Spearman Correlation -0.32 **iRT Error:** 32.11

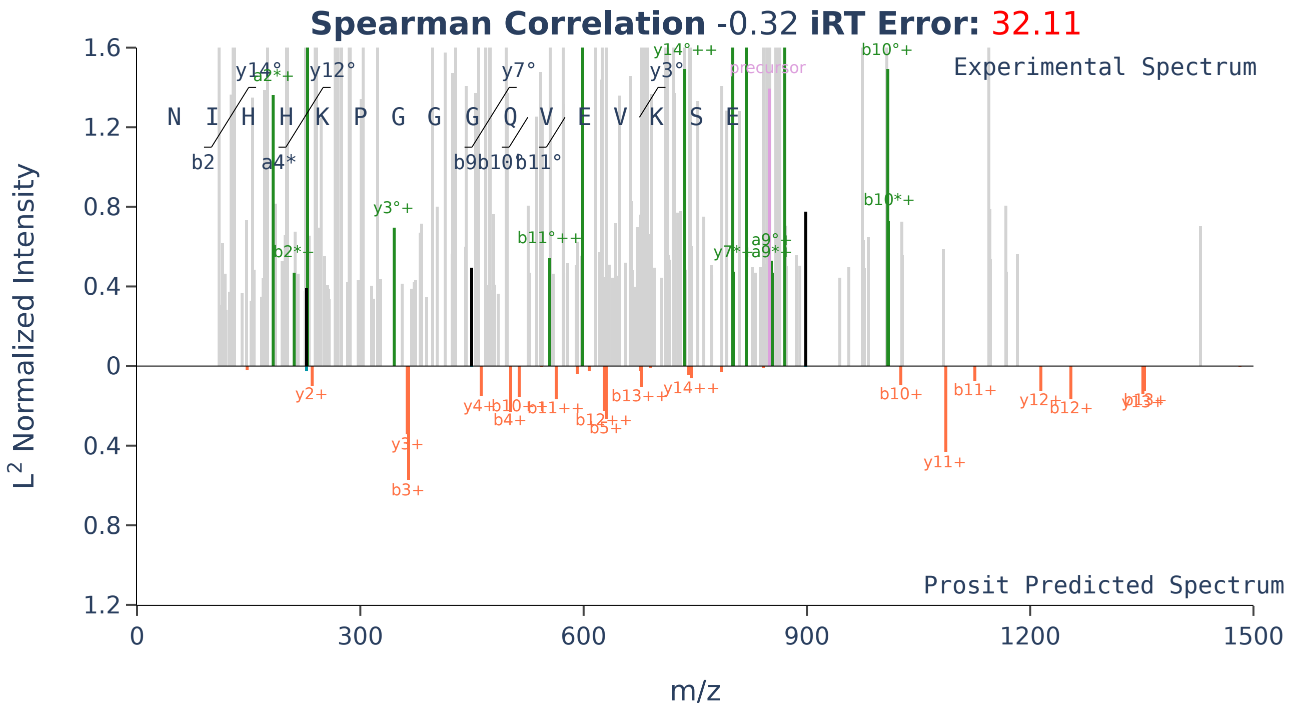

Source WSoh_221121_011221_HFGoe_L1_hTau_2h_R1 **Scan** 6503
Peptide GQTRIPAKT **Charge** 2 **Spectral Angle** 0.91
Spearman Correlation 0.93 **iRT Error:** 3.34

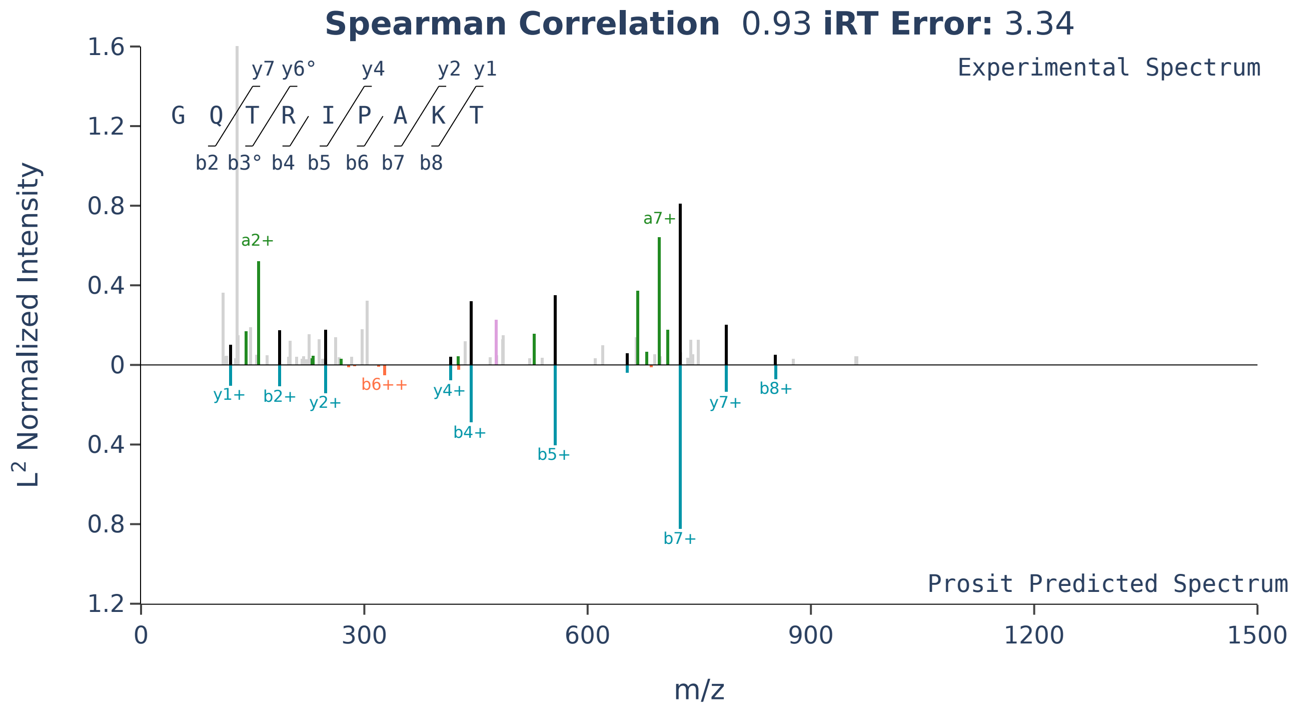

Source WSoh_221121_011221_HFGoe_L1_hTau_2h_R1 **Scan** 6503
Peptide AKSRLQTAP **Charge** 2 **Spectral Angle** 0.05
Spearman Correlation -0.03 **iRT Error:** 13.51

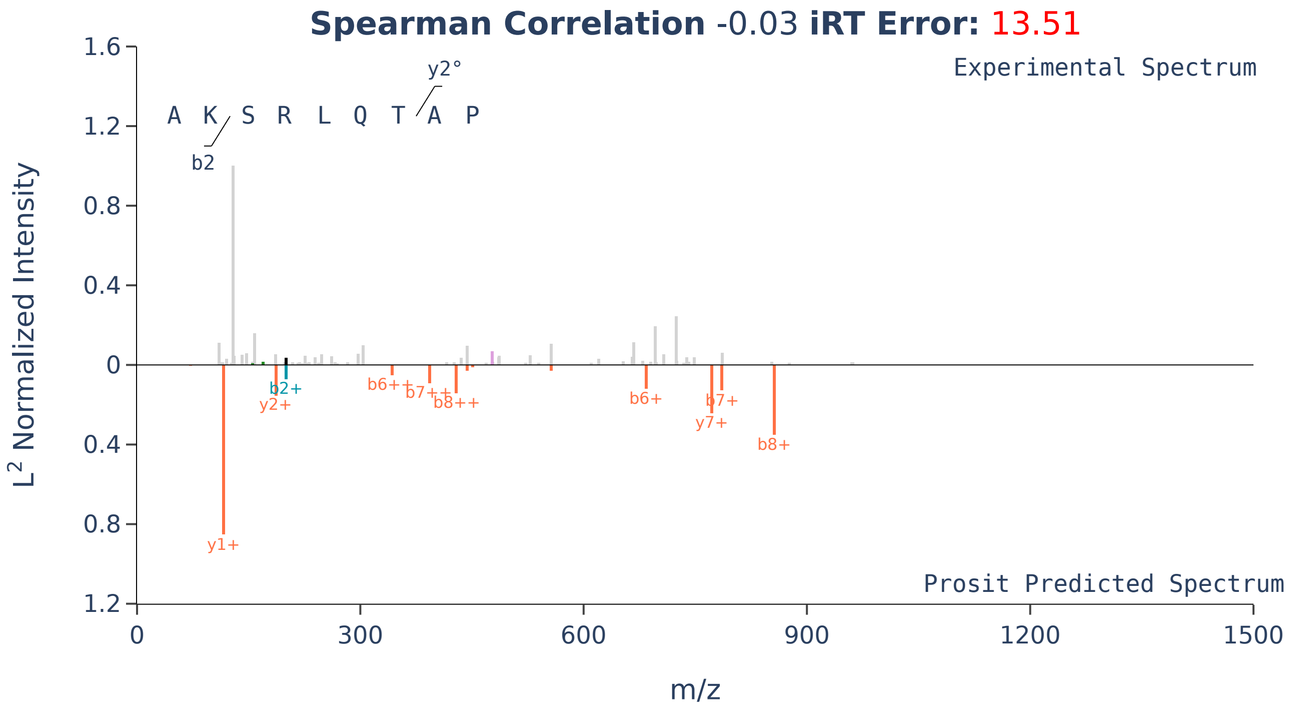

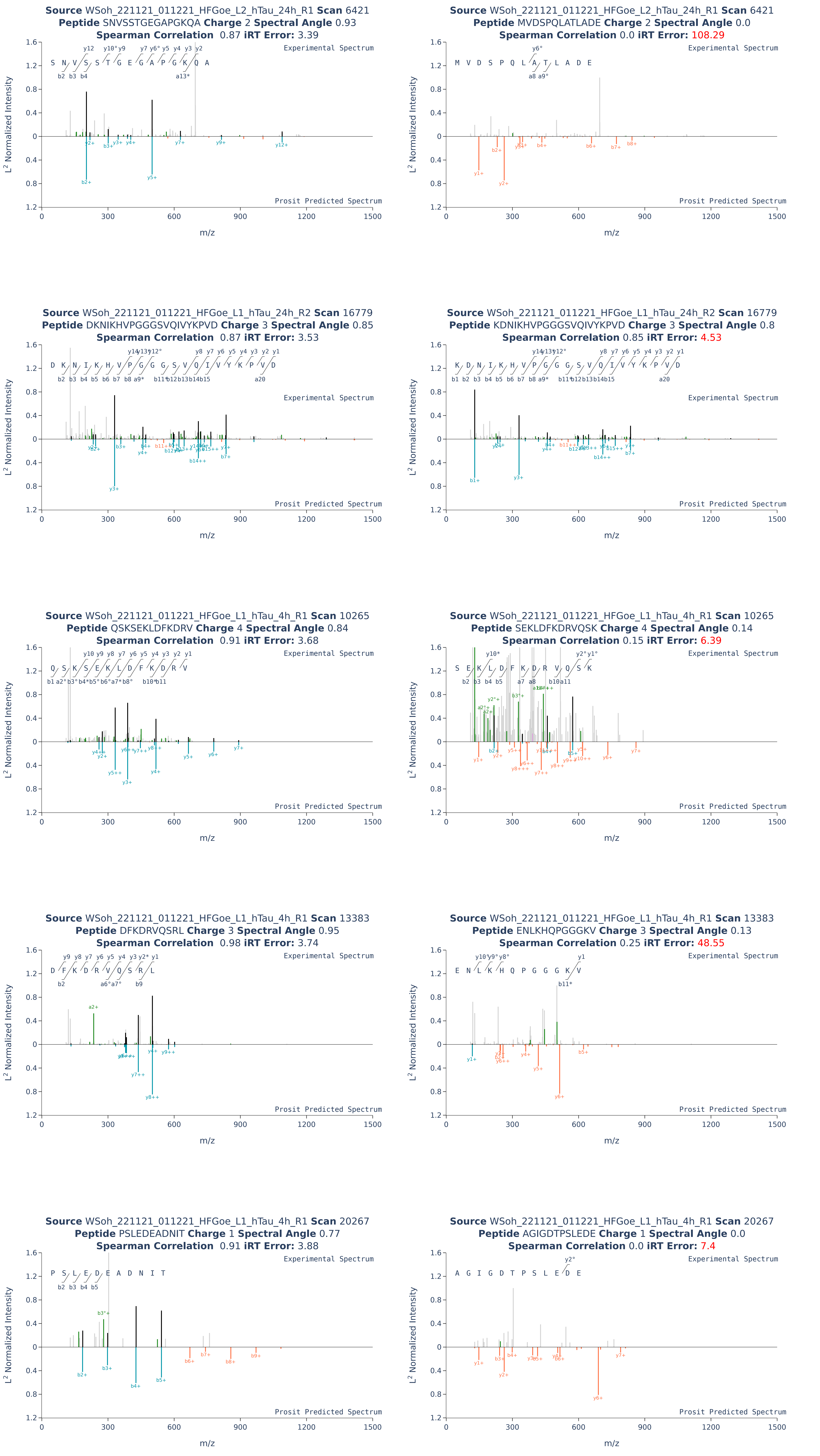
Source WSoh_221121_011221_HFGoe_L2_hTau_24h_R1 Scan 6421
Peptide SNVSSTGEGAPGKQA Charge 2 Spectral Angle 0.93
Spearman Correlation 0.87 iRT Error: 3.39
Source WSoh_221121_011221_HFGoe_L2_hTau_24h_R1 Scan 6421
Peptide MVDSPQLATLADE Charge 2 Spectral Angle 0.0
Spearman Correlation 0.0 iRT Error: 108.29
Source WSoh_221121_011221_HFGoe_L1_hTau_24h_R2 Scan 16779
Peptide DKNIKHVPGGGSVQIVYKPVD Charge 3 Spectral Angle 0.85
Spearman Correlation 0.87 iRT Error: 3.53
Source WSoh_221121_011221_HFGoe_L1_hTau_24h_R2 Scan 16779
Peptide KDNIKHVPGGGSVQIVYKPVD Charge 3 Spectral Angle 0.8
Spearman Correlation 0.85 iRT Error: 4.53
Source WSoh_221121_011221_HFGoe_L1_hTau_4h_R1 Scan 10265
Peptide QSKSEKLDFKDRV Charge 4 Spectral Angle 0.84
Spearman Correlation 0.91 iRT Error: 3.68
Source WSoh_221121_011221_HFGoe_L1_hTau_4h_R1 Scan 10265
Peptide SEKLDFKDRVQSK Charge 4 Spectral Angle 0.14
Spearman Correlation 0.15 iRT Error: 6.39
Source WSoh_221121_011221_HFGoe_L1_hTau_4h_R1 Scan 13383
Peptide DFKDRVQSRL Charge 3 Spectral Angle 0.95
Spearman Correlation 0.98 iRT Error: 3.74
Source WSoh_221121_011221_HFGoe_L1_hTau_4h_R1 Scan 13383
Peptide ENLKHQPGGGKV Charge 3 Spectral Angle 0.13
Spearman Correlation 0.25 iRT Error: 48.55
Source WSoh_221121_011221_HFGoe_L1_hTau_4h_R1 Scan 20267
Peptide PSLEDEADNIT Charge 1 Spectral Angle 0.77
Spearman Correlation 0.91 iRT Error: 3.88
Source WSoh_221121_011221_HFGoe_L1_hTau_4h_R1 Scan 20267
Peptide AGIGDTPSLEDE Charge 1 Spectral Angle 0.0
Spearman Correlation 0.0 iRT Error: 7.4
Experimental Spectrum
Prosit Predicted Spectrum
L² Normalized Intensity
m/z

Source WSoh_221121_011221_HFGoe_L2_hTau_24h_R1 **Scan** 21928
Peptide VYKPVDLSKVTSKL **Charge** 3 **Spectral Angle** 0.83
Spearman Correlation 0.89 **iRT Error:** 4.05

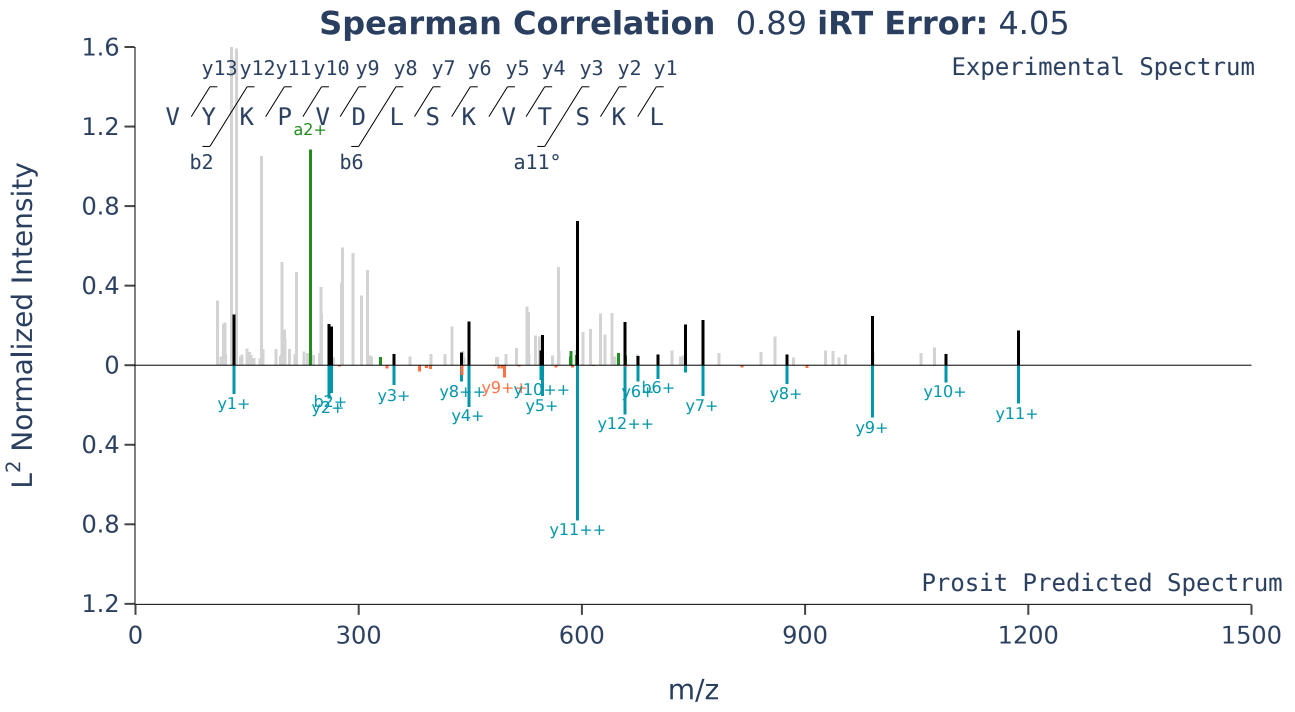

Source WSoh_221121_011221_HFGoe_L2_hTau_24h_R1 **Scan** 21928
Peptide IVYKPVDLSKVTSK **Charge** 3 **Spectral Angle** 0.37
Spearman Correlation 0.32 **iRT Error:** 27.35

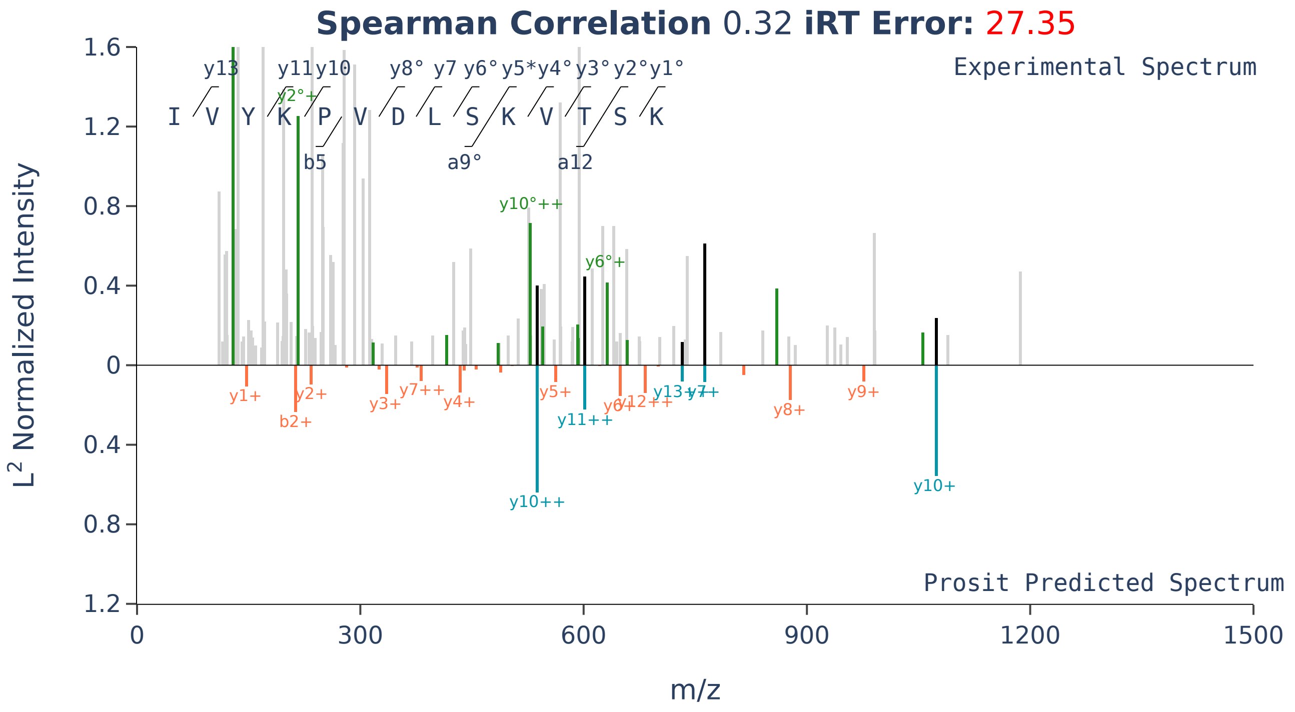

Source WSoh_221121_011221_HFGoe_L1_hTau_2h_R1 **Scan** 9482
Peptide QSKLTFREN **Charge** 3 **Spectral Angle** 0.9
Spearman Correlation 0.85 **iRT Error:** 4.17

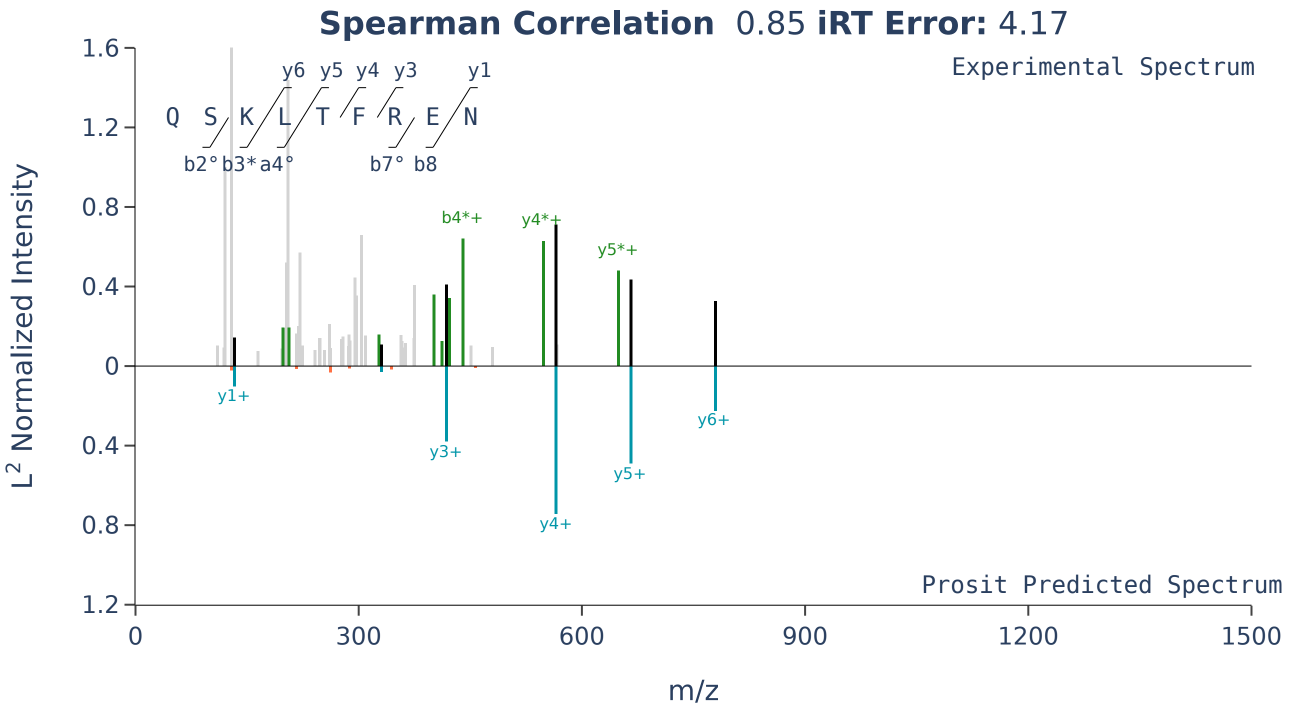

Source WSoh_221121_011221_HFGoe_L1_hTau_2h_R1 **Scan** 9482
Peptide DFKDRVQSK **Charge** 3 **Spectral Angle** 0.0
Spearman Correlation 0.0 **iRT Error:** 6.78

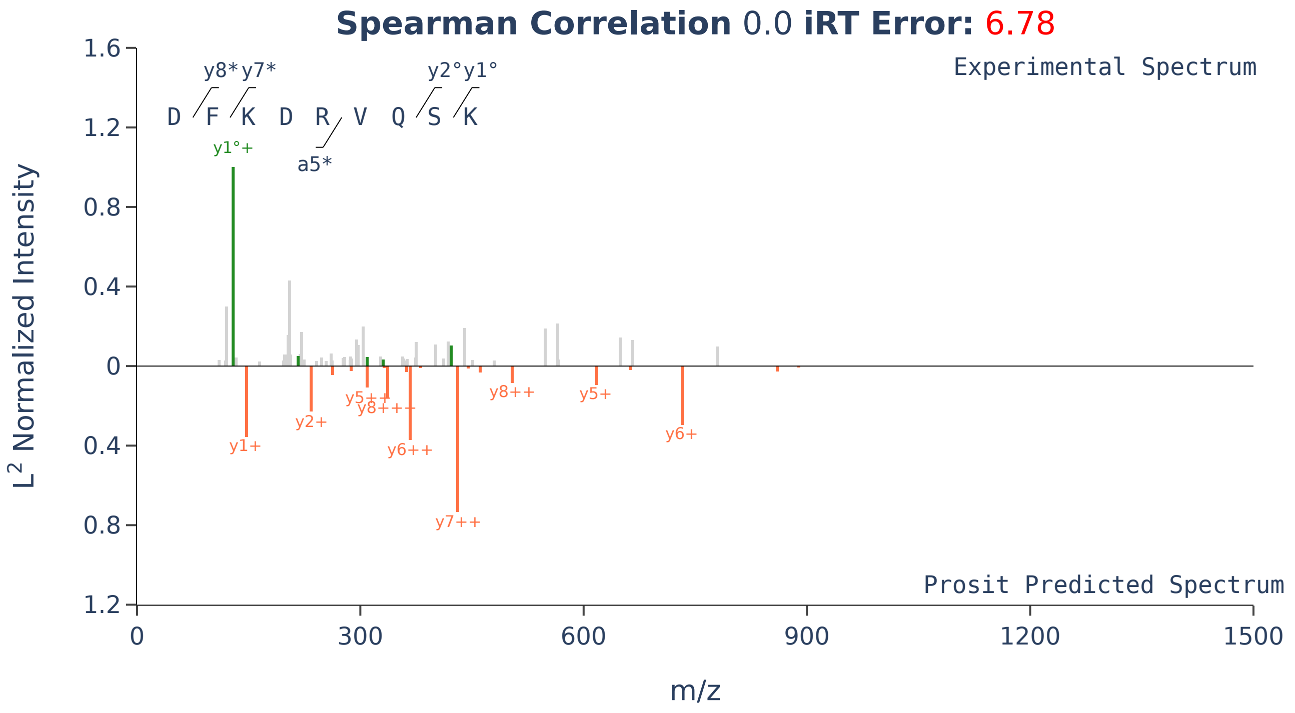

Source WSoh_221121_011221_HFGoe_L1_hTau_4h_R2 **Scan** 12211
Peptide DNITHVPGGGNKKIETHSL **Charge** 3 **Spectral Angle** 0.86
Spearman Correlation 0.87 **iRT Error:** 4.21

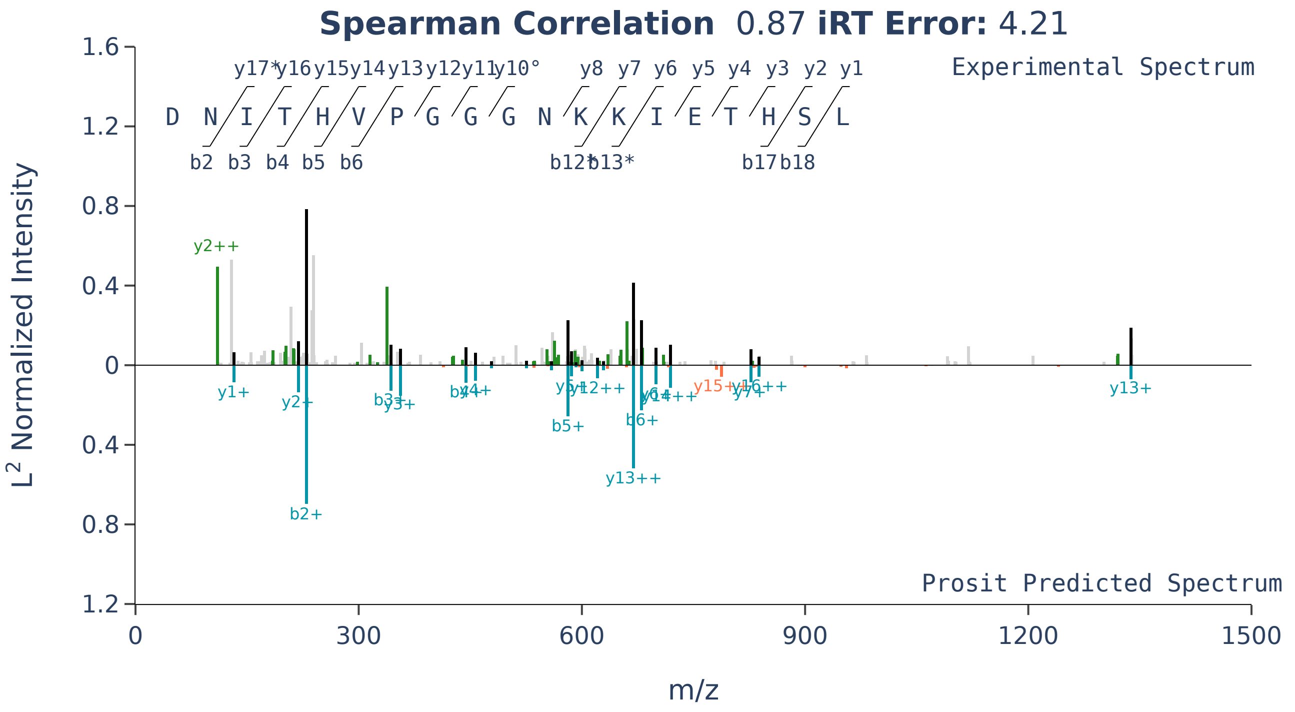

Source WSoh_221121_011221_HFGoe_L1_hTau_4h_R2 **Scan** 12211
Peptide RENAKAKTDHGAEIVYKS **Charge** 3 **Spectral Angle** 0.05
Spearman Correlation 0.17 **iRT Error:** 32.77

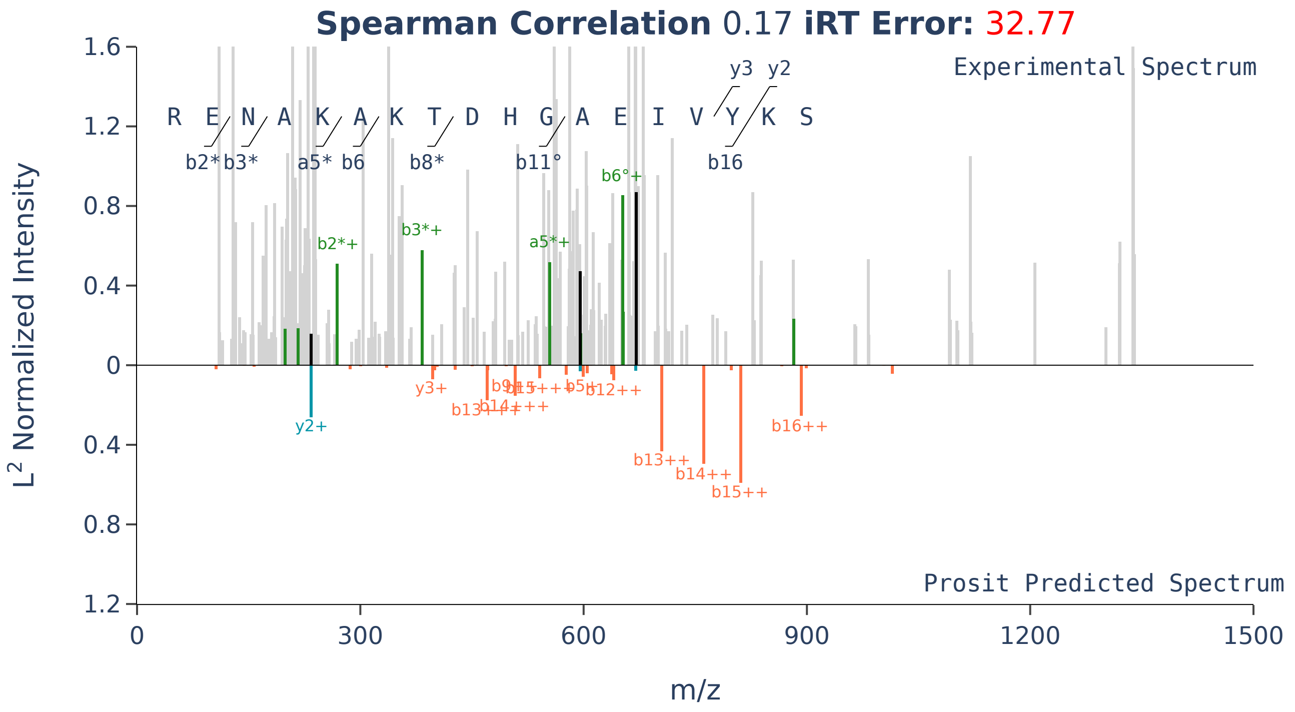

Source WSoh_221121_011221_HFGoe_L2_hTau_24h_R2 **Scan** 22704
Peptide LQTAPVPMPDLKNVKN **Charge** 3 **Spectral Angle** 0.88
Spearman Correlation 0.91 **iRT Error:** 4.31

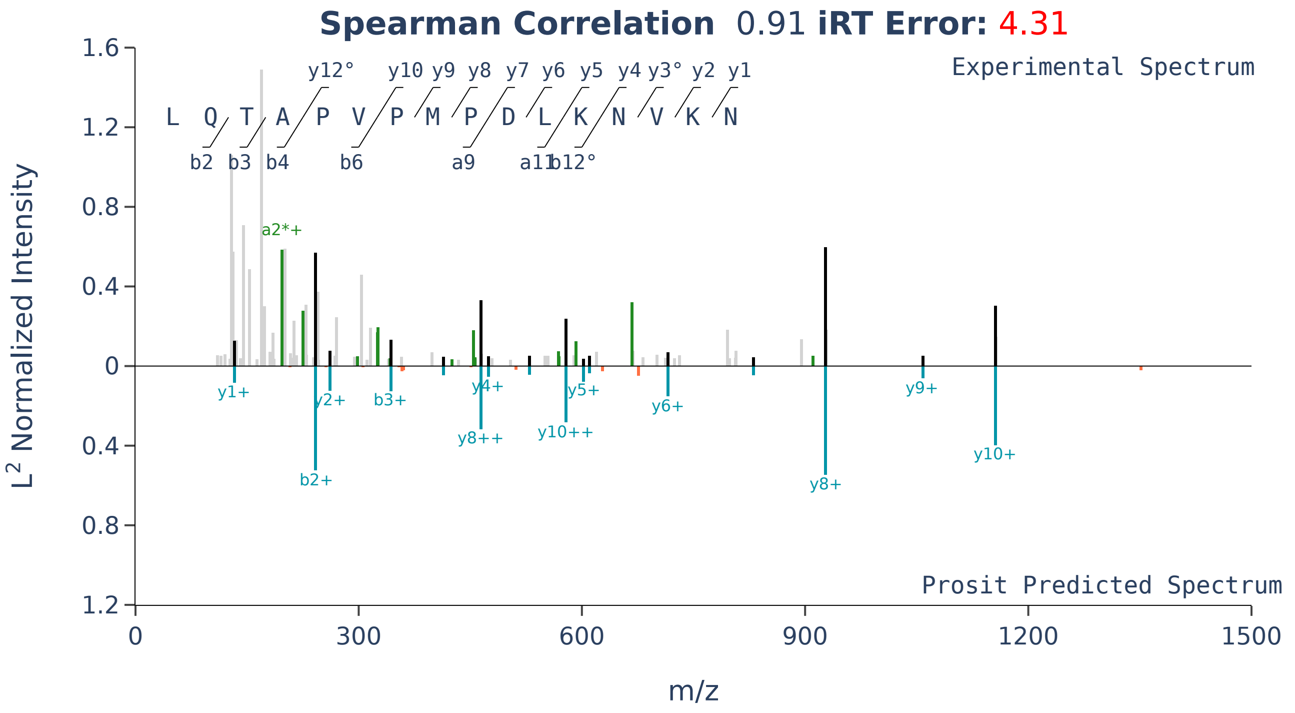

Source WSoh_221121_011221_HFGoe_L2_hTau_24h_R2 **Scan** 22704
Peptide HVPGGGSVQIVYKPVDL **Charge** 3 **Spectral Angle** 0.06
Spearman Correlation 0.11 **iRT Error:** 5.45

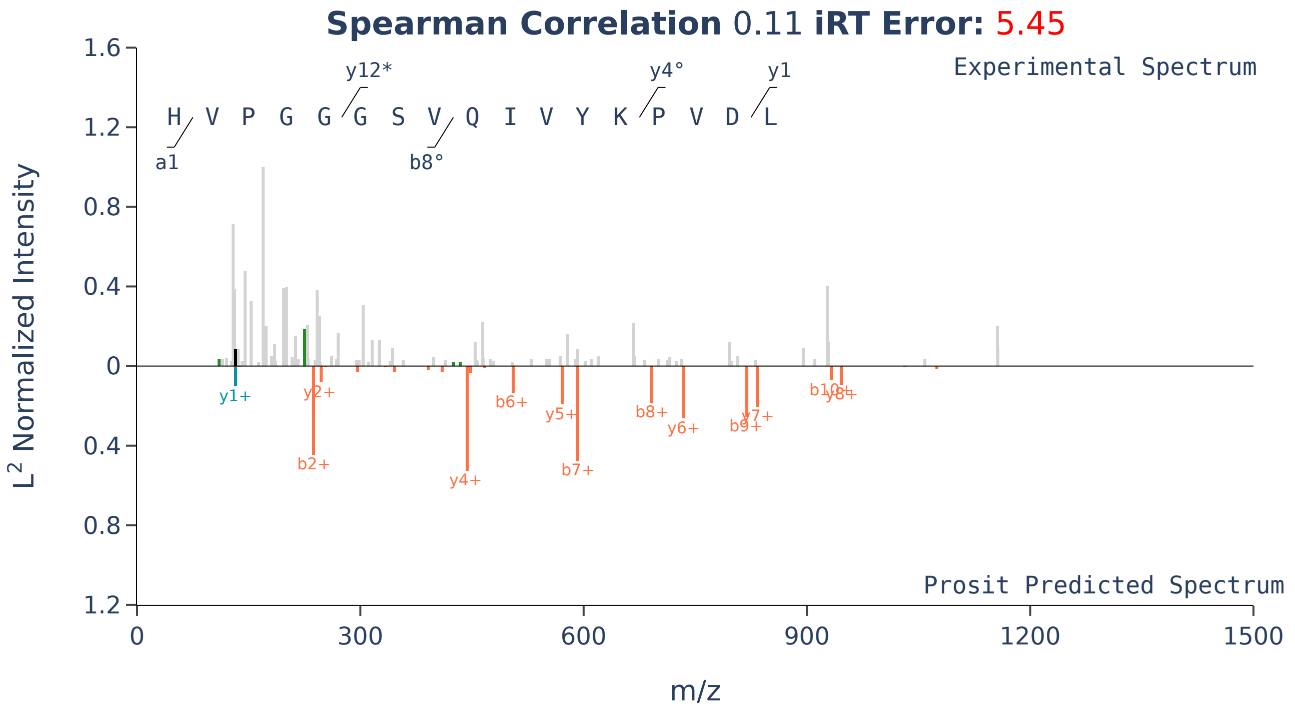

Source WSoh_221121_011221_HFGoe_L1_hTau_24h_R2 **Scan** 15758
Peptide EDTPSLPTPPTREPKKV **Charge** 4 **Spectral Angle** 0.87
Spearman Correlation 0.86 **iRT Error:** 4.48

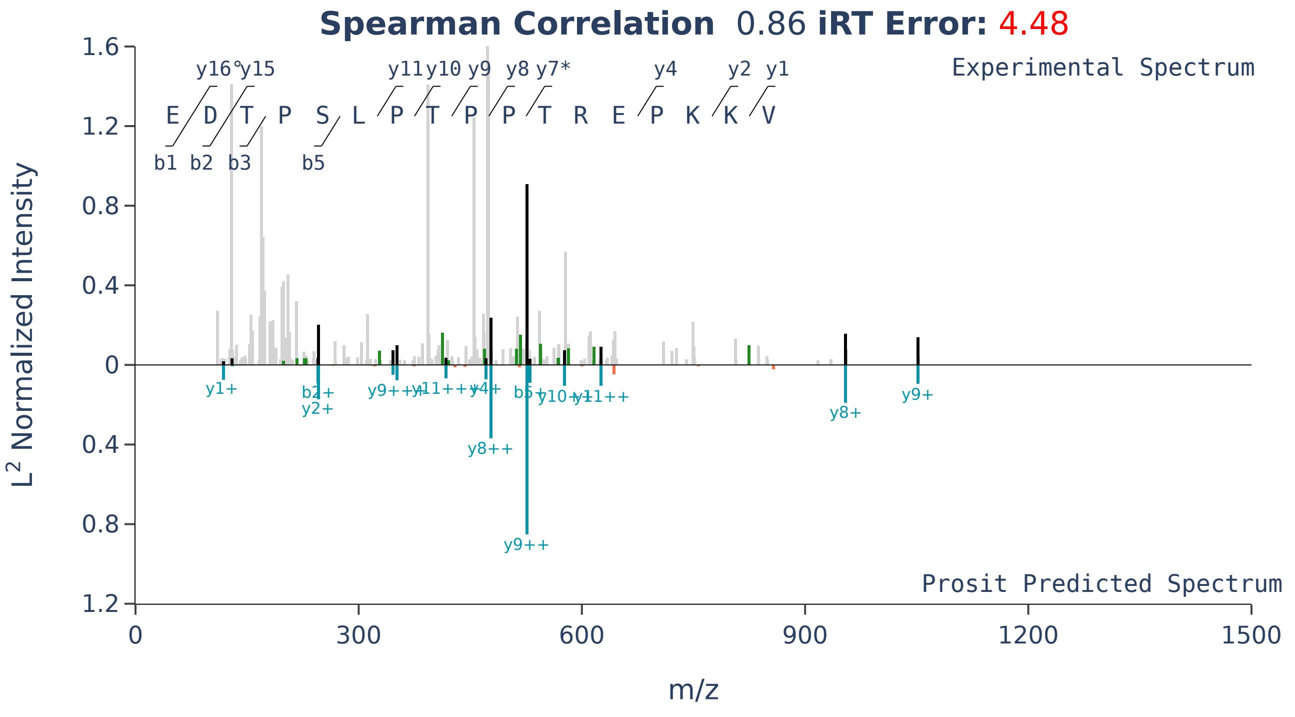

Source WSoh_221121_011221_HFGoe_L1_hTau_24h_R2 **Scan** 15758
Peptide TGSDDKKAKGADGKTKIAT **Charge** 4 **Spectral Angle** 0.0
Spearman Correlation 0.0 **iRT Error:** 70.17

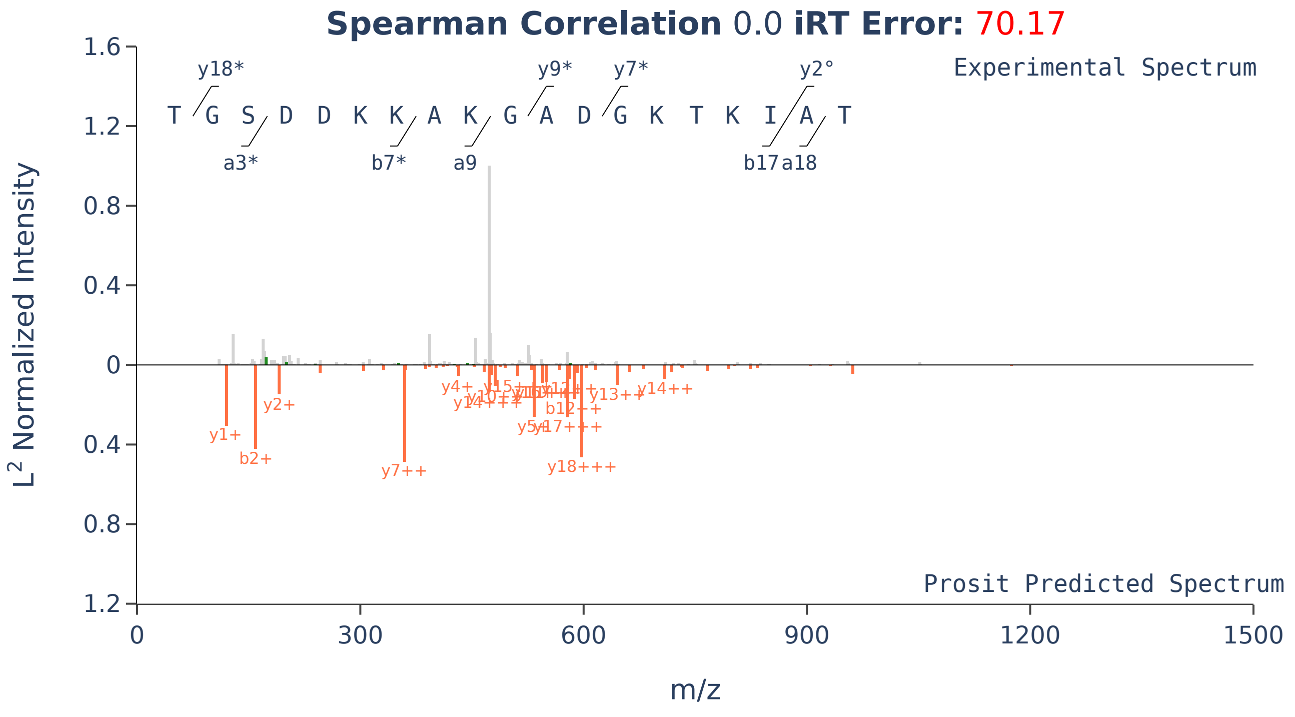

Source WSoh_221121_011221_HFGoe_L1_hTau_4h_R2 **Scan** 14702
Peptide SNVSSKIGSTENL **Charge** 2 **Spectral Angle** 0.92
Spearman Correlation 0.85 **iRT Error:** 4.57

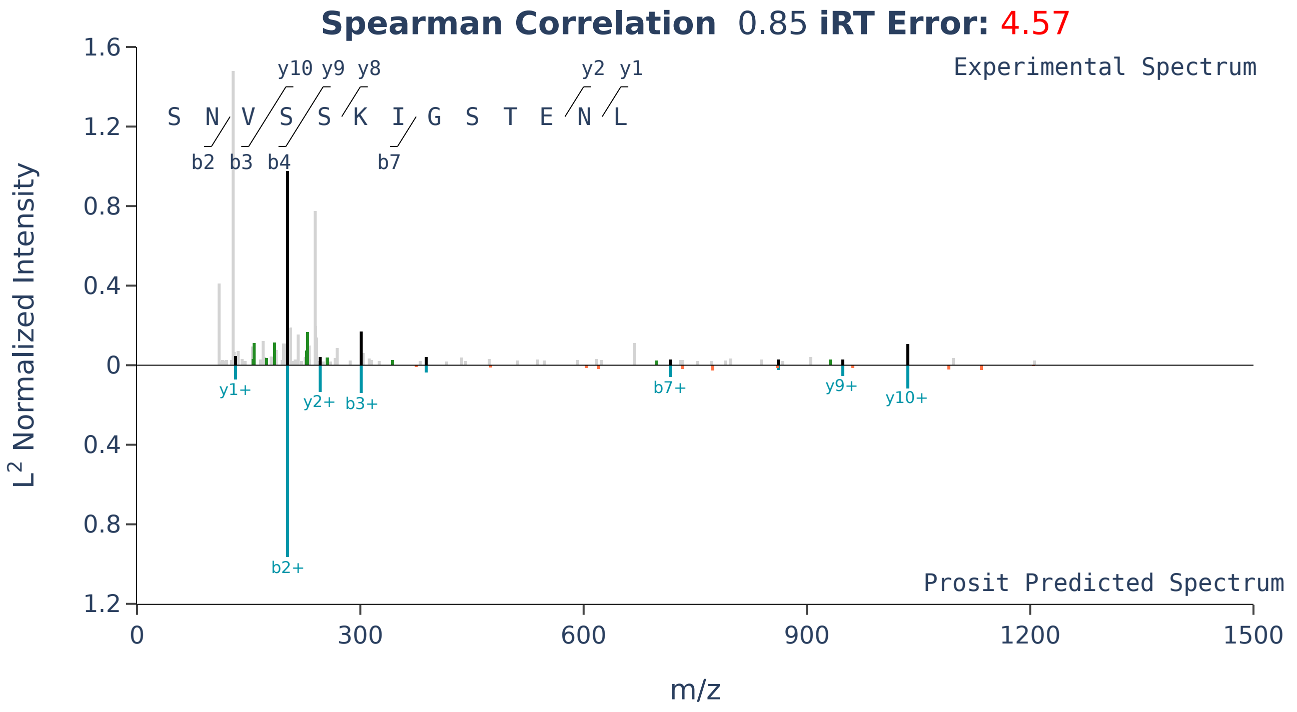

Source WSoh_221121_011221_HFGoe_L1_hTau_4h_R2 **Scan** 14702
Peptide VYKSPVVSGDTSP **Charge** 2 **Spectral Angle** 0.0
Spearman Correlation 0.0 **iRT Error:** 1.17

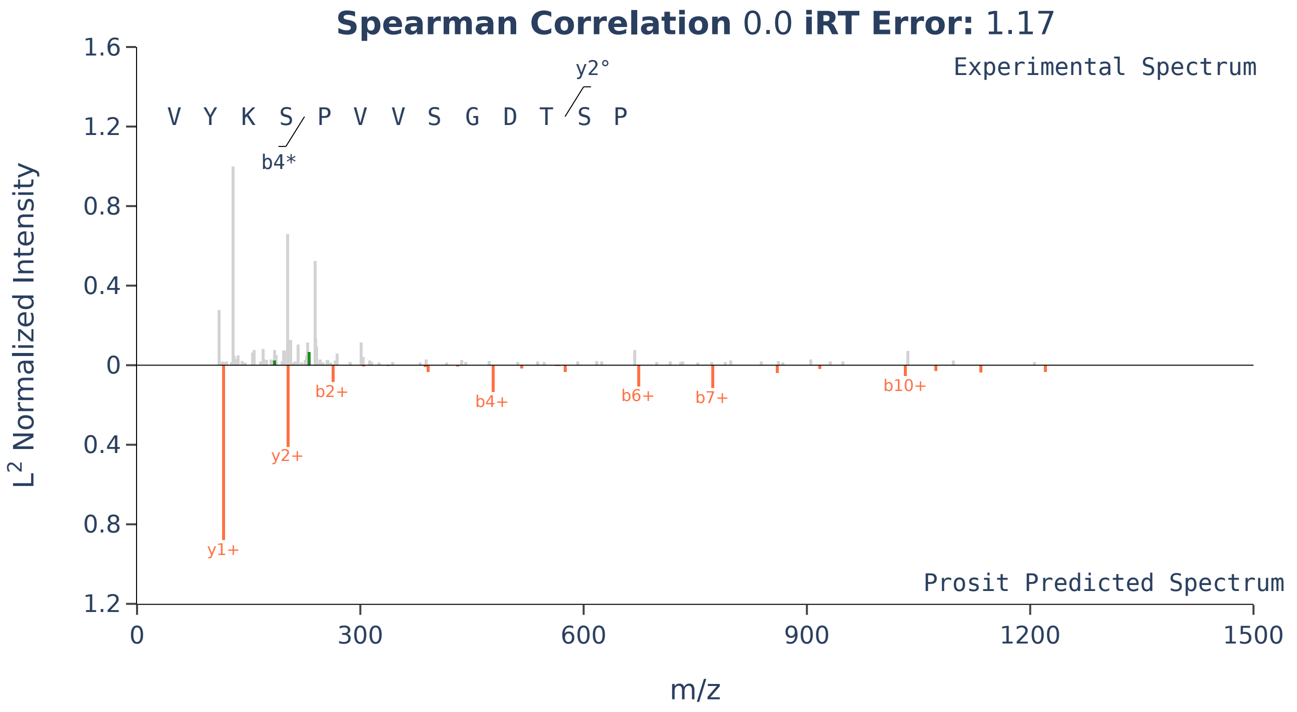

Source WSoh_221121_011221_HFGoe_L1_hTau_24h_R2 **Scan** 9992
Peptide VSLGNIHHKPGGGQVE **Charge** 2 **Spectral Angle** 0.88
Spearman Correlation 0.93 **iRT Error:** 4.71

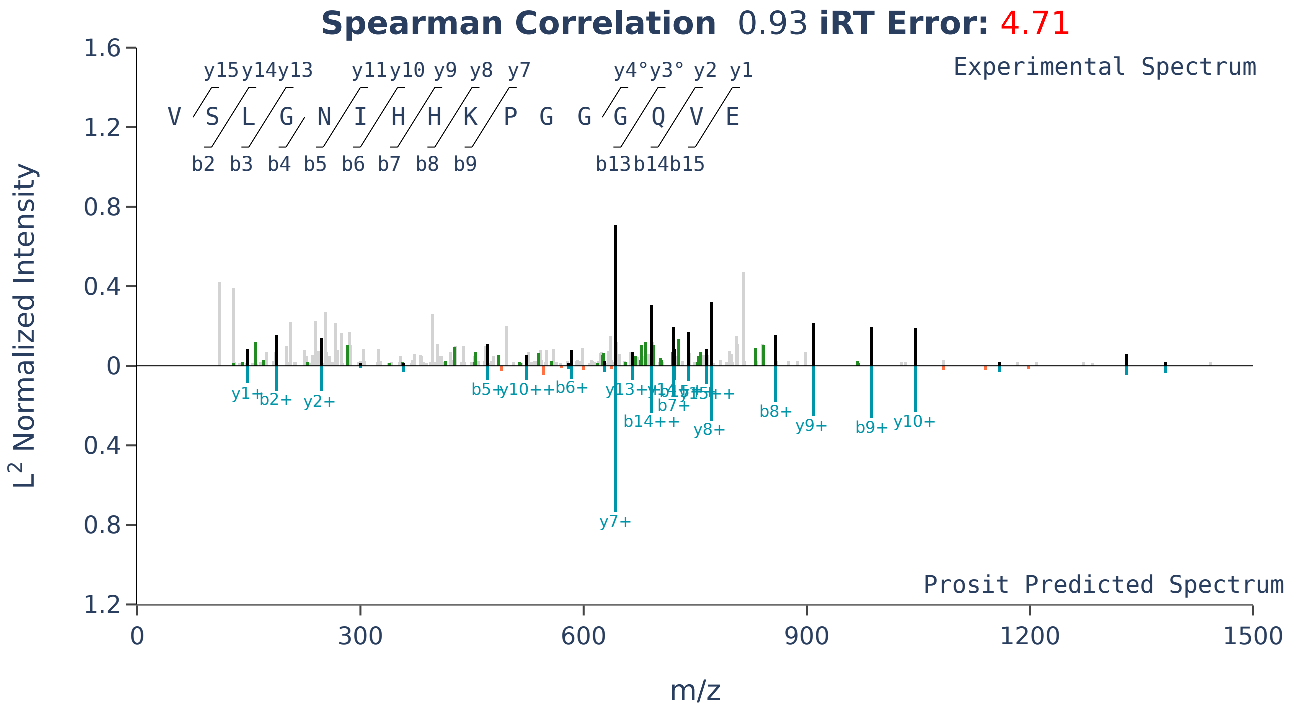

Source WSoh_221121_011221_HFGoe_L1_hTau_24h_R2 **Scan** 9992
Peptide SLGNIHHKPGGGQVEV **Charge** 2 **Spectral Angle** 0.36
Spearman Correlation 0.58 **iRT Error:** 5.48

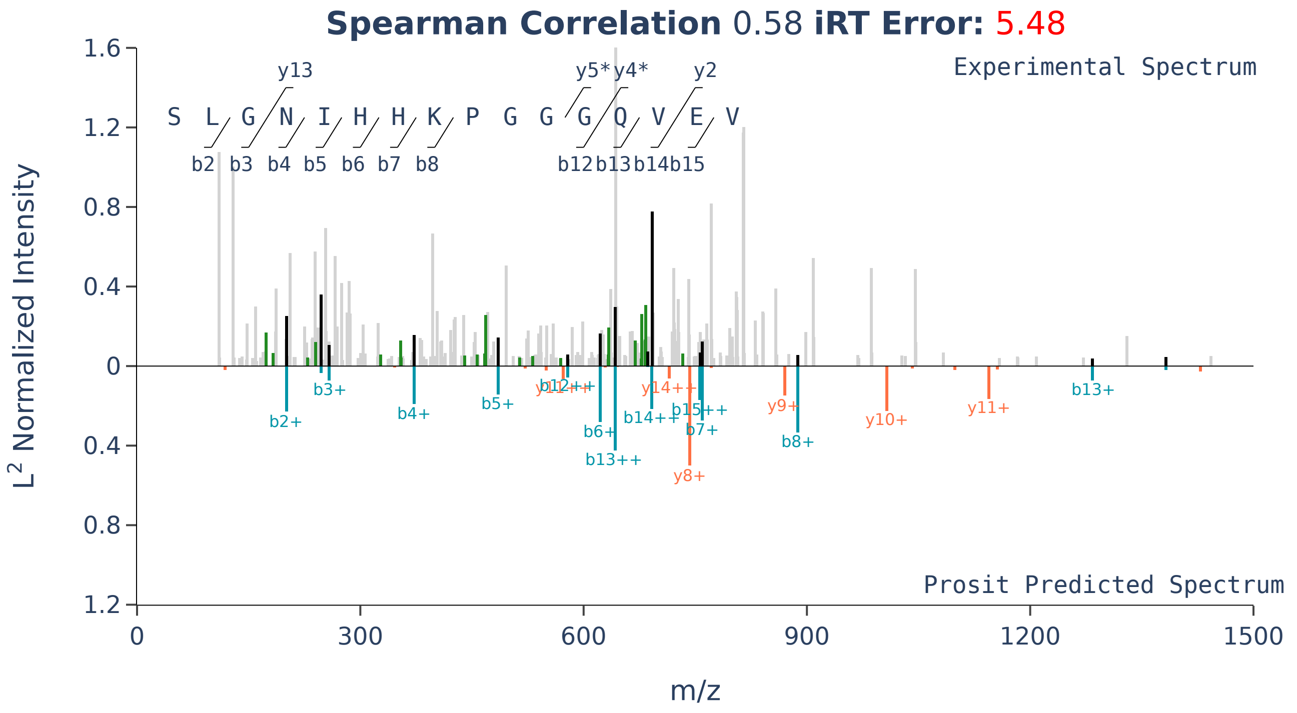

Source WSoh_221121_011221_HFGoe_L2_hTau_24h_R2 **Scan** 6502
Peptide ITHVPGGGNKKIETHGG **Charge** 3 **Spectral Angle** 0.91
Spearman Correlation 0.87 **iRT Error:** 4.92

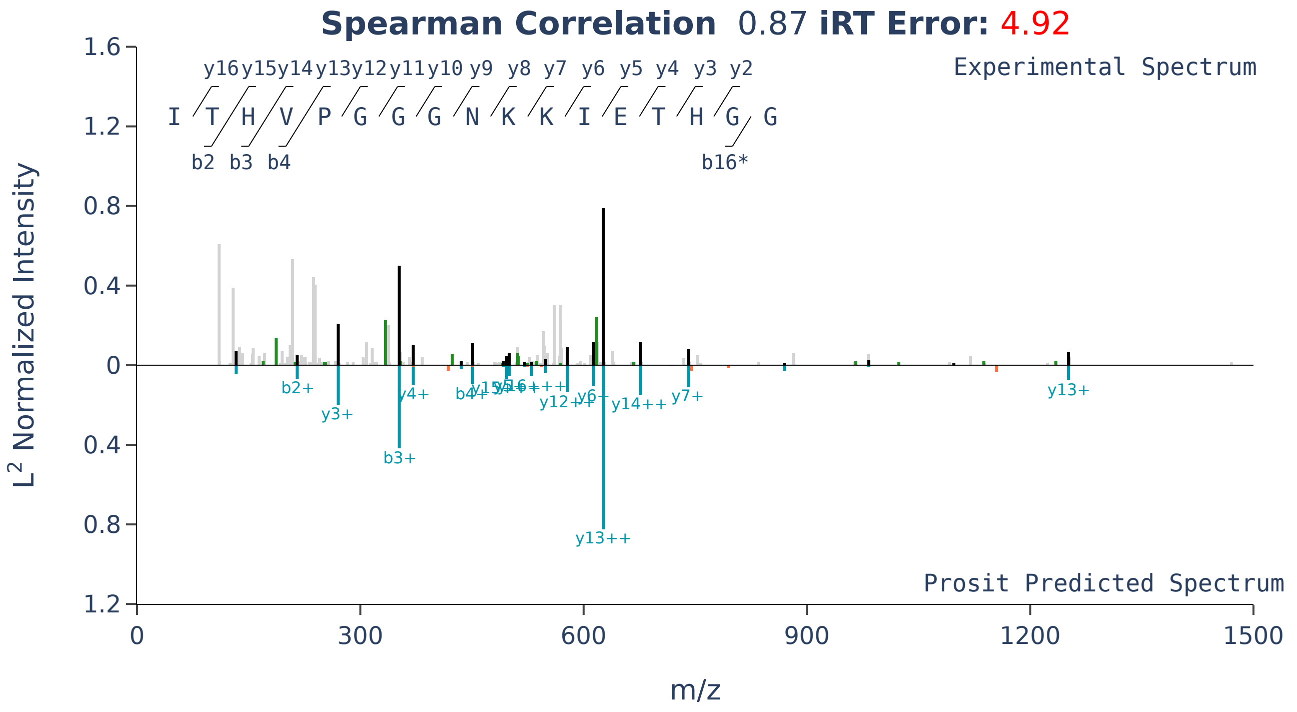

Source WSoh_221121_011221_HFGoe_L2_hTau_24h_R2 **Scan** 6502
Peptide NITHVPGGGNKKIETH **Charge** 3 **Spectral Angle** 0.65
Spearman Correlation 0.29 **iRT Error:** 1.83

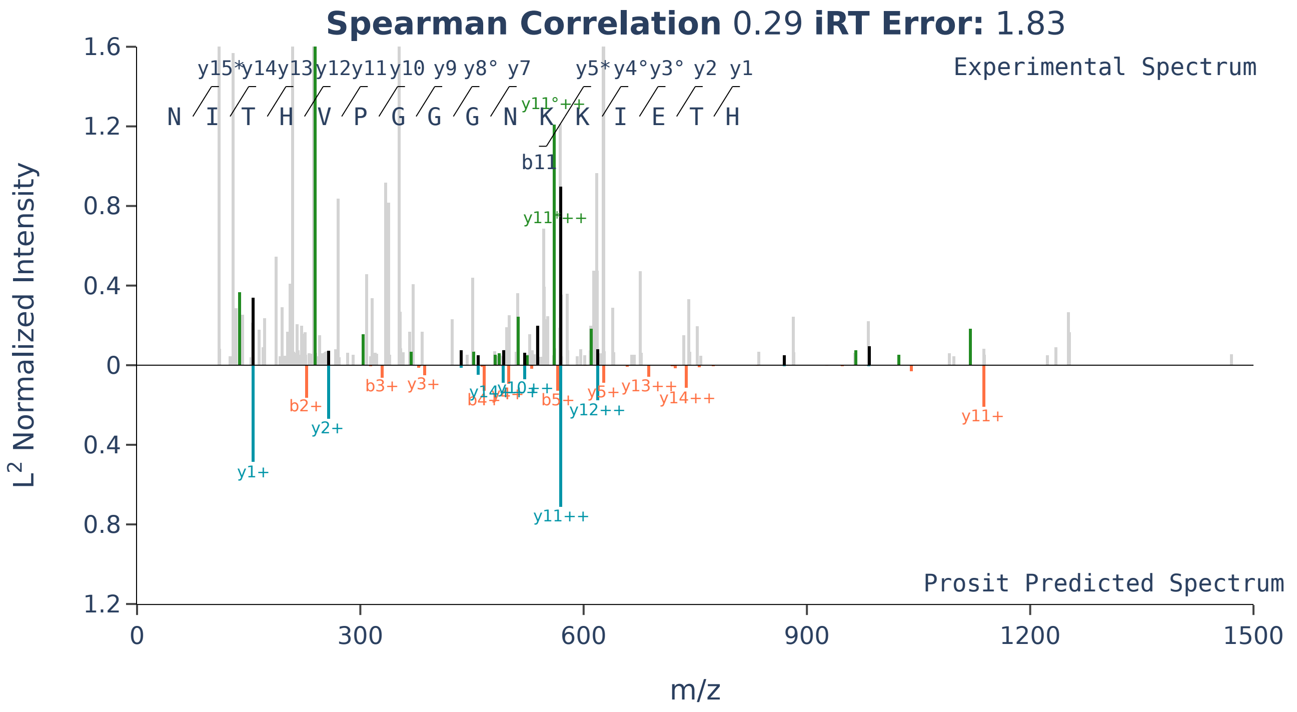

Source WSoh_221121_011221_HFGoe_L1_hTau_24h_R1 **Scan** 19785
Peptide PGGGNVQIVYKPVD **Charge** 2 **Spectral Angle** 0.93
Spearman Correlation 0.97 **iRT Error:** 4.97

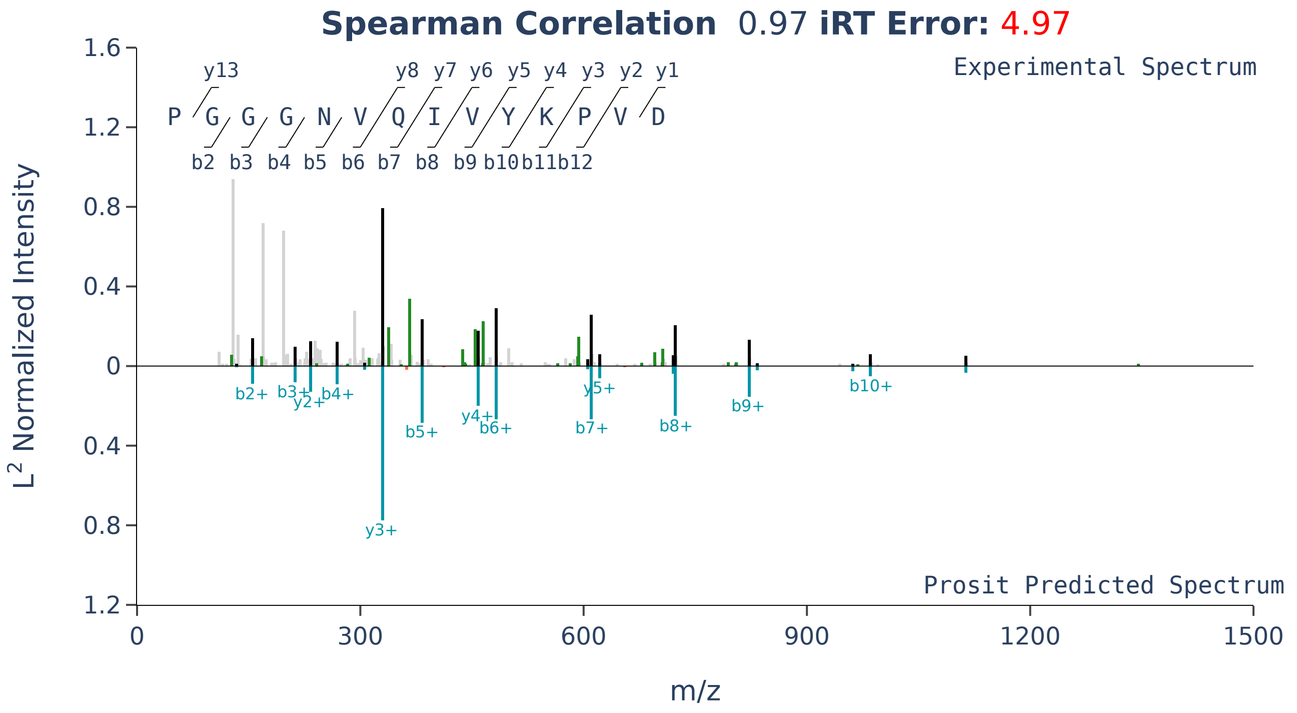

Source WSoh_221121_011221_HFGoe_L1_hTau_24h_R1 **Scan** 19785
Peptide HGAEIVYKSPVVSG **Charge** 2 **Spectral Angle** 0.11
Spearman Correlation 0.32 **iRT Error:** 19.92

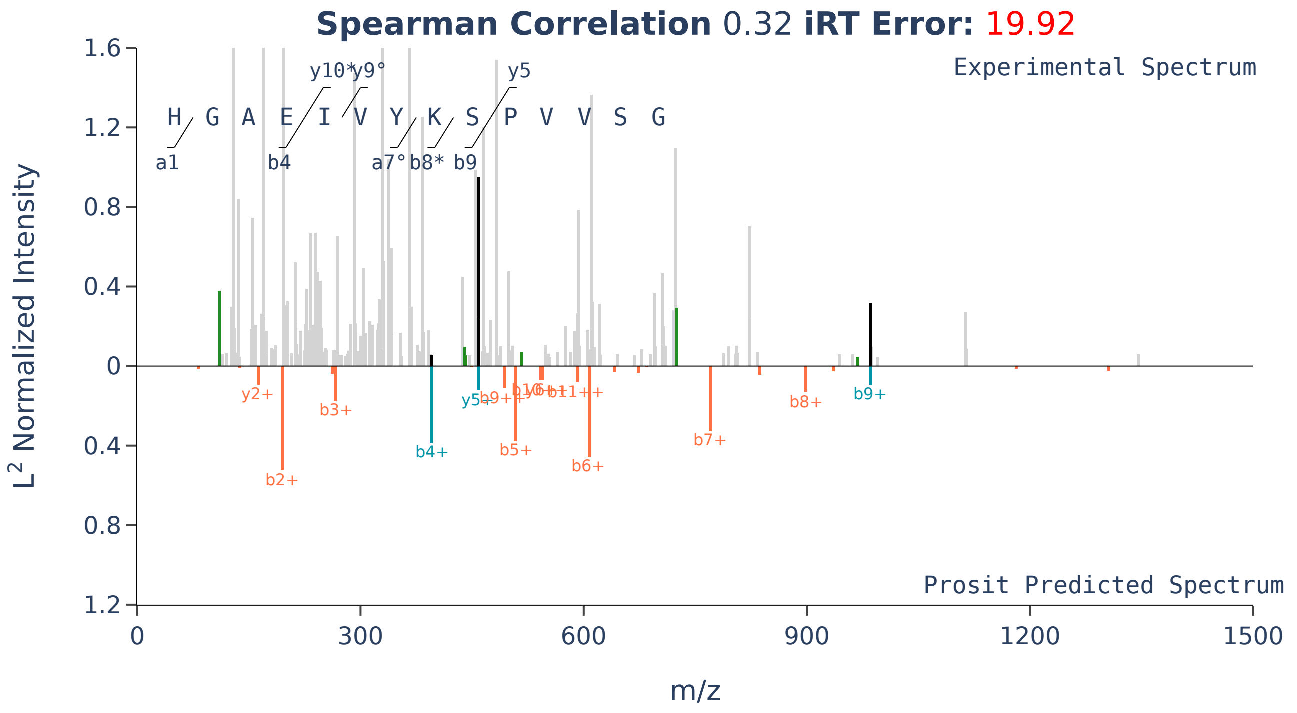

Source WSoh_221121_011221_HFGoe_L1_hTau_24h_R1 **Scan** 13985
Peptide SNVQSKHQPGGGKVQIINKKLDL **Charge** 3 **Spectral Angle** 0.86
Spearman Correlation 0.87 **iRT Error:** 5.02

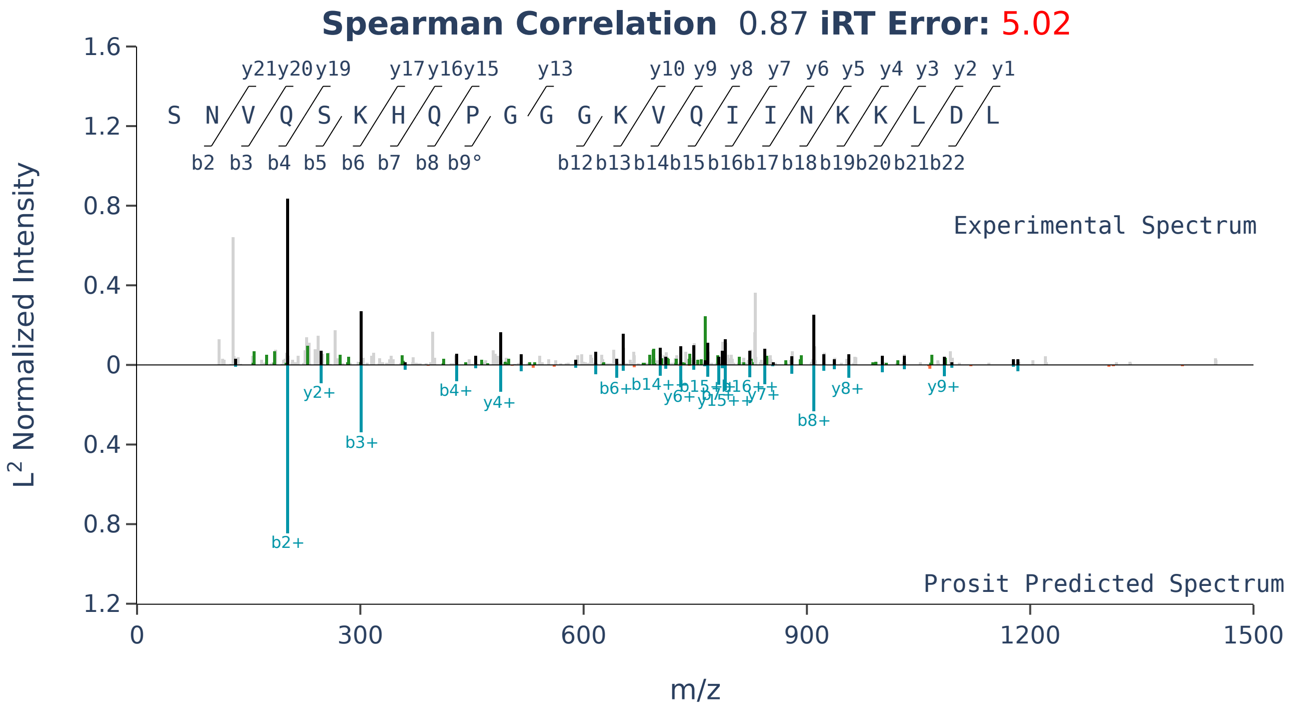

Source WSoh_221121_011221_HFGoe_L1_hTau_24h_R1 **Scan** 13985
Peptide KHQPGGGKVQIINKKLDLSNVQS **Charge** 3 **Spectral Angle** 0.62
Spearman Correlation 0.3 **iRT Error:** 2.82

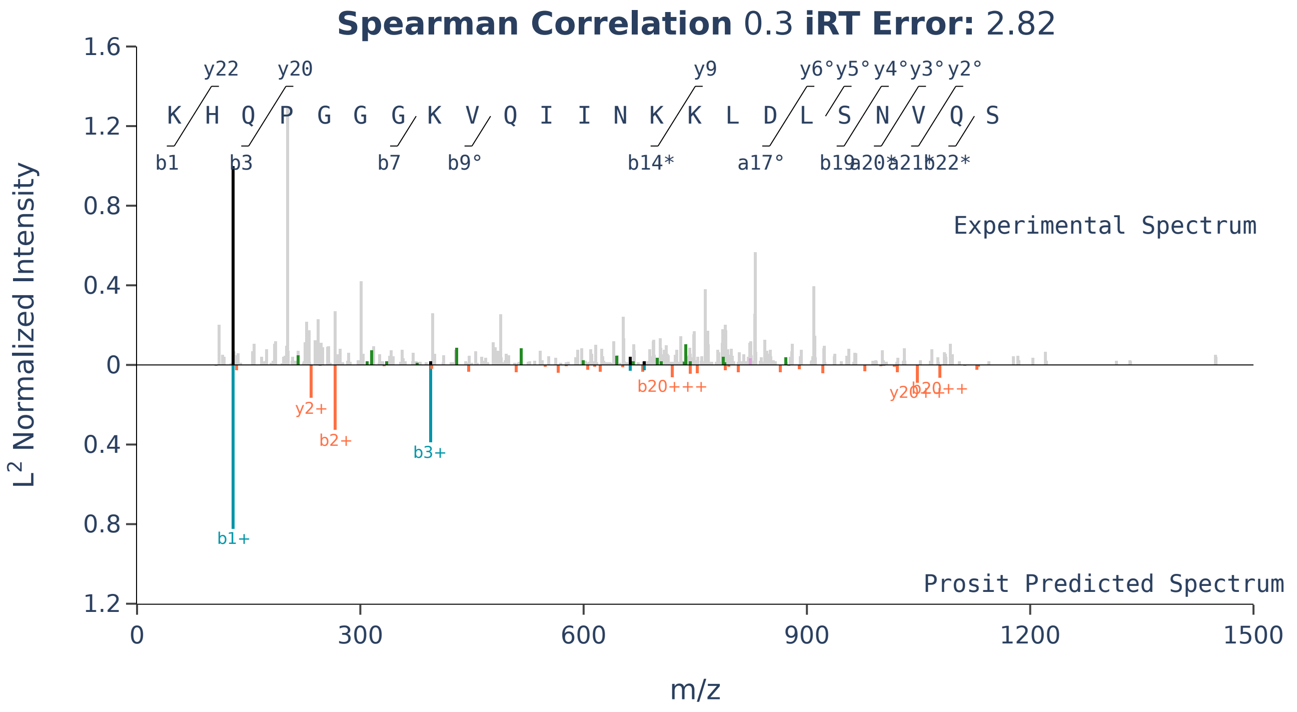

Source WSoh_221121_011221_HFGoe_L1_hTau_4h_R2 **Scan** 14792
Peptide KSVYKPVDL **Charge** 2 **Spectral Angle** 0.75
Spearman Correlation 0.91 **iRT Error:** 5.17

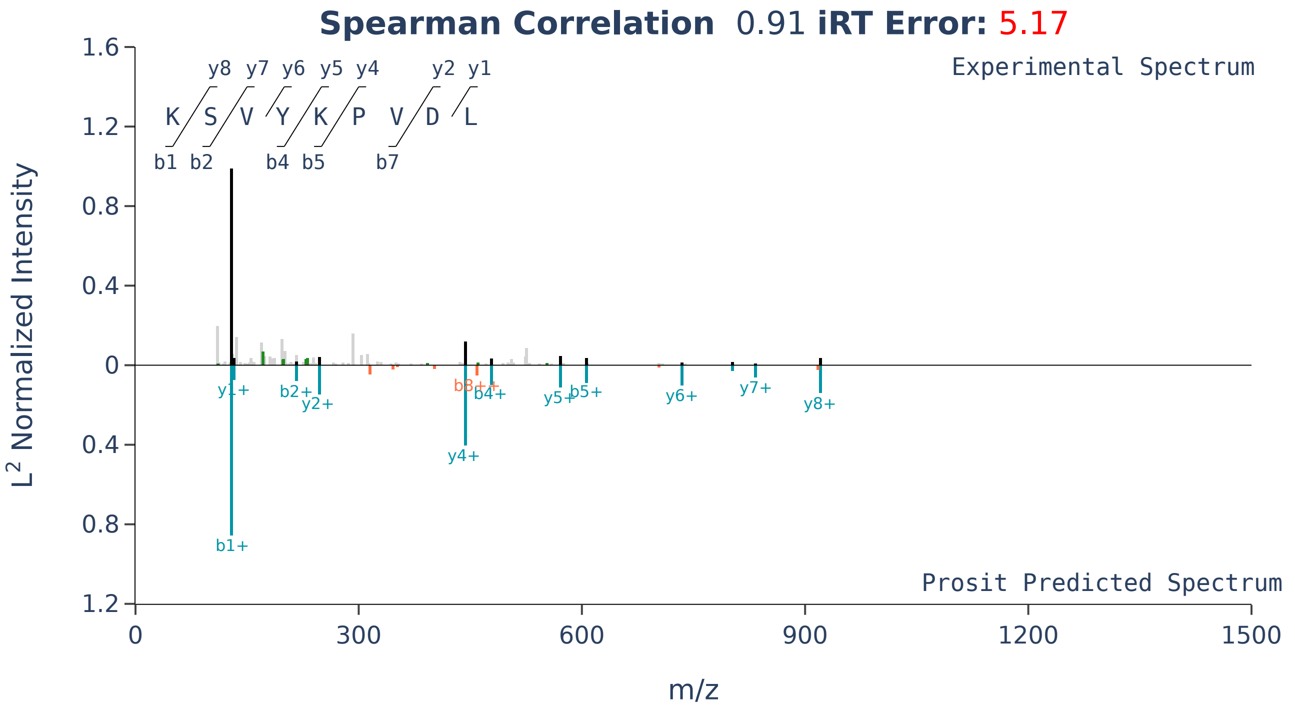

Source WSoh_221121_011221_HFGoe_L1_hTau_4h_R2 **Scan** 14792
Peptide IVYKSPVVSG **Charge** 2 **Spectral Angle** 0.0
Spearman Correlation 0.0 **iRT Error:** 5.7

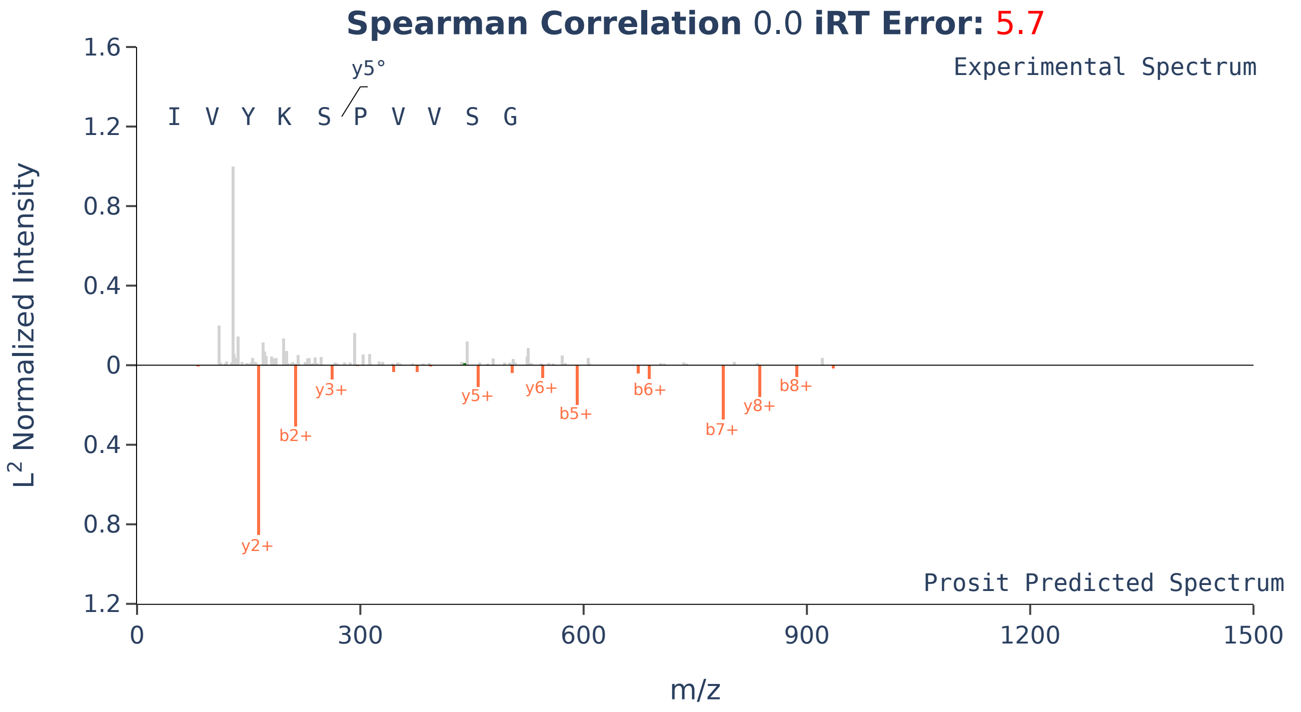

Source WSoh_221121_011221_HFGoe_L2_hTau_24h_R2 **Scan** 24616
Peptide DAAQPHTEIPEGTTAEEAGIGDTPSLE **Charge** 3 **Spectral Angle** 0.9
Spearman Correlation 0.84 **iRT Error:** 5.47

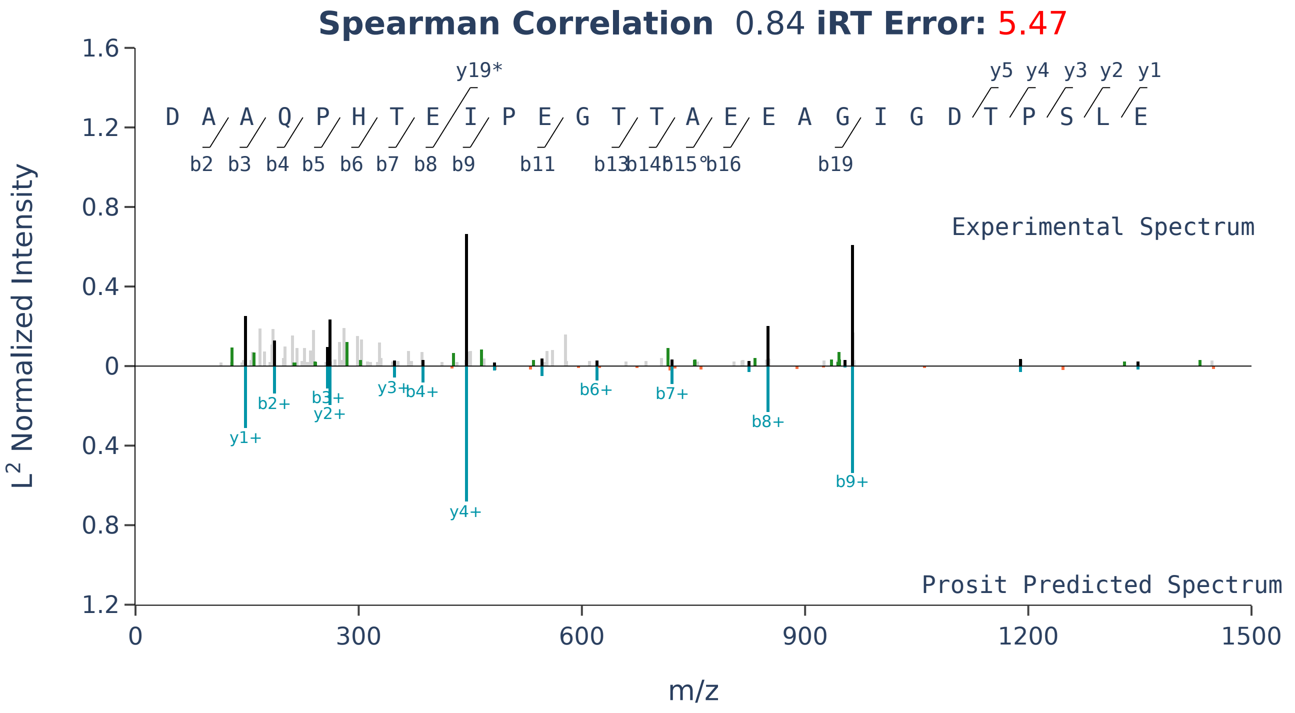

Source WSoh_221121_011221_HFGoe_L2_hTau_24h_R2 **Scan** 24616
Peptide AAQPHTEIPEGTTAEEAGIGDTPSLED **Charge** 3 **Spectral Angle** 0.61
Spearman Correlation 0.38 **iRT Error:** 12.45

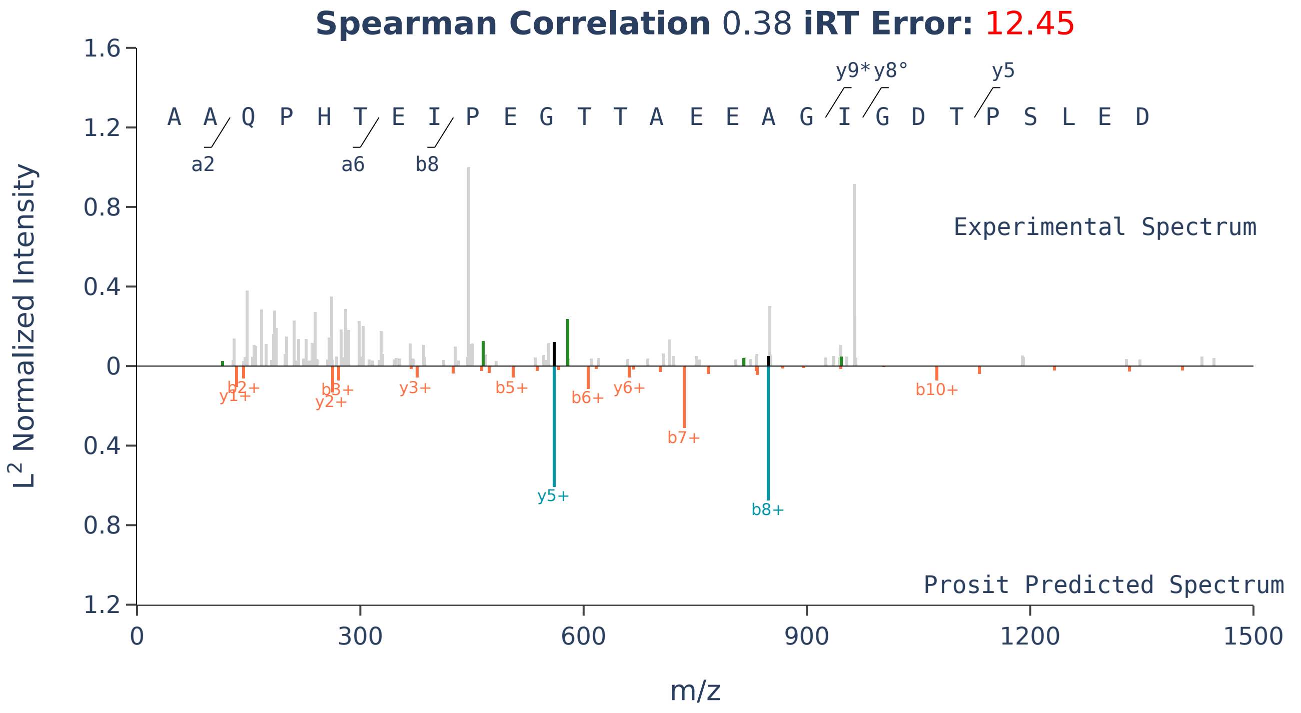

Source WSoh_221121_011221_HFGoe_L1_hTau_24h_R1 **Scan** 22861
Peptide KSQTAPVPMPDLKNV **Charge** 3 **Spectral Angle** 0.86
Spearman Correlation 0.88 **iRT Error:** 5.66

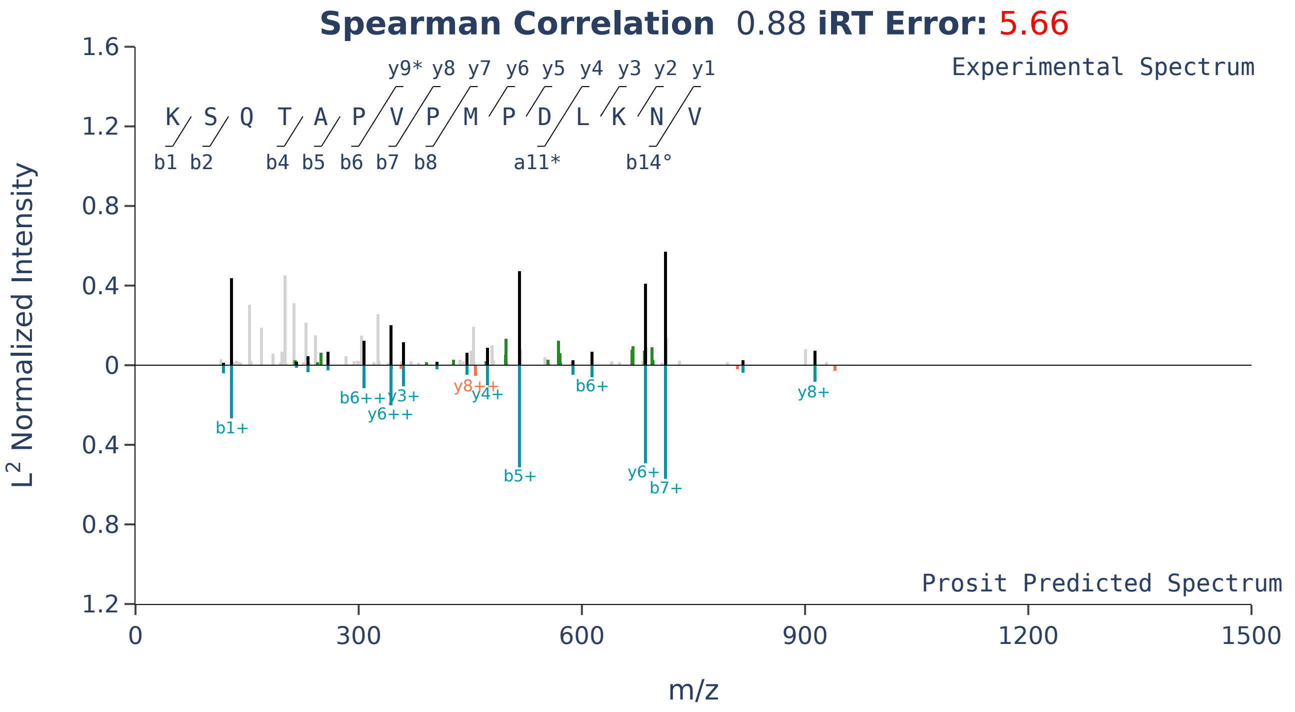

Source WSoh_221121_011221_HFGoe_L1_hTau_24h_R1 **Scan** 22861
Peptide QTAPVPMPDLKNVKS **Charge** 3 **Spectral Angle** 0.18
Spearman Correlation 0.53 **iRT Error:** 14.92

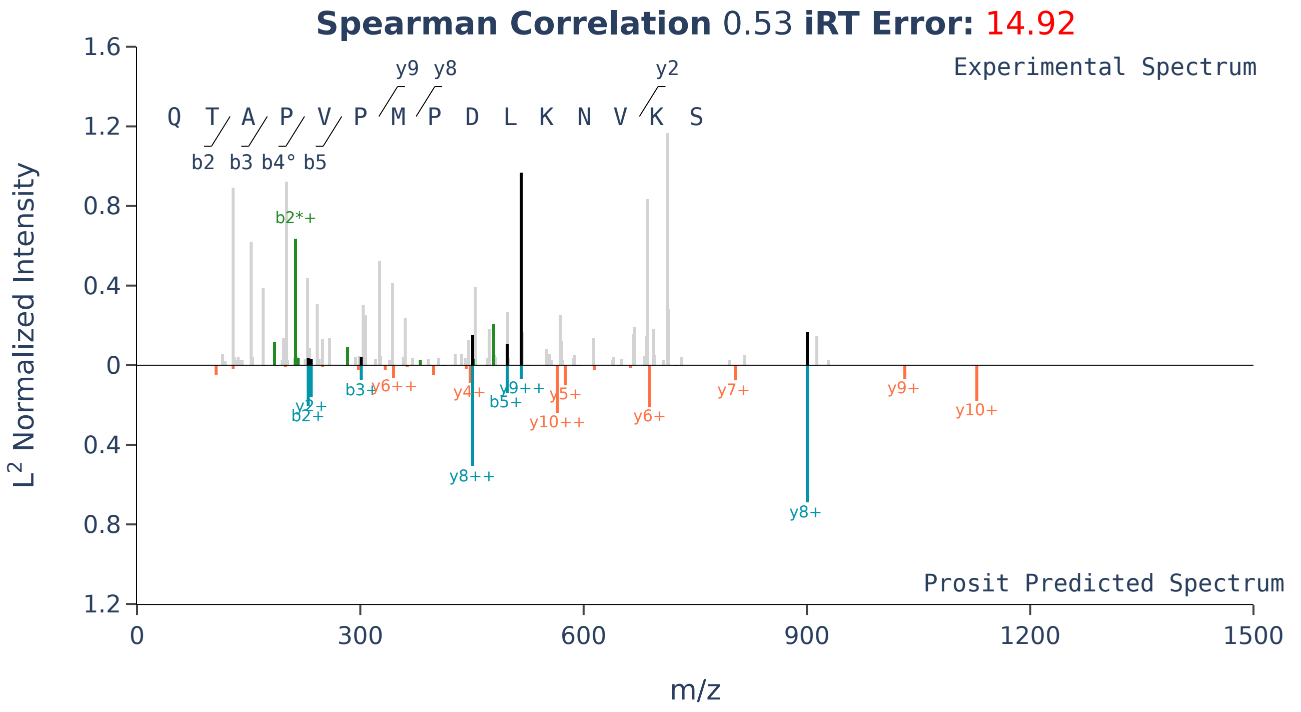

Source WSoh_221121_011221_HFGoe_L2_hTau_4h_R1 **Scan** 9289
Peptide SKPGGGQVEV **Charge** 2 **Spectral Angle** 0.88
Spearman Correlation 0.93 **iRT Error:** 5.86

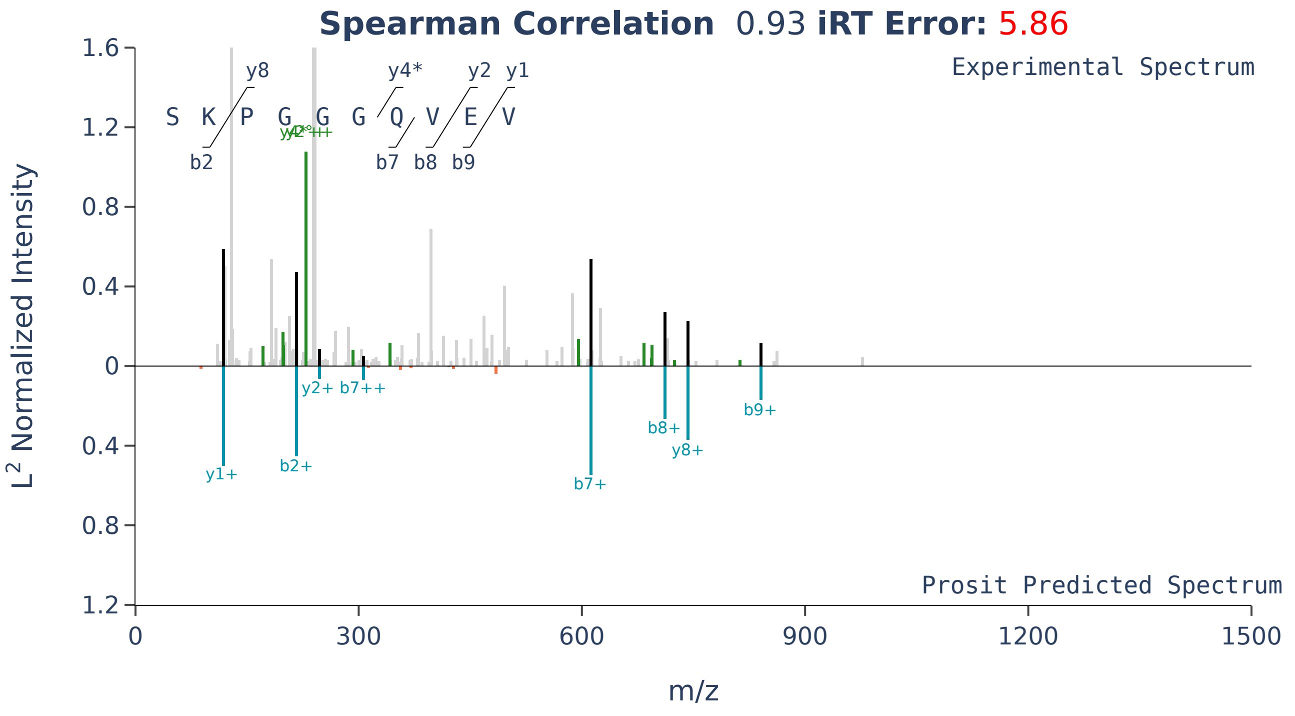

Source WSoh_221121_011221_HFGoe_L2_hTau_4h_R1 **Scan** 9289
Peptide PGGGQVEVKS **Charge** 2 **Spectral Angle** 0.49
Spearman Correlation 0.6 **iRT Error:** 8.0

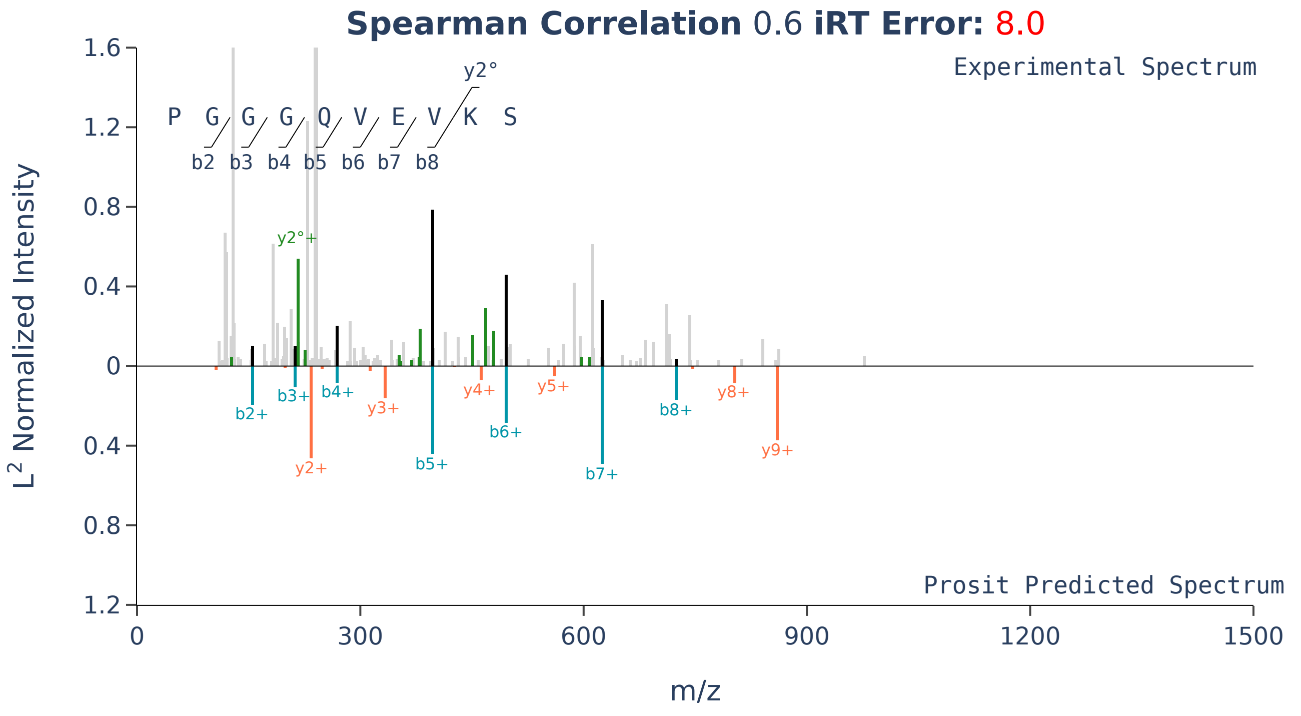

Source WSoh_221121_011221_HFGoe_L2_hTau_4h_R2 **Scan** 11921
Peptide AKIGSTENL **Charge** 2 **Spectral Angle** 0.88
Spearman Correlation 0.95 **iRT Error:** 6.26

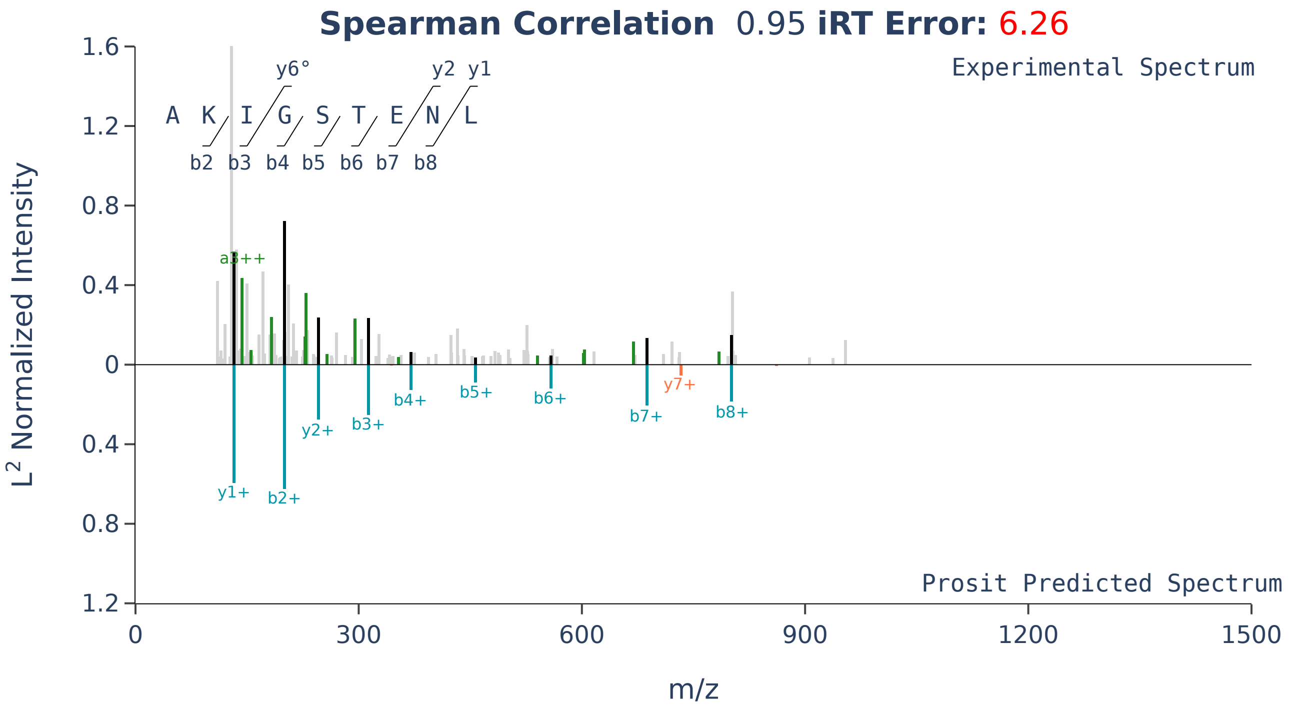

Source WSoh_221121_011221_HFGoe_L2_hTau_4h_R2 **Scan** 11921
Peptide EVSASLAKQ **Charge** 2 **Spectral Angle** 0.11
Spearman Correlation -0.27 **iRT Error:** 12.0

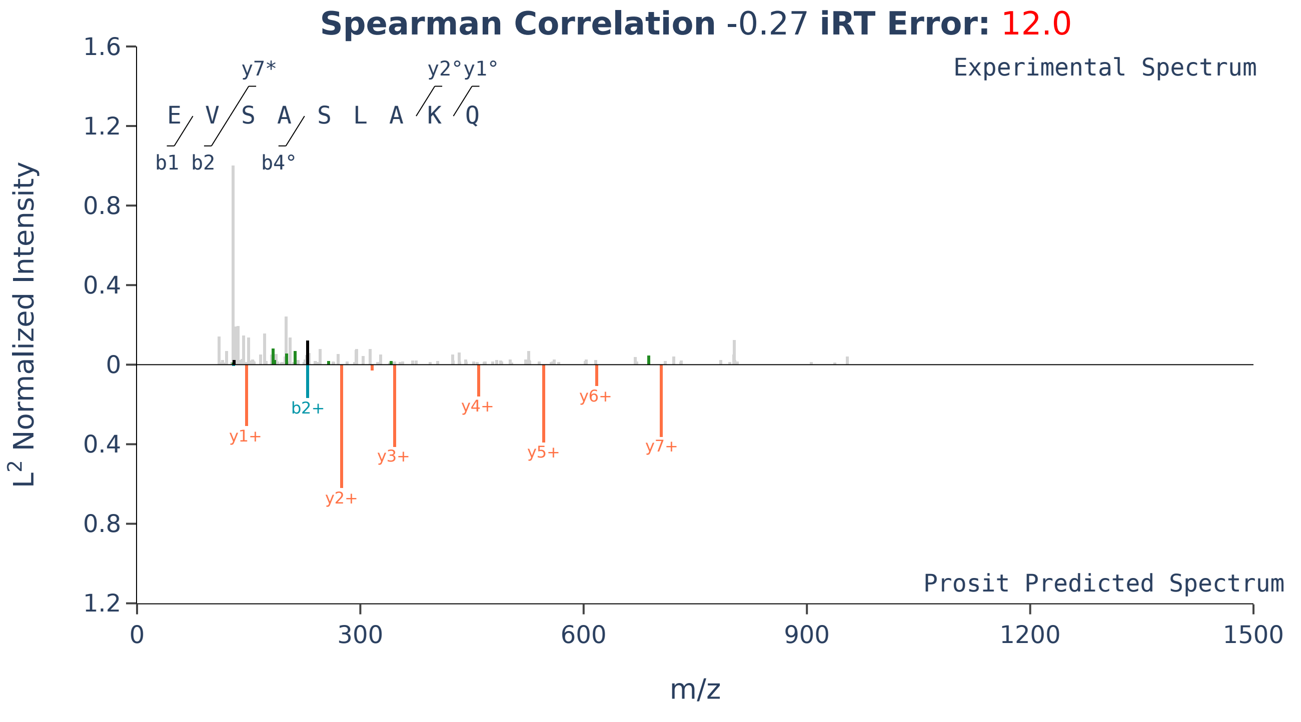

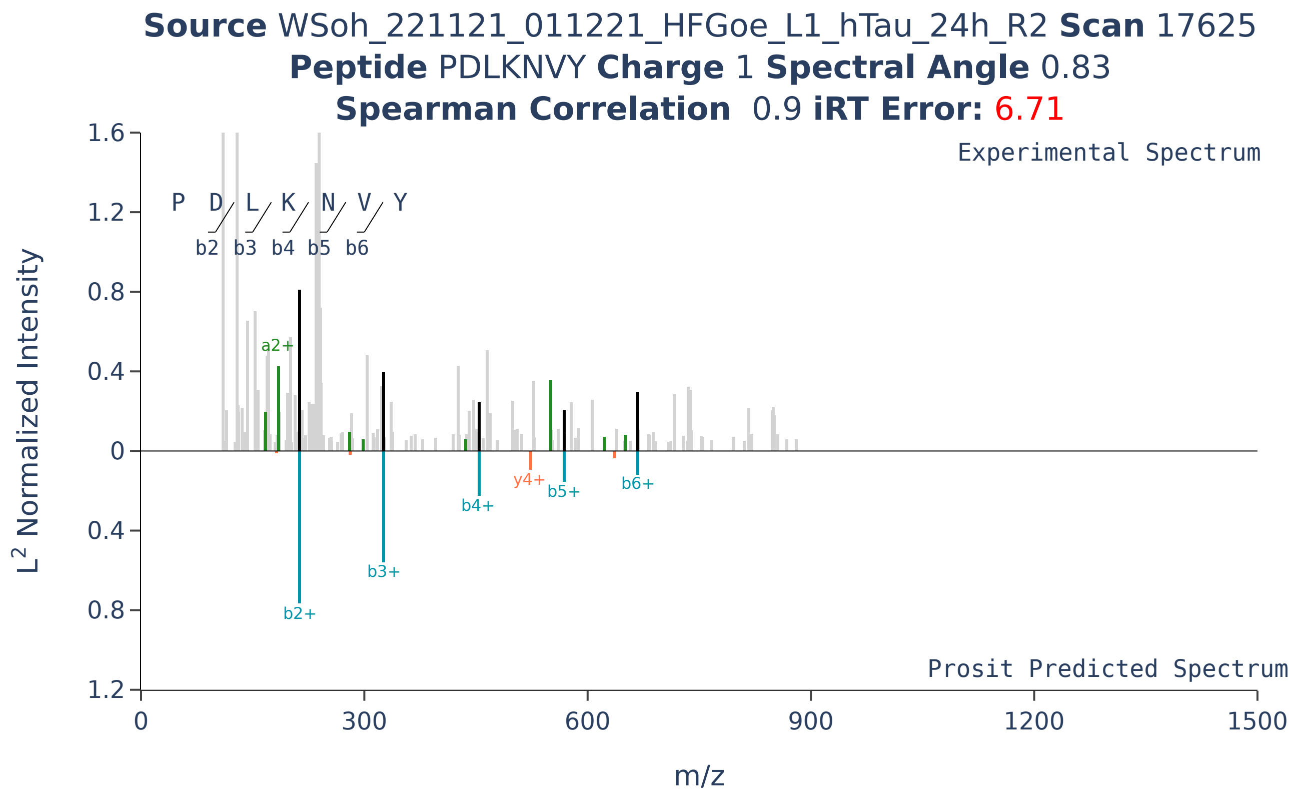
Source WSoh_221121_011221_HFGoe_L1_hTau_24h_R2 **Scan** 17625
Peptide PDLKNVY **Charge** 1 **Spectral Angle** 0.83
Spearman Correlation 0.9 **iRT Error:** 6.71
Experimental Spectrum
Prosit Predicted Spectrum
L² Normalized Intensity
m/z

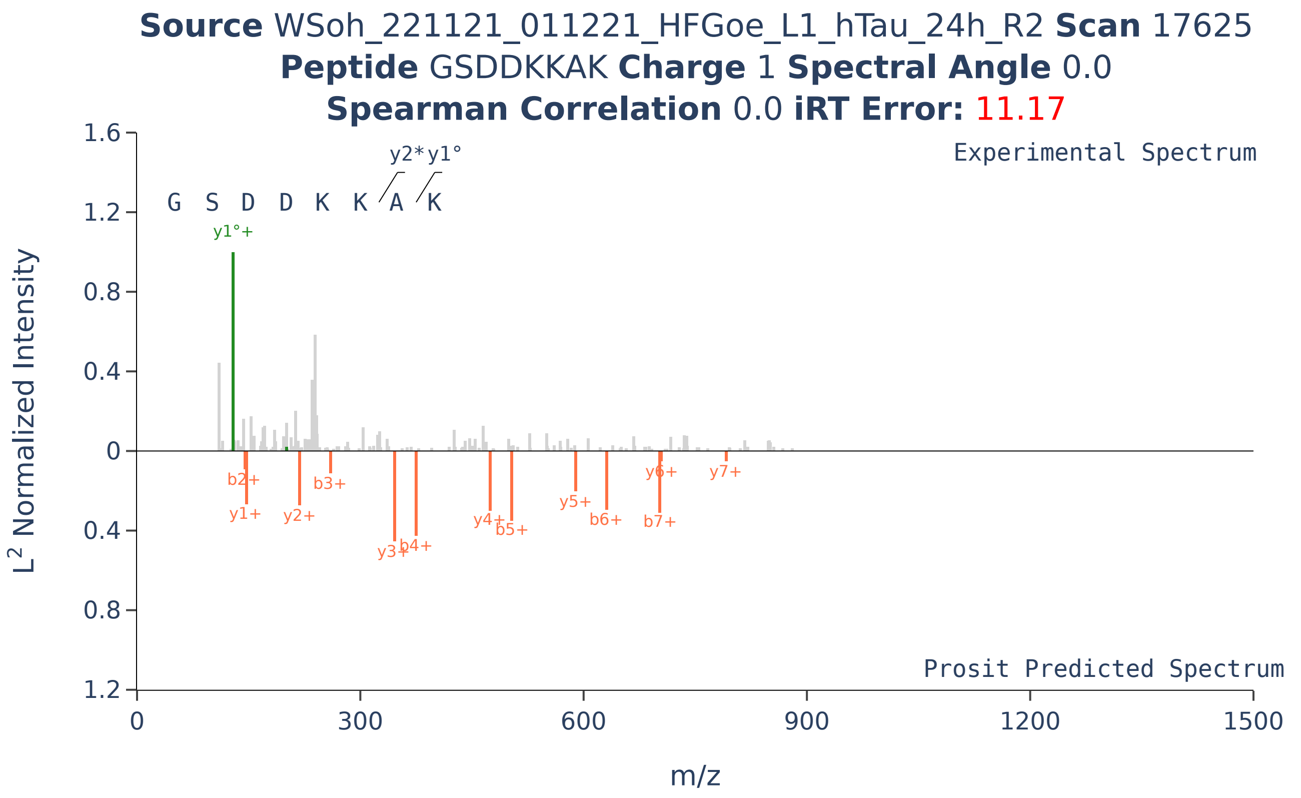
Source WSoh_221121_011221_HFGoe_L1_hTau_24h_R2 **Scan** 17625
Peptide GSDDKKAK **Charge** 1 **Spectral Angle** 0.0
Spearman Correlation 0.0 **iRT Error:** 11.17
Experimental Spectrum
Prosit Predicted Spectrum
L² Normalized Intensity
m/z

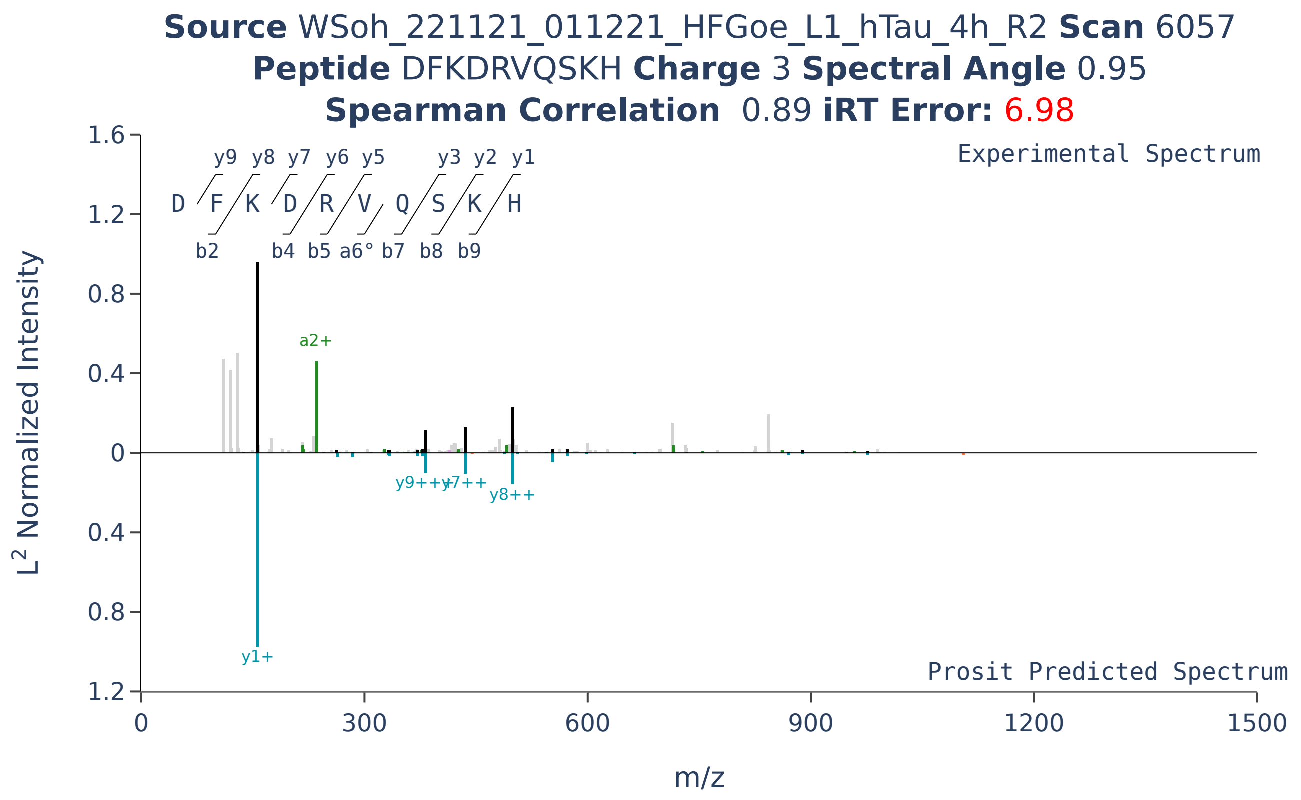
Source WSoh_221121_011221_HFGoe_L1_hTau_4h_R2 **Scan** 6057
Peptide DFKDRVQSKH **Charge** 3 **Spectral Angle** 0.95
Spearman Correlation 0.89 **iRT Error:** 6.98
Experimental Spectrum
Prosit Predicted Spectrum
L² Normalized Intensity
m/z

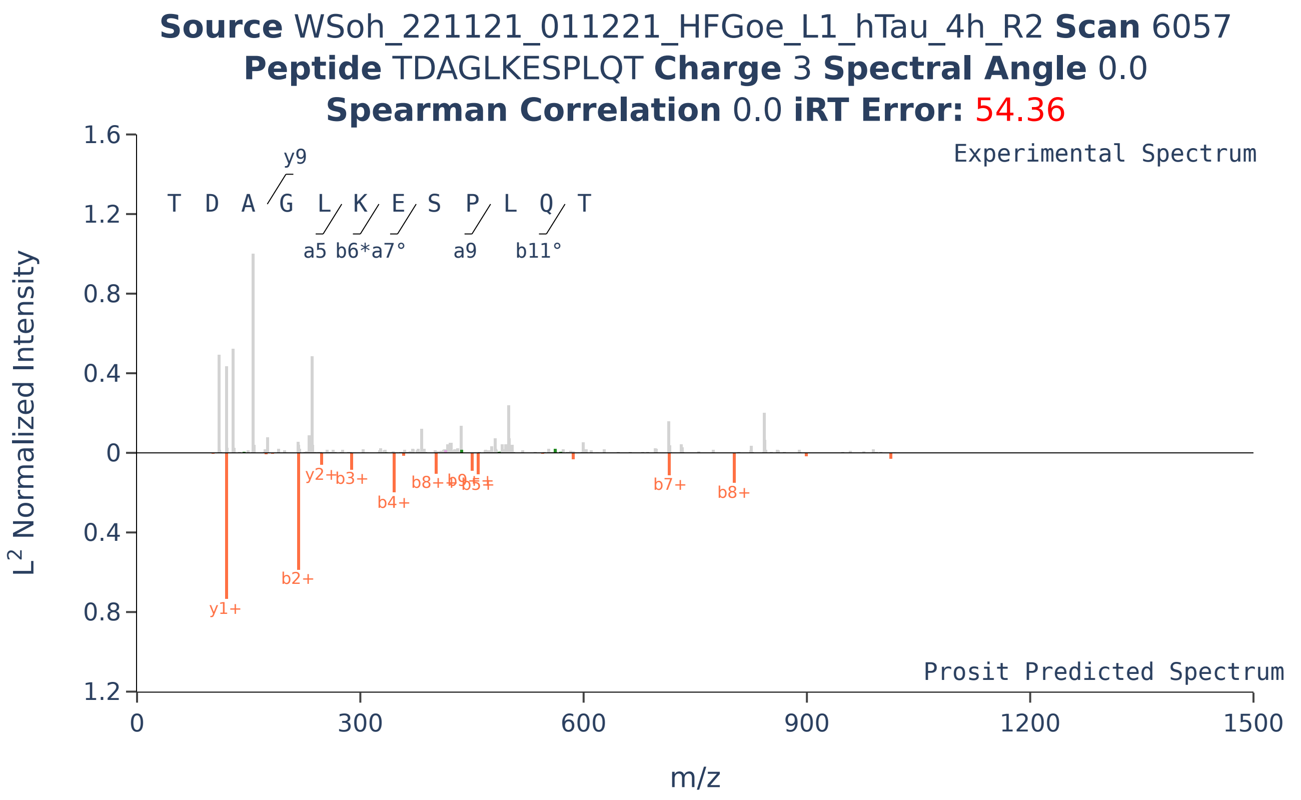
Source WSoh_221121_011221_HFGoe_L1_hTau_4h_R2 **Scan** 6057
Peptide TDAGLKESPLQT **Charge** 3 **Spectral Angle** 0.0
Spearman Correlation 0.0 **iRT Error:** 54.36
Experimental Spectrum
Prosit Predicted Spectrum
L² Normalized Intensity
m/z

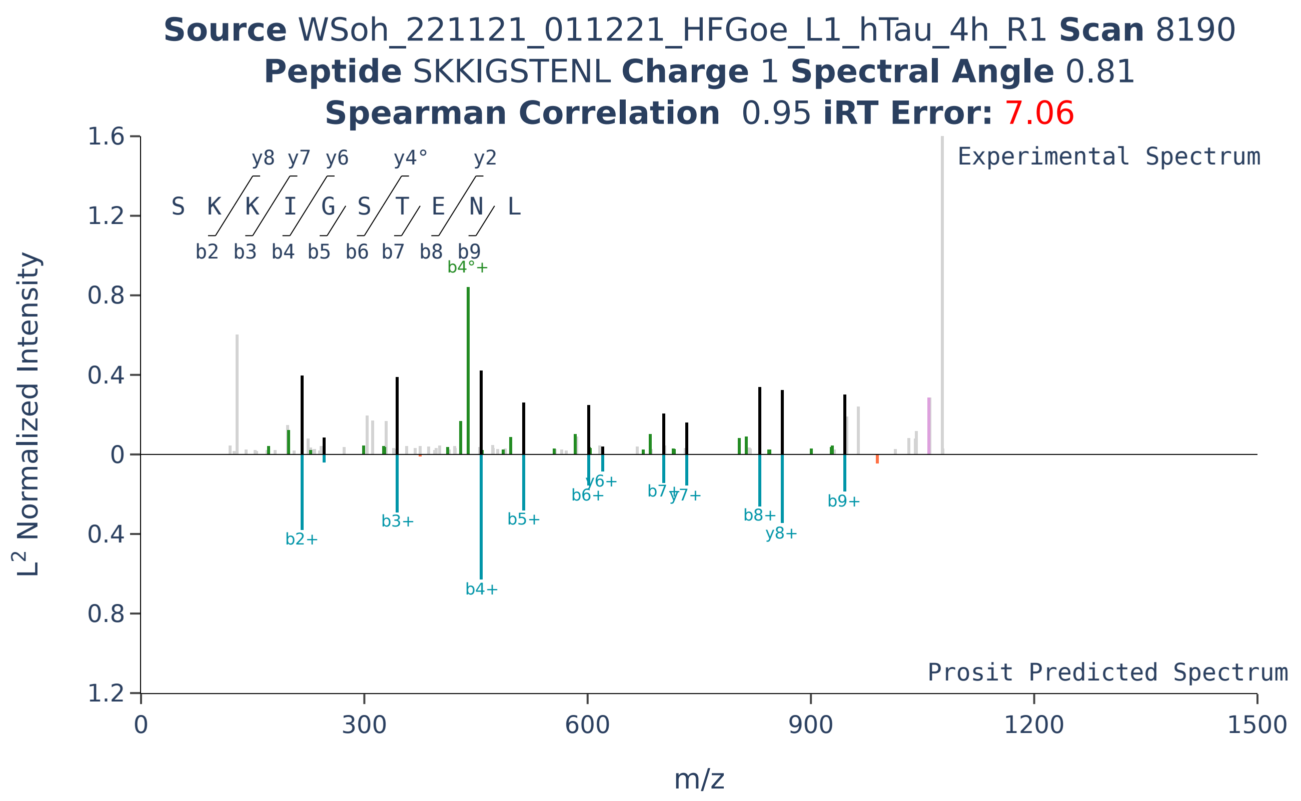
Source WSoh_221121_011221_HFGoe_L1_hTau_4h_R1 **Scan** 8190
Peptide SKKIGSTENL **Charge** 1 **Spectral Angle** 0.81
Spearman Correlation 0.95 **iRT Error:** 7.06
Experimental Spectrum
Prosit Predicted Spectrum
L² Normalized Intensity
m/z

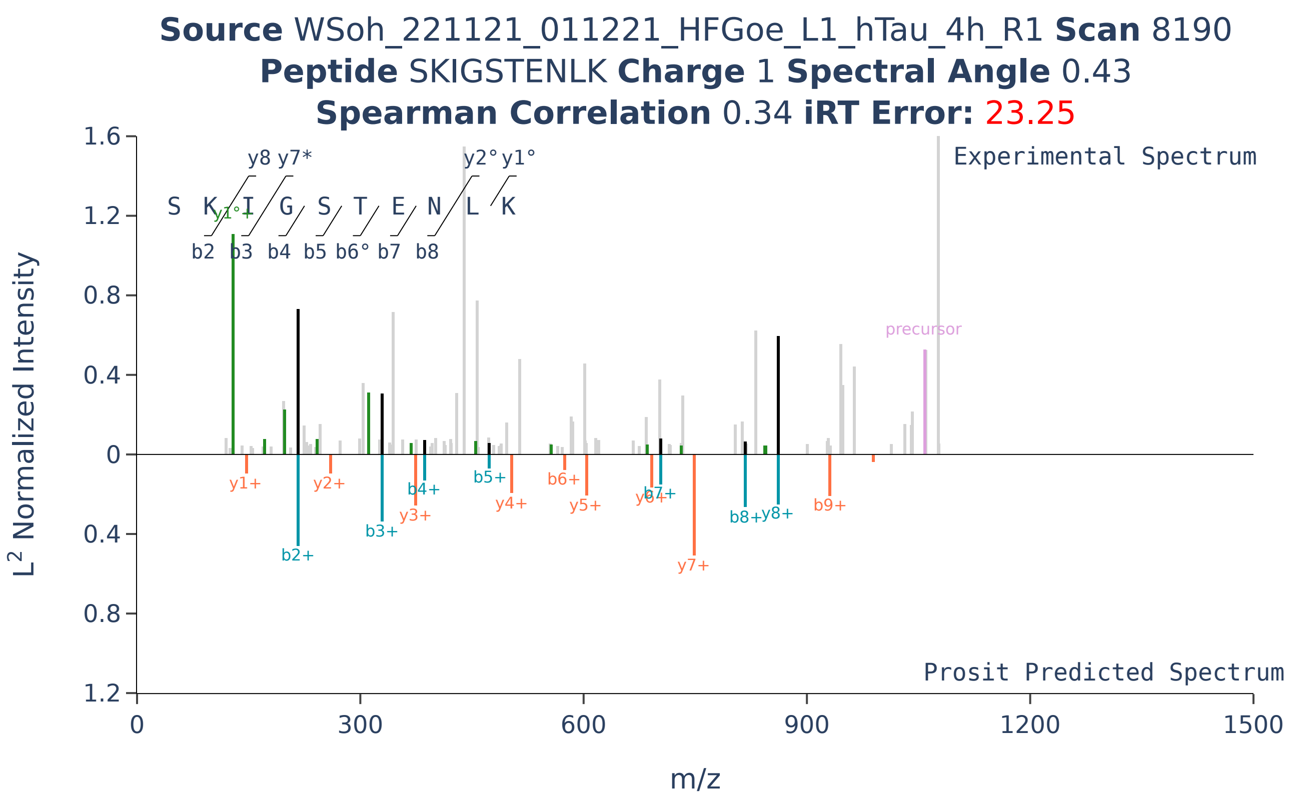
Source WSoh_221121_011221_HFGoe_L1_hTau_4h_R1 **Scan** 8190
Peptide SKIGSTENLK **Charge** 1 **Spectral Angle** 0.43
Spearman Correlation 0.34 **iRT Error:** 23.25
Experimental Spectrum
Prosit Predicted Spectrum
precursor
L² Normalized Intensity
m/z

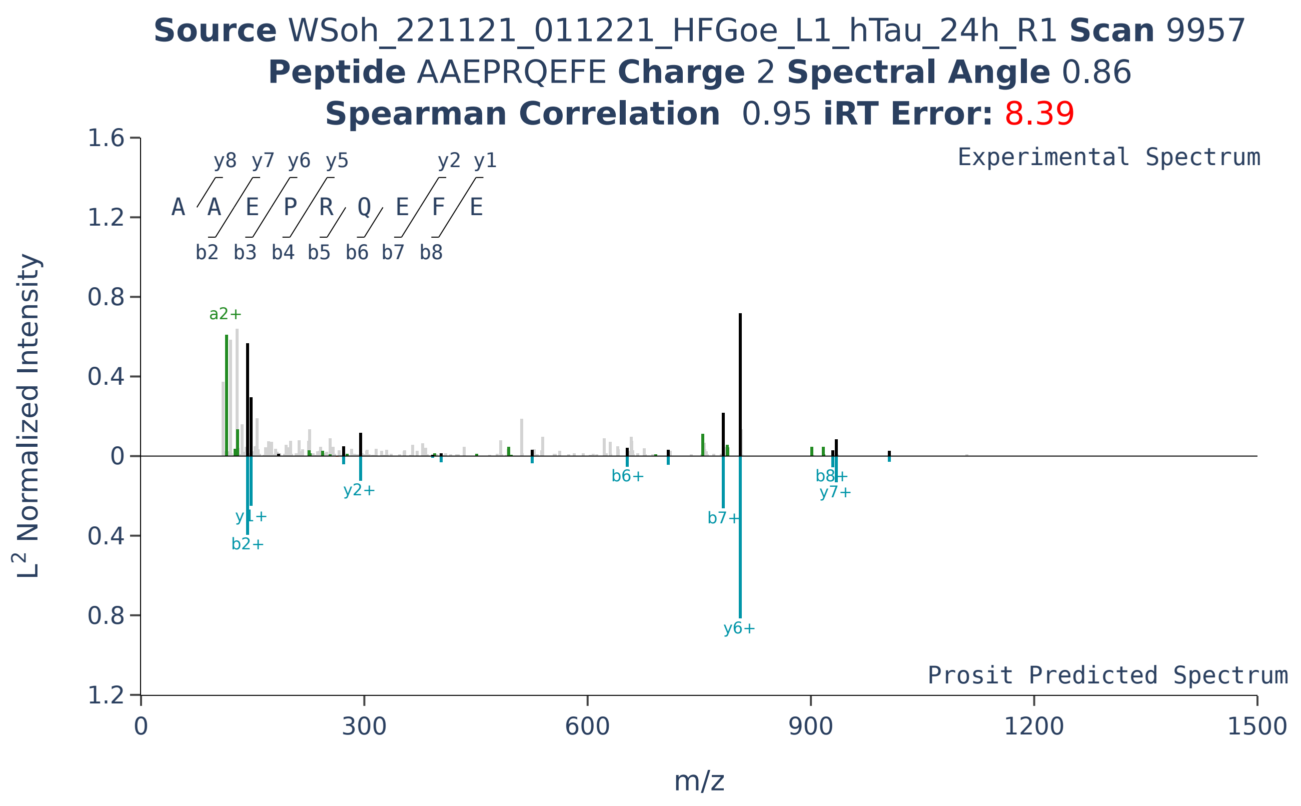
Source WSoh_221121_011221_HFGoe_L1_hTau_24h_R1 **Scan** 9957
Peptide AAEPRQEFE **Charge** 2 **Spectral Angle** 0.86
Spearman Correlation 0.95 **iRT Error:** 8.39
Experimental Spectrum
Prosit Predicted Spectrum
L² Normalized Intensity
m/z

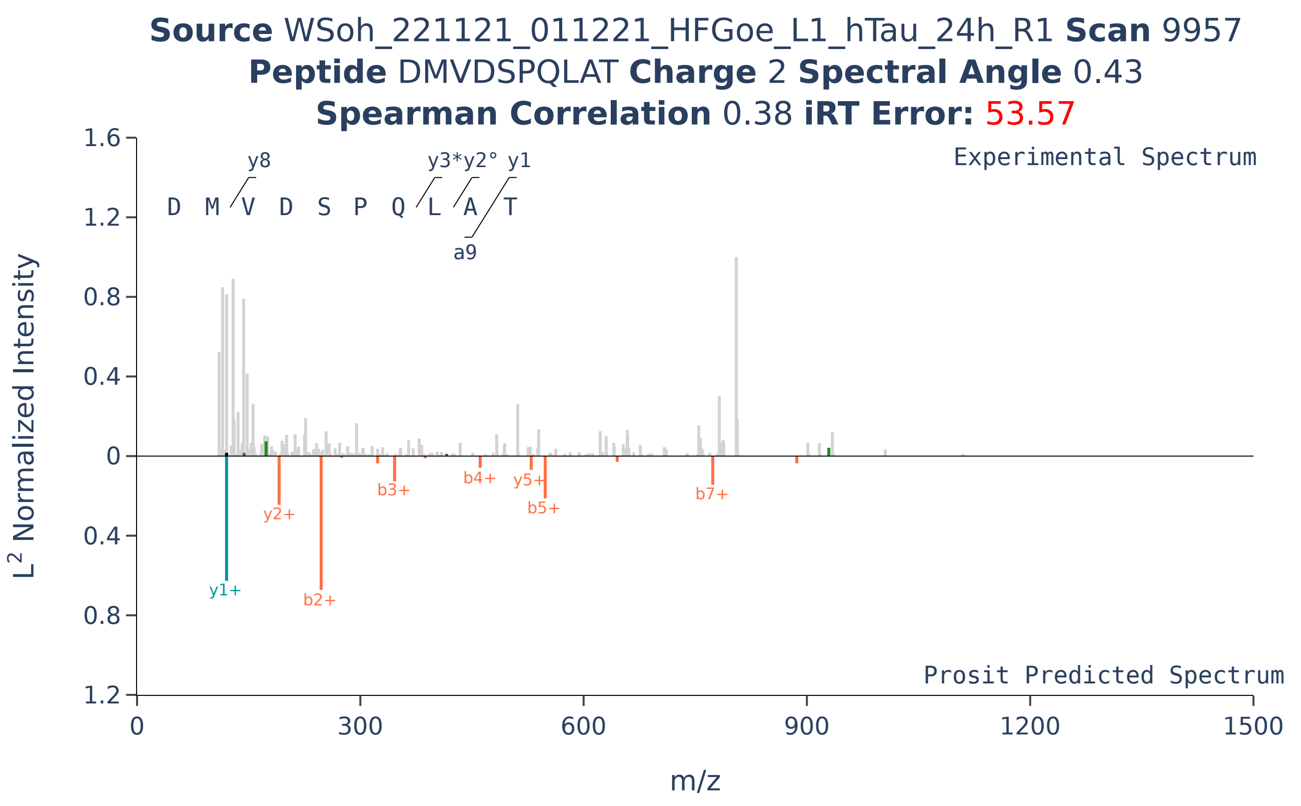
Source WSoh_221121_011221_HFGoe_L1_hTau_24h_R1 **Scan** 9957
Peptide DMVDSPQLAT **Charge** 2 **Spectral Angle** 0.43
Spearman Correlation 0.38 **iRT Error:** 53.57
Experimental Spectrum
Prosit Predicted Spectrum
L² Normalized Intensity
m/z

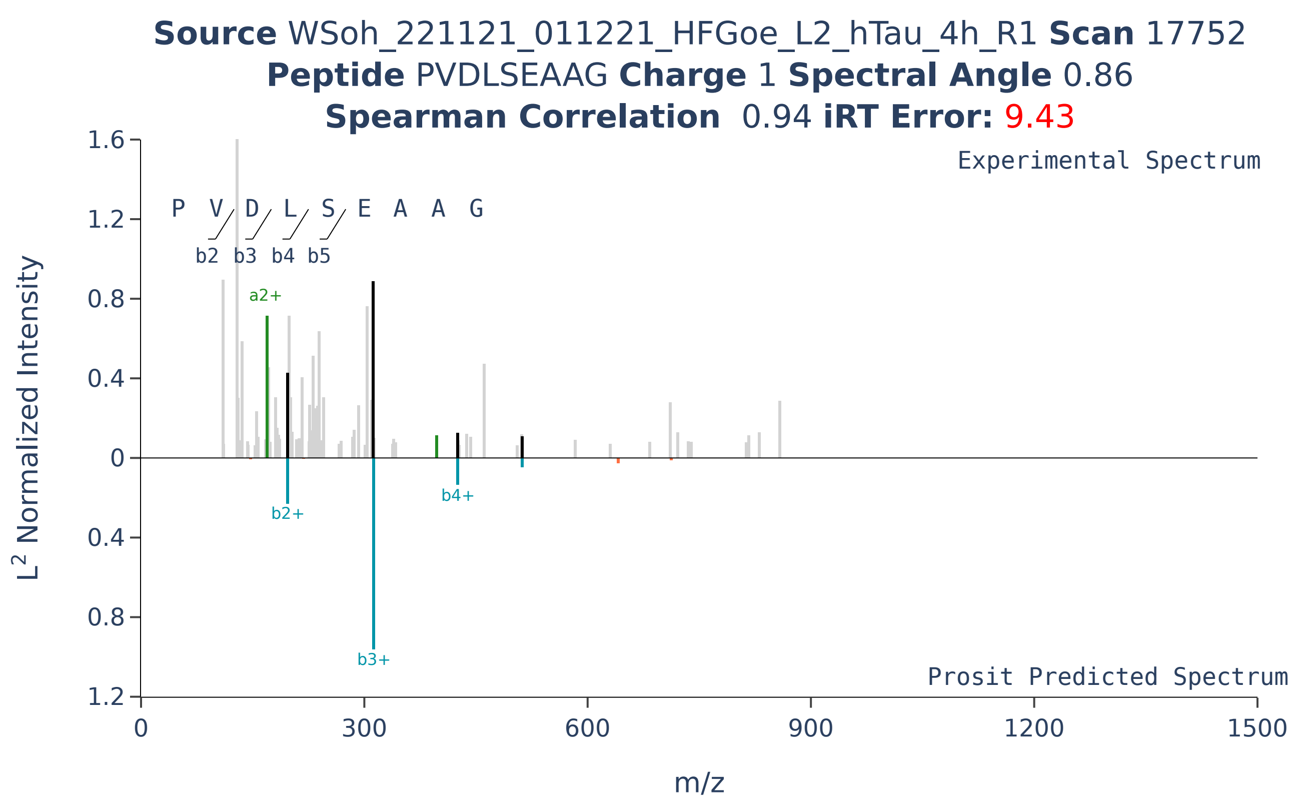
Source WSoh_221121_011221_HFGoe_L2_hTau_4h_R1 **Scan** 17752
Peptide PVDLSEAAG **Charge** 1 **Spectral Angle** 0.86
Spearman Correlation 0.94 **iRT Error:** 9.43
Experimental Spectrum
Prosit Predicted Spectrum
L² Normalized Intensity
m/z

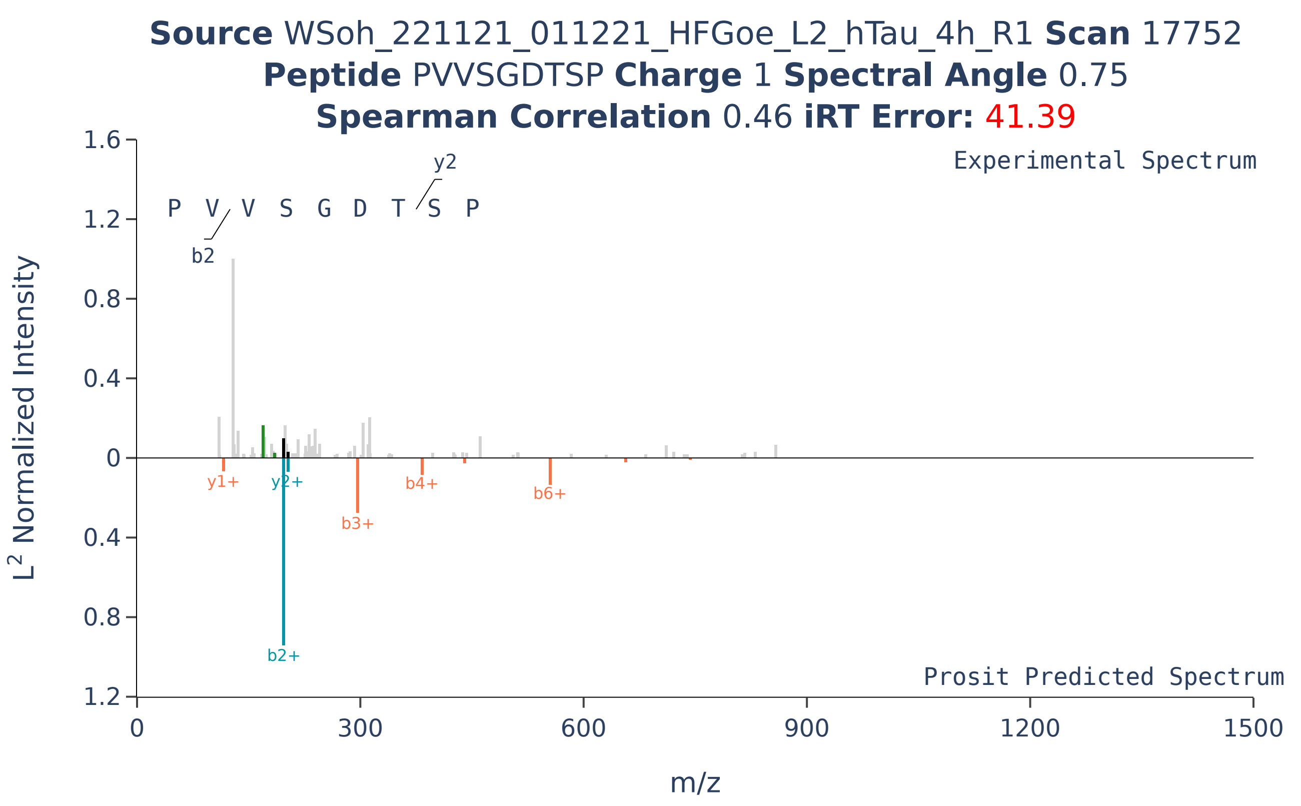
Source WSoh_221121_011221_HFGoe_L2_hTau_4h_R1 **Scan** 17752
Peptide PVVSGDTSP **Charge** 1 **Spectral Angle** 0.75
Spearman Correlation 0.46 **iRT Error:** 41.39
Experimental Spectrum
Prosit Predicted Spectrum
L² Normalized Intensity
m/z

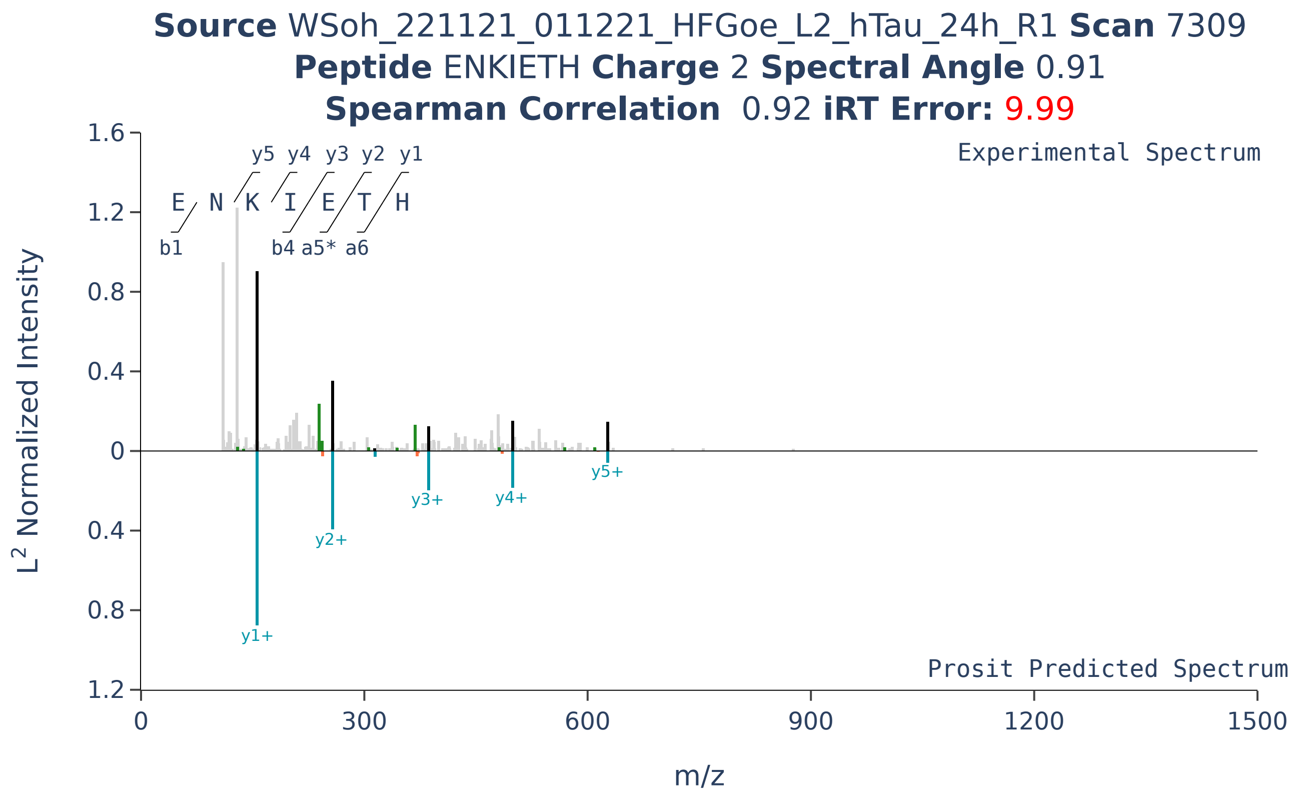

Source WSoh_221121_011221_HFGoe_L2_hTau_24h_R1 Scan 7309
Peptide ENKIETH Charge 2 Spectral Angle 0.91
Spearman Correlation 0.92 iRT Error: 9.99
Experimental Spectrum
Prosit Predicted Spectrum
L2 Normalized Intensity
m/z
y1+
y2+
y3+
y4+
y5+

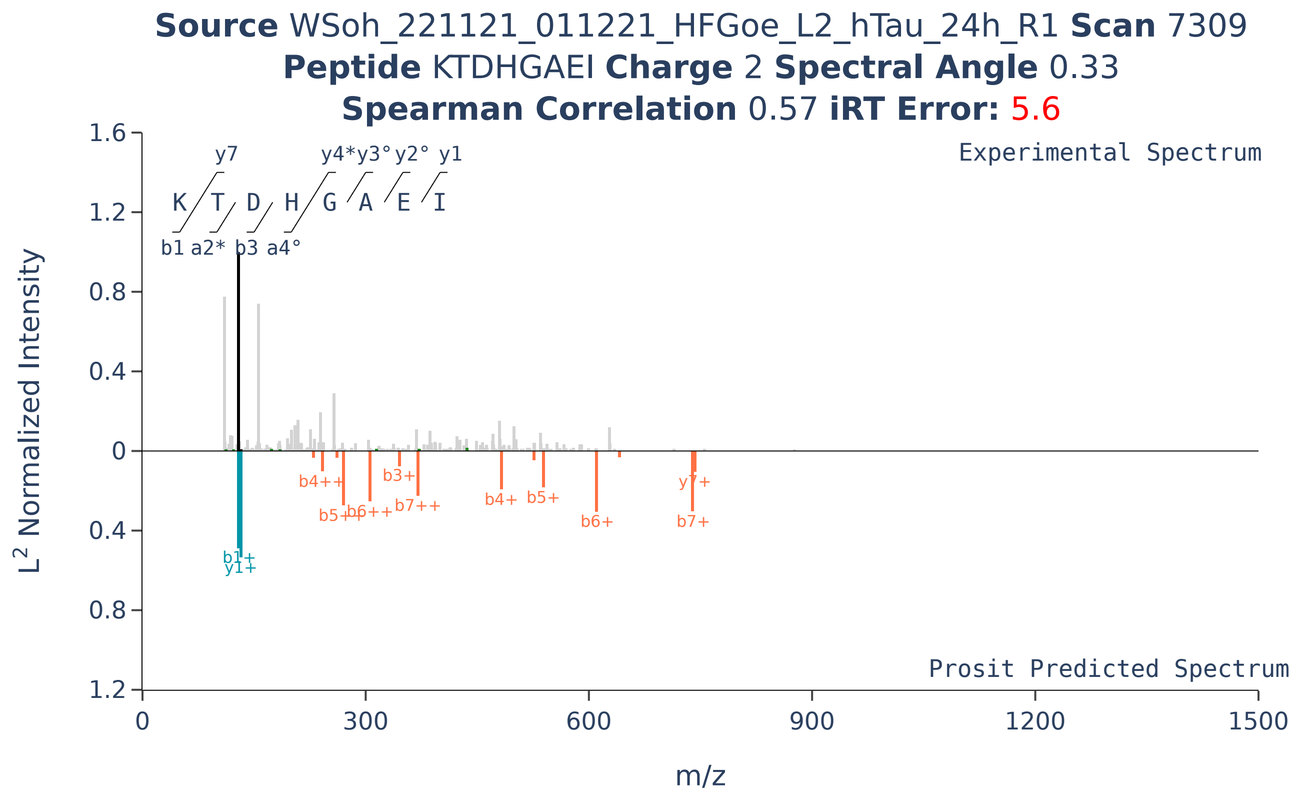

Source WSoh_221121_011221_HFGoe_L2_hTau_24h_R1 Scan 7309
Peptide KTDHGAEI Charge 2 Spectral Angle 0.33
Spearman Correlation 0.57 iRT Error: 5.6
Experimental Spectrum
Prosit Predicted Spectrum
L2 Normalized Intensity
m/z

Source HRoetschke_211222_160123_HFGoe_IF2_b2R1_X **Scan** 13181
Peptide RENLGQEVLE **Charge** 2 **Spectral Angle** 0.77
Spearman Correlation 0.84 **iRT Error:** 0.0

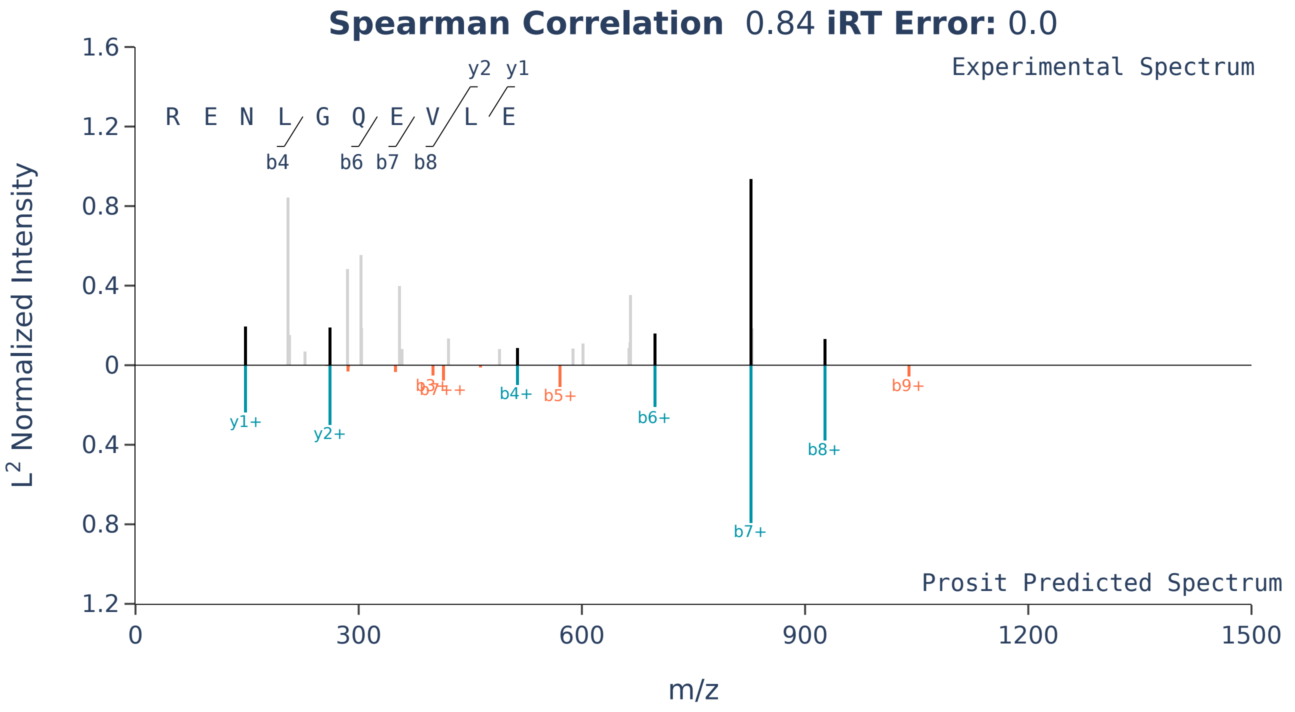

Source HRoetschke_211222_160123_HFGoe_IF2_b2R1_X **Scan** 13181
Peptide EVEGGRGRGRN **Charge** 2 **Spectral Angle** 0.0
Spearman Correlation 0.0 **iRT Error:** 34.37

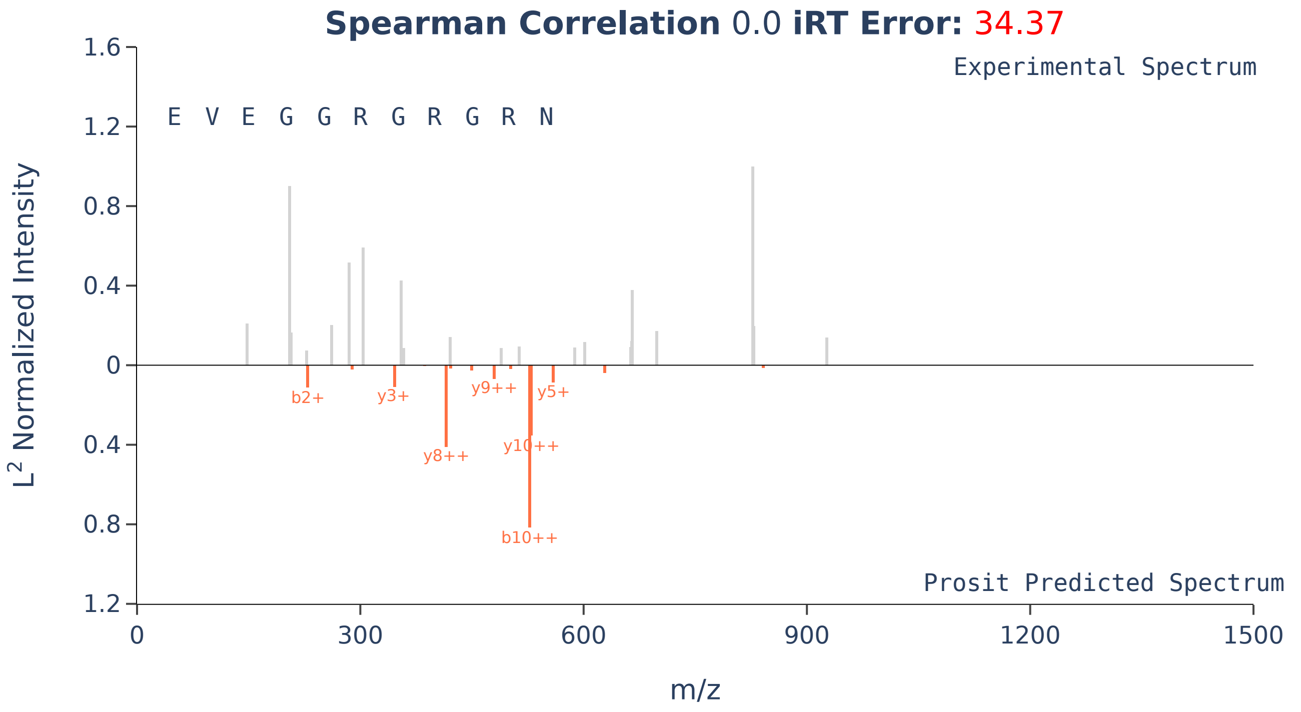

Source HRoetschke_211222_160123_HFGoe_IF2_b2R2_2h **Scan** 11704
Peptide AQLVEAE **Charge** 1 **Spectral Angle** 0.93
Spearman Correlation 0.87 **iRT Error:** 0.11

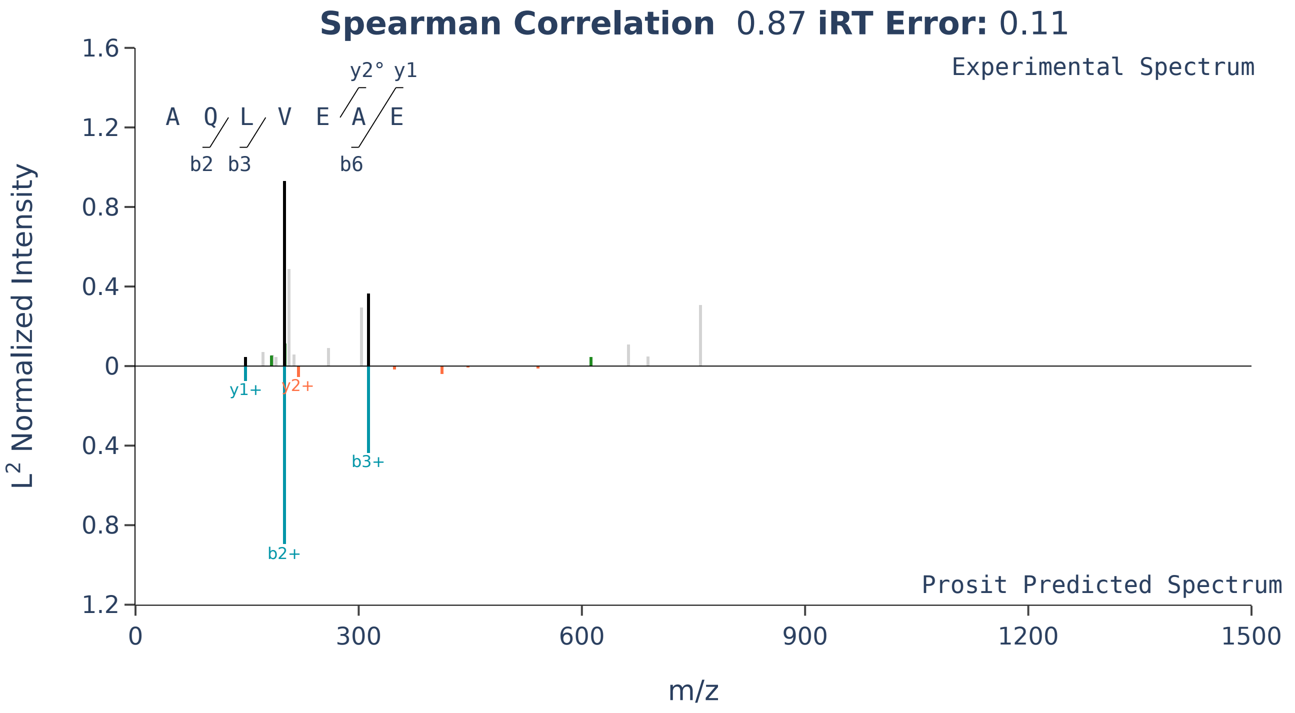

Source HRoetschke_211222_160123_HFGoe_IF2_b2R2_2h **Scan** 11704
Peptide LVVAADDG **Charge** 1 **Spectral Angle** 0.79
Spearman Correlation 0.61 **iRT Error:** 3.98

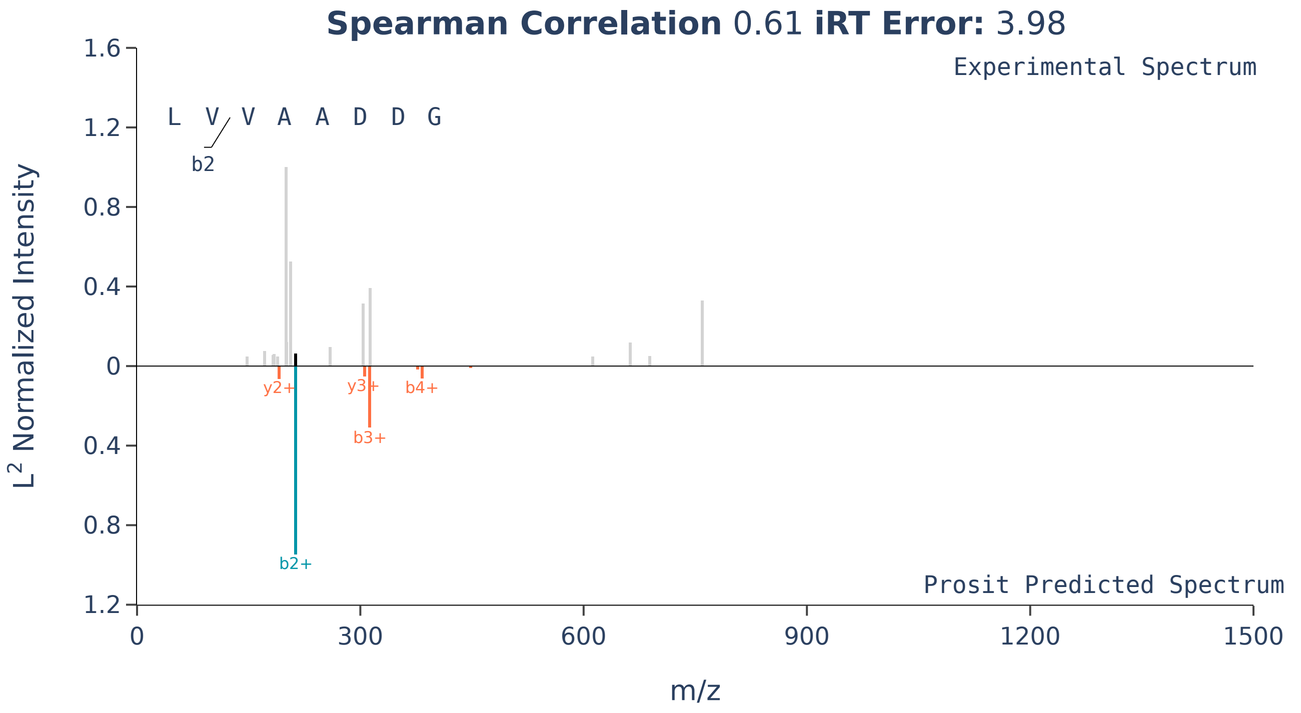

Source HRoetschke_211222_160123_HFGoe_IF2_b1R2_2h **Scan** 16247
Peptide HAIIGLAE **Charge** 2 **Spectral Angle** 0.8
Spearman Correlation 0.89 **iRT Error:** 0.16

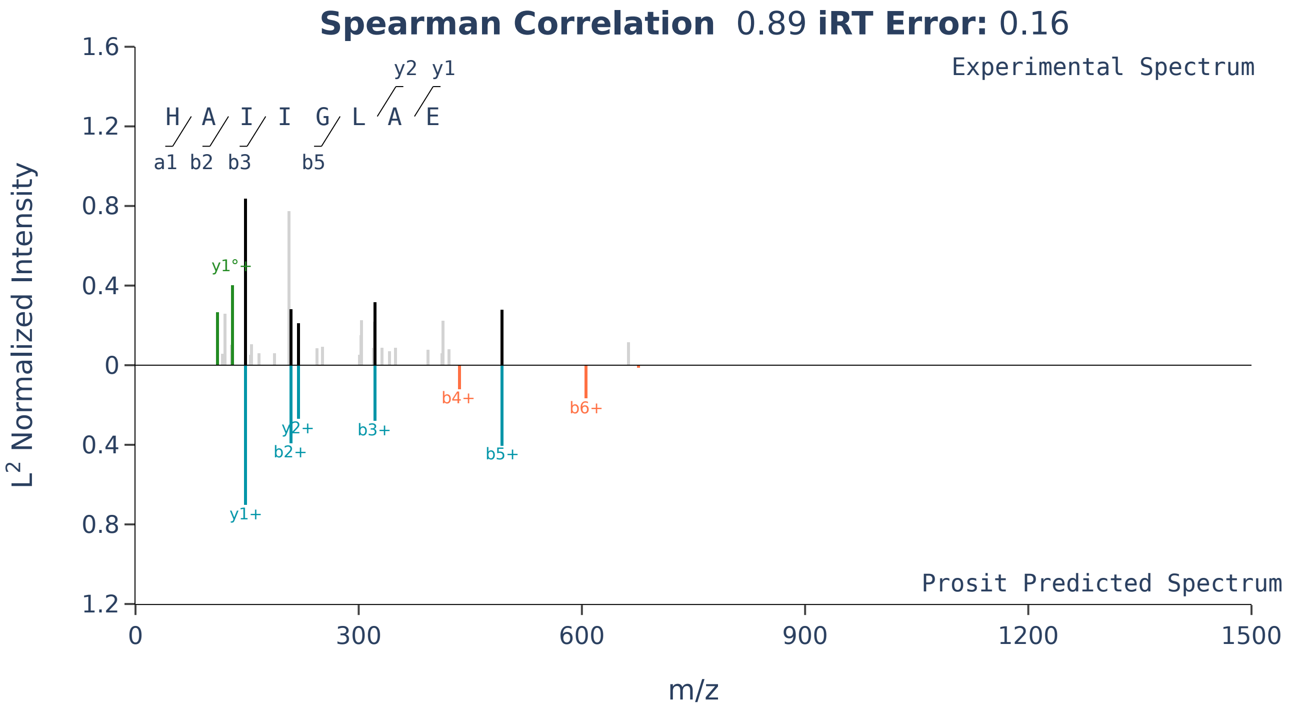

Source HRoetschke_211222_160123_HFGoe_IF2_b1R2_2h **Scan** 16247
Peptide HEVNIVL **Charge** 2 **Spectral Angle** 0.0
Spearman Correlation 0.0 **iRT Error:** 13.39

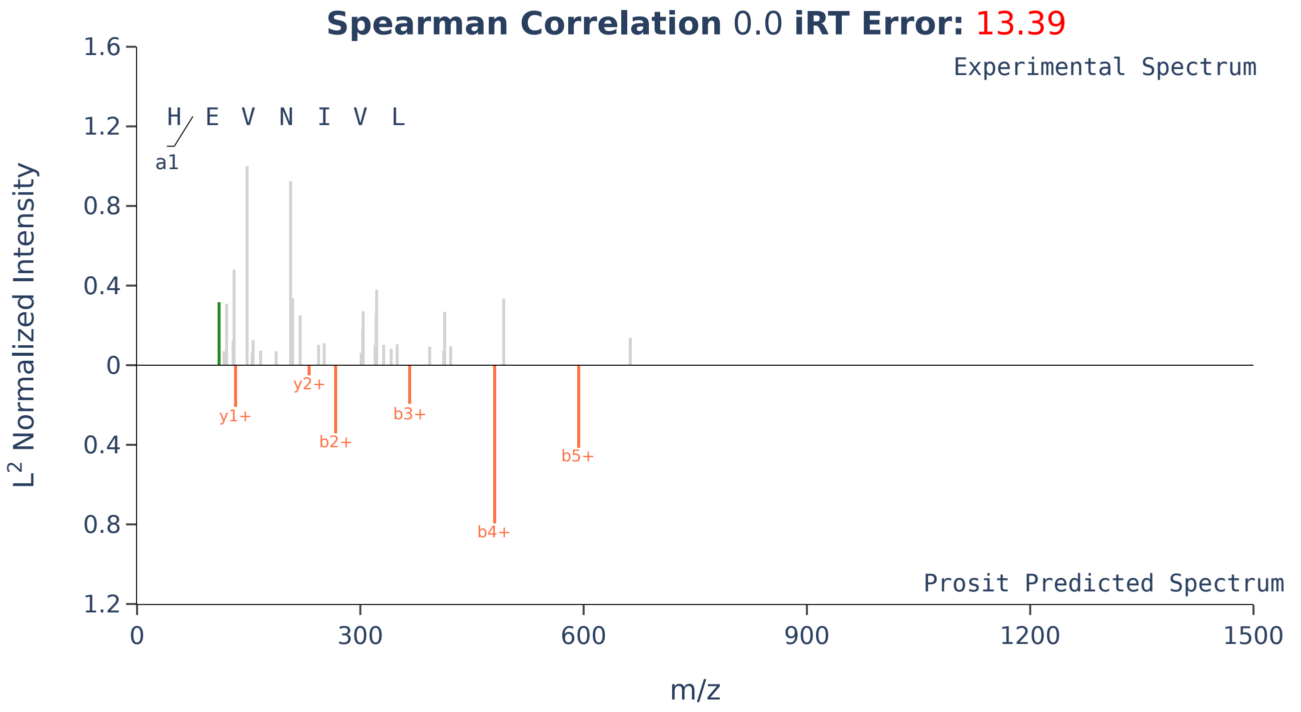

Source HRoetschke_211222_160123_HFGoe_IF2_b1R1_4h **Scan** 18555
Peptide LDAQEVLE **Charge** 2 **Spectral Angle** 0.91
Spearman Correlation 0.96 **iRT Error:** 0.18

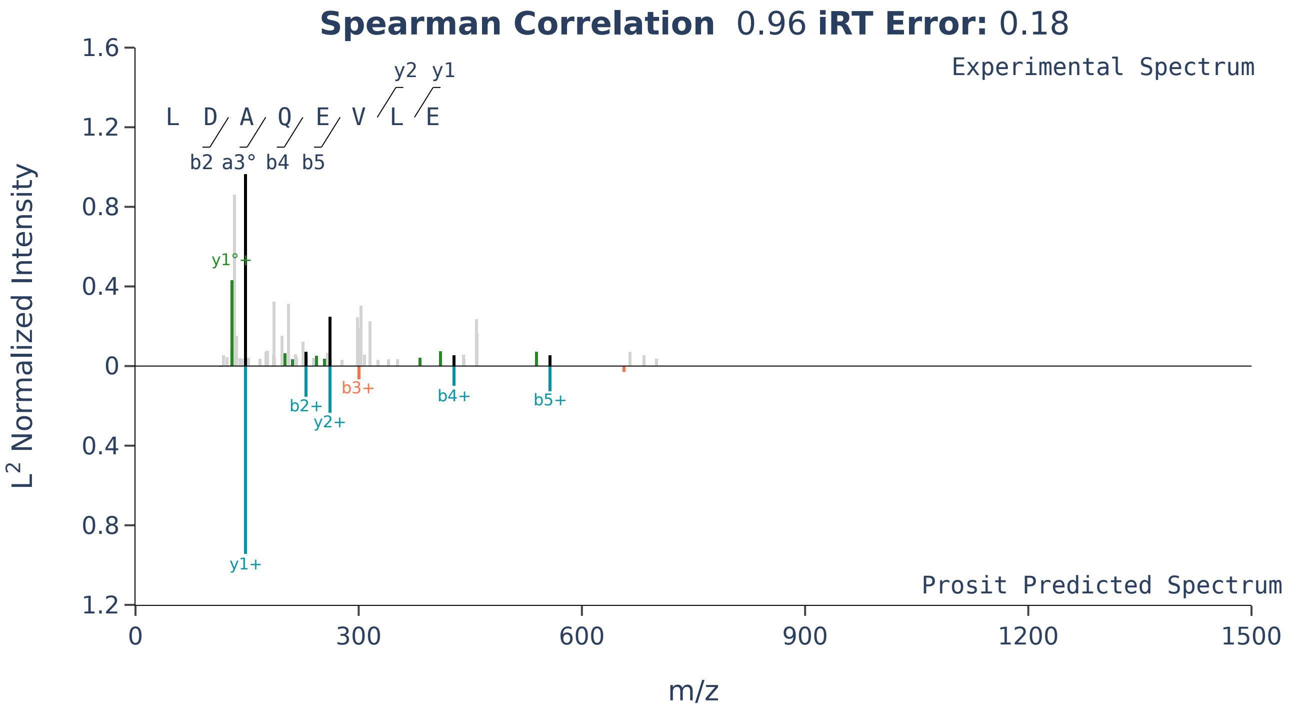

Source HRoetschke_211222_160123_HFGoe_IF2_b1R1_4h **Scan** 18555
Peptide EVEGGRGRG **Charge** 2 **Spectral Angle** 0.02
Spearman Correlation 0.06 **iRT Error:** 71.06

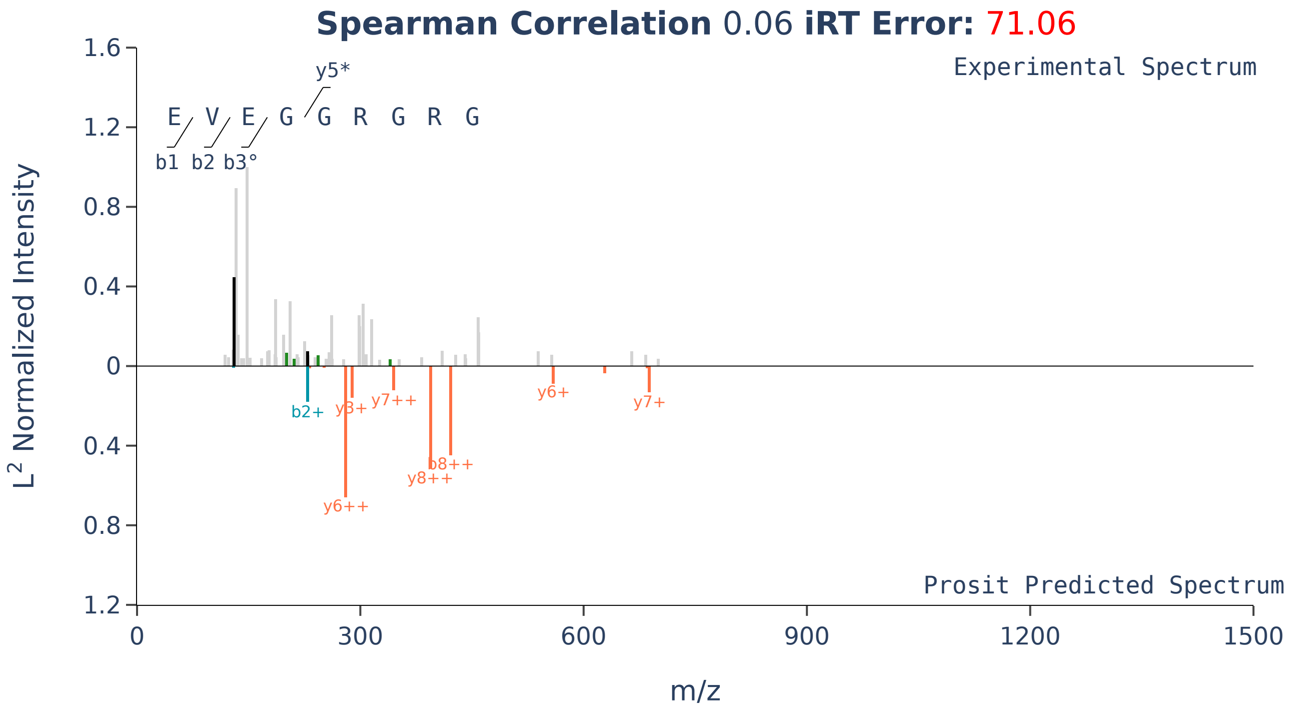

Source HRoetschke_211222_160123_HFGoe_IF2_b2R2_24h **Scan** 20617
Peptide GETILVV **Charge** 1 **Spectral Angle** 0.66
Spearman Correlation 0.93 **iRT Error:** 0.19

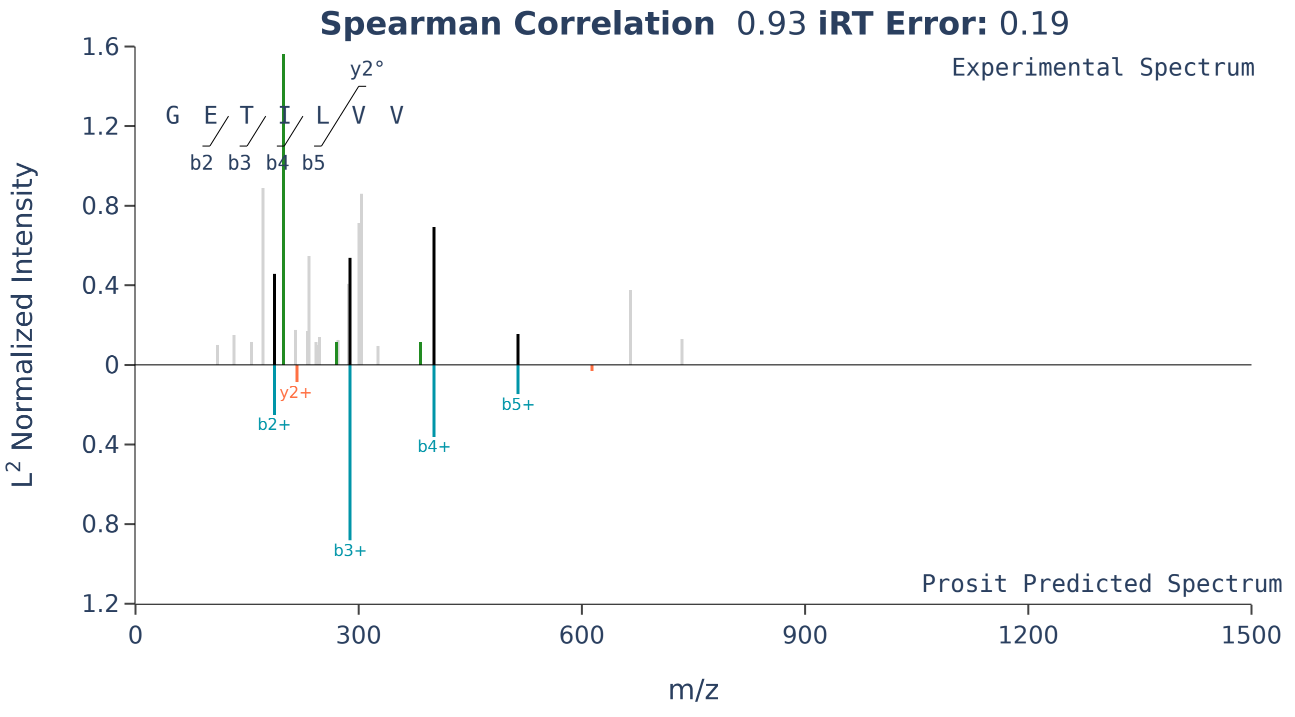

Source HRoetschke_211222_160123_HFGoe_IF2_b2R2_24h **Scan** 20617
Peptide VEILGLS **Charge** 1 **Spectral Angle** 0.0
Spearman Correlation 0.0 **iRT Error:** 7.91

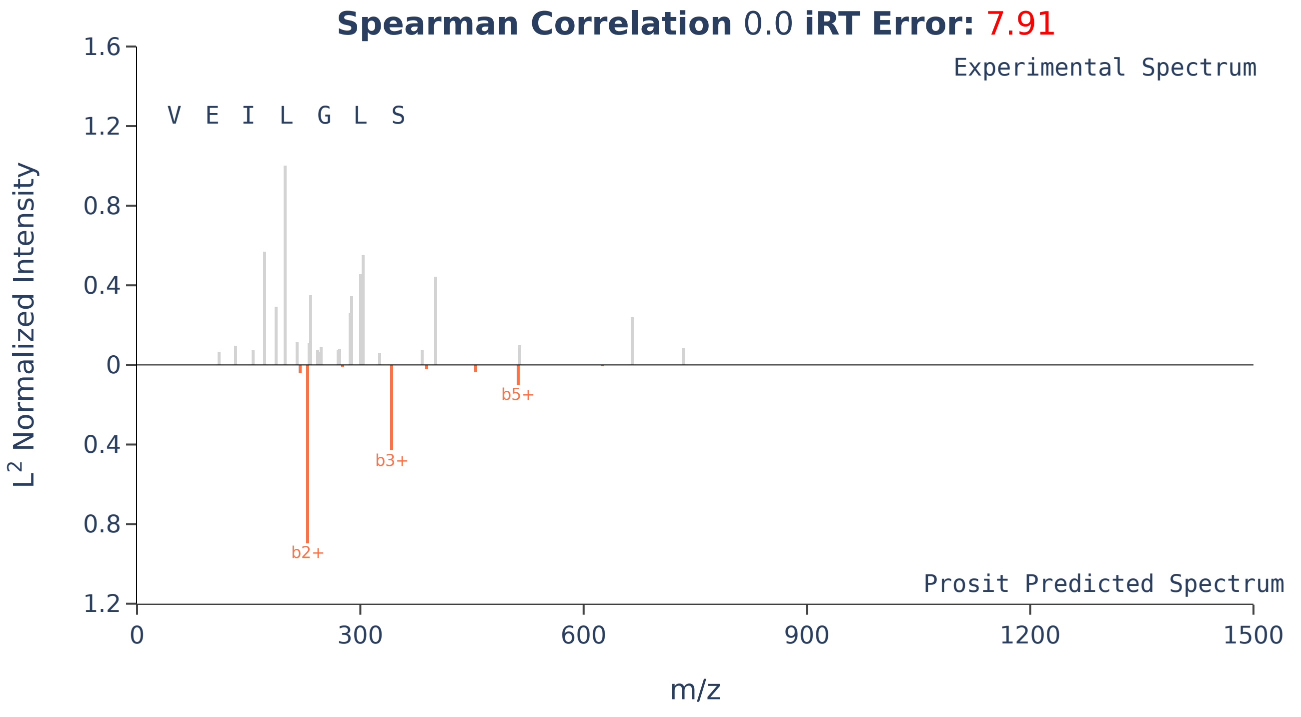

Source HRoetschke_211222_160123_HFGoe_IF2_b1R2_2h **Scan** 13214
Peptide GAYHVETLE **Charge** 2 **Spectral Angle** 0.81
Spearman Correlation 0.82 **iRT Error:** 0.21

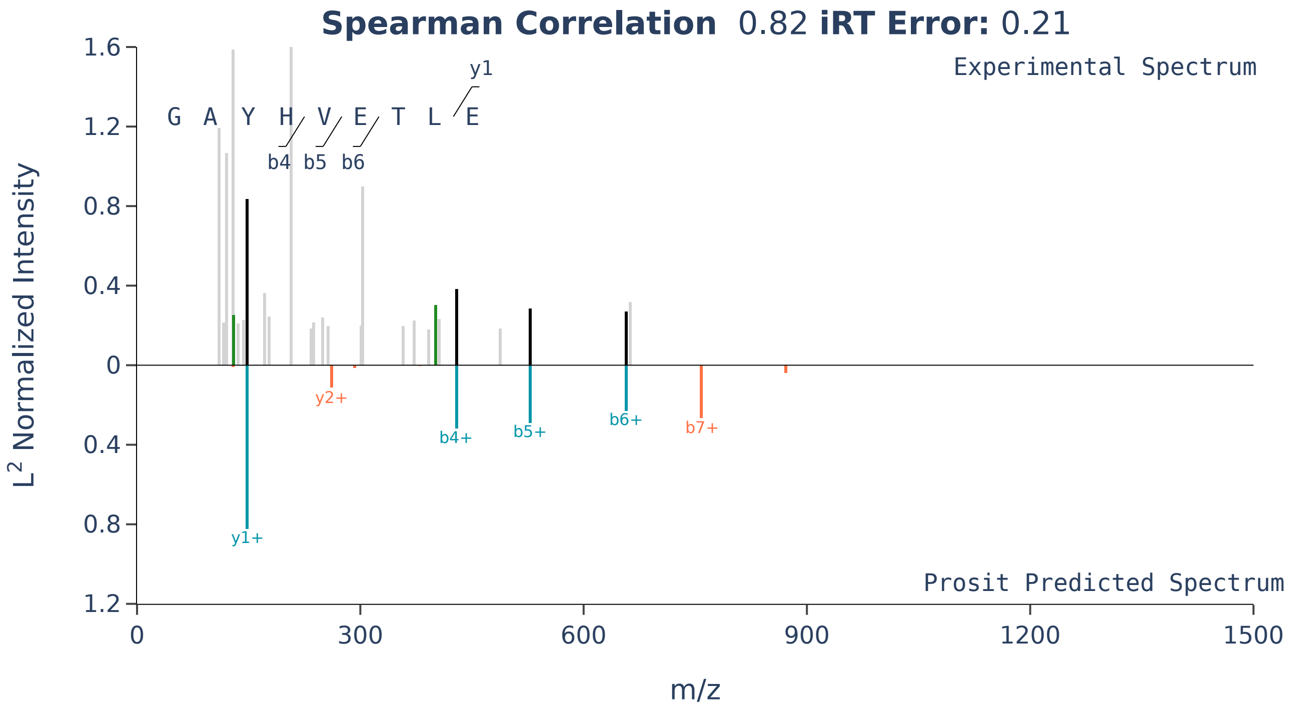

Source HRoetschke_211222_160123_HFGoe_IF2_b1R2_2h **Scan** 13214
Peptide ARREAEESA **Charge** 2 **Spectral Angle** 0.0
Spearman Correlation 0.0 **iRT Error:** 59.13

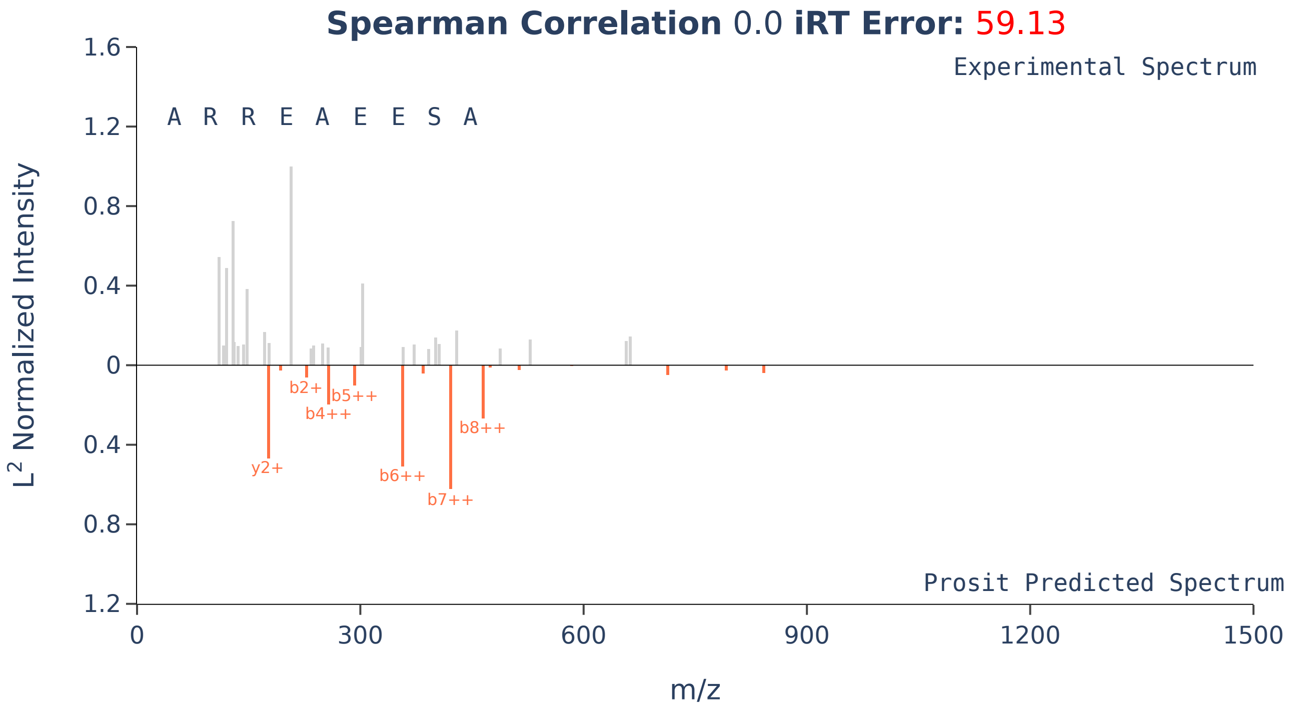

Source HRoetschke_211222_160123_HFGoe_IF2_b2R1_24h **Scan** 11427
Peptide GRDVVIGE **Charge** 2 **Spectral Angle** 0.89
Spearman Correlation 0.95 **iRT Error:** 0.23

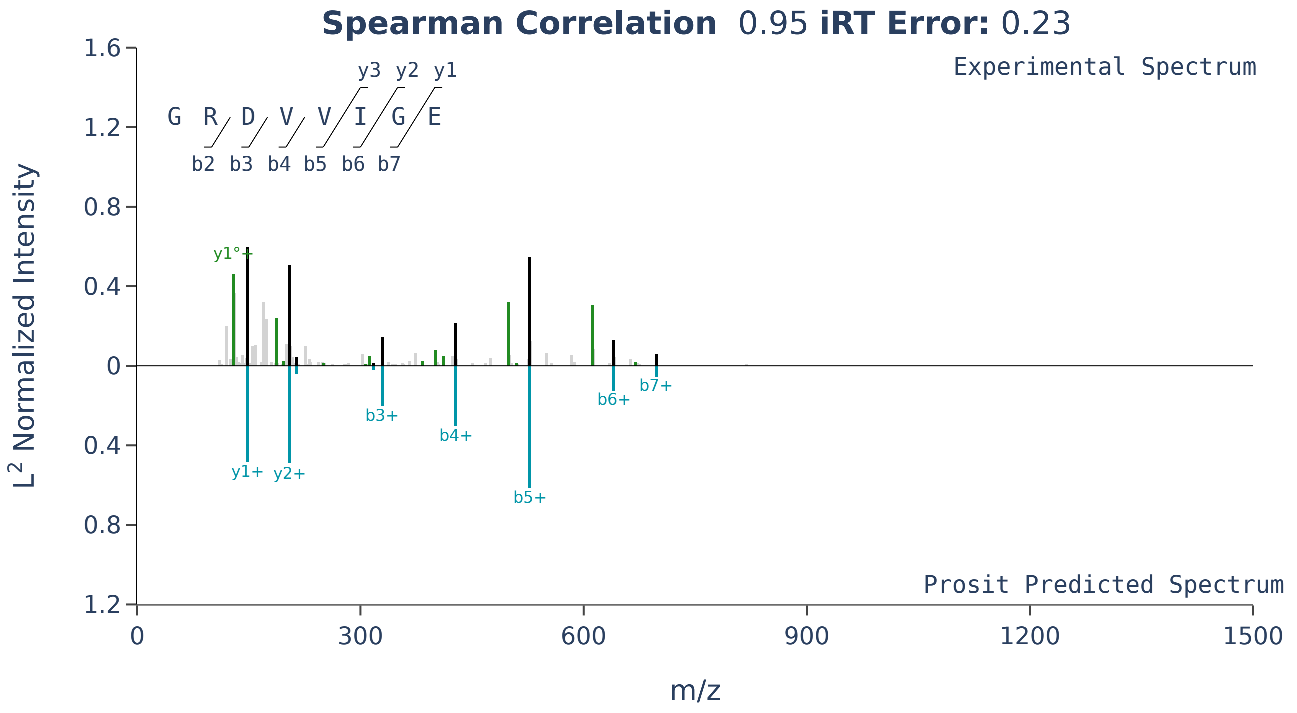

Source HRoetschke_211222_160123_HFGoe_IF2_b2R1_24h **Scan** 11427
Peptide EGGRGRGR **Charge** 2 **Spectral Angle** 0.04
Spearman Correlation -0.08 **iRT Error:** 50.01

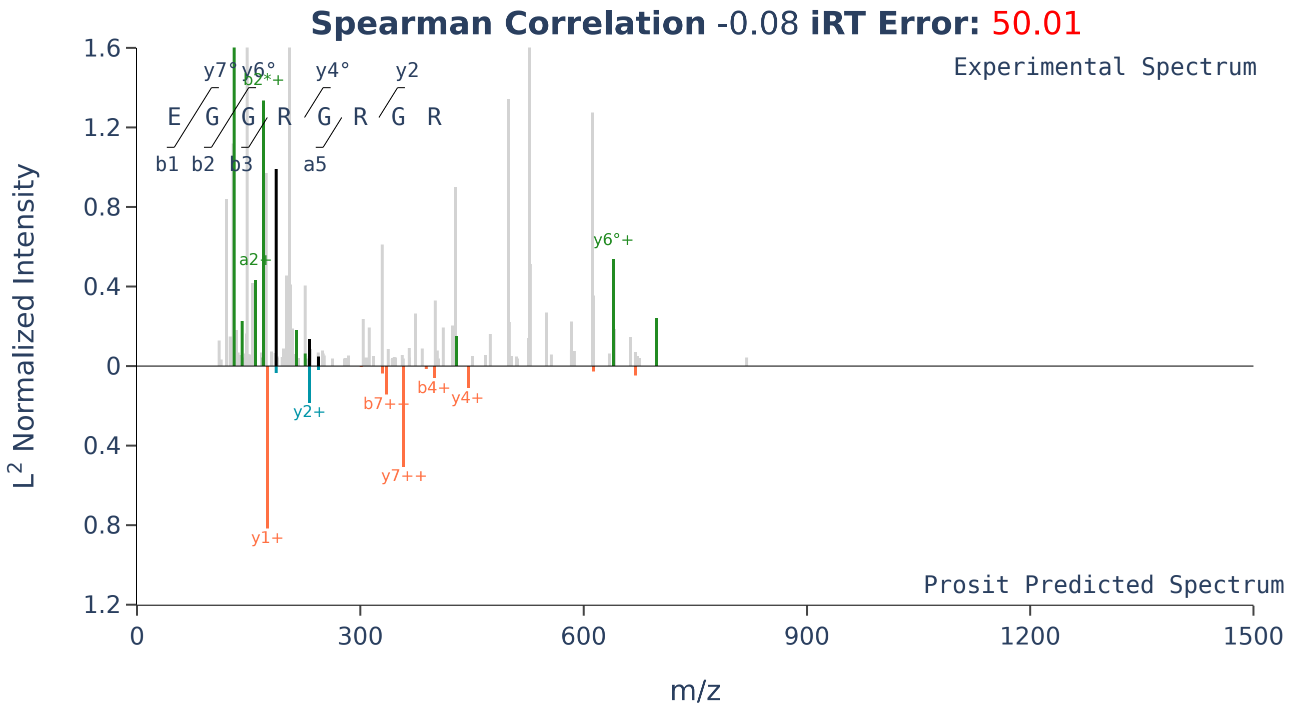

Source HRoetschke_211222_160123_HFGoe_IF2_b1R1_4h **Scan** 13375
Peptide VRADIGAYHVETE **Charge** 3 **Spectral Angle** 0.8
Spearman Correlation 0.83 **iRT Error:** 0.3

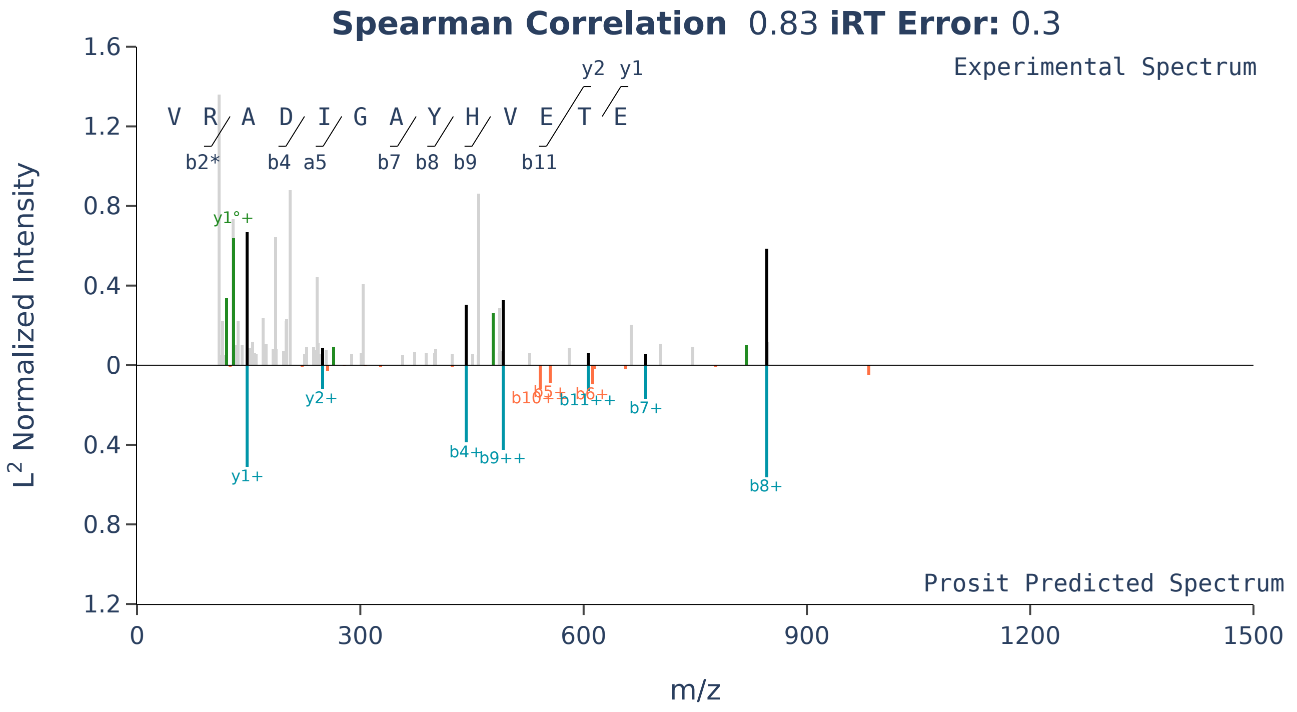

Source HRoetschke_211222_160123_HFGoe_IF2_b1R1_4h **Scan** 13375
Peptide AMRNELGQEVLEA **Charge** 3 **Spectral Angle** 0.0
Spearman Correlation 0.0 **iRT Error:** 41.1

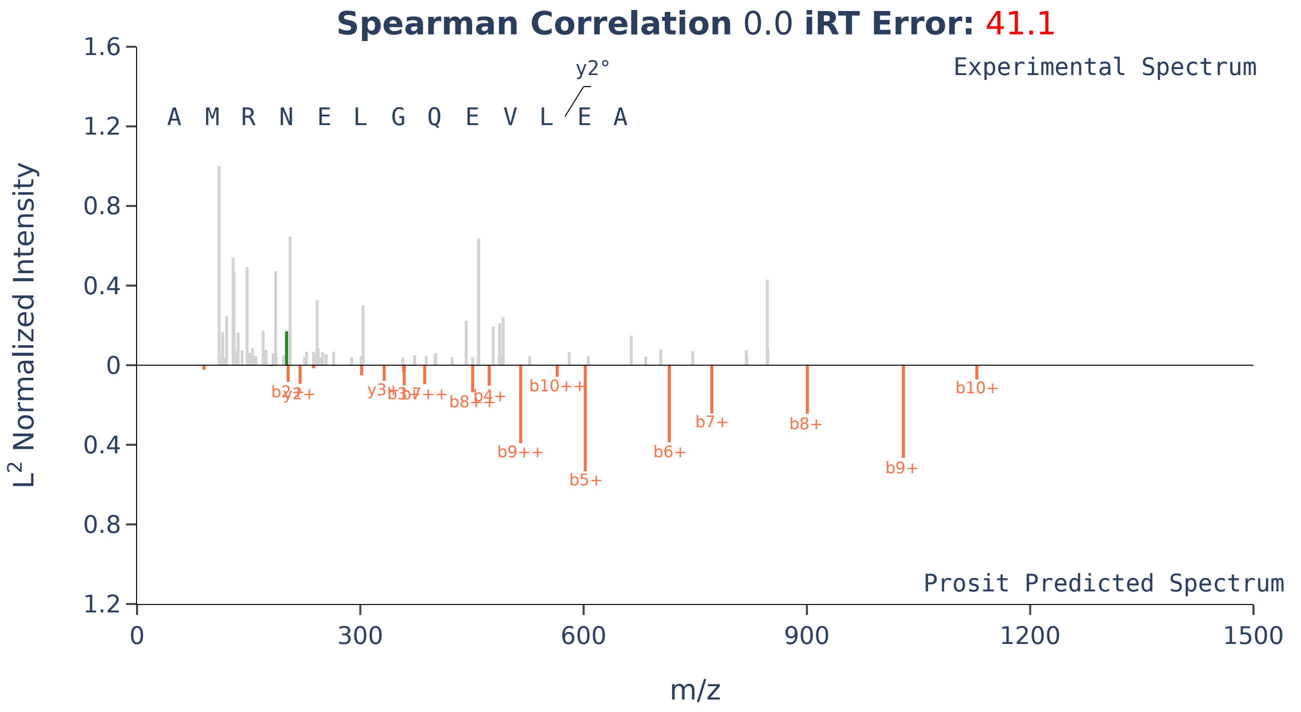

Source HRoetschke_211222_160123_HFGoe_IF2_b1R1_4h **Scan** 11733
Peptide ADRIGAYHVETE **Charge** 2 **Spectral Angle** 0.85
Spearman Correlation 0.87 **iRT Error:** 0.33

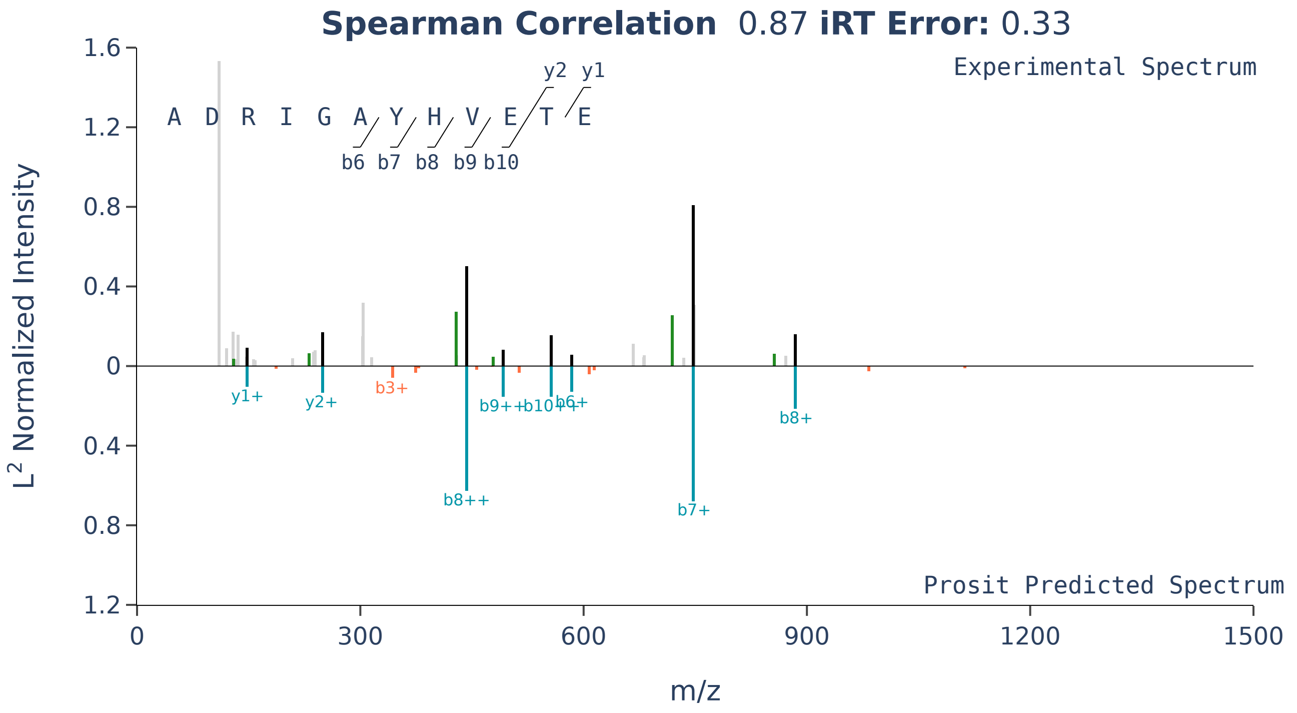

Source HRoetschke_211222_160123_HFGoe_IF2_b1R1_4h **Scan** 11733
Peptide SGEAGGITQHIGAY **Charge** 2 **Spectral Angle** 0.0
Spearman Correlation 0.0 **iRT Error:** 29.4

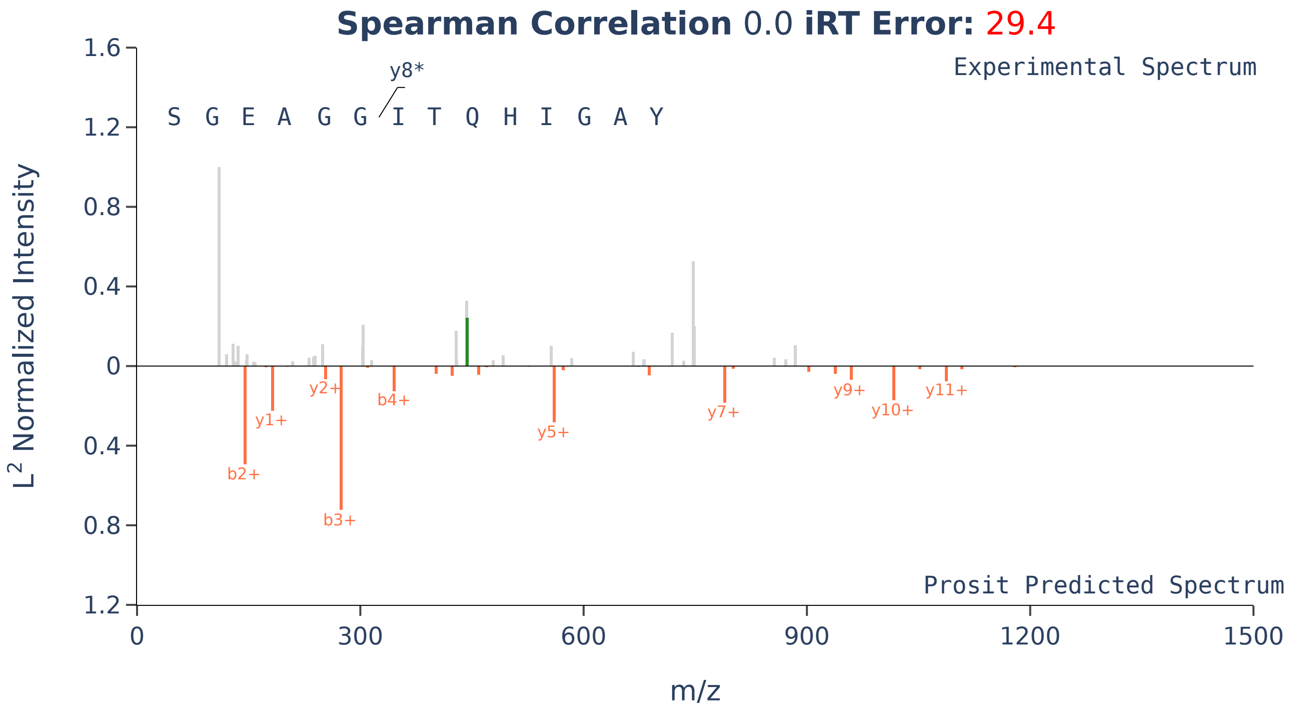

Source HRoetschke_211222_160123_HFGoe_IF2_b1R2_24h **Scan** 15549
Peptide RNGELGQEVLE **Charge** 2 **Spectral Angle** 0.76
Spearman Correlation 0.89 **iRT Error:** 0.35

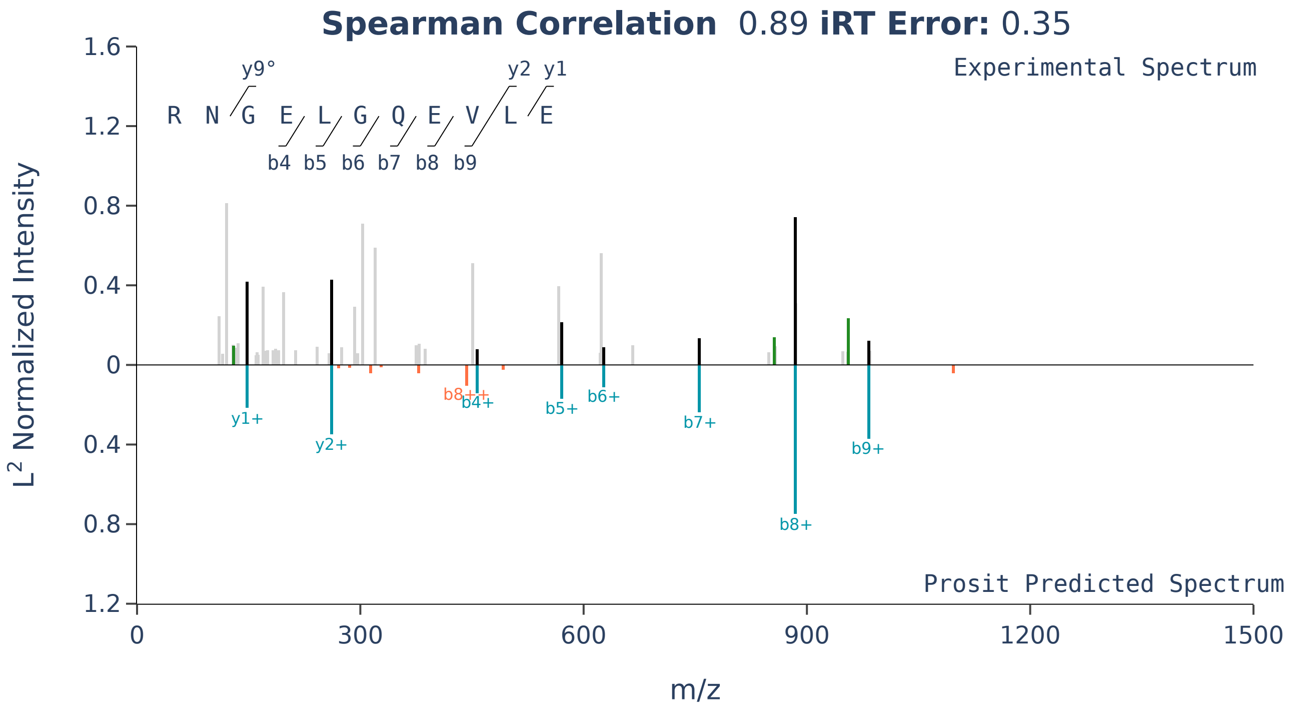

Source HRoetschke_211222_160123_HFGoe_IF2_b1R2_24h **Scan** 15549
Peptide ADPDRVKNELS **Charge** 2 **Spectral Angle** 0.0
Spearman Correlation 0.0 **iRT Error:** 45.48

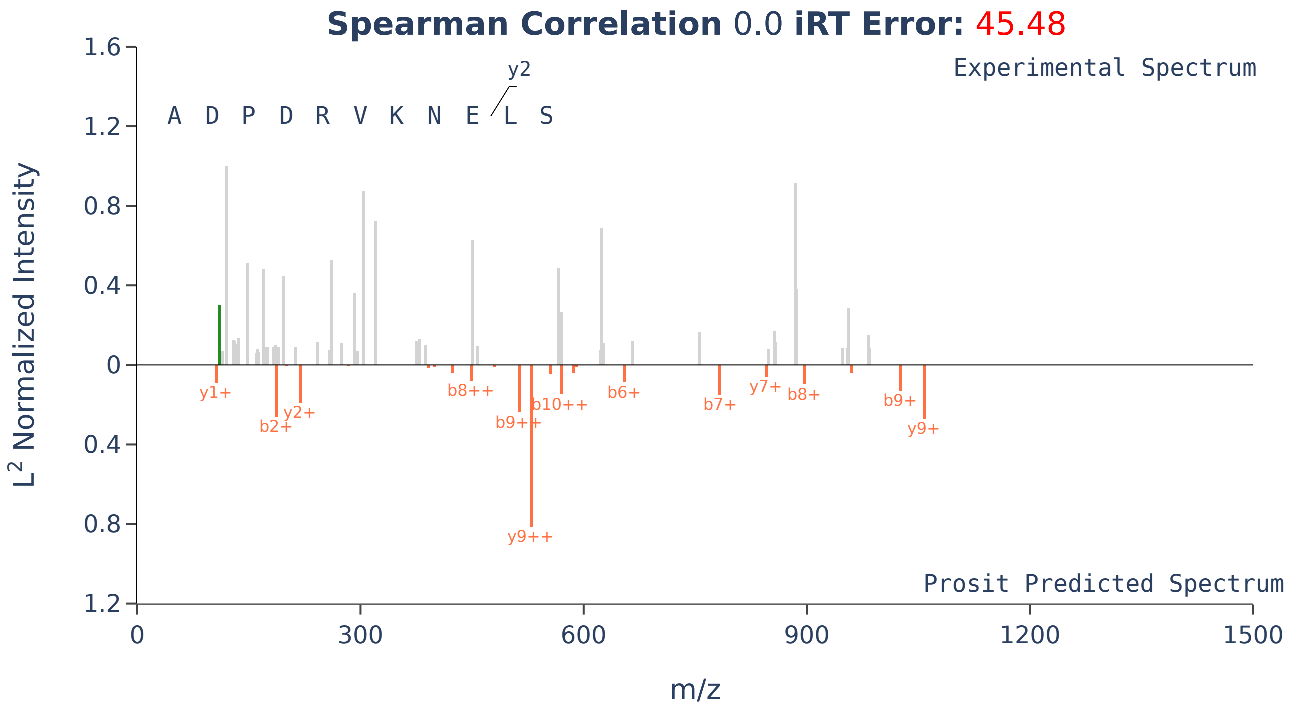

Source HRoetschke_211222_160123_HFGoe_IF2_b1R1_4h **Scan** 15865
Peptide RDNLGQEVLE **Charge** 2 **Spectral Angle** 0.83
Spearman Correlation 0.87 **iRT Error:** 0.35

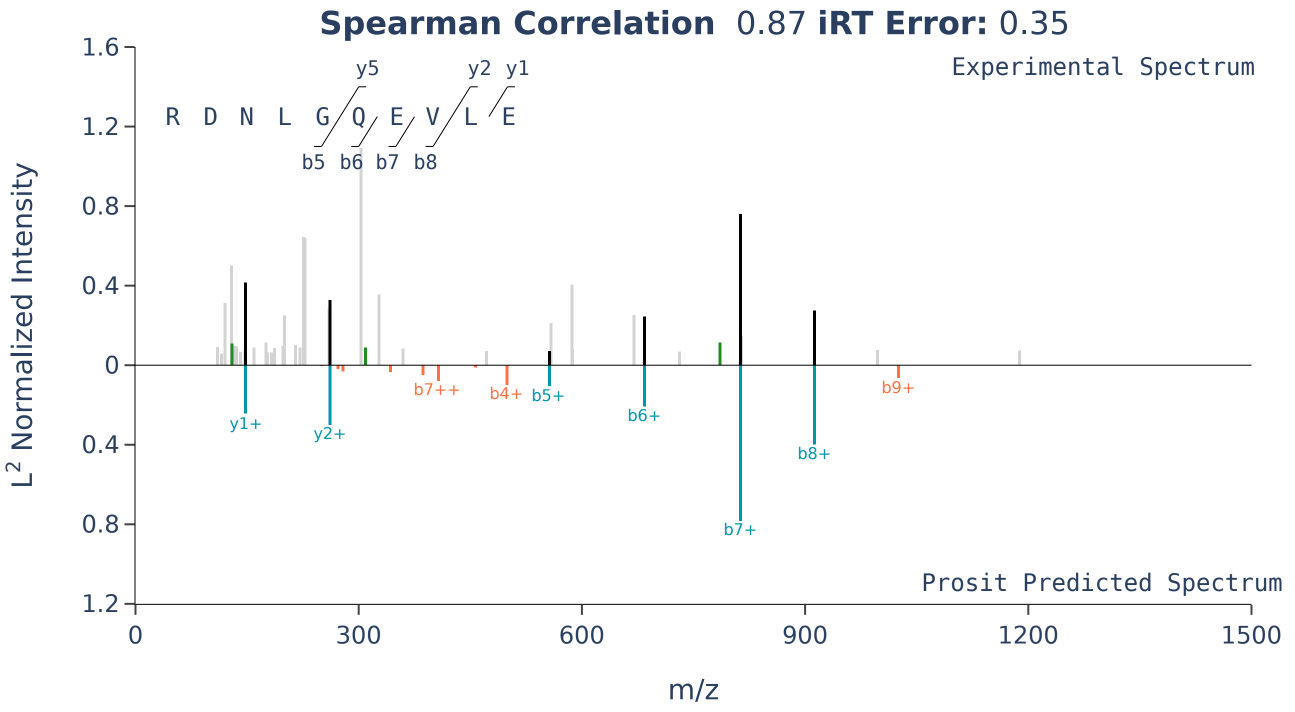

Source HRoetschke_211222_160123_HFGoe_IF2_b1R1_4h **Scan** 15865
Peptide DPDRVKNELS **Charge** 2 **Spectral Angle** 0.0
Spearman Correlation 0.0 **iRT Error:** 48.69

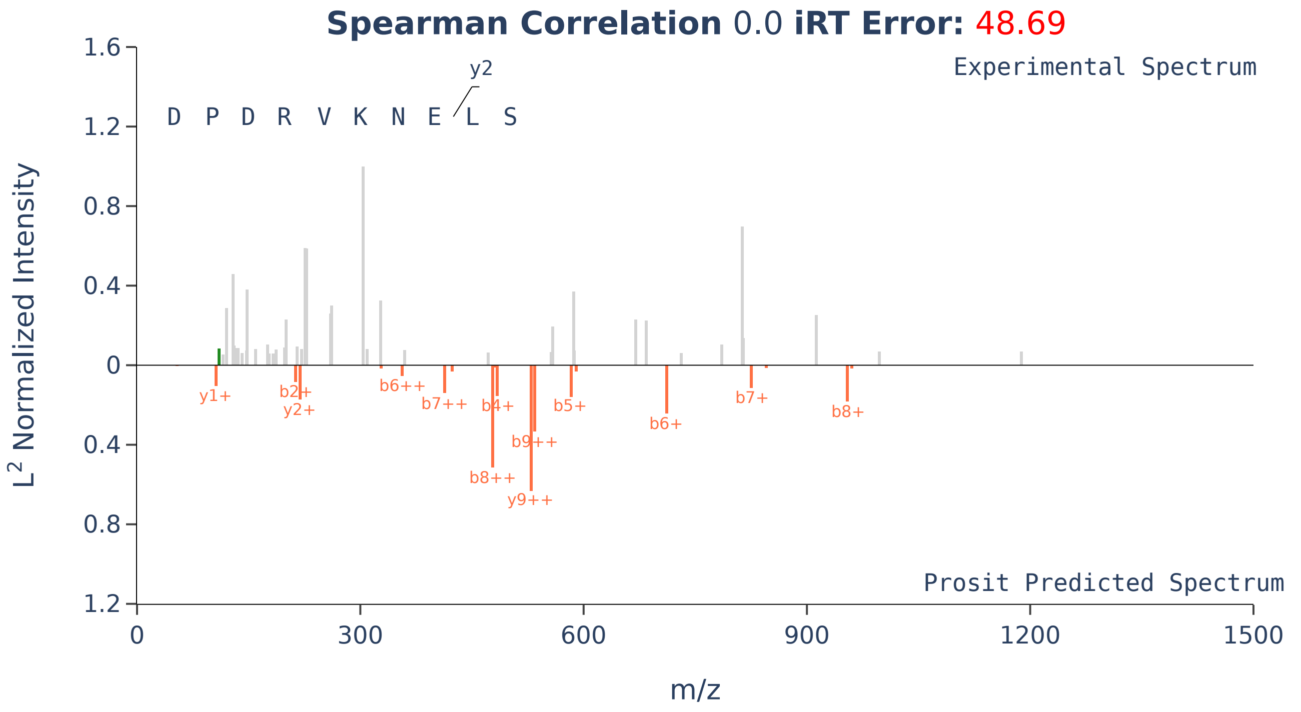

Source HRoetschke_211222_160123_HFGoe_IF2_b2R1_24h **Scan** 11295
Peptide NIPGTIRST **Charge** 2 **Spectral Angle** 0.86
Spearman Correlation 0.95 **iRT Error:** 0.36

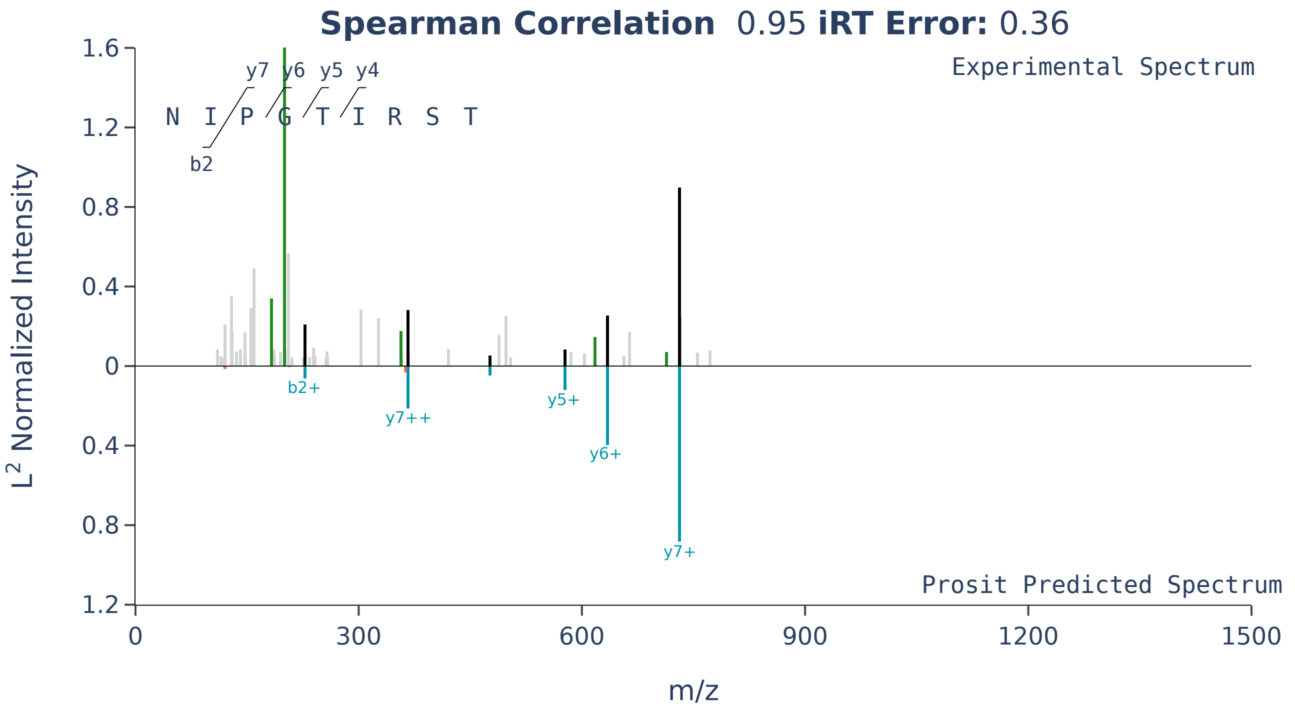

Source HRoetschke_211222_160123_HFGoe_IF2_b2R1_24h **Scan** 11295
Peptide RSTLNIPGT **Charge** 2 **Spectral Angle** 0.0
Spearman Correlation 0.0 **iRT Error:** 27.18

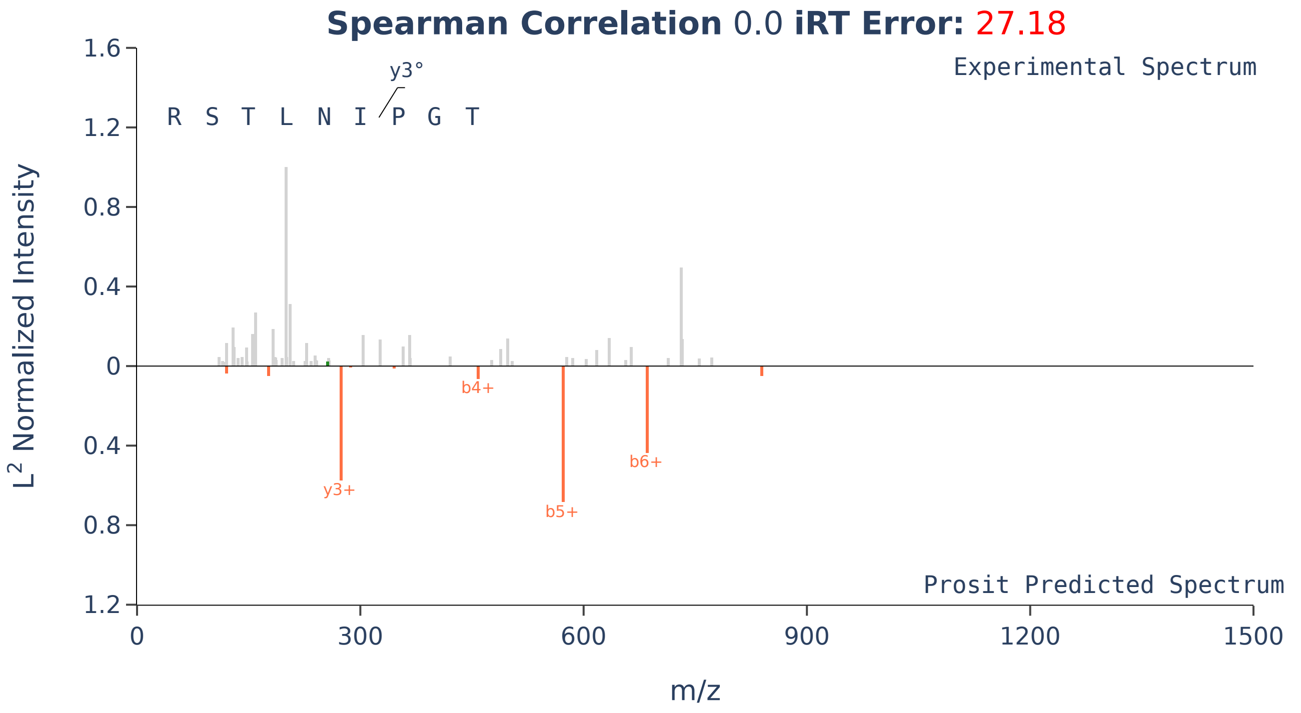

Source HRoetschke_211222_160123_HFGoe_IF2_b1R1_4h **Scan** 11079
Peptide WTDNAVHVSAK **Charge** 3 **Spectral Angle** 0.82
Spearman Correlation 0.84 **iRT Error:** 0.37

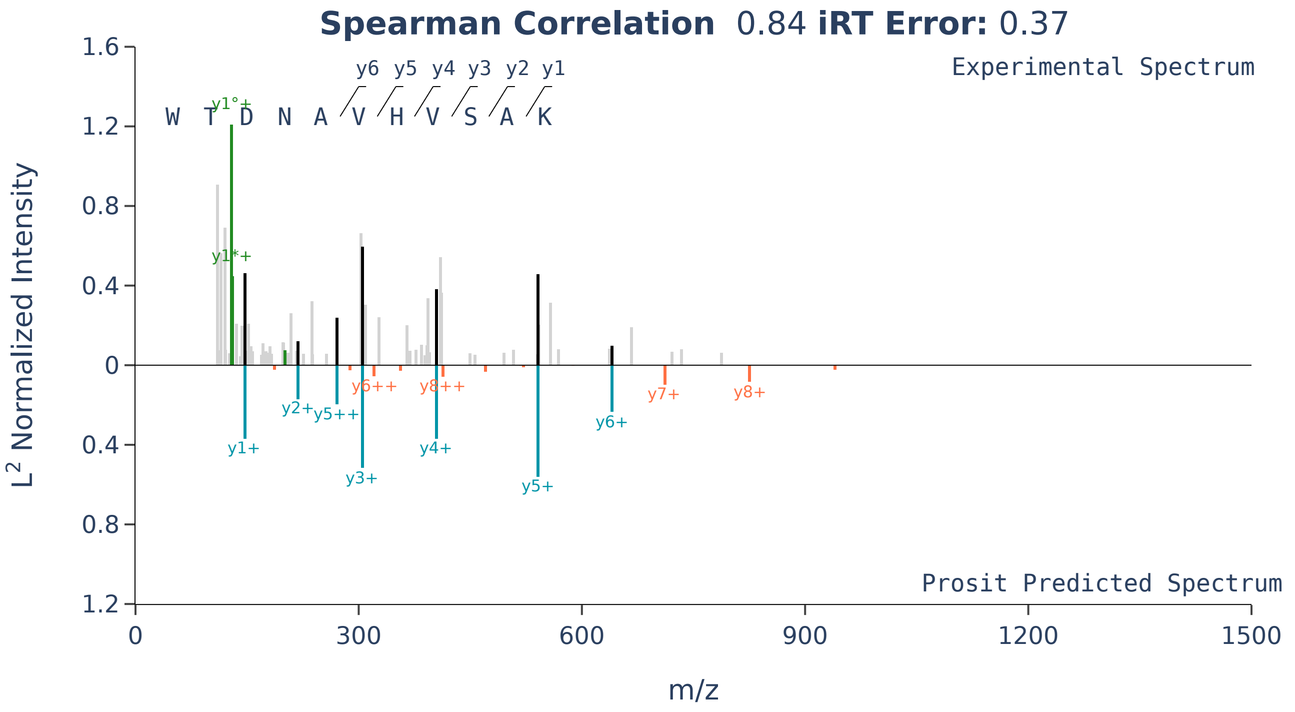

Source HRoetschke_211222_160123_HFGoe_IF2_b1R1_4h **Scan** 11079
Peptide VTTSQHARQAE **Charge** 3 **Spectral Angle** 0.13
Spearman Correlation -0.0 **iRT Error:** 45.8

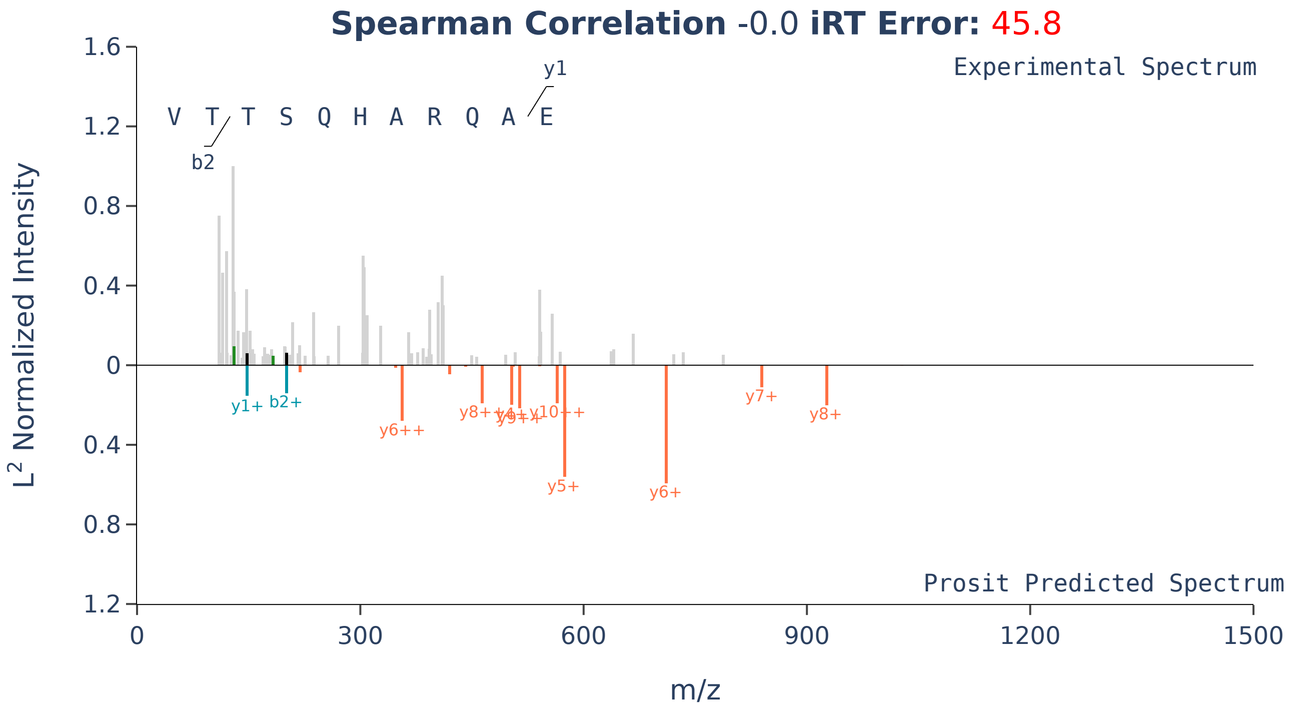

Source HRoetschke_211222_160123_HFGoe_IF2_b1R2_1h **Scan** 11112
Peptide VNRDVVISD **Charge** 2 **Spectral Angle** 0.81
Spearman Correlation 0.89 **iRT Error:** 0.59

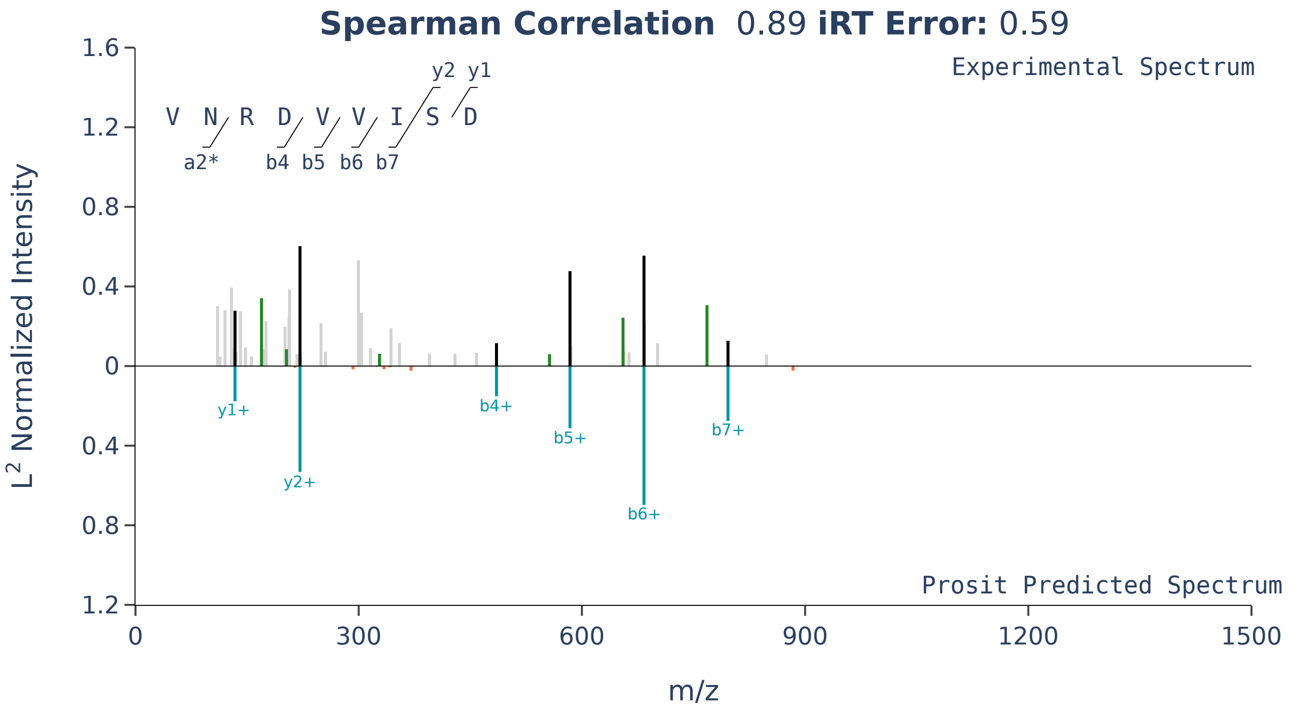

Source HRoetschke_211222_160123_HFGoe_IF2_b1R2_1h **Scan** 11112
Peptide AAGDEVTVVR **Charge** 2 **Spectral Angle** 0.09
Spearman Correlation 0.12 **iRT Error:** 1.46

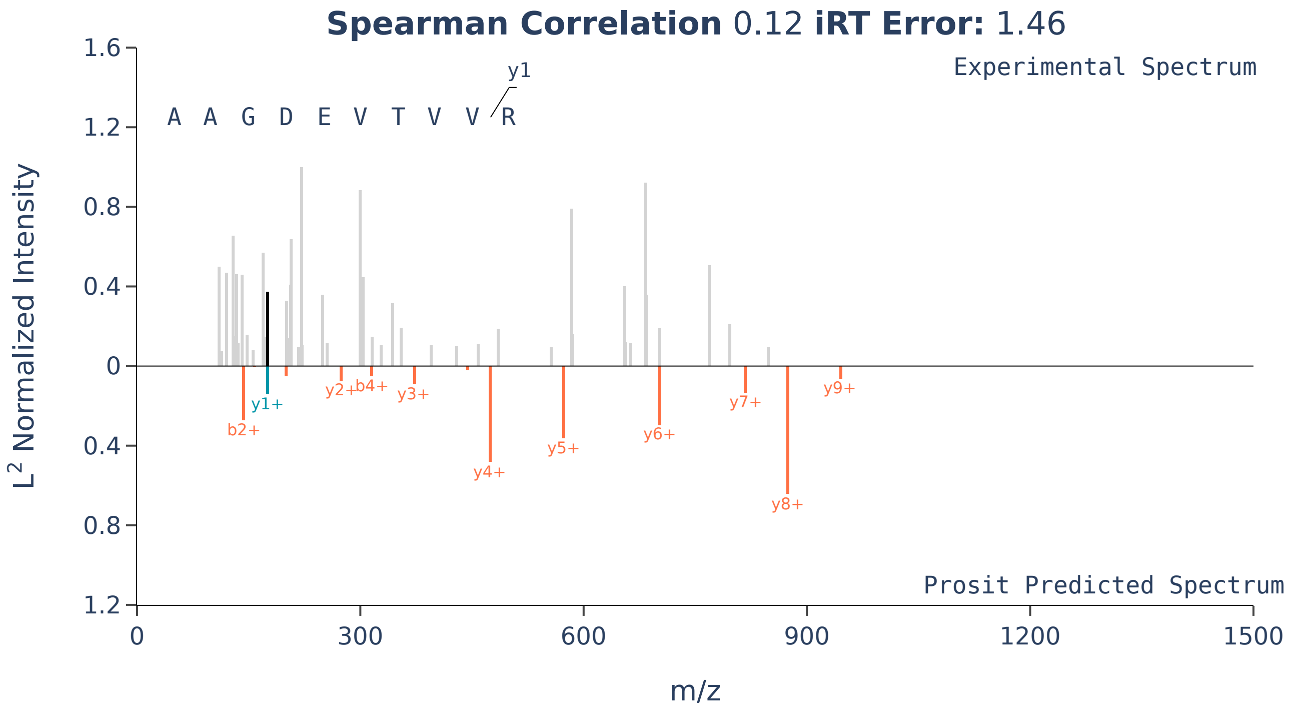

Source HRoetschke_211222_160123_HFGoe_IF2_b1R1_4h **Scan** 16241
Peptide GRNELGQEVLE **Charge** 2 **Spectral Angle** 0.81
Spearman Correlation 0.91 **iRT Error:** 0.6

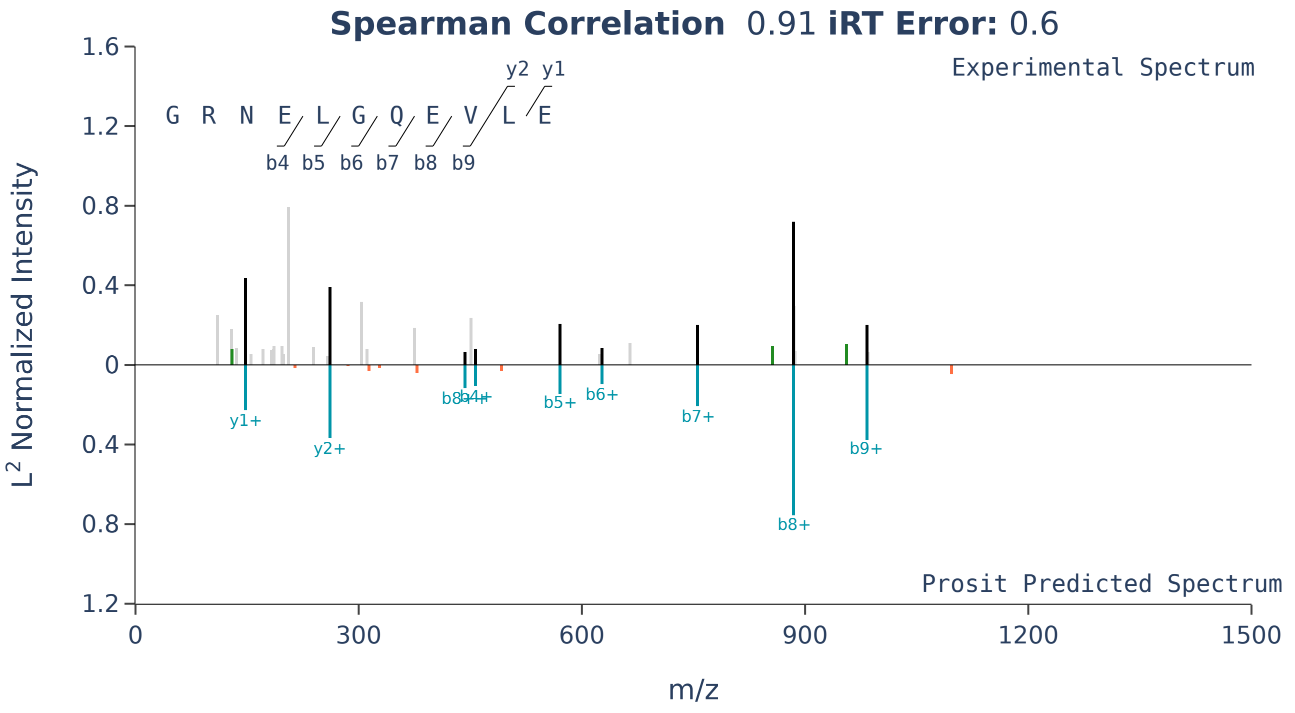

Source HRoetschke_211222_160123_HFGoe_IF2_b1R1_4h **Scan** 16241
Peptide ADPDRVKNELS **Charge** 2 **Spectral Angle** 0.0
Spearman Correlation 0.0 **iRT Error:** 51.34

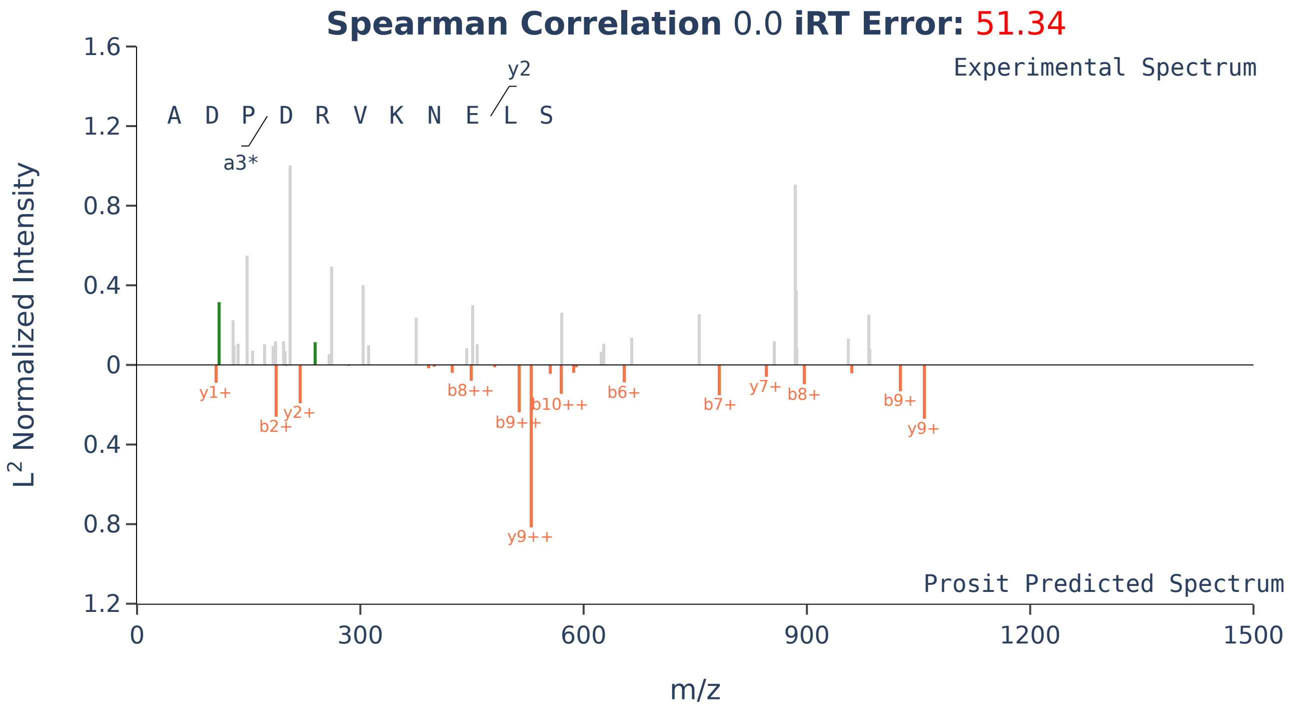

Source HRoetschke_211222_160123_HFGoe_IF2_b1R1_4h **Scan** 11273
Peptide YHGAVIE **Charge** 2 **Spectral Angle** 0.76
Spearman Correlation 0.86 **iRT Error:** 0.61

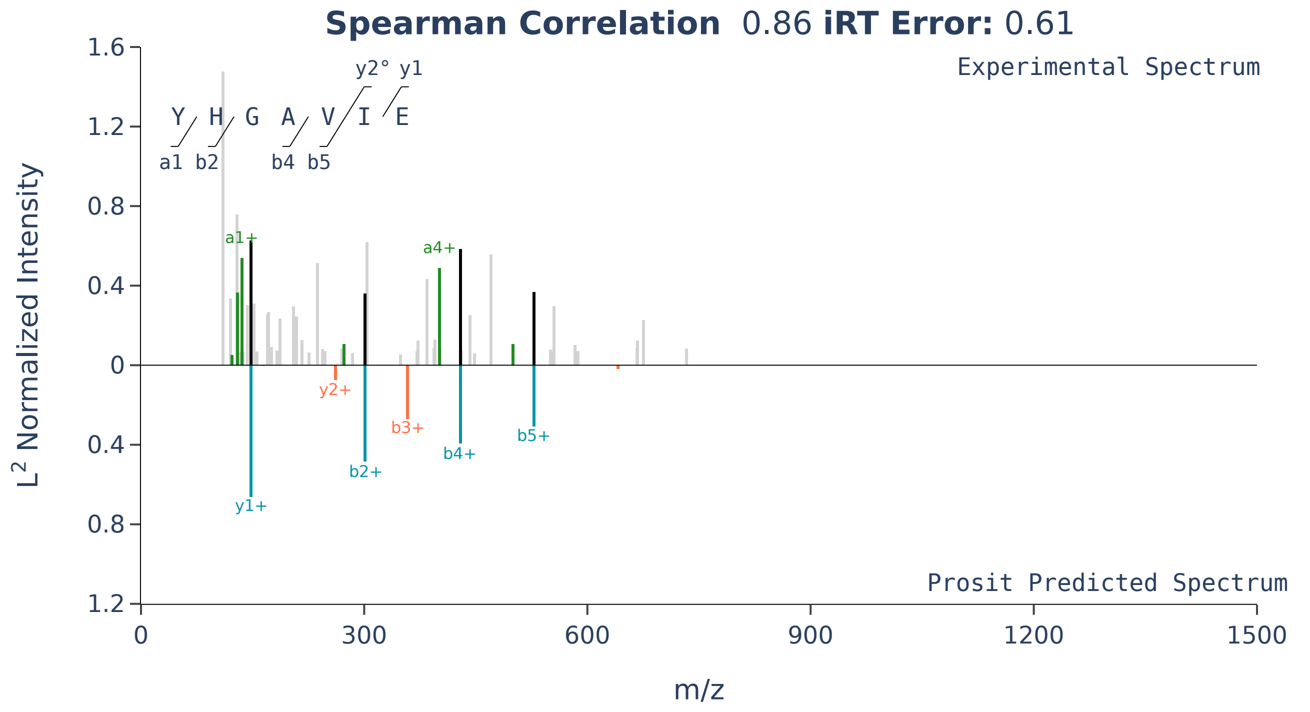

Source HRoetschke_211222_160123_HFGoe_IF2_b1R1_4h **Scan** 11273
Peptide IGAYHVE **Charge** 2 **Spectral Angle** 0.48
Spearman Correlation 0.54 **iRT Error:** 1.81

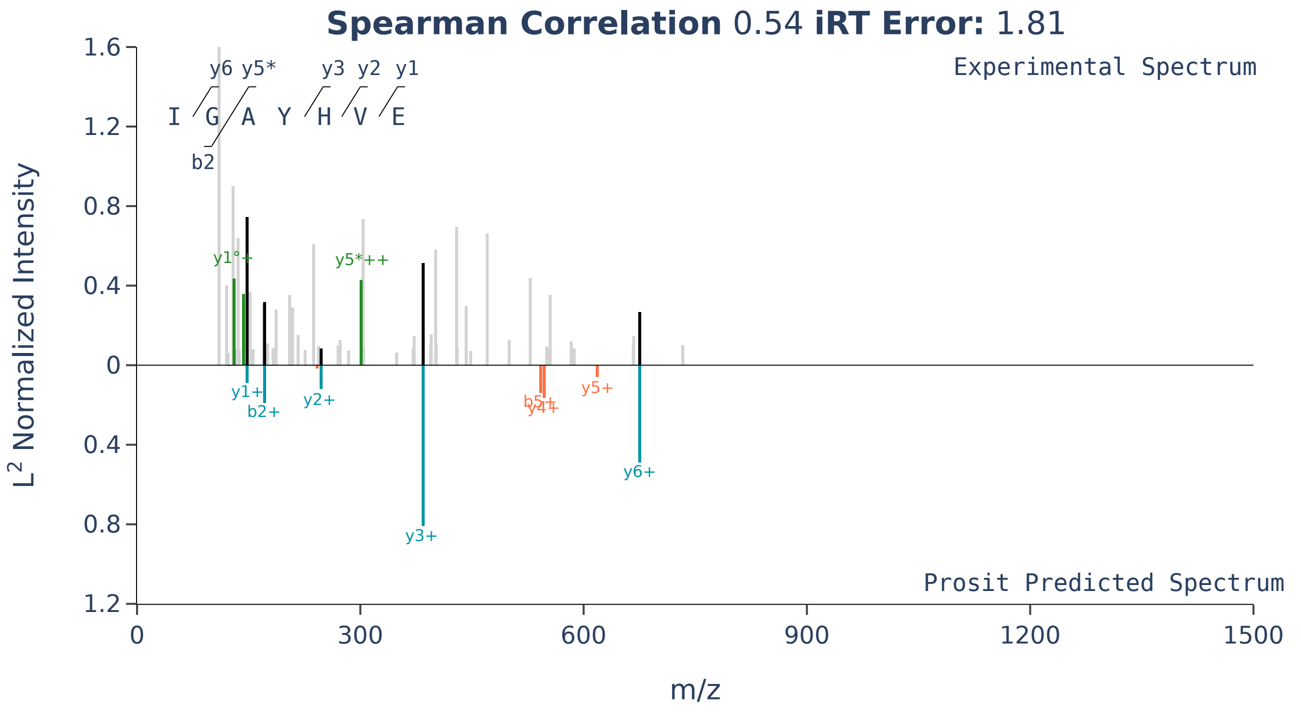

Source HRoetschke_211222_160123_HFGoe_IF2_b2R1_1h **Scan** 11307
Peptide VNRDVVIQE **Charge** 2 **Spectral Angle** 0.85
Spearman Correlation 0.9 **iRT Error:** 0.62

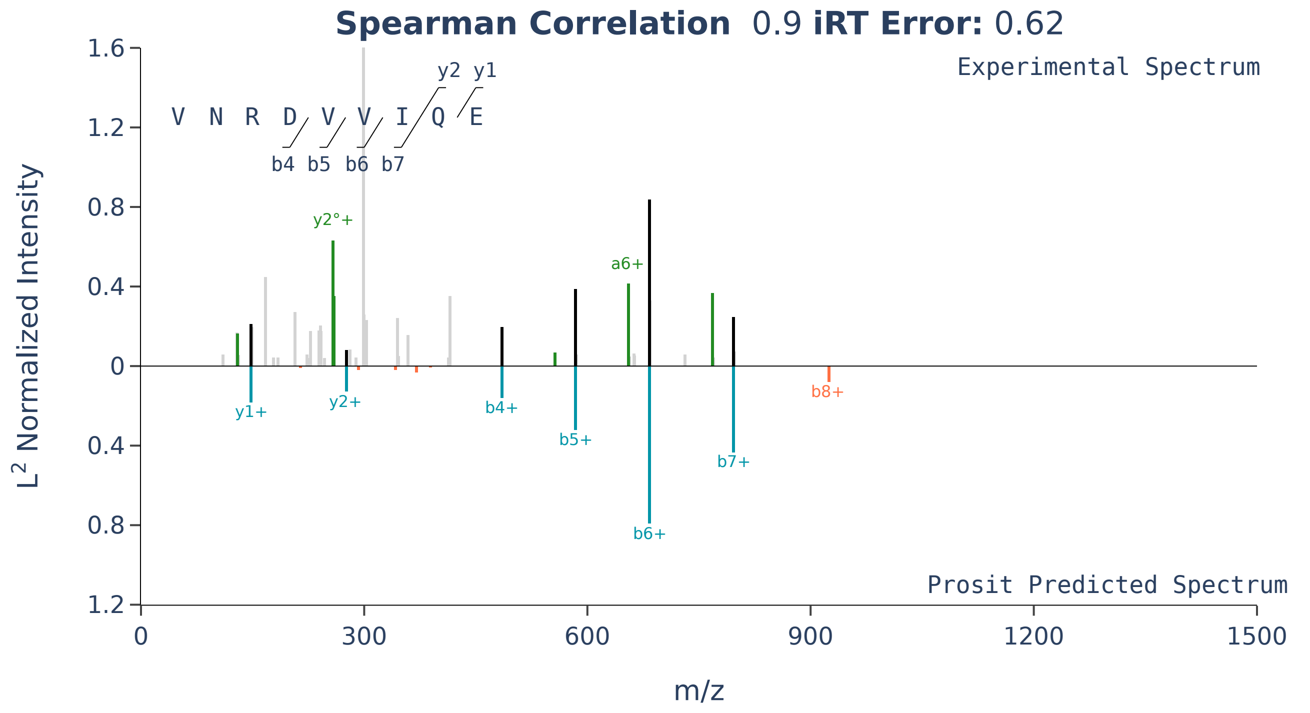

Source HRoetschke_211222_160123_HFGoe_IF2_b2R1_1h **Scan** 11307
Peptide AVNRDVVIGE **Charge** 2 **Spectral Angle** 0.18
Spearman Correlation 0.25 **iRT Error:** 7.78

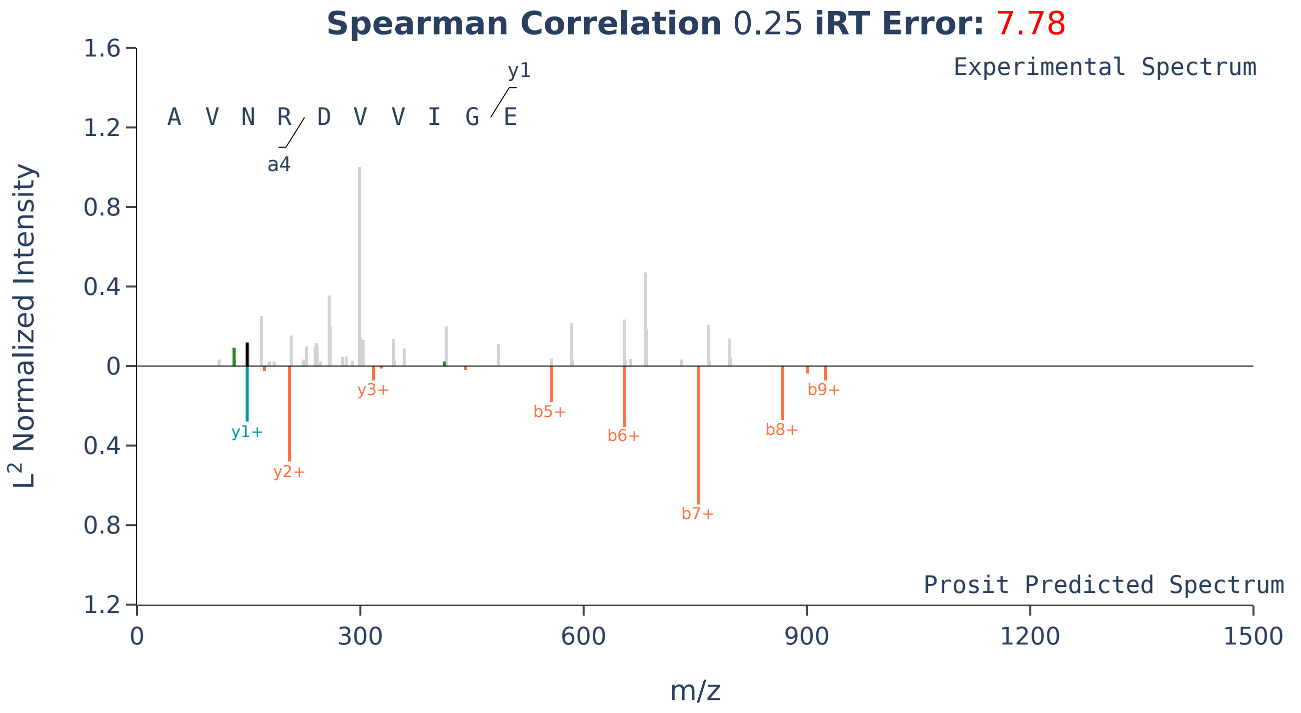

Source HRoetschke_211222_160123_HFGoe_IF2_b1R1_4h **Scan** 6475
Peptide ASGEAGGITQR **Charge** 2 **Spectral Angle** 0.85
Spearman Correlation 0.93 **iRT Error:** 0.63

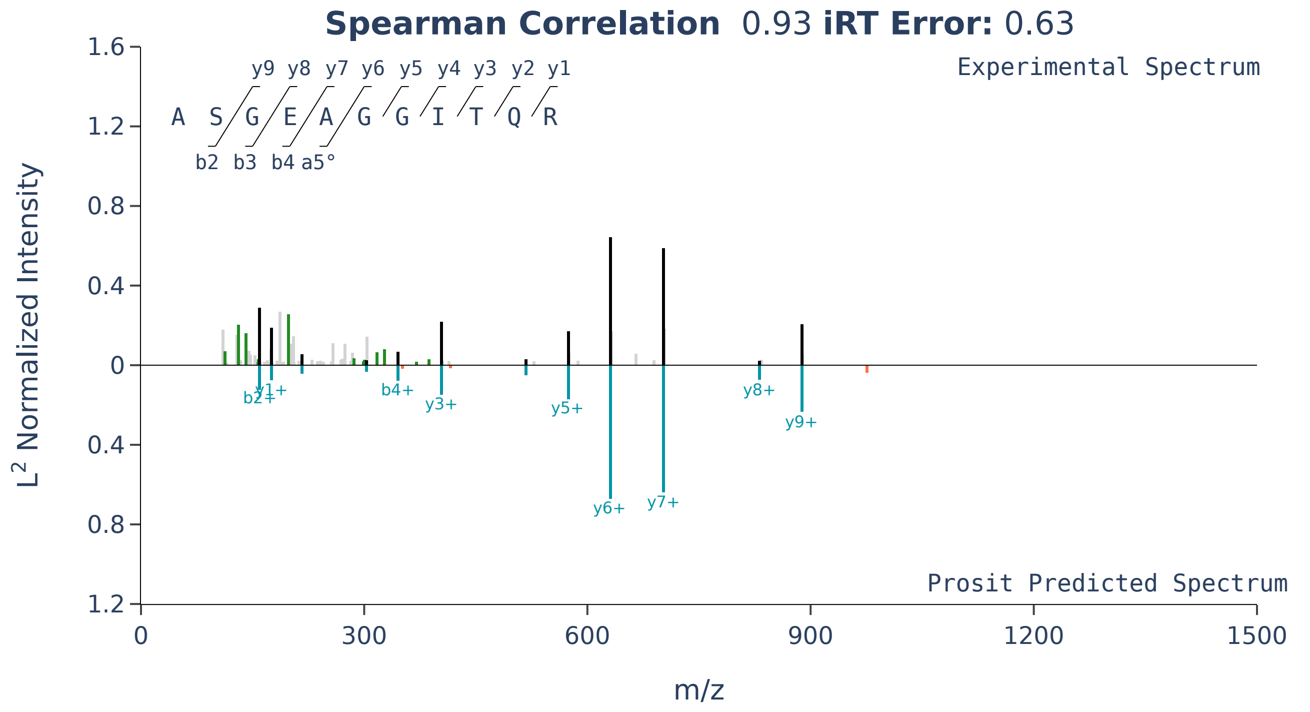

Source HRoetschke_211222_160123_HFGoe_IF2_b1R1_4h **Scan** 6475
Peptide ESAKREAQQ **Charge** 2 **Spectral Angle** 0.54
Spearman Correlation 0.39 **iRT Error:** 9.67

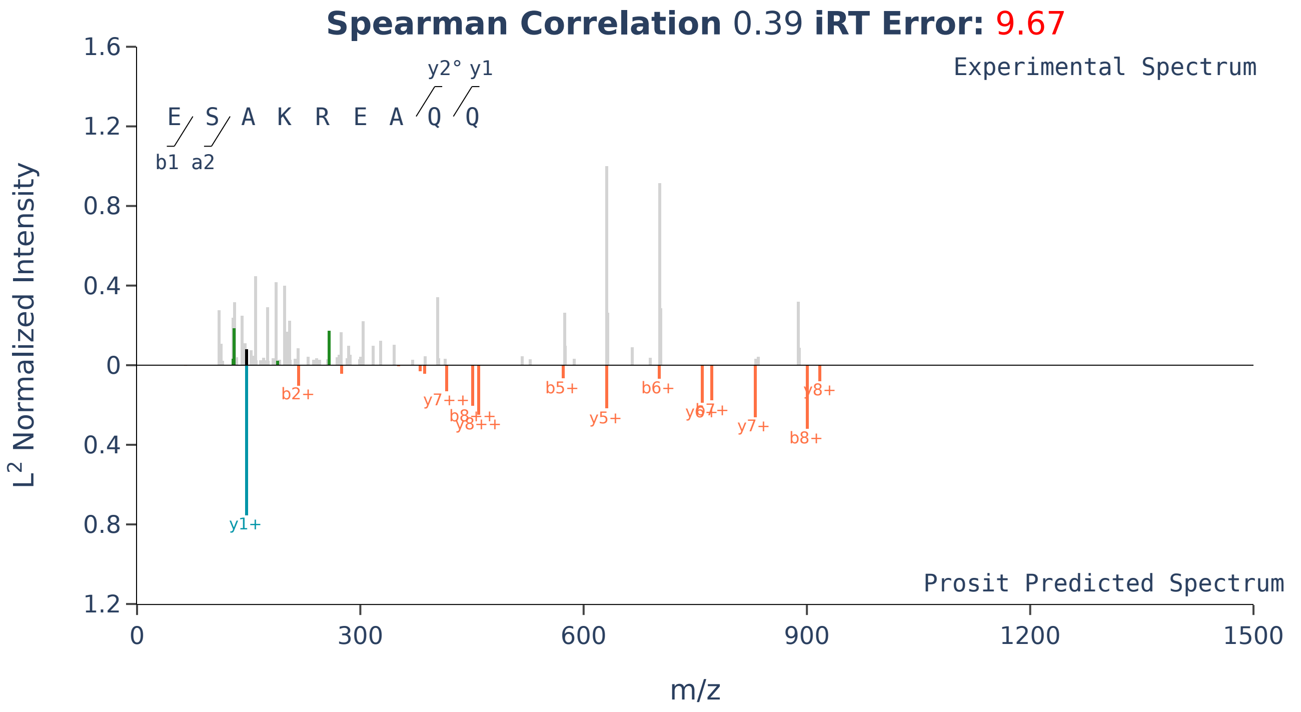

Source HRoetschke_211222_160123_HFGoe_IF2_b2R2_24h **Scan** 6458
Peptide KVASGEAGGIRQT **Charge** 2 **Spectral Angle** 0.83
Spearman Correlation 0.87 **iRT Error:** 0.64

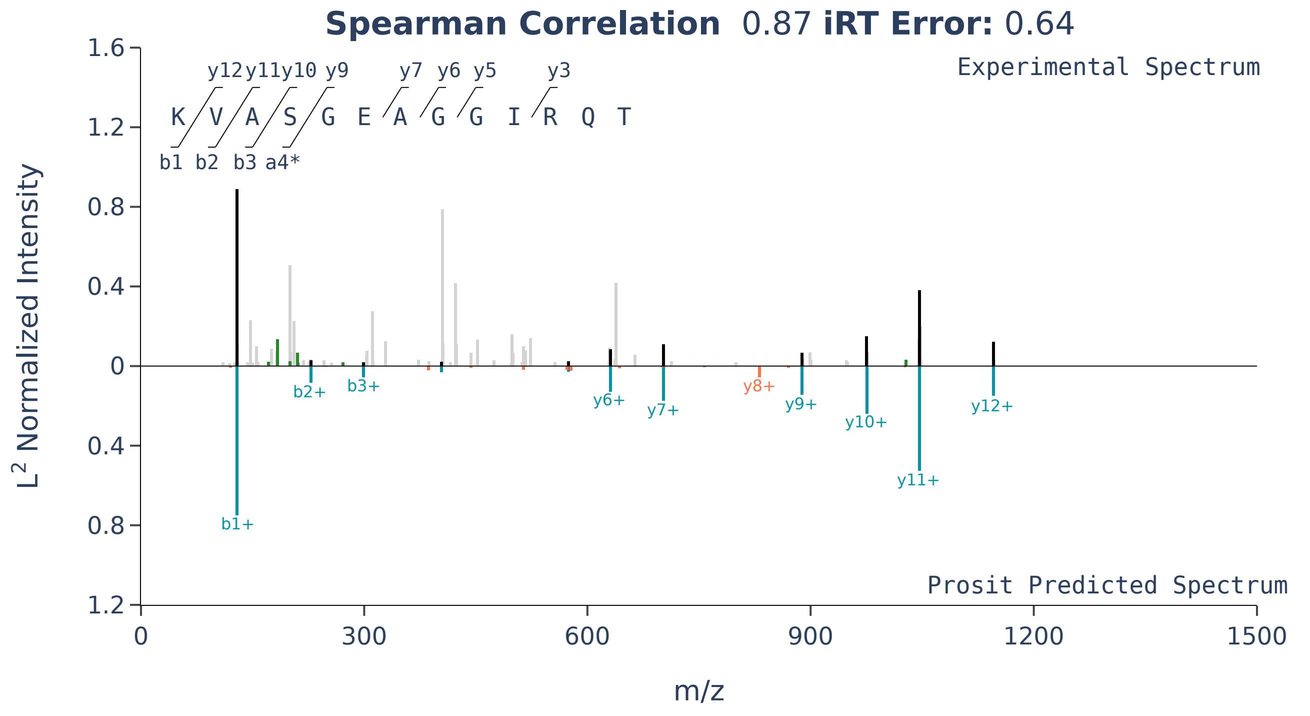

Source HRoetschke_211222_160123_HFGoe_IF2_b2R2_24h **Scan** 6458
Peptide QAKREAAEKDK **Charge** 2 **Spectral Angle** 0.0
Spearman Correlation 0.0 **iRT Error:** 11.71

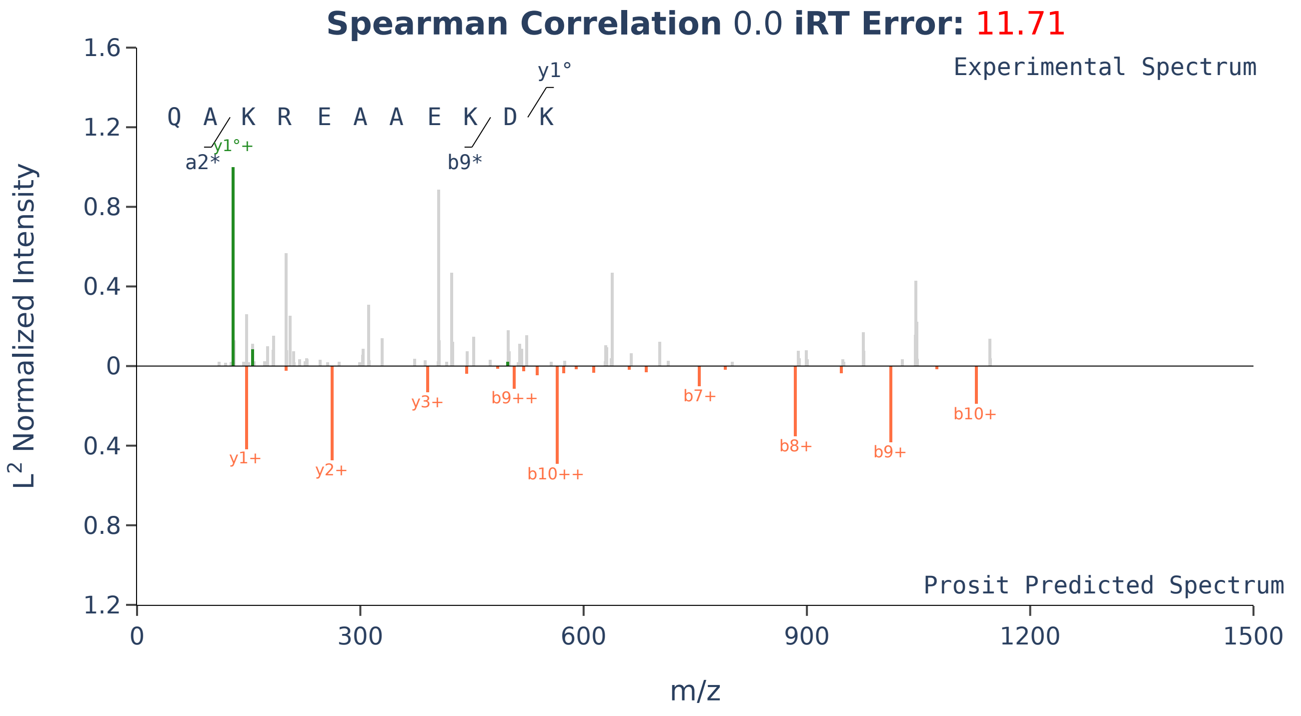

Source HRoetschke_211222_160123_HFGoe_IF2_b2R1_24h **Scan** 11657
Peptide SLQQGFQKPQAE **Charge** 2 **Spectral Angle** 0.85
Spearman Correlation 0.81 **iRT Error:** 0.64

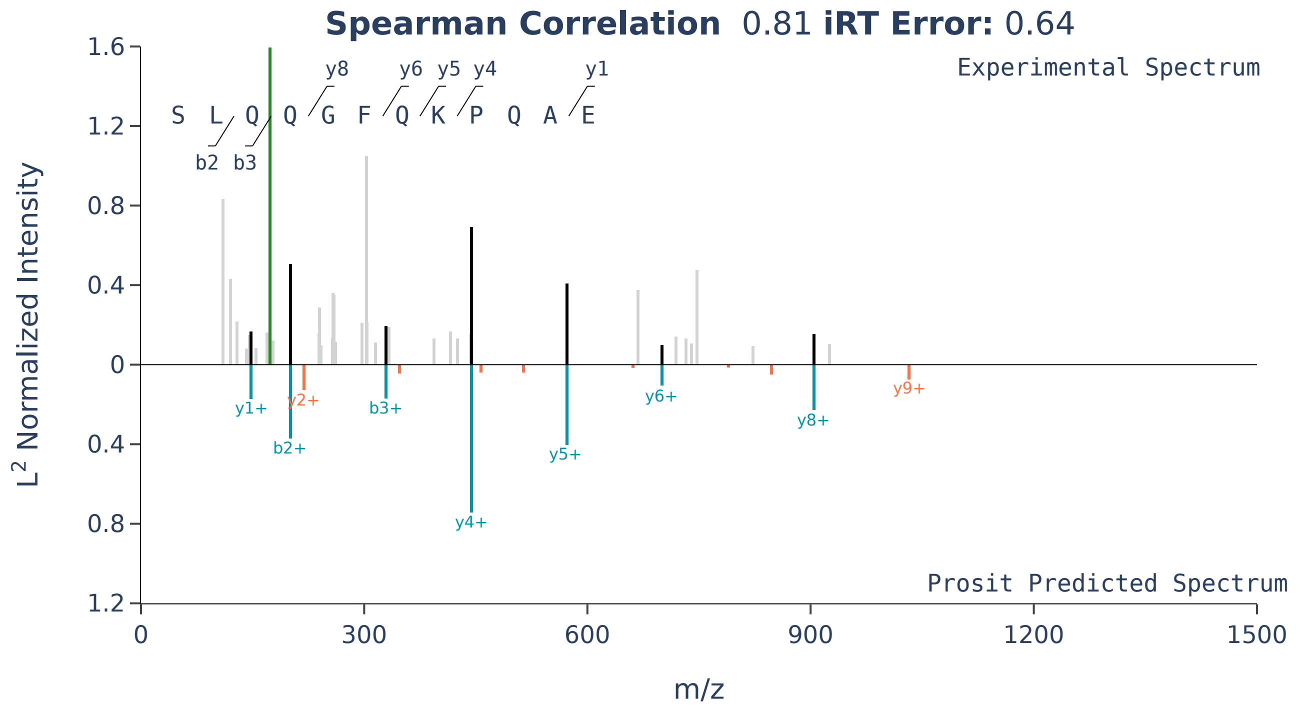

Source HRoetschke_211222_160123_HFGoe_IF2_b2R1_24h **Scan** 11657
Peptide ESQFVHVSAKAGT **Charge** 2 **Spectral Angle** 0.0
Spearman Correlation 0.0 **iRT Error:** 4.74

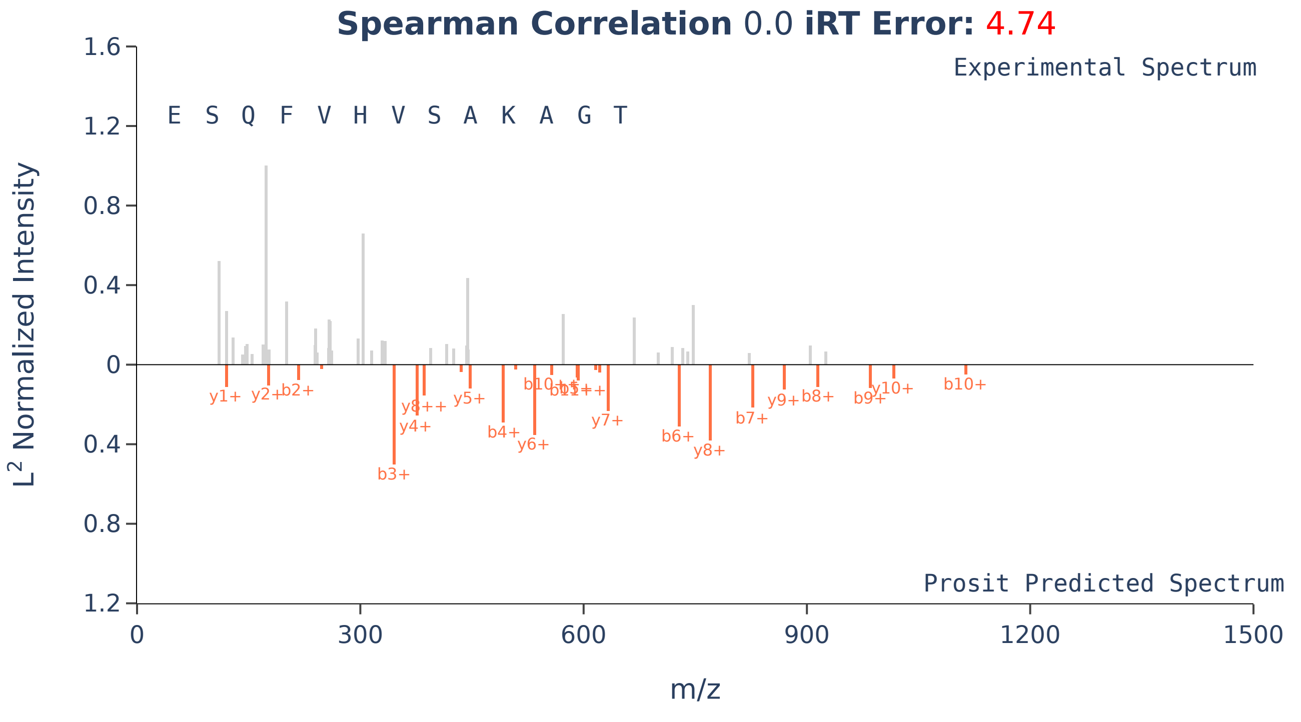

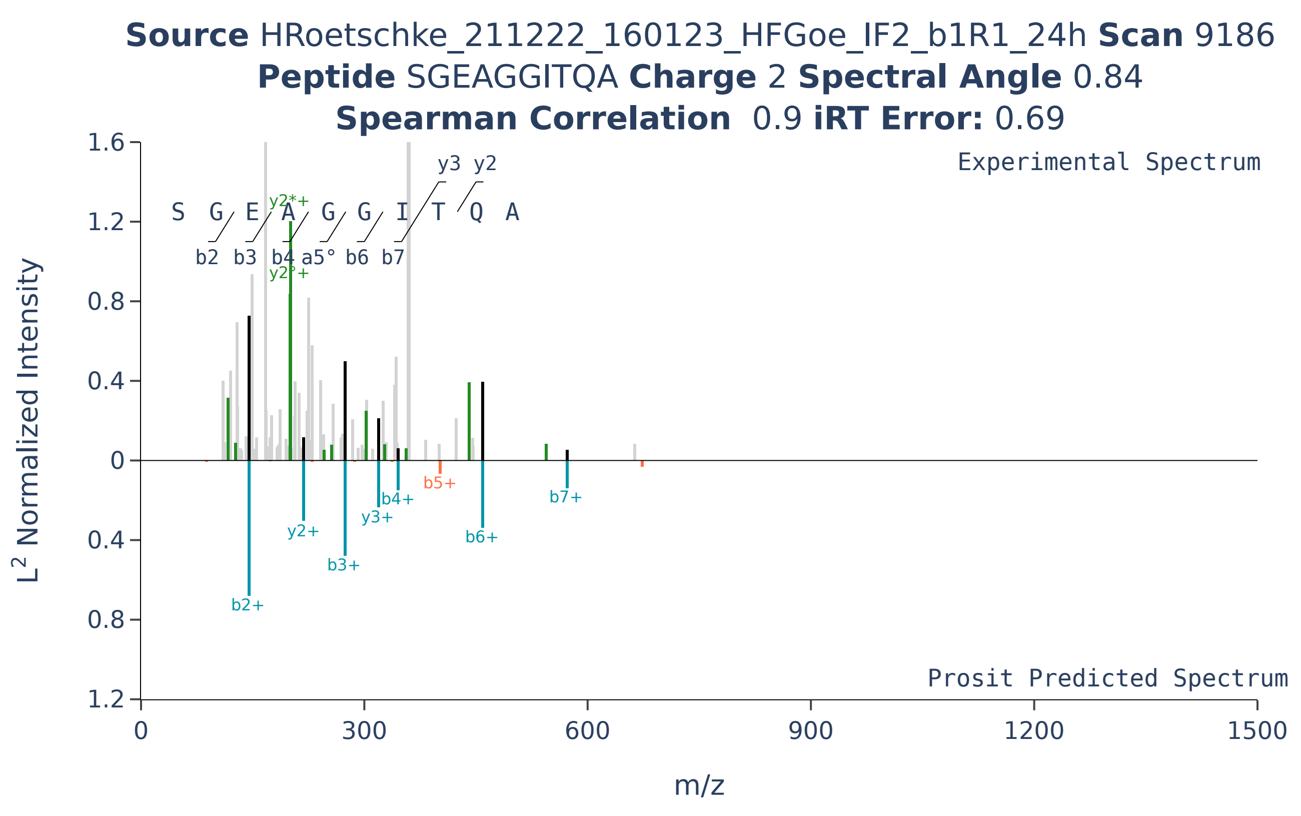
Source HRoetschke_211222_160123_HFGoe_IF2_b1R1_24h Scan 9186
Peptide SGEAGGITQA Charge 2 Spectral Angle 0.84
Spearman Correlation 0.9 iRT Error: 0.69
Experimental Spectrum
Prosit Predicted Spectrum
L² Normalized Intensity
m/z

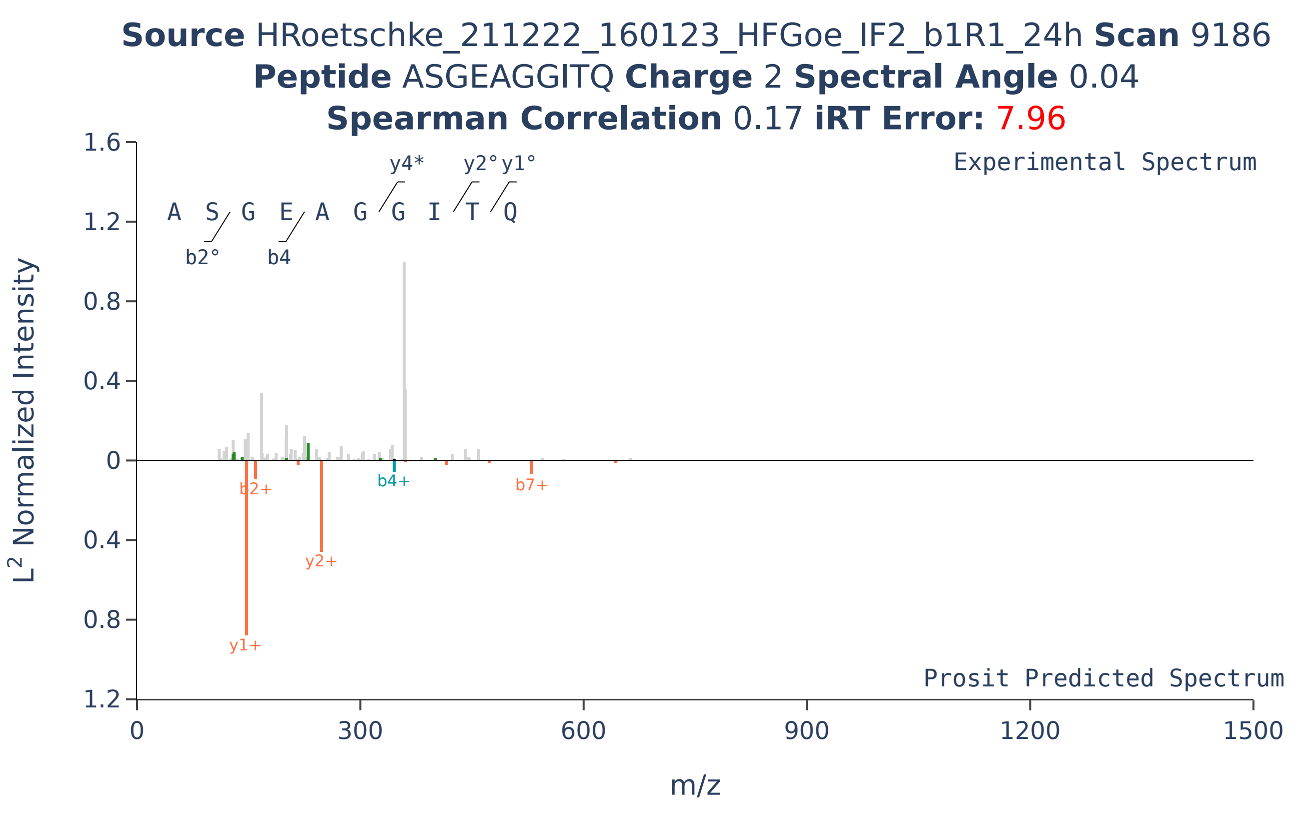
Source HRoetschke_211222_160123_HFGoe_IF2_b1R1_24h Scan 9186
Peptide ASGEAGGITQ Charge 2 Spectral Angle 0.04
Spearman Correlation 0.17 iRT Error: 7.96
Experimental Spectrum
Prosit Predicted Spectrum
L² Normalized Intensity
m/z

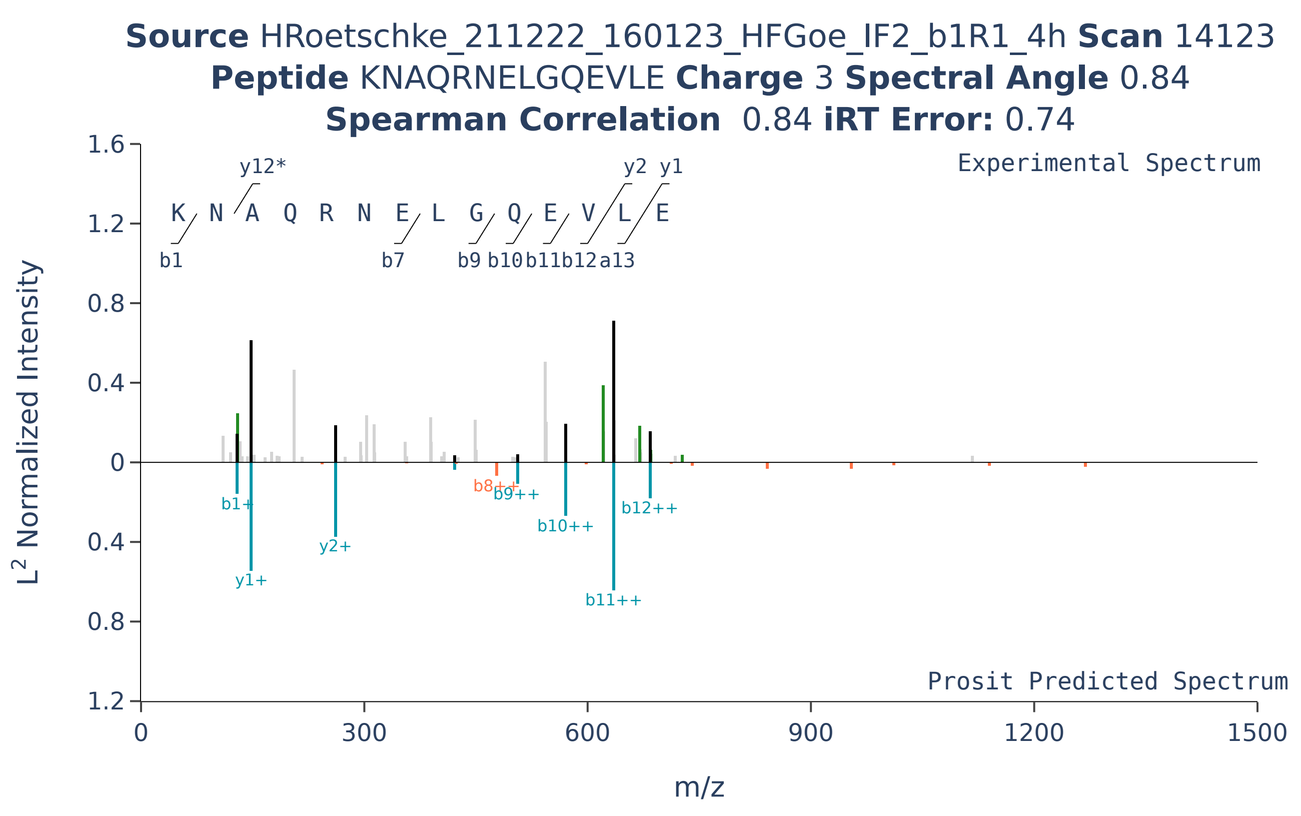
Source HRoetschke_211222_160123_HFGoe_IF2_b1R1_4h Scan 14123
Peptide KNAQRNELGQEVLE Charge 3 Spectral Angle 0.84
Spearman Correlation 0.84 iRT Error: 0.74
Experimental Spectrum
Prosit Predicted Spectrum
L² Normalized Intensity
m/z

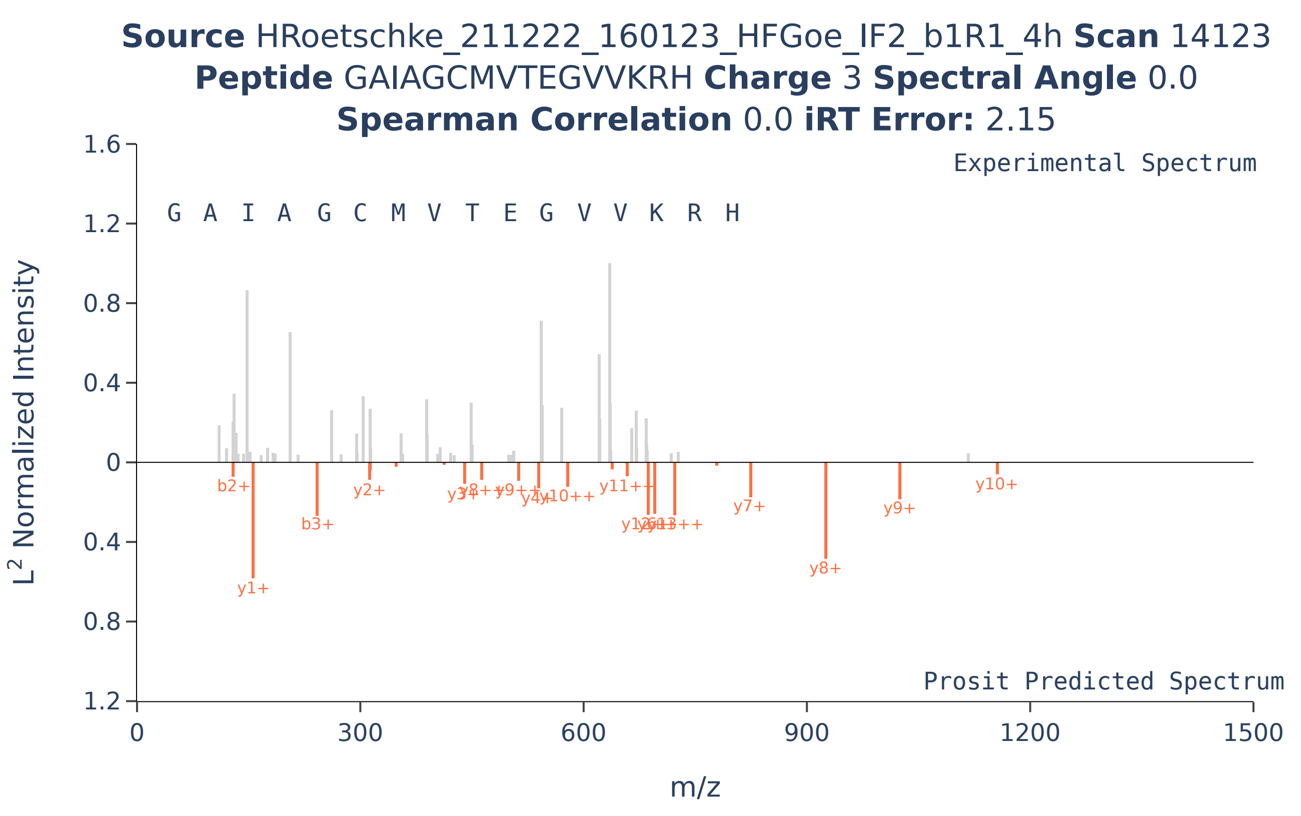
Source HRoetschke_211222_160123_HFGoe_IF2_b1R1_4h Scan 14123
Peptide GAIAGCMVTEGVVKRH Charge 3 Spectral Angle 0.0
Spearman Correlation 0.0 iRT Error: 2.15
Experimental Spectrum
Prosit Predicted Spectrum
L² Normalized Intensity
m/z

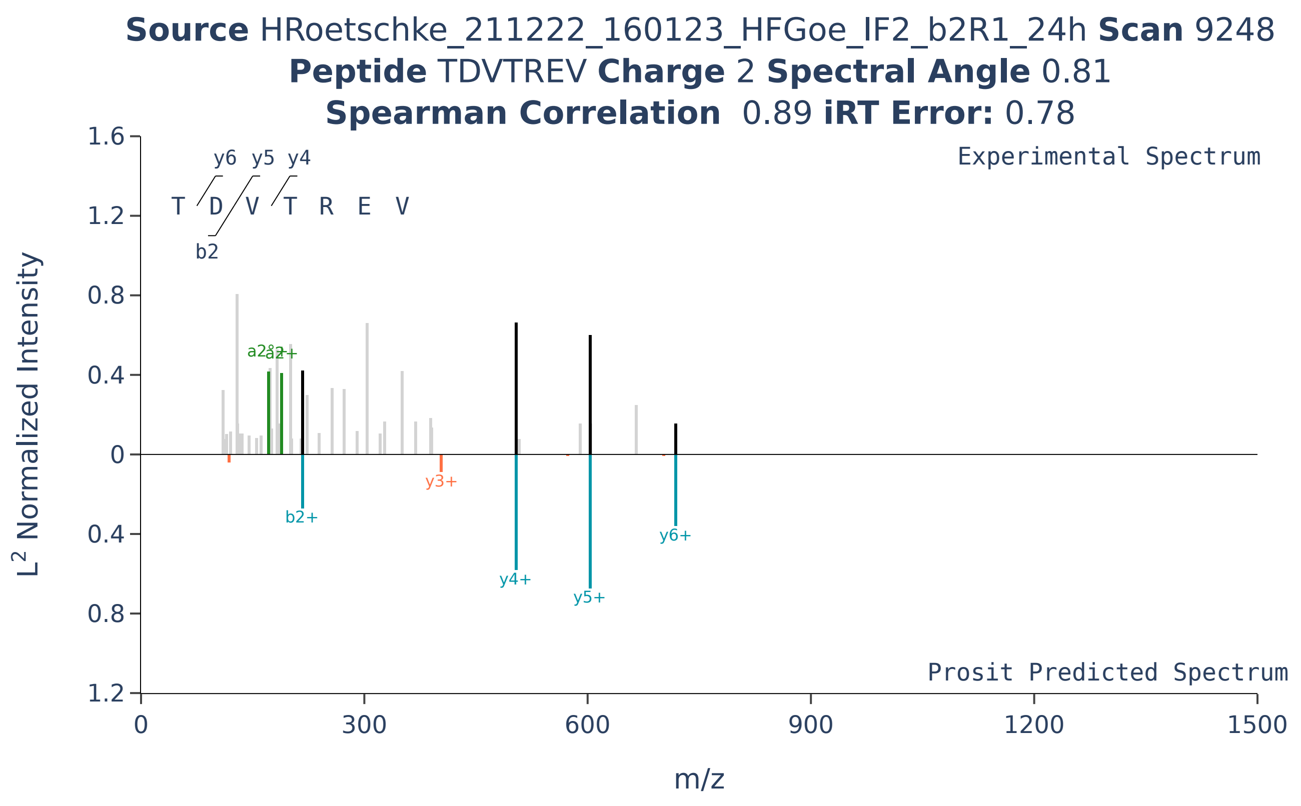
Source HRoetschke_211222_160123_HFGoe_IF2_b2R1_24h Scan 9248
Peptide TDVTREV Charge 2 Spectral Angle 0.81
Spearman Correlation 0.89 iRT Error: 0.78
Experimental Spectrum
Prosit Predicted Spectrum
L² Normalized Intensity
m/z

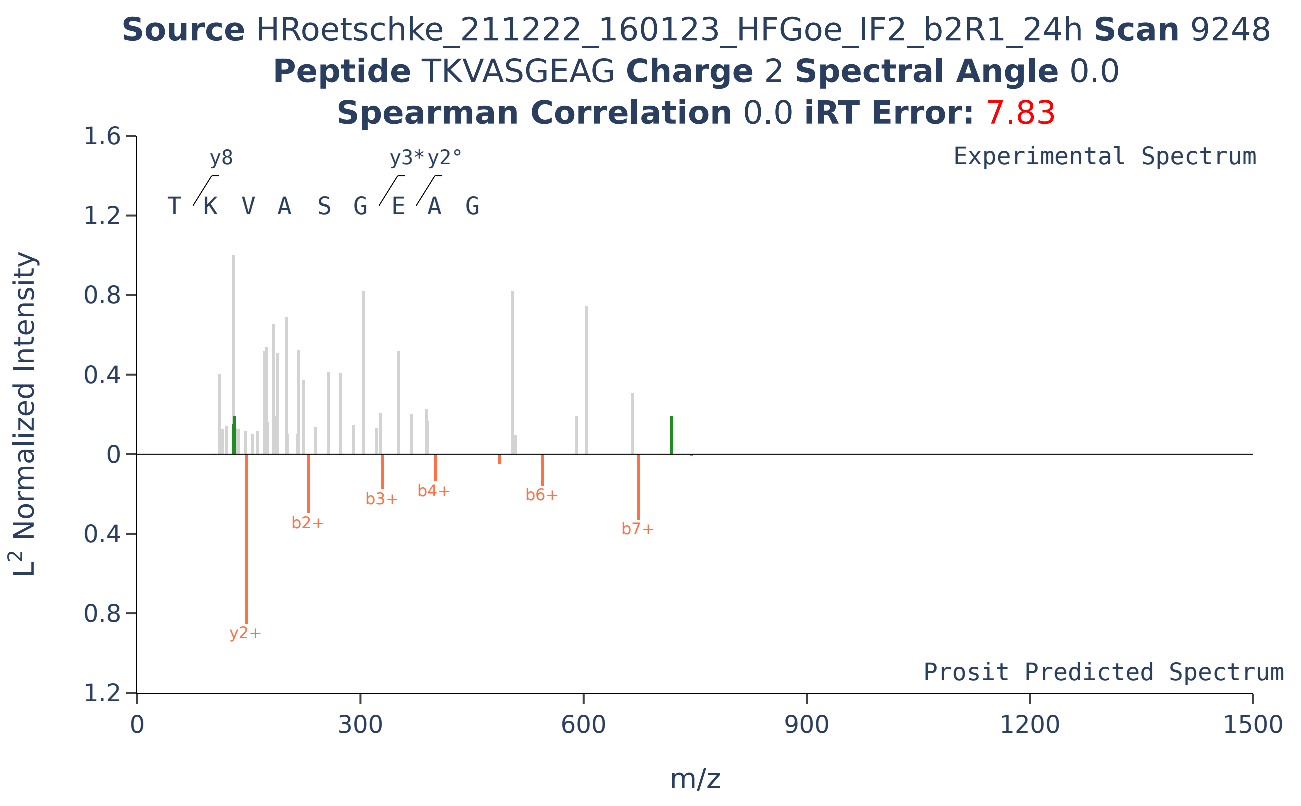
Source HRoetschke_211222_160123_HFGoe_IF2_b2R1_24h Scan 9248
Peptide TKVASGEAG Charge 2 Spectral Angle 0.0
Spearman Correlation 0.0 iRT Error: 7.83
Experimental Spectrum
Prosit Predicted Spectrum
L² Normalized Intensity
m/z

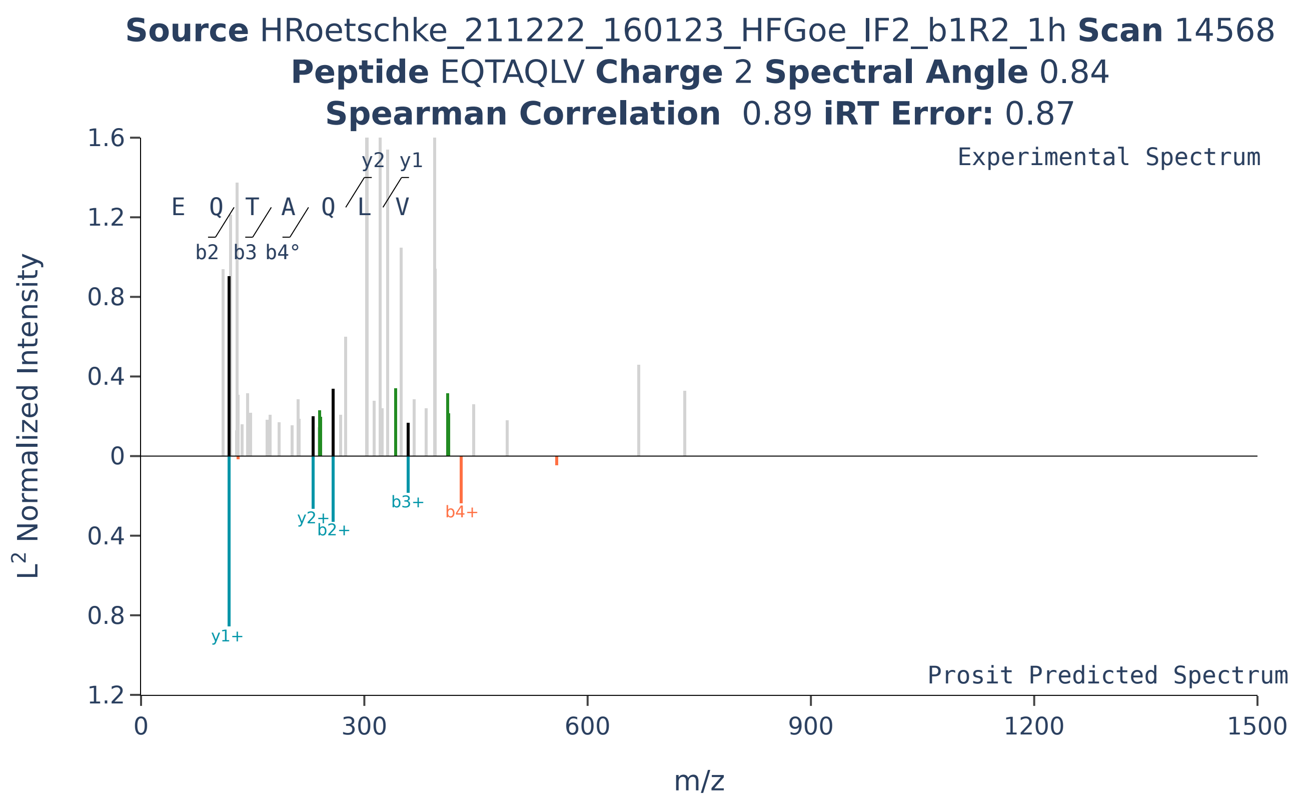
Source HRoetschke_211222_160123_HFGoe_IF2_b1R2_1h Scan 14568
Peptide EQTAQLV Charge 2 Spectral Angle 0.84
Spearman Correlation 0.89 iRT Error: 0.87
Experimental Spectrum
Prosit Predicted Spectrum
L² Normalized Intensity
m/z

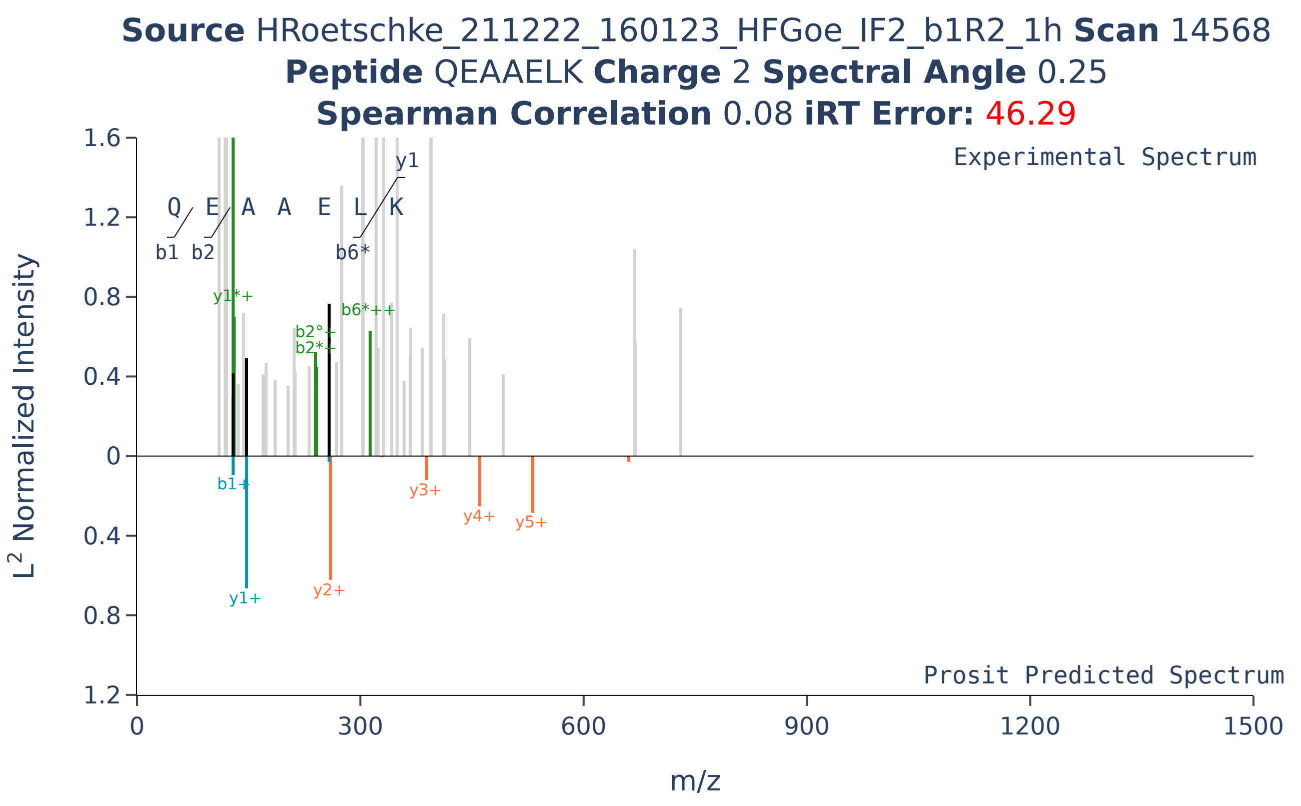
Source HRoetschke_211222_160123_HFGoe_IF2_b1R2_1h Scan 14568
Peptide QEAAELK Charge 2 Spectral Angle 0.25
Spearman Correlation 0.08 iRT Error: 46.29
Experimental Spectrum
Prosit Predicted Spectrum
L² Normalized Intensity
m/z

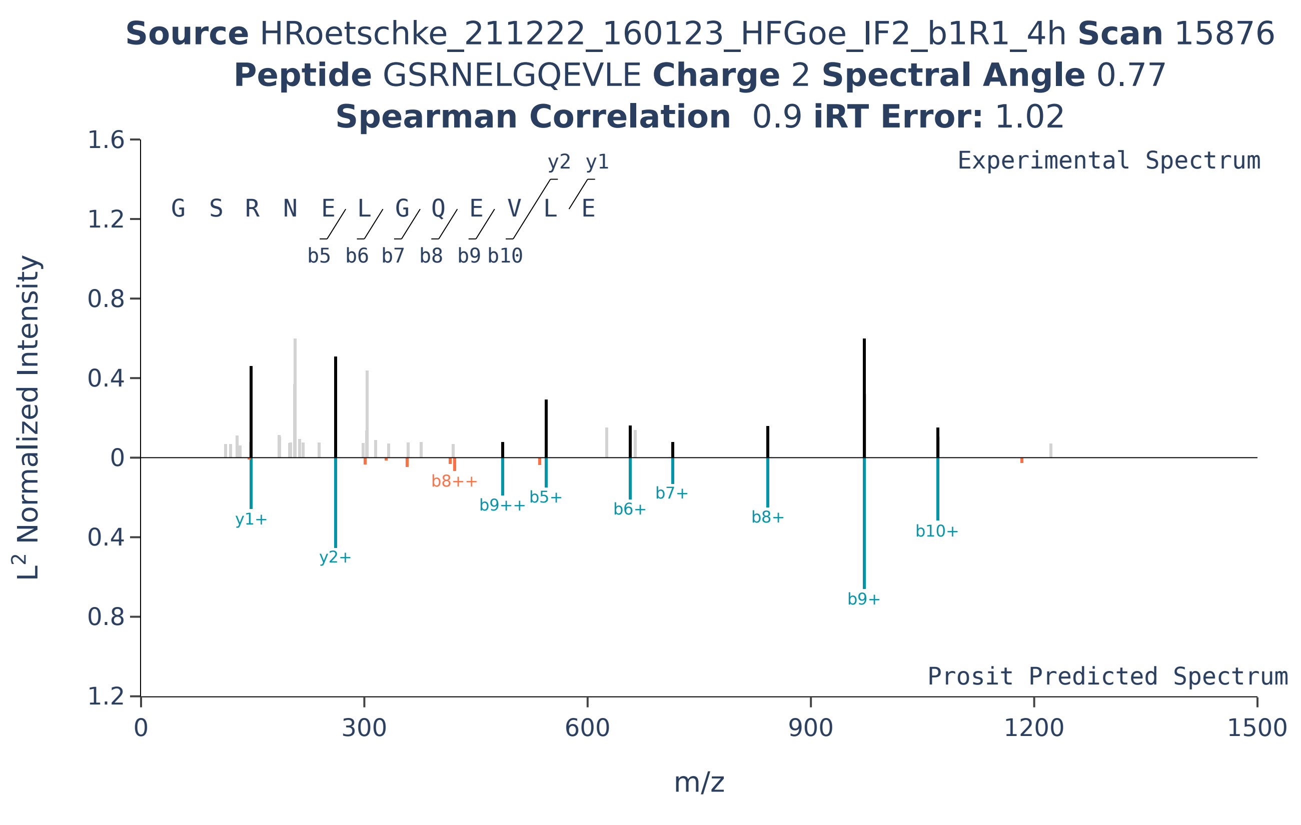
Source HRoetschke_211222_160123_HFGoe_IF2_b1R1_4h Scan 15876
Peptide GSRNELGQEVLE Charge 2 Spectral Angle 0.77
Spearman Correlation 0.9 iRT Error: 1.02
Experimental Spectrum
Prosit Predicted Spectrum
L² Normalized Intensity
m/z

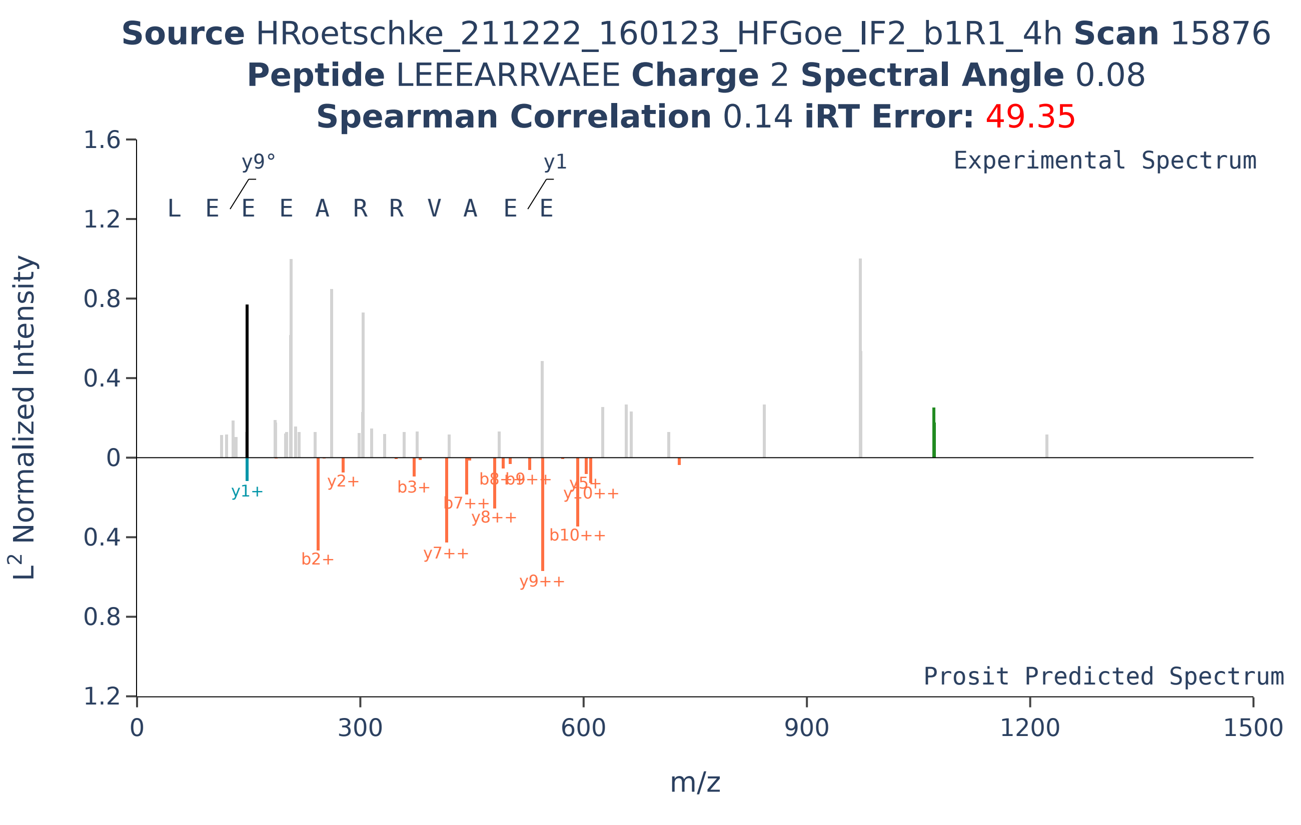
Source HRoetschke_211222_160123_HFGoe_IF2_b1R1_4h Scan 15876
Peptide LEEEARRVAEE Charge 2 Spectral Angle 0.08
Spearman Correlation 0.14 iRT Error: 49.35
Experimental Spectrum
Prosit Predicted Spectrum
L² Normalized Intensity
m/z

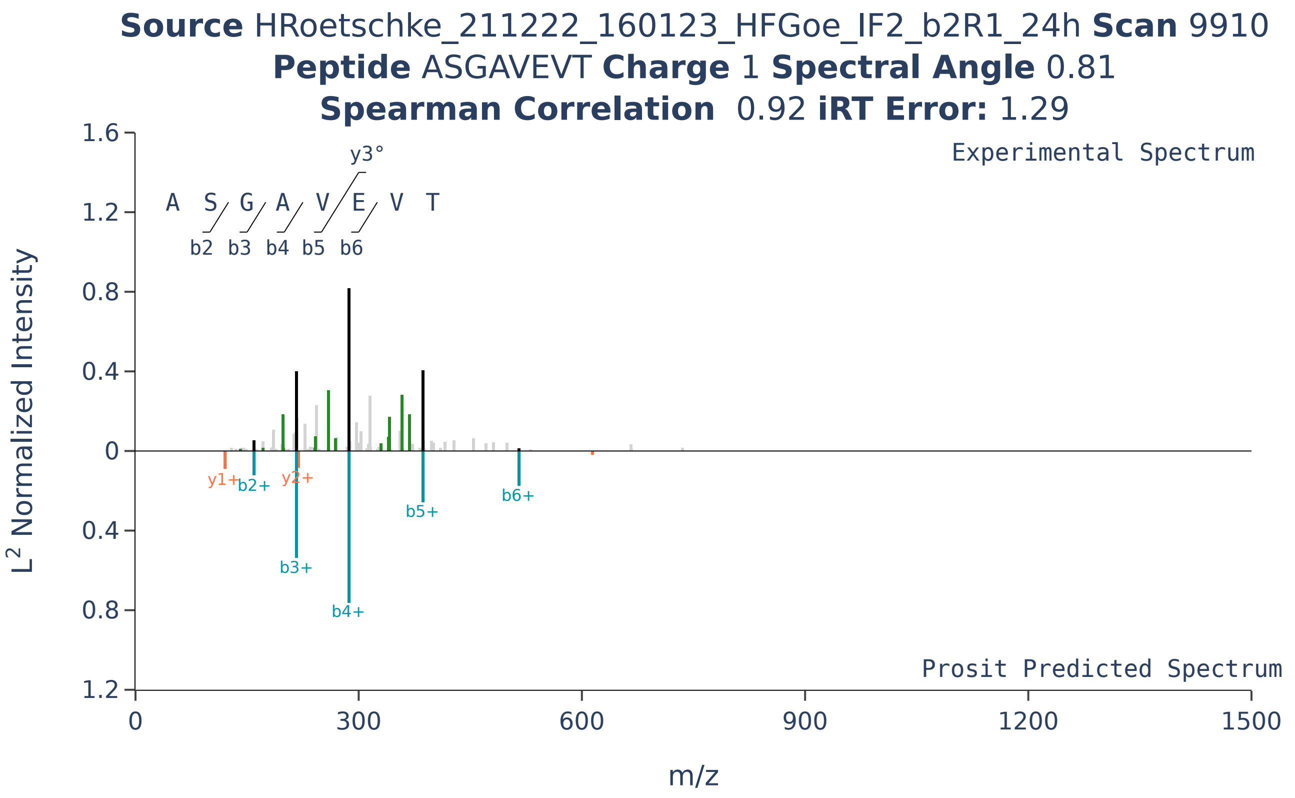
Source HRoetschke_211222_160123_HFGoe_IF2_b2R1_24h Scan 9910
Peptide ASGAVEVT Charge 1 Spectral Angle 0.81
Spearman Correlation 0.92 iRT Error: 1.29
Experimental Spectrum
Prosit Predicted Spectrum
L² Normalized Intensity
m/z

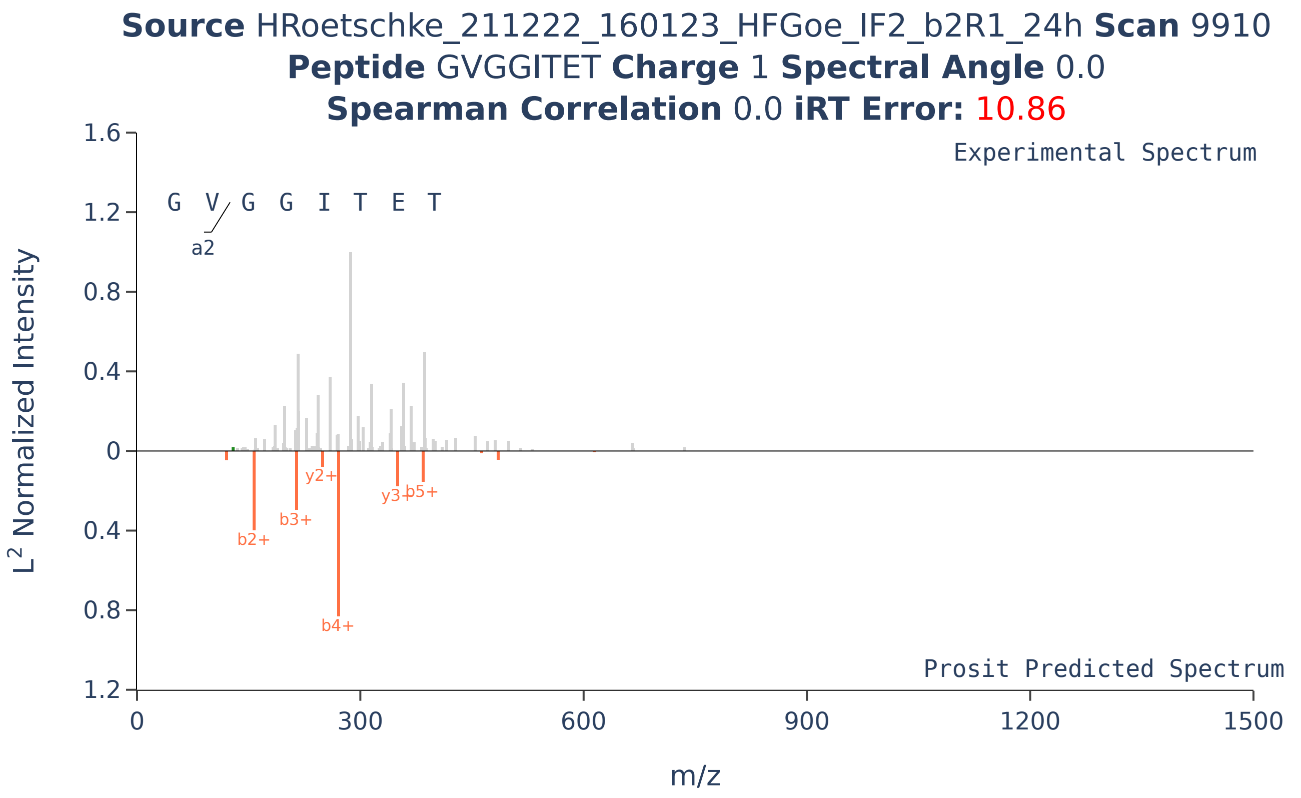
Source HRoetschke_211222_160123_HFGoe_IF2_b2R1_24h Scan 9910
Peptide GVGGITET Charge 1 Spectral Angle 0.0
Spearman Correlation 0.0 iRT Error: 10.86
Experimental Spectrum
Prosit Predicted Spectrum
L² Normalized Intensity
m/z

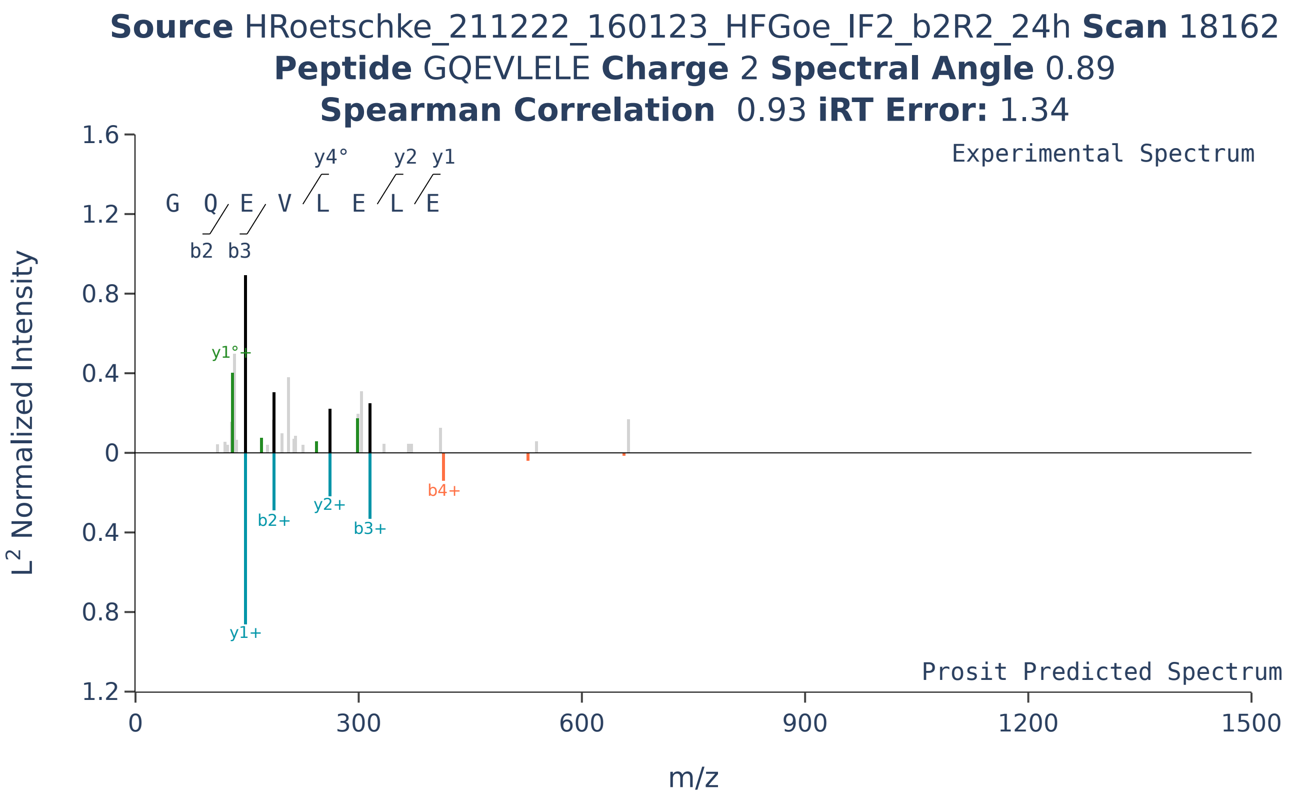
Source HRoetschke_211222_160123_HFGoe_IF2_b2R2_24h Scan 18162
Peptide GQEVLELE Charge 2 Spectral Angle 0.89
Spearman Correlation 0.93 iRT Error: 1.34
Experimental Spectrum
Prosit Predicted Spectrum
L² Normalized Intensity
m/z

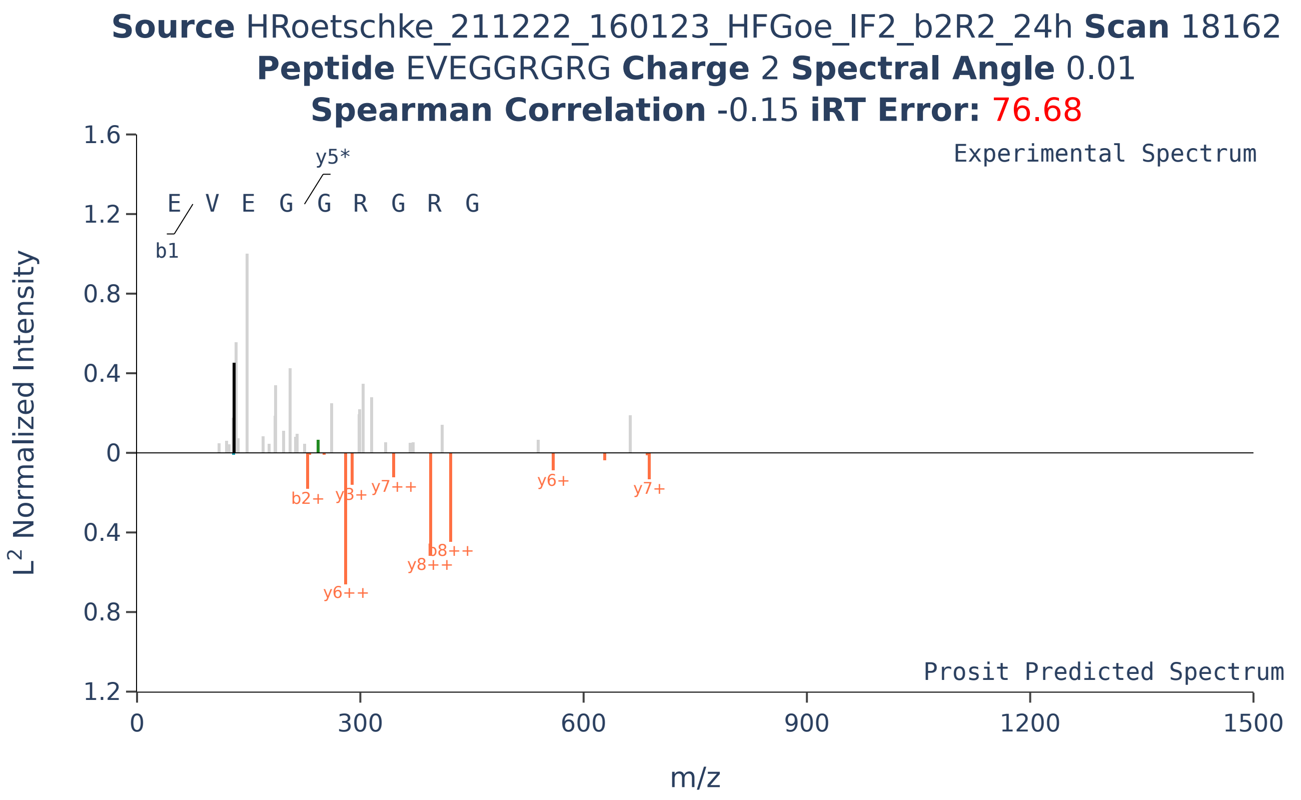
Source HRoetschke_211222_160123_HFGoe_IF2_b2R2_24h Scan 18162
Peptide EVEGGRGRG Charge 2 Spectral Angle 0.01
Spearman Correlation -0.15 iRT Error: 76.68
Experimental Spectrum
Prosit Predicted Spectrum
L² Normalized Intensity
m/z

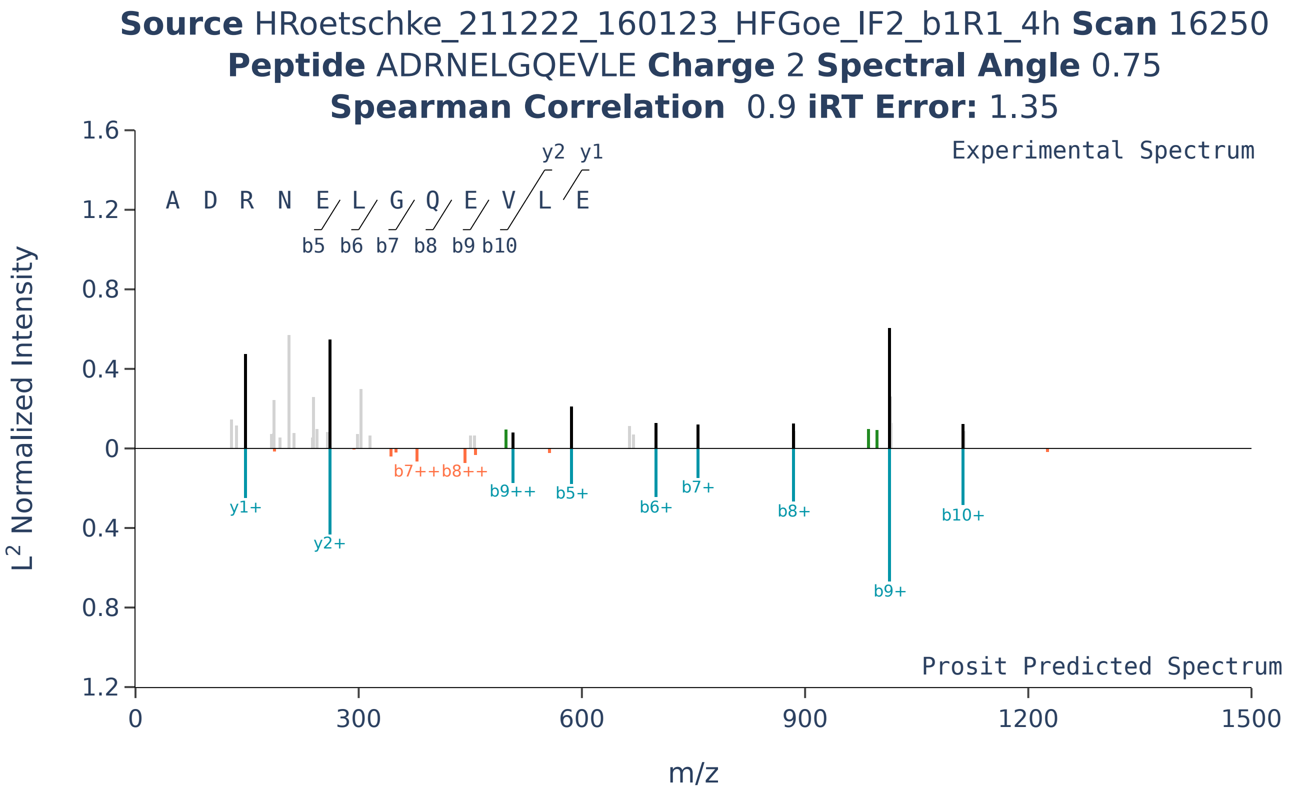
Source HRoetschke_211222_160123_HFGoe_IF2_b1R1_4h Scan 16250
Peptide ADRNELGQEVLE Charge 2 Spectral Angle 0.75
Spearman Correlation 0.9 iRT Error: 1.35
Experimental Spectrum
Prosit Predicted Spectrum
L² Normalized Intensity
m/z

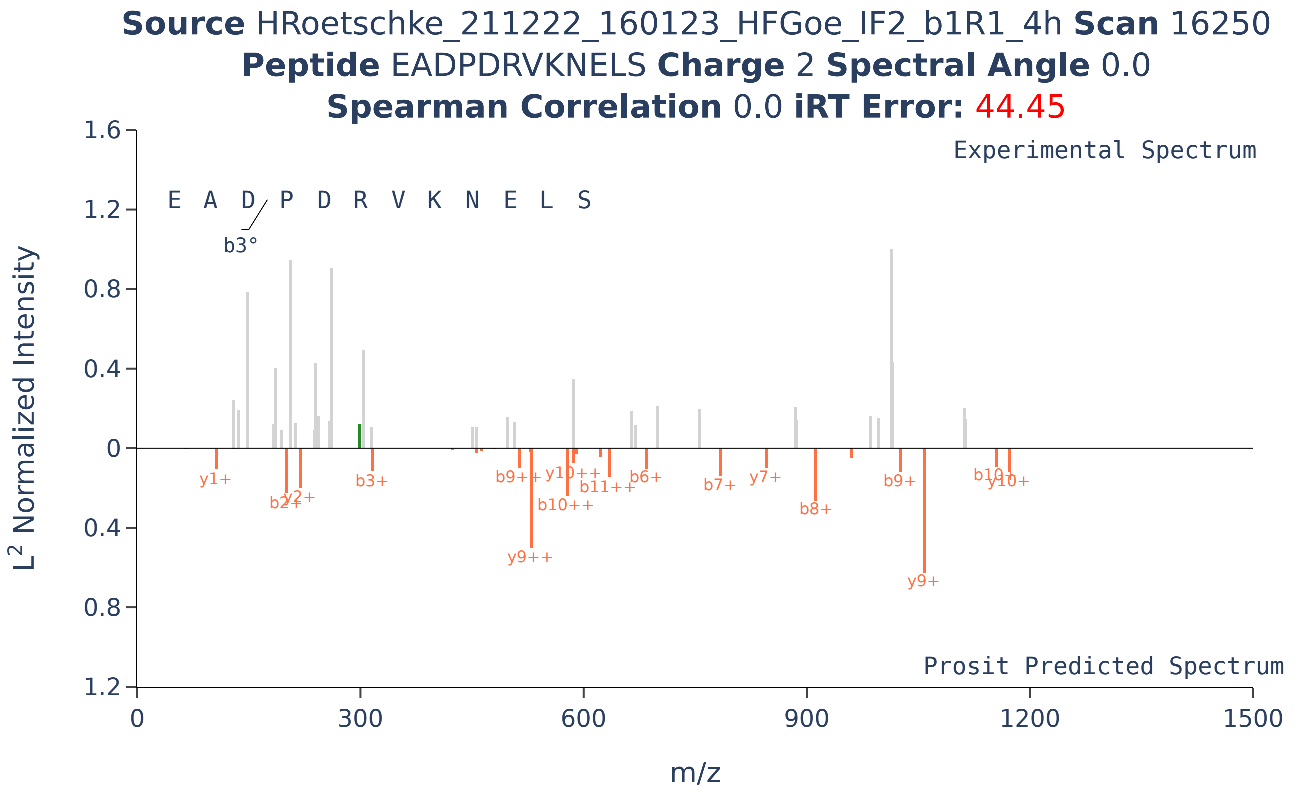
Source HRoetschke_211222_160123_HFGoe_IF2_b1R1_4h Scan 16250
Peptide EADPDRVKNELS Charge 2 Spectral Angle 0.0
Spearman Correlation 0.0 iRT Error: 44.45
Experimental Spectrum
Prosit Predicted Spectrum
L² Normalized Intensity
m/z

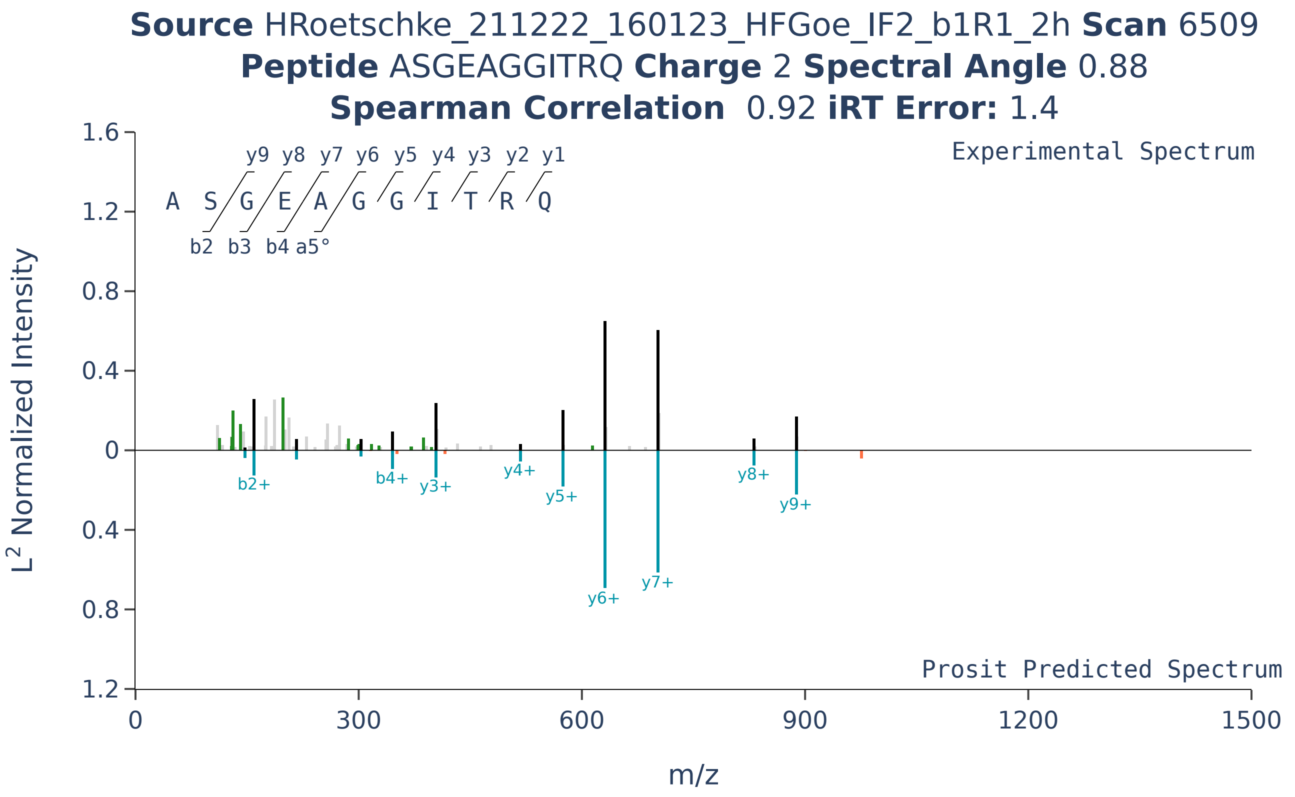
Source HRoetschke_211222_160123_HFGoe_IF2_b1R1_2h Scan 6509
Peptide ASGEAGGITRQ Charge 2 Spectral Angle 0.88
Spearman Correlation 0.92 iRT Error: 1.4
Experimental Spectrum
Prosit Predicted Spectrum
L² Normalized Intensity
m/z

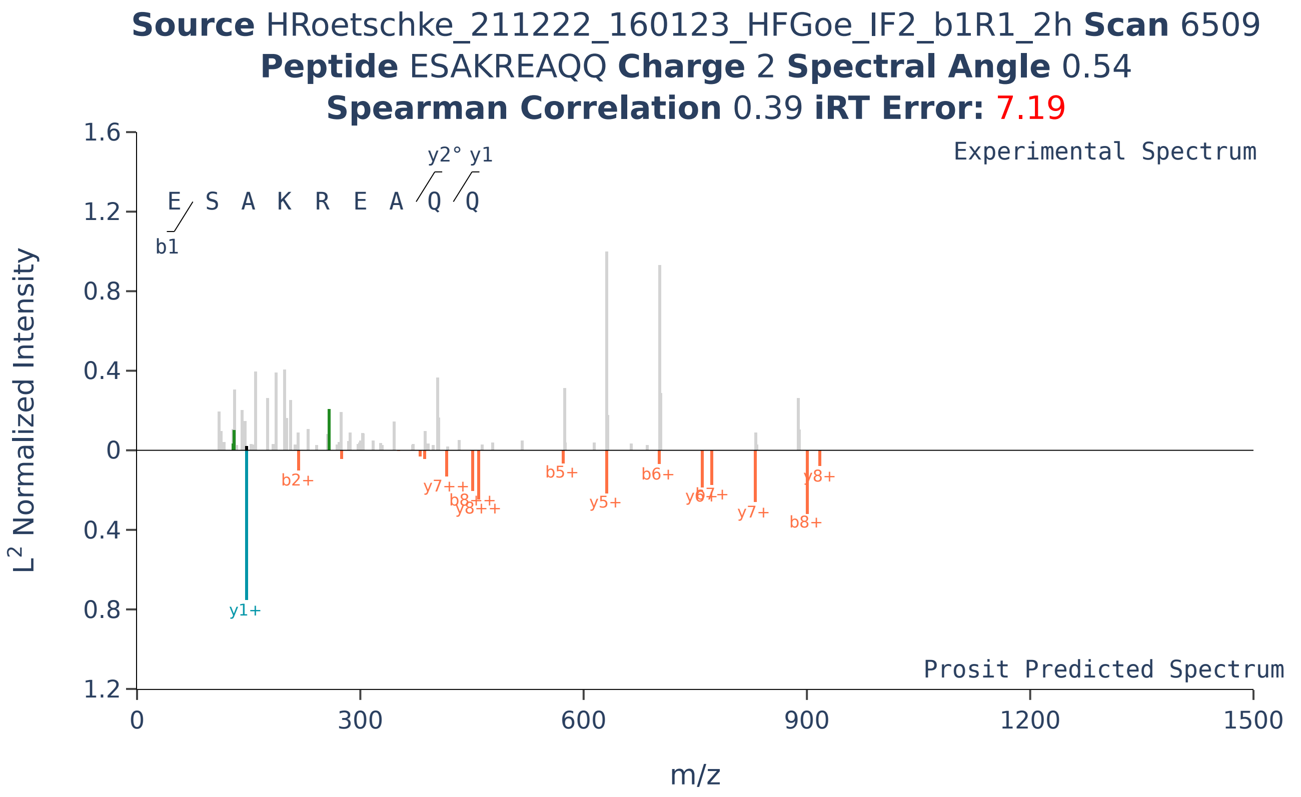
Source HRoetschke_211222_160123_HFGoe_IF2_b1R1_2h Scan 6509
Peptide ESAKREAQQ Charge 2 Spectral Angle 0.54
Spearman Correlation 0.39 iRT Error: 7.19
Experimental Spectrum
Prosit Predicted Spectrum
L² Normalized Intensity
m/z

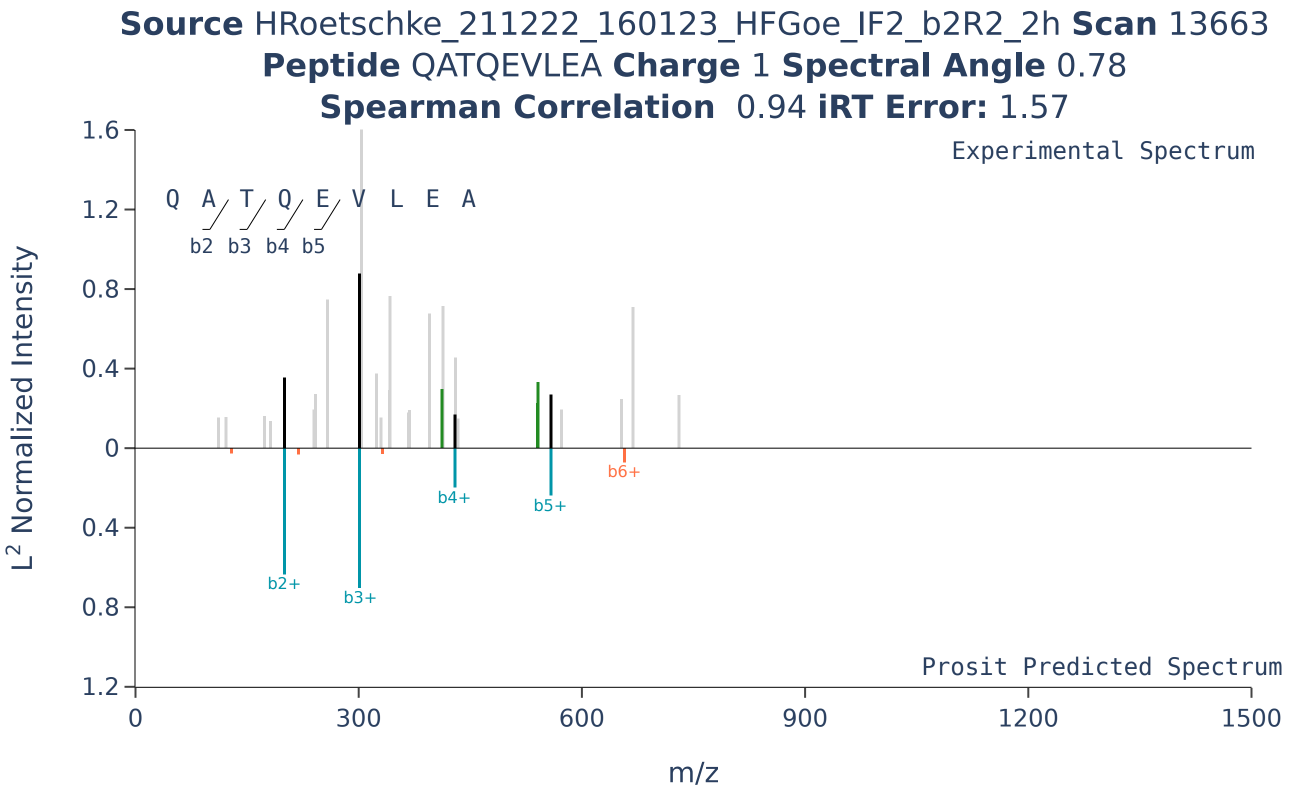
Source HRoetschke_211222_160123_HFGoe_IF2_b2R2_2h Scan 13663
Peptide QATQEVLEA Charge 1 Spectral Angle 0.78
Spearman Correlation 0.94 iRT Error: 1.57
Experimental Spectrum
Prosit Predicted Spectrum
L² Normalized Intensity
m/z

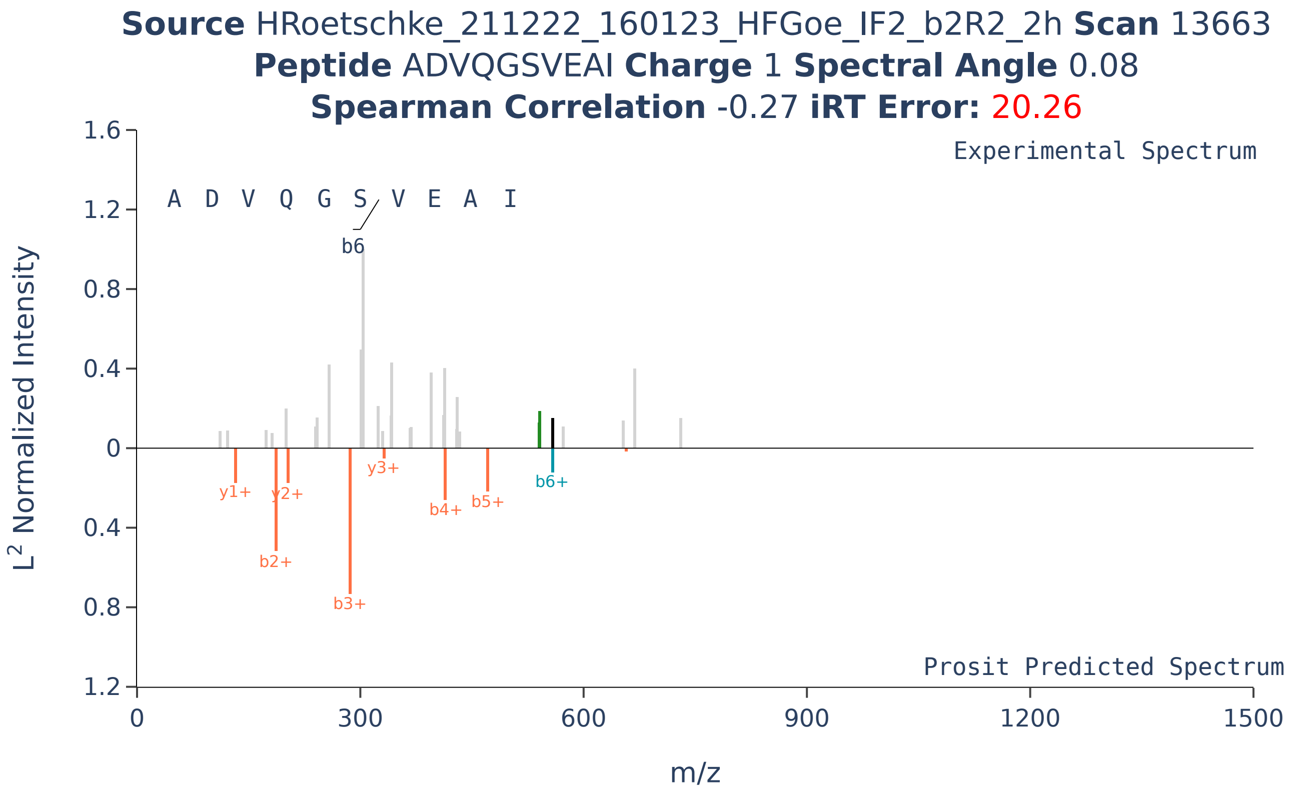
Source HRoetschke_211222_160123_HFGoe_IF2_b2R2_2h Scan 13663
Peptide ADVQGSVEAI Charge 1 Spectral Angle 0.08
Spearman Correlation -0.27 iRT Error: 20.26
Experimental Spectrum
Prosit Predicted Spectrum
L² Normalized Intensity
m/z

Source HRoetschke_211222_160123_HFGoe_IF2_b1R1_1h **Scan** 6417
Peptide ASGEAGGTLQR **Charge** 2 **Spectral Angle** 0.86
Spearman Correlation 0.92 **iRT Error:** 1.63

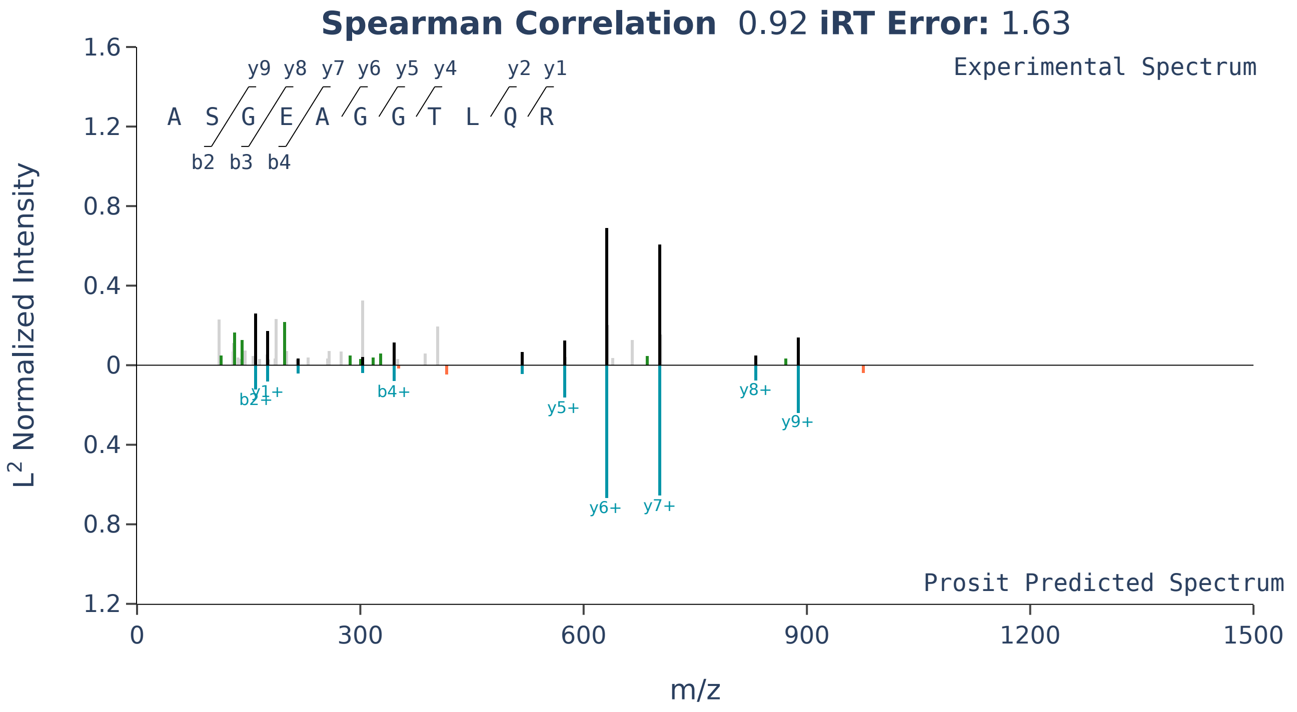

Source HRoetschke_211222_160123_HFGoe_IF2_b1R1_1h **Scan** 6417
Peptide ESAKREAQQ **Charge** 2 **Spectral Angle** 0.0
Spearman Correlation 0.0 **iRT Error:** 6.74

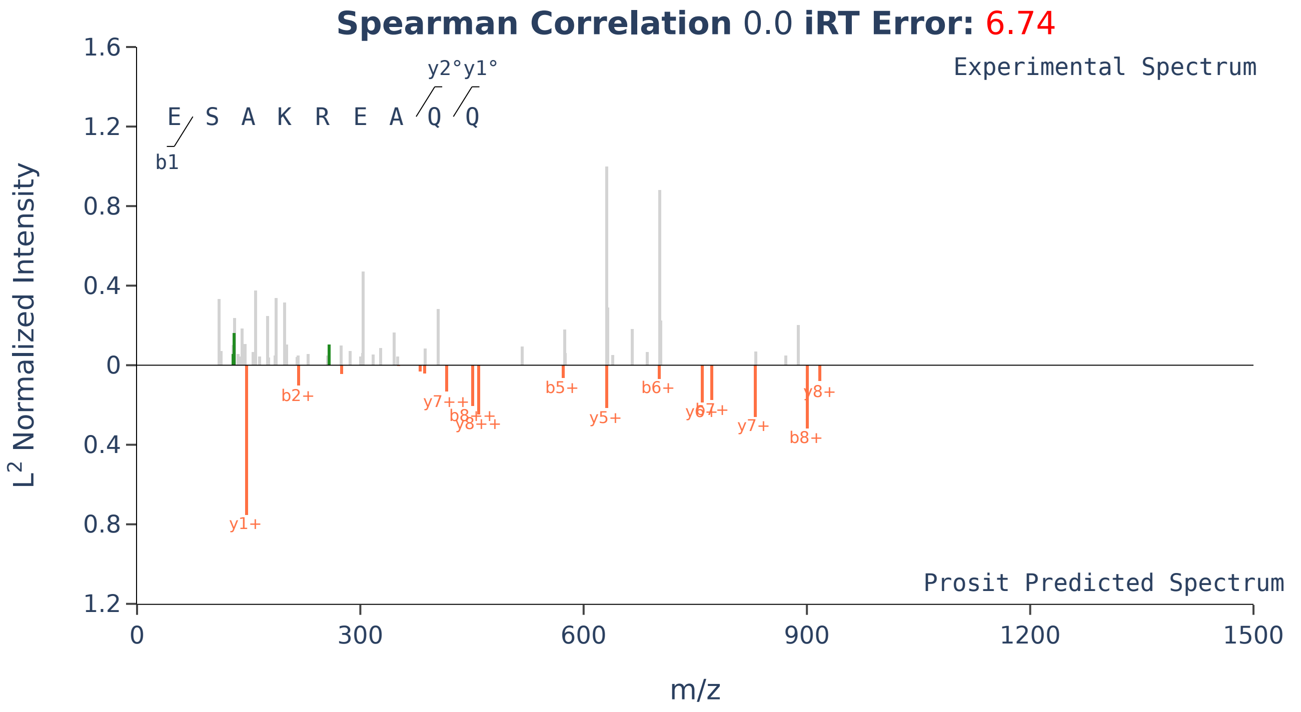

Source HRoetschke_211222_160123_HFGoe_IF2_b2R2_4h **Scan** 11637
Peptide GFEYGRGIR **Charge** 3 **Spectral Angle** 0.8
Spearman Correlation 0.85 **iRT Error:** 1.94

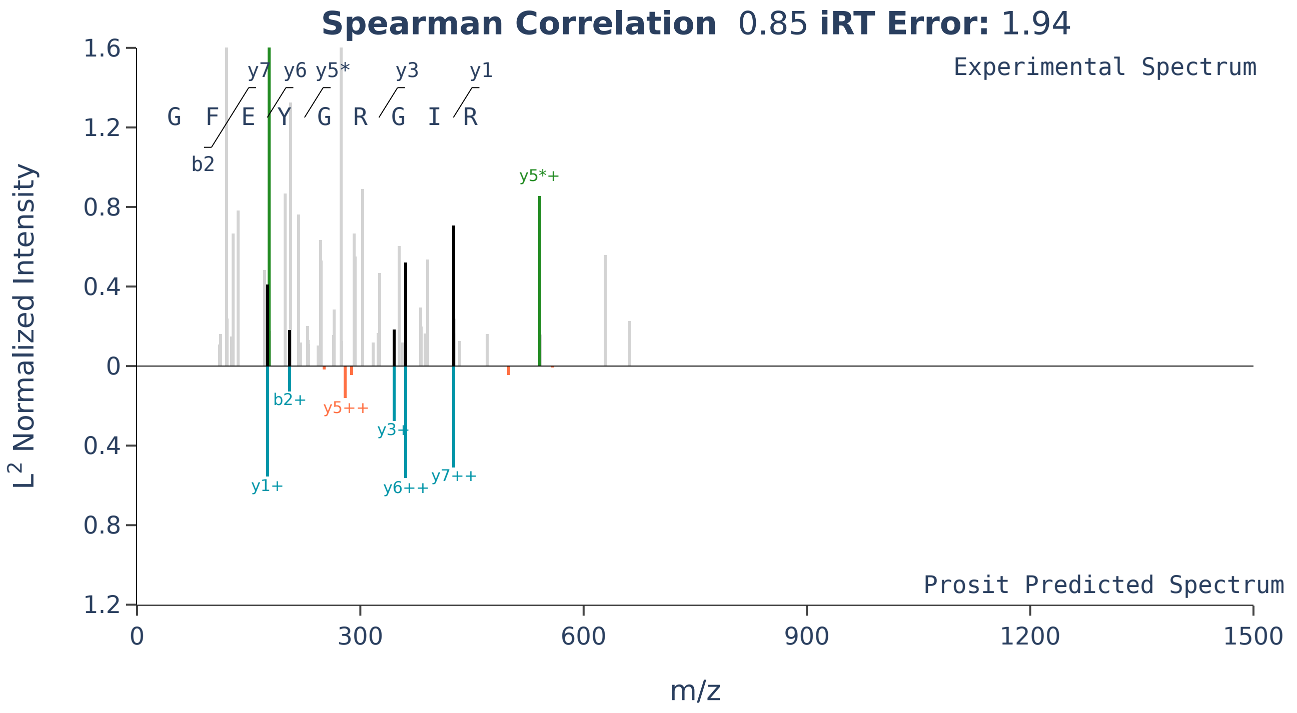

Source HRoetschke_211222_160123_HFGoe_IF2_b2R2_4h **Scan** 11637
Peptide GFEYGRVRA **Charge** 3 **Spectral Angle** 0.88
Spearman Correlation 0.72 **iRT Error:** 4.04

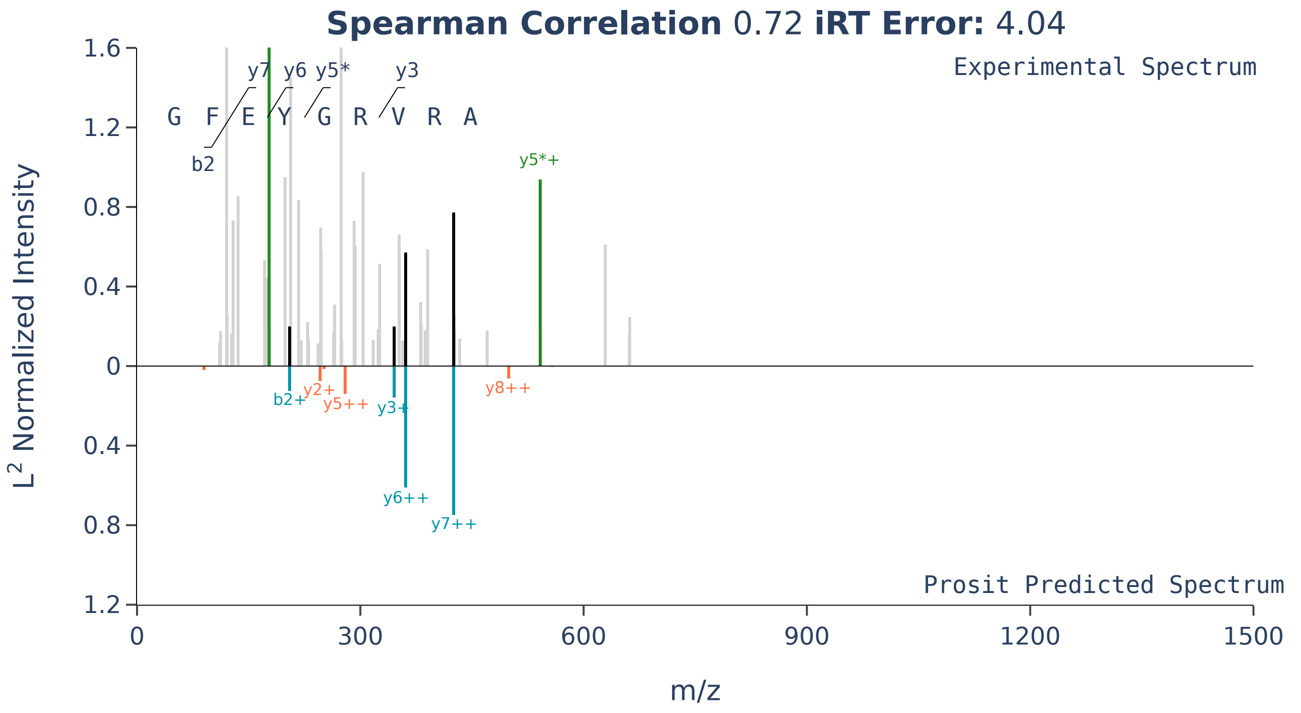

Source HRoetschke_211222_160123_HFGoe_IF2_b1R2_1h **Scan** 15723
Peptide RGNELGQEVLE **Charge** 2 **Spectral Angle** 0.76
Spearman Correlation 0.88 **iRT Error:** 2.0

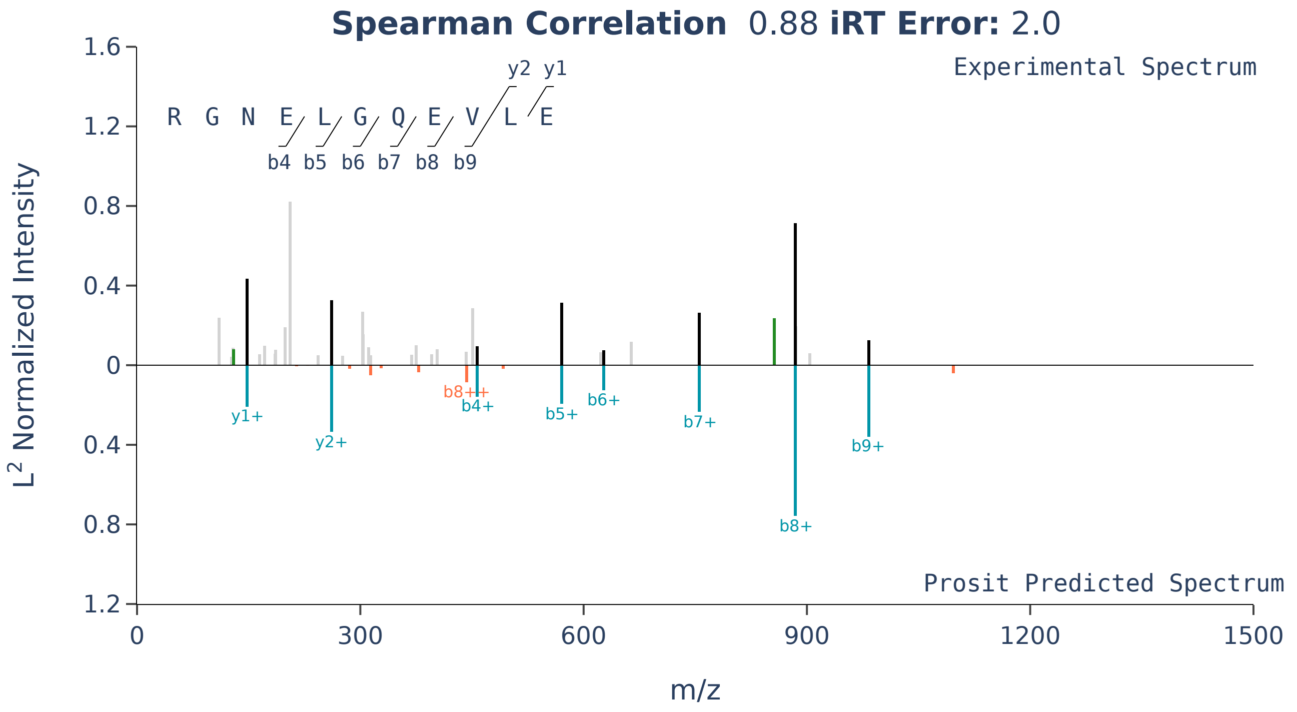

Source HRoetschke_211222_160123_HFGoe_IF2_b1R2_1h **Scan** 15723
Peptide ADPDRVKNELS **Charge** 2 **Spectral Angle** 0.0
Spearman Correlation 0.0 **iRT Error:** 50.37

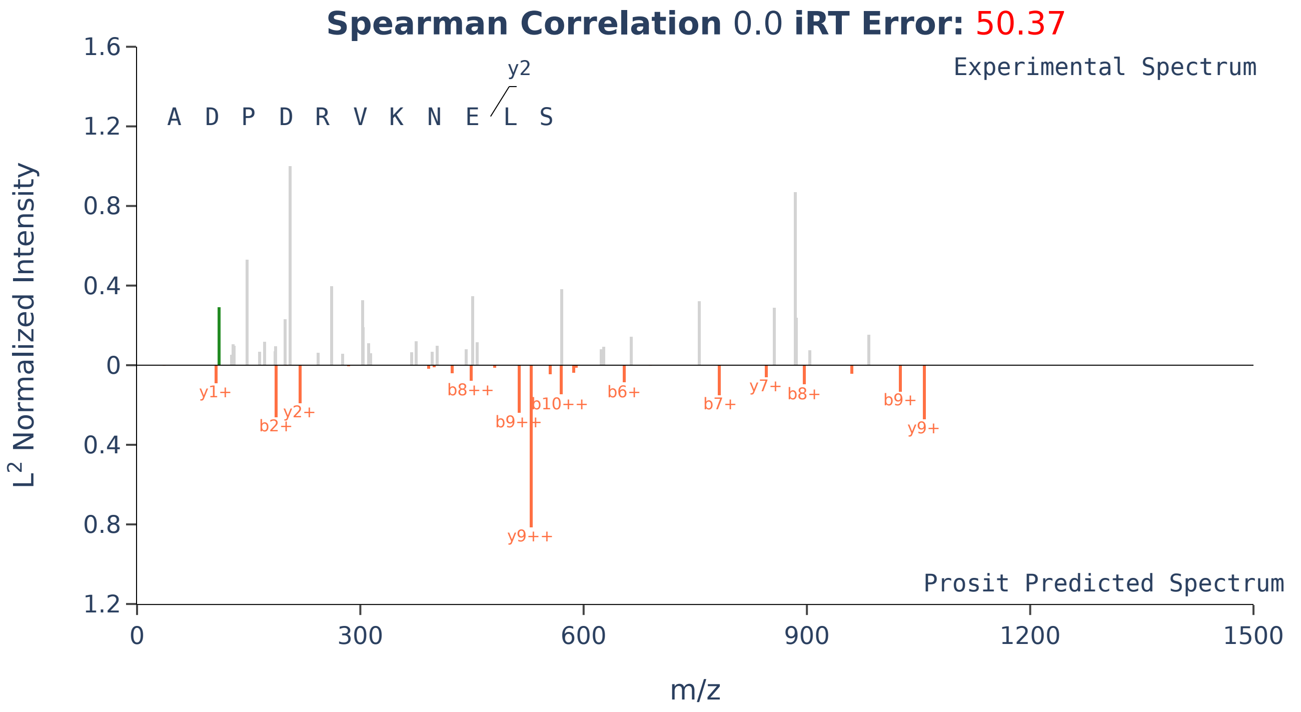

Source HRoetschke_211222_160123_HFGoe_IF2_b1R1_4h **Scan** 10677
Peptide QETAQLVER **Charge** 2 **Spectral Angle** 0.8
Spearman Correlation 0.88 **iRT Error:** 2.06

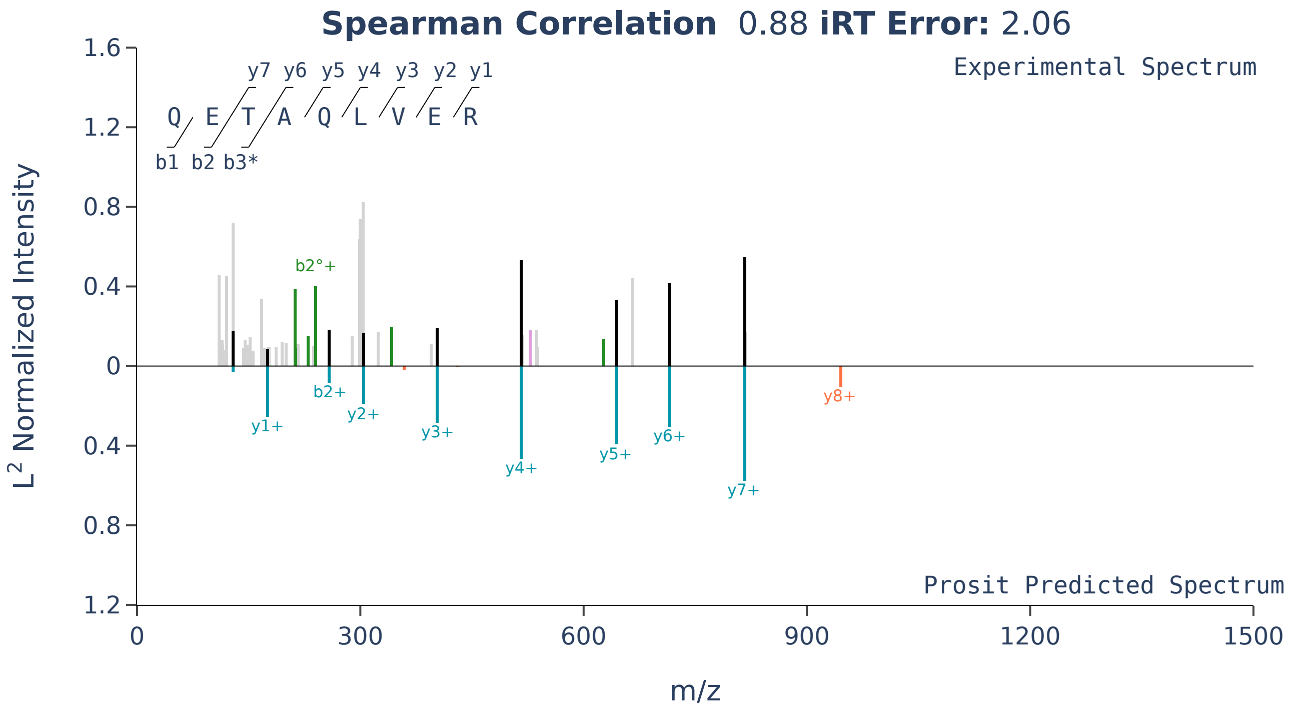

Source HRoetschke_211222_160123_HFGoe_IF2_b1R1_4h **Scan** 10677
Peptide REQEAAELK **Charge** 2 **Spectral Angle** 0.0
Spearman Correlation 0.0 **iRT Error:** 26.6

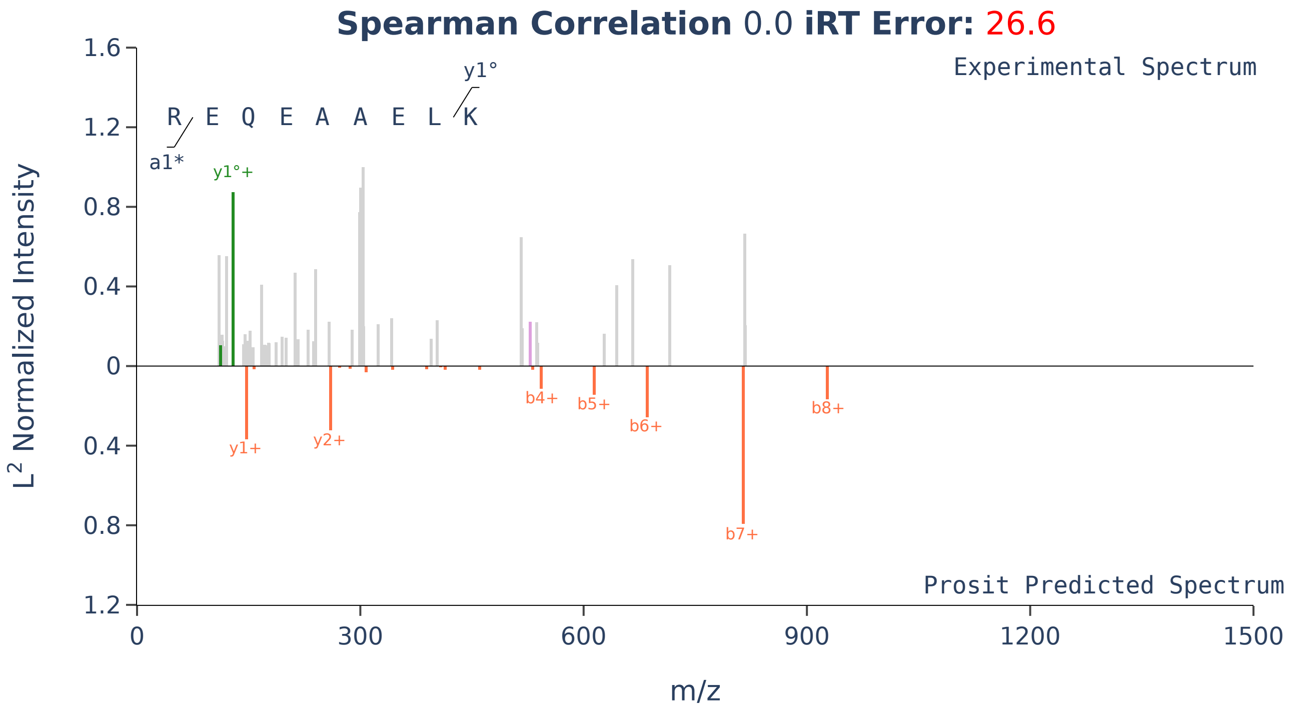

Source HRoetschke_211222_160123_HFGoe_IF2_b1R2_2h **Scan** 13886
Peptide EQARREALGQEVLE **Charge** 3 **Spectral Angle** 0.81
Spearman Correlation 0.9 **iRT Error:** 2.51

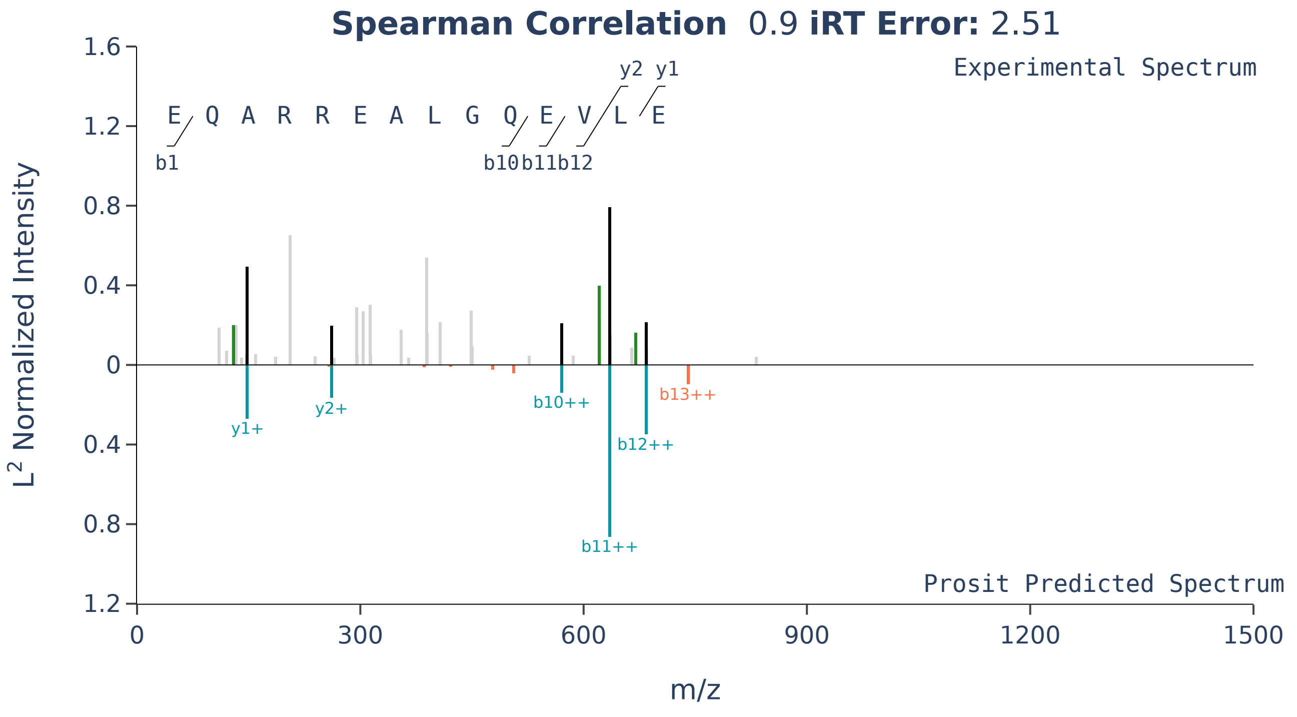

Source HRoetschke_211222_160123_HFGoe_IF2_b1R2_2h **Scan** 13886
Peptide GAIAGCMVTEGVVKRH **Charge** 3 **Spectral Angle** 0.05
Spearman Correlation 0.01 **iRT Error:** 6.81

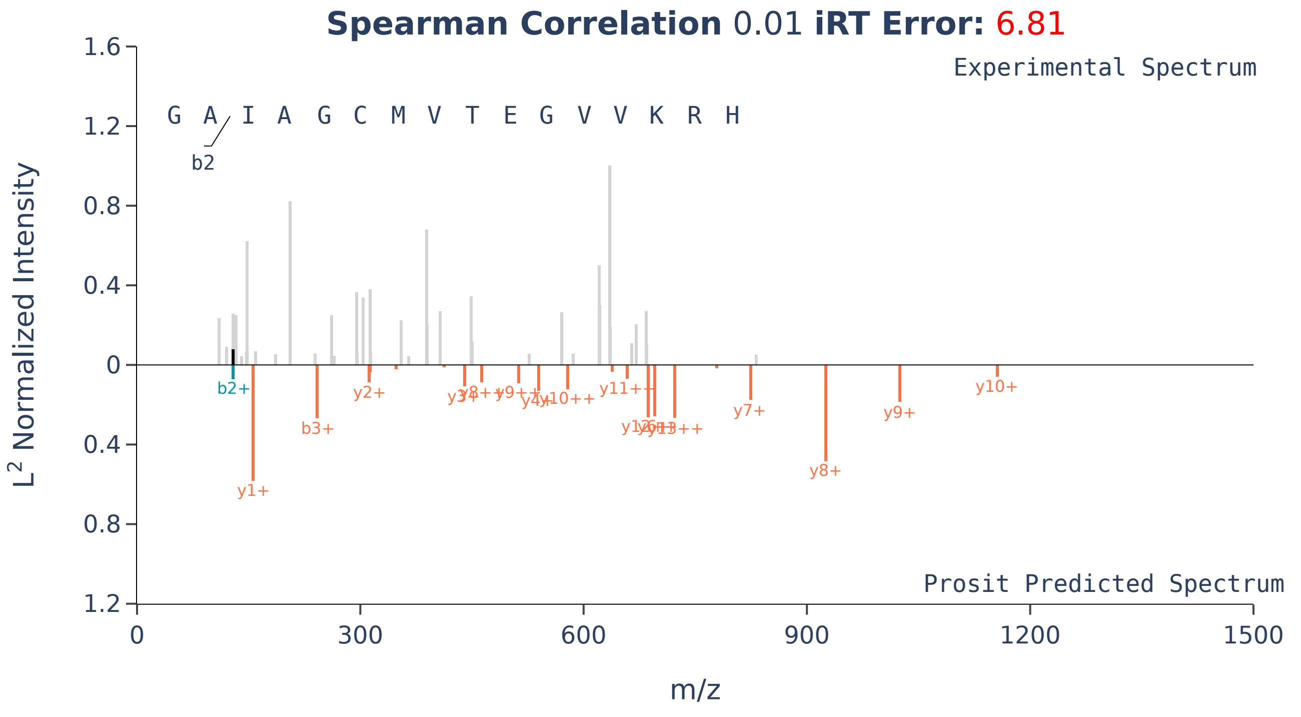

Source HRoetschke_211222_160123_HFGoe_IF2_b1R1_24h **Scan** 9116
Peptide IGAYHVETENDR **Charge** 3 **Spectral Angle** 0.83
Spearman Correlation 0.9 **iRT Error:** 2.52

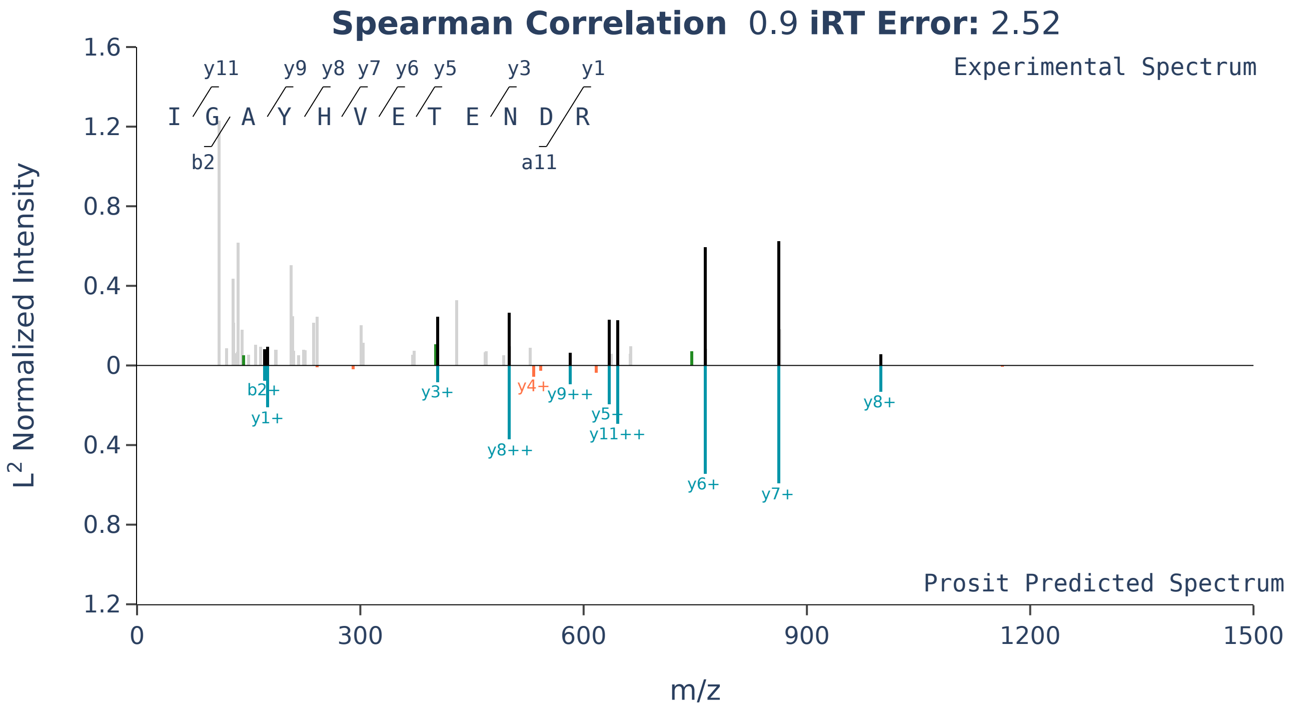

Source HRoetschke_211222_160123_HFGoe_IF2_b1R1_24h **Scan** 9116
Peptide ESDREVEGGRGRG **Charge** 3 **Spectral Angle** 0.01
Spearman Correlation -0.08 **iRT Error:** 37.05

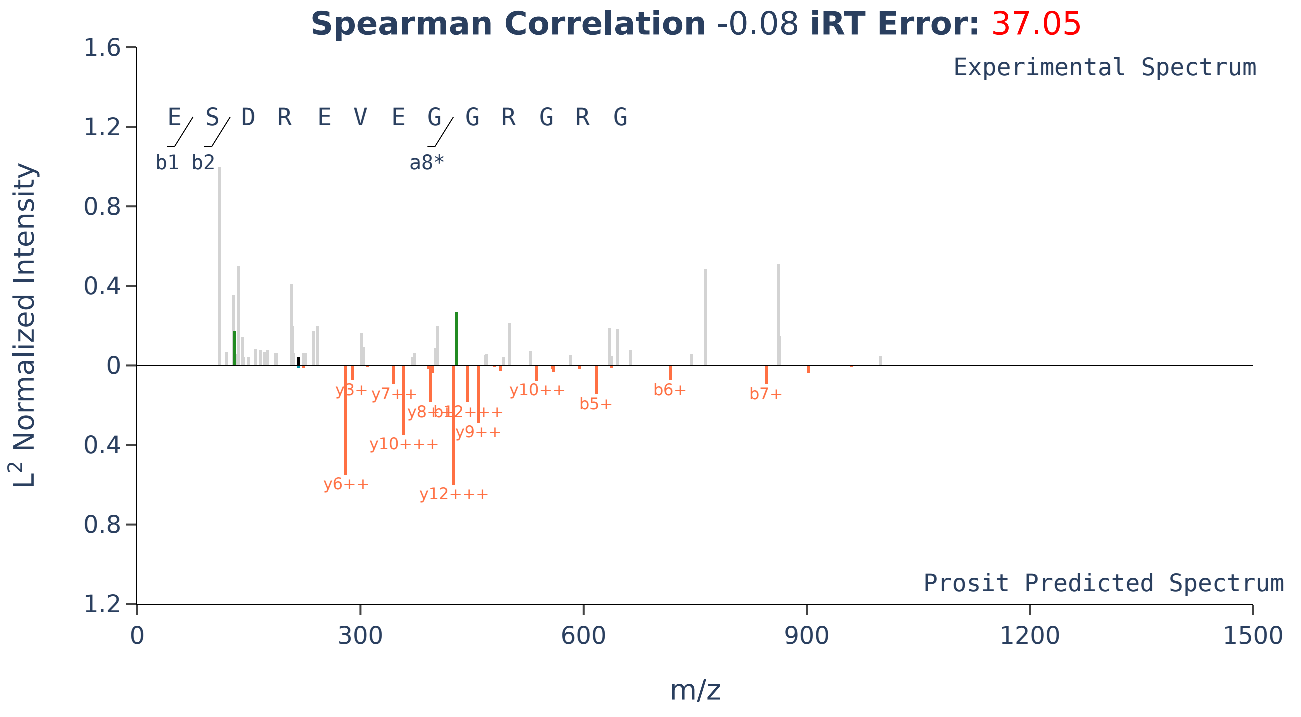

Source HRoetschke_211222_160123_HFGoe_IF2_b1R1_24h **Scan** 17837
Peptide GQEVEIIE **Charge** 2 **Spectral Angle** 0.86
Spearman Correlation 0.93 **iRT Error:** 2.63

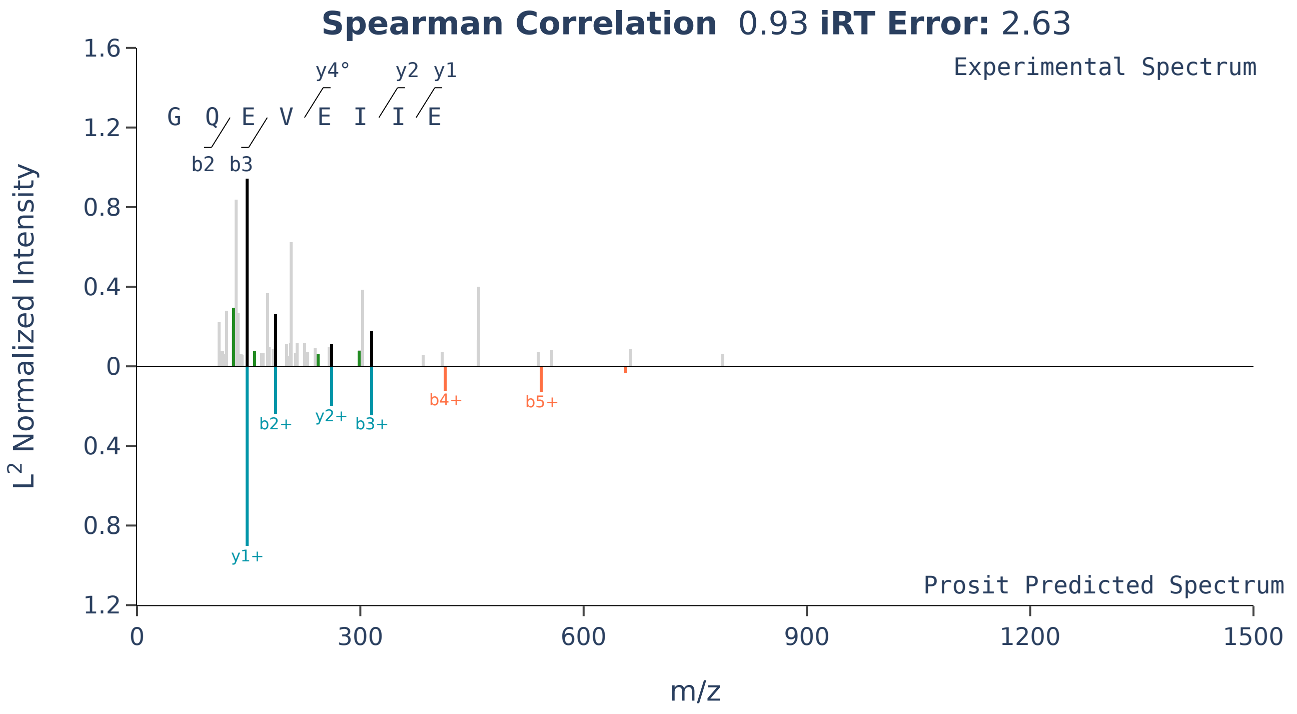

Source HRoetschke_211222_160123_HFGoe_IF2_b1R1_24h **Scan** 17837
Peptide EVEGGRGRG **Charge** 2 **Spectral Angle** 0.03
Spearman Correlation 0.06 **iRT Error:** 71.05

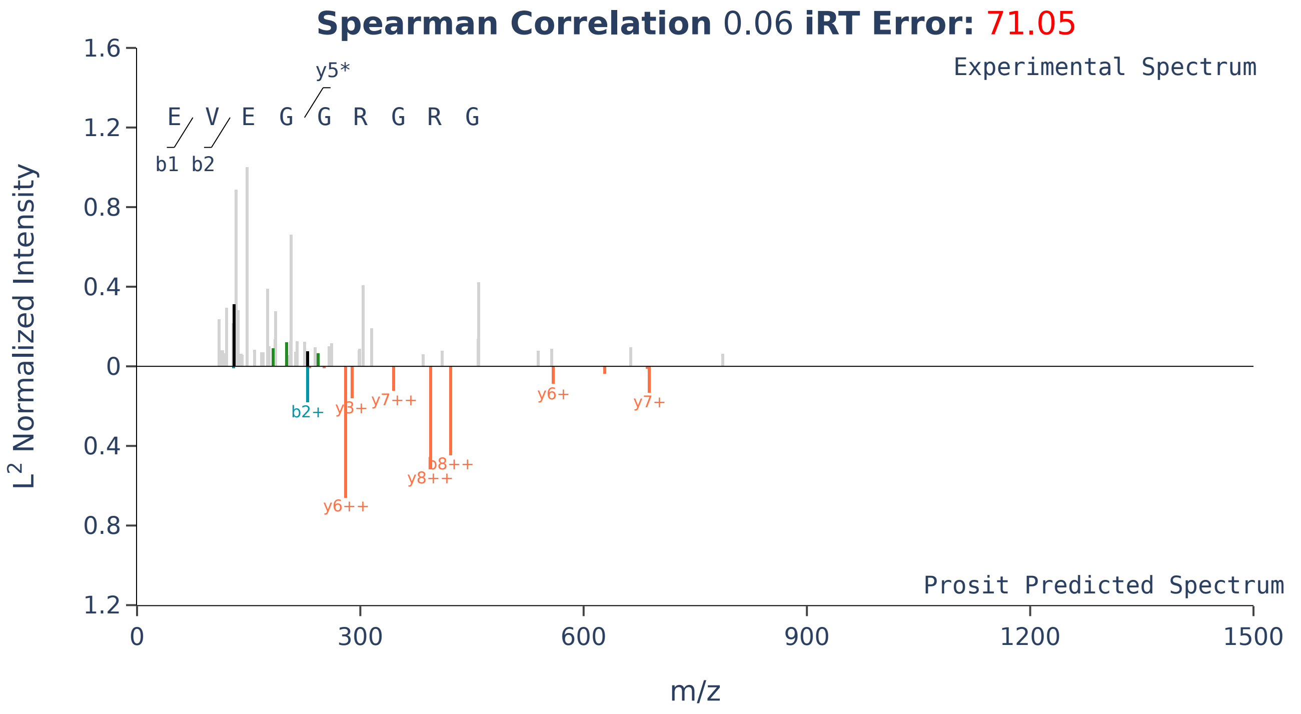

Source HRoetschke_211222_160123_HFGoe_IF2_b1R1_4h **Scan** 13396
Peptide GLGAYHVETE **Charge** 2 **Spectral Angle** 0.75
Spearman Correlation 0.94 **iRT Error:** 2.66

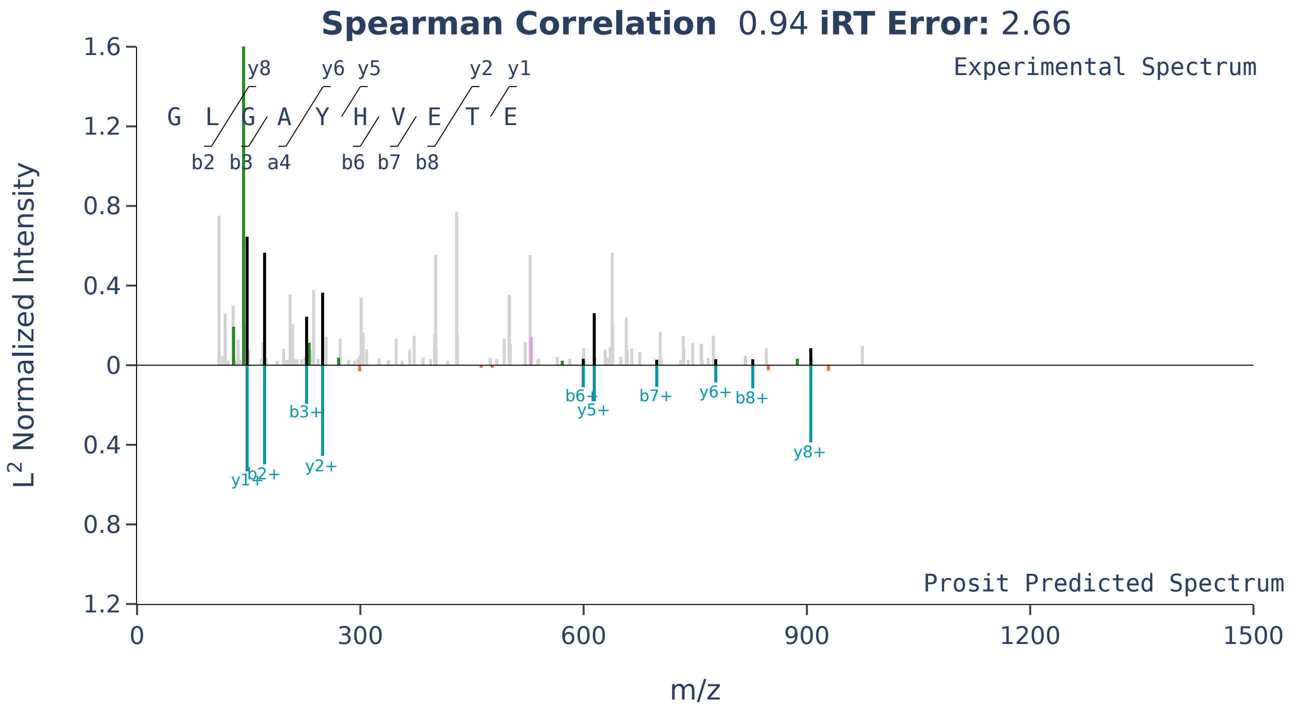

Source HRoetschke_211222_160123_HFGoe_IF2_b1R1_4h **Scan** 13396
Peptide QARREAEES **Charge** 2 **Spectral Angle** 0.0
Spearman Correlation 0.0 **iRT Error:** 57.95

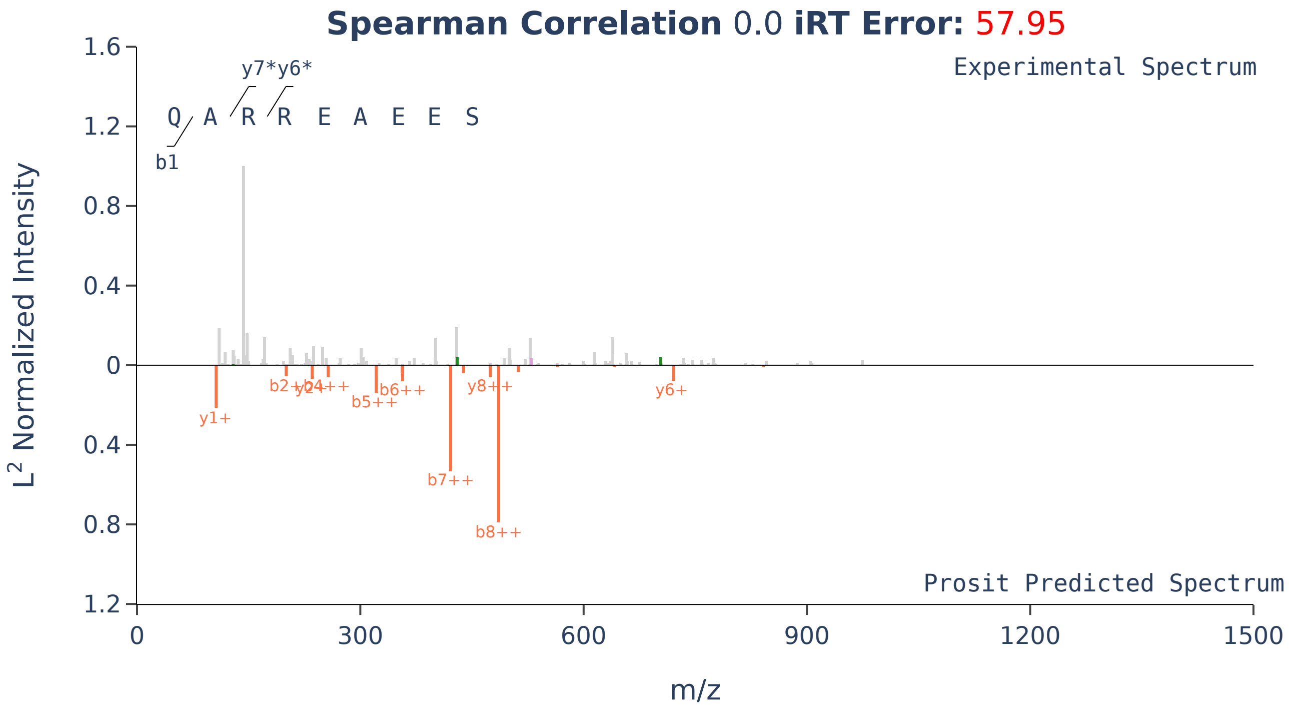

Source HRoetschke_211222_160123_HFGoe_IF2_b1R1_4h **Scan** 21761
Peptide PVVVAVNLAN **Charge** 1 **Spectral Angle** 0.86
Spearman Correlation 0.89 **iRT Error:** 2.83

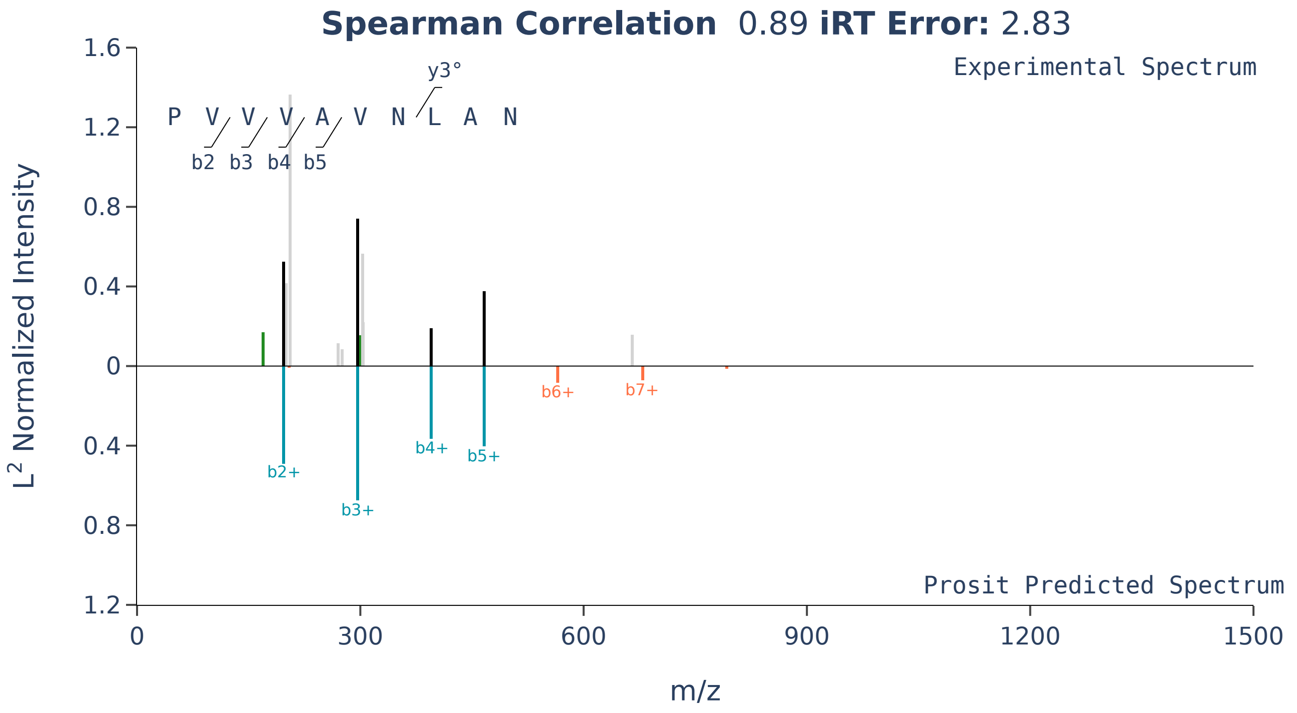

Source HRoetschke_211222_160123_HFGoe_IF2_b1R1_4h **Scan** 21761
Peptide TLHKGDIVL **Charge** 1 **Spectral Angle** 0.0
Spearman Correlation 0.0 **iRT Error:** 56.68

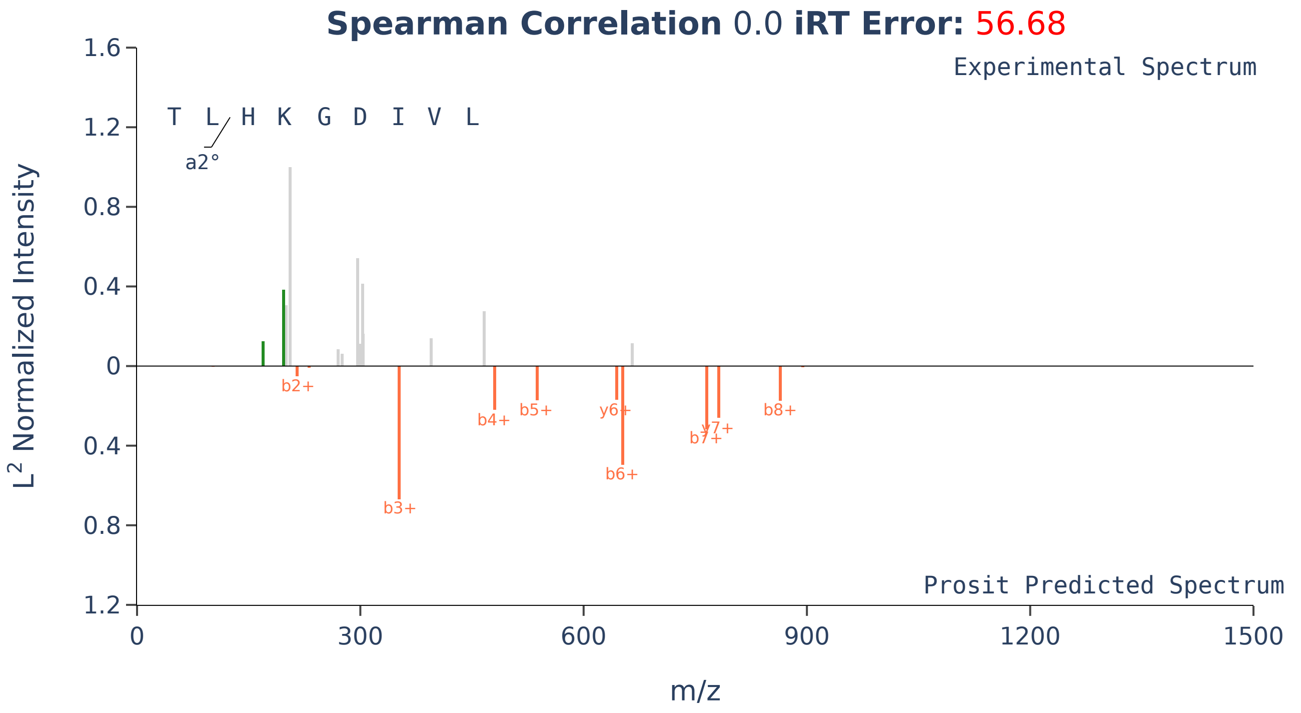

Source HRoetschke_211222_160123_HFGoe_IF2_b1R1_2h **Scan** 14047
Peptide VREGRNELGQEVLE **Charge** 3 **Spectral Angle** 0.78
Spearman Correlation 0.94 **iRT Error:** 2.89

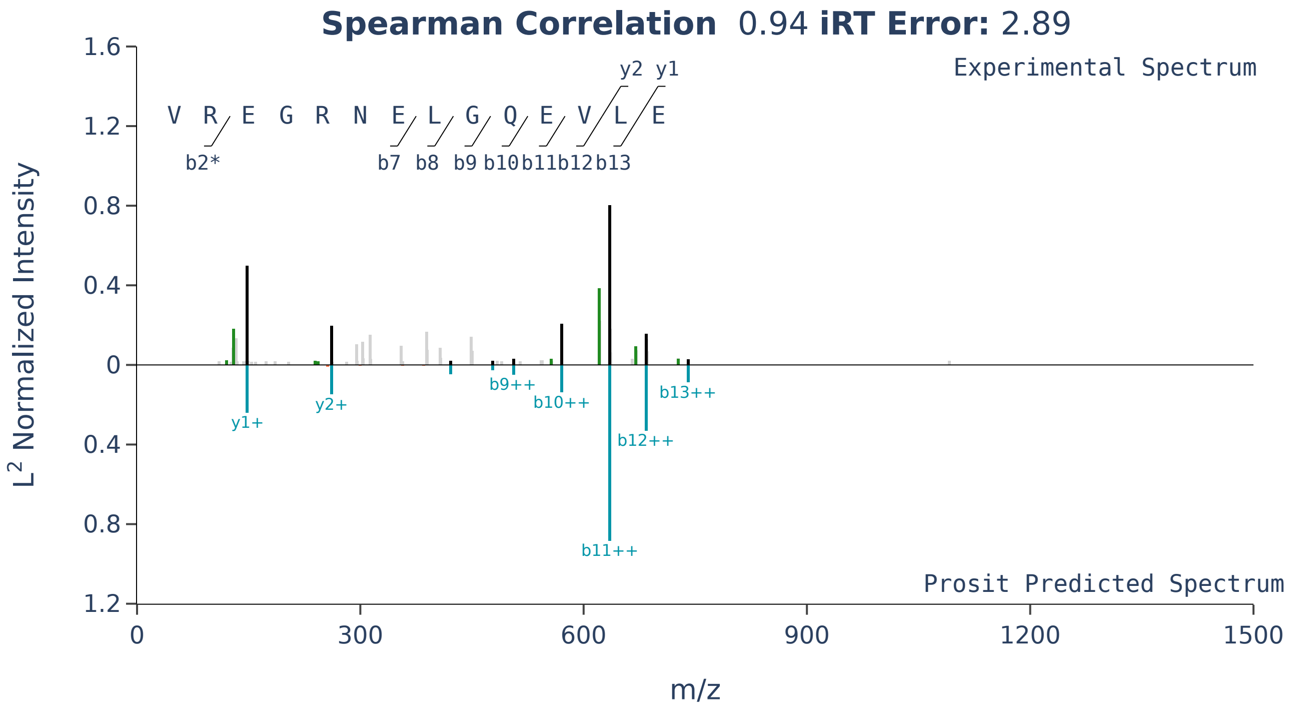

Source HRoetschke_211222_160123_HFGoe_IF2_b1R1_2h **Scan** 14047
Peptide GAIAGCMVTEGVVKRH **Charge** 3 **Spectral Angle** 0.05
Spearman Correlation 0.01 **iRT Error:** 9.79

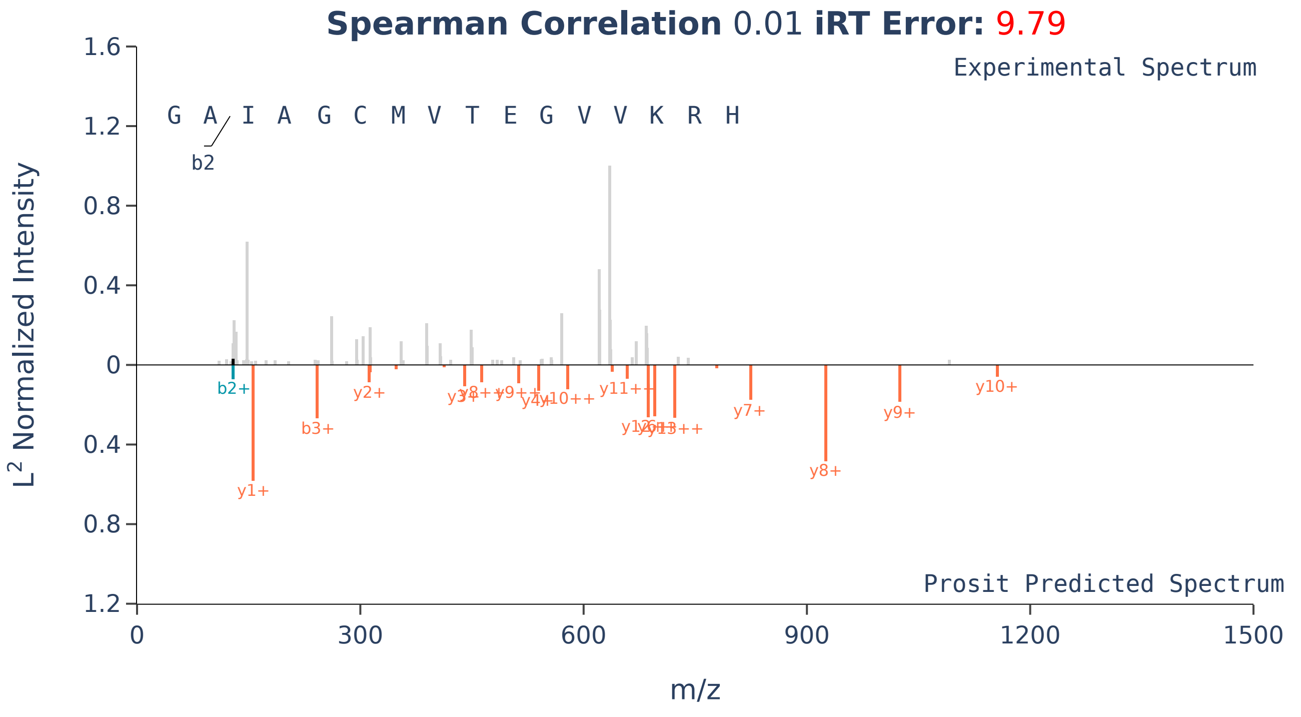

Source HRoetschke_211222_160123_HFGoe_IF2_b1R1_4h **Scan** 21204
Peptide PVVVAVNGGI **Charge** 1 **Spectral Angle** 0.93
Spearman Correlation 0.91 **iRT Error:** 2.97

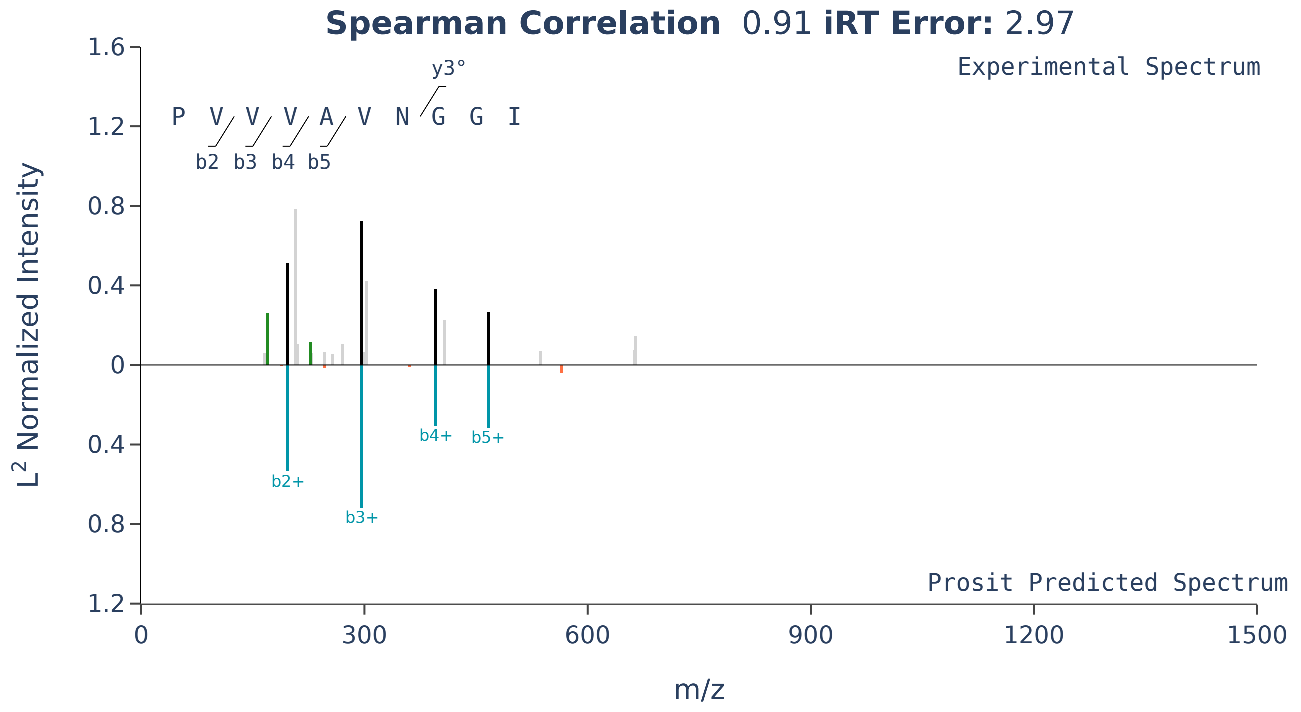

Source HRoetschke_211222_160123_HFGoe_IF2_b1R1_4h **Scan** 21204
Peptide QVPVVVAVN **Charge** 1 **Spectral Angle** 0.51
Spearman Correlation 0.5 **iRT Error:** 21.33

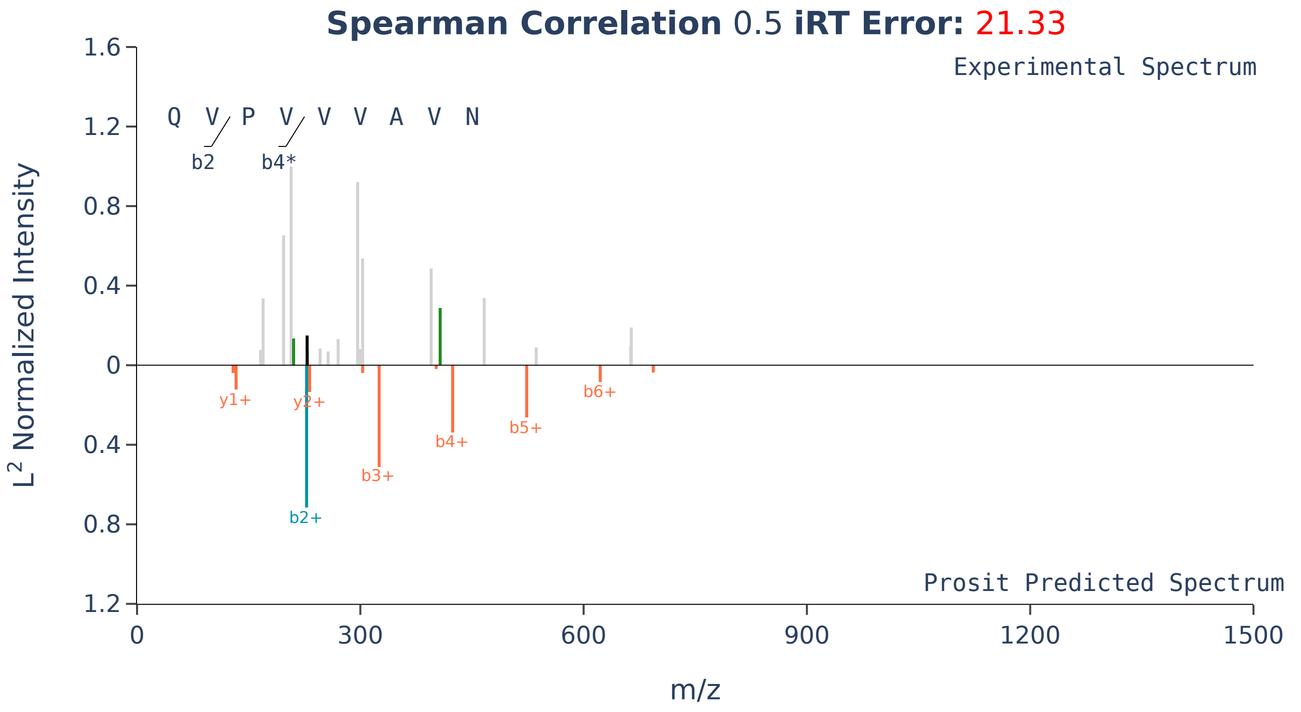

Source HRoetschke_211222_160123_HFGoe_IF2_b2R2_4h **Scan** 13231
Peptide RLVQQFNA **Charge** 2 **Spectral Angle** 0.84
Spearman Correlation 0.86 **iRT Error:** 3.02

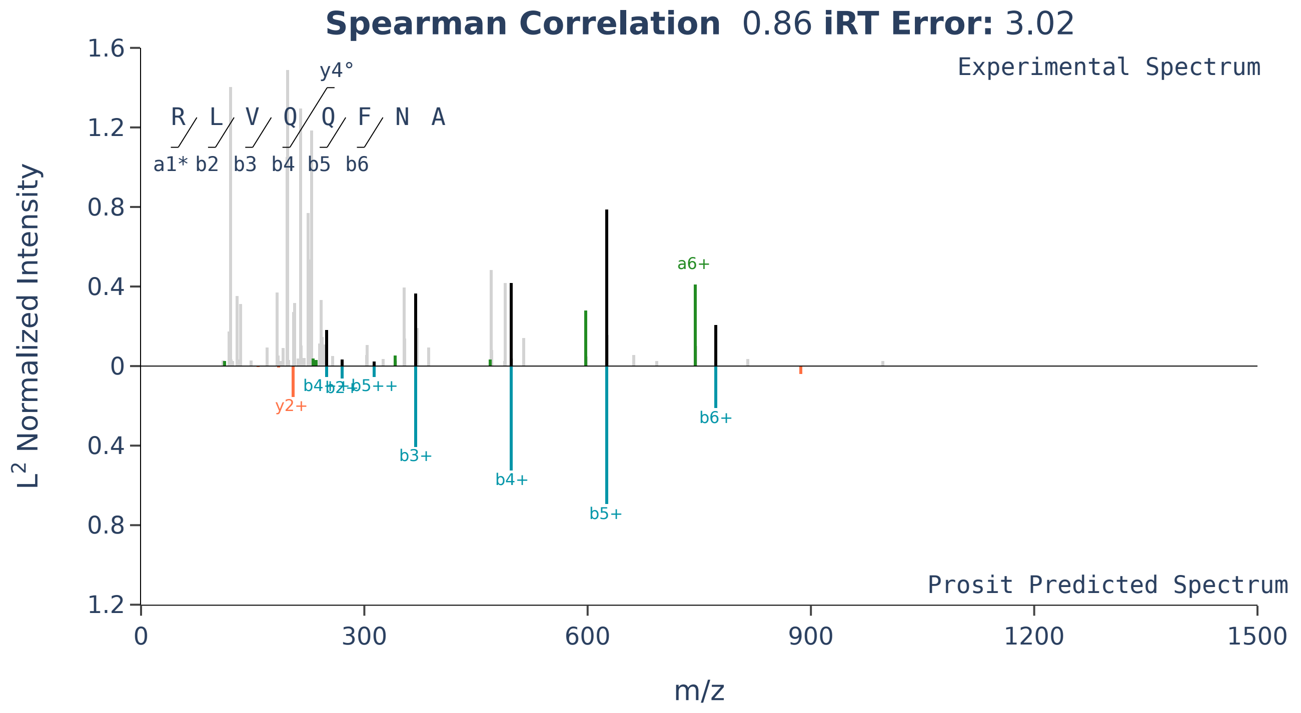

Source HRoetschke_211222_160123_HFGoe_IF2_b2R2_4h **Scan** 13231
Peptide EAISDSLLK **Charge** 2 **Spectral Angle** 0.0
Spearman Correlation -0.31 **iRT Error:** 25.38

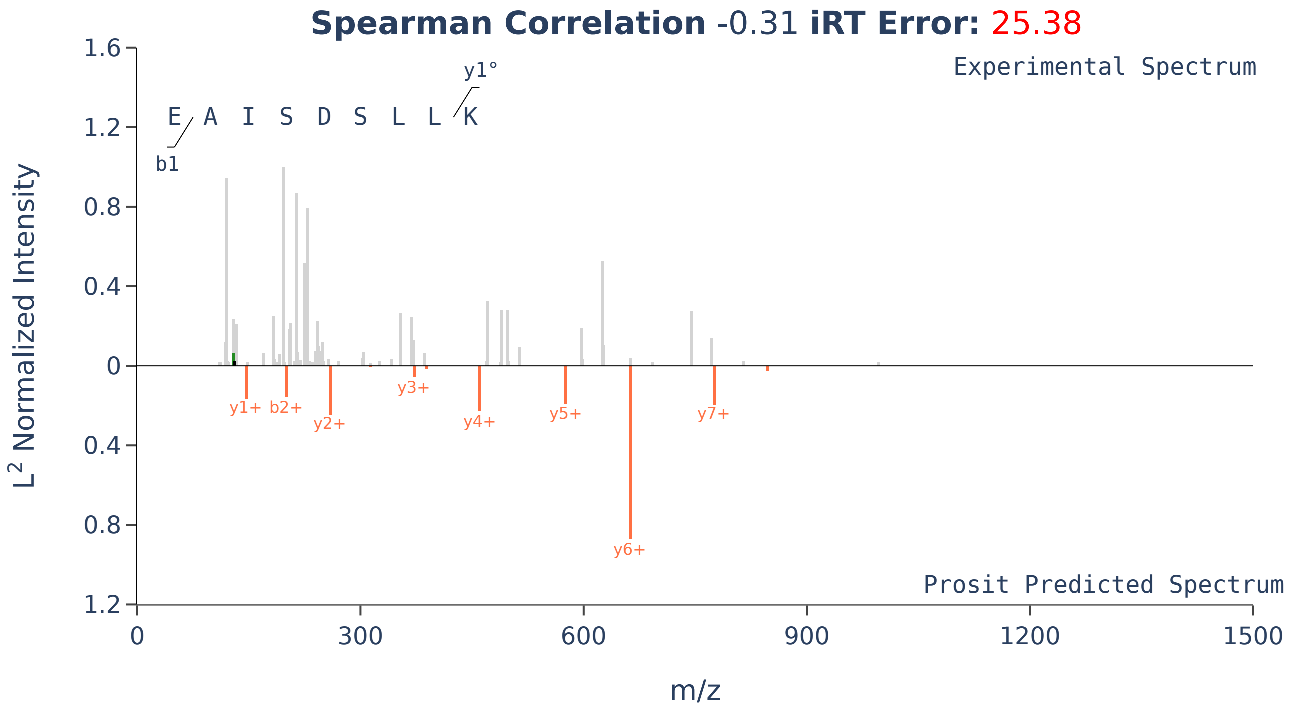

Source HRoetschke_211222_160123_HFGoe_IF2_b1R2_2h **Scan** 11205
Peptide VVTIREAAE **Charge** 2 **Spectral Angle** 0.9
Spearman Correlation 0.83 **iRT Error:** 3.08

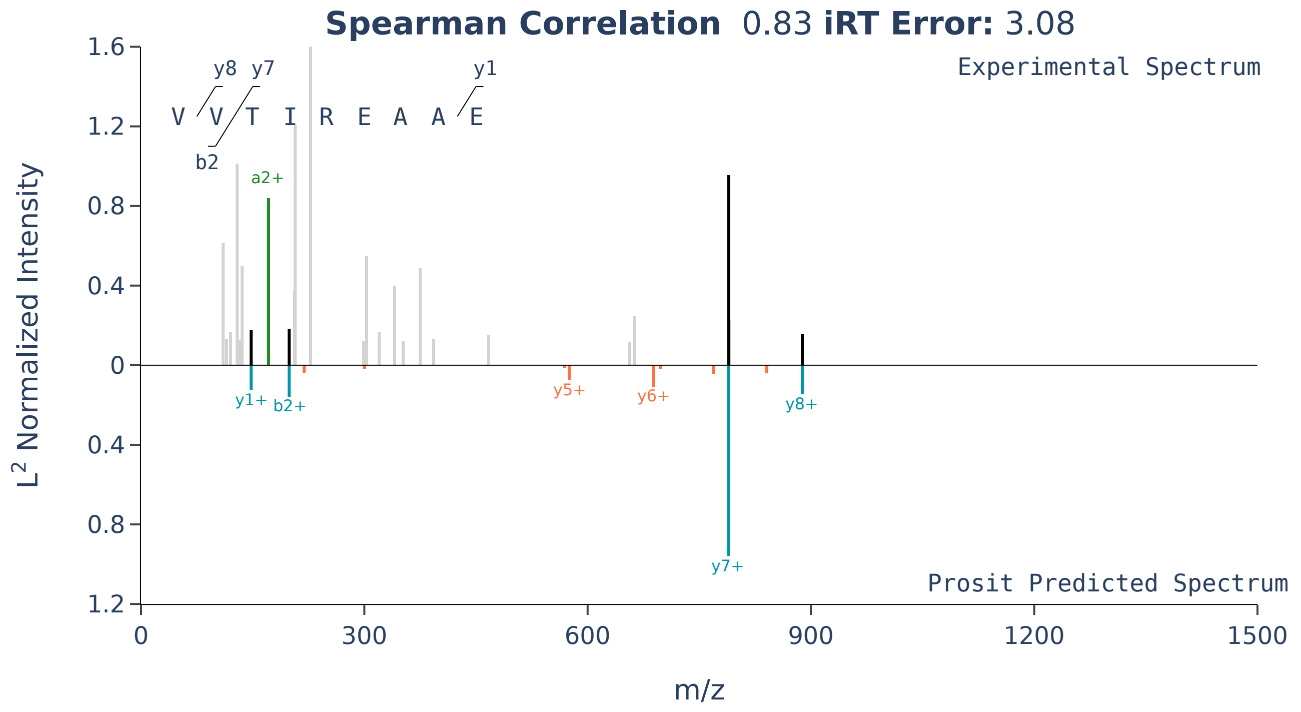

Source HRoetschke_211222_160123_HFGoe_IF2_b1R2_2h **Scan** 11205
Peptide VRTGDVIEV **Charge** 2 **Spectral Angle** 0.0
Spearman Correlation 0.0 **iRT Error:** 34.71

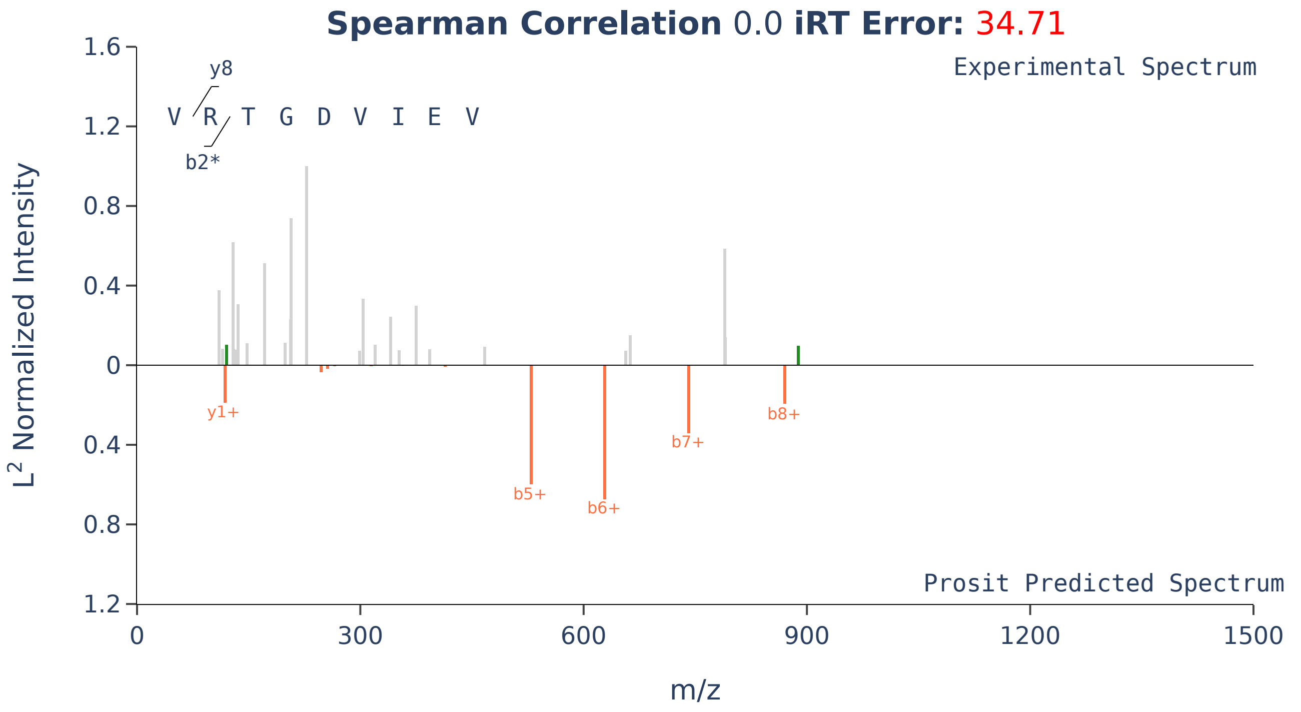

Source HRoetschke_211222_160123_HFGoe_IF2_b2R2_1h **Scan** 11552
Peptide NIPGTRSTL **Charge** 2 **Spectral Angle** 0.88
Spearman Correlation 0.91 **iRT Error:** 3.21

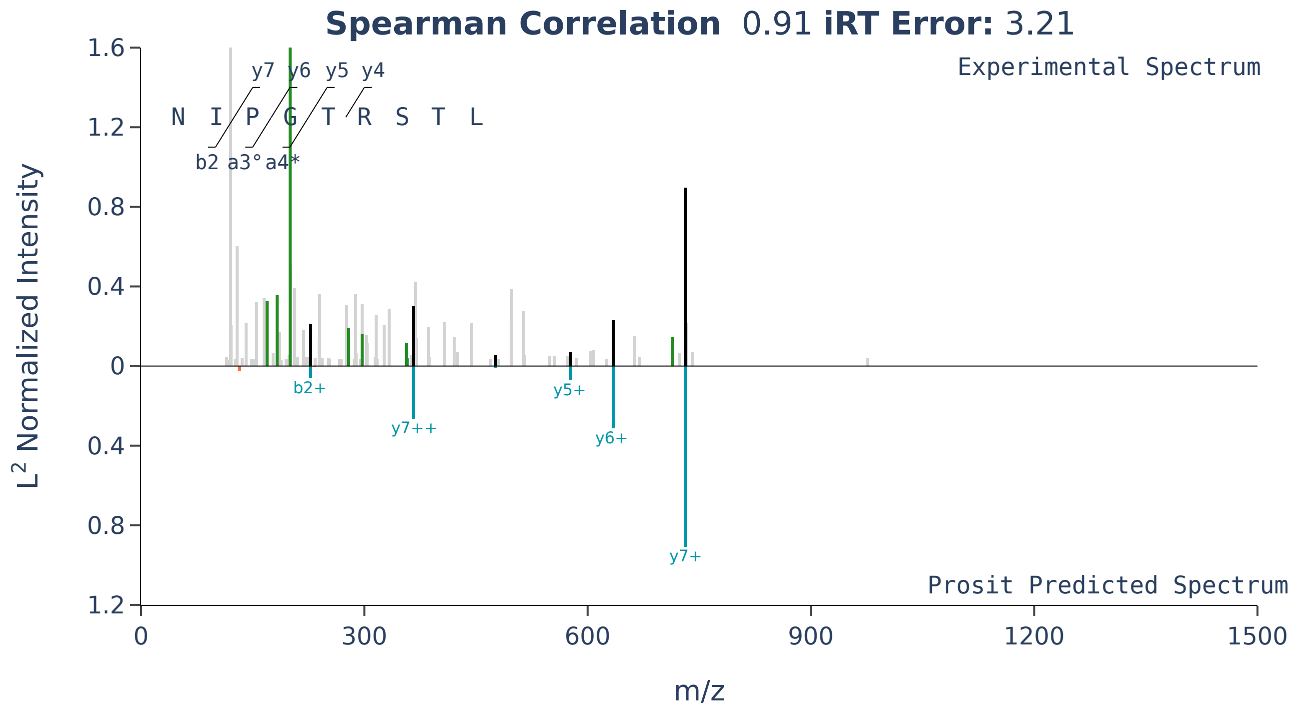

Source HRoetschke_211222_160123_HFGoe_IF2_b2R2_1h **Scan** 11552
Peptide RSTLNIPGT **Charge** 2 **Spectral Angle** 0.0
Spearman Correlation 0.0 **iRT Error:** 18.63

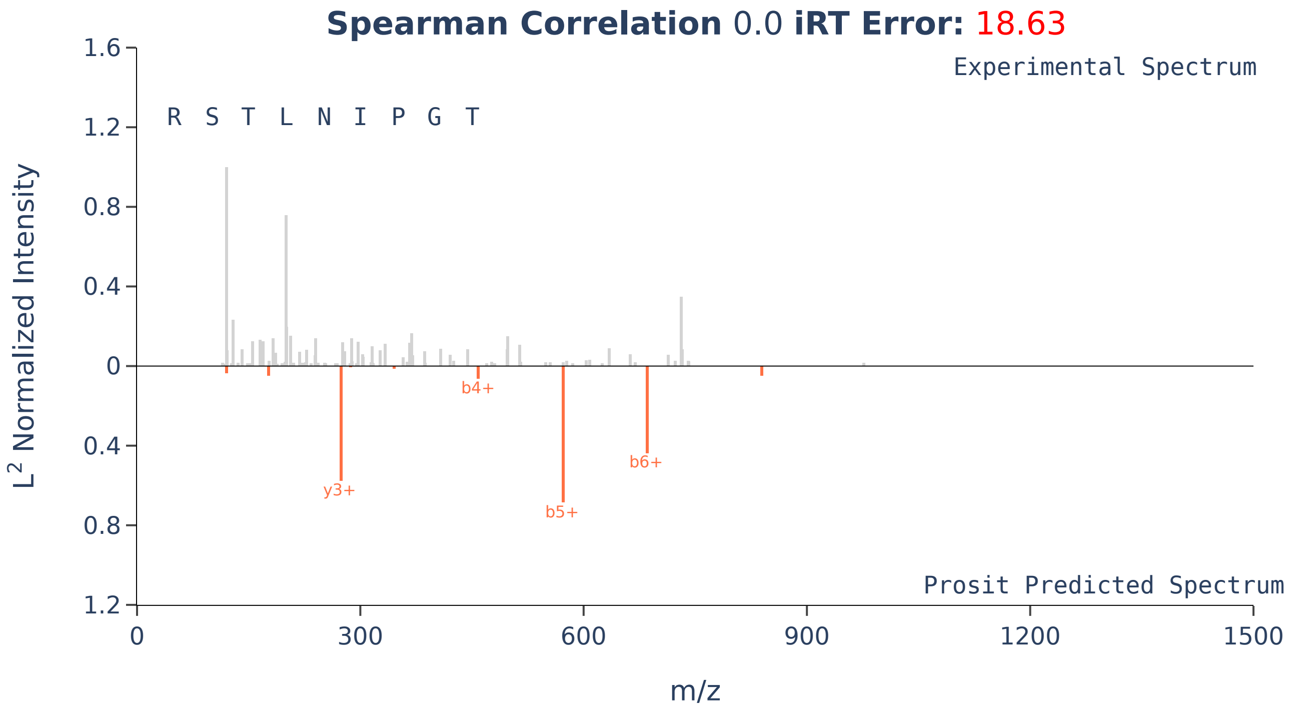

Source HRoetschke_211222_160123_HFGoe_IF2_b1R2_4h **Scan** 8103
Peptide PAAGDEVGS **Charge** 1 **Spectral Angle** 0.77
Spearman Correlation 0.88 **iRT Error:** 3.25

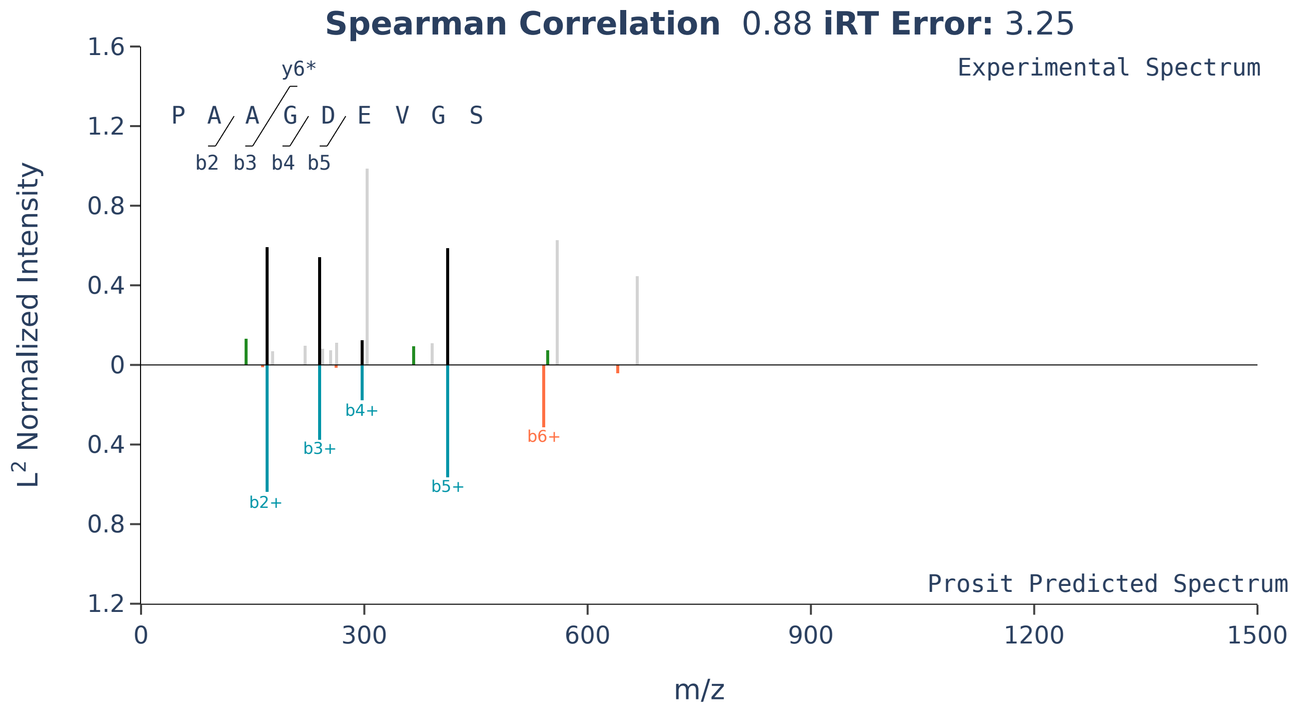

Source HRoetschke_211222_160123_HFGoe_IF2_b1R2_4h **Scan** 8103
Peptide SGVPAAGDE **Charge** 1 **Spectral Angle** 0.78
Spearman Correlation 0.77 **iRT Error:** 4.07

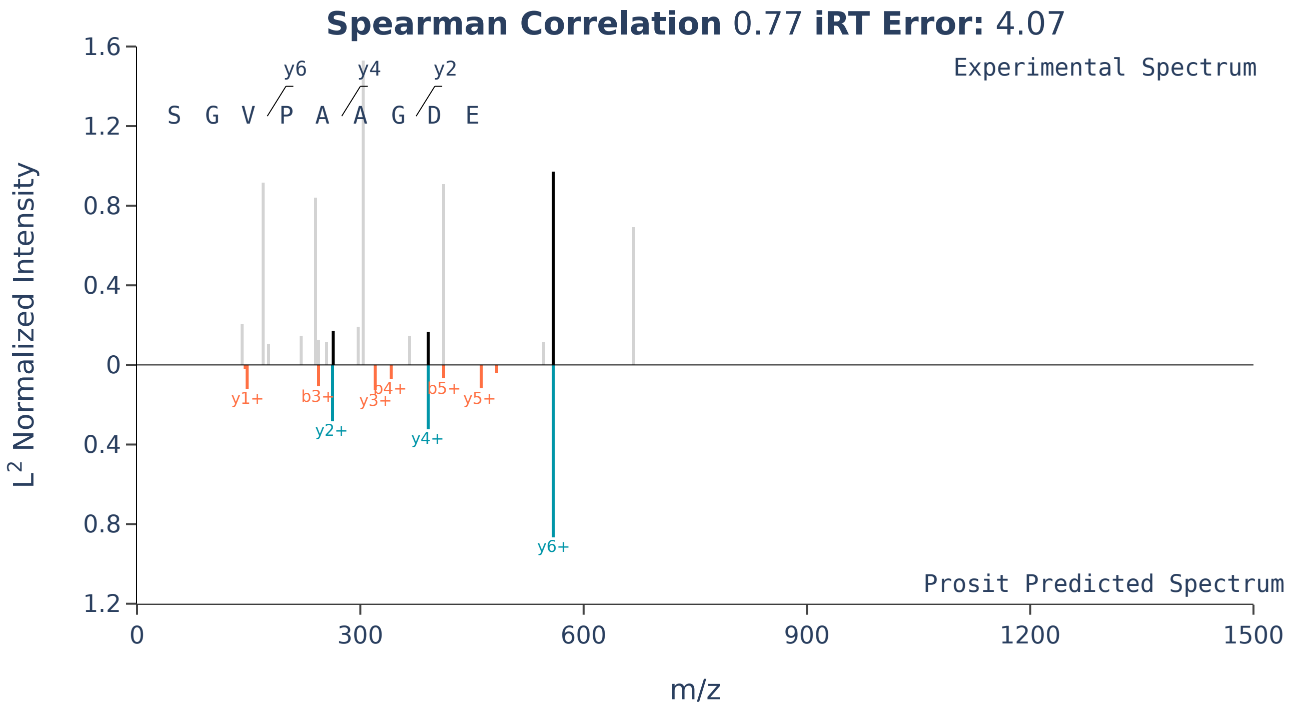

Source HRoetschke_211222_160123_HFGoe_IF2_b1R2_2h **Scan** 11015
Peptide NRDVVLE **Charge** 2 **Spectral Angle** 0.79
Spearman Correlation 0.9 **iRT Error:** 3.57

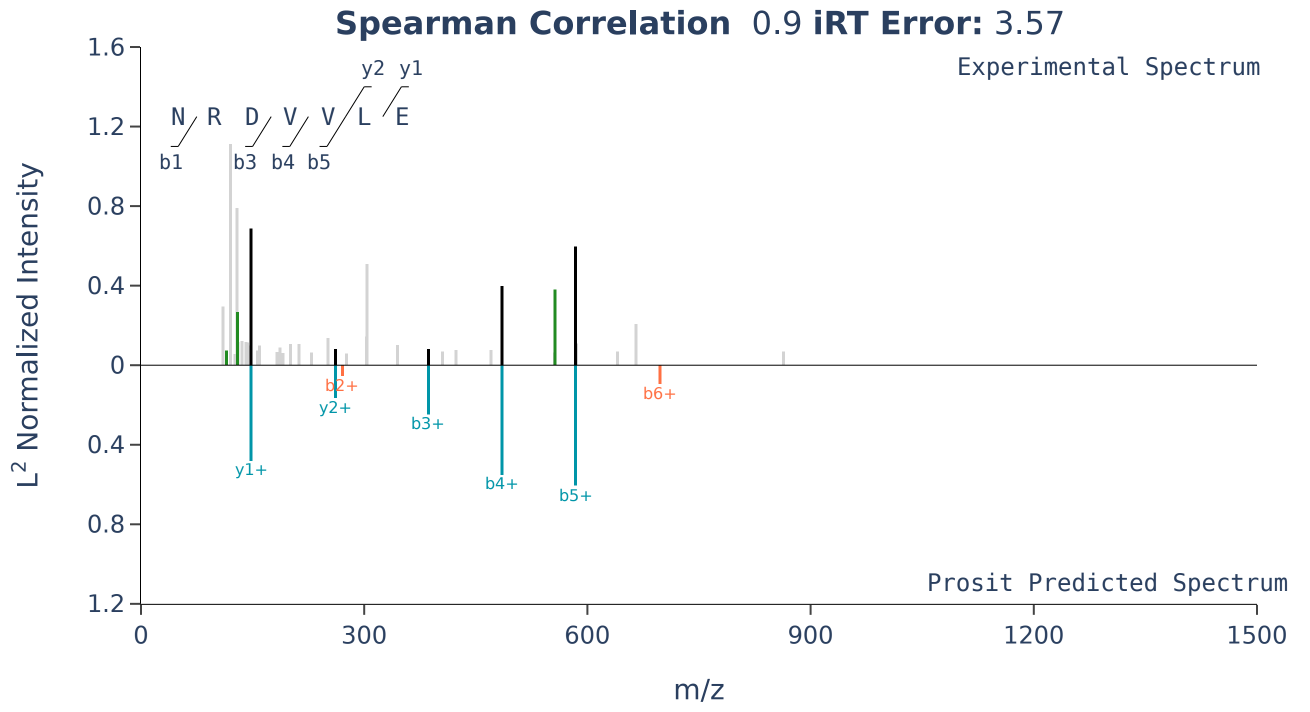

Source HRoetschke_211222_160123_HFGoe_IF2_b1R2_2h **Scan** 11015
Peptide EGGRGRGR **Charge** 2 **Spectral Angle** 0.02
Spearman Correlation -0.15 **iRT Error:** 53.02

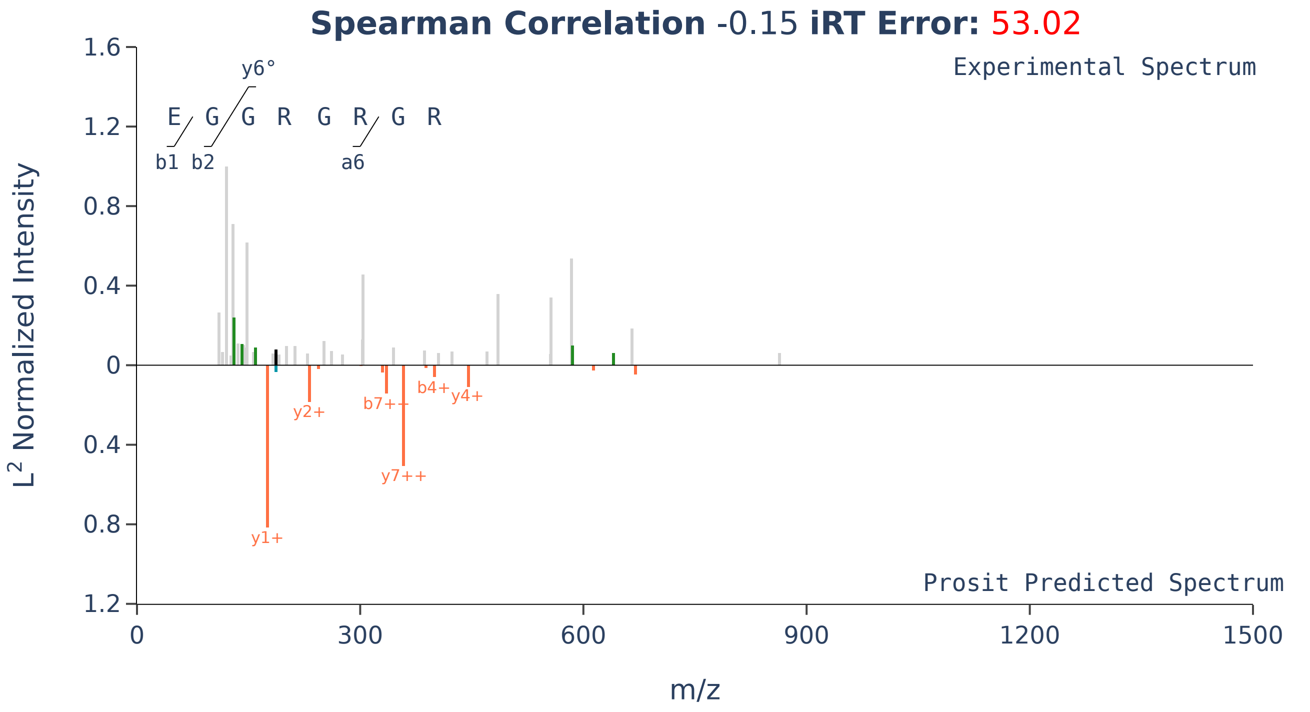

Source HRoetschke_211222_160123_HFGoe_IF2_b2R2_4h **Scan** 11027
Peptide EVHLVQQ **Charge** 2 **Spectral Angle** 0.83
Spearman Correlation 0.87 **iRT Error:** 3.81

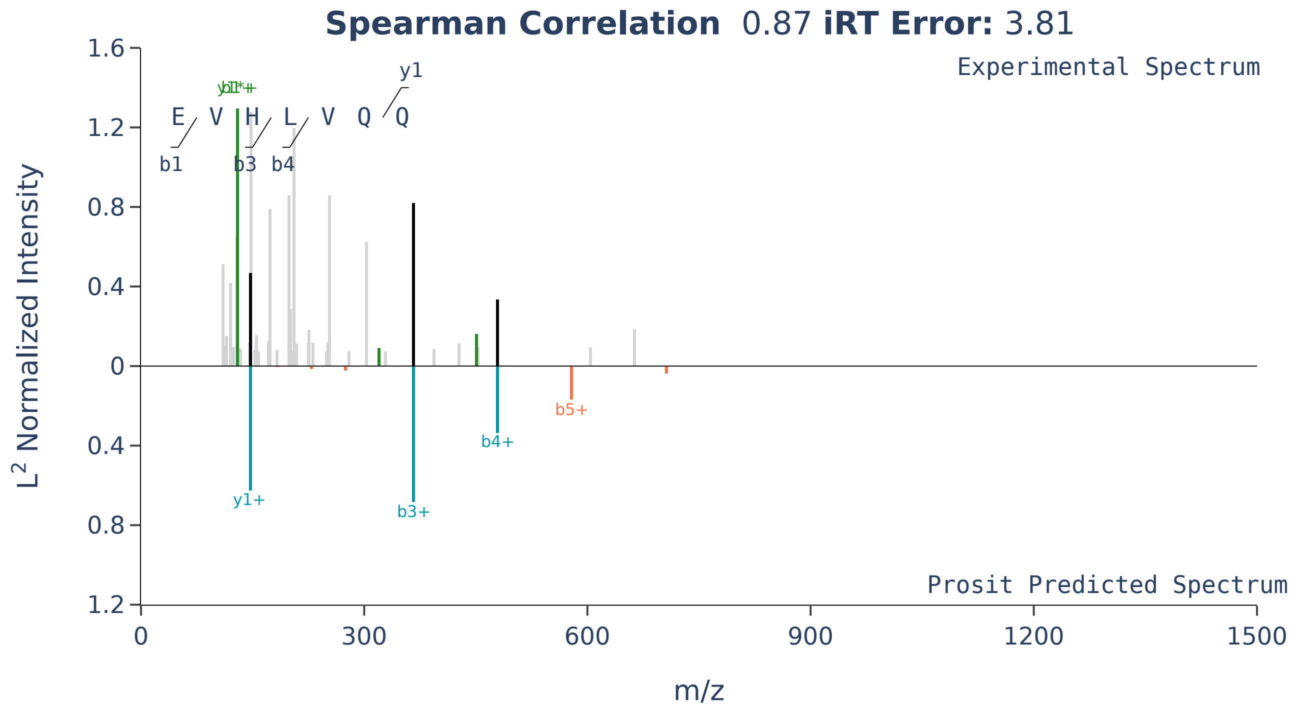

Source HRoetschke_211222_160123_HFGoe_IF2_b2R2_4h **Scan** 11027
Peptide LIDHLNQ **Charge** 2 **Spectral Angle** 0.65
Spearman Correlation 0.62 **iRT Error:** 0.91

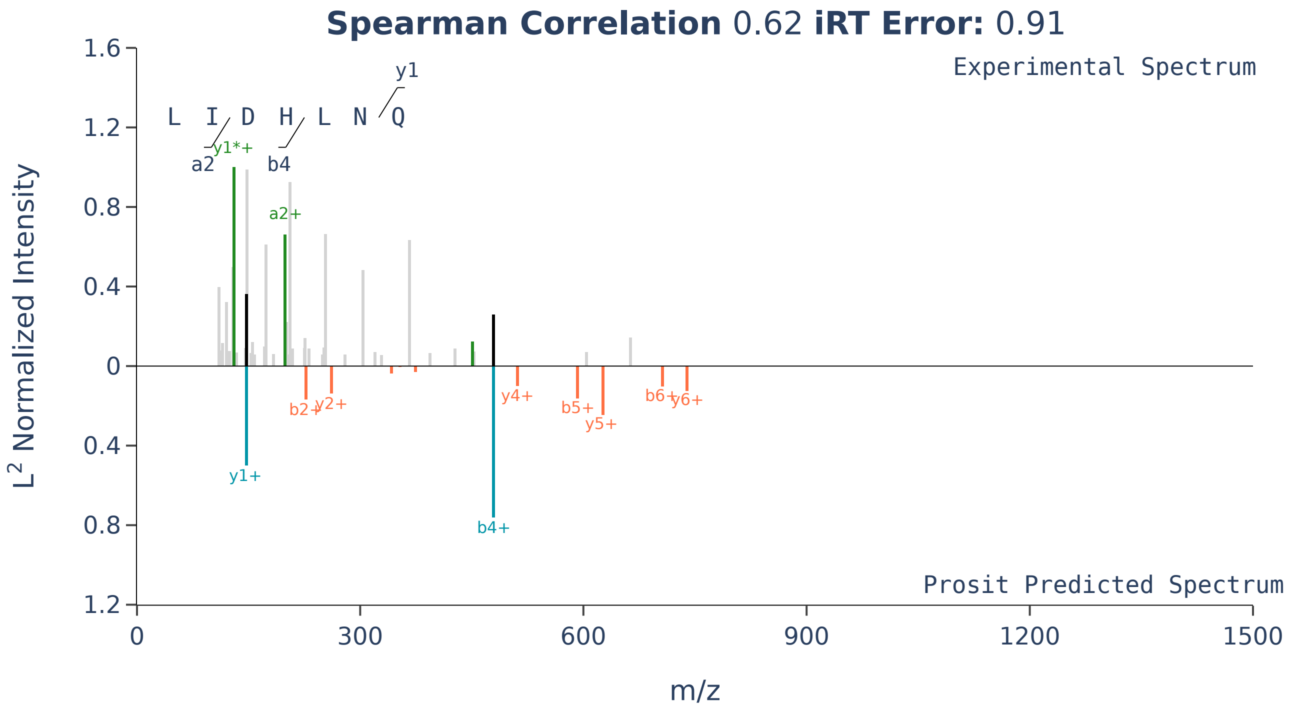

Source HRoetschke_211222_160123_HFGoe_IF2_b1R1_4h **Scan** 15023
Peptide PGHAAFTELS **Charge** 1 **Spectral Angle** 0.86
Spearman Correlation 0.89 **iRT Error:** 4.05

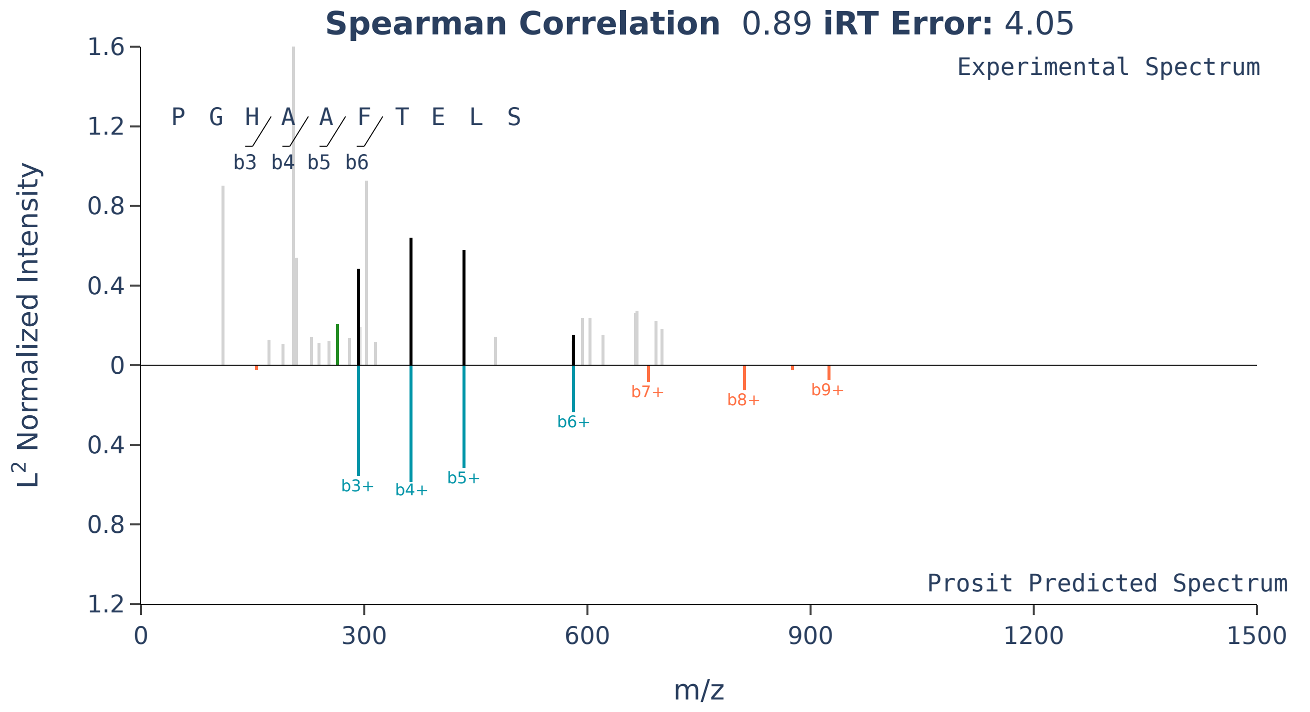

Source HRoetschke_211222_160123_HFGoe_IF2_b1R1_4h **Scan** 15023
Peptide TFLDTPGHAA **Charge** 1 **Spectral Angle** 0.0
Spearman Correlation 0.0 **iRT Error:** 3.19

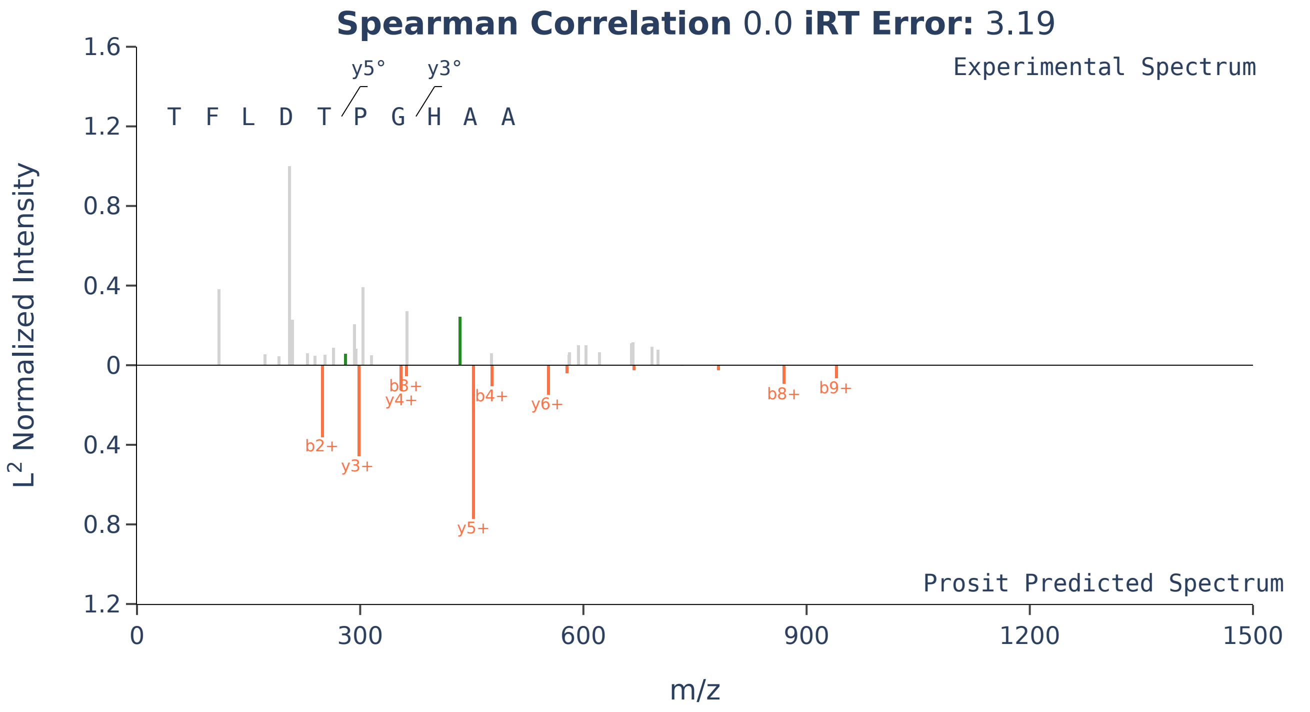

Source HRoetschke_211222_160123_HFGoe_IF2_b1R1_4h **Scan** 5789
Peptide EMGHKVILRER **Charge** 3 **Spectral Angle** 0.85
Spearman Correlation 0.85 **iRT Error:** 4.18

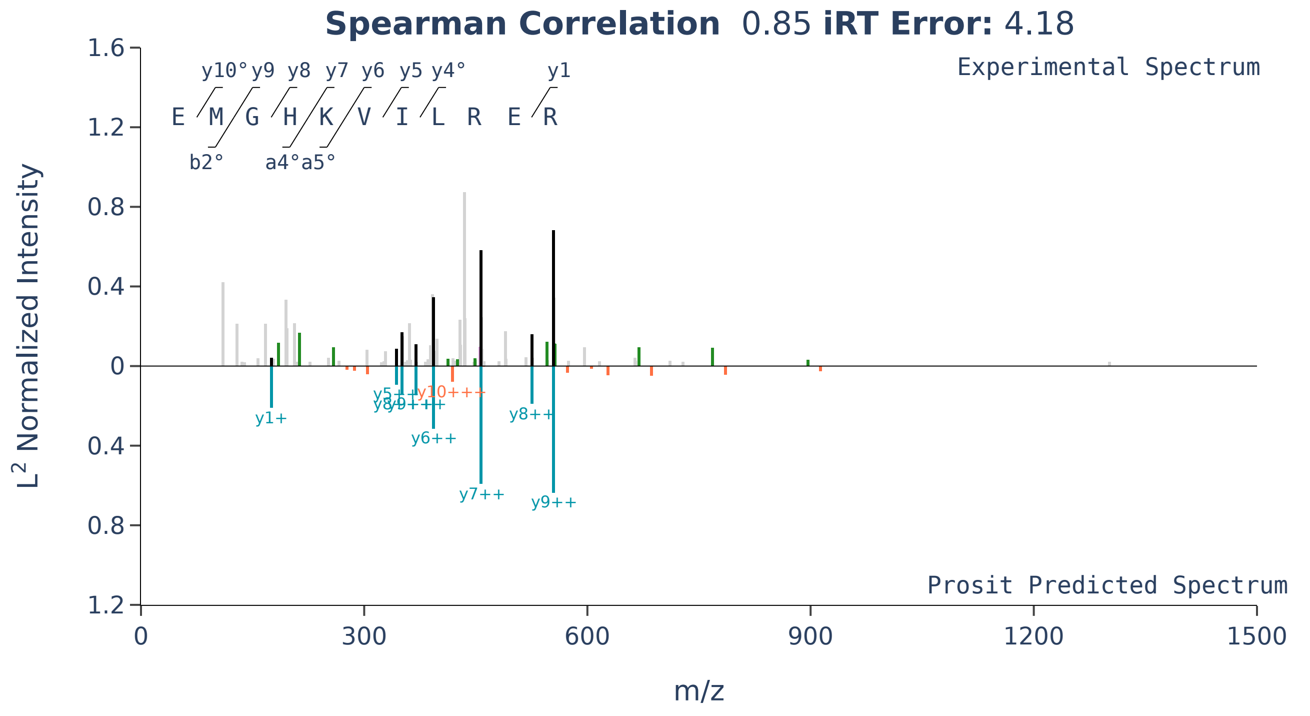

Source HRoetschke_211222_160123_HFGoe_IF2_b1R1_4h **Scan** 5789
Peptide EEMGHKVILRR **Charge** 3 **Spectral Angle** 0.67
Spearman Correlation 0.67 **iRT Error:** 0.01

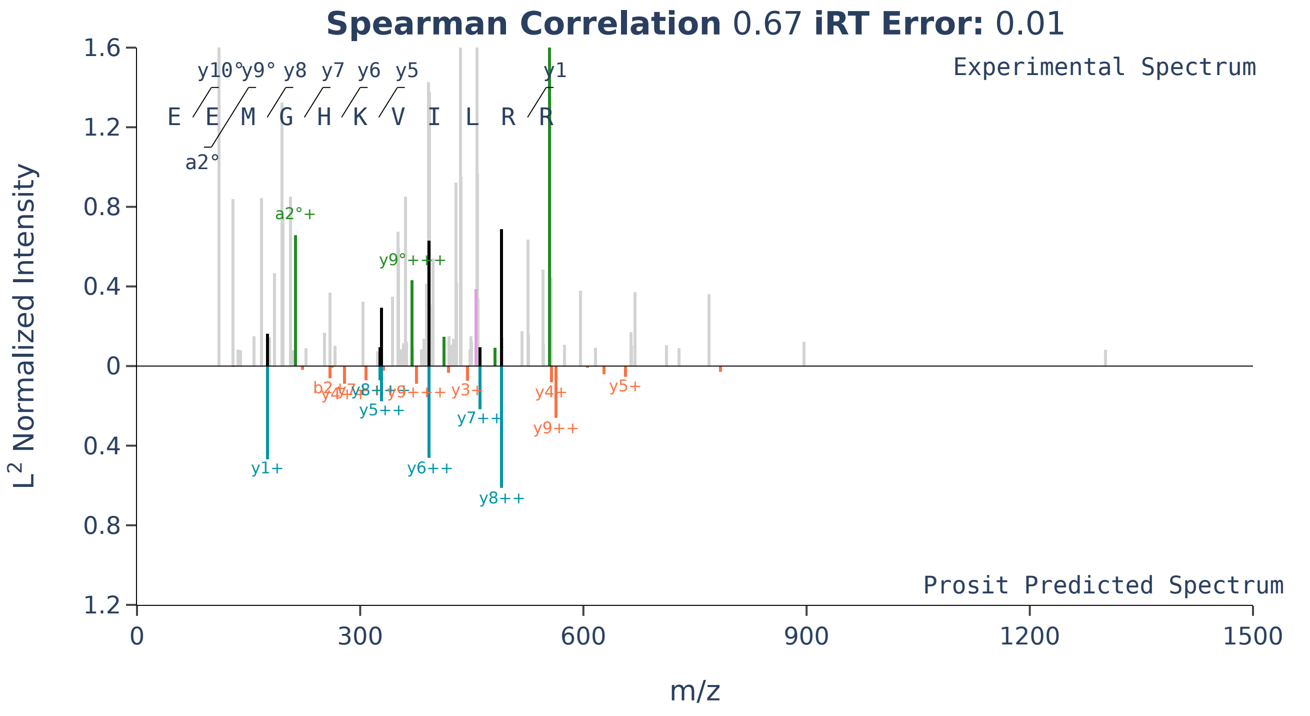

Source HRoetschke_211222_160123_HFGoe_IF2_b1R1_2h **Scan** 8288
Peptide IGAYHVERD **Charge** 3 **Spectral Angle** 0.92
Spearman Correlation 0.94 **iRT Error:** 4.38

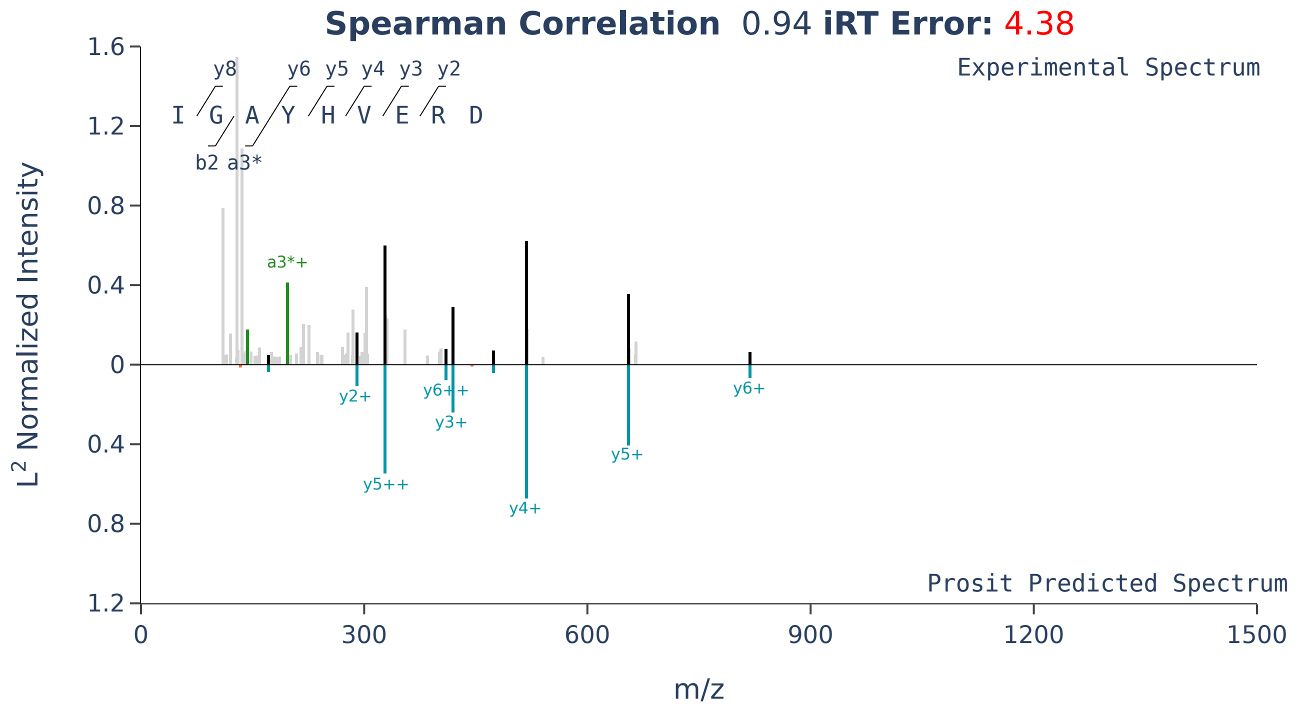

Source HRoetschke_211222_160123_HFGoe_IF2_b1R1_2h **Scan** 8288
Peptide AEEQARREA **Charge** 3 **Spectral Angle** 0.0
Spearman Correlation 0.0 **iRT Error:** 12.82

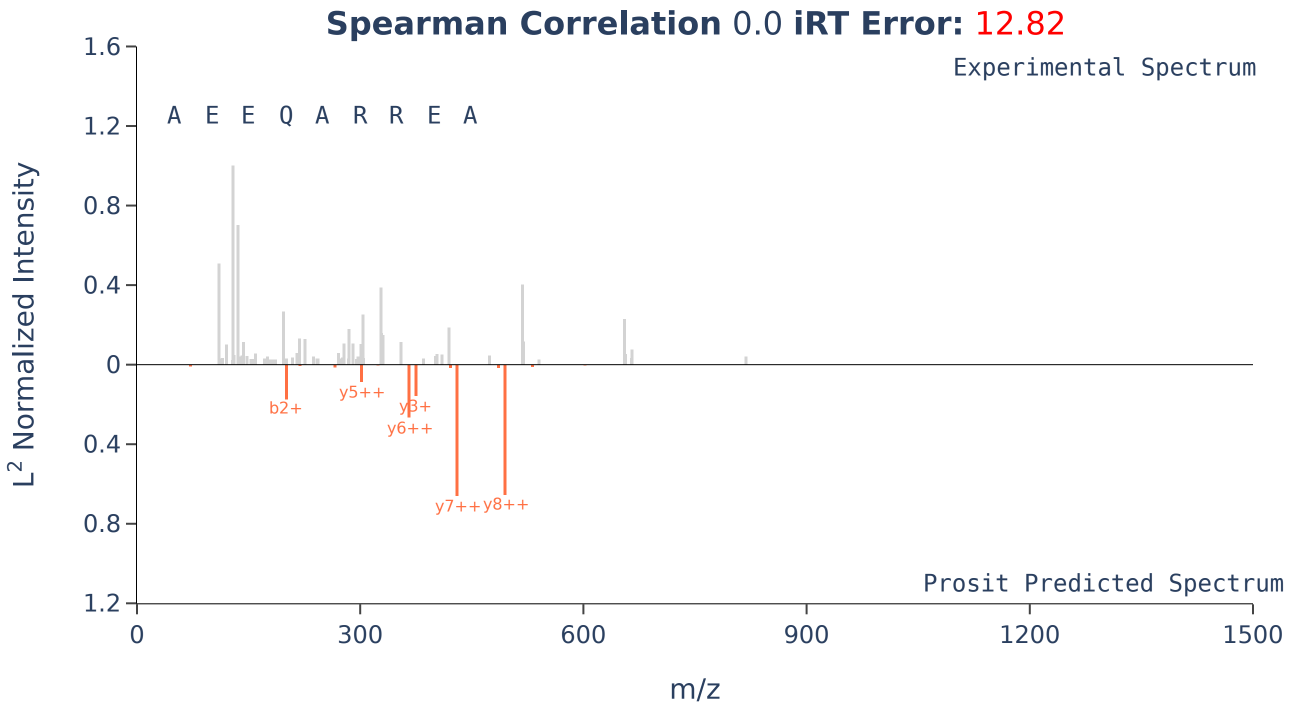

Source HRoetschke_211222_160123_HFGoe_IF2_b2R2_2h **Scan** 7754
Peptide HVETQGS **Charge** 1 **Spectral Angle** 0.9
Spearman Correlation 0.85 **iRT Error:** 4.47

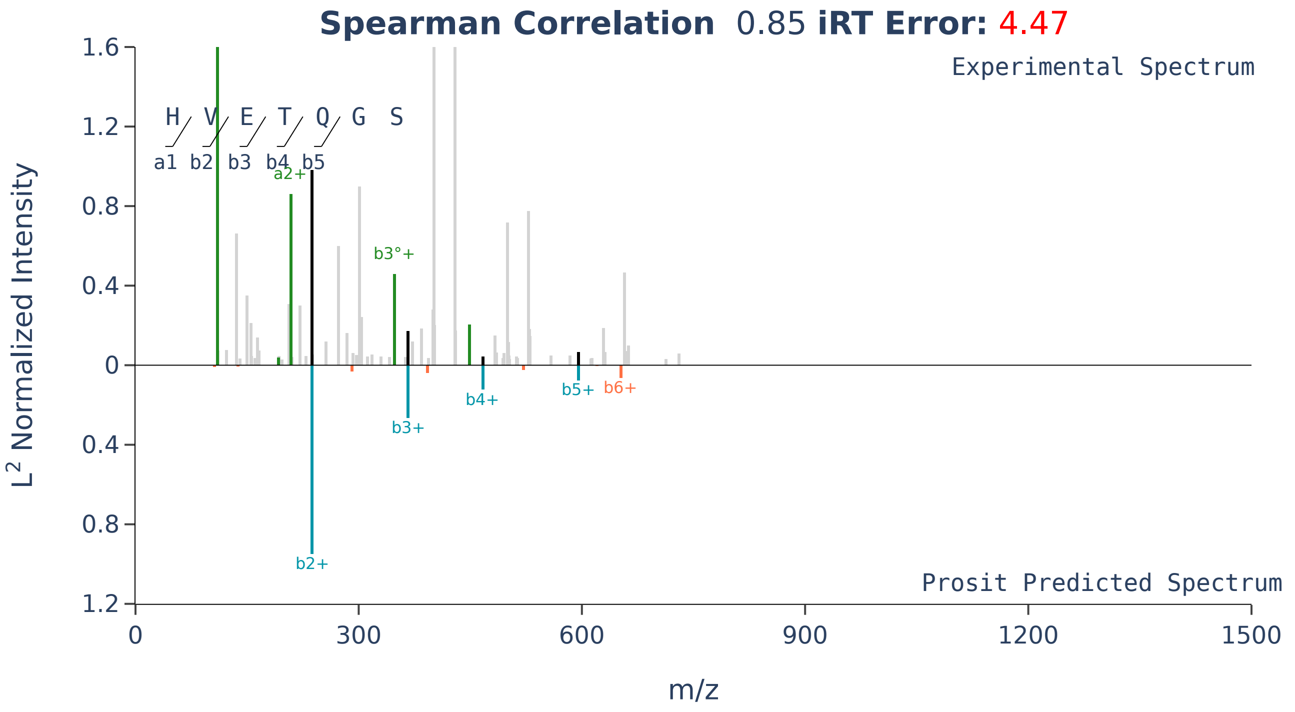

Source HRoetschke_211222_160123_HFGoe_IF2_b2R2_2h **Scan** 7754
Peptide HAESKAD **Charge** 1 **Spectral Angle** 0.0
Spearman Correlation 0.0 **iRT Error:** 12.39

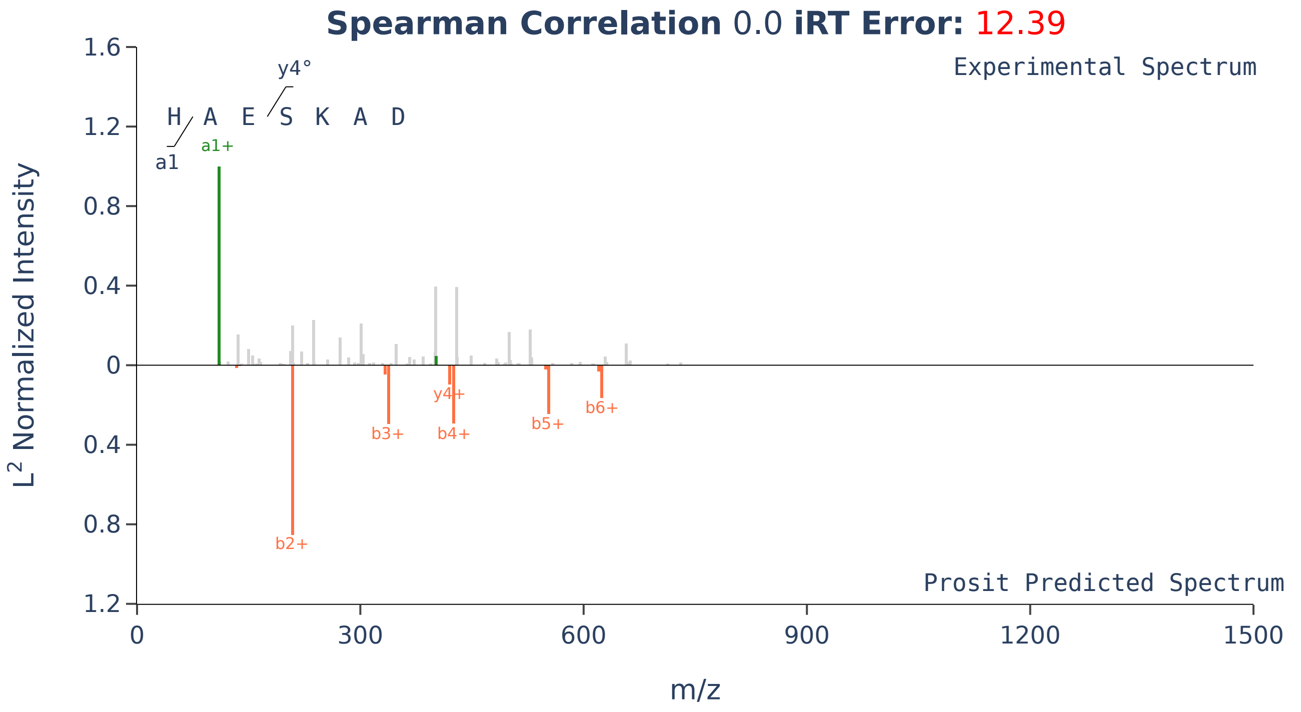

Source HRoetschke_211222_160123_HFGoe_IF2_b1R2_2h **Scan** 9918
Peptide ASGAVITD **Charge** 1 **Spectral Angle** 0.88
Spearman Correlation 0.94 **iRT Error:** 4.47

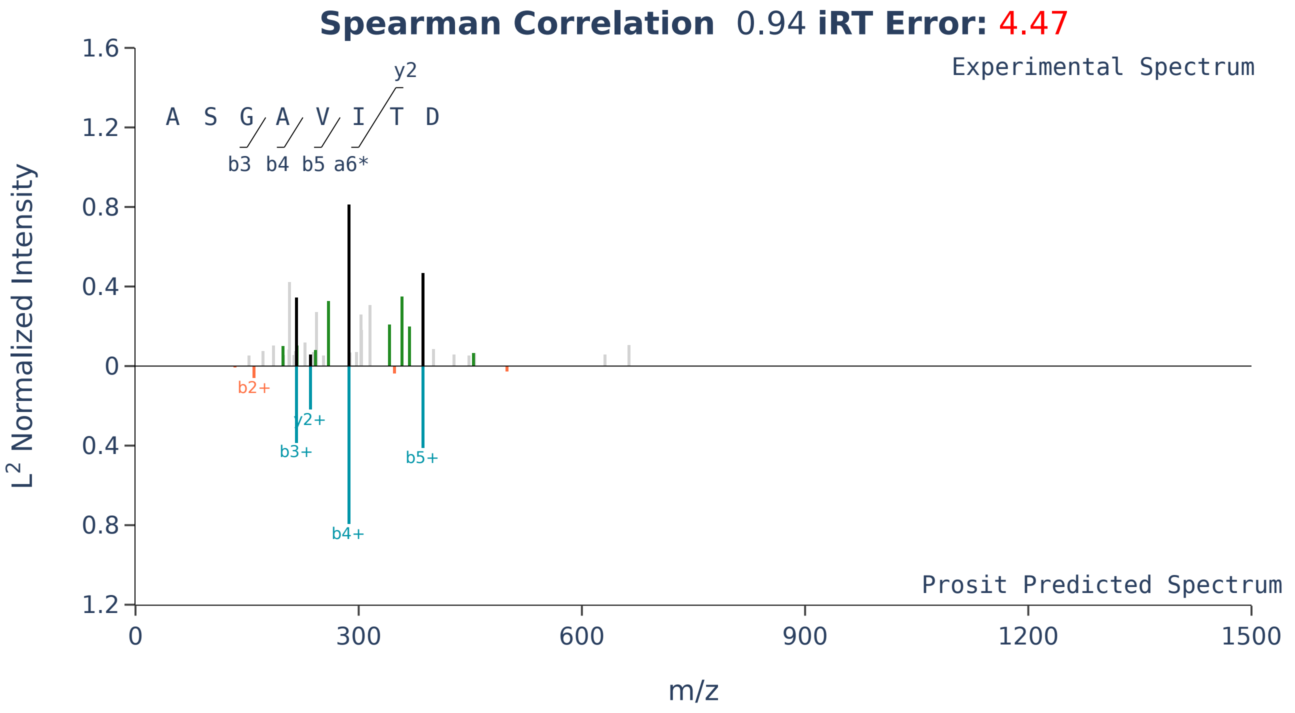

Source HRoetschke_211222_160123_HFGoe_IF2_b1R2_2h **Scan** 9918
Peptide GVGGITET **Charge** 1 **Spectral Angle** 0.0
Spearman Correlation 0.0 **iRT Error:** 10.82

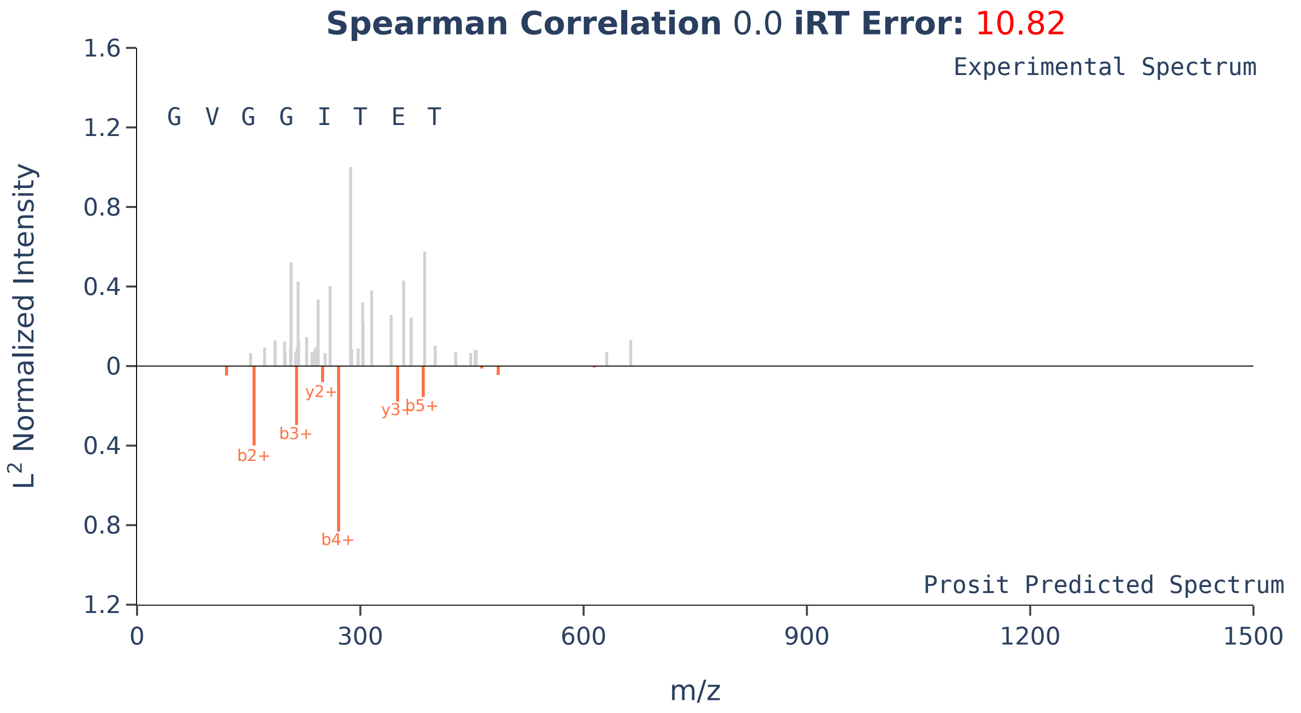

Source HRoetschke_211222_160123_HFGoe_IF2_b1R1_4h **Scan** 19641
Peptide PVVVAGGI **Charge** 1 **Spectral Angle** 0.88
Spearman Correlation 0.84 **iRT Error:** 4.52

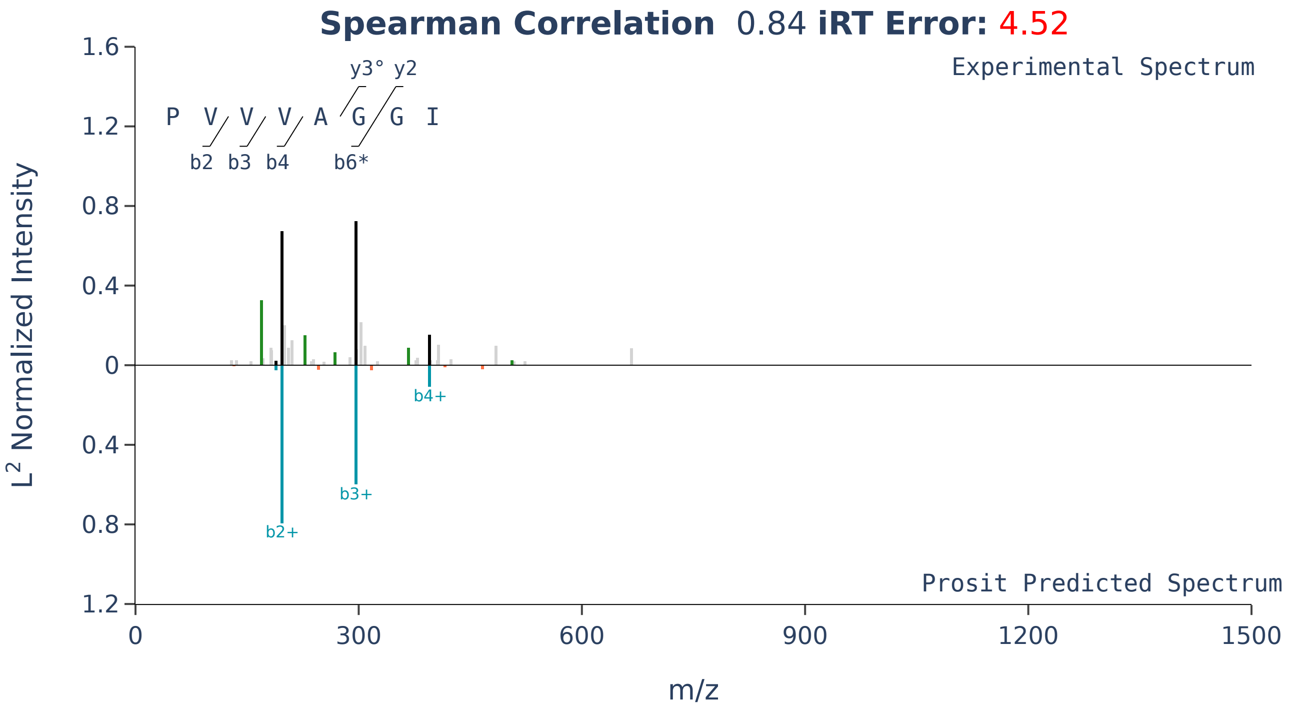

Source HRoetschke_211222_160123_HFGoe_IF2_b1R1_4h **Scan** 19641
Peptide AQVPVVV **Charge** 1 **Spectral Angle** 0.0
Spearman Correlation 0.0 **iRT Error:** 4.16

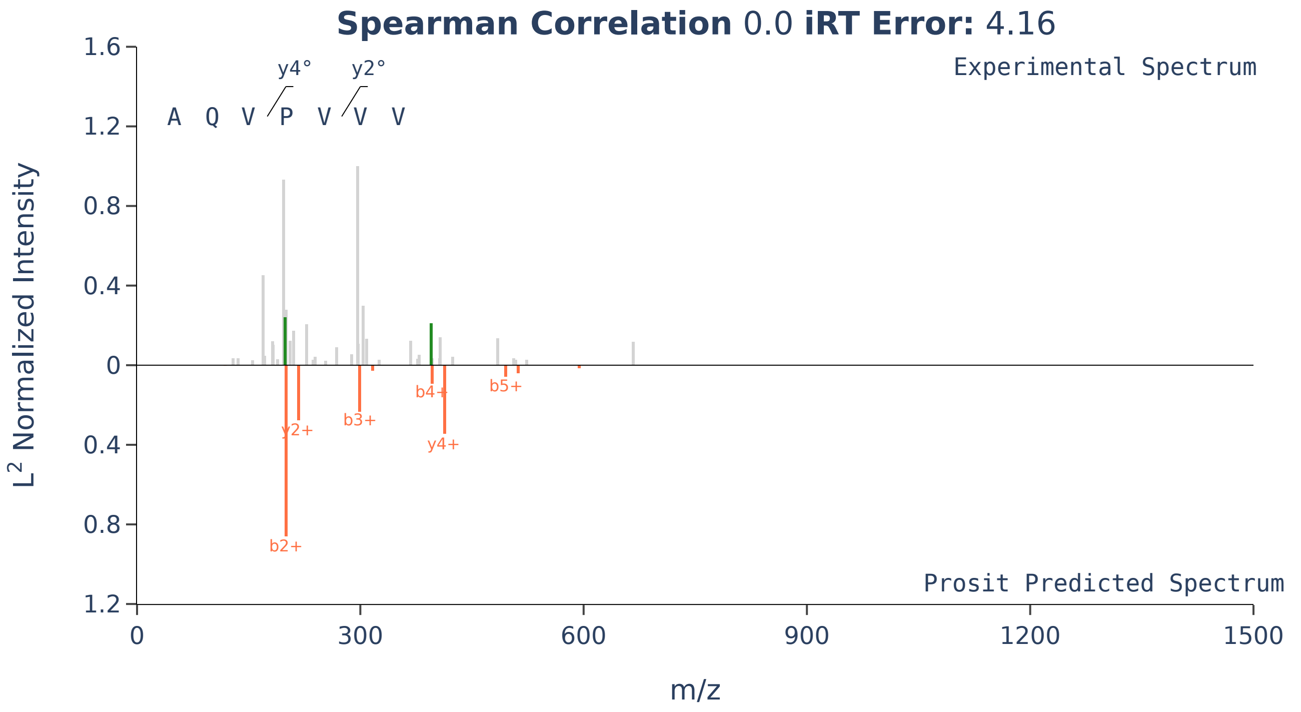

Source HRoetschke_211222_160123_HFGoe_IF2_b1R1_4h **Scan** 13644
Peptide ADRRNELGQEVLE **Charge** 3 **Spectral Angle** 0.76
Spearman Correlation 0.96 **iRT Error:** 4.7

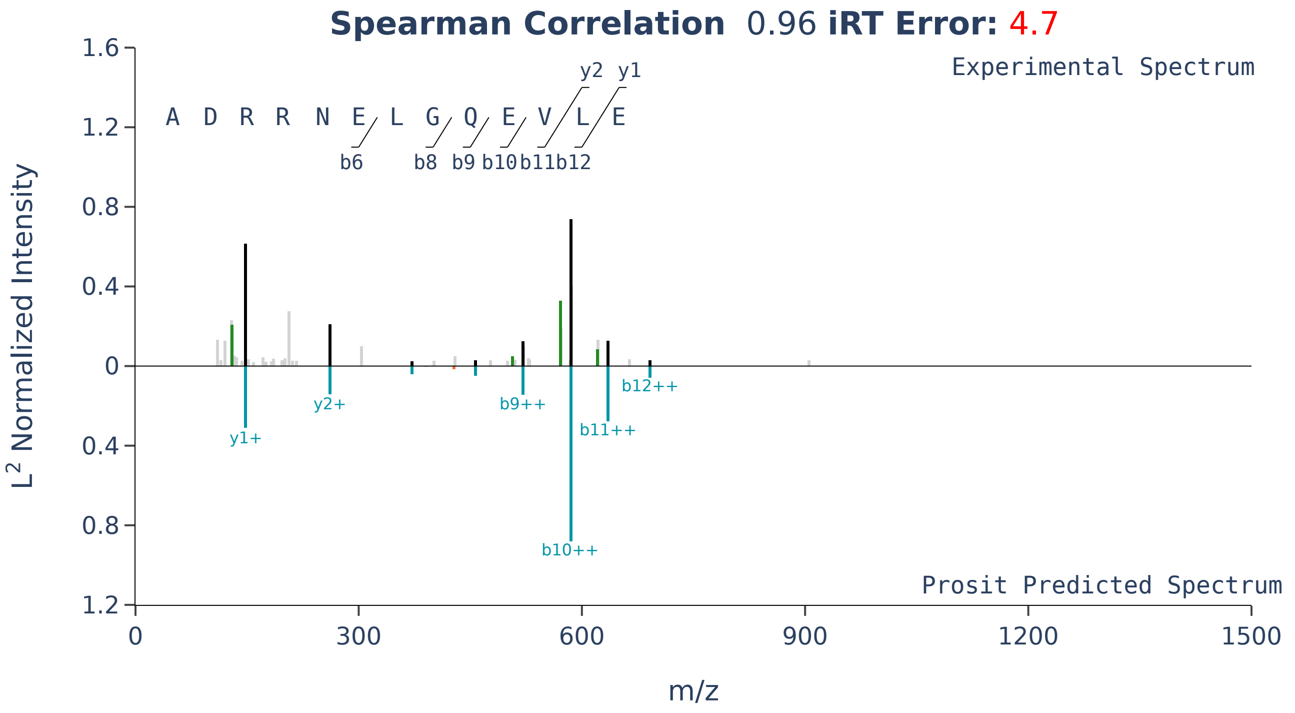

Source HRoetschke_211222_160123_HFGoe_IF2_b1R1_4h **Scan** 13644
Peptide DREVEGGRGRGRNA **Charge** 3 **Spectral Angle** 0.01
Spearman Correlation -0.1 **iRT Error:** 66.01

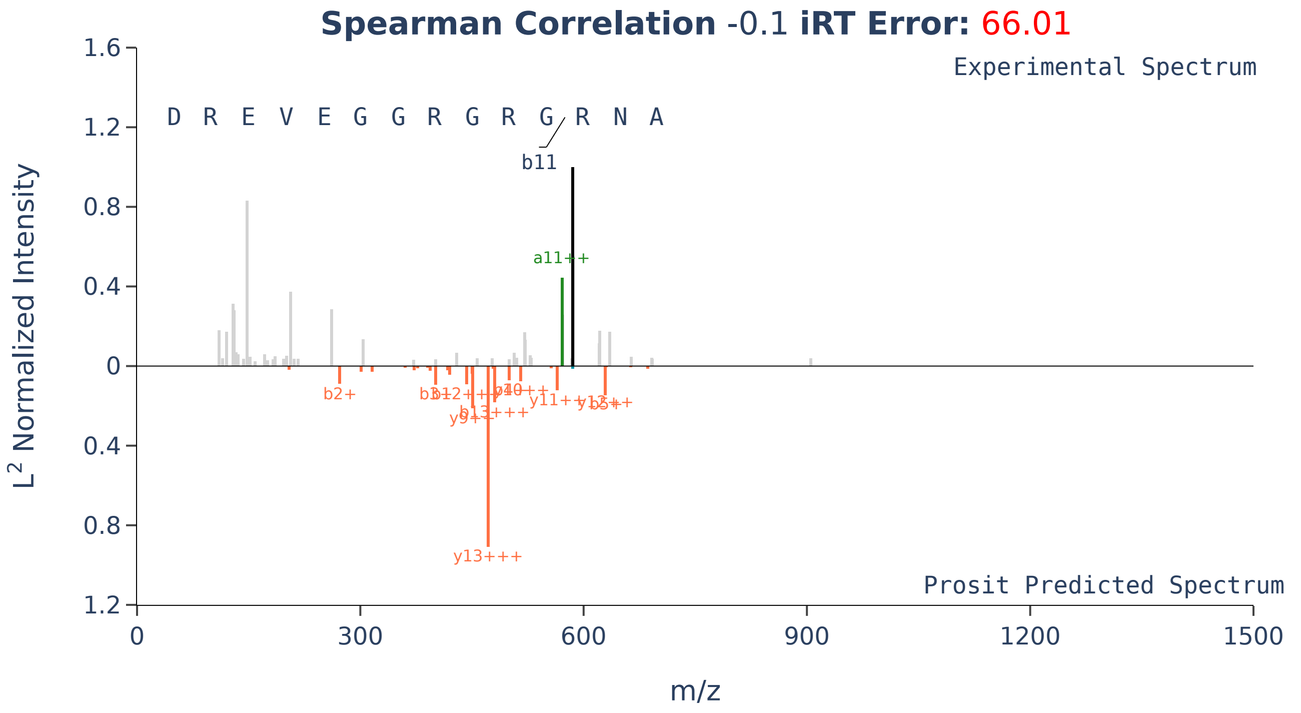

Source HRoetschke_211222_160123_HFGoe_IF2_b1R2_24h **Scan** 8251
Peptide YHVDQET **Charge** 2 **Spectral Angle** 0.86
Spearman Correlation 0.89 **iRT Error:** 5.29

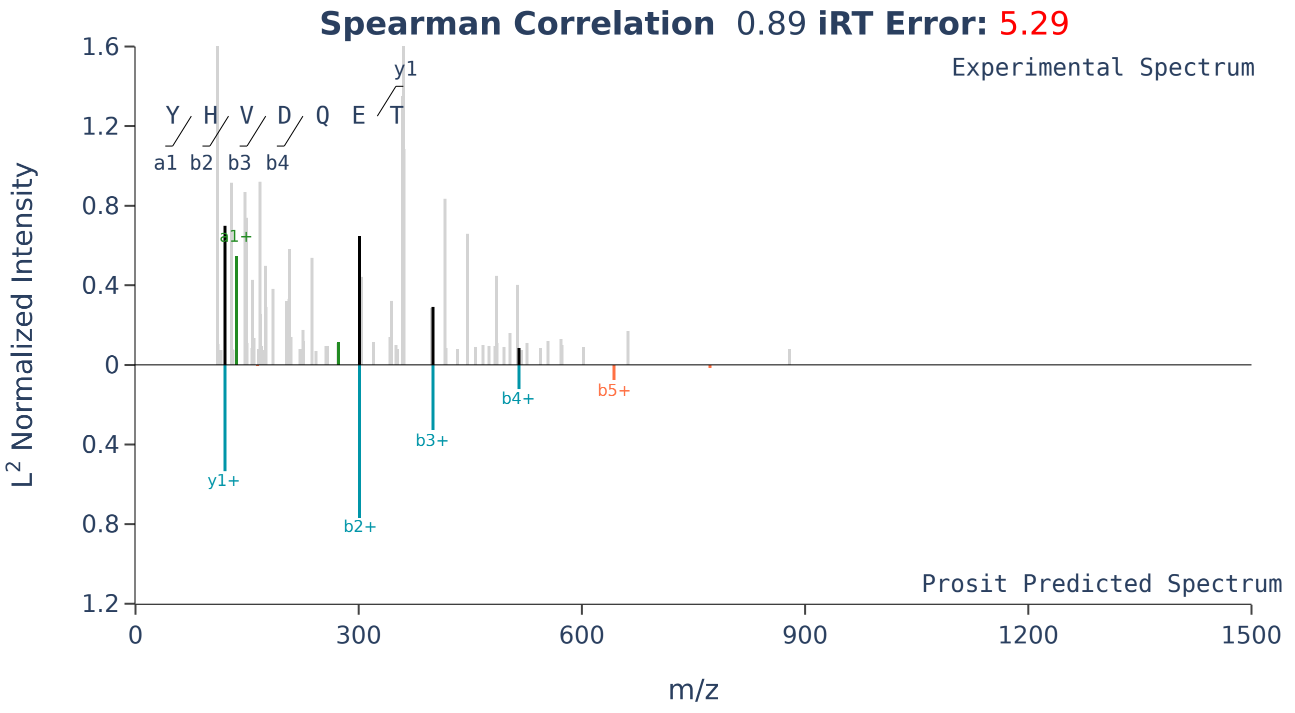

Source HRoetschke_211222_160123_HFGoe_IF2_b1R2_24h **Scan** 8251
Peptide YHVETEN **Charge** 2 **Spectral Angle** 0.57
Spearman Correlation 0.67 **iRT Error:** 5.89

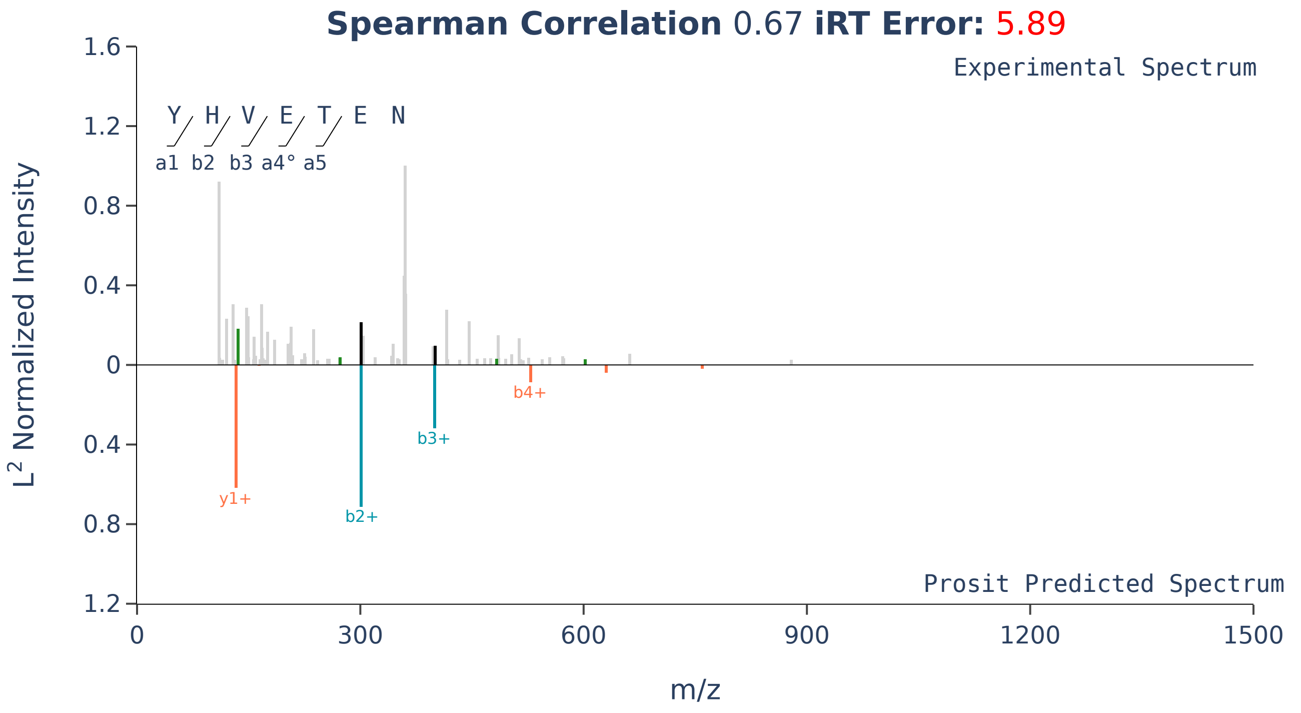

Source HRoetschke_211222_160123_HFGoe_IF2_b2R1_4h **Scan** 15146
Peptide QEVAVIE **Charge** 1 **Spectral Angle** 0.8
Spearman Correlation 0.93 **iRT Error:** 5.35

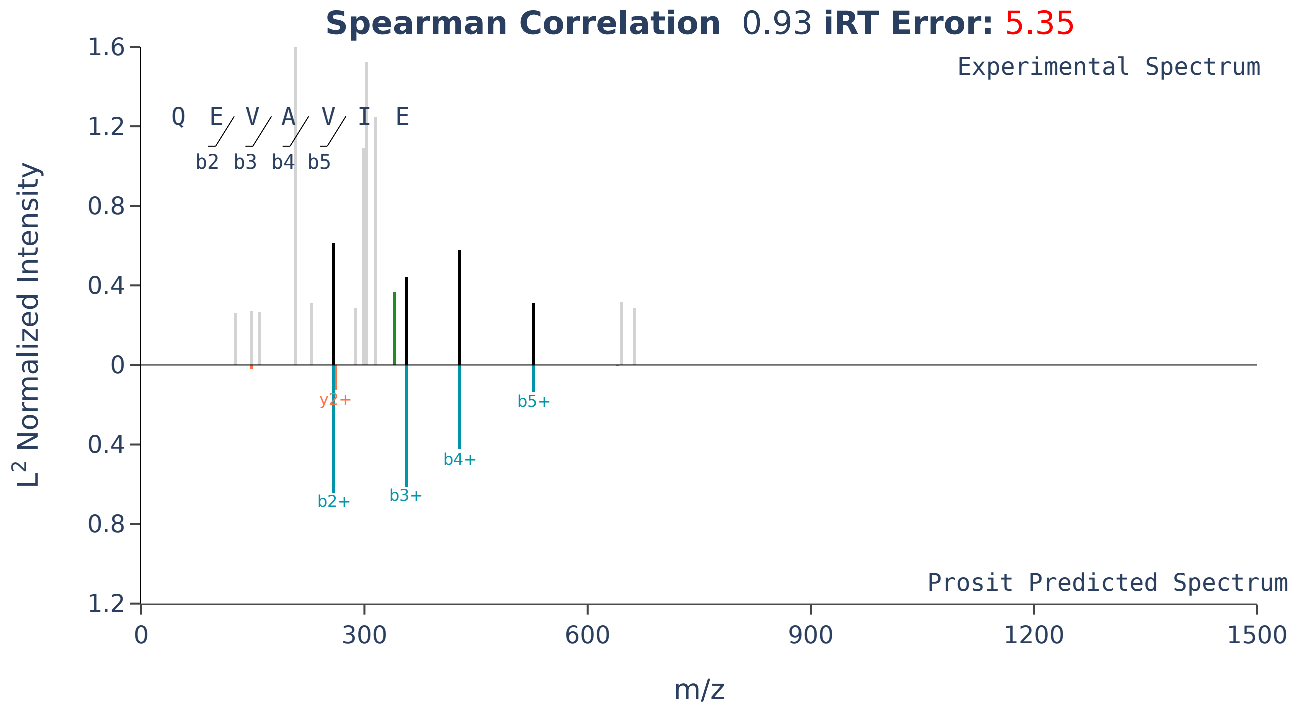

Source HRoetschke_211222_160123_HFGoe_IF2_b2R1_4h **Scan** 15146
Peptide VEGGRGRG **Charge** 1 **Spectral Angle** 0.12
Spearman Correlation 0.08 **iRT Error:** 35.71

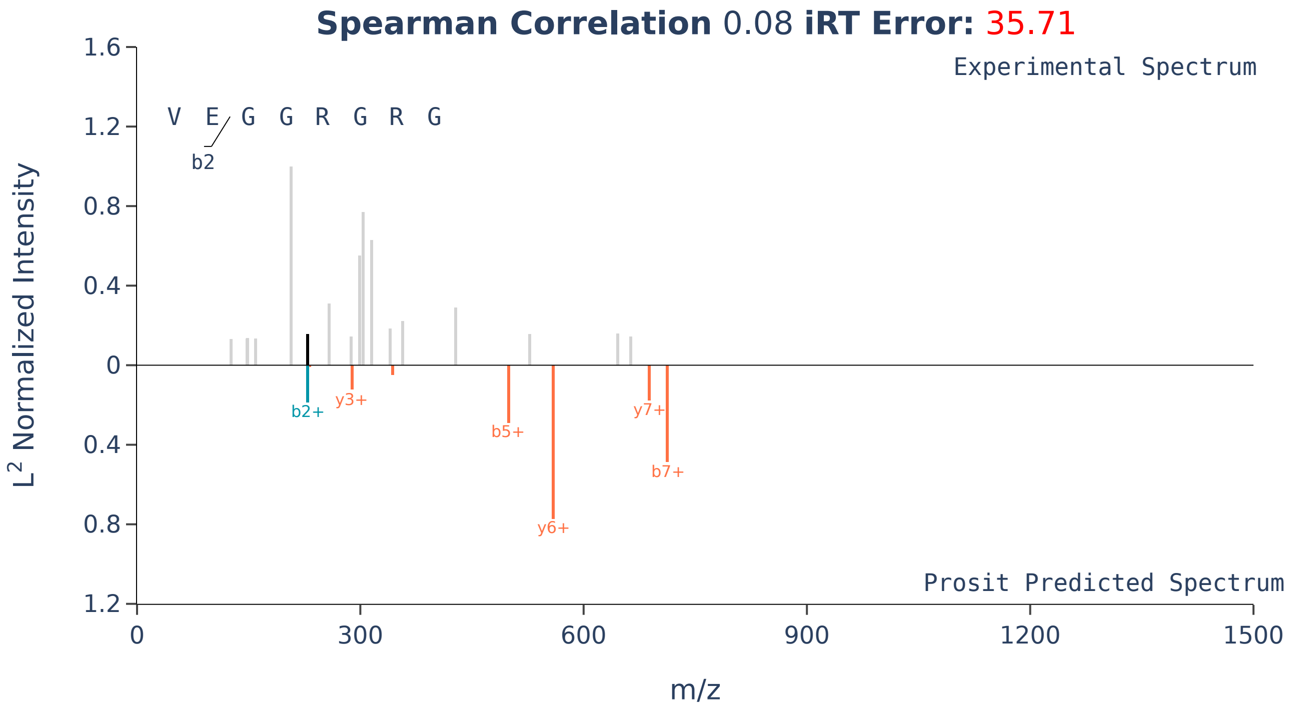

Source HRoetschke_211222_160123_HFGoe_IF2_b1R1_1h **Scan** 8148
Peptide IGAYHVEDR **Charge** 3 **Spectral Angle** 0.76
Spearman Correlation 0.91 **iRT Error:** 5.37

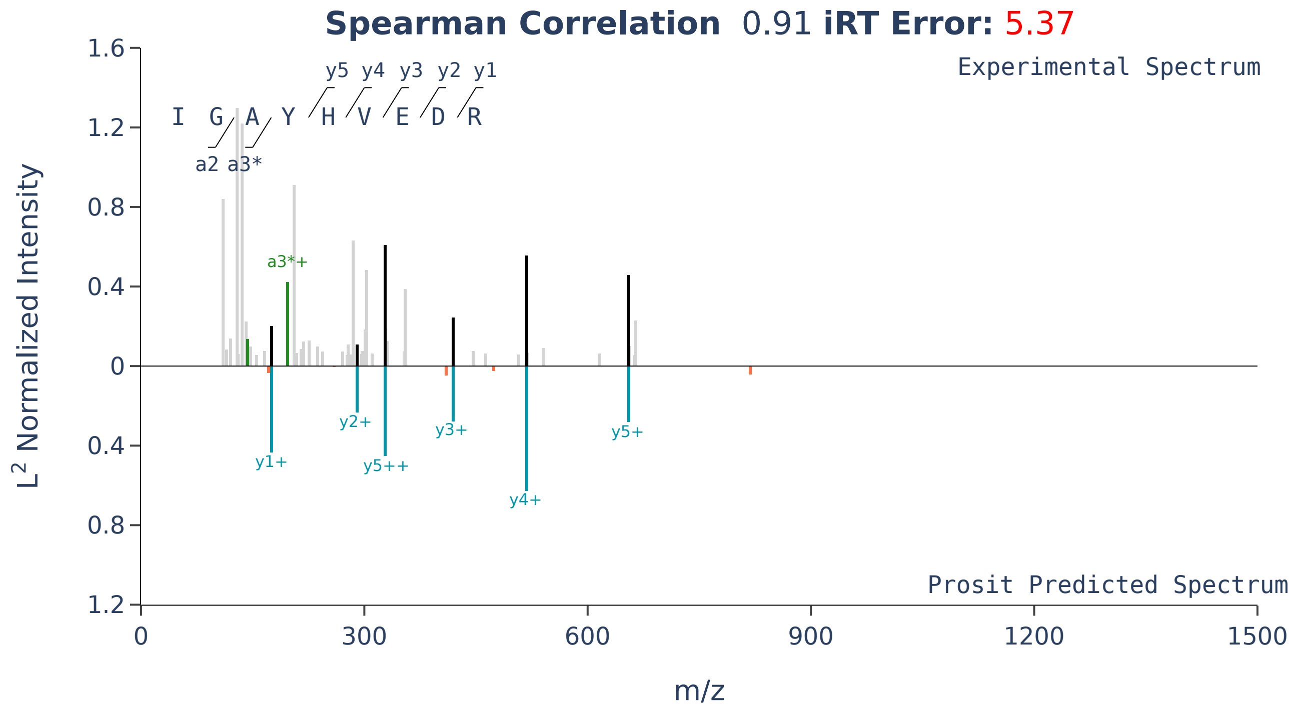

Source HRoetschke_211222_160123_HFGoe_IF2_b1R1_1h **Scan** 8148
Peptide ARREQEAAE **Charge** 3 **Spectral Angle** 0.0
Spearman Correlation 0.0 **iRT Error:** 21.28

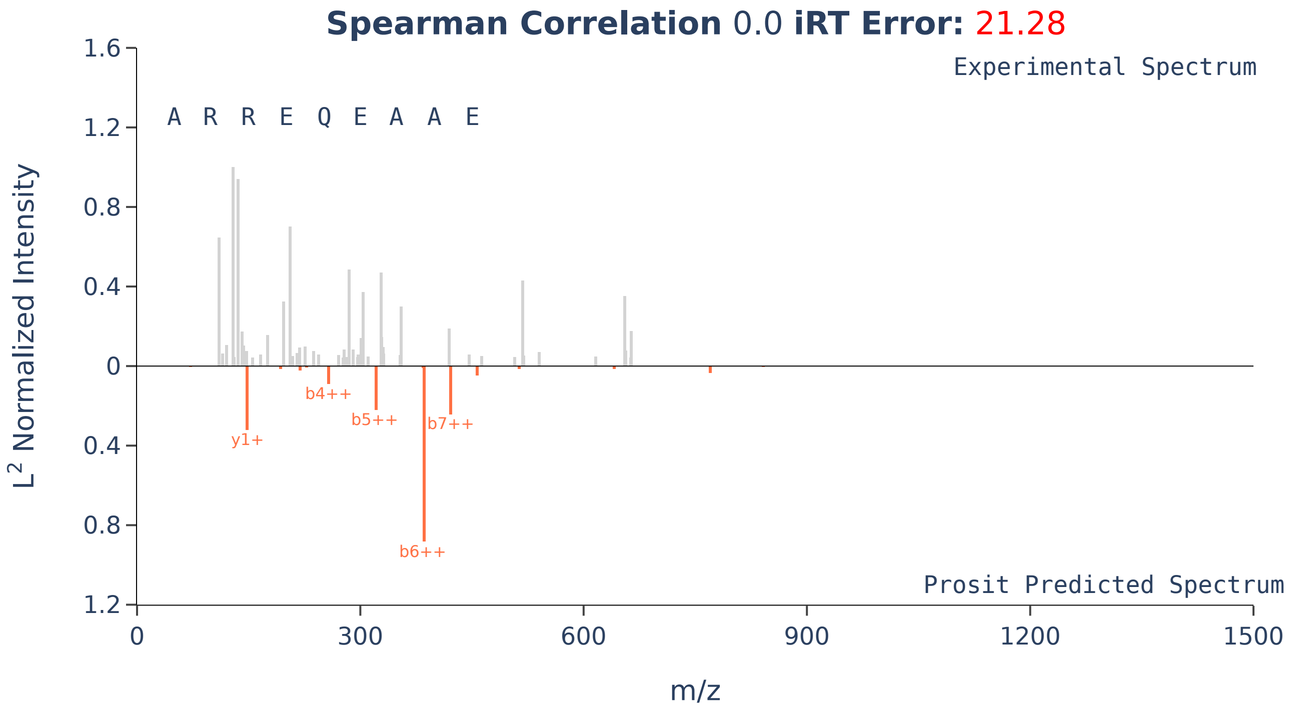

Source HRoetschke_211222_160123_HFGoe_IF2_b1R1_2h **Scan** 13554
Peptide QETAQLVGE **Charge** 2 **Spectral Angle** 0.9
Spearman Correlation 0.88 **iRT Error:** 5.77

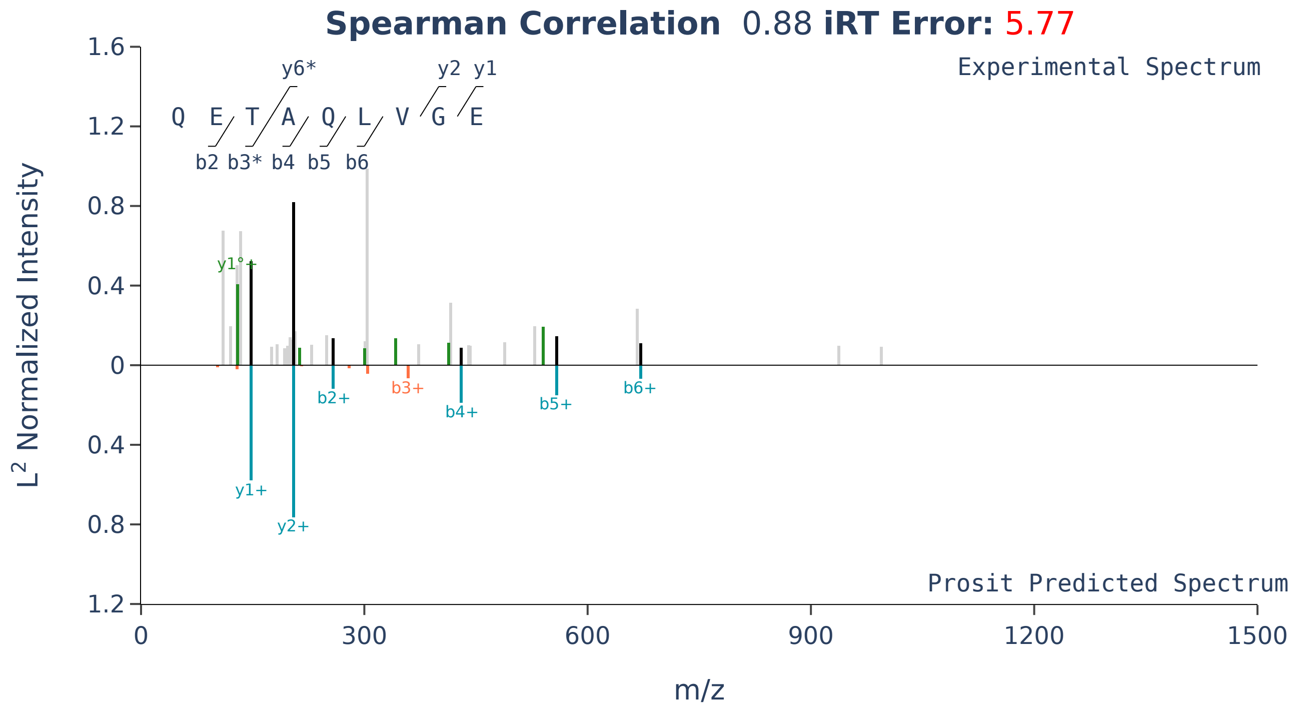

Source HRoetschke_211222_160123_HFGoe_IF2_b1R1_2h **Scan** 13554
Peptide DQETAQLVA **Charge** 2 **Spectral Angle** 0.0
Spearman Correlation 0.0 **iRT Error:** 6.45

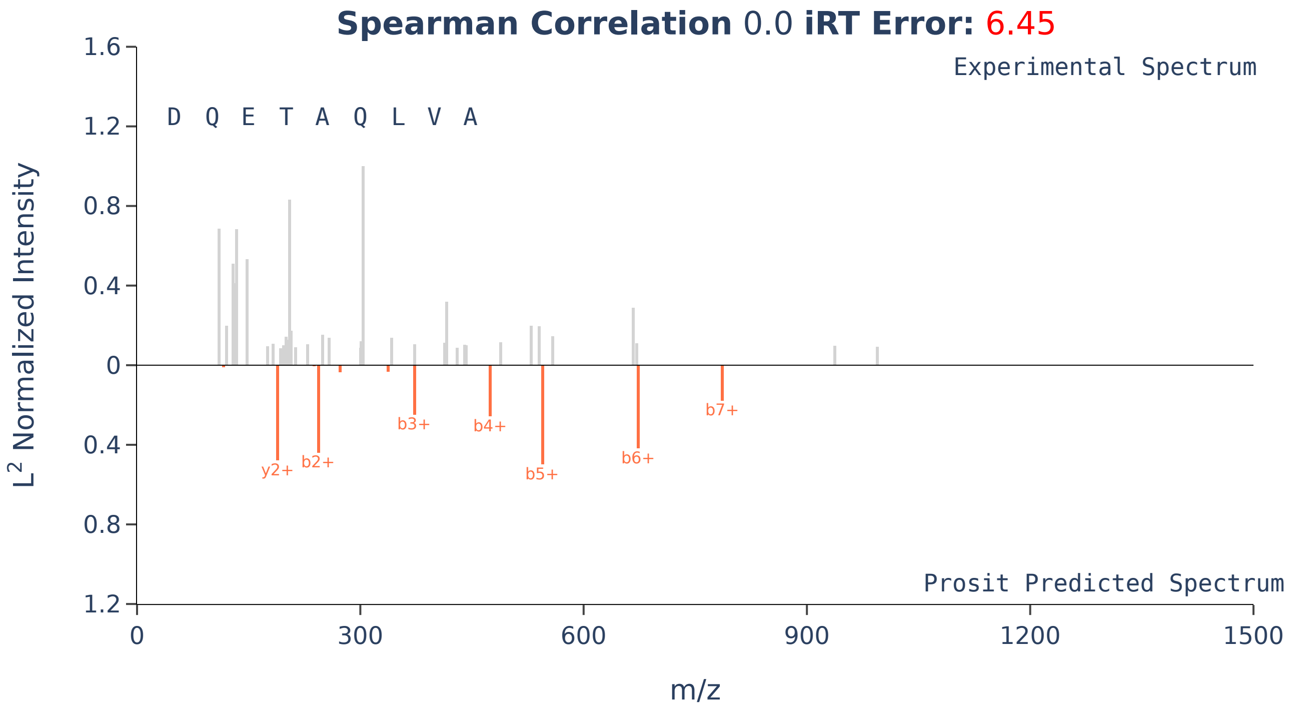

Source HRoetschke_211222_160123_HFGoe_IF2_b2R2_24h **Scan** 16051
Peptide QAVEVLE **Charge** 1 **Spectral Angle** 0.86
Spearman Correlation 0.91 **iRT Error:** 5.83

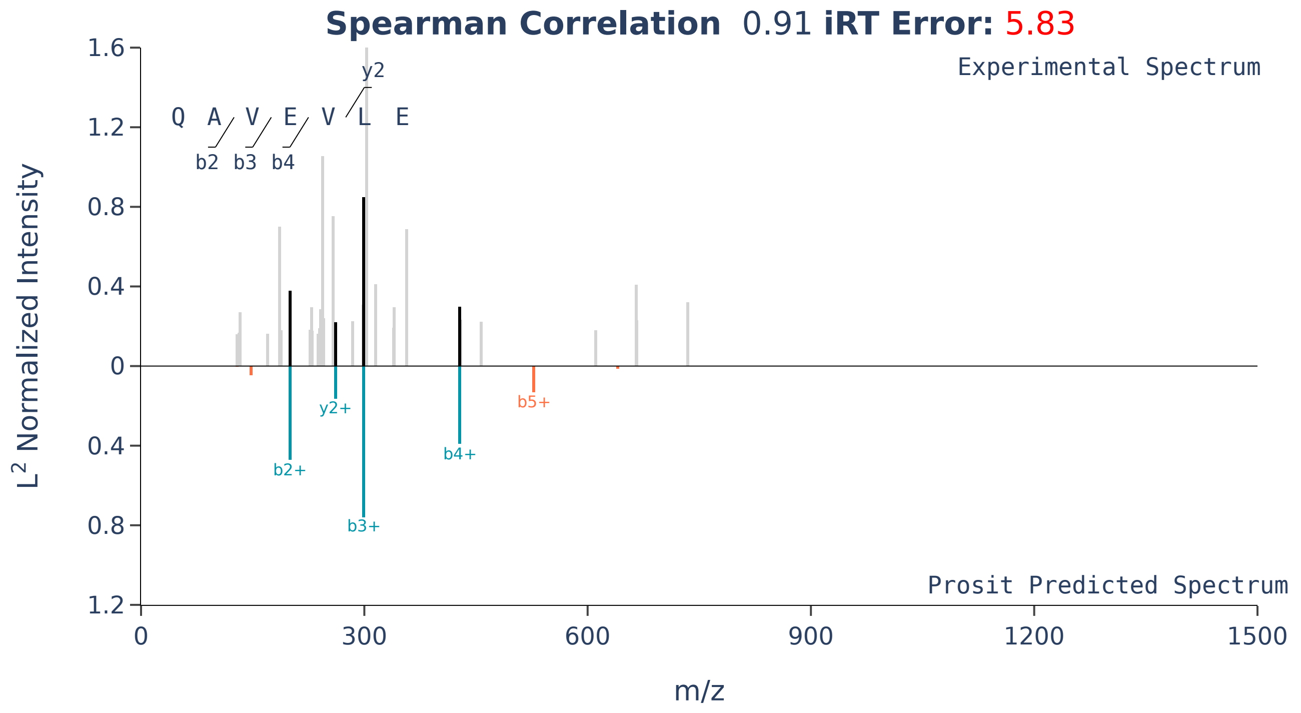

Source HRoetschke_211222_160123_HFGoe_IF2_b2R2_24h **Scan** 16051
Peptide VEGGRGRG **Charge** 1 **Spectral Angle** 0.12
Spearman Correlation 0.08 **iRT Error:** 33.54

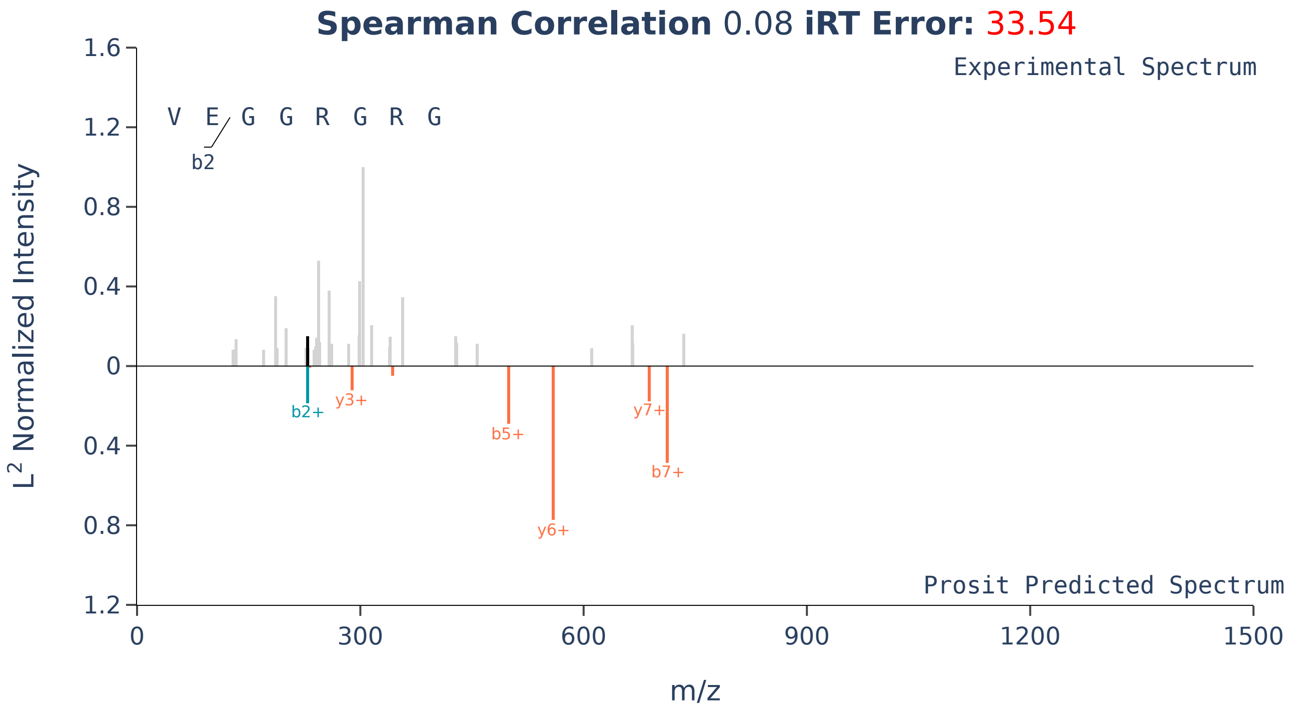

Source HRoetschke_211222_160123_HFGoe_IF2_b1R1_4h **Scan** 23662
Peptide LQAEVLL **Charge** 1 **Spectral Angle** 0.86
Spearman Correlation 0.99 **iRT Error:** 5.91

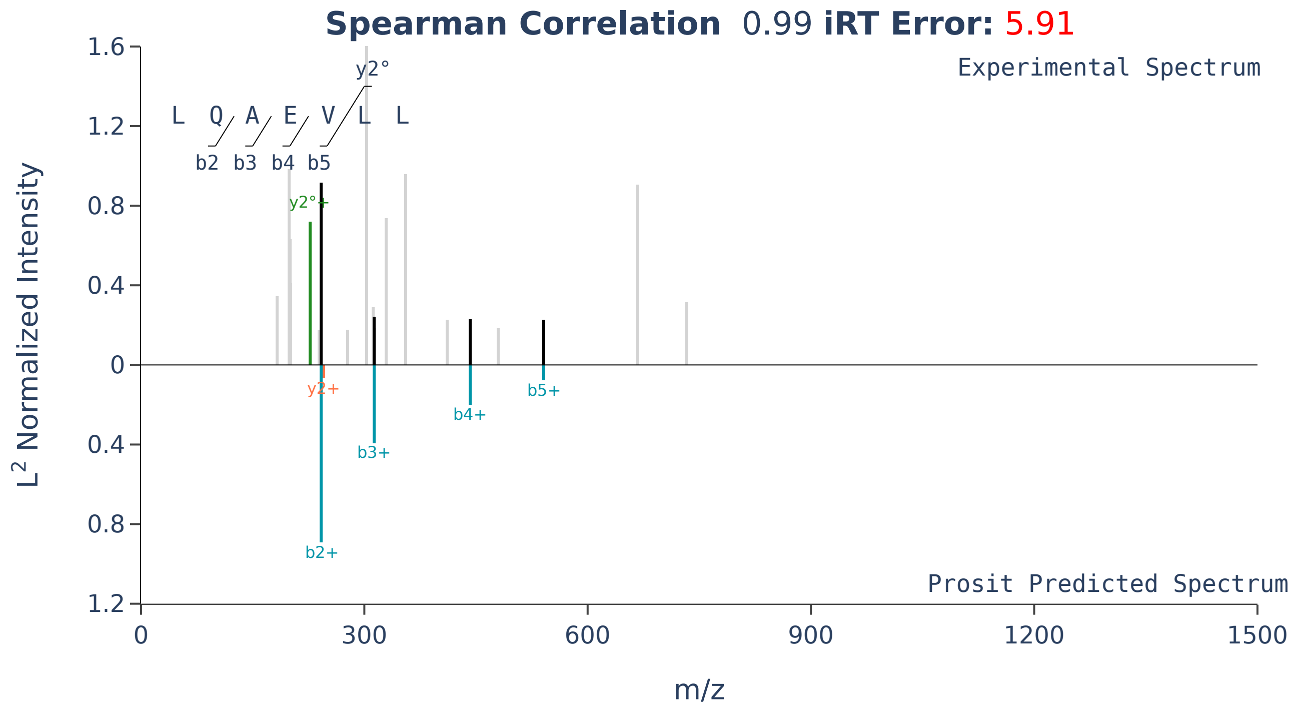

Source HRoetschke_211222_160123_HFGoe_IF2_b1R1_4h **Scan** 23662
Peptide LDAILLQ **Charge** 1 **Spectral Angle** 0.0
Spearman Correlation 0.0 **iRT Error:** 9.48

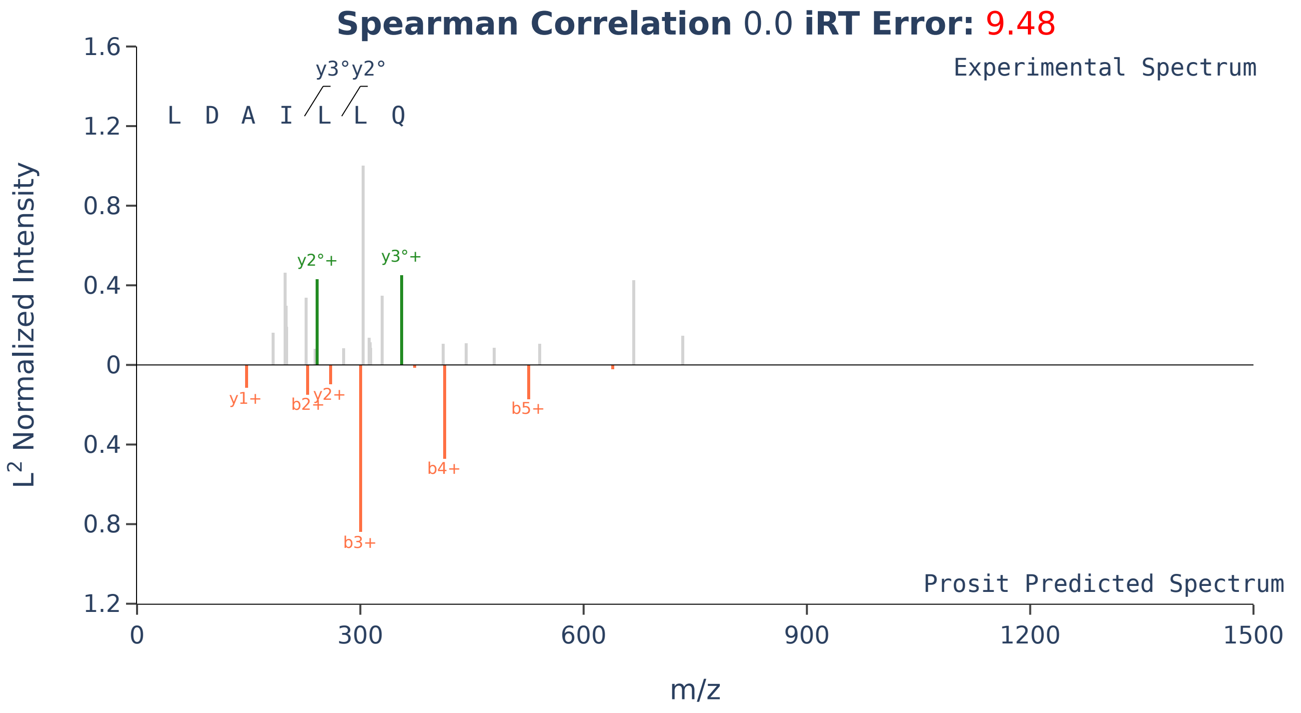

Source HRoetschke_211222_160123_HFGoe_IF2_b1R2_4h **Scan** 20322
Peptide PVVVAVNNL **Charge** 1 **Spectral Angle** 0.88
Spearman Correlation 0.89 **iRT Error:** 7.49

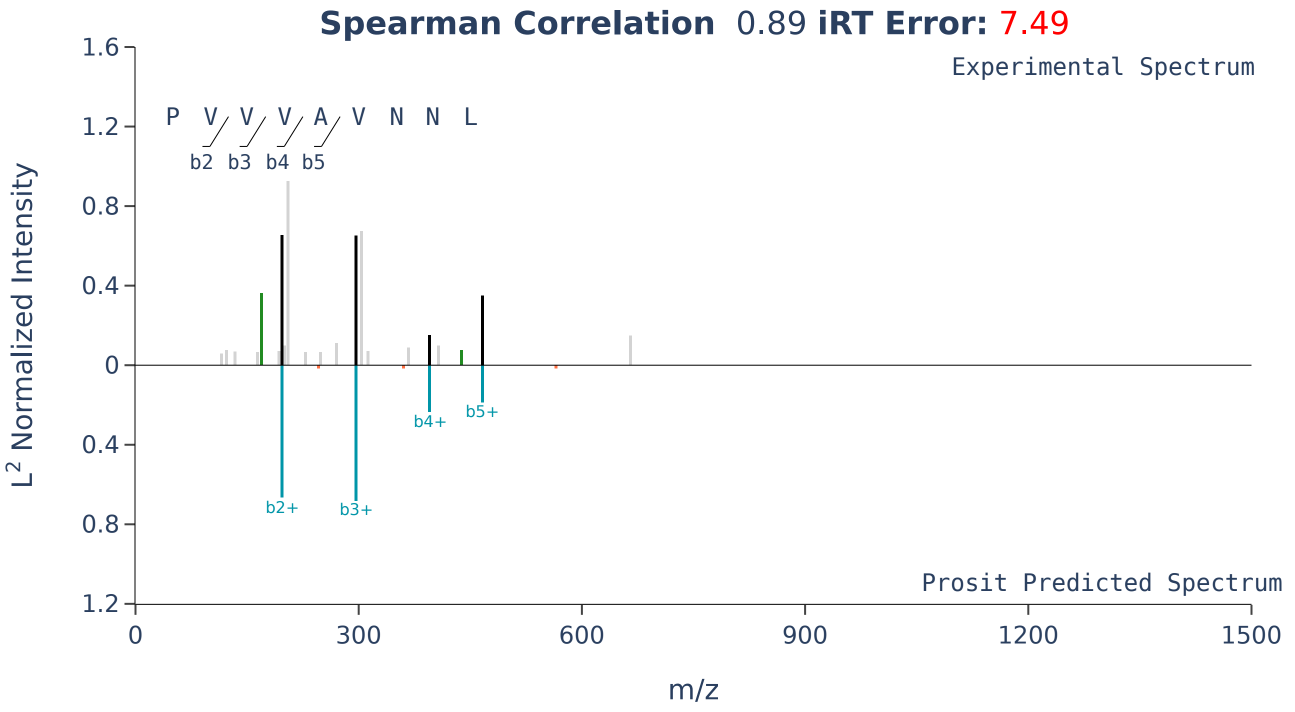

Source HRoetschke_211222_160123_HFGoe_IF2_b1R2_4h **Scan** 20322
Peptide QVPVVVAVN **Charge** 1 **Spectral Angle** 0.0
Spearman Correlation 0.0 **iRT Error:** 30.77

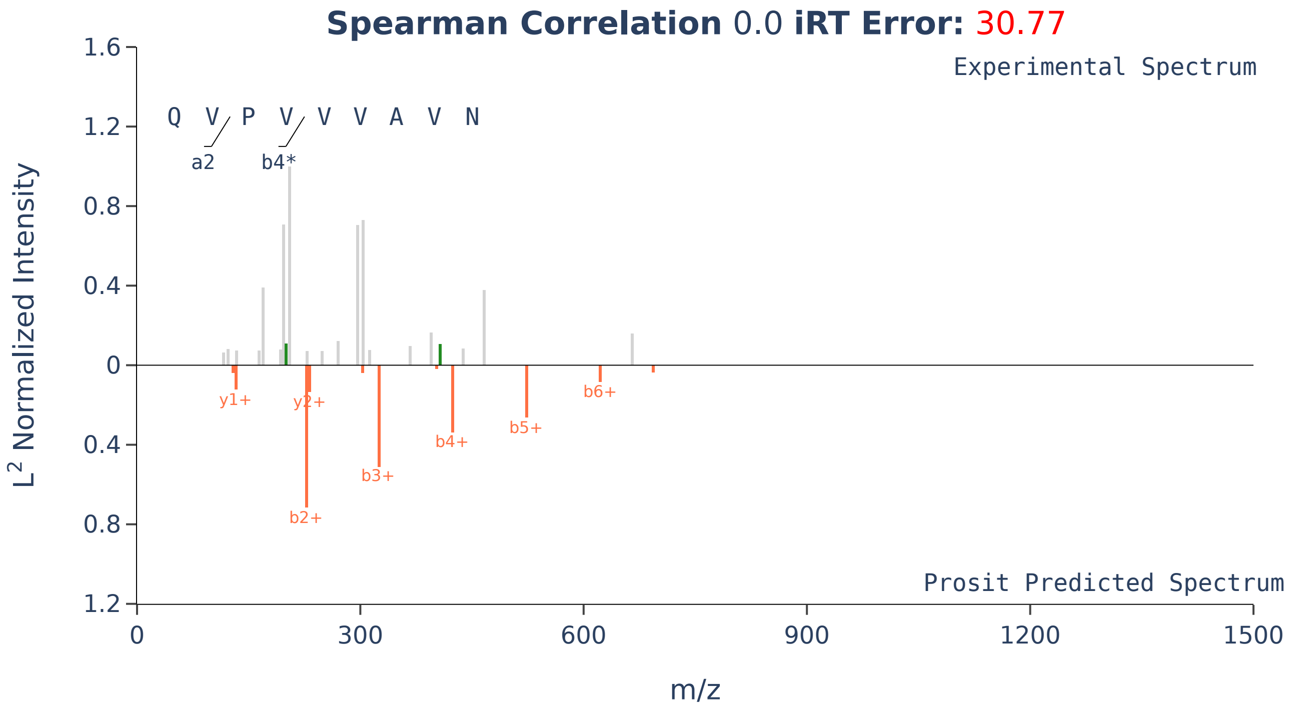

Source HRoetschke_211222_160123_HFGoe_IF2_b1R1_24h **Scan** 16896
Peptide DNESQFVHV **Charge** 2 **Spectral Angle** 0.88
Spearman Correlation 0.91 **iRT Error:** 7.79

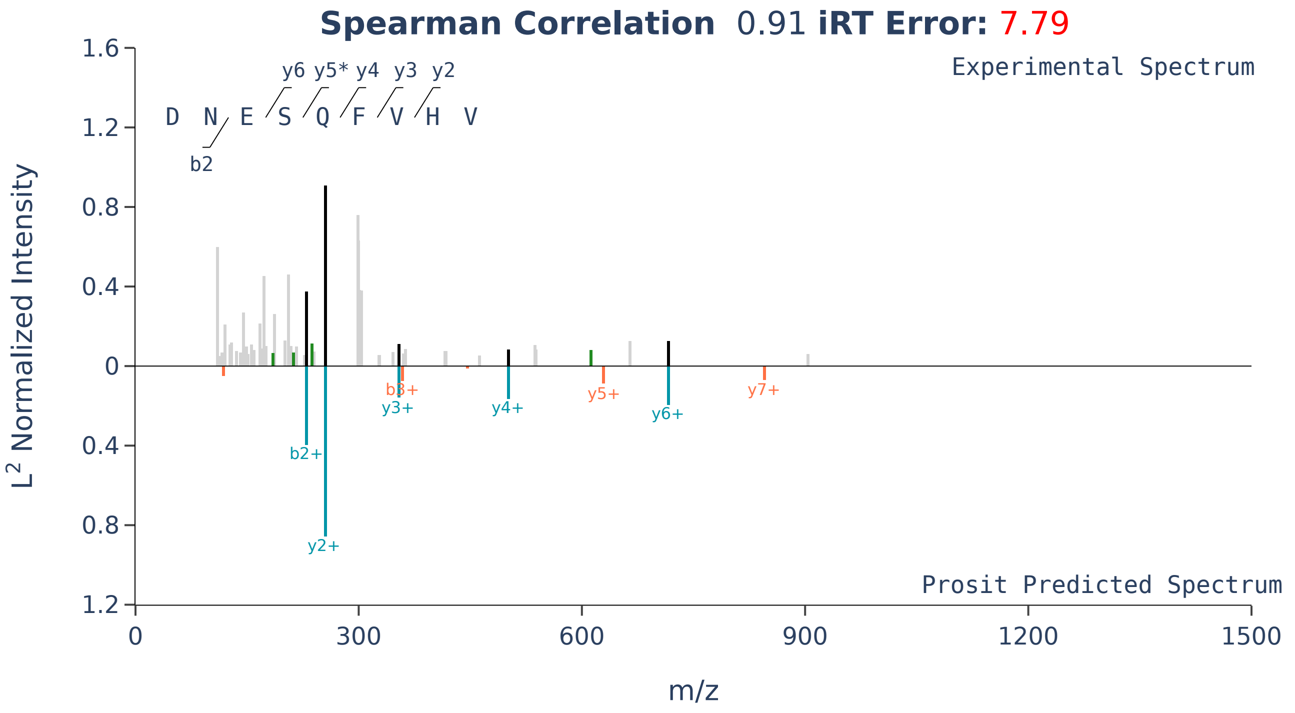

Source HRoetschke_211222_160123_HFGoe_IF2_b1R1_24h **Scan** 16896
Peptide NKWTDNAEP **Charge** 2 **Spectral Angle** 0.88
Spearman Correlation 0.46 **iRT Error:** 53.51

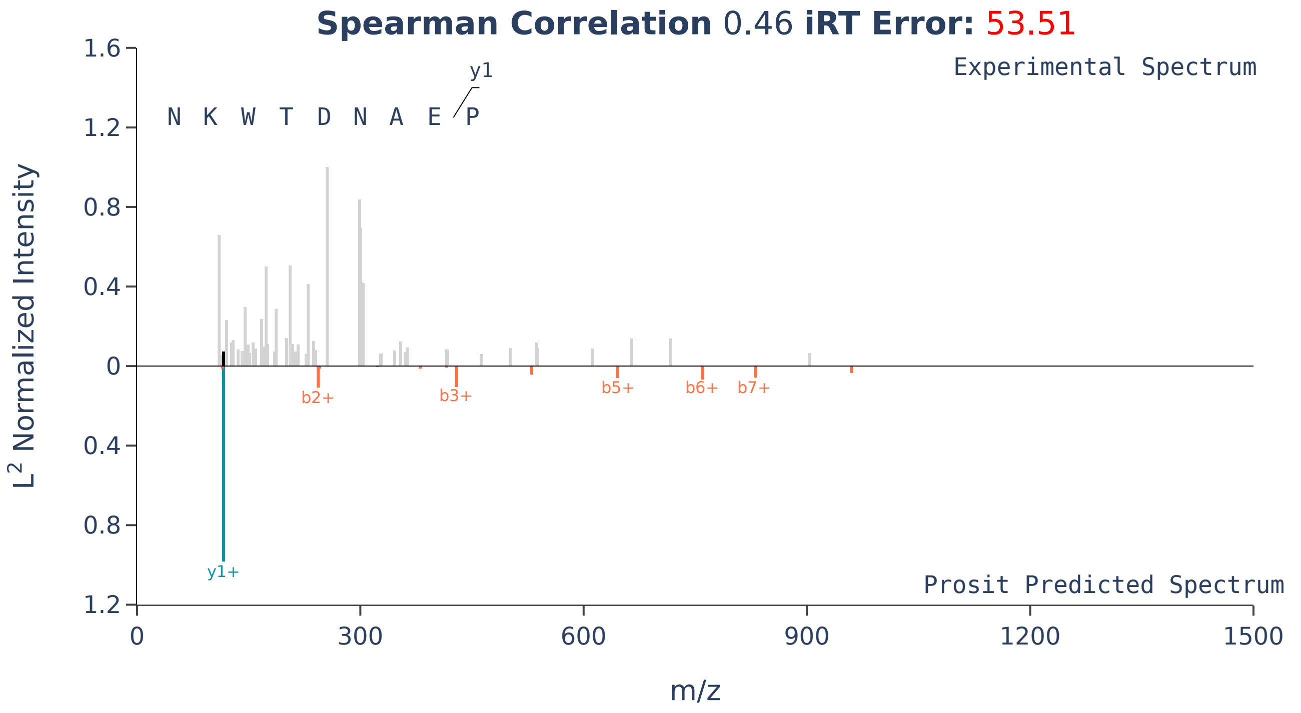

Source HRoetschke_211222_160123_HFGoe_IF2_b2R1_4h **Scan** 9850
Peptide VTEGISD **Charge** 2 **Spectral Angle** 0.84
Spearman Correlation 0.93 **iRT Error:** 7.98

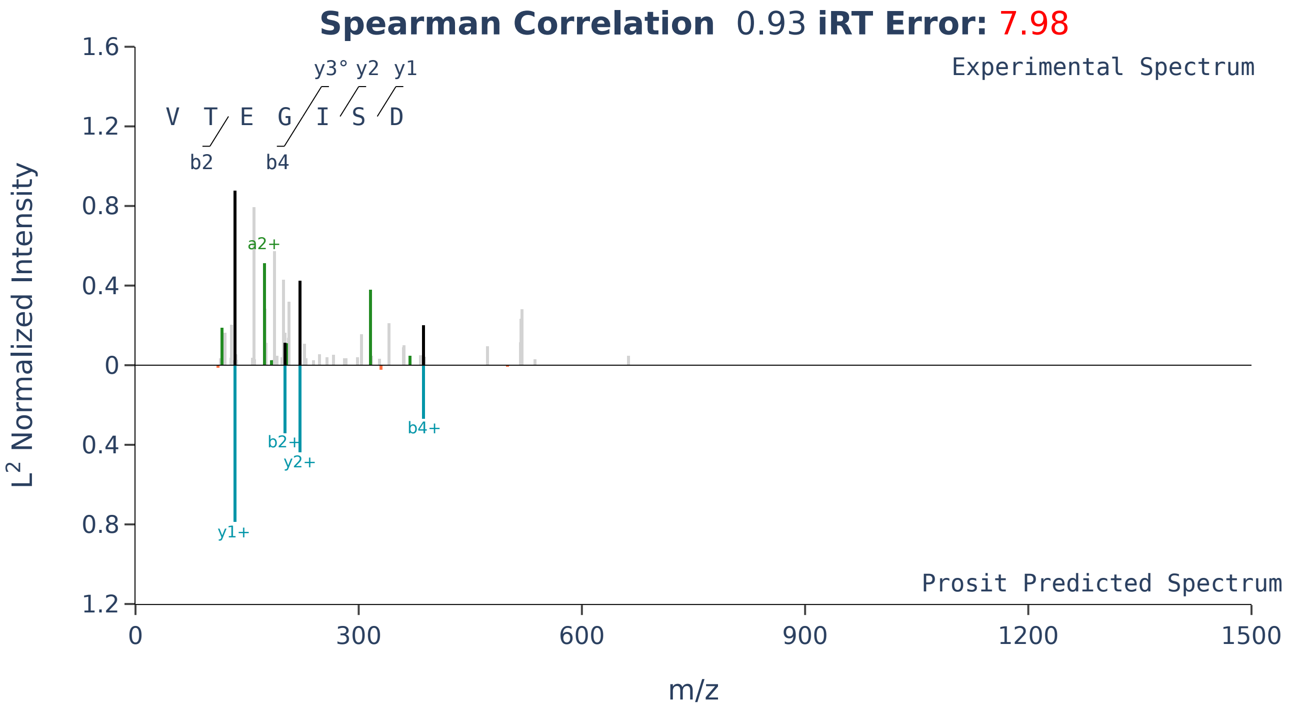

Source HRoetschke_211222_160123_HFGoe_IF2_b2R1_4h **Scan** 9850
Peptide ETDATLA **Charge** 2 **Spectral Angle** 0.0
Spearman Correlation 0.0 **iRT Error:** 0.71

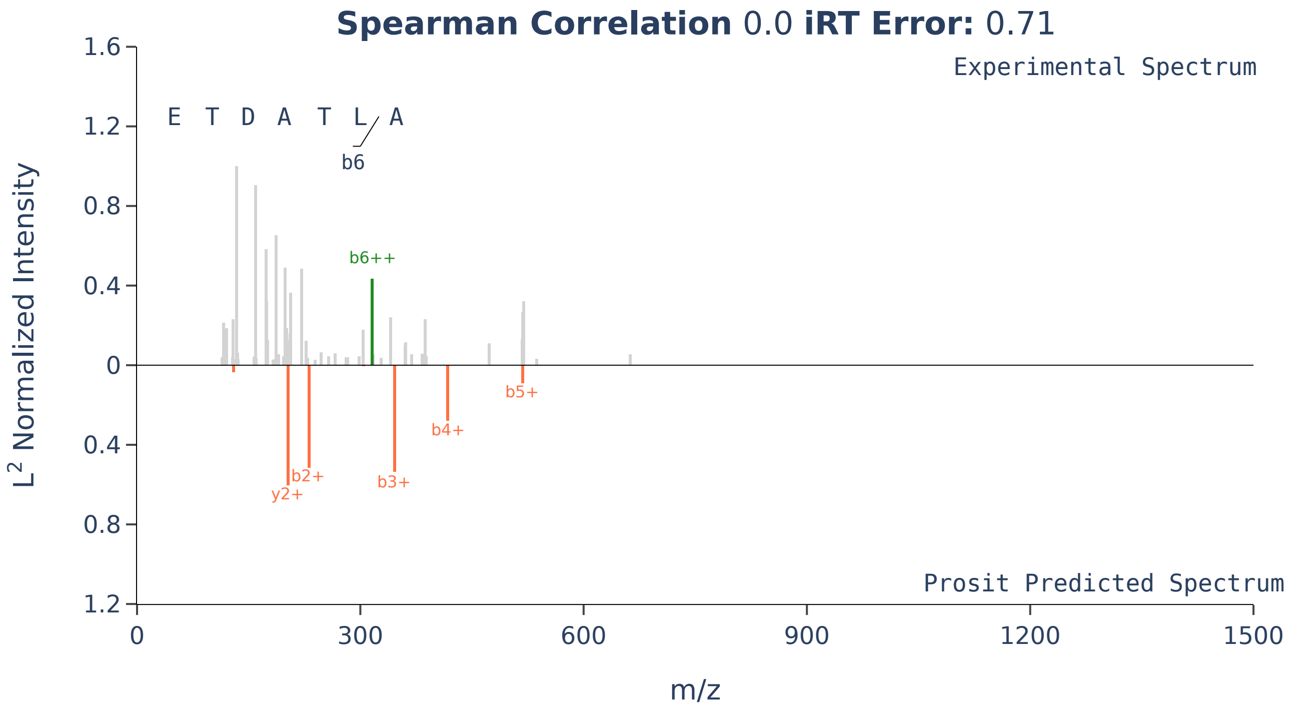

Source HRoetschke_211222_160123_HFGoe_IF2_b1R1_24h **Scan** 13133
Peptide PAAGDEVGSV **Charge** 1 **Spectral Angle** 0.83
Spearman Correlation 0.92 **iRT Error:** 8.41

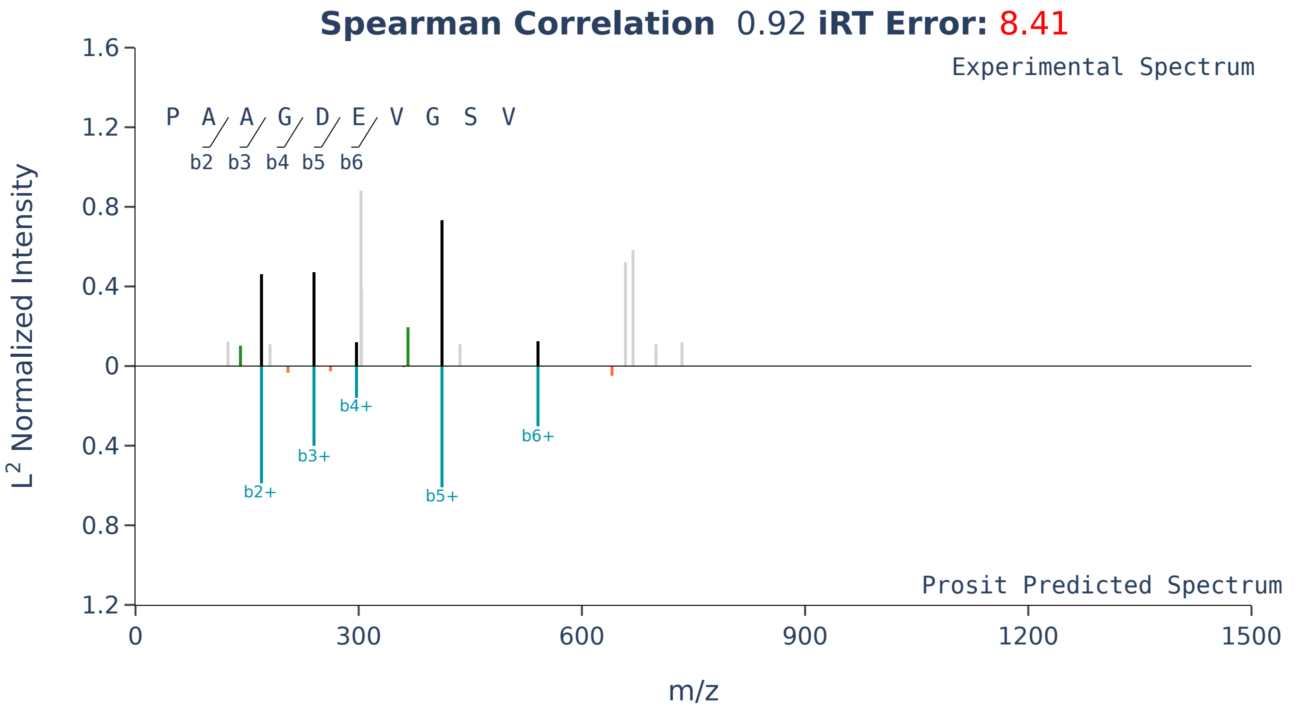

Source HRoetschke_211222_160123_HFGoe_IF2_b1R1_24h **Scan** 13133
Peptide SGVPAAGDEV **Charge** 1 **Spectral Angle** 0.74
Spearman Correlation 0.45 **iRT Error:** 0.31

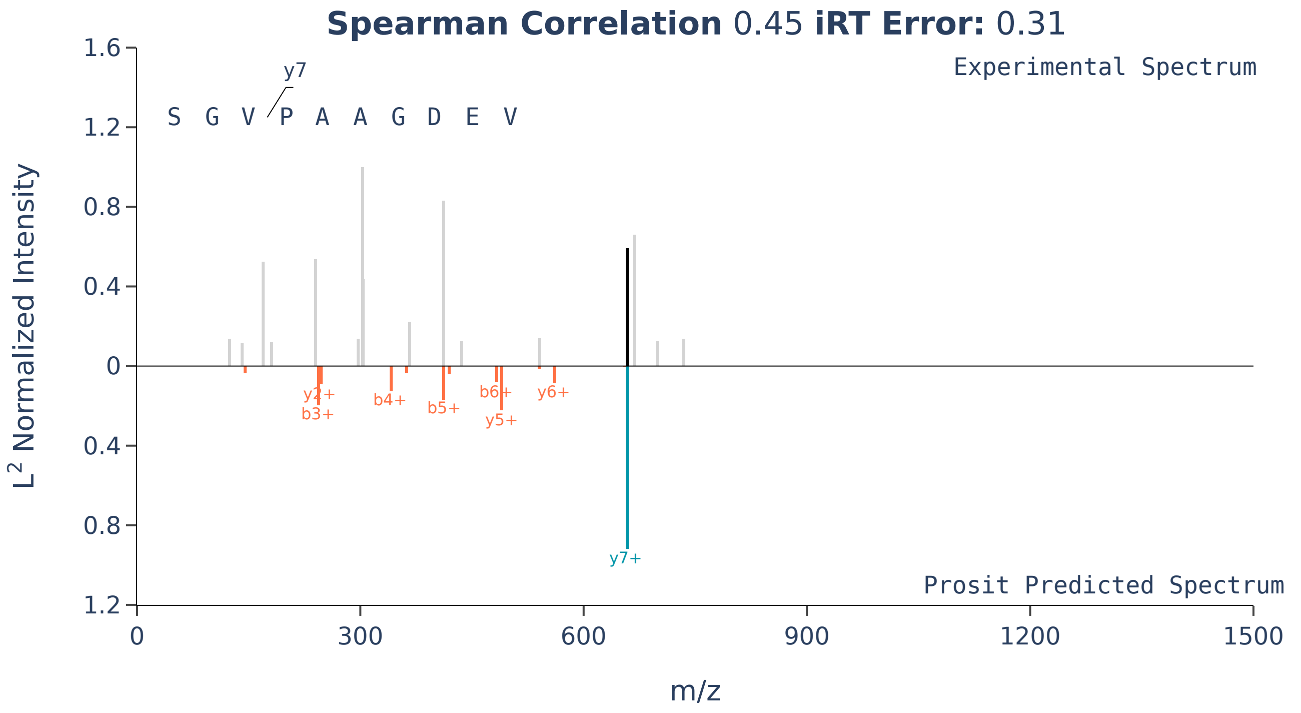

Source HRoetschke_211222_160123_HFGoe_IF2_b1R1_4h **Scan** 13432
Peptide PAAGDEQTL **Charge** 1 **Spectral Angle** 0.9
Spearman Correlation 0.94 **iRT Error:** 11.3

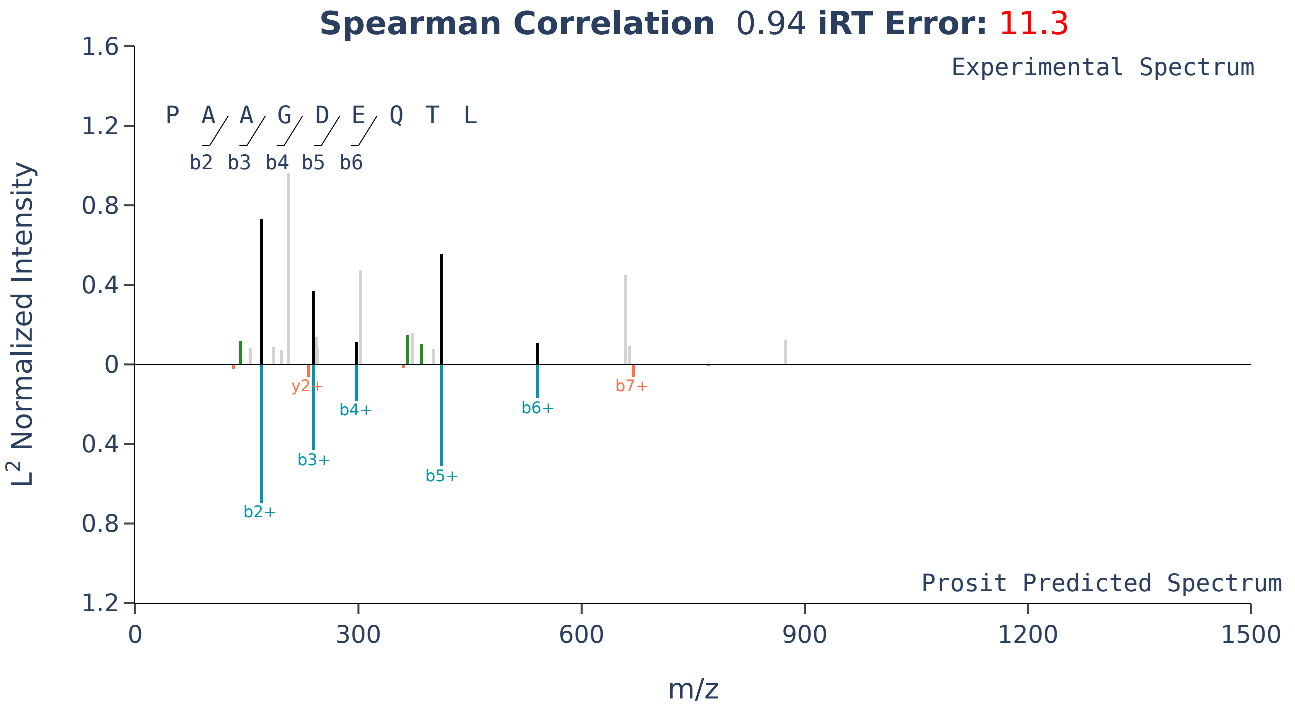

Source HRoetschke_211222_160123_HFGoe_IF2_b1R1_4h **Scan** 13432
Peptide SGVPAAGDEV **Charge** 1 **Spectral Angle** 0.74
Spearman Correlation 0.45 **iRT Error:** 0.32

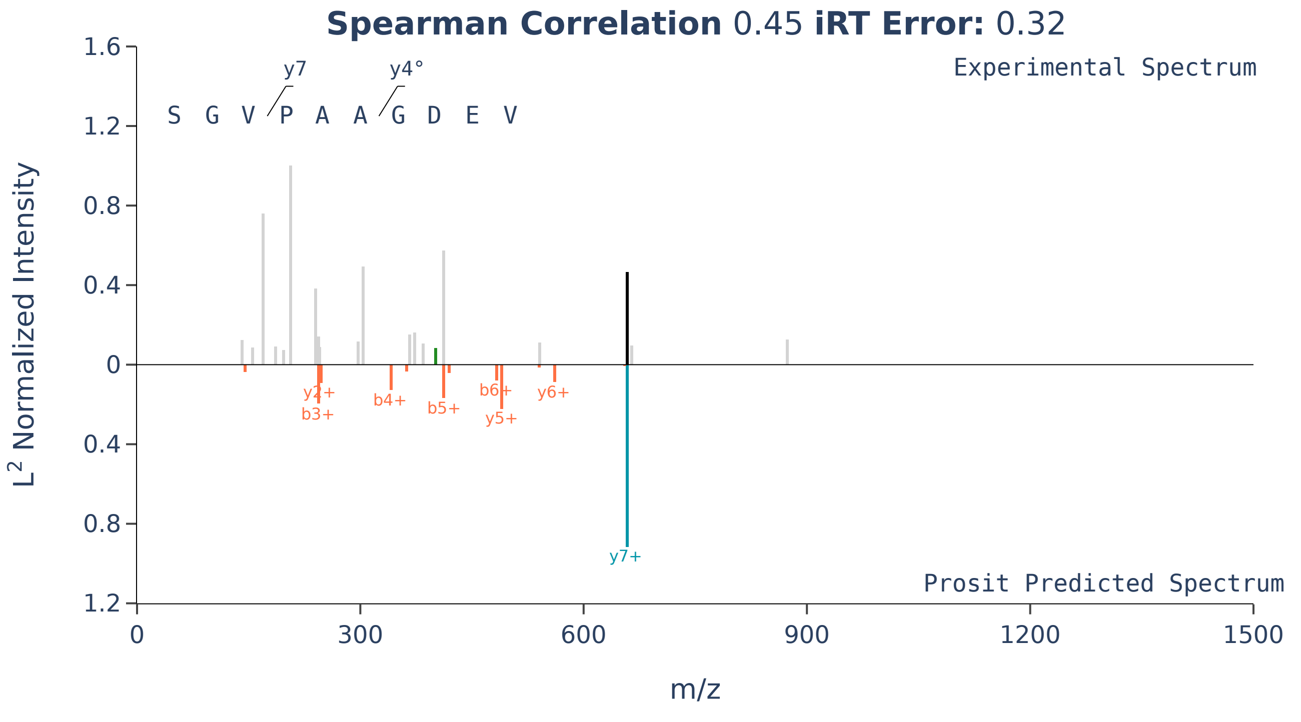

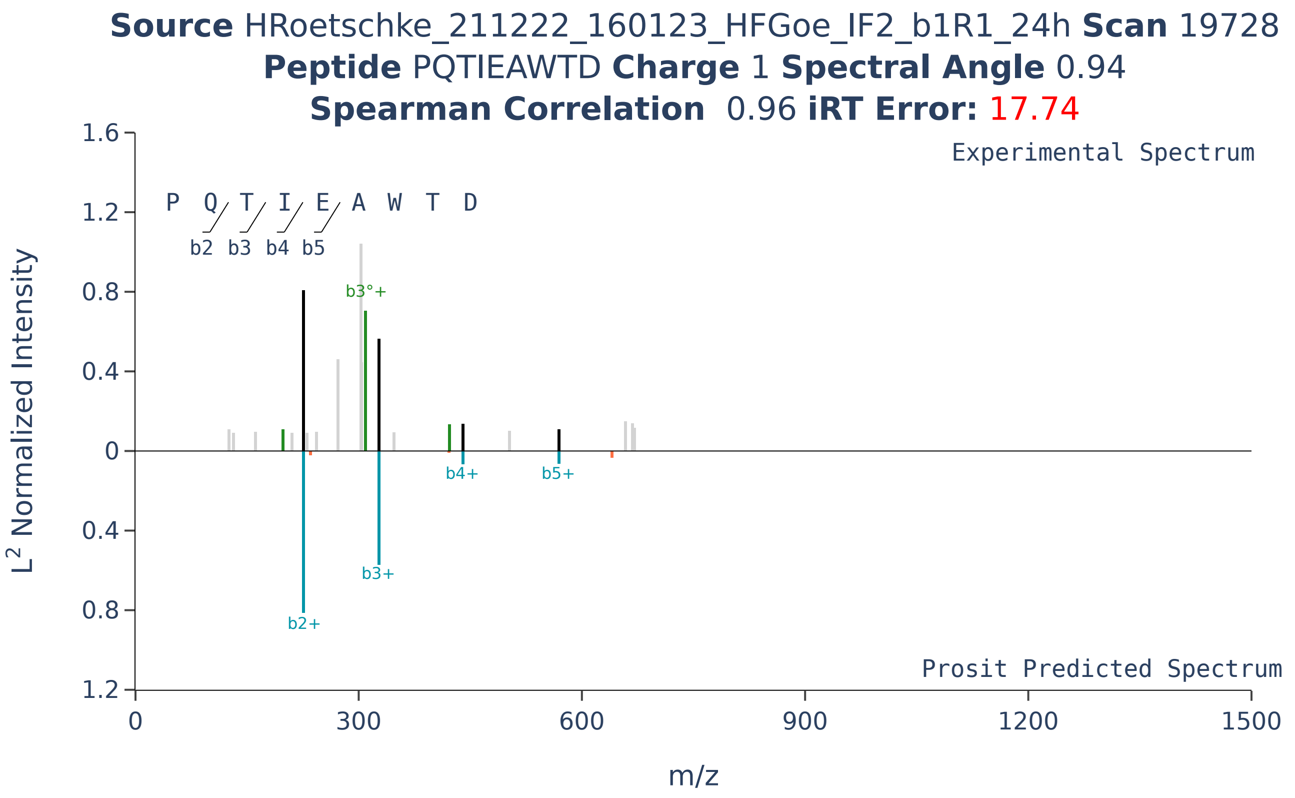
Source HRoetschke_211222_160123_HFGoe_IF2_b1R1_24h Scan 19728
Peptide PQTIEAWTD Charge 1 Spectral Angle 0.94
Spearman Correlation 0.96 iRT Error: 17.74
Experimental Spectrum
Prosit Predicted Spectrum
P Q T I E A W T D
b2 b3 b4 b5
b3°+
b4+
b5+
b3+
b2+
L2 Normalized Intensity
m/z
1.6
1.2
0.8
0.4
0
0.4
0.8
1.2
0
300
600
900
1200
1500

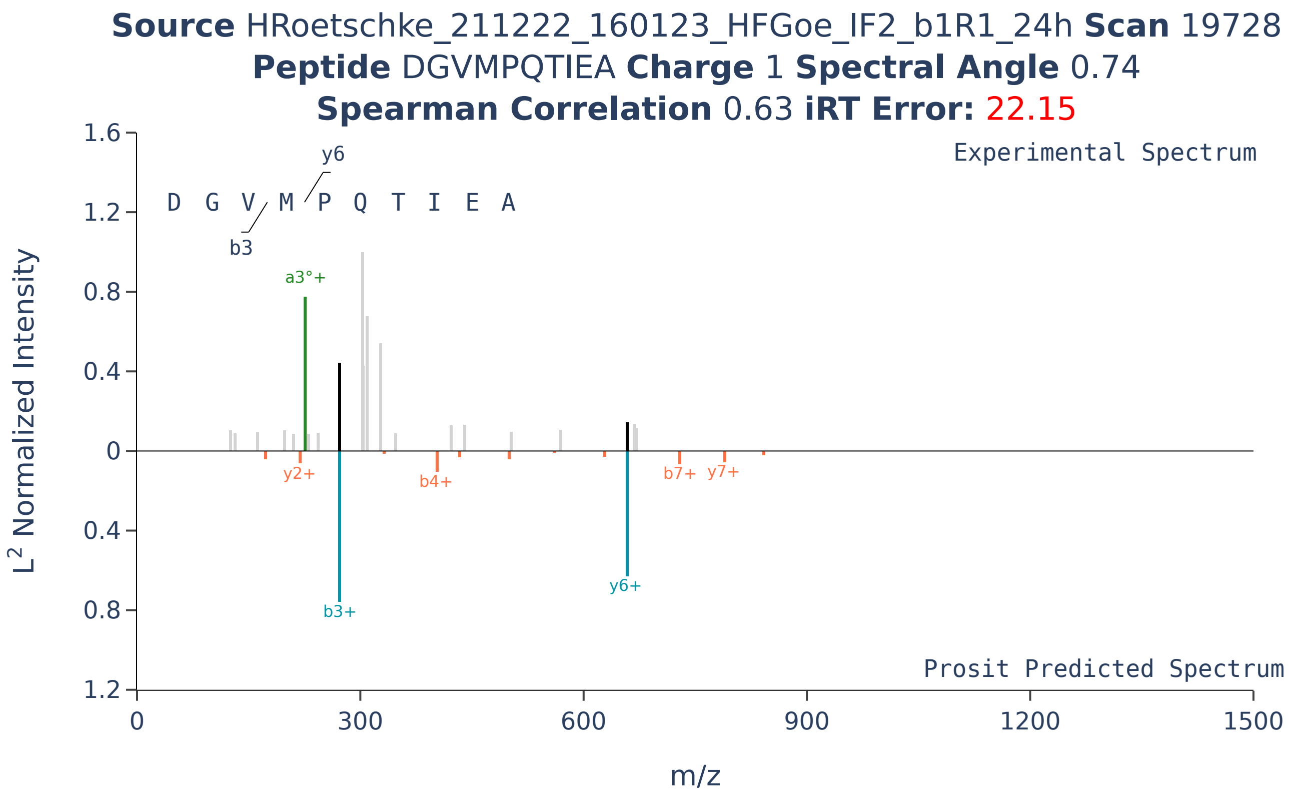
Source HRoetschke_211222_160123_HFGoe_IF2_b1R1_24h Scan 19728
Peptide DGVMPQTIEA Charge 1 Spectral Angle 0.74
Spearman Correlation 0.63 iRT Error: 22.15
Experimental Spectrum
Prosit Predicted Spectrum
y6
D G V M P Q T I E A
b3
a3°+
y2+
b4+
b7+
y7+
b3+
y6+
L2 Normalized Intensity
m/z
1.6
1.2
0.8
0.4
0
0.4
0.8
1.2
0
300
600
900
1200
1500

Source WSoh_101121_151121_HFGoe_G1_IL37b_24h_R1 **Scan** 6752
Peptide DSGDKDKGQSHPSL **Charge** 2 **Spectral Angle** 0.86
Spearman Correlation 0.82 **iRT Error:** 0.02

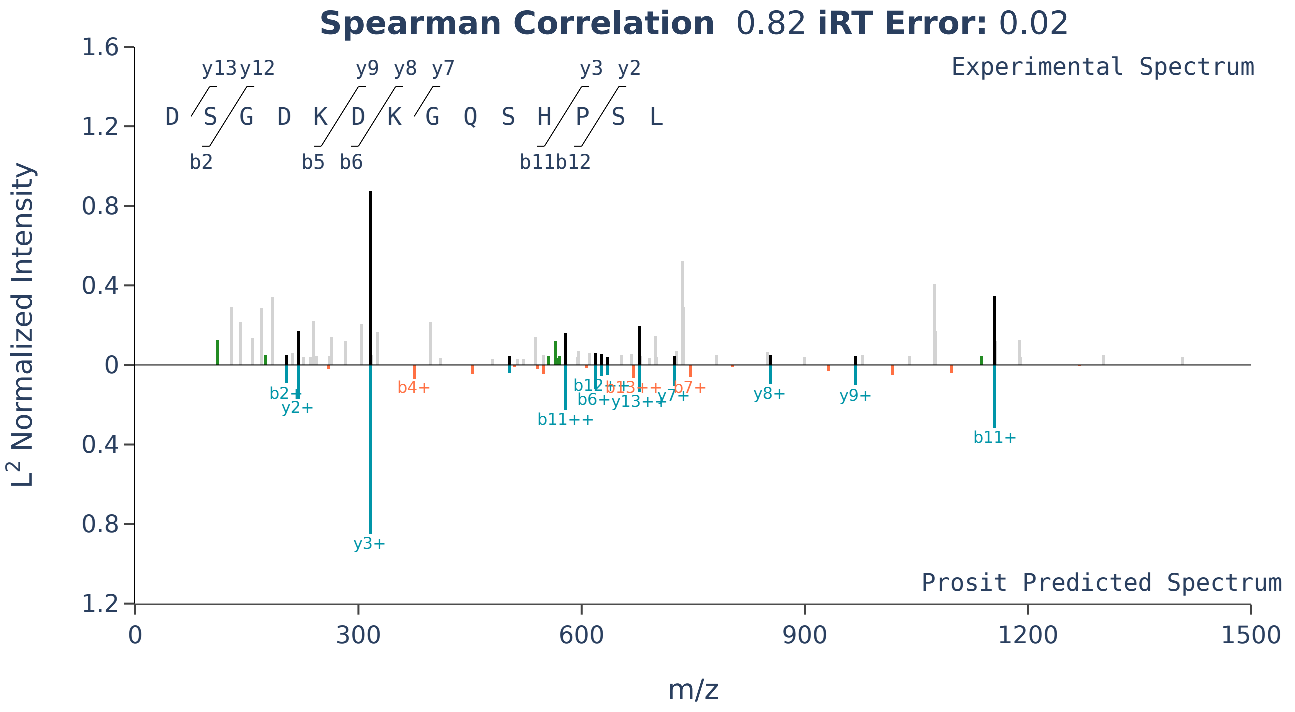

Source WSoh_101121_151121_HFGoe_G1_IL37b_24h_R1 **Scan** 6752
Peptide FSFQPVCKAEMSP **Charge** 2 **Spectral Angle** 0.31
Spearman Correlation 0.33 **iRT Error:** 97.35

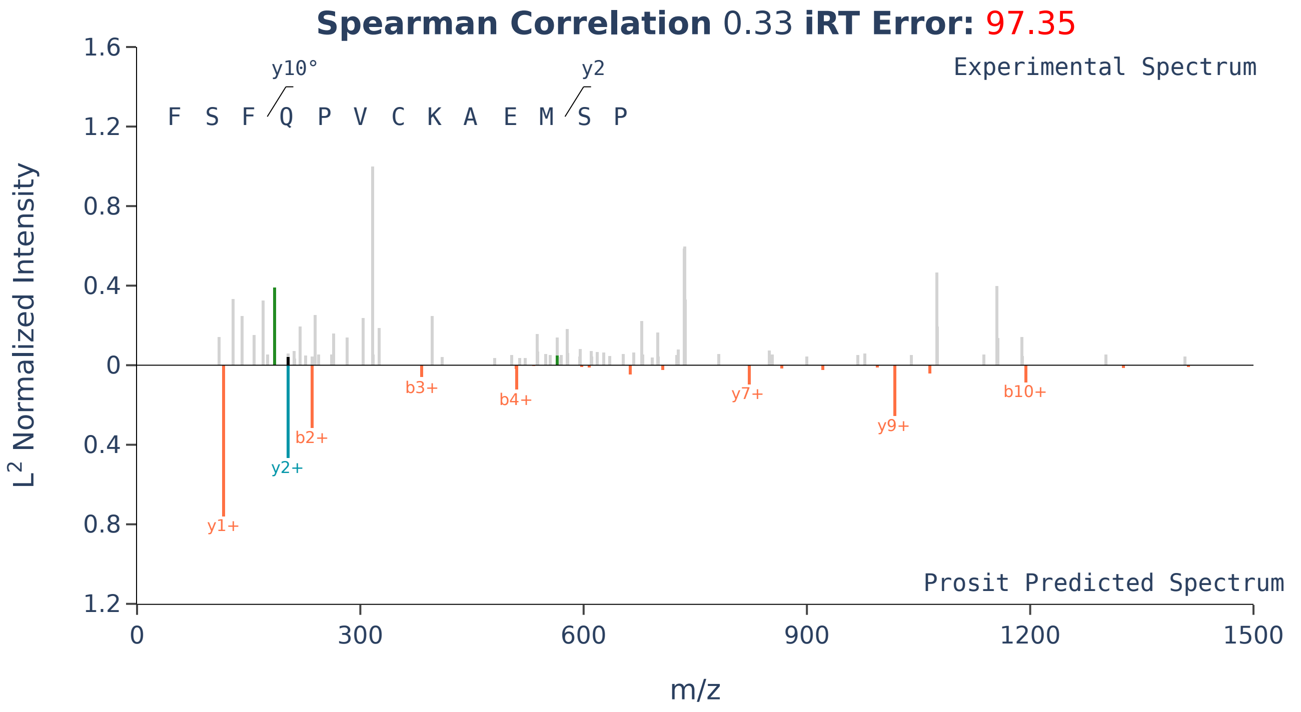

Source WSoh_101121_151121_HFGoe_G2_IL37b_24h_R1 **Scan** 15443
Peptide ASSLHDQDHKVLVL **Charge** 3 **Spectral Angle** 0.86
Spearman Correlation 0.8 **iRT Error:** 0.55

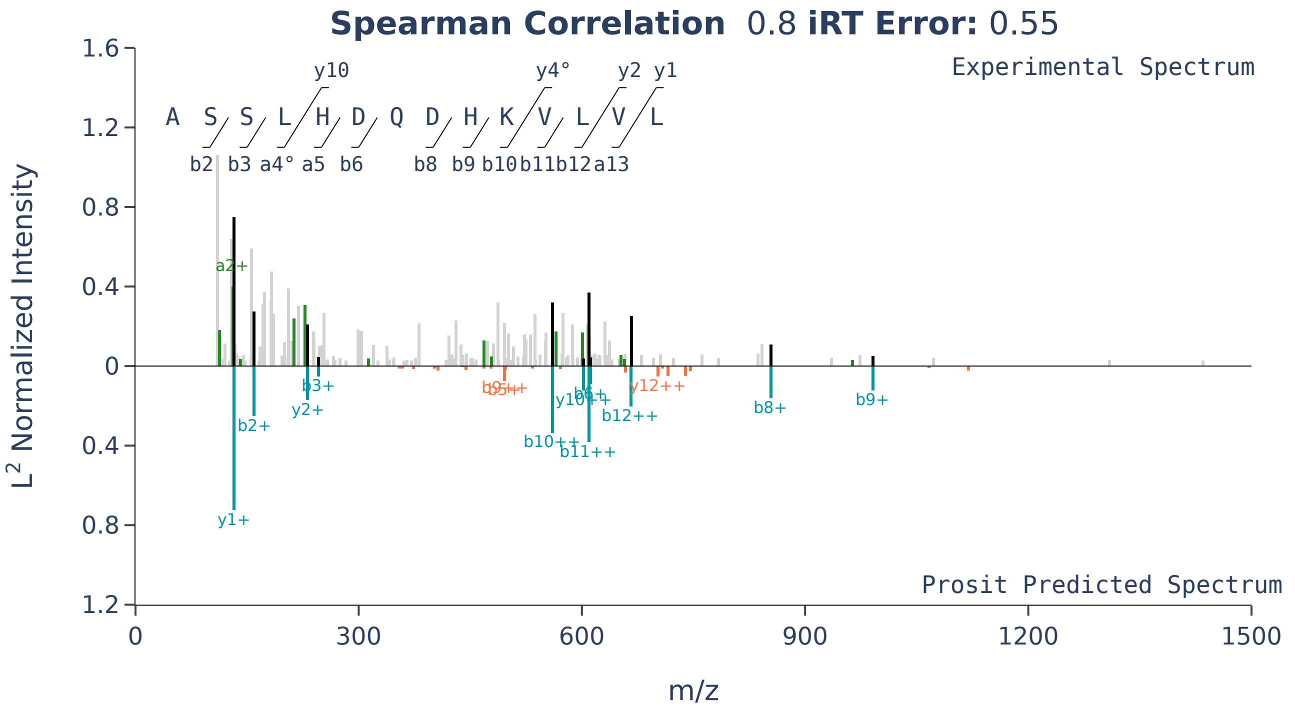

Source WSoh_101121_151121_HFGoe_G2_IL37b_24h_R1 **Scan** 15443
Peptide AVPDKNYIRPEIF **Charge** 3 **Spectral Angle** 0.0
Spearman Correlation 0.0 **iRT Error:** 38.84

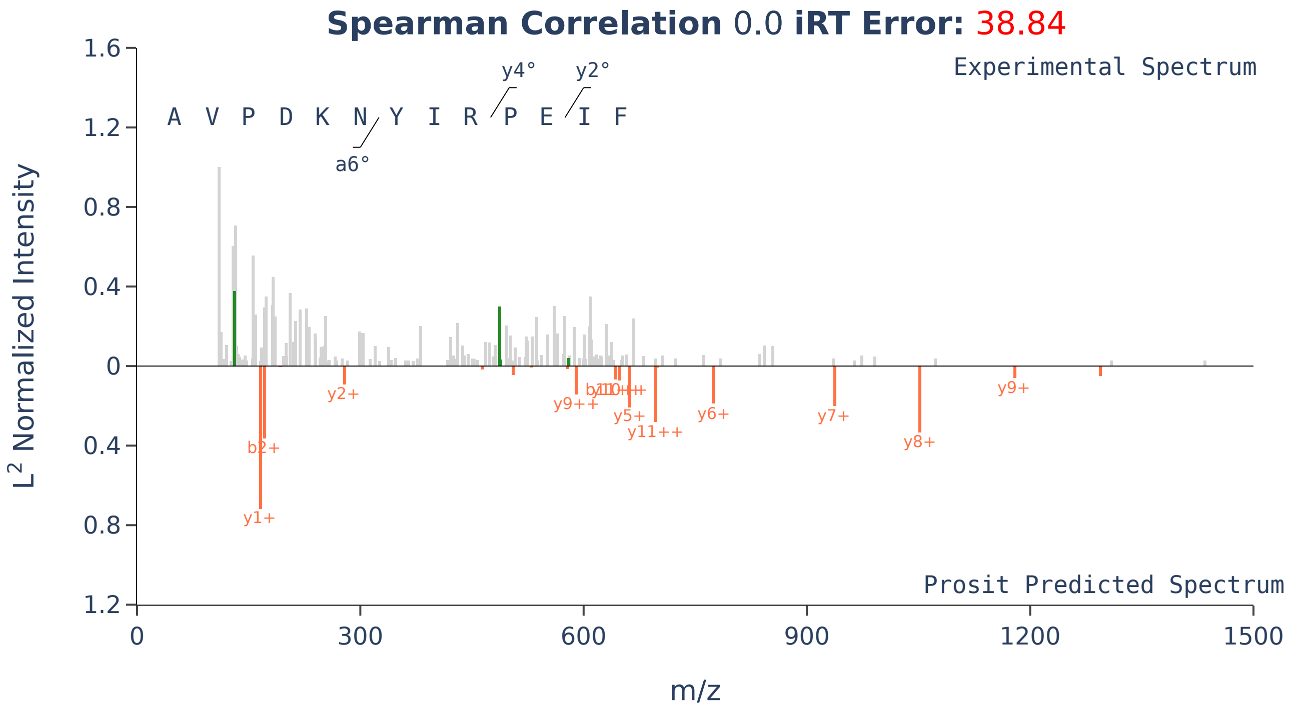

Source WSoh_101121_151121_HFGoe_G2_IL37b_24h_R1 **Scan** 6883
Peptide SSVHTSPKVKNL **Charge** 3 **Spectral Angle** 0.78
Spearman Correlation 0.87 **iRT Error:** 0.89

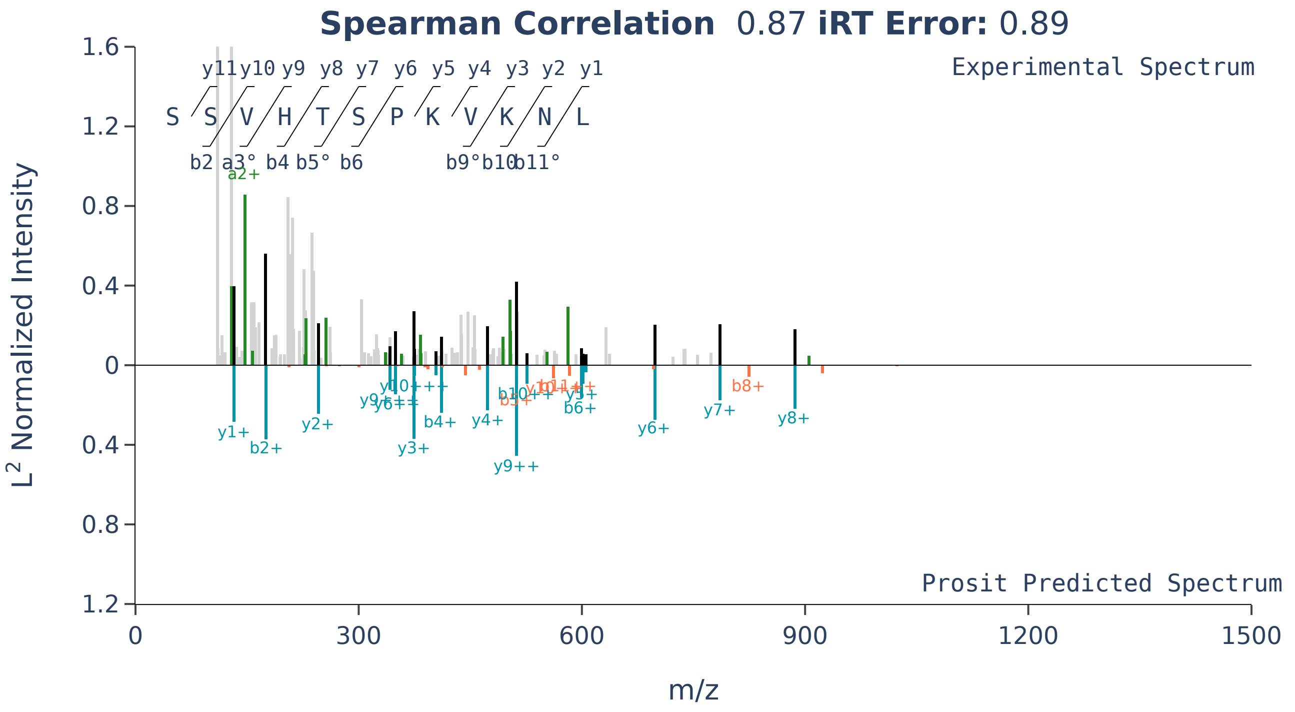

Source WSoh_101121_151121_HFGoe_G2_IL37b_24h_R1 **Scan** 6883
Peptide ARRPFIFYRA **Charge** 3 **Spectral Angle** 0.05
Spearman Correlation 0.13 **iRT Error:** 50.52

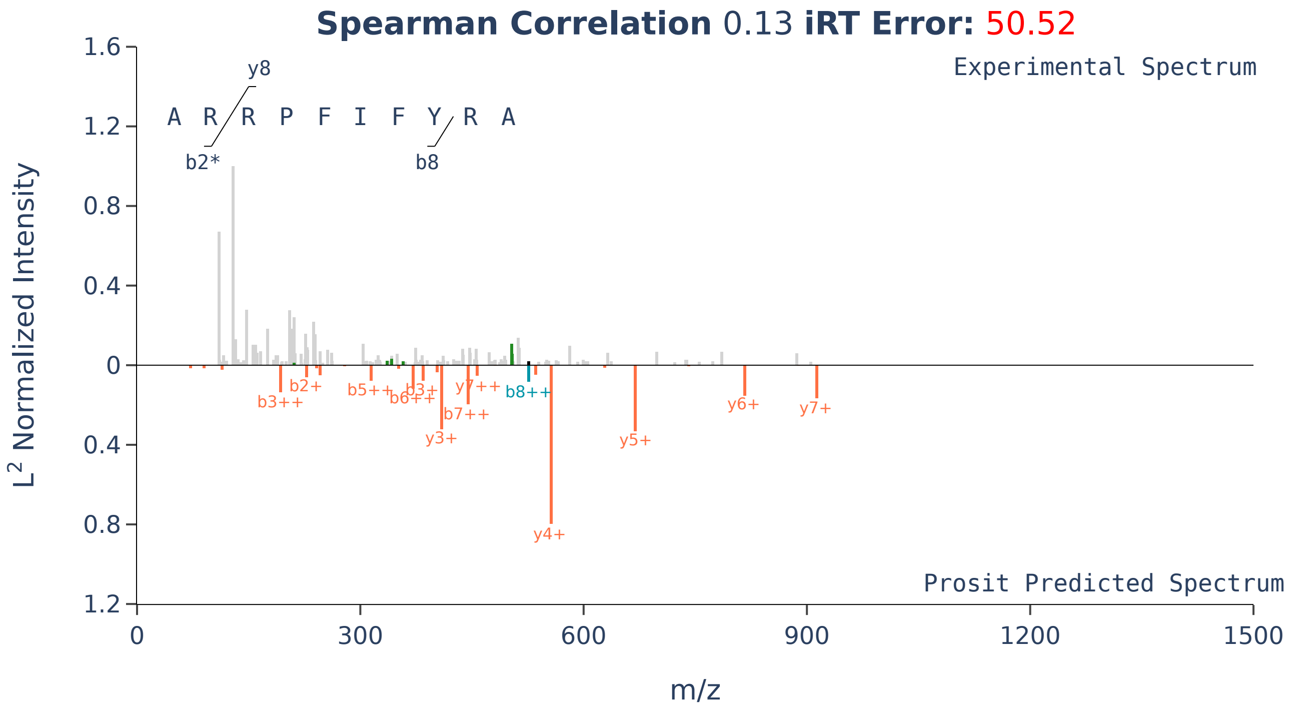

Source WSoh_101121_151121_HFGoe_G2_IL37b_2h_R1 **Scan** 17048
Peptide SAEKGSFCL **Charge** 2 **Spectral Angle** 0.82
Spearman Correlation 0.86 **iRT Error:** 1.07

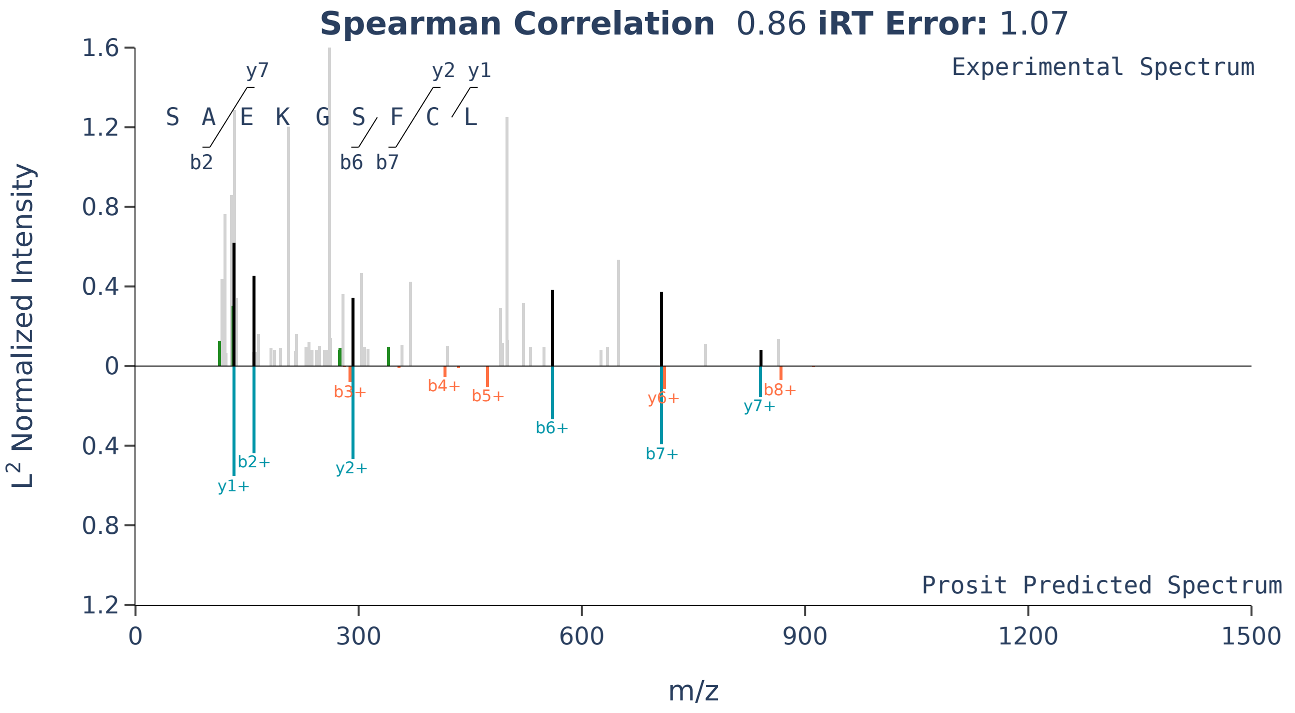

Source WSoh_101121_151121_HFGoe_G2_IL37b_2h_R1 **Scan** 17048
Peptide LYCDKDKG **Charge** 2 **Spectral Angle** 0.0
Spearman Correlation 0.0 **iRT Error:** 57.96

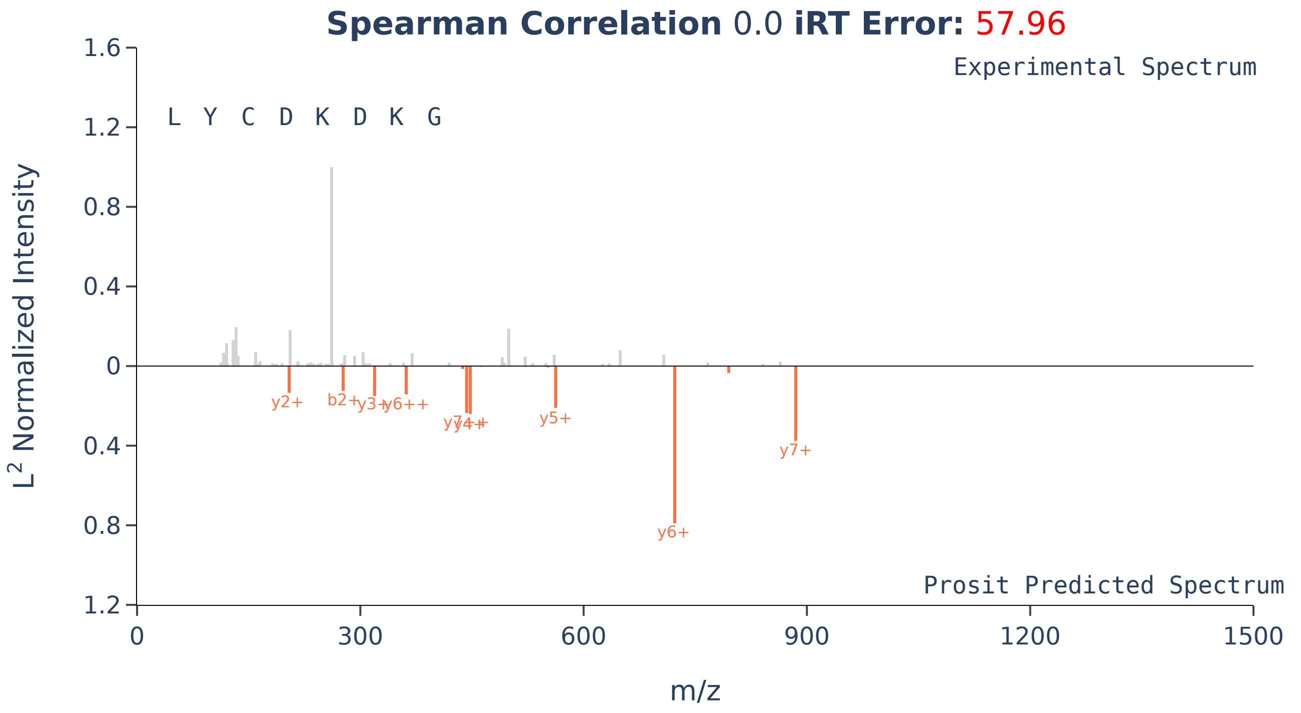

Source WSoh_101121_151121_HFGoe_G2_IL37b_24h_R2 **Scan** 10381
Peptide ALSSLSS **Charge** 1 **Spectral Angle** 0.88
Spearman Correlation 0.89 **iRT Error:** 1.16

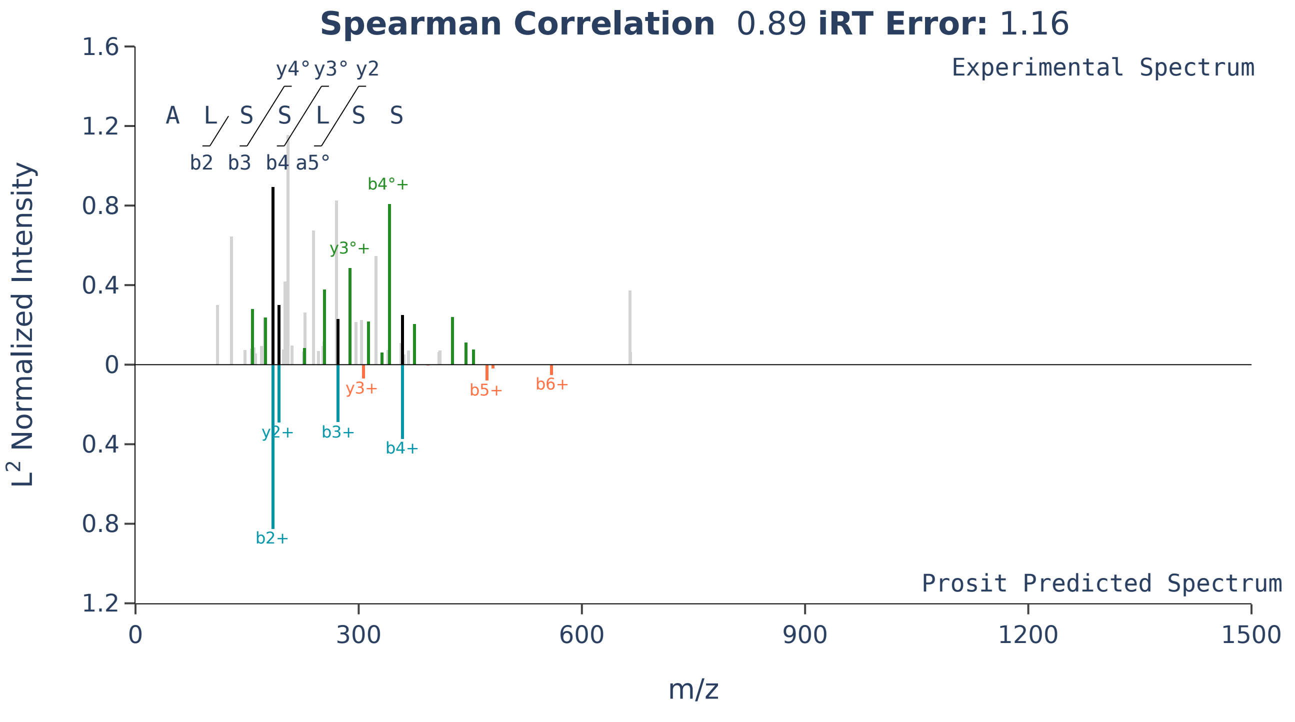

Source WSoh_101121_151121_HFGoe_G2_IL37b_24h_R2 **Scan** 10381
Peptide LASSLSS **Charge** 1 **Spectral Angle** 0.8
Spearman Correlation 0.87 **iRT Error:** 37.32

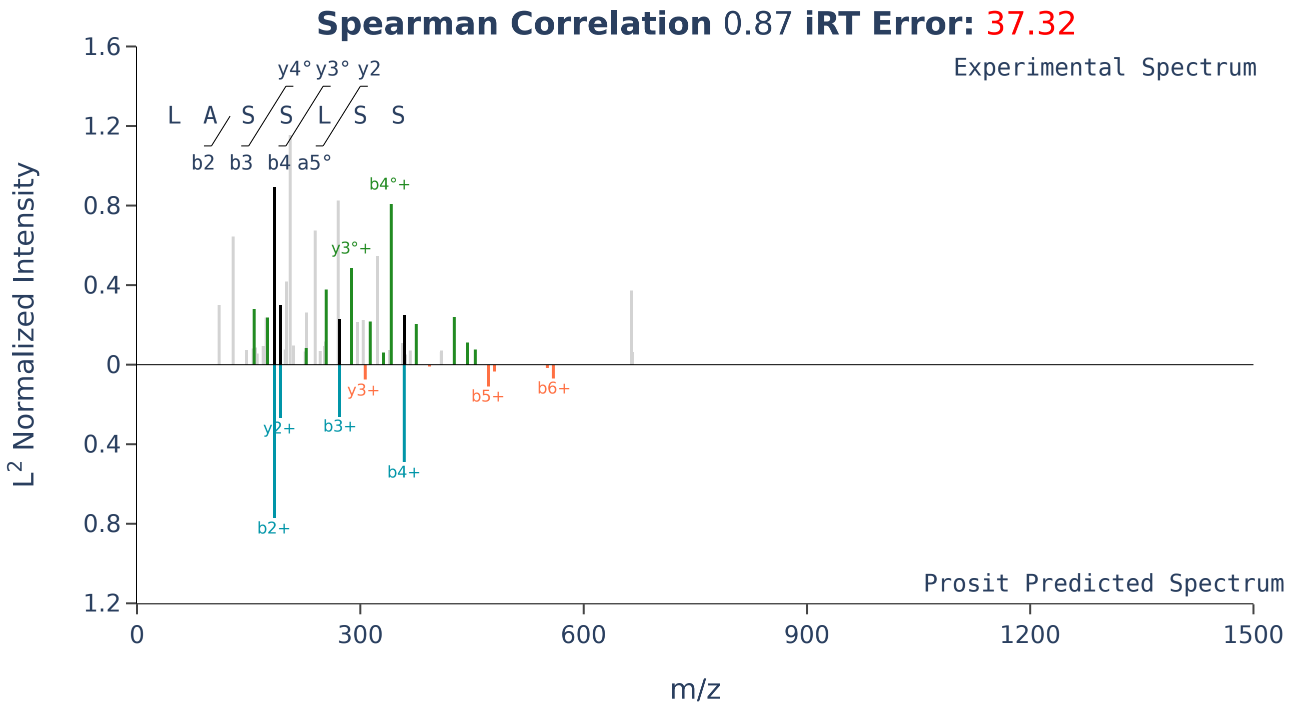

Source WSoh_101121_151121_HFGoe_G2_IL37b_24h_R2 **Scan** 9869
Peptide ESNLNPKKF **Charge** 2 **Spectral Angle** 0.85
Spearman Correlation 0.91 **iRT Error:** 1.26

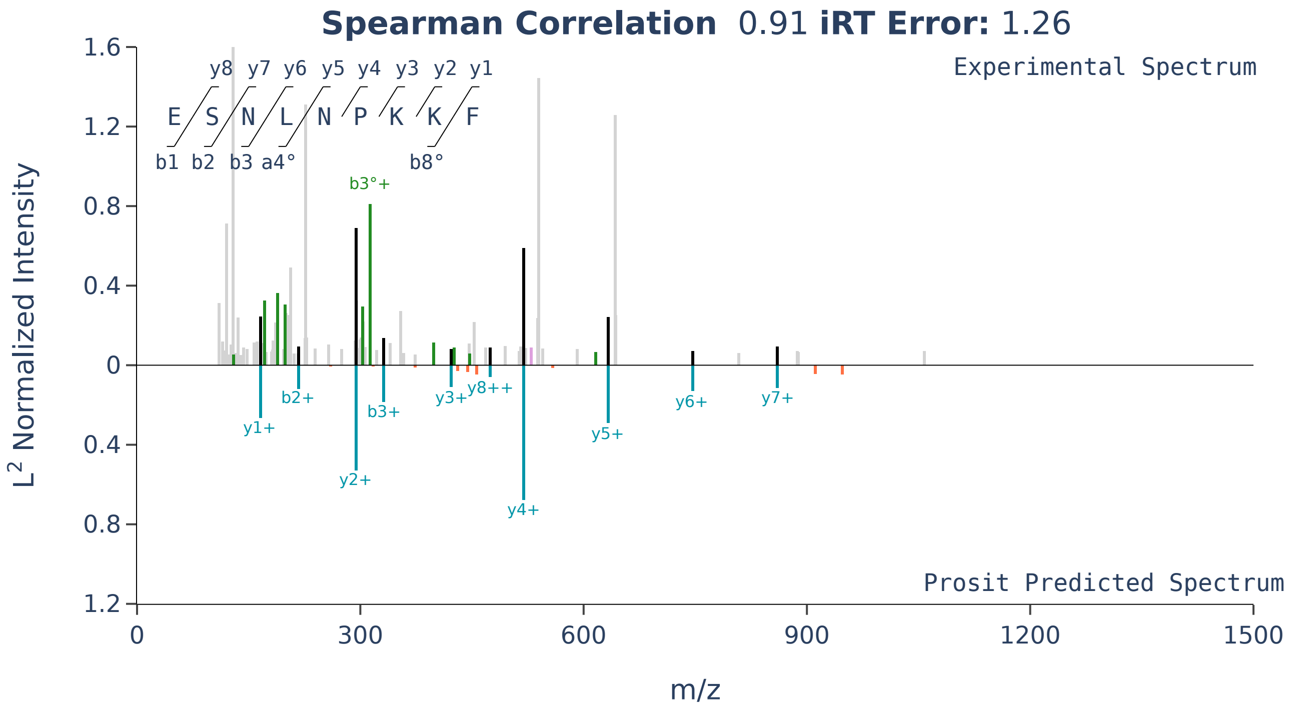

Source WSoh_101121_151121_HFGoe_G2_IL37b_24h_R2 **Scan** 9869
Peptide MKLAAQKESA **Charge** 2 **Spectral Angle** 0.02
Spearman Correlation -0.17 **iRT Error:** 21.09

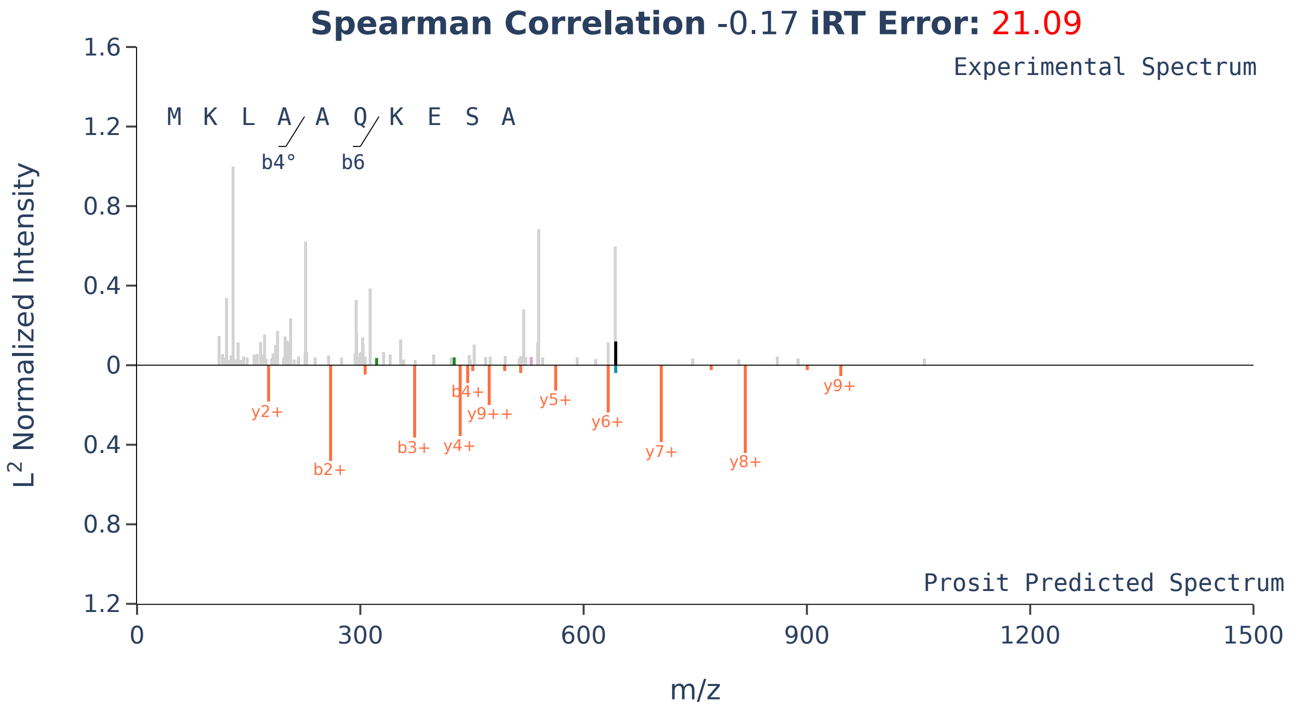

Source WSoh_101121_151121_HFGoe_G1_IL37b_1h_R1 **Scan** 15420
Peptide SSLHDQDHKVLVL **Charge** 3 **Spectral Angle** 0.86
Spearman Correlation 0.84 **iRT Error:** 1.7

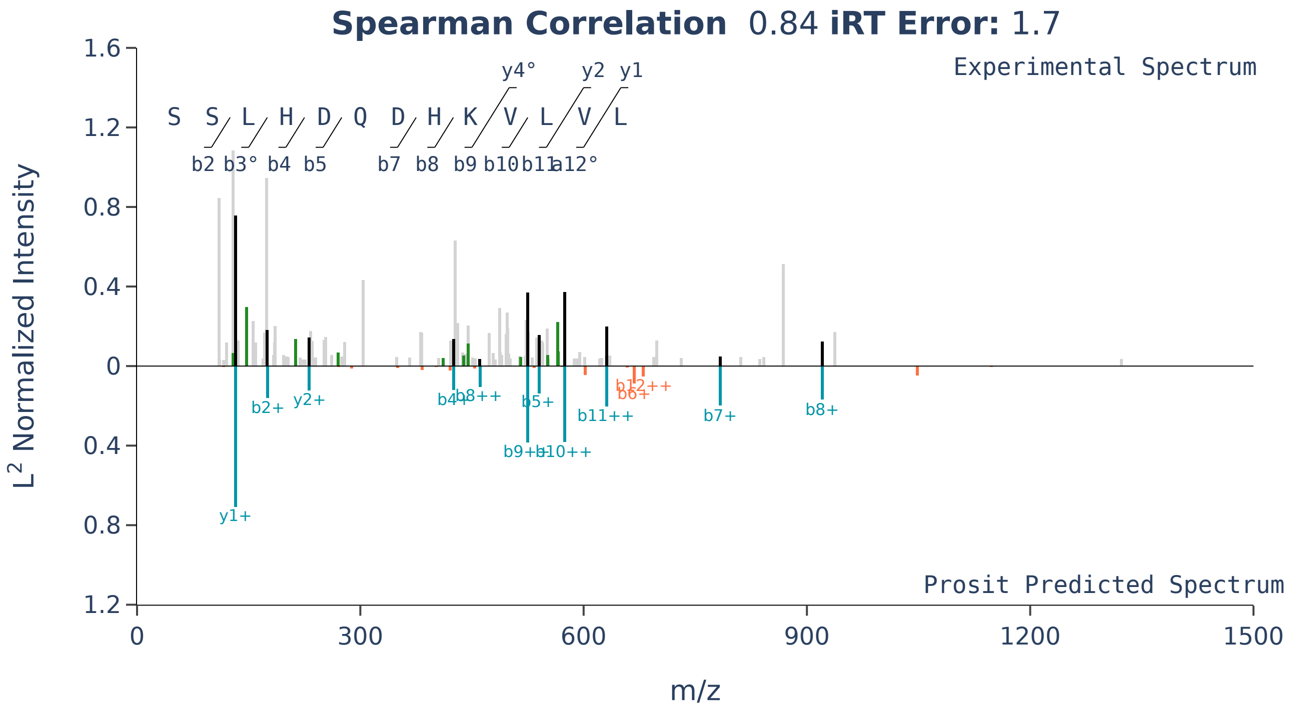

Source WSoh_101121_151121_HFGoe_G1_IL37b_1h_R1 **Scan** 15420
Peptide VPDKNYIRPEIF **Charge** 3 **Spectral Angle** 0.04
Spearman Correlation 0.13 **iRT Error:** 37.68

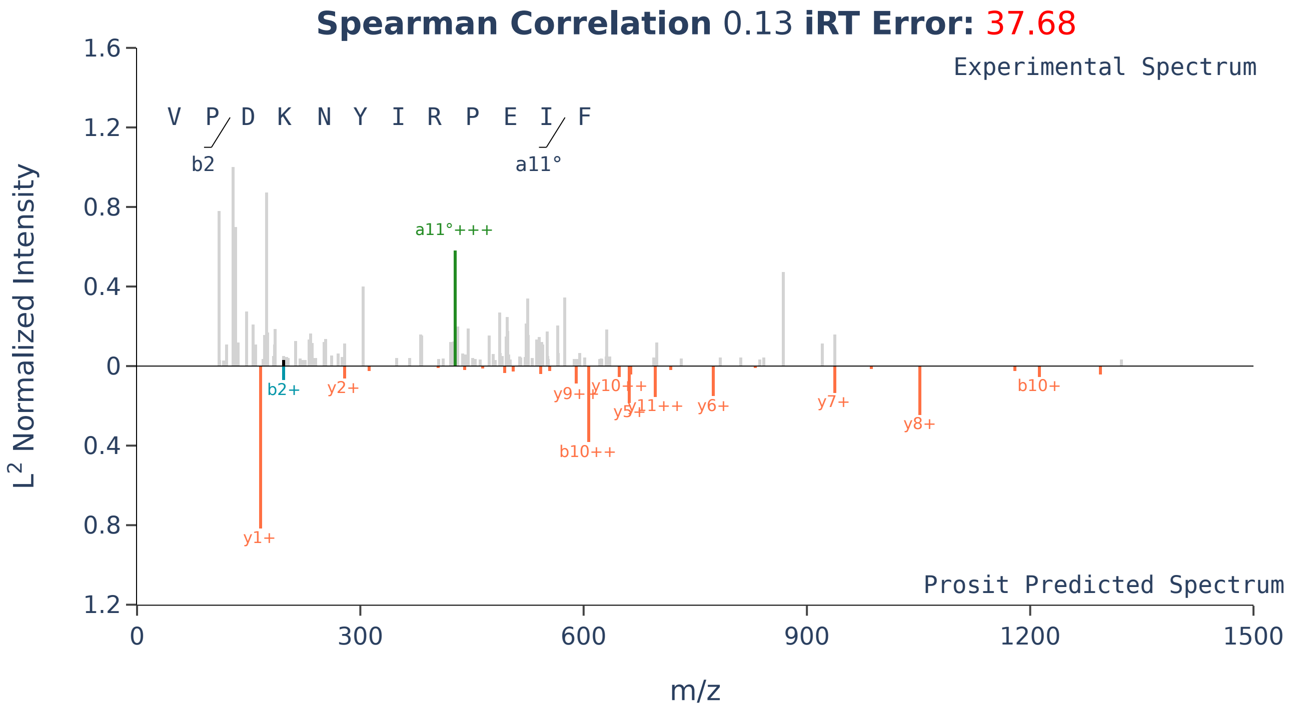

Source WSoh_101121_151121_HFGoe_G2_IL37b_24h_R1 **Scan** 14177
Peptide SHDQDHKVLVL **Charge** 3 **Spectral Angle** 0.82
Spearman Correlation 0.89 **iRT Error:** 2.06

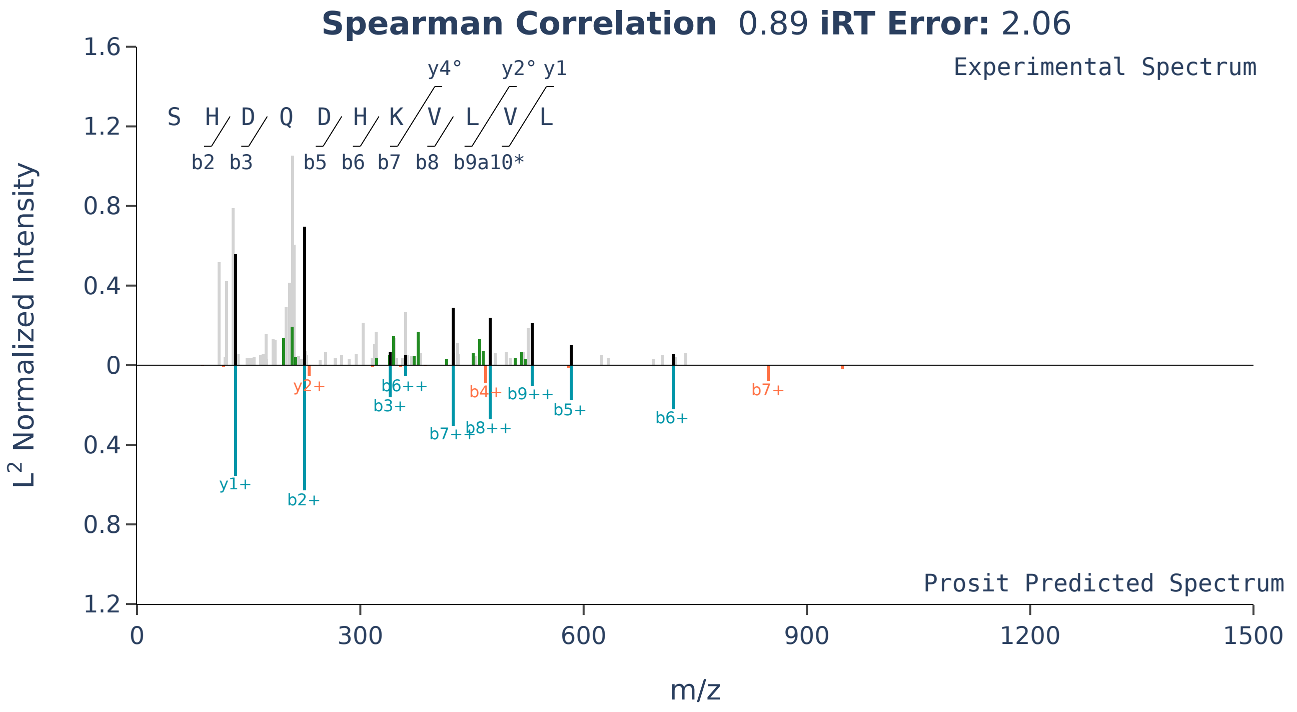

Source WSoh_101121_151121_HFGoe_G2_IL37b_24h_R1 **Scan** 14177
Peptide SIHDQDHKVLV **Charge** 3 **Spectral Angle** 0.28
Spearman Correlation 0.48 **iRT Error:** 27.79

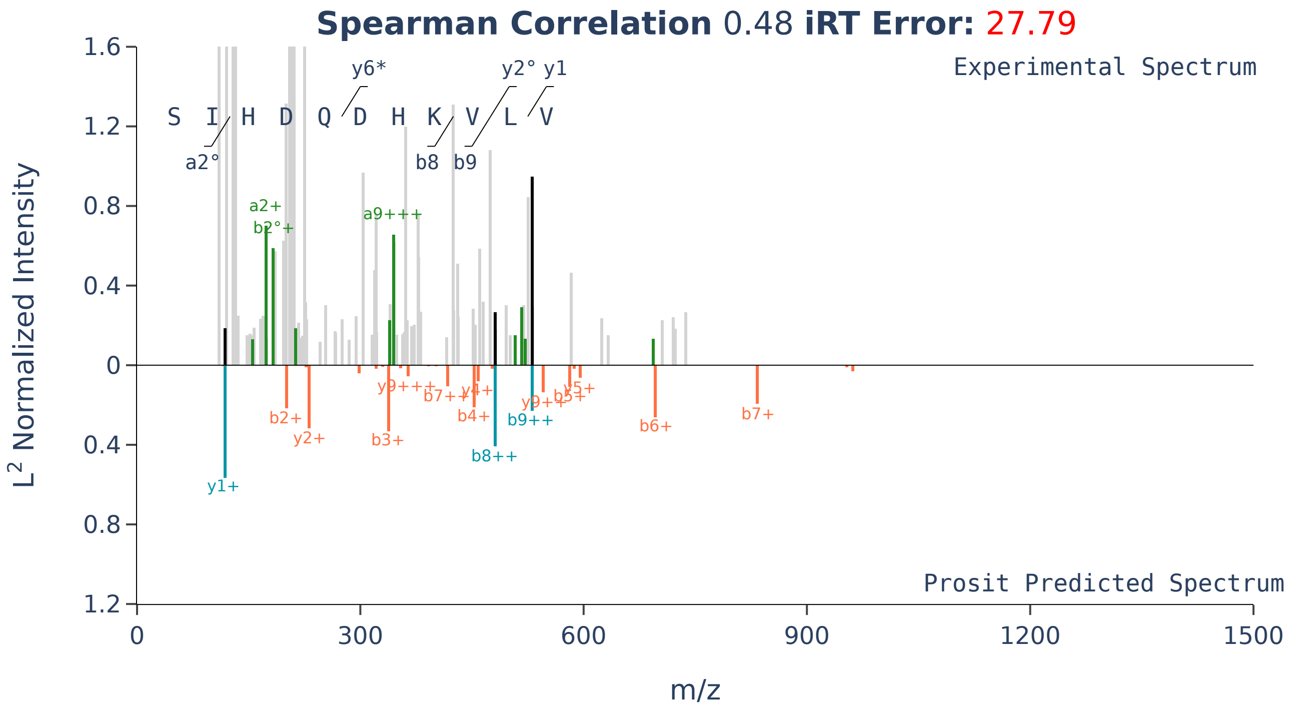

Source WSoh_101121_151121_HFGoe_G1_IL37b_24h_R1 **Scan** 13642
Peptide KEGSPIL **Charge** 1 **Spectral Angle** 0.79
Spearman Correlation 0.89 **iRT Error:** 2.14

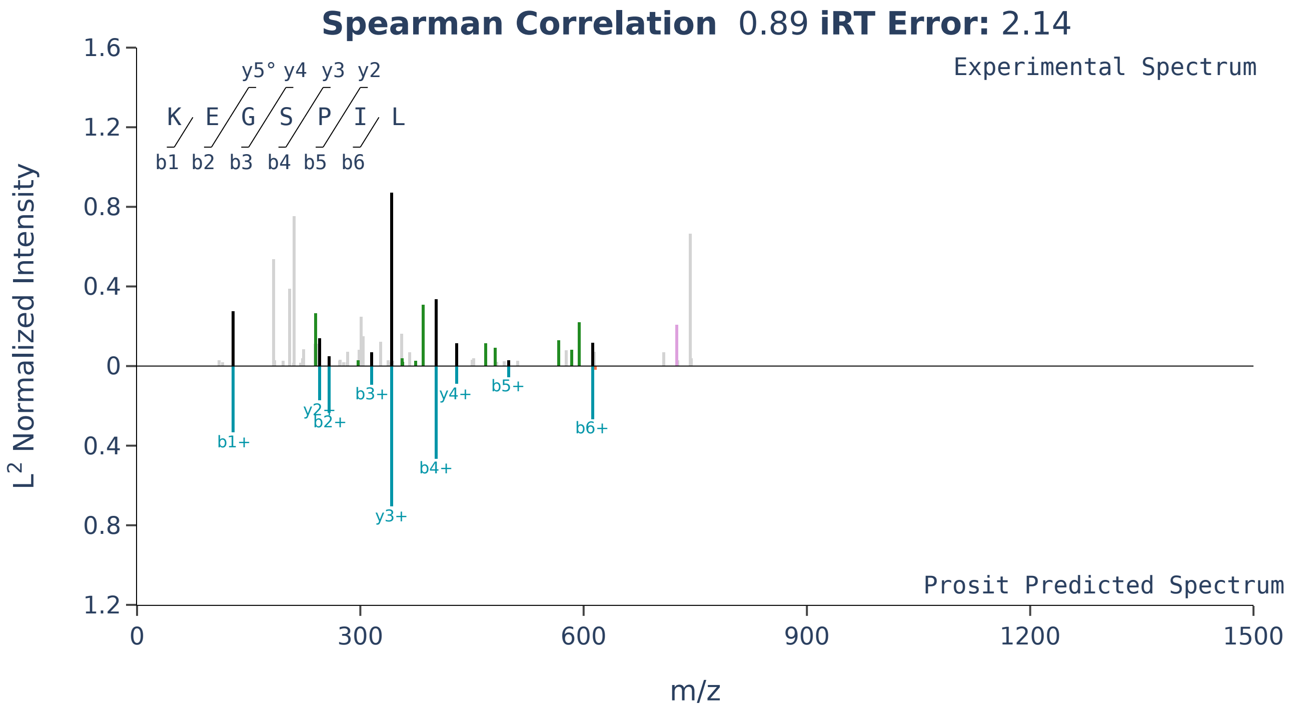

Source WSoh_101121_151121_HFGoe_G1_IL37b_24h_R1 **Scan** 13642
Peptide EKGSPIL **Charge** 1 **Spectral Angle** 0.74
Spearman Correlation 0.45 **iRT Error:** 2.69

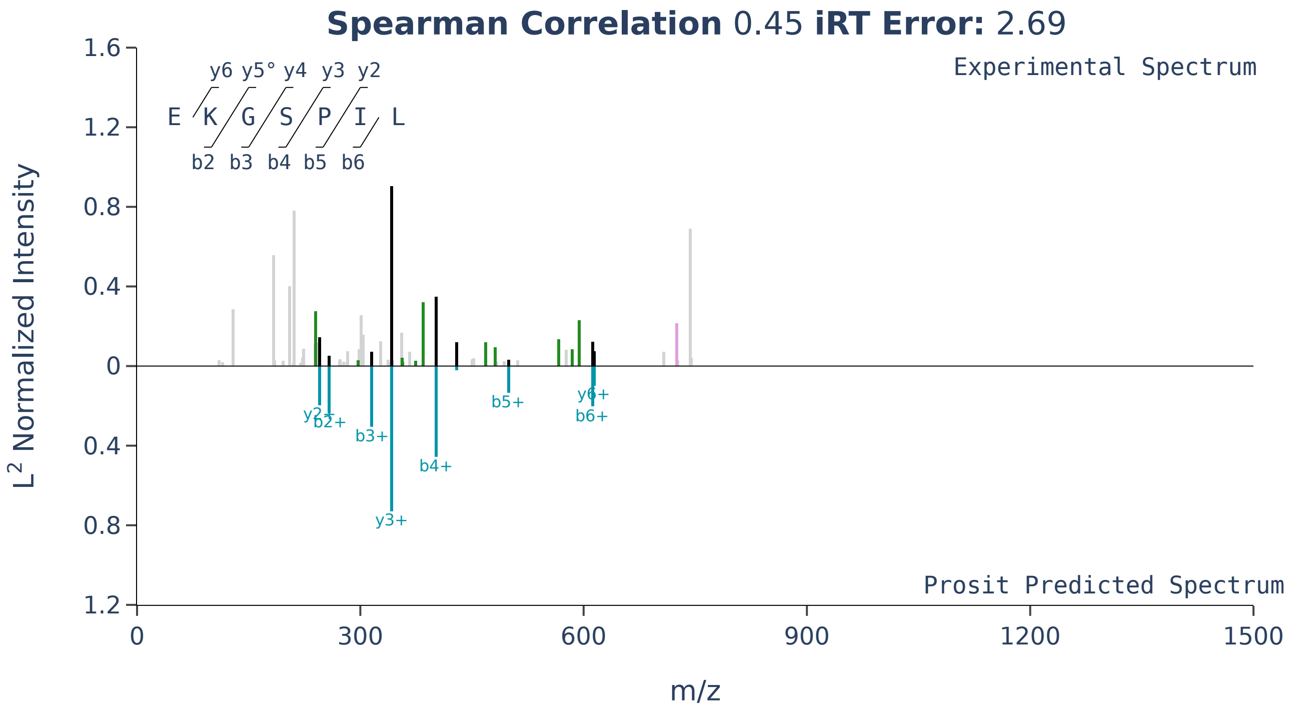

Source WSoh_101121_151121_HFGoe_G2_IL37b_24h_R1 **Scan** 14868
Peptide SQDHKVLVLD **Charge** 2 **Spectral Angle** 0.79
Spearman Correlation 0.91 **iRT Error:** 3.27

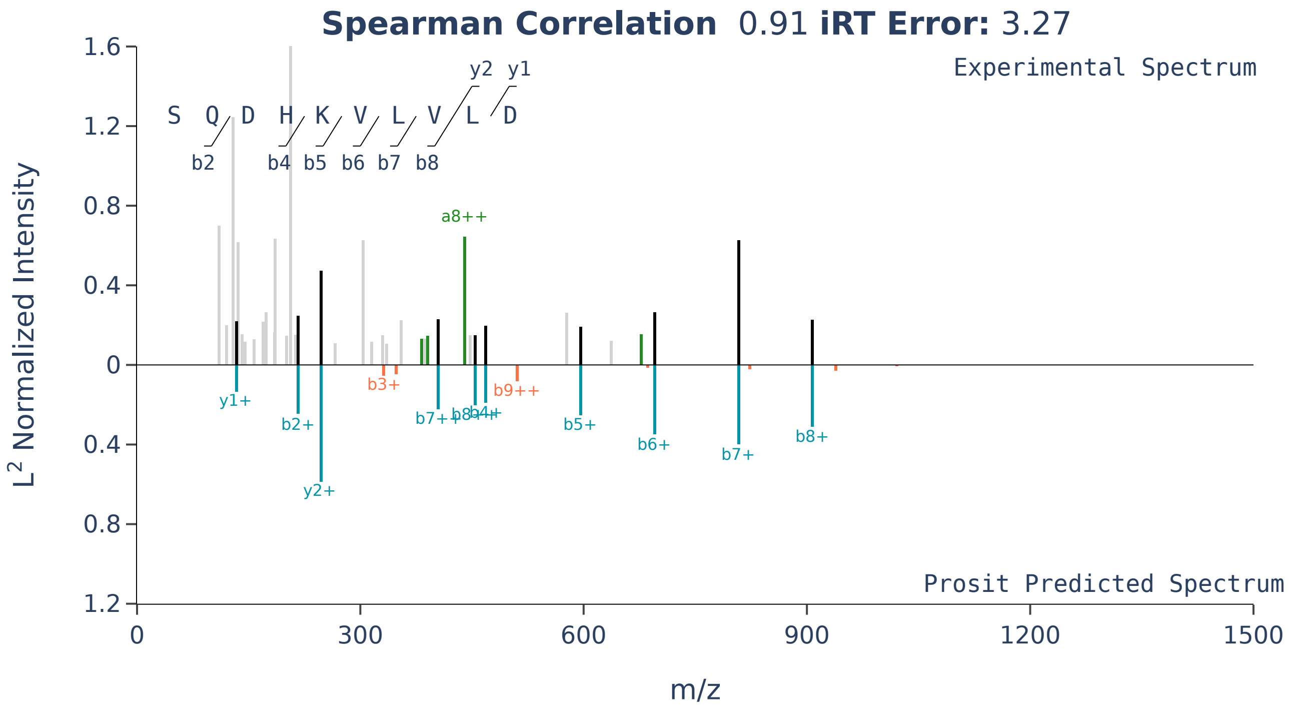

Source WSoh_101121_151121_HFGoe_G2_IL37b_24h_R1 **Scan** 14868
Peptide QDHKVLVLDS **Charge** 2 **Spectral Angle** 0.0
Spearman Correlation 0.0 **iRT Error:** 4.05

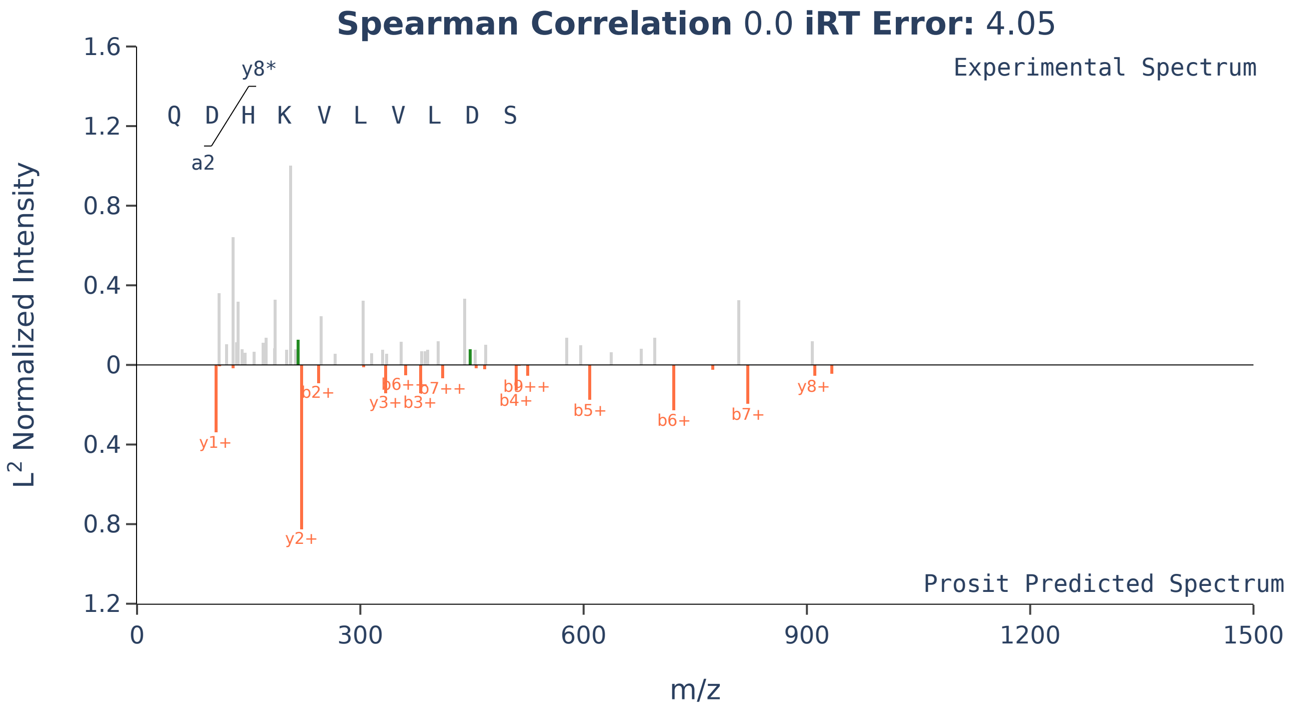

Source WSoh_101121_151121_HFGoe_G1_IL37b_1h_R1 **Scan** 7768
Peptide ASSLSSSAE **Charge** 2 **Spectral Angle** 0.96
Spearman Correlation 0.86 **iRT Error:** 3.46

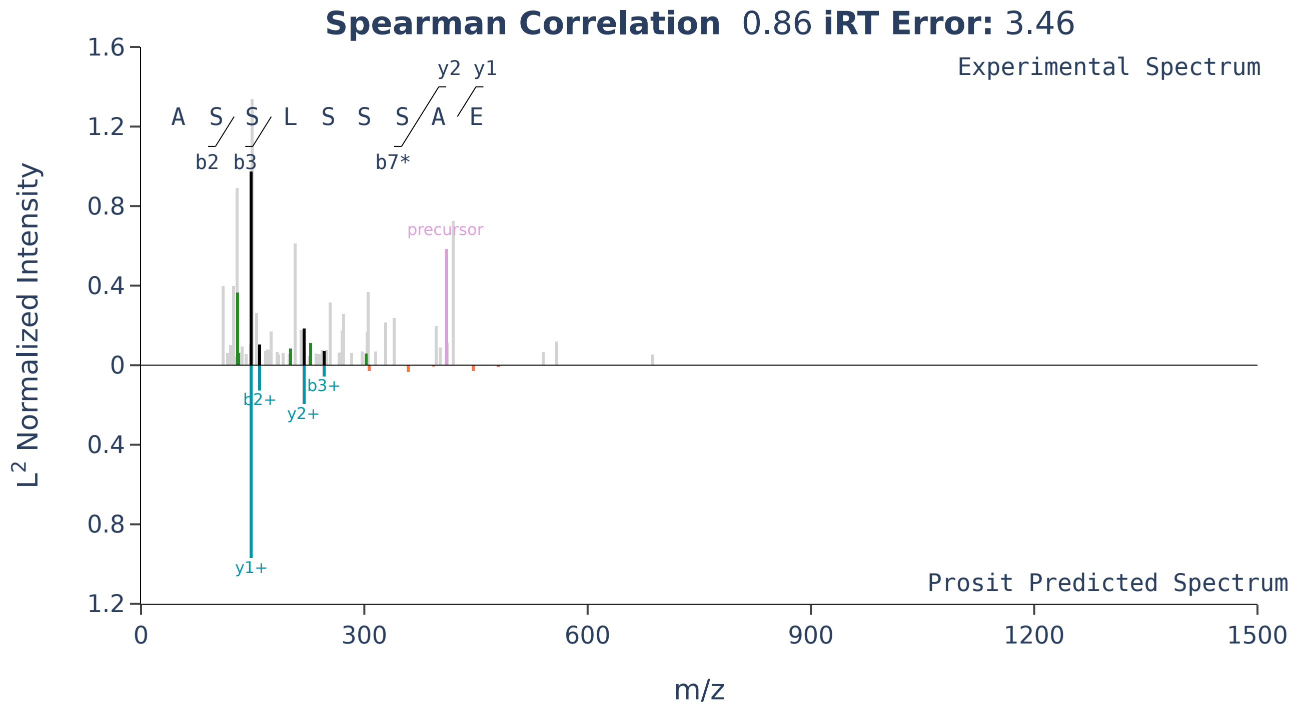

Source WSoh_101121_151121_HFGoe_G1_IL37b_1h_R1 **Scan** 7768
Peptide SSLSSASAE **Charge** 2 **Spectral Angle** 0.95
Spearman Correlation 0.81 **iRT Error:** 7.56

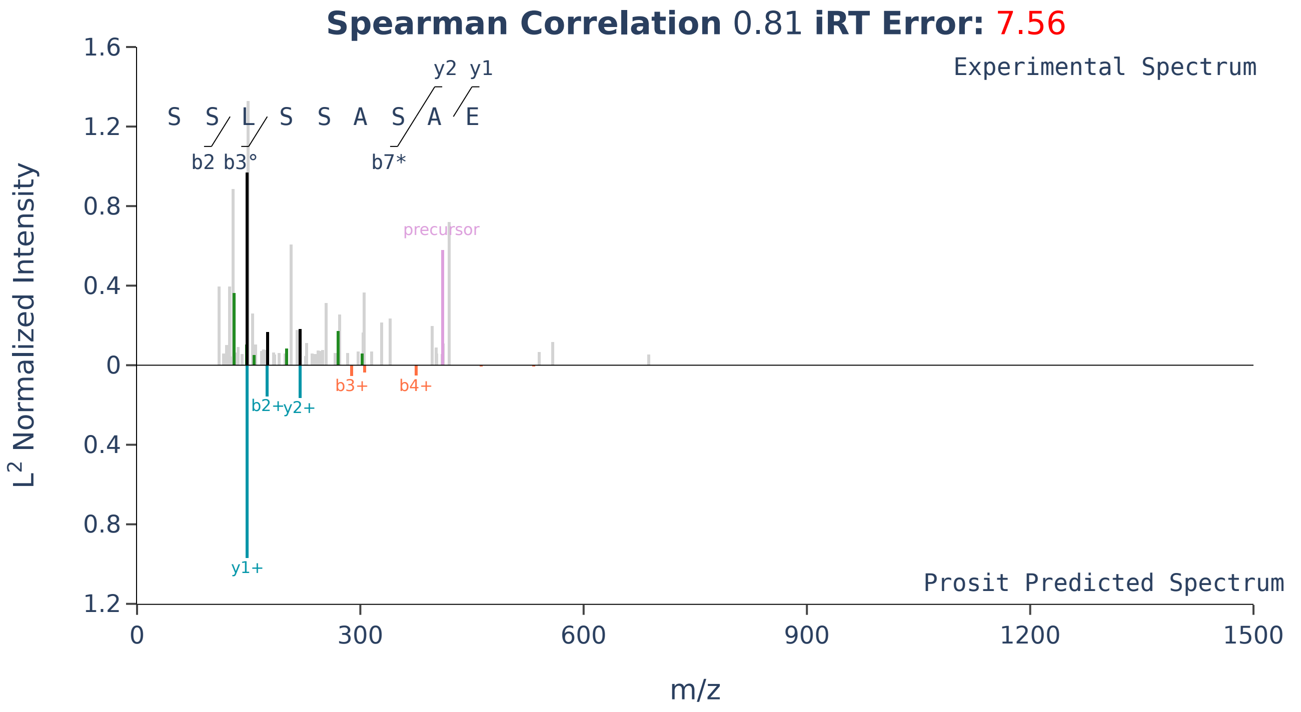

Source WSoh_101121_151121_HFGoe_G2_IL37b_24h_R1 **Scan** 17175
Peptide DSGNLIAVPKN **Charge** 2 **Spectral Angle** 0.92
Spearman Correlation 0.86 **iRT Error:** 3.92

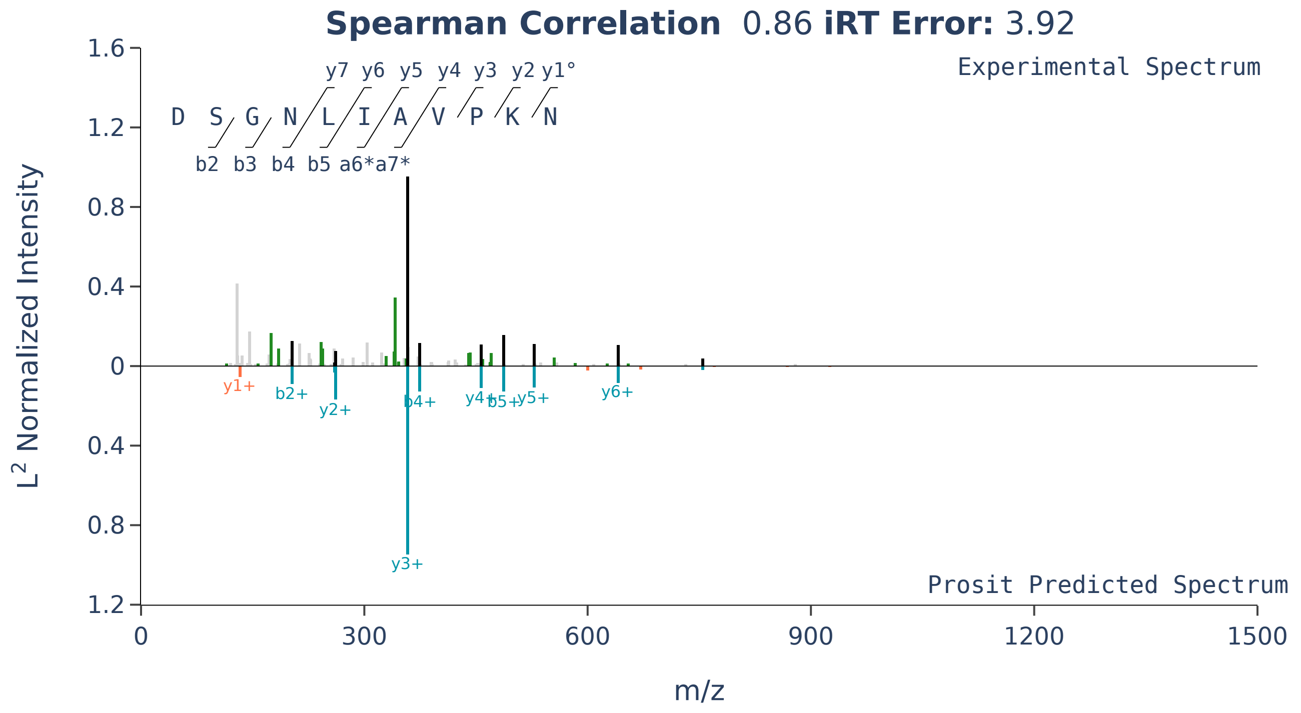

Source WSoh_101121_151121_HFGoe_G2_IL37b_24h_R1 **Scan** 17175
Peptide SGNLIAVPDKN **Charge** 2 **Spectral Angle** 0.34
Spearman Correlation 0.52 **iRT Error:** 10.2

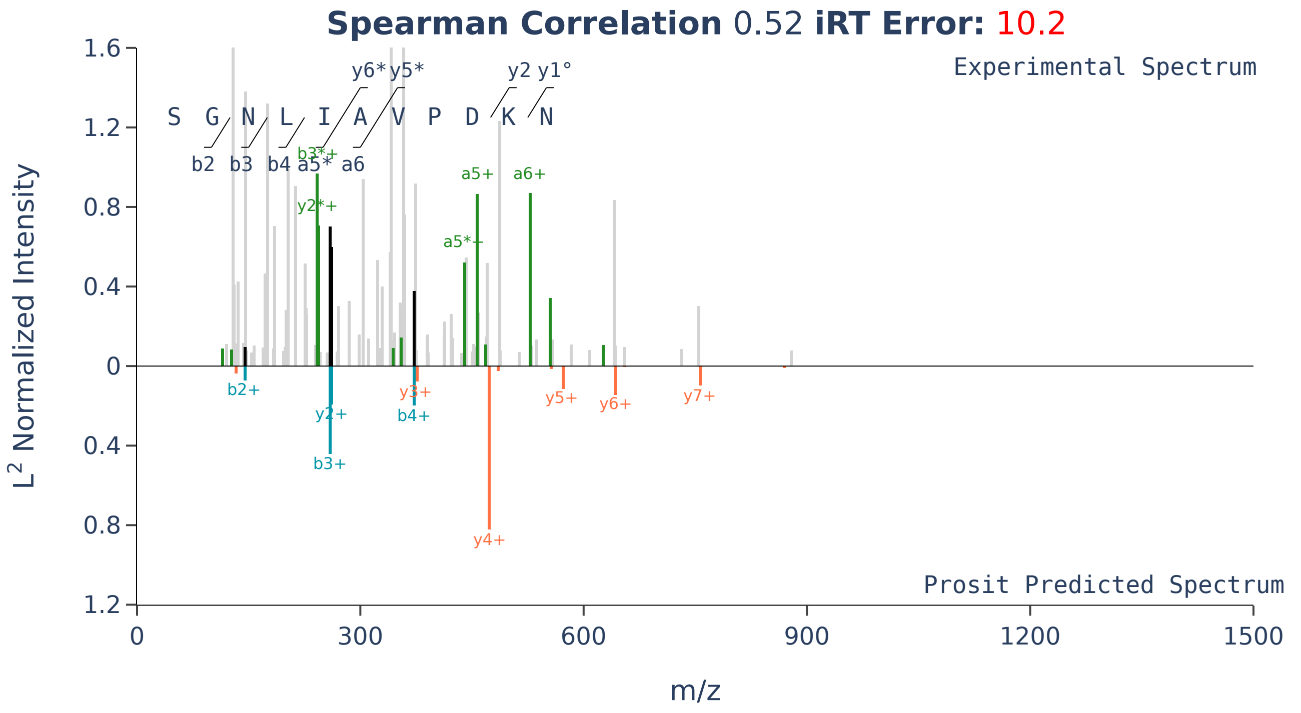

Source WSoh_101121_151121_HFGoe_G2_IL37b_24h_R2 **Scan** 17284
Peptide ASSKGEFCL **Charge** 2 **Spectral Angle** 0.83
Spearman Correlation 0.9 **iRT Error:** 4.18

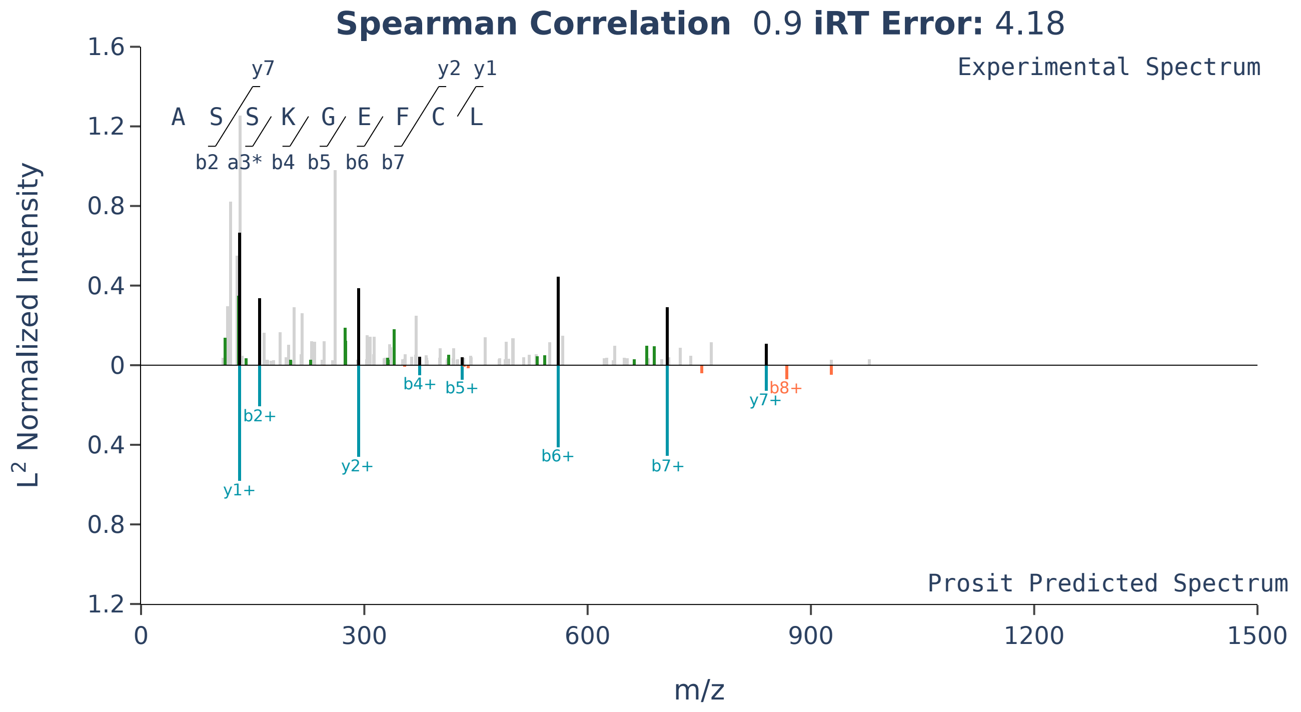

Source WSoh_101121_151121_HFGoe_G2_IL37b_24h_R2 **Scan** 17284
Peptide LYCDKDKG **Charge** 2 **Spectral Angle** 0.0
Spearman Correlation 0.0 **iRT Error:** 63.04

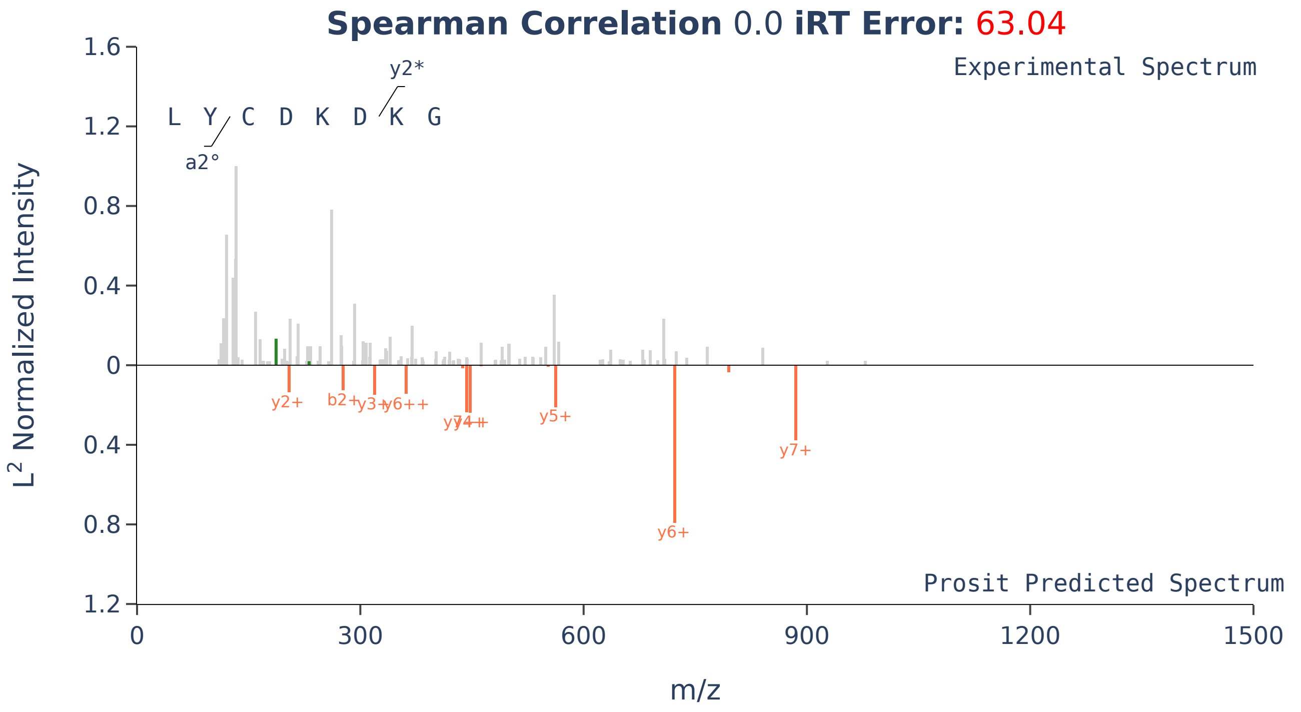

Source WSoh_101121_151121_HFGoe_G2_IL37b_24h_R1 **Scan** 10937
Peptide PSEVSDEMS **Charge** 1 **Spectral Angle** 0.84
Spearman Correlation 0.89 **iRT Error:** 4.34

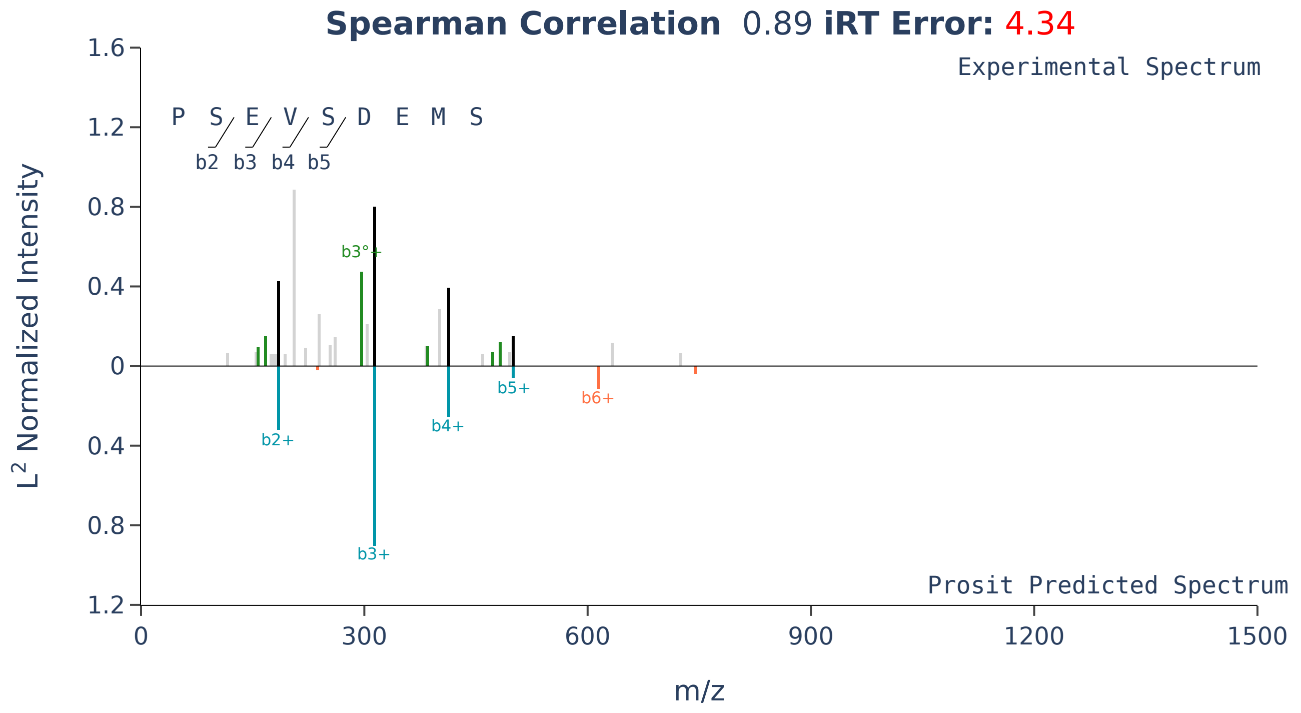

Source WSoh_101121_151121_HFGoe_G2_IL37b_24h_R1 **Scan** 10937
Peptide EMSPSEVSD **Charge** 1 **Spectral Angle** 0.63
Spearman Correlation 0.72 **iRT Error:** 4.98

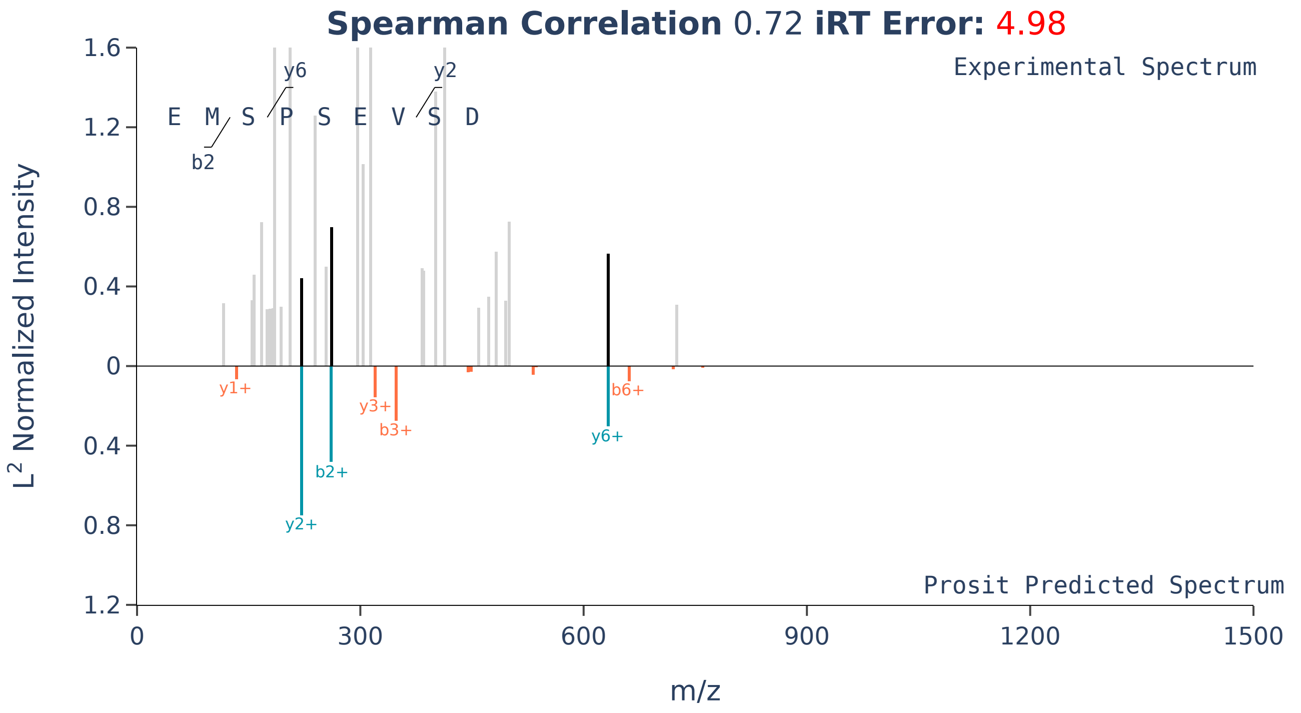

Source WSoh_101121_151121_HFGoe_G1_IL37b_4h_R2 **Scan** 9785
Peptide PSEVSDMS **Charge** 1 **Spectral Angle** 0.87
Spearman Correlation 0.94 **iRT Error:** 5.09

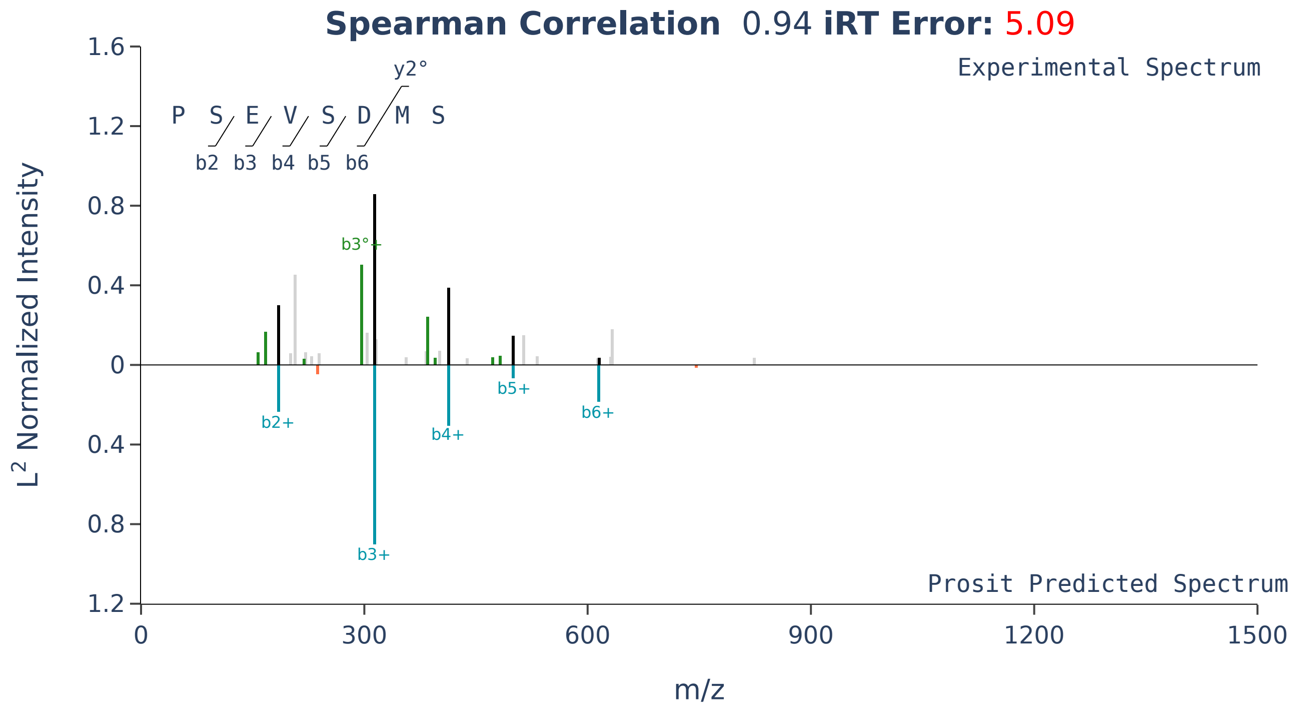

Source WSoh_101121_151121_HFGoe_G1_IL37b_4h_R2 **Scan** 9785
Peptide MSPSEVSD **Charge** 1 **Spectral Angle** 0.49
Spearman Correlation 0.66 **iRT Error:** 5.79

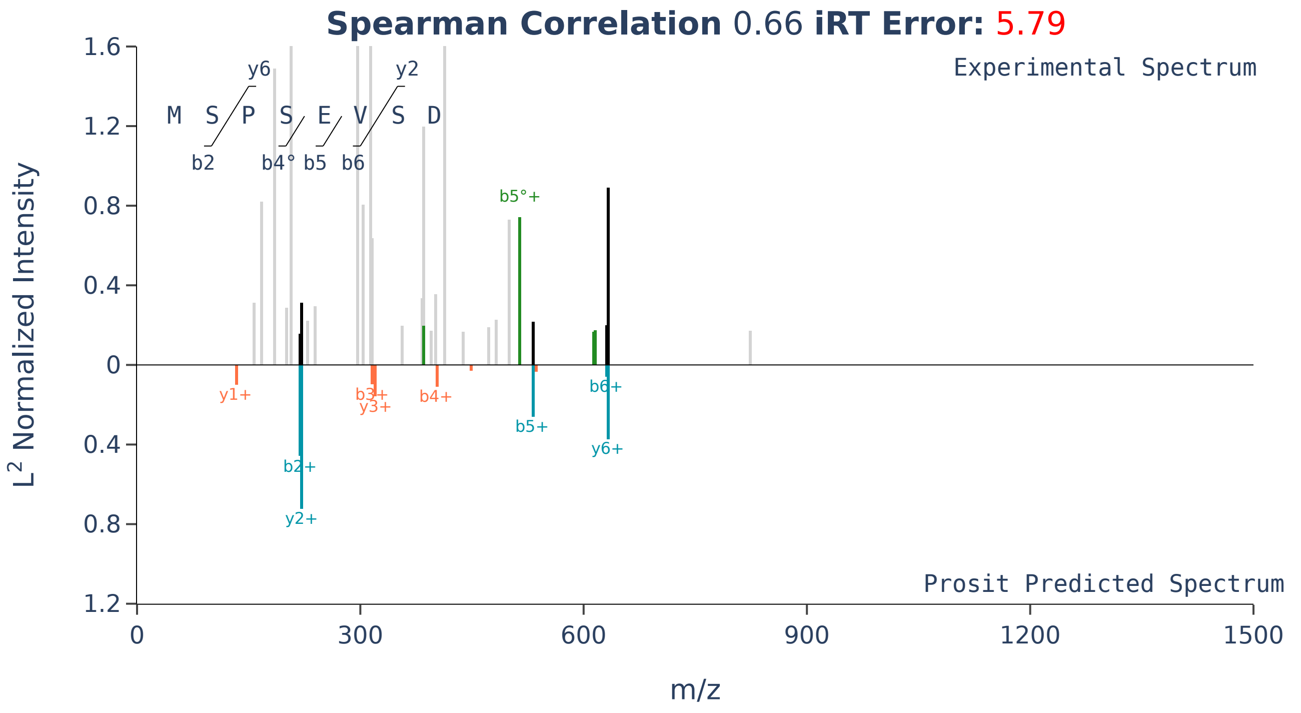

Source WSoh_101121_151121_HFGoe_G2_IL37b_24h_R1 **Scan** 12092
Peptide PSEVSDAEMS **Charge** 1 **Spectral Angle** 0.87
Spearman Correlation 0.95 **iRT Error:** 7.38

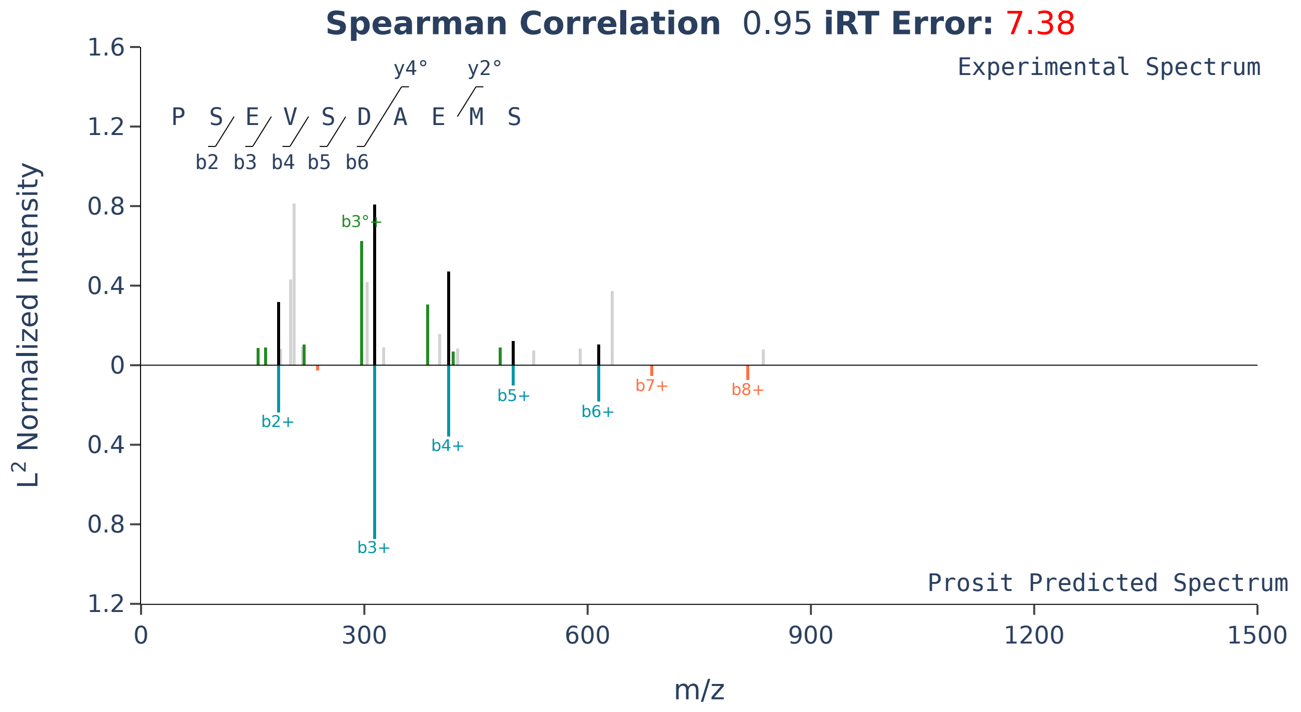

Source WSoh_101121_151121_HFGoe_G2_IL37b_24h_R1 **Scan** 12092
Peptide AEMSPSEVSD **Charge** 1 **Spectral Angle** 0.64
Spearman Correlation 0.61 **iRT Error:** 4.81

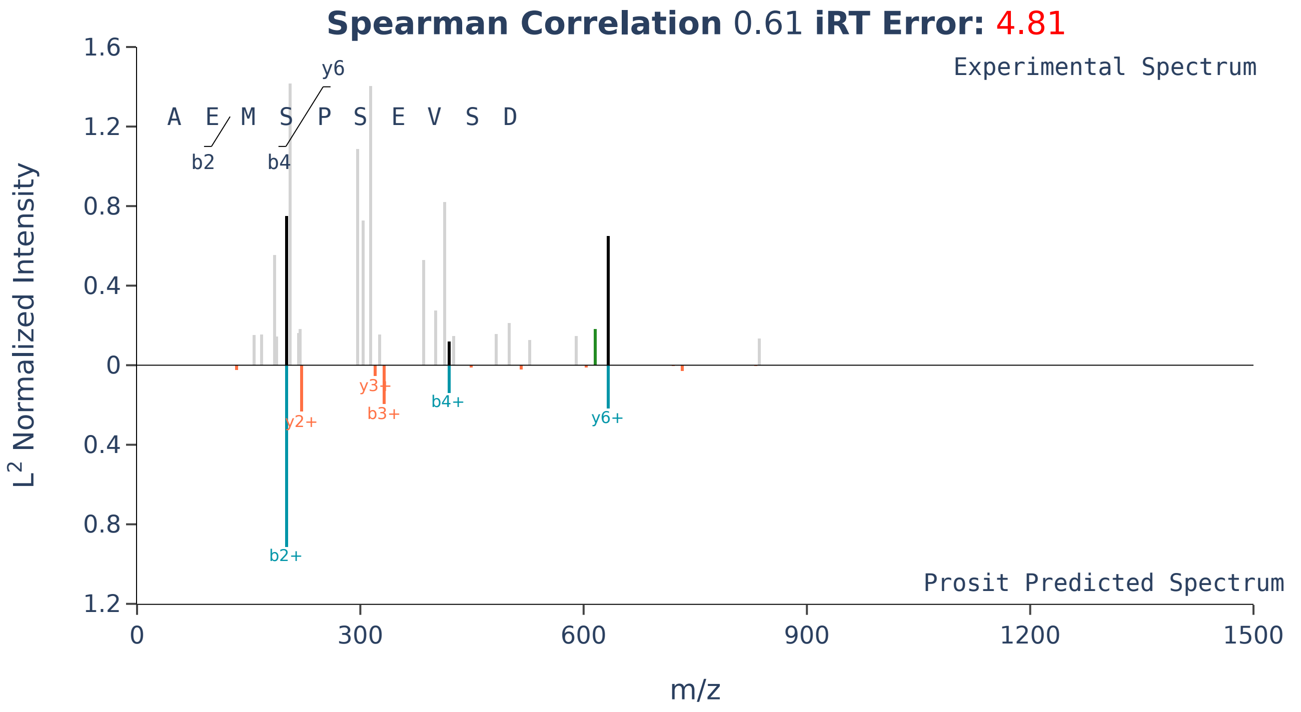

Source WSoh_101121_151121_HFGoe_G2_IL37b_4h_R2 **Scan** 8969
Peptide SNLNPKKF **Charge** 2 **Spectral Angle** 0.87
Spearman Correlation 0.92 **iRT Error:** 8.37

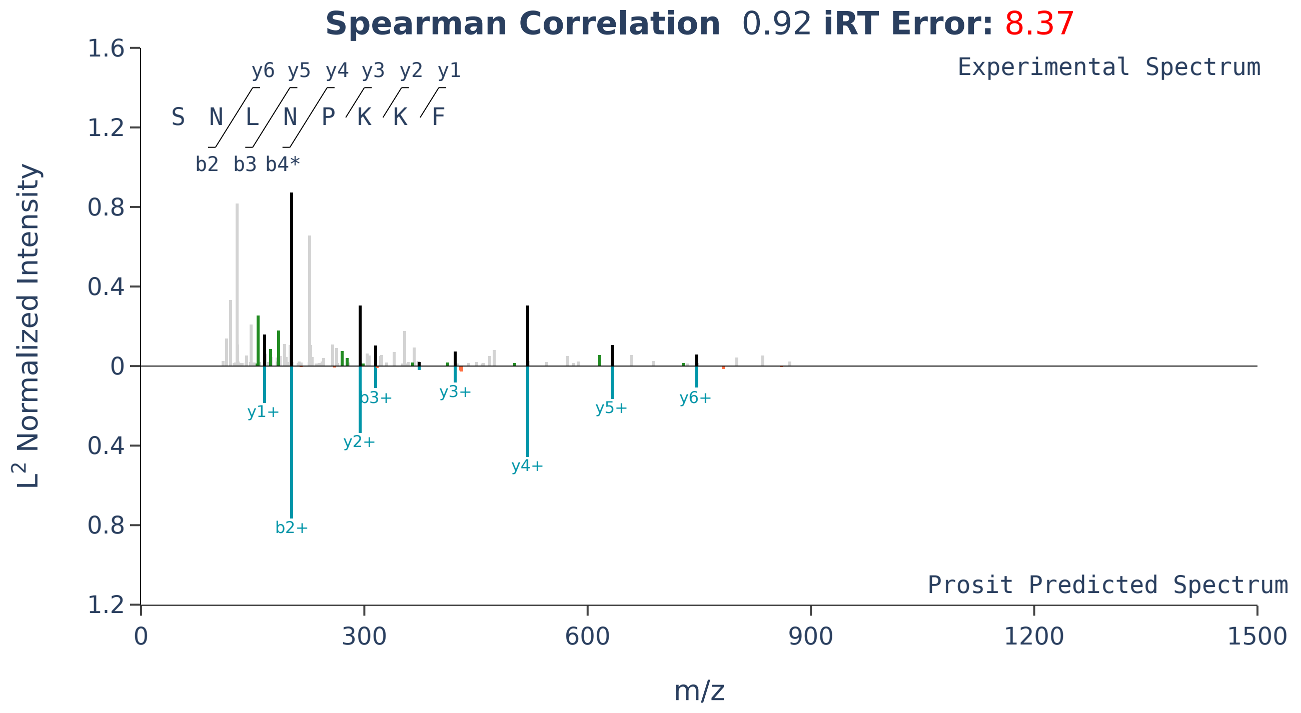

Source WSoh_101121_151121_HFGoe_G2_IL37b_4h_R2 **Scan** 8969
Peptide NLNPKKFS **Charge** 2 **Spectral Angle** 0.23
Spearman Correlation -0.03 **iRT Error:** 3.77

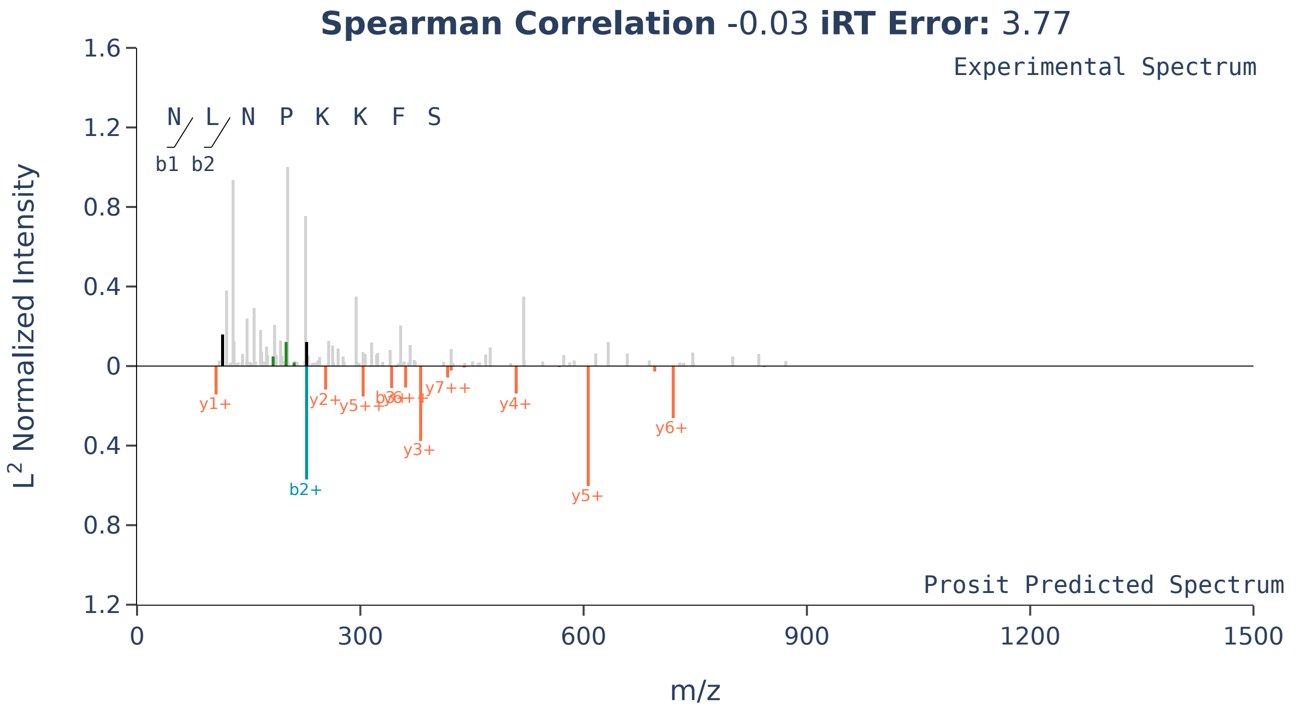

Source HRoetschke_271222_090123_HFGoe_LEDGF_b2R1_24h **Scan** 16678
Peptide LGPKDIFPYSENKEKYGKPNKRAF **Charge** 5 **Spectral Angle** 0.77
Spearman Correlation 0.89 **iRT Error:** 0.09

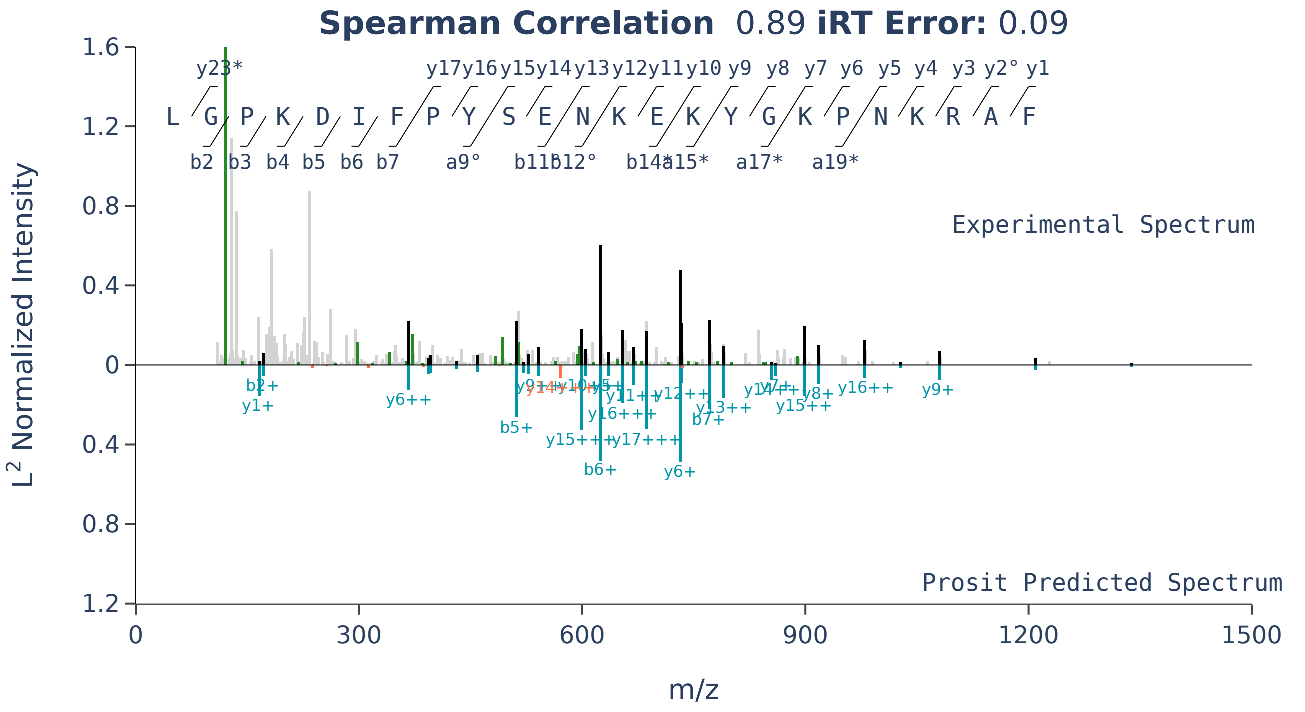

Source HRoetschke_271222_090123_HFGoe_LEDGF_b2R1_24h **Scan** 16678
Peptide EDKSKKKGQEEKQPKKQPKKDEEG **Charge** 5 **Spectral Angle** 0.05
Spearman Correlation -0.12 **iRT Error:** 92.41

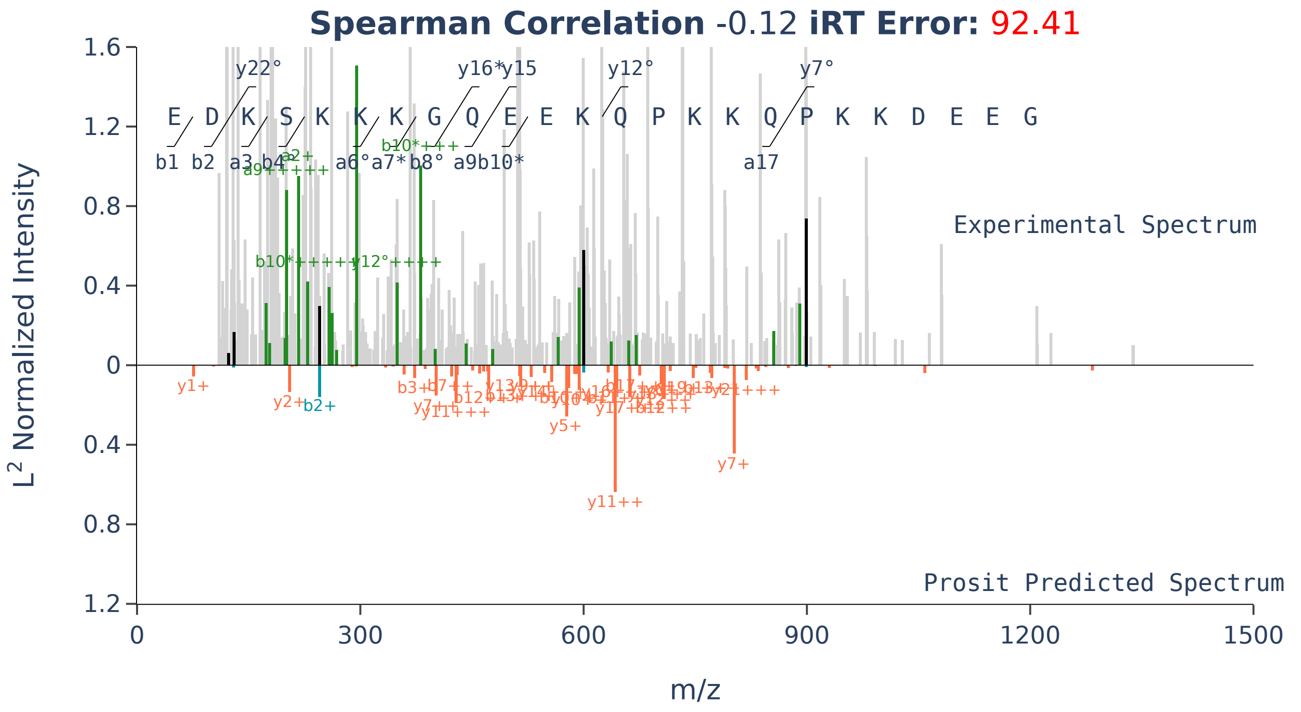

Source HRoetschke_271222_090123_HFGoe_LEDGF_b1R1_4h **Scan** 20820
Peptide TGVKIDNLDVNRCIE **Charge** 2 **Spectral Angle** 0.77
Spearman Correlation 0.86 **iRT Error:** 0.1

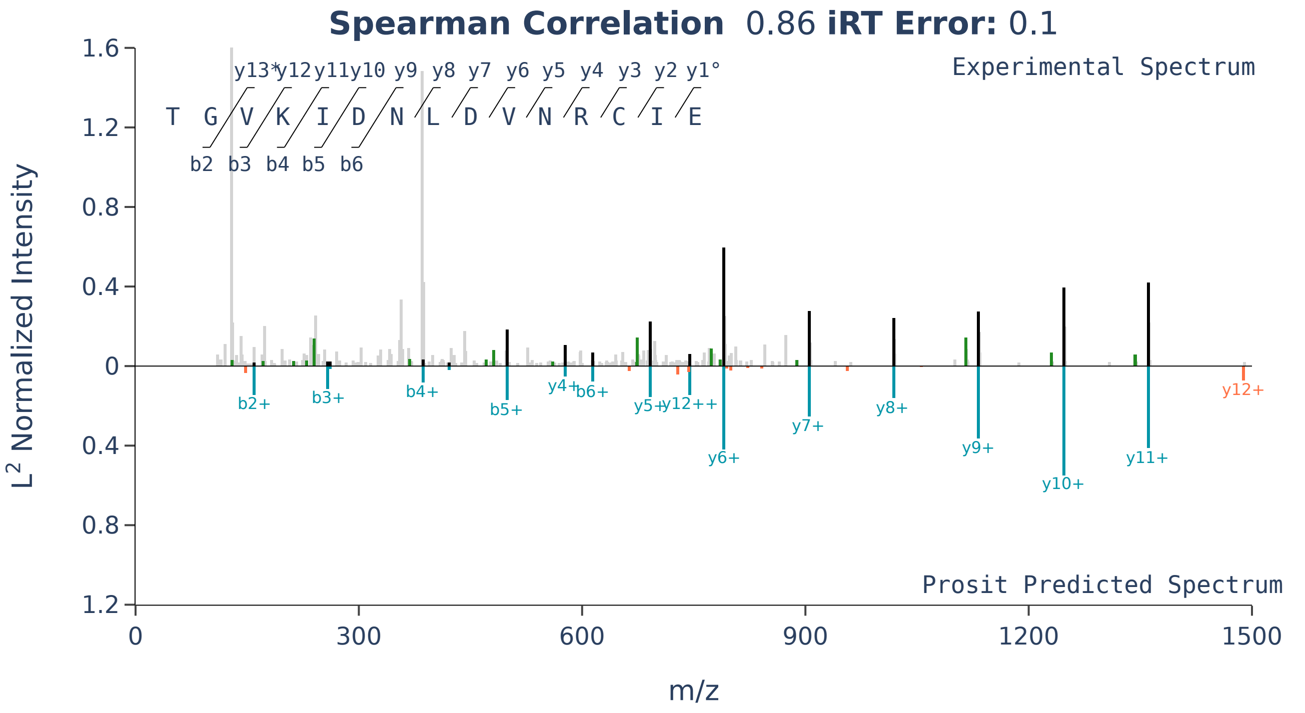

Source HRoetschke_271222_090123_HFGoe_LEDGF_b1R1_4h **Scan** 20820
Peptide SNEDVTKAVDITTPKA **Charge** 2 **Spectral Angle** 0.01
Spearman Correlation -0.18 **iRT Error:** 19.57

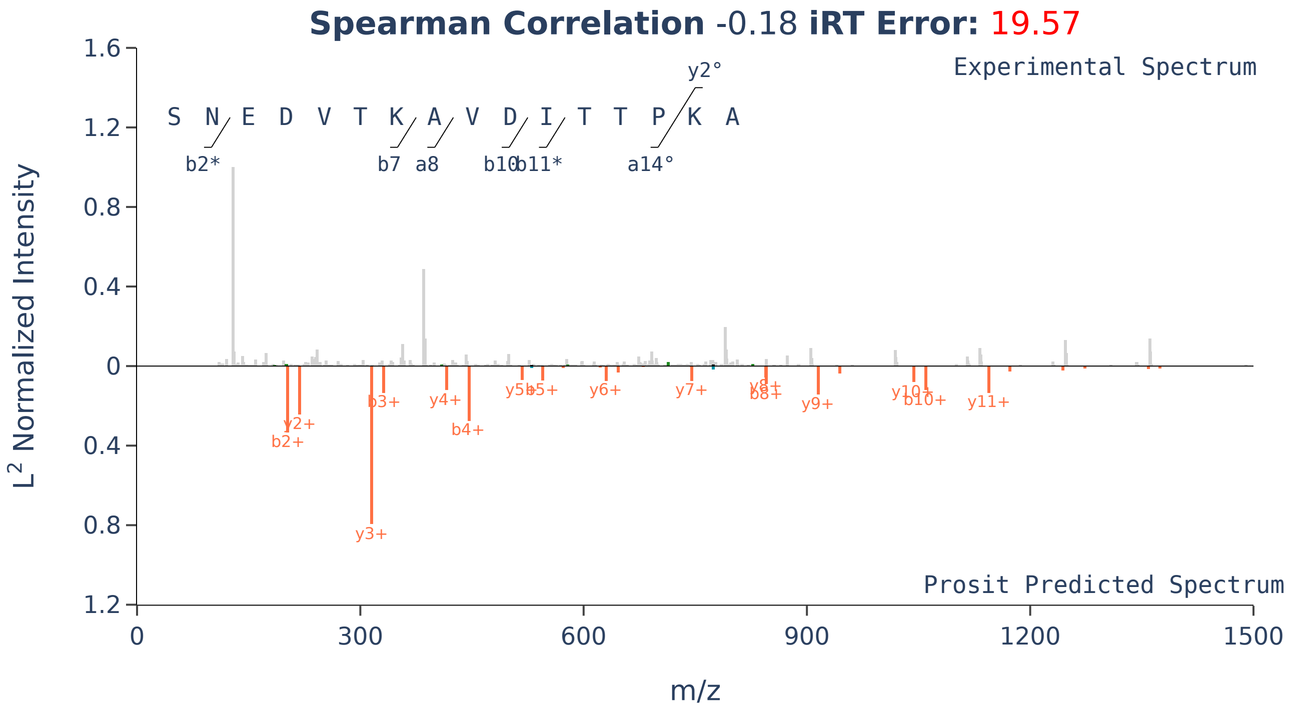

Source HRoetschke_271222_090123_HFGoe_LEDGF_b1R2_24h **Scan** 18399
Peptide TASFLGPKDIFPYSENKEKYGKPNKR **Charge** 5 **Spectral Angle** 0.77
Spearman Correlation 0.87 **iRT Error:** 0.1

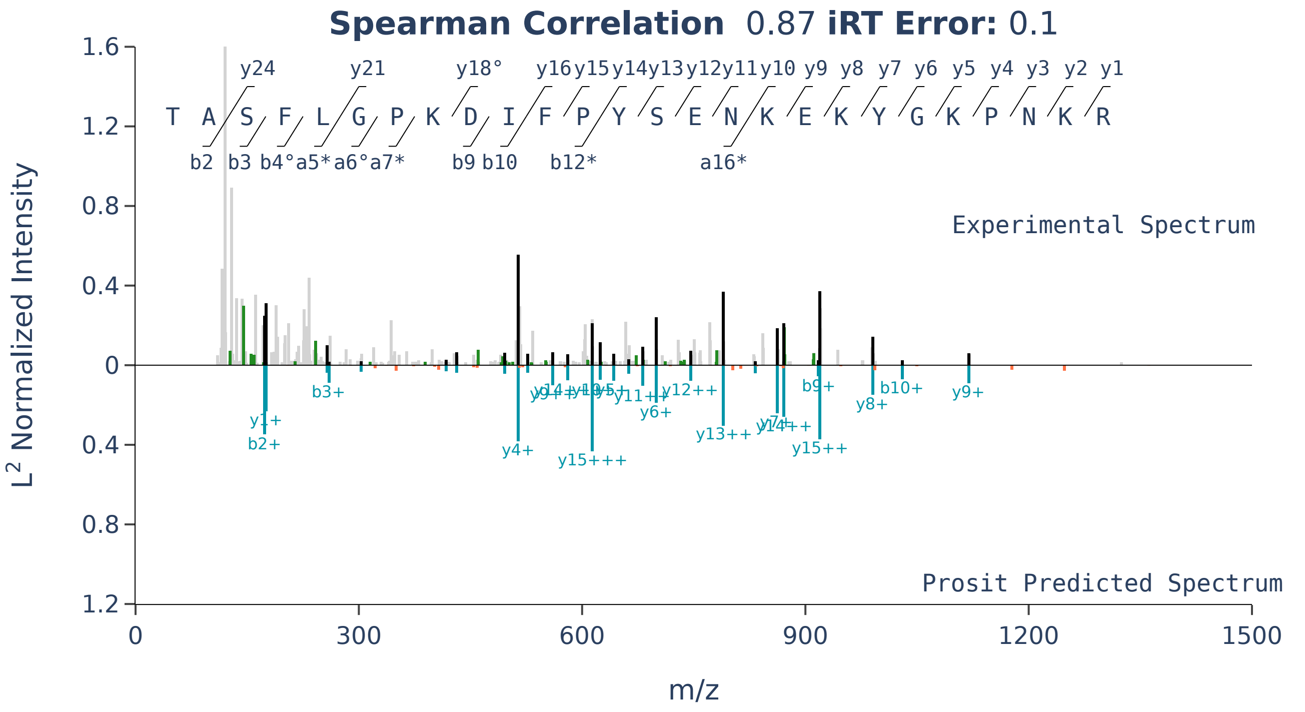

Source HRoetschke_271222_090123_HFGoe_LEDGF_b1R2_24h **Scan** 18399
Peptide KNSLKIDNLDVNRCIEALDELASLQVT **Charge** 5 **Spectral Angle** 0.2
Spearman Correlation 0.06 **iRT Error:** 91.77

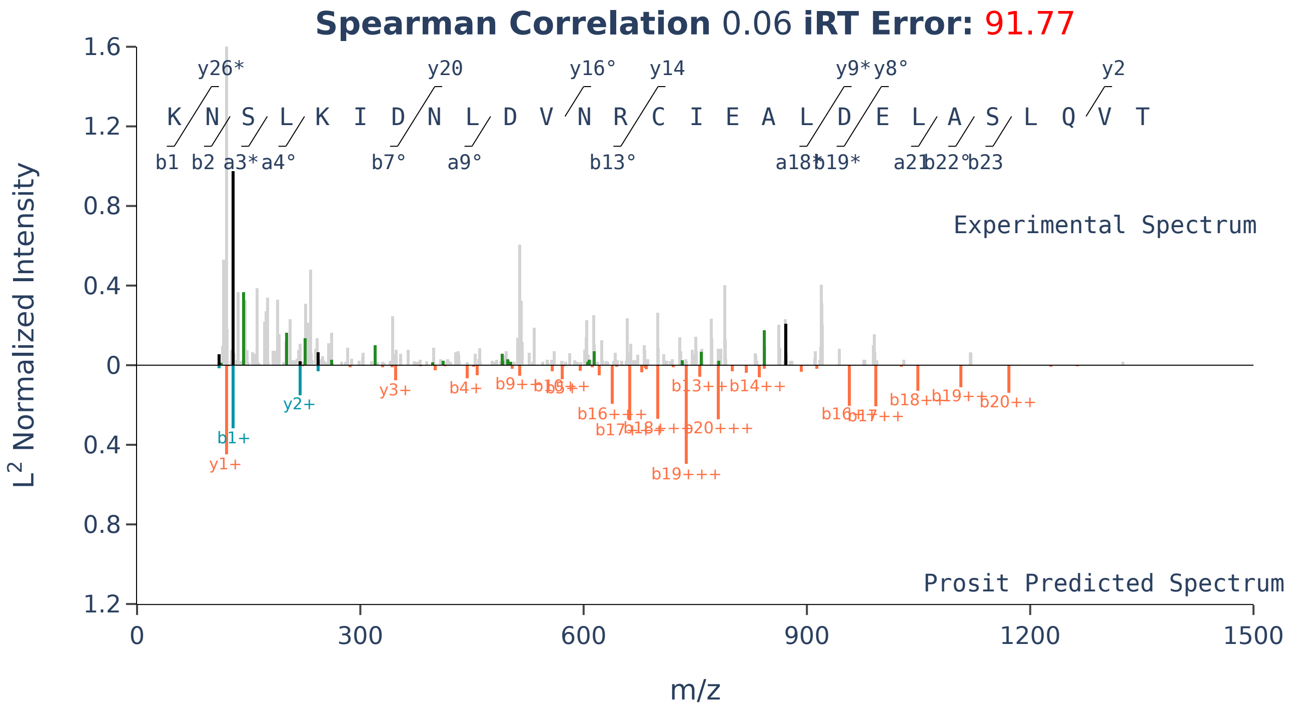

Source HRoetschke_271222_090123_HFGoe_LEDGF_b1R1_4h **Scan** 16428
Peptide QVETEVTTATASVNLKVSPKRGRPAAT **Charge** 4 **Spectral Angle** 0.82
Spearman Correlation 0.85 **iRT Error:** 0.11

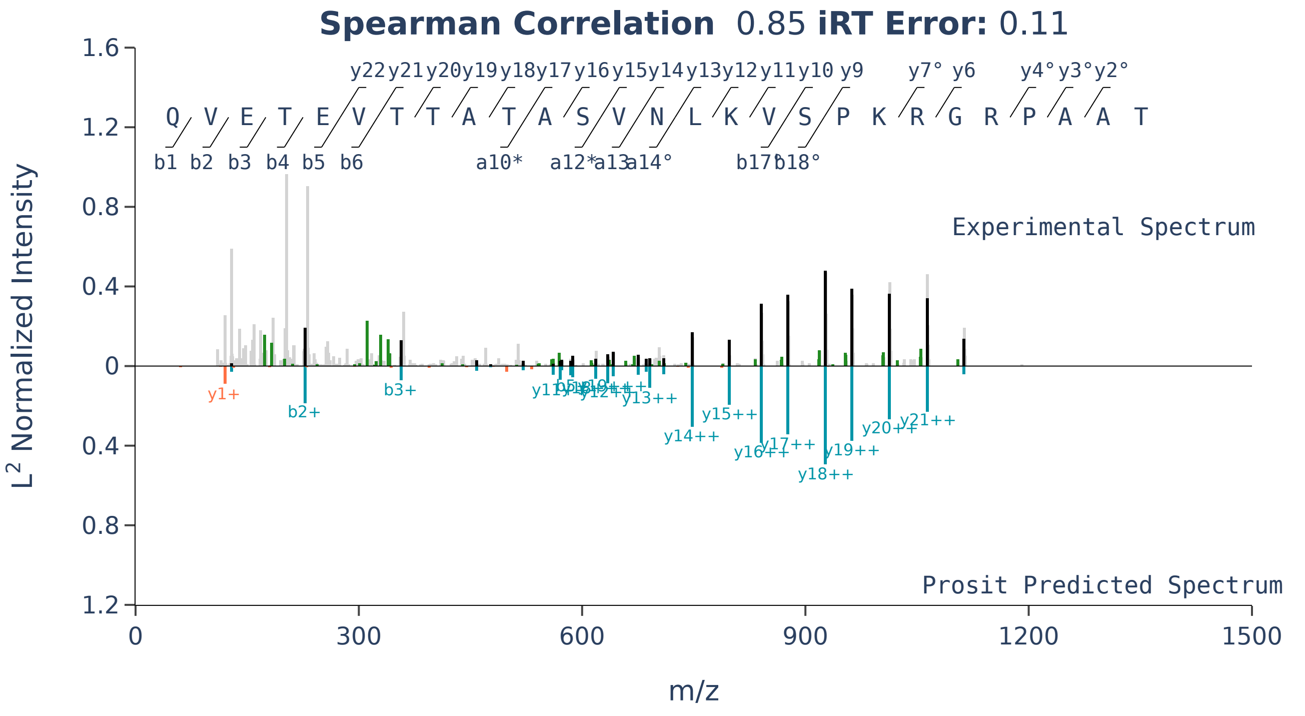

Source HRoetschke_271222_090123_HFGoe_LEDGF_b1R1_4h **Scan** 16428
Peptide TEEAGVVTTATASVNLKVSPKRGRPAAT **Charge** 4 **Spectral Angle** 0.84
Spearman Correlation 0.86 **iRT Error:** 4.95

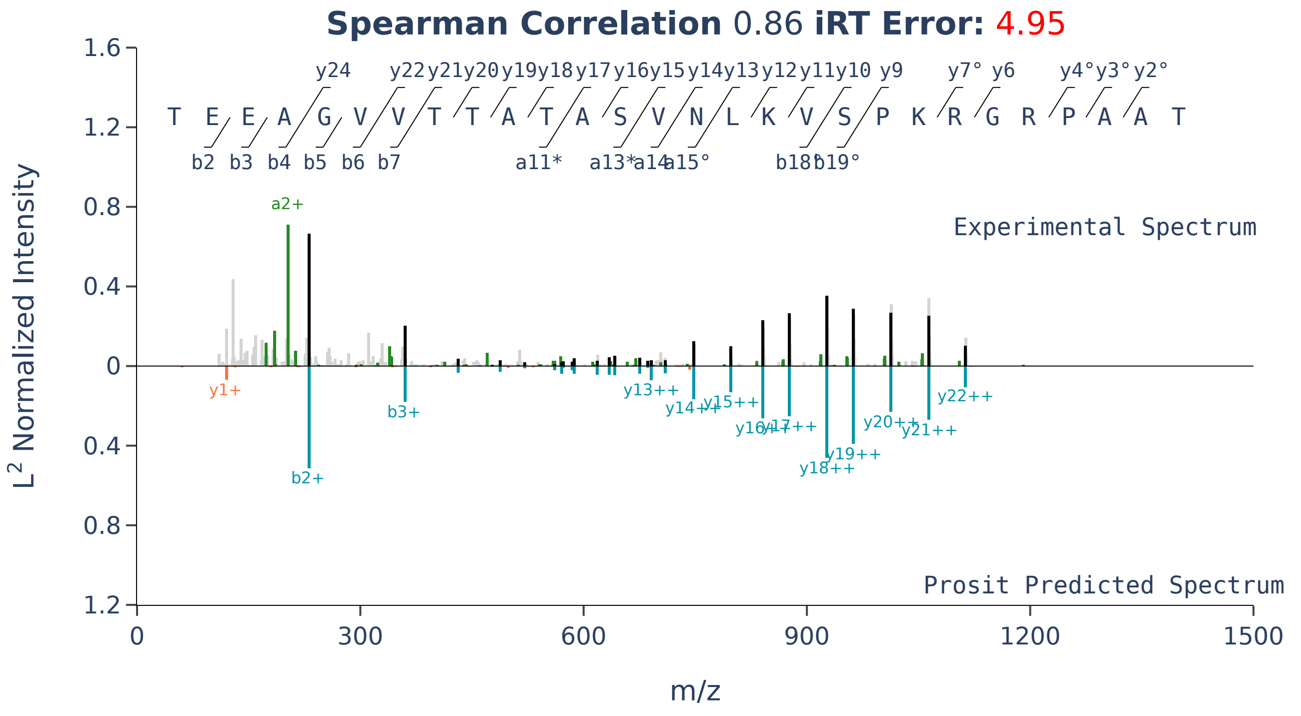

Source HRoetschke_271222_090123_HFGoe_LEDGF_b2R2_2h **Scan** 12081
Peptide SQGPGMTRDFKPGD **Charge** 2 **Spectral Angle** 0.81
Spearman Correlation 0.91 **iRT Error:** 0.12

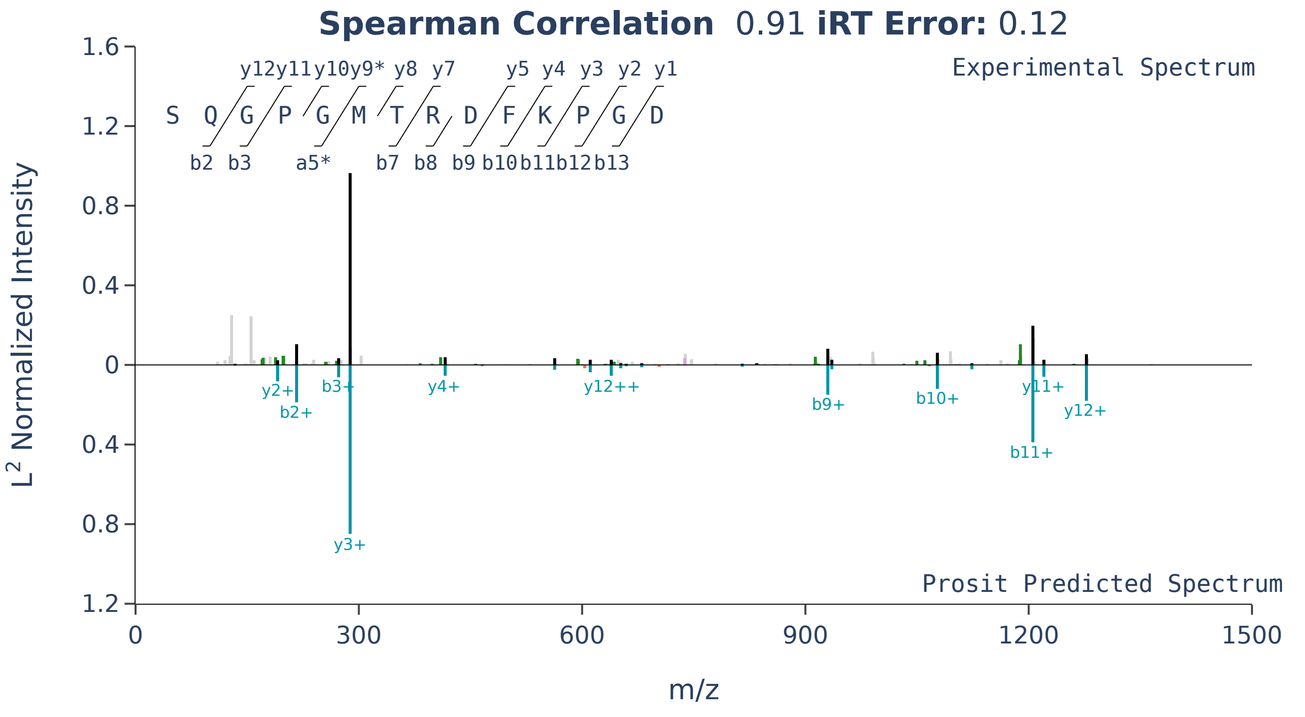

Source HRoetschke_271222_090123_HFGoe_LEDGF_b2R2_2h **Scan** 12081
Peptide EQTGSKTLNGGSDAQ **Charge** 2 **Spectral Angle** 0.01
Spearman Correlation -0.2 **iRT Error:** 38.41

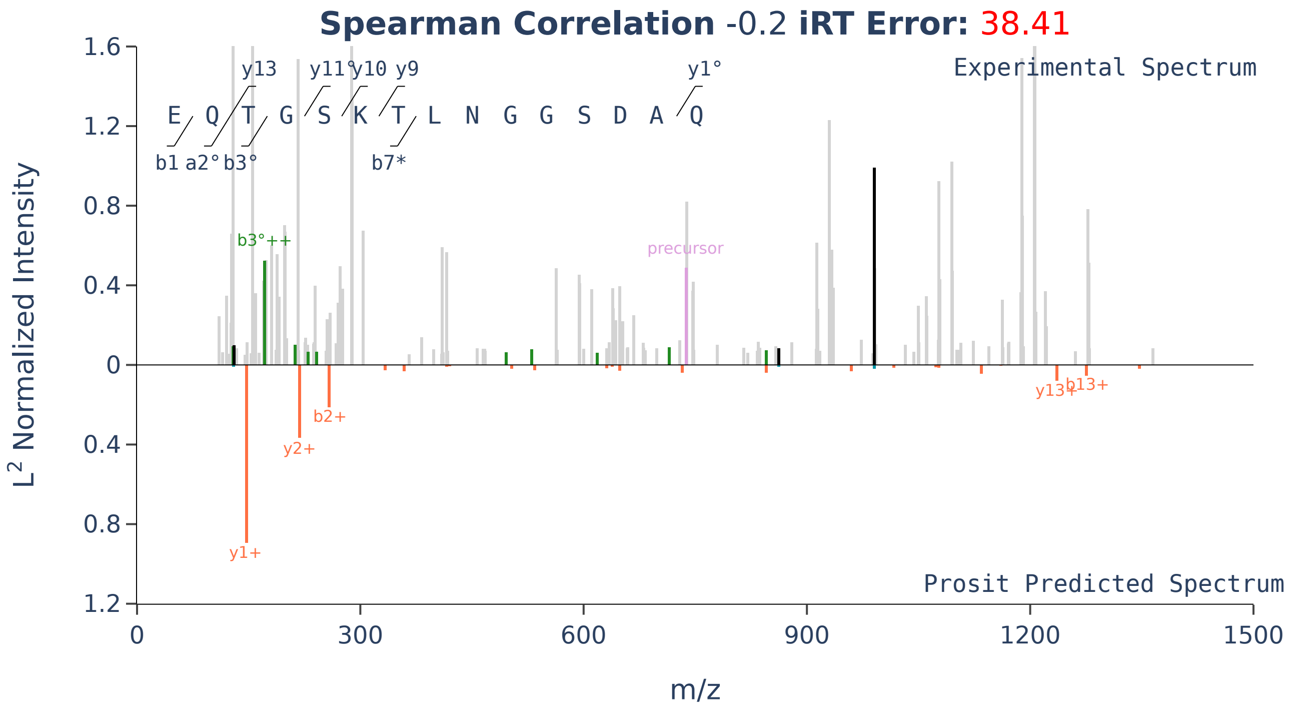

Source HRoetschke_271222_090123_HFGoe_LEDGF_b2R1_4h **Scan** 21199
Peptide ASLKIDNLDVNRCIE **Charge** 3 **Spectral Angle** 0.81
Spearman Correlation 0.89 **iRT Error:** 0.12

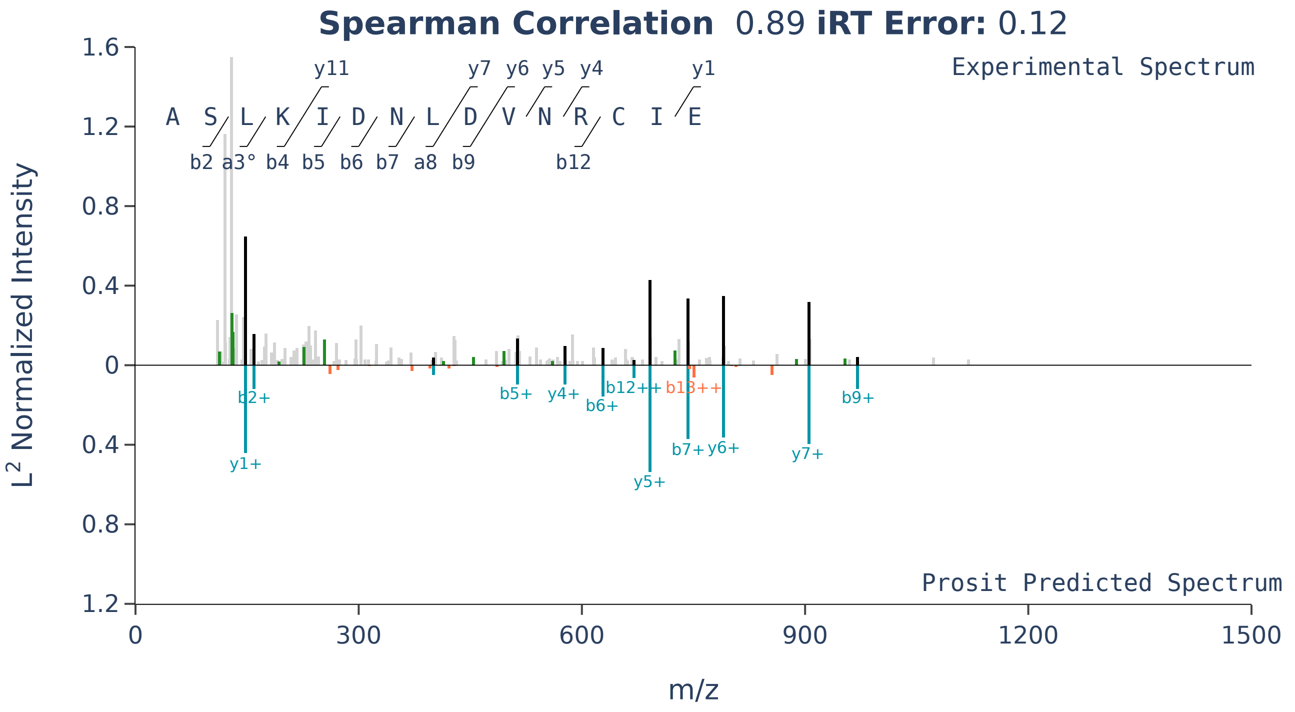

Source HRoetschke_271222_090123_HFGoe_LEDGF_b2R1_4h **Scan** 21199
Peptide SLKIDNLDVNRCIEA **Charge** 3 **Spectral Angle** 0.13
Spearman Correlation 0.14 **iRT Error:** 2.76

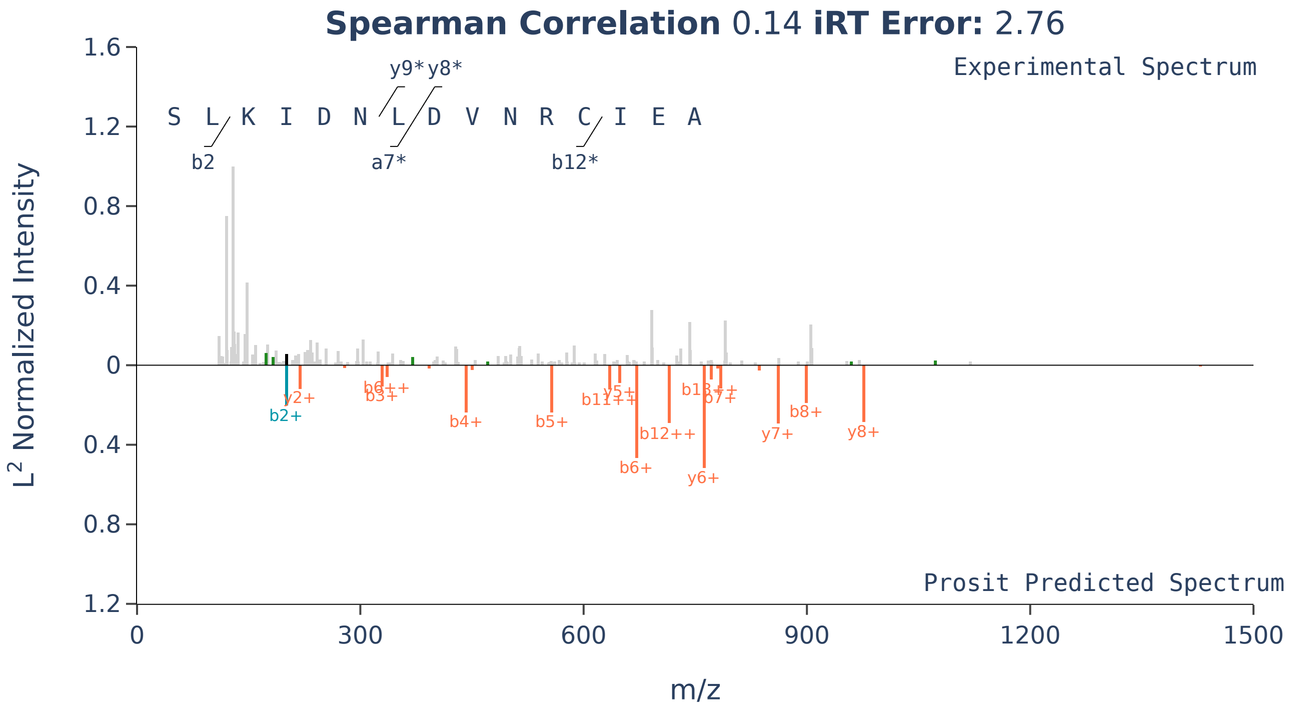

Source HRoetschke_271222_090123_HFGoe_LEDGF_b1R2_4h **Scan** 12670
Peptide LGPKDIFPYSENKEKYGKPNKKR **Charge** 4 **Spectral Angle** 0.75
Spearman Correlation 0.88 **iRT Error:** 0.12

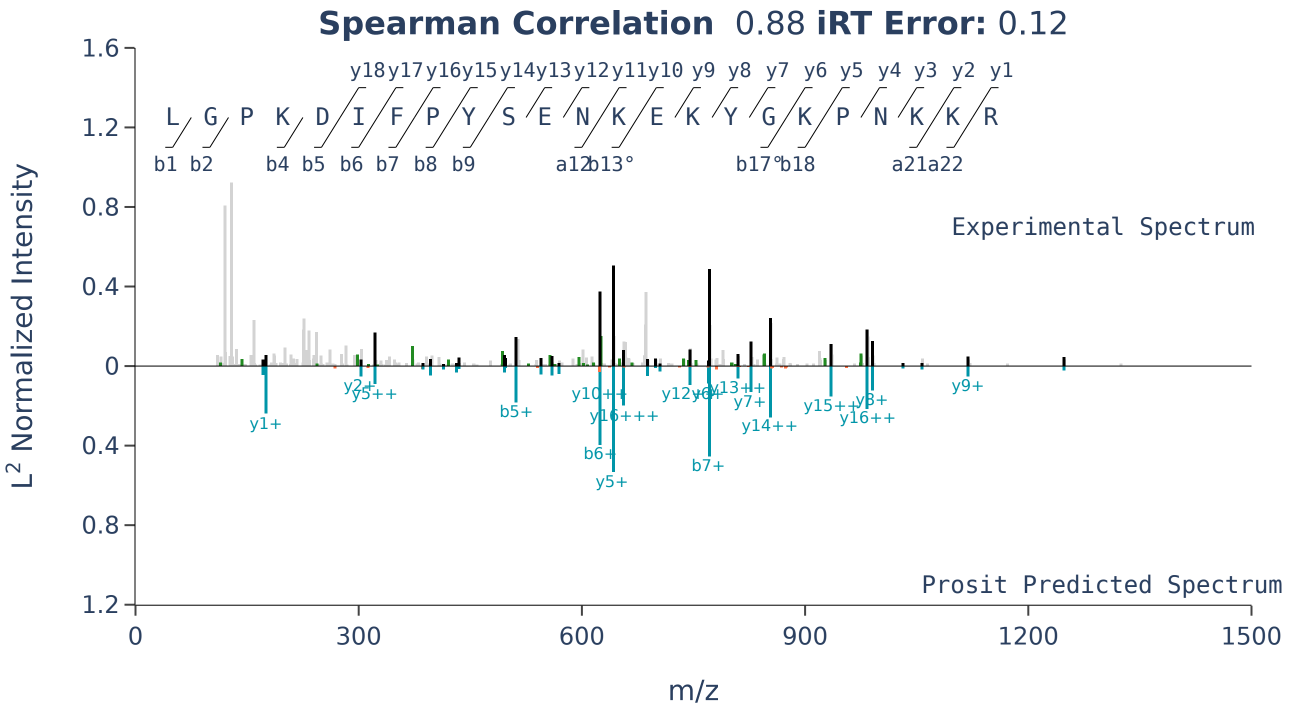

Source HRoetschke_271222_090123_HFGoe_LEDGF_b1R2_4h **Scan** 12670
Peptide LGPKDIFPYSENKEKYGKPNKRK **Charge** 4 **Spectral Angle** 0.76
Spearman Correlation 0.85 **iRT Error:** 0.38

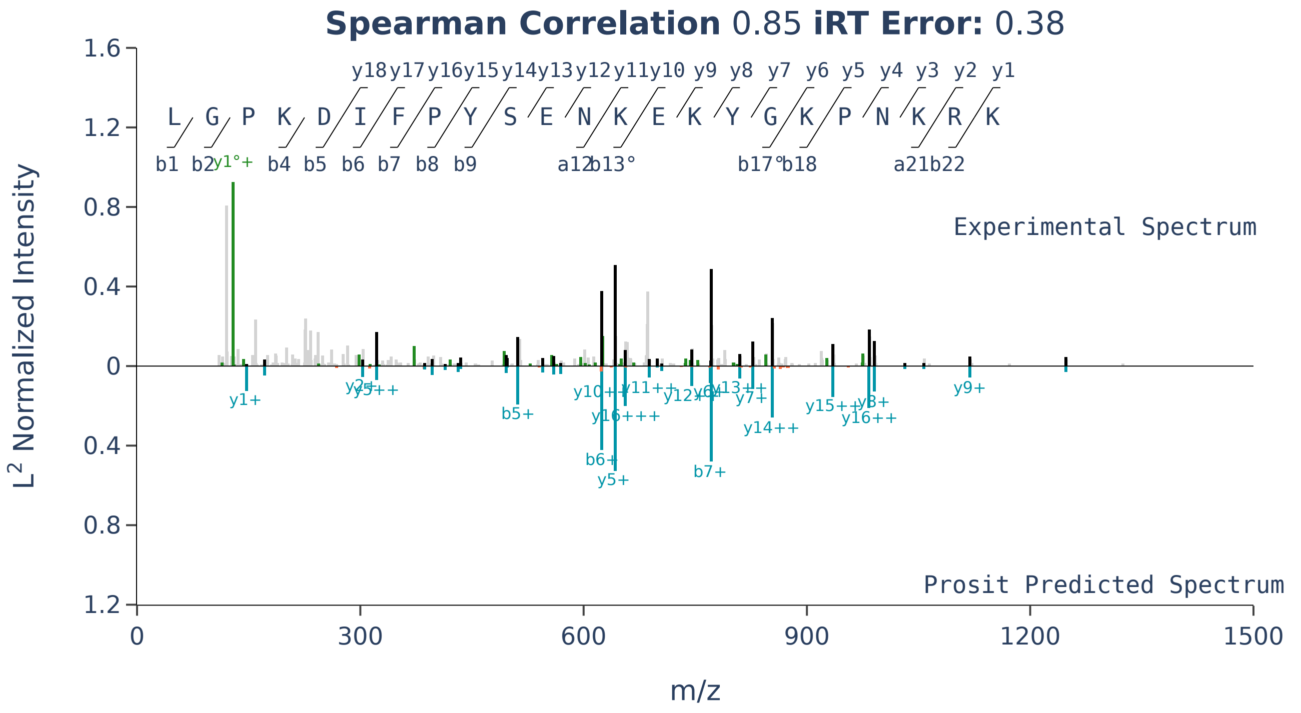

Source HRoetschke_271222_090123_HFGoe_LEDGF_b1R1_2h **Scan** 18708
Peptide TAFLGPKDIFPYSENKEKYGKPNRK **Charge** 4 **Spectral Angle** 0.84
Spearman Correlation 0.83 **iRT Error:** 0.16

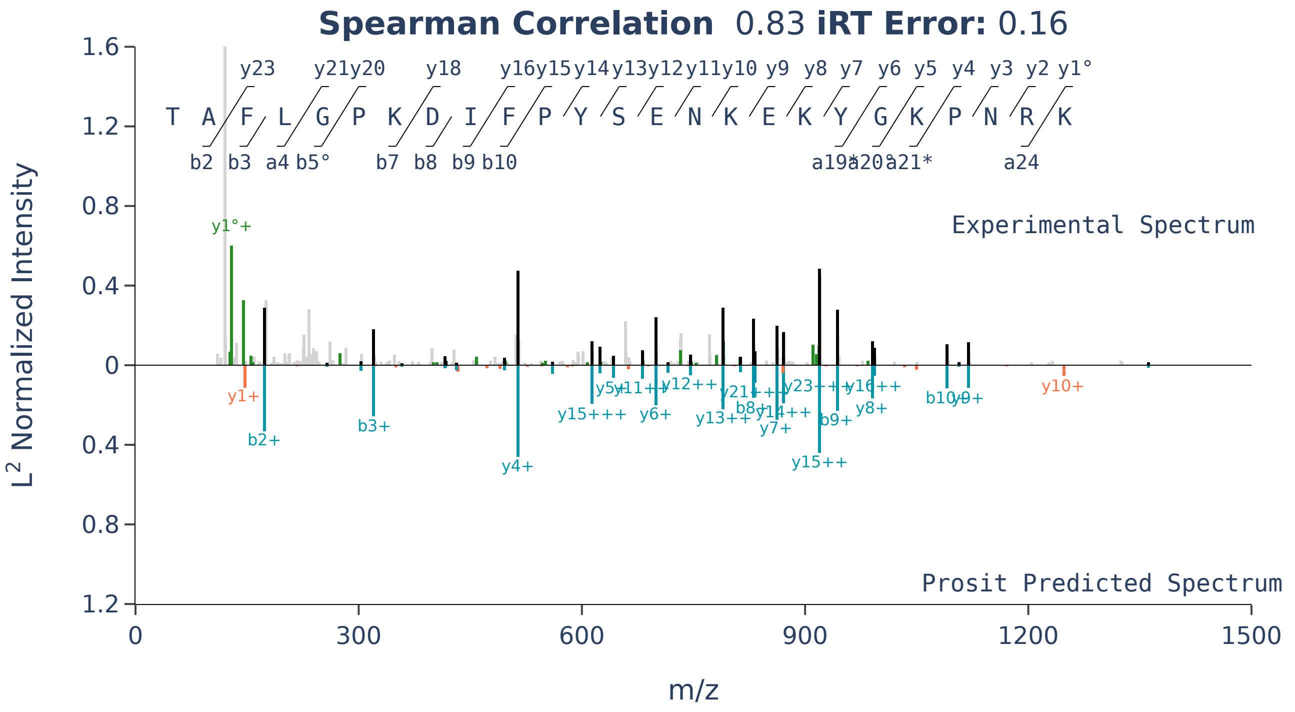

Source HRoetschke_271222_090123_HFGoe_LEDGF_b1R1_2h **Scan** 18708
Peptide TAFLGPKDIFPYSENKEKYGKPNKR **Charge** 4 **Spectral Angle** 0.82
Spearman Correlation 0.81 **iRT Error:** 0.22

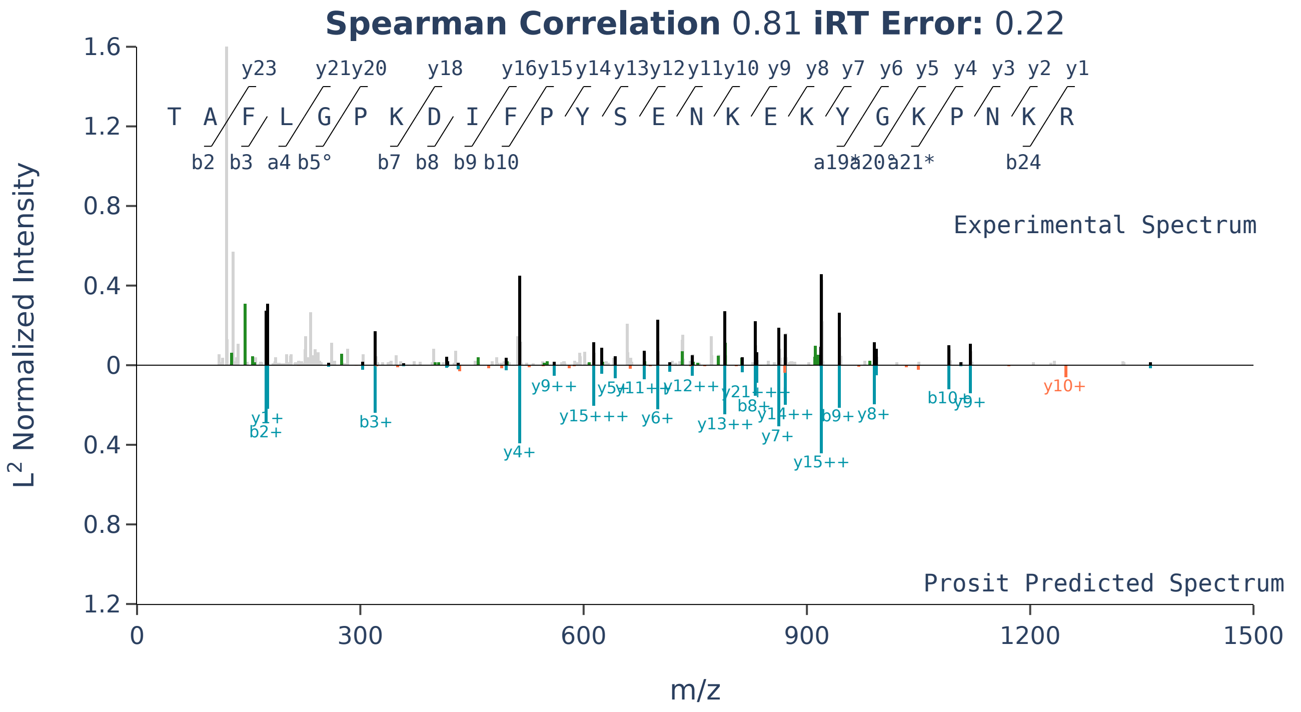

Source HRoetschke_271222_090123_HFGoe_LEDGF_b2R1_4h **Scan** 17472
Peptide AFLGPKDIFPYSENKEKYGKPNKRTA **Charge** 5 **Spectral Angle** 0.74
Spearman Correlation 0.9 **iRT Error:** 0.19

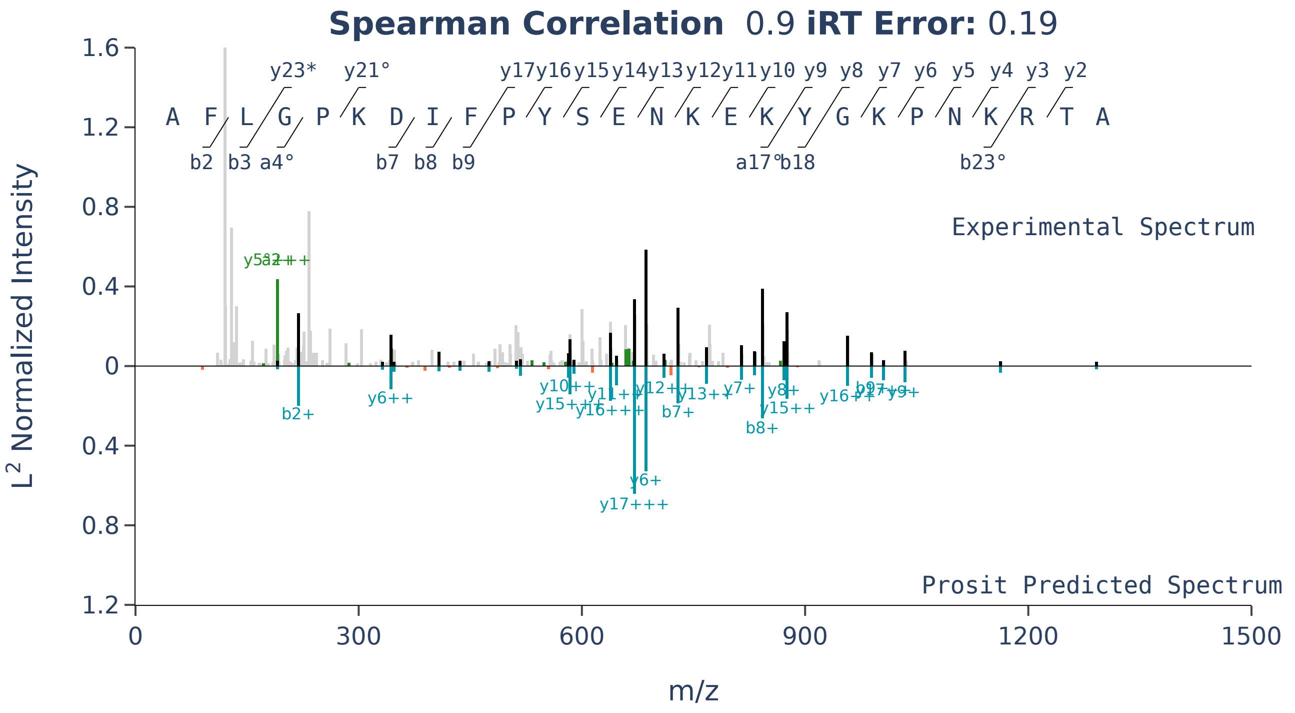

Source HRoetschke_271222_090123_HFGoe_LEDGF_b2R1_4h **Scan** 17472
Peptide WPARVDEVPDGAVKPPTNKLPIFFFGT **Charge** 5 **Spectral Angle** 0.03
Spearman Correlation 0.15 **iRT Error:** 76.54

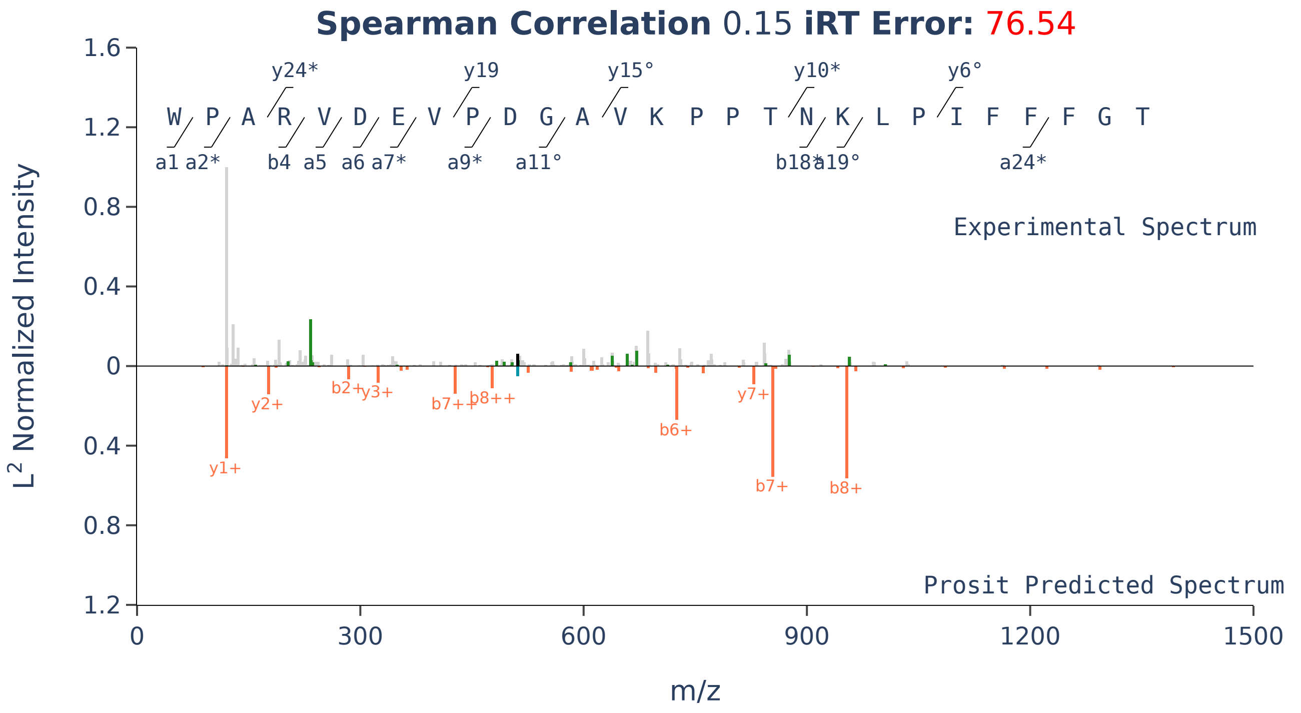

Source HRoetschke_271222_090123_HFGoe_LEDGF_b1R2_1h **Scan** 11974
Peptide TNGPGMTRDFKPGD **Charge** 2 **Spectral Angle** 0.86
Spearman Correlation 0.91 **iRT Error:** 0.24

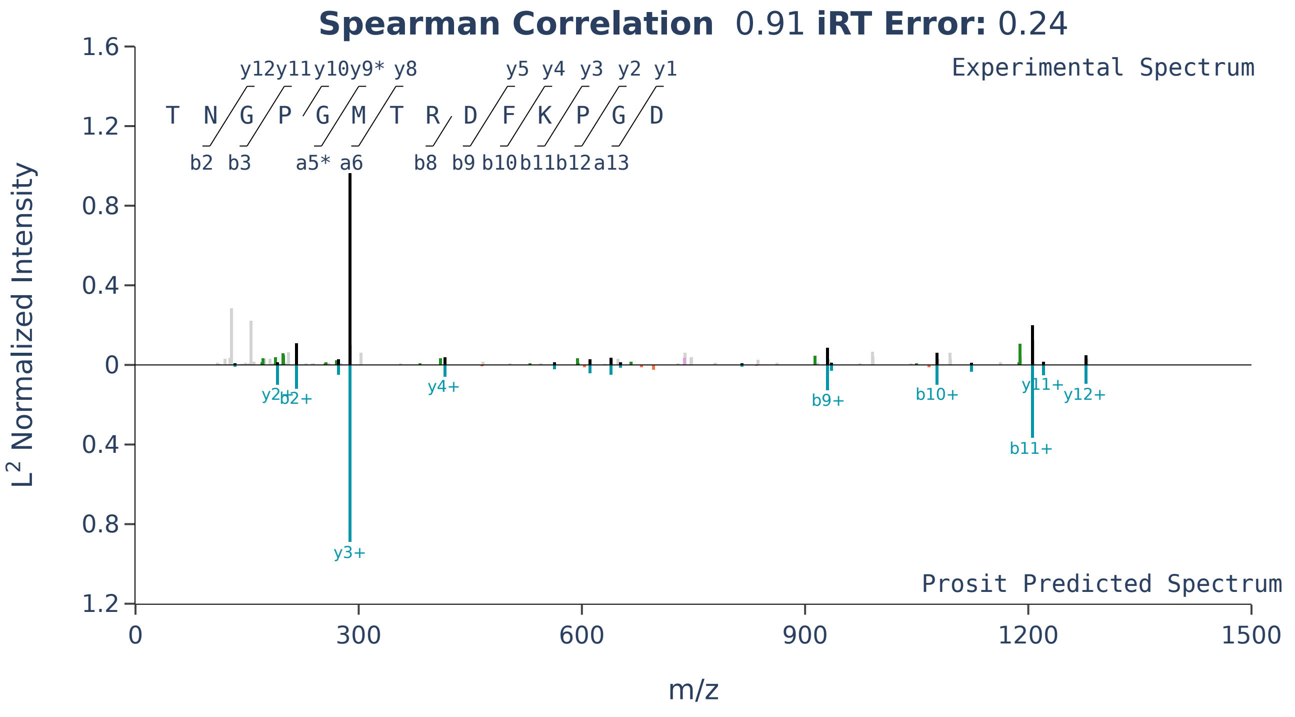

Source HRoetschke_271222_090123_HFGoe_LEDGF_b1R2_1h **Scan** 11974
Peptide EQTGSKTLNGGSDAQ **Charge** 2 **Spectral Angle** 0.01
Spearman Correlation -0.17 **iRT Error:** 38.73

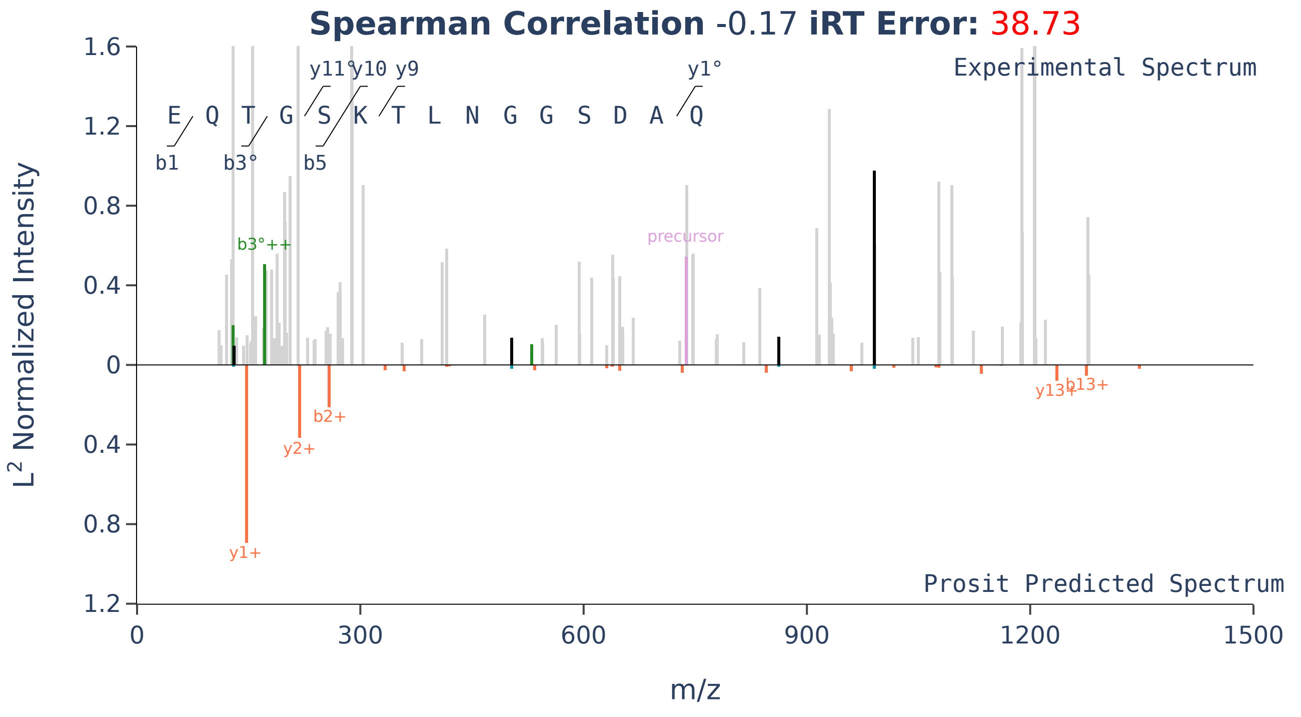

Source HRoetschke_271222_090123_HFGoe_LEDGF_b2R1_2h **Scan** 11805
Peptide AGPGMTRDFKPGD **Charge** 3 **Spectral Angle** 0.9
Spearman Correlation 0.9 **iRT Error:** 0.25

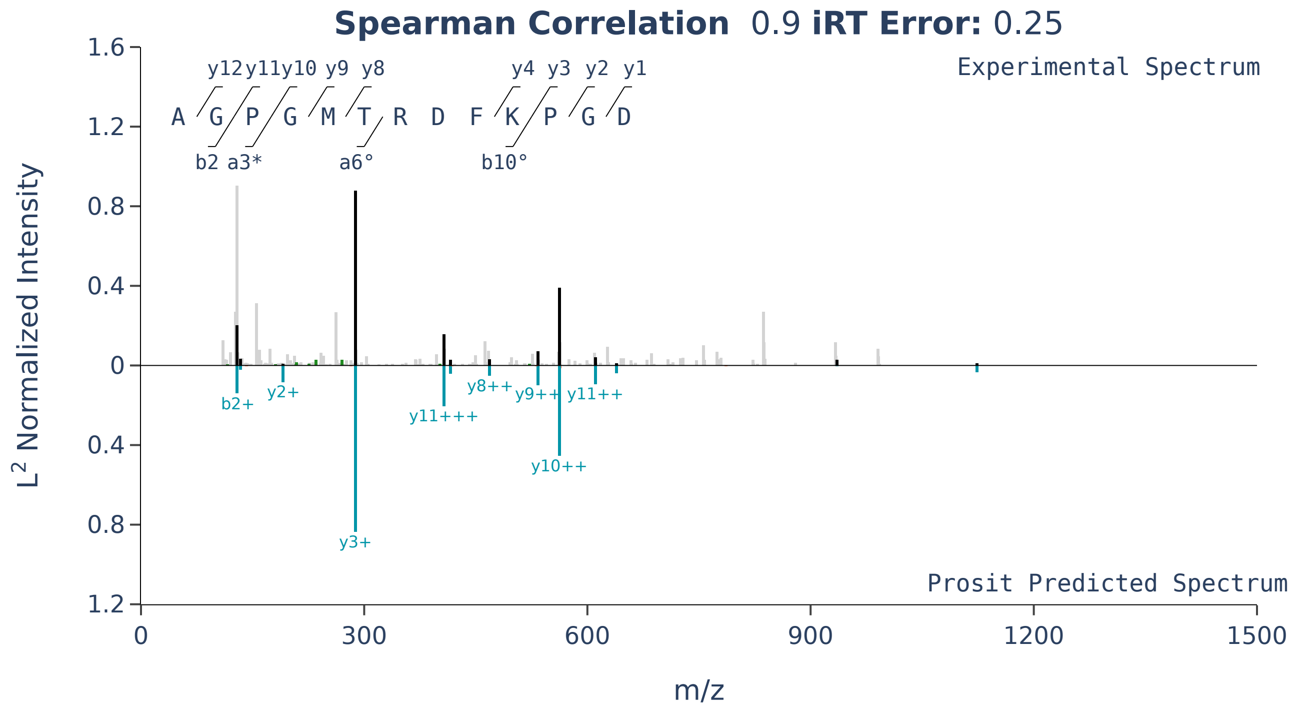

Source HRoetschke_271222_090123_HFGoe_LEDGF_b2R1_2h **Scan** 11805
Peptide KMVKQPCPSESD **Charge** 3 **Spectral Angle** 0.38
Spearman Correlation 0.52 **iRT Error:** 33.36

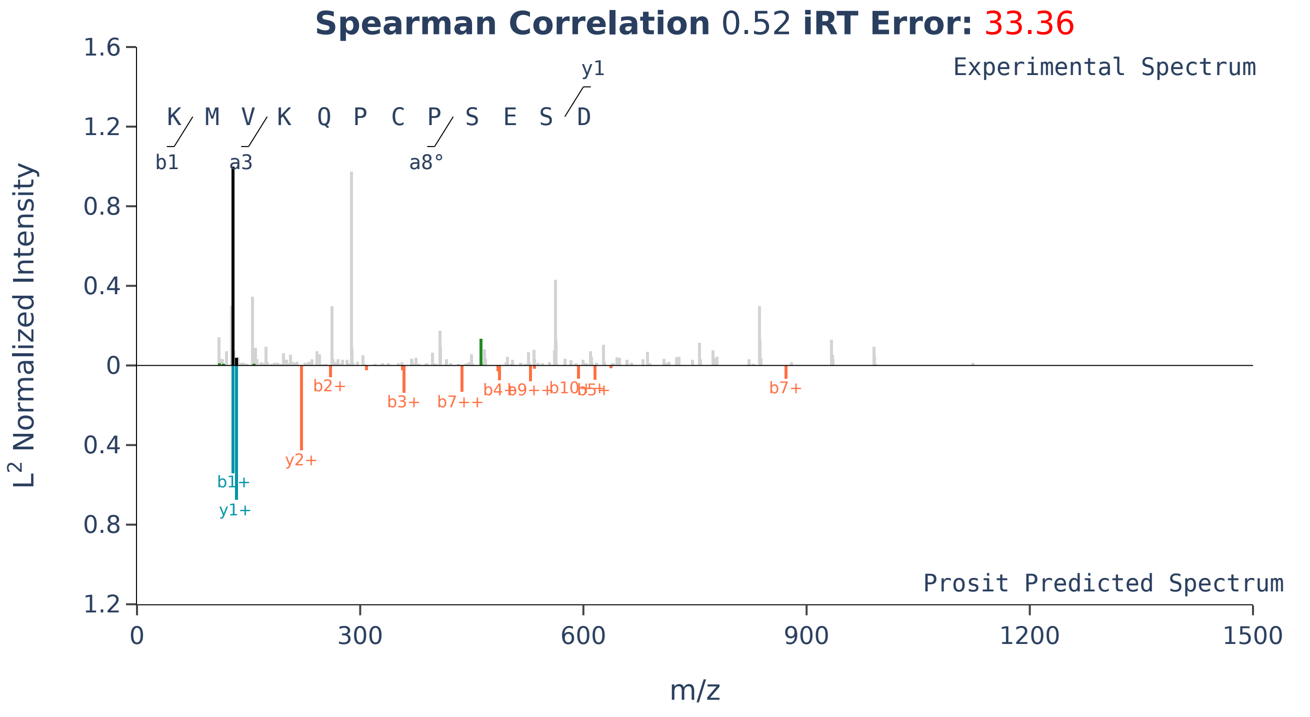

Source HRoetschke_271222_090123_HFGoe_LEDGF_b1R1_24h **Scan** 17501
Peptide NASYPHWPARVDE **Charge** 3 **Spectral Angle** 0.87
Spearman Correlation 0.87 **iRT Error:** 0.27

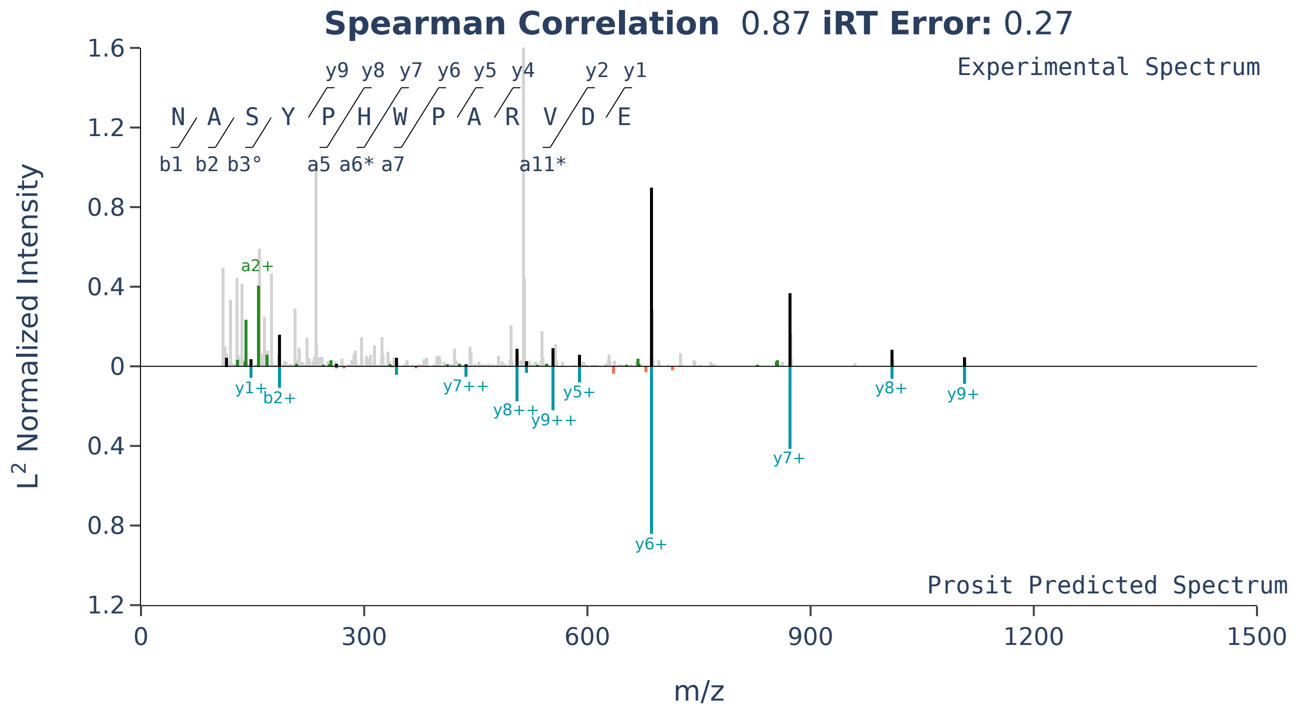

Source HRoetschke_271222_090123_HFGoe_LEDGF_b1R1_24h **Scan** 17501
Peptide FSSQQAATKQSNASS **Charge** 3 **Spectral Angle** 0.01
Spearman Correlation -0.2 **iRT Error:** 73.32

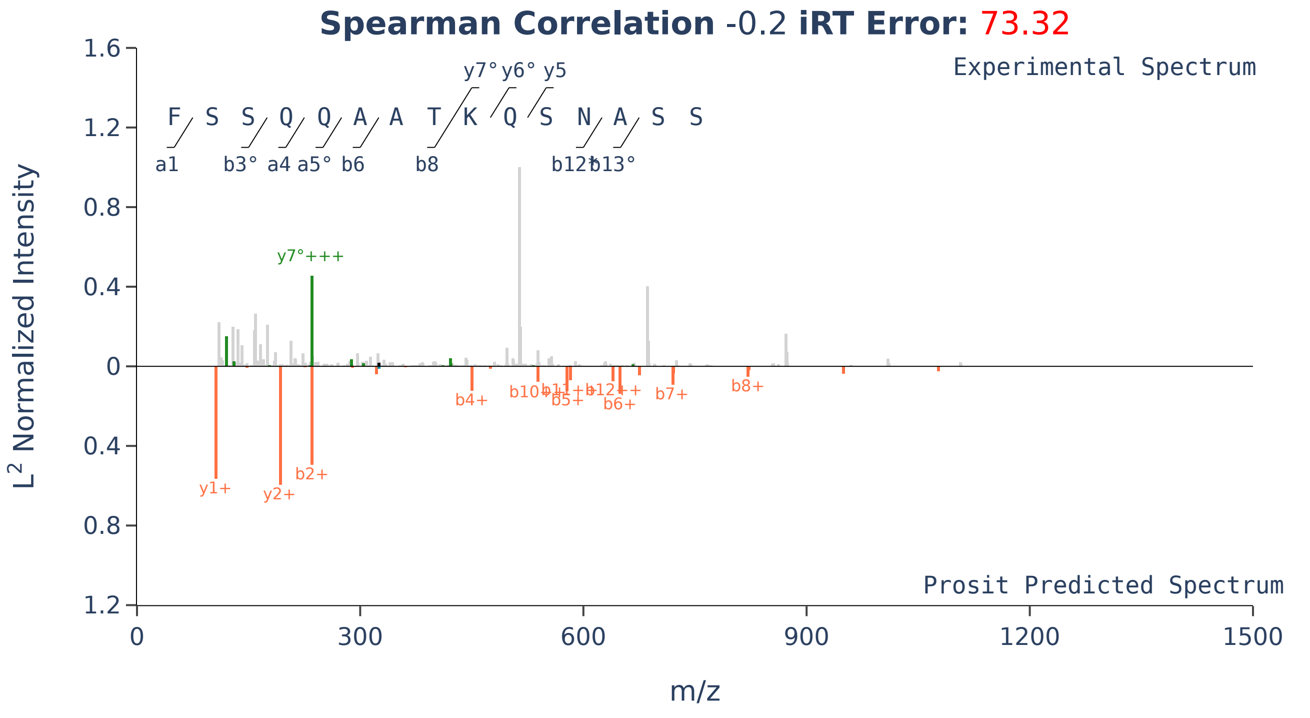

Source HRoetschke_271222_090123_HFGoe_LEDGF_b1R1_4h **Scan** 19813
Peptide DNNPKVKFSAF **Charge** 3 **Spectral Angle** 0.93
Spearman Correlation 0.78 **iRT Error:** 0.33

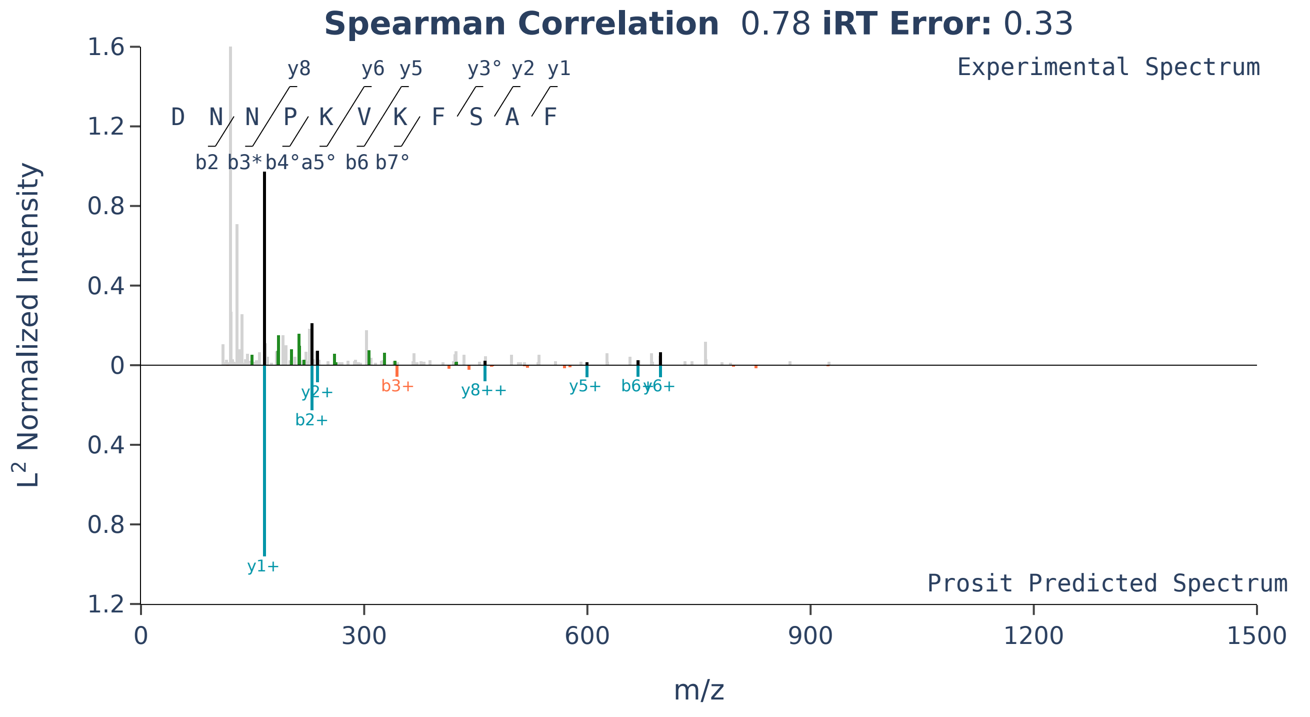

Source HRoetschke_271222_090123_HFGoe_LEDGF_b1R1_4h **Scan** 19813
Peptide SQVIMEKSTML **Charge** 3 **Spectral Angle** 0.22
Spearman Correlation 0.31 **iRT Error:** 7.16

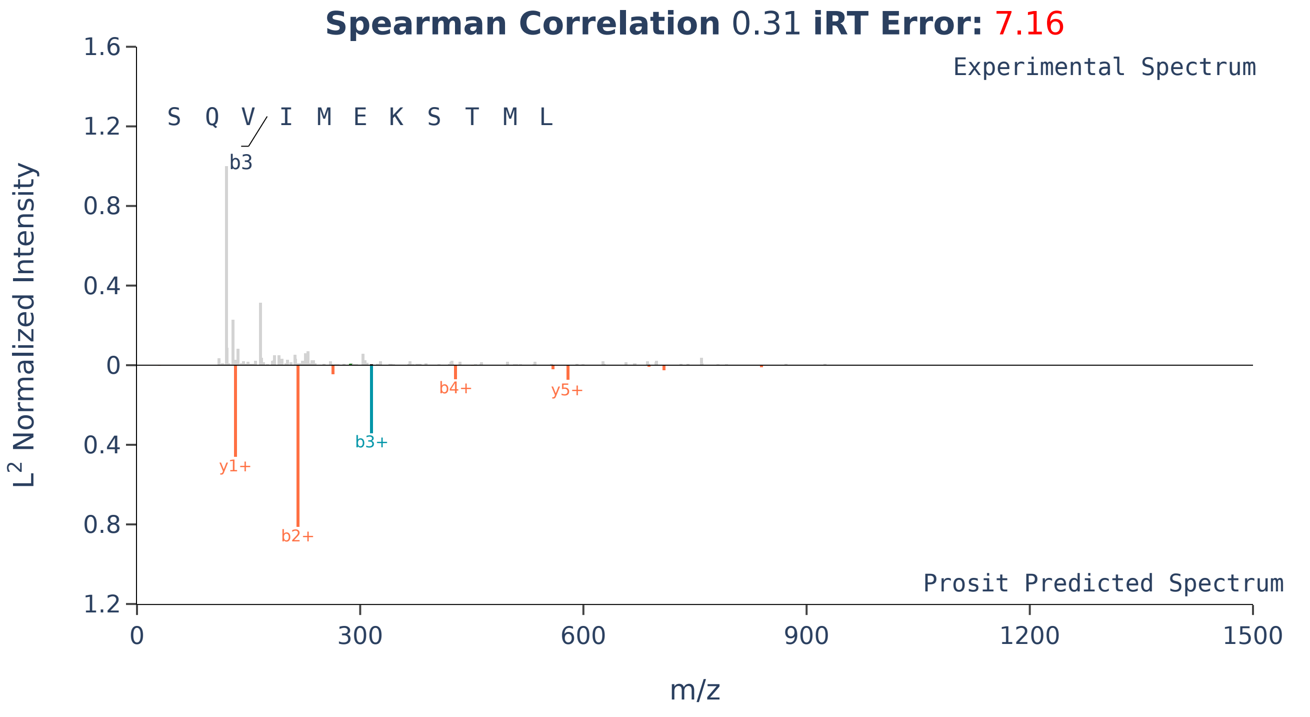

Source HRoetschke_271222_090123_HFGoe_LEDGF_b1R1_4h **Scan** 10790
Peptide SVNLKVSPKRGRPAATATEV **Charge** 5 **Spectral Angle** 0.86
Spearman Correlation 0.82 **iRT Error:** 0.35

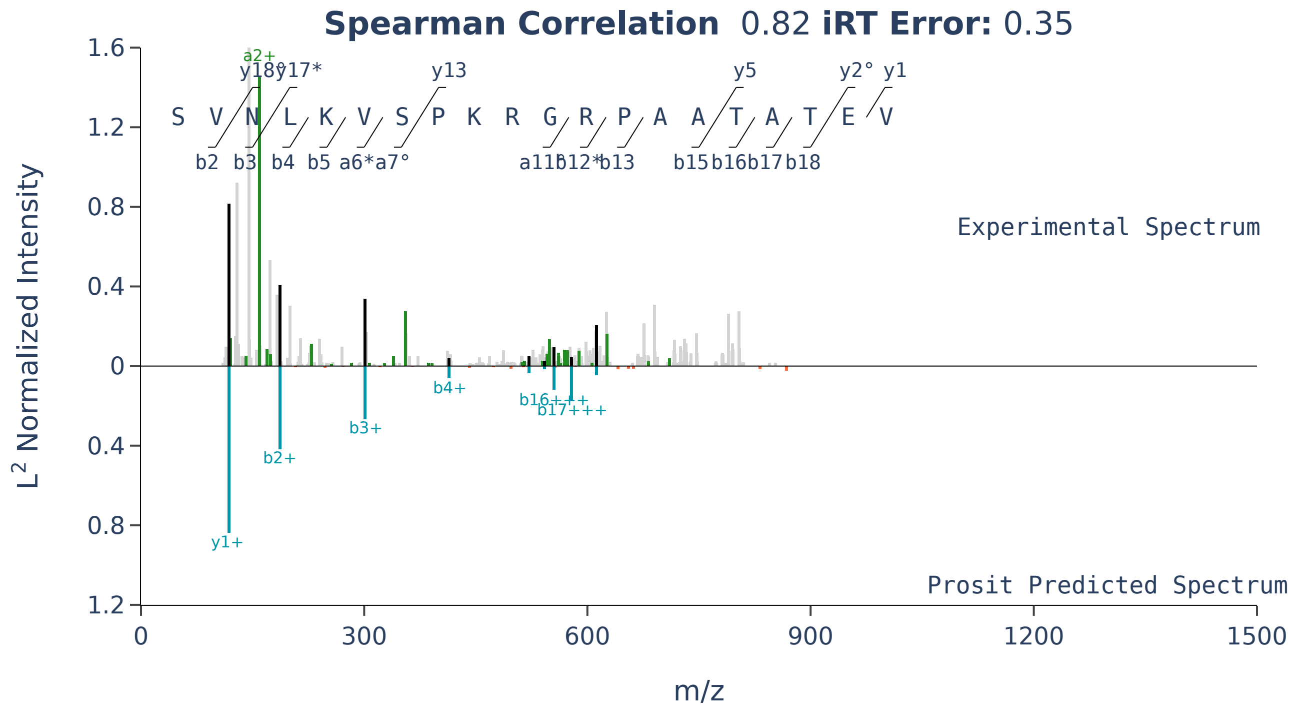

Source HRoetschke_271222_090123_HFGoe_LEDGF_b1R1_4h **Scan** 10790
Peptide TASVNLKVSPKRGRPAATEV **Charge** 5 **Spectral Angle** 0.51
Spearman Correlation 0.32 **iRT Error:** 1.48

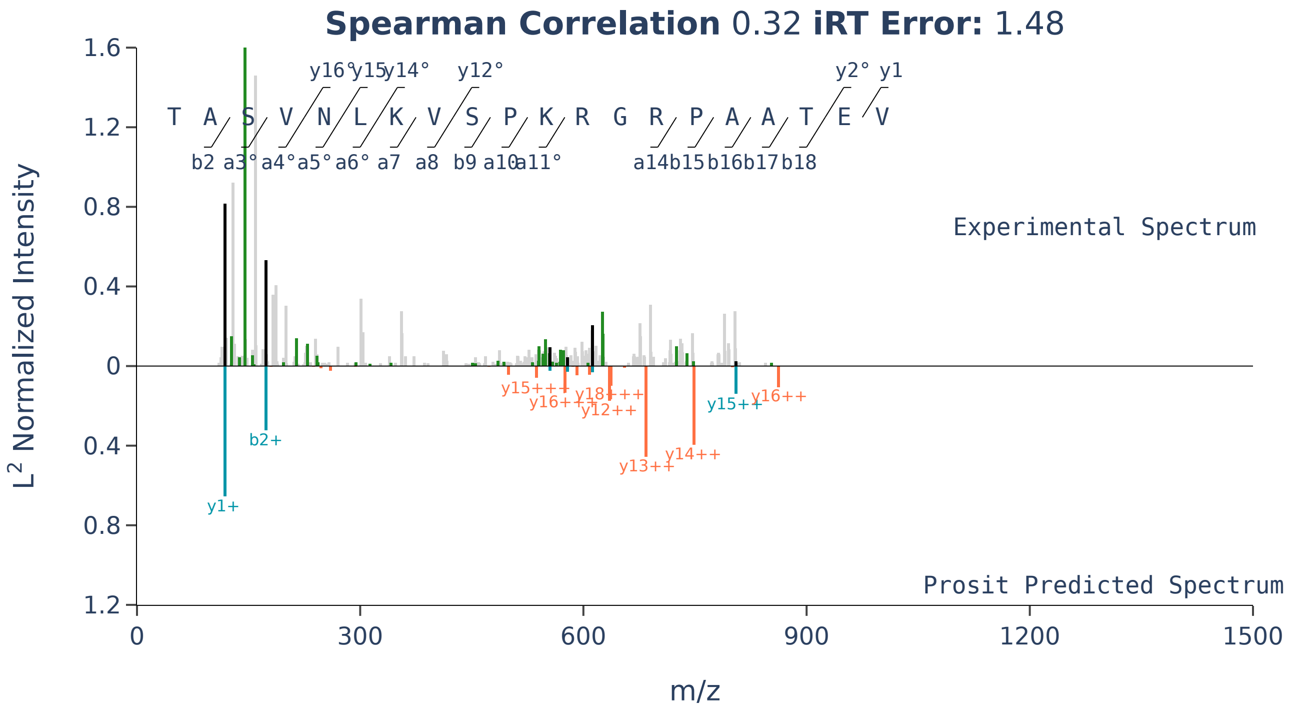

Source HRoetschke_271222_090123_HFGoe_LEDGF_b2R1_2h **Scan** 13330
Peptide QSNPHWPARVDE **Charge** 3 **Spectral Angle** 0.88
Spearman Correlation 0.87 **iRT Error:** 0.39

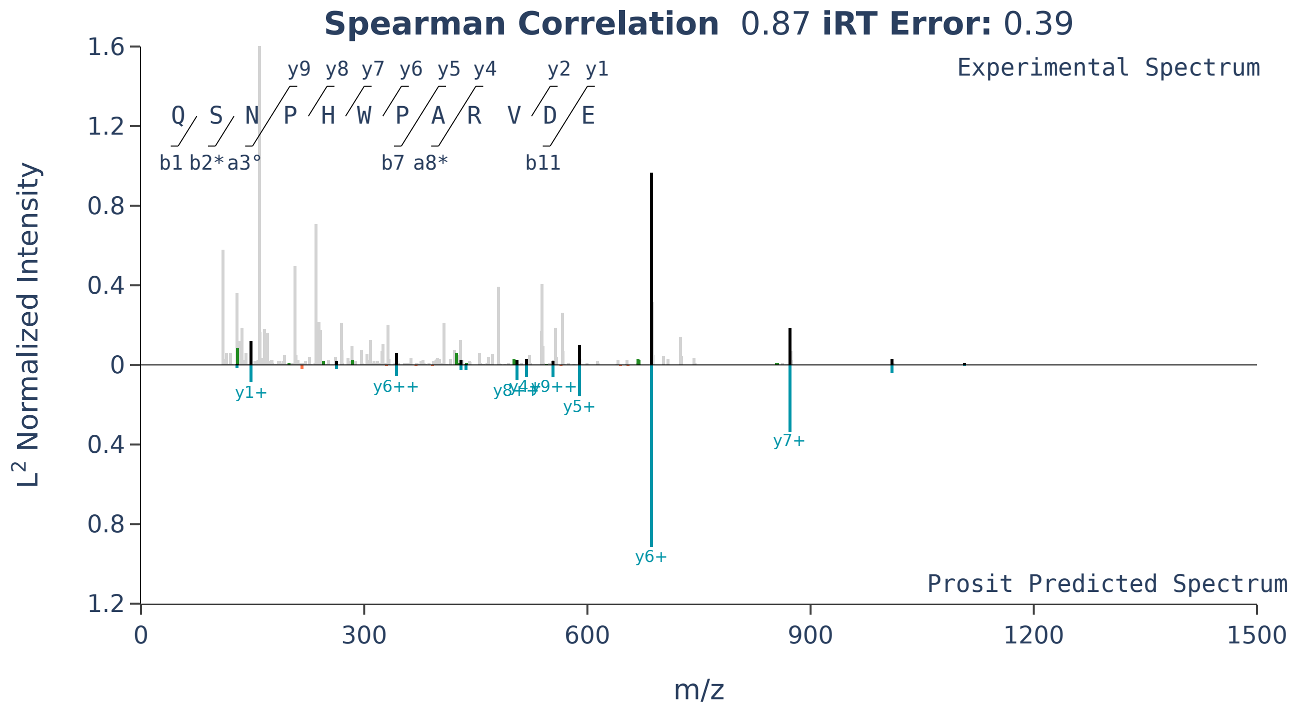

Source HRoetschke_271222_090123_HFGoe_LEDGF_b2R1_2h **Scan** 13330
Peptide QAATKQSNASSDVE **Charge** 3 **Spectral Angle** 0.84
Spearman Correlation 0.29 **iRT Error:** 48.69

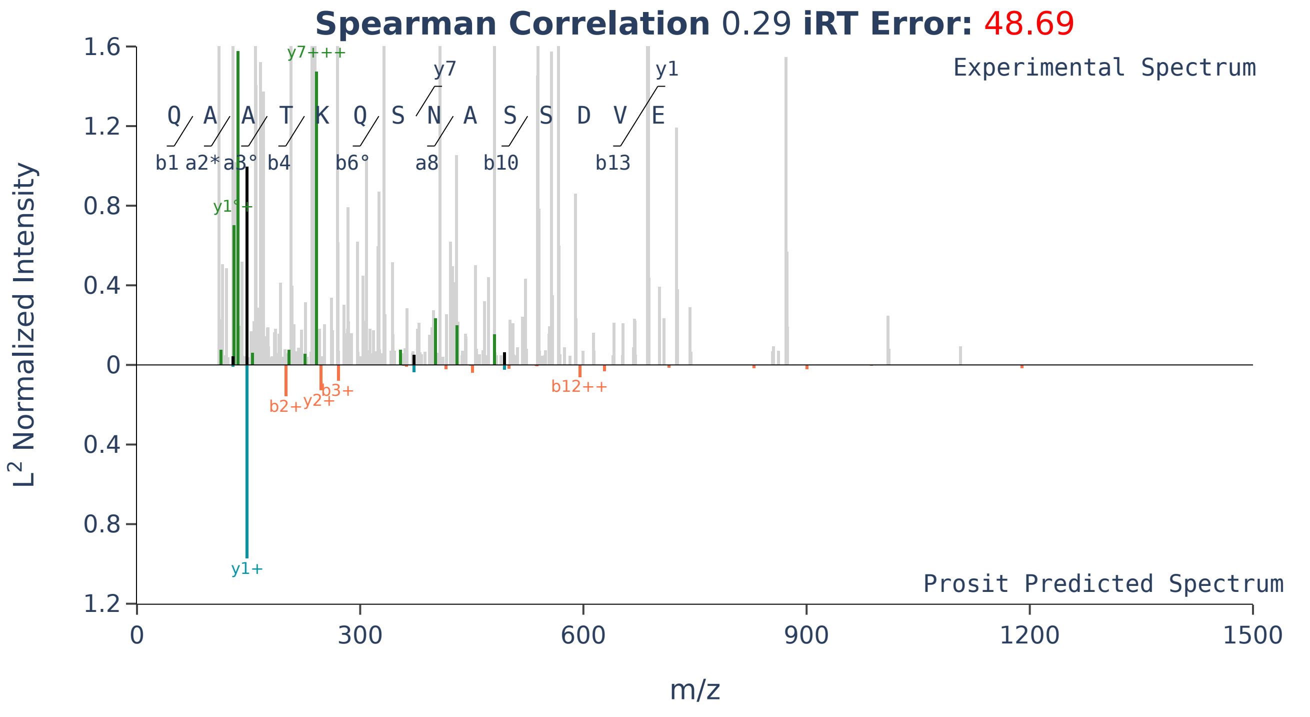

Source HRoetschke_271222_090123_HFGoe_LEDGF_b1R1_2h **Scan** 17236
Peptide VVKIDNLDVNRC **Charge** 2 **Spectral Angle** 0.83
Spearman Correlation 0.85 **iRT Error:** 0.41

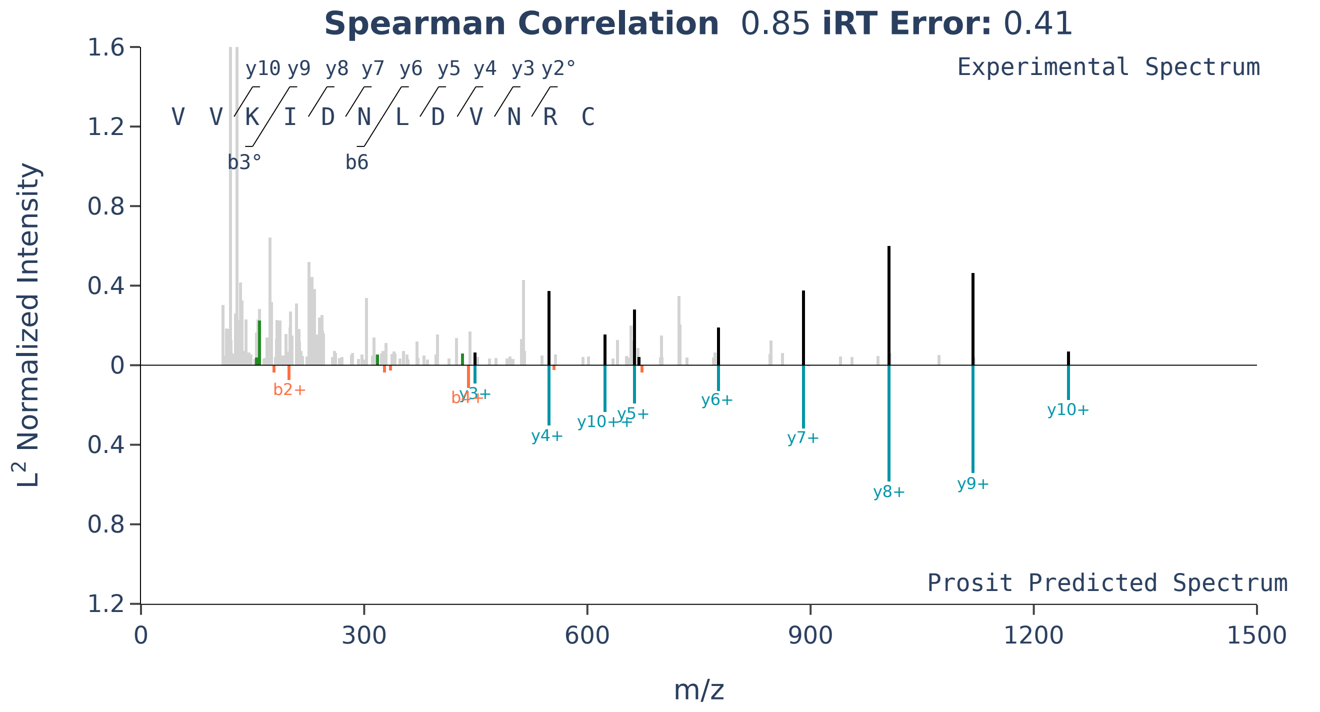

Source HRoetschke_271222_090123_HFGoe_LEDGF_b1R1_2h **Scan** 17236
Peptide DQEGEKKRKGGR **Charge** 2 **Spectral Angle** 0.11
Spearman Correlation 0.4 **iRT Error:** 21.57

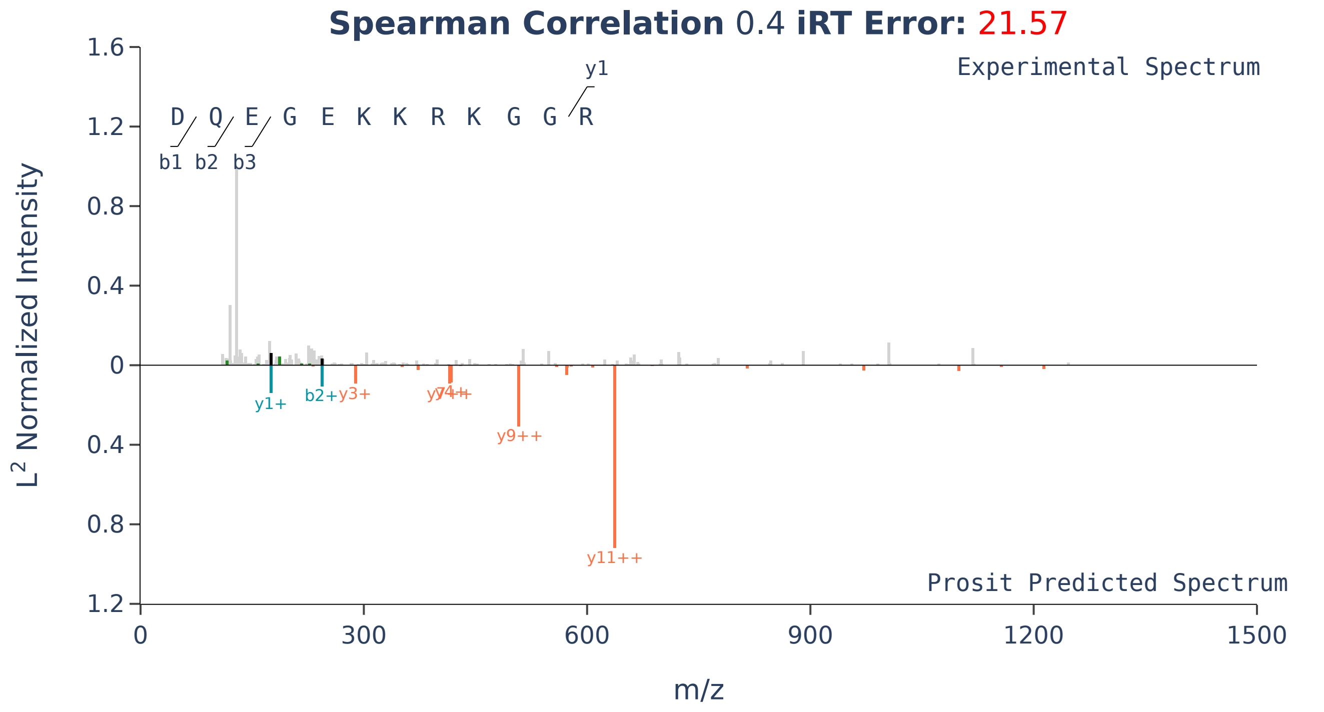

Source HRoetschke_271222_090123_HFGoe_LEDGF_b2R1_24h **Scan** 20873
Peptide SMDSRLQRIEL **Charge** 3 **Spectral Angle** 0.89
Spearman Correlation 0.85 **iRT Error:** 0.46

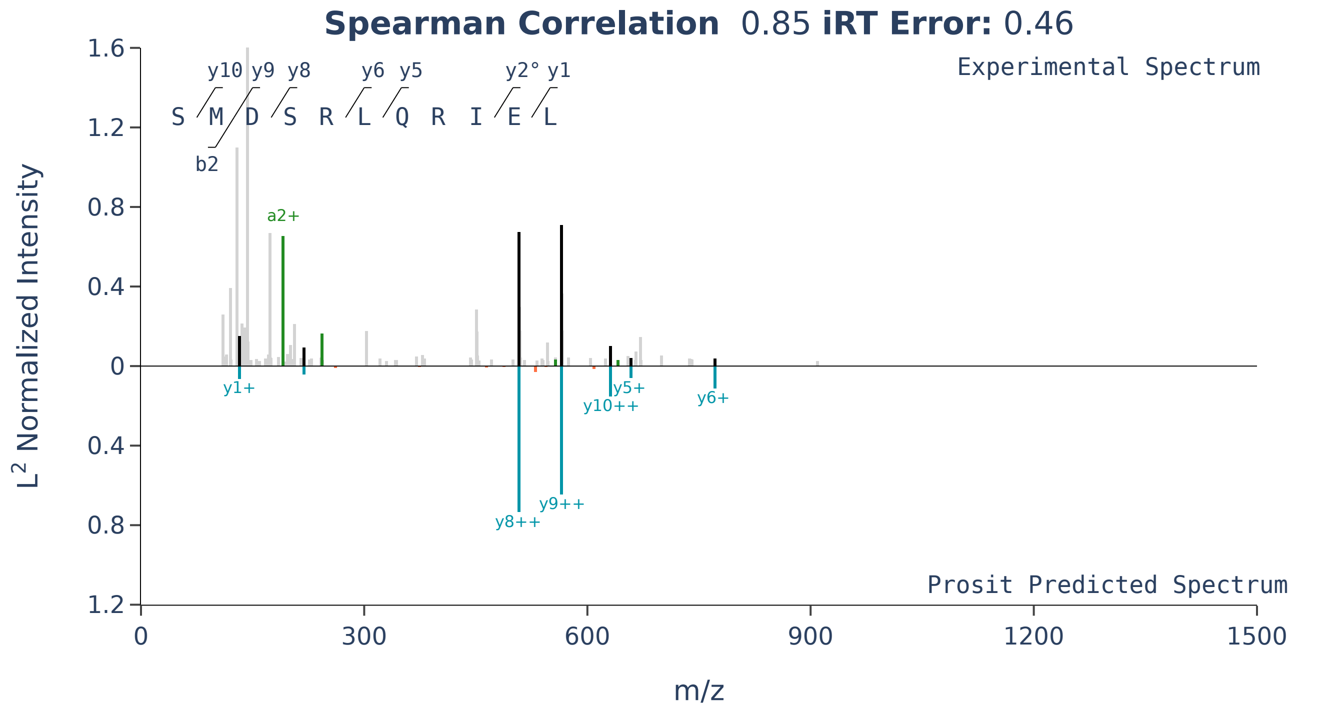

Source HRoetschke_271222_090123_HFGoe_LEDGF_b2R1_24h **Scan** 20873
Peptide LEKEQTGSKTLN **Charge** 3 **Spectral Angle** 0.49
Spearman Correlation 0.46 **iRT Error:** 87.04

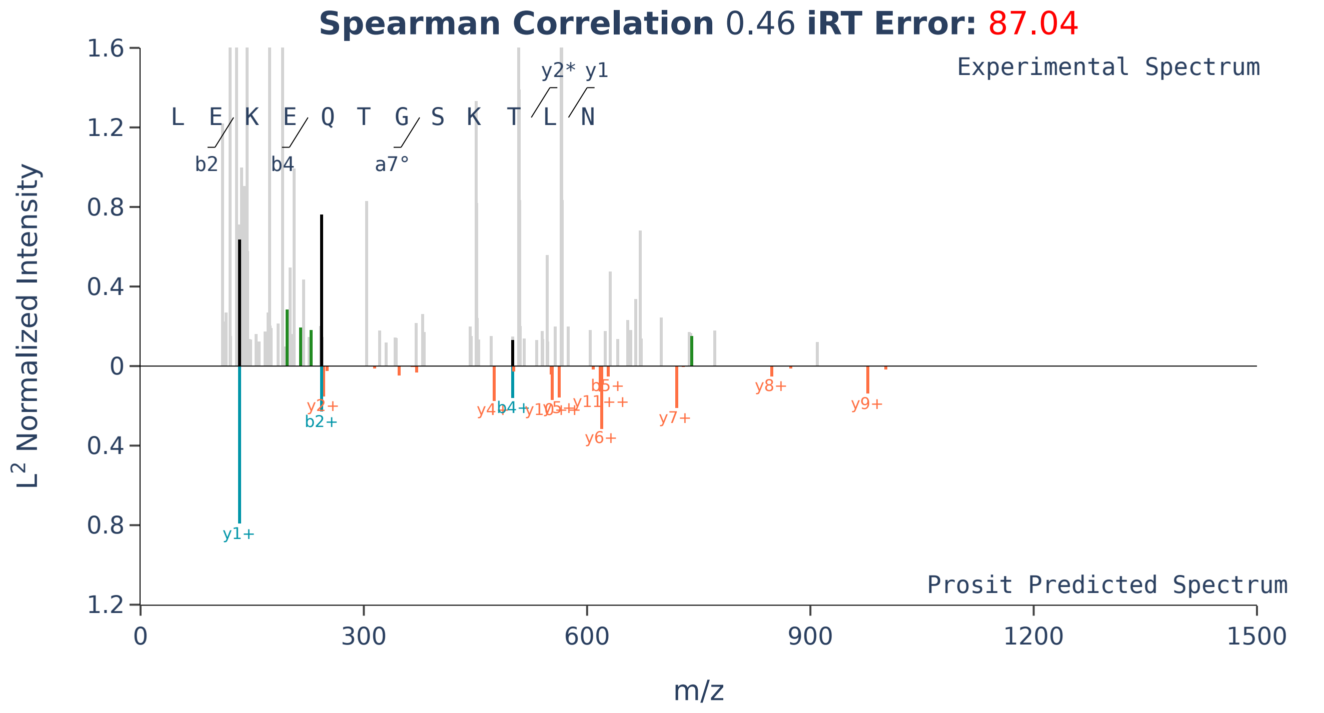

Source HRoetschke_271222_090123_HFGoe_LEDGF_b2R2_1h **Scan** 10913
Peptide SNASSDVETGVT **Charge** 2 **Spectral Angle** 0.86
Spearman Correlation 0.82 **iRT Error:** 0.51

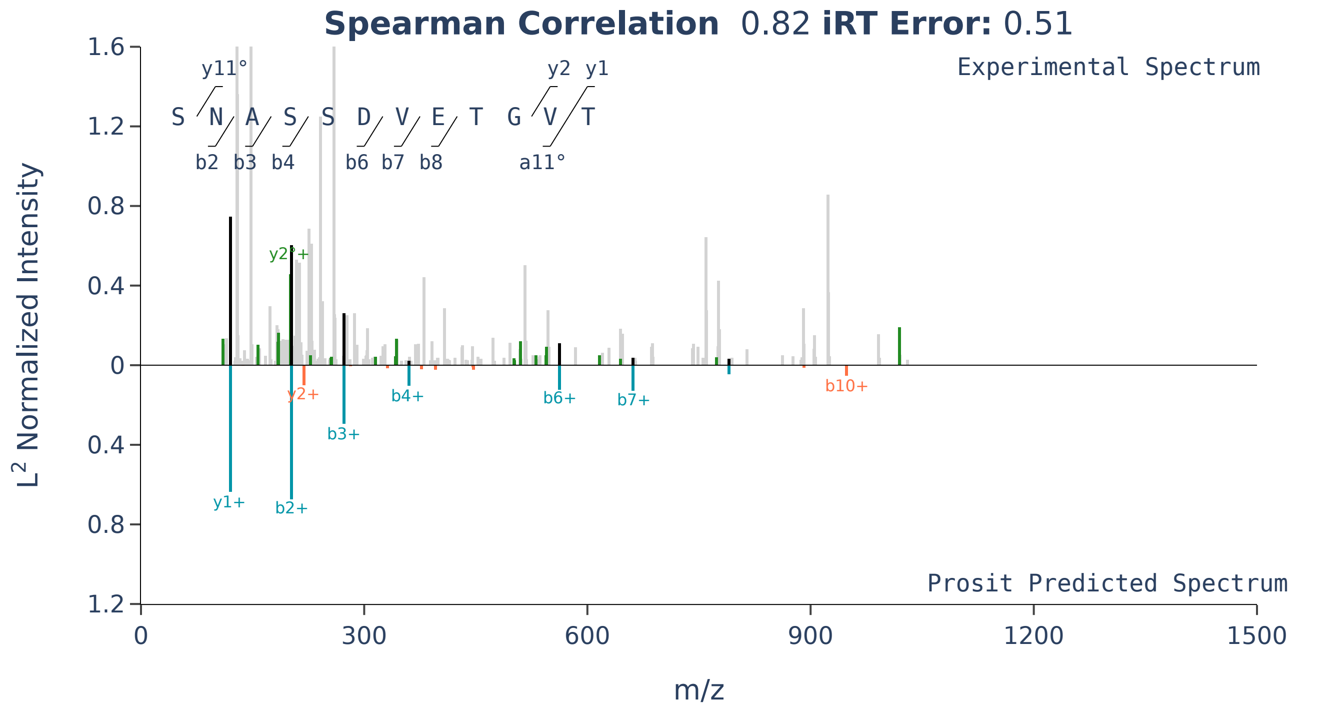

Source HRoetschke_271222_090123_HFGoe_LEDGF_b2R2_1h **Scan** 10913
Peptide SSEERETEIS **Charge** 2 **Spectral Angle** 0.07
Spearman Correlation 0.23 **iRT Error:** 20.24

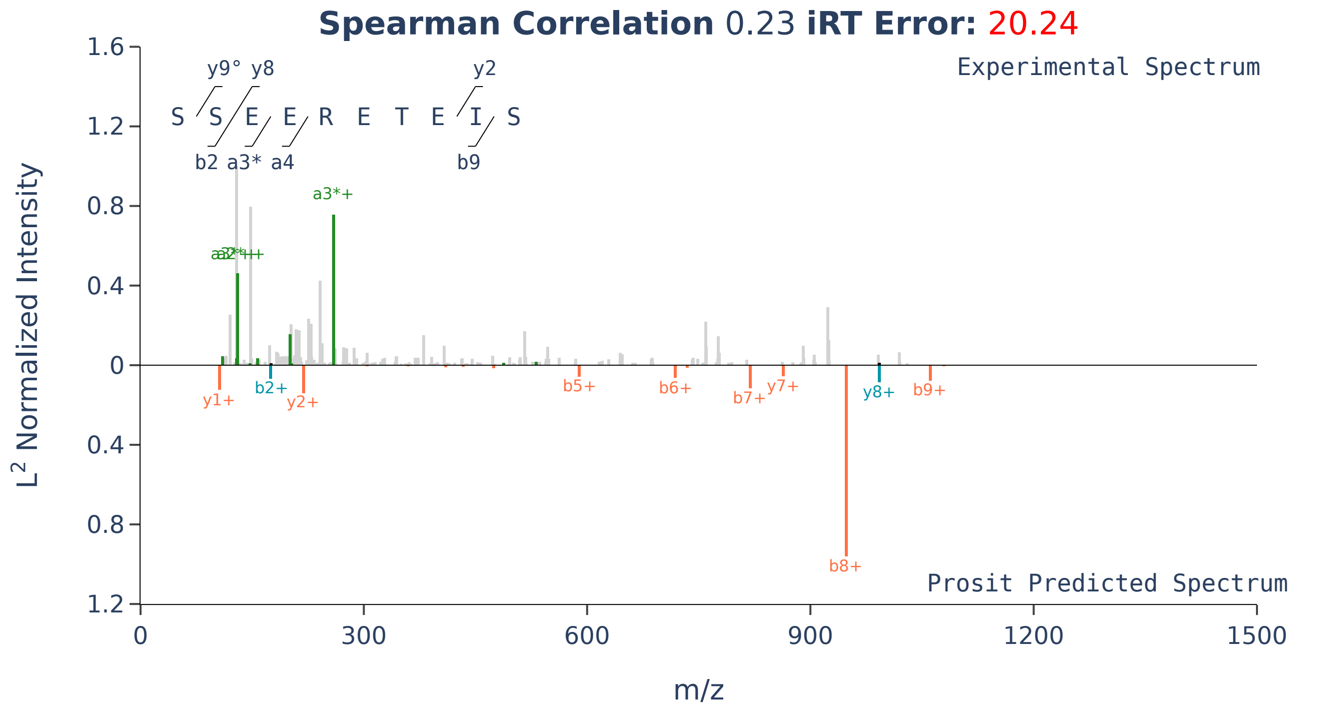

Source HRoetschke_271222_090123_HFGoe_LEDGF_b1R1_2h **Scan** 21180
Peptide KNSLVIMEKSTMLYNKFKNM **Charge** 4 **Spectral Angle** 0.82
Spearman Correlation 0.84 **iRT Error:** 0.52

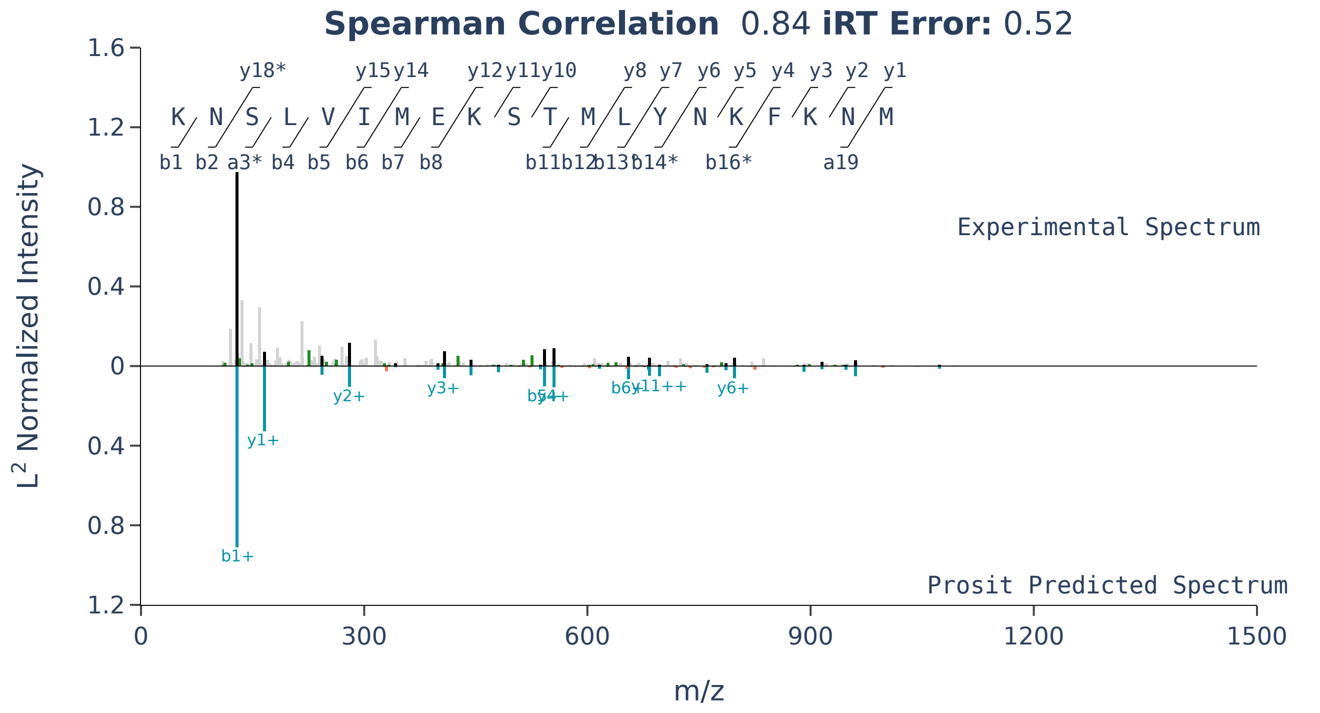

Source HRoetschke_271222_090123_HFGoe_LEDGF_b1R1_2h **Scan** 21180
Peptide KVSQVIMEKSTMLYNKFKNM **Charge** 4 **Spectral Angle** 0.85
Spearman Correlation 0.86 **iRT Error:** 5.39

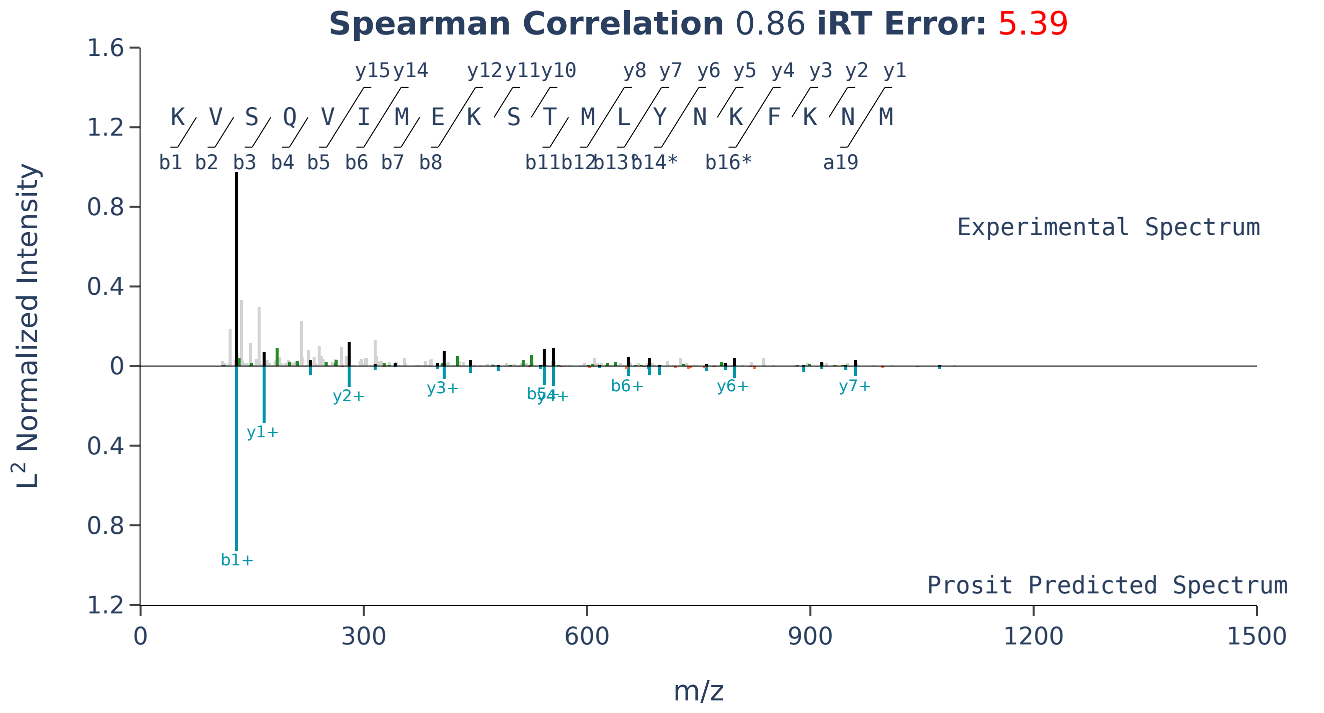

Source HRoetschke_271222_090123_HFGoe_LEDGF_b1R1_24h **Scan** 6873
Peptide SVNLKVSPKRGRAT **Charge** 4 **Spectral Angle** 0.92
Spearman Correlation 0.9 **iRT Error:** 0.55

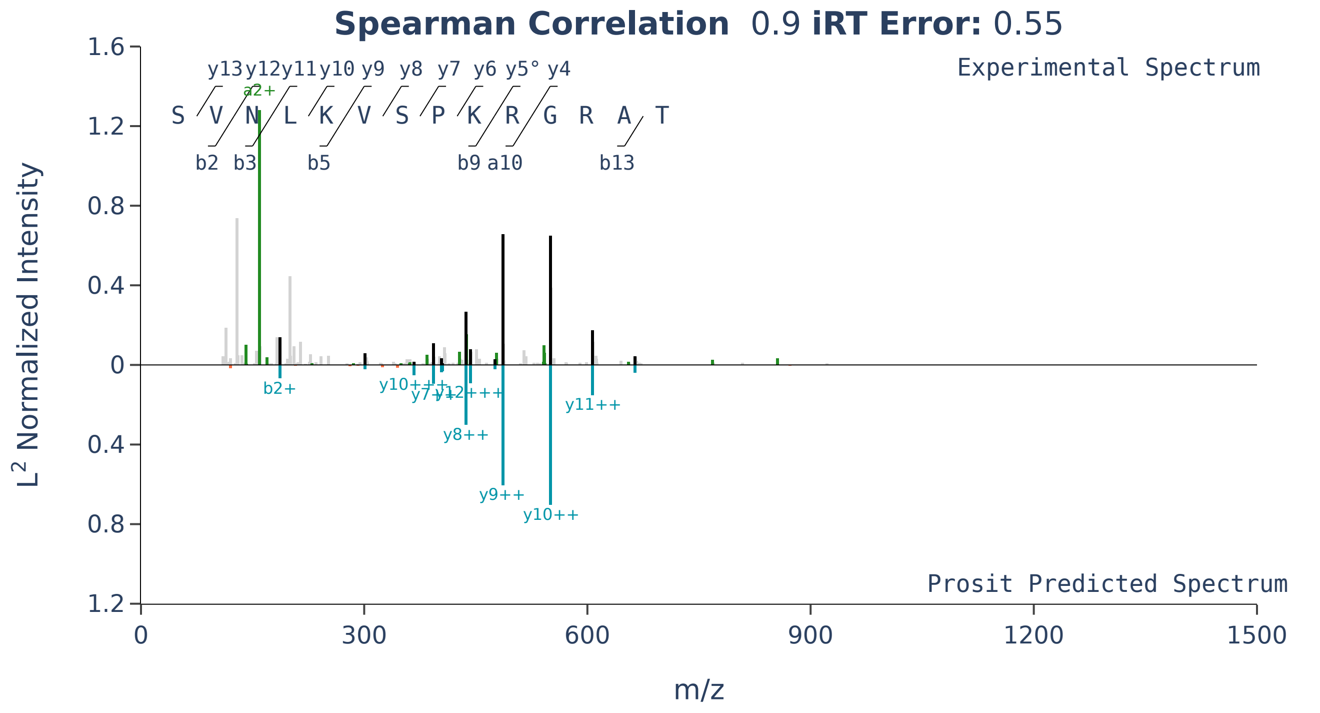

Source HRoetschke_271222_090123_HFGoe_LEDGF_b1R1_24h **Scan** 6873
Peptide TASVNLKVSPKRGR **Charge** 4 **Spectral Angle** 0.23
Spearman Correlation 0.3 **iRT Error:** 6.57

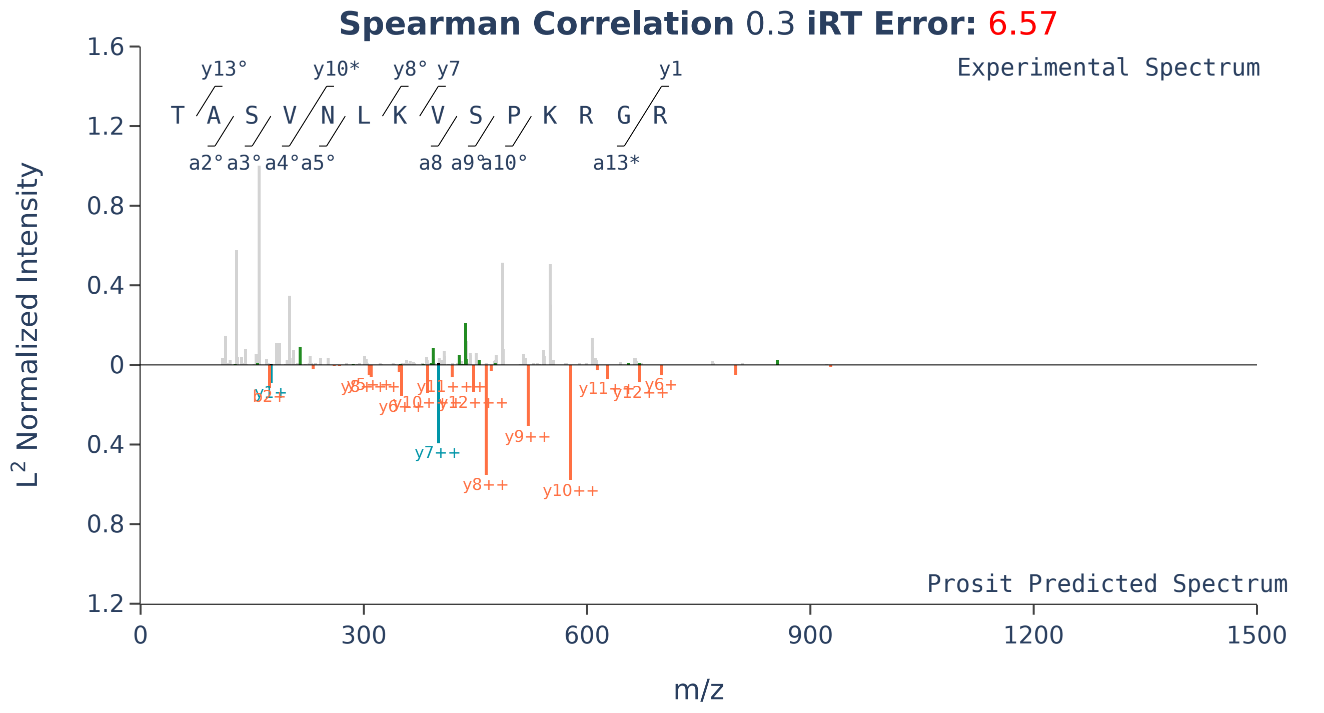

Source HRoetschke_271222_090123_HFGoe_LEDGF_b1R2_24h **Scan** 12400
Peptide RIHAEIKNTAF **Charge** 3 **Spectral Angle** 0.83
Spearman Correlation 0.9 **iRT Error:** 0.56

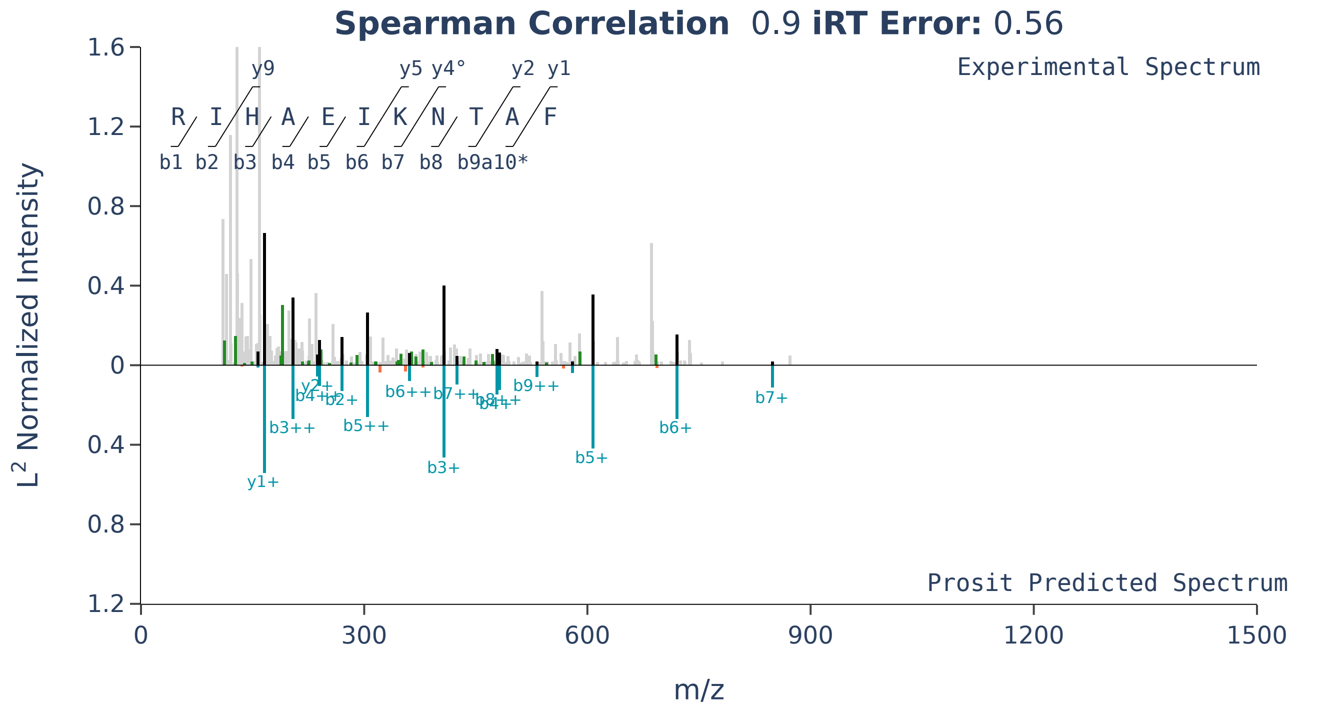

Source HRoetschke_271222_090123_HFGoe_LEDGF_b1R2_24h **Scan** 12400
Peptide RGRPKMVKQPC **Charge** 3 **Spectral Angle** 0.0
Spearman Correlation 0.0 **iRT Error:** 49.27

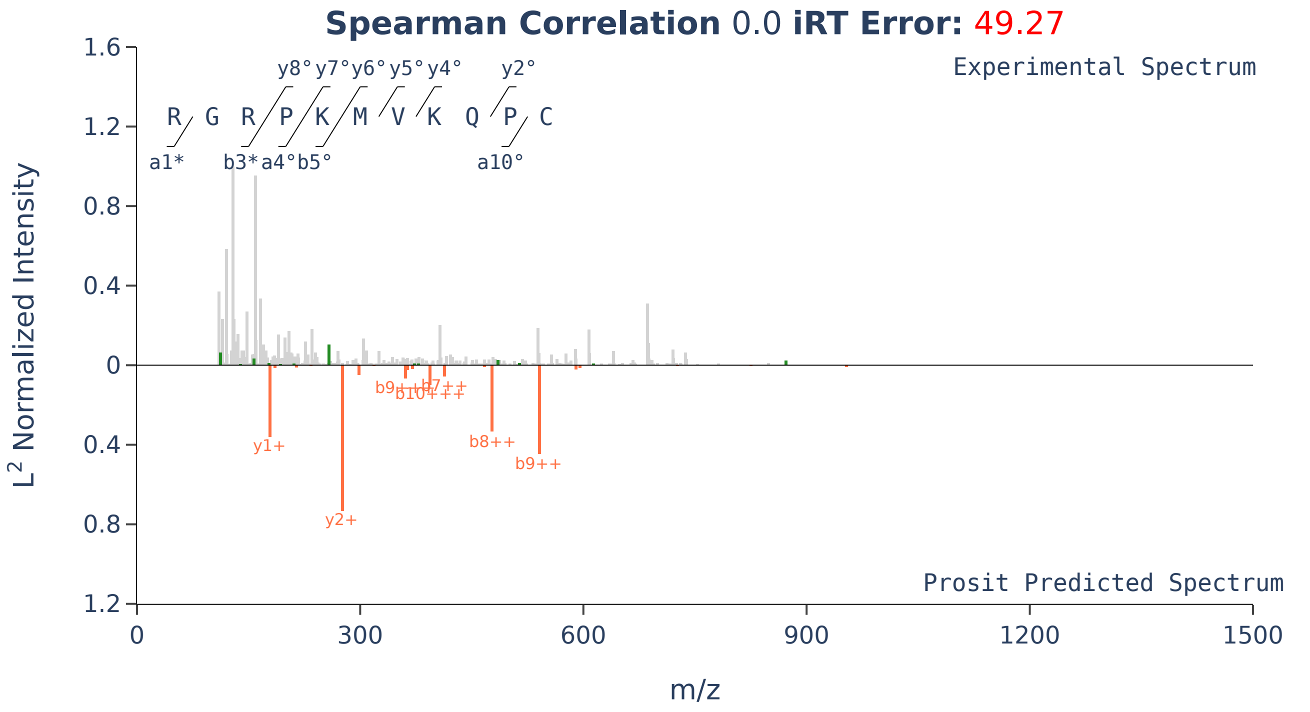

Source HRoetschke_271222_090123_HFGoe_LEDGF_b2R1_4h **Scan** 11714
Peptide GSPGMTRDFKPGD **Charge** 3 **Spectral Angle** 0.9
Spearman Correlation 0.91 **iRT Error:** 0.56

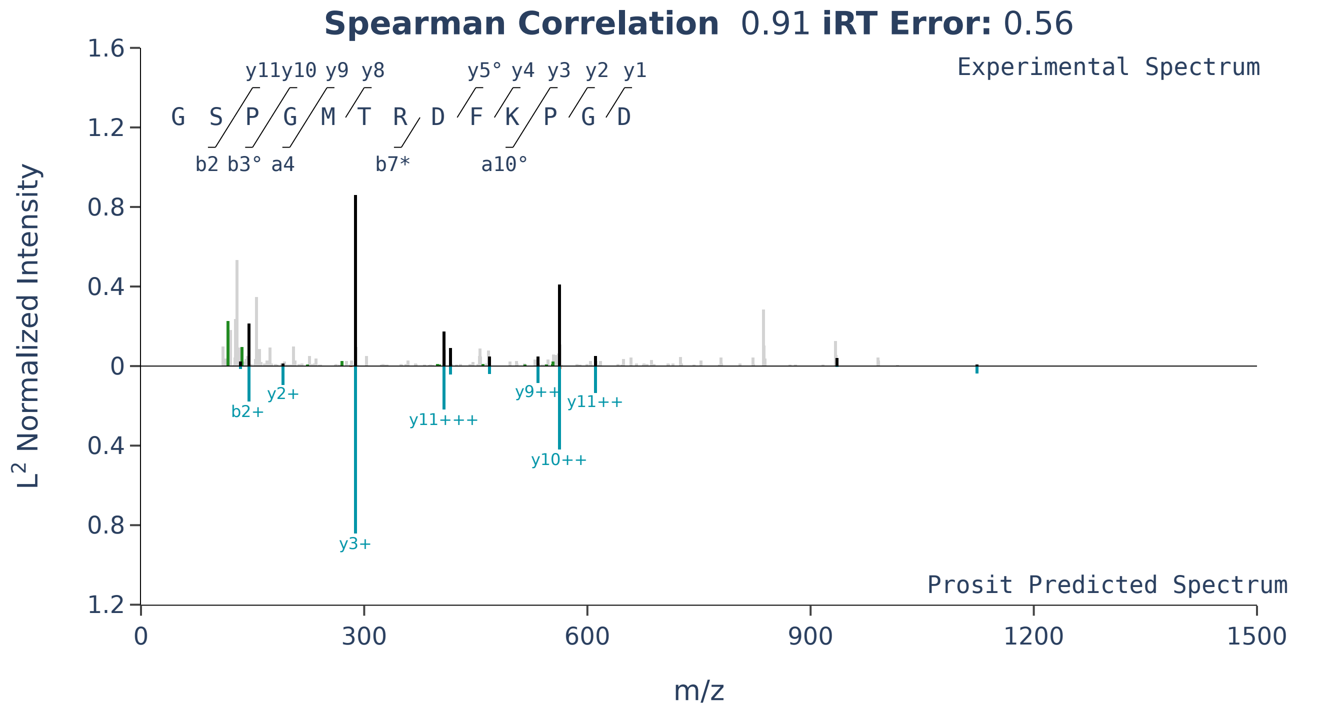

Source HRoetschke_271222_090123_HFGoe_LEDGF_b2R1_4h **Scan** 11714
Peptide EQTGSKTLNGGSDA **Charge** 3 **Spectral Angle** 0.01
Spearman Correlation -0.03 **iRT Error:** 33.99

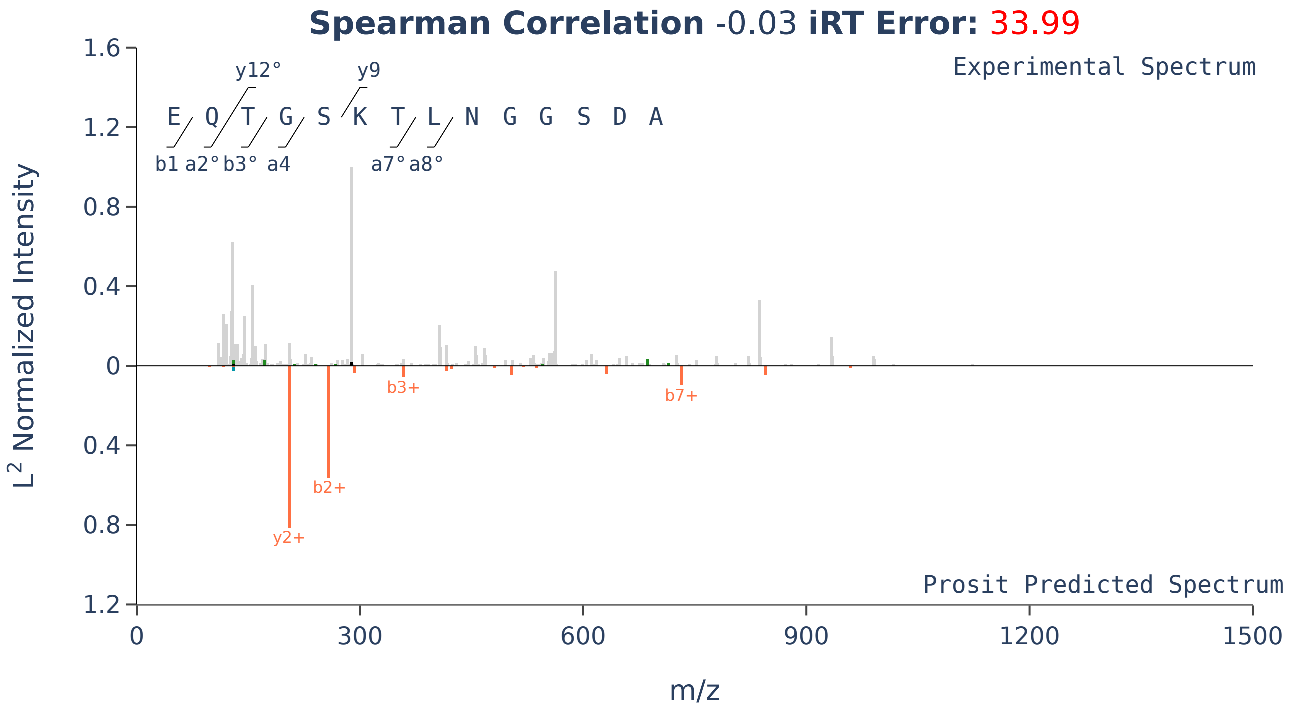

Source HRoetschke_271222_090123_HFGoe_LEDGF_b1R2_24h **Scan** 9473
Peptide PSSEEREITE **Charge** 1 **Spectral Angle** 0.69
Spearman Correlation 0.94 **iRT Error:** 0.59

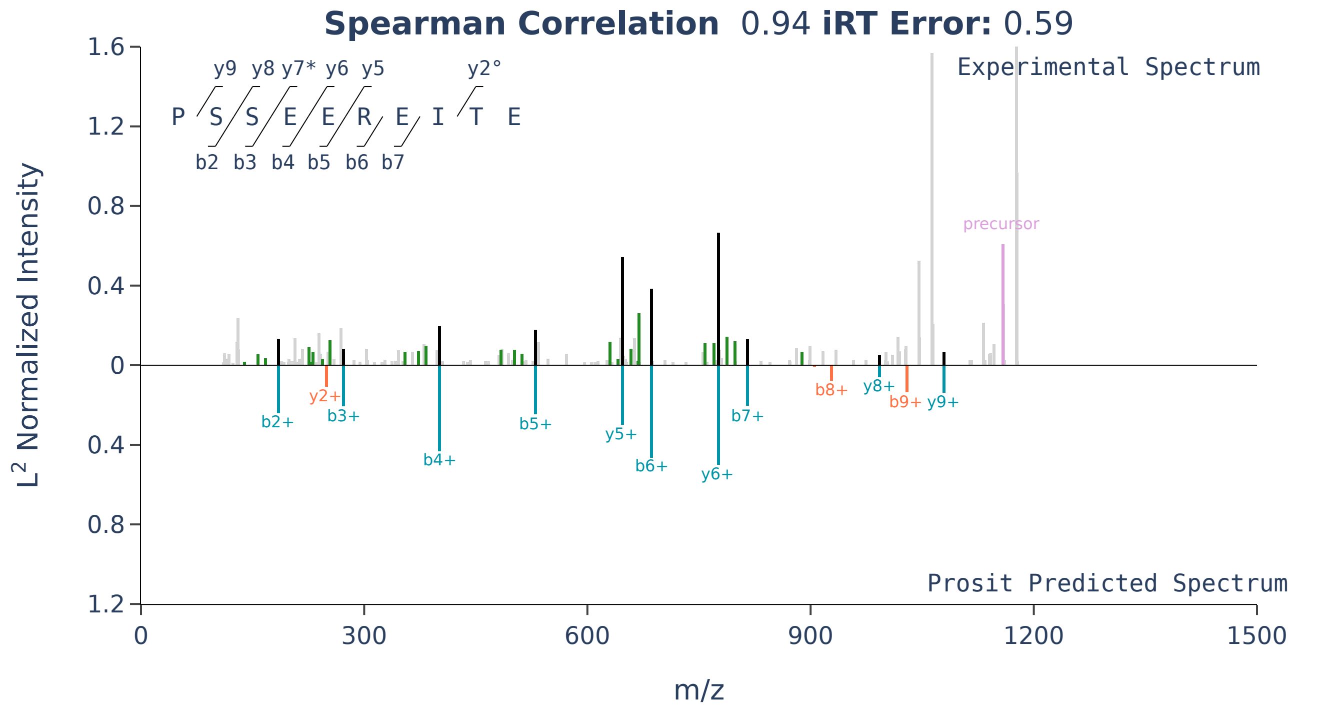

Source HRoetschke_271222_090123_HFGoe_LEDGF_b1R2_24h **Scan** 9473
Peptide PSSEERETEI **Charge** 1 **Spectral Angle** 0.45
Spearman Correlation 0.5 **iRT Error:** 12.51

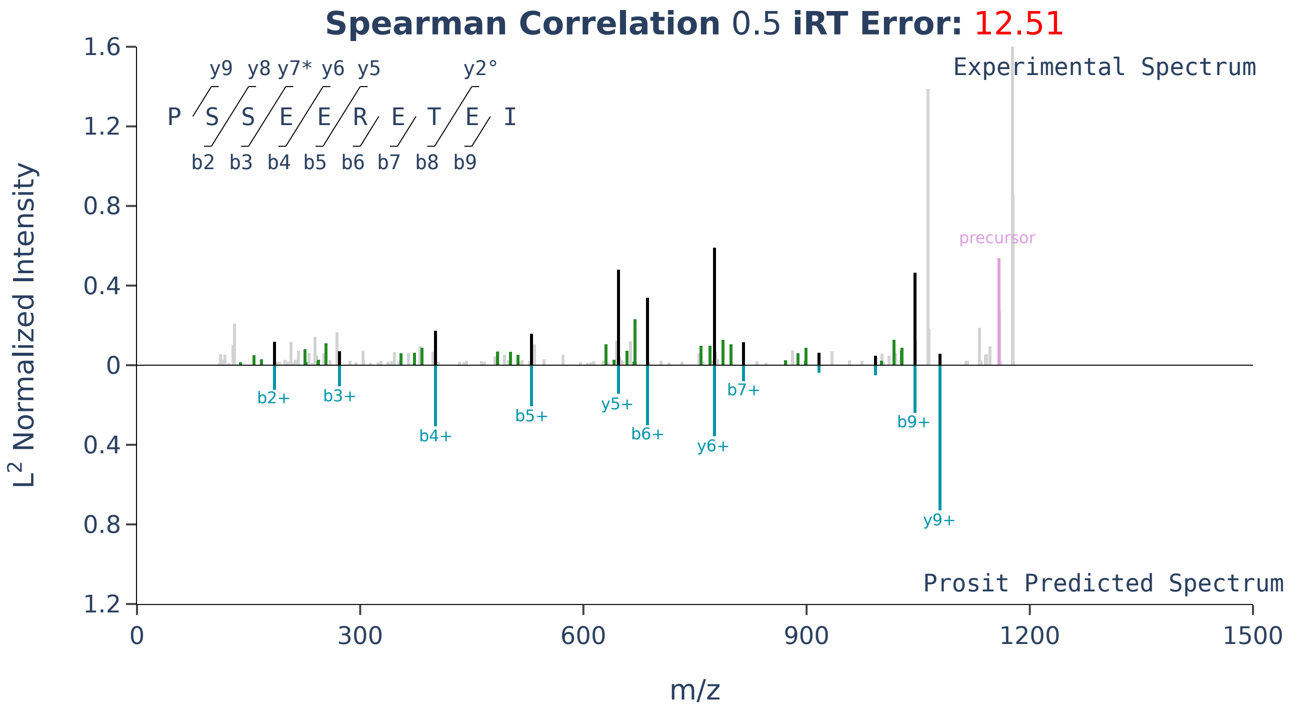

Source HRoetschke_271222_090123_HFGoe_LEDGF_b1R1_24h **Scan** 16050
Peptide KGTAFLGPKDIFPYSENKEKYGKPNKR **Charge** 4 **Spectral Angle** 0.84
Spearman Correlation 0.87 **iRT Error:** 0.59

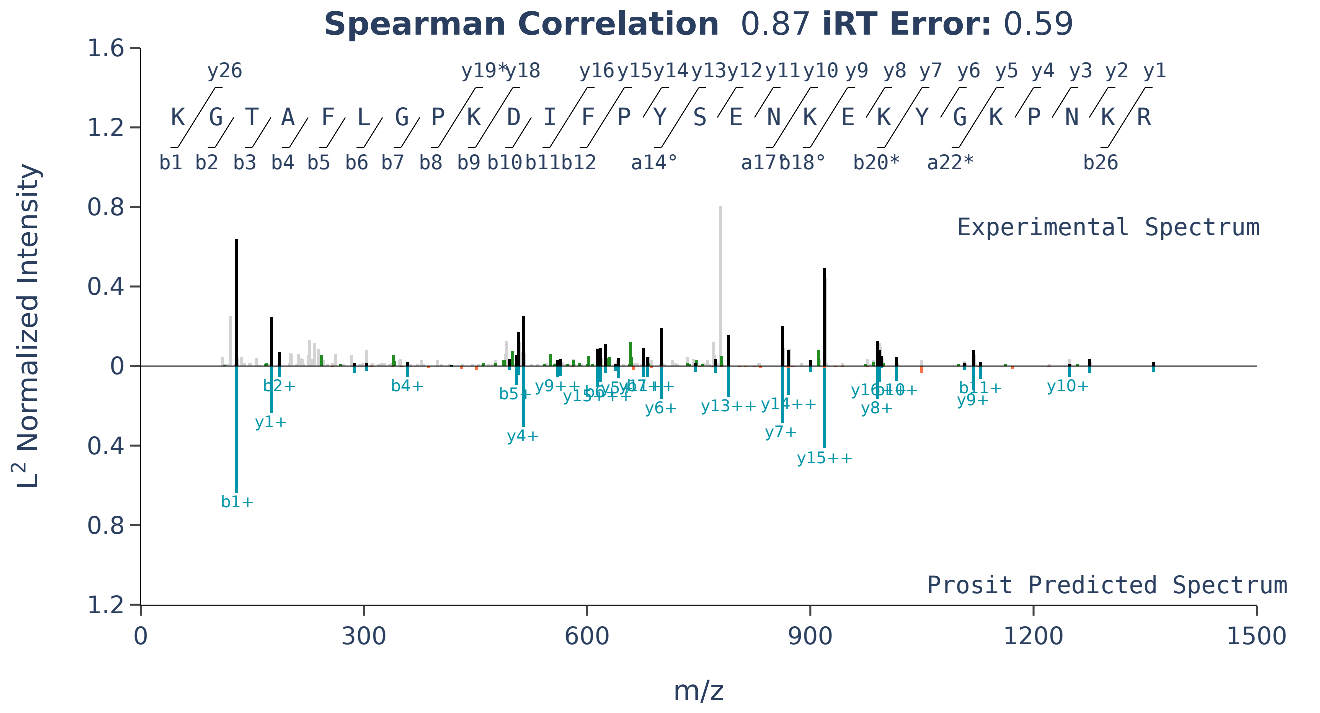

Source HRoetschke_271222_090123_HFGoe_LEDGF_b1R1_24h **Scan** 16050
Peptide TAFLGPKDIFPYSENKEKYGKPNKRKG **Charge** 4 **Spectral Angle** 0.43
Spearman Correlation 0.2 **iRT Error:** 4.27

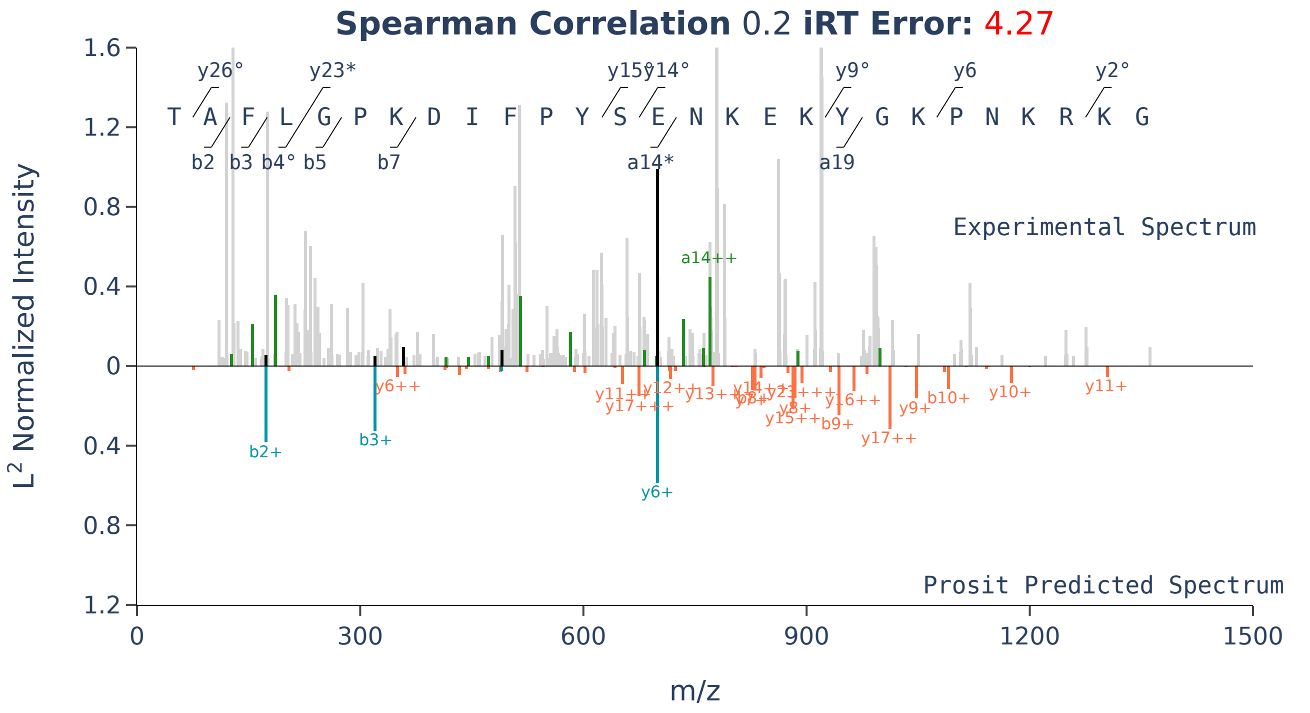

Source HRoetschke_271222_090123_HFGoe_LEDGF_b1R1_4h **Scan** 18915
Peptide QVFLGPKDIFPYSENKEKYGKPNKR **Charge** 4 **Spectral Angle** 0.82
Spearman Correlation 0.85 **iRT Error:** 0.62

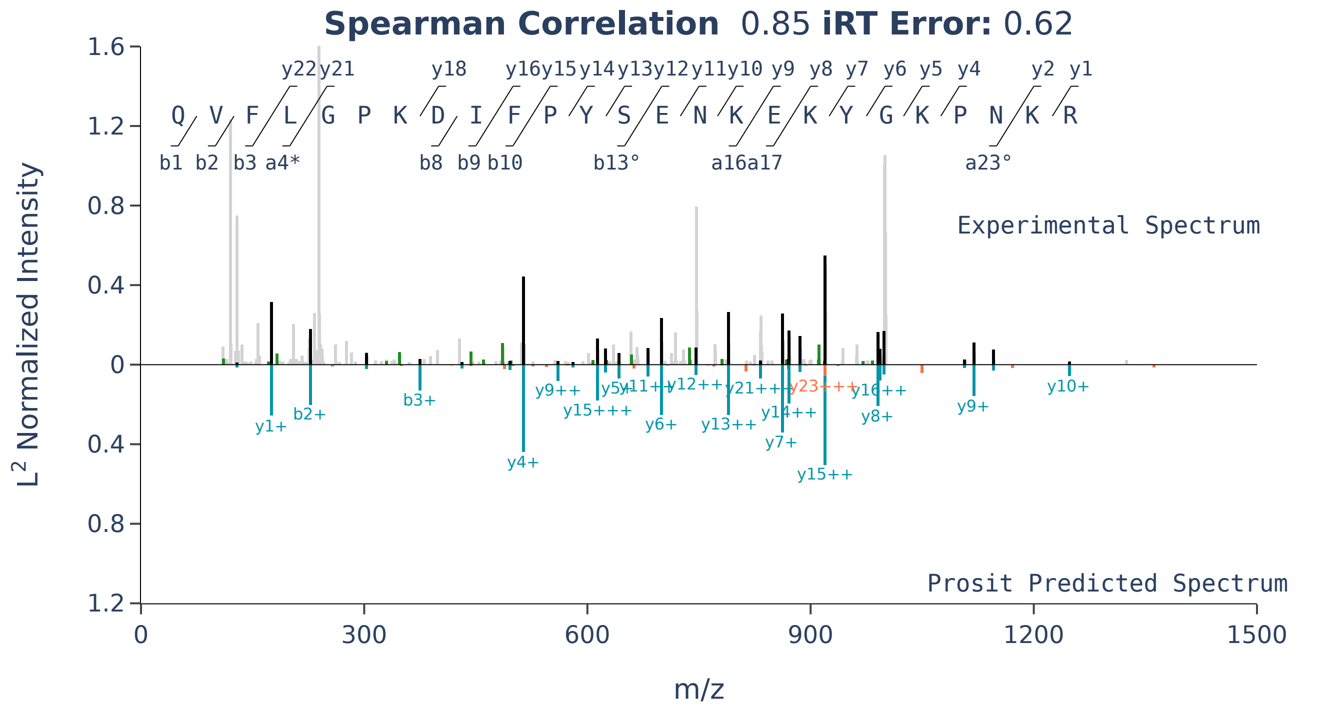

Source HRoetschke_271222_090123_HFGoe_LEDGF_b1R1_4h **Scan** 18915
Peptide KRETSMDSRLQRIHAEIKNSLKIDN **Charge** 4 **Spectral Angle** 0.65
Spearman Correlation 0.26 **iRT Error:** 0.34

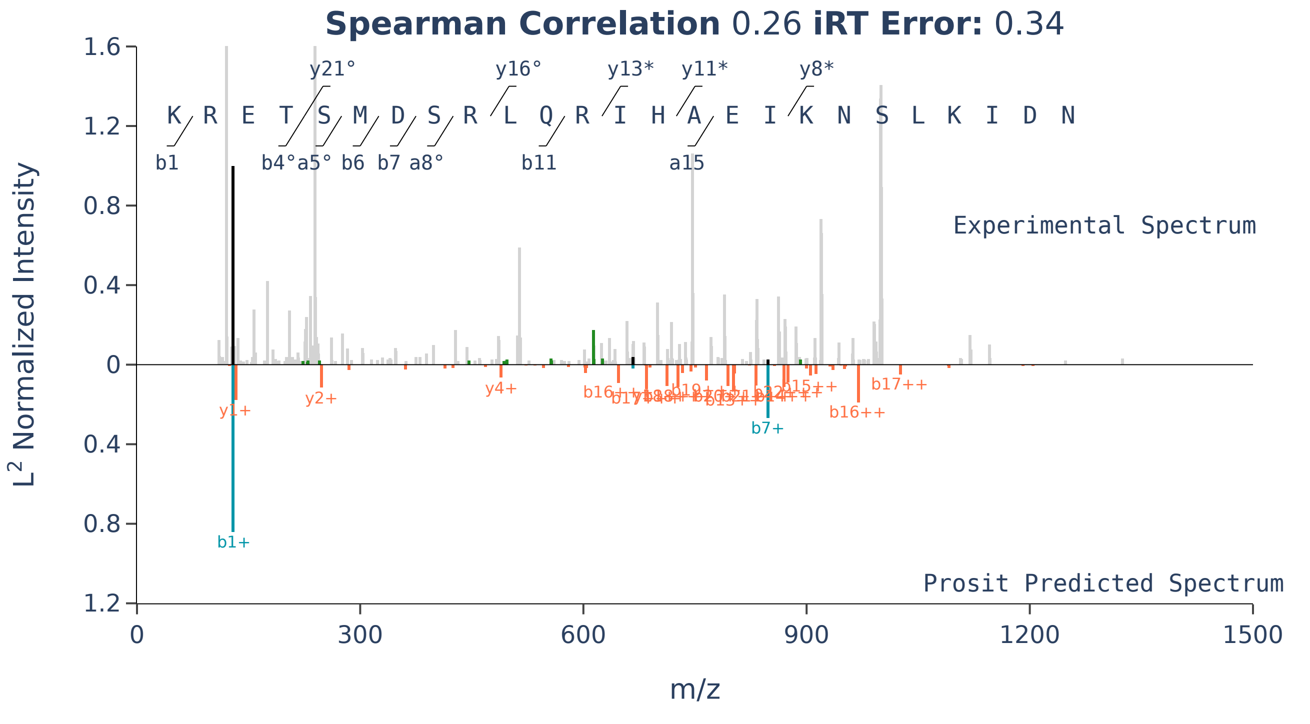

Source HRoetschke_271222_090123_HFGoe_LEDGF_b1R1_24h **Scan** 6553
Peptide LEKEQTGSKTLD **Charge** 3 **Spectral Angle** 0.75
Spearman Correlation 0.9 **iRT Error:** 0.63

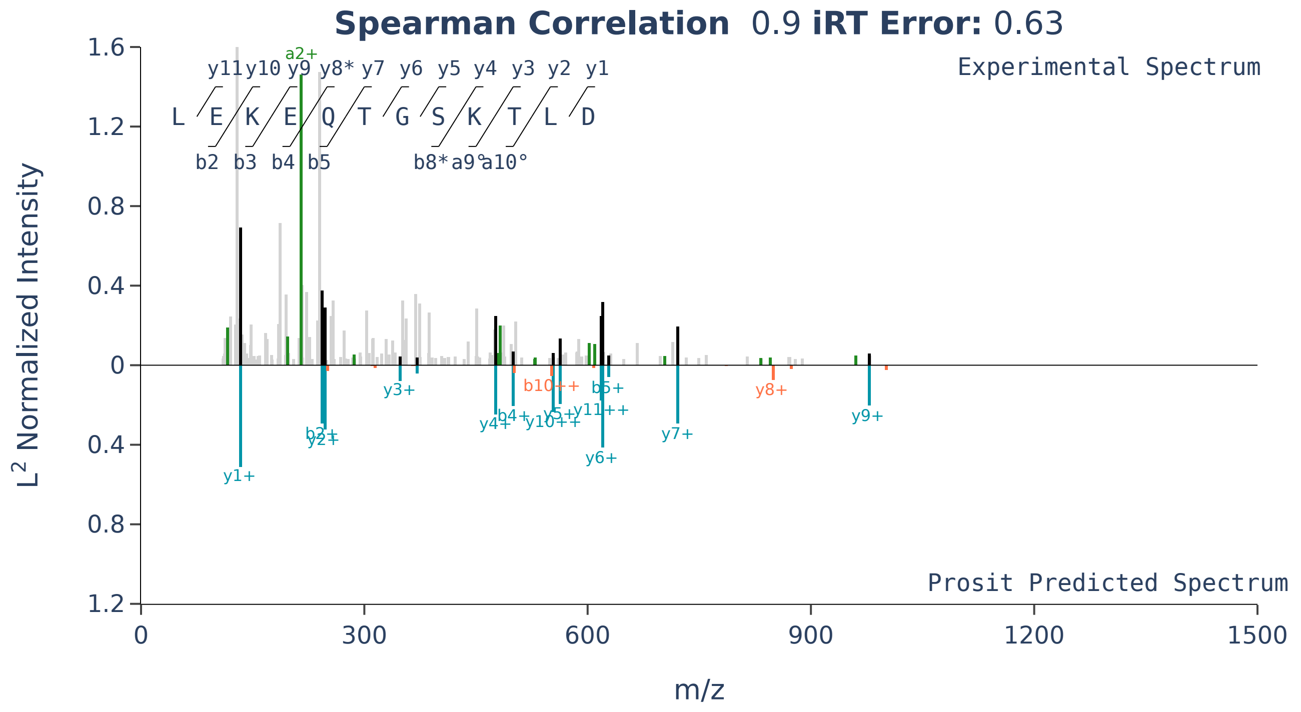

Source HRoetschke_271222_090123_HFGoe_LEDGF_b1R1_24h **Scan** 6553
Peptide RKGFNEGLWEI **Charge** 3 **Spectral Angle** 0.02
Spearman Correlation -0.09 **iRT Error:** 113.4

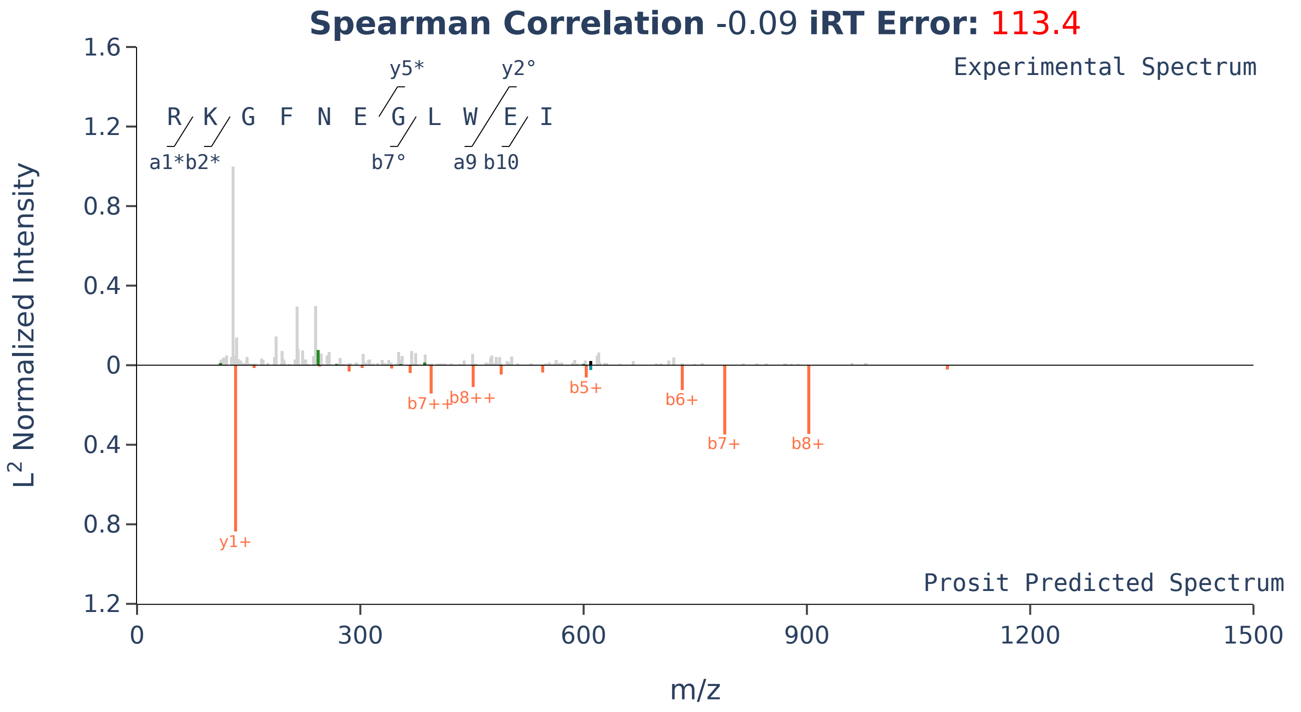

Source HRoetschke_271222_090123_HFGoe_LEDGF_b2R2_24h **Scan** 9419
Peptide TEEAGVVTTKA **Charge** 2 **Spectral Angle** 0.81
Spearman Correlation 0.86 **iRT Error:** 0.63

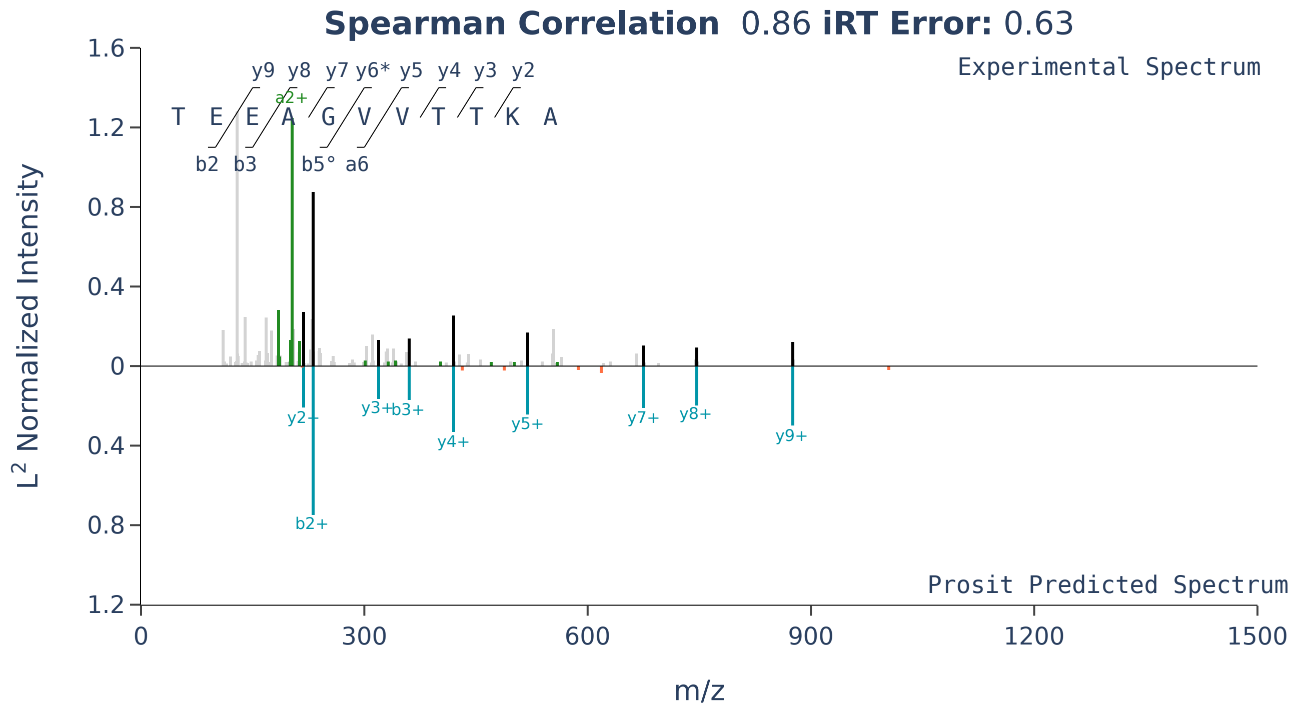

Source HRoetschke_271222_090123_HFGoe_LEDGF_b2R2_24h **Scan** 9419
Peptide SMDSRLQRI **Charge** 2 **Spectral Angle** 0.0
Spearman Correlation 0.0 **iRT Error:** 24.32

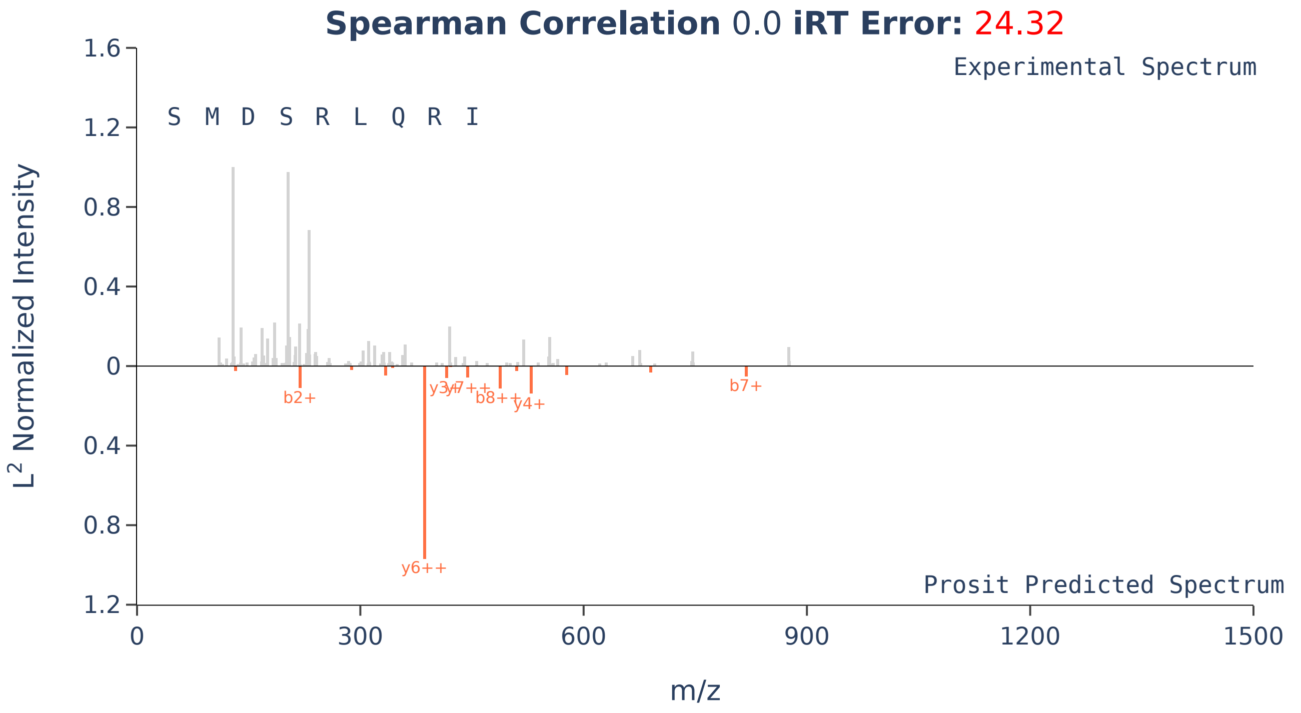

Source HRoetschke_271222_090123_HFGoe_LEDGF_b2R1_4h **Scan** 18121
Peptide SQQAAFLGPKDIFPYSENKEKYGKPNKR **Charge** 5 **Spectral Angle** 0.79
Spearman Correlation 0.84 **iRT Error:** 0.63

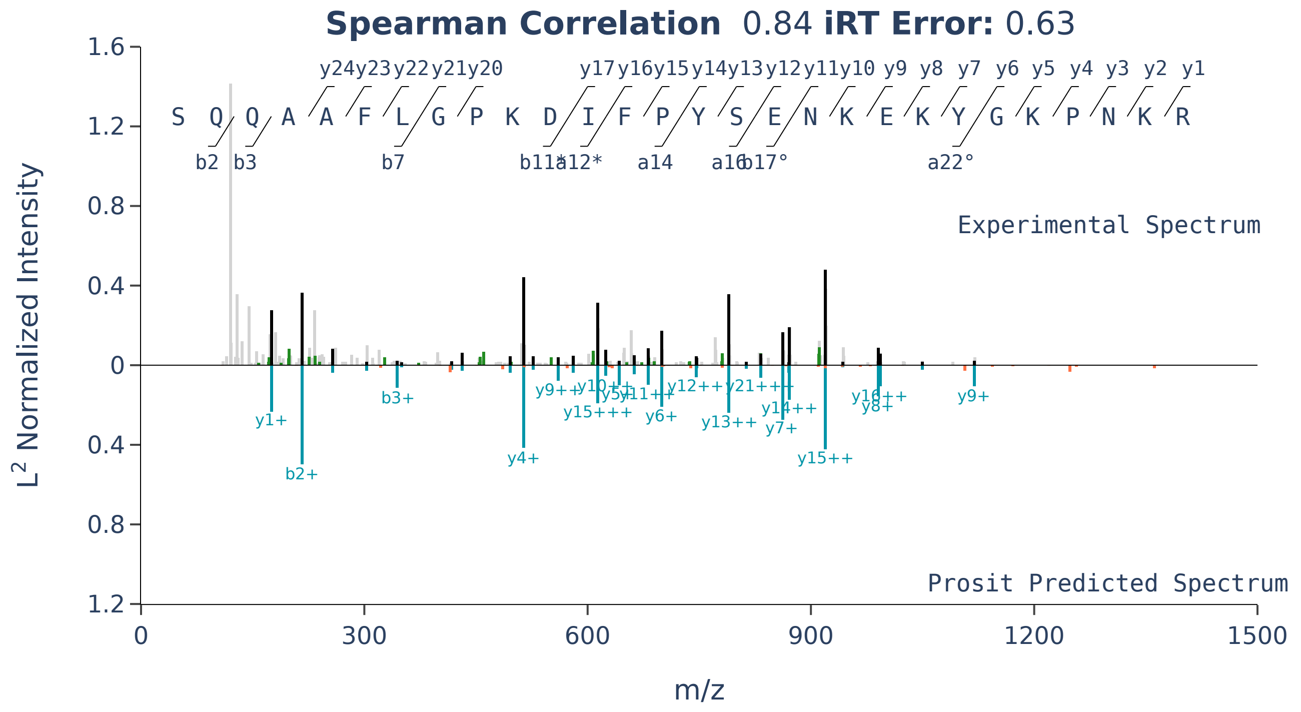

Source HRoetschke_271222_090123_HFGoe_LEDGF_b2R1_4h **Scan** 18121
Peptide EEGQKEEDKPRKEPDKKEGKKEVESKR **Charge** 5 **Spectral Angle** 0.17
Spearman Correlation 0.19 **iRT Error:** 100.32

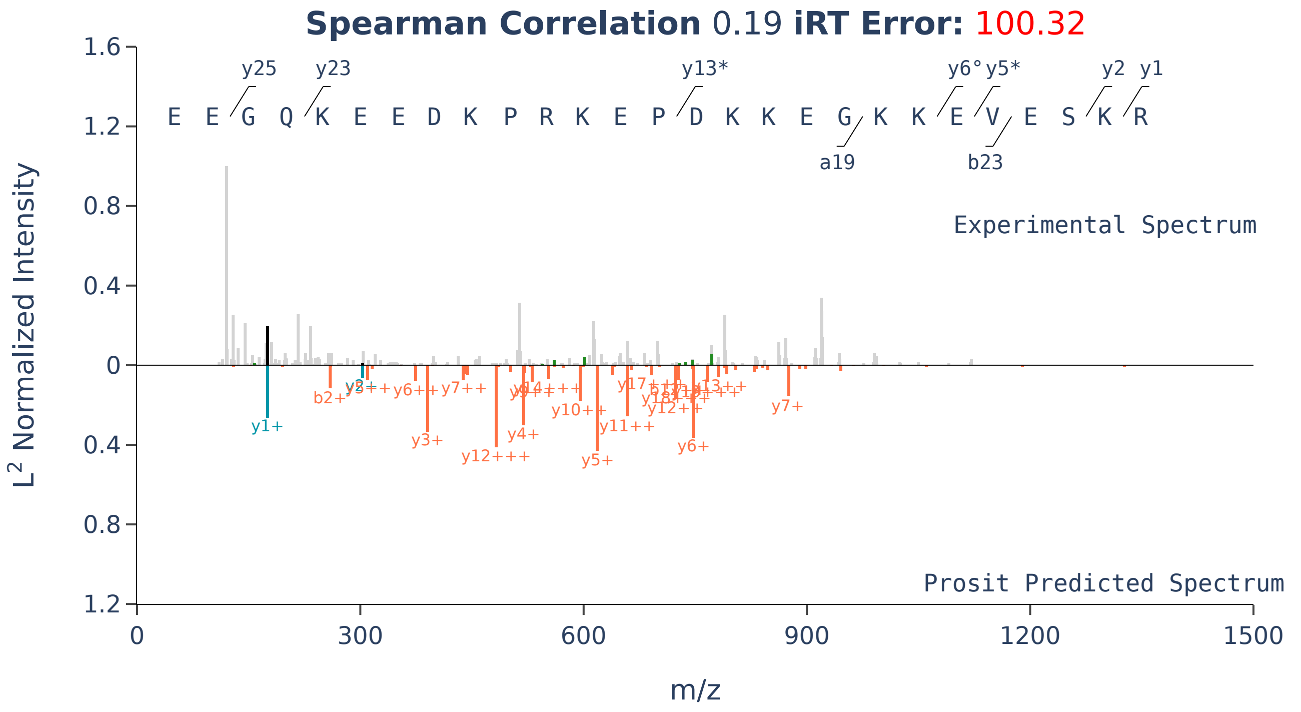

Source HRoetschke_271222_090123_HFGoe_LEDGF_b2R1_2h **Scan** 13805
Peptide LGPKDIFPYSENKEKYGKPNKRKGFNAD **Charge** 5 **Spectral Angle** 0.83
Spearman Correlation 0.82 **iRT Error:** 0.66

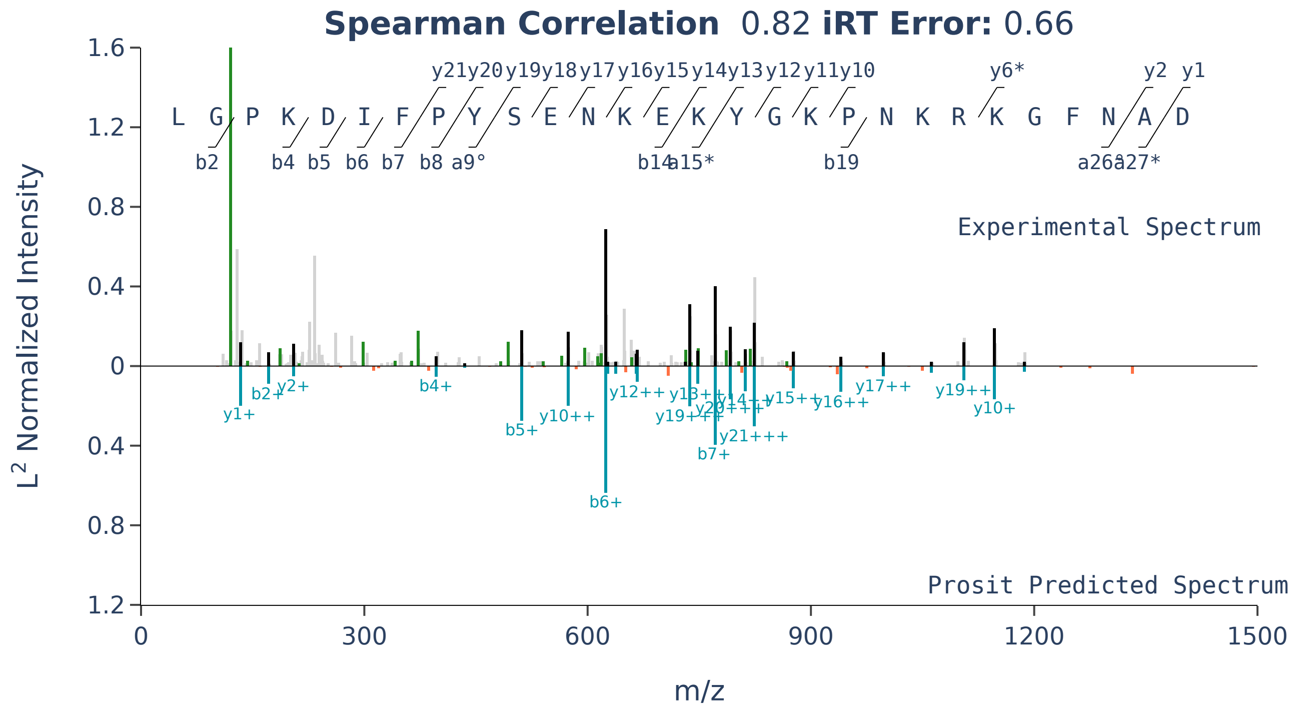

Source HRoetschke_271222_090123_HFGoe_LEDGF_b2R1_2h **Scan** 13805
Peptide EEGQKEEDKPRKEPDKKEGKKEVESKR **Charge** 5 **Spectral Angle** 0.03
Spearman Correlation 0.06 **iRT Error:** 74.46

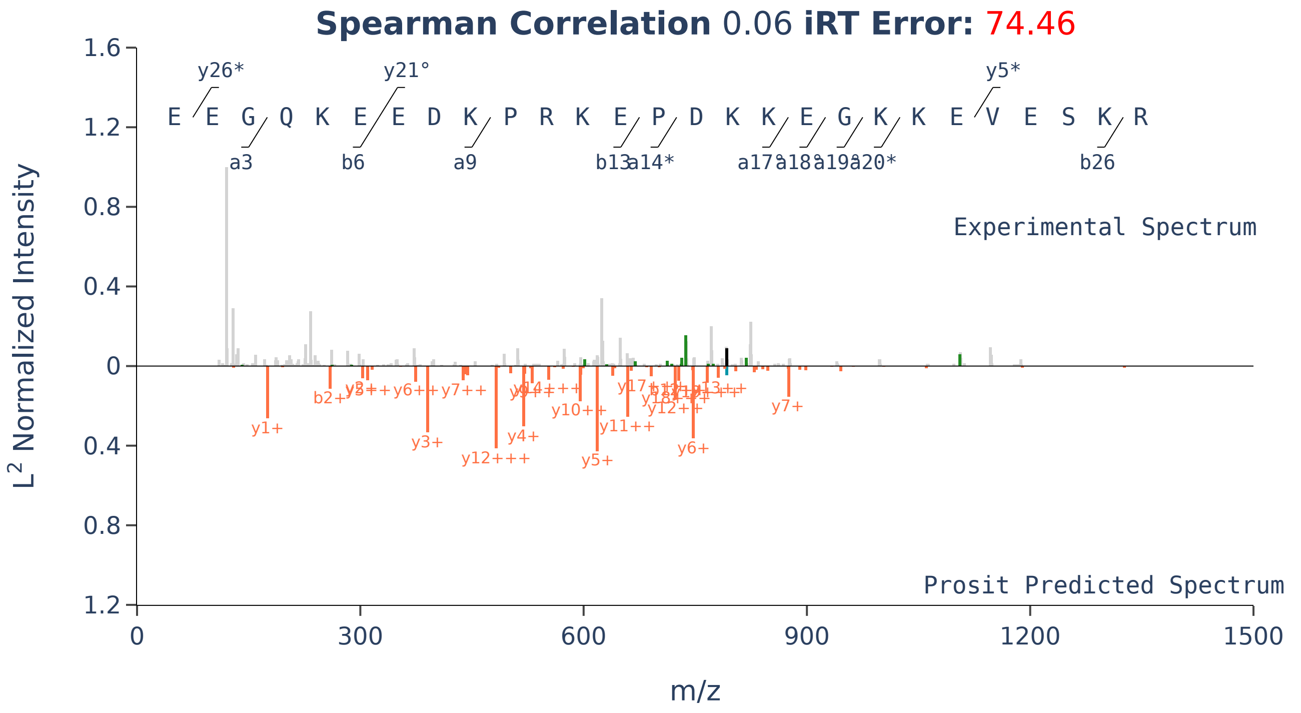

Source HRoetschke_271222_090123_HFGoe_LEDGF_b1R2_4h **Scan** 16243
Peptide MTQQAQKHTEMITTL **Charge** 3 **Spectral Angle** 0.87
Spearman Correlation 0.89 **iRT Error:** 0.73

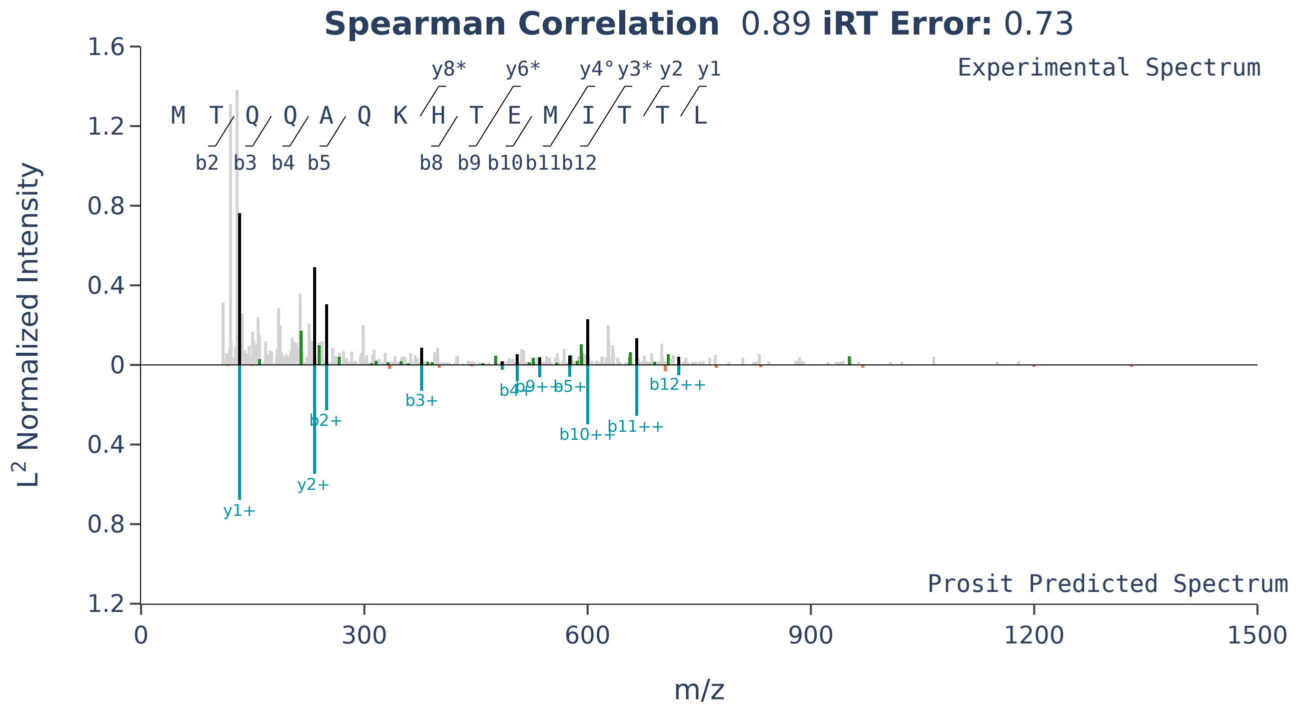

Source HRoetschke_271222_090123_HFGoe_LEDGF_b1R2_4h **Scan** 16243
Peptide TMQQAQKHTEMITTL **Charge** 3 **Spectral Angle** 0.69
Spearman Correlation 0.59 **iRT Error:** 10.96

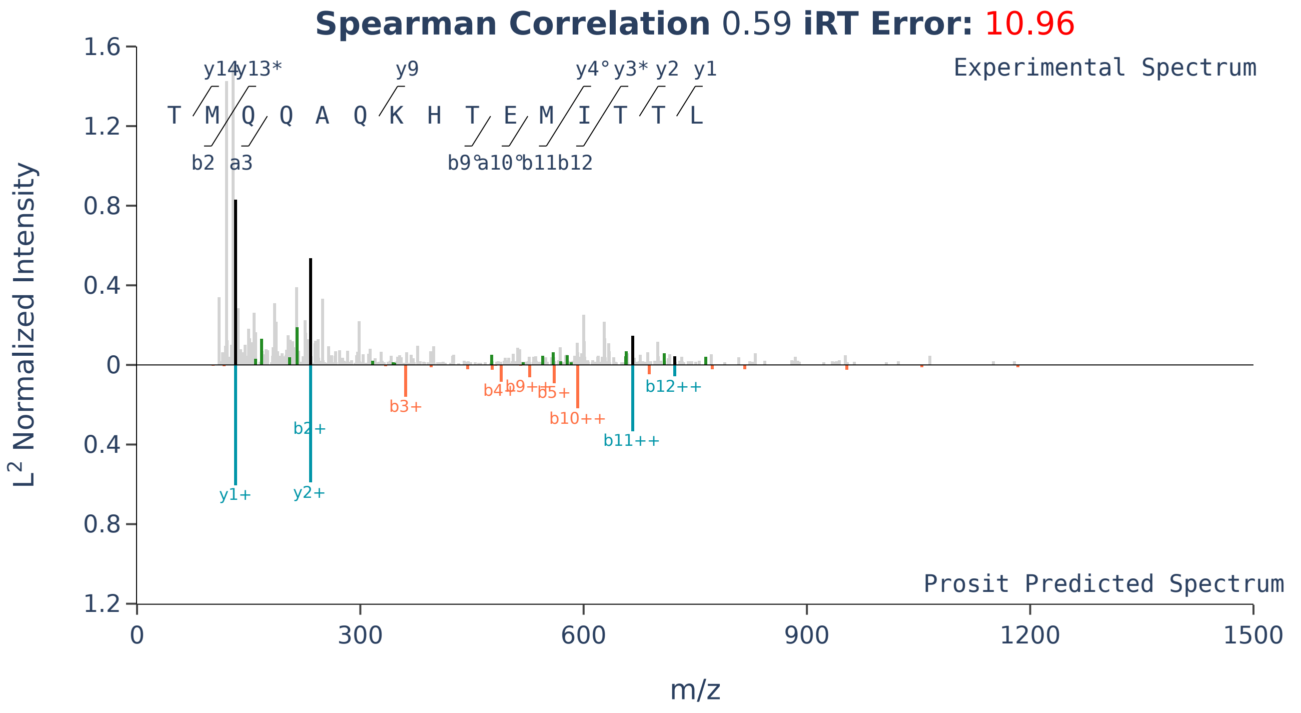

Source HRoetschke_271222_090123_HFGoe_LEDGF_b1R1_4h **Scan** 21258
Peptide SLAKIDNLDVNRCIE **Charge** 3 **Spectral Angle** 0.75
Spearman Correlation 0.89 **iRT Error:** 0.74

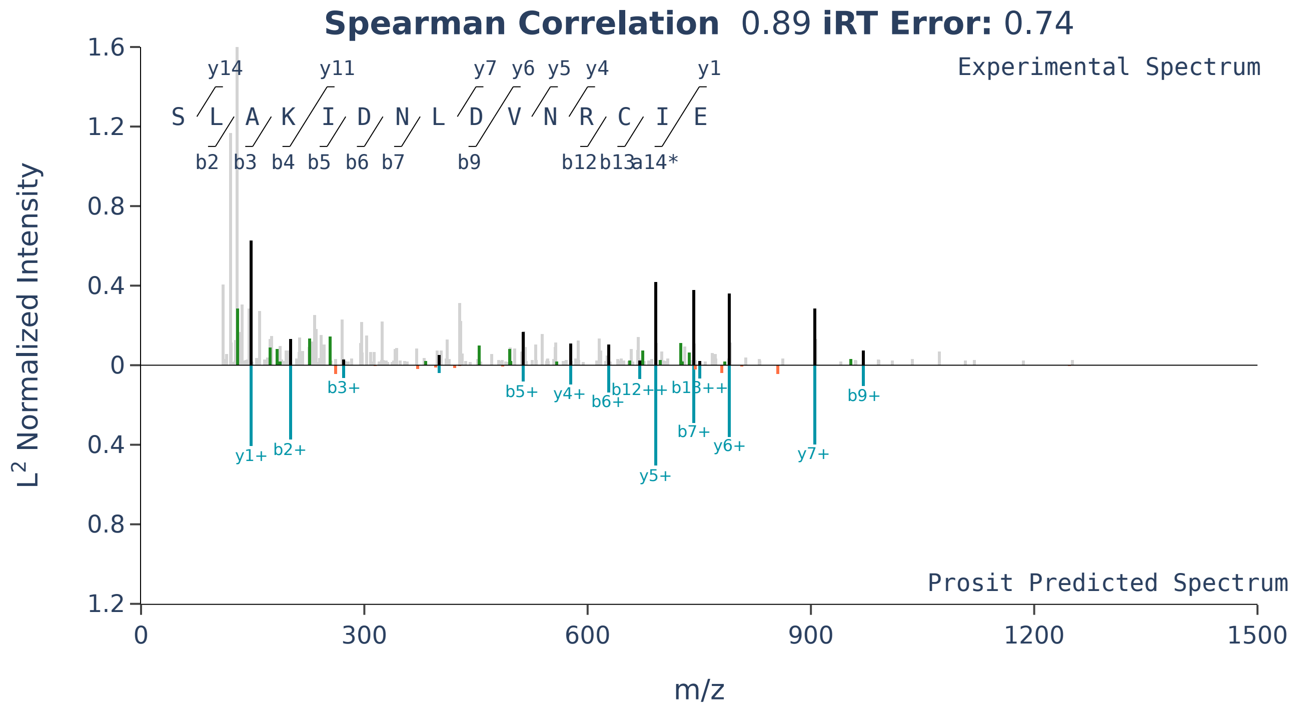

Source HRoetschke_271222_090123_HFGoe_LEDGF_b1R1_4h **Scan** 21258
Peptide SLKIDNLDVNRCIEA **Charge** 3 **Spectral Angle** 0.14
Spearman Correlation 0.11 **iRT Error:** 9.01

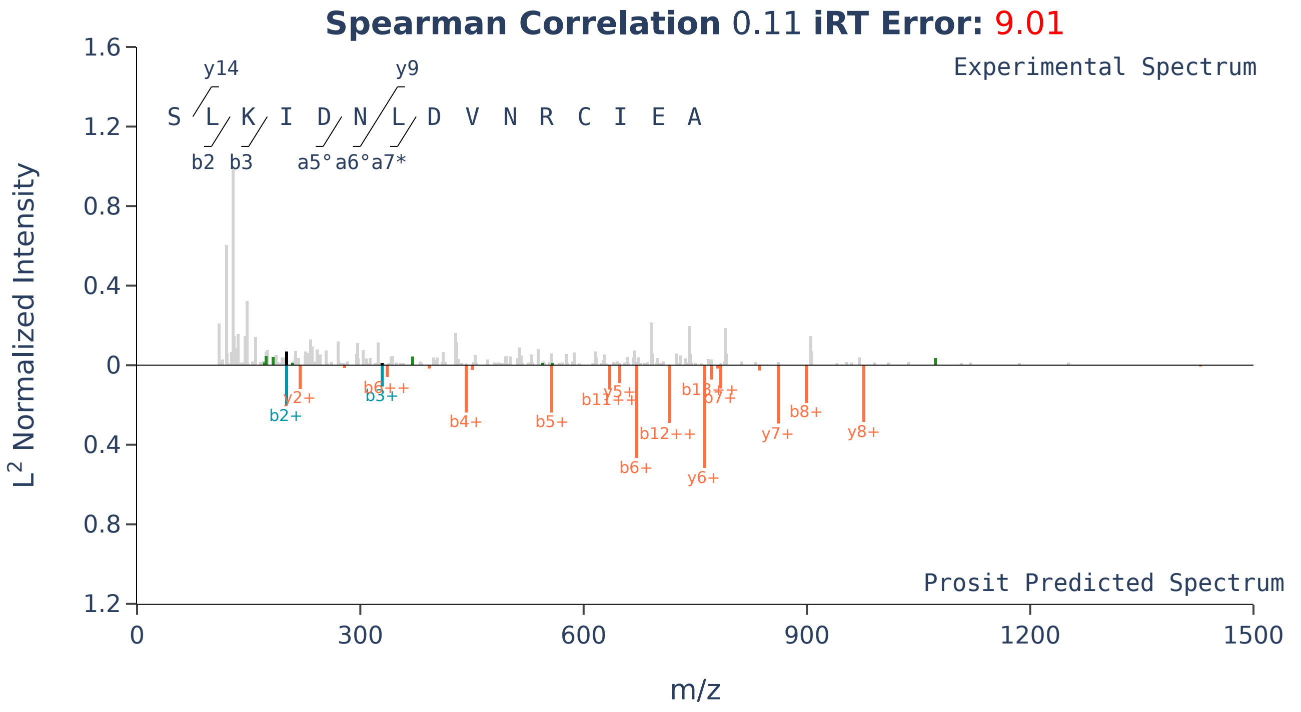

Source HRoetschke_271222_090123_HFGoe_LEDGF_b2R1_24h **Scan** 20377
Peptide ASLKIDNL **Charge** 2 **Spectral Angle** 0.77
Spearman Correlation 0.98 **iRT Error:** 0.75

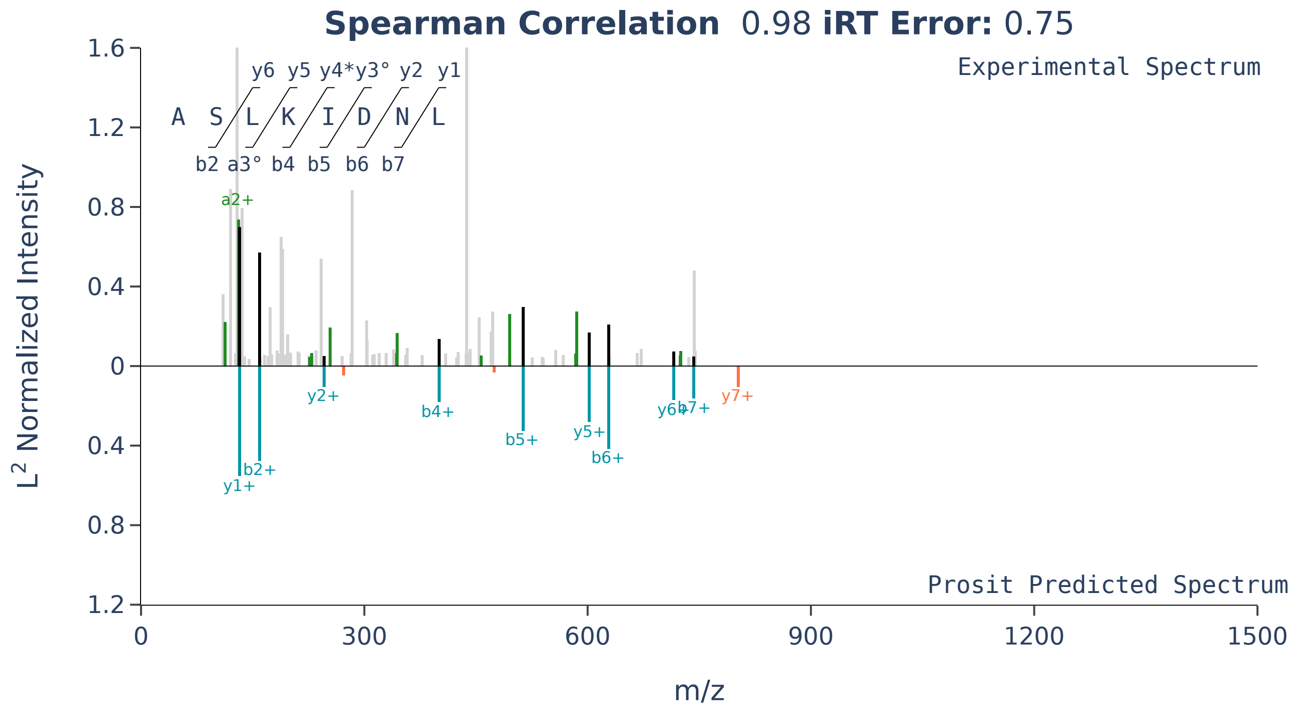

Source HRoetschke_271222_090123_HFGoe_LEDGF_b2R1_24h **Scan** 20377
Peptide VLNKSLAE **Charge** 2 **Spectral Angle** 0.0
Spearman Correlation 0.0 **iRT Error:** 53.5

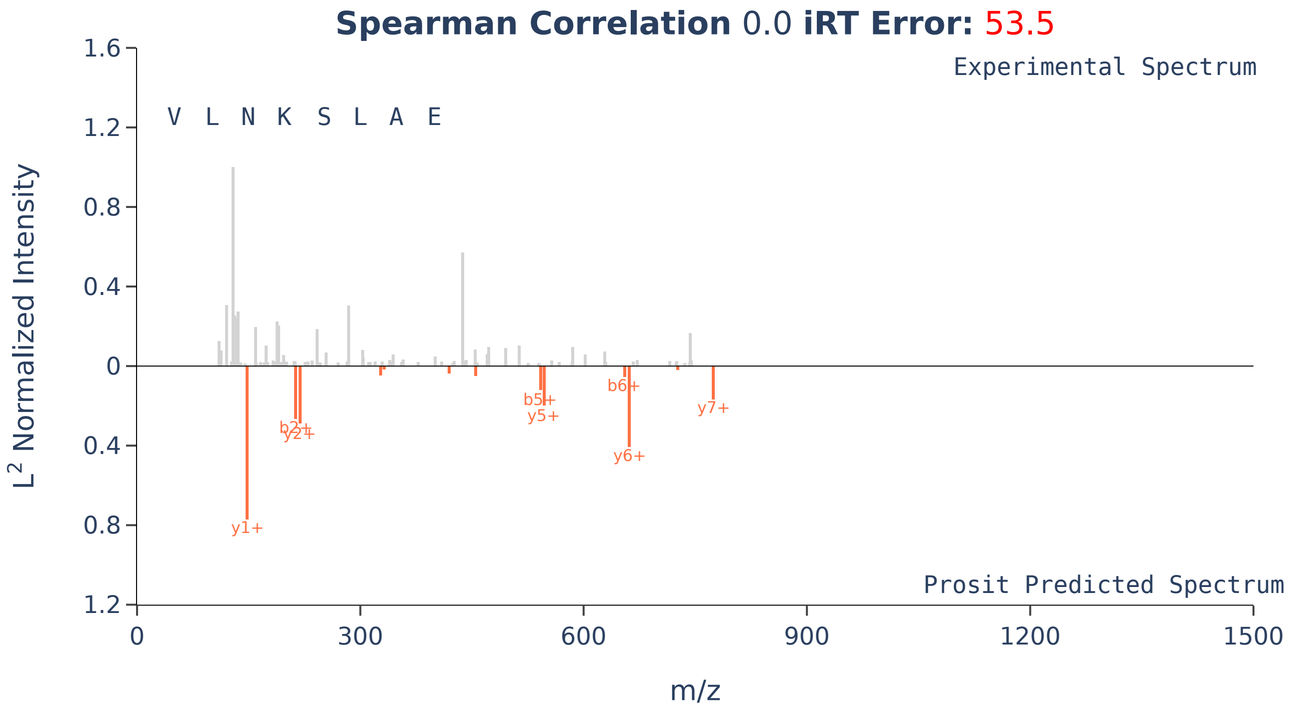

Source HRoetschke_271222_090123_HFGoe_LEDGF_b2R1_4h **Scan** 17002
Peptide ASHETAFLGPKDIFPYSENKEKYGKPNKR **Charge** 4 **Spectral Angle** 0.79
Spearman Correlation 0.84 **iRT Error:** 0.78

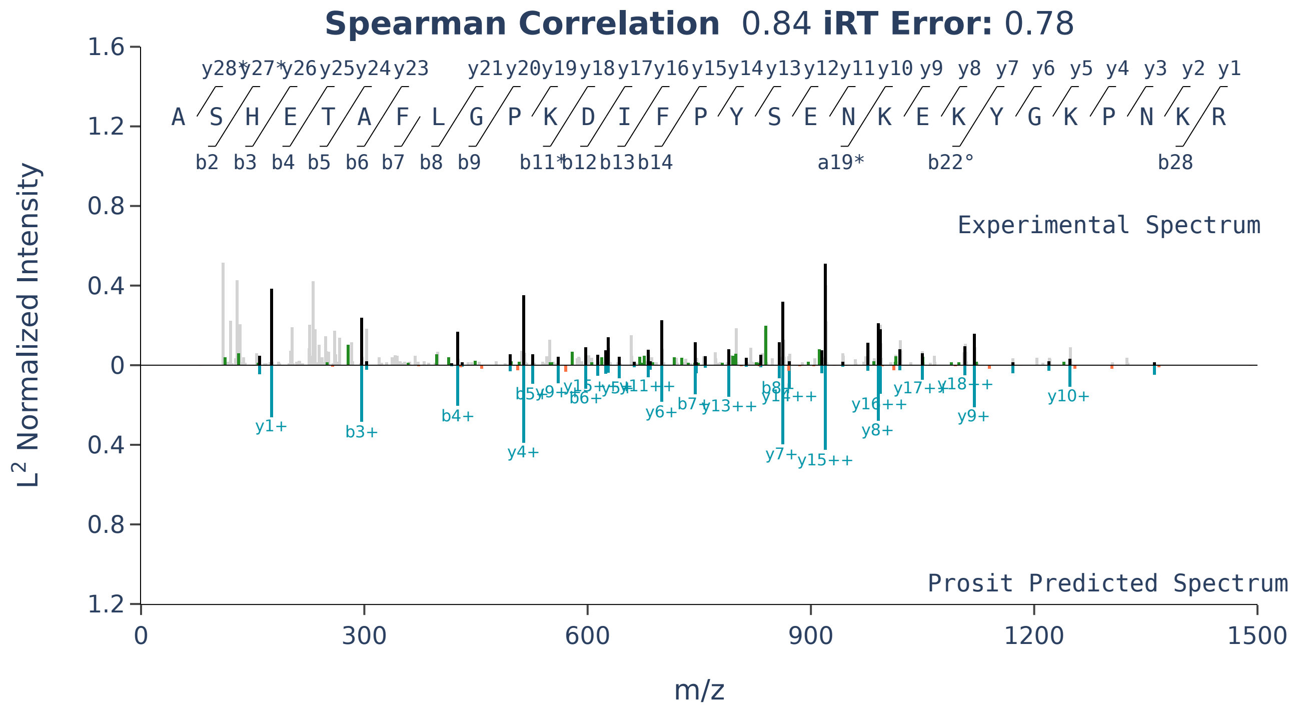

Source HRoetschke_271222_090123_HFGoe_LEDGF_b2R1_4h **Scan** 17002
Peptide GTHETAFLGPKDIFPYSENKEKYGKPNKR **Charge** 4 **Spectral Angle** 0.79
Spearman Correlation 0.84 **iRT Error:** 0.87

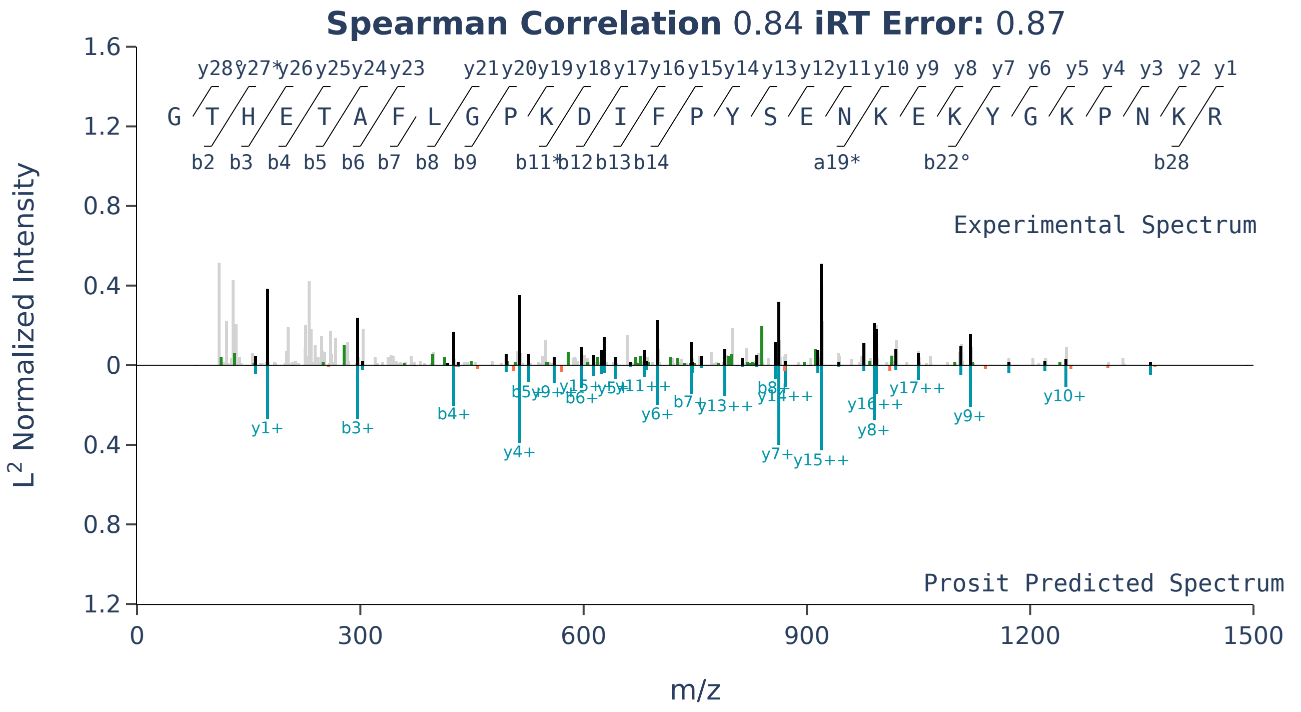

Source HRoetschke_271222_090123_HFGoe_LEDGF_b1R2_4h **Scan** 17592
Peptide ETAFHWPAR **Charge** 3 **Spectral Angle** 0.82
Spearman Correlation 0.95 **iRT Error:** 0.83

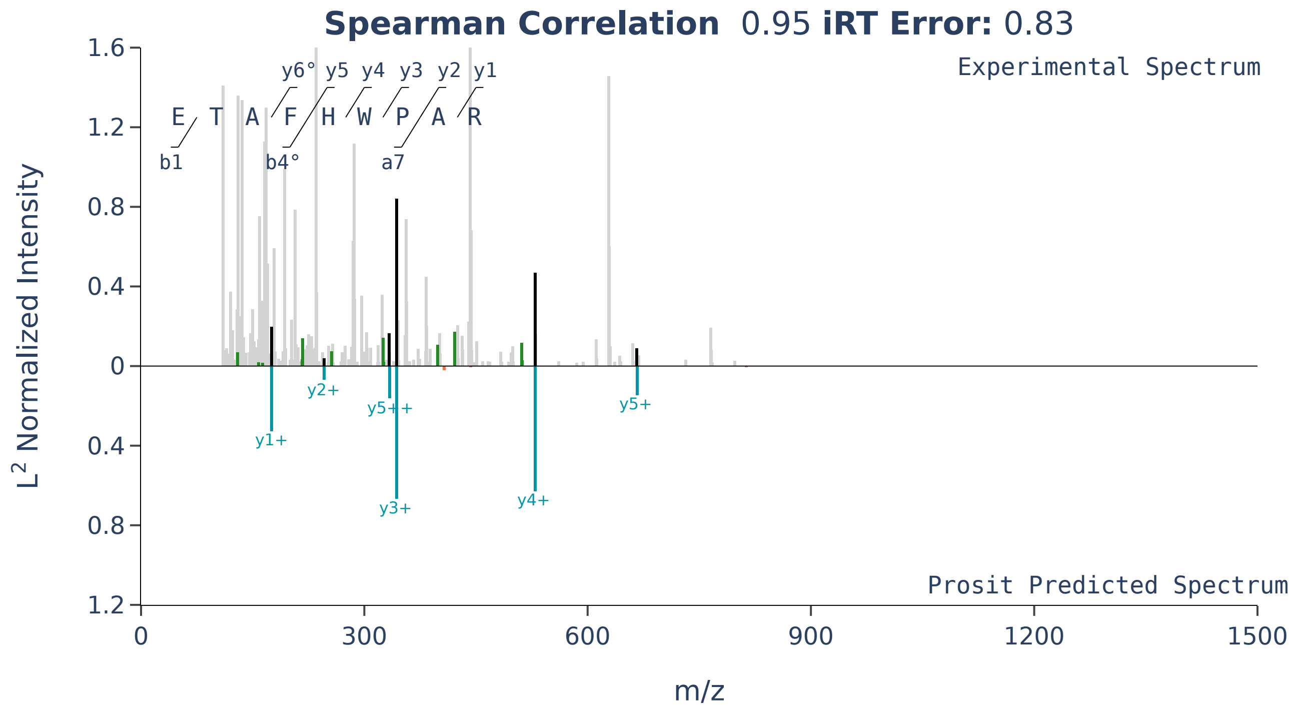

Source HRoetschke_271222_090123_HFGoe_LEDGF_b1R2_4h **Scan** 17592
Peptide WEIDNNPKV **Charge** 3 **Spectral Angle** 0.0
Spearman Correlation 0.0 **iRT Error:** 4.92

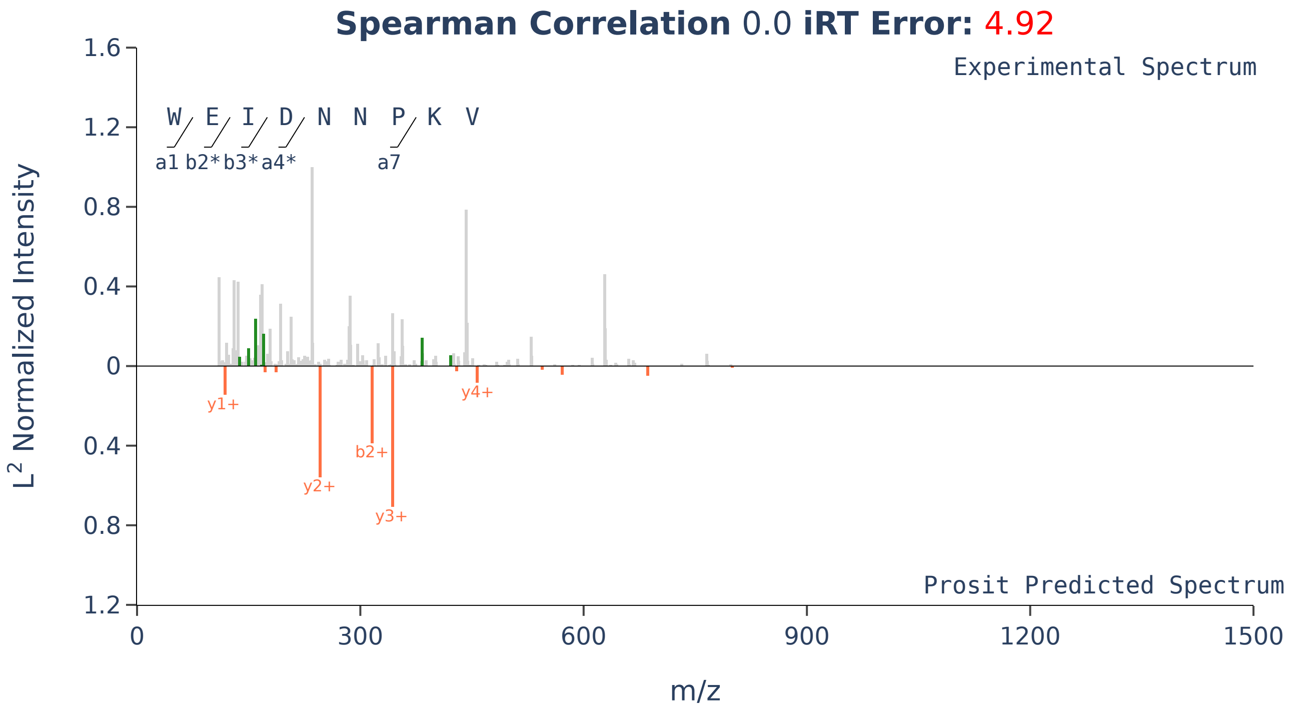

Source HRoetschke_271222_090123_HFGoe_LEDGF_b1R1_24h **Scan** 7533
Peptide PGSVNLKVSPKRGR **Charge** 4 **Spectral Angle** 0.86
Spearman Correlation 0.81 **iRT Error:** 0.88

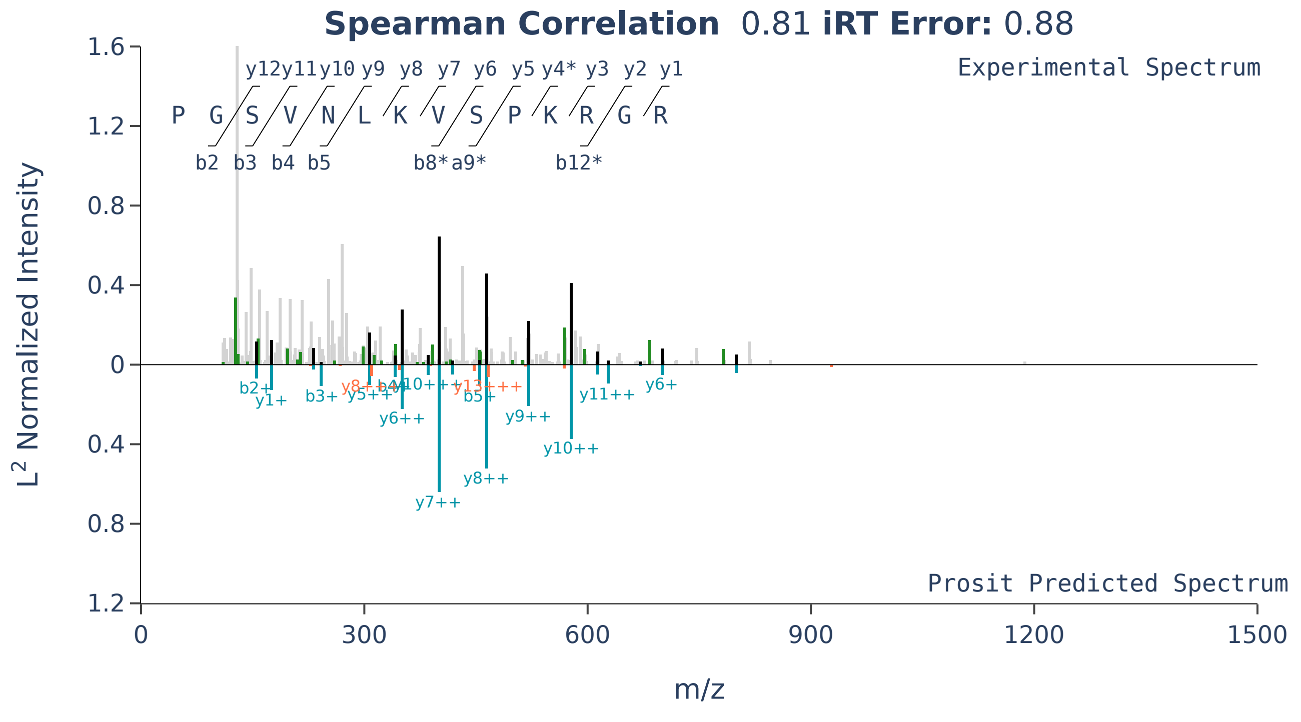

Source HRoetschke_271222_090123_HFGoe_LEDGF_b1R1_24h **Scan** 7533
Peptide NLKVSPKRGRPAAT **Charge** 4 **Spectral Angle** 0.06
Spearman Correlation 0.22 **iRT Error:** 11.58

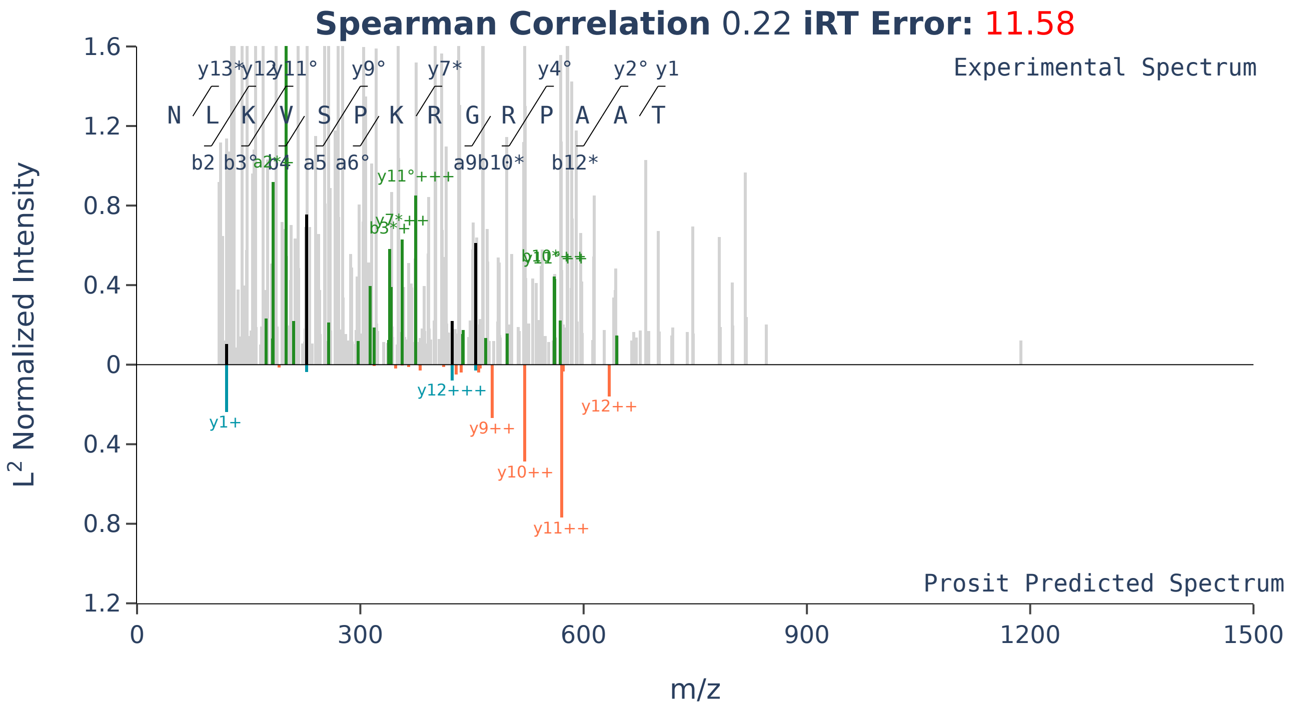

Source HRoetschke_271222_090123_HFGoe_LEDGF_b2R1_4h **Scan** 11970
Peptide QSGPGMTRDFKPGD **Charge** 2 **Spectral Angle** 0.89
Spearman Correlation 0.86 **iRT Error:** 0.89

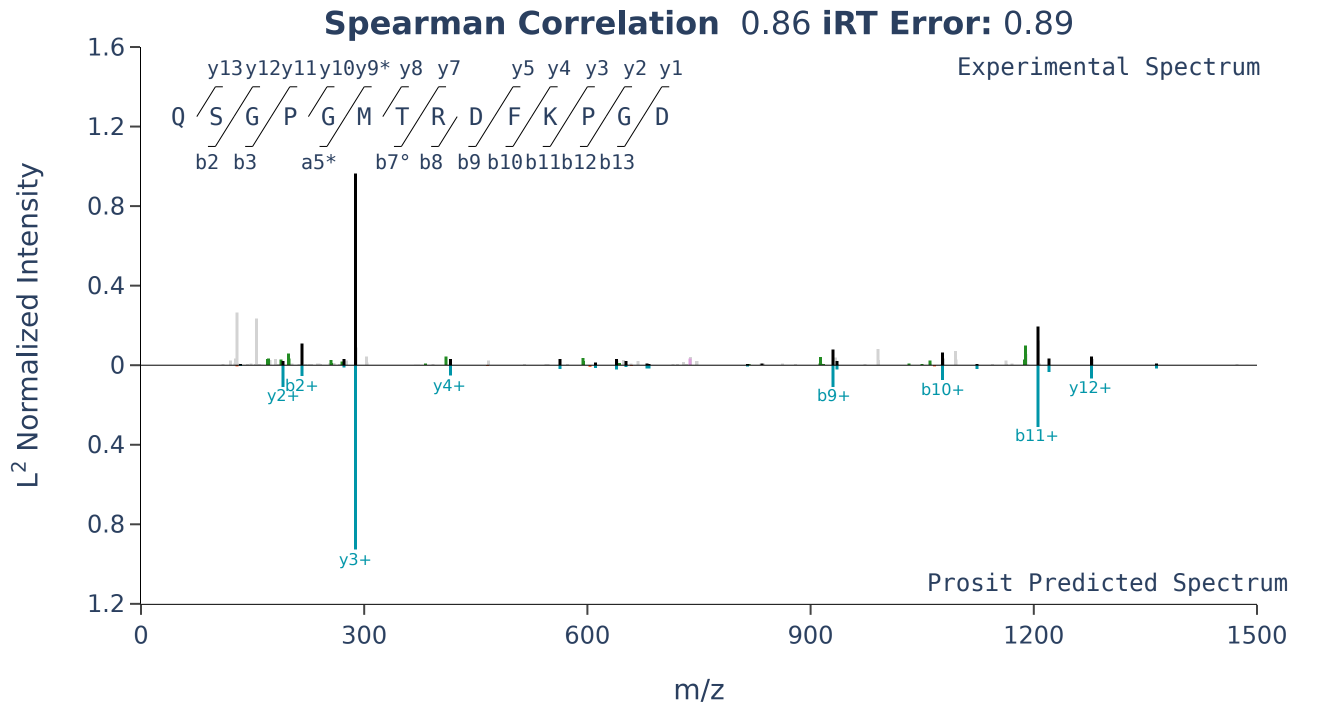

Source HRoetschke_271222_090123_HFGoe_LEDGF_b2R1_4h **Scan** 11970
Peptide EQTGSKTLNGGSDAQ **Charge** 2 **Spectral Angle** 0.01
Spearman Correlation -0.09 **iRT Error:** 38.43

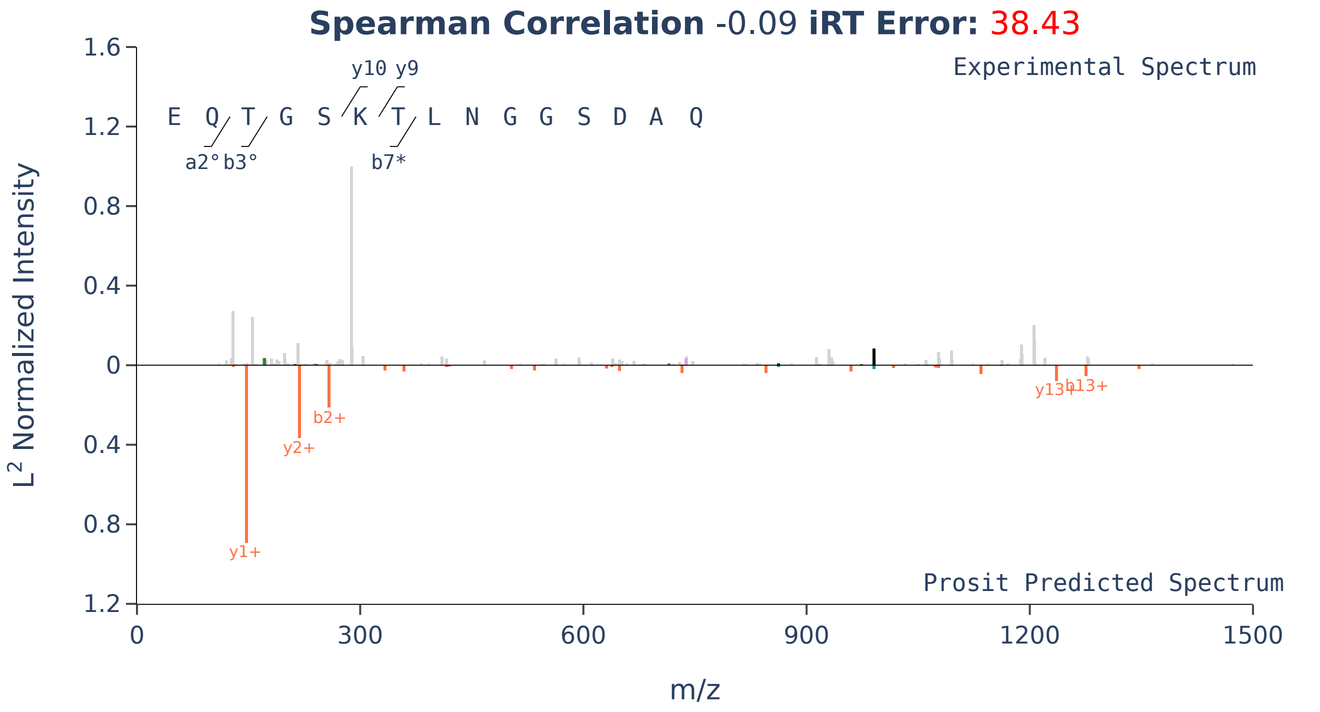

Source HRoetschke_271222_090123_HFGoe_LEDGF_b1R2_24h **Scan** 17255
Peptide SQGYPHWPARVDE **Charge** 3 **Spectral Angle** 0.86
Spearman Correlation 0.82 **iRT Error:** 0.9

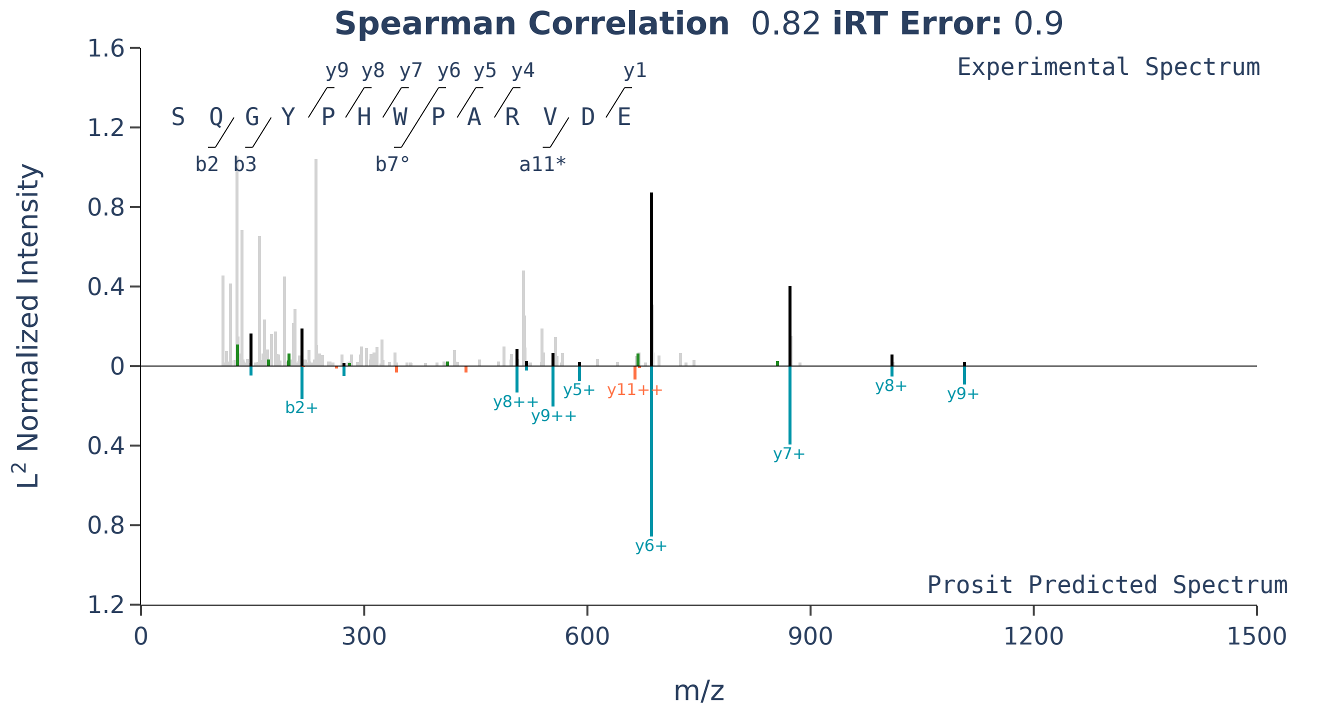

Source HRoetschke_271222_090123_HFGoe_LEDGF_b1R2_24h **Scan** 17255
Peptide FSSQQAATKQSNASS **Charge** 3 **Spectral Angle** 0.01
Spearman Correlation -0.2 **iRT Error:** 69.79

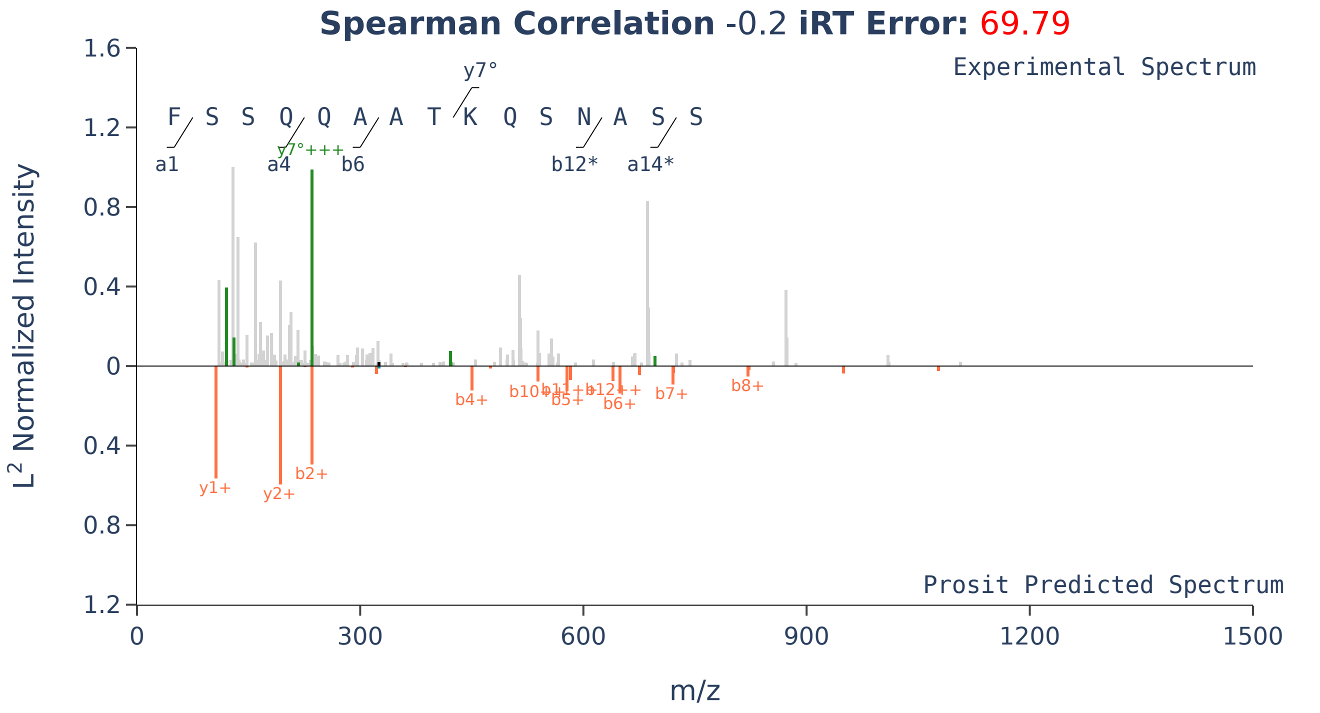

Source HRoetschke_271222_090123_HFGoe_LEDGF_b2R1_4h **Scan** 17529
Peptide AFLGPKDIFPYSENKEKYGKPNKRAT **Charge** 4 **Spectral Angle** 0.87
Spearman Correlation 0.82 **iRT Error:** 0.9

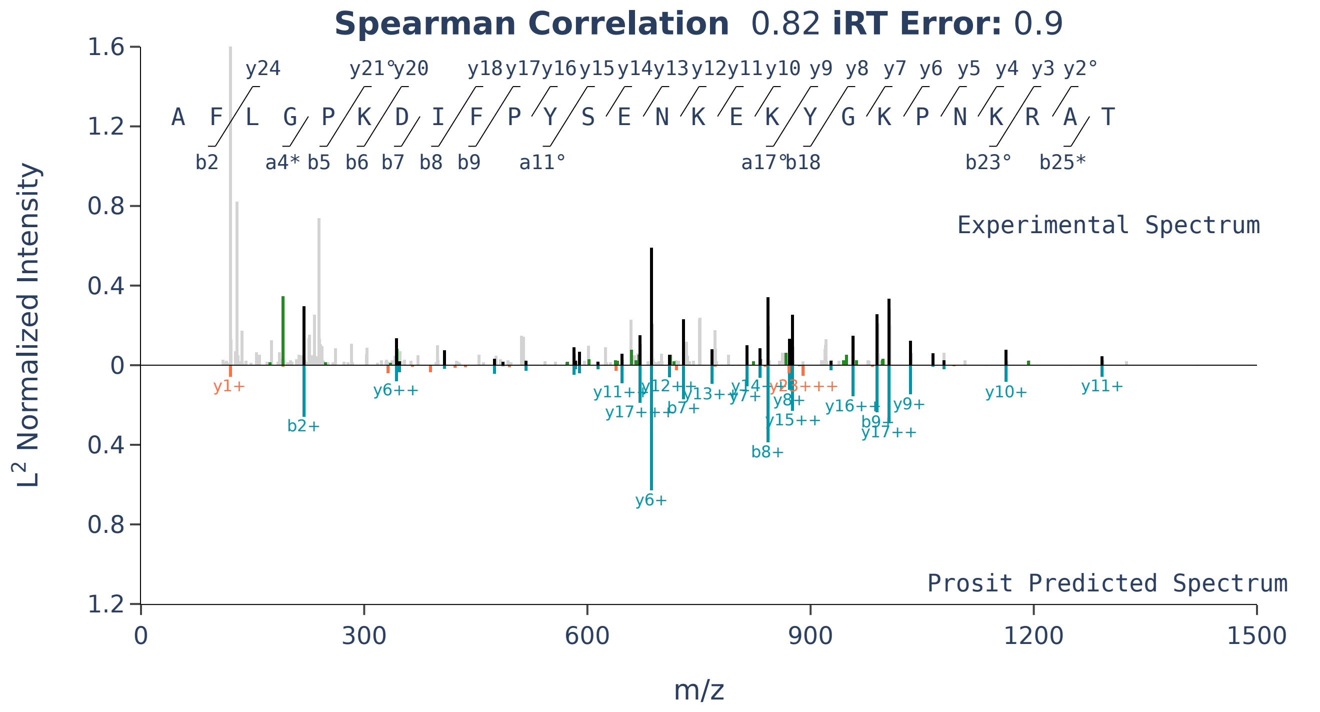

Source HRoetschke_271222_090123_HFGoe_LEDGF_b2R1_4h **Scan** 17529
Peptide WPARVDEVPDGAVKPPTNKLPIFFFGT **Charge** 4 **Spectral Angle** 0.03
Spearman Correlation 0.09 **iRT Error:** 76.19

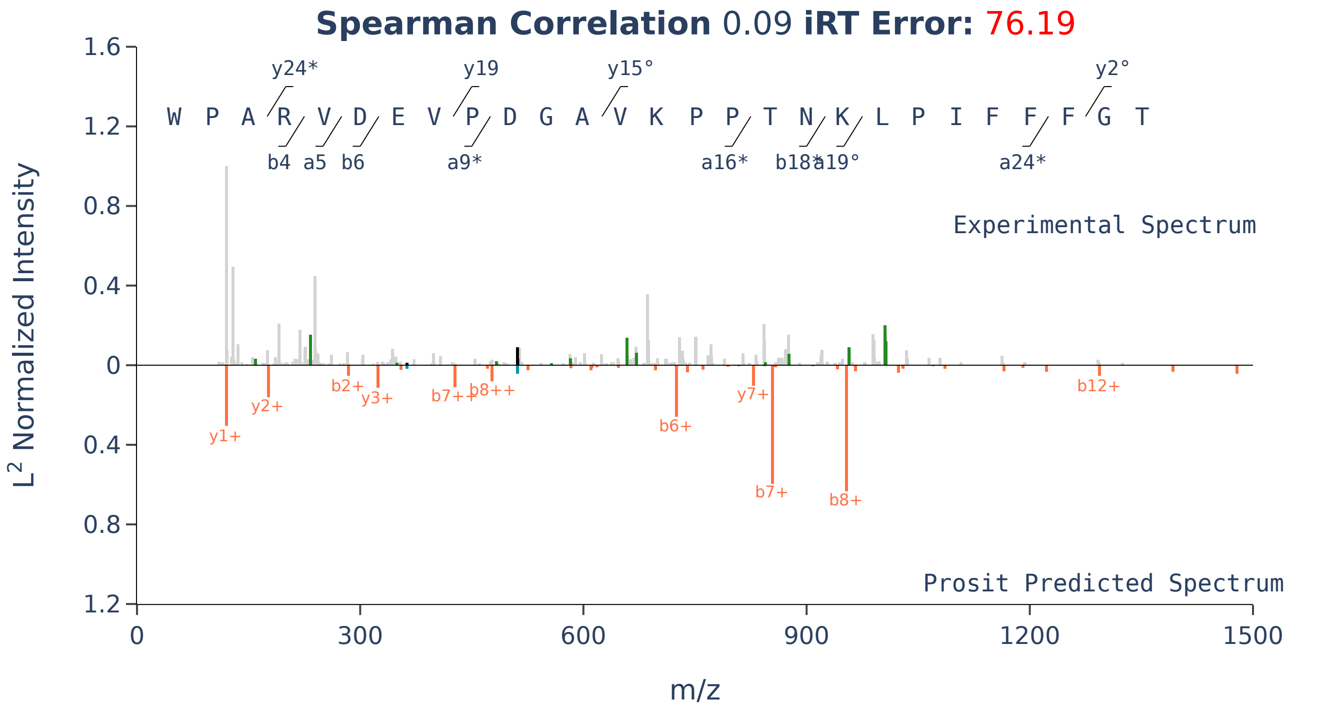

Source HRoetschke_271222_090123_HFGoe_LEDGF_b1R1_24h **Scan** 9355
Peptide PSSEERETLE **Charge** 1 **Spectral Angle** 0.75
Spearman Correlation 0.87 **iRT Error:** 0.91

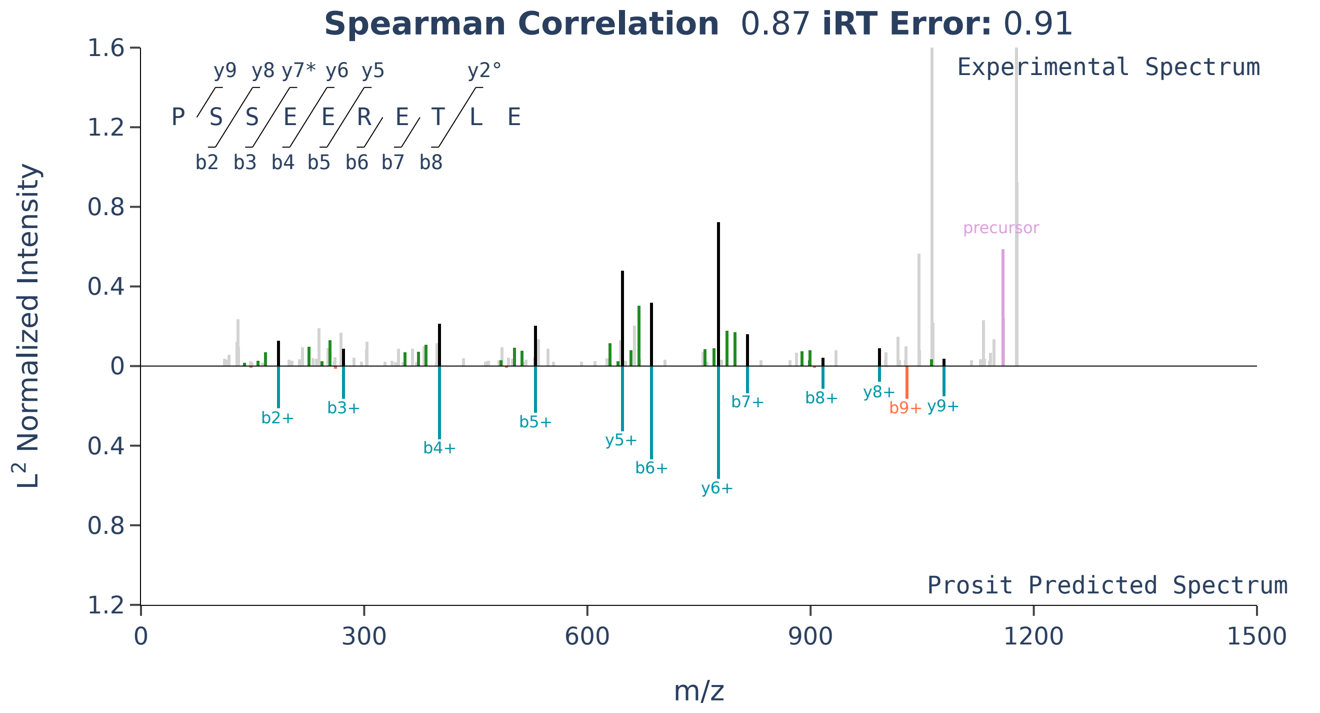

Source HRoetschke_271222_090123_HFGoe_LEDGF_b1R1_24h **Scan** 9355
Peptide PSSEERETEI **Charge** 1 **Spectral Angle** 0.44
Spearman Correlation 0.42 **iRT Error:** 11.53

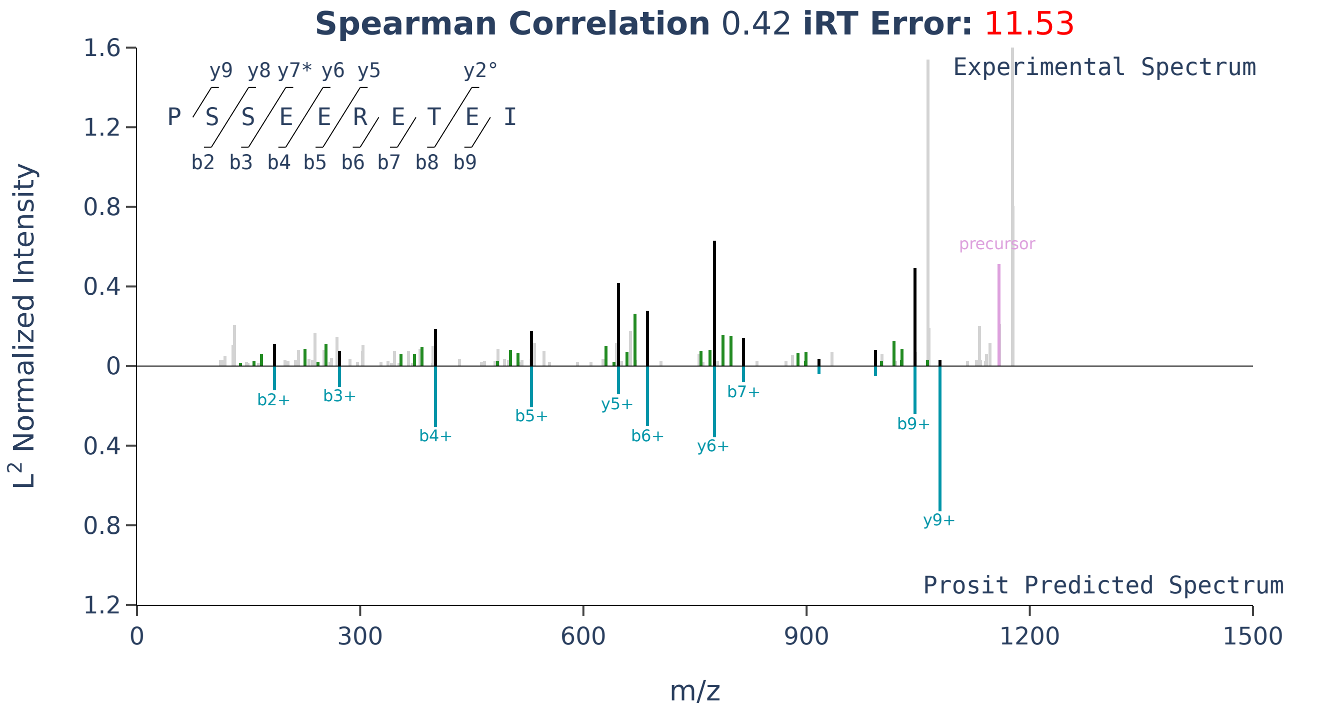

Source HRoetschke_271222_090123_HFGoe_LEDGF_b2R2_2h **Scan** 17273
Peptide FLGPKDIFPYSENKEKYGKPNKRA **Charge** 4 **Spectral Angle** 0.79
Spearman Correlation 0.86 **iRT Error:** 0.97

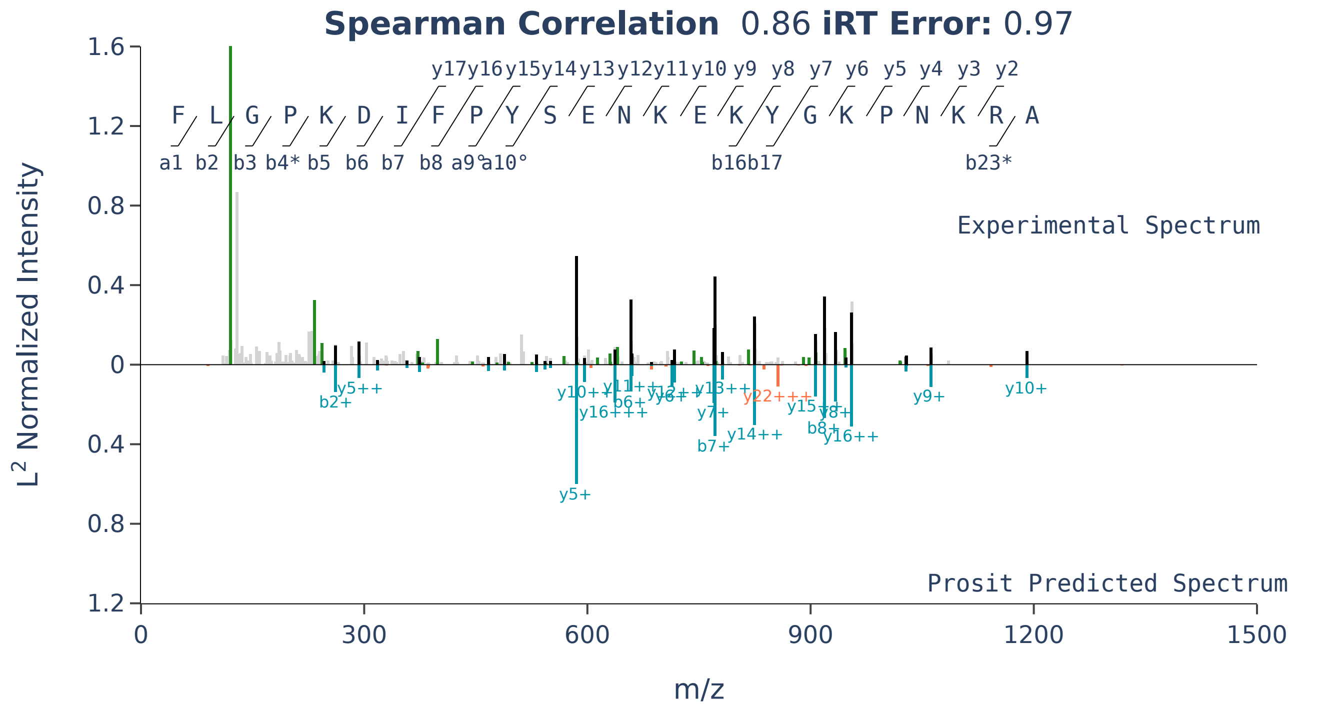

Source HRoetschke_271222_090123_HFGoe_LEDGF_b2R2_2h **Scan** 17273
Peptide GQKEEDKPRKEPDKKEGKKEVESK **Charge** 4 **Spectral Angle** 0.0
Spearman Correlation 0.0 **iRT Error:** 93.11

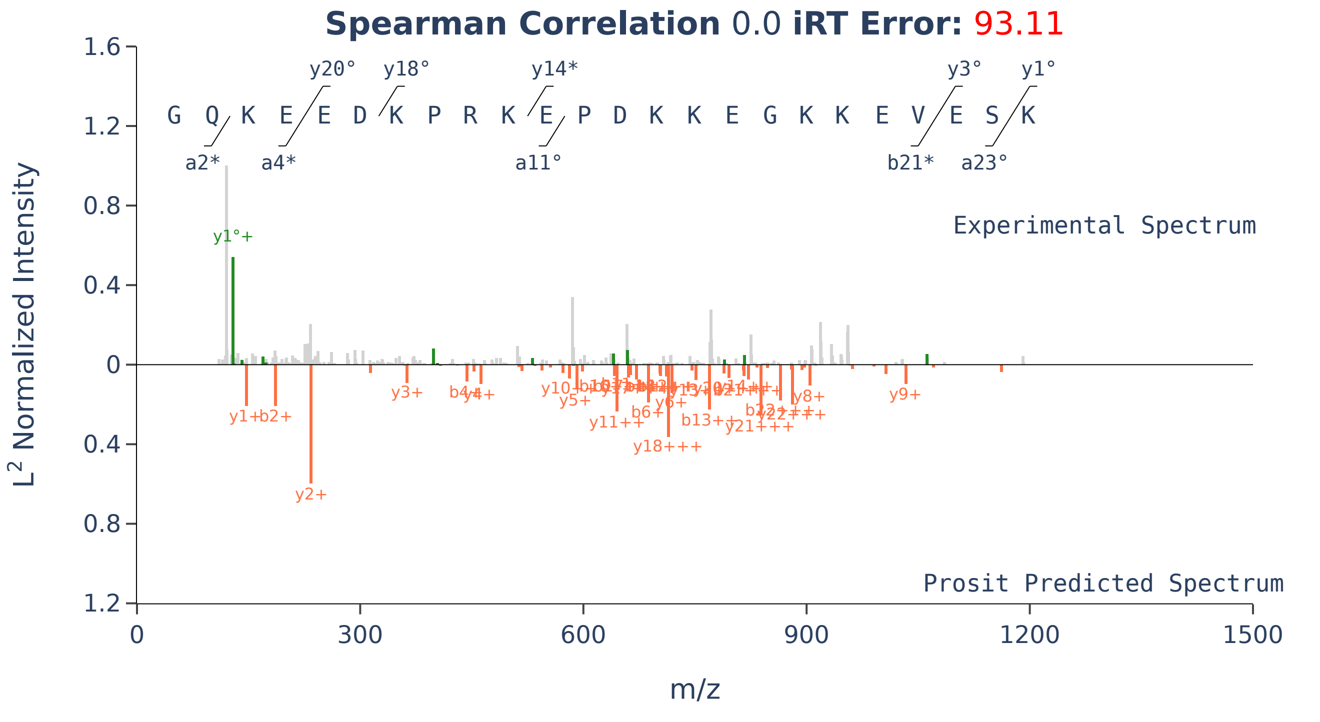

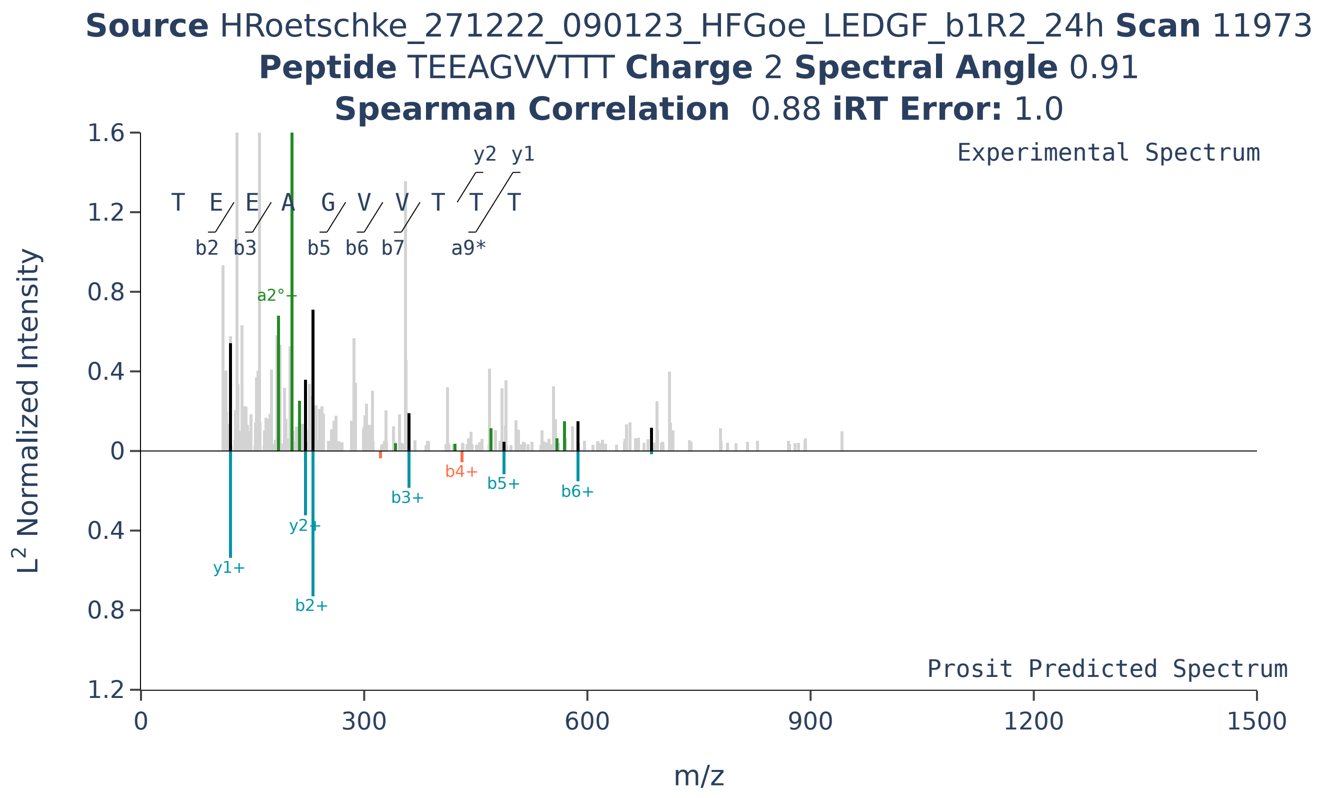
Source HRoetschke_271222_090123_HFGoe_LEDGF_b1R2_24h Scan 11973
Peptide TEEAGVVTTT Charge 2 Spectral Angle 0.91
Spearman Correlation 0.88 iRT Error: 1.0
Experimental Spectrum
Prosit Predicted Spectrum
L² Normalized Intensity
m/z

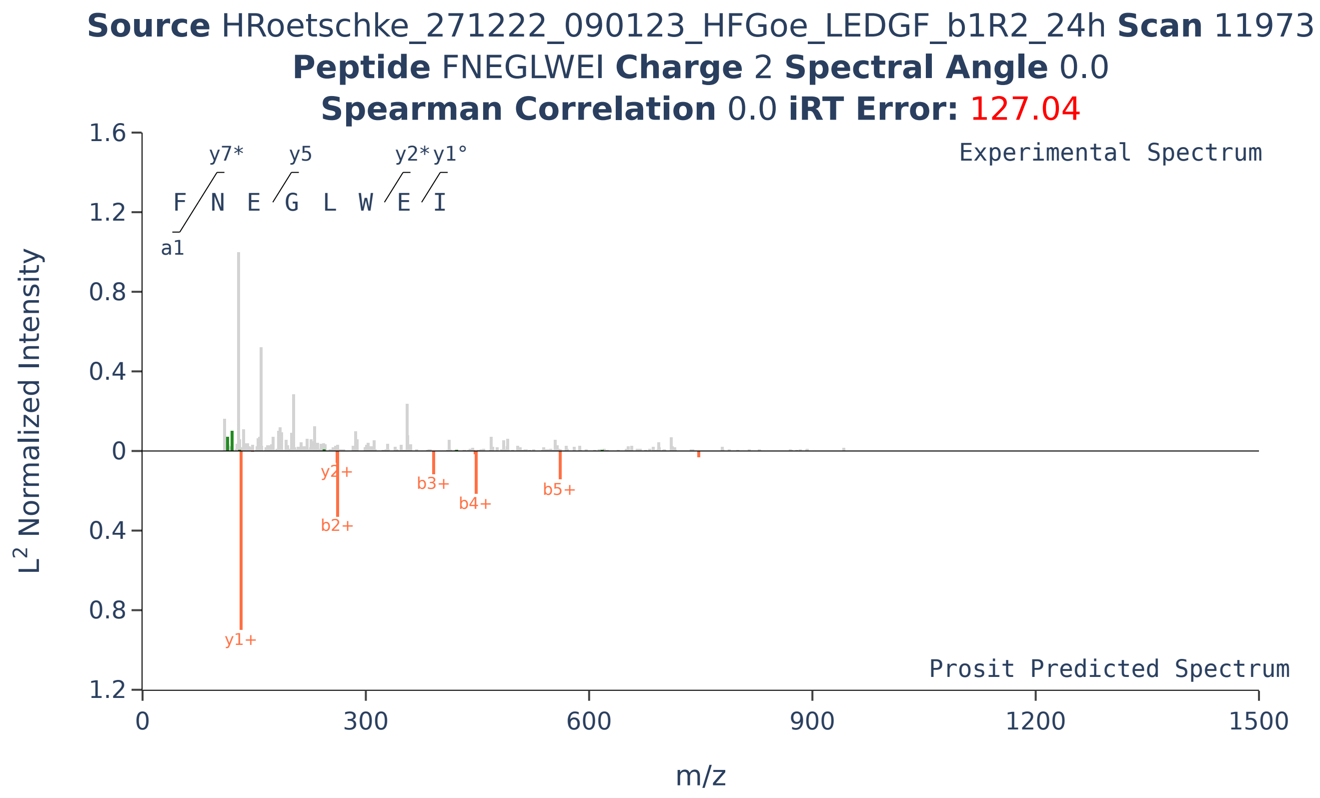
Source HRoetschke_271222_090123_HFGoe_LEDGF_b1R2_24h Scan 11973
Peptide FNEGLWEI Charge 2 Spectral Angle 0.0
Spearman Correlation 0.0 iRT Error: 127.04
Experimental Spectrum
Prosit Predicted Spectrum
L² Normalized Intensity
m/z

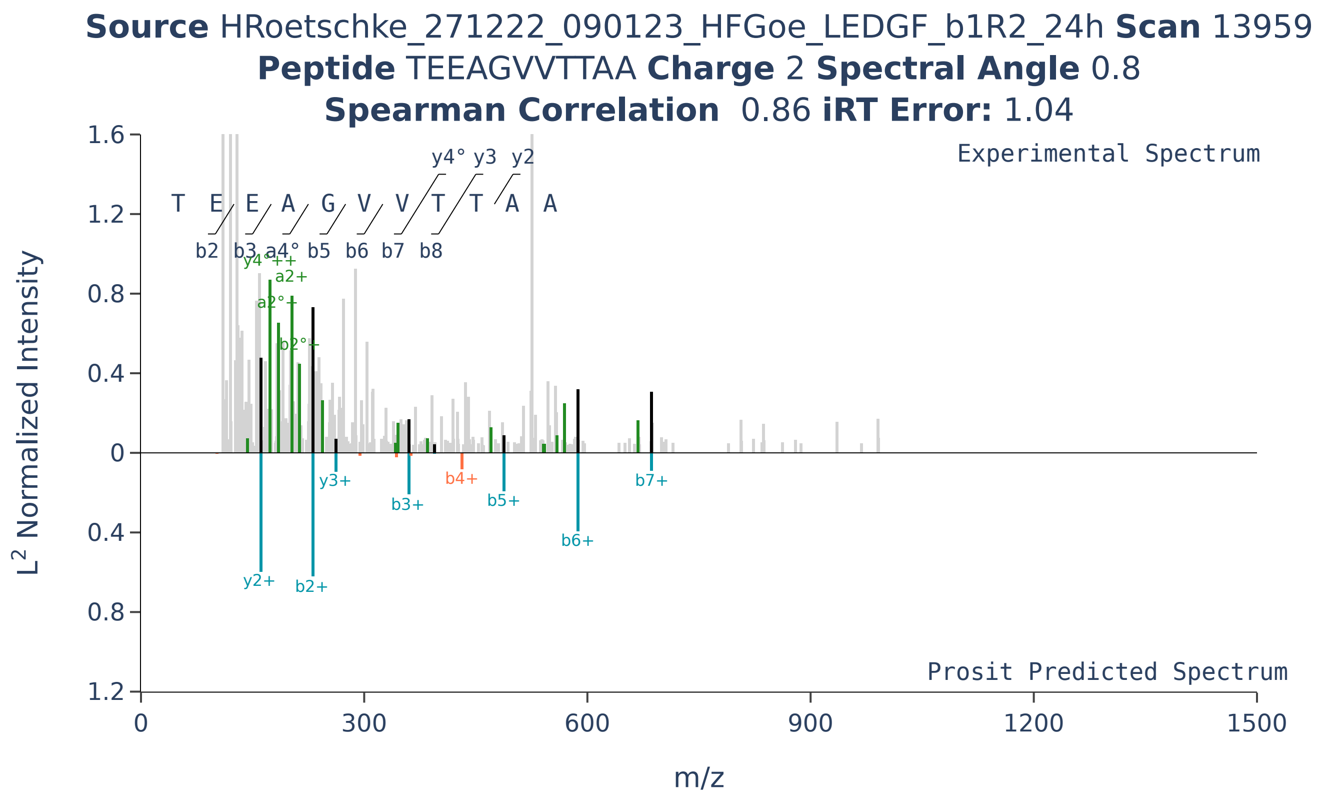
Source HRoetschke_271222_090123_HFGoe_LEDGF_b1R2_24h Scan 13959
Peptide TEEAGVVTTAA Charge 2 Spectral Angle 0.8
Spearman Correlation 0.86 iRT Error: 1.04
Experimental Spectrum
Prosit Predicted Spectrum
L² Normalized Intensity
m/z

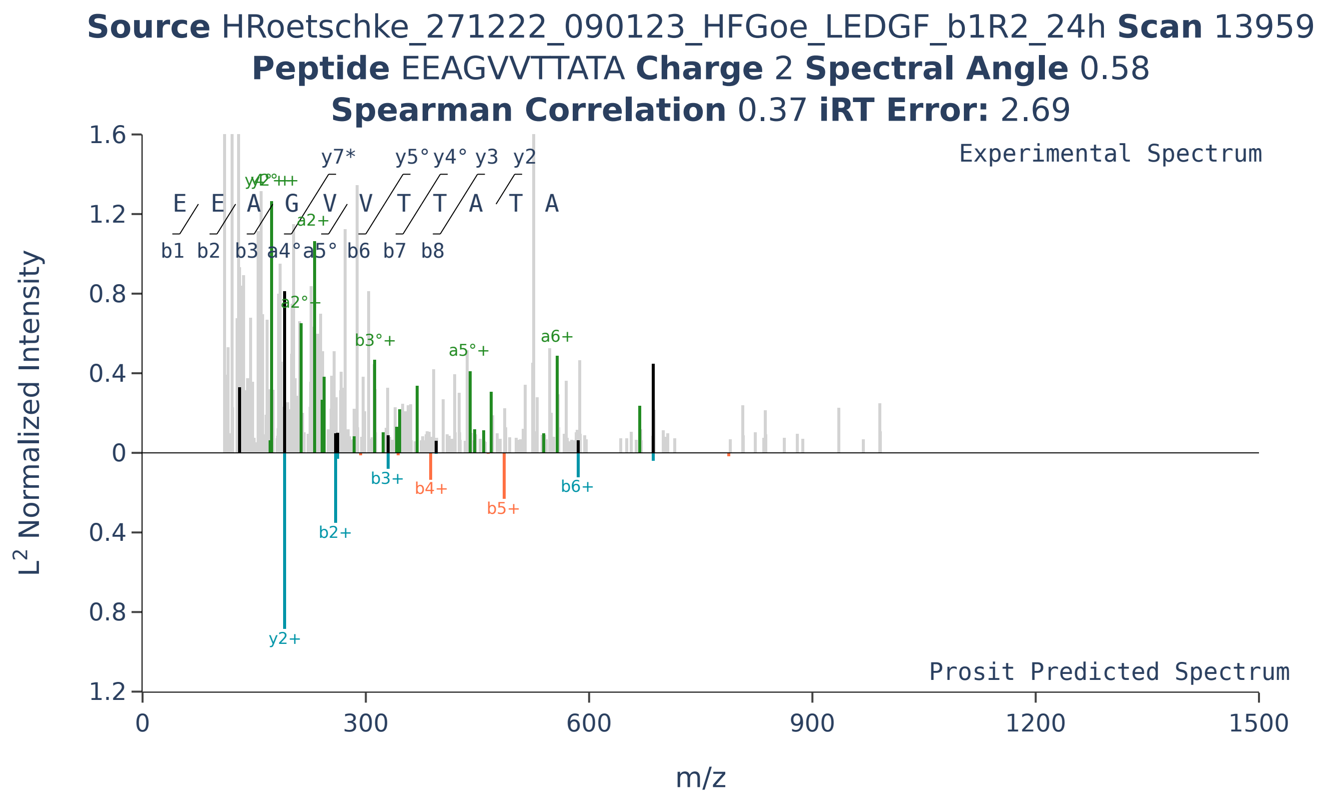
Source HRoetschke_271222_090123_HFGoe_LEDGF_b1R2_24h Scan 13959
Peptide EEAGVVTTATA Charge 2 Spectral Angle 0.58
Spearman Correlation 0.37 iRT Error: 2.69
Experimental Spectrum
Prosit Predicted Spectrum
L² Normalized Intensity
m/z

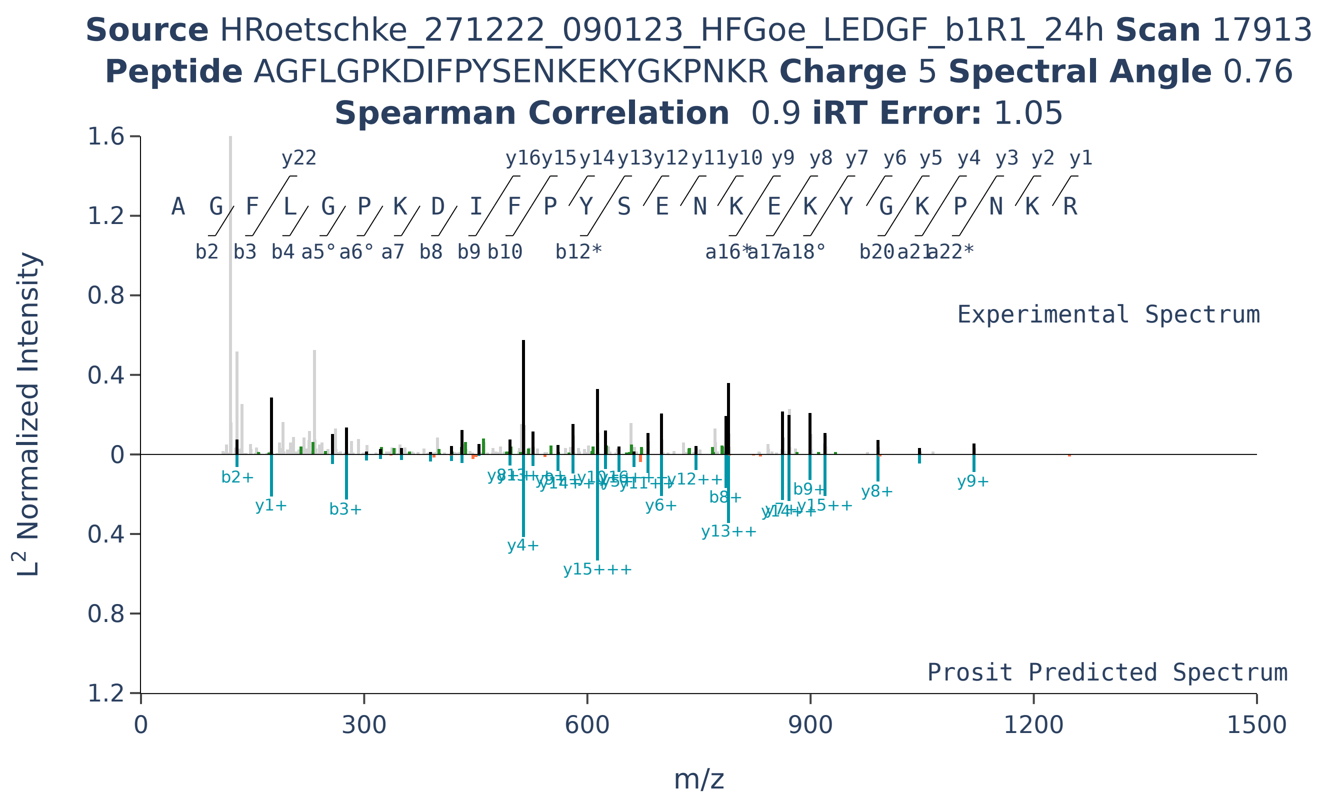
Source HRoetschke_271222_090123_HFGoe_LEDGF_b1R1_24h Scan 17913
Peptide AGFLGPKDIFPYSENKEKYGKPNKR Charge 5 Spectral Angle 0.76
Spearman Correlation 0.9 iRT Error: 1.05
Experimental Spectrum
Prosit Predicted Spectrum
L² Normalized Intensity
m/z

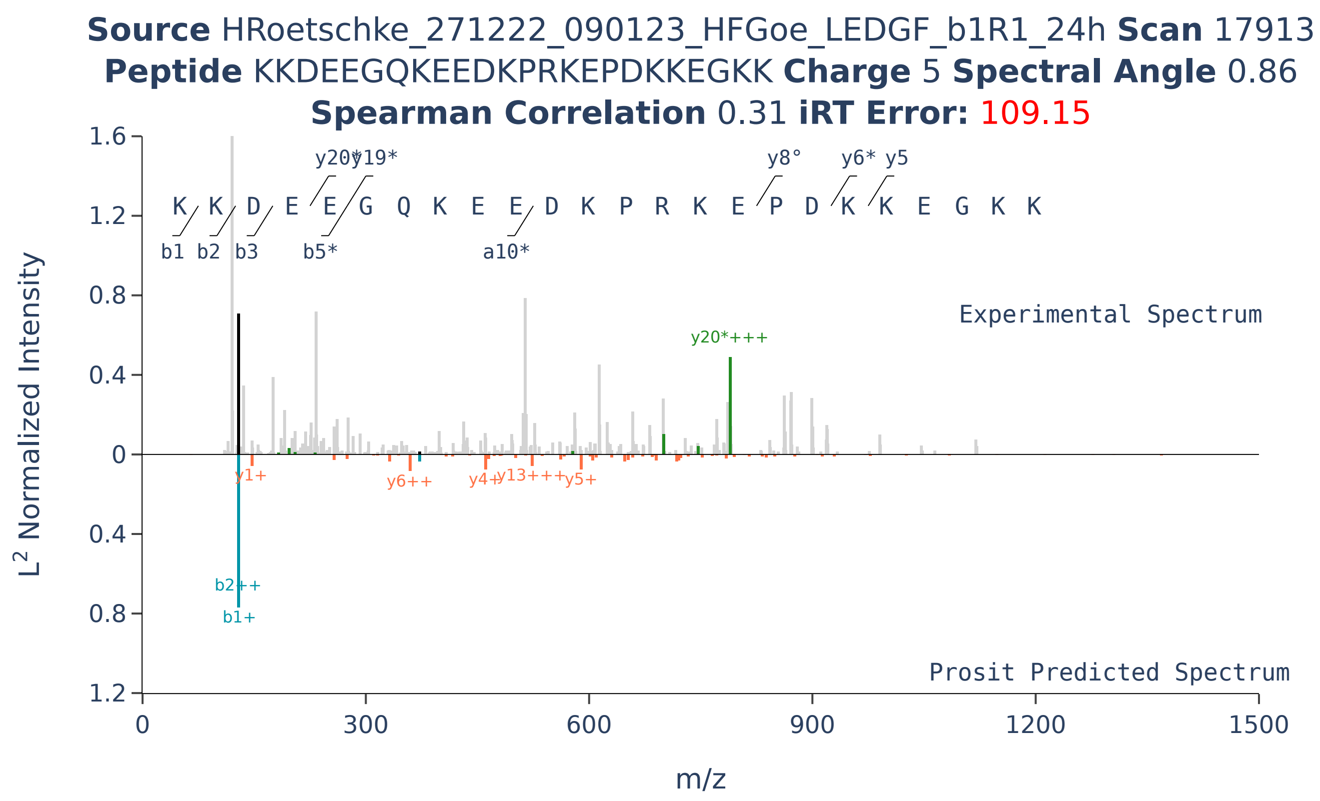
Source HRoetschke_271222_090123_HFGoe_LEDGF_b1R1_24h Scan 17913
Peptide KKDEEGQKEEDKPRKEPDKKEGKK Charge 5 Spectral Angle 0.86
Spearman Correlation 0.31 iRT Error: 109.15
Experimental Spectrum
Prosit Predicted Spectrum
L² Normalized Intensity
m/z

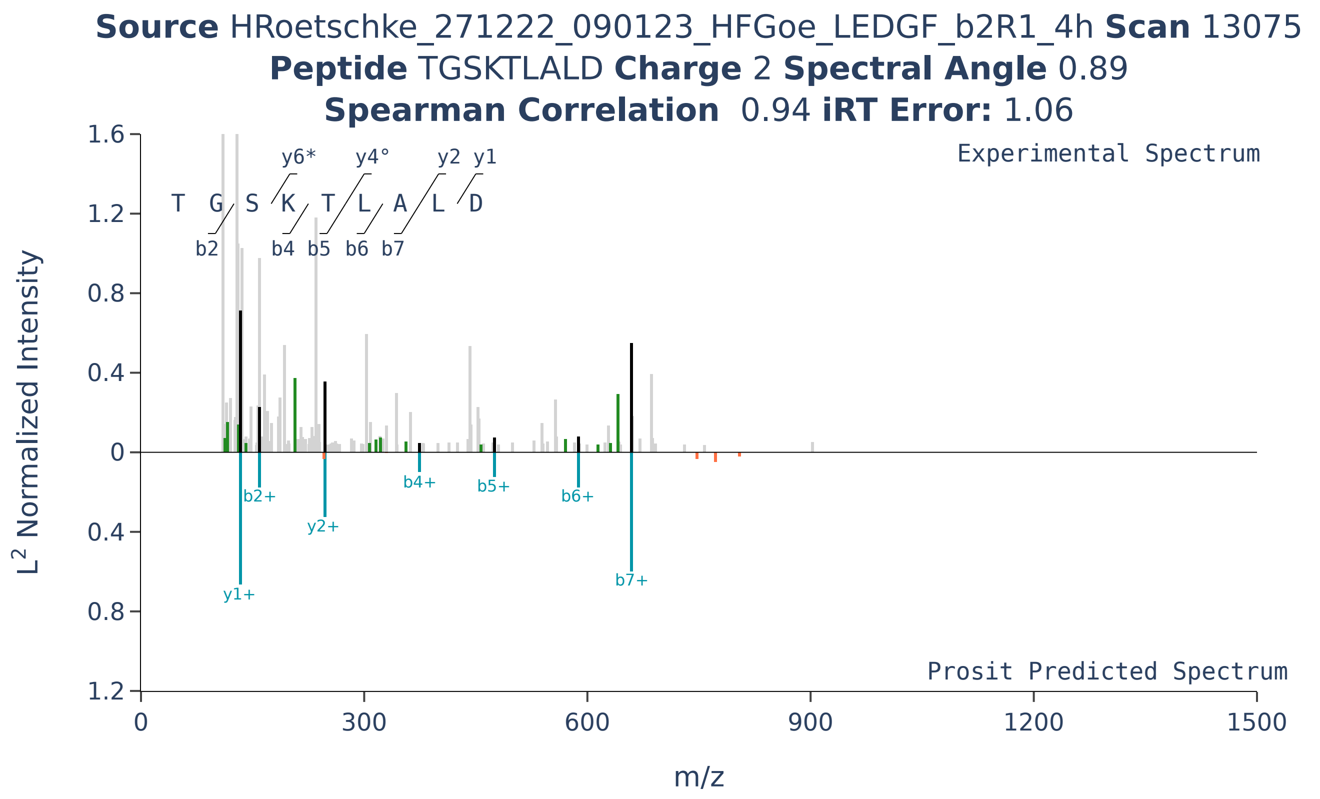
Source HRoetschke_271222_090123_HFGoe_LEDGF_b2R1_4h Scan 13075
Peptide TGSKTLALD Charge 2 Spectral Angle 0.89
Spearman Correlation 0.94 iRT Error: 1.06
Experimental Spectrum
Prosit Predicted Spectrum
L² Normalized Intensity
m/z

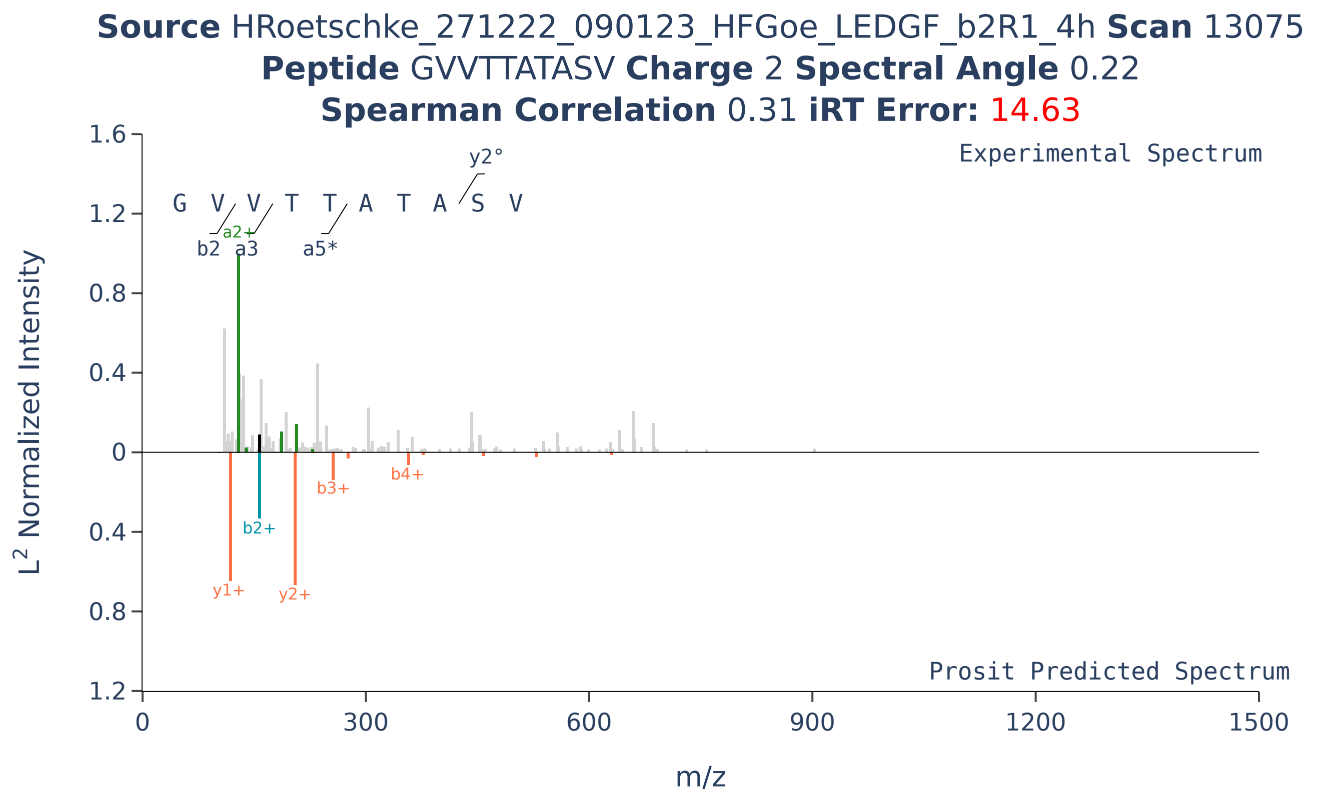
Source HRoetschke_271222_090123_HFGoe_LEDGF_b2R1_4h Scan 13075
Peptide GVVTTATASV Charge 2 Spectral Angle 0.22
Spearman Correlation 0.31 iRT Error: 14.63
Experimental Spectrum
Prosit Predicted Spectrum
L² Normalized Intensity
m/z

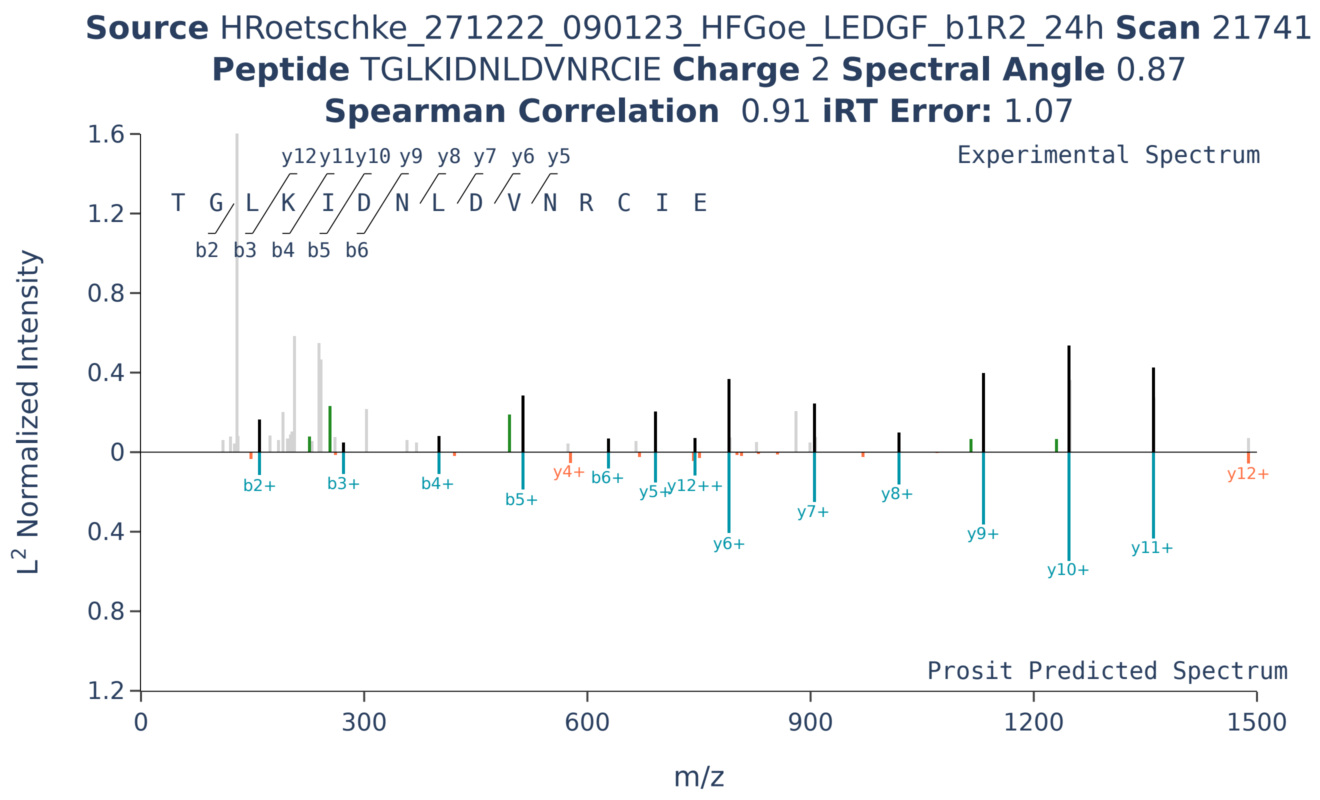
Source HRoetschke_271222_090123_HFGoe_LEDGF_b1R2_24h Scan 21741
Peptide TGLKIDNLDVNRCIE Charge 2 Spectral Angle 0.87
Spearman Correlation 0.91 iRT Error: 1.07
Experimental Spectrum
Prosit Predicted Spectrum
L² Normalized Intensity
m/z

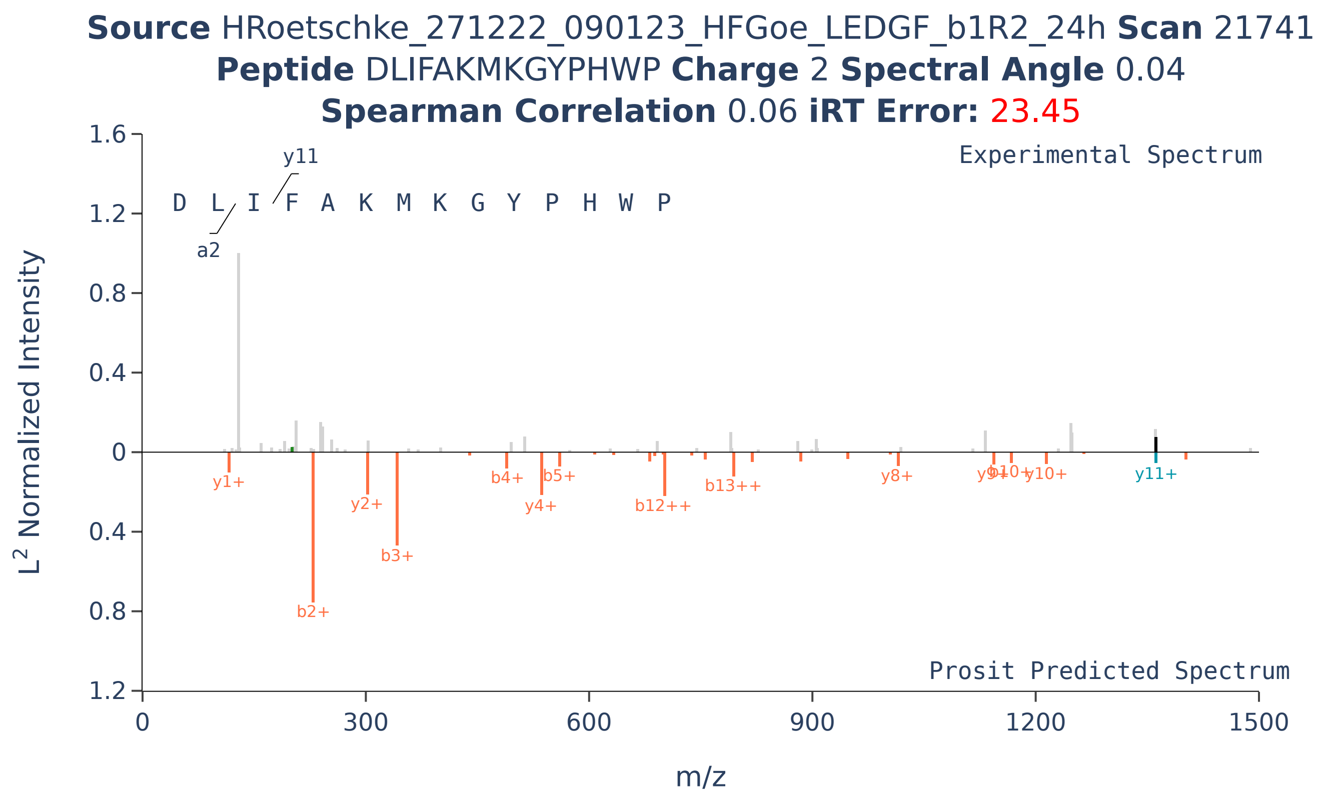
Source HRoetschke_271222_090123_HFGoe_LEDGF_b1R2_24h Scan 21741
Peptide DLIFAKMKGYPHWP Charge 2 Spectral Angle 0.04
Spearman Correlation 0.06 iRT Error: 23.45
Experimental Spectrum
Prosit Predicted Spectrum
L² Normalized Intensity
m/z

Source HRoetschke_271222_090123_HFGoe_LEDGF_b1R2_24h **Scan** 16677
Peptide KVSQVIMEKSEL **Charge** 2 **Spectral Angle** 0.89
Spearman Correlation 0.88 **iRT Error:** 1.07

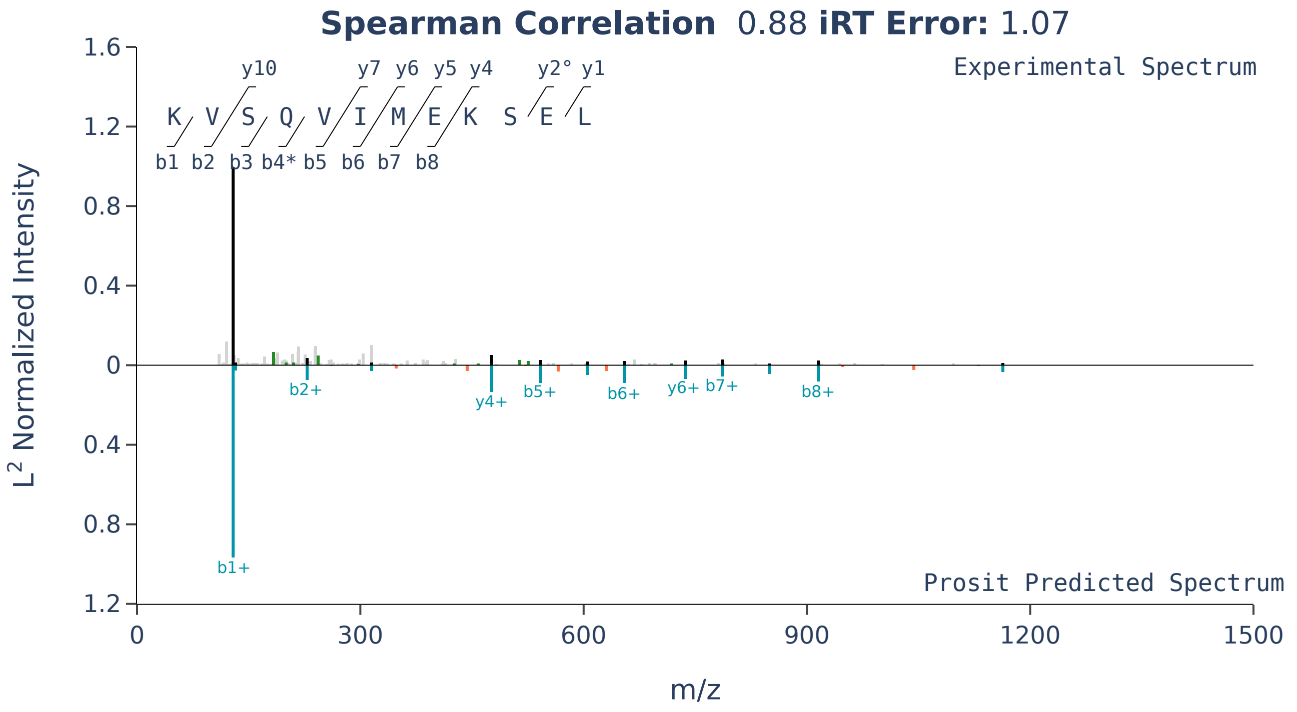

Source HRoetschke_271222_090123_HFGoe_LEDGF_b1R2_24h **Scan** 16677
Peptide ITEEDKSKKKGQ **Charge** 2 **Spectral Angle** 0.0
Spearman Correlation 0.0 **iRT Error:** 77.44

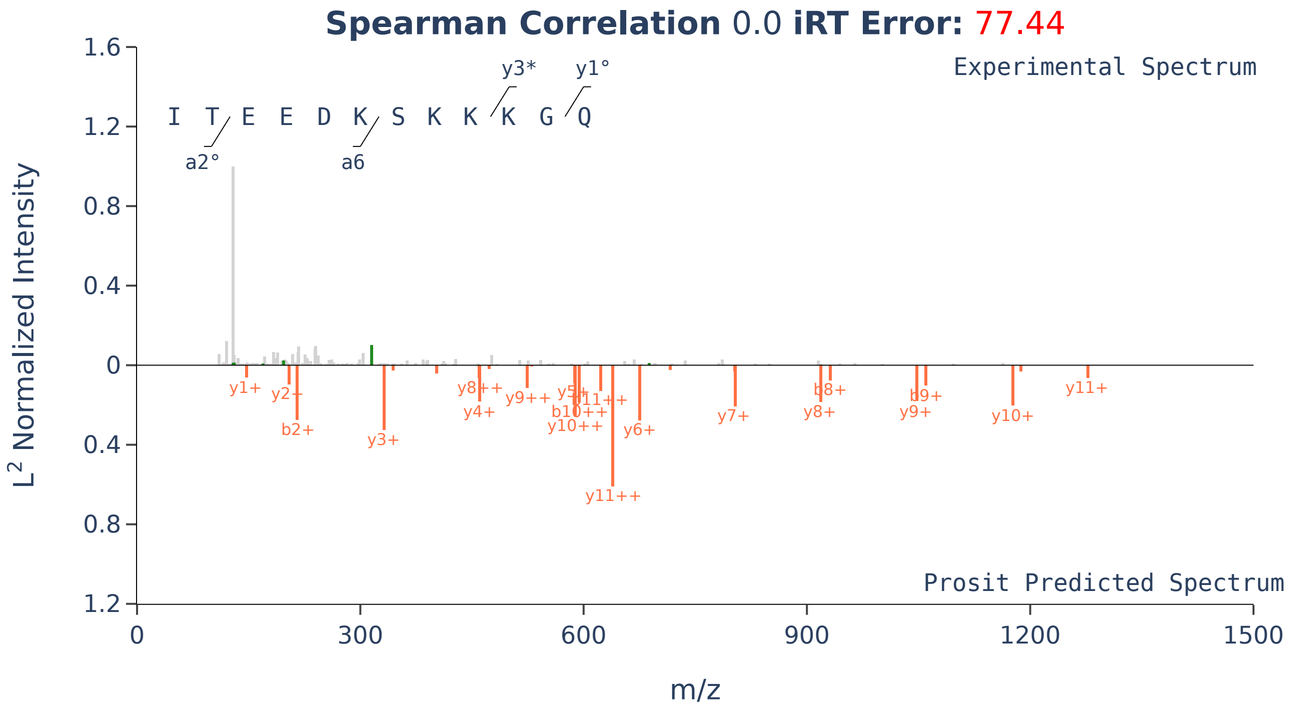

Source HRoetschke_271222_090123_HFGoe_LEDGF_b1R2_24h **Scan** 12144
Peptide TKVSQVIMEKS **Charge** 2 **Spectral Angle** 0.81
Spearman Correlation 0.92 **iRT Error:** 1.1

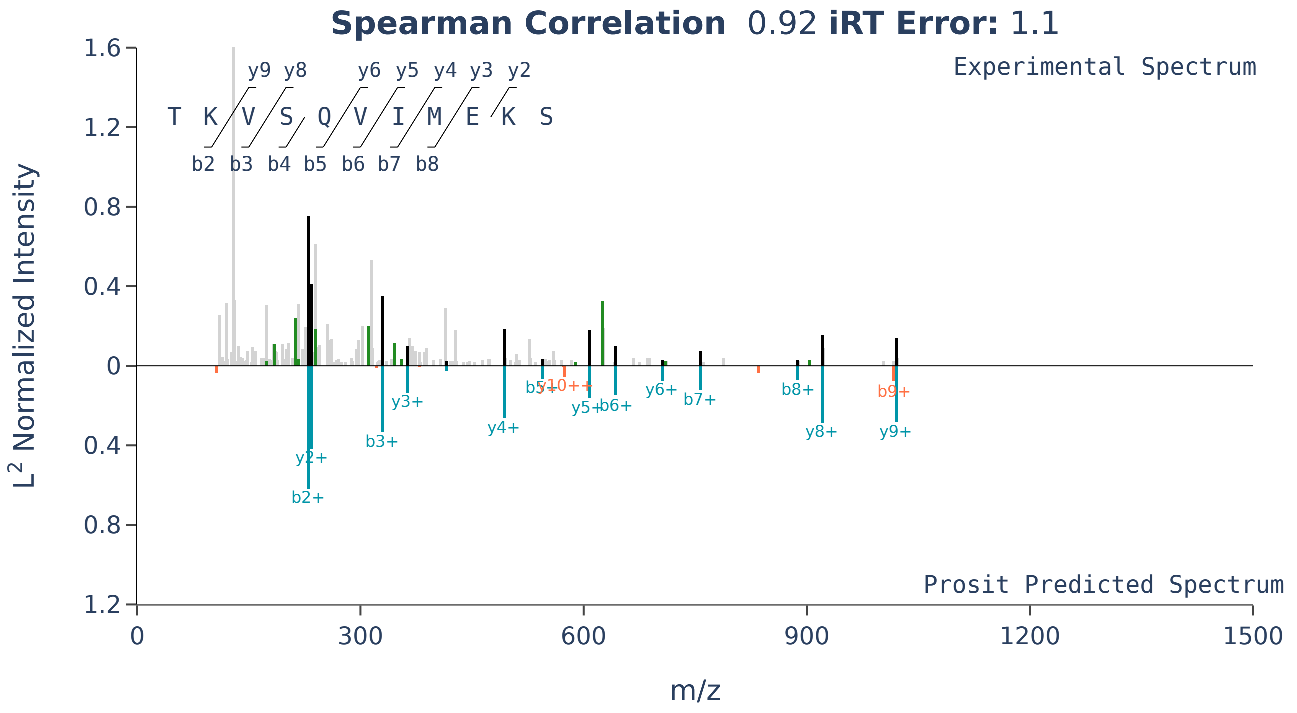

Source HRoetschke_271222_090123_HFGoe_LEDGF_b1R2_24h **Scan** 12144
Peptide KVSQVIMEKST **Charge** 2 **Spectral Angle** 0.87
Spearman Correlation 0.28 **iRT Error:** 8.01

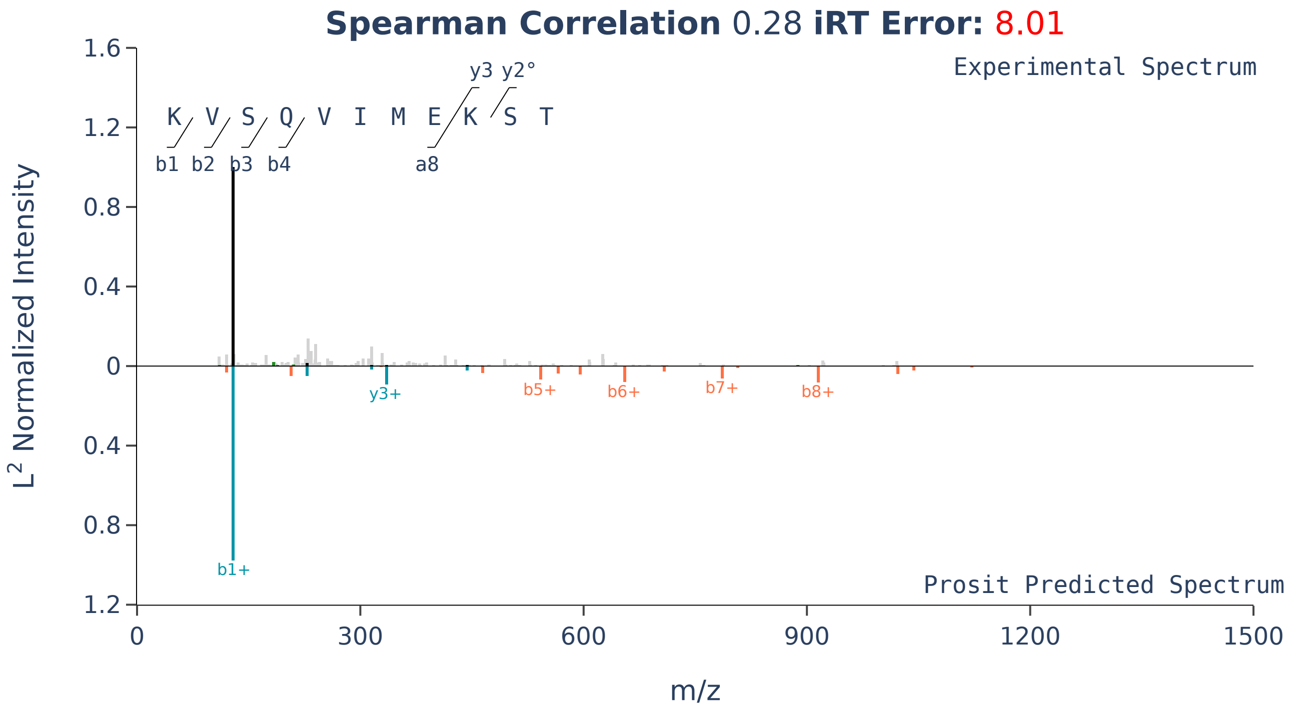

Source HRoetschke_271222_090123_HFGoe_LEDGF_b2R2_1h **Scan** 17360
Peptide SQYPHWPARV **Charge** 3 **Spectral Angle** 0.87
Spearman Correlation 0.85 **iRT Error:** 1.13

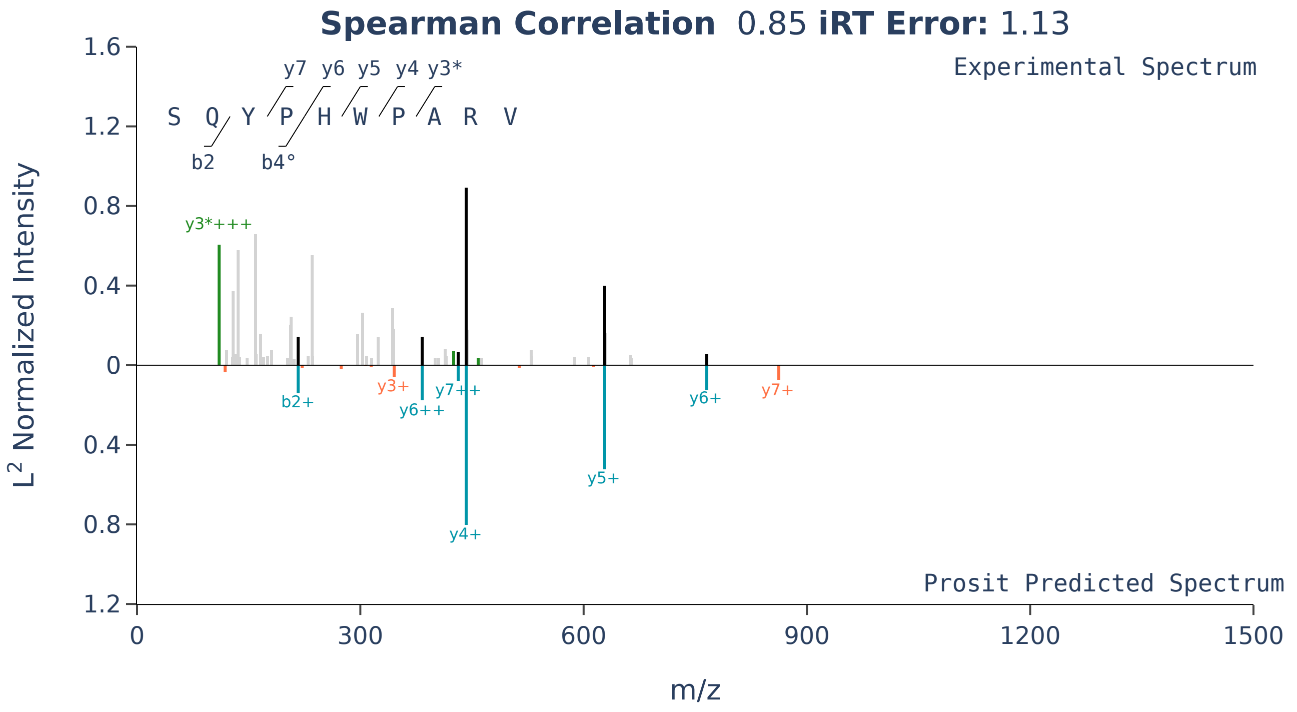

Source HRoetschke_271222_090123_HFGoe_LEDGF_b2R2_1h **Scan** 17360
Peptide KDIFPYSENK **Charge** 3 **Spectral Angle** 0.3
Spearman Correlation 0.39 **iRT Error:** 13.83

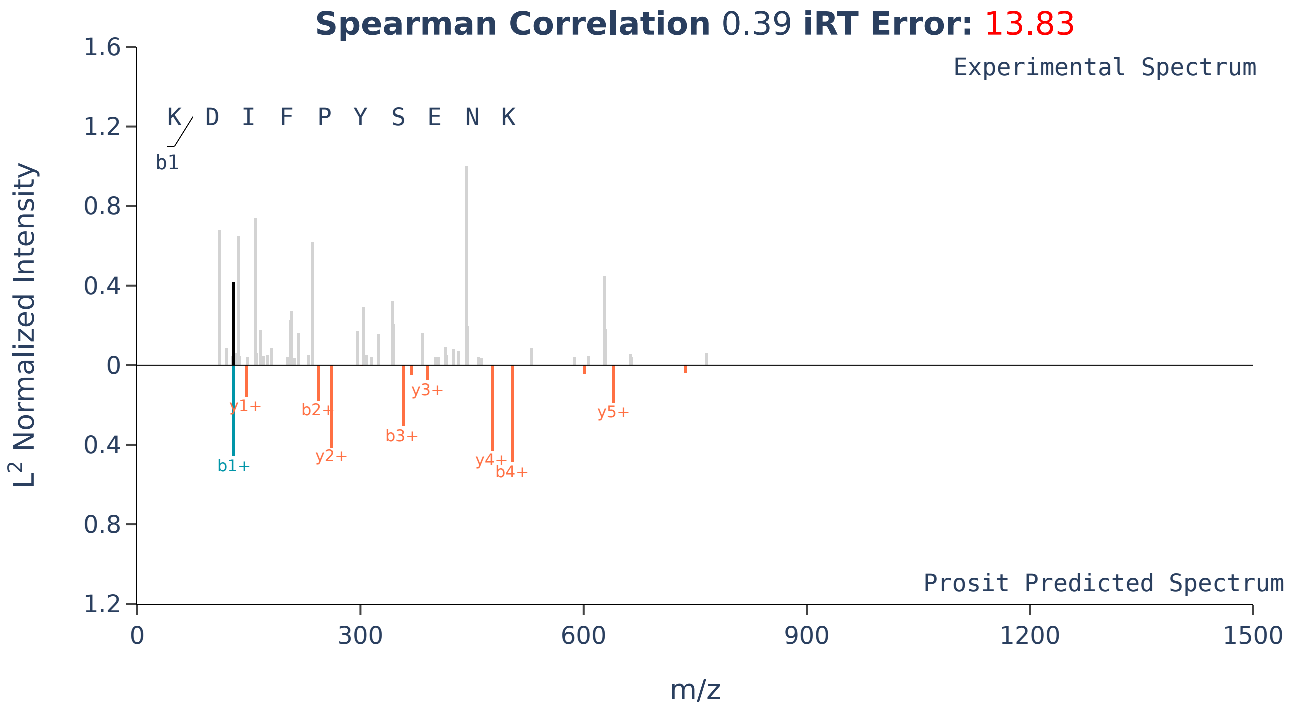

Source HRoetschke_271222_090123_HFGoe_LEDGF_b1R2_24h **Scan** 18138
Peptide QTGFLGPKDIFPYSENKEKYGKPNKR **Charge** 5 **Spectral Angle** 0.83
Spearman Correlation 0.88 **iRT Error:** 1.13

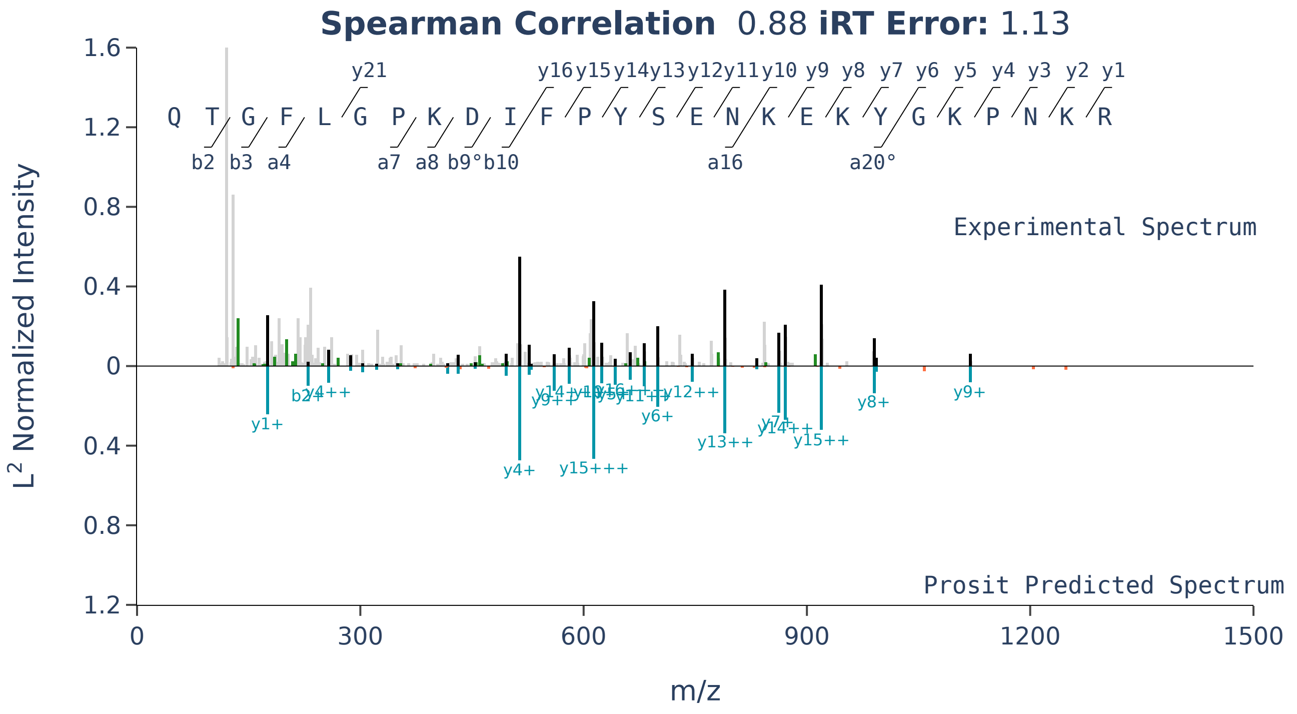

Source HRoetschke_271222_090123_HFGoe_LEDGF_b1R2_24h **Scan** 18138
Peptide KNMFLVGEGDSVITQVLNKSLAEQRQH **Charge** 5 **Spectral Angle** 0.2
Spearman Correlation 0.15 **iRT Error:** 60.19

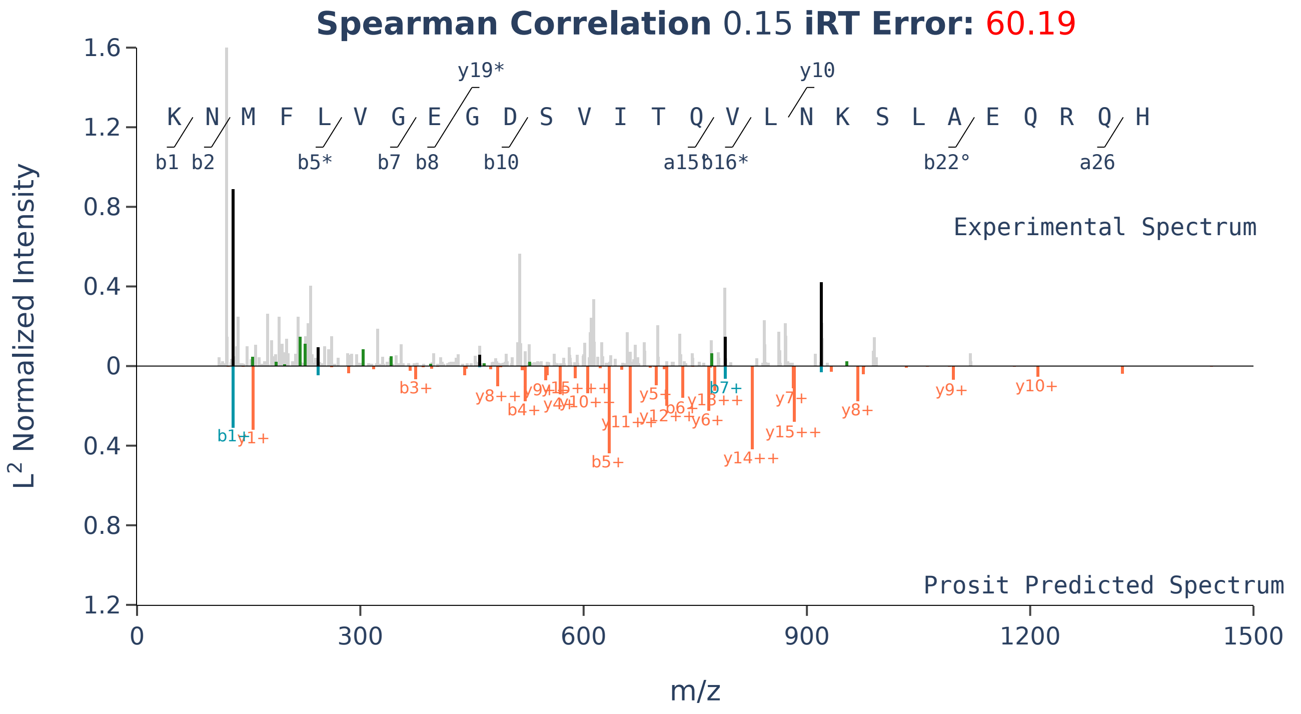

Source HRoetschke_271222_090123_HFGoe_LEDGF_b2R1_24h **Scan** 11661
Peptide TEEAGVVTTQQA **Charge** 2 **Spectral Angle** 0.89
Spearman Correlation 0.89 **iRT Error:** 1.17

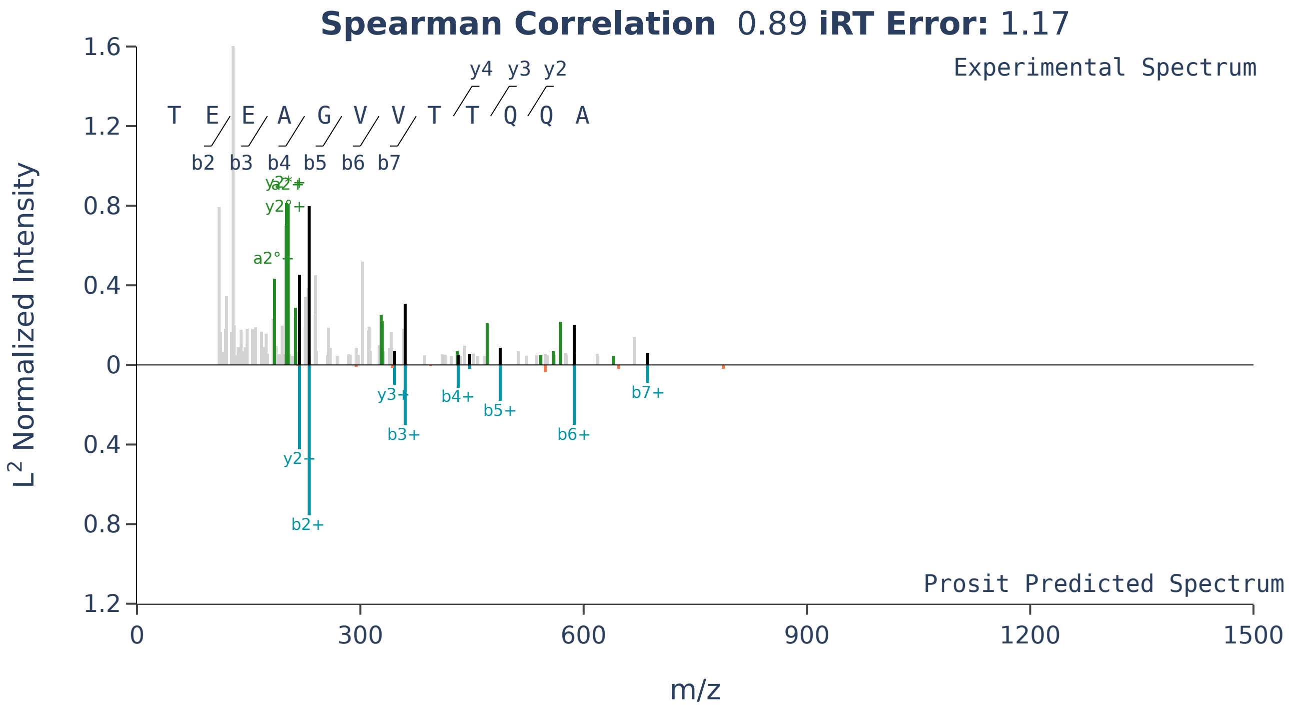

Source HRoetschke_271222_090123_HFGoe_LEDGF_b2R1_24h **Scan** 11661
Peptide KMVKQPCPSES **Charge** 2 **Spectral Angle** 0.58
Spearman Correlation 0.33 **iRT Error:** 33.92

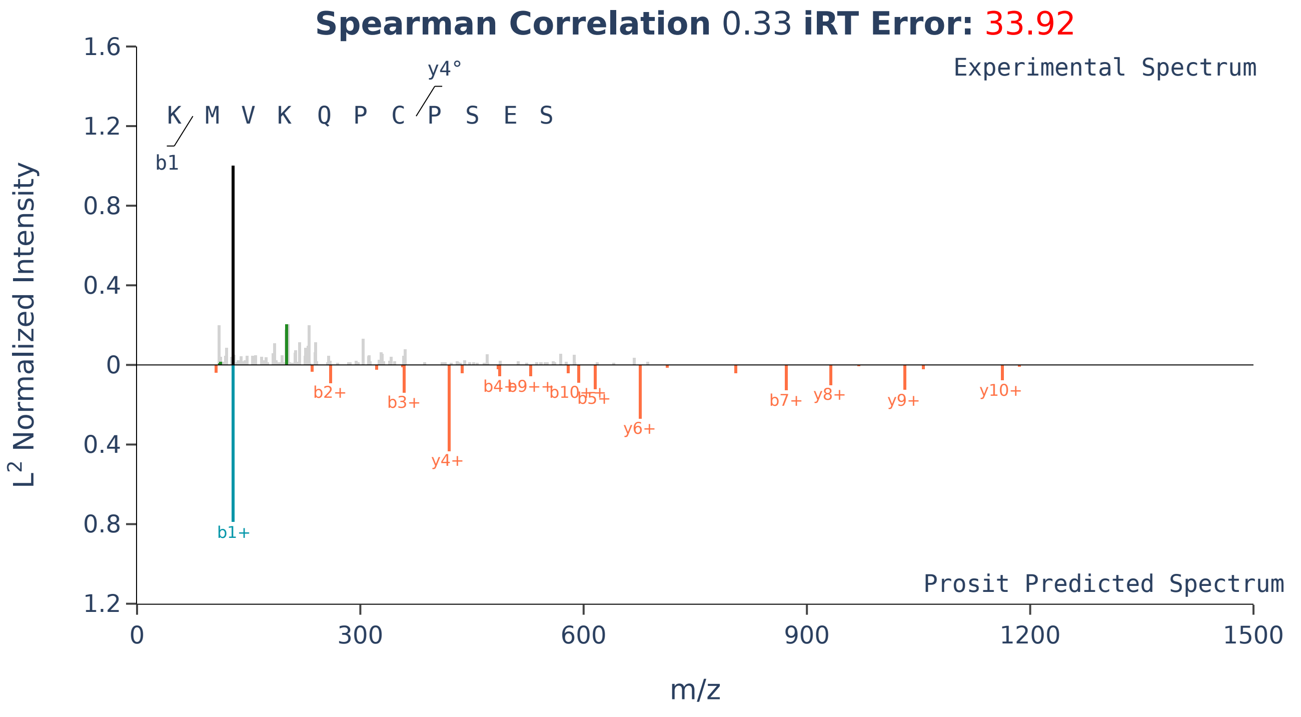

Source HRoetschke_271222_090123_HFGoe_LEDGF_b1R1_24h **Scan** 13697
Peptide GDEVPDGAVKPPTN **Charge** 2 **Spectral Angle** 0.85
Spearman Correlation 0.95 **iRT Error:** 1.18

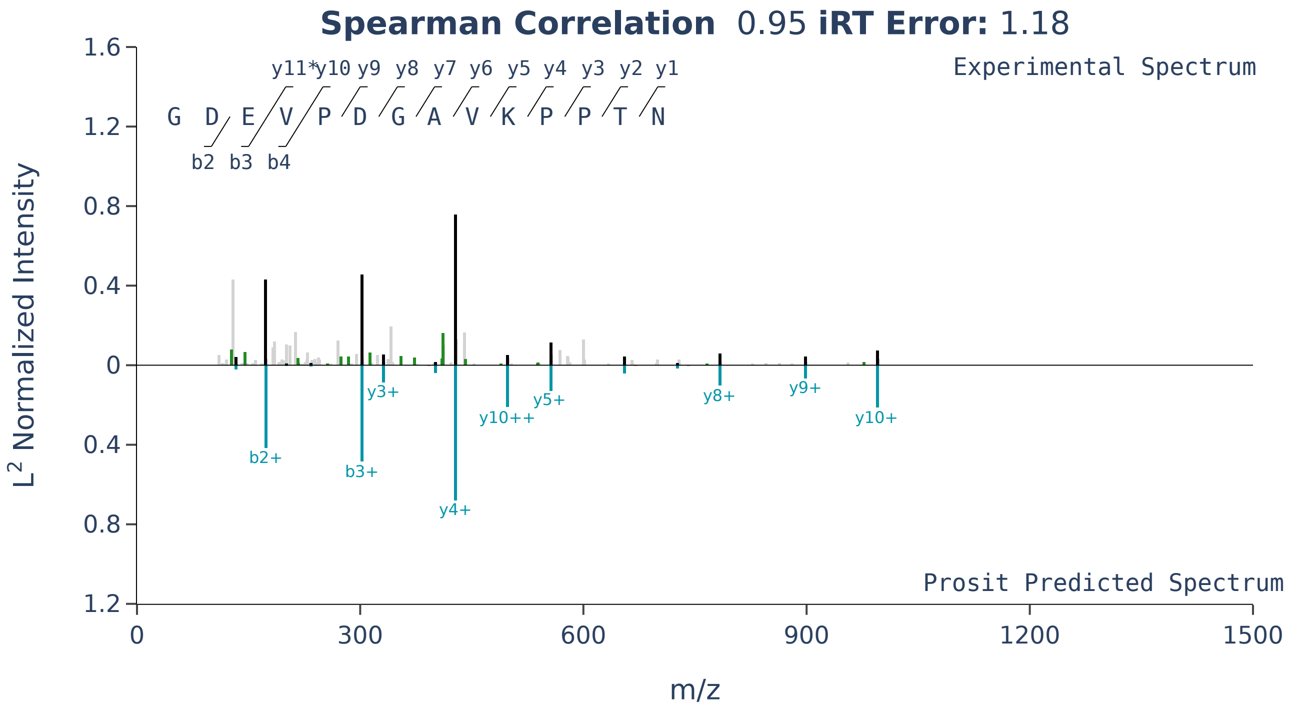

Source HRoetschke_271222_090123_HFGoe_LEDGF_b1R1_24h **Scan** 13697
Peptide MFLVGEGDSVITQ **Charge** 2 **Spectral Angle** 0.0
Spearman Correlation 0.0 **iRT Error:** 86.61

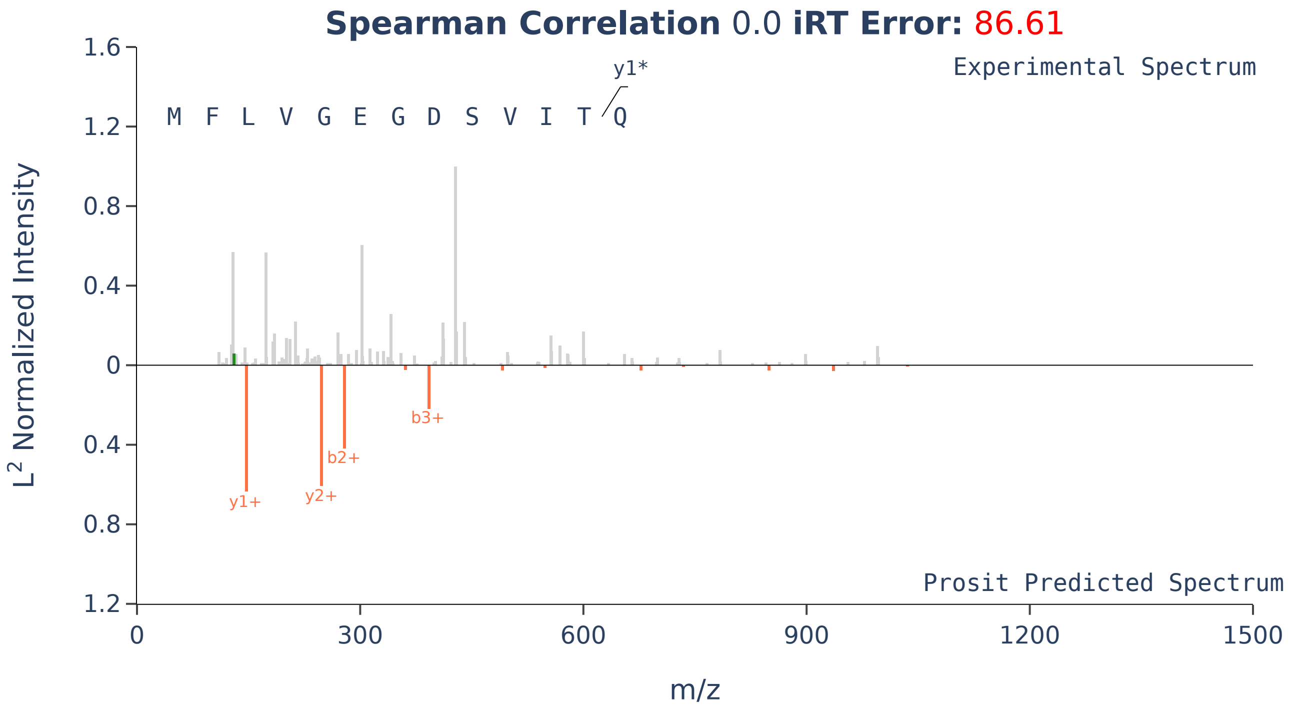

Source HRoetschke_271222_090123_HFGoe_LEDGF_b2R2_2h **Scan** 17185
Peptide FLGPKDIFPYSENKEKYGKPNKRTA **Charge** 5 **Spectral Angle** 0.8
Spearman Correlation 0.95 **iRT Error:** 1.19

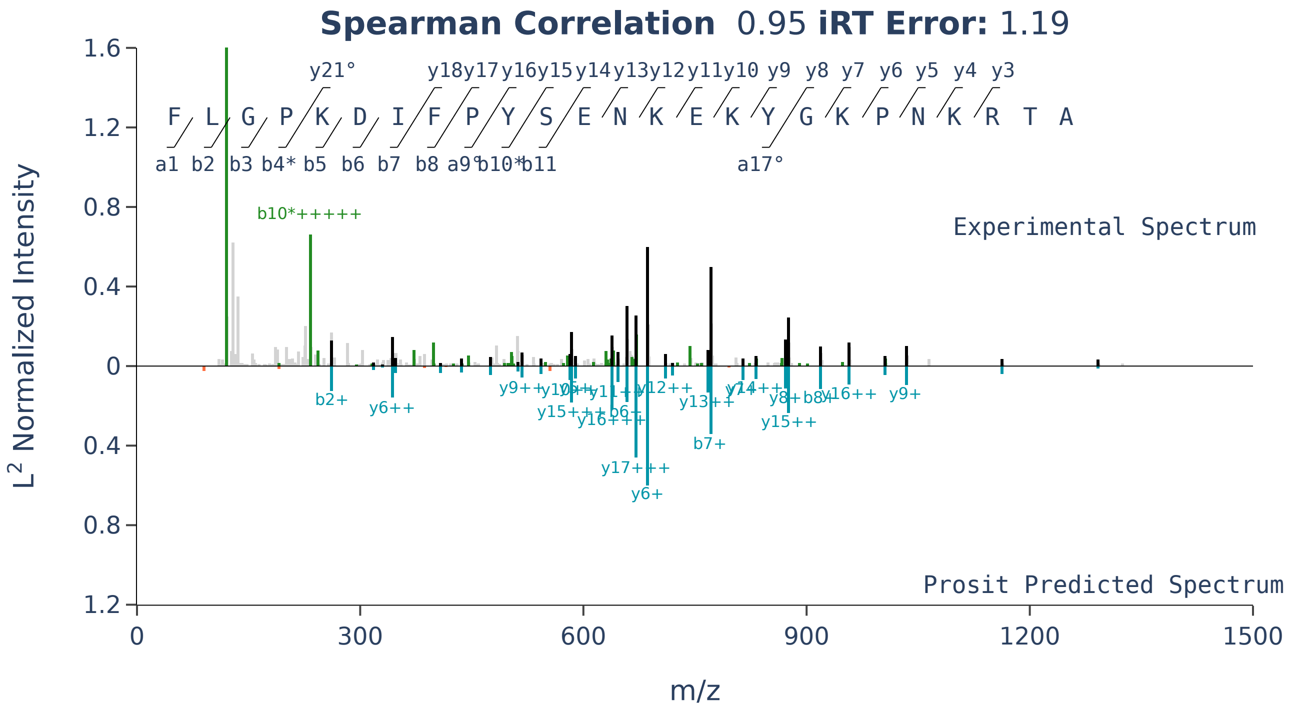

Source HRoetschke_271222_090123_HFGoe_LEDGF_b2R2_2h **Scan** 17185
Peptide TAFLGPKDIFPYSENKEKYGKPNKR **Charge** 5 **Spectral Angle** 0.04
Spearman Correlation 0.09 **iRT Error:** 7.43

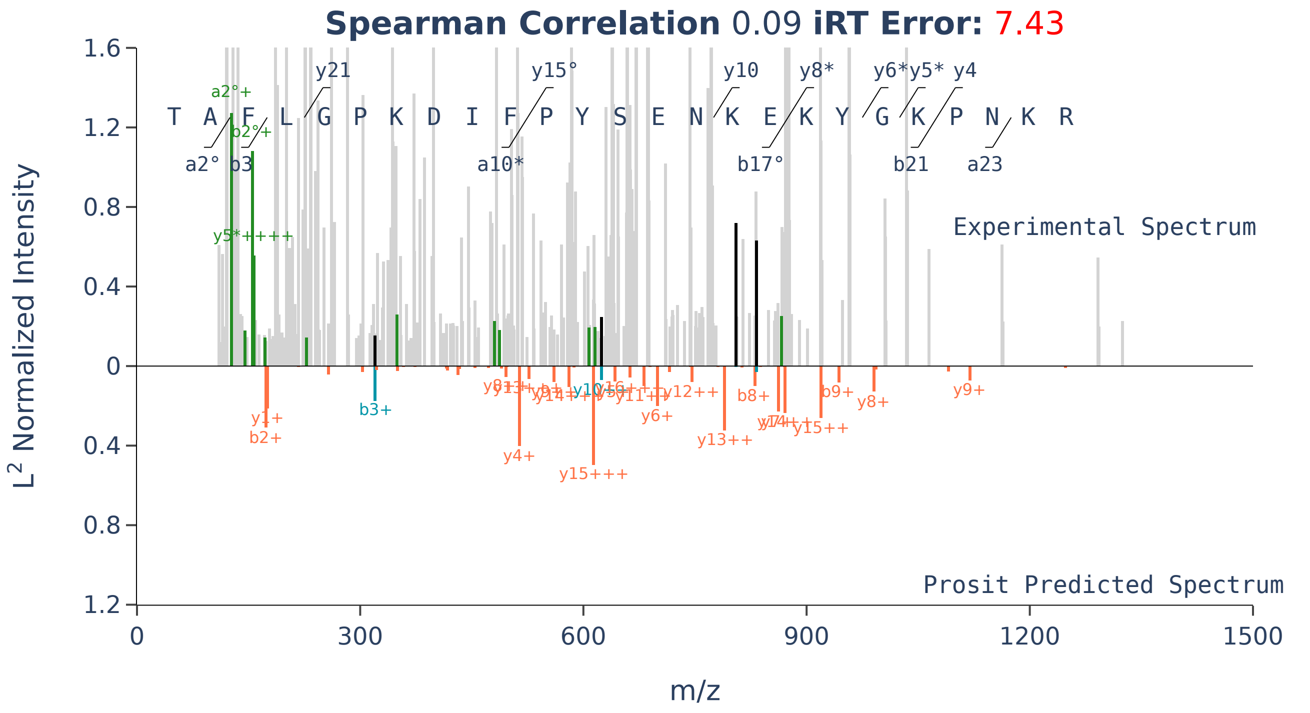

Source HRoetschke_271222_090123_HFGoe_LEDGF_b1R2_24h **Scan** 16275
Peptide KFKNMFLVGKETSVSKE **Charge** 3 **Spectral Angle** 0.88
Spearman Correlation 0.8 **iRT Error:** 1.22

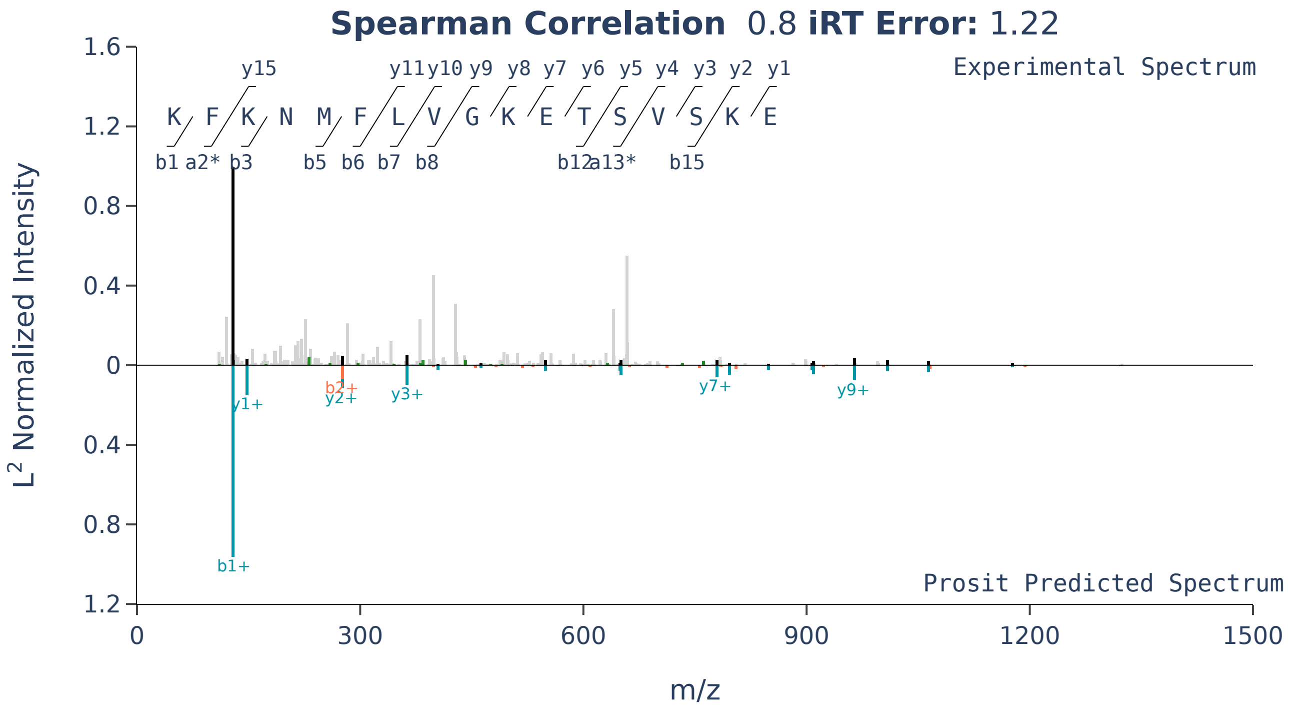

Source HRoetschke_271222_090123_HFGoe_LEDGF_b1R2_24h **Scan** 16275
Peptide LVGEGDSVITQVLNKSLAE **Charge** 3 **Spectral Angle** 0.42
Spearman Correlation 0.31 **iRT Error:** 86.58

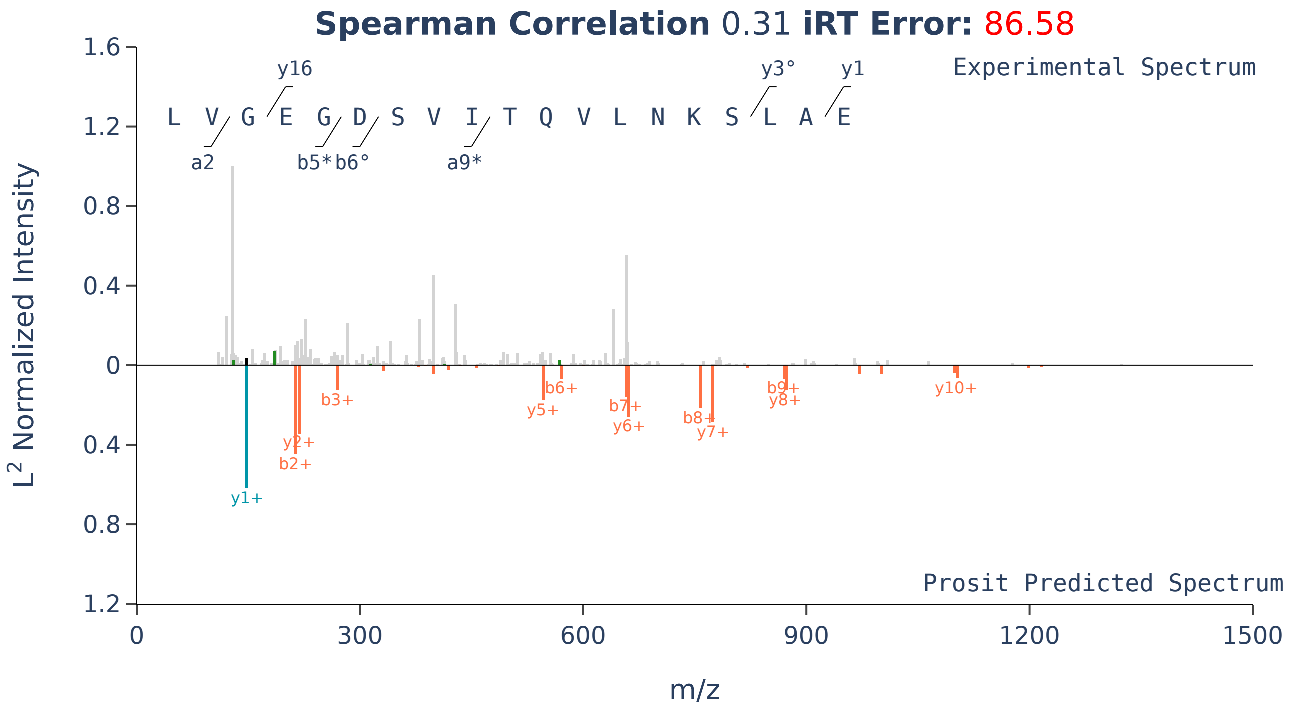

Source HRoetschke_271222_090123_HFGoe_LEDGF_b2R1_2h **Scan** 8326
Peptide AQDGNQPQHEL **Charge** 2 **Spectral Angle** 0.71
Spearman Correlation 0.89 **iRT Error:** 1.22

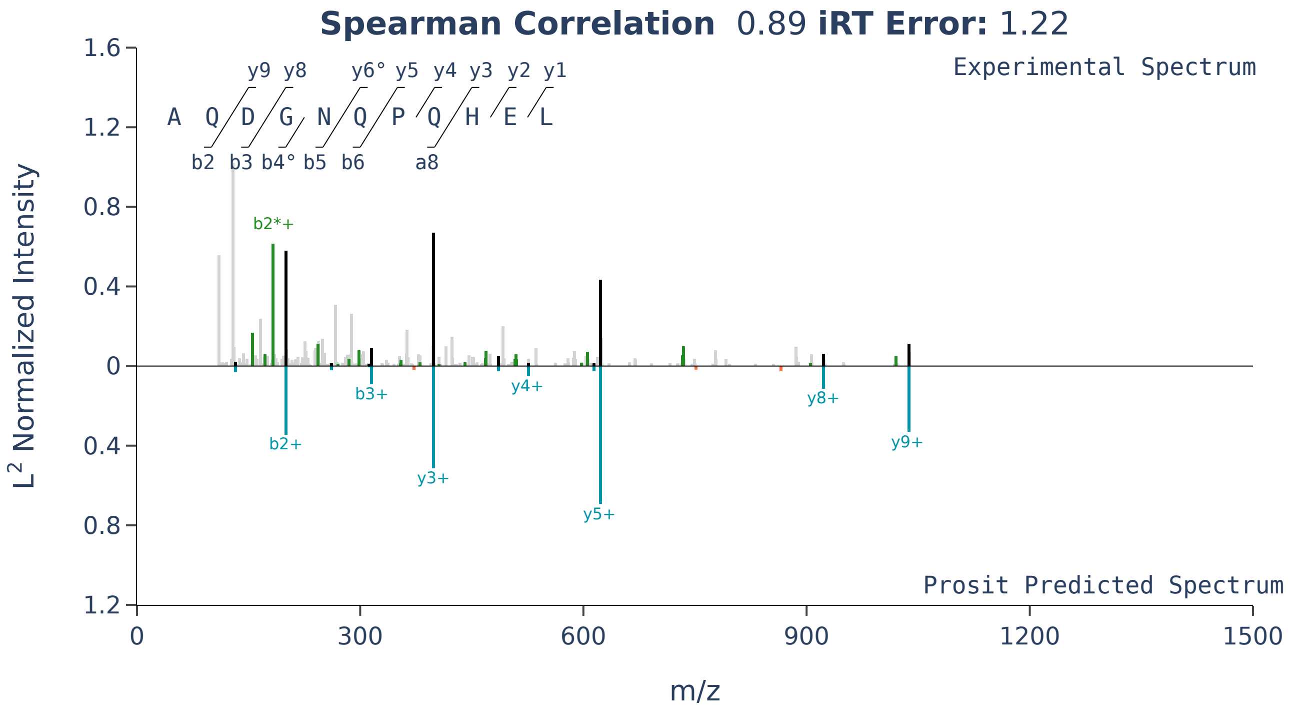

Source HRoetschke_271222_090123_HFGoe_LEDGF_b2R1_2h **Scan** 8326
Peptide FNEGLWEIDN **Charge** 2 **Spectral Angle** 0.0
Spearman Correlation 0.0 **iRT Error:** 115.26

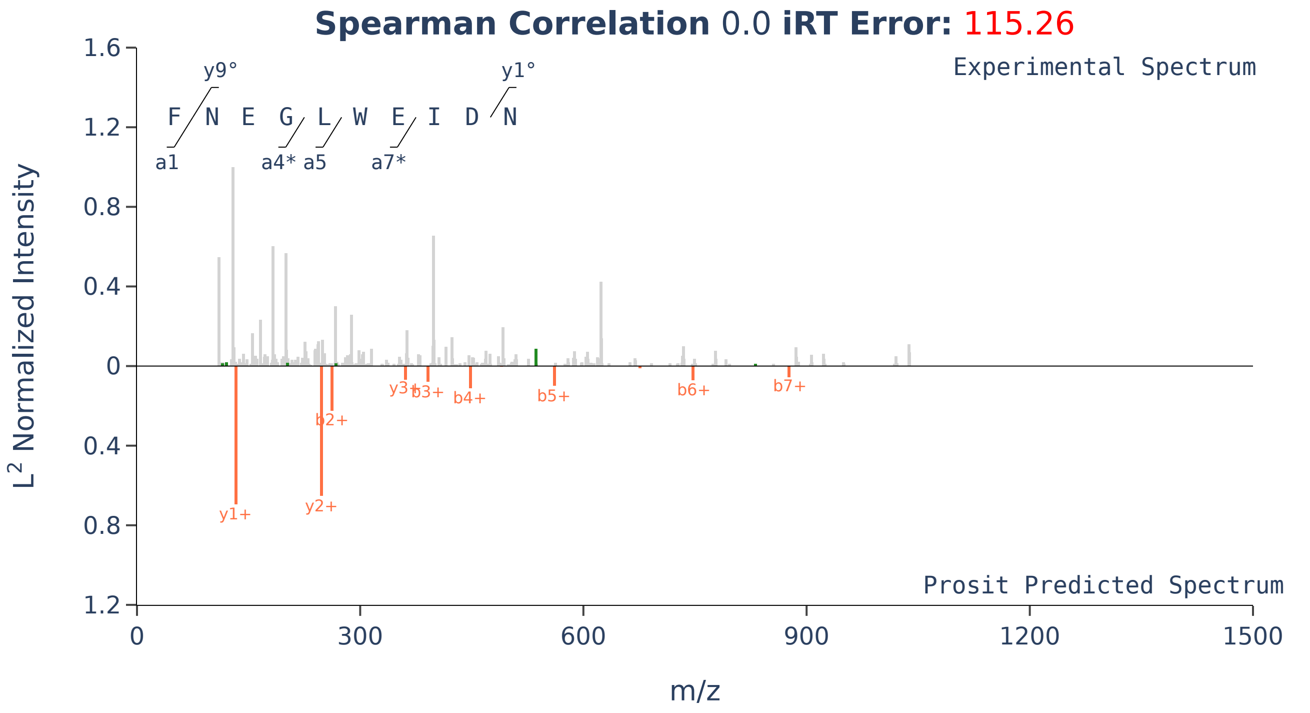

Source HRoetschke_271222_090123_HFGoe_LEDGF_b2R2_2h **Scan** 6799
Peptide PKMVKQPCTEE **Charge** 3 **Spectral Angle** 0.89
Spearman Correlation 0.9 **iRT Error:** 1.22

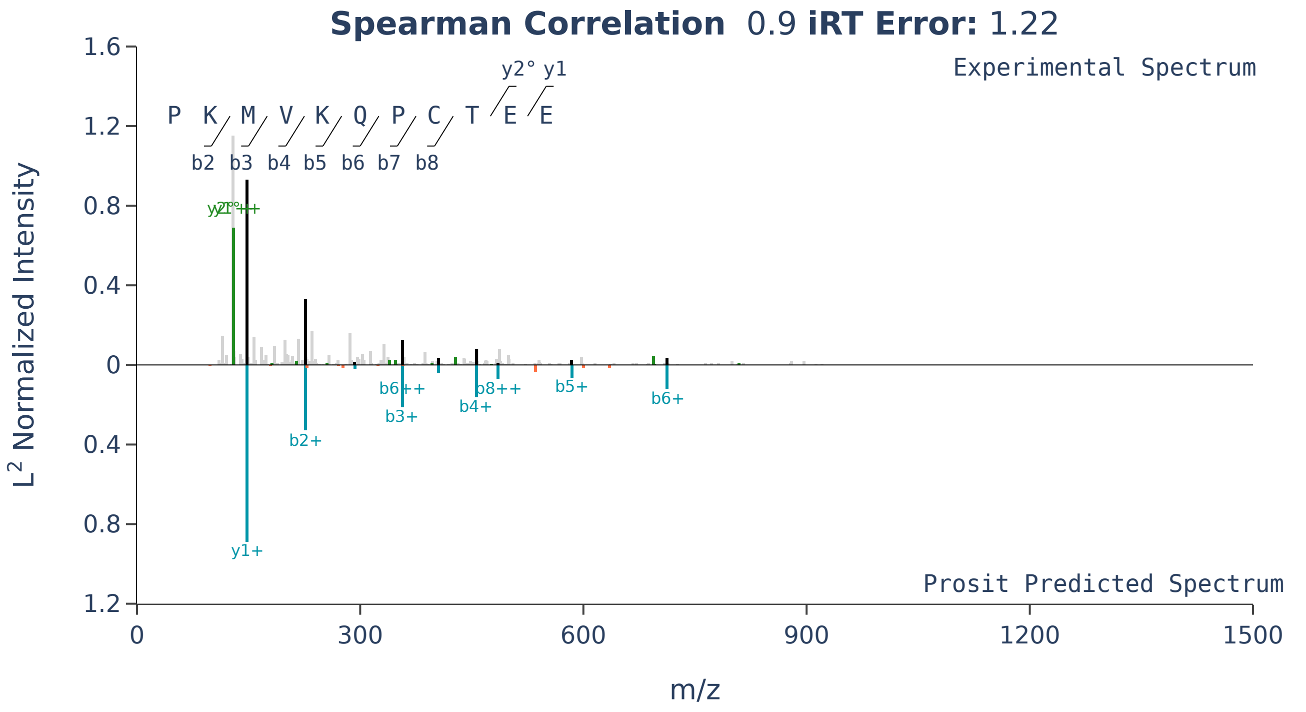

Source HRoetschke_271222_090123_HFGoe_LEDGF_b2R2_2h **Scan** 6799
Peptide AEKQVETEEAGV **Charge** 3 **Spectral Angle** 0.19
Spearman Correlation 0.41 **iRT Error:** 19.89

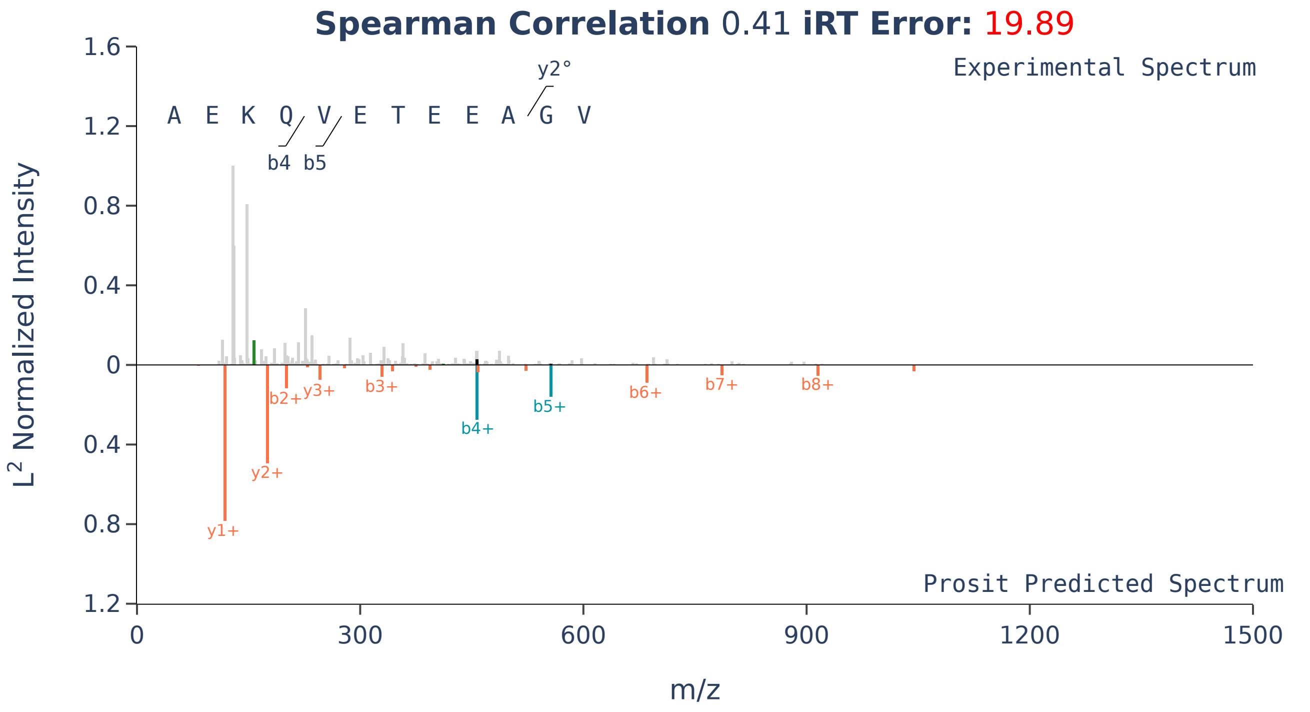

Source HRoetschke_271222_090123_HFGoe_LEDGF_b2R1_24h **Scan** 17050
Peptide FLGPKDIFPYSENKEKYGKPNKRAT **Charge** 4 **Spectral Angle** 0.86
Spearman Correlation 0.84 **iRT Error:** 1.23

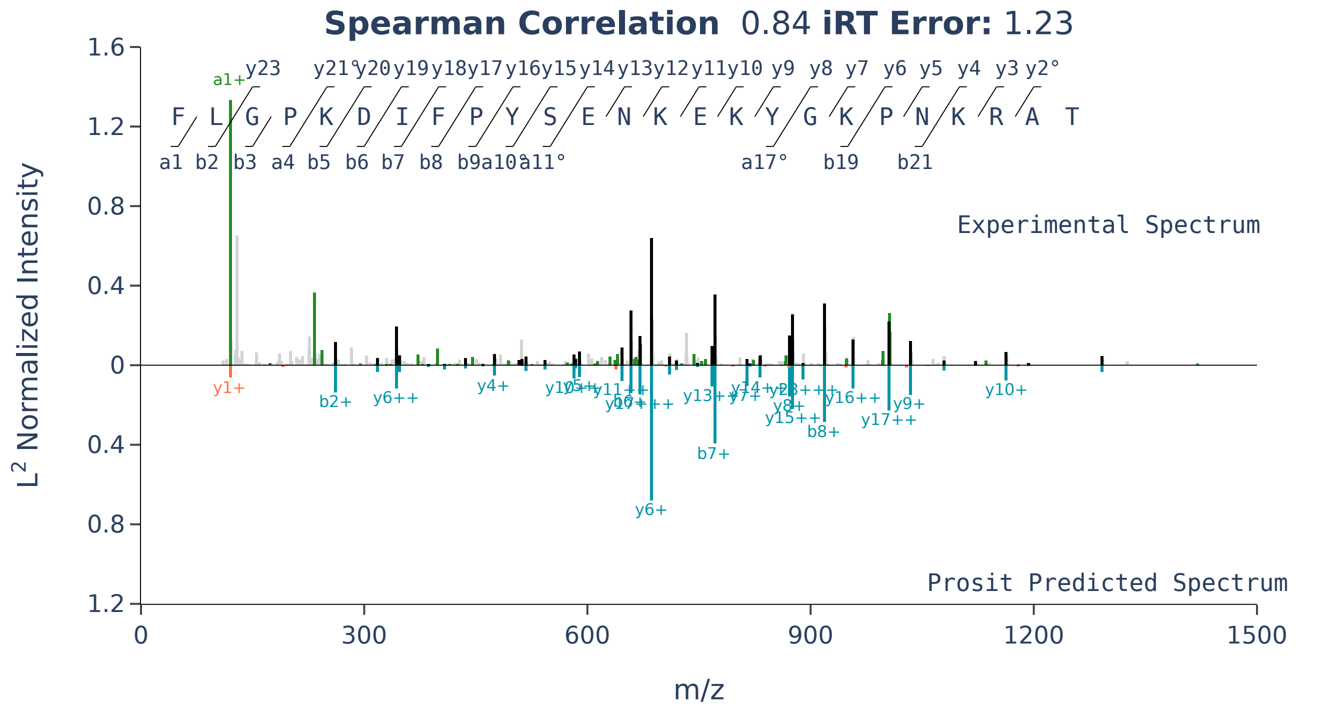

Source HRoetschke_271222_090123_HFGoe_LEDGF_b2R1_24h **Scan** 17050
Peptide TAFLGPKDIFPYSENKEKYGKPNKR **Charge** 4 **Spectral Angle** 0.21
Spearman Correlation 0.35 **iRT Error:** 8.14

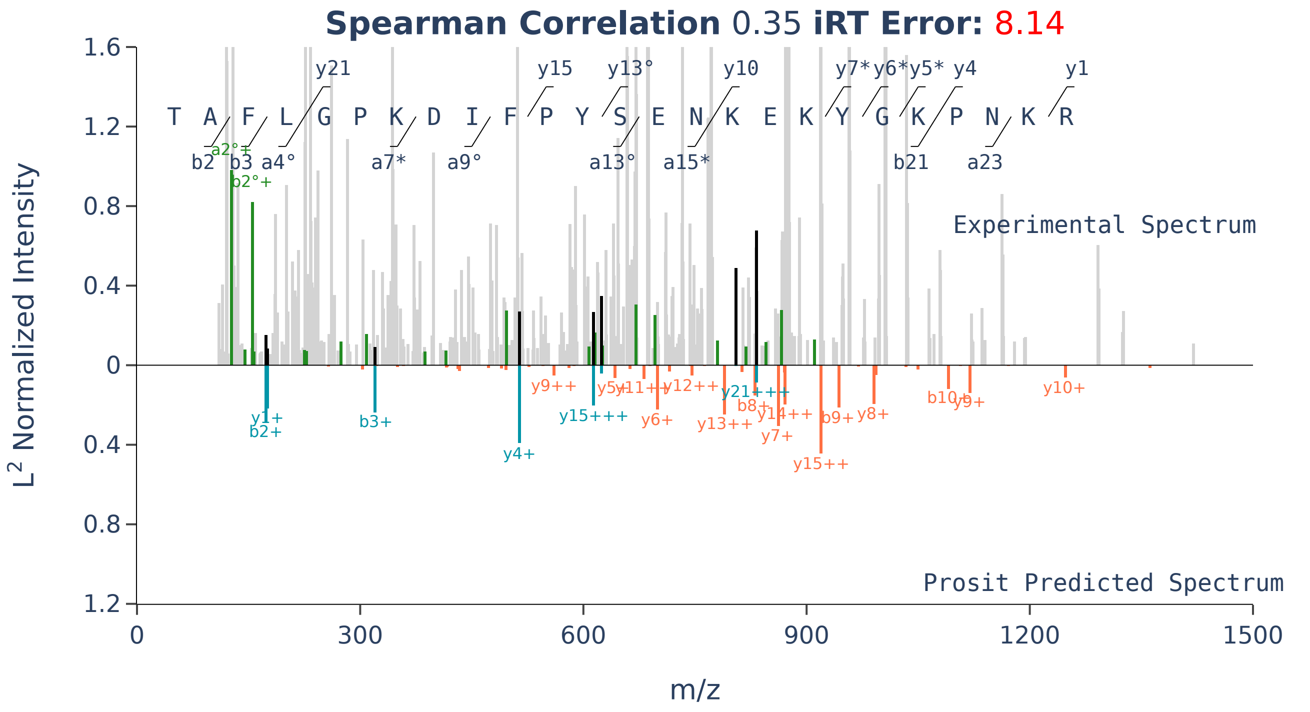

Source HRoetschke_271222_090123_HFGoe_LEDGF_b2R2_2h **Scan** 18885
Peptide VGYPHWPARVDE **Charge** 3 **Spectral Angle** 0.84
Spearman Correlation 0.85 **iRT Error:** 1.26

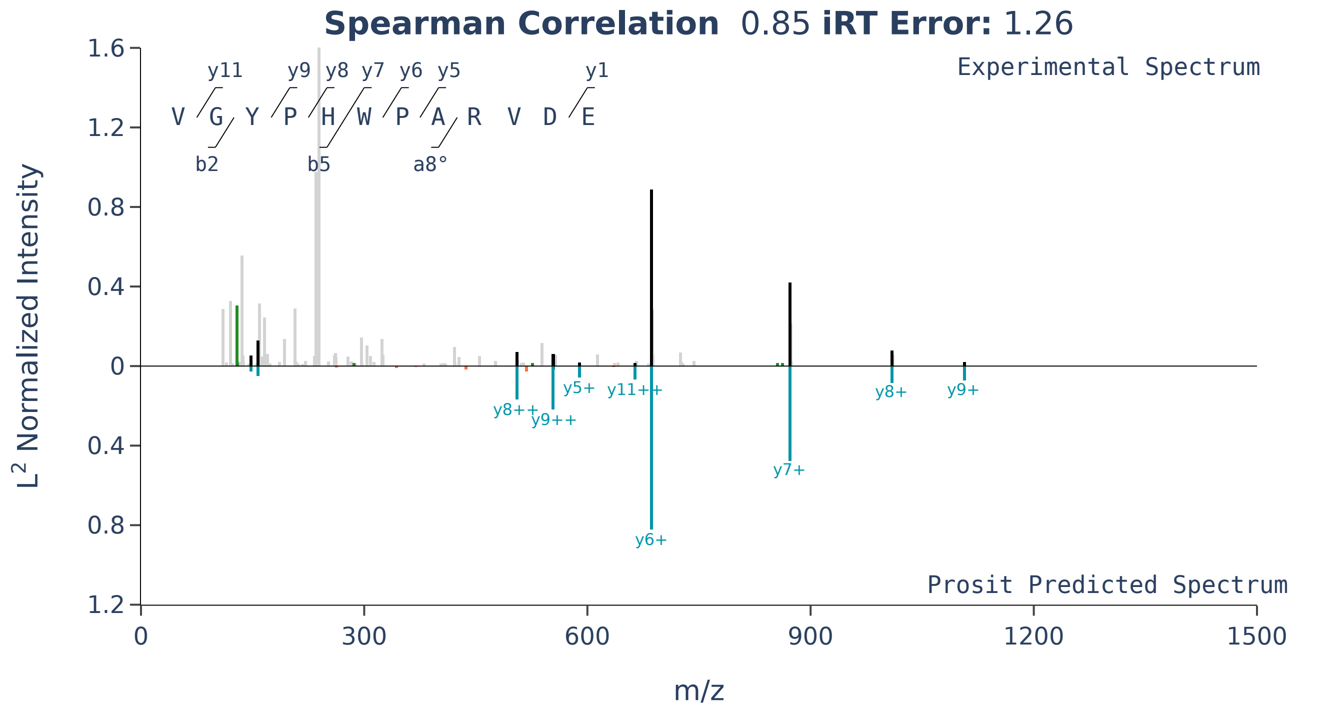

Source HRoetschke_271222_090123_HFGoe_LEDGF_b2R2_2h **Scan** 18885
Peptide GYPHWPARVDEV **Charge** 3 **Spectral Angle** 0.05
Spearman Correlation 0.26 **iRT Error:** 9.71

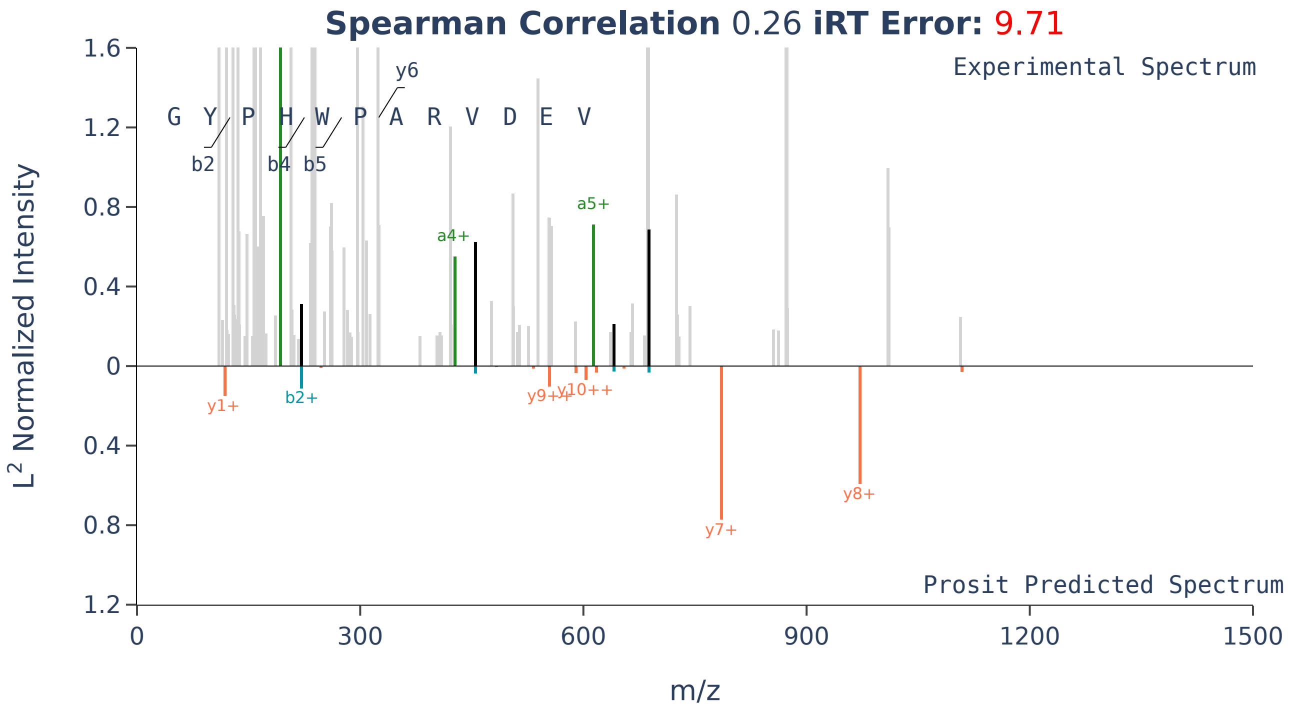

Source HRoetschke_271222_090123_HFGoe_LEDGF_b2R1_24h **Scan** 18083
Peptide ASHETAFLGPKDIFPYSENKEKYGKPNK **Charge** 3 **Spectral Angle** 0.84
Spearman Correlation 0.83 **iRT Error:** 1.28

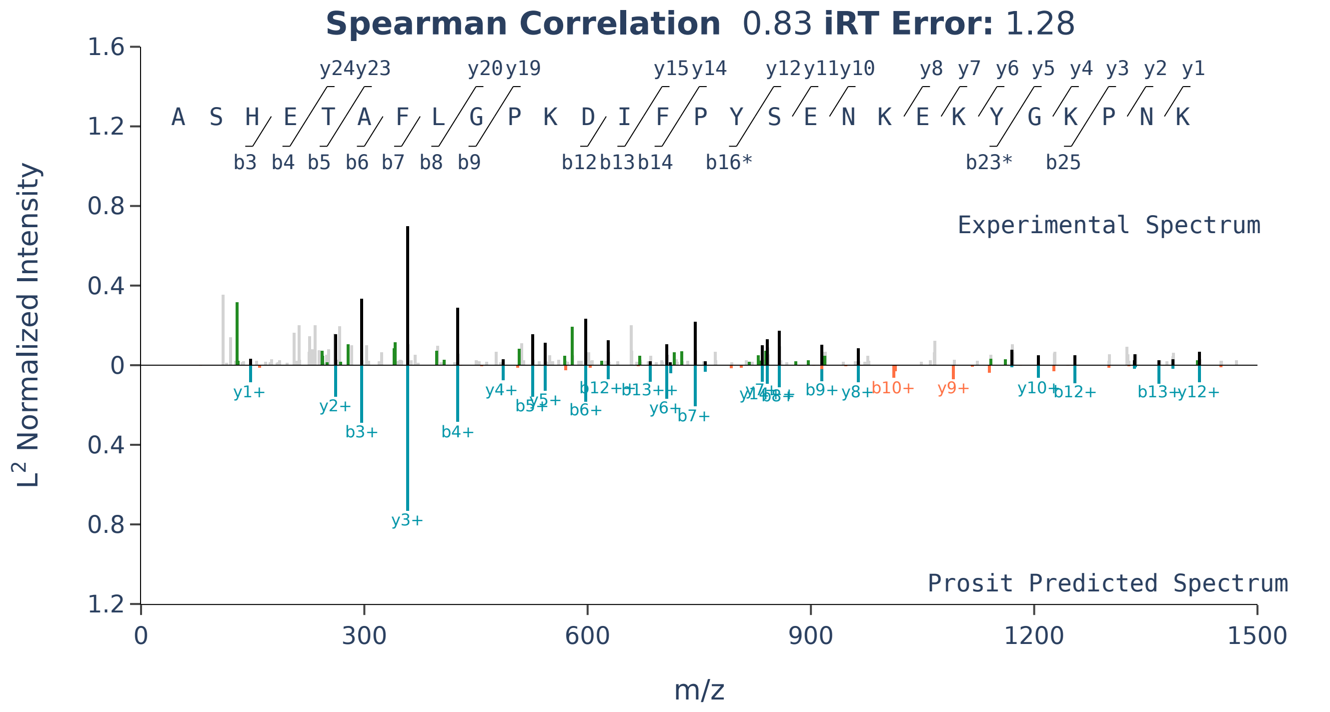

Source HRoetschke_271222_090123_HFGoe_LEDGF_b2R1_24h **Scan** 18083
Peptide GTHETAFLGPKDIFPYSENKEKYGKPNK **Charge** 3 **Spectral Angle** 0.84
Spearman Correlation 0.8 **iRT Error:** 1.96

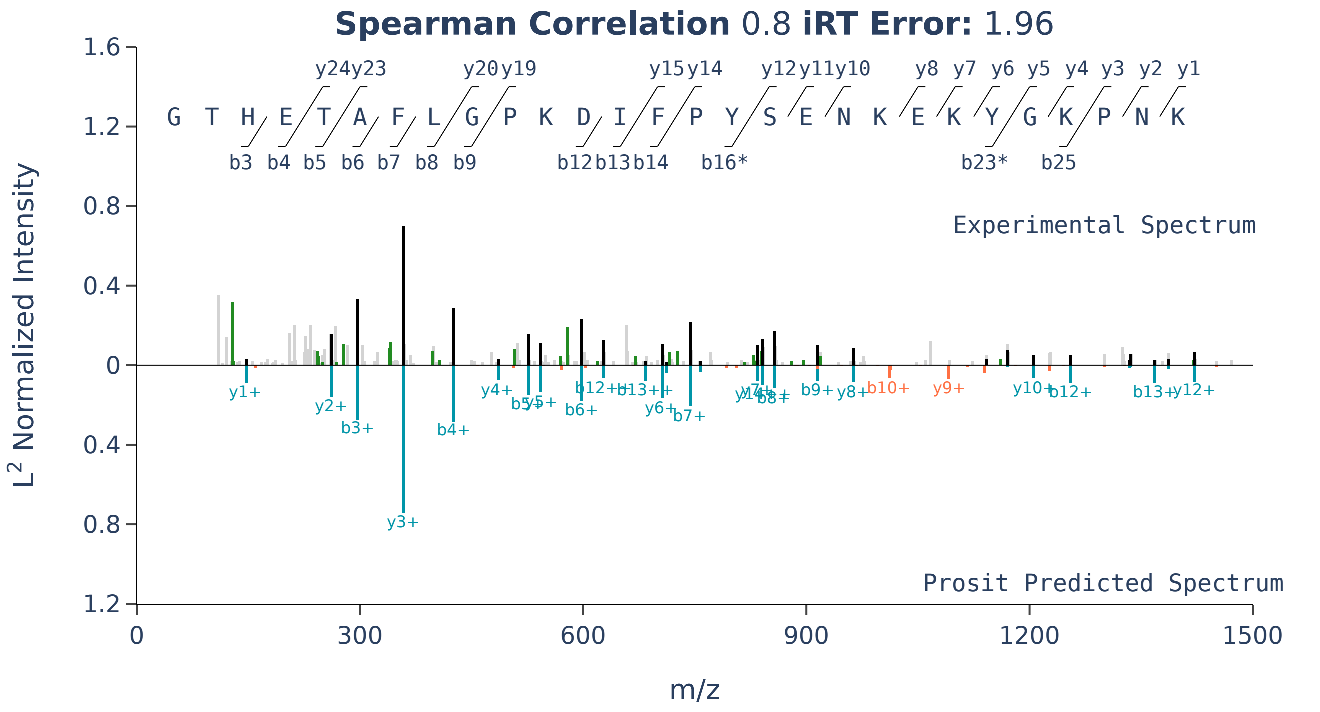

Source HRoetschke_271222_090123_HFGoe_LEDGF_b2R2_2h **Scan** 13277
Peptide EGDVPDGAVKPPTN **Charge** 2 **Spectral Angle** 0.82
Spearman Correlation 0.85 **iRT Error:** 1.3

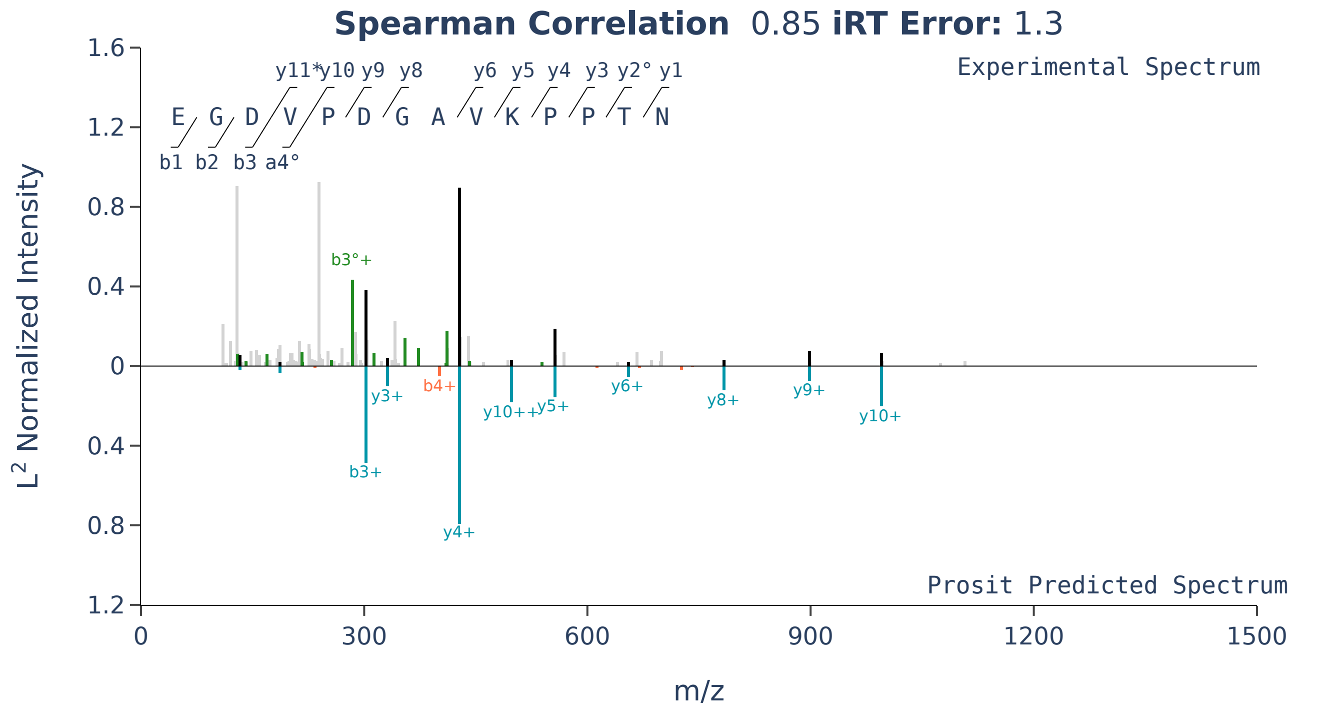

Source HRoetschke_271222_090123_HFGoe_LEDGF_b2R2_2h **Scan** 13277
Peptide MFLVGEGDSVITQ **Charge** 2 **Spectral Angle** 0.0
Spearman Correlation 0.0 **iRT Error:** 90.15

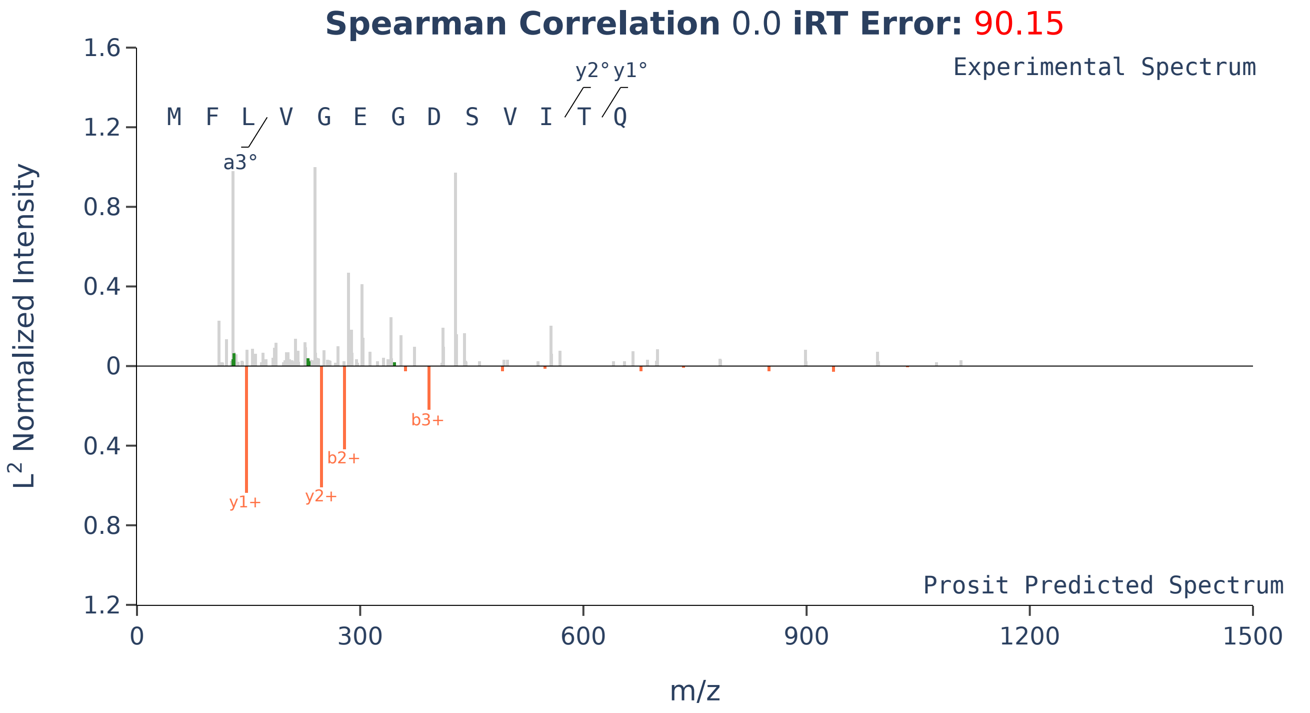

Source HRoetschke_271222_090123_HFGoe_LEDGF_b2R1_4h **Scan** 18290
Peptide ATAFLGPKDIFPYSENKEKYGKPNKR **Charge** 4 **Spectral Angle** 0.79
Spearman Correlation 0.86 **iRT Error:** 1.3

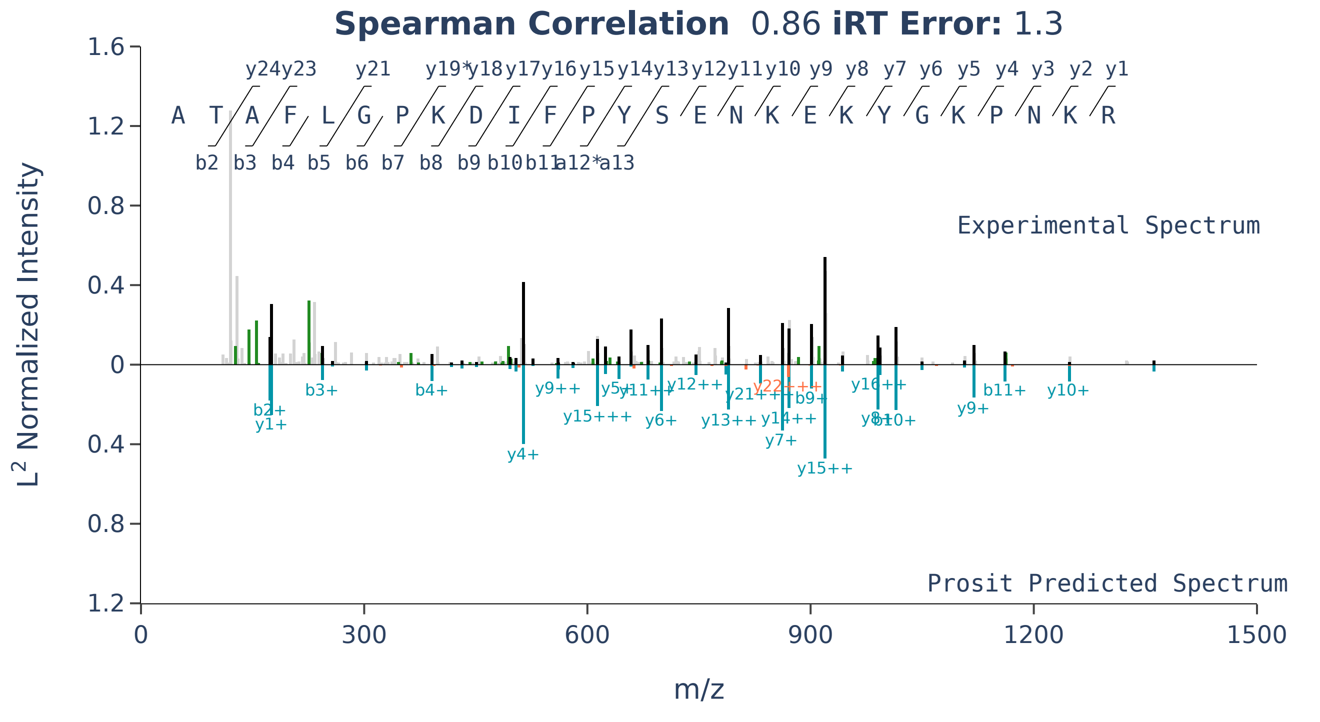

Source HRoetschke_271222_090123_HFGoe_LEDGF_b2R1_4h **Scan** 18290
Peptide WPARVDEVPDGAVKPPTNKLPIFFFGT **Charge** 4 **Spectral Angle** 0.03
Spearman Correlation 0.02 **iRT Error:** 71.32

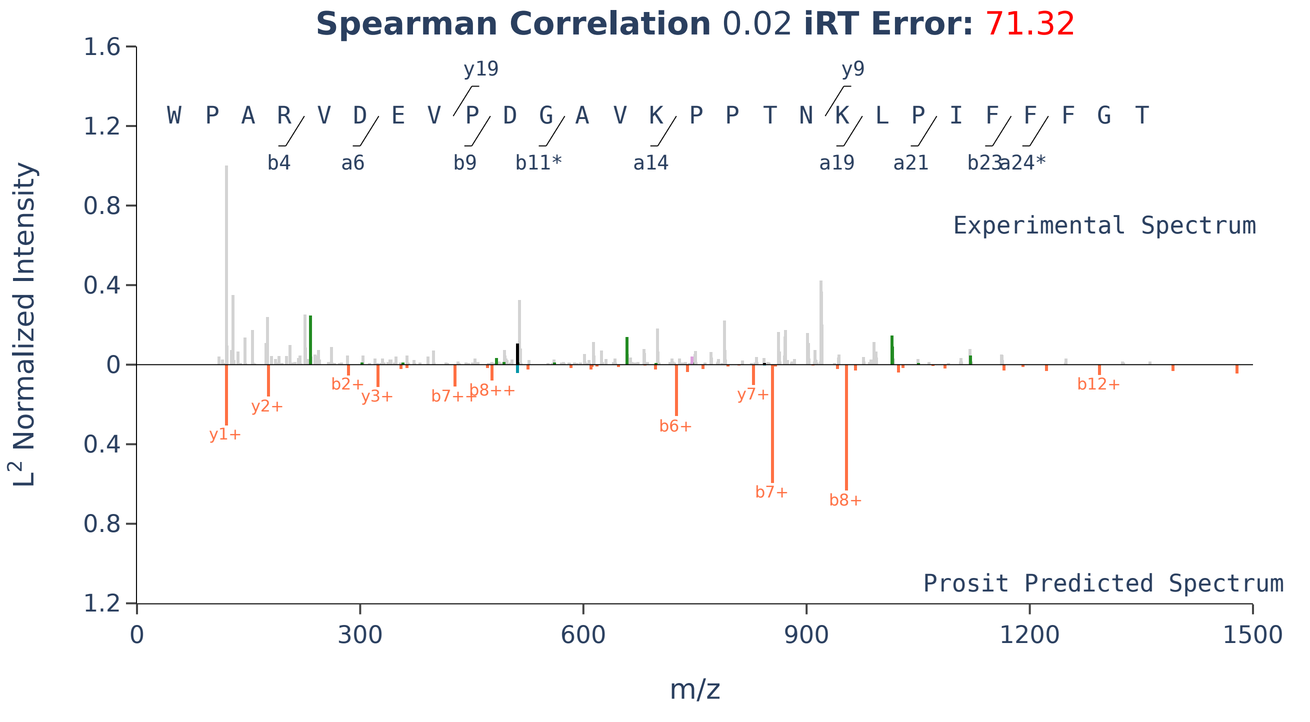

Source HRoetschke_271222_090123_HFGoe_LEDGF_b1R2_24h **Scan** 16736
Peptide TAFLGPKDIFPYSENKEKYGKPKRKGG **Charge** 5 **Spectral Angle** 0.81
Spearman Correlation 0.88 **iRT Error:** 1.36

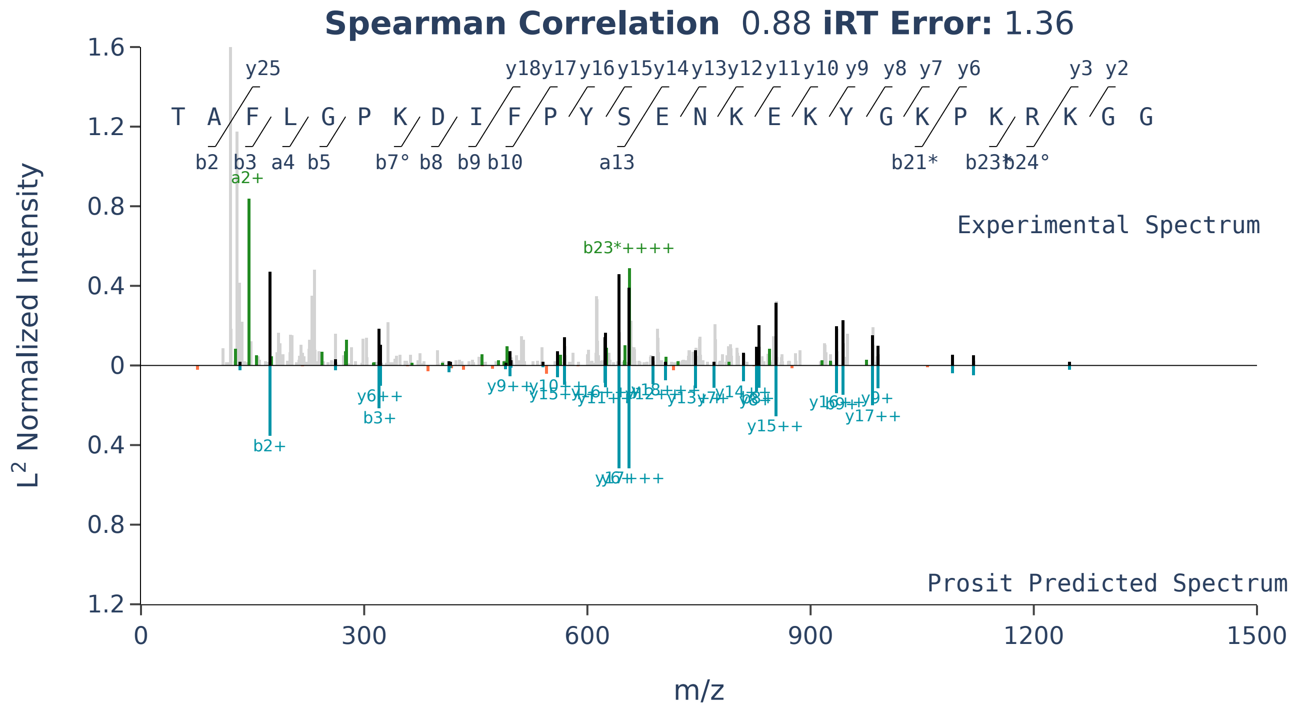

Source HRoetschke_271222_090123_HFGoe_LEDGF_b1R2_24h **Scan** 16736
Peptide TAFLGPKDIFPYSENKEKYGKPNKRK **Charge** 5 **Spectral Angle** 0.79
Spearman Correlation 0.87 **iRT Error:** 1.99

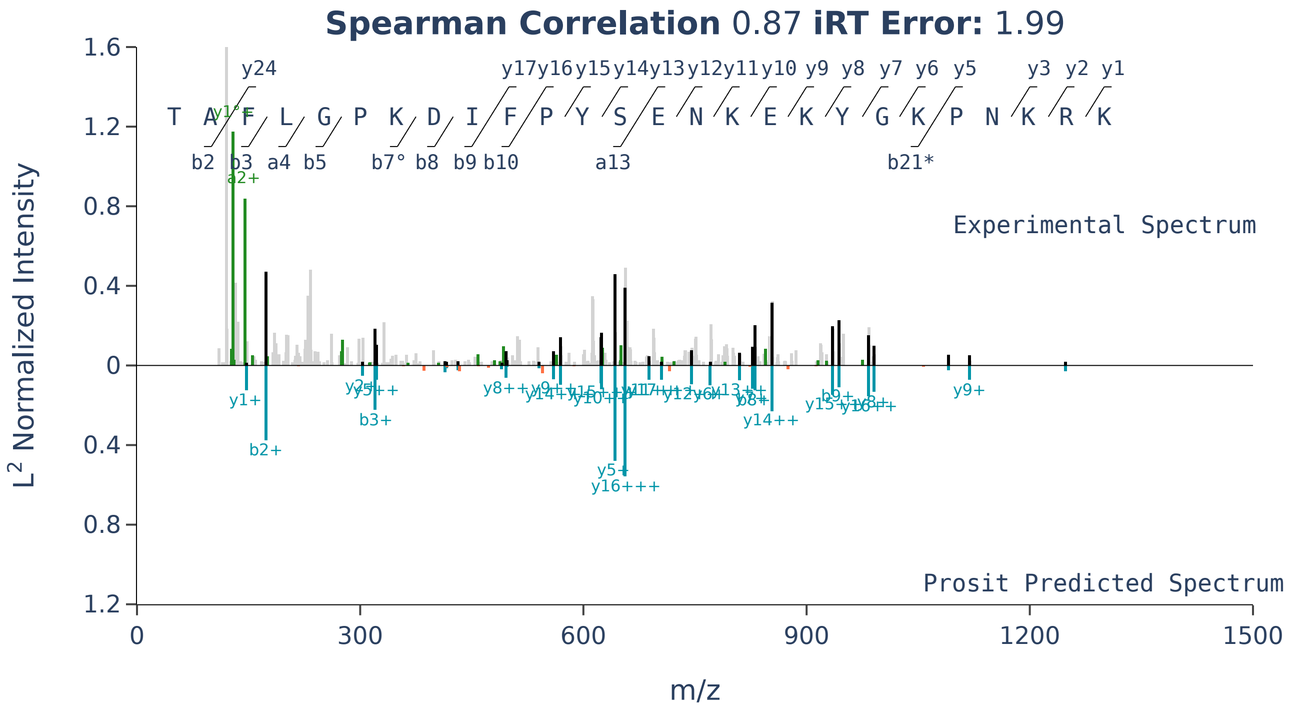

Source HRoetschke_271222_090123_HFGoe_LEDGF_b1R1_24h **Scan** 14036
Peptide TPHWPARVD **Charge** 3 **Spectral Angle** 0.94
Spearman Correlation 0.93 **iRT Error:** 1.36

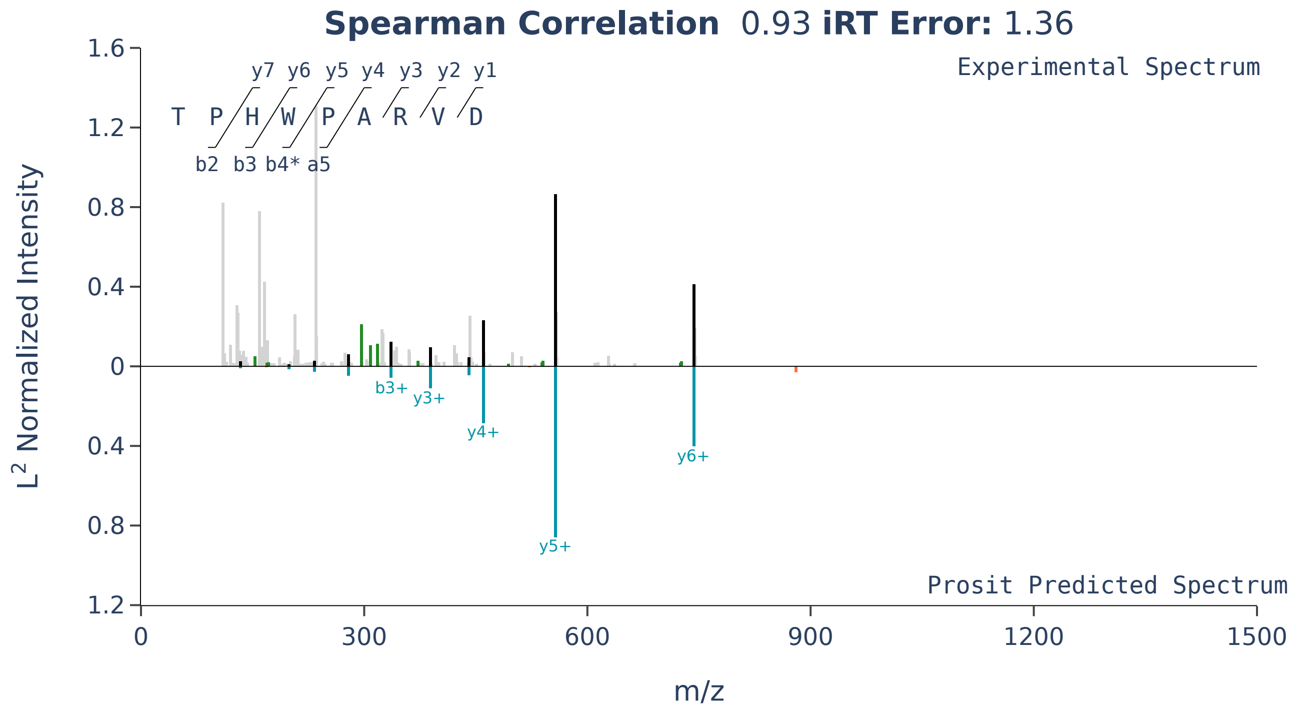

Source HRoetschke_271222_090123_HFGoe_LEDGF_b1R1_24h **Scan** 14036
Peptide FAKMKGYPH **Charge** 3 **Spectral Angle** 0.0
Spearman Correlation 0.0 **iRT Error:** 35.44

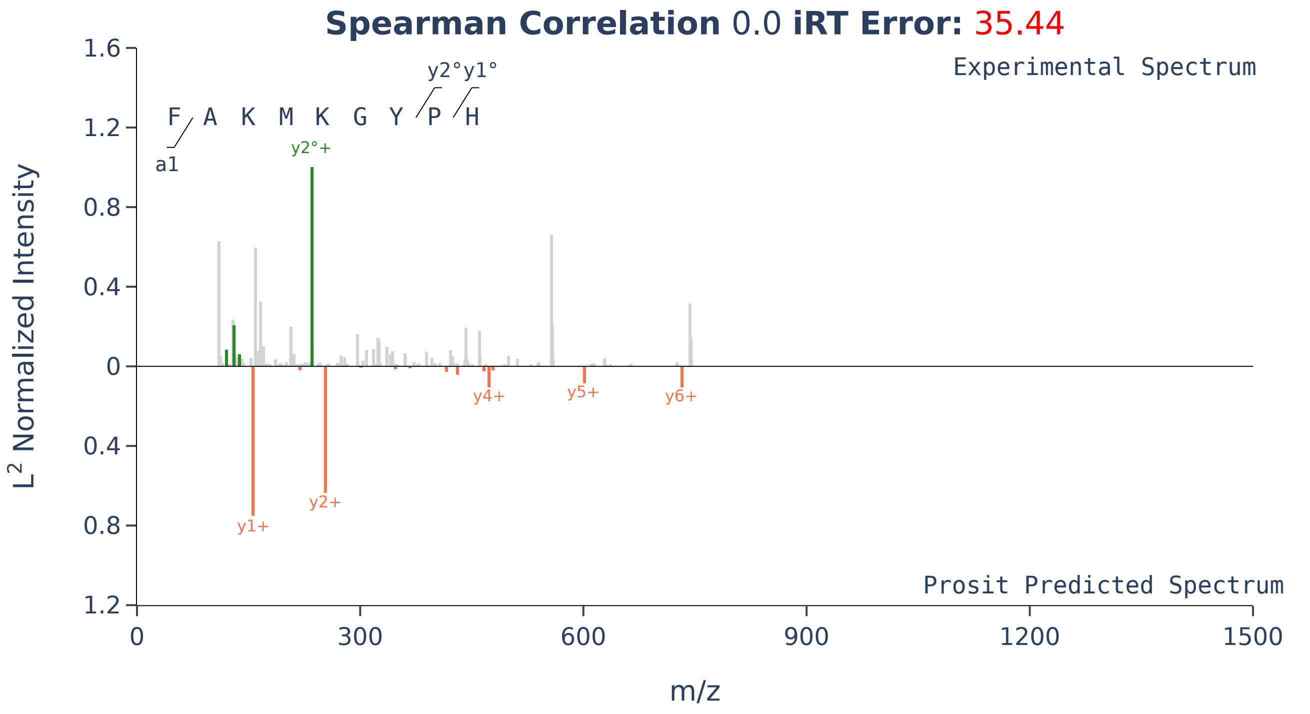

Source HRoetschke_271222_090123_HFGoe_LEDGF_b2R2_2h **Scan** 11378
Peptide IDNNPKVKFSSQQAATE **Charge** 2 **Spectral Angle** 0.82
Spearman Correlation 0.9 **iRT Error:** 1.39

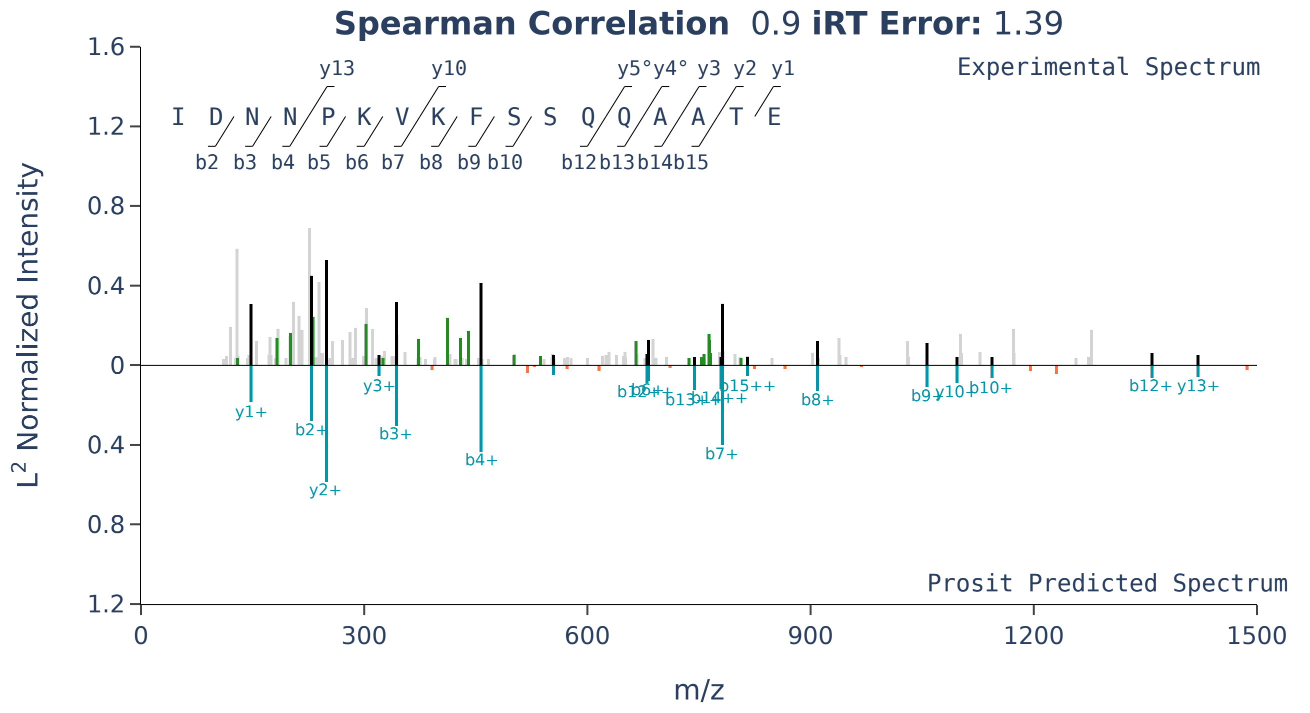

Source HRoetschke_271222_090123_HFGoe_LEDGF_b2R2_2h **Scan** 11378
Peptide EIDNNPKVKFSSQQAAT **Charge** 2 **Spectral Angle** 0.0
Spearman Correlation 0.0 **iRT Error:** 6.14

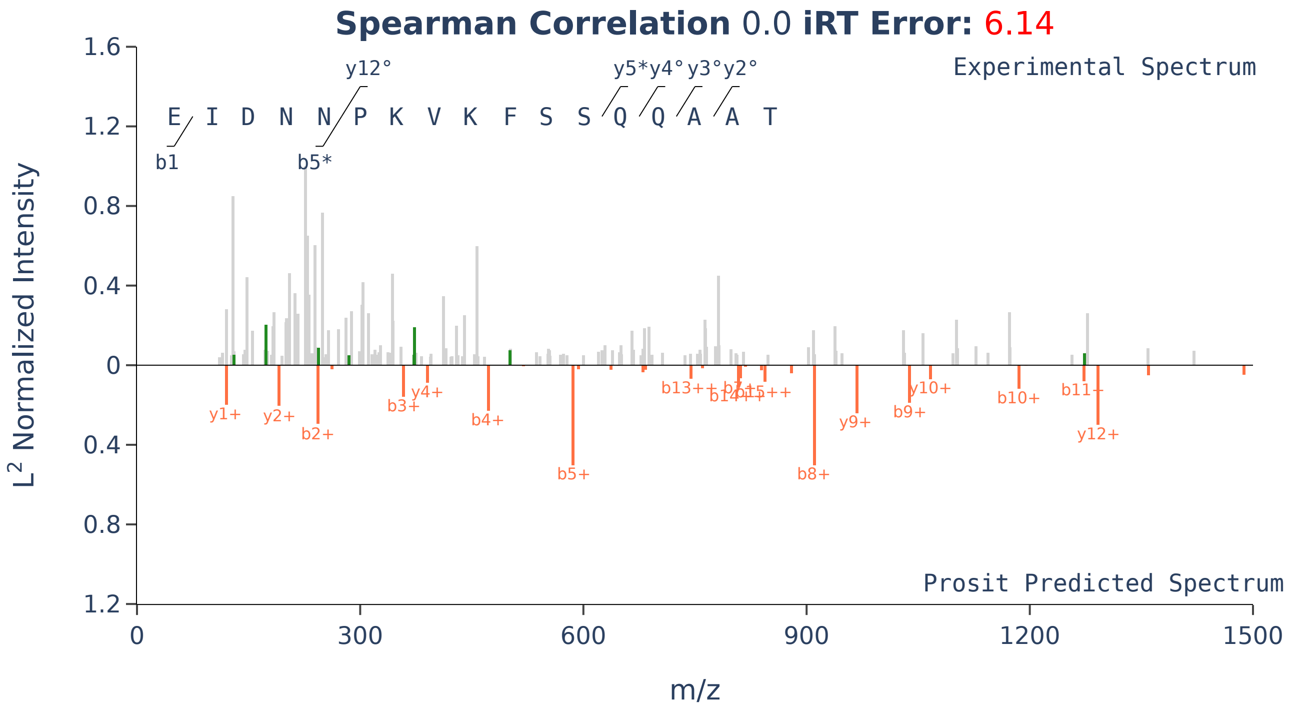

Source HRoetschke_271222_090123_HFGoe_LEDGF_b2R1_2h **Scan** 13226
Peptide VGGPGMTRDFKPGD **Charge** 3 **Spectral Angle** 0.82
Spearman Correlation 0.86 **iRT Error:** 1.4

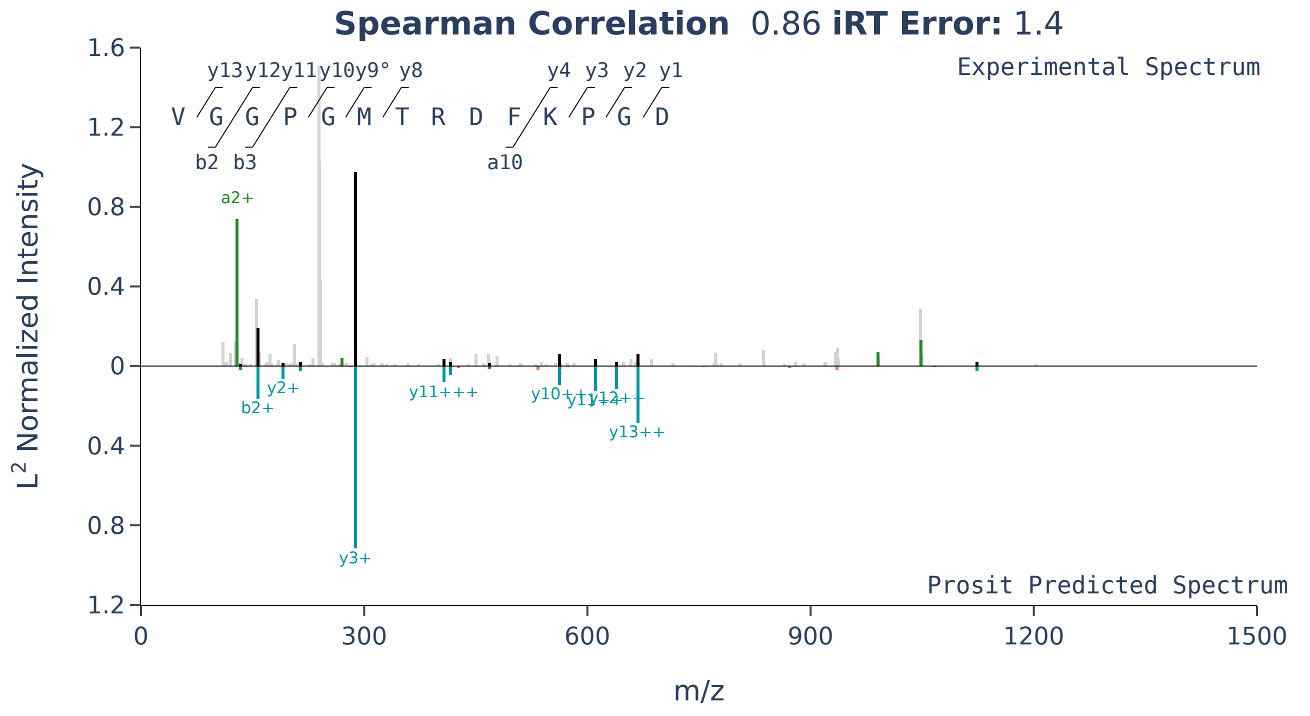

Source HRoetschke_271222_090123_HFGoe_LEDGF_b2R1_2h **Scan** 13226
Peptide ETEQQNKDEGKK **Charge** 3 **Spectral Angle** 0.27
Spearman Correlation 0.16 **iRT Error:** 62.25

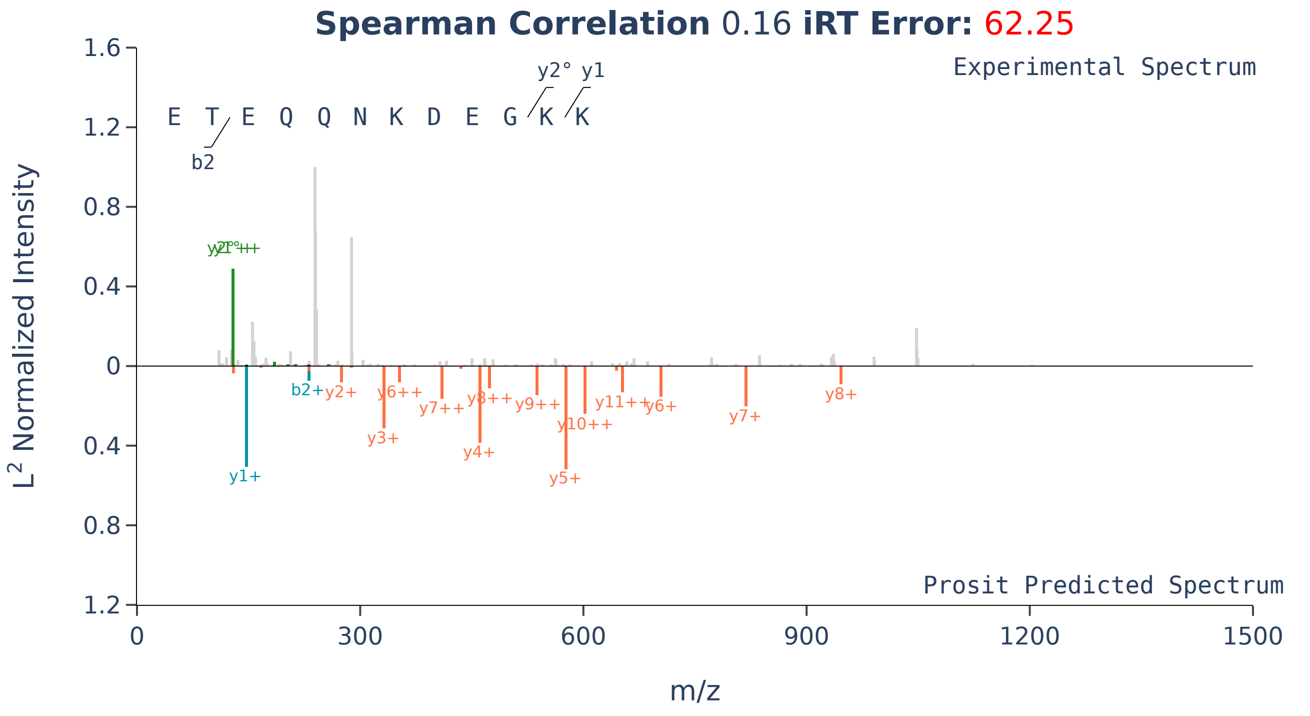

Source HRoetschke_271222_090123_HFGoe_LEDGF_b1R2_24h **Scan** 16291
Peptide ELSMDSRLQRIHA **Charge** 4 **Spectral Angle** 0.89
Spearman Correlation 0.83 **iRT Error:** 1.43

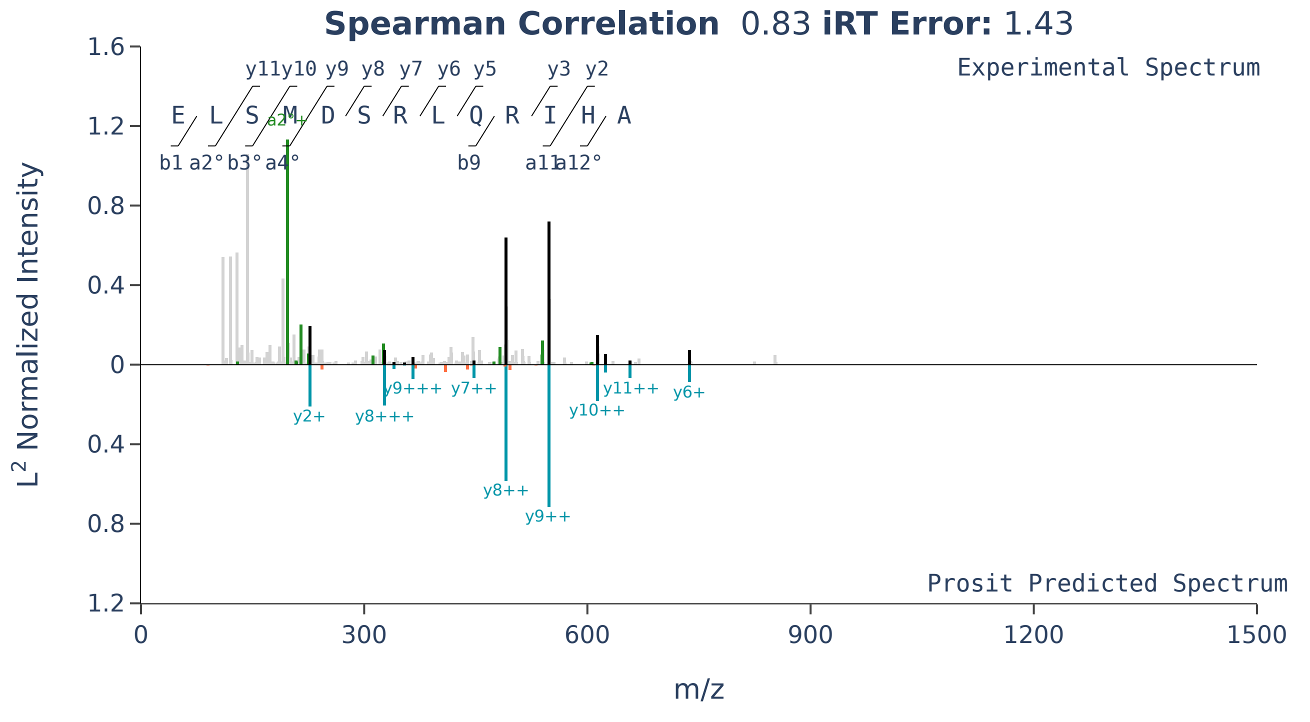

Source HRoetschke_271222_090123_HFGoe_LEDGF_b1R2_24h **Scan** 16291
Peptide SMDSRLQRIHAEI **Charge** 4 **Spectral Angle** 0.48
Spearman Correlation 0.35 **iRT Error:** 3.8

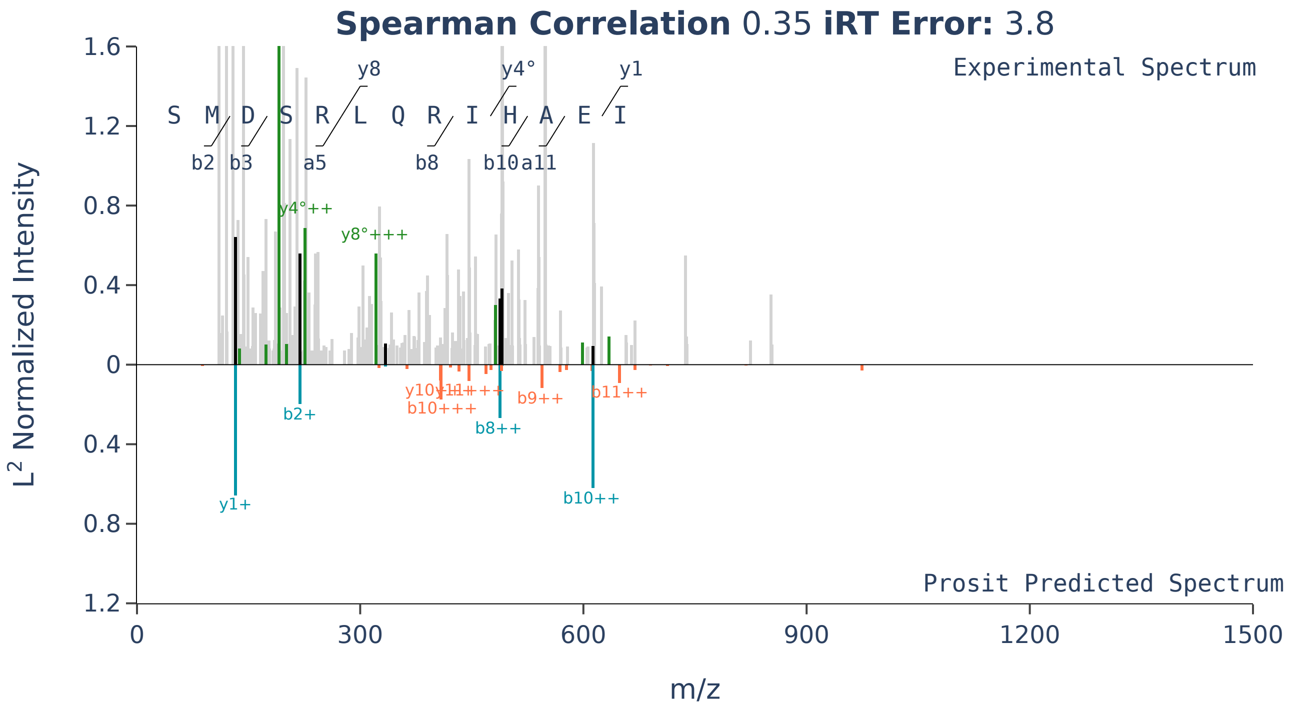

Source HRoetschke_271222_090123_HFGoe_LEDGF_b2R1_24h **Scan** 23405
Peptide GALWEIDNNPKVKFSSQQA **Charge** 3 **Spectral Angle** 0.75
Spearman Correlation 0.87 **iRT Error:** 1.5

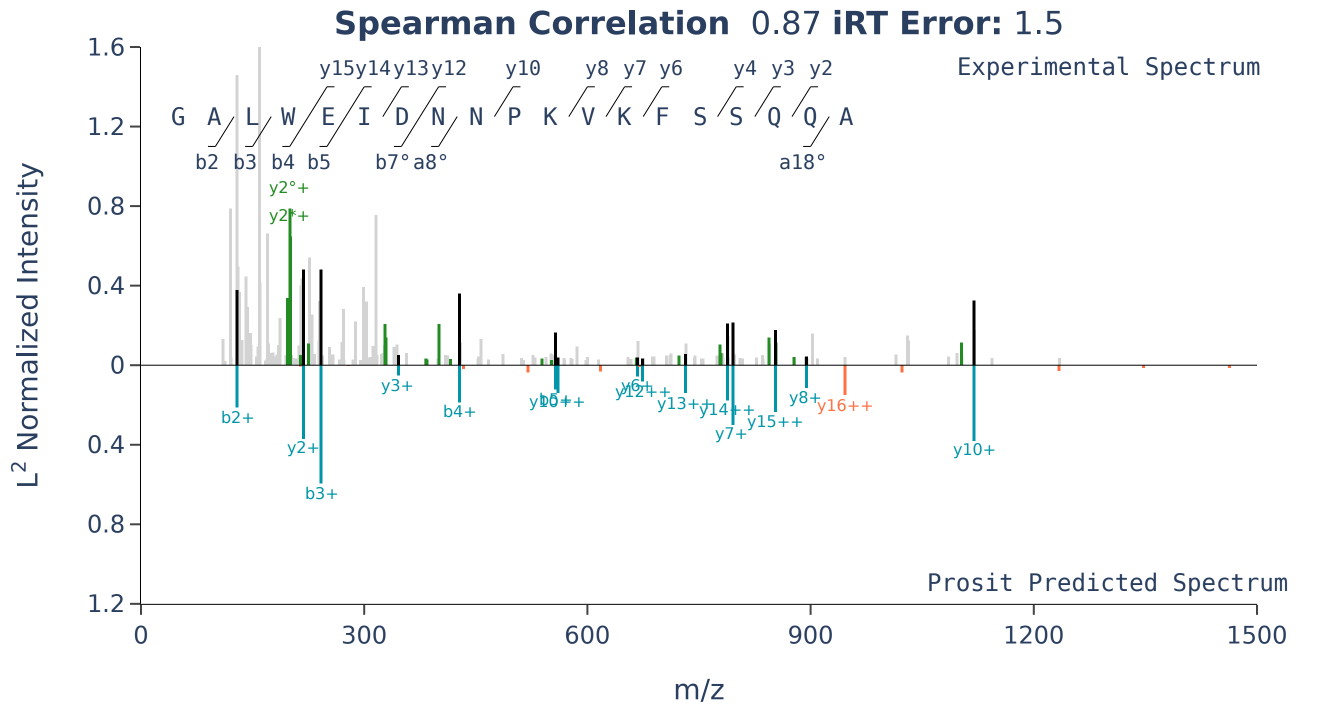

Source HRoetschke_271222_090123_HFGoe_LEDGF_b2R1_24h **Scan** 23405
Peptide GLWEIDNNPKVKFSSQQAA **Charge** 3 **Spectral Angle** 0.52
Spearman Correlation 0.46 **iRT Error:** 2.13

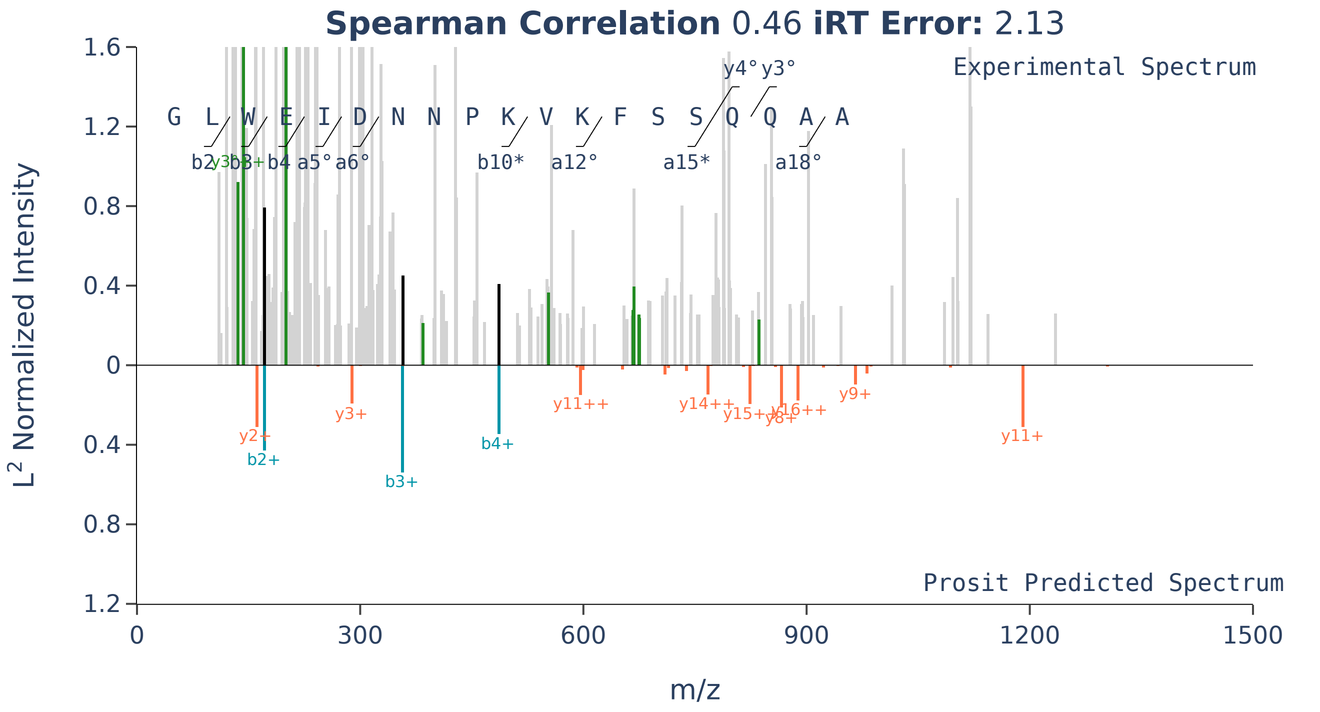

Source HRoetschke_271222_090123_HFGoe_LEDGF_b1R1_2h **Scan** 8447
Peptide GPKDIFPYSENKEKYGKPNKKR **Charge** 6 **Spectral Angle** 0.79
Spearman Correlation 0.89 **iRT Error:** 1.53

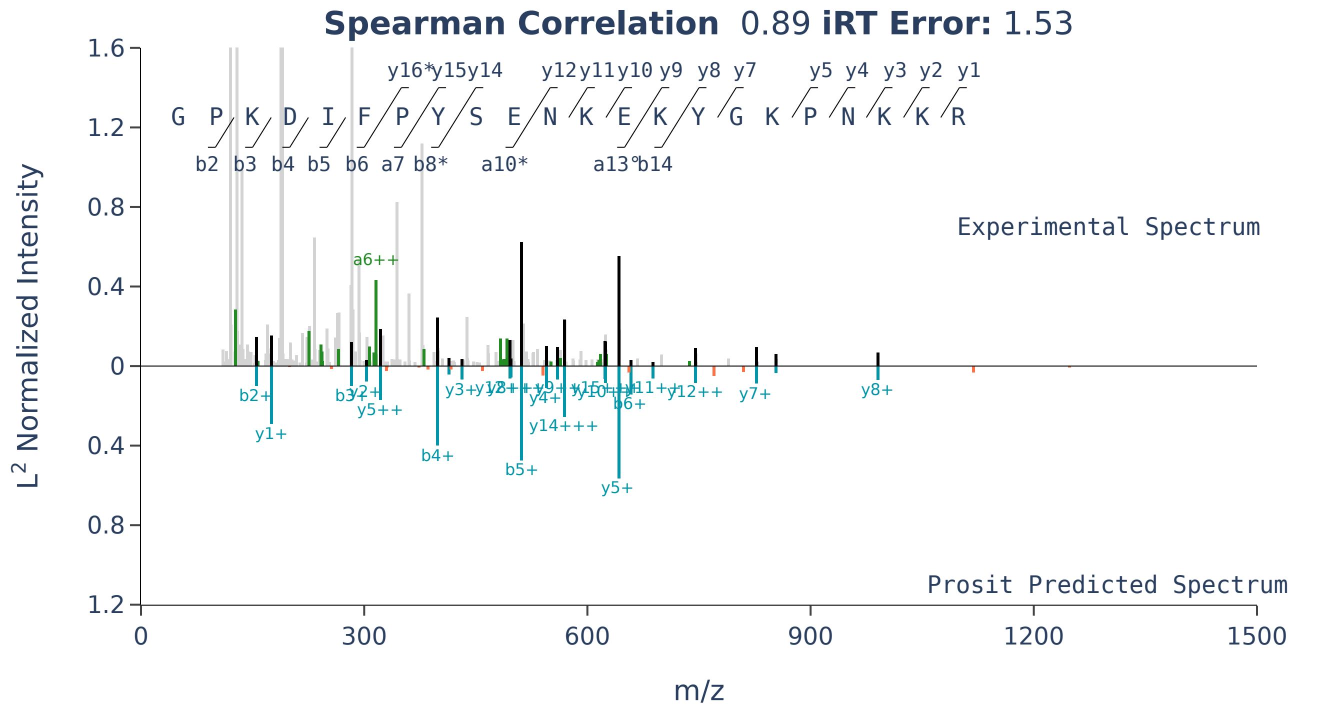

Source HRoetschke_271222_090123_HFGoe_LEDGF_b1R1_2h **Scan** 8447
Peptide PKDIFPYSENKEKYGKPNKRKG **Charge** 6 **Spectral Angle** 0.28
Spearman Correlation 0.31 **iRT Error:** 1.06

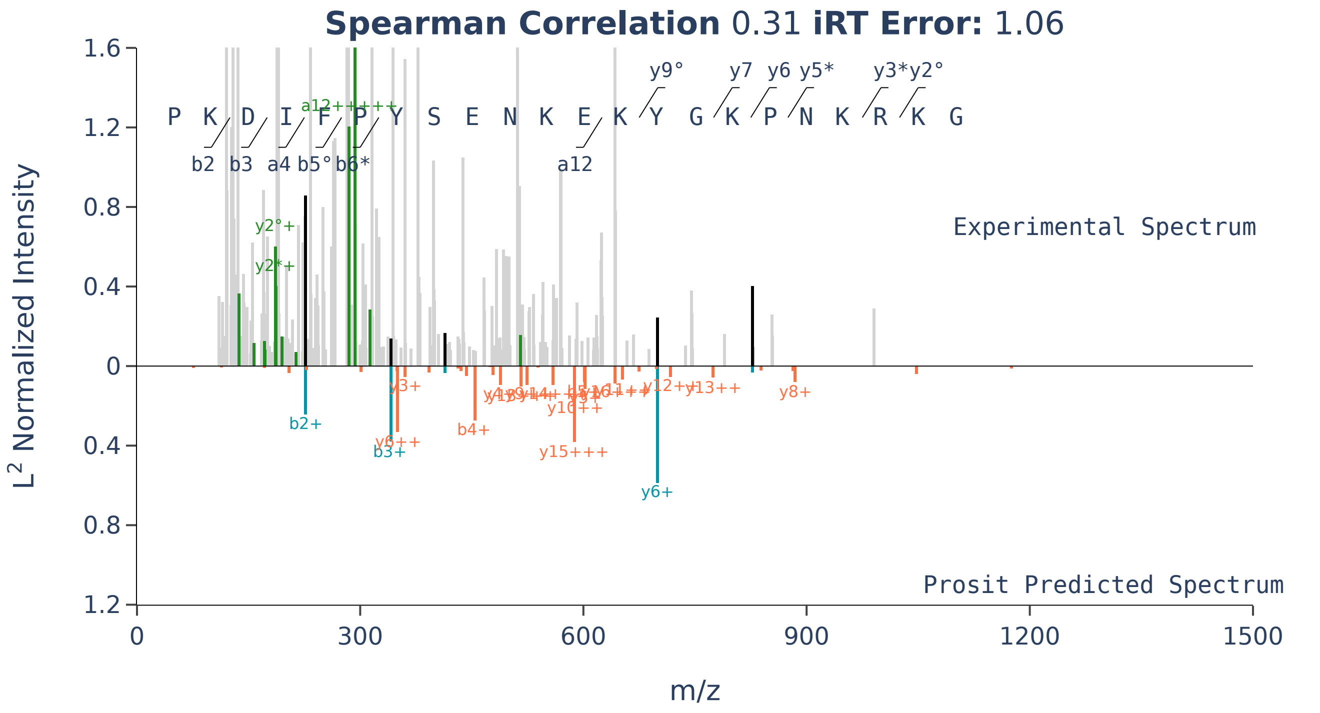

Source HRoetschke_271222_090123_HFGoe_LEDGF_b1R1_2h **Scan** 18526
Peptide LWEIDNNPKVKFSSQQAATKSQ **Charge** 3 **Spectral Angle** 0.89
Spearman Correlation 0.81 **iRT Error:** 1.53

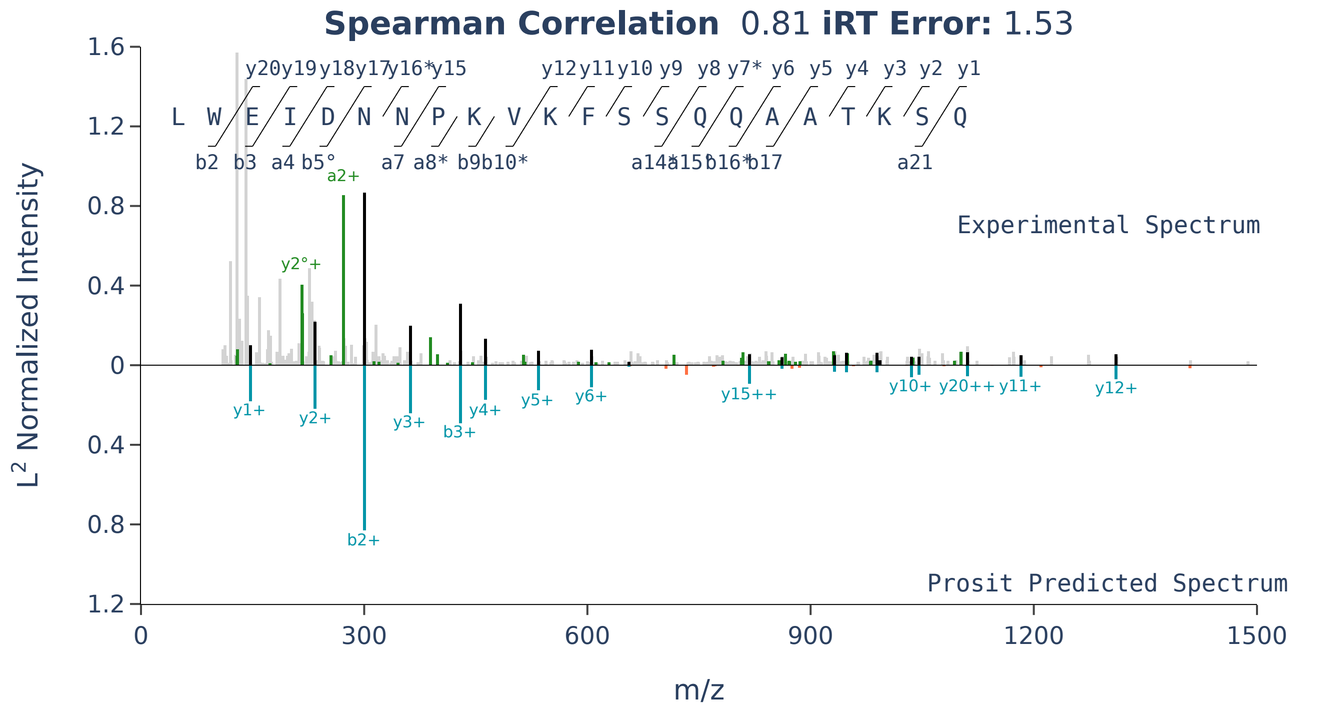

Source HRoetschke_271222_090123_HFGoe_LEDGF_b1R1_2h **Scan** 18526
Peptide LWEIDNNPKVKFSSQQAATKQS **Charge** 3 **Spectral Angle** 0.93
Spearman Correlation 0.77 **iRT Error:** 1.73

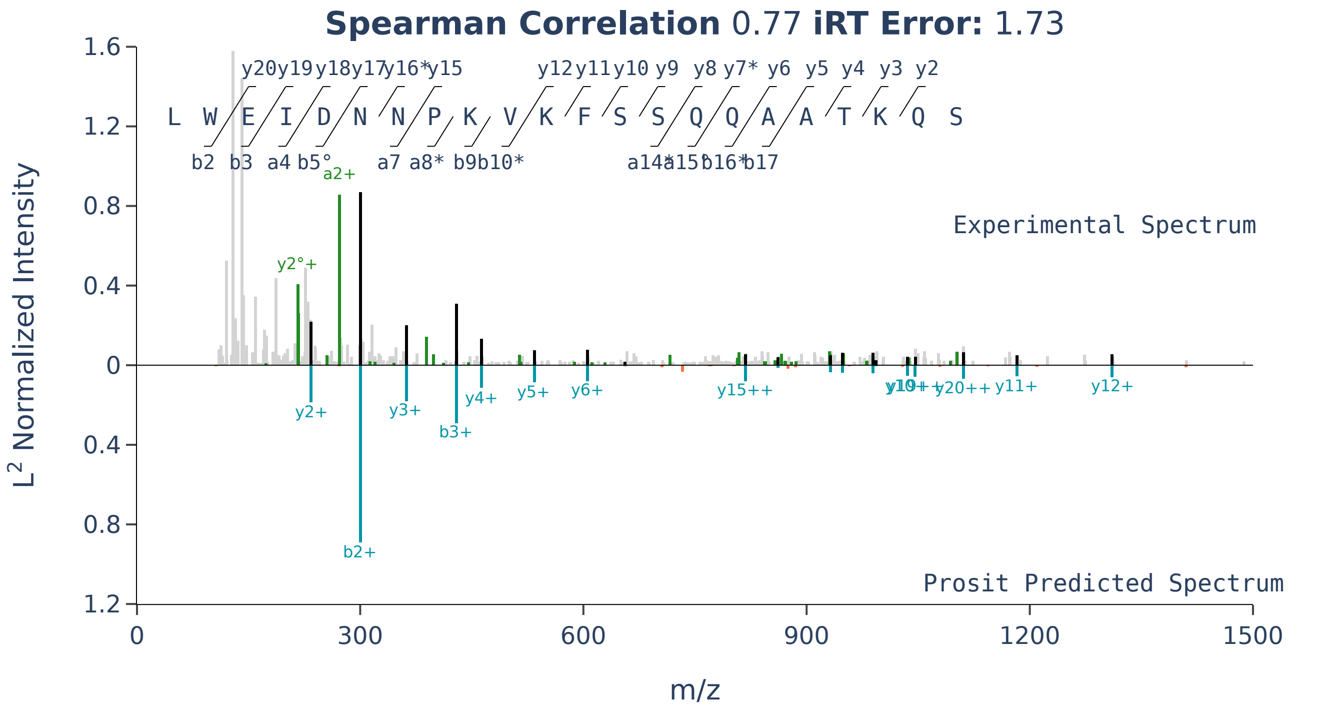

Source HRoetschke_271222_090123_HFGoe_LEDGF_b1R2_4h **Scan** 21069
Peptide NGGSDELASL **Charge** 1 **Spectral Angle** 0.84
Spearman Correlation 0.96 **iRT Error:** 1.54

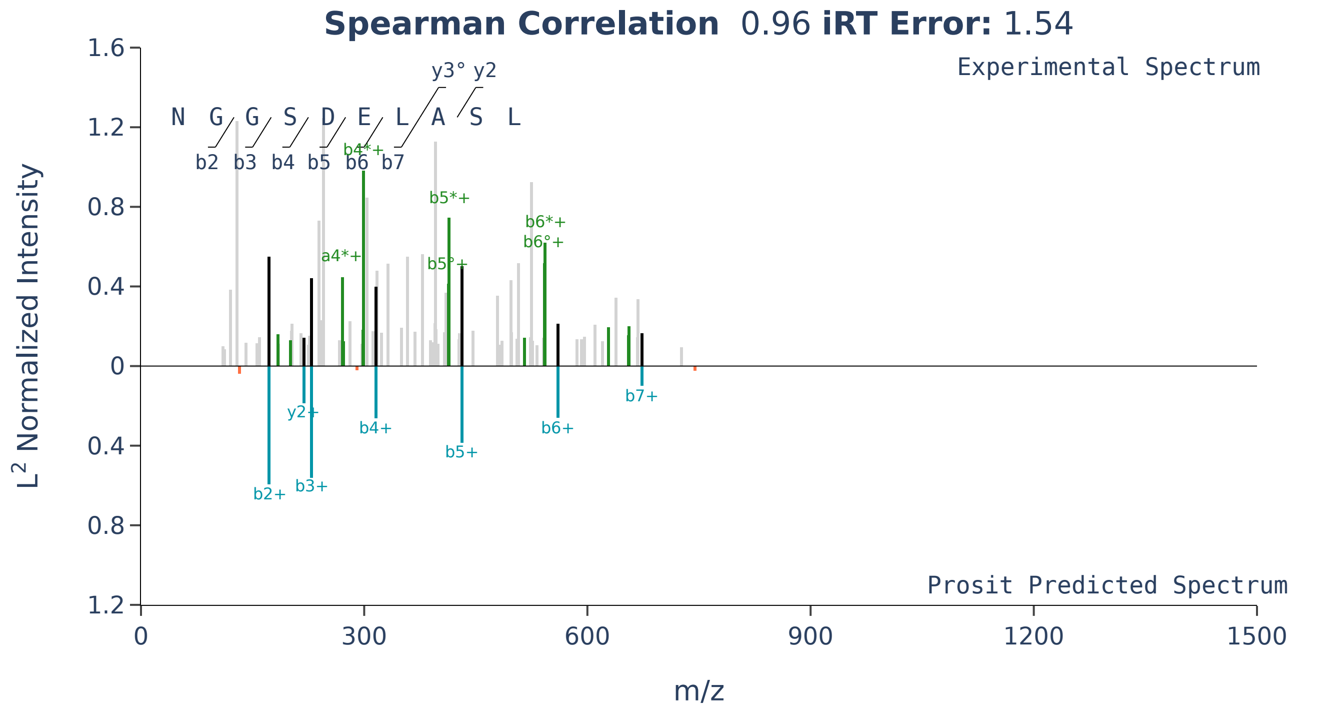

Source HRoetschke_271222_090123_HFGoe_LEDGF_b1R2_4h **Scan** 21069
Peptide EGQKEEDK **Charge** 1 **Spectral Angle** 0.0
Spearman Correlation 0.0 **iRT Error:** 96.69

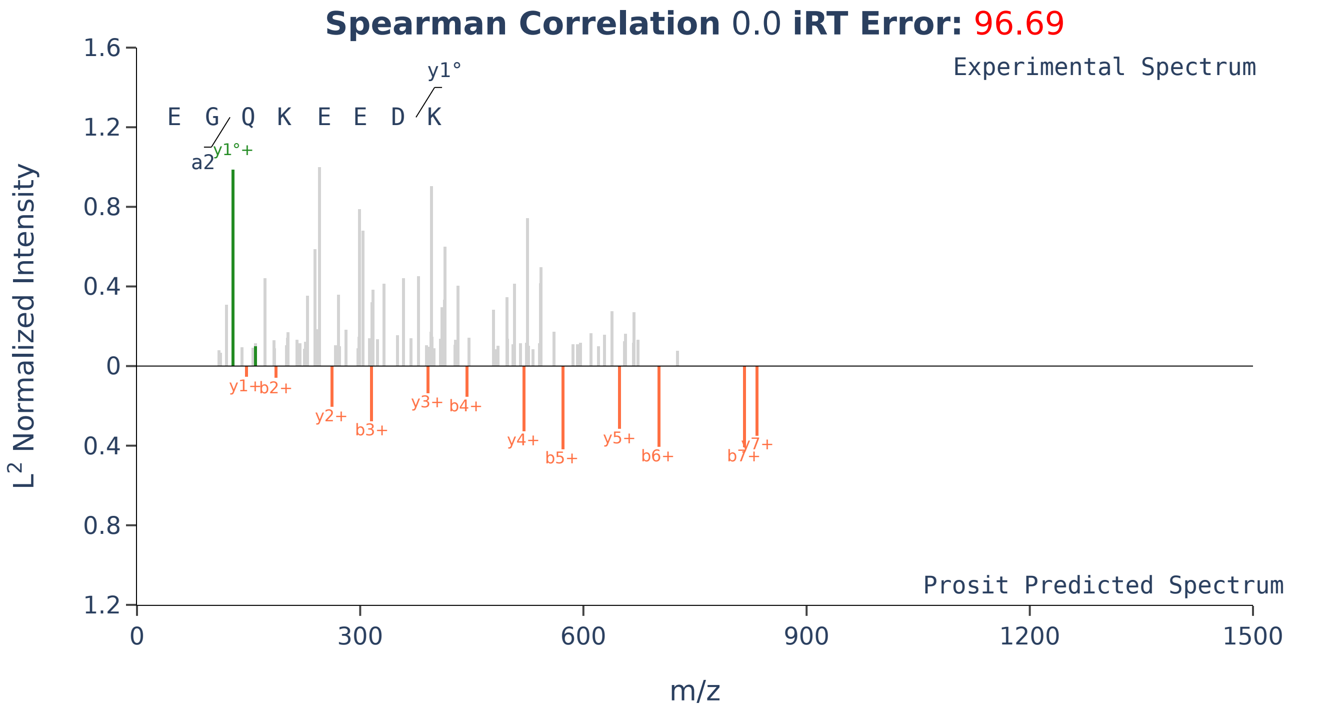

Source HRoetschke_271222_090123_HFGoe_LEDGF_b2R1_24h **Scan** 18285
Peptide EISLMDSRLQR **Charge** 3 **Spectral Angle** 0.79
Spearman Correlation 0.87 **iRT Error:** 1.6

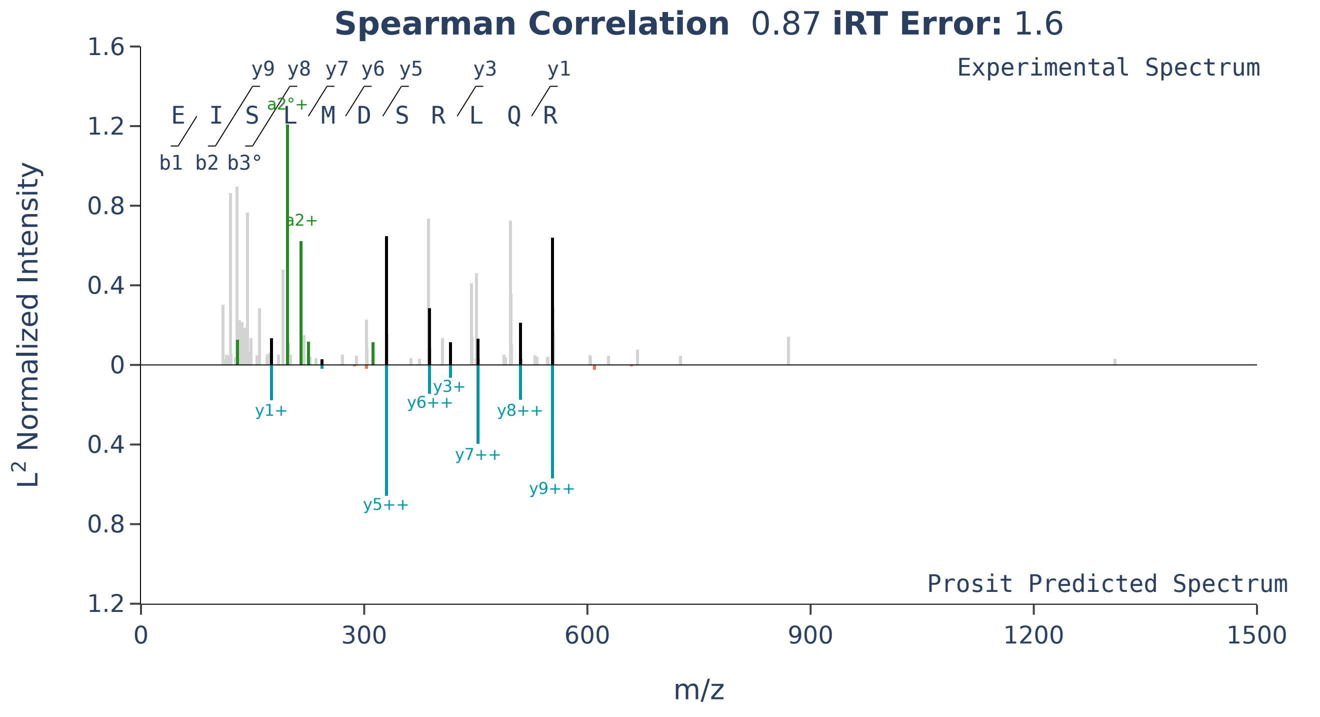

Source HRoetschke_271222_090123_HFGoe_LEDGF_b2R1_24h **Scan** 18285
Peptide LEKEQTGSKTLN **Charge** 3 **Spectral Angle** 0.2
Spearman Correlation 0.47 **iRT Error:** 72.87

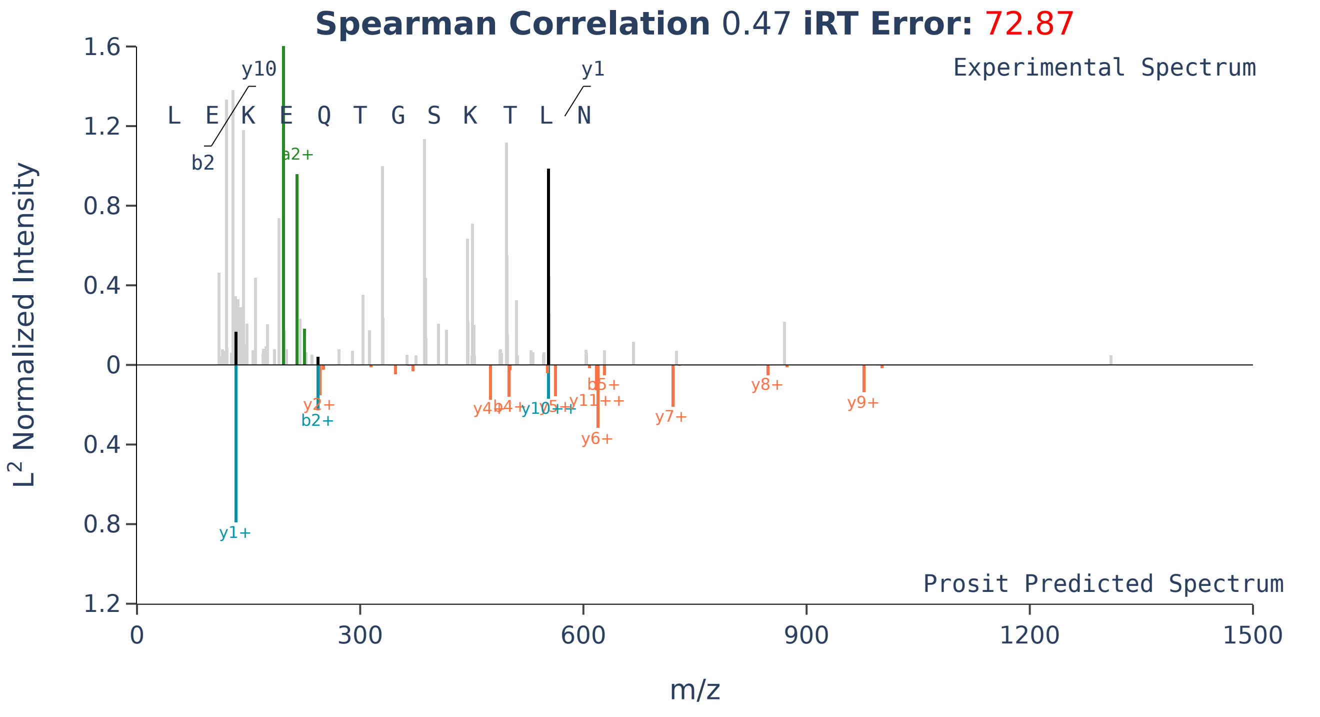

Source HRoetschke_271222_090123_HFGoe_LEDGF_b1R1_4h **Scan** 15738
Peptide FLGPKDIFPYSENKEKYGKPNKKR **Charge** 4 **Spectral Angle** 0.76
Spearman Correlation 0.87 **iRT Error:** 1.63

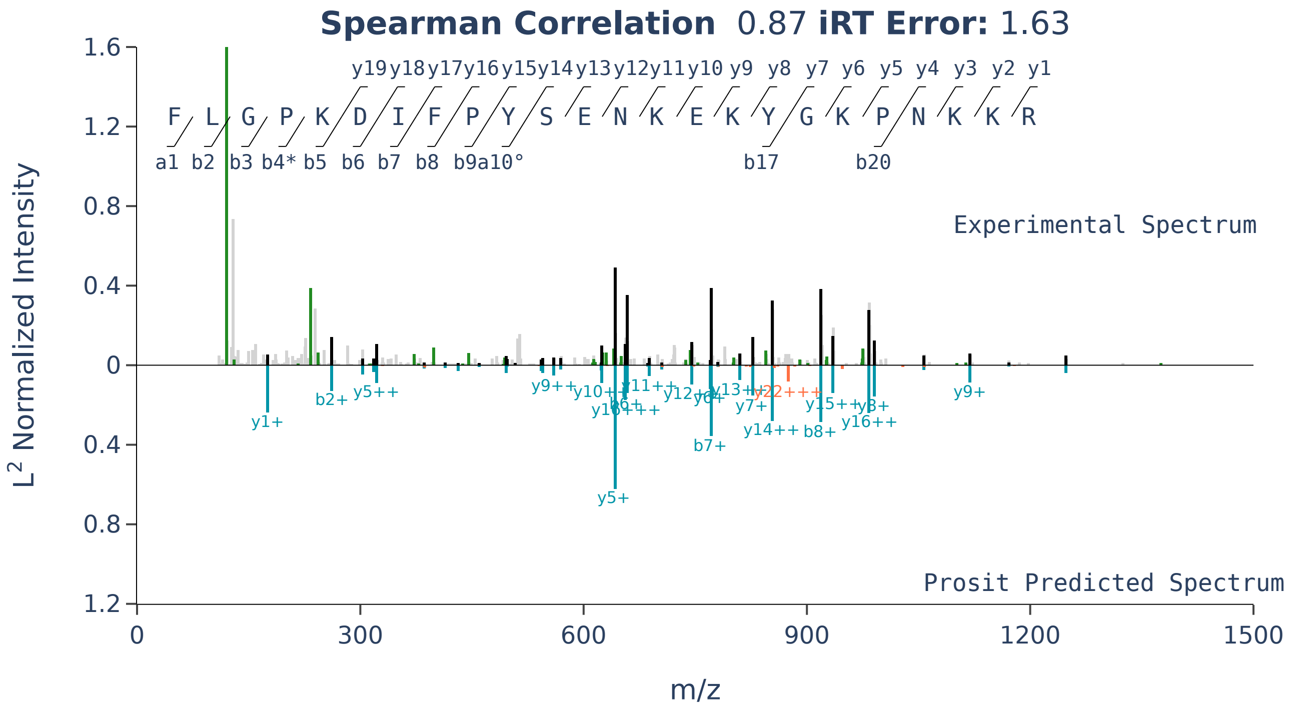

Source HRoetschke_271222_090123_HFGoe_LEDGF_b1R1_4h **Scan** 15738
Peptide FLGPKDIFPYSENKEKYGKPNKRK **Charge** 4 **Spectral Angle** 0.78
Spearman Correlation 0.81 **iRT Error:** 2.09

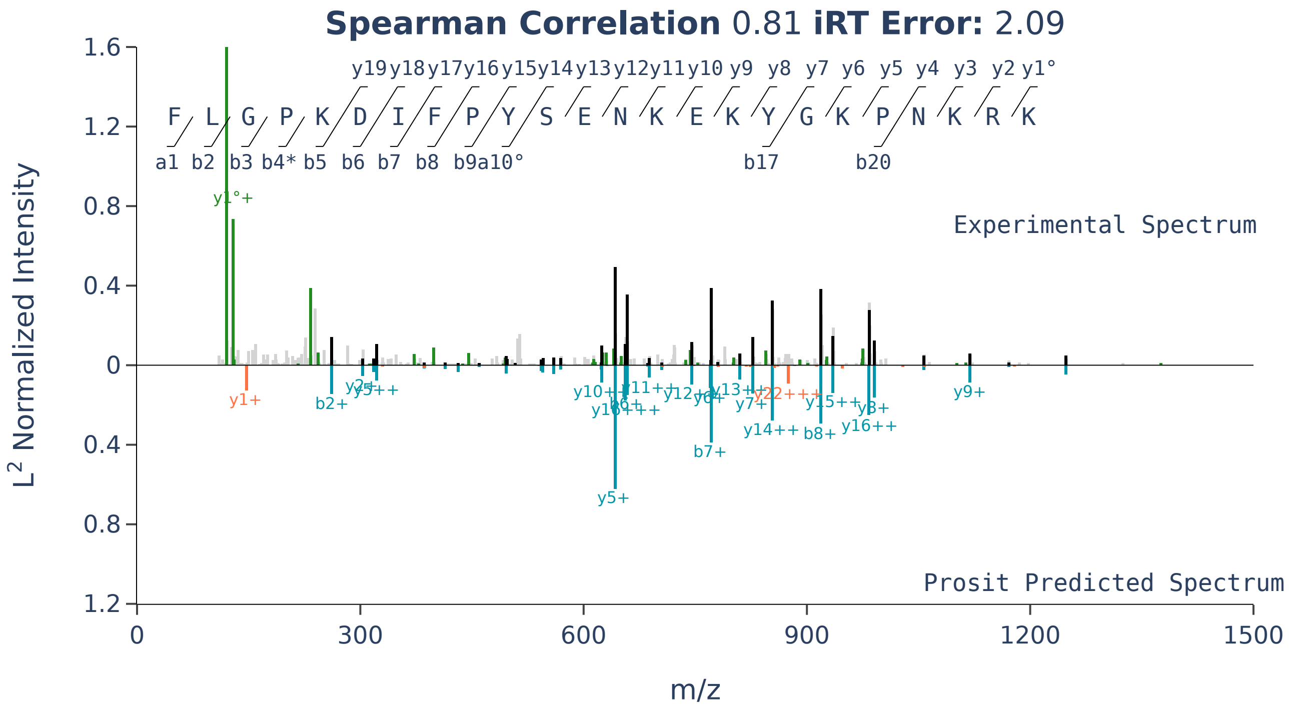

Source HRoetschke_271222_090123_HFGoe_LEDGF_b1R1_4h **Scan** 18073
Peptide GPKDIFIFPYSENKEKYGKPNKR **Charge** 4 **Spectral Angle** 0.79
Spearman Correlation 0.89 **iRT Error:** 1.65

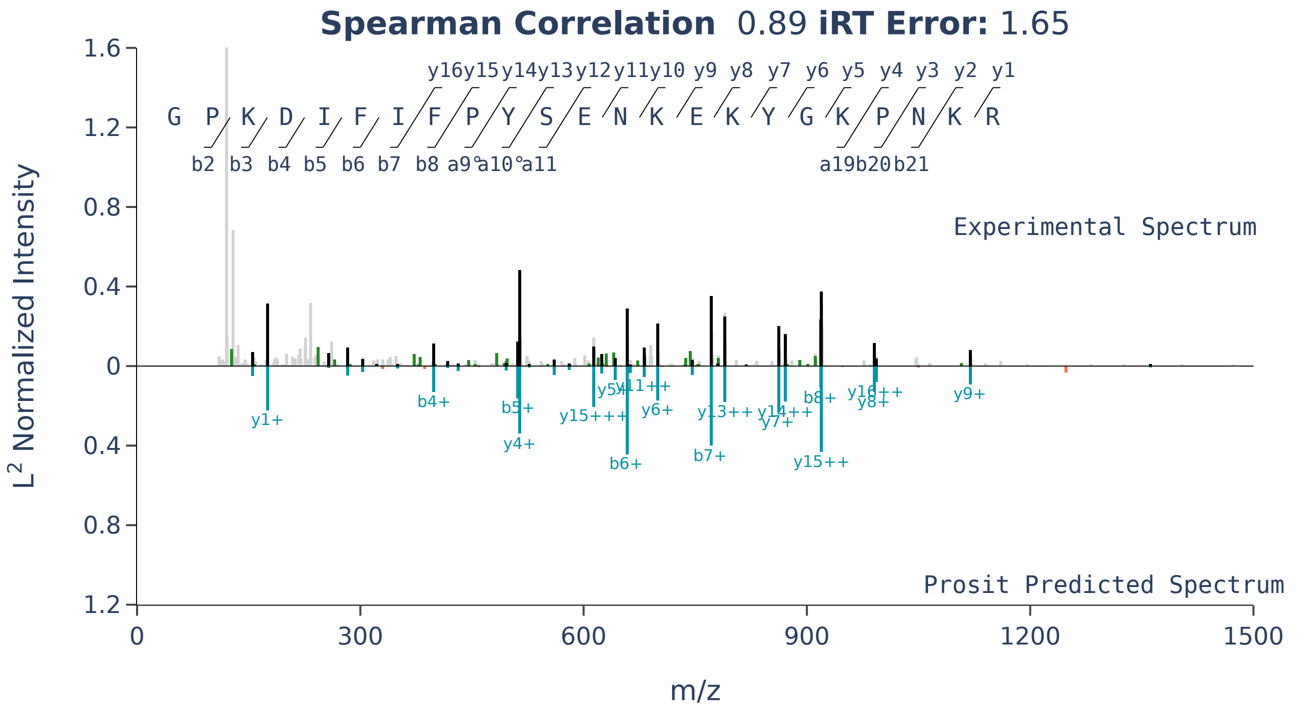

Source HRoetschke_271222_090123_HFGoe_LEDGF_b1R1_4h **Scan** 18073
Peptide FLGPKDIFPYSENKEKYGKPNKR **Charge** 4 **Spectral Angle** 0.84
Spearman Correlation 0.83 **iRT Error:** 3.13

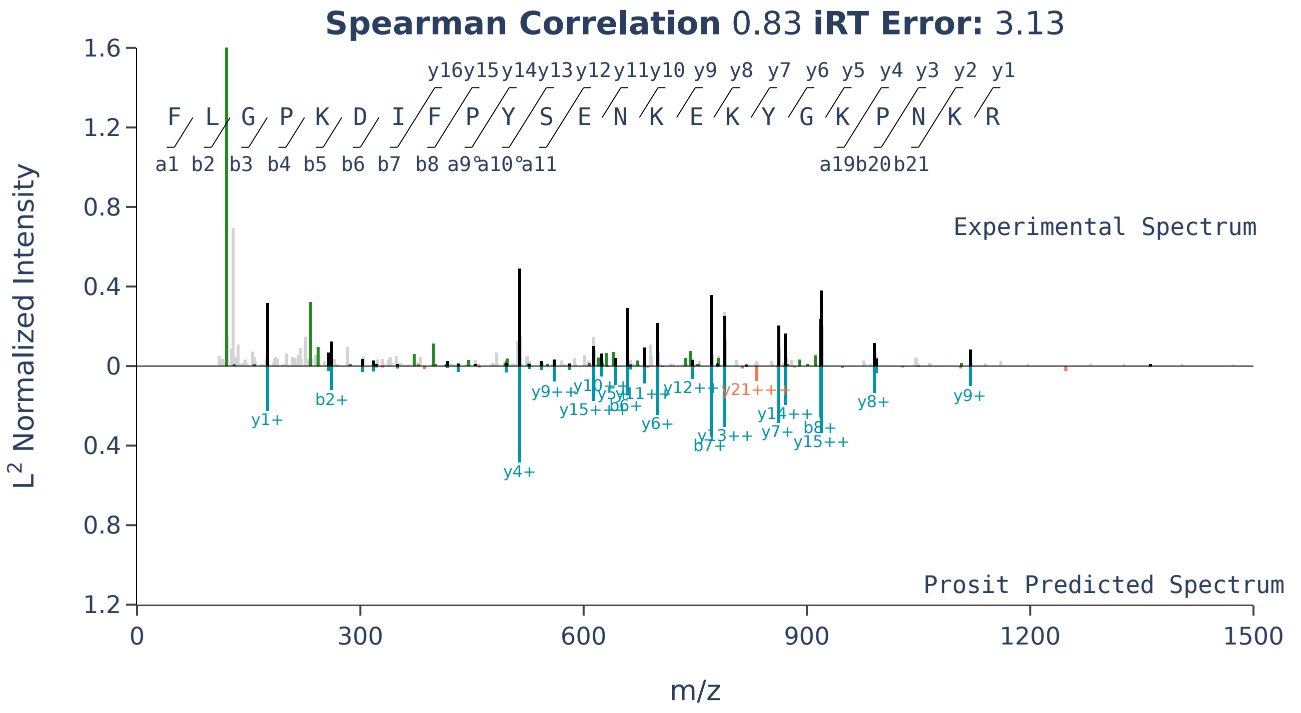

Source HRoetschke_271222_090123_HFGoe_LEDGF_b1R1_1h **Scan** 11816
Peptide GAPGMTRDFKPGD **Charge** 3 **Spectral Angle** 0.86
Spearman Correlation 0.91 **iRT Error:** 1.72

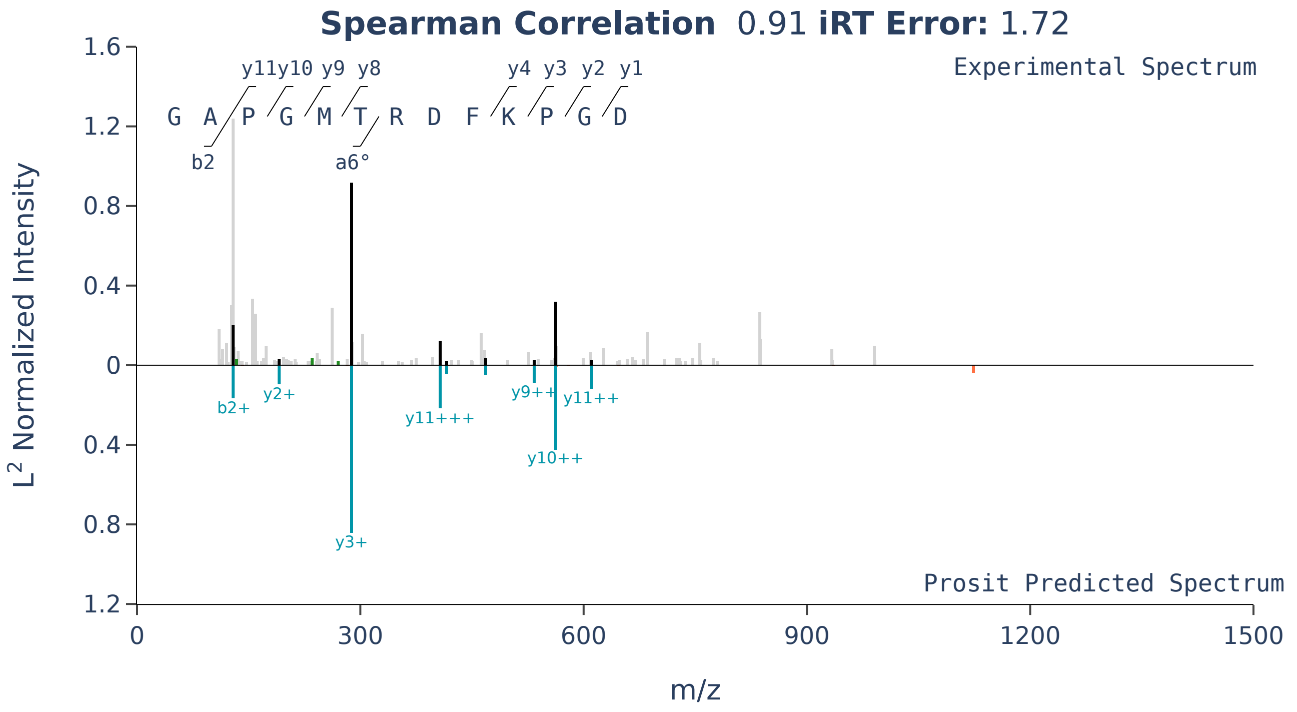

Source HRoetschke_271222_090123_HFGoe_LEDGF_b1R1_1h **Scan** 11816
Peptide KMVKQPCPSESD **Charge** 3 **Spectral Angle** 0.38
Spearman Correlation 0.52 **iRT Error:** 33.71

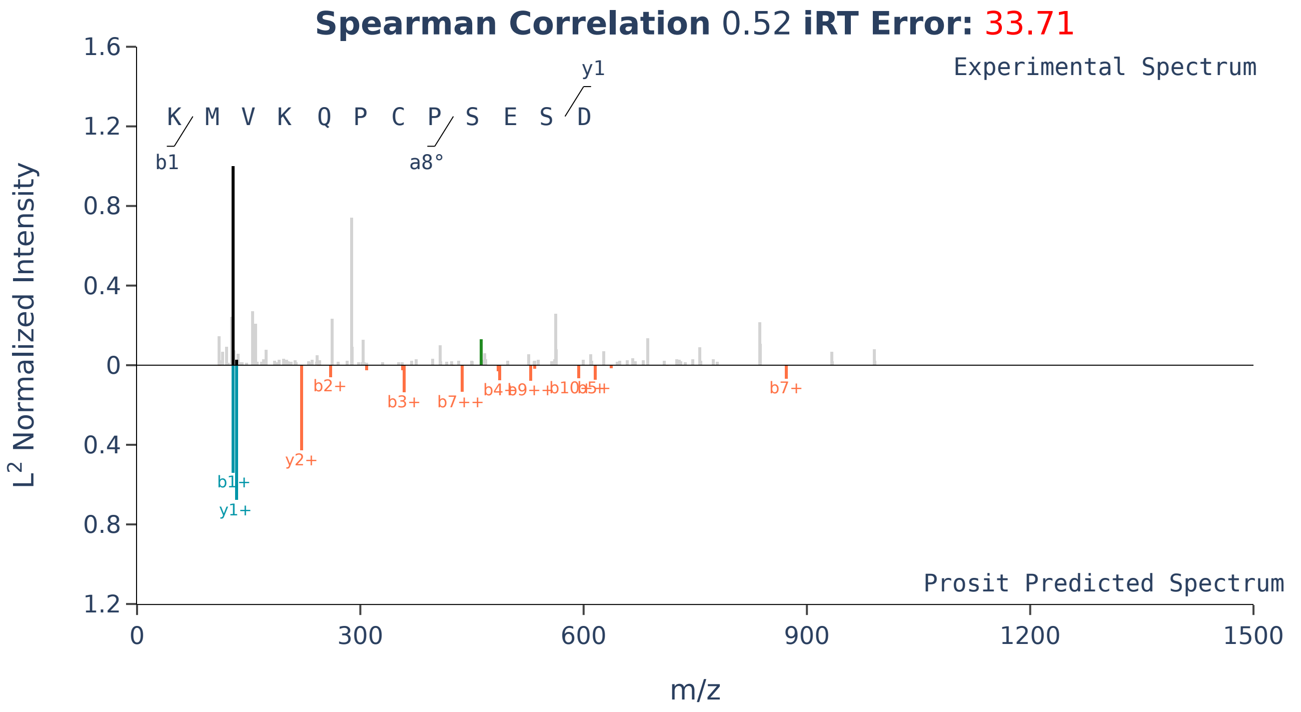

Source HRoetschke_271222_090123_HFGoe_LEDGF_b1R1_24h **Scan** 14597
Peptide TEEAGVVTTATQQA **Charge** 2 **Spectral Angle** 0.9
Spearman Correlation 0.87 **iRT Error:** 1.76

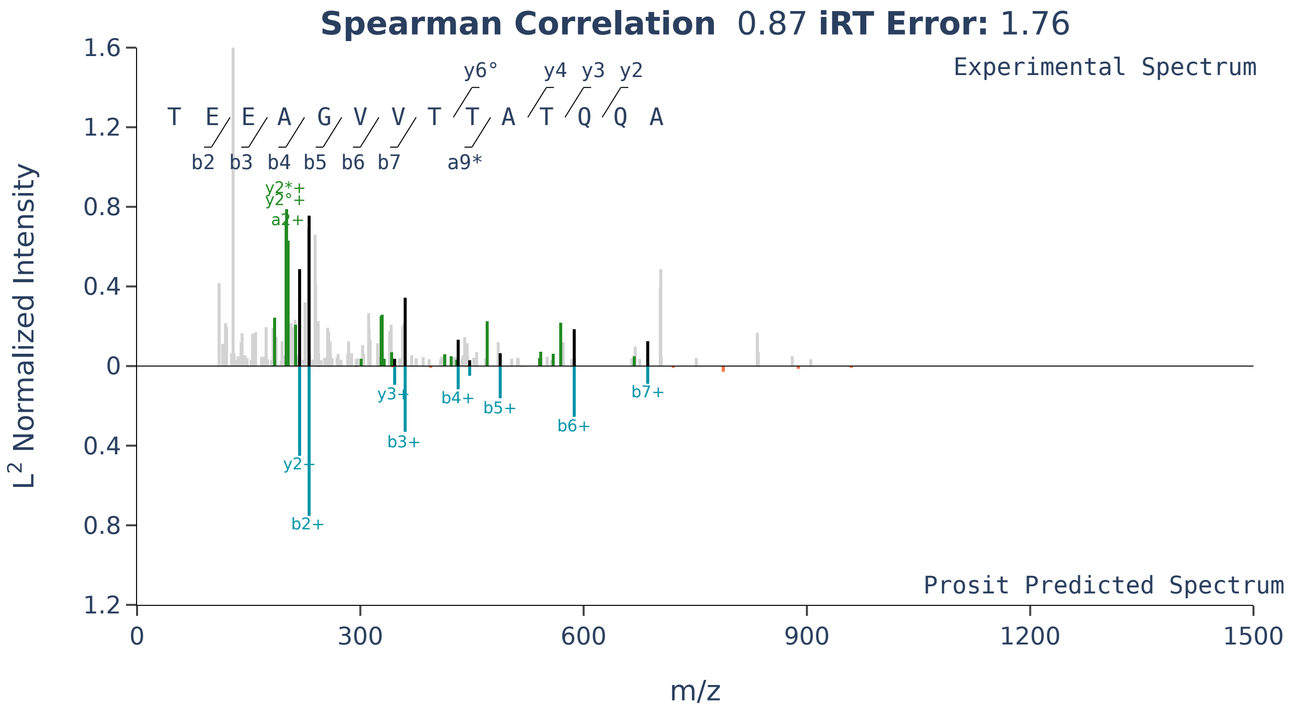

Source HRoetschke_271222_090123_HFGoe_LEDGF_b1R1_24h **Scan** 14597
Peptide EKASNEDVTKAVD **Charge** 2 **Spectral Angle** 0.39
Spearman Correlation 0.46 **iRT Error:** 39.93

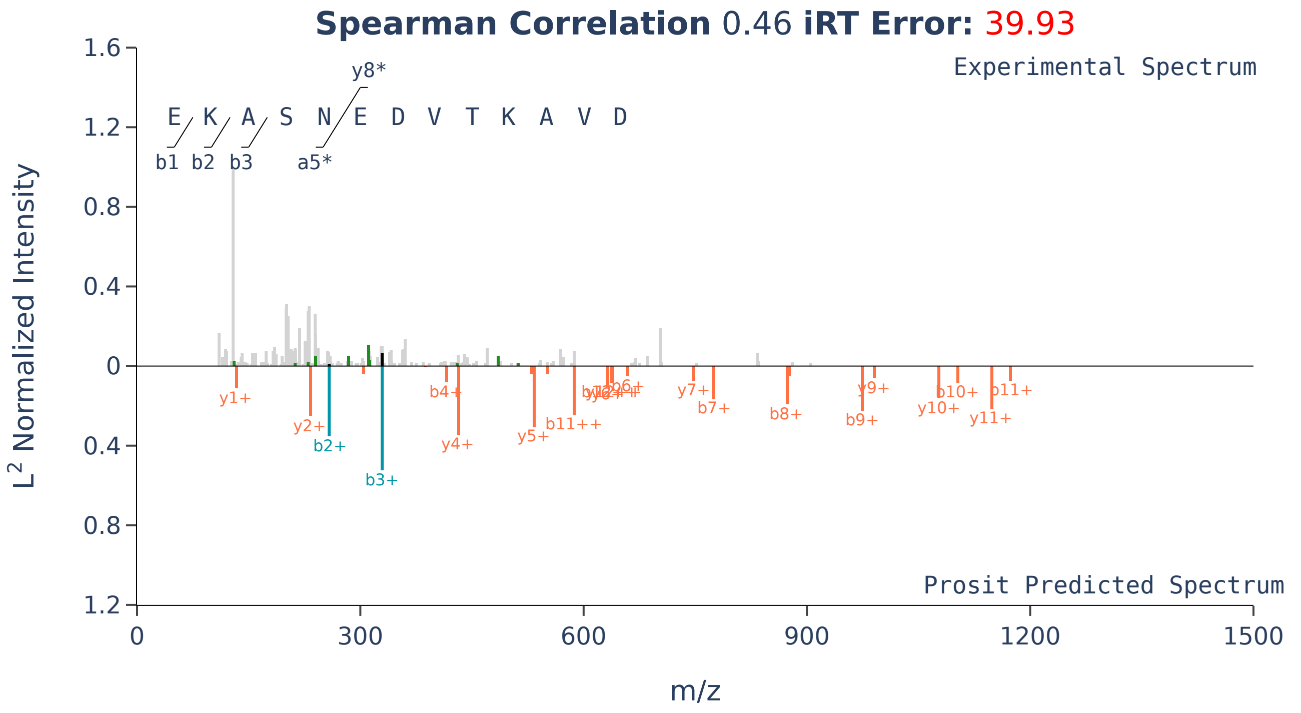

Source HRoetschke_271222_090123_HFGoe_LEDGF_b2R1_4h **Scan** 19287
Peptide EDGYPHWPARV **Charge** 3 **Spectral Angle** 0.93
Spearman Correlation 0.87 **iRT Error:** 1.85

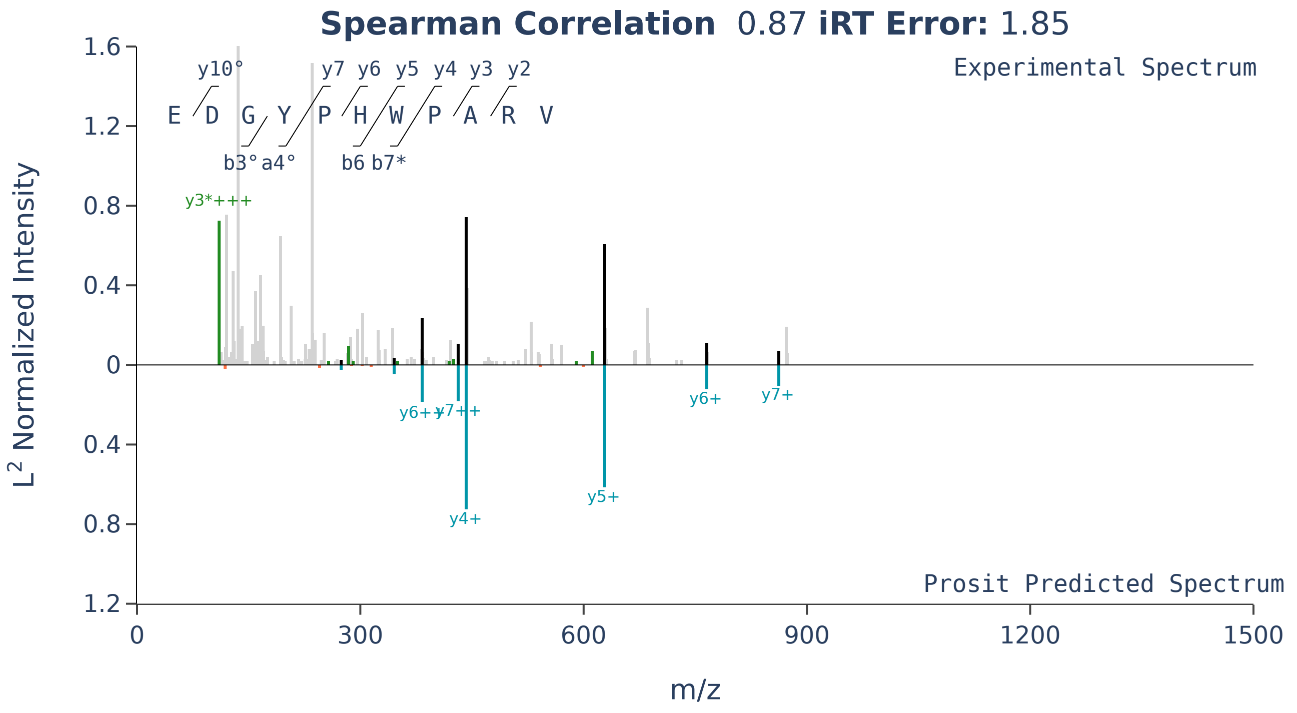

Source HRoetschke_271222_090123_HFGoe_LEDGF_b2R1_4h **Scan** 19287
Peptide GYPHWPARVDE **Charge** 3 **Spectral Angle** 0.9
Spearman Correlation 0.64 **iRT Error:** 13.18

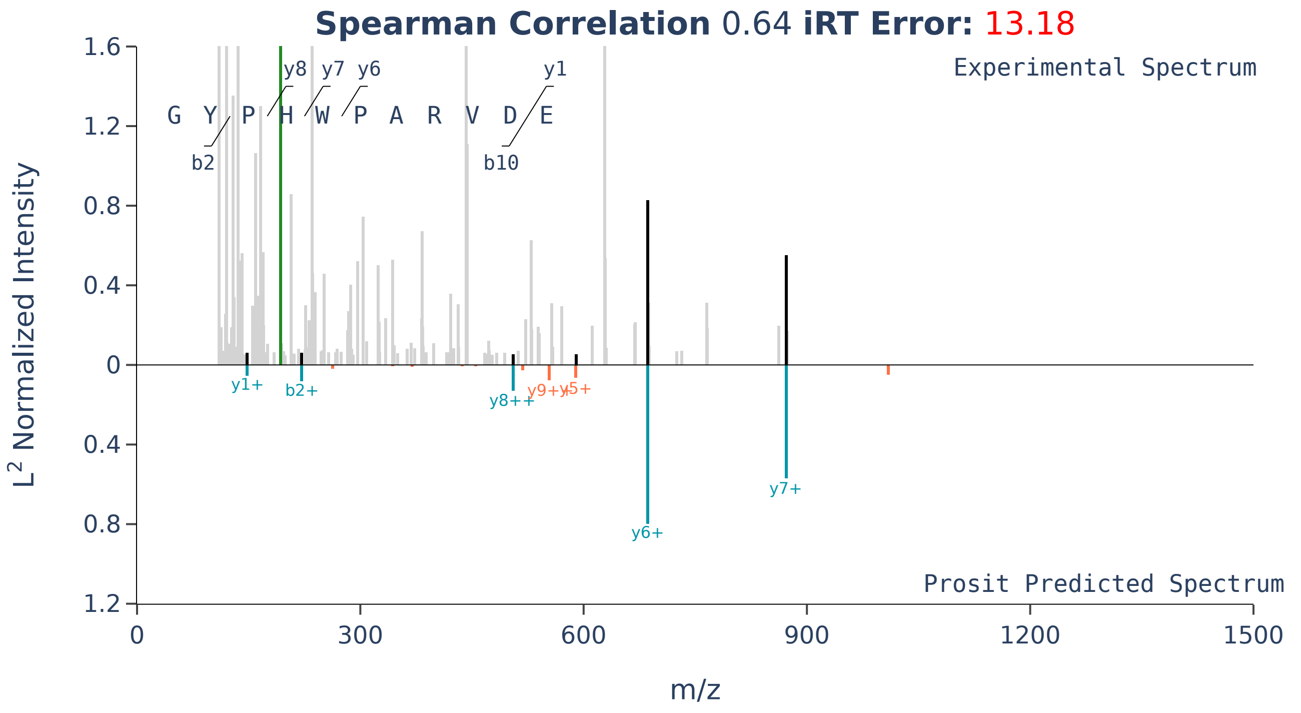

Source HRoetschke_271222_090123_HFGoe_LEDGF_b1R1_4h **Scan** 18025
Peptide QVETEVTTATASVNLKVSPKRGRPAATEV **Charge** 5 **Spectral Angle** 0.77
Spearman Correlation 0.89 **iRT Error:** 1.86

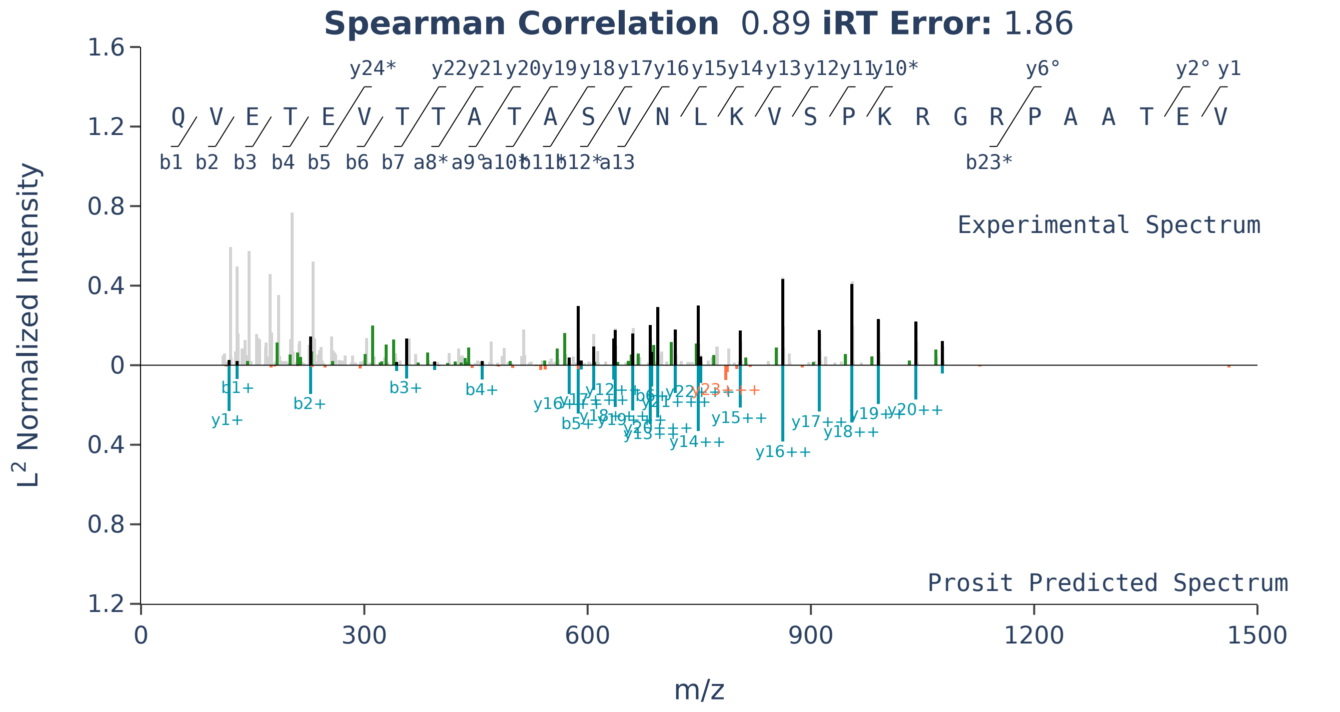

Source HRoetschke_271222_090123_HFGoe_LEDGF_b1R1_4h **Scan** 18025
Peptide VETEEAGVVTTATASVNLKVSPKRGRPAAT **Charge** 5 **Spectral Angle** 0.17
Spearman Correlation 0.14 **iRT Error:** 3.93

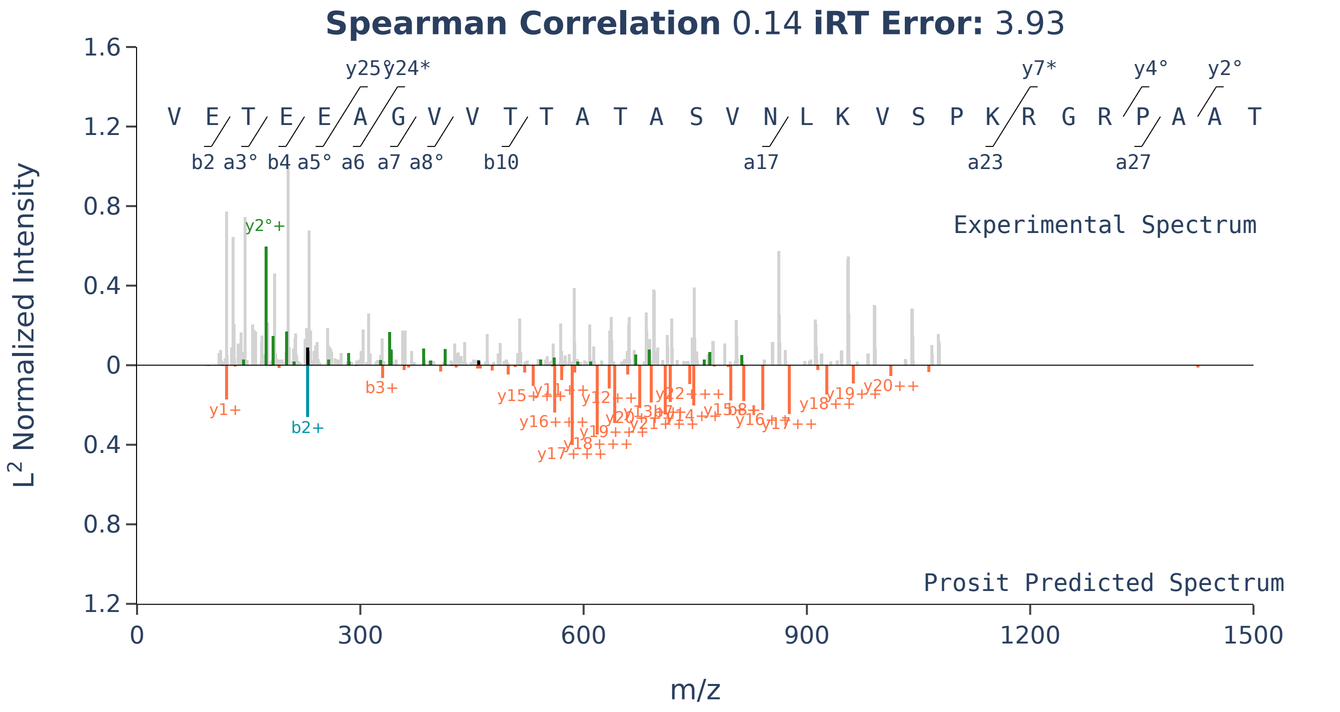

Source HRoetschke_271222_090123_HFGoe_LEDGF_b2R1_24h **Scan** 18809
Peptide LGPKDIFFPYSENKEKYGKPNKR **Charge** 4 **Spectral Angle** 0.77
Spearman Correlation 0.9 **iRT Error:** 1.87

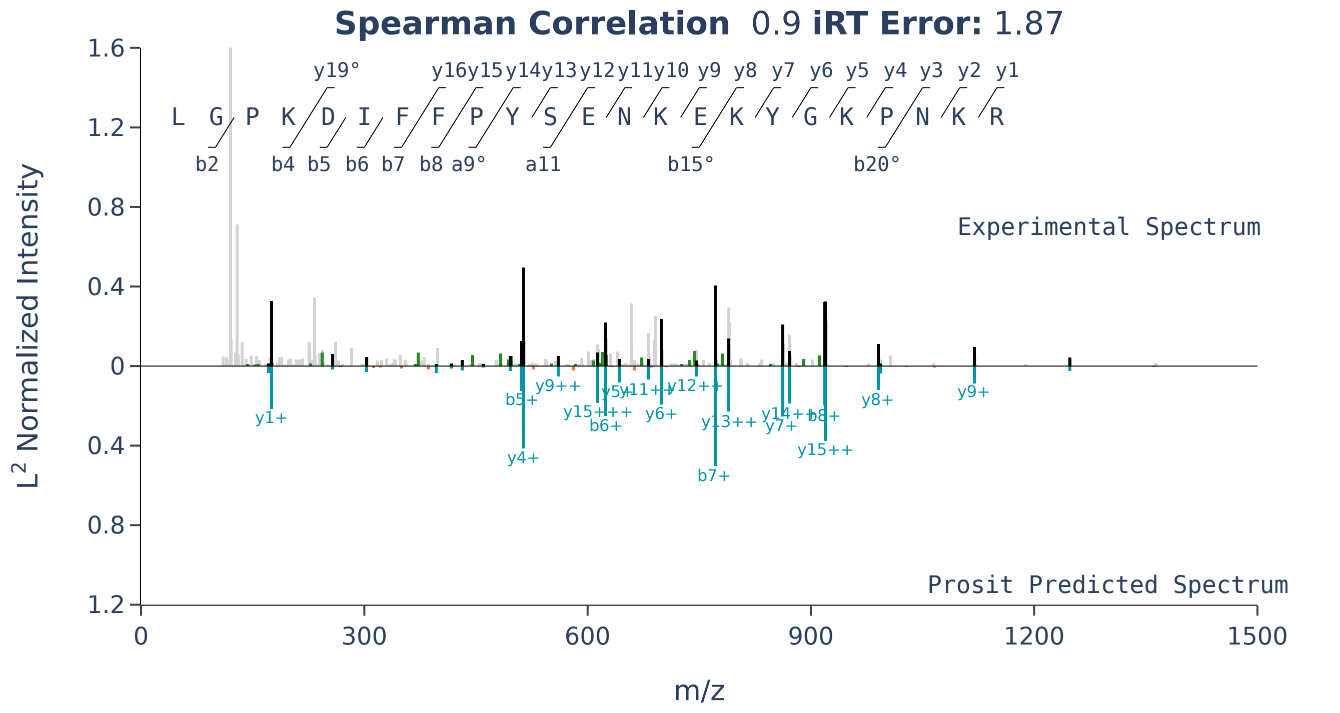

Source HRoetschke_271222_090123_HFGoe_LEDGF_b2R1_24h **Scan** 18809
Peptide FLGPKDIFFPYSENKEKYGKPNKR **Charge** 4 **Spectral Angle** 0.75
Spearman Correlation 0.86 **iRT Error:** 7.98

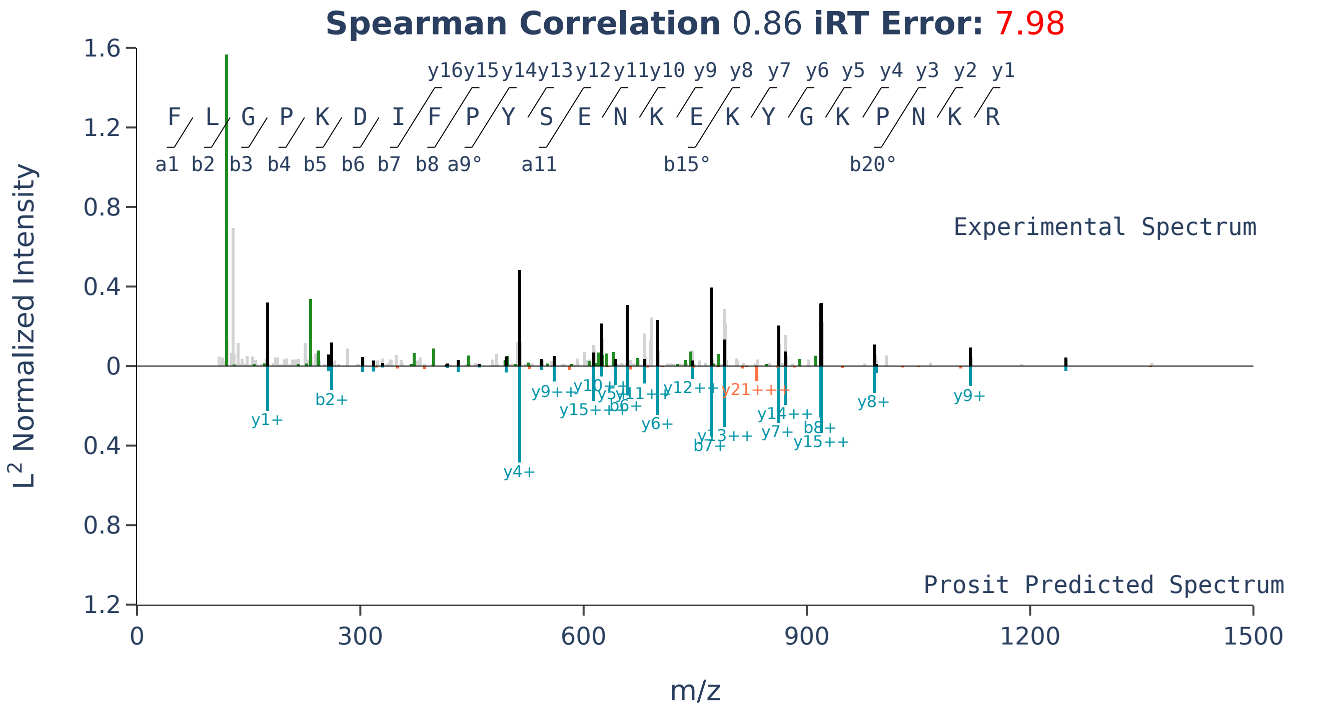

Source HRoetschke_271222_090123_HFGoe_LEDGF_b2R2_1h **Scan** 12812
Peptide TEISLKE **Charge** 2 **Spectral Angle** 0.94
Spearman Correlation 0.92 **iRT Error:** 1.88

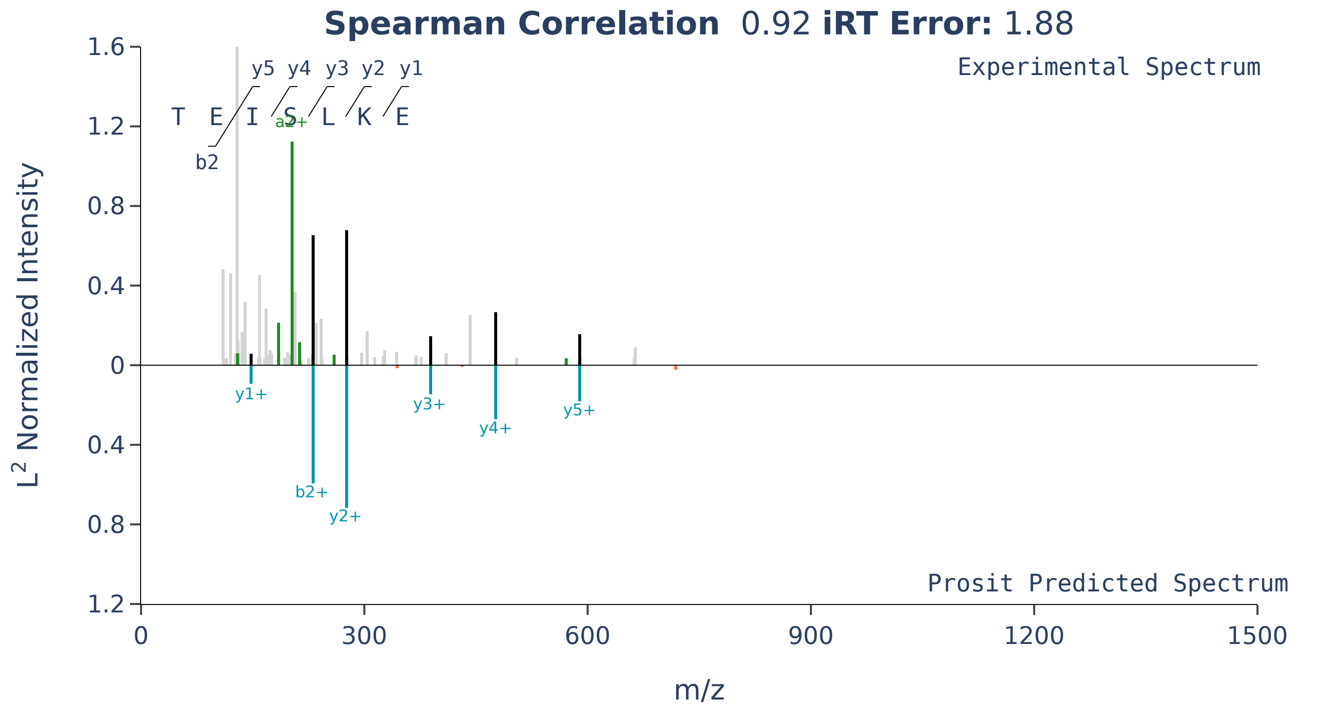

Source HRoetschke_271222_090123_HFGoe_LEDGF_b2R2_1h **Scan** 12812
Peptide ETEISLK **Charge** 2 **Spectral Angle** 0.03
Spearman Correlation -0.41 **iRT Error:** 2.16

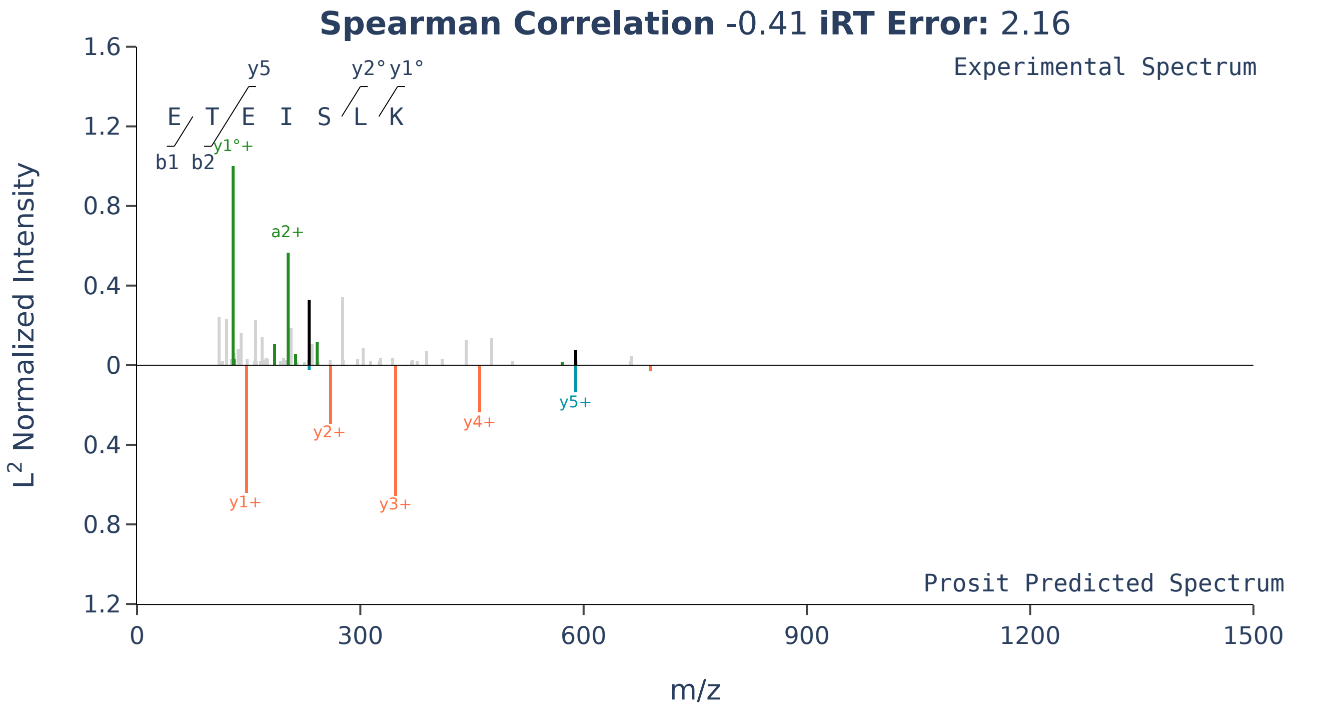

Source HRoetschke_271222_090123_HFGoe_LEDGF_b1R2_24h **Scan** 25047
Peptide TQVLSVNL **Charge** 1 **Spectral Angle** 0.87
Spearman Correlation 0.93 **iRT Error:** 1.95

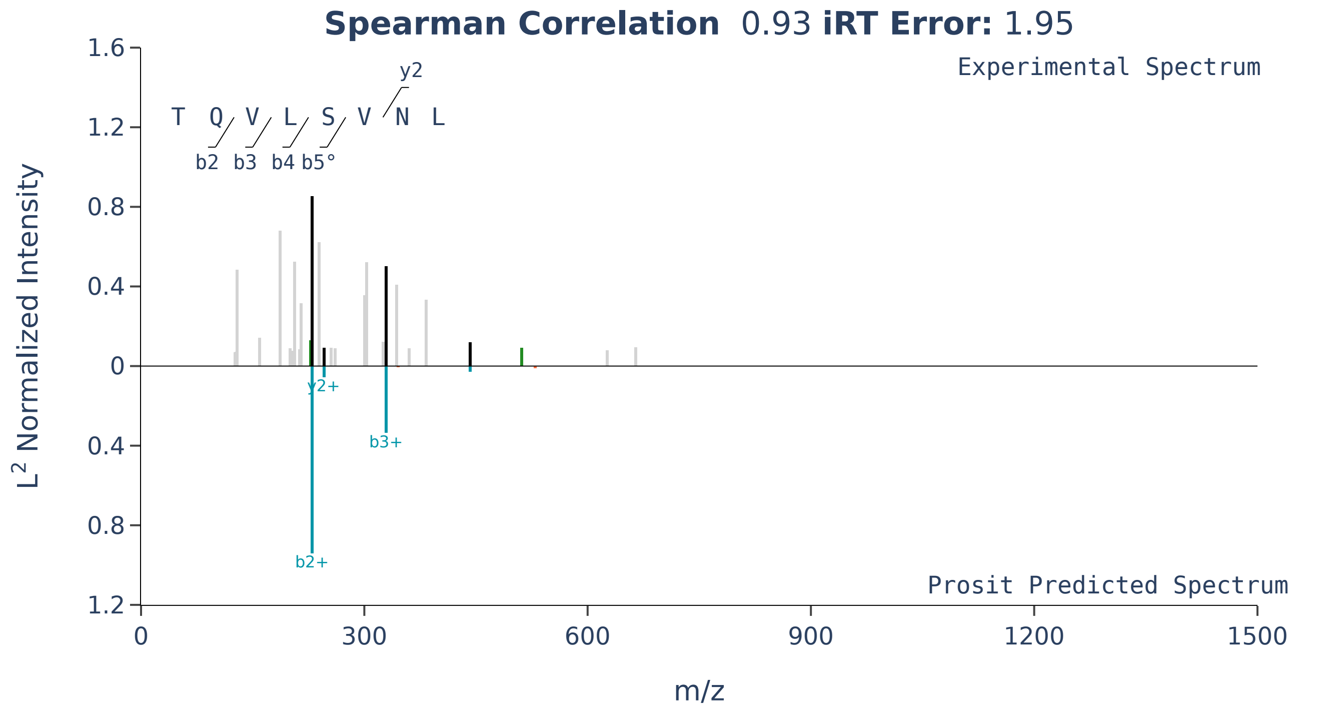

Source HRoetschke_271222_090123_HFGoe_LEDGF_b1R2_24h **Scan** 25047
Peptide VLNKSLAE **Charge** 1 **Spectral Angle** 0.4
Spearman Correlation 0.4 **iRT Error:** 88.9

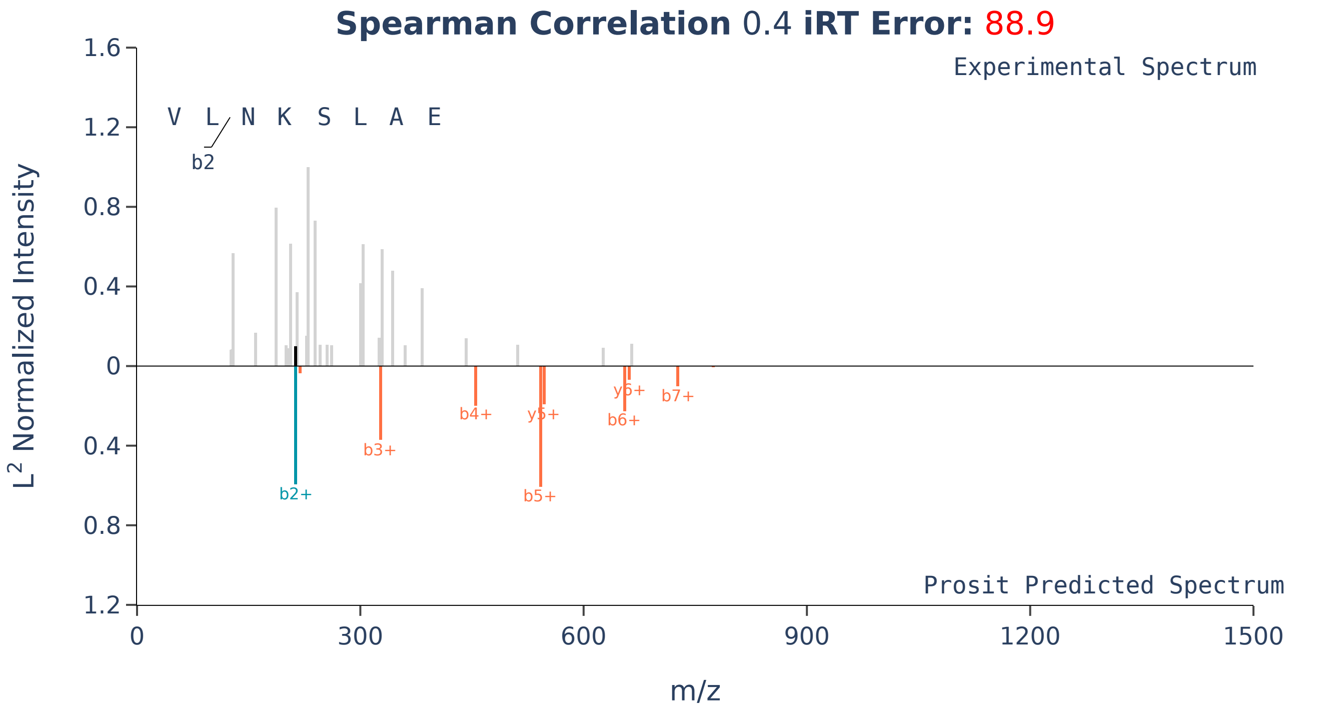

Source HRoetschke_271222_090123_HFGoe_LEDGF_b1R2_4h **Scan** 15906
Peptide MLEKSTMLYNKFKNM **Charge** 2 **Spectral Angle** 0.81
Spearman Correlation 0.95 **iRT Error:** 2.06

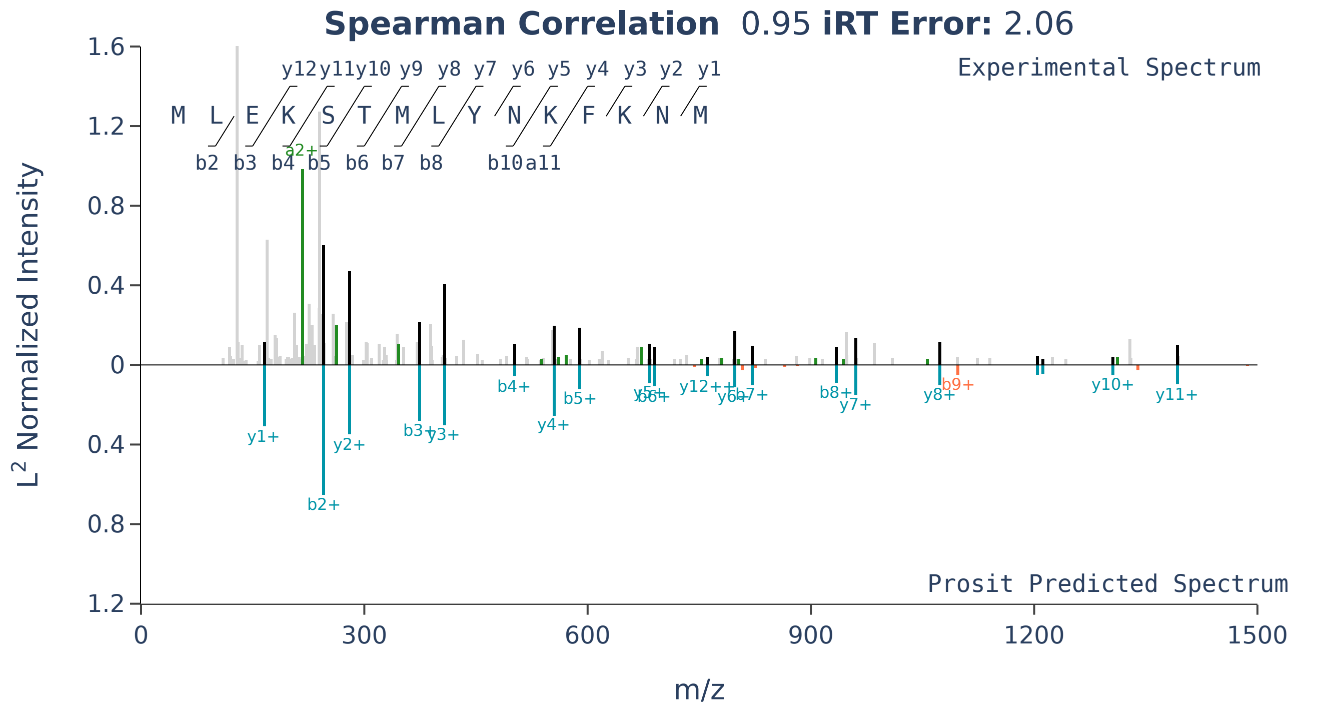

Source HRoetschke_271222_090123_HFGoe_LEDGF_b1R2_4h **Scan** 15906
Peptide IMEKSTMLYNKFKNM **Charge** 2 **Spectral Angle** 0.11
Spearman Correlation 0.2 **iRT Error:** 8.8

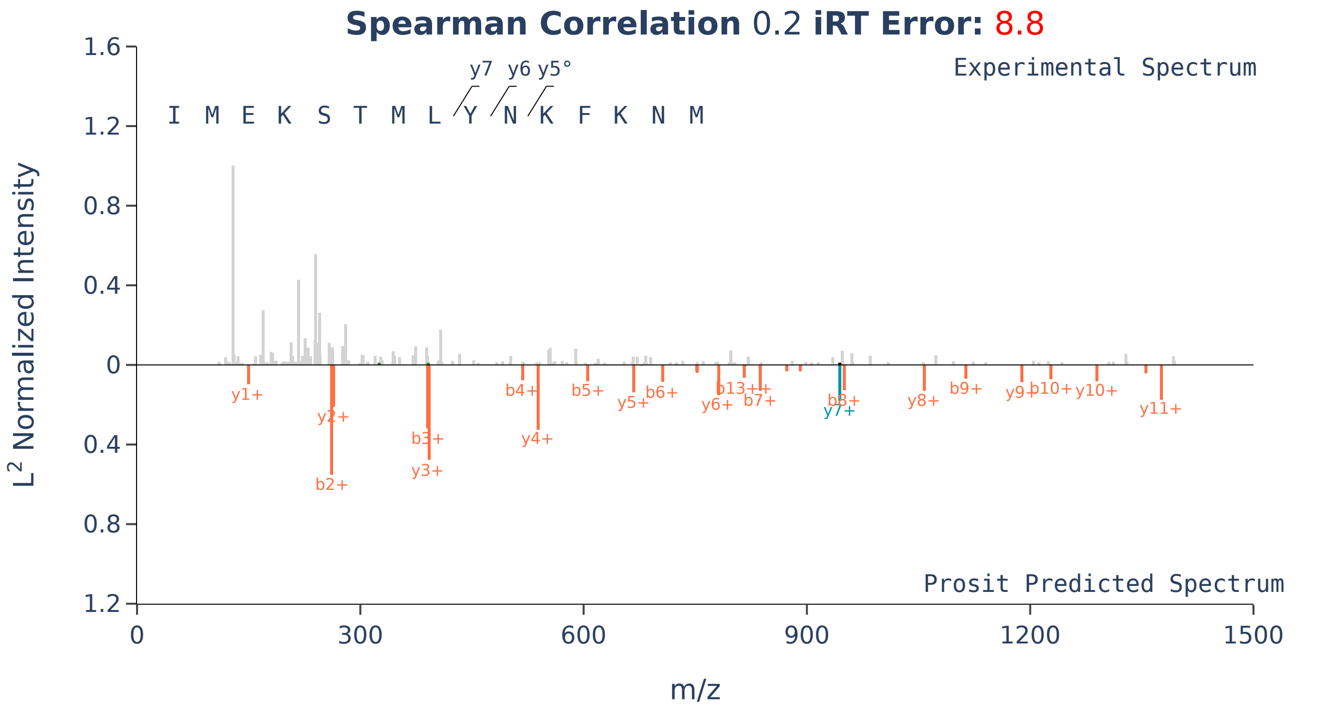

Source HRoetschke_271222_090123_HFGoe_LEDGF_b1R2_24h **Scan** 6939
Peptide SVNLKVSPKRGRTA **Charge** 3 **Spectral Angle** 0.85
Spearman Correlation 0.82 **iRT Error:** 2.08

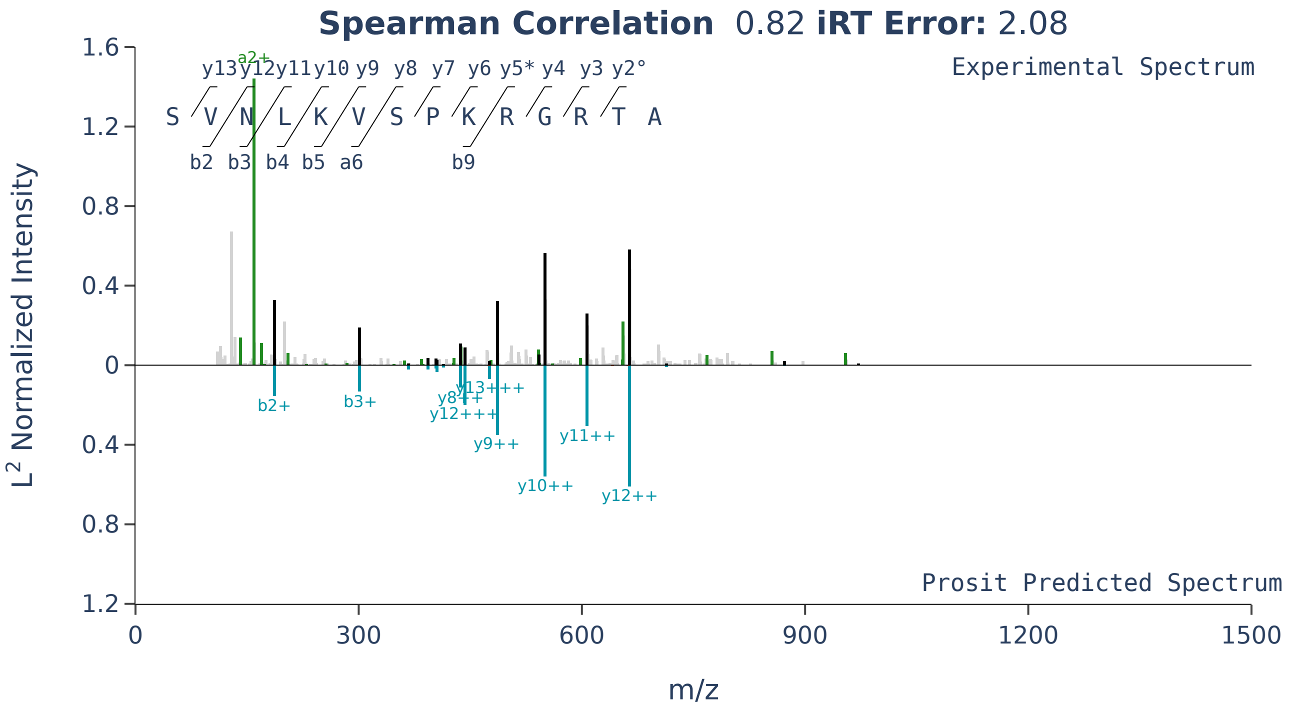

Source HRoetschke_271222_090123_HFGoe_LEDGF_b1R2_24h **Scan** 6939
Peptide TASVNLKVSPKRGR **Charge** 3 **Spectral Angle** 0.17
Spearman Correlation 0.3 **iRT Error:** 7.57

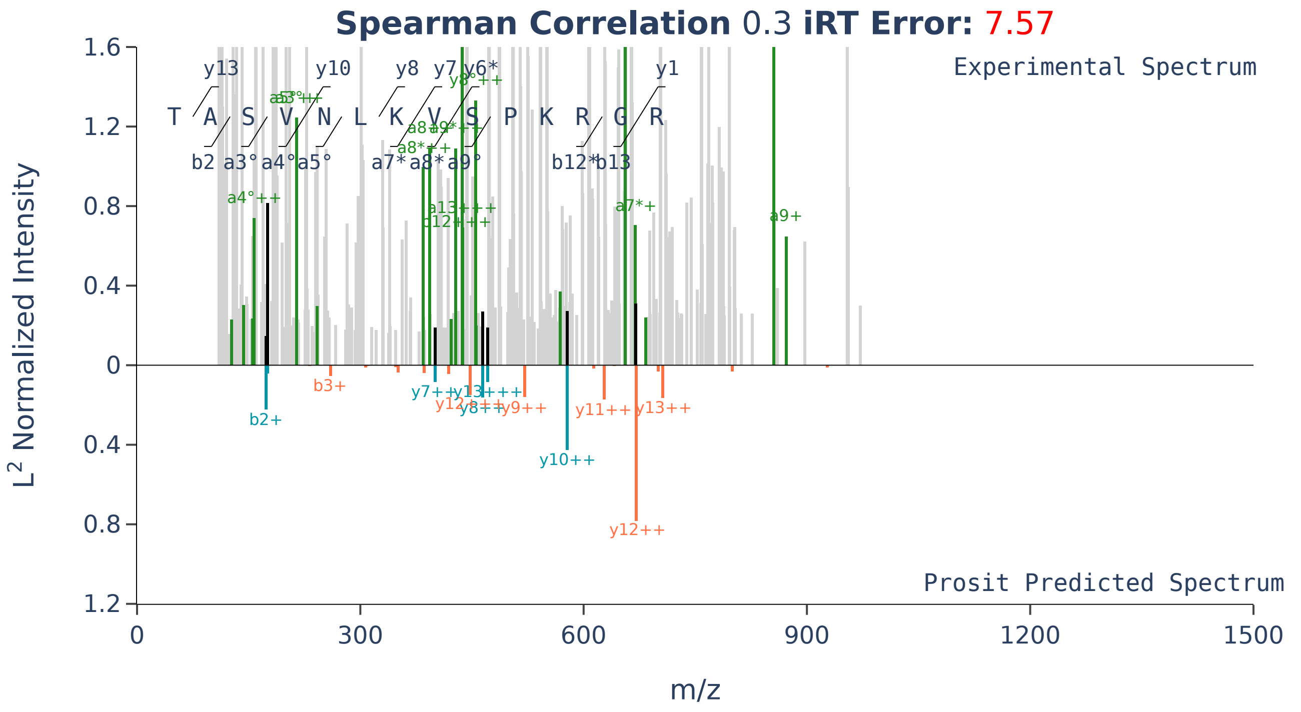

Source HRoetschke_271222_090123_HFGoe_LEDGF_b2R1_4h **Scan** 13443
Peptide ASLMDSRLQR **Charge** 3 **Spectral Angle** 0.82
Spearman Correlation 0.84 **iRT Error:** 2.11

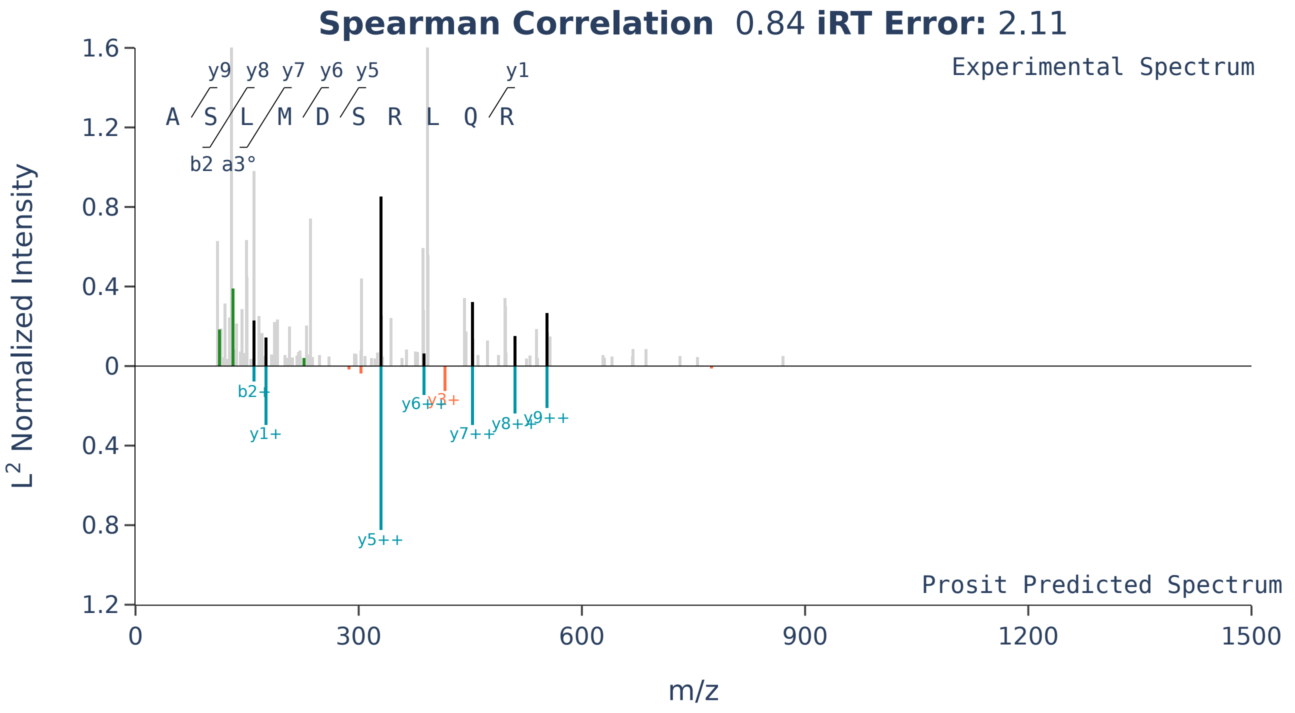

Source HRoetschke_271222_090123_HFGoe_LEDGF_b2R1_4h **Scan** 13443
Peptide EEDKSKKKGQ **Charge** 3 **Spectral Angle** 0.01
Spearman Correlation -0.16 **iRT Error:** 29.3

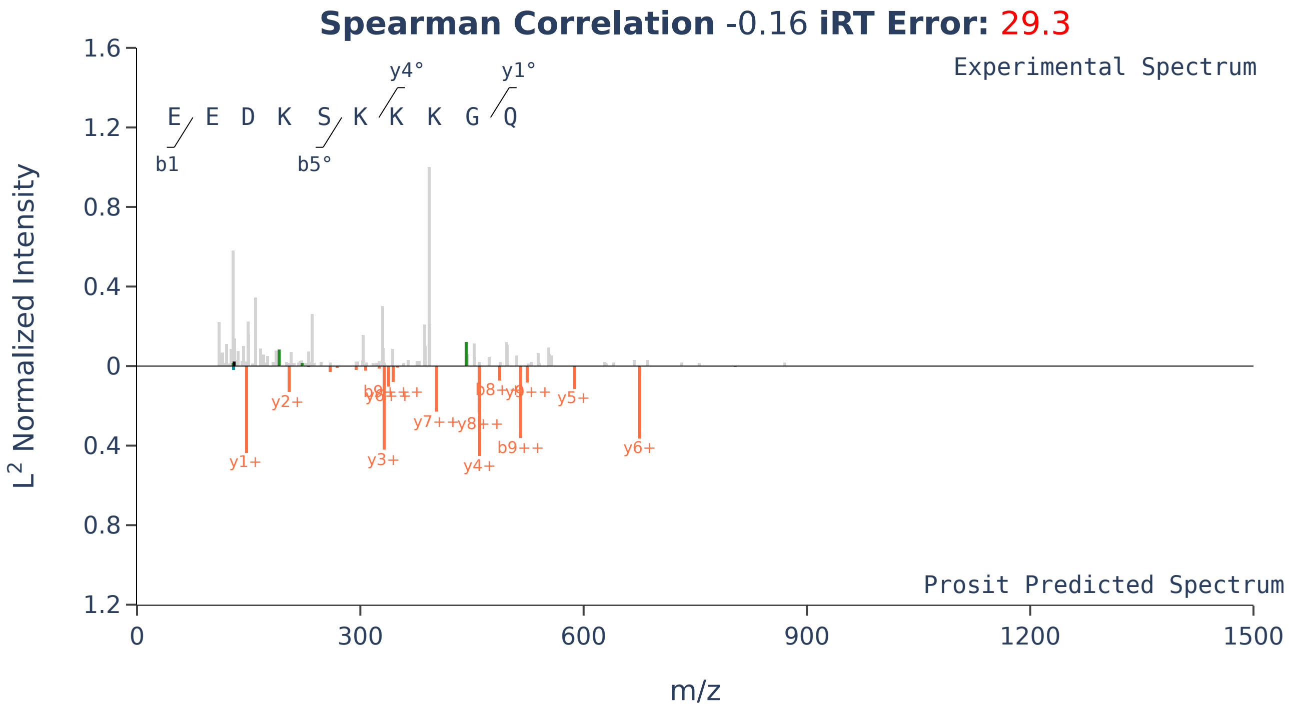

Source HRoetschke_271222_090123_HFGoe_LEDGF_b1R2_2h **Scan** 17238
Peptide QSGYPHWPARVDE **Charge** 3 **Spectral Angle** 0.96
Spearman Correlation 0.88 **iRT Error:** 2.2

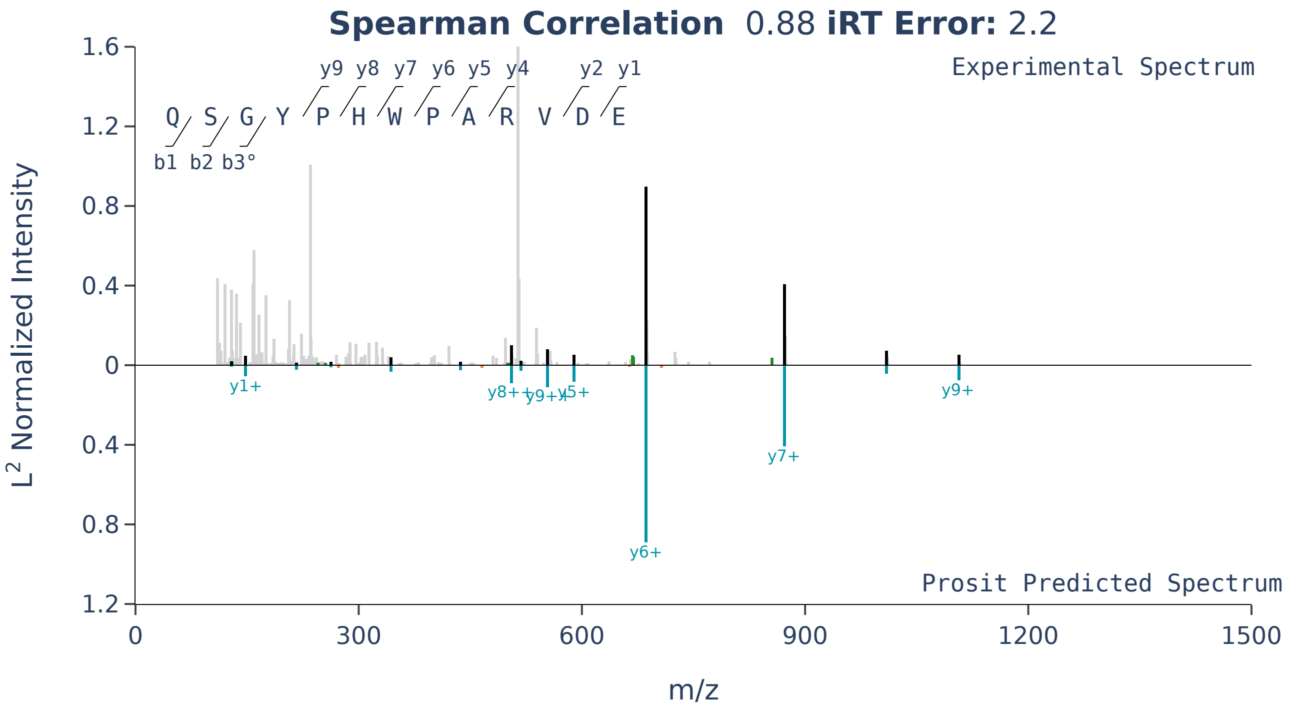

Source HRoetschke_271222_090123_HFGoe_LEDGF_b1R2_2h **Scan** 17238
Peptide FSSQQAATKQSNASS **Charge** 3 **Spectral Angle** 0.05
Spearman Correlation -0.01 **iRT Error:** 68.68

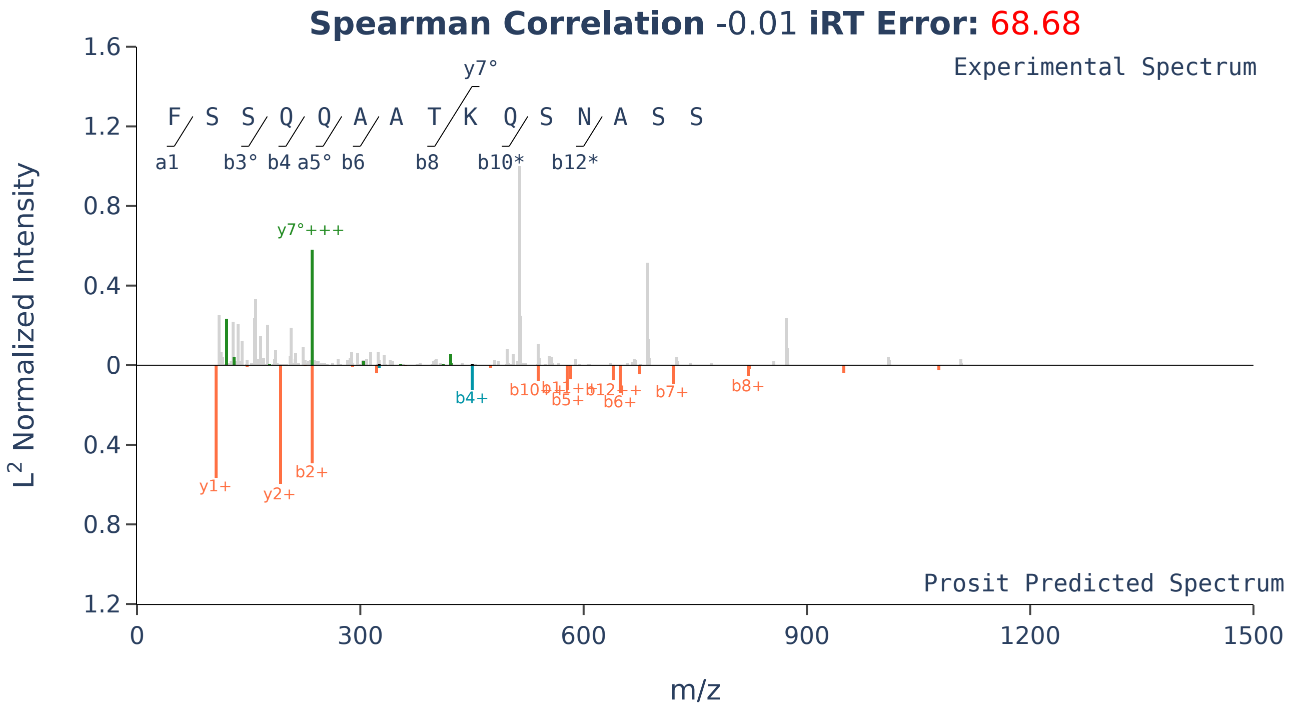

Source HRoetschke_271222_090123_HFGoe_LEDGF_b2R1_24h **Scan** 18806
Peptide FLGPKDIFPYSENKEKYGKPNKREL **Charge** 5 **Spectral Angle** 0.81
Spearman Correlation 0.86 **iRT Error:** 2.28

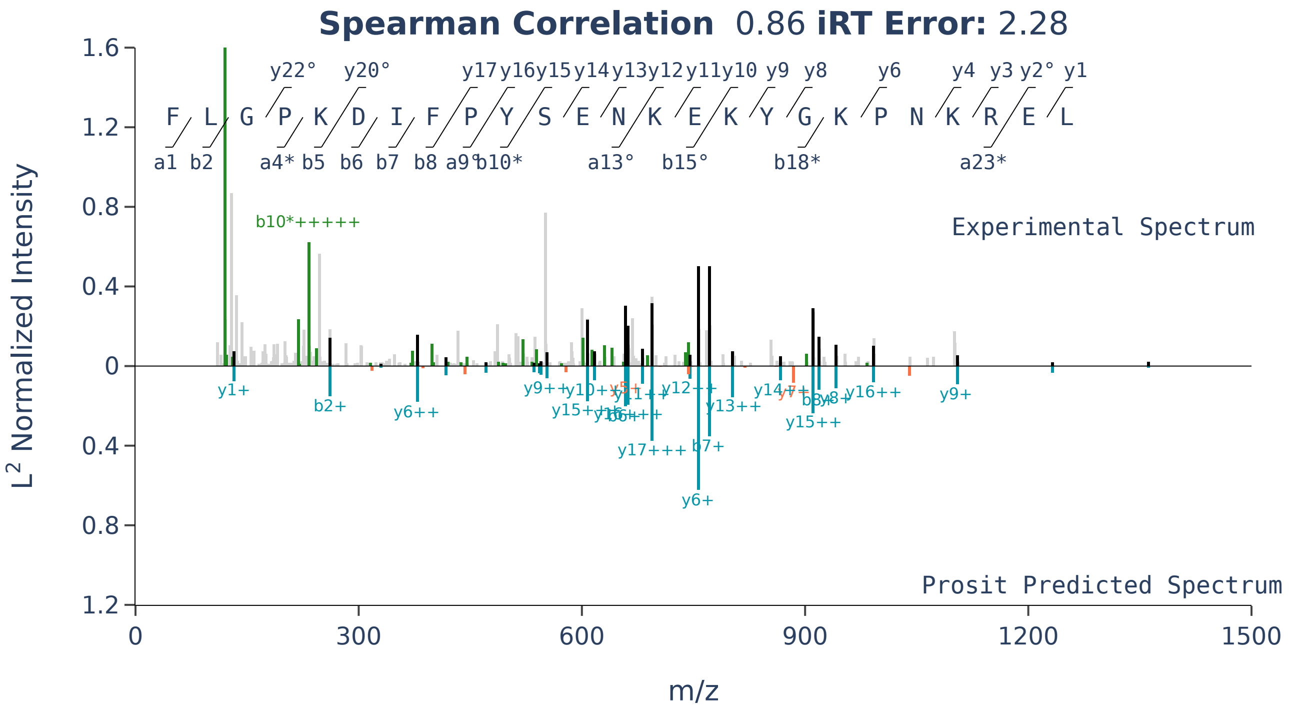

Source HRoetschke_271222_090123_HFGoe_LEDGF_b2R1_24h **Scan** 18806
Peptide VPDGAVKPPTNKLPIFFFGTHETAFLGP **Charge** 5 **Spectral Angle** 0.46
Spearman Correlation 0.26 **iRT Error:** 81.38

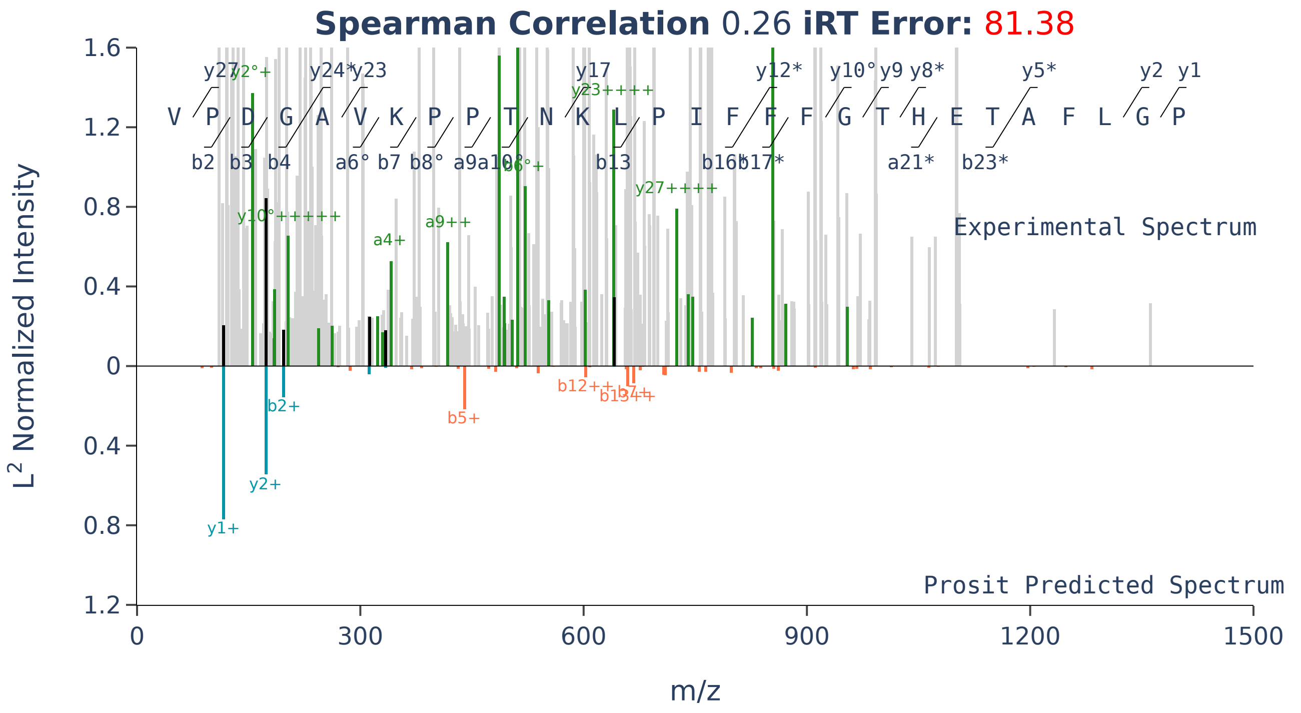

Source HRoetschke_271222_090123_HFGoe_LEDGF_b1R1_24h **Scan** 16533
Peptide ALDELKKIRRF **Charge** 3 **Spectral Angle** 0.86
Spearman Correlation 0.85 **iRT Error:** 2.33

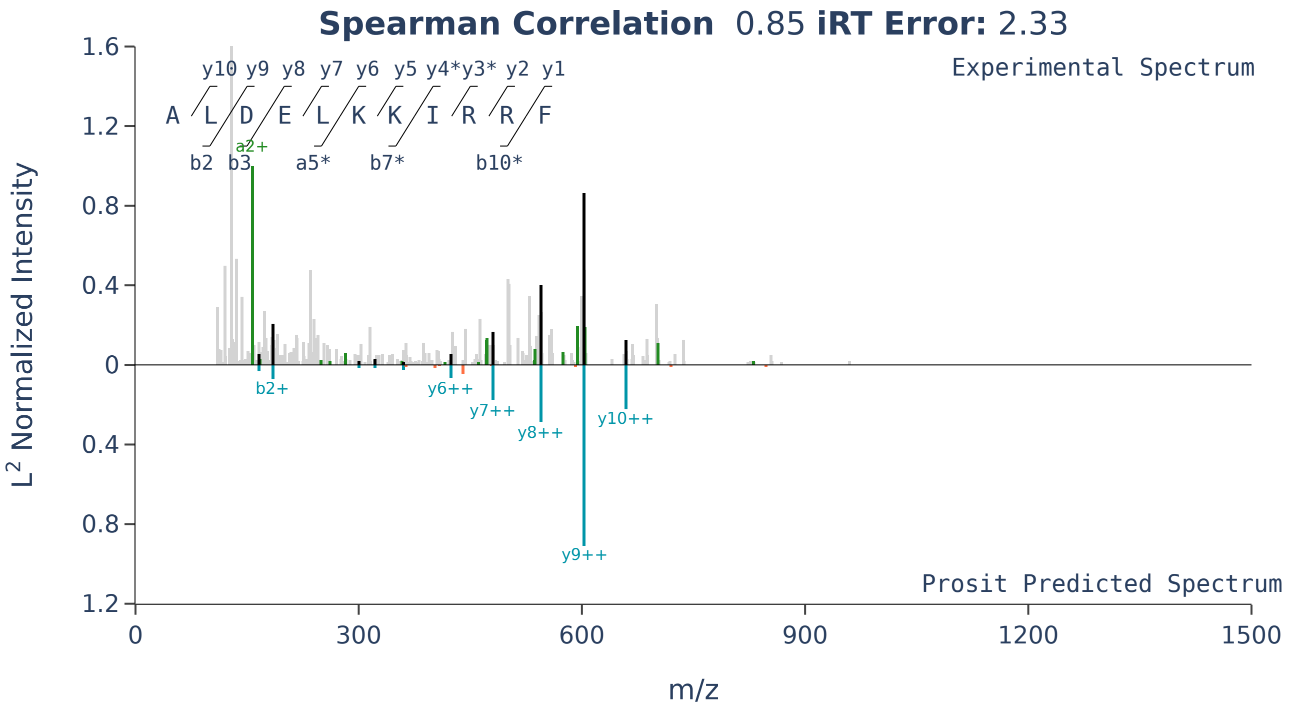

Source HRoetschke_271222_090123_HFGoe_LEDGF_b1R1_24h **Scan** 16533
Peptide EMITTLKKIRR **Charge** 3 **Spectral Angle** 0.15
Spearman Correlation 0.28 **iRT Error:** 30.71

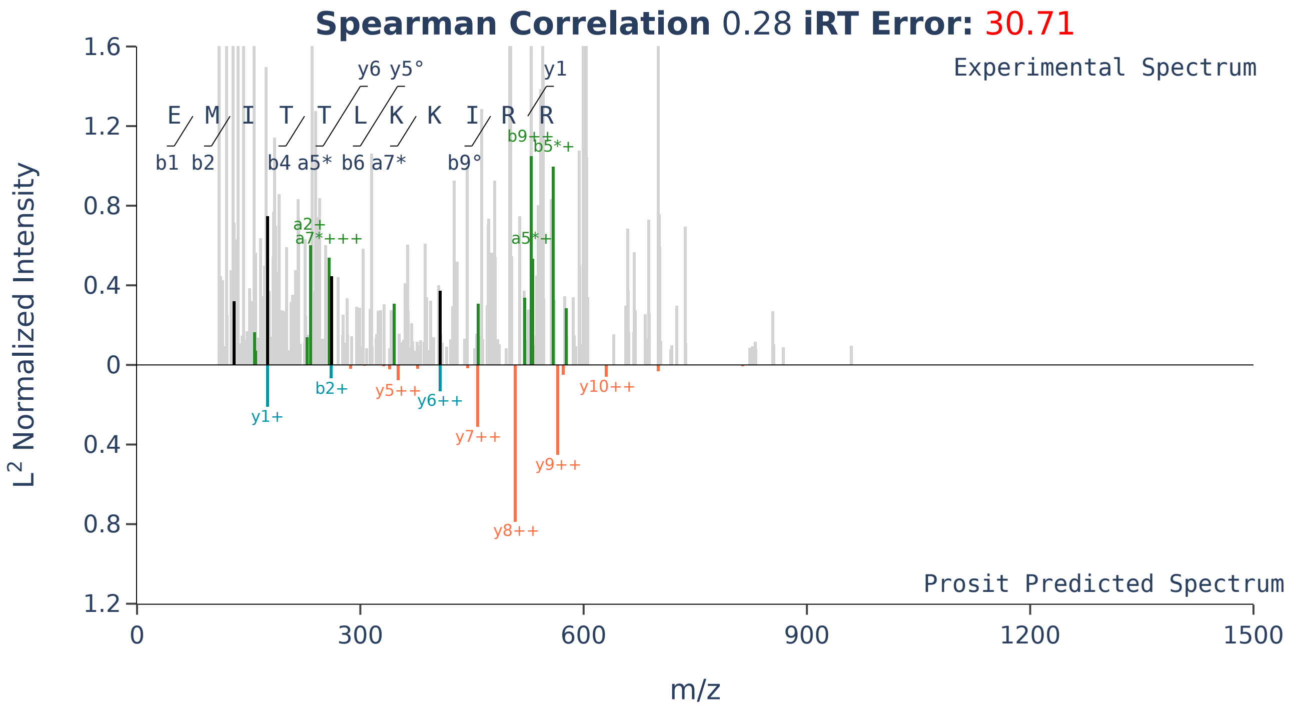

Source HRoetschke_271222_090123_HFGoe_LEDGF_b2R1_2h **Scan** 17574
Peptide STAFLGPKDIFPYSENKEKYGKPNKR **Charge** 5 **Spectral Angle** 0.77
Spearman Correlation 0.9 **iRT Error:** 2.35

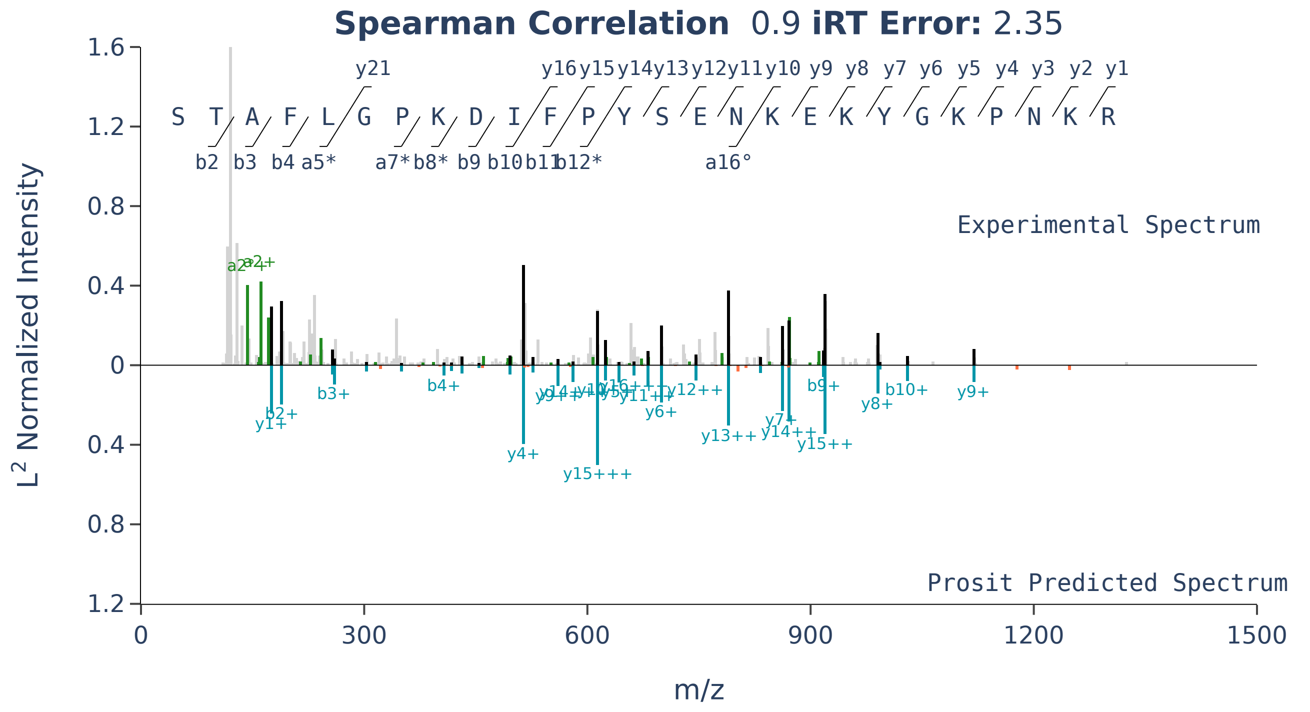

Source HRoetschke_271222_090123_HFGoe_LEDGF_b2R1_2h **Scan** 17574
Peptide KNSLKIDNLDVNRCIEALDELASLQVT **Charge** 5 **Spectral Angle** 0.19
Spearman Correlation -0.05 **iRT Error:** 95.75

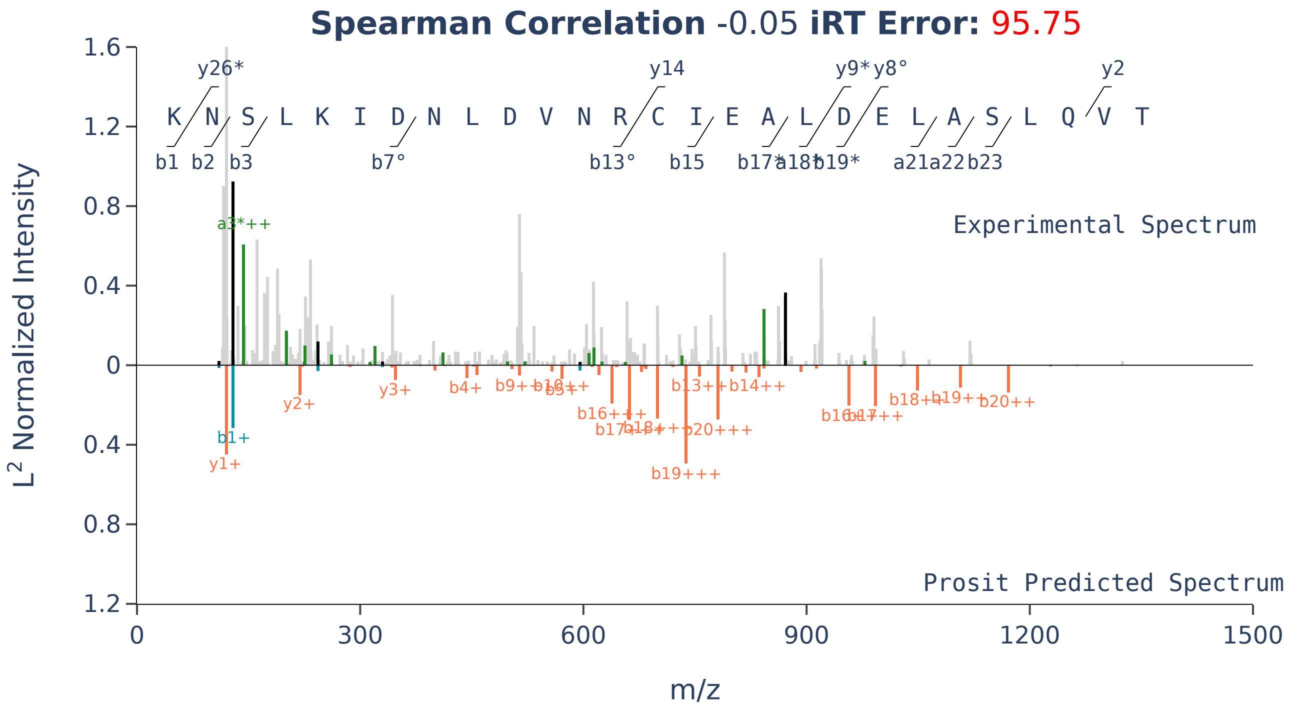

Source HRoetschke_271222_090123_HFGoe_LEDGF_b2R1_1h **Scan** 9621
Peptide KMKGTEISLKD **Charge** 3 **Spectral Angle** 0.93
Spearman Correlation 0.8 **iRT Error:** 2.35

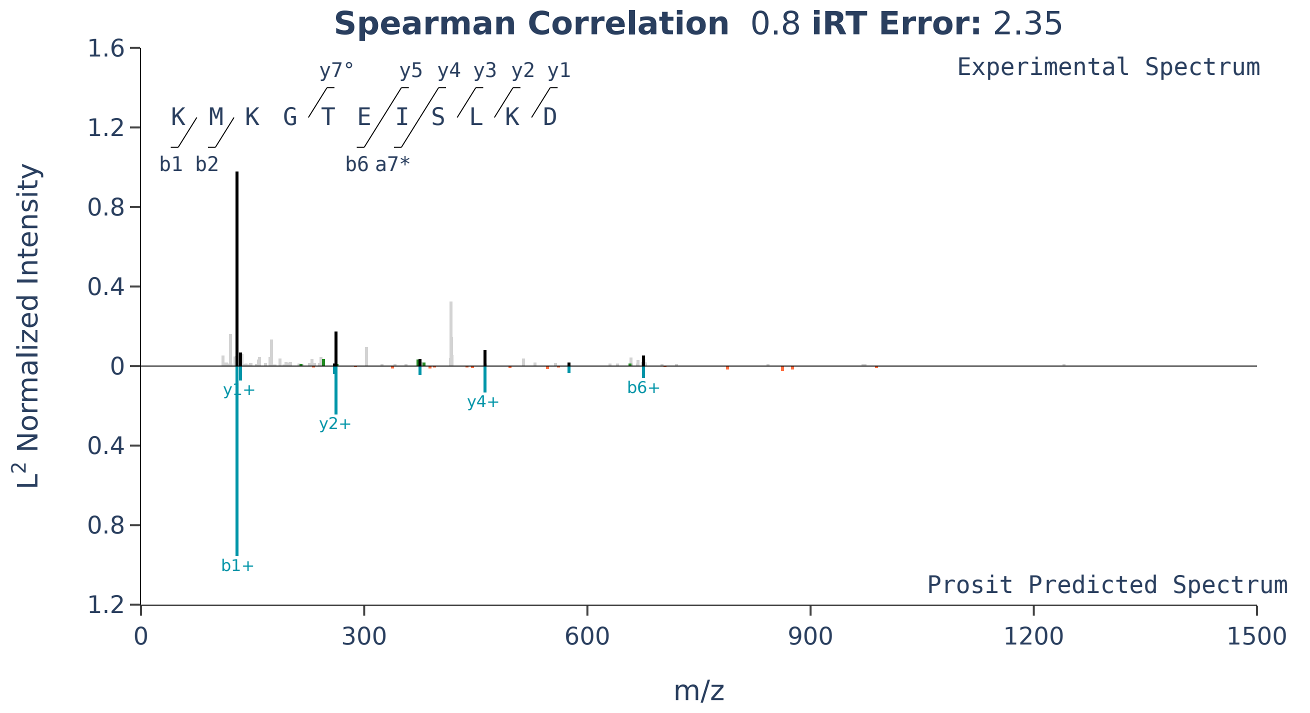

Source HRoetschke_271222_090123_HFGoe_LEDGF_b2R1_1h **Scan** 9621
Peptide KVSQVIMEKST **Charge** 3 **Spectral Angle** 0.67
Spearman Correlation 0.39 **iRT Error:** 2.22

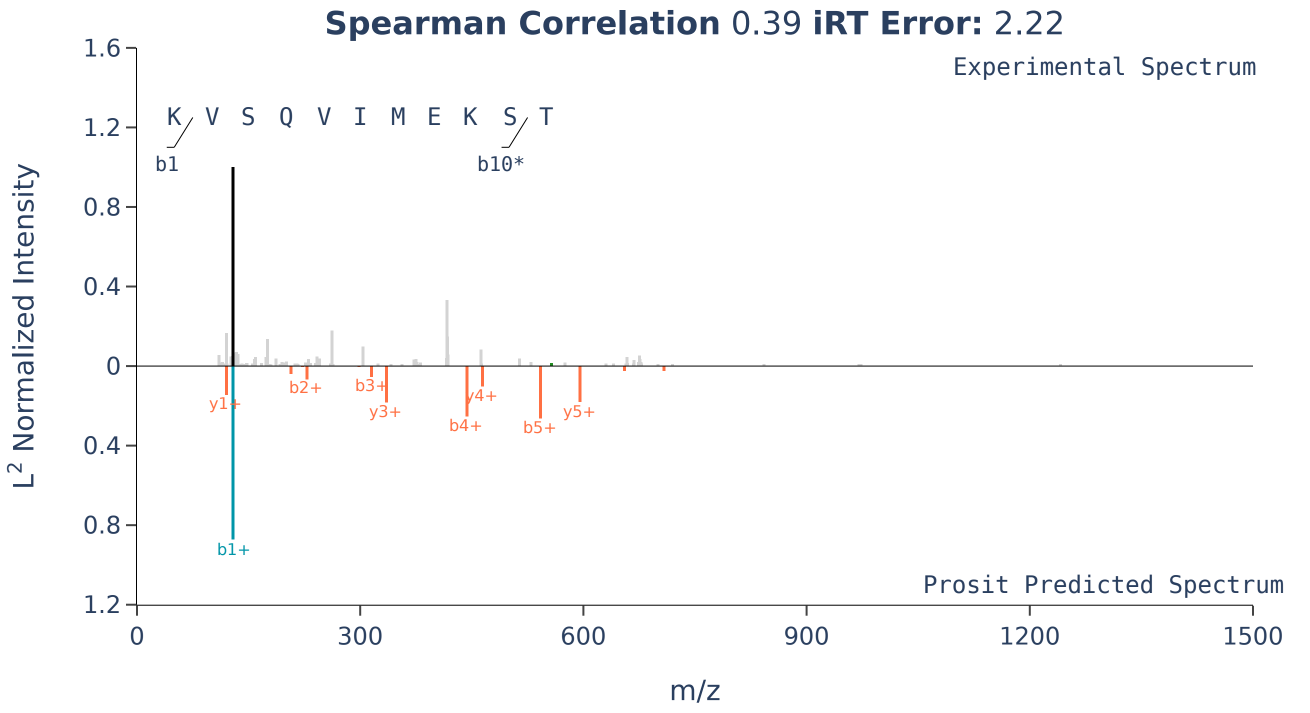

Source HRoetschke_271222_090123_HFGoe_LEDGF_b1R1_4h **Scan** 18884
Peptide VTTATASVNLEPD **Charge** 2 **Spectral Angle** 0.94
Spearman Correlation 0.83 **iRT Error:** 2.37

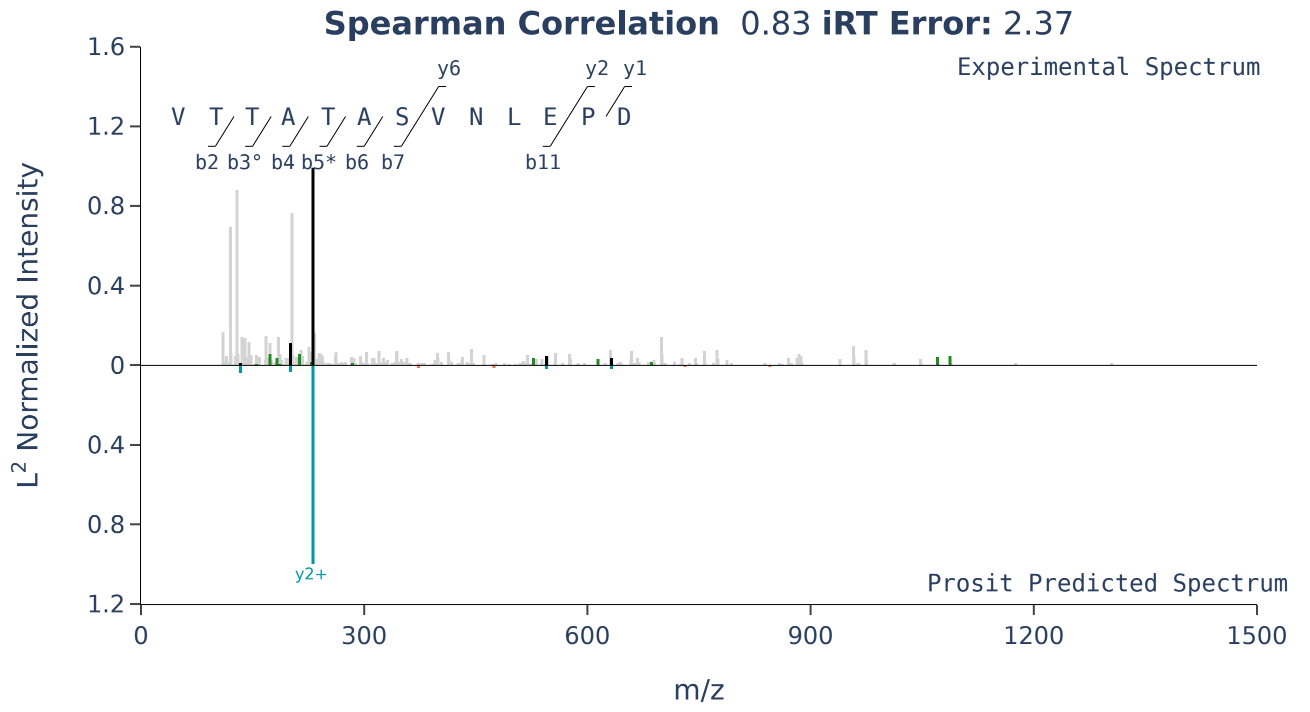

Source HRoetschke_271222_090123_HFGoe_LEDGF_b1R1_4h **Scan** 18884
Peptide EKQVETEEAGVV **Charge** 2 **Spectral Angle** 0.06
Spearman Correlation -0.15 **iRT Error:** 27.43

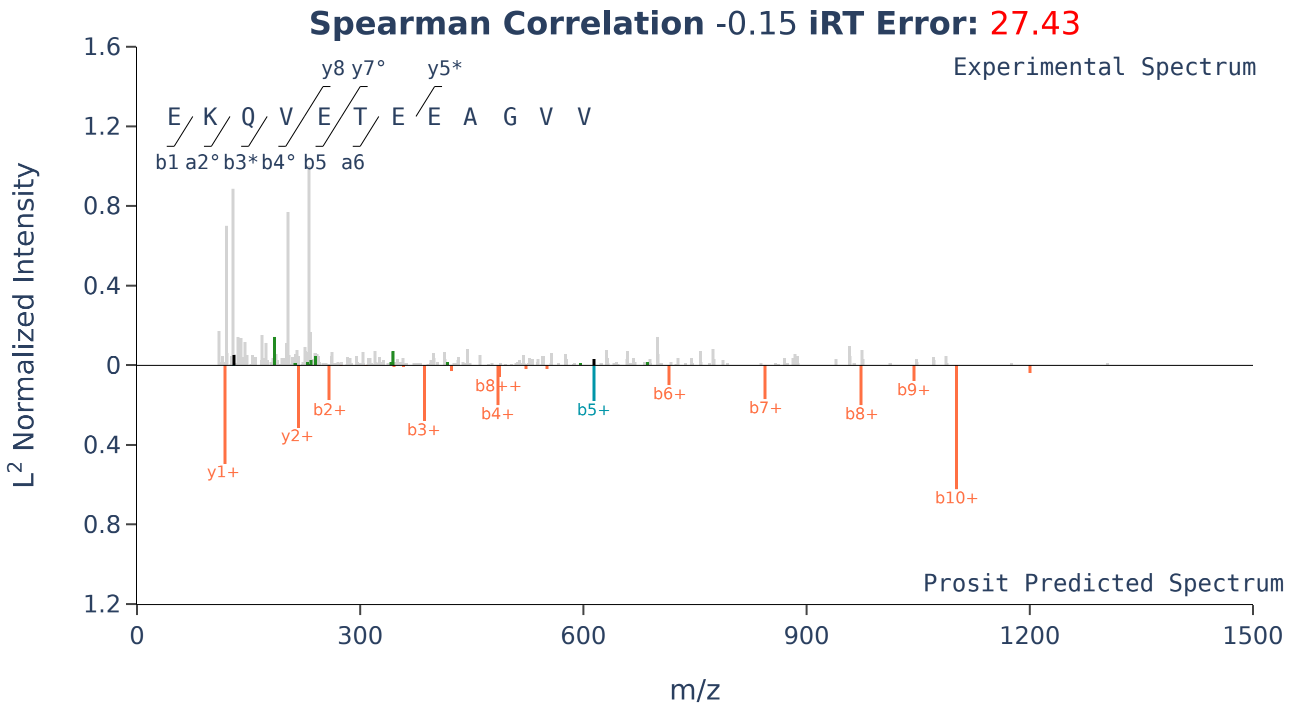

Source HRoetschke_271222_090123_HFGoe_LEDGF_b1R2_2h **Scan** 24045
Peptide GPKDLIFA **Charge** 2 **Spectral Angle** 0.89
Spearman Correlation 1.0 **iRT Error:** 2.47

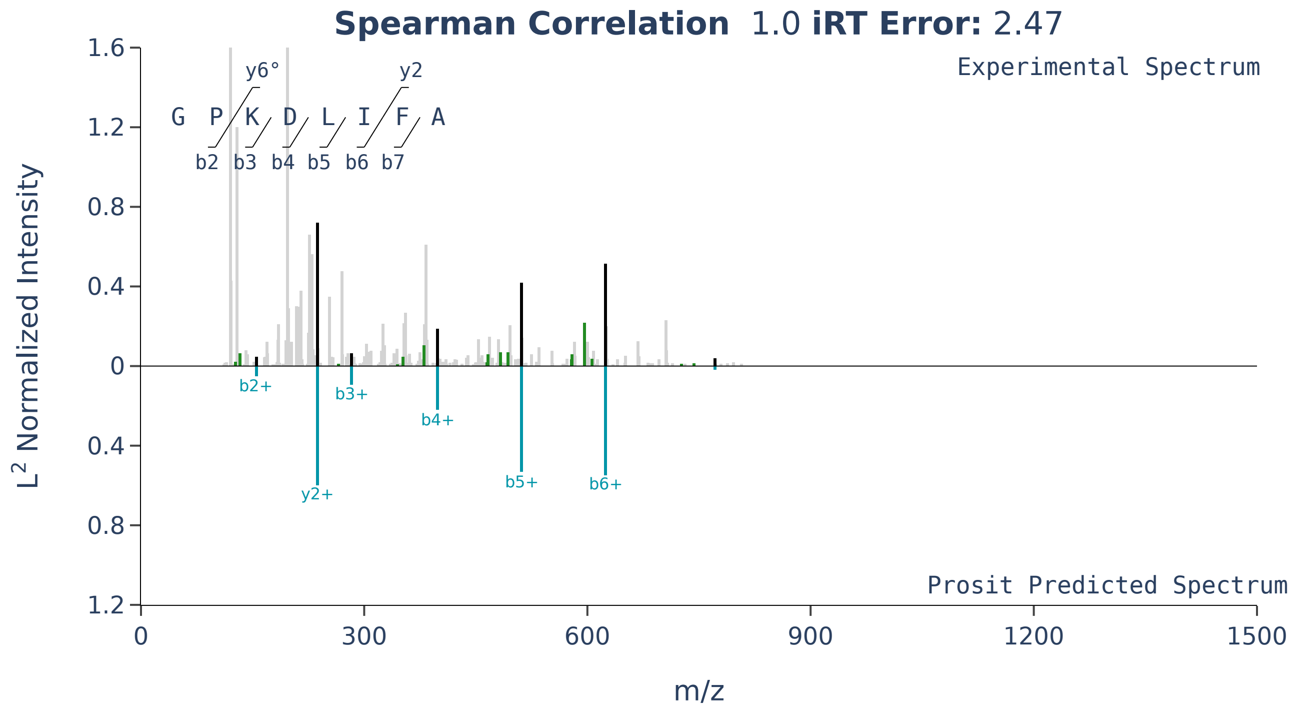

Source HRoetschke_271222_090123_HFGoe_LEDGF_b1R2_2h **Scan** 24045
Peptide AFLGPKDI **Charge** 2 **Spectral Angle** 0.0
Spearman Correlation 0.0 **iRT Error:** 10.91

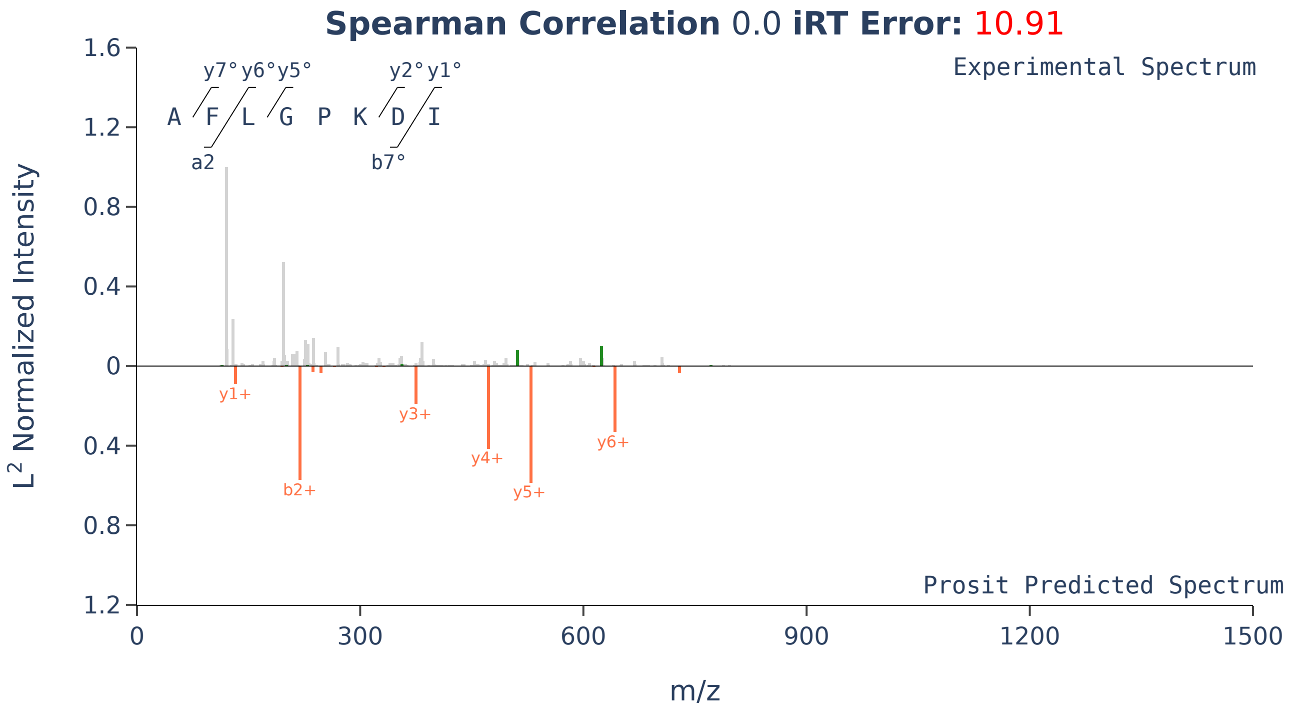

Source HRoetschke_271222_090123_HFGoe_LEDGF_b2R1_24h **Scan** 17068
Peptide FLGPKDIFPYSENKEKYGKPNRKA **Charge** 5 **Spectral Angle** 0.81
Spearman Correlation 0.91 **iRT Error:** 2.5

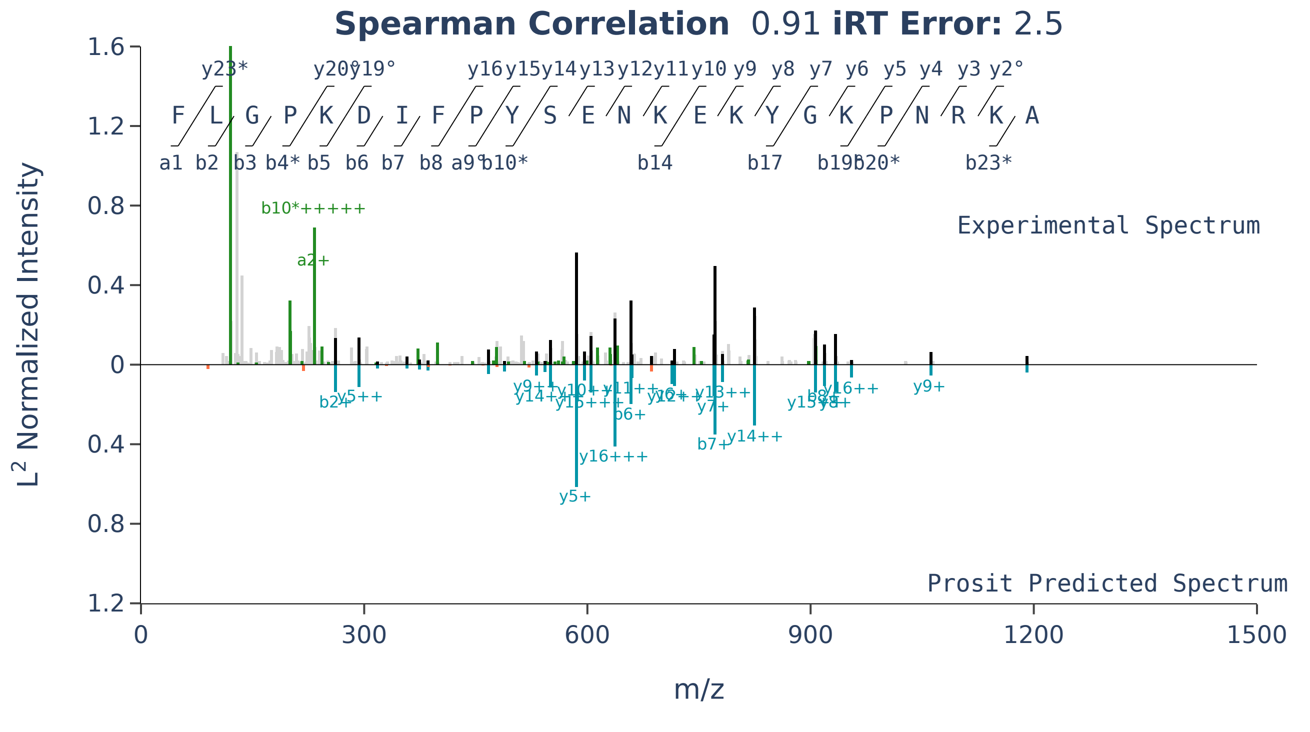

Source HRoetschke_271222_090123_HFGoe_LEDGF_b2R1_24h **Scan** 17068
Peptide EDKSKKKGQEEKQPKKQPKKDEEG **Charge** 5 **Spectral Angle** 0.01
Spearman Correlation -0.11 **iRT Error:** 94.6

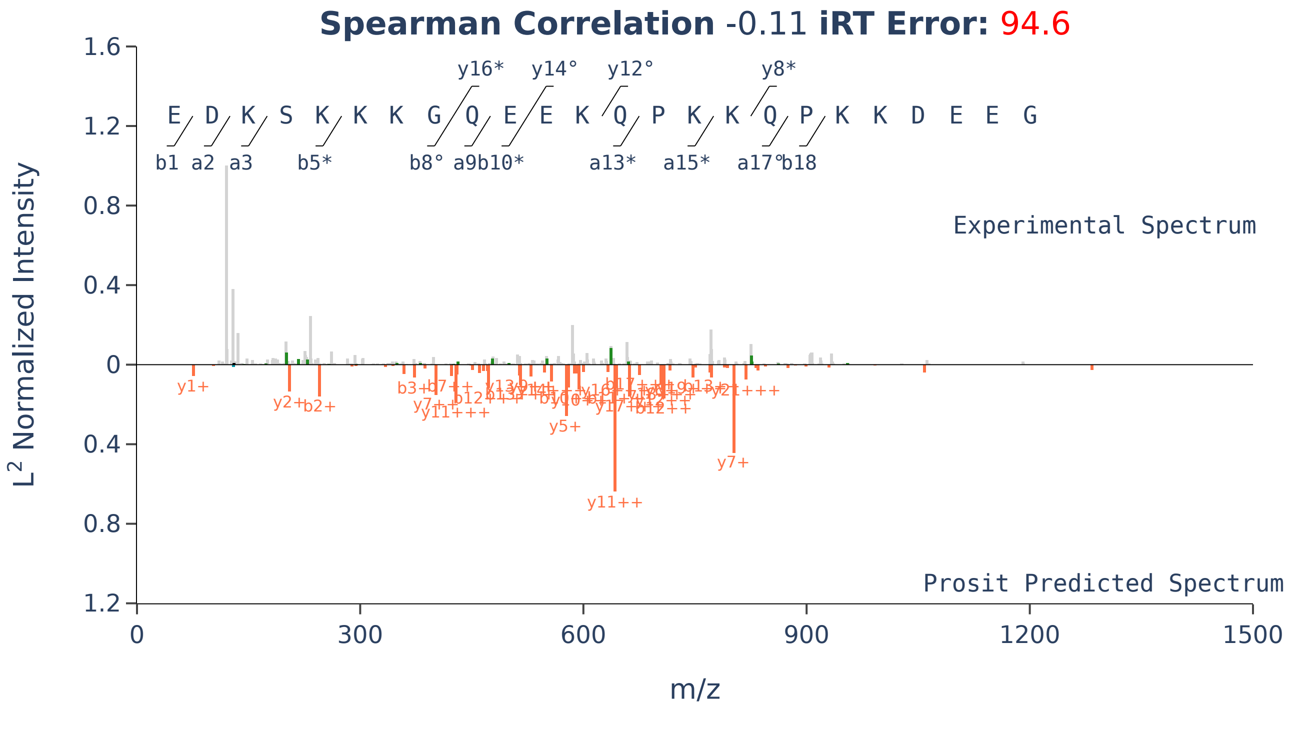

Source HRoetschke_271222_090123_HFGoe_LEDGF_b2R1_2h **Scan** 11924
Peptide QHNGAKMKGYPHWPARVD **Charge** 5 **Spectral Angle** 0.94
Spearman Correlation 0.84 **iRT Error:** 2.61

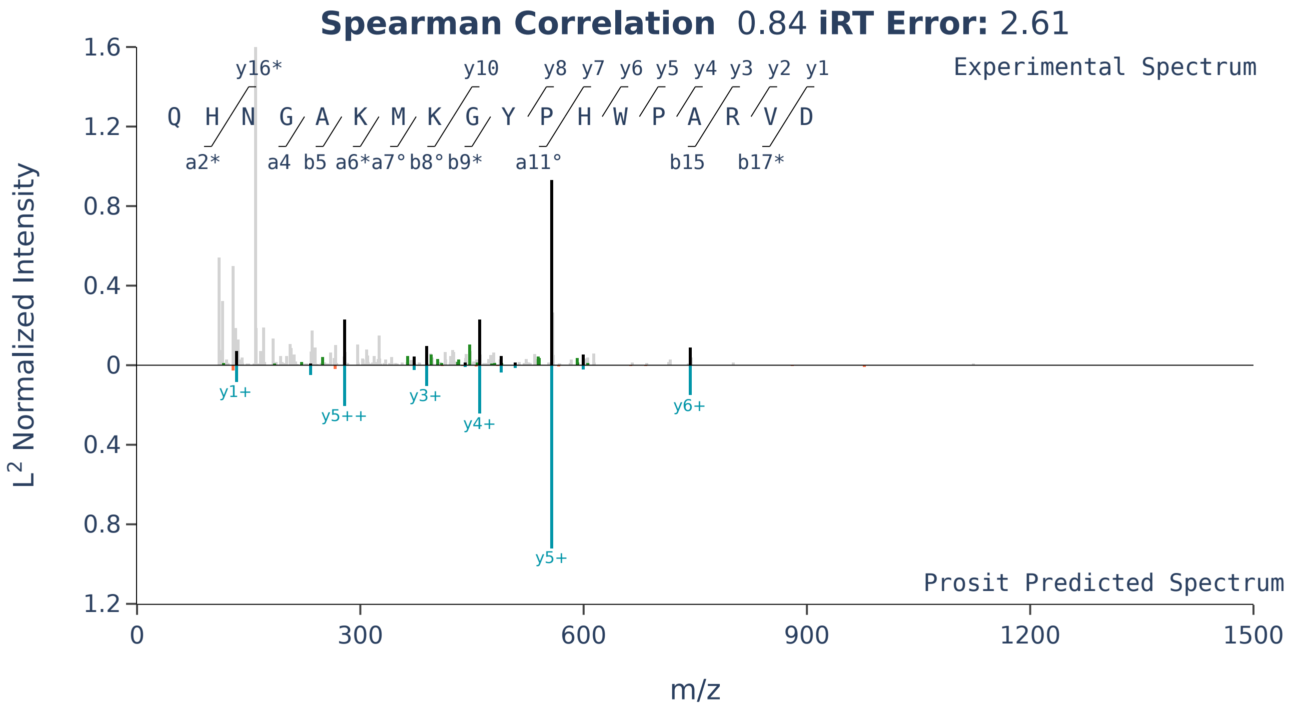

Source HRoetschke_271222_090123_HFGoe_LEDGF_b2R1_2h **Scan** 11924
Peptide MLYNKFKNMFLVGEGDSV **Charge** 5 **Spectral Angle** 0.0
Spearman Correlation 0.0 **iRT Error:** 70.0

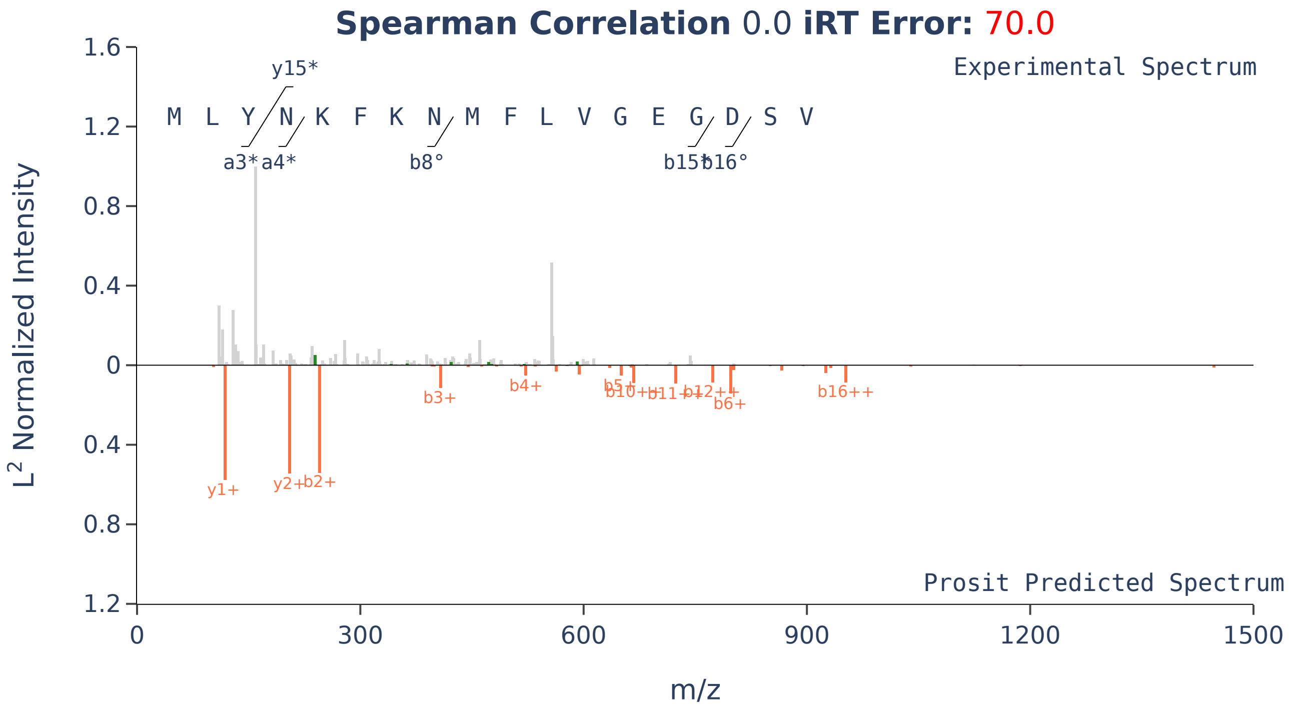

Source HRoetschke_271222_090123_HFGoe_LEDGF_b1R1_4h **Scan** 17051
Peptide FLGPKDIFPYSENKEKYGKPNKAR **Charge** 4 **Spectral Angle** 0.81
Spearman Correlation 0.85 **iRT Error:** 2.64

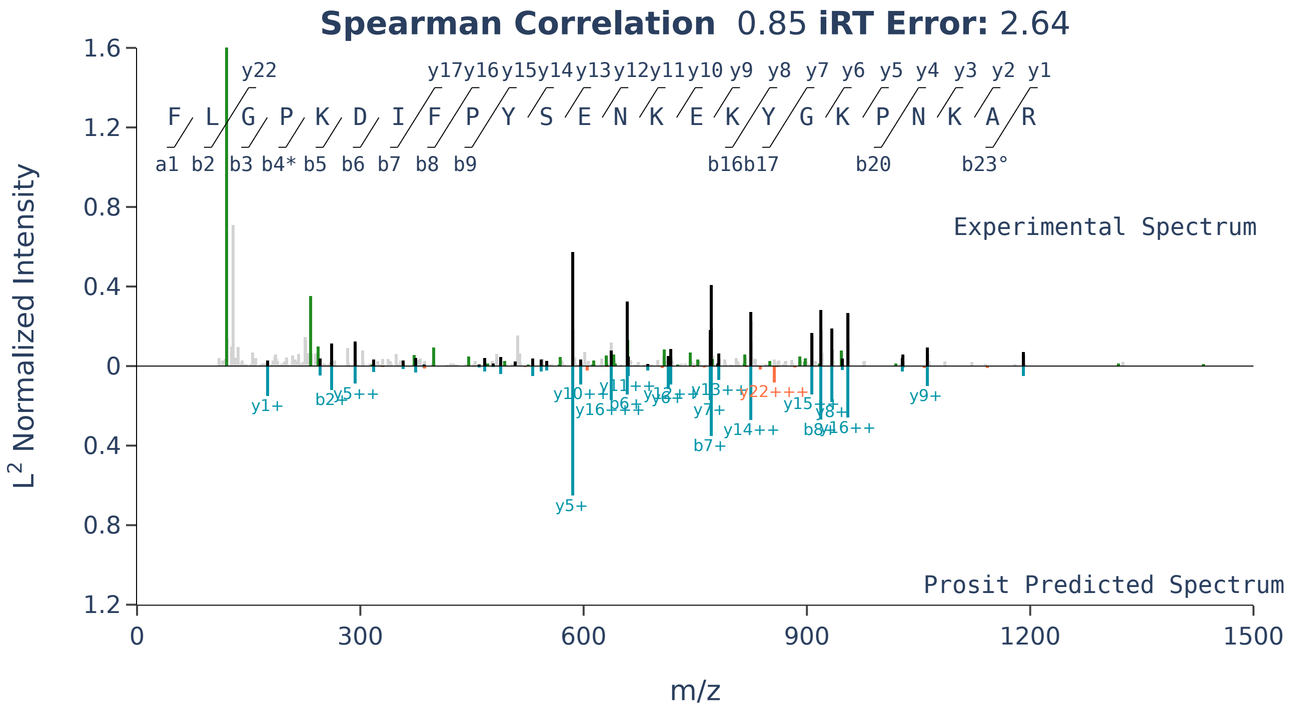

Source HRoetschke_271222_090123_HFGoe_LEDGF_b1R1_4h **Scan** 17051
Peptide GQKEEDKPRKEPDKKEGKKEVESK **Charge** 4 **Spectral Angle** 0.06
Spearman Correlation 0.08 **iRT Error:** 91.35

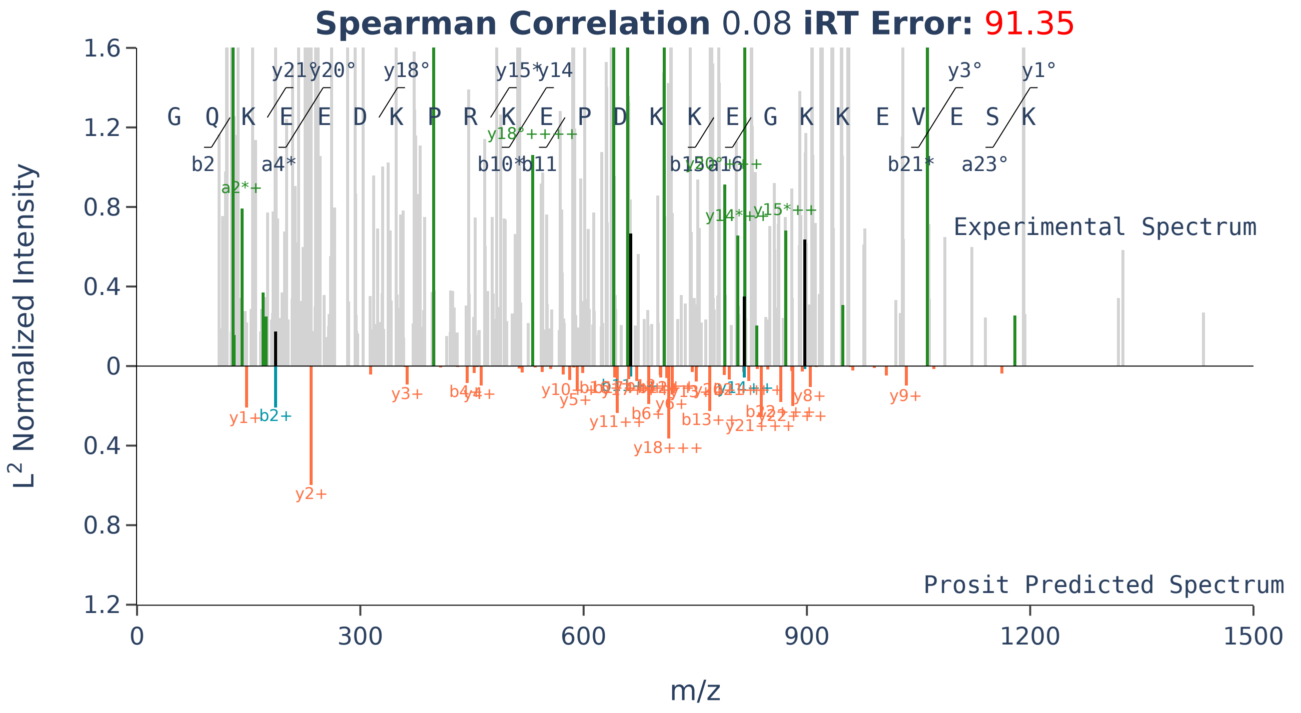

Source HRoetschke_271222_090123_HFGoe_LEDGF_b1R2_24h **Scan** 21246
Peptide GSLKIDNLDVNRCIE **Charge** 3 **Spectral Angle** 0.75
Spearman Correlation 0.9 **iRT Error:** 2.64

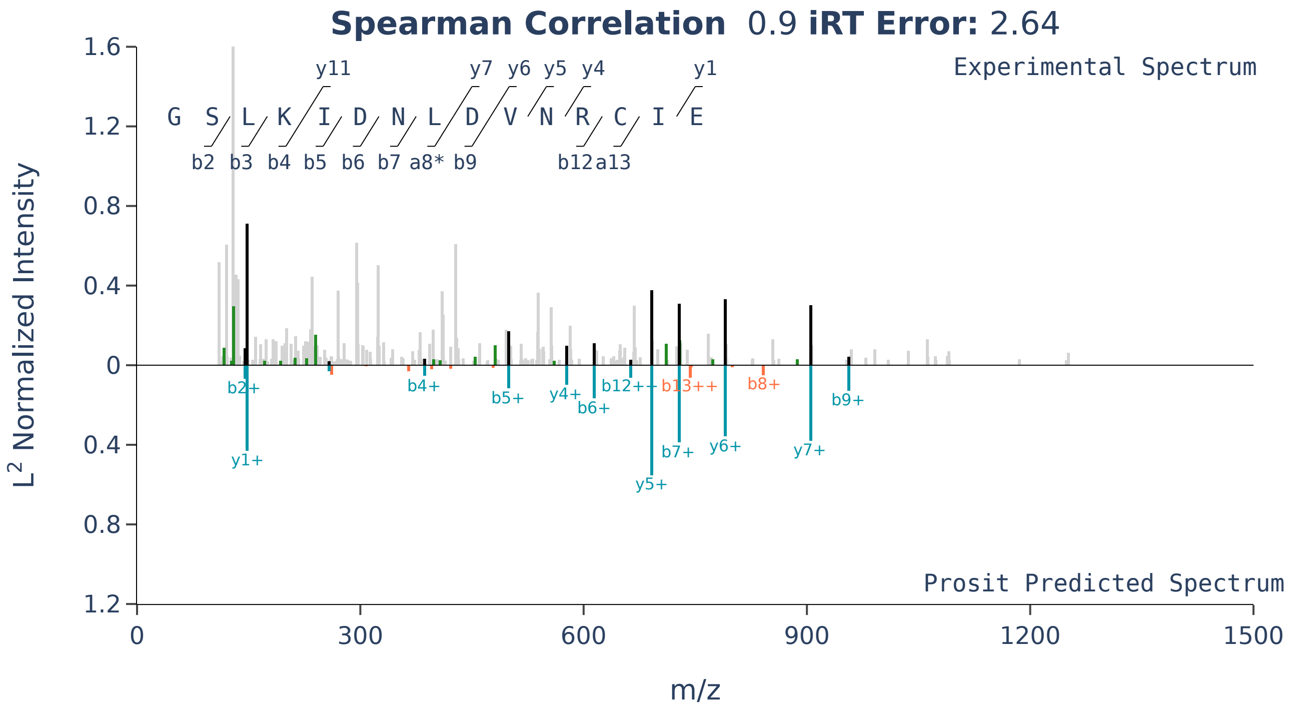

Source HRoetschke_271222_090123_HFGoe_LEDGF_b1R2_24h **Scan** 21246
Peptide ASNEDVTKAVDITTPK **Charge** 3 **Spectral Angle** 0.0
Spearman Correlation 0.0 **iRT Error:** 31.87

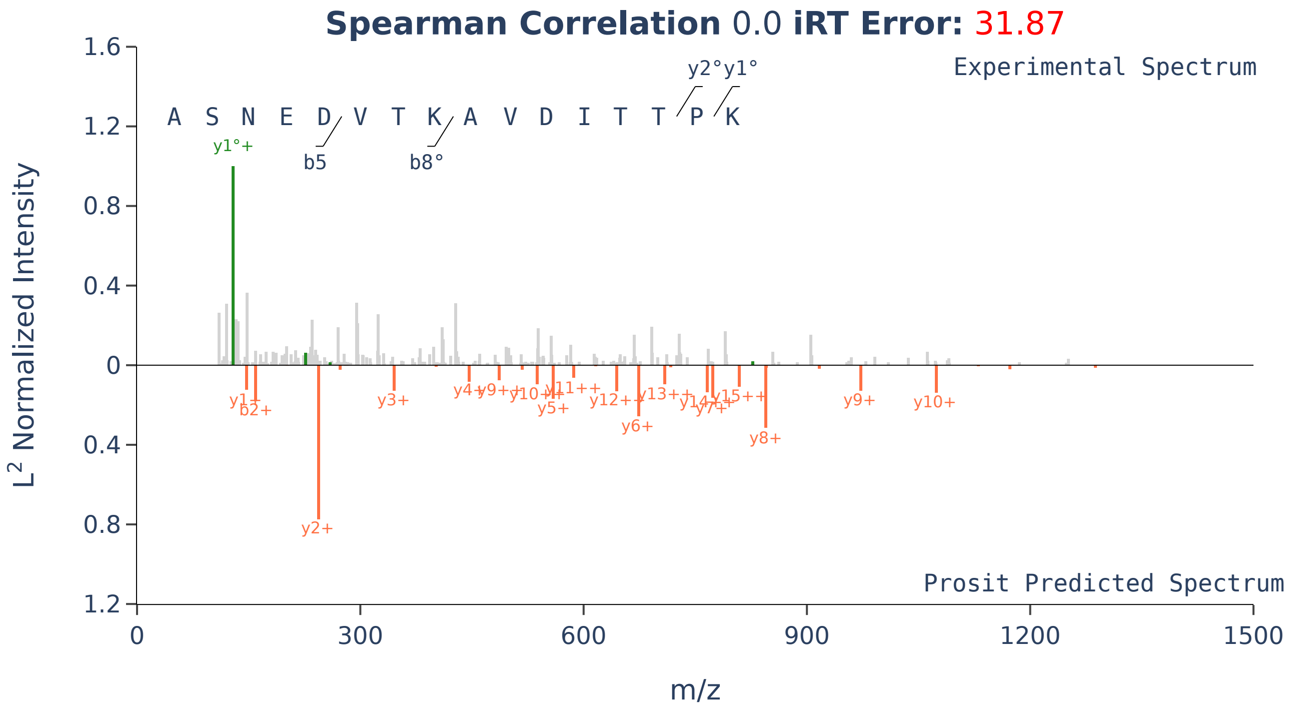

Source HRoetschke_271222_090123_HFGoe_LEDGF_b1R2_24h **Scan** 9974
Peptide PKHWPARVDE **Charge** 3 **Spectral Angle** 0.85
Spearman Correlation 0.89 **iRT Error:** 2.65

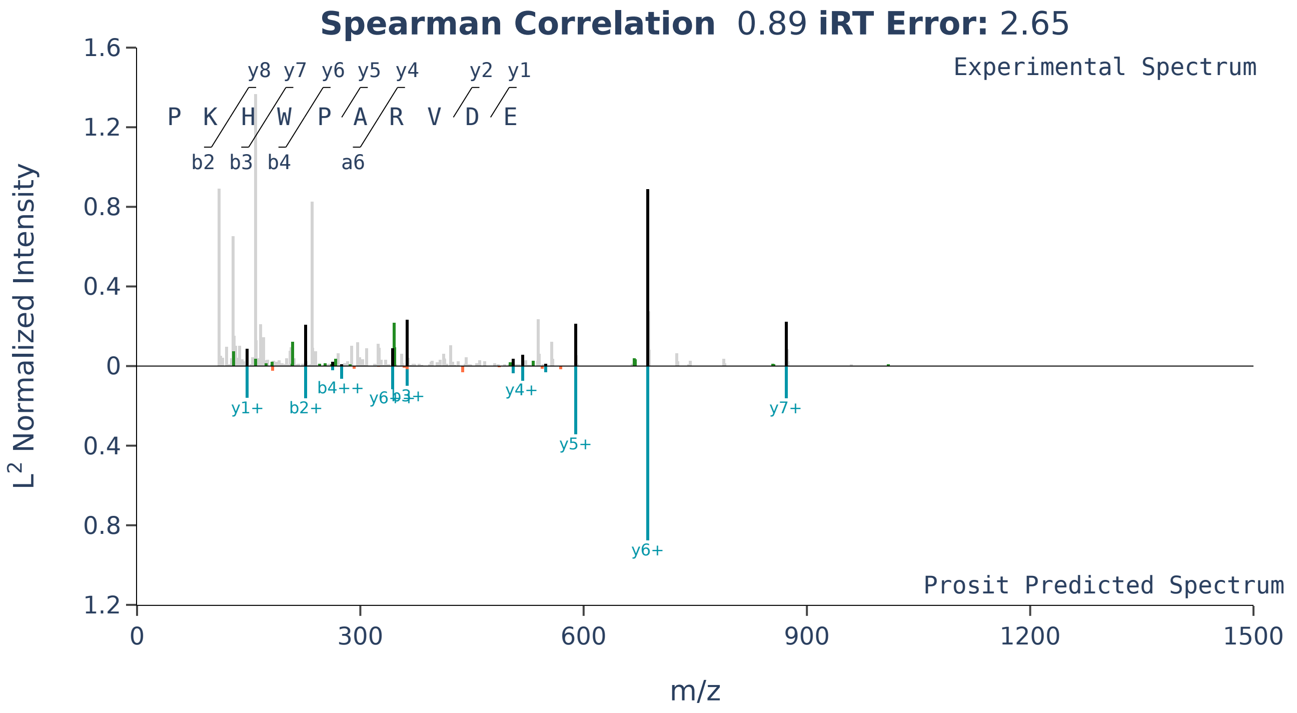

Source HRoetschke_271222_090123_HFGoe_LEDGF_b1R2_24h **Scan** 9974
Peptide EKEQTGSKTLN **Charge** 3 **Spectral Angle** 0.02
Spearman Correlation 0.17 **iRT Error:** 23.0

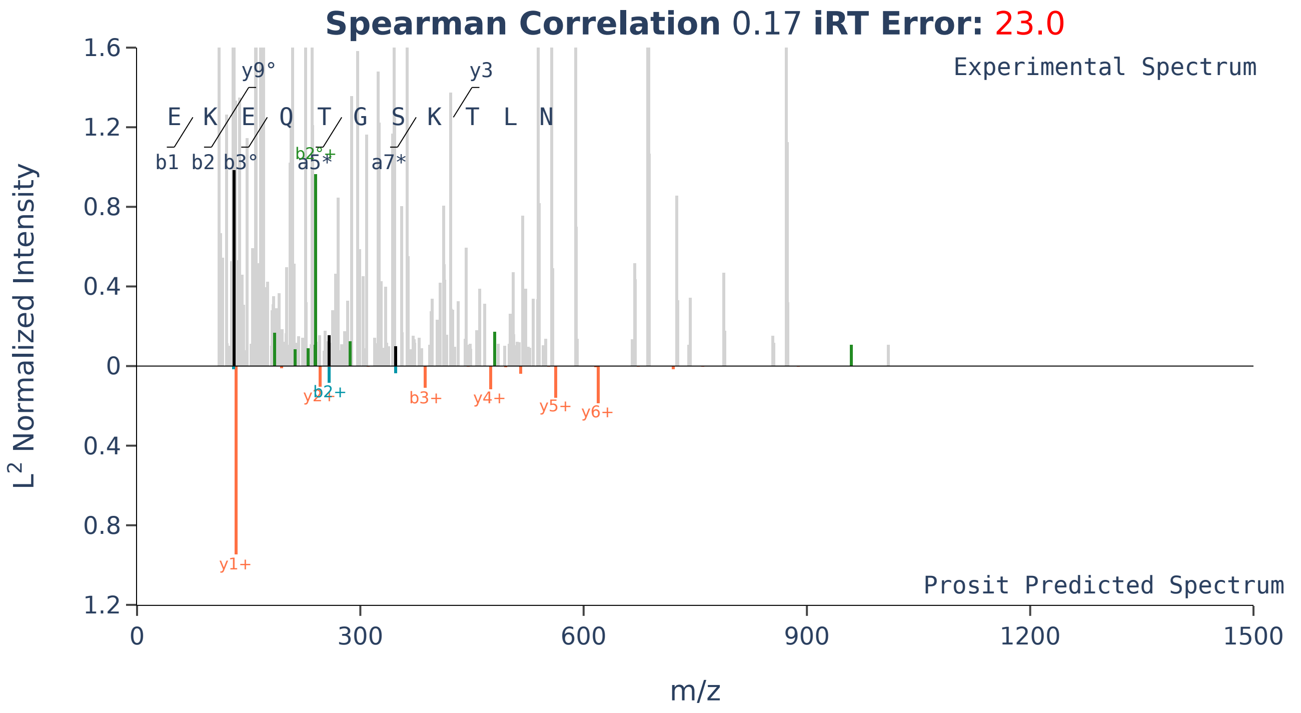

Source HRoetschke_271222_090123_HFGoe_LEDGF_b1R2_24h **Scan** 6989
Peptide SQQAATKQSALD **Charge** 2 **Spectral Angle** 0.82
Spearman Correlation 0.86 **iRT Error:** 2.7

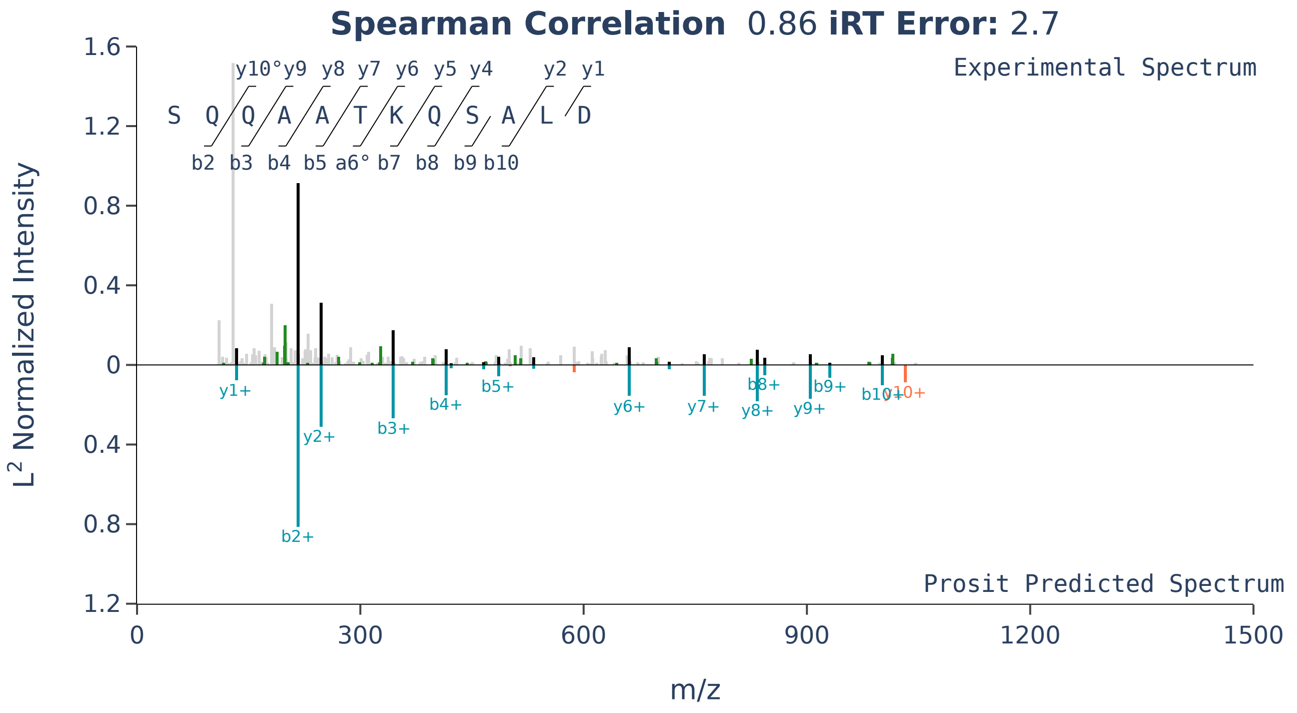

Source HRoetschke_271222_090123_HFGoe_LEDGF_b1R2_24h **Scan** 6989
Peptide EEANKTKDQGK **Charge** 2 **Spectral Angle** 0.0
Spearman Correlation 0.0 **iRT Error:** 21.63

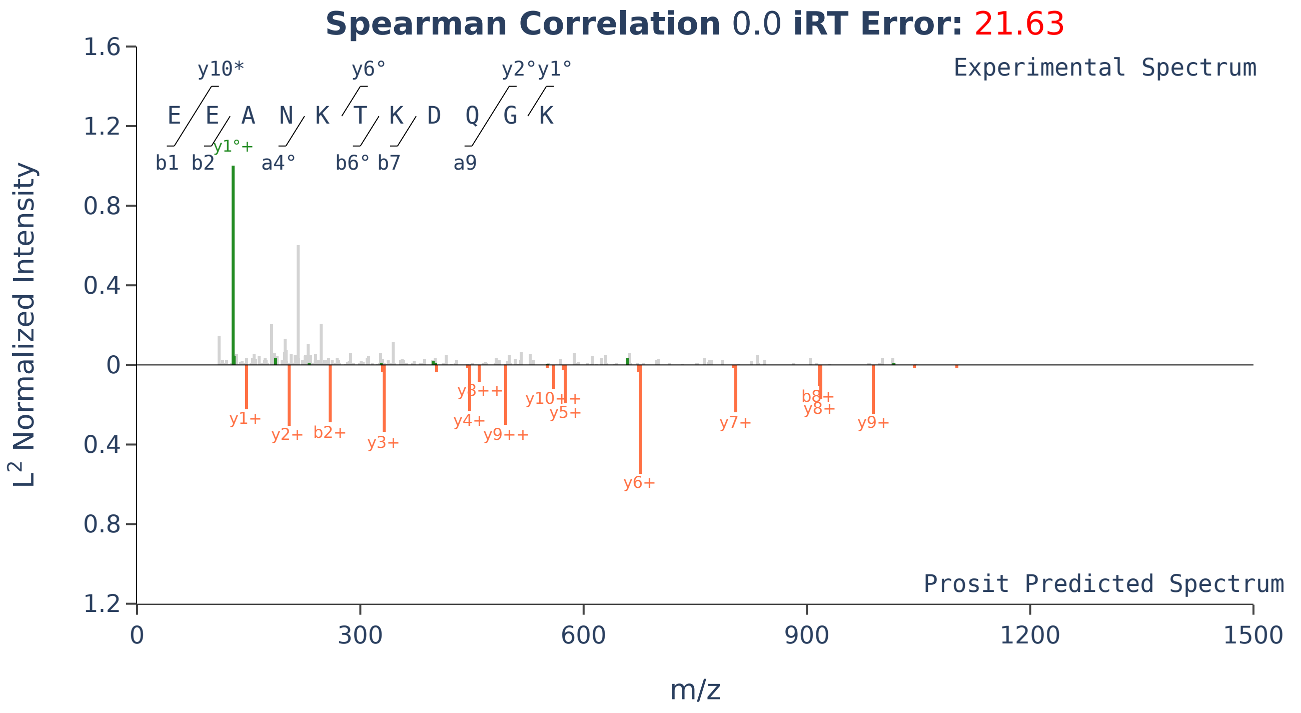

Source HRoetschke_271222_090123_HFGoe_LEDGF_b1R1_4h **Scan** 17687
Peptide GAFLGPKDIFPYSENKEKYGKPNKR **Charge** 4 **Spectral Angle** 0.84
Spearman Correlation 0.9 **iRT Error:** 2.79

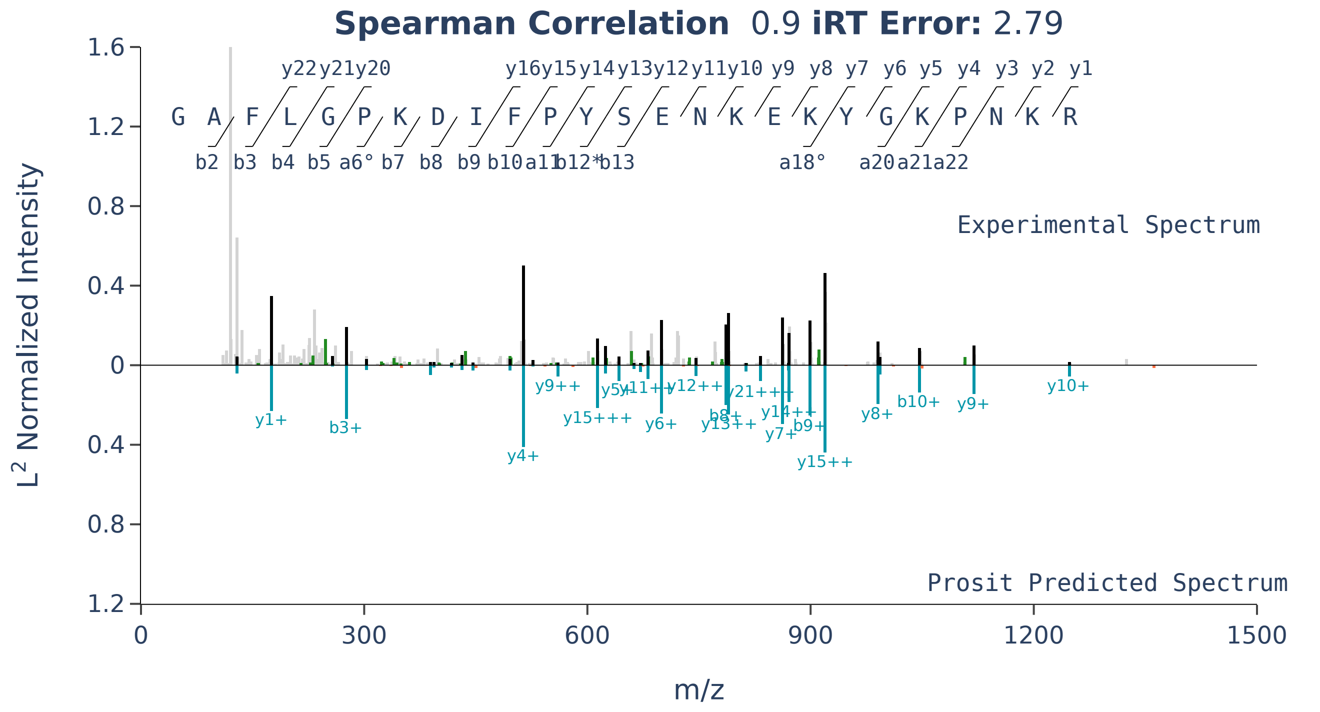

Source HRoetschke_271222_090123_HFGoe_LEDGF_b1R1_4h **Scan** 17687
Peptide KKDEEGQKEEDKPRKEPDKKEGKK **Charge** 4 **Spectral Angle** 0.82
Spearman Correlation 0.23 **iRT Error:** 103.12

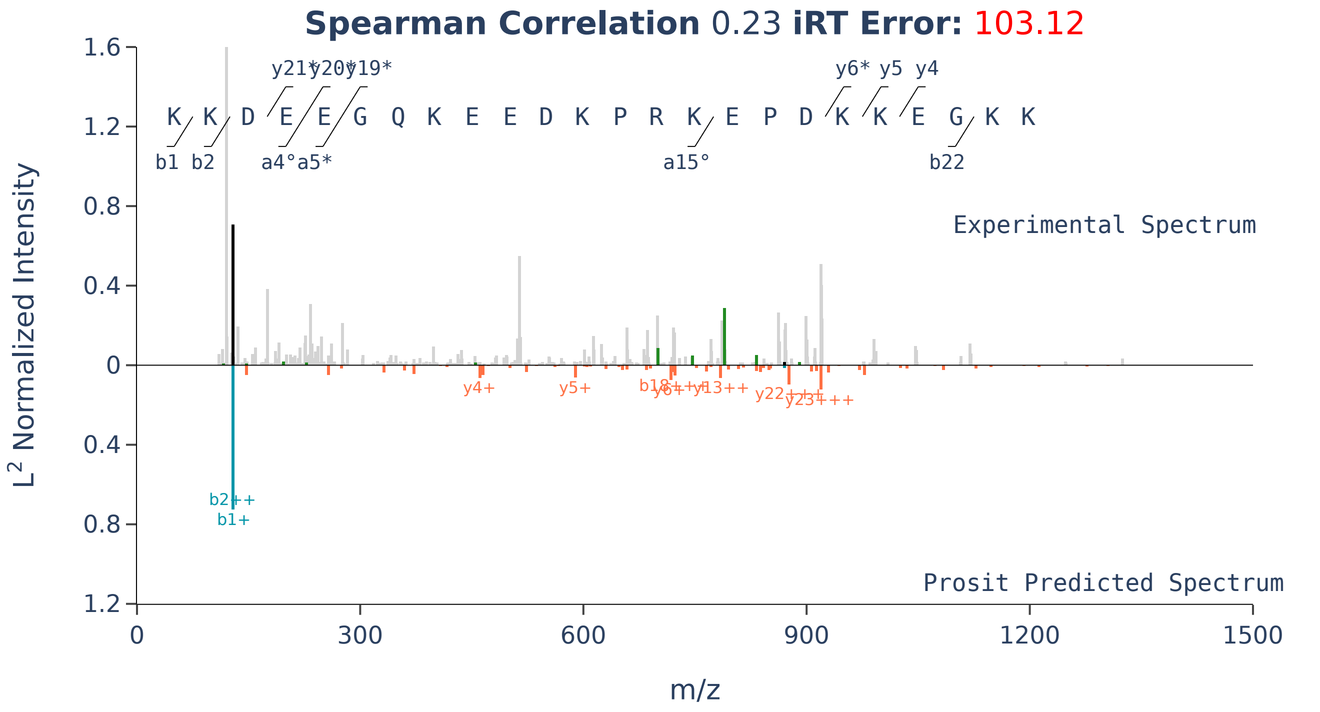

Source HRoetschke_271222_090123_HFGoe_LEDGF_b1R1_24h **Scan** 16040
Peptide FLGPKDIFPYSENKEKYGKPNKRKA **Charge** 5 **Spectral Angle** 0.79
Spearman Correlation 0.86 **iRT Error:** 2.84

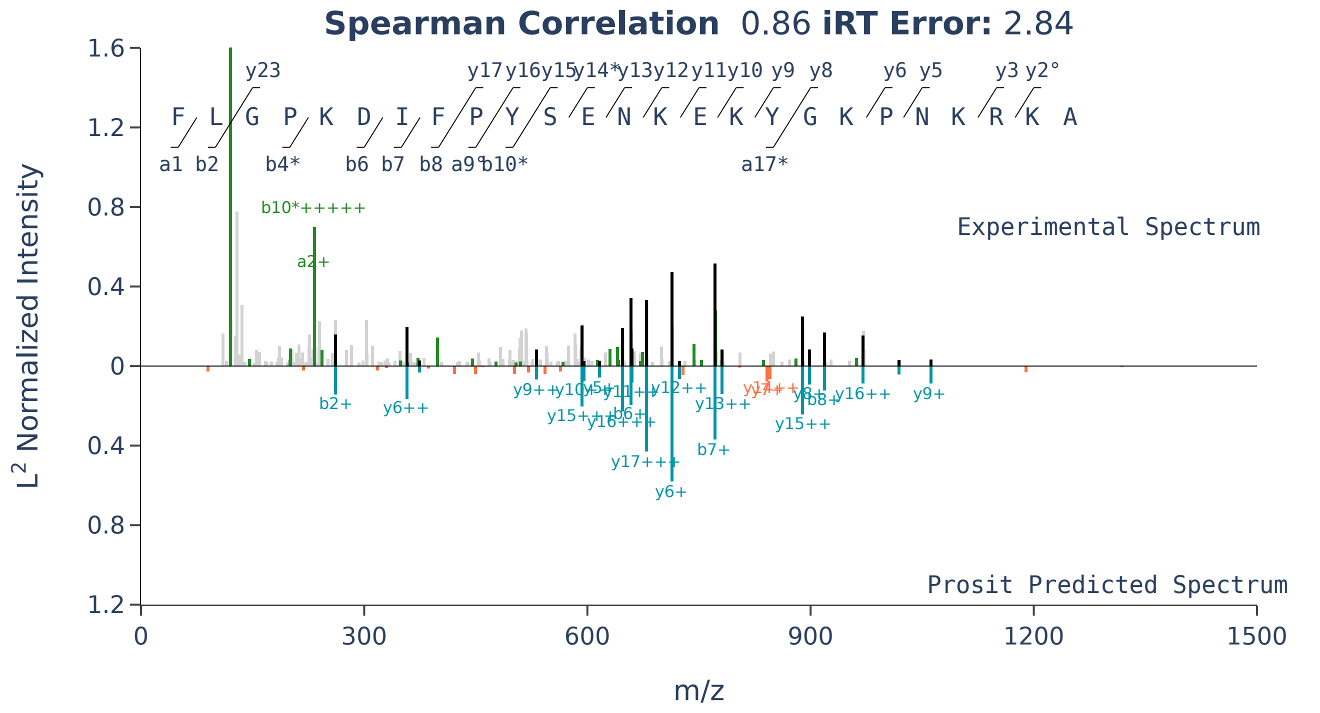

Source HRoetschke_271222_090123_HFGoe_LEDGF_b1R1_24h **Scan** 16040
Peptide AFLGPKDIFPYSENKEKYGKPNKRK **Charge** 5 **Spectral Angle** 0.0
Spearman Correlation 0.0 **iRT Error:** 1.38

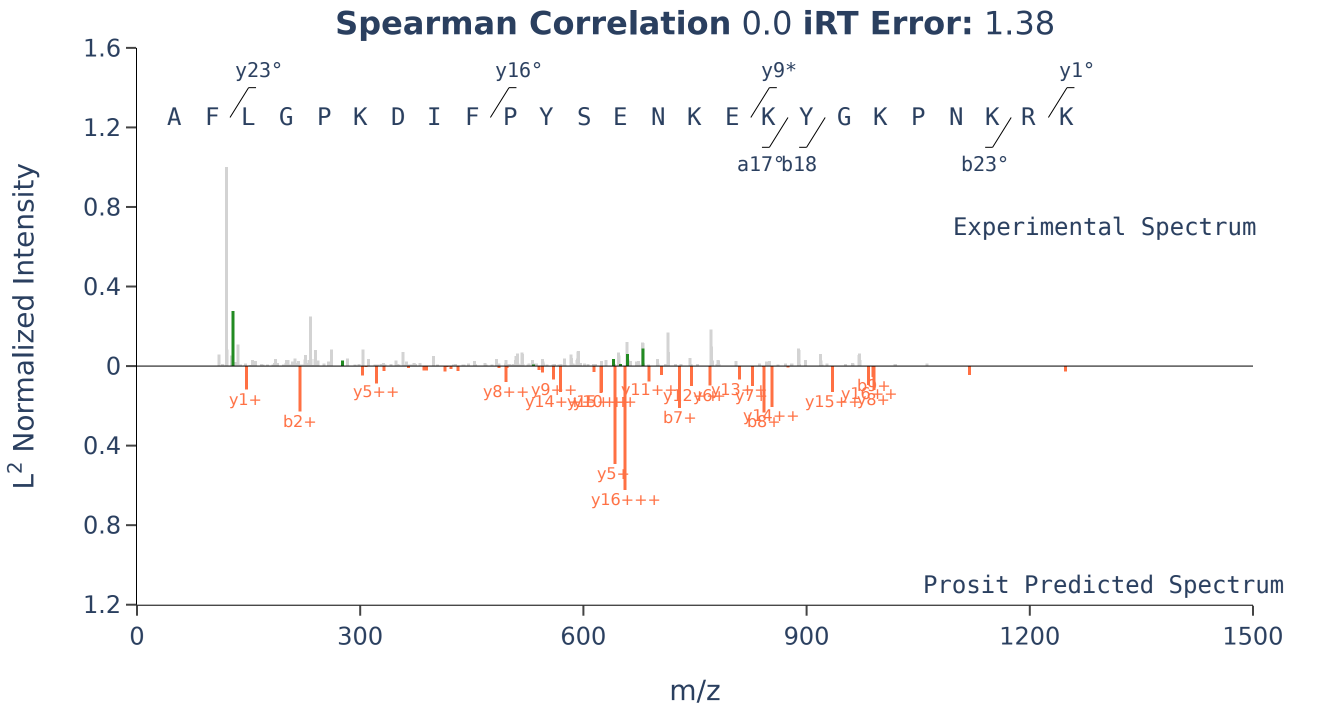

Source HRoetschke_271222_090123_HFGoe_LEDGF_b1R1_24h **Scan** 10081
Peptide KVSQVIMEKSMT **Charge** 2 **Spectral Angle** 0.89
Spearman Correlation 0.92 **iRT Error:** 2.95

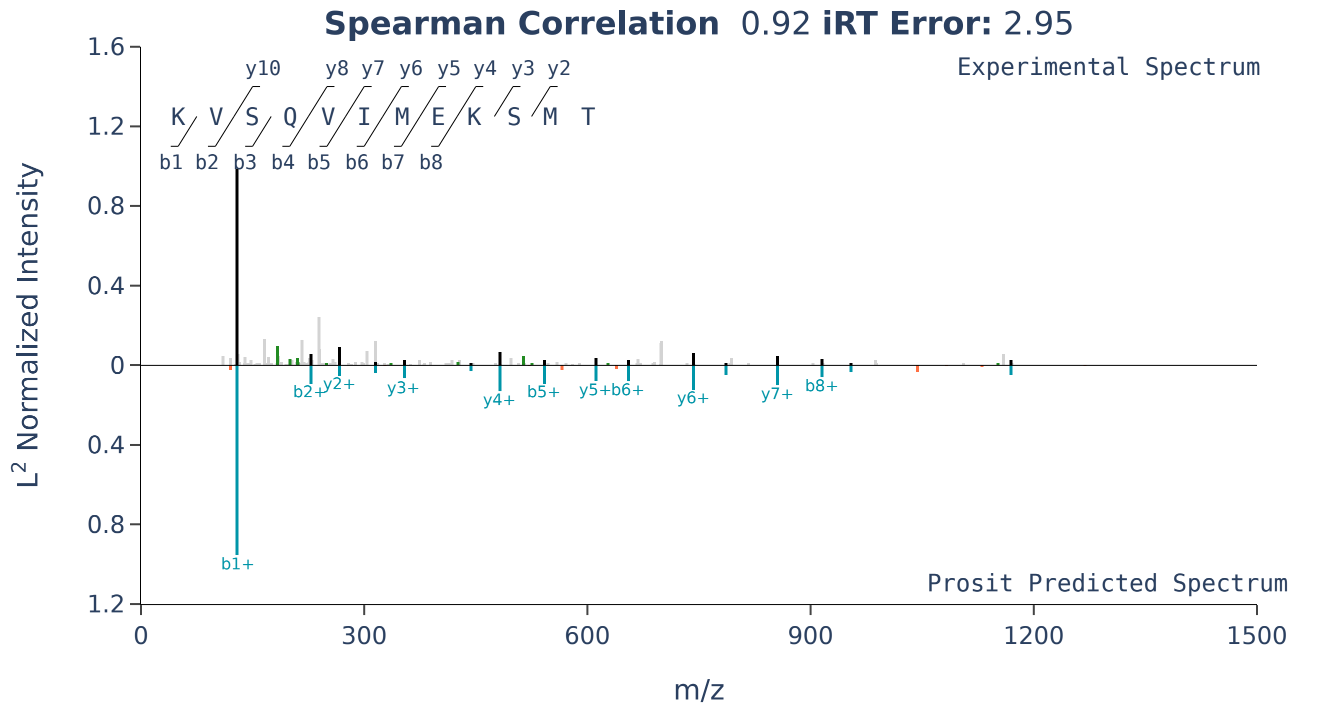

Source HRoetschke_271222_090123_HFGoe_LEDGF_b1R1_24h **Scan** 10081
Peptide KVSQVIMEKSTM **Charge** 2 **Spectral Angle** 0.81
Spearman Correlation 0.95 **iRT Error:** 3.16

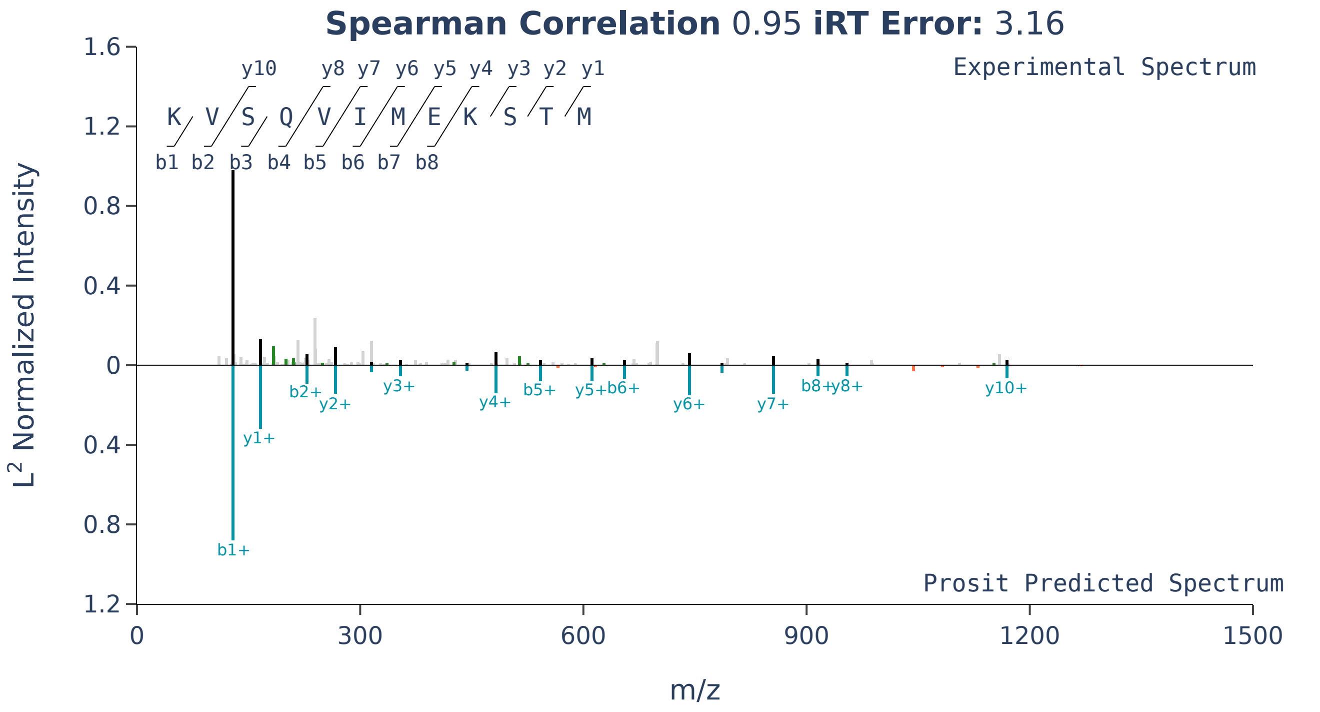

Source HRoetschke_271222_090123_HFGoe_LEDGF_b2R1_24h **Scan** 25803
Peptide ELSLKIDNLDVNRCIE **Charge** 2 **Spectral Angle** 0.86
Spearman Correlation 0.87 **iRT Error:** 3.01

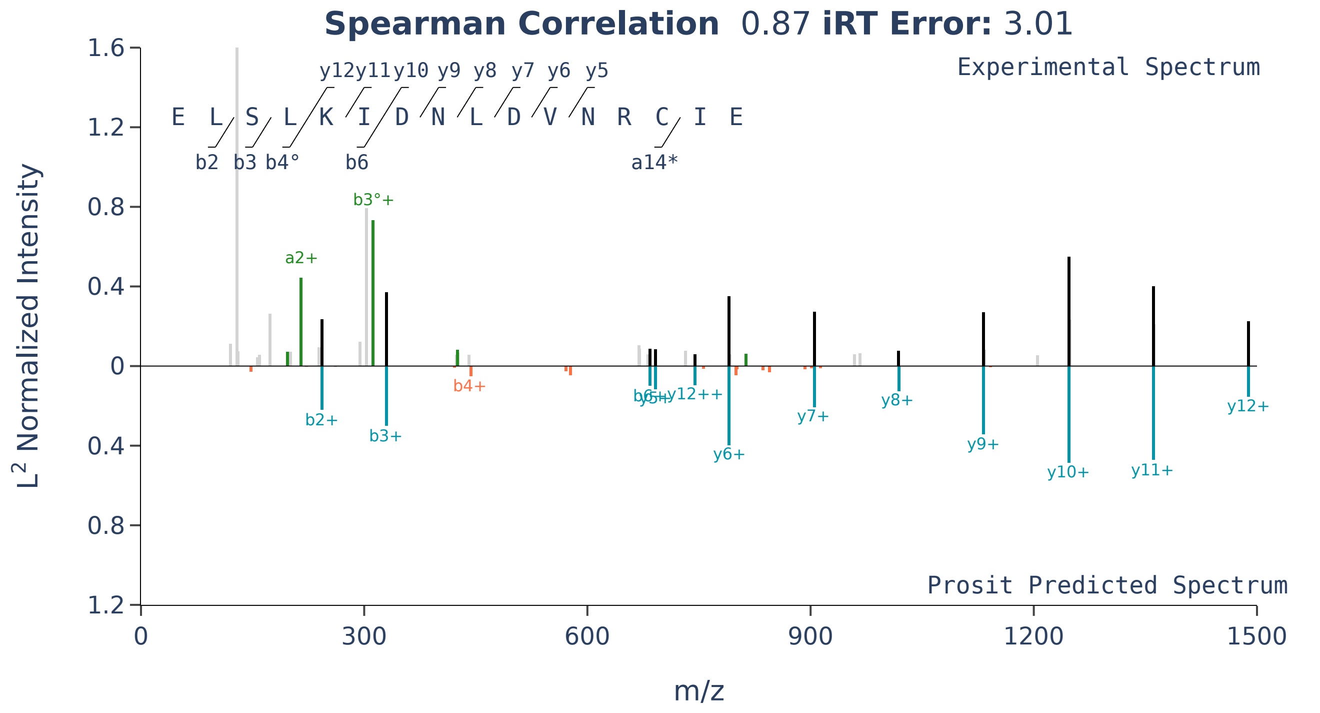

Source HRoetschke_271222_090123_HFGoe_LEDGF_b2R1_24h **Scan** 25803
Peptide VNRCIEALDELASLQVT **Charge** 2 **Spectral Angle** 0.0
Spearman Correlation 0.0 **iRT Error:** 32.8

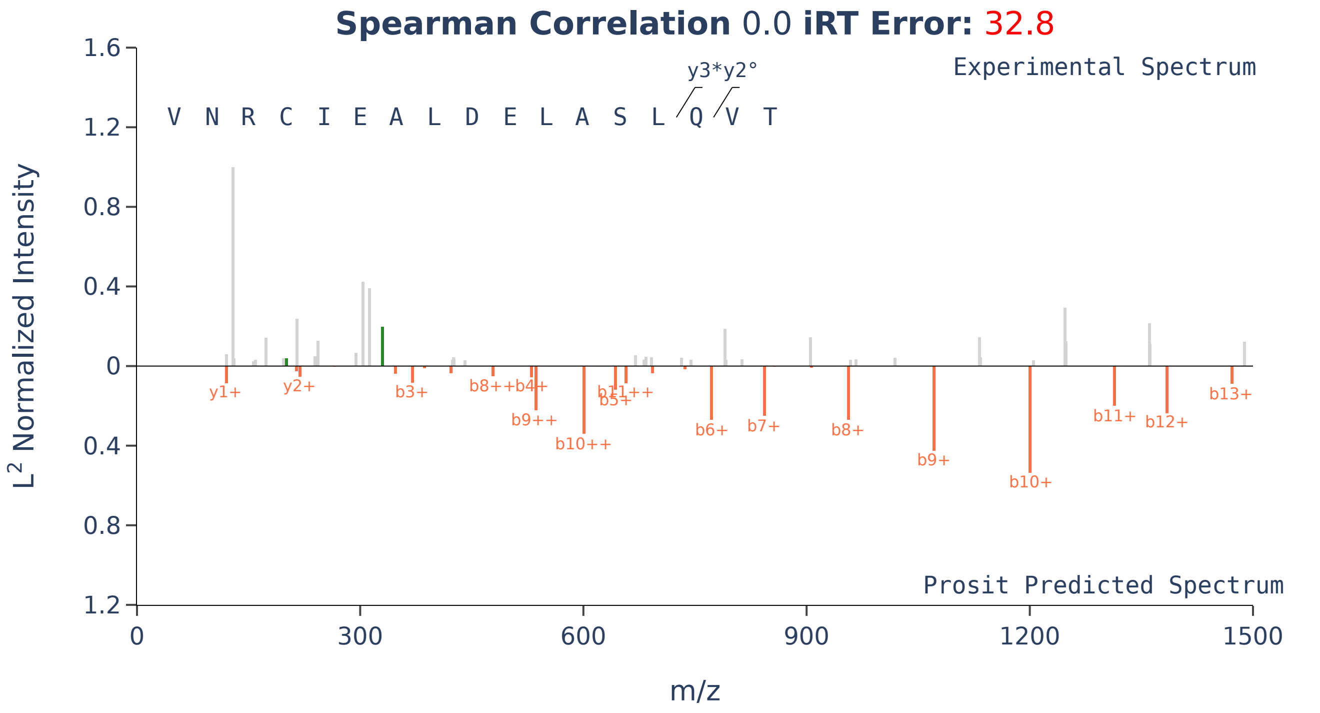

Source HRoetschke_271222_090123_HFGoe_LEDGF_b2R1_24h **Scan** 17977
Peptide TSAFLGPKDIFPYSENKEKYGKPNKR **Charge** 4 **Spectral Angle** 0.79
Spearman Correlation 0.87 **iRT Error:** 3.03

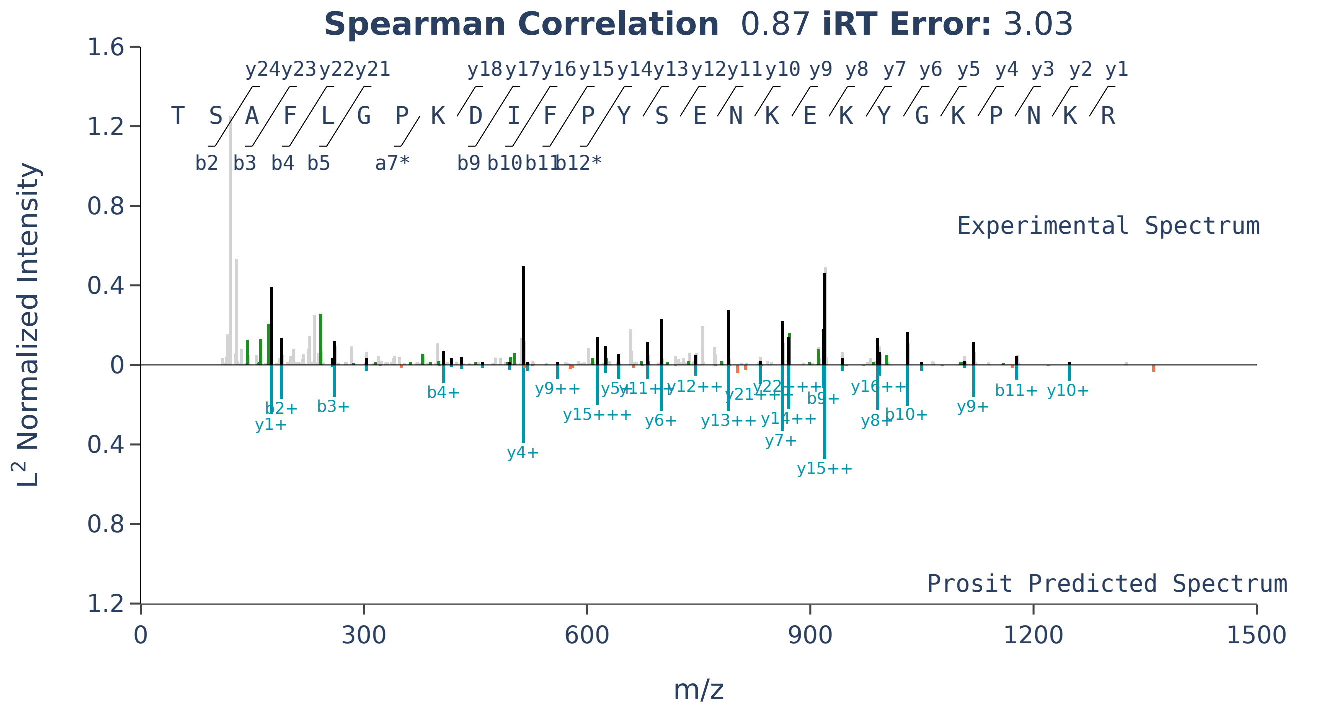

Source HRoetschke_271222_090123_HFGoe_LEDGF_b2R1_24h **Scan** 17977
Peptide KNSLKIDNLDVNRCIEALDELASLQVT **Charge** 4 **Spectral Angle** 0.19
Spearman Correlation 0.02 **iRT Error:** 96.49

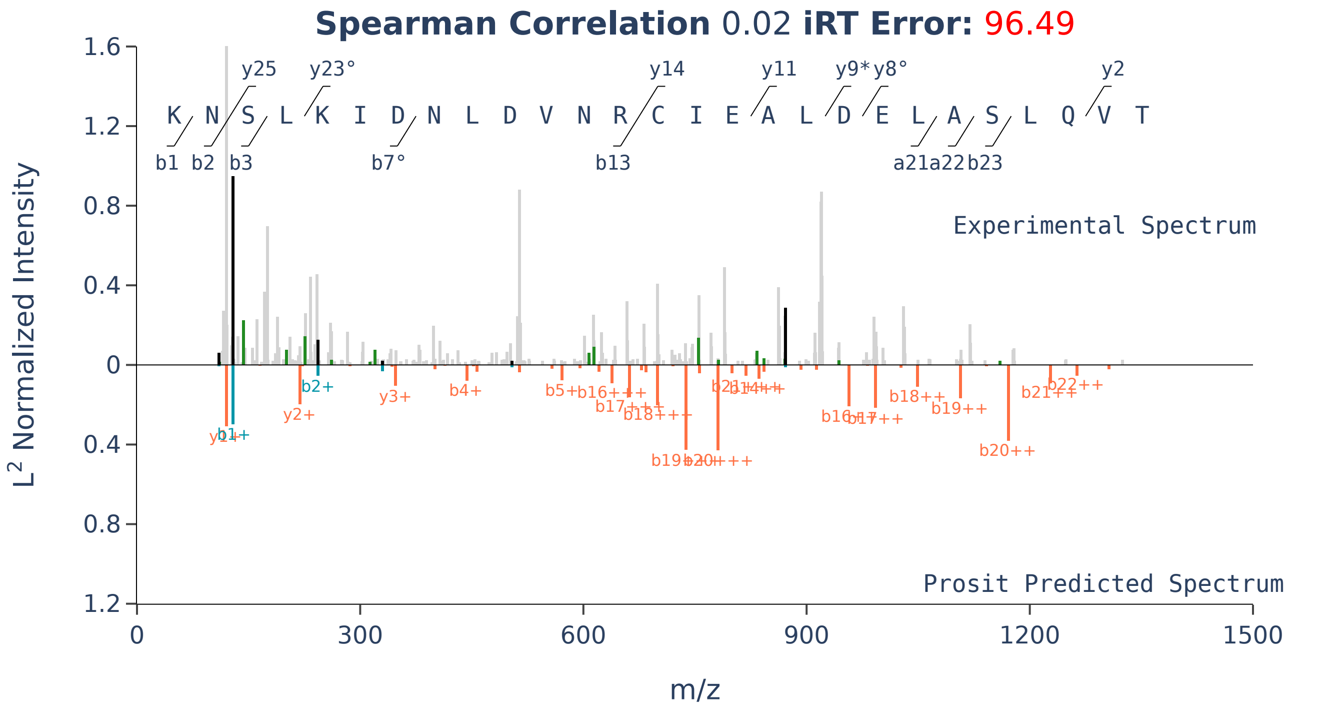

Source HRoetschke_271222_090123_HFGoe_LEDGF_b1R1_4h **Scan** 9168
Peptide GGNPKVKF **Charge** 1 **Spectral Angle** 0.84
Spearman Correlation 0.88 **iRT Error:** 3.09

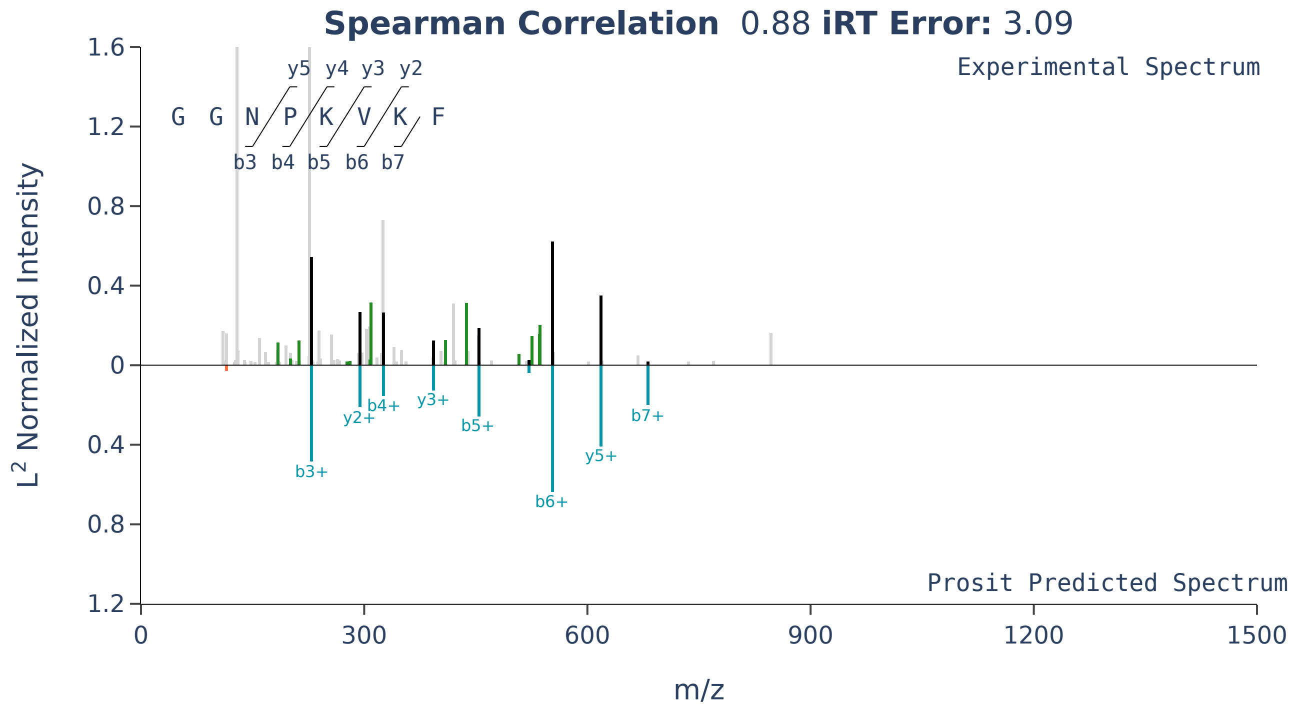

Source HRoetschke_271222_090123_HFGoe_LEDGF_b1R1_4h **Scan** 9168
Peptide NNPKVKF **Charge** 1 **Spectral Angle** 0.78
Spearman Correlation 0.81 **iRT Error:** 32.1

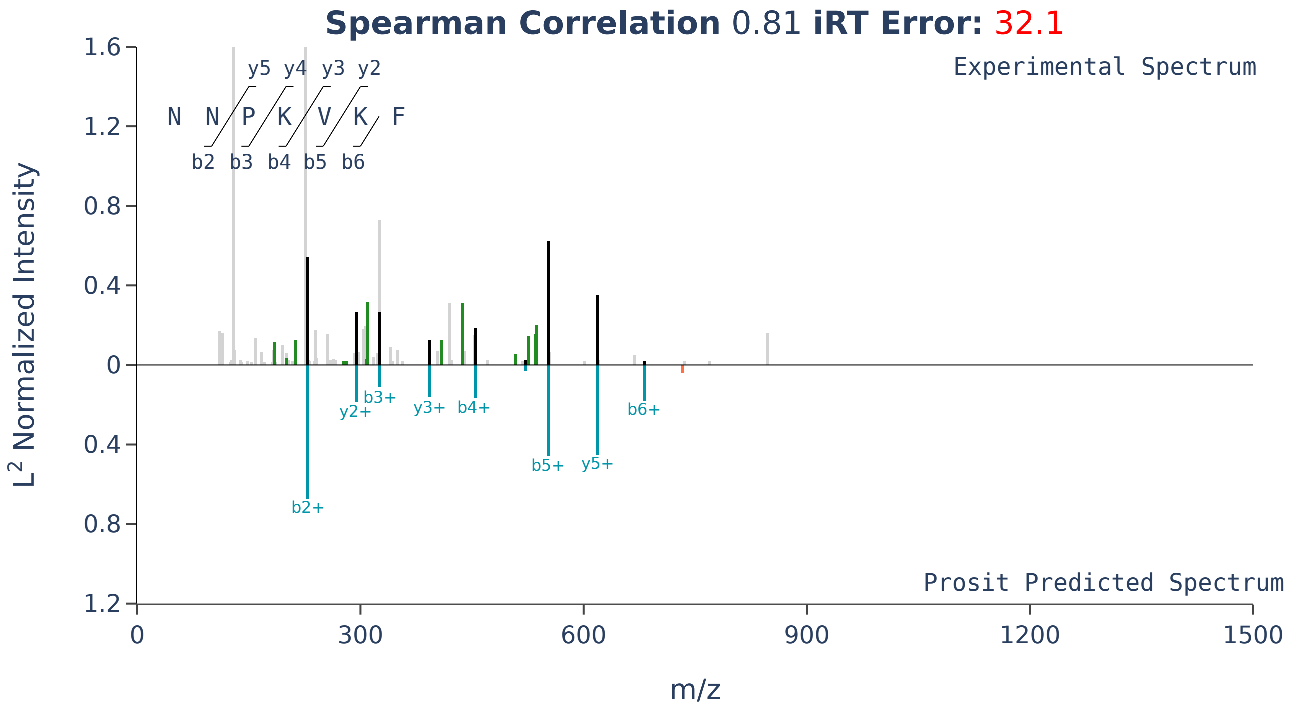

Source HRoetschke_271222_090123_HFGoe_LEDGF_b1R1_4h **Scan** 18055
Peptide TGHETAFLGPKDIFPYSENKEKYGKPNK **Charge** 3 **Spectral Angle** 0.84
Spearman Correlation 0.85 **iRT Error:** 3.11

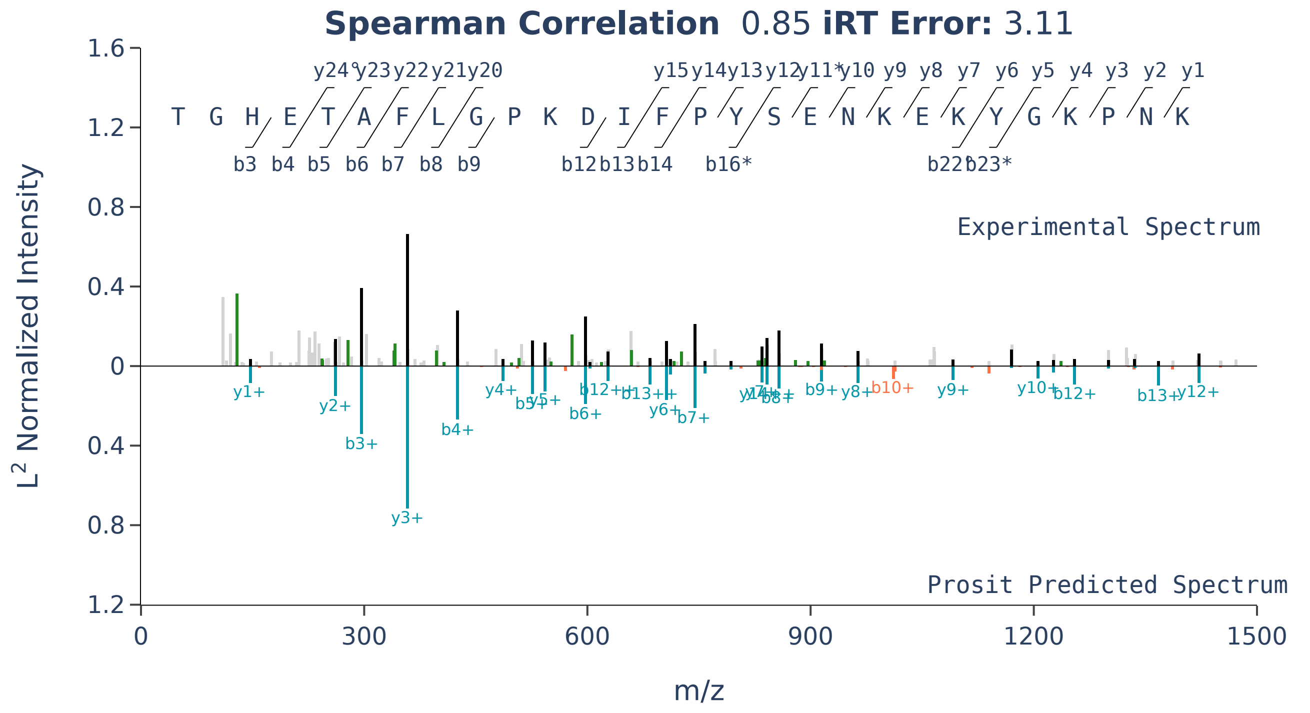

Source HRoetschke_271222_090123_HFGoe_LEDGF_b1R1_4h **Scan** 18055
Peptide GTHETAFLGPKDIFPYSENKEKYGKPNK **Charge** 3 **Spectral Angle** 0.82
Spearman Correlation 0.85 **iRT Error:** 2.94

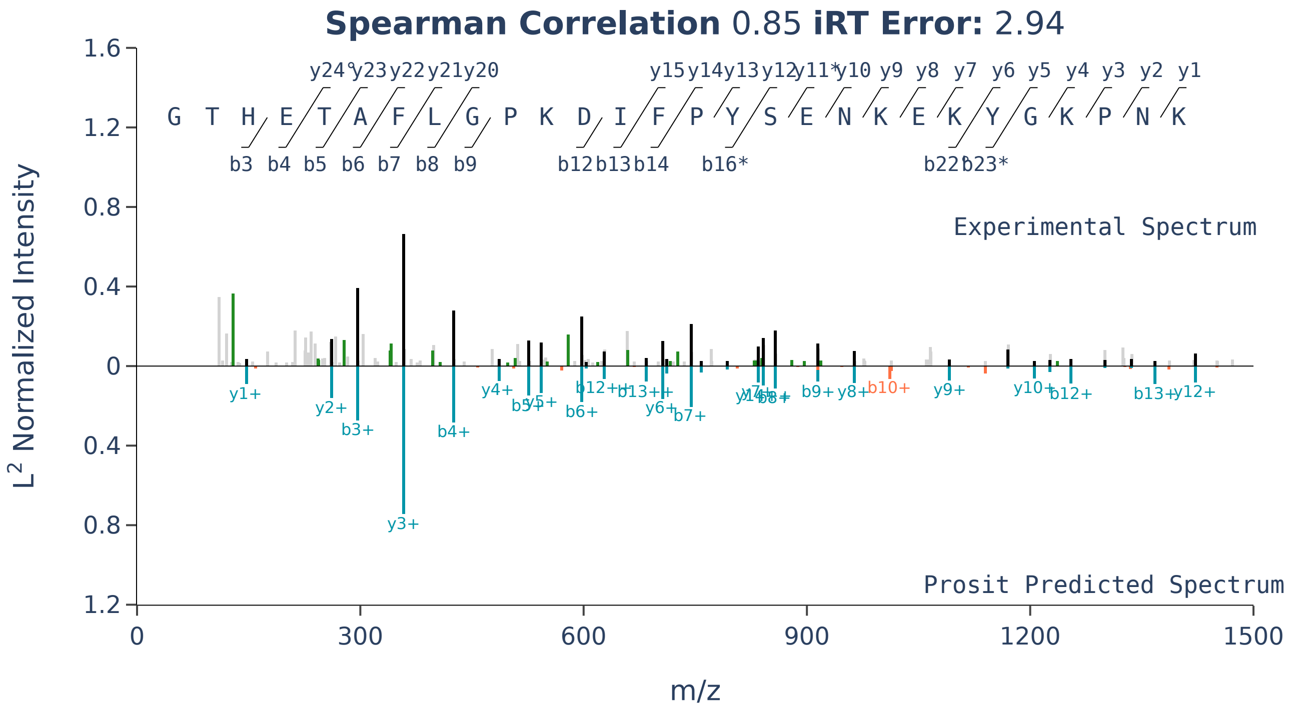

Source HRoetschke_271222_090123_HFGoe_LEDGF_b2R1_1h **Scan** 17893
Peptide TAFLGPKDIFPYSENKEKYGKPRKGG **Charge** 3 **Spectral Angle** 0.86
Spearman Correlation 0.83 **iRT Error:** 3.11

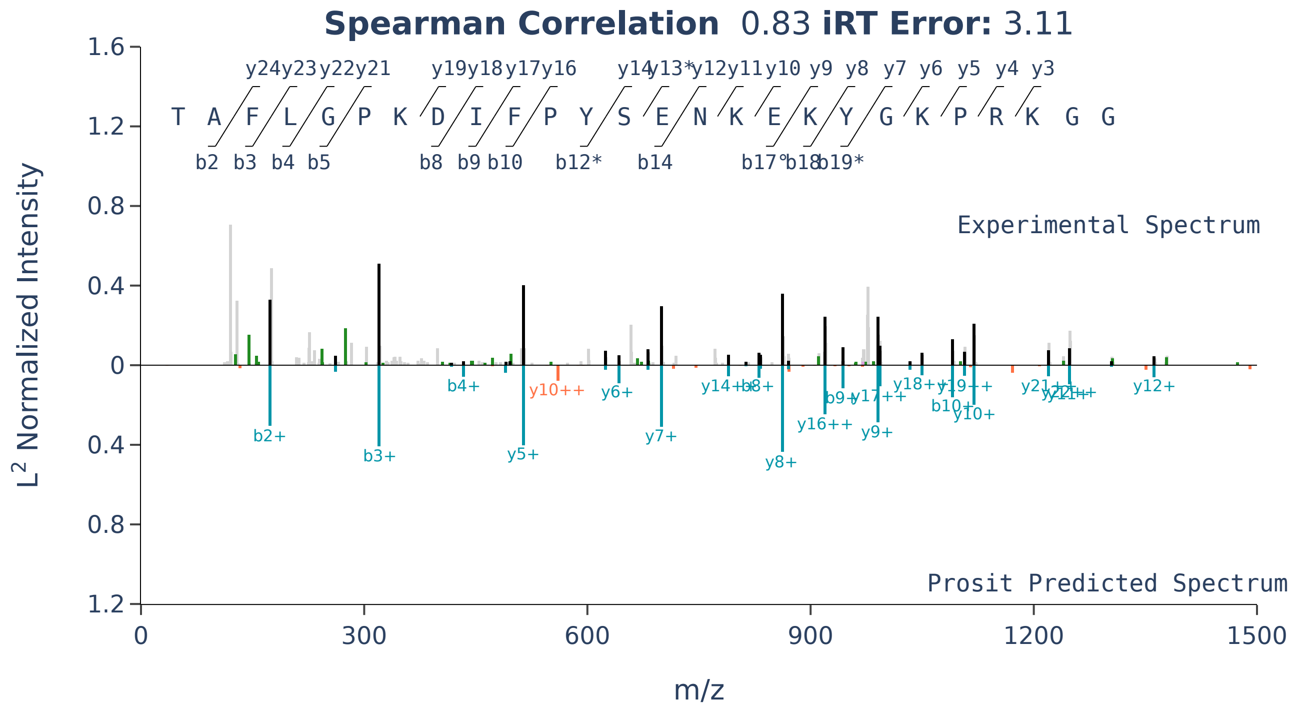

Source HRoetschke_271222_090123_HFGoe_LEDGF_b2R1_1h **Scan** 17893
Peptide TAFLGPKDIFPYSENKEKYGKPNKR **Charge** 3 **Spectral Angle** 0.78
Spearman Correlation 0.82 **iRT Error:** 3.11

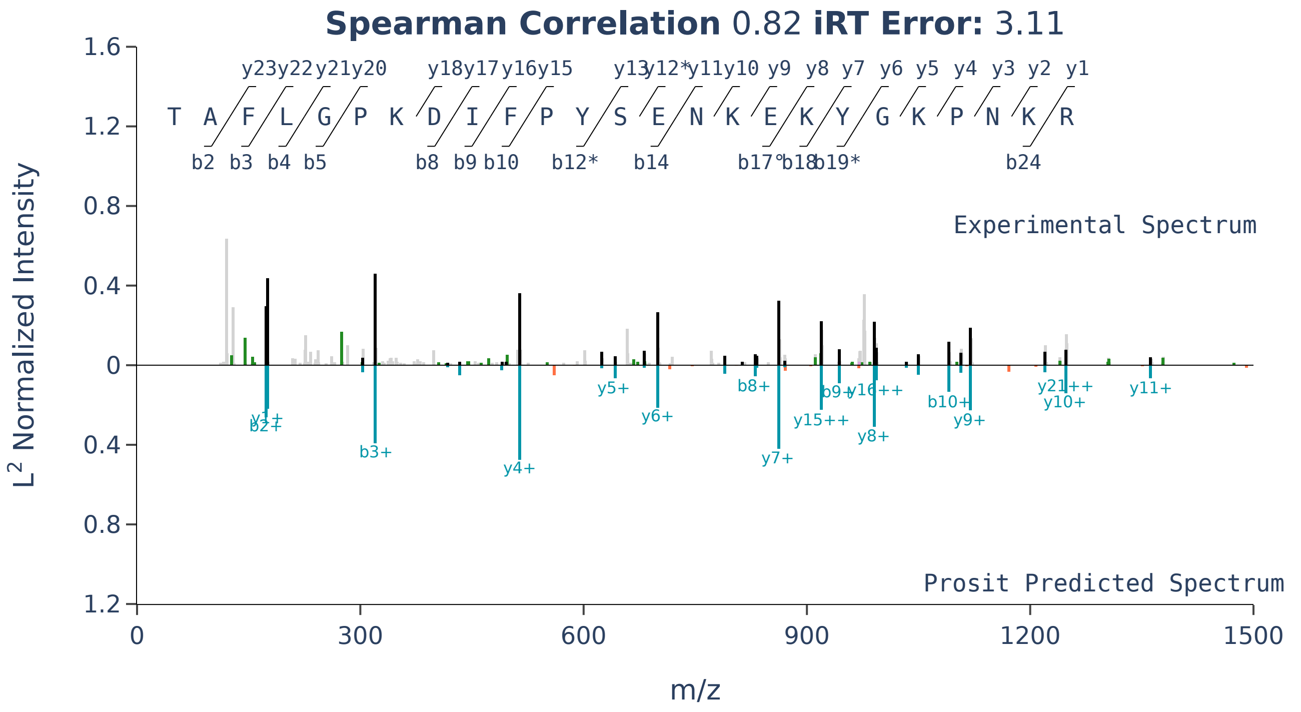

Source HRoetschke_271222_090123_HFGoe_LEDGF_b1R2_24h **Scan** 10959
Peptide SKTMLYNKFKNM **Charge** 4 **Spectral Angle** 0.76
Spearman Correlation 0.92 **iRT Error:** 3.25

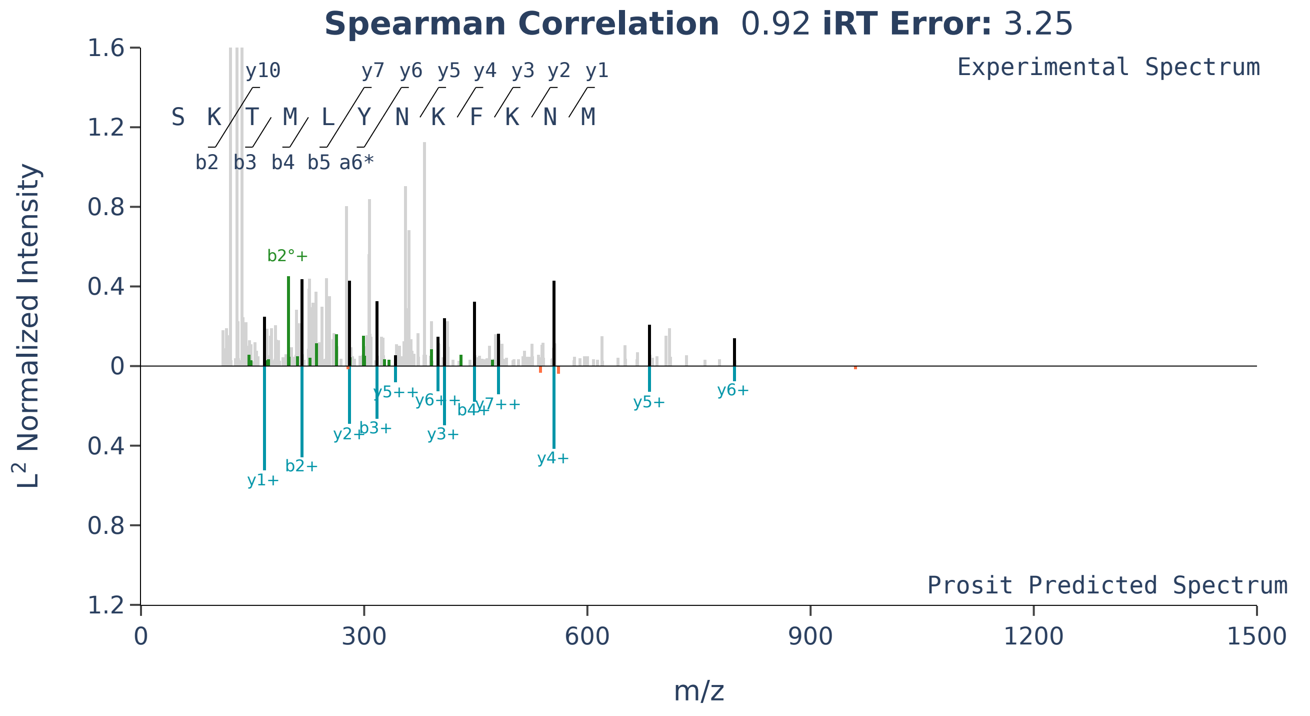

Source HRoetschke_271222_090123_HFGoe_LEDGF_b1R2_24h **Scan** 10959
Peptide KSTMLYNKFKNM **Charge** 4 **Spectral Angle** 0.8
Spearman Correlation 0.88 **iRT Error:** 3.32

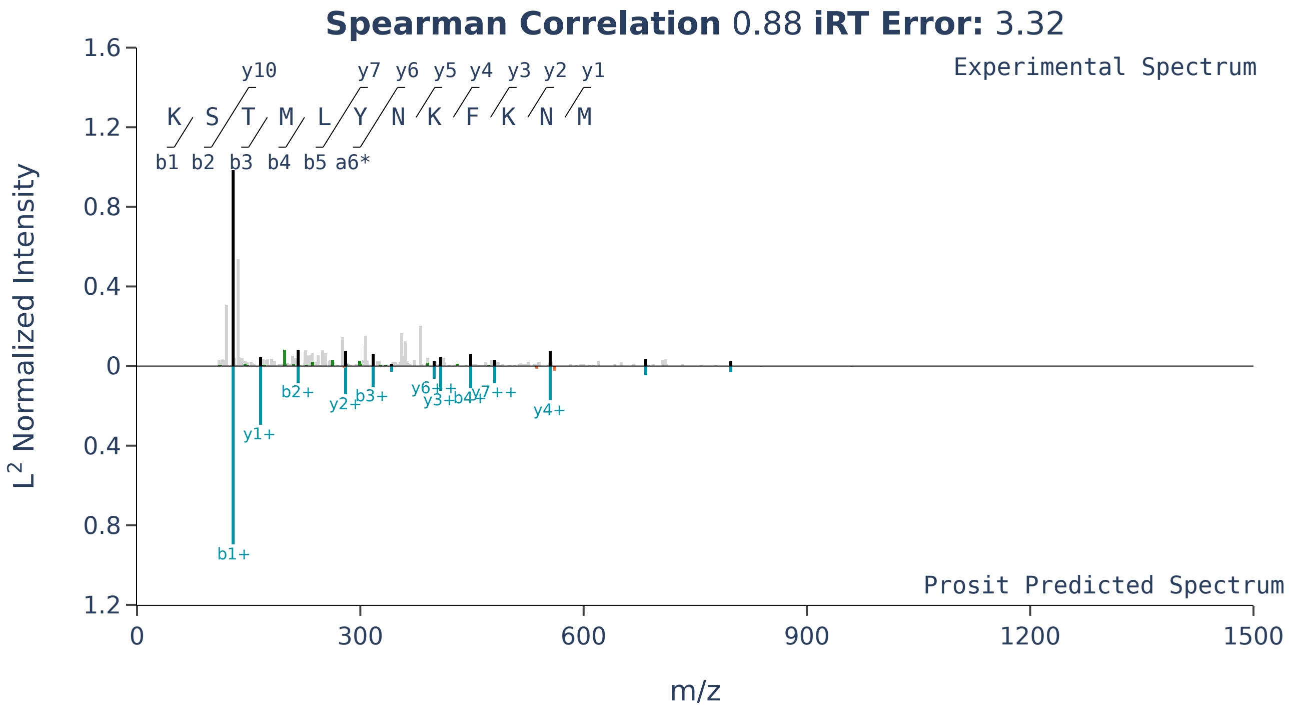

Source HRoetschke_271222_090123_HFGoe_LEDGF_b1R1_24h **Scan** 27943
Peptide DELALASL **Charge** 2 **Spectral Angle** 0.77
Spearman Correlation 0.89 **iRT Error:** 3.29

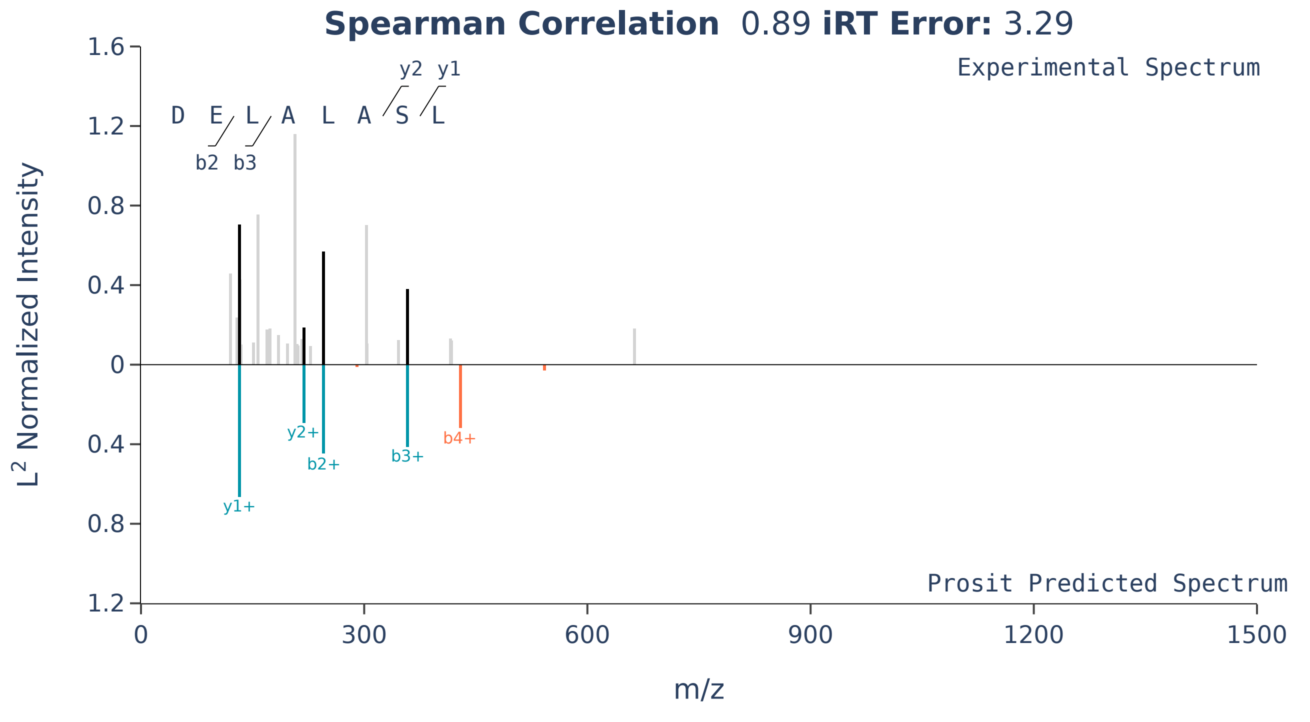

Source HRoetschke_271222_090123_HFGoe_LEDGF_b1R1_24h **Scan** 27943
Peptide ALDELASL **Charge** 2 **Spectral Angle** 0.77
Spearman Correlation 0.71 **iRT Error:** 19.74

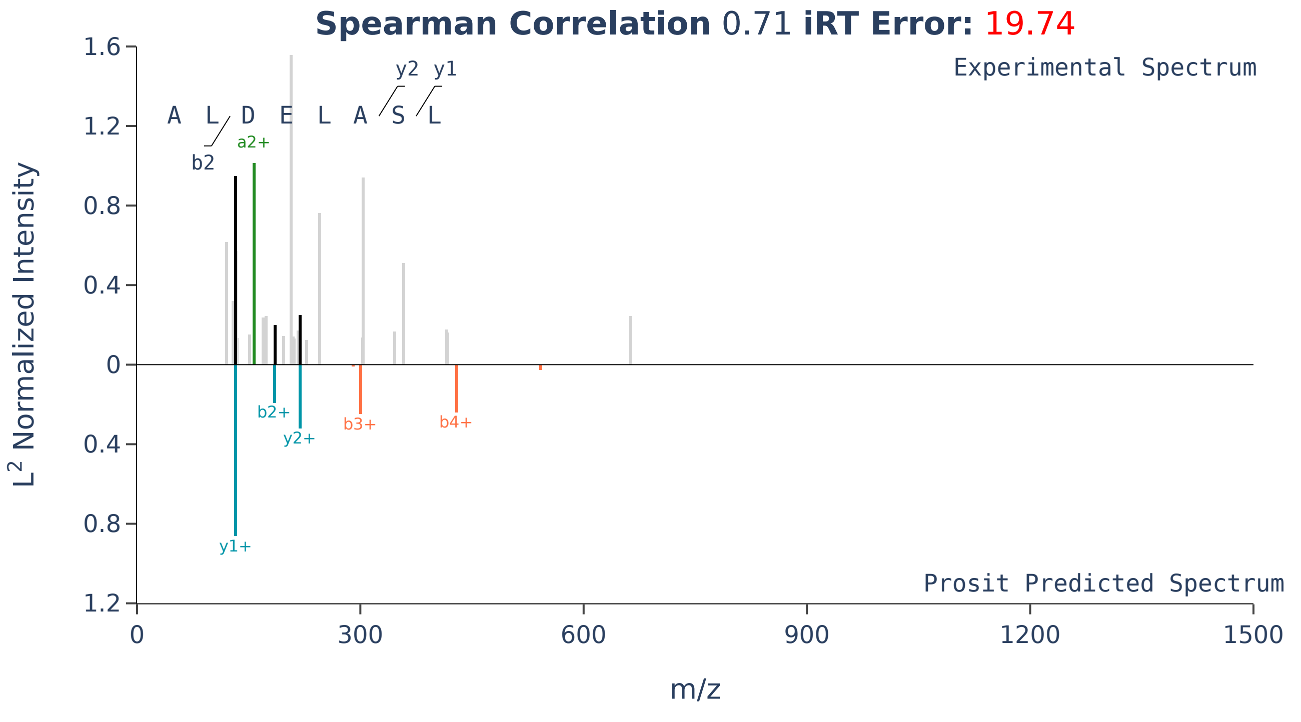

Source HRoetschke_271222_090123_HFGoe_LEDGF_b2R1_24h **Scan** 19445
Peptide KLENSLKIDNL **Charge** 3 **Spectral Angle** 0.86
Spearman Correlation 0.86 **iRT Error:** 3.37

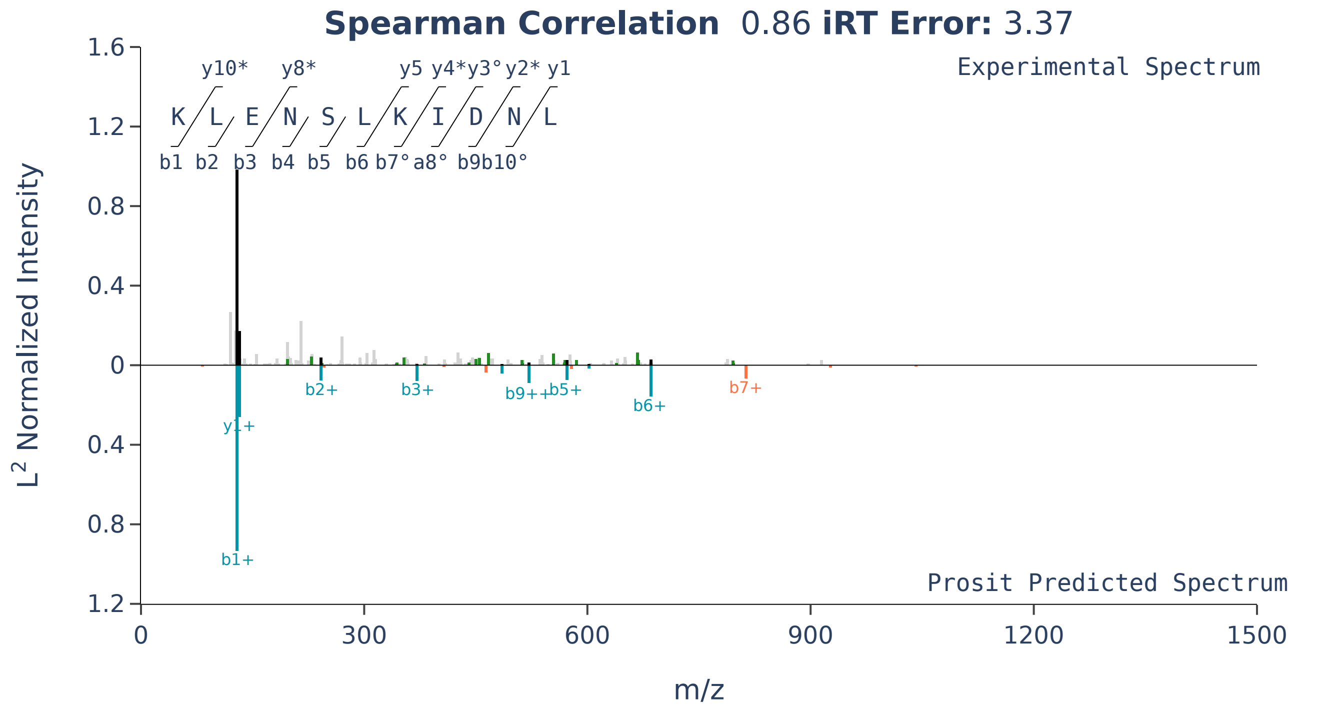

Source HRoetschke_271222_090123_HFGoe_LEDGF_b2R1_24h **Scan** 19445
Peptide EIKNSLKIDNL **Charge** 3 **Spectral Angle** 0.87
Spearman Correlation 0.76 **iRT Error:** 1.34

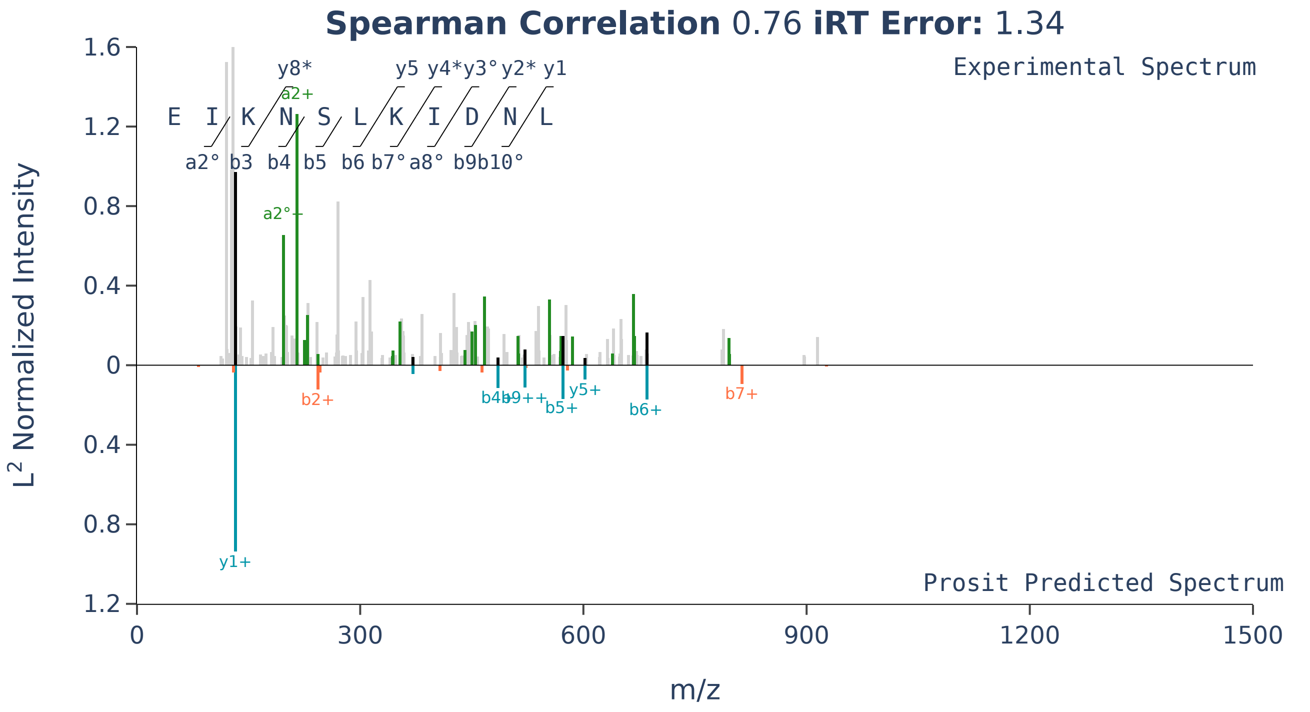

Source HRoetschke_271222_090123_HFGoe_LEDGF_b2R2_4h **Scan** 8390
Peptide GKSNASSDVEVEE **Charge** 2 **Spectral Angle** 0.9
Spearman Correlation 0.92 **iRT Error:** 3.37

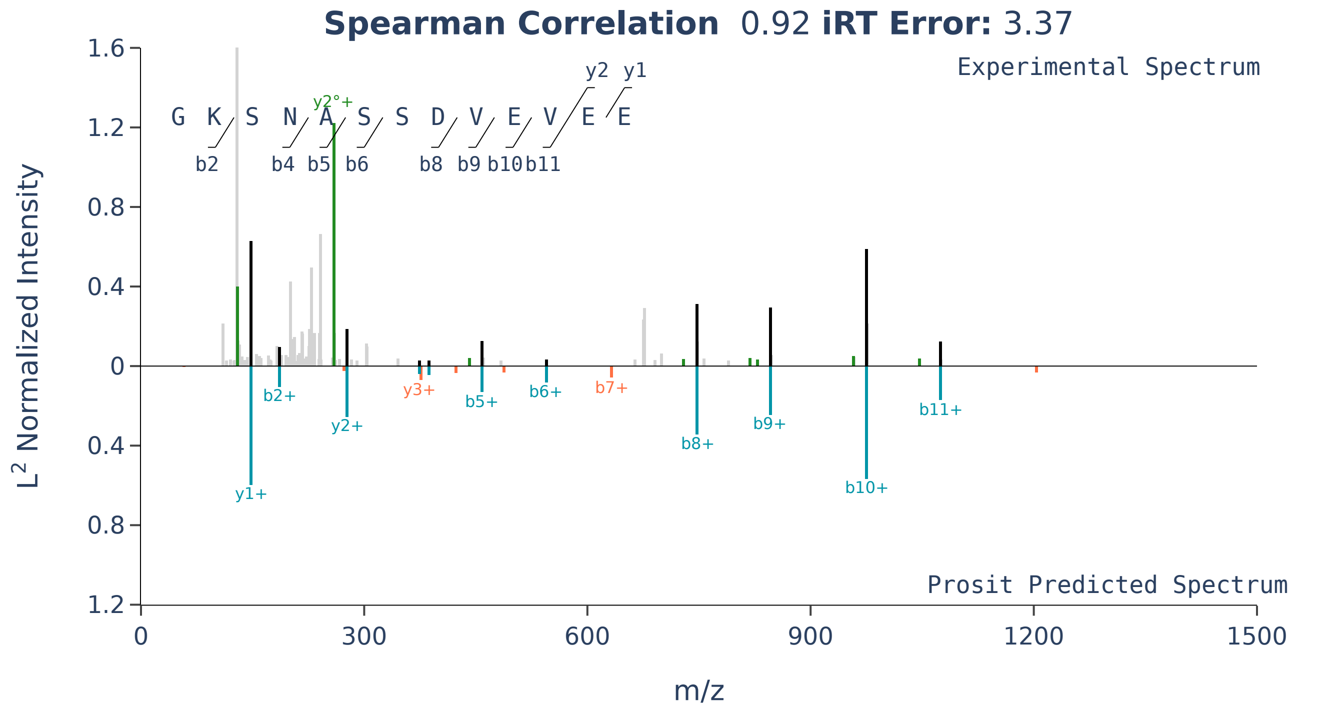

Source HRoetschke_271222_090123_HFGoe_LEDGF_b2R2_4h **Scan** 8390
Peptide FNEGLWEIDNN **Charge** 2 **Spectral Angle** 0.0
Spearman Correlation 0.0 **iRT Error:** 110.38

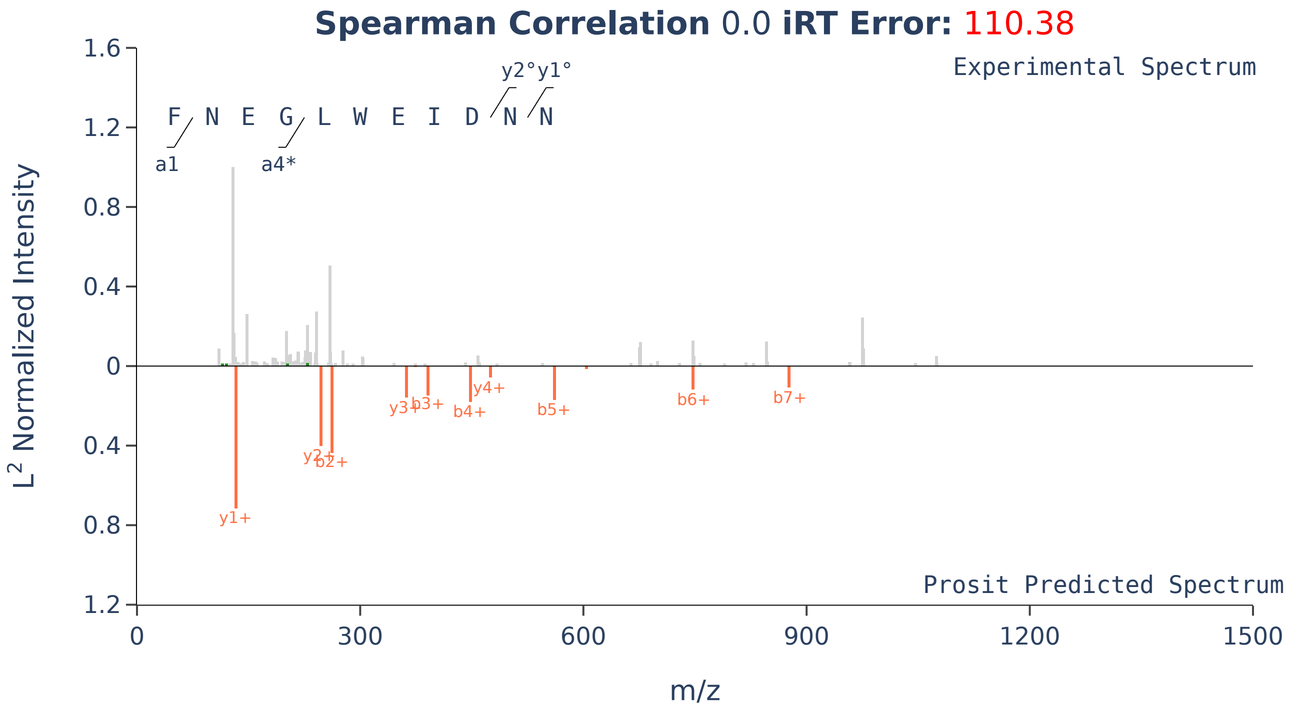

Source HRoetschke_271222_090123_HFGoe_LEDGF_b1R1_1h **Scan** 16231
Peptide YPHWPAQQAAT **Charge** 1 **Spectral Angle** 0.79
Spearman Correlation 0.87 **iRT Error:** 3.38

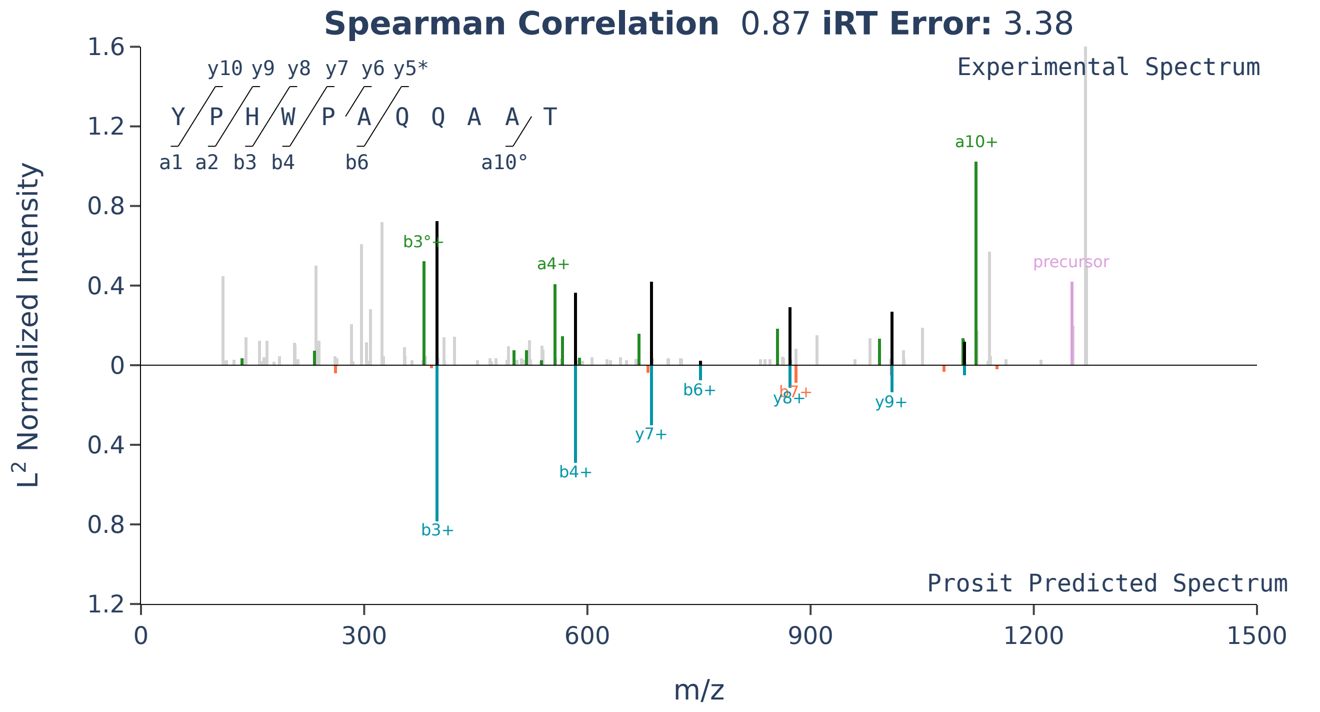

Source HRoetschke_271222_090123_HFGoe_LEDGF_b1R1_1h **Scan** 16231
Peptide YPHWPARVDE **Charge** 1 **Spectral Angle** 0.4
Spearman Correlation 0.63 **iRT Error:** 3.93

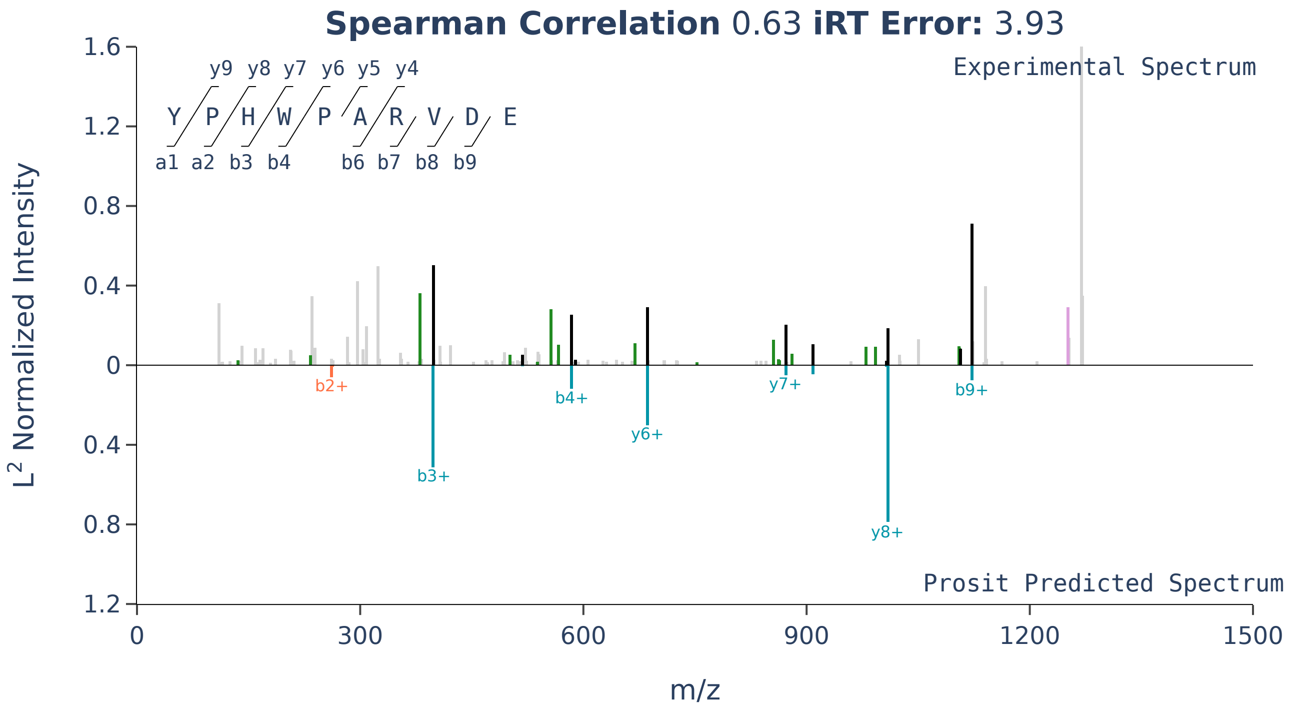

Source HRoetschke_271222_090123_HFGoe_LEDGF_b2R2_24h **Scan** 17536
Peptide IFGPKDIFPYSENKEKYGKPNKR **Charge** 6 **Spectral Angle** 0.81
Spearman Correlation 0.92 **iRT Error:** 3.56

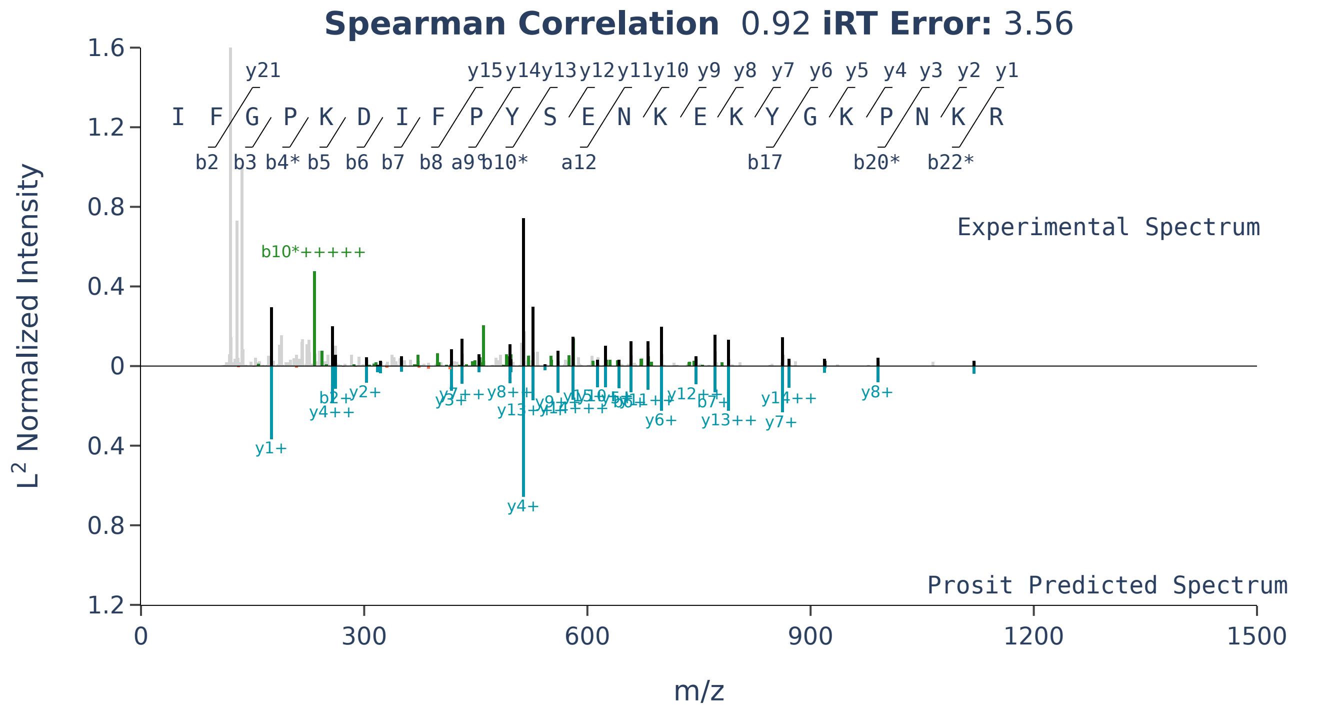

Source HRoetschke_271222_090123_HFGoe_LEDGF_b2R2_24h **Scan** 17536
Peptide FLGPKDIFPYSENKEKYGKPNKR **Charge** 6 **Spectral Angle** 0.81
Spearman Correlation 0.92 **iRT Error:** 3.13

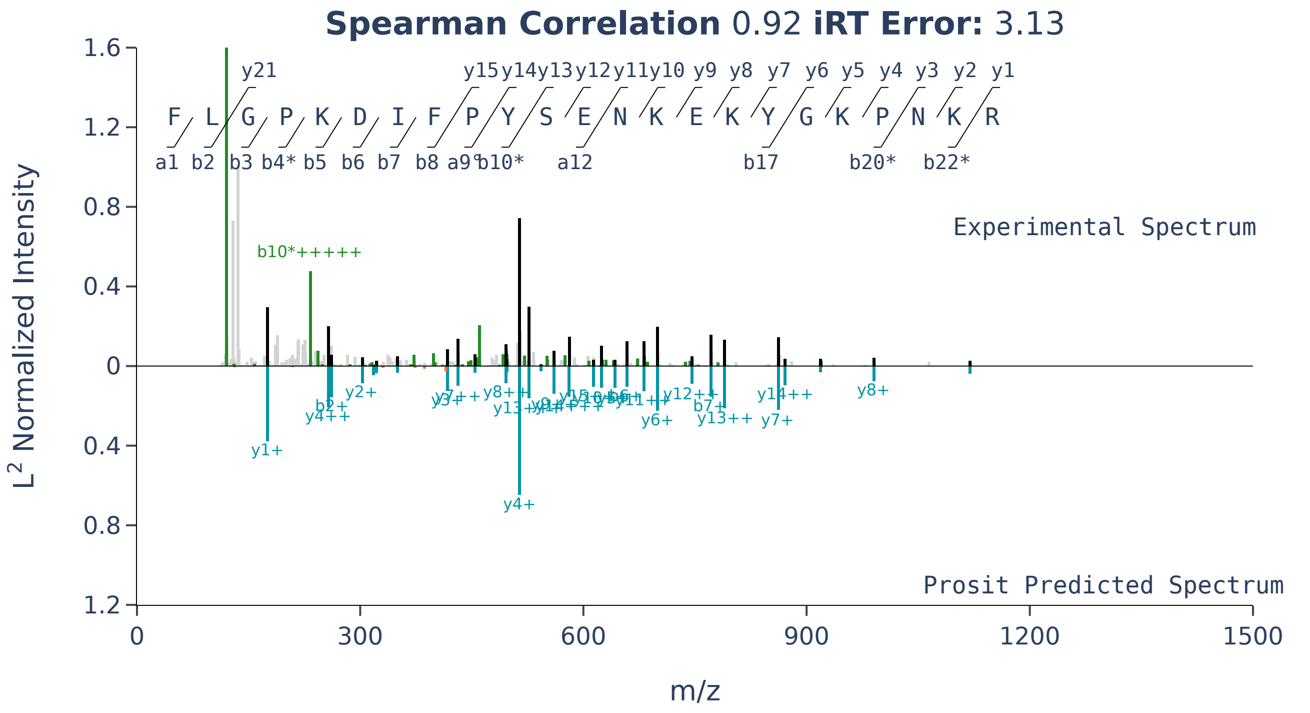

Source HRoetschke_271222_090123_HFGoe_LEDGF_b2R1_4h **Scan** 7512
Peptide TNVIMEKS **Charge** 2 **Spectral Angle** 0.81
Spearman Correlation 0.93 **iRT Error:** 3.76

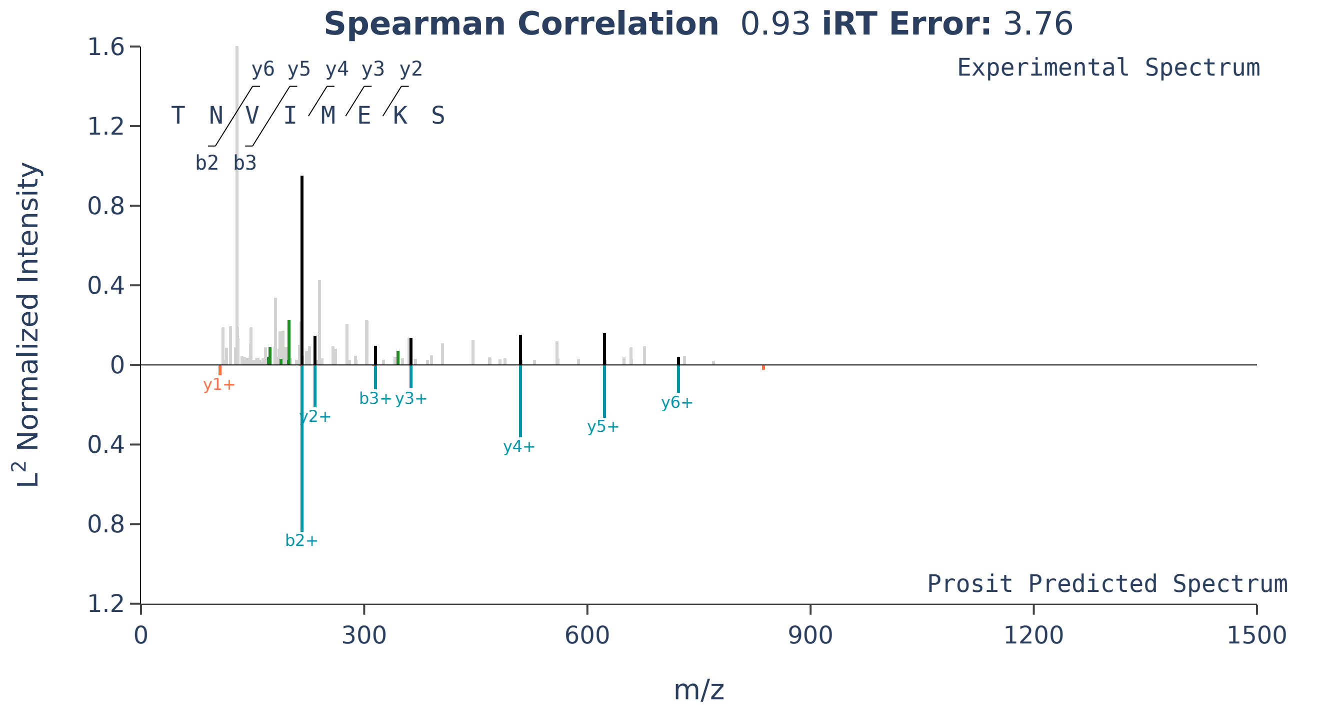

Source HRoetschke_271222_090123_HFGoe_LEDGF_b2R1_4h **Scan** 7512
Peptide SQVIMEKS **Charge** 2 **Spectral Angle** 0.82
Spearman Correlation 0.86 **iRT Error:** 34.07

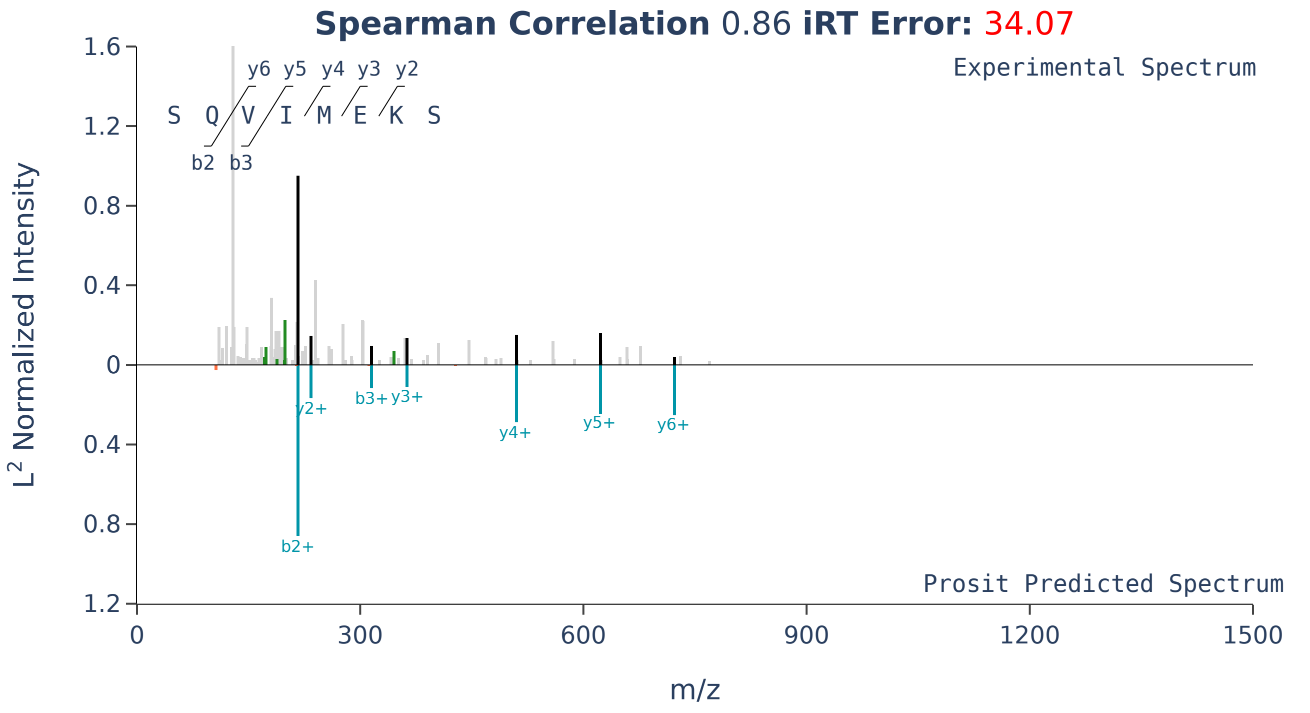

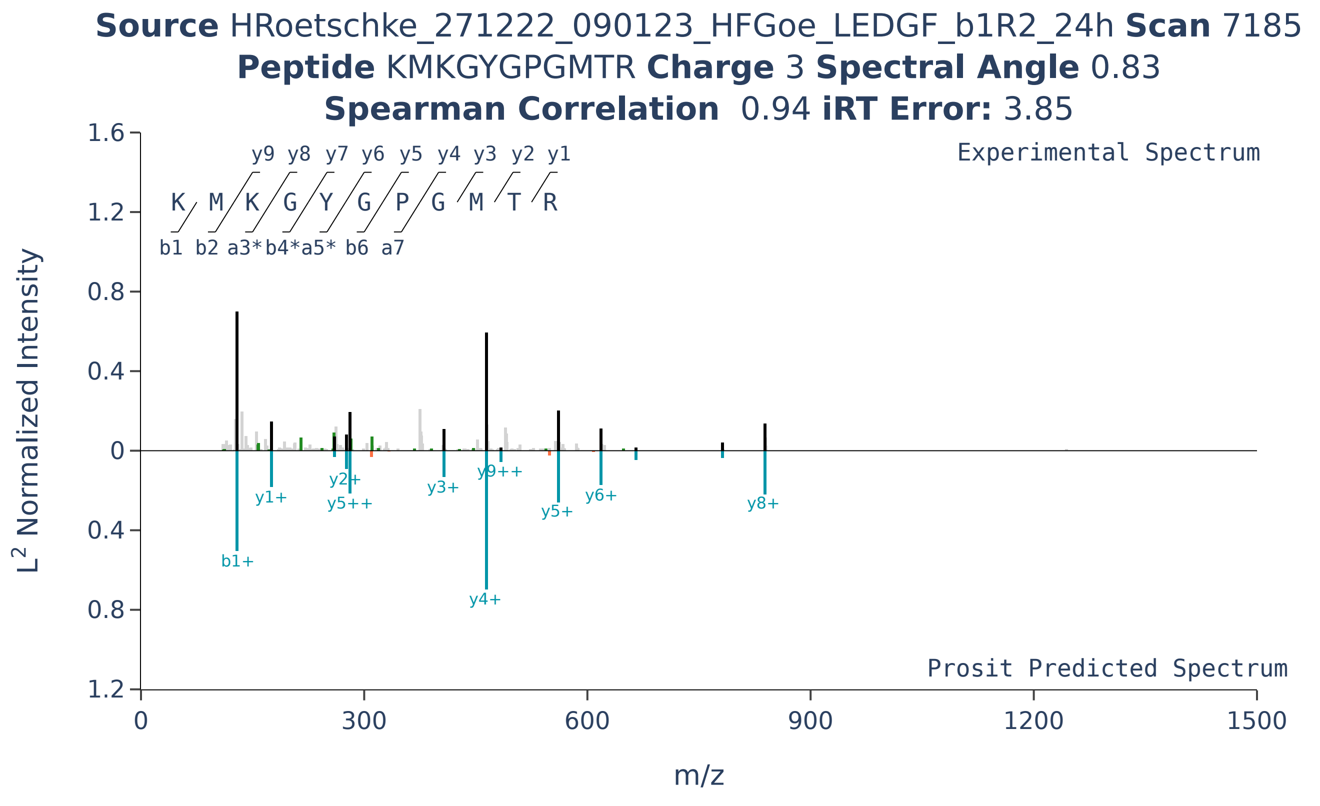
Source HRoetschke_271222_090123_HFGoe_LEDGF_b1R2_24h **Scan** 7185
Peptide KMKGYGPGMTR **Charge** 3 **Spectral Angle** 0.83
Spearman Correlation 0.94 **iRT Error:** 3.85
Experimental Spectrum
Prosit Predicted Spectrum
L² Normalized Intensity
m/z

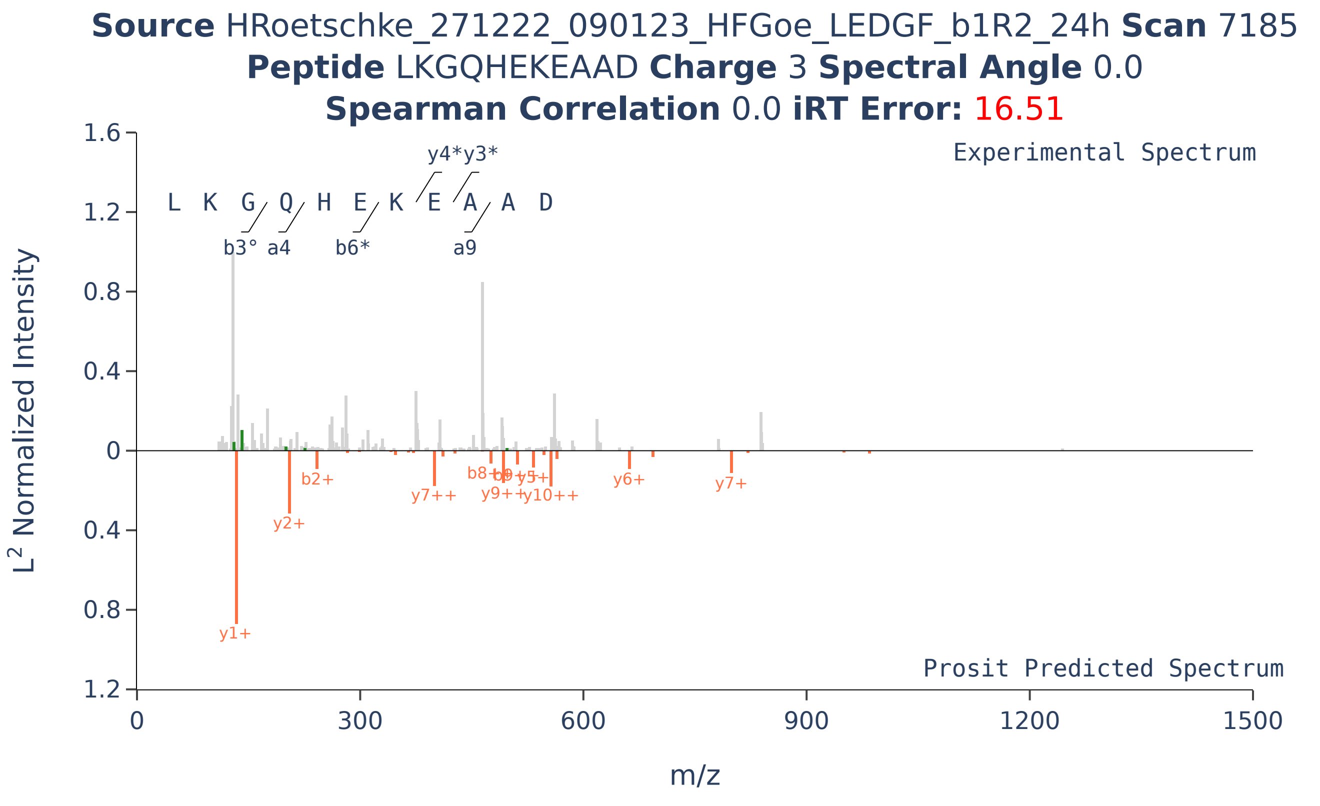
Source HRoetschke_271222_090123_HFGoe_LEDGF_b1R2_24h **Scan** 7185
Peptide LKGQHEKEAAD **Charge** 3 **Spectral Angle** 0.0
Spearman Correlation 0.0 **iRT Error:** 16.51
Experimental Spectrum
Prosit Predicted Spectrum
L² Normalized Intensity
m/z

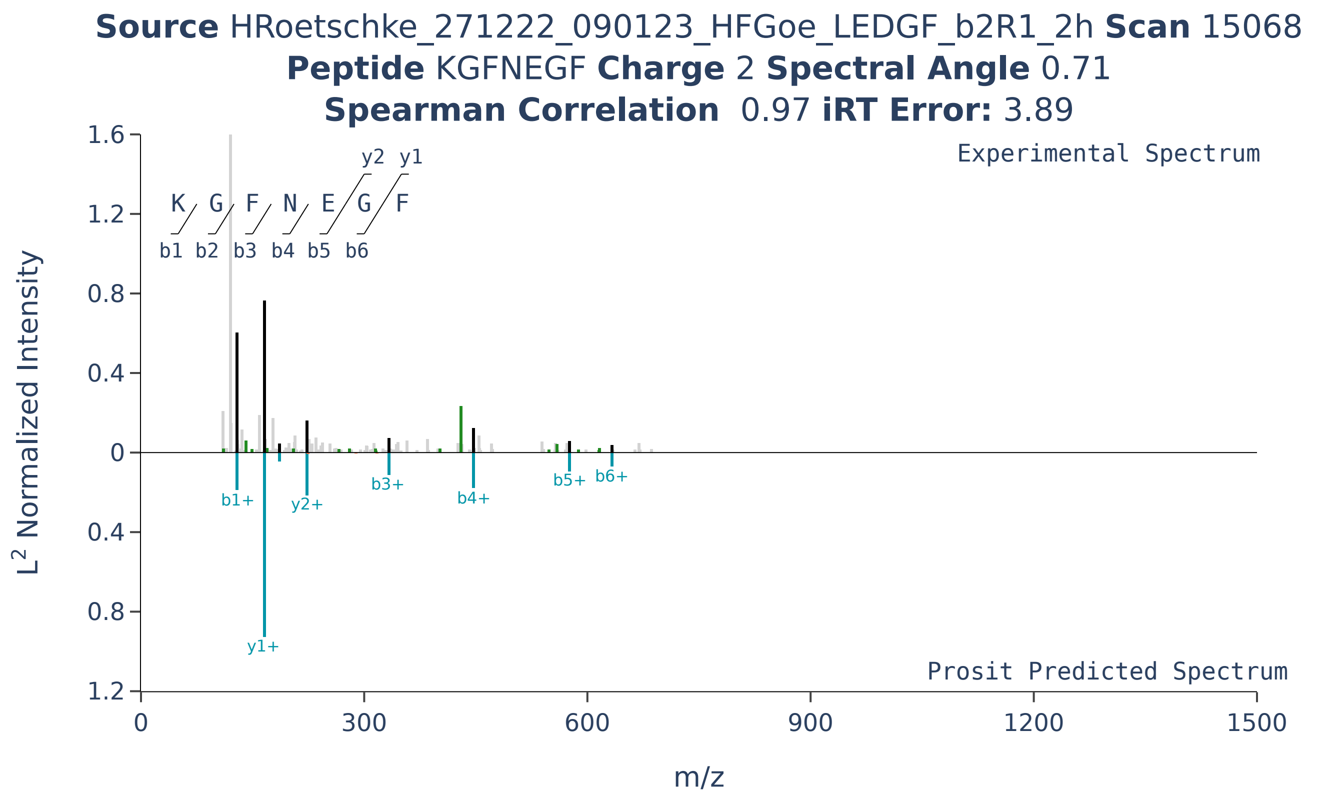
Source HRoetschke_271222_090123_HFGoe_LEDGF_b2R1_2h **Scan** 15068
Peptide KGFNEGF **Charge** 2 **Spectral Angle** 0.71
Spearman Correlation 0.97 **iRT Error:** 3.89
Experimental Spectrum
Prosit Predicted Spectrum
L² Normalized Intensity
m/z

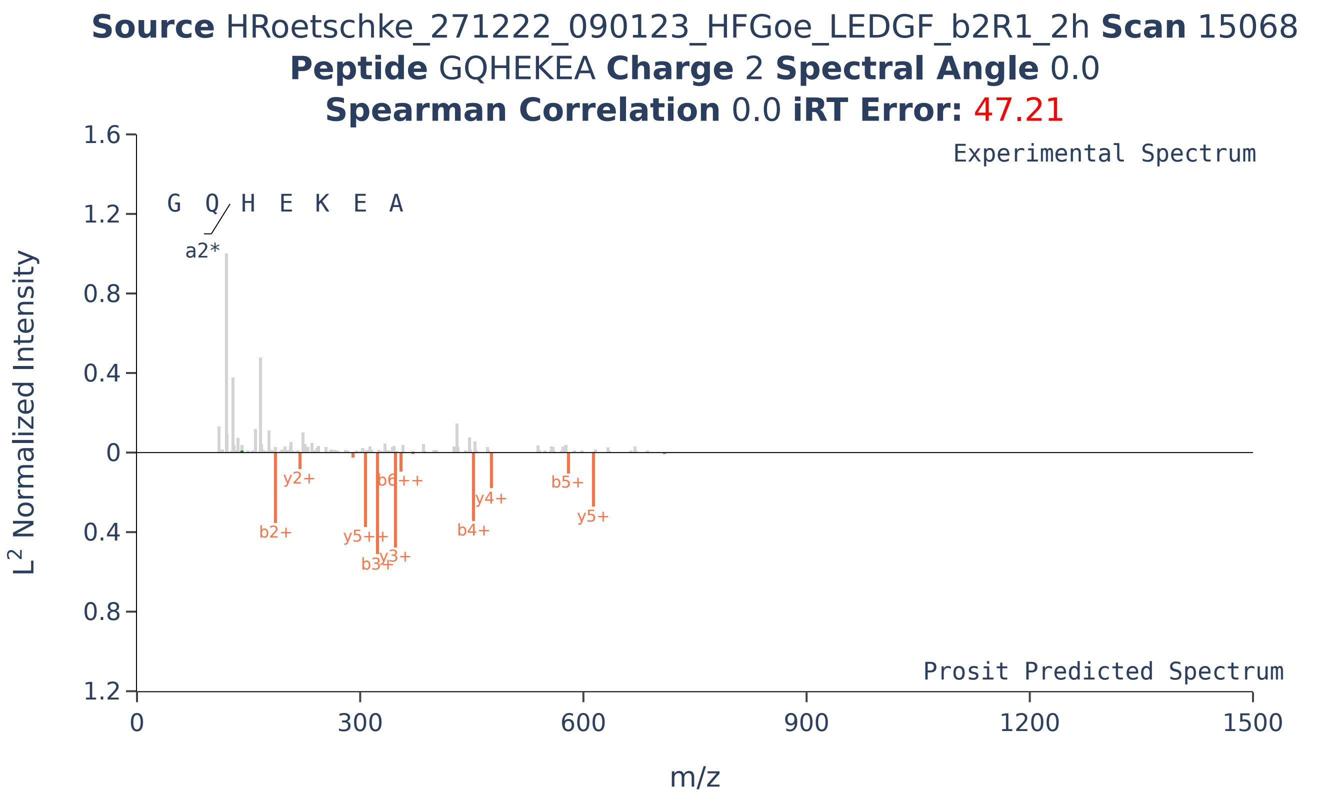
Source HRoetschke_271222_090123_HFGoe_LEDGF_b2R1_2h **Scan** 15068
Peptide GQHEKEA **Charge** 2 **Spectral Angle** 0.0
Spearman Correlation 0.0 **iRT Error:** 47.21
Experimental Spectrum
Prosit Predicted Spectrum
L² Normalized Intensity
m/z

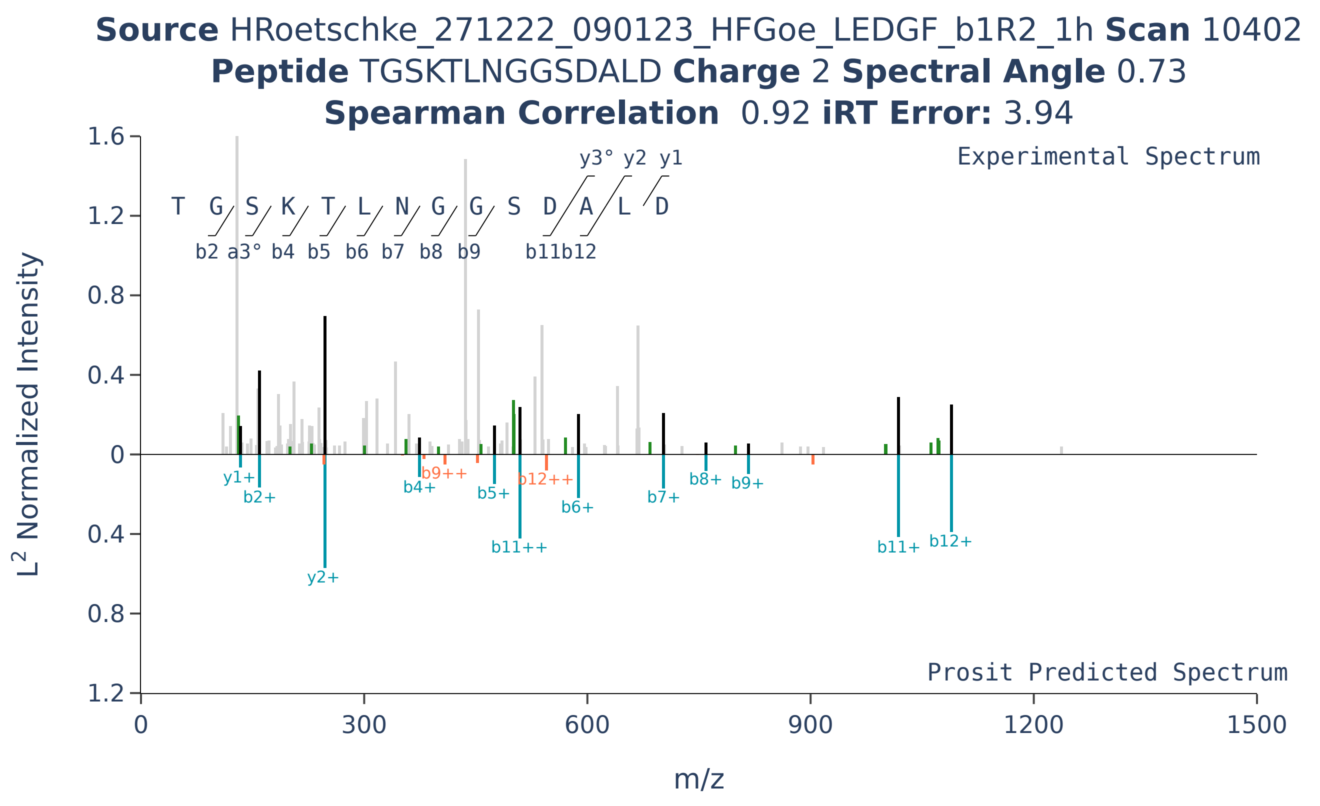
Source HRoetschke_271222_090123_HFGoe_LEDGF_b1R2_1h **Scan** 10402
Peptide TGSKTLNGGSDALD **Charge** 2 **Spectral Angle** 0.73
Spearman Correlation 0.92 **iRT Error:** 3.94
Experimental Spectrum
Prosit Predicted Spectrum
L² Normalized Intensity
m/z

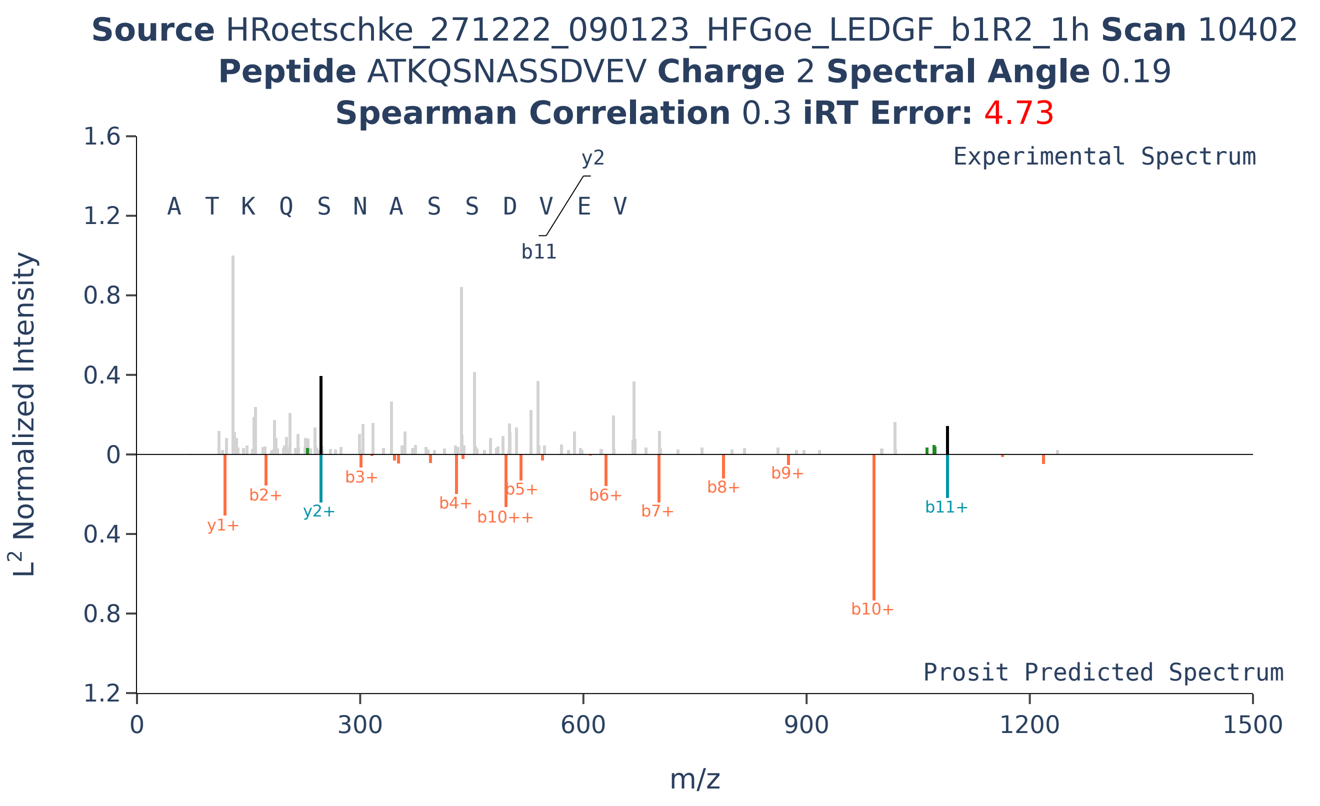
Source HRoetschke_271222_090123_HFGoe_LEDGF_b1R2_1h **Scan** 10402
Peptide ATKQSNASSDVEV **Charge** 2 **Spectral Angle** 0.19
Spearman Correlation 0.3 **iRT Error:** 4.73
Experimental Spectrum
Prosit Predicted Spectrum
L² Normalized Intensity
m/z

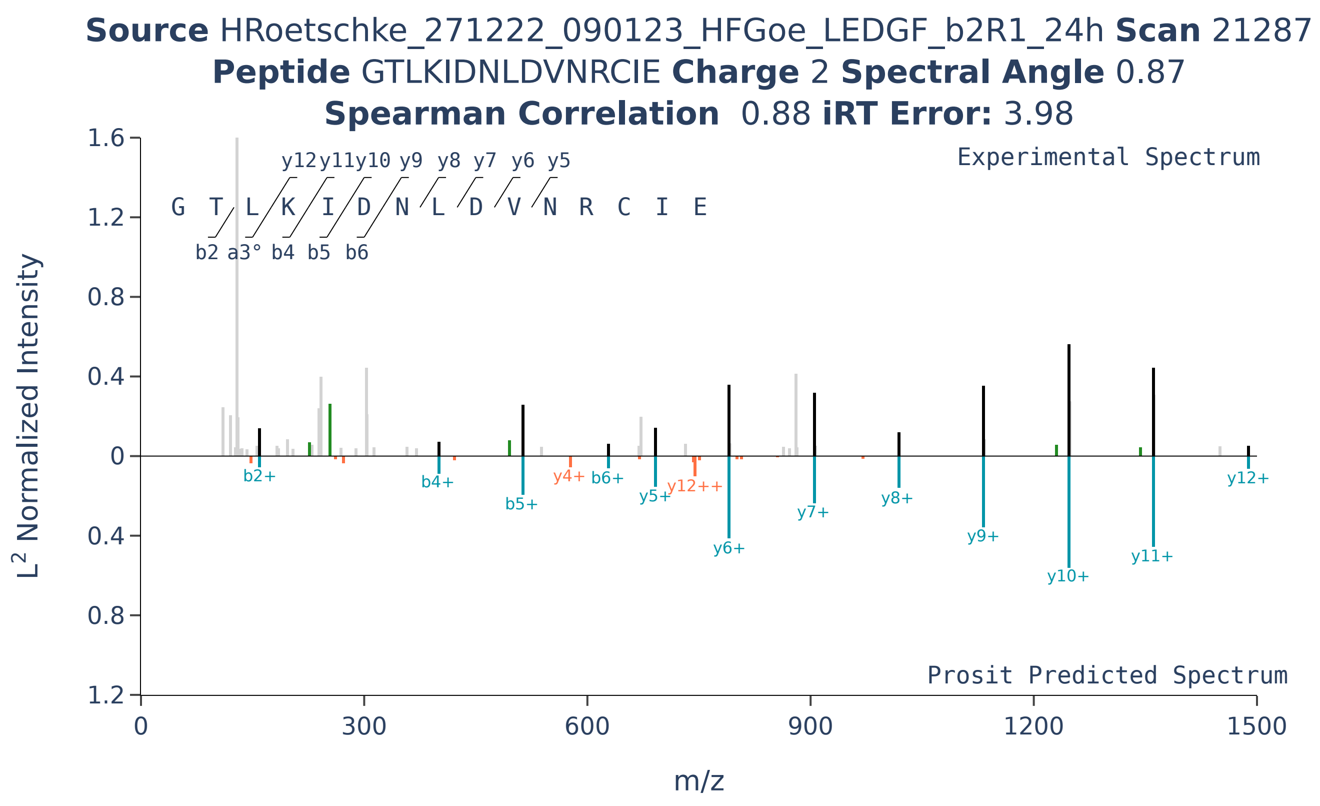
Source HRoetschke_271222_090123_HFGoe_LEDGF_b2R1_24h **Scan** 21287
Peptide GTLKIDNLDVNRCIE **Charge** 2 **Spectral Angle** 0.87
Spearman Correlation 0.88 **iRT Error:** 3.98
Experimental Spectrum
Prosit Predicted Spectrum
L² Normalized Intensity
m/z

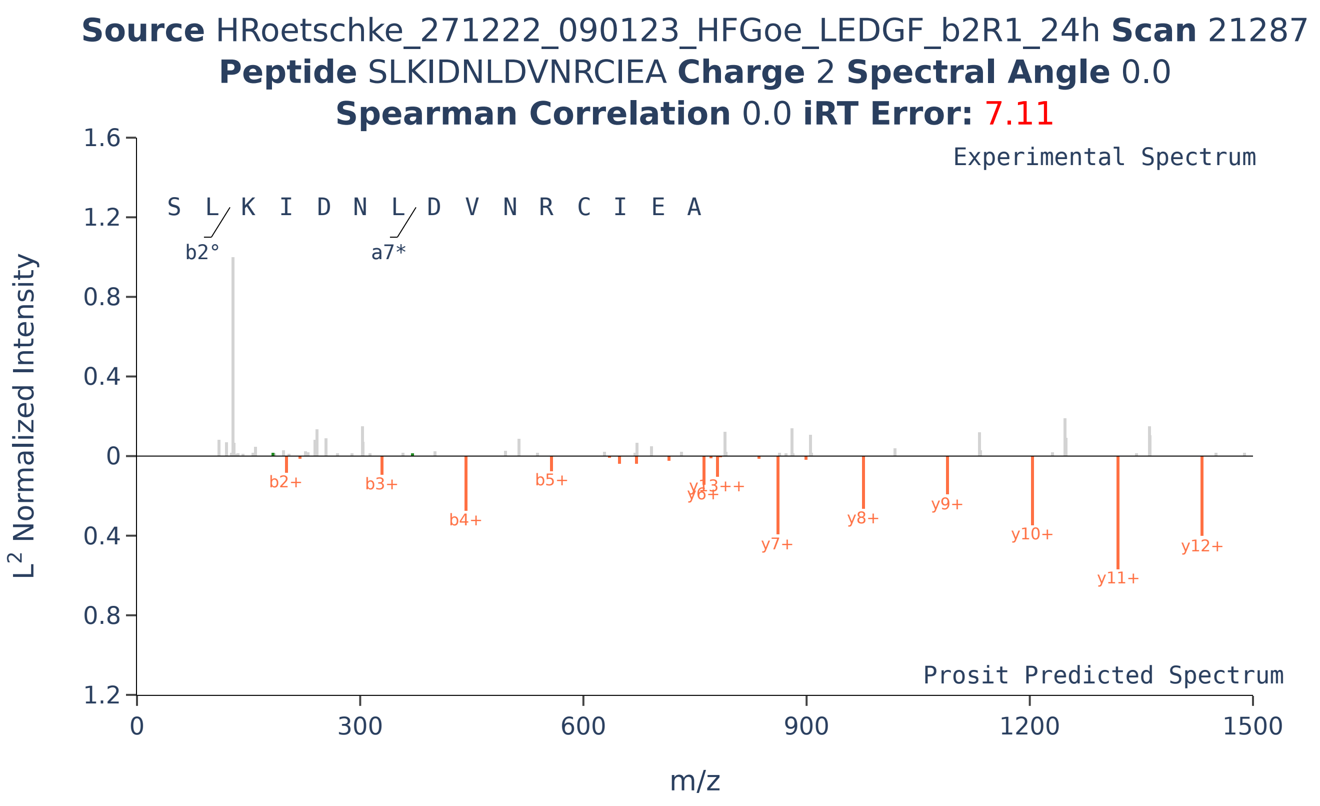
Source HRoetschke_271222_090123_HFGoe_LEDGF_b2R1_24h **Scan** 21287
Peptide SLKIDNLDVNRCIEA **Charge** 2 **Spectral Angle** 0.0
Spearman Correlation 0.0 **iRT Error:** 7.11
Experimental Spectrum
Prosit Predicted Spectrum
L² Normalized Intensity
m/z

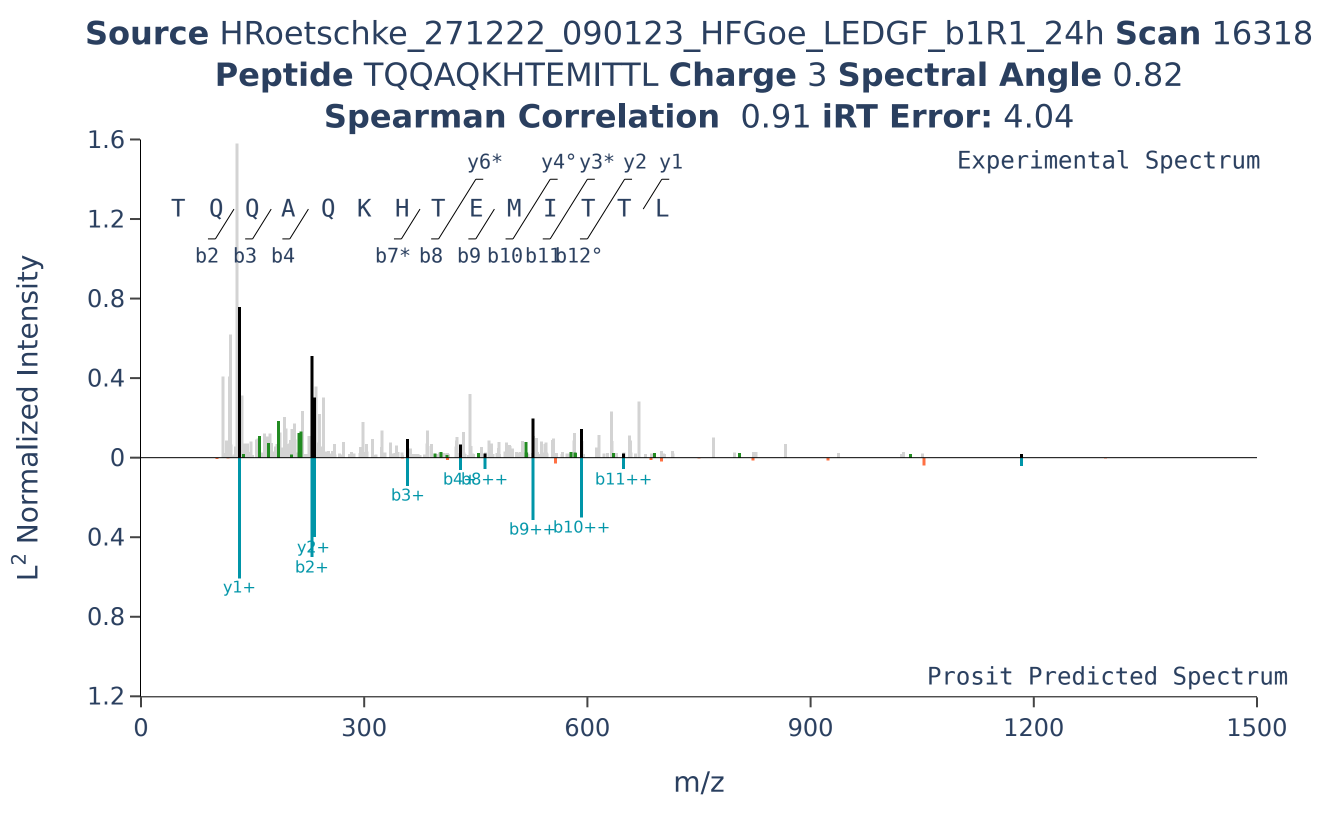
Source HRoetschke_271222_090123_HFGoe_LEDGF_b1R1_24h **Scan** 16318
Peptide TQQAQKHTEMITTL **Charge** 3 **Spectral Angle** 0.82
Spearman Correlation 0.91 **iRT Error:** 4.04
Experimental Spectrum
Prosit Predicted Spectrum
L² Normalized Intensity
m/z

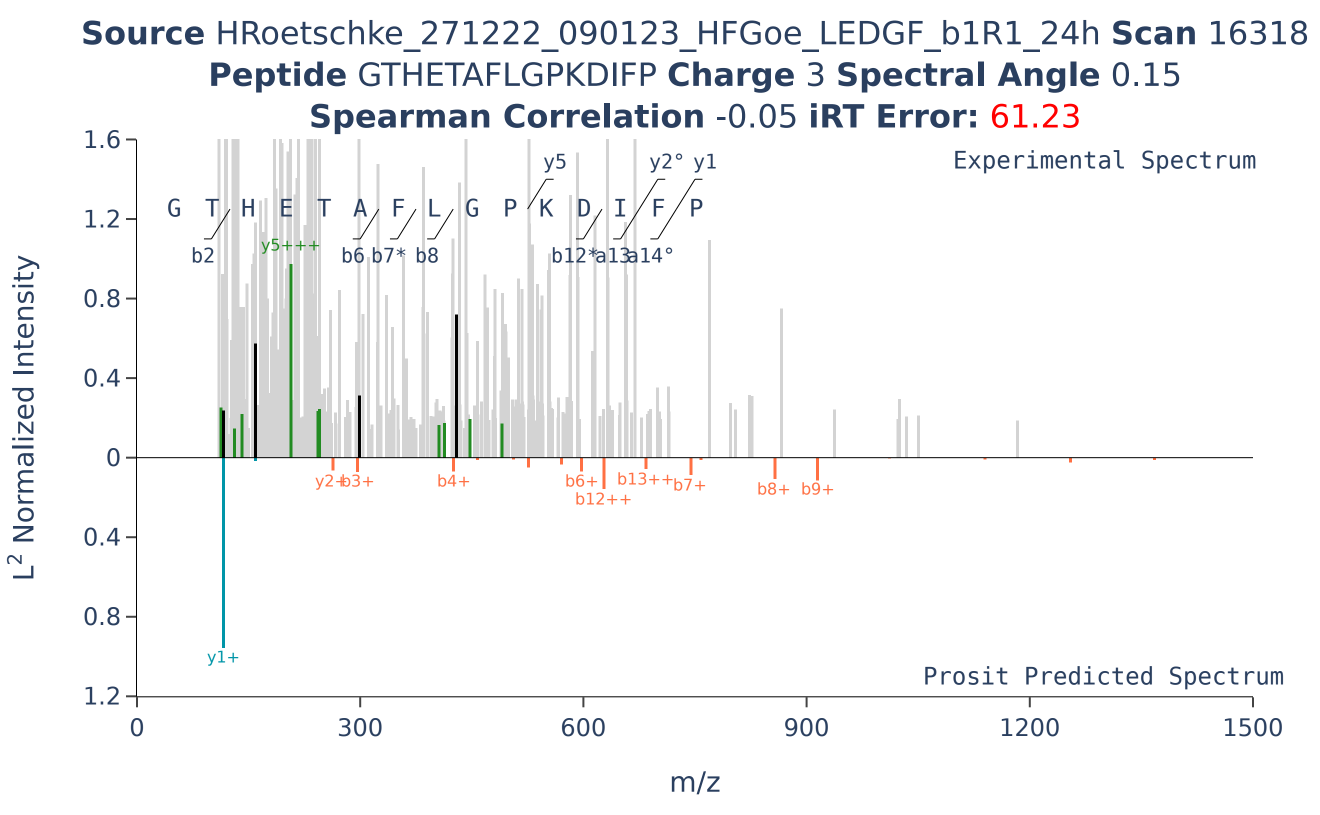
Source HRoetschke_271222_090123_HFGoe_LEDGF_b1R1_24h **Scan** 16318
Peptide GTHETAFLGPKDIFP **Charge** 3 **Spectral Angle** 0.15
Spearman Correlation -0.05 **iRT Error:** 61.23
Experimental Spectrum
Prosit Predicted Spectrum
L² Normalized Intensity
m/z

Source HRoetschke_271222_090123_HFGoe_LEDGF_b1R1_2h **Scan** 19202
Peptide TAFLGPKDIFPYSENKEKYGKPNKRLE **Charge** 5 **Spectral Angle** 0.81
Spearman Correlation 0.91 **iRT Error:** 4.13

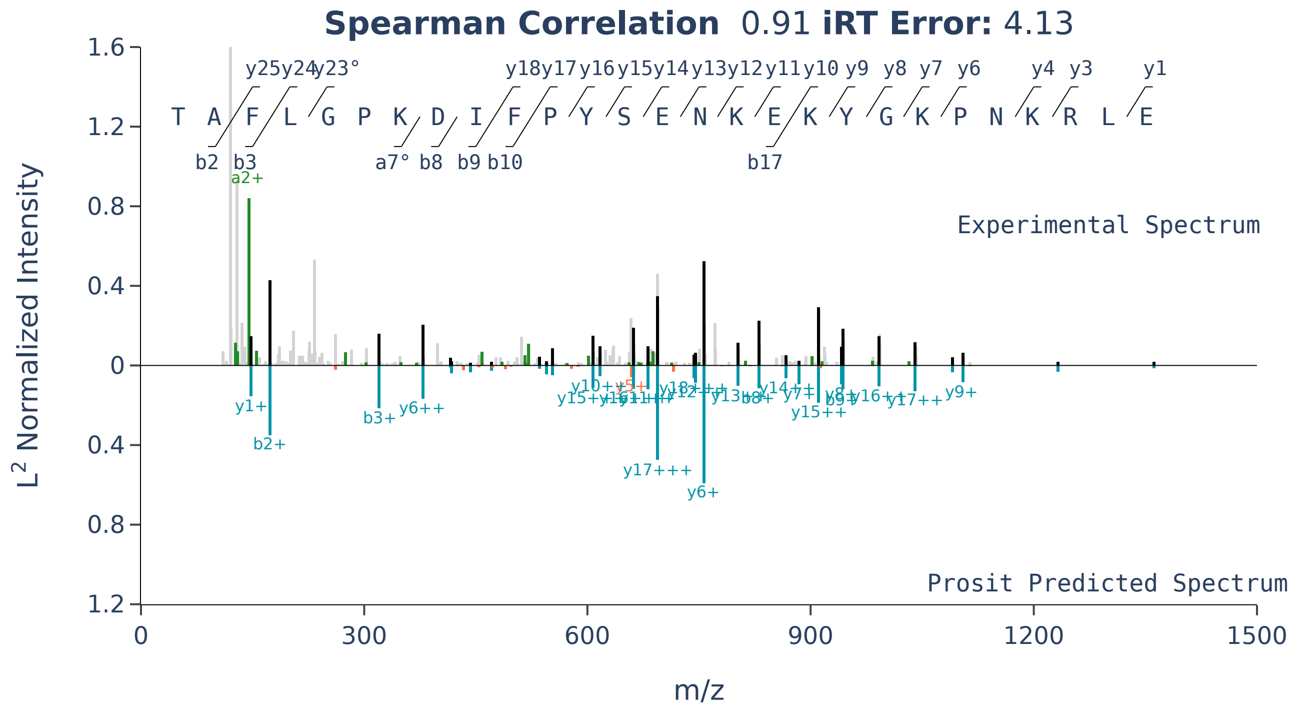

Source HRoetschke_271222_090123_HFGoe_LEDGF_b1R1_2h **Scan** 19202
Peptide ITEEDKSKKKGQEEKQPKKQPKKDEEG **Charge** 5 **Spectral Angle** 0.09
Spearman Correlation 0.21 **iRT Error:** 102.38

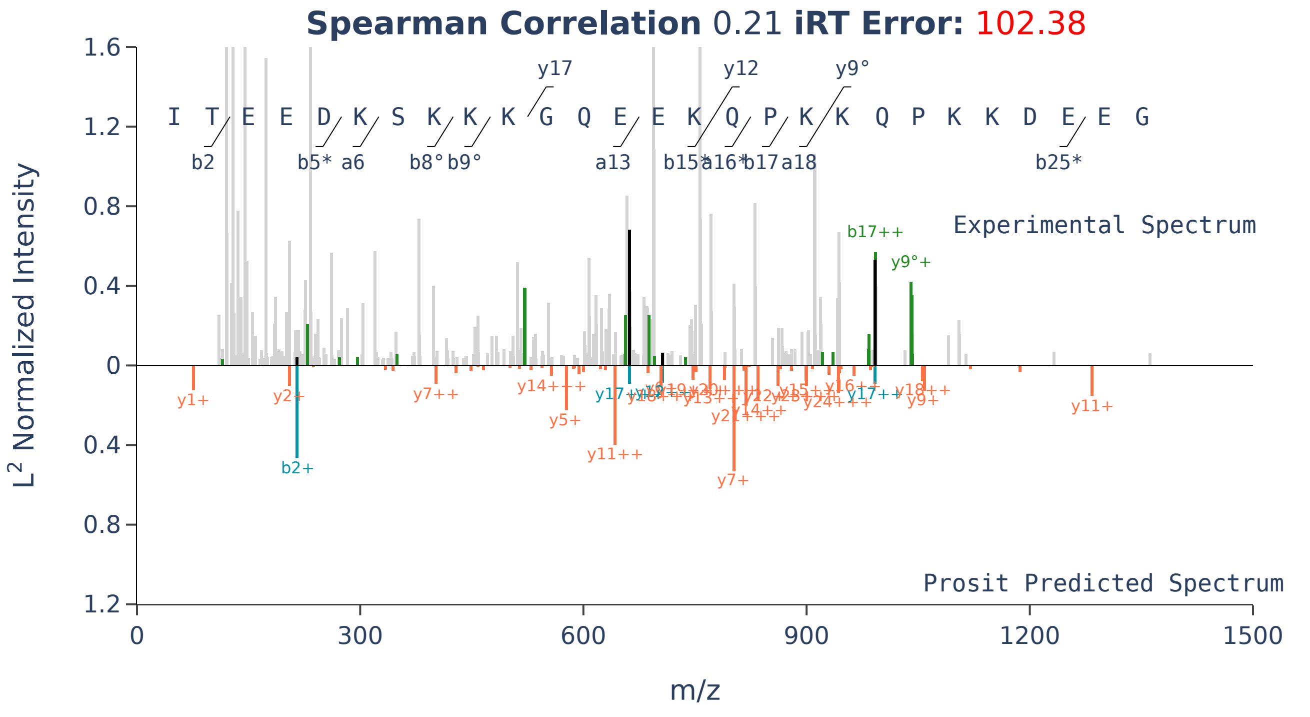

Source HRoetschke_271222_090123_HFGoe_LEDGF_b1R2_2h **Scan** 17307
Peptide AFLGPKDIFPYSENKEKYGKPNKRKFG **Charge** 5 **Spectral Angle** 0.83
Spearman Correlation 0.89 **iRT Error:** 4.21

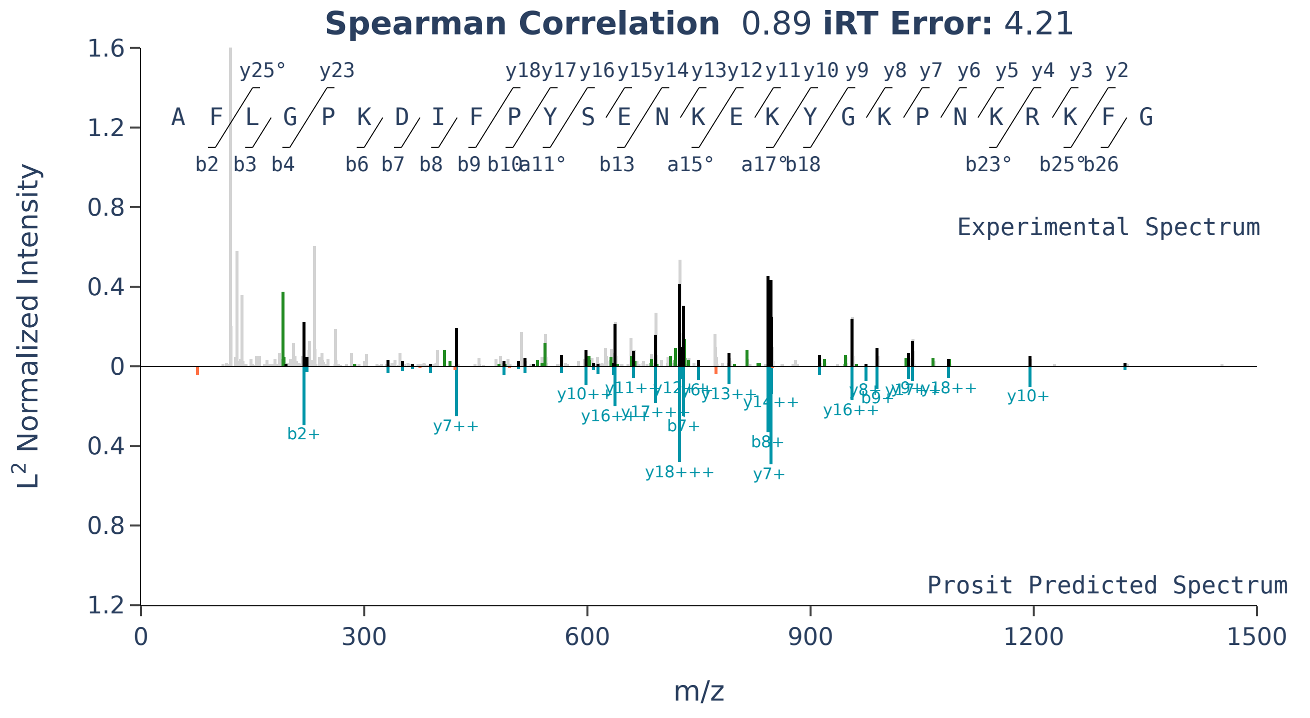

Source HRoetschke_271222_090123_HFGoe_LEDGF_b1R2_2h **Scan** 17307
Peptide AFLGPKDIFPYSENKEKYGKPNKRKGF **Charge** 5 **Spectral Angle** 0.81
Spearman Correlation 0.9 **iRT Error:** 7.27

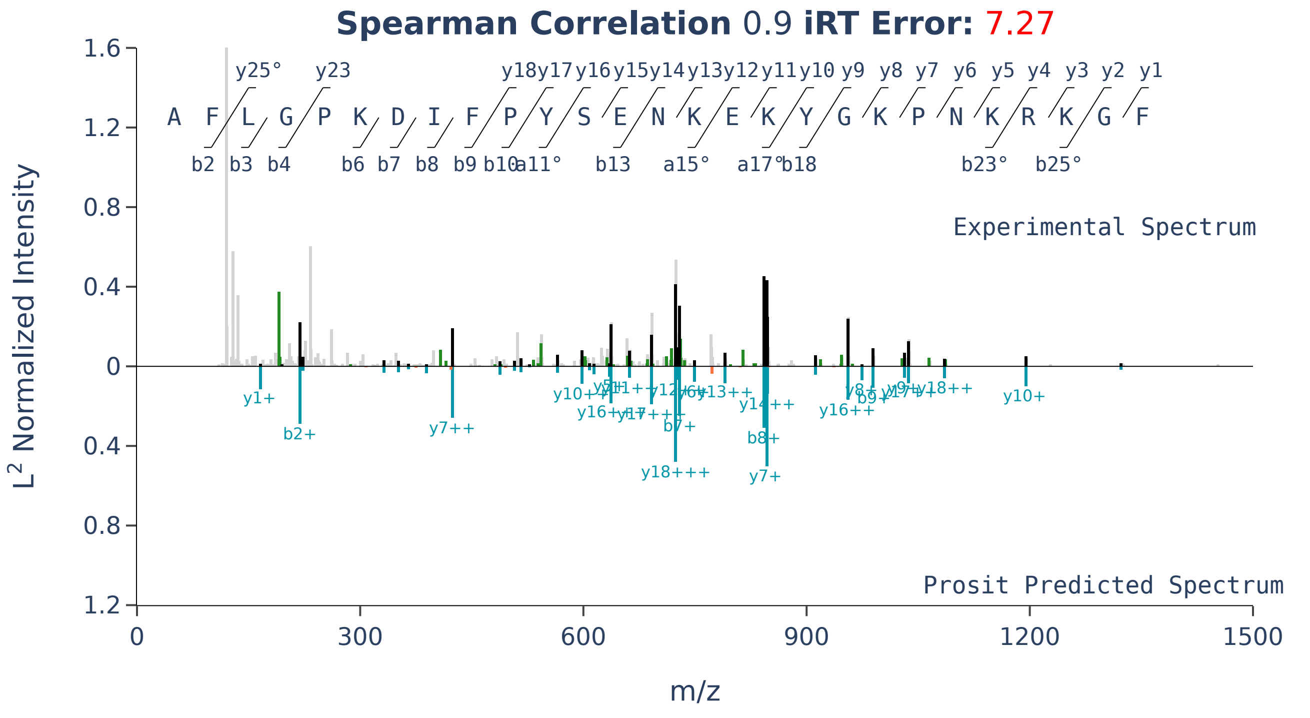

Source HRoetschke_271222_090123_HFGoe_LEDGF_b1R1_24h **Scan** 16523
Peptide NLVTTATASVNLKVSPKRGRPAATEV **Charge** 5 **Spectral Angle** 0.8
Spearman Correlation 0.89 **iRT Error:** 4.27

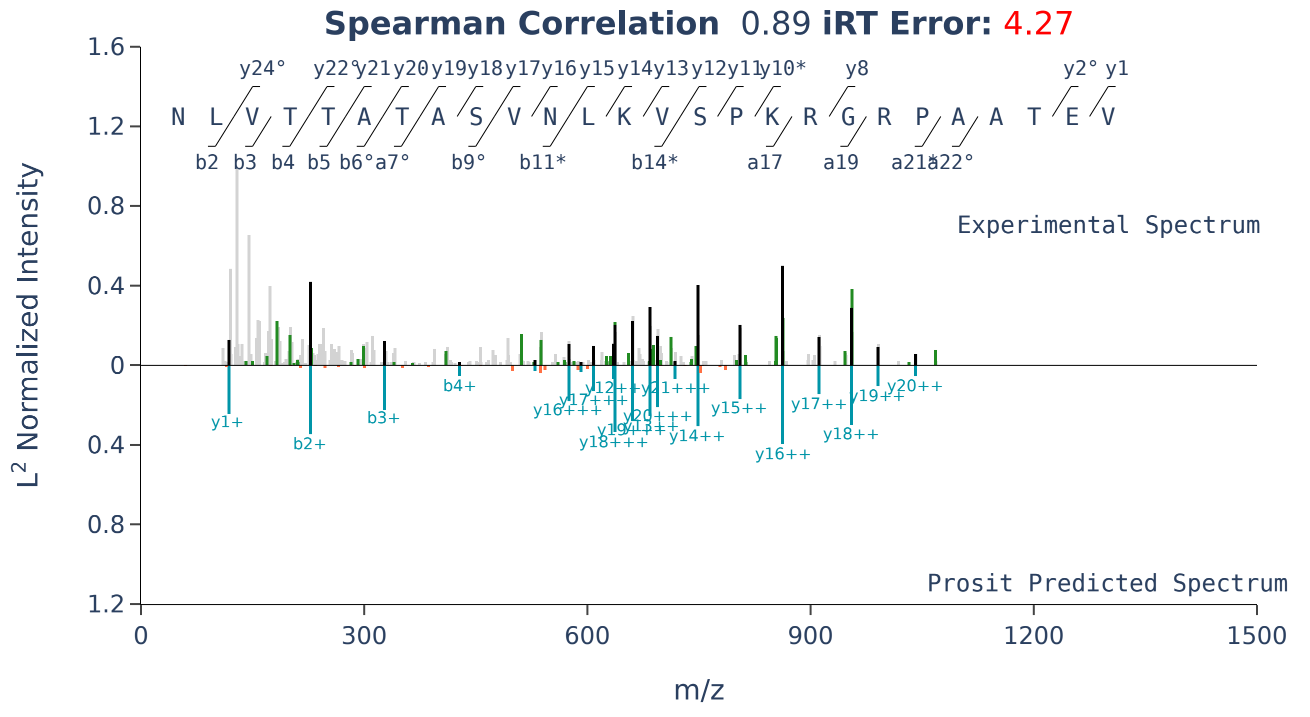

Source HRoetschke_271222_090123_HFGoe_LEDGF_b1R1_24h **Scan** 16523
Peptide AGVVTTATASVNLKVSPKRGRPAATEV **Charge** 5 **Spectral Angle** 0.78
Spearman Correlation 0.86 **iRT Error:** 3.73

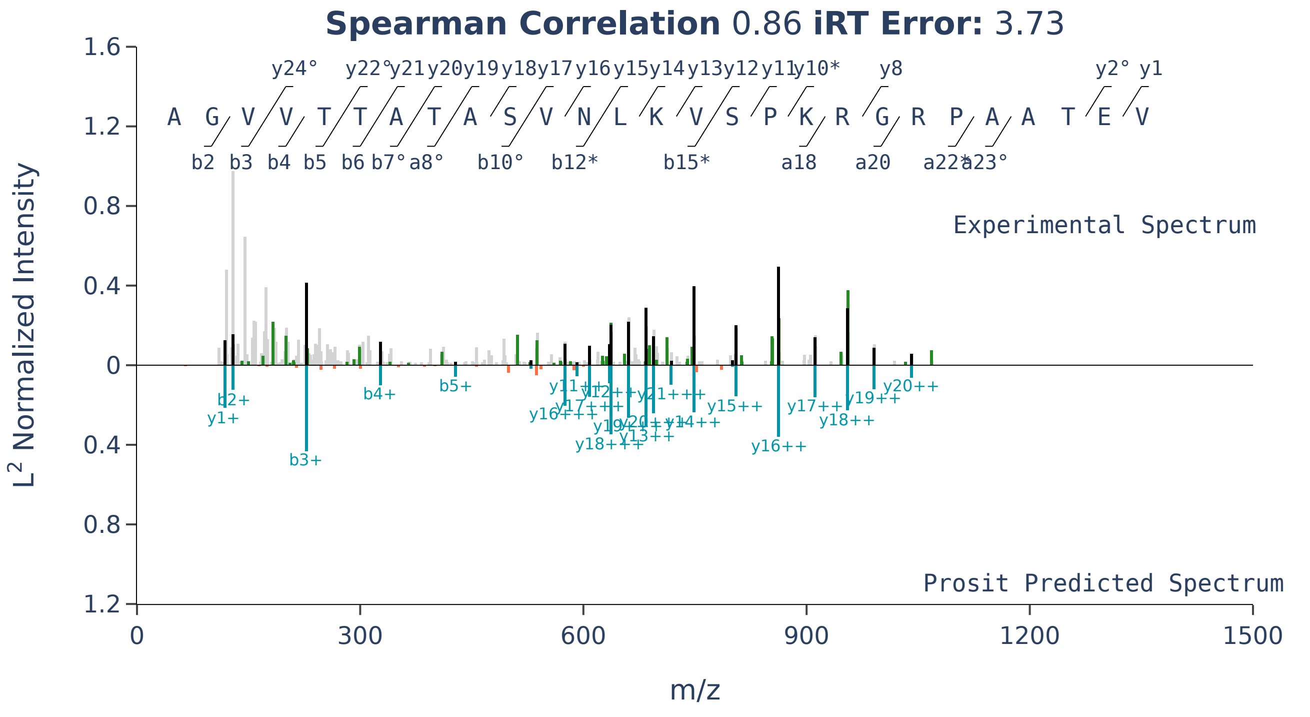

Source HRoetschke_271222_090123_HFGoe_LEDGF_b2R2_24h **Scan** 5941
Peptide FPYSENKEKYGKPNKKR **Charge** 3 **Spectral Angle** 0.83
Spearman Correlation 0.86 **iRT Error:** 4.52

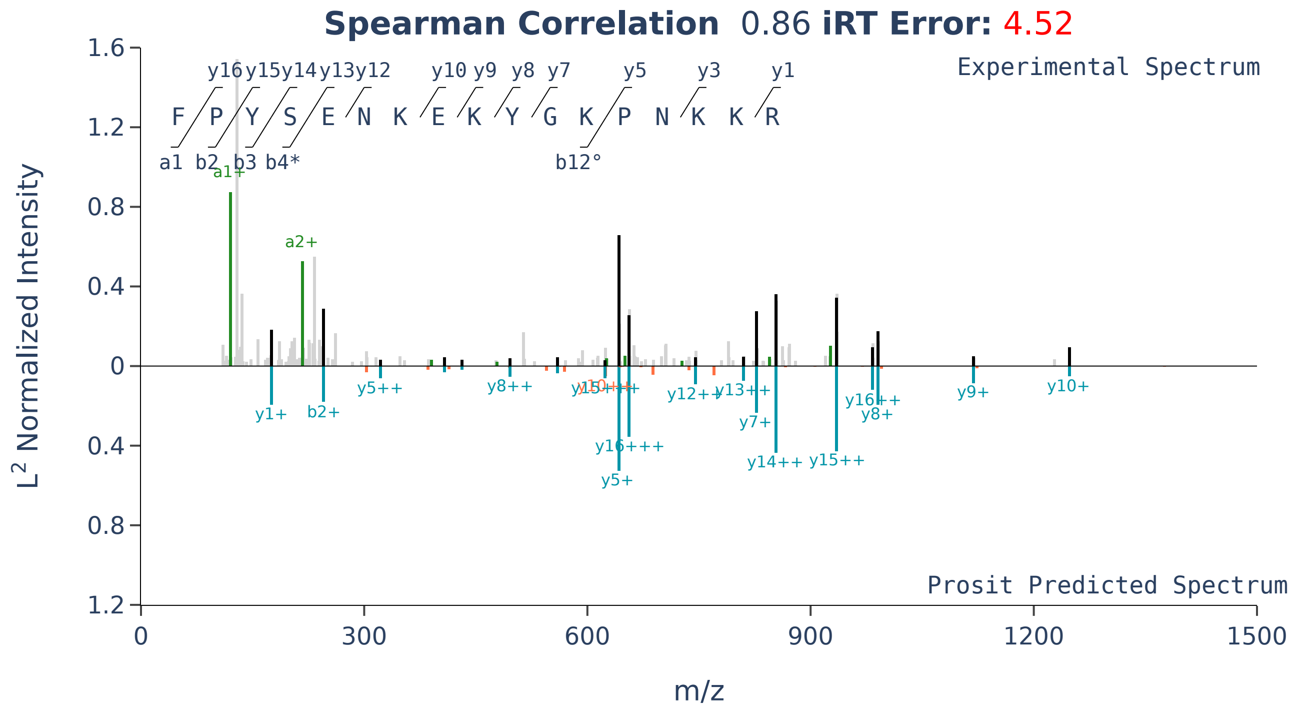

Source HRoetschke_271222_090123_HFGoe_LEDGF_b2R2_24h **Scan** 5941
Peptide FPYSENKEKYGKPNKRK **Charge** 3 **Spectral Angle** 0.74
Spearman Correlation 0.73 **iRT Error:** 5.2

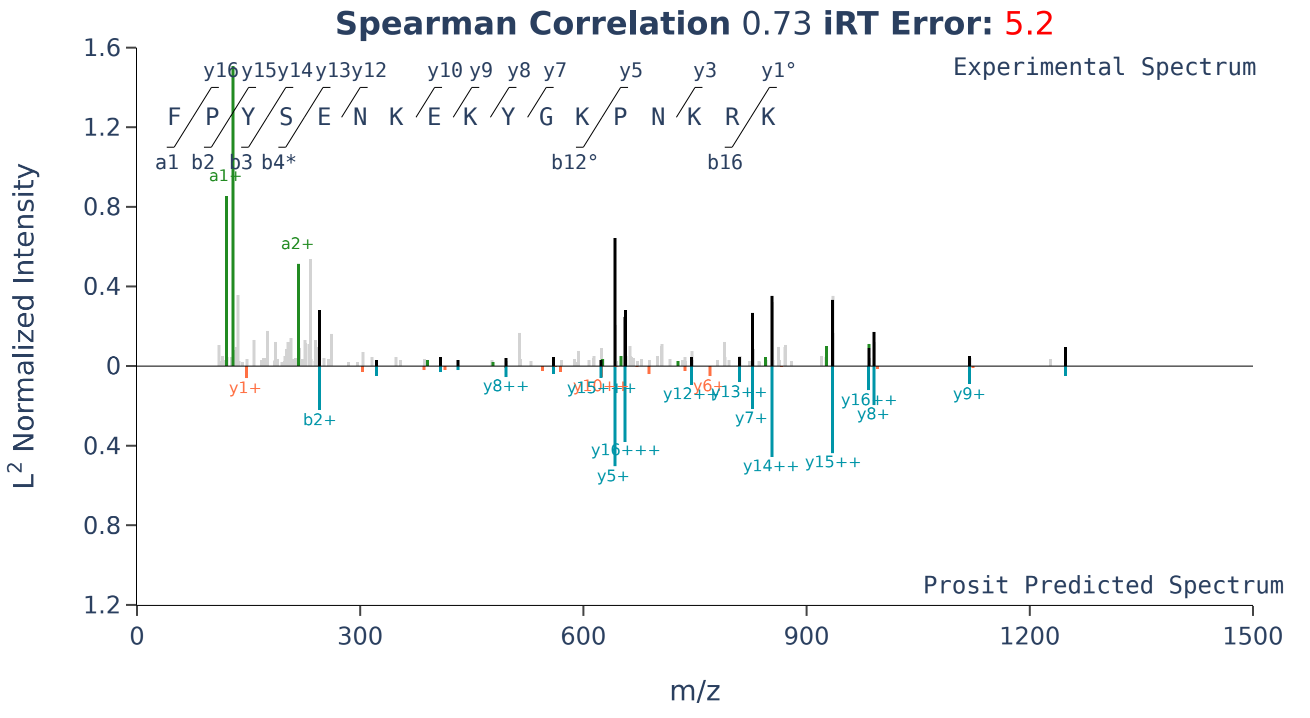

Source HRoetschke_271222_090123_HFGoe_LEDGF_b1R1_4h **Scan** 24843
Peptide NPMTRDFKPGDLIFAKMKGY **Charge** 3 **Spectral Angle** 0.82
Spearman Correlation 0.88 **iRT Error:** 4.64

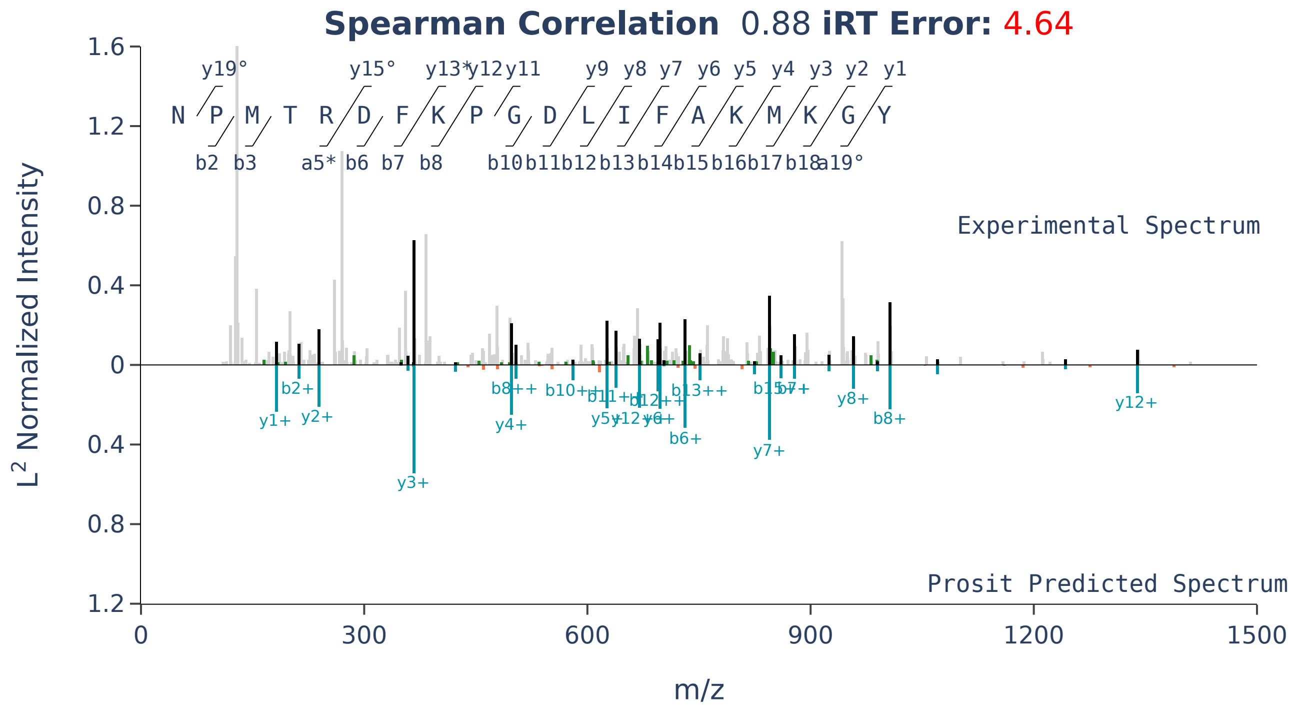

Source HRoetschke_271222_090123_HFGoe_LEDGF_b1R1_4h **Scan** 24843
Peptide GPGMTRDFKPGDLIFAKMKGY **Charge** 3 **Spectral Angle** 0.25
Spearman Correlation 0.56 **iRT Error:** 18.12

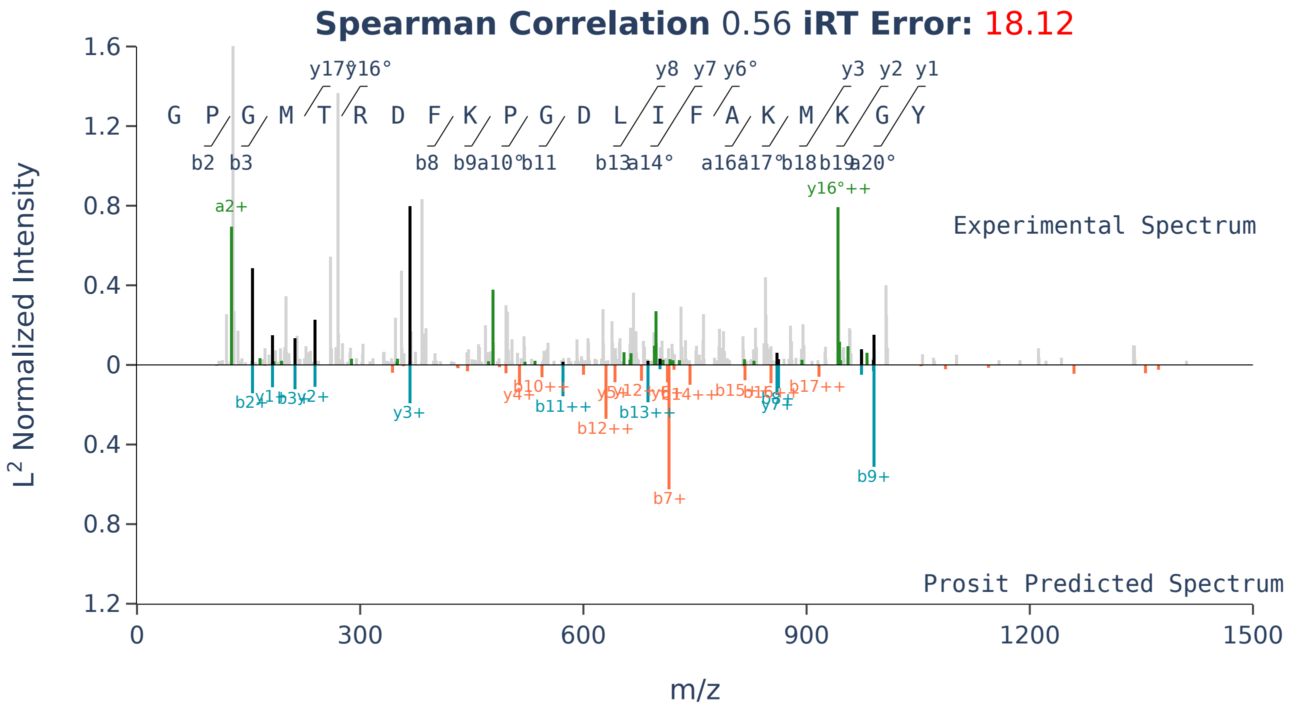

Source HRoetschke_271222_090123_HFGoe_LEDGF_b2R1_4h **Scan** 13184
Peptide GPHWPARVDE **Charge** 2 **Spectral Angle** 0.87
Spearman Correlation 0.87 **iRT Error:** 4.76

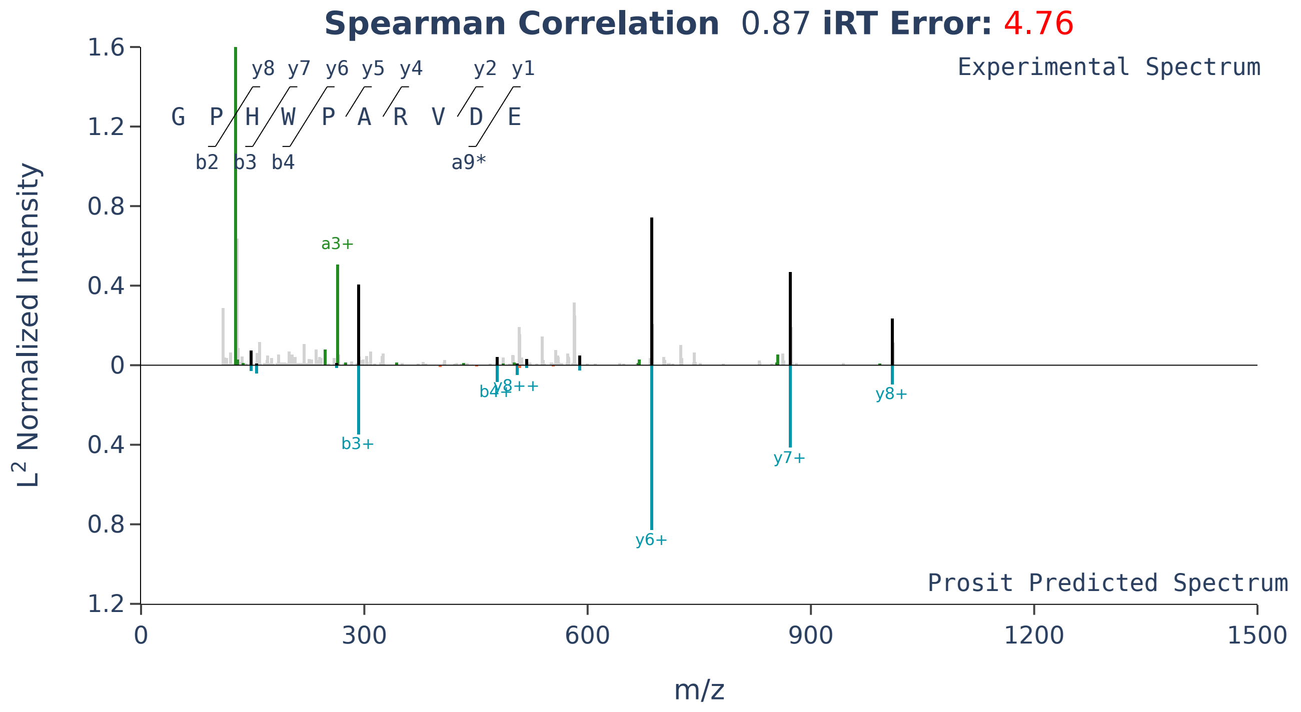

Source HRoetschke_271222_090123_HFGoe_LEDGF_b2R1_4h **Scan** 13184
Peptide KQSNASSDVEV **Charge** 2 **Spectral Angle** 0.12
Spearman Correlation 0.26 **iRT Error:** 19.97

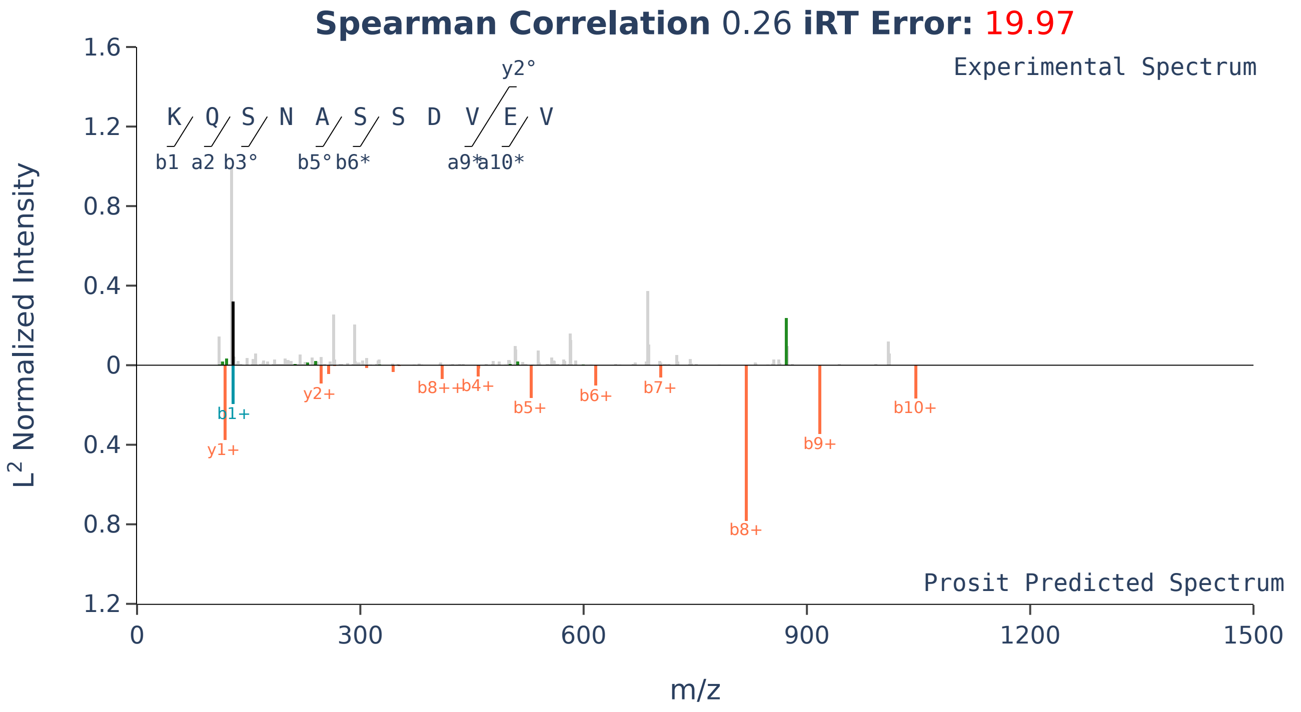

Source HRoetschke_271222_090123_HFGoe_LEDGF_b1R1_4h **Scan** 21483
Peptide NPMTRDFKPGDLIFAKMK **Charge** 3 **Spectral Angle** 0.74
Spearman Correlation 0.91 **iRT Error:** 4.77

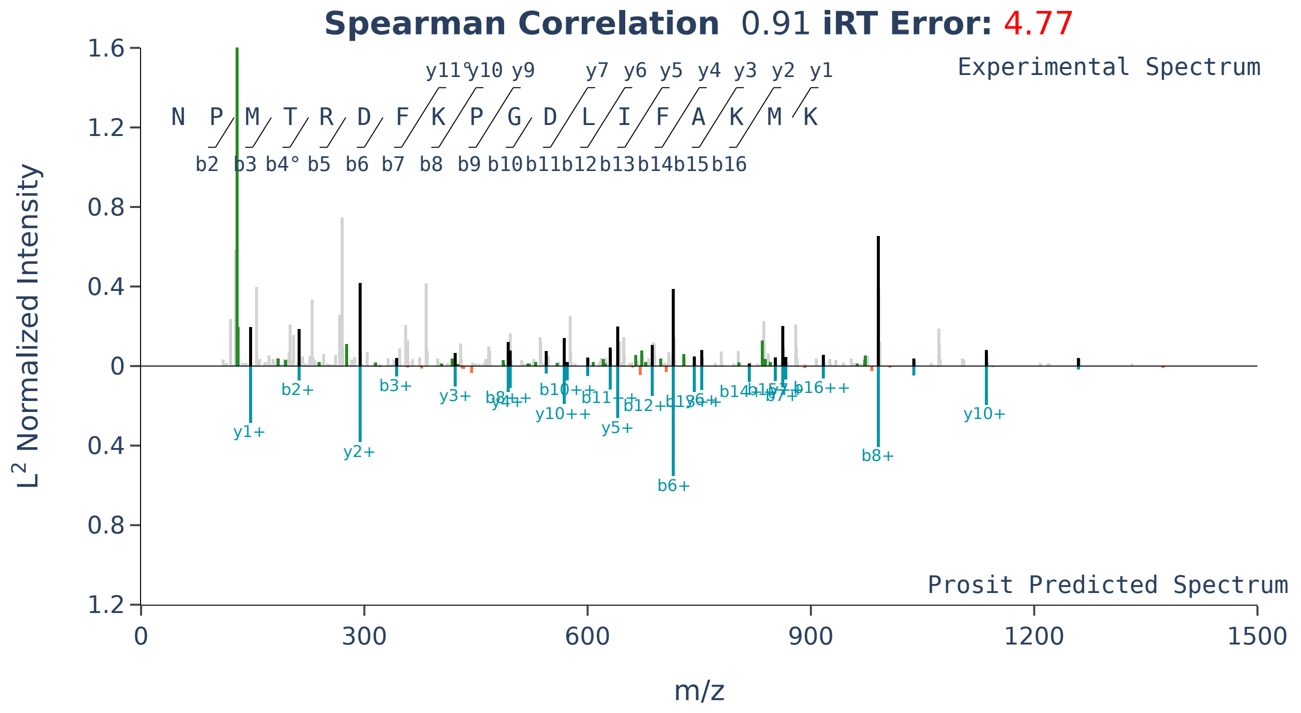

Source HRoetschke_271222_090123_HFGoe_LEDGF_b1R1_4h **Scan** 21483
Peptide PGMTRDFKPGDLIFAKMKG **Charge** 3 **Spectral Angle** 0.06
Spearman Correlation -0.13 **iRT Error:** 2.51

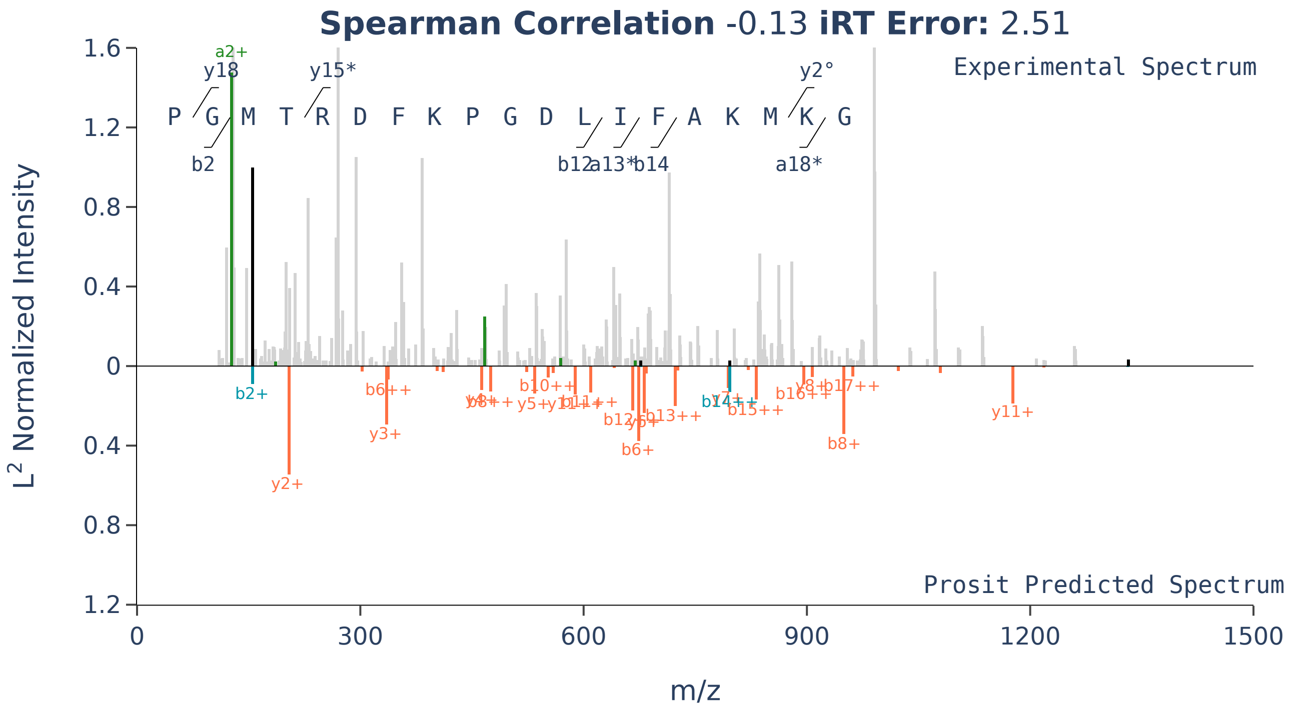

Source HRoetschke_271222_090123_HFGoe_LEDGF_b2R1_2h **Scan** 8218
Peptide GKNASSDVEVEE **Charge** 2 **Spectral Angle** 0.86
Spearman Correlation 0.97 **iRT Error:** 4.81

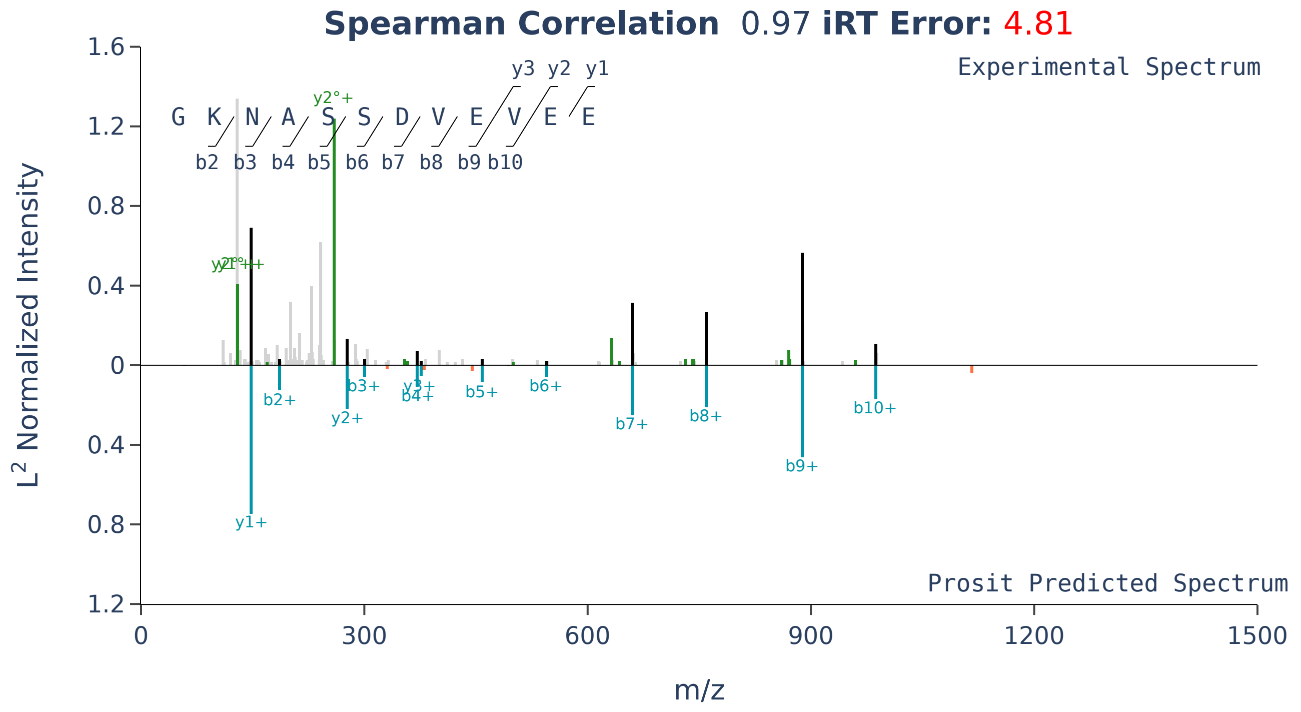

Source HRoetschke_271222_090123_HFGoe_LEDGF_b2R1_2h **Scan** 8218
Peptide PSSEERETEIS **Charge** 2 **Spectral Angle** 0.14
Spearman Correlation 0.22 **iRT Error:** 0.71

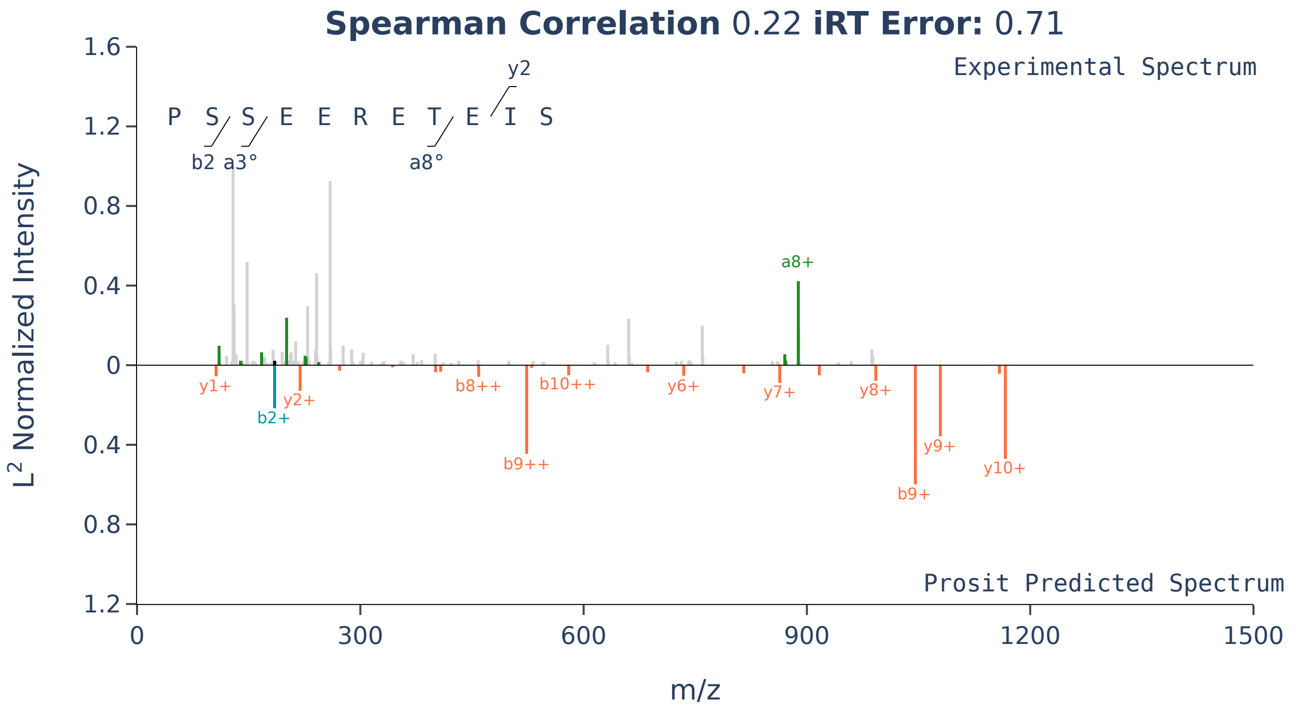

Source HRoetschke_271222_090123_HFGoe_LEDGF_b2R1_24h **Scan** 20879
Peptide SSLKIDNLDVNRCIE **Charge** 2 **Spectral Angle** 0.85
Spearman Correlation 0.91 **iRT Error:** 4.83

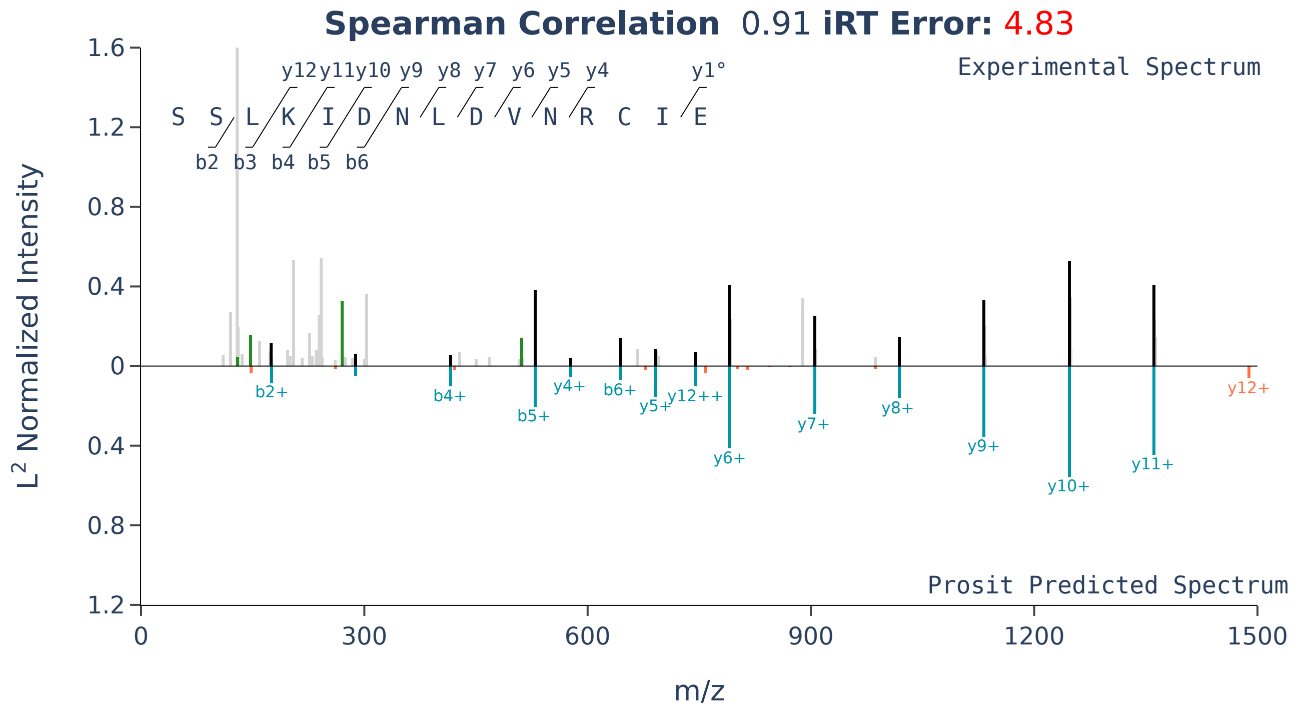

Source HRoetschke_271222_090123_HFGoe_LEDGF_b2R1_24h **Scan** 20879
Peptide KAEKQVETEEAGVVTT **Charge** 2 **Spectral Angle** 0.26
Spearman Correlation 0.28 **iRT Error:** 54.49

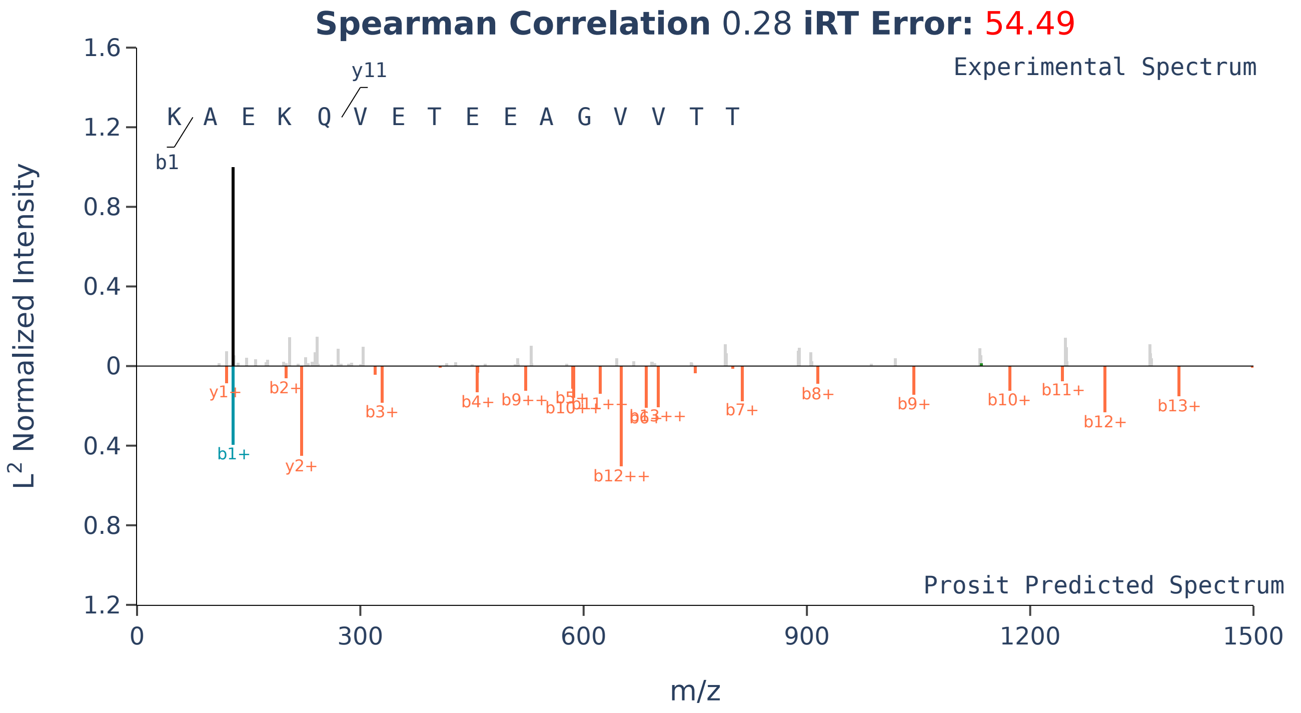

Source HRoetschke_271222_090123_HFGoe_LEDGF_b2R1_2h **Scan** 9542
Peptide PKHWPARVD **Charge** 3 **Spectral Angle** 0.83
Spearman Correlation 0.88 **iRT Error:** 4.9

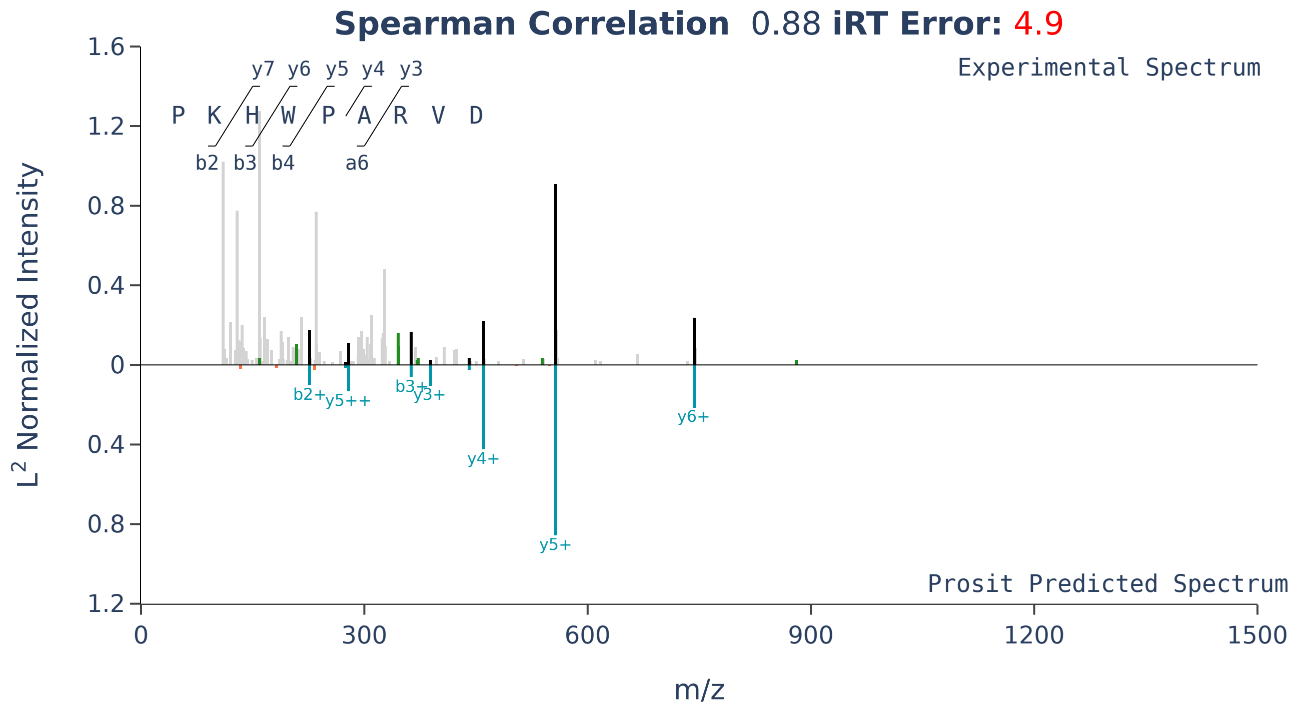

Source HRoetschke_271222_090123_HFGoe_LEDGF_b2R1_2h **Scan** 9542
Peptide KEQTGSKTLN **Charge** 3 **Spectral Angle** 0.71
Spearman Correlation 0.39 **iRT Error:** 19.15

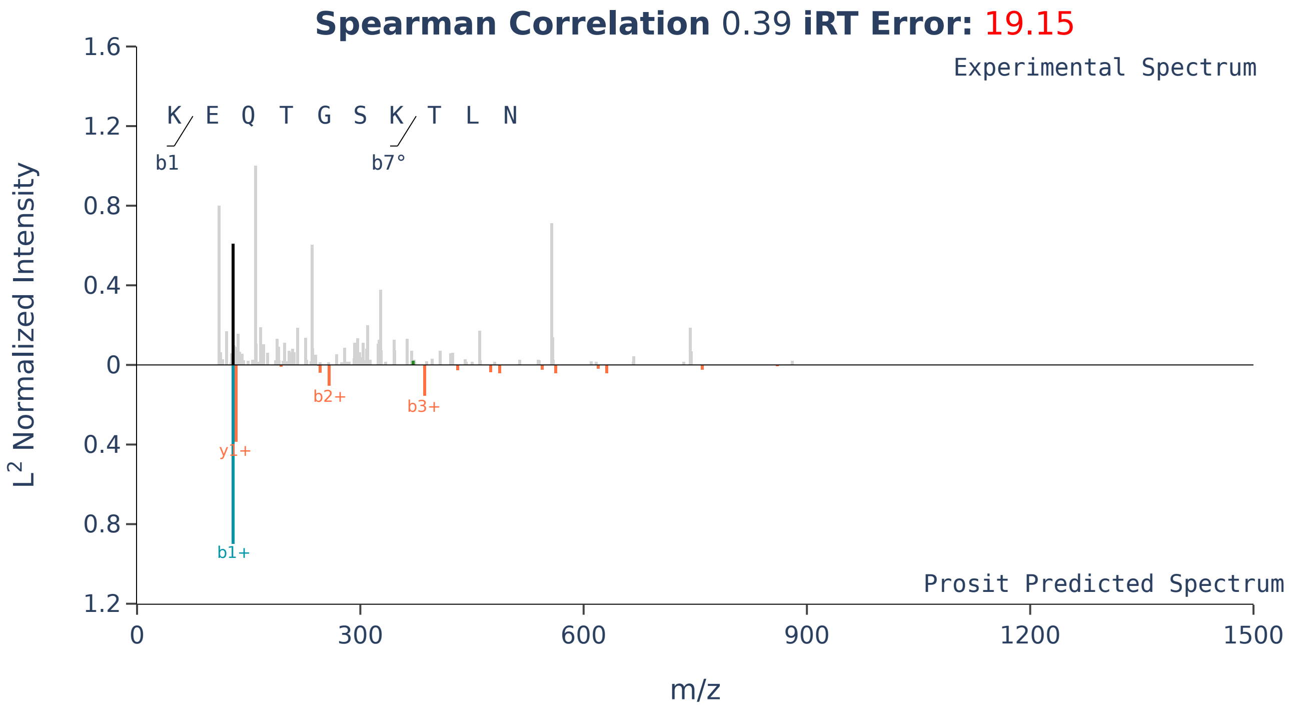

Source HRoetschke_271222_090123_HFGoe_LEDGF_b2R2_4h **Scan** 12550
Peptide GTHETKGYPHWPARV **Charge** 4 **Spectral Angle** 0.93
Spearman Correlation 0.9 **iRT Error:** 4.94

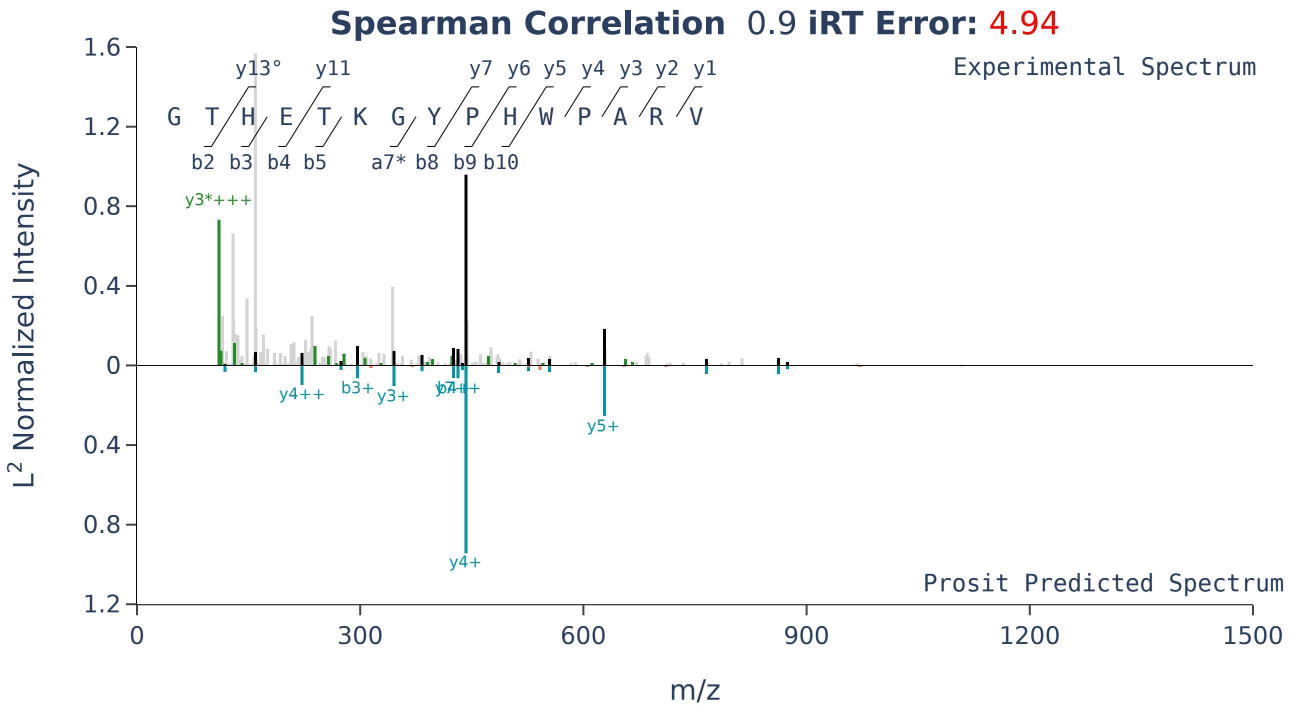

Source HRoetschke_271222_090123_HFGoe_LEDGF_b2R2_4h **Scan** 12550
Peptide THETAFLGPKDIFPY **Charge** 4 **Spectral Angle** 0.35
Spearman Correlation 0.17 **iRT Error:** 93.58

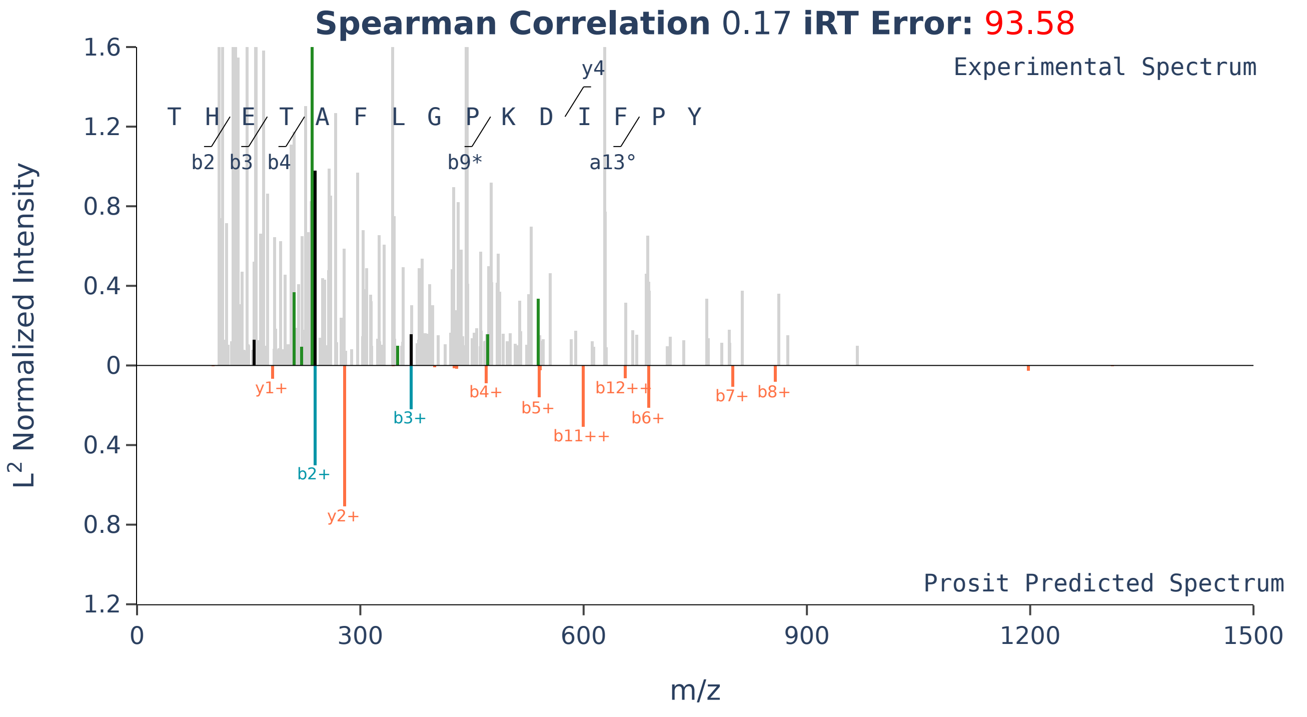

Source HRoetschke_271222_090123_HFGoe_LEDGF_b1R1_24h **Scan** 10544
Peptide LDNVETEE **Charge** 2 **Spectral Angle** 0.91
Spearman Correlation 0.87 **iRT Error:** 5.01

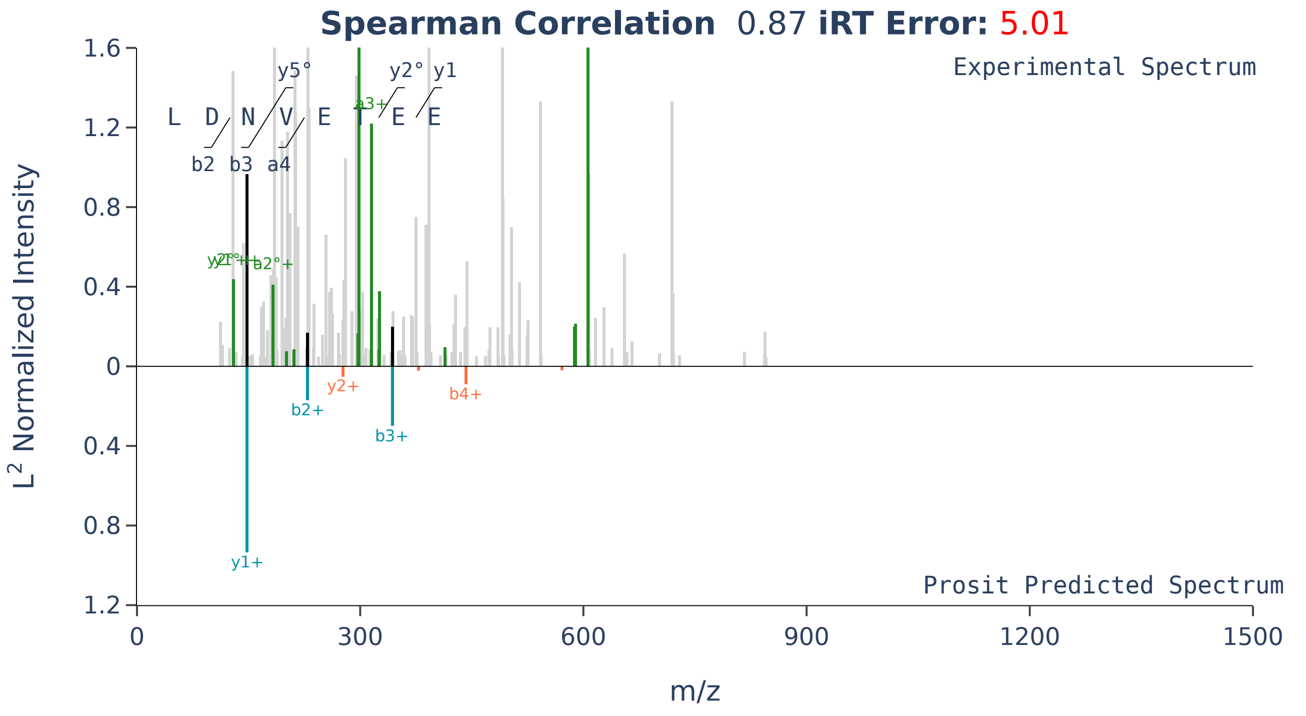

Source HRoetschke_271222_090123_HFGoe_LEDGF_b1R1_24h **Scan** 10544
Peptide DNLDVNRC **Charge** 2 **Spectral Angle** 0.35
Spearman Correlation 0.19 **iRT Error:** 2.2

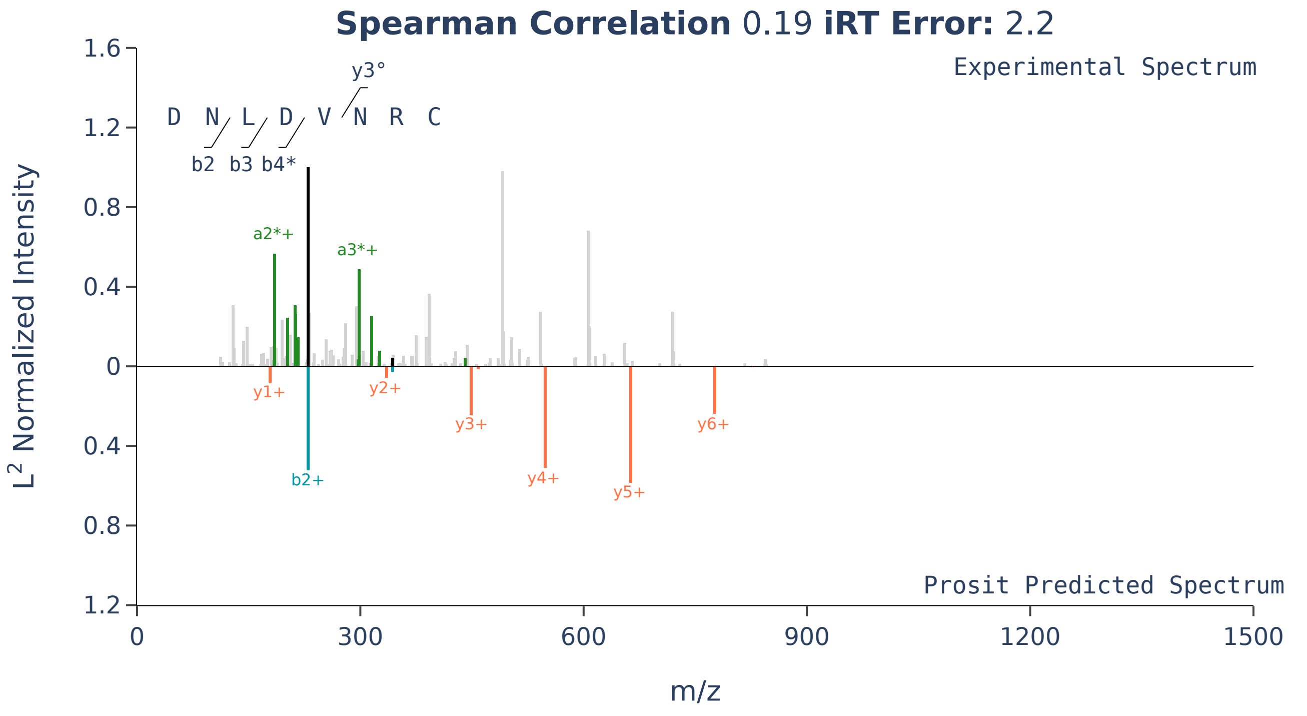

Source HRoetschke_271222_090123_HFGoe_LEDGF_b1R2_24h **Scan** 14122
Peptide NKYPHWPARV **Charge** 3 **Spectral Angle** 0.92
Spearman Correlation 0.84 **iRT Error:** 5.08

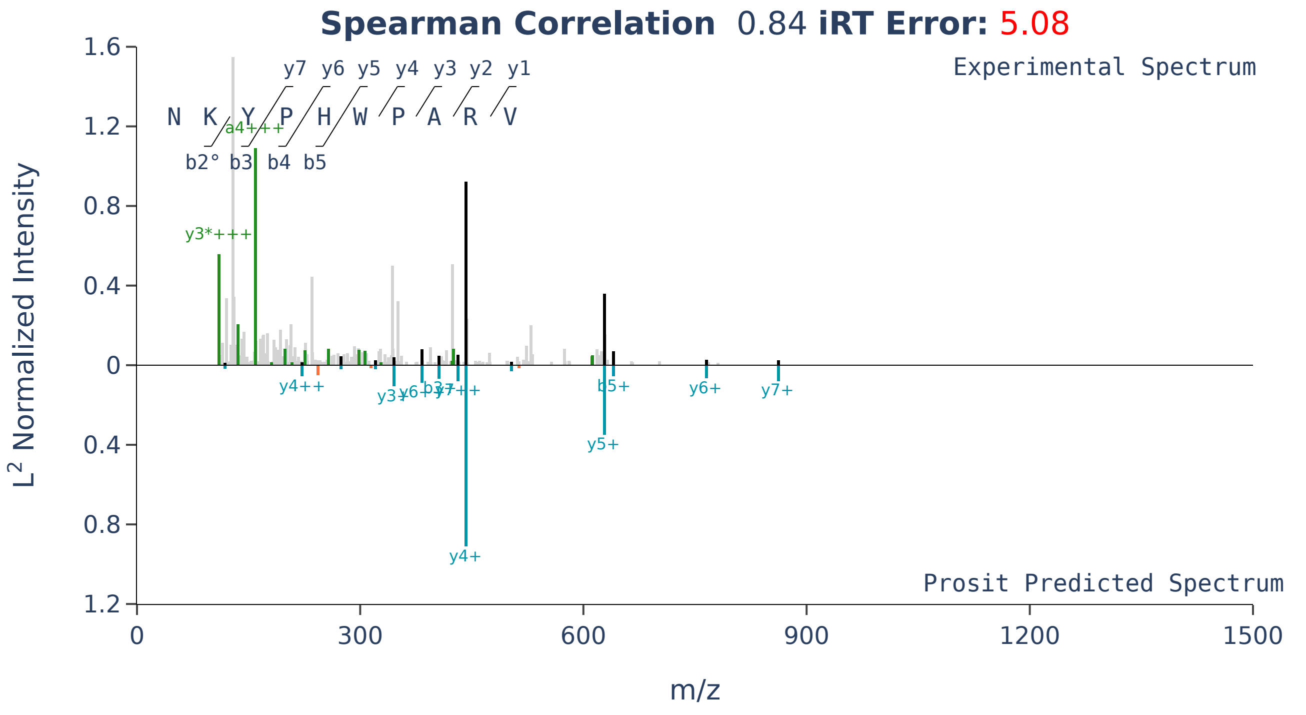

Source HRoetschke_271222_090123_HFGoe_LEDGF_b1R2_24h **Scan** 14122
Peptide AFLGPKDIFPY **Charge** 3 **Spectral Angle** 0.0
Spearman Correlation -0.09 **iRT Error:** 102.47

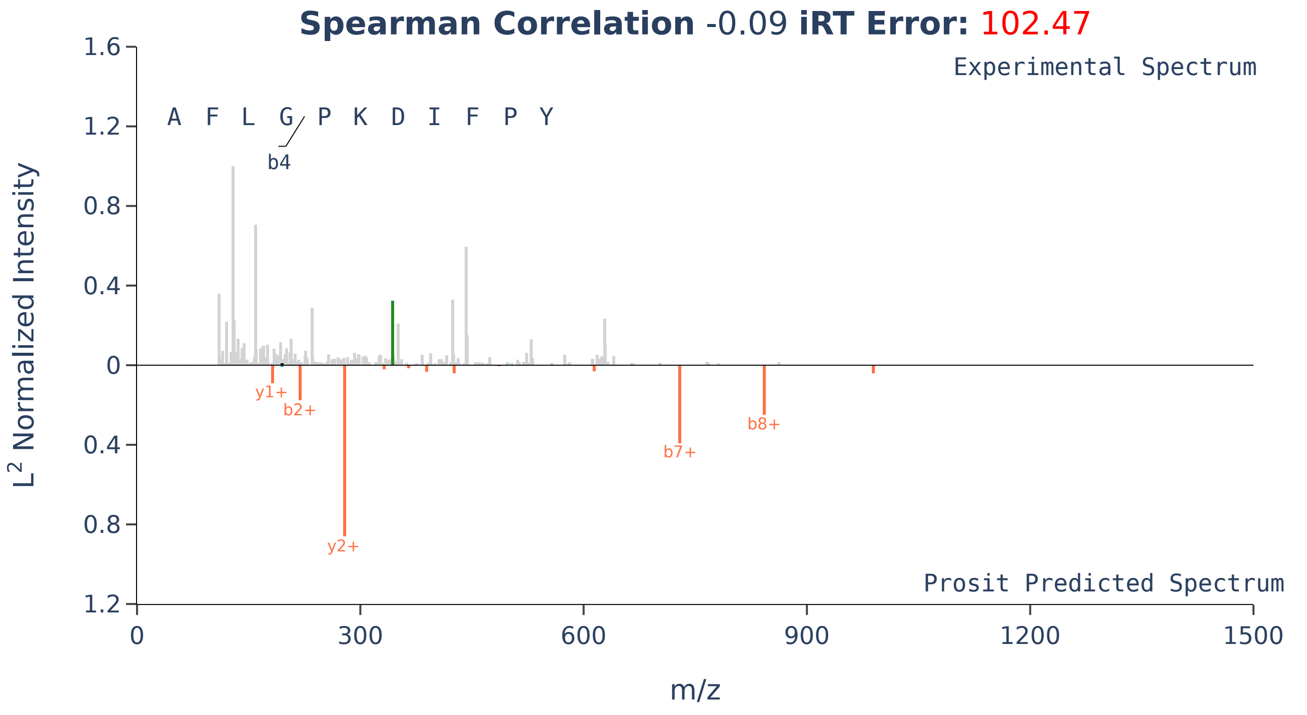

Source HRoetschke_271222_090123_HFGoe_LEDGF_b2R1_1h **Scan** 27742
Peptide PGDLIFEA **Charge** 1 **Spectral Angle** 0.82
Spearman Correlation 0.93 **iRT Error:** 5.11

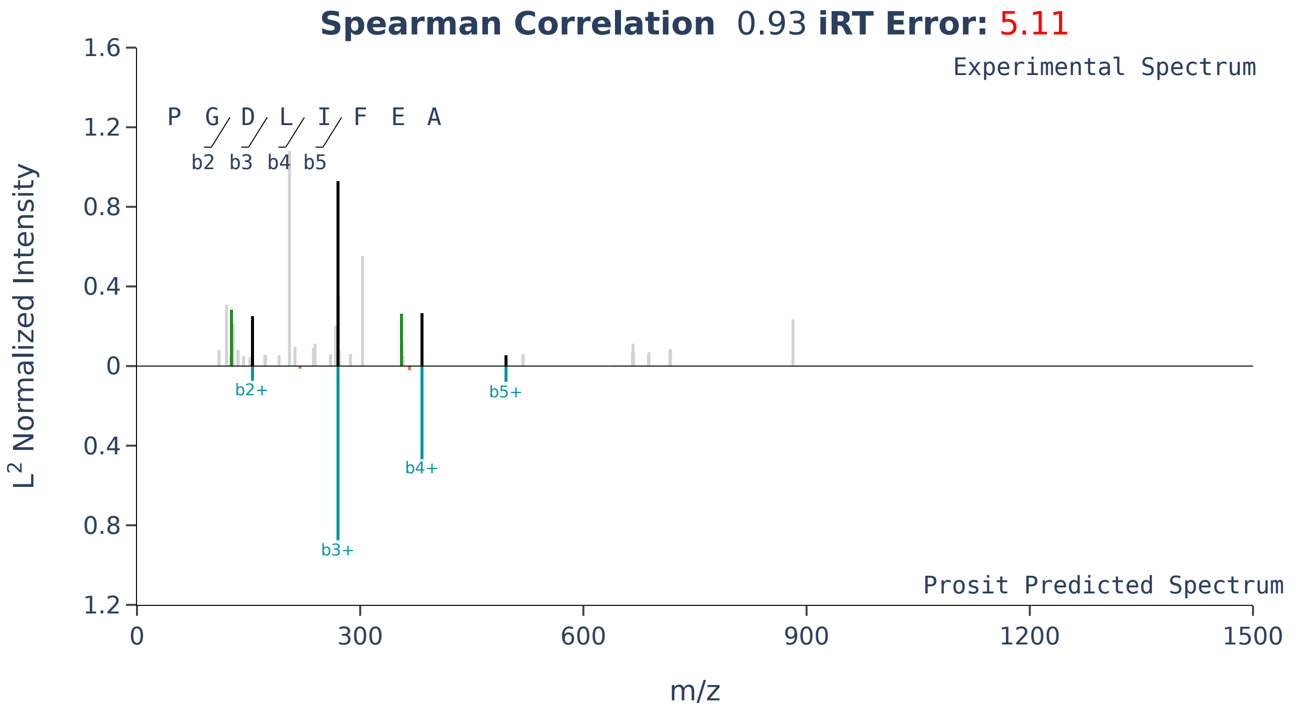

Source HRoetschke_271222_090123_HFGoe_LEDGF_b2R1_1h **Scan** 27742
Peptide SQQAATKQ **Charge** 1 **Spectral Angle** 0.0
Spearman Correlation 0.0 **iRT Error:** 120.25

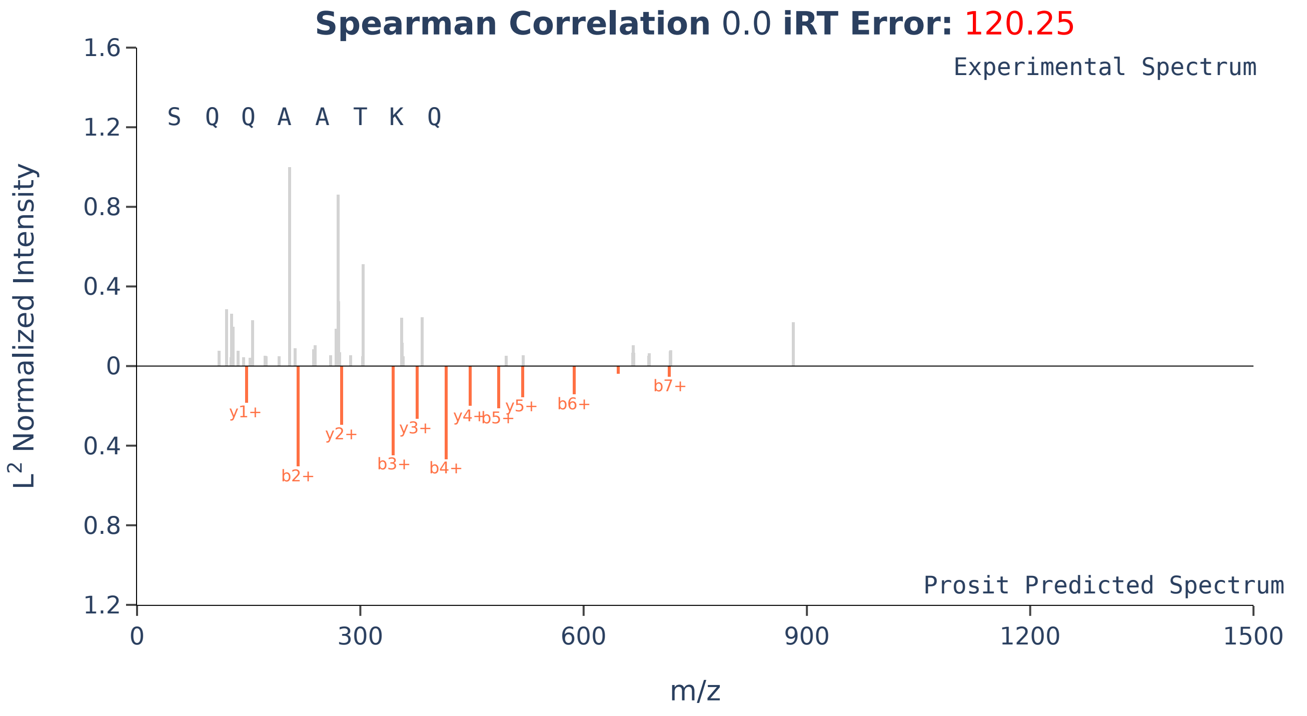

Source HRoetschke_271222_090123_HFGoe_LEDGF_b1R1_24h **Scan** 6117
Peptide RLQHAEIKN **Charge** 2 **Spectral Angle** 0.78
Spearman Correlation 0.93 **iRT Error:** 5.23

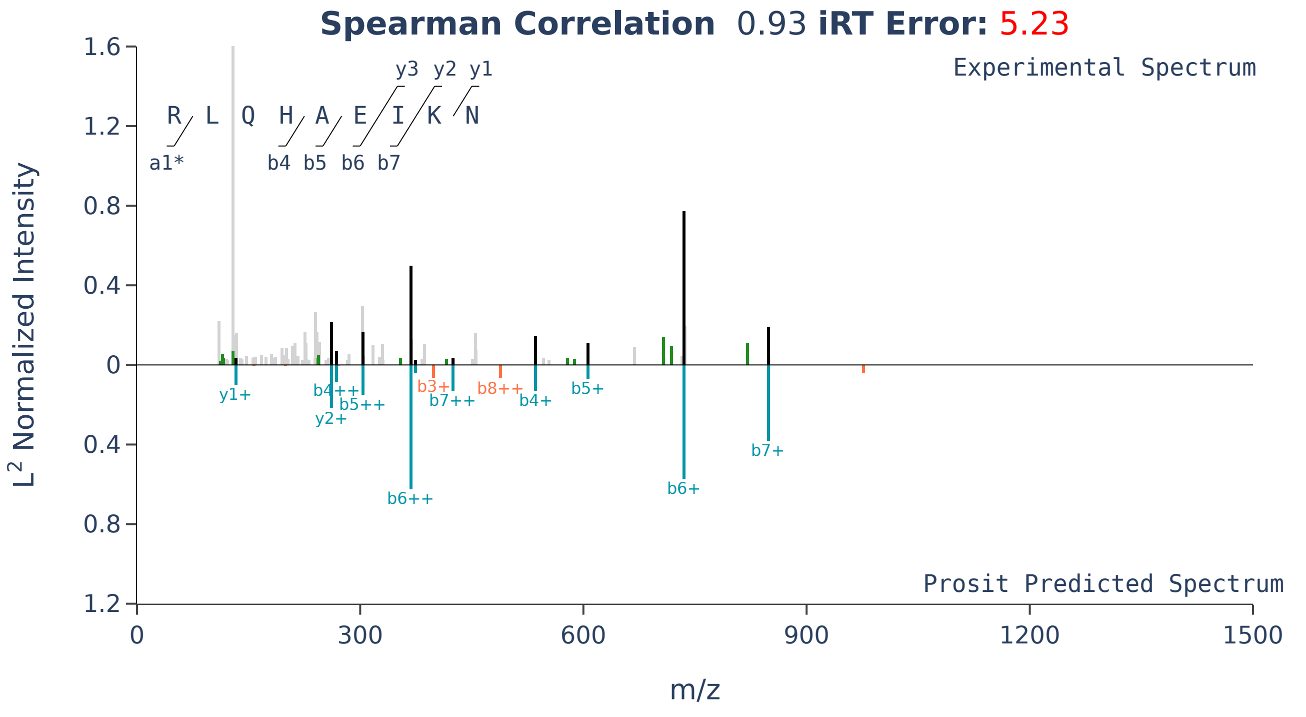

Source HRoetschke_271222_090123_HFGoe_LEDGF_b1R1_24h **Scan** 6117
Peptide QRIHAEIKN **Charge** 2 **Spectral Angle** 0.78
Spearman Correlation 0.84 **iRT Error:** 37.73

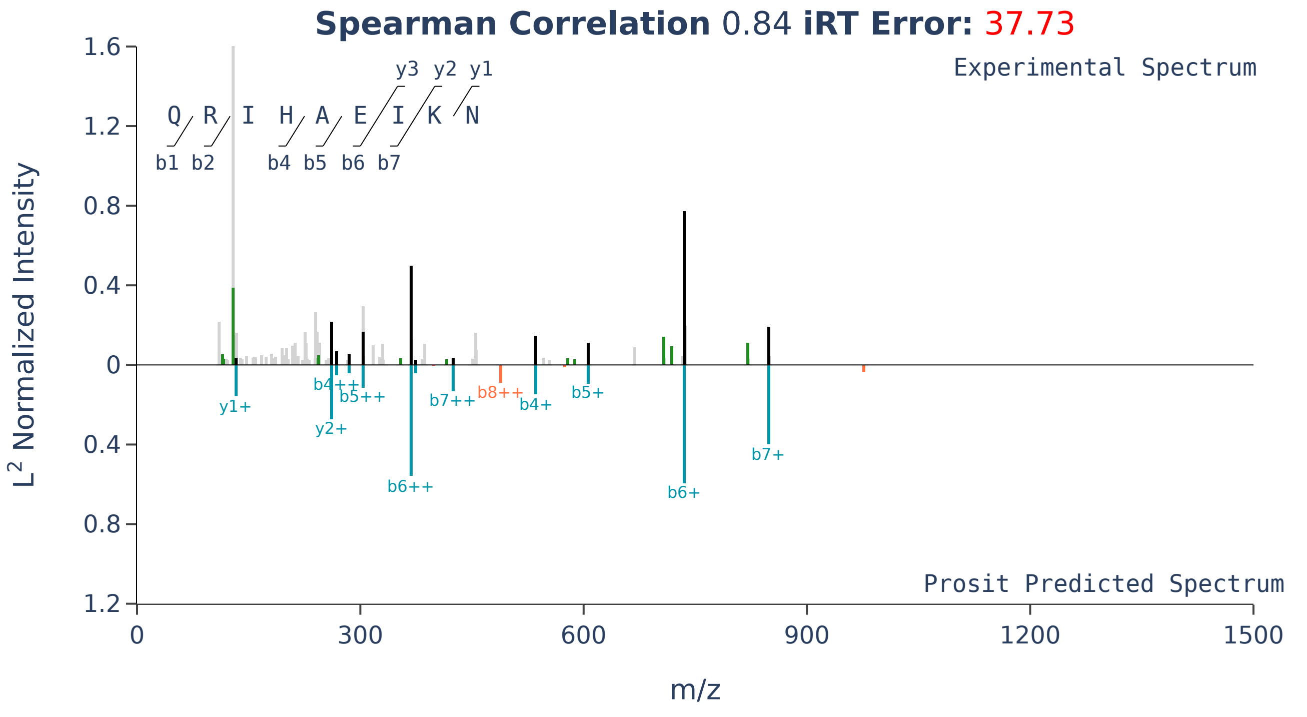

Source HRoetschke_271222_090123_HFGoe_LEDGF_b1R1_2h **Scan** 11036
Peptide GEDSVITQ **Charge** 2 **Spectral Angle** 0.84
Spearman Correlation 0.93 **iRT Error:** 5.61

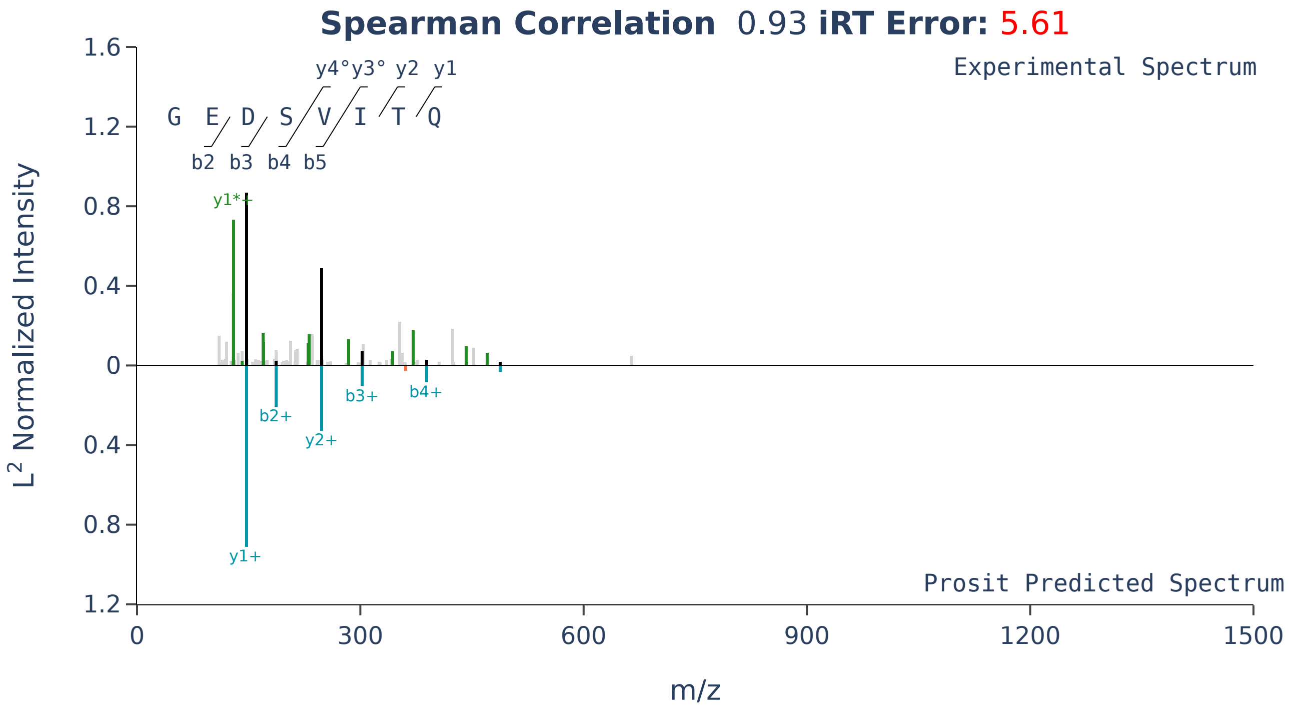

Source HRoetschke_271222_090123_HFGoe_LEDGF_b1R1_2h **Scan** 11036
Peptide DVNRCIE **Charge** 2 **Spectral Angle** 0.3
Spearman Correlation 0.38 **iRT Error:** 6.72

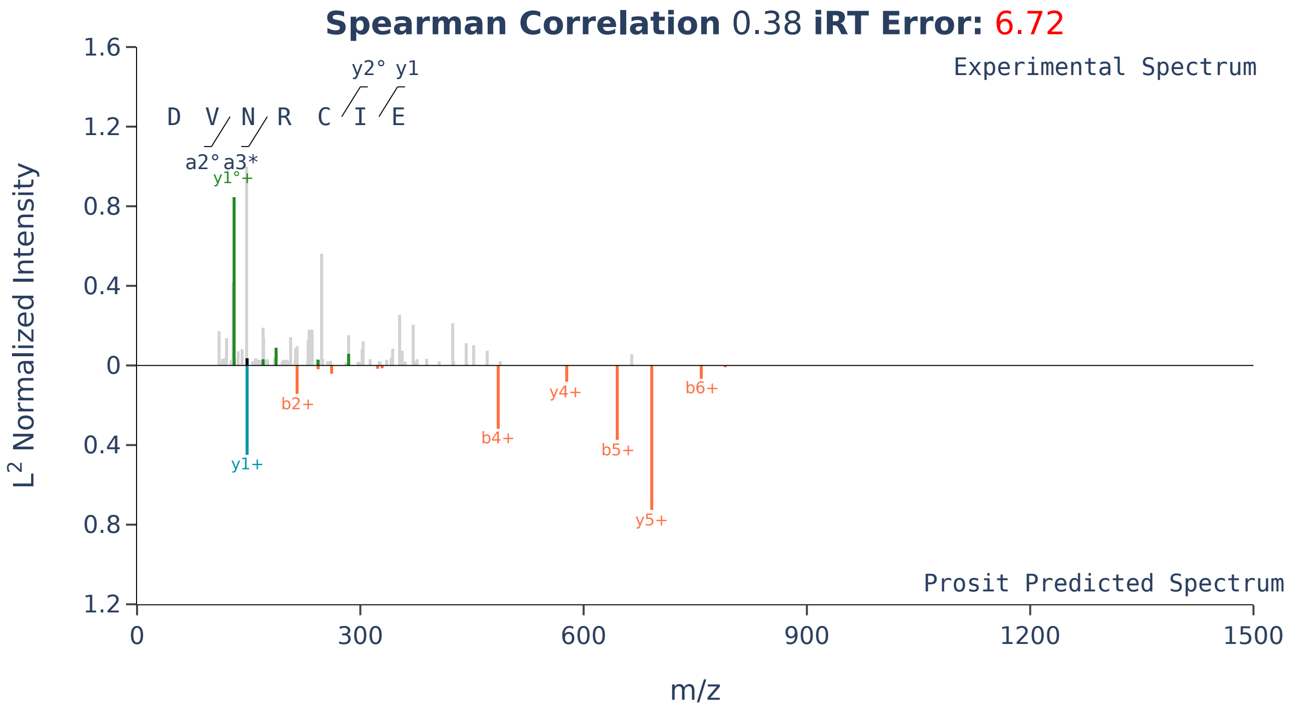

Source HRoetschke_271222_090123_HFGoe_LEDGF_b1R1_4h **Scan** 19324
Peptide TAFLGPKDIFPYSENKEKYGKPNKREL **Charge** 5 **Spectral Angle** 0.84
Spearman Correlation 0.89 **iRT Error:** 5.7

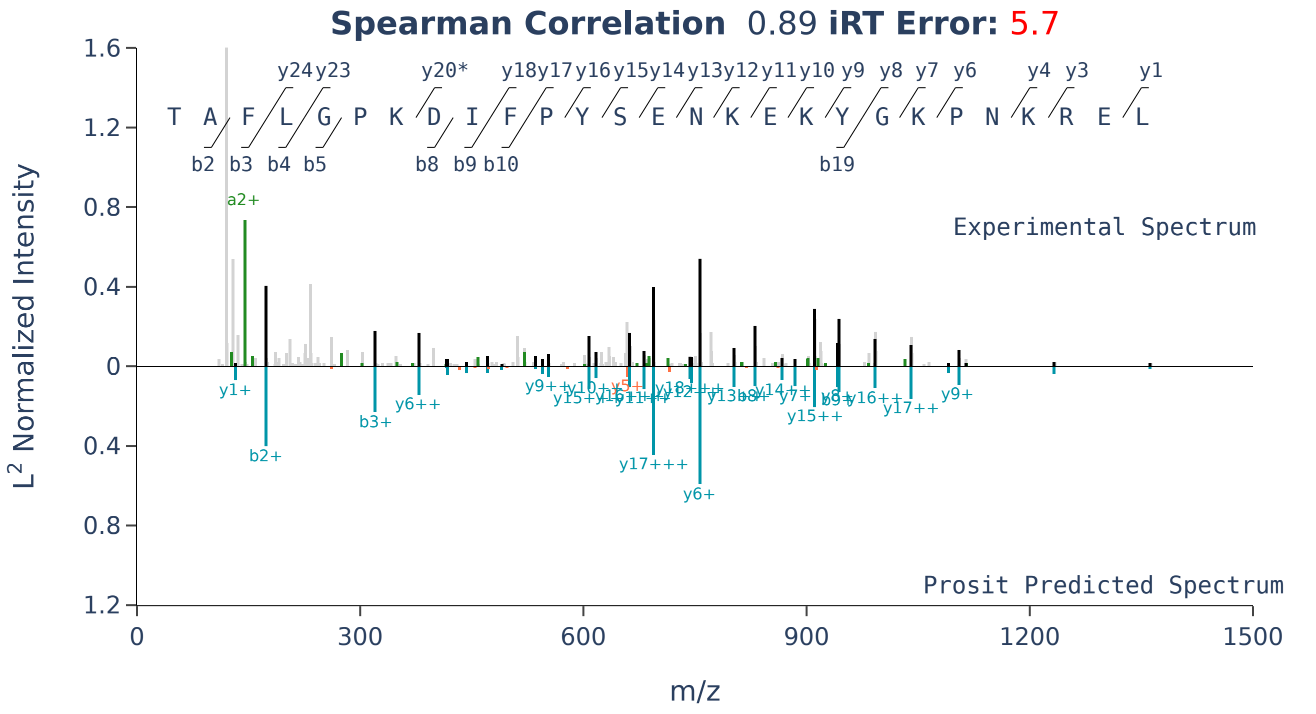

Source HRoetschke_271222_090123_HFGoe_LEDGF_b1R1_4h **Scan** 19324
Peptide ITEEDKSKKKGQEEKQPKKQPKKDEEG **Charge** 5 **Spectral Angle** 0.07
Spearman Correlation 0.1 **iRT Error:** 103.29

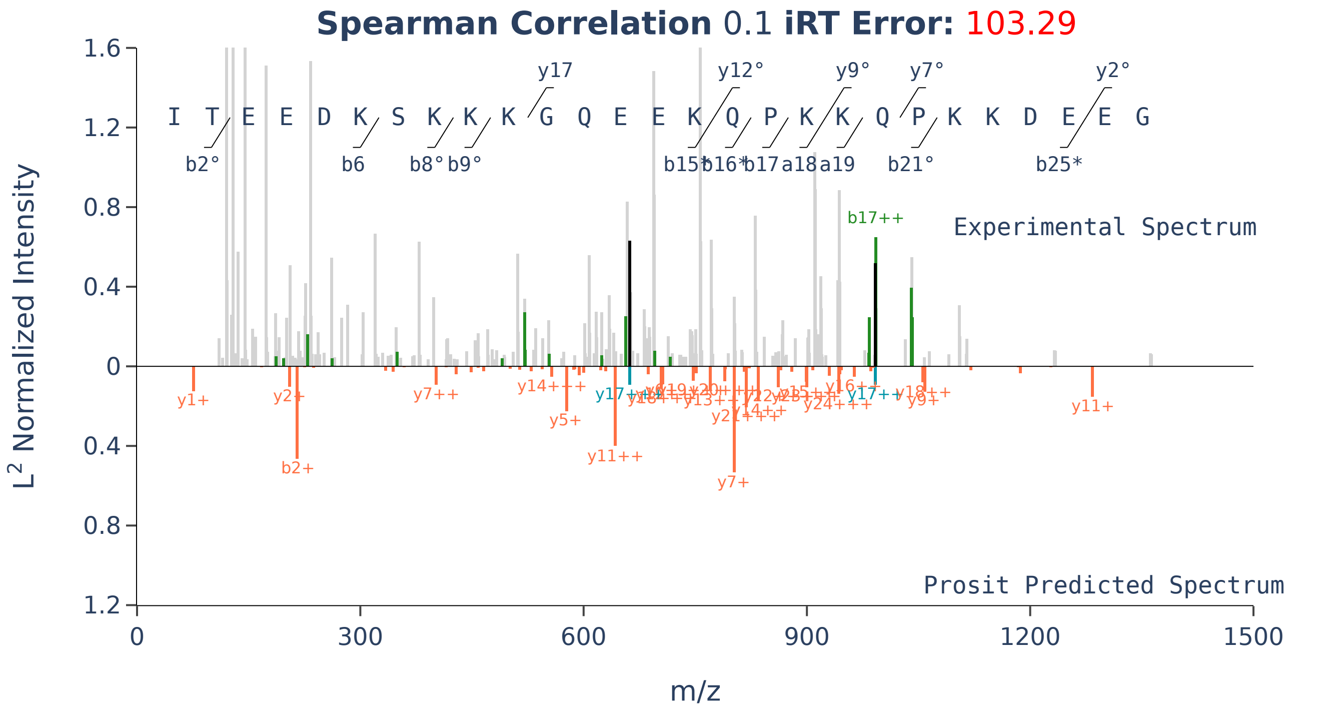

Source HRoetschke_271222_090123_HFGoe_LEDGF_b1R2_1h **Scan** 6581
Peptide SVITQQAQKH **Charge** 3 **Spectral Angle** 0.88
Spearman Correlation 0.89 **iRT Error:** 5.7

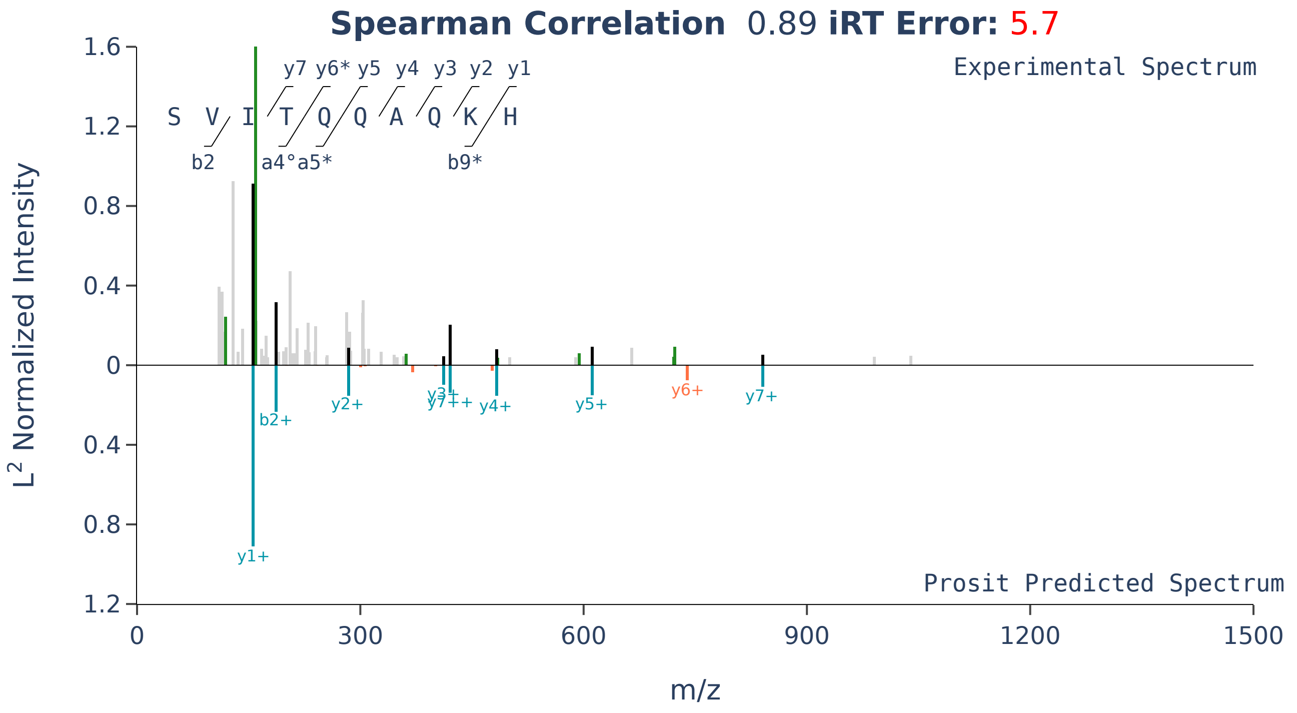

Source HRoetschke_271222_090123_HFGoe_LEDGF_b1R2_1h **Scan** 6581
Peptide HRRNMLKGQ **Charge** 3 **Spectral Angle** 0.0
Spearman Correlation 0.0 **iRT Error:** 0.57

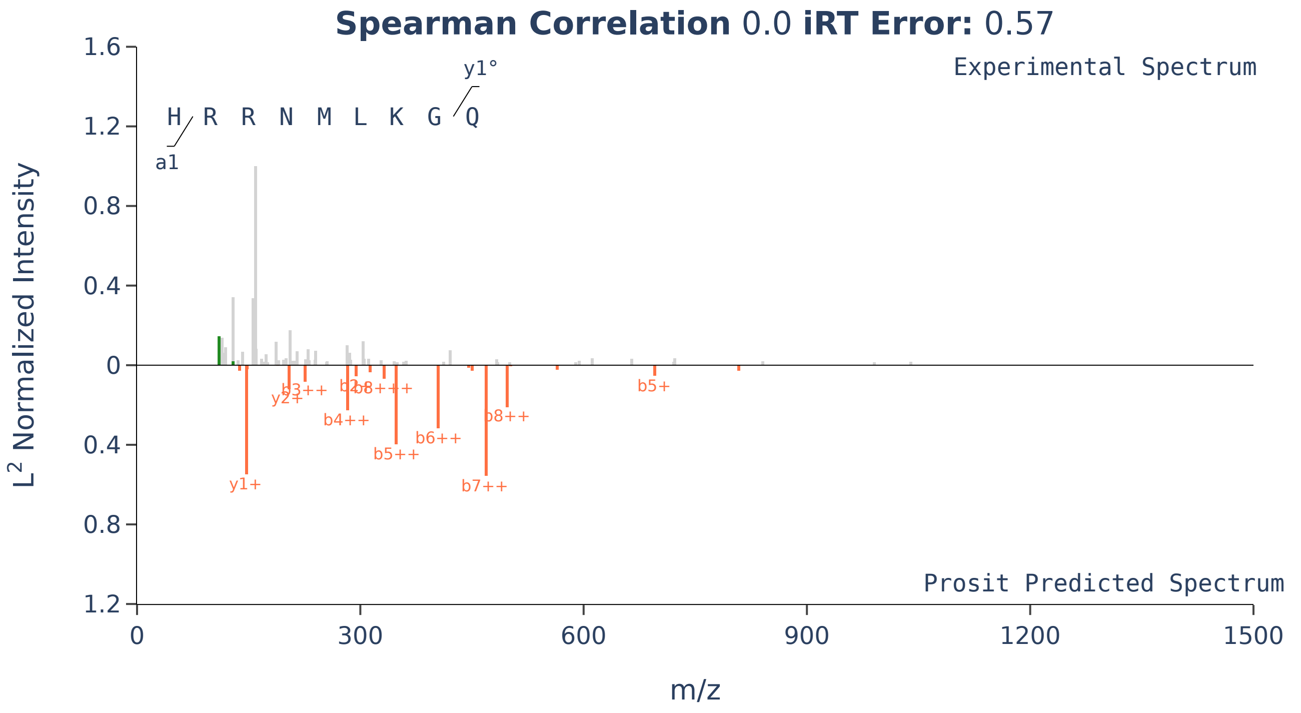

Source HRoetschke_271222_090123_HFGoe_LEDGF_b2R1_24h **Scan** 13419
Peptide ASMDSRLQRI **Charge** 3 **Spectral Angle** 0.9
Spearman Correlation 0.93 **iRT Error:** 5.95

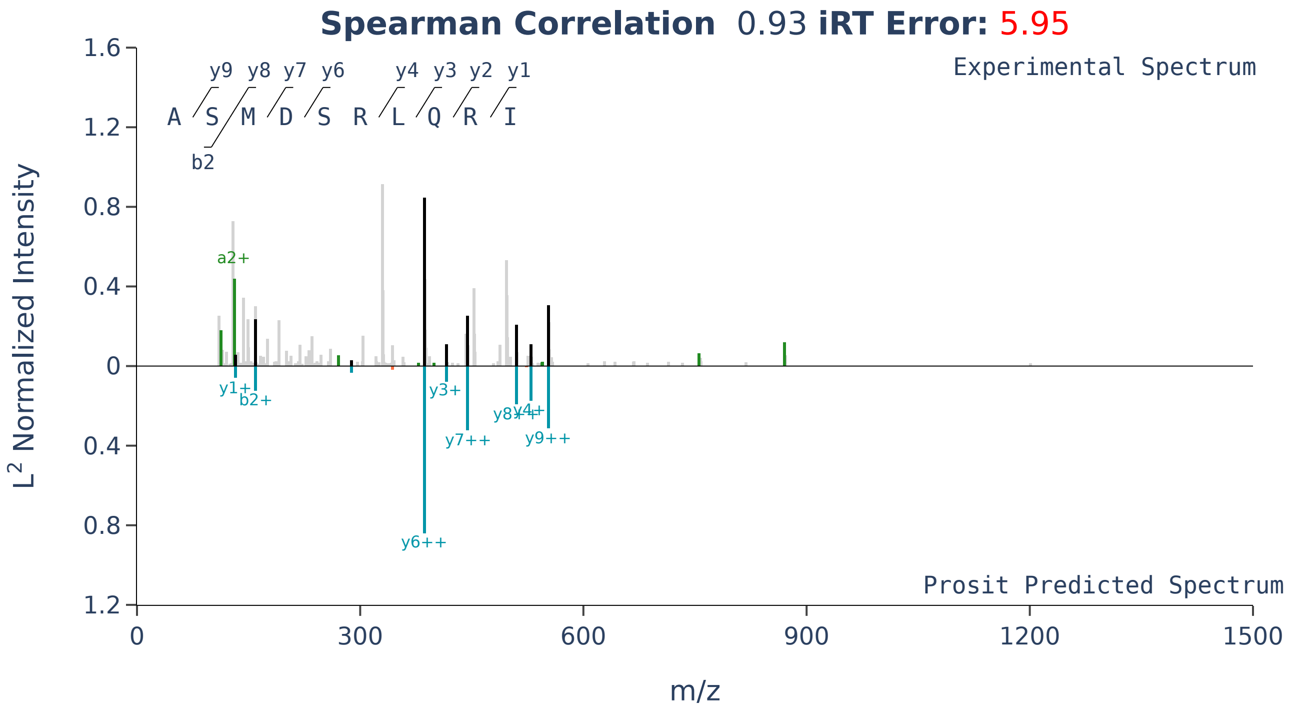

Source HRoetschke_271222_090123_HFGoe_LEDGF_b2R1_24h **Scan** 13419
Peptide EDKSKKKGQE **Charge** 3 **Spectral Angle** 0.01
Spearman Correlation -0.23 **iRT Error:** 51.22

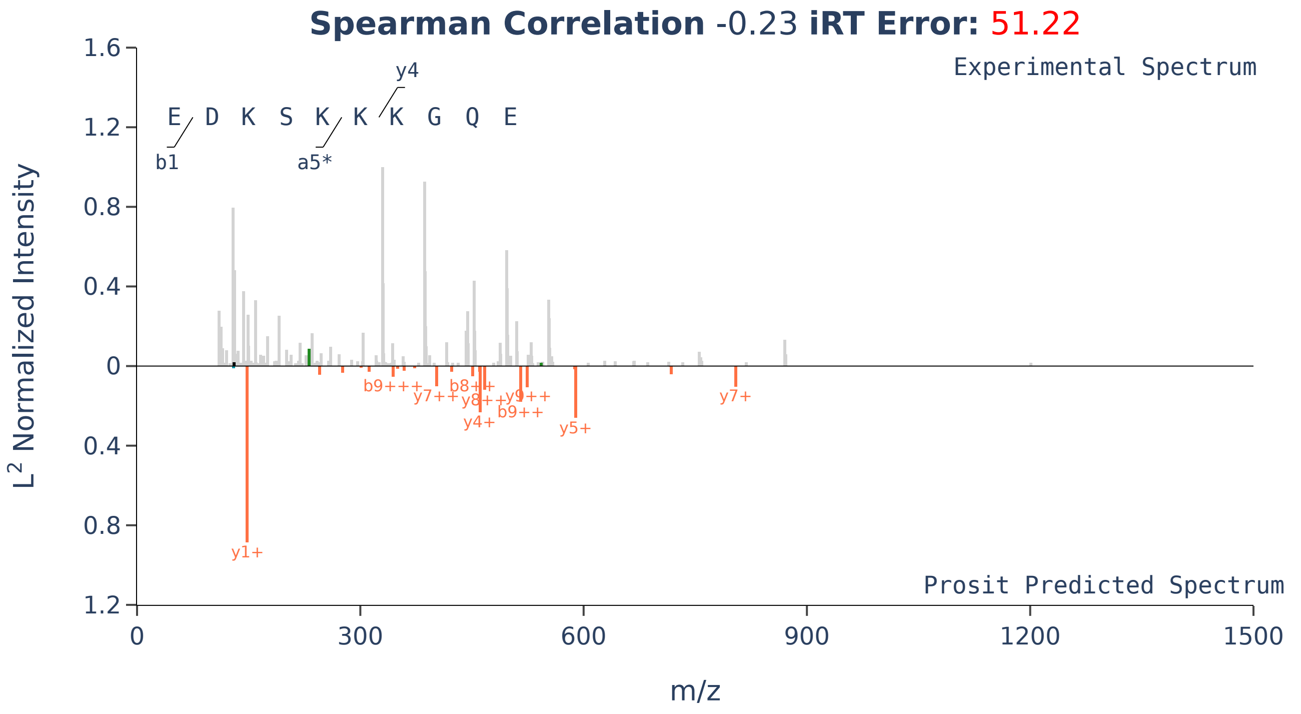

Source HRoetschke_271222_090123_HFGoe_LEDGF_b1R1_24h **Scan** 17859
Peptide WYPHWPAR **Charge** 3 **Spectral Angle** 0.81
Spearman Correlation 0.97 **iRT Error:** 6.23

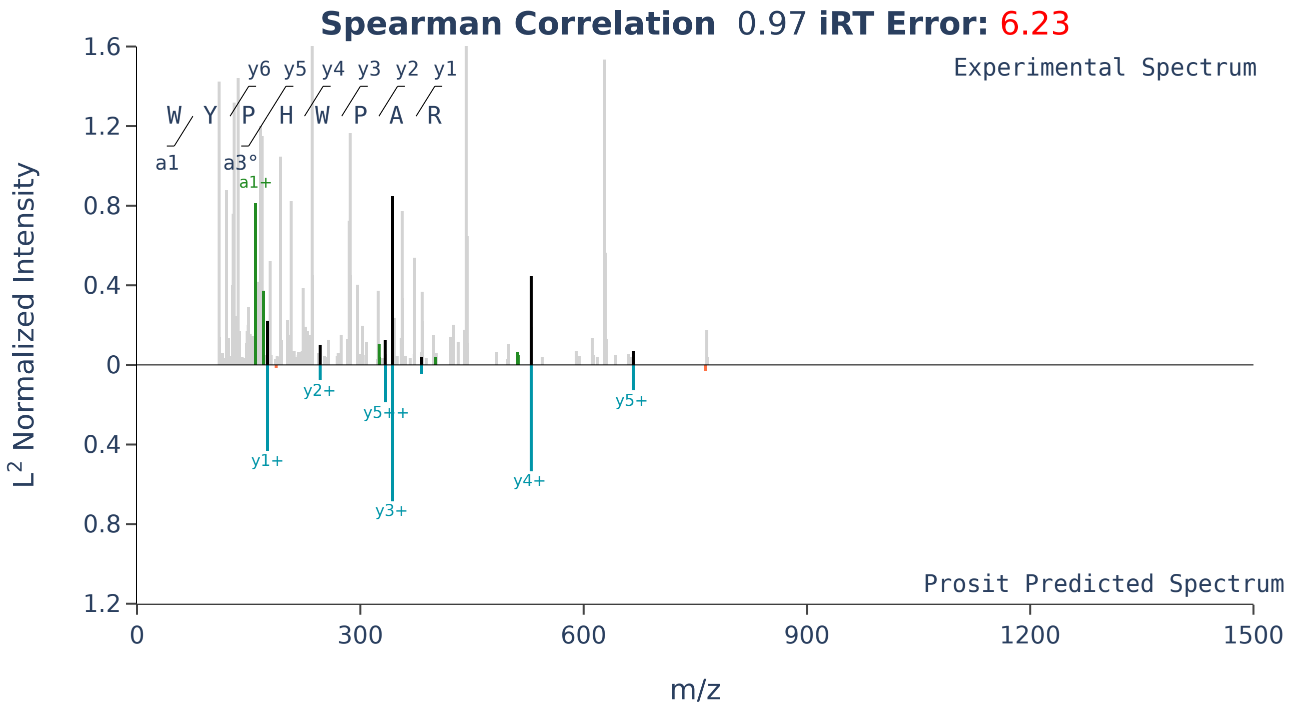

Source HRoetschke_271222_090123_HFGoe_LEDGF_b1R1_24h **Scan** 17859
Peptide KGQHEKEAAD **Charge** 3 **Spectral Angle** 0.17
Spearman Correlation 0.2 **iRT Error:** 91.09

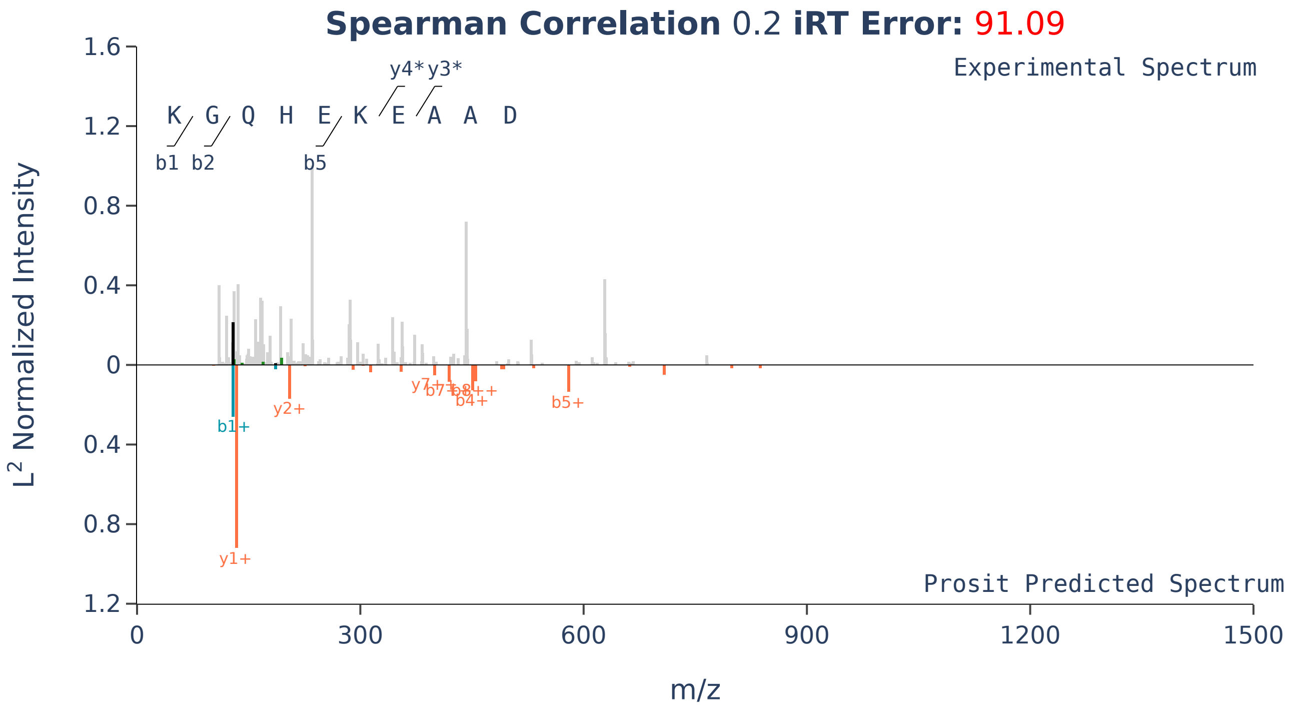

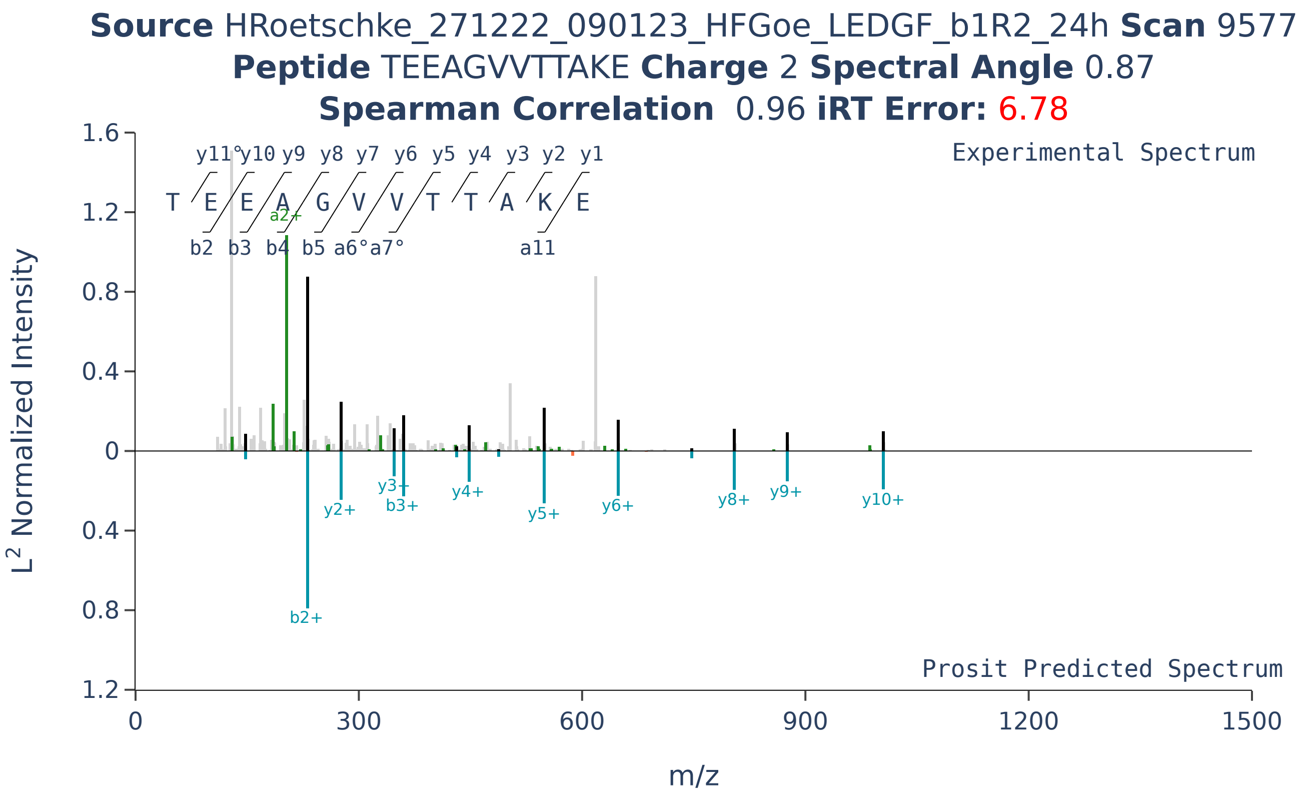
Source HRoetschke_271222_090123_HFGoe_LEDGF_b1R2_24h Scan 9577
Peptide TEEAGVVTTAKE Charge 2 Spectral Angle 0.87
Spearman Correlation 0.96 iRT Error: 6.78
Experimental Spectrum
Prosit Predicted Spectrum
L2 Normalized Intensity
m/z

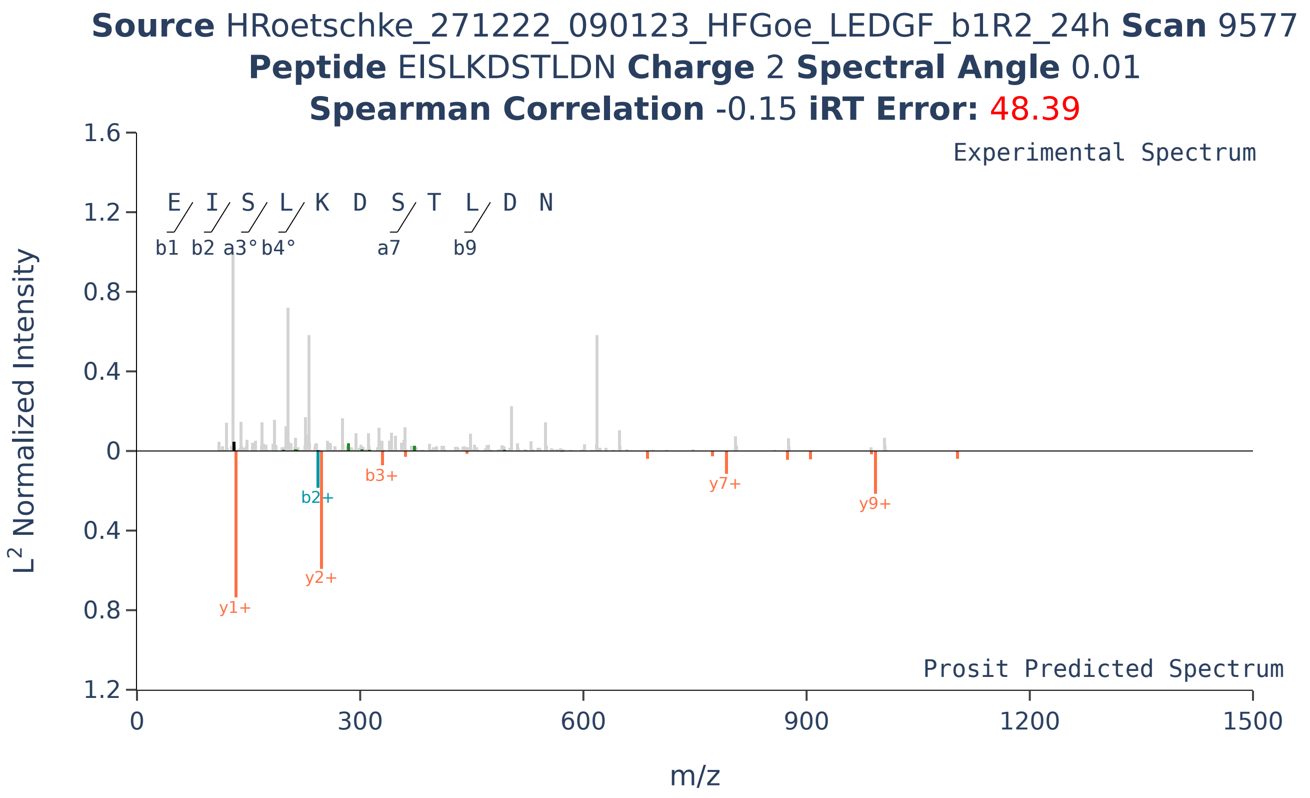
Source HRoetschke_271222_090123_HFGoe_LEDGF_b1R2_24h Scan 9577
Peptide EISLKDSTLDN Charge 2 Spectral Angle 0.01
Spearman Correlation -0.15 iRT Error: 48.39
Experimental Spectrum
Prosit Predicted Spectrum
L2 Normalized Intensity
m/z

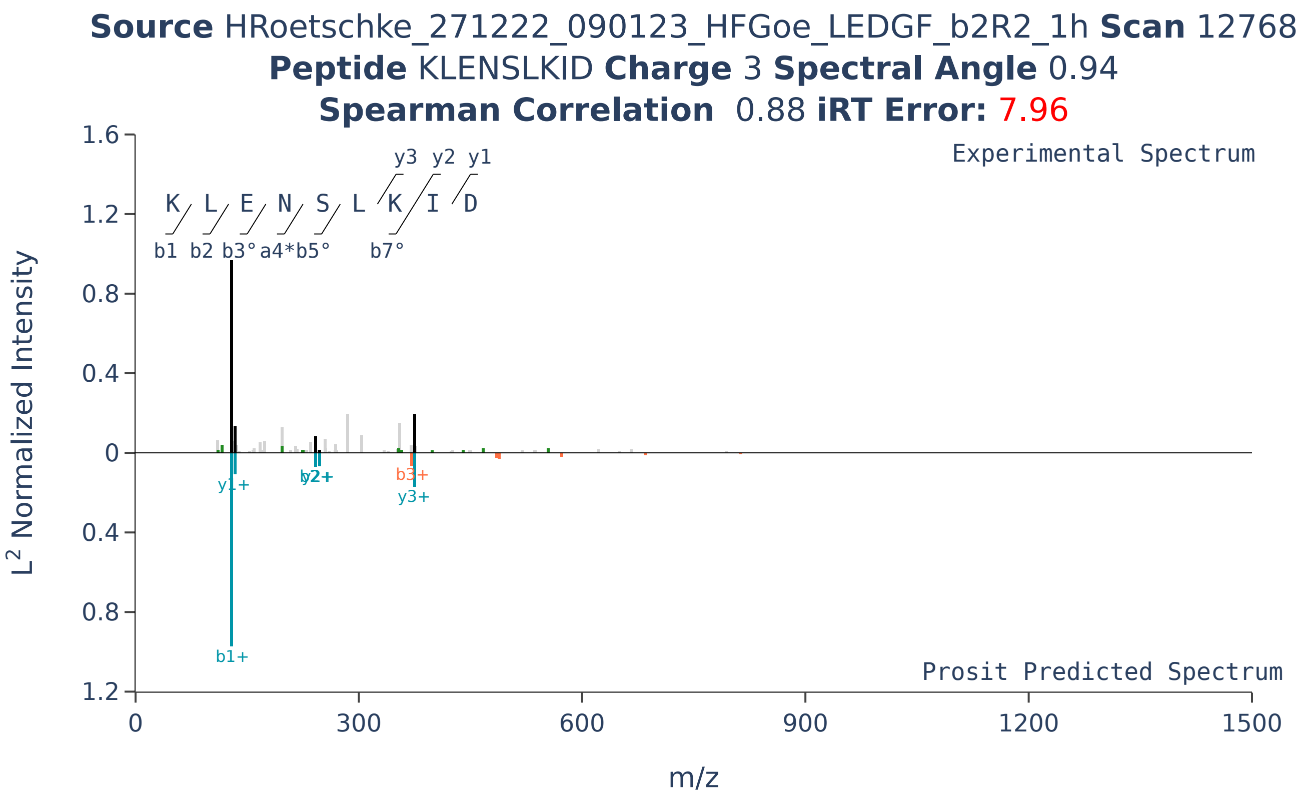
Source HRoetschke_271222_090123_HFGoe_LEDGF_b2R2_1h Scan 12768
Peptide KLENSLKID Charge 3 Spectral Angle 0.94
Spearman Correlation 0.88 iRT Error: 7.96
Experimental Spectrum
Prosit Predicted Spectrum
L2 Normalized Intensity
m/z

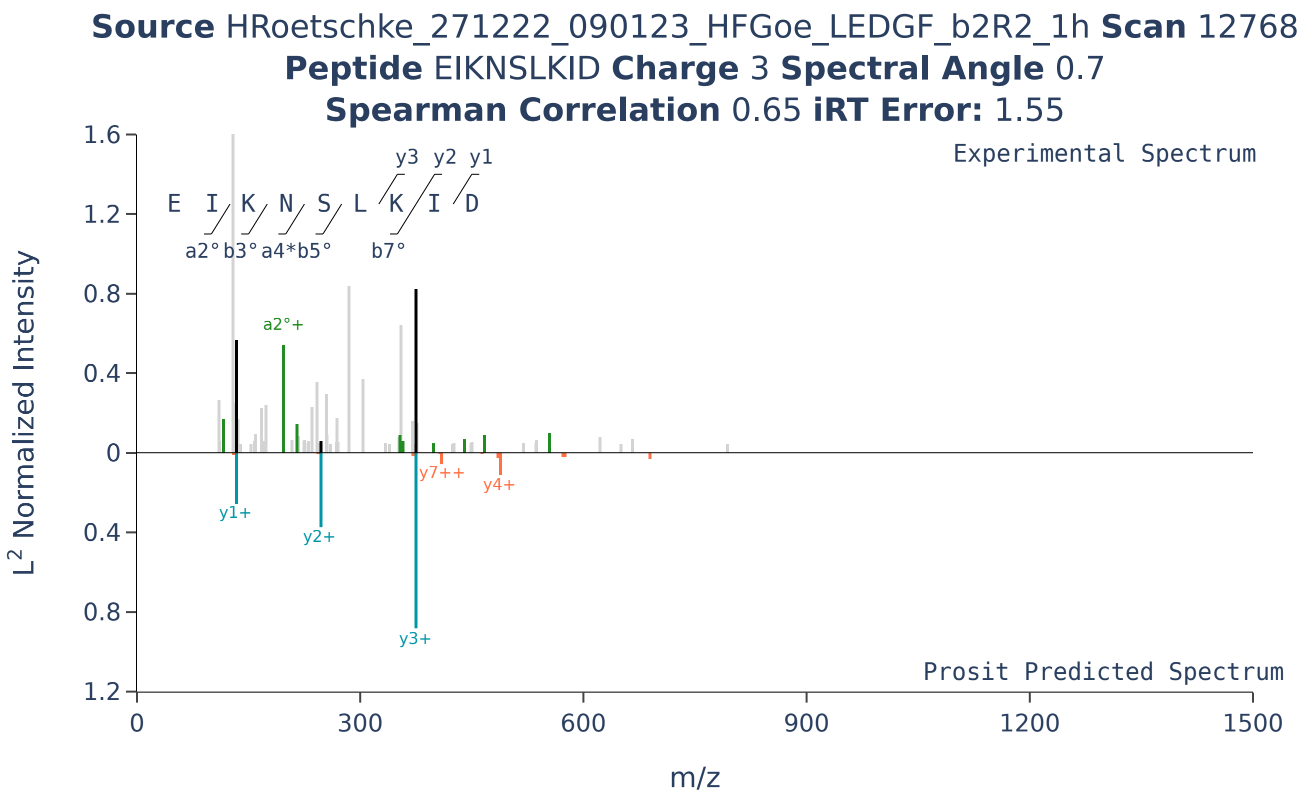
Source HRoetschke_271222_090123_HFGoe_LEDGF_b2R2_1h Scan 12768
Peptide EIKNSLKID Charge 3 Spectral Angle 0.7
Spearman Correlation 0.65 iRT Error: 1.55
Experimental Spectrum
Prosit Predicted Spectrum
L2 Normalized Intensity
m/z

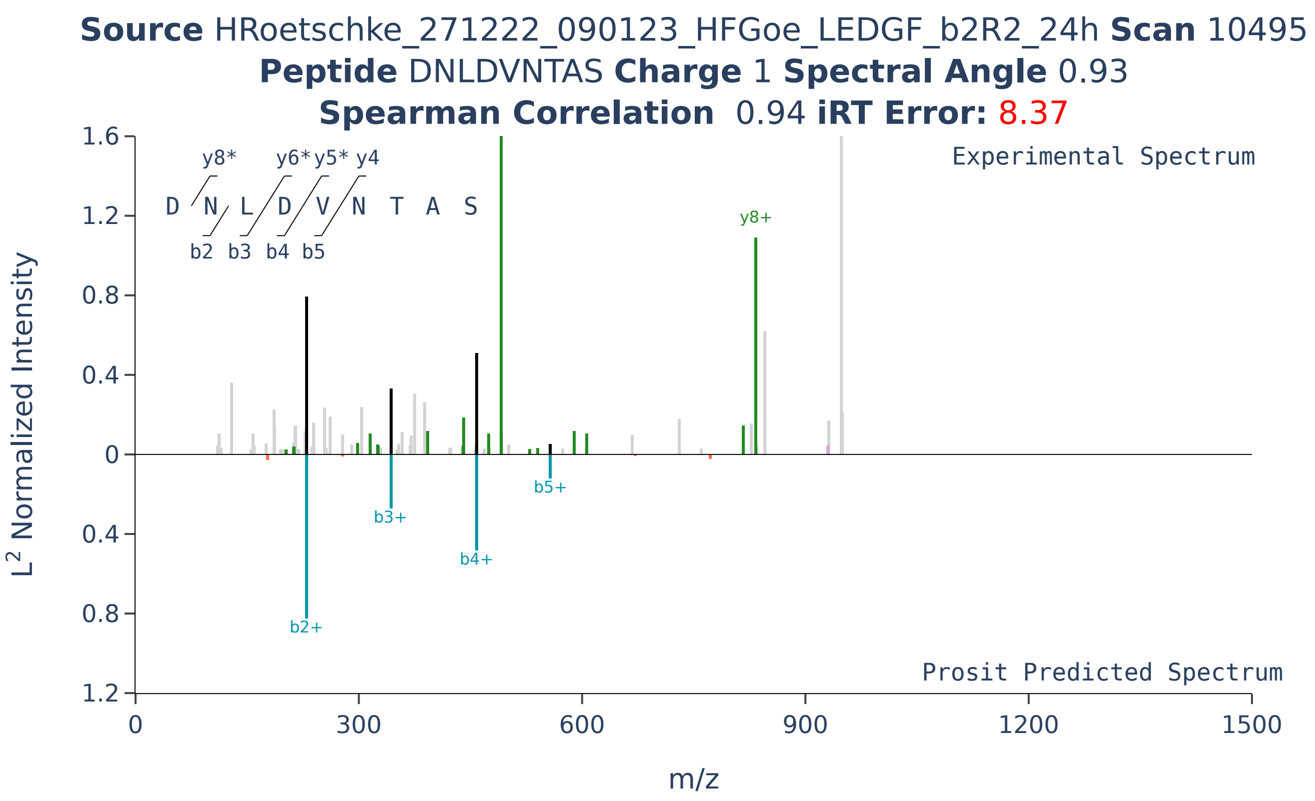
Source HRoetschke_271222_090123_HFGoe_LEDGF_b2R2_24h Scan 10495
Peptide DNLDVNTAS Charge 1 Spectral Angle 0.93
Spearman Correlation 0.94 iRT Error: 8.37
Experimental Spectrum
Prosit Predicted Spectrum
L2 Normalized Intensity
m/z

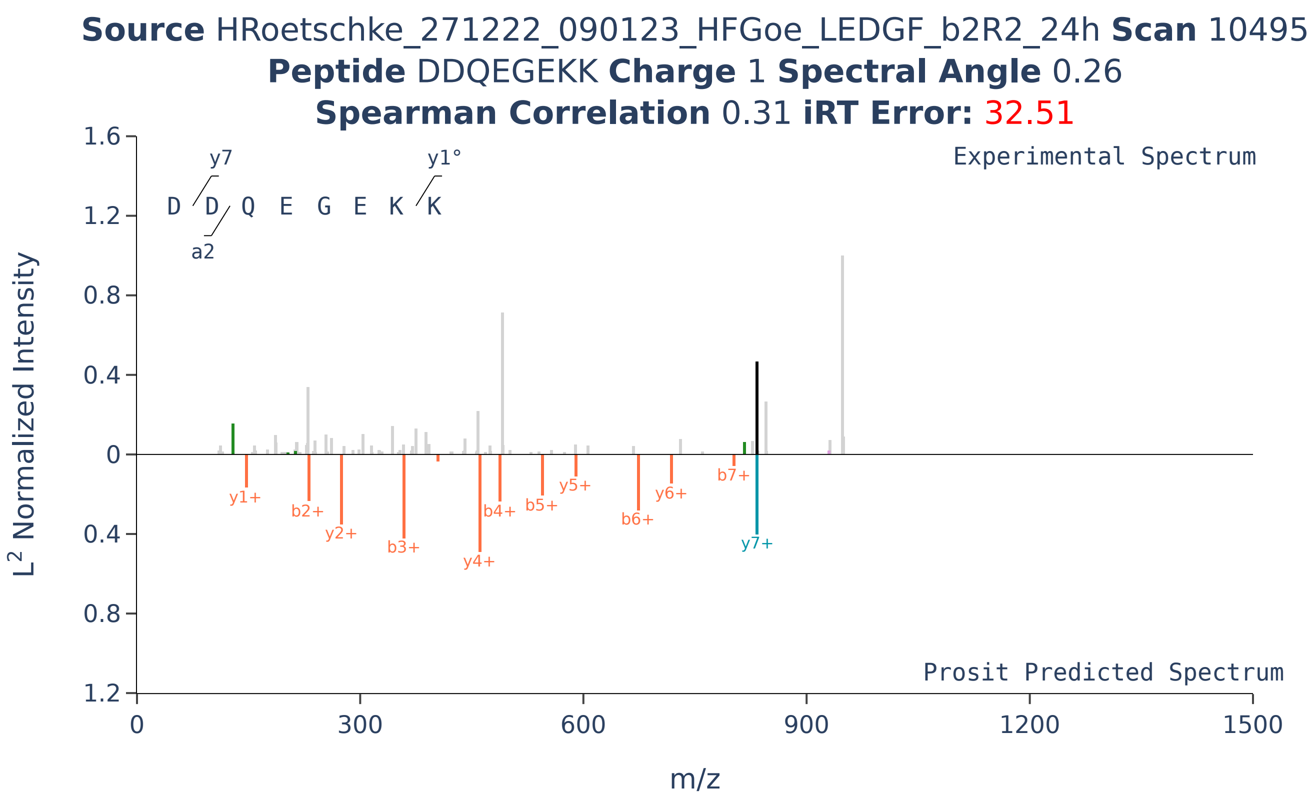
Source HRoetschke_271222_090123_HFGoe_LEDGF_b2R2_24h Scan 10495
Peptide DDQEGEKK Charge 1 Spectral Angle 0.26
Spearman Correlation 0.31 iRT Error: 32.51
Experimental Spectrum
Prosit Predicted Spectrum
L2 Normalized Intensity
m/z

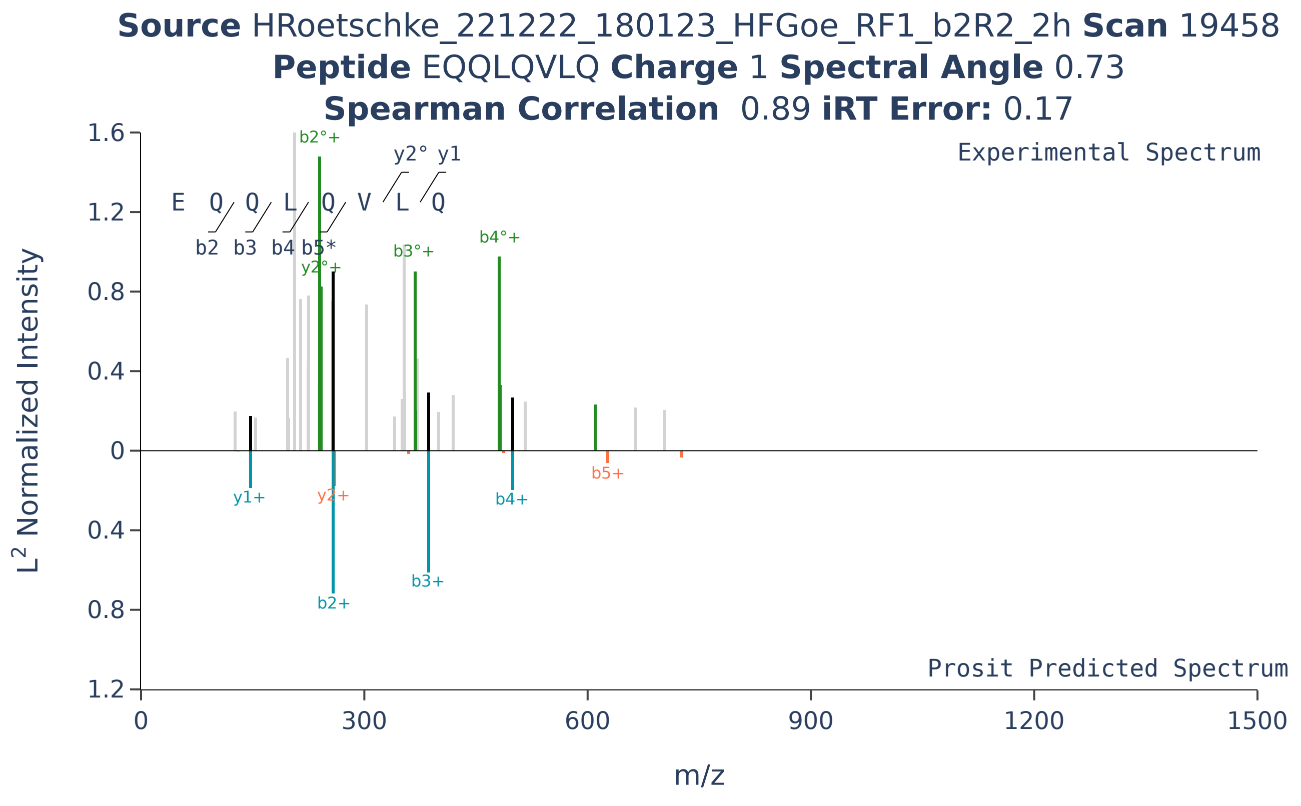
Source HRoetschke_221222_180123_HFGoe_RF1_b2R2_2h **Scan** 19458
Peptide EQQLQVLQ **Charge** 1 **Spectral Angle** 0.73
Spearman Correlation 0.89 **iRT Error:** 0.17
Experimental Spectrum
Prosit Predicted Spectrum
L² Normalized Intensity
m/z

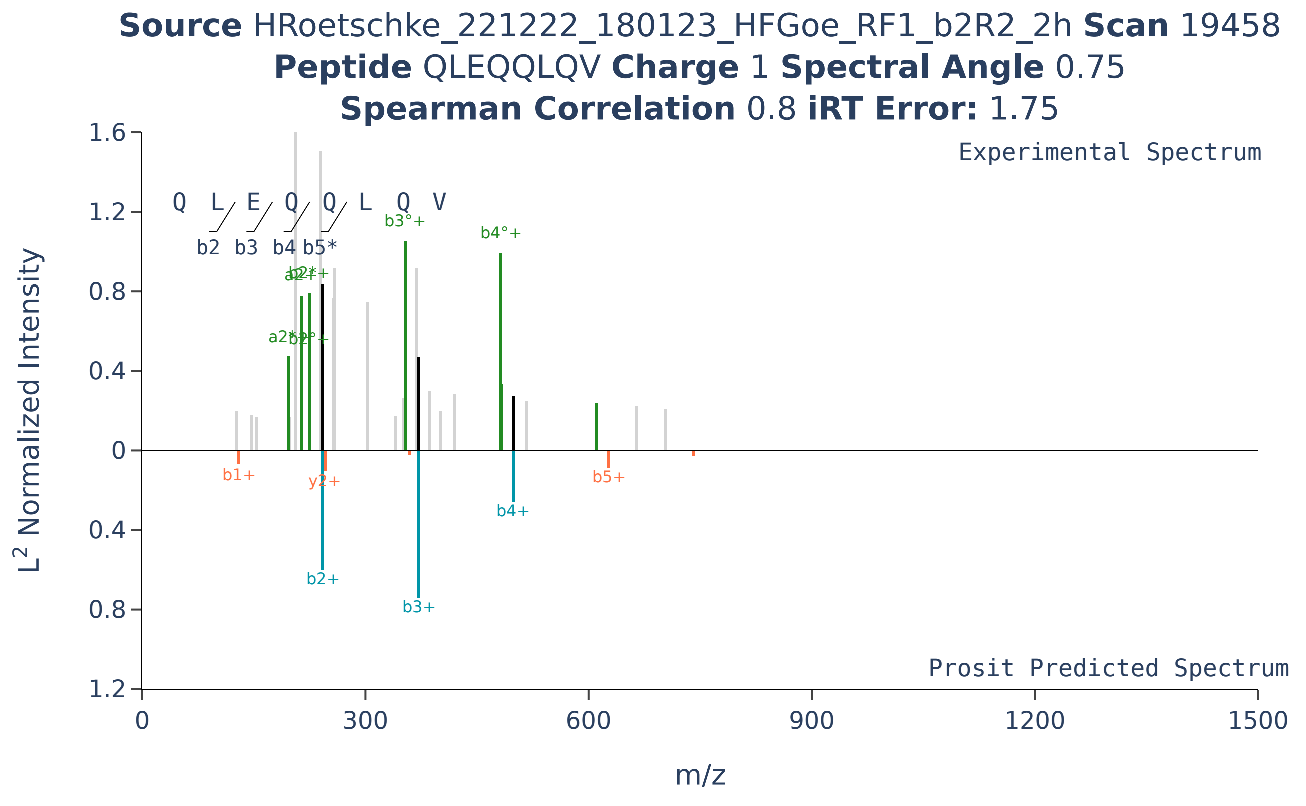
Source HRoetschke_221222_180123_HFGoe_RF1_b2R2_2h **Scan** 19458
Peptide QLEQQLQV **Charge** 1 **Spectral Angle** 0.75
Spearman Correlation 0.8 **iRT Error:** 1.75
Experimental Spectrum
Prosit Predicted Spectrum
L² Normalized Intensity
m/z

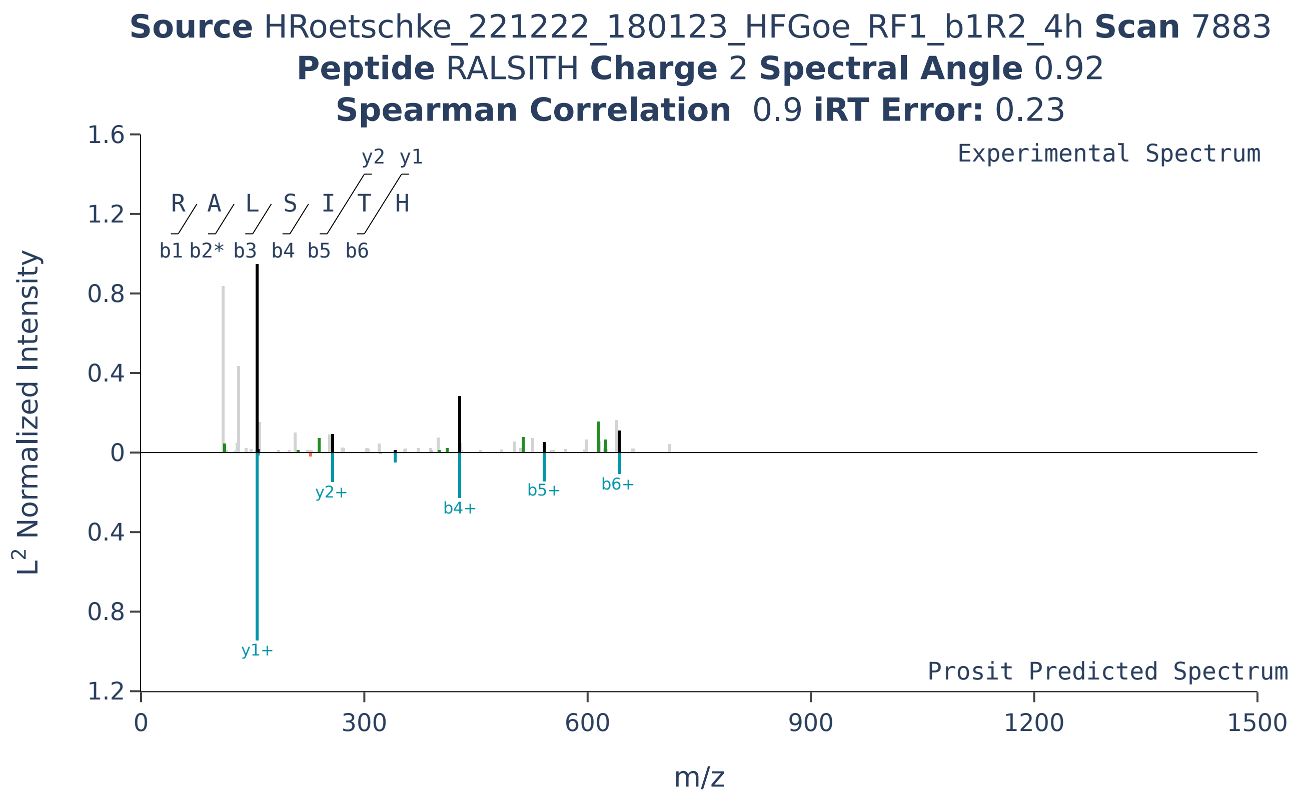
Source HRoetschke_221222_180123_HFGoe_RF1_b1R2_4h **Scan** 7883
Peptide RALSITH **Charge** 2 **Spectral Angle** 0.92
Spearman Correlation 0.9 **iRT Error:** 0.23
Experimental Spectrum
Prosit Predicted Spectrum
L² Normalized Intensity
m/z

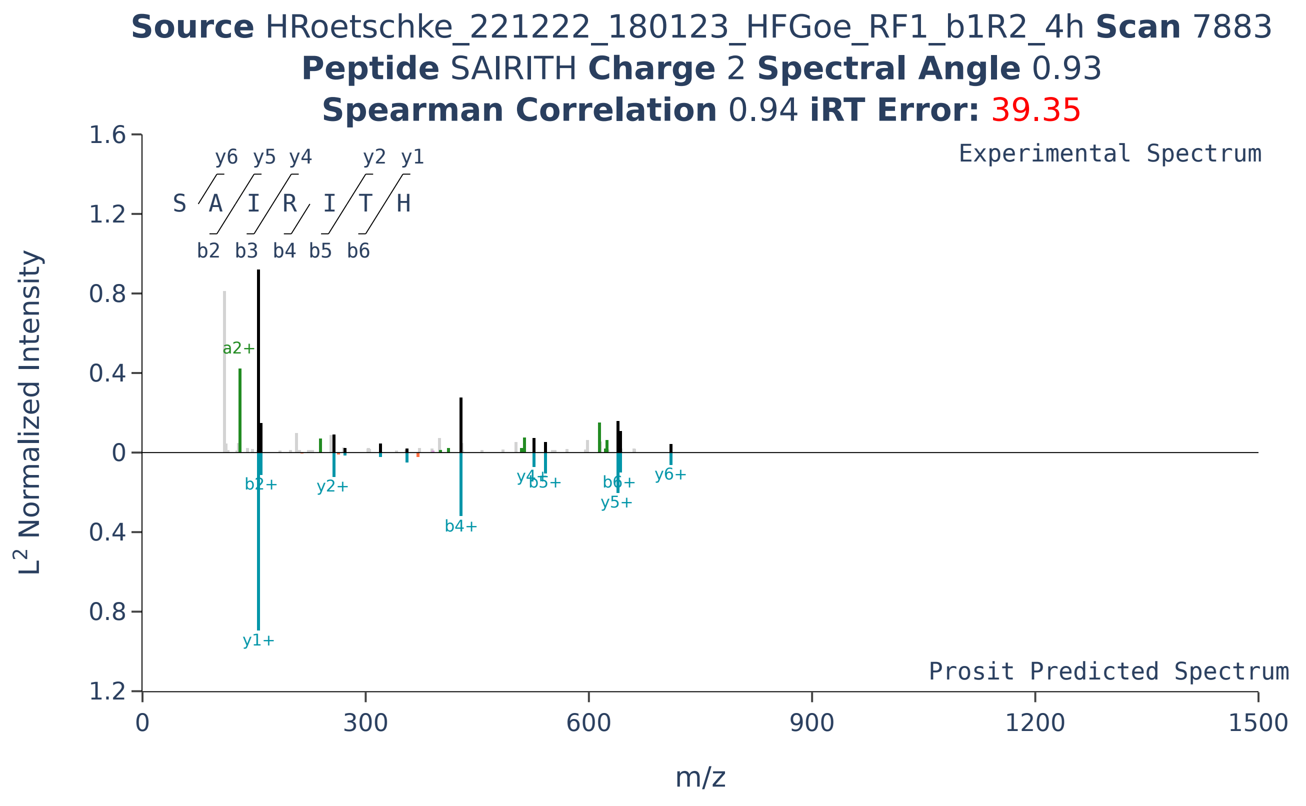
Source HRoetschke_221222_180123_HFGoe_RF1_b1R2_4h **Scan** 7883
Peptide SAIRITH **Charge** 2 **Spectral Angle** 0.93
Spearman Correlation 0.94 **iRT Error:** 39.35
Experimental Spectrum
Prosit Predicted Spectrum
L² Normalized Intensity
m/z

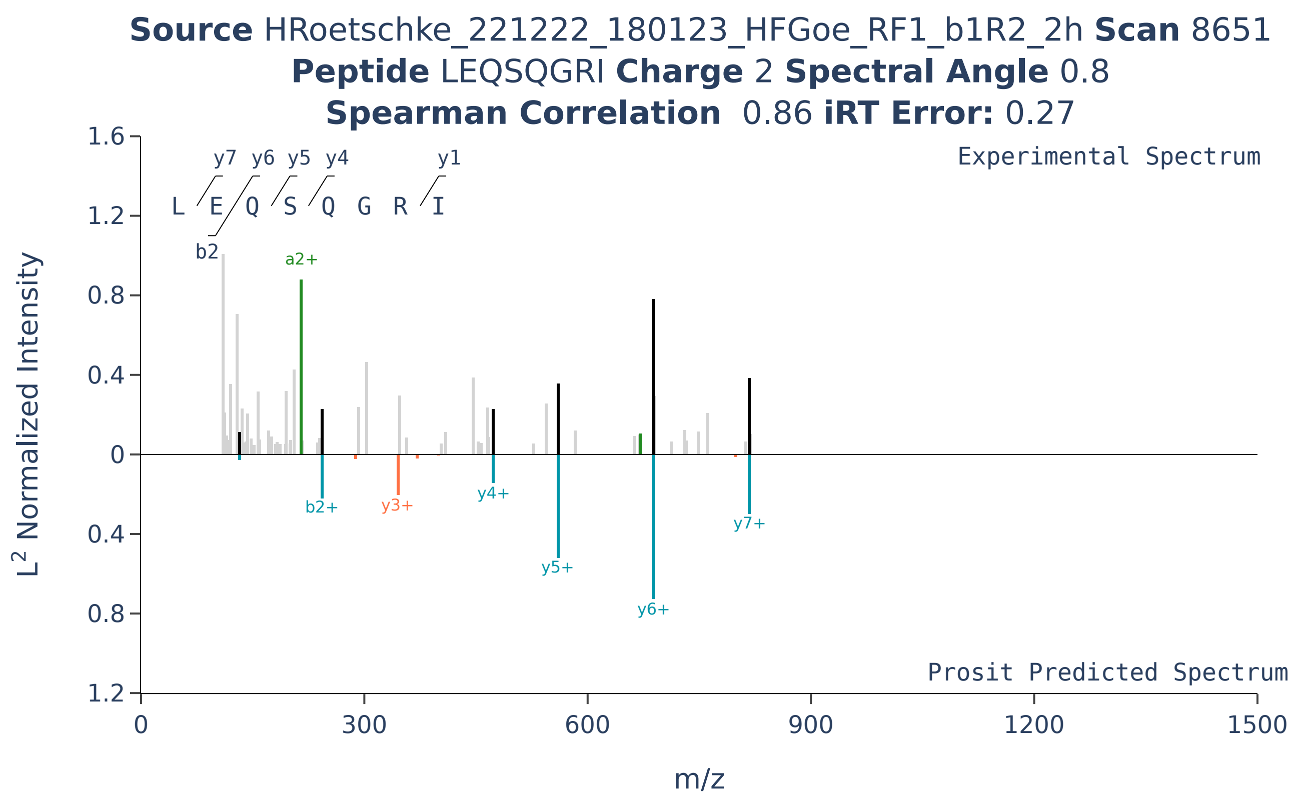
Source HRoetschke_221222_180123_HFGoe_RF1_b1R2_2h **Scan** 8651
Peptide LEQSQGRI **Charge** 2 **Spectral Angle** 0.8
Spearman Correlation 0.86 **iRT Error:** 0.27
Experimental Spectrum
Prosit Predicted Spectrum
L² Normalized Intensity
m/z

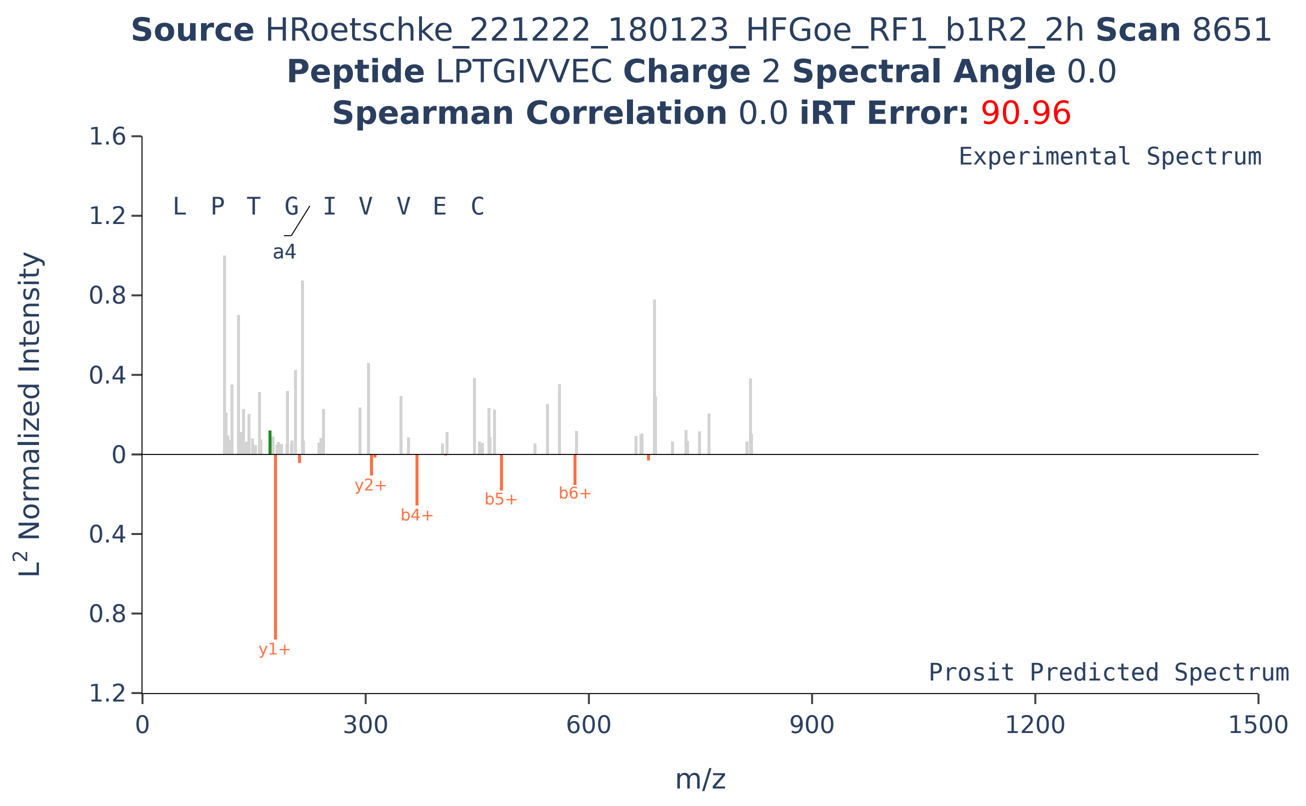
Source HRoetschke_221222_180123_HFGoe_RF1_b1R2_2h **Scan** 8651
Peptide LPTGIVVEC **Charge** 2 **Spectral Angle** 0.0
Spearman Correlation 0.0 **iRT Error:** 90.96
Experimental Spectrum
Prosit Predicted Spectrum
L² Normalized Intensity
m/z

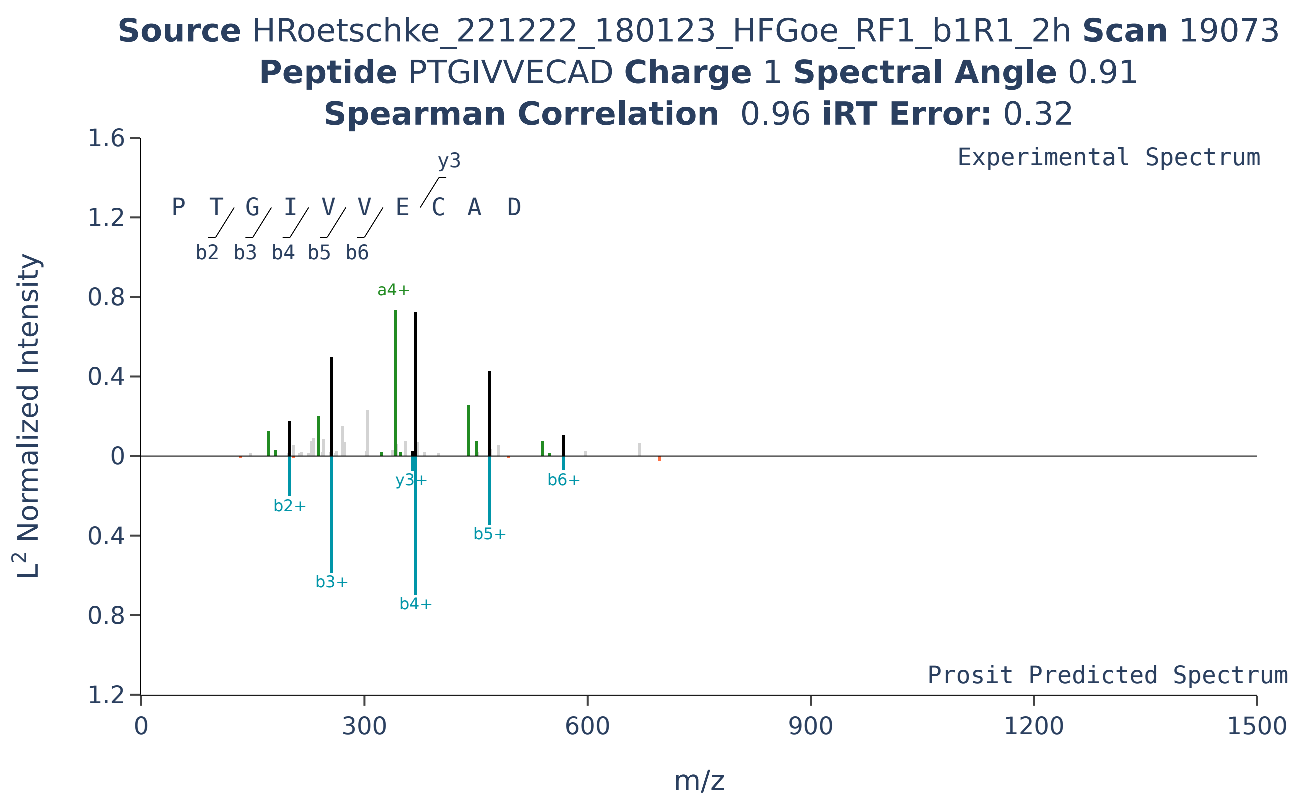
Source HRoetschke_221222_180123_HFGoe_RF1_b1R1_2h **Scan** 19073
Peptide PTGIVVECAD **Charge** 1 **Spectral Angle** 0.91
Spearman Correlation 0.96 **iRT Error:** 0.32
Experimental Spectrum
Prosit Predicted Spectrum
L² Normalized Intensity
m/z

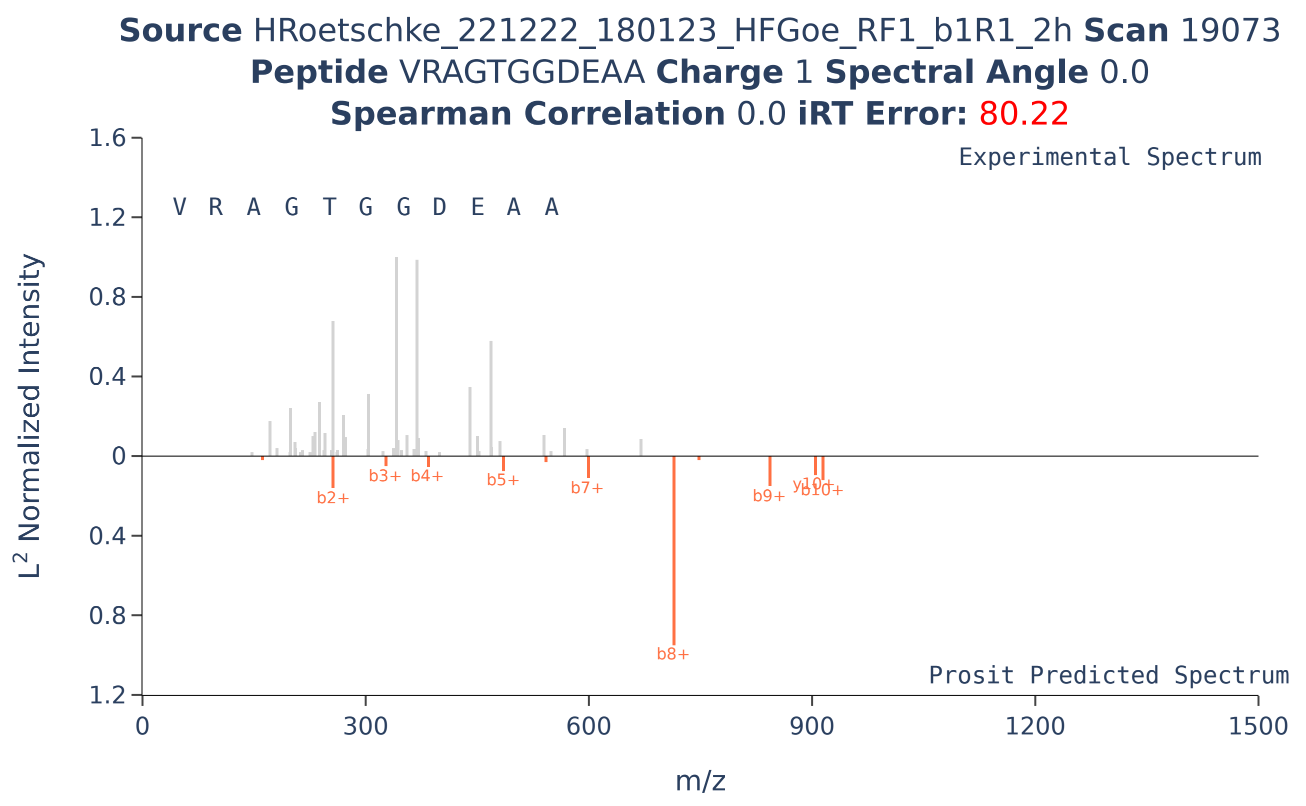
Source HRoetschke_221222_180123_HFGoe_RF1_b1R1_2h **Scan** 19073
Peptide VRAGTGGDEAA **Charge** 1 **Spectral Angle** 0.0
Spearman Correlation 0.0 **iRT Error:** 80.22
Experimental Spectrum
Prosit Predicted Spectrum
L² Normalized Intensity
m/z

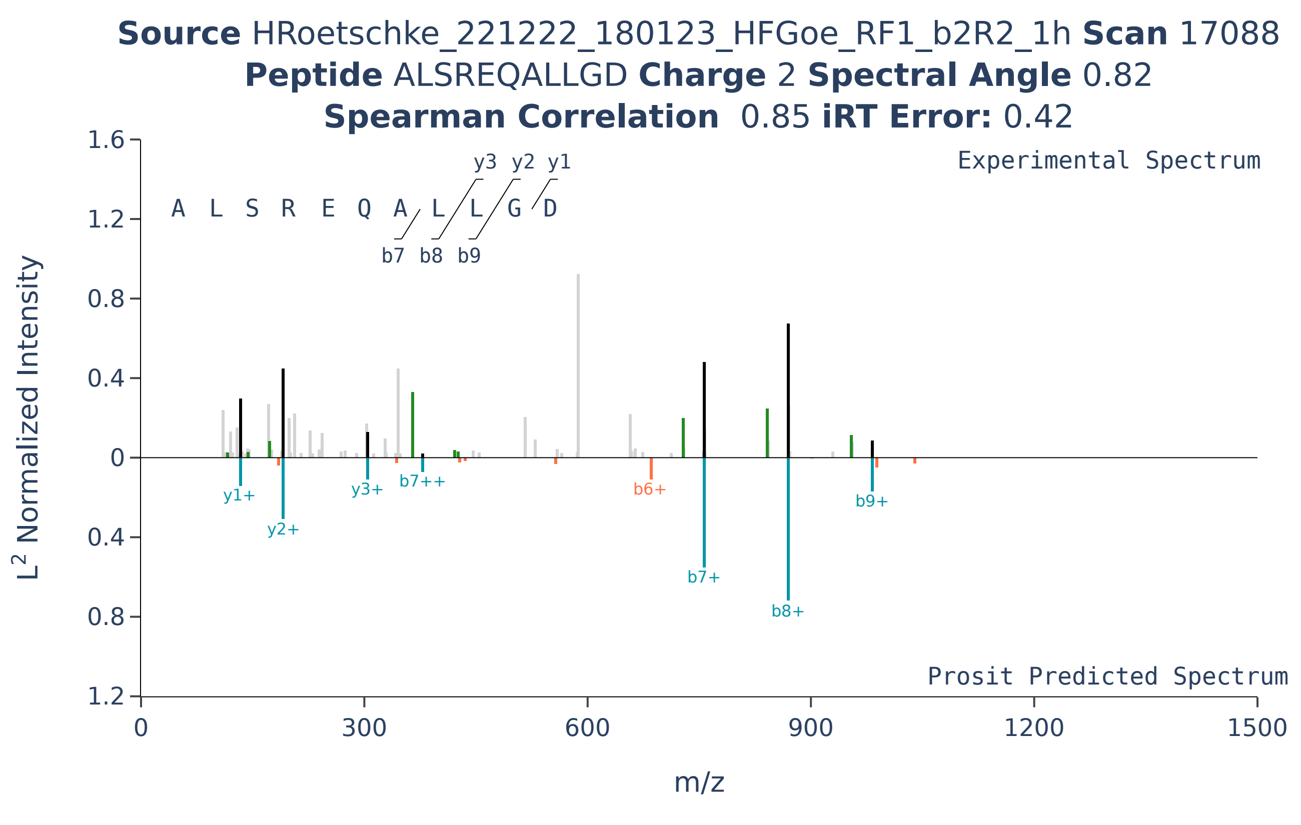
Source HRoetschke_221222_180123_HFGoe_RF1_b2R2_1h **Scan** 17088
Peptide ALSREQALLGD **Charge** 2 **Spectral Angle** 0.82
Spearman Correlation 0.85 **iRT Error:** 0.42
Experimental Spectrum
Prosit Predicted Spectrum
L² Normalized Intensity
m/z

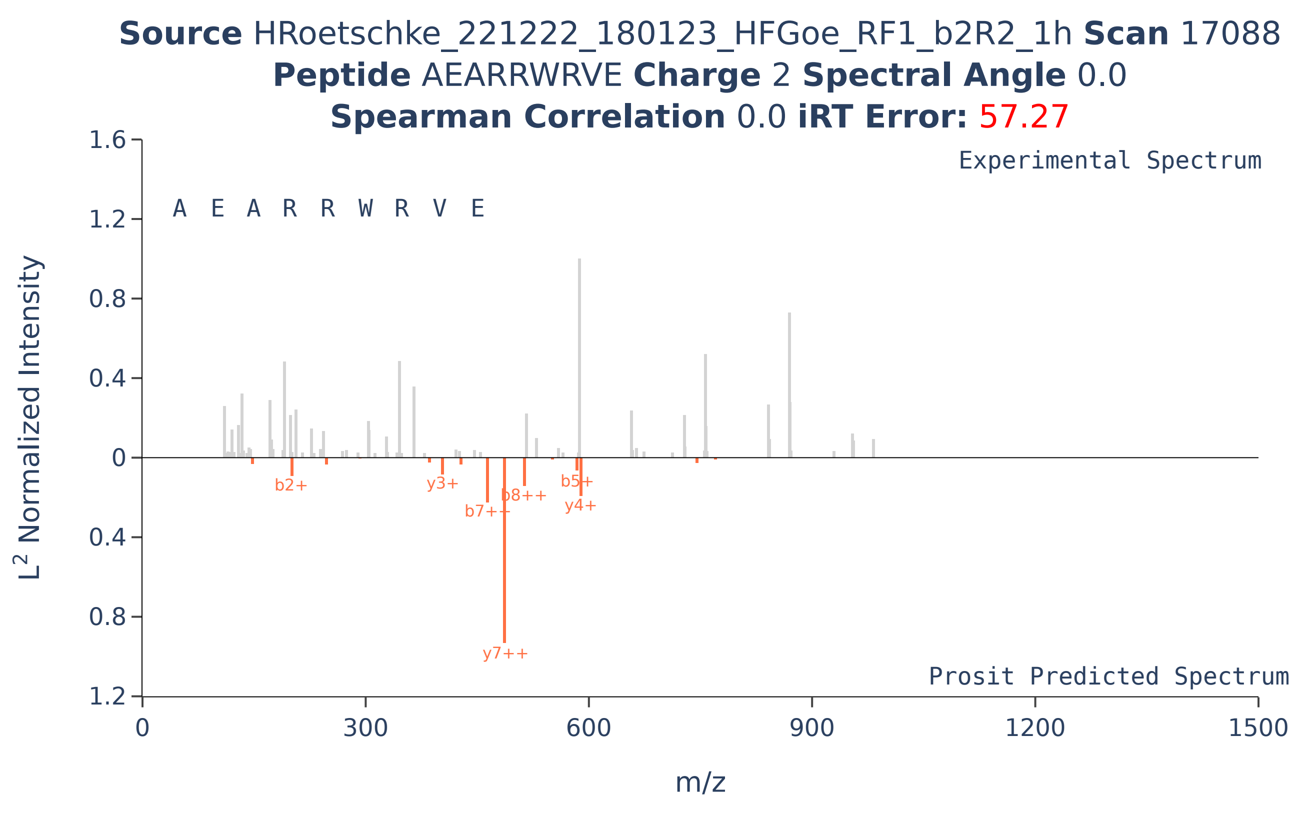
Source HRoetschke_221222_180123_HFGoe_RF1_b2R2_1h **Scan** 17088
Peptide AEARRWRVE **Charge** 2 **Spectral Angle** 0.0
Spearman Correlation 0.0 **iRT Error:** 57.27
Experimental Spectrum
Prosit Predicted Spectrum
L² Normalized Intensity
m/z

Source HRoetschke_221222_180123_HFGoe_RF1_b1R1_24h **Scan** 8287
Peptide RNLLGSGSDR **Charge** 3 **Spectral Angle** 0.82
Spearman Correlation 0.82 **iRT Error:** 0.46

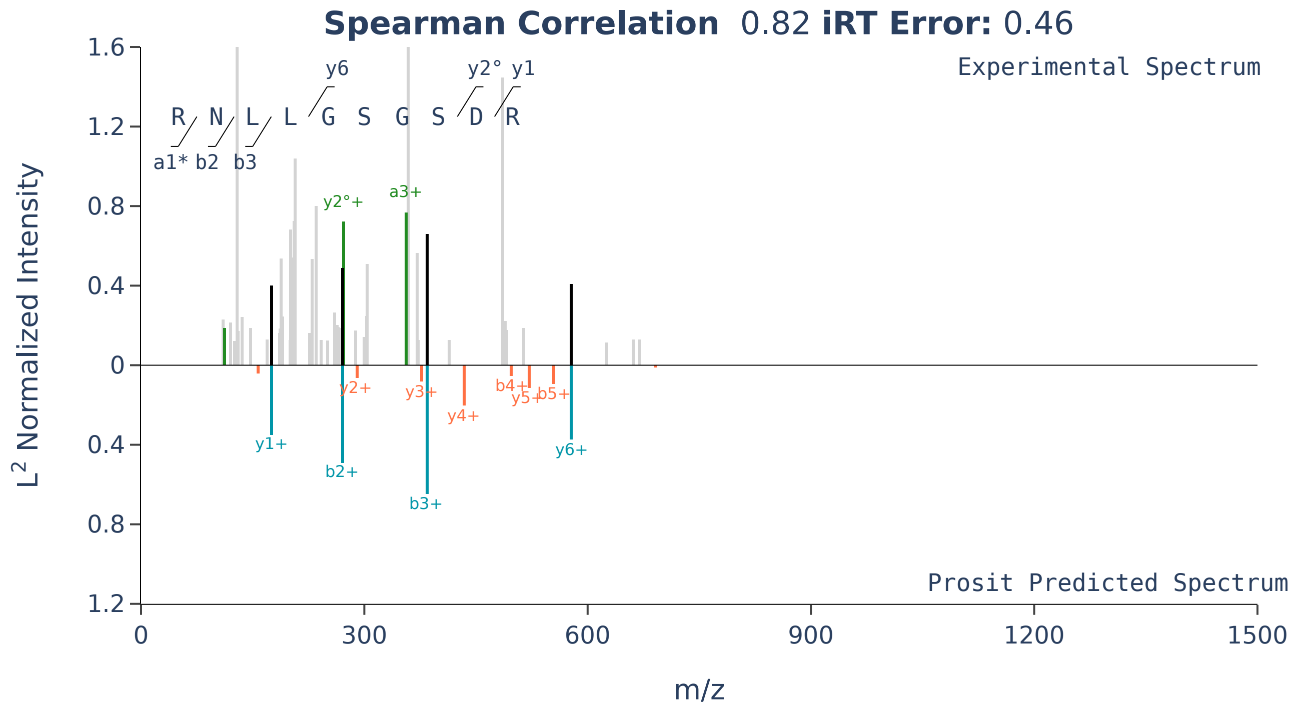

Source HRoetschke_221222_180123_HFGoe_RF1_b1R1_24h **Scan** 8287
Peptide RNLLGSGDRS **Charge** 3 **Spectral Angle** 0.84
Spearman Correlation 0.67 **iRT Error:** 0.14

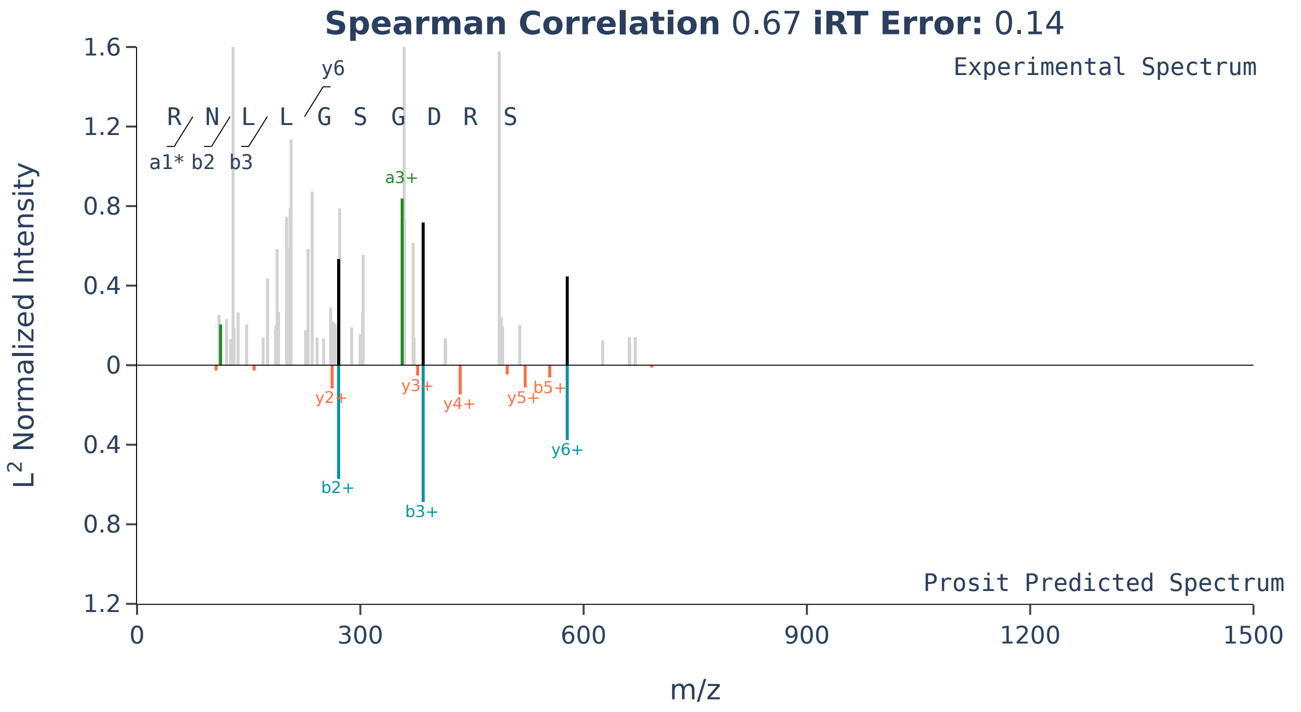

Source HRoetschke_221222_180123_HFGoe_RF1_b2R1_2h **Scan** 18521
Peptide PTGIVVECEG **Charge** 1 **Spectral Angle** 0.89
Spearman Correlation 0.91 **iRT Error:** 0.68

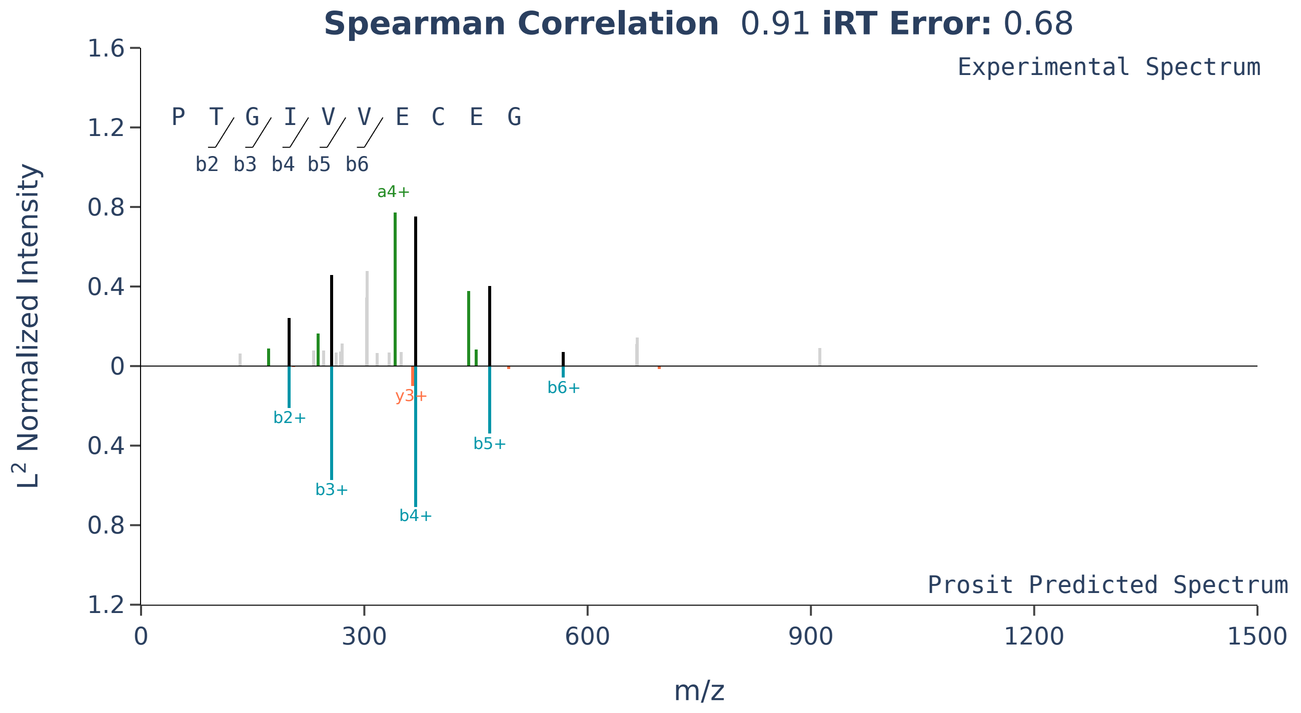

Source HRoetschke_221222_180123_HFGoe_RF1_b2R1_2h **Scan** 18521
Peptide VRAGTGGDEAA **Charge** 1 **Spectral Angle** 0.0
Spearman Correlation 0.0 **iRT Error:** 78.89

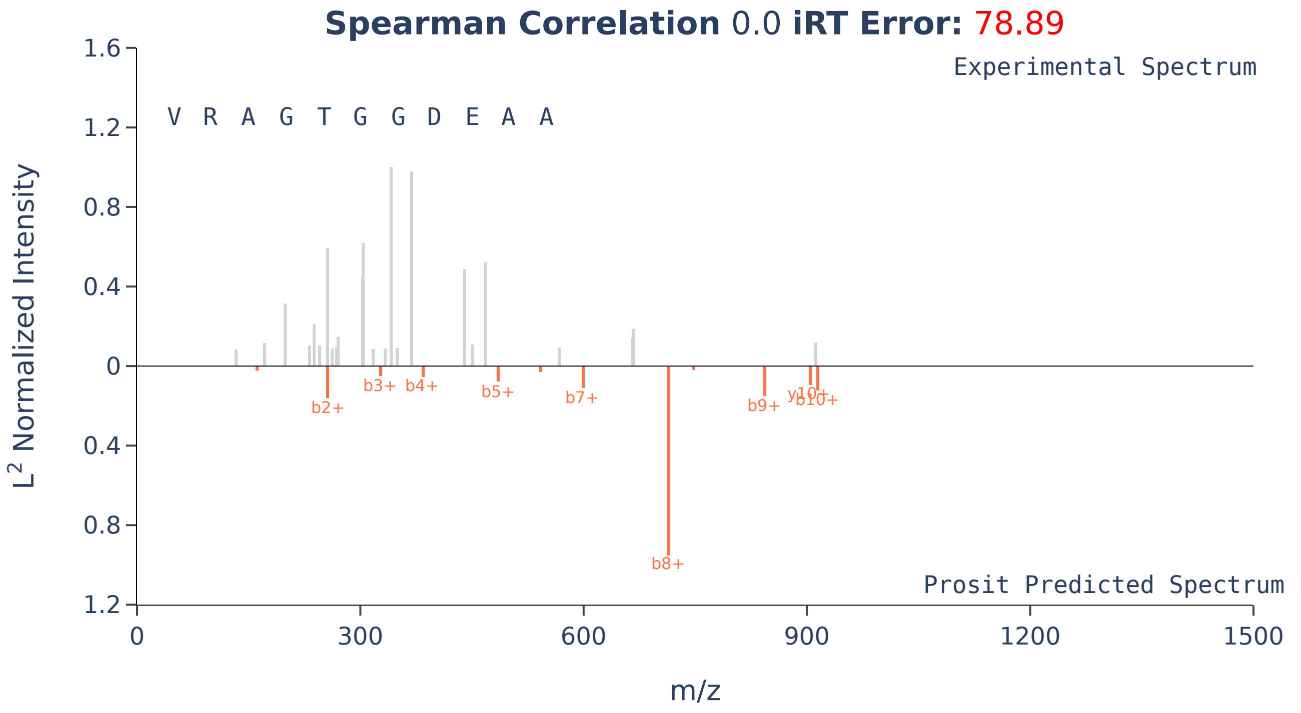

Source HRoetschke_221222_180123_HFGoe_RF1_b2R2_2h **Scan** 11142
Peptide GGDEAQTI **Charge** 1 **Spectral Angle** 0.79
Spearman Correlation 0.89 **iRT Error:** 0.73

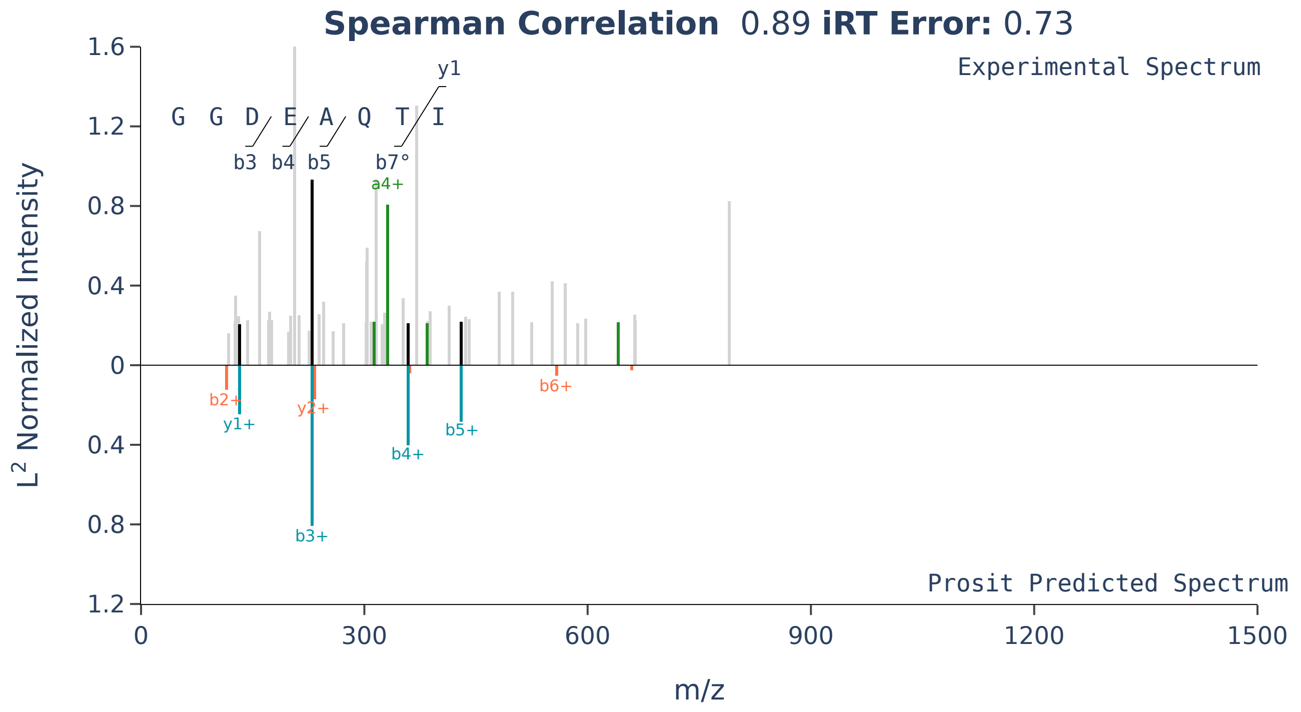

Source HRoetschke_221222_180123_HFGoe_RF1_b2R2_2h **Scan** 11142
Peptide GDAQTIAD **Charge** 1 **Spectral Angle** 0.19
Spearman Correlation 0.25 **iRT Error:** 15.58

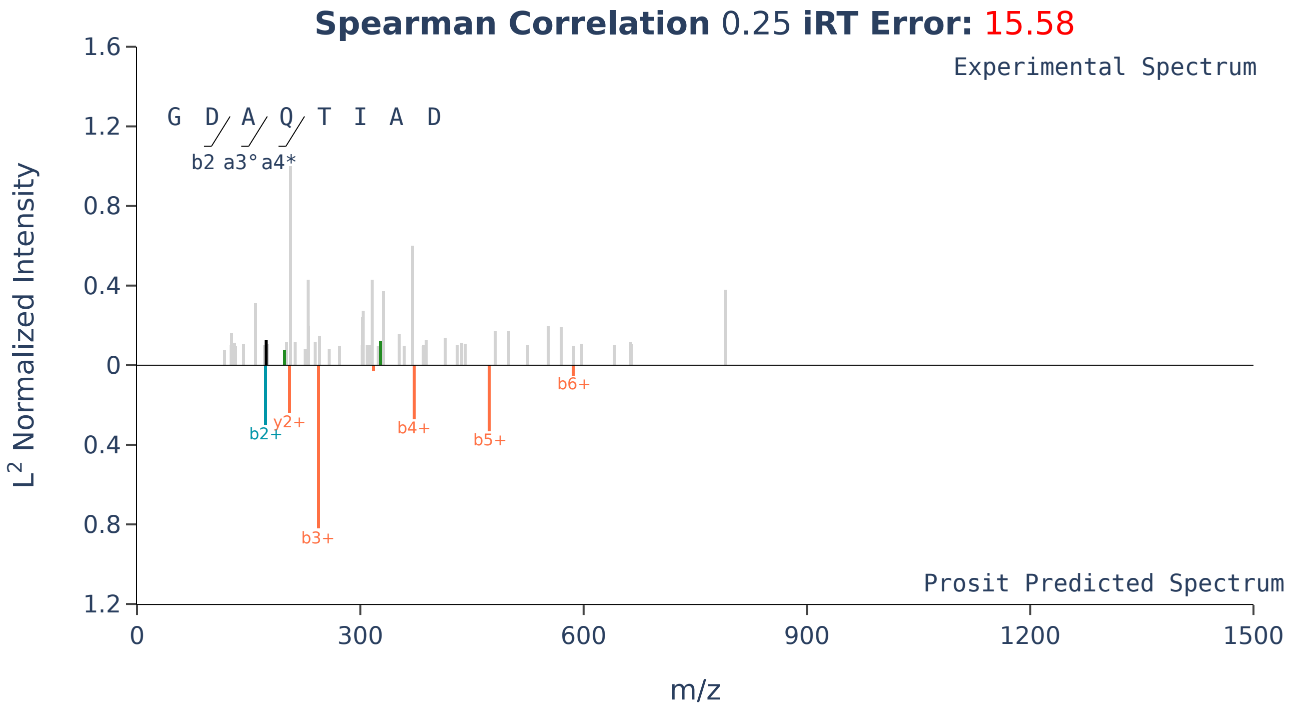

Source HRoetschke_221222_180123_HFGoe_RF1_b2R2_1h **Scan** 18593
Peptide PTGIVVECDA **Charge** 1 **Spectral Angle** 0.88
Spearman Correlation 0.91 **iRT Error:** 0.8

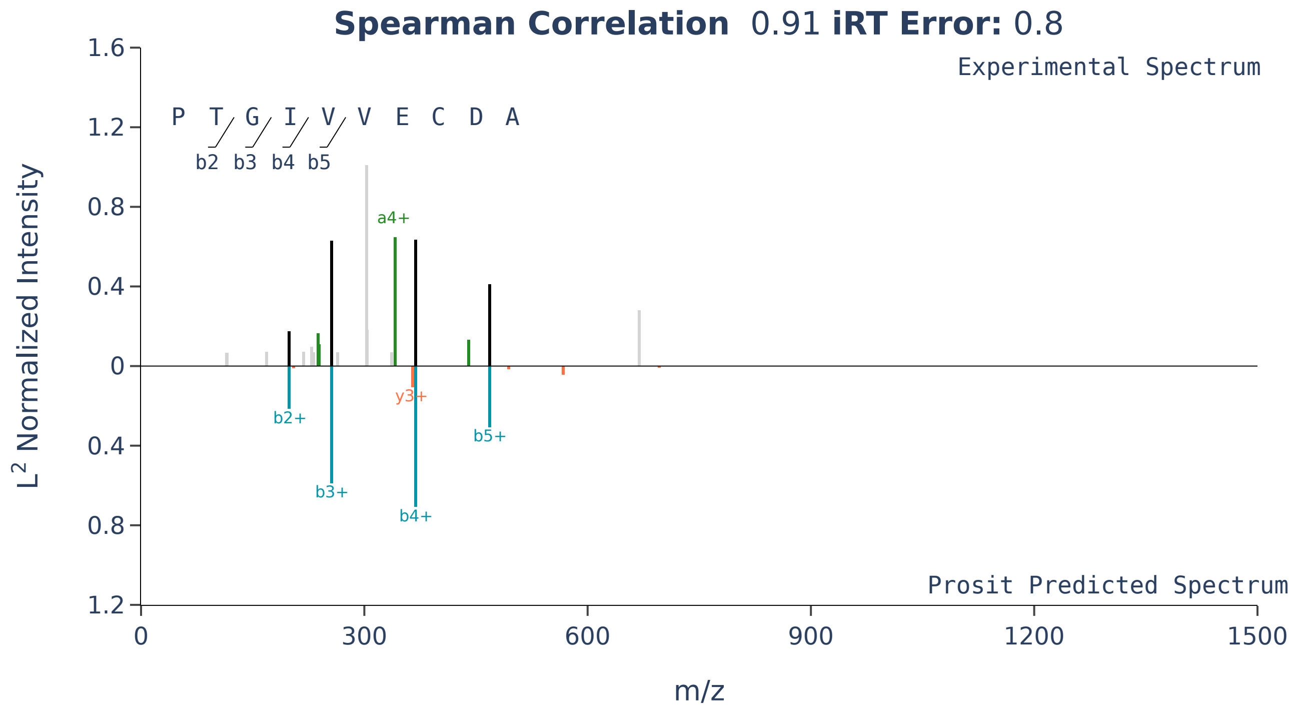

Source HRoetschke_221222_180123_HFGoe_RF1_b2R2_1h **Scan** 18593
Peptide VRAGTGGDEAA **Charge** 1 **Spectral Angle** 0.0
Spearman Correlation 0.0 **iRT Error:** 79.86

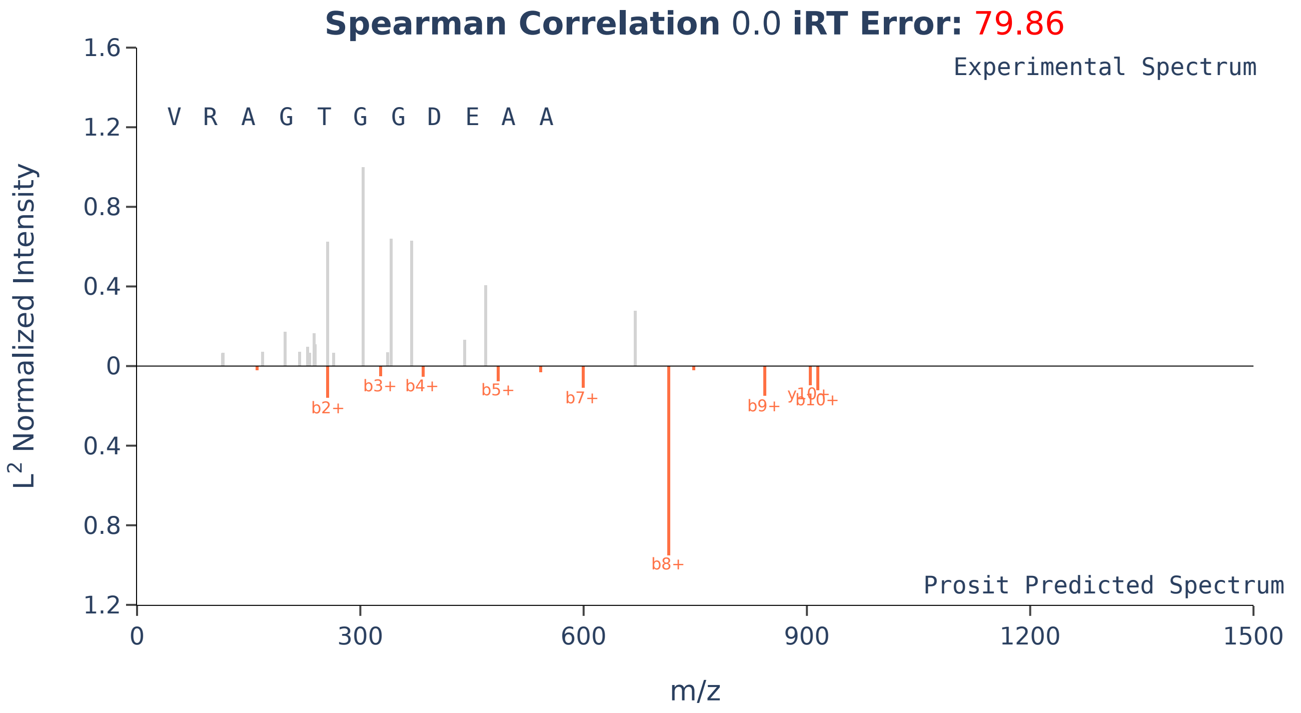

Source HRoetschke_221222_180123_HFGoe_RF1_b1R1_24h **Scan** 17486
Peptide AALFADG **Charge** 1 **Spectral Angle** 0.96
Spearman Correlation 0.97 **iRT Error:** 0.84

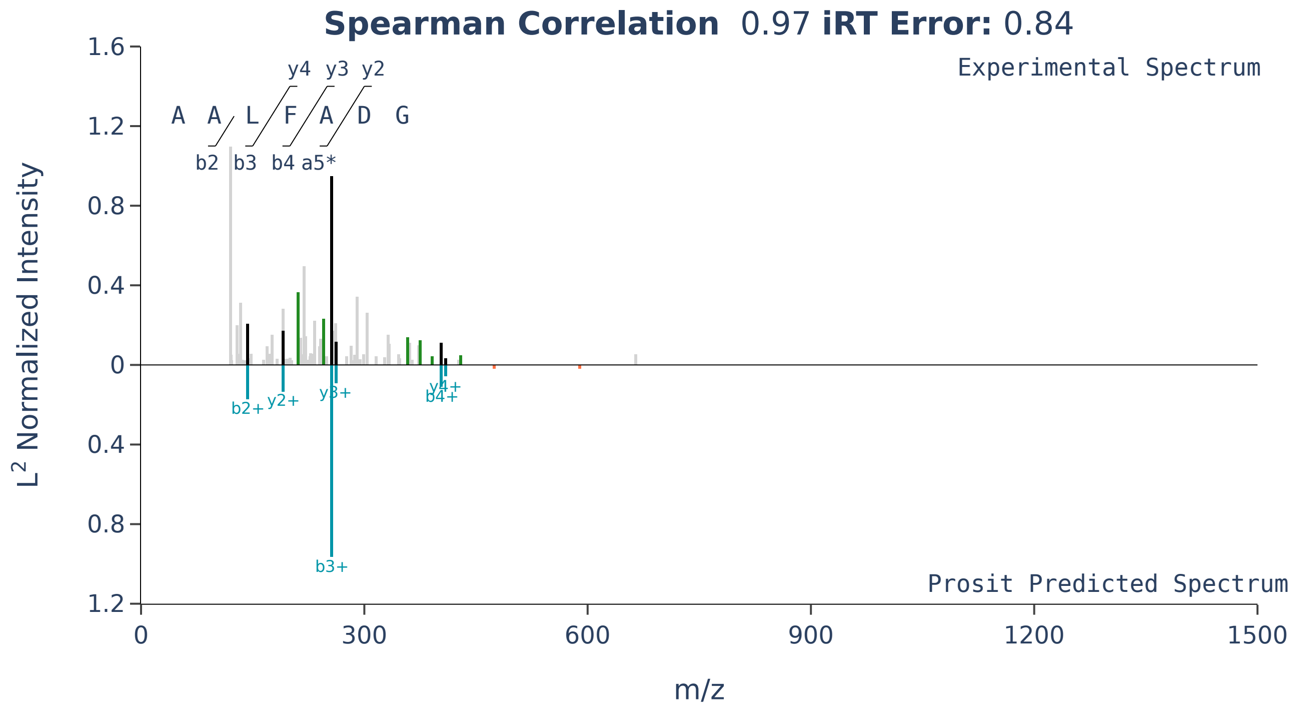

Source HRoetschke_221222_180123_HFGoe_RF1_b1R1_24h **Scan** 17486
Peptide AALFAGD **Charge** 1 **Spectral Angle** 0.8
Spearman Correlation 0.53 **iRT Error:** 2.82

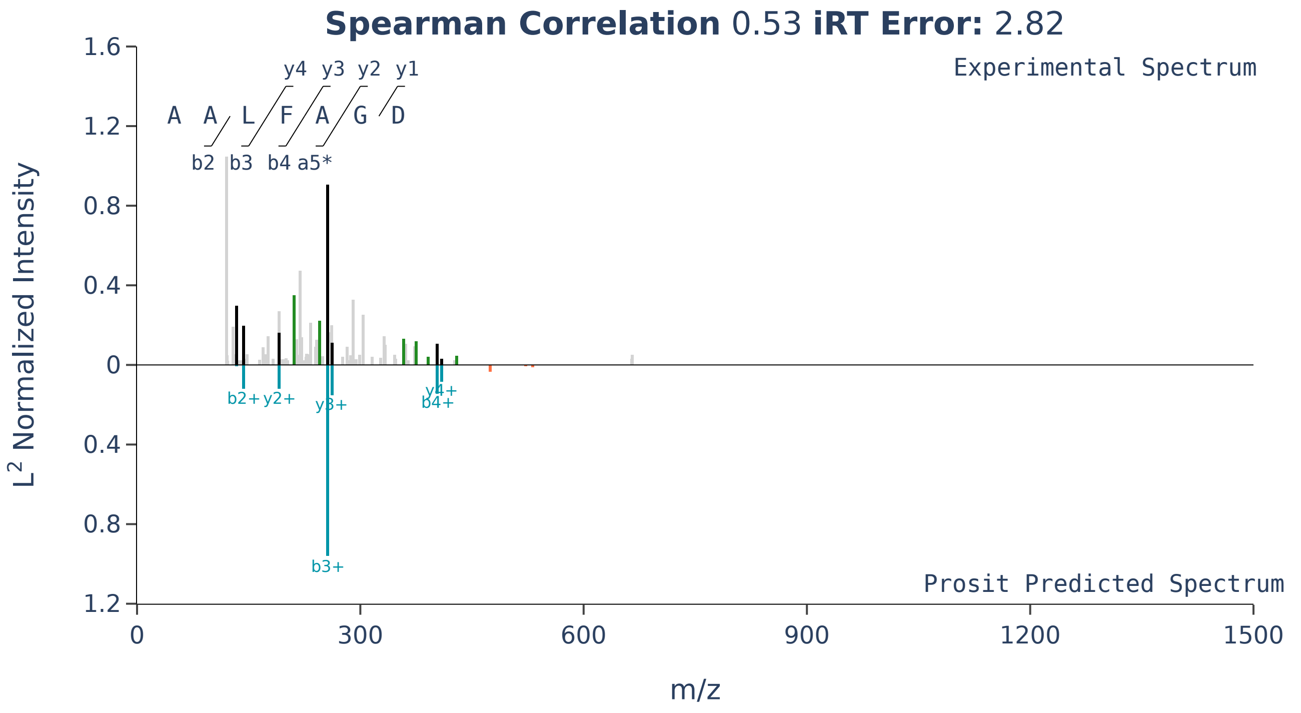

Source HRoetschke_221222_180123_HFGoe_RF1_b1R1_24h **Scan** 7869
Peptide GTIRITH **Charge** 2 **Spectral Angle** 0.91
Spearman Correlation 0.87 **iRT Error:** 0.85

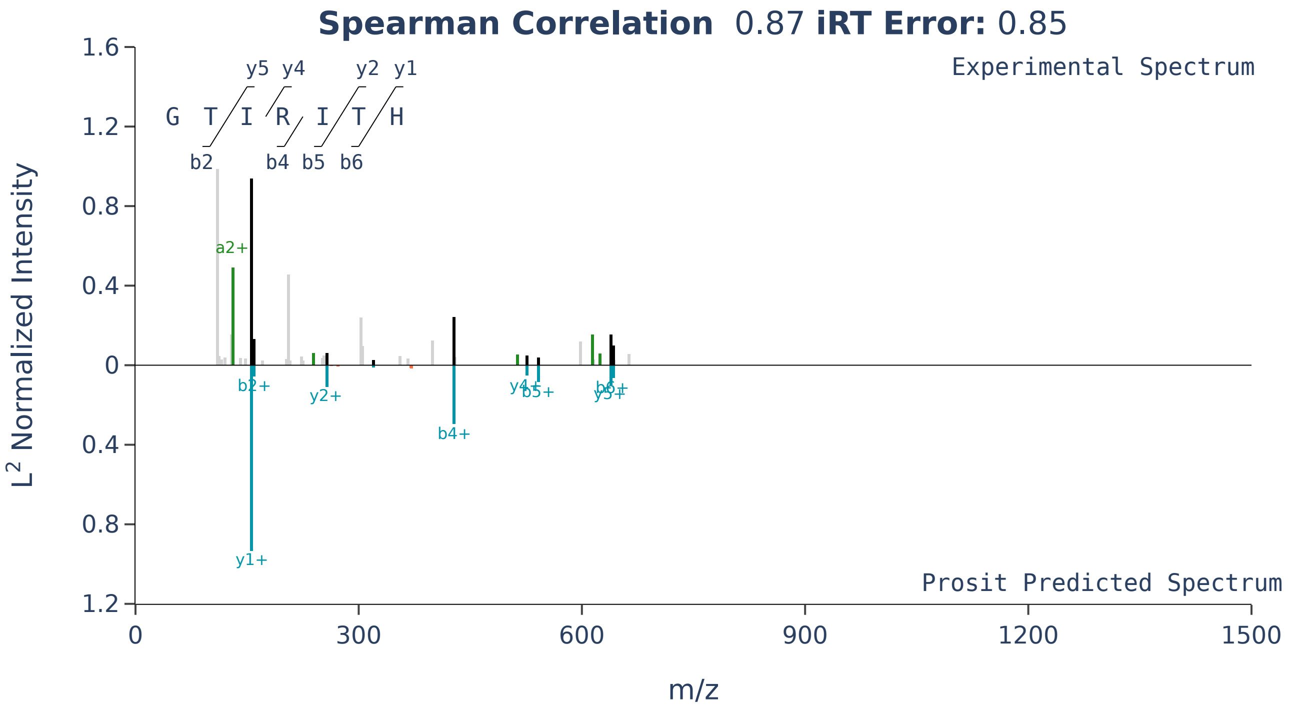

Source HRoetschke_221222_180123_HFGoe_RF1_b1R1_24h **Scan** 7869
Peptide SAIRITH **Charge** 2 **Spectral Angle** 0.9
Spearman Correlation 0.92 **iRT Error:** 40.22

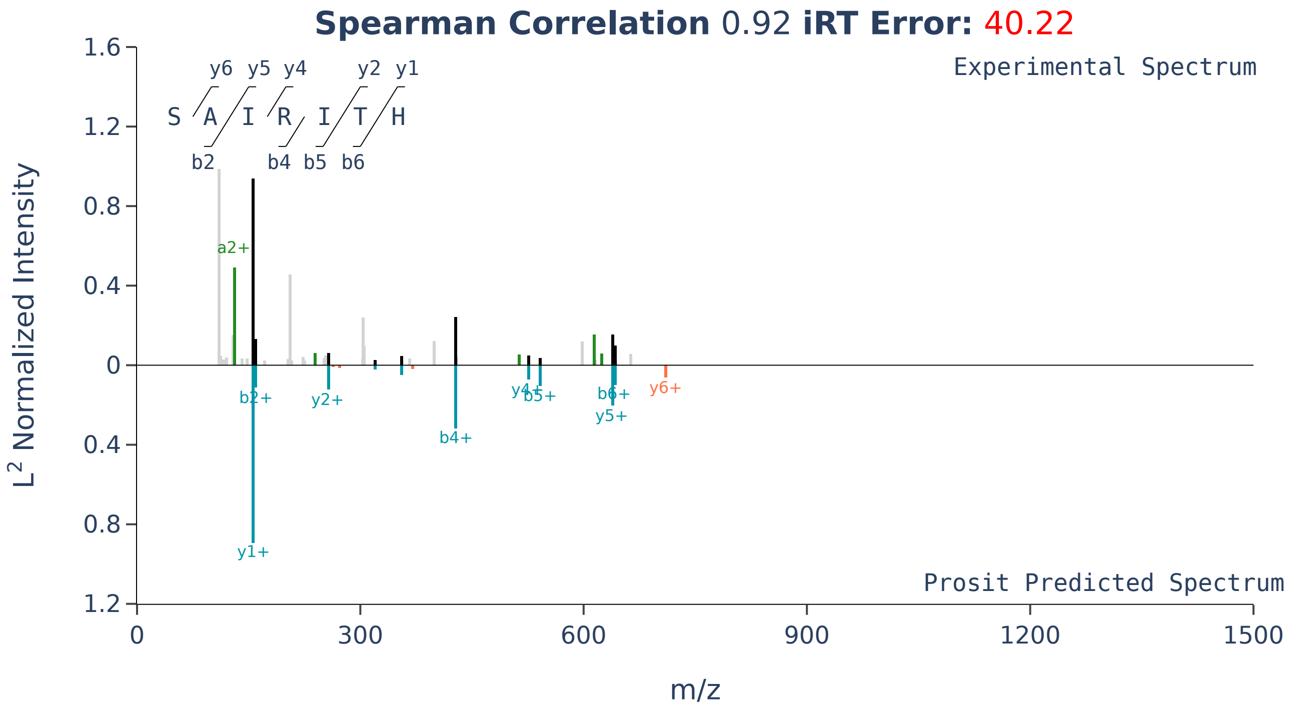

Source HRoetschke_221222_180123_HFGoe_RF1_b1R1_24h **Scan** 14815
Peptide GTLYRLDE **Charge** 2 **Spectral Angle** 0.86
Spearman Correlation 0.87 **iRT Error:** 0.94

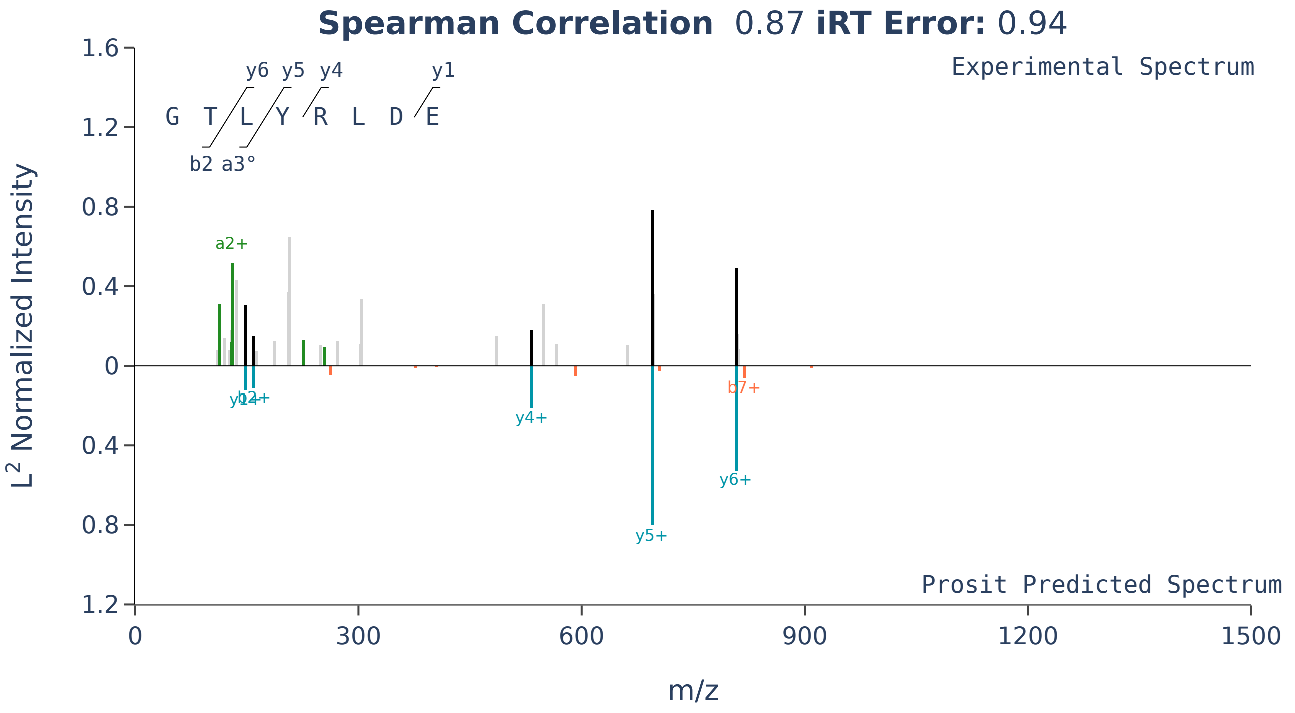

Source HRoetschke_221222_180123_HFGoe_RF1_b1R1_24h **Scan** 14815
Peptide AKISGDGVYG **Charge** 2 **Spectral Angle** 0.0
Spearman Correlation 0.0 **iRT Error:** 29.97

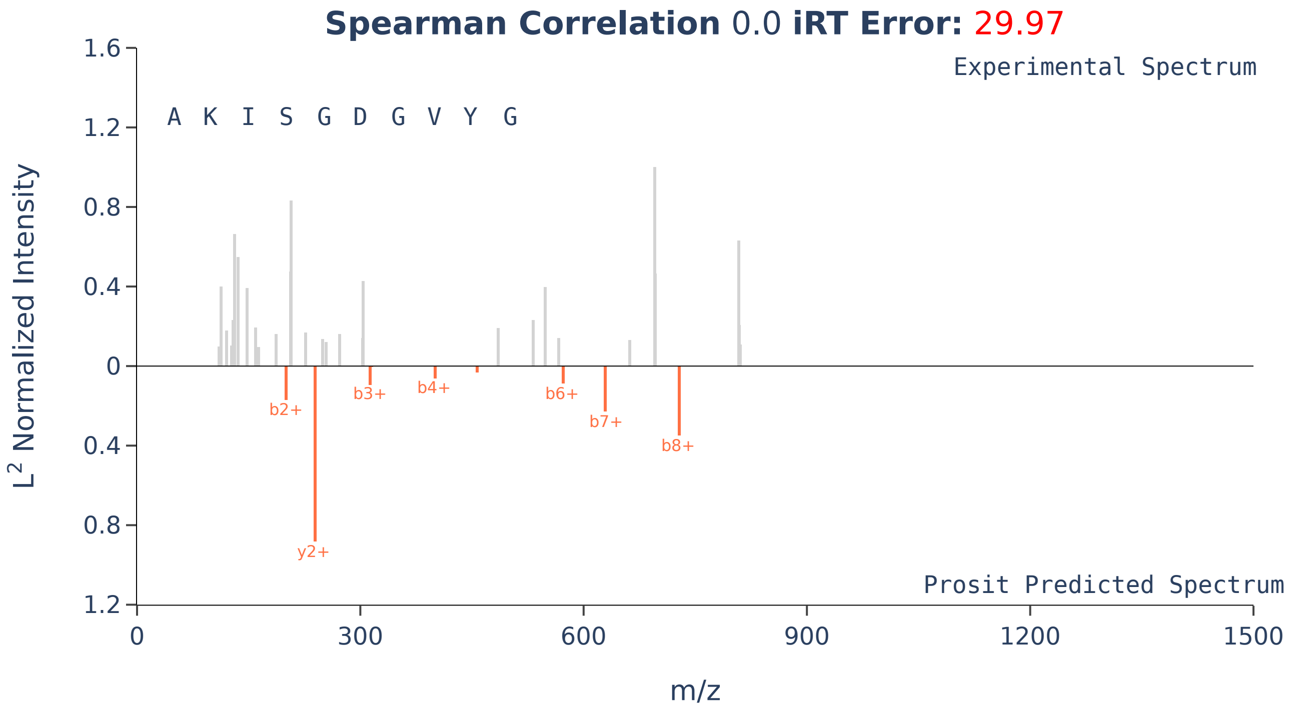

Source HRoetschke_221222_180123_HFGoe_RF1_b1R1_2h **Scan** 13369
Peptide STRGAGGQHVNTTDSAIRI **Charge** 4 **Spectral Angle** 0.81
Spearman Correlation 0.85 **iRT Error:** 1.22

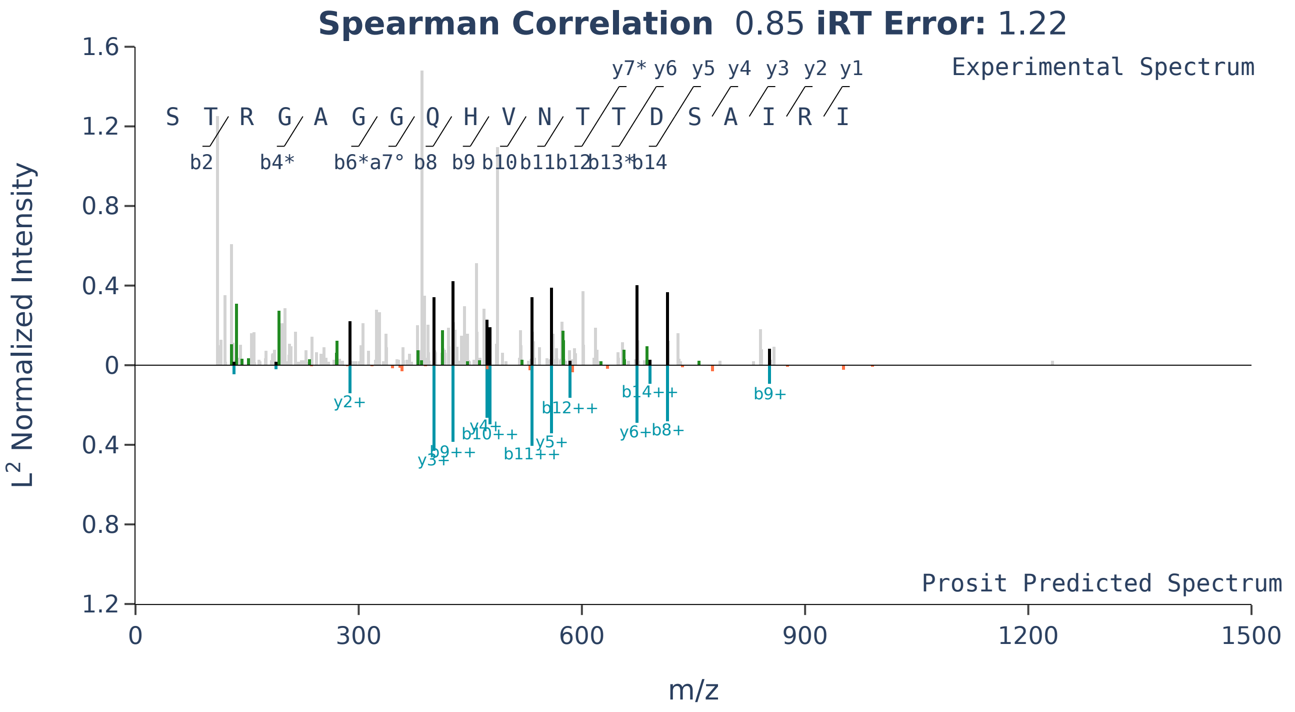

Source HRoetschke_221222_180123_HFGoe_RF1_b1R1_2h **Scan** 13369
Peptide HAAEMAKRQQAEASTRR **Charge** 4 **Spectral Angle** 0.19
Spearman Correlation 0.12 **iRT Error:** 61.32

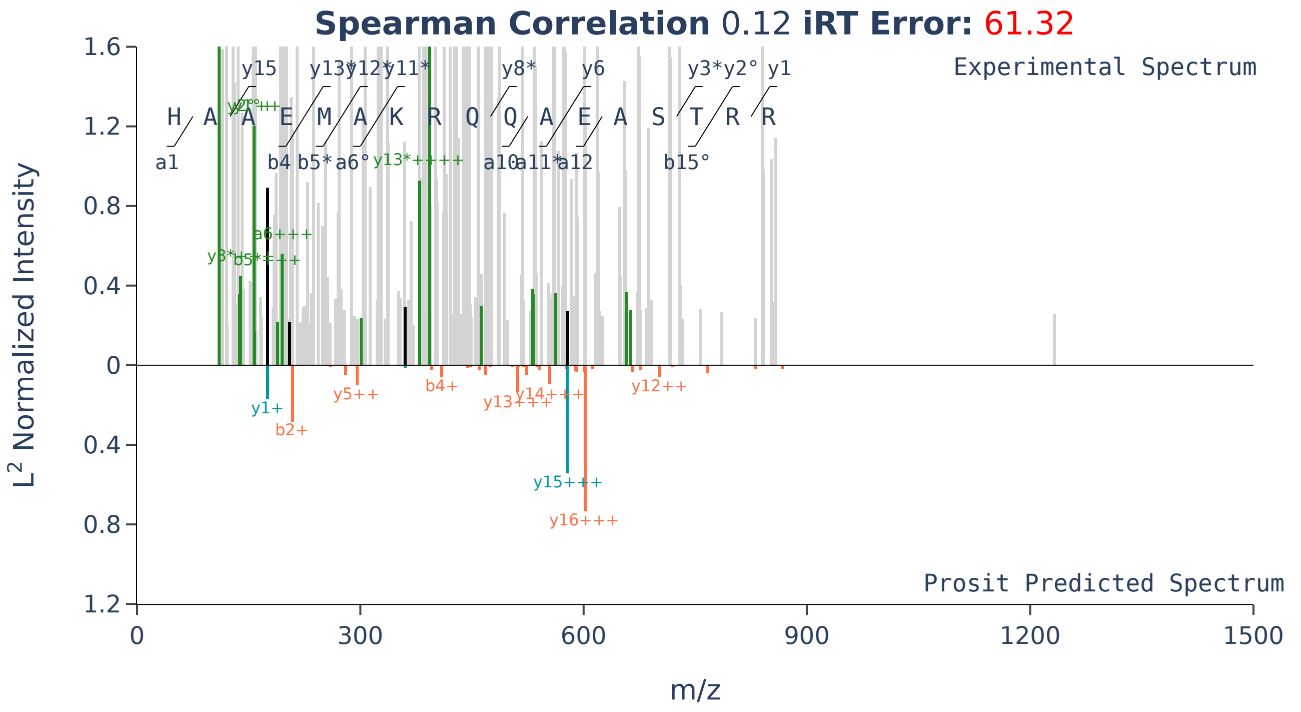

Source HRoetschke_221222_180123_HFGoe_RF1_b1R2_2h **Scan** 7983
Peptide DQQTIAD **Charge** 2 **Spectral Angle** 0.69
Spearman Correlation 0.96 **iRT Error:** 1.29

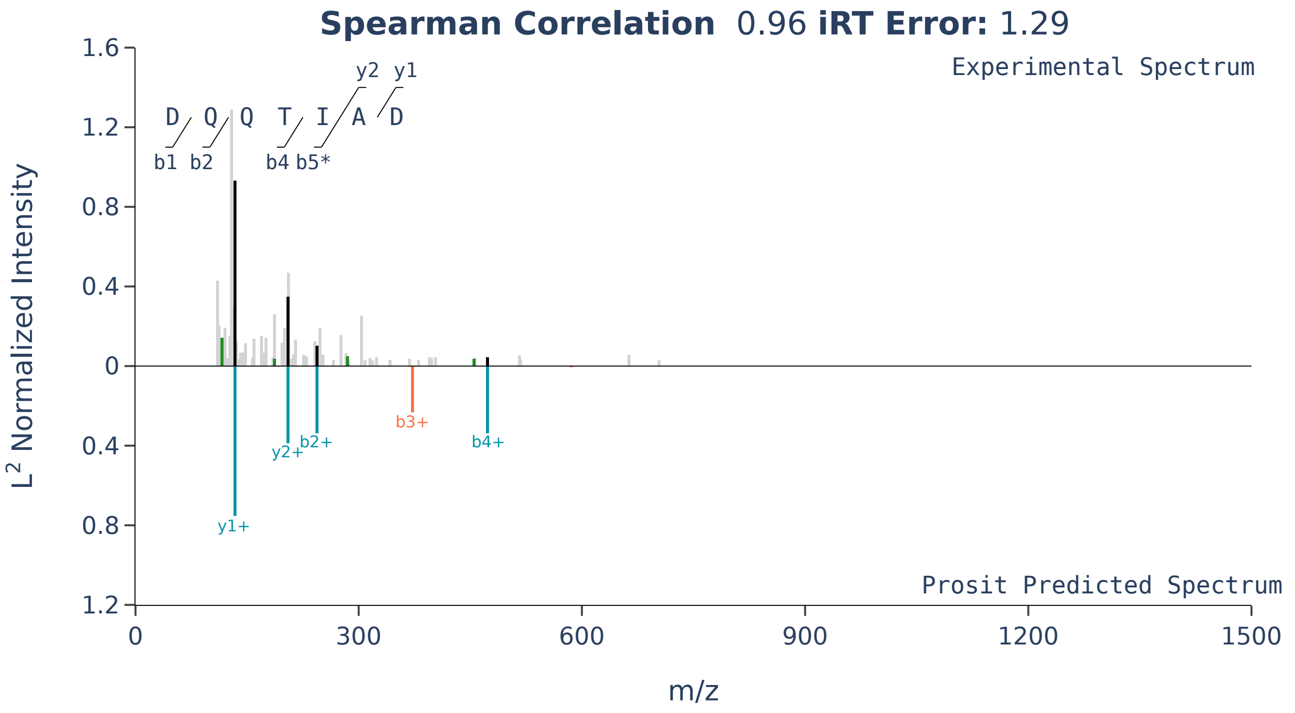

Source HRoetschke_221222_180123_HFGoe_RF1_b1R2_2h **Scan** 7983
Peptide GTGGDEAAL **Charge** 2 **Spectral Angle** 0.09
Spearman Correlation 0.31 **iRT Error:** 26.08

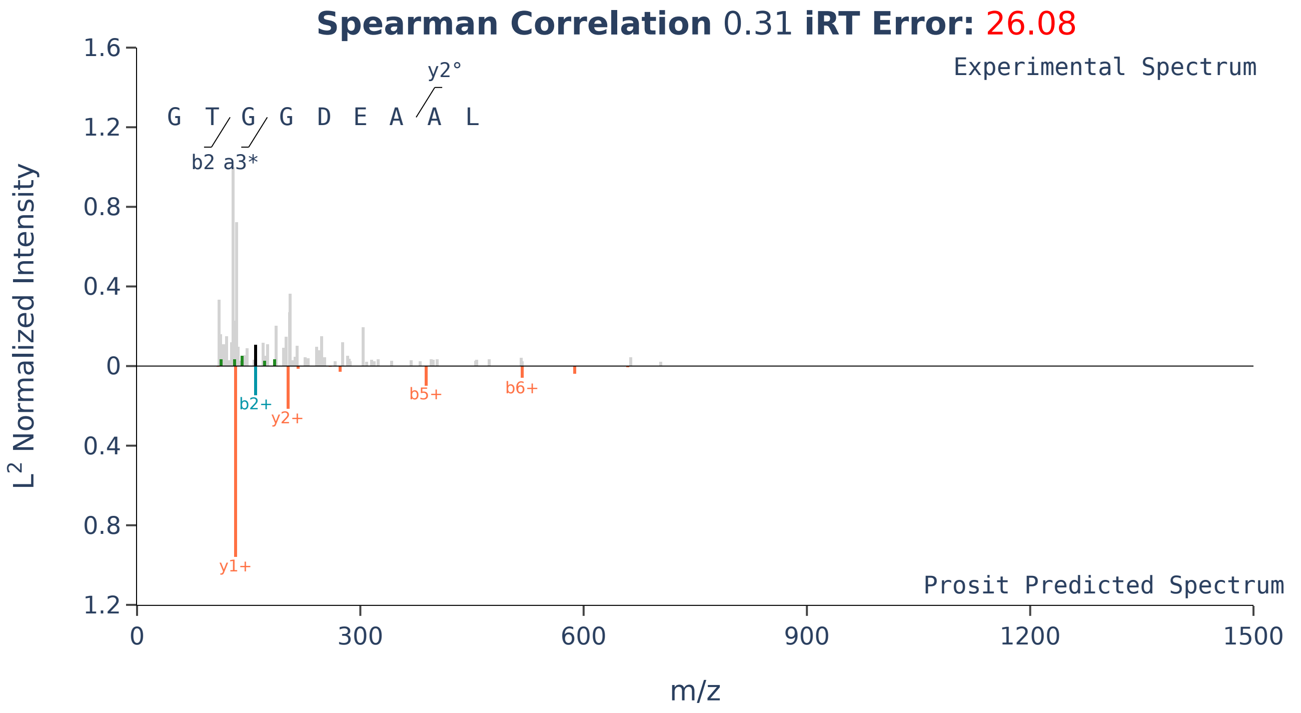

Source HRoetschke_221222_180123_HFGoe_RF1_b2R2_4h **Scan** 8058
Peptide VTDHRINLTR **Charge** 3 **Spectral Angle** 0.77
Spearman Correlation 0.89 **iRT Error:** 1.33

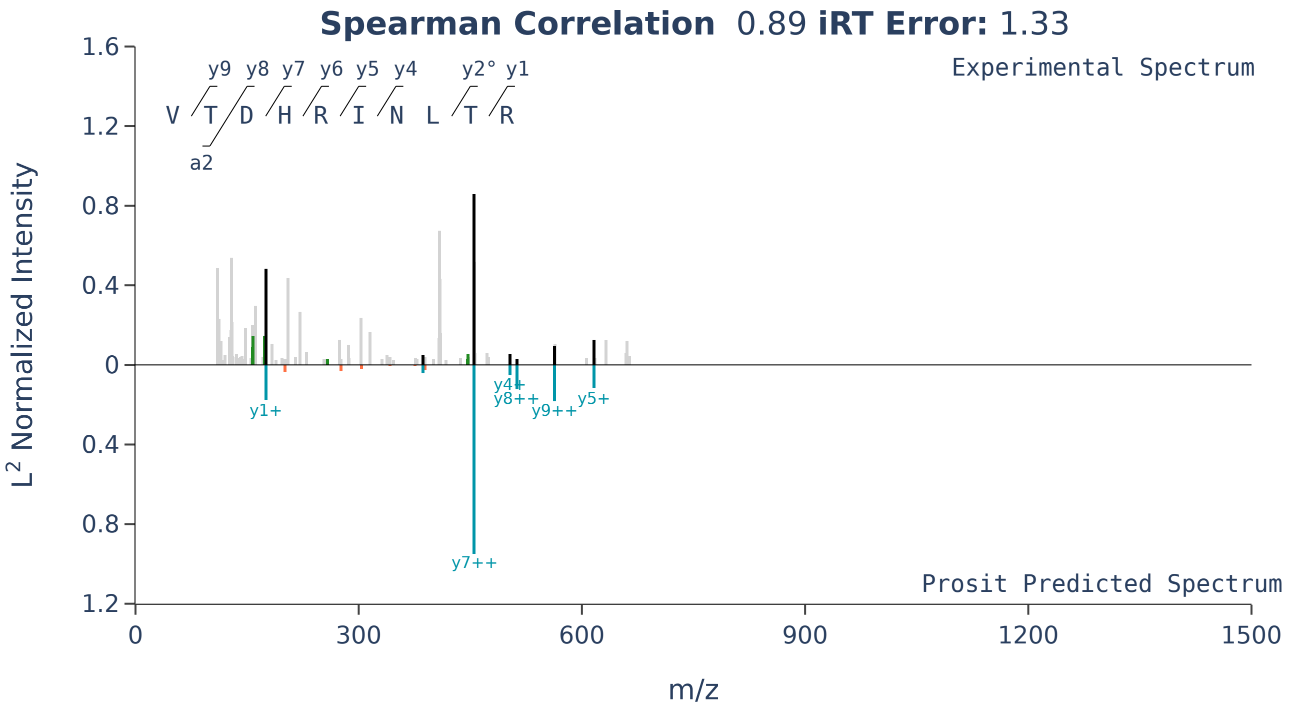

Source HRoetschke_221222_180123_HFGoe_RF1_b2R2_4h **Scan** 8058
Peptide RVTDHRINLT **Charge** 3 **Spectral Angle** 0.02
Spearman Correlation -0.27 **iRT Error:** 7.82

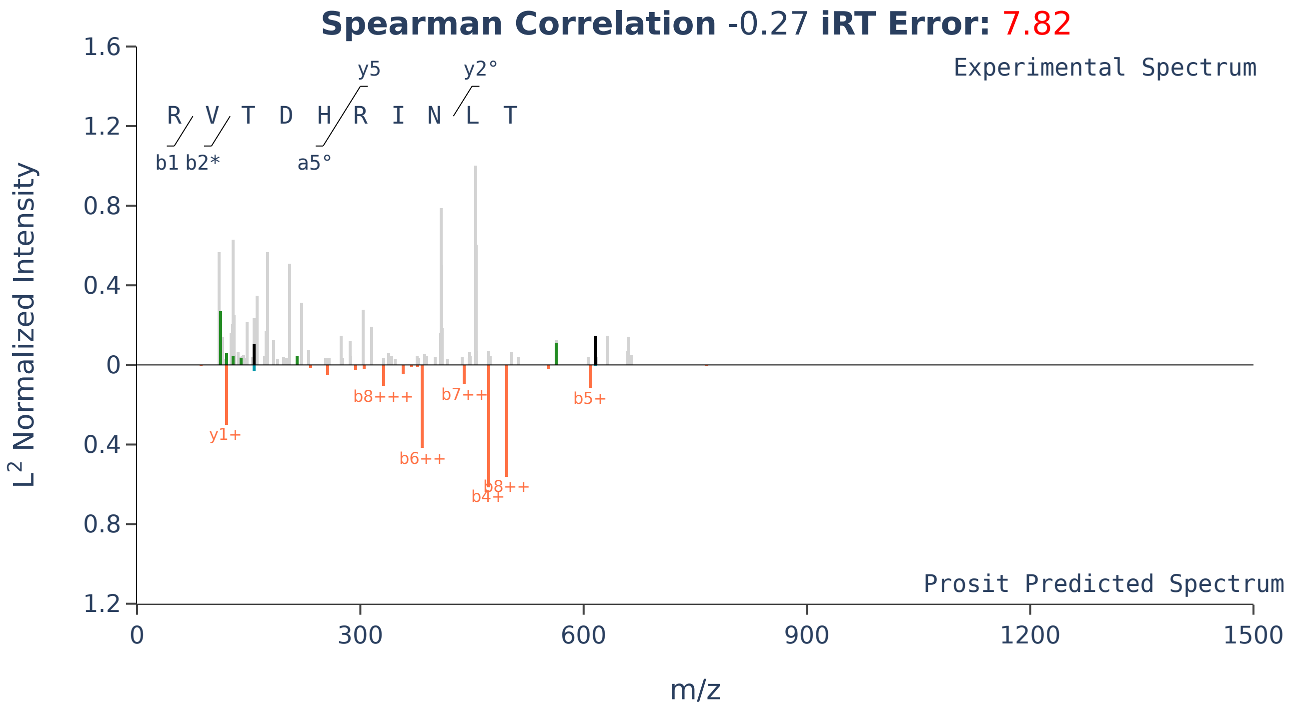

Source HRoetschke_221222_180123_HFGoe_RF1_b2R1_24h **Scan** 17570
Peptide SEGEHGGYKEIIAKIC **Charge** 3 **Spectral Angle** 0.85
Spearman Correlation 0.88 **iRT Error:** 1.35

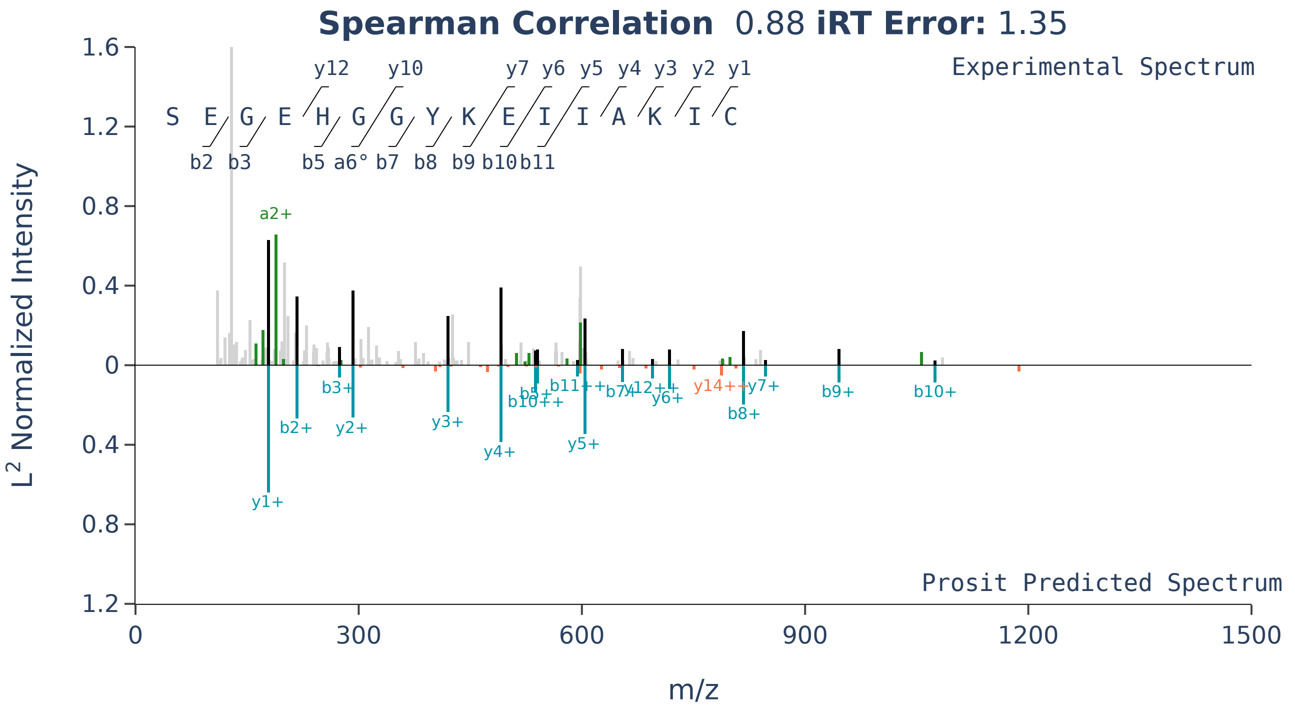

Source HRoetschke_221222_180123_HFGoe_RF1_b2R1_24h **Scan** 17570
Peptide EASTRRNLLGSGDRSD **Charge** 3 **Spectral Angle** 0.0
Spearman Correlation 0.0 **iRT Error:** 59.71

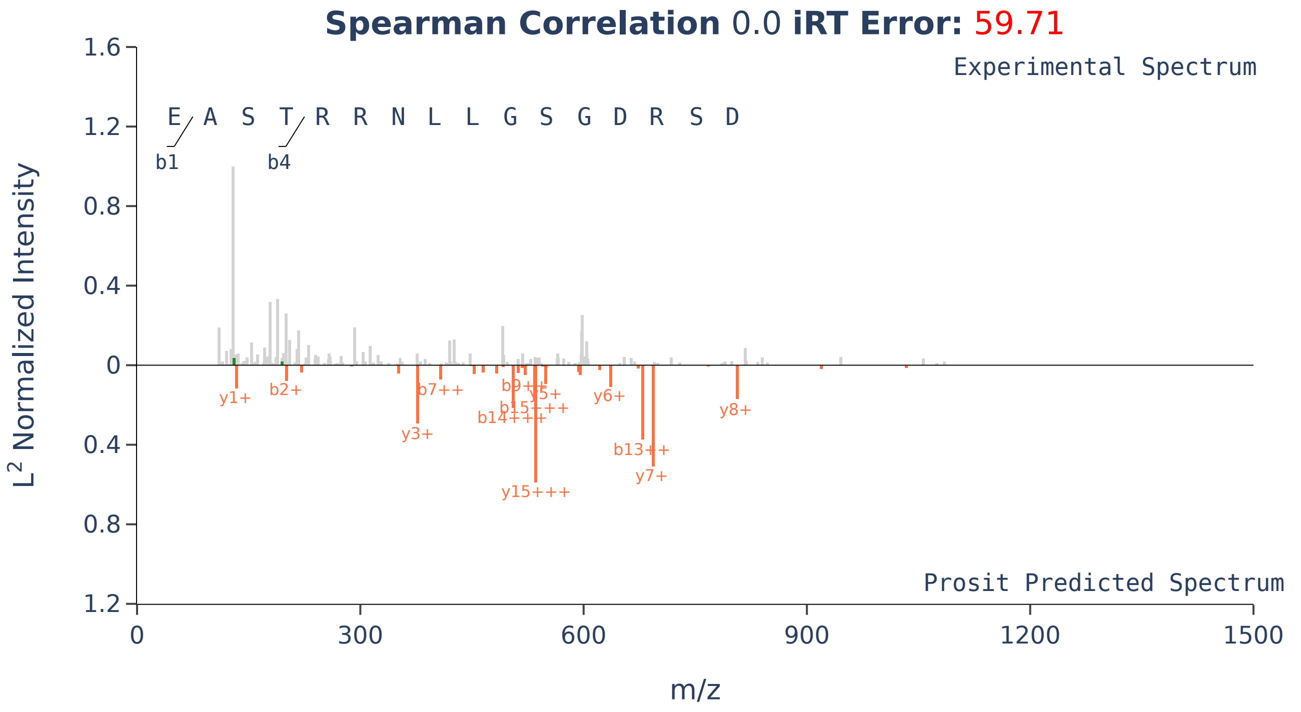

Source HRoetschke_221222_180123_HFGoe_RF1_b1R1_24h **Scan** 14588
Peptide DYNFPQGRVT **Charge** 2 **Spectral Angle** 0.68
Spearman Correlation 0.91 **iRT Error:** 1.7

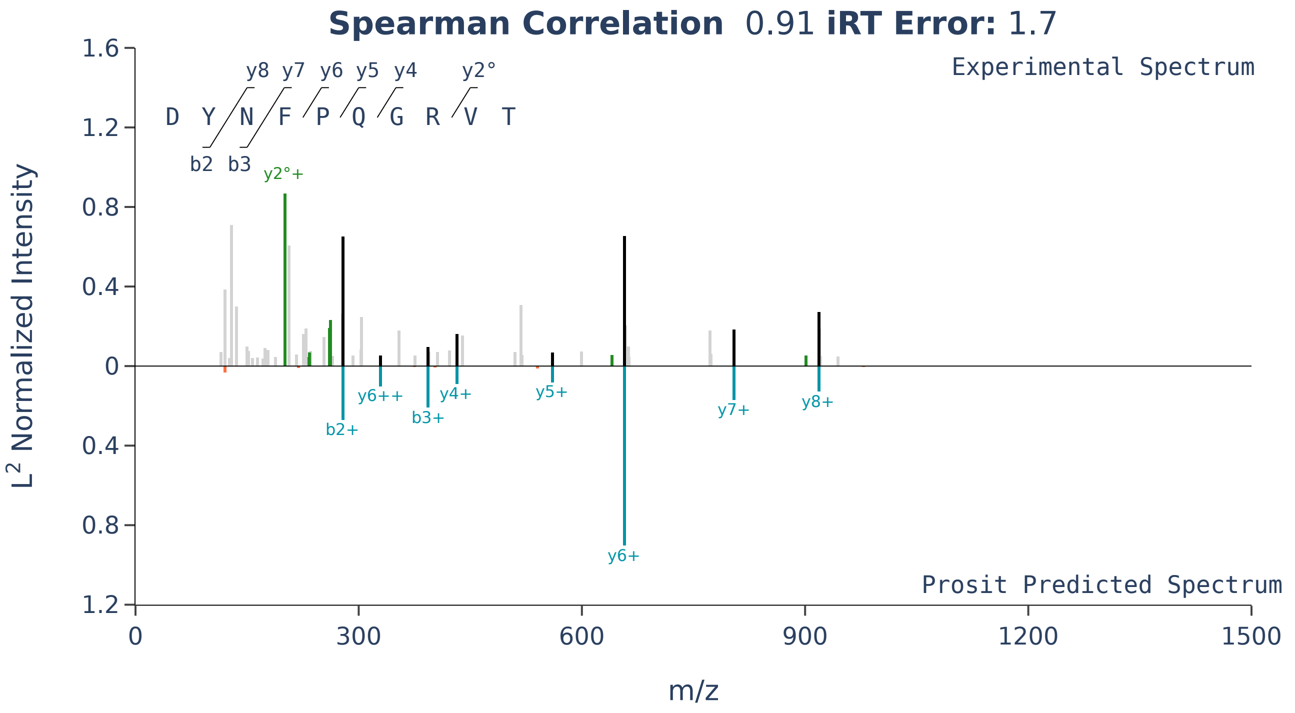

Source HRoetschke_221222_180123_HFGoe_RF1_b1R1_24h **Scan** 14588
Peptide YNFPQGRVTD **Charge** 2 **Spectral Angle** 0.65
Spearman Correlation 0.67 **iRT Error:** 5.14

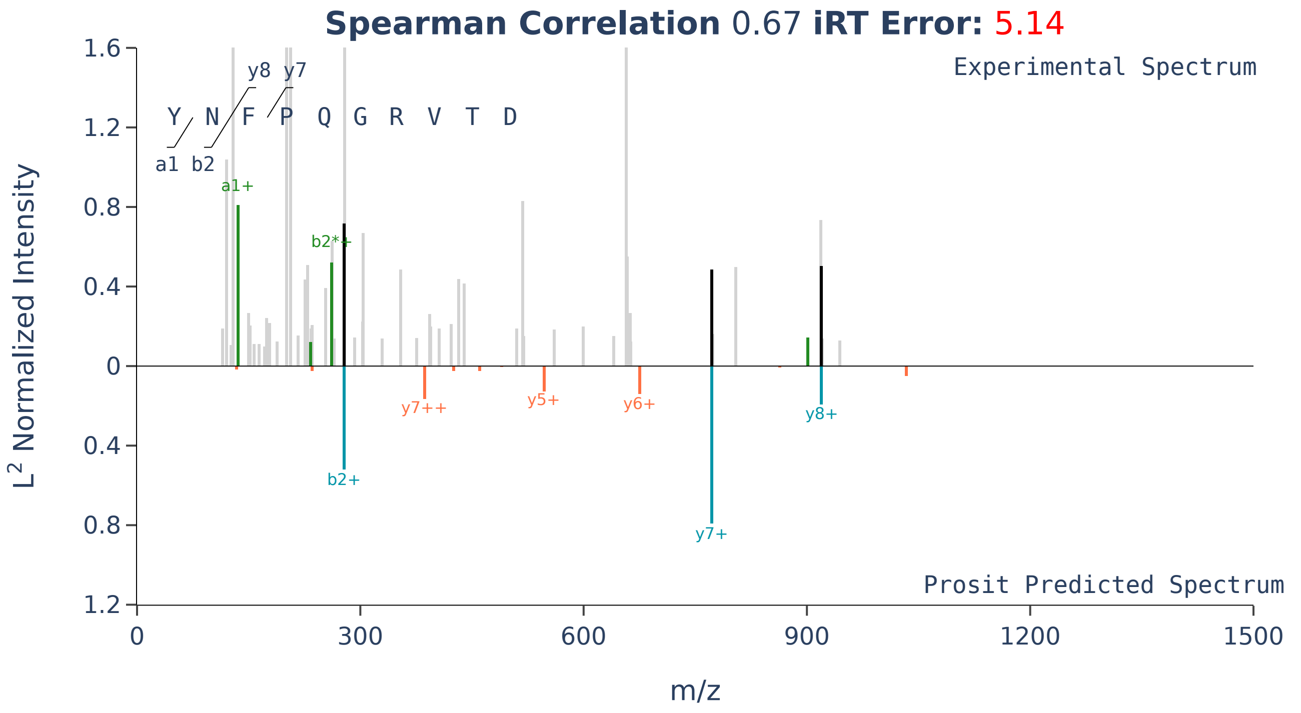

Source HRoetschke_221222_180123_HFGoe_RF1_b1R2_2h **Scan** 19892
Peptide FALEVRA **Charge** 1 **Spectral Angle** 0.83
Spearman Correlation 0.86 **iRT Error:** 1.88

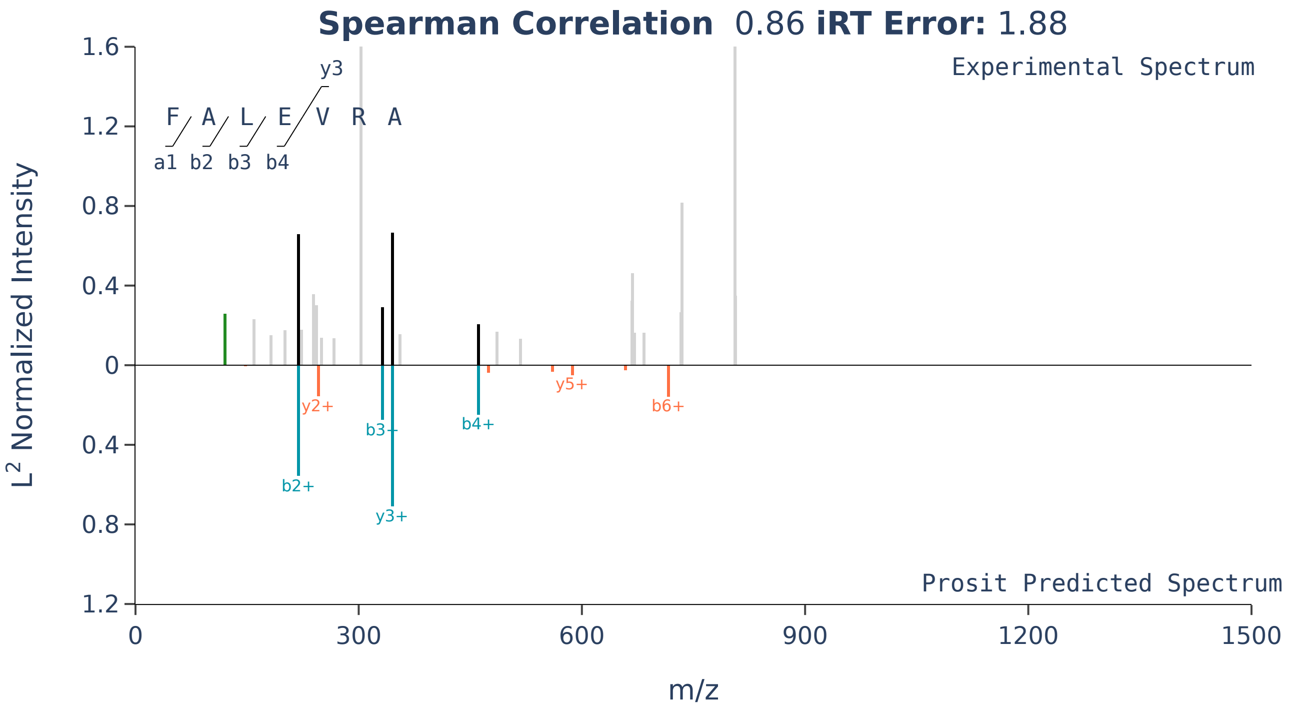

Source HRoetschke_221222_180123_HFGoe_RF1_b1R2_2h **Scan** 19892
Peptide AFLEVRA **Charge** 1 **Spectral Angle** 0.55
Spearman Correlation 0.36 **iRT Error:** 3.4

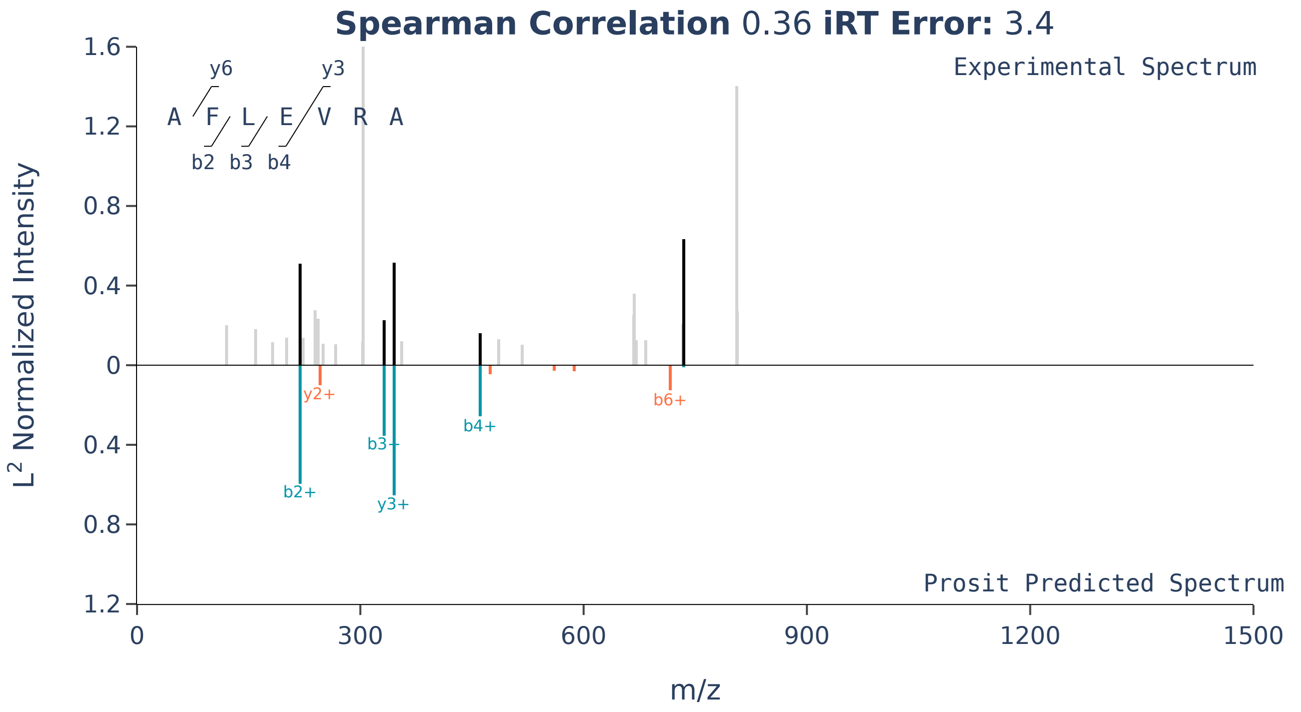

Source HRoetschke_221222_180123_HFGoe_RF1_b2R1_4h **Scan** 20495
Peptide PTGIVAEM **Charge** 1 **Spectral Angle** 0.88
Spearman Correlation 0.87 **iRT Error:** 1.96

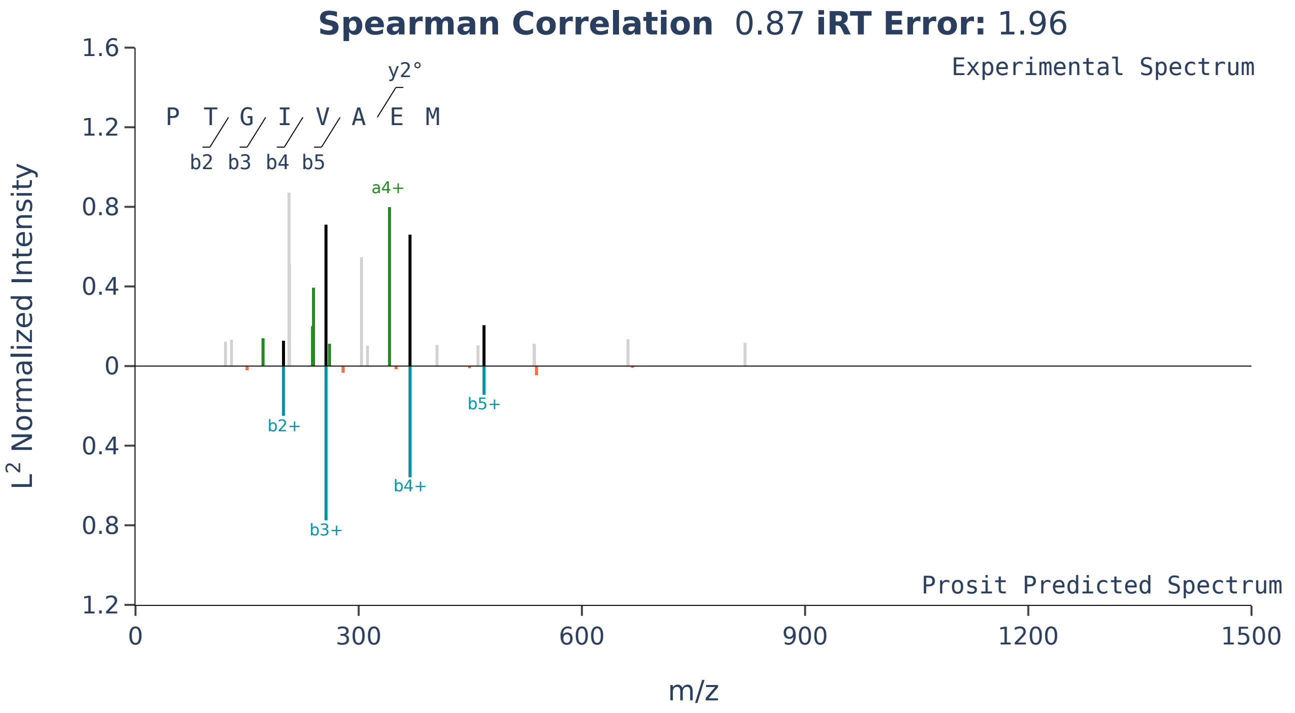

Source HRoetschke_221222_180123_HFGoe_RF1_b2R1_4h **Scan** 20495
Peptide PTGIVVEC **Charge** 1 **Spectral Angle** 0.86
Spearman Correlation 0.87 **iRT Error:** 17.98

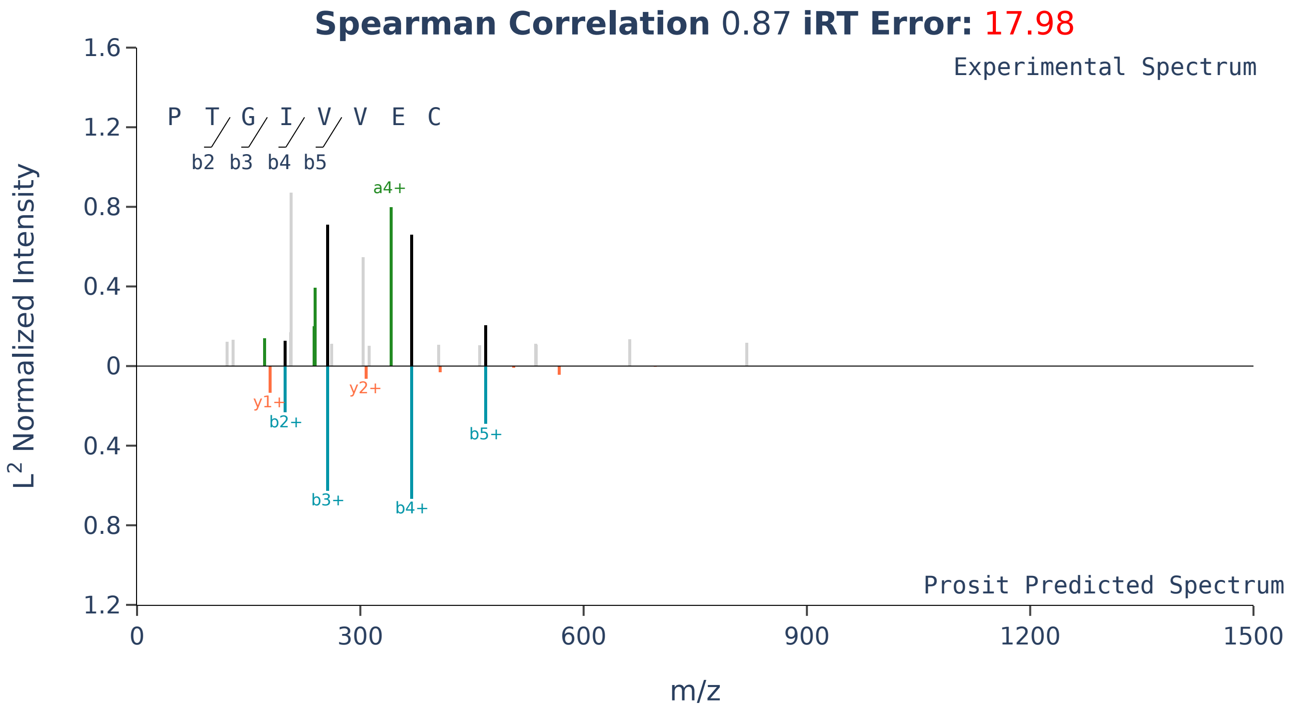

Source HRoetschke_221222_180123_HFGoe_RF1_b2R2_1h **Scan** 11379
Peptide NFPQGRVSE **Charge** 2 **Spectral Angle** 0.9
Spearman Correlation 0.84 **iRT Error:** 1.98

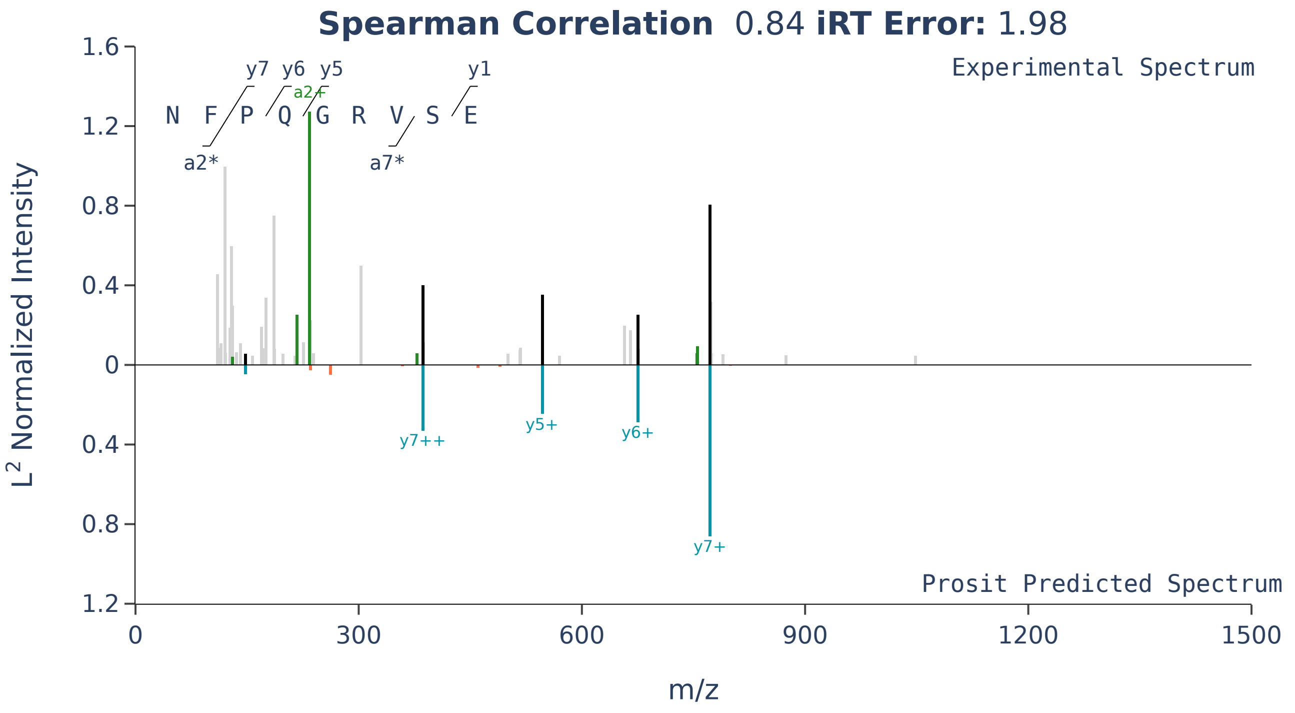

Source HRoetschke_221222_180123_HFGoe_RF1_b2R2_1h **Scan** 11379
Peptide ACTVAVMPEL **Charge** 2 **Spectral Angle** 0.0
Spearman Correlation 0.0 **iRT Error:** 98.87

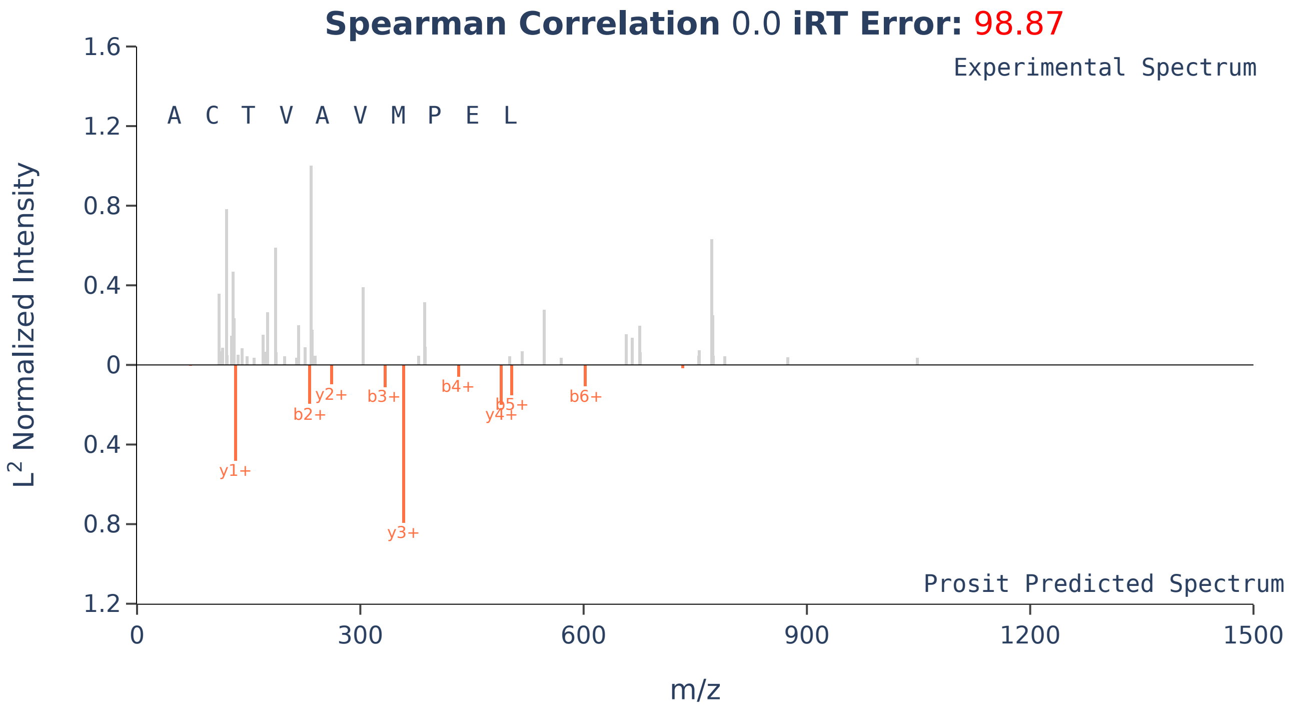

Source HRoetschke_221222_180123_HFGoe_RF1_b2R2_2h **Scan** 11407
Peptide ALSRREYAQLSD **Charge** 3 **Spectral Angle** 0.8
Spearman Correlation 0.85 **iRT Error:** 2.17

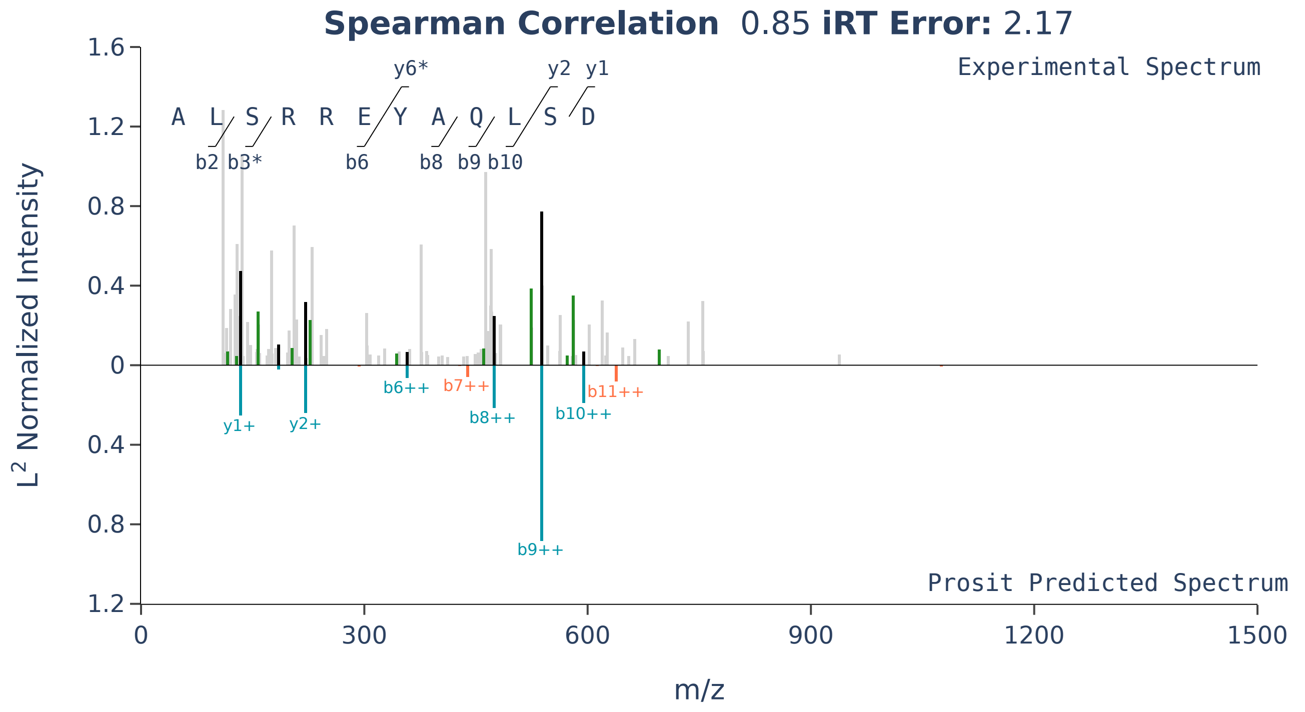

Source HRoetschke_221222_180123_HFGoe_RF1_b2R2_2h **Scan** 11407
Peptide ADLRIDTFRSSGA **Charge** 3 **Spectral Angle** 0.0
Spearman Correlation 0.0 **iRT Error:** 33.54

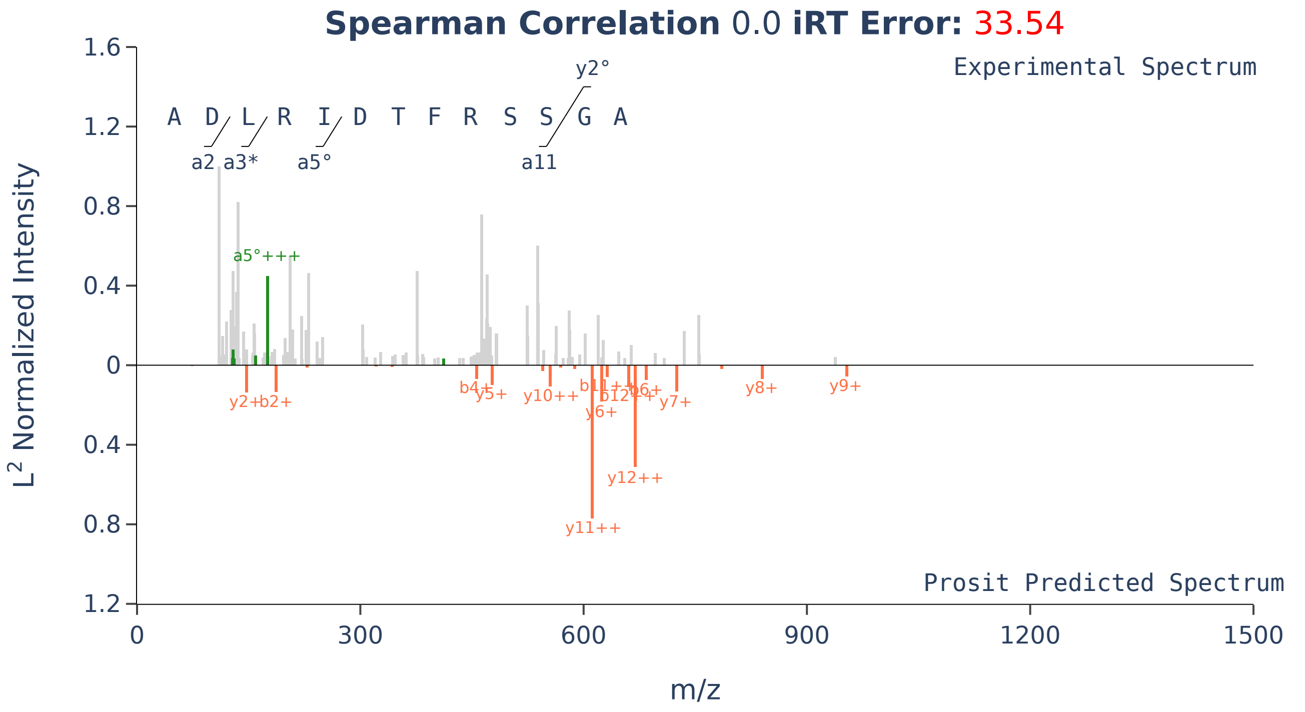

Source HRoetschke_221222_180123_HFGoe_RF1_b2R2_24h **Scan** 15815
Peptide SLYRLDE **Charge** 2 **Spectral Angle** 0.92
Spearman Correlation 0.91 **iRT Error:** 2.34

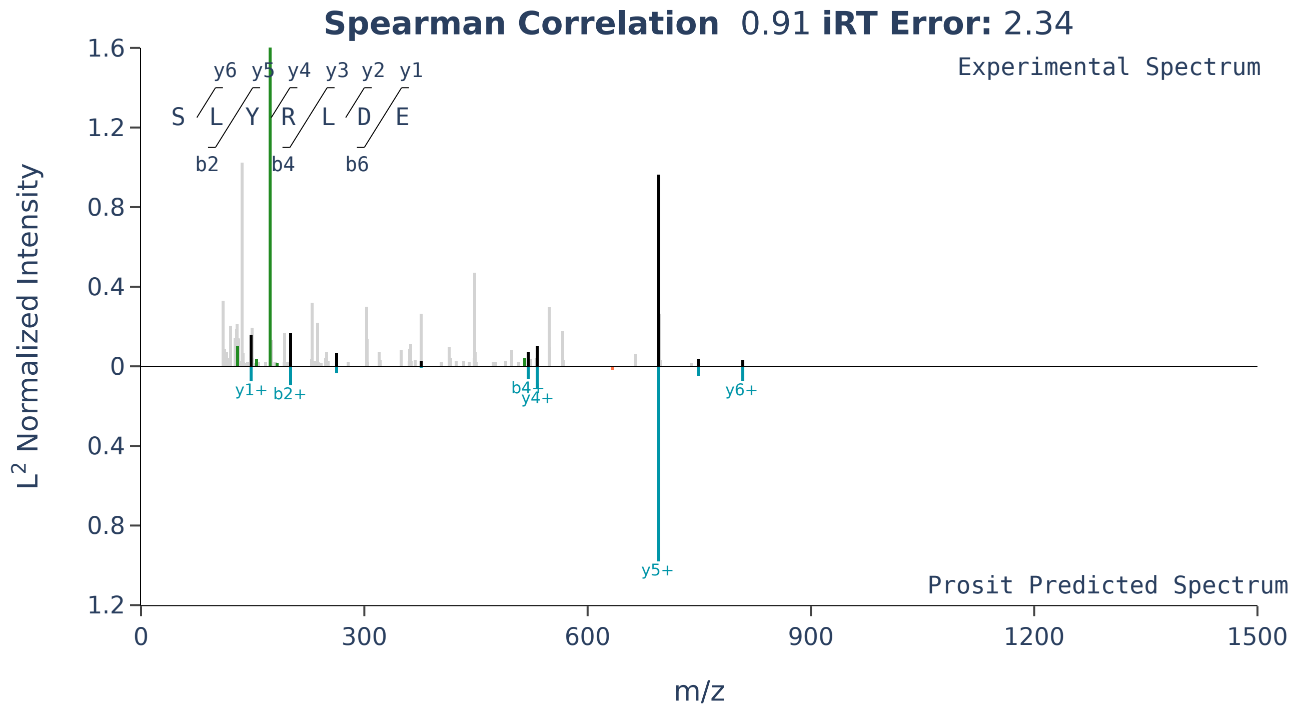

Source HRoetschke_221222_180123_HFGoe_RF1_b2R2_24h **Scan** 15815
Peptide KISGDGVYG **Charge** 2 **Spectral Angle** 0.23
Spearman Correlation 0.27 **iRT Error:** 31.25

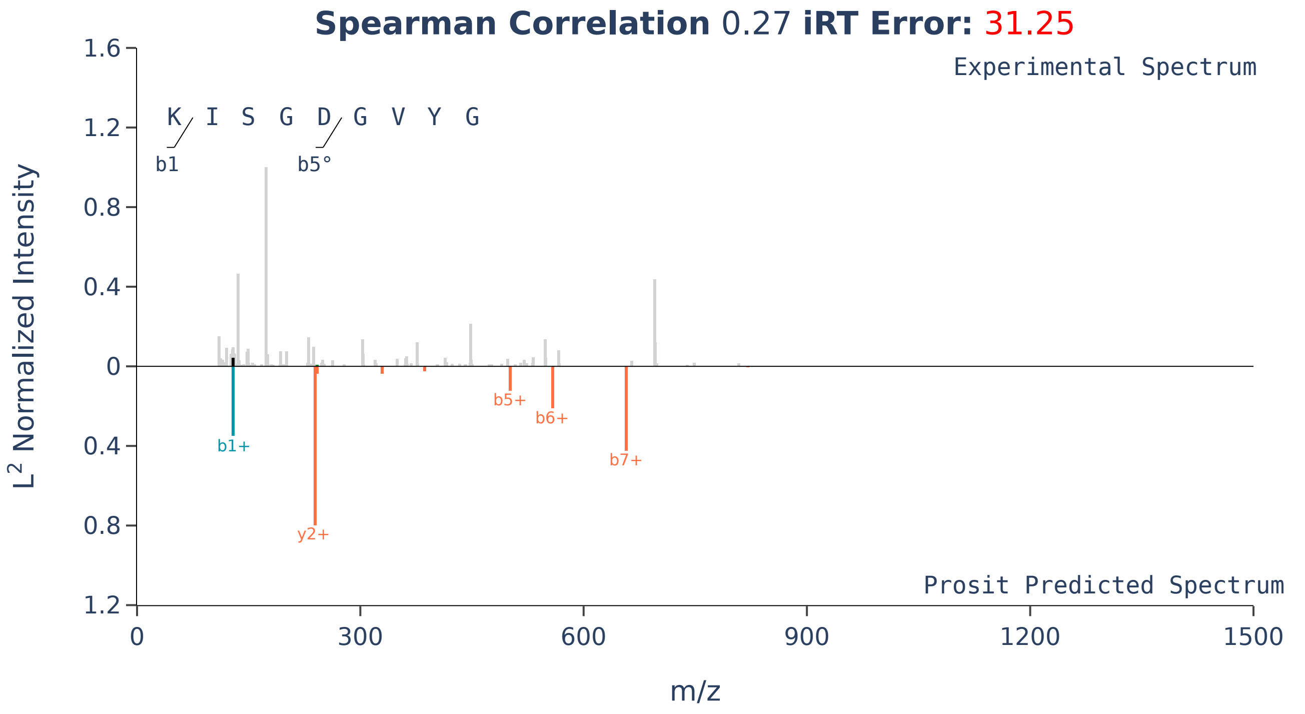

Source HRoetschke_221222_180123_HFGoe_RF1_b2R1_24h **Scan** 11325
Peptide RAGTGGDEQVL **Charge** 2 **Spectral Angle** 0.8
Spearman Correlation 0.91 **iRT Error:** 2.96

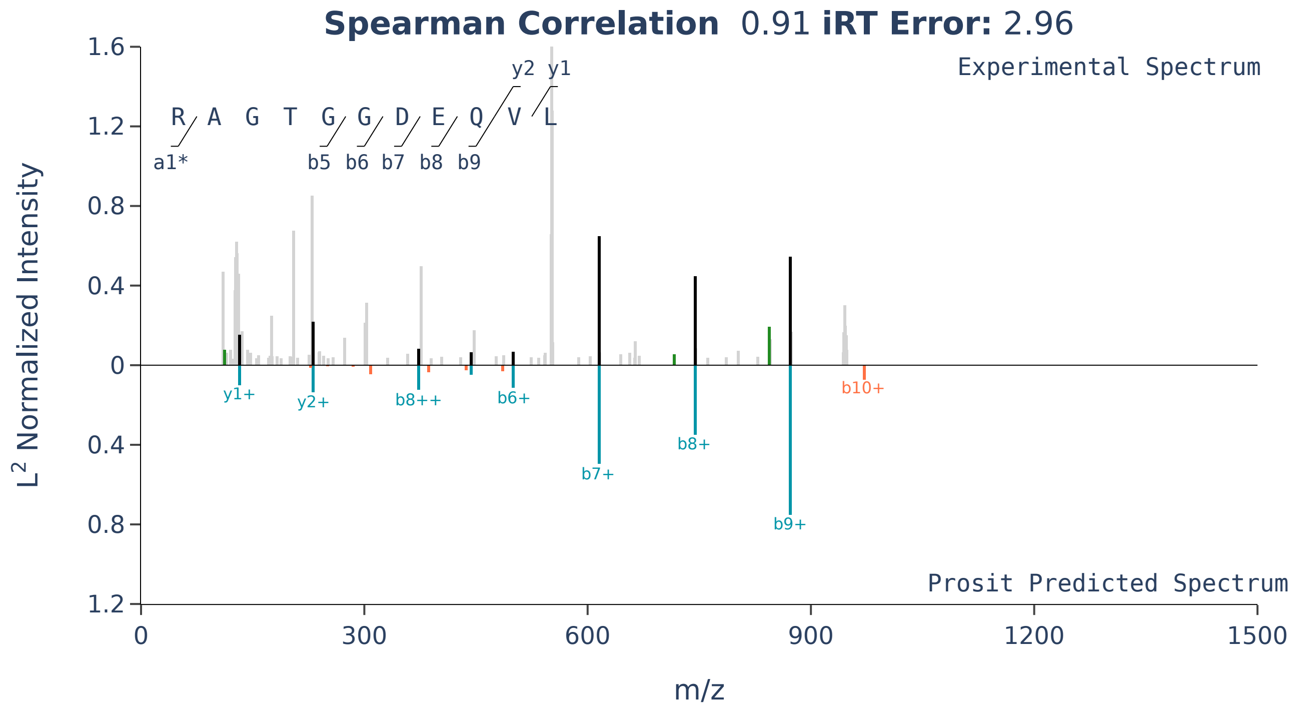

Source HRoetschke_221222_180123_HFGoe_RF1_b2R1_24h **Scan** 11325
Peptide GEHGGYKEII **Charge** 2 **Spectral Angle** 0.15
Spearman Correlation 0.16 **iRT Error:** 10.92

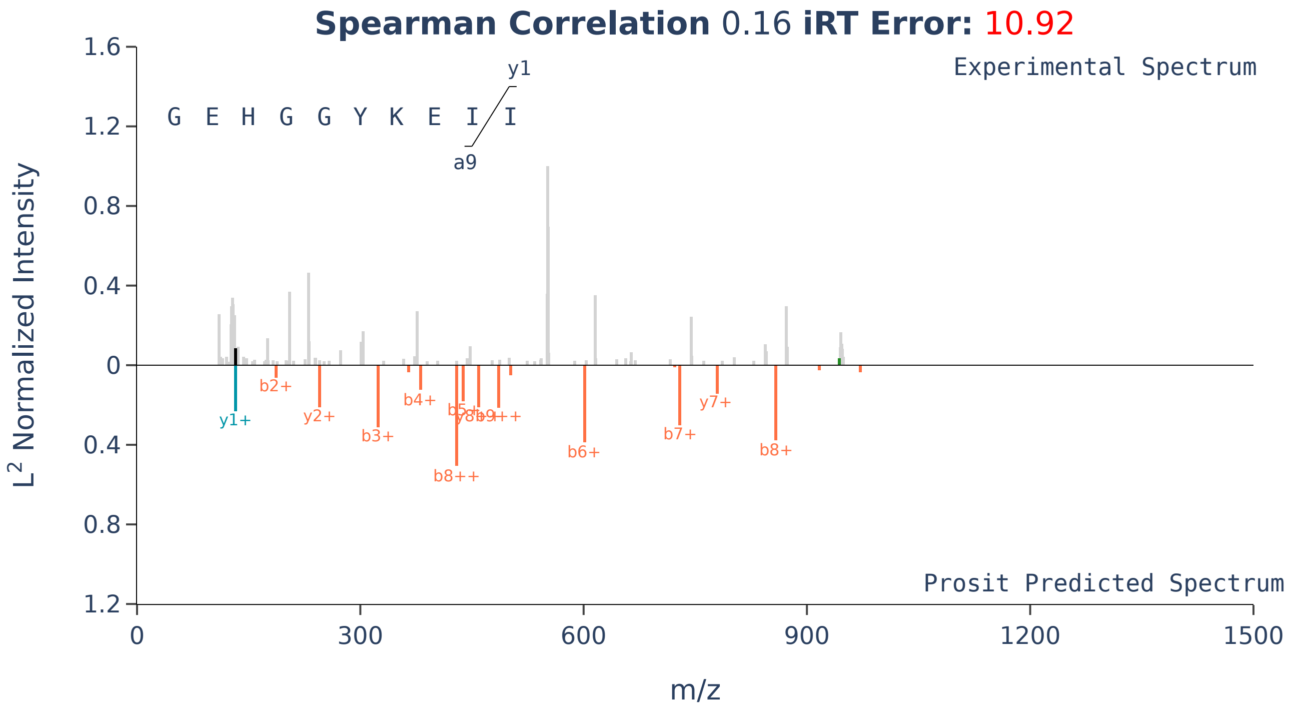

Source HRoetschke_221222_180123_HFGoe_RF1_b2R1_24h **Scan** 19051
Peptide ELLLPKD **Charge** 2 **Spectral Angle** 0.97
Spearman Correlation 0.98 **iRT Error:** 3.36

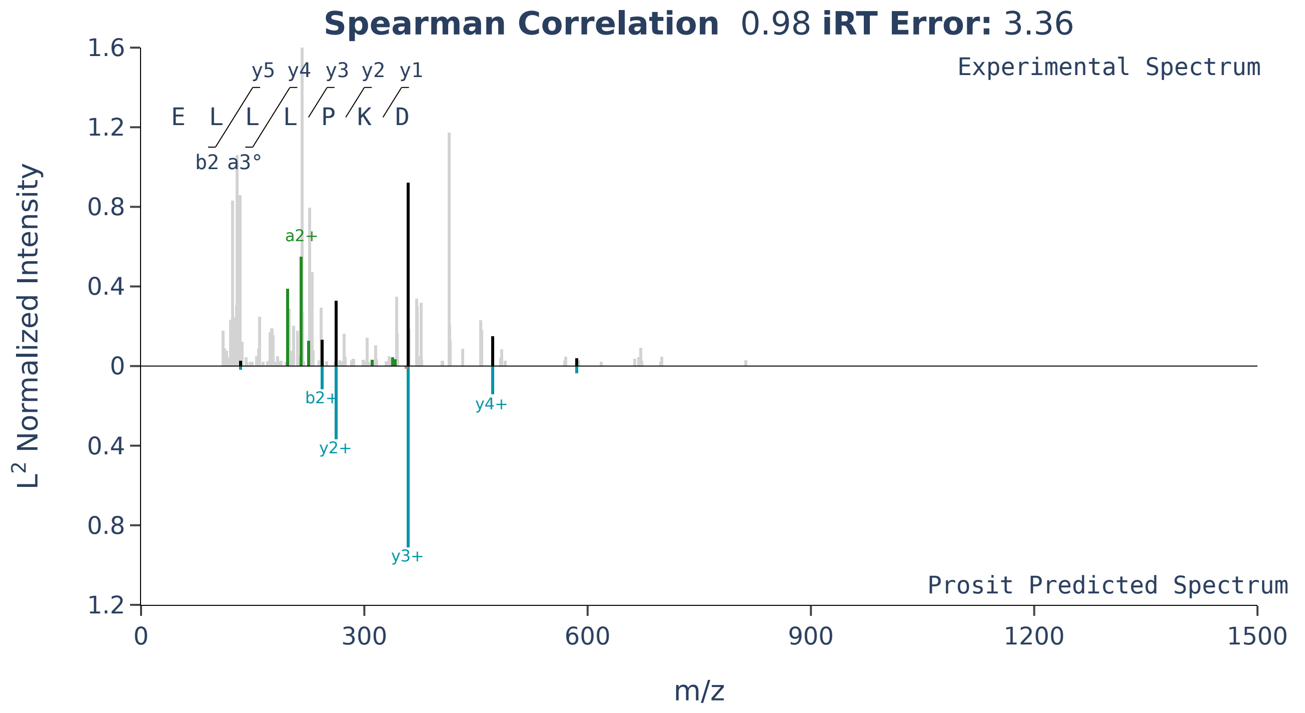

Source HRoetschke_221222_180123_HFGoe_RF1_b2R1_24h **Scan** 19051
Peptide LPTGIVVE **Charge** 2 **Spectral Angle** 0.04
Spearman Correlation -0.41 **iRT Error:** 19.29

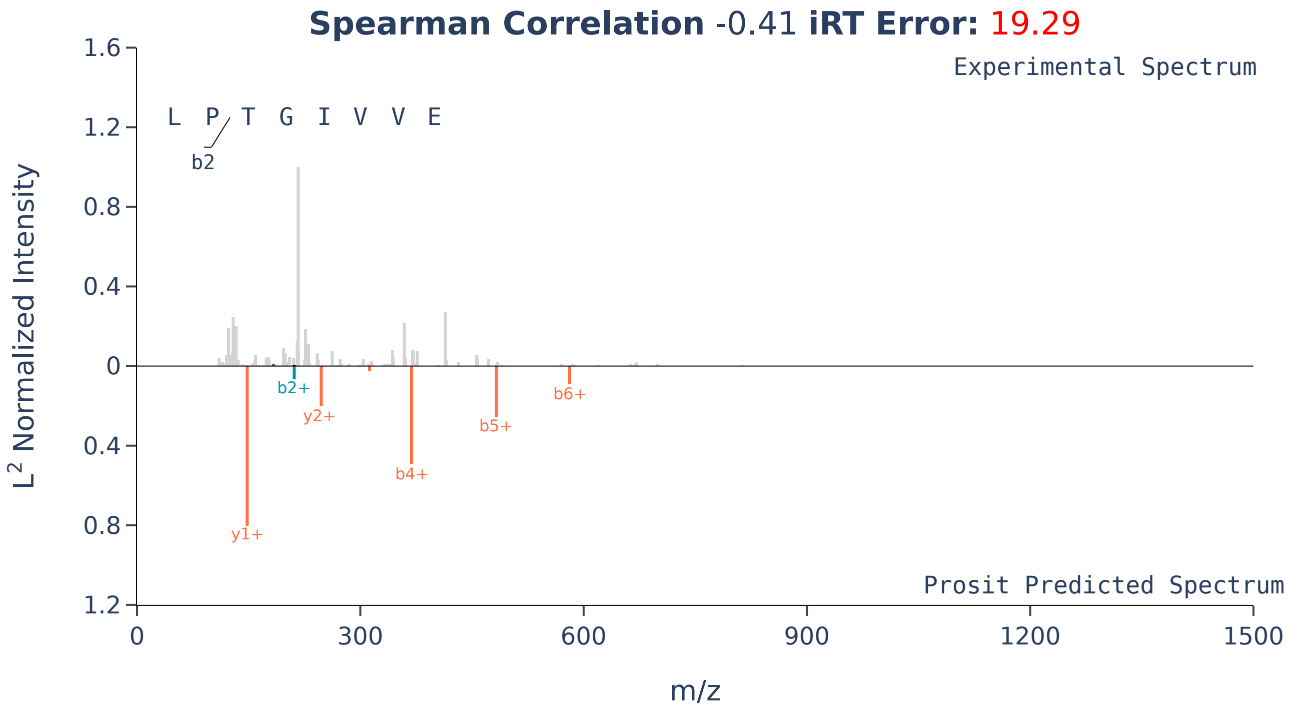

Source HRoetschke_221222_180123_HFGoe_RF1_b1R2_0h **Scan** 20883
Peptide PTGIIVVE **Charge** 1 **Spectral Angle** 0.8
Spearman Correlation 0.93 **iRT Error:** 3.88

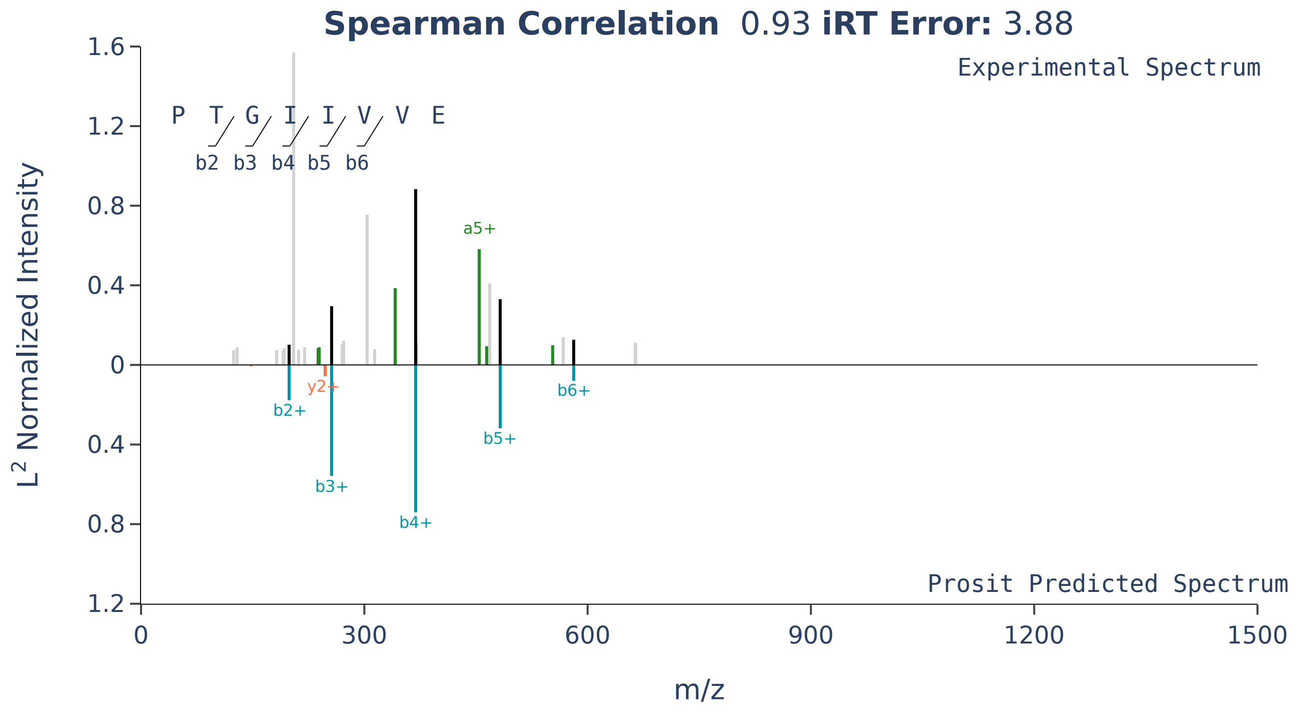

Source HRoetschke_221222_180123_HFGoe_RF1_b1R2_0h **Scan** 20883
Peptide LPTGIVVE **Charge** 1 **Spectral Angle** 0.85
Spearman Correlation 0.75 **iRT Error:** 7.54

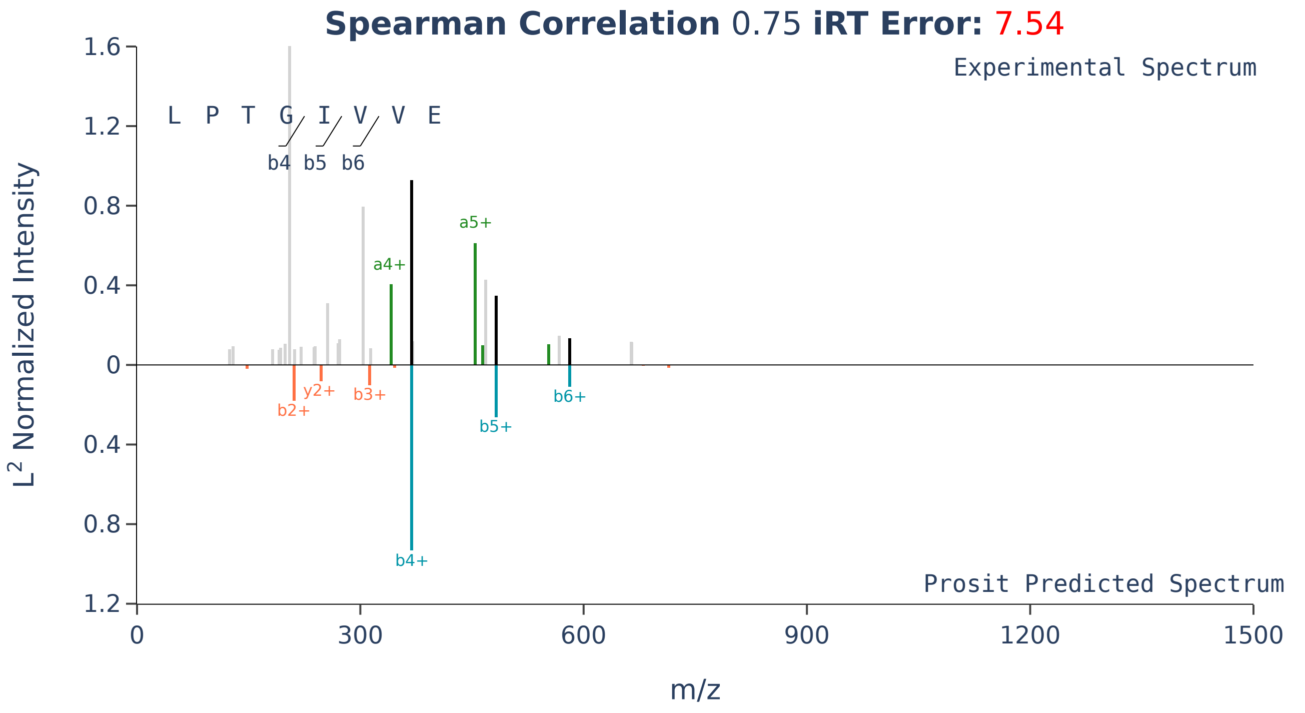

Source HRoetschke_221222_180123_HFGoe_RF1_b1R2_2h **Scan** 12898
Peptide DTFRSSQQL **Charge** 2 **Spectral Angle** 0.73
Spearman Correlation 0.94 **iRT Error:** 4.29

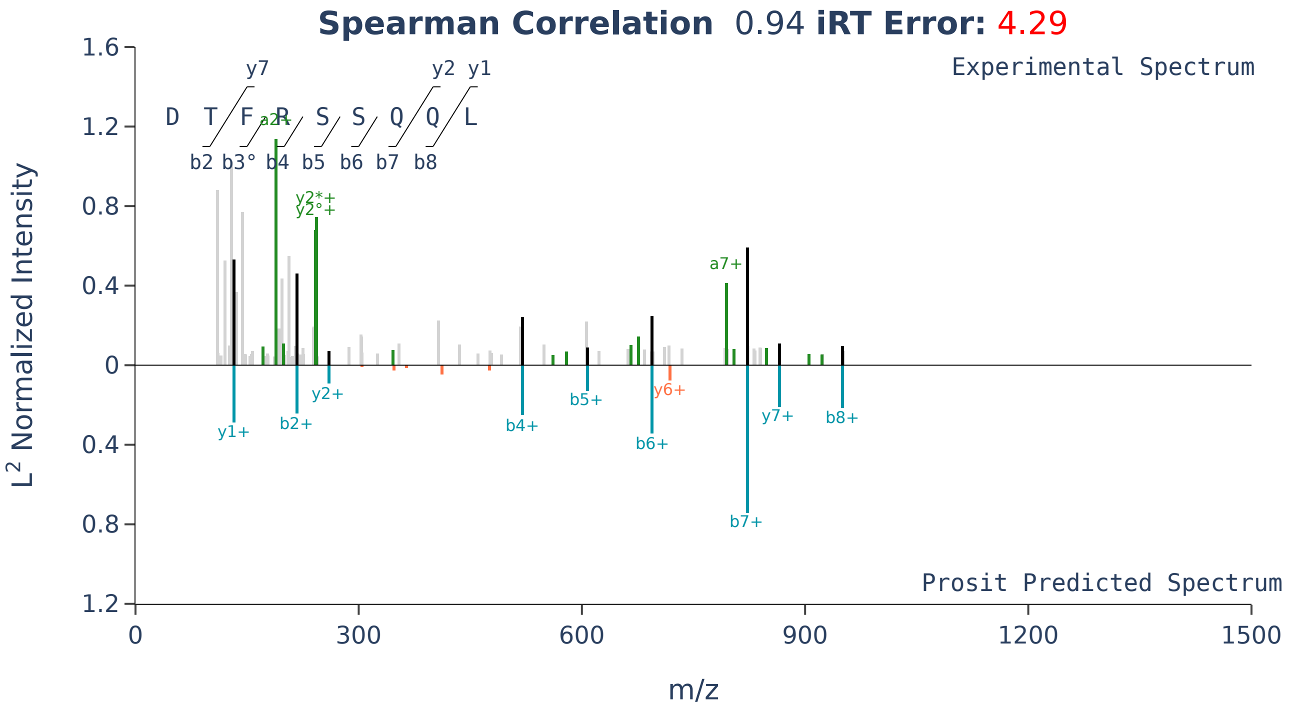

Source HRoetschke_221222_180123_HFGoe_RF1_b1R2_2h **Scan** 12898
Peptide IIQEHQADQ **Charge** 2 **Spectral Angle** 0.42
Spearman Correlation 0.41 **iRT Error:** 42.15

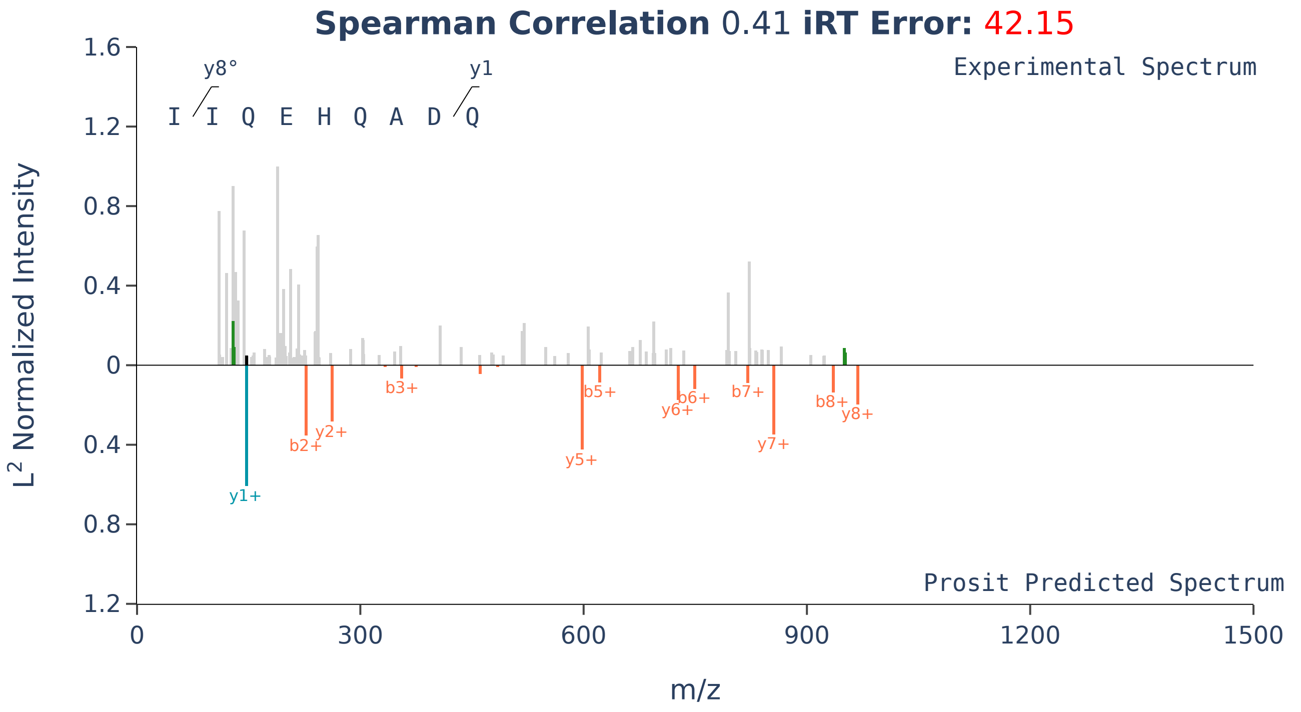

Source HRoetschke_221222_180123_HFGoe_RF1_b2R2_24h **Scan** 11452
Peptide DRSNRTYNFPQ **Charge** 3 **Spectral Angle** 0.81
Spearman Correlation 0.89 **iRT Error:** 4.8

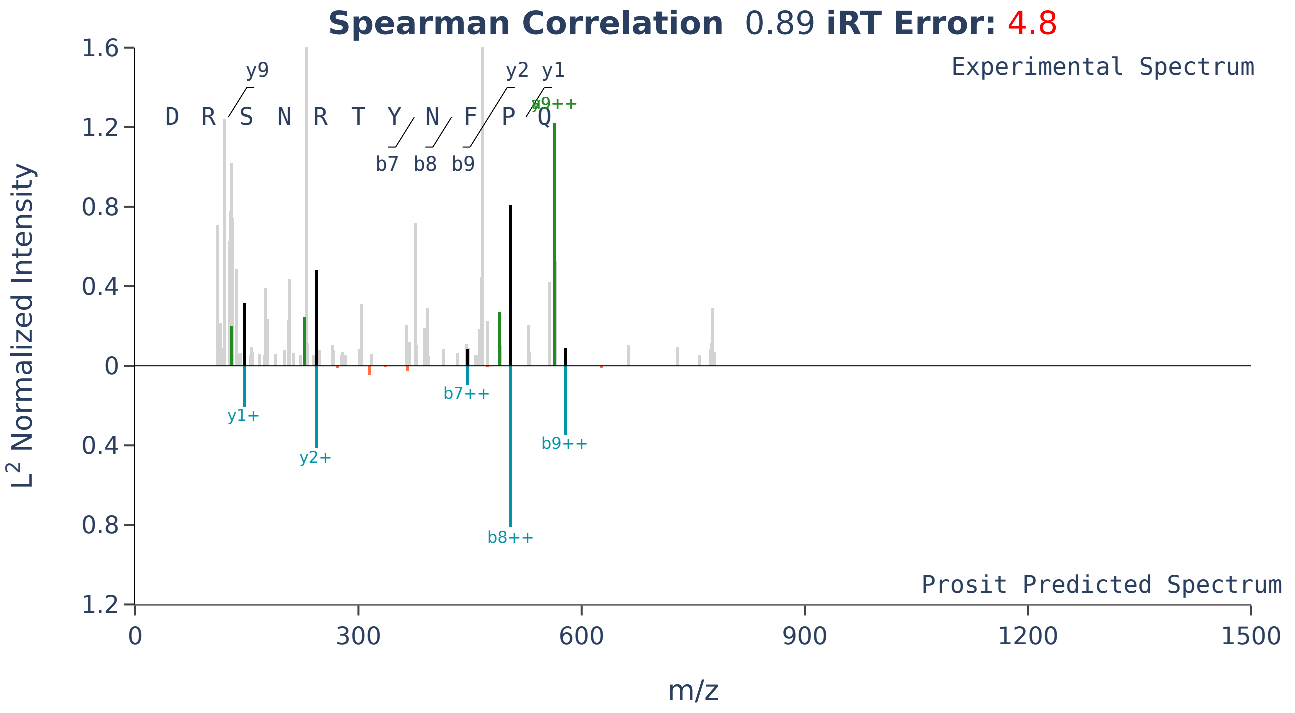

Source HRoetschke_221222_180123_HFGoe_RF1_b2R2_24h **Scan** 11452
Peptide SDRNRTYNFPQ **Charge** 3 **Spectral Angle** 0.78
Spearman Correlation 0.85 **iRT Error:** 1.55

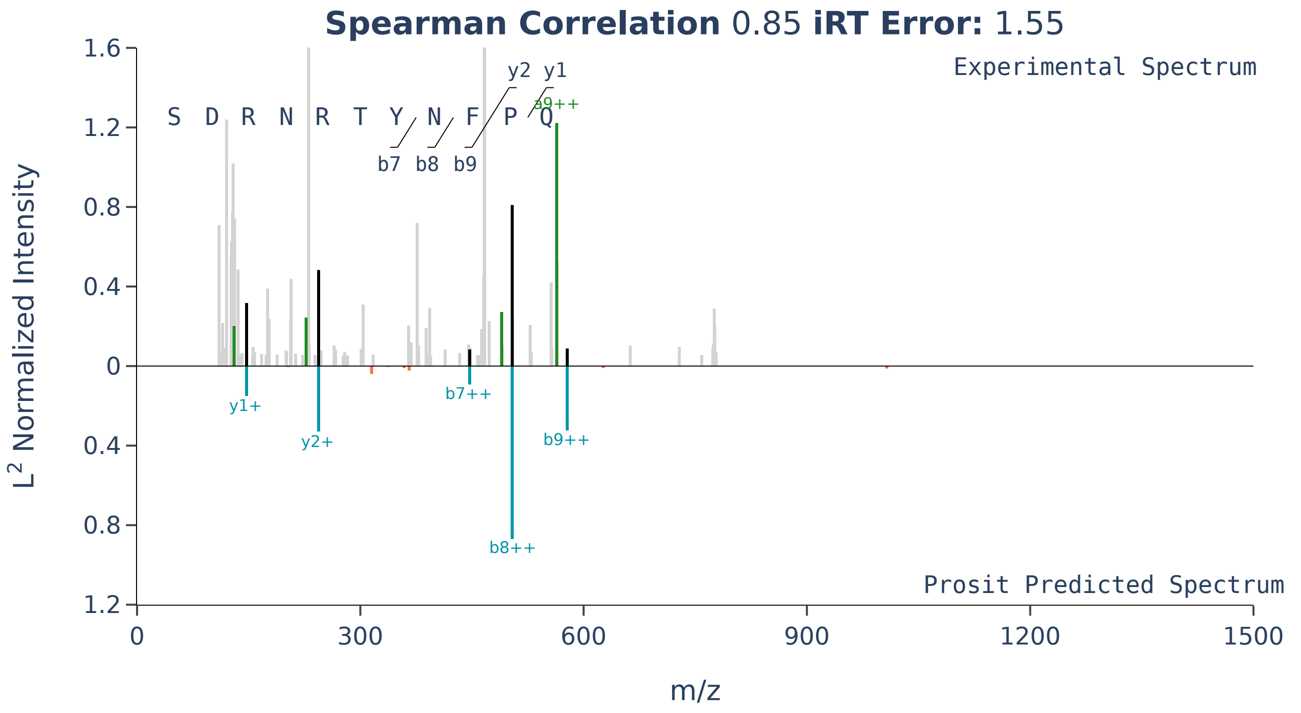

Source HRoetschke_221222_180123_HFGoe_RF1_b2R1_2h **Scan** 19332
Peptide TDWQQVAG **Charge** 1 **Spectral Angle** 0.93
Spearman Correlation 0.98 **iRT Error:** 16.66

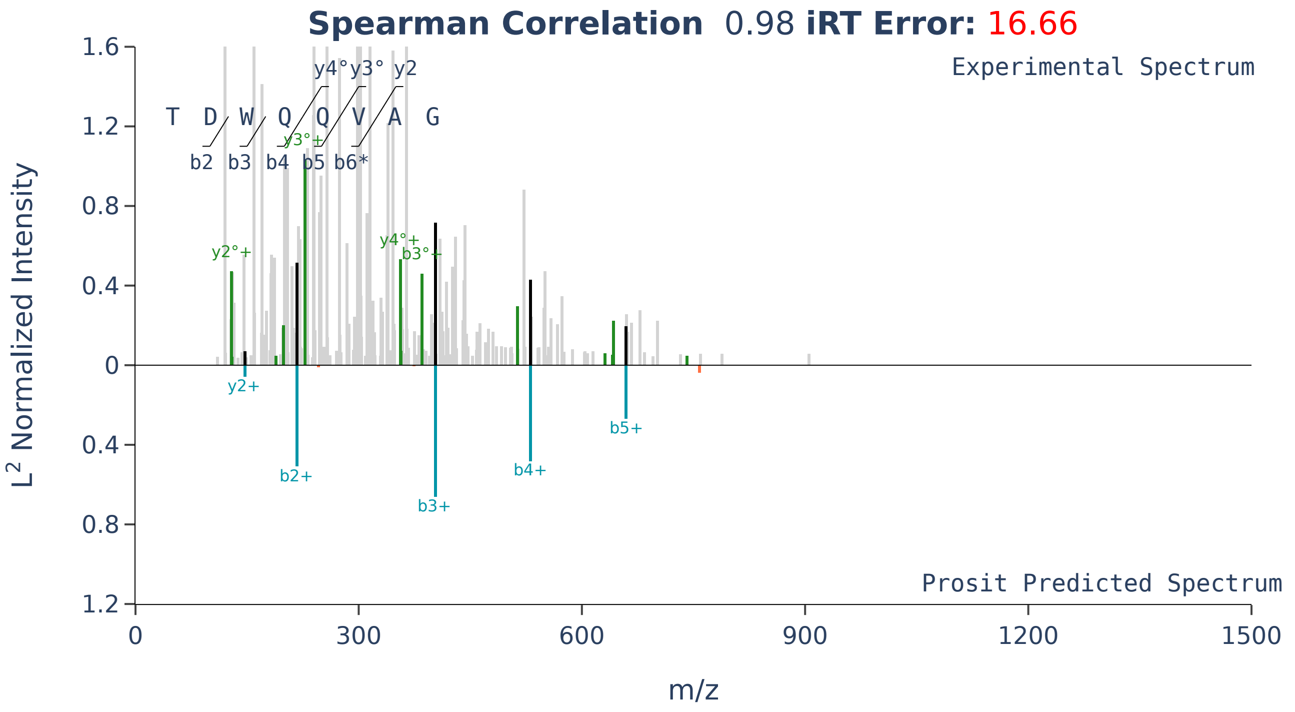

Source HRoetschke_221222_180123_HFGoe_RF1_b2R1_2h **Scan** 19332
Peptide TDWQQVQ **Charge** 1 **Spectral Angle** 0.61
Spearman Correlation 0.71 **iRT Error:** 27.4

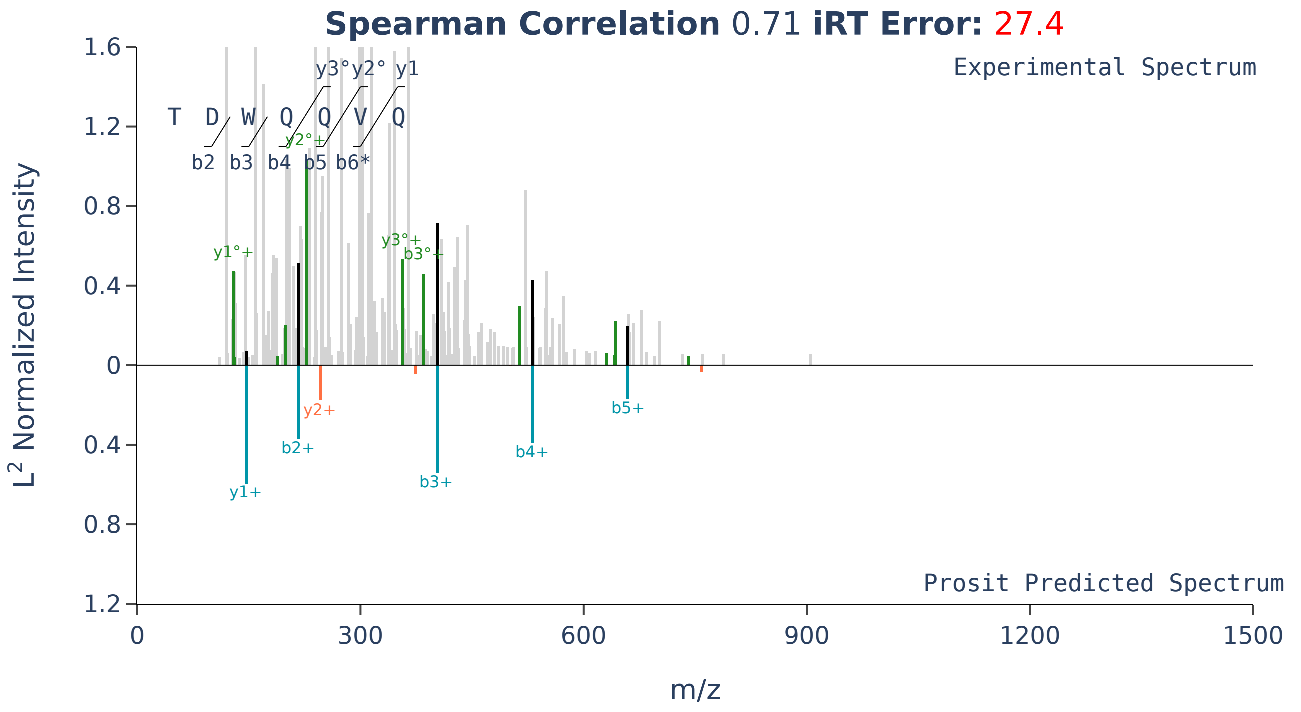

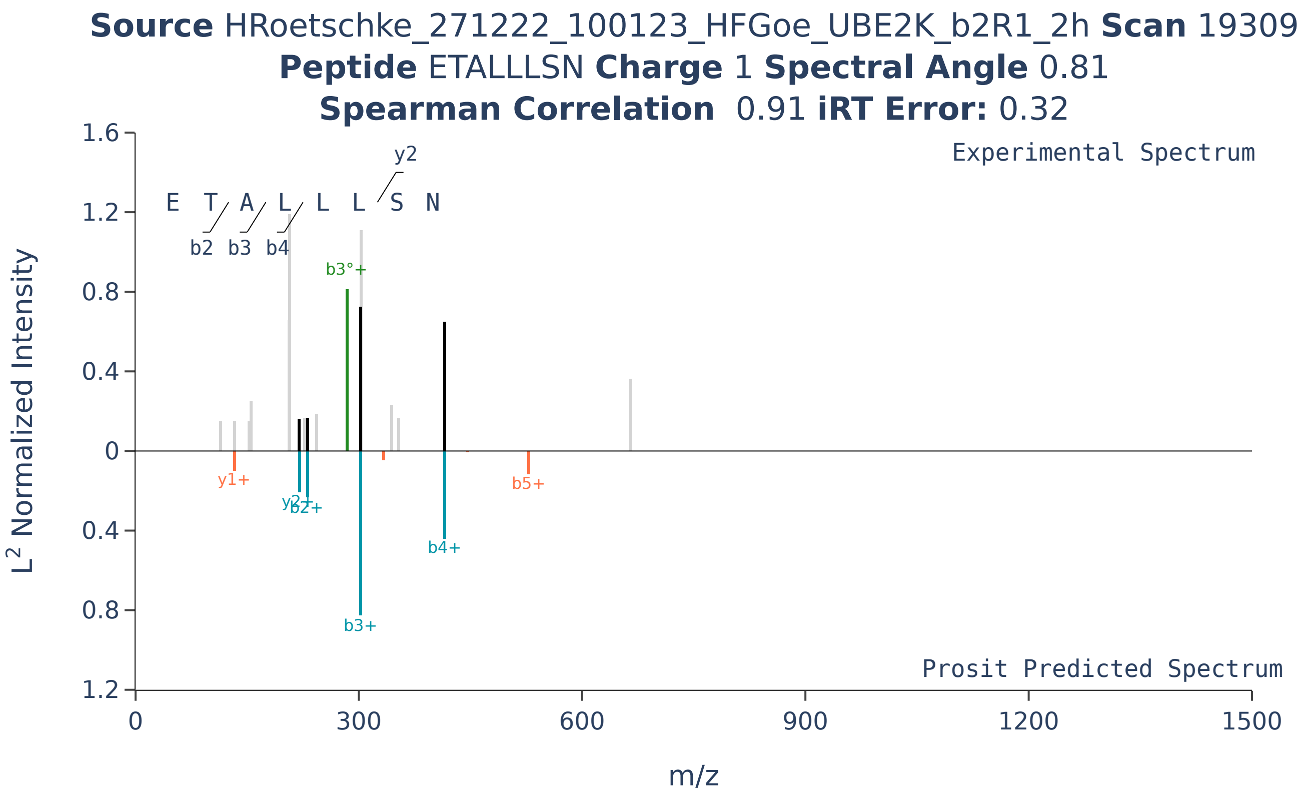
Source HRoetschke_271222_100123_HFGoe_UBE2K_b2R1_2h Scan 19309
Peptide ETALLLSN Charge 1 Spectral Angle 0.81
Spearman Correlation 0.91 iRT Error: 0.32
Experimental Spectrum
Prosit Predicted Spectrum
L2 Normalized Intensity
m/z

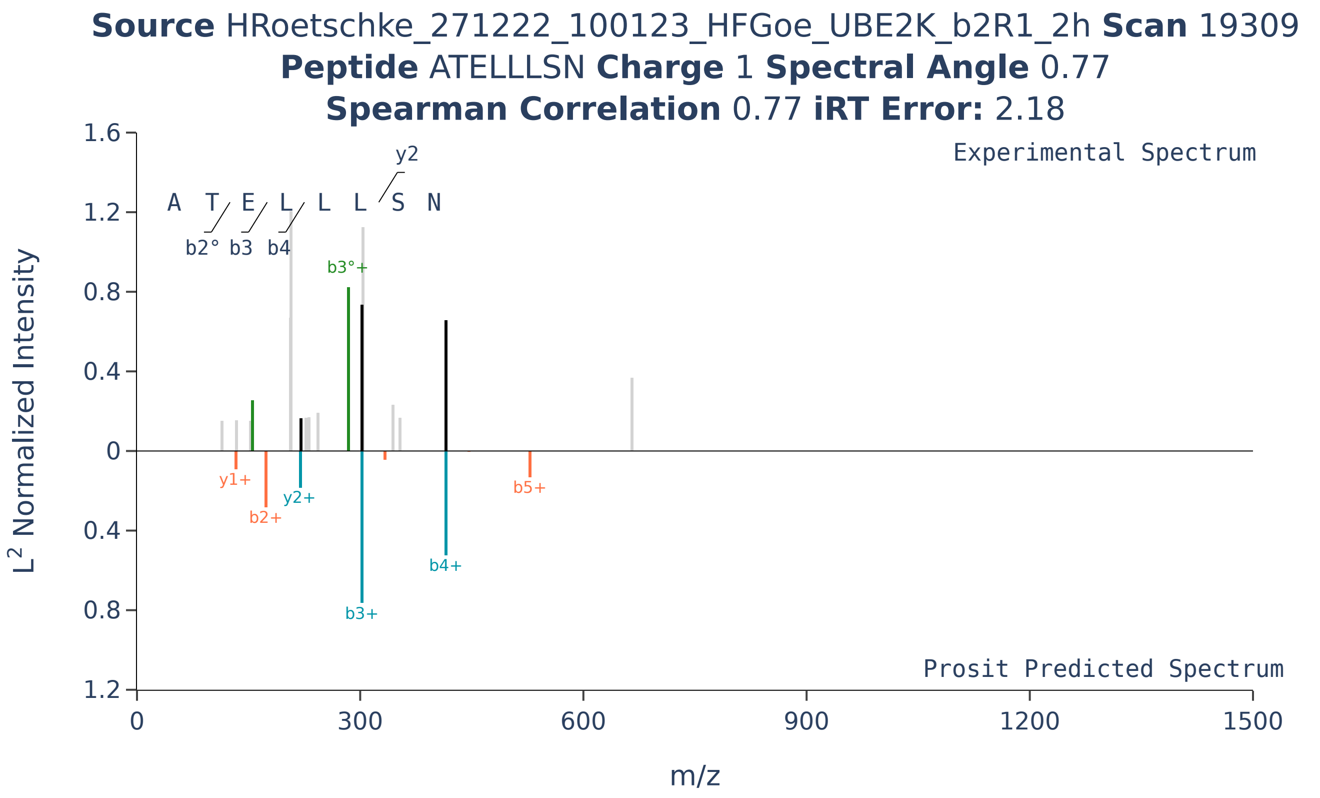
Source HRoetschke_271222_100123_HFGoe_UBE2K_b2R1_2h Scan 19309
Peptide ATELLLSN Charge 1 Spectral Angle 0.77
Spearman Correlation 0.77 iRT Error: 2.18
Experimental Spectrum
Prosit Predicted Spectrum
L2 Normalized Intensity
m/z

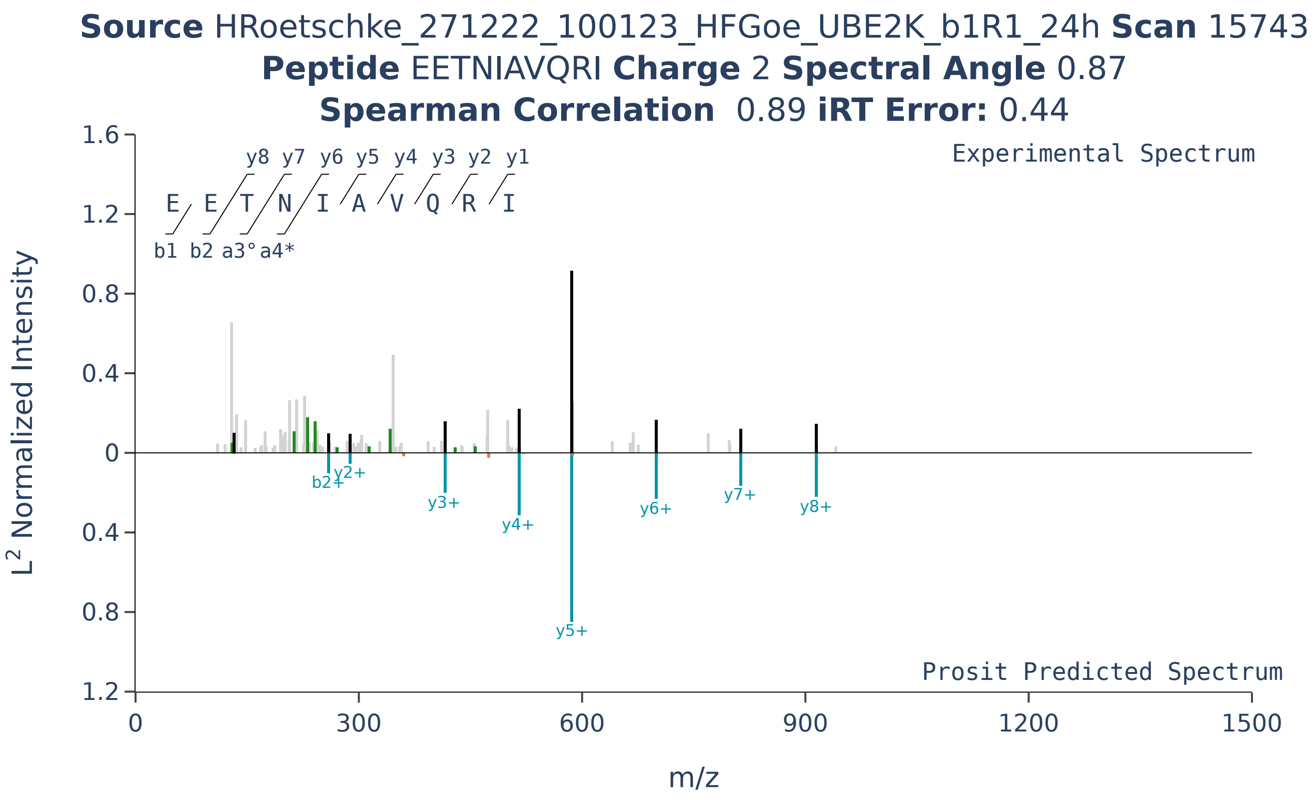
Source HRoetschke_271222_100123_HFGoe_UBE2K_b1R1_24h Scan 15743
Peptide EETNIAVQRI Charge 2 Spectral Angle 0.87
Spearman Correlation 0.89 iRT Error: 0.44
Experimental Spectrum
Prosit Predicted Spectrum
L2 Normalized Intensity
m/z

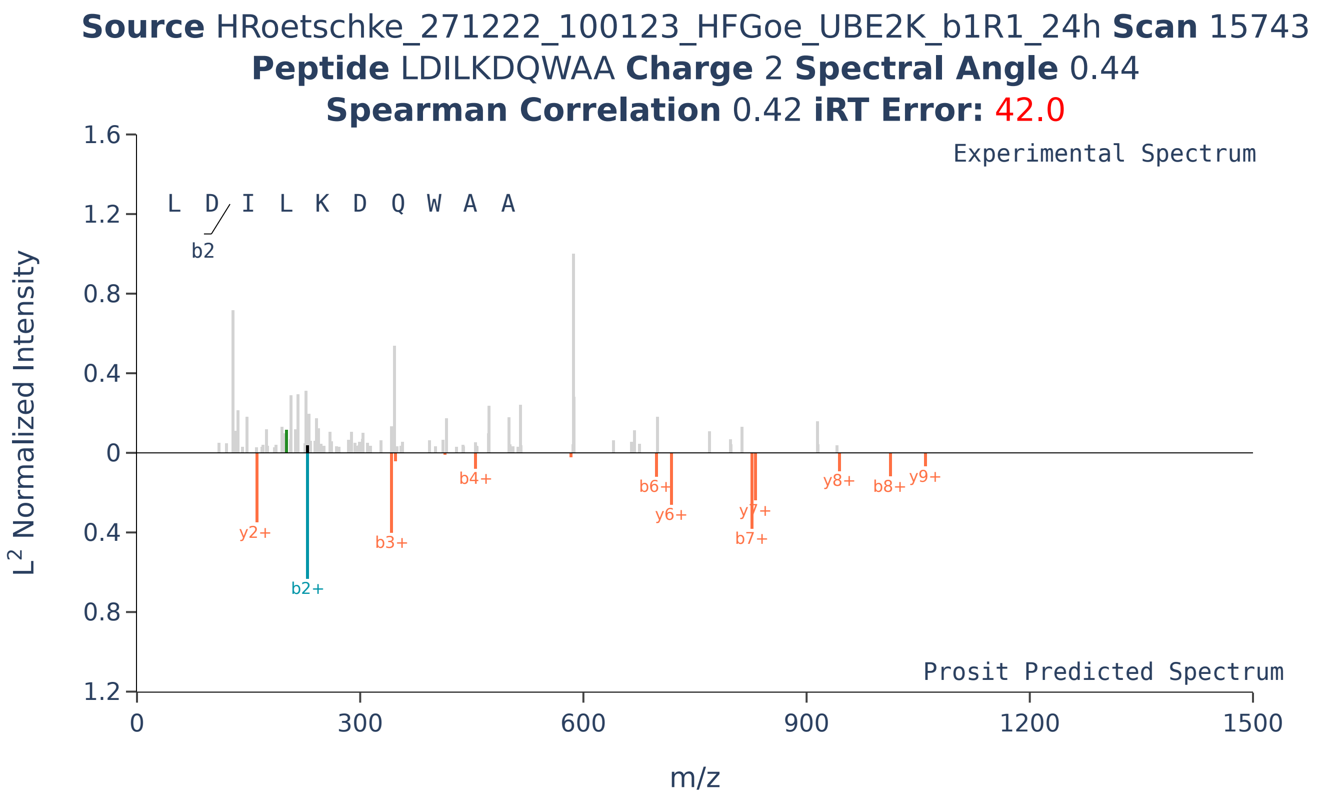
Source HRoetschke_271222_100123_HFGoe_UBE2K_b1R1_24h Scan 15743
Peptide LDILKDQWAA Charge 2 Spectral Angle 0.44
Spearman Correlation 0.42 iRT Error: 42.0
Experimental Spectrum
Prosit Predicted Spectrum
L2 Normalized Intensity
m/z

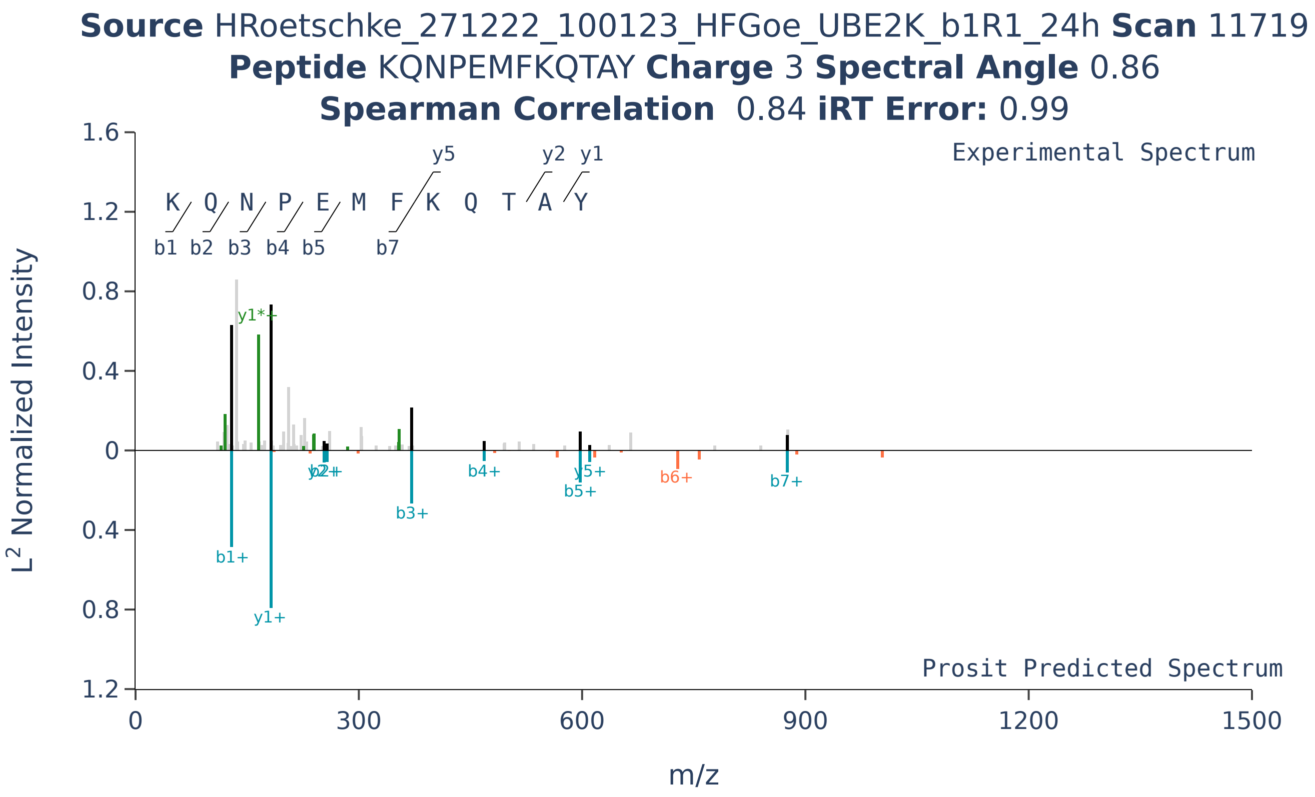
Source HRoetschke_271222_100123_HFGoe_UBE2K_b1R1_24h Scan 11719
Peptide KQNPEMFKQTAY Charge 3 Spectral Angle 0.86
Spearman Correlation 0.84 iRT Error: 0.99
Experimental Spectrum
Prosit Predicted Spectrum
L2 Normalized Intensity
m/z

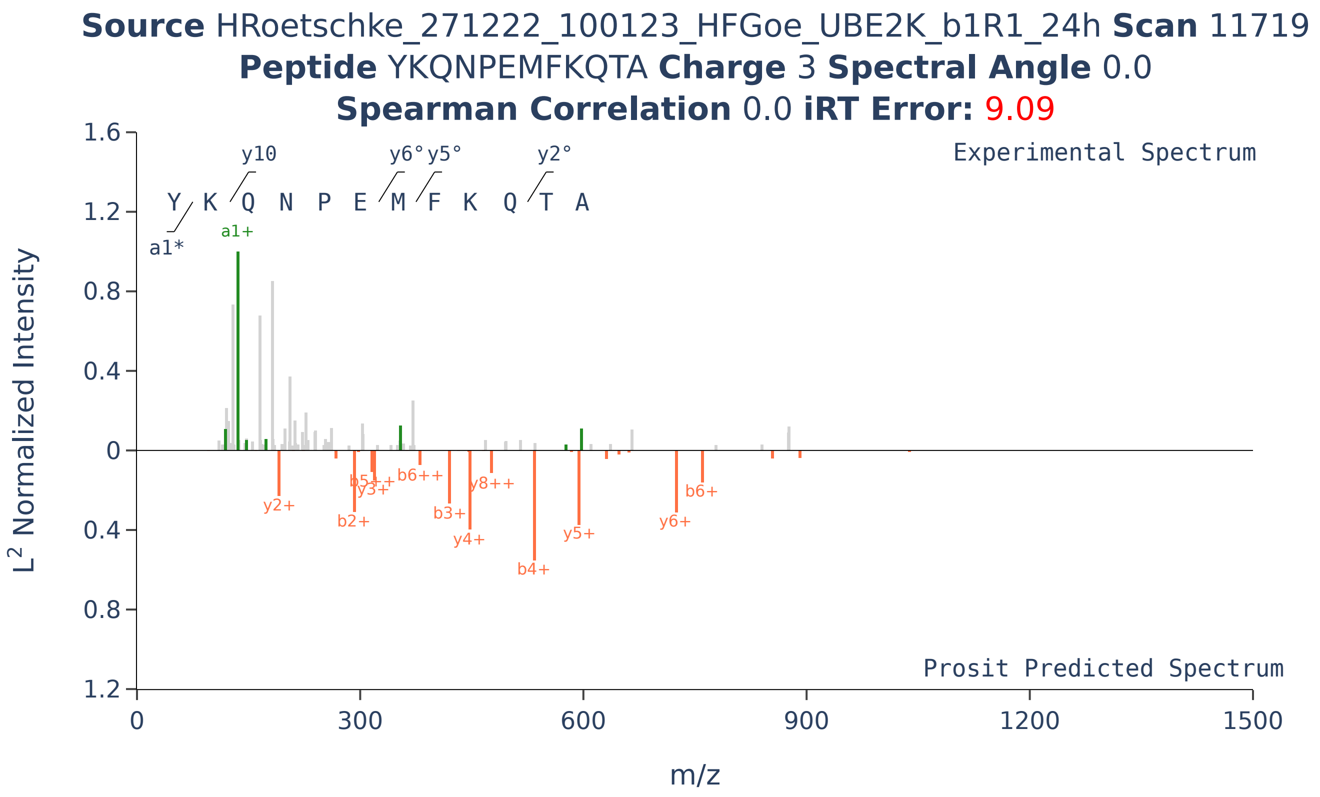
Source HRoetschke_271222_100123_HFGoe_UBE2K_b1R1_24h Scan 11719
Peptide YKQNPEMFKQTA Charge 3 Spectral Angle 0.0
Spearman Correlation 0.0 iRT Error: 9.09
Experimental Spectrum
Prosit Predicted Spectrum
L2 Normalized Intensity
m/z

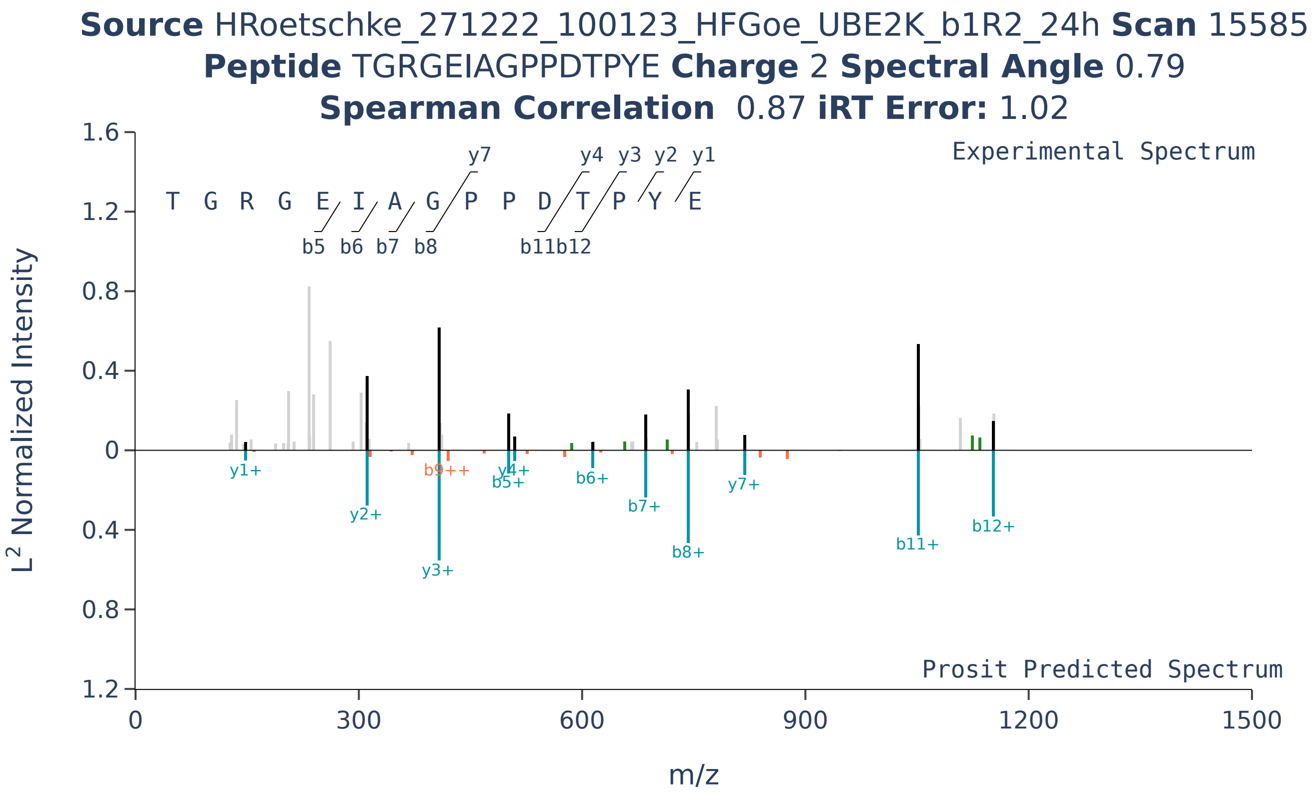
Source HRoetschke_271222_100123_HFGoe_UBE2K_b1R2_24h Scan 15585
Peptide TGRGEIAGPPDTPYE Charge 2 Spectral Angle 0.79
Spearman Correlation 0.87 iRT Error: 1.02
Experimental Spectrum
Prosit Predicted Spectrum
L2 Normalized Intensity
m/z

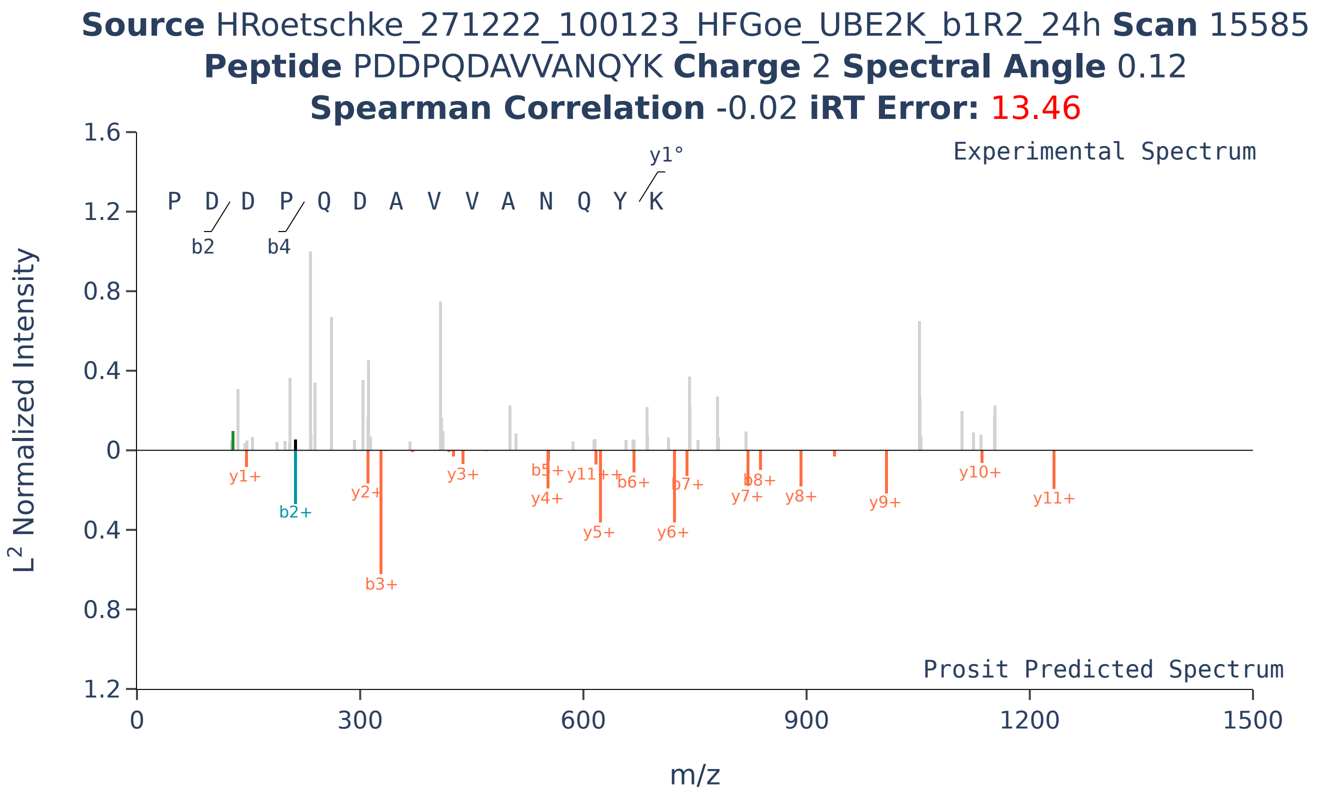
Source HRoetschke_271222_100123_HFGoe_UBE2K_b1R2_24h Scan 15585
Peptide PDDPQDAVVANQYK Charge 2 Spectral Angle 0.12
Spearman Correlation -0.02 iRT Error: 13.46
Experimental Spectrum
Prosit Predicted Spectrum
L2 Normalized Intensity
m/z

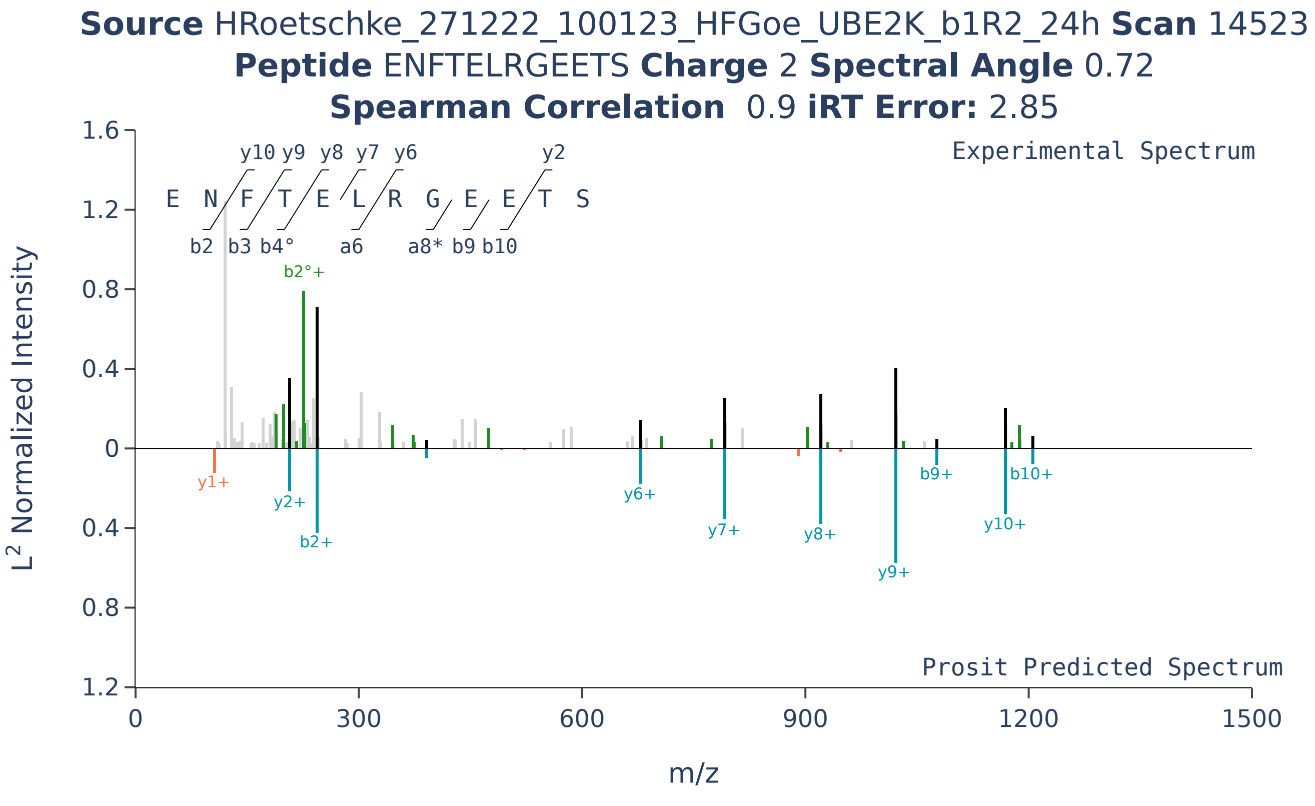
Source HRoetschke_271222_100123_HFGoe_UBE2K_b1R2_24h Scan 14523
Peptide ENFTELRGEETS Charge 2 Spectral Angle 0.72
Spearman Correlation 0.9 iRT Error: 2.85
Experimental Spectrum
Prosit Predicted Spectrum
L2 Normalized Intensity
m/z

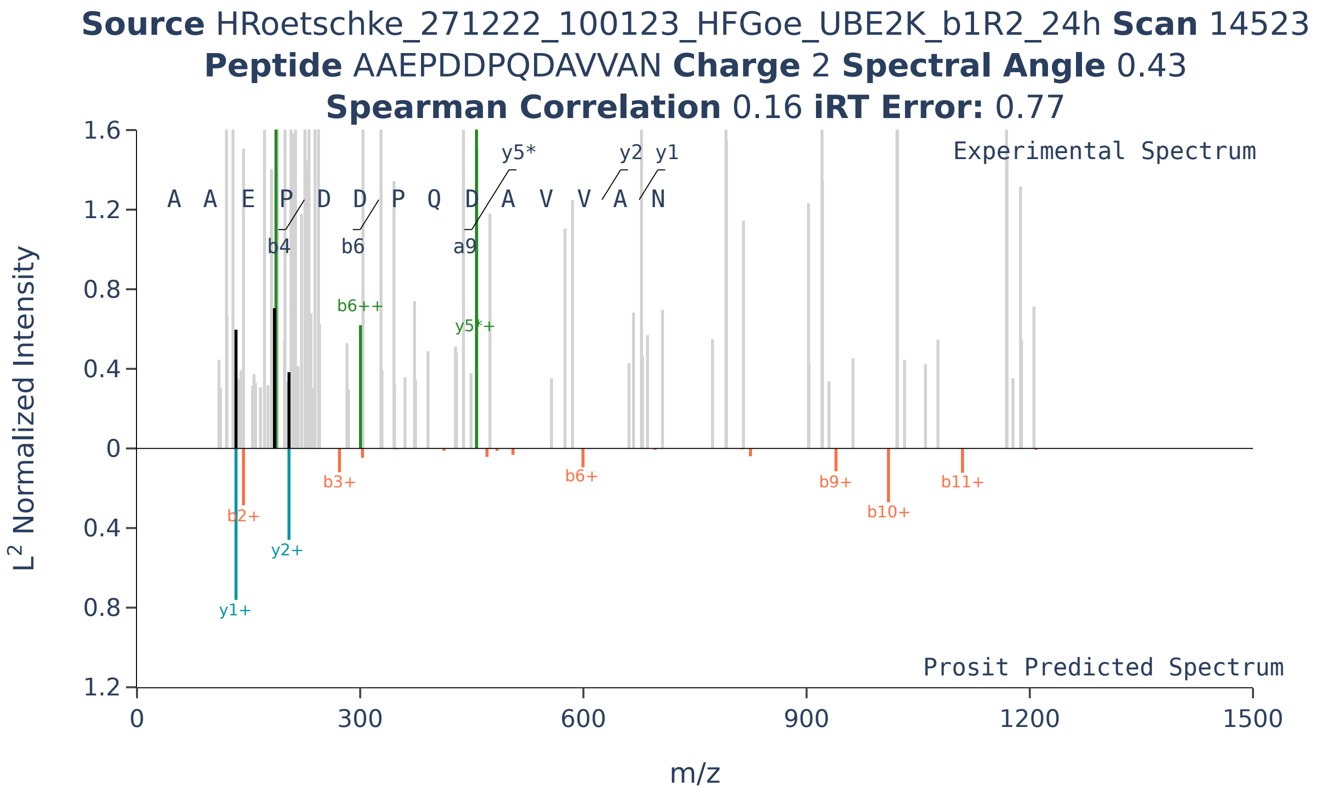
Source HRoetschke_271222_100123_HFGoe_UBE2K_b1R2_24h Scan 14523
Peptide AAEPDDPQDAVVAN Charge 2 Spectral Angle 0.43
Spearman Correlation 0.16 iRT Error: 0.77
Experimental Spectrum
Prosit Predicted Spectrum
L2 Normalized Intensity
m/z

Source HRoetschke_271222_100123_HFGoe_UBE2K_b1R2_4h **Scan** 12606
Peptide ENFTELRGD **Charge** 2 **Spectral Angle** 0.91
Spearman Correlation 0.94 **iRT Error:** 2.86

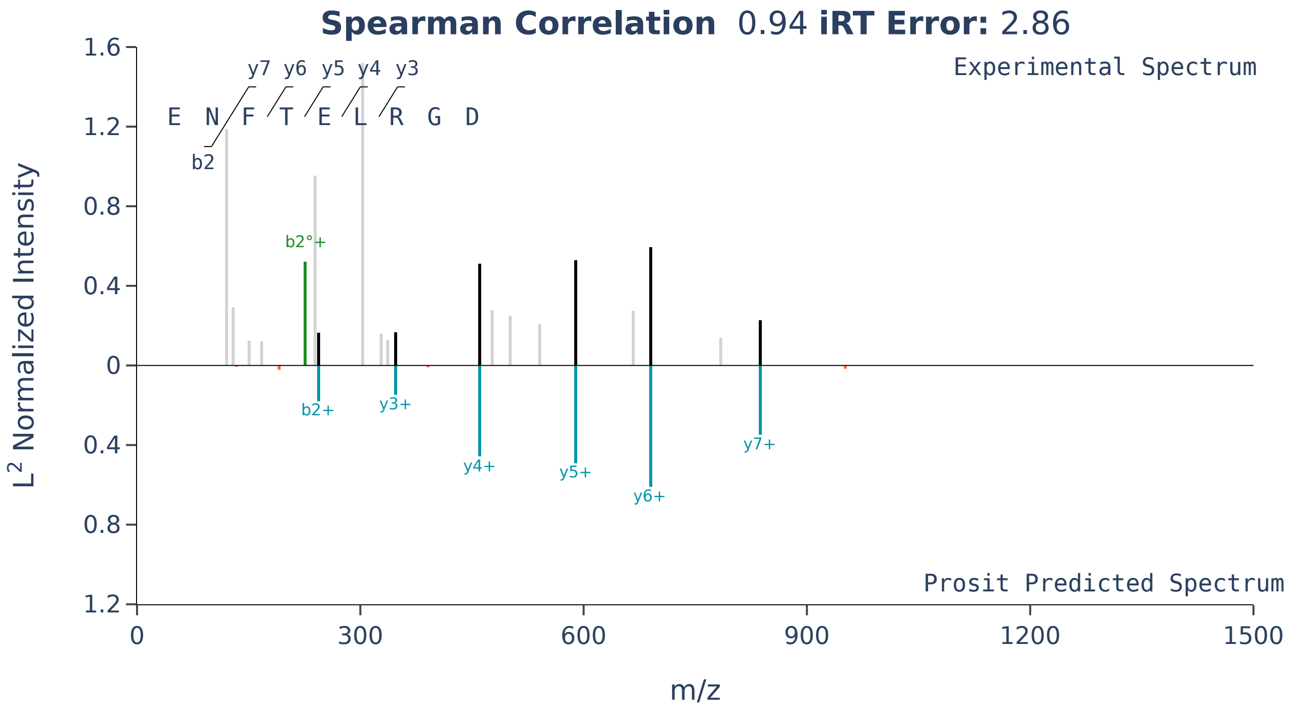

Source HRoetschke_271222_100123_HFGoe_UBE2K_b1R2_4h **Scan** 12606
Peptide DENFTELRG **Charge** 2 **Spectral Angle** 0.0
Spearman Correlation 0.0 **iRT Error:** 7.08

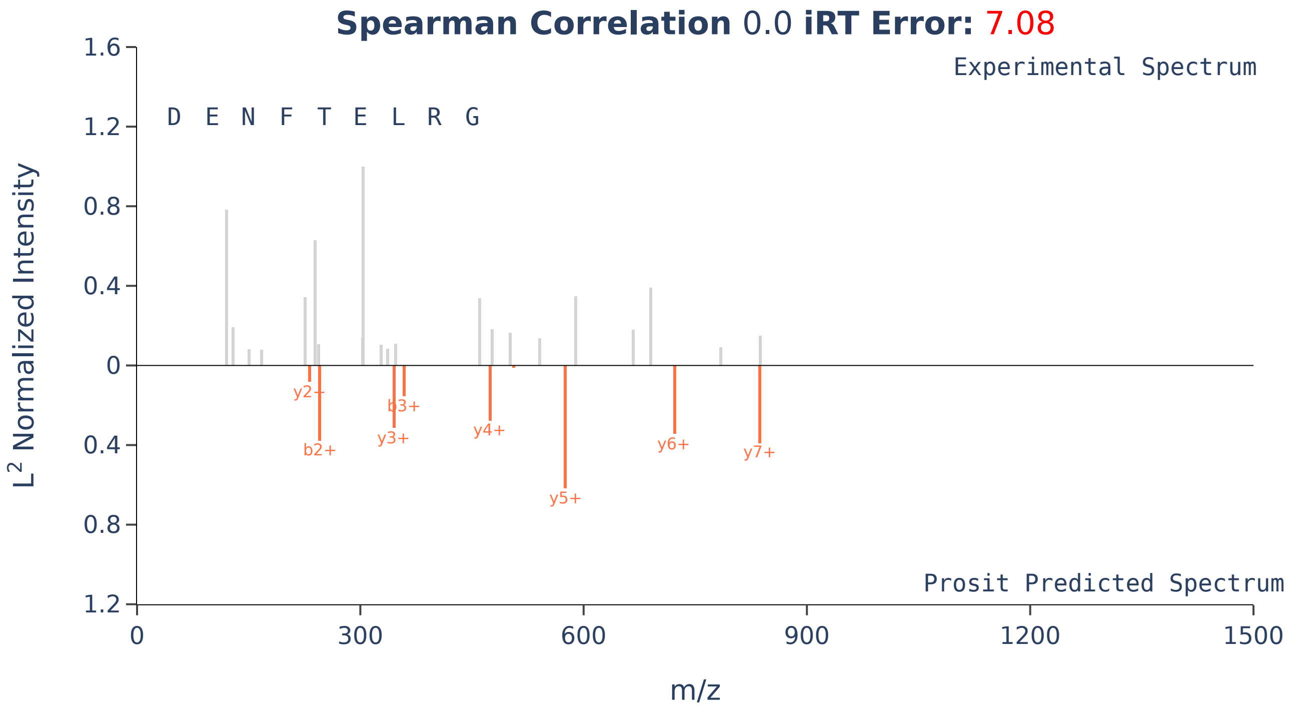

Source HRoetschke_271222_100123_HFGoe_UBE2K_b1R2_24h **Scan** 20057
Peptide TGEIKIPETY **Charge** 2 **Spectral Angle** 0.8
Spearman Correlation 0.9 **iRT Error:** 3.35

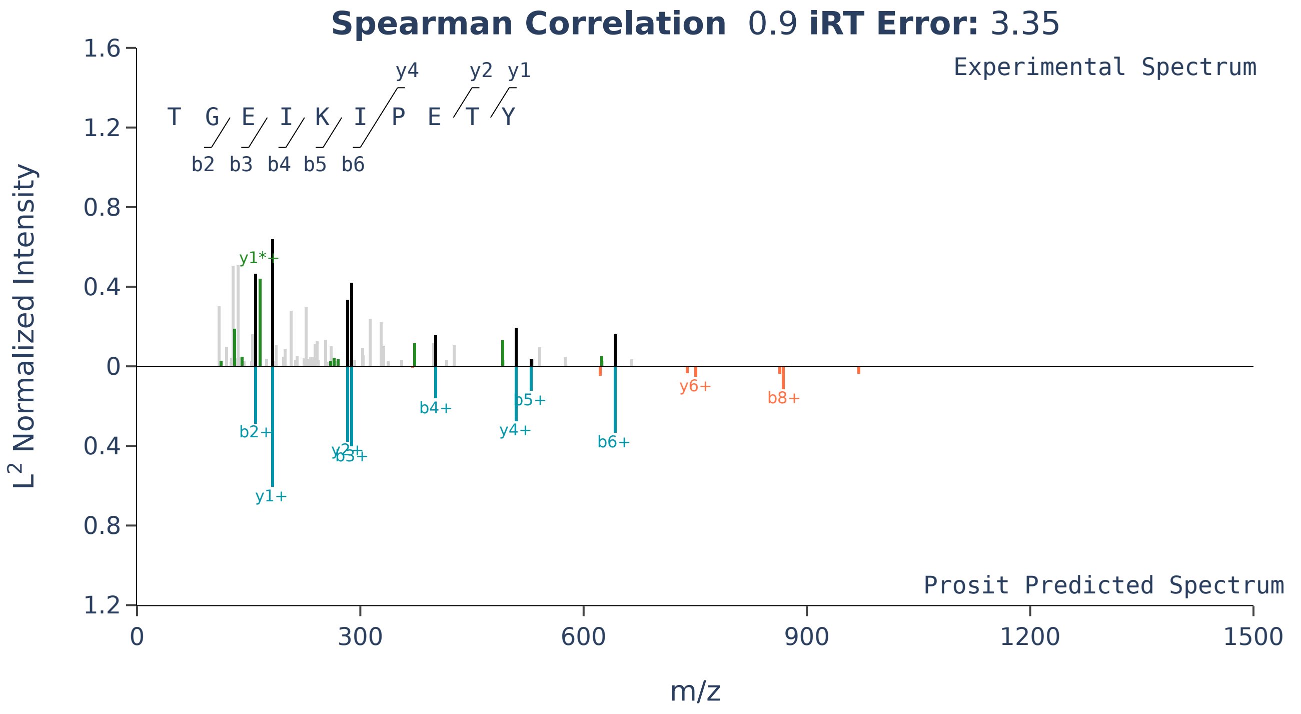

Source HRoetschke_271222_100123_HFGoe_UBE2K_b1R2_24h **Scan** 20057
Peptide TKKIENLCAM **Charge** 2 **Spectral Angle** 0.0
Spearman Correlation 0.0 **iRT Error:** 38.59

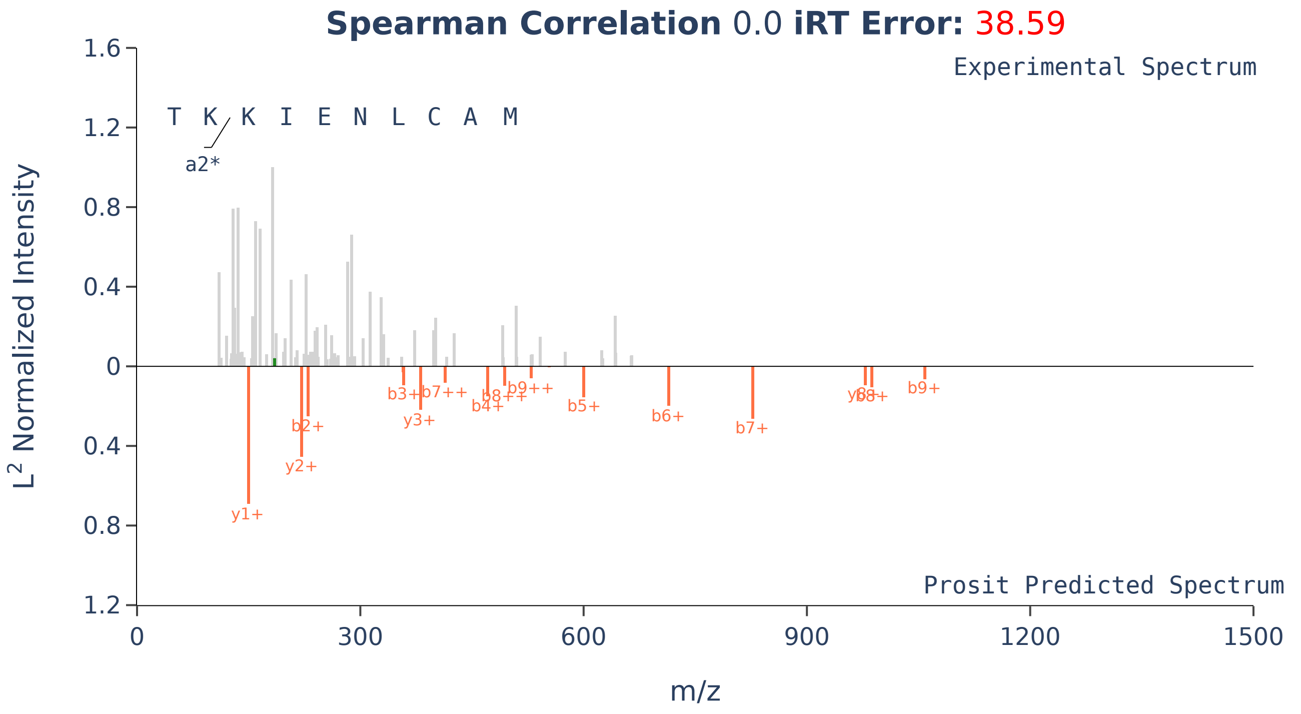

Source HRoetschke_271222_100123_HFGoe_UBE2K_b1R2_24h **Scan** 11086
Peptide MAGFDRNA **Charge** 1 **Spectral Angle** 0.9
Spearman Correlation 0.95 **iRT Error:** 4.29

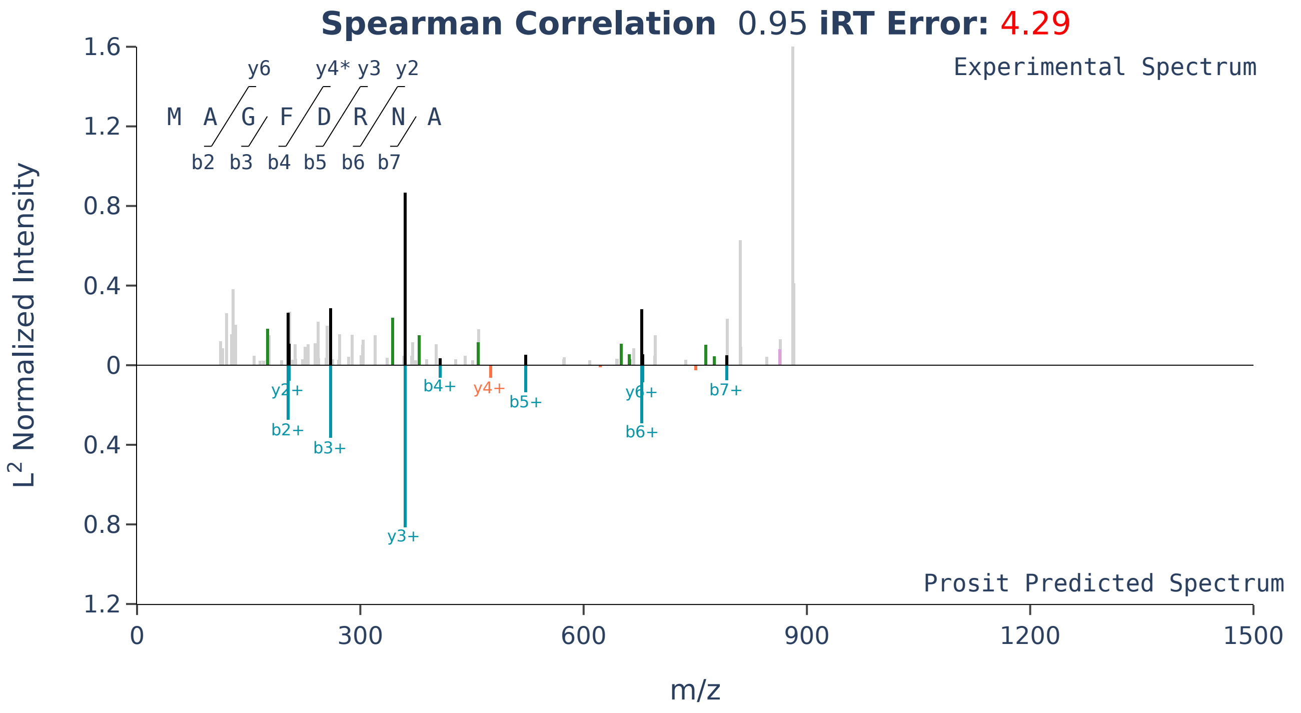

Source HRoetschke_271222_100123_HFGoe_UBE2K_b1R2_24h **Scan** 11086
Peptide AMGFDRNA **Charge** 1 **Spectral Angle** 0.63
Spearman Correlation 0.51 **iRT Error:** 0.77

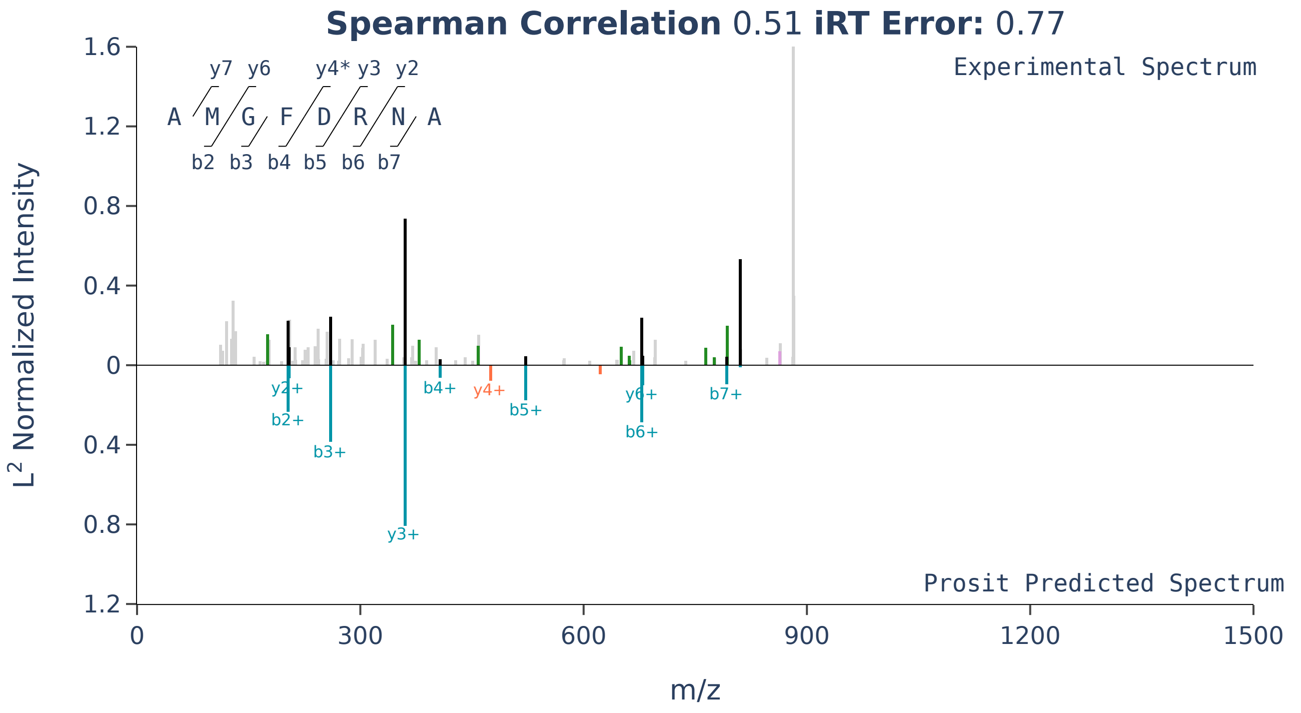

Source HRoetschke_271222_100123_HFGoe_UBE2K_b1R2_24h **Scan** 16628
Peptide ENFTELRDA **Charge** 2 **Spectral Angle** 0.85
Spearman Correlation 0.93 **iRT Error:** 4.88

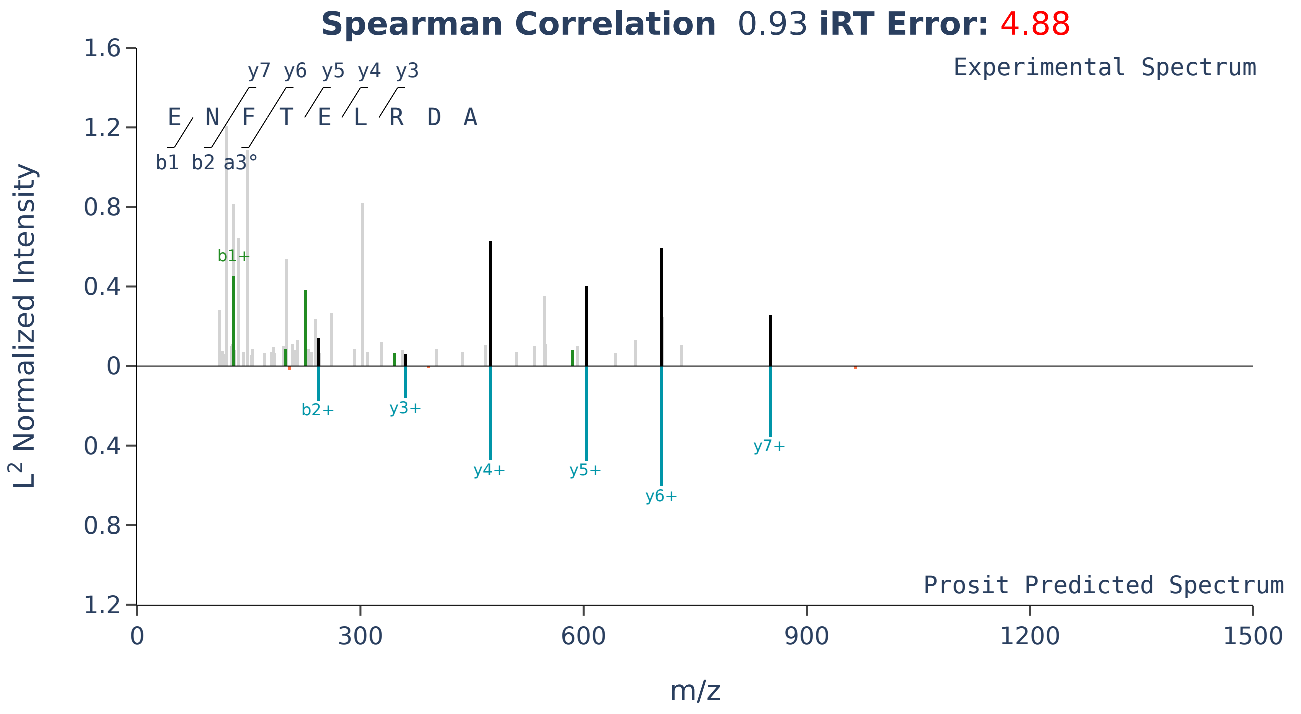

Source HRoetschke_271222_100123_HFGoe_UBE2K_b1R2_24h **Scan** 16628
Peptide ENFTELRGE **Charge** 2 **Spectral Angle** 0.47
Spearman Correlation 0.67 **iRT Error:** 10.34

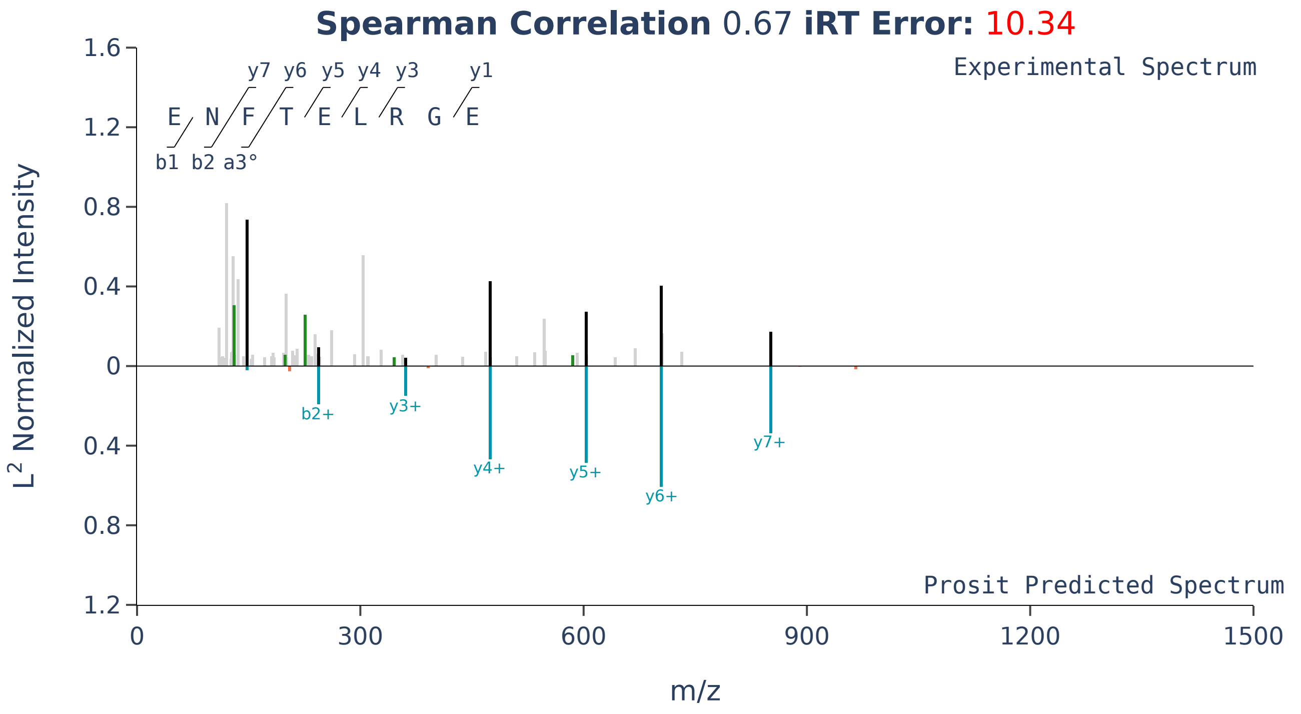

Source HRoetschke_271222_100123_HFGoe_UBE2K_b1R1_24h **Scan** 11219
Peptide SSKVDLVD **Charge** 2 **Spectral Angle** 0.83
Spearman Correlation 0.99 **iRT Error:** 6.02

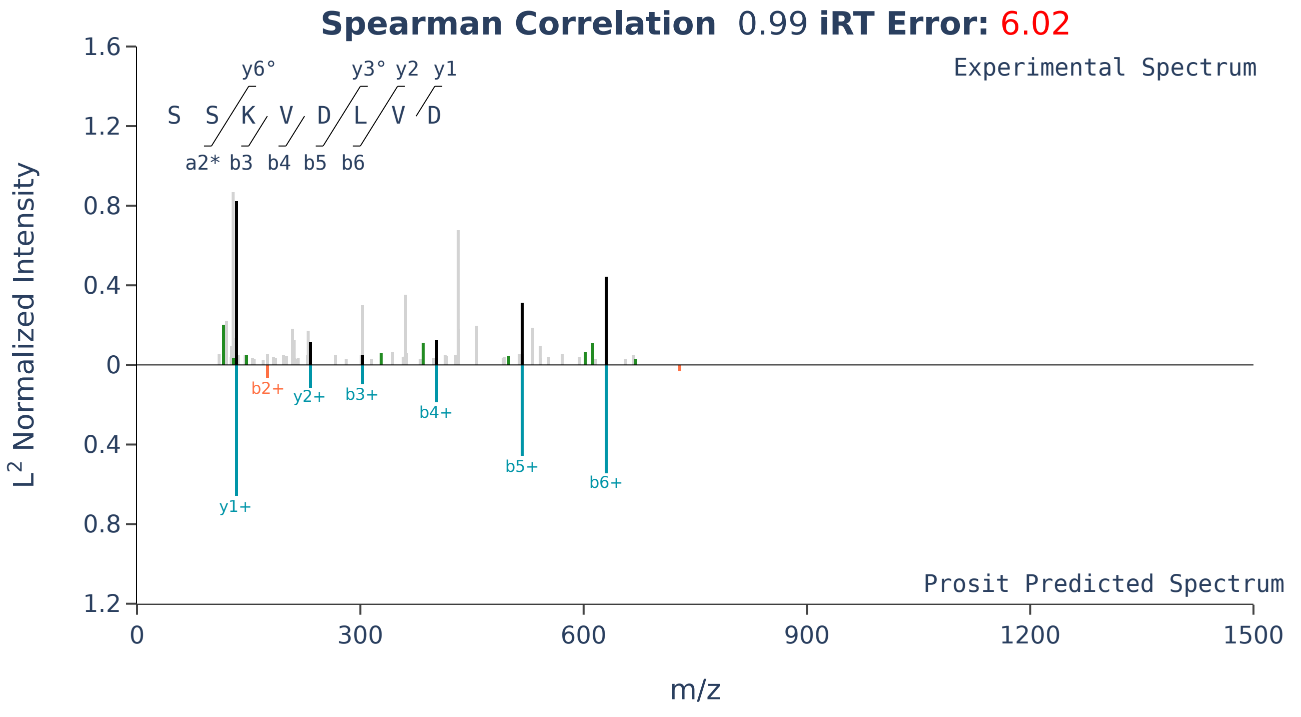

Source HRoetschke_271222_100123_HFGoe_UBE2K_b1R1_24h **Scan** 11219
Peptide YPFNPPK **Charge** 2 **Spectral Angle** 0.0
Spearman Correlation 0.0 **iRT Error:** 14.91

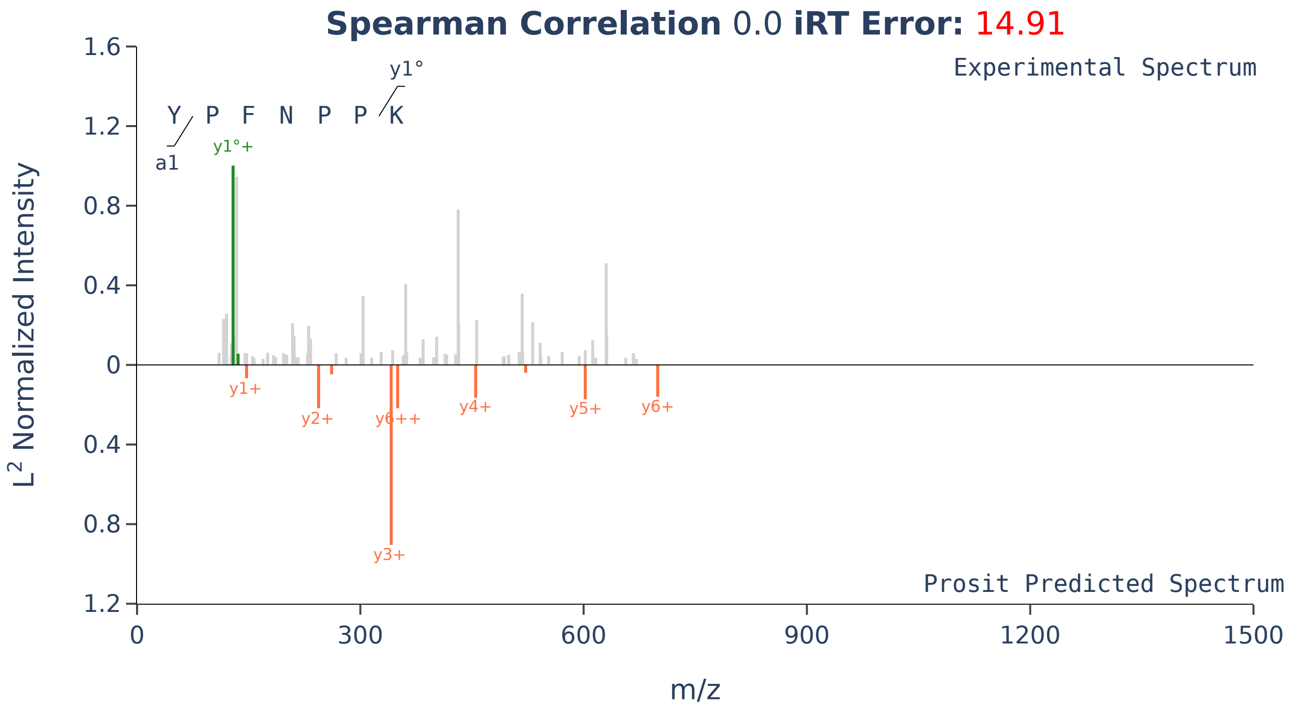

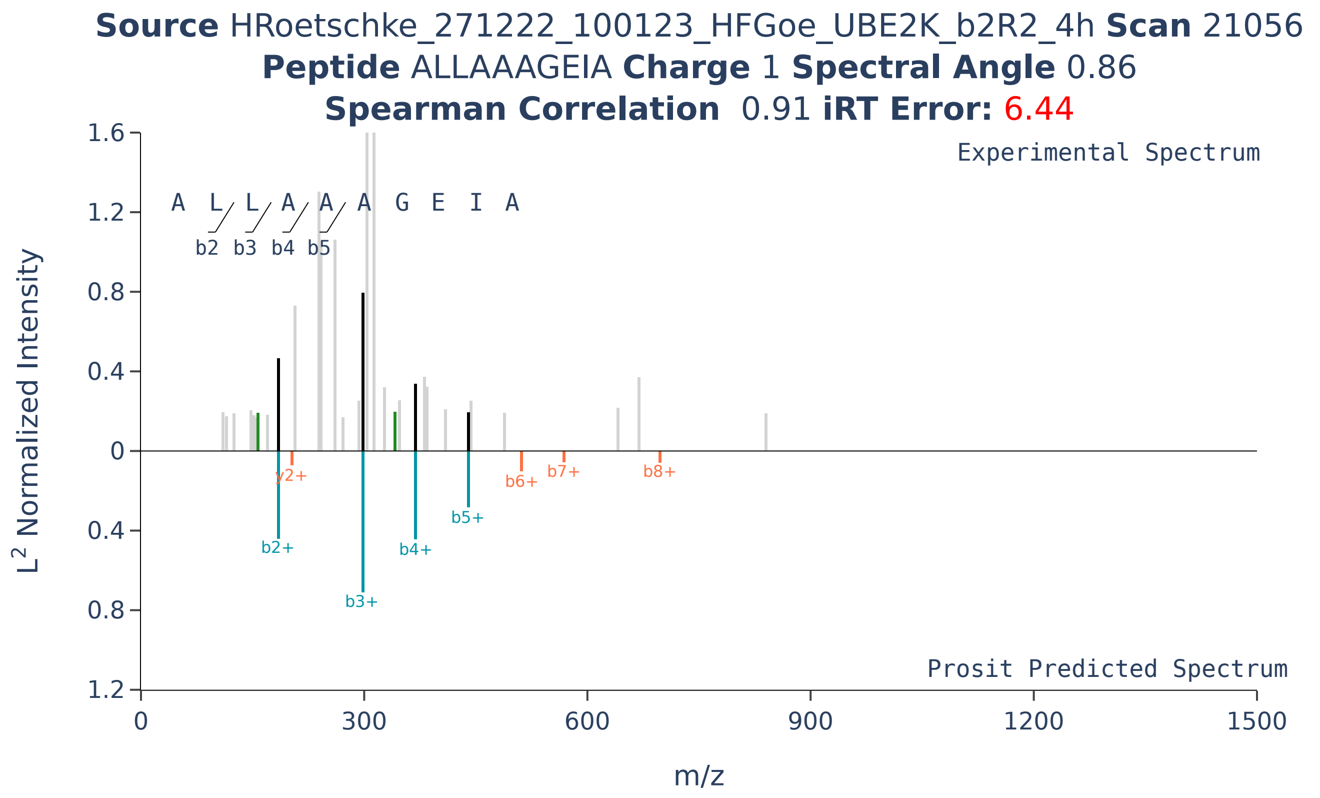
Source HRoetschke_271222_100123_HFGoe_UBE2K_b2R2_4h Scan 21056
Peptide ALLAAAGEIA Charge 1 Spectral Angle 0.86
Spearman Correlation 0.91 iRT Error: 6.44
Experimental Spectrum
Prosit Predicted Spectrum
L2 Normalized Intensity
m/z

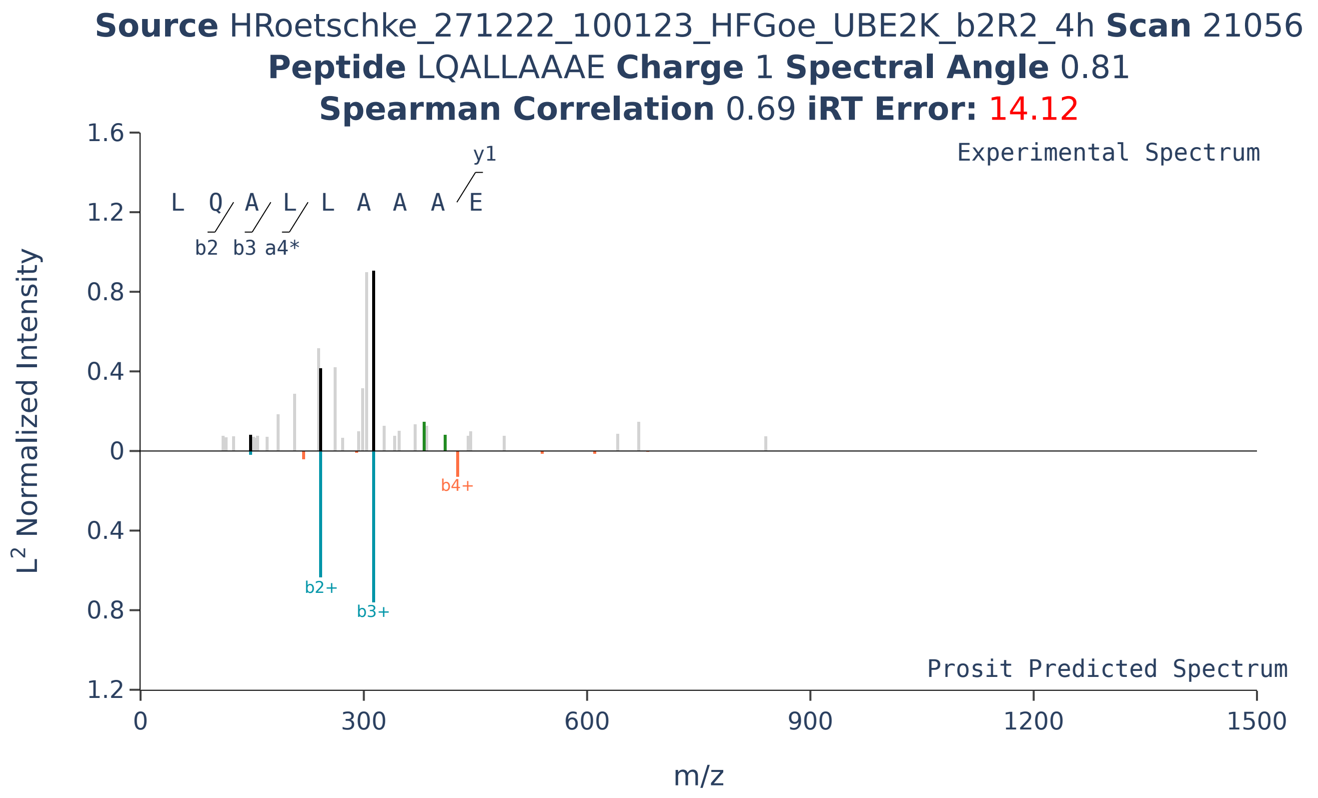
Source HRoetschke_271222_100123_HFGoe_UBE2K_b2R2_4h Scan 21056
Peptide LQALLAAAE Charge 1 Spectral Angle 0.81
Spearman Correlation 0.69 iRT Error: 14.12
Experimental Spectrum
Prosit Predicted Spectrum
L2 Normalized Intensity
m/z

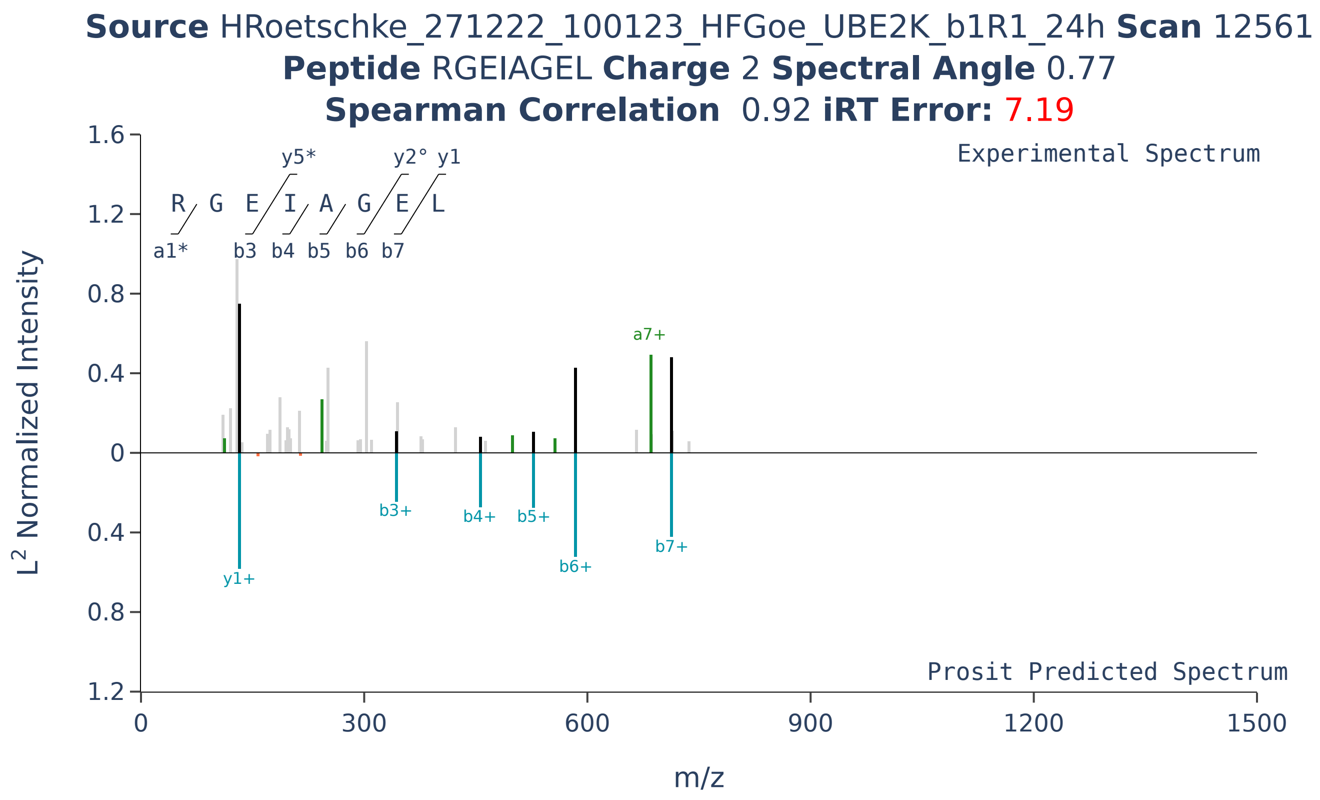
Source HRoetschke_271222_100123_HFGoe_UBE2K_b1R1_24h Scan 12561
Peptide RGEIAGEL Charge 2 Spectral Angle 0.77
Spearman Correlation 0.92 iRT Error: 7.19
Experimental Spectrum
Prosit Predicted Spectrum
L2 Normalized Intensity
m/z

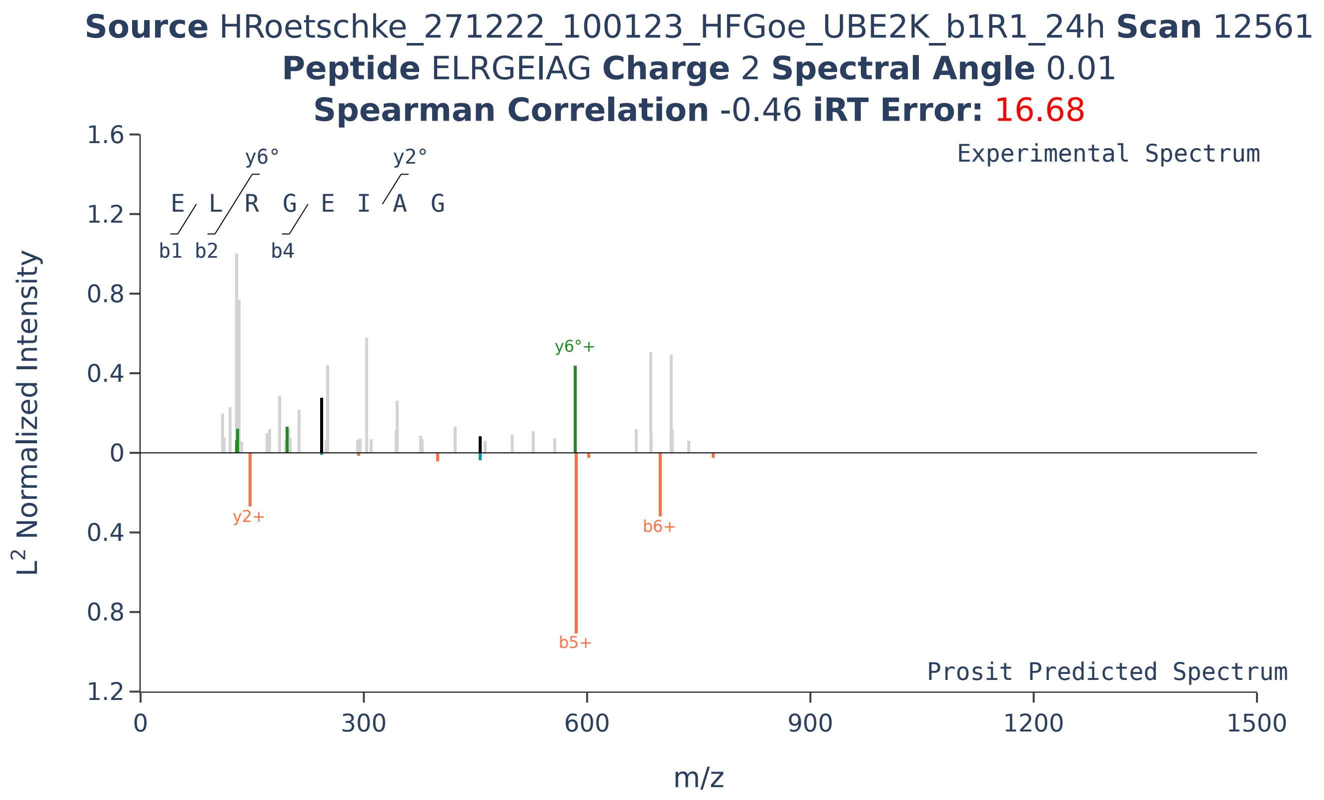
Source HRoetschke_271222_100123_HFGoe_UBE2K_b1R1_24h Scan 12561
Peptide ELRGEIAG Charge 2 Spectral Angle 0.01
Spearman Correlation -0.46 iRT Error: 16.68
Experimental Spectrum
Prosit Predicted Spectrum
L2 Normalized Intensity
m/z

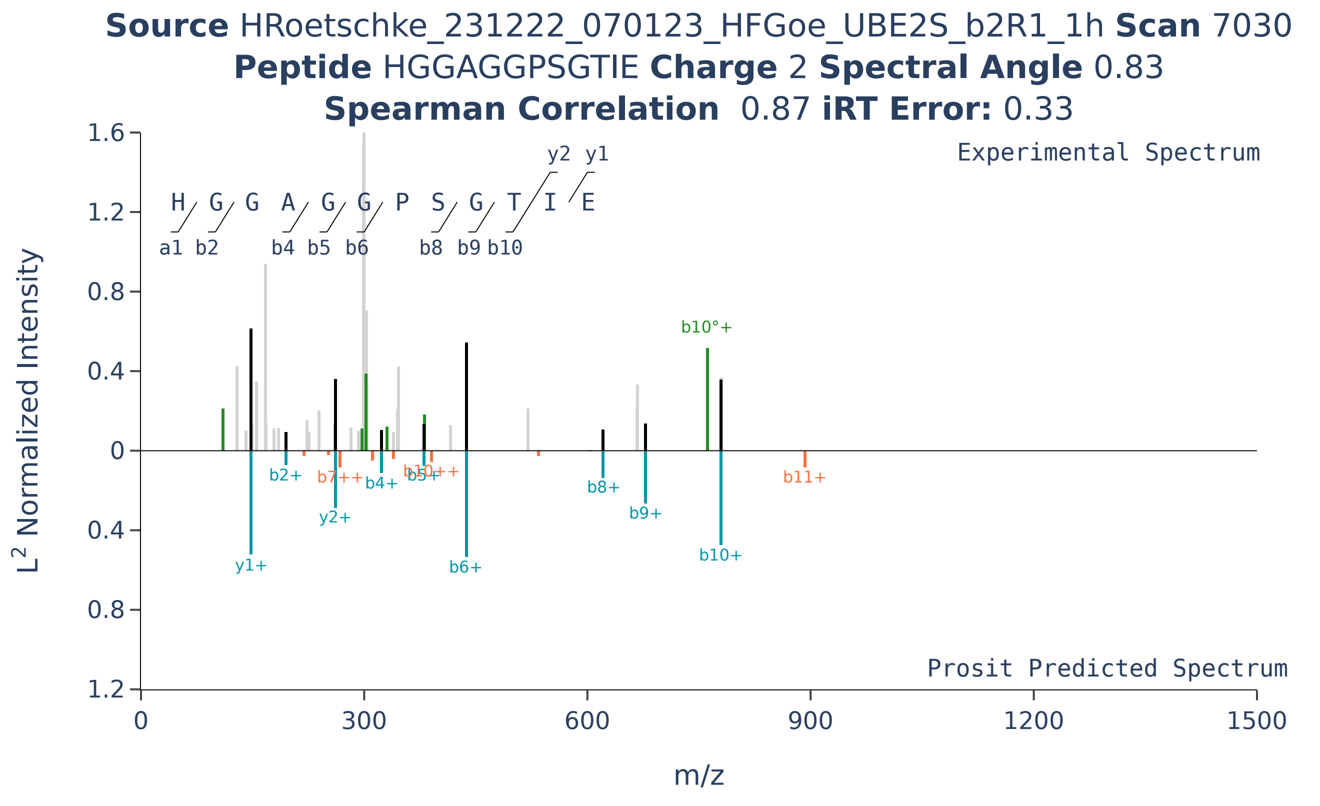
Source HRoetschke_231222_070123_HFGoe_UBE2S_b2R1_1h Scan 7030
Peptide HGGAGGPSGTIE Charge 2 Spectral Angle 0.83
Spearman Correlation 0.87 iRT Error: 0.33
Experimental Spectrum
Prosit Predicted Spectrum
L² Normalized Intensity
m/z

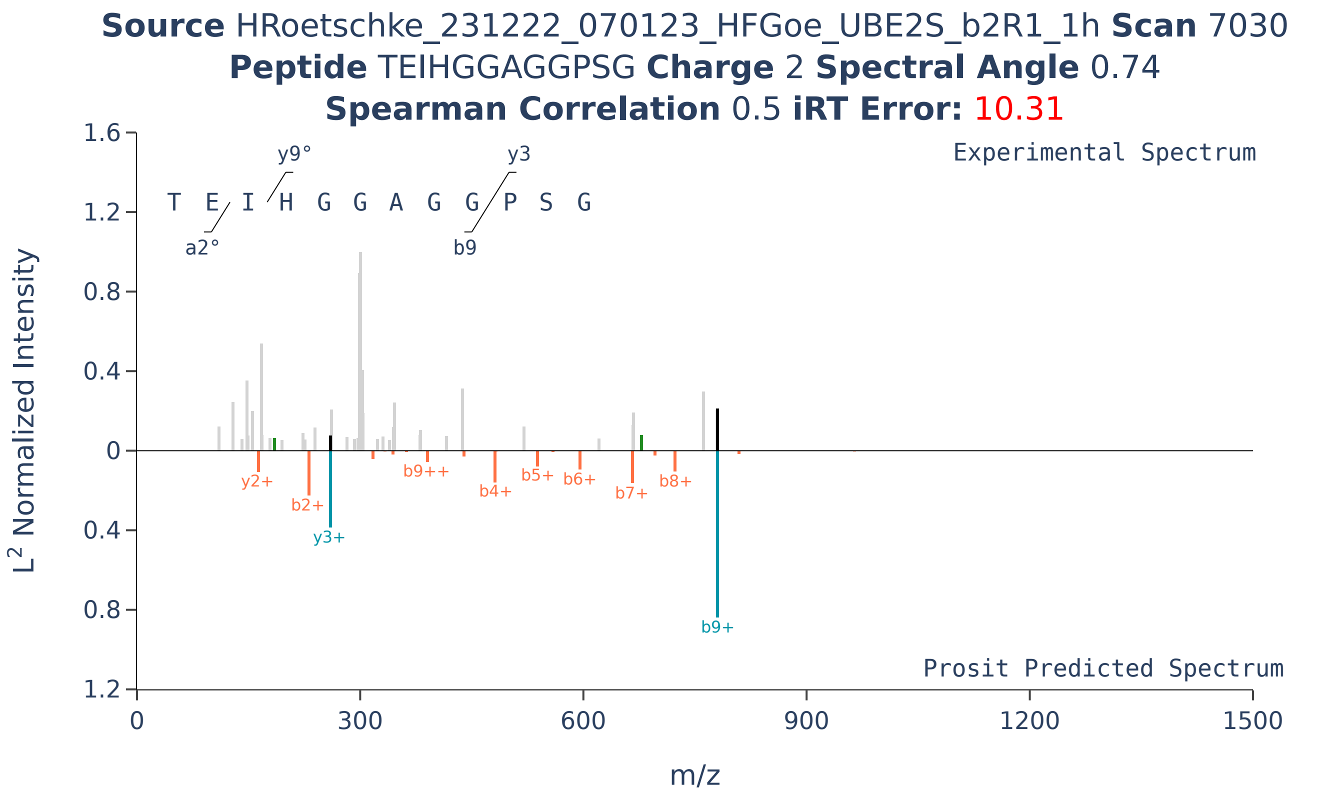
Source HRoetschke_231222_070123_HFGoe_UBE2S_b2R1_1h Scan 7030
Peptide TEIHGGAGGPSG Charge 2 Spectral Angle 0.74
Spearman Correlation 0.5 iRT Error: 10.31
Experimental Spectrum
Prosit Predicted Spectrum
L² Normalized Intensity
m/z

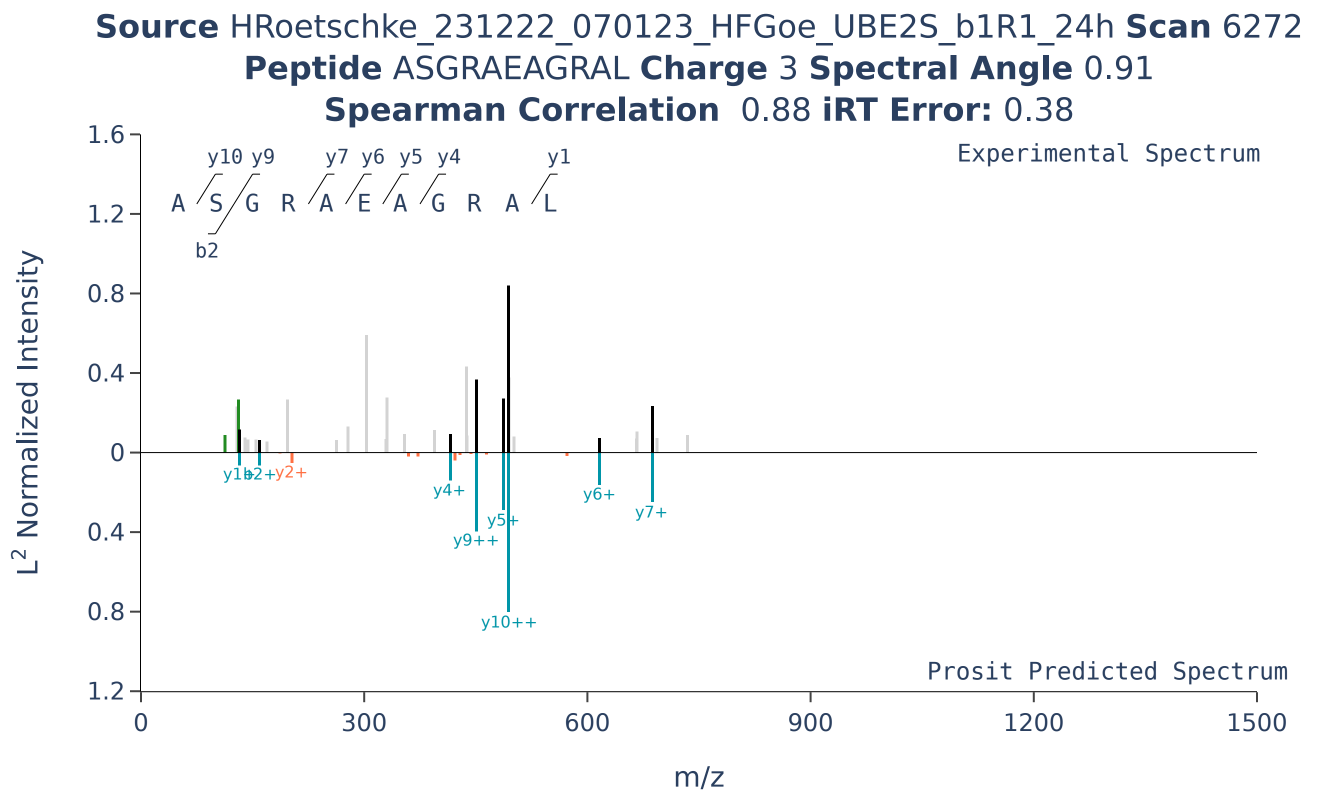
Source HRoetschke_231222_070123_HFGoe_UBE2S_b1R1_24h Scan 6272
Peptide ASGRAEAGRAL Charge 3 Spectral Angle 0.91
Spearman Correlation 0.88 iRT Error: 0.38
Experimental Spectrum
Prosit Predicted Spectrum
L² Normalized Intensity
m/z

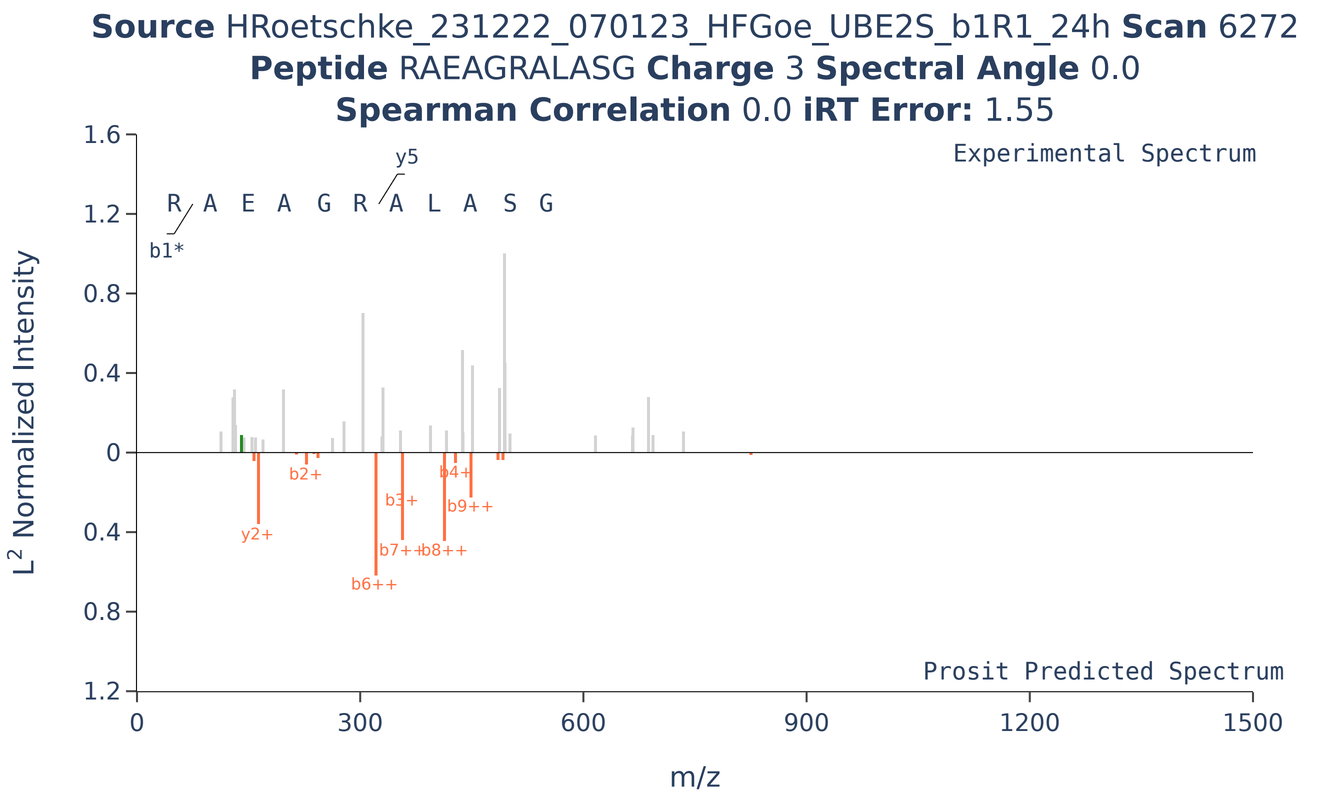
Source HRoetschke_231222_070123_HFGoe_UBE2S_b1R1_24h Scan 6272
Peptide RAEAGRALASG Charge 3 Spectral Angle 0.0
Spearman Correlation 0.0 iRT Error: 1.55
Experimental Spectrum
Prosit Predicted Spectrum
L² Normalized Intensity
m/z

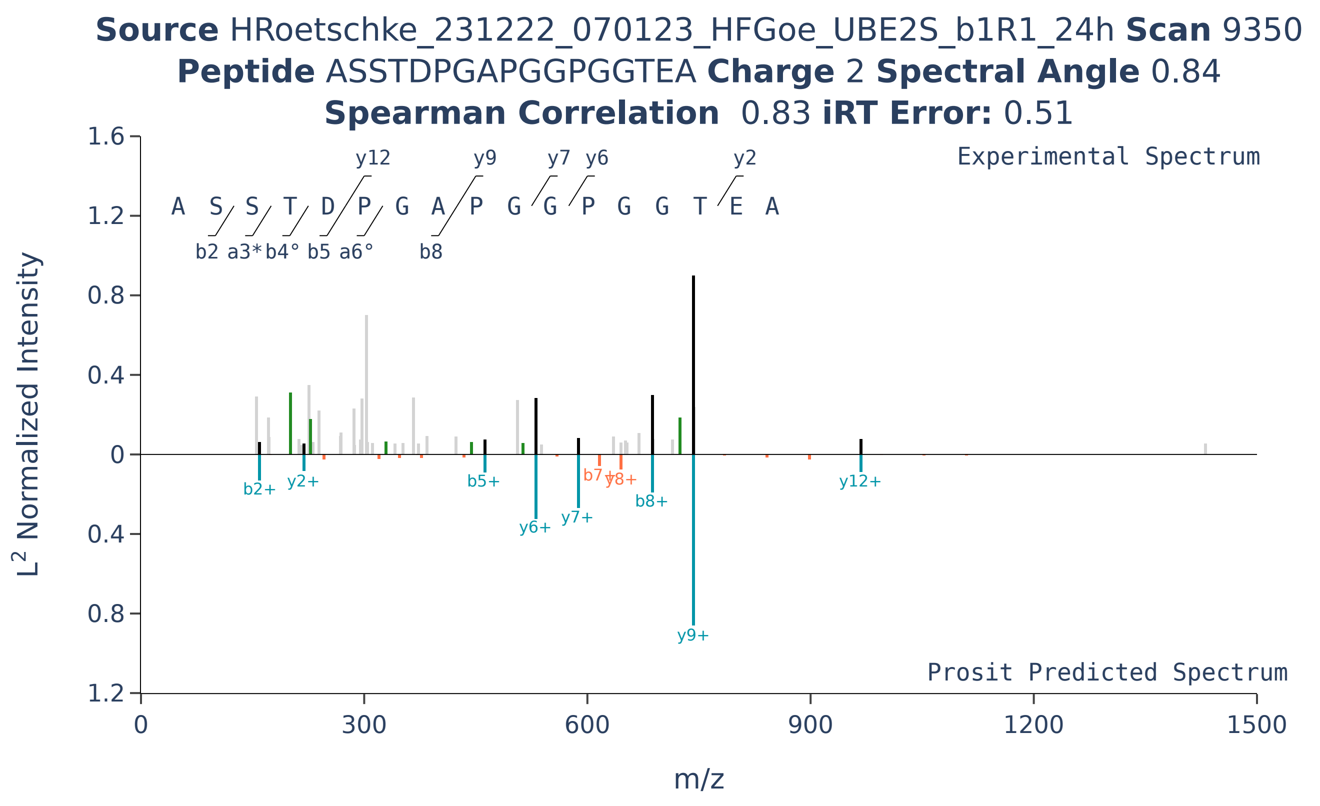
Source HRoetschke_231222_070123_HFGoe_UBE2S_b1R1_24h Scan 9350
Peptide ASSTDPGAPGGPGGTEA Charge 2 Spectral Angle 0.84
Spearman Correlation 0.83 iRT Error: 0.51
Experimental Spectrum
Prosit Predicted Spectrum
L² Normalized Intensity
m/z

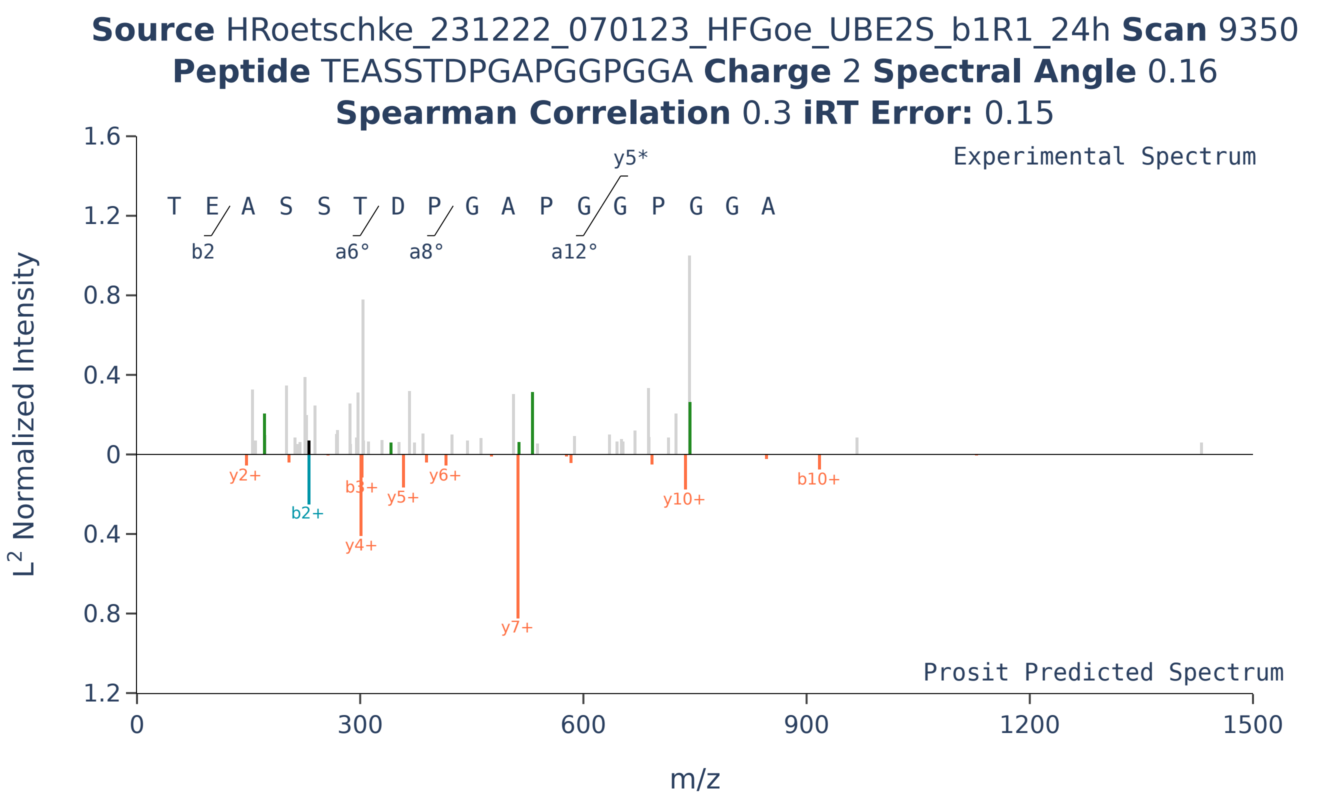
Source HRoetschke_231222_070123_HFGoe_UBE2S_b1R1_24h Scan 9350
Peptide TEASSTDPGAPGGPGGA Charge 2 Spectral Angle 0.16
Spearman Correlation 0.3 iRT Error: 0.15
Experimental Spectrum
Prosit Predicted Spectrum
L² Normalized Intensity
m/z

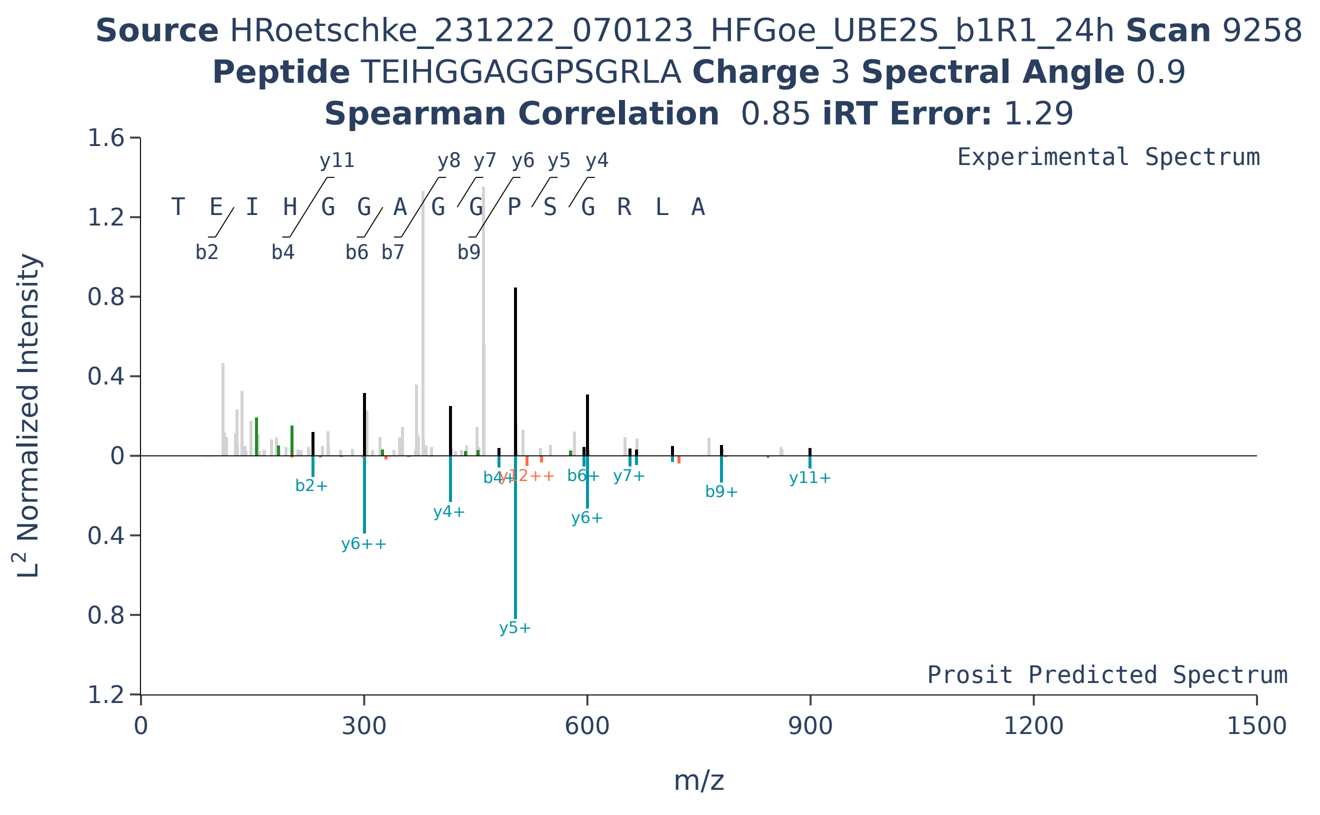
Source HRoetschke_231222_070123_HFGoe_UBE2S_b1R1_24h Scan 9258
Peptide TEIHGGAGGPSGRLA Charge 3 Spectral Angle 0.9
Spearman Correlation 0.85 iRT Error: 1.29
Experimental Spectrum
Prosit Predicted Spectrum
L² Normalized Intensity
m/z

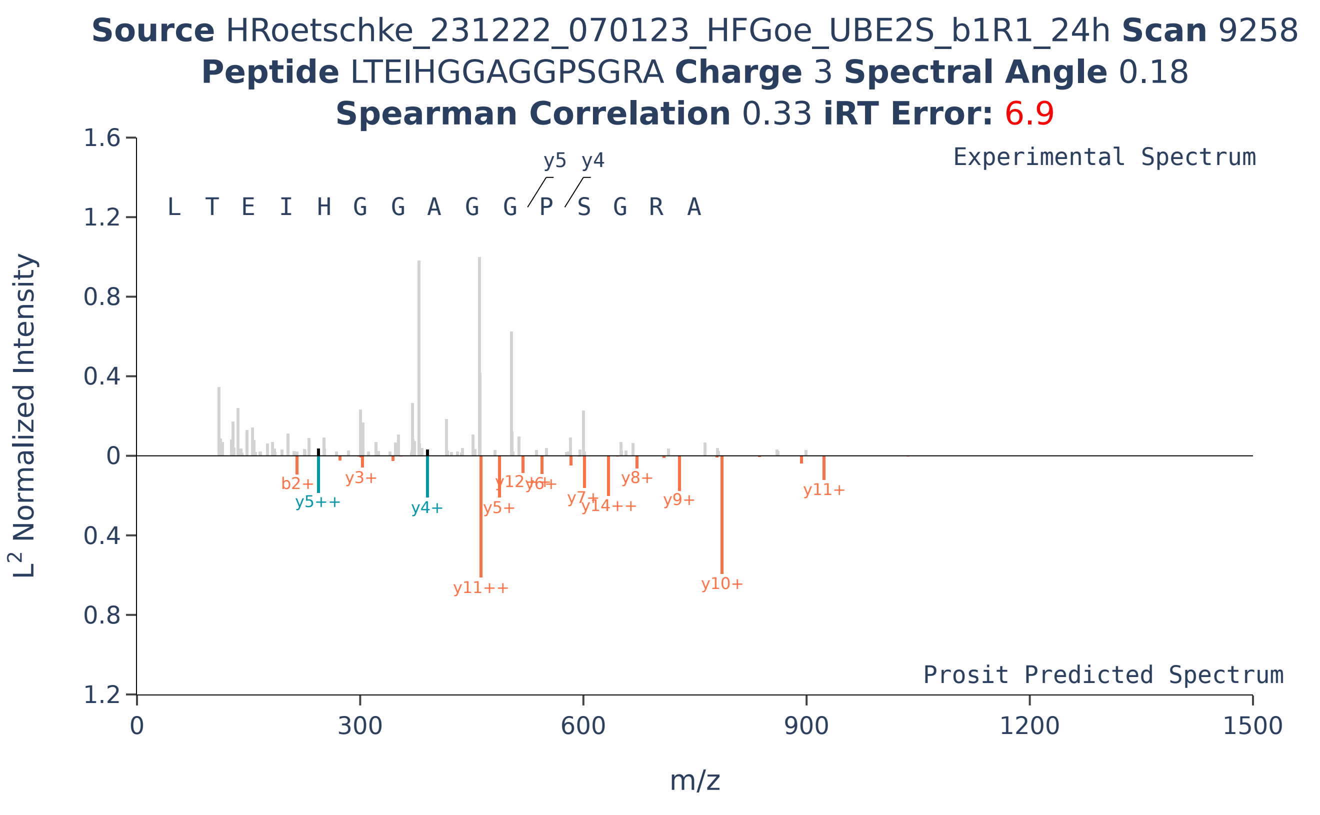
Source HRoetschke_231222_070123_HFGoe_UBE2S_b1R1_24h Scan 9258
Peptide LTEIHGGAGGPSGRA Charge 3 Spectral Angle 0.18
Spearman Correlation 0.33 iRT Error: 6.9
Experimental Spectrum
Prosit Predicted Spectrum
L² Normalized Intensity
m/z

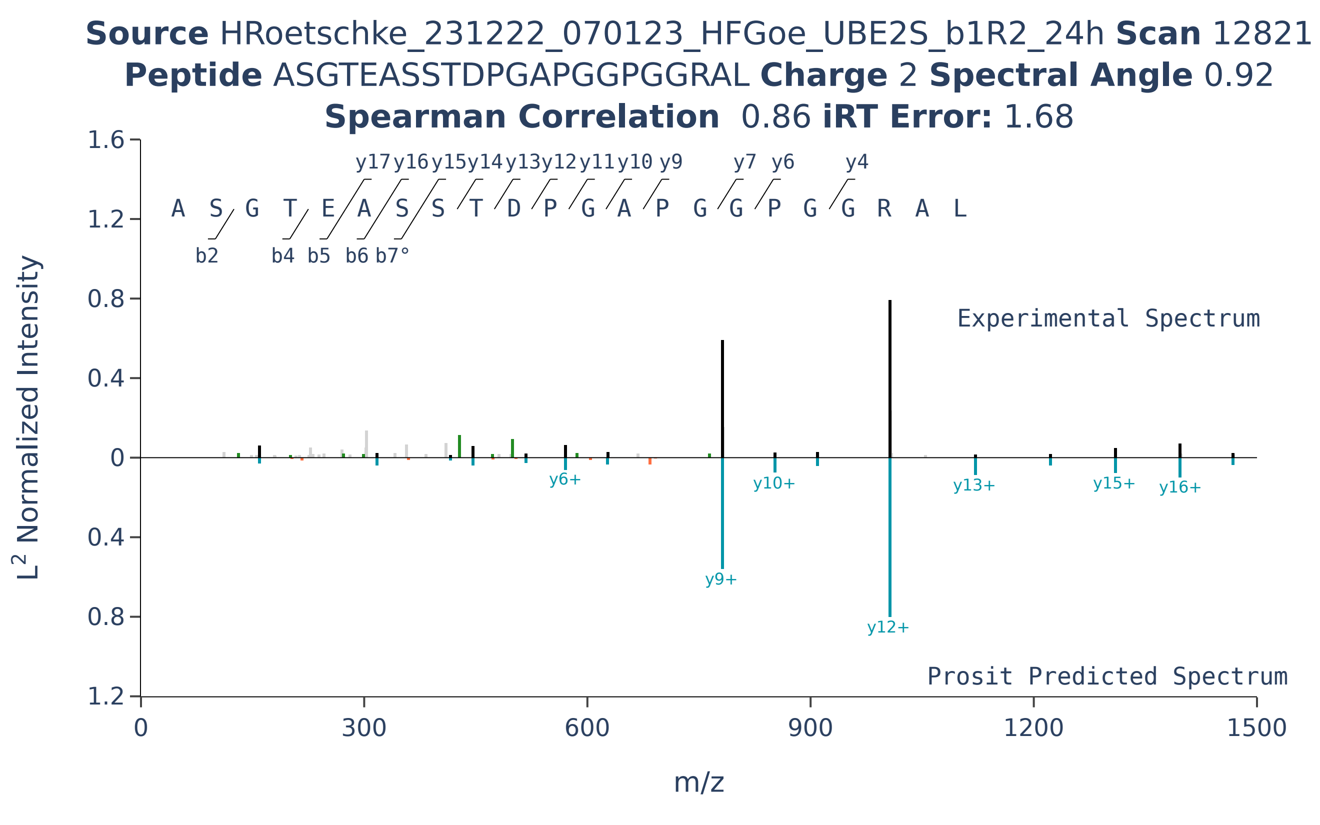
Source HRoetschke_231222_070123_HFGoe_UBE2S_b1R2_24h Scan 12821
Peptide ASGTEASSTDPGAPGGPGGRAL Charge 2 Spectral Angle 0.92
Spearman Correlation 0.86 iRT Error: 1.68
Experimental Spectrum
Prosit Predicted Spectrum
L² Normalized Intensity
m/z

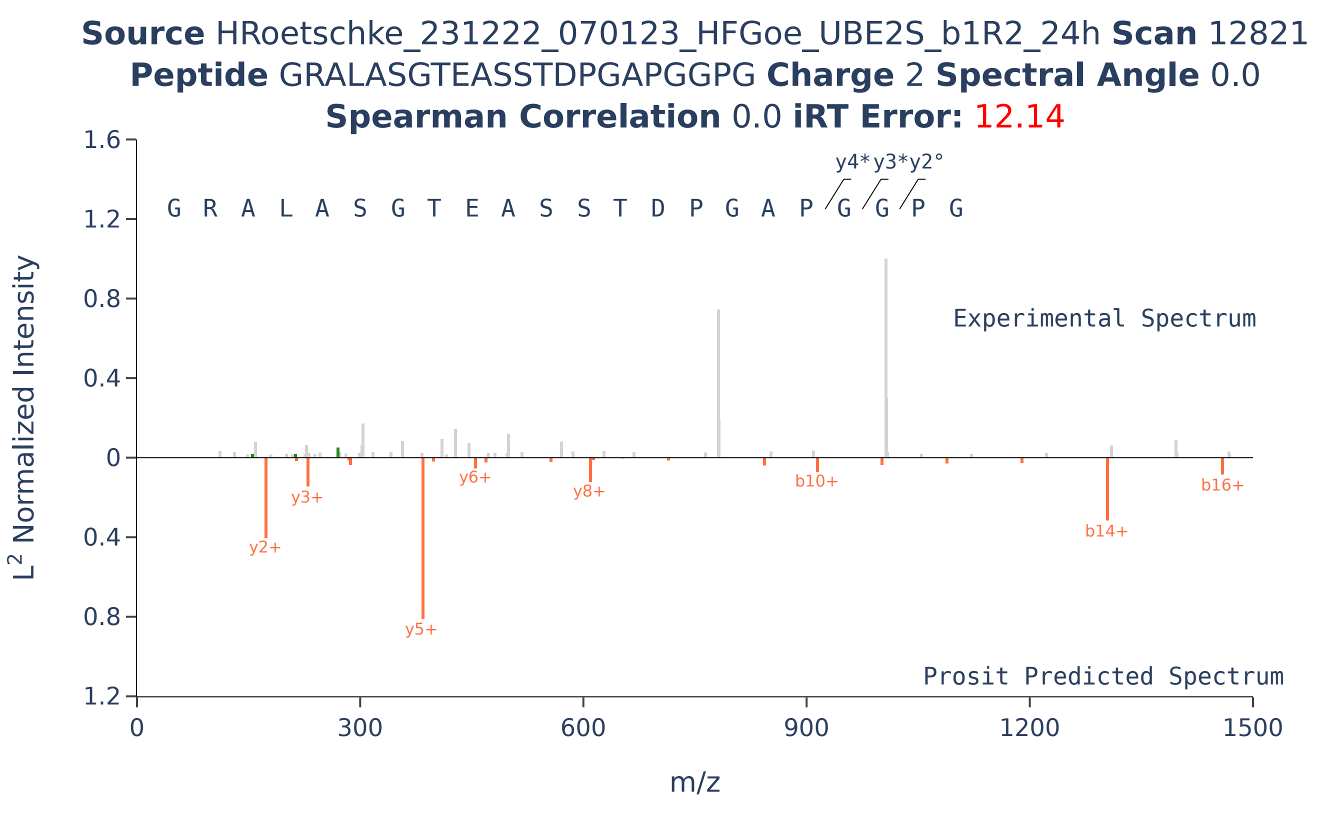
Source HRoetschke_231222_070123_HFGoe_UBE2S_b1R2_24h Scan 12821
Peptide GRALASGTEASSTDPGAPGGPG Charge 2 Spectral Angle 0.0
Spearman Correlation 0.0 iRT Error: 12.14
Experimental Spectrum
Prosit Predicted Spectrum
L² Normalized Intensity
m/z

Source HRoetschke_231222_070123_HFGoe_UBE2S_b2R1_2h **Scan** 8075
Peptide GASSTDPGAPGGPGGAE **Charge** 2 **Spectral Angle** 0.86
Spearman Correlation 0.83 **iRT Error:** 2.28

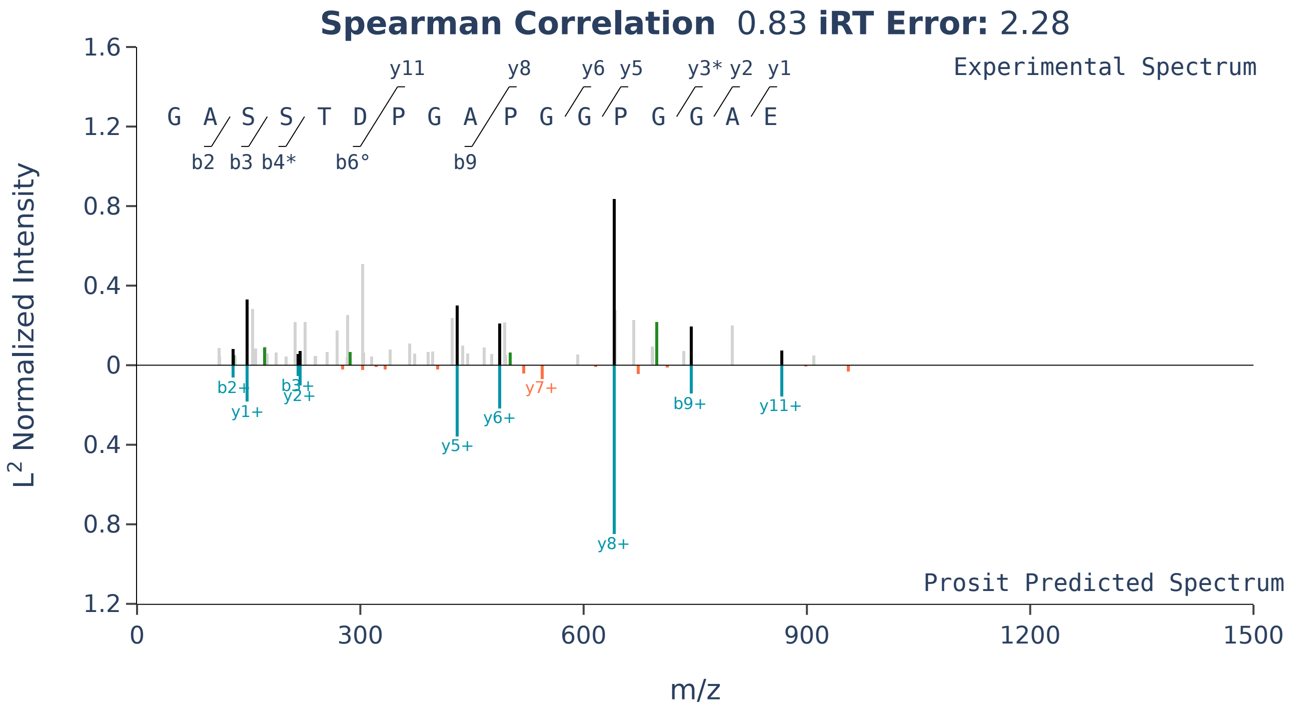

Source HRoetschke_231222_070123_HFGoe_UBE2S_b2R1_2h **Scan** 8075
Peptide ASSTDPGAPGGPGGAEG **Charge** 2 **Spectral Angle** 0.64
Spearman Correlation 0.52 **iRT Error:** 0.68

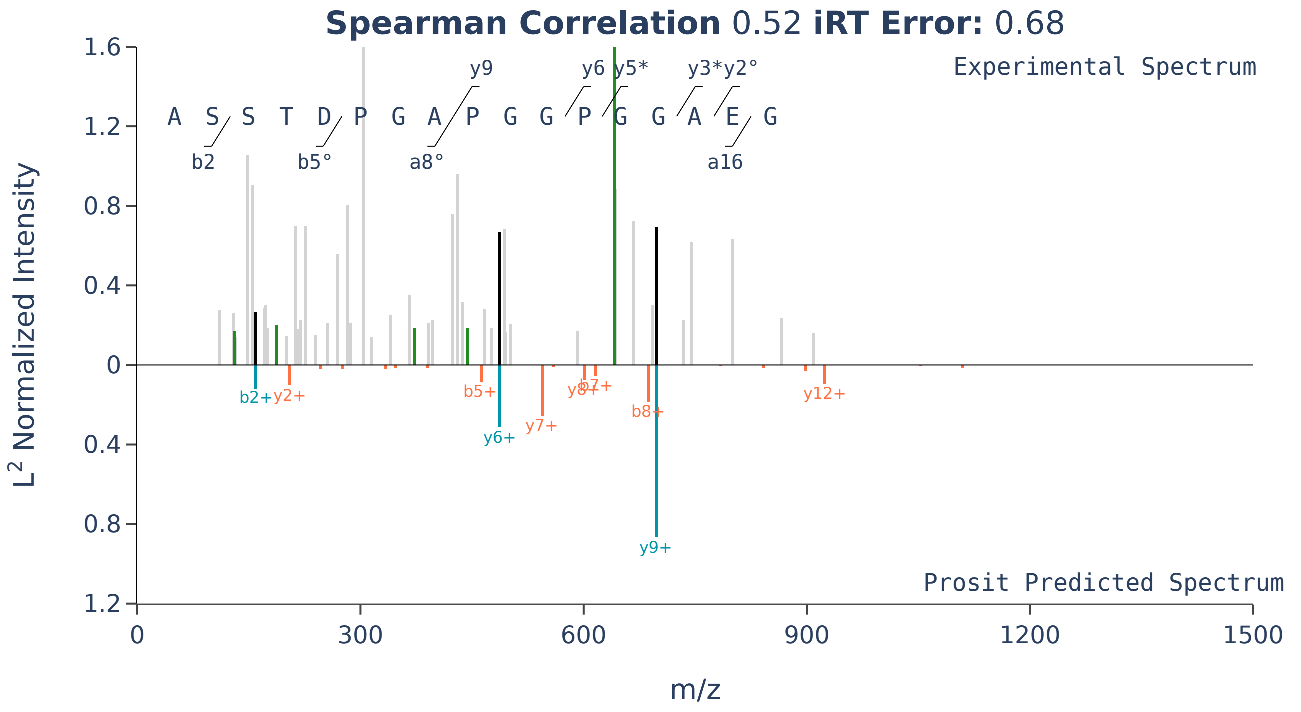

Source HRoetschke_231222_070123_HFGoe_UBE2S_b2R1_24h **Scan** 8154
Peptide ASGTEARL **Charge** 2 **Spectral Angle** 0.8
Spearman Correlation 0.92 **iRT Error:** 2.63

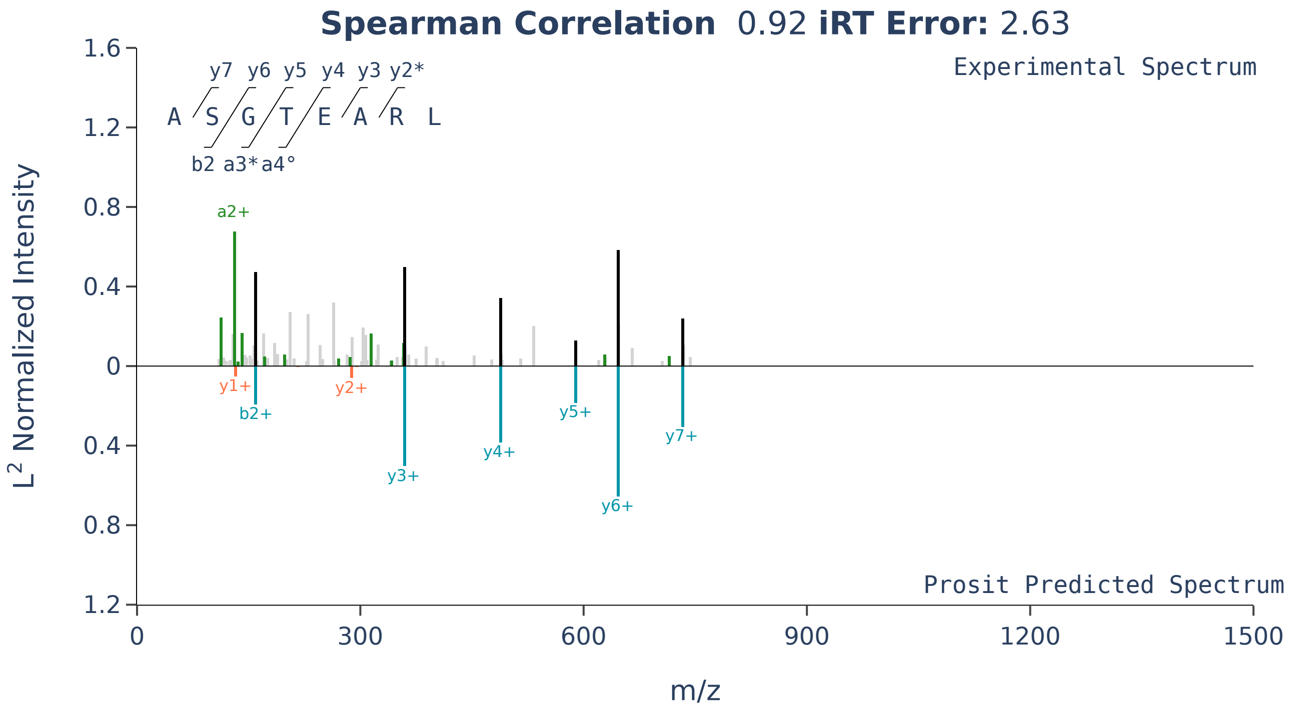

Source HRoetschke_231222_070123_HFGoe_UBE2S_b2R1_24h **Scan** 8154
Peptide RALASGTE **Charge** 2 **Spectral Angle** 0.0
Spearman Correlation 0.0 **iRT Error:** 30.02

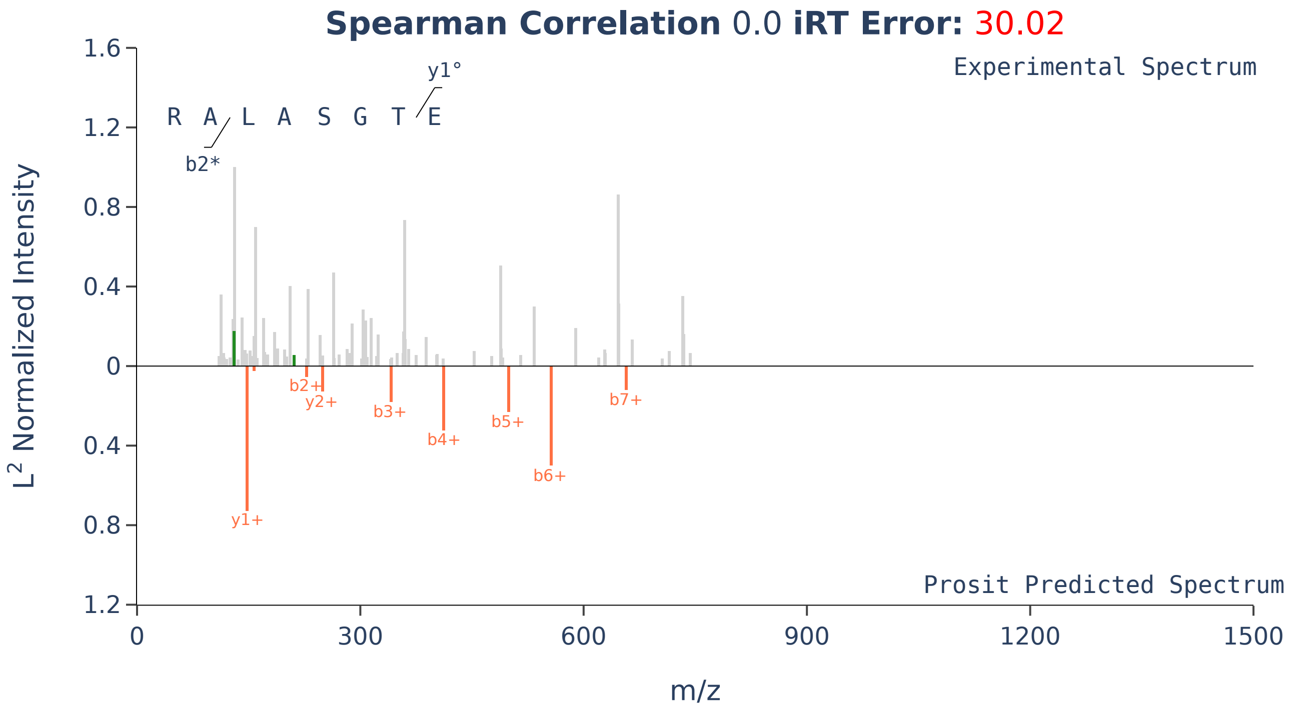

Source HRoetschke_231222_070123_HFGoe_UBE2S_b1R2_2h **Scan** 8050
Peptide HGGAGGPSGL **Charge** 2 **Spectral Angle** 0.93
Spearman Correlation 0.93 **iRT Error:** 3.02

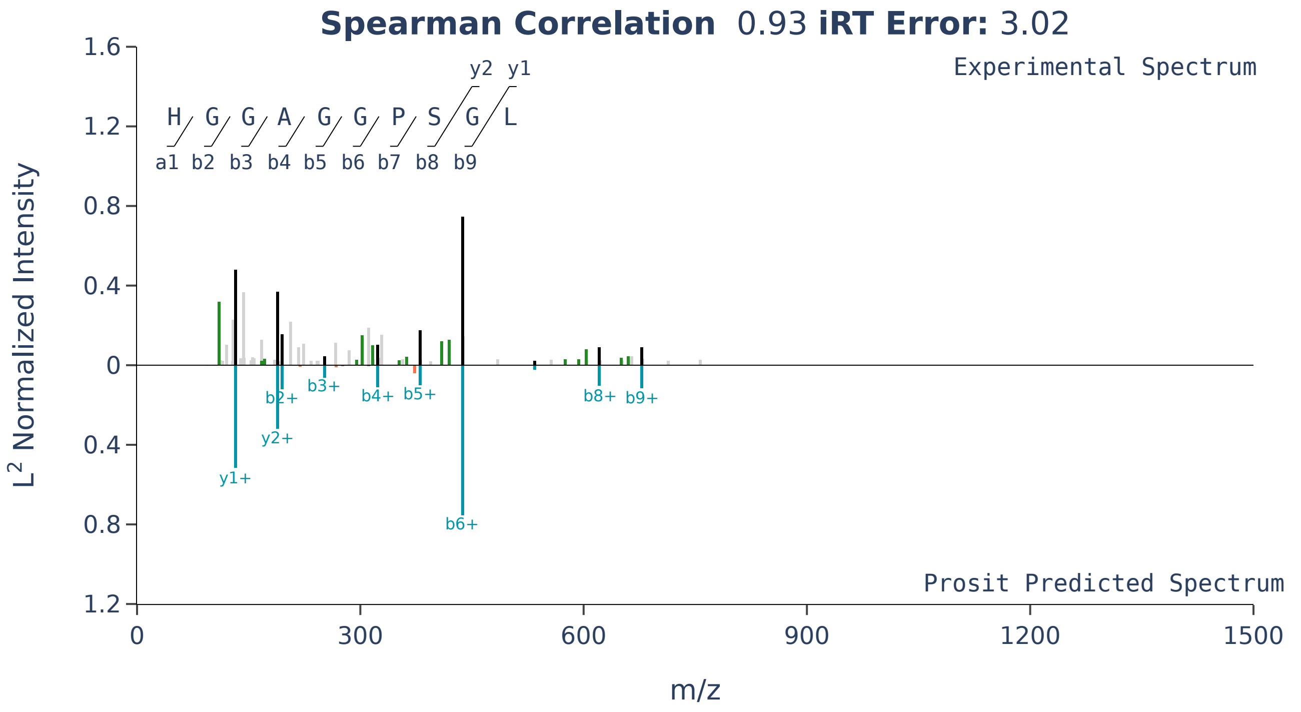

Source HRoetschke_231222_070123_HFGoe_UBE2S_b1R2_2h **Scan** 8050
Peptide IHGGAGGPSG **Charge** 2 **Spectral Angle** 0.0
Spearman Correlation 0.0 **iRT Error:** 17.64

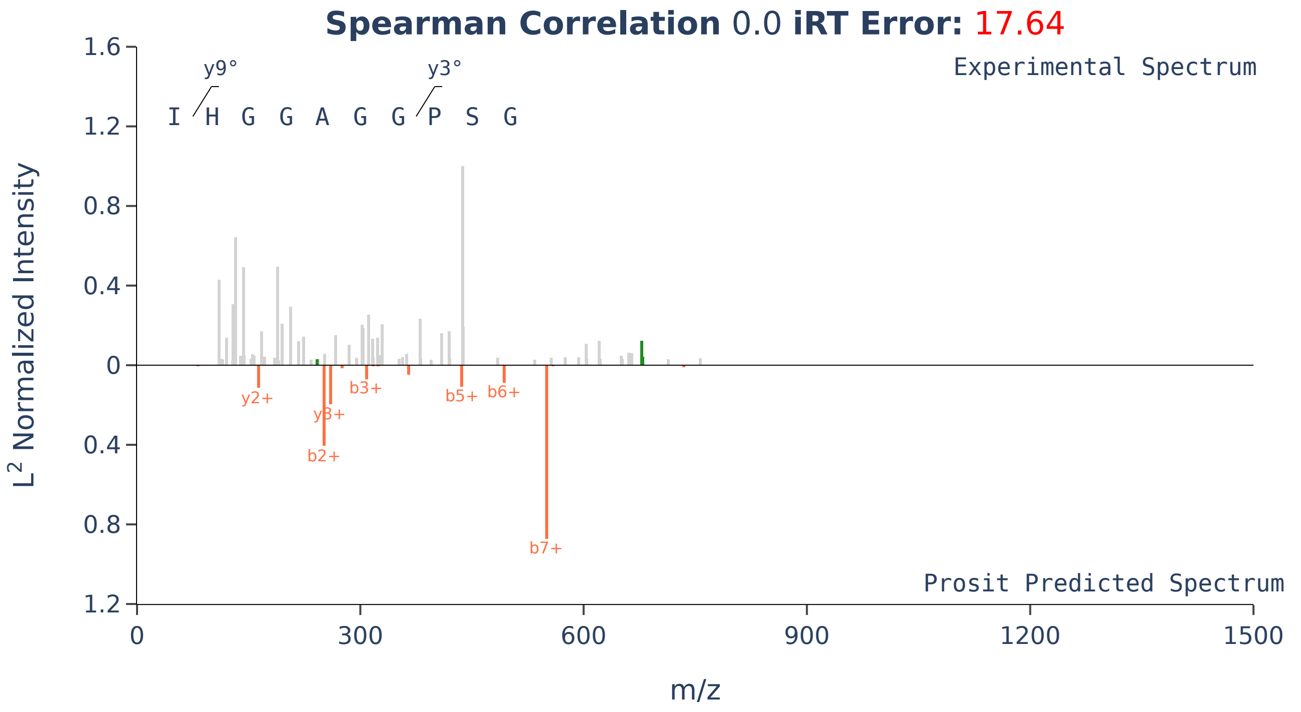

Source HRoetschke_231222_070123_HFGoe_UBE2S_b2R2_24h **Scan** 8293
Peptide ASSTDPGAPGGPGGTE **Charge** 2 **Spectral Angle** 0.91
Spearman Correlation 0.91 **iRT Error:** 3.31

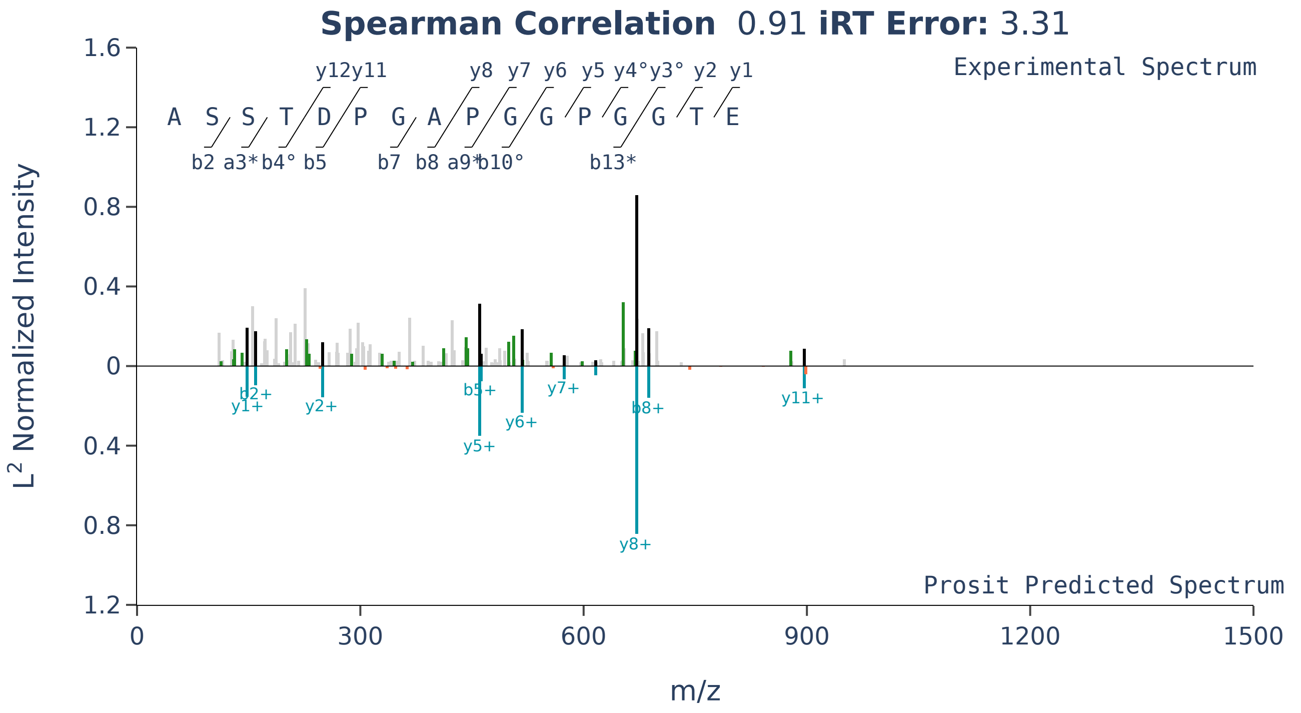

Source HRoetschke_231222_070123_HFGoe_UBE2S_b2R2_24h **Scan** 8293
Peptide GTEASSTDPGAPGGPG **Charge** 2 **Spectral Angle** 0.35
Spearman Correlation 0.65 **iRT Error:** 6.05

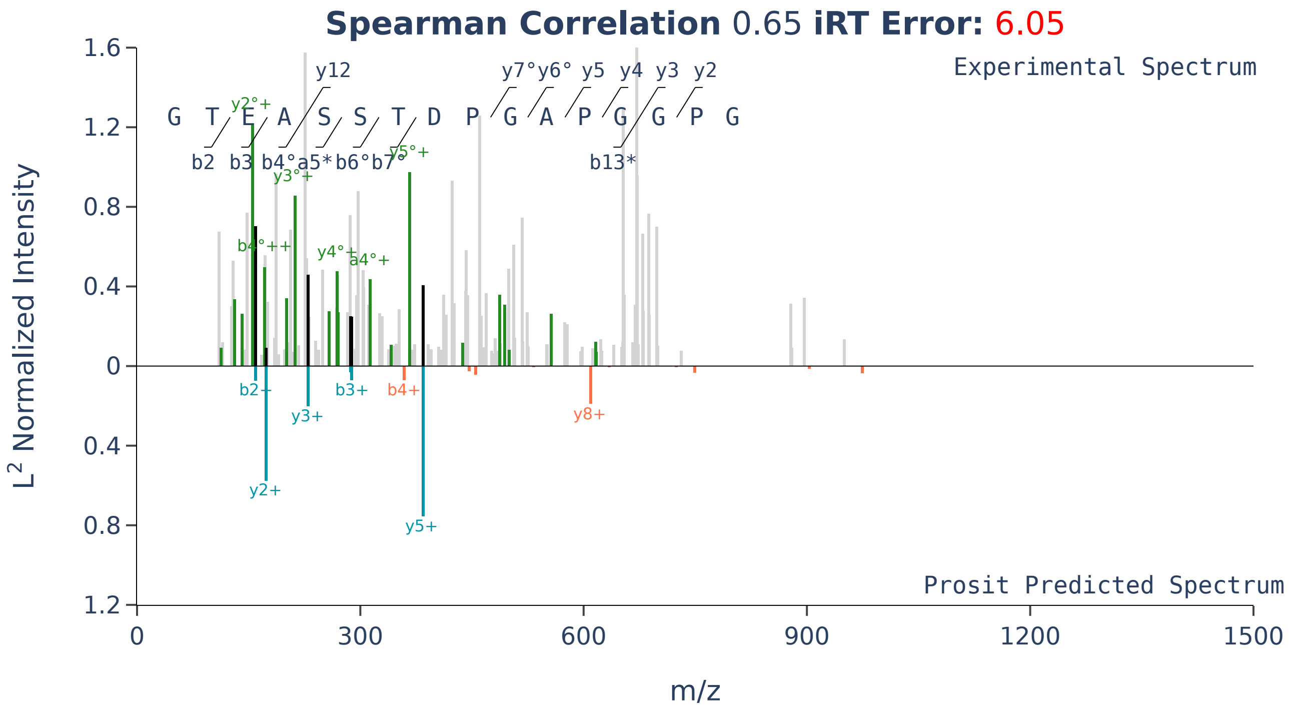

Source HRoetschke_231222_070123_HFGoe_UBE2S_b1R2_2h **Scan** 6698
Peptide GPEGTPGGPGG **Charge** 1 **Spectral Angle** 0.91
Spearman Correlation 0.85 **iRT Error:** 3.36

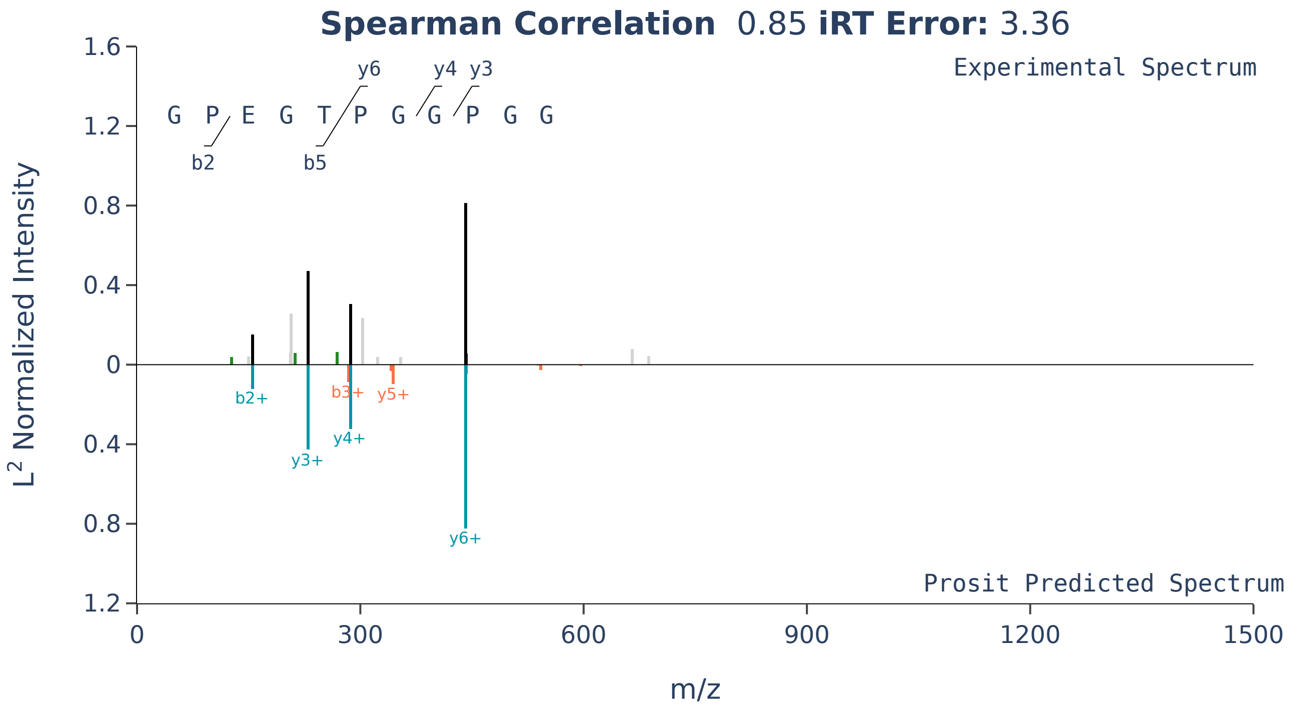

Source HRoetschke_231222_070123_HFGoe_UBE2S_b1R2_2h **Scan** 6698
Peptide TDPGAPGGPGG **Charge** 1 **Spectral Angle** 0.91
Spearman Correlation 0.79 **iRT Error:** 0.44

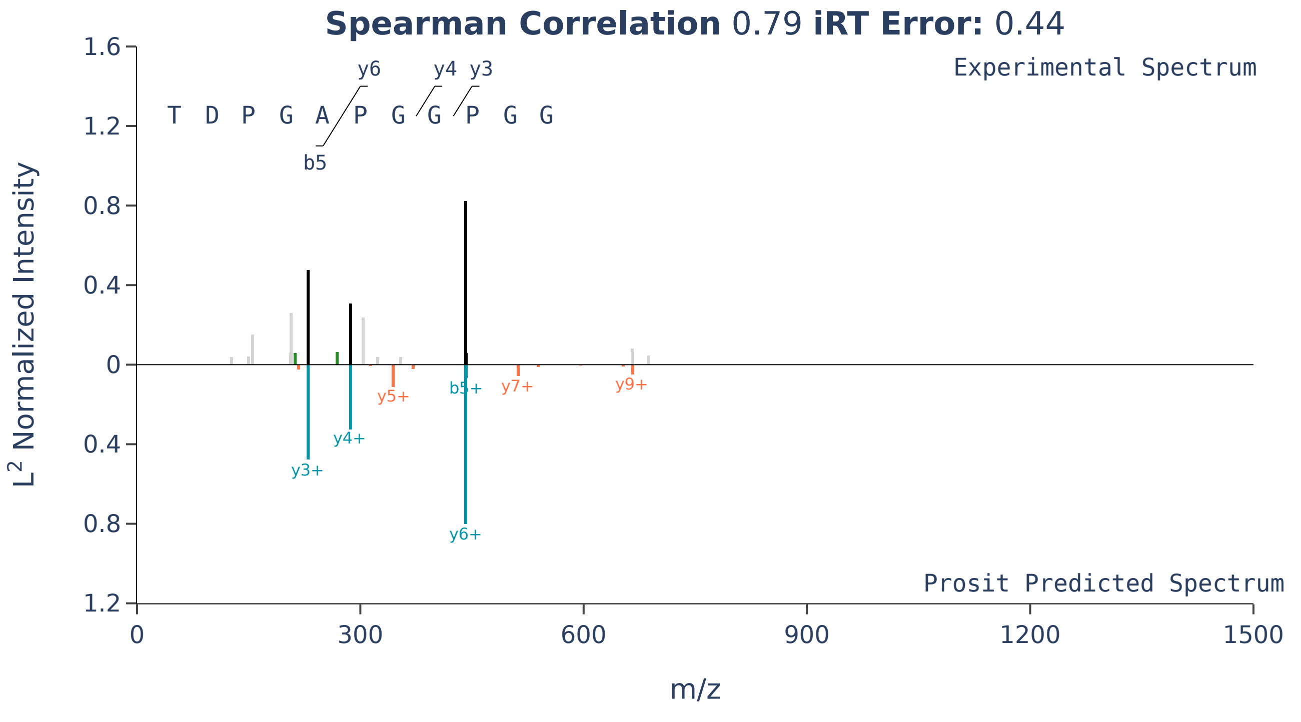

Source HRoetschke_231222_070123_HFGoe_UBE2S_b1R1_24h **Scan** 7818
Peptide VYKEVTA **Charge** 2 **Spectral Angle** 0.84
Spearman Correlation 0.85 **iRT Error:** 3.49

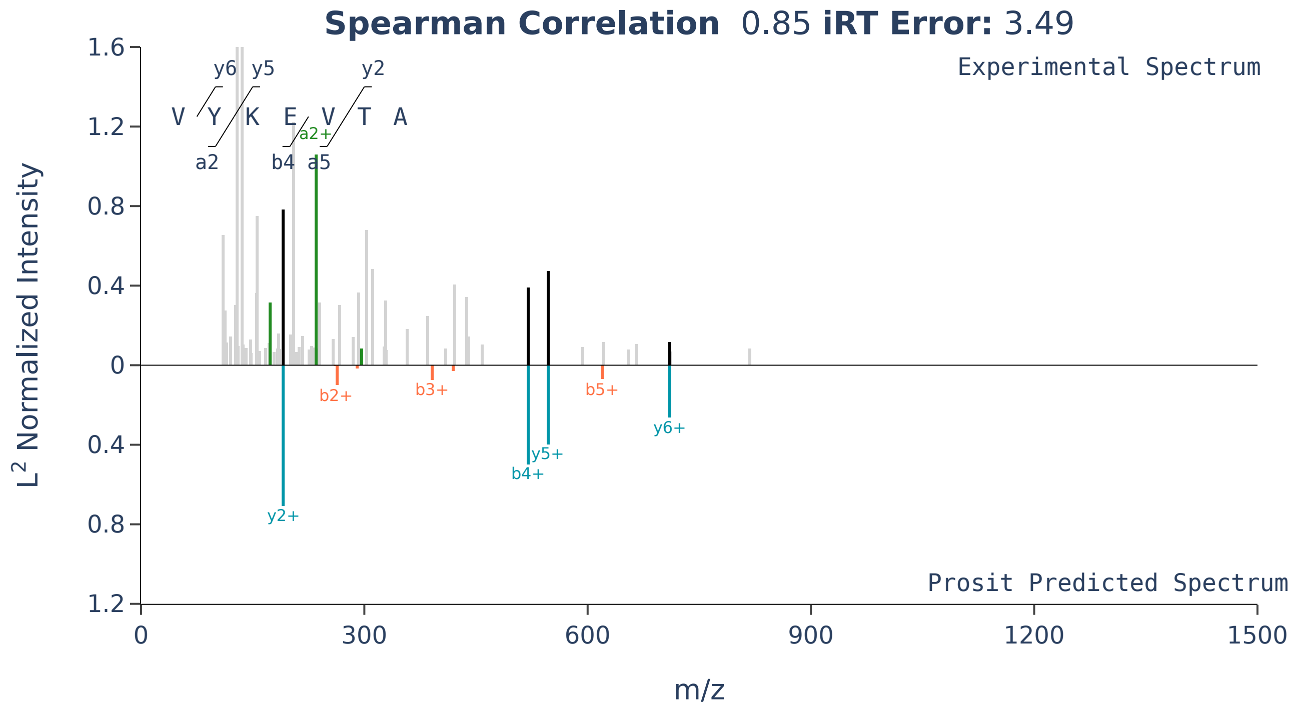

Source HRoetschke_231222_070123_HFGoe_UBE2S_b1R1_24h **Scan** 7818
Peptide CLLIHPN **Charge** 2 **Spectral Angle** 0.11
Spearman Correlation 0.22 **iRT Error:** 54.45

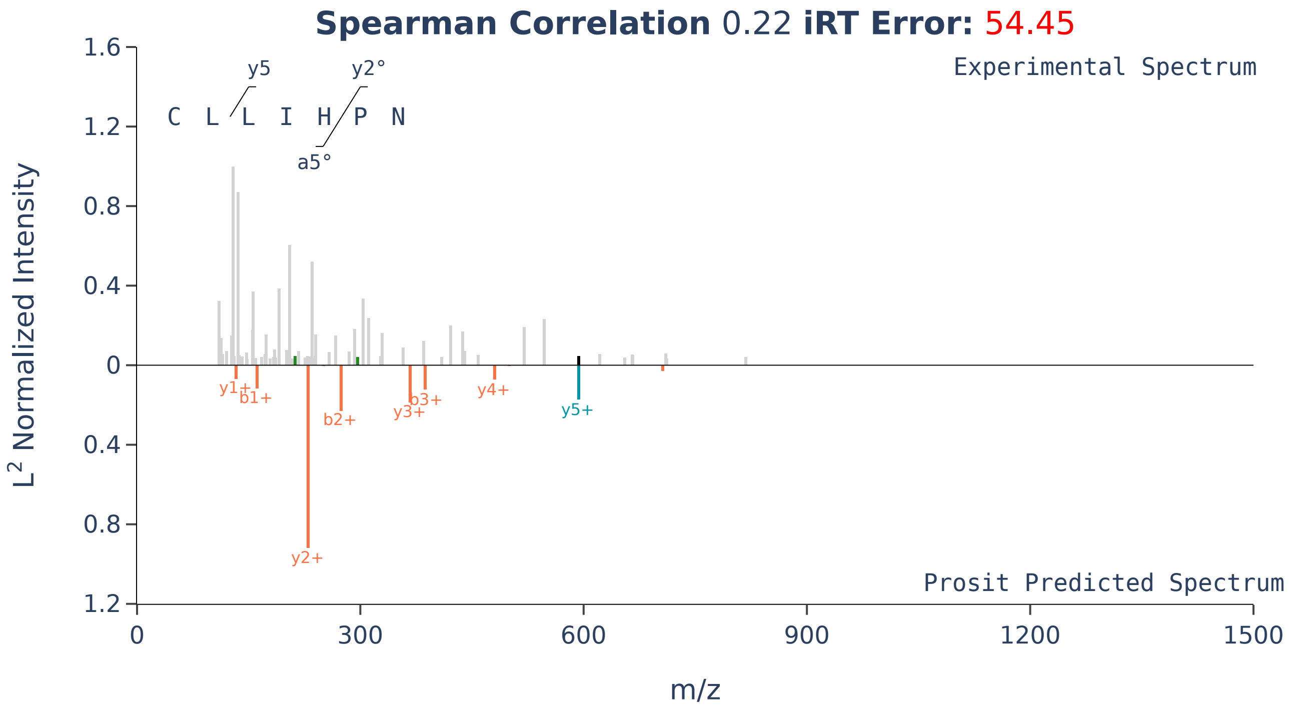

Source HRoetschke_231222_070123_HFGoe_UBE2S_b2R1_24h **Scan** 17285
Peptide DGEICVNVLKR **Charge** 3 **Spectral Angle** 0.82
Spearman Correlation 0.95 **iRT Error:** 7.66

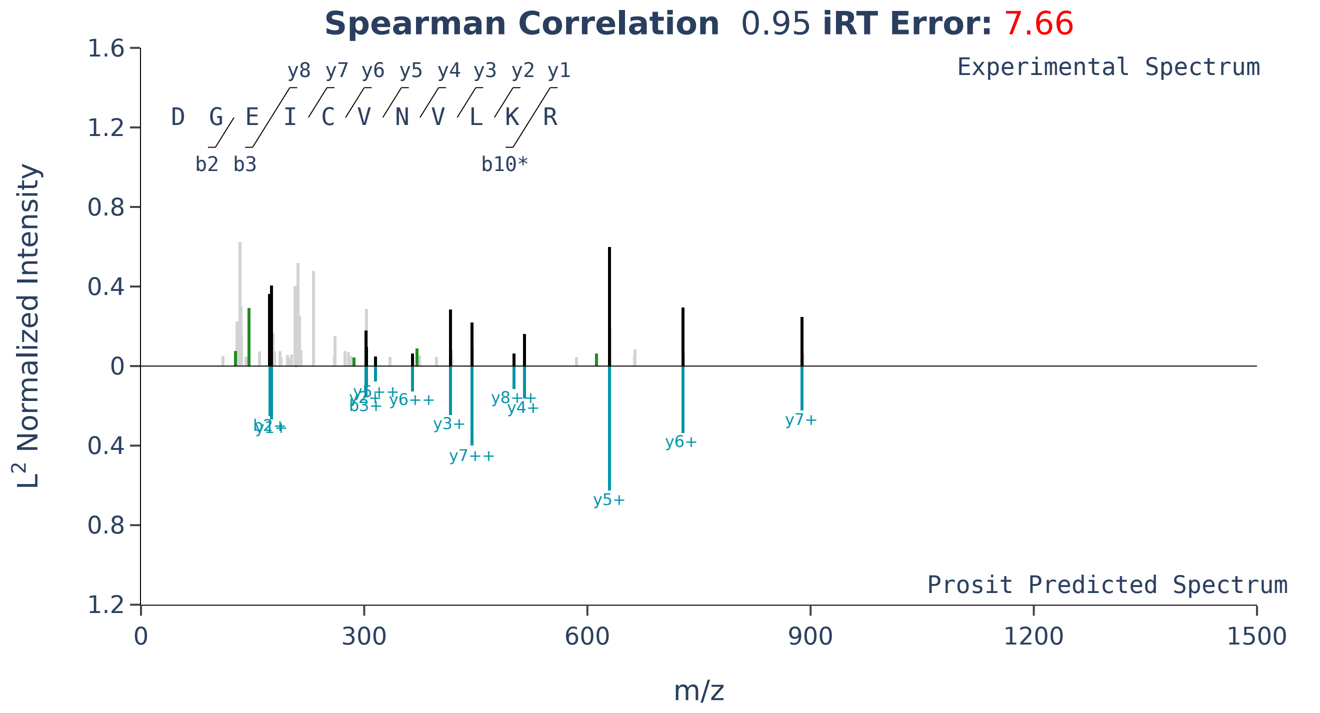

Source HRoetschke_231222_070123_HFGoe_UBE2S_b2R1_24h **Scan** 17285
Peptide GEICVNVLKRD **Charge** 3 **Spectral Angle** 0.17
Spearman Correlation 0.22 **iRT Error:** 13.95

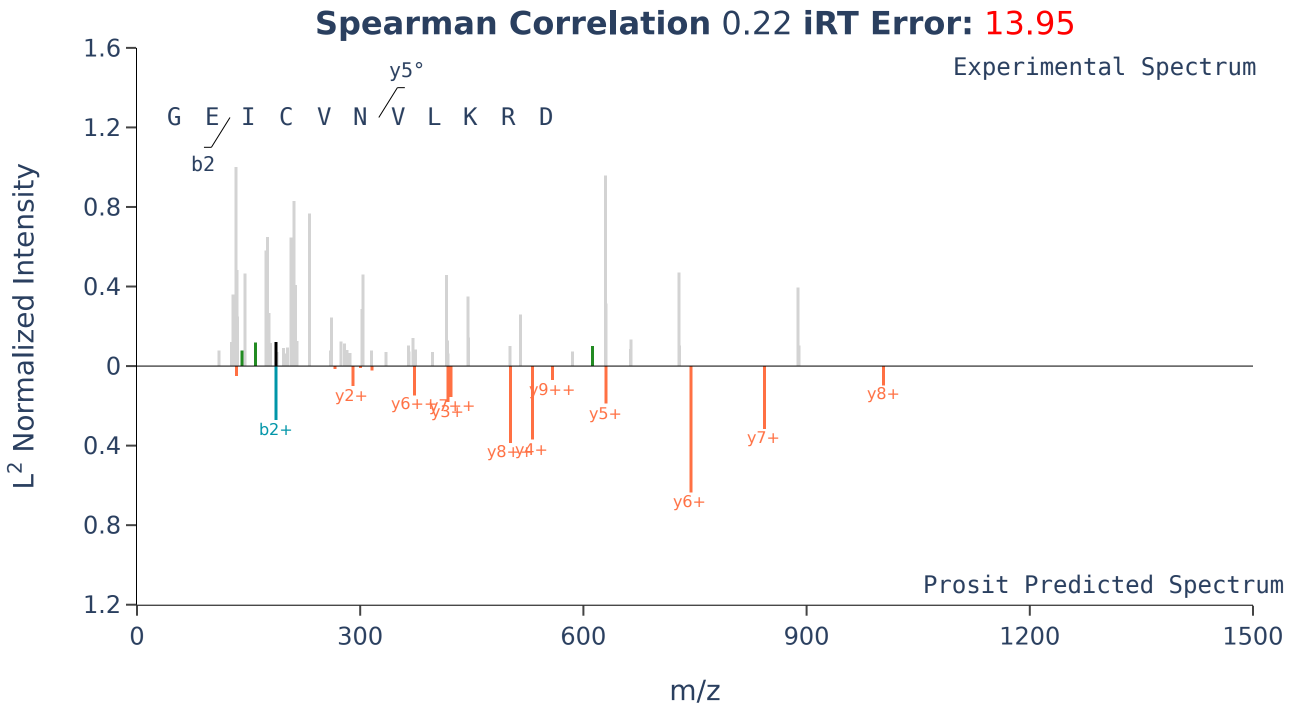

Source HRoetschke_231222_070123_HFGoe_UBE2S_b1R1_24h **Scan** 13862
Peptide PNVGEDLTD **Charge** 2 **Spectral Angle** 0.84
Spearman Correlation 0.96 **iRT Error:** 8.97

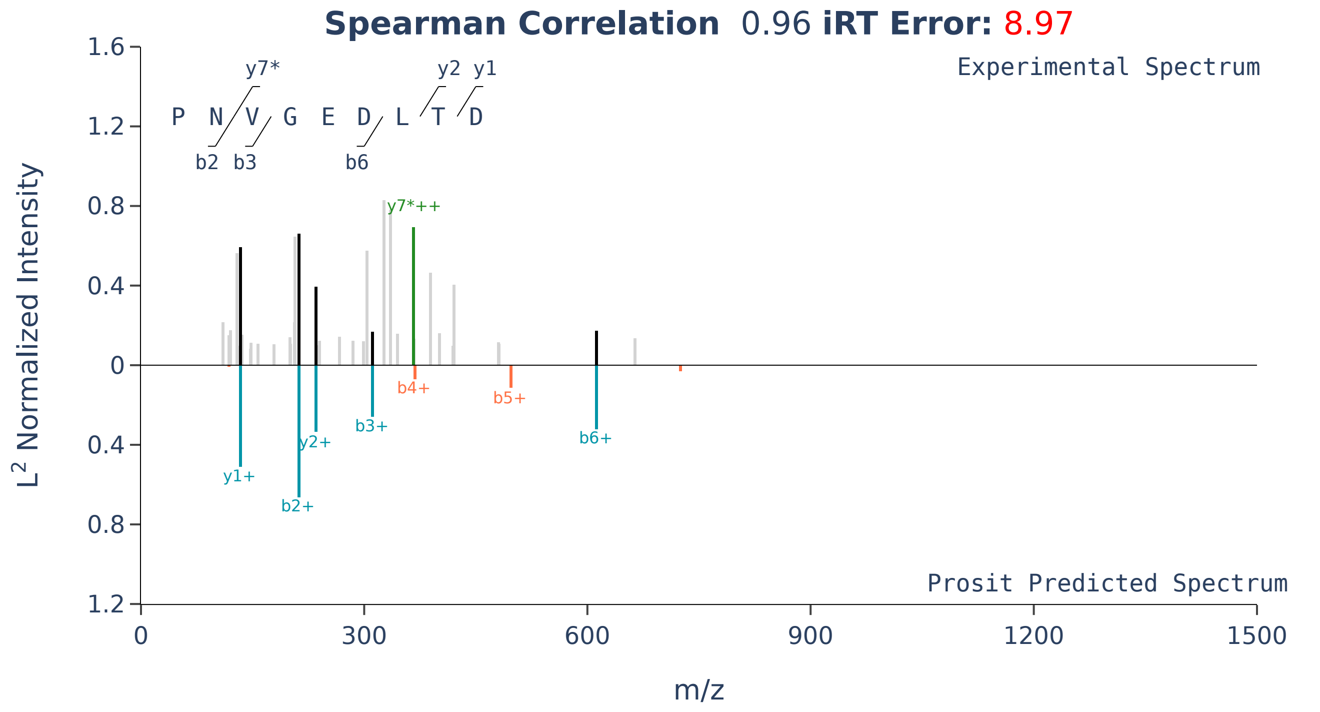

Source HRoetschke_231222_070123_HFGoe_UBE2S_b1R1_24h **Scan** 13862
Peptide PESALNEEA **Charge** 2 **Spectral Angle** 0.0
Spearman Correlation 0.0 **iRT Error:** 22.75

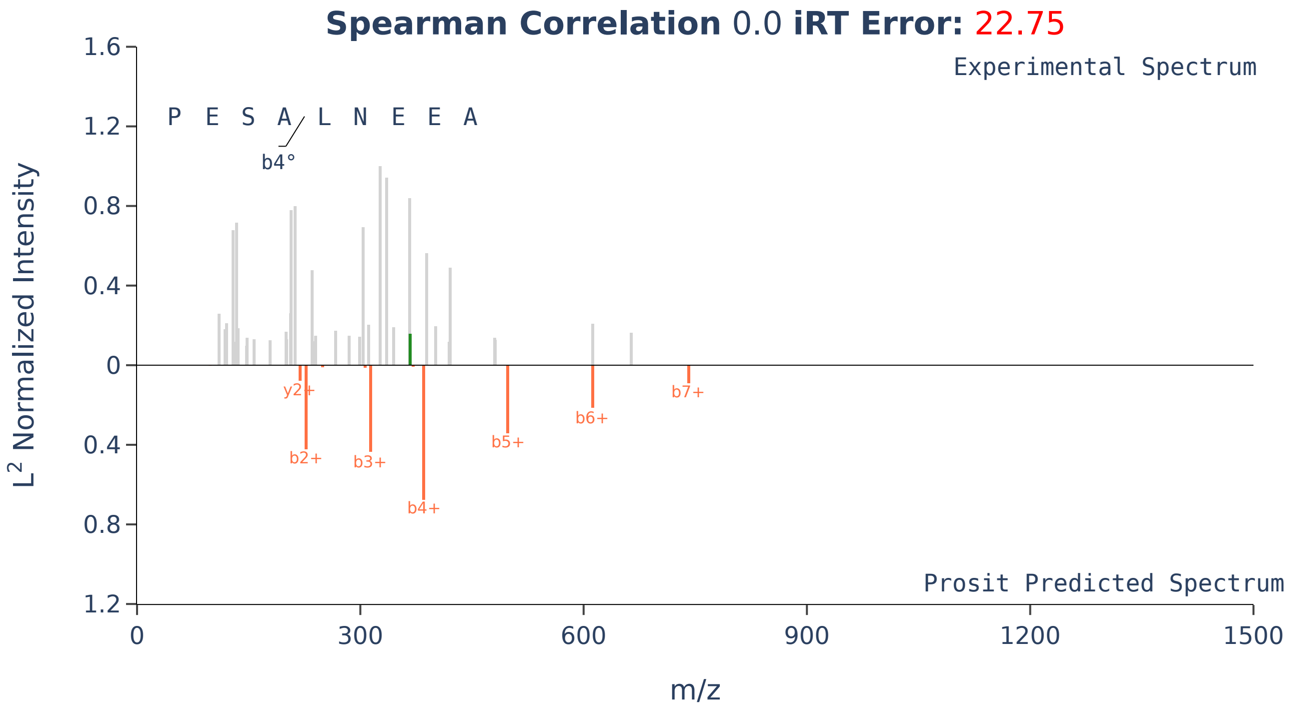

Supplement: Supplementary file 11 — Supplementary Data 8 [file 41467_2024_45339_MOESM11_ESM.pdf]
