# Supplementary material for: Protein degradation by human 20S proteasomes elucidates the interplay between peptide hydrolysis and splicing
Source: Nat Commun. 2024 Feb 7;15:1147. doi: 10.1038/s41467-024-45339-3 (PMC10850103; doi:10.1038/s41467-024-45339-3)
Supplement: Supplementary file 6 — Supplementary Data 3 [file 41467_2024_45339_MOESM6_ESM.pdf]

**Supplementary Data 3. Experimental vs. Prosit-predicted MS2 spectra for all identified non-spliced peptides.** Plots comparing the experimental spectrum for an identified peptide on the positive y-axis against the Prosit predicted spectrum for that peptide on the negative y-axis. Comparisons are shown for the best scoring spectrum of all non-spliced peptides identified at 1% FDR.

# inSPIRE Spectral Plotting for experimental vs. prosit comparisons of non-spliced assignments

## Experimental Spectrum Colour Code:

- Experimental peak matched to a Prosit predicted peak.
- Possible ion unknown to Prosit.
- Precursor matched peak.
- Experimental peak not matched to any potential ion.

## Prosit Spectrum Colour Code:

- Prosit predicted peak matched to experimental spectrum.
- Prosit predicted peak not matched to experimental spectrum.

## Additional Notes:

° indicates an ion with loss of $H_2O$.

* indicates an ion with loss of $NH_3$.

Loading [MathJax]/extensions/MathMenu.js

**Source** WSoh_301121_021221_HFGoe_M1_AnnexinA1_2h_R1 **Scan** 9327
**Peptide** EILASRTN **Charge** 2 **Spectral Angle** 0.97

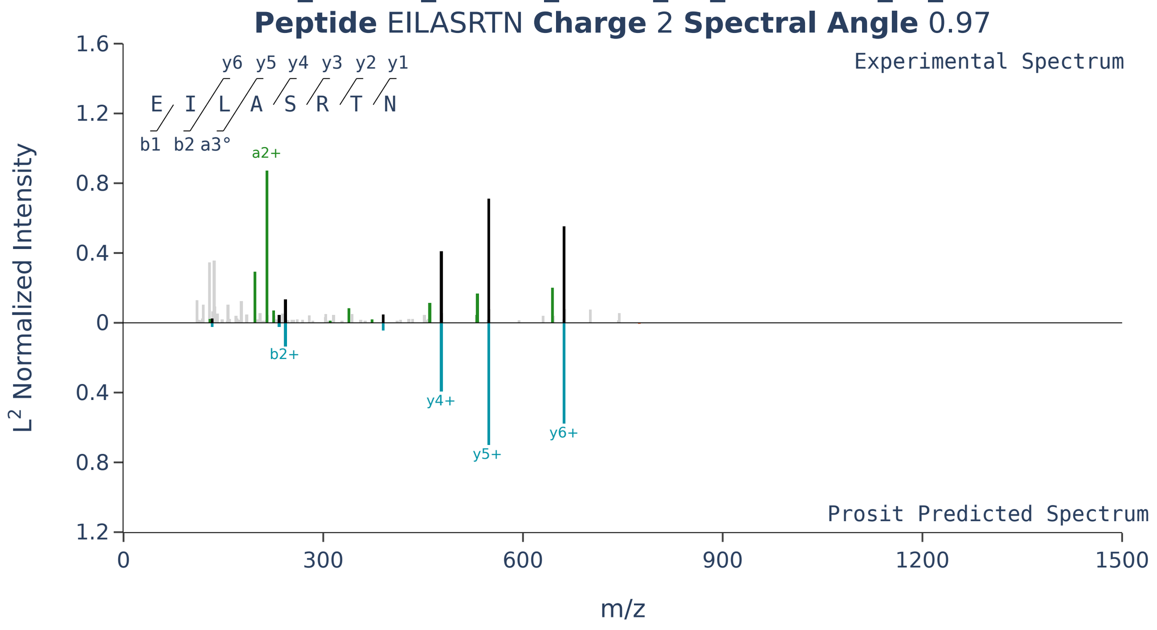


**Source** WSoh_301121_021221_HFGoe_M1_AnnexinA1_4h_R2 **Scan** 11693
**Peptide** SDARALY **Charge** 2 **Spectral Angle** 0.94

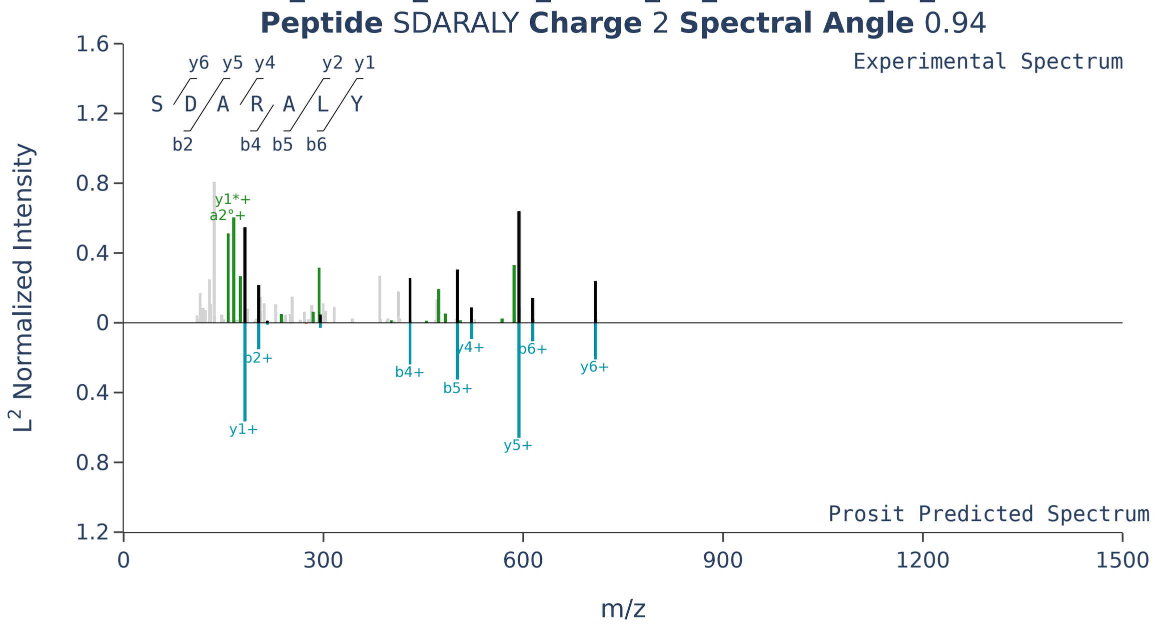


**Source** WSoh_301121_021221_HFGoe_M2_AnnexinA1_24h_R1 **Scan** 11598
**Peptide** NTILTTR **Charge** 2 **Spectral Angle** 0.95

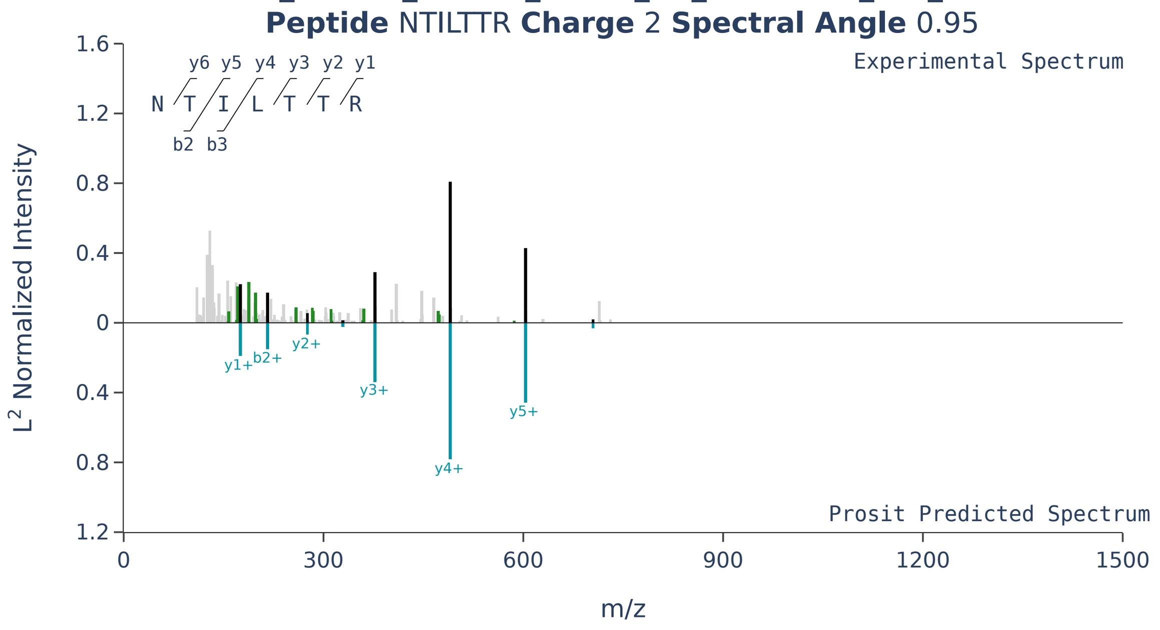


**Source** WSoh_301121_021221_HFGoe_M1_AnnexinA1_2h_R1 **Scan** 8964
**Peptide** AYLQETGK **Charge** 2 **Spectral Angle** 0.93

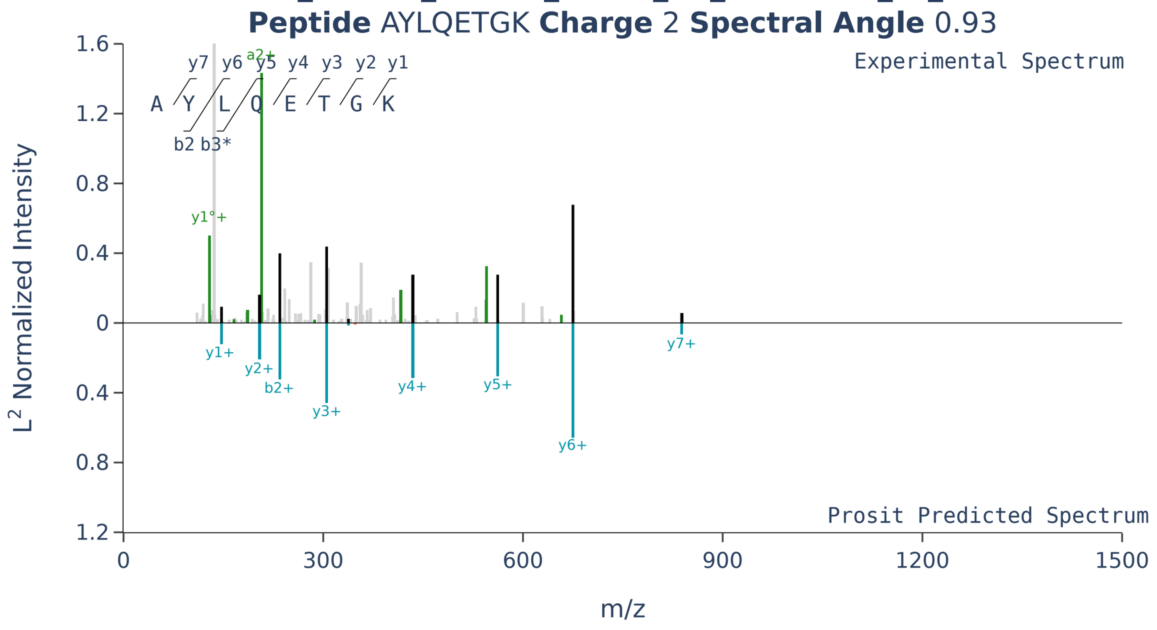


**Source** WSoh_301121_021221_HFGoe_M2_AnnexinA1_24h_R1 **Scan** 9799
**Peptide** ADELRAAMK **Charge** 2 **Spectral Angle** 0.95

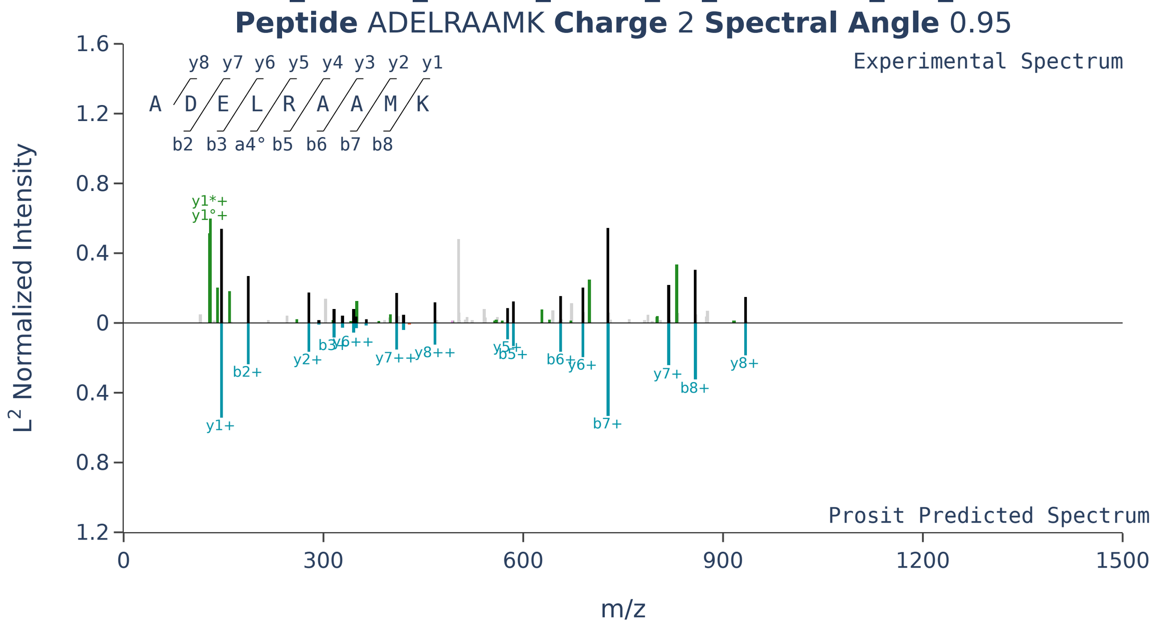


**Source** WSoh_301121_021221_HFGoe_M2_AnnexinA1_4h_R2 **Scan** 20833
**Peptide** HLEEVVL **Charge** 2 **Spectral Angle** 0.94

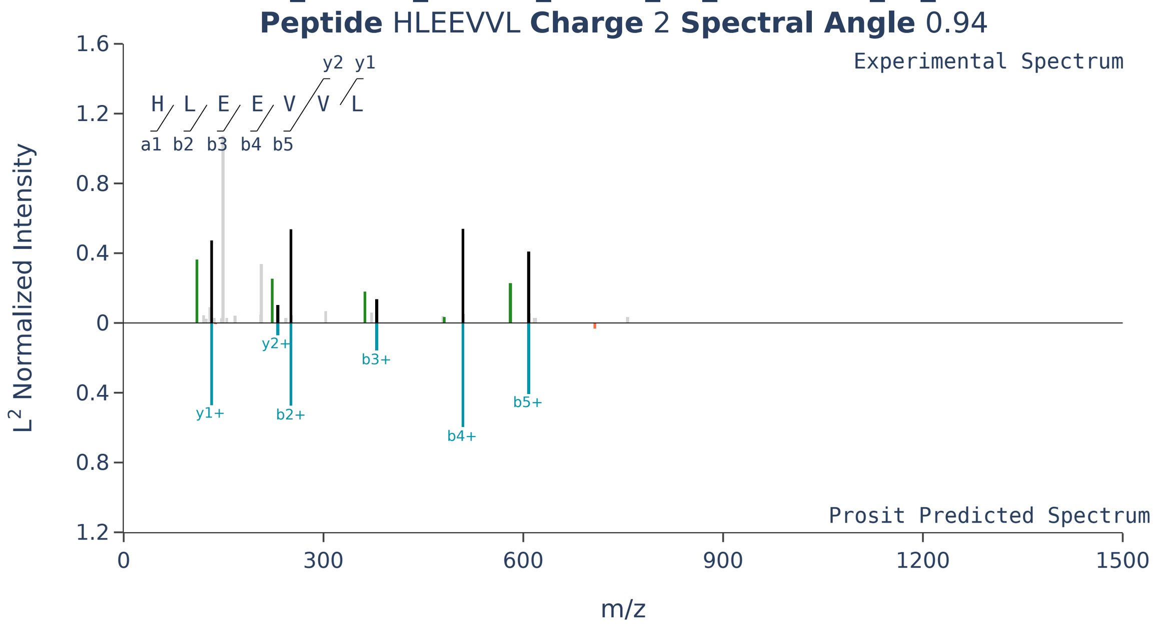


**Source** WSoh_301121_021221_HFGoe_M2_AnnexinA1_2h_R1 **Scan** 19161
**Peptide** ITSDTSGDFRNAL **Charge** 2 **Spectral Angle** 0.93

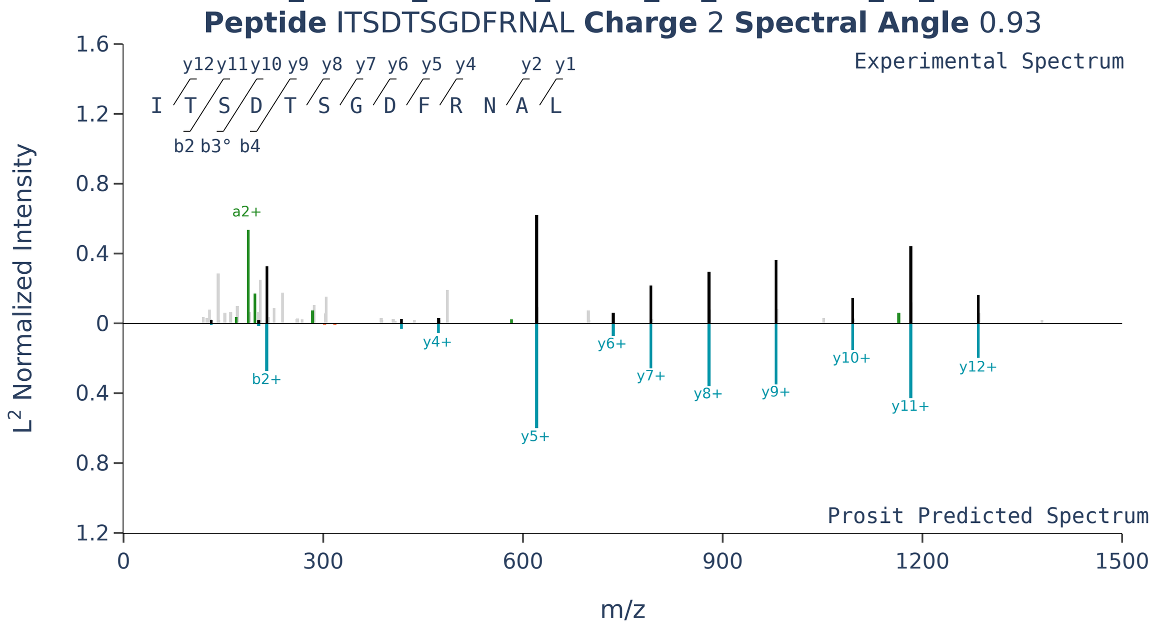


**Source** WSoh_301121_021221_HFGoe_M2_AnnexinA1_24h_R1 **Scan** 12631
**Peptide** DARALYE **Charge** 2 **Spectral Angle** 0.95

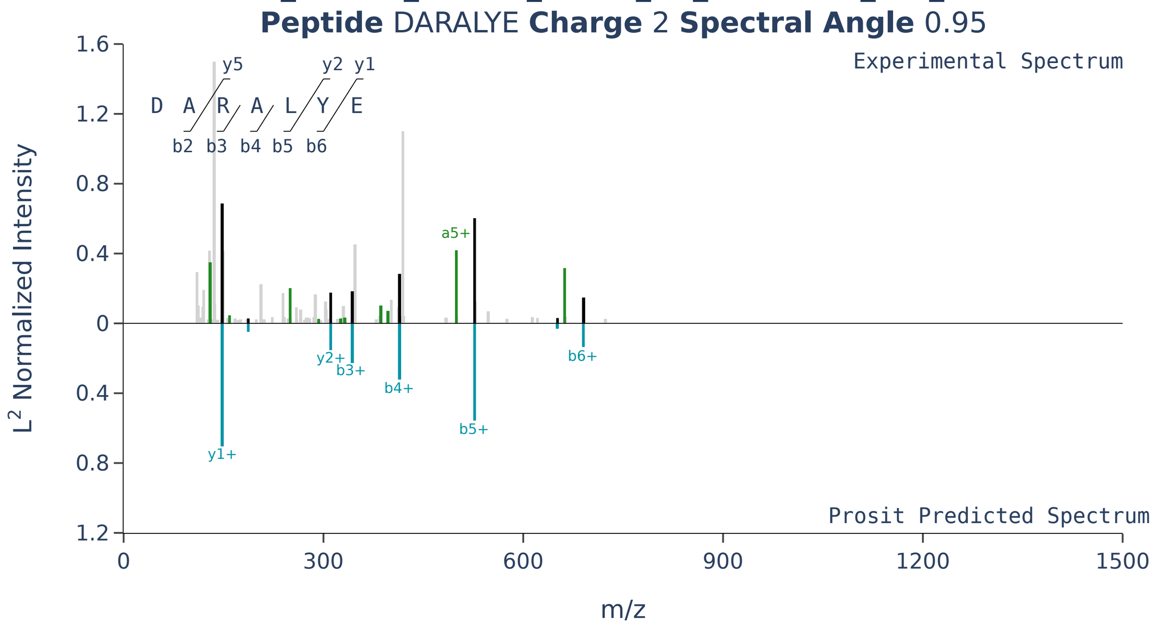


**Source** WSoh_301121_021221_HFGoe_M1_AnnexinA1_4h_R2 **Scan** 12849
**Peptide** IMVKGVD **Charge** 2 **Spectral Angle** 0.92

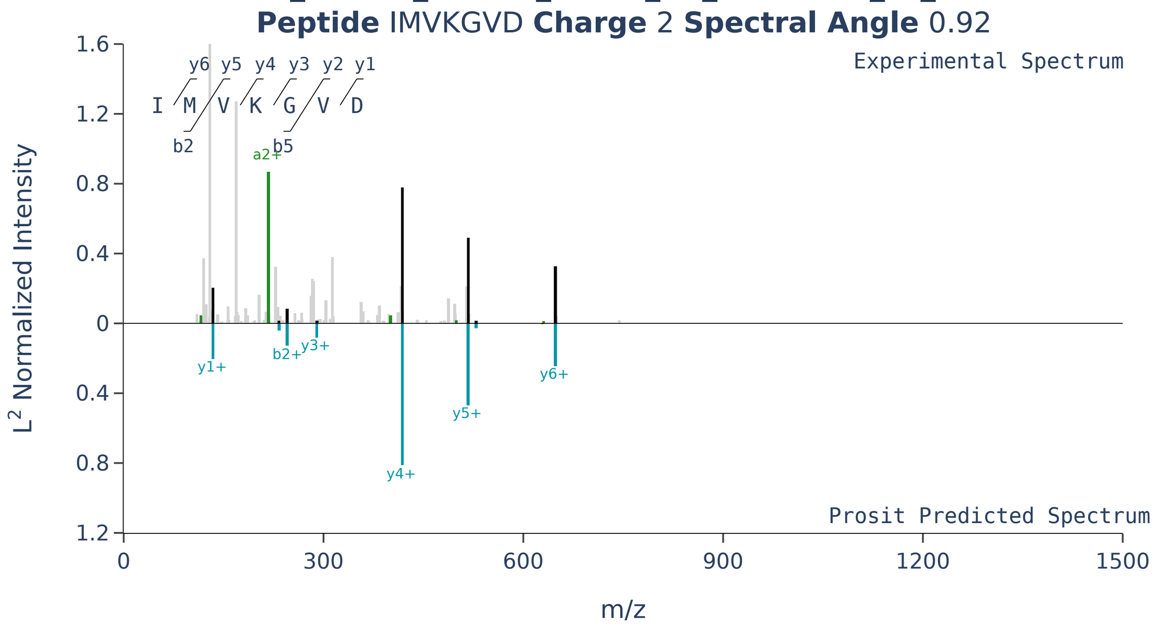


**Source** WSoh_301121_021221_HFGoe_M2_AnnexinA1_24h_R2 **Scan** 7117
**Peptide** TSGDFRNA **Charge** 2 **Spectral Angle** 0.94

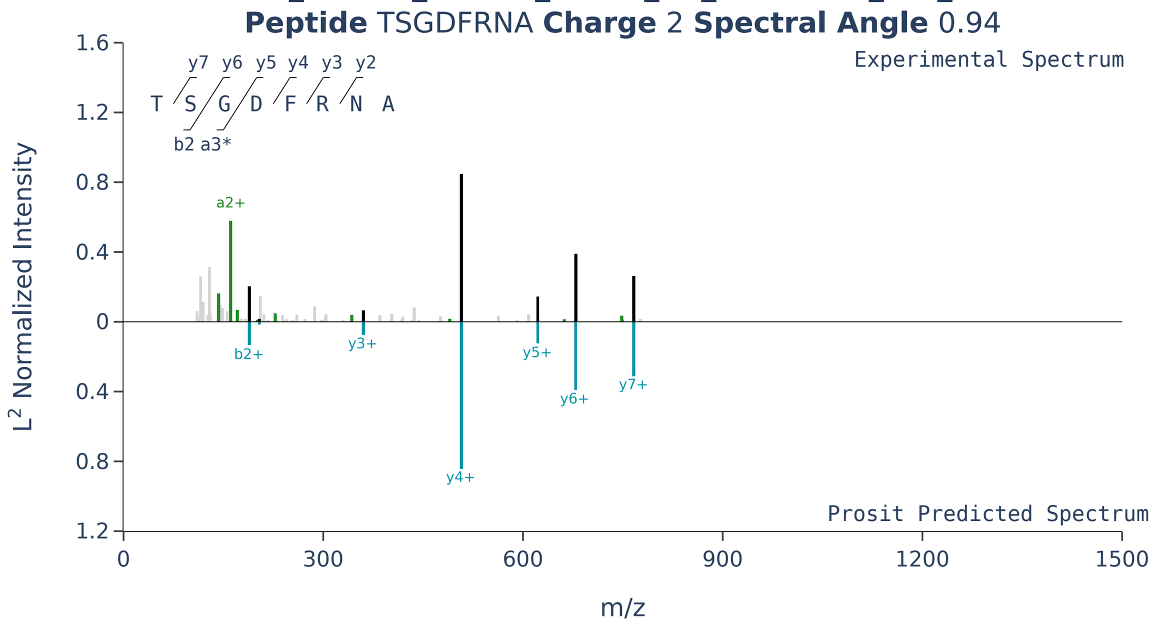


**Source** WSoh_301121_021221_HFGoe_M2_AnnexinA1_2h_R1 **Scan** 12708
**Peptide** LLSLAKGDRSE **Charge** 3 **Spectral Angle** 0.94

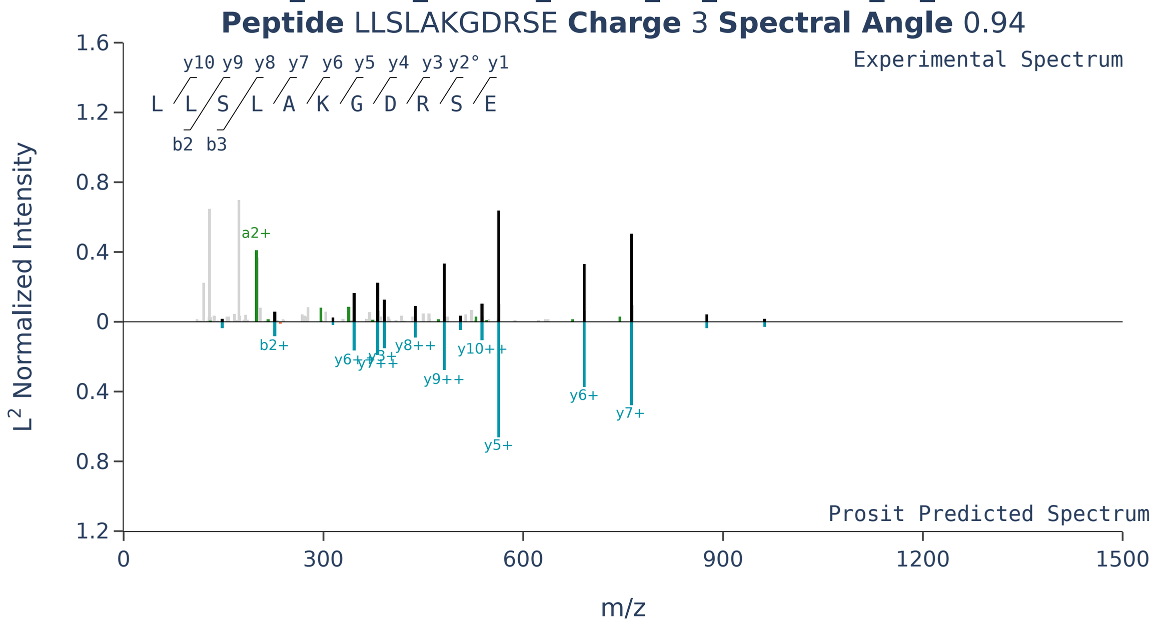


**Source** WSoh_301121_021221_HFGoe_M1_AnnexinA1_24h_R1 **Scan** 11620
**Peptide** SDARALYE **Charge** 2 **Spectral Angle** 0.94

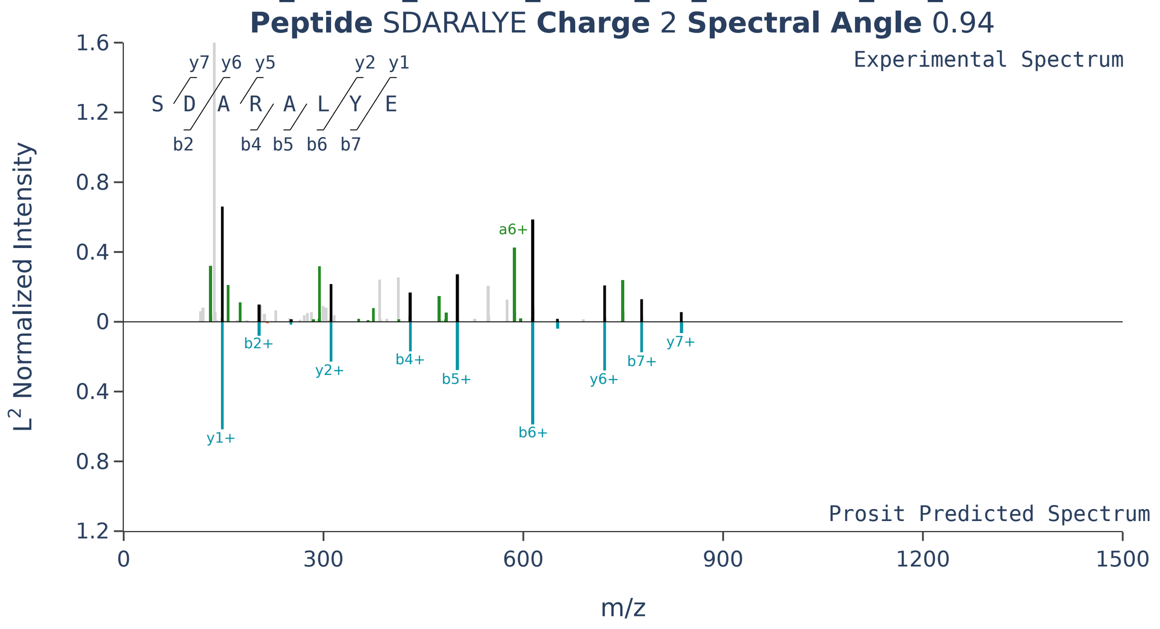


**Source** WSoh_301121_021221_HFGoe_M2_AnnexinA1_2h_R2 **Scan** 13103
**Peptide** ALLKTPAQ **Charge** 2 **Spectral Angle** 0.94

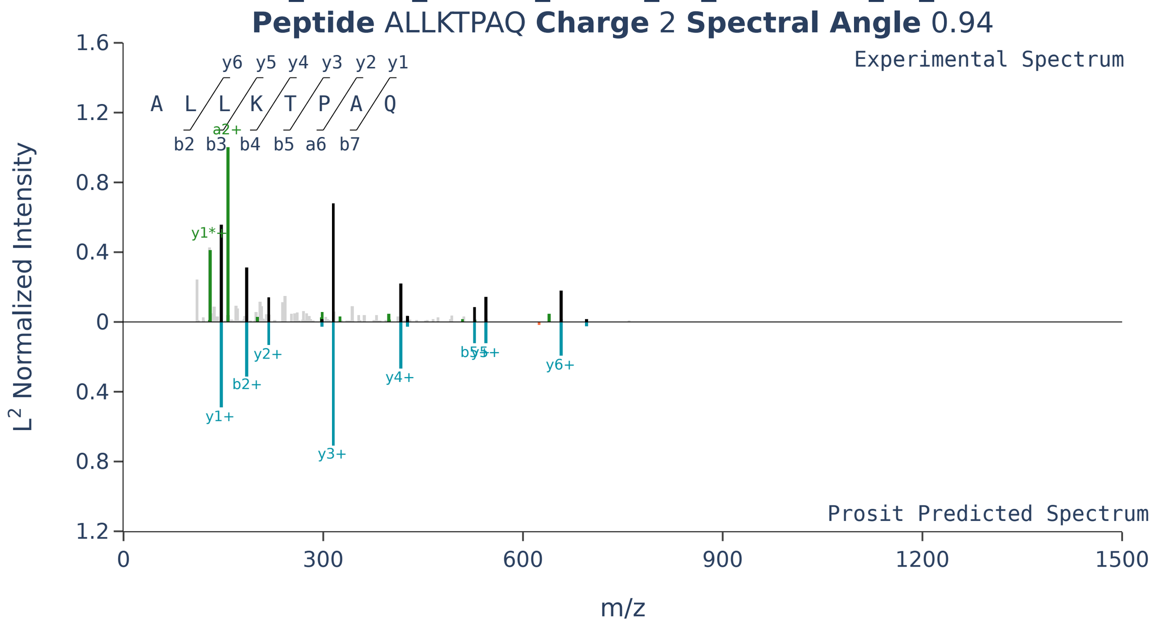


**Source** WSoh_301121_021221_HFGoe_M1_AnnexinA1_1h_R2 **Scan** 15999
**Peptide** LADSDARALYE **Charge** 2 **Spectral Angle** 0.94

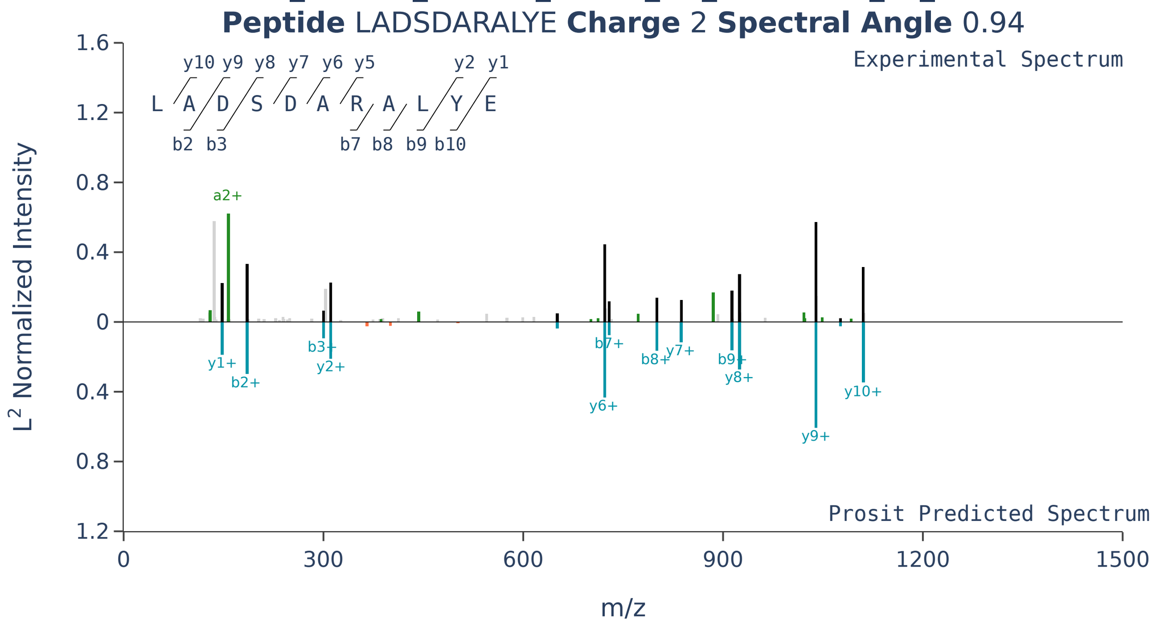


**Source** WSoh_301121_021221_HFGoe_M2_AnnexinA1_4h_R1 **Scan** 21038
**Peptide** GHLEEVVL **Charge** 2 **Spectral Angle** 0.94

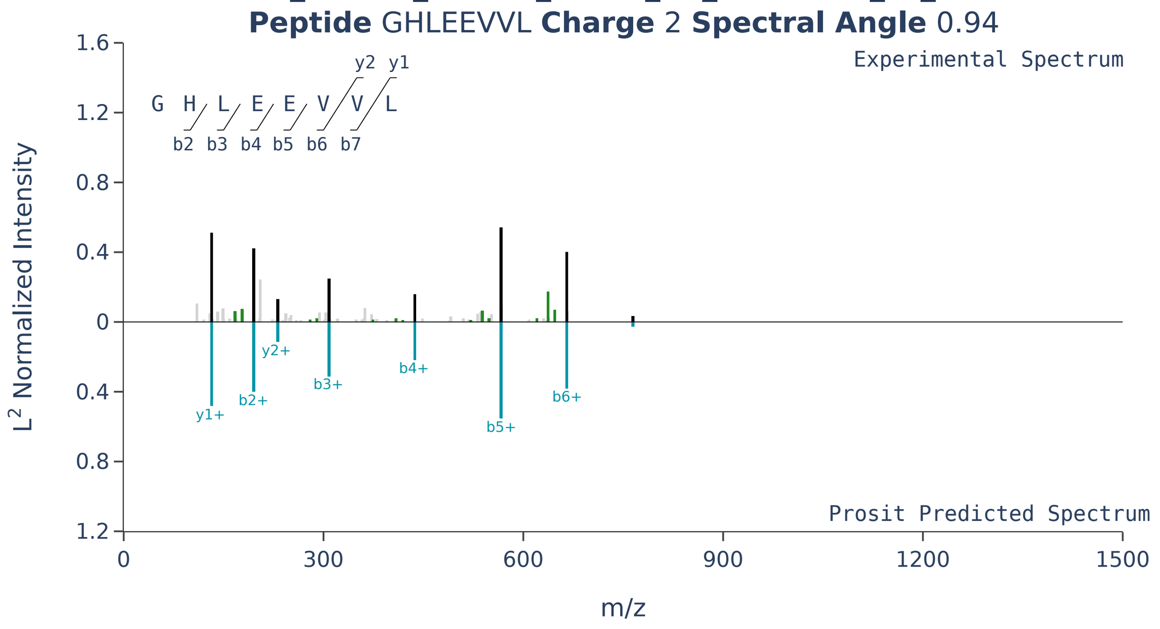

**Source** WSoh_301121_021221_HFGoe_M2_AnnexinA1_2h_R2 **Scan** 15784
**Peptide** NTILTTRSY **Charge** 2 **Spectral Angle** 0.92

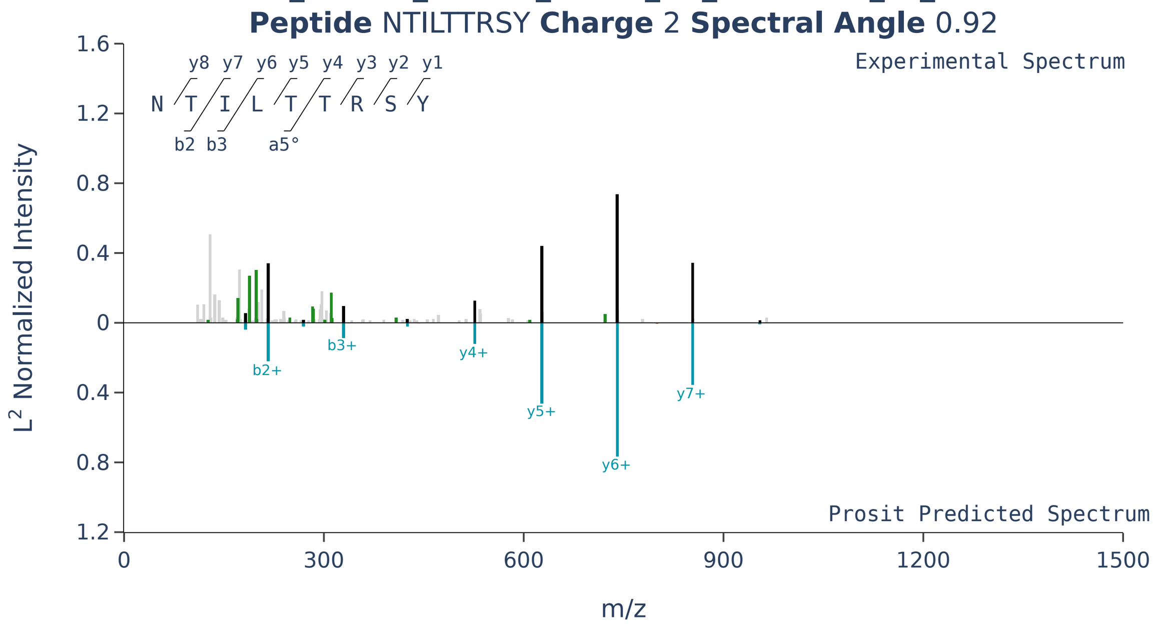


**Source** WSoh_301121_021221_HFGoe_M2_AnnexinA1_2h_R1 **Scan** 8159
**Peptide** DVAALHKA **Charge** 1 **Spectral Angle** 0.92

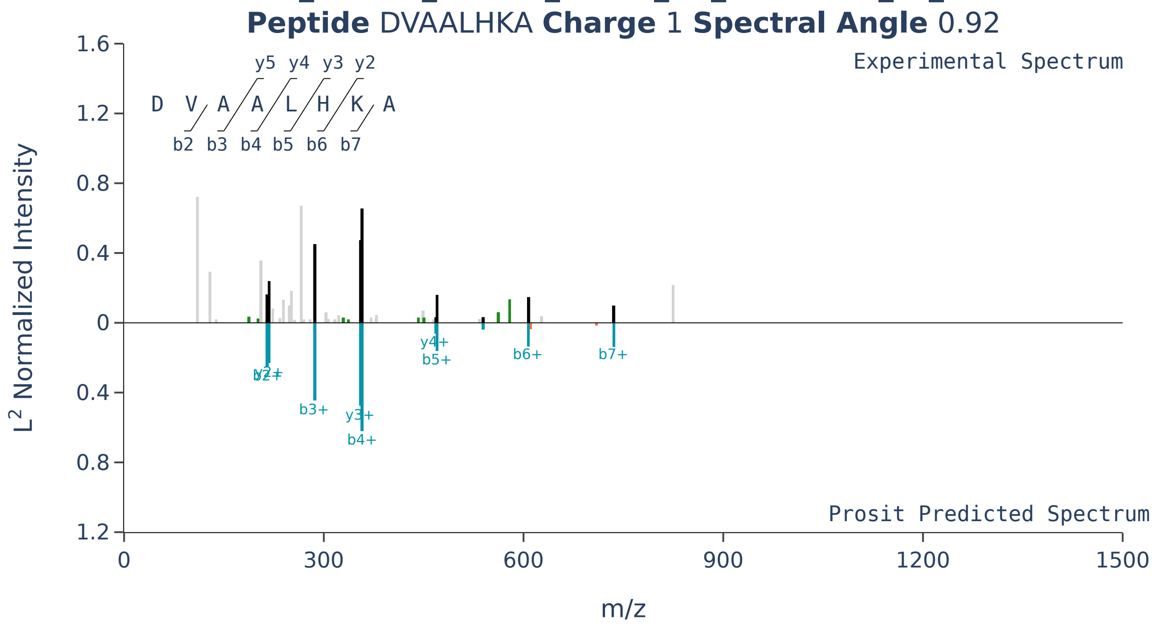


**Source** WSoh_301121_021221_HFGoe_M1_AnnexinA1_24h_R1 **Scan** 11564
**Peptide** AKDITSDTSGDFRNA **Charge** 2 **Spectral Angle** 0.93

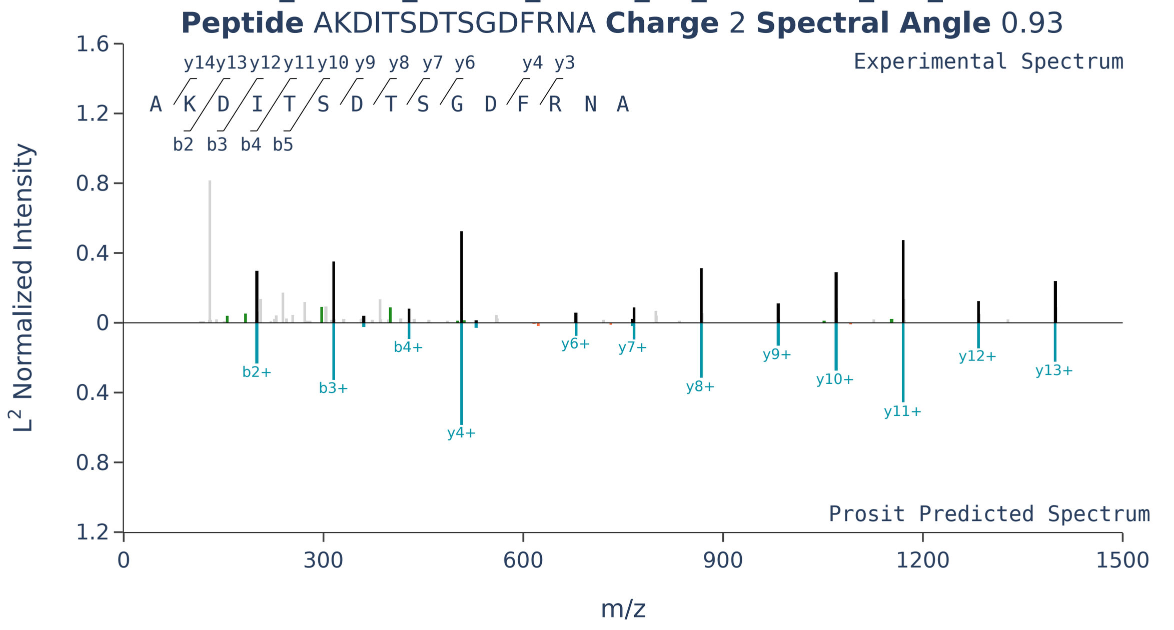


**Source** WSoh_301121_021221_HFGoe_M1_AnnexinA1_2h_R2 **Scan** 11069
**Peptide** ITSDTSGDFRNA **Charge** 2 **Spectral Angle** 0.93

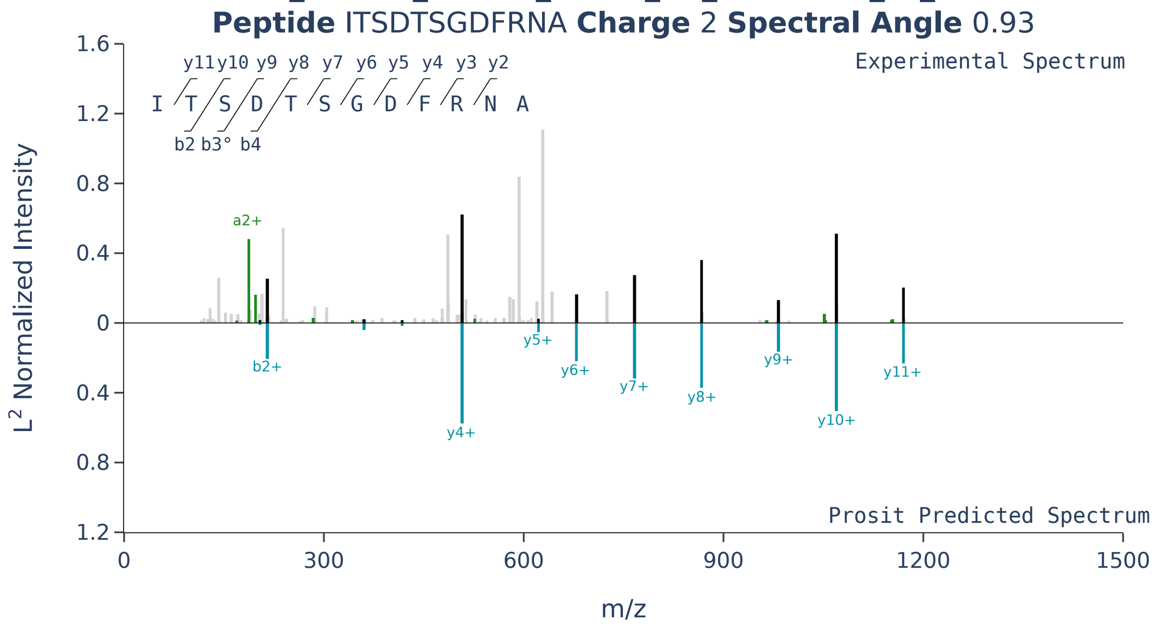


**Source** WSoh_301121_021221_HFGoe_M2_AnnexinA1_24h_R1 **Scan** 21437
**Peptide** DMNKVLDL **Charge** 2 **Spectral Angle** 0.92

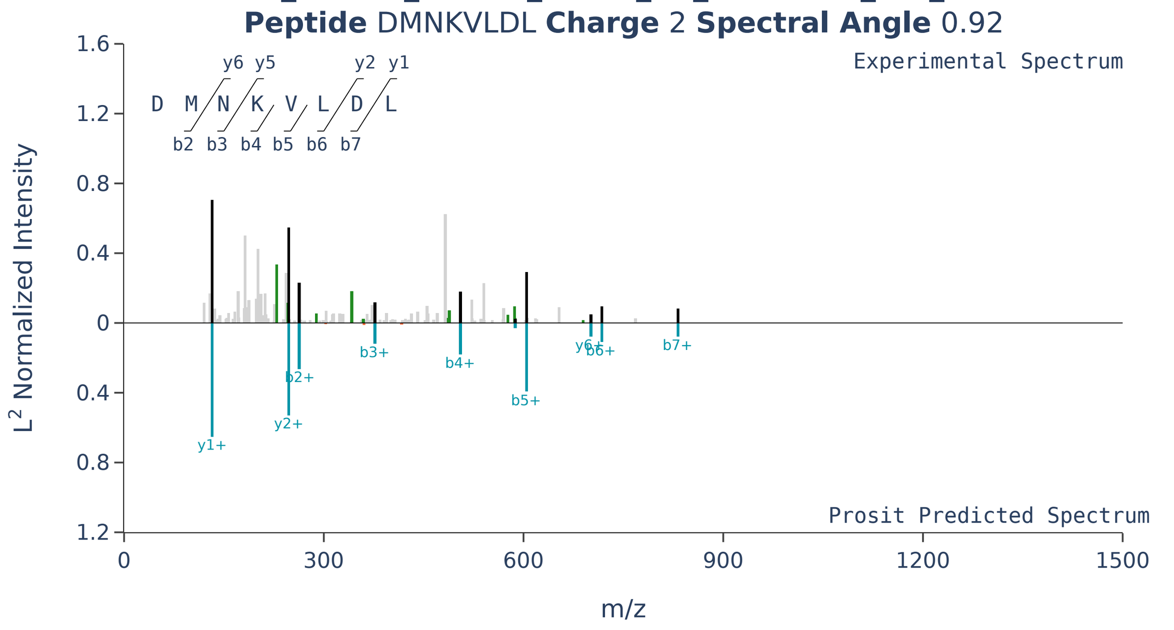


**Source** WSoh_301121_021221_HFGoe_M1_AnnexinA1_24h_R1 **Scan** 21636
**Peptide** TGHLEEVVL **Charge** 2 **Spectral Angle** 0.9

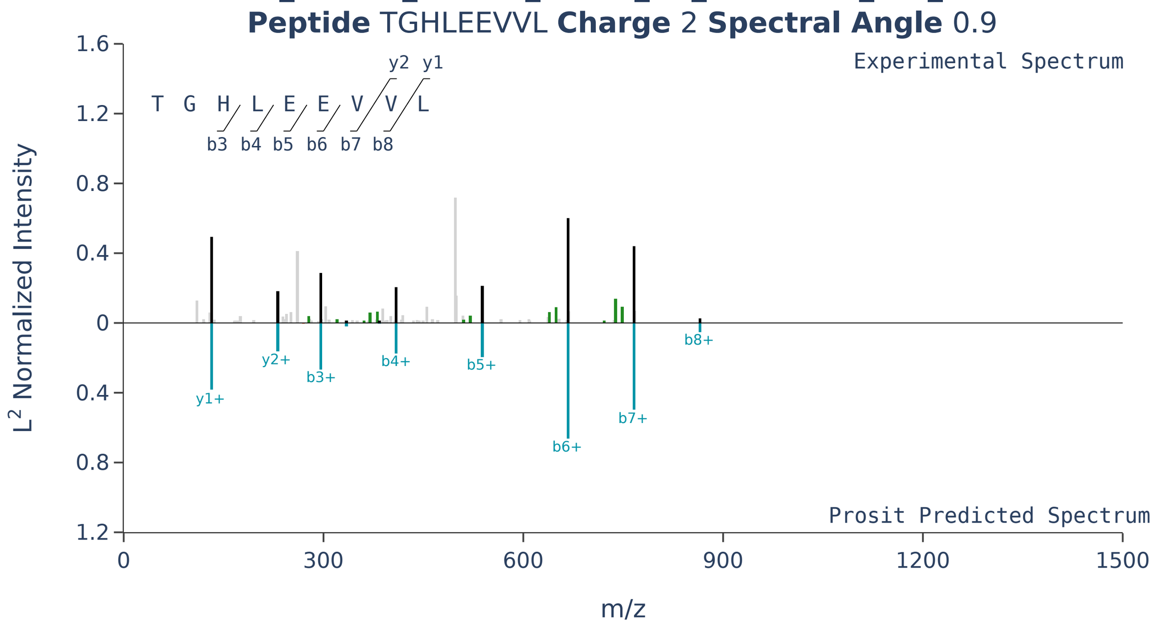


**Source** WSoh_301121_021221_HFGoe_M2_AnnexinA1_24h_R2 **Scan** 11461
**Peptide** KDITSDTSGDFRNA **Charge** 2 **Spectral Angle** 0.94

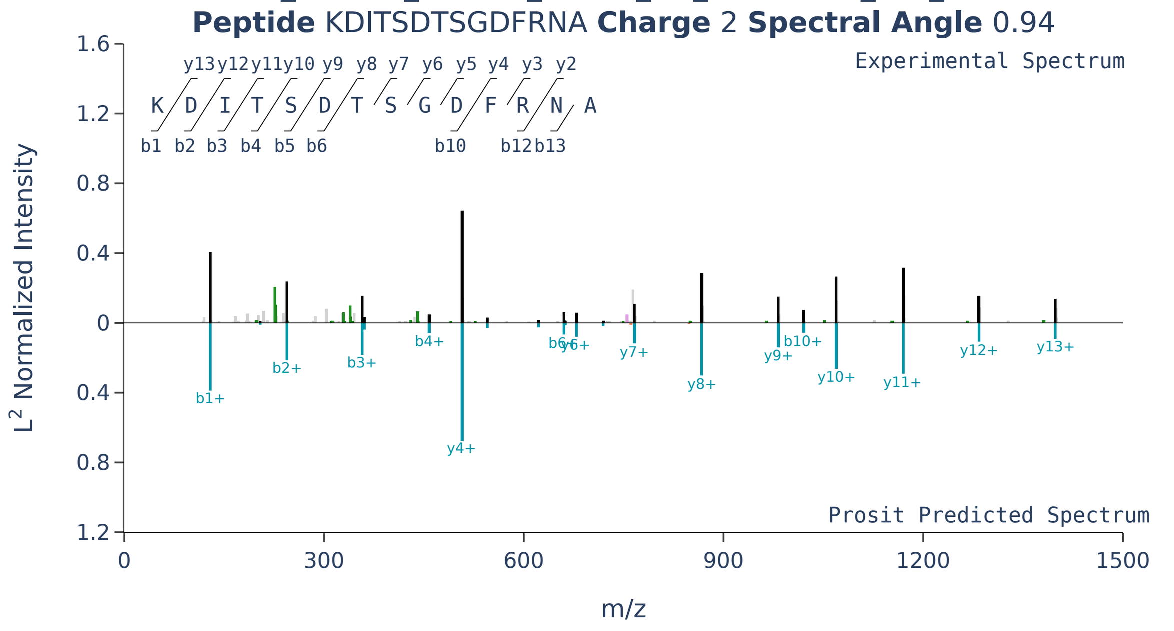


**Source** WSoh_301121_021221_HFGoe_M2_AnnexinA1_2h_R1 **Scan** 12913
**Peptide** LLSLAKGDRSED **Charge** 3 **Spectral Angle** 0.93

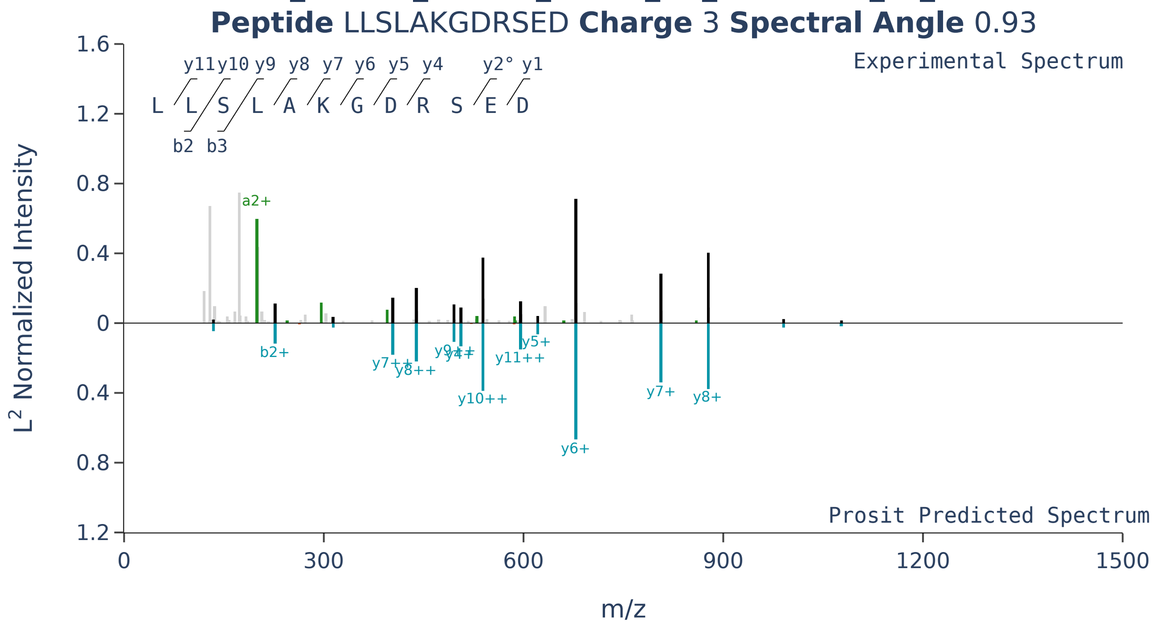


**Source** WSoh_301121_021221_HFGoe_M2_AnnexinA1_2h_R1 **Scan** 10012
**Peptide** AMKGLGTDE **Charge** 2 **Spectral Angle** 0.94

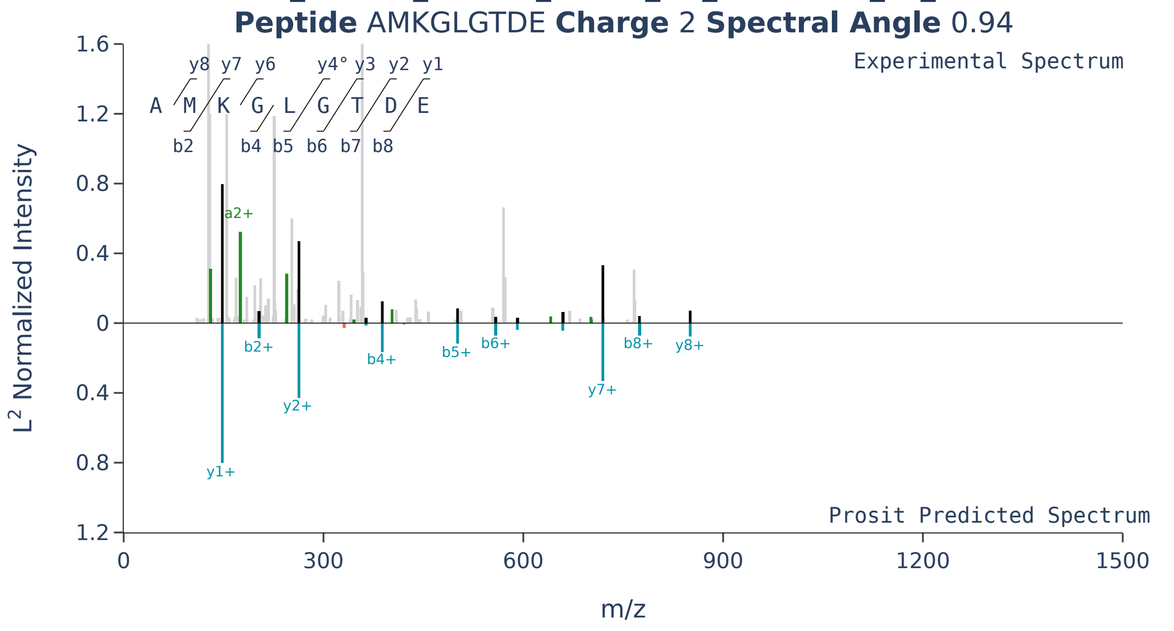


**Source** WSoh_301121_021221_HFGoe_M1_AnnexinA1_24h_R1 **Scan** 11360
**Peptide** AILDETKG **Charge** 1 **Spectral Angle** 0.92

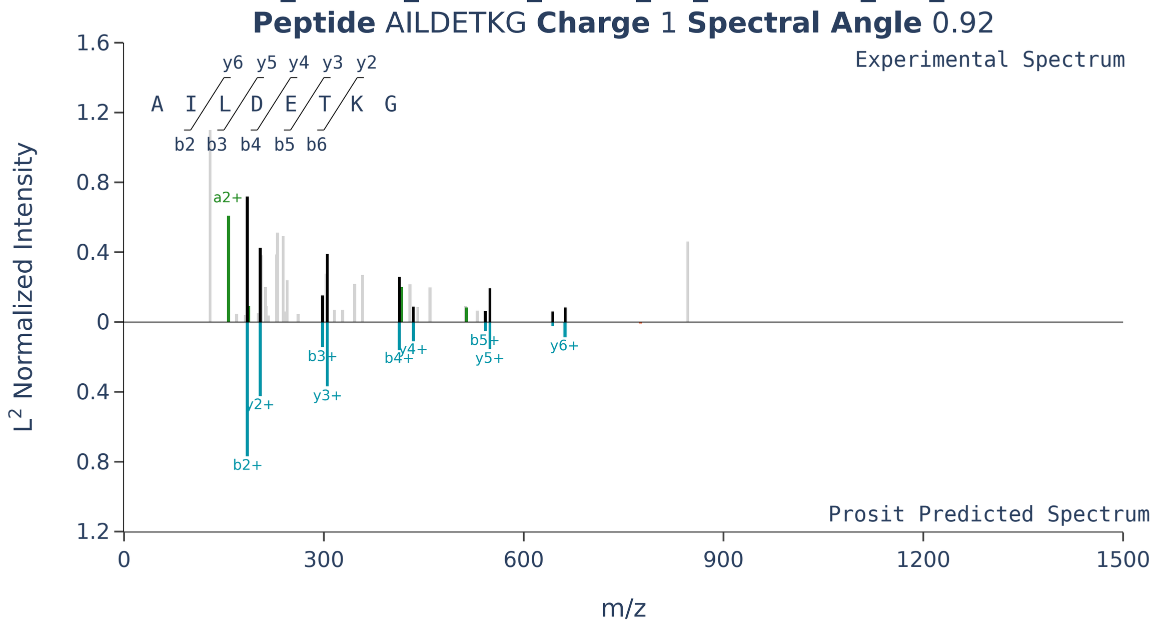


**Source** WSoh_301121_021221_HFGoe_M1_AnnexinA1_2h_R2 **Scan** 11264
**Peptide** SDARALYEAGER **Charge** 3 **Spectral Angle** 0.93

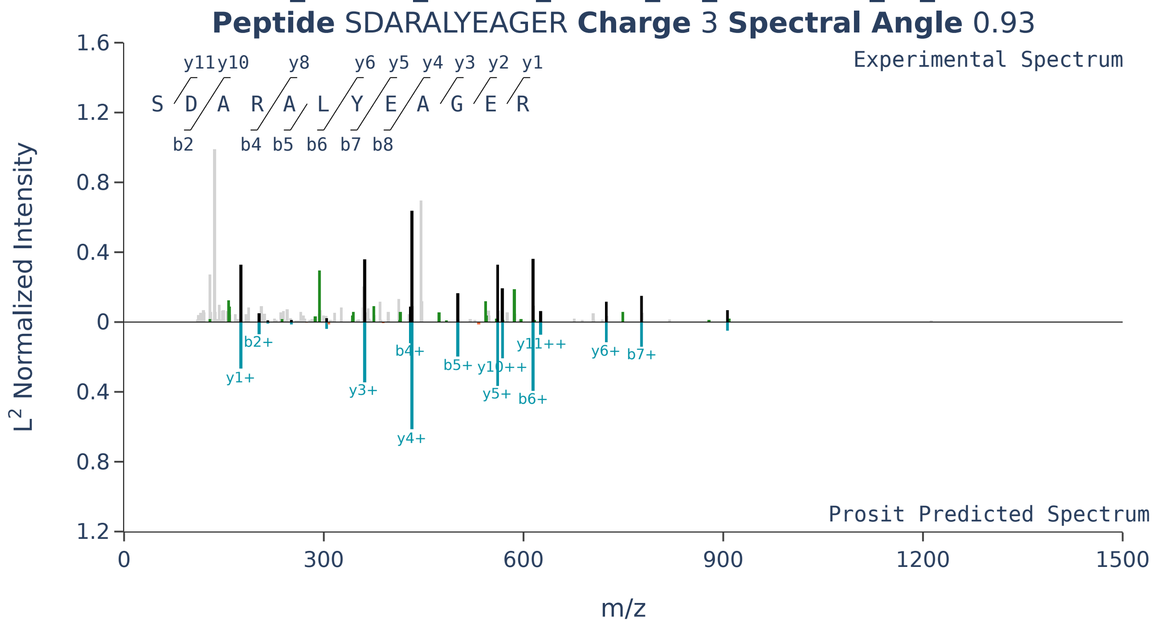


**Source** WSoh_301121_021221_HFGoe_M1_AnnexinA1_24h_R2 **Scan** 14954
**Peptide** TTRSYPQL **Charge** 2 **Spectral Angle** 0.95

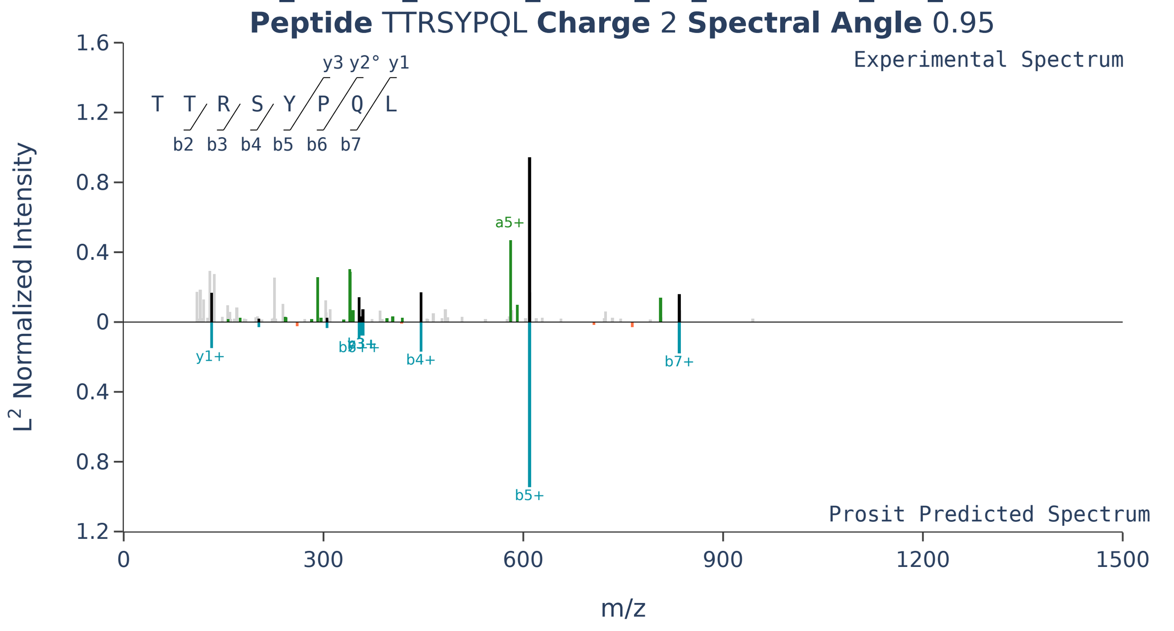


**Source** WSoh_301121_021221_HFGoe_M2_AnnexinA1_4h_R1 **Scan** 18638
**Peptide** SRSEIDMNDI **Charge** 1 **Spectral Angle** 0.89

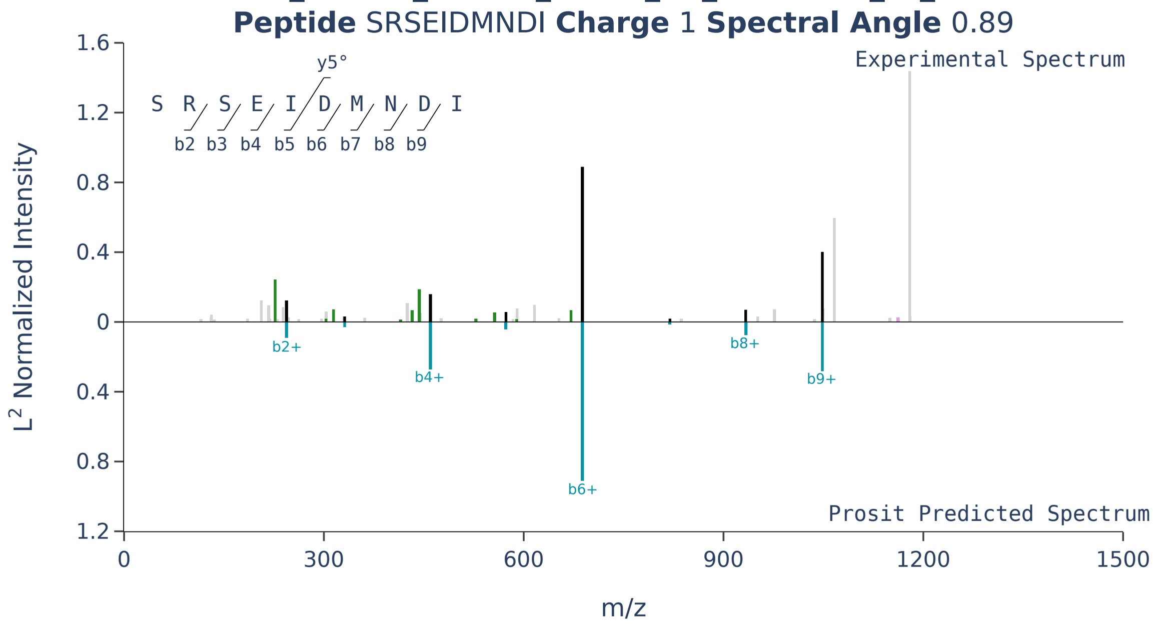


**Source** WSoh_301121_021221_HFGoe_M2_AnnexinA1_2h_R1 **Scan** 19447
**Peptide** VSRSEIDMNDI **Charge** 2 **Spectral Angle** 0.95

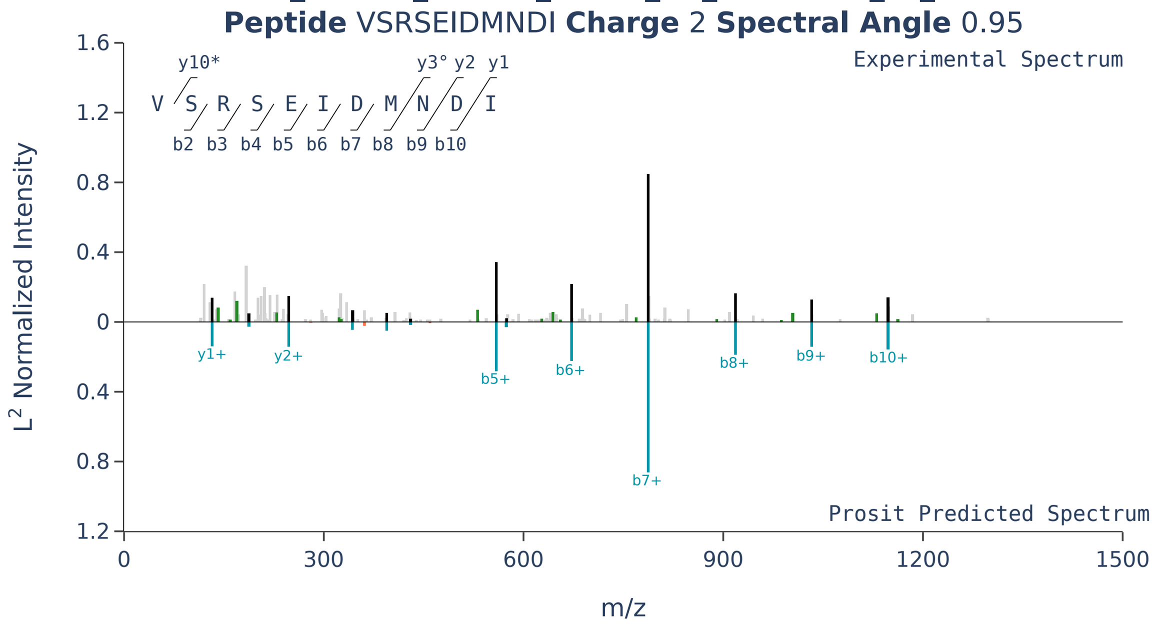


**Source** WSoh_301121_021221_HFGoe_M2_AnnexinA1_2h_R1 **Scan** 8245
**Peptide** SDTSGDFRNA **Charge** 2 **Spectral Angle** 0.91

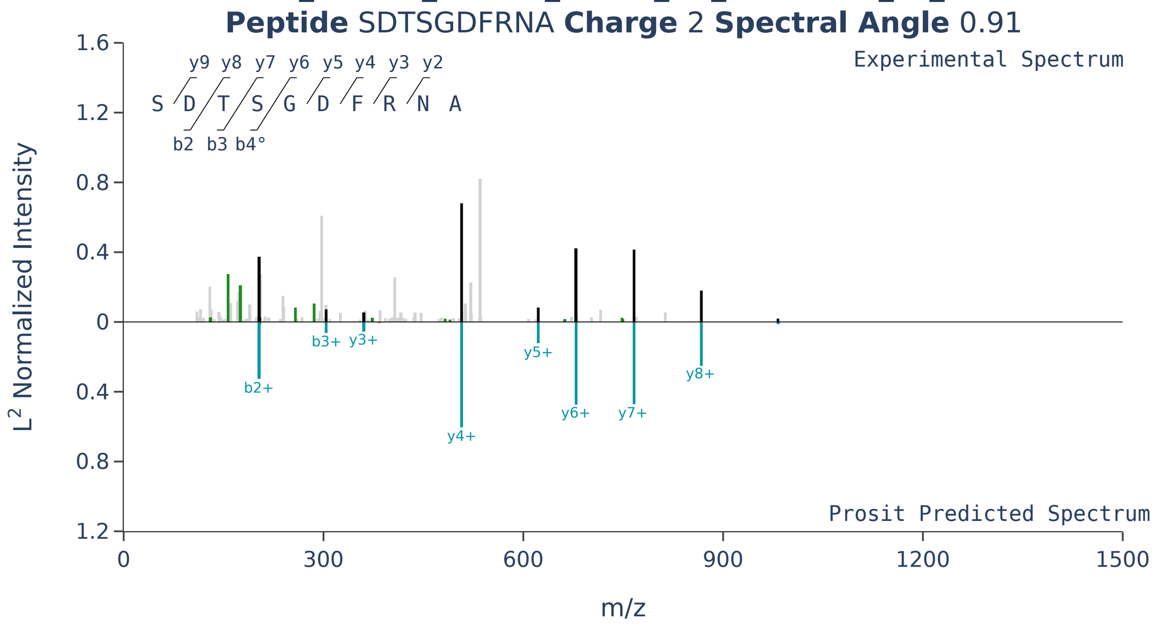

**Source** WSoh_301121_021221_HFGoe_M2_AnnexinA1_2h_R1 **Scan** 16385
**Peptide** LLSLAKGD **Charge** 2 **Spectral Angle** 0.92

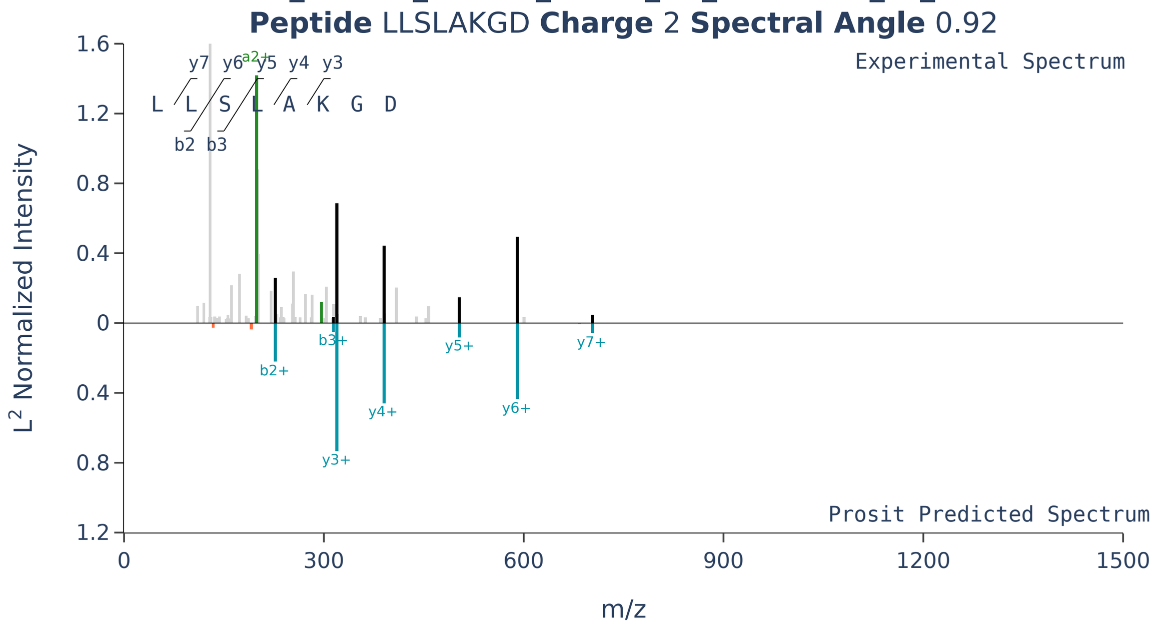


**Source** WSoh_301121_021221_HFGoe_M1_AnnexinA1_4h_R1 **Scan** 11996
**Peptide** RSEDFGVNED **Charge** 1 **Spectral Angle** 0.94

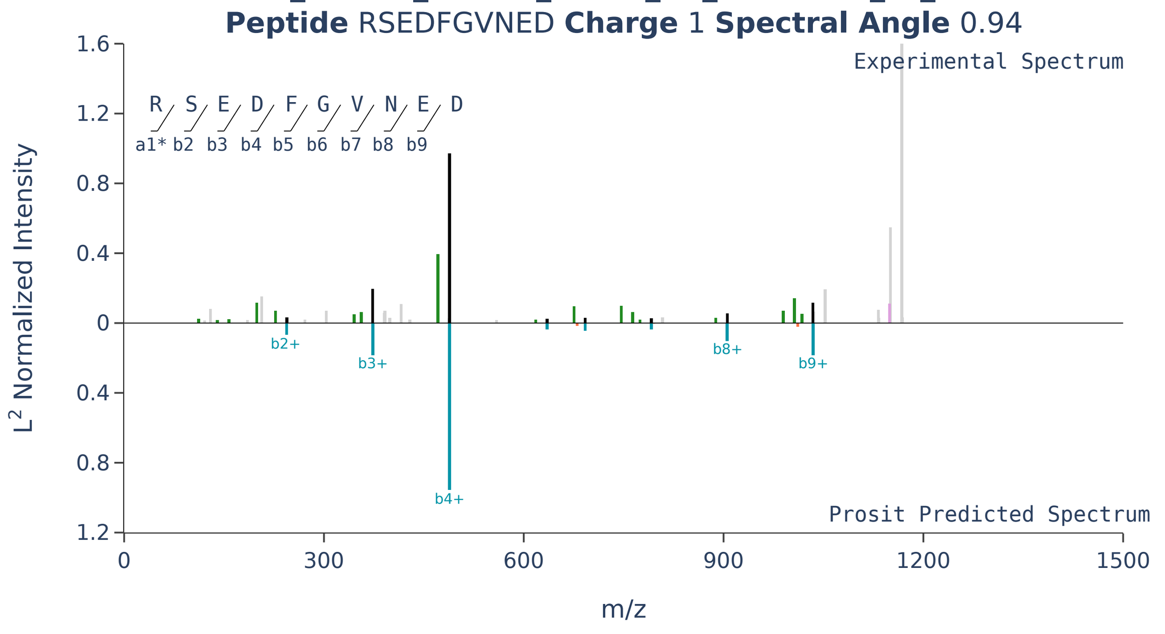


**Source** WSoh_301121_021221_HFGoe_M1_AnnexinA1_24h_R1 **Scan** 10103
**Peptide** EILASRT **Charge** 2 **Spectral Angle** 0.95

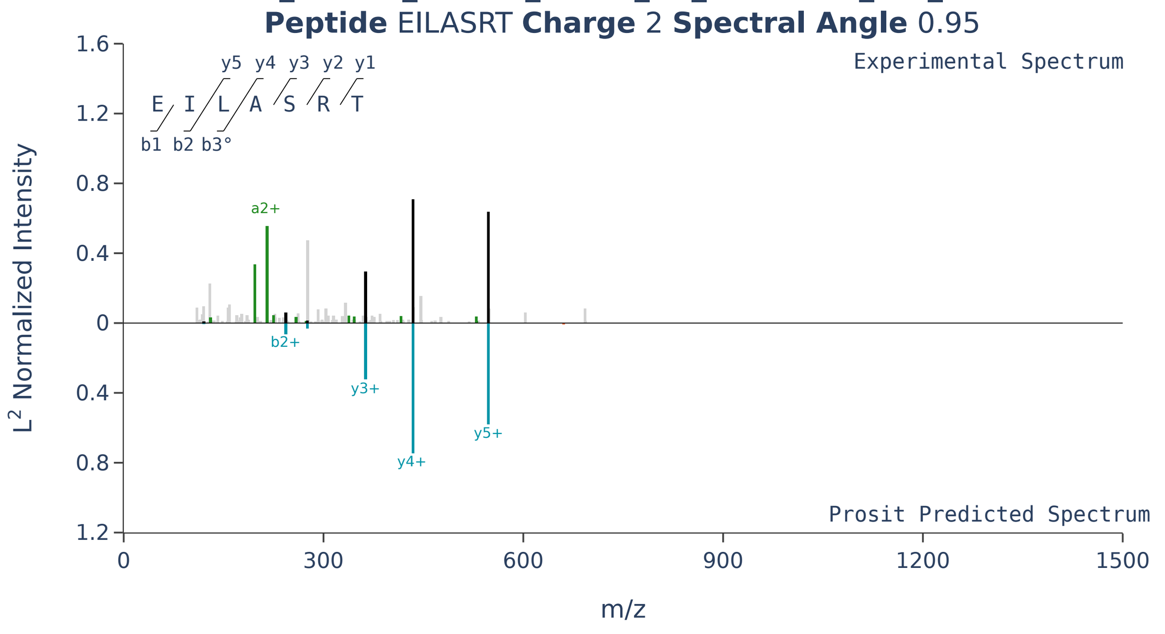


**Source** WSoh_301121_021221_HFGoe_M2_AnnexinA1_24h_R1 **Scan** 10512
**Peptide** ALHKAIMV **Charge** 1 **Spectral Angle** 0.9

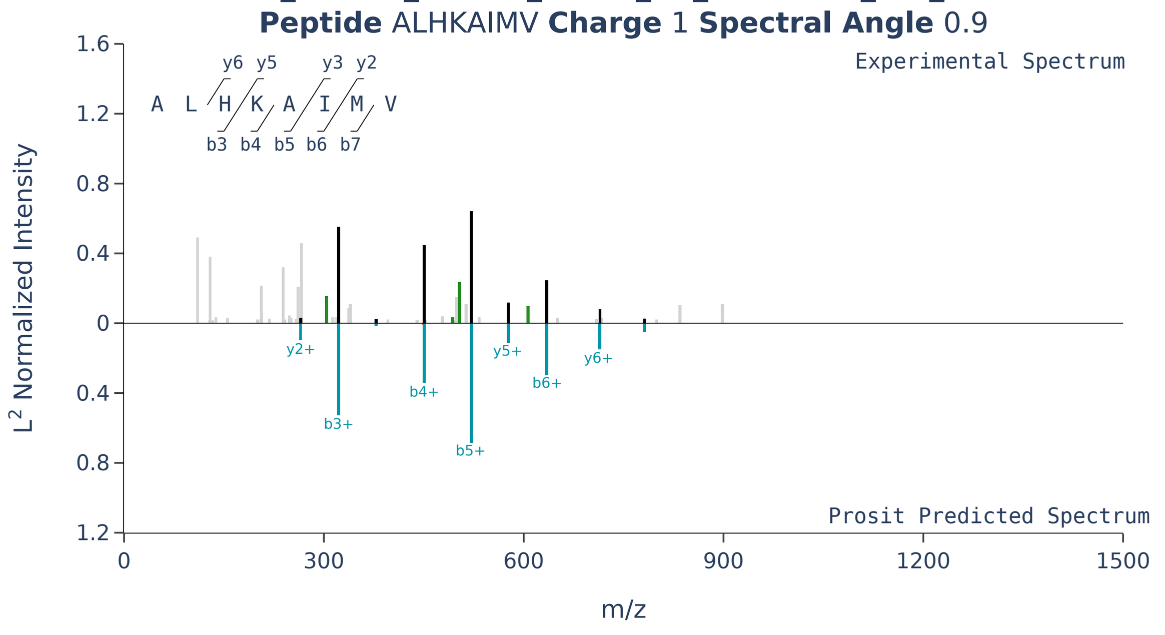


**Source** WSoh_301121_021221_HFGoe_M1_AnnexinA1_1h_R1 **Scan** 13915
**Peptide** ADELRAAM **Charge** 2 **Spectral Angle** 0.92

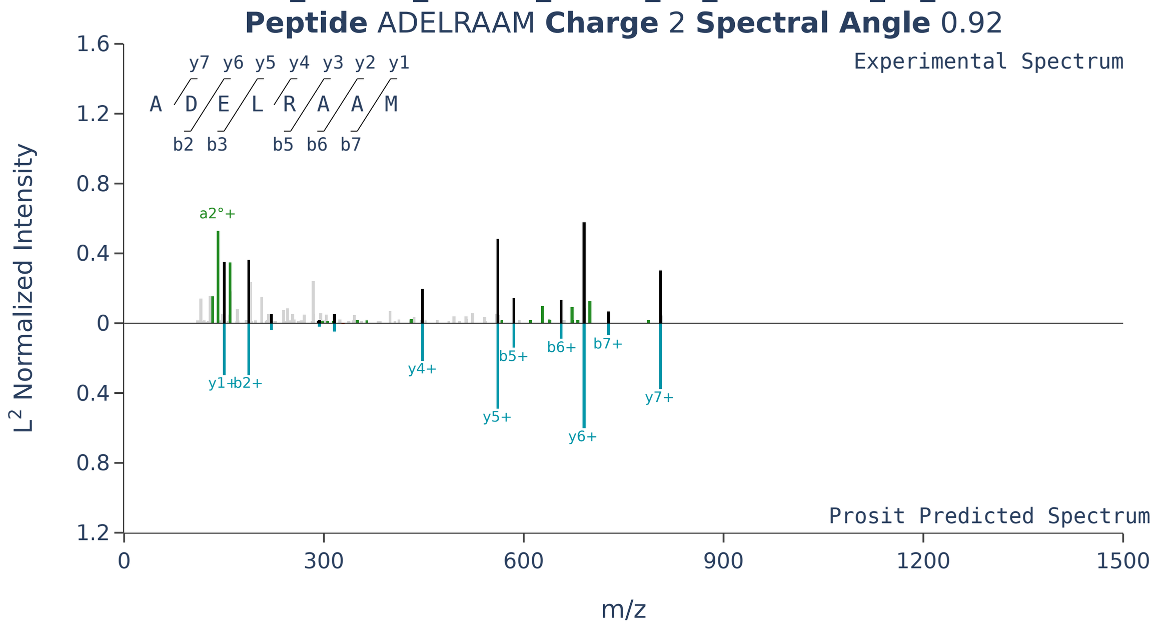


**Source** WSoh_301121_021221_HFGoe_M2_AnnexinA1_24h_R2 **Scan** 9201
**Peptide** DINRVYRE **Charge** 2 **Spectral Angle** 0.95

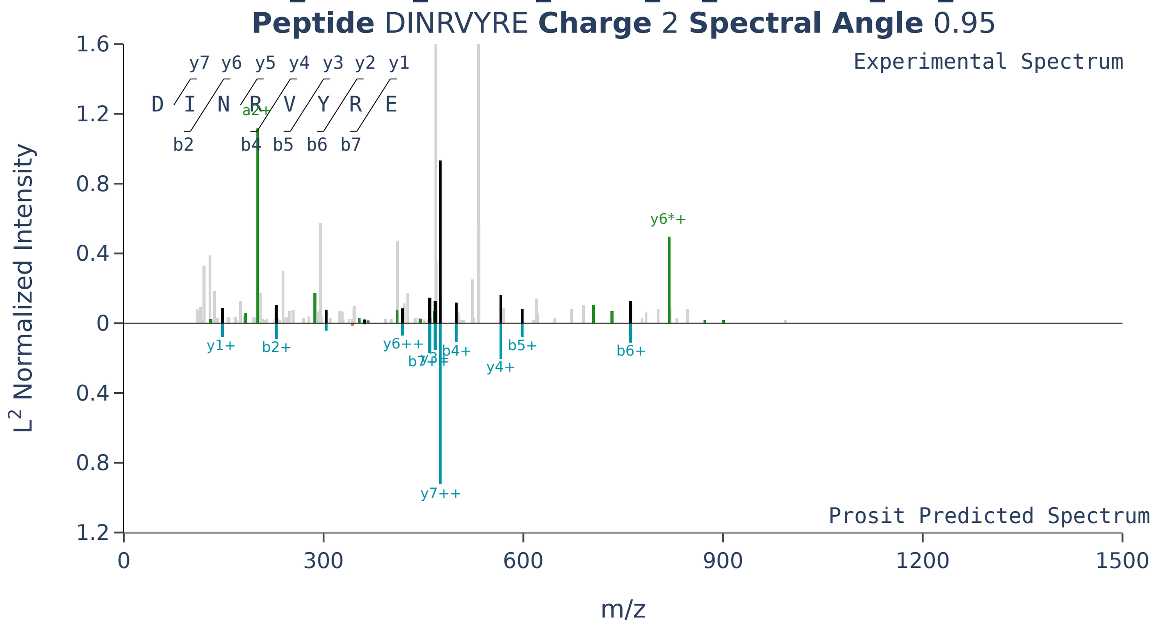


**Source** WSoh_301121_021221_HFGoe_M2_AnnexinA1_2h_R1 **Scan** 9414
**Peptide** TRSYPQLR **Charge** 2 **Spectral Angle** 0.94

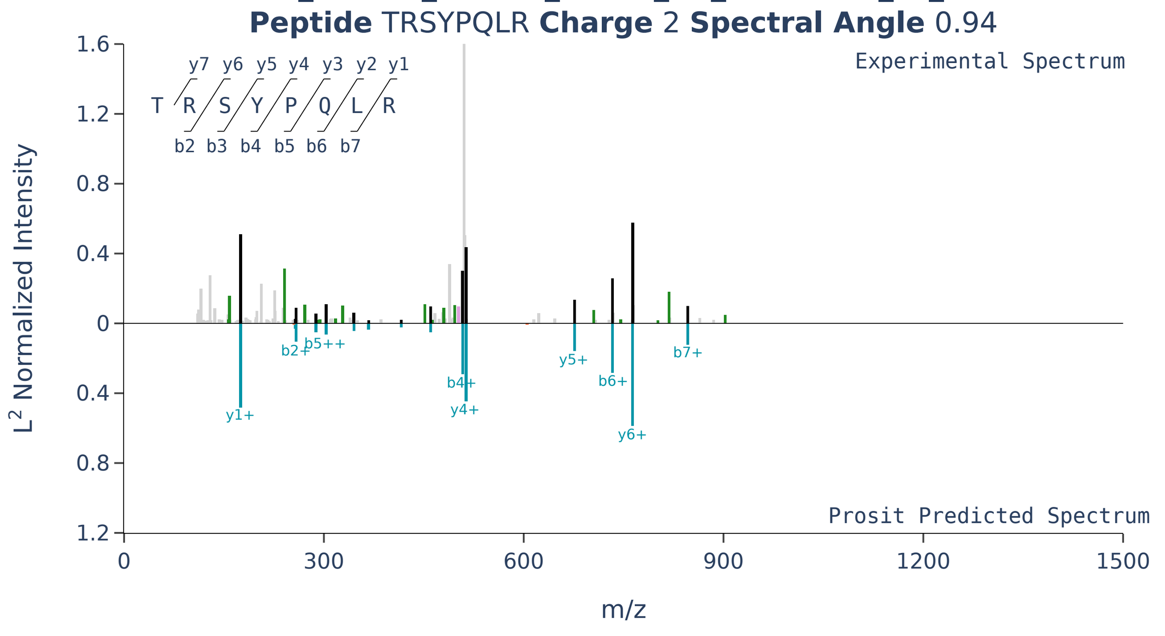


**Source** WSoh_301121_021221_HFGoe_M2_AnnexinA1_4h_R1 **Scan** 6264
**Peptide** KKALTGHLE **Charge** 2 **Spectral Angle** 0.94

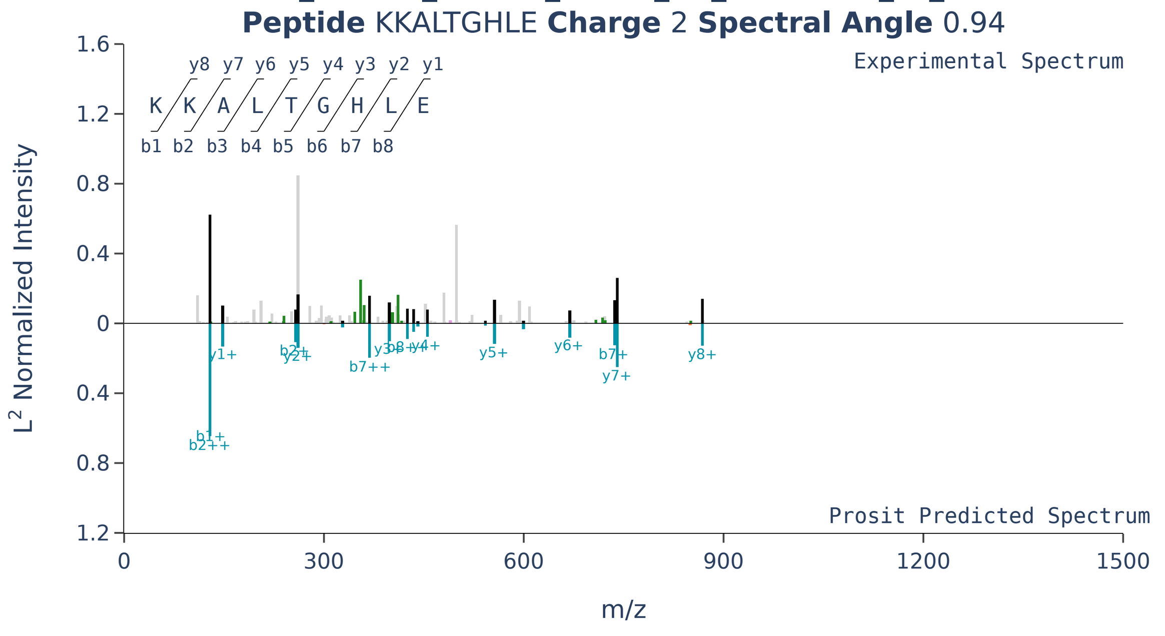


**Source** WSoh_301121_021221_HFGoe_M2_AnnexinA1_24h_R2 **Scan** 7976
**Peptide** ILDETKG **Charge** 2 **Spectral Angle** 0.9

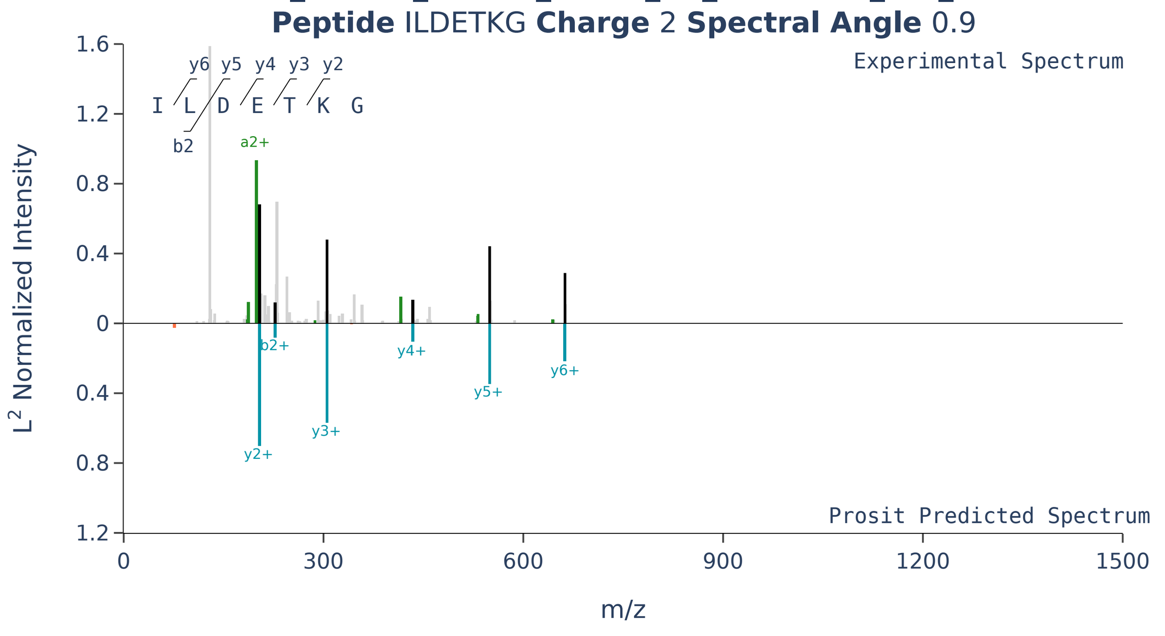


**Source** WSoh_301121_021221_HFGoe_M2_AnnexinA1_24h_R1 **Scan** 9620
**Peptide** ILDETKGDYEK **Charge** 2 **Spectral Angle** 0.91

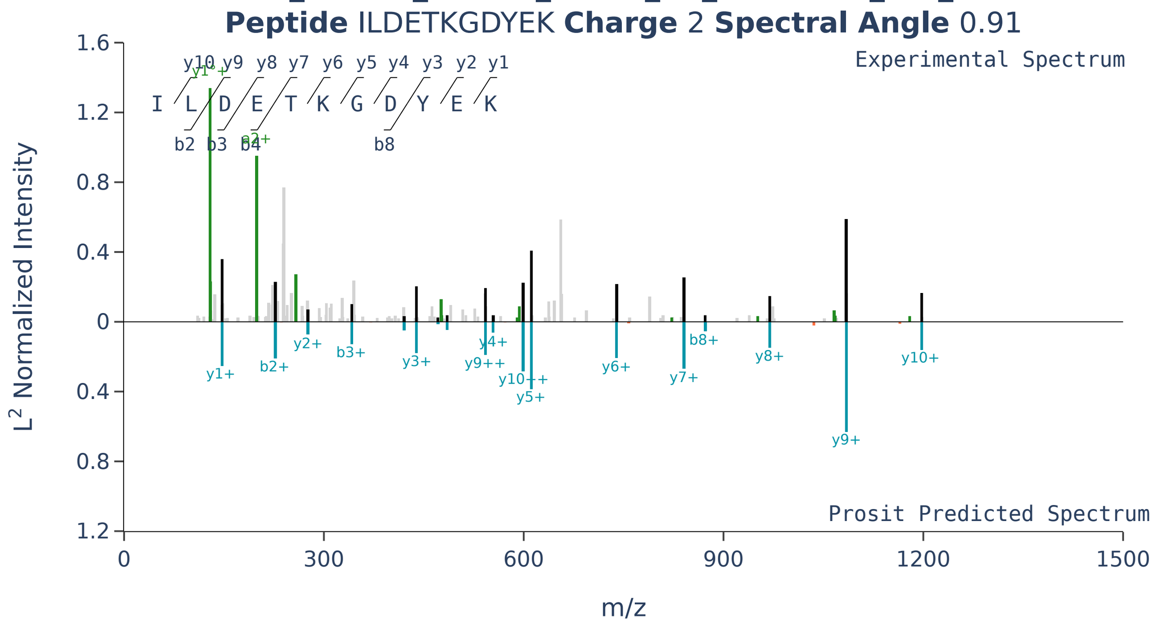


**Source** WSoh_301121_021221_HFGoe_M1_AnnexinA1_2h_R2 **Scan** 16924
**Peptide** NDIKAFYQ **Charge** 2 **Spectral Angle** 0.93

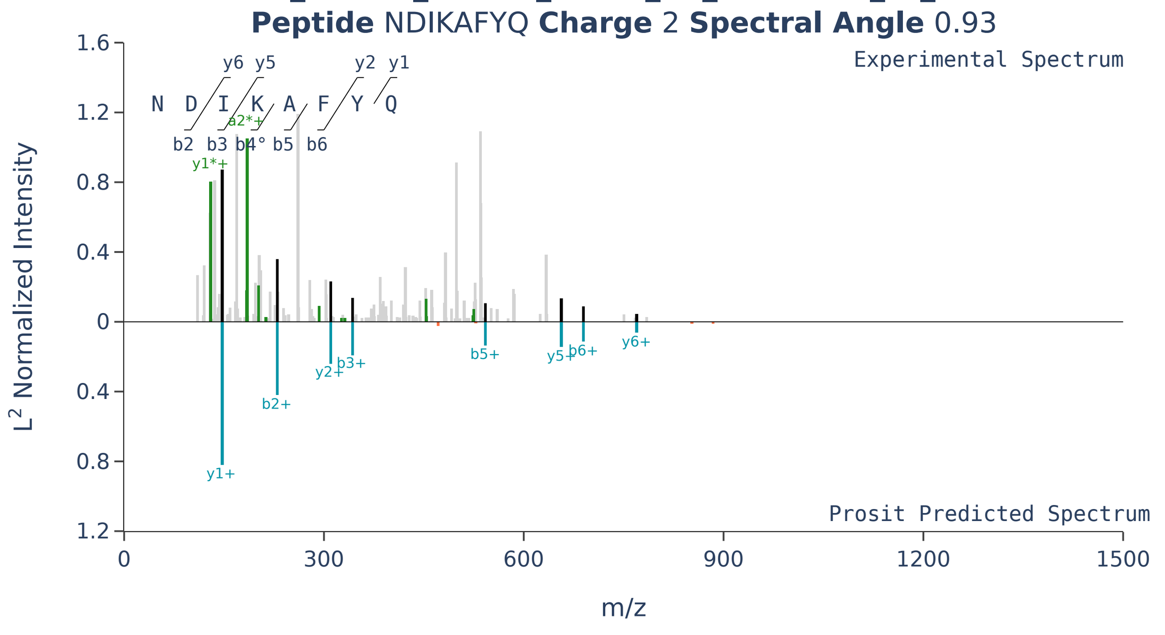


**Source** WSoh_301121_021221_HFGoe_M1_AnnexinA1_2h_R1 **Scan** 13987
**Peptide** VSEFLKQ **Charge** 2 **Spectral Angle** 0.93

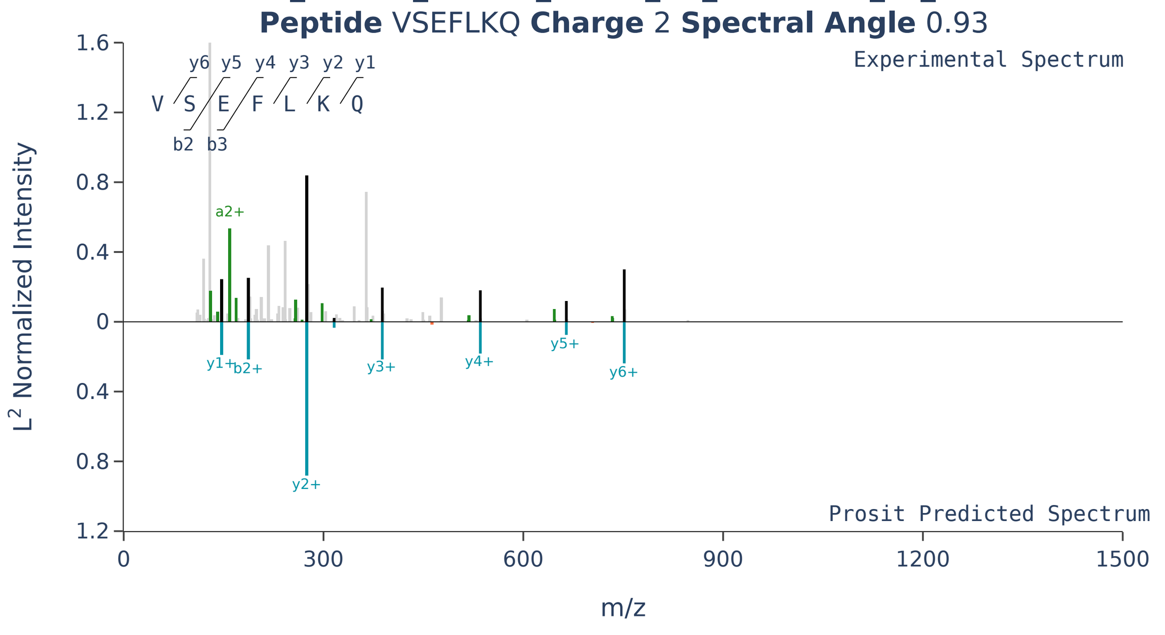


**Source** WSoh_301121_021221_HFGoe_M1_AnnexinA1_24h_R2 **Scan** 8726
**Peptide** DTSGDFRNA **Charge** 2 **Spectral Angle** 0.93

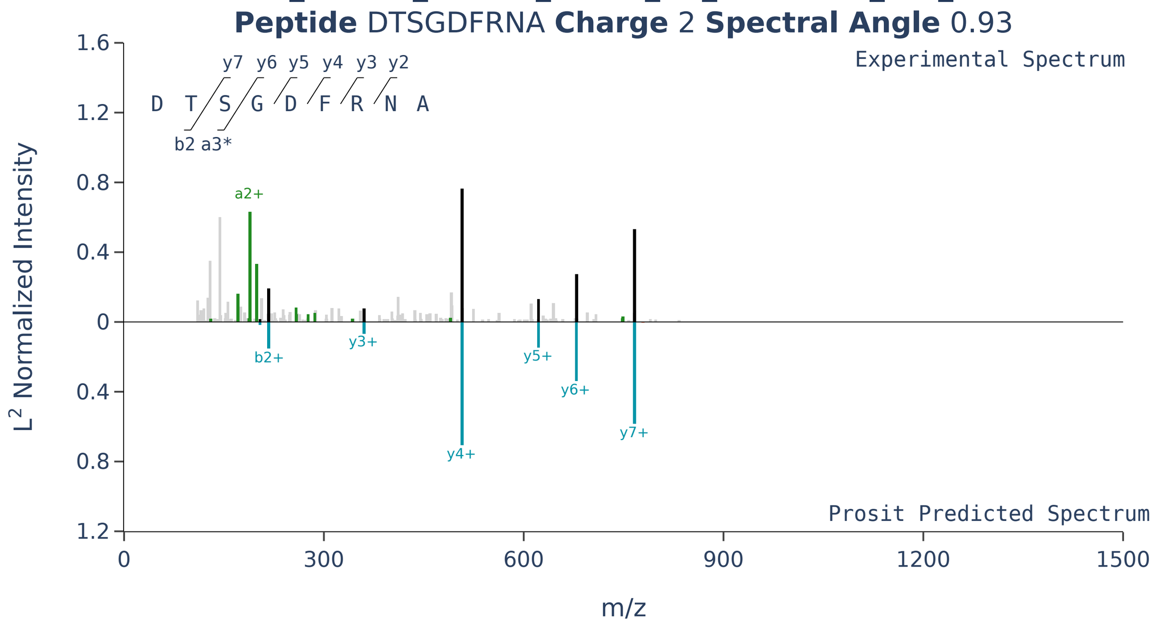


**Source** WSoh_301121_021221_HFGoe_M2_AnnexinA1_2h_R1 **Scan** 10310
**Peptide** AKGDRSEDFGVNE **Charge** 2 **Spectral Angle** 0.95

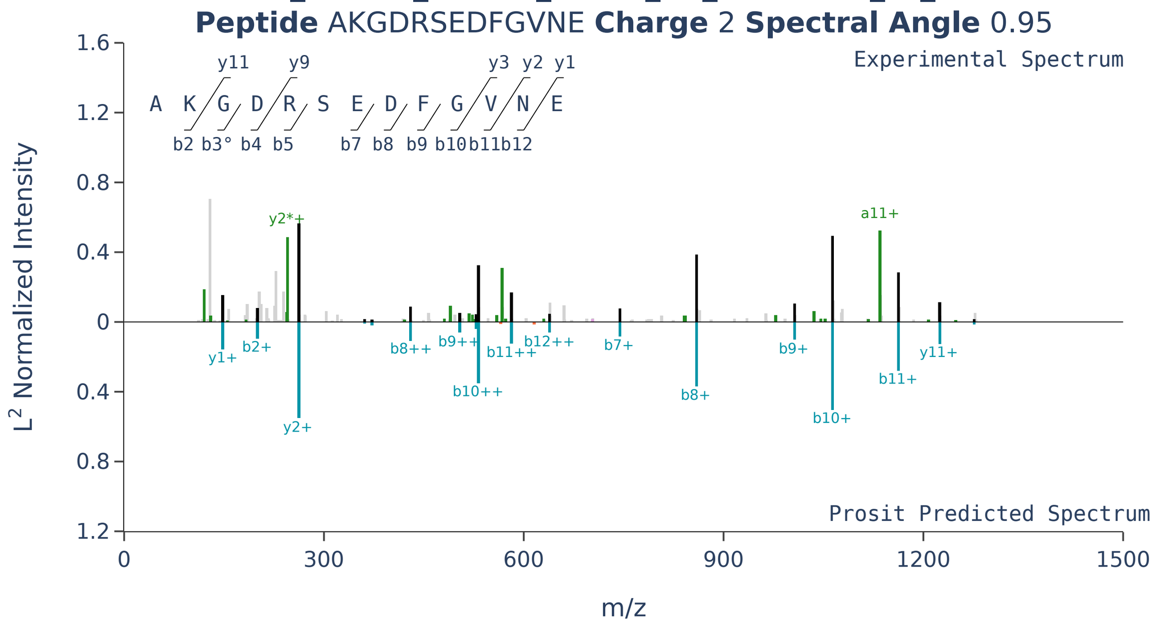


**Source** WSoh_301121_021221_HFGoe_M2_AnnexinA1_2h_R2 **Scan** 12599
**Peptide** SEFLKQA **Charge** 2 **Spectral Angle** 0.95

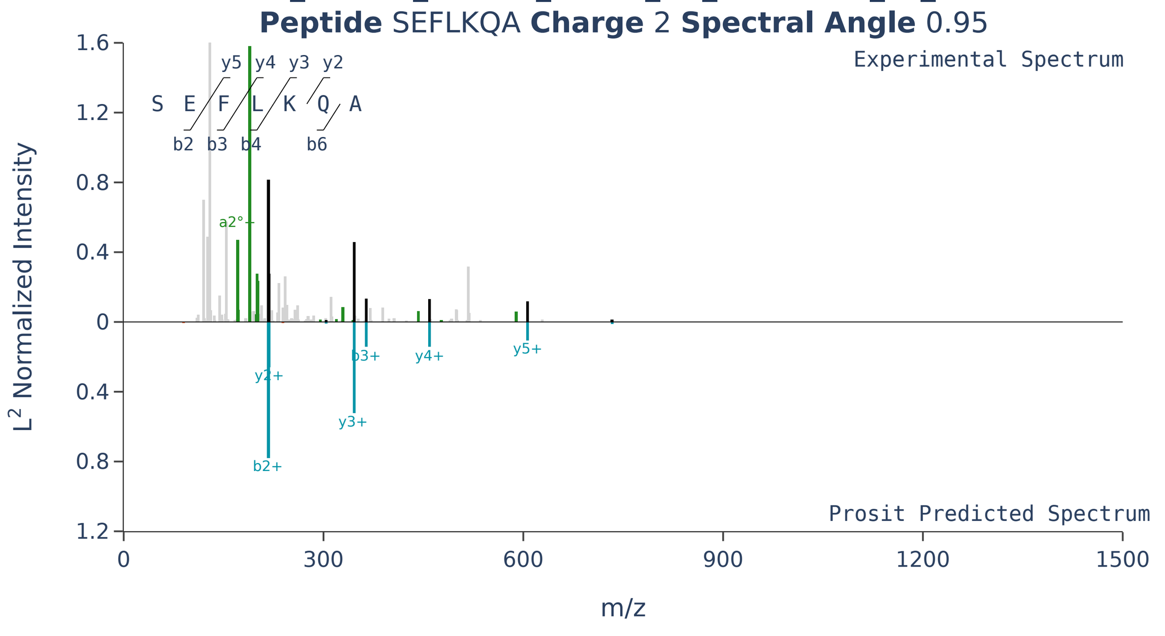

**Source** WSoh_301121_021221_HFGoe_M2_AnnexinA1_4h_R1 **Scan** 15529
**Peptide** SGDFRNAL **Charge** 2 **Spectral Angle** 0.89

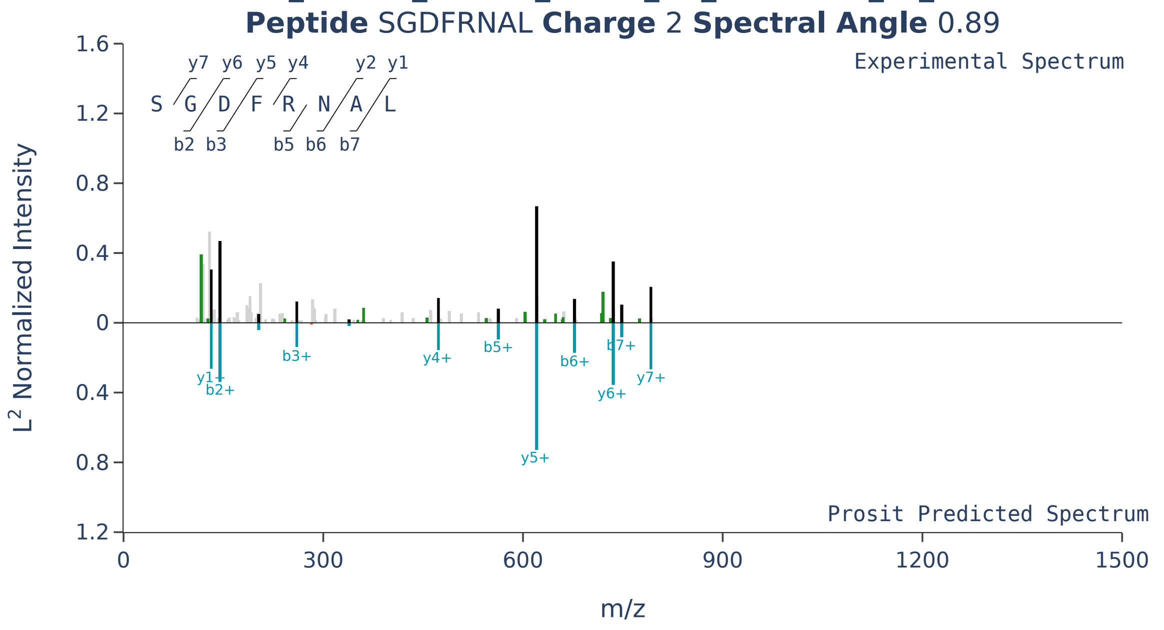


**Source** WSoh_301121_021221_HFGoe_M1_AnnexinA1_1h_R2 **Scan** 14380
**Peptide** TRSYPQL **Charge** 2 **Spectral Angle** 0.97

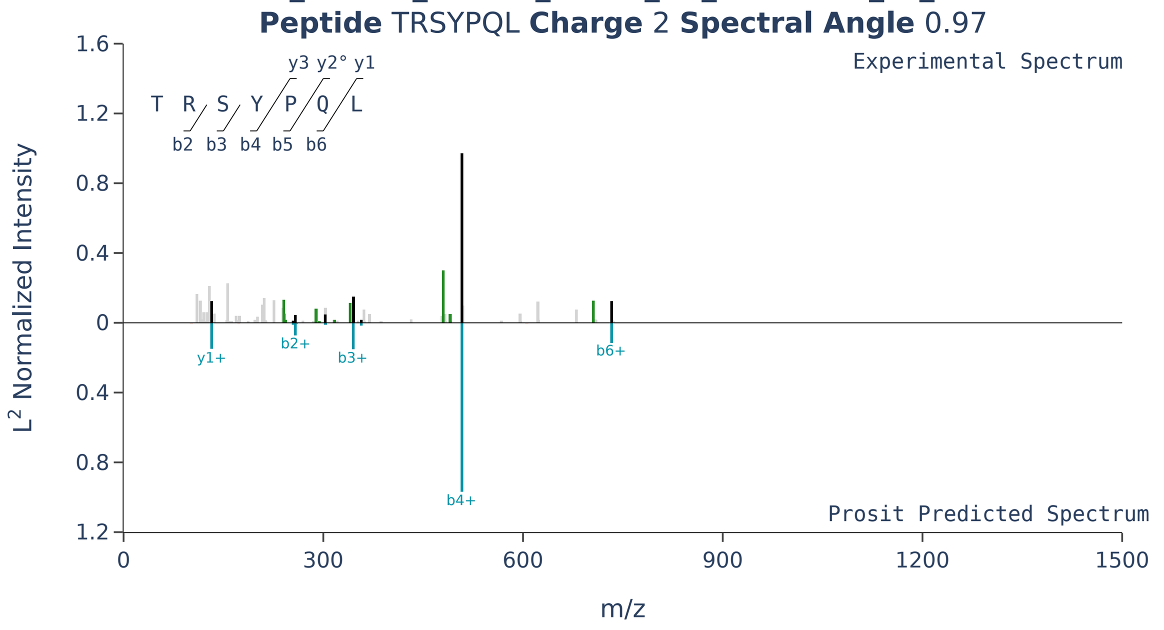


**Source** WSoh_301121_021221_HFGoe_M1_AnnexinA1_2h_R2 **Scan** 20317
**Peptide** GHLEEVVLA **Charge** 2 **Spectral Angle** 0.9

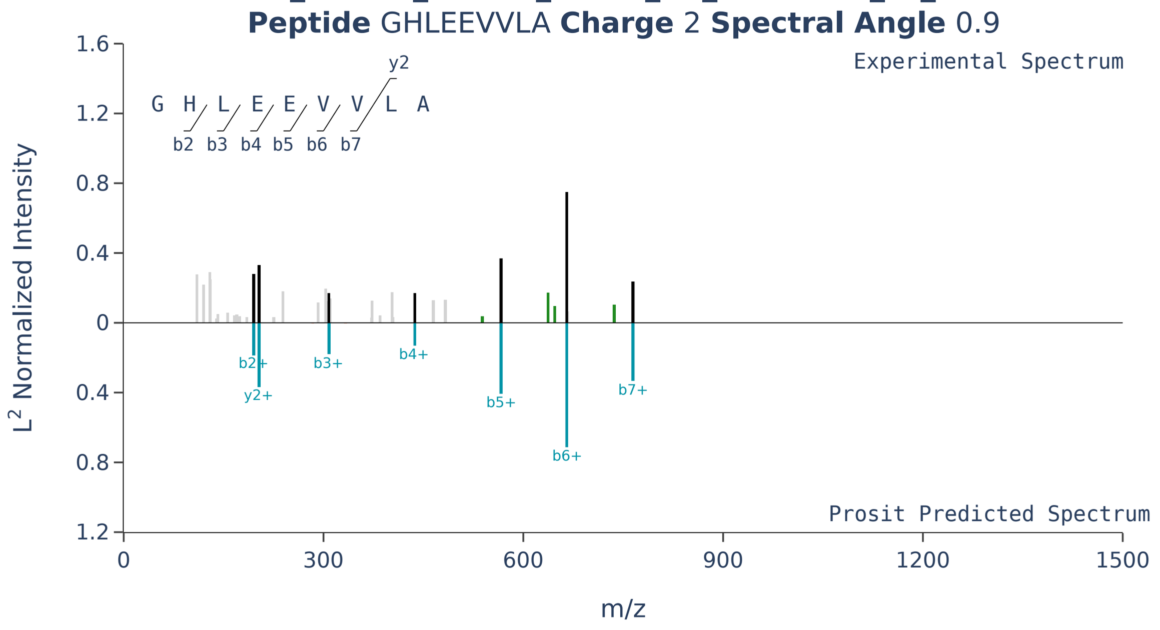


**Source** WSoh_301121_021221_HFGoe_M1_AnnexinA1_24h_R2 **Scan** 18722
**Peptide** SRSEIDMNDL **Charge** 1 **Spectral Angle** 0.89

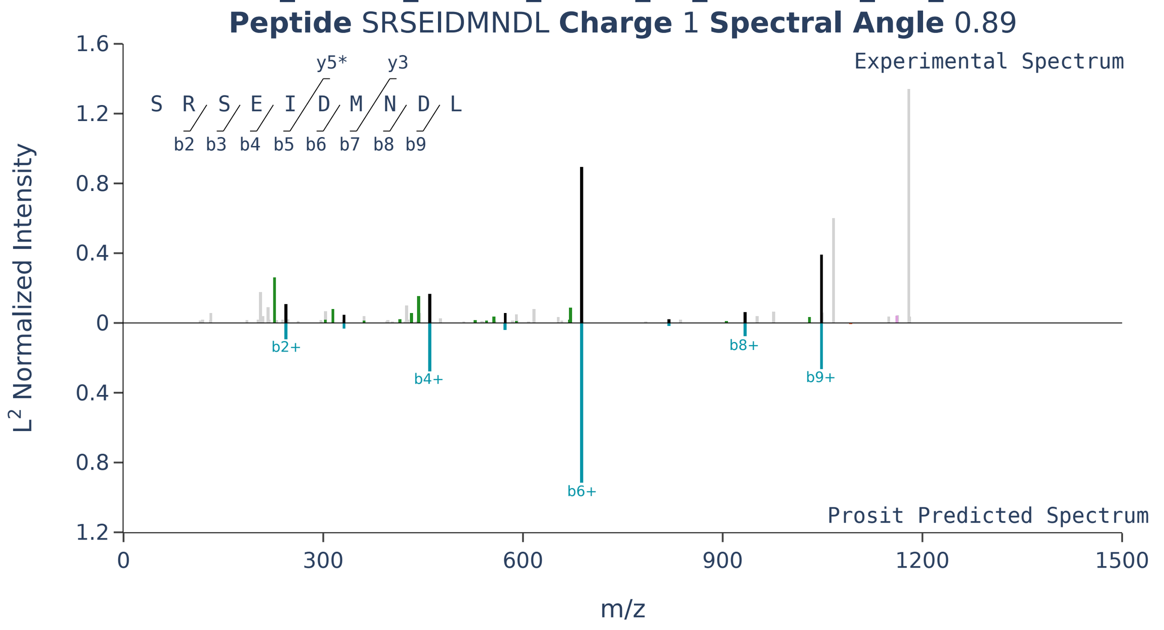


**Source** WSoh_301121_021221_HFGoe_M1_AnnexinA1_24h_R1 **Scan** 10121
**Peptide** LLKTPAQ **Charge** 2 **Spectral Angle** 0.93

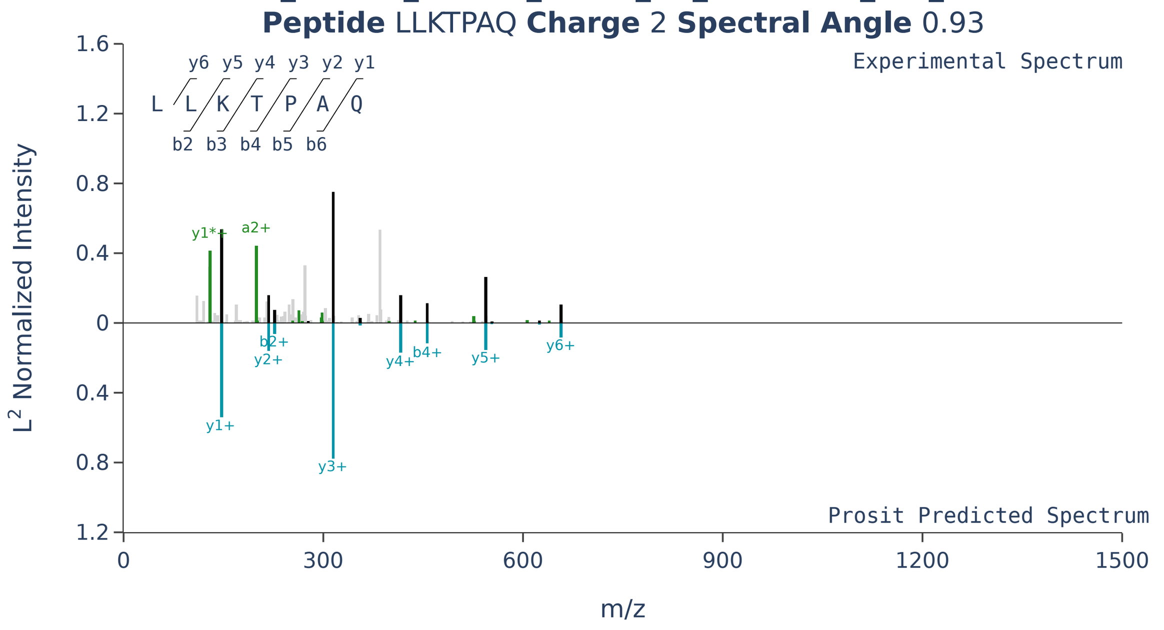


**Source** WSoh_301121_021221_HFGoe_M2_AnnexinA1_4h_R1 **Scan** 12567
**Peptide** ADSDARALYE **Charge** 2 **Spectral Angle** 0.89

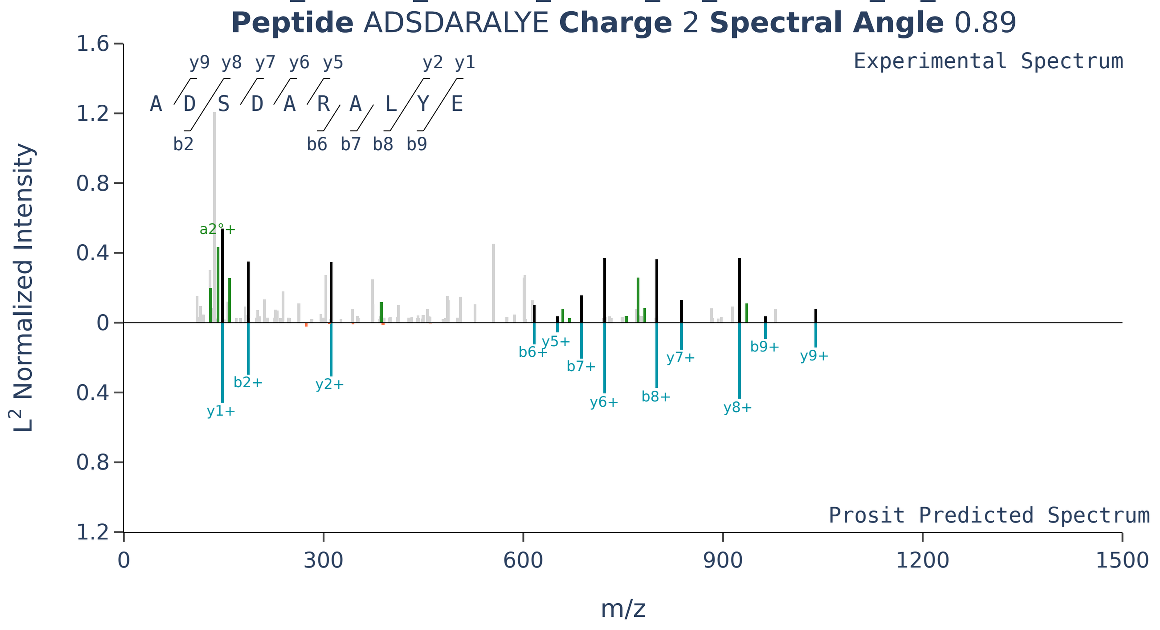


**Source** WSoh_301121_021221_HFGoe_M2_AnnexinA1_24h_R1 **Scan** 9445
**Peptide** AMKGLGTD **Charge** 2 **Spectral Angle** 0.93

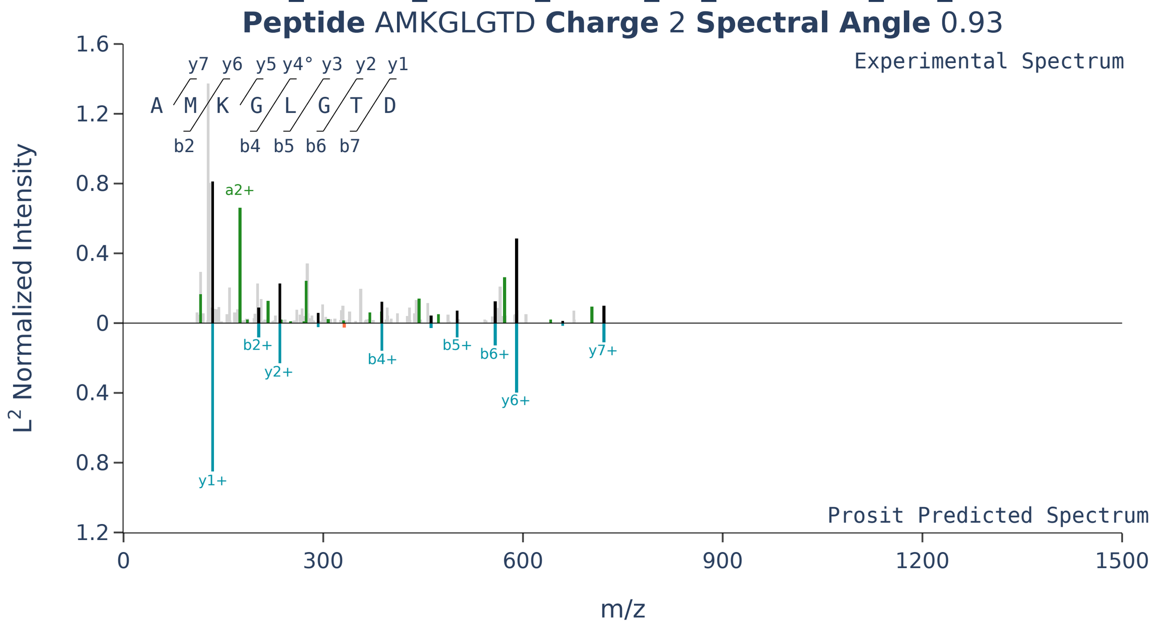


**Source** WSoh_301121_021221_HFGoe_M2_AnnexinA1_4h_R1 **Scan** 8453
**Peptide** FQKYTKY **Charge** 1 **Spectral Angle** 0.88

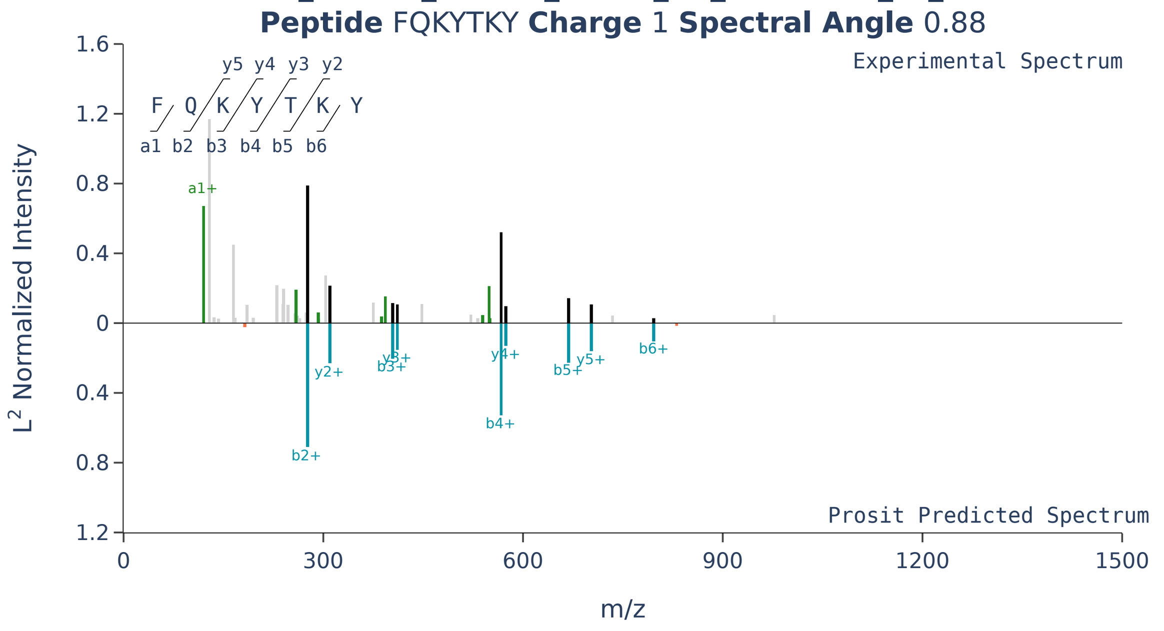


**Source** WSoh_301121_021221_HFGoe_M2_AnnexinA1_4h_R1 **Scan** 10183
**Peptide** KGDRSEDFGVNE **Charge** 2 **Spectral Angle** 0.92

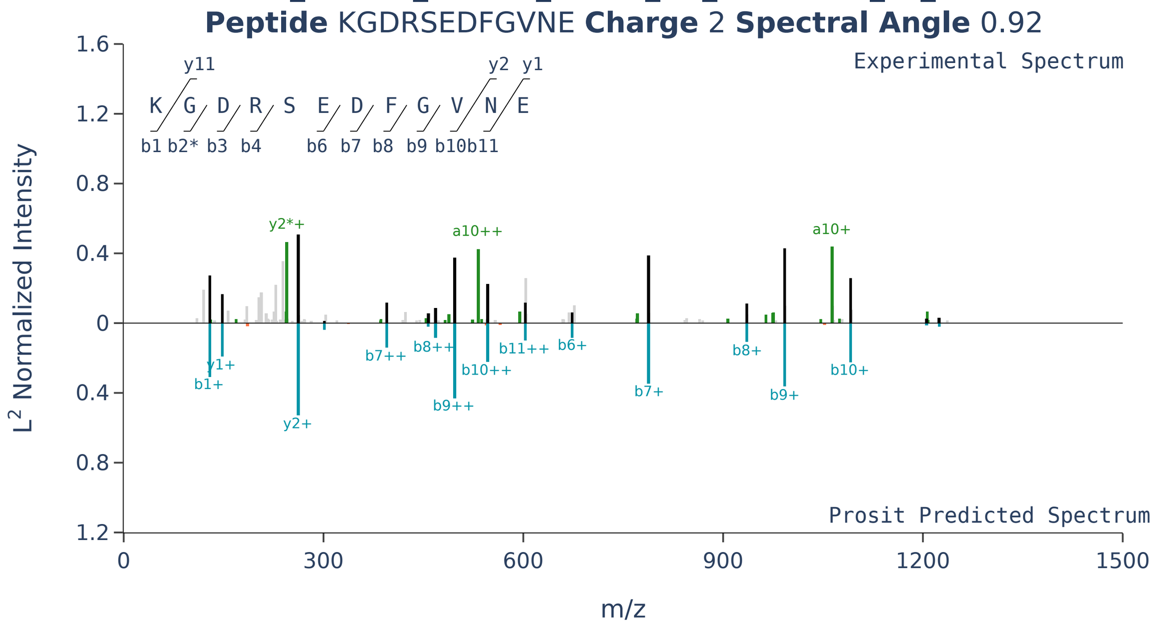


**Source** WSoh_301121_021221_HFGoe_M2_AnnexinA1_2h_R1 **Scan** 19692
**Peptide** ATSKPAFF **Charge** 2 **Spectral Angle** 0.91

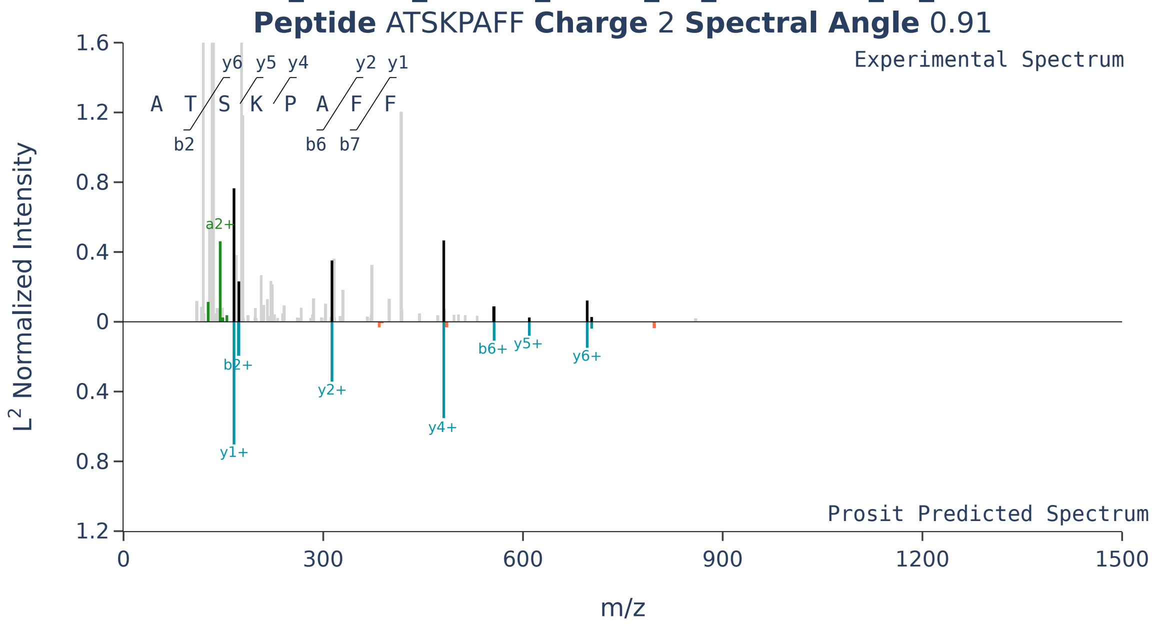


**Source** WSoh_301121_021221_HFGoe_M2_AnnexinA1_4h_R1 **Scan** 9221
**Peptide** LTGHLEE **Charge** 2 **Spectral Angle** 0.95

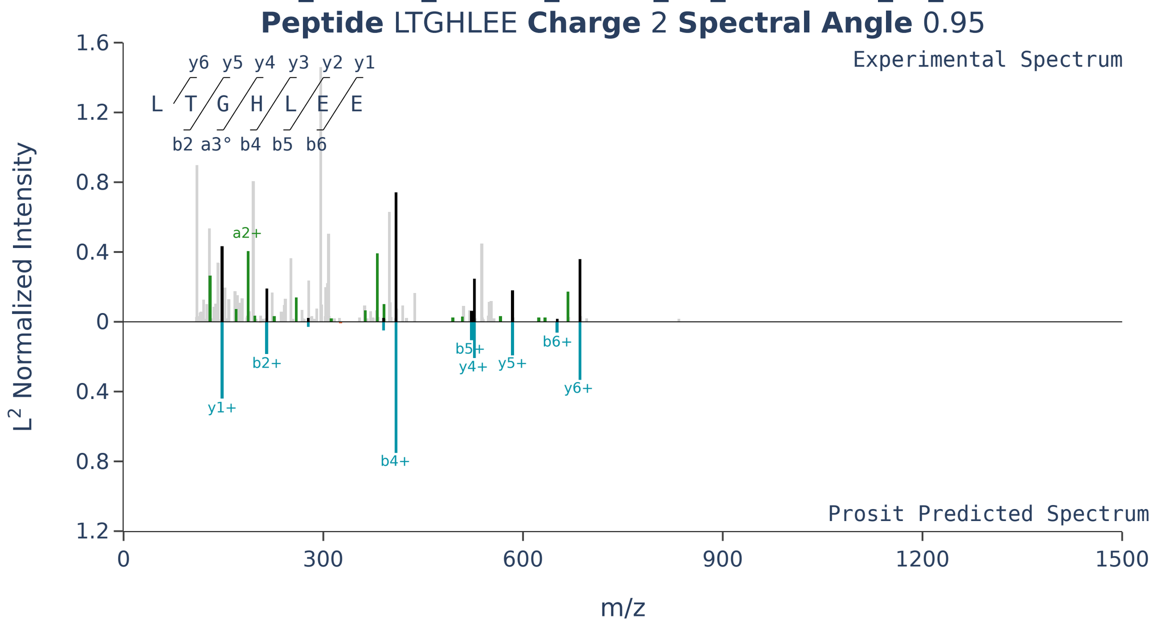


**Source** WSoh_301121_021221_HFGoe_M1_AnnexinA1_4h_R1 **Scan** 15950
**Peptide** TGKPLDETL **Charge** 2 **Spectral Angle** 0.92

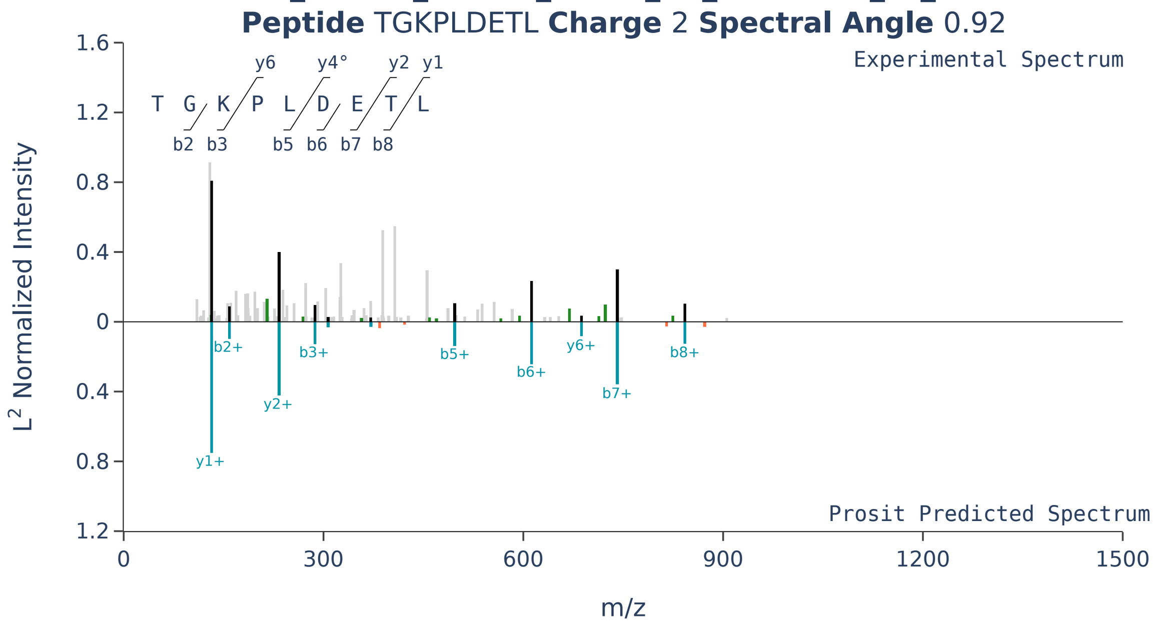


**Source** WSoh_301121_021221_HFGoe_M2_AnnexinA1_24h_R2 **Scan** 13781
**Peptide** RSEDFGV **Charge** 2 **Spectral Angle** 0.93

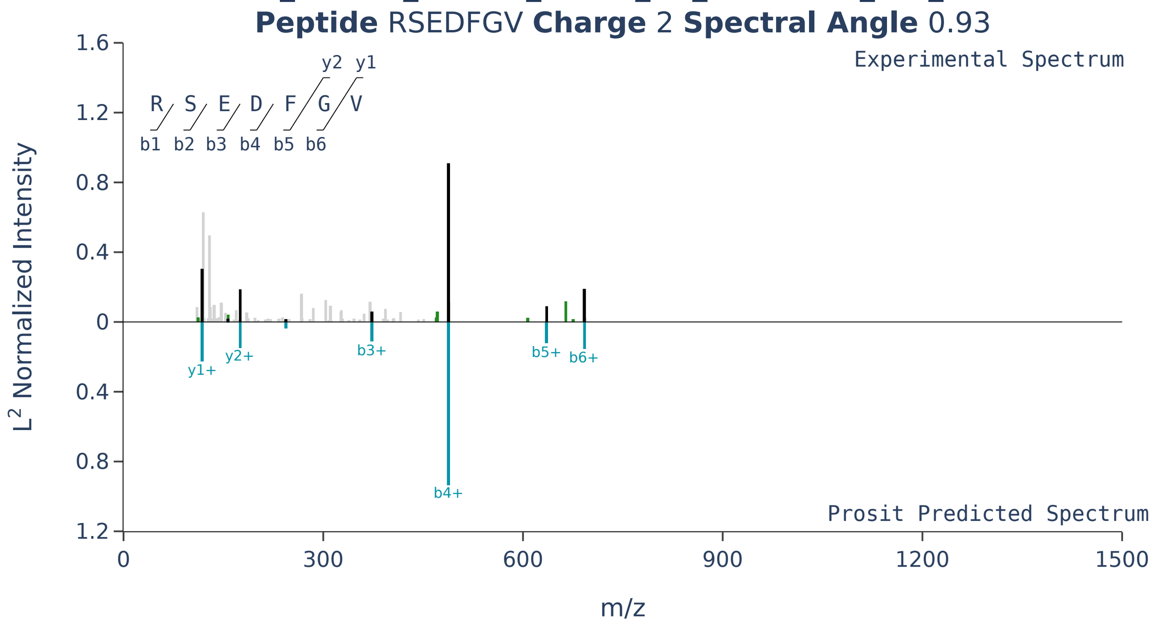


**Source** WSoh_301121_021221_HFGoe_M2_AnnexinA1_24h_R1 **Scan** 16172
**Peptide** TSGDFRNAL **Charge** 2 **Spectral Angle** 0.89

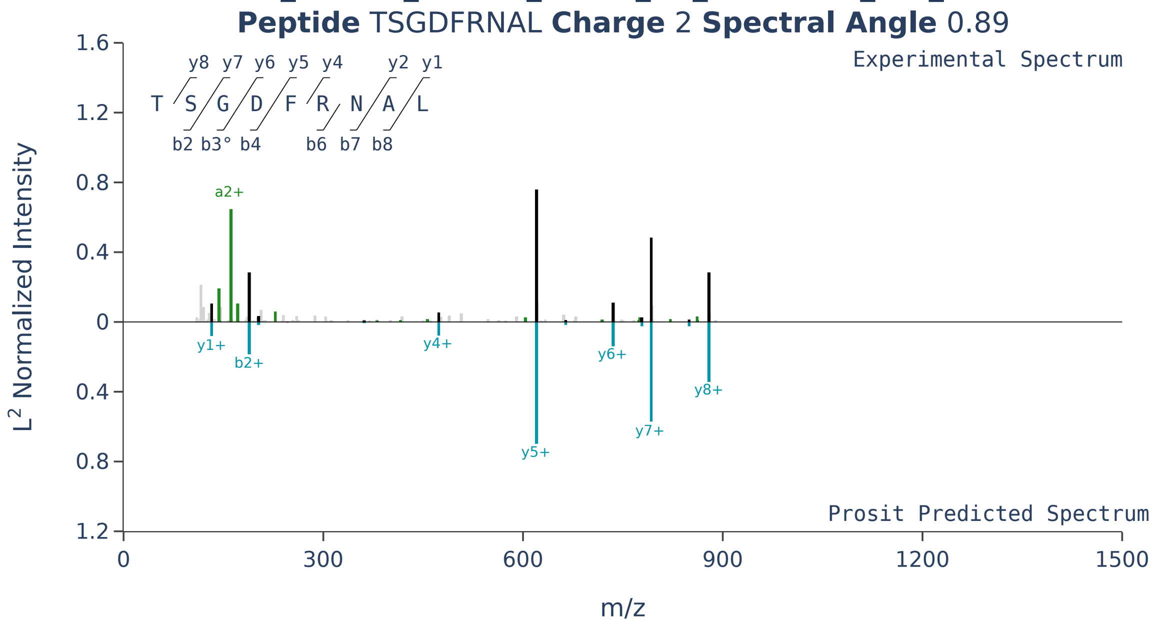


**Source** WSoh_301121_021221_HFGoe_M2_AnnexinA1_2h_R1 **Scan** 9188
**Peptide** RVFQKYTKY **Charge** 3 **Spectral Angle** 0.91

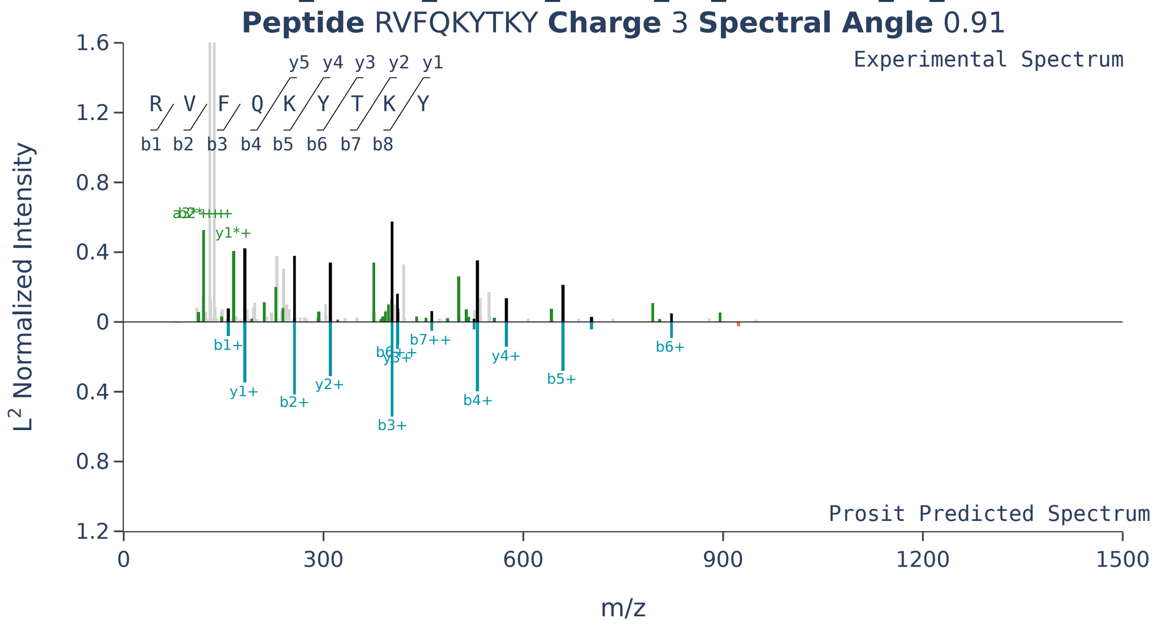

**Source** WSoh_301121_021221_HFGoe_M2_AnnexinA1_2h_R2 **Scan** 9627
**Peptide** DADELRA **Charge** 2 **Spectral Angle** 0.93

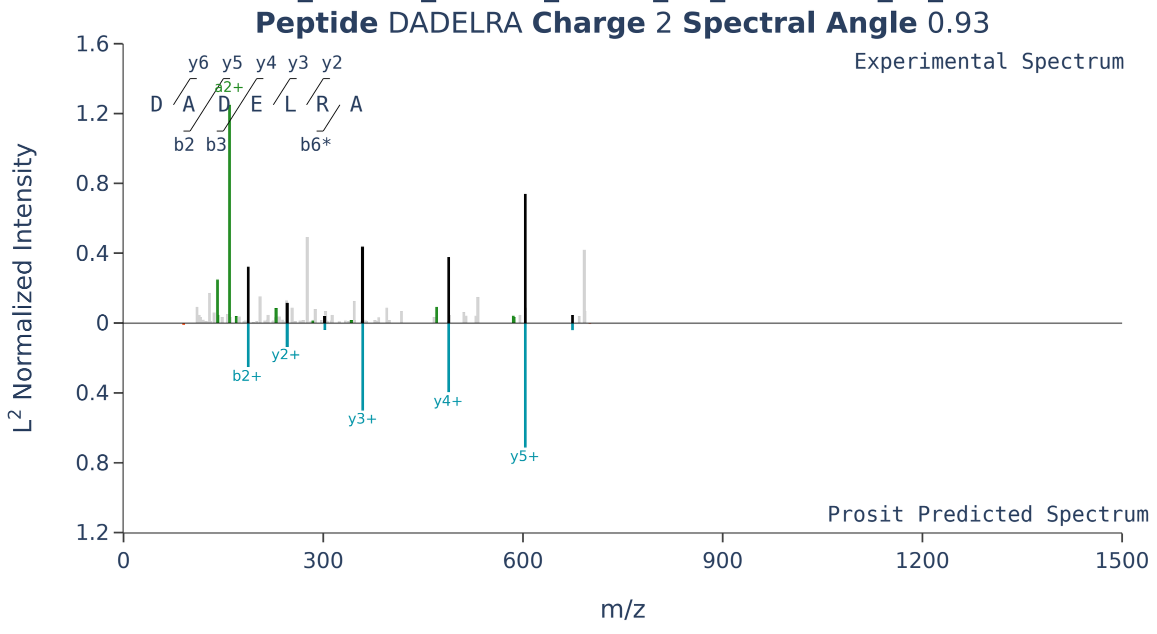


**Source** WSoh_301121_021221_HFGoe_M2_AnnexinA1_4h_R1 **Scan** 12946
**Peptide** IDILTKRN **Charge** 2 **Spectral Angle** 0.9

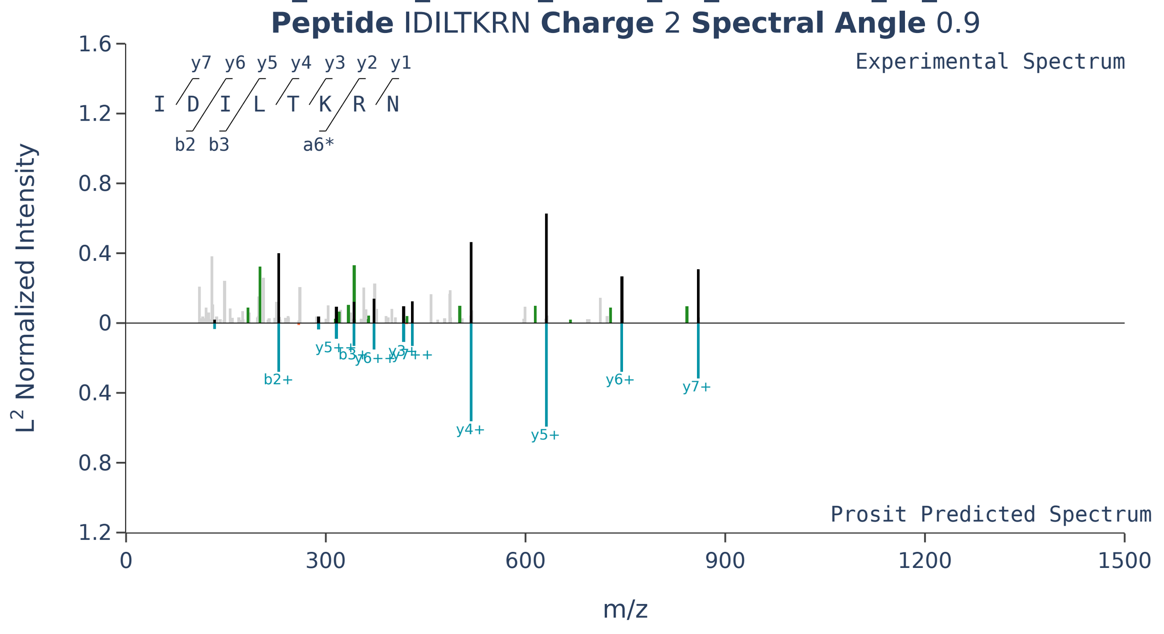


**Source** WSoh_301121_021221_HFGoe_M1_AnnexinA1_1h_R2 **Scan** 11869
**Peptide** RSEDFGVNE **Charge** 2 **Spectral Angle** 0.9

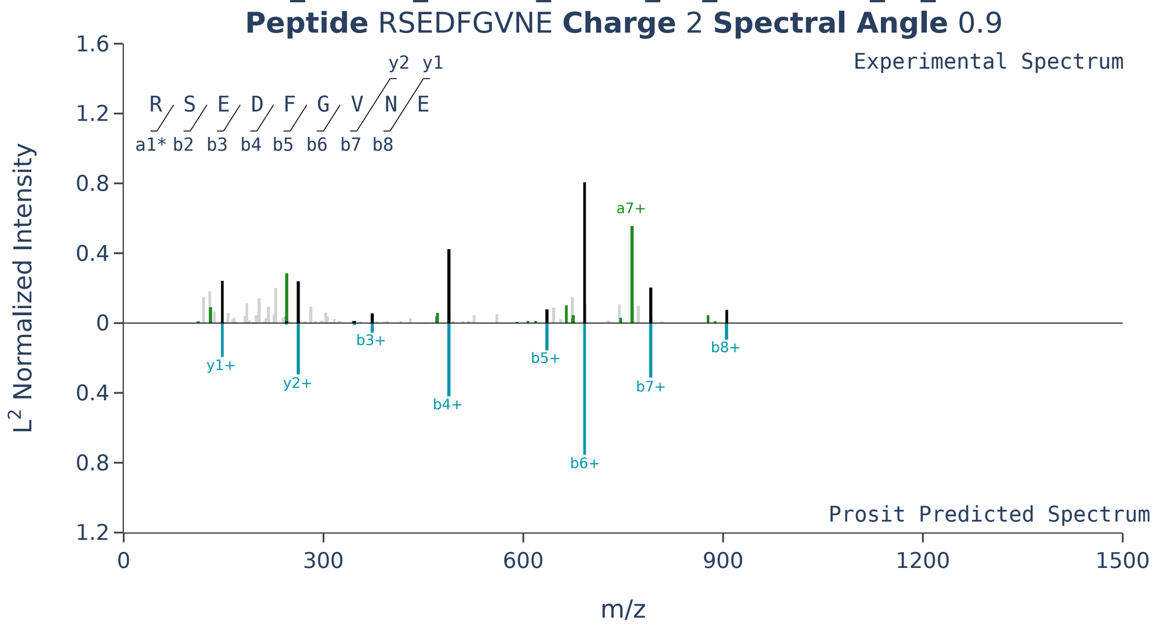


**Source** WSoh_301121_021221_HFGoe_M1_AnnexinA1_24h_R2 **Scan** 9400
**Peptide** ETLKKALTG **Charge** 1 **Spectral Angle** 0.89

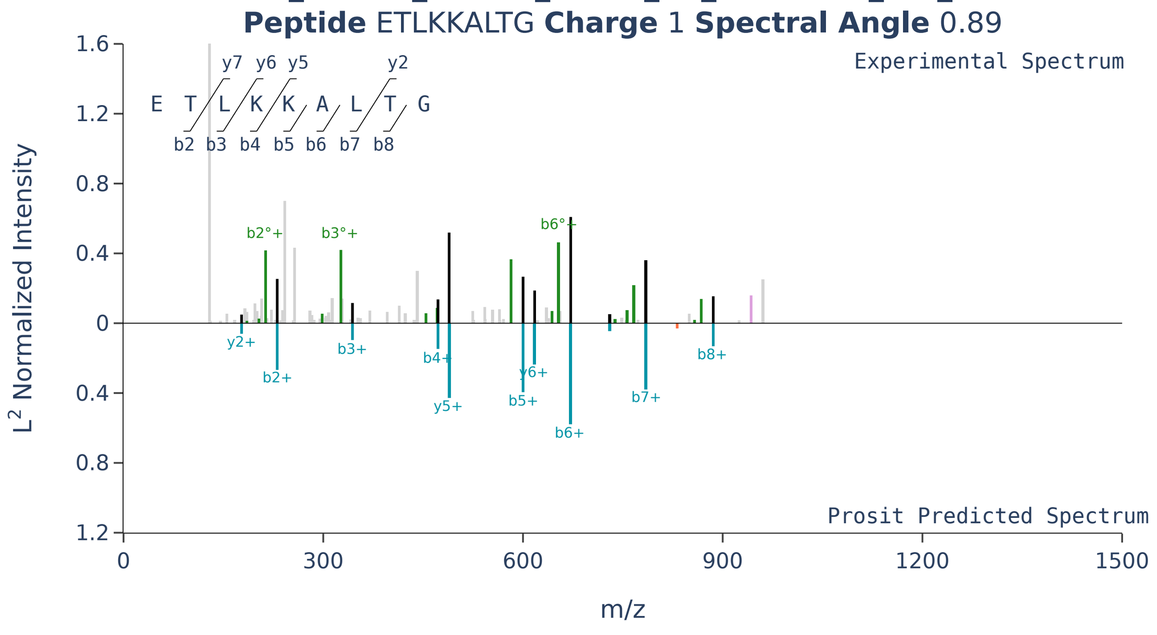


**Source** WSoh_301121_021221_HFGoe_M1_AnnexinA1_24h_R1 **Scan** 12846
**Peptide** GDRSEDFGVNE **Charge** 2 **Spectral Angle** 0.91

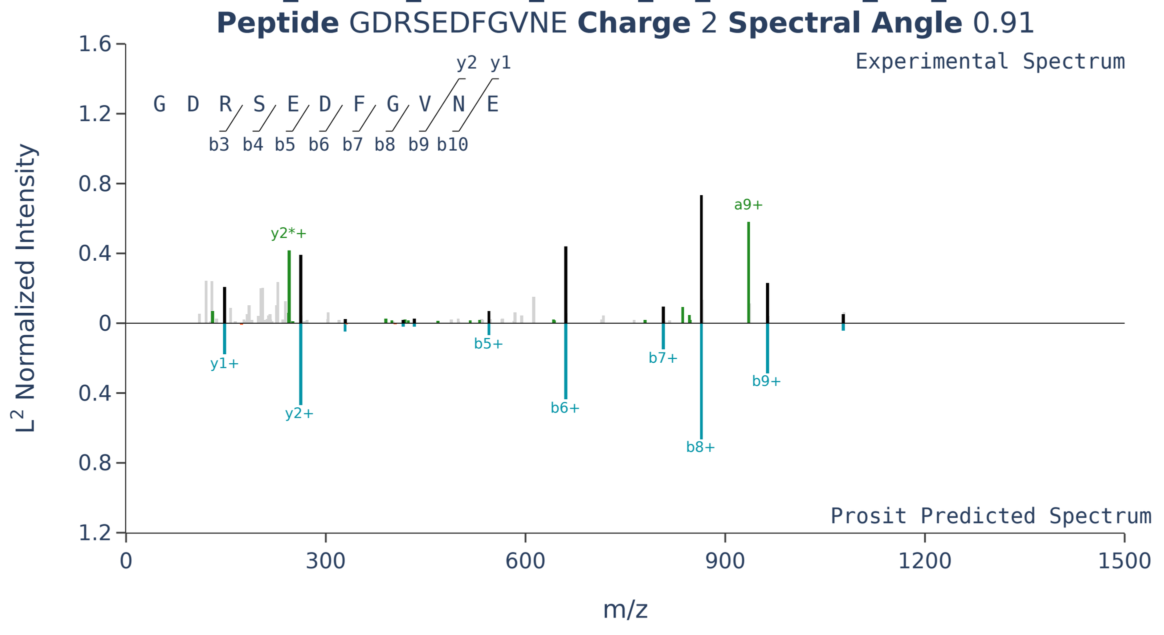


**Source** WSoh_301121_021221_HFGoe_M1_AnnexinA1_24h_R1 **Scan** 18286
**Peptide** AKDITSDTSGDFRNAL **Charge** 2 **Spectral Angle** 0.92

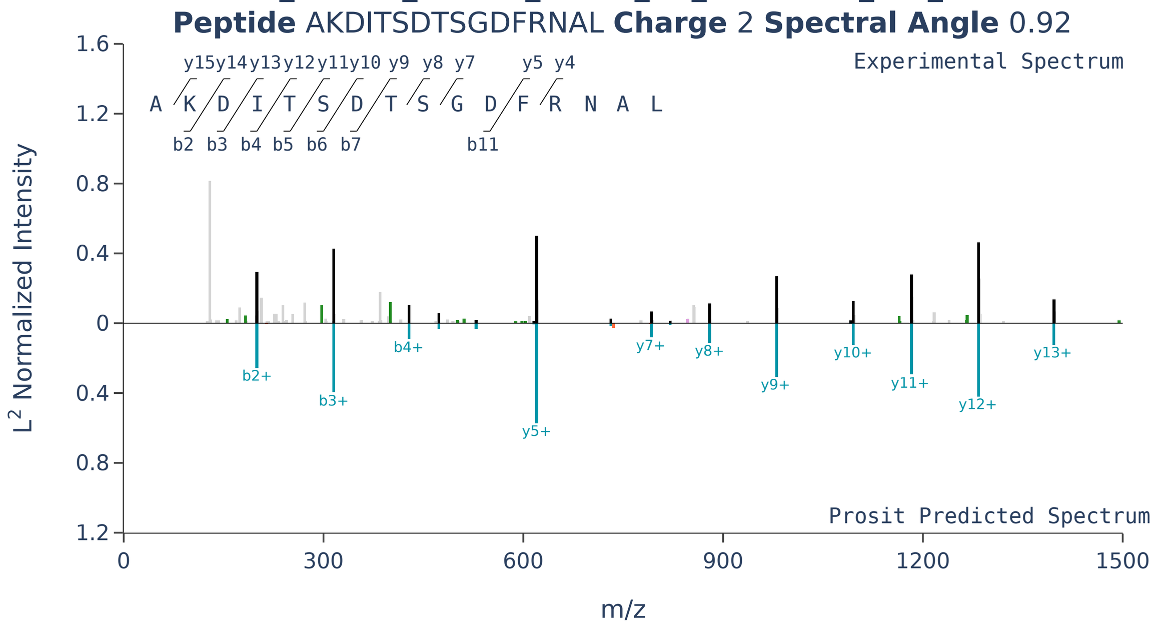


**Source** WSoh_301121_021221_HFGoe_M1_AnnexinA1_2h_R2 **Scan** 8252
**Peptide** DILTKRNNA **Charge** 2 **Spectral Angle** 0.93

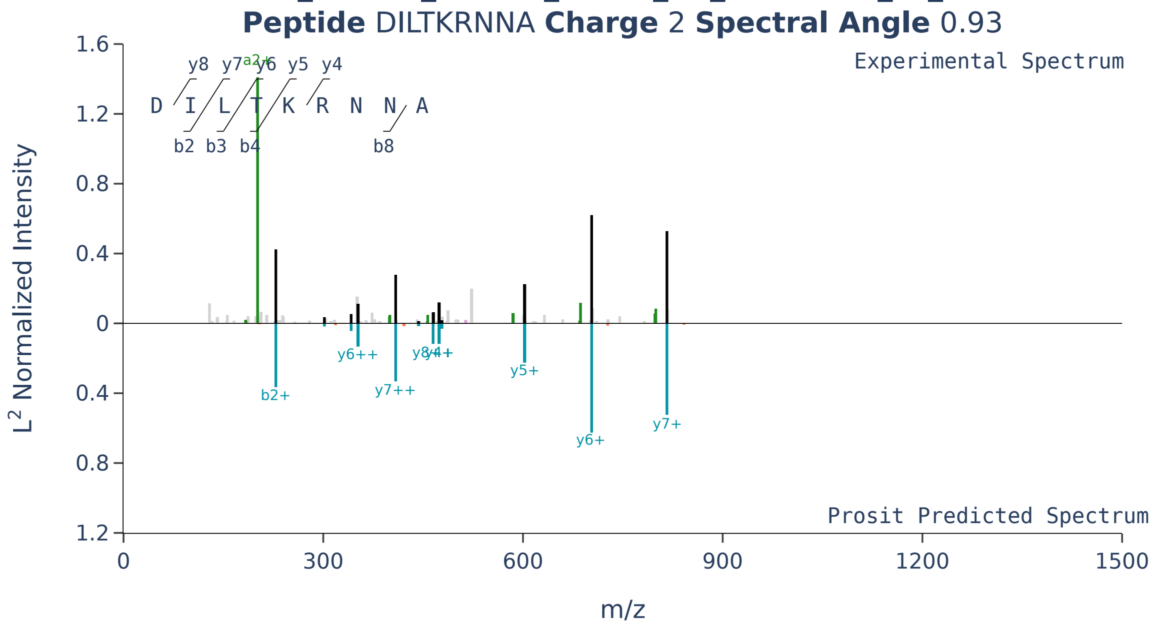


**Source** WSoh_301121_021221_HFGoe_M1_AnnexinA1_1h_R1 **Scan** 10504
**Peptide** ADELRAAMKG **Charge** 2 **Spectral Angle** 0.93

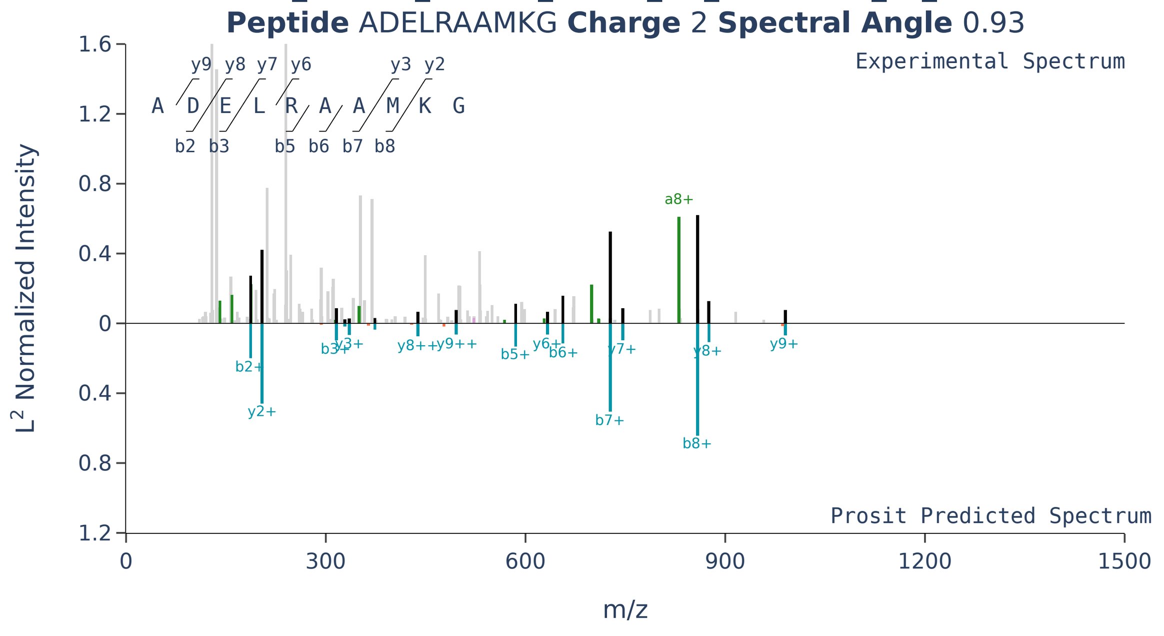


**Source** WSoh_301121_021221_HFGoe_M2_AnnexinA1_24h_R2 **Scan** 12302
**Peptide** LLSLAKGDRS **Charge** 2 **Spectral Angle** 0.9

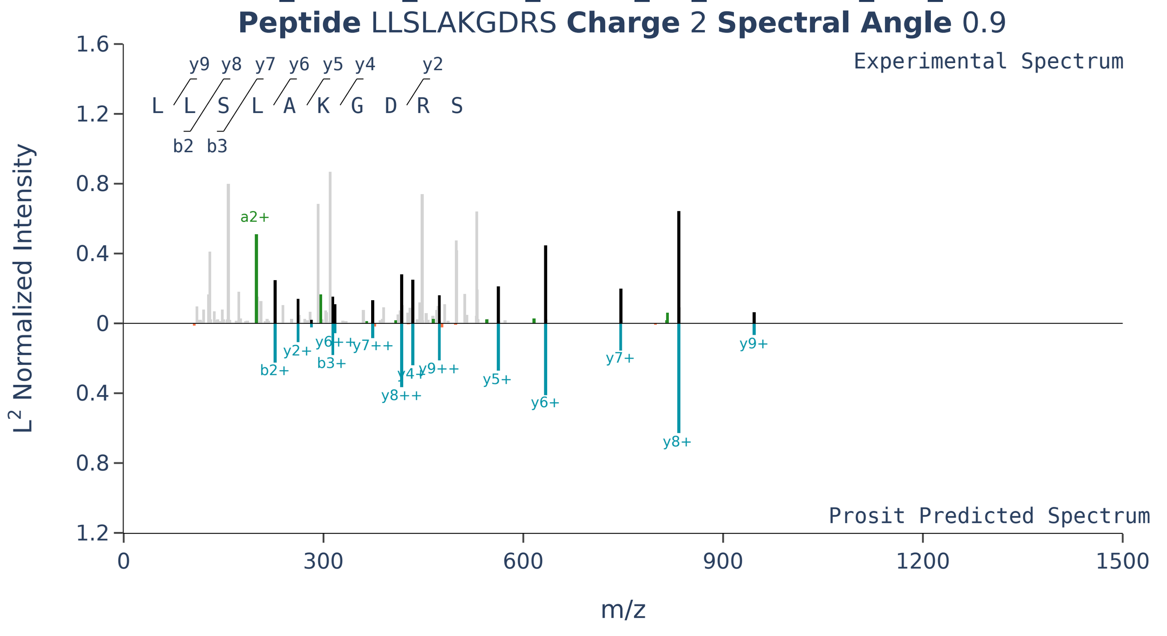


**Source** WSoh_301121_021221_HFGoe_M2_AnnexinA1_4h_R1 **Scan** 8346
**Peptide** TSDTSGDFRNA **Charge** 2 **Spectral Angle** 0.9

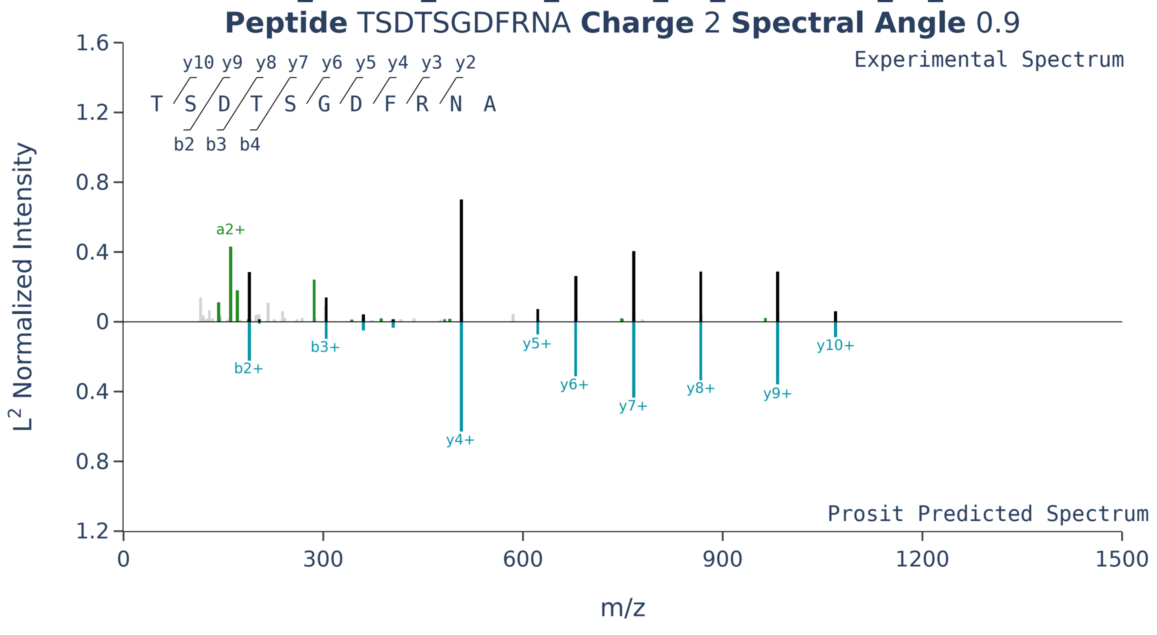


**Source** WSoh_301121_021221_HFGoe_M1_AnnexinA1_4h_R2 **Scan** 8113
**Peptide** LAKDITSD **Charge** 2 **Spectral Angle** 0.93

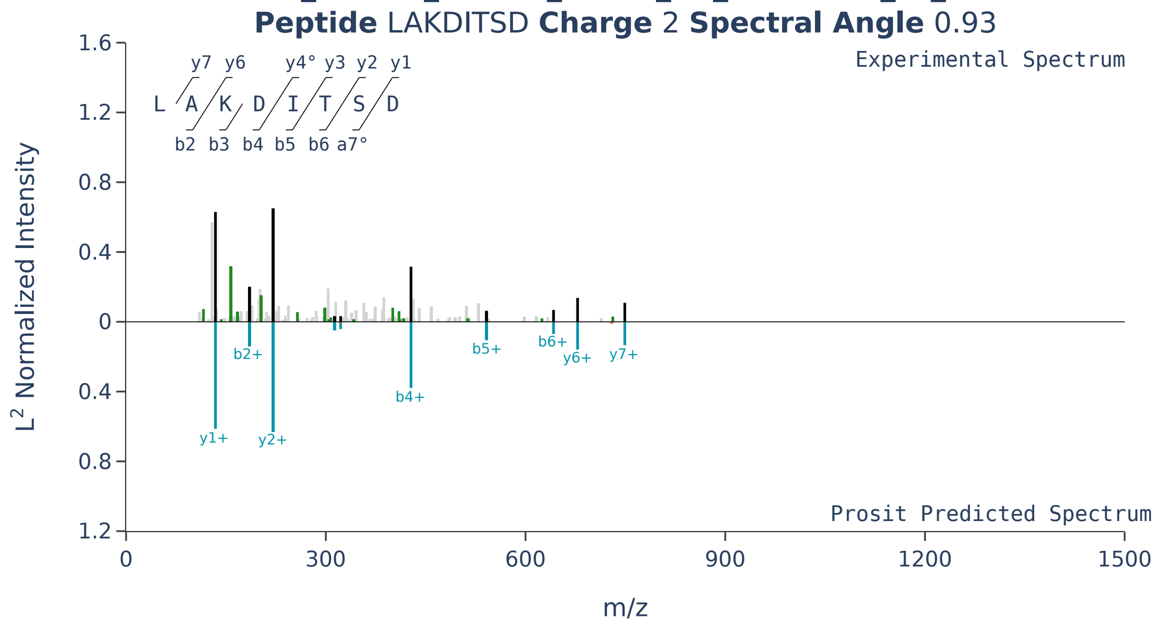


**Source** WSoh_301121_021221_HFGoe_M2_AnnexinA1_24h_R1 **Scan** 18669
**Peptide** IIDILTKRN **Charge** 2 **Spectral Angle** 0.92

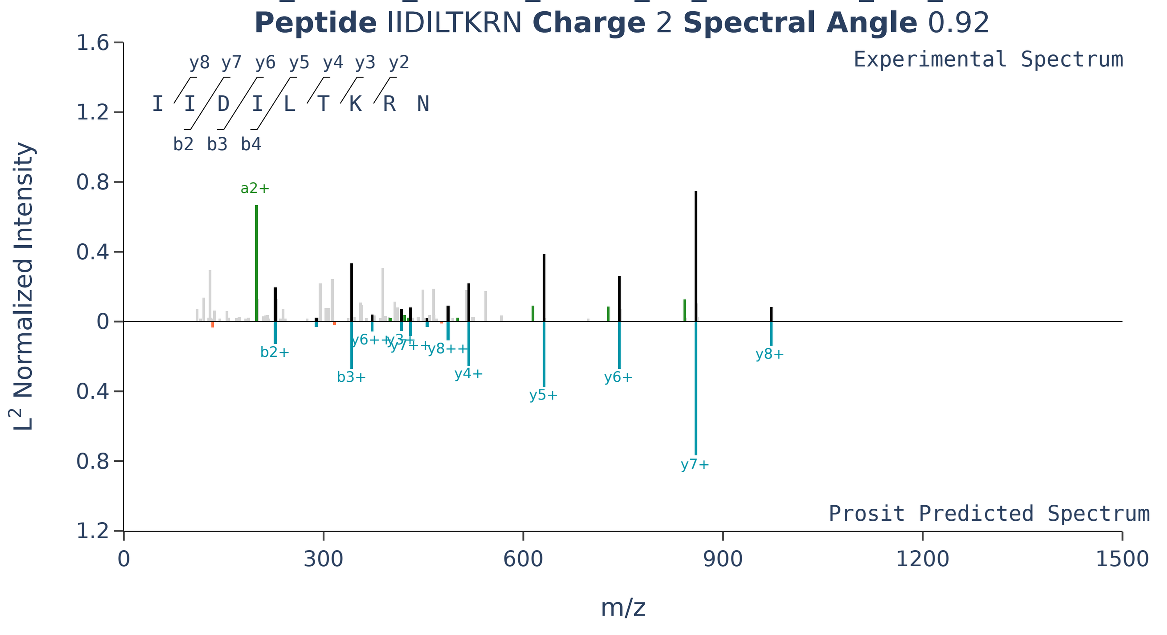


**Source** WSoh_301121_021221_HFGoe_M1_AnnexinA1_4h_R1 **Scan** 14402
**Peptide** KGDYEKIL **Charge** 2 **Spectral Angle** 0.91

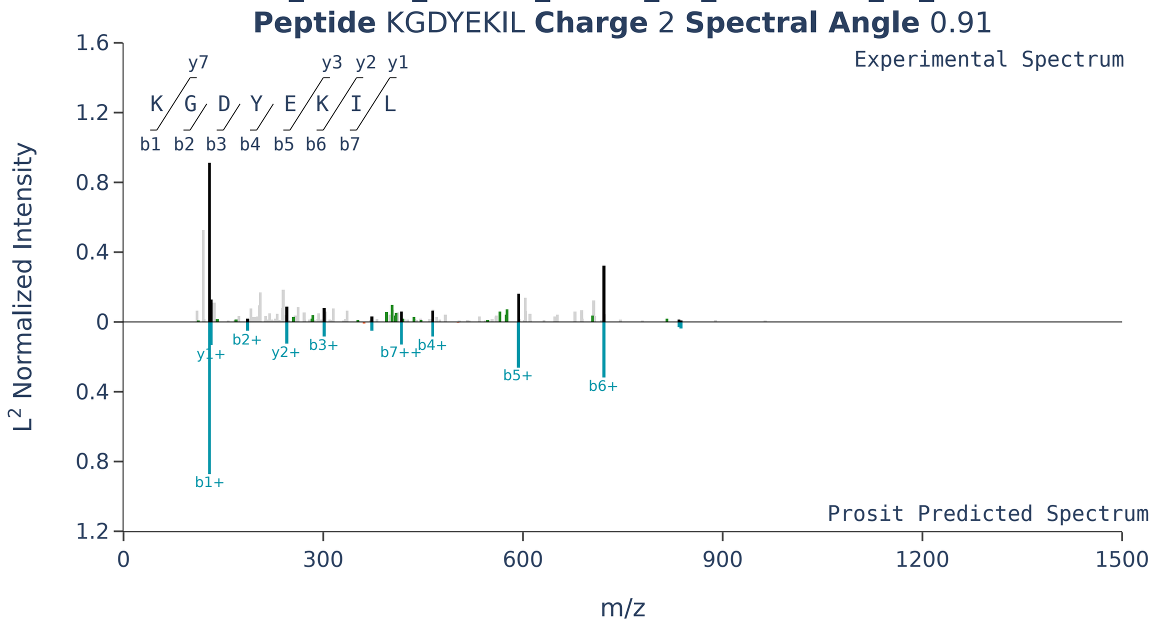


**Source** WSoh_301121_021221_HFGoe_M2_AnnexinA1_24h_R1 **Scan** 18528
**Peptide** KGVDEATII **Charge** 1 **Spectral Angle** 0.9

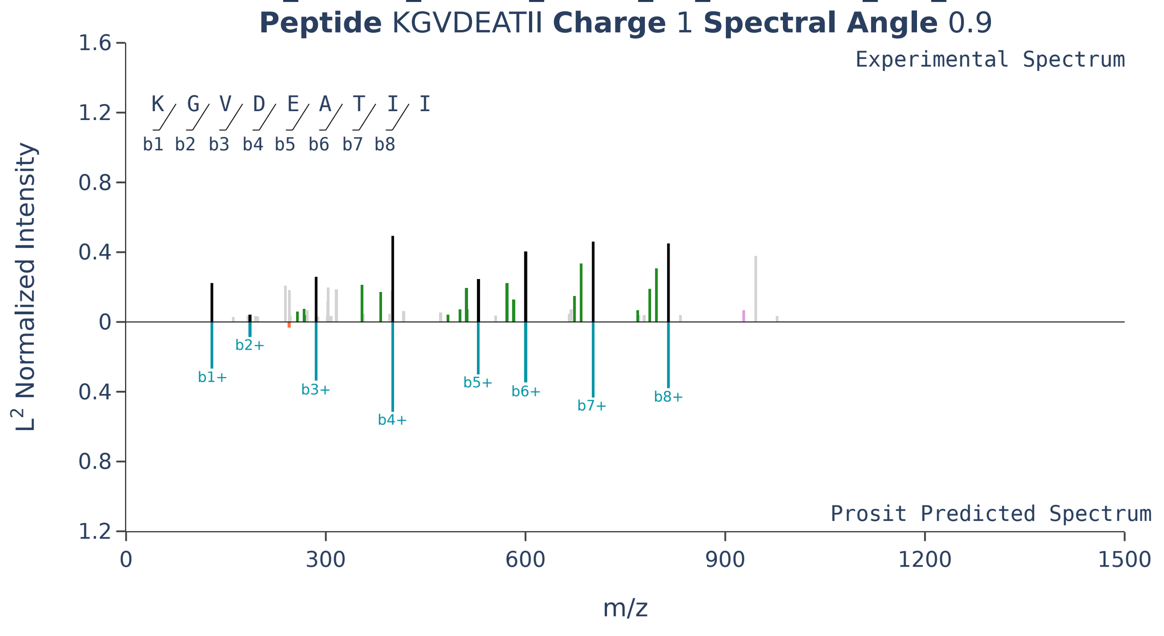


**Source** WSoh_301121_021221_HFGoe_M1_AnnexinA1_24h_R1 **Scan** 8891
**Peptide** RSYPQLR **Charge** 2 **Spectral Angle** 0.94

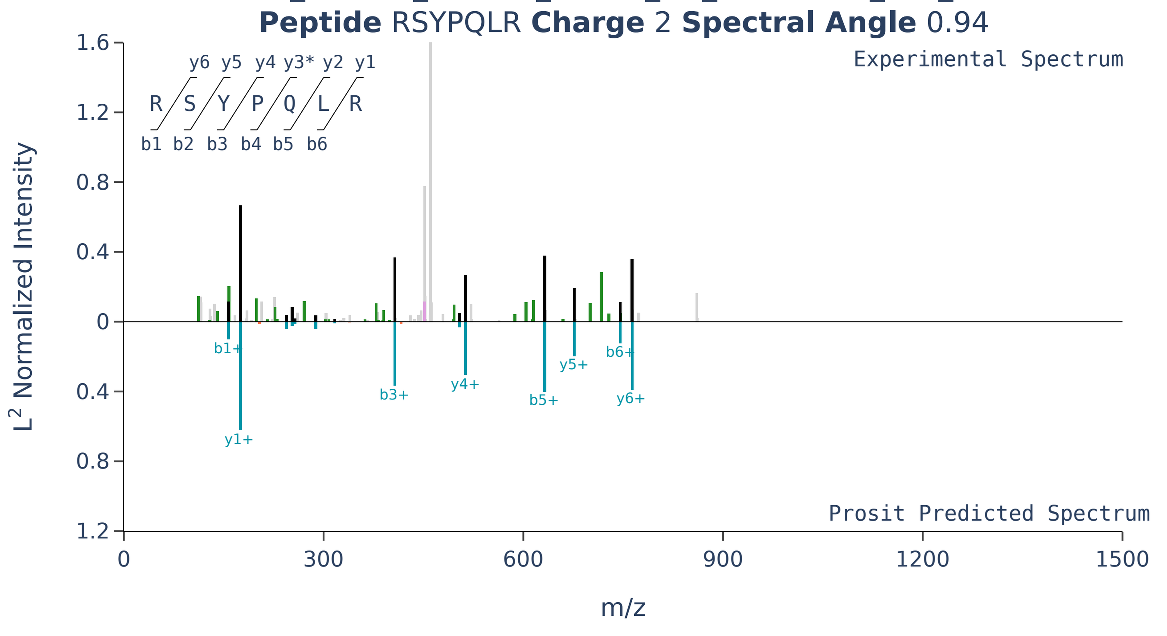

**Source** WSoh_301121_021221_HFGoe_M1_AnnexinA1_4h_R2 **Scan** 9675
**Peptide** DELRAAMK **Charge** 2 **Spectral Angle** 0.94

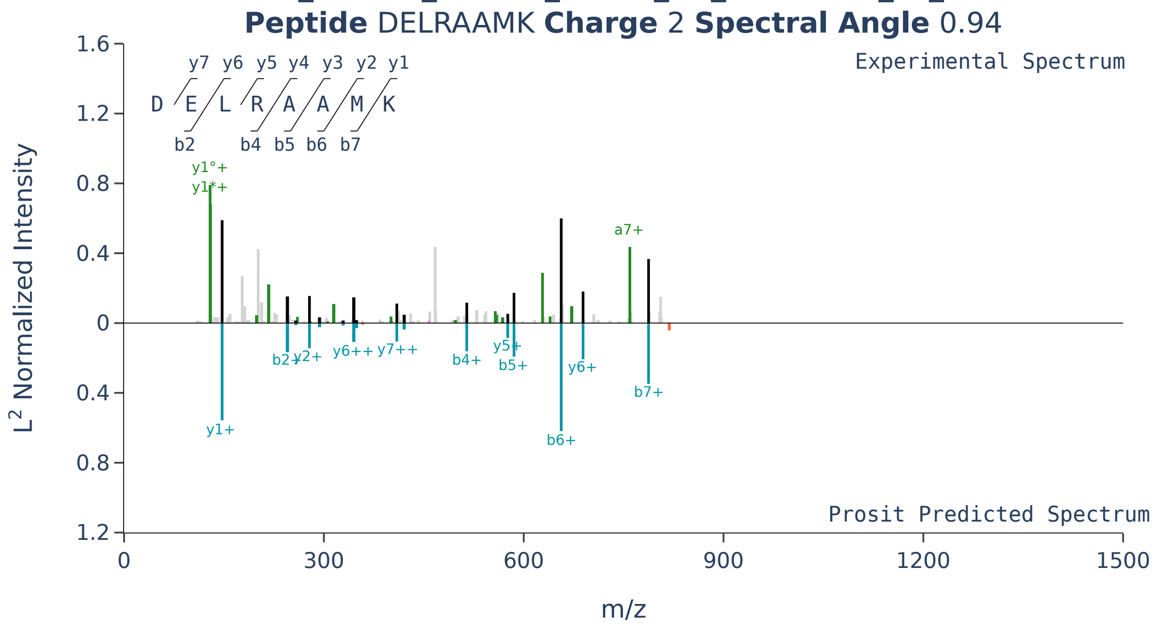


**Source** WSoh_301121_021221_HFGoe_M2_AnnexinA1_4h_R1 **Scan** 7500
**Peptide** ELKRDLA **Charge** 2 **Spectral Angle** 0.88

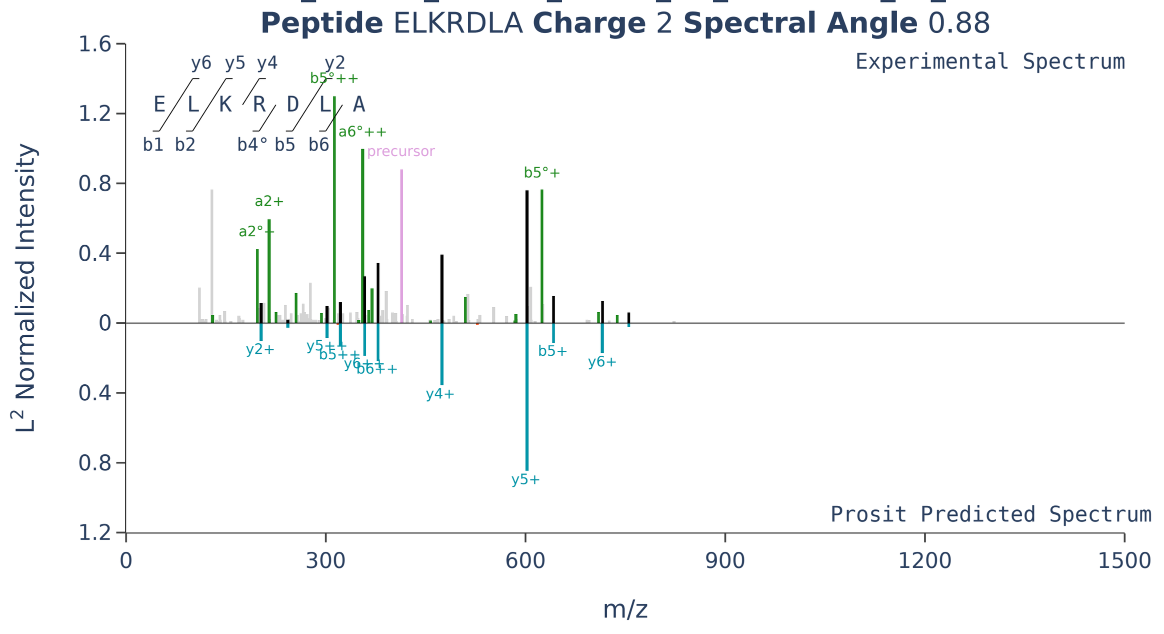


**Source** WSoh_301121_021221_HFGoe_M2_AnnexinA1_24h_R1 **Scan** 8225
**Peptide** LSLAKGDRSED **Charge** 2 **Spectral Angle** 0.93

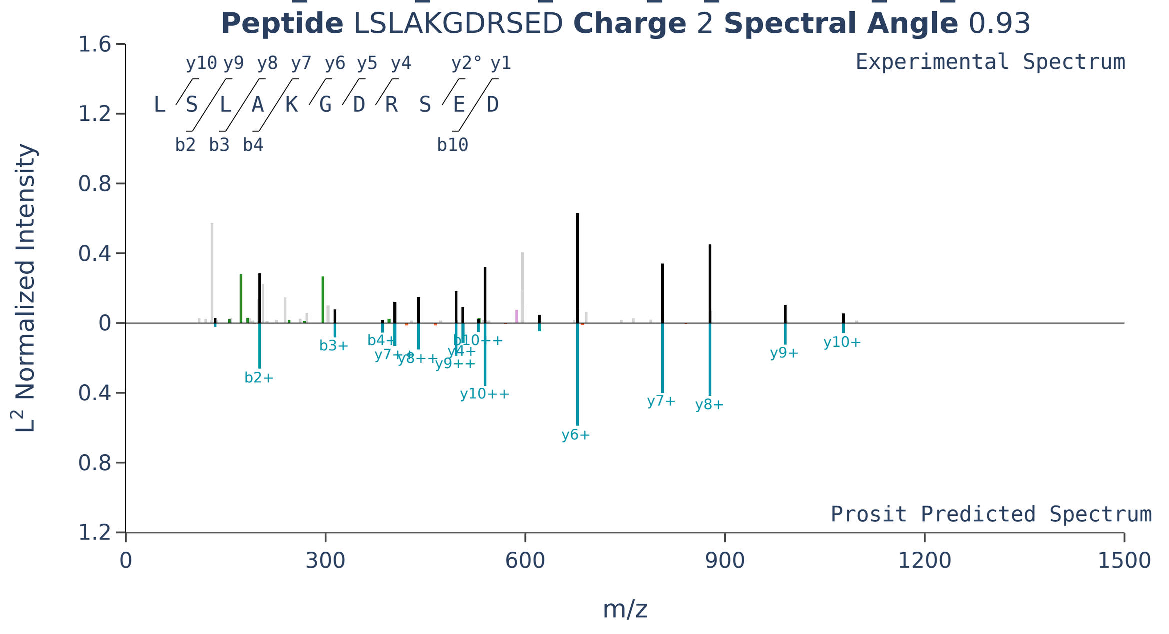


**Source** WSoh_301121_021221_HFGoe_M2_AnnexinA1_24h_R1 **Scan** 12848
**Peptide** ADSDARALY **Charge** 2 **Spectral Angle** 0.88

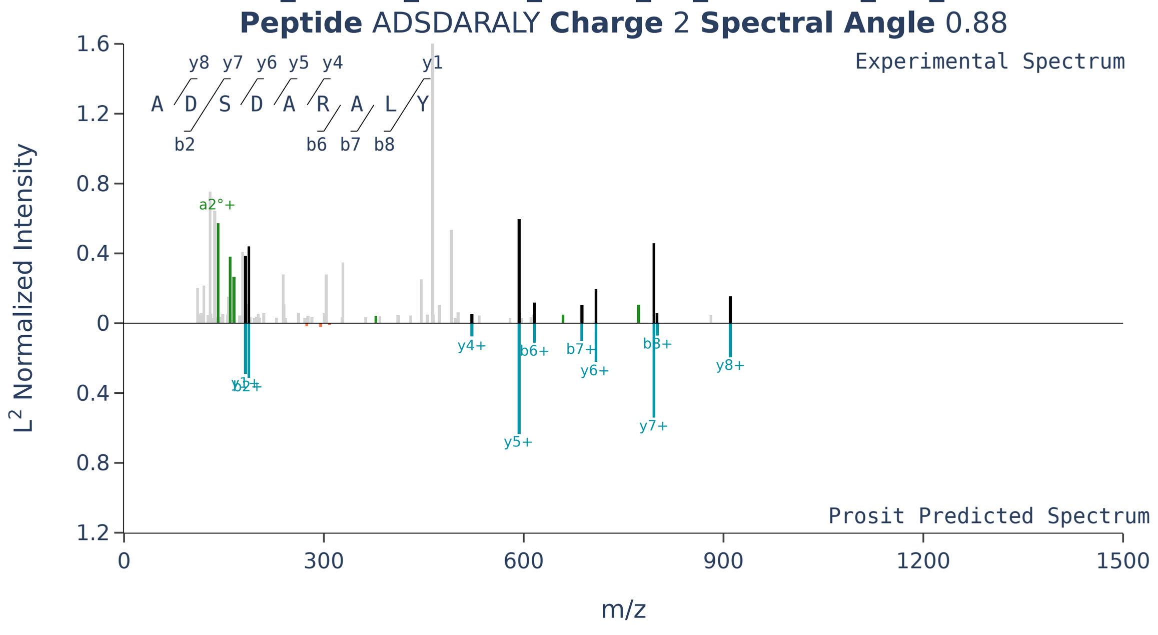


**Source** WSoh_301121_021221_HFGoe_M2_AnnexinA1_2h_R1 **Scan** 10529
**Peptide** EQEYVQTVKS **Charge** 1 **Spectral Angle** 0.92

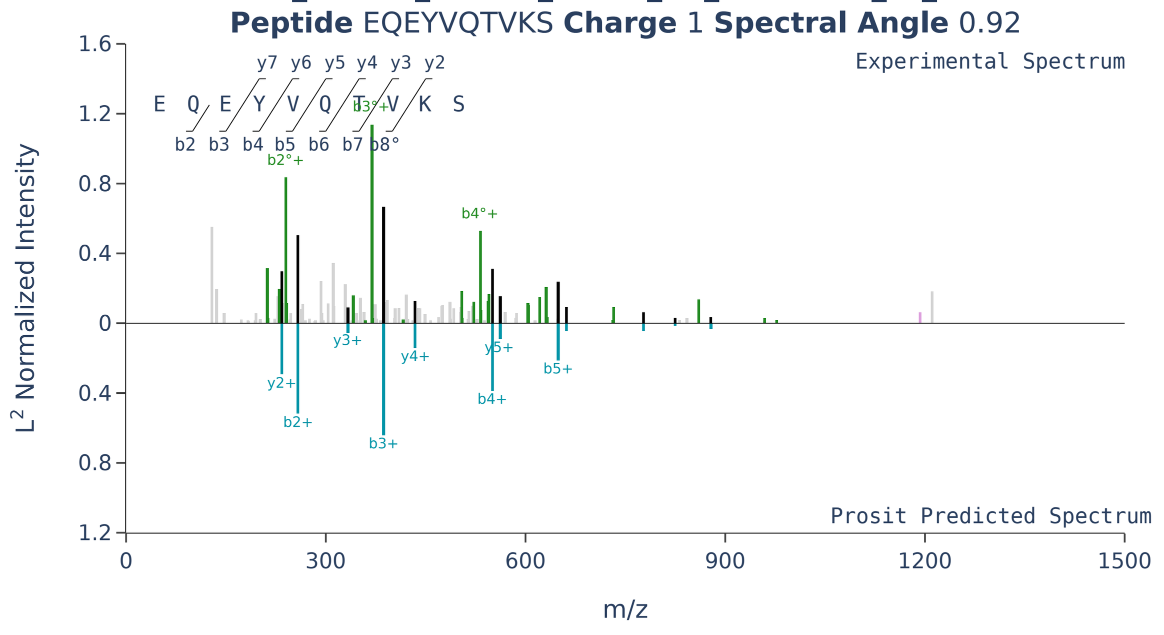


**Source** WSoh_301121_021221_HFGoe_M1_AnnexinA1_24h_R2 **Scan** 11841
**Peptide** QAILDETKG **Charge** 1 **Spectral Angle** 0.91

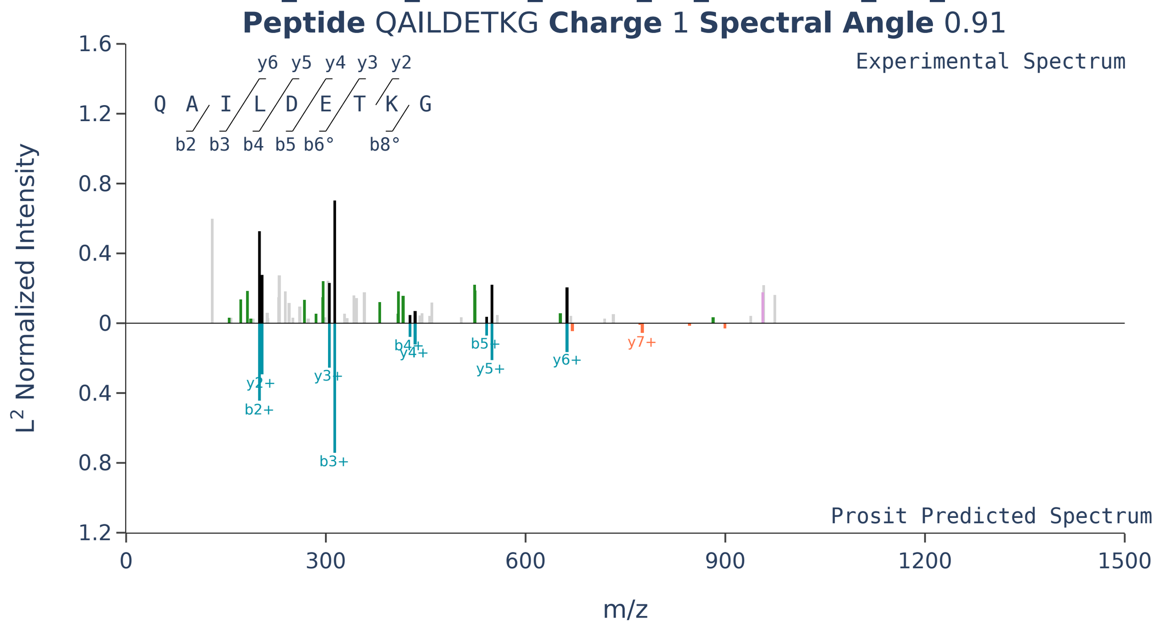


**Source** WSoh_301121_021221_HFGoe_M2_AnnexinA1_1h_R1 **Scan** 10321
**Peptide** EQEYVQTVKSS **Charge** 2 **Spectral Angle** 0.95

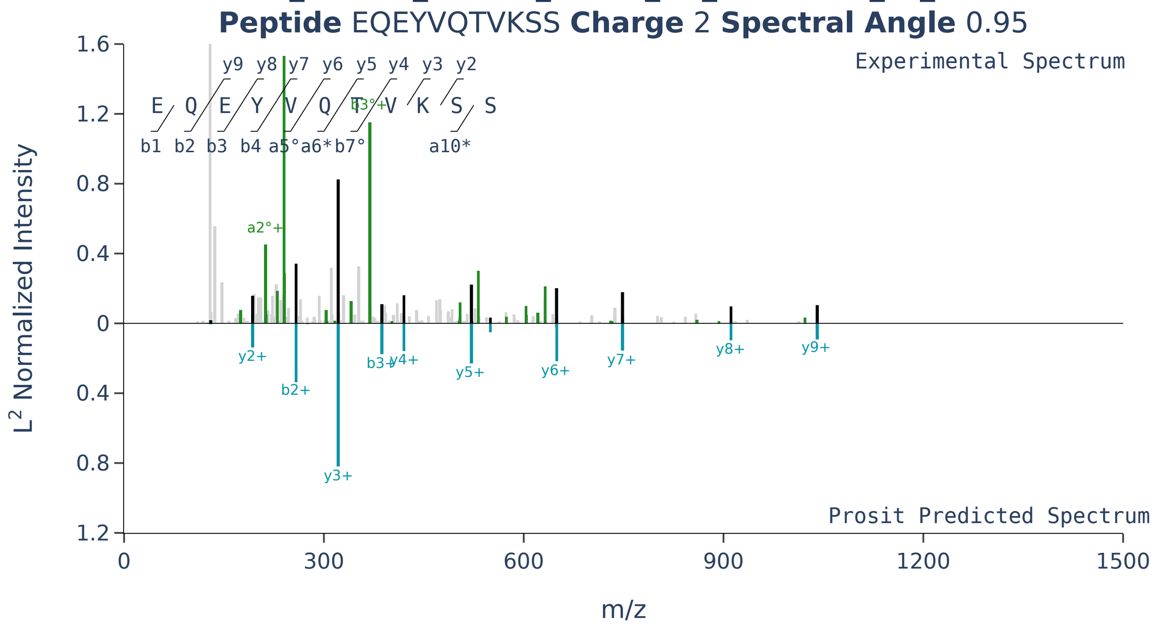


**Source** WSoh_301121_021221_HFGoe_M2_AnnexinA1_24h_R2 **Scan** 5836
**Peptide** RSEIDMN **Charge** 2 **Spectral Angle** 0.95

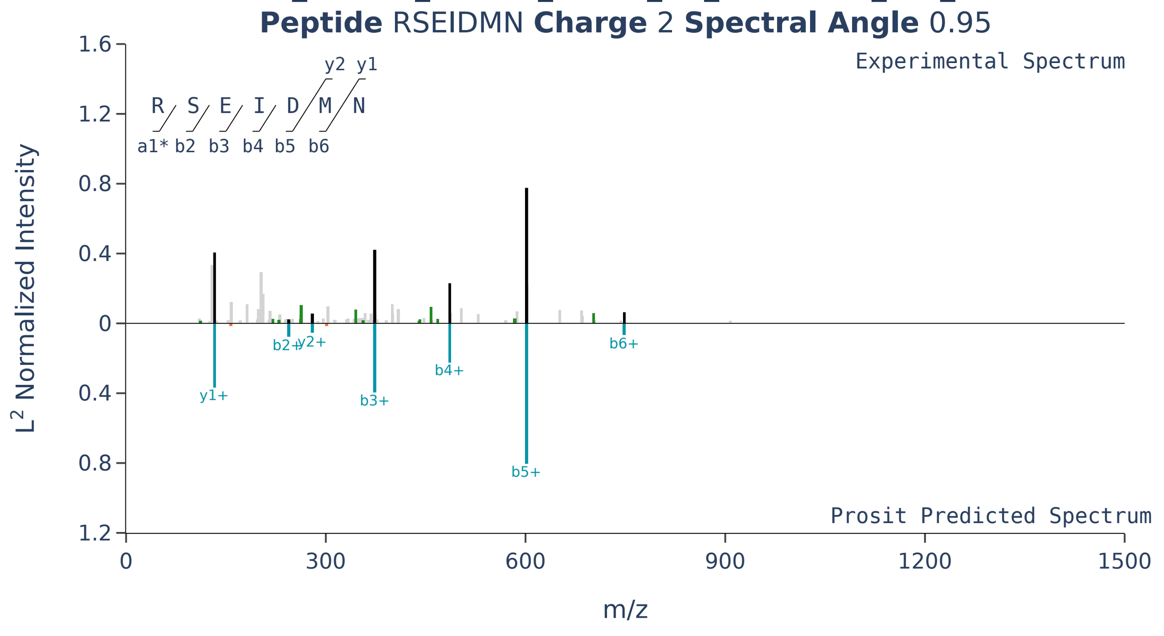


**Source** WSoh_301121_021221_HFGoe_M1_AnnexinA1_24h_R2 **Scan** 15260
**Peptide** DIEKCLTA **Charge** 2 **Spectral Angle** 0.91

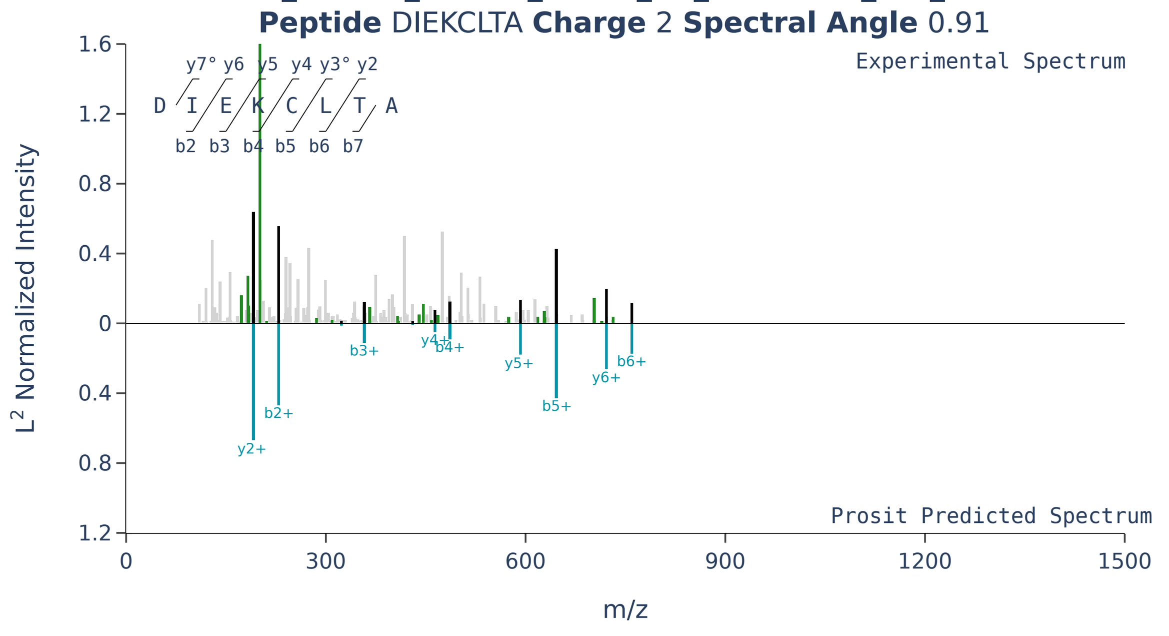


**Source** WSoh_301121_021221_HFGoe_M2_AnnexinA1_24h_R1 **Scan** 9774
**Peptide** DMNKVLD **Charge** 2 **Spectral Angle** 0.91

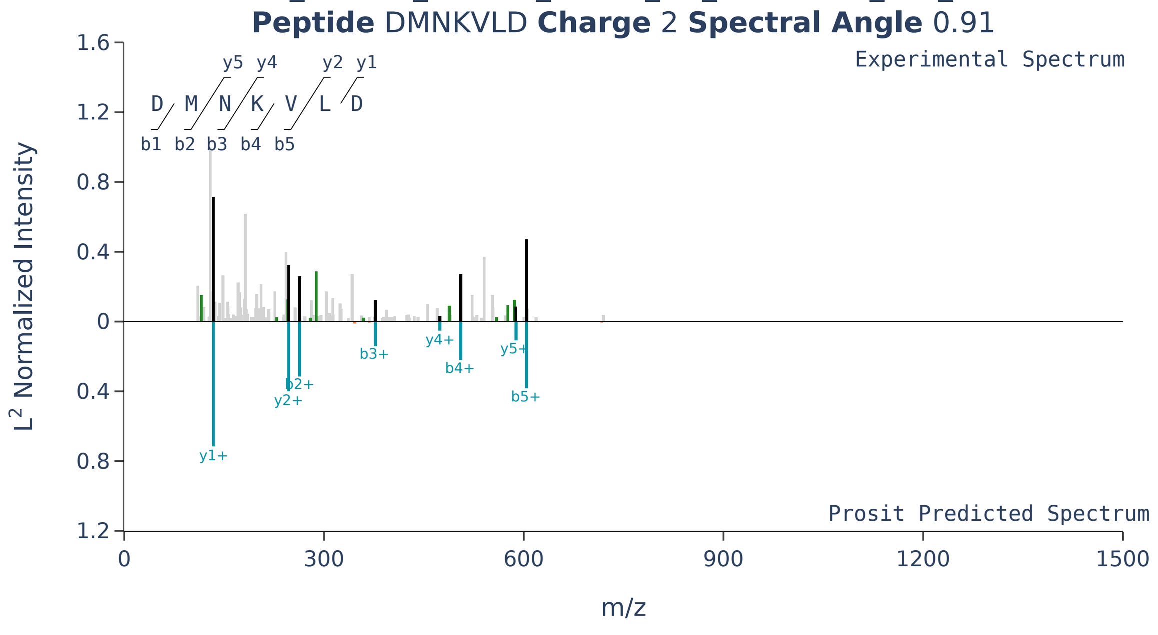


**Source** WSoh_301121_021221_HFGoe_M2_AnnexinA1_4h_R1 **Scan** 10193
**Peptide** KGDRSEDFGVNED **Charge** 2 **Spectral Angle** 0.9

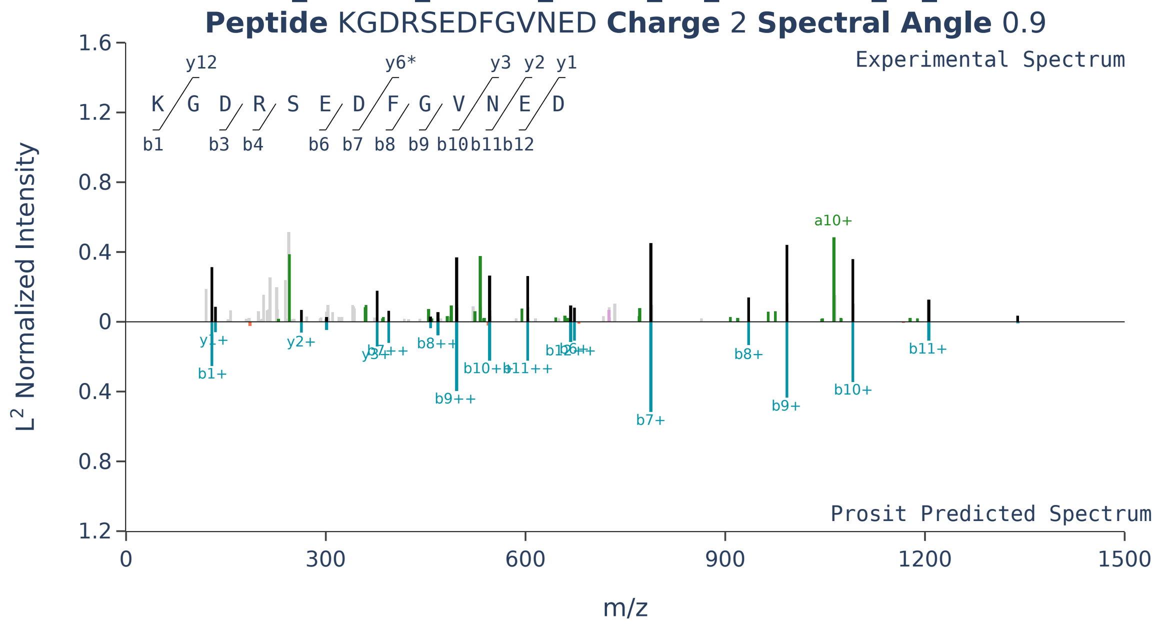


**Source** WSoh_301121_021221_HFGoe_M1_AnnexinA1_24h_R1 **Scan** 12496
**Peptide** INRVYREEL **Charge** 2 **Spectral Angle** 0.93

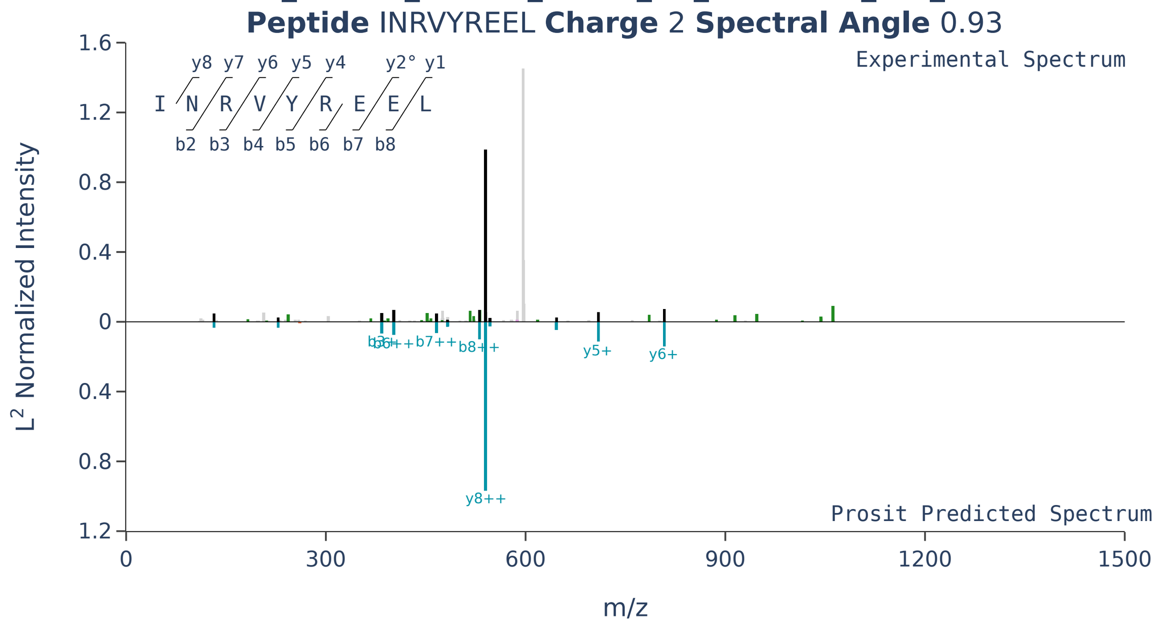


**Source** WSoh_301121_021221_HFGoe_M2_AnnexinA1_2h_R1 **Scan** 24518
**Peptide** DMNKVLDLELKG **Charge** 2 **Spectral Angle** 0.88

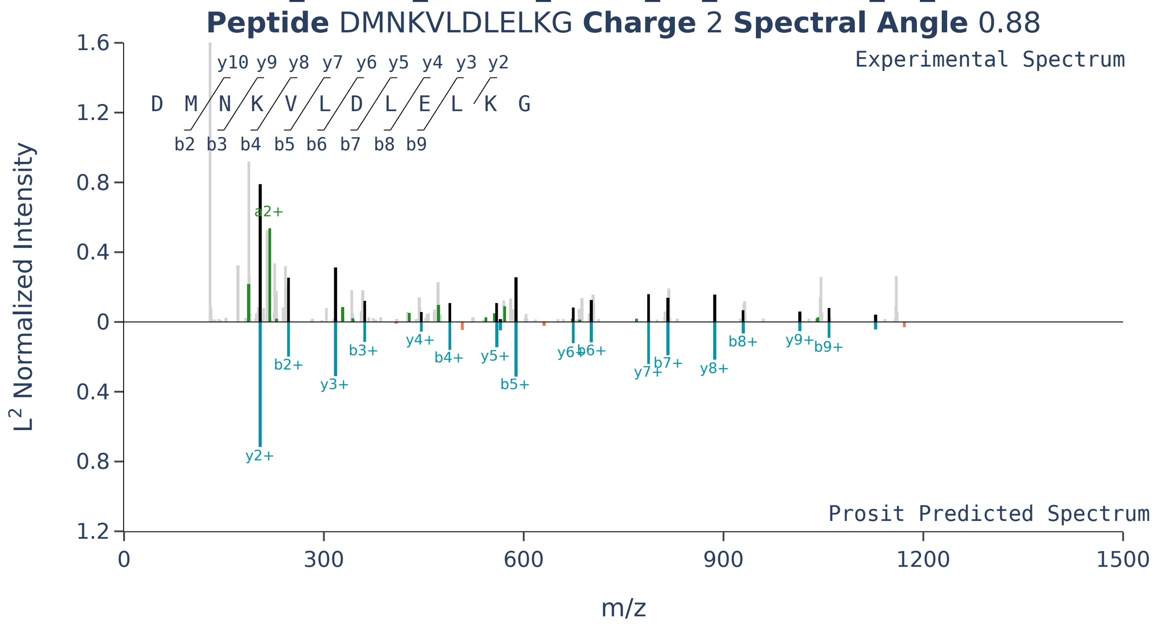


**Source** WSoh_301121_021221_HFGoe_M2_AnnexinA1_24h_R2 **Scan** 12640
**Peptide** IDILTKRNN **Charge** 2 **Spectral Angle** 0.89

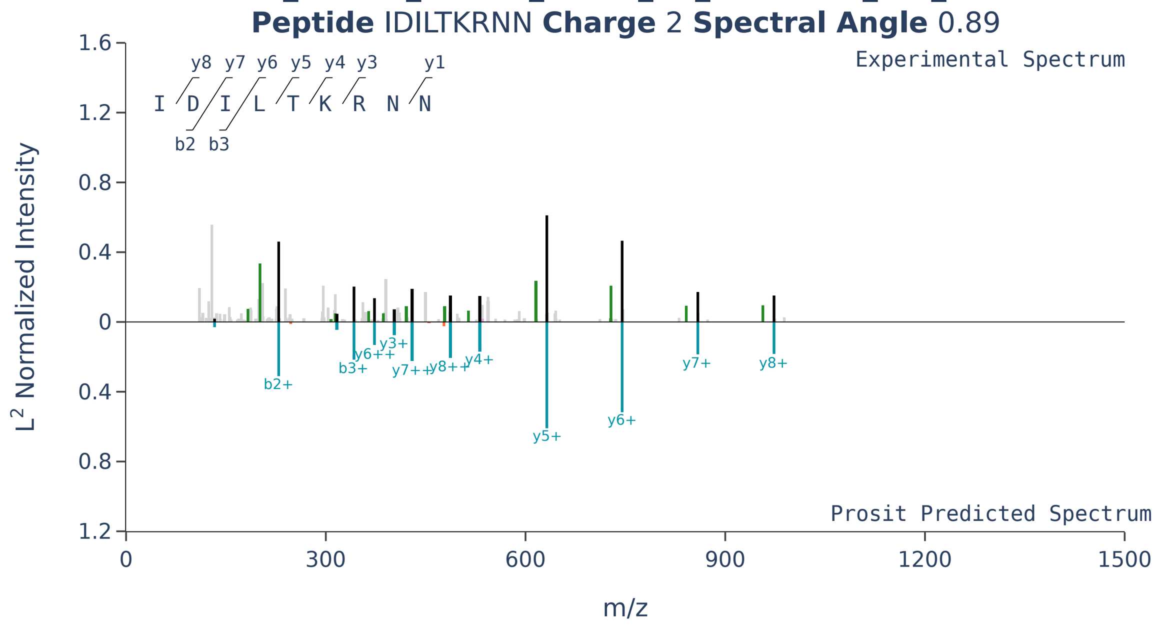


**Source** WSoh_301121_021221_HFGoe_M2_AnnexinA1_24h_R1 **Scan** 20625
**Peptide** SKPAFFAEKL **Charge** 2 **Spectral Angle** 0.91

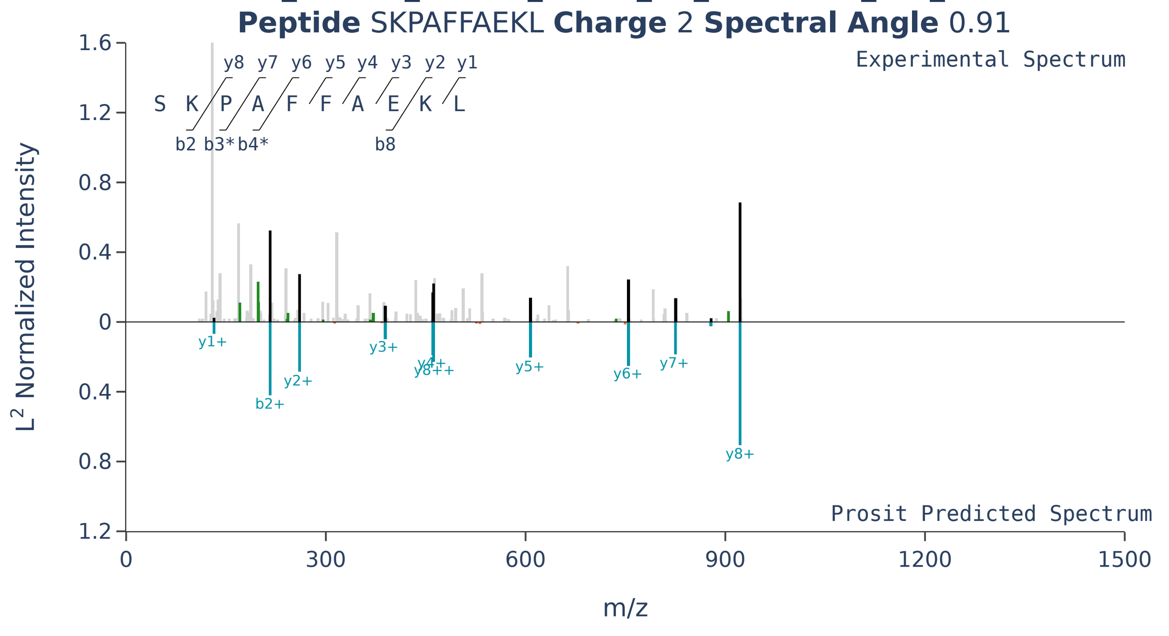

**Source** WSoh_301121_021221_HFGoe_M1_AnnexinA1_4h_R1 **Scan** 12936
**Peptide** DSDARALYE **Charge** 2 **Spectral Angle** 0.9

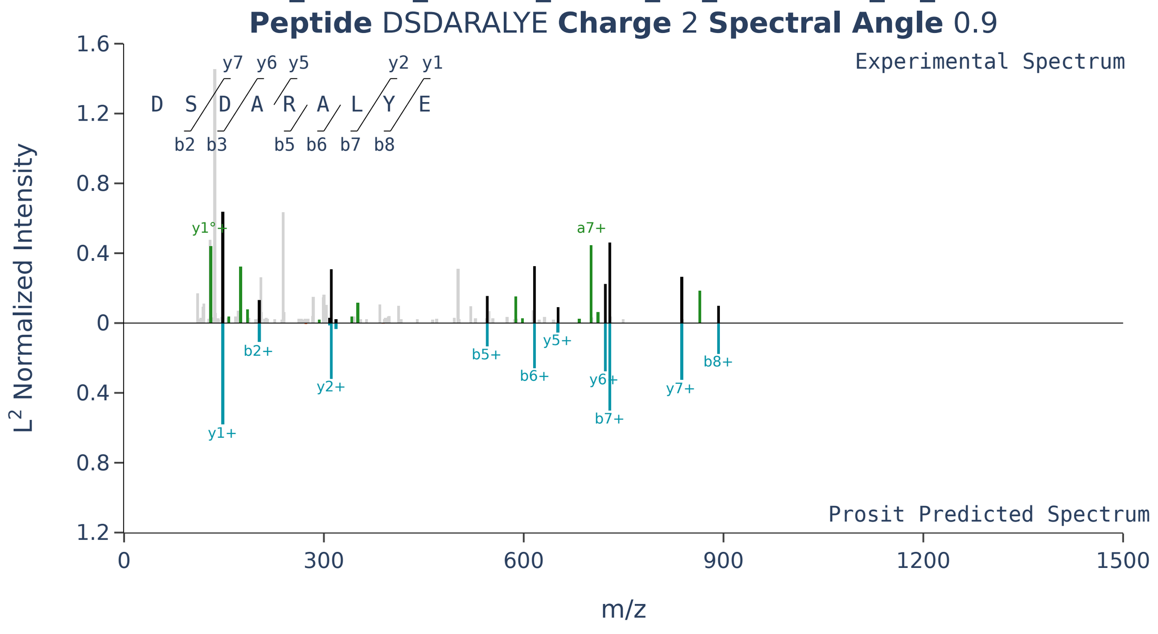


**Source** WSoh_301121_021221_HFGoe_M2_AnnexinA1_2h_R2 **Scan** 17761
**Peptide** AMKGLGTDEDTL **Charge** 2 **Spectral Angle** 0.9

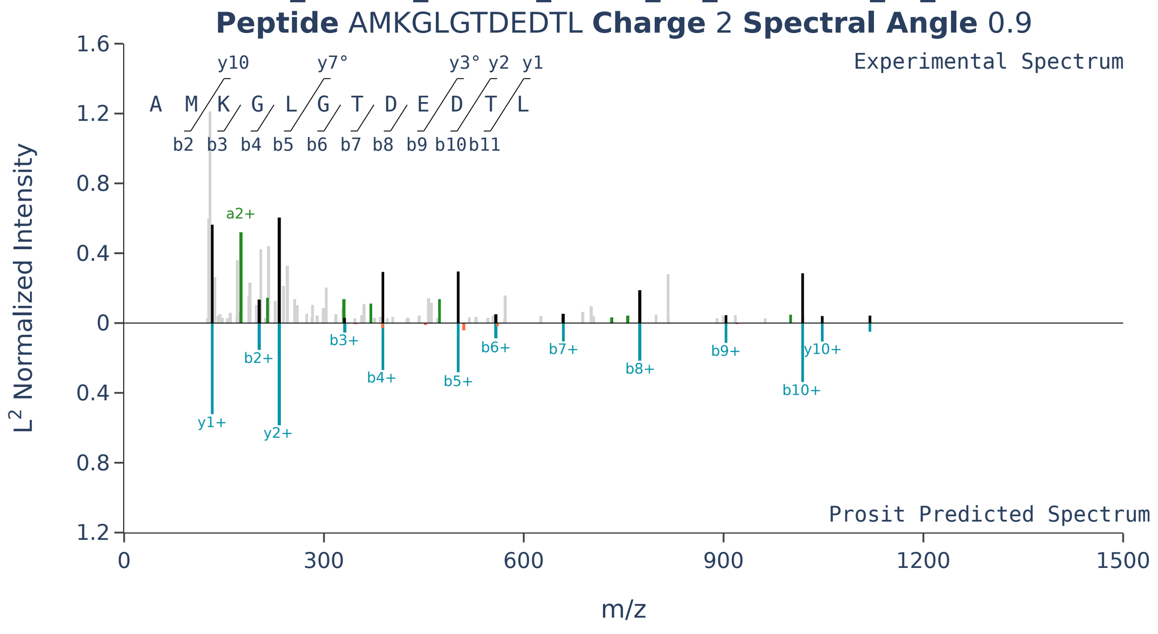


**Source** WSoh_301121_021221_HFGoe_M2_AnnexinA1_1h_R1 **Scan** 15636
**Peptide** SRSEIDMNDIKA **Charge** 3 **Spectral Angle** 0.94

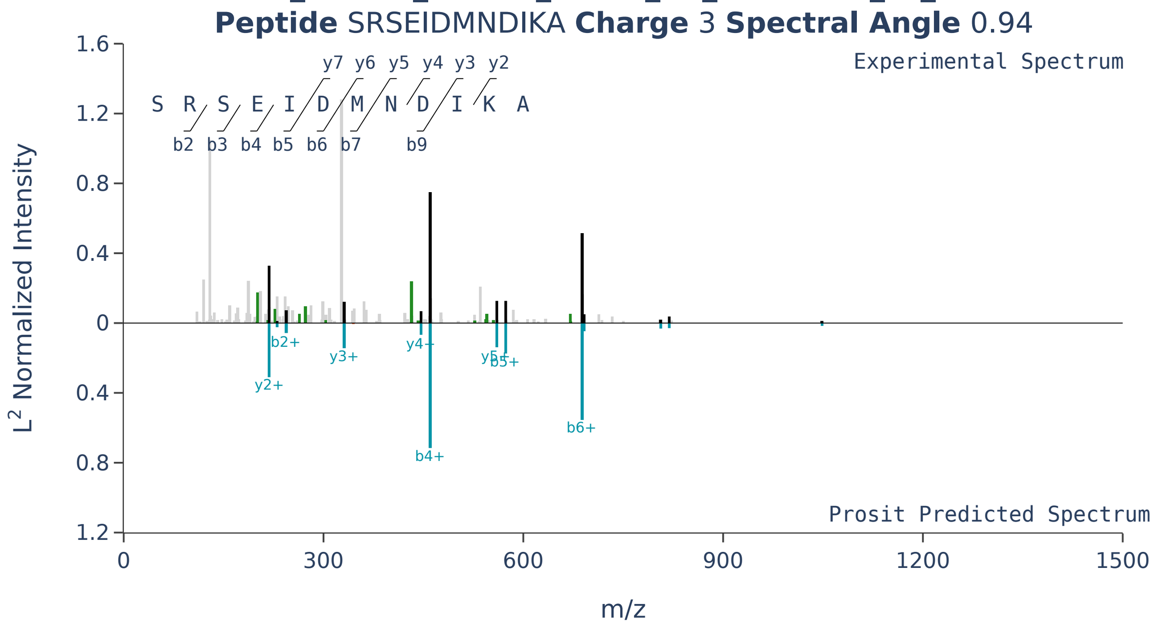


**Source** WSoh_301121_021221_HFGoe_M2_AnnexinA1_24h_R1 **Scan** 9426
**Peptide** IRDINRVYRE **Charge** 3 **Spectral Angle** 0.9

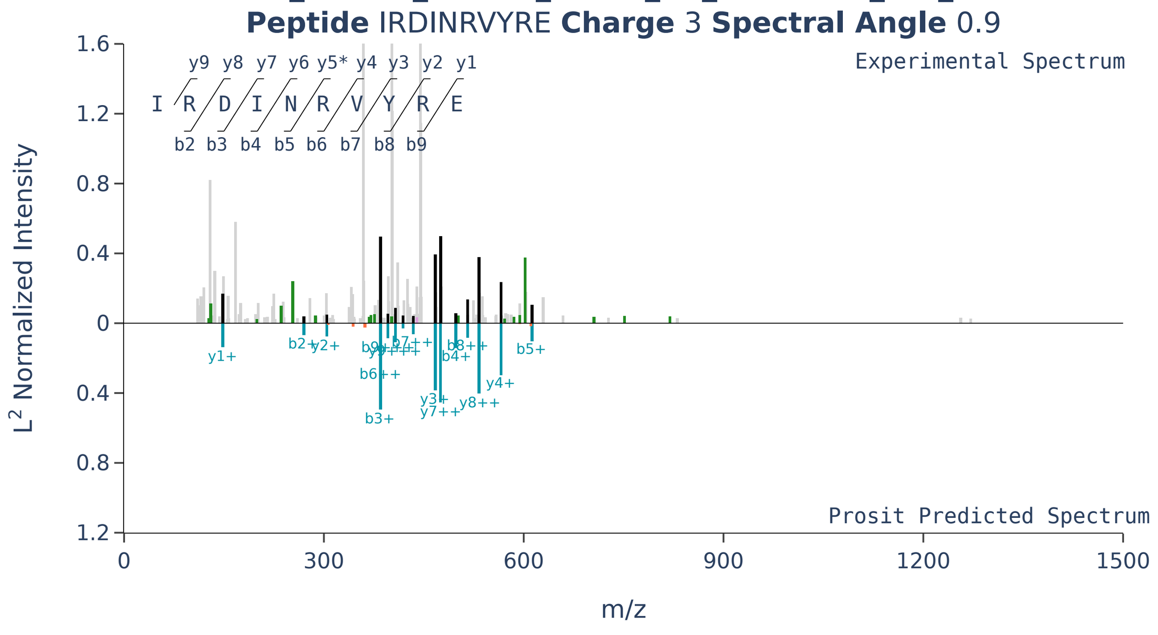


**Source** WSoh_301121_021221_HFGoe_M2_AnnexinA1_24h_R1 **Scan** 12756
**Peptide** KPAFFAEK **Charge** 2 **Spectral Angle** 0.82

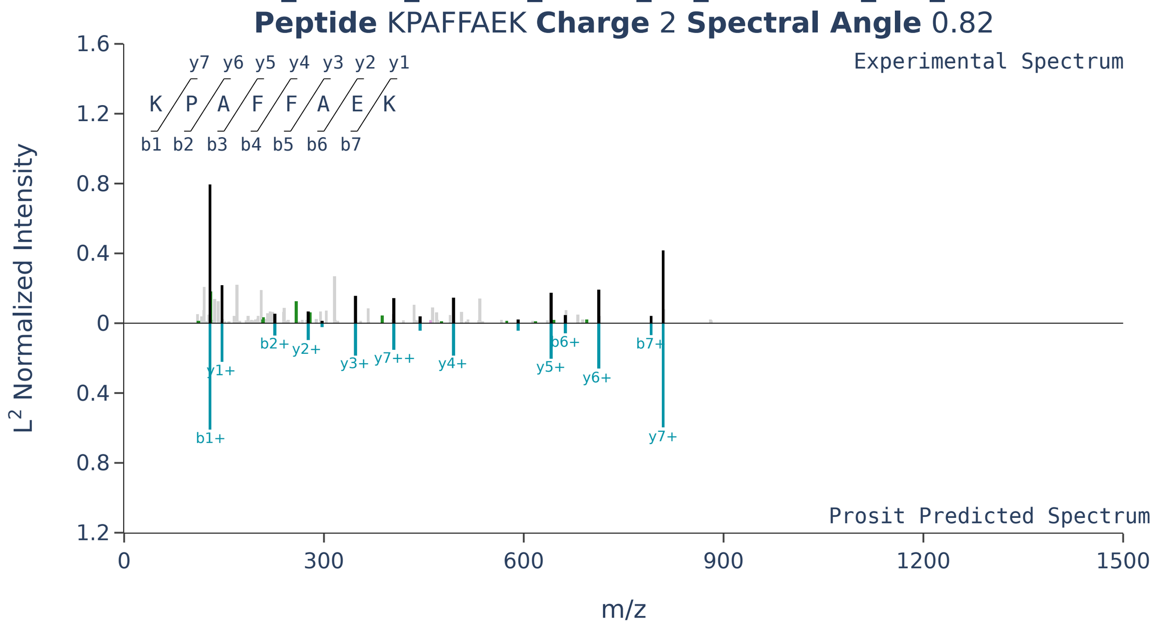


**Source** WSoh_301121_021221_HFGoe_M2_AnnexinA1_24h_R2 **Scan** 11895
**Peptide** FIENEEQE **Charge** 2 **Spectral Angle** 0.88

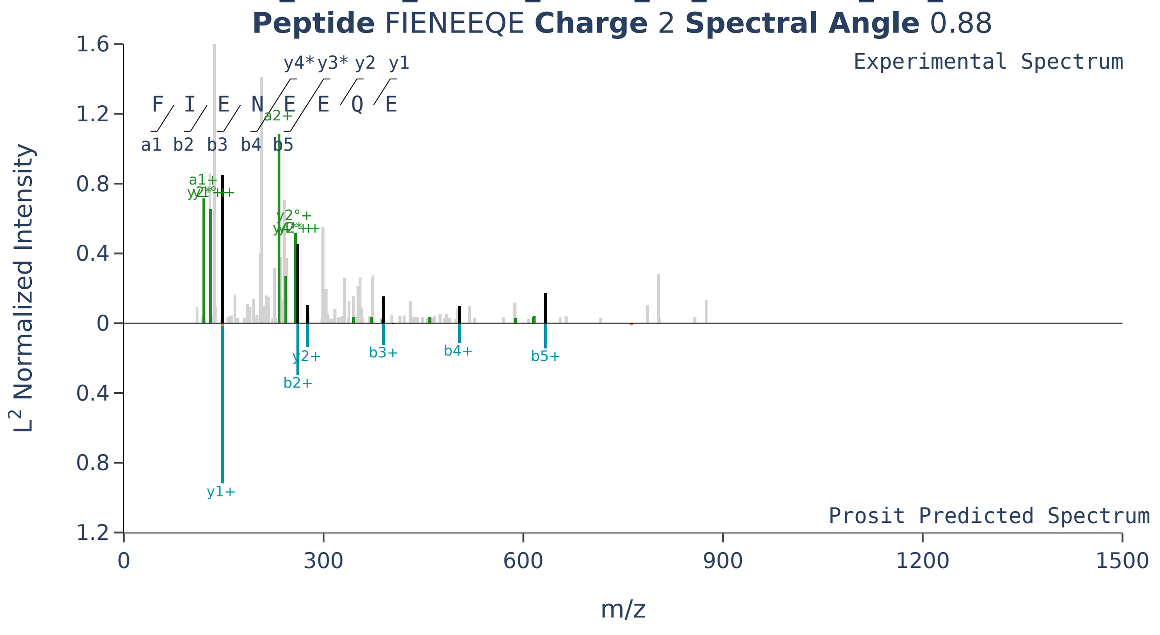


**Source** WSoh_301121_021221_HFGoe_M2_AnnexinA1_2h_R2 **Scan** 18247
**Peptide** KKALTGHLEEVVL **Charge** 2 **Spectral Angle** 0.91

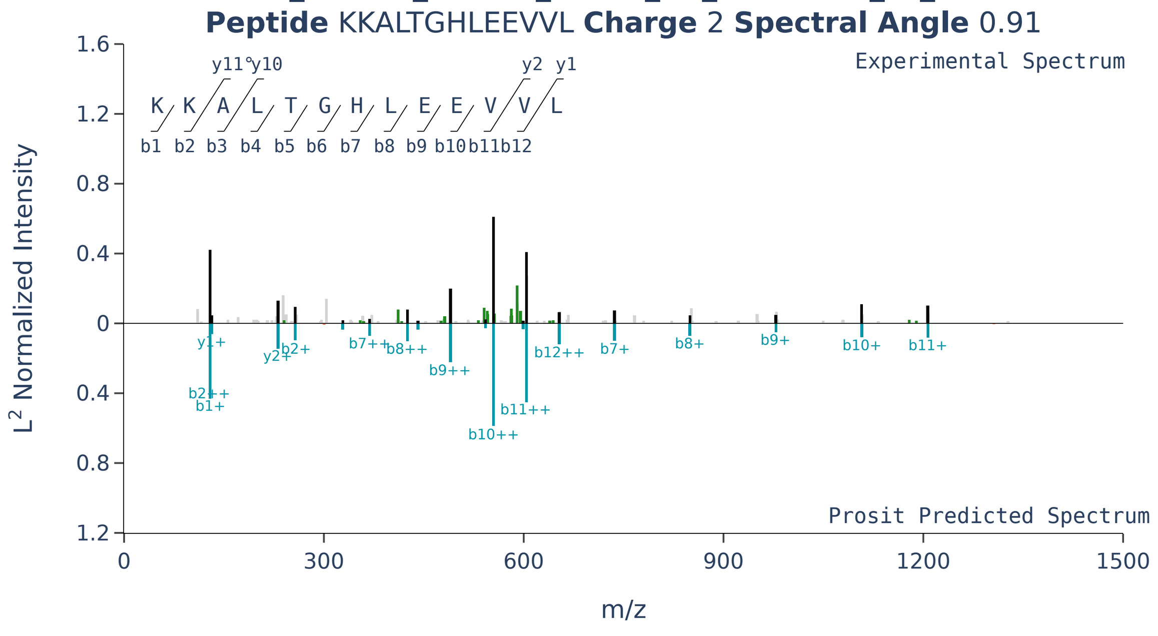


**Source** WSoh_301121_021221_HFGoe_M1_AnnexinA1_24h_R2 **Scan** 19828
**Peptide** DLADSDARALYE **Charge** 2 **Spectral Angle** 0.94

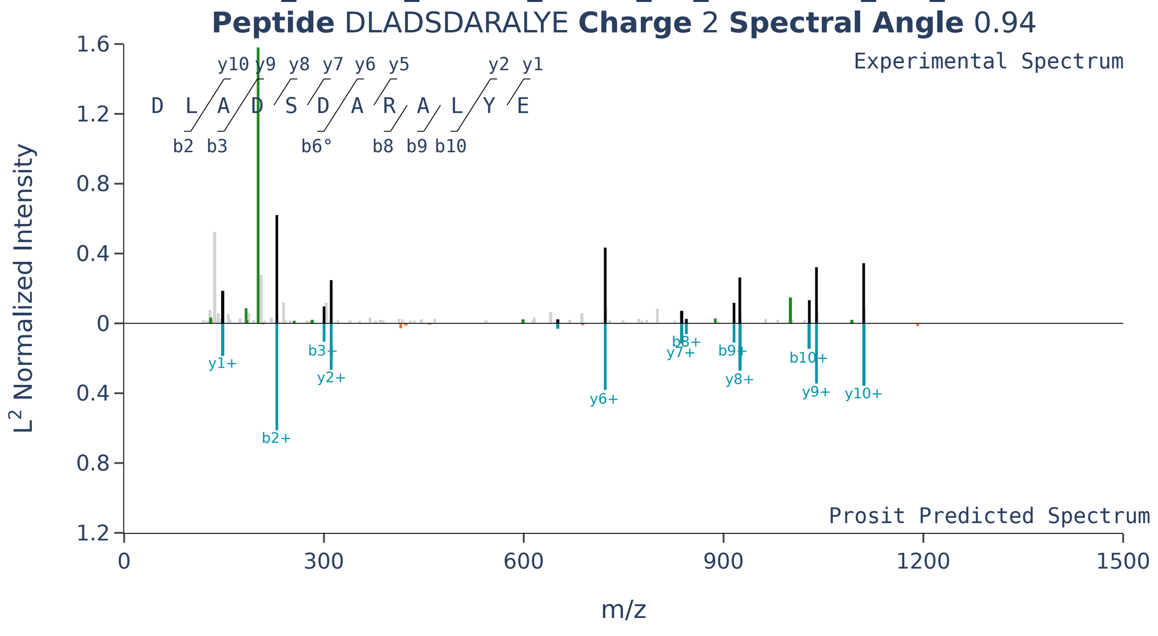


**Source** WSoh_301121_021221_HFGoe_M2_AnnexinA1_24h_R1 **Scan** 10609
**Peptide** AKDITSDTSGDFR **Charge** 2 **Spectral Angle** 0.93

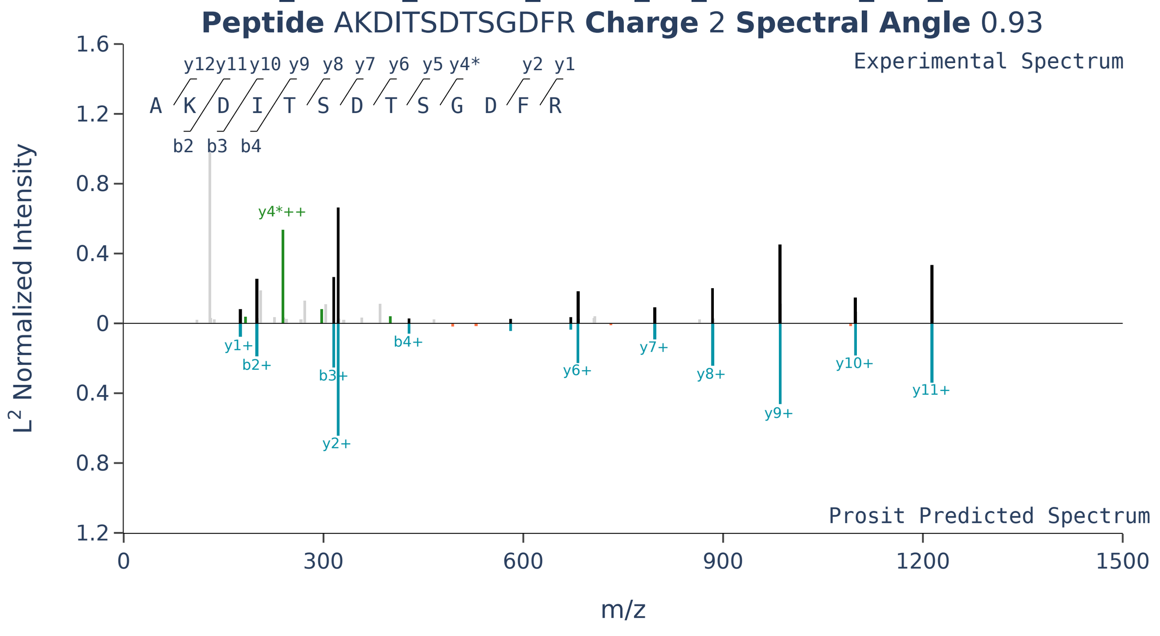


**Source** WSoh_301121_021221_HFGoe_M2_AnnexinA1_2h_R1 **Scan** 20930
**Peptide** KGLGTDEDTLI **Charge** 2 **Spectral Angle** 0.9

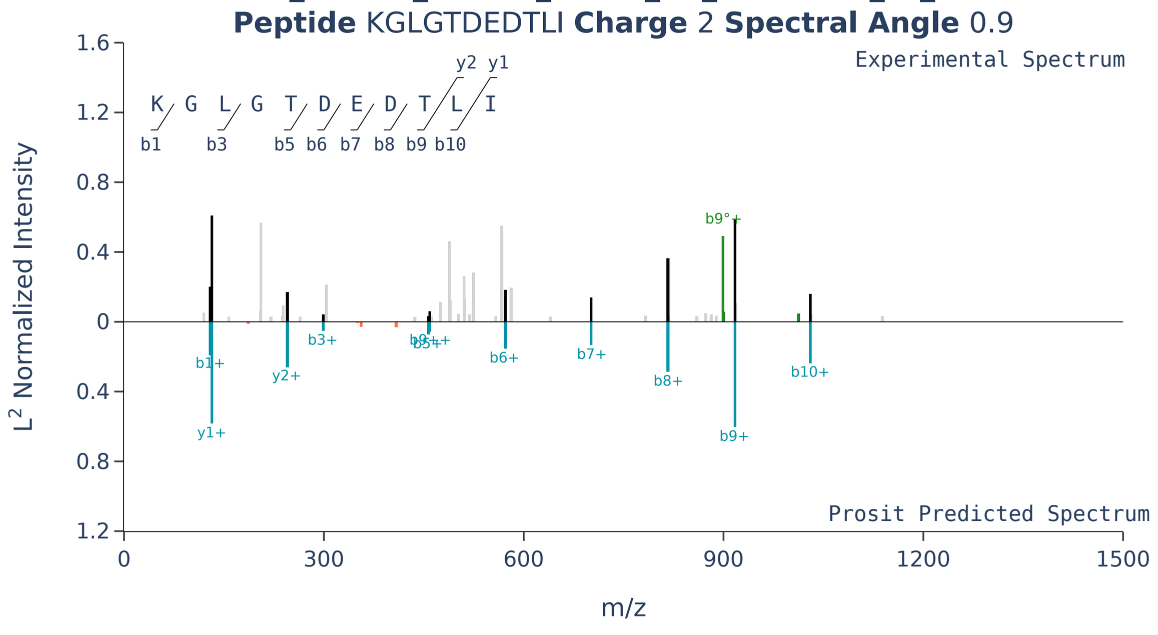


**Source** WSoh_301121_021221_HFGoe_M2_AnnexinA1_1h_R2 **Scan** 12492
**Peptide** CQAILDETKG **Charge** 2 **Spectral Angle** 0.91

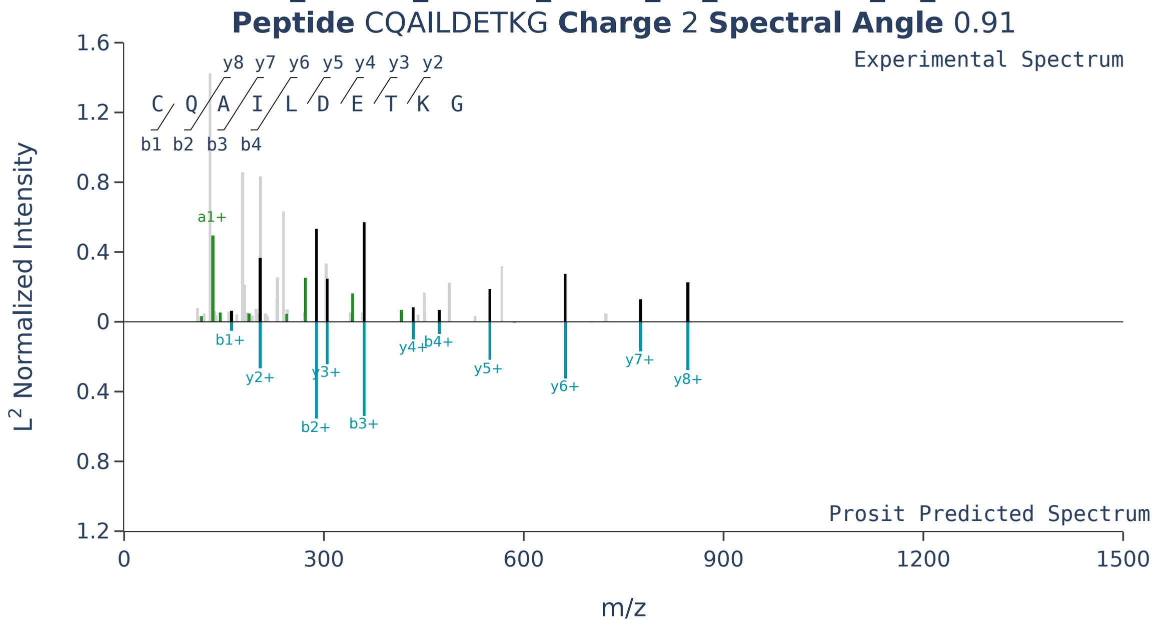


**Source** WSoh_301121_021221_HFGoe_M2_AnnexinA1_24h_R1 **Scan** 15874
**Peptide** DITSDTSGDFRNA **Charge** 2 **Spectral Angle** 0.92

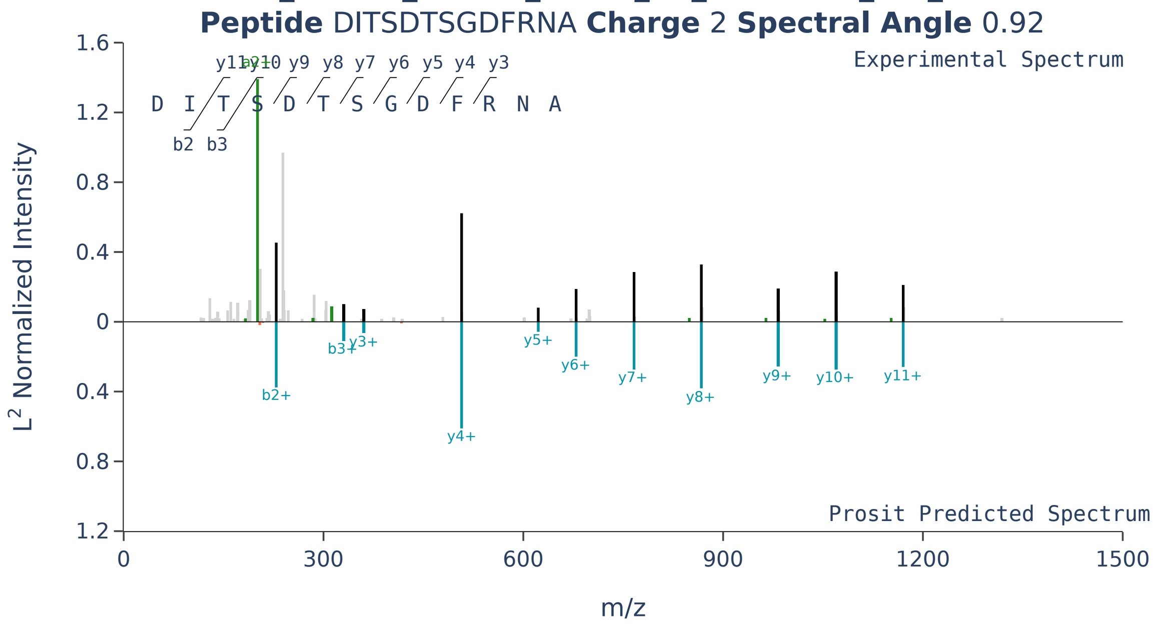


**Source** WSoh_301121_021221_HFGoe_M1_AnnexinA1_2h_R1 **Scan** 9154
**Peptide** MVSRSEID **Charge** 2 **Spectral Angle** 0.91

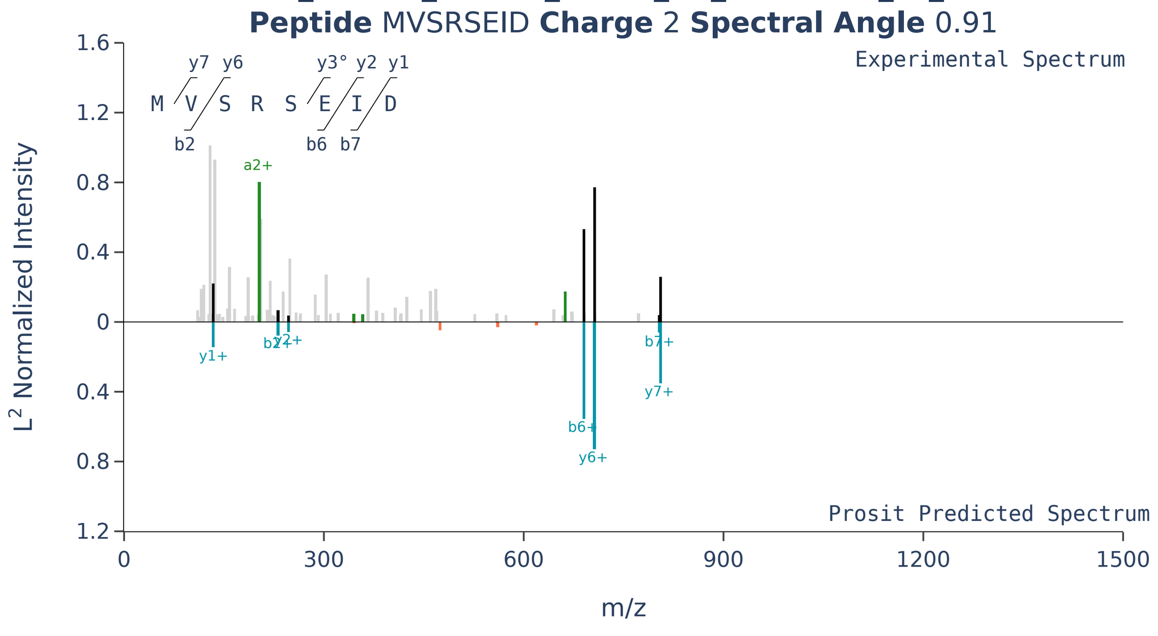


**Source** WSoh_301121_021221_HFGoe_M2_AnnexinA1_4h_R1 **Scan** 17247
**Peptide** TKGDYEKILV **Charge** 2 **Spectral Angle** 0.89

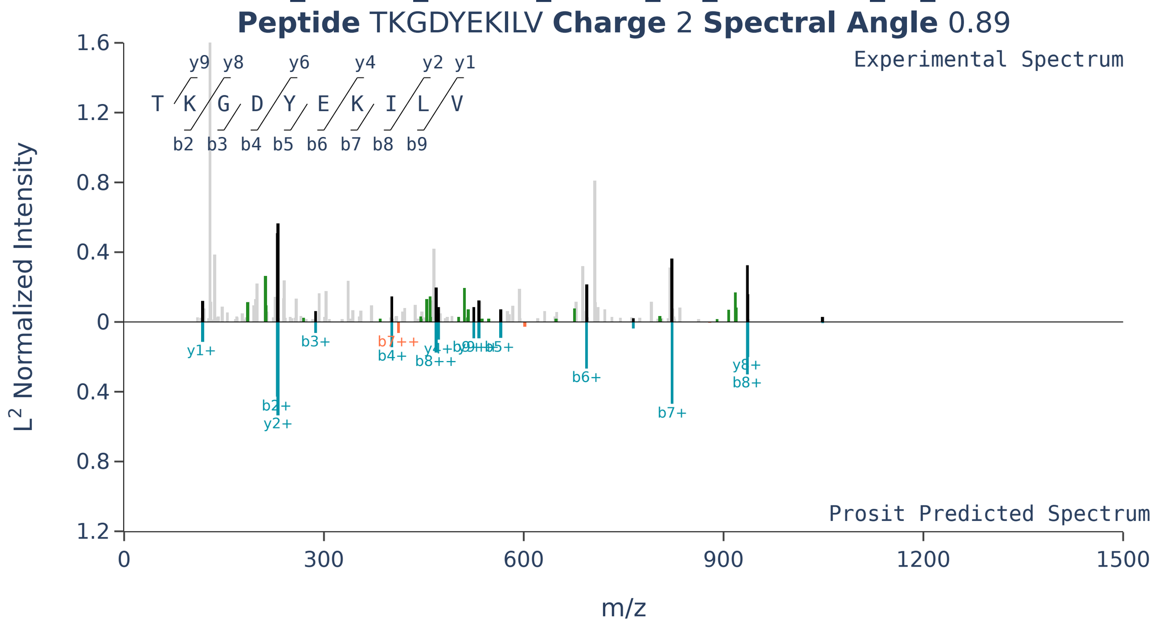


**Source** WSoh_301121_021221_HFGoe_M2_AnnexinA1_24h_R1 **Scan** 18524
**Peptide** KDITSDTSGDFRNAL **Charge** 2 **Spectral Angle** 0.89

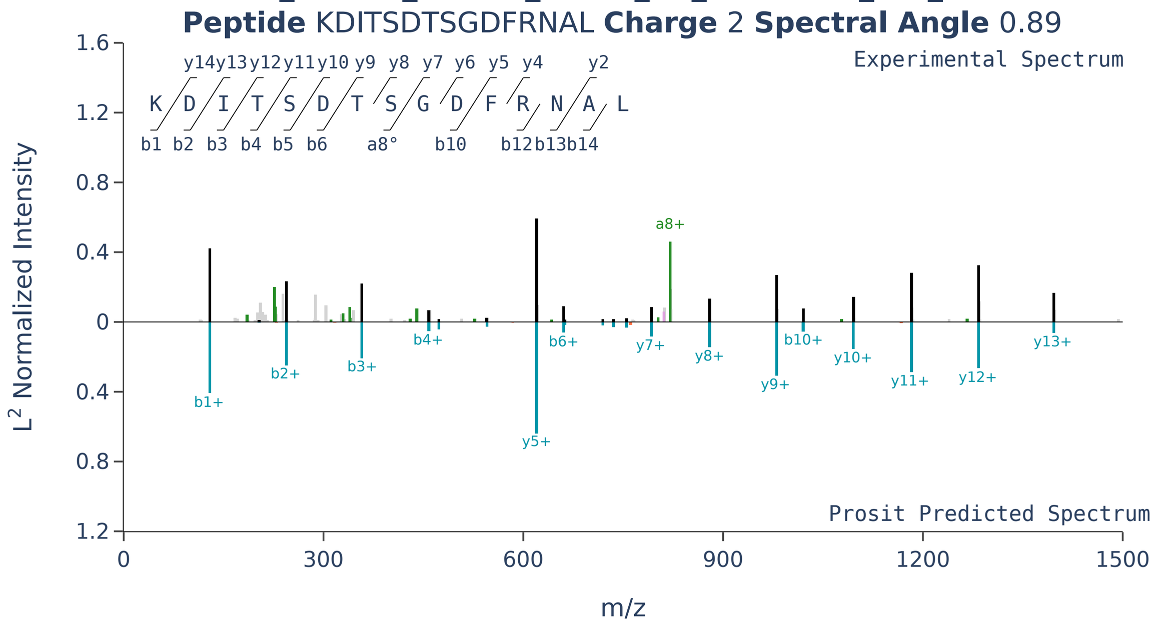

**Source** WSoh_301121_021221_HFGoe_M2_AnnexinA1_4h_R2 **Scan** 17054
**Peptide** TSDTSGDFRNAL **Charge** 2 **Spectral Angle** 0.87

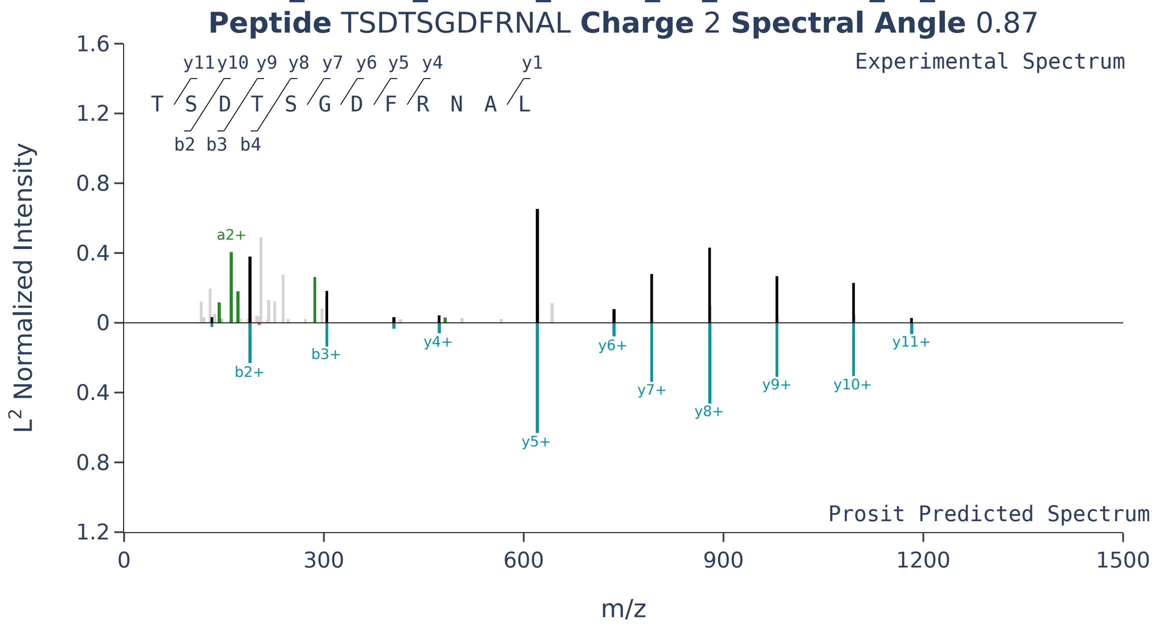


**Source** WSoh_301121_021221_HFGoe_M1_AnnexinA1_24h_R1 **Scan** 18230
**Peptide** IIDILTKRNNA **Charge** 3 **Spectral Angle** 0.93

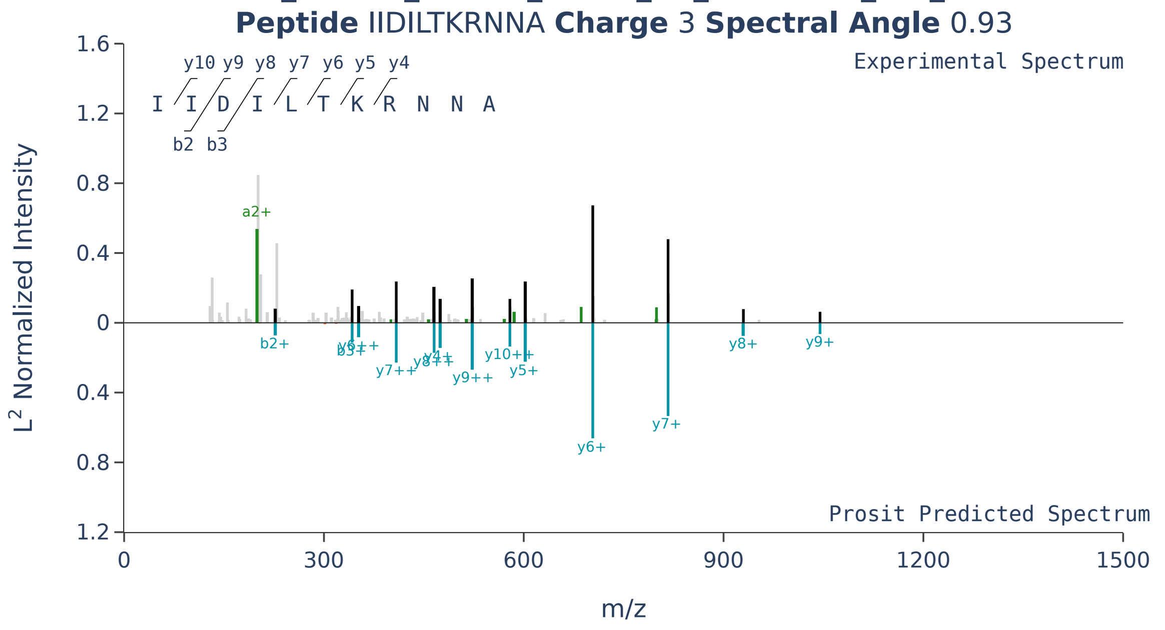


**Source** WSoh_301121_021221_HFGoe_M1_AnnexinA1_4h_R2 **Scan** 6798
**Peptide** RDINRVYRE **Charge** 3 **Spectral Angle** 0.89

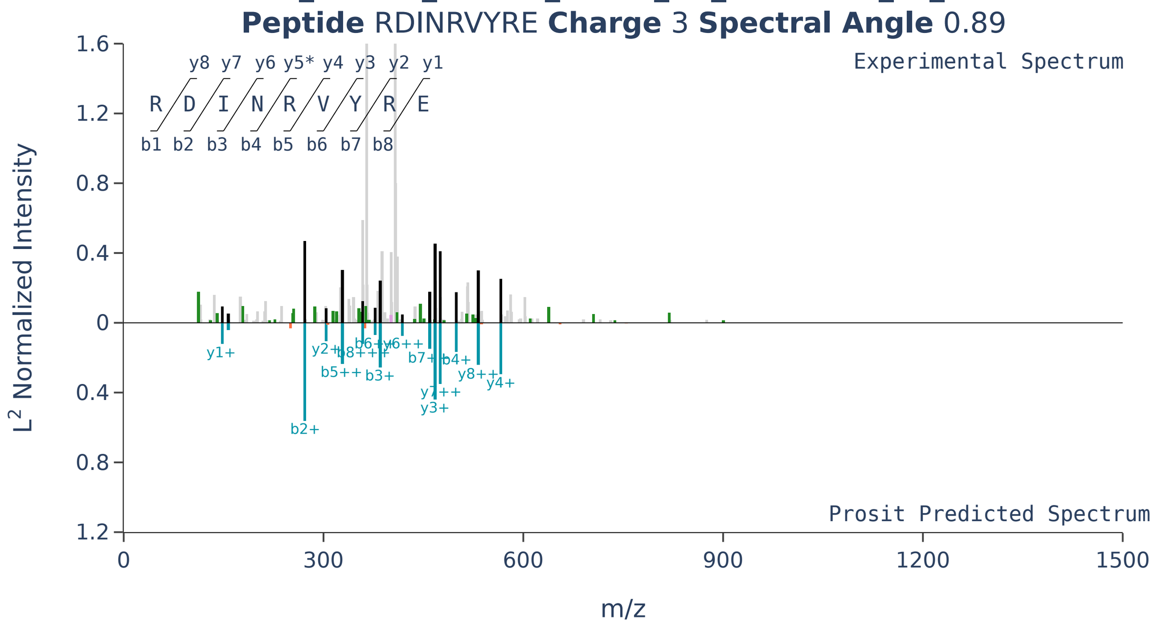


**Source** WSoh_301121_021221_HFGoe_M2_AnnexinA1_2h_R2 **Scan** 17195
**Peptide** KGDYEKILVA **Charge** 2 **Spectral Angle** 0.86

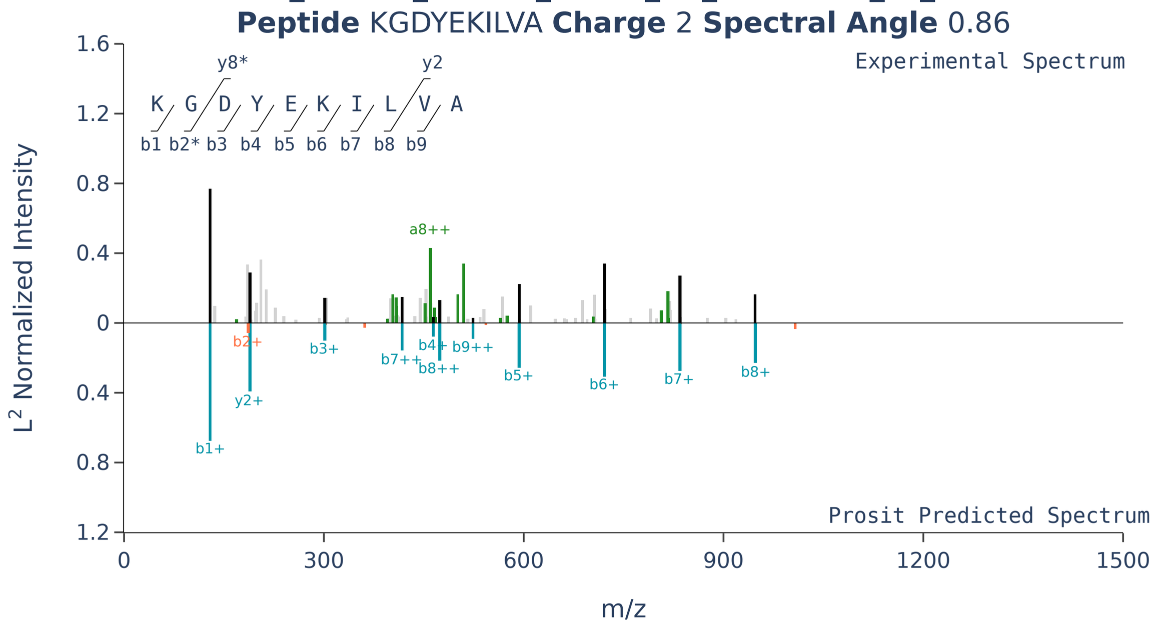


**Source** WSoh_301121_021221_HFGoe_M2_AnnexinA1_4h_R1 **Scan** 8832
**Peptide** YLQETGKP **Charge** 2 **Spectral Angle** 0.95

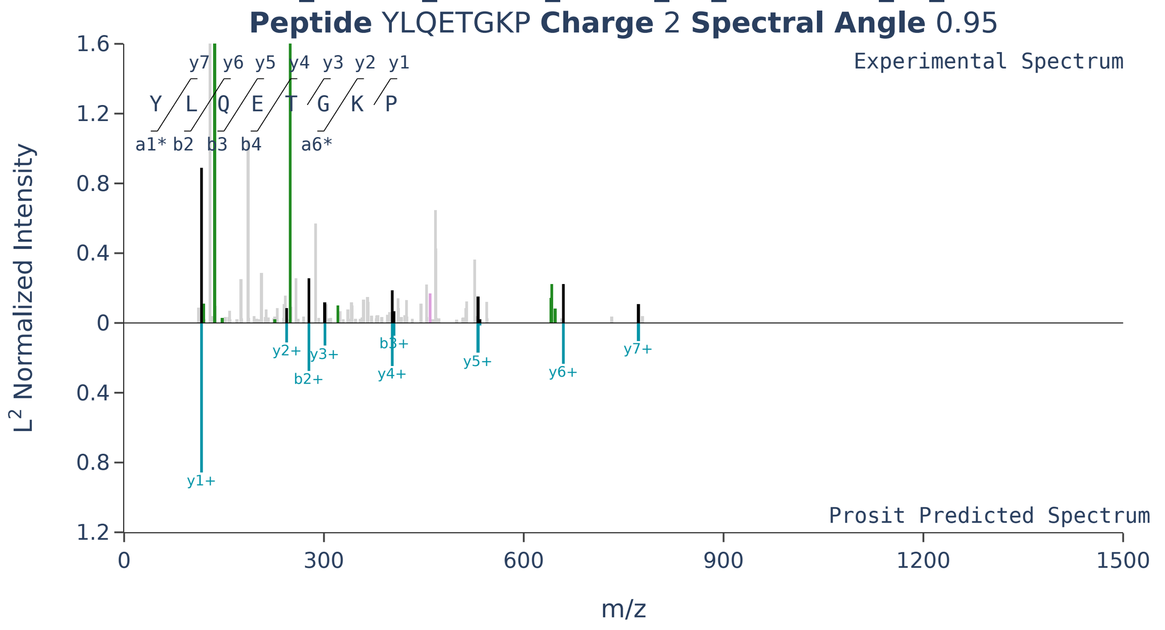


**Source** WSoh_301121_021221_HFGoe_M2_AnnexinA1_24h_R1 **Scan** 21122
**Peptide** TGHLEEVVLA **Charge** 2 **Spectral Angle** 0.86

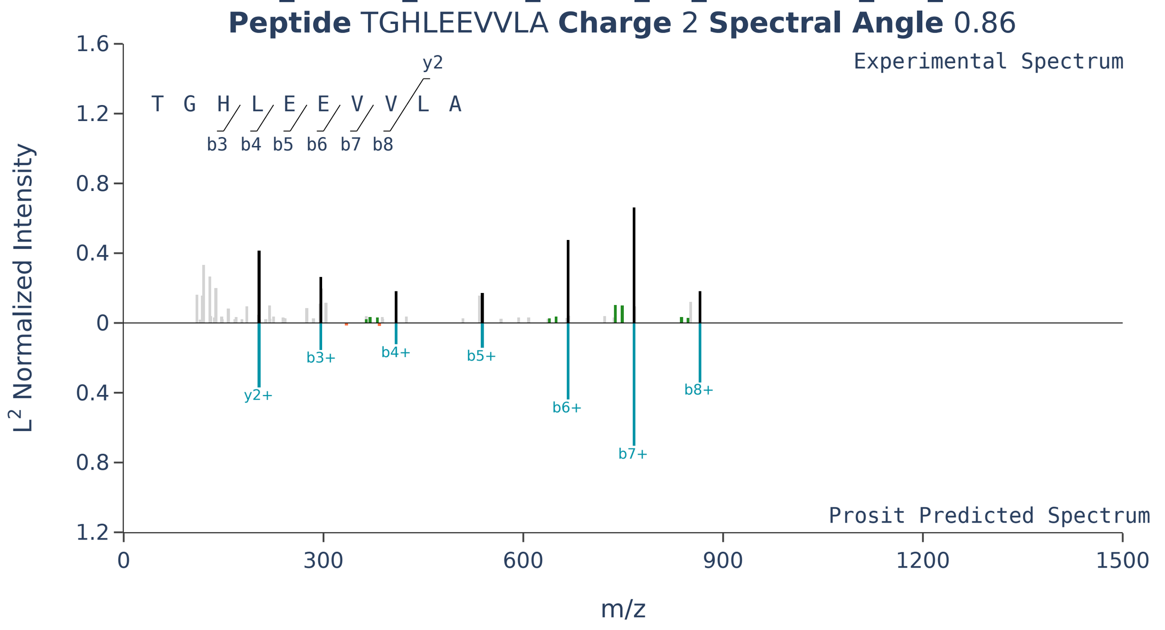


**Source** WSoh_301121_021221_HFGoe_M1_AnnexinA1_24h_R1 **Scan** 21924
**Peptide** DYEKILVA **Charge** 2 **Spectral Angle** 0.9

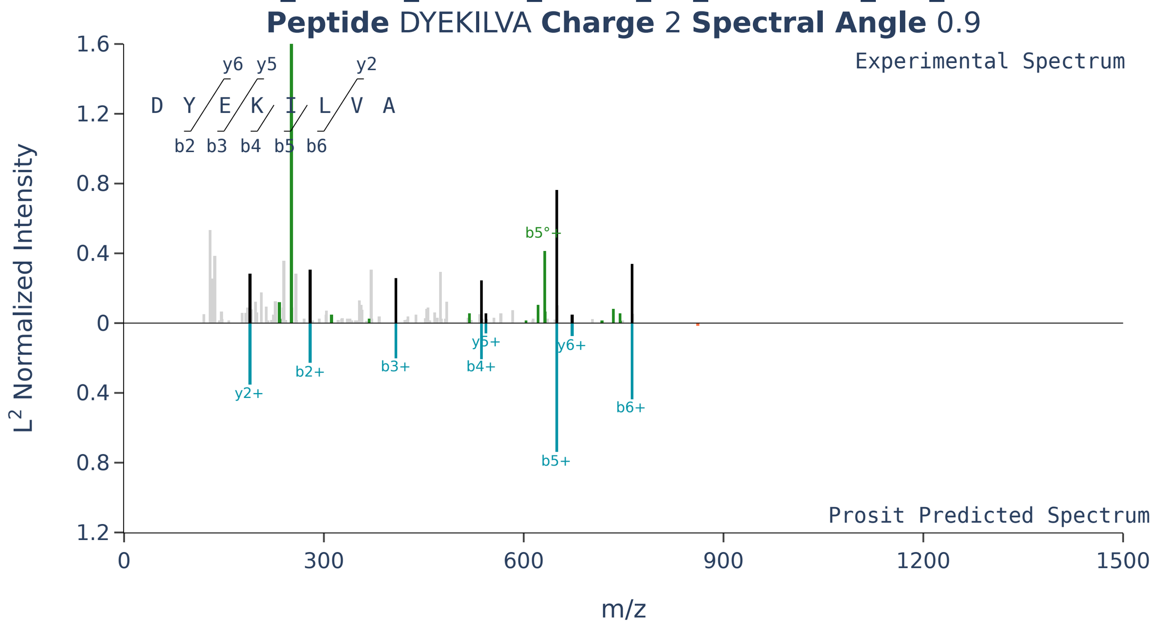


**Source** WSoh_301121_021221_HFGoe_M1_AnnexinA1_4h_R2 **Scan** 14203
**Peptide** KGLGTDEDTL **Charge** 2 **Spectral Angle** 0.9

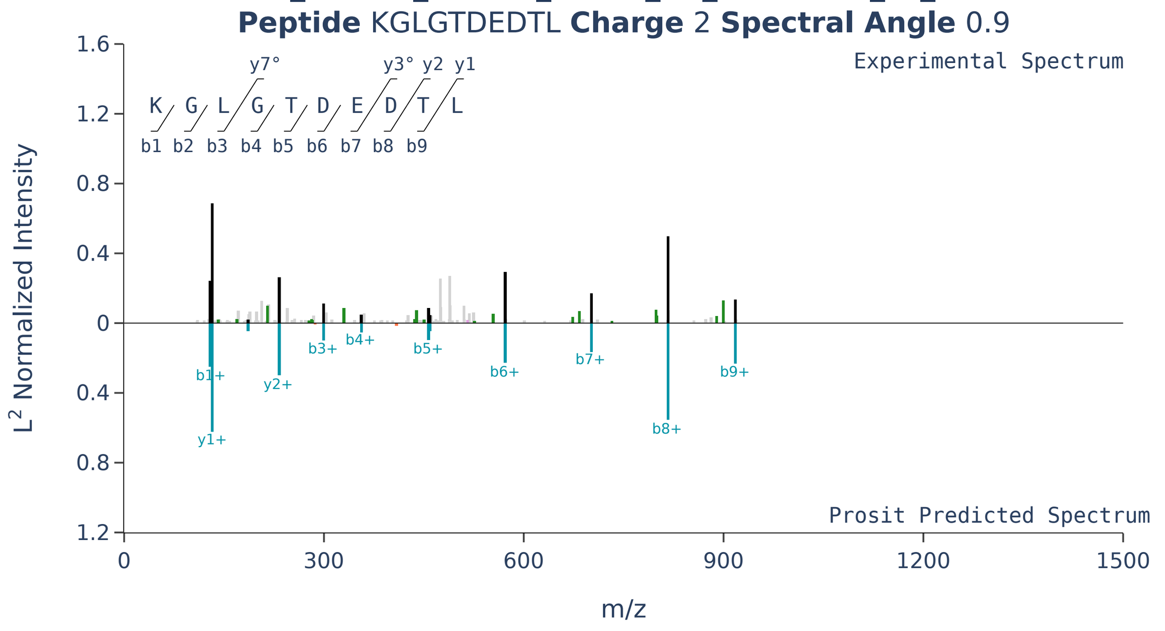


**Source** WSoh_301121_021221_HFGoe_M2_AnnexinA1_24h_R1 **Scan** 14486
**Peptide** LAKDITSDTSGDFRNA **Charge** 2 **Spectral Angle** 0.92

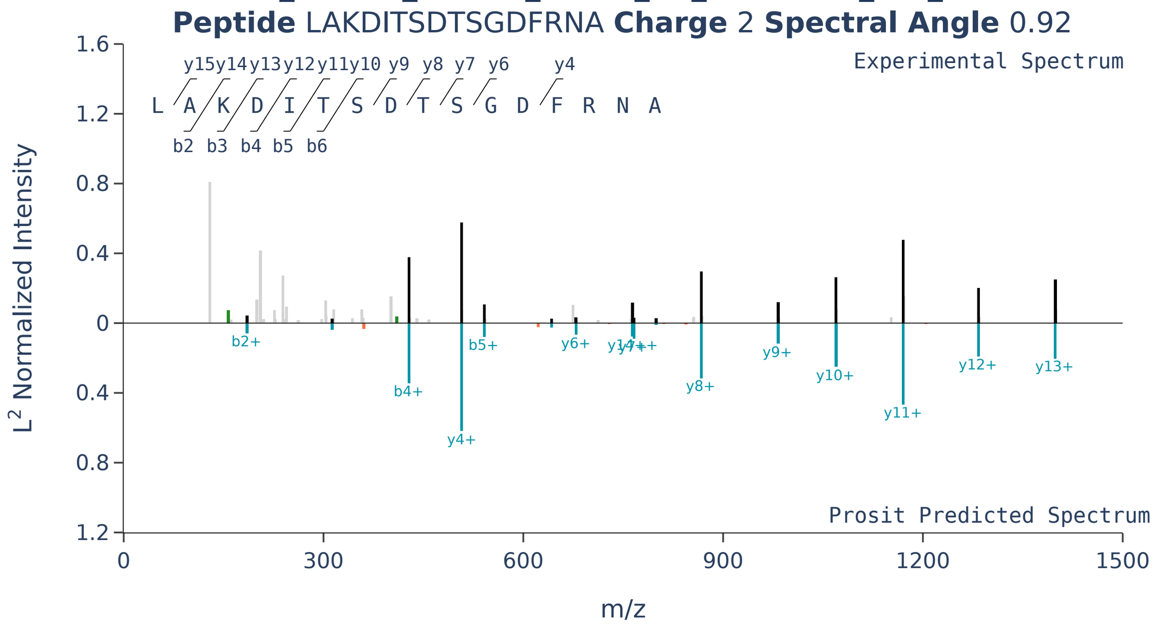


**Source** WSoh_301121_021221_HFGoe_M2_AnnexinA1_24h_R1 **Scan** 6256
**Peptide** ELKRDLAKD **Charge** 2 **Spectral Angle** 0.94

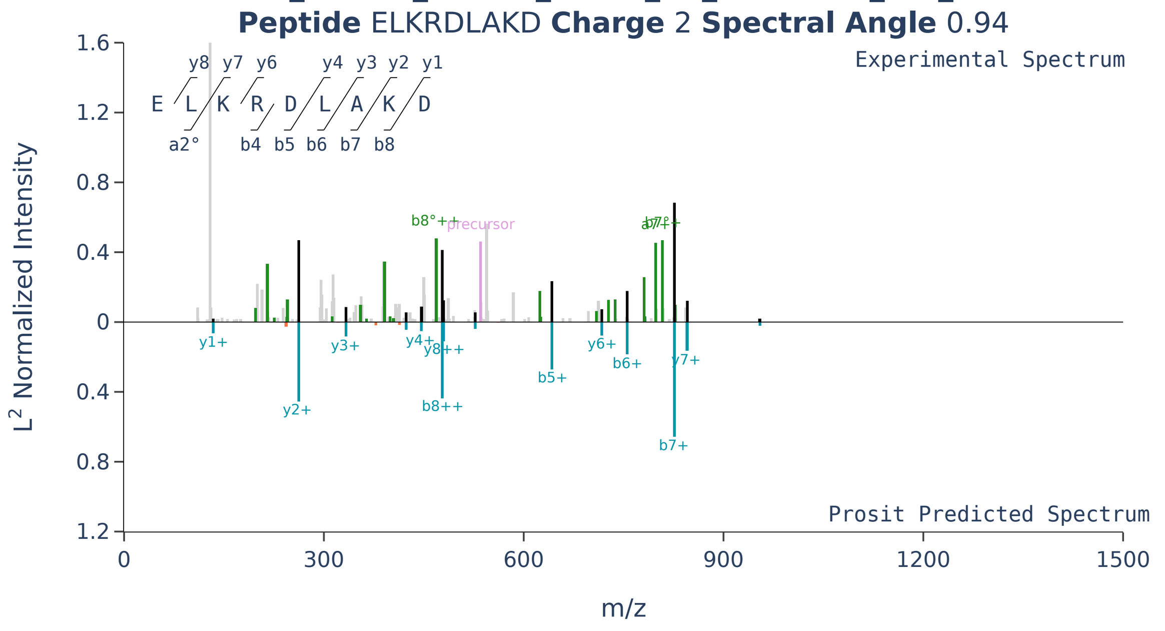


**Source** WSoh_301121_021221_HFGoe_M1_AnnexinA1_4h_R2 **Scan** 8722
**Peptide** TNKEIRDINRV **Charge** 3 **Spectral Angle** 0.91

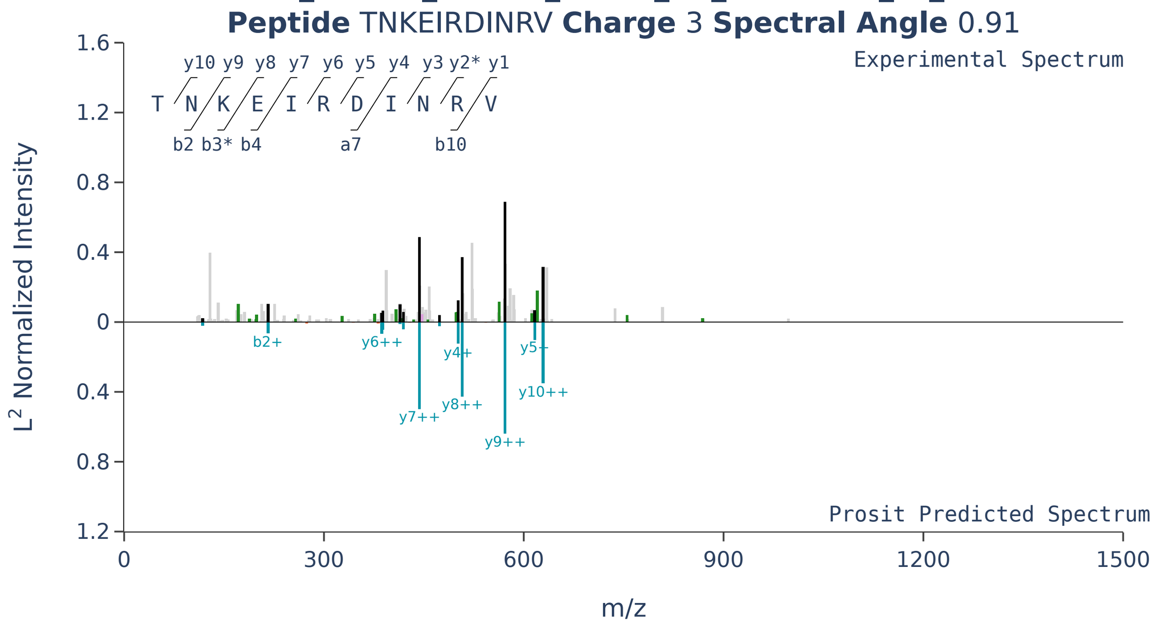


**Source** WSoh_301121_021221_HFGoe_M1_AnnexinA1_24h_R2 **Scan** 16978
**Peptide** KGTDVNVF **Charge** 2 **Spectral Angle** 0.9

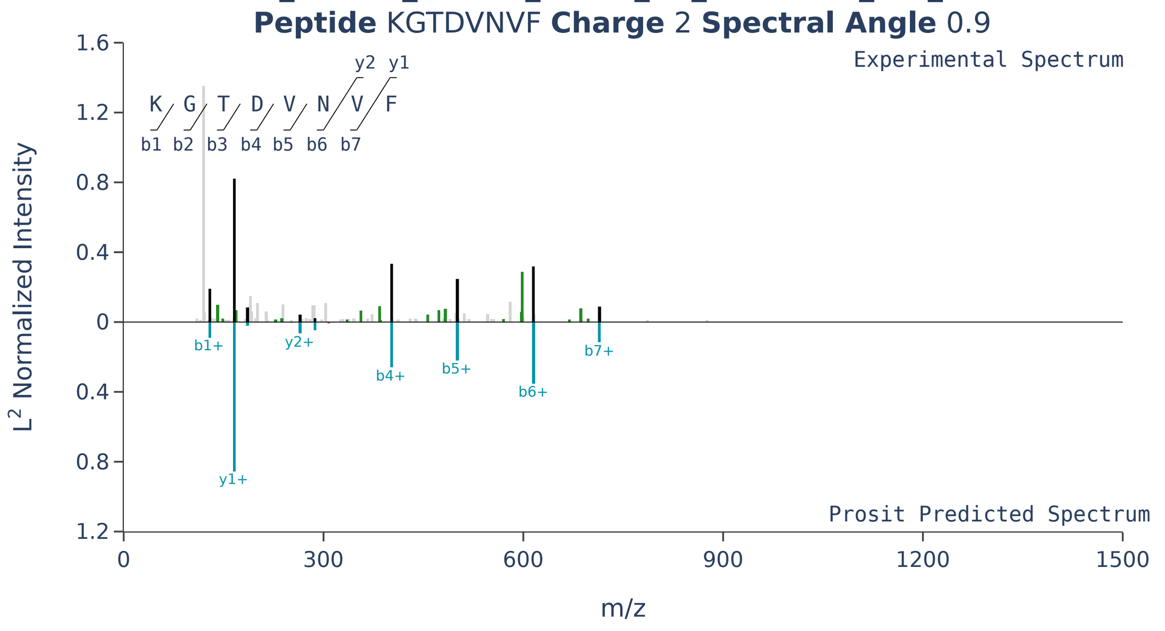


**Source** WSoh_301121_021221_HFGoe_M2_AnnexinA1_4h_R1 **Scan** 13301
**Peptide** LELKGDIEKC **Charge** 3 **Spectral Angle** 0.85

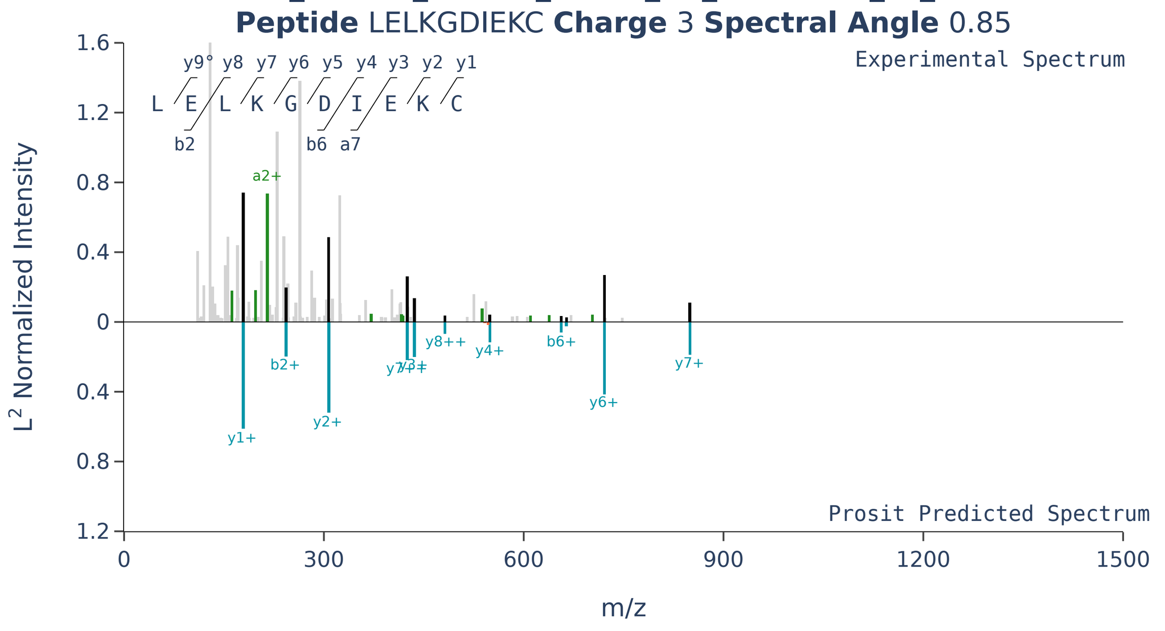


**Source** WSoh_301121_021221_HFGoe_M2_AnnexinA1_4h_R2 **Scan** 17539
**Peptide** GISLCQA **Charge** 1 **Spectral Angle** 0.93

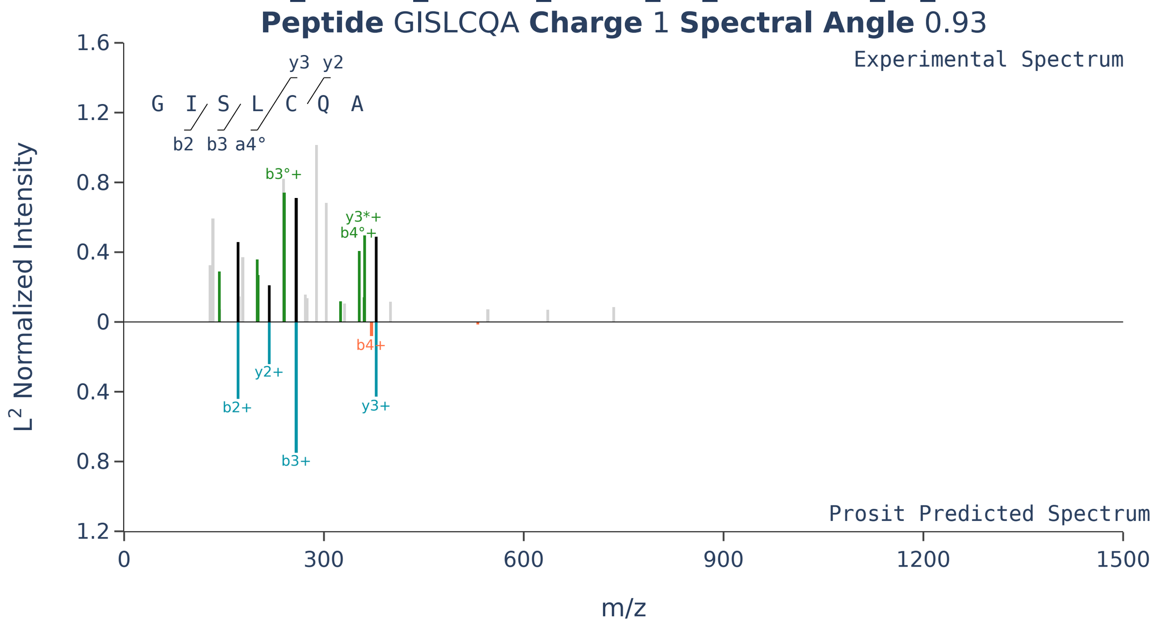


**Source** WSoh_301121_021221_HFGoe_M2_AnnexinA1_24h_R1 **Scan** 12728
**Peptide** GDRSEDFGVNED **Charge** 2 **Spectral Angle** 0.92

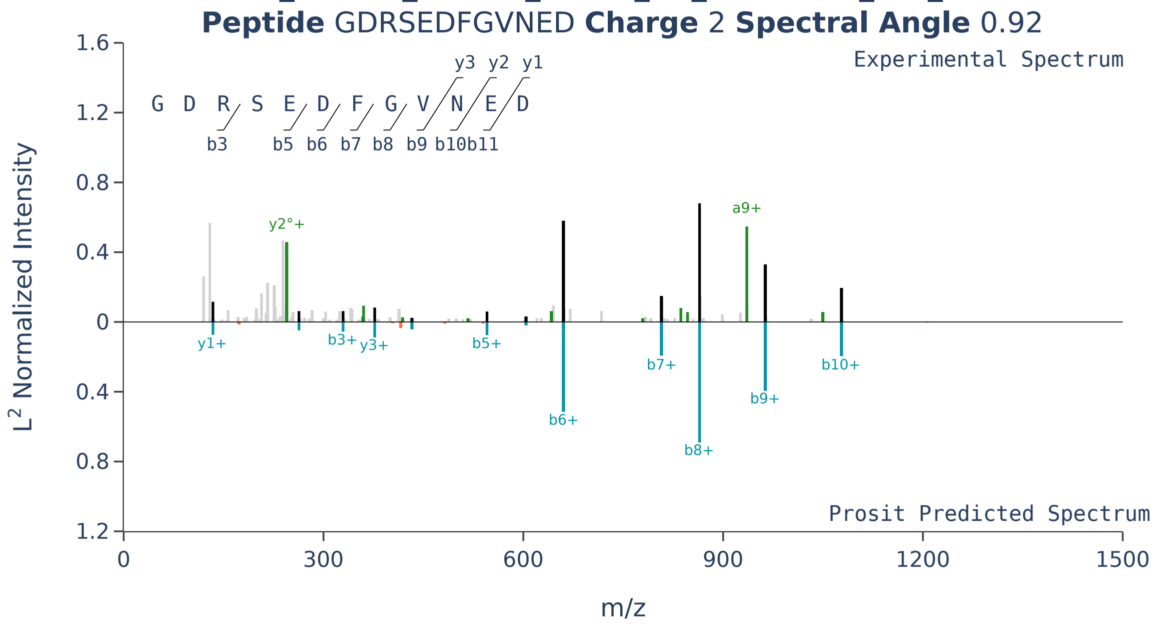

**Source** WSoh_301121_021221_HFGoe_M1_AnnexinA1_24h_R2 **Scan** 8424
**Peptide** DLADSDARA **Charge** 2 **Spectral Angle** 0.92

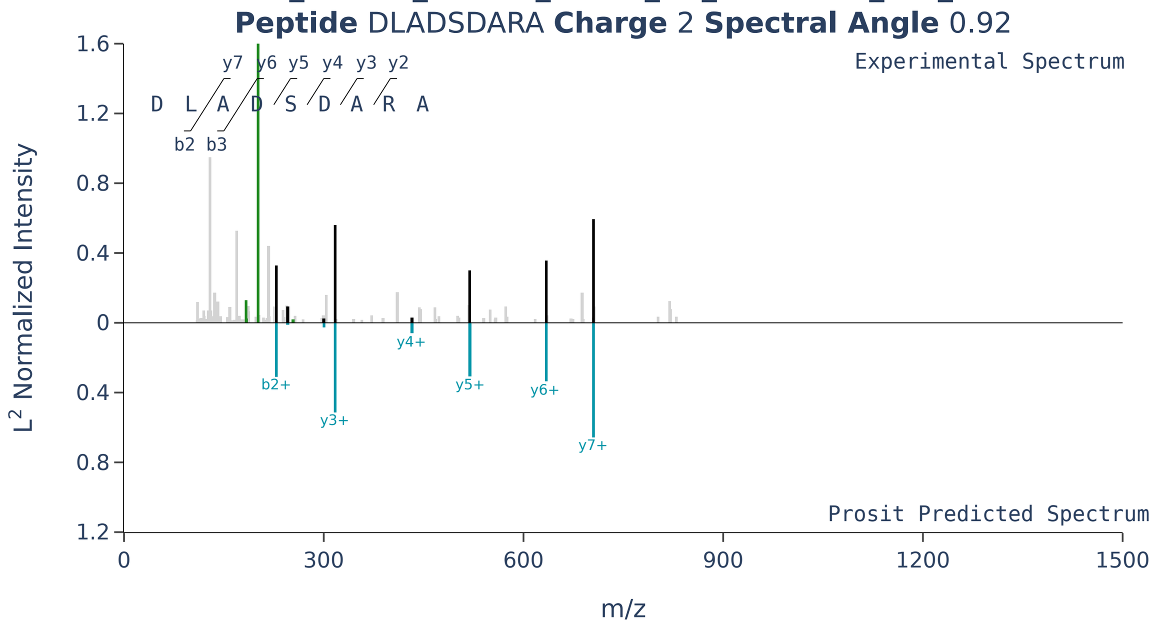


**Source** WSoh_301121_021221_HFGoe_M1_AnnexinA1_4h_R1 **Scan** 17307
**Peptide** YQKMYGI **Charge** 2 **Spectral Angle** 0.88

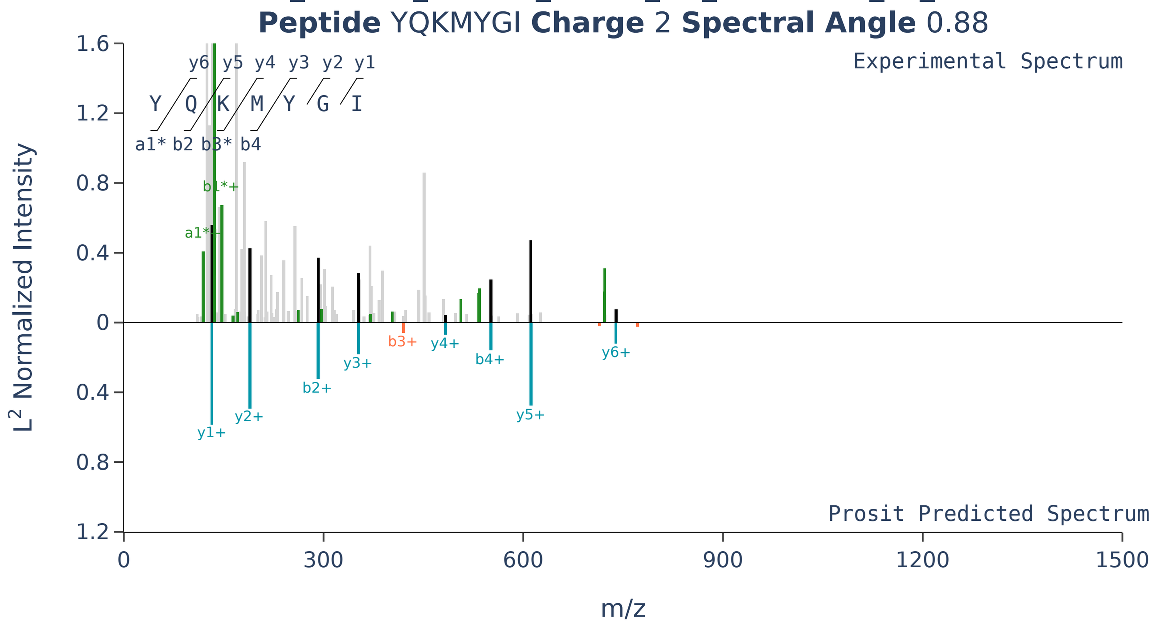


**Source** WSoh_301121_021221_HFGoe_M2_AnnexinA1_24h_R2 **Scan** 10140
**Peptide** AKGDRSEDFGVNED **Charge** 2 **Spectral Angle** 0.88

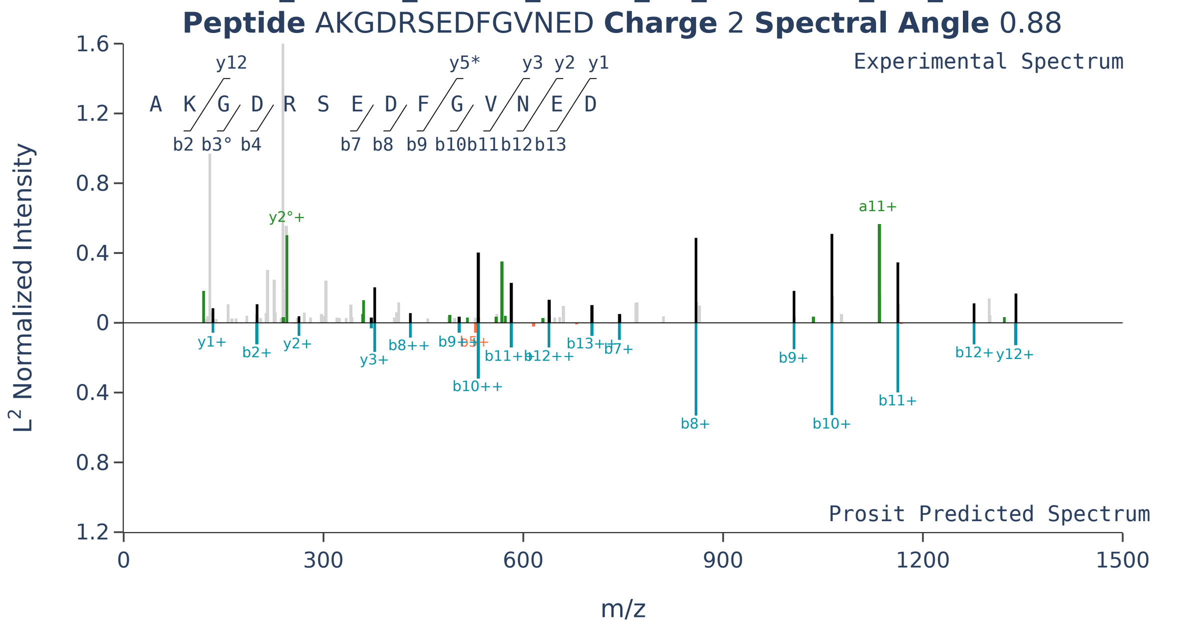


**Source** WSoh_301121_021221_HFGoe_M1_AnnexinA1_4h_R1 **Scan** 13036
**Peptide** RSEIDMNDI **Charge** 2 **Spectral Angle** 0.88

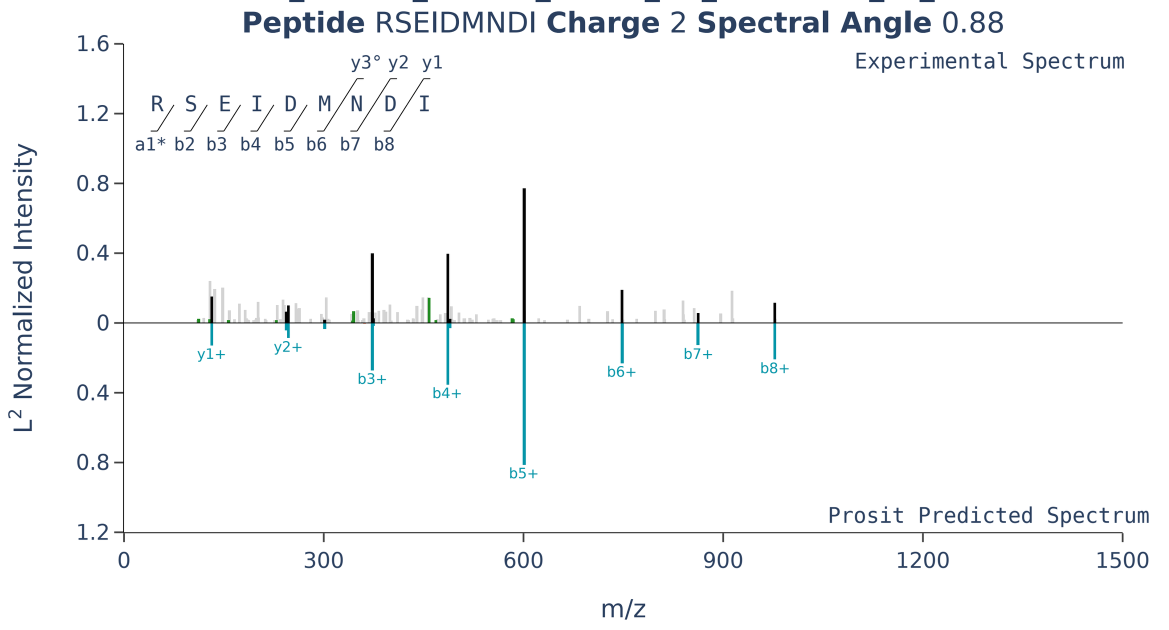


**Source** WSoh_301121_021221_HFGoe_M1_AnnexinA1_24h_R1 **Scan** 15829
**Peptide** RSEIDMNDIKA **Charge** 3 **Spectral Angle** 0.93

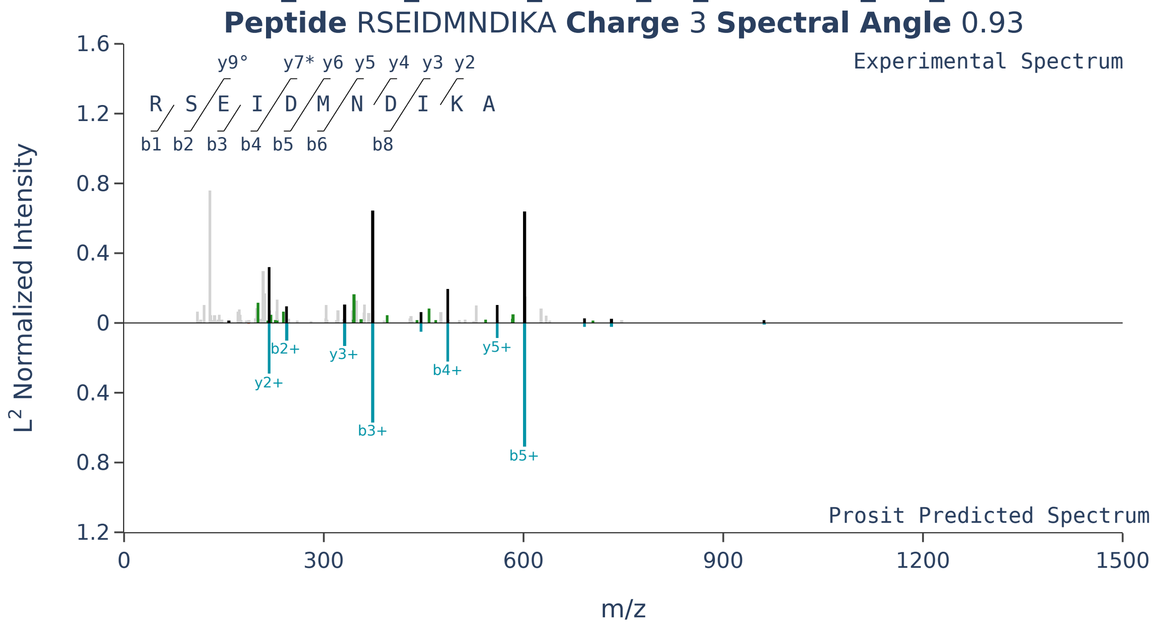


**Source** WSoh_301121_021221_HFGoe_M2_AnnexinA1_24h_R2 **Scan** 10470
**Peptide** DELRAAMKG **Charge** 2 **Spectral Angle** 0.93

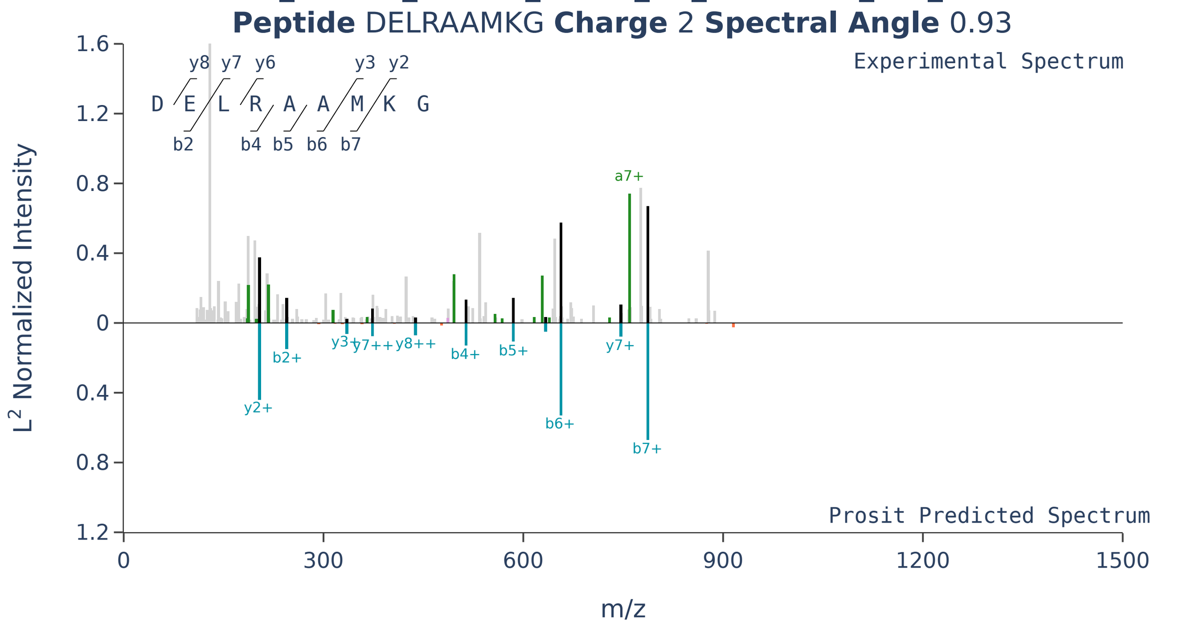


**Source** WSoh_301121_021221_HFGoe_M2_AnnexinA1_2h_R1 **Scan** 22373
**Peptide** AMVSEFLK **Charge** 2 **Spectral Angle** 0.88

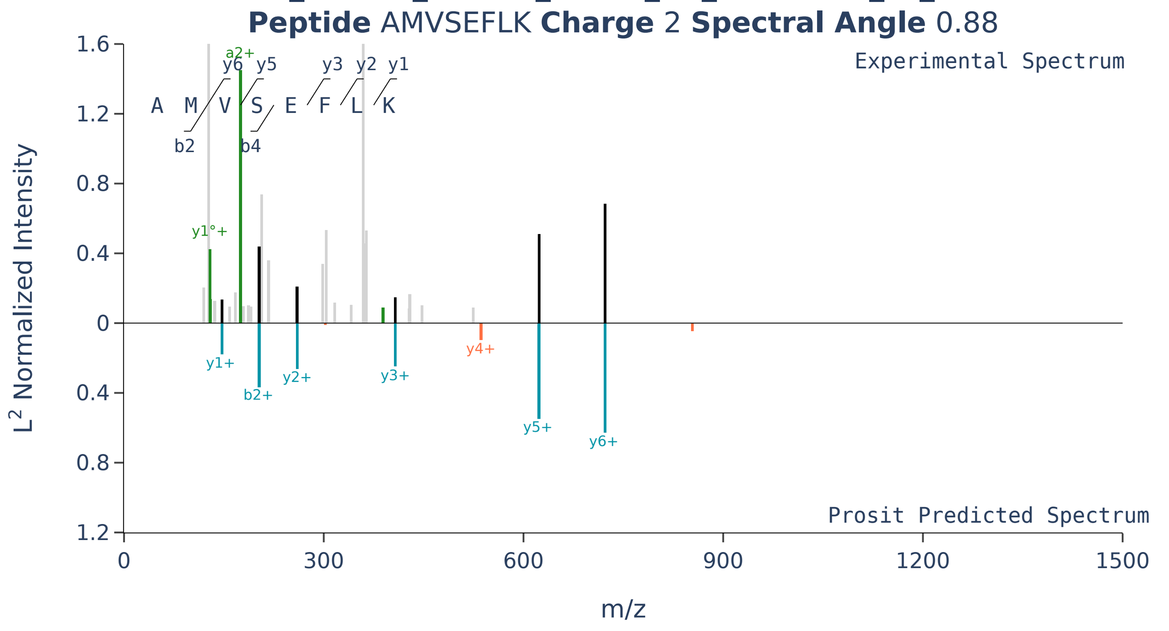


**Source** WSoh_301121_021221_HFGoe_M1_AnnexinA1_24h_R1 **Scan** 13318
**Peptide** SKPAFFAEK **Charge** 2 **Spectral Angle** 0.89

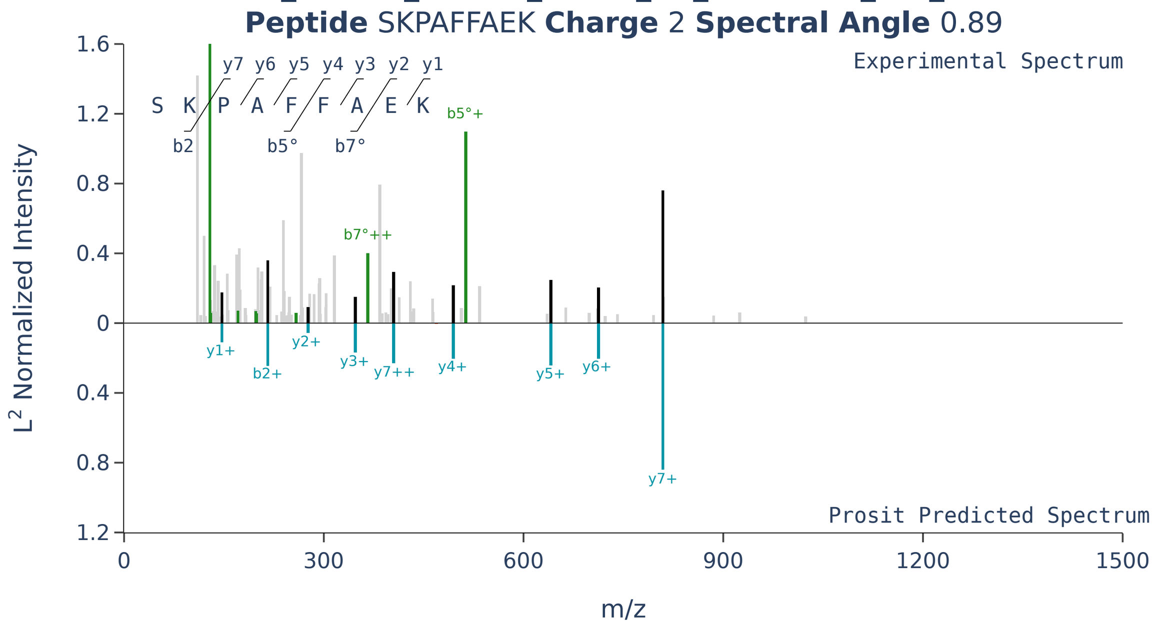


**Source** WSoh_301121_021221_HFGoe_M2_AnnexinA1_1h_R1 **Scan** 19016
**Peptide** PAFFAEKLH **Charge** 3 **Spectral Angle** 0.89

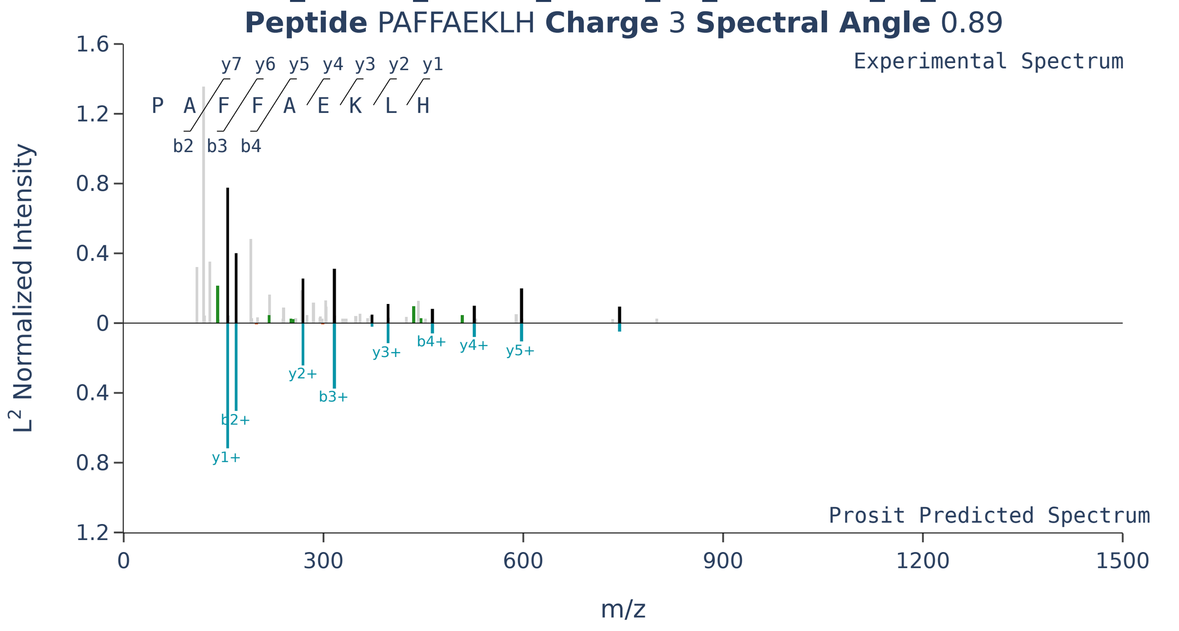


**Source** WSoh_301121_021221_HFGoe_M2_AnnexinA1_24h_R2 **Scan** 7827
**Peptide** DILTKRN **Charge** 2 **Spectral Angle** 0.95

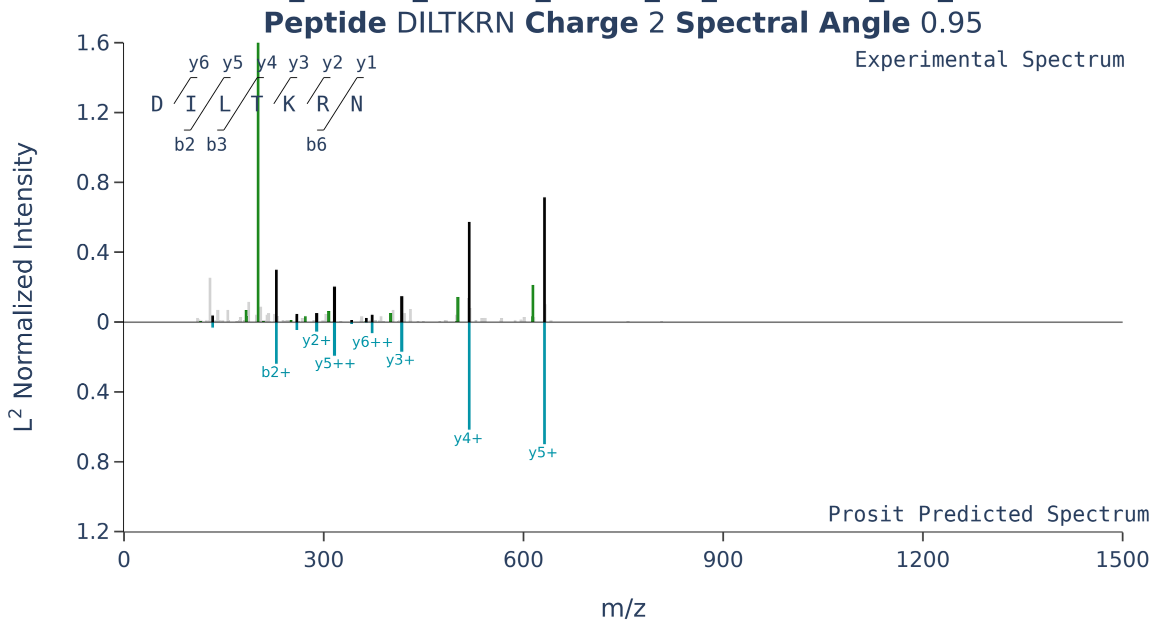


**Source** WSoh_301121_021221_HFGoe_M1_AnnexinA1_2h_R1 **Scan** 6962
**Peptide** RTNKEIRDI **Charge** 3 **Spectral Angle** 0.9

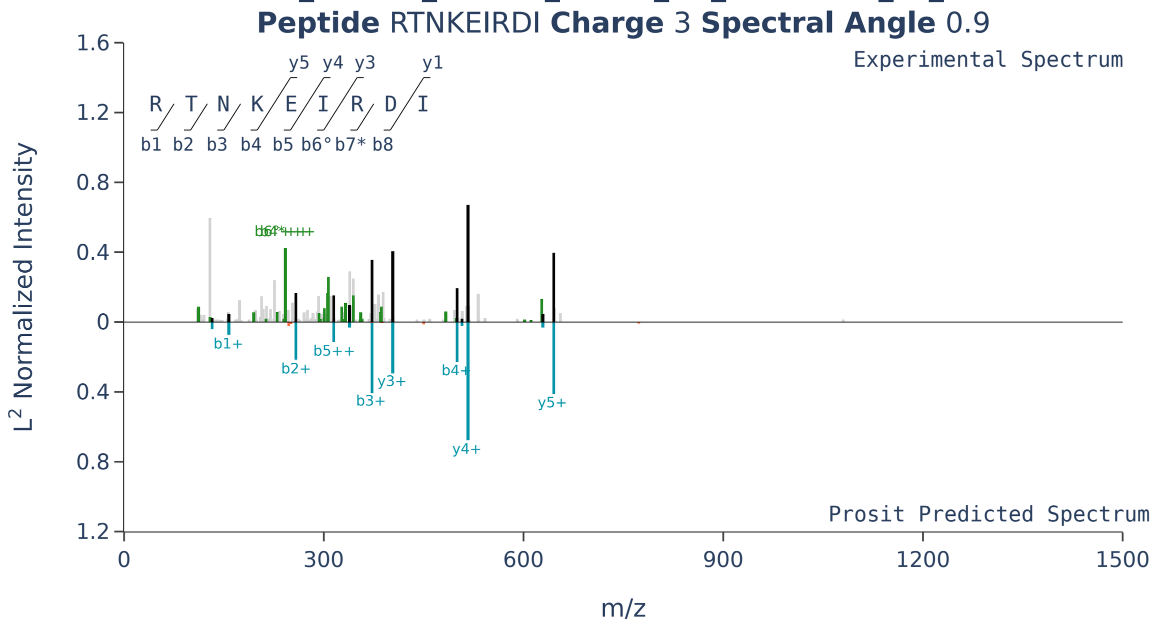


**Source** WSoh_301121_021221_HFGoe_M2_AnnexinA1_2h_R1 **Scan** 18405
**Peptide** NTILTTRSYPQLR **Charge** 3 **Spectral Angle** 0.94

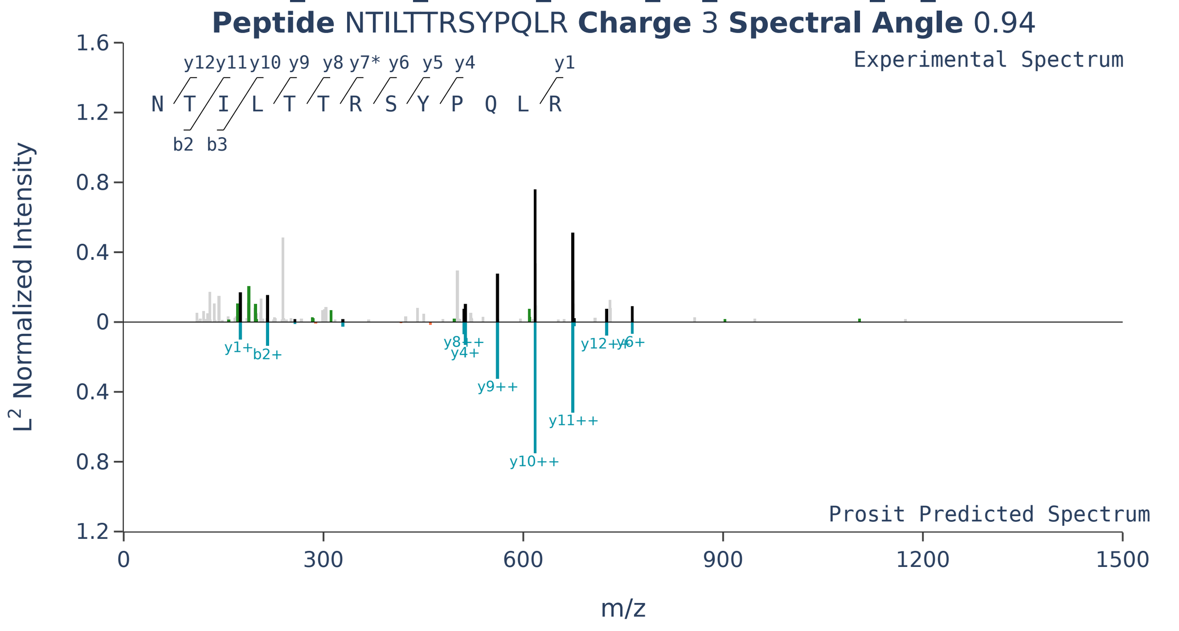


**Source** WSoh_301121_021221_HFGoe_M2_AnnexinA1_24h_R1 **Scan** 9184
**Peptide** SYPQLRR **Charge** 2 **Spectral Angle** 0.84

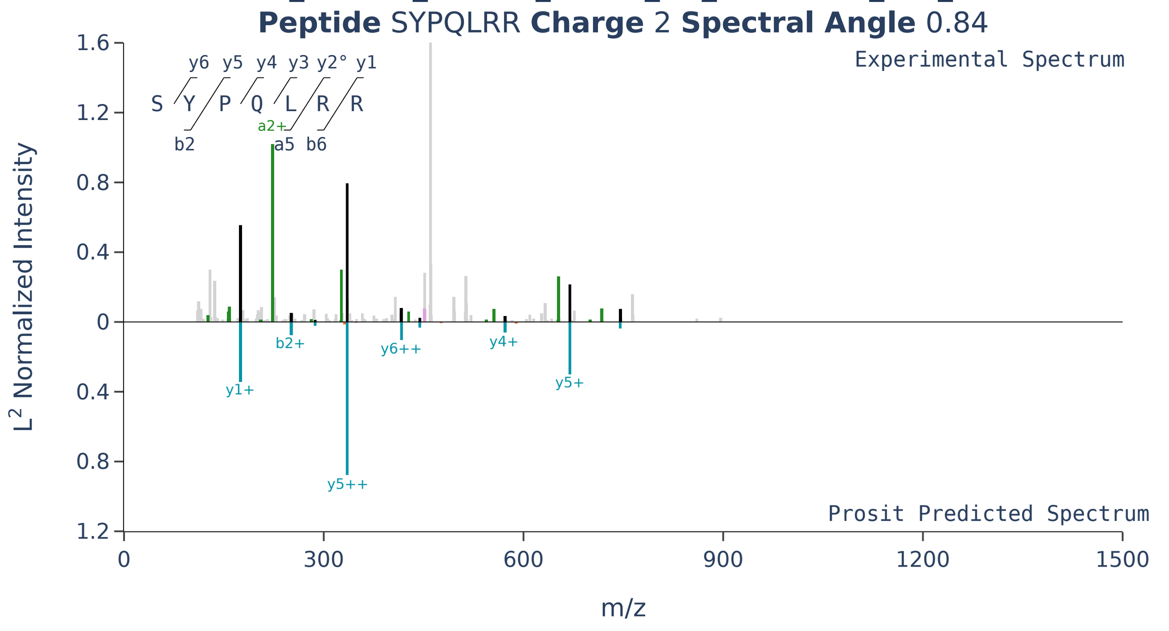


**Source** WSoh_301121_021221_HFGoe_M2_AnnexinA1_2h_R1 **Scan** 15759
**Peptide** AYLQETGKPLD **Charge** 1 **Spectral Angle** 0.91

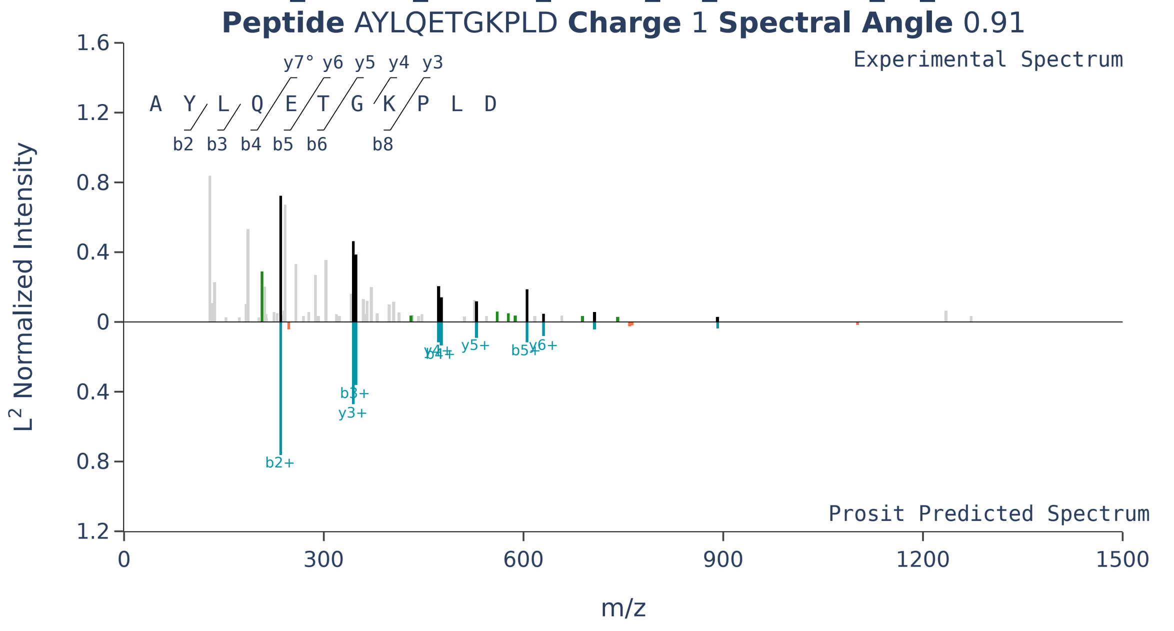


**Source** WSoh_301121_021221_HFGoe_M2_AnnexinA1_24h_R1 **Scan** 21104
**Peptide** ATSKPAFFAEKL **Charge** 3 **Spectral Angle** 0.89

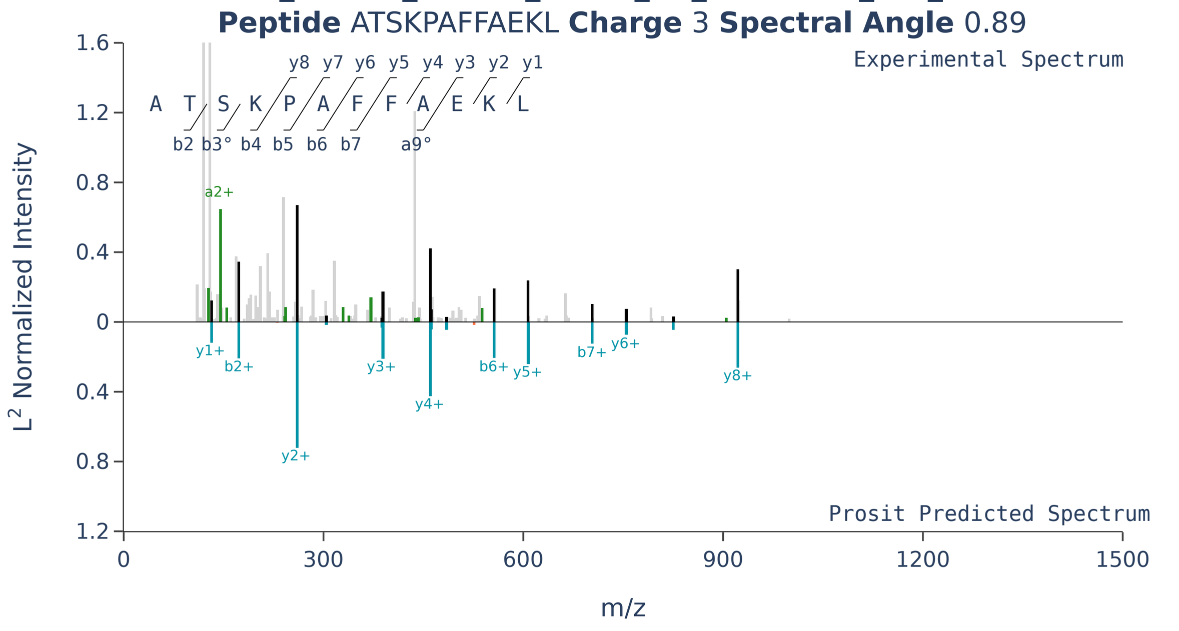

**Source** WSoh_301121_021221_HFGoe_M2_AnnexinA1_1h_R1 **Scan** 9721
**Peptide** QEYVQTVK **Charge** 2 **Spectral Angle** 0.84

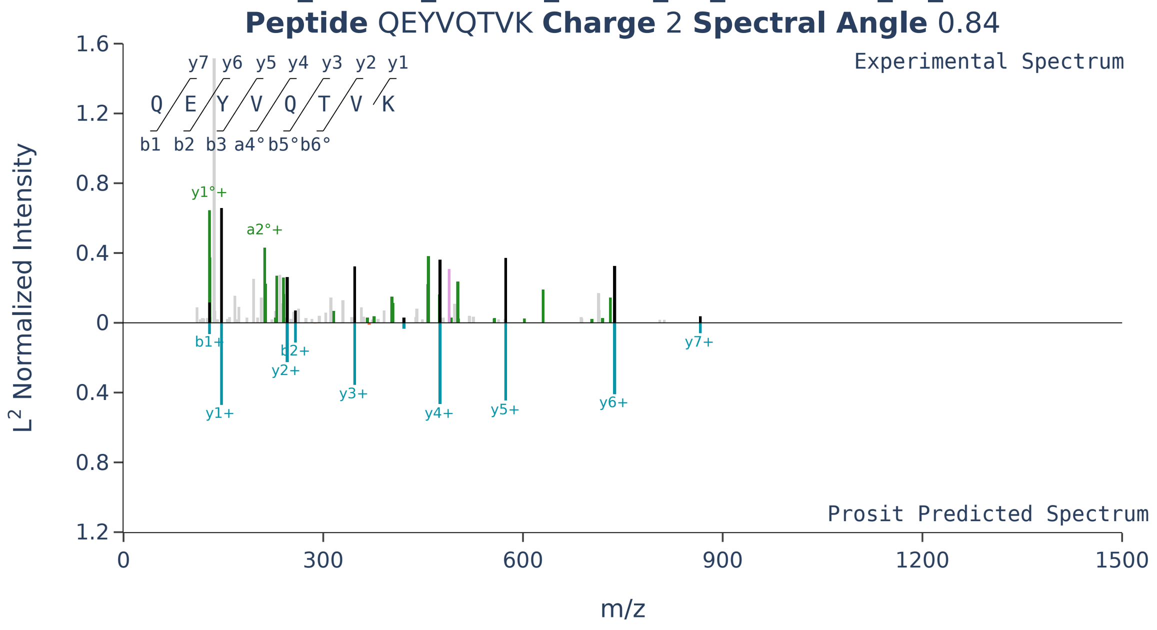


**Source** WSoh_301121_021221_HFGoe_M2_AnnexinA1_4h_R1 **Scan** 15642
**Peptide** GDFRNAL **Charge** 2 **Spectral Angle** 0.88

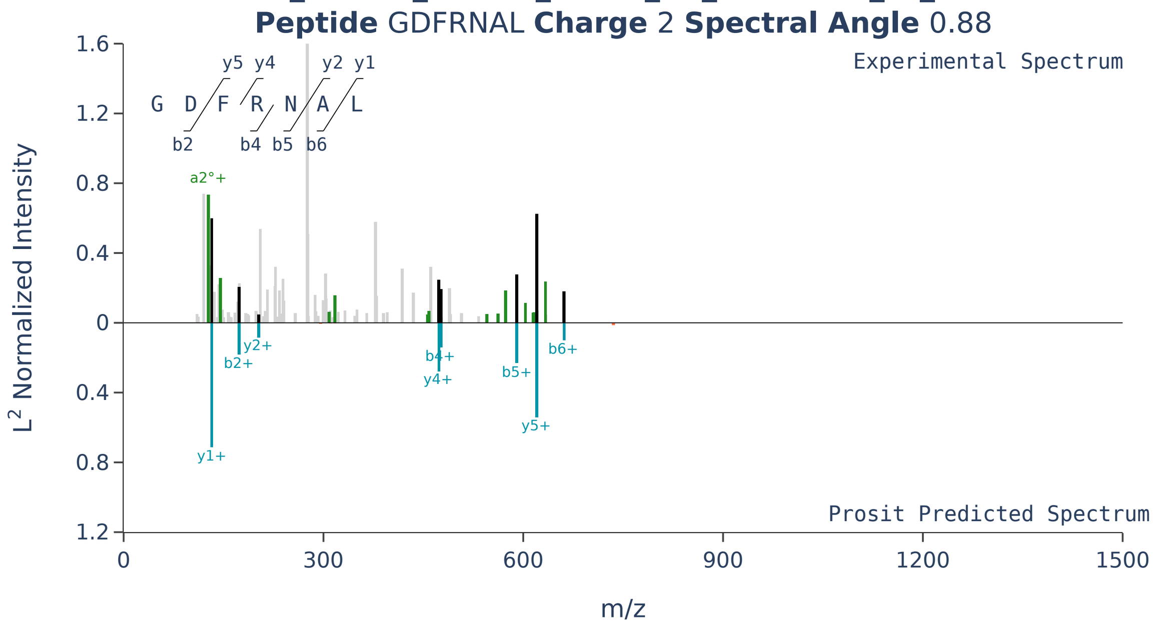


**Source** WSoh_301121_021221_HFGoe_M2_AnnexinA1_2h_R2 **Scan** 13889
**Peptide** IMVKGVDEA **Charge** 1 **Spectral Angle** 0.94

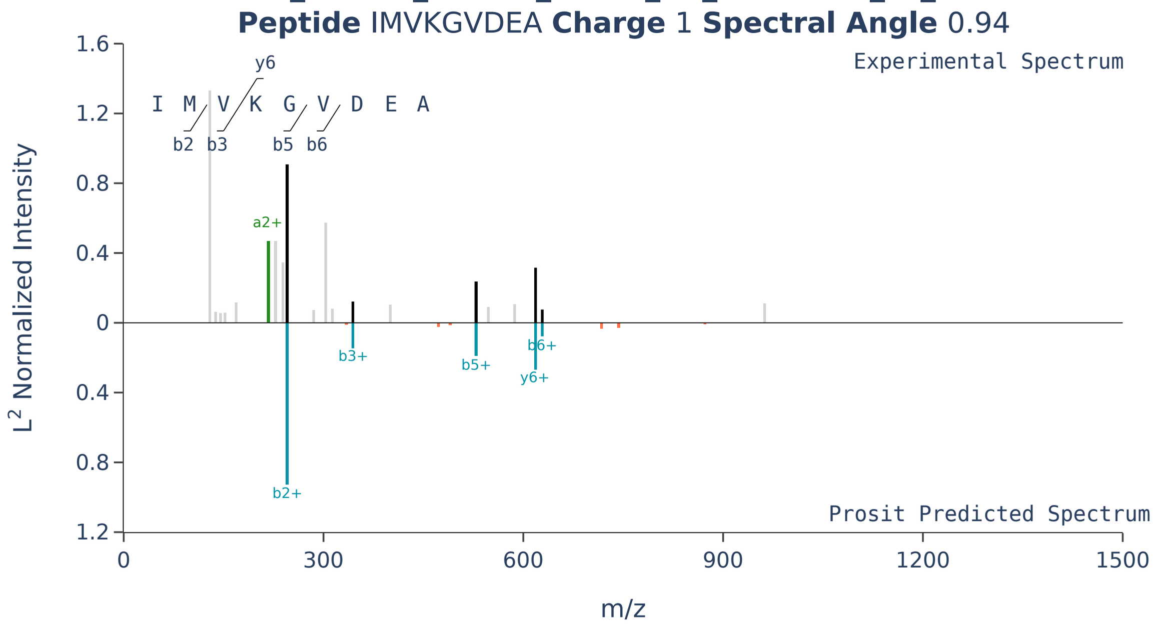


**Source** WSoh_301121_021221_HFGoe_M2_AnnexinA1_24h_R1 **Scan** 14845
**Peptide** TGHLEEVV **Charge** 1 **Spectral Angle** 0.91

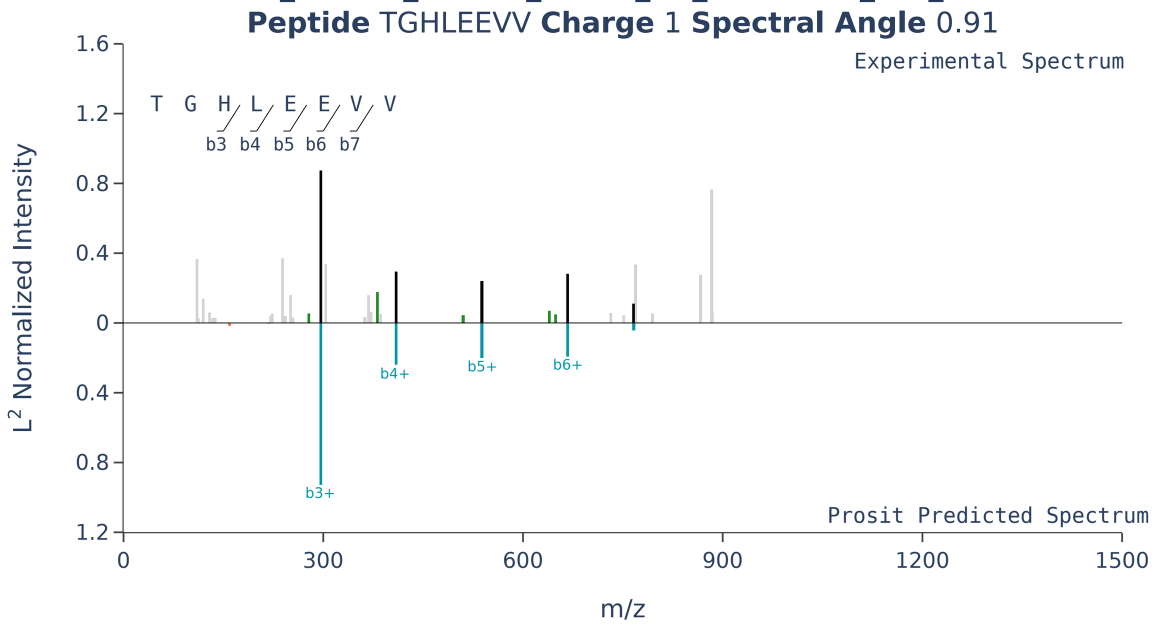


**Source** WSoh_301121_021221_HFGoe_M2_AnnexinA1_4h_R1 **Scan** 9436
**Peptide** MKGLGTDE **Charge** 2 **Spectral Angle** 0.9

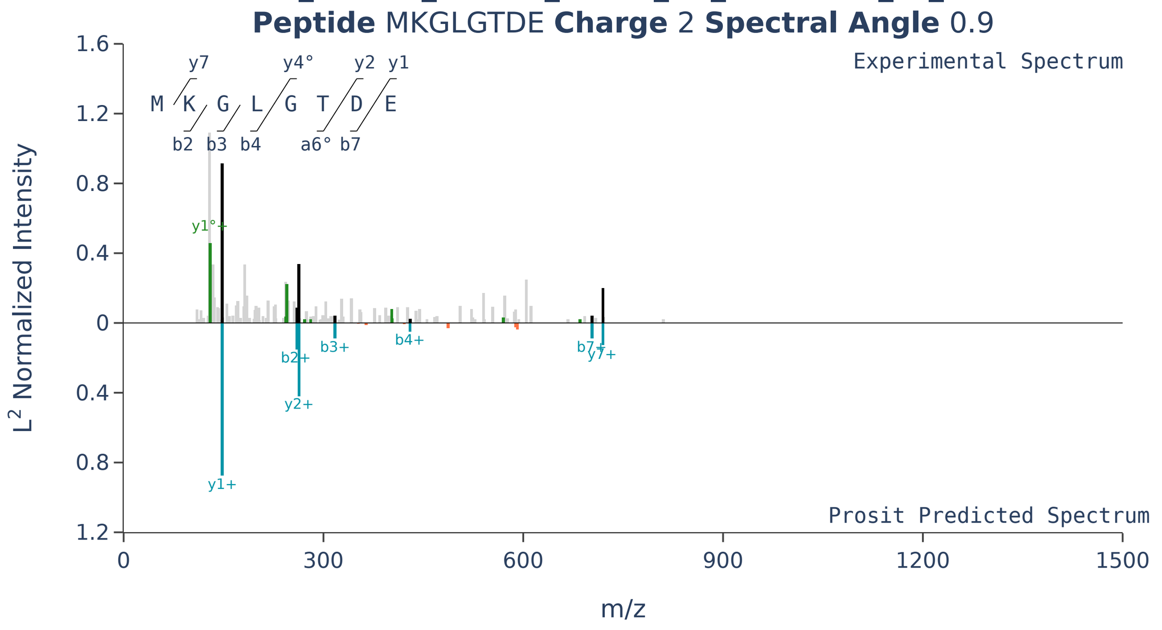


**Source** WSoh_301121_021221_HFGoe_M2_AnnexinA1_24h_R2 **Scan** 16427
**Peptide** DLADSDARAL **Charge** 2 **Spectral Angle** 0.89

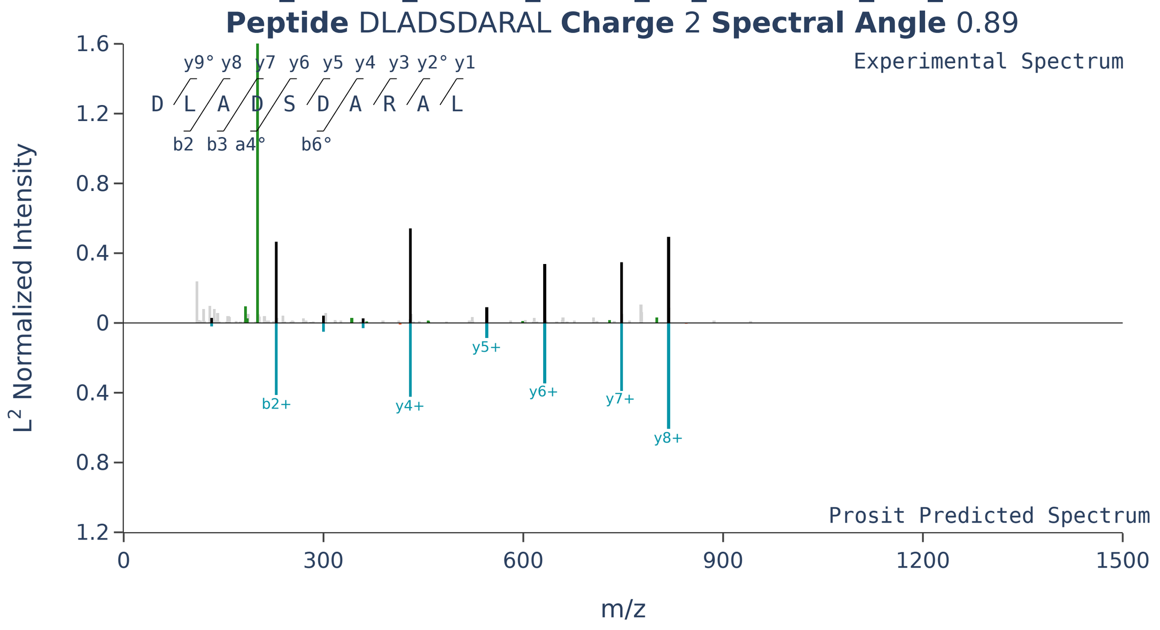


**Source** WSoh_301121_021221_HFGoe_M2_AnnexinA1_1h_R2 **Scan** 19167
**Peptide** FIENEEQEYVQTVK **Charge** 2 **Spectral Angle** 0.9

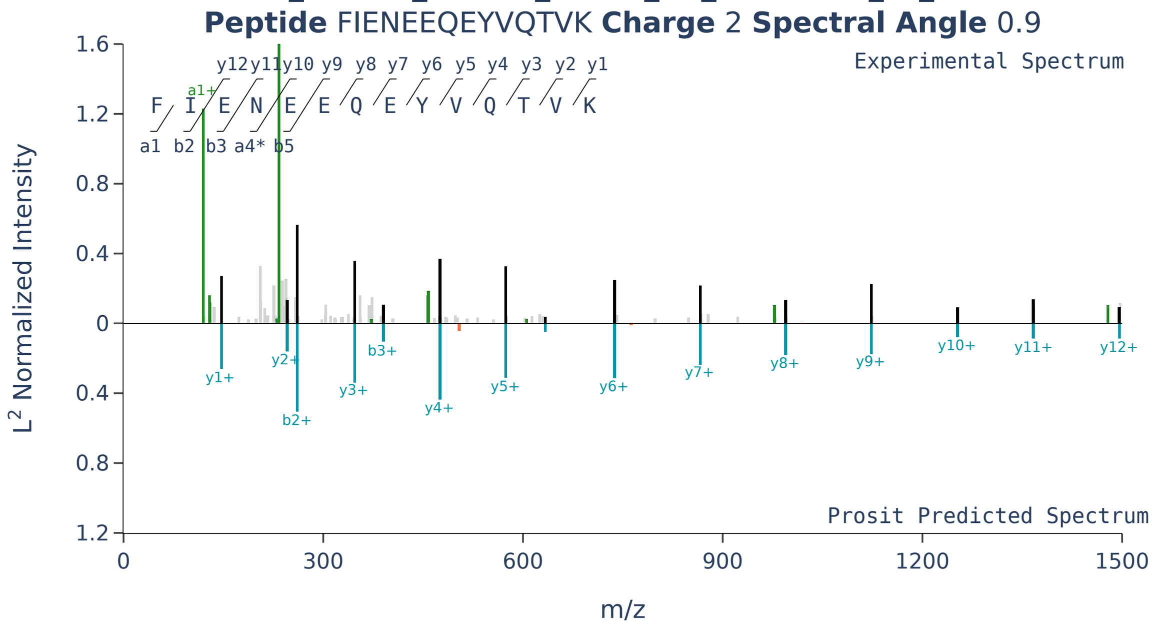


**Source** WSoh_301121_021221_HFGoe_M1_AnnexinA1_2h_R2 **Scan** 13445
**Peptide** YLQETGKPLD **Charge** 1 **Spectral Angle** 0.9

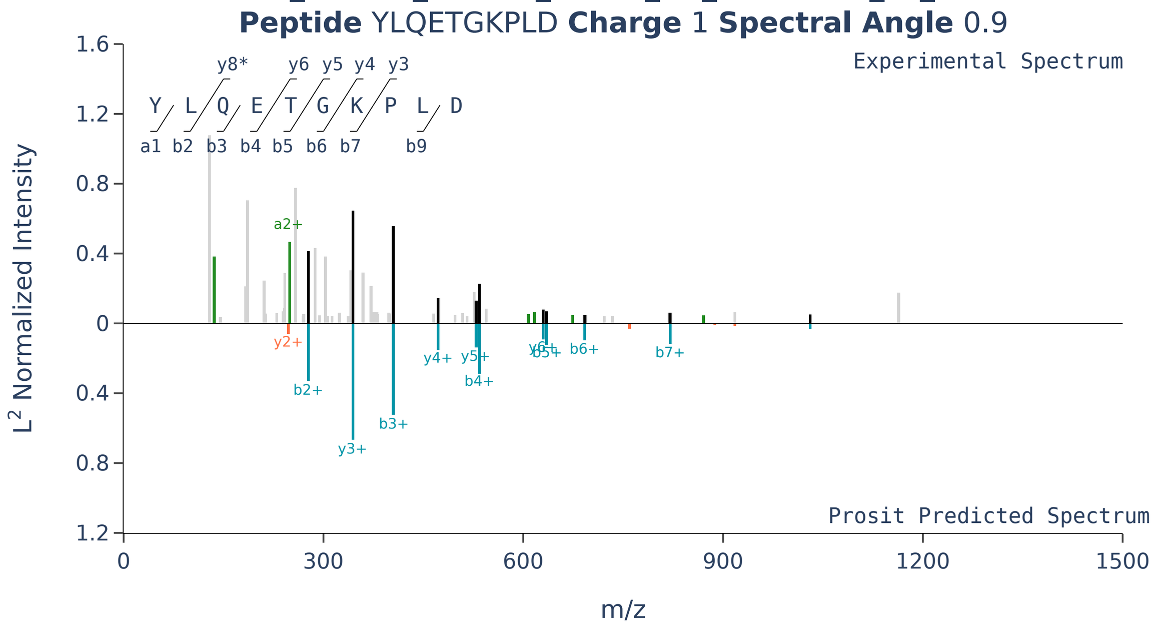


**Source** WSoh_301121_021221_HFGoe_M1_AnnexinA1_2h_R2 **Scan** 15167
**Peptide** ALMVKGVDE **Charge** 2 **Spectral Angle** 0.87

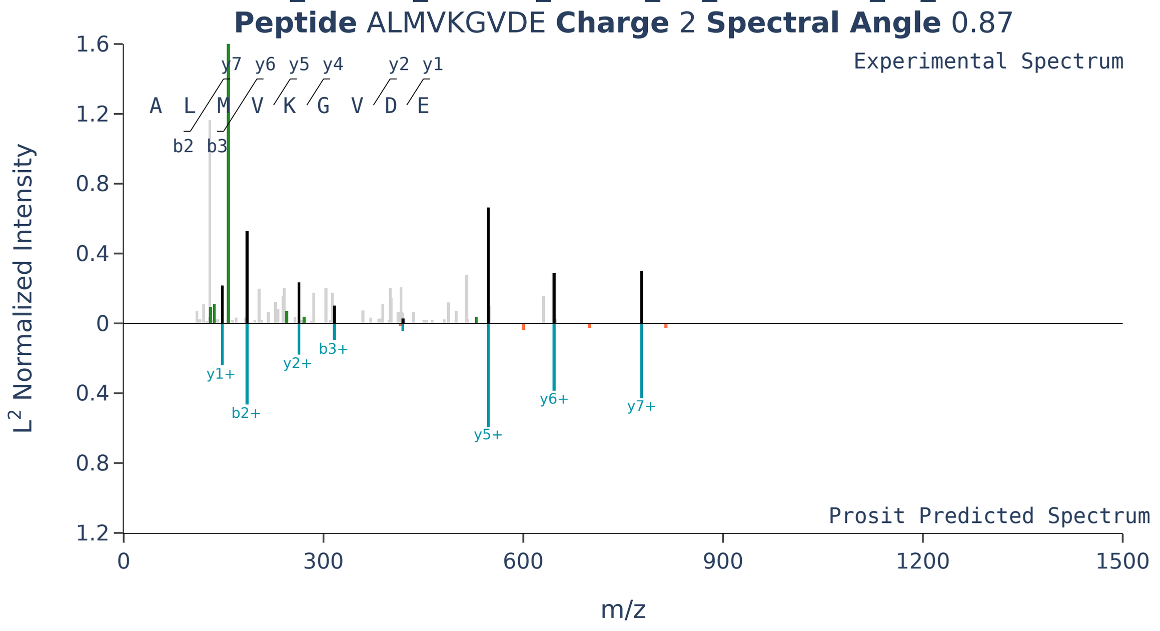


**Source** WSoh_301121_021221_HFGoe_M2_AnnexinA1_4h_R1 **Scan** 16634
**Peptide** CQAILDETKGDYE **Charge** 2 **Spectral Angle** 0.92

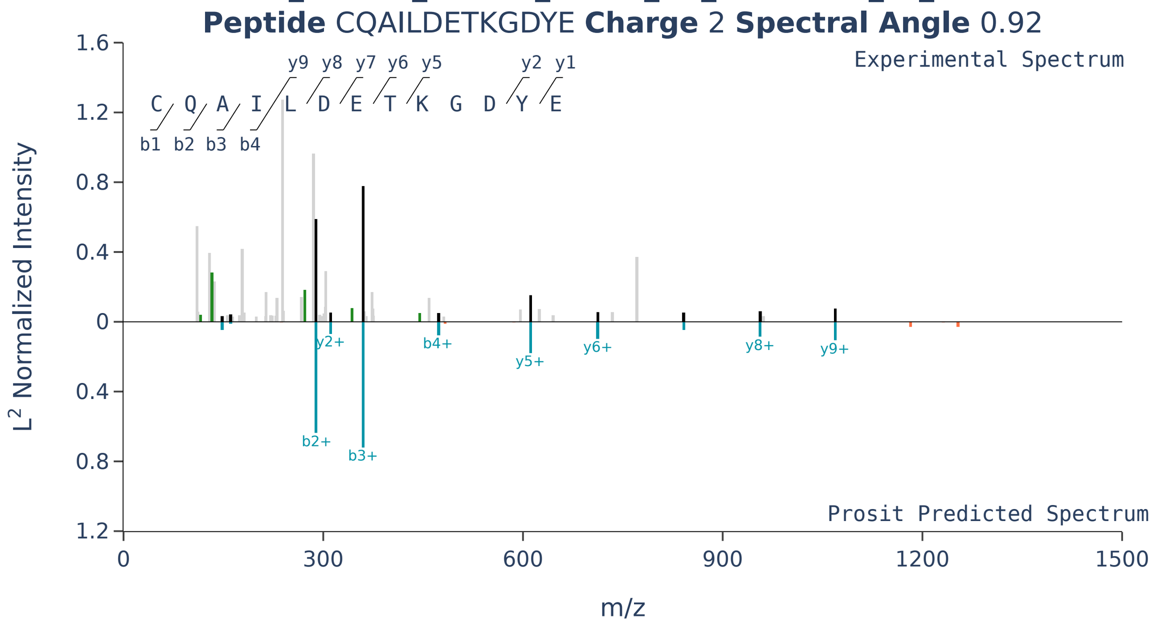


**Source** WSoh_301121_021221_HFGoe_M1_AnnexinA1_1h_R1 **Scan** 9661
**Peptide** KKALTGHLEEV **Charge** 2 **Spectral Angle** 0.91

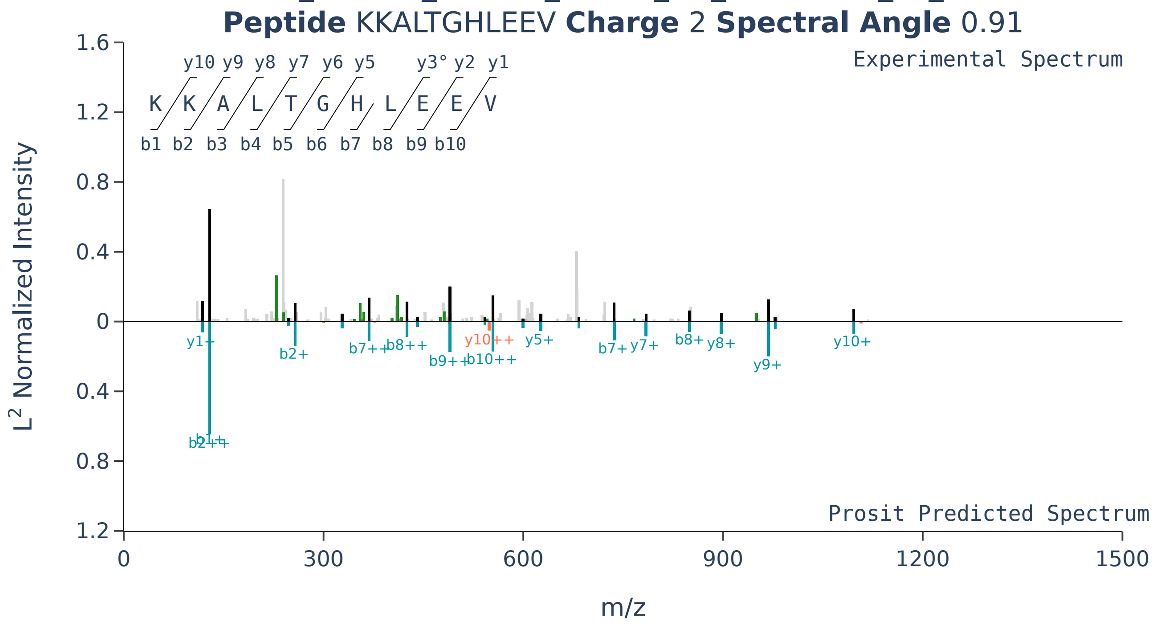


**Source** WSoh_301121_021221_HFGoe_M2_AnnexinA1_2h_R2 **Scan** 17349
**Peptide** AKGDRSEDFGVNEDL **Charge** 3 **Spectral Angle** 0.91

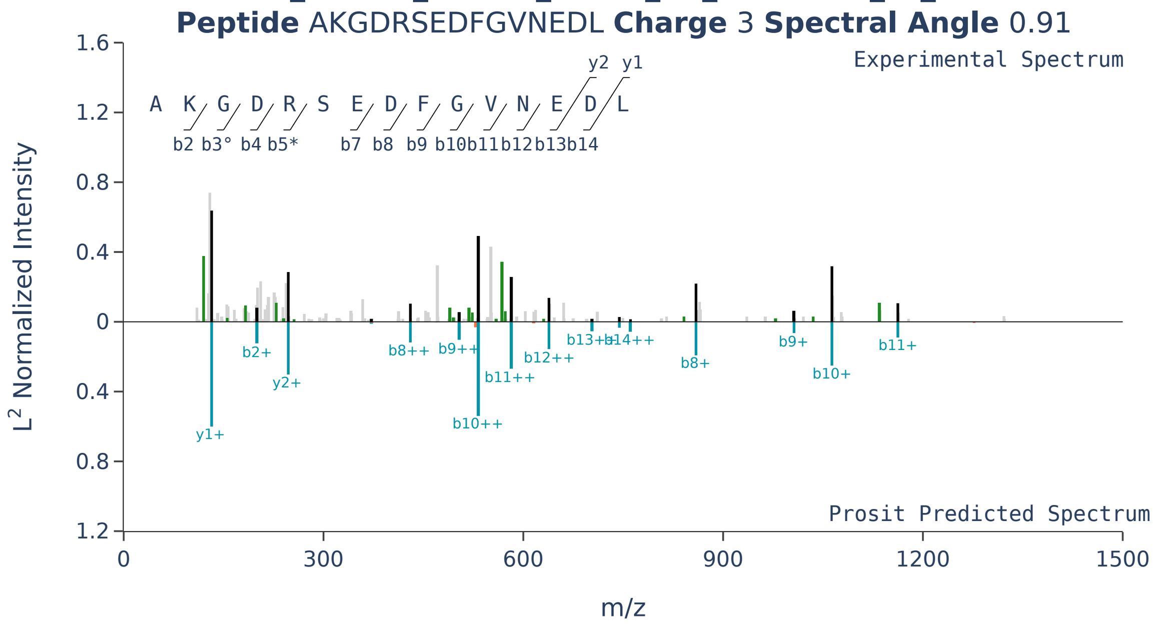


**Source** WSoh_301121_021221_HFGoe_M1_AnnexinA1_2h_R1 **Scan** 9947
**Peptide** LQETGKPLD **Charge** 2 **Spectral Angle** 0.89

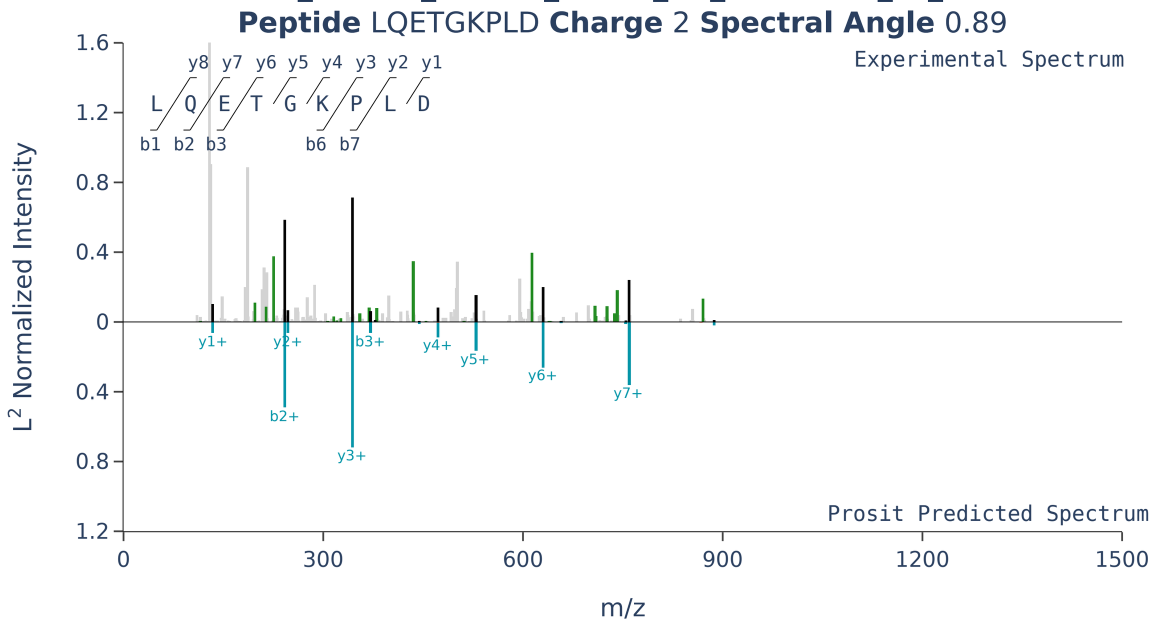


**Source** WSoh_301121_021221_HFGoe_M2_AnnexinA1_24h_R2 **Scan** 11491
**Peptide** AKGDRSEDFGV **Charge** 2 **Spectral Angle** 0.89

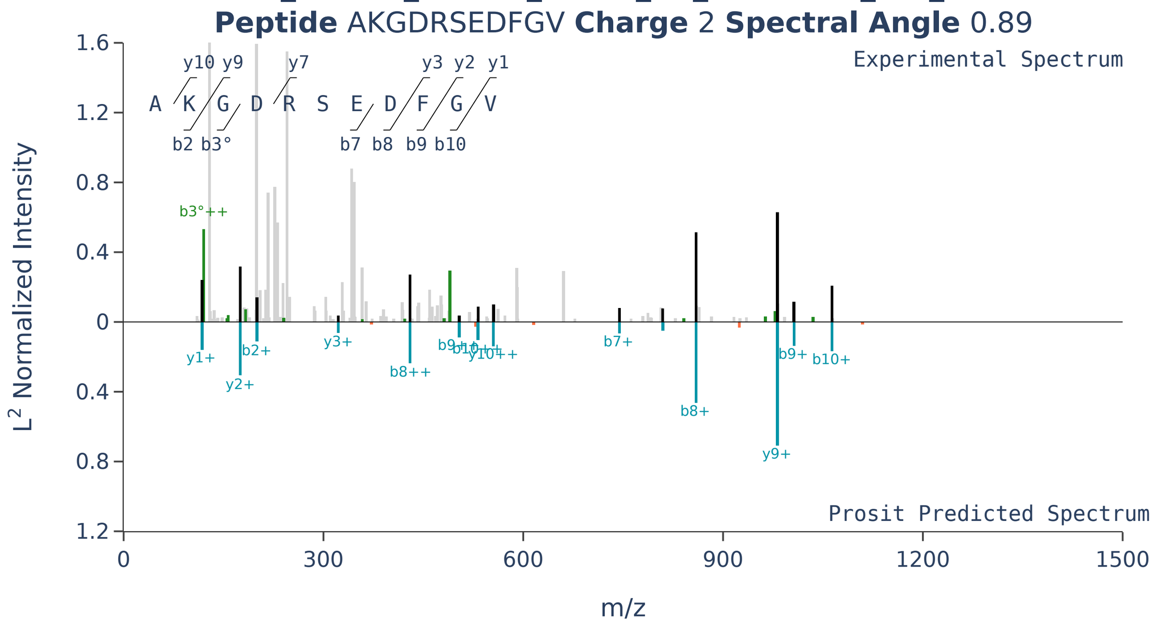


**Source** WSoh_301121_021221_HFGoe_M1_AnnexinA1_24h_R2 **Scan** 9848
**Peptide** KGDIEKCL **Charge** 2 **Spectral Angle** 0.91

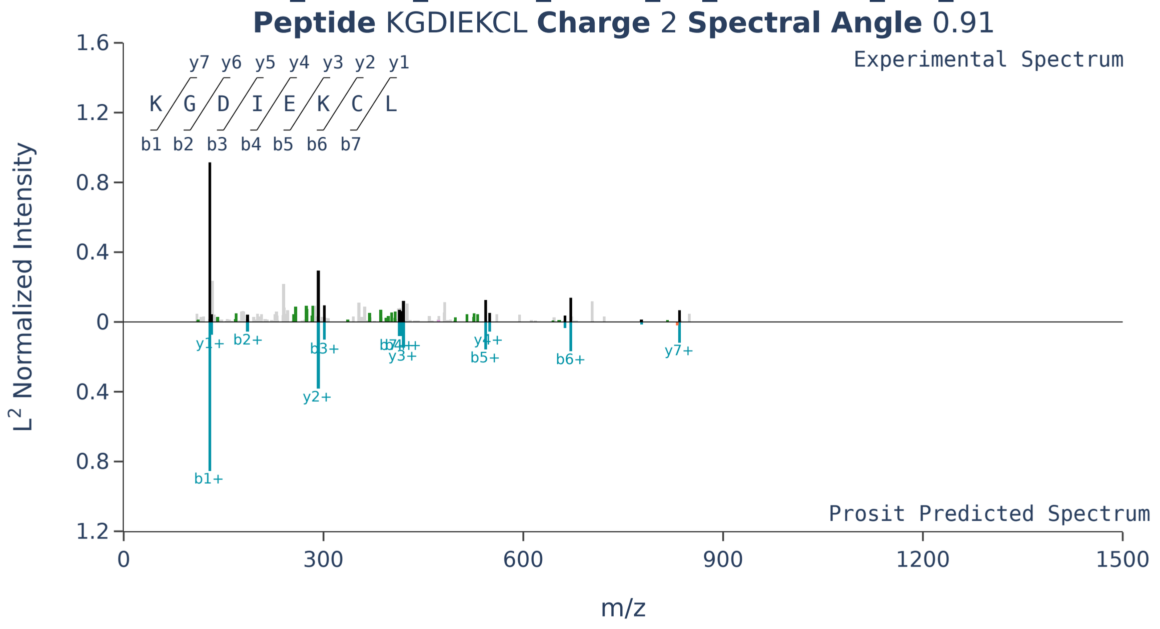

**Source** WSoh_301121_021221_HFGoe_M1_AnnexinA1_4h_R1 **Scan** 13955
**Peptide** GHLEEVV **Charge** 2 **Spectral Angle** 0.92

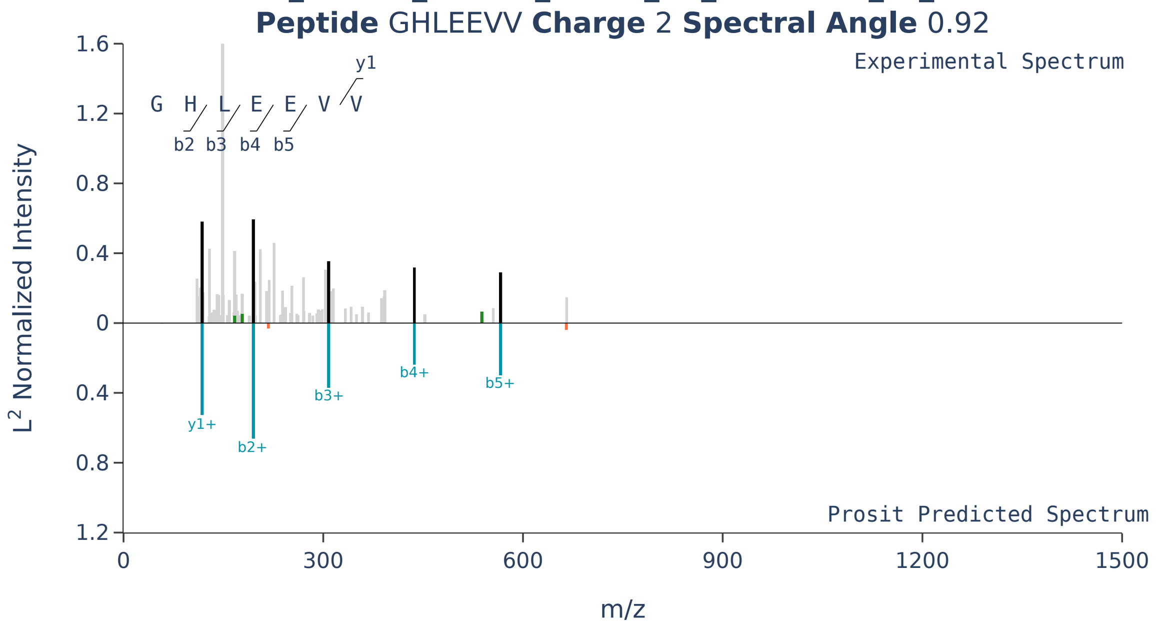


**Source** WSoh_301121_021221_HFGoe_M2_AnnexinA1_24h_R1 **Scan** 8124
**Peptide** LSLAKGDRSE **Charge** 2 **Spectral Angle** 0.89

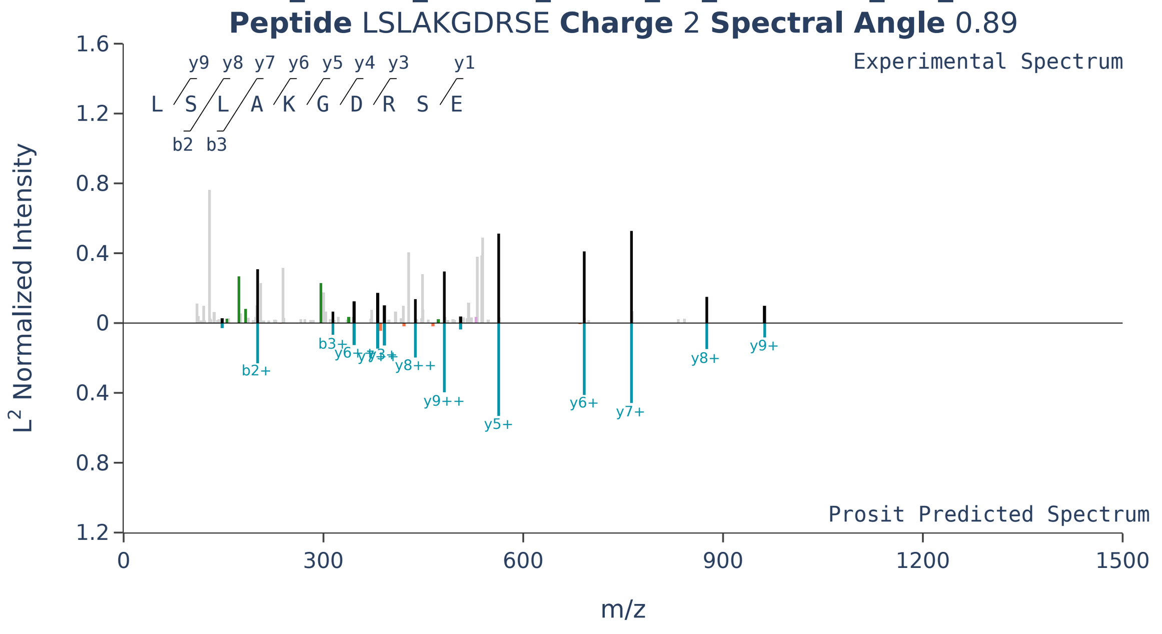


**Source** WSoh_301121_021221_HFGoe_M2_AnnexinA1_4h_R2 **Scan** 6568
**Peptide** KKALTGHLEE **Charge** 2 **Spectral Angle** 0.92

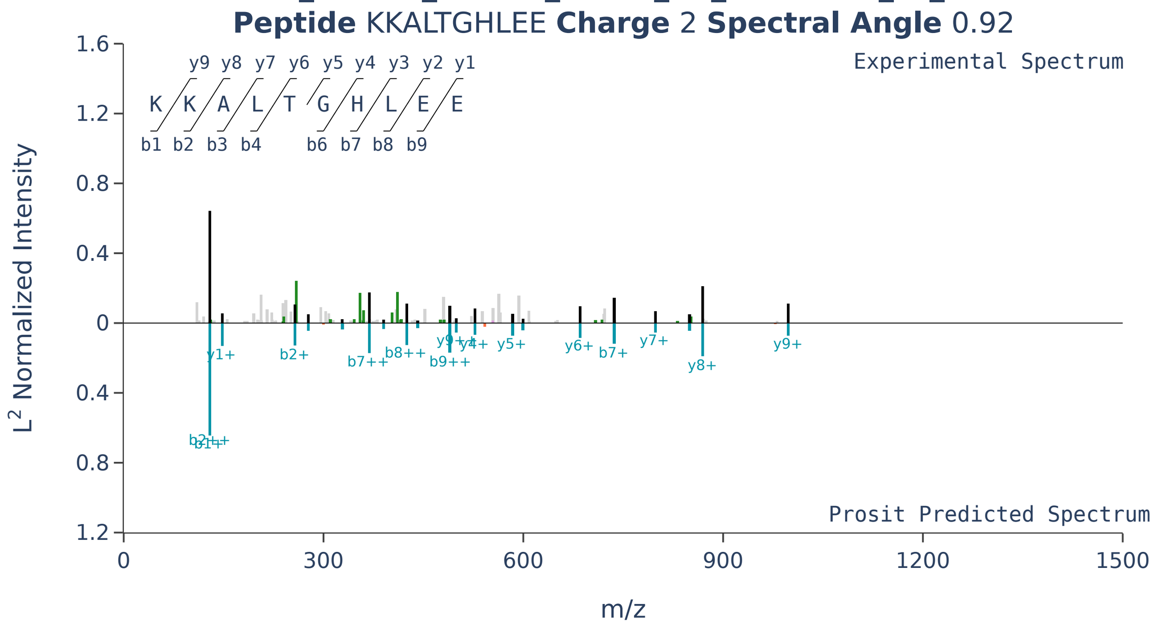


**Source** WSoh_301121_021221_HFGoe_M2_AnnexinA1_4h_R1 **Scan** 13389
**Peptide** KAAYLQETGKPLD **Charge** 2 **Spectral Angle** 0.94

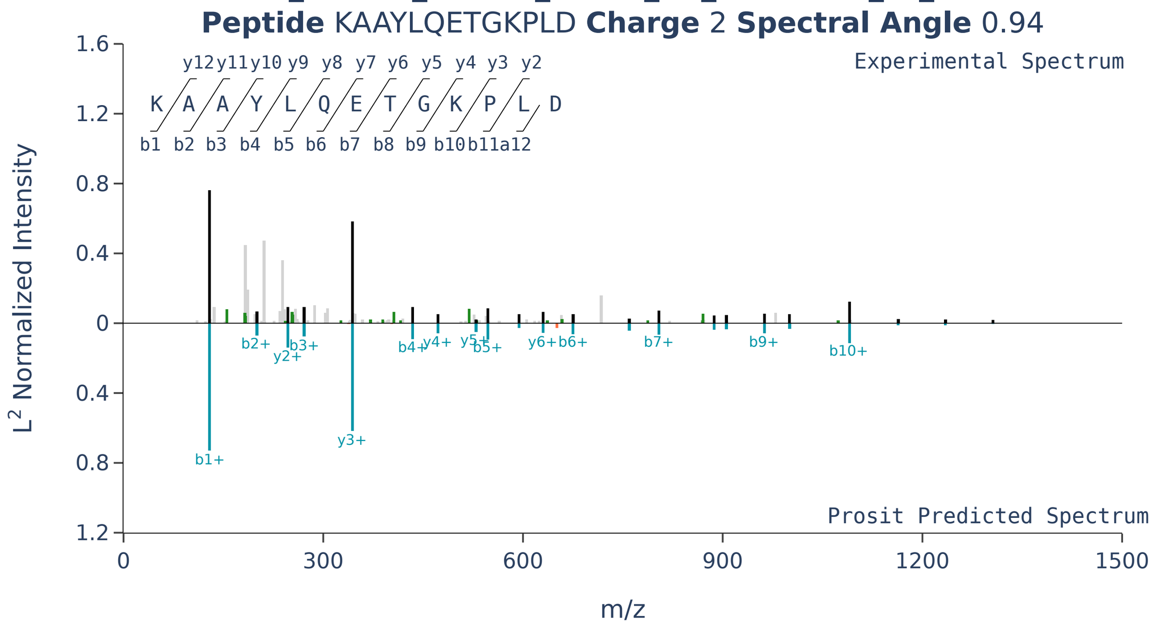


**Source** WSoh_301121_021221_HFGoe_M2_AnnexinA1_4h_R1 **Scan** 18619
**Peptide** LLKTPAQFD **Charge** 2 **Spectral Angle** 0.9

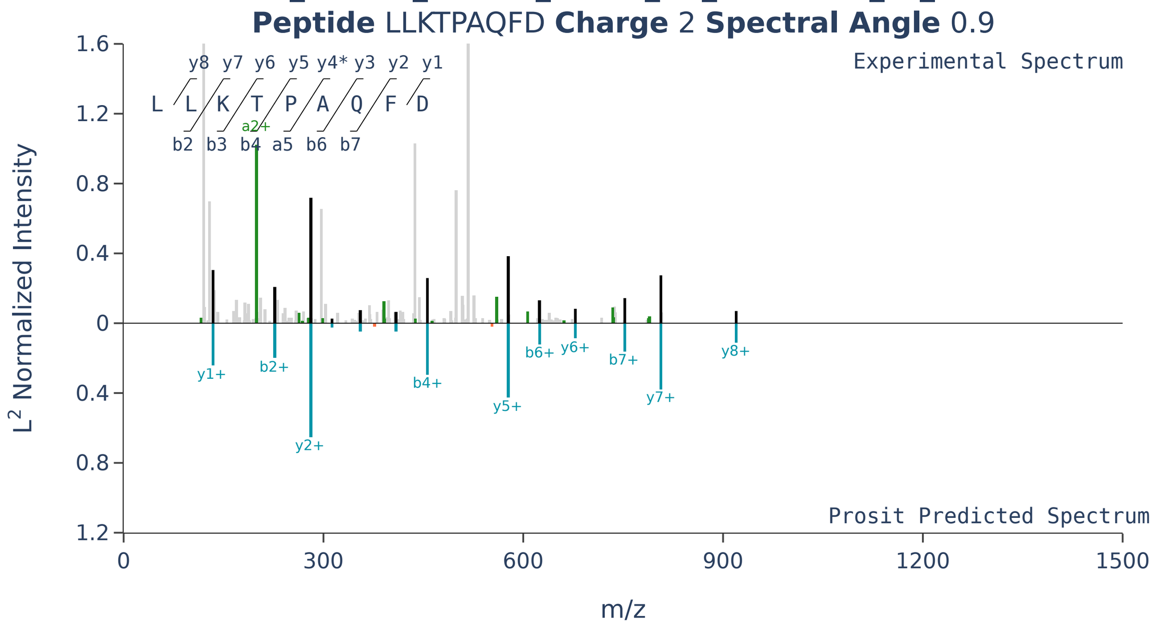


**Source** WSoh_301121_021221_HFGoe_M2_AnnexinA1_4h_R1 **Scan** 12483
**Peptide** NEEQEYVQTVKS **Charge** 2 **Spectral Angle** 0.91

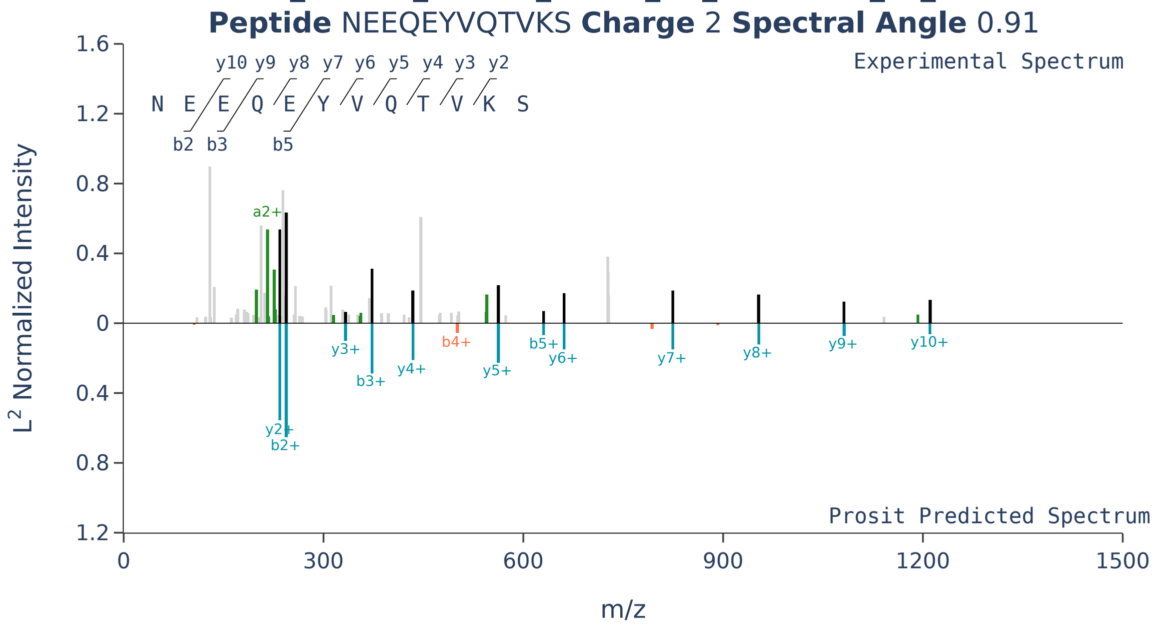


**Source** WSoh_301121_021221_HFGoe_M1_AnnexinA1_4h_R1 **Scan** 9910
**Peptide** AKDITSDTSGDFRN **Charge** 3 **Spectral Angle** 0.92

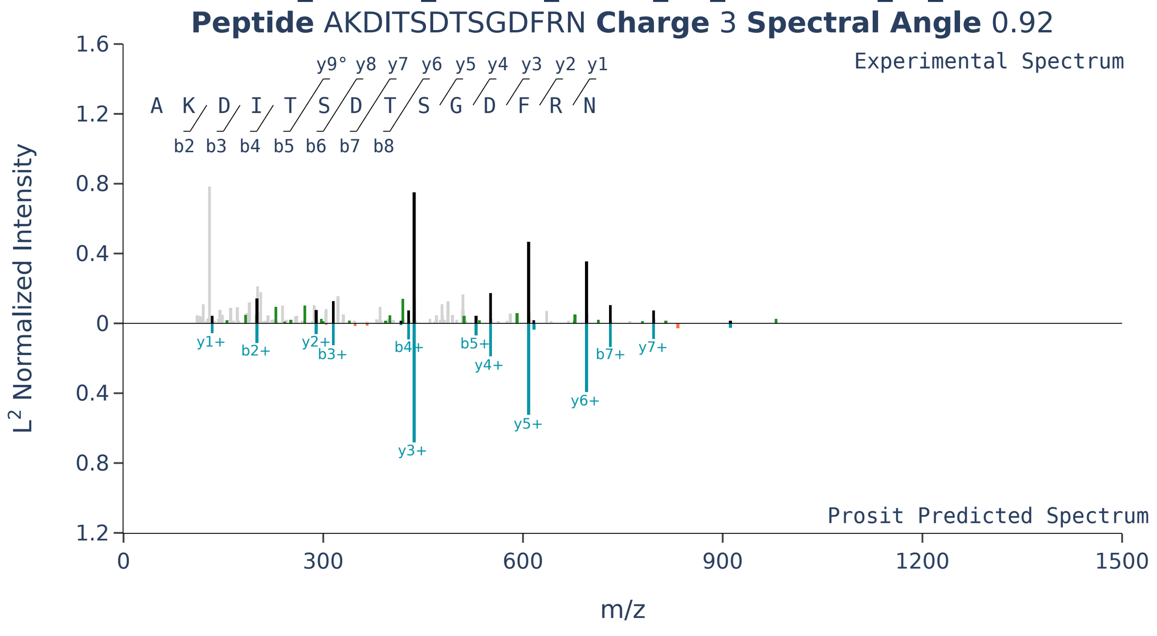


**Source** WSoh_301121_021221_HFGoe_M2_AnnexinA1_24h_R2 **Scan** 6577
**Peptide** INRVYRE **Charge** 2 **Spectral Angle** 0.86

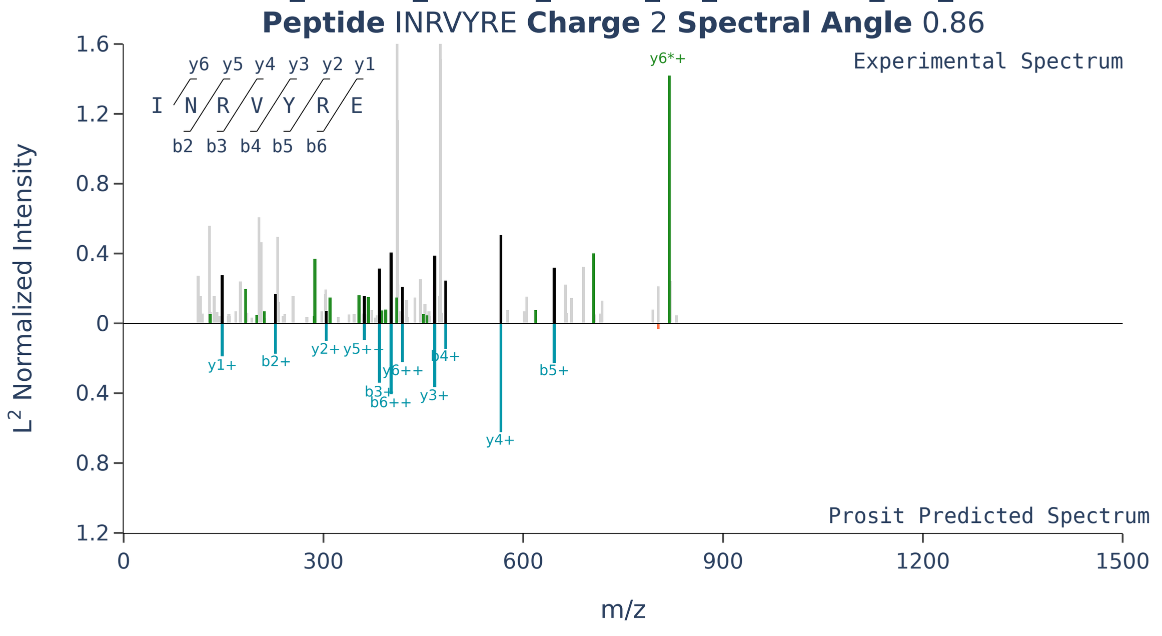


**Source** WSoh_301121_021221_HFGoe_M2_AnnexinA1_24h_R2 **Scan** 10419
**Peptide** EIRDINRV **Charge** 2 **Spectral Angle** 0.81

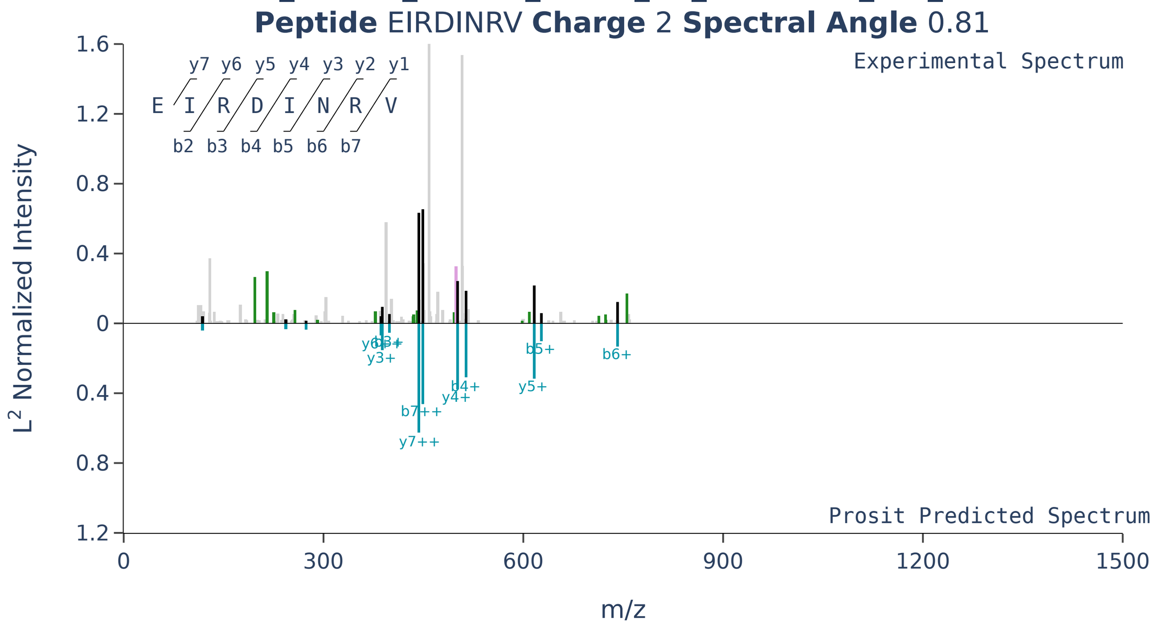


**Source** WSoh_301121_021221_HFGoe_M2_AnnexinA1_4h_R1 **Scan** 18609
**Peptide** LTGHLEEVV **Charge** 2 **Spectral Angle** 0.87

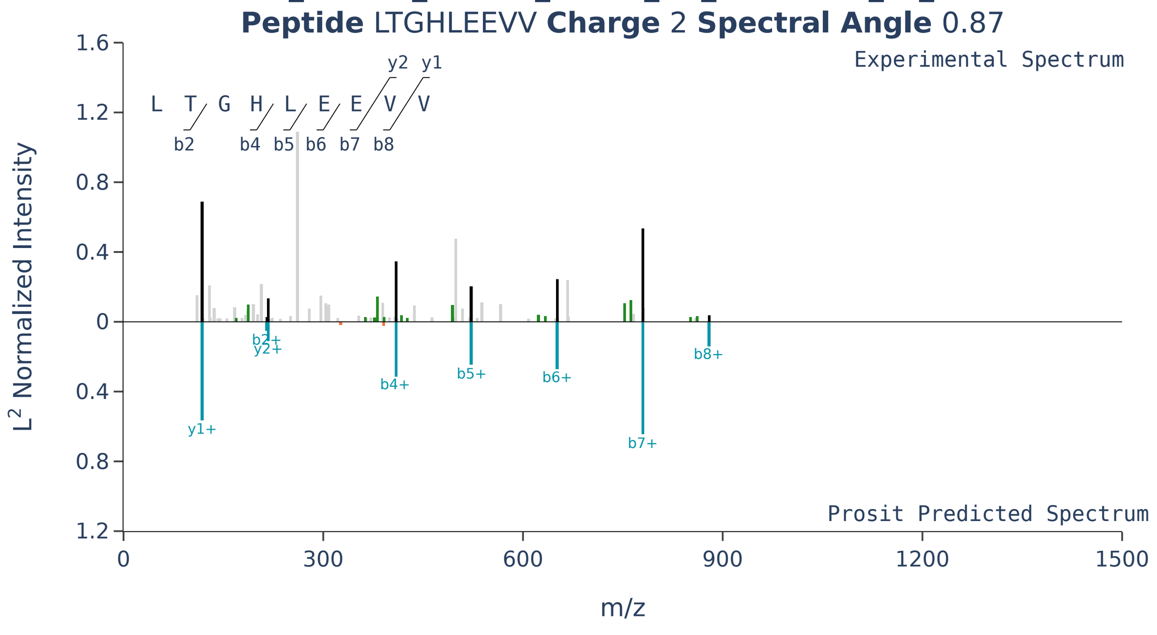


**Source** WSoh_301121_021221_HFGoe_M2_AnnexinA1_1h_R1 **Scan** 17659
**Peptide** LDMNDIKA **Charge** 2 **Spectral Angle** 0.91

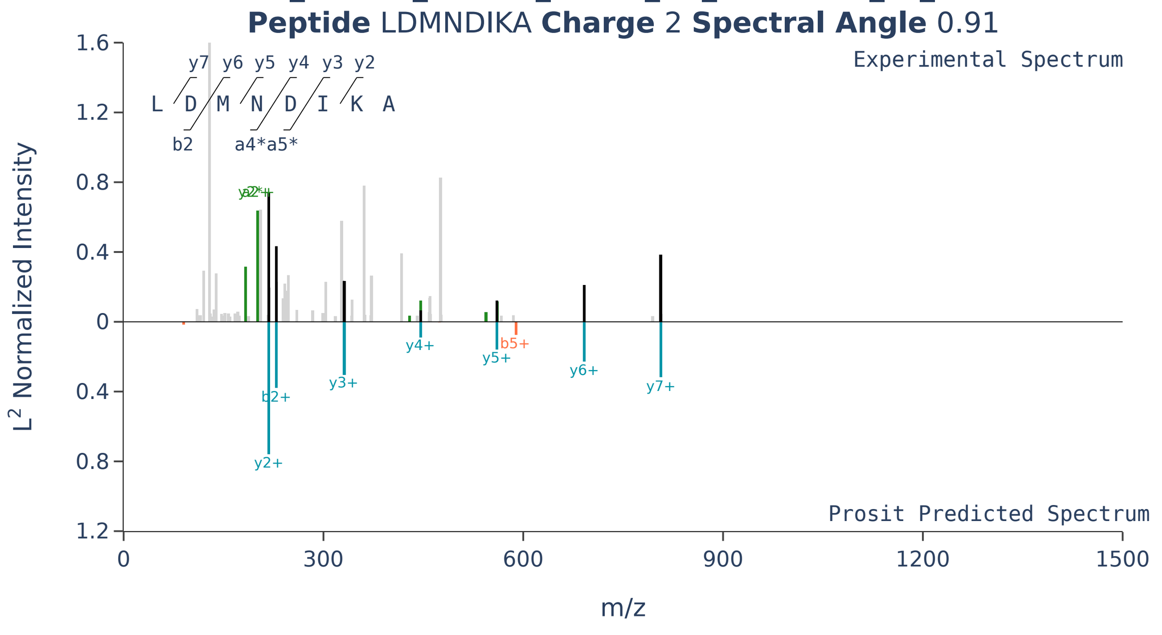


**Source** WSoh_301121_021221_HFGoe_M2_AnnexinA1_24h_R1 **Scan** 13585
**Peptide** SDARALYEA **Charge** 2 **Spectral Angle** 0.91

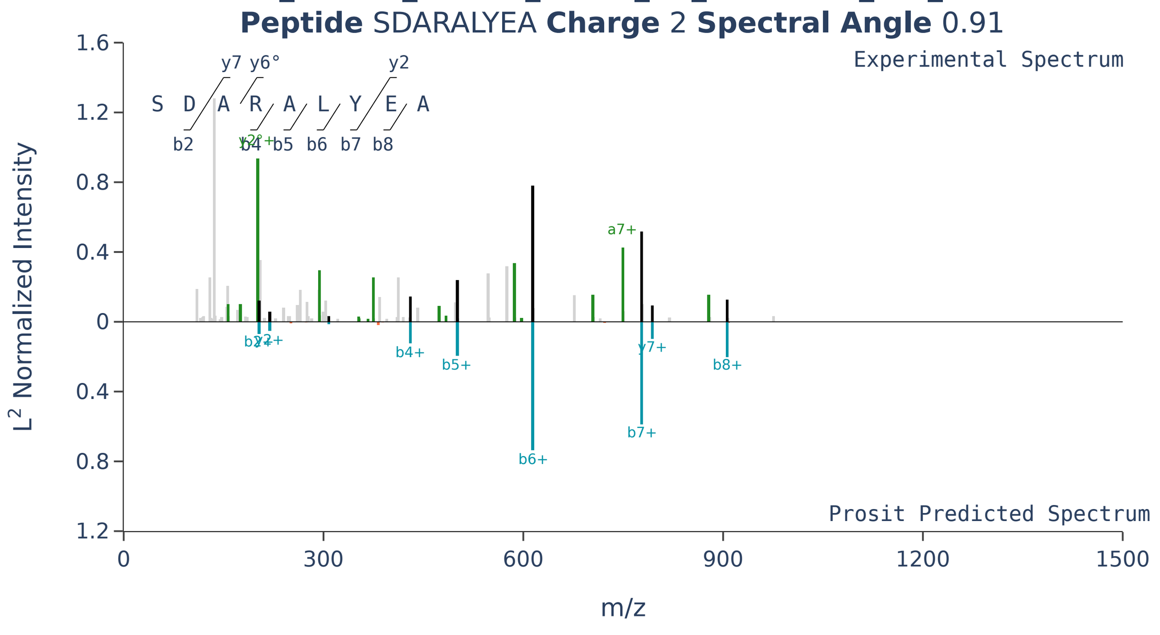


**Source** WSoh_301121_021221_HFGoe_M2_AnnexinA1_2h_R1 **Scan** 16071
**Peptide** ATSKPAFFAEKLH **Charge** 4 **Spectral Angle** 0.92

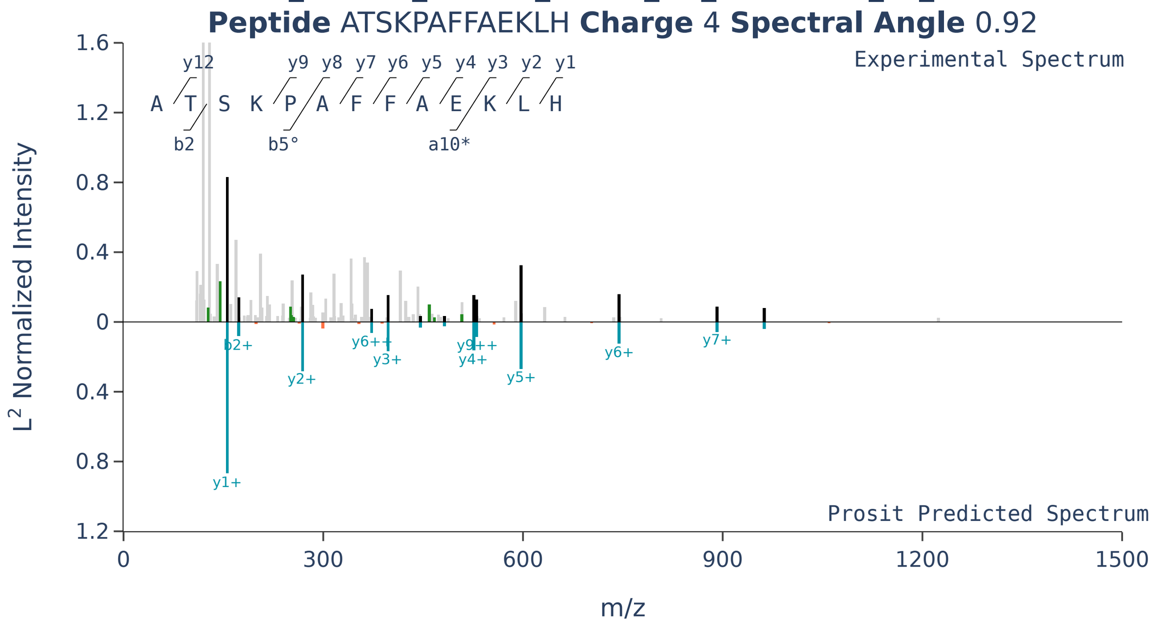


**Source** WSoh_301121_021221_HFGoe_M2_AnnexinA1_2h_R2 **Scan** 22162
**Peptide** DYEKILV **Charge** 2 **Spectral Angle** 0.87

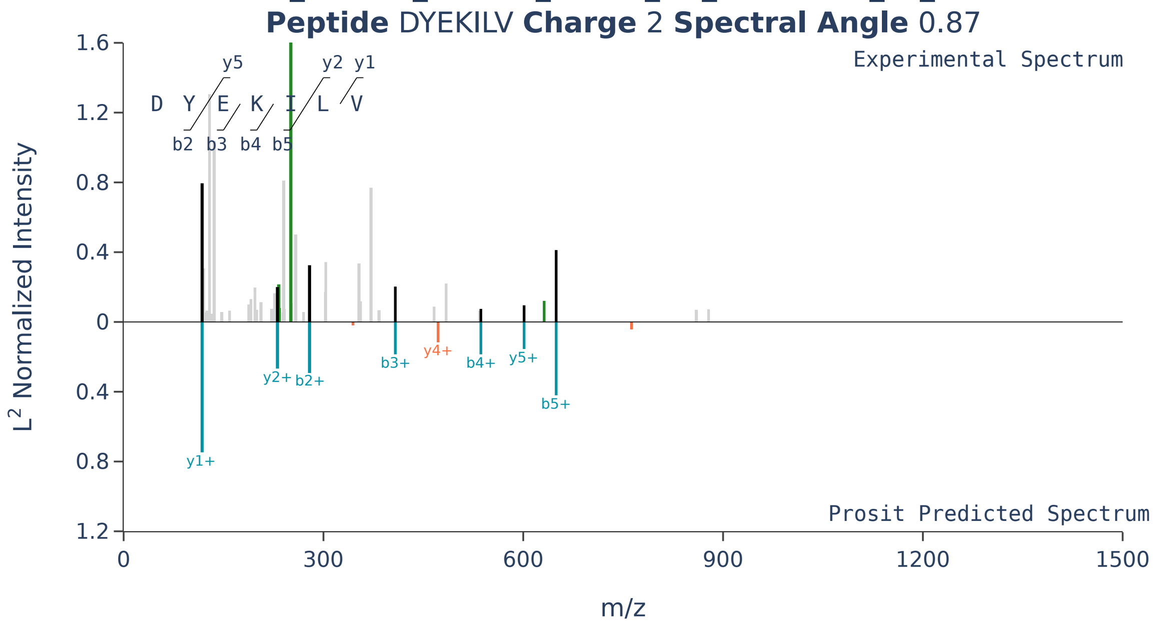


**Source** WSoh_301121_021221_HFGoe_M1_AnnexinA1_4h_R1 **Scan** 10679
**Peptide** KEIRDINRVY **Charge** 2 **Spectral Angle** 0.87

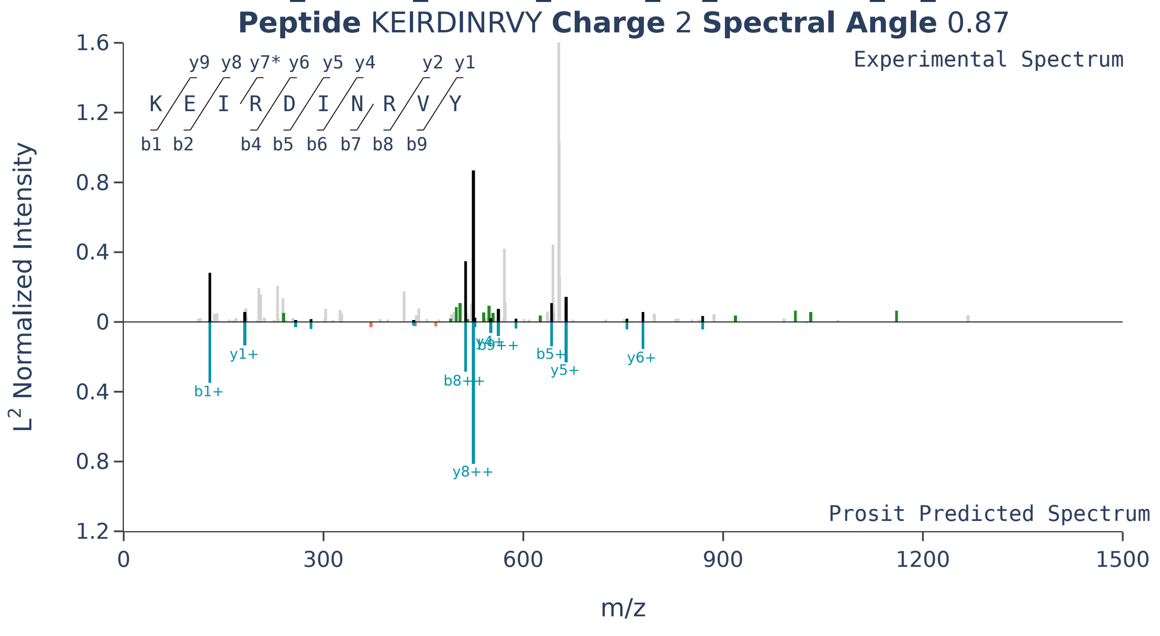

**Source** WSoh_301121_021221_HFGoe_M2_AnnexinA1_4h_R2 **Scan** 18515
**Peptide** SKPAFFAE **Charge** 2 **Spectral Angle** 0.95

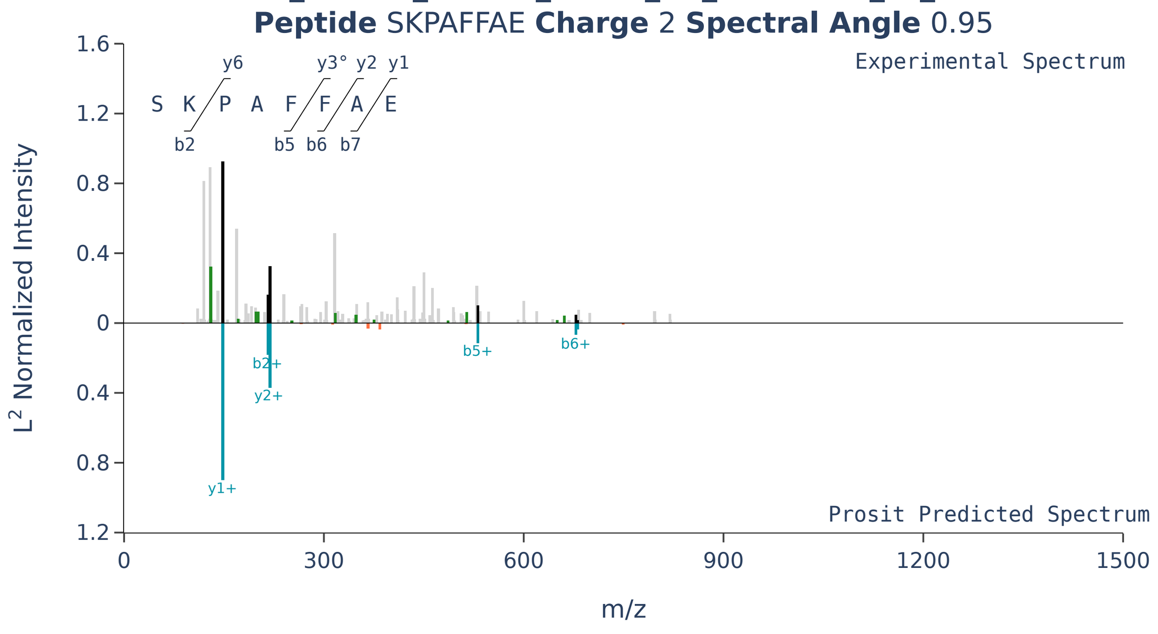


**Source** WSoh_301121_021221_HFGoe_M1_AnnexinA1_24h_R1 **Scan** 9686
**Peptide** MKGLGTDED **Charge** 2 **Spectral Angle** 0.91

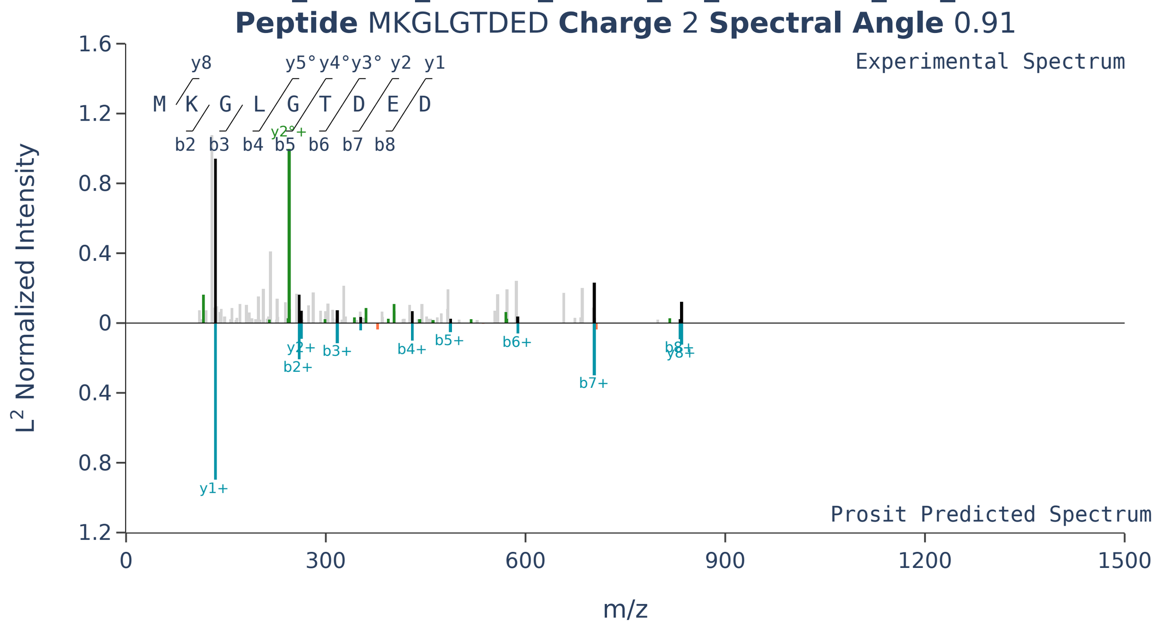


**Source** WSoh_301121_021221_HFGoe_M1_AnnexinA1_4h_R2 **Scan** 10975
**Peptide** AGERRKGTDVNVF **Charge** 3 **Spectral Angle** 0.9

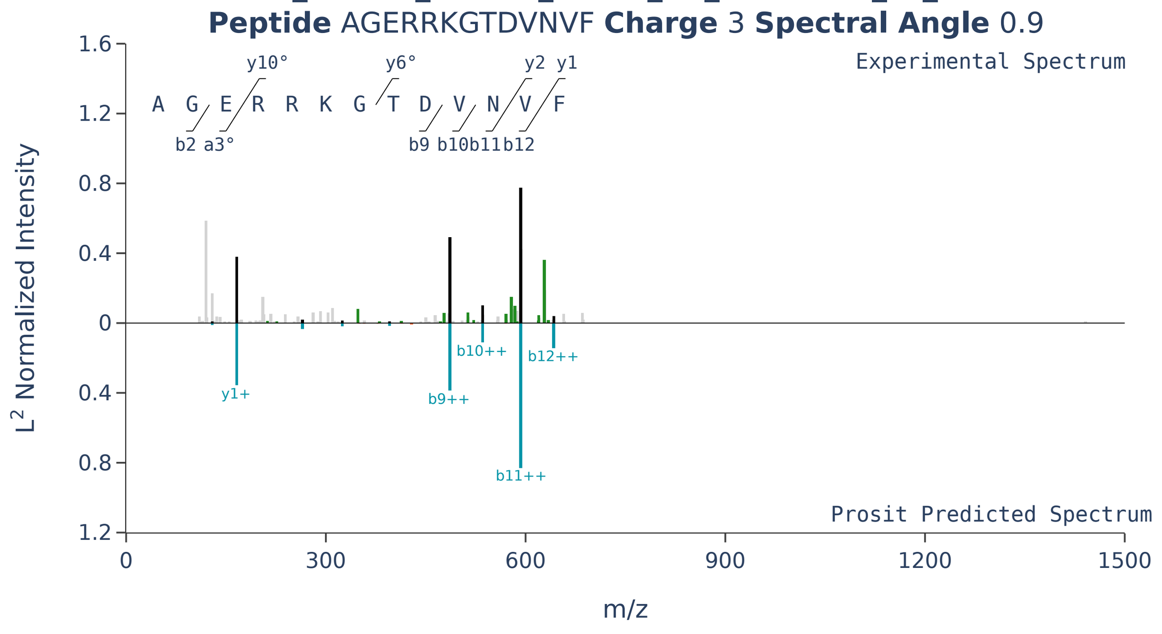


**Source** WSoh_301121_021221_HFGoe_M2_AnnexinA1_4h_R1 **Scan** 12131
**Peptide** SYPQLRRV **Charge** 2 **Spectral Angle** 0.94

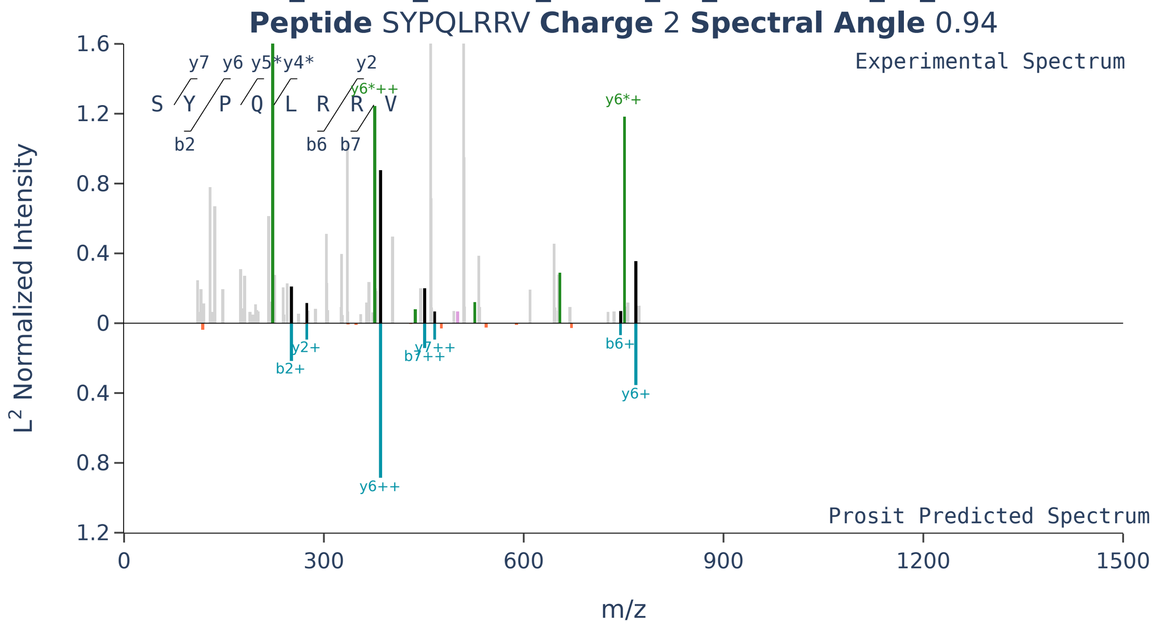


**Source** WSoh_301121_021221_HFGoe_M1_AnnexinA1_2h_R1 **Scan** 9637
**Peptide** RSEIDMND **Charge** 2 **Spectral Angle** 0.95

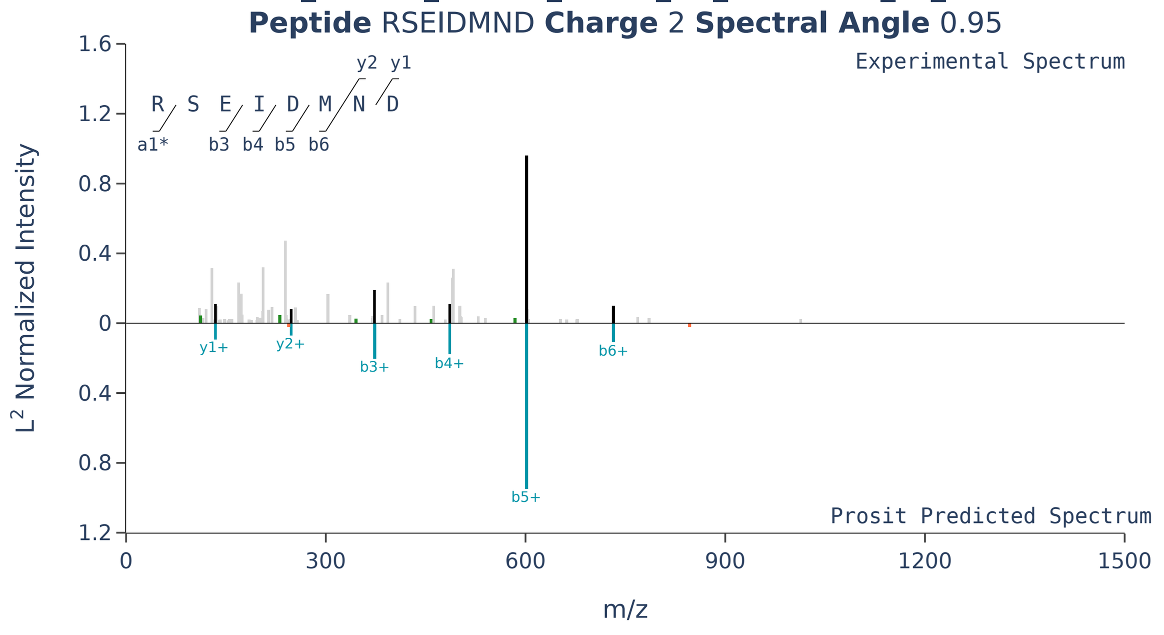


**Source** WSoh_301121_021221_HFGoe_M2_AnnexinA1_24h_R1 **Scan** 19049
**Peptide** KGDIEKCLTAI **Charge** 3 **Spectral Angle** 0.92

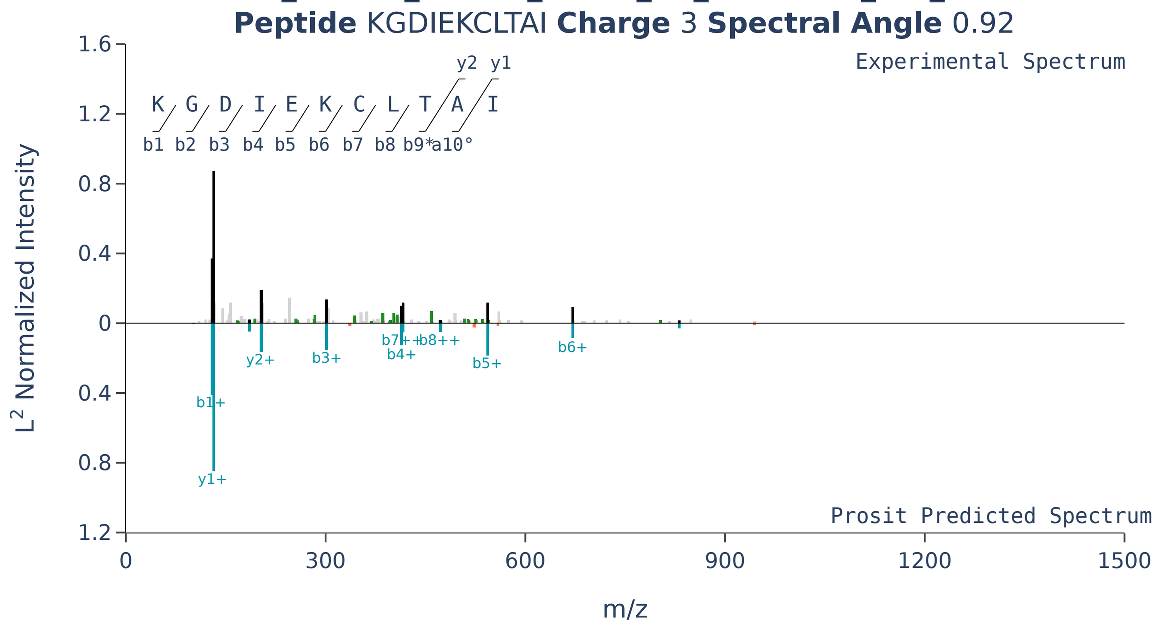


**Source** WSoh_301121_021221_HFGoe_M2_AnnexinA1_24h_R2 **Scan** 9922
**Peptide** KAAYLQE **Charge** 1 **Spectral Angle** 0.91

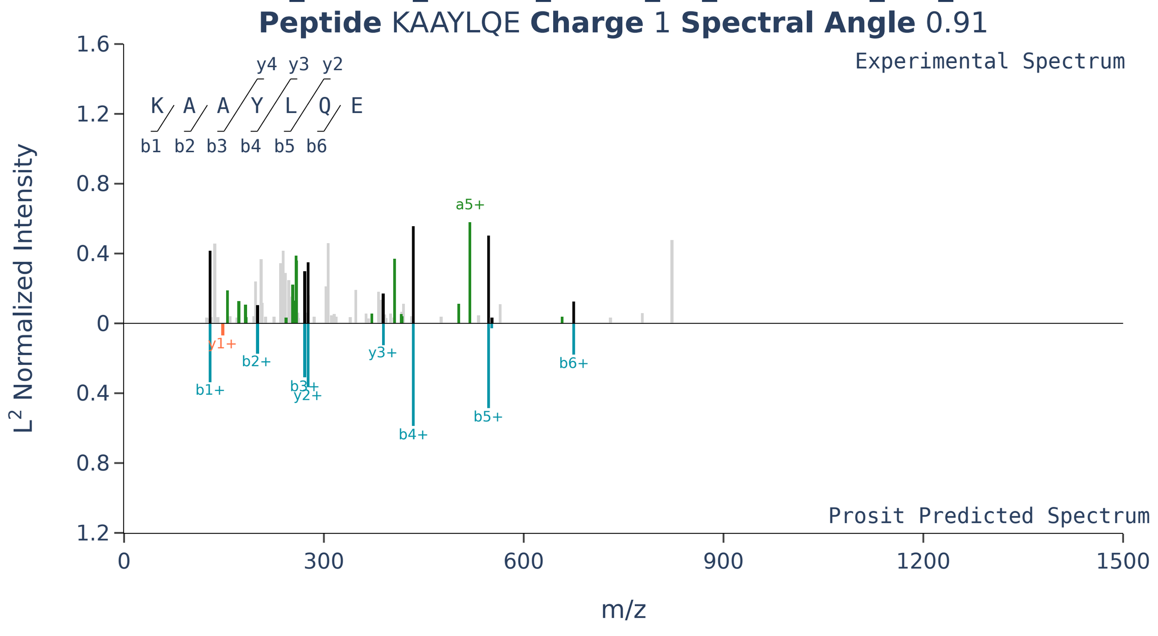


**Source** WSoh_301121_021221_HFGoe_M2_AnnexinA1_4h_R1 **Scan** 12301
**Peptide** LNRVYREEL **Charge** 2 **Spectral Angle** 0.93

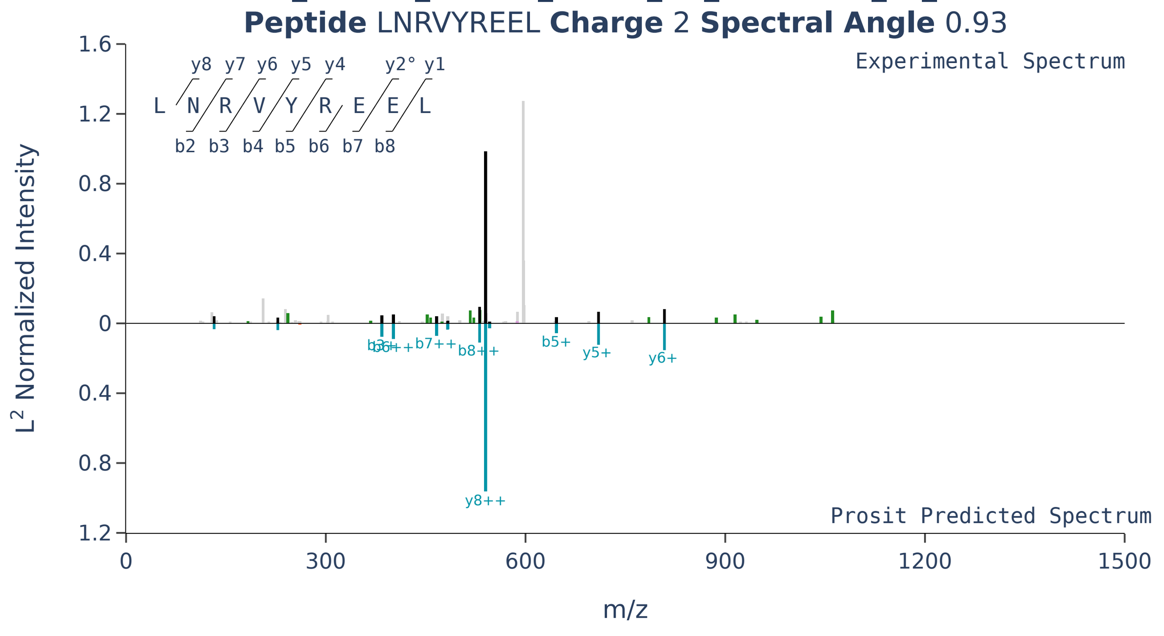


**Source** WSoh_301121_021221_HFGoe_M2_AnnexinA1_24h_R1 **Scan** 19324
**Peptide** TDVNVFN **Charge** 1 **Spectral Angle** 0.94

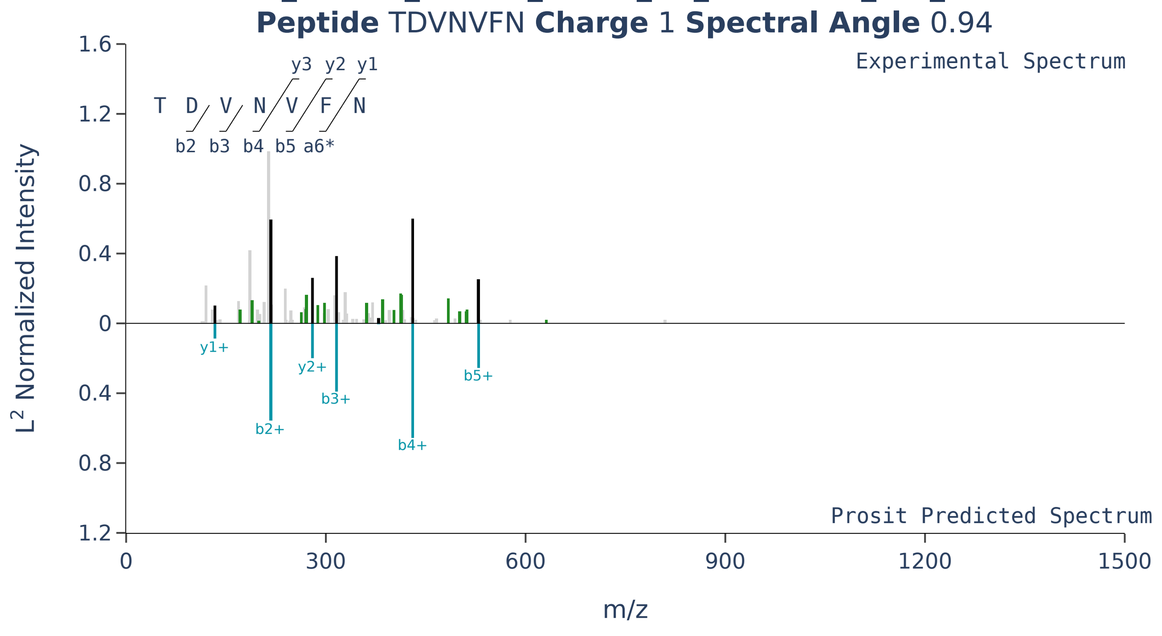


**Source** WSoh_301121_021221_HFGoe_M1_AnnexinA1_1h_R1 **Scan** 9218
**Peptide** KTPAQFD **Charge** 2 **Spectral Angle** 0.83

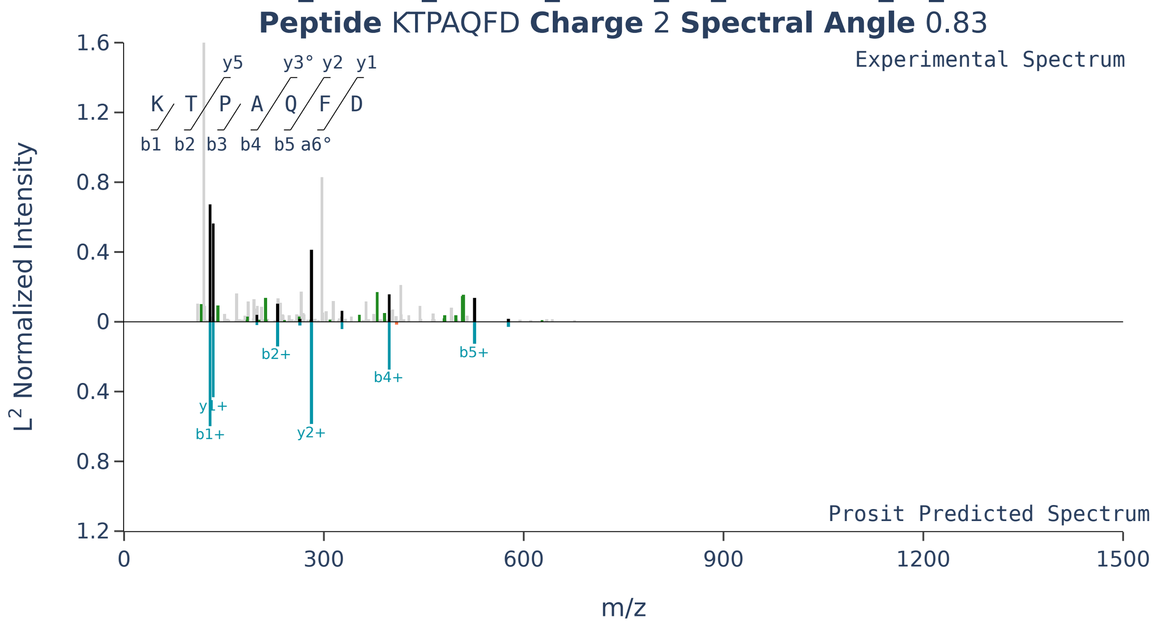


**Source** WSoh_301121_021221_HFGoe_M2_AnnexinA1_24h_R2 **Scan** 11661
**Peptide** LAKGDRSEDFGVNE **Charge** 2 **Spectral Angle** 0.89

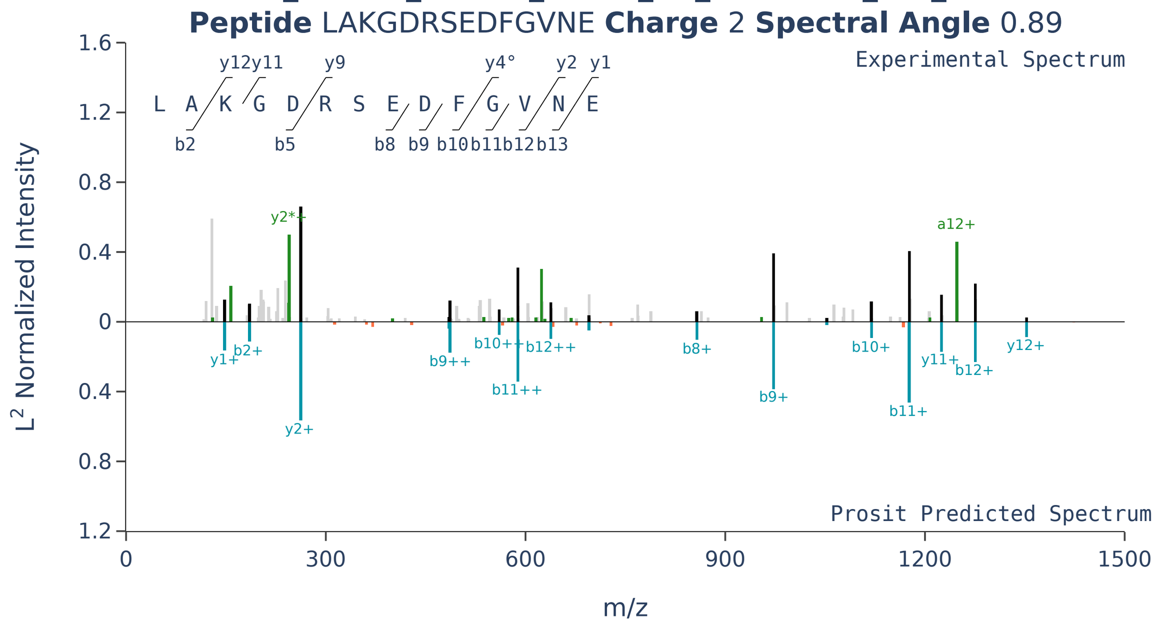


**Source** WSoh_301121_021221_HFGoe_M2_AnnexinA1_24h_R1 **Scan** 19837
**Peptide** DELRAAMKGL **Charge** 2 **Spectral Angle** 0.88

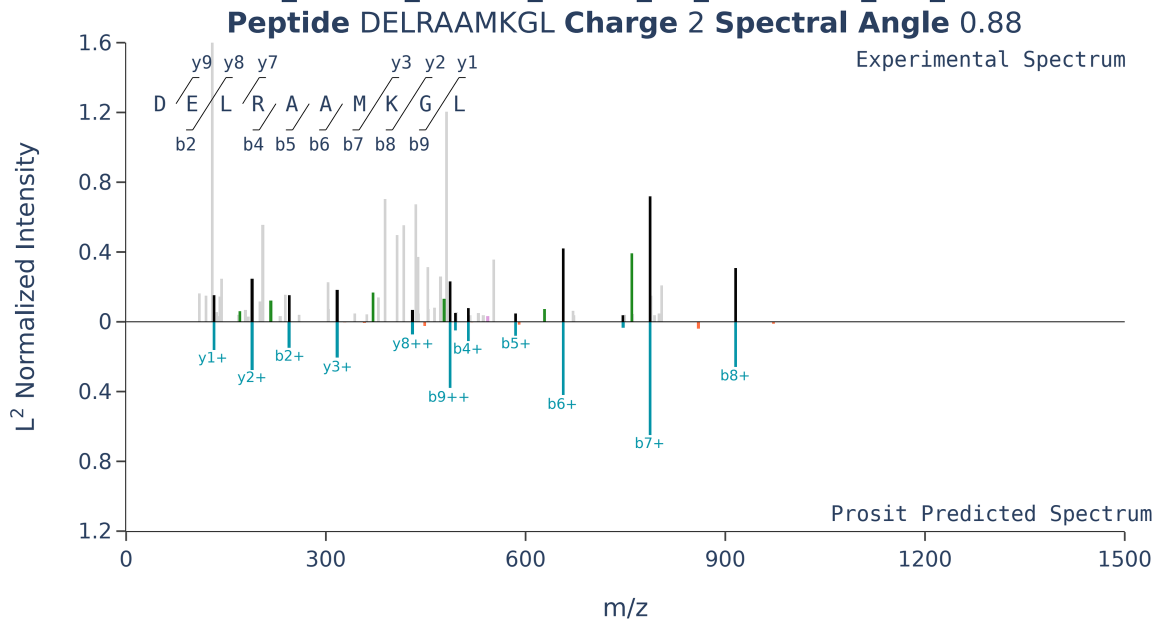


**Source** WSoh_301121_021221_HFGoe_M2_AnnexinA1_24h_R1 **Scan** 10694
**Peptide** AMKGLGTDEDT **Charge** 2 **Spectral Angle** 0.84

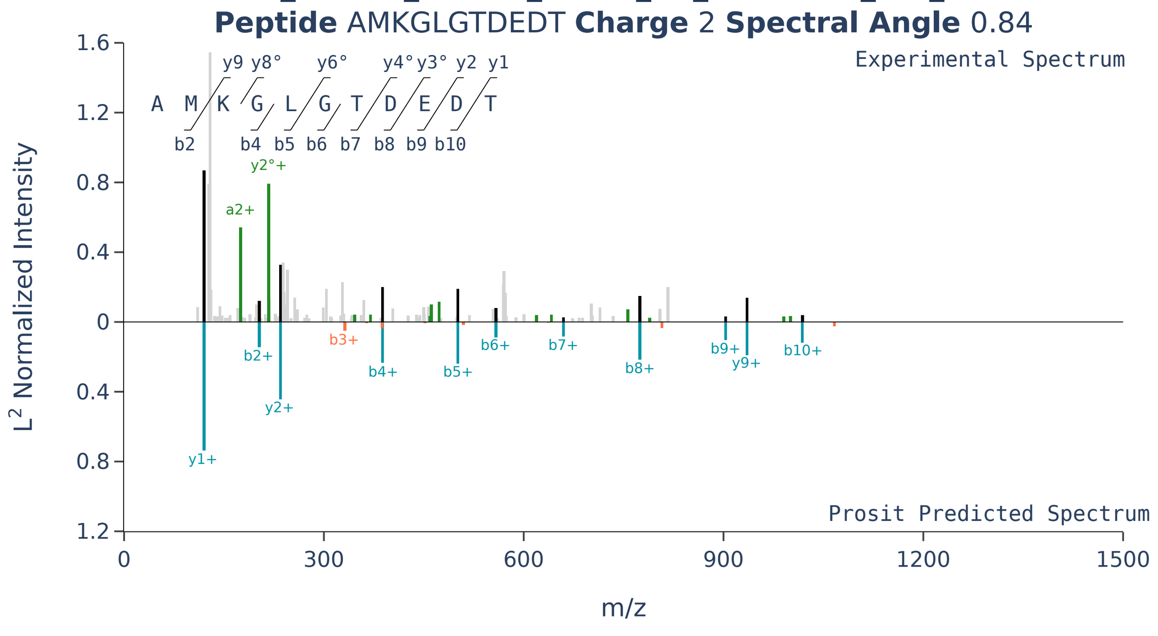


**Source** WSoh_301121_021221_HFGoe_M1_AnnexinA1_4h_R2 **Scan** 8086
**Peptide** ETLKKALT **Charge** 2 **Spectral Angle** 0.91

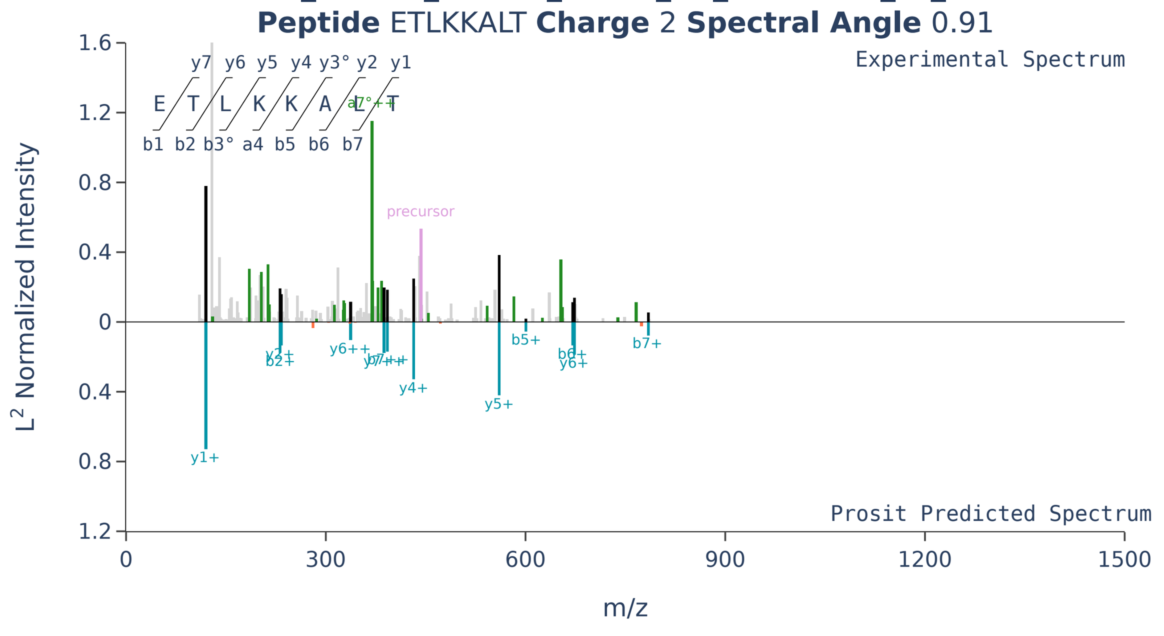


**Source** WSoh_301121_021221_HFGoe_M1_AnnexinA1_2h_R2 **Scan** 8142
**Peptide** EQEYVQTVKSSK **Charge** 2 **Spectral Angle** 0.89

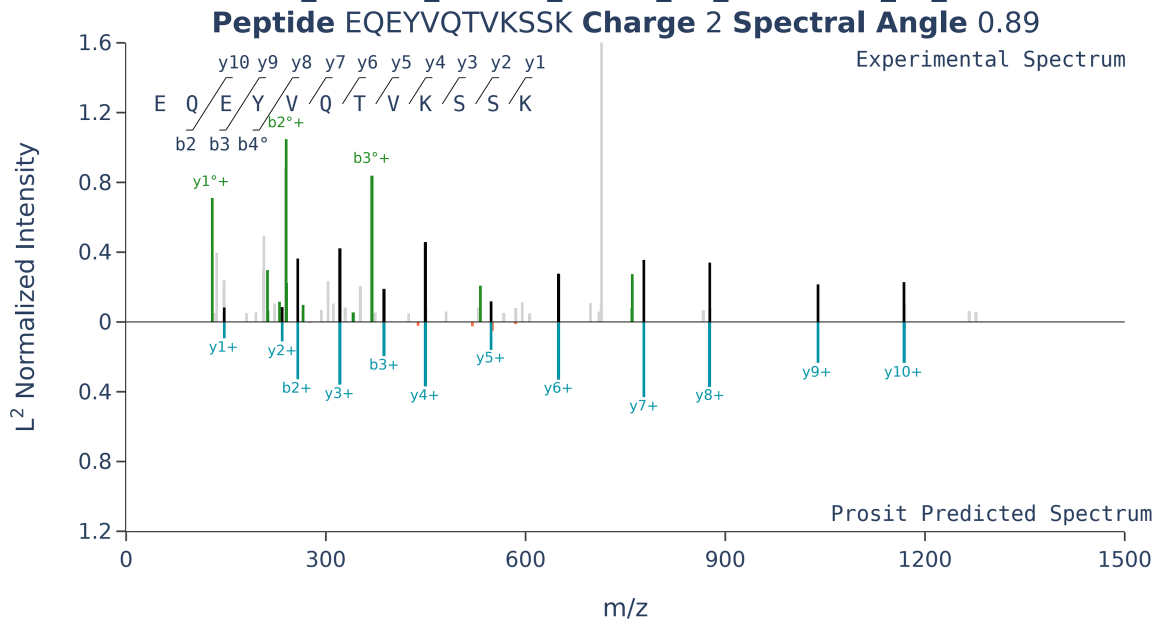

**Source** WSoh_301121_021221_HFGoe_M2_AnnexinA1_24h_R1 **Scan** 11106
**Peptide** RSEDFGVN **Charge** 2 **Spectral Angle** 0.87

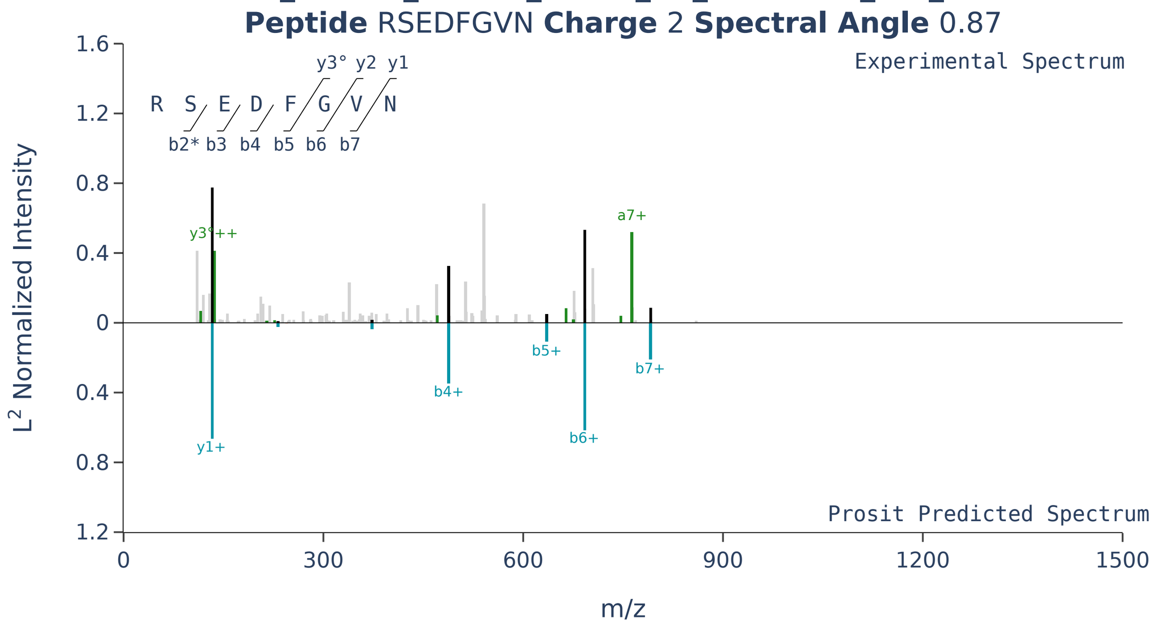


**Source** WSoh_301121_021221_HFGoe_M2_AnnexinA1_2h_R1 **Scan** 14780
**Peptide** KPAFFAEKLHQA **Charge** 3 **Spectral Angle** 0.84

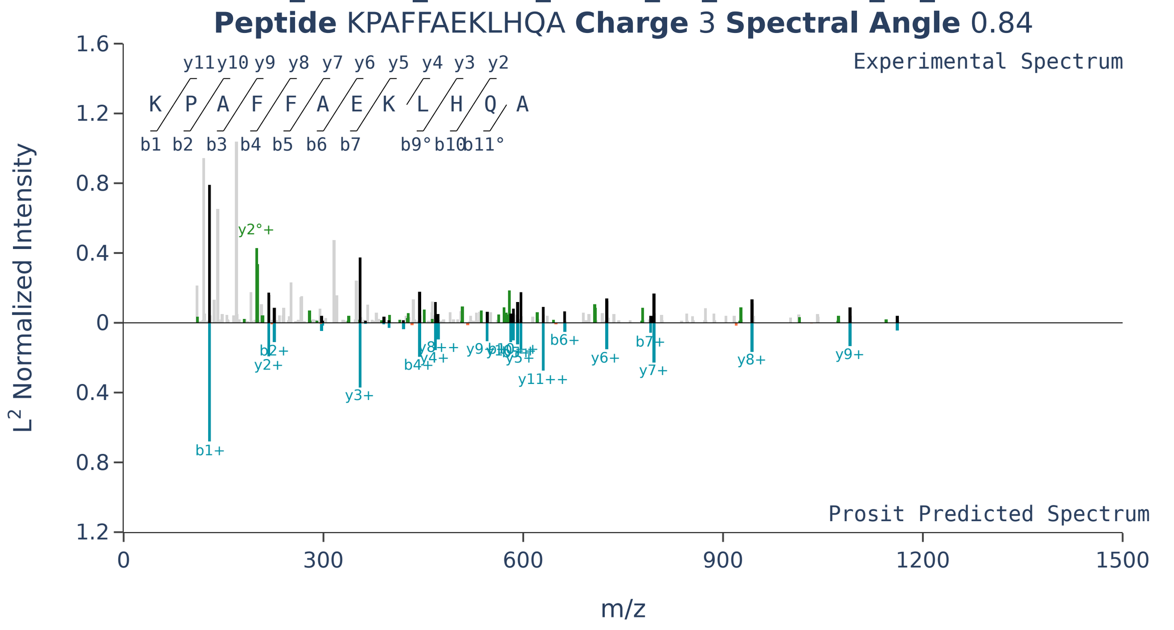


**Source** WSoh_301121_021221_HFGoe_M2_AnnexinA1_4h_R2 **Scan** 9881
**Peptide** TGHLEEV **Charge** 1 **Spectral Angle** 0.95

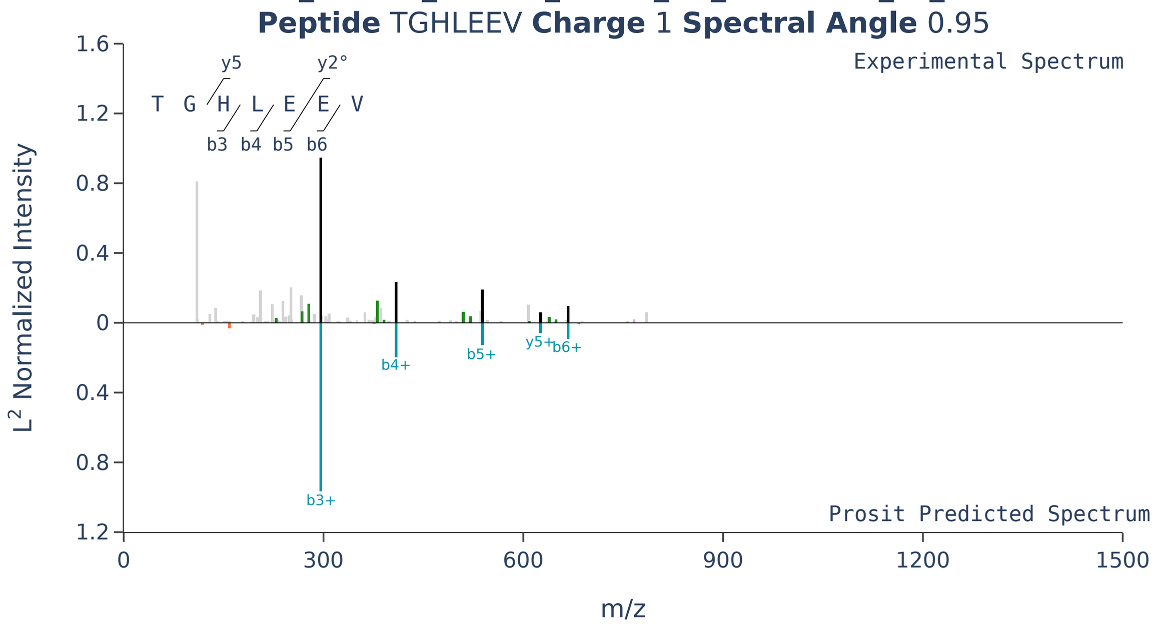


**Source** WSoh_301121_021221_HFGoe_M2_AnnexinA1_24h_R1 **Scan** 20127
**Peptide** LDLELKGD **Charge** 2 **Spectral Angle** 0.88

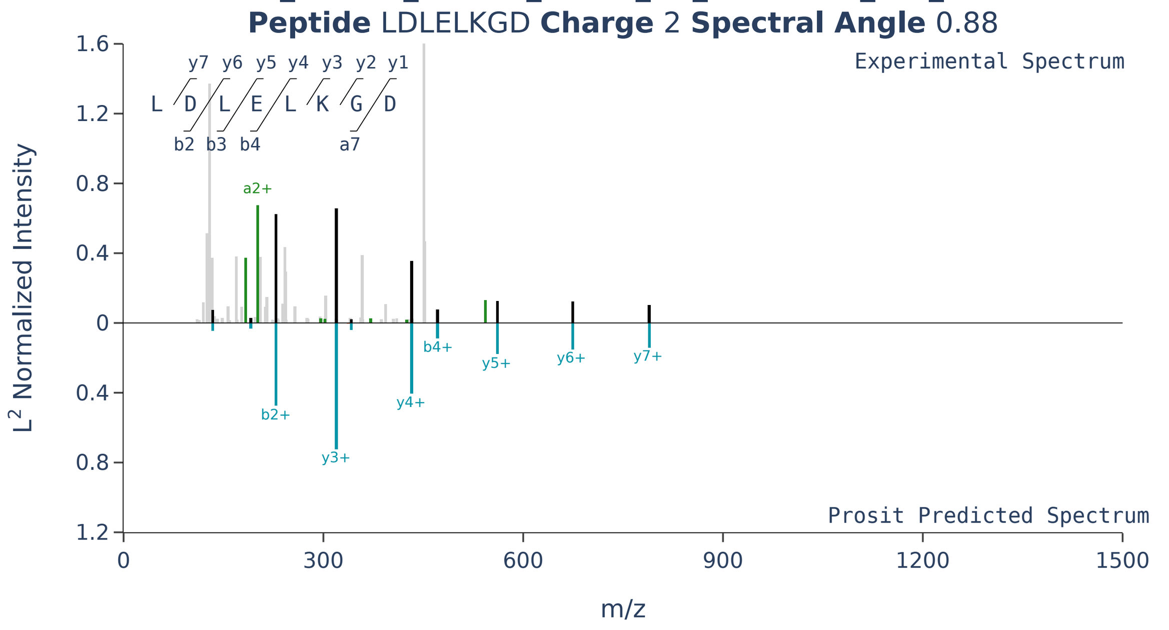


**Source** WSoh_301121_021221_HFGoe_M2_AnnexinA1_24h_R2 **Scan** 11149
**Peptide** ERRKGTDVNVF **Charge** 3 **Spectral Angle** 0.94

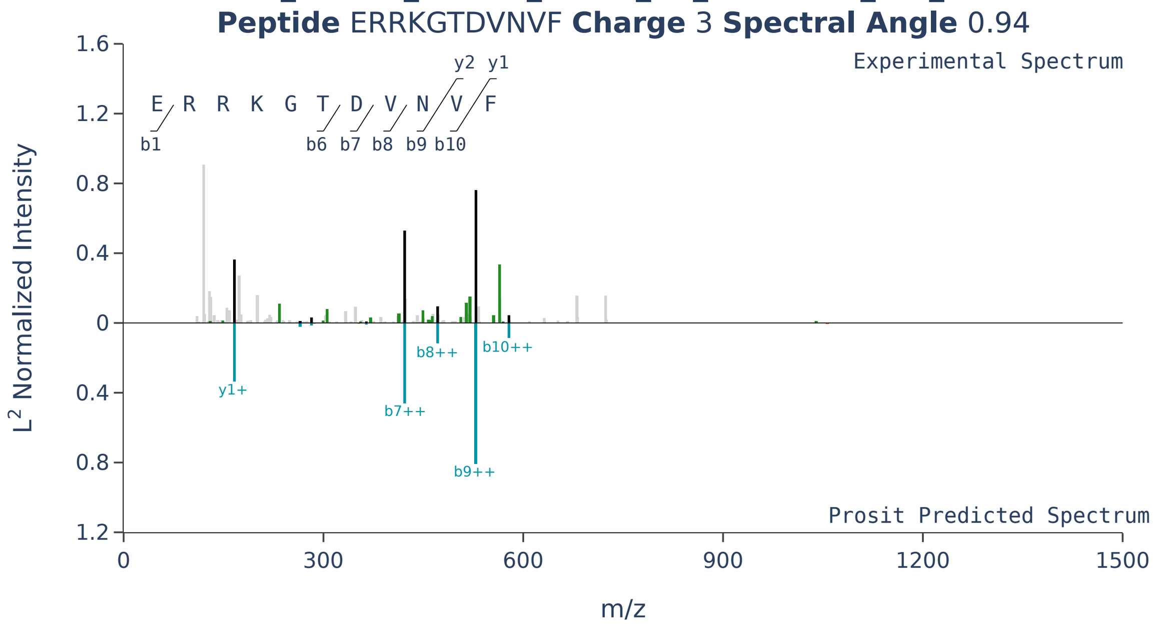


**Source** WSoh_301121_021221_HFGoe_M2_AnnexinA1_4h_R1 **Scan** 13512
**Peptide** IDILTKR **Charge** 2 **Spectral Angle** 0.93

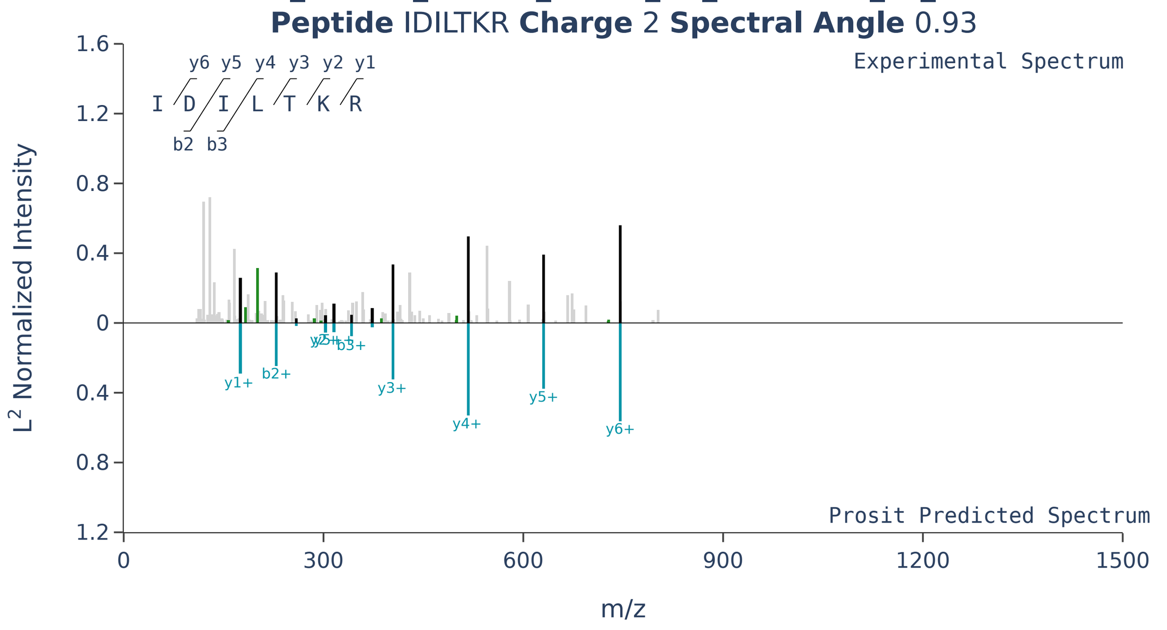


**Source** WSoh_301121_021221_HFGoe_M1_AnnexinA1_24h_R2 **Scan** 17874
**Peptide** YEKILVA **Charge** 2 **Spectral Angle** 0.89

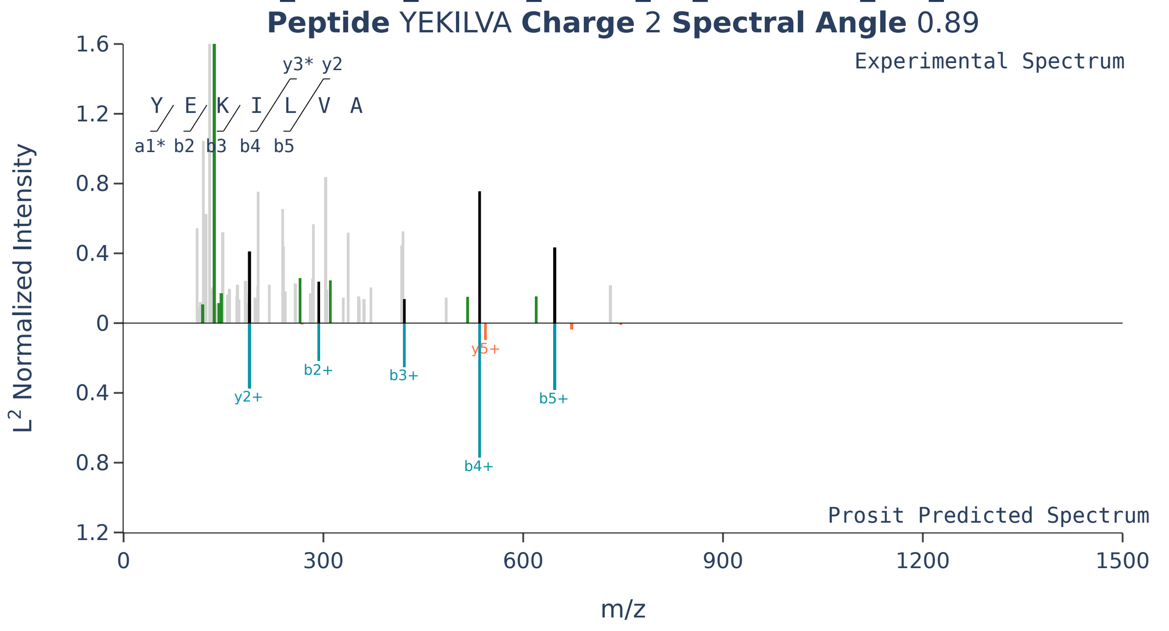


**Source** WSoh_301121_021221_HFGoe_M2_AnnexinA1_4h_R2 **Scan** 7633
**Peptide** MVKGVDE **Charge** 2 **Spectral Angle** 0.91

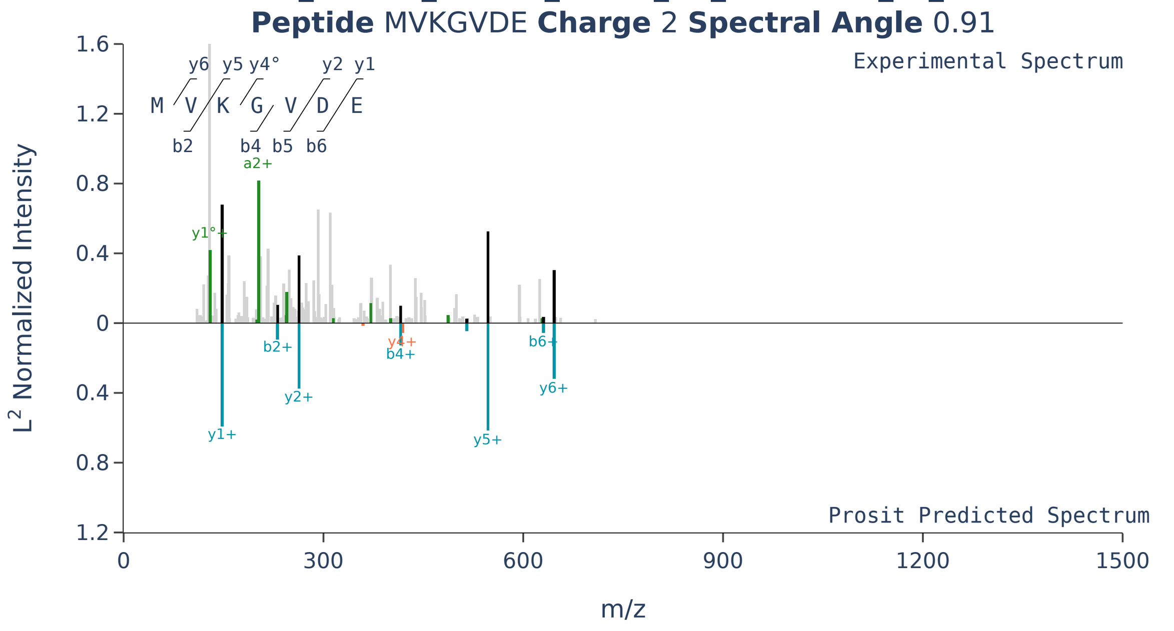


**Source** WSoh_301121_021221_HFGoe_M1_AnnexinA1_24h_R2 **Scan** 6263
**Peptide** SRTNKEIRDIN **Charge** 3 **Spectral Angle** 0.93

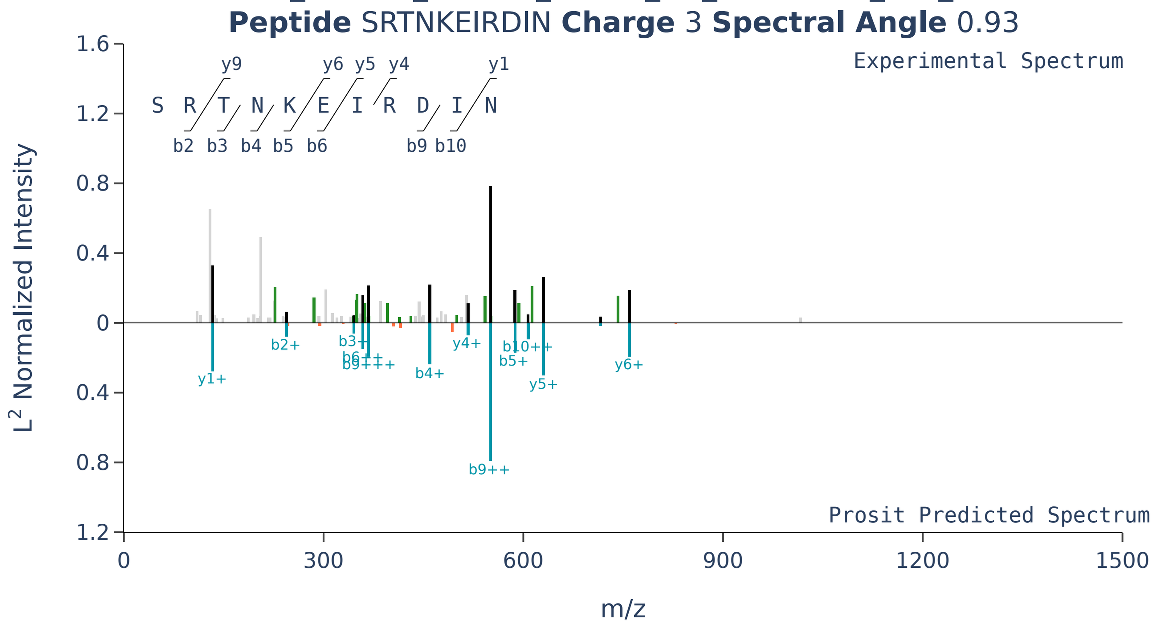


**Source** WSoh_301121_021221_HFGoe_M2_AnnexinA1_1h_R1 **Scan** 19272
**Peptide** DELRAAMKGLGTD **Charge** 2 **Spectral Angle** 0.86

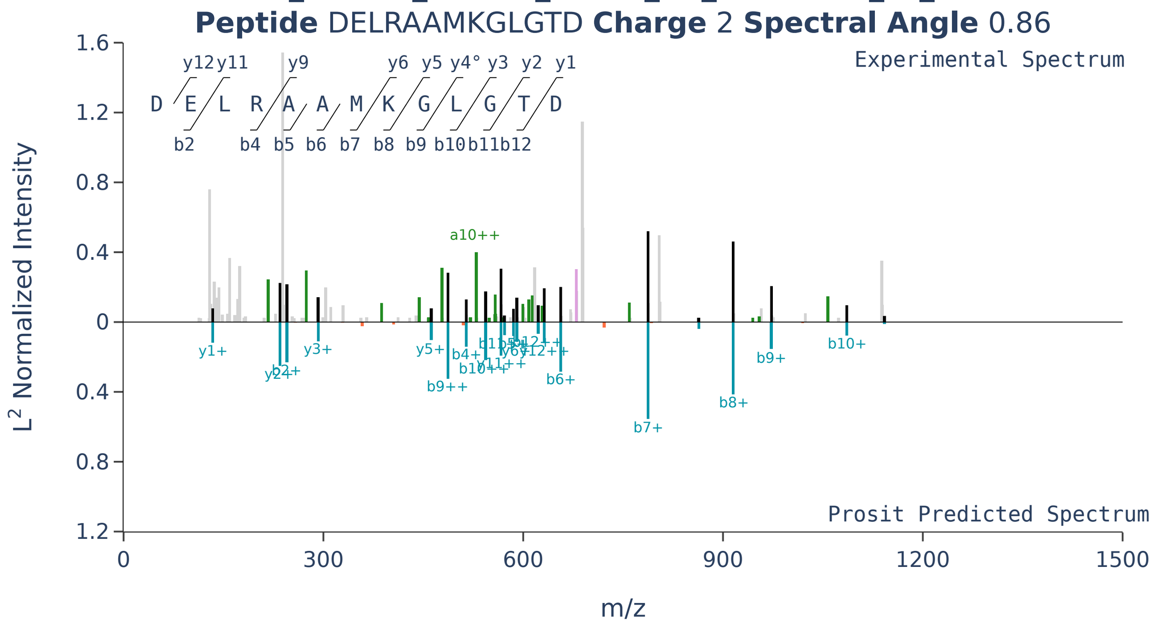


**Source** WSoh_301121_021221_HFGoe_M1_AnnexinA1_4h_R2 **Scan** 5727
**Peptide** FQKYTKYSKH **Charge** 3 **Spectral Angle** 0.96

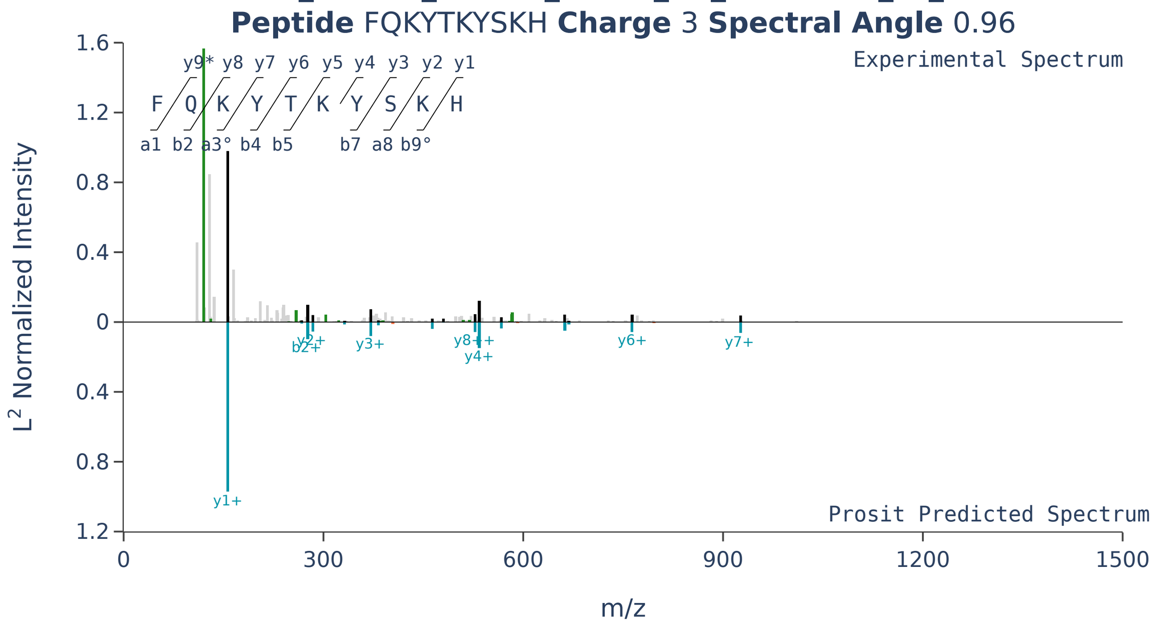


**Source** WSoh_301121_021221_HFGoe_M2_AnnexinA1_24h_R2 **Scan** 8195
**Peptide** NKEIRDINRV **Charge** 2 **Spectral Angle** 0.88

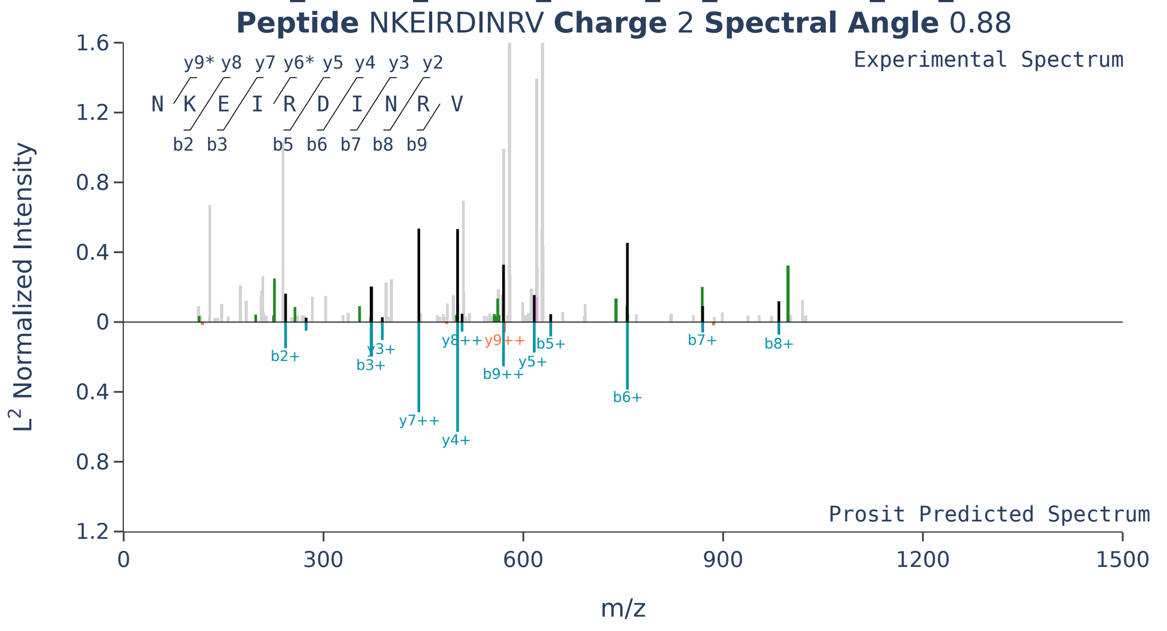


**Source** WSoh_301121_021221_HFGoe_M2_AnnexinA1_24h_R1 **Scan** 17264
**Peptide** KGDYEKILV **Charge** 2 **Spectral Angle** 0.85

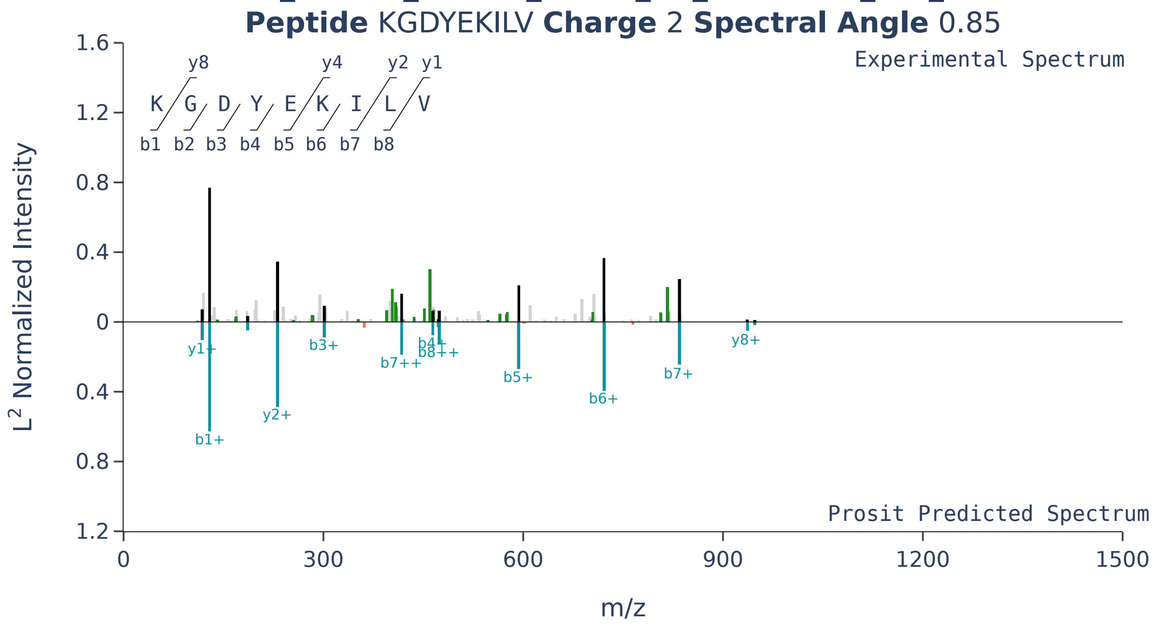


**Source** WSoh_301121_021221_HFGoe_M2_AnnexinA1_24h_R1 **Scan** 17386
**Peptide** SDTSGDFRNAL **Charge** 2 **Spectral Angle** 0.91

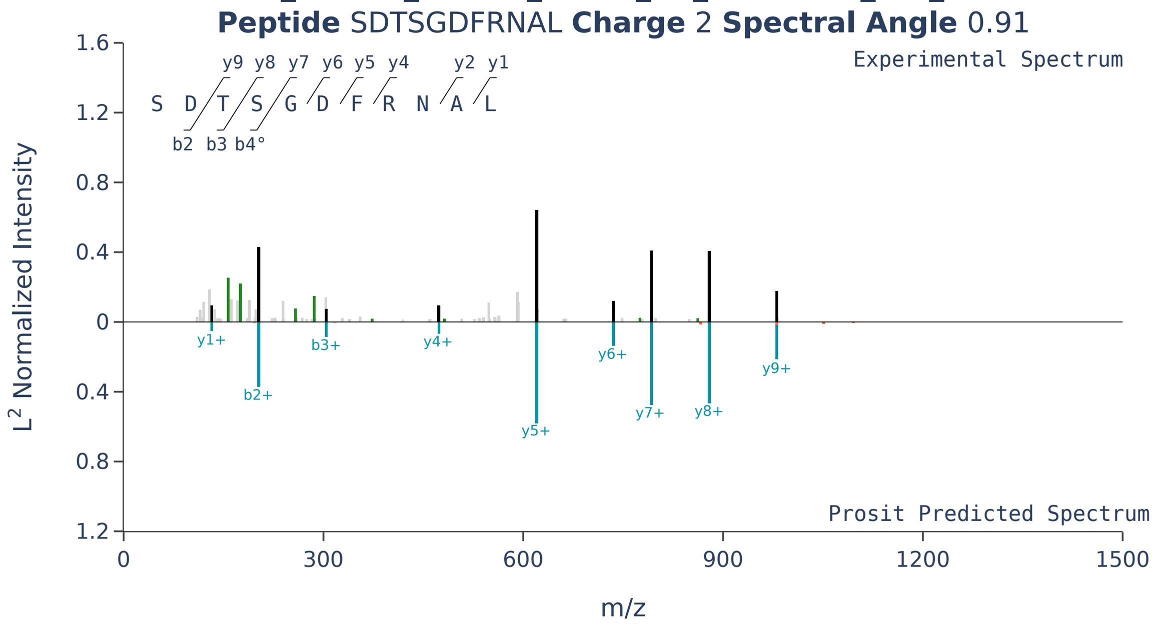


**Source** WSoh_301121_021221_HFGoe_M2_AnnexinA1_24h_R1 **Scan** 9635
**Peptide** QEYVQTVKSS **Charge** 1 **Spectral Angle** 0.97

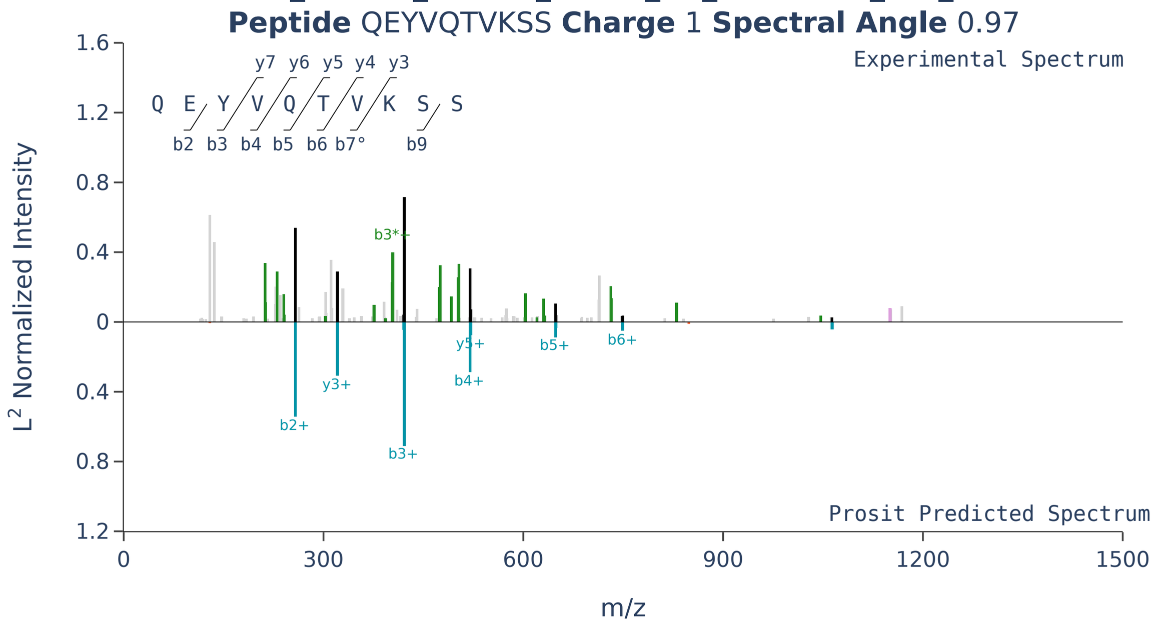

**Source** WSoh_301121_021221_HFGoe_M2_AnnexinA1_24h_R1 **Scan** 17700
**Peptide** TKGDYEKILVA **Charge** 2 **Spectral Angle** 0.84

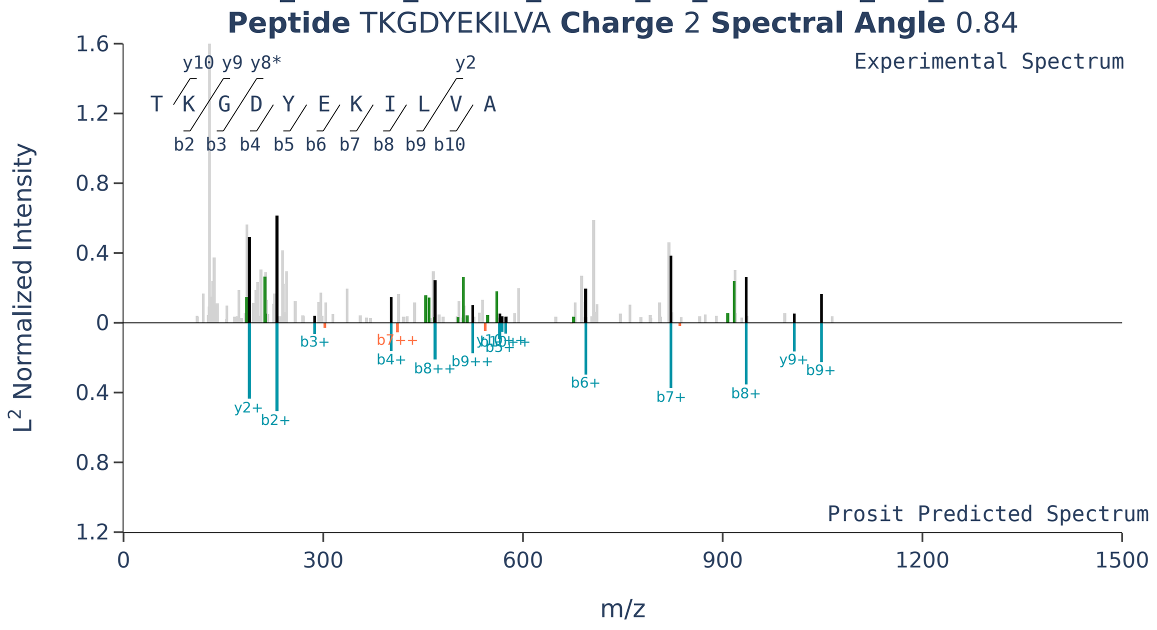


**Source** WSoh_301121_021221_HFGoe_M1_AnnexinA1_24h_R2 **Scan** 17337
**Peptide** MNDIKAF **Charge** 2 **Spectral Angle** 0.88

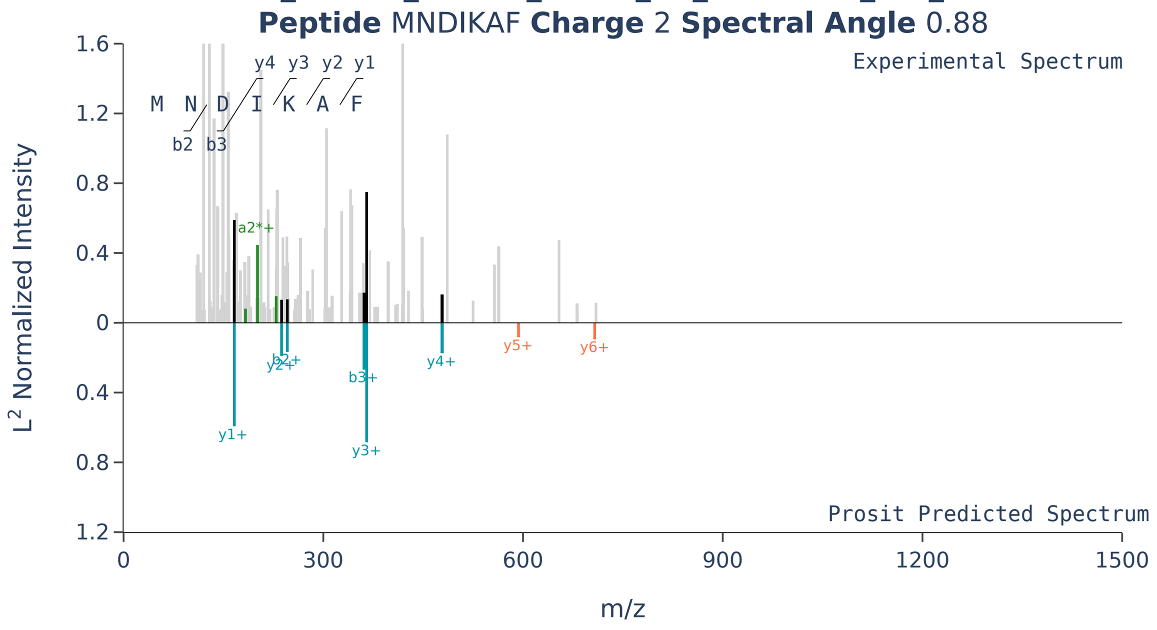


**Source** WSoh_301121_021221_HFGoe_M2_AnnexinA1_24h_R1 **Scan** 15396
**Peptide** IENEEQEYVQTVKS **Charge** 2 **Spectral Angle** 0.89

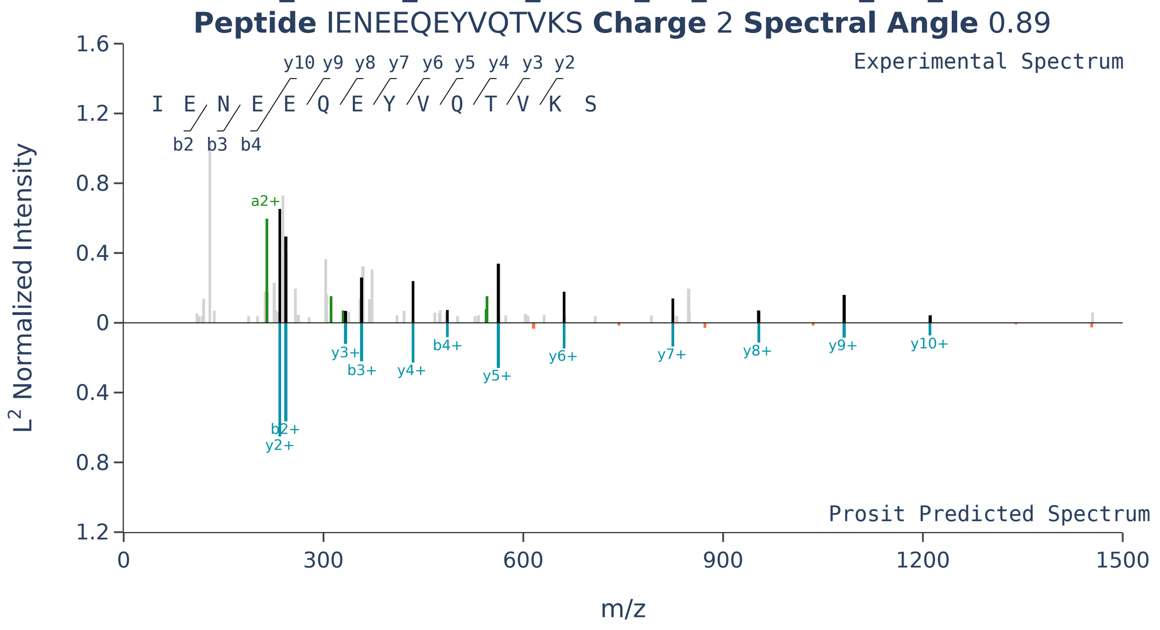


**Source** WSoh_301121_021221_HFGoe_M1_AnnexinA1_24h_R1 **Scan** 13832
**Peptide** ENEEQEYVQTVKS **Charge** 2 **Spectral Angle** 0.91

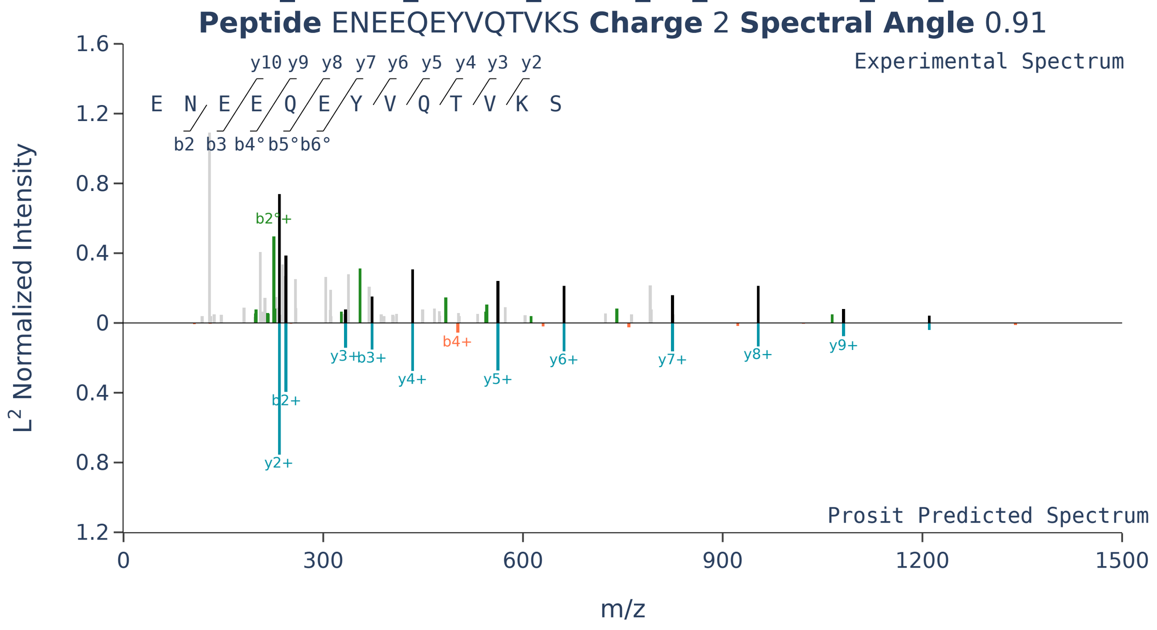


**Source** WSoh_301121_021221_HFGoe_M2_AnnexinA1_2h_R1 **Scan** 13952
**Peptide** KAFYQKMY **Charge** 2 **Spectral Angle** 0.91

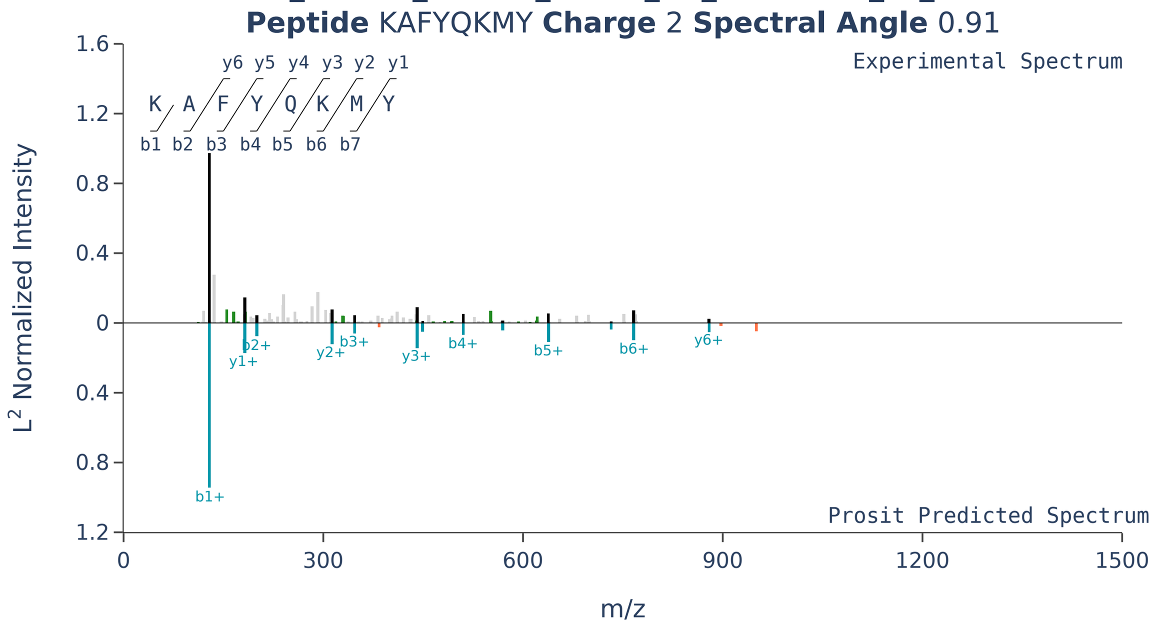


**Source** WSoh_301121_021221_HFGoe_M1_AnnexinA1_4h_R2 **Scan** 14580
**Peptide** IMVSRSEID **Charge** 2 **Spectral Angle** 0.92

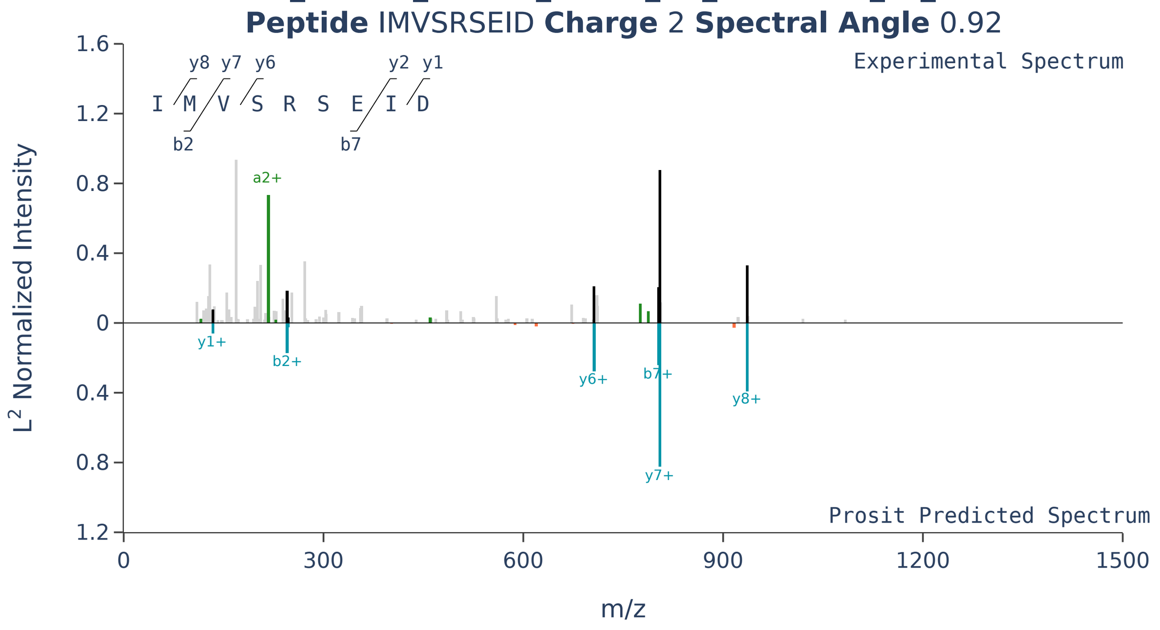


**Source** WSoh_301121_021221_HFGoe_M2_AnnexinA1_1h_R1 **Scan** 20658
**Peptide** DLAKDITSDTSGDFRNA **Charge** 3 **Spectral Angle** 0.88

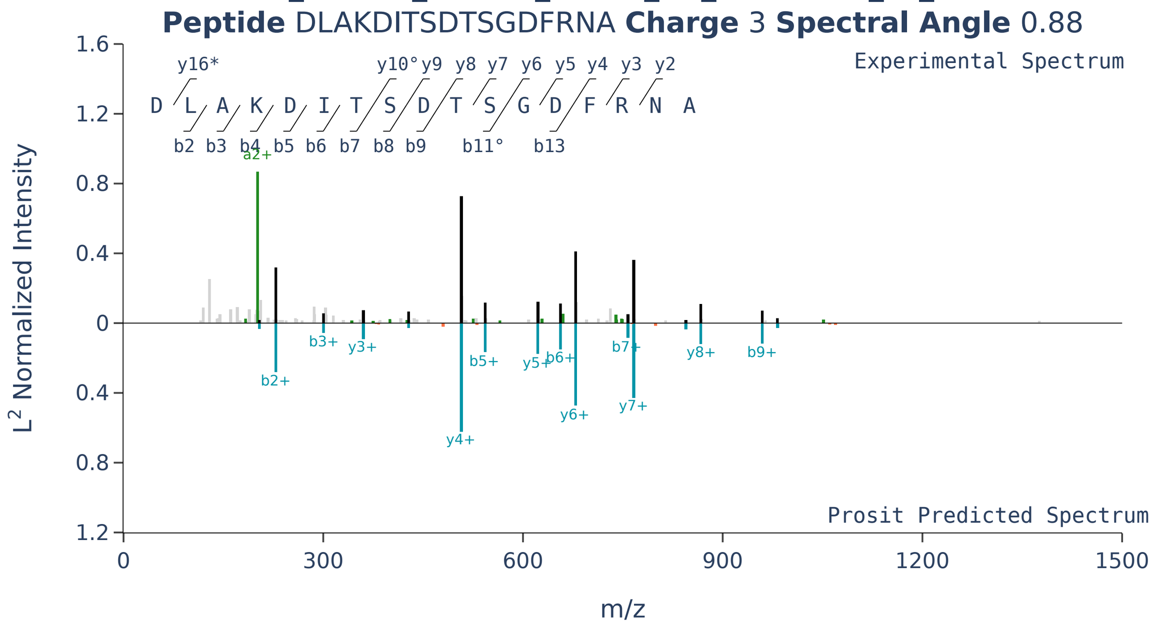


**Source** WSoh_301121_021221_HFGoe_M2_AnnexinA1_24h_R2 **Scan** 9272
**Peptide** KGDRSEDFGVN **Charge** 2 **Spectral Angle** 0.82

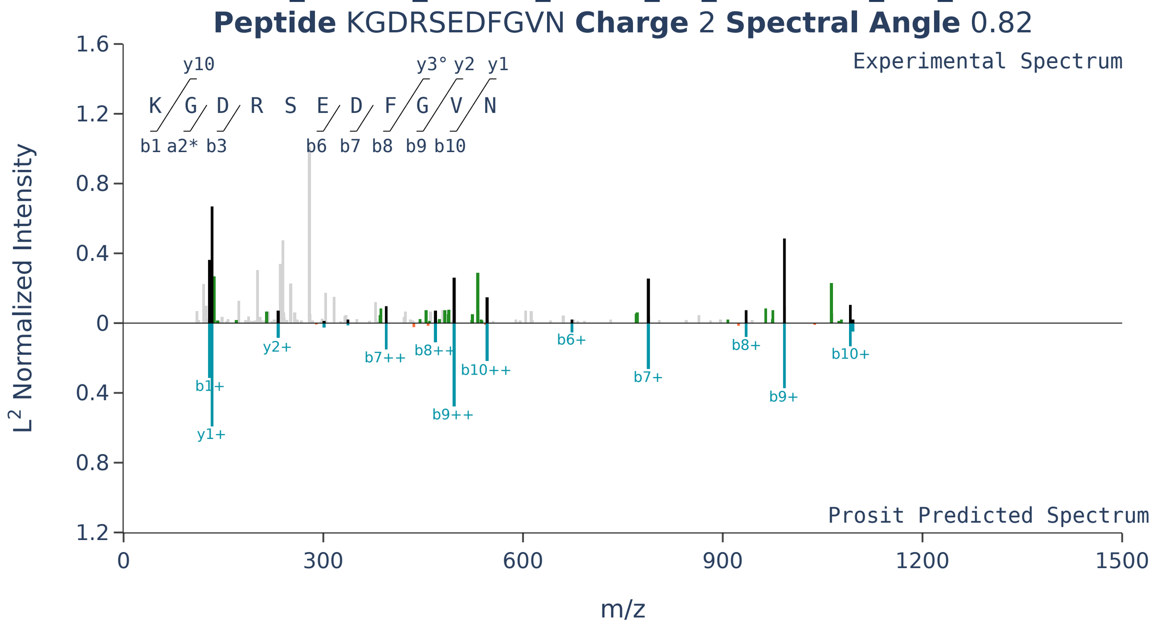


**Source** WSoh_301121_021221_HFGoe_M2_AnnexinA1_2h_R1 **Scan** 8410
**Peptide** QEYVQTVKSSKGGPGS **Charge** 2 **Spectral Angle** 0.92

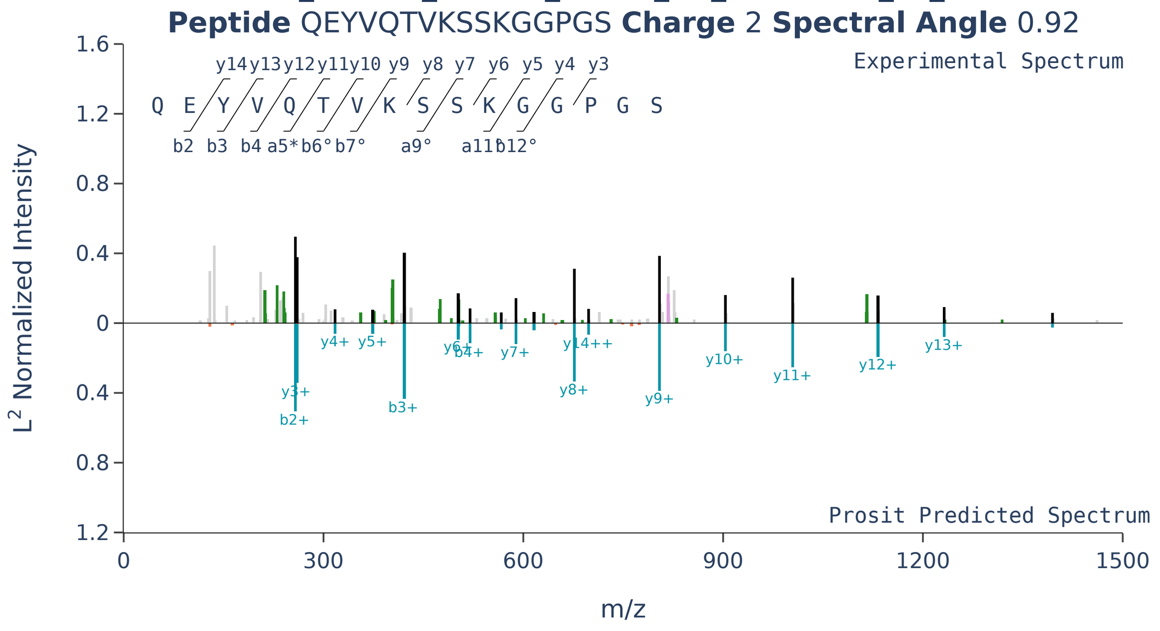


**Source** WSoh_301121_021221_HFGoe_M2_AnnexinA1_2h_R1 **Scan** 16073
**Peptide** ATSKPAFFAEKLHQA **Charge** 4 **Spectral Angle** 0.89

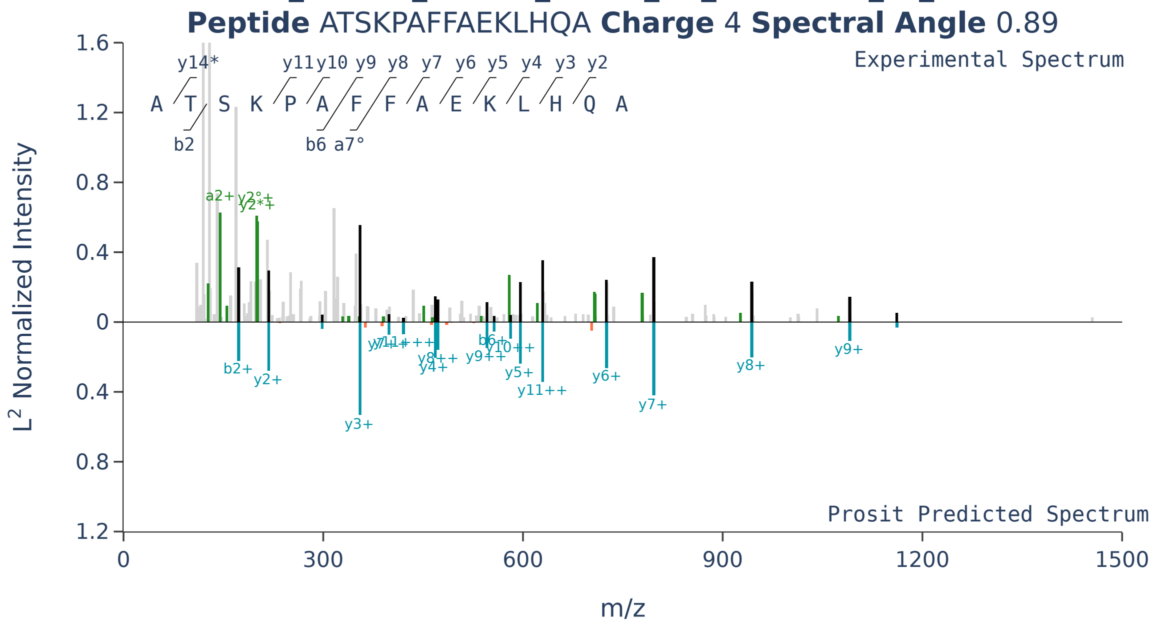


**Source** WSoh_301121_021221_HFGoe_M1_AnnexinA1_4h_R1 **Scan** 13510
**Peptide** LTAIVKC **Charge** 2 **Spectral Angle** 0.87

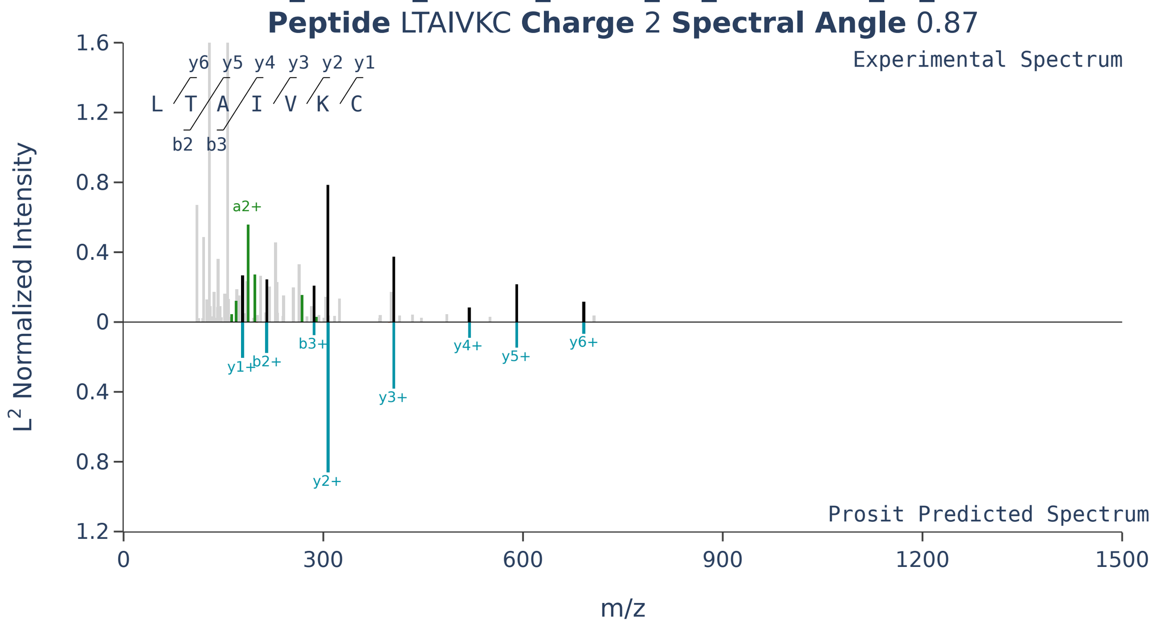


**Source** WSoh_301121_021221_HFGoe_M2_AnnexinA1_2h_R2 **Scan** 24187
**Peptide** LTGHLEEVVL **Charge** 2 **Spectral Angle** 0.88

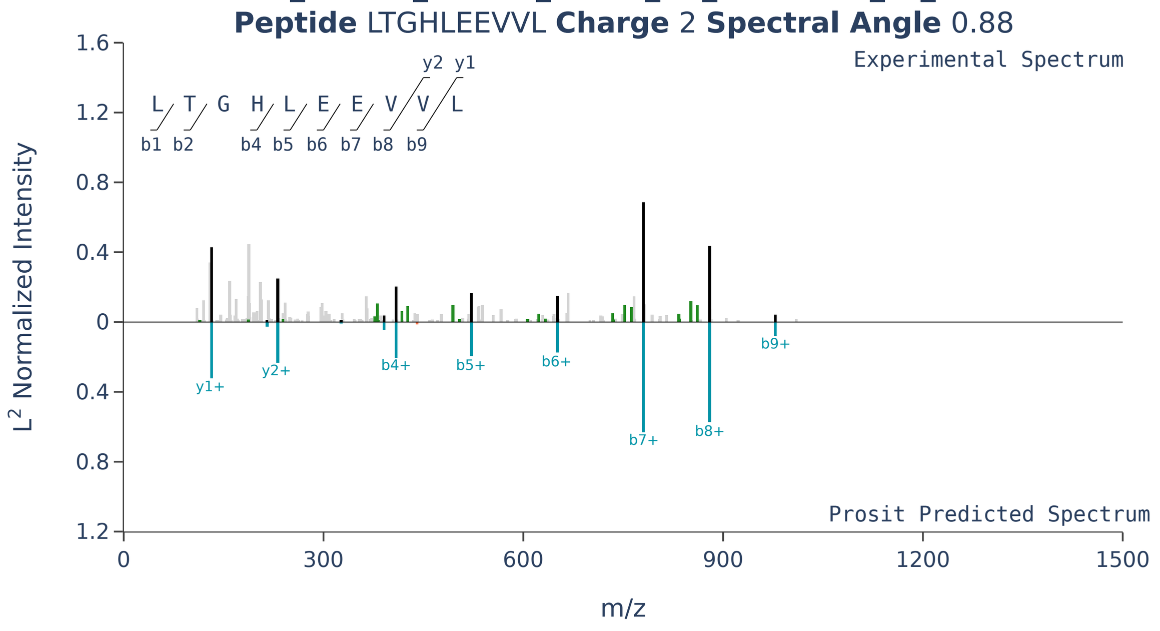


**Source** WSoh_301121_021221_HFGoe_M2_AnnexinA1_2h_R2 **Scan** 19366
**Peptide** VSRSEIDMNDL **Charge** 2 **Spectral Angle** 0.89

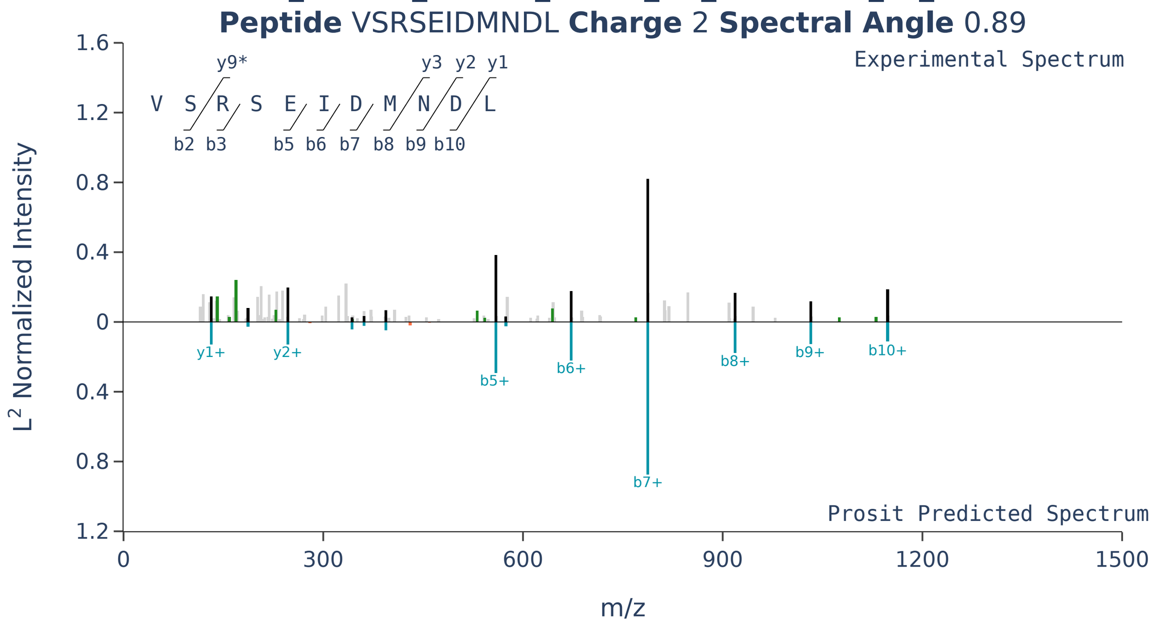


**Source** WSoh_301121_021221_HFGoe_M2_AnnexinA1_24h_R1 **Scan** 19258
**Peptide** GTDVNVFN **Charge** 1 **Spectral Angle** 0.83

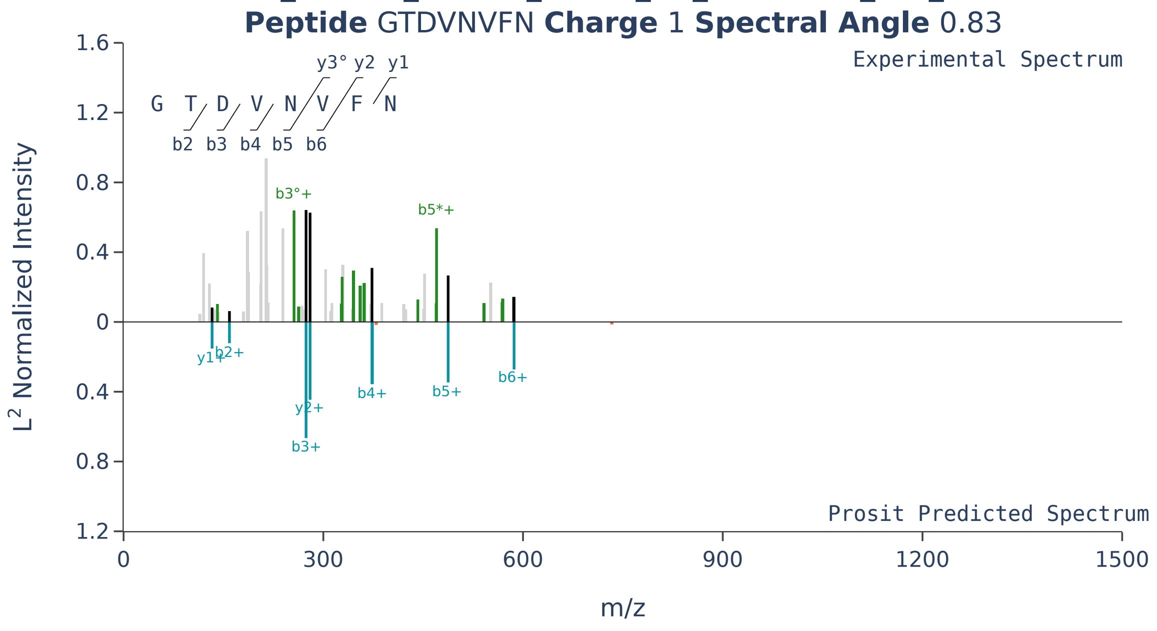


**Source** WSoh_301121_021221_HFGoe_M2_AnnexinA1_2h_R2 **Scan** 9183
**Peptide** EQEYVQTVKSSKGGPGS **Charge** 2 **Spectral Angle** 0.91

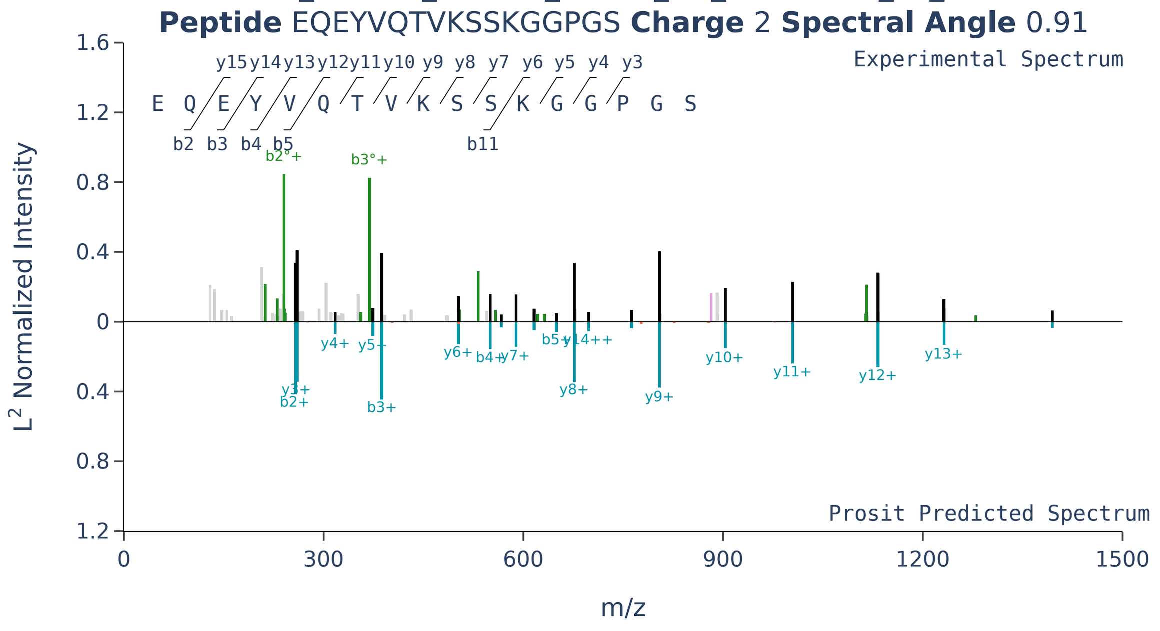

**Source** WSoh_301121_021221_HFGoe_M2_AnnexinA1_2h_R2 **Scan** 12366
**Peptide** QRQQIKAAYLQE **Charge** 2 **Spectral Angle** 0.86

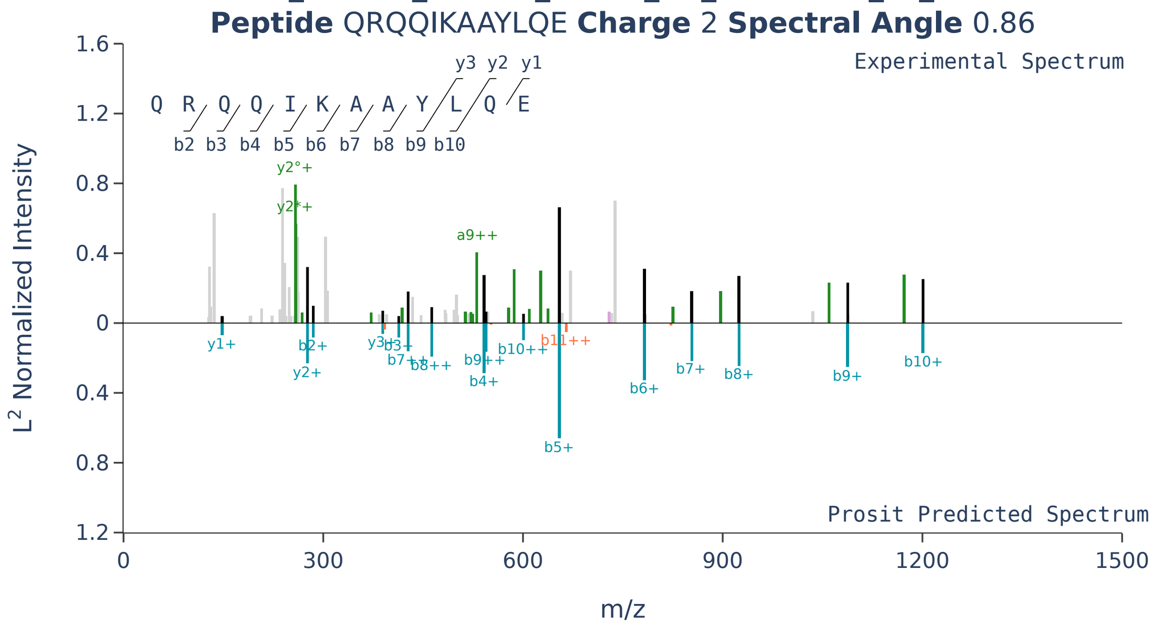


**Source** WSoh_301121_021221_HFGoe_M2_AnnexinA1_24h_R2 **Scan** 16302
**Peptide** LKTPAQF **Charge** 2 **Spectral Angle** 0.95

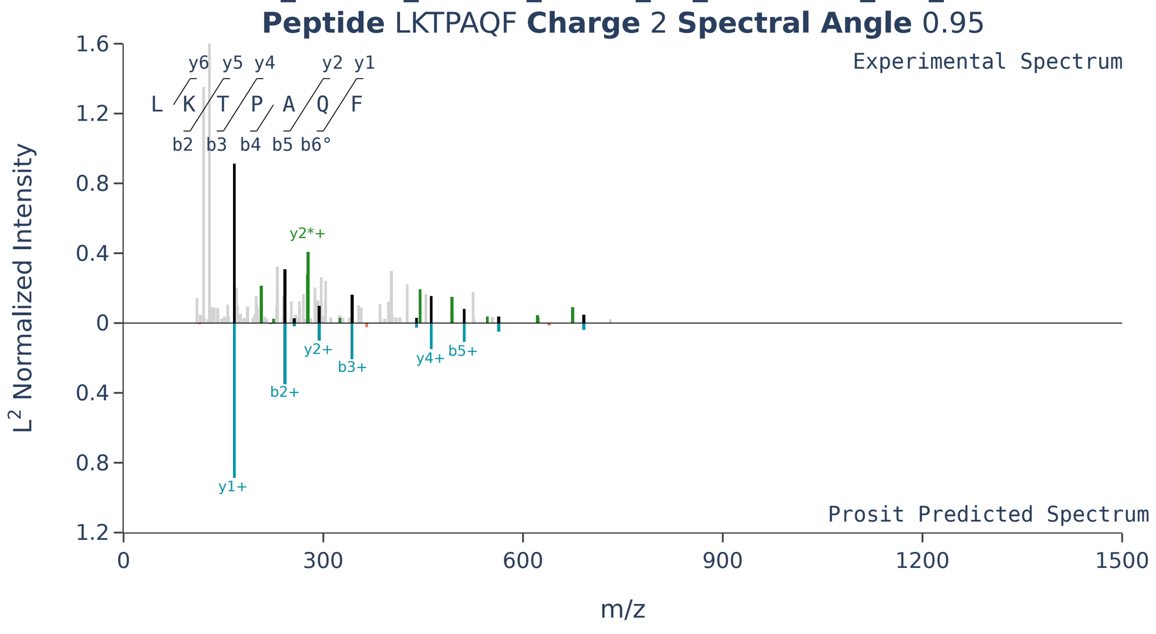


**Source** WSoh_301121_021221_HFGoe_M2_AnnexinA1_24h_R1 **Scan** 20230
**Peptide** HLEEVVLA **Charge** 2 **Spectral Angle** 0.87

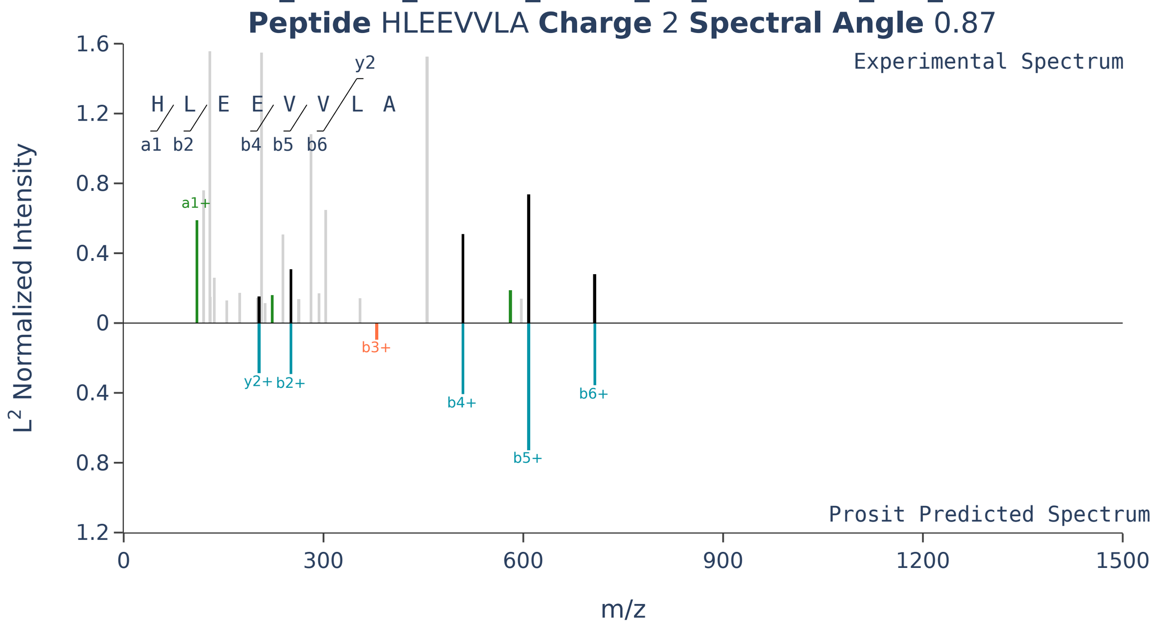


**Source** WSoh_301121_021221_HFGoe_M2_AnnexinA1_24h_R2 **Scan** 12213
**Peptide** SLAKGDRSEDFGVN **Charge** 3 **Spectral Angle** 0.88

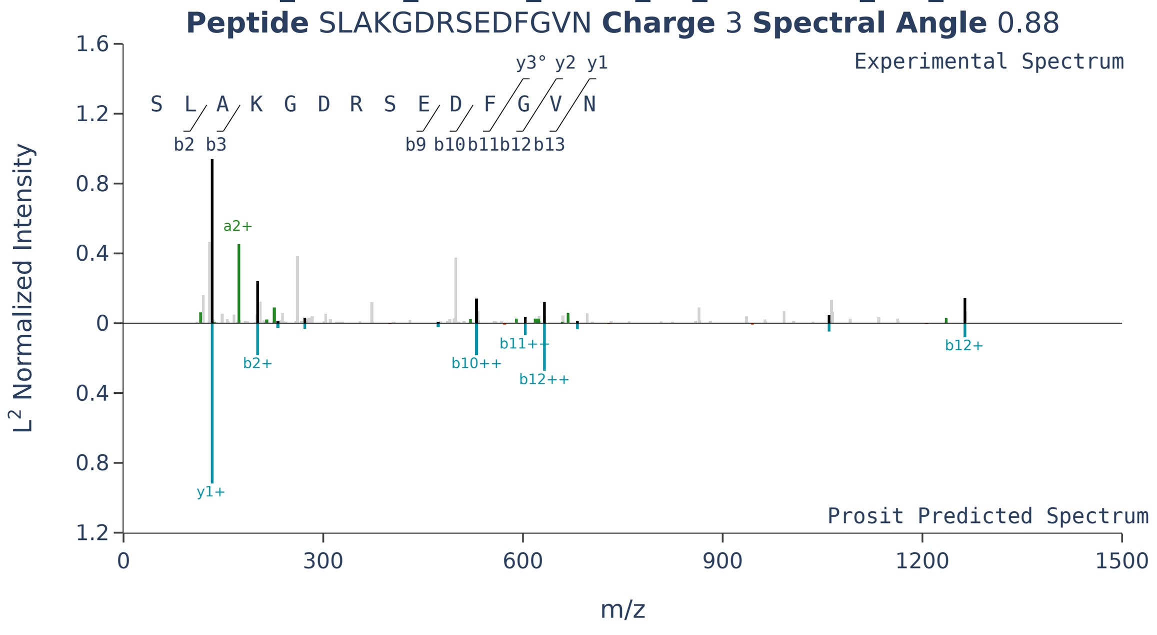


**Source** WSoh_301121_021221_HFGoe_M1_AnnexinA1_24h_R2 **Scan** 6828
**Peptide** EIRDINR **Charge** 2 **Spectral Angle** 0.84

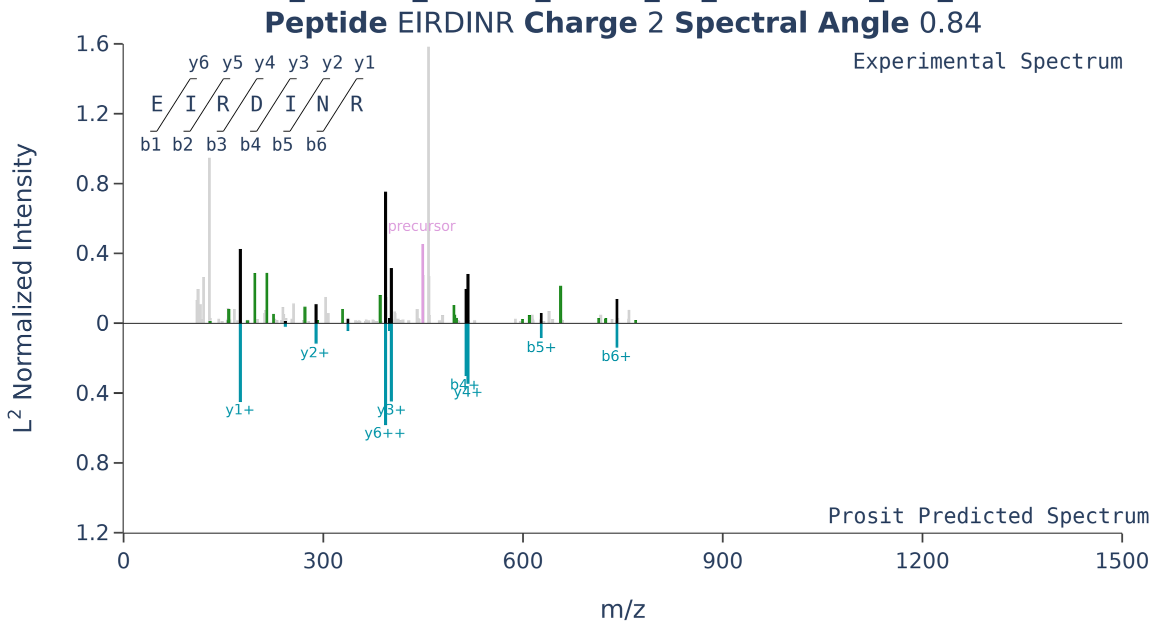


**Source** WSoh_301121_021221_HFGoe_M2_AnnexinA1_24h_R1 **Scan** 21212
**Peptide** GDYEKILVA **Charge** 2 **Spectral Angle** 0.87

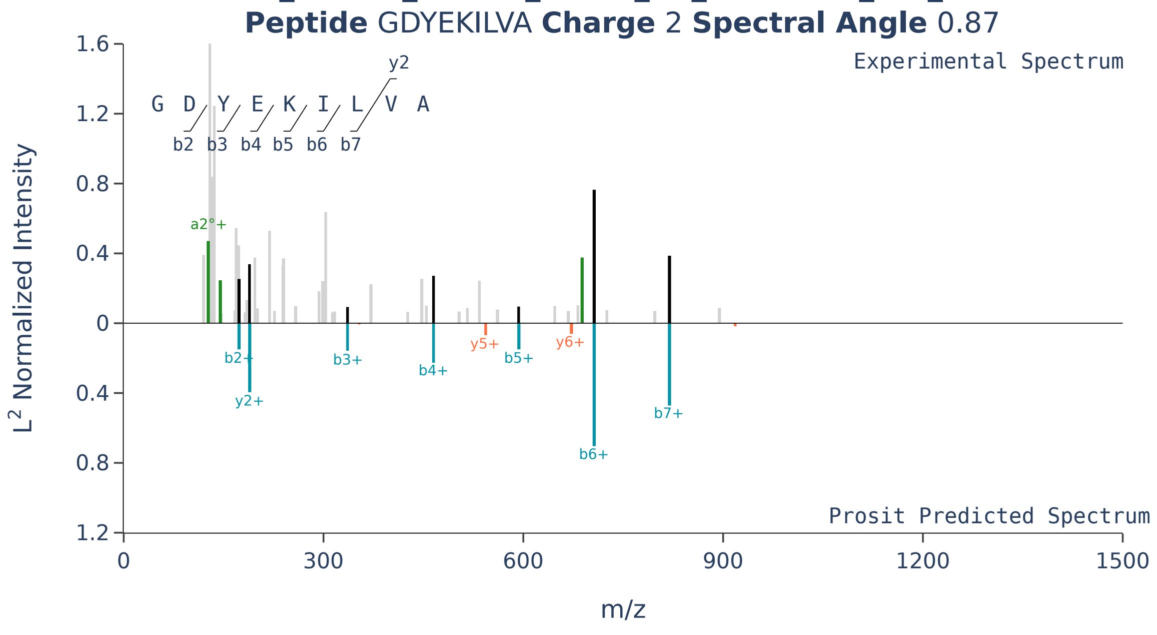


**Source** WSoh_301121_021221_HFGoe_M1_AnnexinA1_24h_R2 **Scan** 12875
**Peptide** SLAKGDRSEDFGVNE **Charge** 2 **Spectral Angle** 0.88

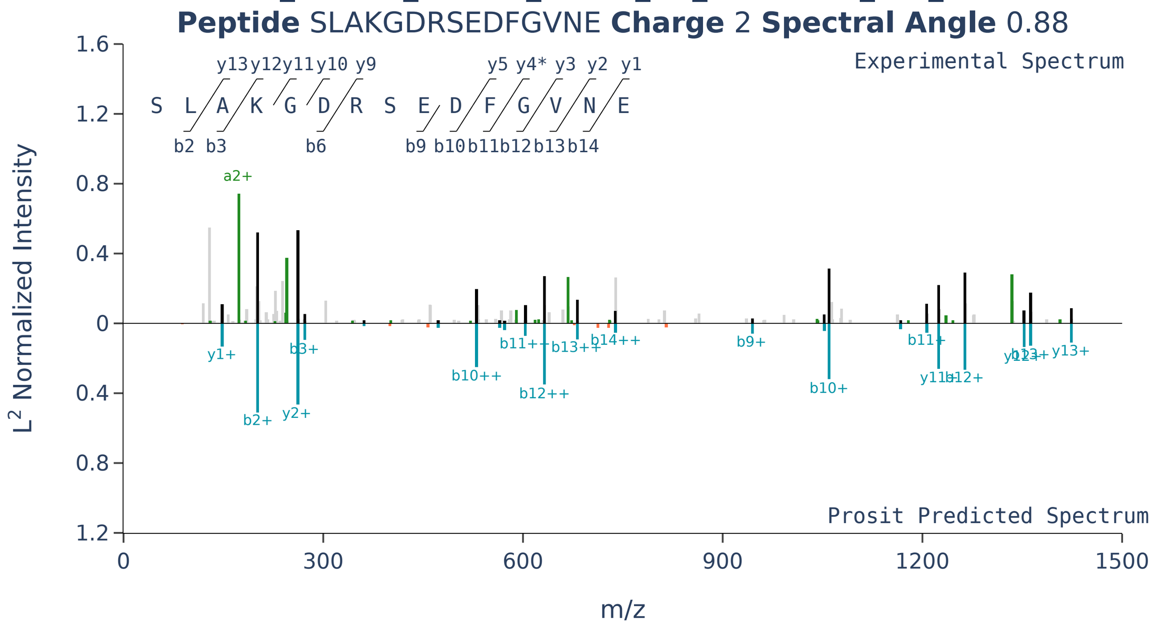


**Source** WSoh_301121_021221_HFGoe_M1_AnnexinA1_24h_R2 **Scan** 13465
**Peptide** DRSEDFGVNED **Charge** 2 **Spectral Angle** 0.87

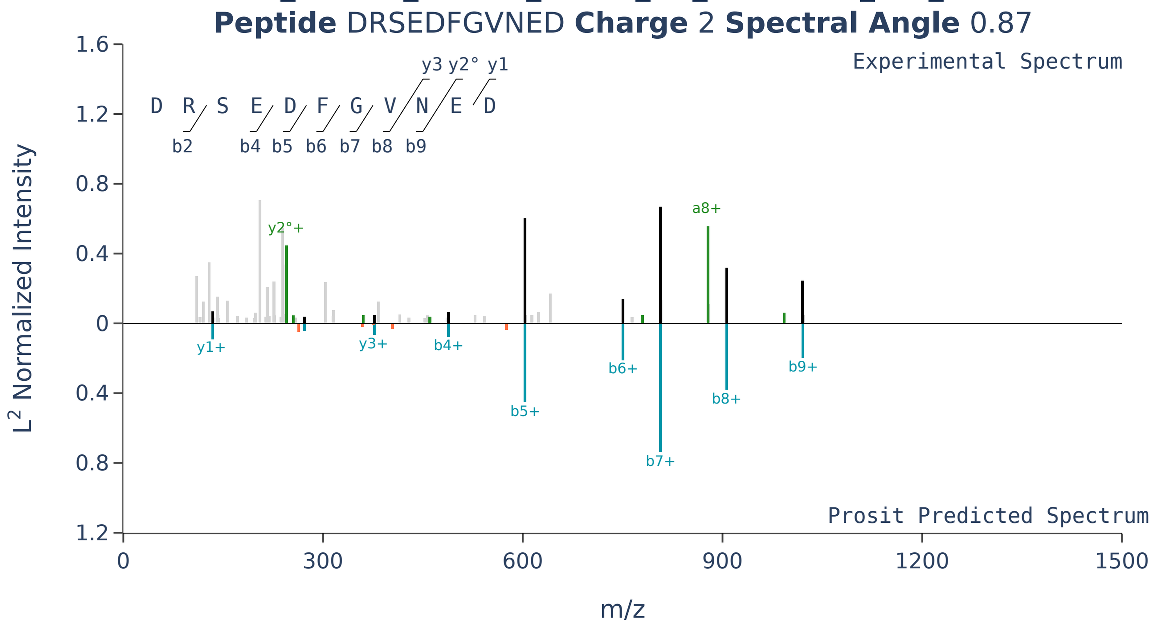


**Source** WSoh_301121_021221_HFGoe_M2_AnnexinA1_2h_R1 **Scan** 16688
**Peptide** AKGDRSEDFGVNEDLAD **Charge** 3 **Spectral Angle** 0.85

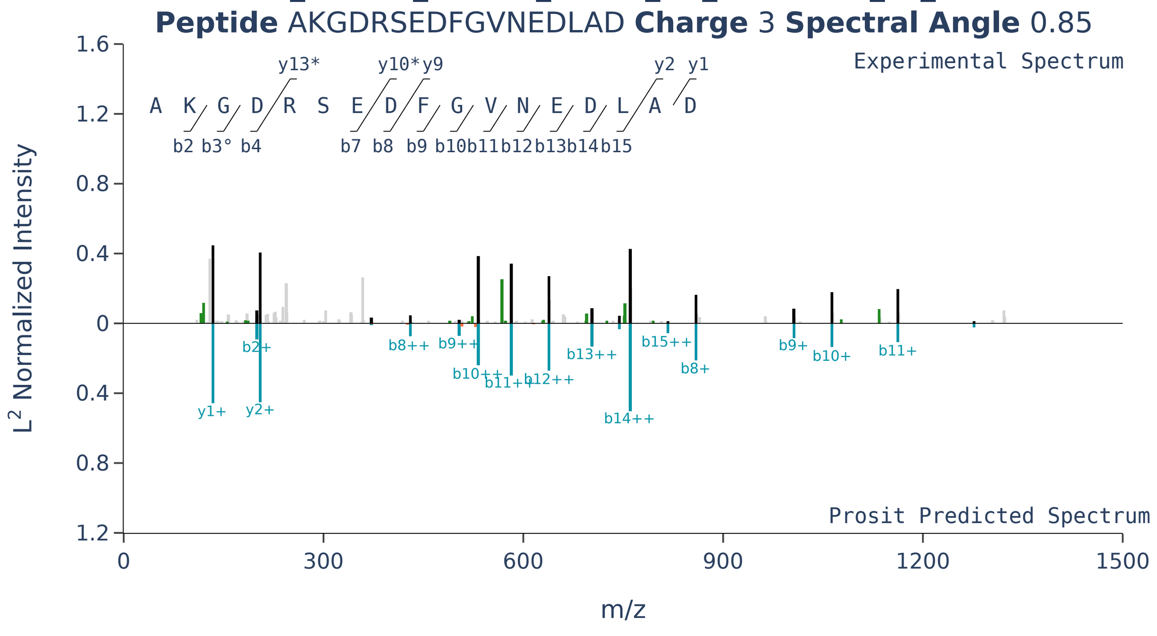


**Source** WSoh_301121_021221_HFGoe_M2_AnnexinA1_24h_R2 **Scan** 15194
**Peptide** DFGVNED **Charge** 1 **Spectral Angle** 0.85

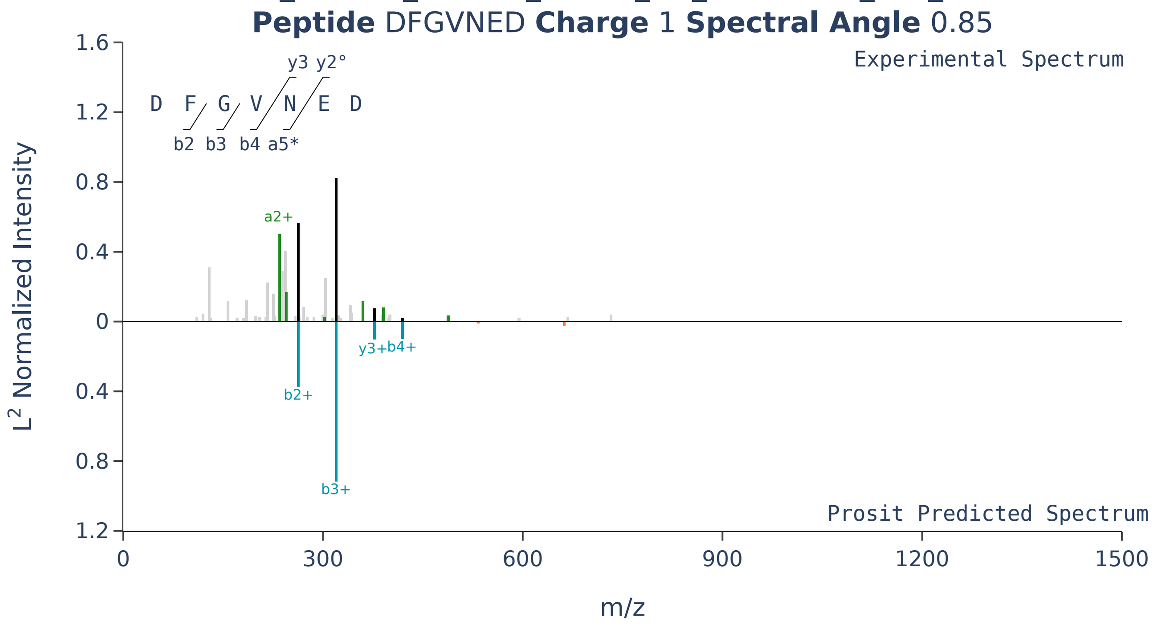


**Source** WSoh_301121_021221_HFGoe_M1_AnnexinA1_2h_R2 **Scan** 20083
**Peptide** LDLELKG **Charge** 1 **Spectral Angle** 0.92

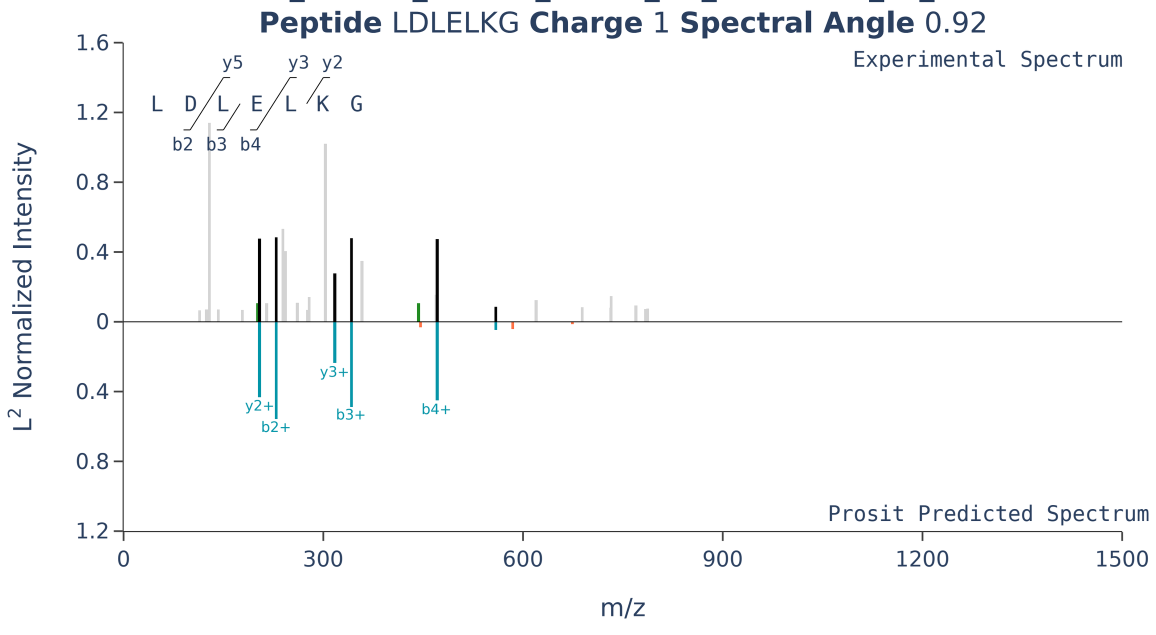


**Source** WSoh_301121_021221_HFGoe_M2_AnnexinA1_24h_R2 **Scan** 14395
**Peptide** ADSDARALYEA **Charge** 2 **Spectral Angle** 0.87

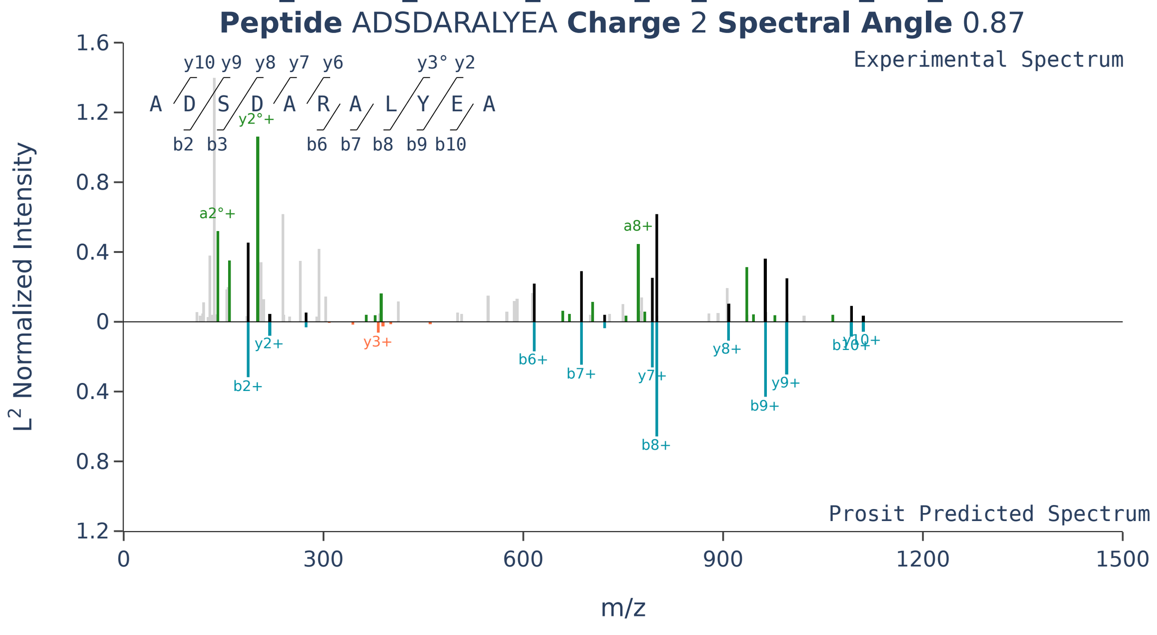


**Source** WSoh_301121_021221_HFGoe_M2_AnnexinA1_24h_R1 **Scan** 15365
**Peptide** SEDFGVNED **Charge** 1 **Spectral Angle** 0.9

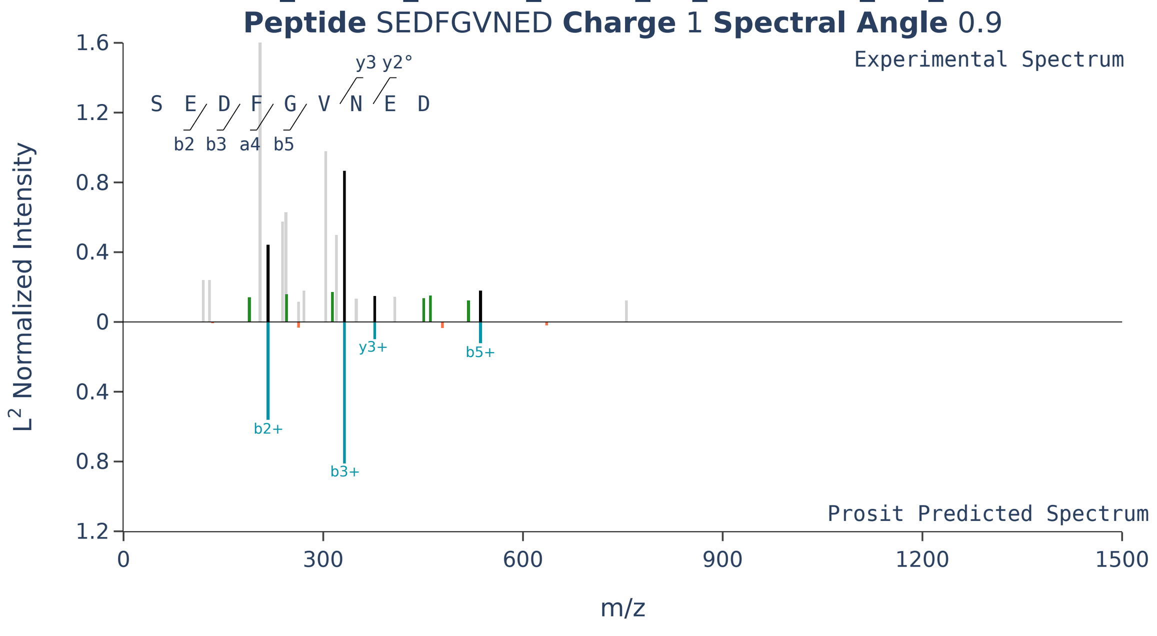


**Source** WSoh_301121_021221_HFGoe_M2_AnnexinA1_24h_R2 **Scan** 12620
**Peptide** AFYQKMY **Charge** 2 **Spectral Angle** 0.88

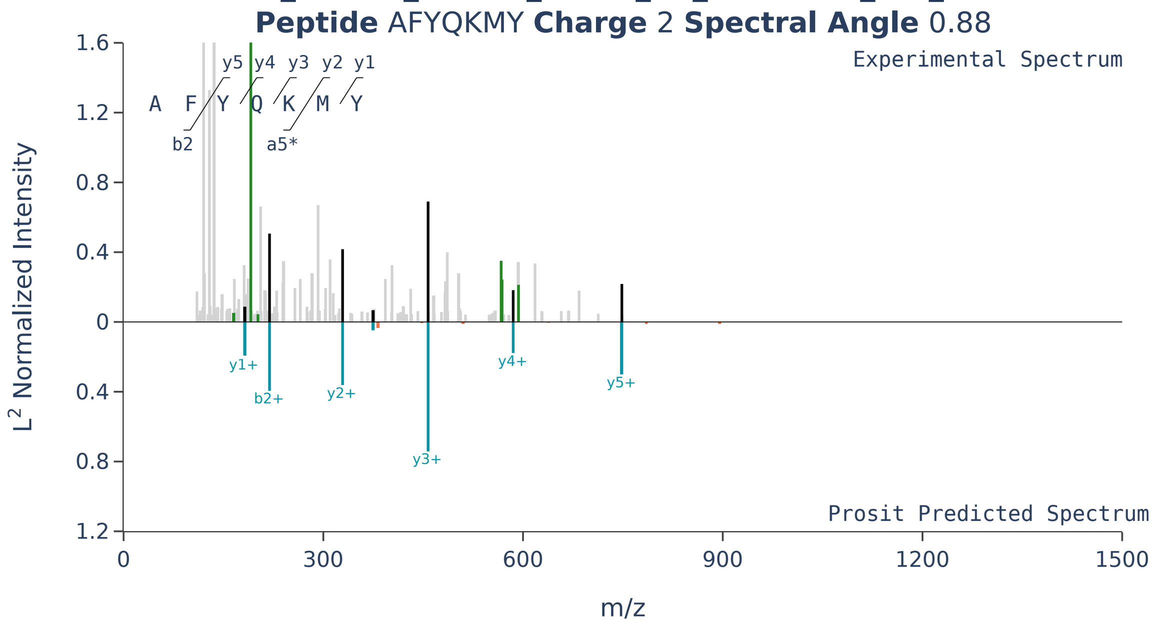


**Source** WSoh_301121_021221_HFGoe_M2_AnnexinA1_4h_R1 **Scan** 9290
**Peptide** KGDIEKCLT **Charge** 3 **Spectral Angle** 0.93

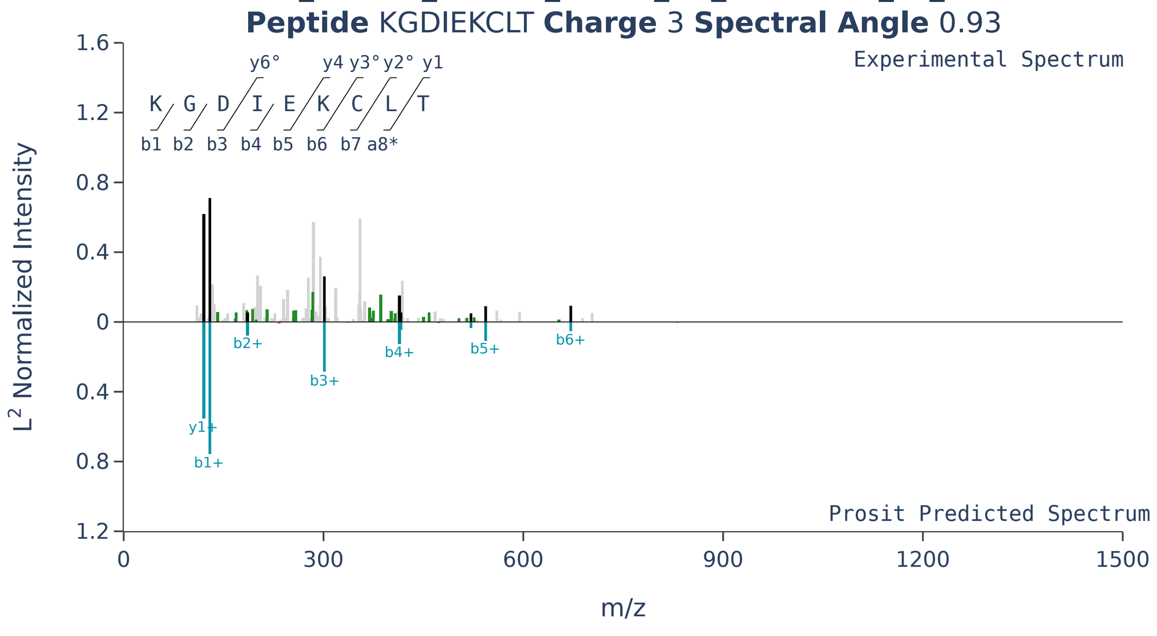

**Source** WSoh_301121_021221_HFGoe_M2_AnnexinA1_4h_R1 **Scan** 19108
**Peptide** ATSKPAFFAE **Charge** 2 **Spectral Angle** 0.89

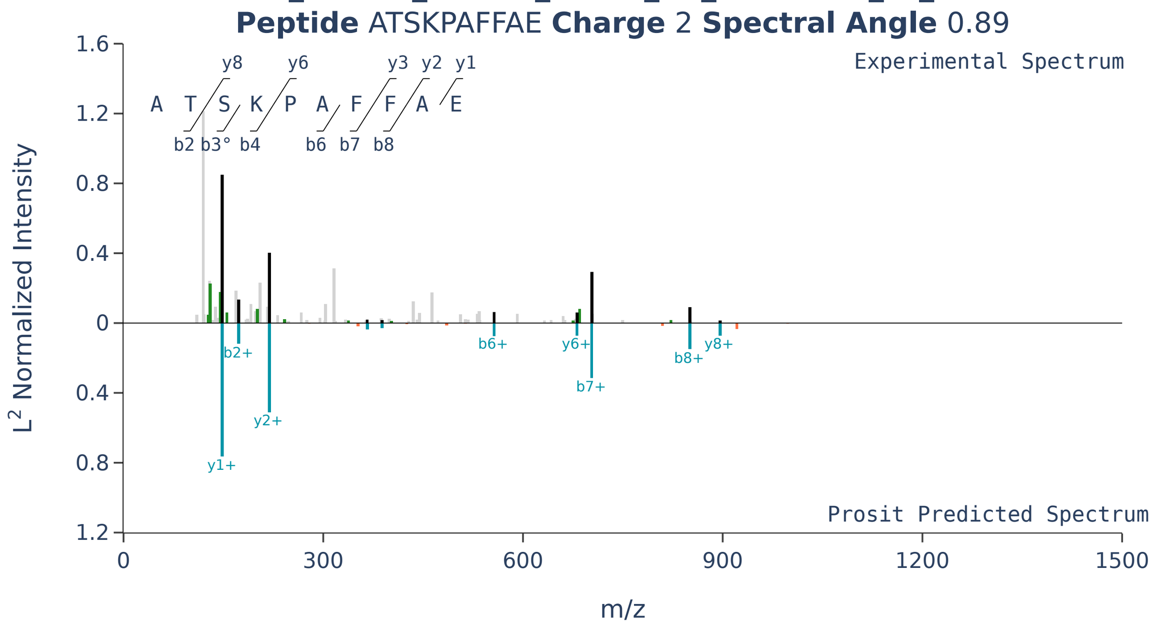


**Source** WSoh_301121_021221_HFGoe_M1_AnnexinA1_24h_R1 **Scan** 10391
**Peptide** KAFYQKM **Charge** 2 **Spectral Angle** 0.93

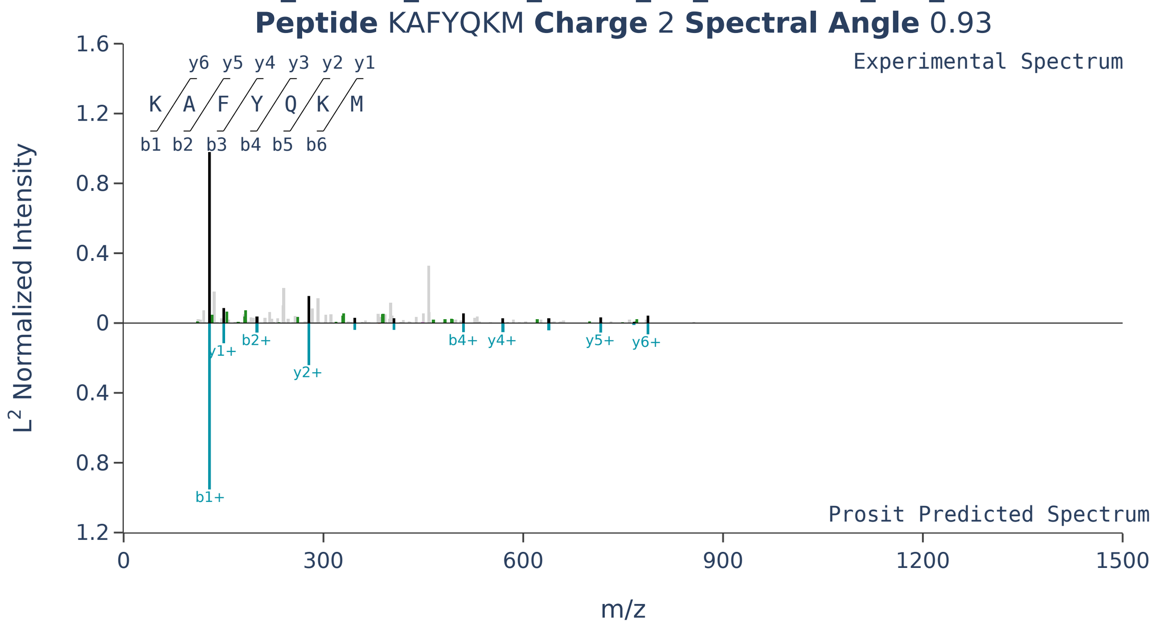


**Source** WSoh_301121_021221_HFGoe_M2_AnnexinA1_24h_R1 **Scan** 6926
**Peptide** QETGKPLD **Charge** 2 **Spectral Angle** 0.91

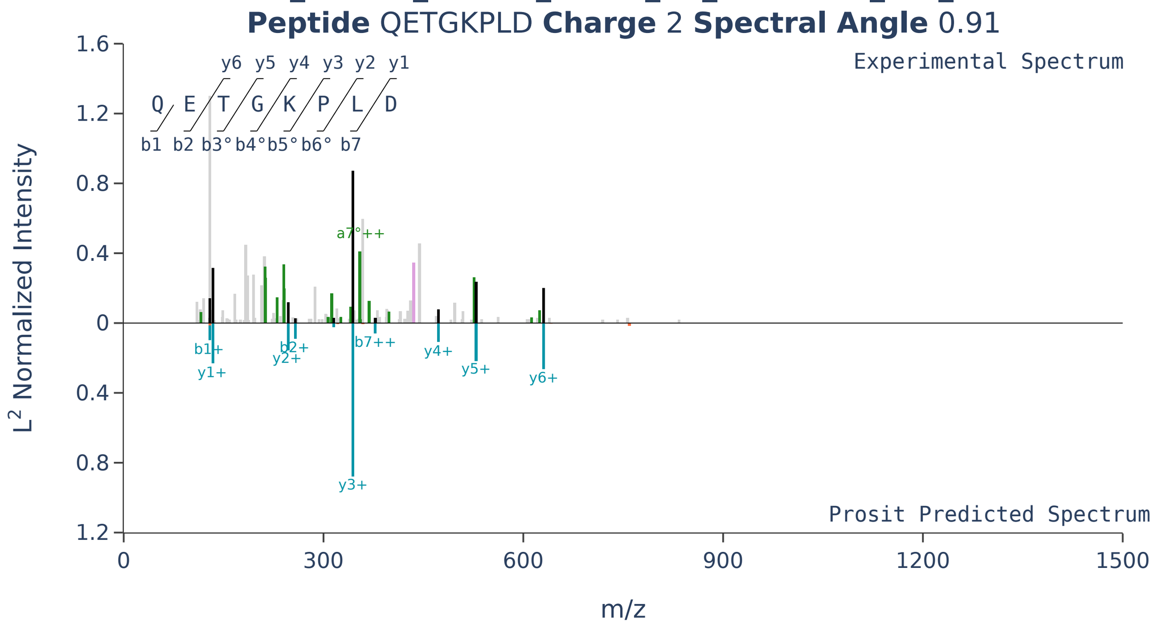


**Source** WSoh_301121_021221_HFGoe_M2_AnnexinA1_24h_R1 **Scan** 7921
**Peptide** TKGDYEKI **Charge** 2 **Spectral Angle** 0.9

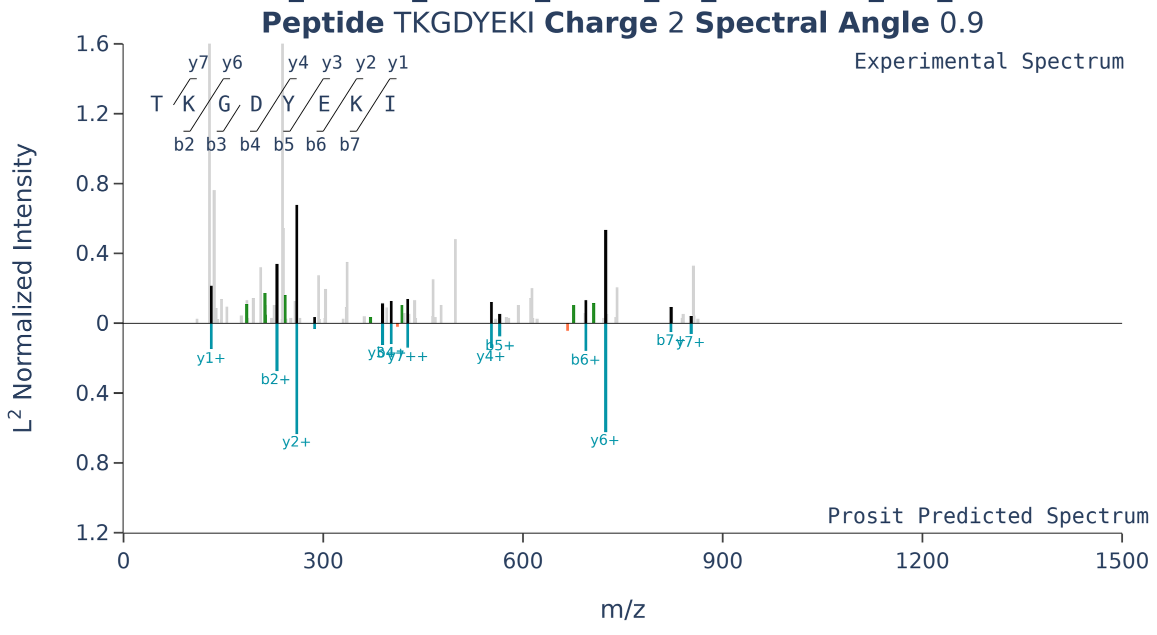


**Source** WSoh_301121_021221_HFGoe_M2_AnnexinA1_2h_R2 **Scan** 12601
**Peptide** SLAKGDRSEDFGVNED **Charge** 3 **Spectral Angle** 0.91

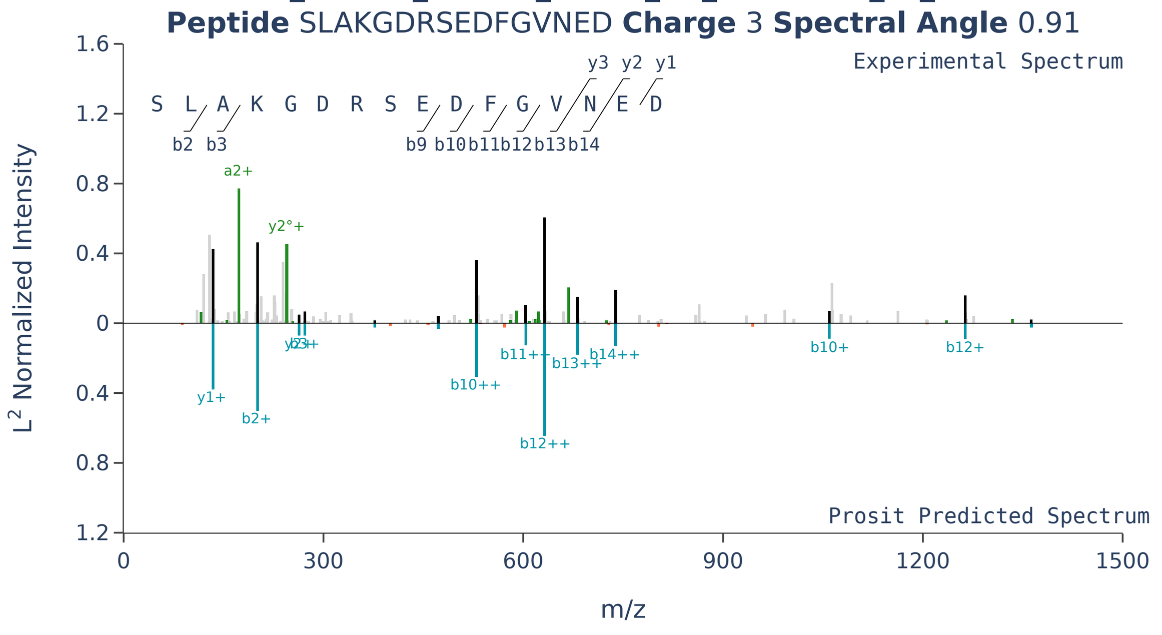


**Source** WSoh_301121_021221_HFGoe_M1_AnnexinA1_24h_R1 **Scan** 10970
**Peptide** SRSEIDM **Charge** 2 **Spectral Angle** 0.95

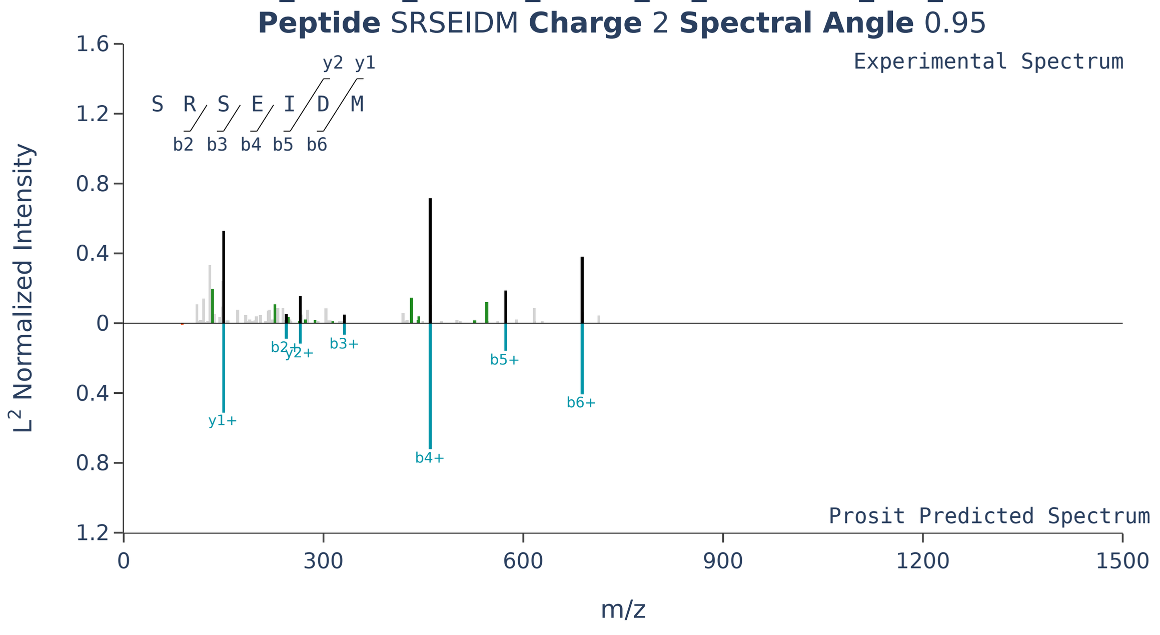


**Source** WSoh_301121_021221_HFGoe_M2_AnnexinA1_24h_R2 **Scan** 11715
**Peptide** IRDINRVY **Charge** 2 **Spectral Angle** 0.78

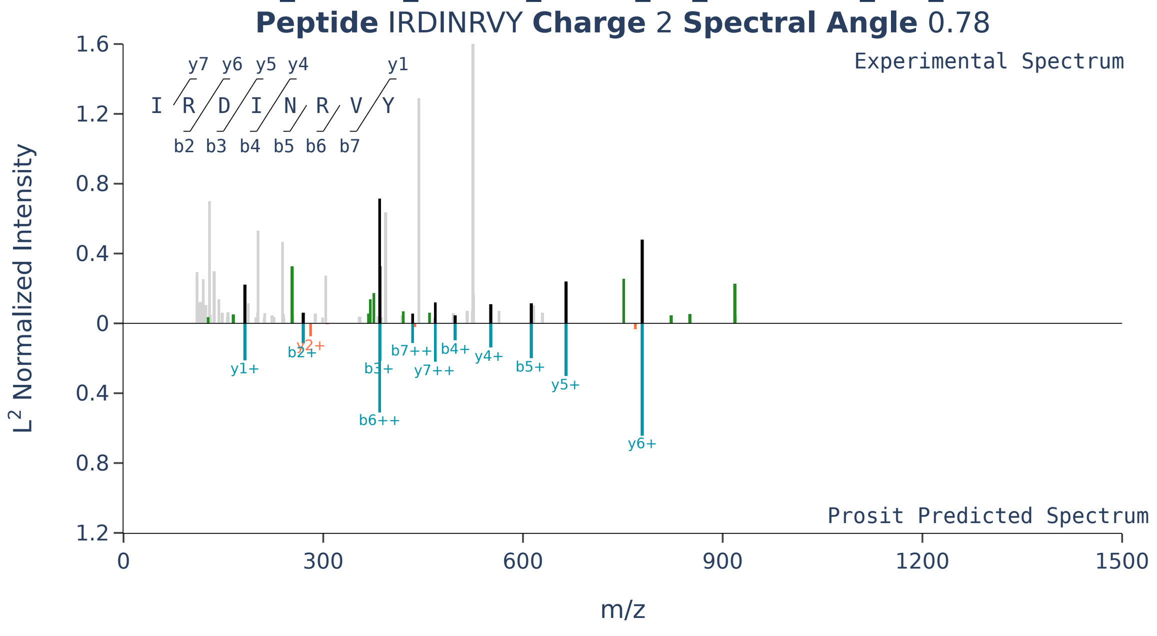


**Source** WSoh_301121_021221_HFGoe_M1_AnnexinA1_24h_R1 **Scan** 11305
**Peptide** EEQEYVQTVKS **Charge** 2 **Spectral Angle** 0.94

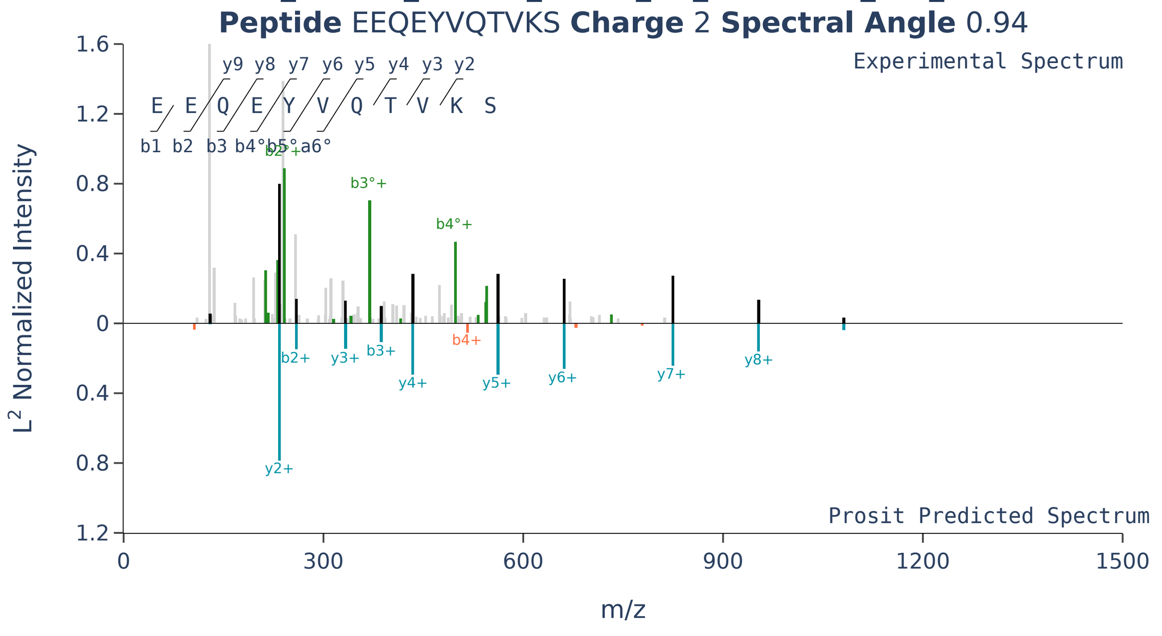


**Source** WSoh_301121_021221_HFGoe_M1_AnnexinA1_4h_R1 **Scan** 9816
**Peptide** KDITSDTSGDFRN **Charge** 2 **Spectral Angle** 0.88

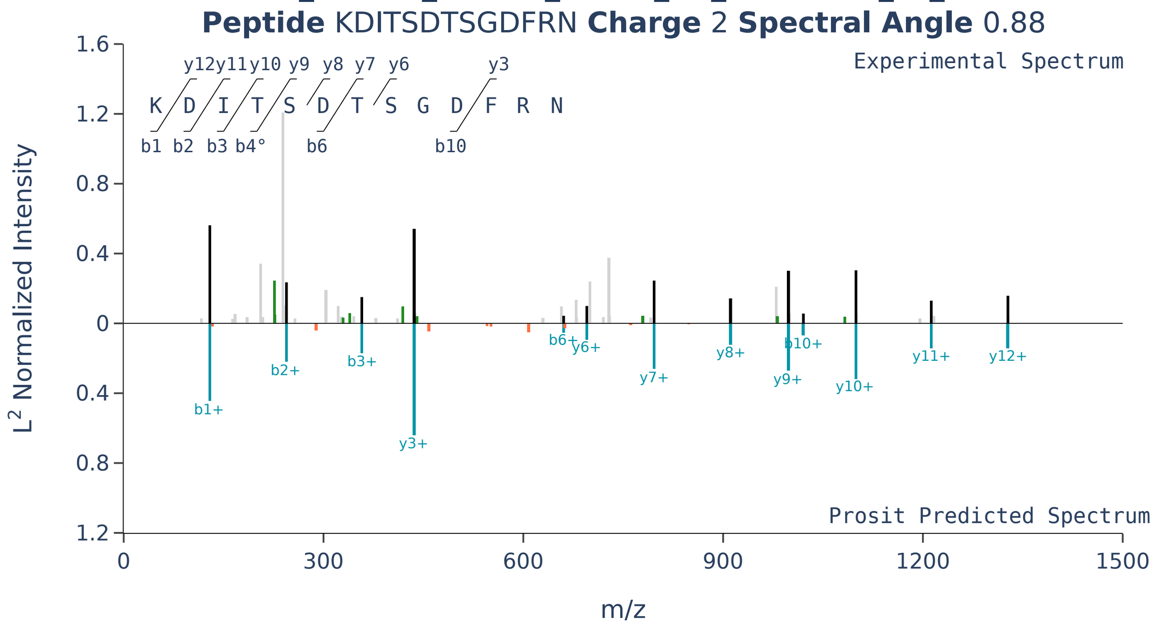


**Source** WSoh_301121_021221_HFGoe_M1_AnnexinA1_2h_R2 **Scan** 9393
**Peptide** AKGDRSEDFGVN **Charge** 2 **Spectral Angle** 0.83

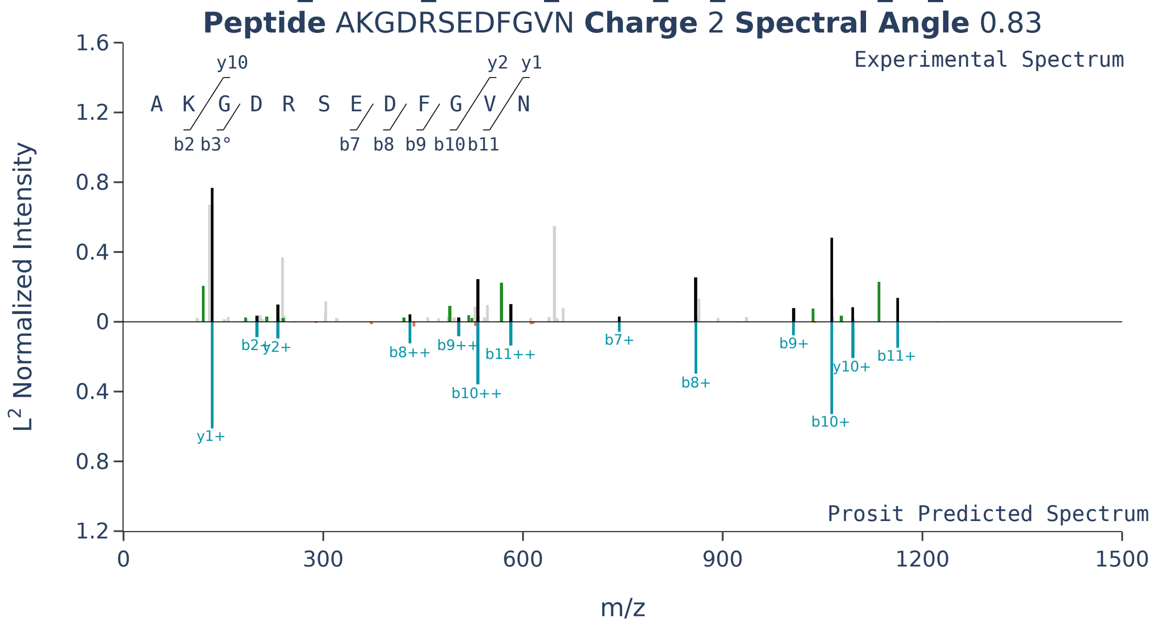


**Source** WSoh_301121_021221_HFGoe_M2_AnnexinA1_2h_R1 **Scan** 22006
**Peptide** AMVSEFLKQ **Charge** 2 **Spectral Angle** 0.85

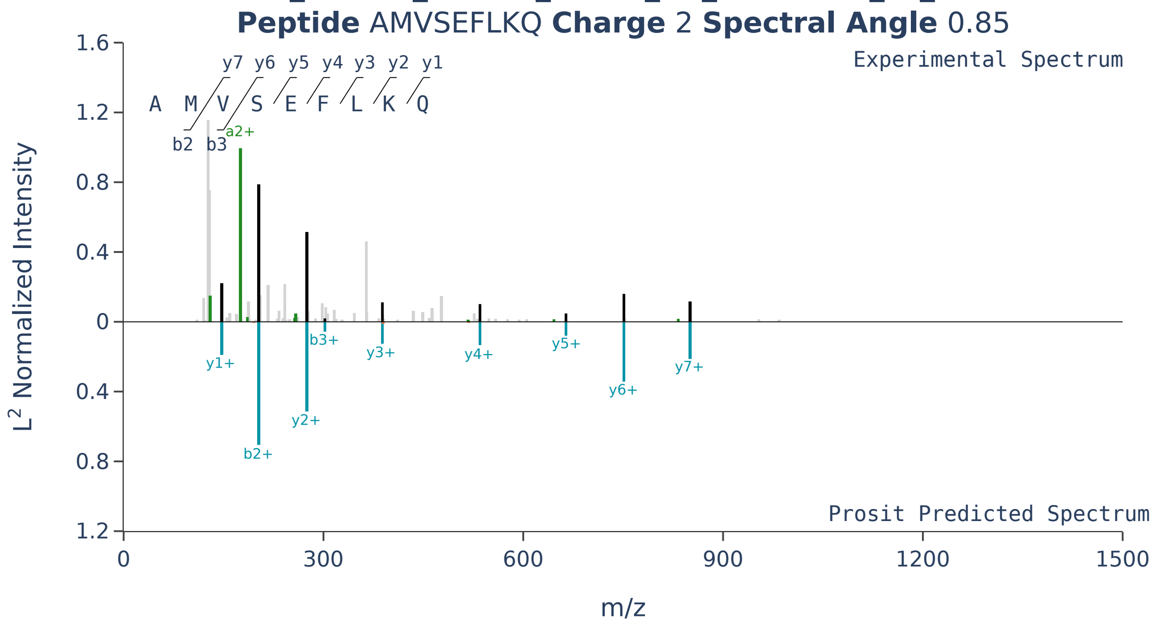


**Source** WSoh_301121_021221_HFGoe_M1_AnnexinA1_2h_R2 **Scan** 11659
**Peptide** TNKEIRDINRVY **Charge** 3 **Spectral Angle** 0.92

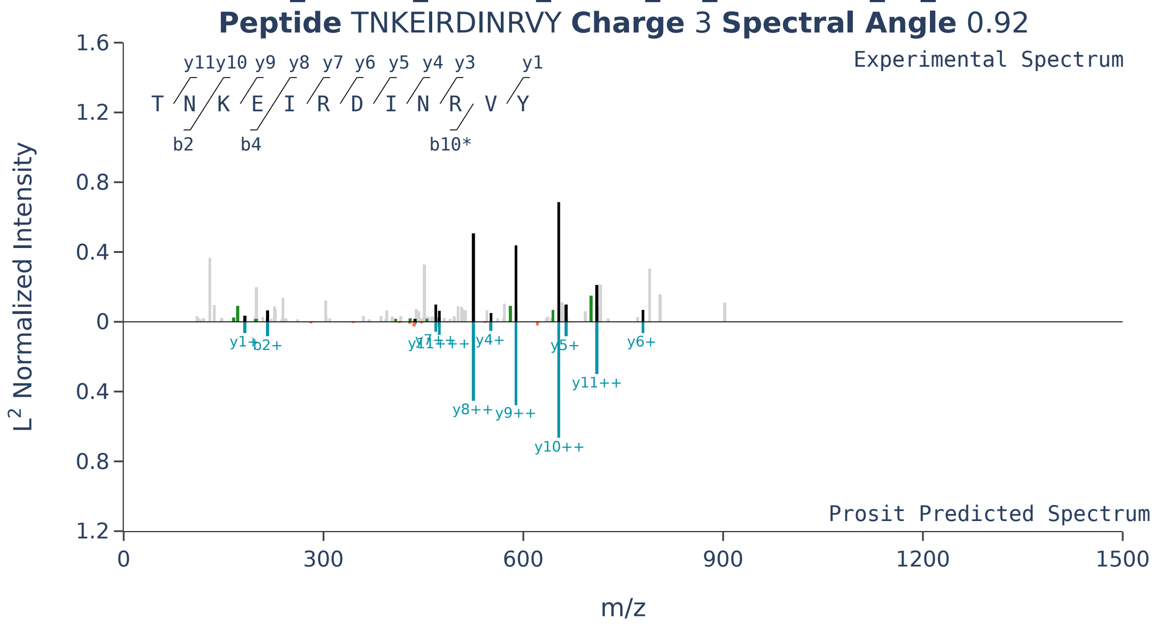


**Source** WSoh_301121_021221_HFGoe_M2_AnnexinA1_2h_R1 **Scan** 6124
**Peptide** RTNKEIRDIN **Charge** 3 **Spectral Angle** 0.91

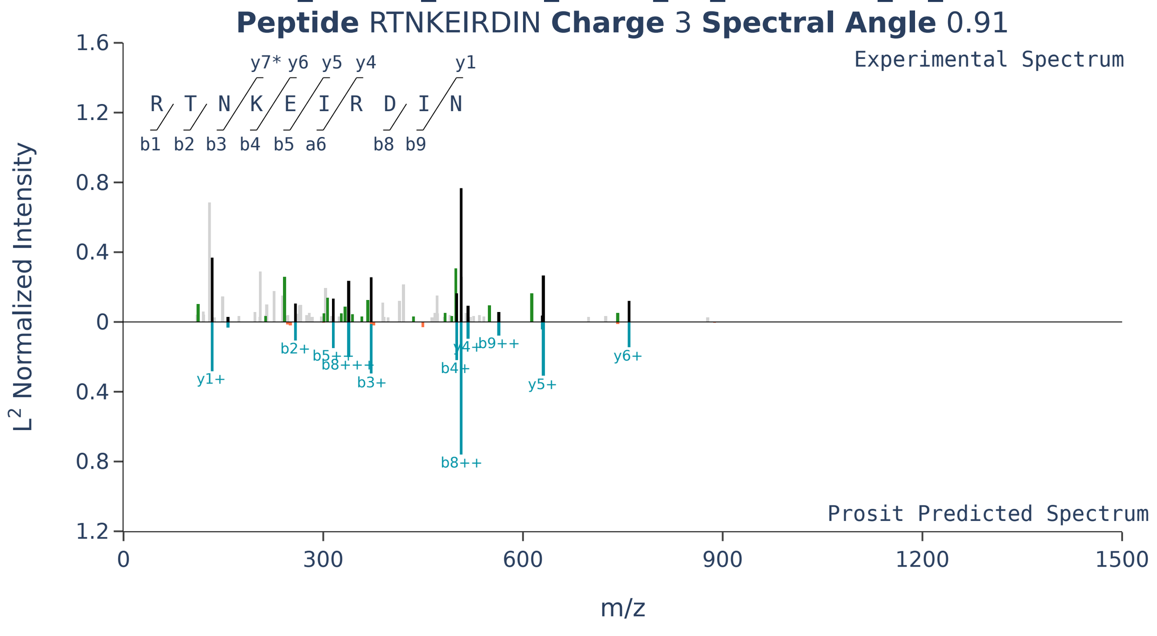


**Source** WSoh_301121_021221_HFGoe_M2_AnnexinA1_24h_R1 **Scan** 16304
**Peptide** EQEYVQTV **Charge** 1 **Spectral Angle** 0.92

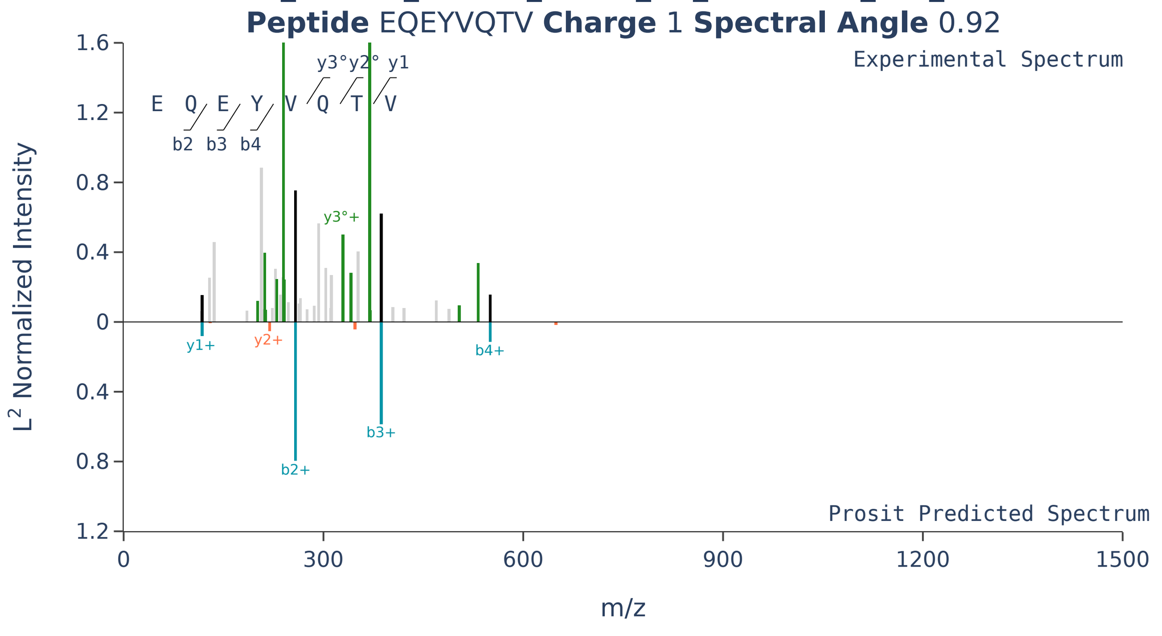


**Source** WSoh_301121_021221_HFGoe_M1_AnnexinA1_2h_R1 **Scan** 9049
**Peptide** ETLKKALTGH **Charge** 2 **Spectral Angle** 0.93

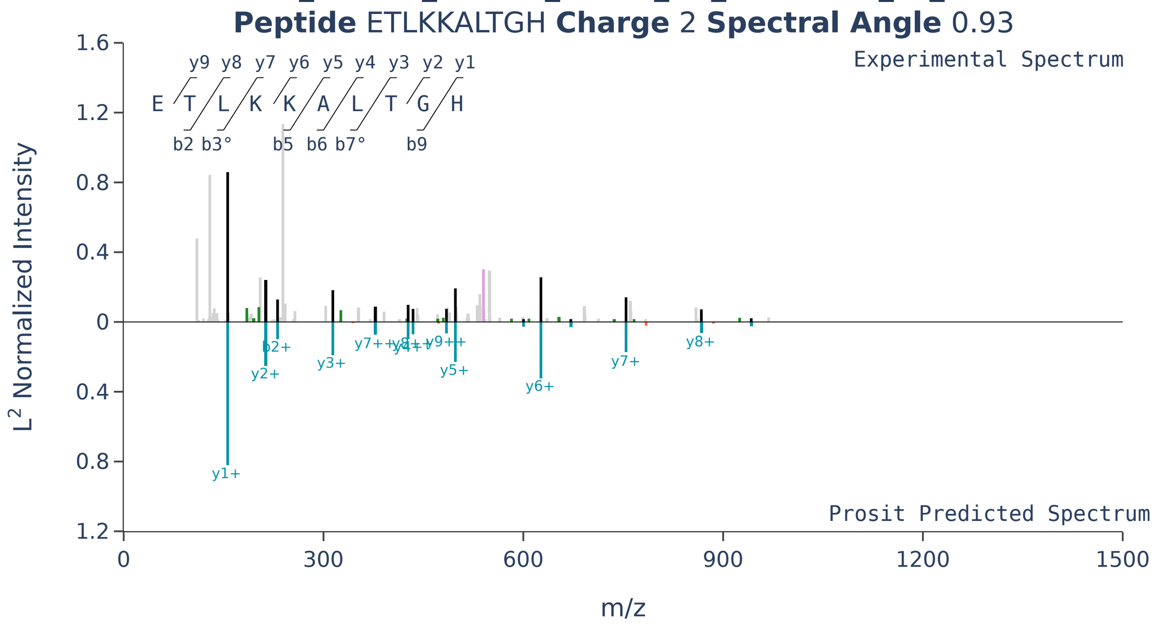

**Source** WSoh_301121_021221_HFGoe_M1_AnnexinA1_4h_R2 **Scan** 11100
**Peptide** KGDIEKCLTA **Charge** 3 **Spectral Angle** 0.89

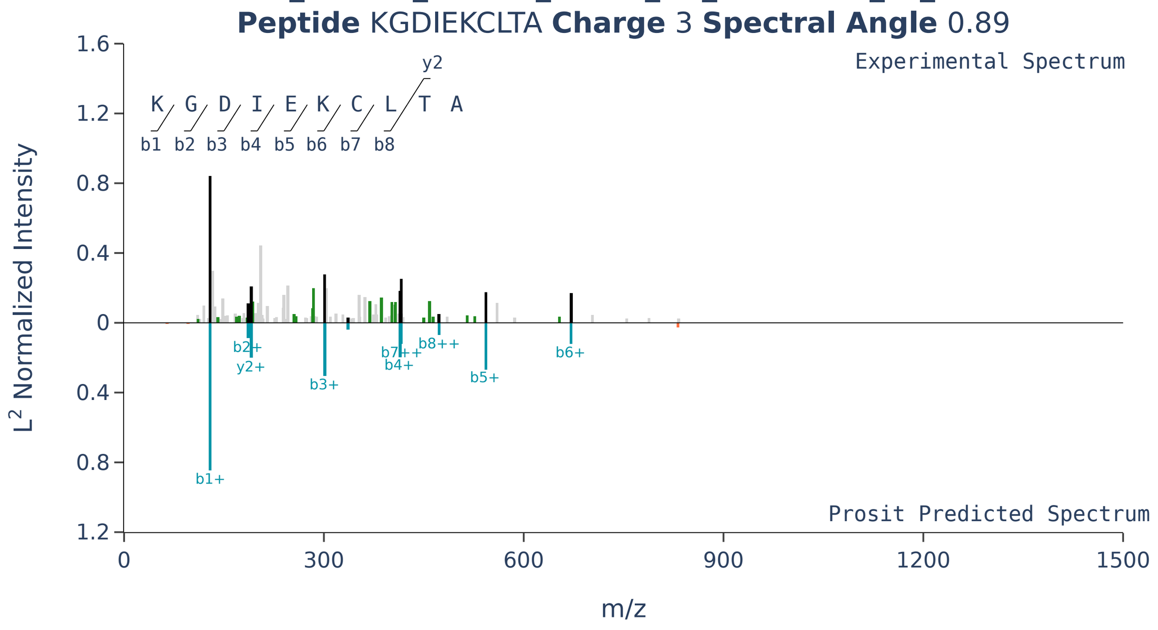


**Source** WSoh_301121_021221_HFGoe_M1_AnnexinA1_24h_R2 **Scan** 8699
**Peptide** TNKEIRDI **Charge** 2 **Spectral Angle** 0.91

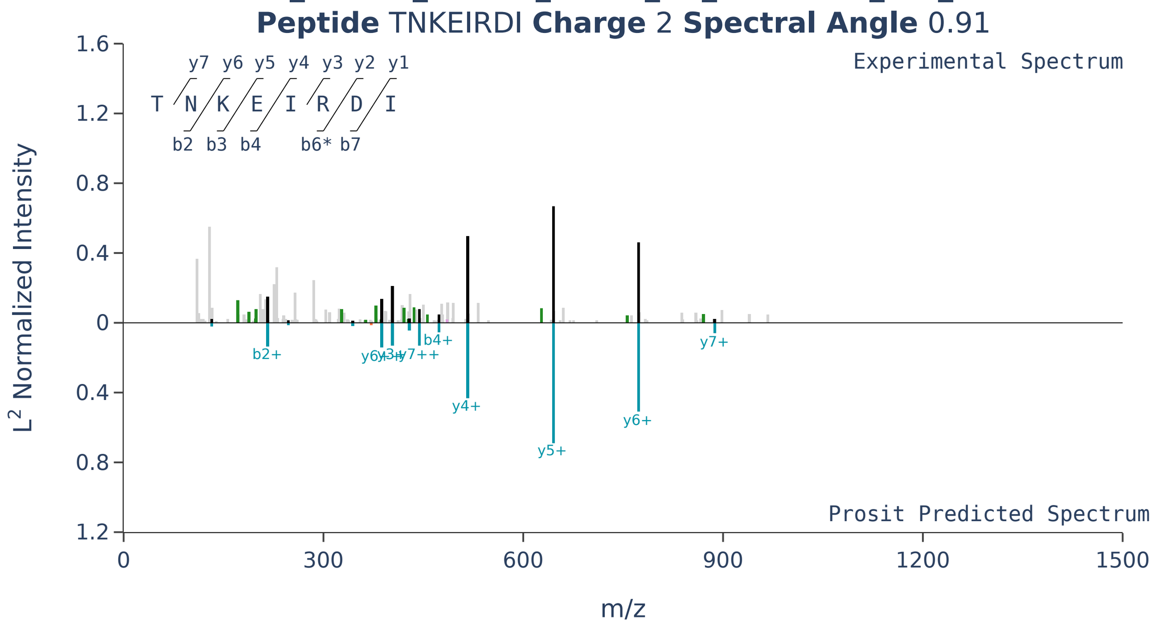


**Source** WSoh_301121_021221_HFGoe_M1_AnnexinA1_24h_R2 **Scan** 12282
**Peptide** LADSDARAL **Charge** 2 **Spectral Angle** 0.9

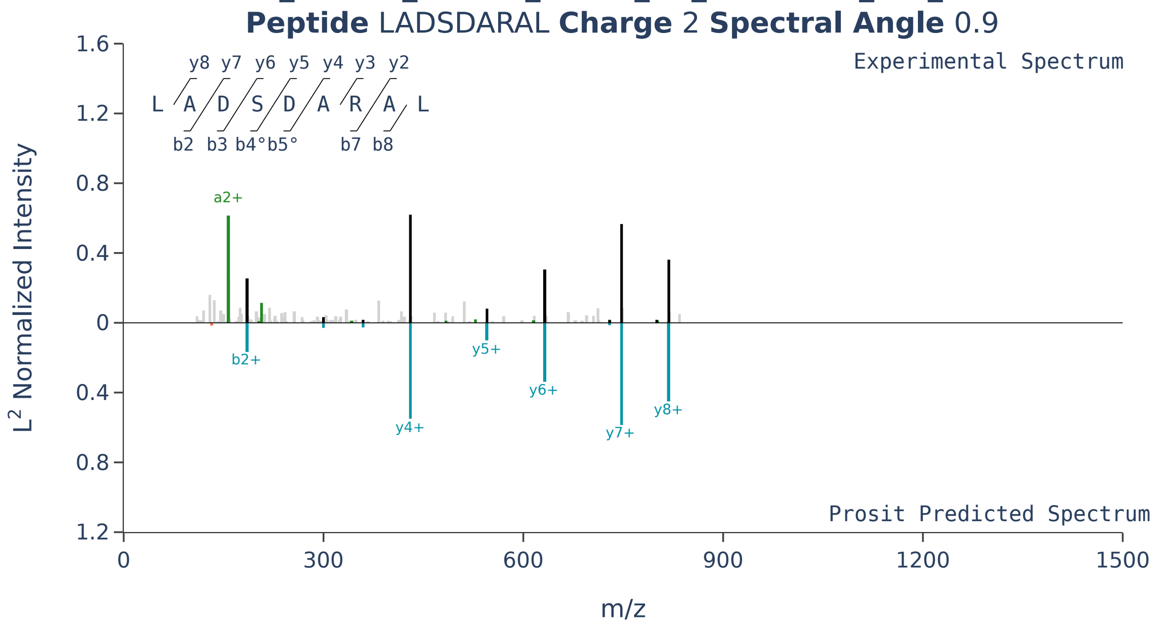


**Source** WSoh_301121_021221_HFGoe_M2_AnnexinA1_2h_R2 **Scan** 8630
**Peptide** RVFQKYTKYS **Charge** 2 **Spectral Angle** 0.83

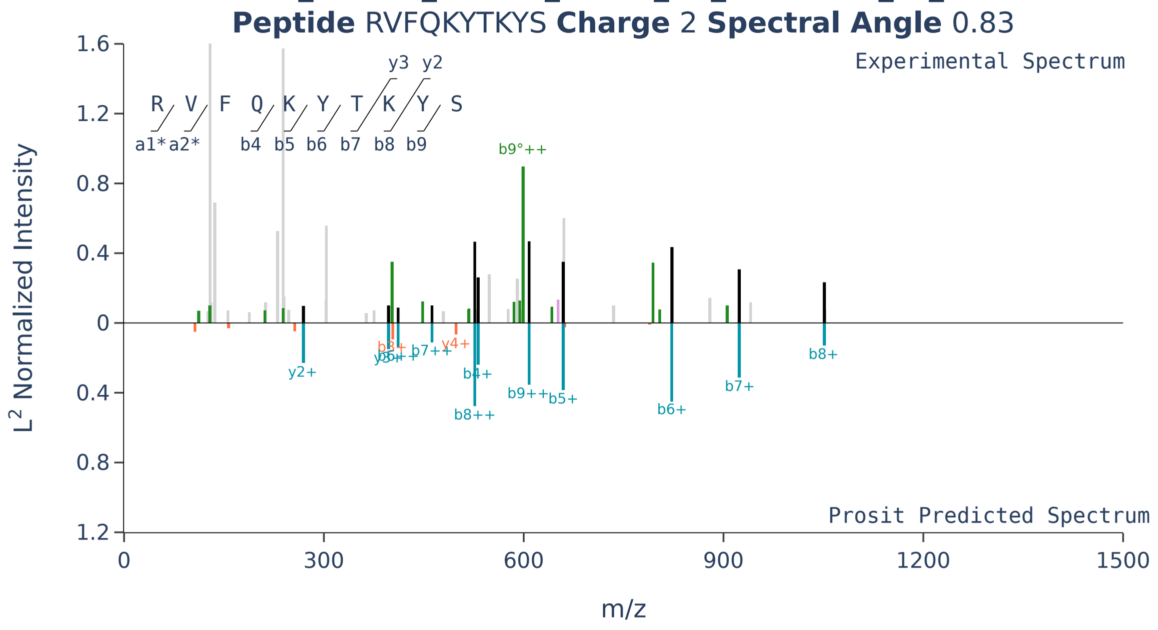


**Source** WSoh_301121_021221_HFGoe_M1_AnnexinA1_2h_R1 **Scan** 12912
**Peptide** IDILTKRNNA **Charge** 3 **Spectral Angle** 0.83

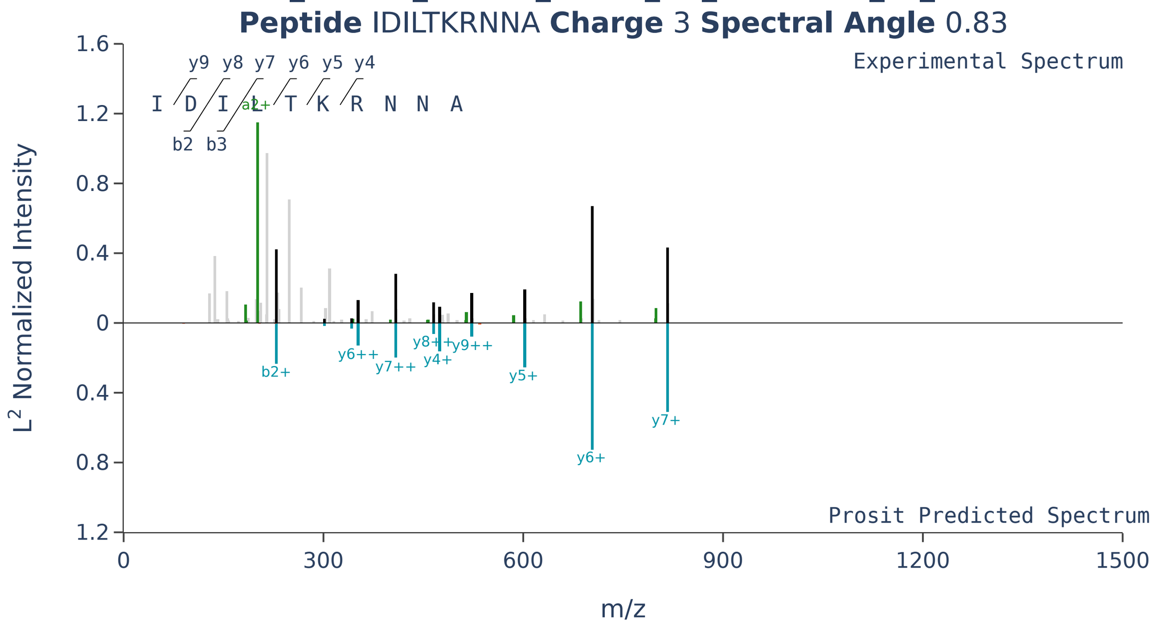


**Source** WSoh_301121_021221_HFGoe_M2_AnnexinA1_24h_R1 **Scan** 19699
**Peptide** ADELRAAMKGLGTD **Charge** 2 **Spectral Angle** 0.87

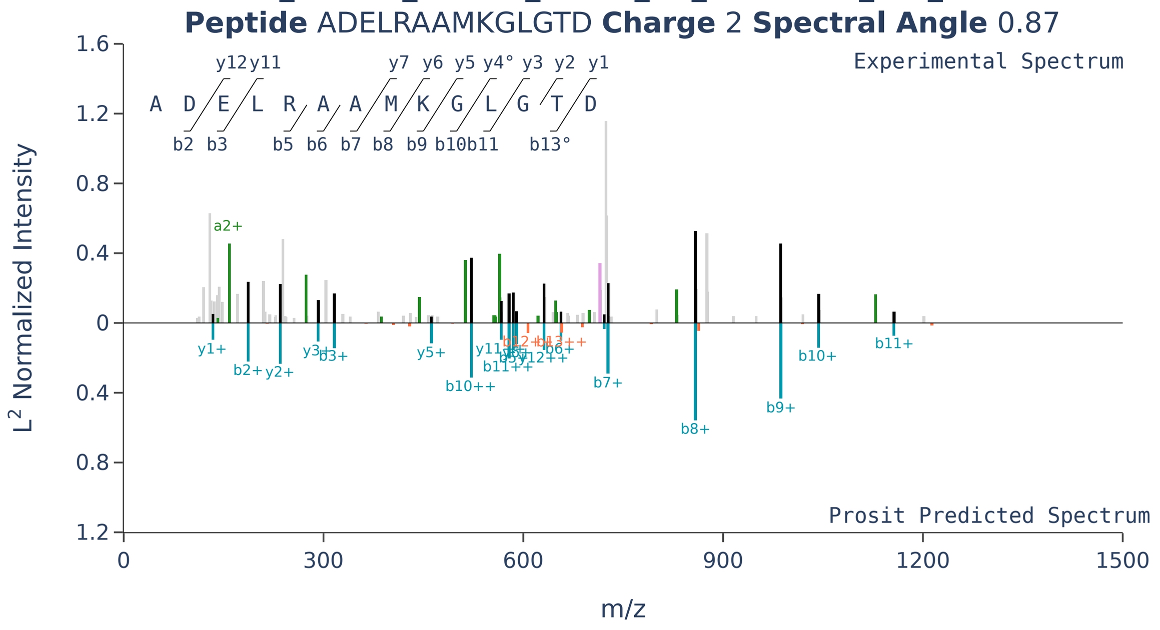


**Source** WSoh_301121_021221_HFGoe_M1_AnnexinA1_1h_R1 **Scan** 10789
**Peptide** GERRKGTDVNVF **Charge** 3 **Spectral Angle** 0.94

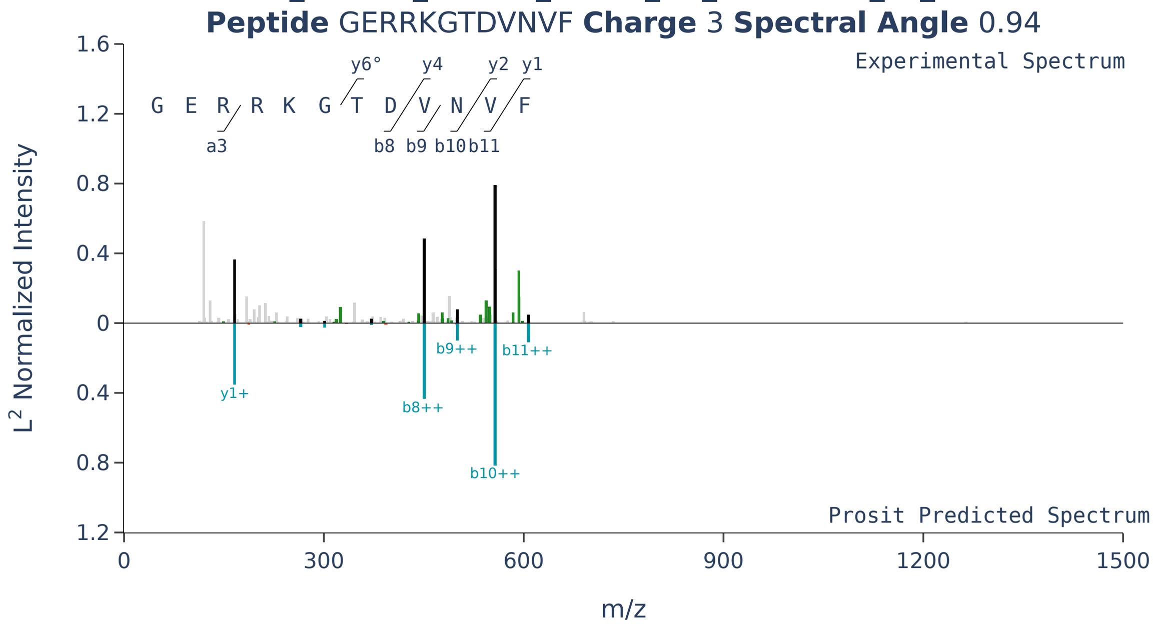


**Source** WSoh_301121_021221_HFGoe_M1_AnnexinA1_2h_R2 **Scan** 10434
**Peptide** LSLAKGD **Charge** 1 **Spectral Angle** 0.91

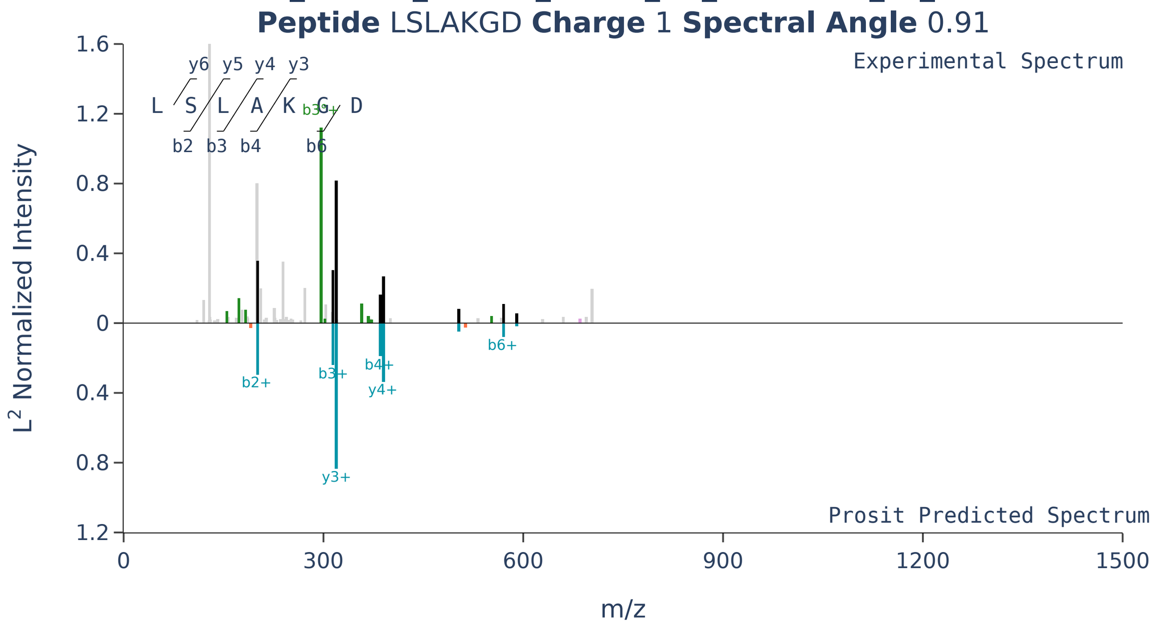


**Source** WSoh_301121_021221_HFGoe_M2_AnnexinA1_4h_R2 **Scan** 11031
**Peptide** LTAIVKCATSK **Charge** 2 **Spectral Angle** 0.87

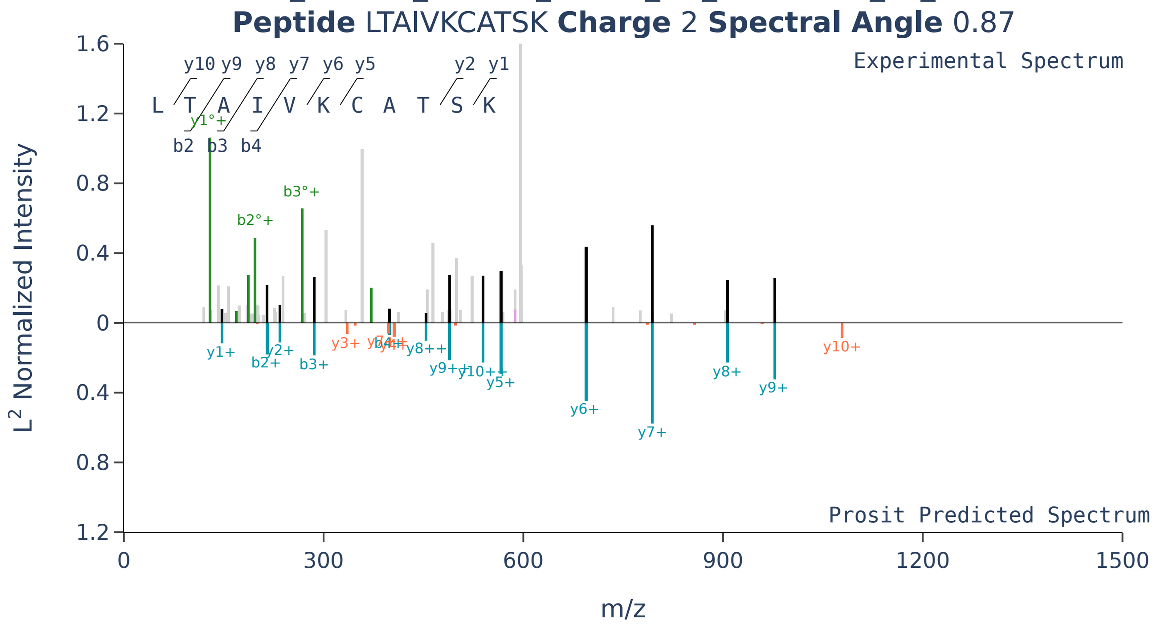


**Source** WSoh_301121_021221_HFGoe_M2_AnnexinA1_4h_R1 **Scan** 9222
**Peptide** HDMNKVLD **Charge** 1 **Spectral Angle** 0.95

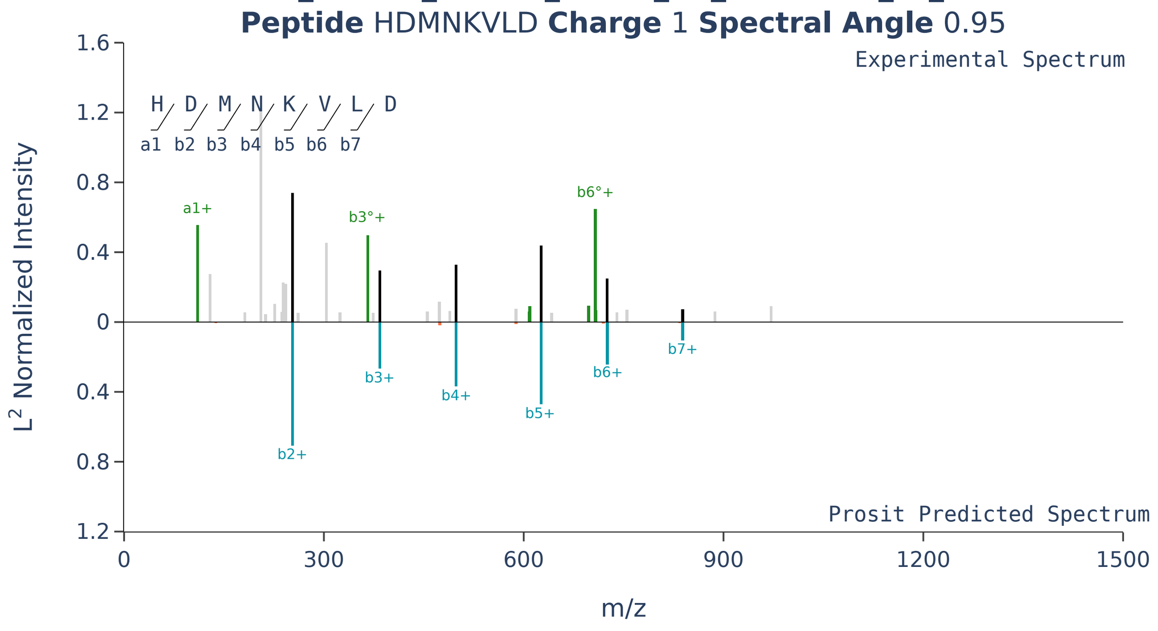


**Source** WSoh_301121_021221_HFGoe_M1_AnnexinA1_4h_R1 **Scan** 8481
**Peptide** KALTGHLEE **Charge** 2 **Spectral Angle** 0.85

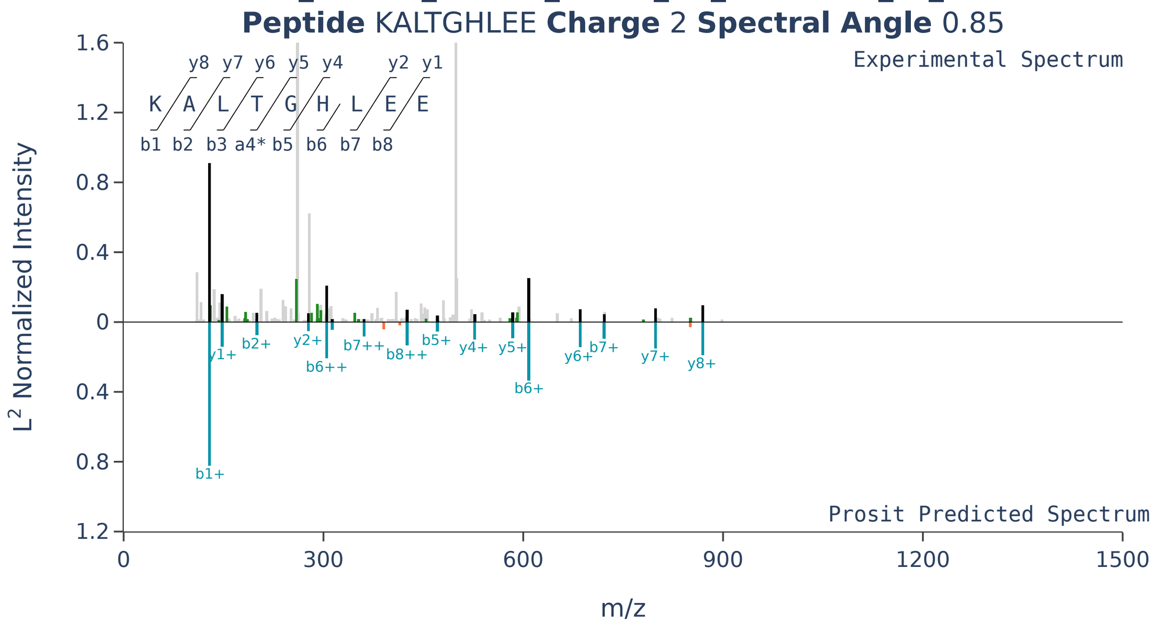


**Source** WSoh_301121_021221_HFGoe_M2_AnnexinA1_4h_R1 **Scan** 8142
**Peptide** FAEKLHQ **Charge** 2 **Spectral Angle** 0.92

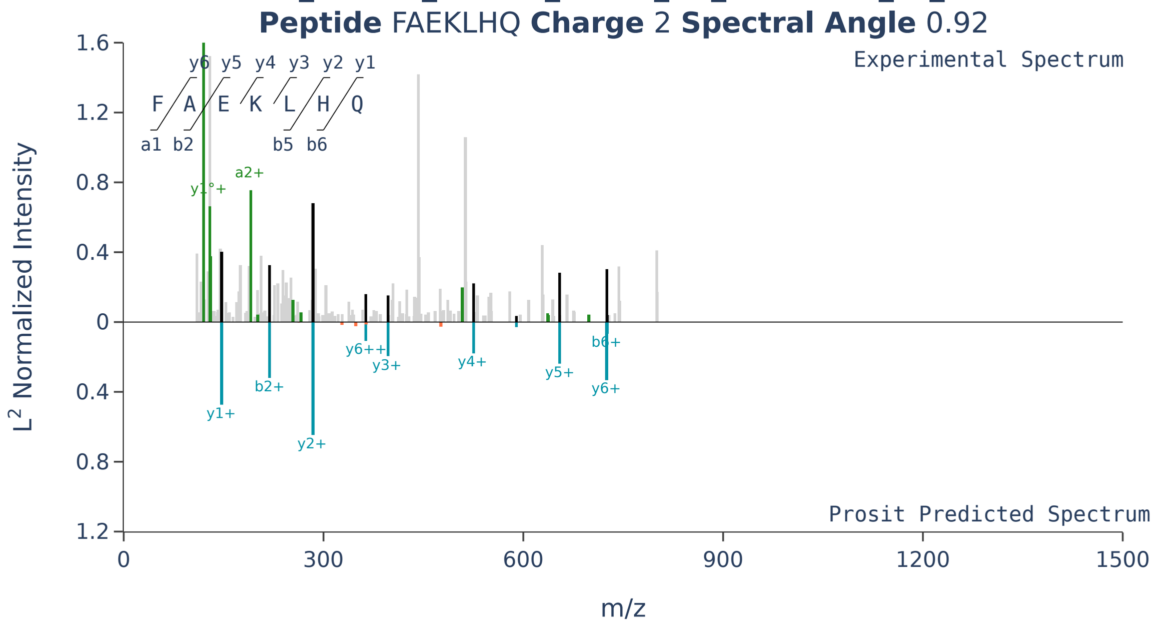


**Source** WSoh_301121_021221_HFGoe_M1_AnnexinA1_1h_R1 **Scan** 19551
**Peptide** KPAFFAEKL **Charge** 3 **Spectral Angle** 0.84

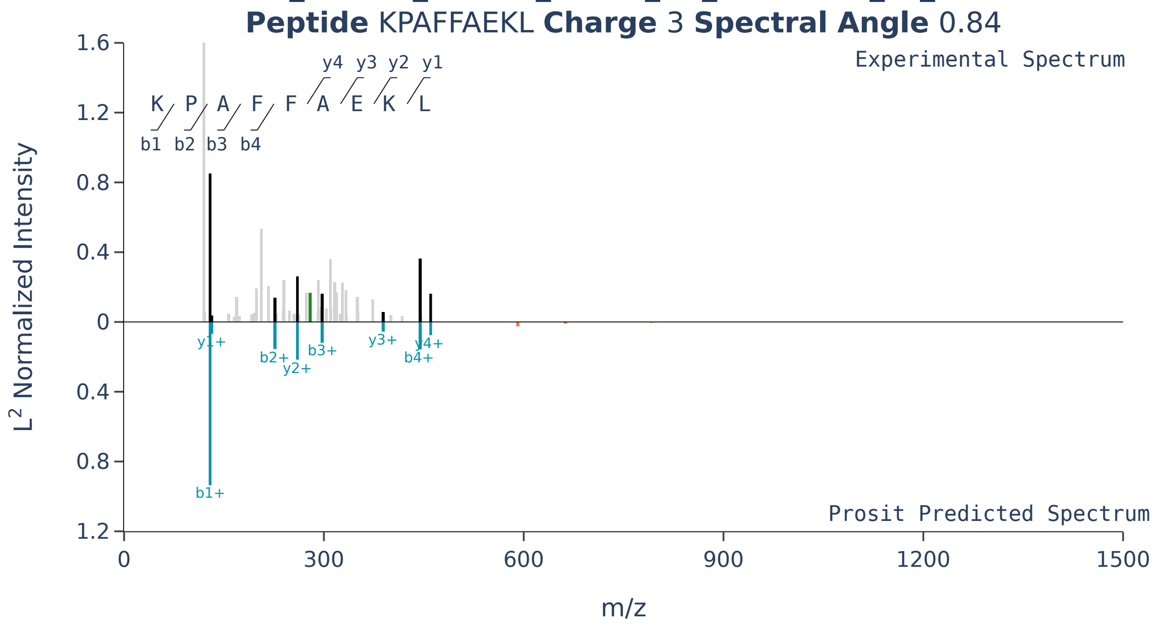


**Source** WSoh_301121_021221_HFGoe_M2_AnnexinA1_4h_R1 **Scan** 16835
**Peptide** QQIKAAYL **Charge** 2 **Spectral Angle** 0.87

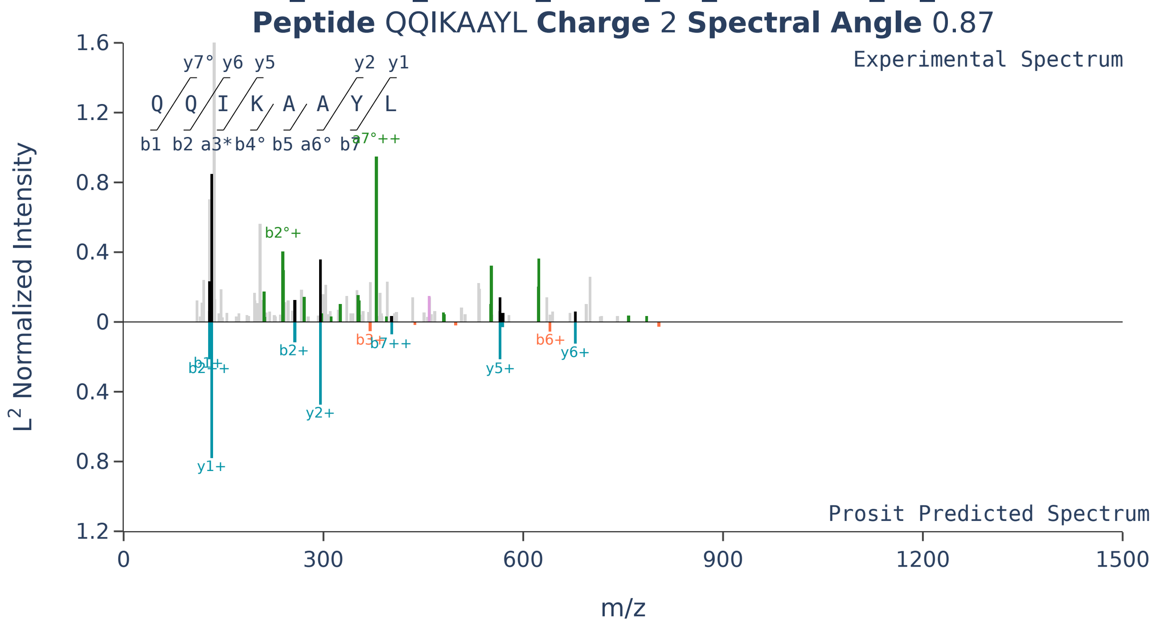


**Source** WSoh_301121_021221_HFGoe_M2_AnnexinA1_2h_R1 **Scan** 19016
**Peptide** MVSEFLKQ **Charge** 2 **Spectral Angle** 0.86

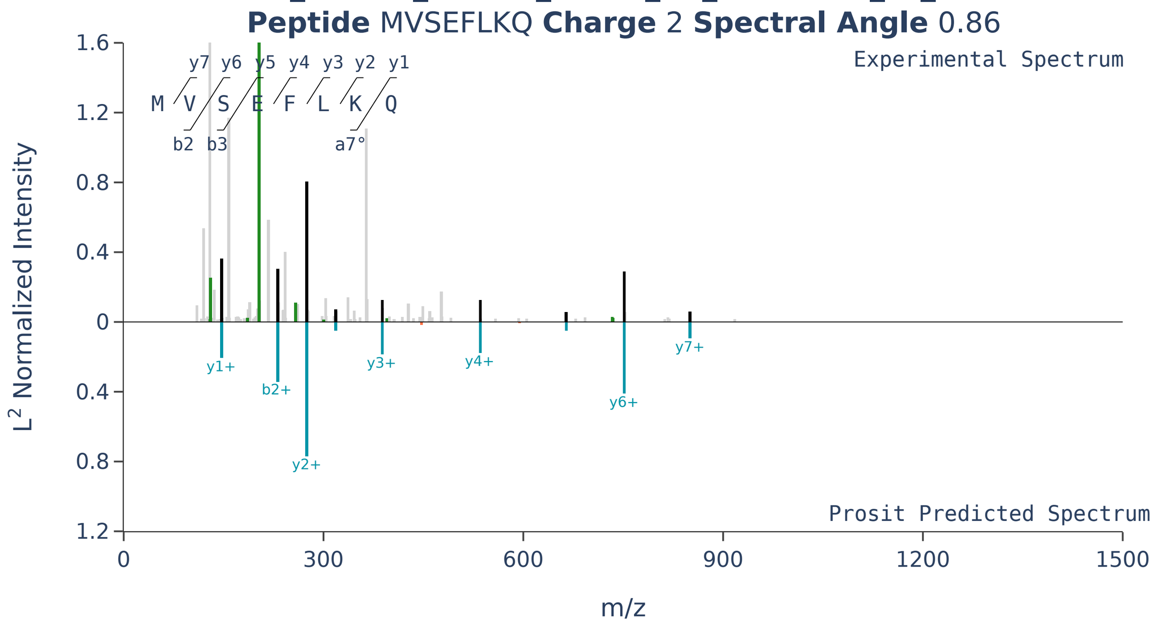

**Source** WSoh_301121_021221_HFGoe_M2_AnnexinA1_24h_R2 **Scan** 14840
**Peptide** AIMVKGVD **Charge** 1 **Spectral Angle** 0.82

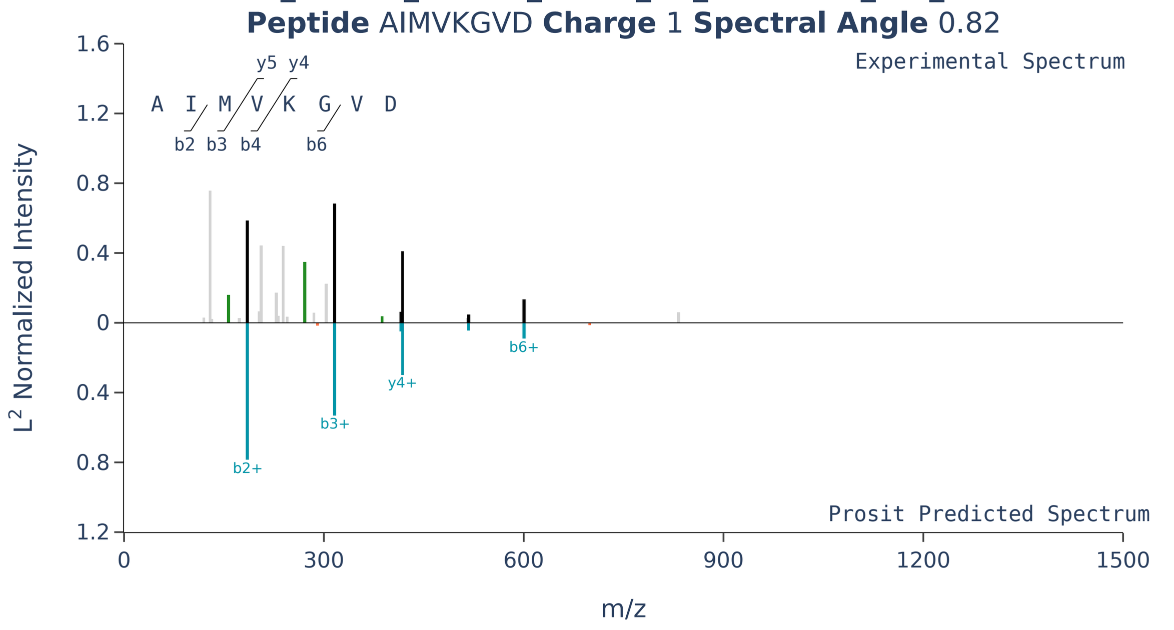


**Source** WSoh_301121_021221_HFGoe_M1_AnnexinA1_24h_R2 **Scan** 14547
**Peptide** IKAAYLQE **Charge** 2 **Spectral Angle** 0.92

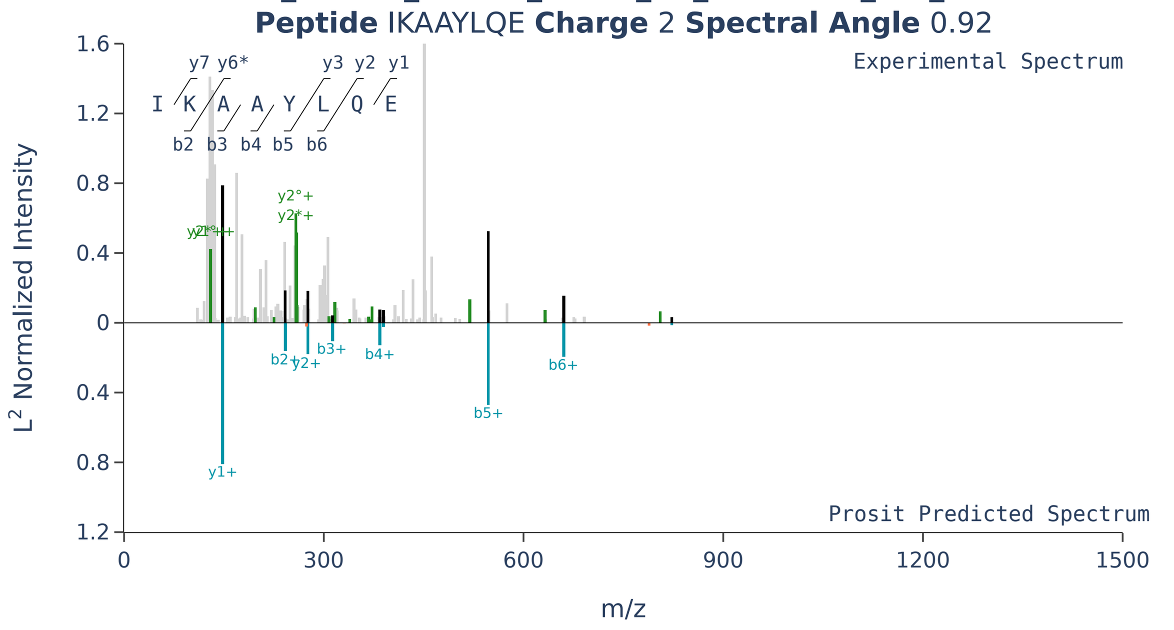


**Source** WSoh_301121_021221_HFGoe_M2_AnnexinA1_24h_R2 **Scan** 11197
**Peptide** AYLQETGKP **Charge** 2 **Spectral Angle** 0.85

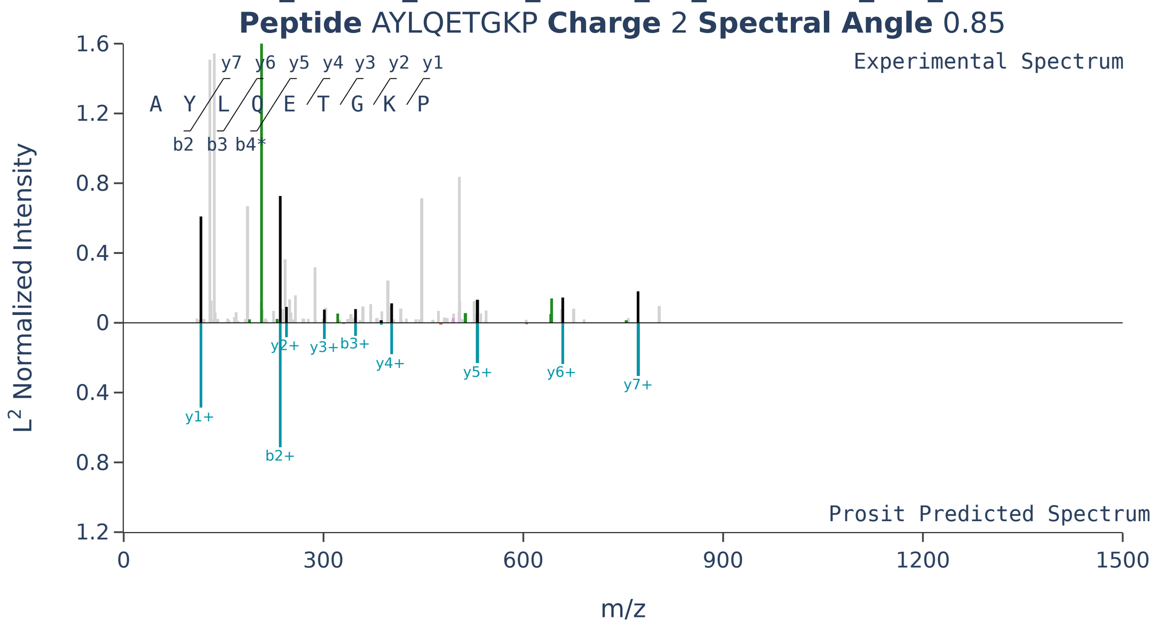


**Source** WSoh_301121_021221_HFGoe_M1_AnnexinA1_2h_R1 **Scan** 8560
**Peptide** KPLDETLKKA **Charge** 2 **Spectral Angle** 0.82

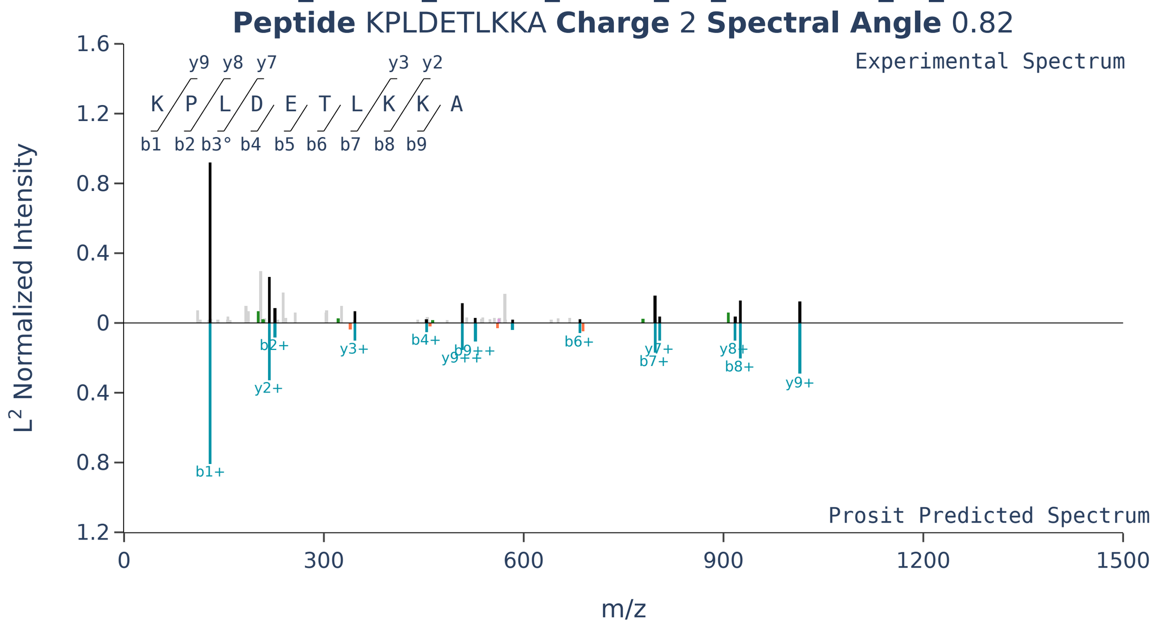


**Source** WSoh_301121_021221_HFGoe_M2_AnnexinA1_2h_R1 **Scan** 20844
**Peptide** TSKPAFFAEKL **Charge** 3 **Spectral Angle** 0.91

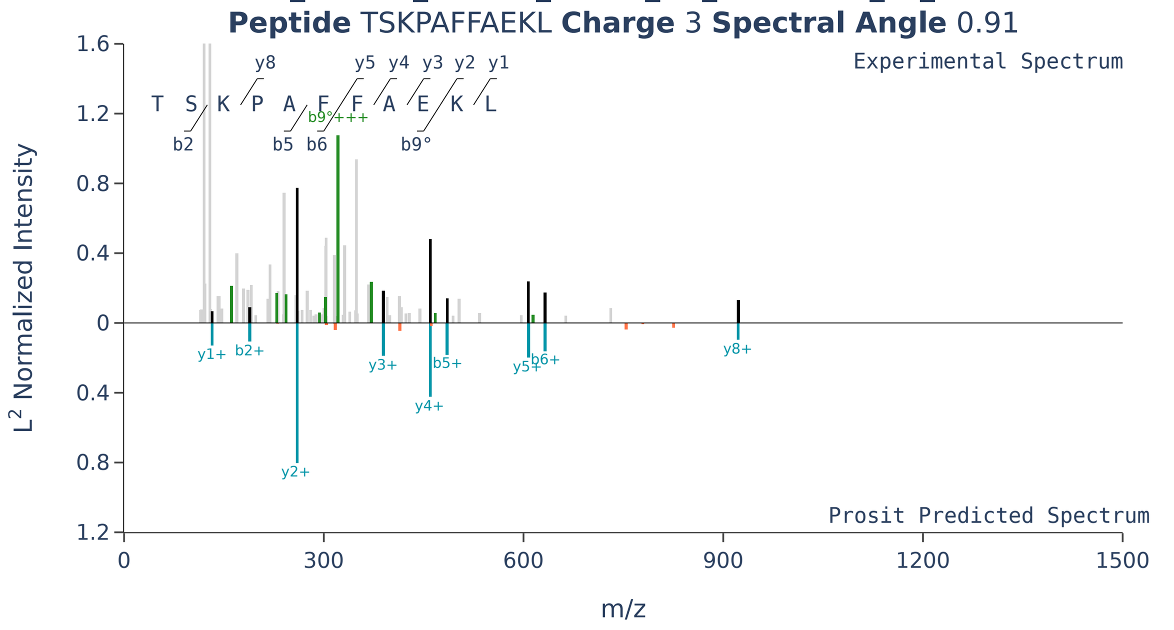


**Source** WSoh_301121_021221_HFGoe_M2_AnnexinA1_24h_R1 **Scan** 26336
**Peptide** QAWFIENE **Charge** 2 **Spectral Angle** 0.96

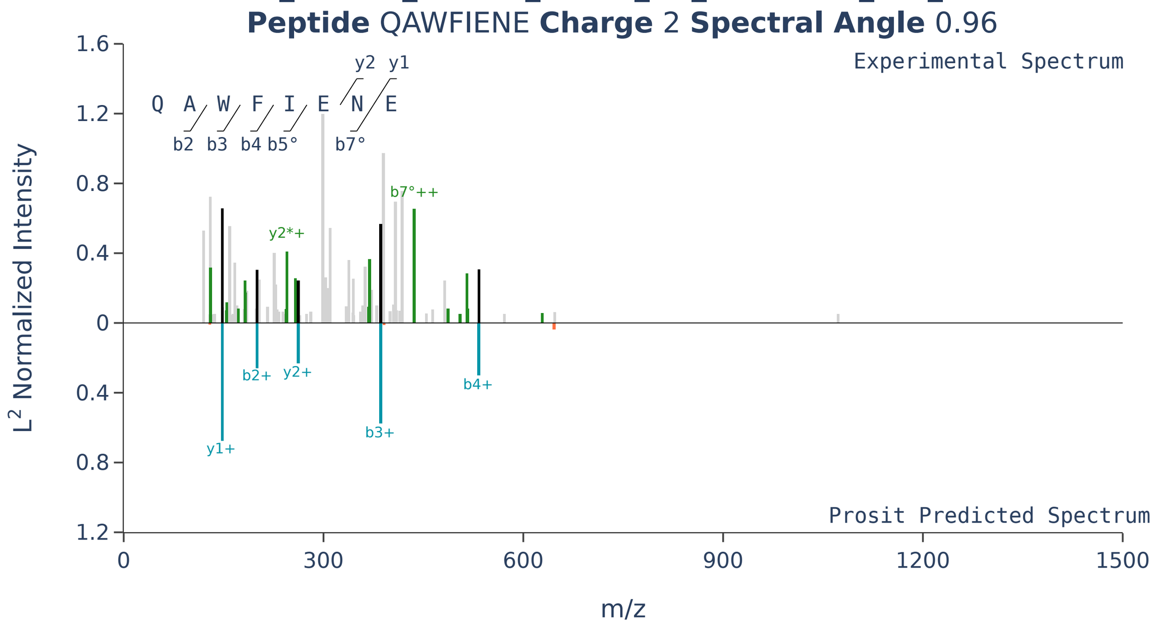


**Source** WSoh_301121_021221_HFGoe_M1_AnnexinA1_24h_R1 **Scan** 7827
**Peptide** KEIRDINRV **Charge** 2 **Spectral Angle** 0.91

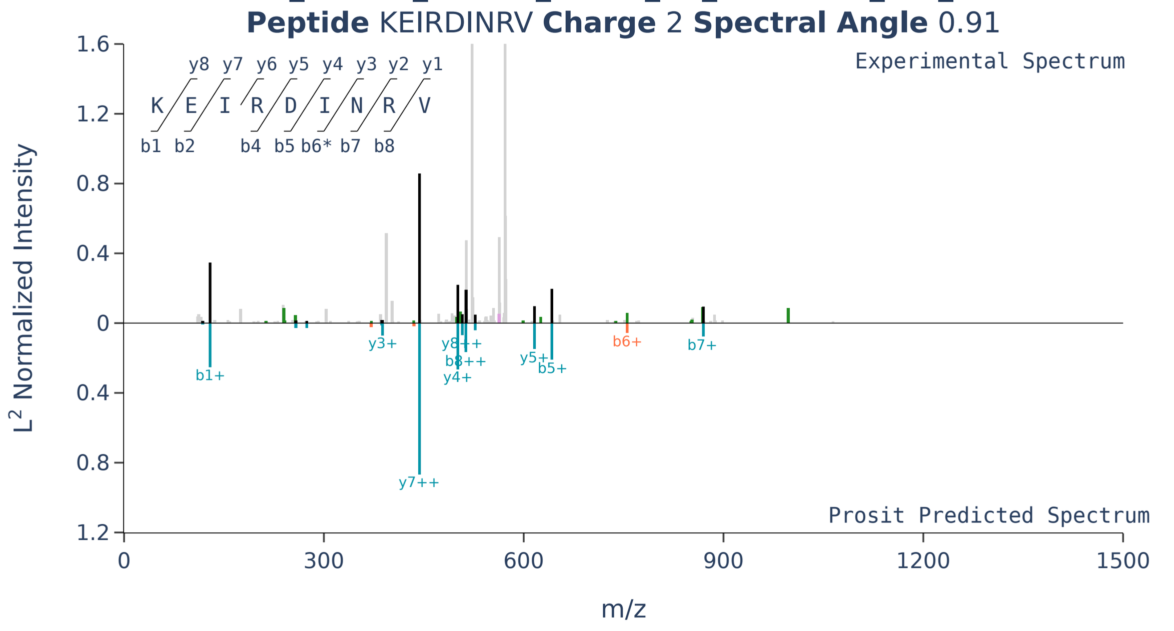


**Source** WSoh_301121_021221_HFGoe_M2_AnnexinA1_24h_R1 **Scan** 19308
**Peptide** TSKPAFFAE **Charge** 2 **Spectral Angle** 0.92

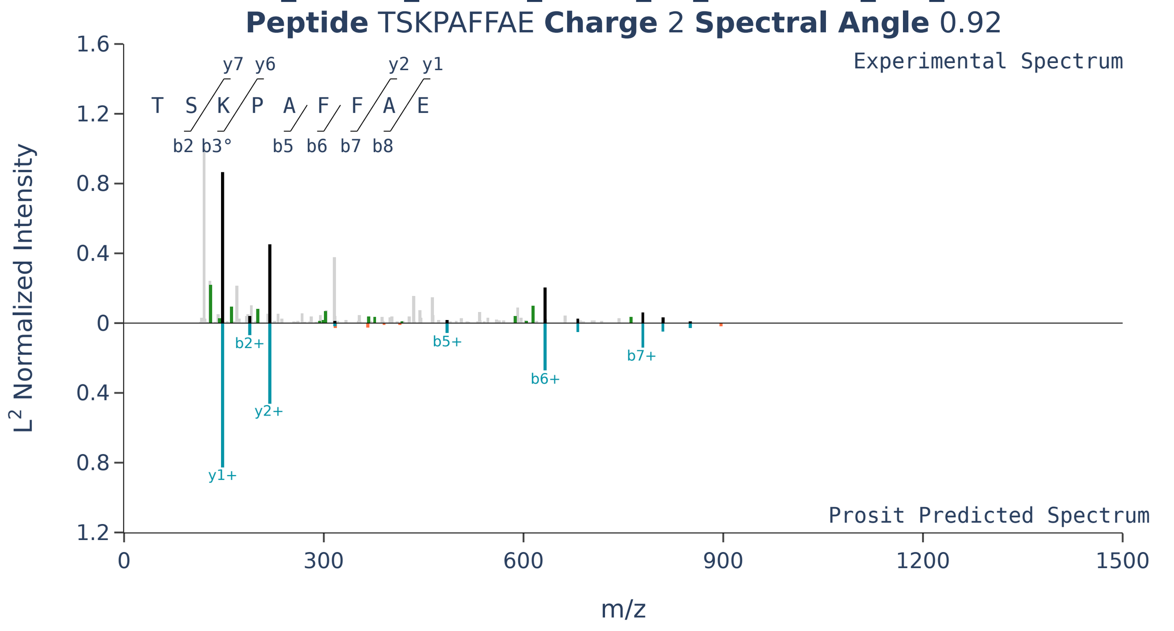


**Source** WSoh_301121_021221_HFGoe_M2_AnnexinA1_24h_R1 **Scan** 18176
**Peptide** KALIRIM **Charge** 2 **Spectral Angle** 0.91

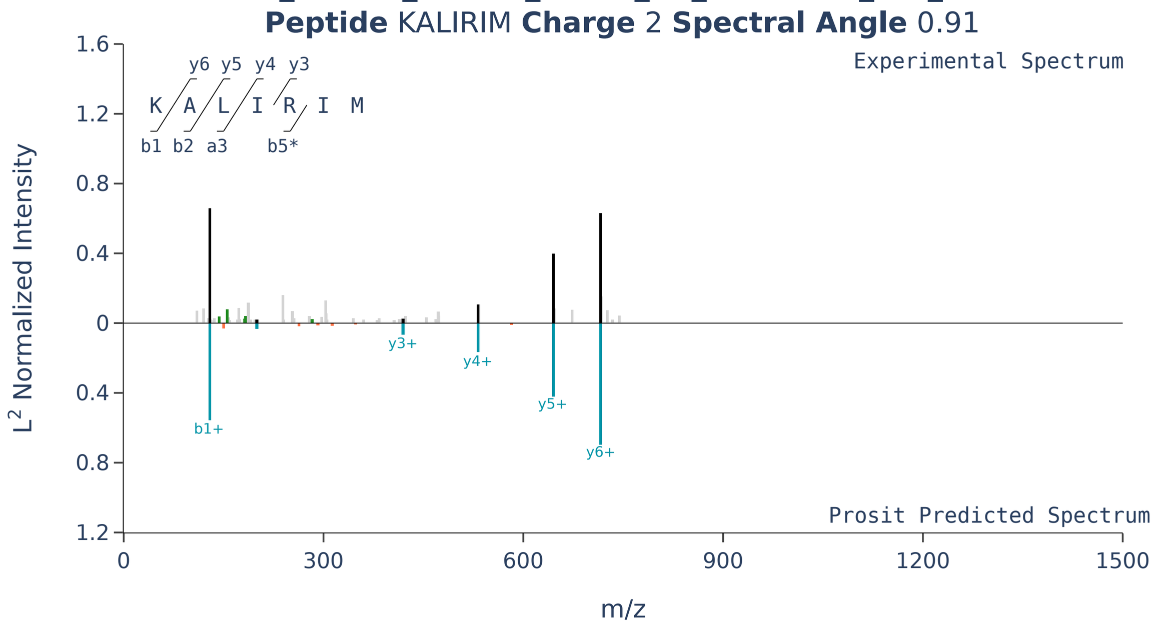


**Source** WSoh_301121_021221_HFGoe_M2_AnnexinA1_1h_R2 **Scan** 14077
**Peptide** KRDLAKDITSDTSGDFRNA **Charge** 3 **Spectral Angle** 0.85

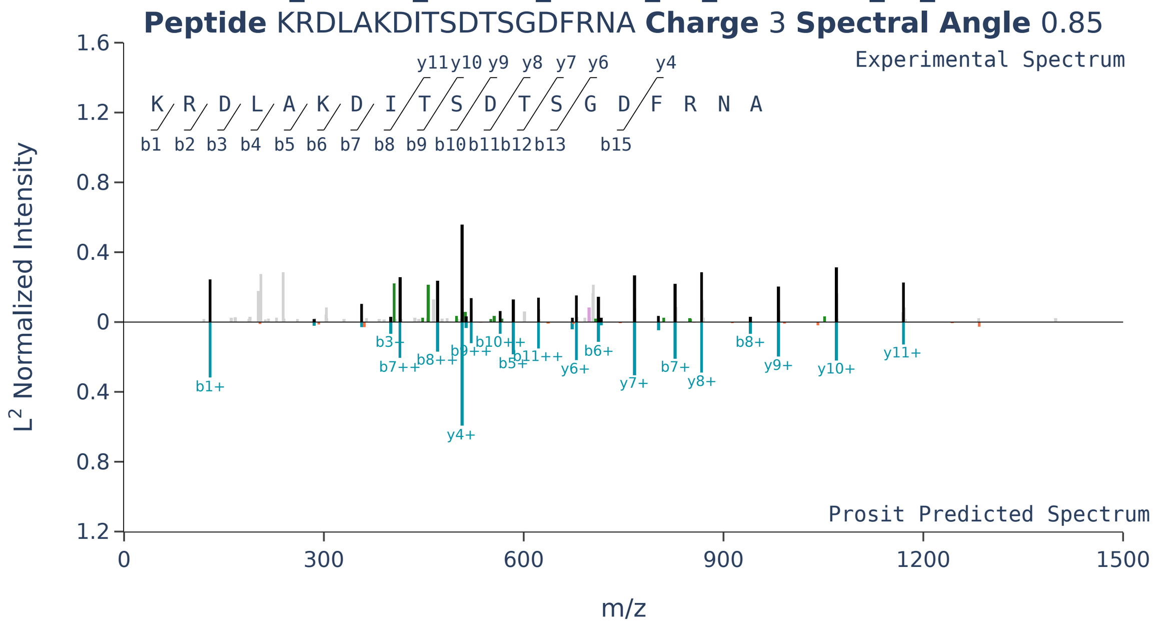


**Source** WSoh_301121_021221_HFGoe_M2_AnnexinA1_2h_R2 **Scan** 21152
**Peptide** MVSRSEIDMNDL **Charge** 2 **Spectral Angle** 0.91

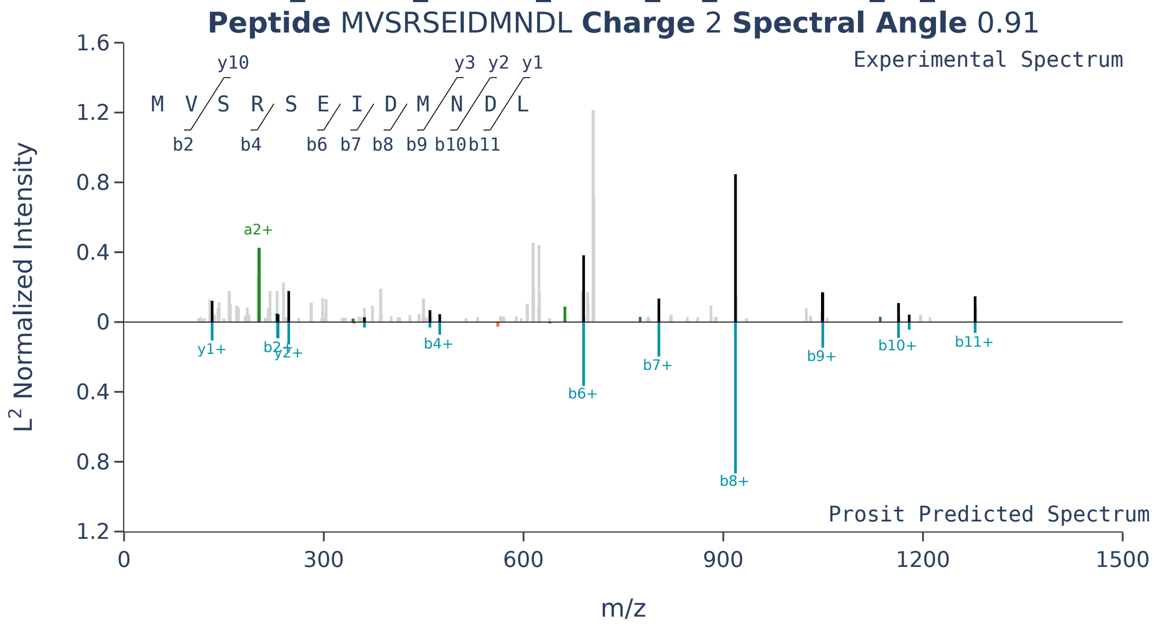


**Source** WSoh_301121_021221_HFGoe_M1_AnnexinA1_4h_R2 **Scan** 7600
**Peptide** KGDYEKI **Charge** 2 **Spectral Angle** 0.96

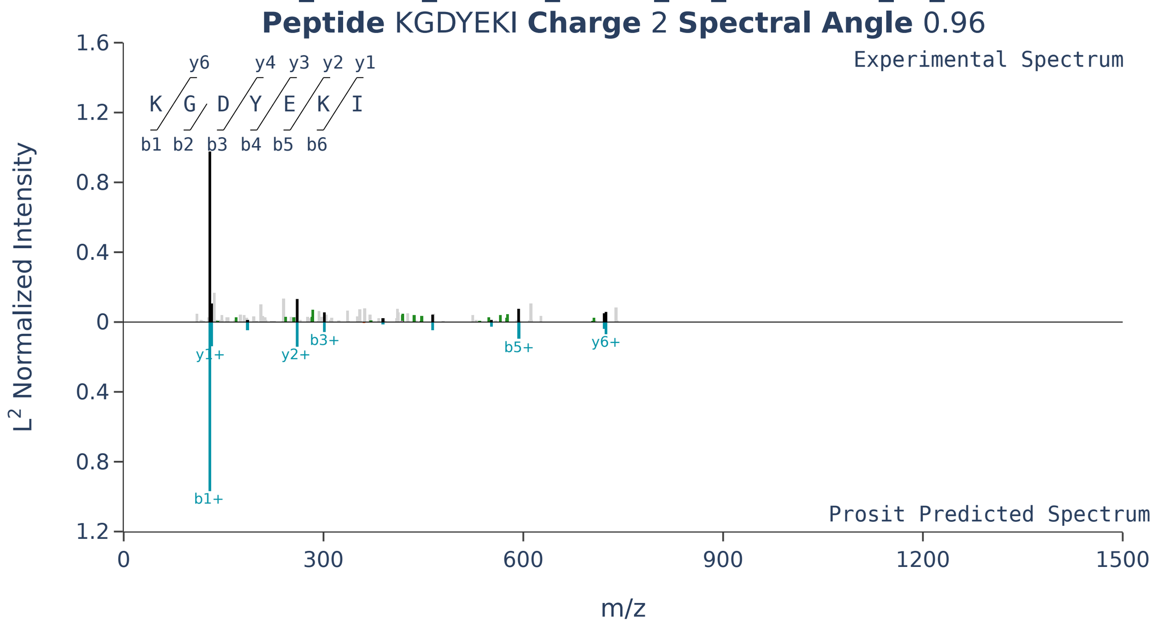


**Source** WSoh_301121_021221_HFGoe_M2_AnnexinA1_24h_R1 **Scan** 16257
**Peptide** QAILDETKGDYE **Charge** 2 **Spectral Angle** 0.86

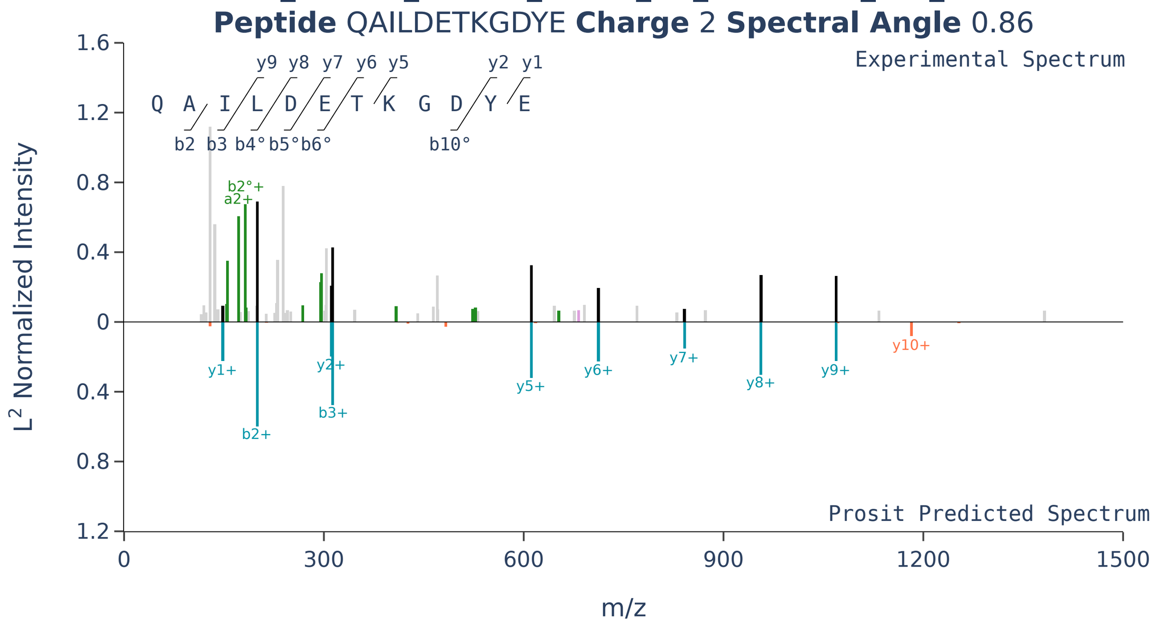


**Source** WSoh_301121_021221_HFGoe_M2_AnnexinA1_2h_R1 **Scan** 19572
**Peptide** ADELRAAMKGL **Charge** 2 **Spectral Angle** 0.87

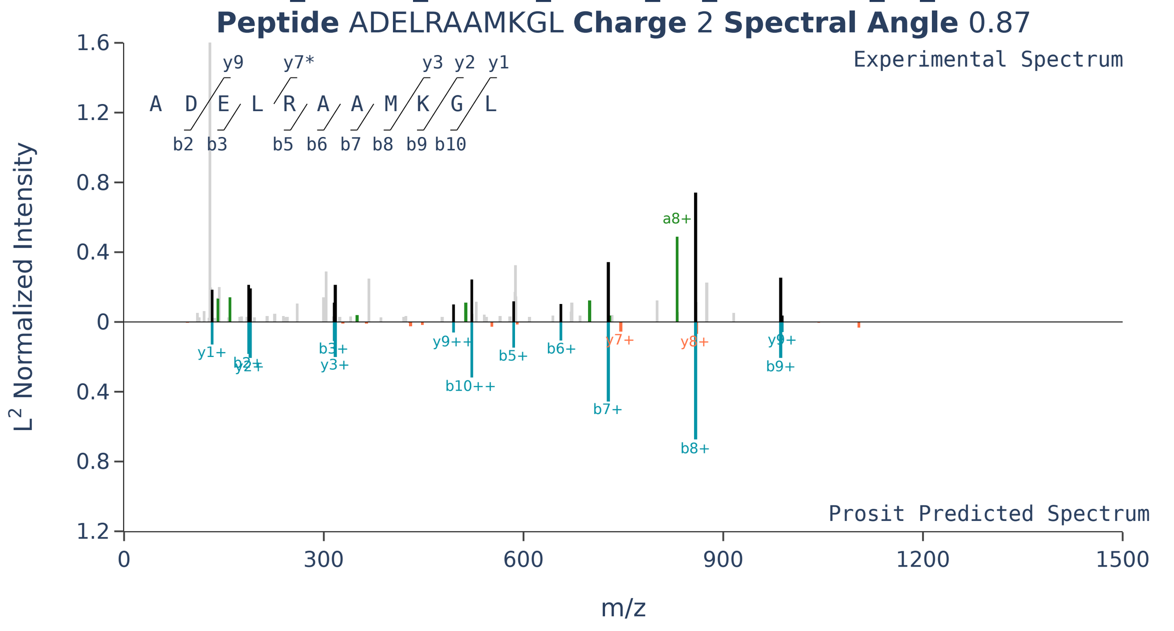


**Source** WSoh_301121_021221_HFGoe_M2_AnnexinA1_2h_R1 **Scan** 16674
**Peptide** AAYLQETGKPLD **Charge** 2 **Spectral Angle** 0.87

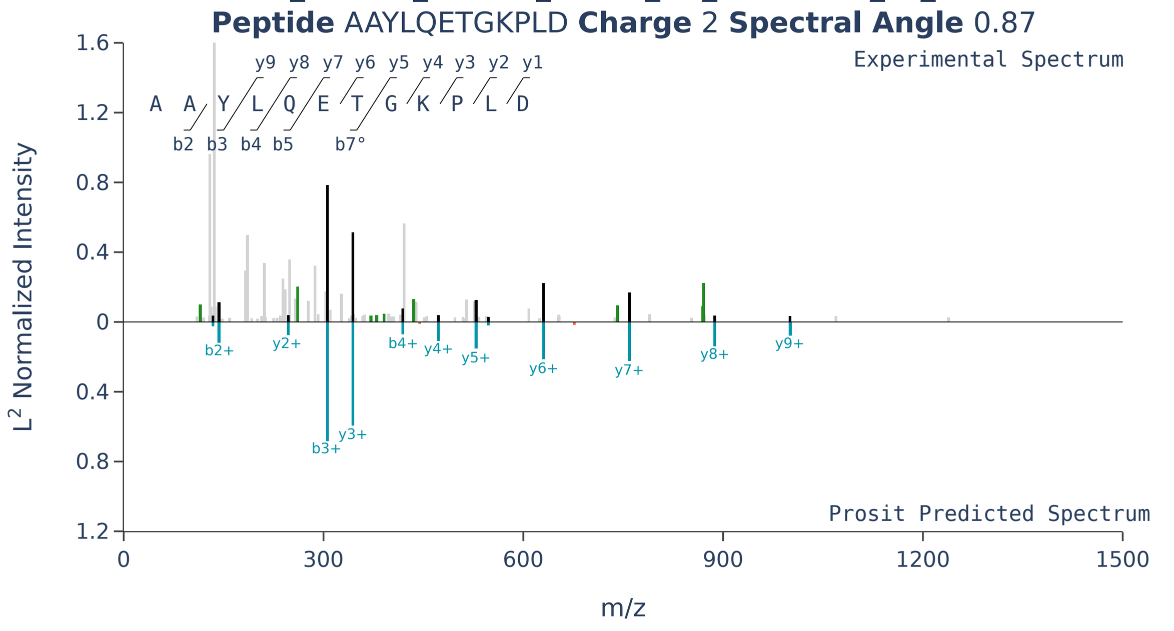

**Source** WSoh_301121_021221_HFGoe_M2_AnnexinA1_2h_R2 **Scan** 19729
**Peptide** RSEDFGVNEDL **Charge** 2 **Spectral Angle** 0.87

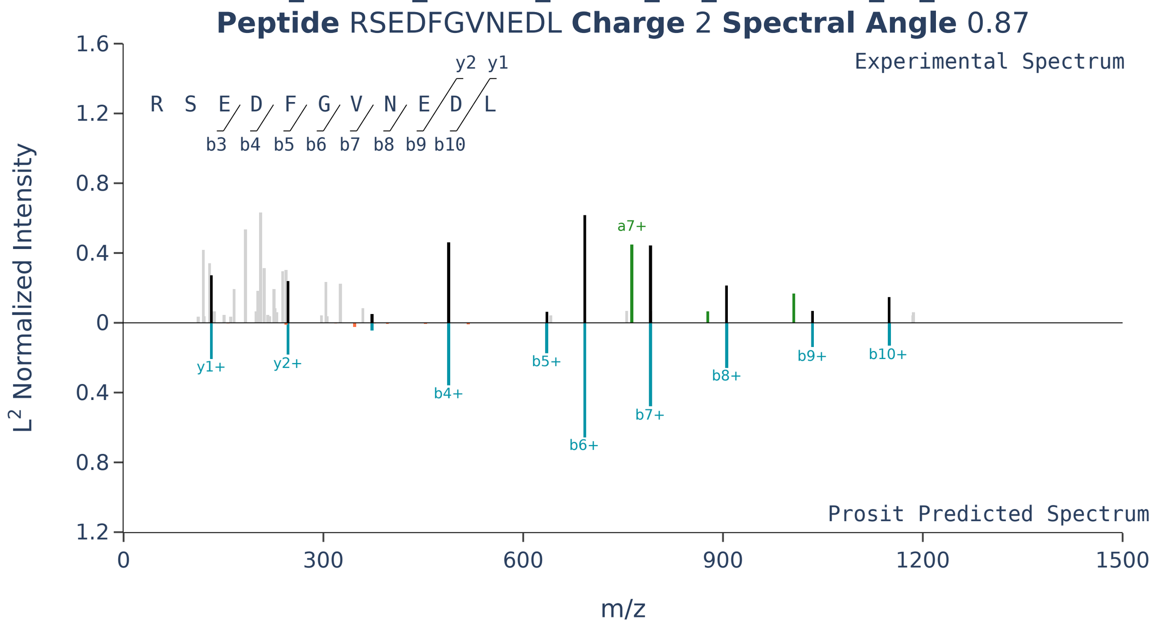


**Source** WSoh_301121_021221_HFGoe_M2_AnnexinA1_24h_R1 **Scan** 23375
**Peptide** MKGLGTDEDTLI **Charge** 2 **Spectral Angle** 0.88

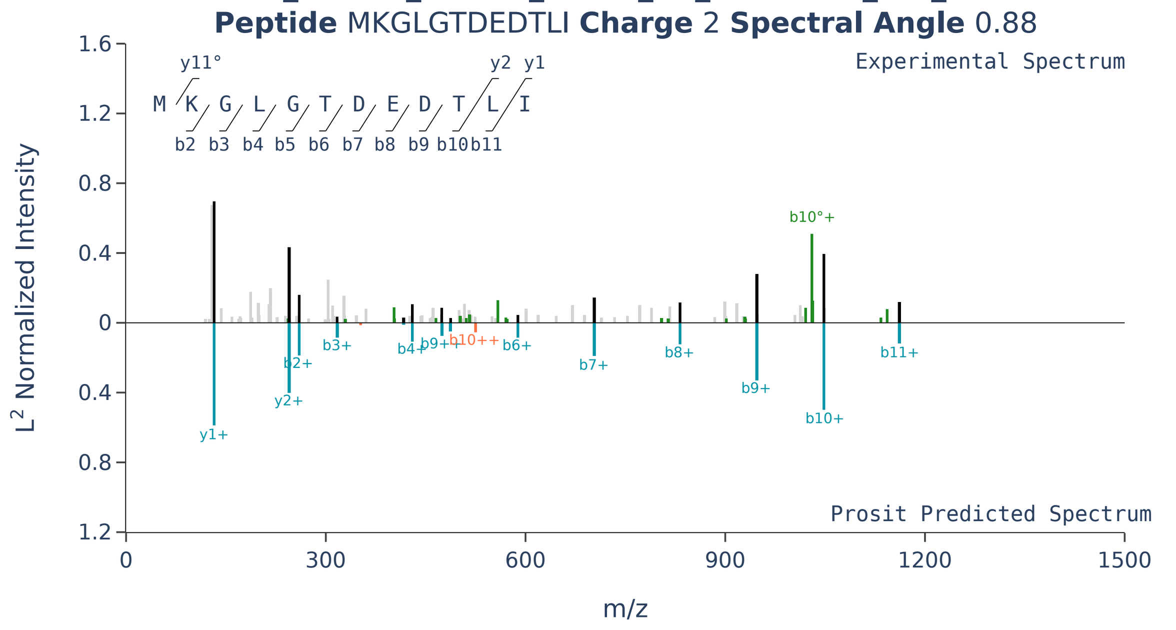


**Source** WSoh_301121_021221_HFGoe_M2_AnnexinA1_24h_R2 **Scan** 10060
**Peptide** MKGLGTDEDT **Charge** 2 **Spectral Angle** 0.87

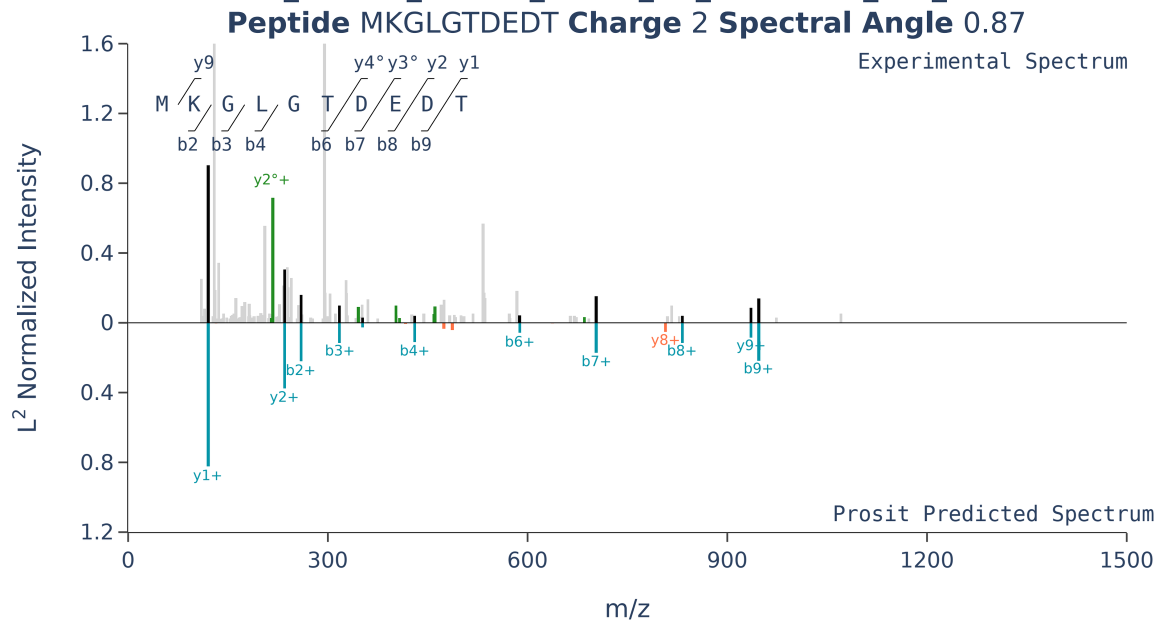


**Source** WSoh_301121_021221_HFGoe_M2_AnnexinA1_2h_R2 **Scan** 7182
**Peptide** ASRTNKEIRDI **Charge** 3 **Spectral Angle** 0.83

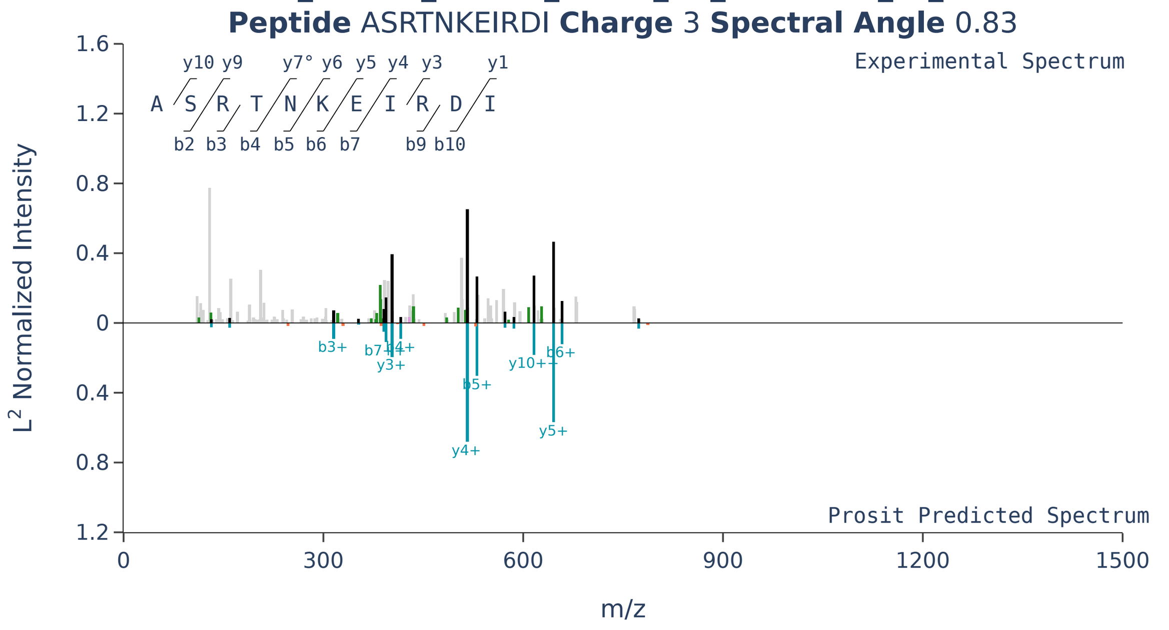


**Source** WSoh_301121_021221_HFGoe_M1_AnnexinA1_24h_R1 **Scan** 18331
**Peptide** NDIKAFY **Charge** 2 **Spectral Angle** 0.9

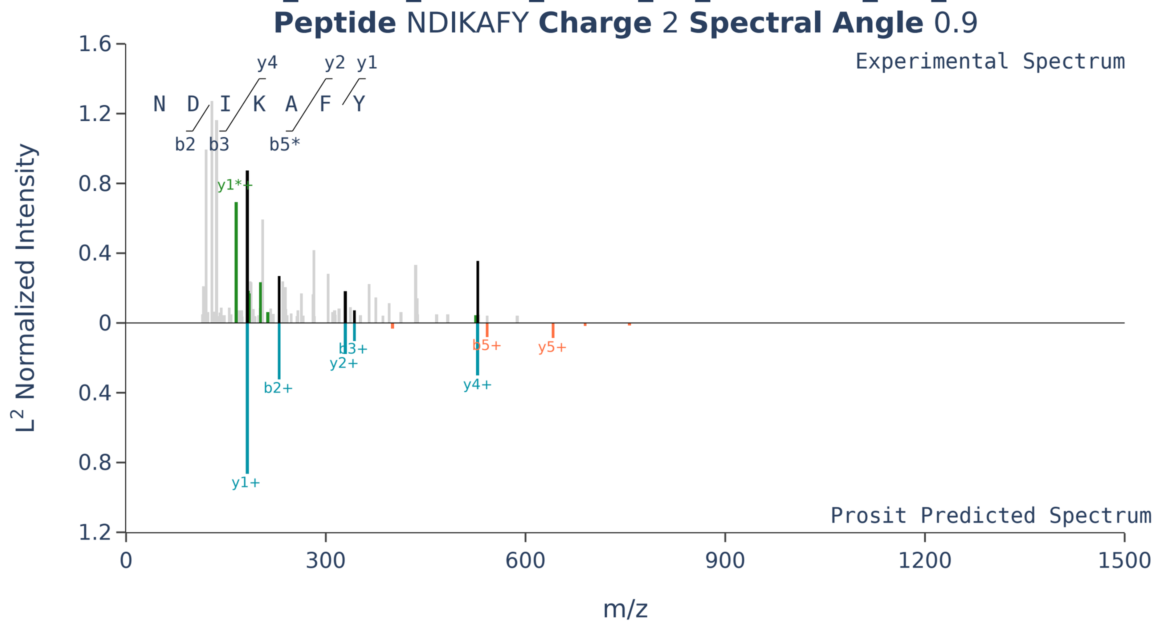


**Source** WSoh_301121_021221_HFGoe_M2_AnnexinA1_24h_R1 **Scan** 10536
**Peptide** EQEYVQTVK **Charge** 1 **Spectral Angle** 0.84

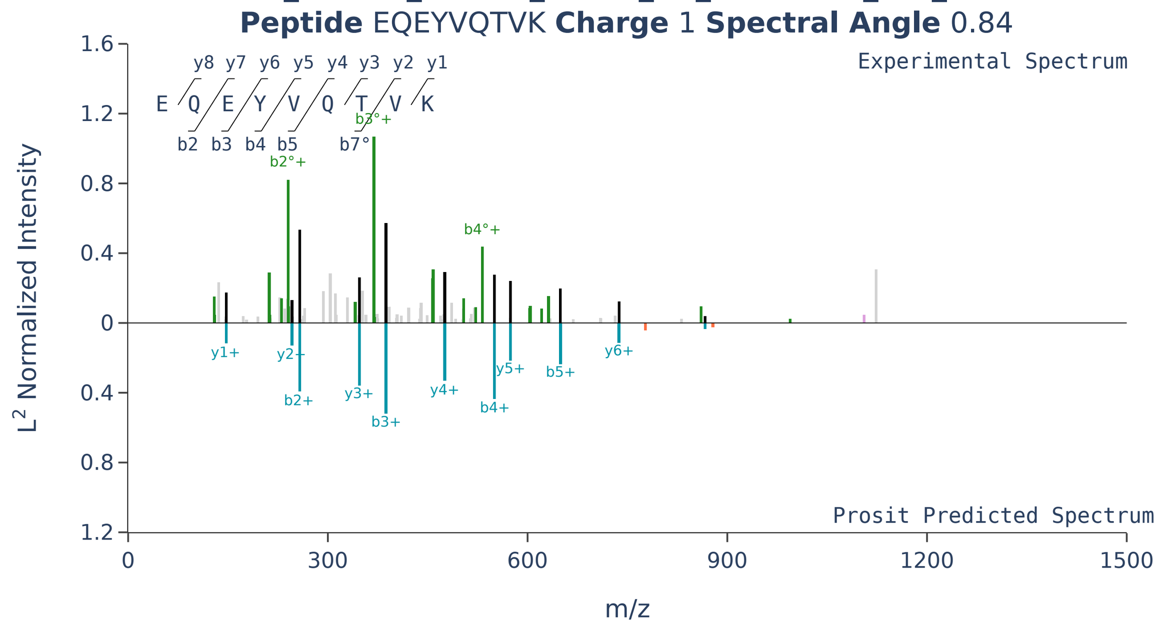


**Source** WSoh_301121_021221_HFGoe_M2_AnnexinA1_2h_R1 **Scan** 16644
**Peptide** RDLAKDITSDTSGDFRNA **Charge** 3 **Spectral Angle** 0.86

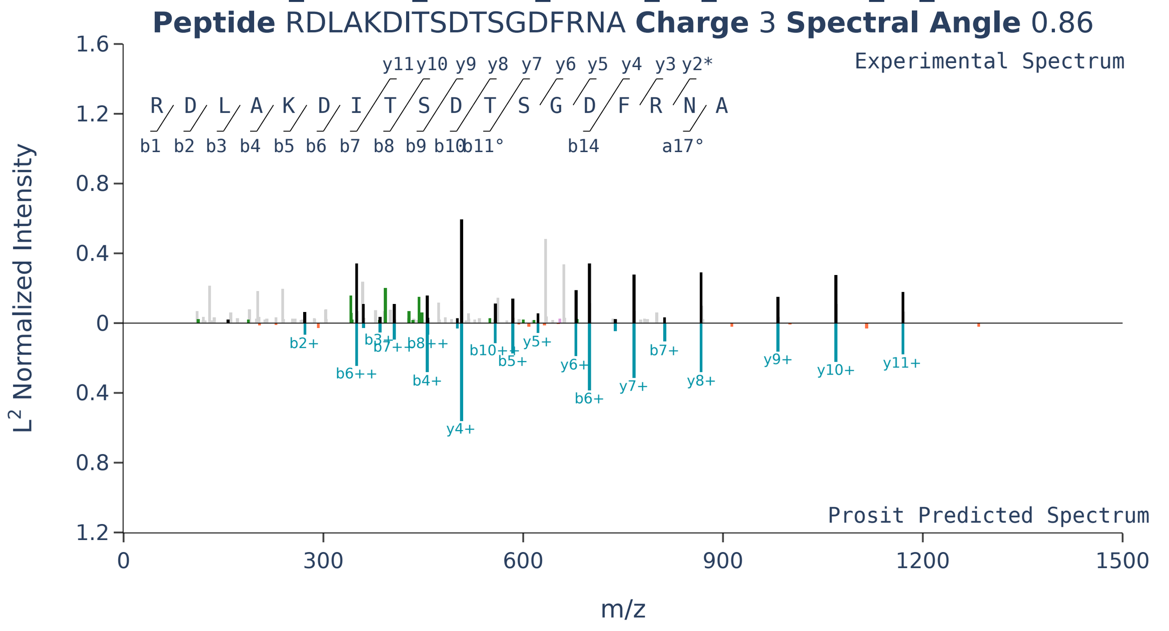


**Source** WSoh_301121_021221_HFGoe_M2_AnnexinA1_24h_R1 **Scan** 24616
**Peptide** TIIDILTKRN **Charge** 3 **Spectral Angle** 0.91

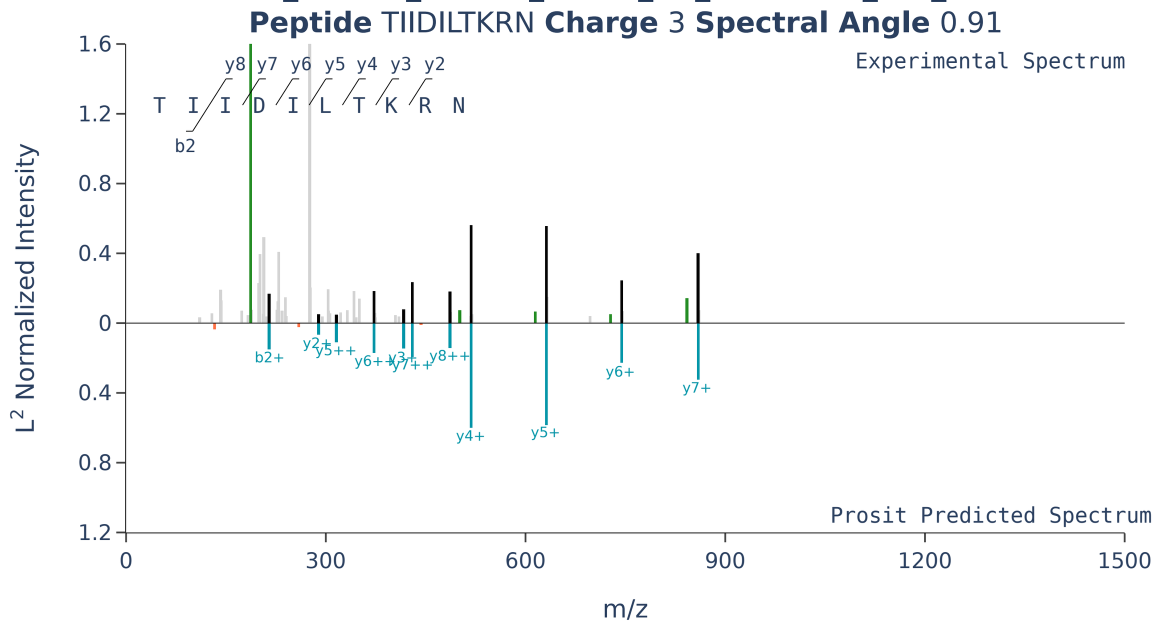


**Source** WSoh_301121_021221_HFGoe_M1_AnnexinA1_4h_R2 **Scan** 17299
**Peptide** PAFFAEK **Charge** 2 **Spectral Angle** 0.87

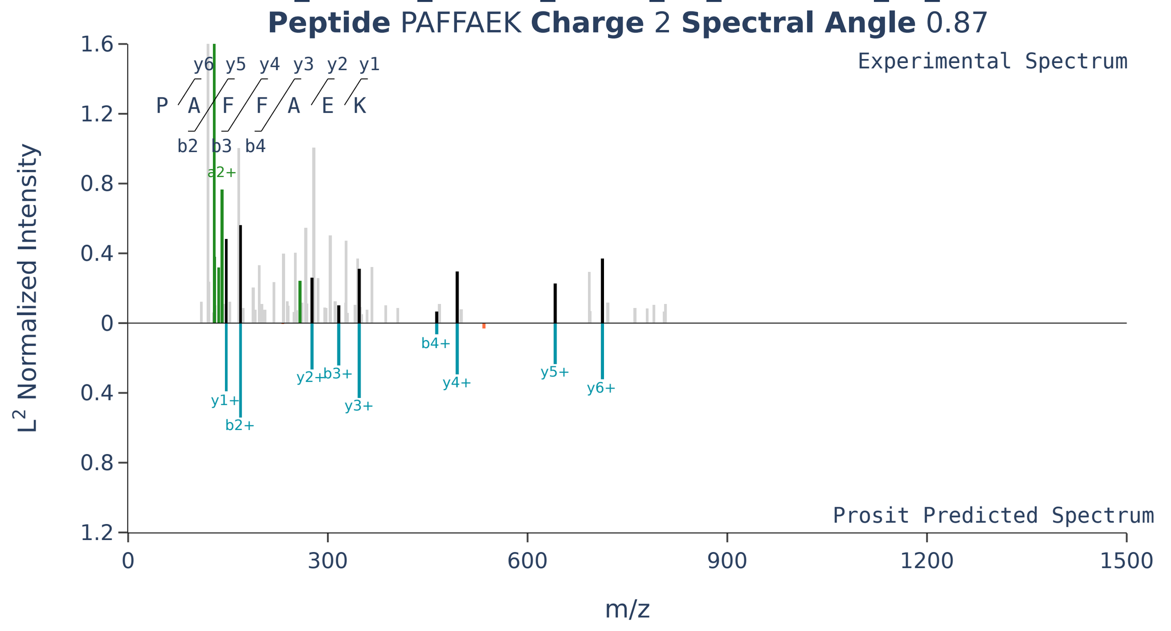


**Source** WSoh_301121_021221_HFGoe_M2_AnnexinA1_24h_R2 **Scan** 9903
**Peptide** AMKGLGTDED **Charge** 2 **Spectral Angle** 0.84

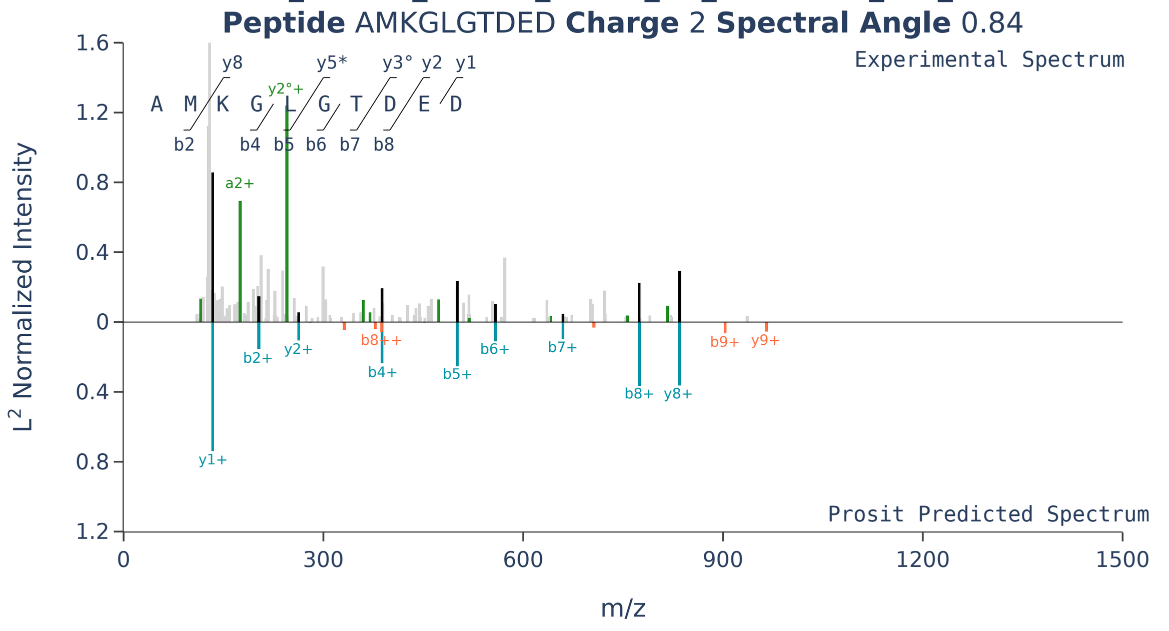


**Source** WSoh_301121_021221_HFGoe_M1_AnnexinA1_2h_R1 **Scan** 15935
**Peptide** TSKPAFFAEKLH **Charge** 3 **Spectral Angle** 0.9

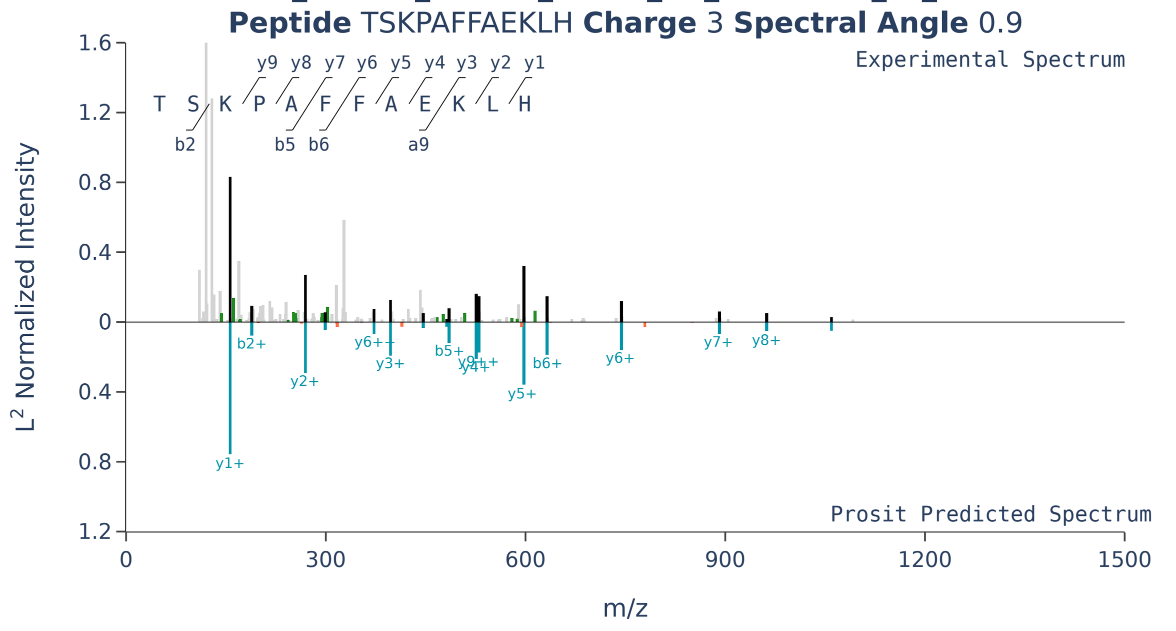


**Source** WSoh_301121_021221_HFGoe_M1_AnnexinA1_2h_R1 **Scan** 17537
**Peptide** MKGLGTDEDTL **Charge** 2 **Spectral Angle** 0.86

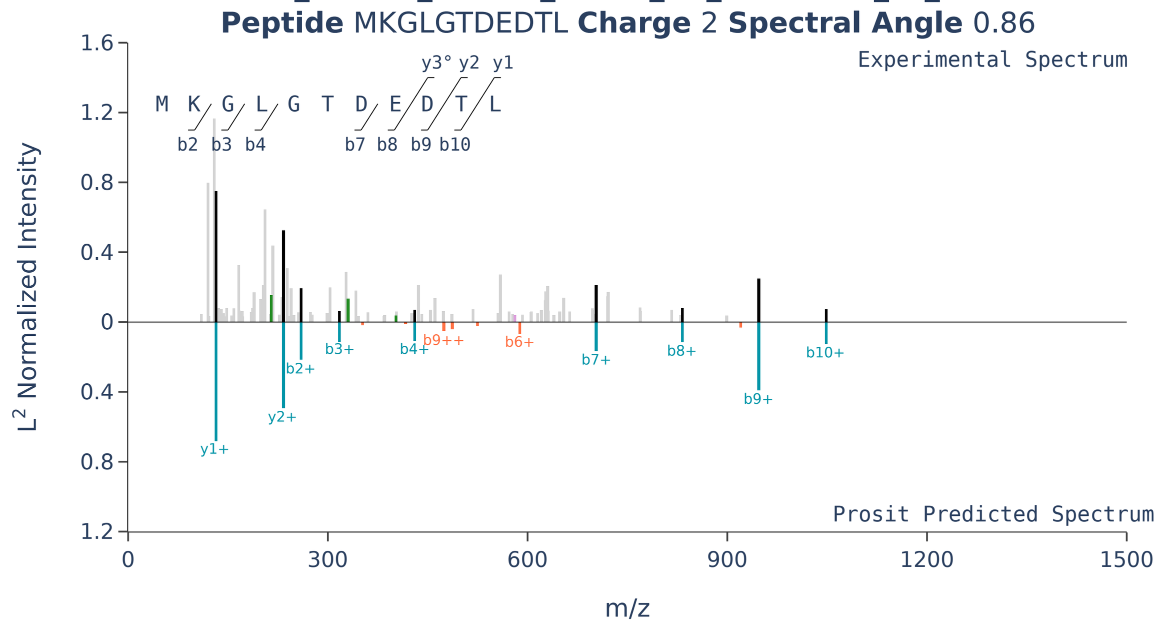


**Source** WSoh_301121_021221_HFGoe_M2_AnnexinA1_24h_R2 **Scan** 13083
**Peptide** NDIKAFYQK **Charge** 2 **Spectral Angle** 0.83

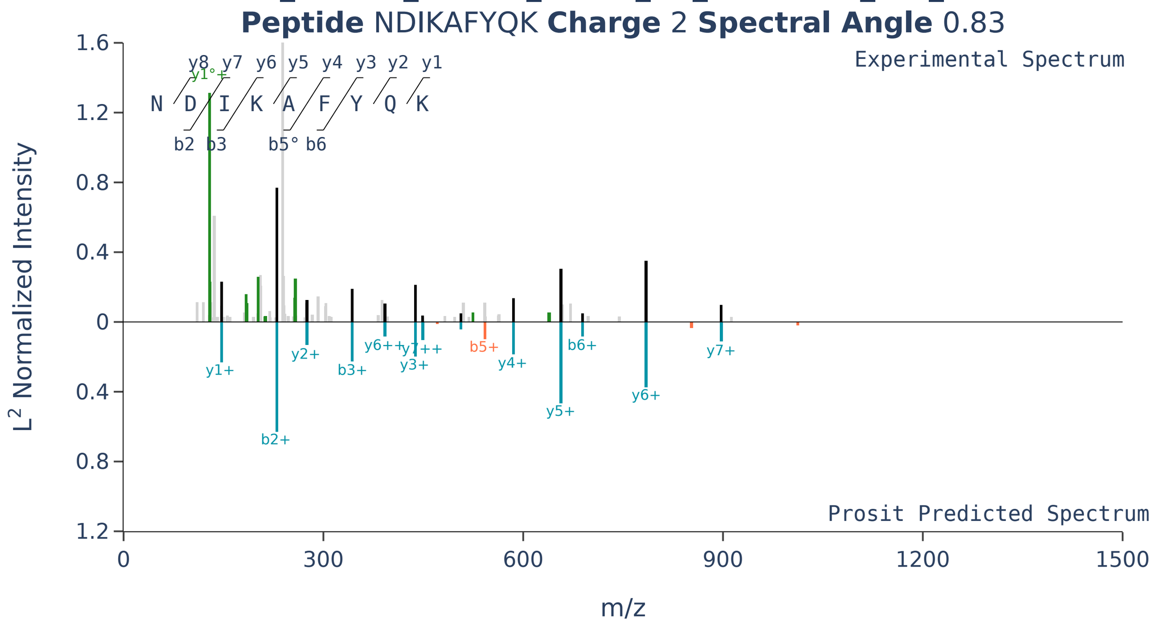


**Source** WSoh_301121_021221_HFGoe_M1_AnnexinA1_24h_R1 **Scan** 10018
**Peptide** TFNPSSD **Charge** 1 **Spectral Angle** 0.88

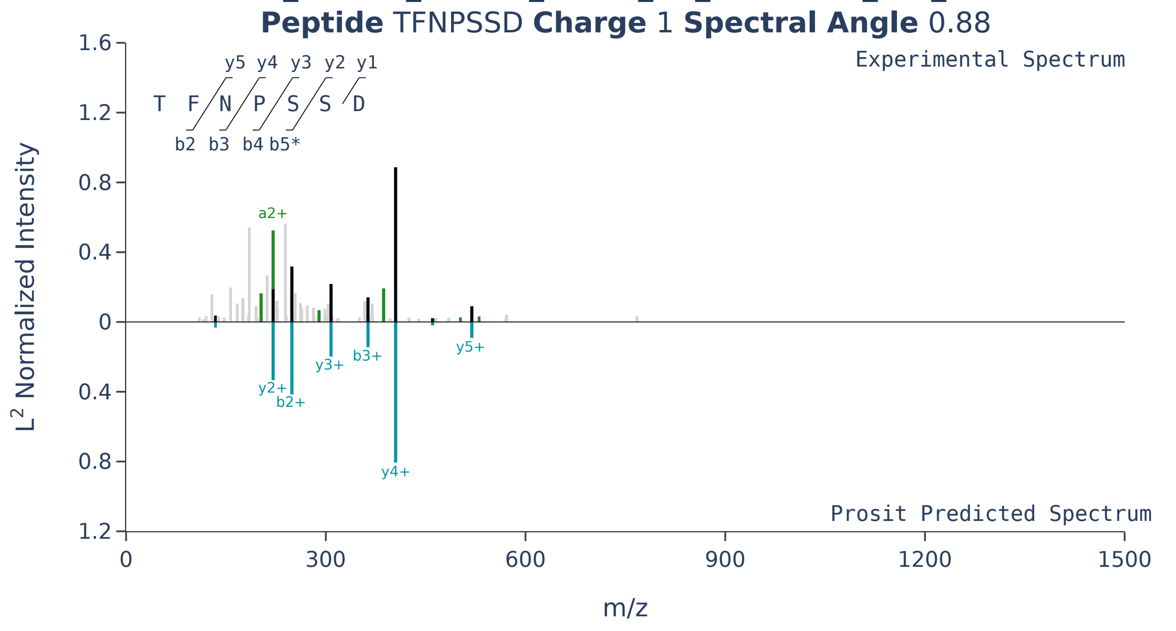


**Source** WSoh_301121_021221_HFGoe_M2_AnnexinA1_24h_R1 **Scan** 11258
**Peptide** NKEIRDINRVY **Charge** 2 **Spectral Angle** 0.82

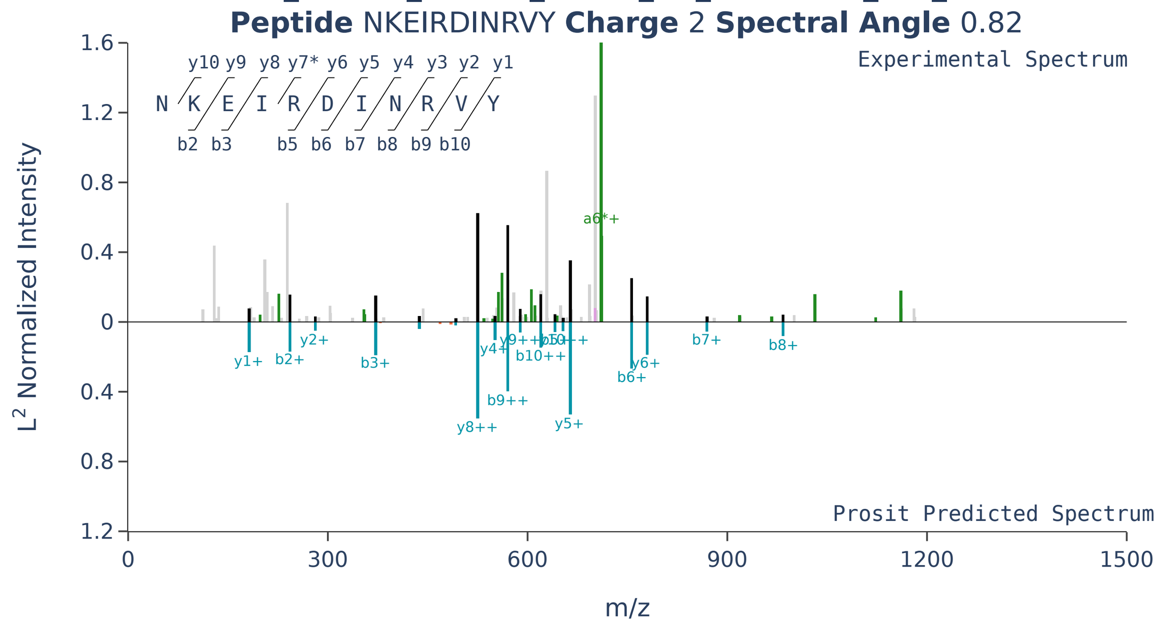

**Source** WSoh_301121_021221_HFGoe_M1_AnnexinA1_24h_R2 **Scan** 13223
**Peptide** DIEKCLT **Charge** 2 **Spectral Angle** 0.93

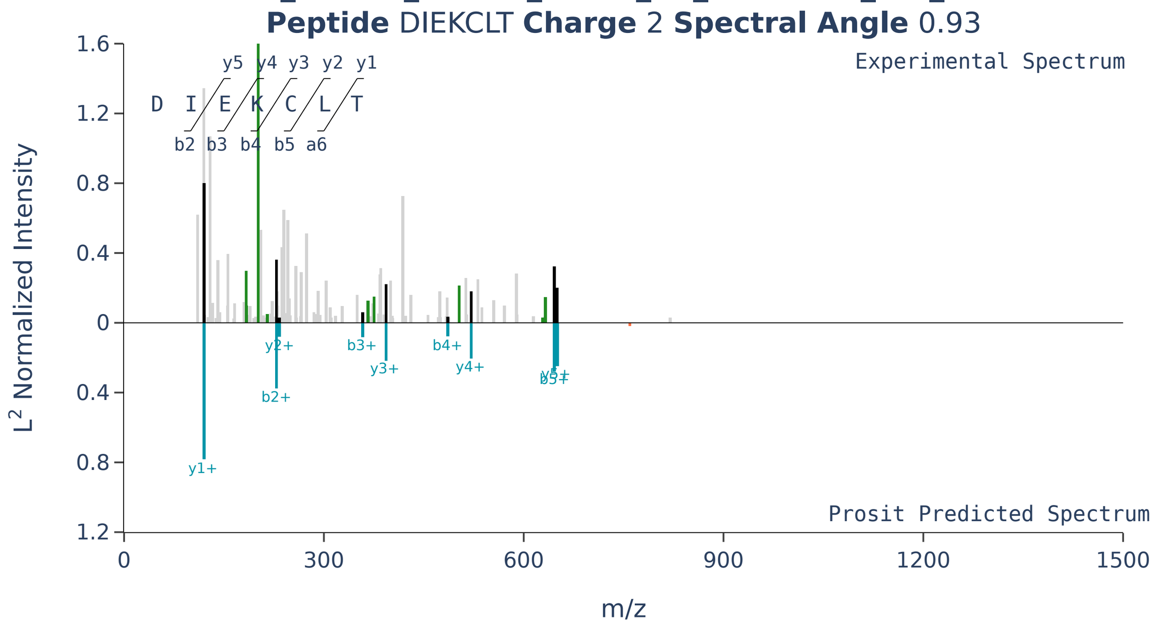


**Source** WSoh_301121_021221_HFGoe_M2_AnnexinA1_2h_R1 **Scan** 21179
**Peptide** MVSRSEIDMNDI **Charge** 2 **Spectral Angle** 0.91

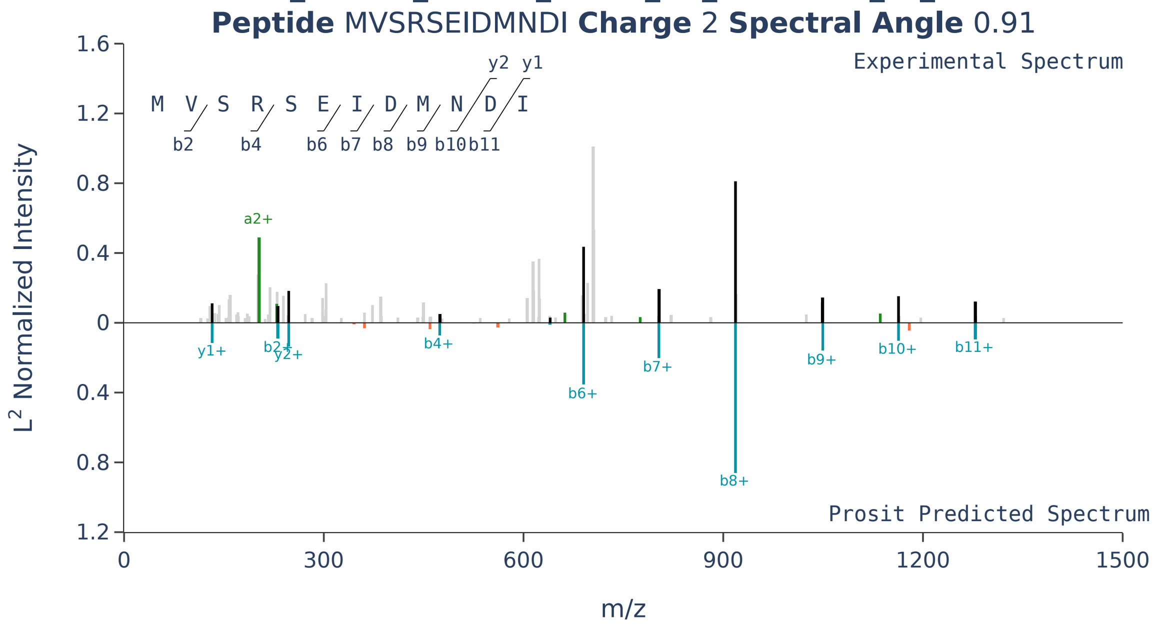


**Source** WSoh_301121_021221_HFGoe_M2_AnnexinA1_4h_R1 **Scan** 19571
**Peptide** DLAKDITSDTSGDFRN **Charge** 2 **Spectral Angle** 0.89

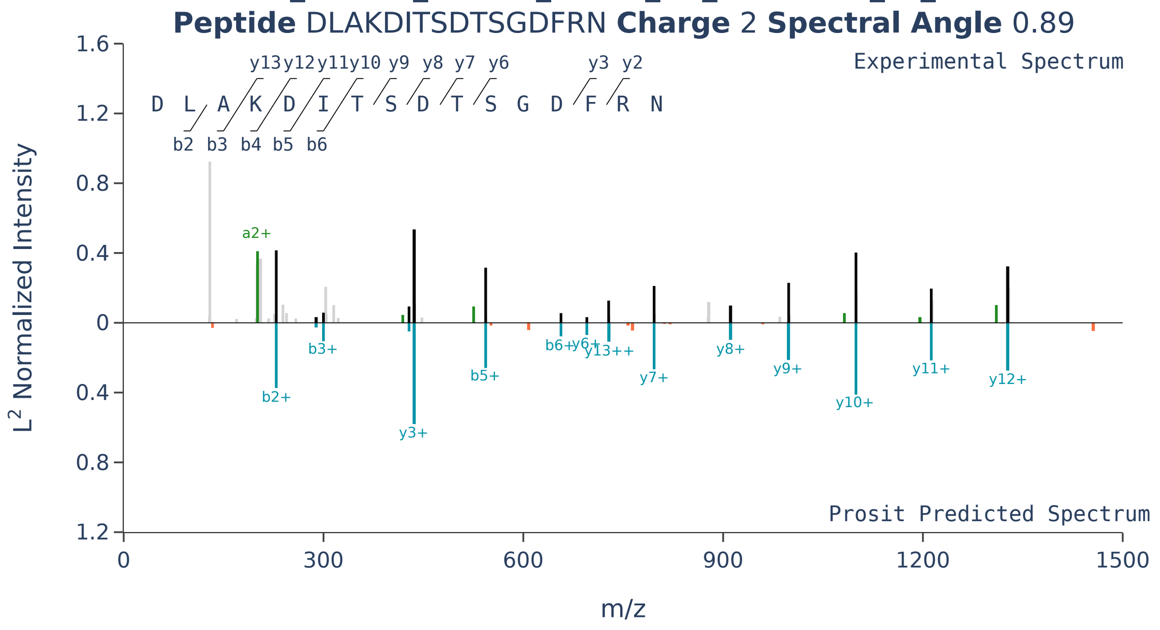


**Source** WSoh_301121_021221_HFGoe_M2_AnnexinA1_24h_R1 **Scan** 17856
**Peptide** FDADELRA **Charge** 2 **Spectral Angle** 0.93

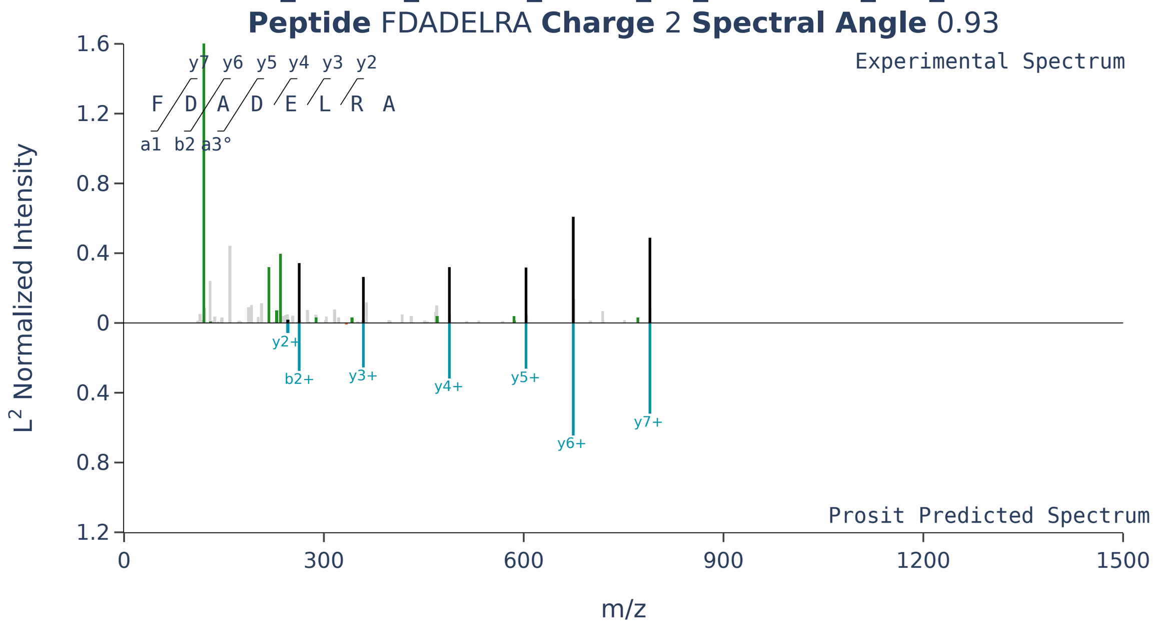


**Source** WSoh_301121_021221_HFGoe_M1_AnnexinA1_24h_R1 **Scan** 7956
**Peptide** DILTKRNNAQ **Charge** 2 **Spectral Angle** 0.88

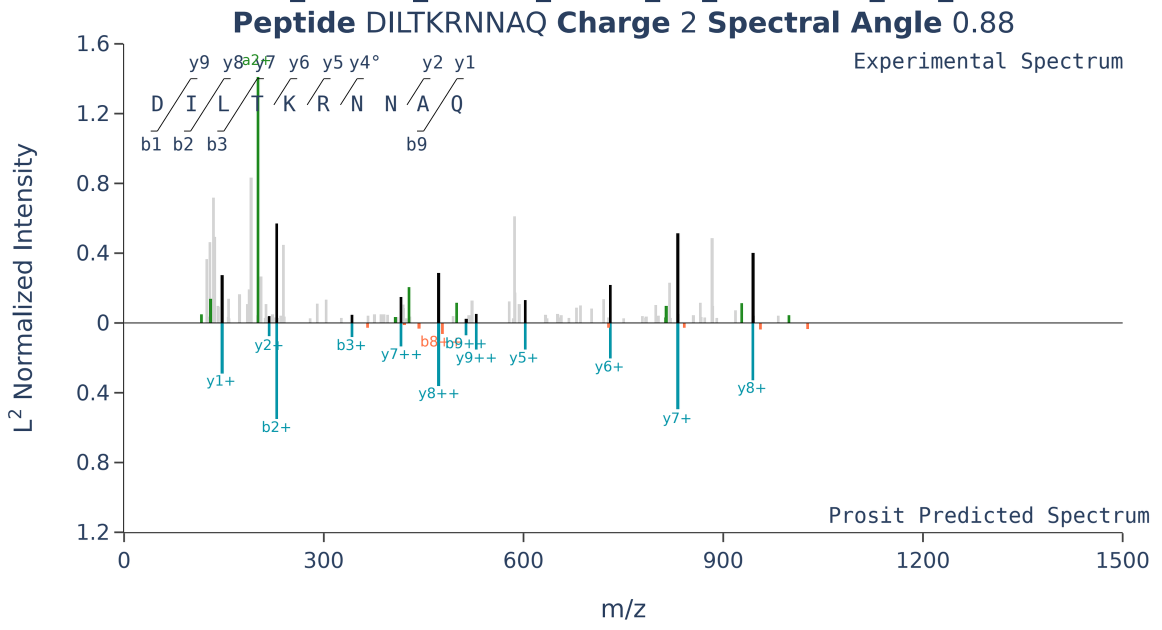


**Source** WSoh_301121_021221_HFGoe_M2_AnnexinA1_24h_R2 **Scan** 19618
**Peptide** TSKPAFF **Charge** 2 **Spectral Angle** 0.88

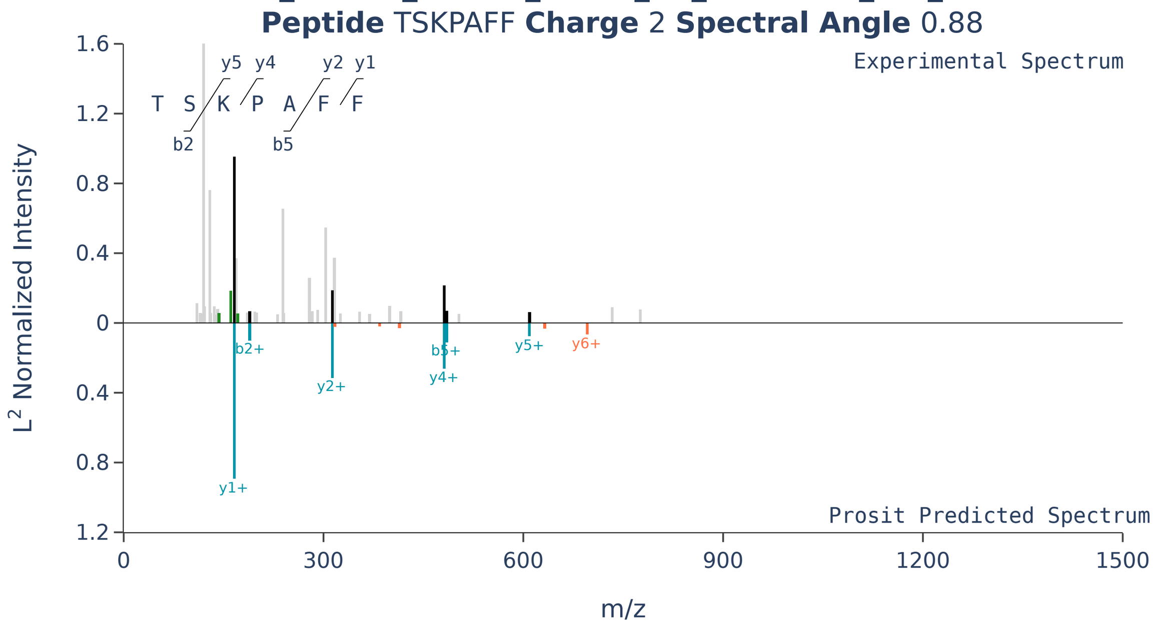


**Source** WSoh_301121_021221_HFGoe_M2_AnnexinA1_4h_R1 **Scan** 10372
**Peptide** DILTKRNNAQRQQI **Charge** 3 **Spectral Angle** 0.91

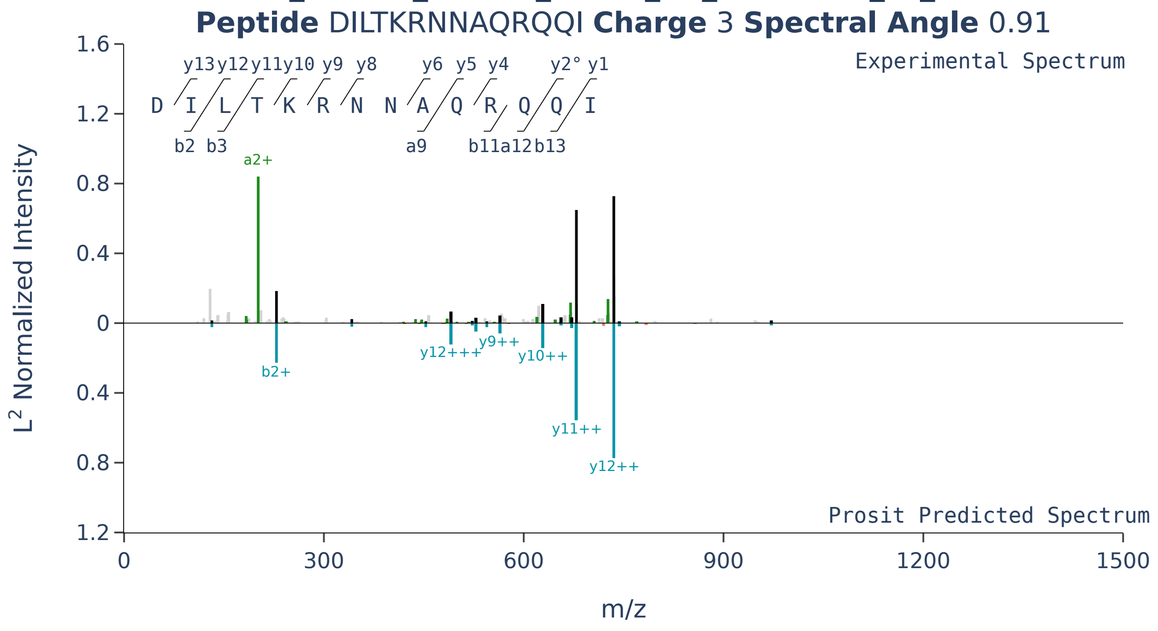


**Source** WSoh_301121_021221_HFGoe_M2_AnnexinA1_2h_R1 **Scan** 16708
**Peptide** IKAAYLQETGKPLD **Charge** 3 **Spectral Angle** 0.88

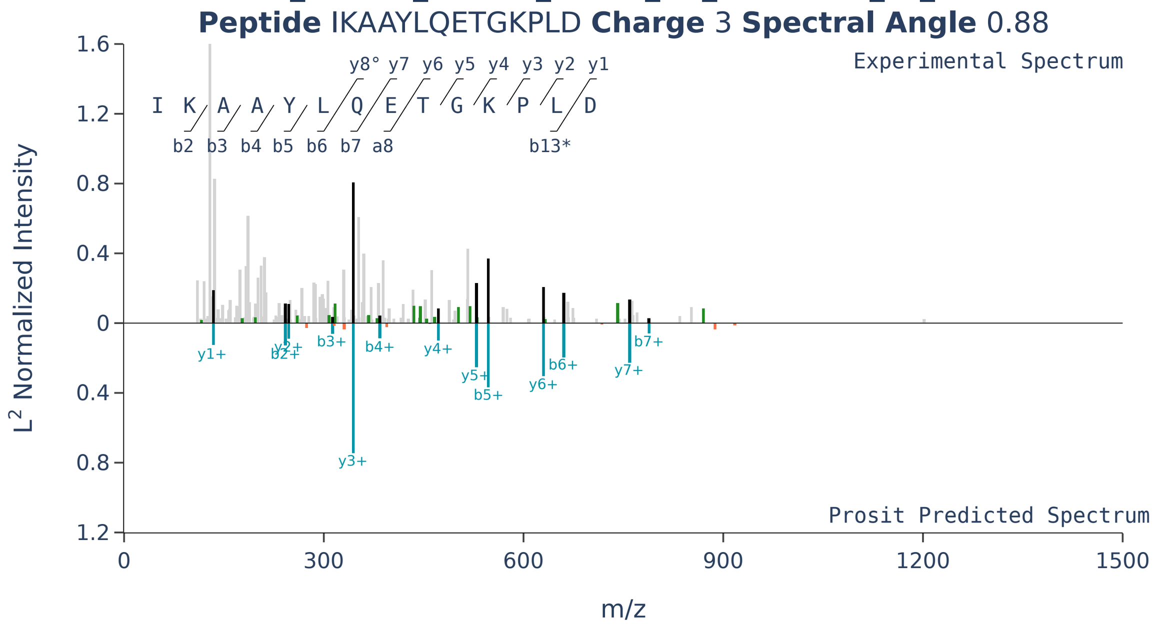


**Source** WSoh_301121_021221_HFGoe_M2_AnnexinA1_4h_R1 **Scan** 21841
**Peptide** KQAWFIENE **Charge** 2 **Spectral Angle** 0.88

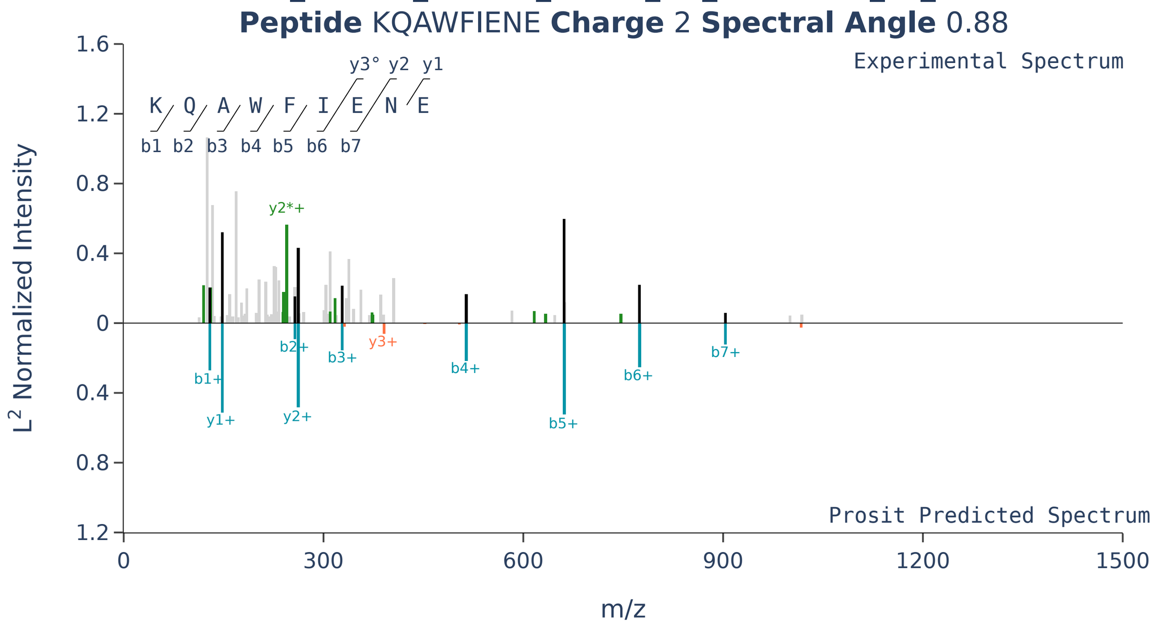


**Source** WSoh_301121_021221_HFGoe_M2_AnnexinA1_24h_R1 **Scan** 5889
**Peptide** YSKHDMNKV **Charge** 2 **Spectral Angle** 0.83

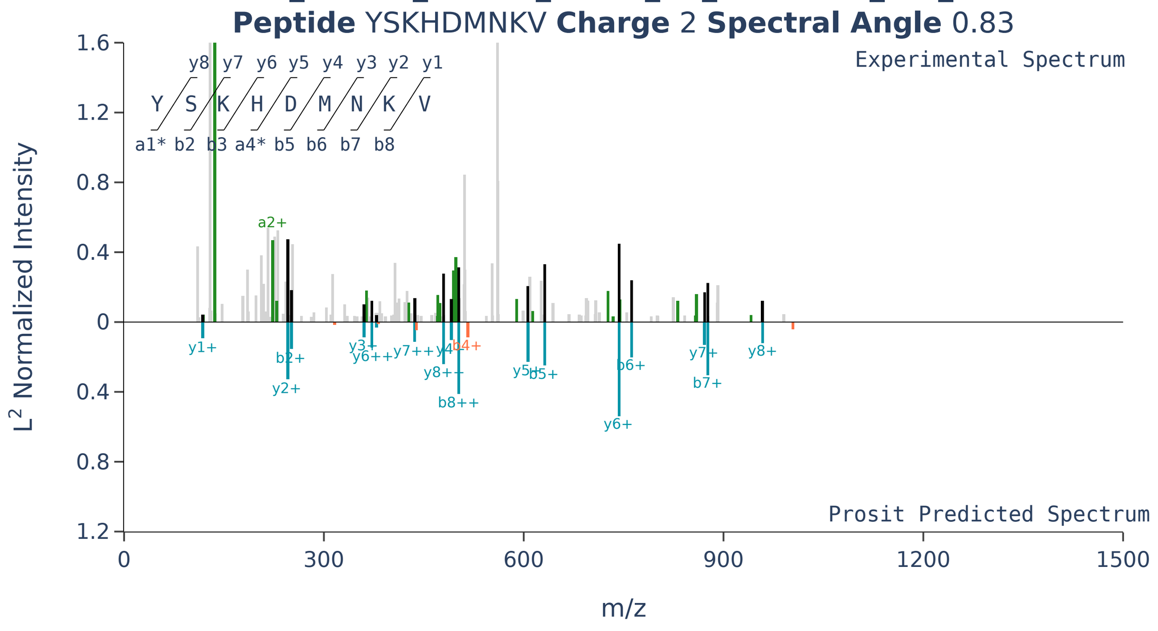


**Source** WSoh_301121_021221_HFGoe_M2_AnnexinA1_24h_R1 **Scan** 22234
**Peptide** KQAWFIENEE **Charge** 2 **Spectral Angle** 0.86

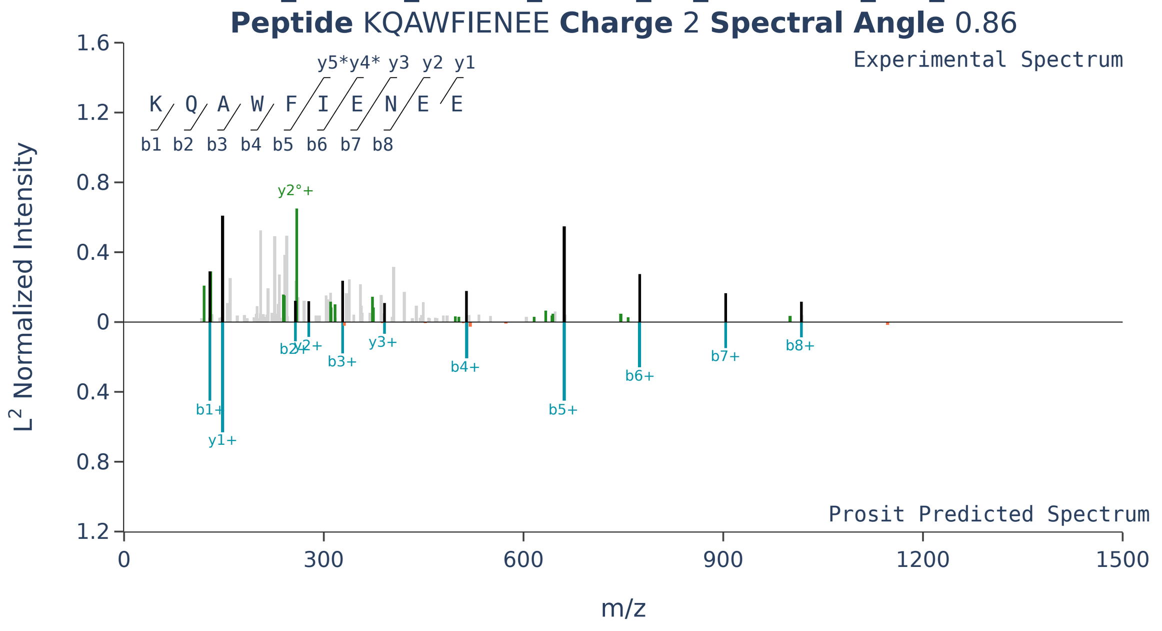


**Source** WSoh_301121_021221_HFGoe_M1_AnnexinA1_1h_R1 **Scan** 10142
**Peptide** NPSSDVAALHKA **Charge** 3 **Spectral Angle** 0.92

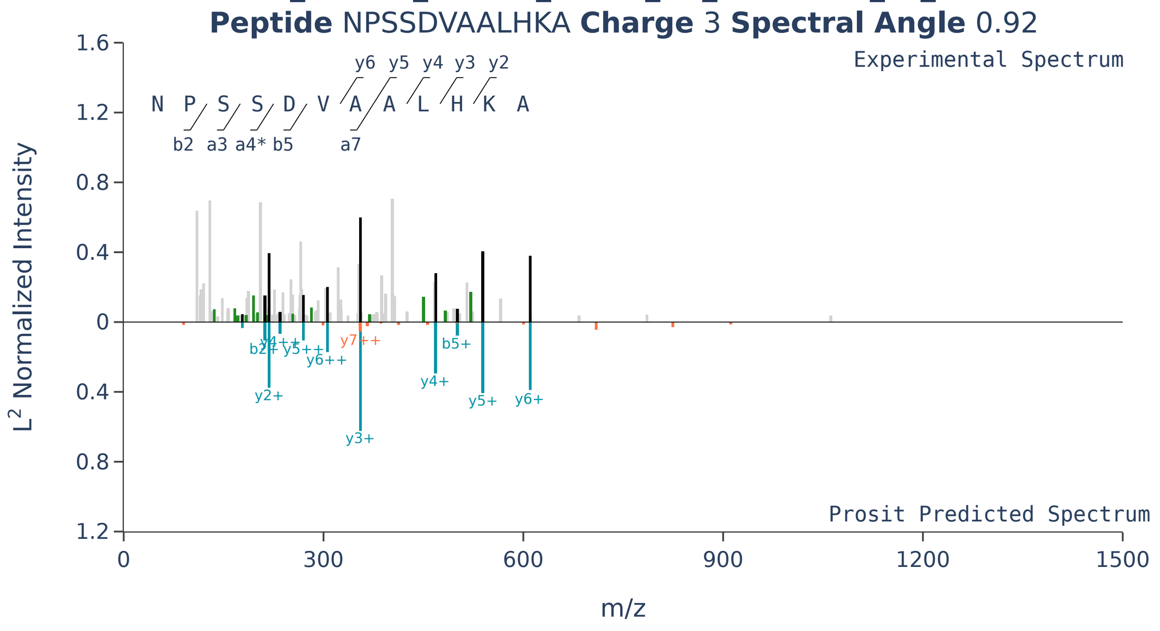


**Source** WSoh_301121_021221_HFGoe_M2_AnnexinA1_4h_R2 **Scan** 7608
**Peptide** ALHKAIMVK **Charge** 2 **Spectral Angle** 0.88

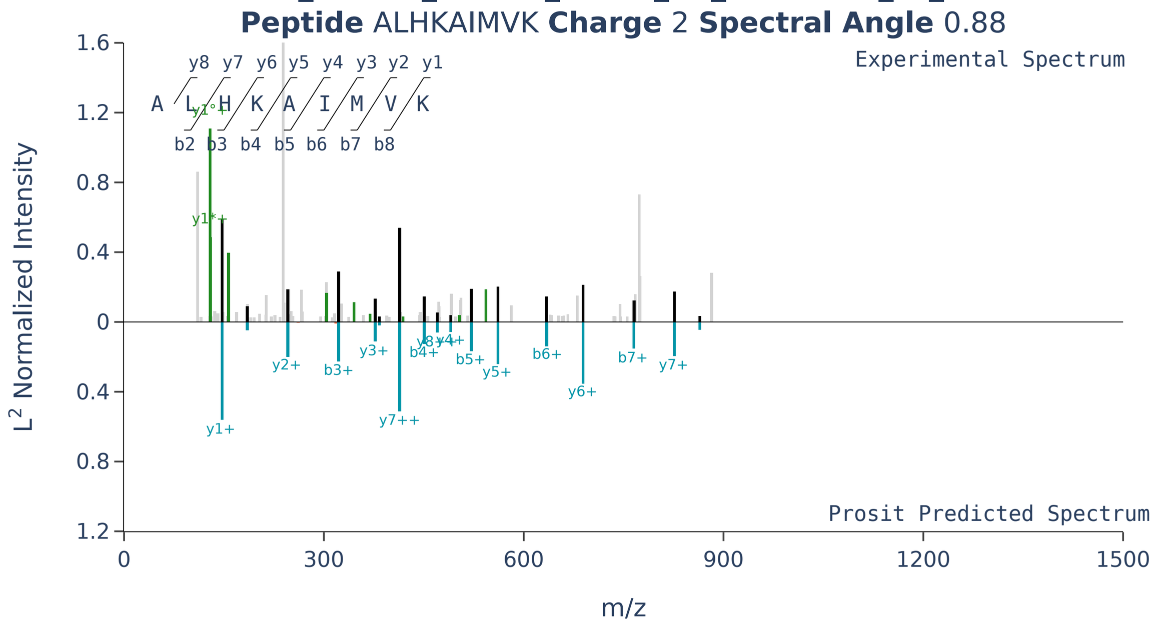


**Source** WSoh_301121_021221_HFGoe_M2_AnnexinA1_24h_R2 **Scan** 11058
**Peptide** DMNDIKA **Charge** 2 **Spectral Angle** 0.95

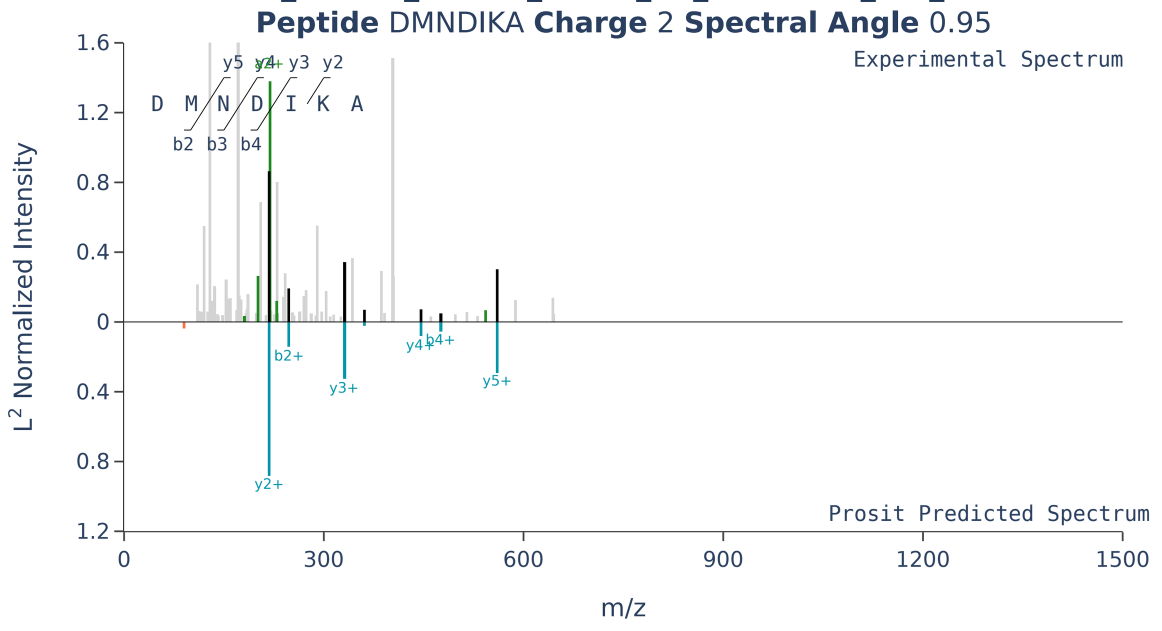


**Source** WSoh_301121_021221_HFGoe_M2_AnnexinA1_4h_R2 **Scan** 15105
**Peptide** AIMVKGVDE **Charge** 2 **Spectral Angle** 0.87

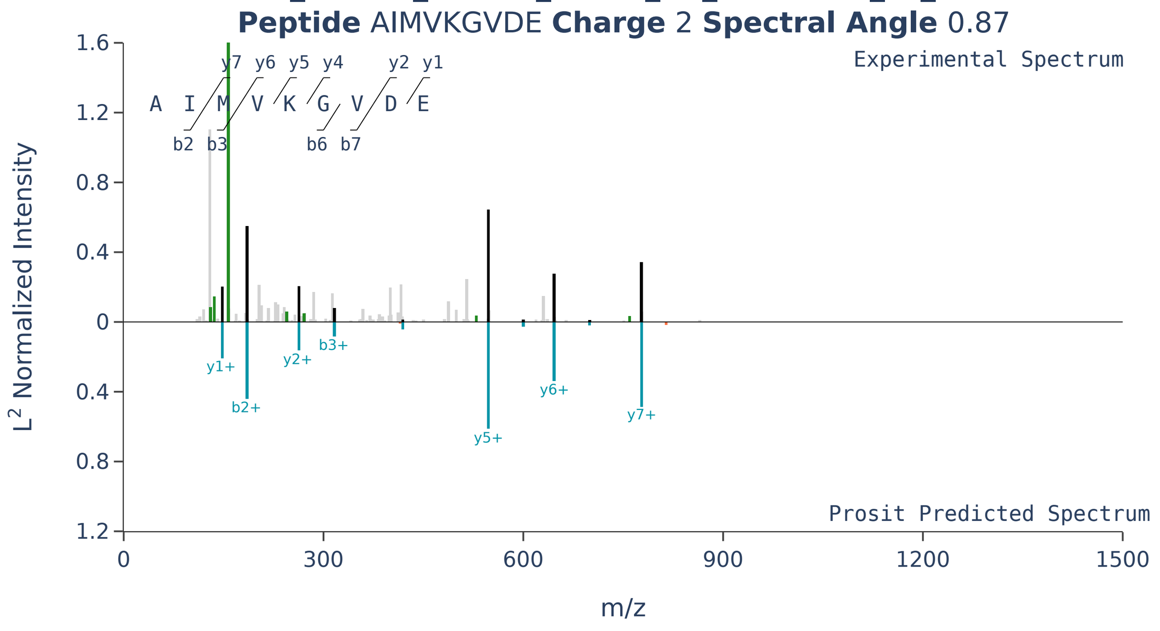

**Source** WSoh_301121_021221_HFGoe_M2_AnnexinA1_24h_R2 **Scan** 9683
**Peptide** QEYVQTVKS **Charge** 1 **Spectral Angle** 0.89

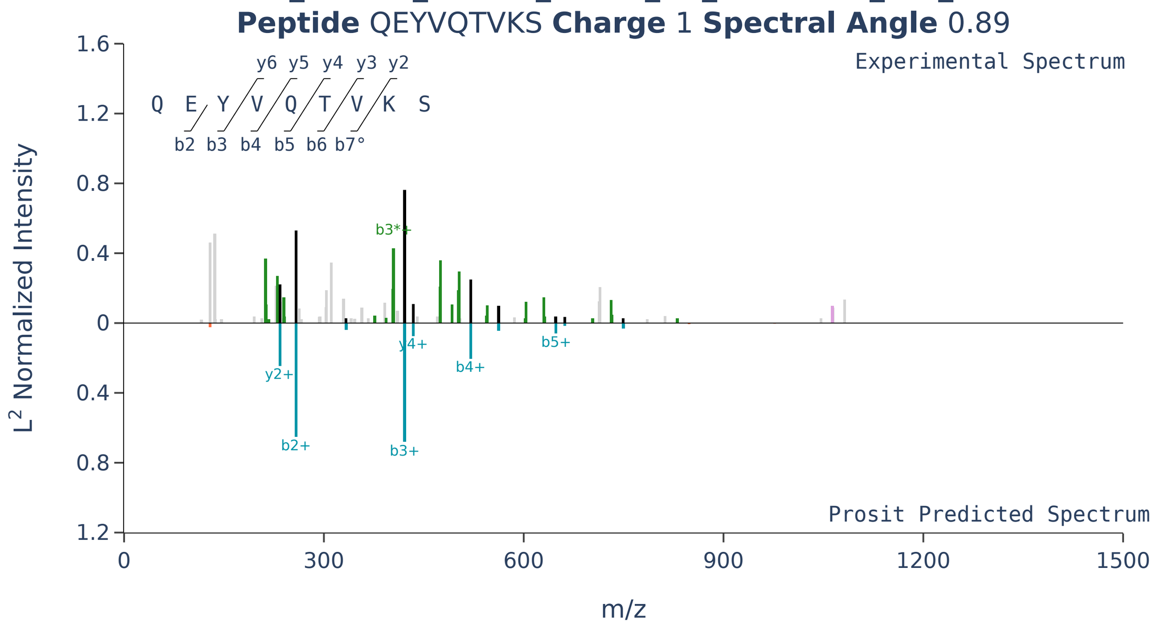


**Source** WSoh_301121_021221_HFGoe_M2_AnnexinA1_24h_R1 **Scan** 18964
**Peptide** AILDETKGDYEKI **Charge** 3 **Spectral Angle** 0.86

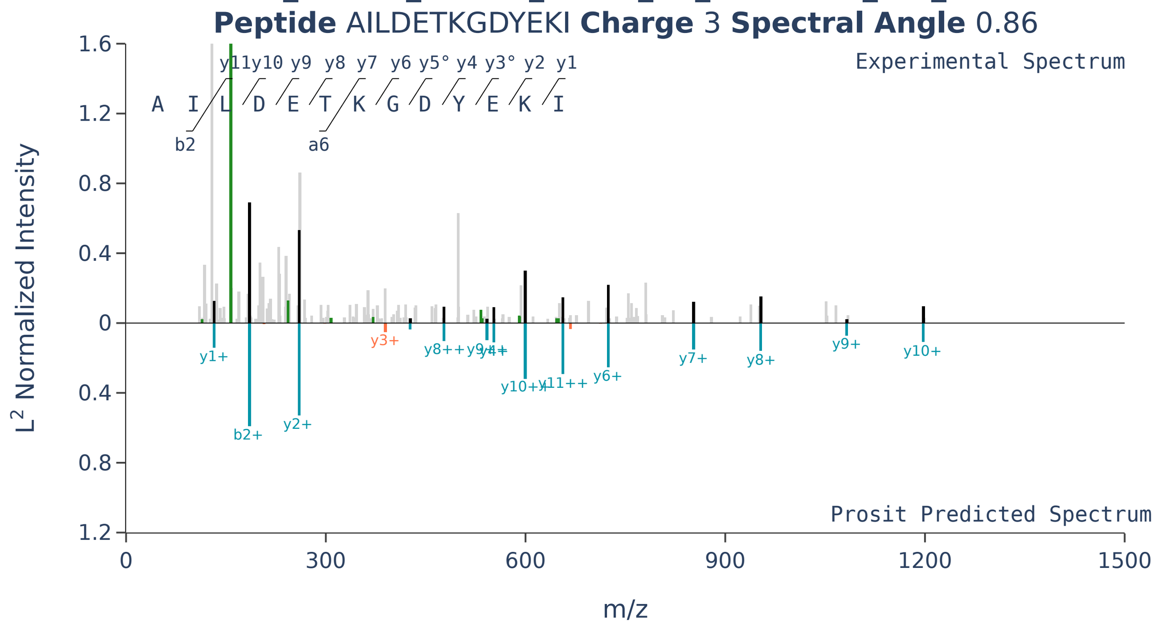


**Source** WSoh_301121_021221_HFGoe_M2_AnnexinA1_24h_R1 **Scan** 14088
**Peptide** ATSKPAFFAEK **Charge** 3 **Spectral Angle** 0.87

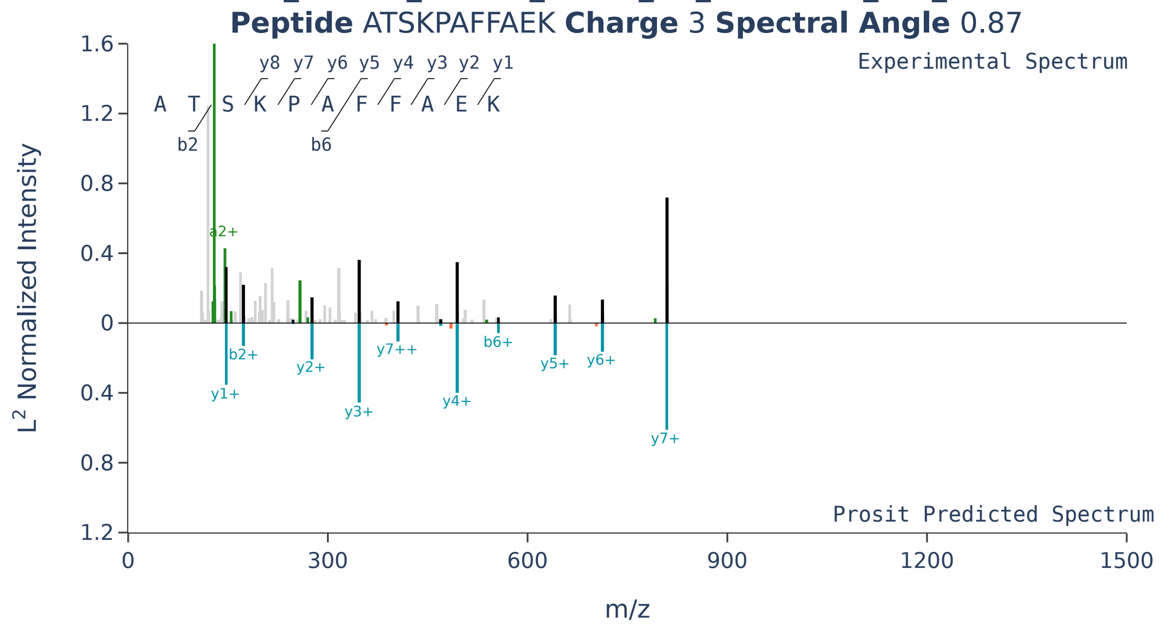


**Source** WSoh_301121_021221_HFGoe_M1_AnnexinA1_24h_R2 **Scan** 16126
**Peptide** ILDETKGDYEKI **Charge** 2 **Spectral Angle** 0.83

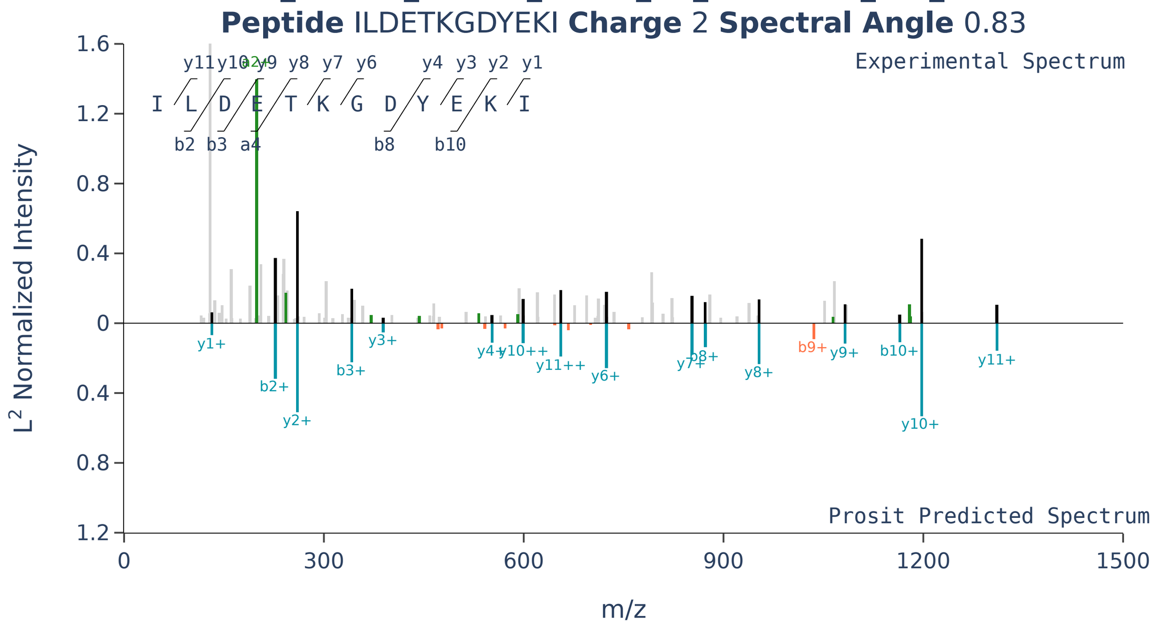


**Source** WSoh_301121_021221_HFGoe_M2_AnnexinA1_24h_R2 **Scan** 8940
**Peptide** SLAKGDRSEDFG **Charge** 2 **Spectral Angle** 0.86

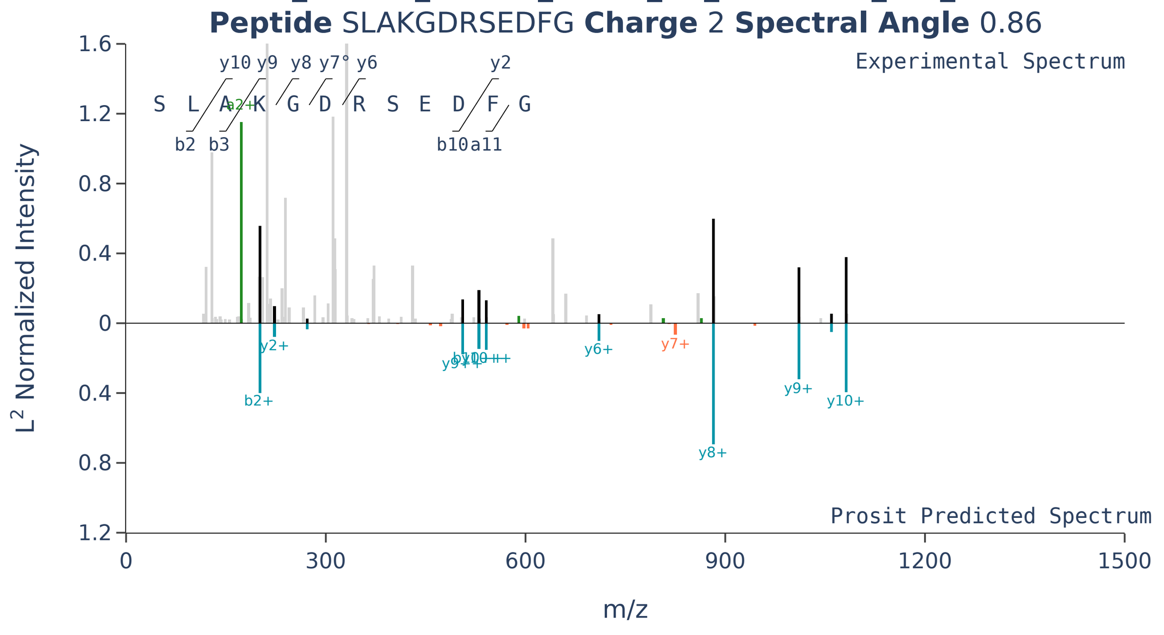


**Source** WSoh_301121_021221_HFGoe_M1_AnnexinA1_2h_R2 **Scan** 15097
**Peptide** RAAMKGLGTDEDTL **Charge** 3 **Spectral Angle** 0.86

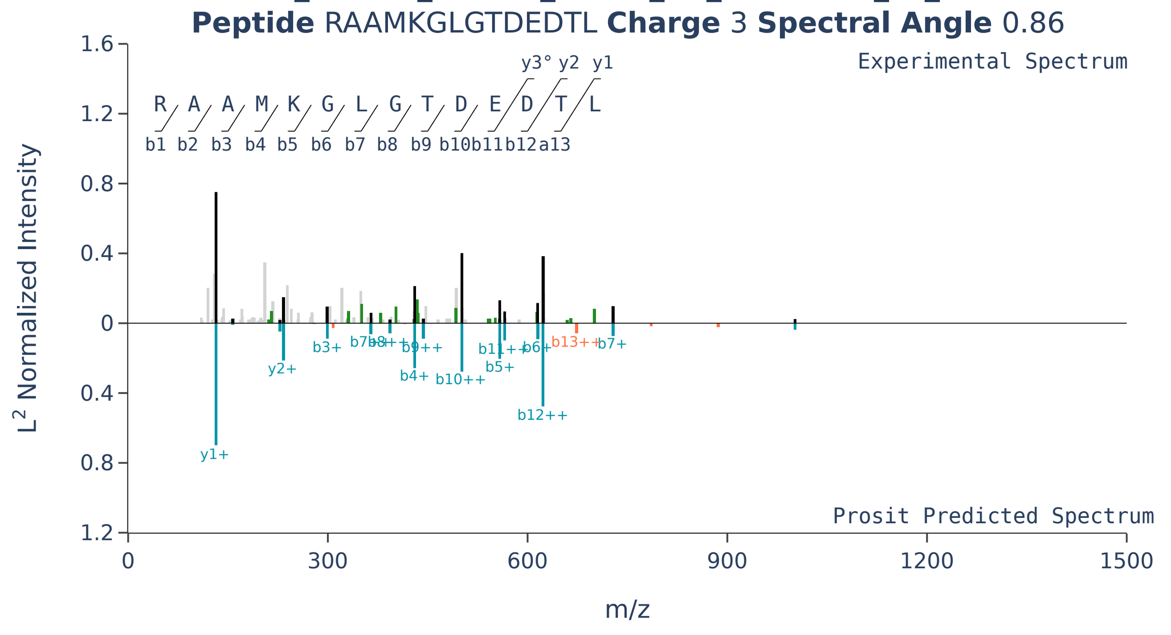


**Source** WSoh_301121_021221_HFGoe_M2_AnnexinA1_2h_R1 **Scan** 19054
**Peptide** LQETGKPLDETL **Charge** 2 **Spectral Angle** 0.9

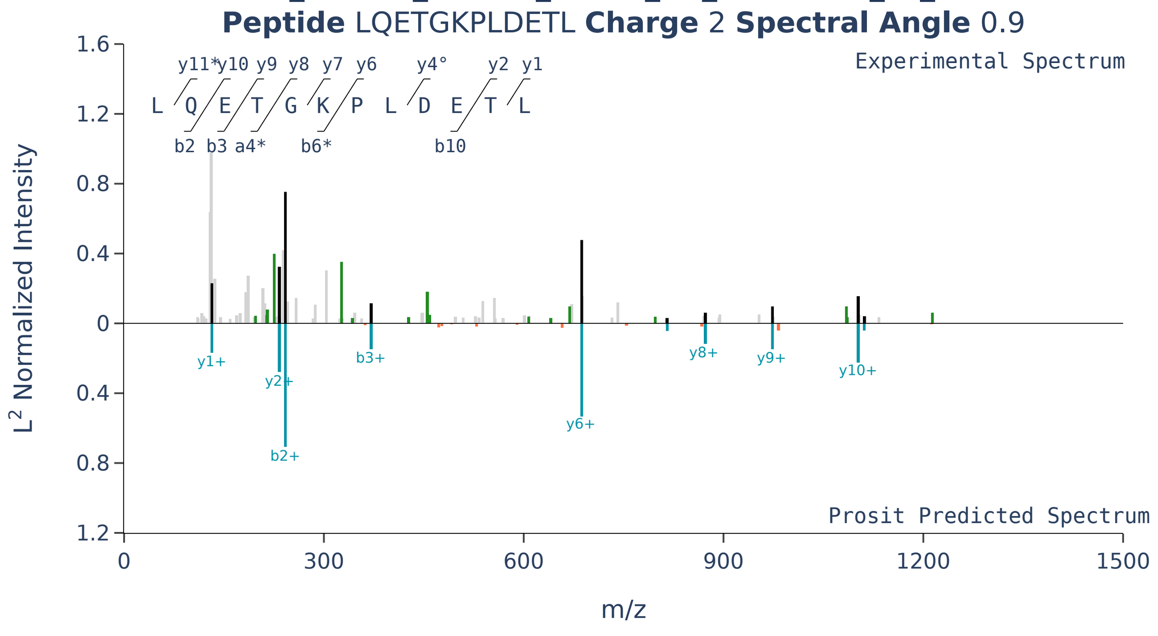


**Source** WSoh_301121_021221_HFGoe_M1_AnnexinA1_4h_R2 **Scan** 15112
**Peptide** LKTPAQFD **Charge** 2 **Spectral Angle** 0.89

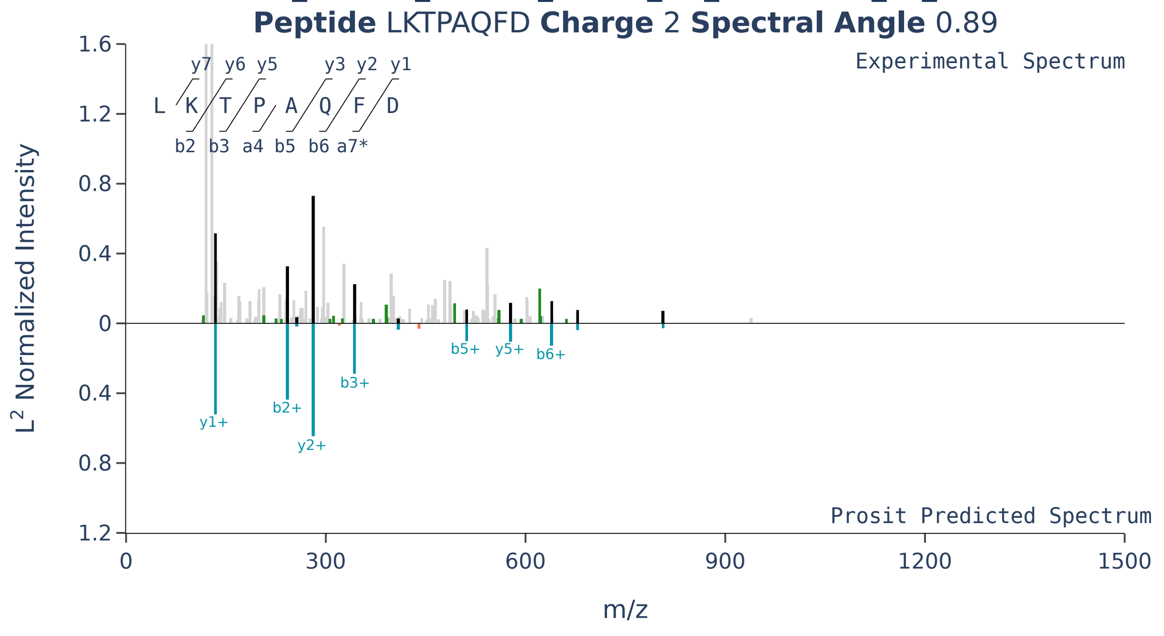


**Source** WSoh_301121_021221_HFGoe_M2_AnnexinA1_4h_R1 **Scan** 12744
**Peptide** DADELRAAMK **Charge** 2 **Spectral Angle** 0.81

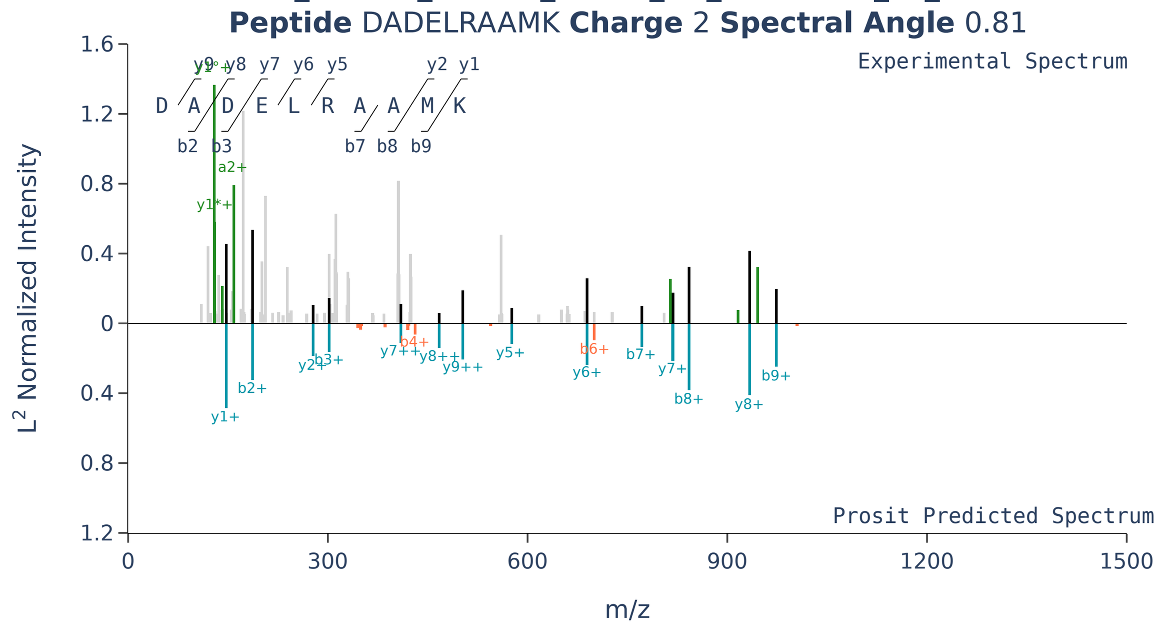


**Source** WSoh_301121_021221_HFGoe_M2_AnnexinA1_1h_R2 **Scan** 7116
**Peptide** RTNKEIRDINRV **Charge** 4 **Spectral Angle** 0.82

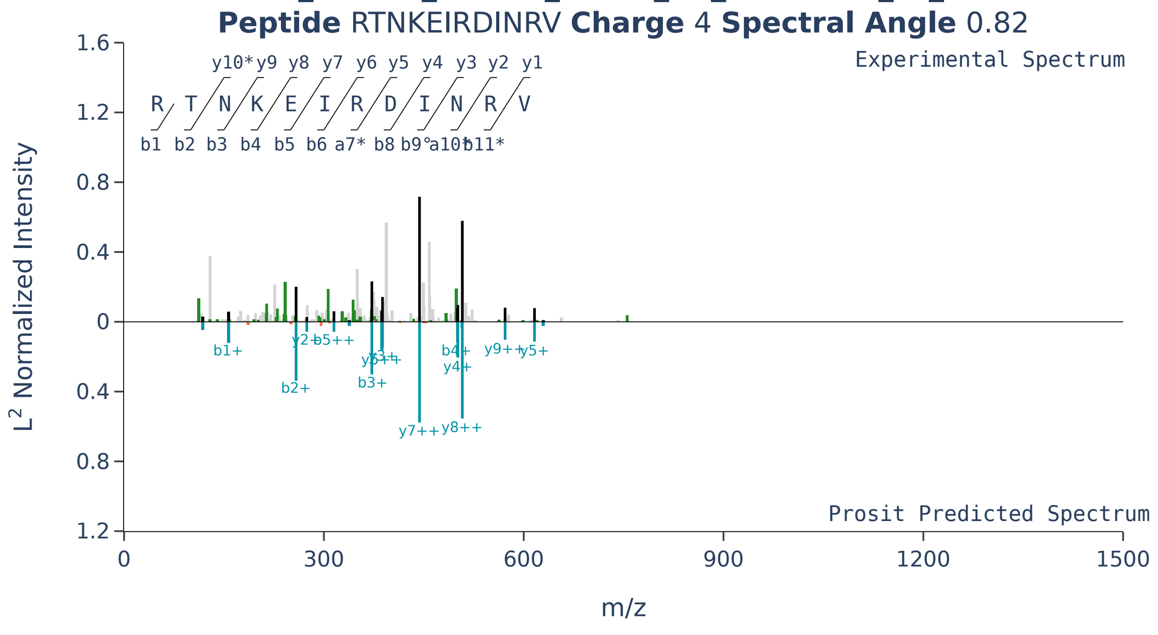


**Source** WSoh_301121_021221_HFGoe_M2_AnnexinA1_24h_R1 **Scan** 9736
**Peptide** TTRSYPQLR **Charge** 3 **Spectral Angle** 0.83

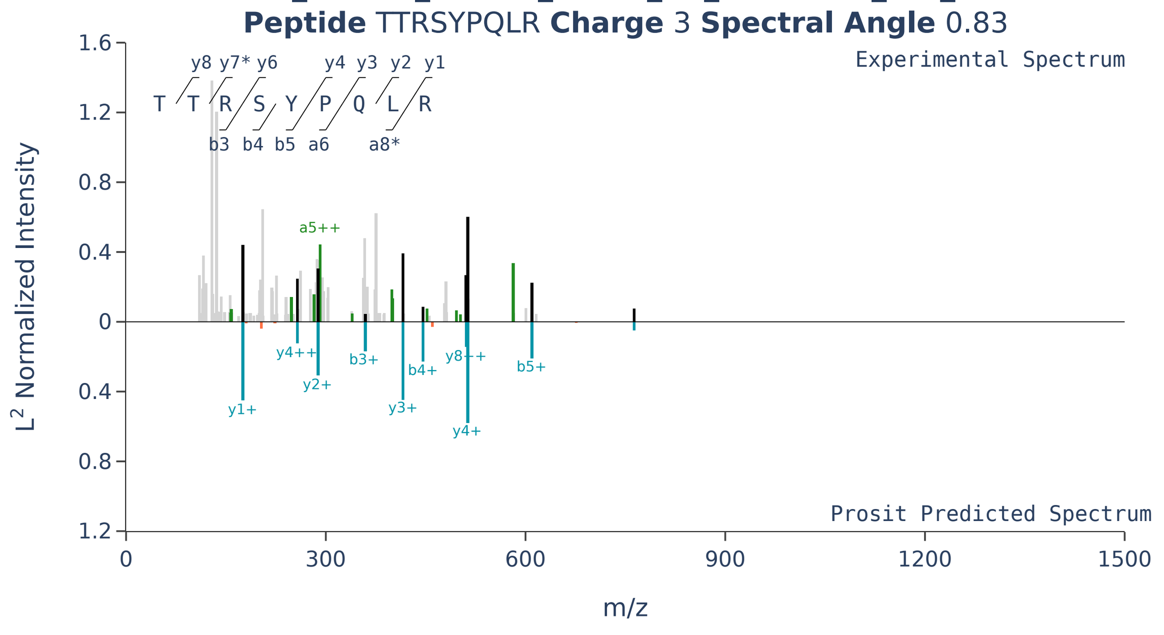


**Source** WSoh_301121_021221_HFGoe_M1_AnnexinA1_24h_R2 **Scan** 9498
**Peptide** VAALHKAI **Charge** 2 **Spectral Angle** 0.83

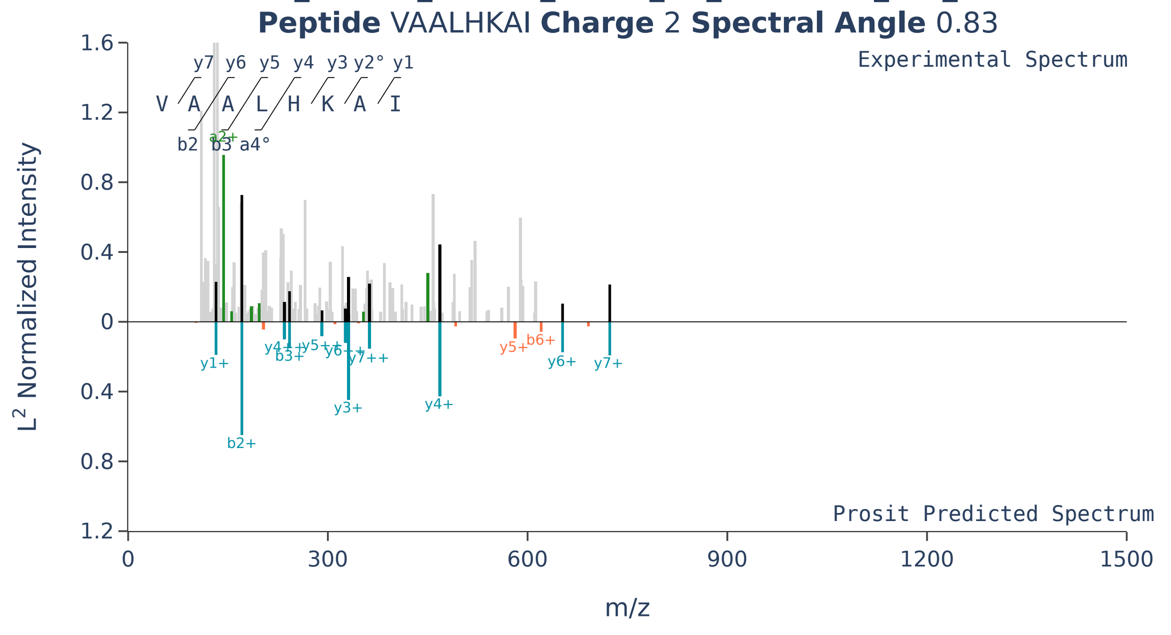


**Source** WSoh_301121_021221_HFGoe_M2_AnnexinA1_4h_R1 **Scan** 7248
**Peptide** SKHDMNKVLD **Charge** 3 **Spectral Angle** 0.9

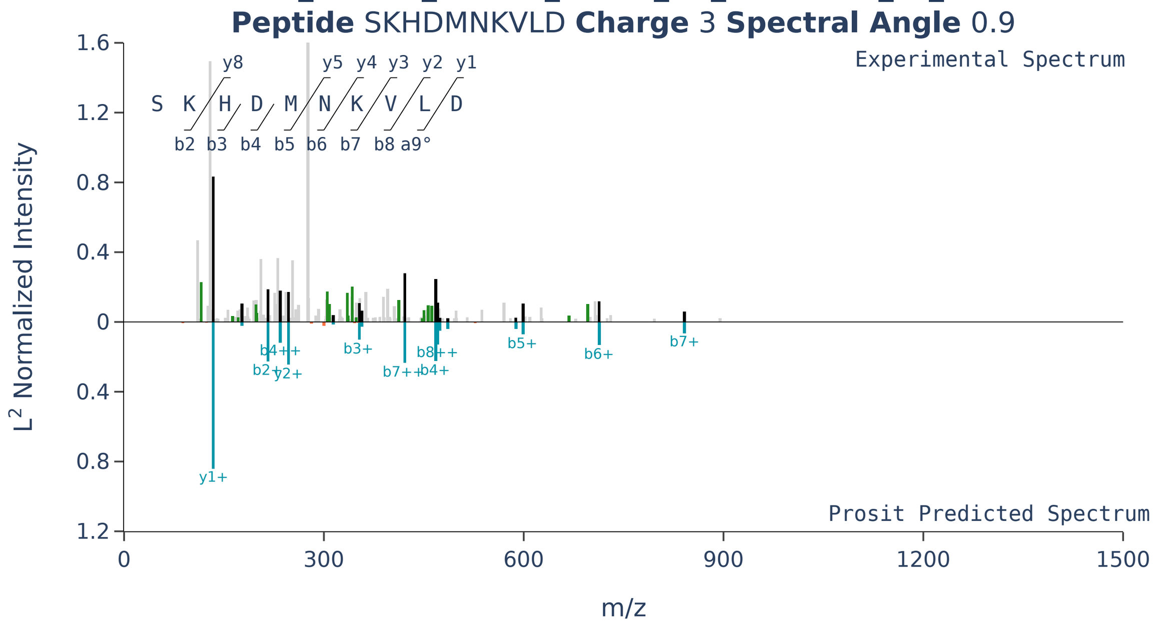


**Source** WSoh_301121_021221_HFGoe_M2_AnnexinA1_2h_R2 **Scan** 13506
**Peptide** NAQRQQIKAAYL **Charge** 3 **Spectral Angle** 0.84

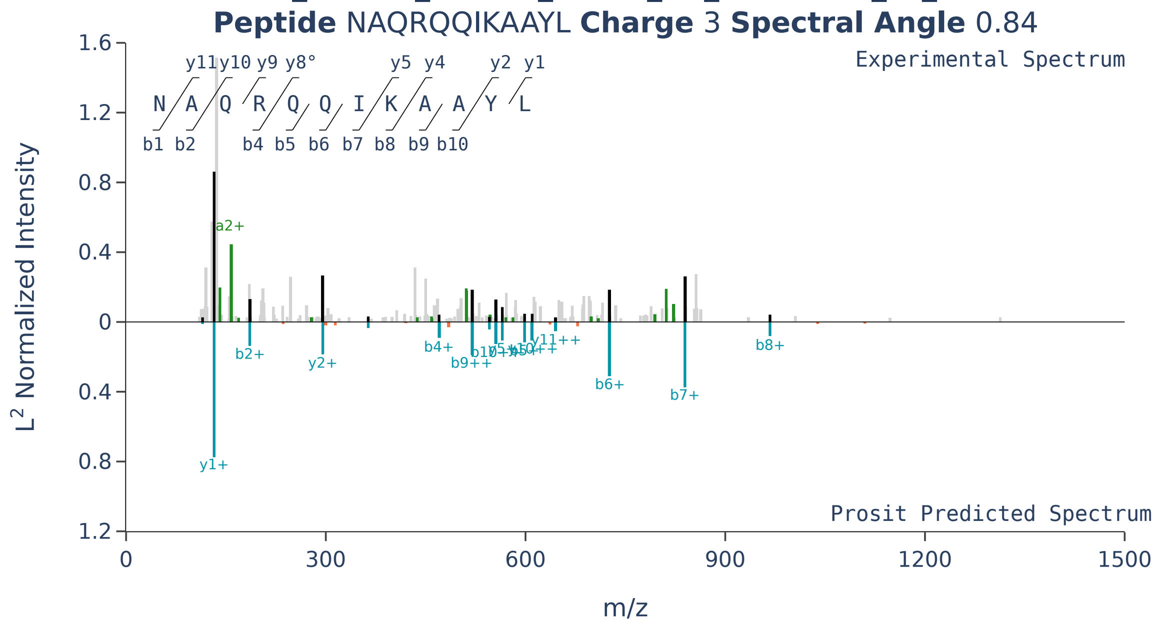


**Source** WSoh_301121_021221_HFGoe_M1_AnnexinA1_2h_R2 **Scan** 17642
**Peptide** IDMNDIKA **Charge** 2 **Spectral Angle** 0.91

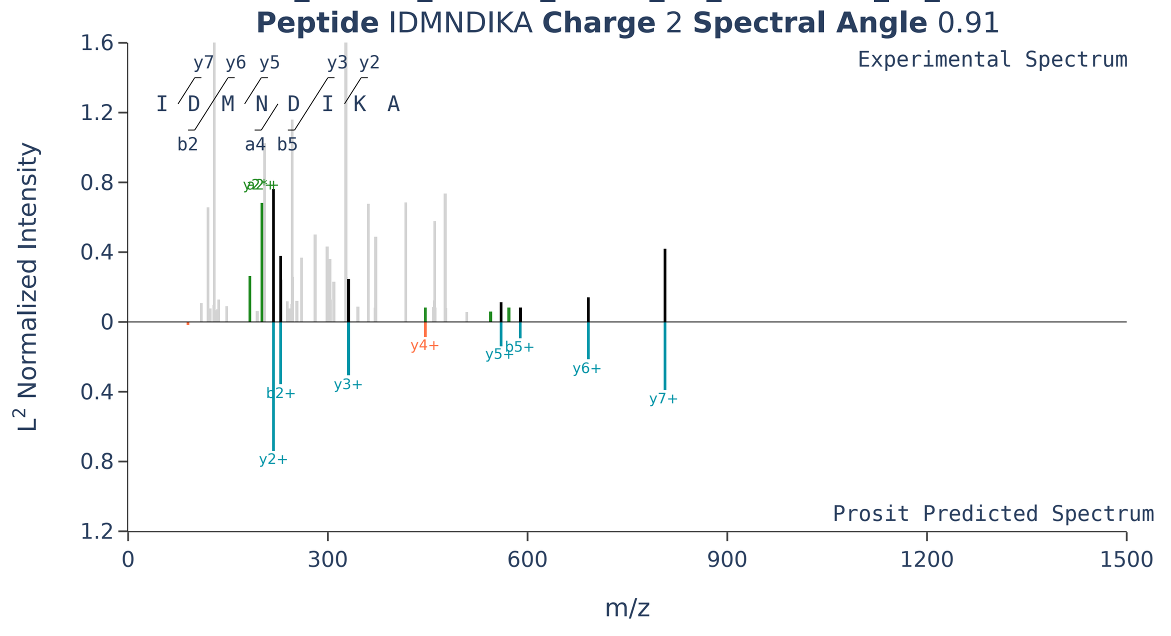

**Source** WSoh_301121_021221_HFGoe_M1_AnnexinA1_24h_R2 **Scan** 13666
**Peptide** DRSEDFGVNE **Charge** 2 **Spectral Angle** 0.86

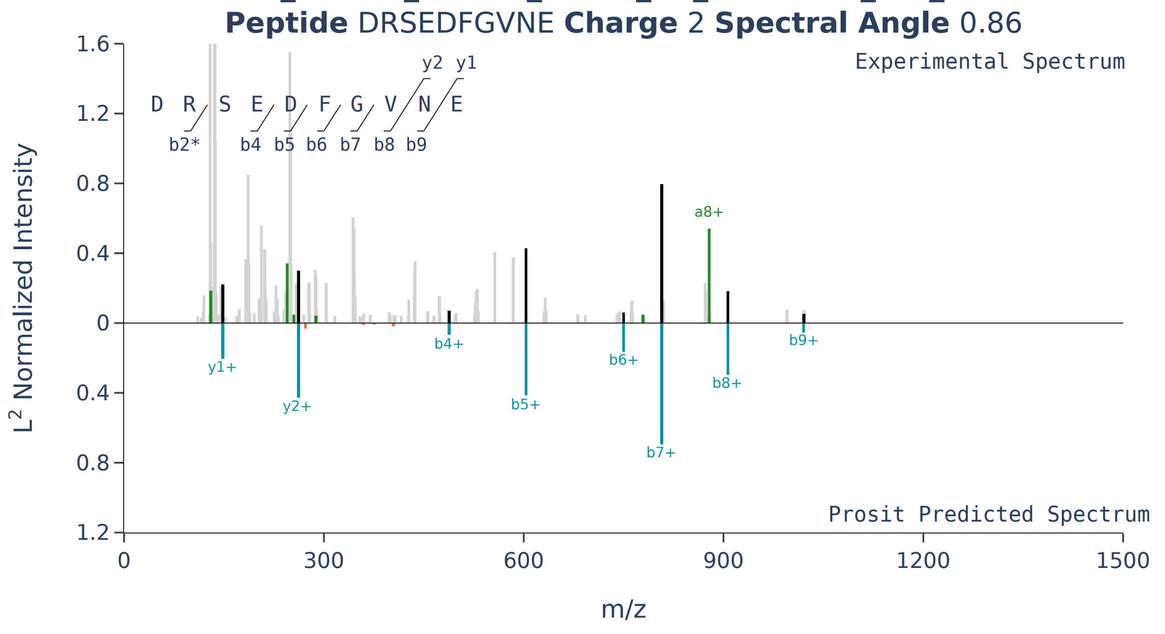


**Source** WSoh_301121_021221_HFGoe_M2_AnnexinA1_24h_R1 **Scan** 7791
**Peptide** LDETLKKA **Charge** 2 **Spectral Angle** 0.84

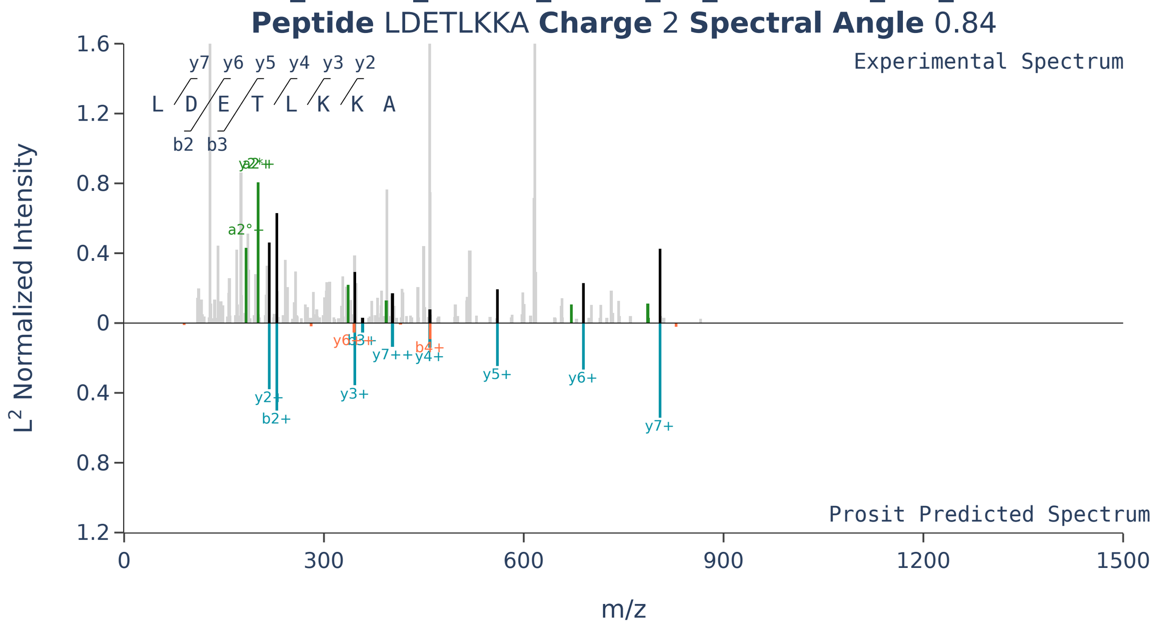


**Source** WSoh_301121_021221_HFGoe_M1_AnnexinA1_4h_R2 **Scan** 19444
**Peptide** KTPAQFDADEL **Charge** 2 **Spectral Angle** 0.89

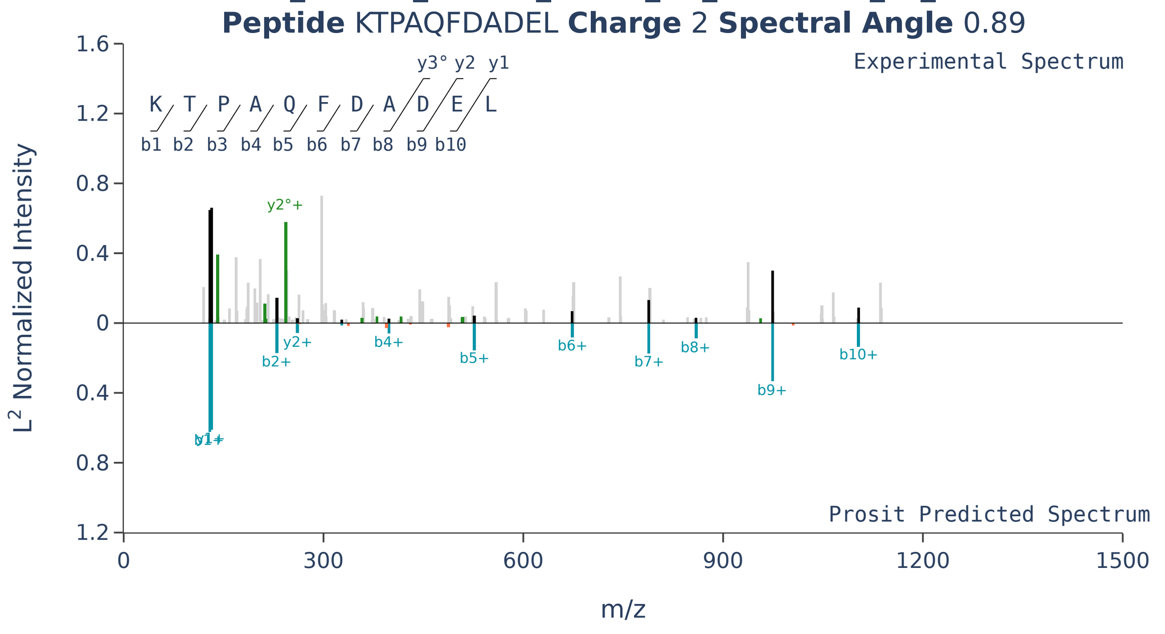


**Source** WSoh_301121_021221_HFGoe_M1_AnnexinA1_24h_R2 **Scan** 7725
**Peptide** QRQQIKAAY **Charge** 3 **Spectral Angle** 0.8

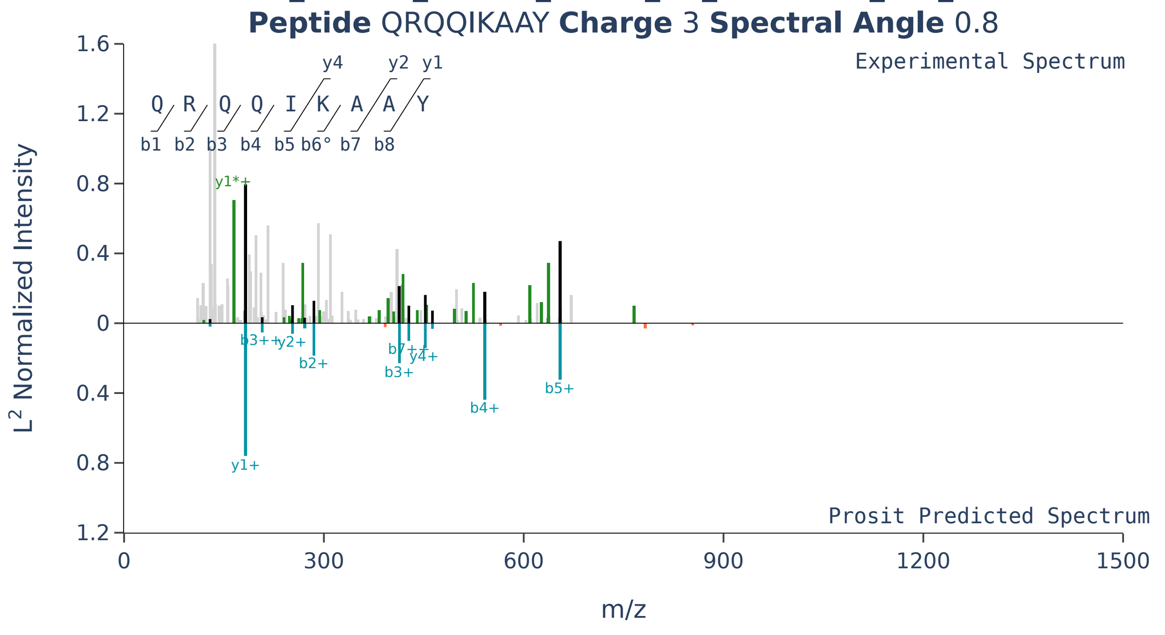


**Source** WSoh_301121_021221_HFGoe_M2_AnnexinA1_24h_R2 **Scan** 12922
**Peptide** LAKDITSDTSGDFRN **Charge** 2 **Spectral Angle** 0.89

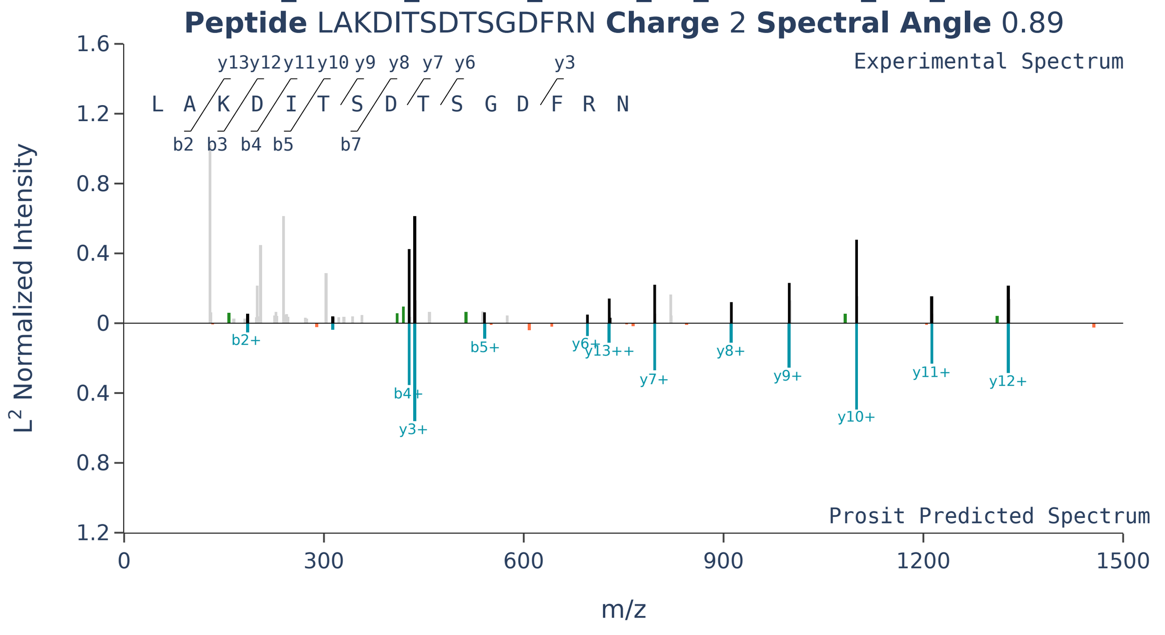


**Source** WSoh_301121_021221_HFGoe_M2_AnnexinA1_24h_R2 **Scan** 13160
**Peptide** QRQQIKAAYL **Charge** 3 **Spectral Angle** 0.84

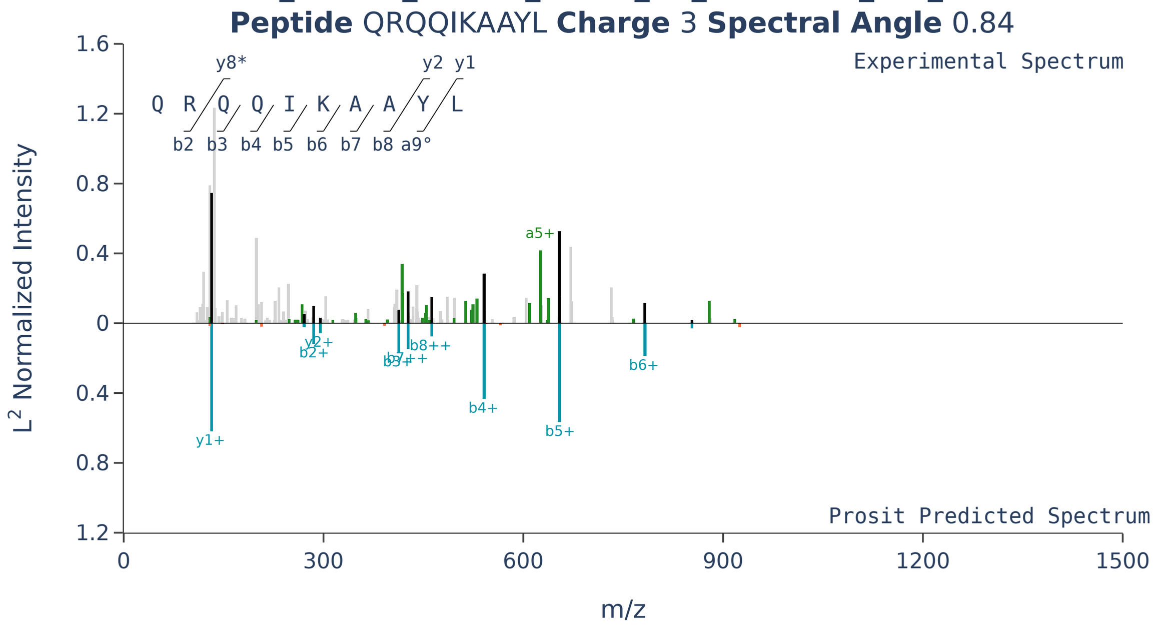


**Source** WSoh_301121_021221_HFGoe_M1_AnnexinA1_24h_R2 **Scan** 9876
**Peptide** NRVYREEL **Charge** 2 **Spectral Angle** 0.84

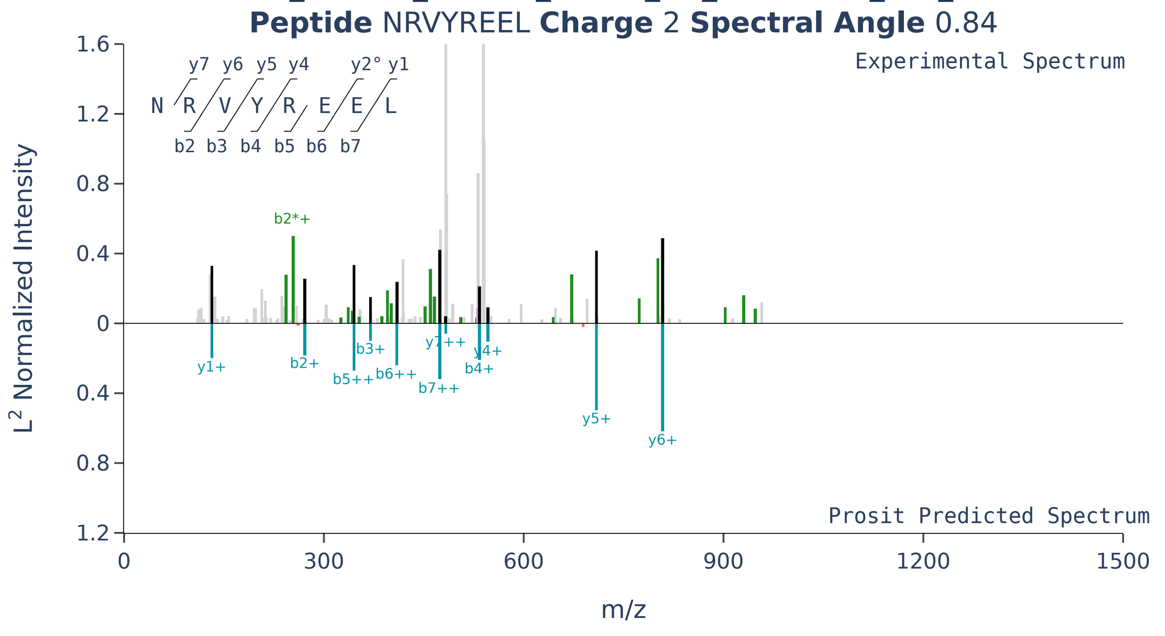


**Source** WSoh_301121_021221_HFGoe_M2_AnnexinA1_2h_R1 **Scan** 14784
**Peptide** DVAALHKAI **Charge** 2 **Spectral Angle** 0.85

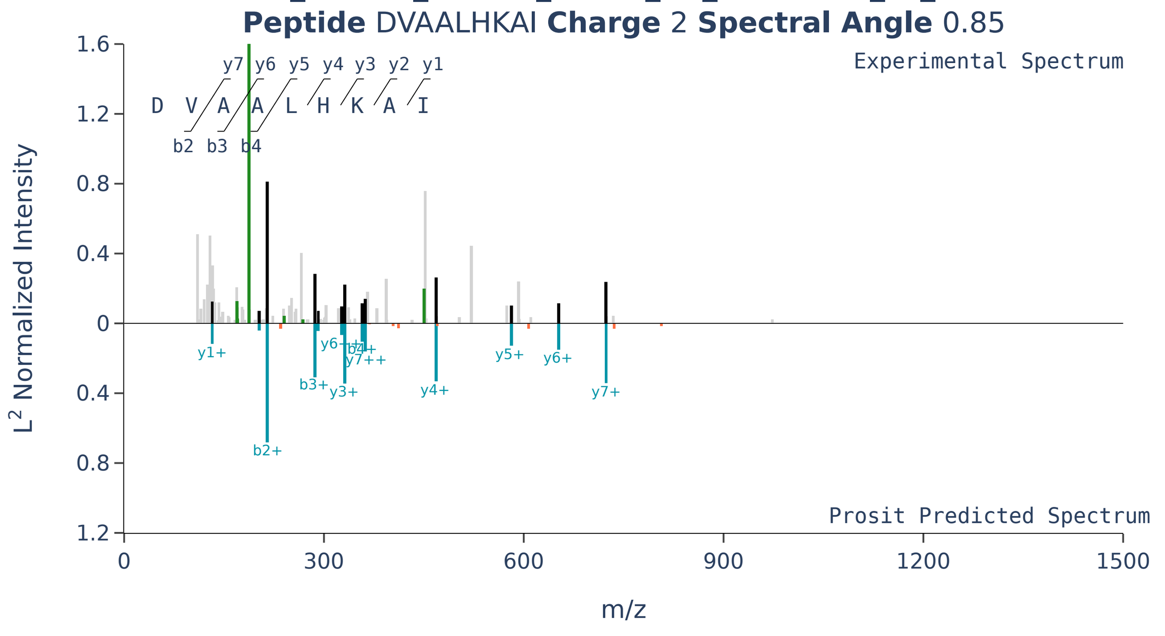


**Source** WSoh_301121_021221_HFGoe_M2_AnnexinA1_24h_R1 **Scan** 15082
**Peptide** DIKAFYQK **Charge** 2 **Spectral Angle** 0.86

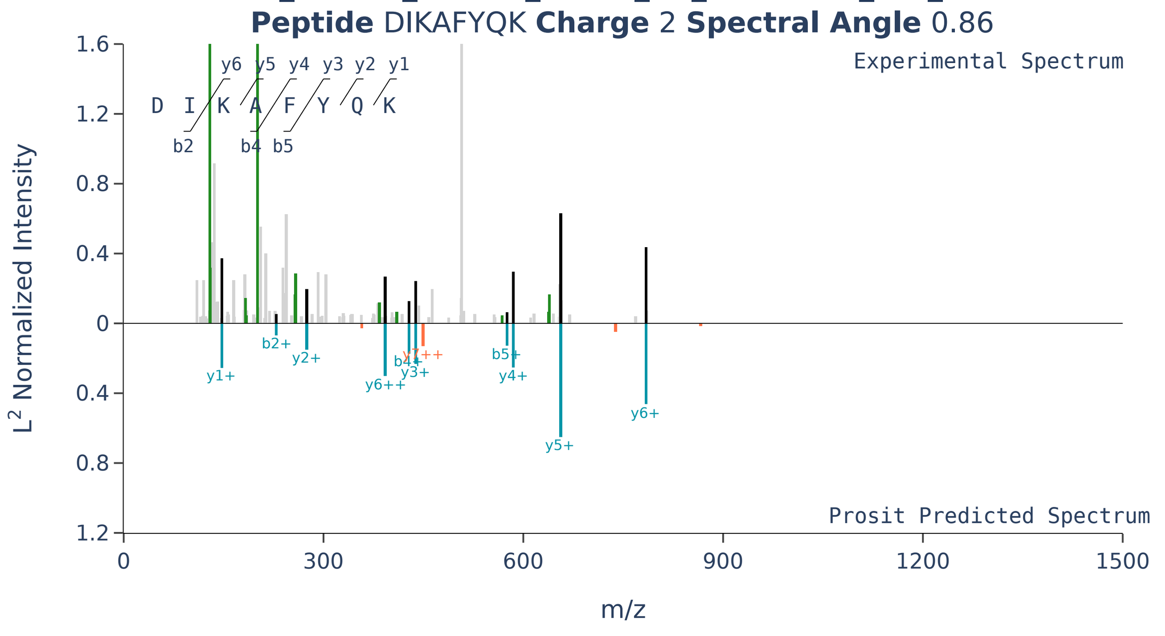


**Source** WSoh_301121_021221_HFGoe_M2_AnnexinA1_4h_R2 **Scan** 16094
**Peptide** KHDMNKVLDL **Charge** 3 **Spectral Angle** 0.95

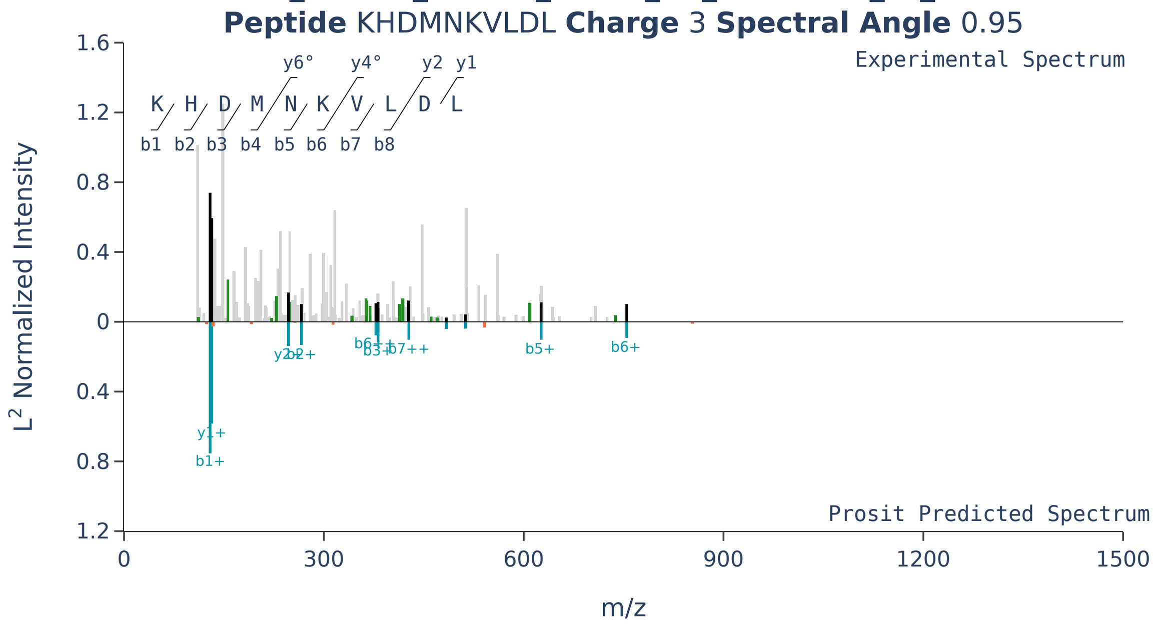


**Source** WSoh_301121_021221_HFGoe_M2_AnnexinA1_1h_R1 **Scan** 9717
**Peptide** RSYPQLRRV **Charge** 3 **Spectral Angle** 0.93

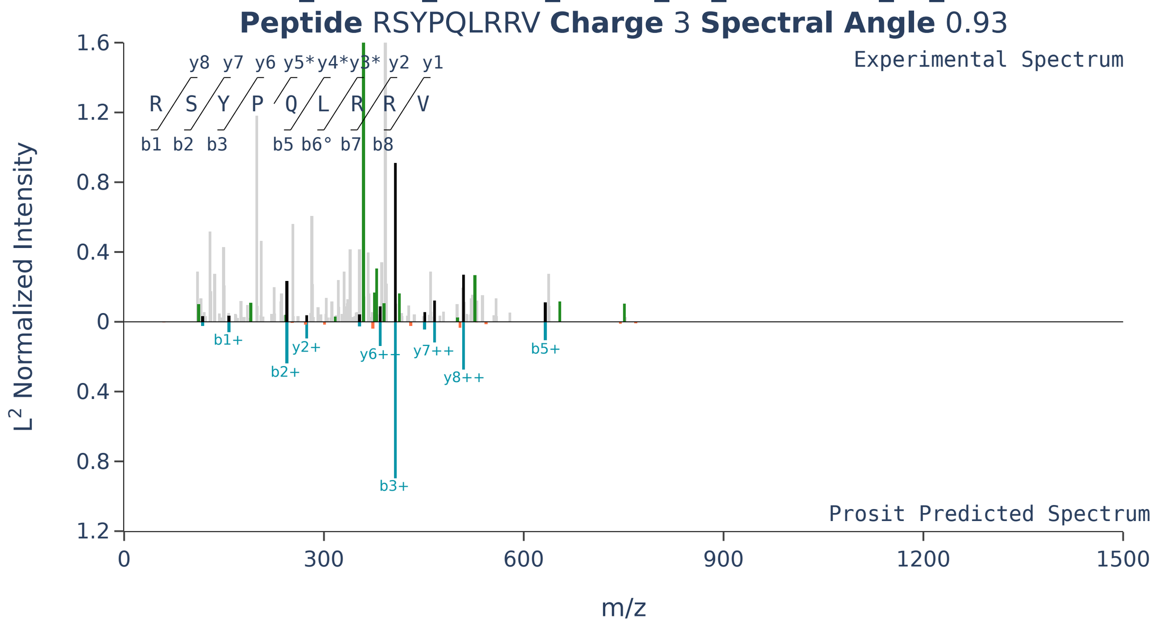


**Source** WSoh_301121_021221_HFGoe_M1_AnnexinA1_24h_R2 **Scan** 14700
**Peptide** DARALYEA **Charge** 2 **Spectral Angle** 0.92

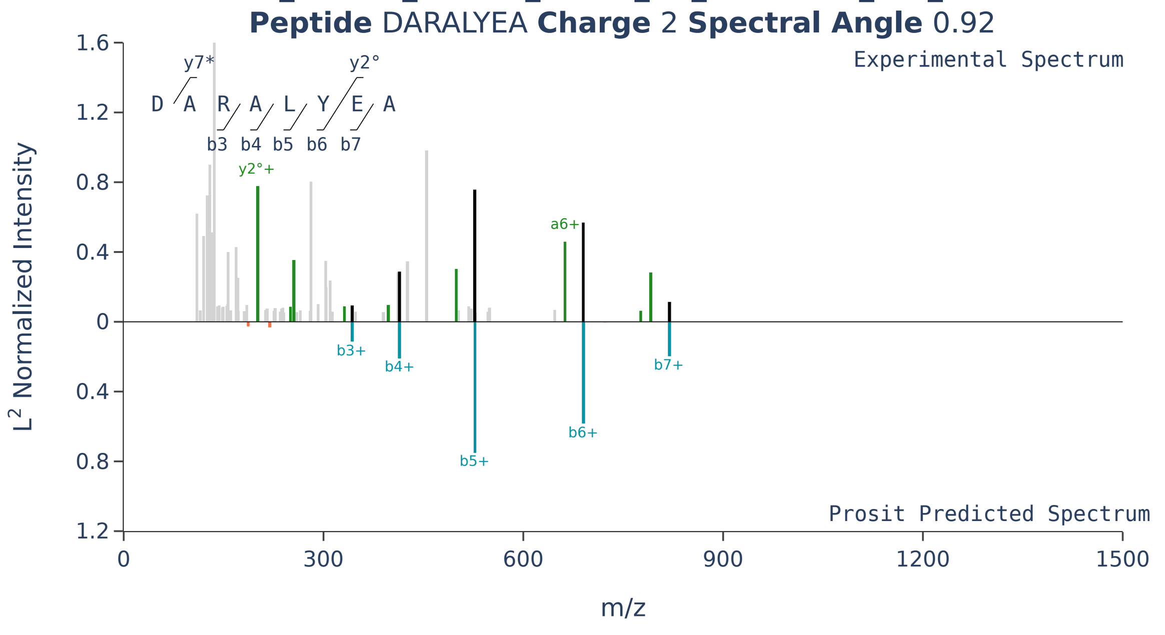


**Source** WSoh_301121_021221_HFGoe_M1_AnnexinA1_4h_R1 **Scan** 7492
**Peptide** SRTNKEIRDINRV **Charge** 4 **Spectral Angle** 0.9

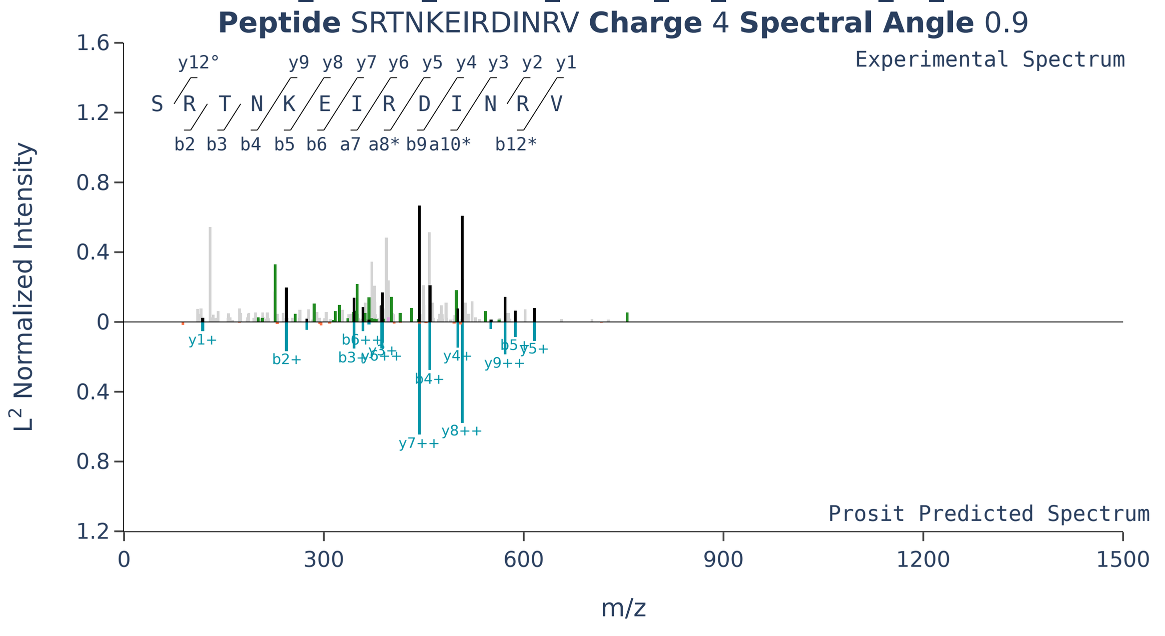


**Source** WSoh_301121_021221_HFGoe_M2_AnnexinA1_2h_R2 **Scan** 19114
**Peptide** DIKAFYQ **Charge** 2 **Spectral Angle** 0.94

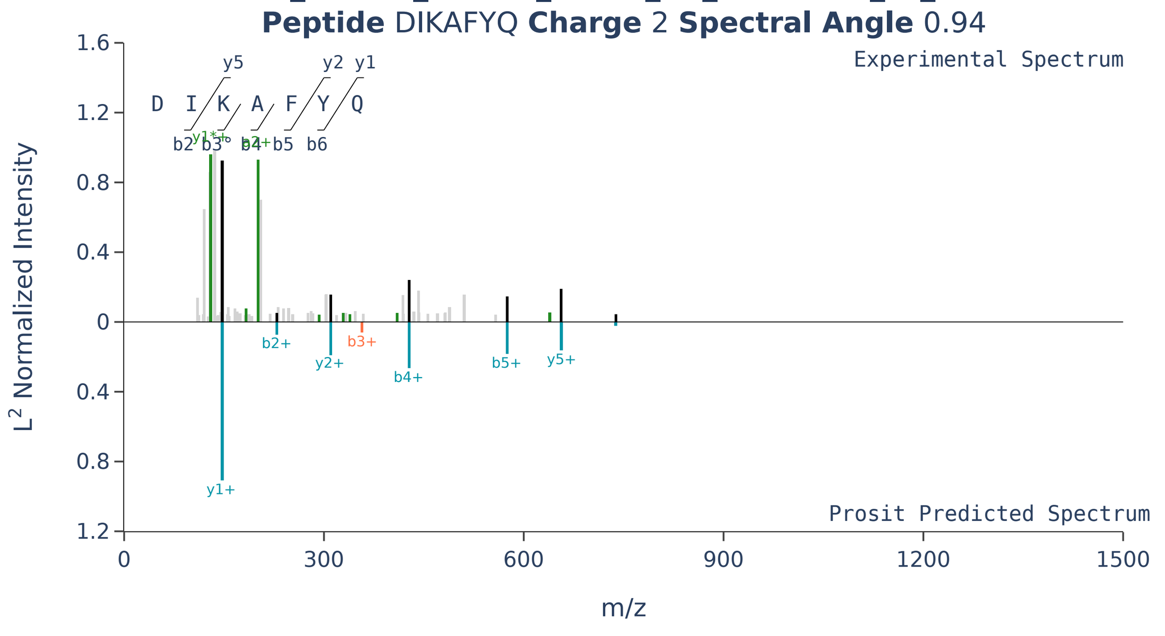


**Source** WSoh_301121_021221_HFGoe_M1_AnnexinA1_24h_R1 **Scan** 7145
**Peptide** RDINRVYREE **Charge** 3 **Spectral Angle** 0.88

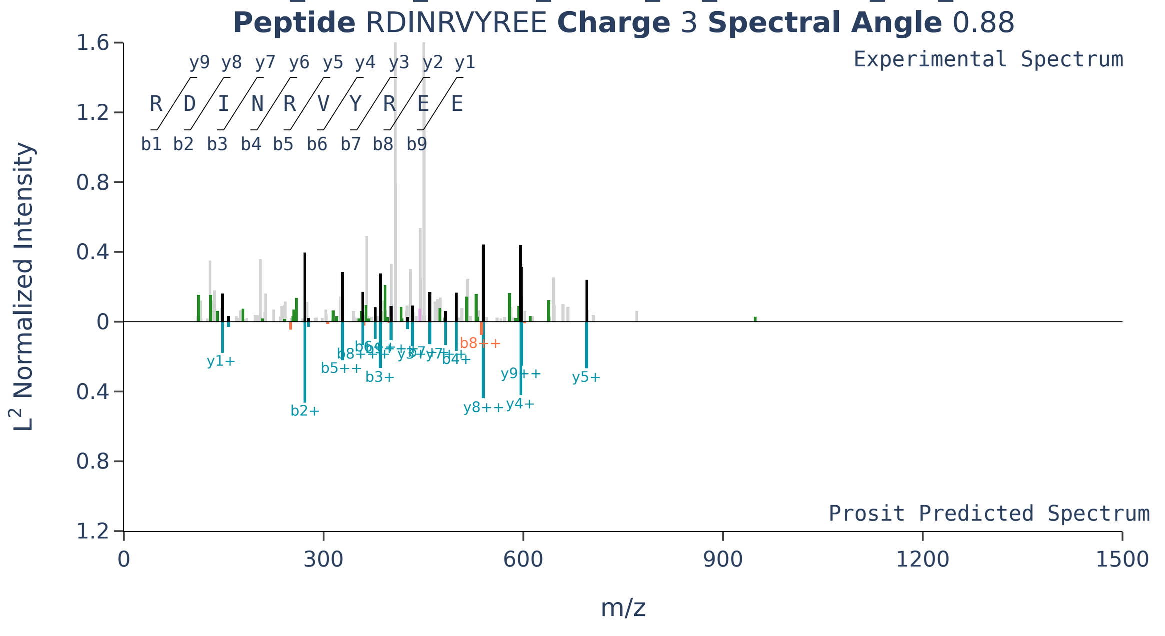

**Source** WSoh_301121_021221_HFGoe_M1_AnnexinA1_24h_R2 **Scan** 14367
**Peptide** FFAEKLHQA **Charge** 2 **Spectral Angle** 0.85

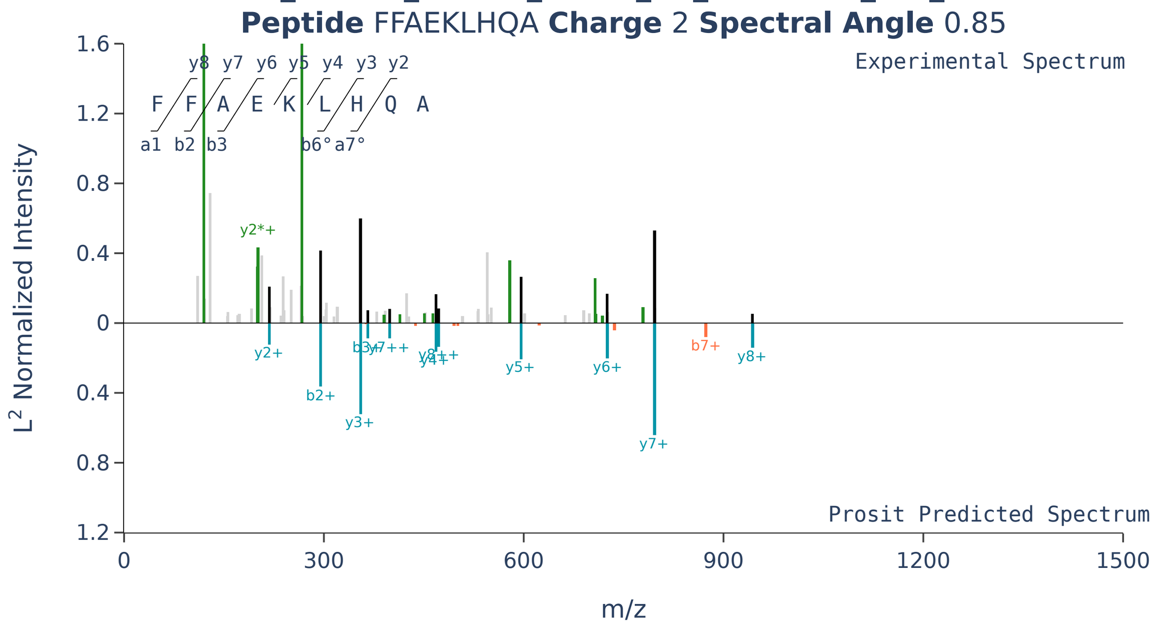


**Source** WSoh_301121_021221_HFGoe_M1_AnnexinA1_24h_R2 **Scan** 6337
**Peptide** ALYEAGERRKG **Charge** 3 **Spectral Angle** 0.95

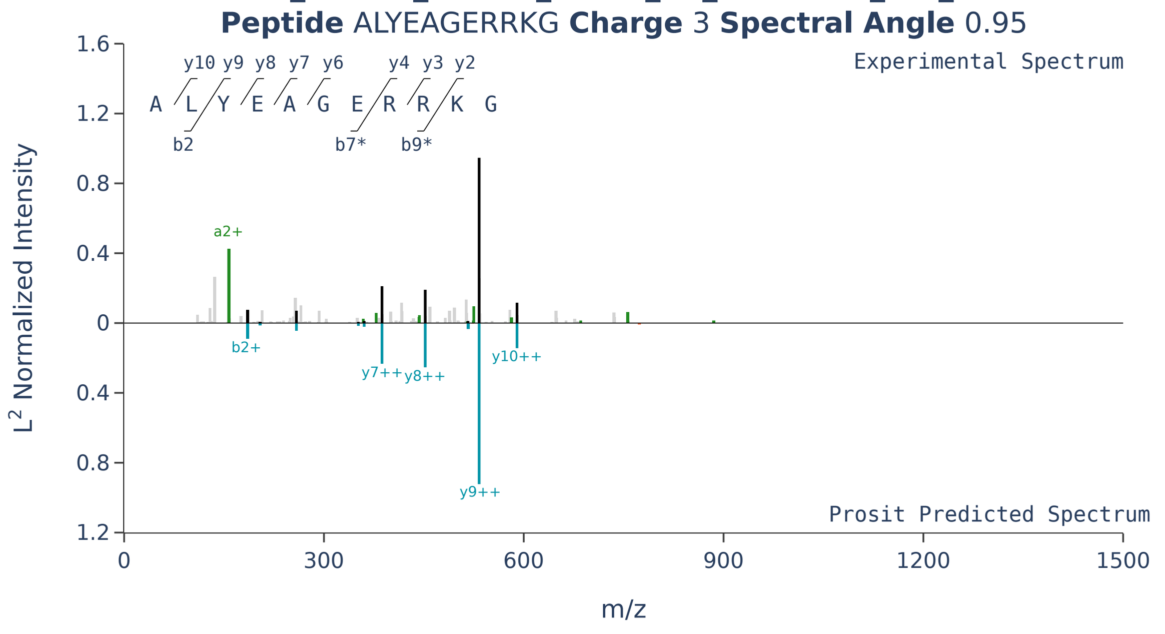


**Source** WSoh_301121_021221_HFGoe_M2_AnnexinA1_4h_R1 **Scan** 8458
**Peptide** RDINRVY **Charge** 2 **Spectral Angle** 0.83

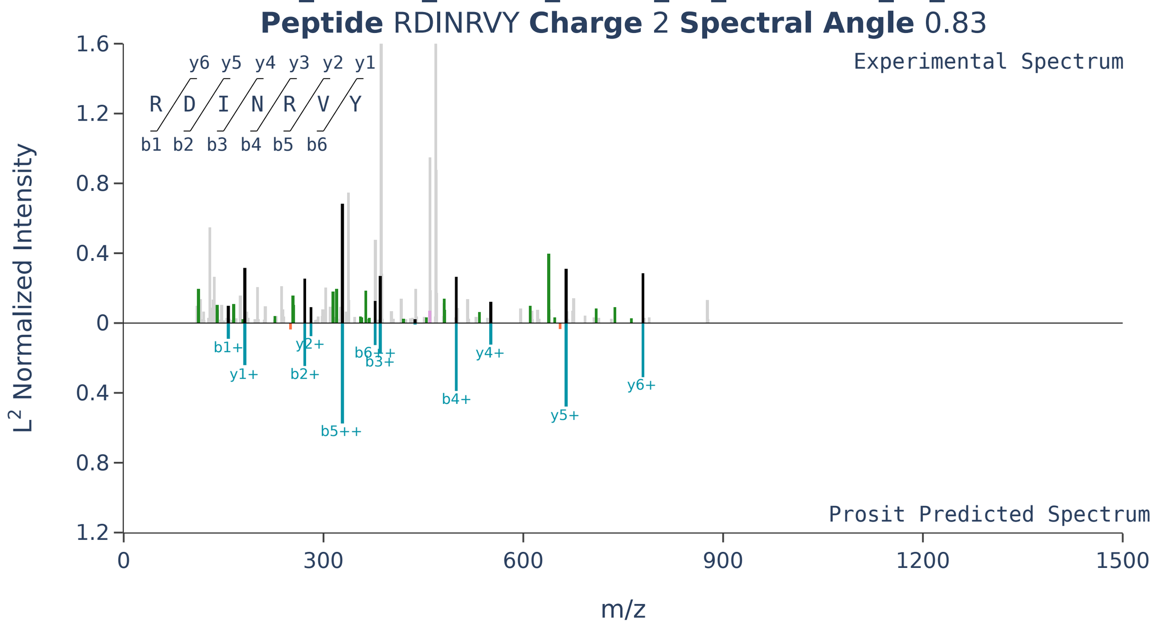


**Source** WSoh_301121_021221_HFGoe_M1_AnnexinA1_24h_R1 **Scan** 7257
**Peptide** REELKRDLA **Charge** 2 **Spectral Angle** 0.84

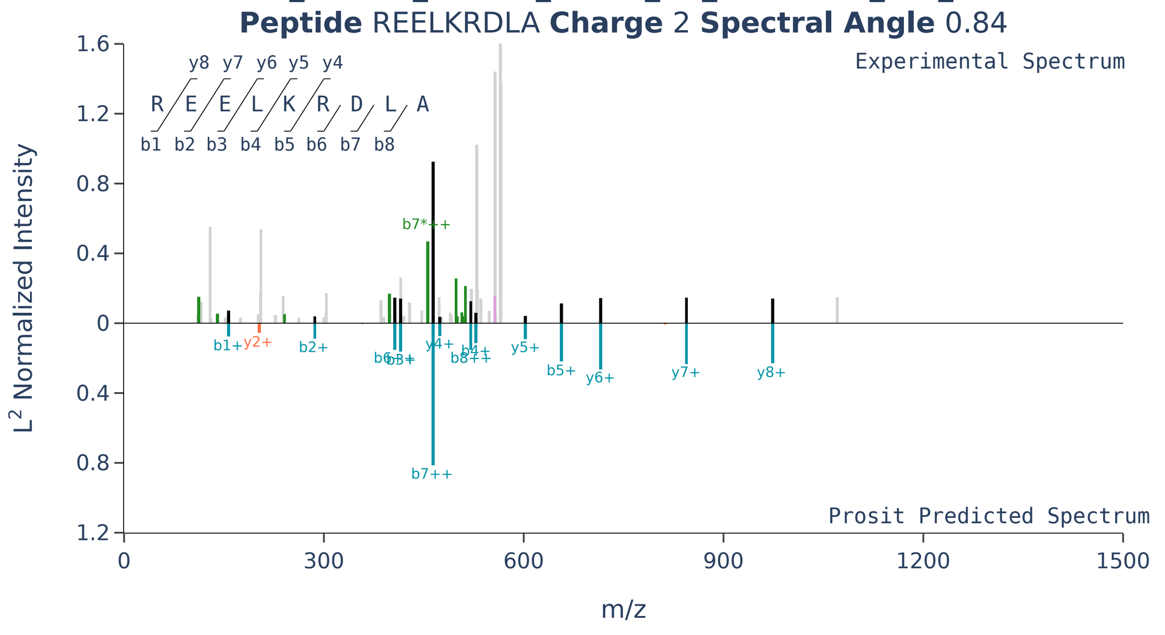


**Source** WSoh_301121_021221_HFGoe_M1_AnnexinA1_4h_R2 **Scan** 14997
**Peptide** KPAFFAEKLH **Charge** 3 **Spectral Angle** 0.78

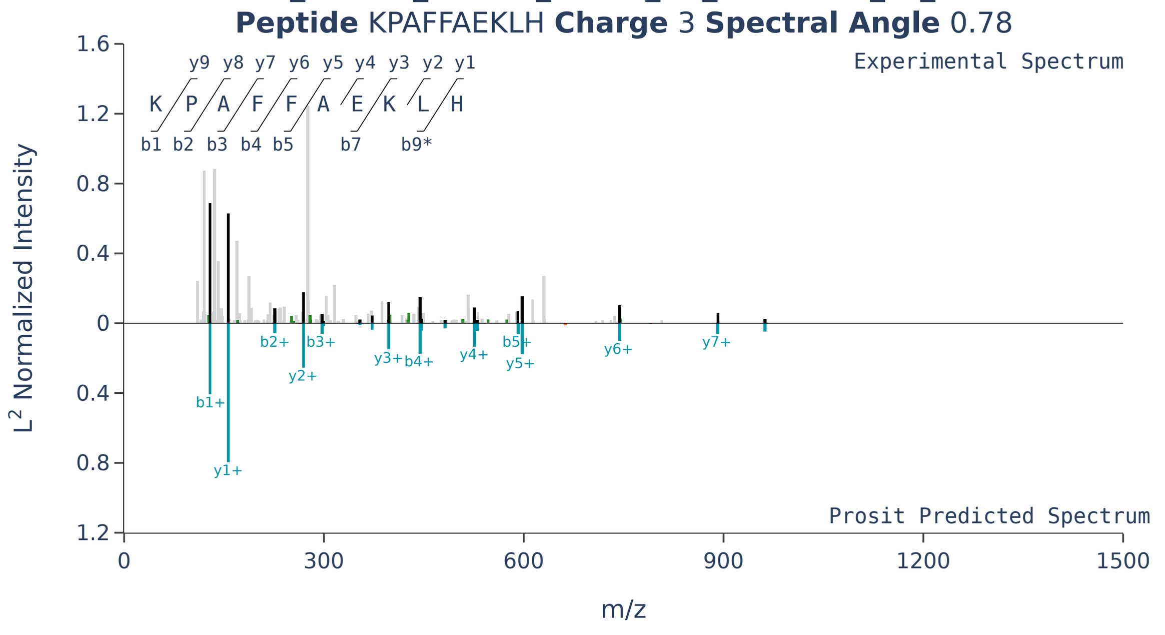


**Source** WSoh_301121_021221_HFGoe_M2_AnnexinA1_1h_R2 **Scan** 9535
**Peptide** NKEIRDINRVYRE **Charge** 4 **Spectral Angle** 0.87

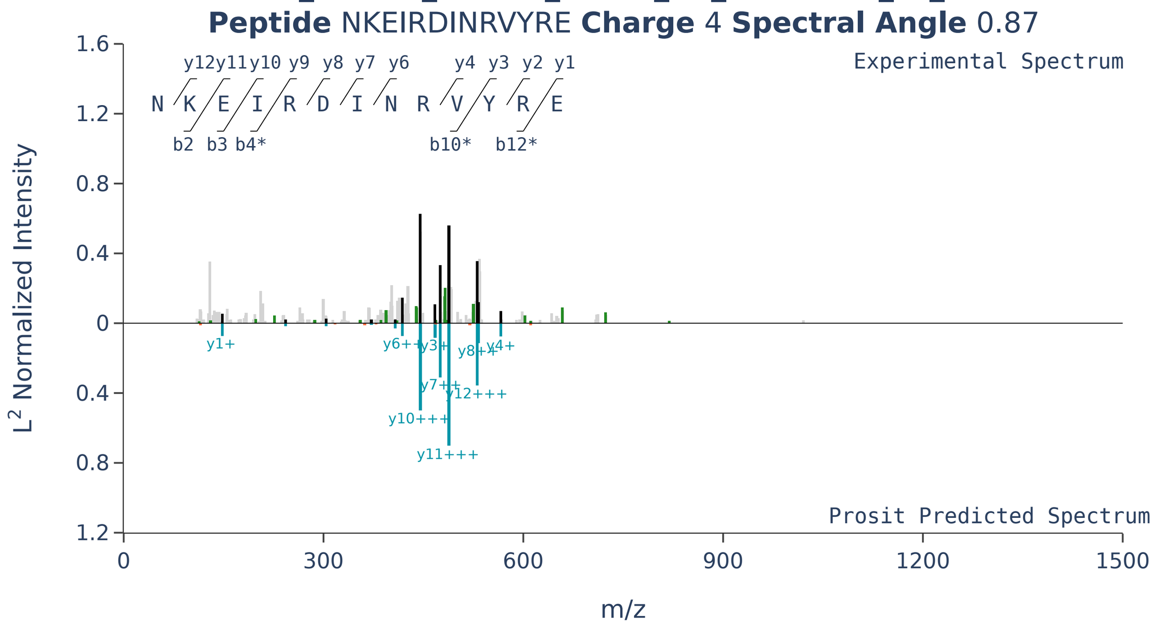


**Source** WSoh_301121_021221_HFGoe_M2_AnnexinA1_24h_R2 **Scan** 9145
**Peptide** MKGLGTD **Charge** 2 **Spectral Angle** 0.9

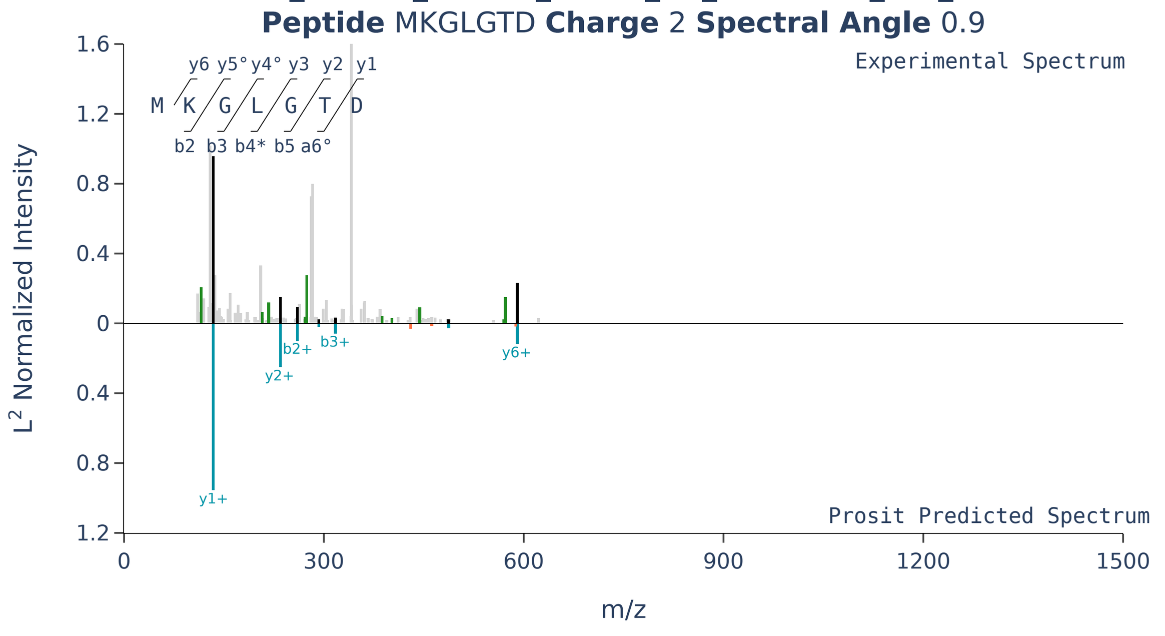


**Source** WSoh_301121_021221_HFGoe_M2_AnnexinA1_24h_R2 **Scan** 9336
**Peptide** KRDLAKDITSDT **Charge** 2 **Spectral Angle** 0.88

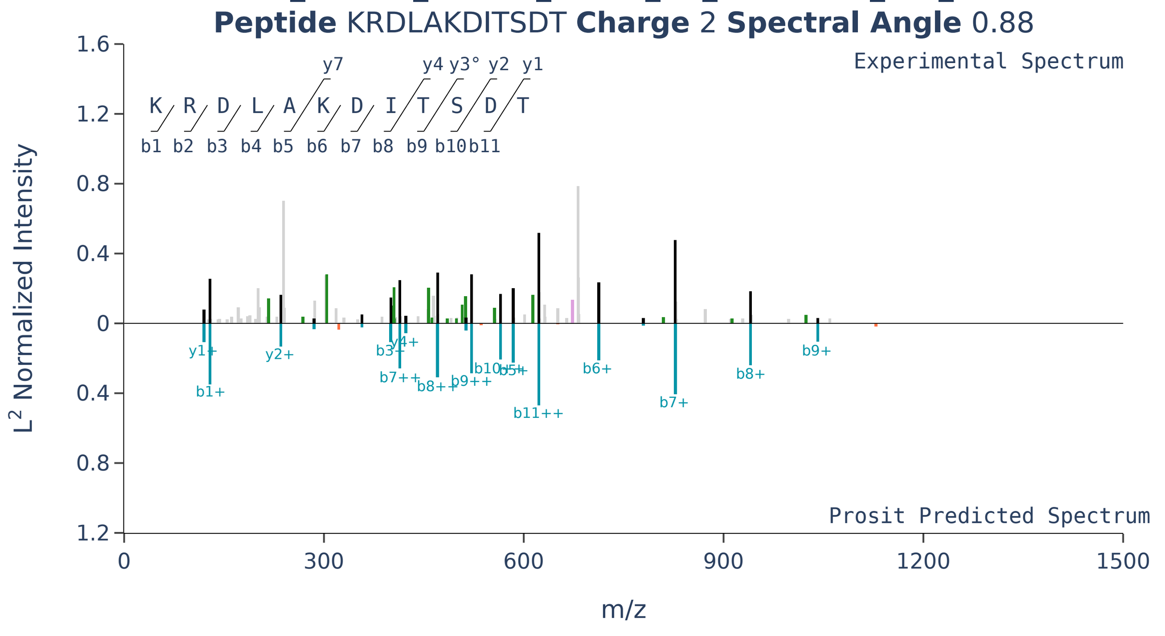


**Source** WSoh_301121_021221_HFGoe_M1_AnnexinA1_2h_R2 **Scan** 7425
**Peptide** QEYVQTVKSSK **Charge** 3 **Spectral Angle** 0.9

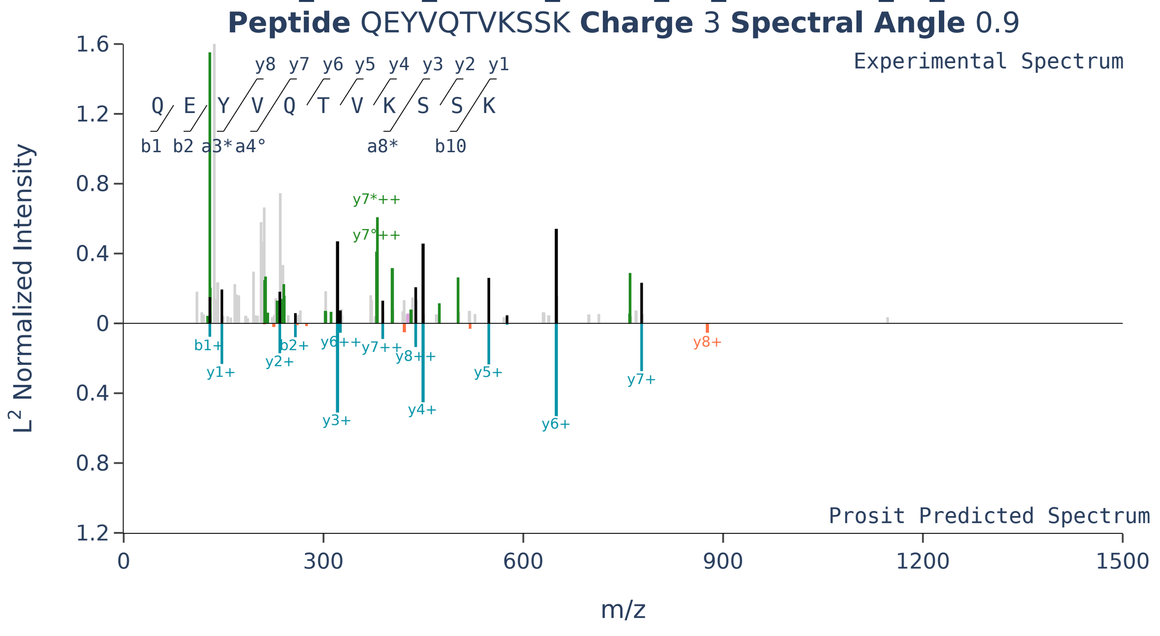


**Source** WSoh_301121_021221_HFGoe_M2_AnnexinA1_2h_R1 **Scan** 15916
**Peptide** TSKPAFFAEKLHQA **Charge** 2 **Spectral Angle** 0.88

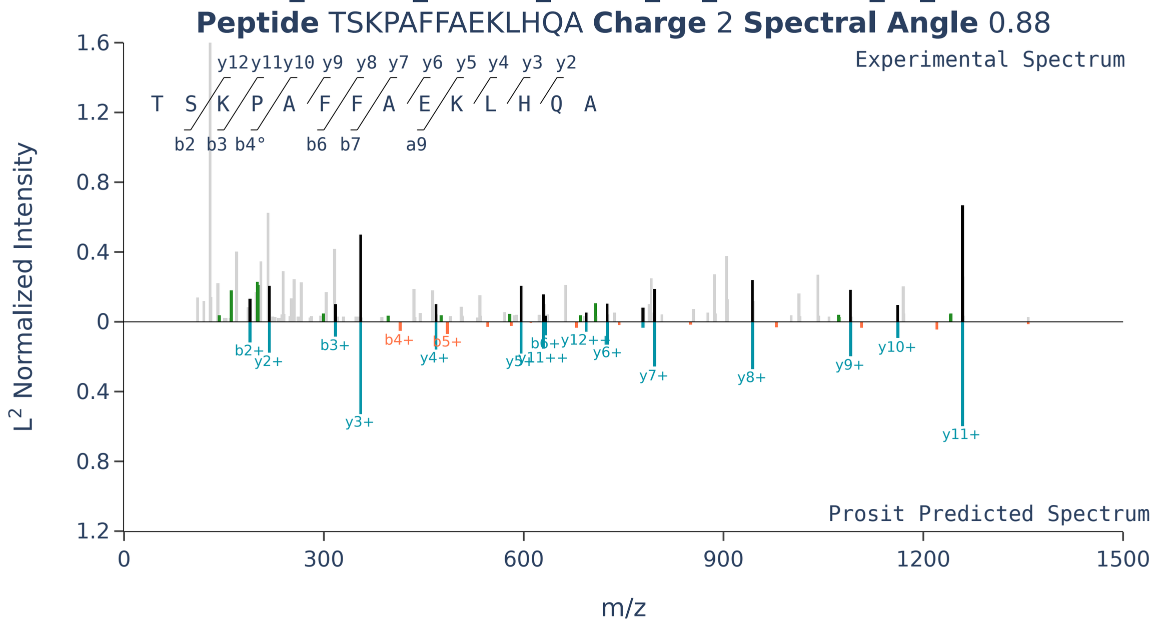


**Source** WSoh_301121_021221_HFGoe_M1_AnnexinA1_24h_R1 **Scan** 14600
**Peptide** FFAEKLH **Charge** 2 **Spectral Angle** 0.83

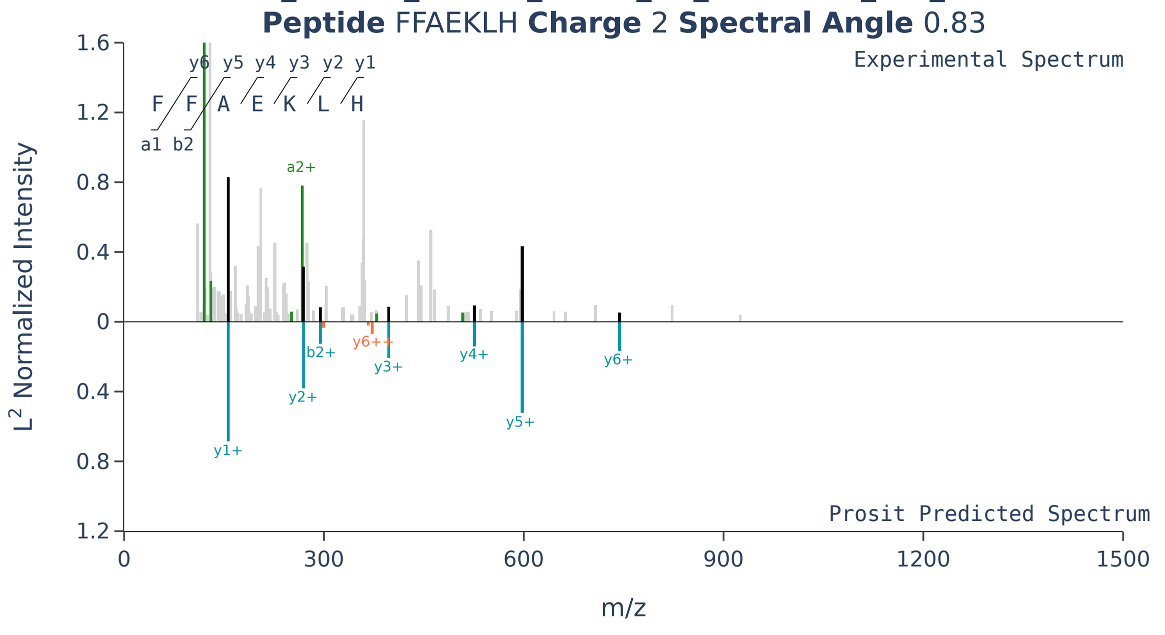


**Source** WSoh_301121_021221_HFGoe_M2_AnnexinA1_2h_R2 **Scan** 12759
**Peptide** NAQRQQIKAAYLQE **Charge** 3 **Spectral Angle** 0.86

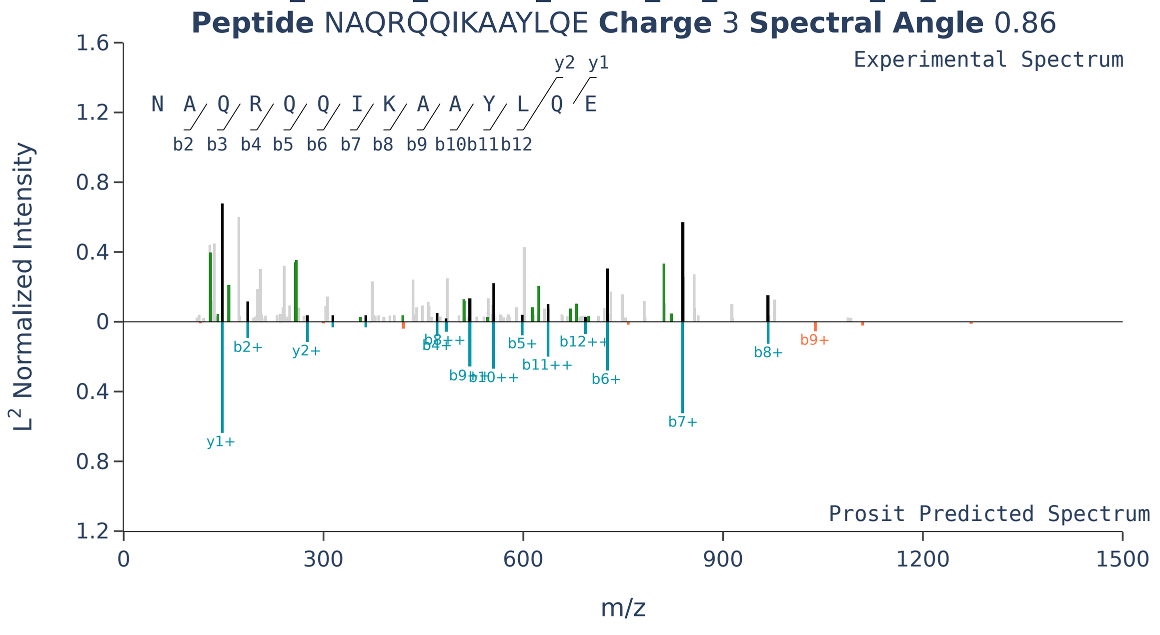


**Source** WSoh_301121_021221_HFGoe_M1_AnnexinA1_24h_R1 **Scan** 9396
**Peptide** RDLAKDIT **Charge** 2 **Spectral Angle** 0.88

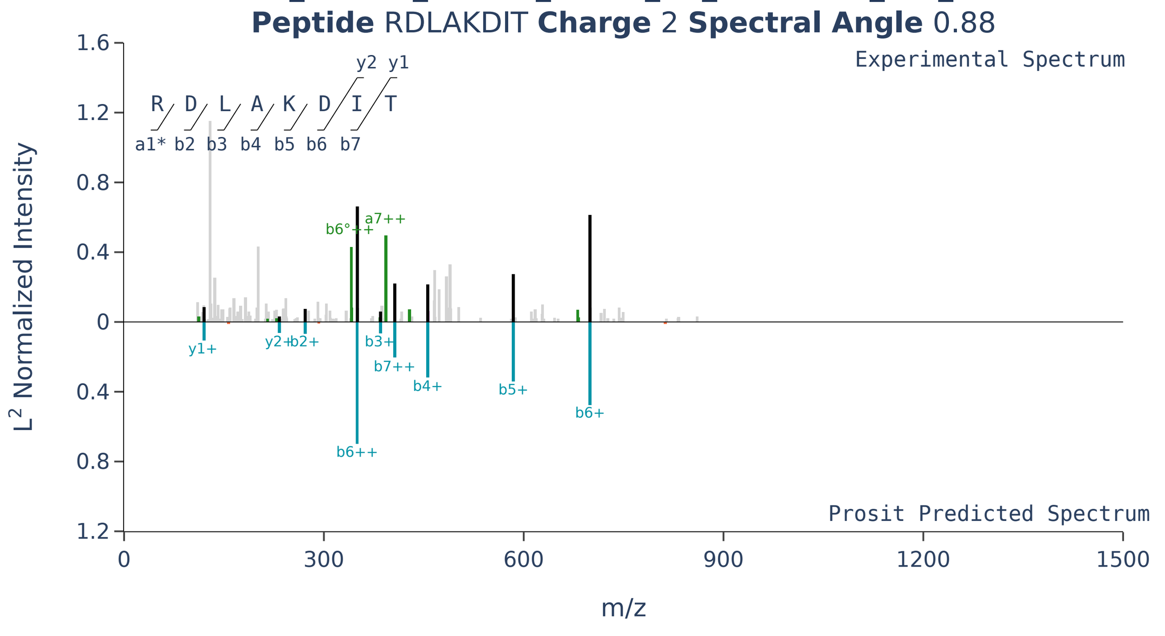


**Source** WSoh_301121_021221_HFGoe_M1_AnnexinA1_24h_R1 **Scan** 10165
**Peptide** AGERRKGTDVNVFN **Charge** 3 **Spectral Angle** 0.85

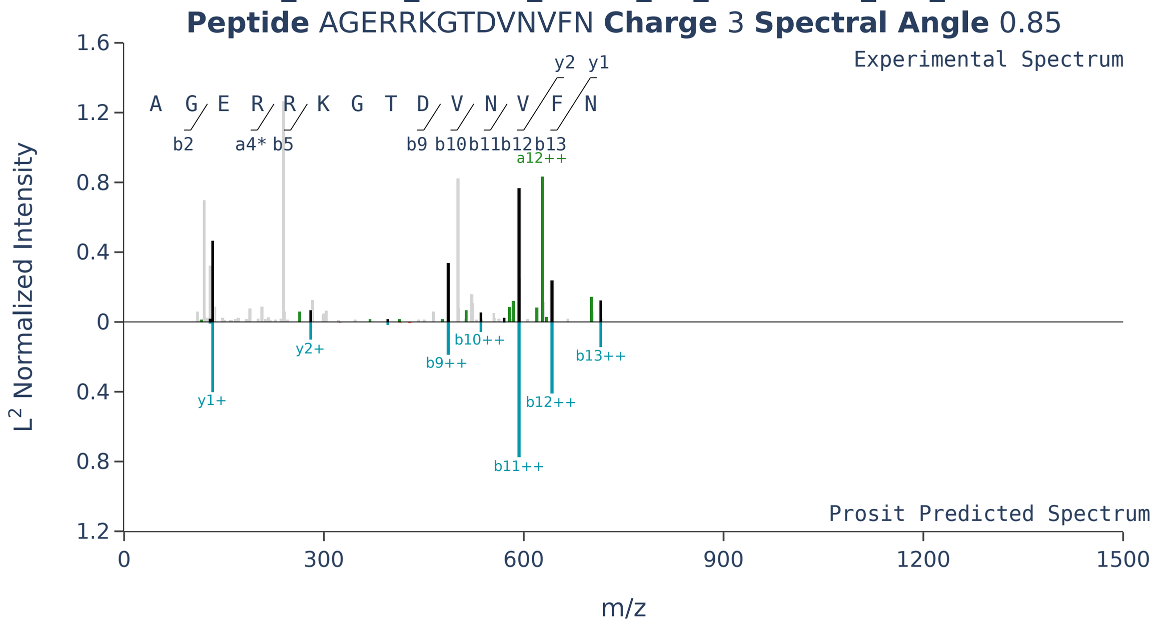


**Source** WSoh_301121_021221_HFGoe_M2_AnnexinA1_1h_R1 **Scan** 21948
**Peptide** SGDFRNALL **Charge** 2 **Spectral Angle** 0.76

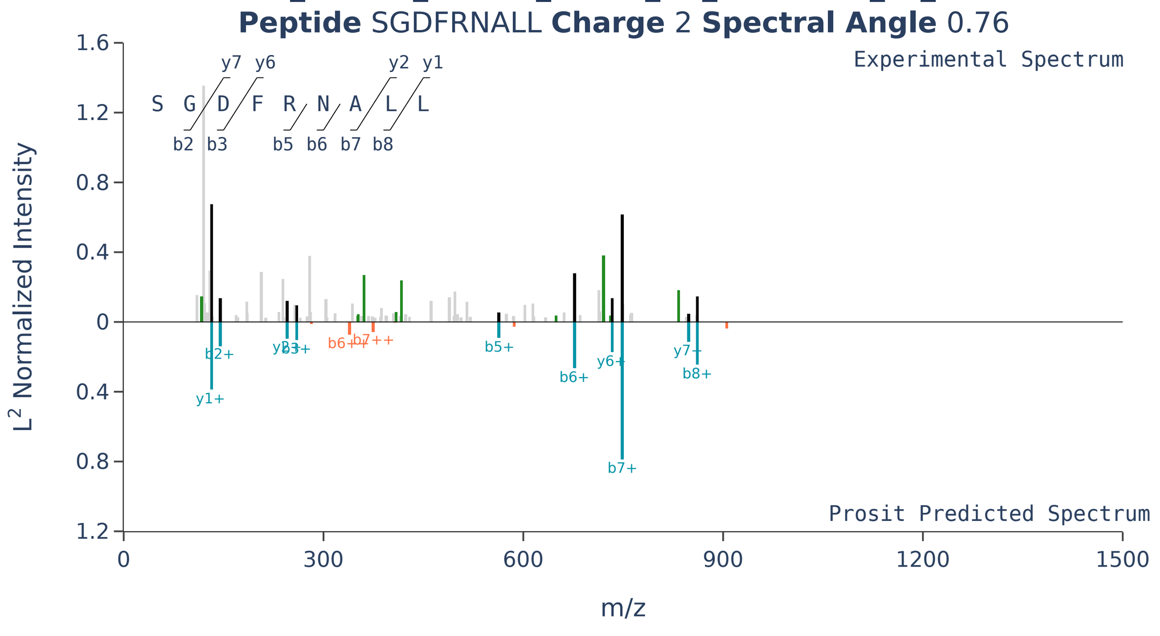

**Source** WSoh_301121_021221_HFGoe_M2_AnnexinA1_24h_R2 **Scan** 8696
**Peptide** GLGTDEDT **Charge** 1 **Spectral Angle** 0.87

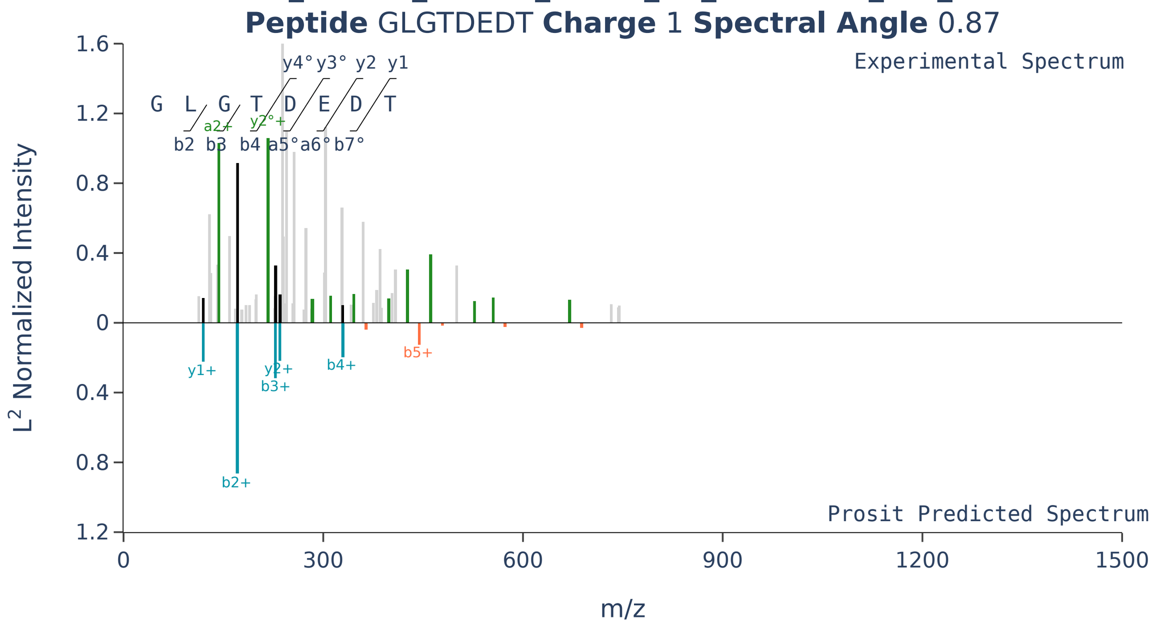


**Source** WSoh_301121_021221_HFGoe_M2_AnnexinA1_4h_R2 **Scan** 12777
**Peptide** SDARALYEAG **Charge** 2 **Spectral Angle** 0.88

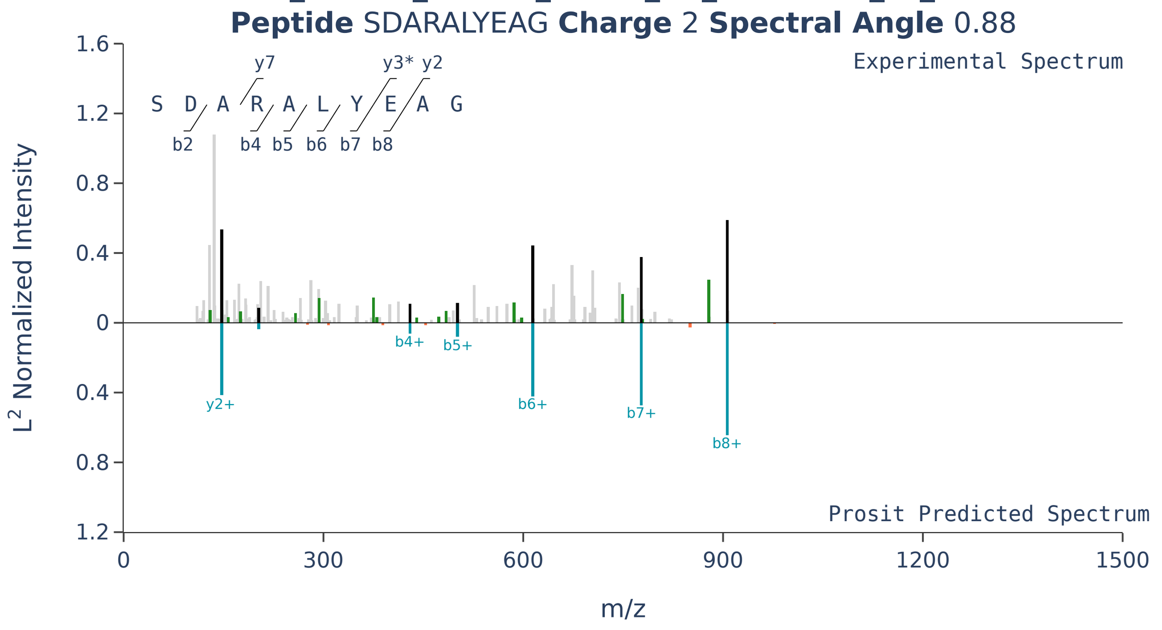


**Source** WSoh_301121_021221_HFGoe_M2_AnnexinA1_24h_R2 **Scan** 7692
**Peptide** SKHDMNKVL **Charge** 2 **Spectral Angle** 0.82

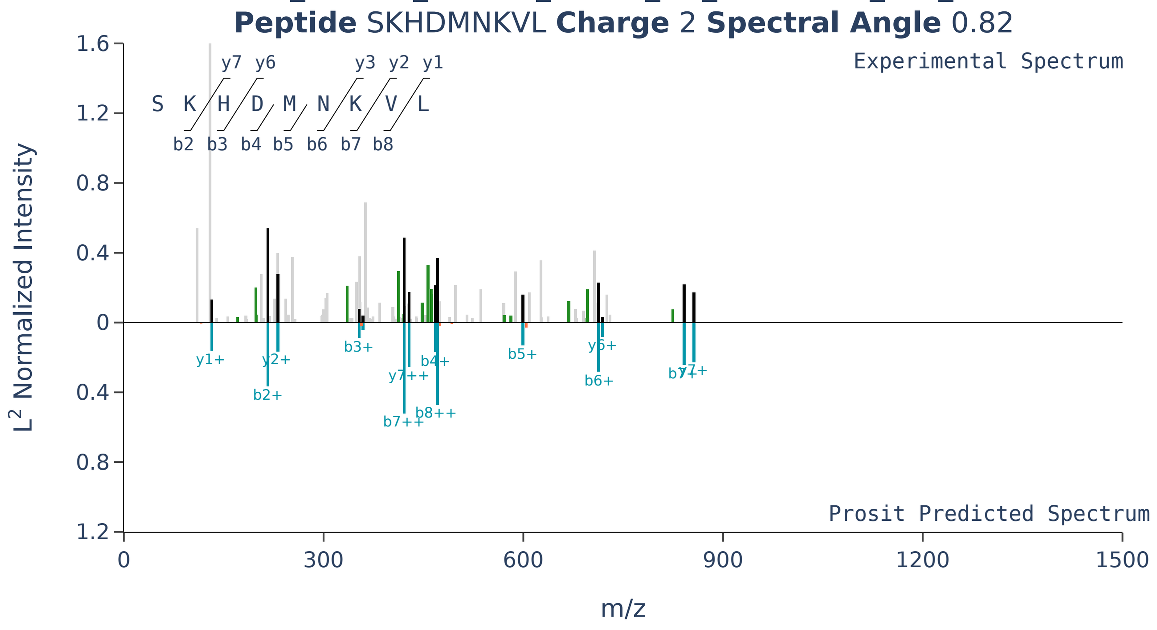


**Source** WSoh_301121_021221_HFGoe_M2_AnnexinA1_24h_R1 **Scan** 7320
**Peptide** RAAMKGLG **Charge** 2 **Spectral Angle** 0.83

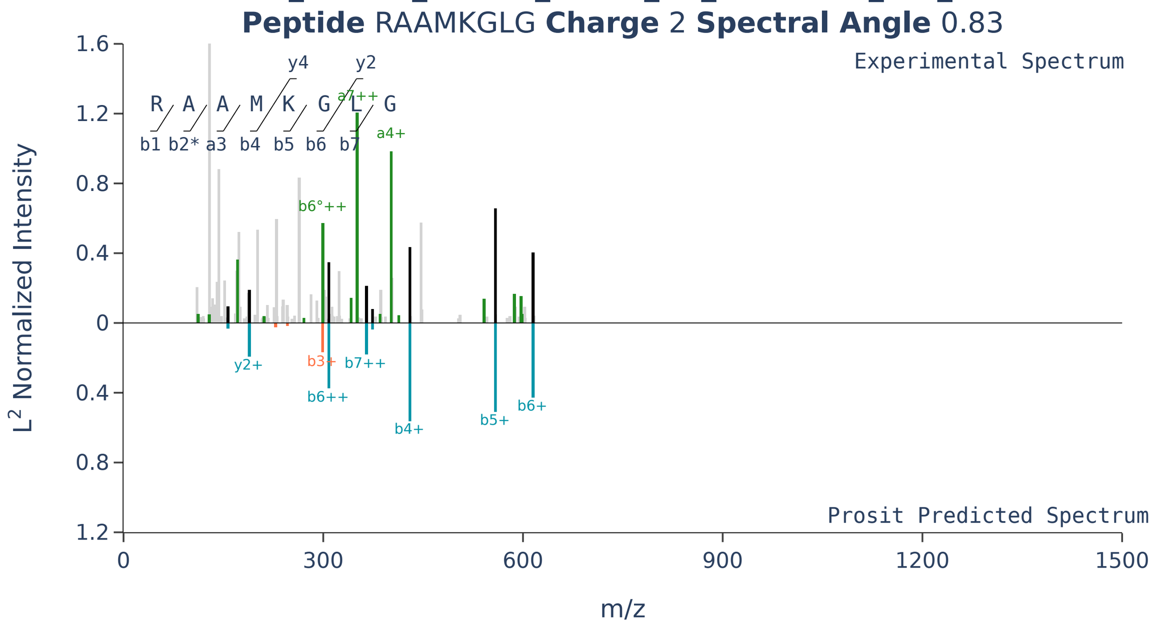


**Source** WSoh_301121_021221_HFGoe_M1_AnnexinA1_24h_R2 **Scan** 6826
**Peptide** LNRVYREE **Charge** 2 **Spectral Angle** 0.87

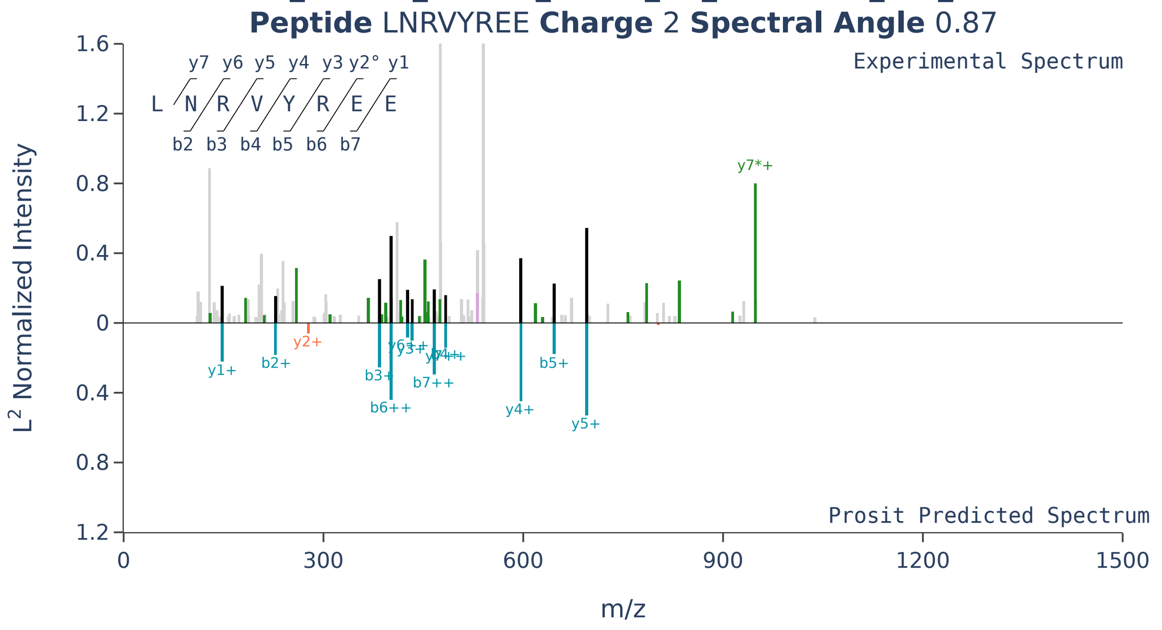


**Source** WSoh_301121_021221_HFGoe_M1_AnnexinA1_4h_R1 **Scan** 9289
**Peptide** ITSDTSGDFRN **Charge** 2 **Spectral Angle** 0.86

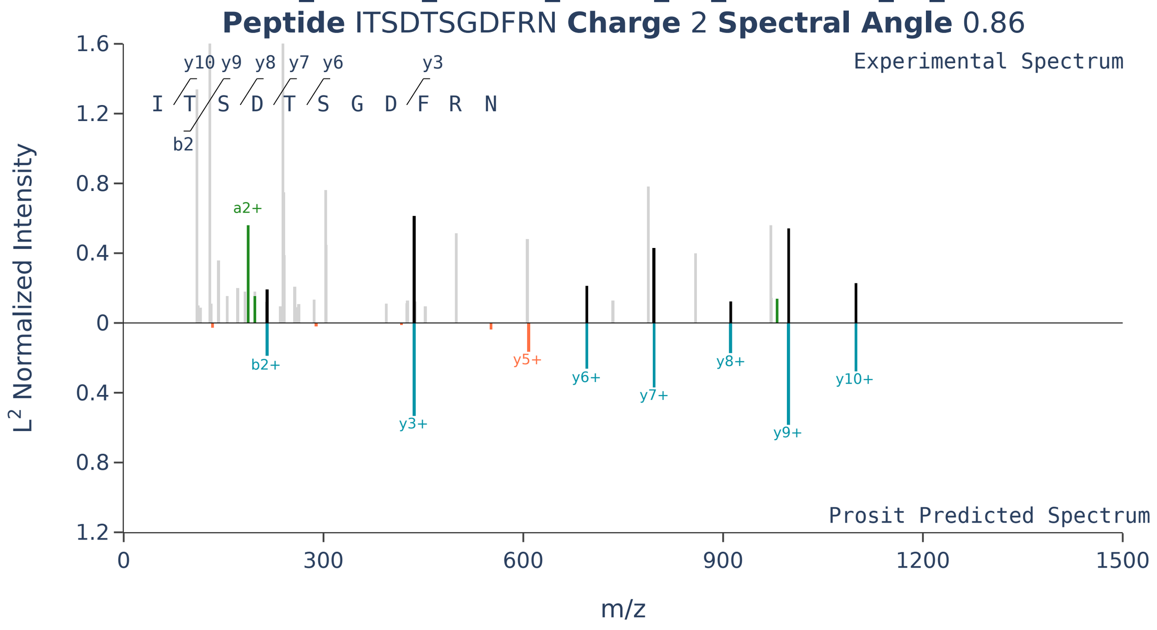


**Source** WSoh_301121_021221_HFGoe_M2_AnnexinA1_2h_R2 **Scan** 10302
**Peptide** LKRDLAKDITSD **Charge** 2 **Spectral Angle** 0.8

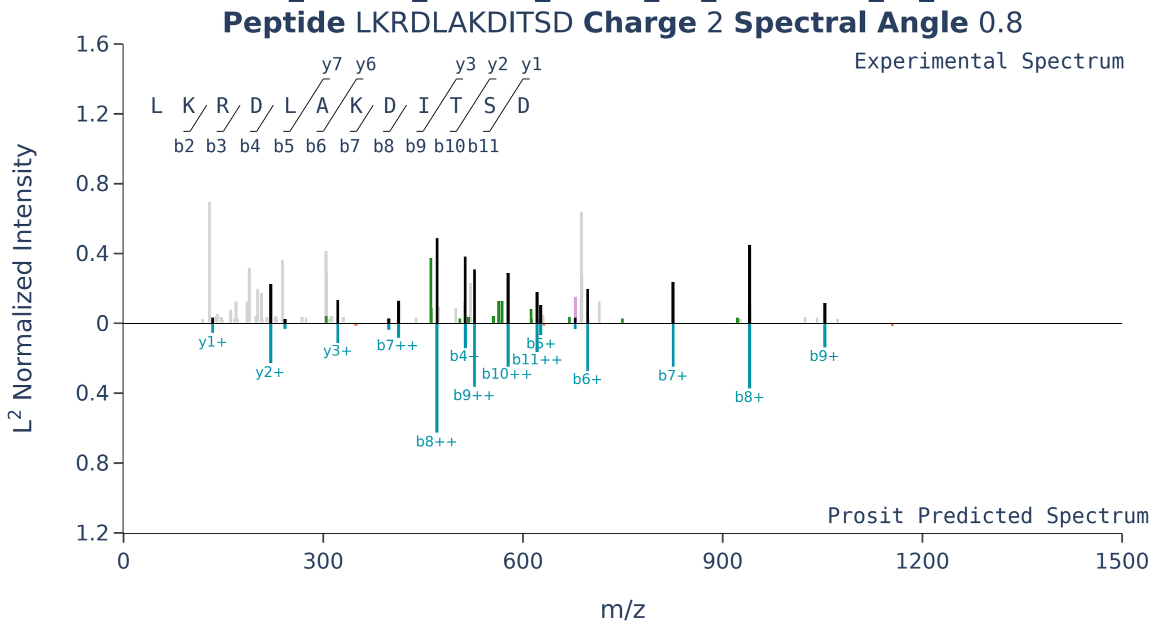


**Source** WSoh_301121_021221_HFGoe_M2_AnnexinA1_4h_R2 **Scan** 20060
**Peptide** RAAMKGLGTDEDTLI **Charge** 3 **Spectral Angle** 0.9

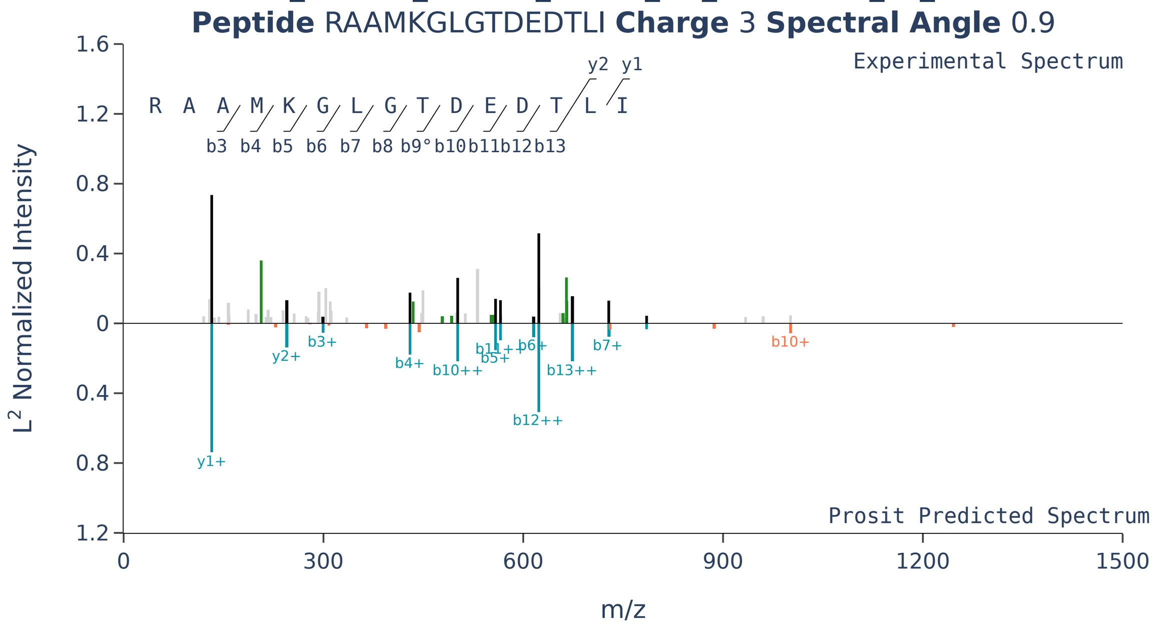


**Source** WSoh_301121_021221_HFGoe_M2_AnnexinA1_2h_R2 **Scan** 19177
**Peptide** FIENEEQEYVQTVKS **Charge** 3 **Spectral Angle** 0.87

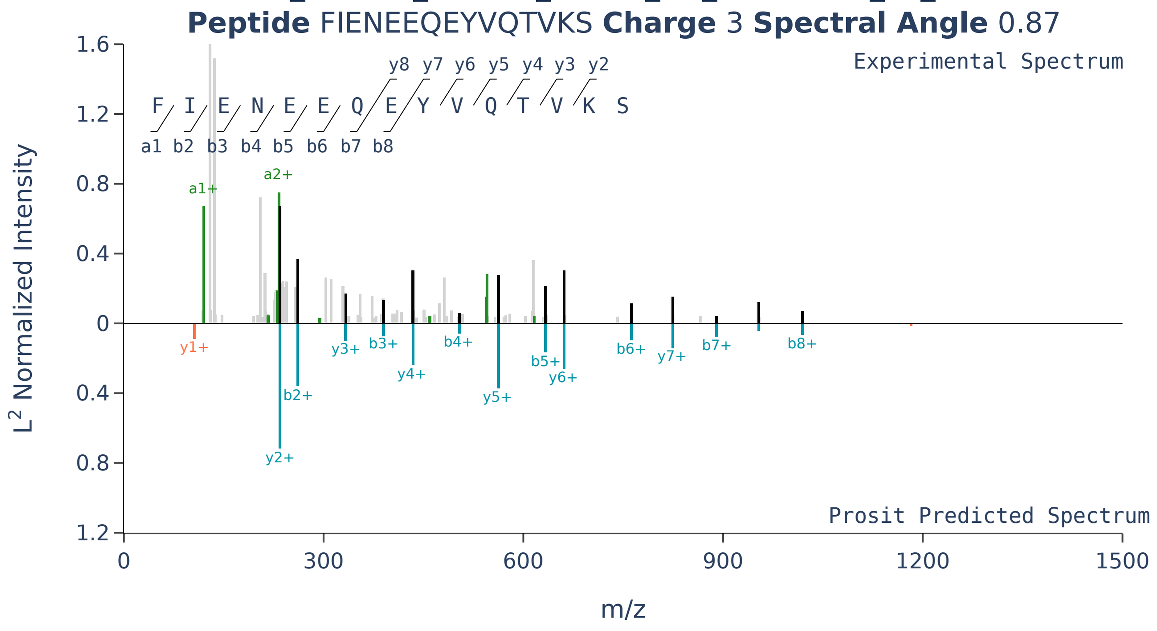


**Source** WSoh_301121_021221_HFGoe_M2_AnnexinA1_24h_R2 **Scan** 15559
**Peptide** SEDFGVNE **Charge** 2 **Spectral Angle** 0.81

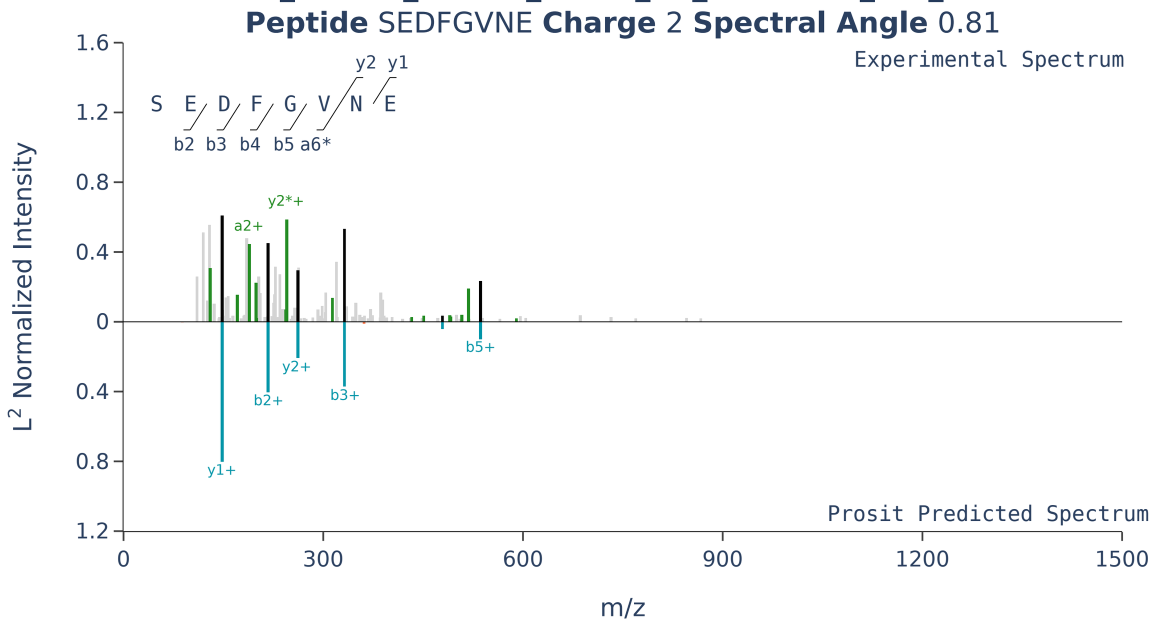


**Source** WSoh_301121_021221_HFGoe_M2_AnnexinA1_24h_R1 **Scan** 17223
**Peptide** KTPAQFDADELRA **Charge** 2 **Spectral Angle** 0.9

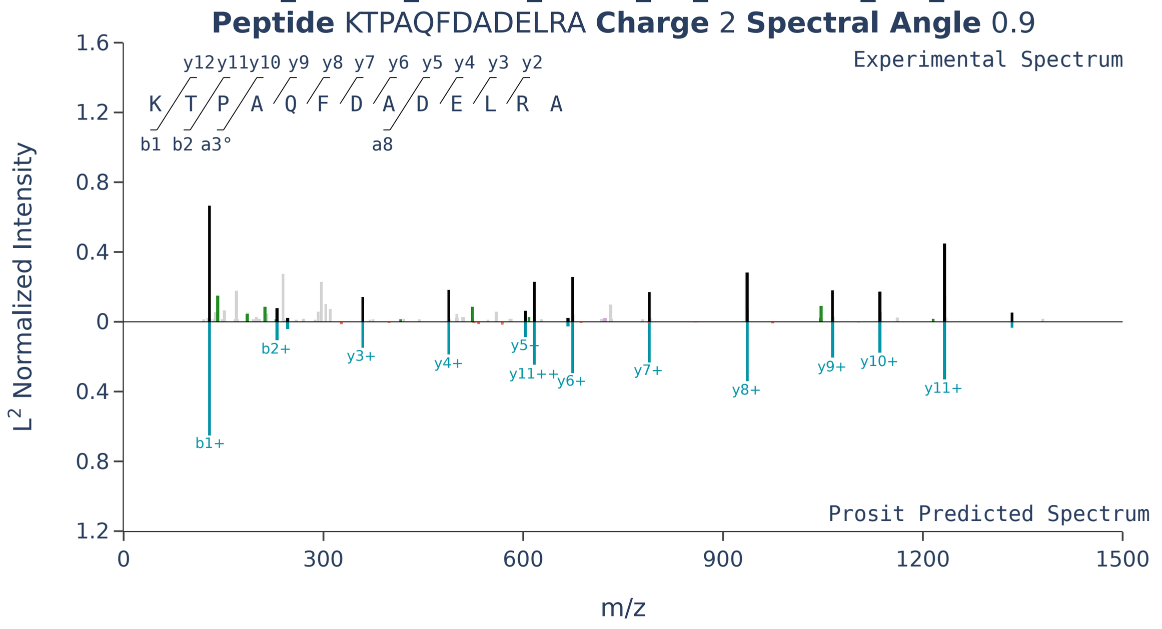


**Source** WSoh_301121_021221_HFGoe_M1_AnnexinA1_24h_R1 **Scan** 13827
**Peptide** ETLKKALTGHLE **Charge** 2 **Spectral Angle** 0.85

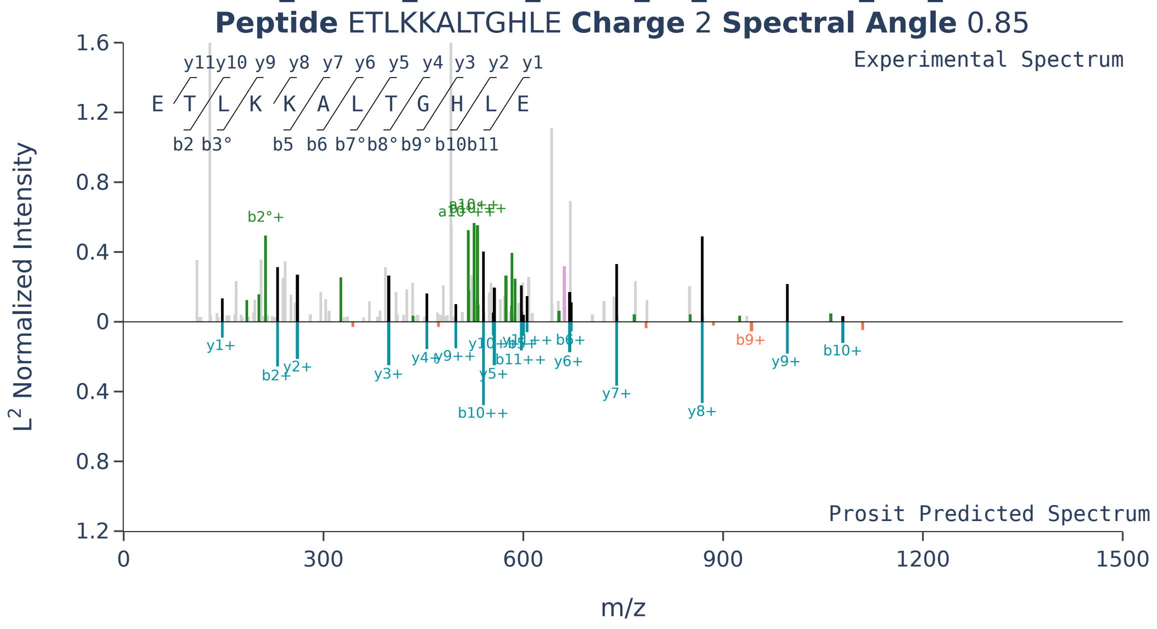


**Source** WSoh_301121_021221_HFGoe_M2_AnnexinA1_24h_R2 **Scan** 18083
**Peptide** FDADELRAA **Charge** 2 **Spectral Angle** 0.88

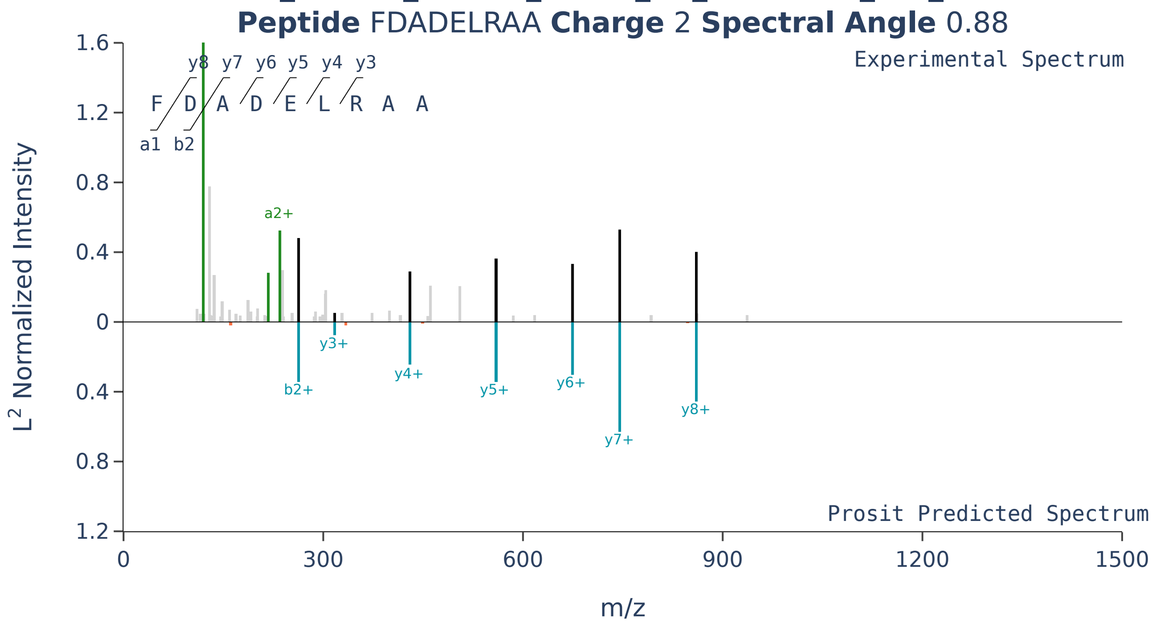


**Source** WSoh_301121_021221_HFGoe_M1_AnnexinA1_24h_R1 **Scan** 6861
**Peptide** INRVYREE **Charge** 2 **Spectral Angle** 0.87

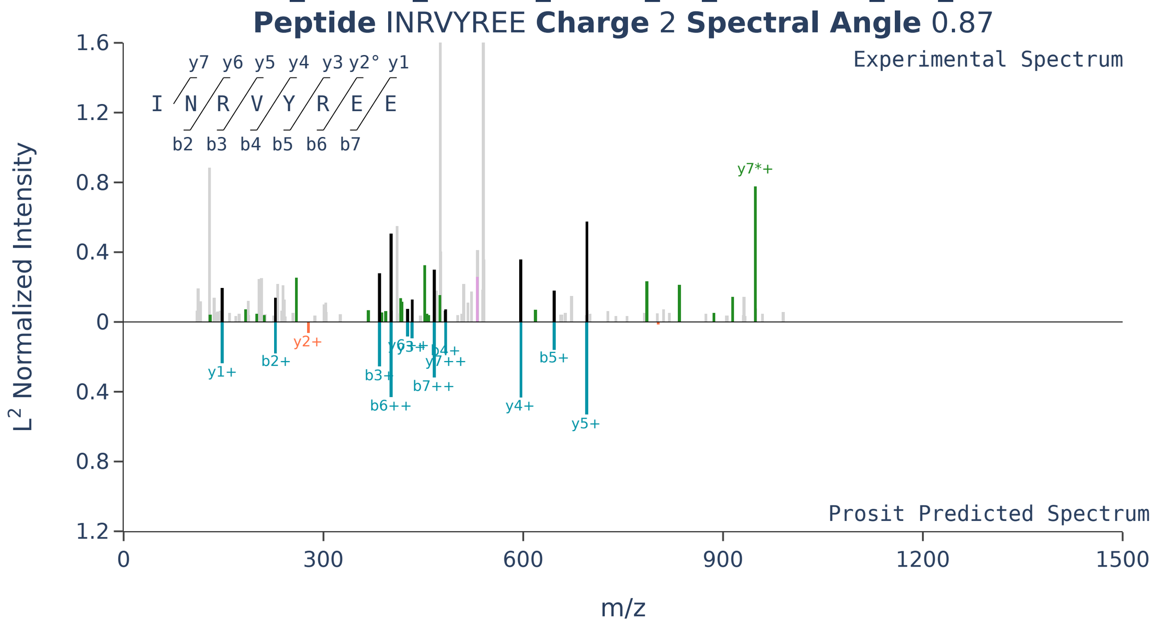


**Source** WSoh_301121_021221_HFGoe_M2_AnnexinA1_4h_R1 **Scan** 18341
**Peptide** KSSKGGPGSAVSPYPTF **Charge** 3 **Spectral Angle** 0.86

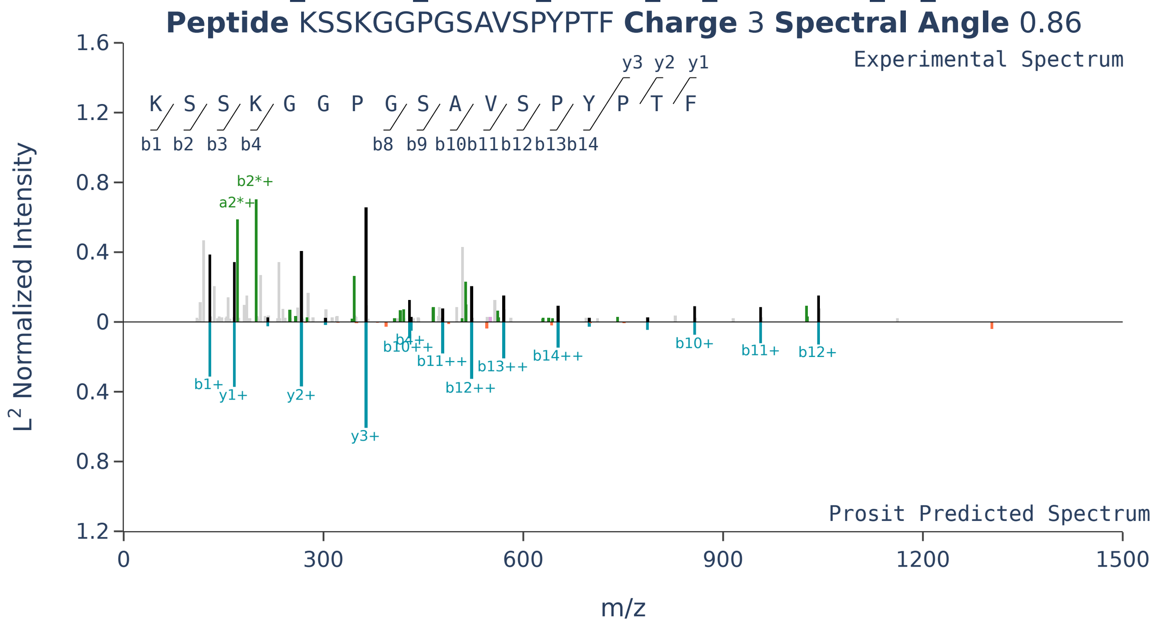

**Source** WSoh_301121_021221_HFGoe_M1_AnnexinA1_24h_R2 **Scan** 15415
**Peptide** SKPAFFAEKLHQA **Charge** 3 **Spectral Angle** 0.86

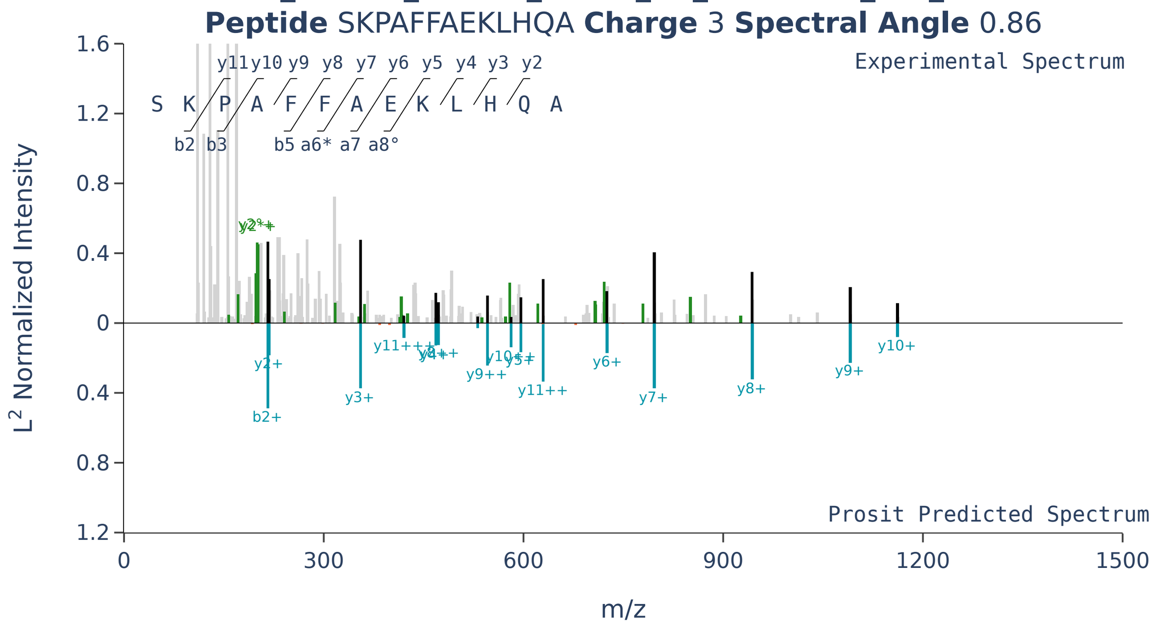


**Source** WSoh_301121_021221_HFGoe_M2_AnnexinA1_24h_R1 **Scan** 13883
**Peptide** ENEEQEYVQTVK **Charge** 2 **Spectral Angle** 0.8

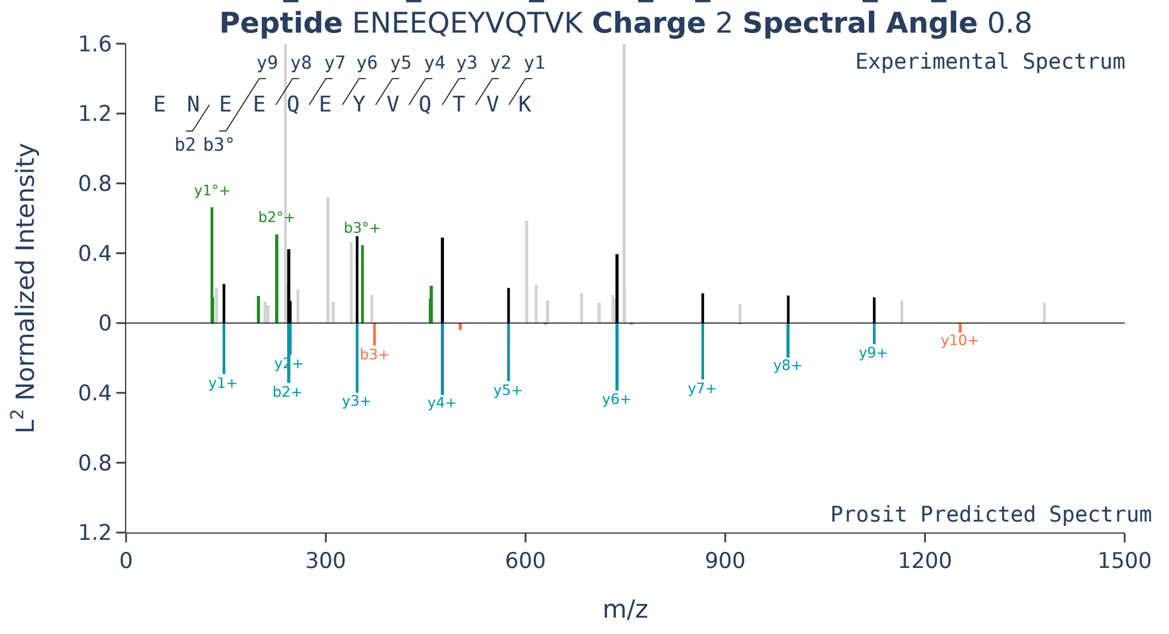


**Source** WSoh_301121_021221_HFGoe_M2_AnnexinA1_24h_R1 **Scan** 18759
**Peptide** PAFFAEKLHQA **Charge** 2 **Spectral Angle** 0.89

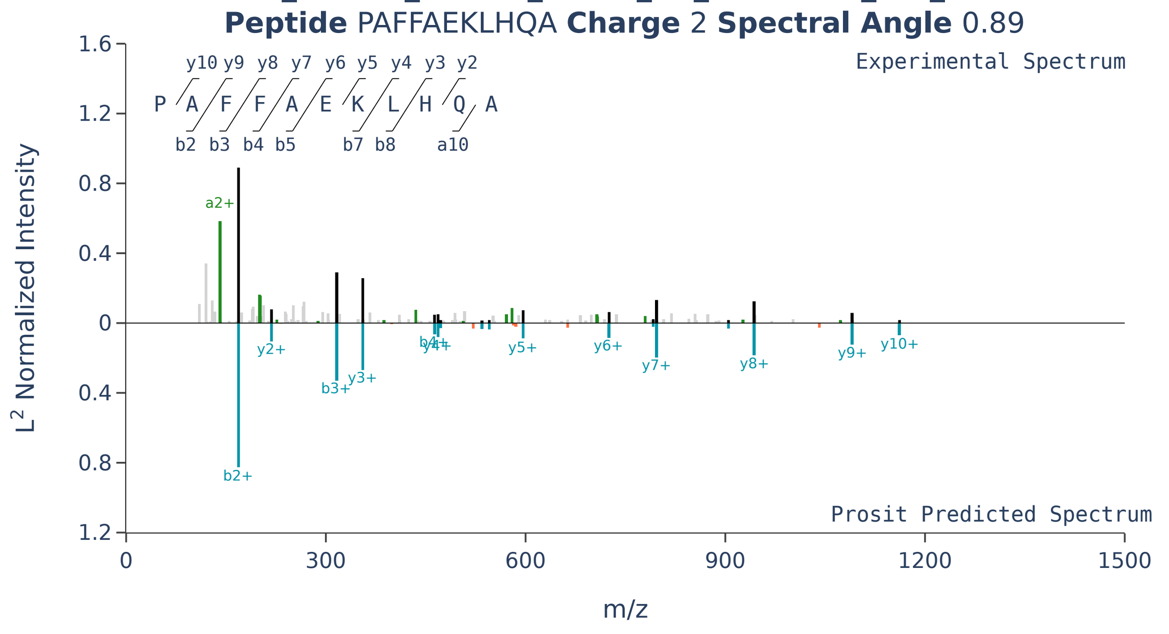


**Source** WSoh_301121_021221_HFGoe_M2_AnnexinA1_2h_R1 **Scan** 15155
**Peptide** IRDINRVYREEL **Charge** 3 **Spectral Angle** 0.86

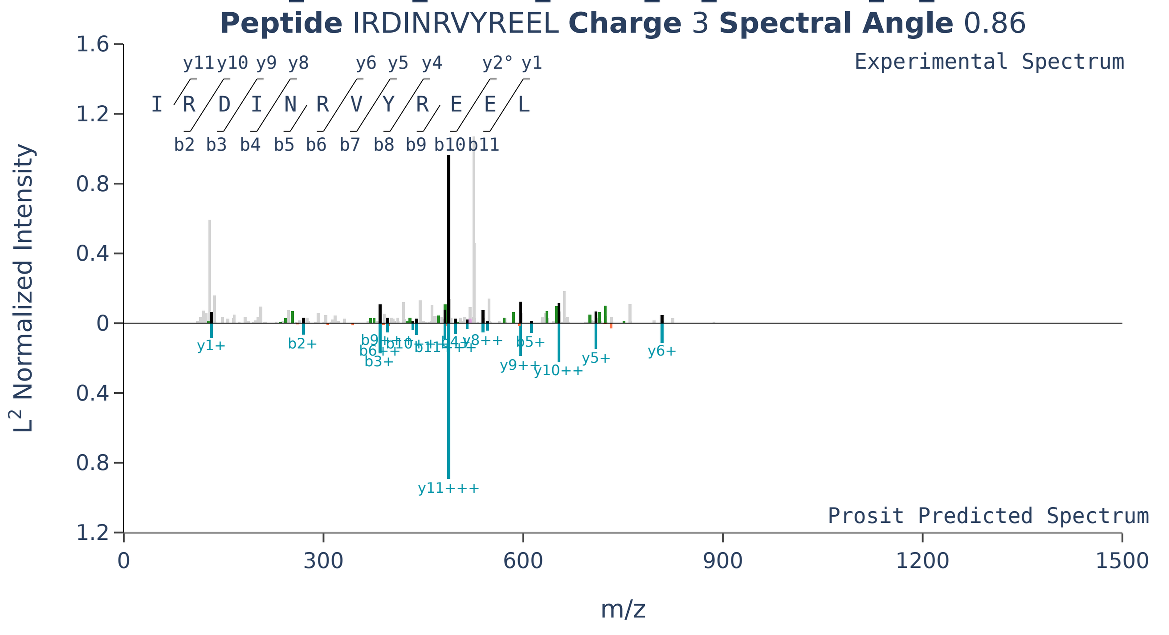


**Source** WSoh_301121_021221_HFGoe_M2_AnnexinA1_24h_R1 **Scan** 20518
**Peptide** PYPTFNPSSD **Charge** 2 **Spectral Angle** 0.92

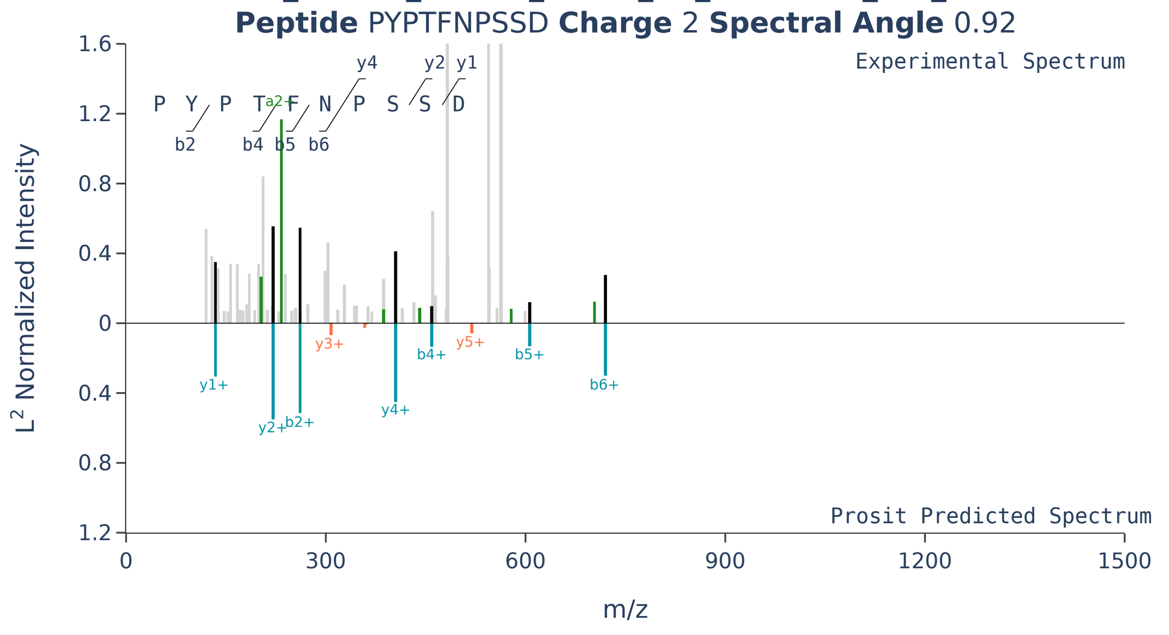


**Source** WSoh_301121_021221_HFGoe_M1_AnnexinA1_2h_R2 **Scan** 16705
**Peptide** IEILASRTN **Charge** 2 **Spectral Angle** 0.88

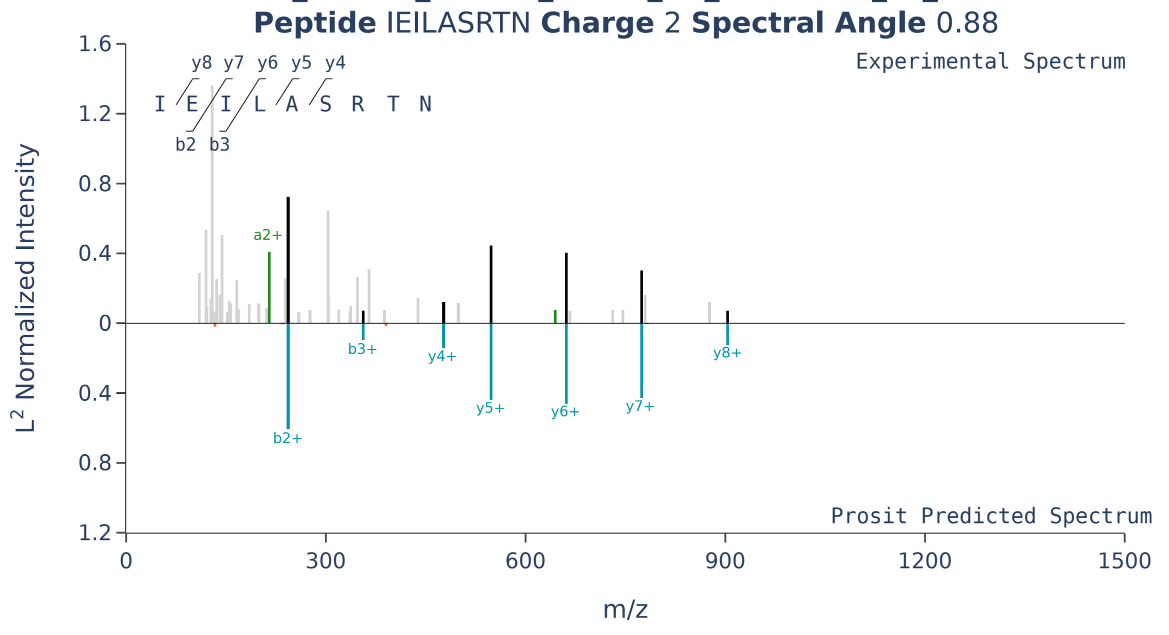


**Source** WSoh_301121_021221_HFGoe_M1_AnnexinA1_1h_R1 **Scan** 12298
**Peptide** RDINRVYREEL **Charge** 3 **Spectral Angle** 0.83

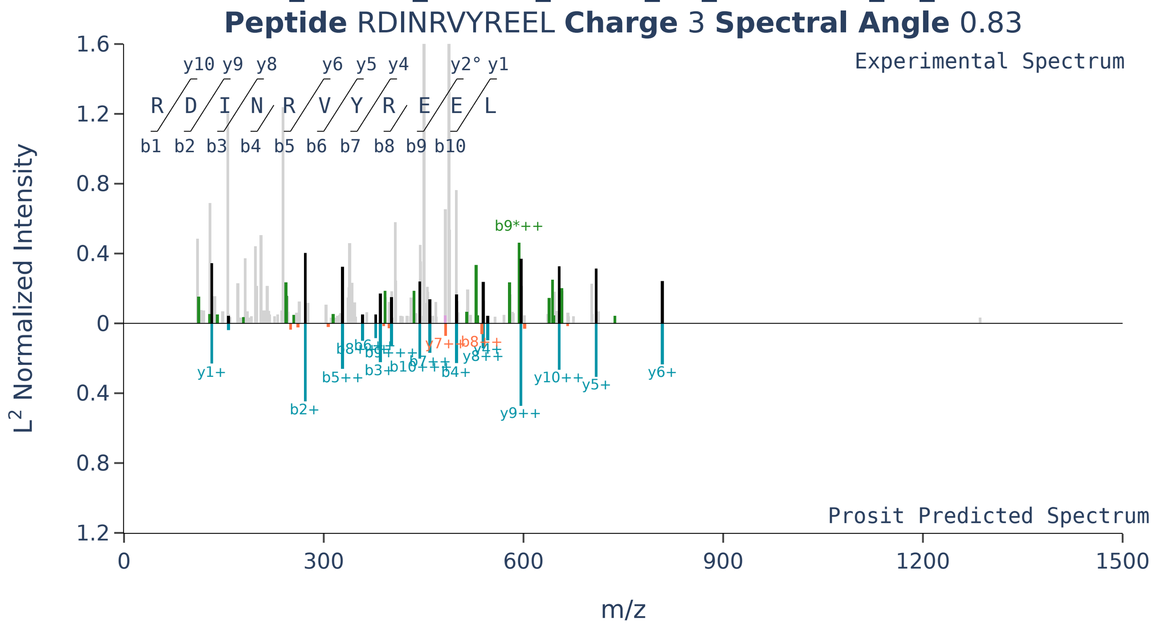


**Source** WSoh_301121_021221_HFGoe_M2_AnnexinA1_24h_R2 **Scan** 7051
**Peptide** SRTNKEIRDI **Charge** 3 **Spectral Angle** 0.84

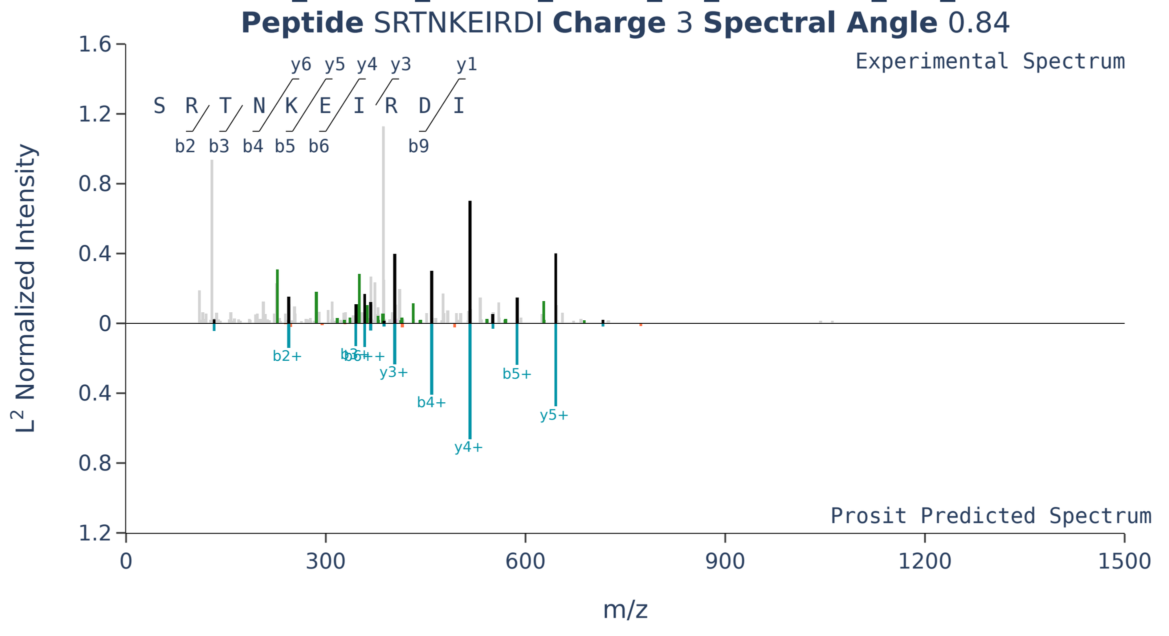


**Source** WSoh_301121_021221_HFGoe_M2_AnnexinA1_24h_R1 **Scan** 12431
**Peptide** LDETKGDYEKI **Charge** 3 **Spectral Angle** 0.88

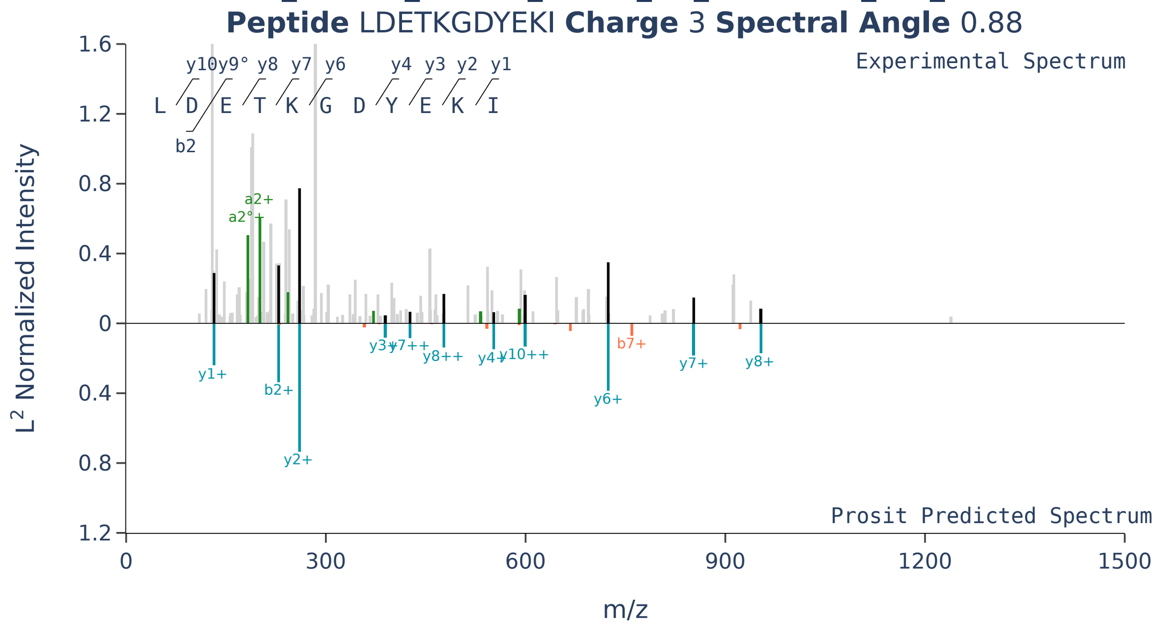


**Source** WSoh_301121_021221_HFGoe_M1_AnnexinA1_4h_R1 **Scan** 8327
**Peptide** SDARALYEAGERRKG **Charge** 4 **Spectral Angle** 0.87

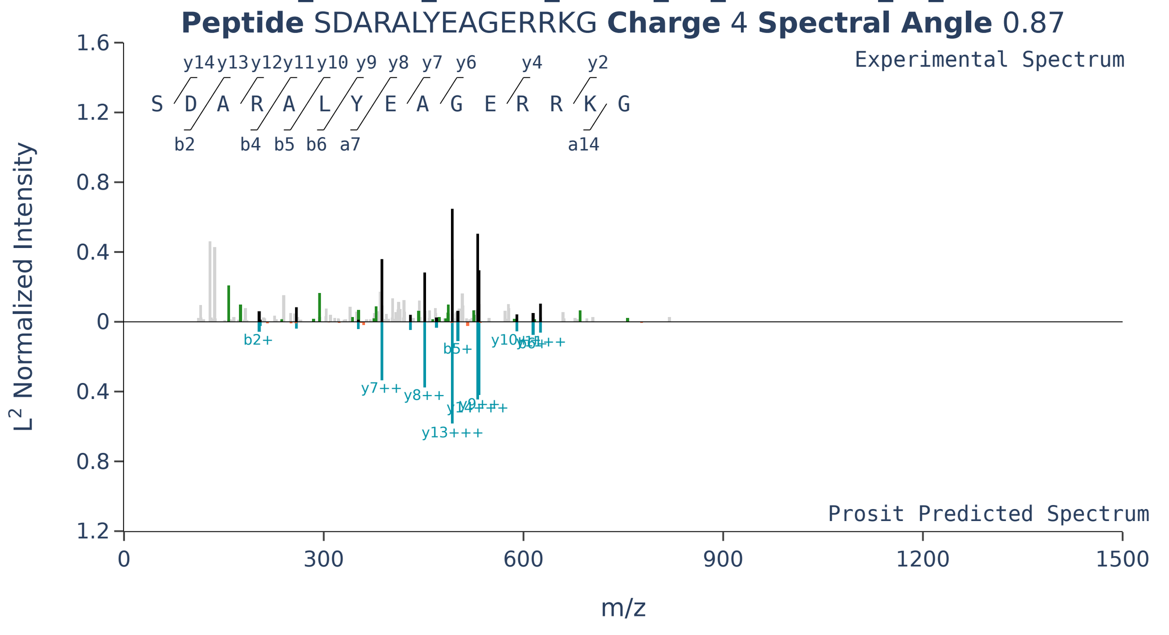


**Source** WSoh_301121_021221_HFGoe_M2_AnnexinA1_2h_R1 **Scan** 24289
**Peptide** RSEIDMNDIKAFYQ **Charge** 3 **Spectral Angle** 0.93

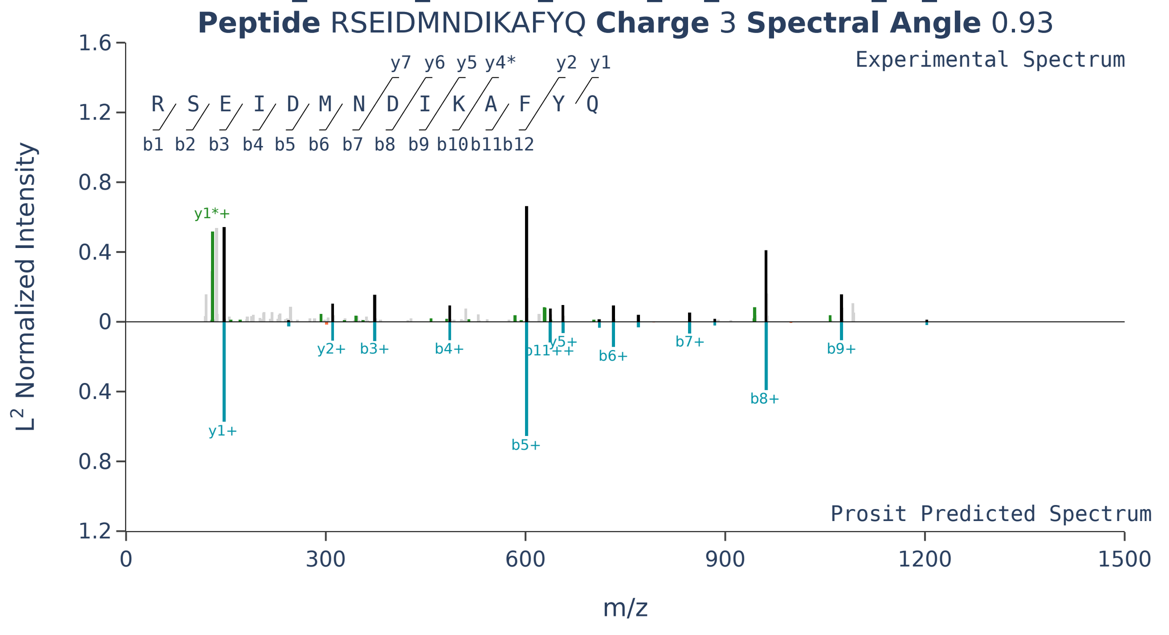


**Source** WSoh_301121_021221_HFGoe_M2_AnnexinA1_4h_R1 **Scan** 18682
**Peptide** RSEDFGVNEDLAD **Charge** 2 **Spectral Angle** 0.87

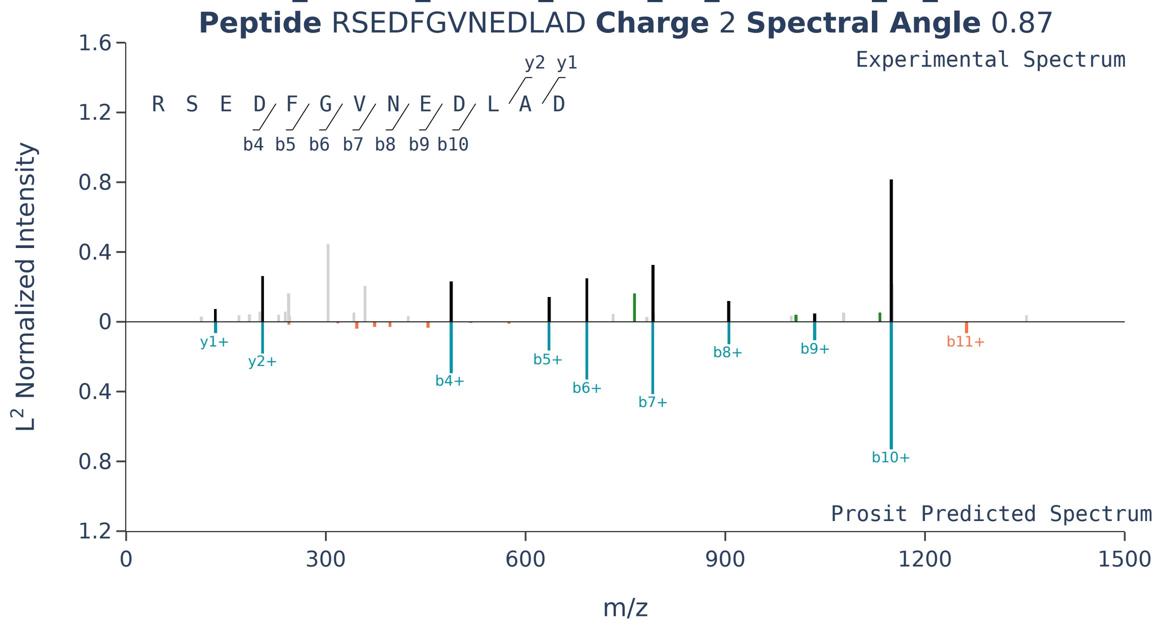


**Source** WSoh_301121_021221_HFGoe_M2_AnnexinA1_2h_R2 **Scan** 11698
**Peptide** ALTGHLEE **Charge** 2 **Spectral Angle** 0.84

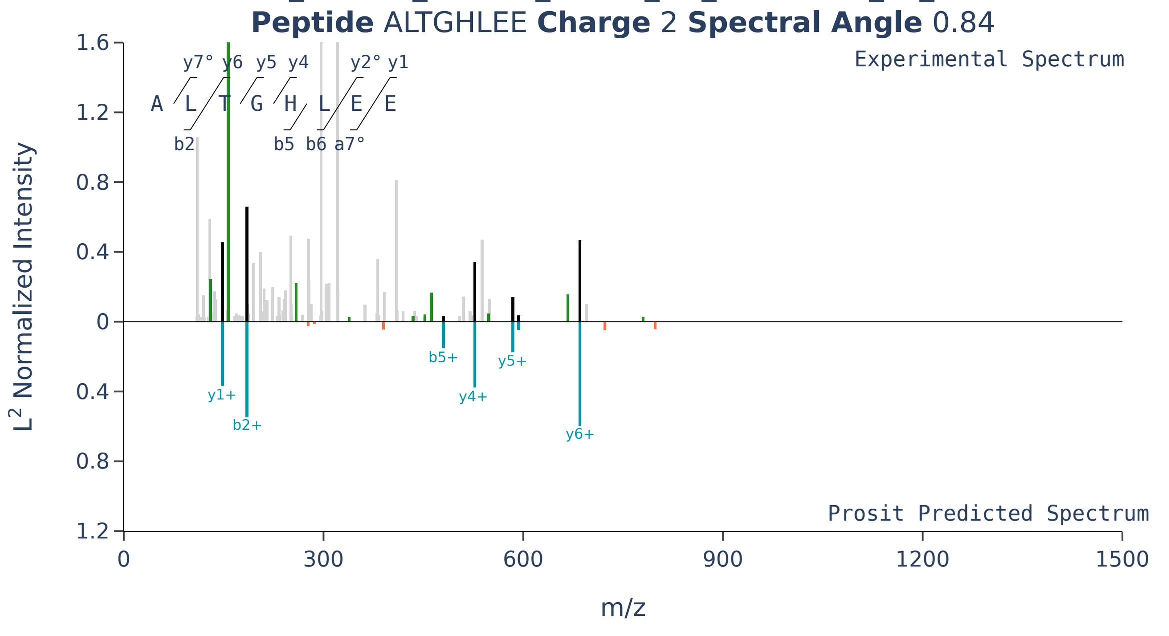


**Source** WSoh_301121_021221_HFGoe_M2_AnnexinA1_24h_R2 **Scan** 20277
**Peptide** AMVSEFLKQA **Charge** 2 **Spectral Angle** 0.9

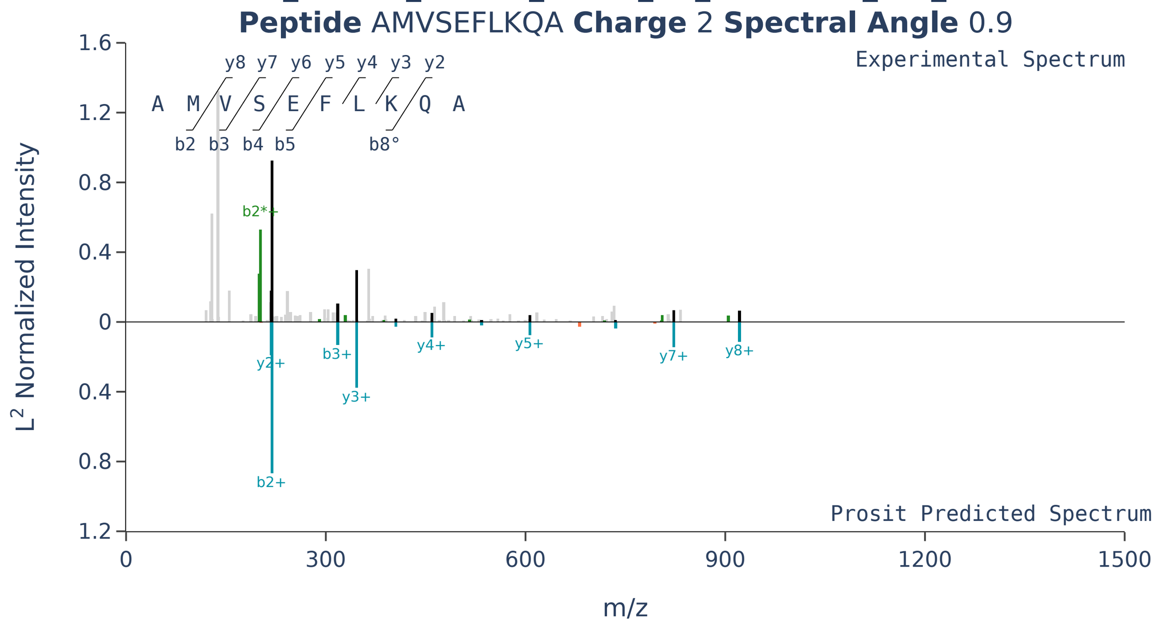


**Source** WSoh_301121_021221_HFGoe_M1_AnnexinA1_24h_R2 **Scan** 17122
**Peptide** DLAKDITSDT **Charge** 1 **Spectral Angle** 0.88

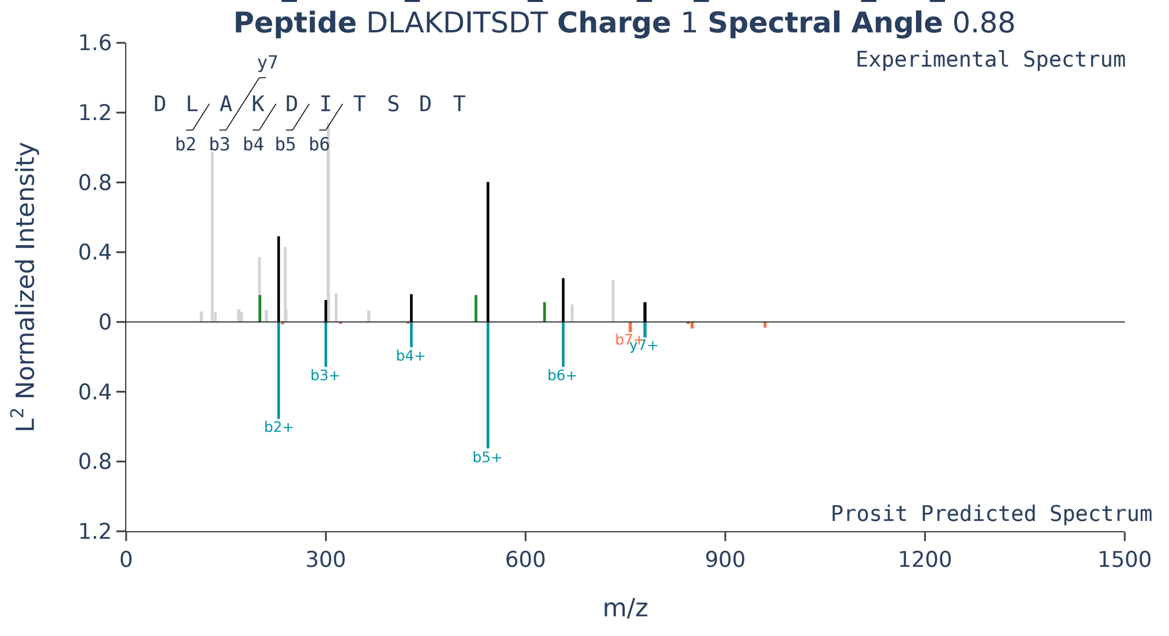

**Source** WSoh_301121_021221_HFGoe_M2_AnnexinA1_2h_R2 **Scan** 8358
**Peptide** RAAMKGLGTDE **Charge** 3 **Spectral Angle** 0.86

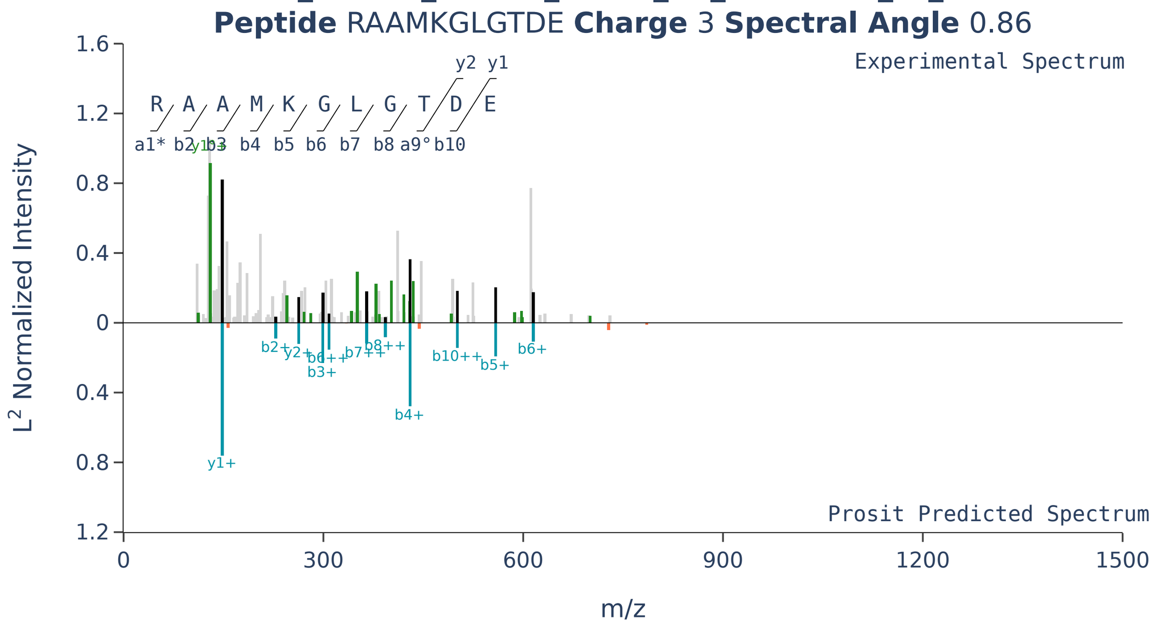


**Source** WSoh_301121_021221_HFGoe_M1_AnnexinA1_4h_R2 **Scan** 10196
**Peptide** RDLAKDITSD **Charge** 2 **Spectral Angle** 0.88

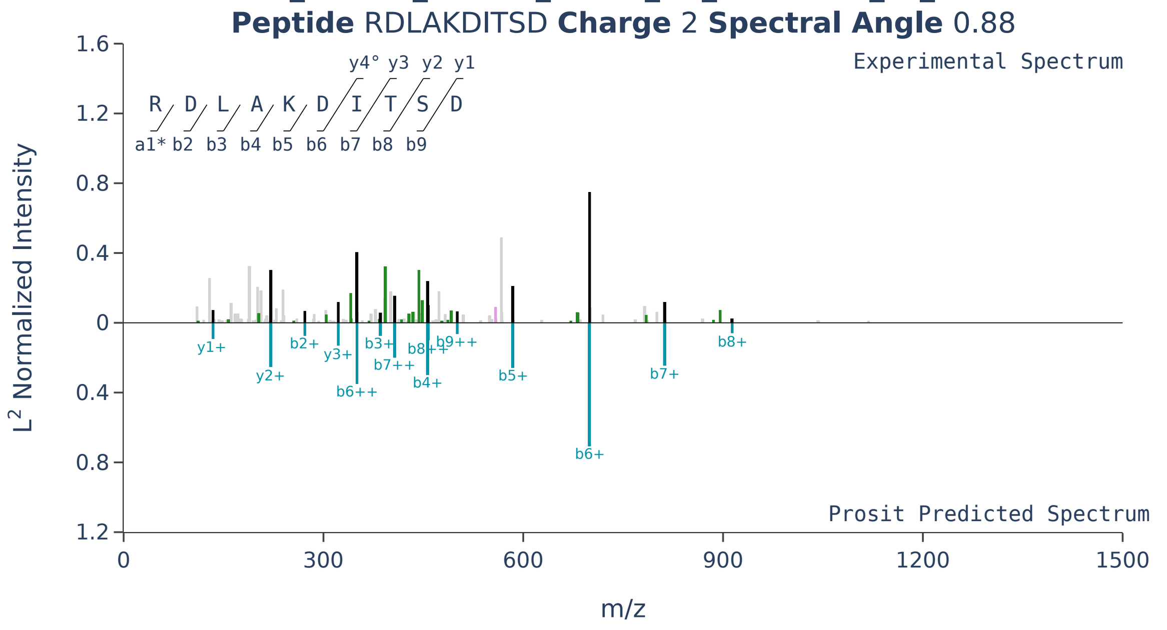


**Source** WSoh_301121_021221_HFGoe_M1_AnnexinA1_24h_R2 **Scan** 13363
**Peptide** SKGGPGSAVSPYPT **Charge** 2 **Spectral Angle** 0.97

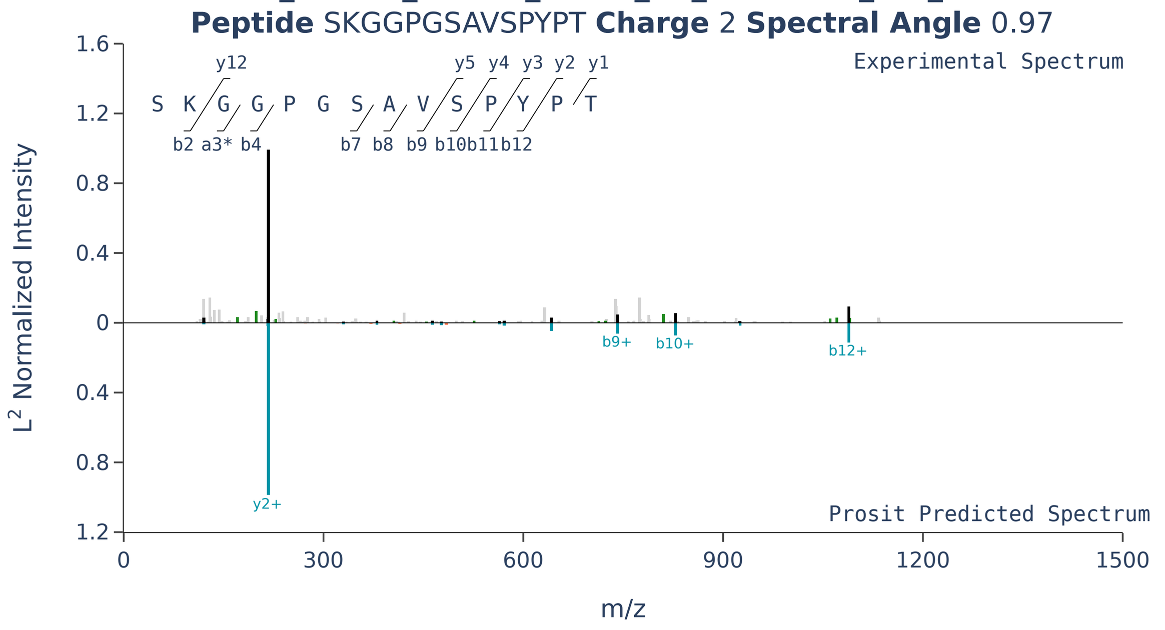


**Source** WSoh_301121_021221_HFGoe_M2_AnnexinA1_2h_R1 **Scan** 13808
**Peptide** RKGTDVNVFNT **Charge** 2 **Spectral Angle** 0.81

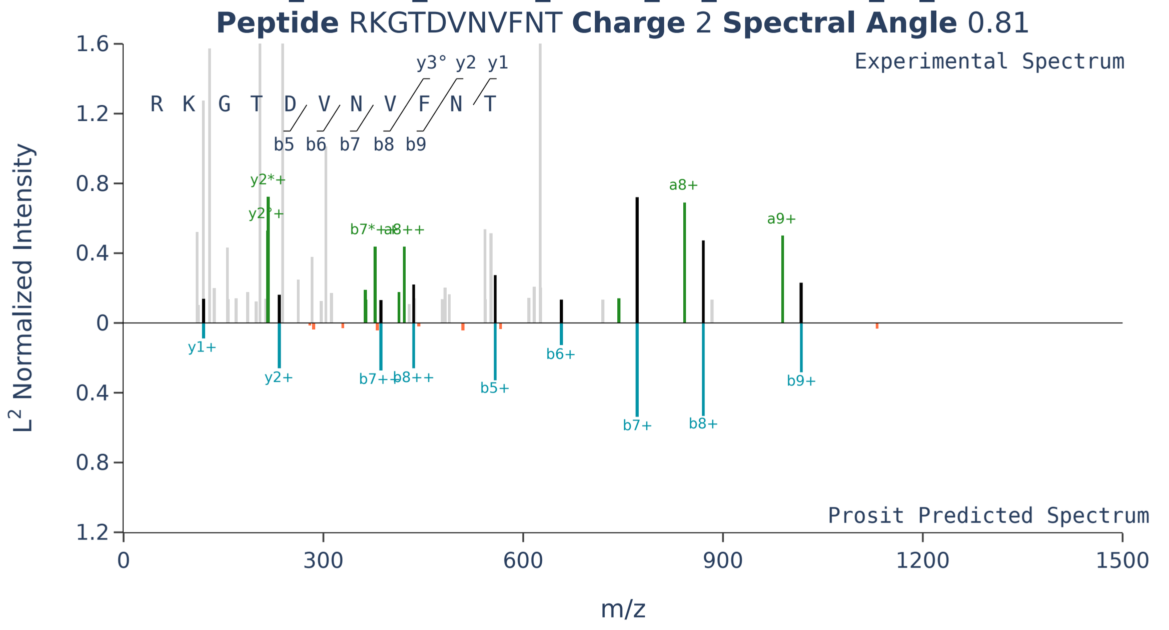


**Source** WSoh_301121_021221_HFGoe_M2_AnnexinA1_24h_R1 **Scan** 17688
**Peptide** KGDRSEDFGVNEDL **Charge** 2 **Spectral Angle** 0.75

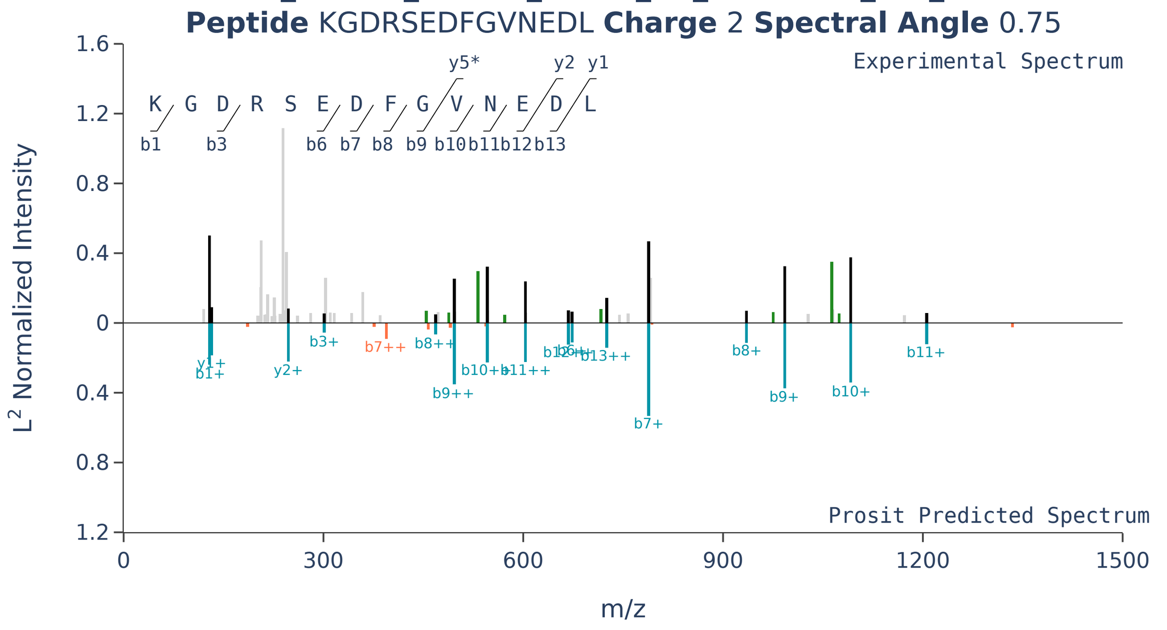


**Source** WSoh_301121_021221_HFGoe_M2_AnnexinA1_24h_R2 **Scan** 7542
**Peptide** KHDMNKVL **Charge** 2 **Spectral Angle** 0.92

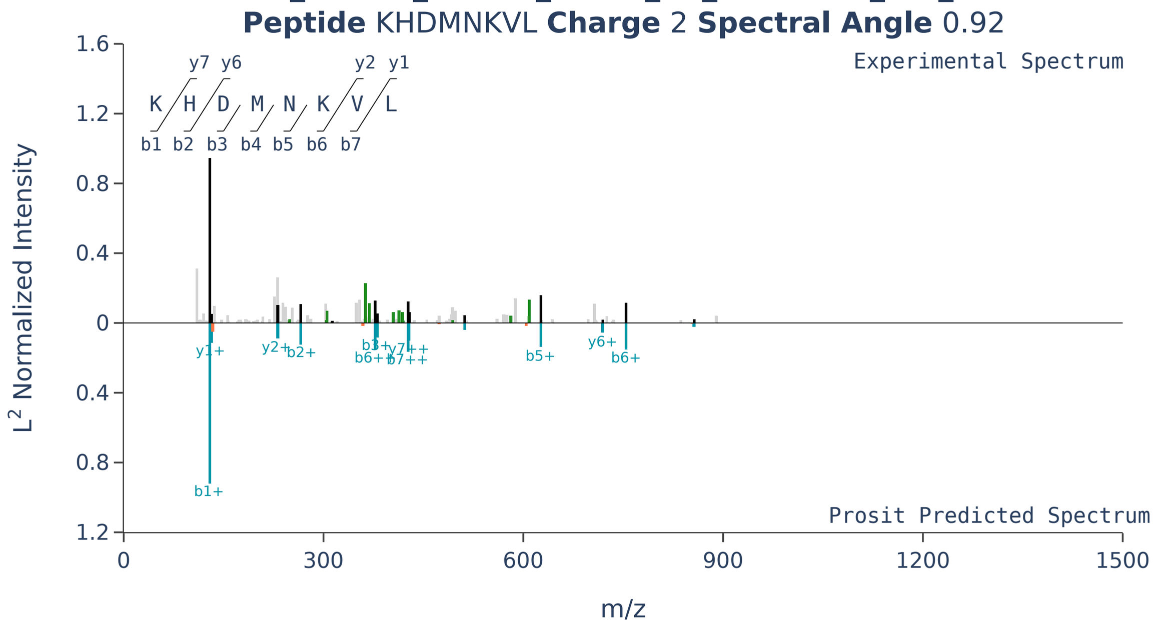


**Source** WSoh_301121_021221_HFGoe_M2_AnnexinA1_4h_R1 **Scan** 9352
**Peptide** TGKPLDETLKKA **Charge** 2 **Spectral Angle** 0.91

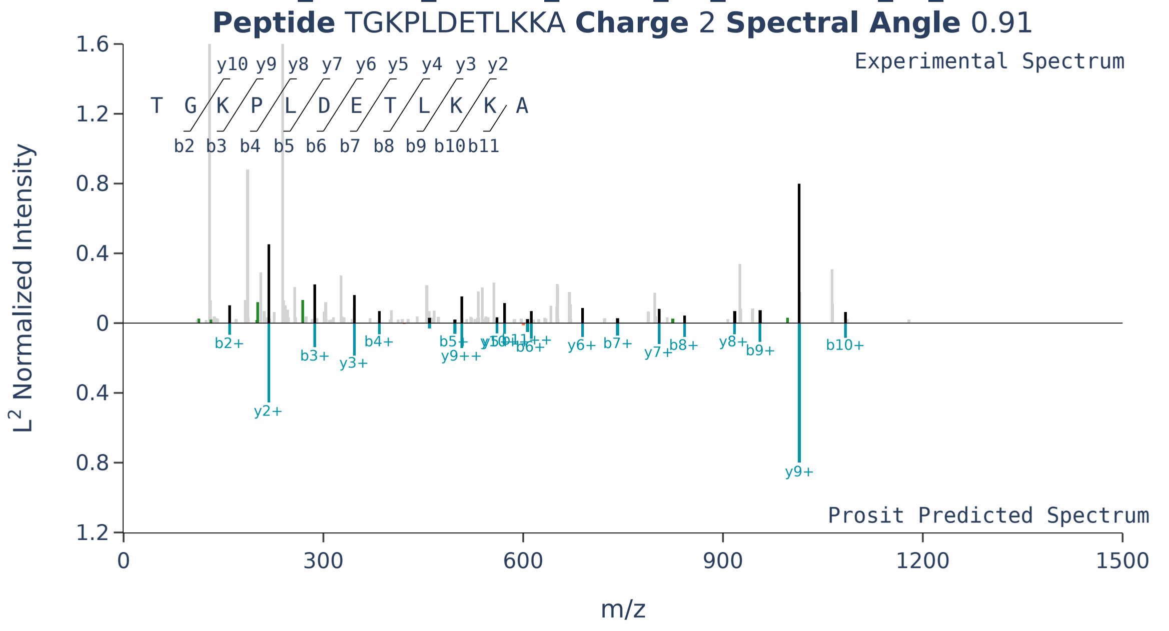


**Source** WSoh_301121_021221_HFGoe_M1_AnnexinA1_4h_R1 **Scan** 8240
**Peptide** FAEKLHQA **Charge** 2 **Spectral Angle** 0.89

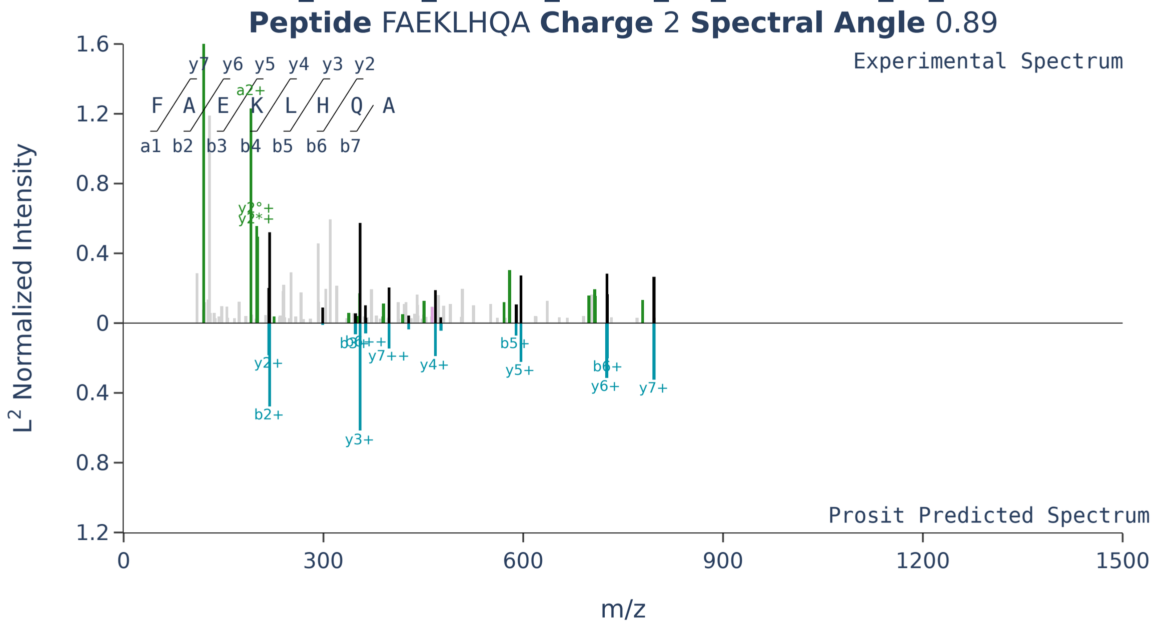


**Source** WSoh_301121_021221_HFGoe_M2_AnnexinA1_24h_R1 **Scan** 12718
**Peptide** KALTGHLEEV **Charge** 2 **Spectral Angle** 0.86

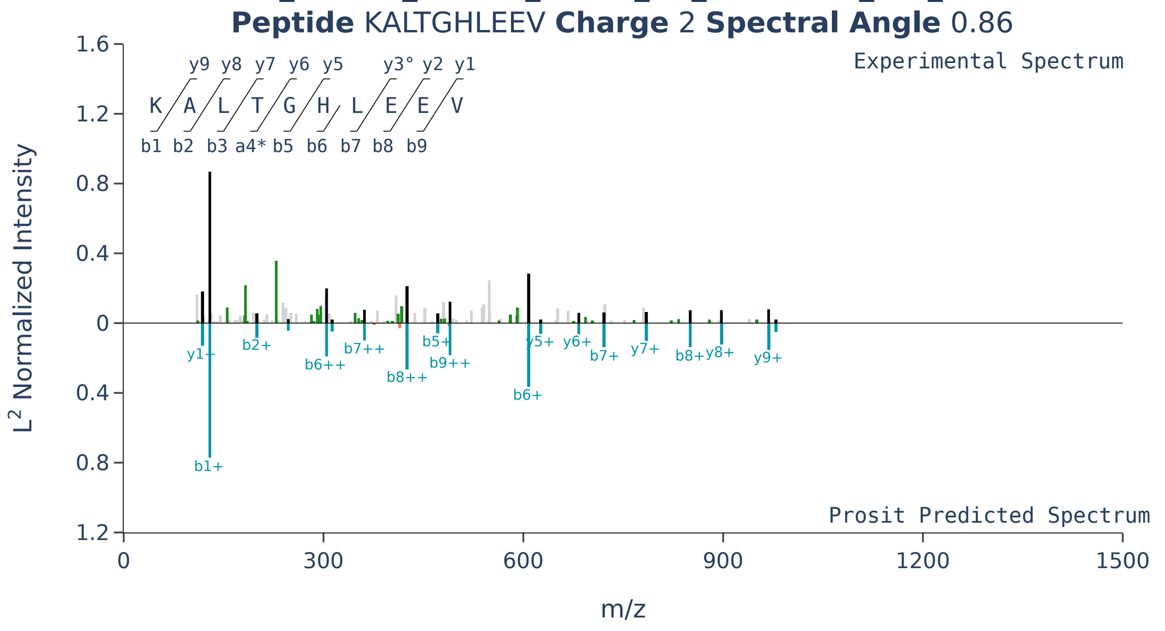


**Source** WSoh_301121_021221_HFGoe_M1_AnnexinA1_24h_R2 **Scan** 16224
**Peptide** SKHDMNKVLDL **Charge** 3 **Spectral Angle** 0.88

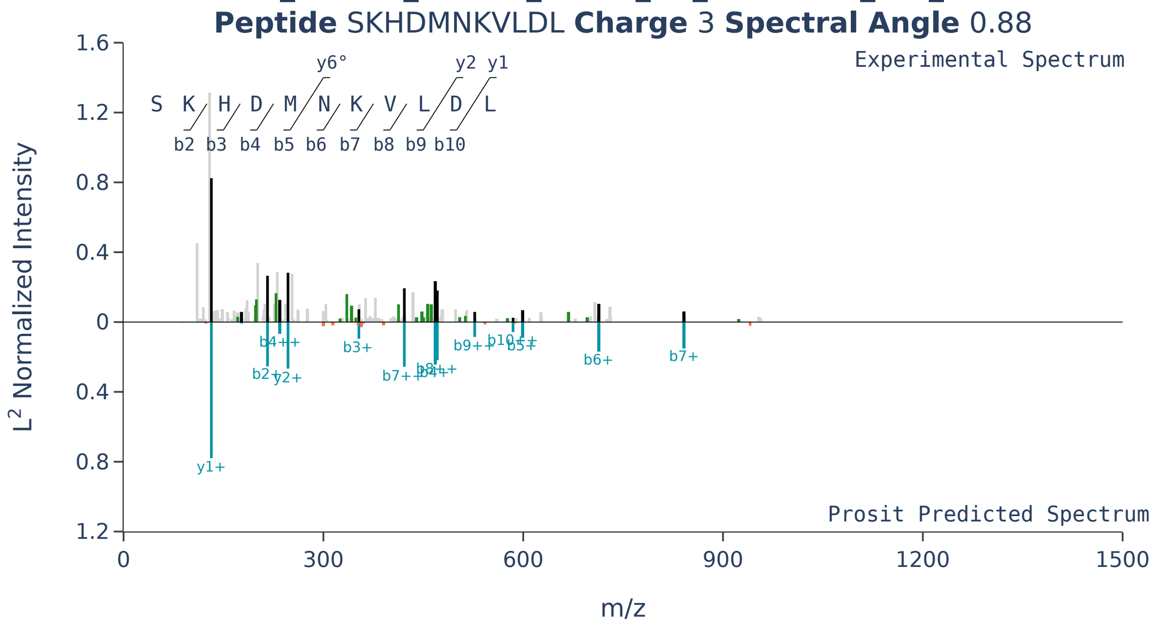


**Source** WSoh_301121_021221_HFGoe_M2_AnnexinA1_1h_R1 **Scan** 16650
**Peptide** KGDRSEDFGVNEDLAD **Charge** 3 **Spectral Angle** 0.88

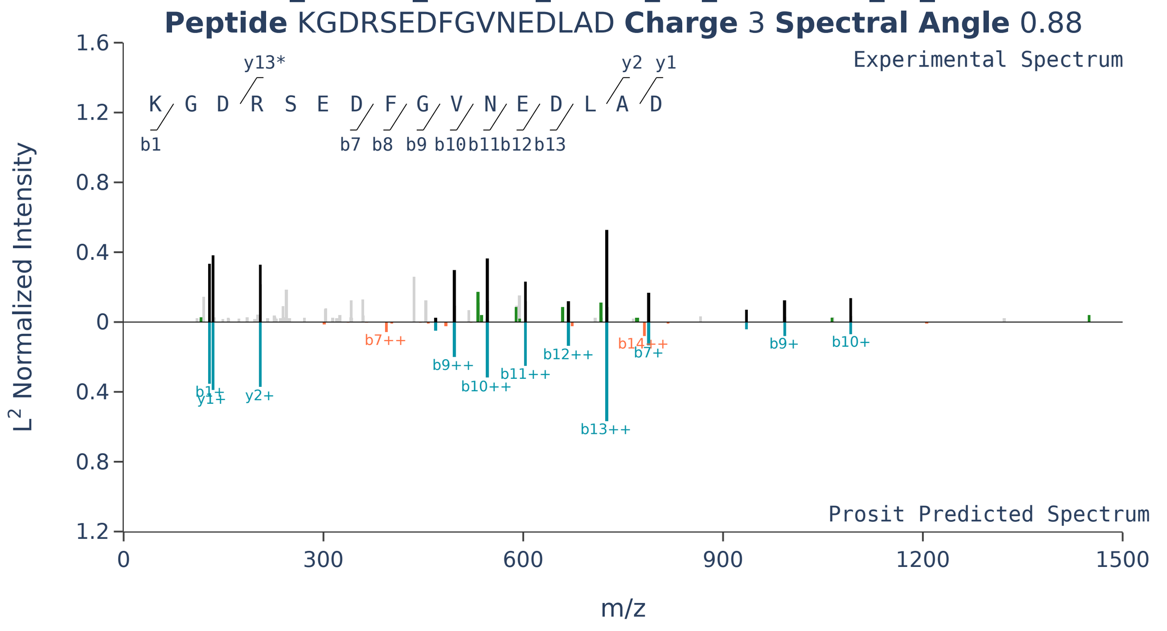


**Source** WSoh_301121_021221_HFGoe_M2_AnnexinA1_24h_R1 **Scan** 11820
**Peptide** AGERRKGTDVNVFNT **Charge** 3 **Spectral Angle** 0.83

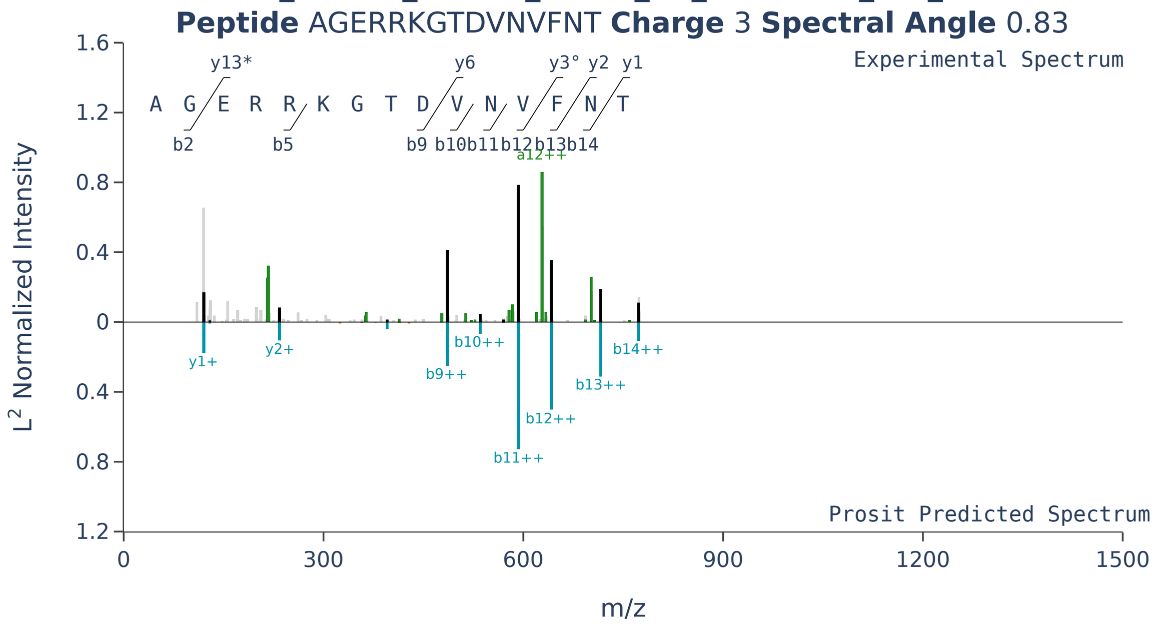


**Source** WSoh_301121_021221_HFGoe_M2_AnnexinA1_24h_R1 **Scan** 7315
**Peptide** REELKRDL **Charge** 2 **Spectral Angle** 0.81

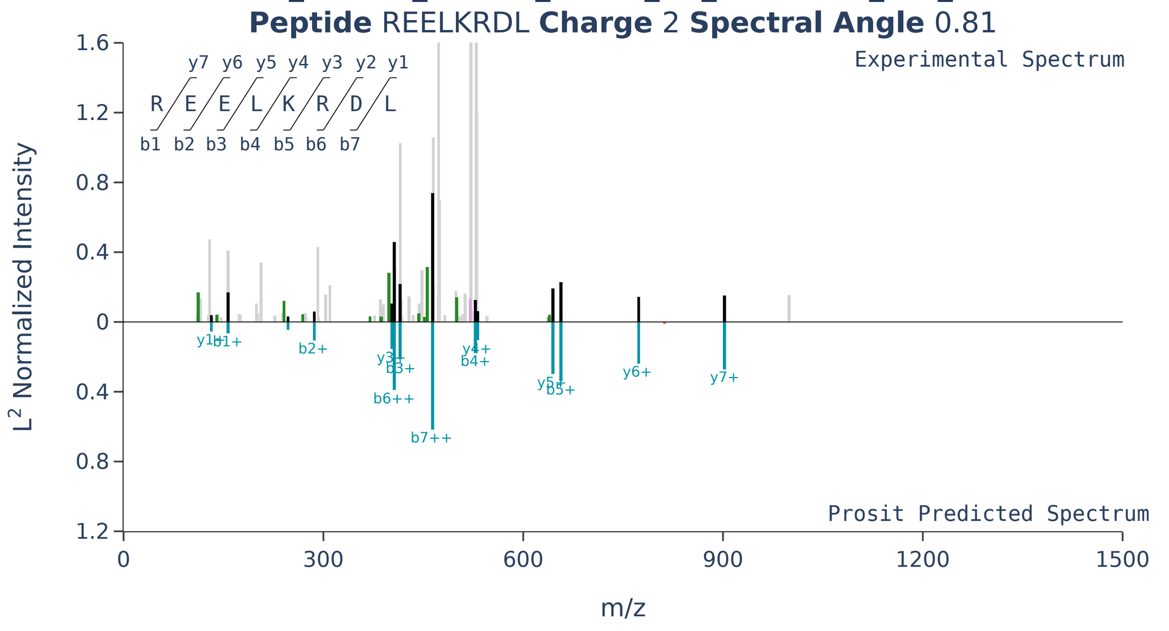


**Source** WSoh_301121_021221_HFGoe_M1_AnnexinA1_4h_R1 **Scan** 13828
**Peptide** TSKPAFFAEK **Charge** 2 **Spectral Angle** 0.92

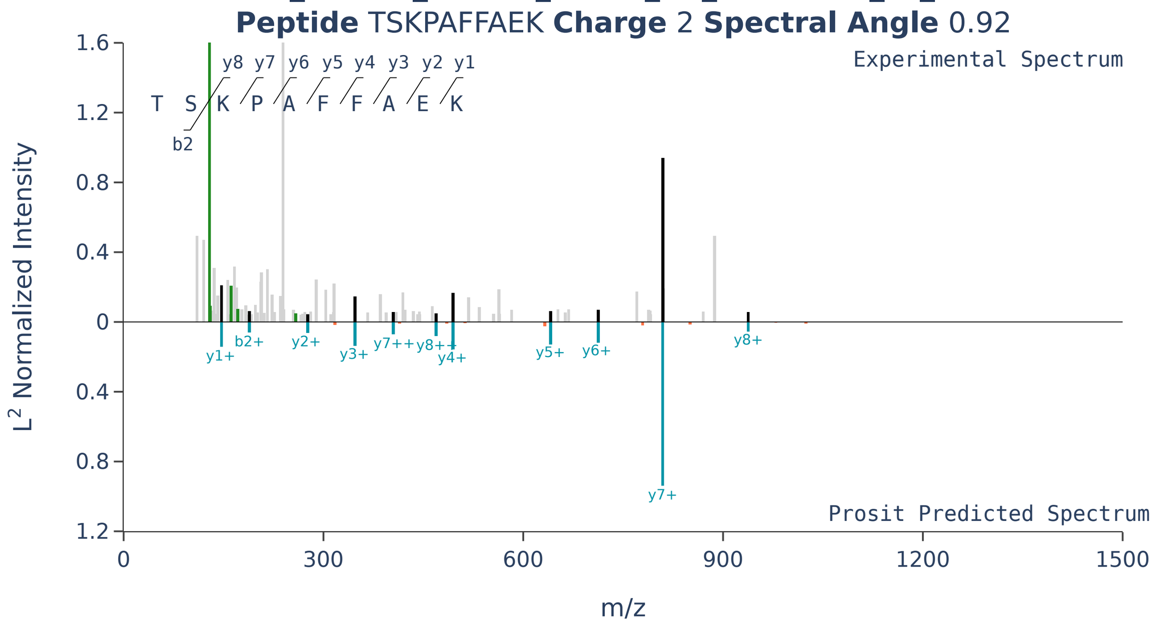


**Source** WSoh_301121_021221_HFGoe_M1_AnnexinA1_1h_R1 **Scan** 8225
**Peptide** ETLKKAL **Charge** 1 **Spectral Angle** 0.86

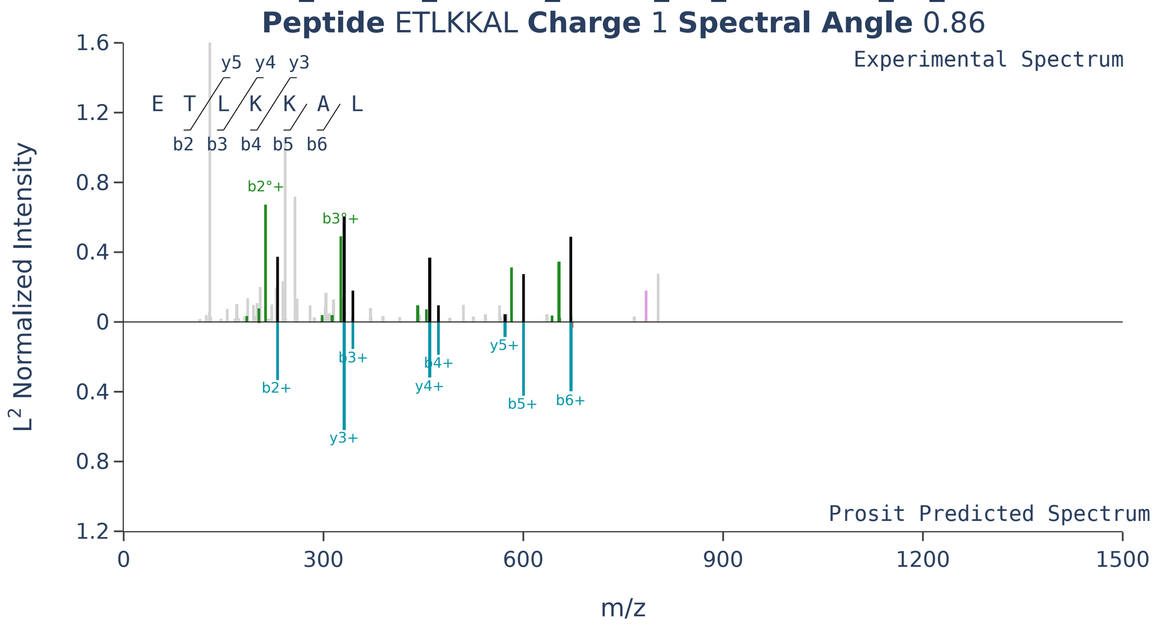

**Source** WSoh_301121_021221_HFGoe_M1_AnnexinA1_4h_R2 **Scan** 19428
**Peptide** KAFYQKMYGI **Charge** 3 **Spectral Angle** 0.92

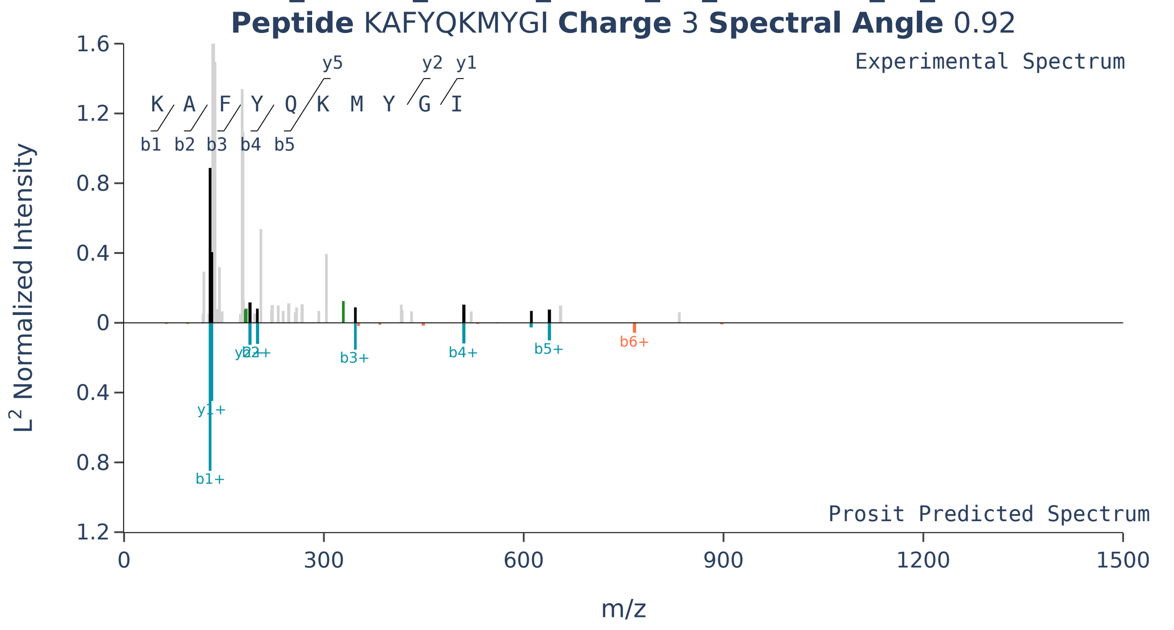


**Source** WSoh_301121_021221_HFGoe_M2_AnnexinA1_4h_R1 **Scan** 19409
**Peptide** ADELRAAMKGLGTDE **Charge** 3 **Spectral Angle** 0.88

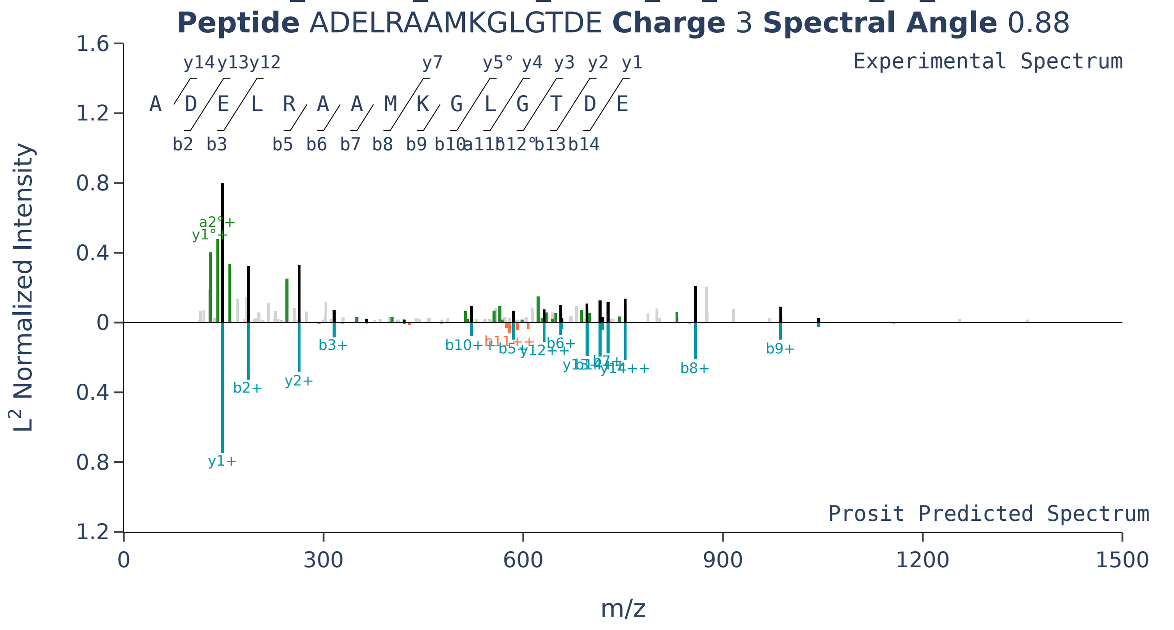


**Source** WSoh_301121_021221_HFGoe_M2_AnnexinA1_24h_R2 **Scan** 18217
**Peptide** KPAFFAE **Charge** 1 **Spectral Angle** 0.84

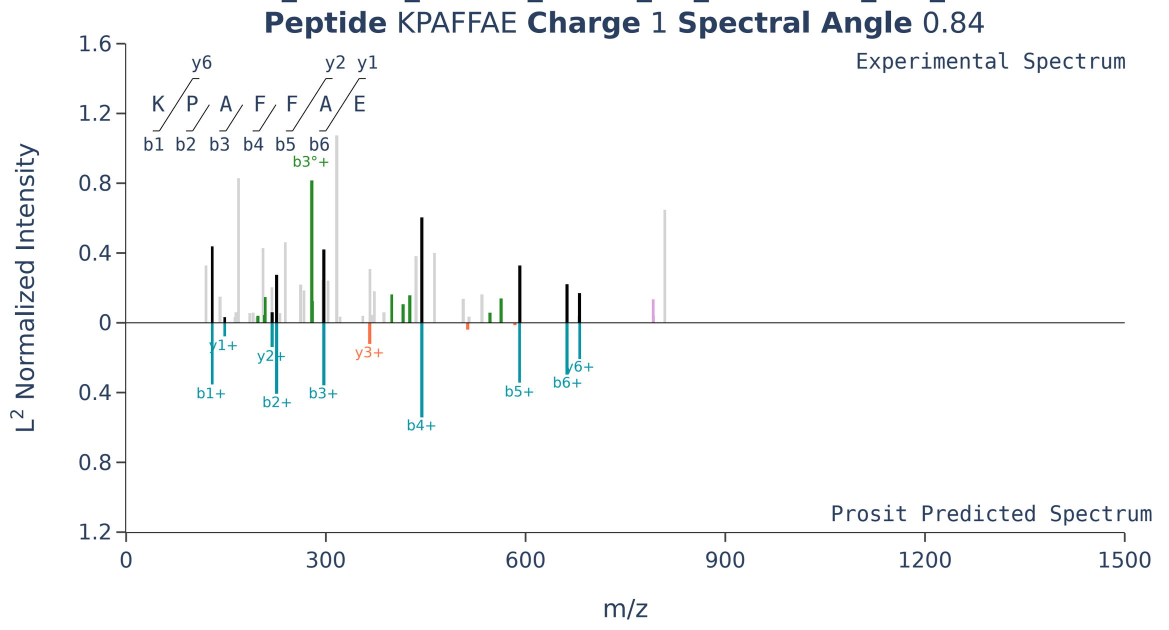


**Source** WSoh_301121_021221_HFGoe_M1_AnnexinA1_24h_R2 **Scan** 14014
**Peptide** KDITSDTSGDF **Charge** 2 **Spectral Angle** 0.85

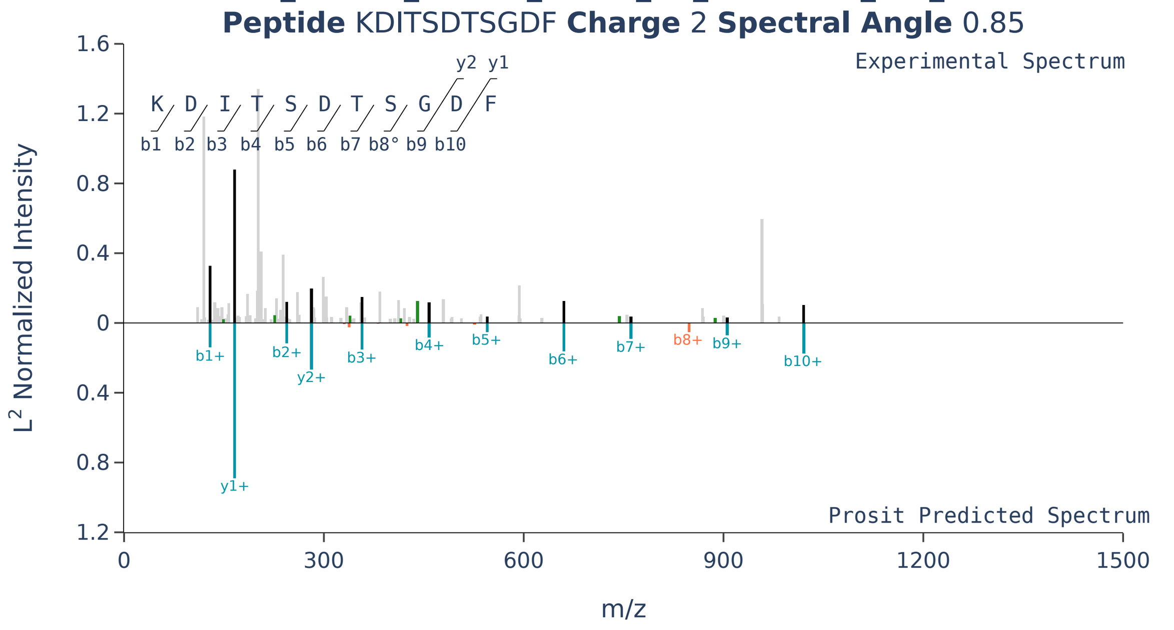


**Source** WSoh_301121_021221_HFGoe_M2_AnnexinA1_2h_R1 **Scan** 11723
**Peptide** LAKGDRSEDFGVNED **Charge** 3 **Spectral Angle** 0.85

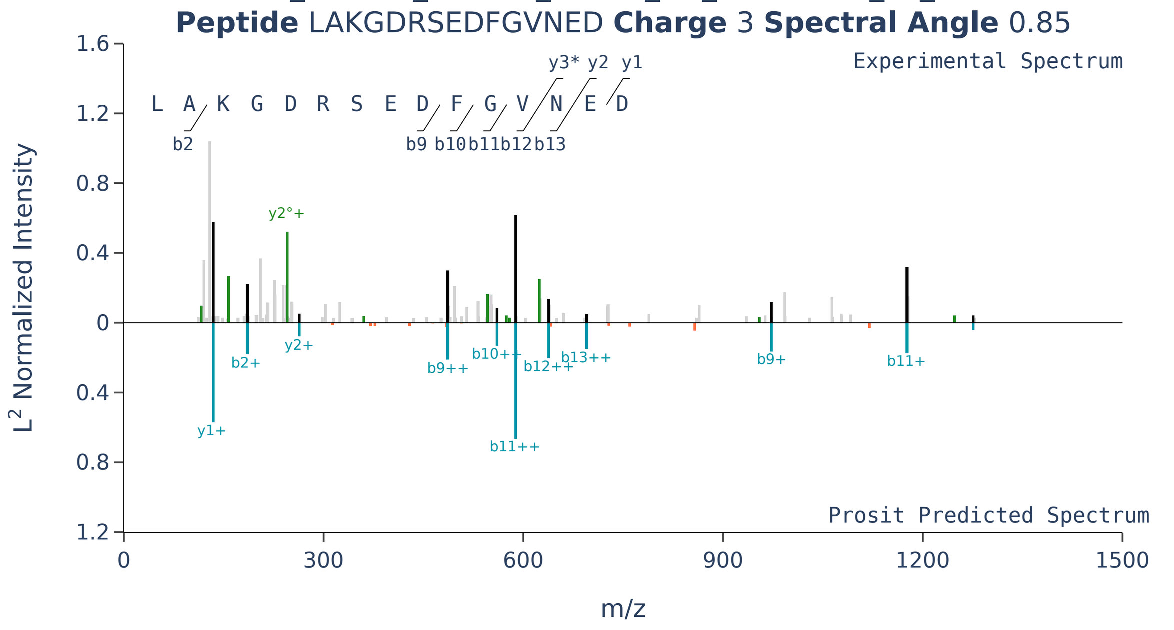


**Source** WSoh_301121_021221_HFGoe_M1_AnnexinA1_24h_R1 **Scan** 10268
**Peptide** DETKGDYEKI **Charge** 2 **Spectral Angle** 0.83

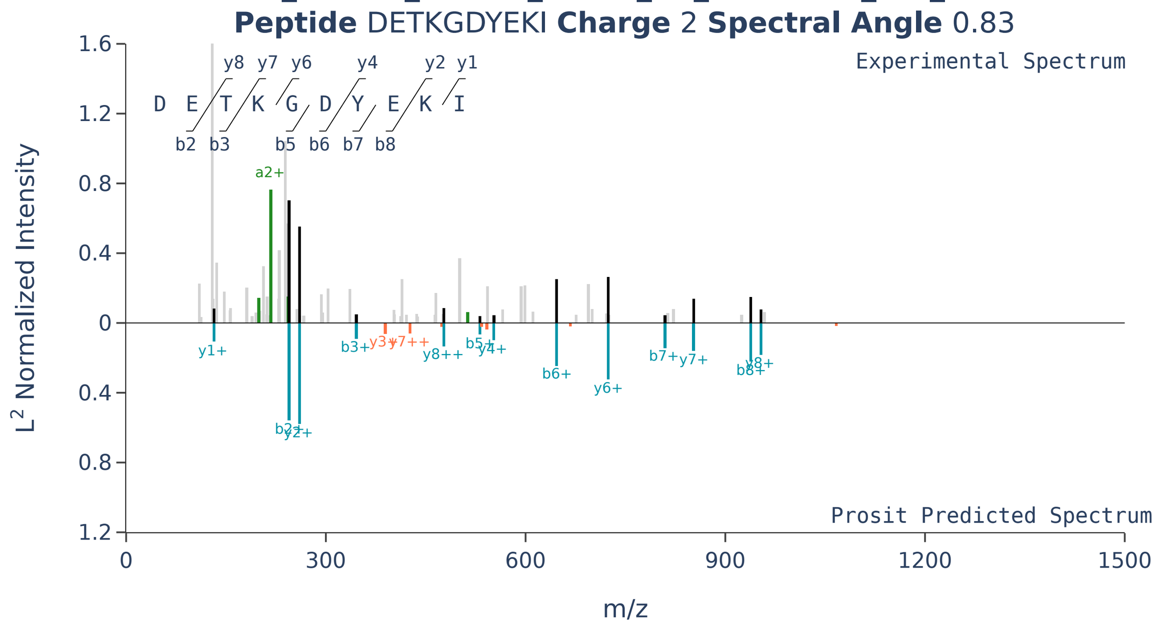


**Source** WSoh_301121_021221_HFGoe_M1_AnnexinA1_2h_R1 **Scan** 17000
**Peptide** CQAILDET **Charge** 2 **Spectral Angle** 0.91

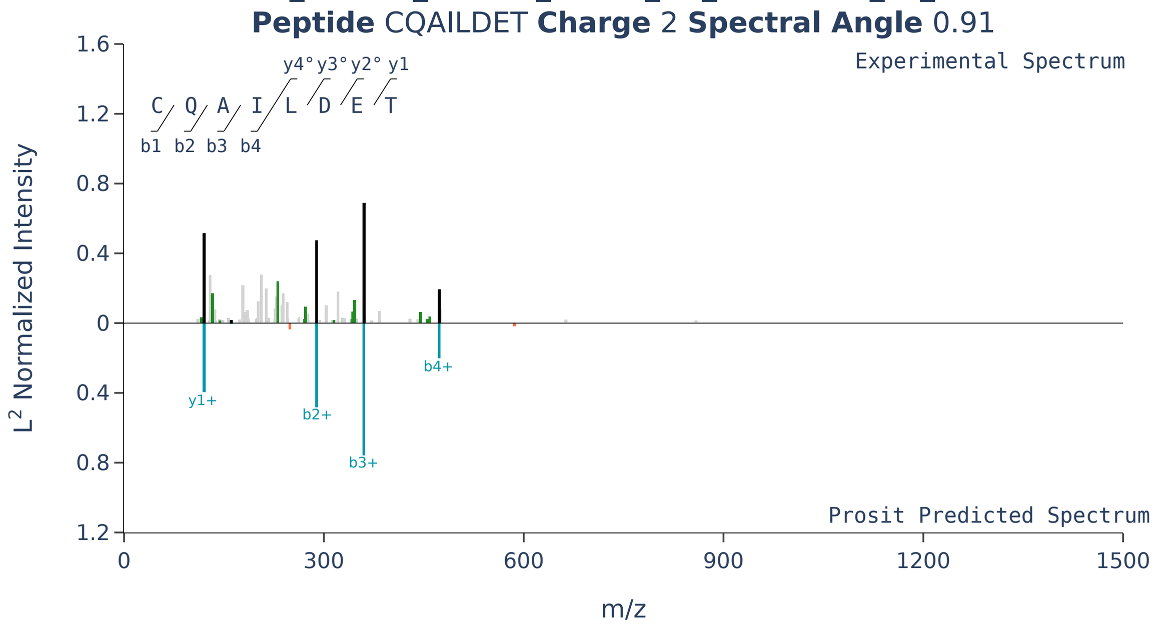


**Source** WSoh_301121_021221_HFGoe_M2_AnnexinA1_4h_R1 **Scan** 15491
**Peptide** VSEFLKQA **Charge** 1 **Spectral Angle** 0.84

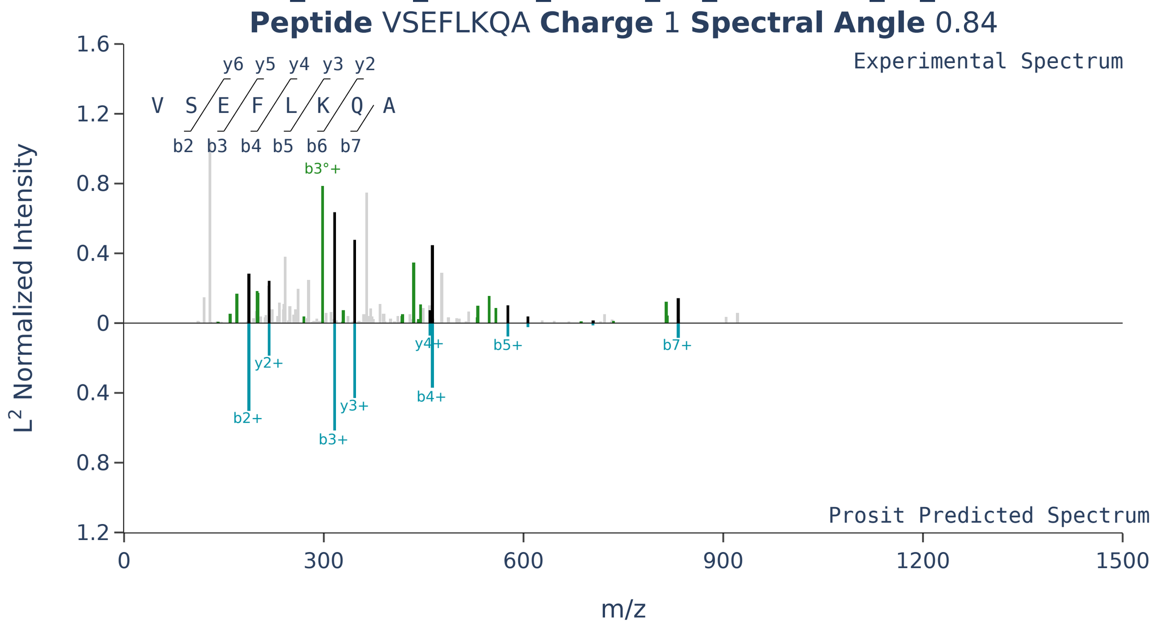


**Source** WSoh_301121_021221_HFGoe_M1_AnnexinA1_2h_R1 **Scan** 13039
**Peptide** KRDLAKDITSDTSGDFRN **Charge** 3 **Spectral Angle** 0.85

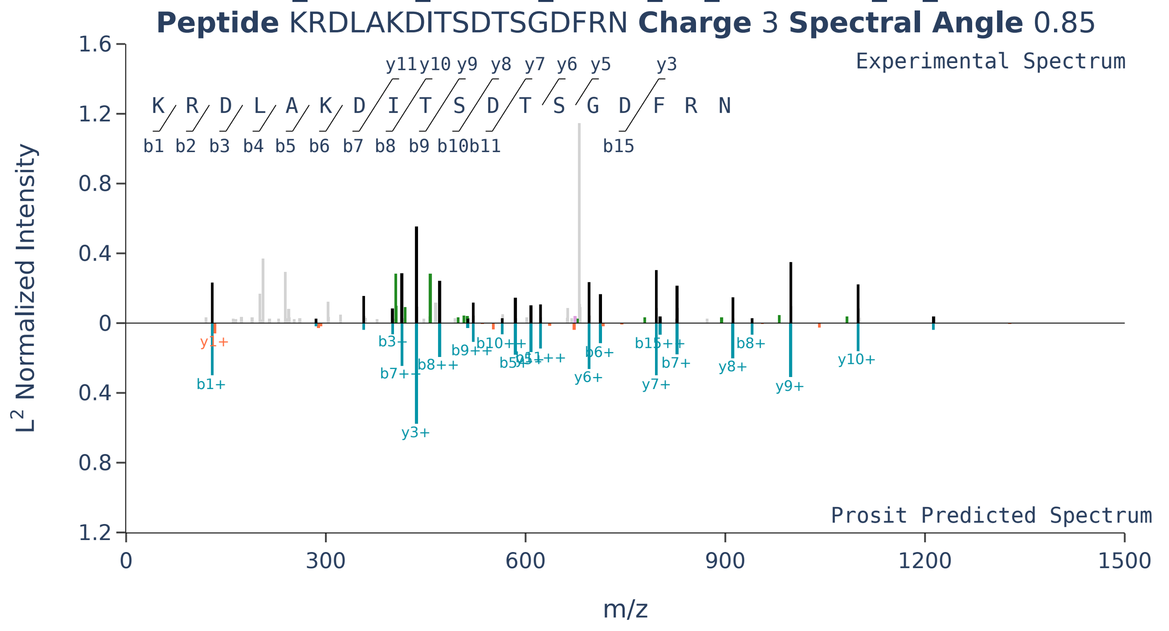


**Source** WSoh_301121_021221_HFGoe_M2_AnnexinA1_1h_R2 **Scan** 9480
**Peptide** RTNKEIRDINRVY **Charge** 4 **Spectral Angle** 0.87

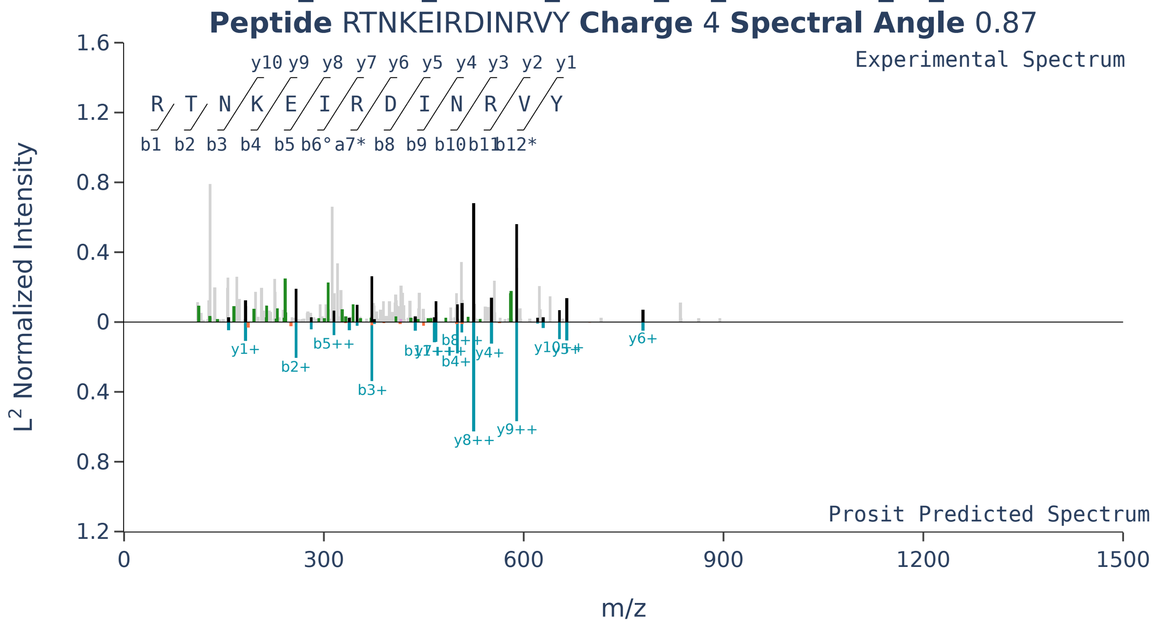


**Source** WSoh_301121_021221_HFGoe_M2_AnnexinA1_4h_R1 **Scan** 17601
**Peptide** MVSEFLKQA **Charge** 2 **Spectral Angle** 0.82

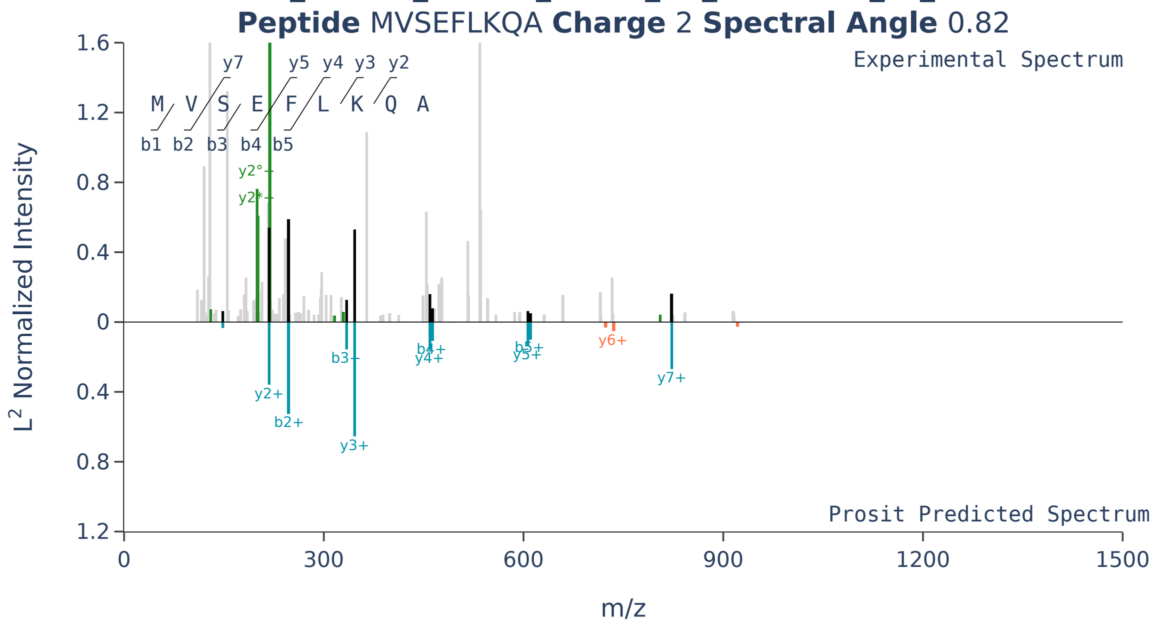


**Source** WSoh_301121_021221_HFGoe_M2_AnnexinA1_2h_R1 **Scan** 22601
**Peptide** TSGDFRNALL **Charge** 2 **Spectral Angle** 0.81

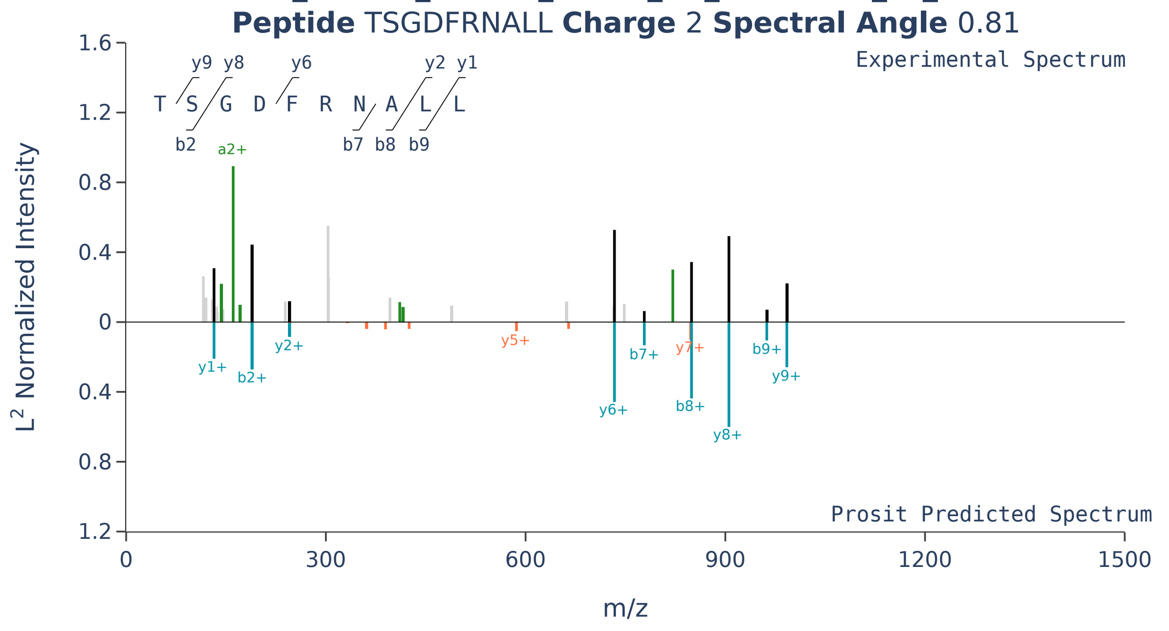


**Source** WSoh_301121_021221_HFGoe_M2_AnnexinA1_2h_R1 **Scan** 19380
**Peptide** PAQFDADELRA **Charge** 2 **Spectral Angle** 0.88

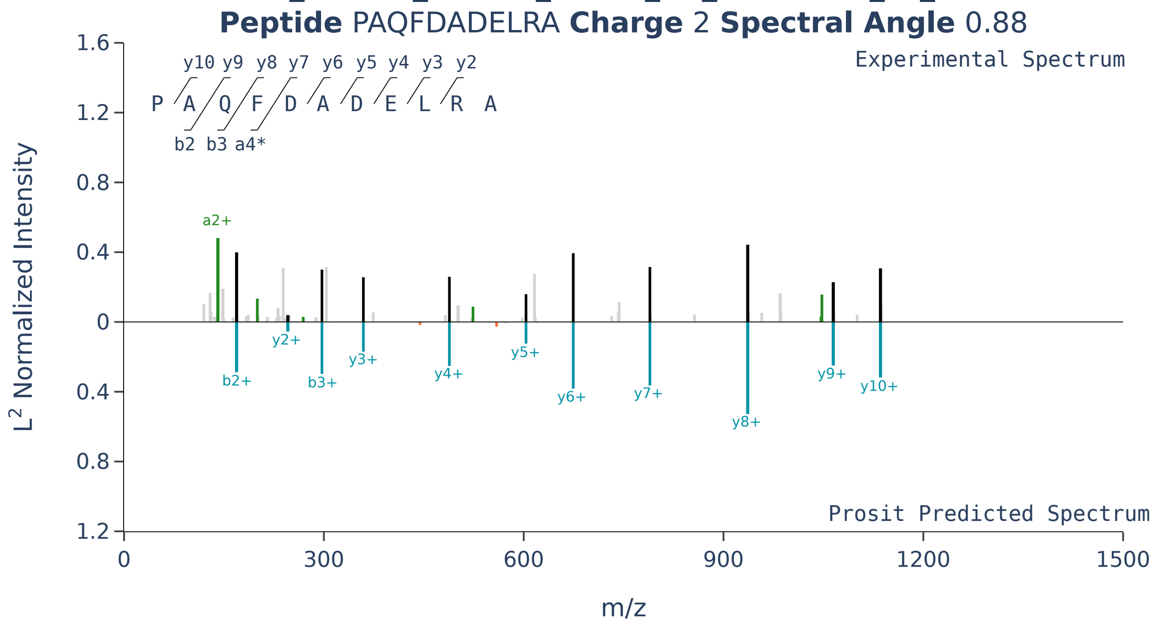


**Source** WSoh_301121_021221_HFGoe_M2_AnnexinA1_24h_R2 **Scan** 7017
**Peptide** RRVFQKYT **Charge** 3 **Spectral Angle** 0.85

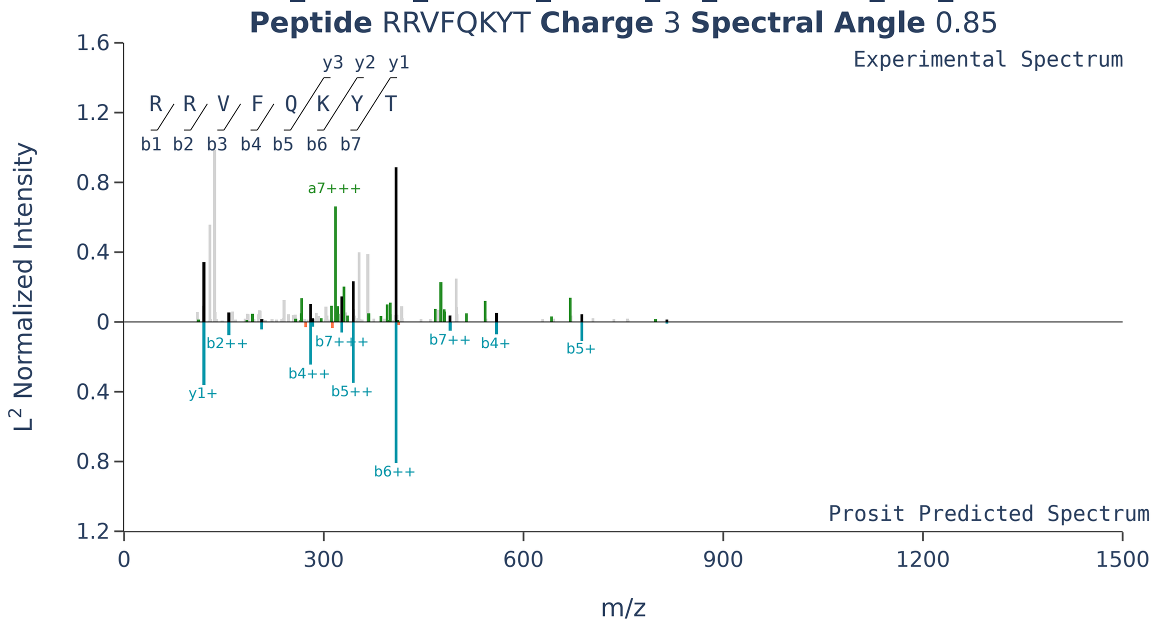


**Source** WSoh_301121_021221_HFGoe_M2_AnnexinA1_24h_R1 **Scan** 7234
**Peptide** KHDMNKVLD **Charge** 3 **Spectral Angle** 0.93

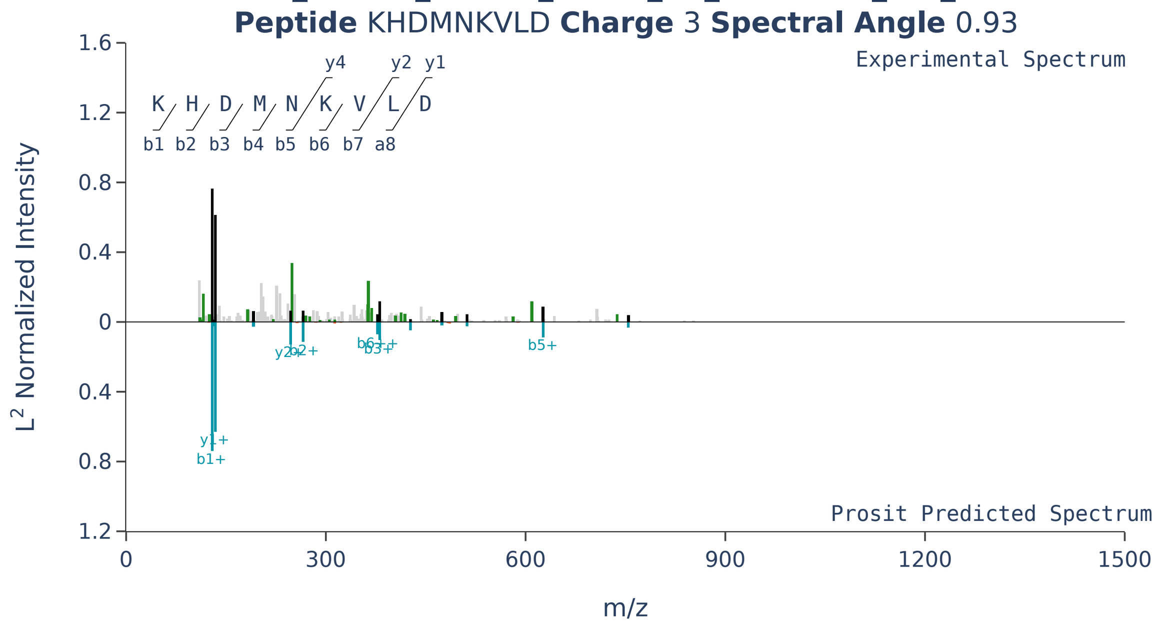

**Source** WSoh_301121_021221_HFGoe_M2_AnnexinA1_2h_R2 **Scan** 5731
**Peptide** FQKYTKYSKHD **Charge** 3 **Spectral Angle** 0.89

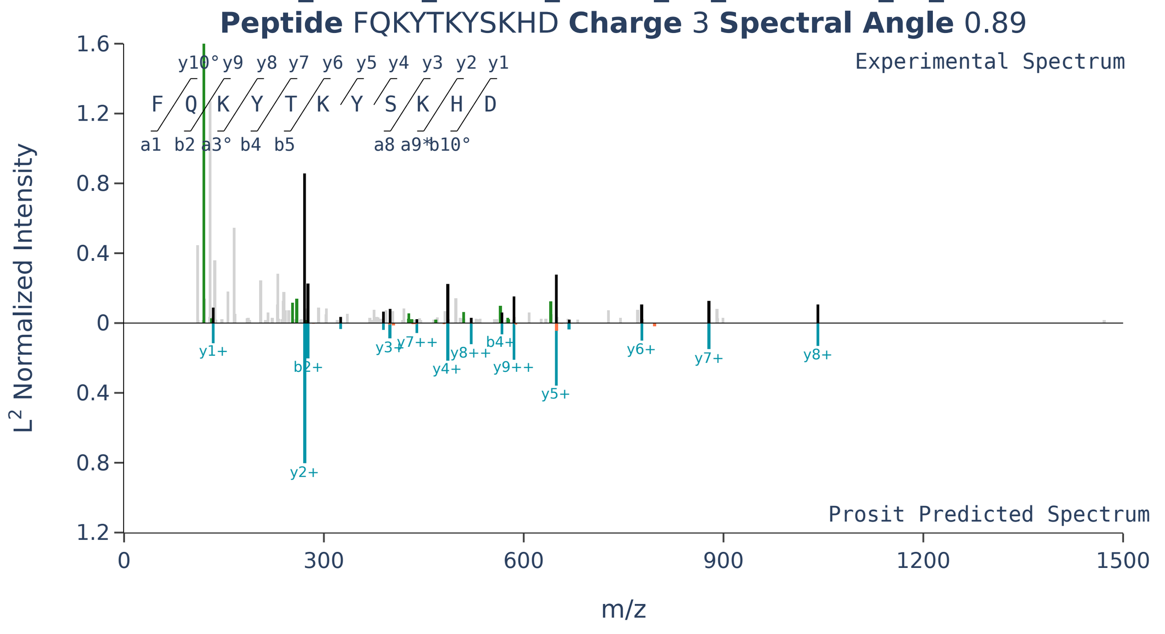


**Source** WSoh_301121_021221_HFGoe_M2_AnnexinA1_24h_R1 **Scan** 13368
**Peptide** IMVKGVDE **Charge** 2 **Spectral Angle** 0.79

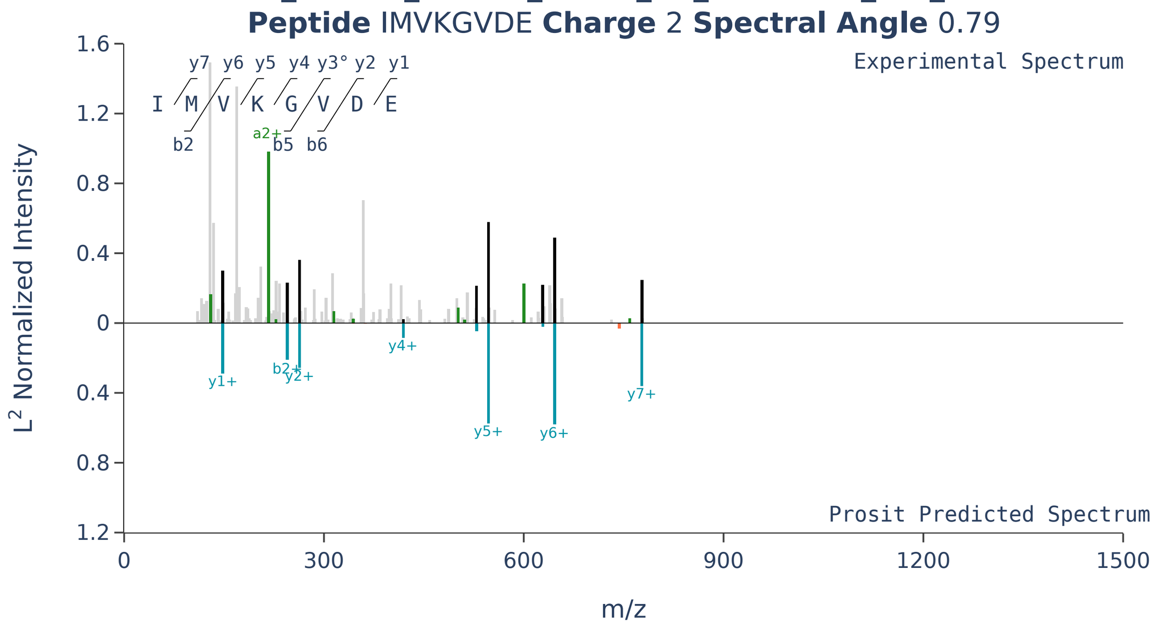


**Source** WSoh_301121_021221_HFGoe_M2_AnnexinA1_24h_R1 **Scan** 9561
**Peptide** NPSSDVAALHK **Charge** 3 **Spectral Angle** 0.87

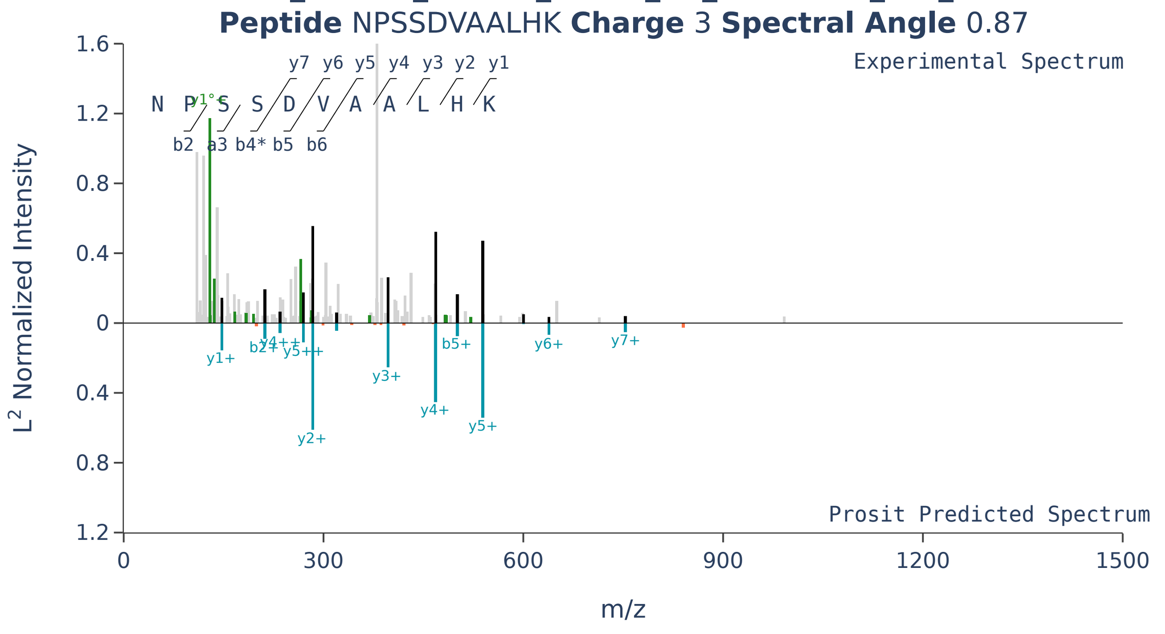


**Source** WSoh_301121_021221_HFGoe_M2_AnnexinA1_24h_R1 **Scan** 8946
**Peptide** KEIRDINRVYRE **Charge** 4 **Spectral Angle** 0.79

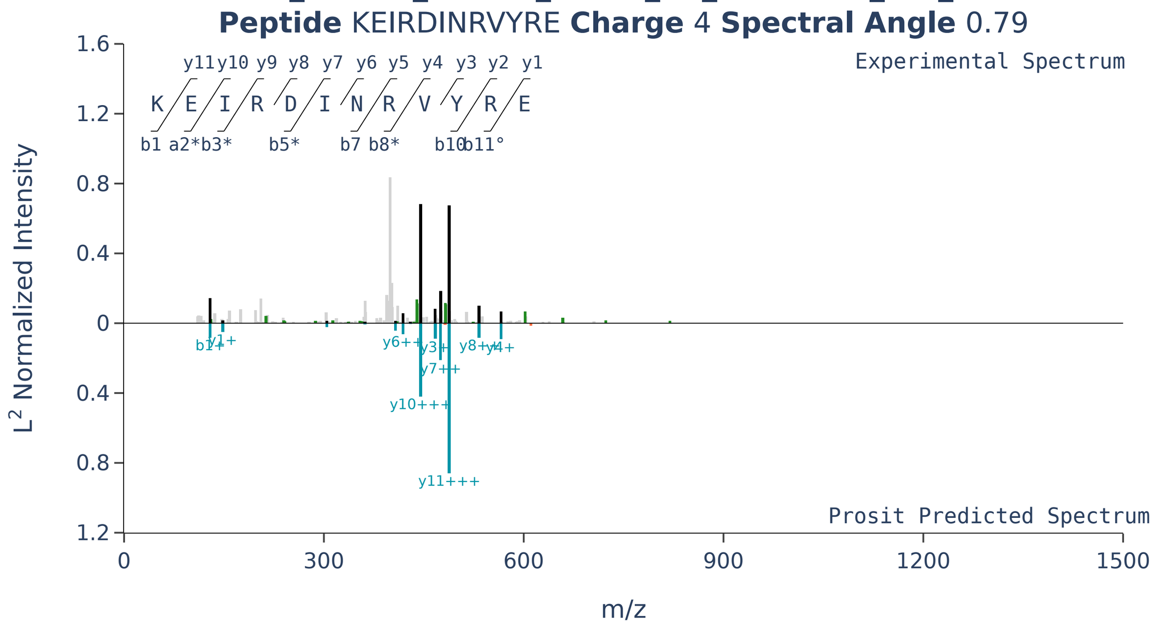


**Source** WSoh_301121_021221_HFGoe_M2_AnnexinA1_2h_R1 **Scan** 25541
**Peptide** ALTGHLEEVVL **Charge** 2 **Spectral Angle** 0.83

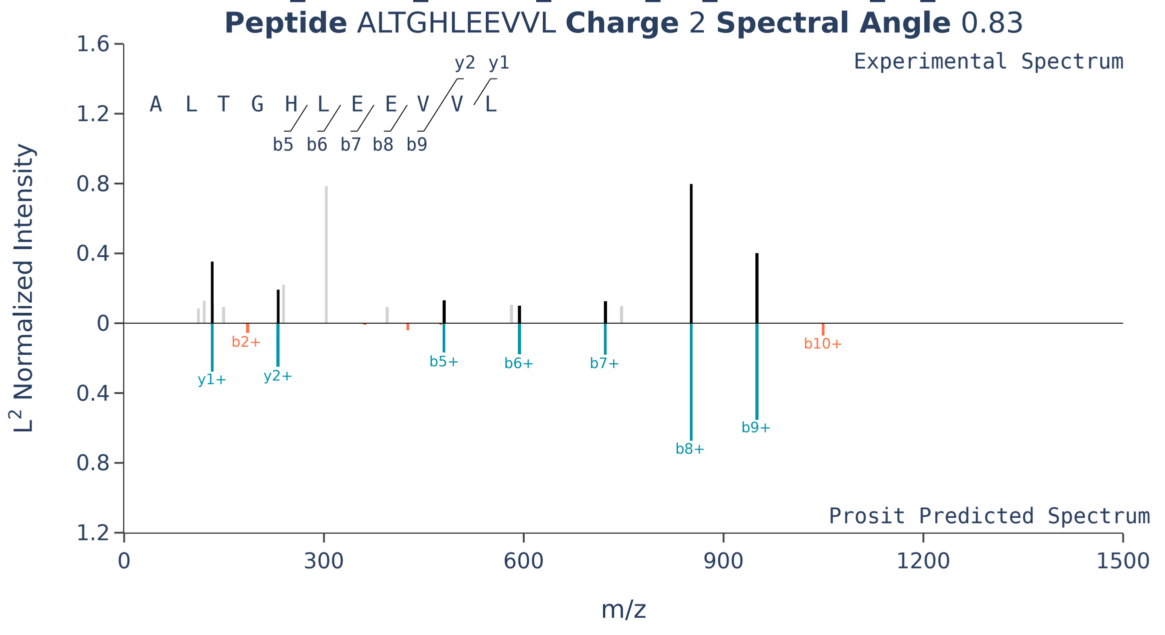


**Source** WSoh_301121_021221_HFGoe_M2_AnnexinA1_24h_R1 **Scan** 12612
**Peptide** AILDETKGDYEK **Charge** 2 **Spectral Angle** 0.81

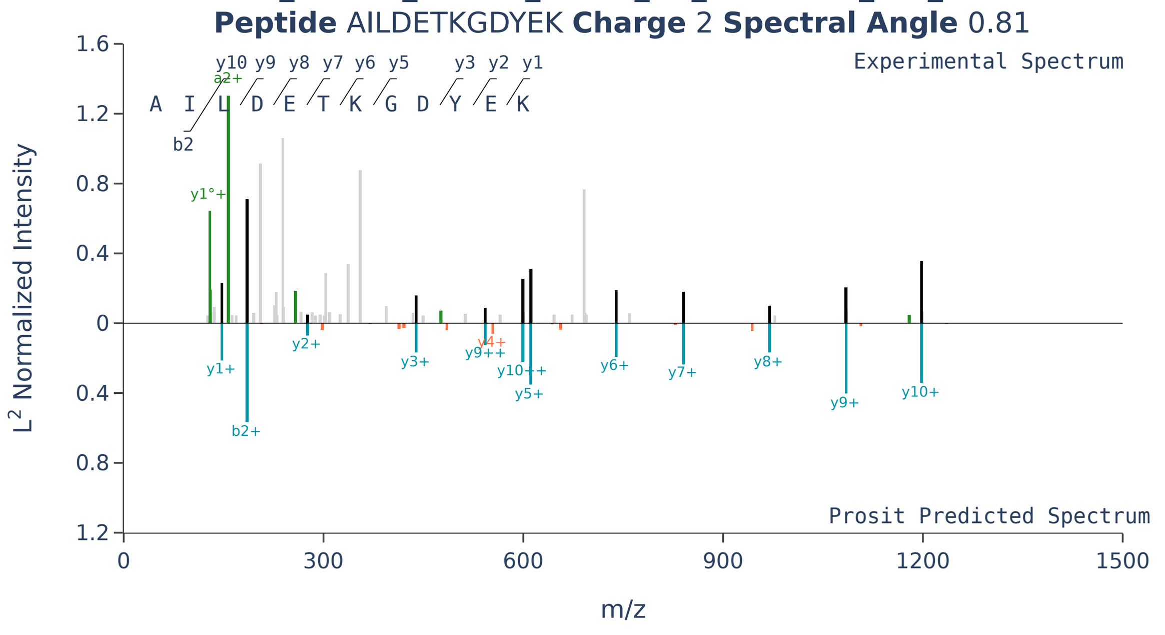


**Source** WSoh_301121_021221_HFGoe_M2_AnnexinA1_24h_R2 **Scan** 16197
**Peptide** KGVDEATIID **Charge** 2 **Spectral Angle** 0.77

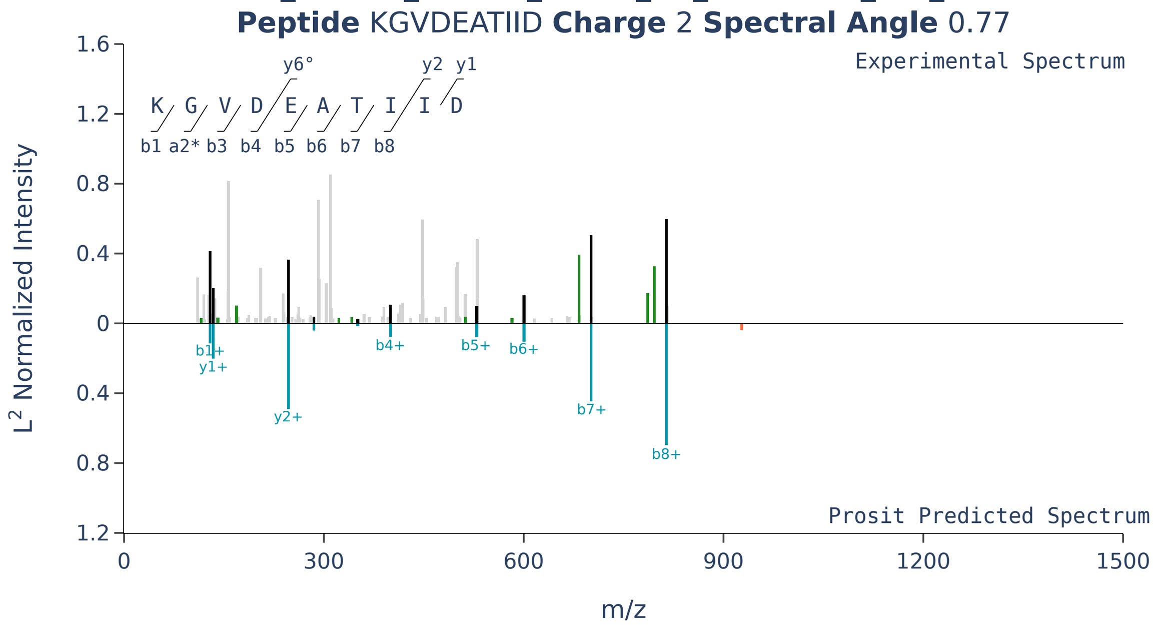


**Source** WSoh_301121_021221_HFGoe_M2_AnnexinA1_2h_R2 **Scan** 20856
**Peptide** RKGTDVNVFNTI **Charge** 3 **Spectral Angle** 0.81

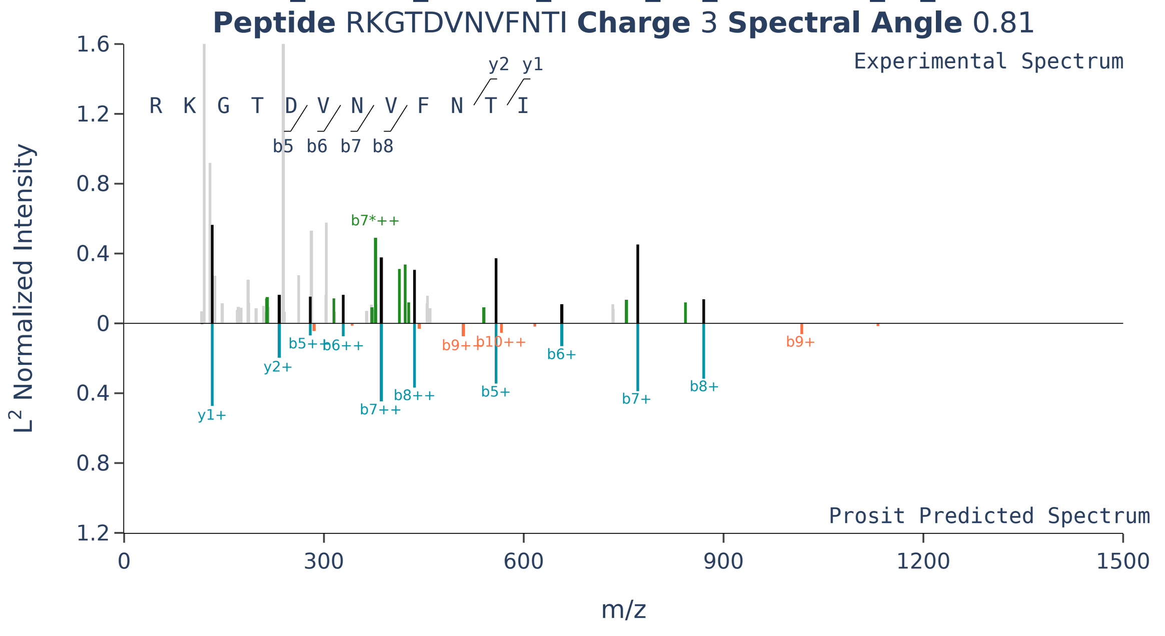


**Source** WSoh_301121_021221_HFGoe_M1_AnnexinA1_24h_R1 **Scan** 9943
**Peptide** TRSYPQLRRV **Charge** 3 **Spectral Angle** 0.86

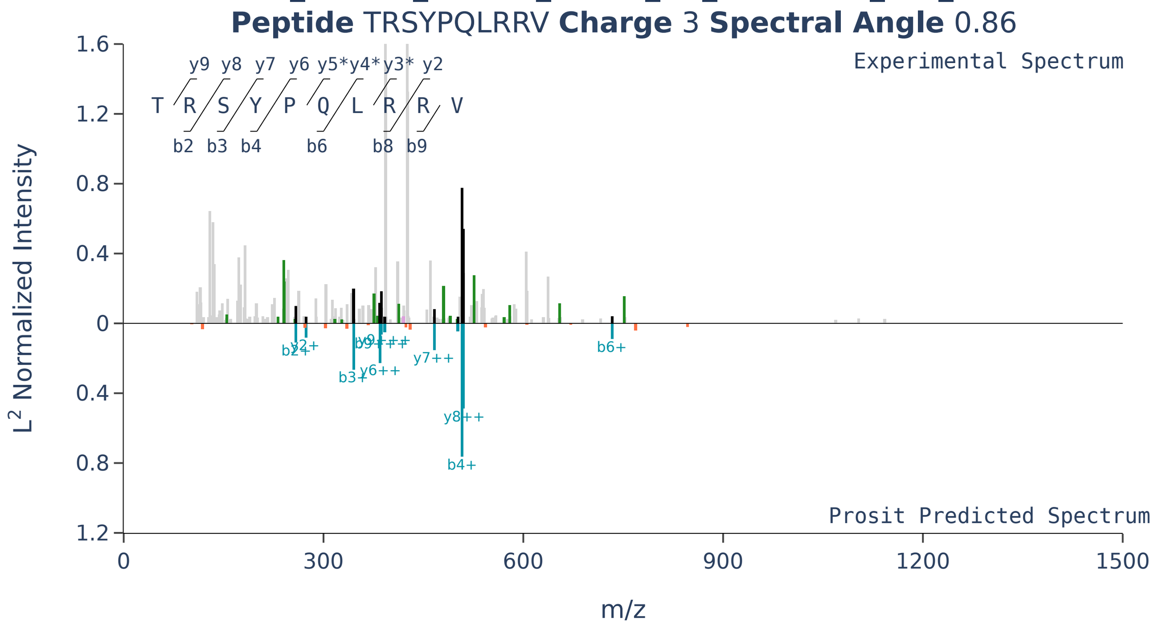


**Source** WSoh_301121_021221_HFGoe_M2_AnnexinA1_2h_R1 **Scan** 21919
**Peptide** KGGPGSAVSPYPTF **Charge** 2 **Spectral Angle** 0.95

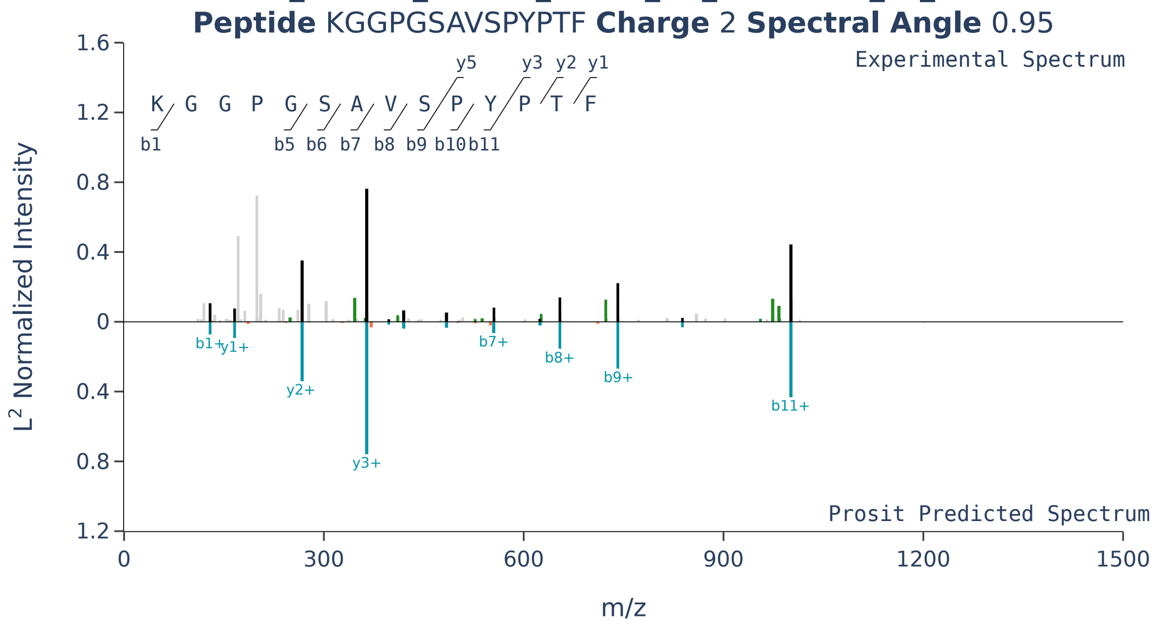


**Source** WSoh_301121_021221_HFGoe_M2_AnnexinA1_4h_R1 **Scan** 9264
**Peptide** YSKHDMNKVLD **Charge** 2 **Spectral Angle** 0.83

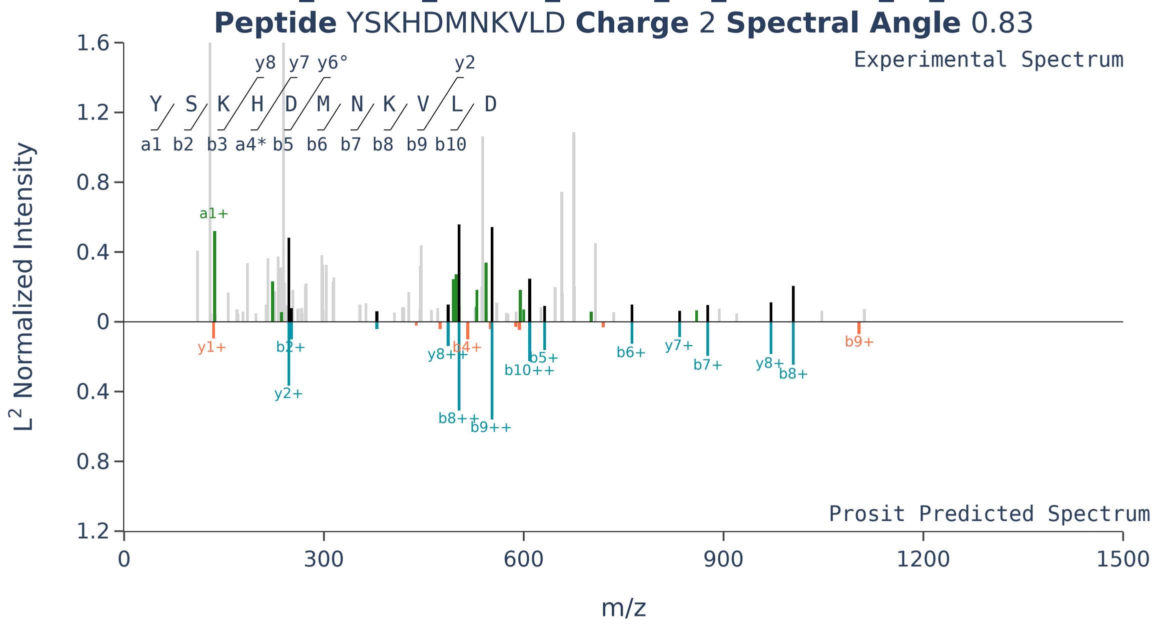


**Source** WSoh_301121_021221_HFGoe_M2_AnnexinA1_2h_R1 **Scan** 14005
**Peptide** IDILTKRNNAQRQQI **Charge** 4 **Spectral Angle** 0.86

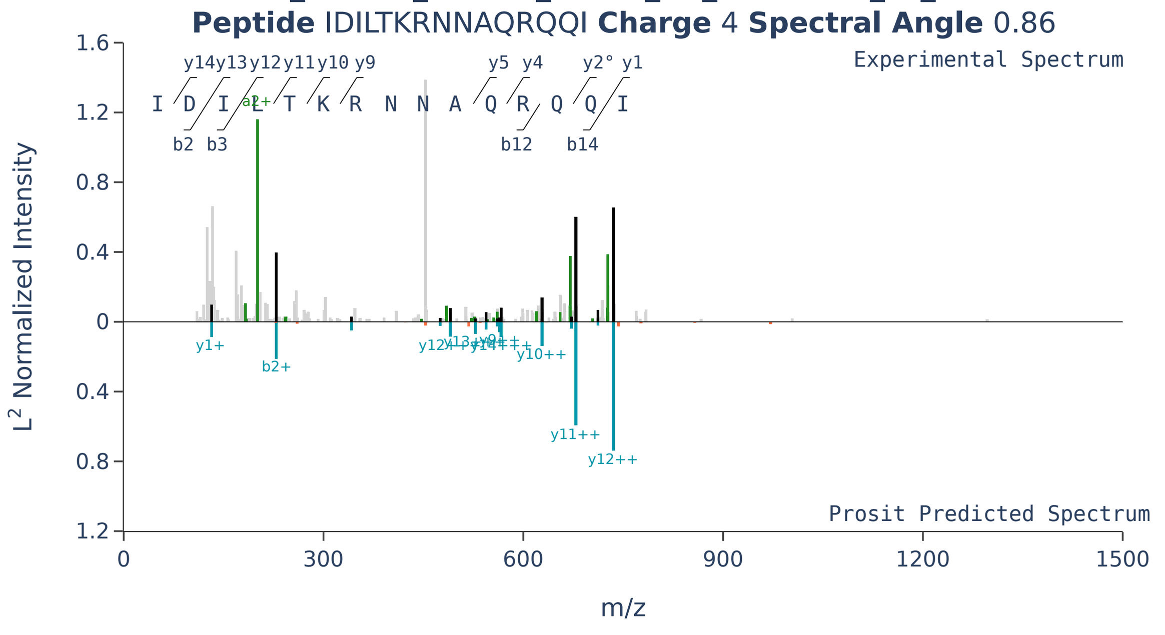


**Source** WSoh_301121_021221_HFGoe_M1_AnnexinA1_24h_R2 **Scan** 21840
**Peptide** GTDVNVF **Charge** 1 **Spectral Angle** 0.87

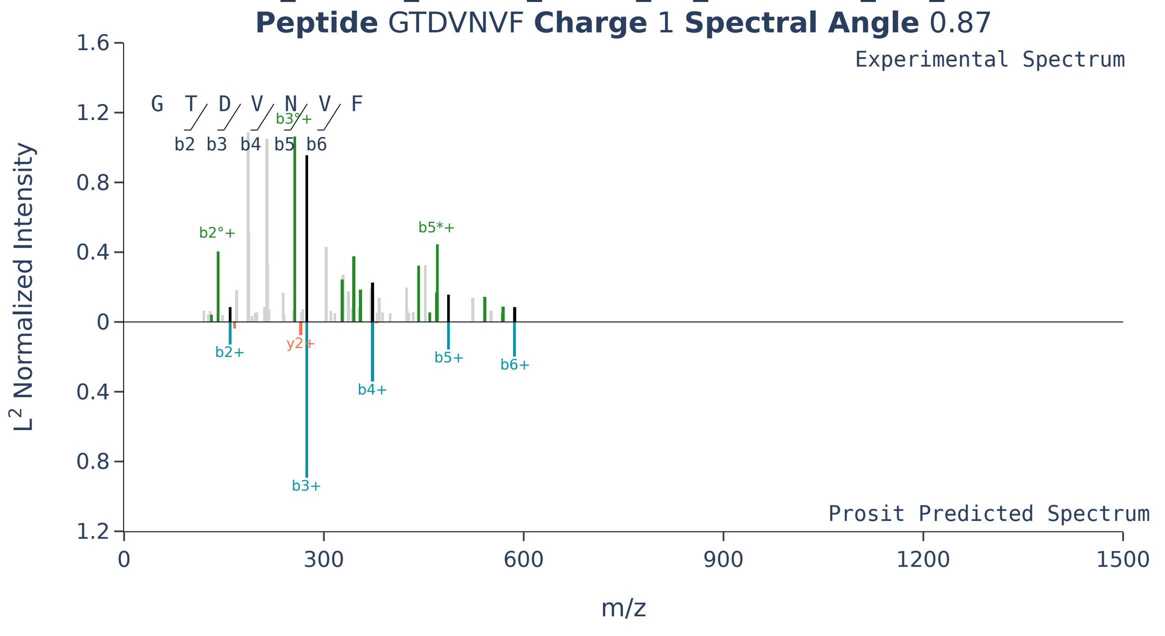


**Source** WSoh_301121_021221_HFGoe_M1_AnnexinA1_4h_R2 **Scan** 11342
**Peptide** KAIMVKGVDE **Charge** 2 **Spectral Angle** 0.87

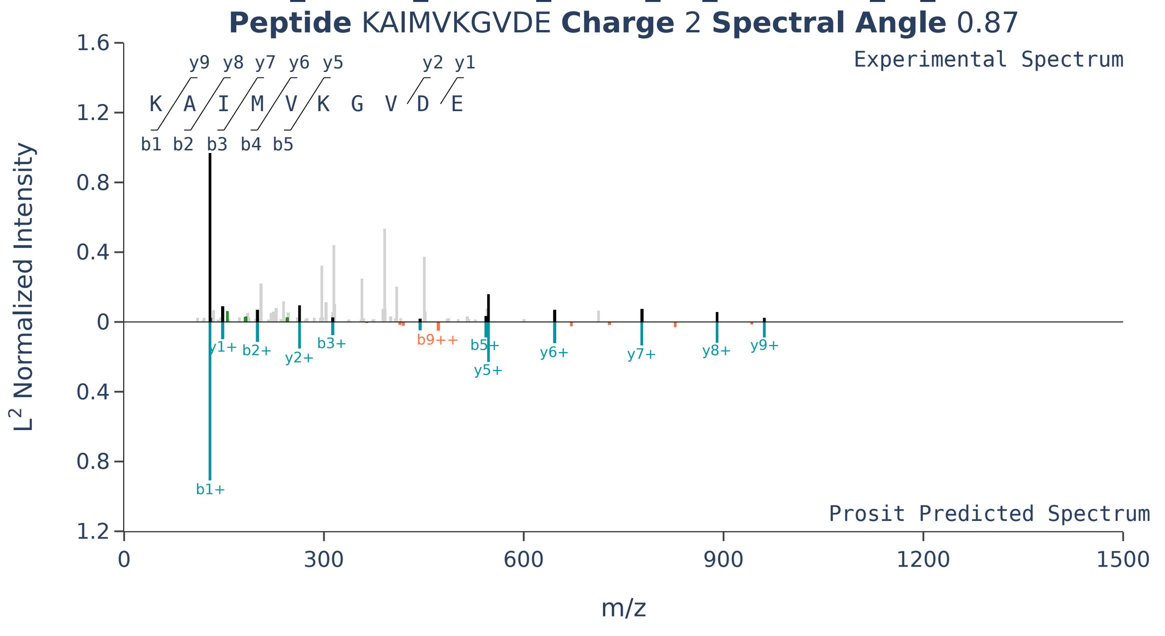


**Source** WSoh_301121_021221_HFGoe_M2_AnnexinA1_24h_R1 **Scan** 14746
**Peptide** TKGDYEKIL **Charge** 2 **Spectral Angle** 0.82

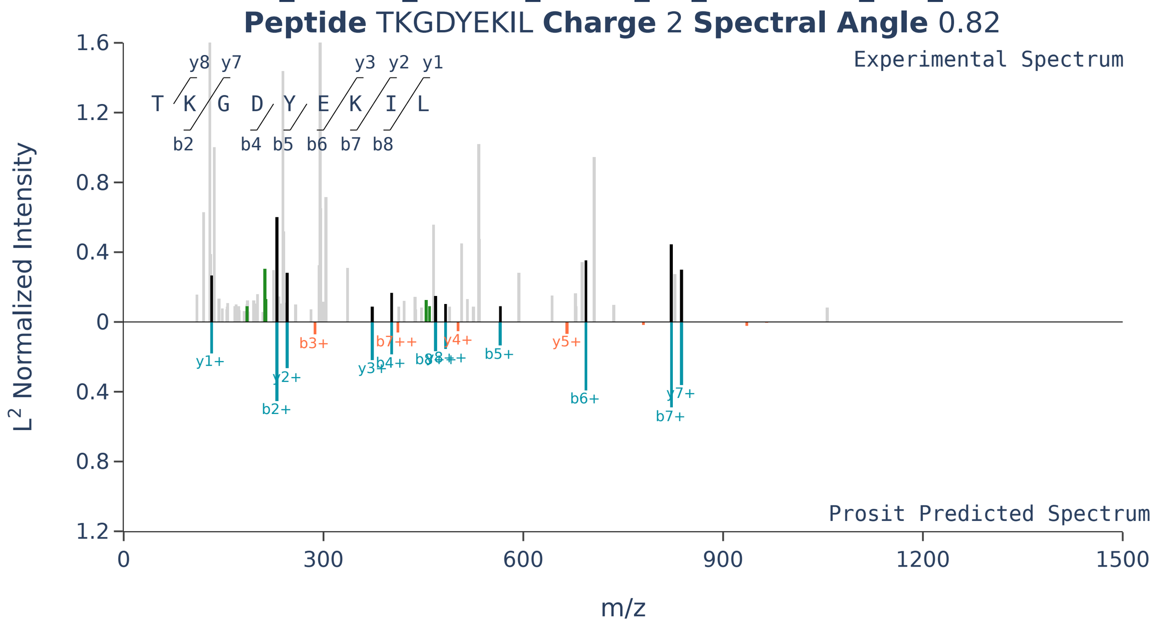

**Source** WSoh_301121_021221_HFGoe_M2_AnnexinA1_4h_R2 **Scan** 7917
**Peptide** RAAMKGLGTD **Charge** 2 **Spectral Angle** 0.83

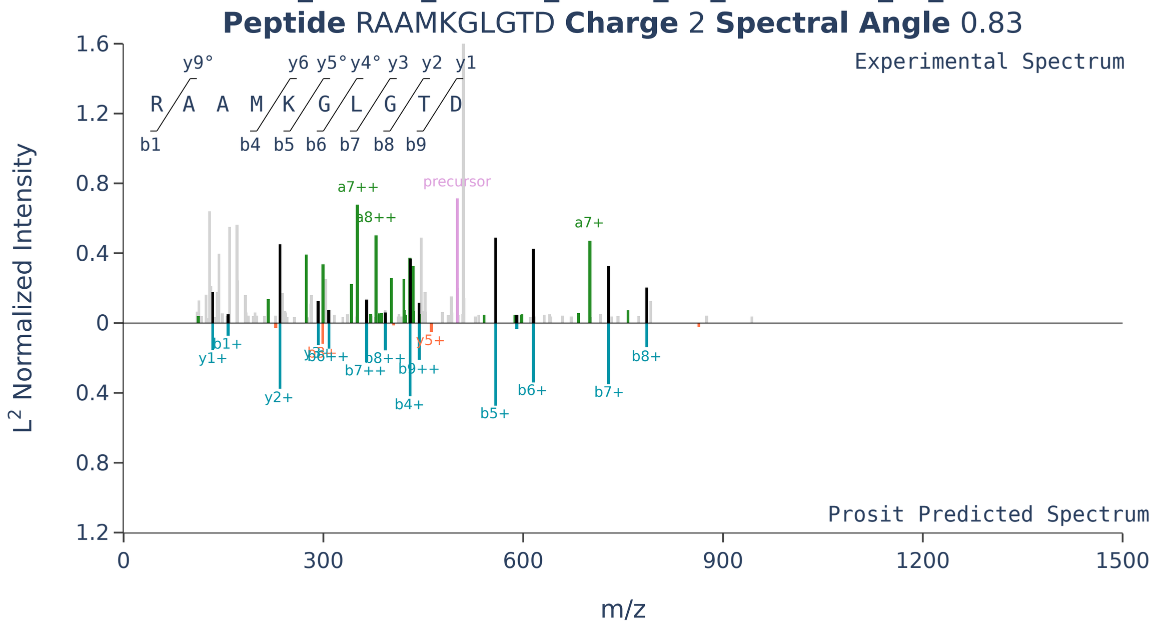


**Source** WSoh_301121_021221_HFGoe_M1_AnnexinA1_4h_R2 **Scan** 8127
**Peptide** KRDLAKDITSD **Charge** 2 **Spectral Angle** 0.83

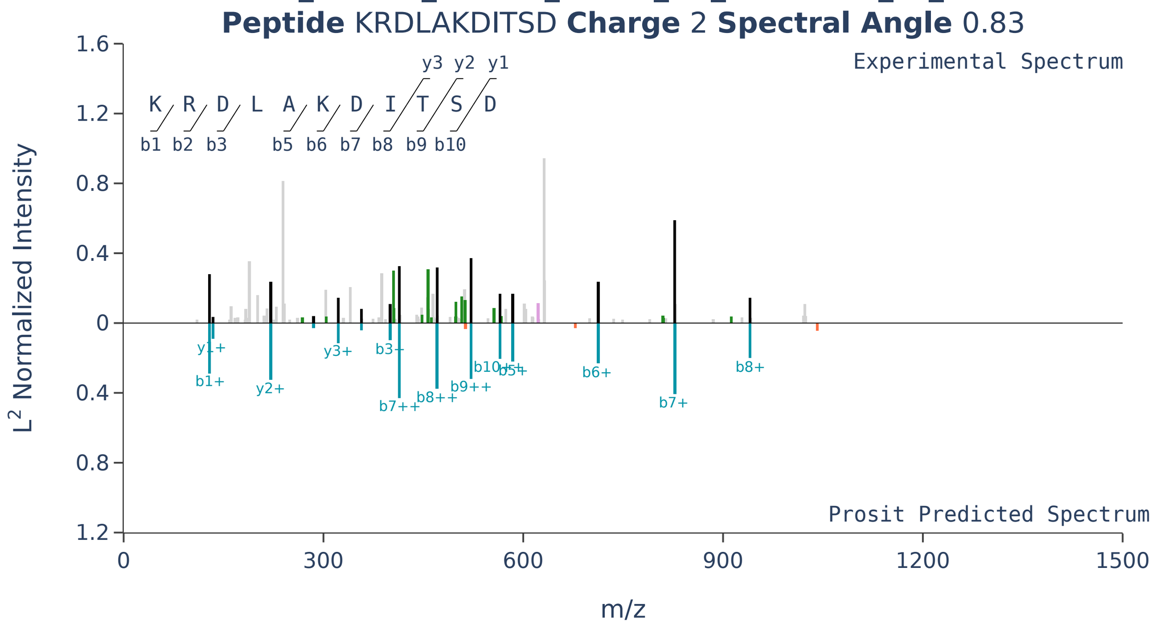


**Source** WSoh_301121_021221_HFGoe_M2_AnnexinA1_24h_R2 **Scan** 14373
**Peptide** LTGHLEEV **Charge** 1 **Spectral Angle** 0.91

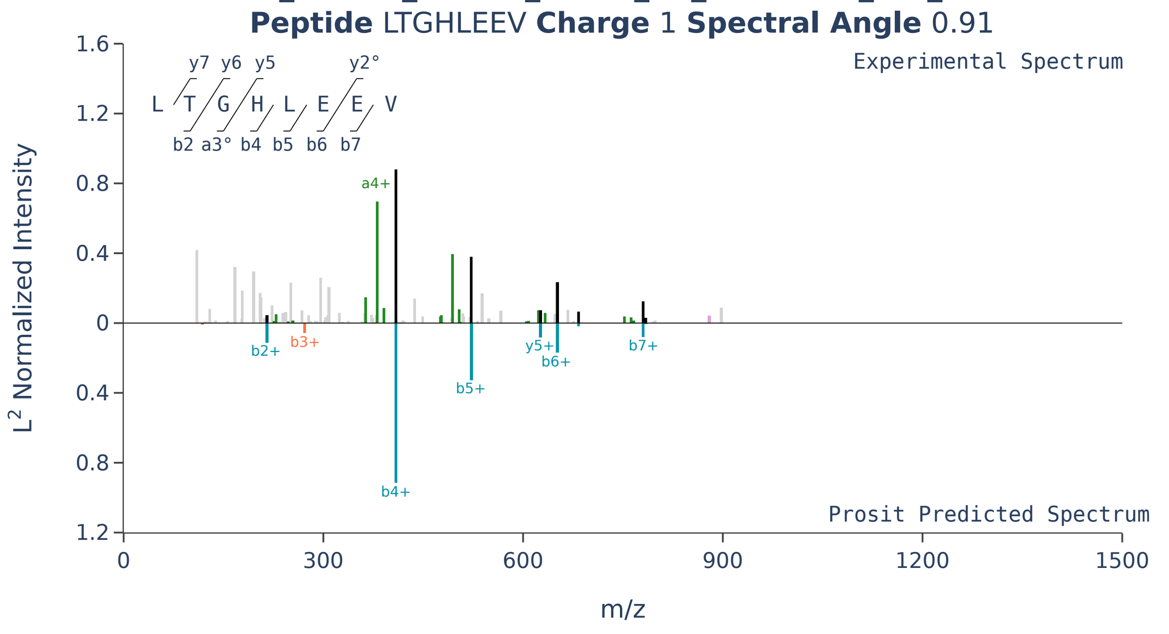


**Source** WSoh_301121_021221_HFGoe_M1_AnnexinA1_24h_R2 **Scan** 13261
**Peptide** RKGTDVNVF **Charge** 2 **Spectral Angle** 0.88

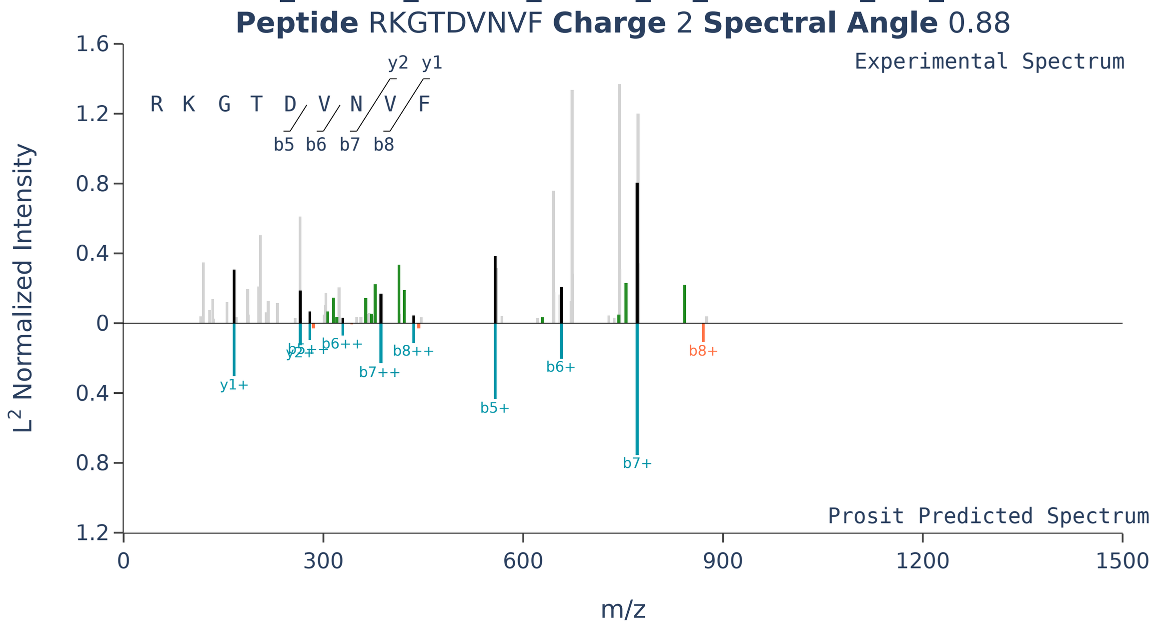


**Source** WSoh_301121_021221_HFGoe_M2_AnnexinA1_1h_R1 **Scan** 8687
**Peptide** HKAIMVKGVD **Charge** 3 **Spectral Angle** 0.9

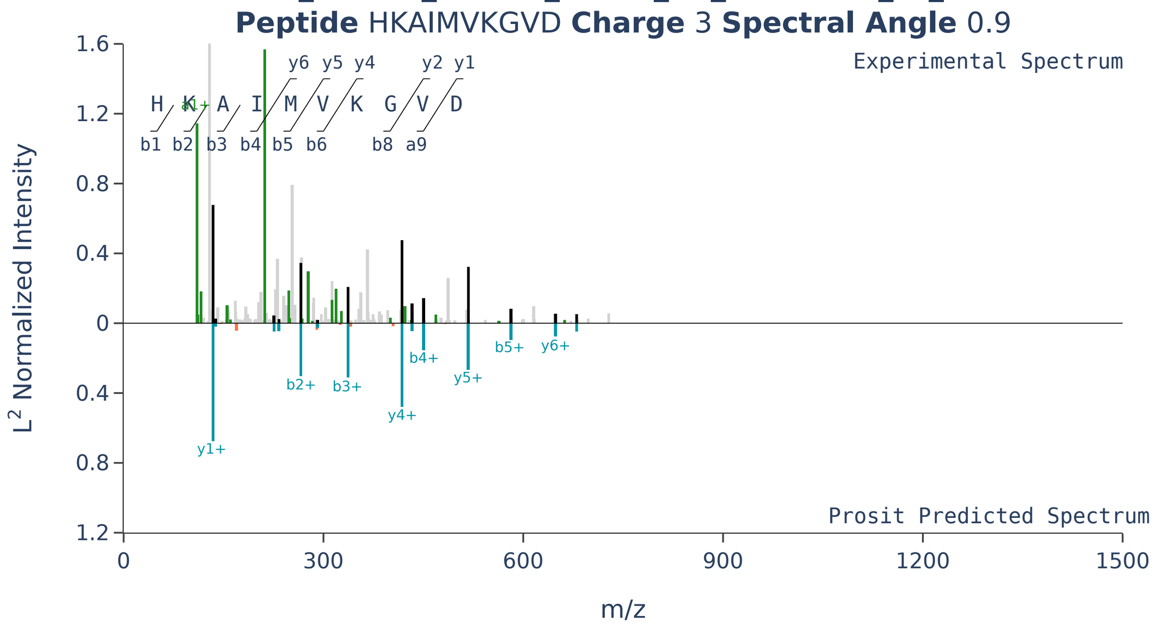


**Source** WSoh_301121_021221_HFGoe_M1_AnnexinA1_4h_R2 **Scan** 19606
**Peptide** HDMNKVLDL **Charge** 2 **Spectral Angle** 0.84

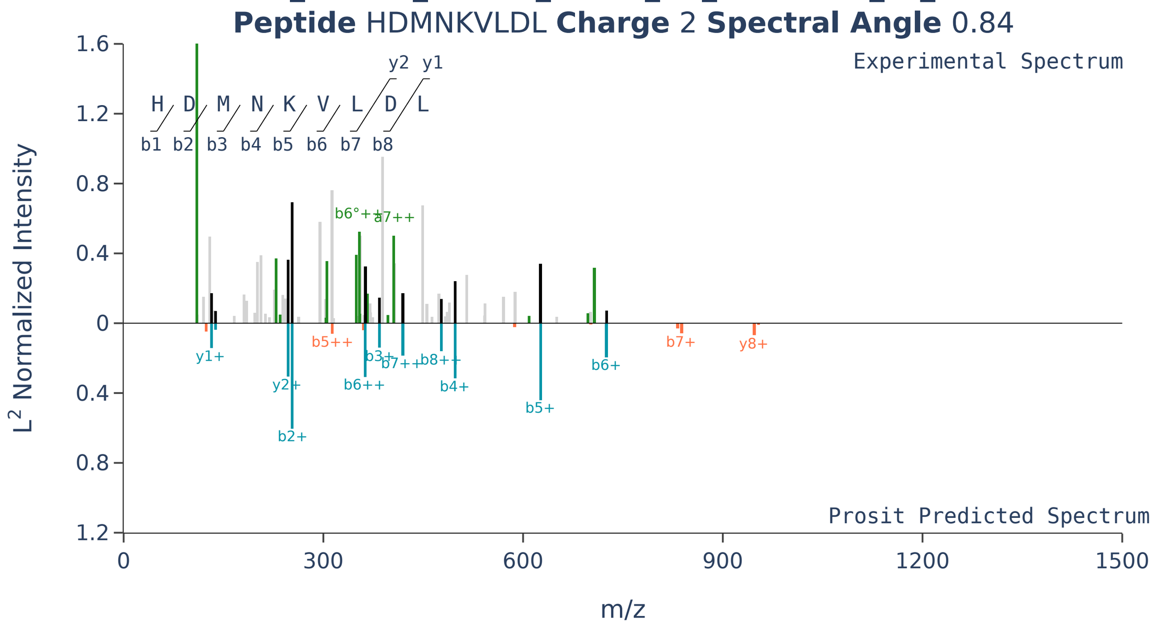


**Source** WSoh_301121_021221_HFGoe_M2_AnnexinA1_2h_R2 **Scan** 18798
**Peptide** EIDMNDIKA **Charge** 2 **Spectral Angle** 0.86

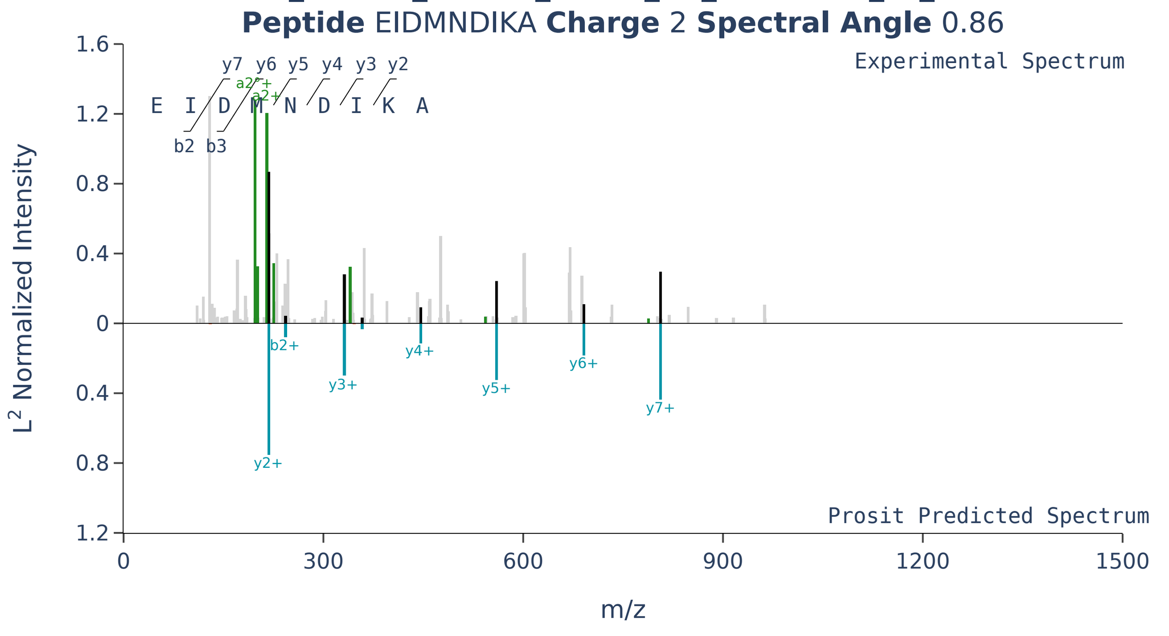


**Source** WSoh_301121_021221_HFGoe_M2_AnnexinA1_24h_R2 **Scan** 7305
**Peptide** KYSKHDMNKVLD **Charge** 2 **Spectral Angle** 0.9

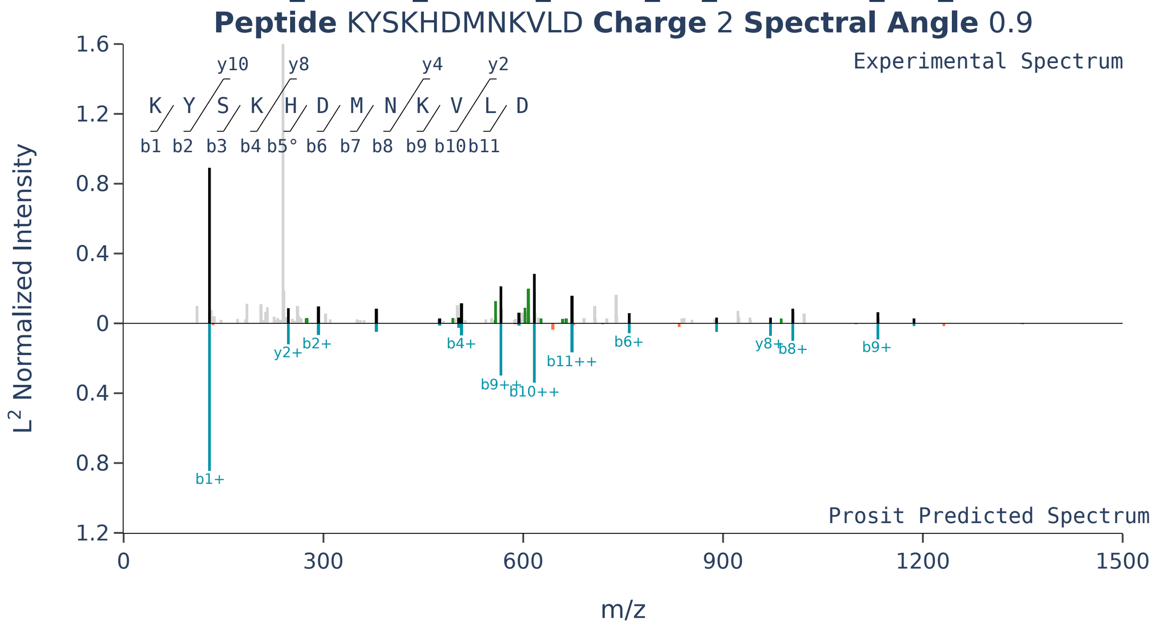


**Source** WSoh_301121_021221_HFGoe_M2_AnnexinA1_4h_R1 **Scan** 11353
**Peptide** ALHKAIMVKGVD **Charge** 3 **Spectral Angle** 0.9

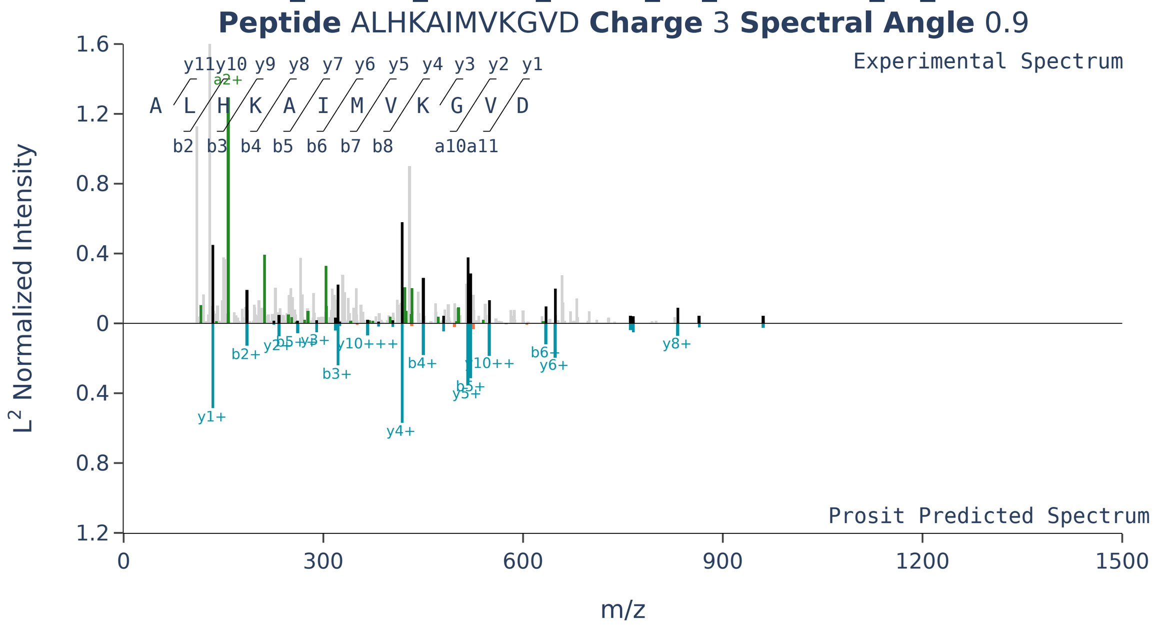


**Source** WSoh_301121_021221_HFGoe_M2_AnnexinA1_24h_R2 **Scan** 12894
**Peptide** YEAGERRKGTDVNVF **Charge** 3 **Spectral Angle** 0.87

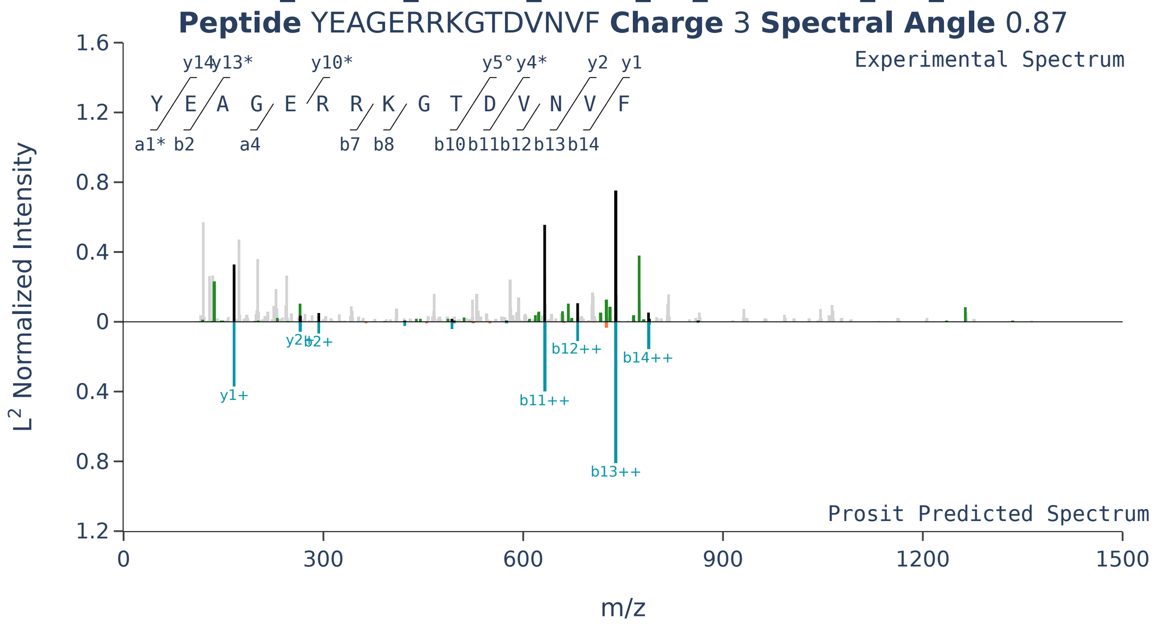


**Source** WSoh_301121_021221_HFGoe_M2_AnnexinA1_4h_R1 **Scan** 12409
**Peptide** LTTRSYPQLR **Charge** 3 **Spectral Angle** 0.86

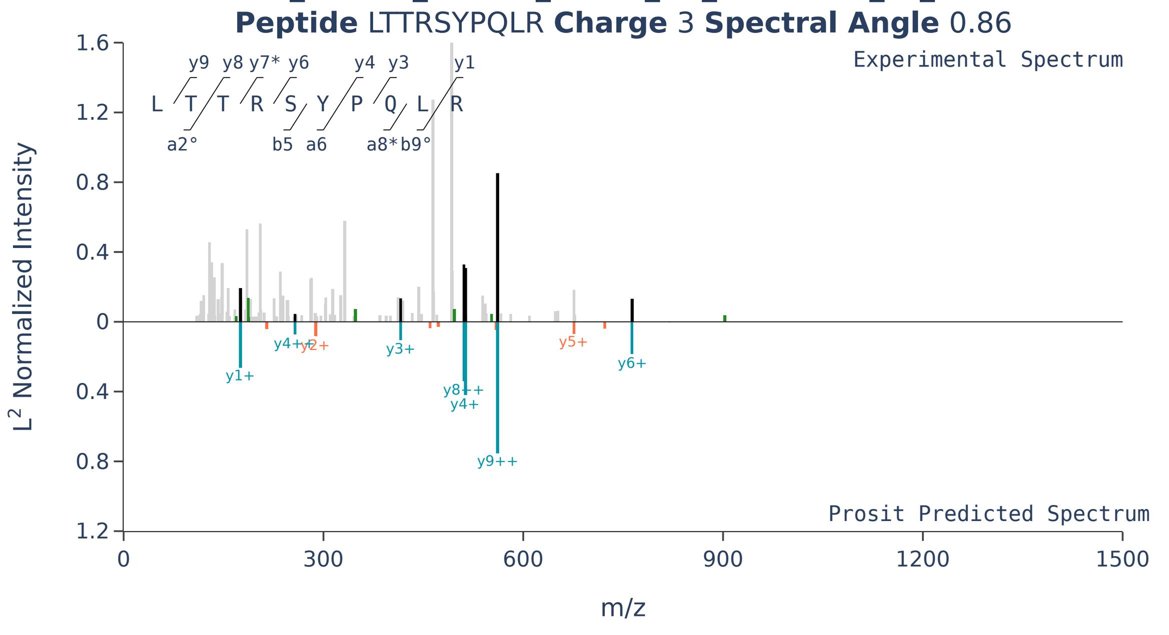


**Source** WSoh_301121_021221_HFGoe_M1_AnnexinA1_24h_R1 **Scan** 17568
**Peptide** DETKGDYEKIL **Charge** 2 **Spectral Angle** 0.84

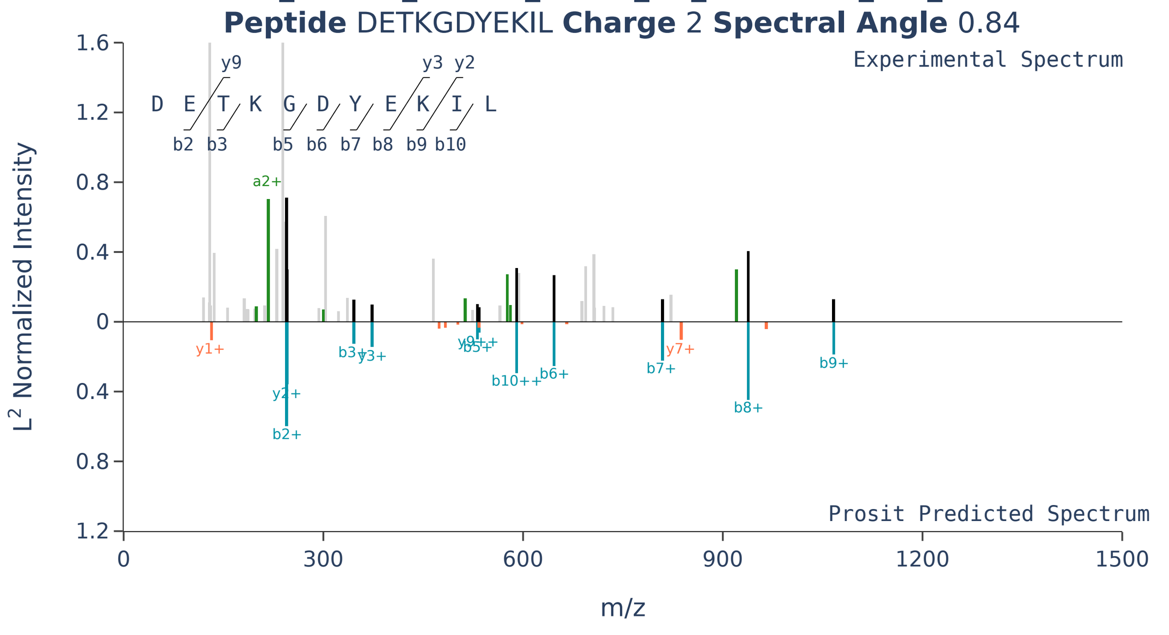


**Source** WSoh_301121_021221_HFGoe_M1_AnnexinA1_24h_R1 **Scan** 11475
**Peptide** KGVDEATI **Charge** 1 **Spectral Angle** 0.88

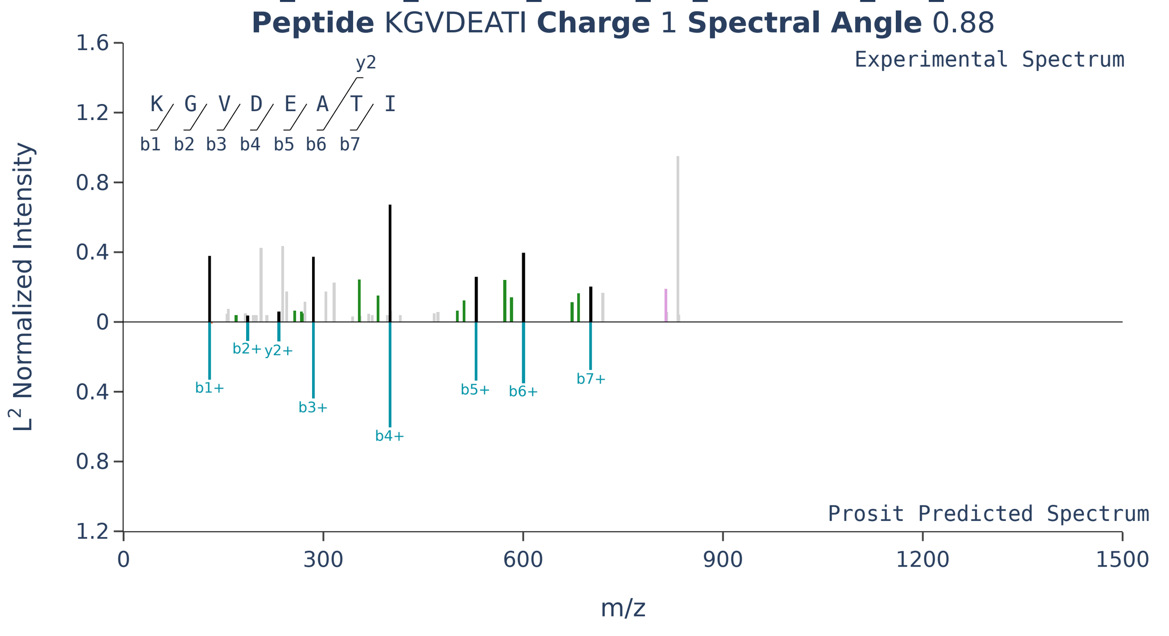


**Source** WSoh_301121_021221_HFGoe_M1_AnnexinA1_24h_R1 **Scan** 11666
**Peptide** HKALIRI **Charge** 2 **Spectral Angle** 0.92

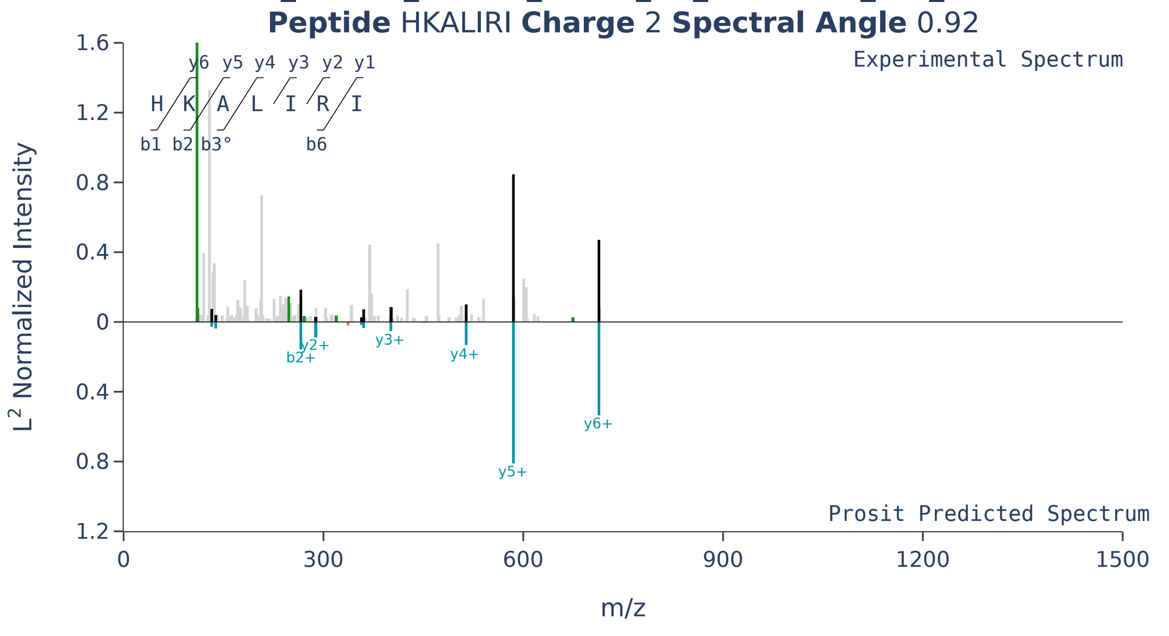


**Source** WSoh_301121_021221_HFGoe_M2_AnnexinA1_2h_R1 **Scan** 15156
**Peptide** YLQETGKPLDETLKKA **Charge** 4 **Spectral Angle** 0.87

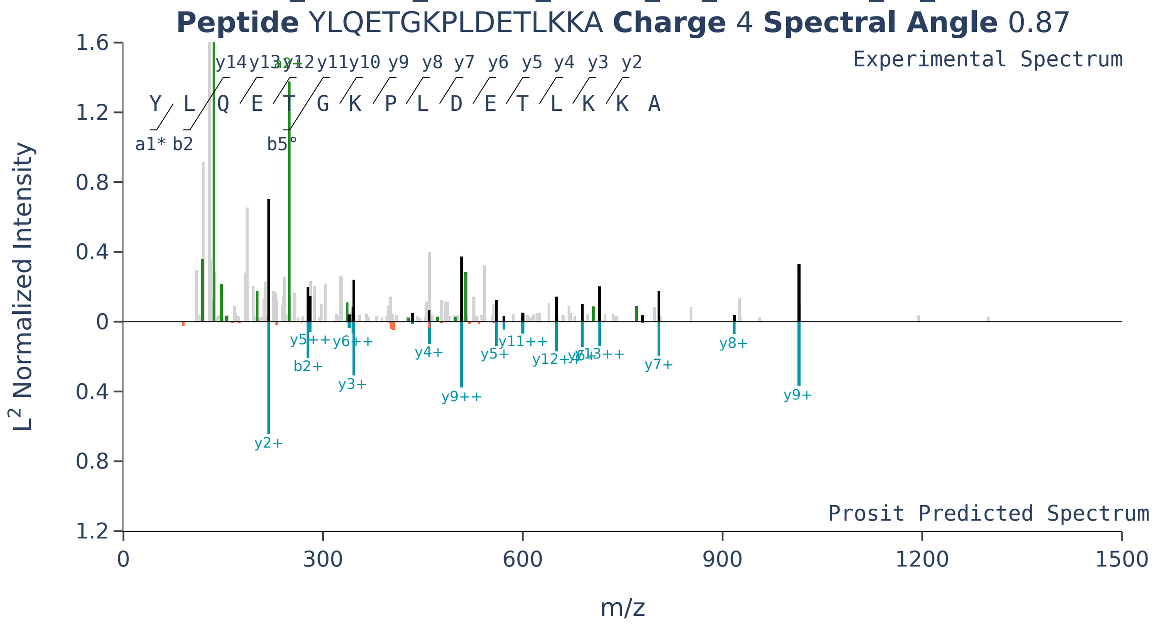

**Source** WSoh_301121_021221_HFGoe_M1_AnnexinA1_24h_R2 **Scan** 8292
**Peptide** SDARALYEAGERRK **Charge** 4 **Spectral Angle** 0.86

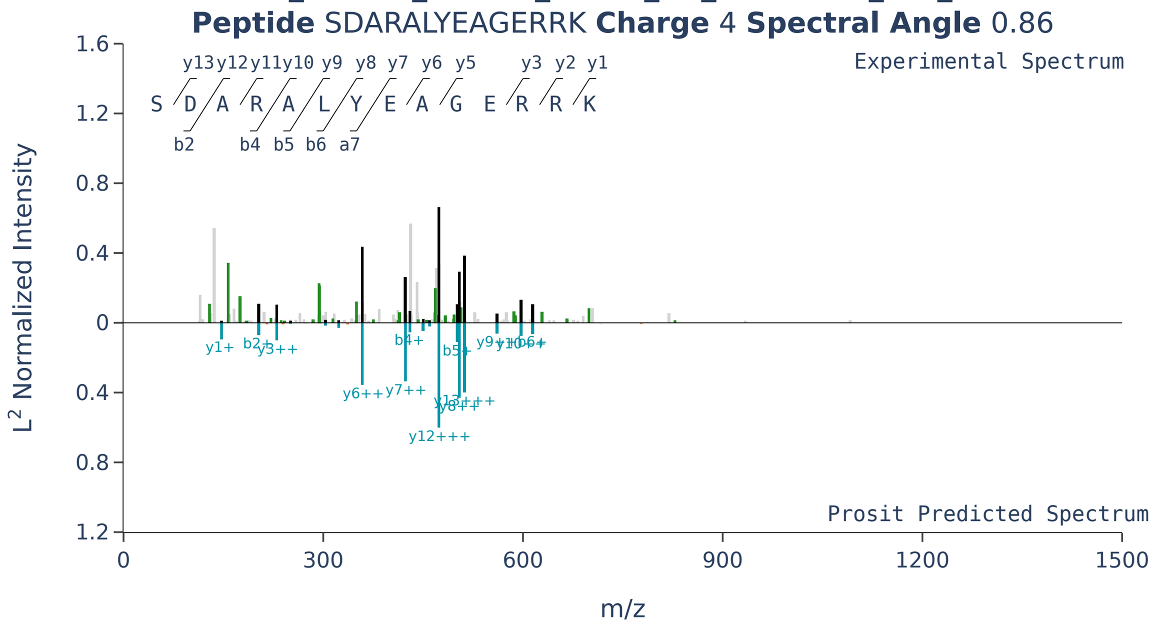


**Source** WSoh_301121_021221_HFGoe_M2_AnnexinA1_2h_R2 **Scan** 13861
**Peptide** ETLKKALTGHLEE **Charge** 2 **Spectral Angle** 0.83

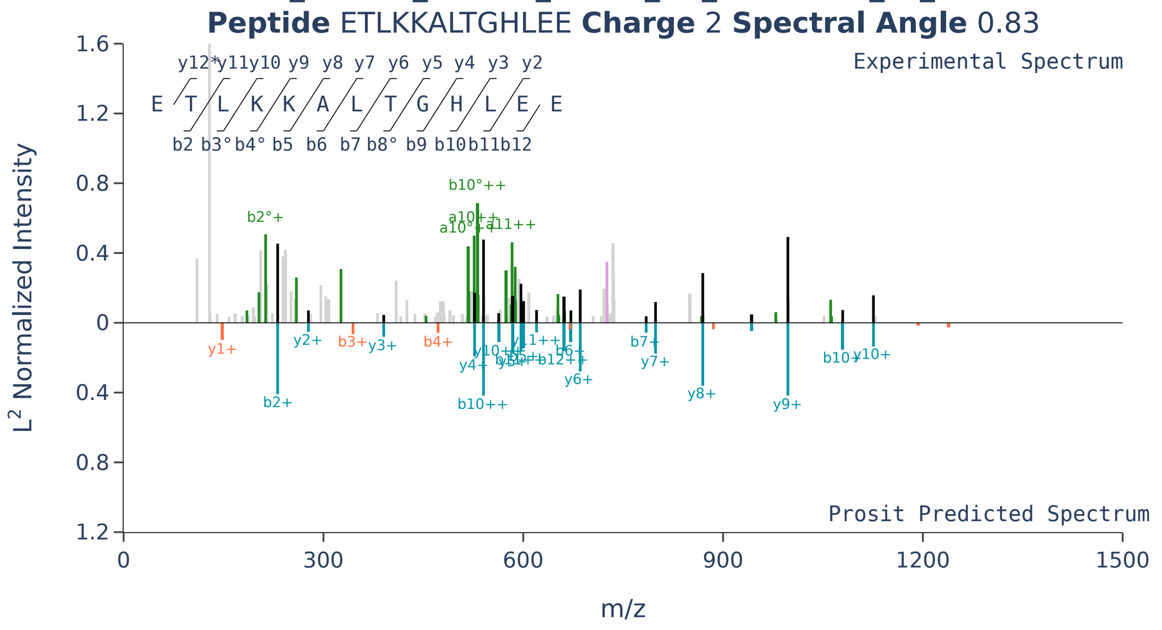


**Source** WSoh_301121_021221_HFGoe_M2_AnnexinA1_2h_R1 **Scan** 18994
**Peptide** SLAKGDRSEDFGVNEDL **Charge** 3 **Spectral Angle** 0.84

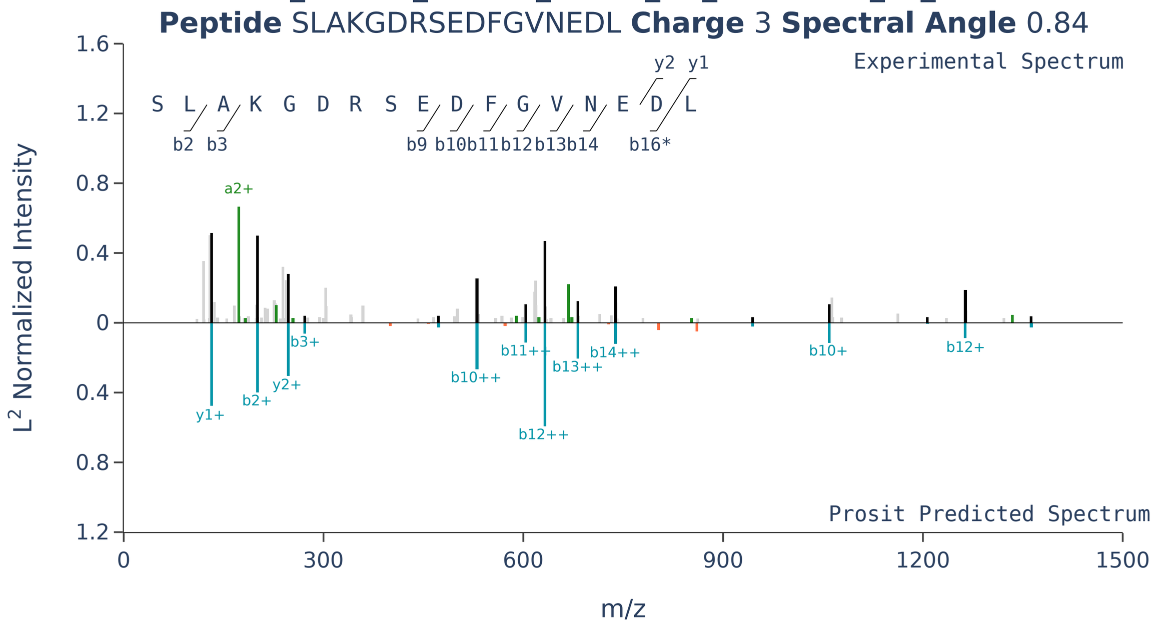


**Source** WSoh_301121_021221_HFGoe_M2_AnnexinA1_24h_R2 **Scan** 13971
**Peptide** AKDITSDTSGDF **Charge** 2 **Spectral Angle** 0.9

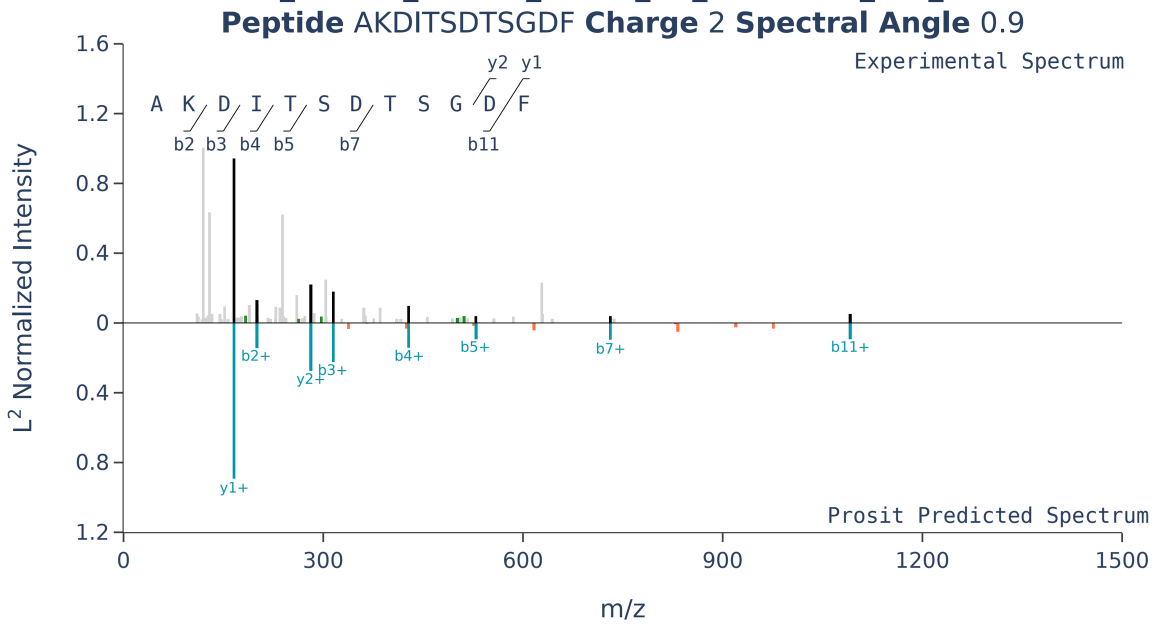


**Source** WSoh_301121_021221_HFGoe_M2_AnnexinA1_24h_R2 **Scan** 10858
**Peptide** EQEYVQT **Charge** 1 **Spectral Angle** 0.94

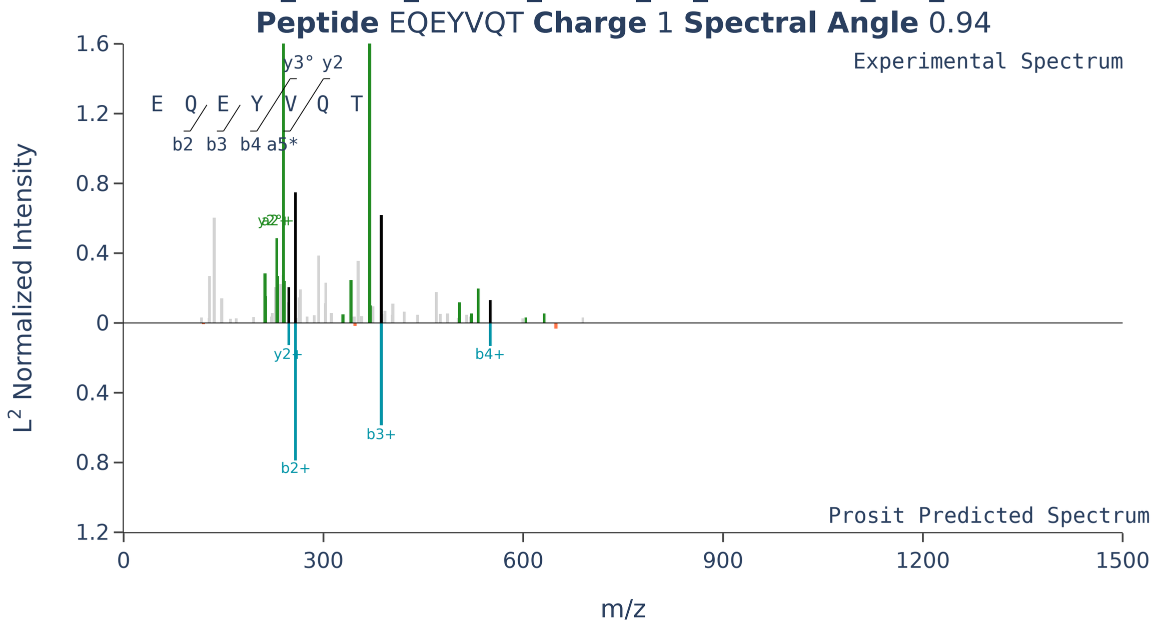


**Source** WSoh_301121_021221_HFGoe_M2_AnnexinA1_2h_R2 **Scan** 19757
**Peptide** MNDIKAFYQ **Charge** 2 **Spectral Angle** 0.84

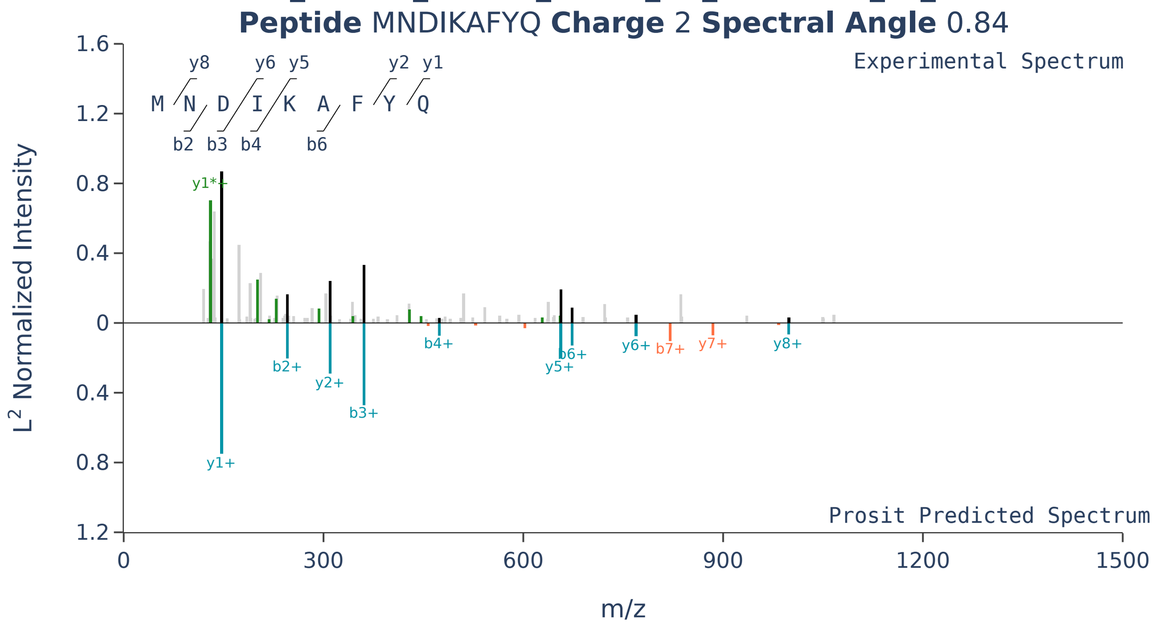


**Source** WSoh_301121_021221_HFGoe_M2_AnnexinA1_24h_R2 **Scan** 7375
**Peptide** KRDLAKDI **Charge** 2 **Spectral Angle** 0.83

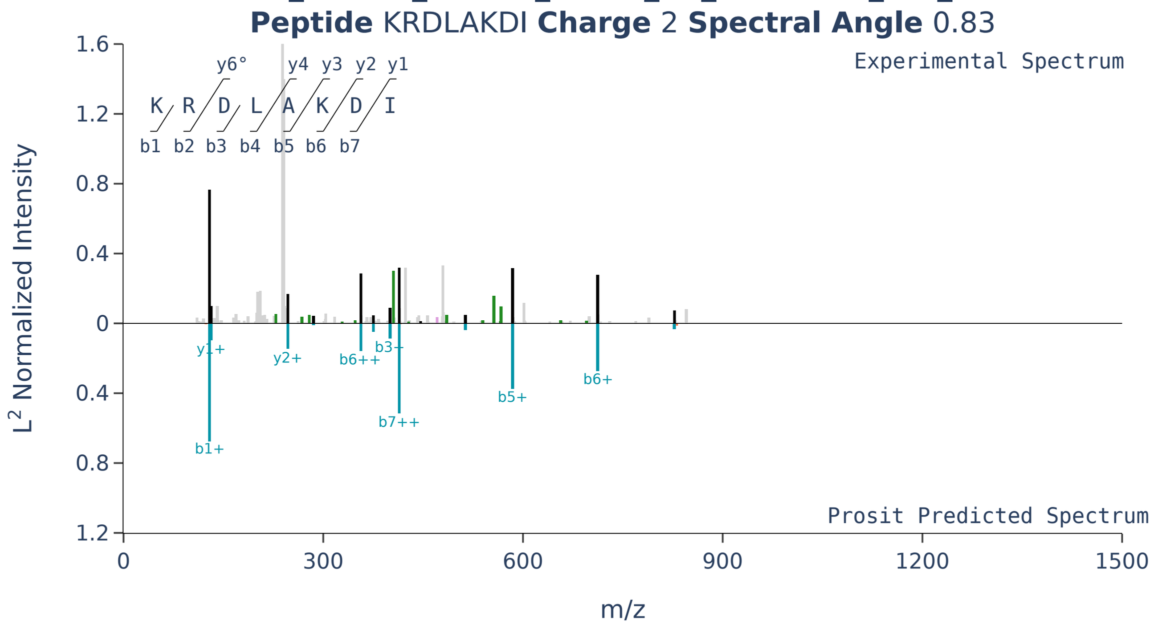


**Source** WSoh_301121_021221_HFGoe_M2_AnnexinA1_1h_R2 **Scan** 12785
**Peptide** ELKRDLAKDITSD **Charge** 2 **Spectral Angle** 0.77

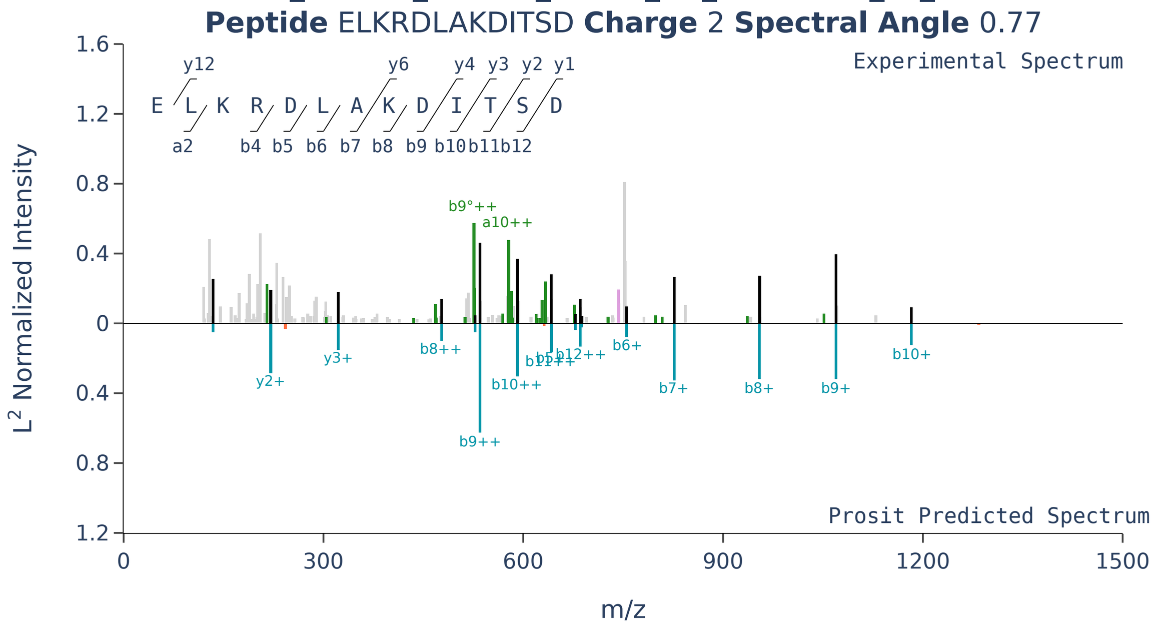


**Source** WSoh_301121_021221_HFGoe_M2_AnnexinA1_2h_R2 **Scan** 7729
**Peptide** RAAMKGL **Charge** 2 **Spectral Angle** 0.83

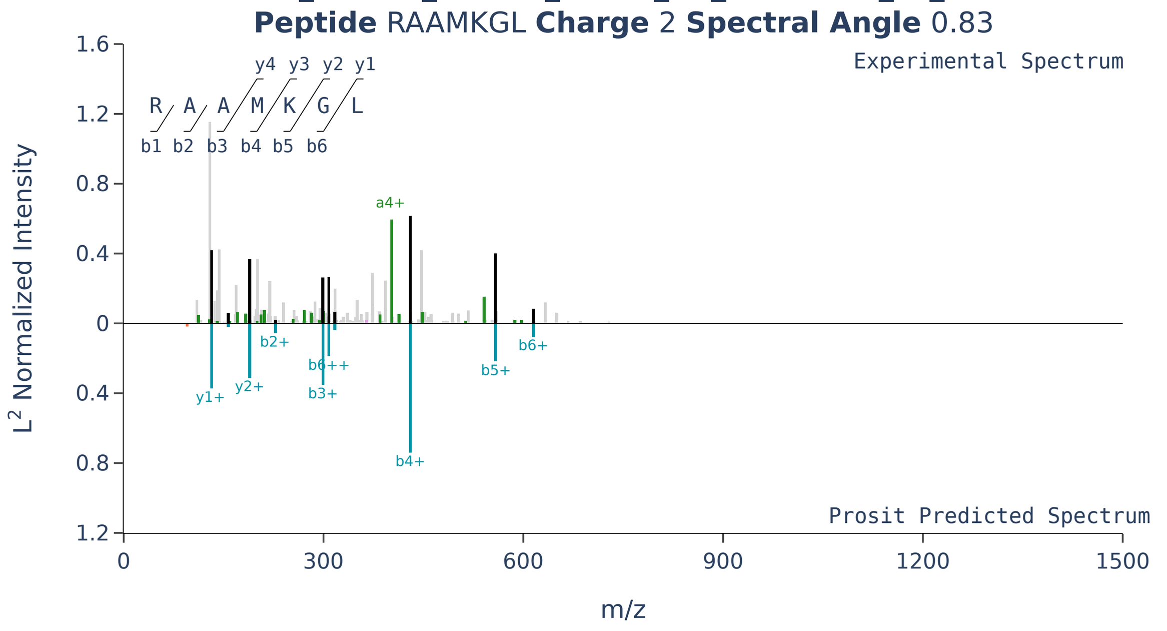


**Source** WSoh_301121_021221_HFGoe_M2_AnnexinA1_2h_R2 **Scan** 12675
**Peptide** PQLRRVFQKY **Charge** 3 **Spectral Angle** 0.88

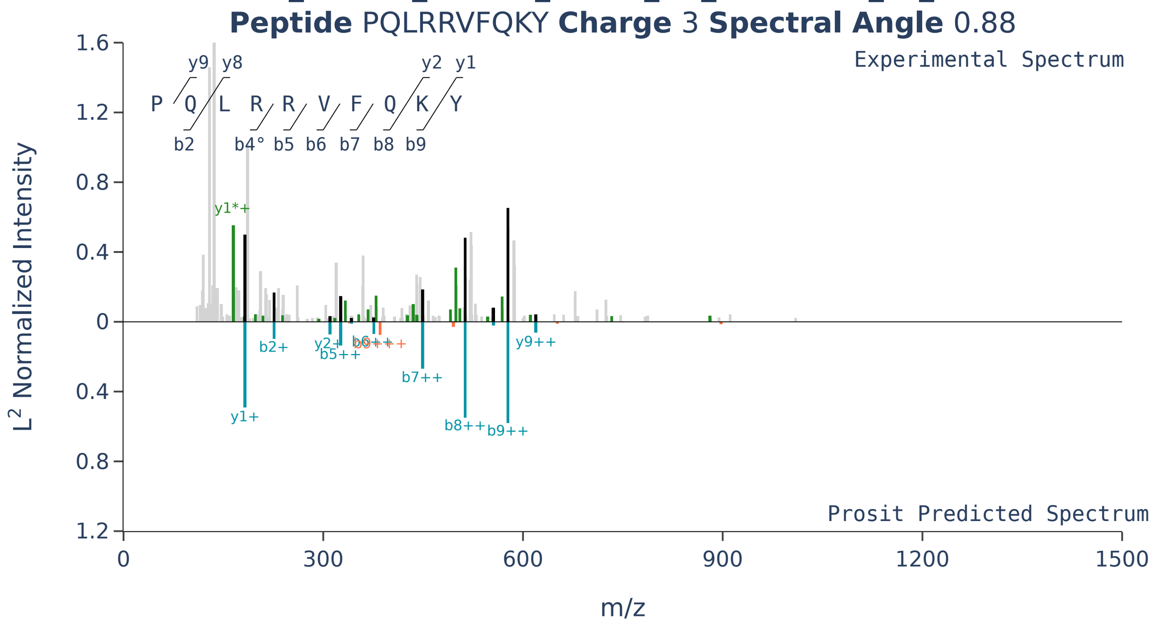


**Source** WSoh_301121_021221_HFGoe_M2_AnnexinA1_2h_R1 **Scan** 17669
**Peptide** NTILTTRSYPQLRRV **Charge** 4 **Spectral Angle** 0.95

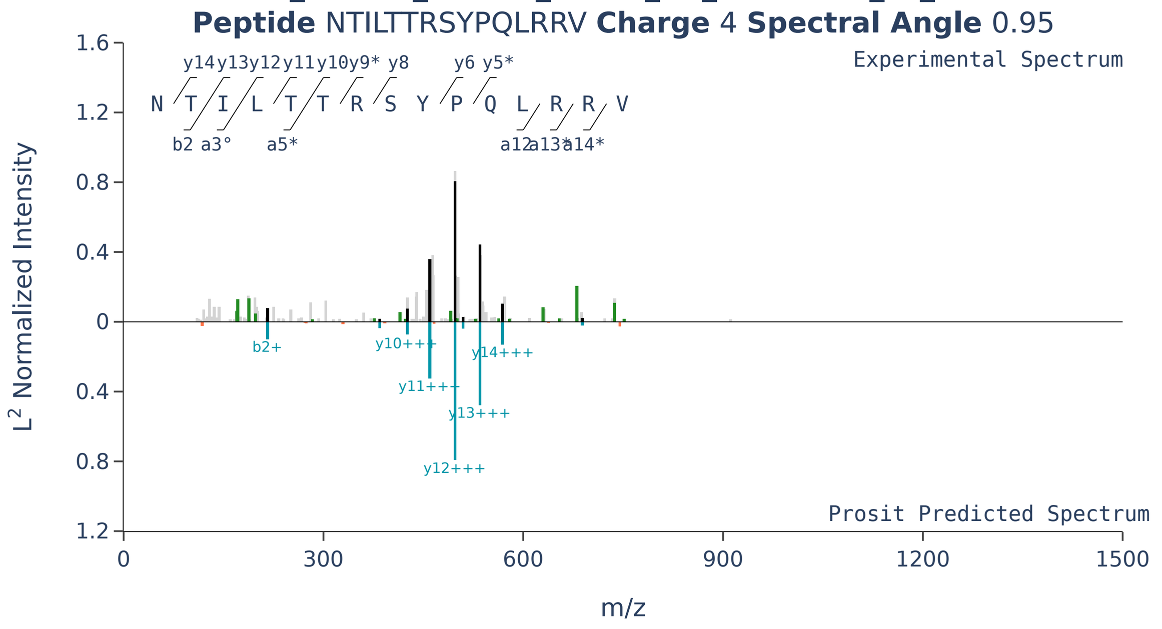


**Source** WSoh_301121_021221_HFGoe_M1_AnnexinA1_24h_R2 **Scan** 10573
**Peptide** QETGKPLDETLKKA **Charge** 4 **Spectral Angle** 0.79

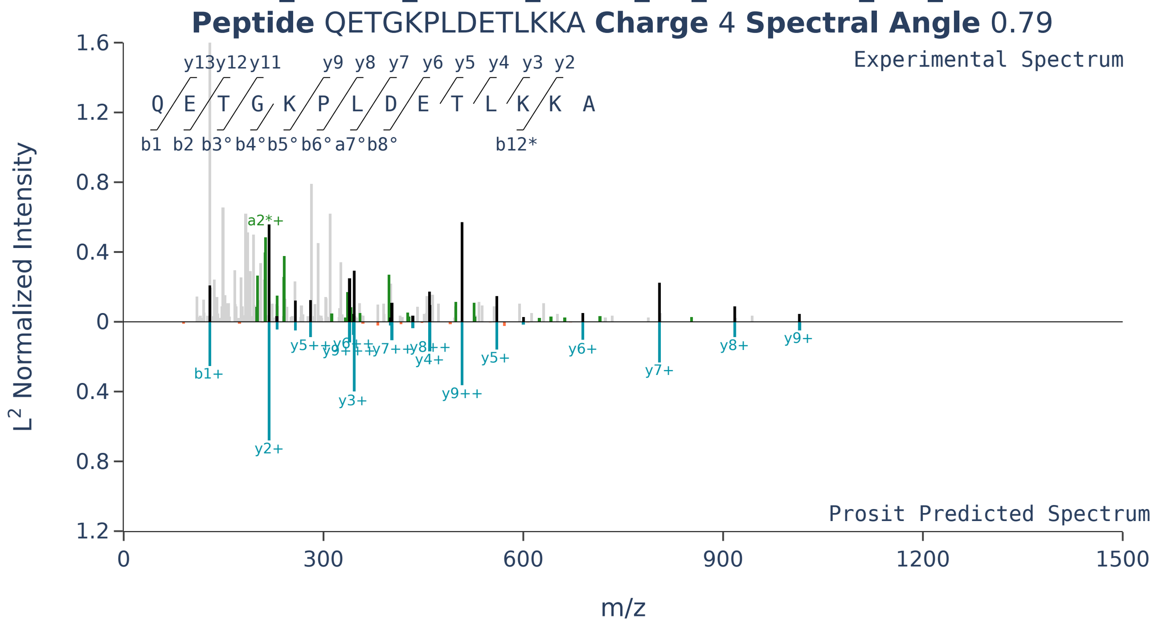


**Source** WSoh_301121_021221_HFGoe_M2_AnnexinA1_24h_R2 **Scan** 16934
**Peptide** DINRVYREEL **Charge** 3 **Spectral Angle** 0.92

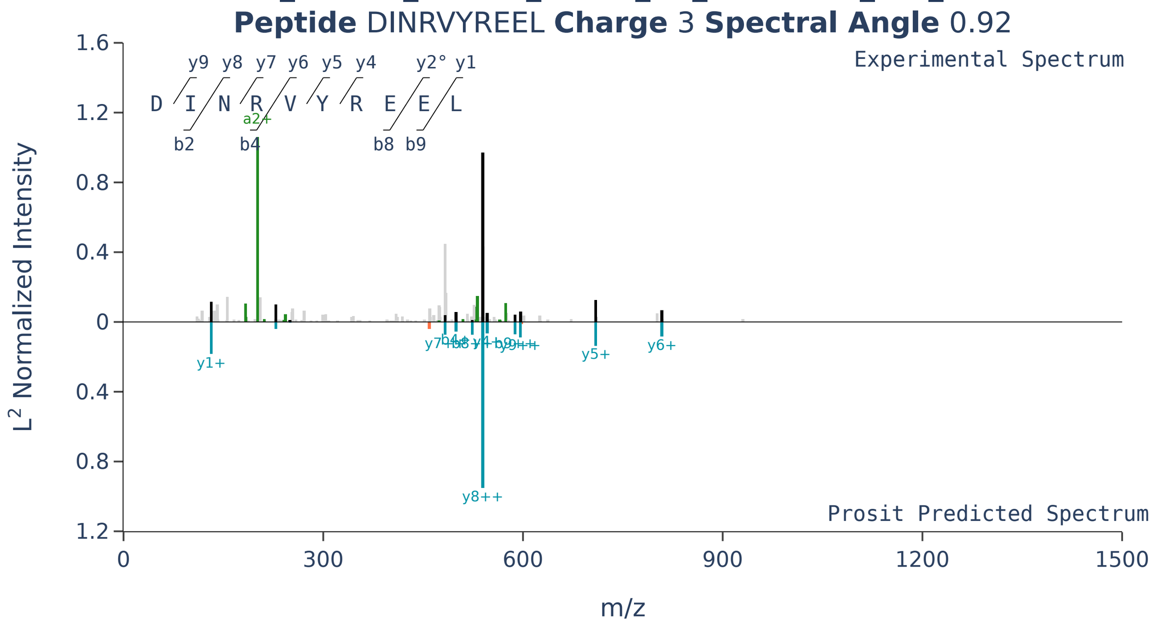


**Source** WSoh_301121_021221_HFGoe_M1_AnnexinA1_24h_R2 **Scan** 11866
**Peptide** GDRSEDFGVN **Charge** 2 **Spectral Angle** 0.79

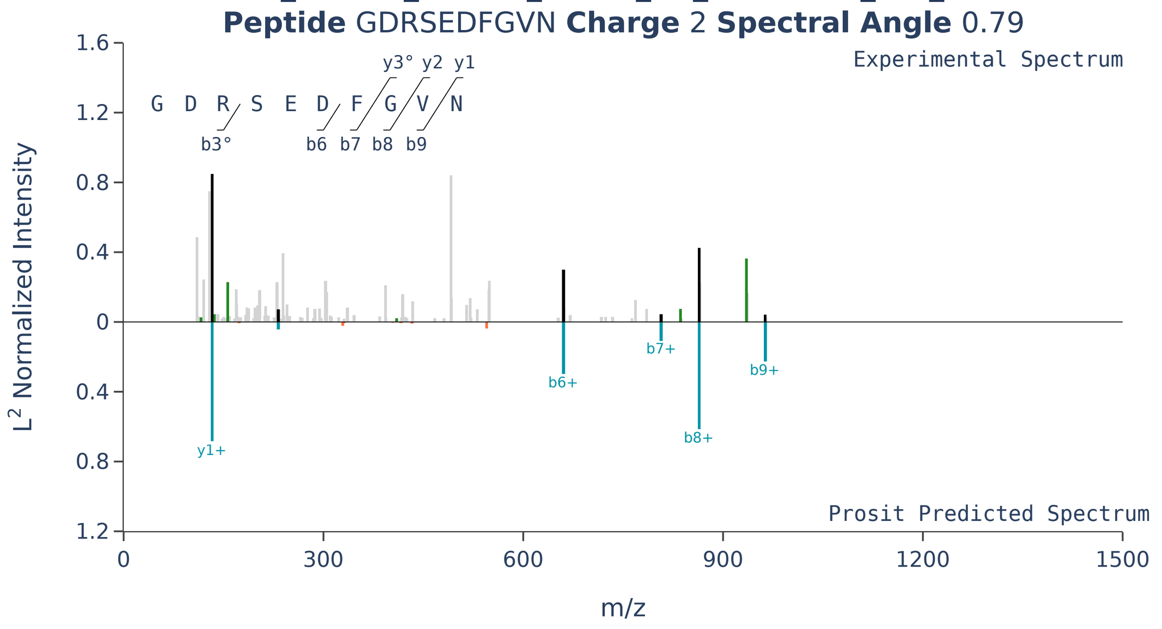


**Source** WSoh_301121_021221_HFGoe_M2_AnnexinA1_2h_R2 **Scan** 15068
**Peptide** LRDINRVYREEL **Charge** 3 **Spectral Angle** 0.82

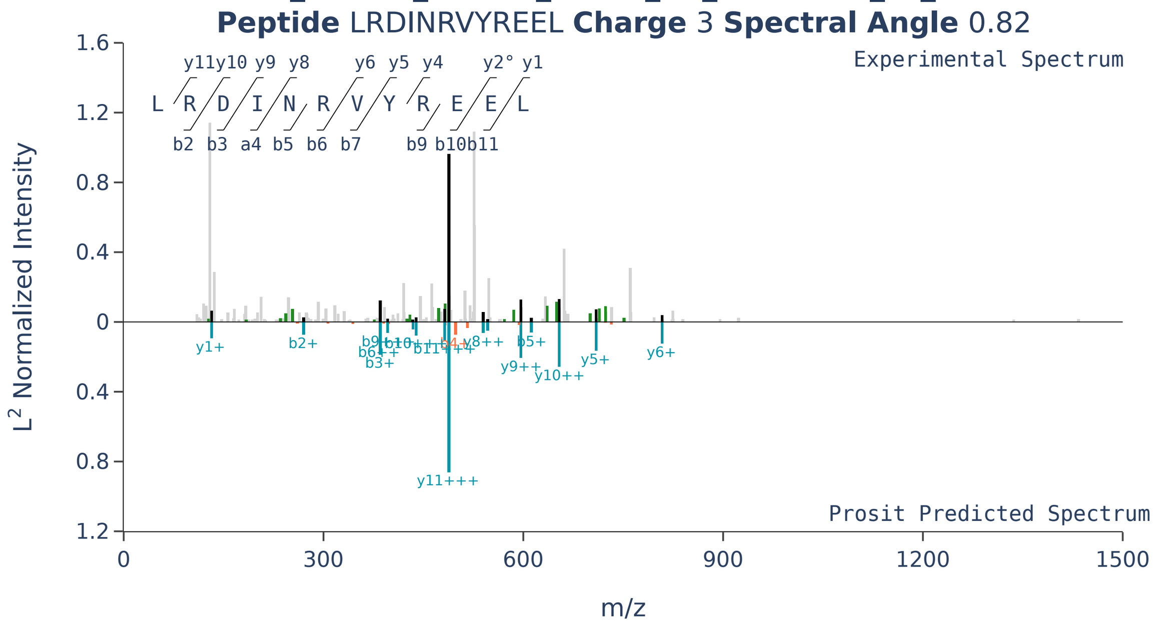

**Source** WSoh_301121_021221_HFGoe_M2_AnnexinA1_2h_R1 **Scan** 18821
**Peptide** PAFFAEKLHQ **Charge** 3 **Spectral Angle** 0.86

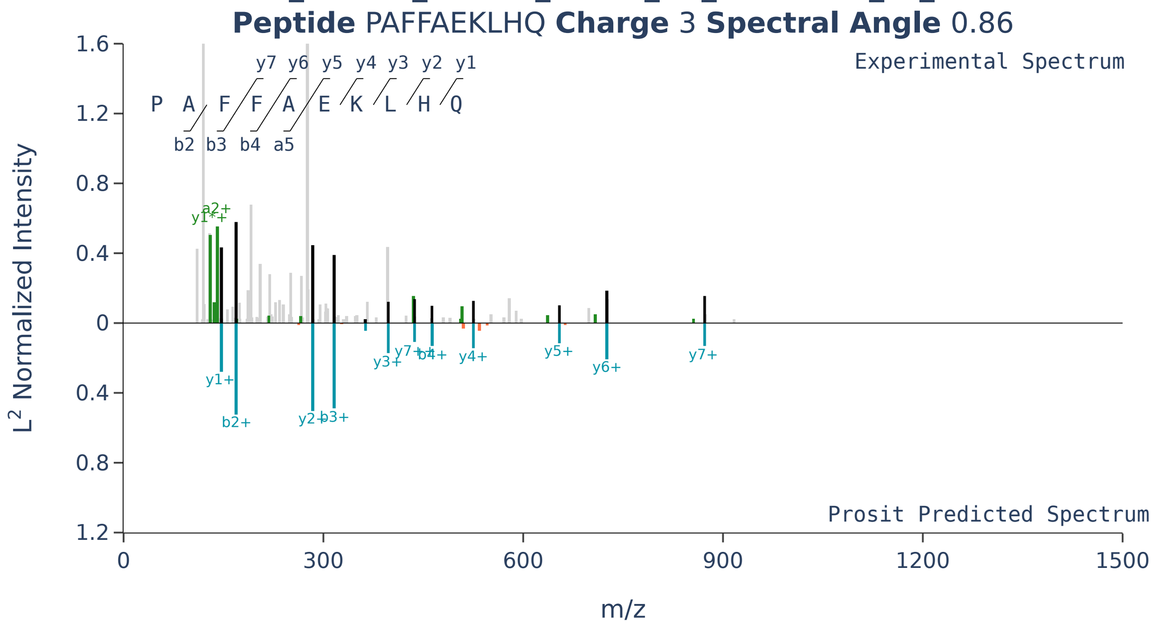


**Source** WSoh_301121_021221_HFGoe_M1_AnnexinA1_24h_R2 **Scan** 7172
**Peptide** ELRDINR **Charge** 2 **Spectral Angle** 0.76

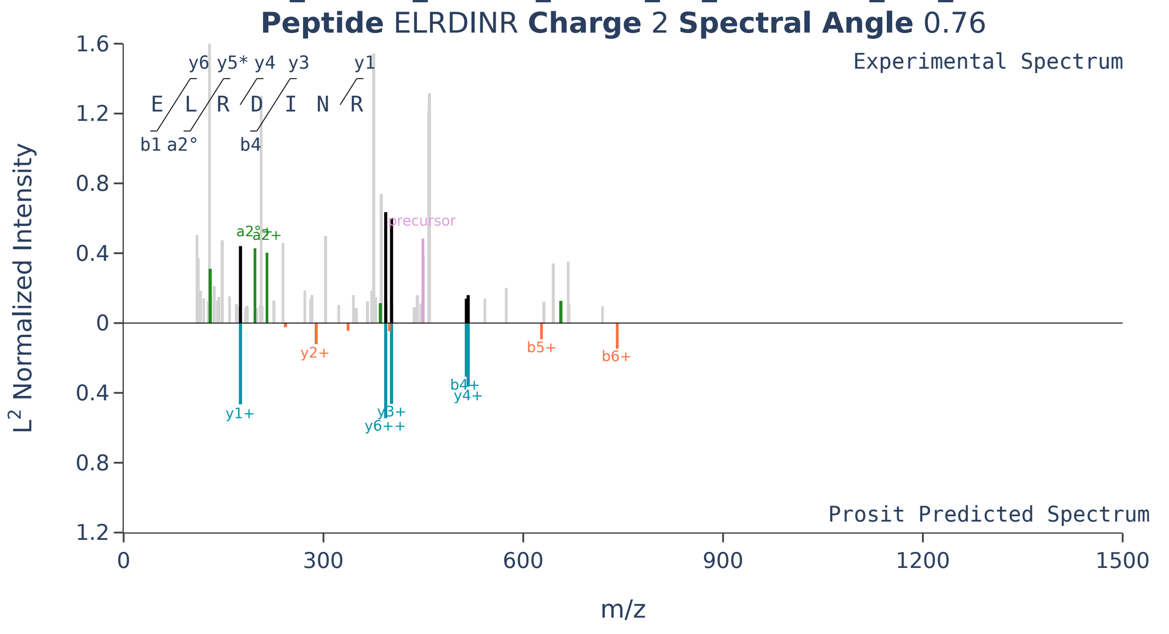


**Source** WSoh_301121_021221_HFGoe_M2_AnnexinA1_24h_R1 **Scan** 19892
**Peptide** PAQFDADELRAA **Charge** 2 **Spectral Angle** 0.88

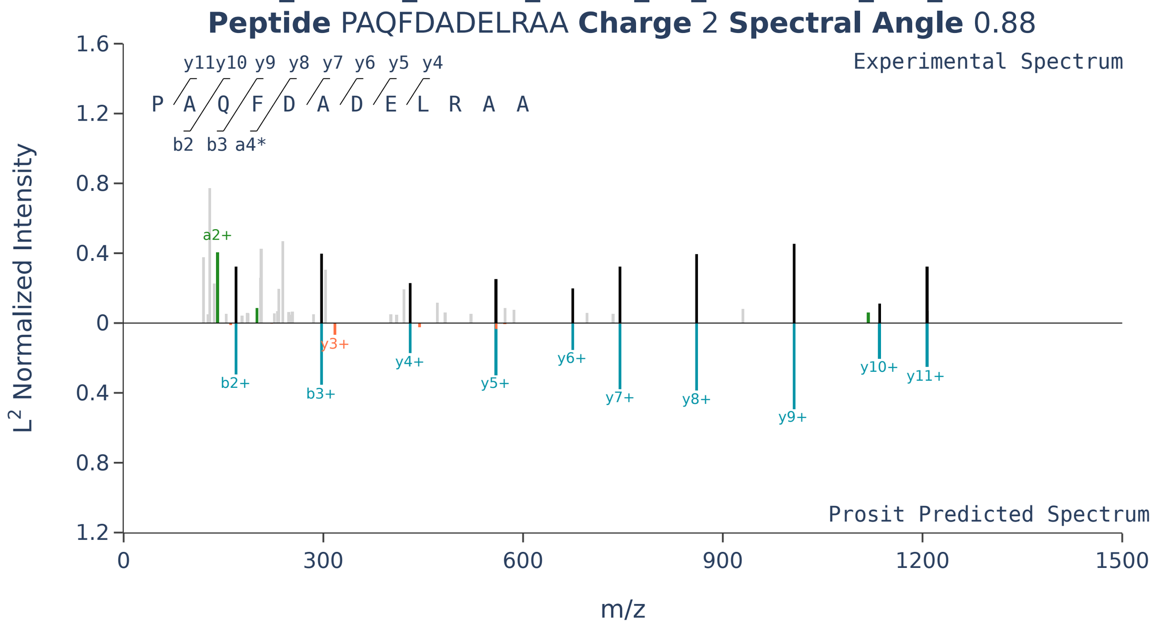


**Source** WSoh_301121_021221_HFGoe_M1_AnnexinA1_4h_R1 **Scan** 6329
**Peptide** ASRTNKEIRDIN **Charge** 3 **Spectral Angle** 0.85

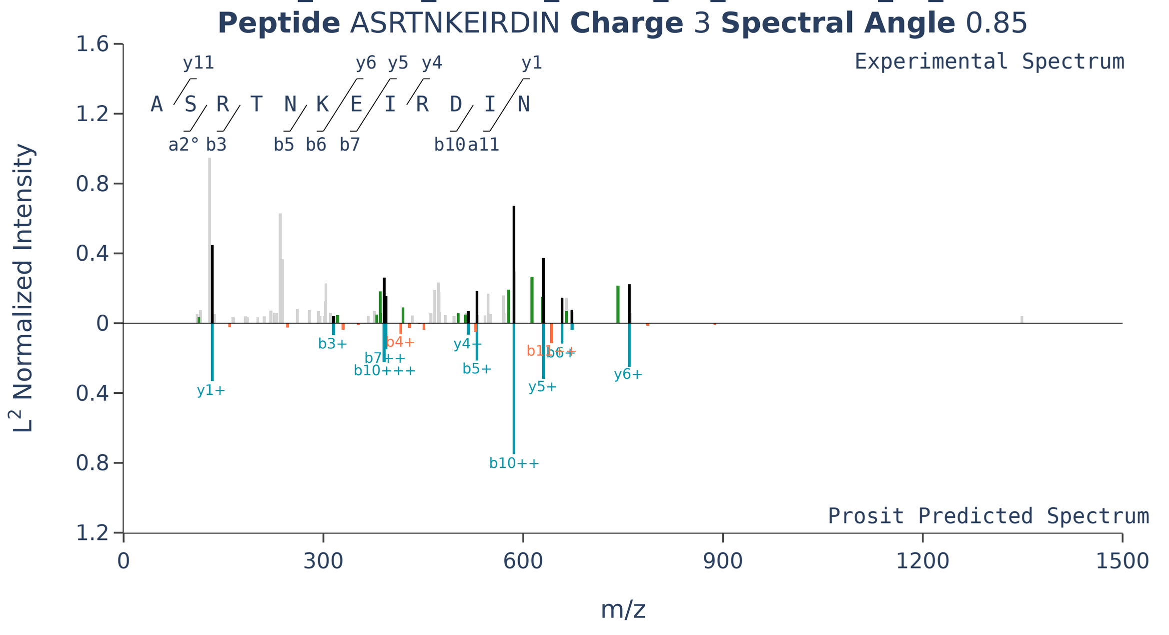


**Source** WSoh_301121_021221_HFGoe_M2_AnnexinA1_4h_R2 **Scan** 11403
**Peptide** AALHKAIMV **Charge** 2 **Spectral Angle** 0.77

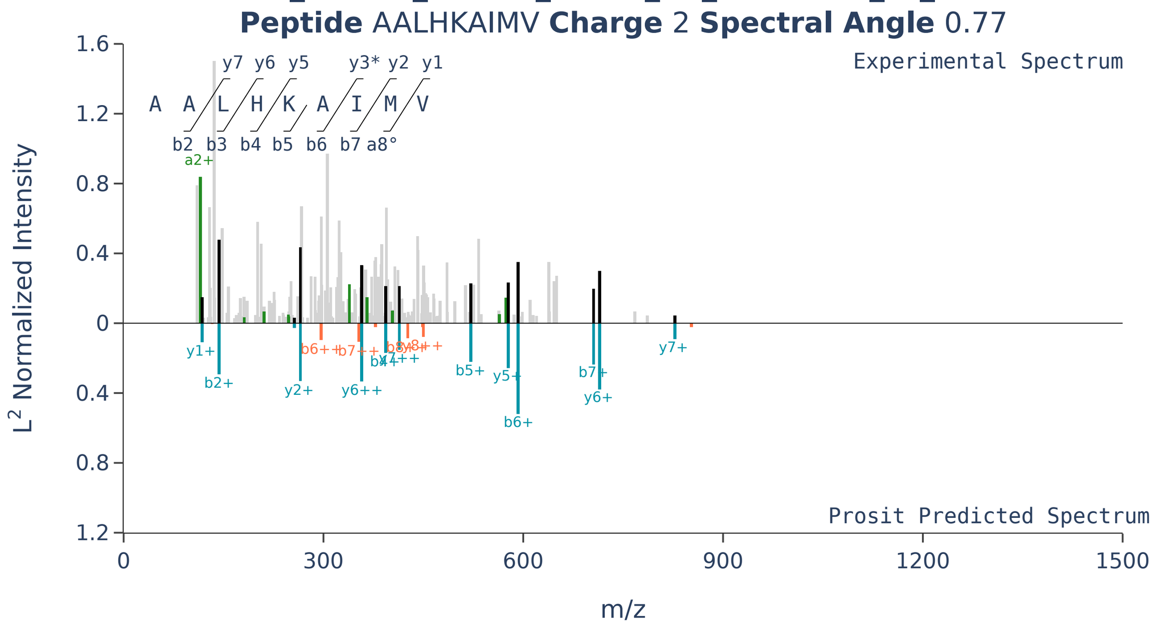


**Source** WSoh_301121_021221_HFGoe_M1_AnnexinA1_1h_R1 **Scan** 9935
**Peptide** ATSKPAF **Charge** 2 **Spectral Angle** 0.89

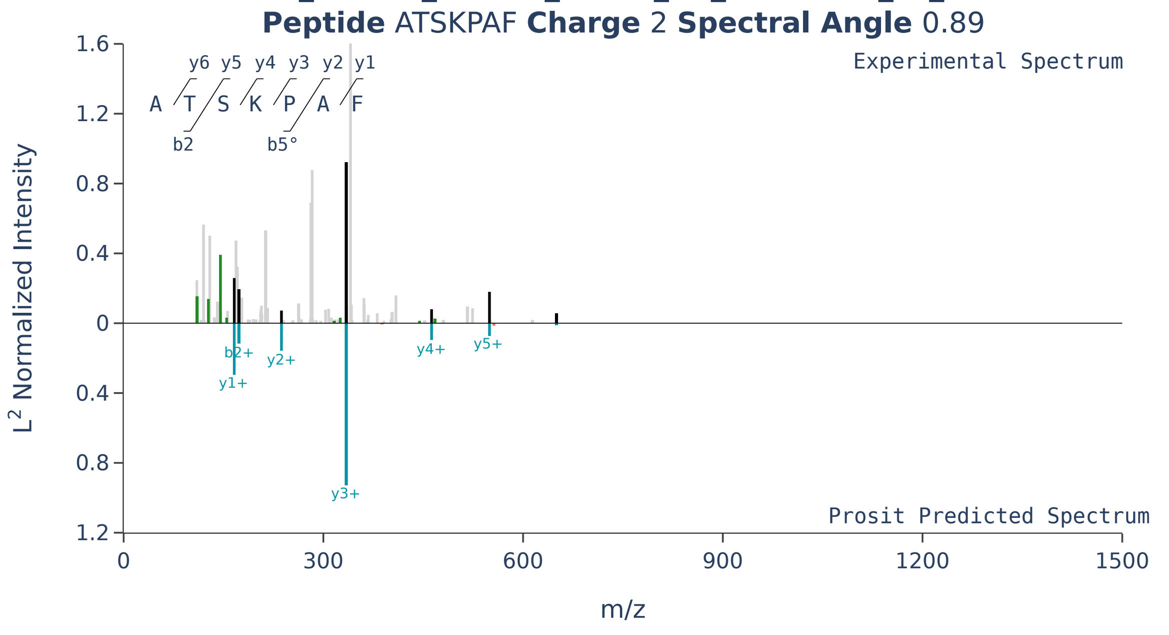


**Source** WSoh_301121_021221_HFGoe_M1_AnnexinA1_24h_R1 **Scan** 21022
**Peptide** LDETKGDYEKILV **Charge** 2 **Spectral Angle** 0.87

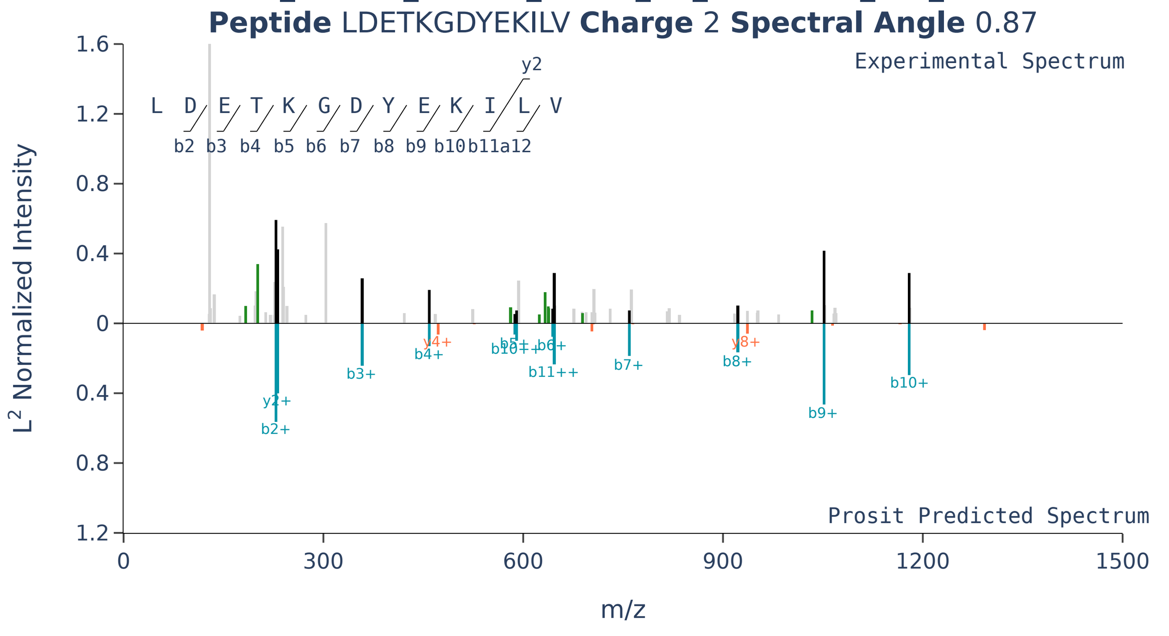


**Source** WSoh_301121_021221_HFGoe_M1_AnnexinA1_24h_R1 **Scan** 13417
**Peptide** KGGPGSAVSPYPT **Charge** 2 **Spectral Angle** 0.94

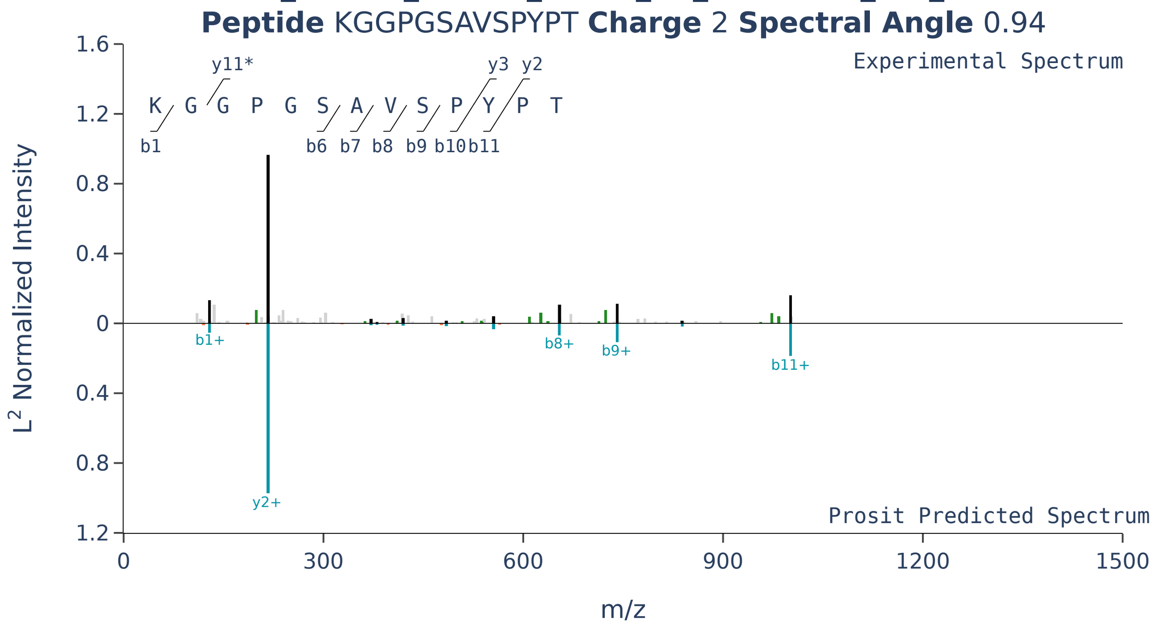


**Source** WSoh_301121_021221_HFGoe_M2_AnnexinA1_2h_R1 **Scan** 17351
**Peptide** LSLAKGDRSEDFGVNED **Charge** 3 **Spectral Angle** 0.83

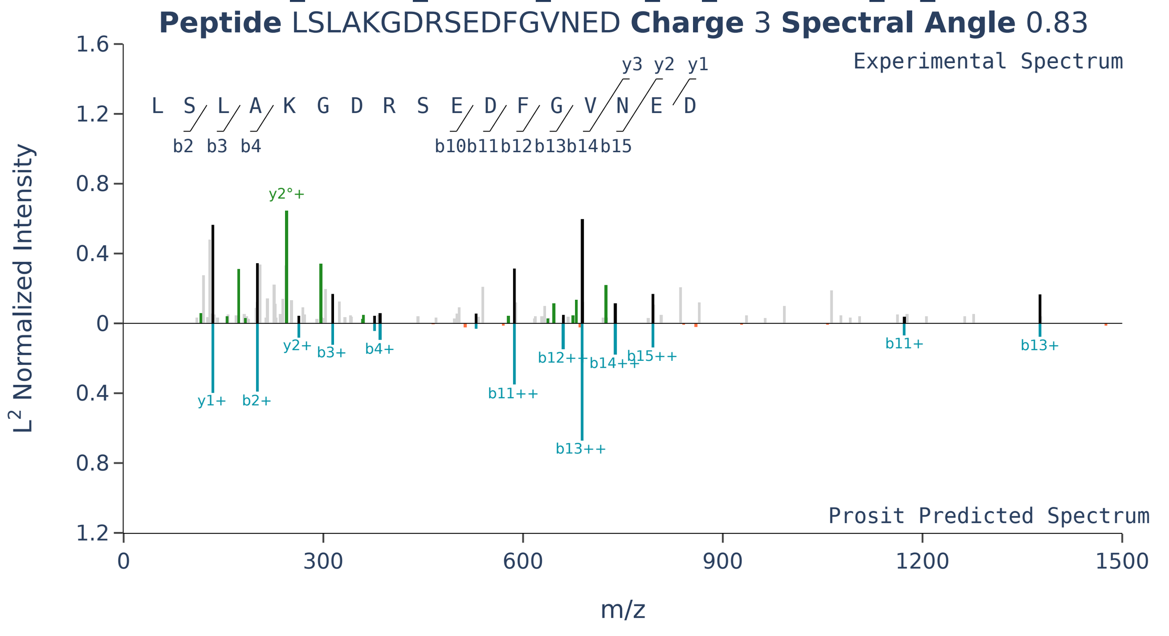


**Source** WSoh_301121_021221_HFGoe_M2_AnnexinA1_2h_R2 **Scan** 20644
**Peptide** LKTPAQFDADELRAA **Charge** 2 **Spectral Angle** 0.84

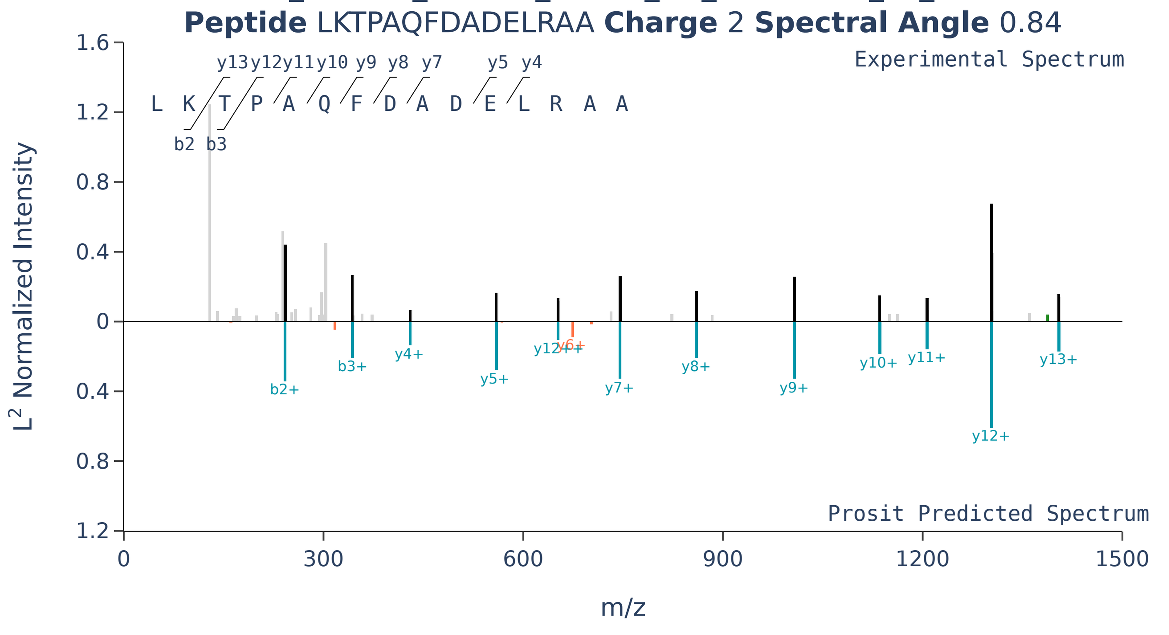


**Source** WSoh_301121_021221_HFGoe_M1_AnnexinA1_24h_R1 **Scan** 13904
**Peptide** NNAQRQQIKAAYL **Charge** 3 **Spectral Angle** 0.86

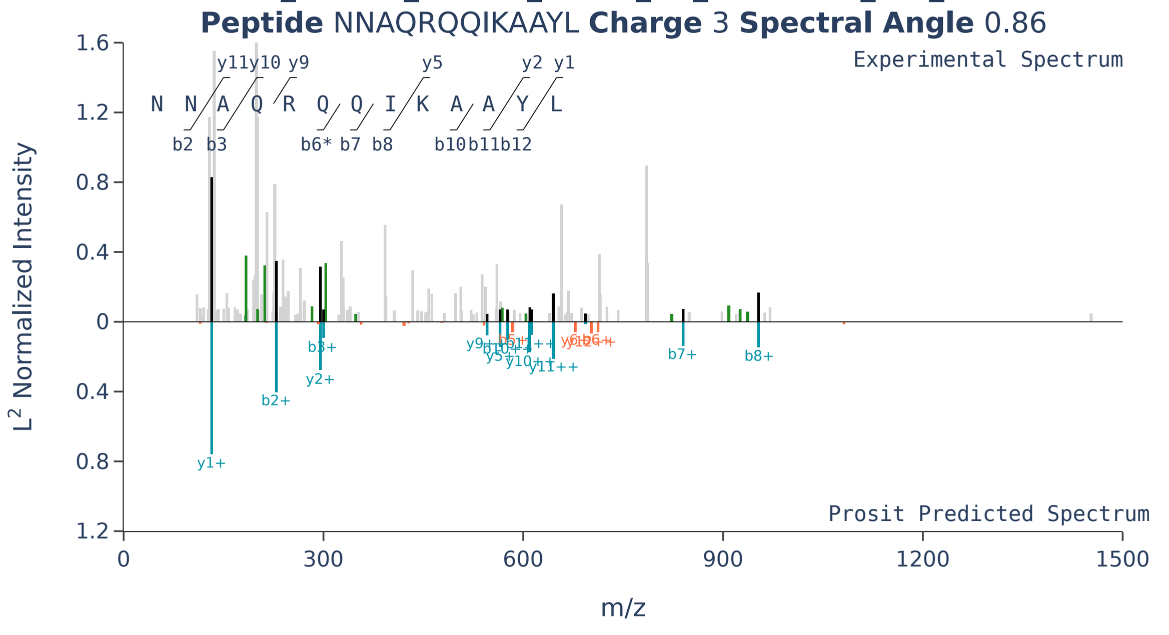


**Source** WSoh_301121_021221_HFGoe_M2_AnnexinA1_24h_R1 **Scan** 8304
**Peptide** NKEIRDI **Charge** 2 **Spectral Angle** 0.93

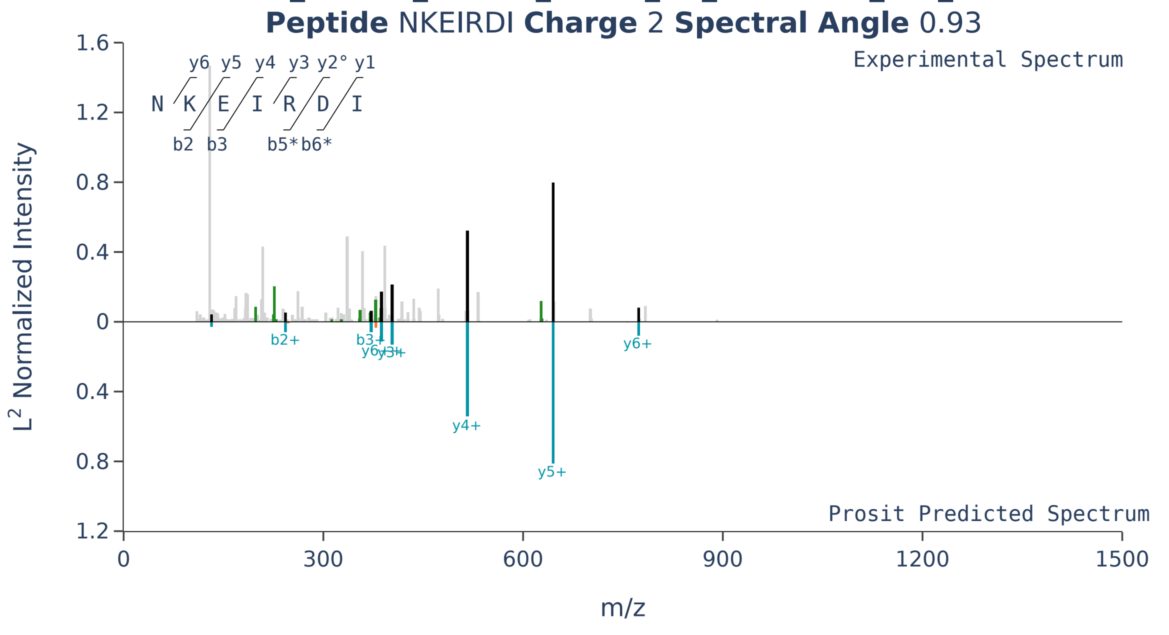


**Source** WSoh_301121_021221_HFGoe_M2_AnnexinA1_2h_R2 **Scan** 5785
**Peptide** MKGVGTRHKAL **Charge** 2 **Spectral Angle** 0.91

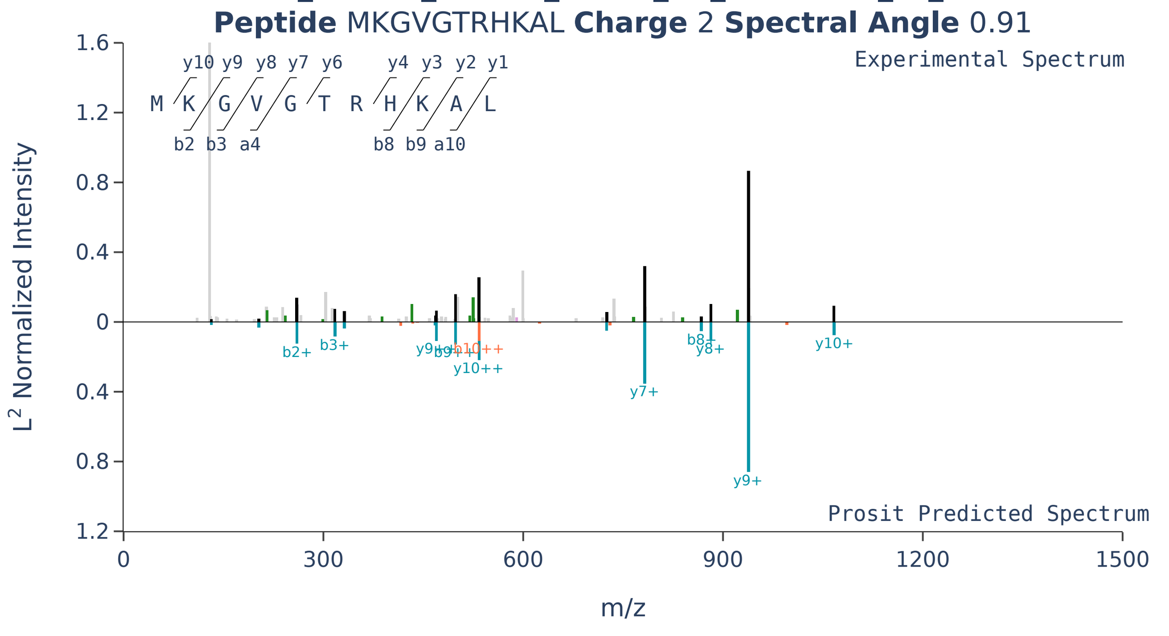


**Source** WSoh_301121_021221_HFGoe_M2_AnnexinA1_24h_R1 **Scan** 7558
**Peptide** QETGKPLDE **Charge** 2 **Spectral Angle** 0.8

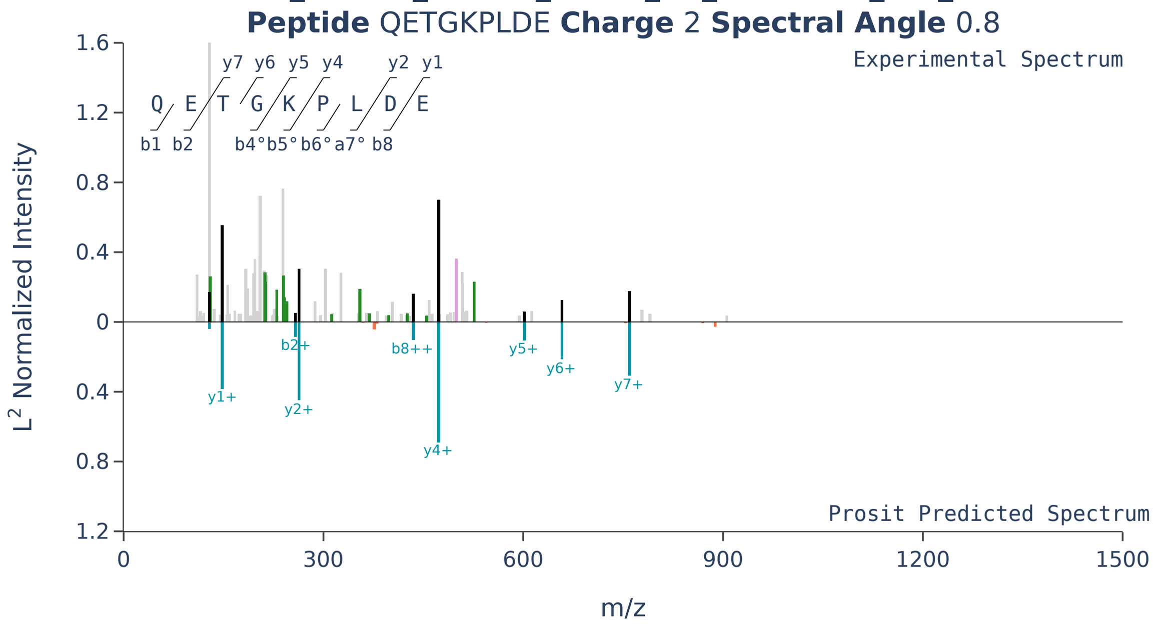


**Source** WSoh_301121_021221_HFGoe_M2_AnnexinA1_4h_R1 **Scan** 23771
**Peptide** ITSDTSGDFRNALL **Charge** 2 **Spectral Angle** 0.89

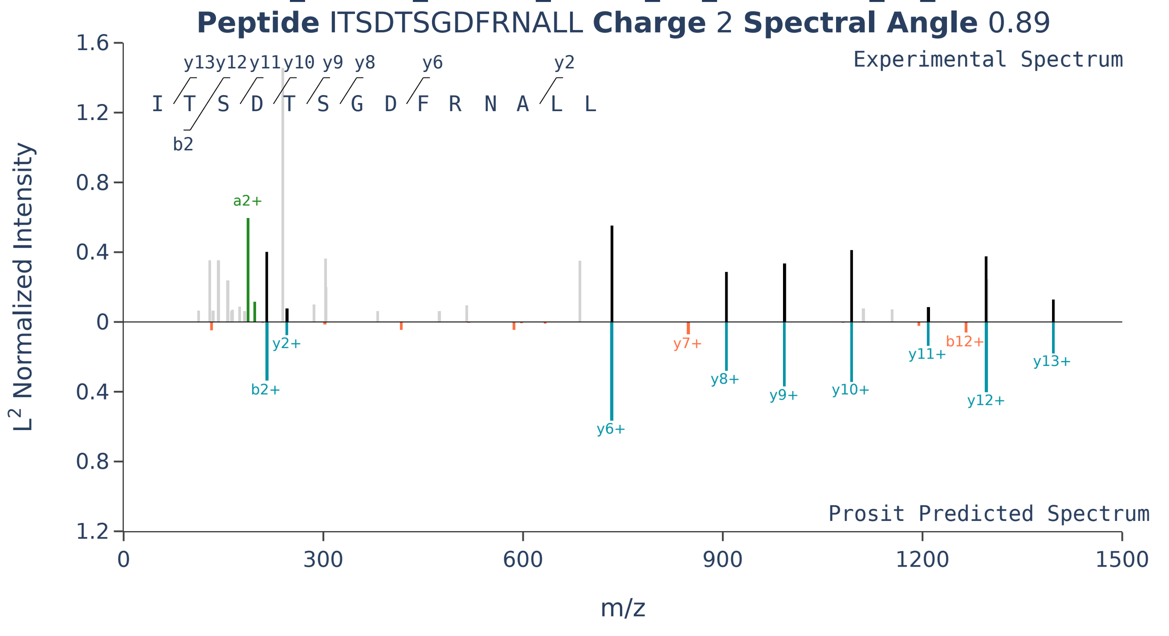

**Source** WSoh_301121_021221_HFGoe_M2_AnnexinA1_24h_R2 **Scan** 9038
**Peptide** EILASRTNKEIRD **Charge** 3 **Spectral Angle** 0.89

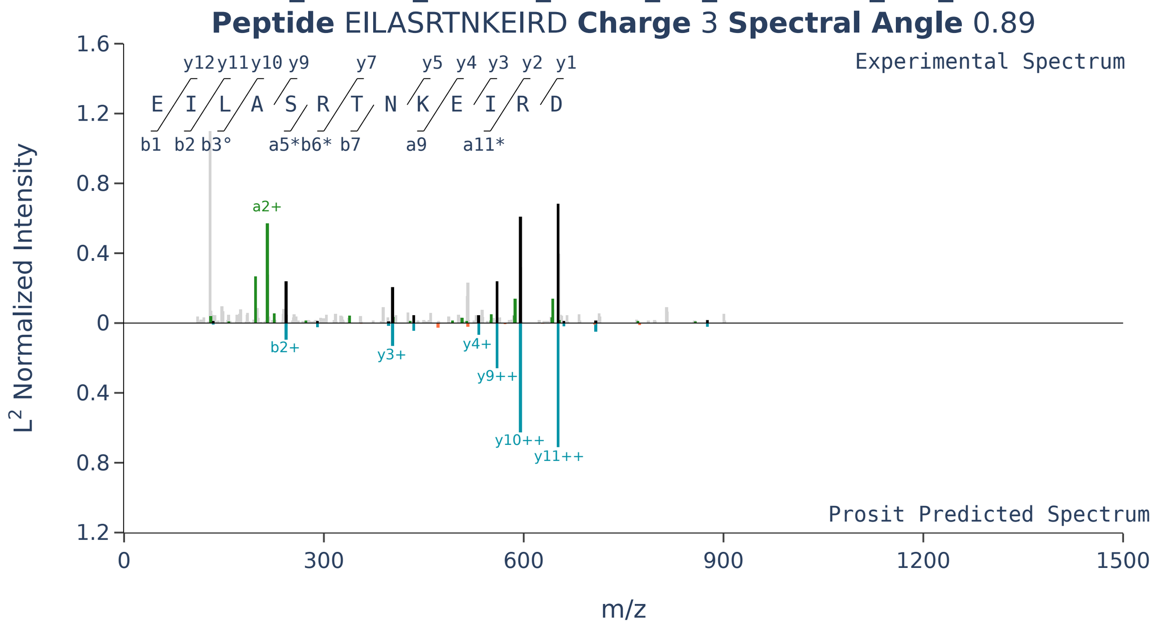


**Source** WSoh_301121_021221_HFGoe_M2_AnnexinA1_2h_R2 **Scan** 15875
**Peptide** LADSDARALYEAGER **Charge** 3 **Spectral Angle** 0.91

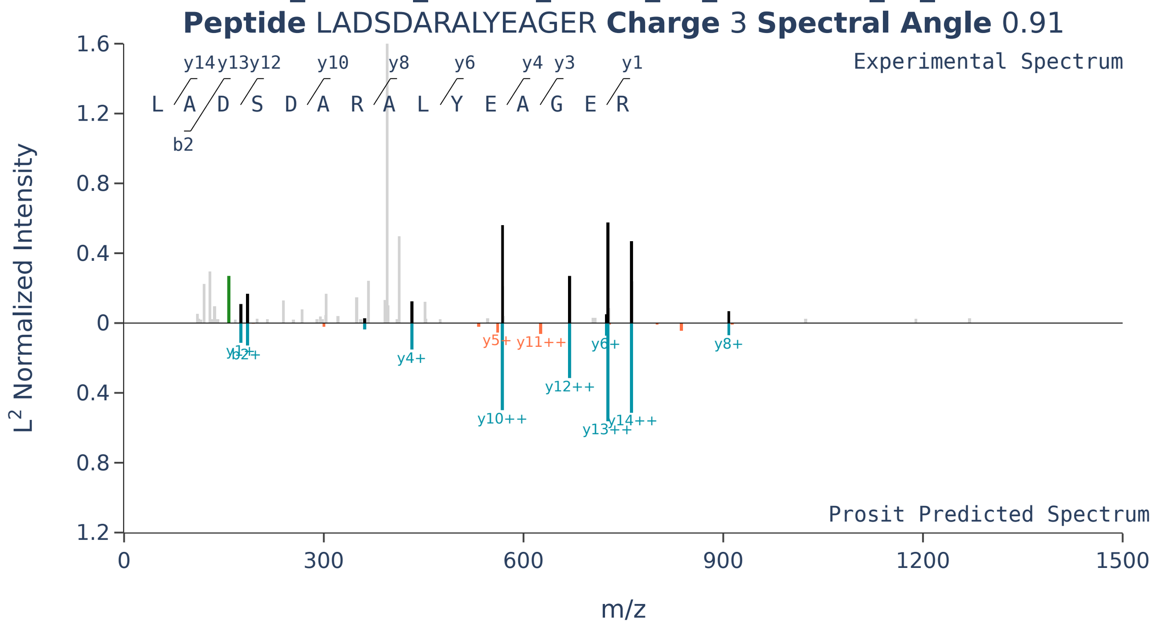


**Source** WSoh_301121_021221_HFGoe_M2_AnnexinA1_2h_R1 **Scan** 14143
**Peptide** ETLKKALTGHL **Charge** 3 **Spectral Angle** 0.84

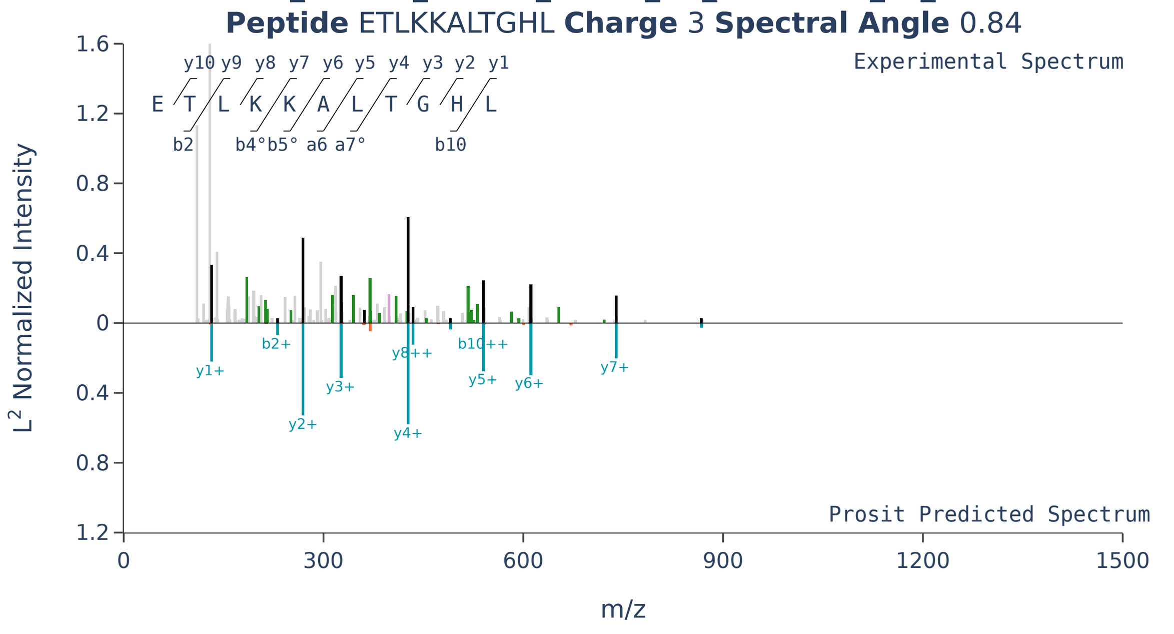


**Source** WSoh_301121_021221_HFGoe_M1_AnnexinA1_24h_R2 **Scan** 14992
**Peptide** DLAKDITSD **Charge** 2 **Spectral Angle** 0.88

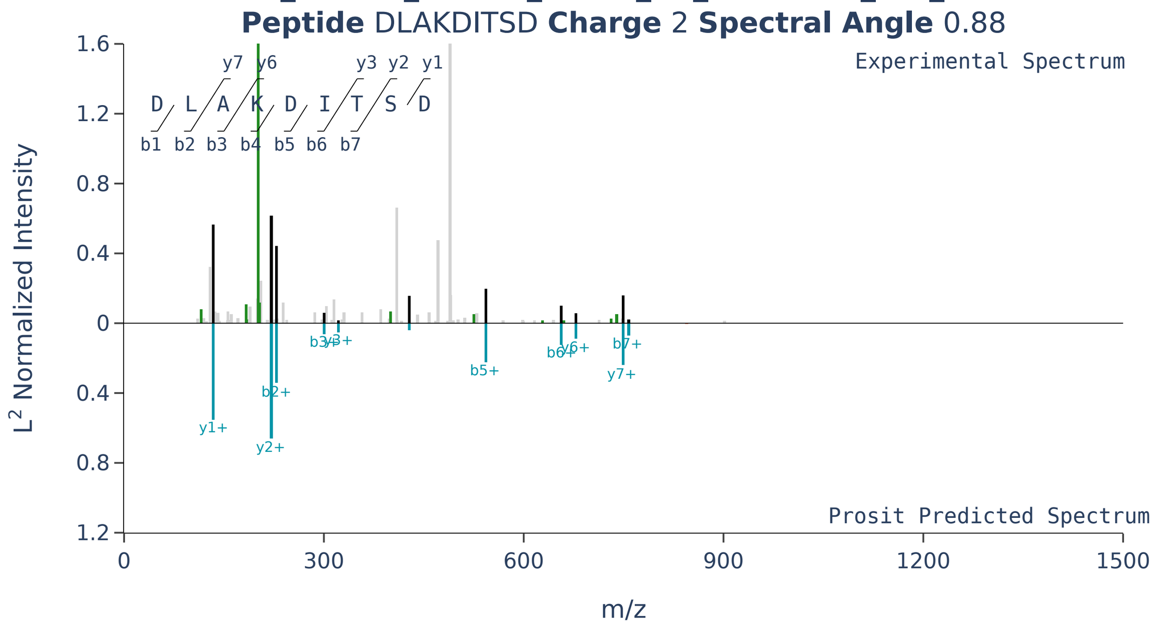


**Source** WSoh_301121_021221_HFGoe_M1_AnnexinA1_4h_R1 **Scan** 13552
**Peptide** KAFYQKMYG **Charge** 2 **Spectral Angle** 0.87

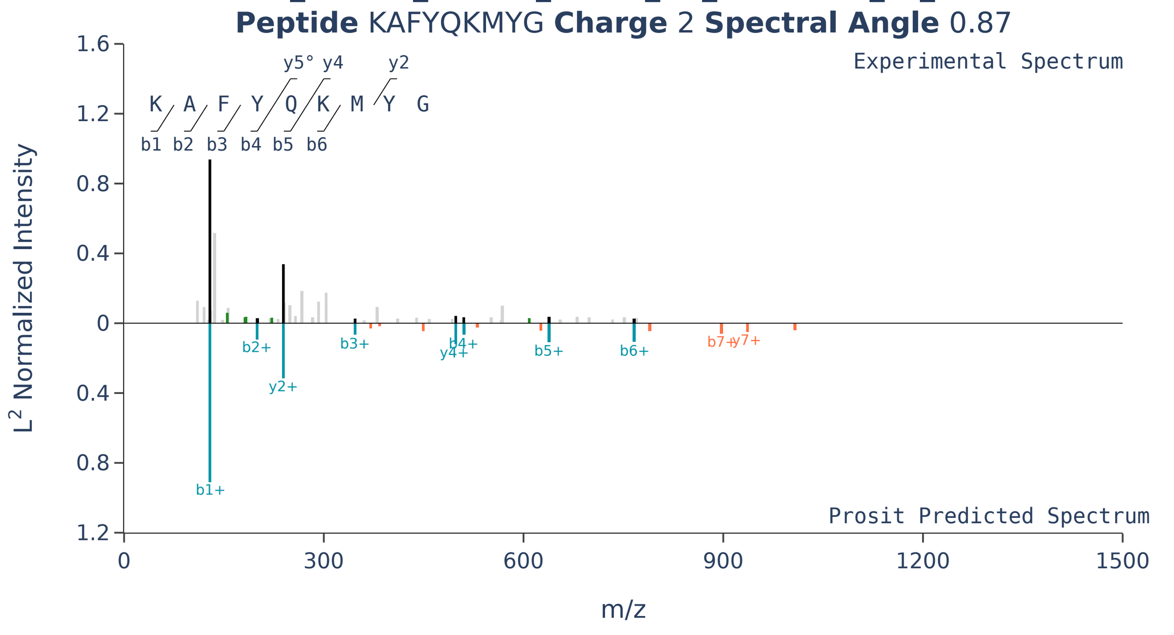


**Source** WSoh_301121_021221_HFGoe_M2_AnnexinA1_4h_R2 **Scan** 11678
**Peptide** QEYVQTVKSSKGGPGSAV **Charge** 2 **Spectral Angle** 0.89

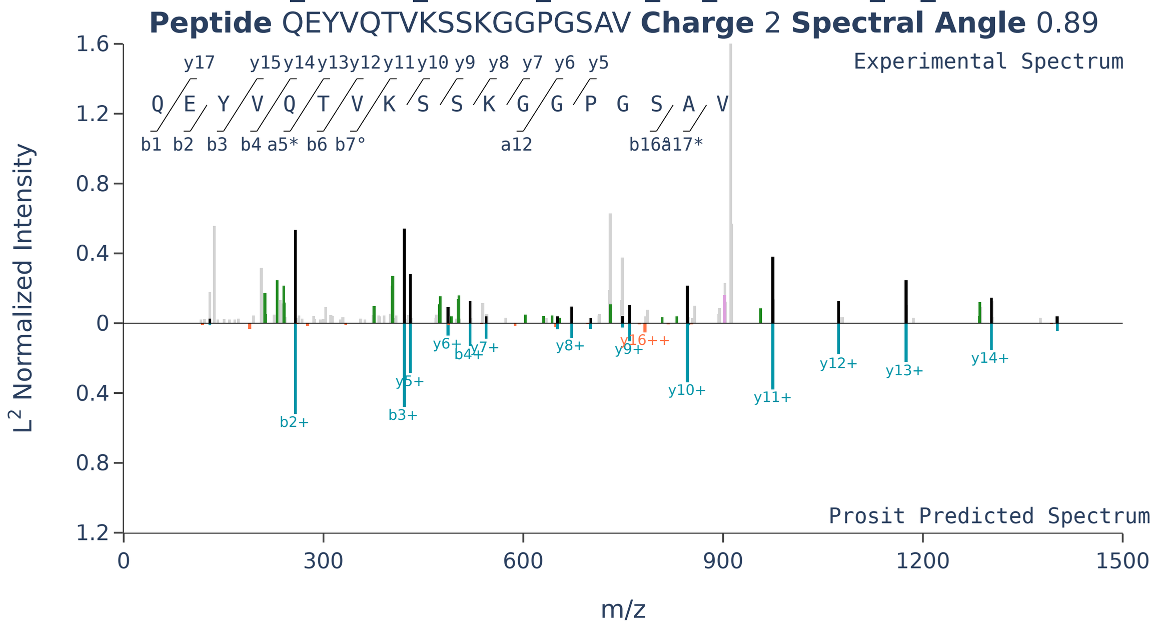


**Source** WSoh_301121_021221_HFGoe_M2_AnnexinA1_2h_R1 **Scan** 20347
**Peptide** LKTPAQFDADELRA **Charge** 2 **Spectral Angle** 0.87

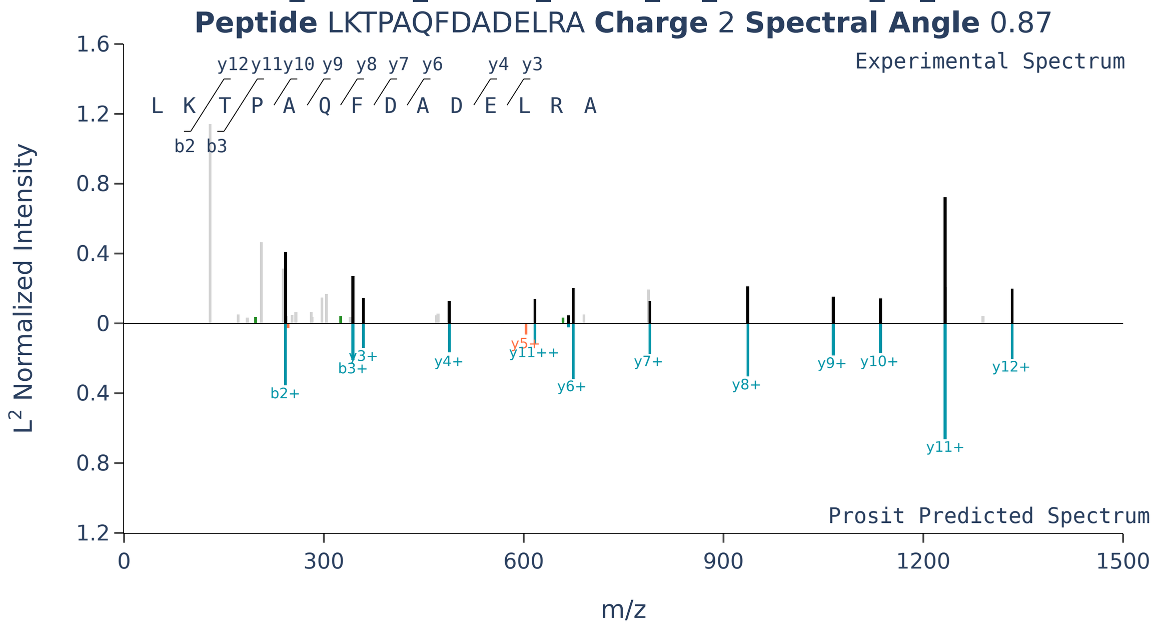


**Source** WSoh_301121_021221_HFGoe_M1_AnnexinA1_24h_R2 **Scan** 15433
**Peptide** QEYVQTV **Charge** 2 **Spectral Angle** 0.86

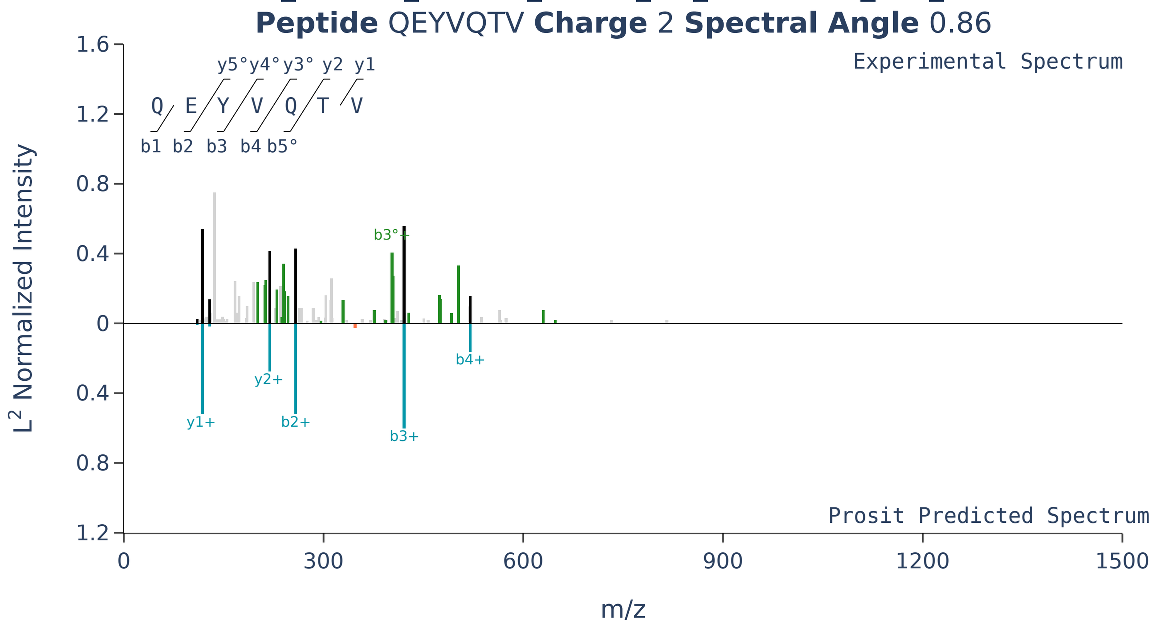


**Source** WSoh_301121_021221_HFGoe_M2_AnnexinA1_4h_R1 **Scan** 22162
**Peptide** GDFRNALL **Charge** 2 **Spectral Angle** 0.84

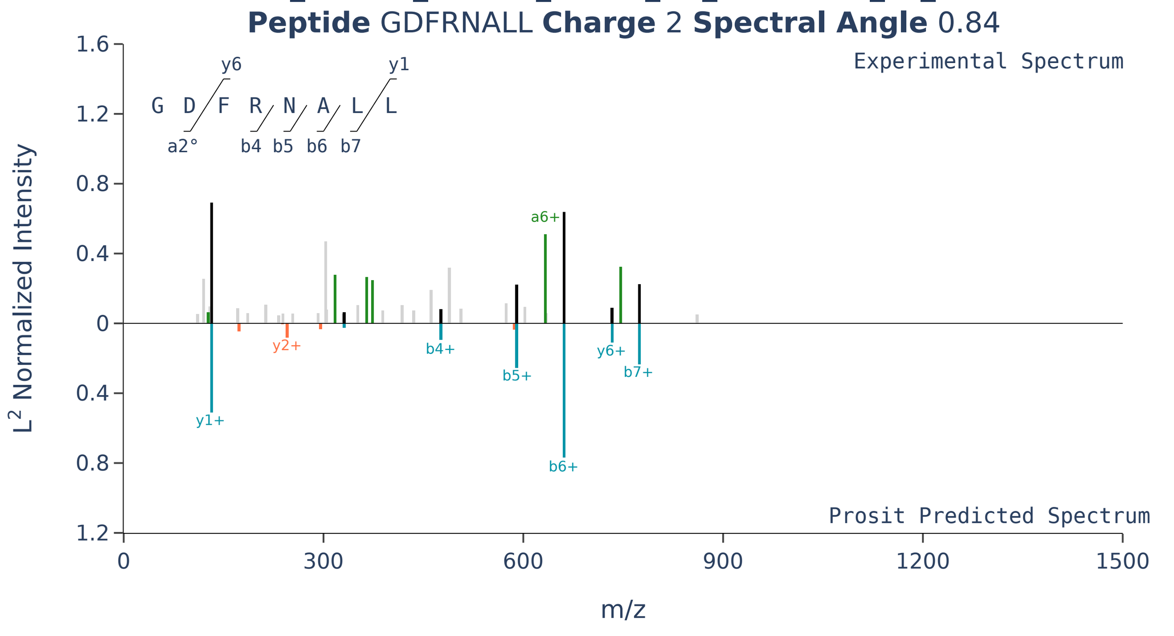


**Source** WSoh_301121_021221_HFGoe_M2_AnnexinA1_2h_R1 **Scan** 17492
**Peptide** KTPAQFDADELRAA **Charge** 2 **Spectral Angle** 0.8

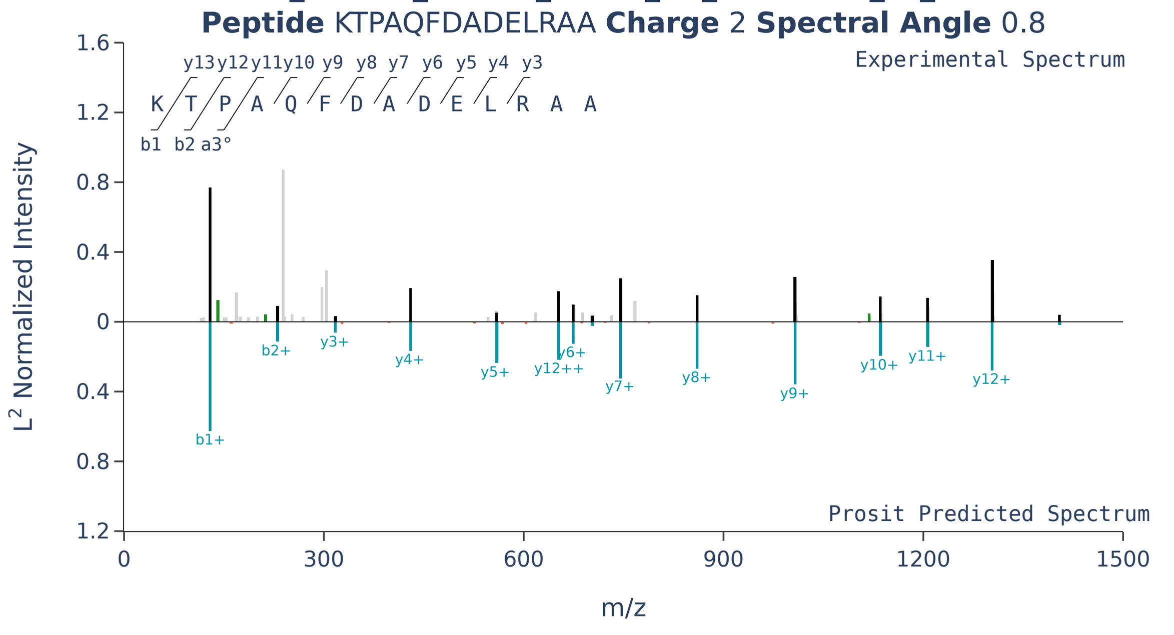


**Source** WSoh_301121_021221_HFGoe_M1_AnnexinA1_24h_R2 **Scan** 22377
**Peptide** SEFLKQAW **Charge** 2 **Spectral Angle** 0.88

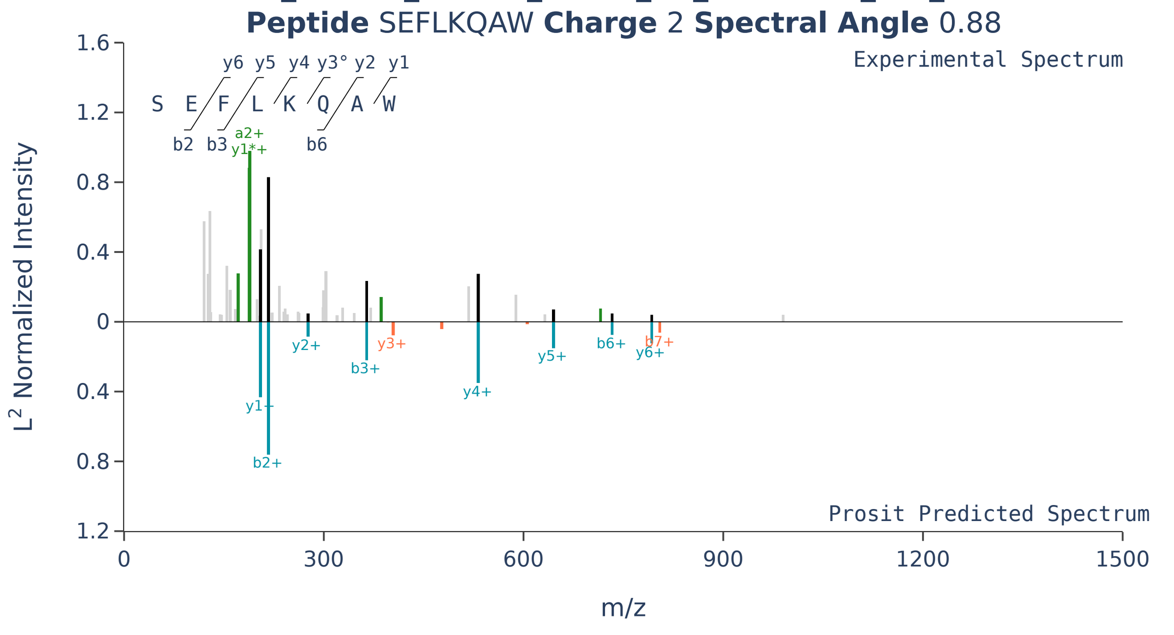


**Source** WSoh_301121_021221_HFGoe_M2_AnnexinA1_2h_R1 **Scan** 18115
**Peptide** KSSKGGPGSAVSPYPTFNPSSD **Charge** 3 **Spectral Angle** 0.81

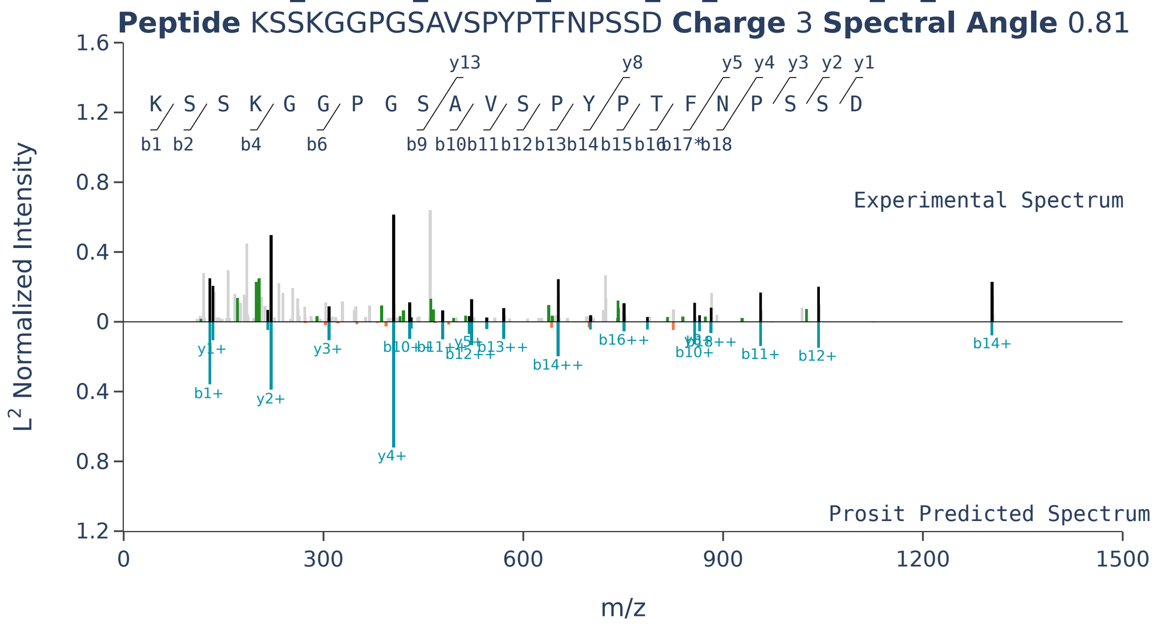


**Source** WSoh_301121_021221_HFGoe_M2_AnnexinA1_4h_R1 **Scan** 16380
**Peptide** VSRSEIDMNDIKA **Charge** 3 **Spectral Angle** 0.89

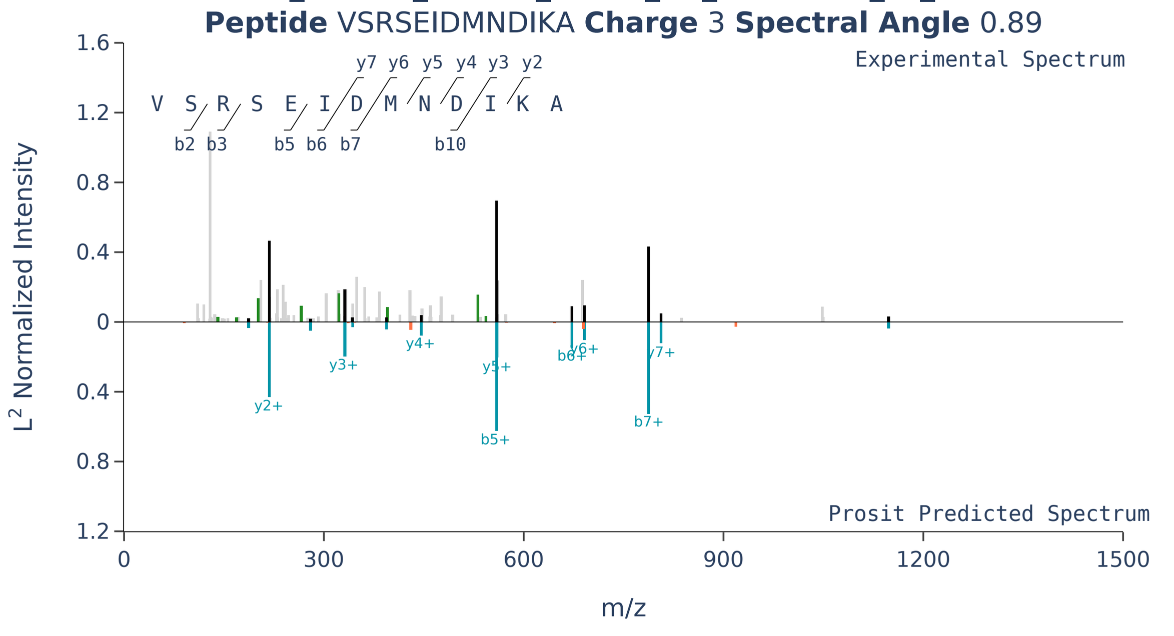


**Source** WSoh_301121_021221_HFGoe_M1_AnnexinA1_24h_R1 **Scan** 14049
**Peptide** EIRDINRVY **Charge** 3 **Spectral Angle** 0.88

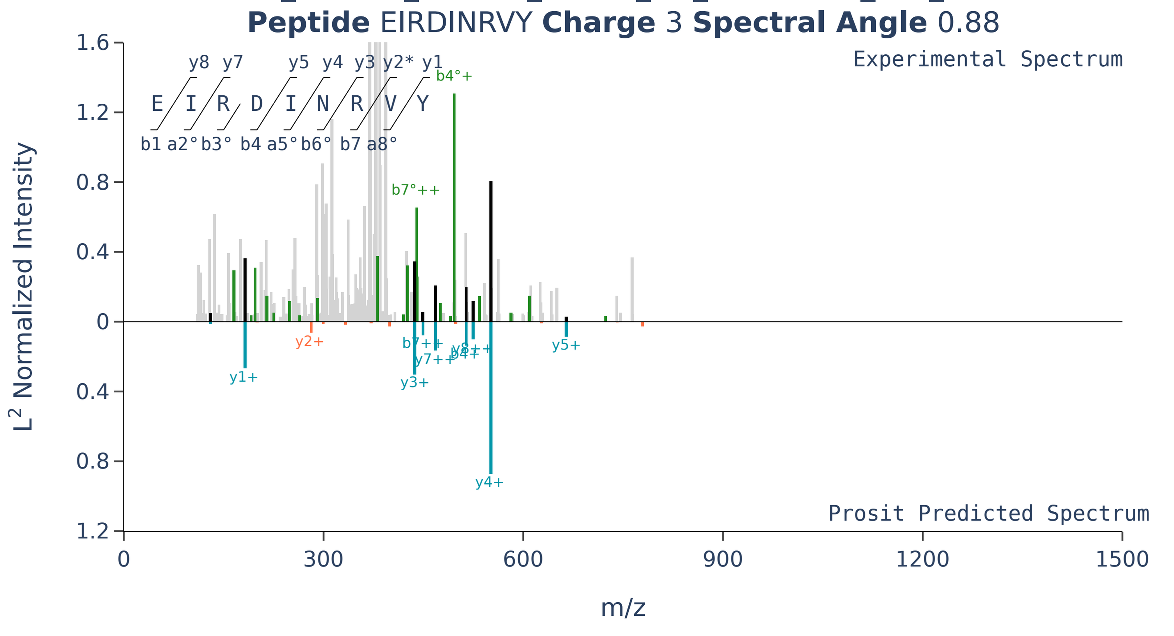


**Source** WSoh_301121_021221_HFGoe_M1_AnnexinA1_1h_R2 **Scan** 10760
**Peptide** GTDEDTL **Charge** 1 **Spectral Angle** 0.79

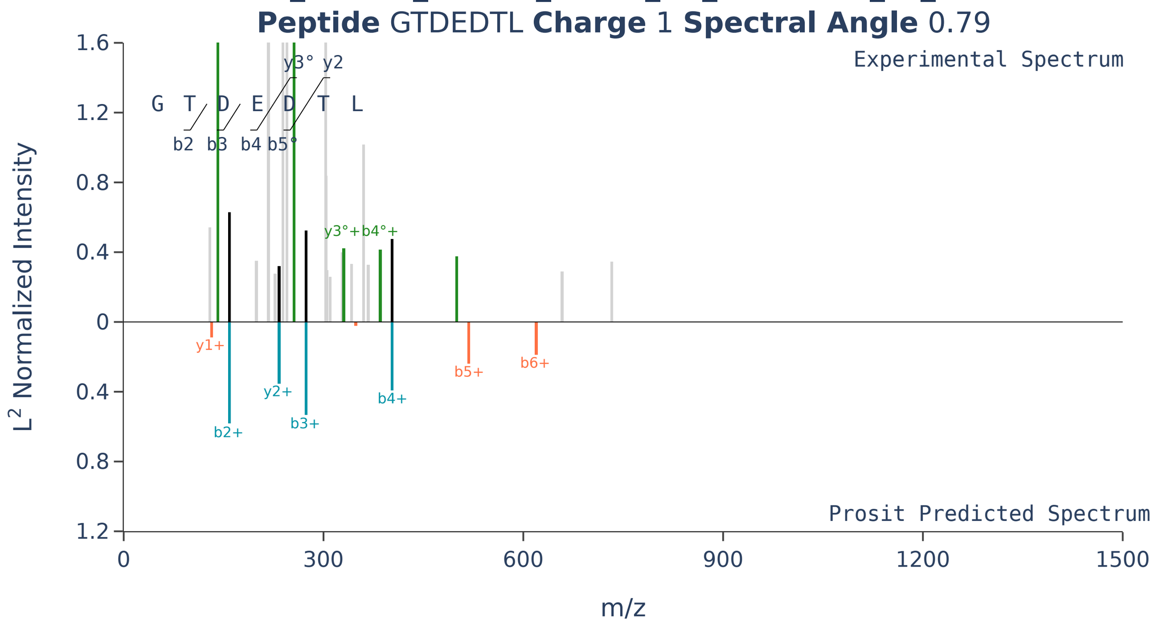

**Source** WSoh_301121_021221_HFGoe_M2_AnnexinA1_24h_R1 **Scan** 10789
**Peptide** KAIMVKGVD **Charge** 2 **Spectral Angle** 0.89

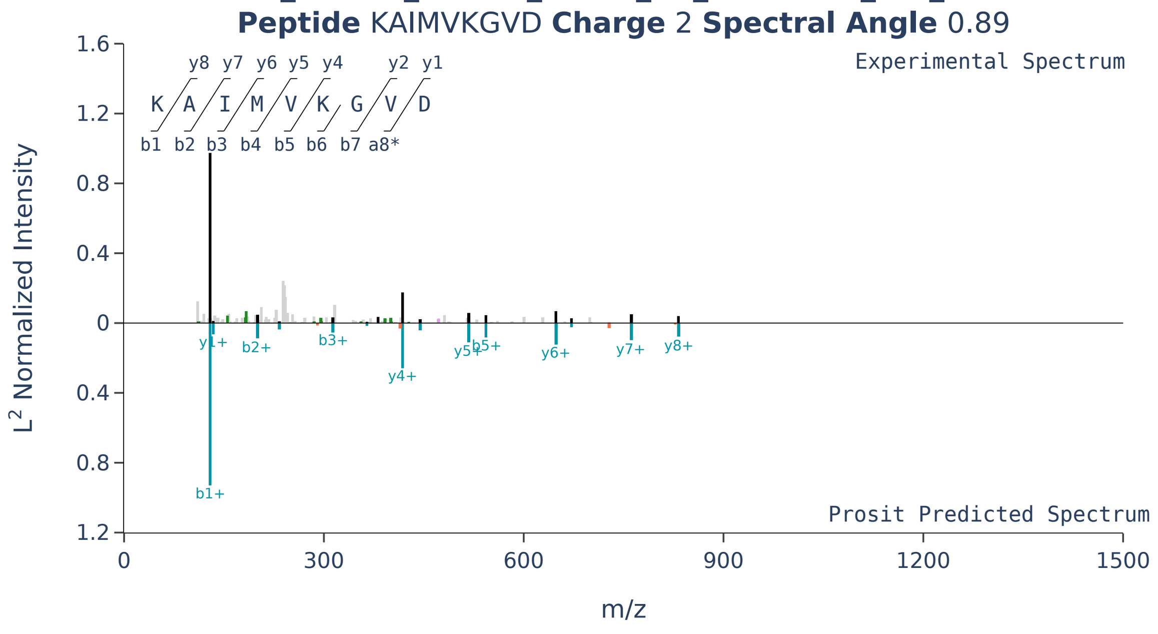


**Source** WSoh_301121_021221_HFGoe_M2_AnnexinA1_2h_R2 **Scan** 10682
**Peptide** VFQKYTKY **Charge** 3 **Spectral Angle** 0.82

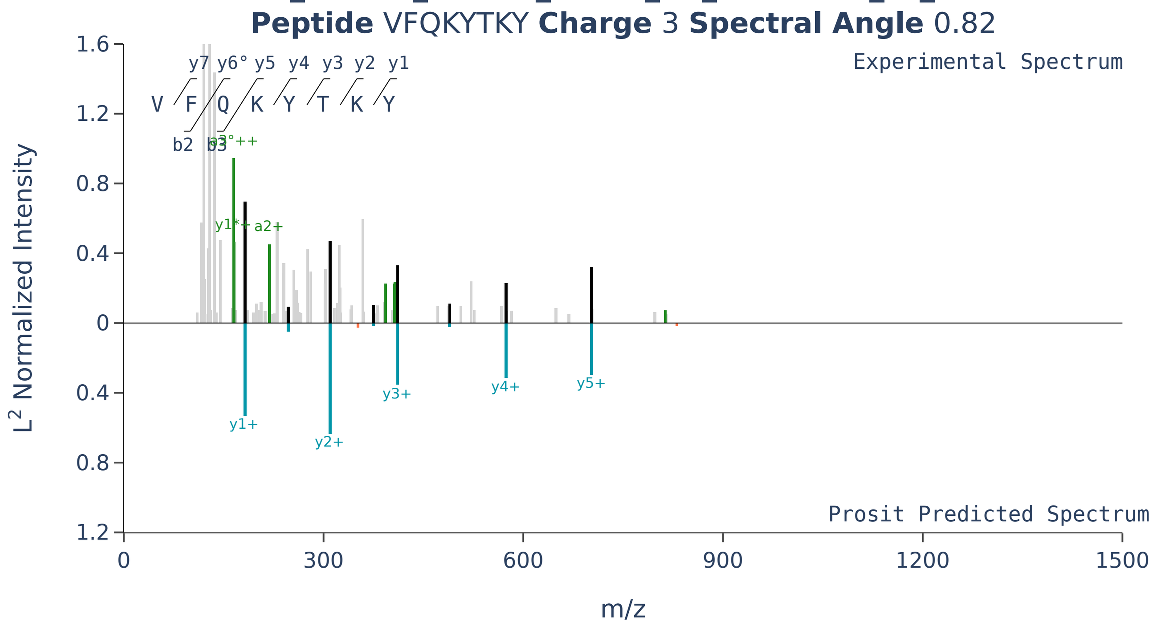


**Source** WSoh_301121_021221_HFGoe_M2_AnnexinA1_24h_R1 **Scan** 26798
**Peptide** AWFIENE **Charge** 1 **Spectral Angle** 0.95

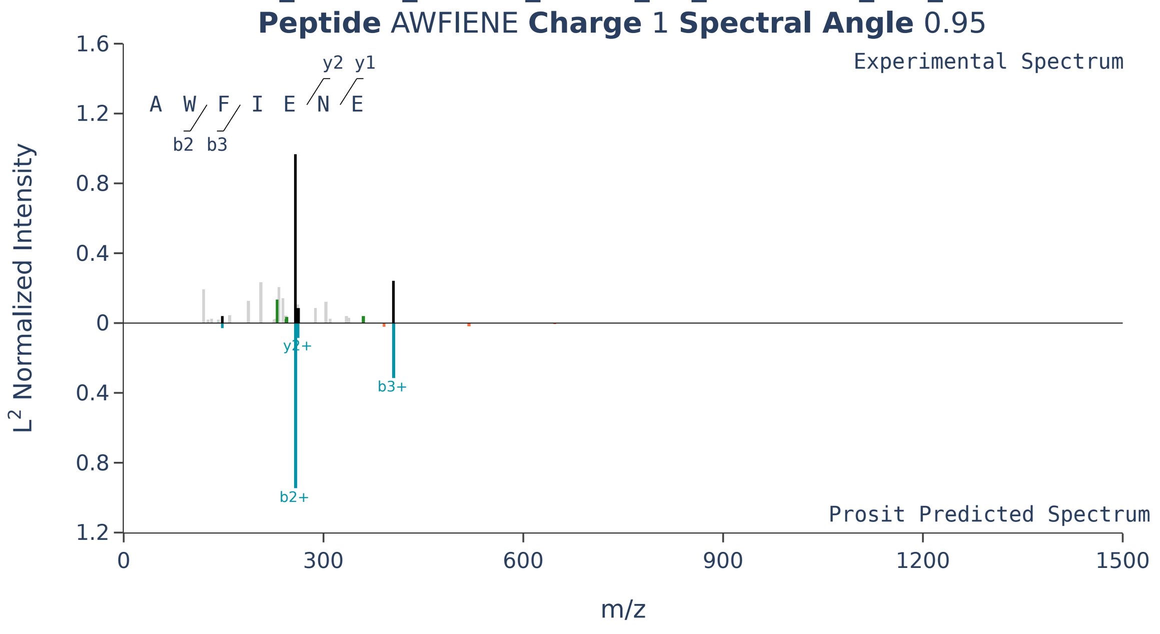


**Source** WSoh_301121_021221_HFGoe_M2_AnnexinA1_4h_R2 **Scan** 16500
**Peptide** QETGKPLDETL **Charge** 1 **Spectral Angle** 0.86

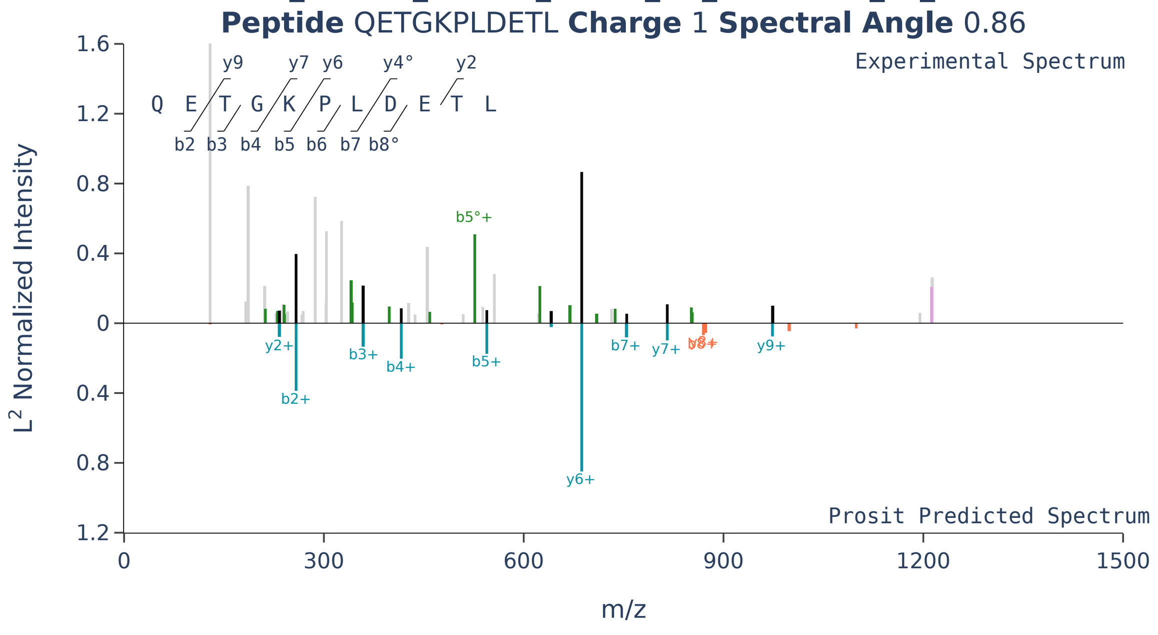


**Source** WSoh_301121_021221_HFGoe_M2_AnnexinA1_2h_R1 **Scan** 18284
**Peptide** SLAKGDRSEDFGVNEDLAD **Charge** 3 **Spectral Angle** 0.84

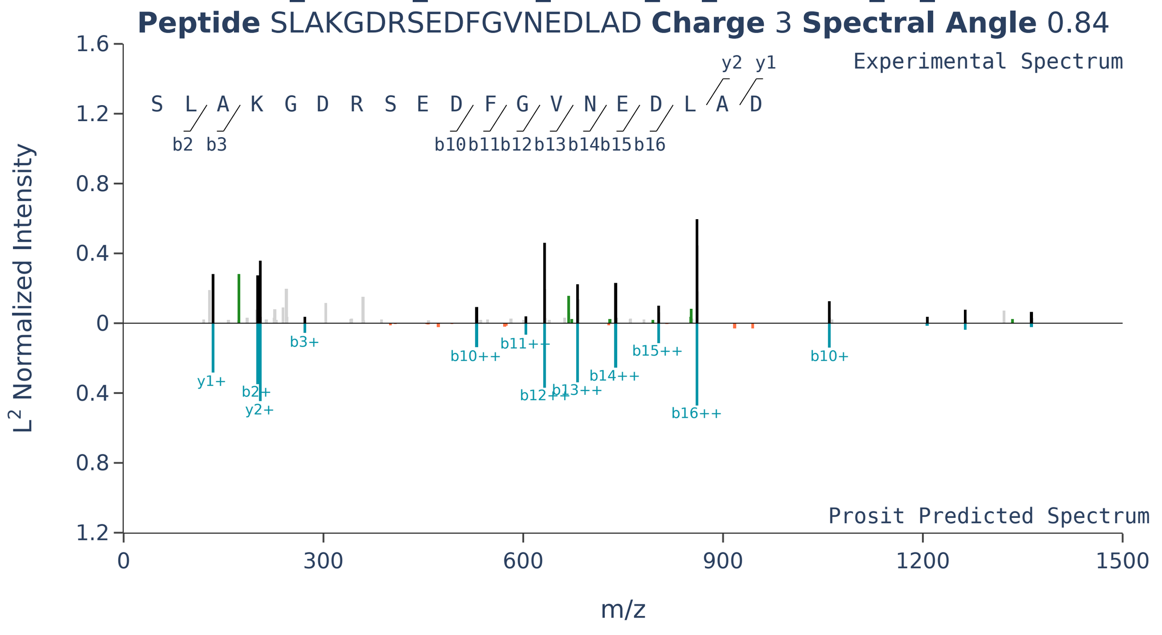


**Source** WSoh_301121_021221_HFGoe_M1_AnnexinA1_24h_R2 **Scan** 10258
**Peptide** TNKEIRDINRVYRE **Charge** 4 **Spectral Angle** 0.94

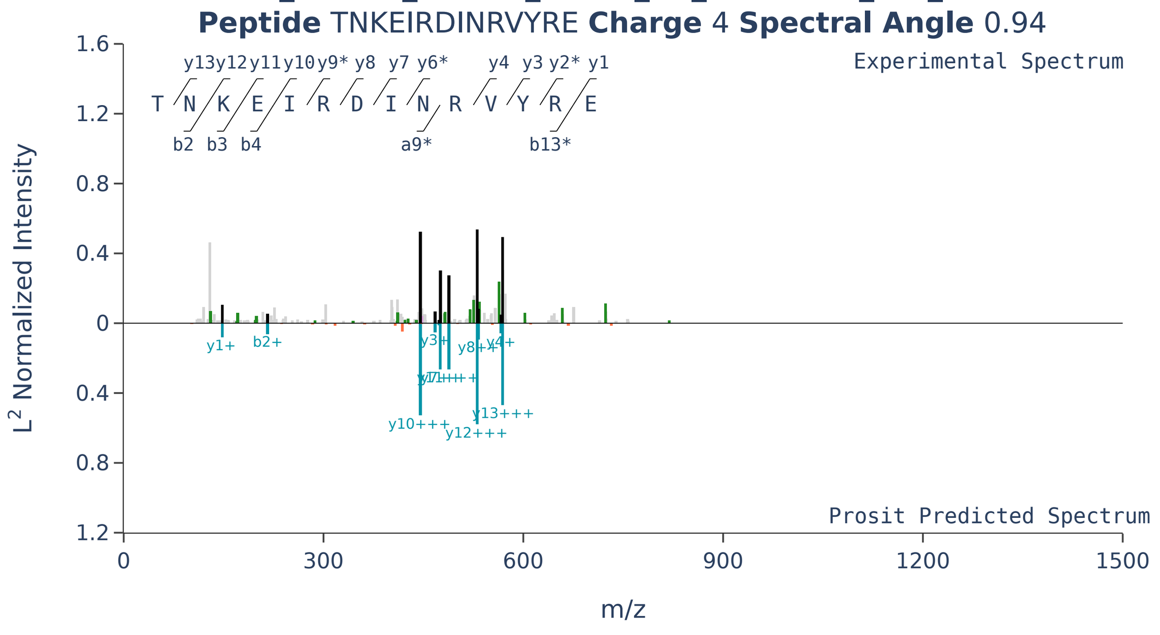


**Source** WSoh_301121_021221_HFGoe_M1_AnnexinA1_24h_R2 **Scan** 11671
**Peptide** EAGERRKGTDVNVF **Charge** 3 **Spectral Angle** 0.94

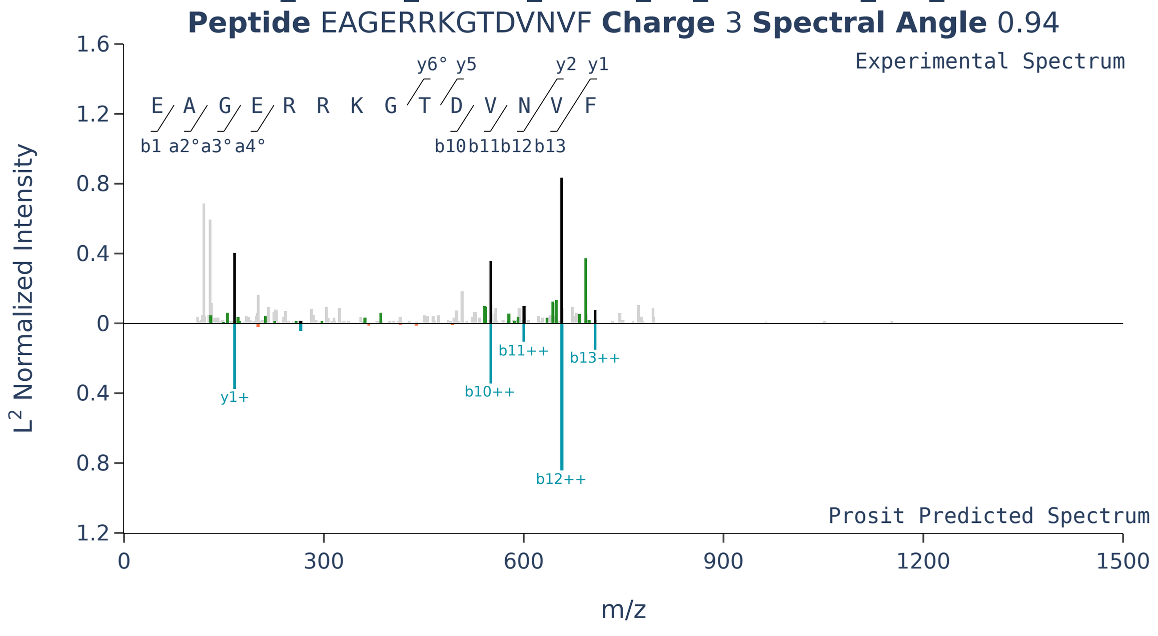


**Source** WSoh_301121_021221_HFGoe_M2_AnnexinA1_24h_R2 **Scan** 14497
**Peptide** SLAKGDRSEDFGV **Charge** 2 **Spectral Angle** 0.84

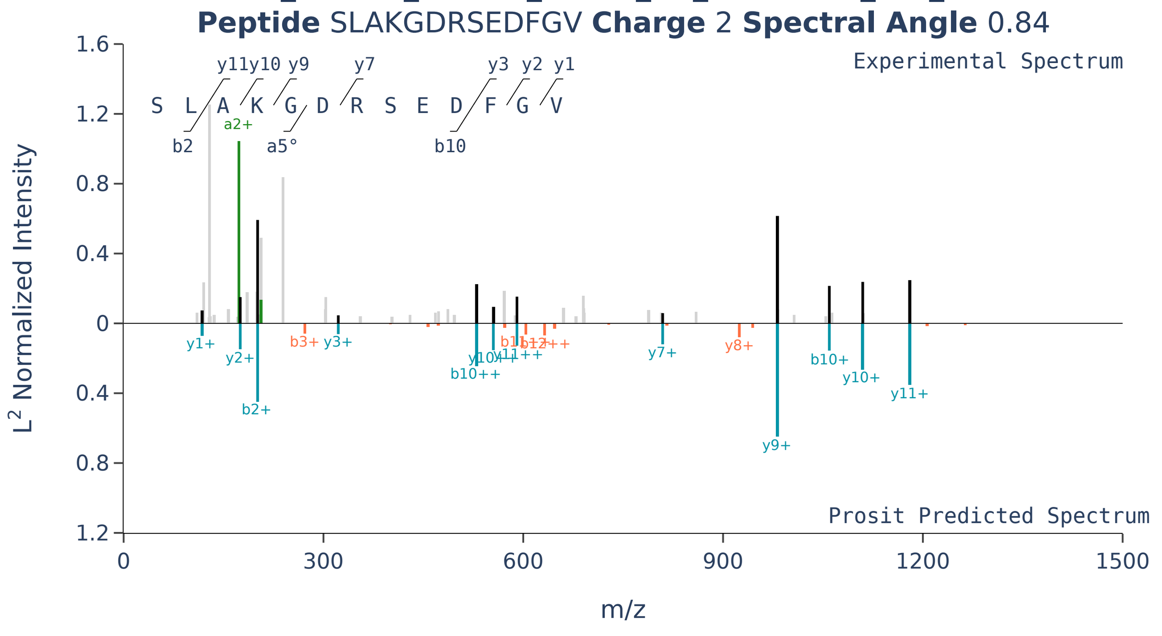


**Source** WSoh_301121_021221_HFGoe_M1_AnnexinA1_24h_R2 **Scan** 7671
**Peptide** ELKGDIEKC **Charge** 3 **Spectral Angle** 0.91

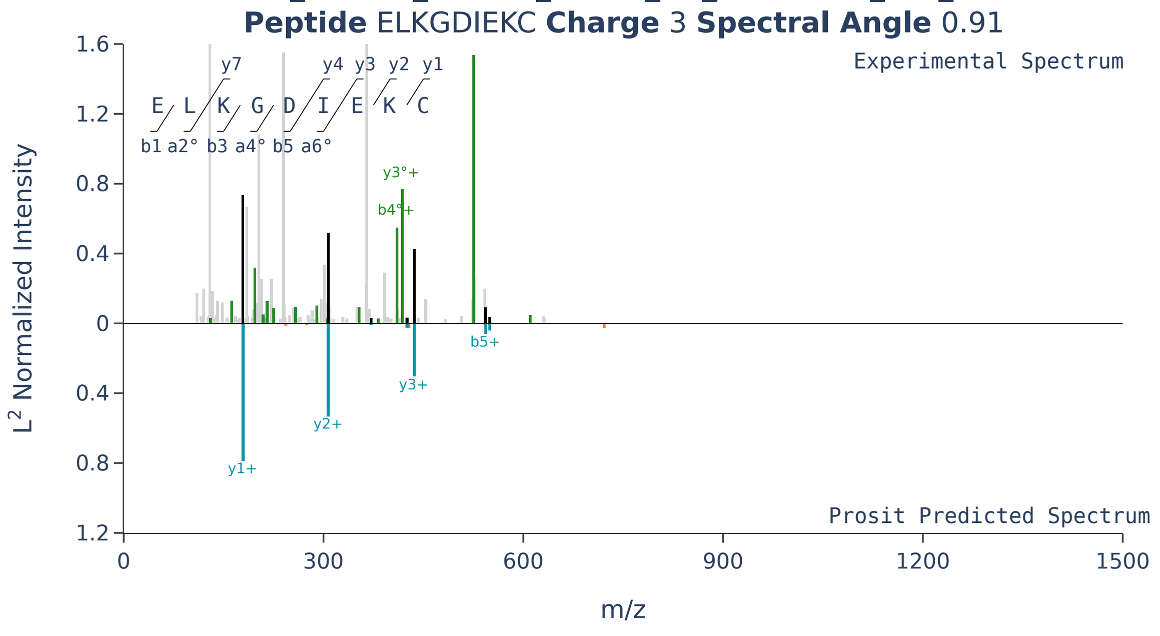


**Source** WSoh_301121_021221_HFGoe_M2_AnnexinA1_4h_R1 **Scan** 6586
**Peptide** LASRTNKEI **Charge** 2 **Spectral Angle** 0.81

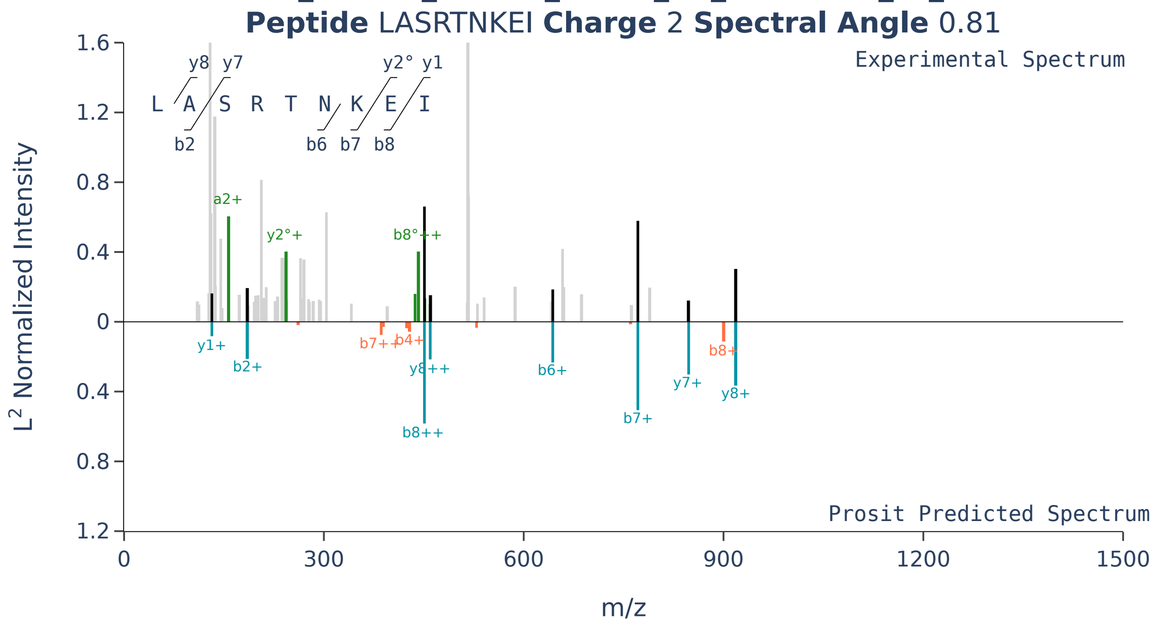


**Source** WSoh_301121_021221_HFGoe_M2_AnnexinA1_4h_R1 **Scan** 13353
**Peptide** AQRQQIKAAYL **Charge** 2 **Spectral Angle** 0.81

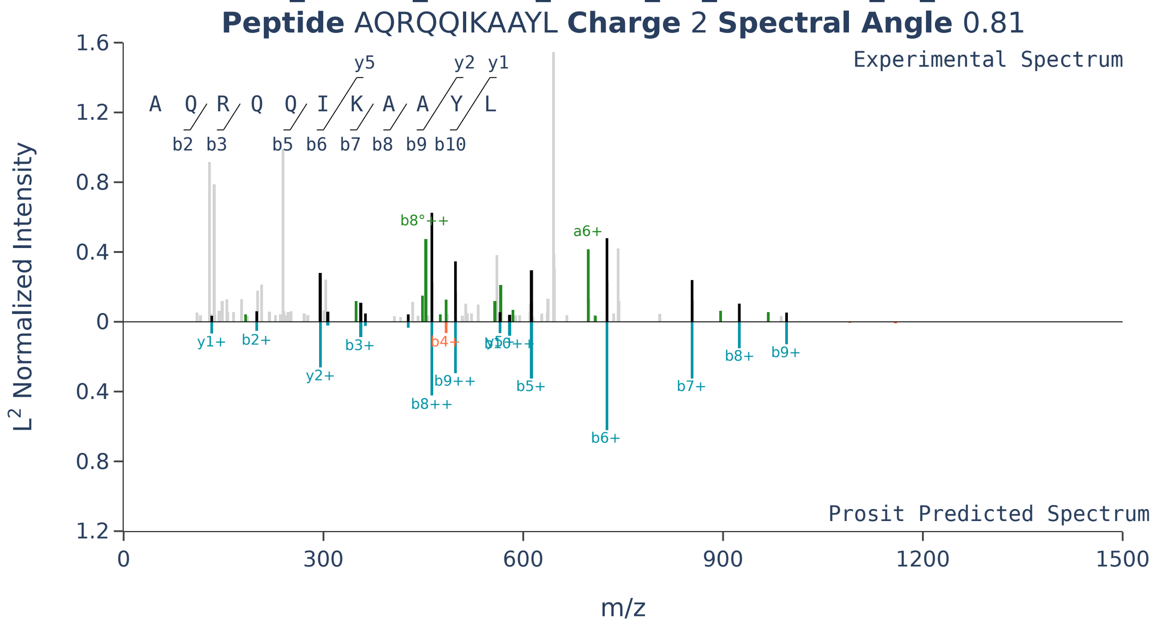


**Source** WSoh_301121_021221_HFGoe_M2_AnnexinA1_2h_R2 **Scan** 19159
**Peptide** ADELRAAMKGLGT **Charge** 2 **Spectral Angle** 0.83

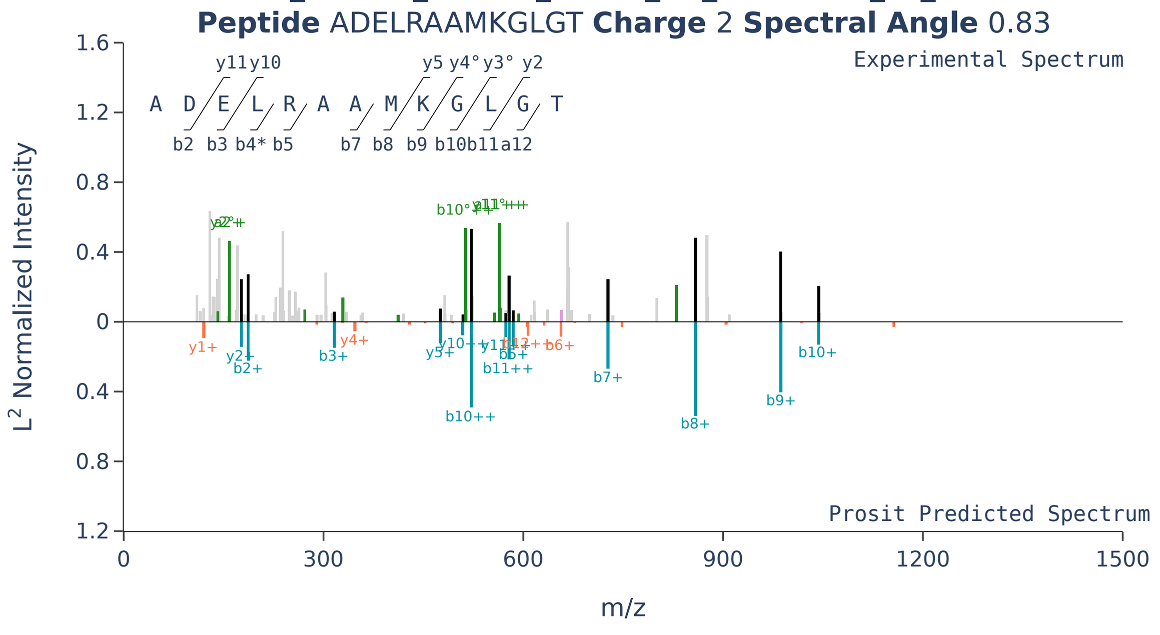


**Source** WSoh_301121_021221_HFGoe_M1_AnnexinA1_24h_R1 **Scan** 5684
**Peptide** LTKRNNAQRQQI **Charge** 3 **Spectral Angle** 0.92

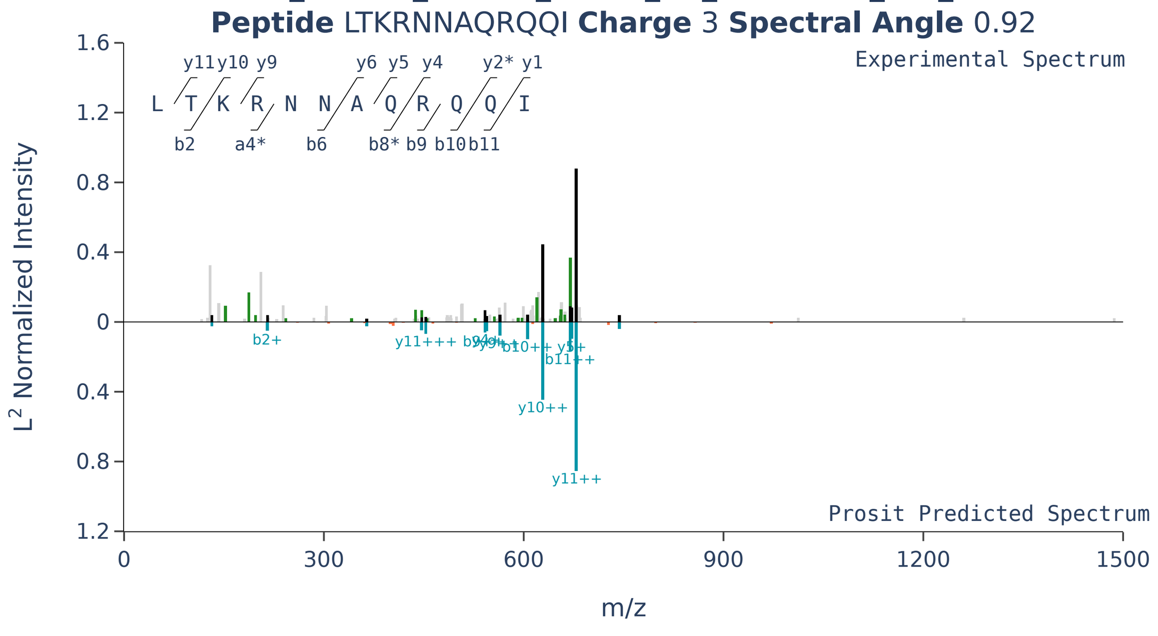


**Source** WSoh_301121_021221_HFGoe_M2_AnnexinA1_2h_R1 **Scan** 22705
**Peptide** PAQFDADEL **Charge** 2 **Spectral Angle** 0.83

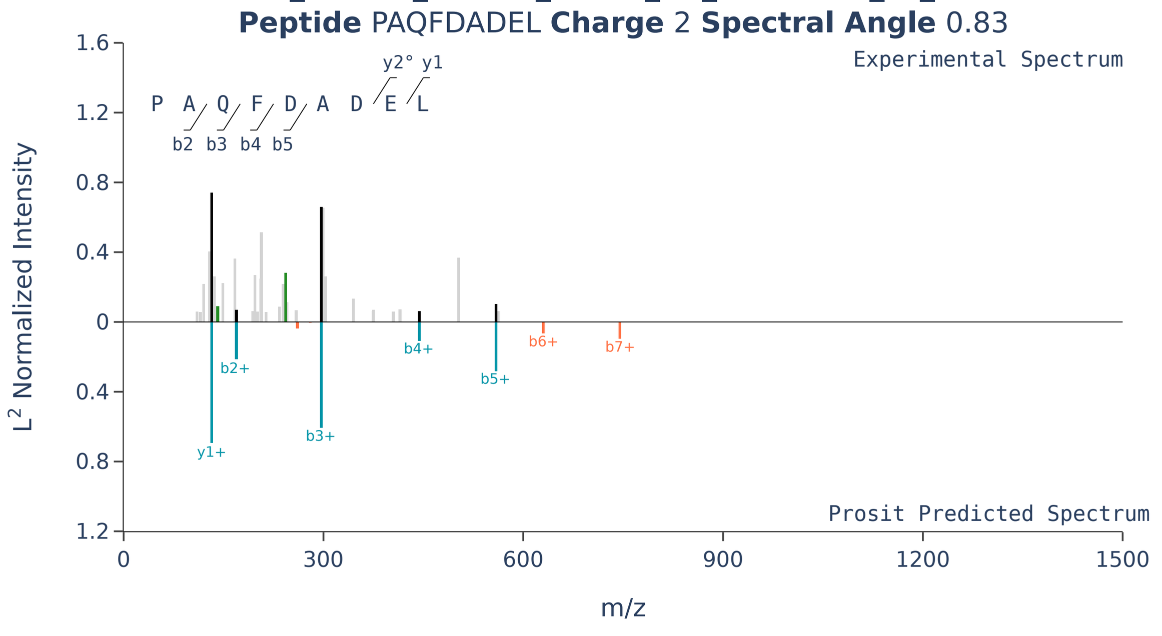


**Source** WSoh_301121_021221_HFGoe_M1_AnnexinA1_24h_R2 **Scan** 15121
**Peptide** EDFGVNED **Charge** 2 **Spectral Angle** 0.8

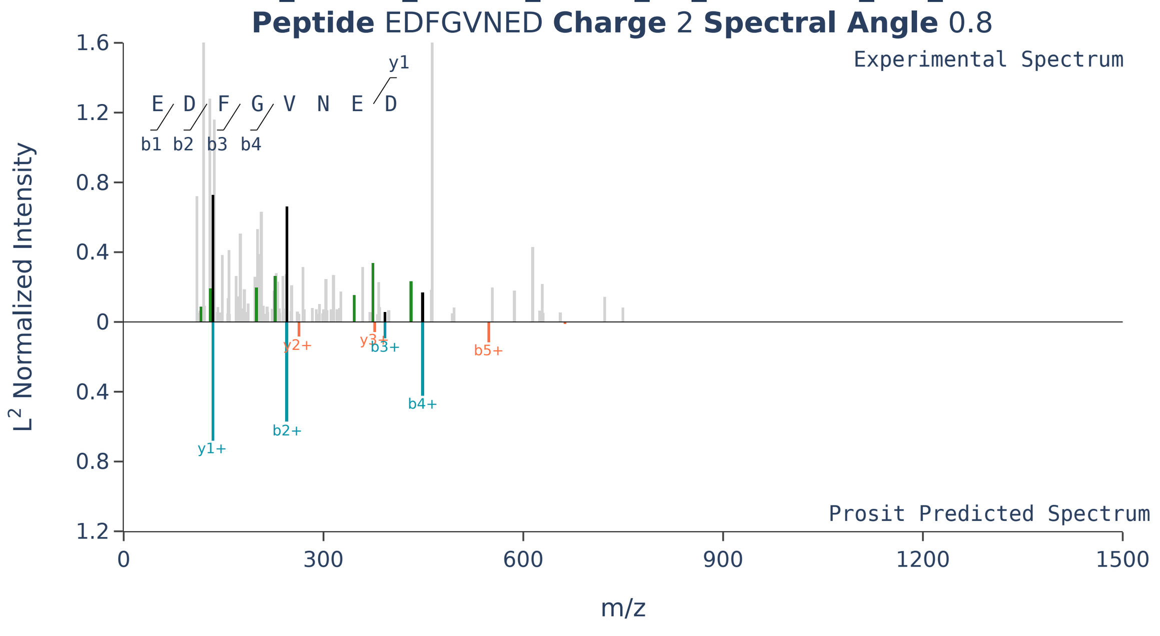

**Source** WSoh_301121_021221_HFGoe_M1_AnnexinA1_24h_R2 **Scan** 15129
**Peptide** FNPSSDV **Charge** 1 **Spectral Angle** 0.87

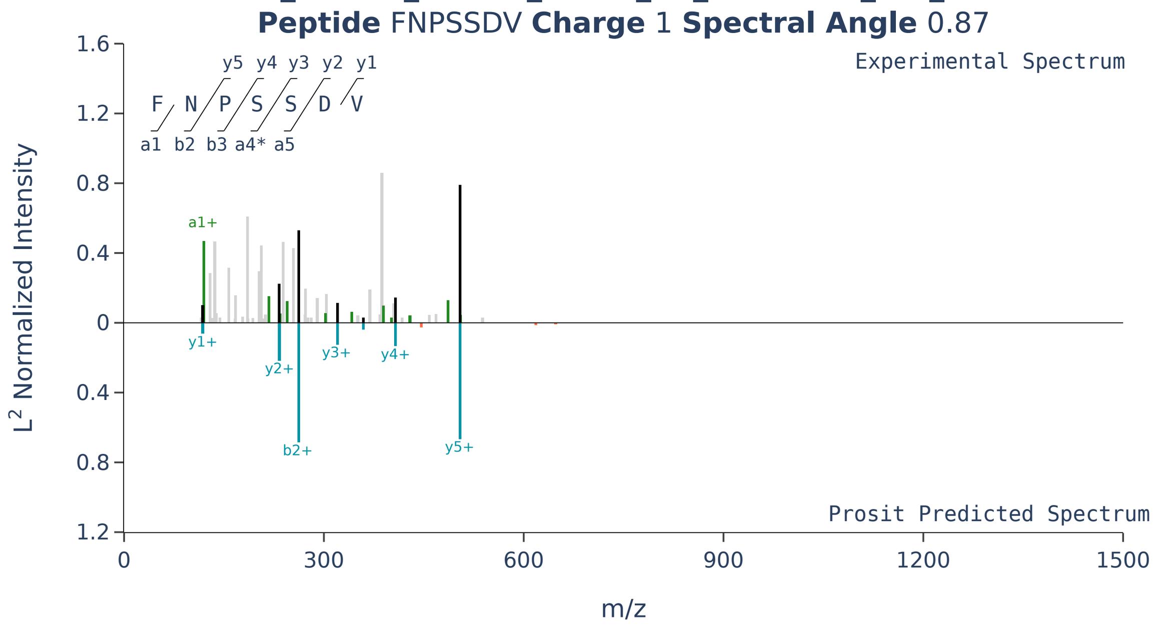


**Source** WSoh_301121_021221_HFGoe_M2_AnnexinA1_2h_R2 **Scan** 20233
**Peptide** DETKGDYEKILVA **Charge** 3 **Spectral Angle** 0.86

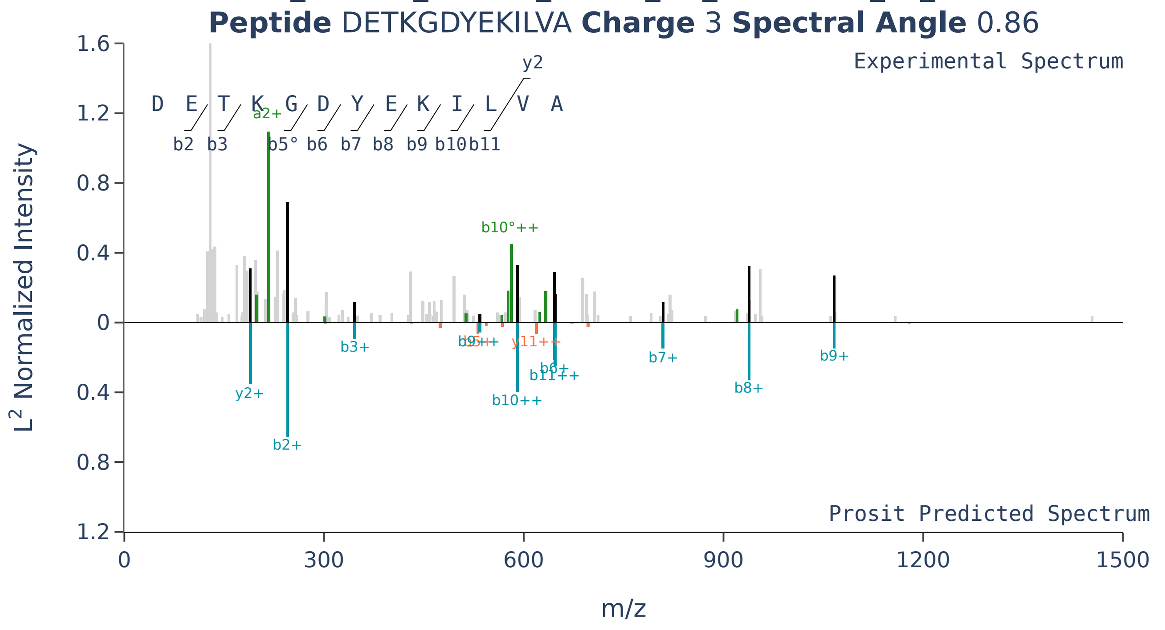


**Source** WSoh_301121_021221_HFGoe_M1_AnnexinA1_4h_R2 **Scan** 6856
**Peptide** RRVFQKY **Charge** 2 **Spectral Angle** 0.76

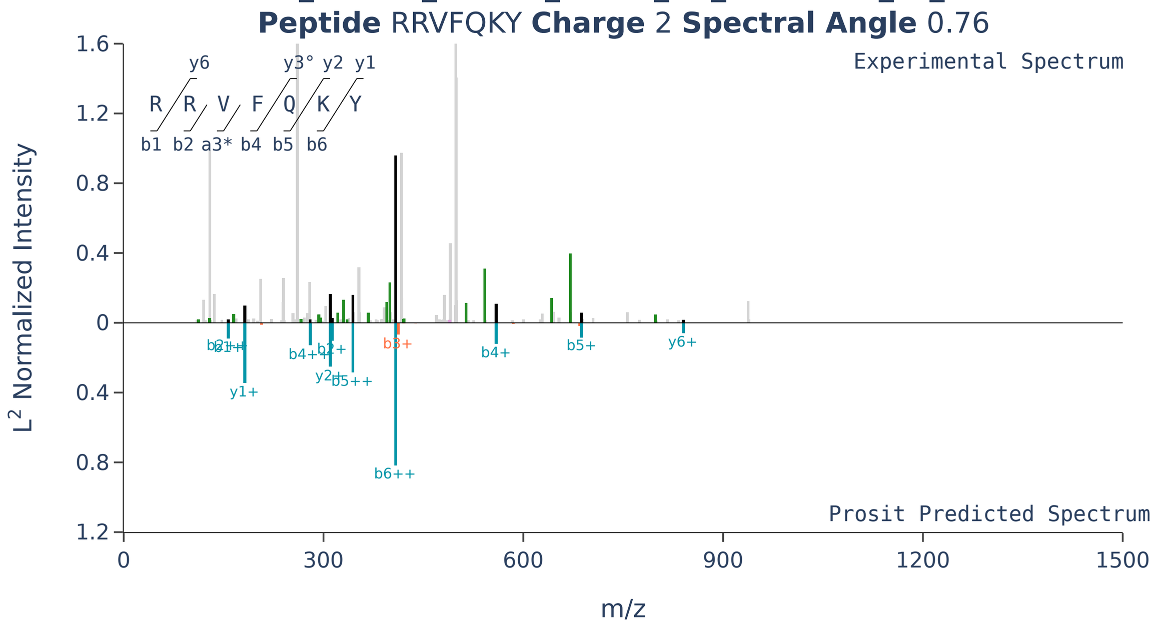


**Source** WSoh_301121_021221_HFGoe_M1_AnnexinA1_4h_R1 **Scan** 16610
**Peptide** TGKPLDETLKKAL **Charge** 3 **Spectral Angle** 0.86

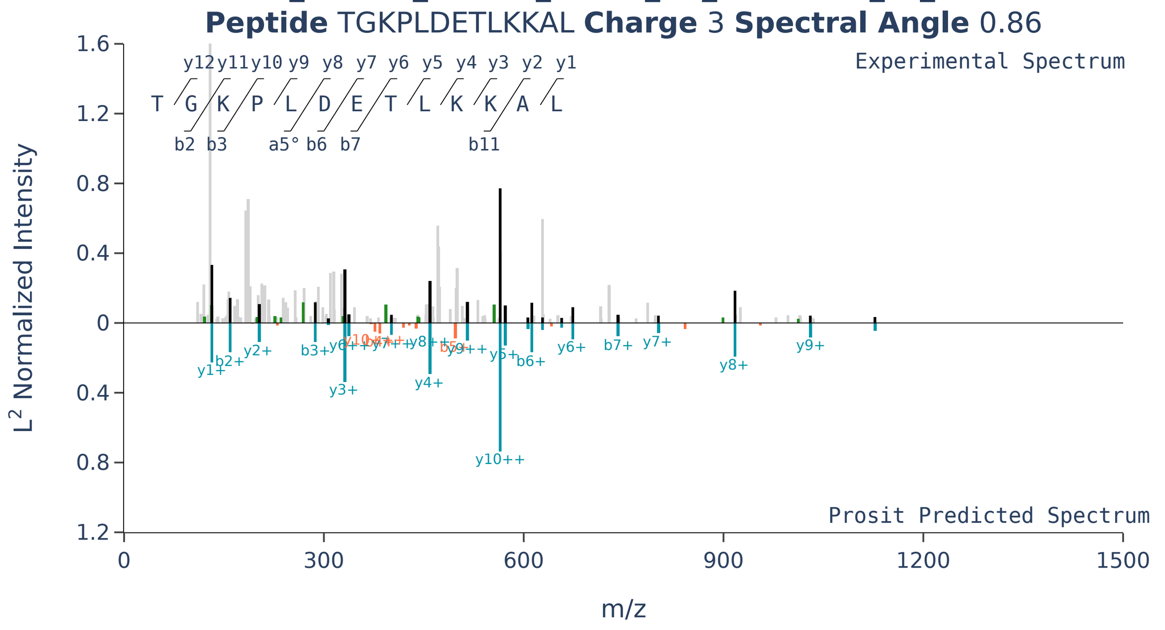


**Source** WSoh_301121_021221_HFGoe_M1_AnnexinA1_2h_R2 **Scan** 22299
**Peptide** RSEIDMNDIKAF **Charge** 3 **Spectral Angle** 0.88

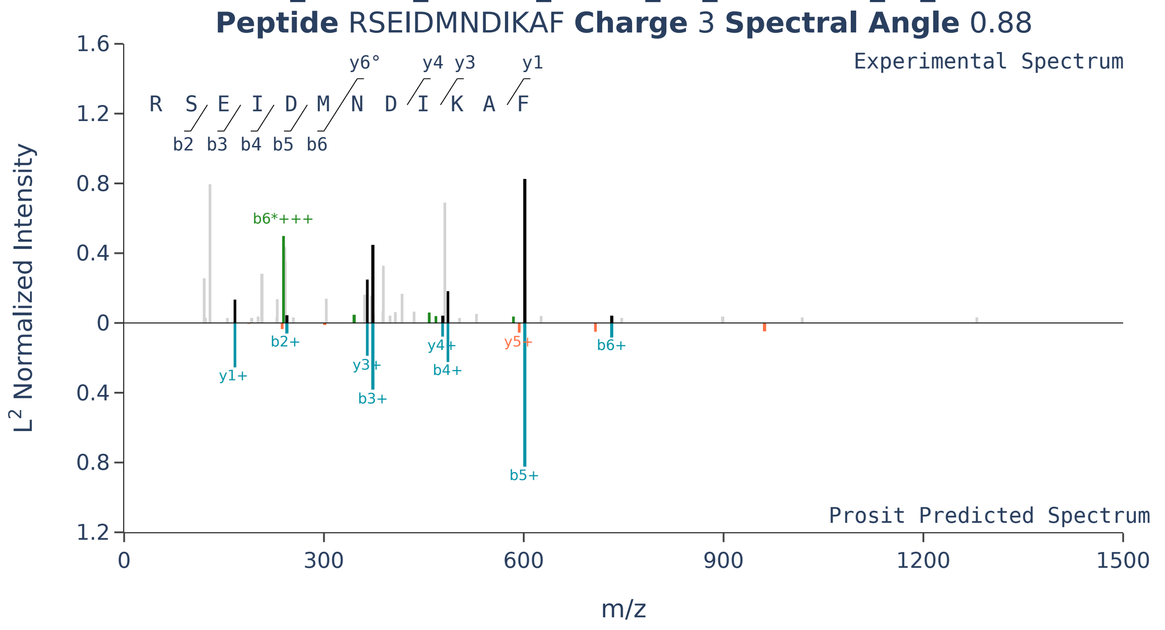


**Source** WSoh_301121_021221_HFGoe_M2_AnnexinA1_2h_R2 **Scan** 6333
**Peptide** RVFQKYTK **Charge** 3 **Spectral Angle** 0.9

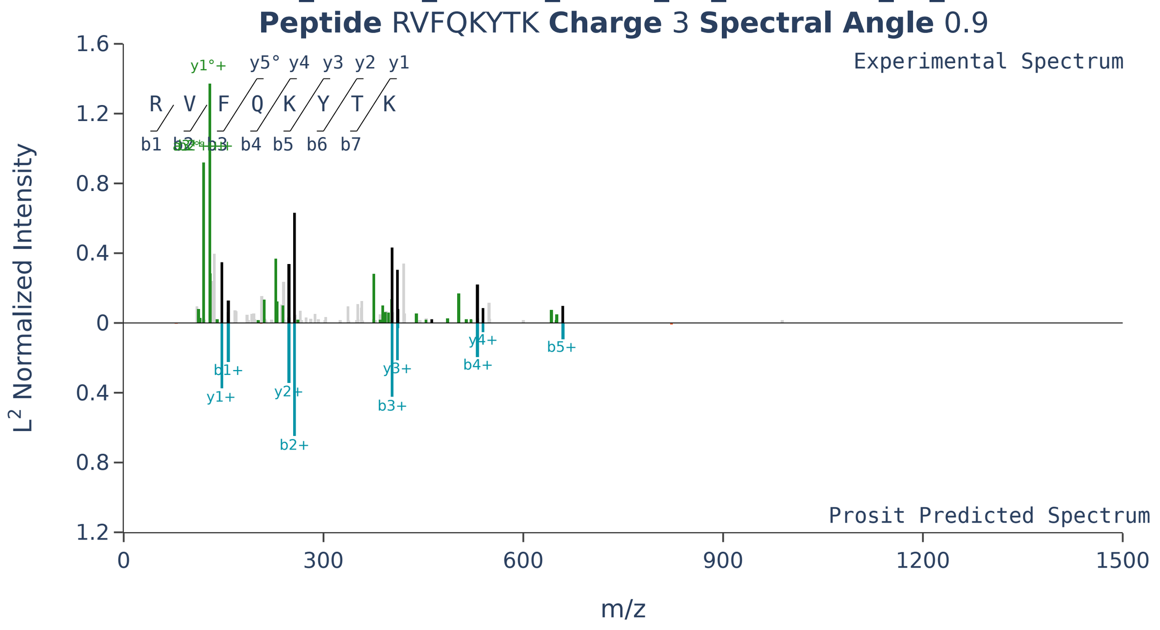


**Source** WSoh_301121_021221_HFGoe_M1_AnnexinA1_24h_R1 **Scan** 21074
**Peptide** SKGGPGSAVSPYPTFNPSSD **Charge** 2 **Spectral Angle** 0.9

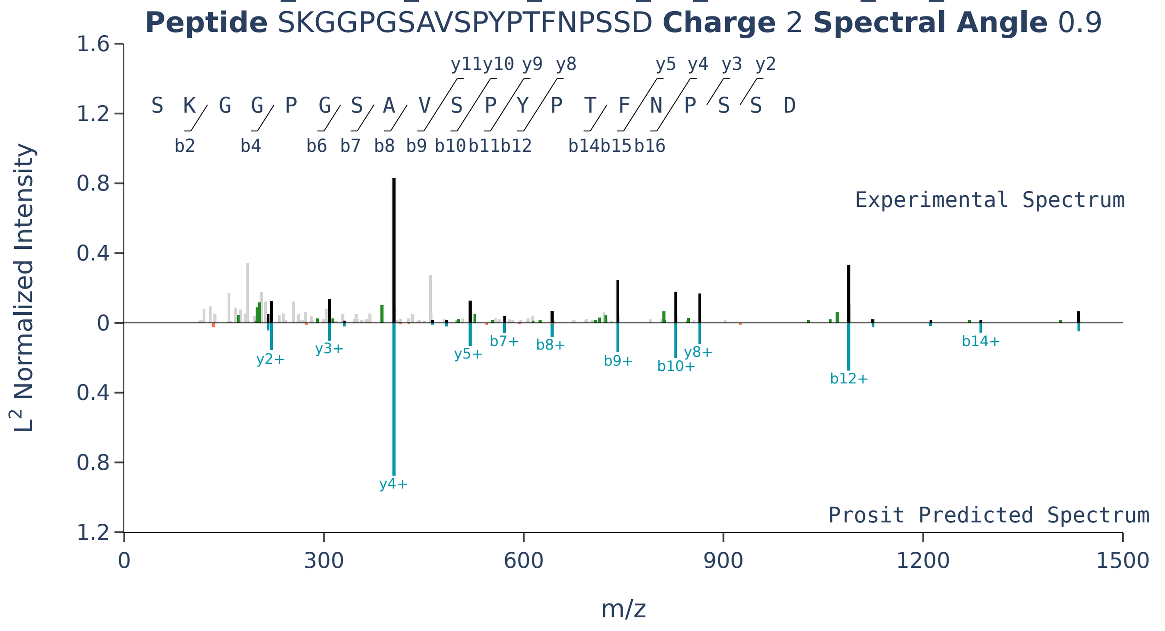


**Source** WSoh_301121_021221_HFGoe_M1_AnnexinA1_1h_R1 **Scan** 7160
**Peptide** RSYPQLRR **Charge** 3 **Spectral Angle** 0.8

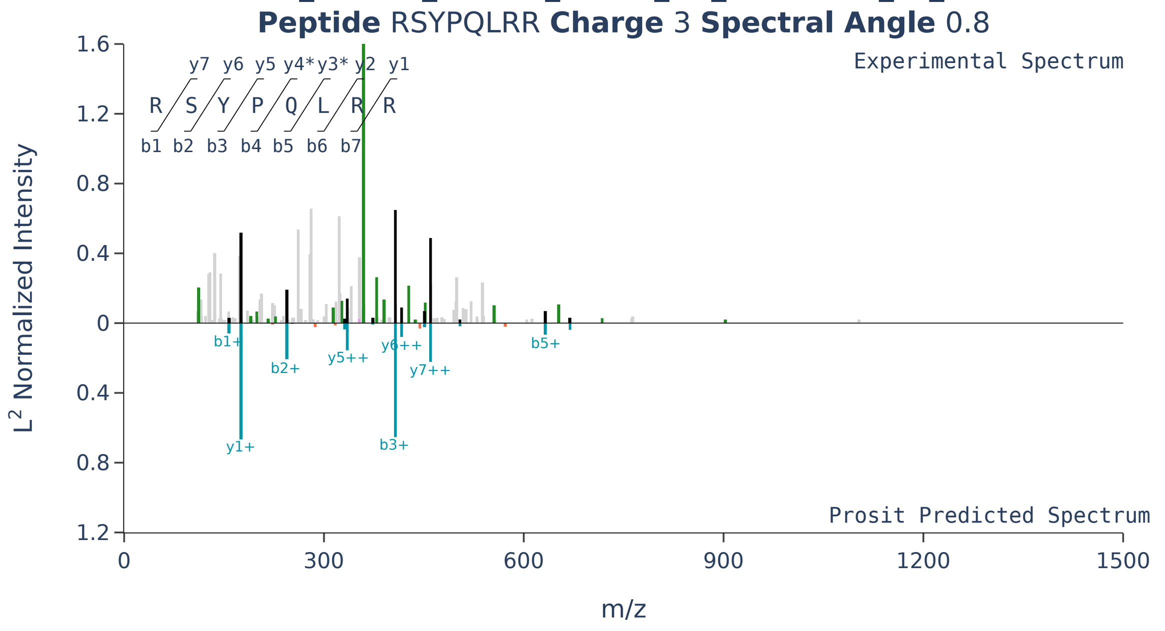


**Source** WSoh_301121_021221_HFGoe_M1_AnnexinA1_2h_R2 **Scan** 6237
**Peptide** YVQTVKSSKGGPGS **Charge** 3 **Spectral Angle** 0.87

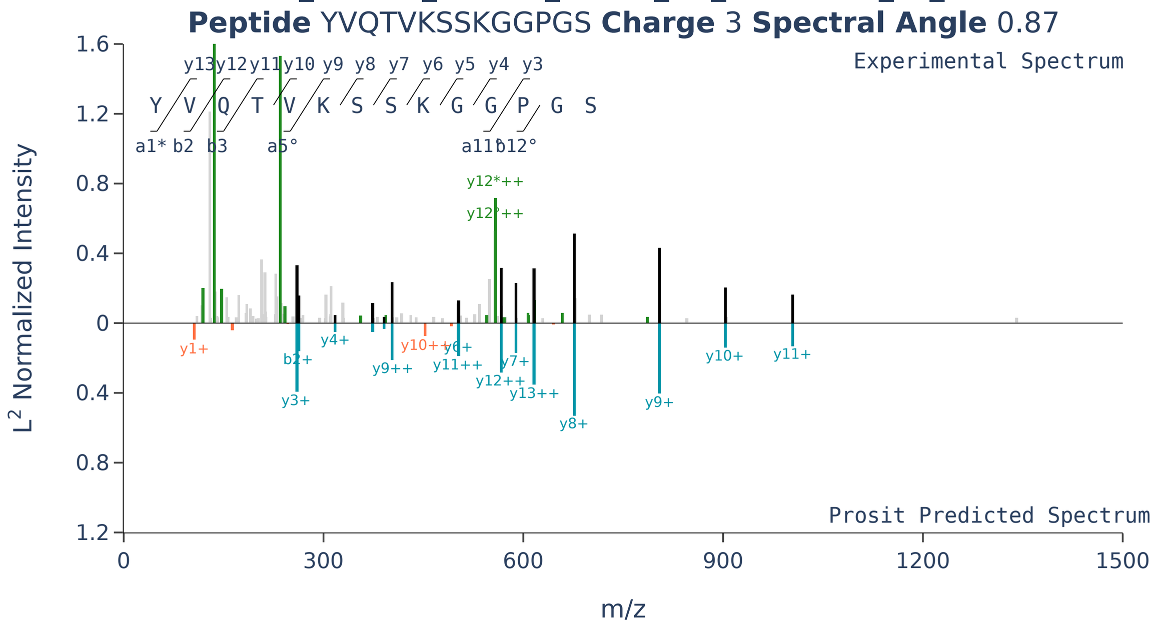


**Source** WSoh_301121_021221_HFGoe_M1_AnnexinA1_24h_R2 **Scan** 10508
**Peptide** AAMKGLGTD **Charge** 2 **Spectral Angle** 0.85

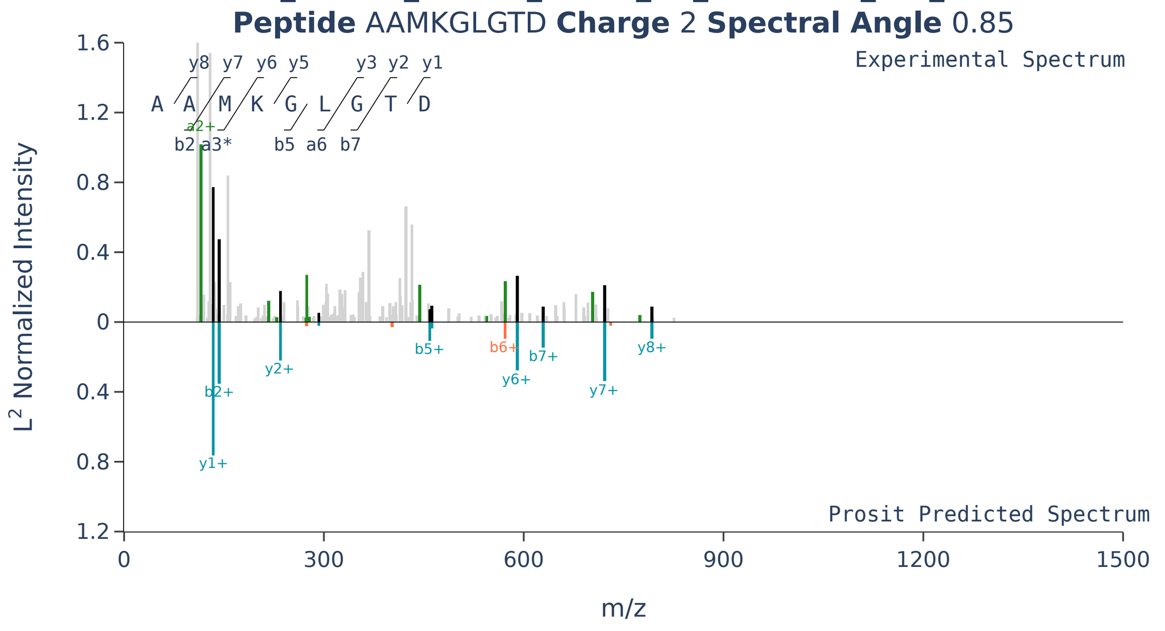


**Source** WSoh_301121_021221_HFGoe_M2_AnnexinA1_2h_R2 **Scan** 7480
**Peptide** ASRTNKEIRDINRV **Charge** 4 **Spectral Angle** 0.89

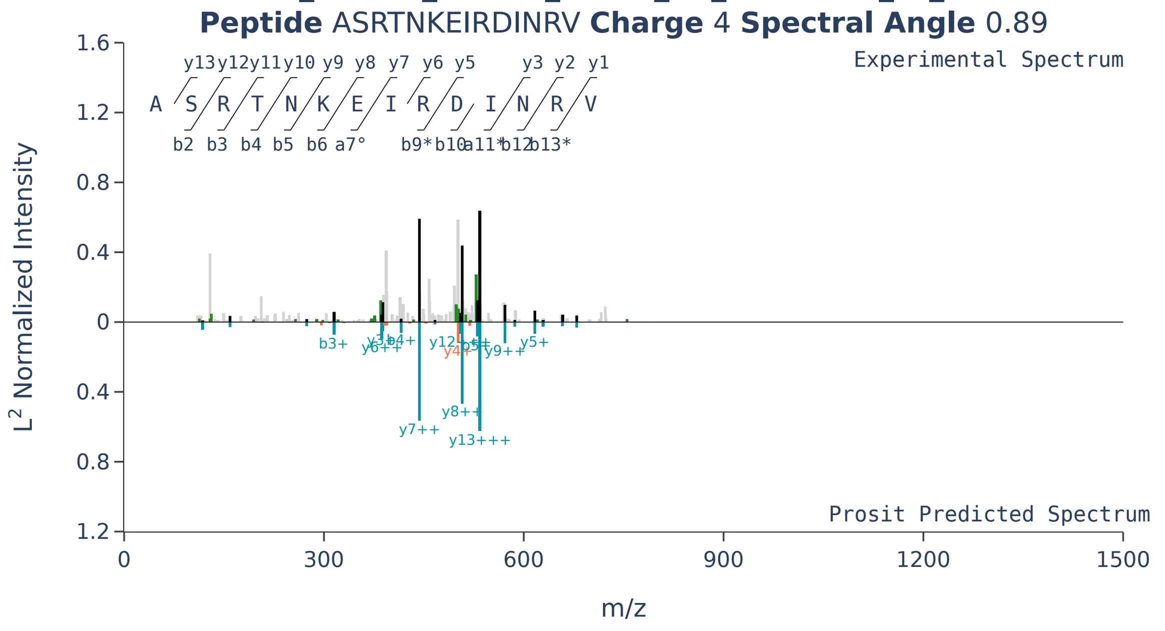


**Source** WSoh_301121_021221_HFGoe_M2_AnnexinA1_4h_R1 **Scan** 16589
**Peptide** KALTGHLEEVV **Charge** 3 **Spectral Angle** 0.85

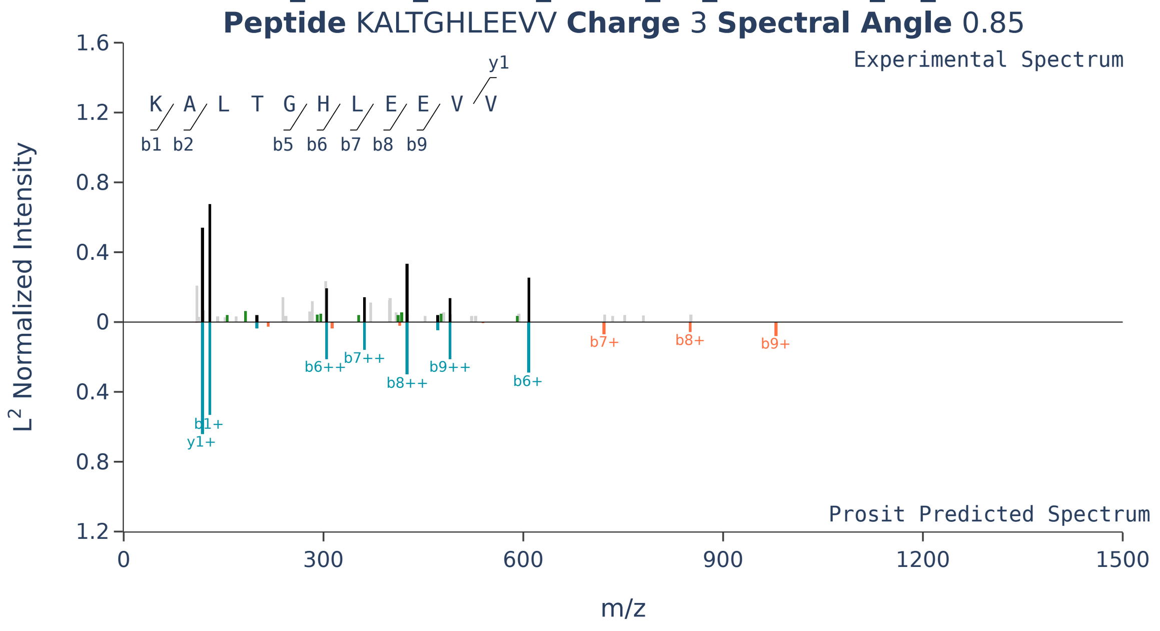


**Source** WSoh_301121_021221_HFGoe_M2_AnnexinA1_24h_R1 **Scan** 18552
**Peptide** AGERRKGTDVNVFNTI **Charge** 3 **Spectral Angle** 0.81

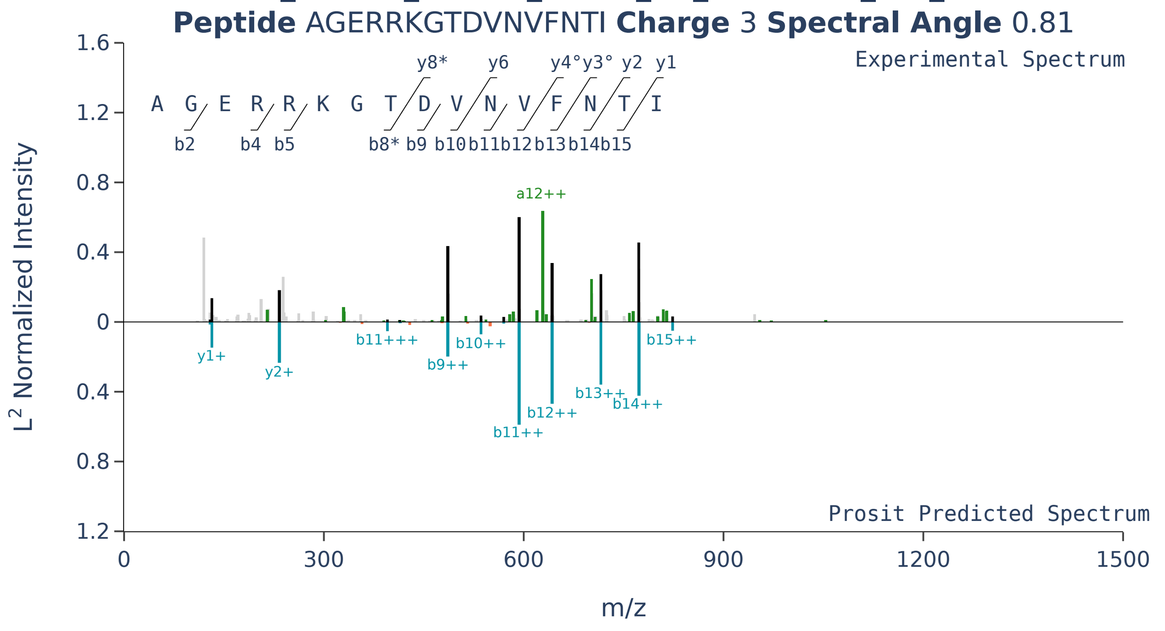


**Source** WSoh_301121_021221_HFGoe_M2_AnnexinA1_2h_R2 **Scan** 26358
**Peptide** ATIIDILTKRN **Charge** 3 **Spectral Angle** 0.89

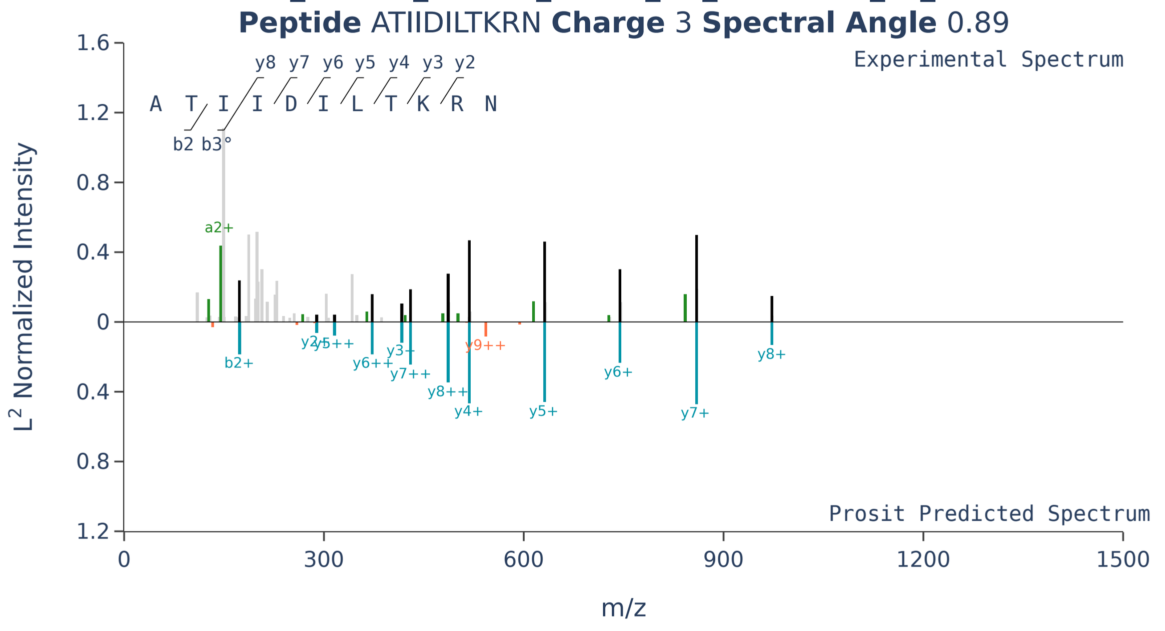


**Source** WSoh_301121_021221_HFGoe_M2_AnnexinA1_2h_R2 **Scan** 11458
**Peptide** IDILTKRNNAQRQQIKA **Charge** 3 **Spectral Angle** 0.85

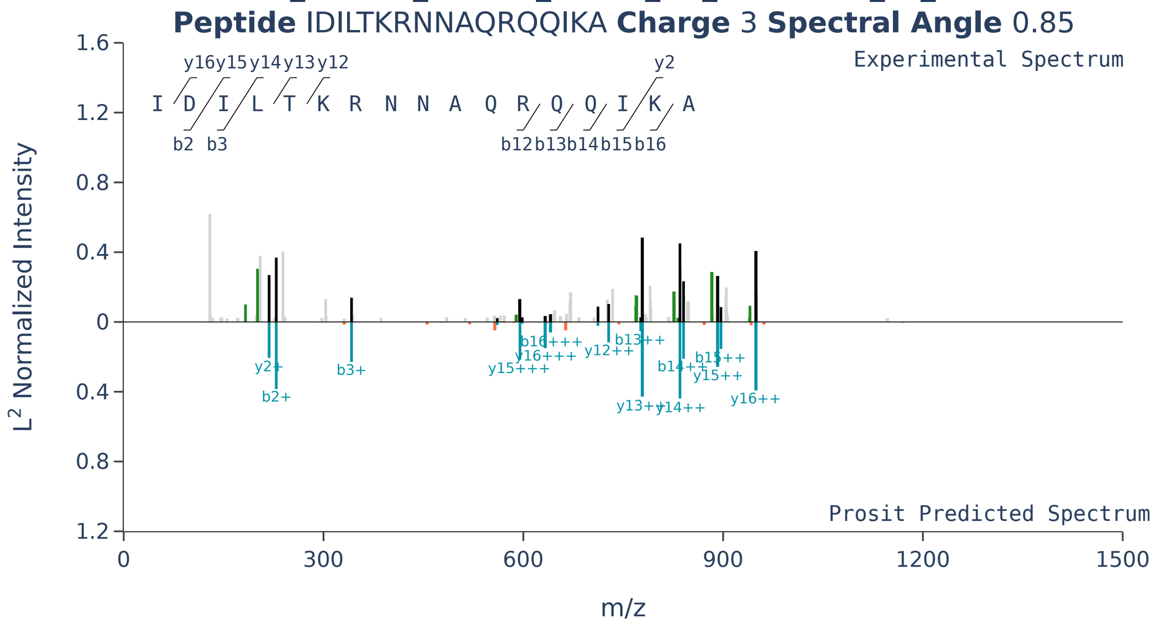

**Source** WSoh_301121_021221_HFGoe_M2_AnnexinA1_2h_R1 **Scan** 23085
**Peptide** GERRKGTDVNVFNTILT **Charge** 3 **Spectral Angle** 0.8

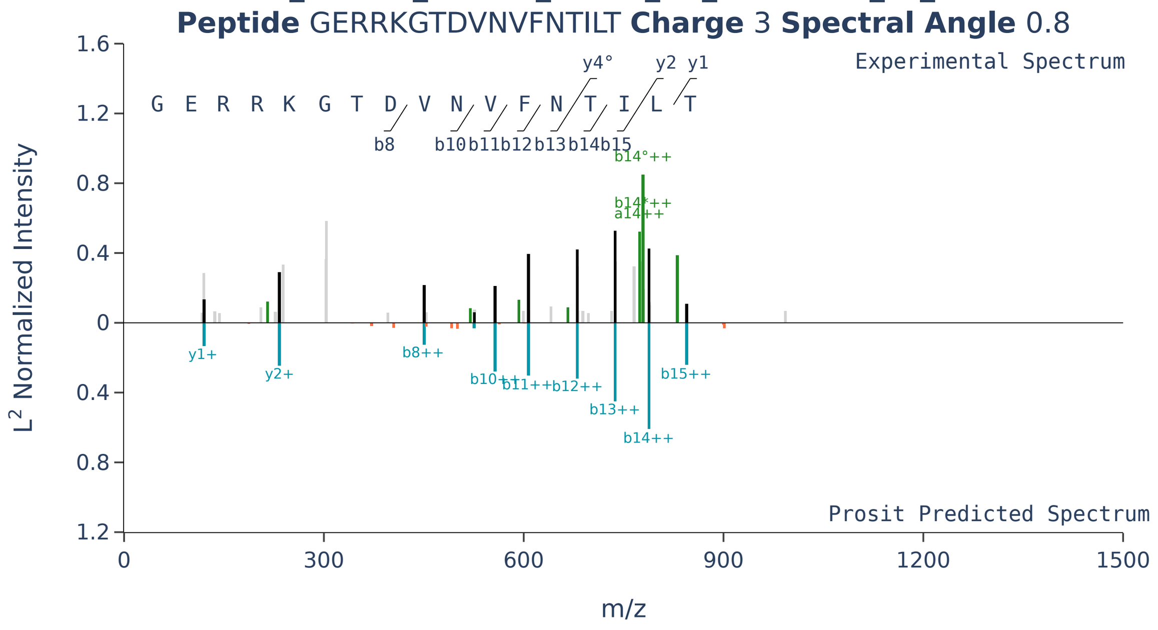


**Source** WSoh_301121_021221_HFGoe_M2_AnnexinA1_2h_R2 **Scan** 8571
**Peptide** RAAMKGLGTDED **Charge** 2 **Spectral Angle** 0.81

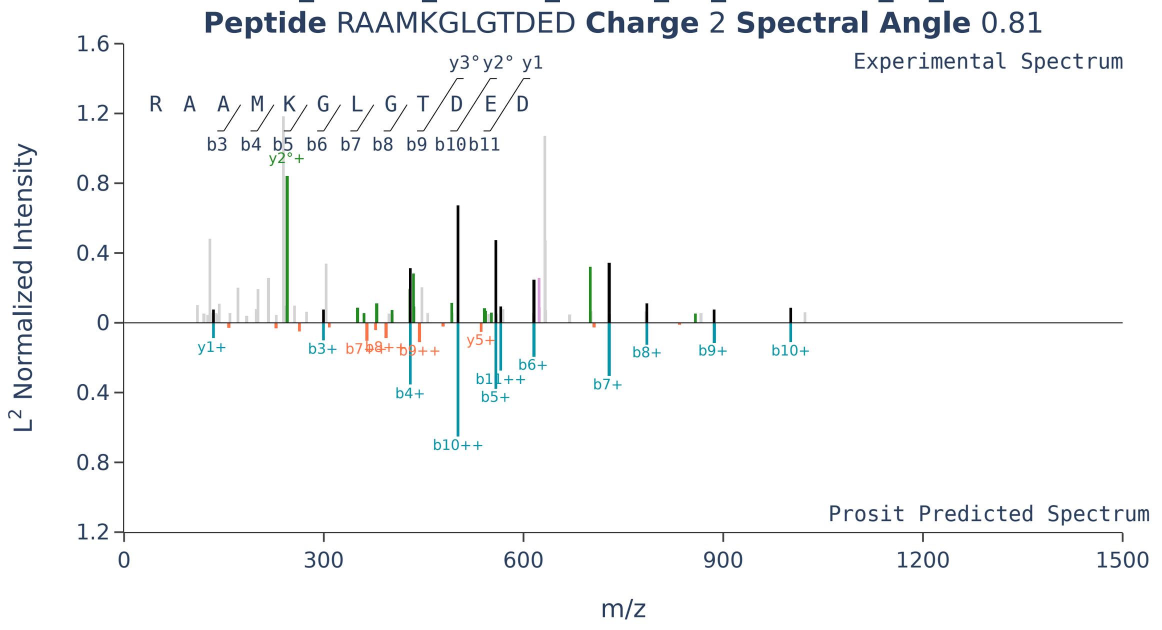


**Source** WSoh_301121_021221_HFGoe_M2_AnnexinA1_2h_R1 **Scan** 19113
**Peptide** KRDLAKDITSDTSGDFRNAL **Charge** 3 **Spectral Angle** 0.82

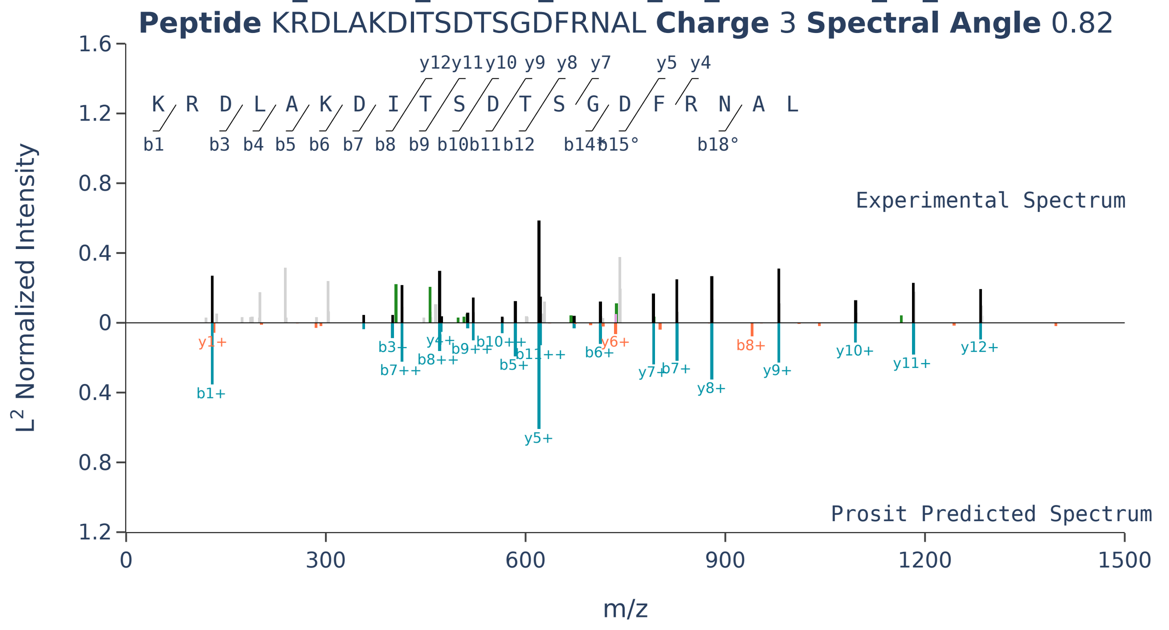


**Source** WSoh_301121_021221_HFGoe_M2_AnnexinA1_24h_R1 **Scan** 9081
**Peptide** RAAMKGLGTDEDT **Charge** 2 **Spectral Angle** 0.77

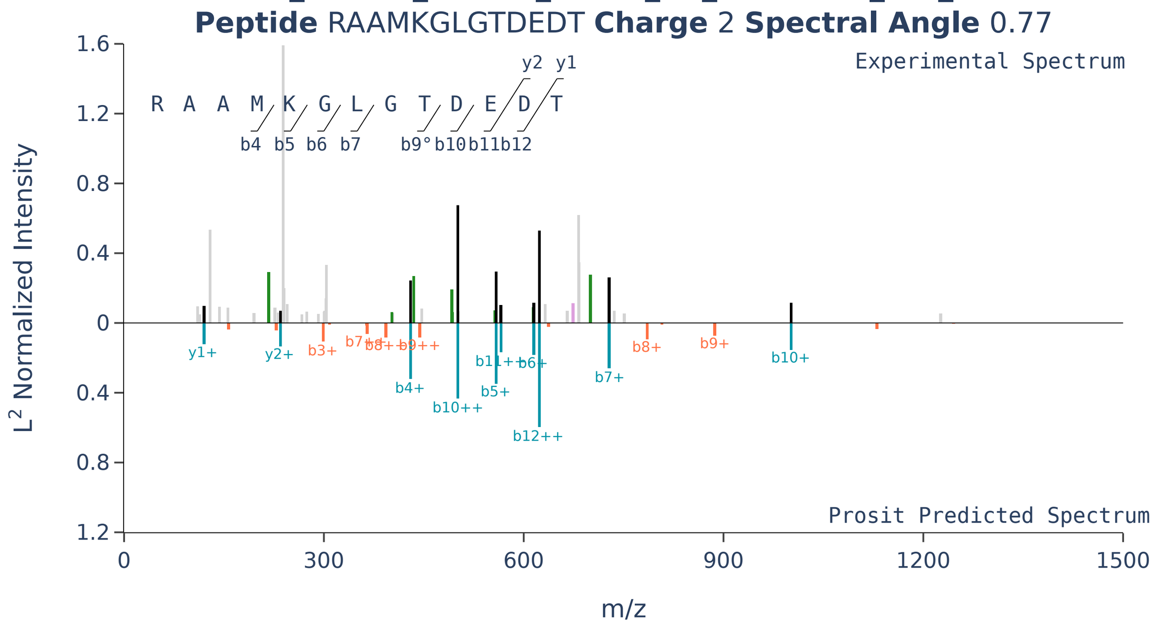


**Source** WSoh_301121_021221_HFGoe_M2_AnnexinA1_1h_R2 **Scan** 9374
**Peptide** YREELKRDL **Charge** 3 **Spectral Angle** 0.82

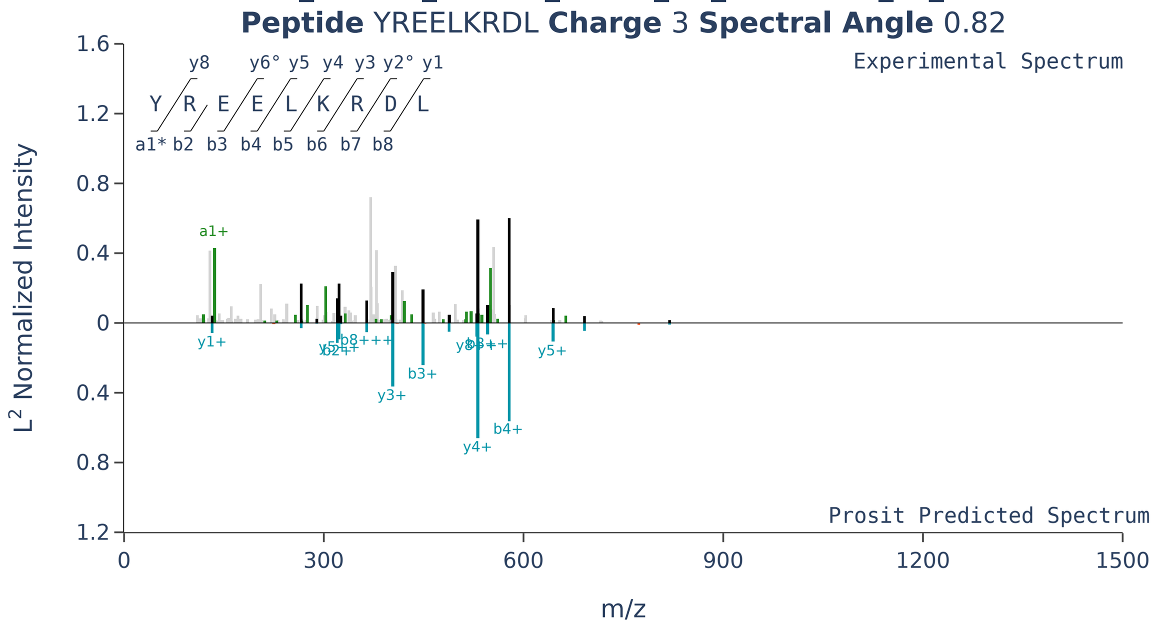


**Source** WSoh_301121_021221_HFGoe_M2_AnnexinA1_2h_R2 **Scan** 8460
**Peptide** MVKGVDEA **Charge** 2 **Spectral Angle** 0.79

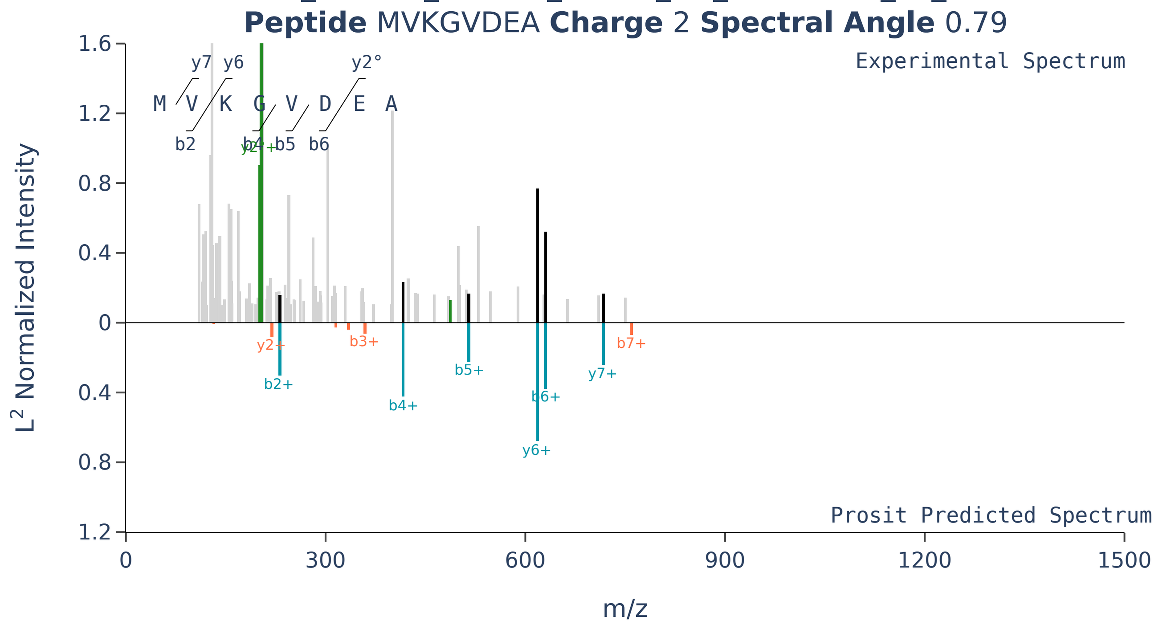


**Source** WSoh_301121_021221_HFGoe_M2_AnnexinA1_24h_R1 **Scan** 7170
**Peptide** KRDLAKDIT **Charge** 2 **Spectral Angle** 0.7

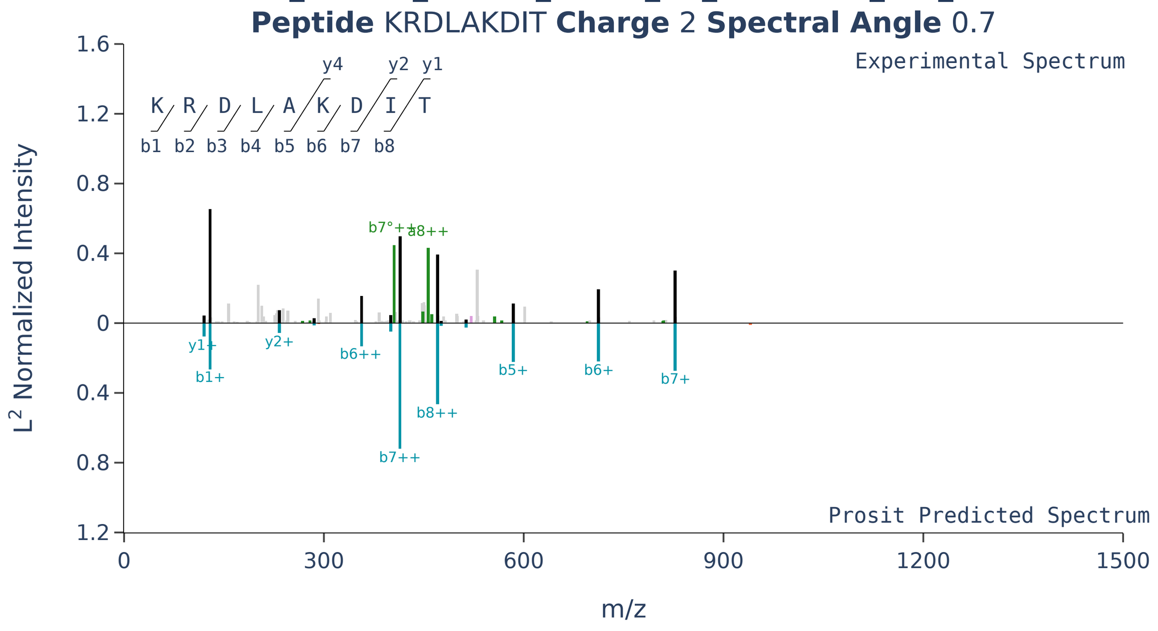


**Source** WSoh_301121_021221_HFGoe_M2_AnnexinA1_1h_R2 **Scan** 18174
**Peptide** ETKGDYEKILVA **Charge** 3 **Spectral Angle** 0.83

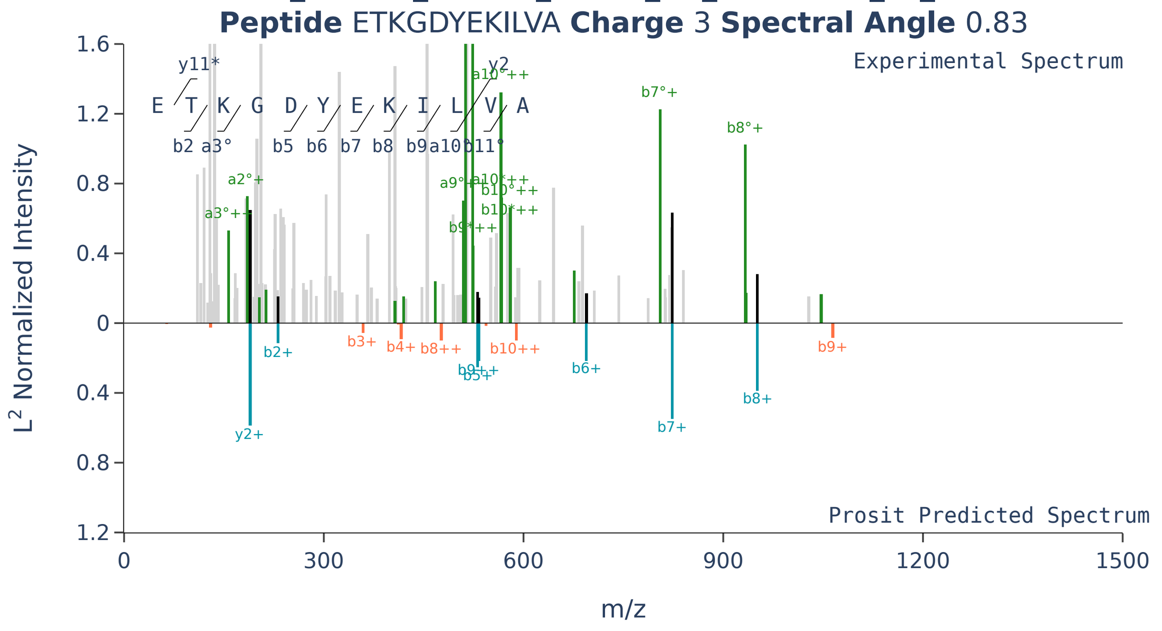


**Source** WSoh_301121_021221_HFGoe_M1_AnnexinA1_24h_R1 **Scan** 10172
**Peptide** ASRTNKEIRDINRVY **Charge** 4 **Spectral Angle** 0.87

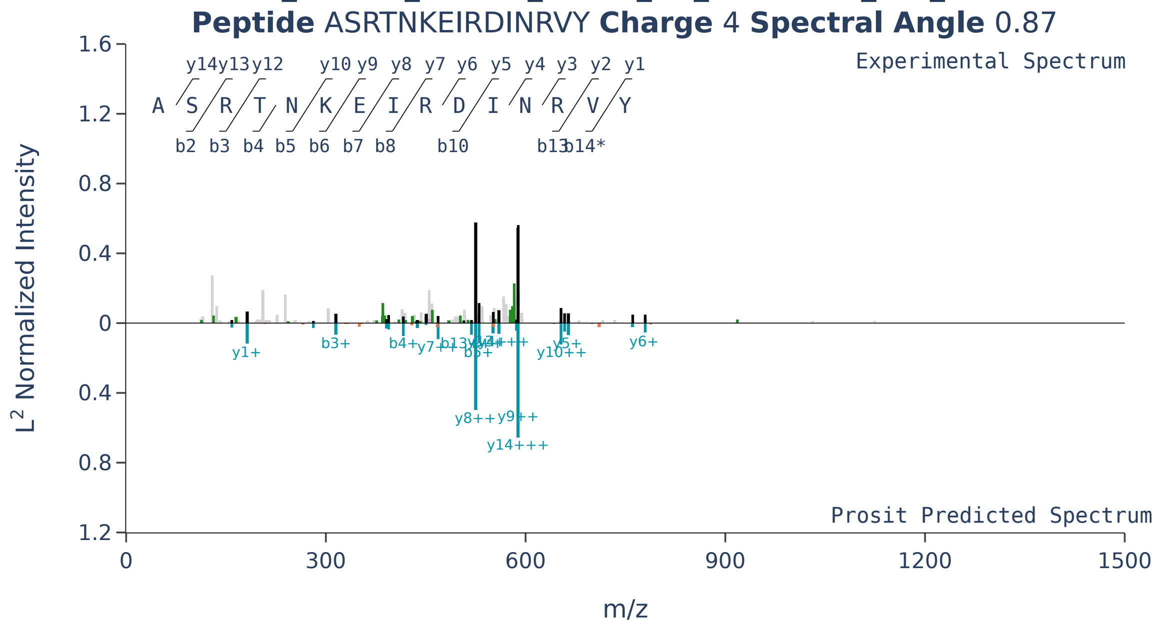


**Source** WSoh_301121_021221_HFGoe_M2_AnnexinA1_4h_R1 **Scan** 16637
**Peptide** LKAAYLQETGKPLD **Charge** 3 **Spectral Angle** 0.84

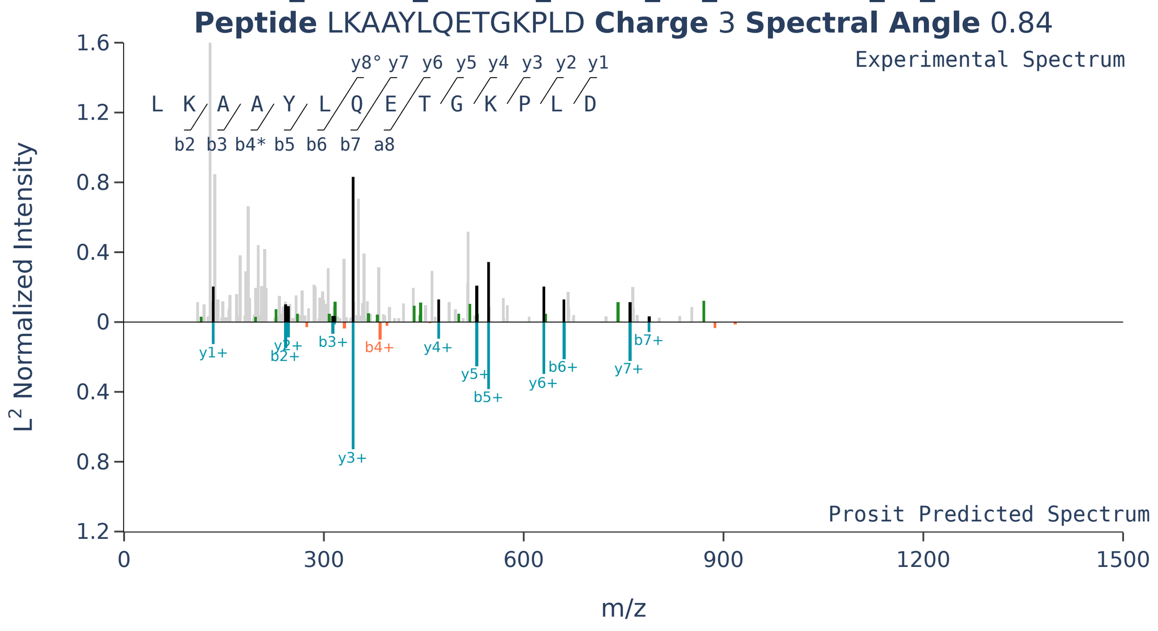


**Source** WSoh_301121_021221_HFGoe_M2_AnnexinA1_2h_R1 **Scan** 21771
**Peptide** SKGGPGSAVSPYPTF **Charge** 2 **Spectral Angle** 0.93

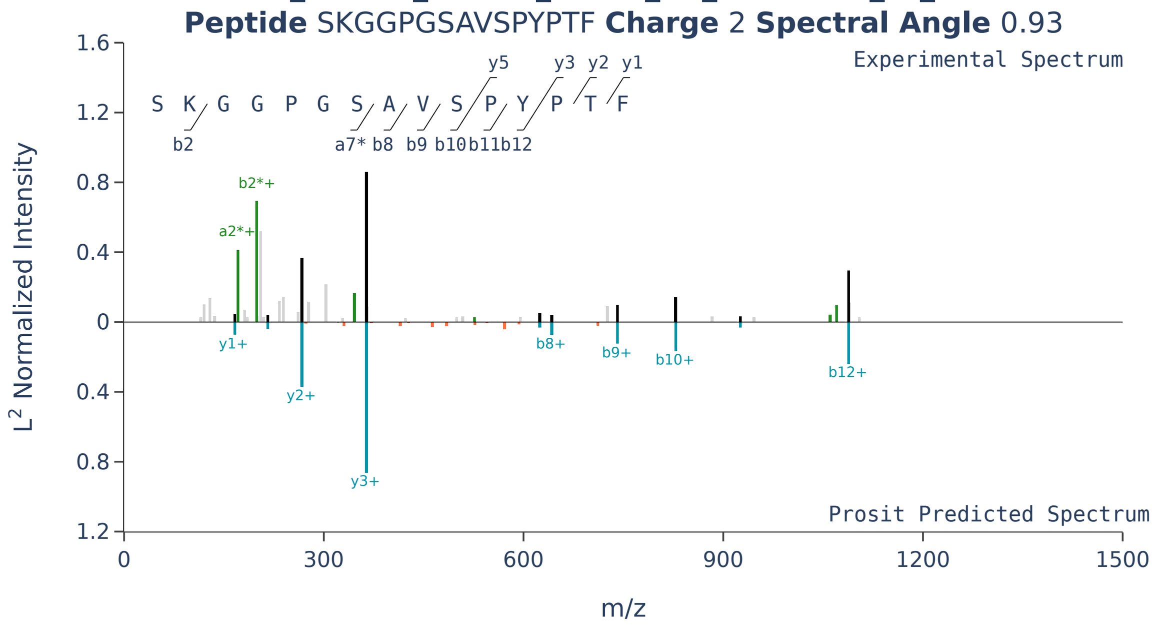


**Source** WSoh_301121_021221_HFGoe_M2_AnnexinA1_24h_R2 **Scan** 9887
**Peptide** SRTNKEIRDINRVY **Charge** 4 **Spectral Angle** 0.85

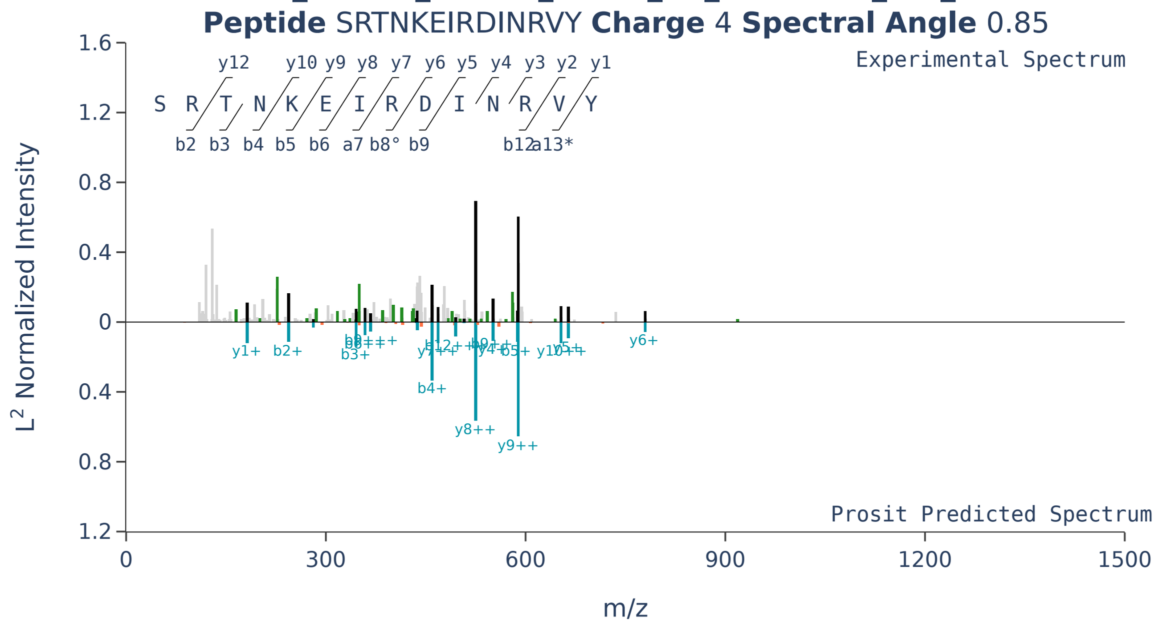


**Source** WSoh_301121_021221_HFGoe_M2_AnnexinA1_24h_R2 **Scan** 8780
**Peptide** KRDLAKDITSDTS **Charge** 2 **Spectral Angle** 0.84

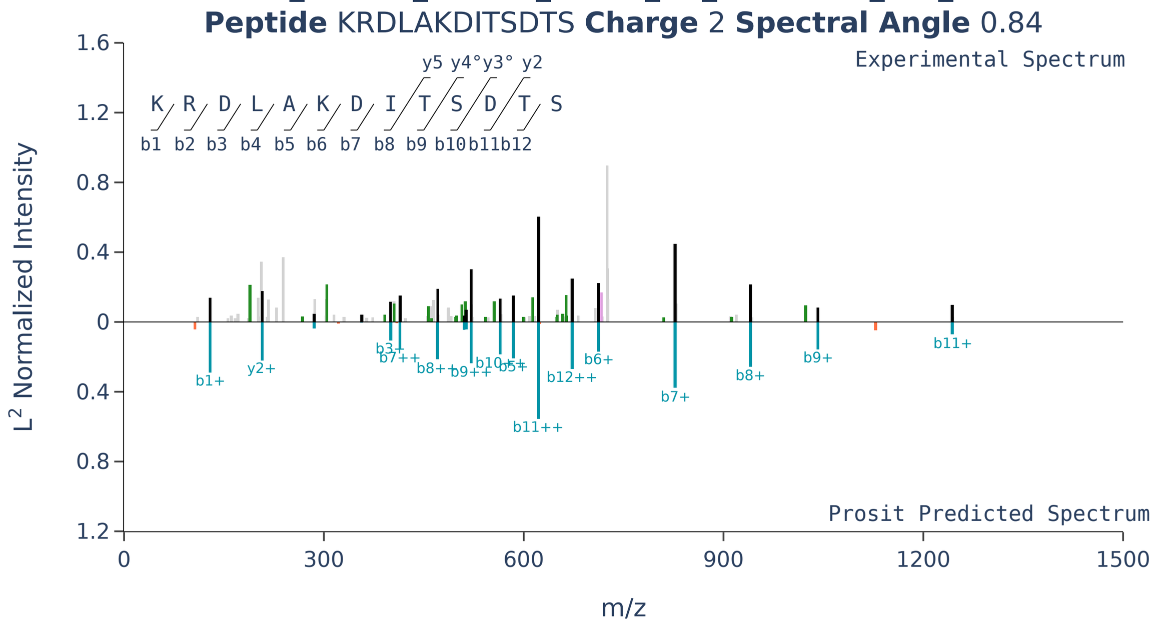


**Source** WSoh_301121_021221_HFGoe_M2_AnnexinA1_2h_R2 **Scan** 20468
**Peptide** SKGGPGSAVSPYPTFNPSS **Charge** 2 **Spectral Angle** 0.93

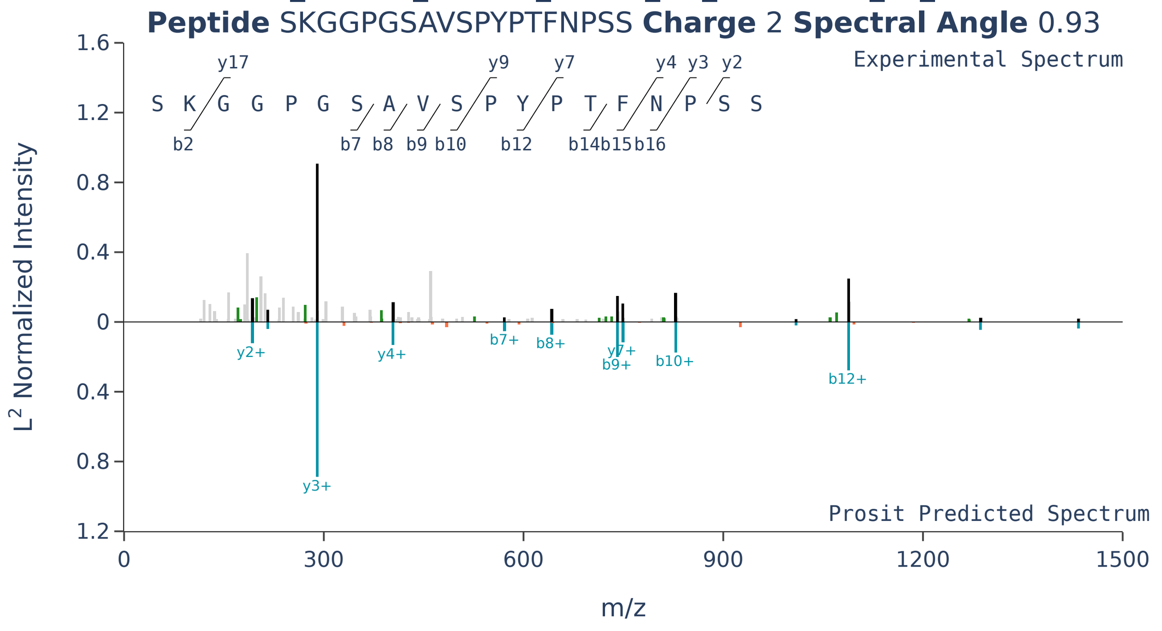


**Source** WSoh_301121_021221_HFGoe_M2_AnnexinA1_24h_R1 **Scan** 19062
**Peptide** QAILDETKGDYEKI **Charge** 2 **Spectral Angle** 0.9

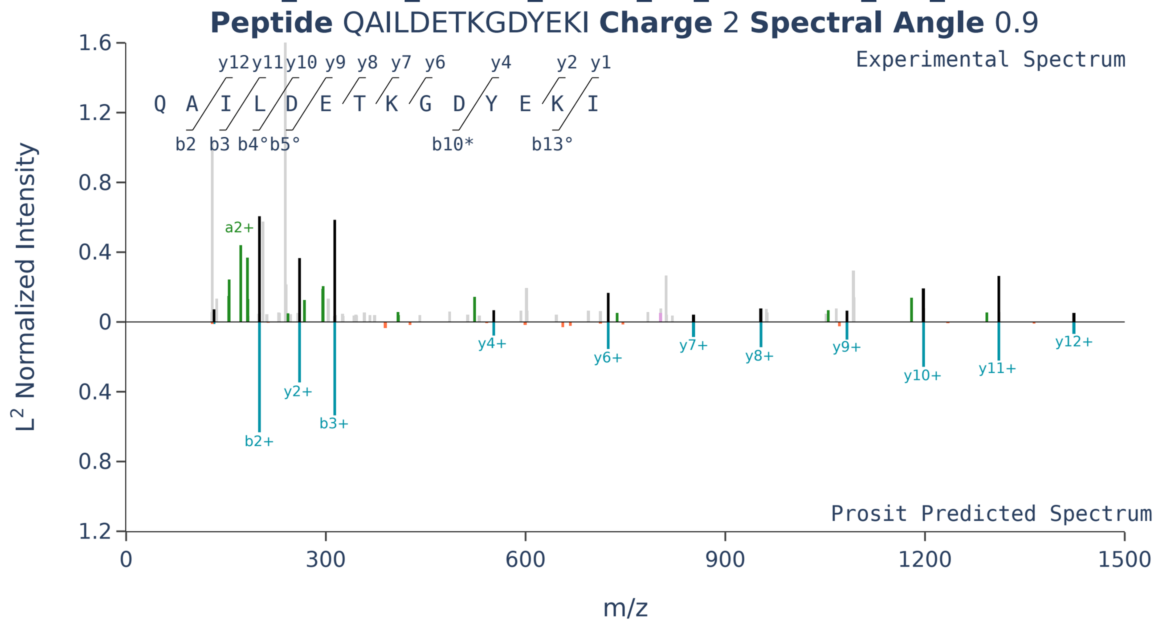

**Source** WSoh_301121_021221_HFGoe_M2_AnnexinA1_2h_R2 **Scan** 21147
**Peptide** TDVNVFNT **Charge** 1 **Spectral Angle** 0.85

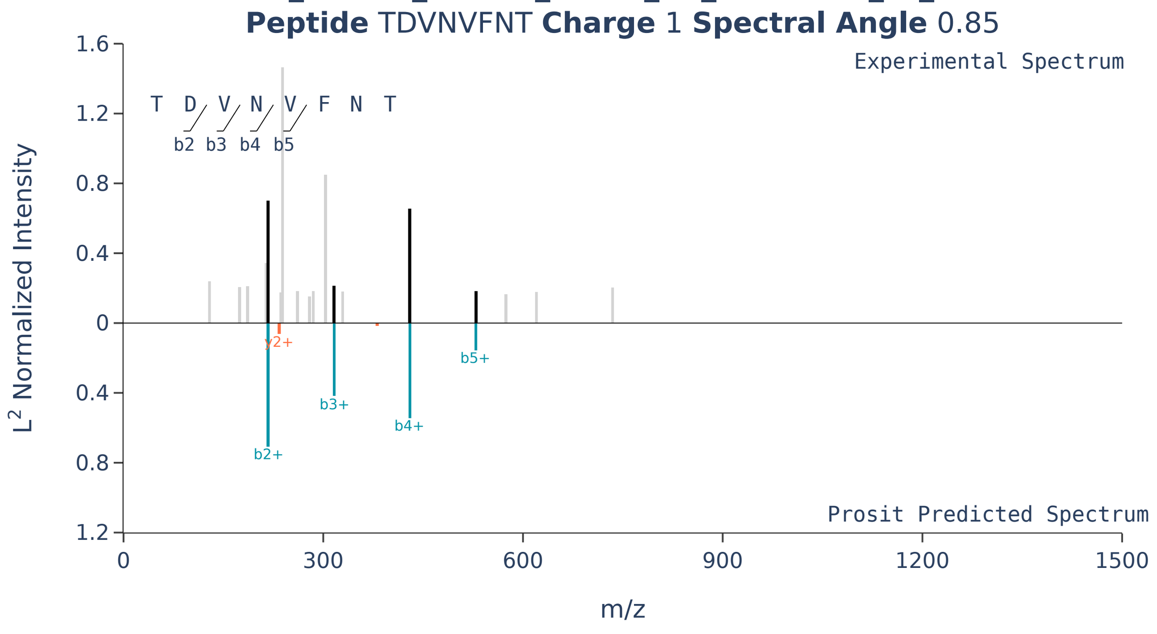

**Source** WSoh_301121_021221_HFGoe_M2_AnnexinA1_24h_R2 **Scan** 12484
**Peptide** IDILTKRNNAQ **Charge** 3 **Spectral Angle** 0.81

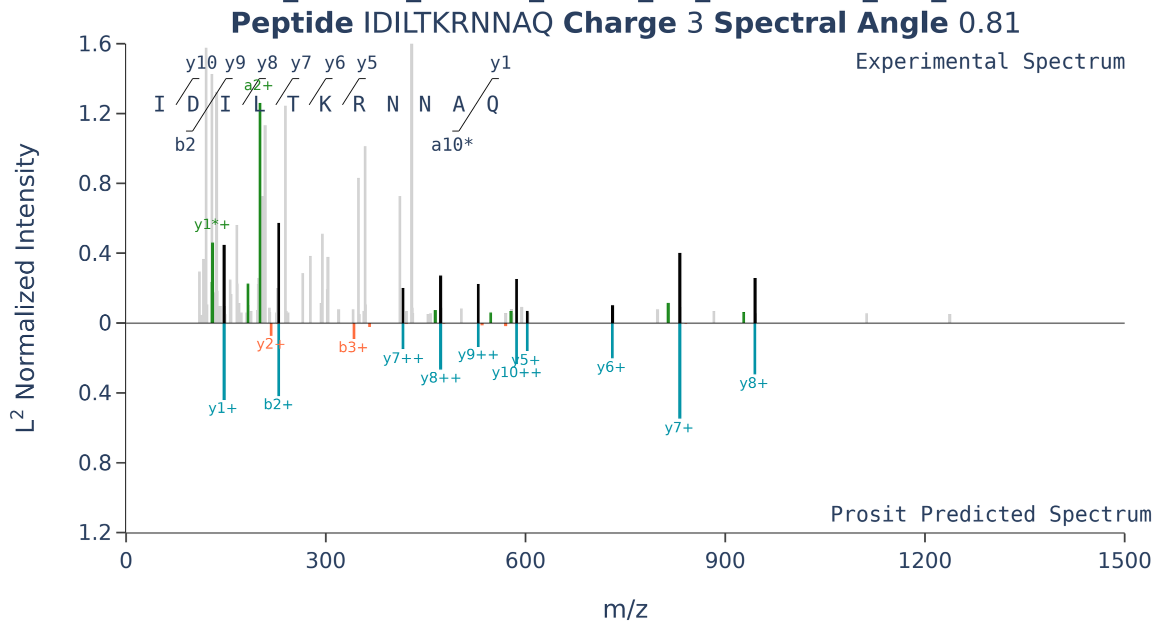

**Source** WSoh_301121_021221_HFGoe_M2_AnnexinA1_2h_R2 **Scan** 22774
**Peptide** KMYGISL **Charge** 2 **Spectral Angle** 0.81

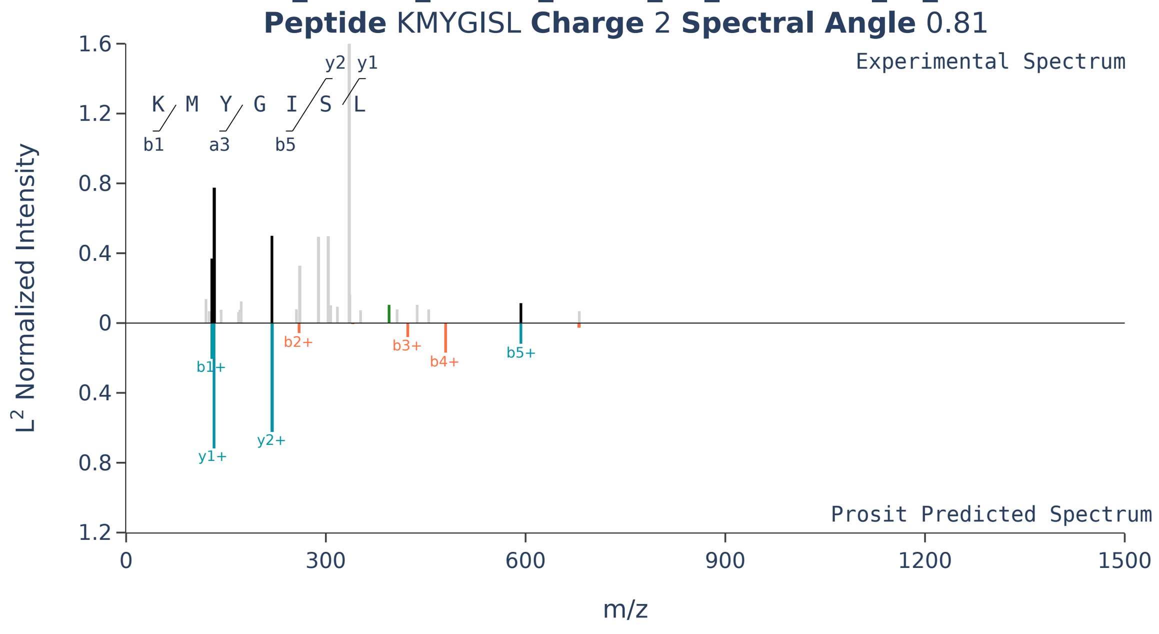

**Source** WSoh_301121_021221_HFGoe_M1_AnnexinA1_24h_R1 **Scan** 14005
**Peptide** AIMVKGVDEA **Charge** 2 **Spectral Angle** 0.83

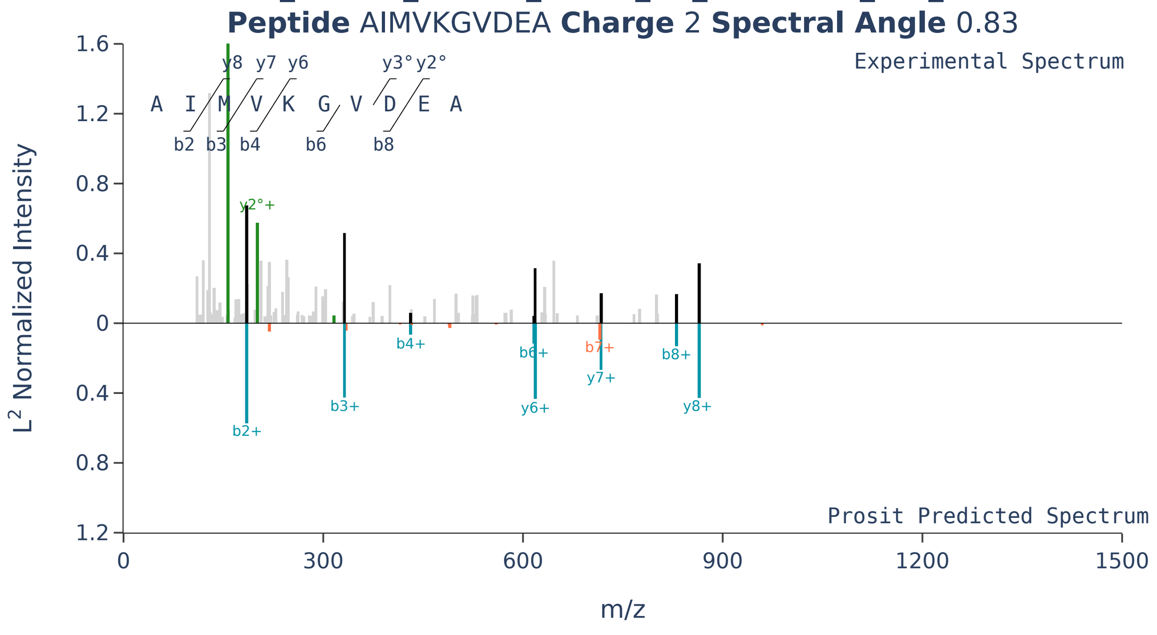

**Source** WSoh_301121_021221_HFGoe_M2_AnnexinA1_24h_R1 **Scan** 12746
**Peptide** EQEYVQTVKSSKGGPGSAV **Charge** 2 **Spectral Angle** 0.83

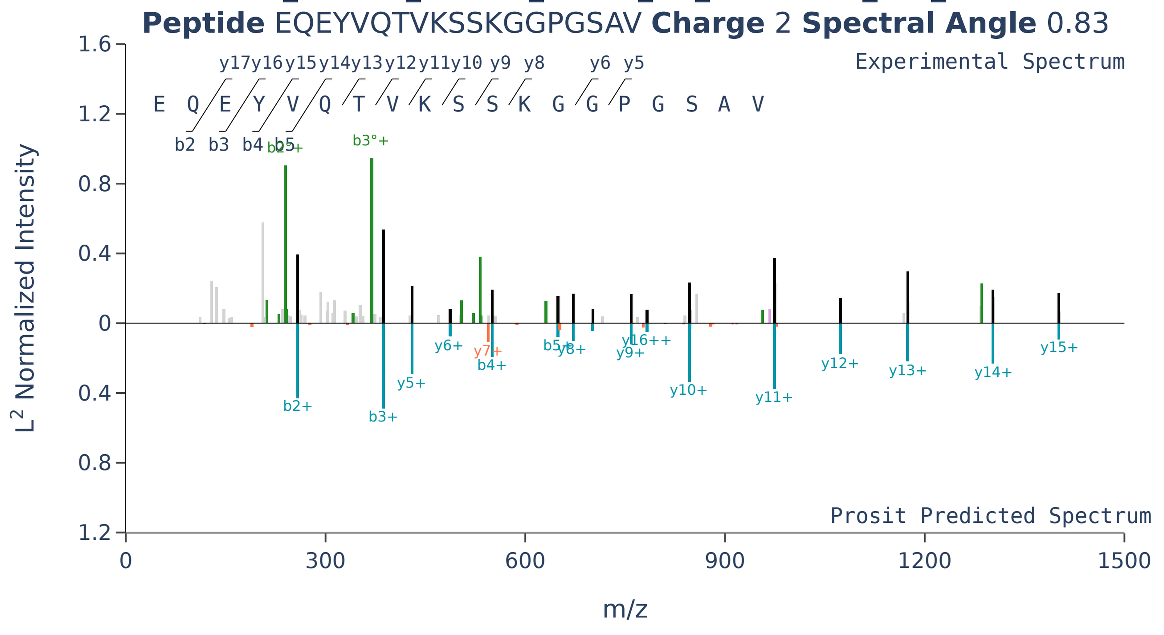

**Source** WSoh_301121_021221_HFGoe_M2_AnnexinA1_2h_R1 **Scan** 15886
**Peptide** LKRDLAKDITSDTSGDFRNA **Charge** 3 **Spectral Angle** 0.79

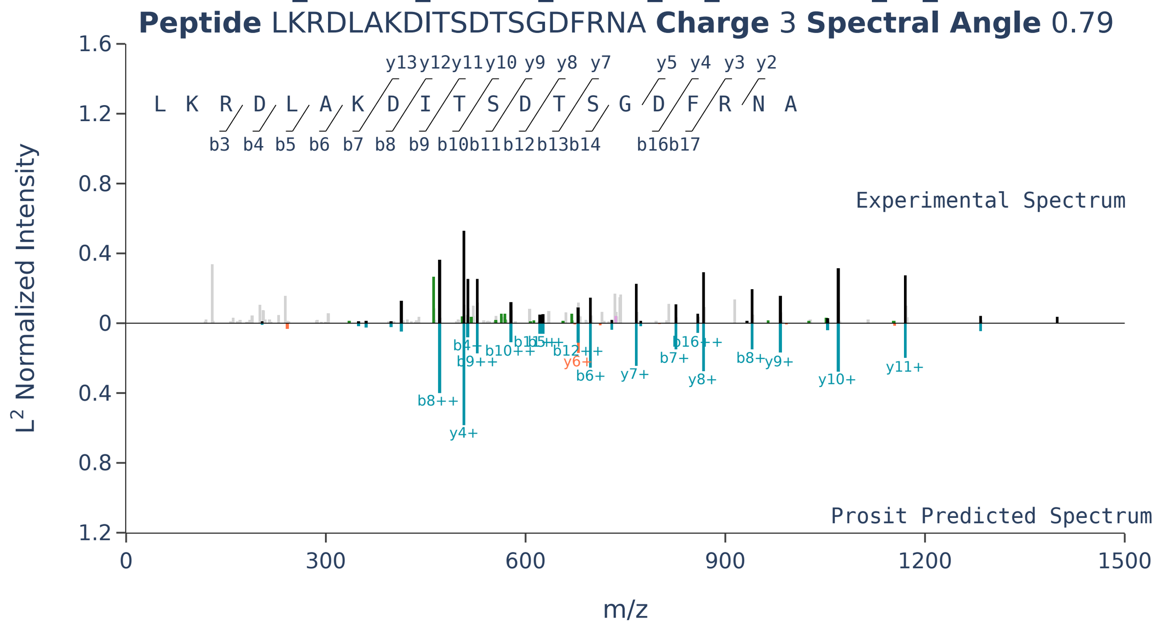

**Source** WSoh_301121_021221_HFGoe_M2_AnnexinA1_2h_R2 **Scan** 16710
**Peptide** DVAALHKAIMVKG **Charge** 3 **Spectral Angle** 0.82

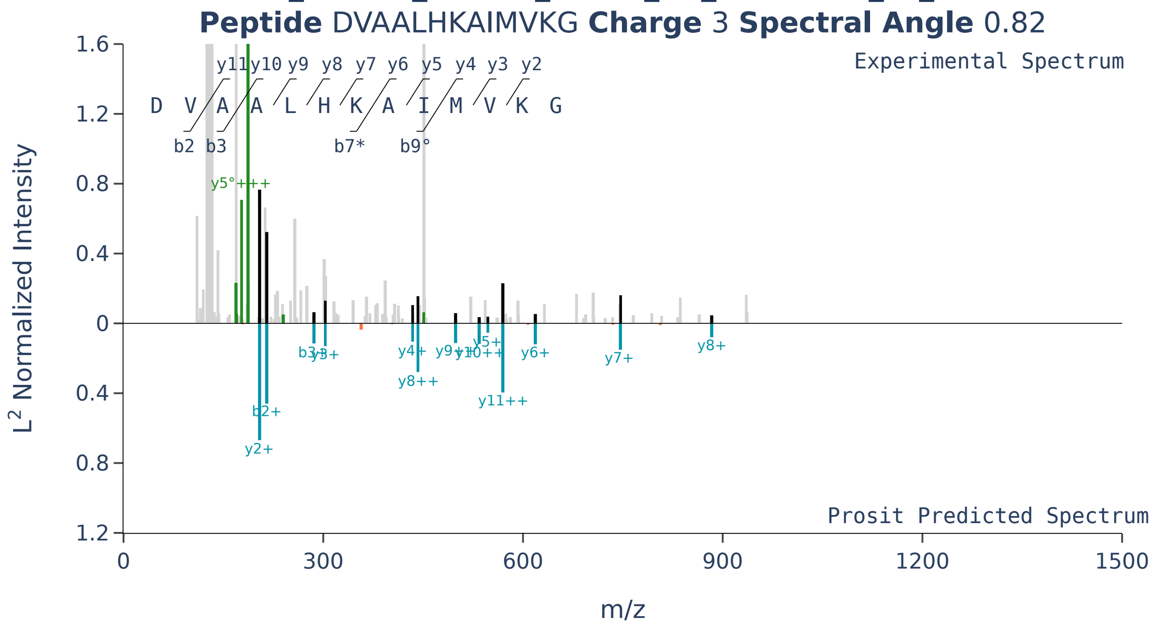

**Source** WSoh_301121_021221_HFGoe_M2_AnnexinA1_2h_R1 **Scan** 22891
**Peptide** AKDITSDTSGDFRNALL **Charge** 3 **Spectral Angle** 0.81

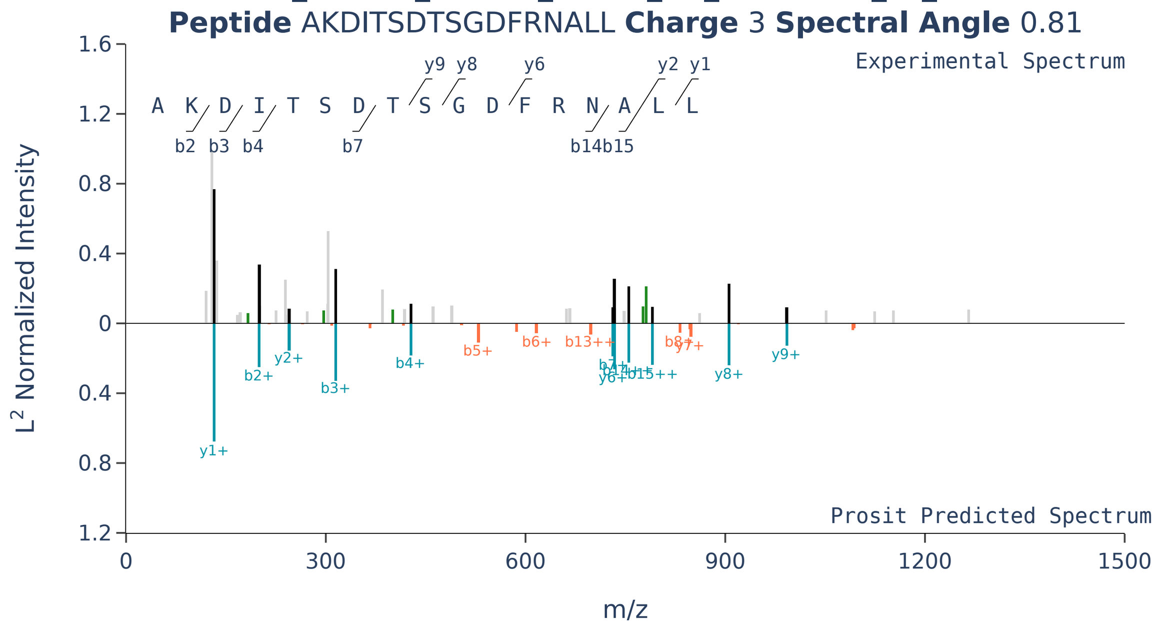

**Source** WSoh_301121_021221_HFGoe_M1_AnnexinA1_4h_R2 **Scan** 15227
**Peptide** KPLDETL **Charge** 2 **Spectral Angle** 0.84

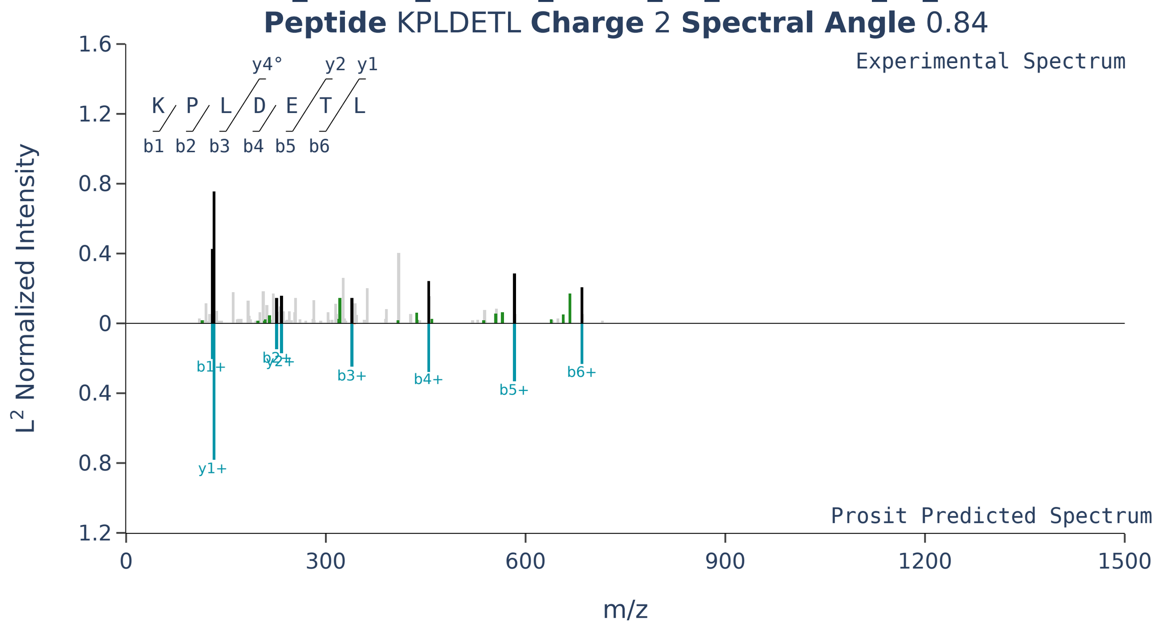

**Source** WSoh_301121_021221_HFGoe_M2_AnnexinA1_2h_R1 **Scan** 17388
**Peptide** LSLAKGDRSEDFGVNE **Charge** 2 **Spectral Angle** 0.85

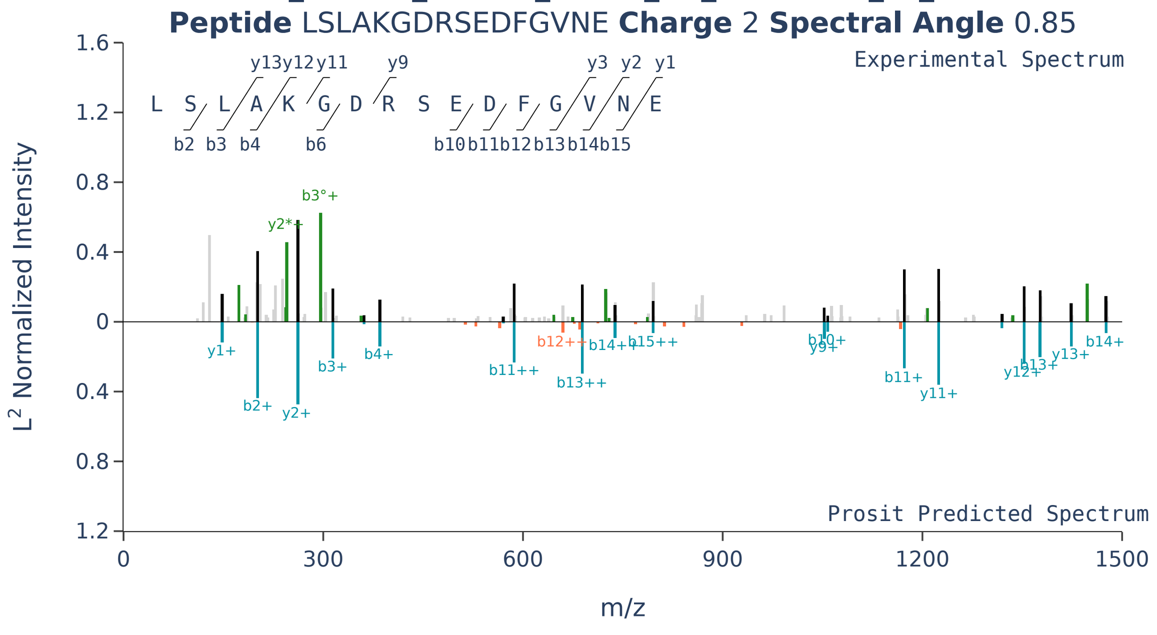

**Source** WSoh_301121_021221_HFGoe_M2_AnnexinA1_2h_R1 **Scan** 22007
**Peptide** AGERRKGTDVNVFNTILTTR **Charge** 4 **Spectral Angle** 0.83

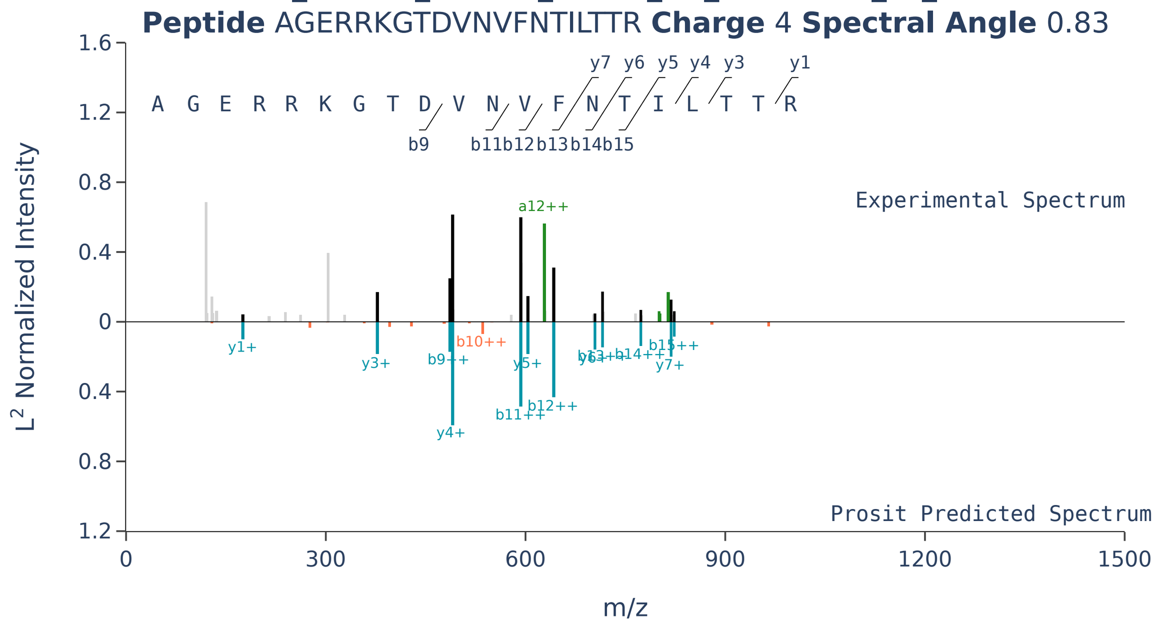

**Source** WSoh_301121_021221_HFGoe_M1_AnnexinA1_4h_R2 **Scan** 8734
**Peptide** SDARALYEAGERRKGTD **Charge** 4 **Spectral Angle** 0.82

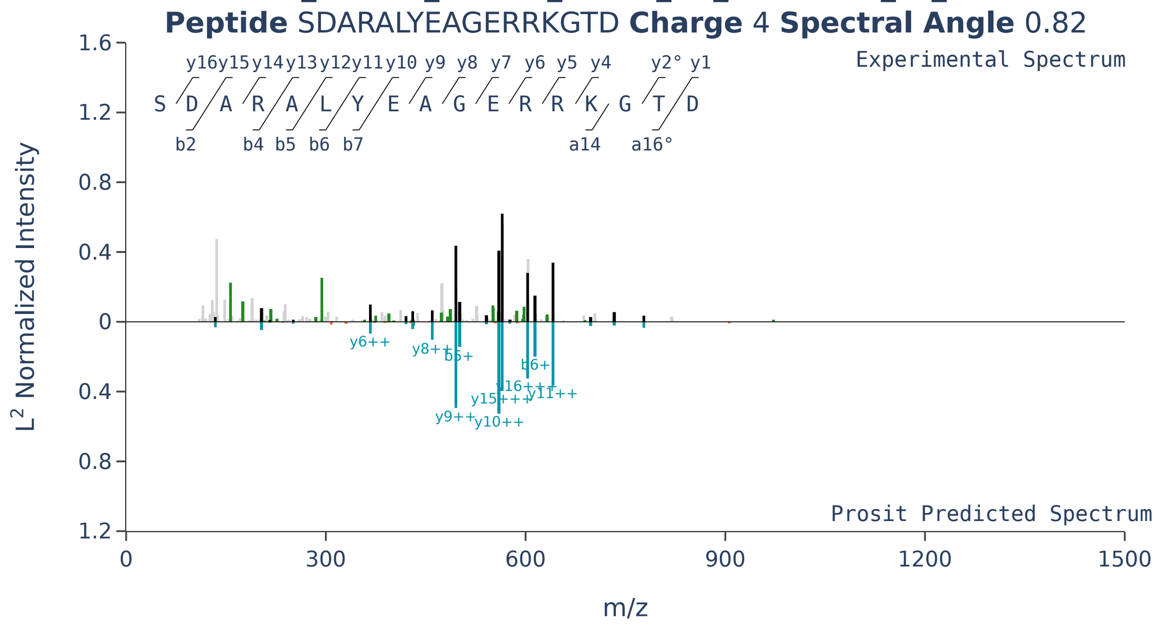

**Source** WSoh_301121_021221_HFGoe_M2_AnnexinA1_2h_R2 **Scan** 16591
**Peptide** DVAALHKAIMVK **Charge** 3 **Spectral Angle** 0.86

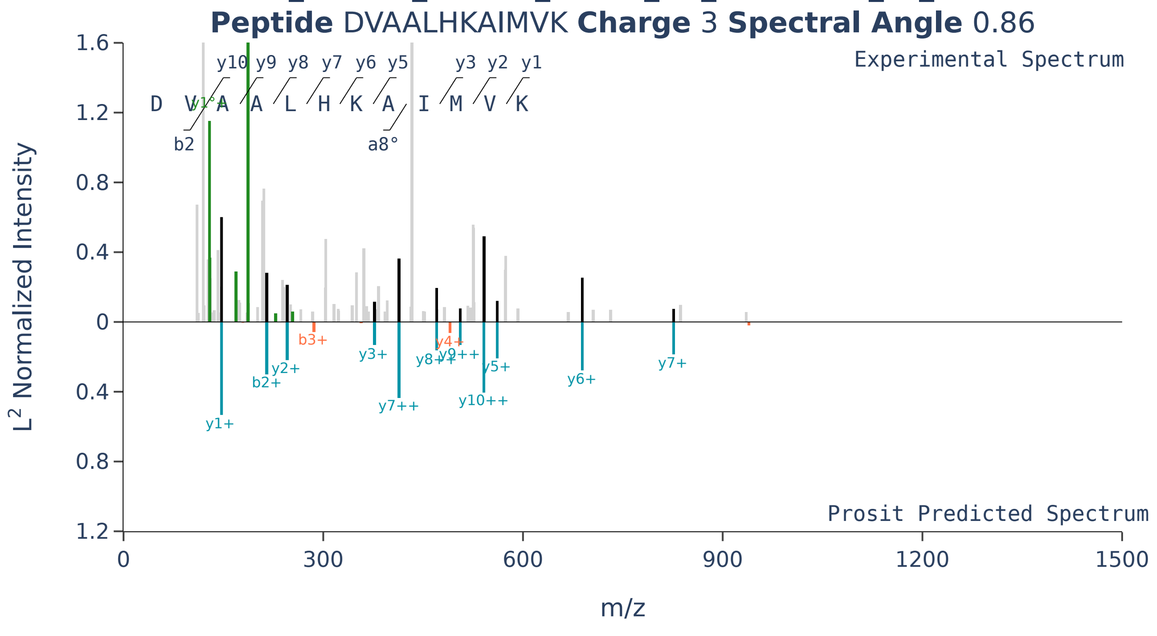

**Source** WSoh_301121_021221_HFGoe_M2_AnnexinA1_24h_R1 **Scan** 22744
**Peptide** SEIDMNDI **Charge** 2 **Spectral Angle** 0.79

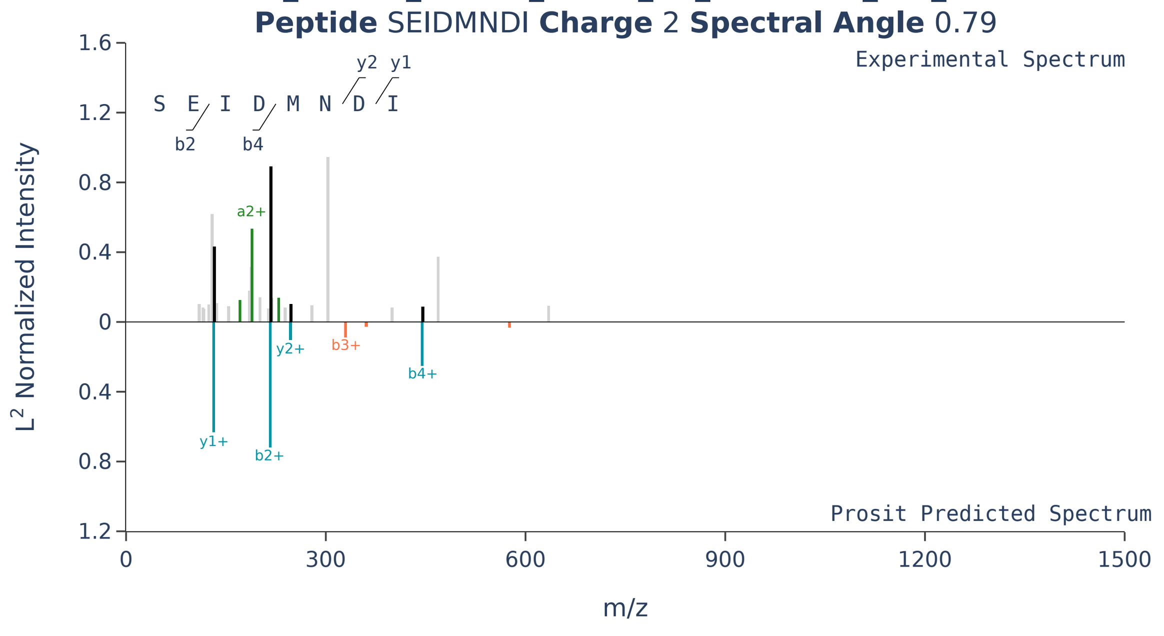

**Source** WSoh_301121_021221_HFGoe_M2_AnnexinA1_4h_R2 **Scan** 15973
**Peptide** QAILDET **Charge** 1 **Spectral Angle** 0.97

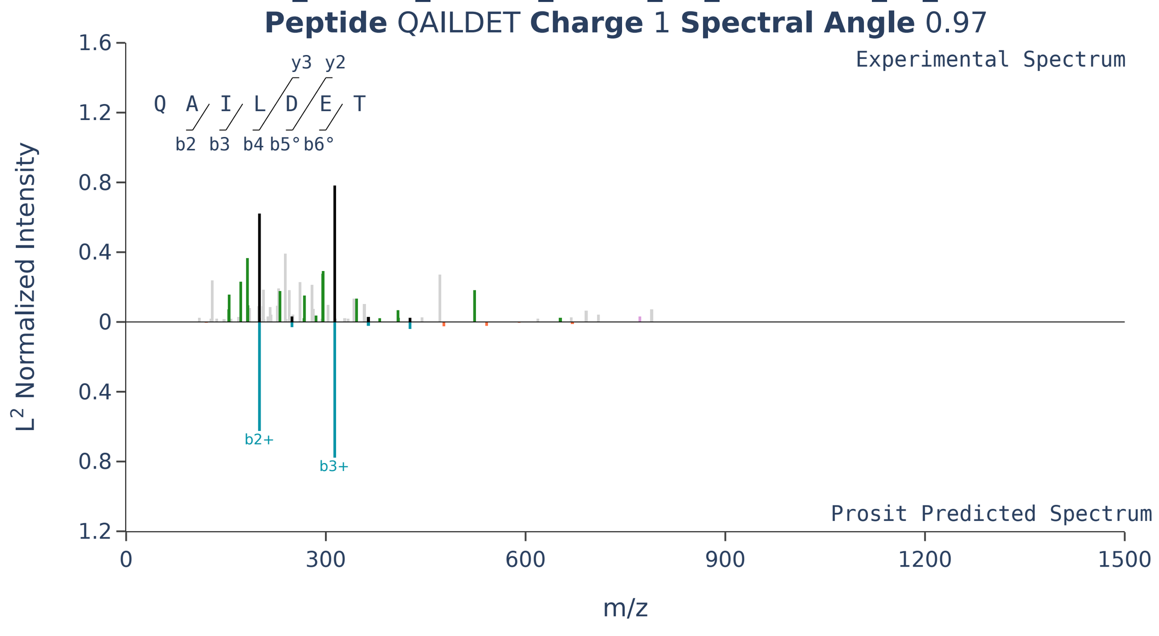

**Source** WSoh_301121_021221_HFGoe_M2_AnnexinA1_24h_R2 **Scan** 13709
**Peptide** SKGGPGSAVSPYP **Charge** 2 **Spectral Angle** 0.82

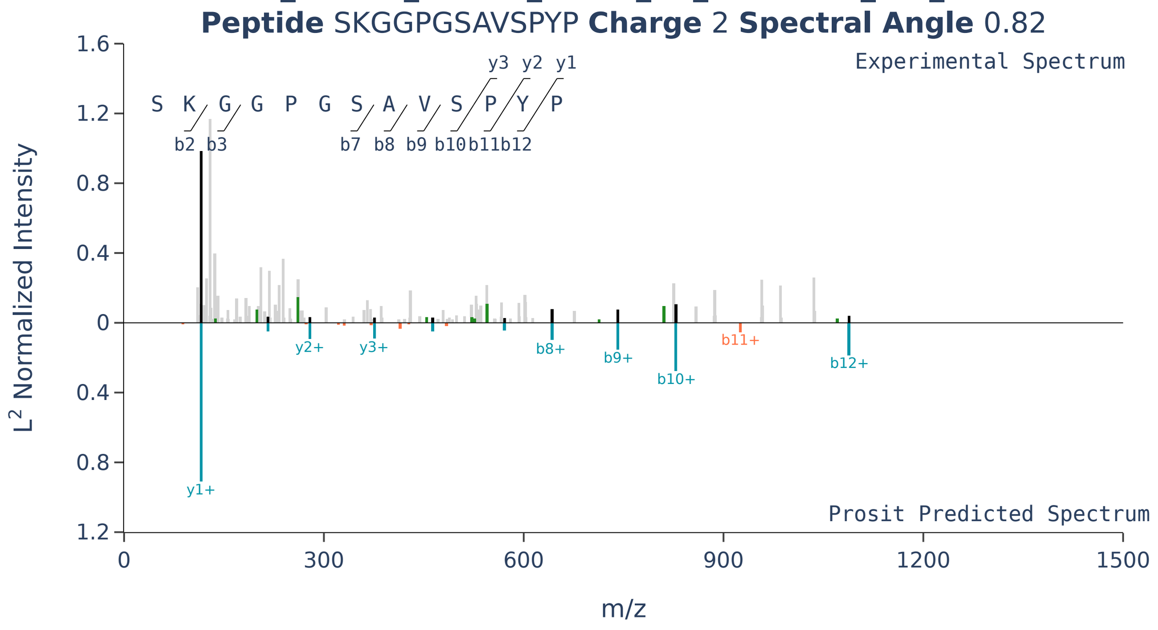


**Source** WSoh_301121_021221_HFGoe_M2_AnnexinA1_24h_R2 **Scan** 8458
**Peptide** ETKGDYEKI **Charge** 2 **Spectral Angle** 0.77

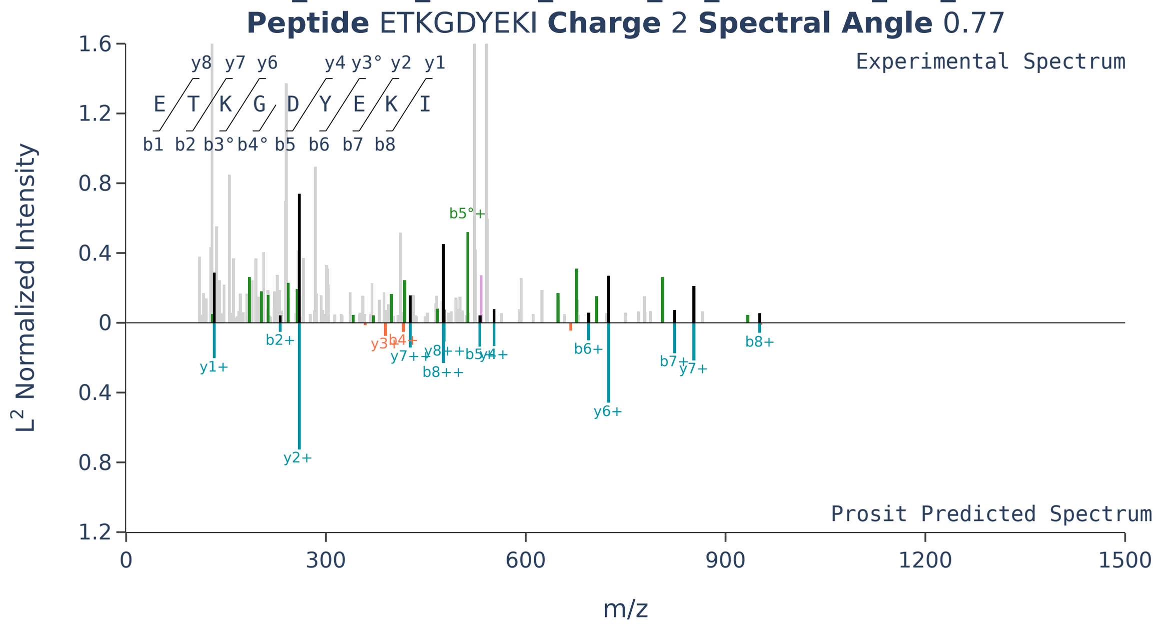


**Source** WSoh_301121_021221_HFGoe_M2_AnnexinA1_2h_R2 **Scan** 21292
**Peptide** ALLKTPAQFD **Charge** 2 **Spectral Angle** 0.84

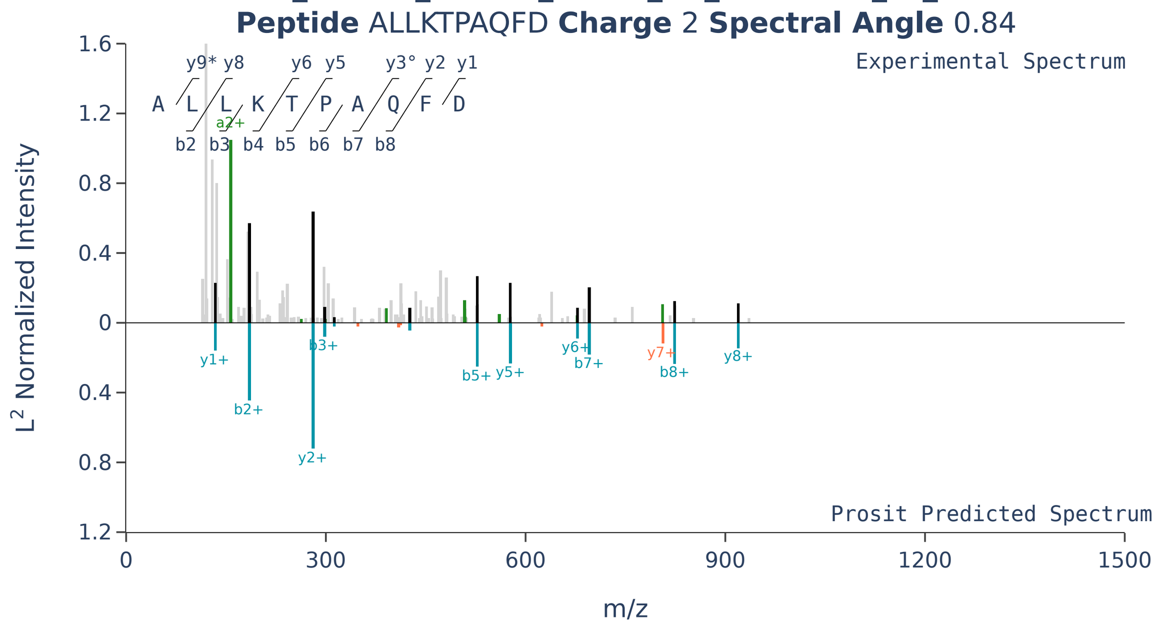


**Source** WSoh_301121_021221_HFGoe_M2_AnnexinA1_2h_R1 **Scan** 16739
**Peptide** AYLQETGKPLDETLKKA **Charge** 3 **Spectral Angle** 0.85

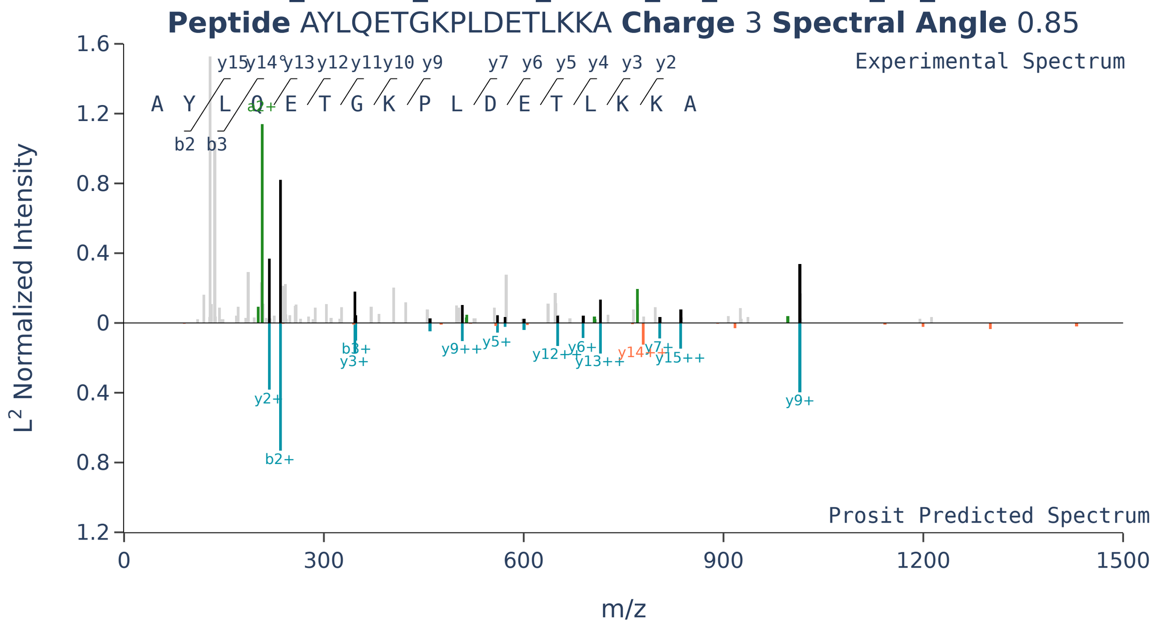


**Source** WSoh_301121_021221_HFGoe_M2_AnnexinA1_2h_R2 **Scan** 11340
**Peptide** IDILTKRNNAQRQQIKAA **Charge** 3 **Spectral Angle** 0.76

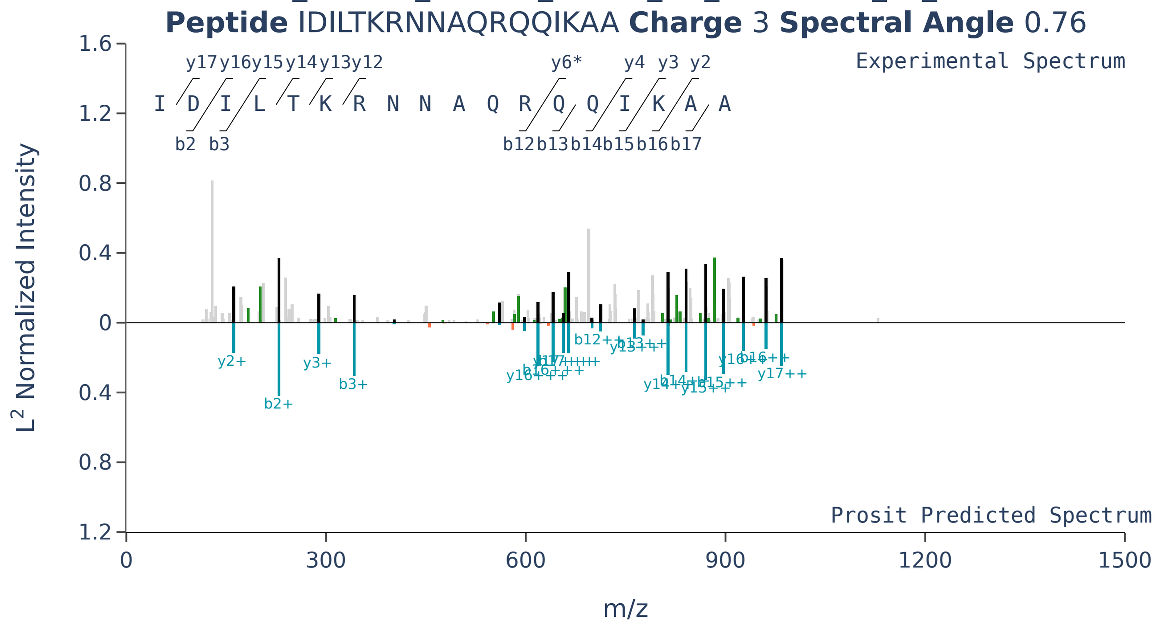


**Source** WSoh_301121_021221_HFGoe_M2_AnnexinA1_1h_R1 **Scan** 14175
**Peptide** KEIRDINRVYREEL **Charge** 4 **Spectral Angle** 0.86

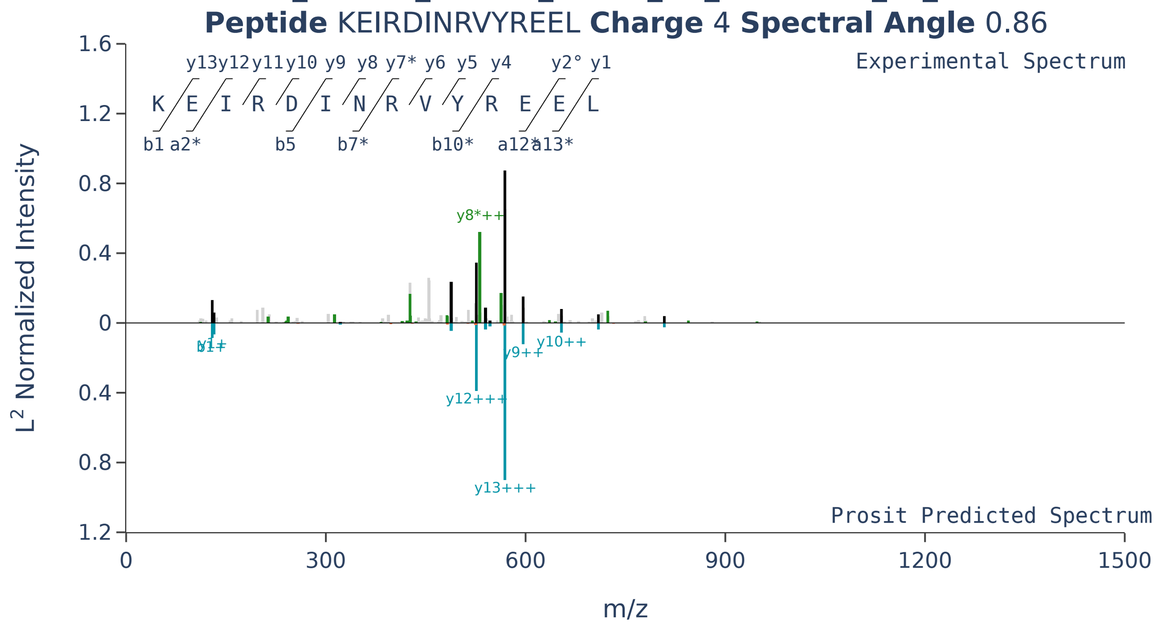


**Source** WSoh_301121_021221_HFGoe_M2_AnnexinA1_4h_R1 **Scan** 19265
**Peptide** ATSKPAFFA **Charge** 2 **Spectral Angle** 0.78

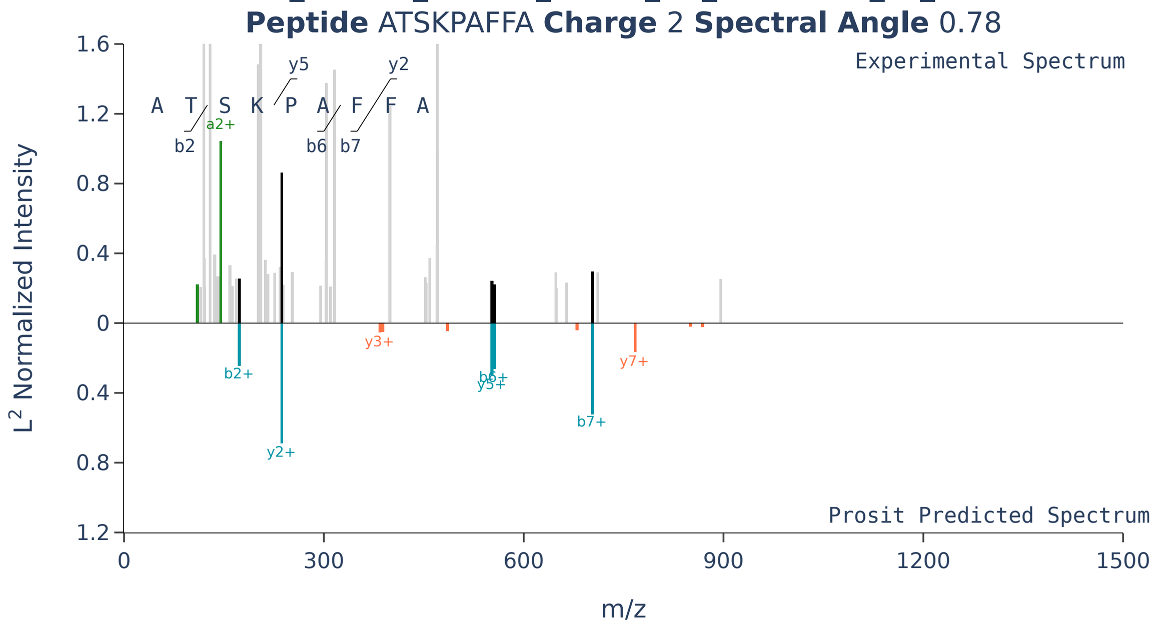


**Source** WSoh_301121_021221_HFGoe_M2_AnnexinA1_24h_R2 **Scan** 16814
**Peptide** LGTDEDTL **Charge** 1 **Spectral Angle** 0.8

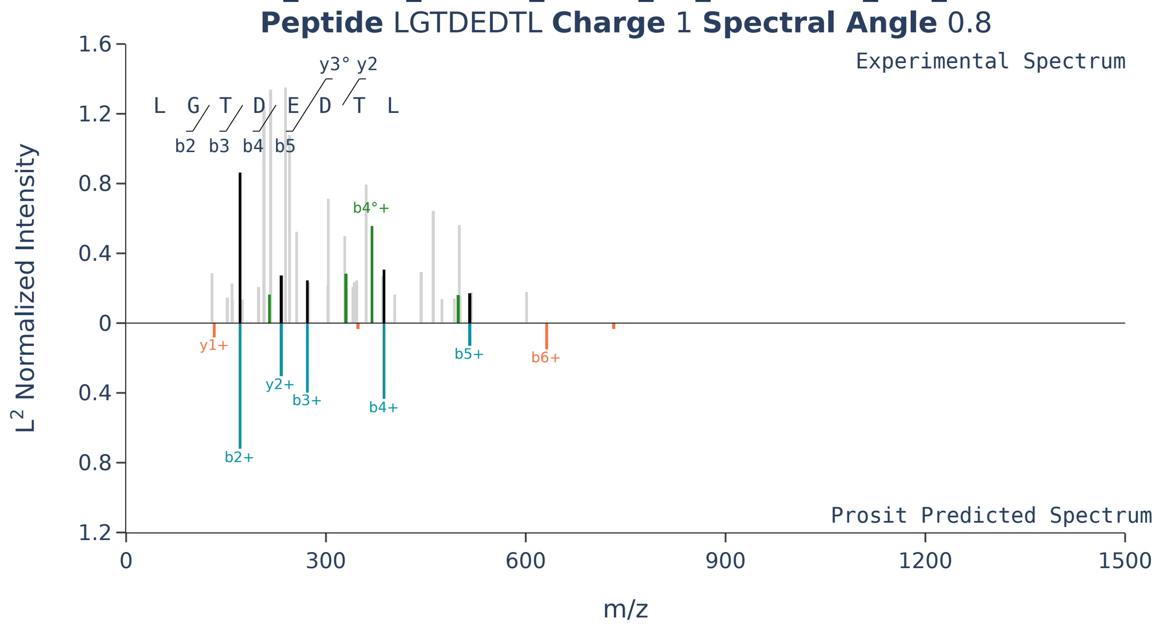


**Source** WSoh_301121_021221_HFGoe_M1_AnnexinA1_4h_R1 **Scan** 14797
**Peptide** AKDITSD **Charge** 1 **Spectral Angle** 0.92

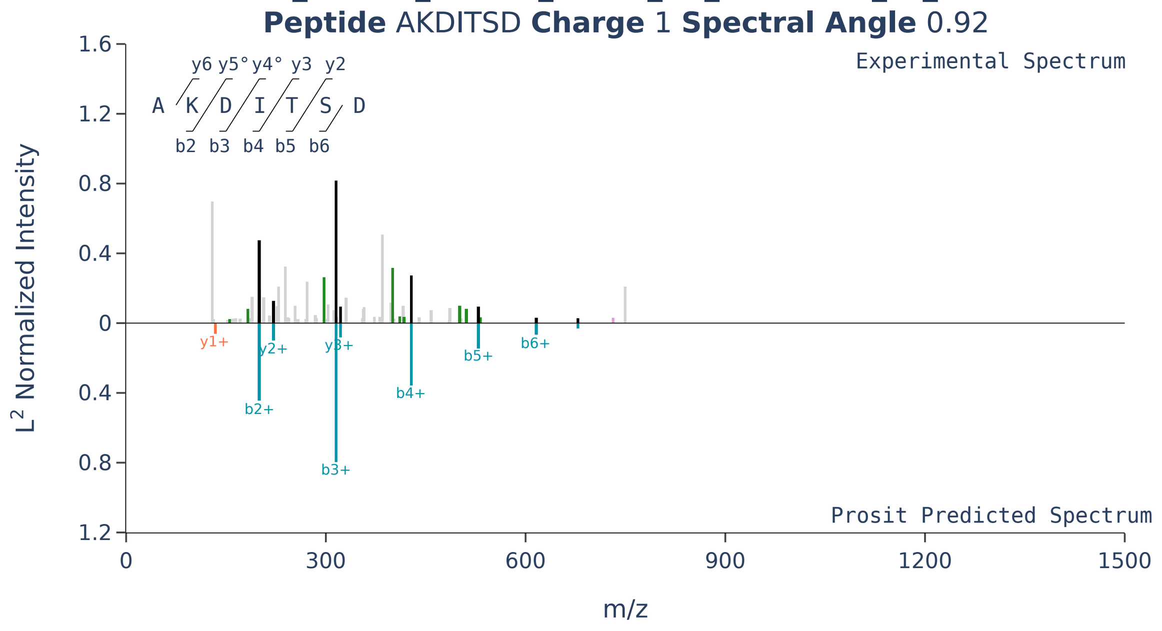


**Source** WSoh_301121_021221_HFGoe_M2_AnnexinA1_24h_R1 **Scan** 13129
**Peptide** NEEQEYVQTVKSS **Charge** 2 **Spectral Angle** 0.81

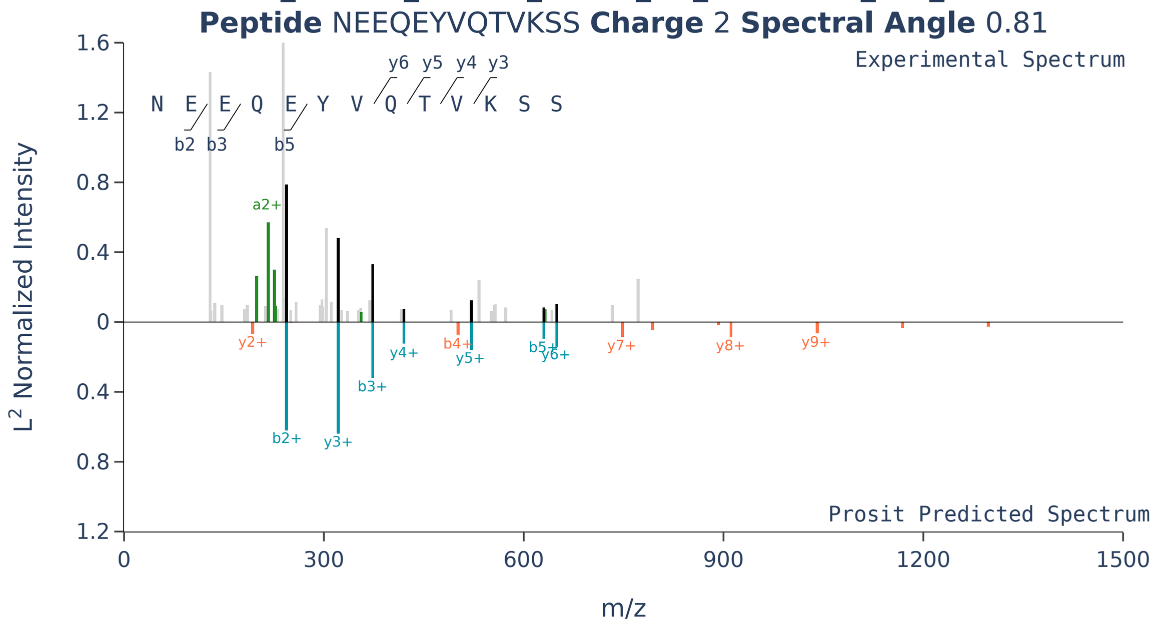


**Source** WSoh_301121_021221_HFGoe_M1_AnnexinA1_24h_R1 **Scan** 5733
**Peptide** RTNKEIRDINR **Charge** 3 **Spectral Angle** 0.85

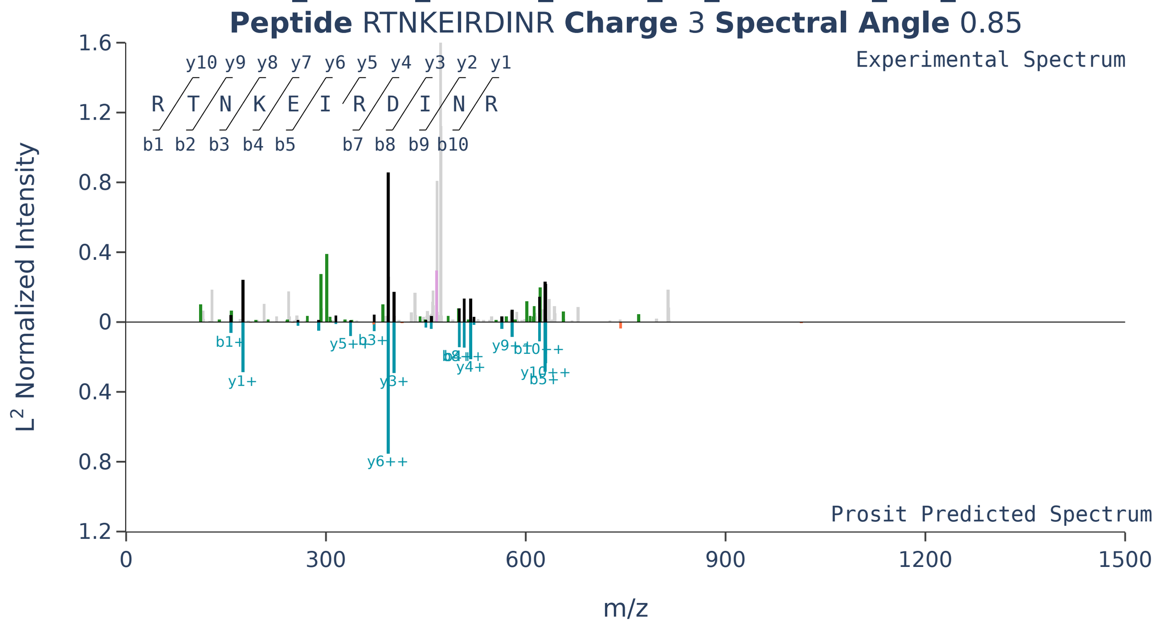


**Source** WSoh_301121_021221_HFGoe_M2_AnnexinA1_2h_R1 **Scan** 19527
**Peptide** DLADSDARALYEAGER **Charge** 3 **Spectral Angle** 0.83

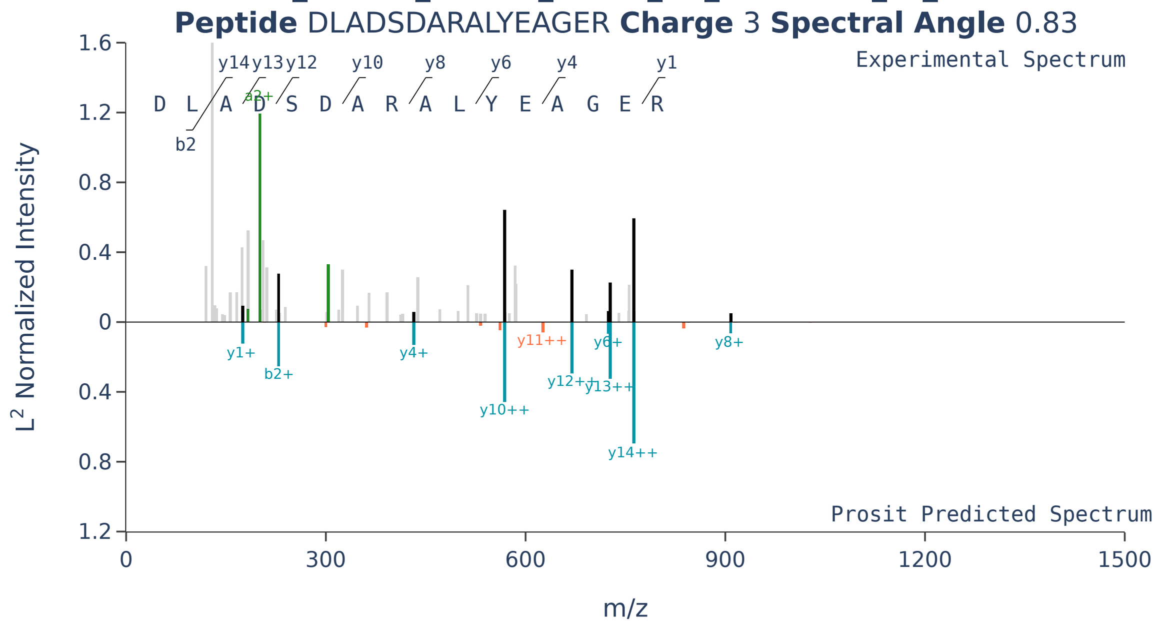


**Source** WSoh_301121_021221_HFGoe_M2_AnnexinA1_2h_R1 **Scan** 24701
**Peptide** PAFFAEKL **Charge** 2 **Spectral Angle** 0.82

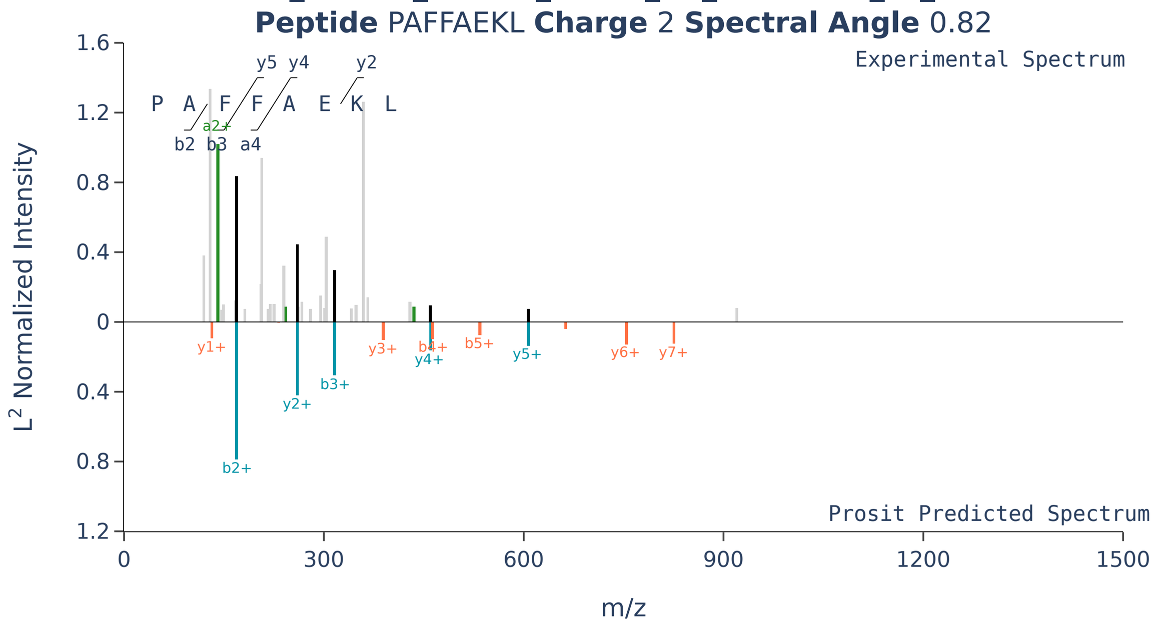


**Source** WSoh_301121_021221_HFGoe_M2_AnnexinA1_24h_R1 **Scan** 10764
**Peptide** KSSKGGPGSAVSPYPT **Charge** 2 **Spectral Angle** 0.92

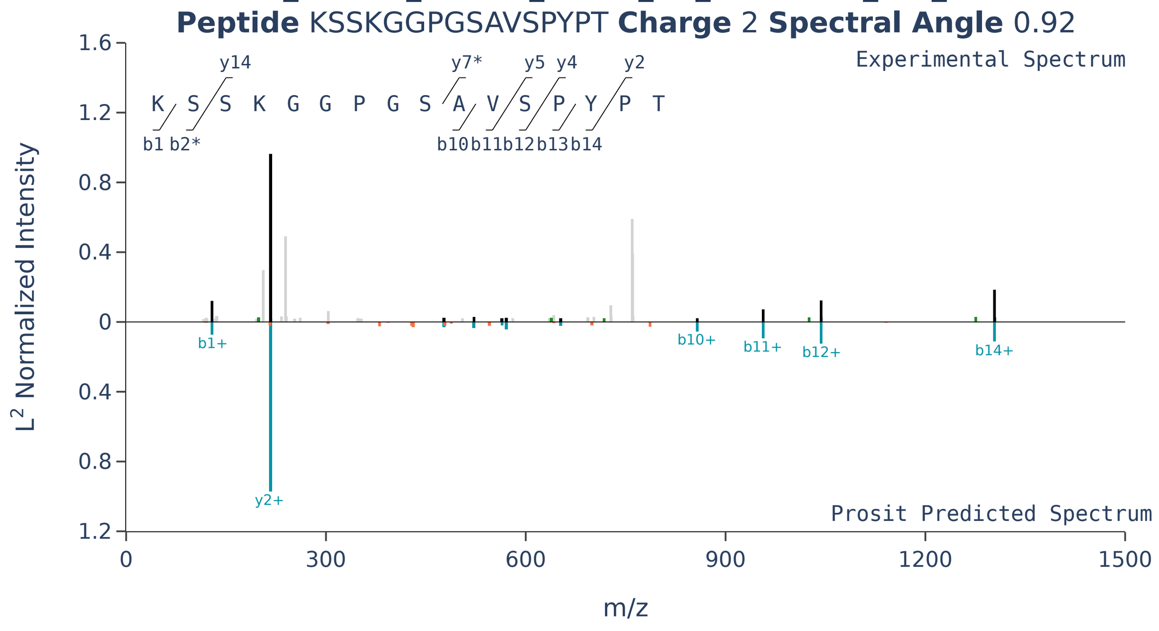


**Source** WSoh_301121_021221_HFGoe_M2_AnnexinA1_24h_R1 **Scan** 12037
**Peptide** RKGTDVNVFN **Charge** 2 **Spectral Angle** 0.75

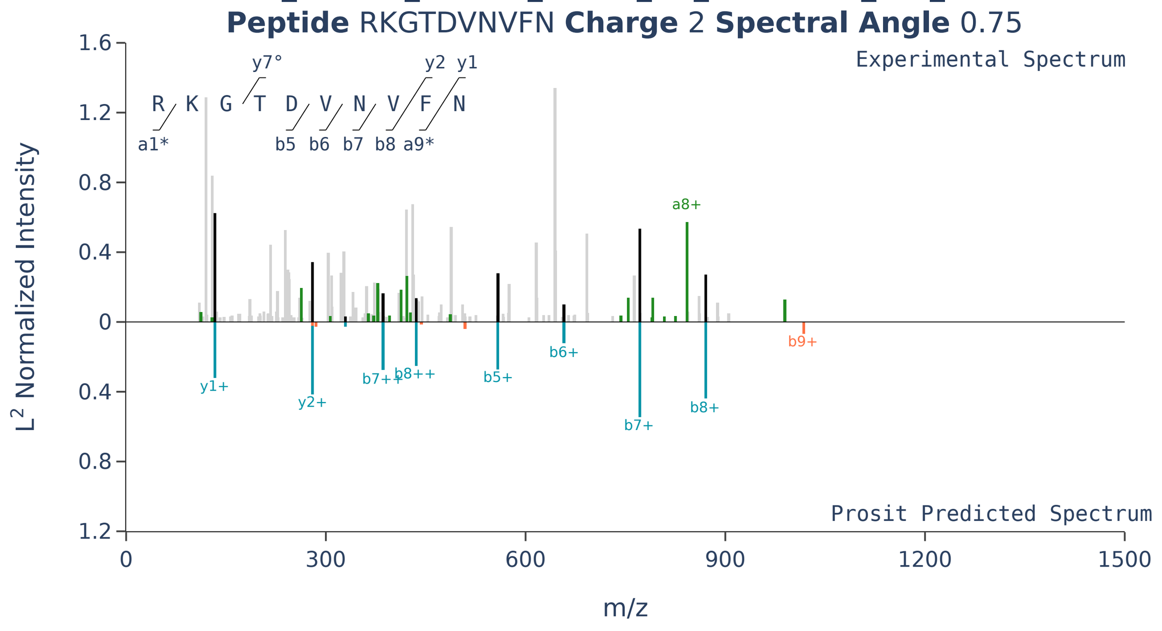

**Source** WSoh_301121_021221_HFGoe_M2_AnnexinA1_2h_R1 **Scan** 16365
**Peptide** TRSYPQLRRVF **Charge** 3 **Spectral Angle** 0.82

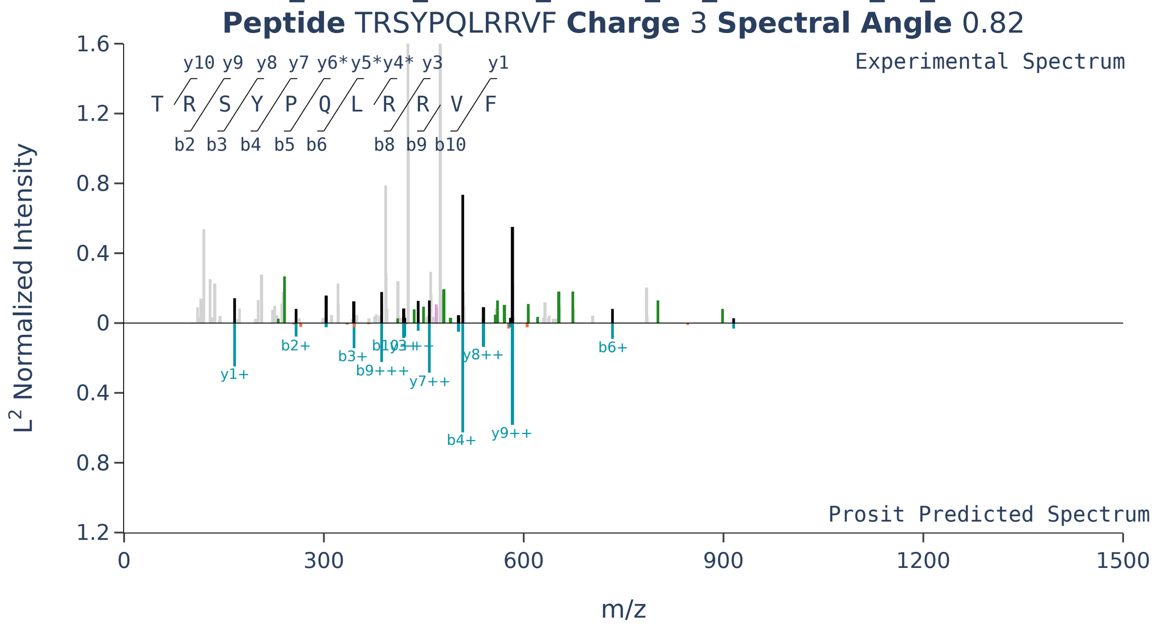


**Source** WSoh_301121_021221_HFGoe_M2_AnnexinA1_2h_R2 **Scan** 7874
**Peptide** TGKPLDETLKK **Charge** 3 **Spectral Angle** 0.8

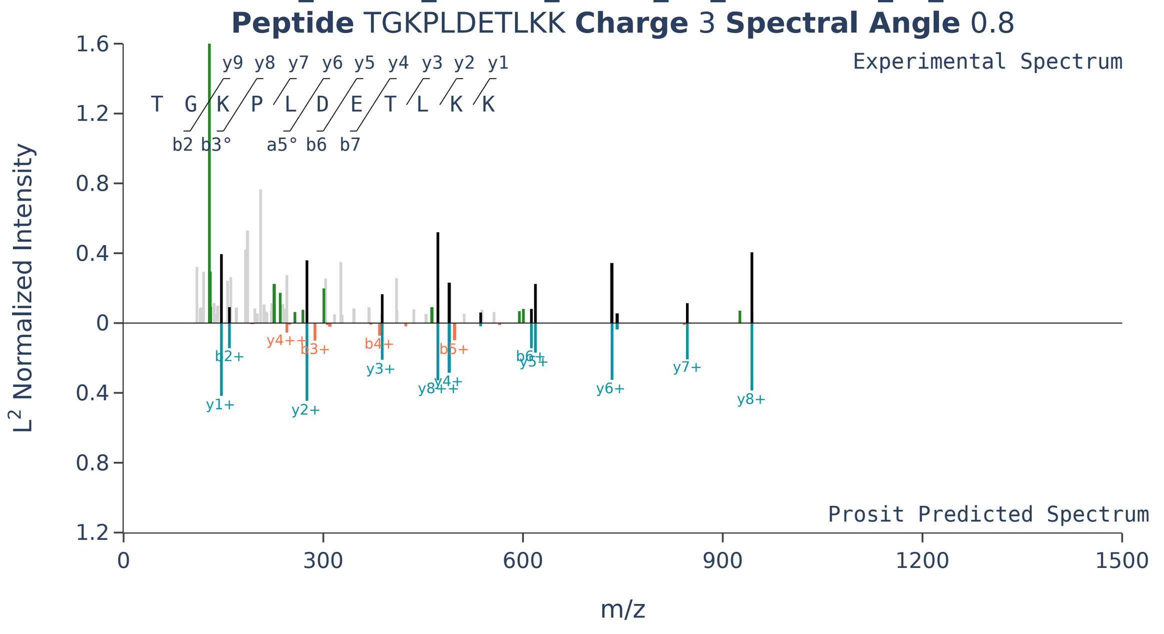


**Source** WSoh_301121_021221_HFGoe_M1_AnnexinA1_24h_R2 **Scan** 16990
**Peptide** DADELRAAM **Charge** 2 **Spectral Angle** 0.72

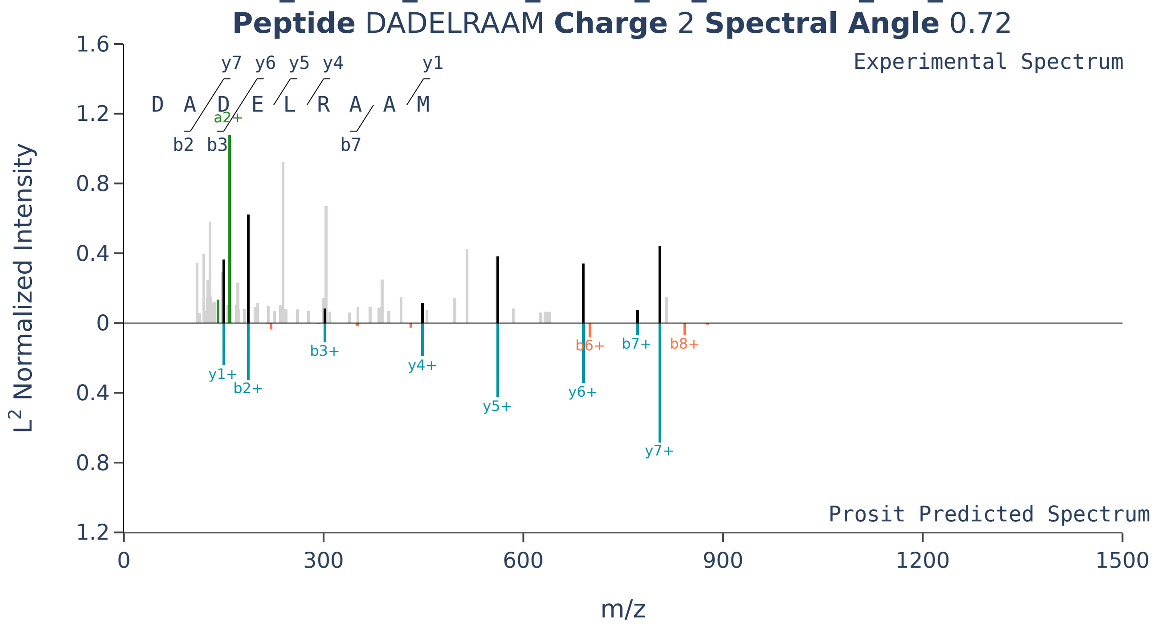


**Source** WSoh_301121_021221_HFGoe_M2_AnnexinA1_2h_R2 **Scan** 32173
**Peptide** EDTLIEIL **Charge** 2 **Spectral Angle** 0.81

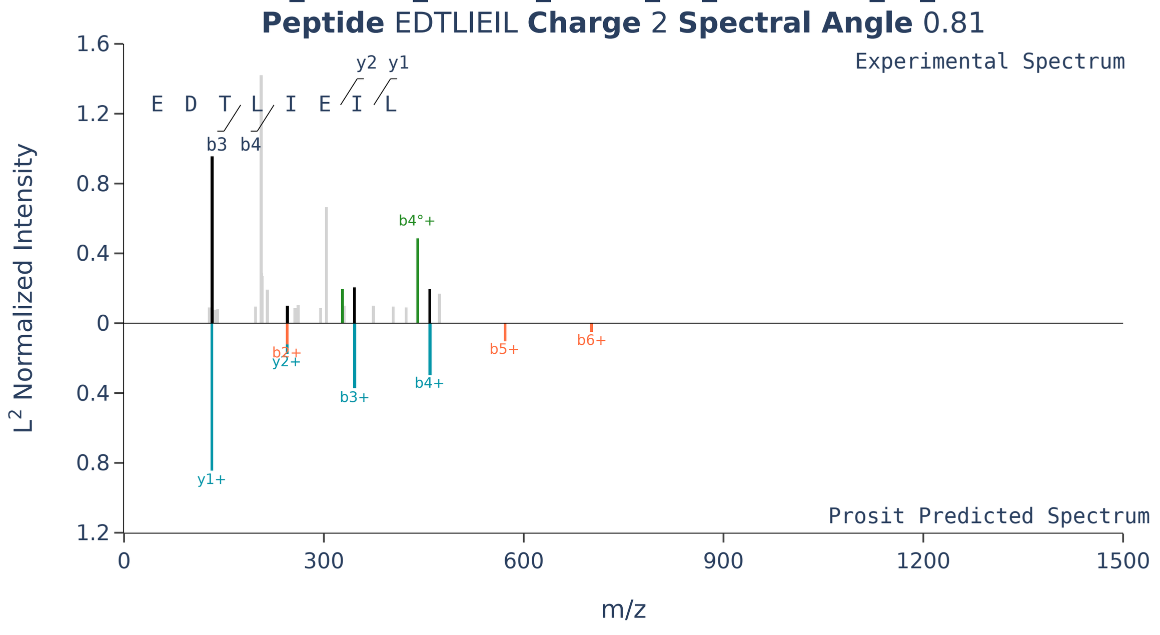


**Source** WSoh_301121_021221_HFGoe_M2_AnnexinA1_2h_R2 **Scan** 19002
**Peptide** TGKPLDETLKKALTG **Charge** 3 **Spectral Angle** 0.79

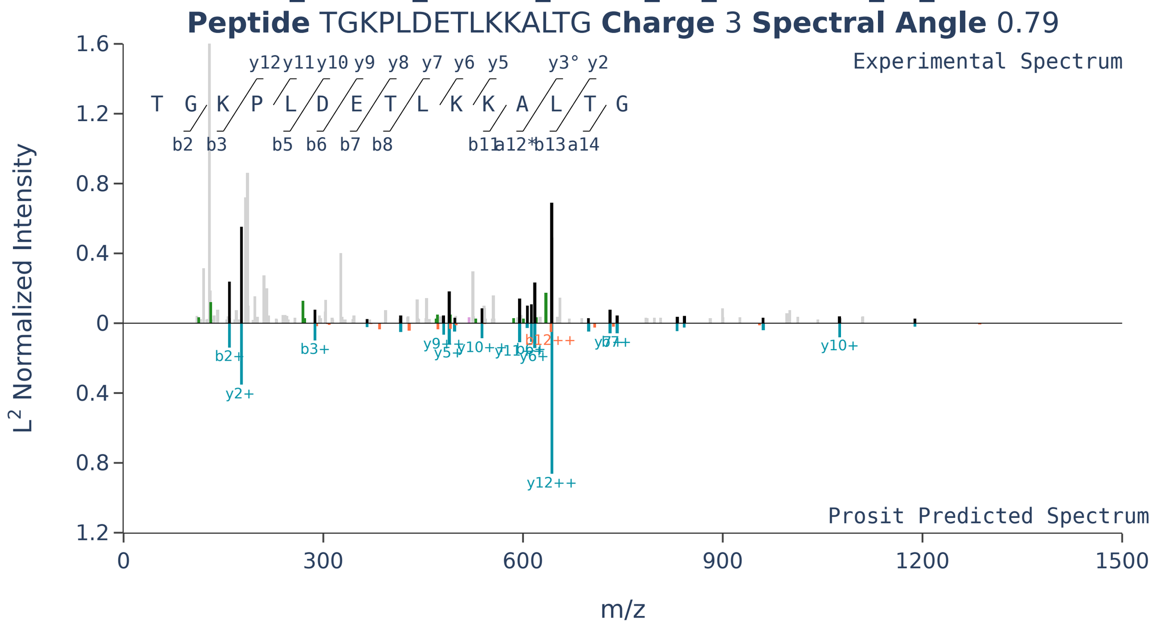


**Source** WSoh_301121_021221_HFGoe_M2_AnnexinA1_24h_R1 **Scan** 12370
**Peptide** EILASRTNKEI **Charge** 2 **Spectral Angle** 0.8

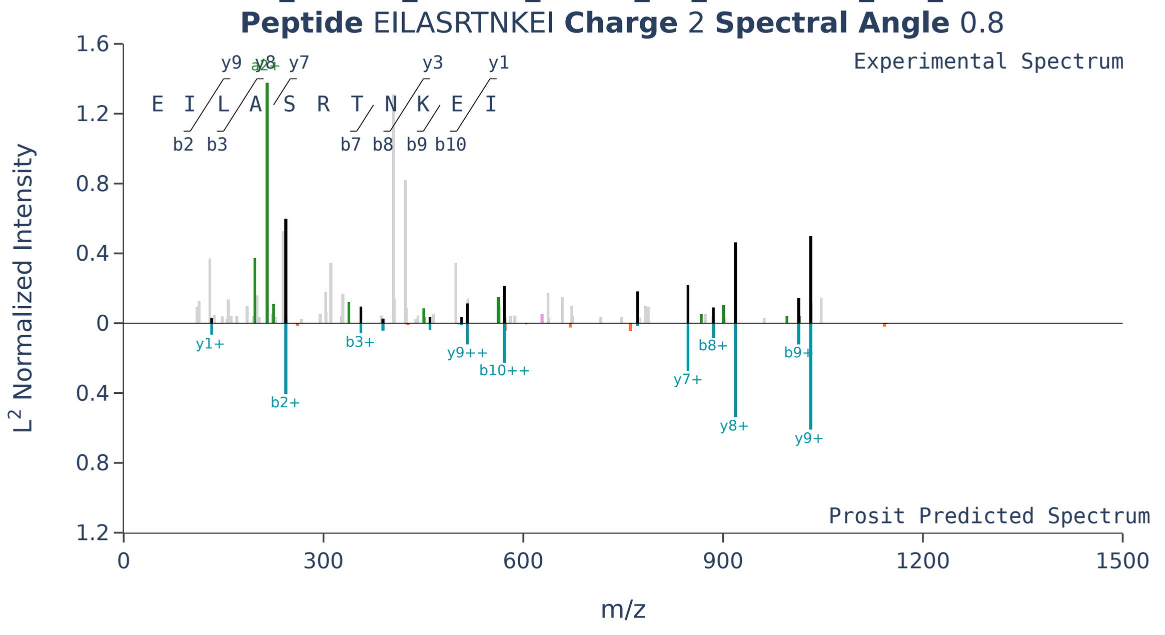


**Source** WSoh_301121_021221_HFGoe_M2_AnnexinA1_2h_R1 **Scan** 21943
**Peptide** DVAALHKAIMV **Charge** 2 **Spectral Angle** 0.85

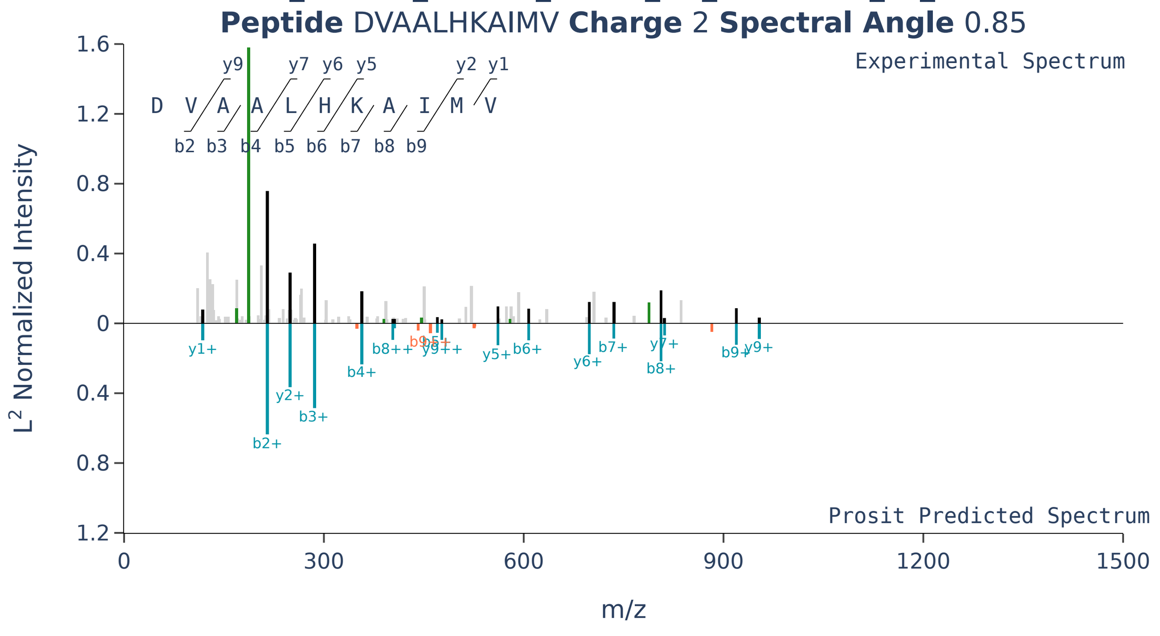


**Source** WSoh_301121_021221_HFGoe_M2_AnnexinA1_2h_R2 **Scan** 6012
**Peptide** AGERRKGTDVNV **Charge** 3 **Spectral Angle** 0.95

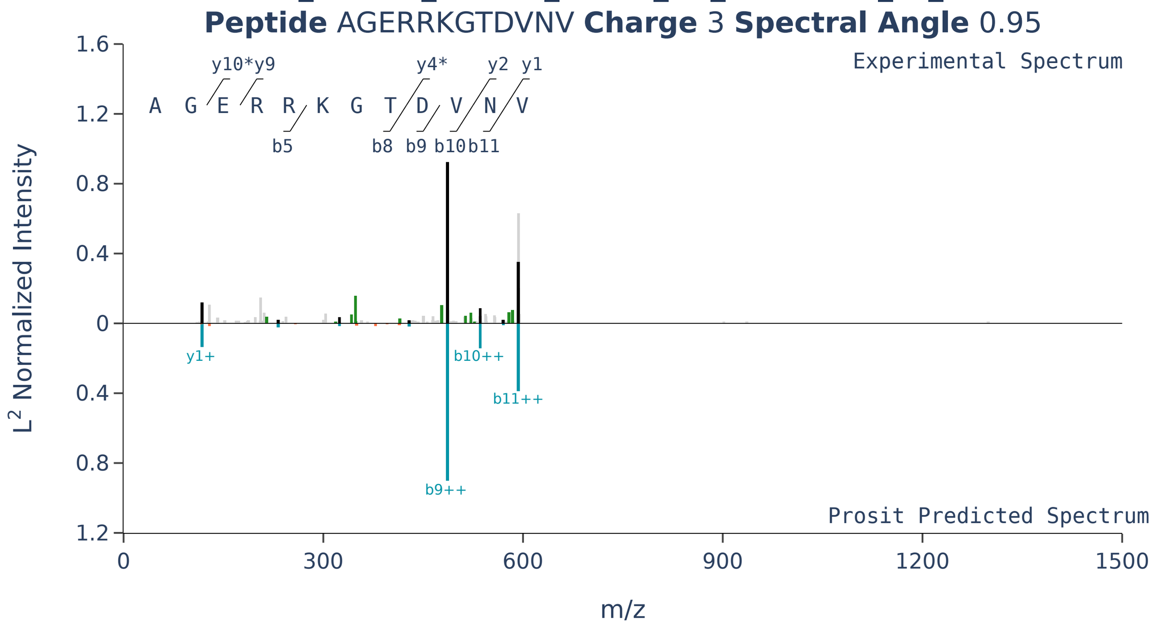


**Source** WSoh_301121_021221_HFGoe_M2_AnnexinA1_24h_R1 **Scan** 26592
**Peptide** AWFIENEE **Charge** 2 **Spectral Angle** 0.84

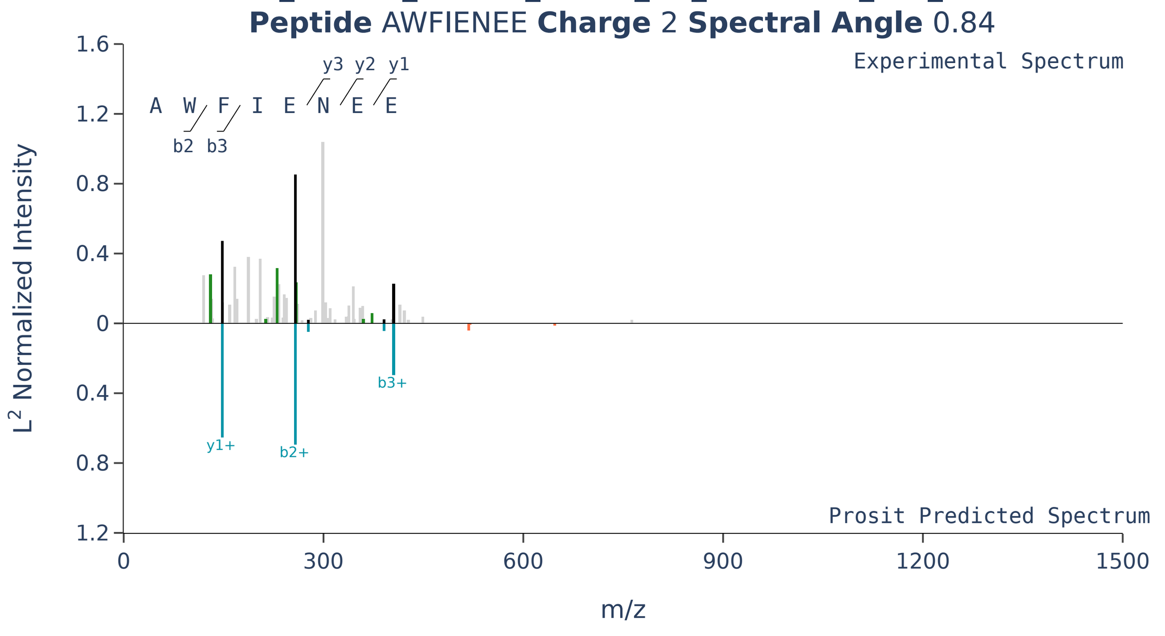


**Source** WSoh_301121_021221_HFGoe_M2_AnnexinA1_4h_R1 **Scan** 14258
**Peptide** VAALHKAIMV **Charge** 3 **Spectral Angle** 0.86

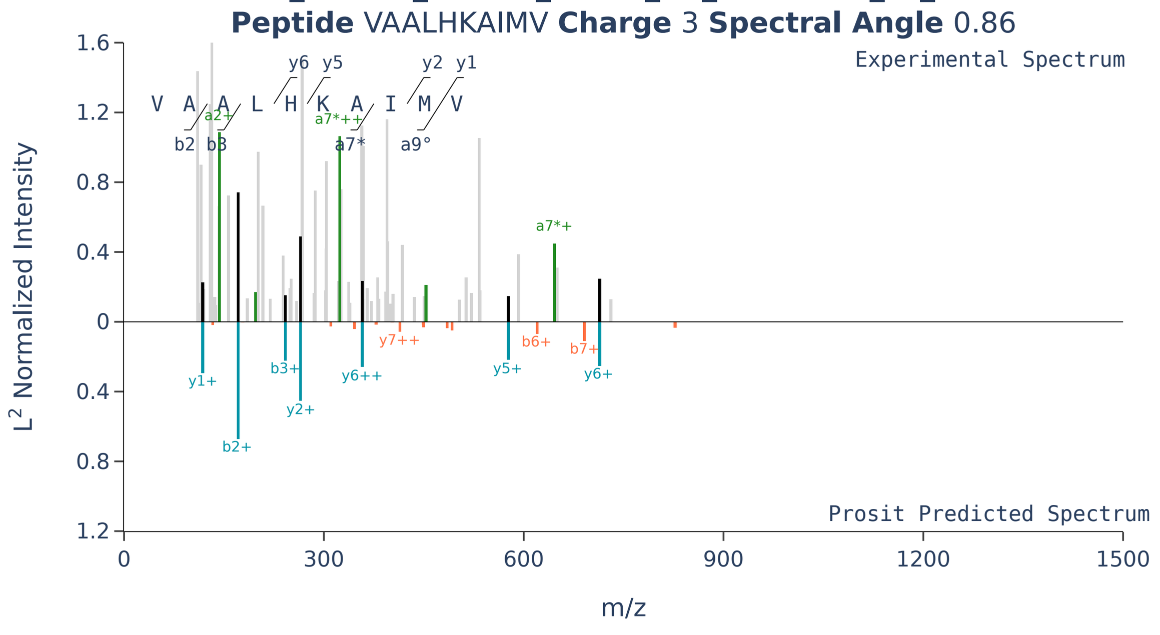


**Source** WSoh_301121_021221_HFGoe_M2_AnnexinA1_24h_R2 **Scan** 17975
**Peptide** GTDEDTLIE **Charge** 2 **Spectral Angle** 0.92

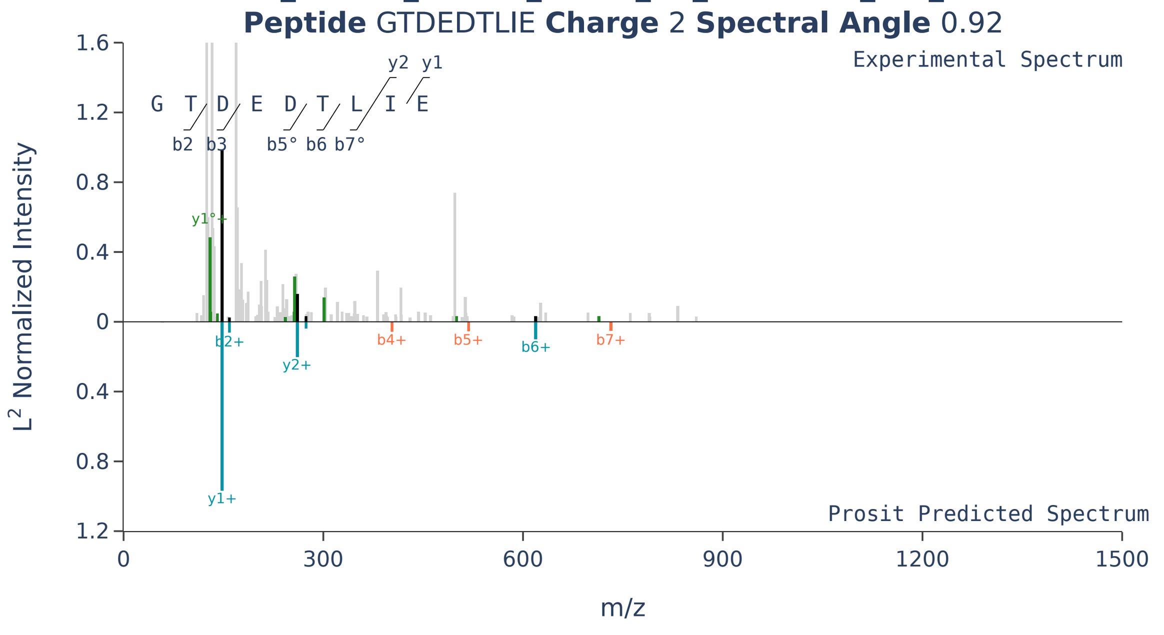


**Source** WSoh_301121_021221_HFGoe_M2_AnnexinA1_4h_R1 **Scan** 12283
**Peptide** AALHKAIMVKGVD **Charge** 3 **Spectral Angle** 0.85

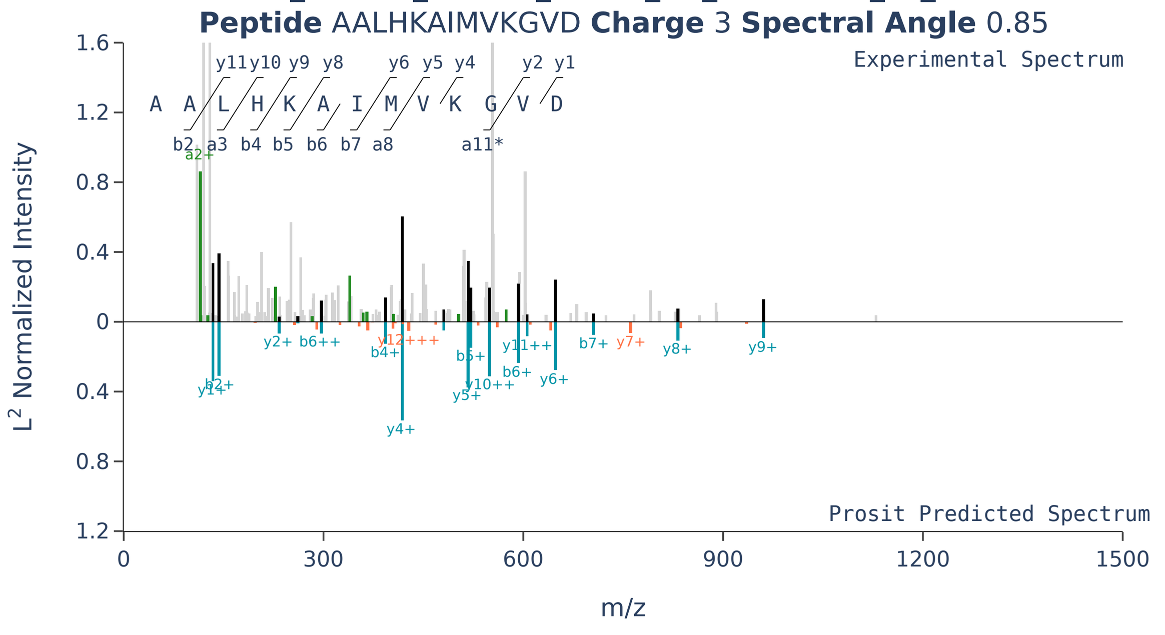


**Source** WSoh_301121_021221_HFGoe_M2_AnnexinA1_24h_R2 **Scan** 19866
**Peptide** SPYPTFN **Charge** 1 **Spectral Angle** 0.87

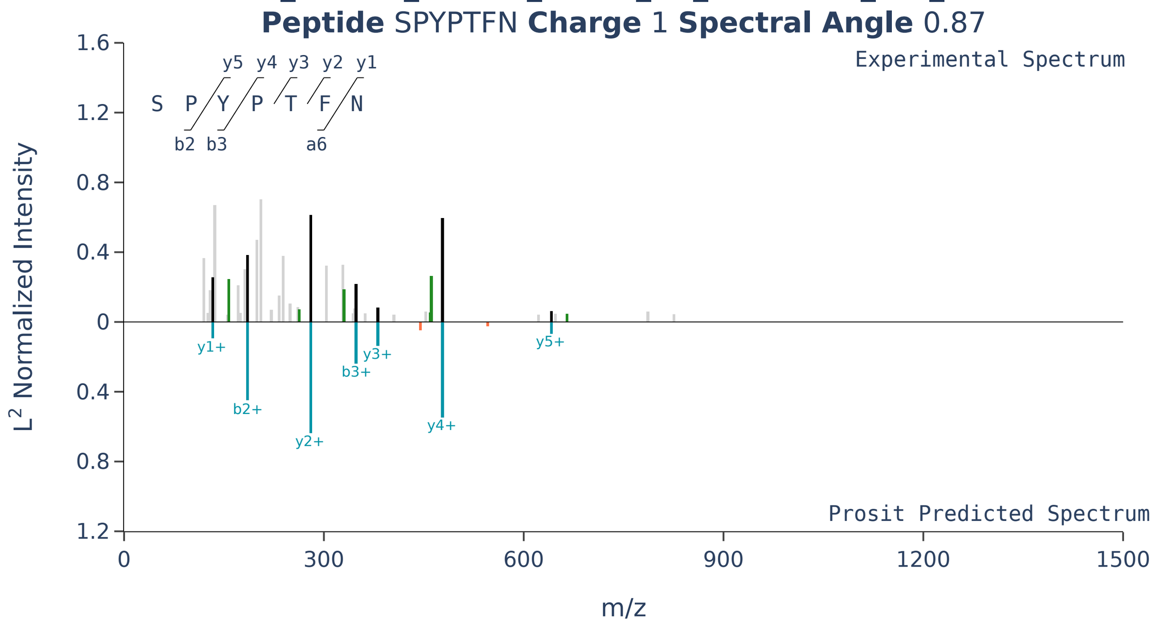


**Source** WSoh_301121_021221_HFGoe_M2_AnnexinA1_1h_R2 **Scan** 23892
**Peptide** TIIDILTKRNNA **Charge** 3 **Spectral Angle** 0.89

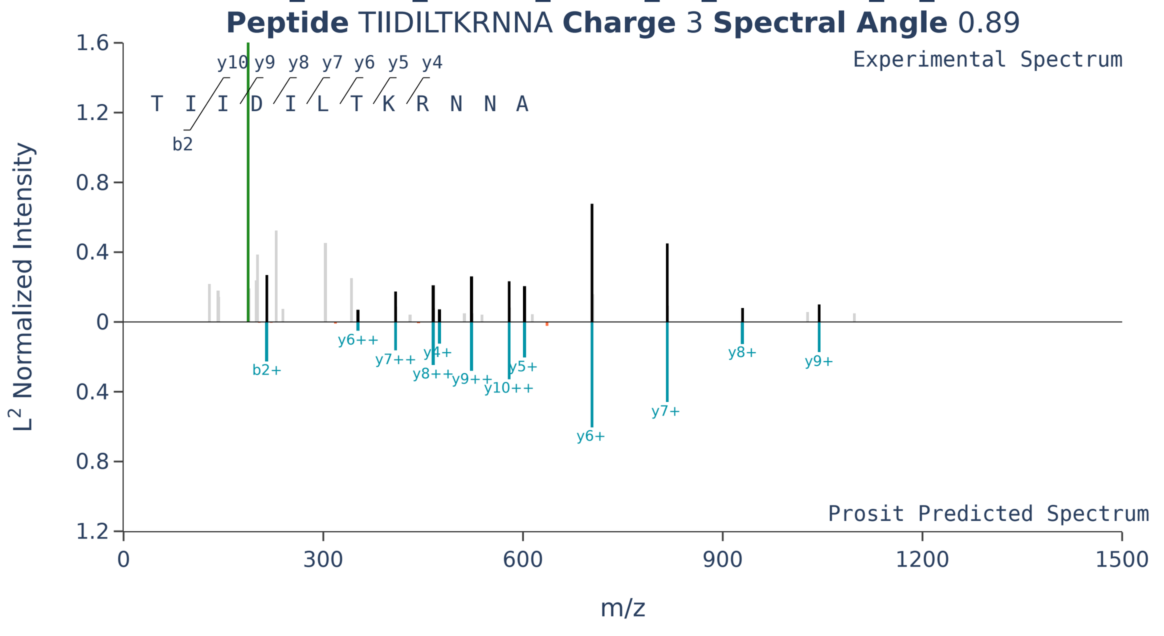


**Source** WSoh_301121_021221_HFGoe_M1_AnnexinA1_24h_R1 **Scan** 28532
**Peptide** DYEKILVAL **Charge** 2 **Spectral Angle** 0.8

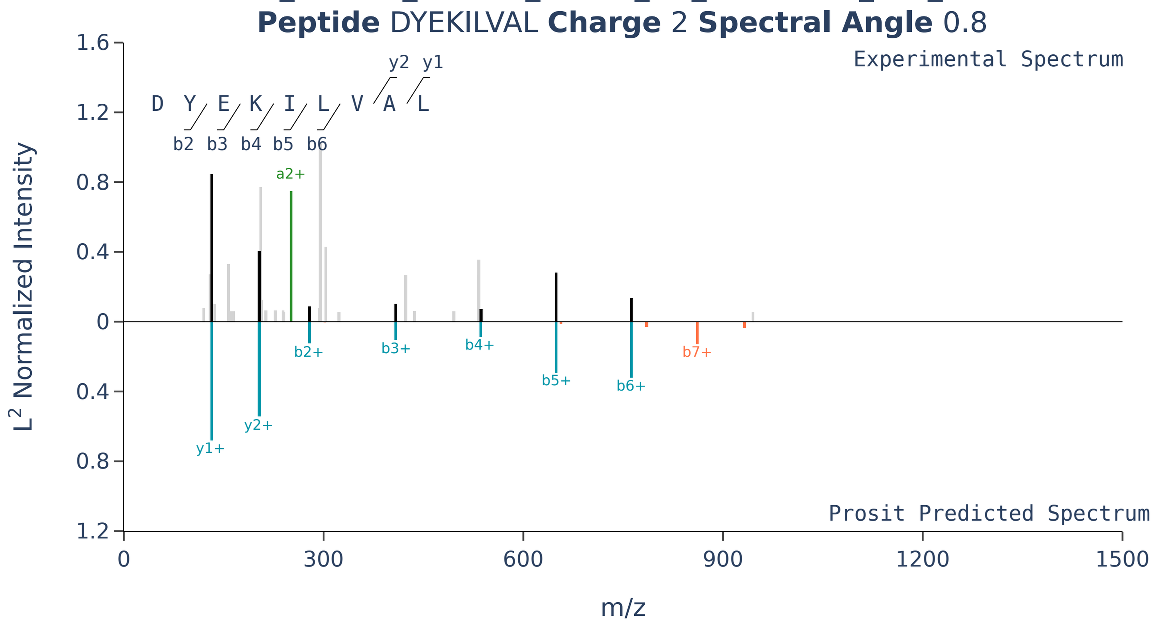

**Source** WSoh_301121_021221_HFGoe_M2_AnnexinA1_2h_R2 **Scan** 20881
**Peptide** KGGPGSAVSPYPTFNPSSD **Charge** 2 **Spectral Angle** 0.91

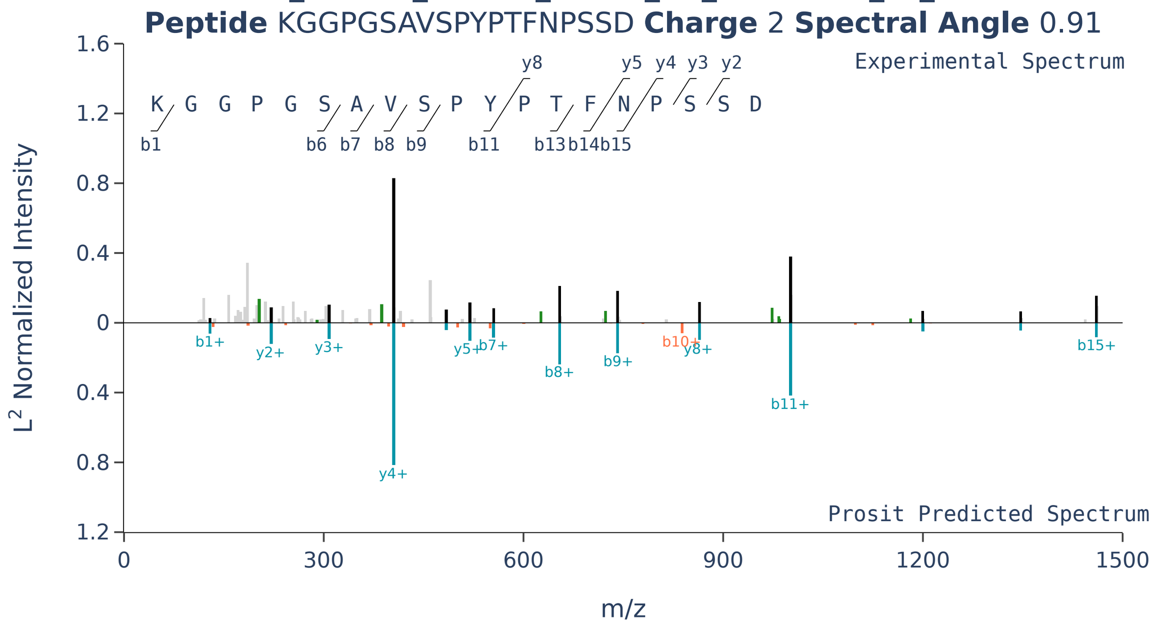


**Source** WSoh_301121_021221_HFGoe_M2_AnnexinA1_24h_R2 **Scan** 14376
**Peptide** ENEEQEYVQTVKSS **Charge** 2 **Spectral Angle** 0.87

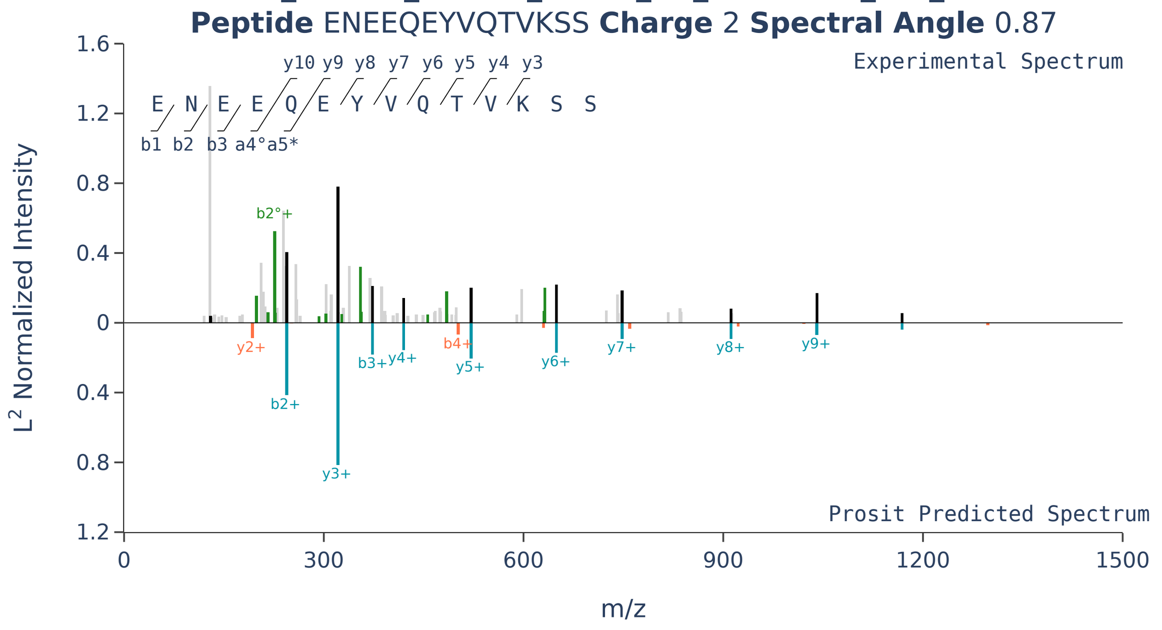


**Source** WSoh_301121_021221_HFGoe_M2_AnnexinA1_24h_R2 **Scan** 8014
**Peptide** HKAIMVKGVDE **Charge** 3 **Spectral Angle** 0.87

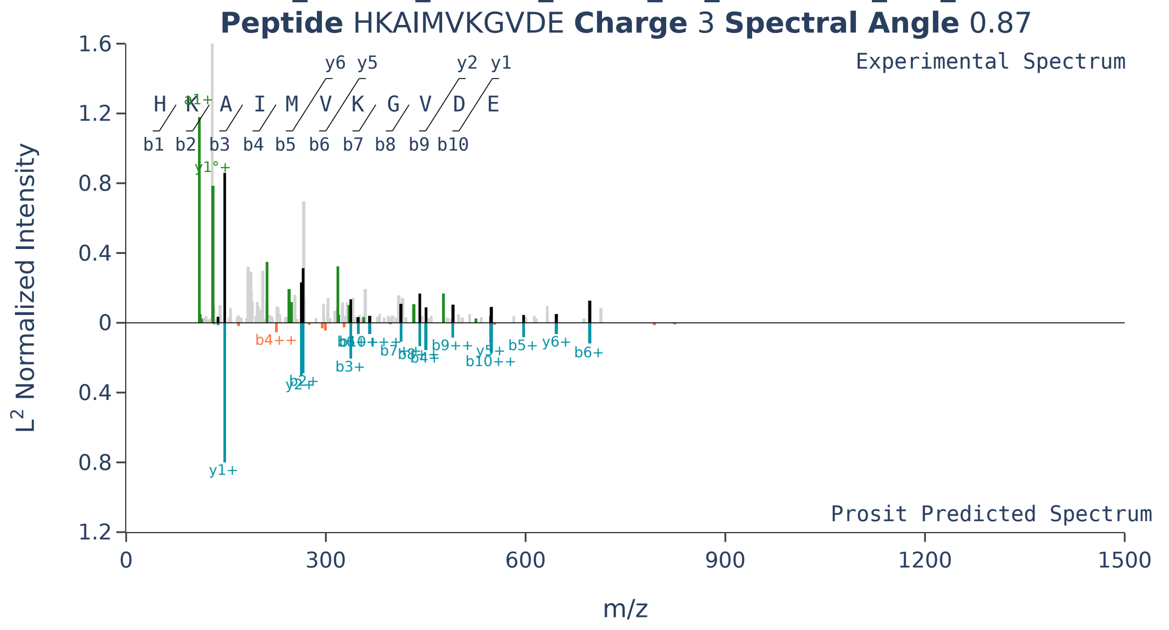


**Source** WSoh_301121_021221_HFGoe_M2_AnnexinA1_2h_R1 **Scan** 22301
**Peptide** KTPAQFDADELRAAM **Charge** 3 **Spectral Angle** 0.84

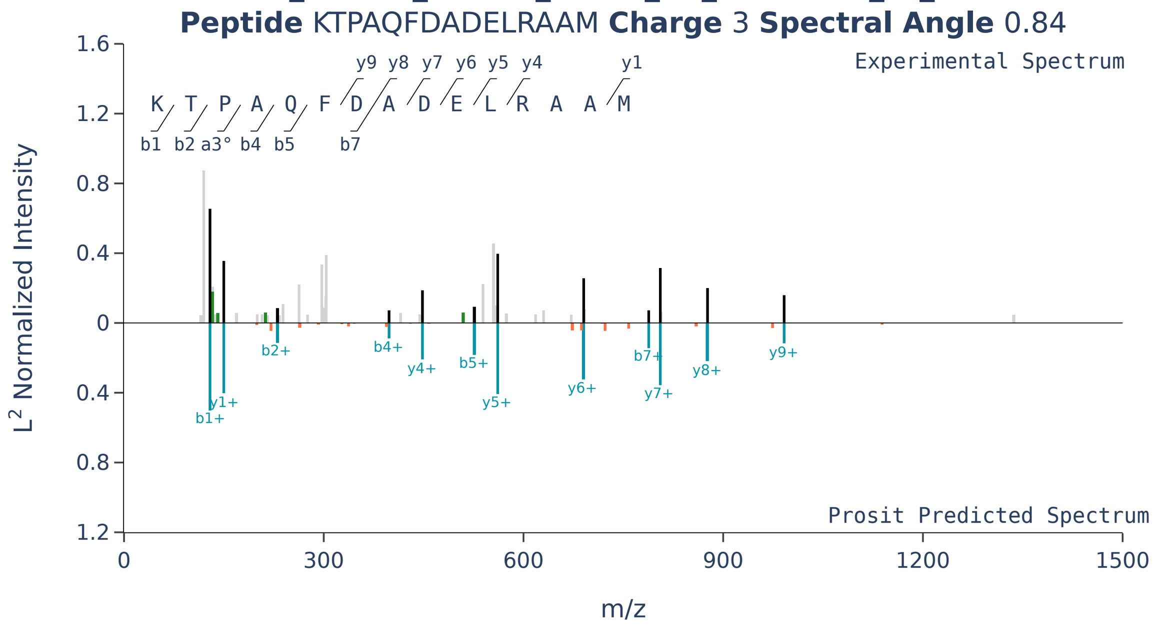


**Source** WSoh_301121_021221_HFGoe_M2_AnnexinA1_2h_R1 **Scan** 26297
**Peptide** ATIIDILTKRNNA **Charge** 3 **Spectral Angle** 0.89

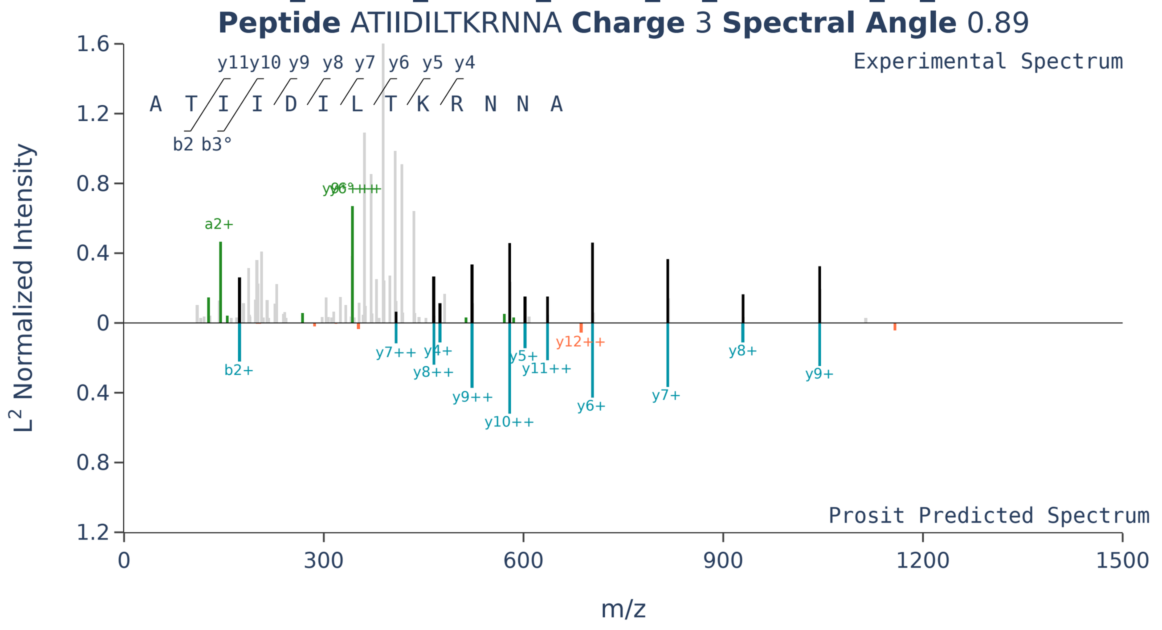


**Source** WSoh_301121_021221_HFGoe_M2_AnnexinA1_2h_R1 **Scan** 15912
**Peptide** RSYPQLRRVF **Charge** 3 **Spectral Angle** 0.88

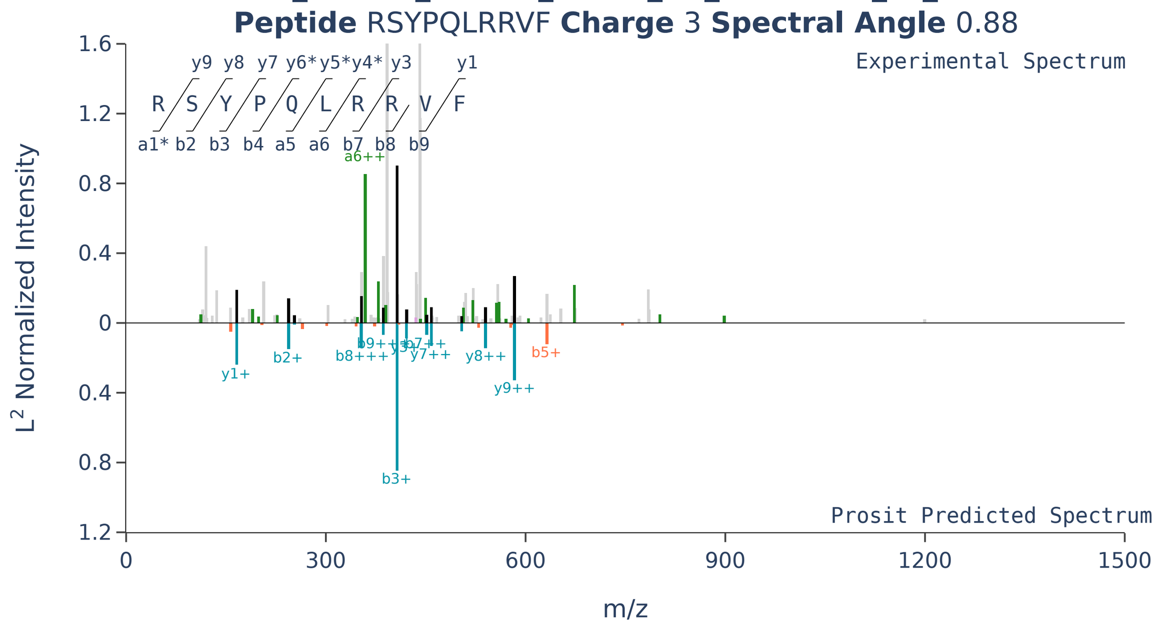


**Source** WSoh_301121_021221_HFGoe_M2_AnnexinA1_24h_R1 **Scan** 7209
**Peptide** TLKKALTG **Charge** 2 **Spectral Angle** 0.83

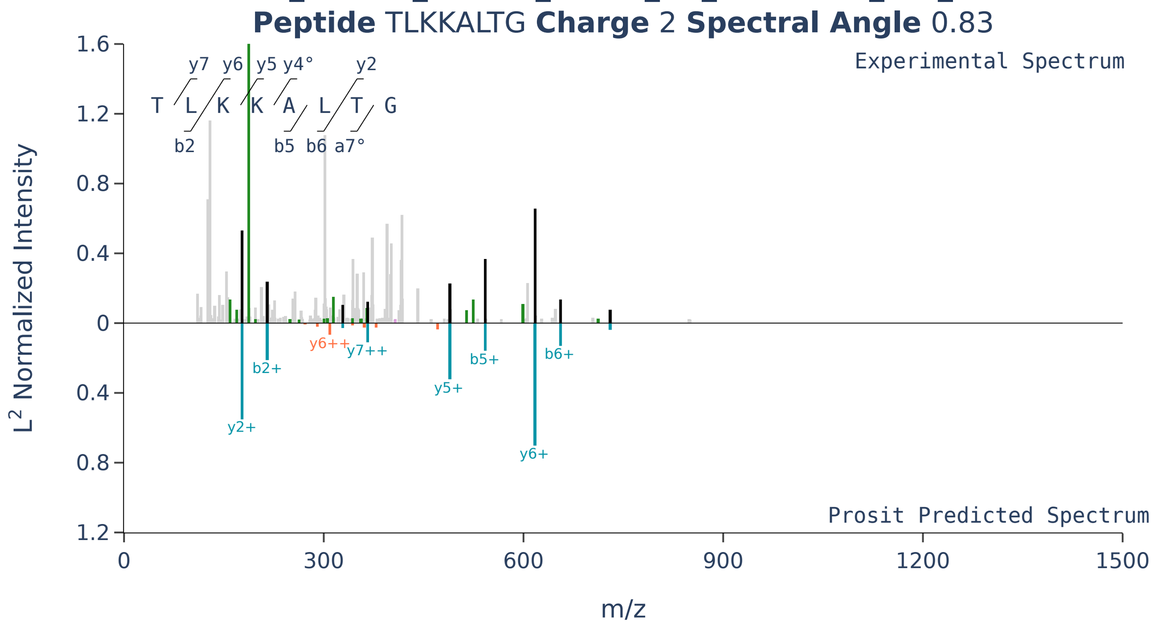


**Source** WSoh_301121_021221_HFGoe_M2_AnnexinA1_24h_R2 **Scan** 15724
**Peptide** KPLDETLKKAL **Charge** 3 **Spectral Angle** 0.8

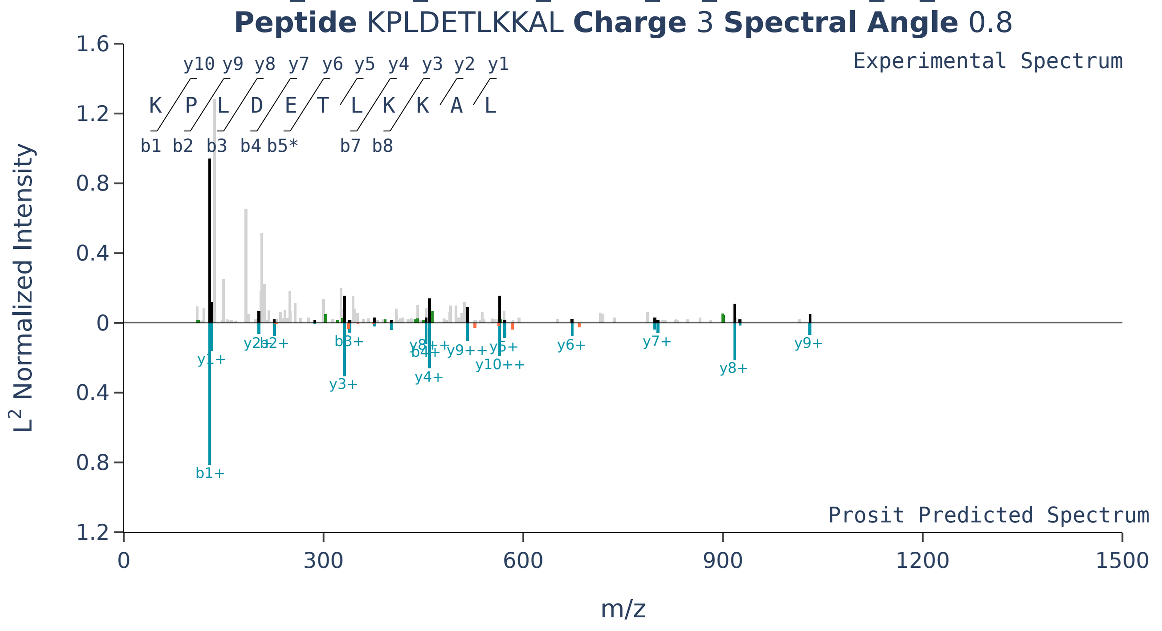


**Source** WSoh_301121_021221_HFGoe_M2_AnnexinA1_24h_R1 **Scan** 12769
**Peptide** SEIDMNDIKA **Charge** 2 **Spectral Angle** 0.83

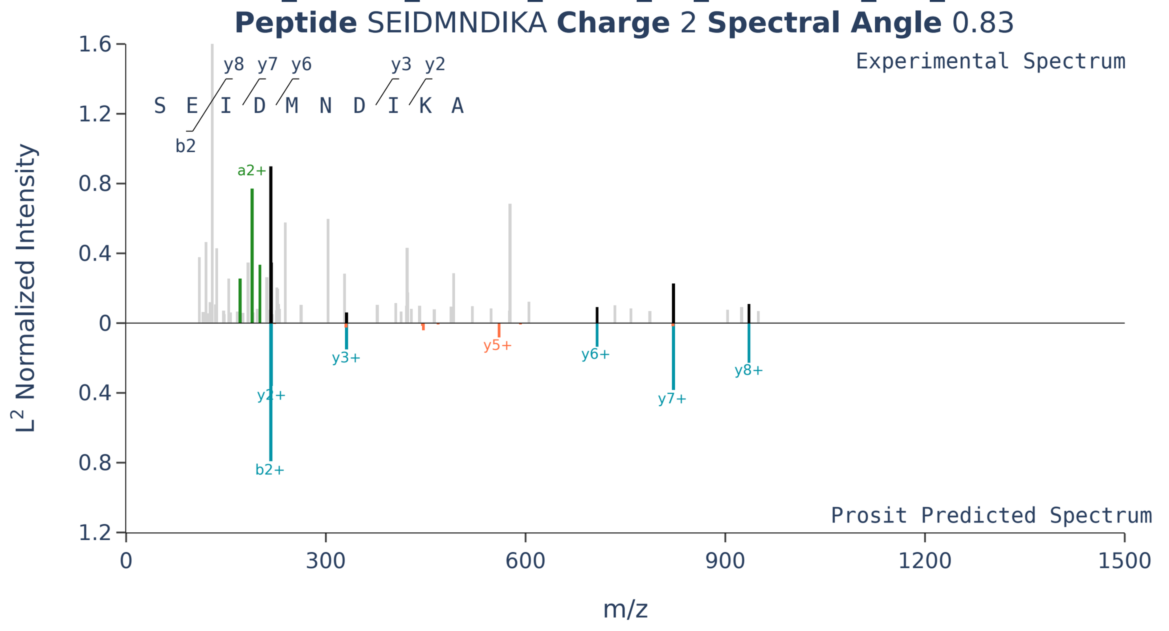


**Source** WSoh_301121_021221_HFGoe_M2_AnnexinA1_24h_R1 **Scan** 12054
**Peptide** REELKRDLAKDITSD **Charge** 3 **Spectral Angle** 0.79

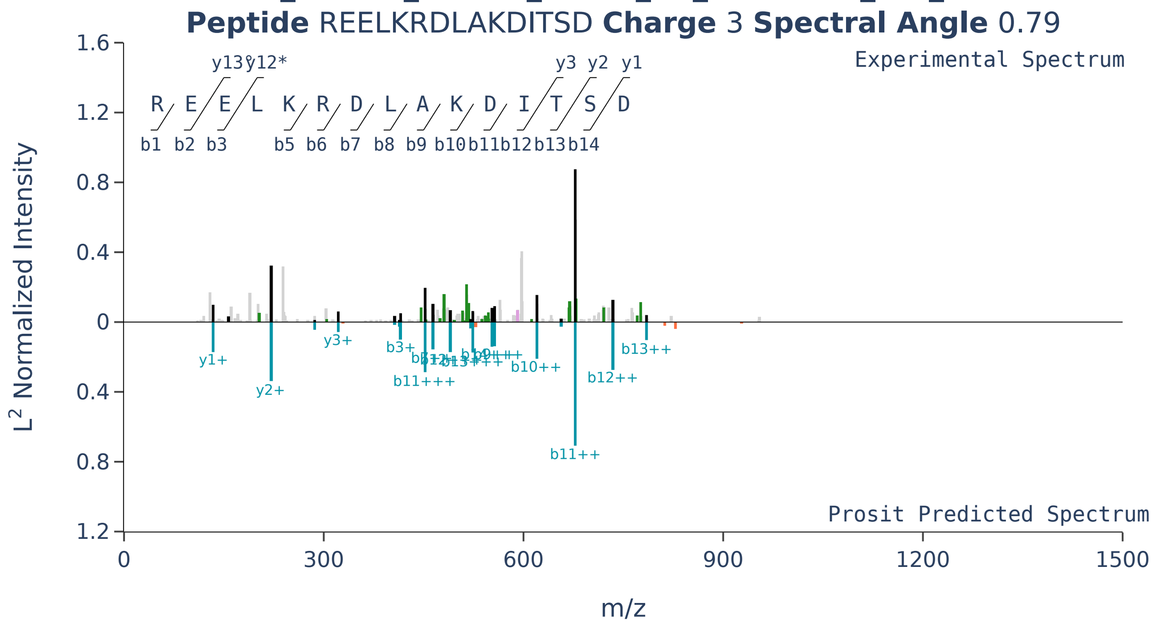


**Source** WSoh_301121_021221_HFGoe_M1_AnnexinA1_2h_R1 **Scan** 13538
**Peptide** KKALTGHLEEVV **Charge** 3 **Spectral Angle** 0.68

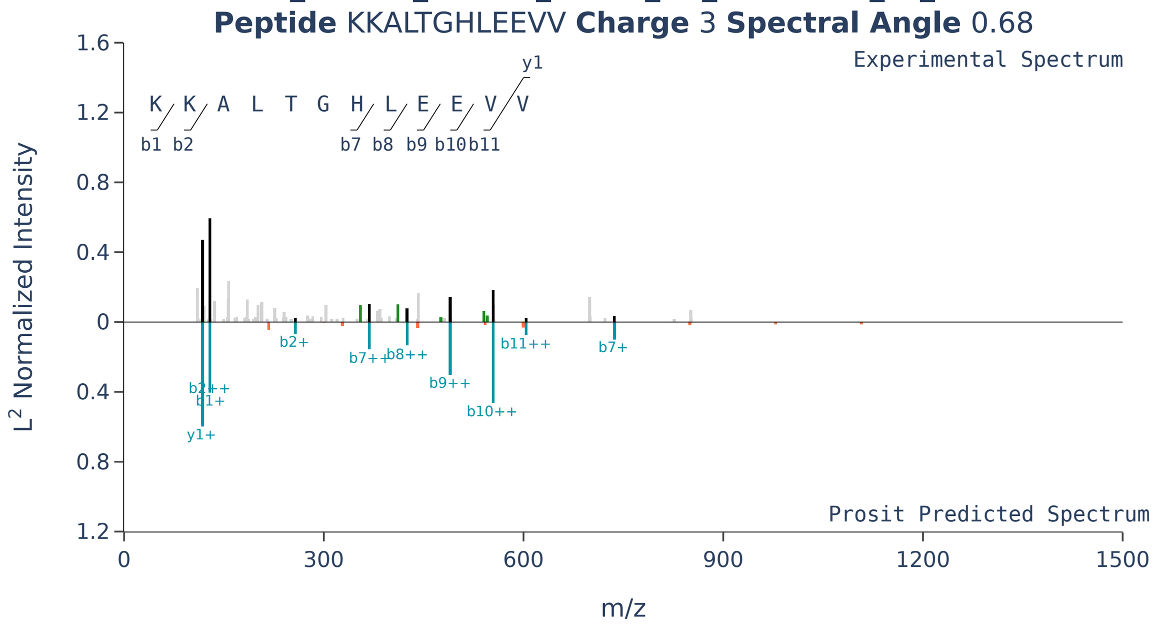


**Source** WSoh_301121_021221_HFGoe_M2_AnnexinA1_24h_R1 **Scan** 13839
**Peptide** DLAKDIT **Charge** 1 **Spectral Angle** 0.85

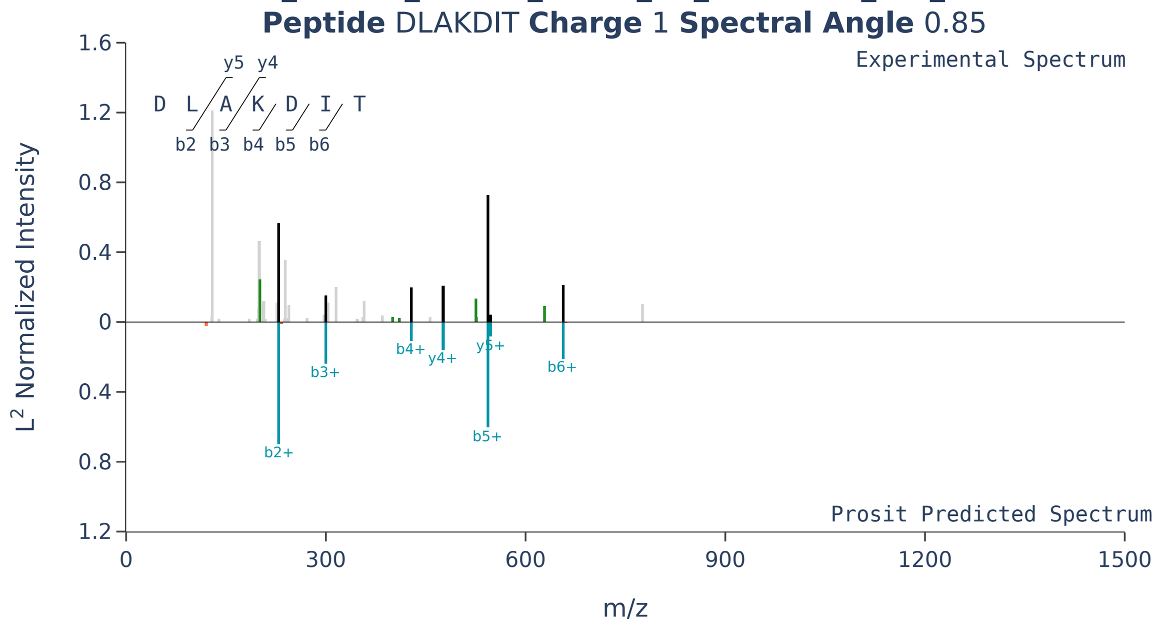


**Source** WSoh_301121_021221_HFGoe_M2_AnnexinA1_24h_R2 **Scan** 18660
**Peptide** MNKVLDL **Charge** 2 **Spectral Angle** 0.84

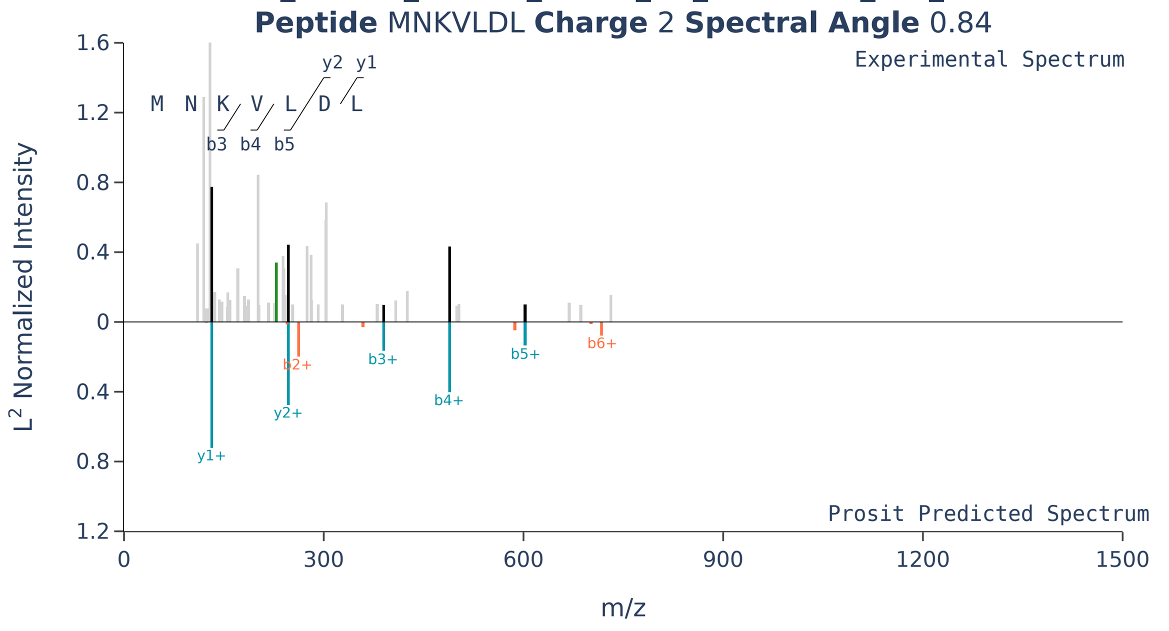


**Source** WSoh_301121_021221_HFGoe_M2_AnnexinA1_2h_R1 **Scan** 17843
**Peptide** ELKRDLAKDITSDTSGDFRNA **Charge** 4 **Spectral Angle** 0.85

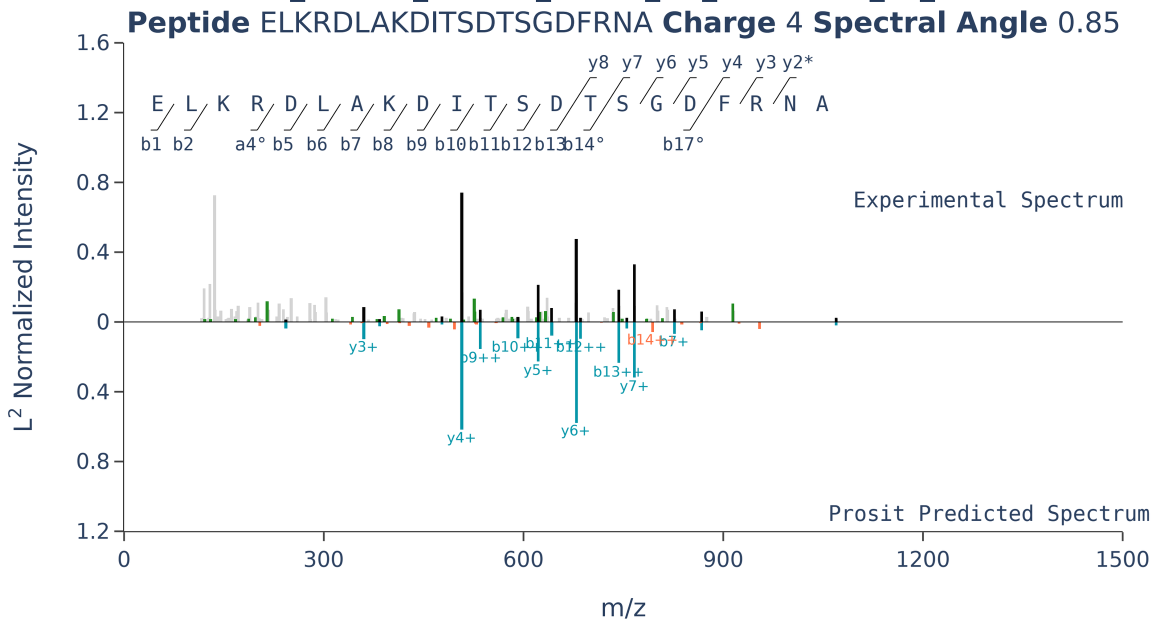


**Source** WSoh_301121_021221_HFGoe_M2_AnnexinA1_24h_R1 **Scan** 24448
**Peptide** TIIDILTKRNN **Charge** 3 **Spectral Angle** 0.85

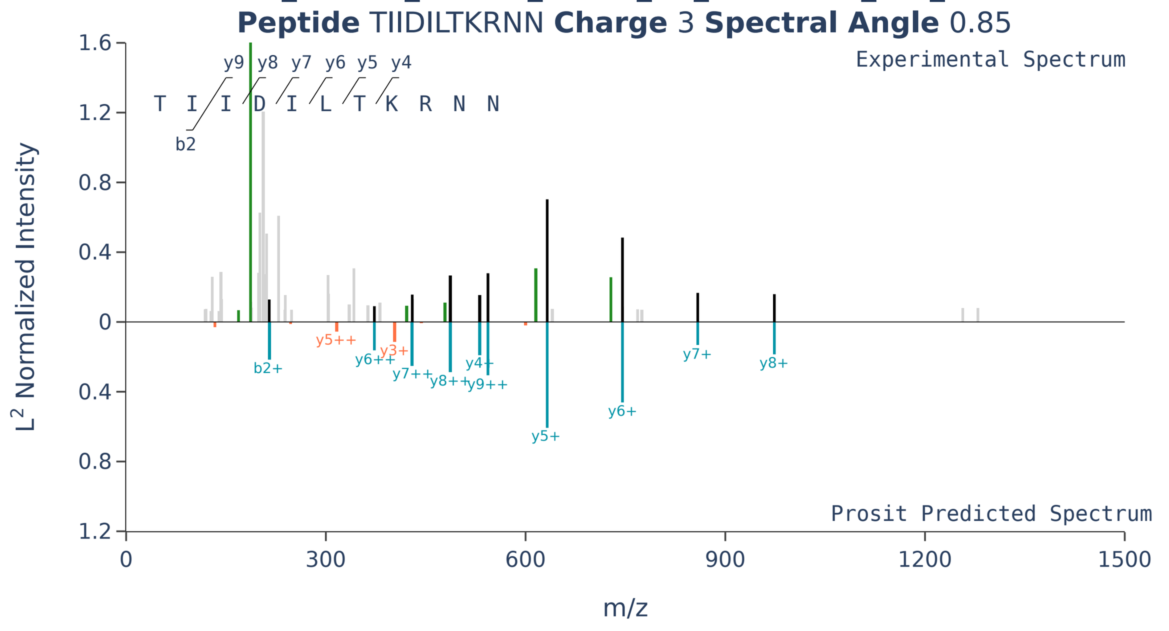

**Source** WSoh_301121_021221_HFGoe_M2_AnnexinA1_4h_R1 **Scan** 8552
**Peptide** AALHKAIMVK **Charge** 3 **Spectral Angle** 0.85

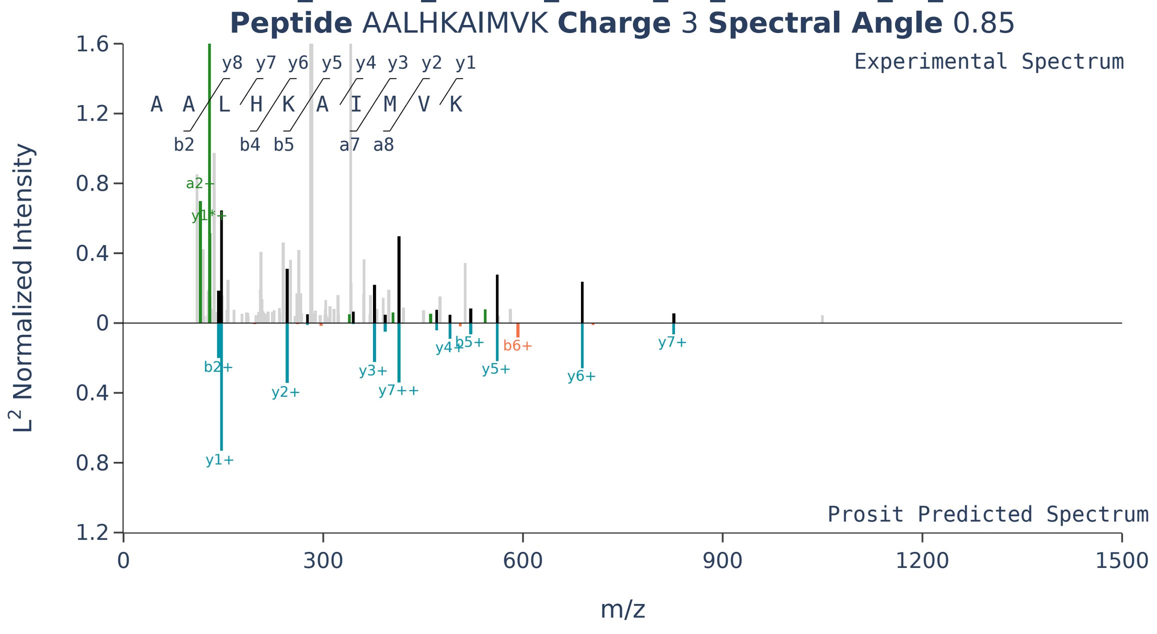


**Source** WSoh_301121_021221_HFGoe_M2_AnnexinA1_2h_R2 **Scan** 18786
**Peptide** QAILDETKGDYEKL **Charge** 3 **Spectral Angle** 0.78

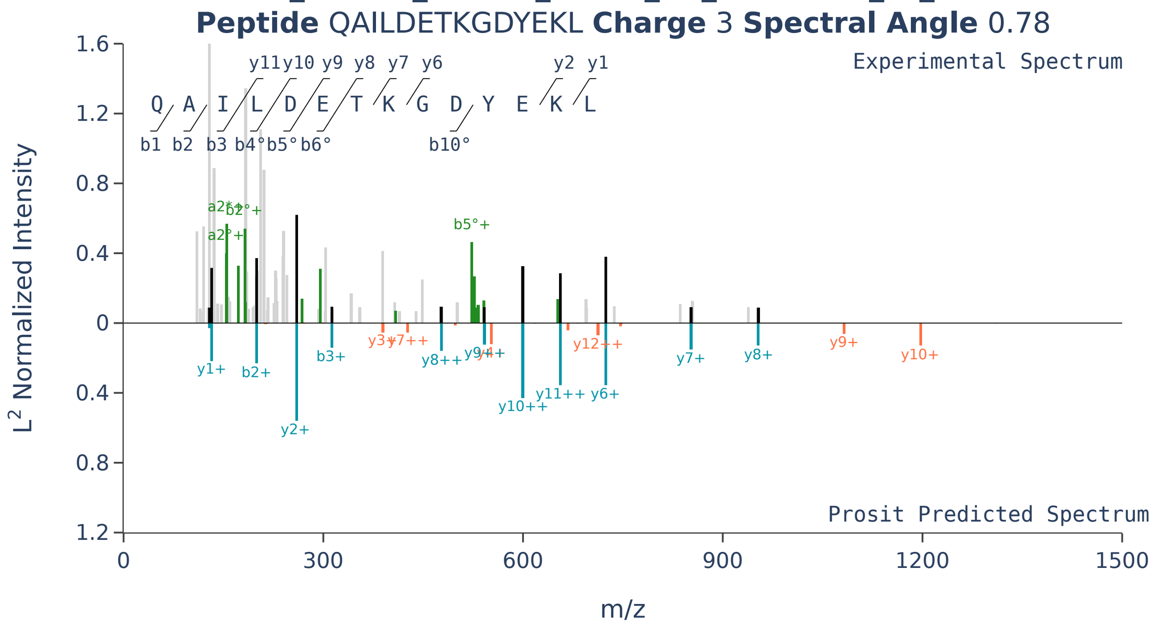


**Source** WSoh_301121_021221_HFGoe_M2_AnnexinA1_2h_R2 **Scan** 19710
**Peptide** KGDIEKCLTAIVK **Charge** 3 **Spectral Angle** 0.8

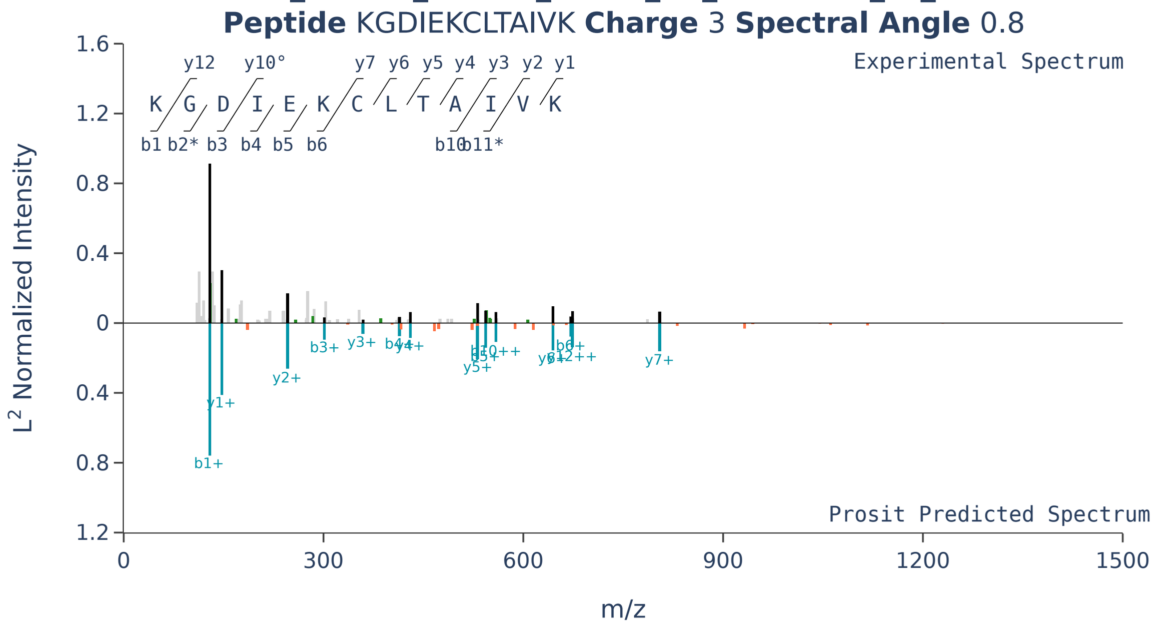


**Source** WSoh_301121_021221_HFGoe_M2_AnnexinA1_2h_R2 **Scan** 16049
**Peptide** ALLSLAKGDRSED **Charge** 2 **Spectral Angle** 0.79

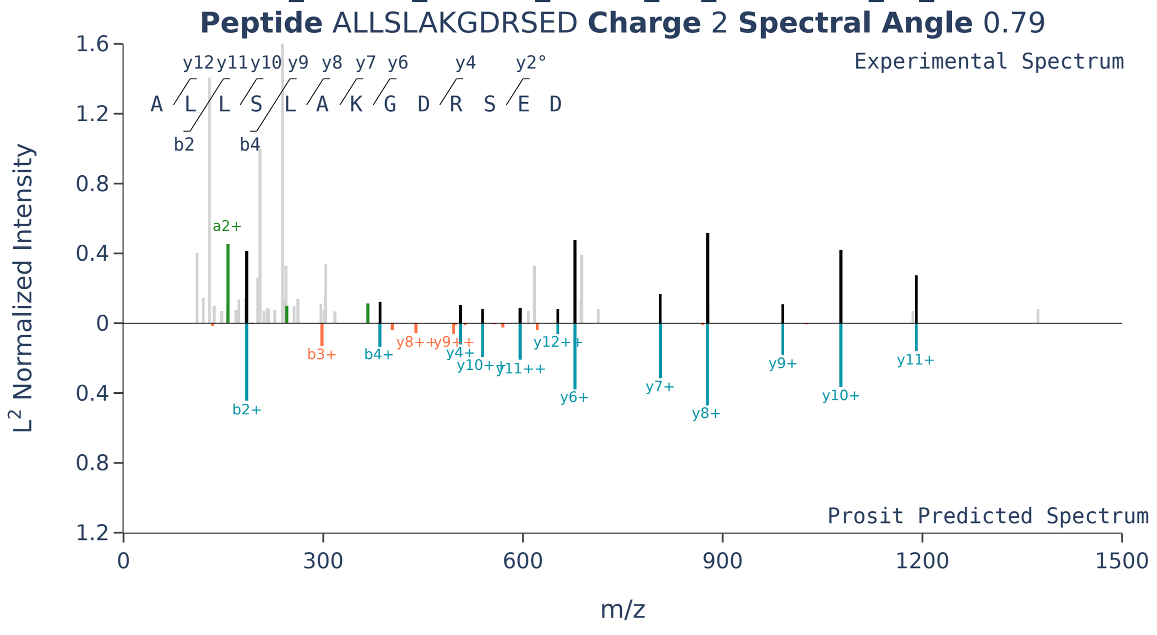


**Source** WSoh_301121_021221_HFGoe_M2_AnnexinA1_2h_R2 **Scan** 23910
**Peptide** GERRKGTDVNVFNTILTT **Charge** 3 **Spectral Angle** 0.84

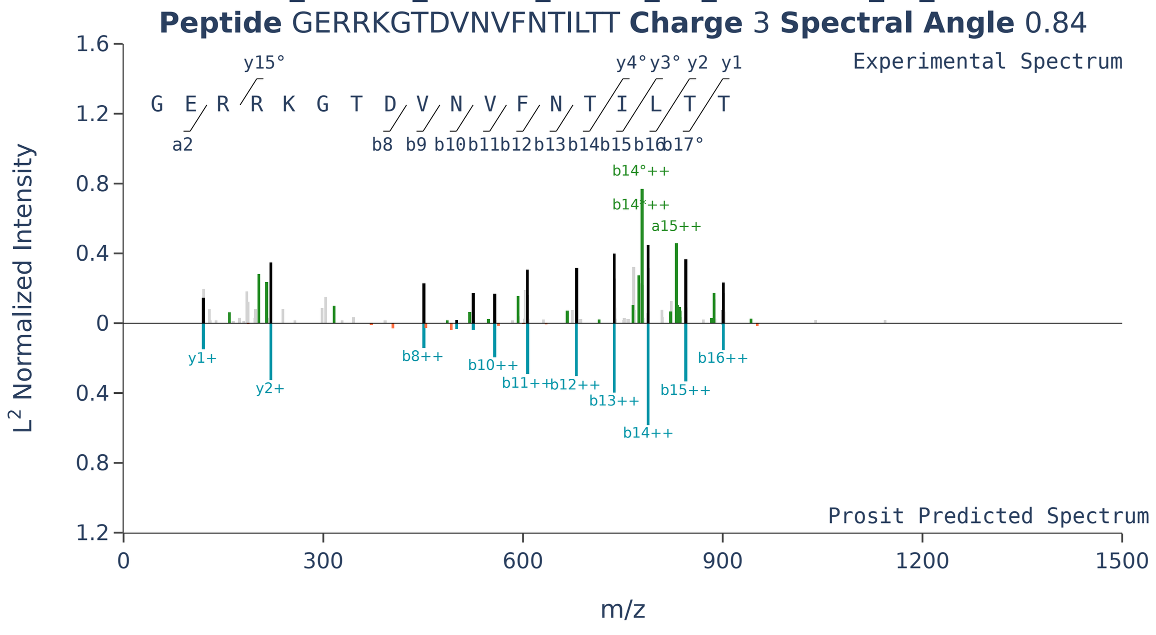


**Source** WSoh_301121_021221_HFGoe_M1_AnnexinA1_4h_R2 **Scan** 18704
**Peptide** RSEIDMNDL **Charge** 1 **Spectral Angle** 0.87

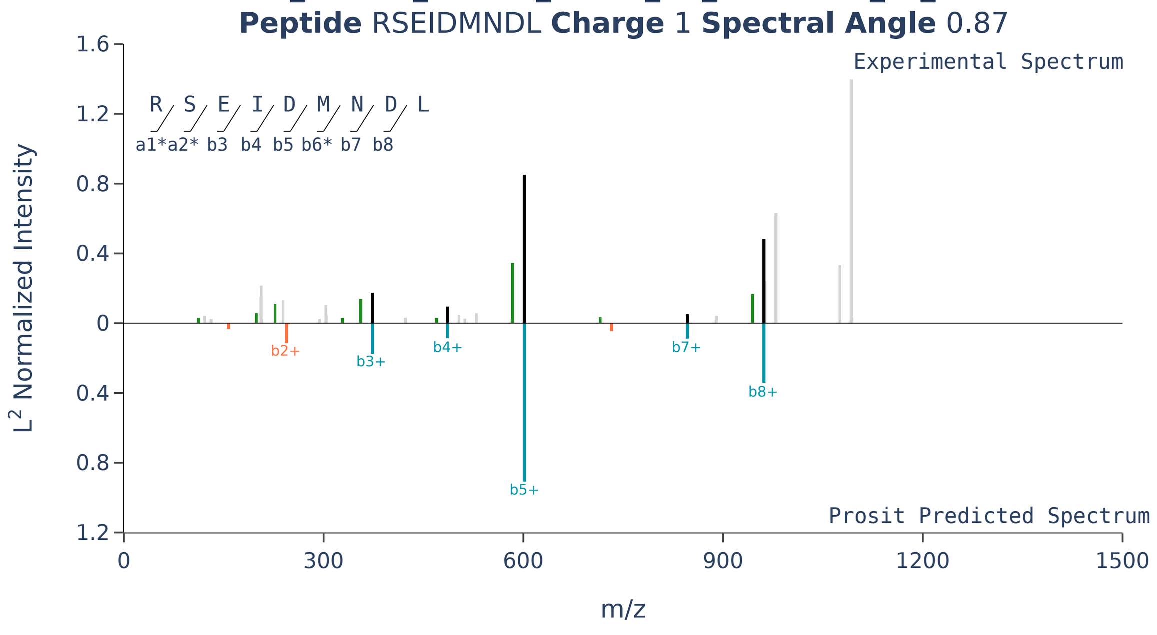


**Source** WSoh_301121_021221_HFGoe_M2_AnnexinA1_2h_R1 **Scan** 12101
**Peptide** ALHKAIMVKGVDE **Charge** 2 **Spectral Angle** 0.8

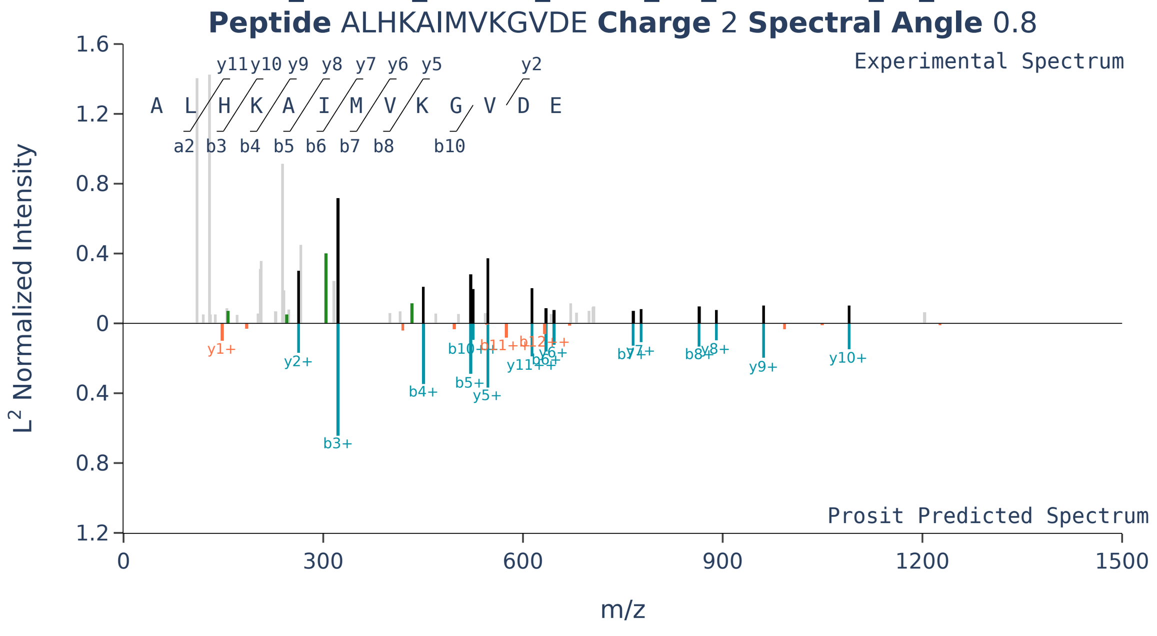


**Source** WSoh_301121_021221_HFGoe_M2_AnnexinA1_1h_R1 **Scan** 8267
**Peptide** MKGVGTRHKALI **Charge** 3 **Spectral Angle** 0.82

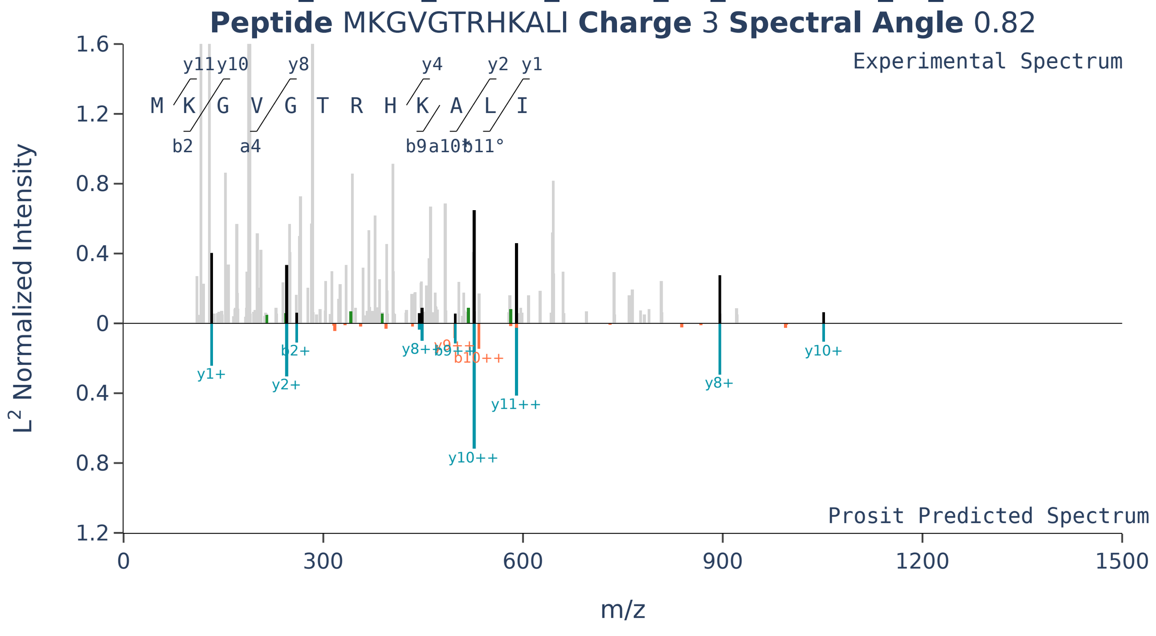


**Source** WSoh_301121_021221_HFGoe_M1_AnnexinA1_24h_R1 **Scan** 24803
**Peptide** TIIDILTKR **Charge** 3 **Spectral Angle** 0.93

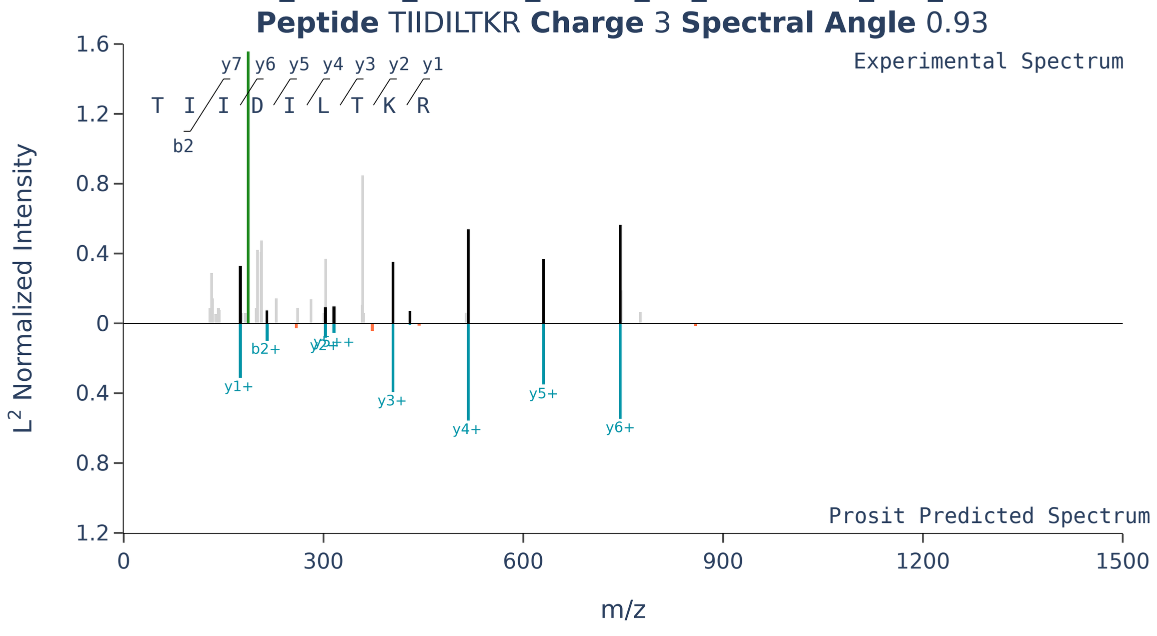


**Source** WSoh_301121_021221_HFGoe_M2_AnnexinA1_4h_R1 **Scan** 20970
**Peptide** GDYEKILV **Charge** 2 **Spectral Angle** 0.75

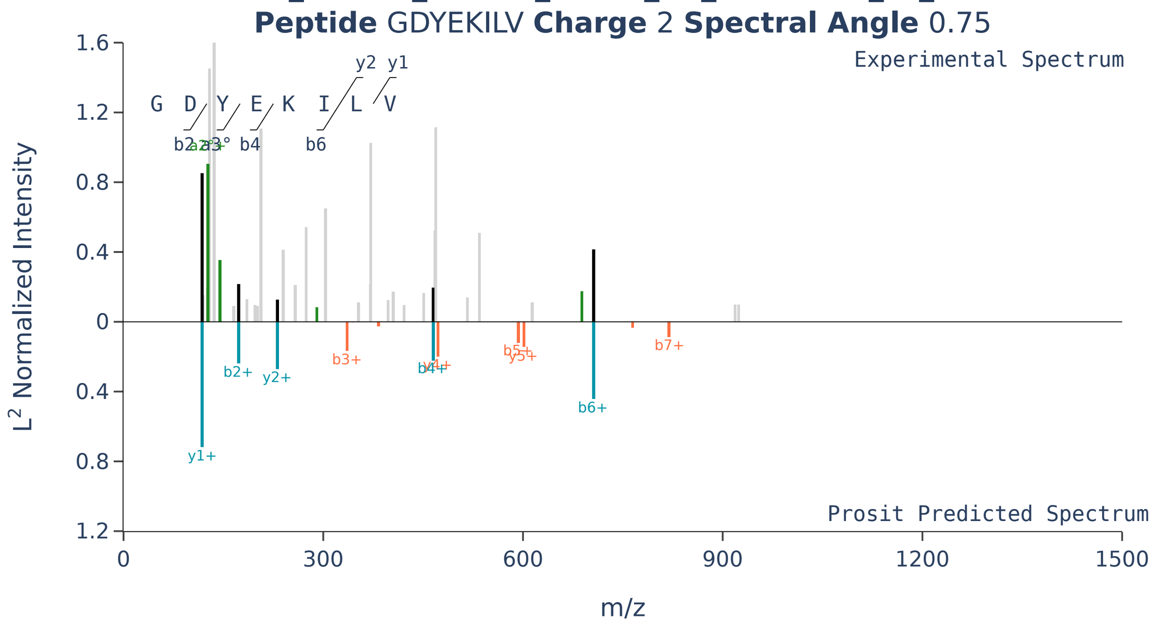


**Source** WSoh_301121_021221_HFGoe_M2_AnnexinA1_2h_R1 **Scan** 21677
**Peptide** KALTGHLEEVVL **Charge** 3 **Spectral Angle** 0.68

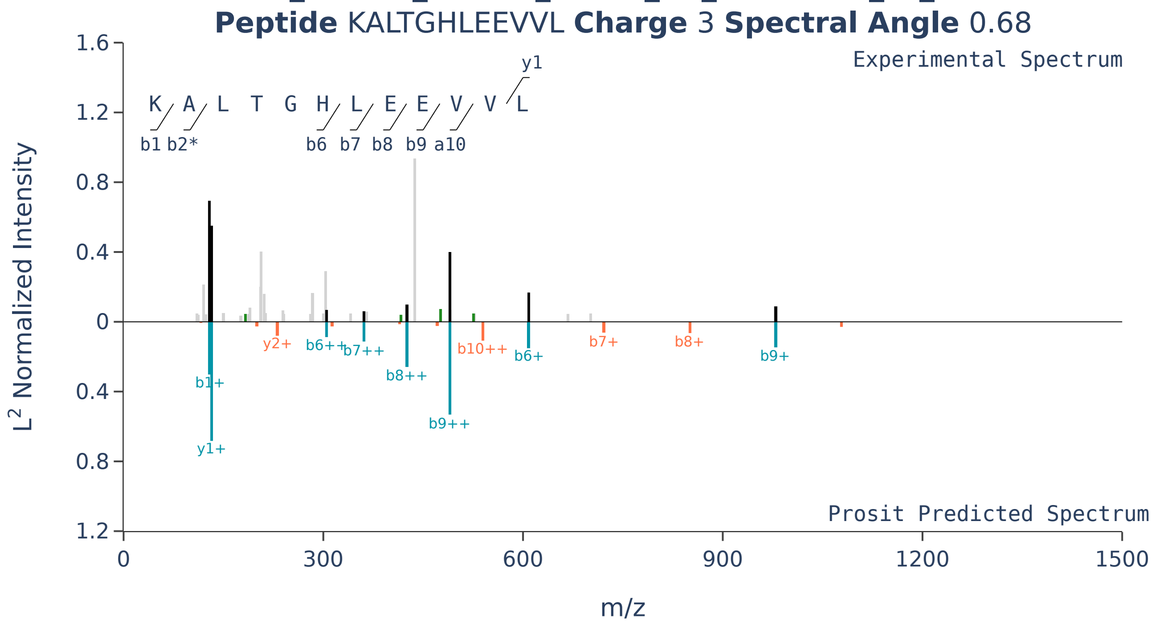


**Source** WSoh_301121_021221_HFGoe_M2_AnnexinA1_1h_R1 **Scan** 24270
**Peptide** RSEIDMNDIKAFY **Charge** 3 **Spectral Angle** 0.9

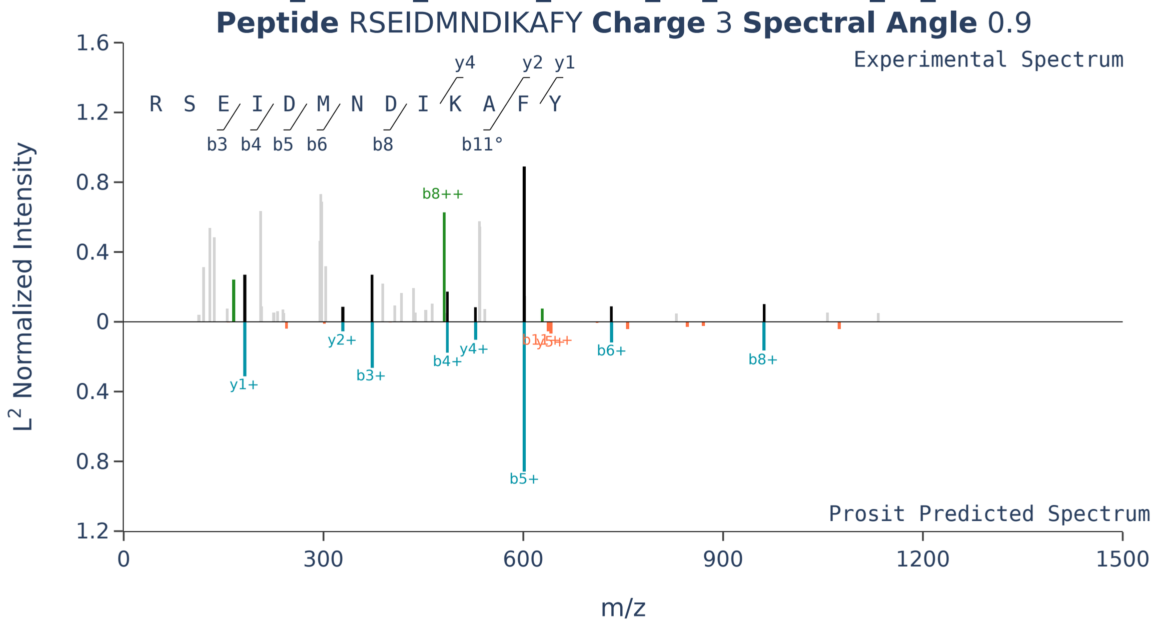


**Source** WSoh_301121_021221_HFGoe_M2_AnnexinA1_4h_R1 **Scan** 8139
**Peptide** DLLTKRNNA **Charge** 1 **Spectral Angle** 0.84

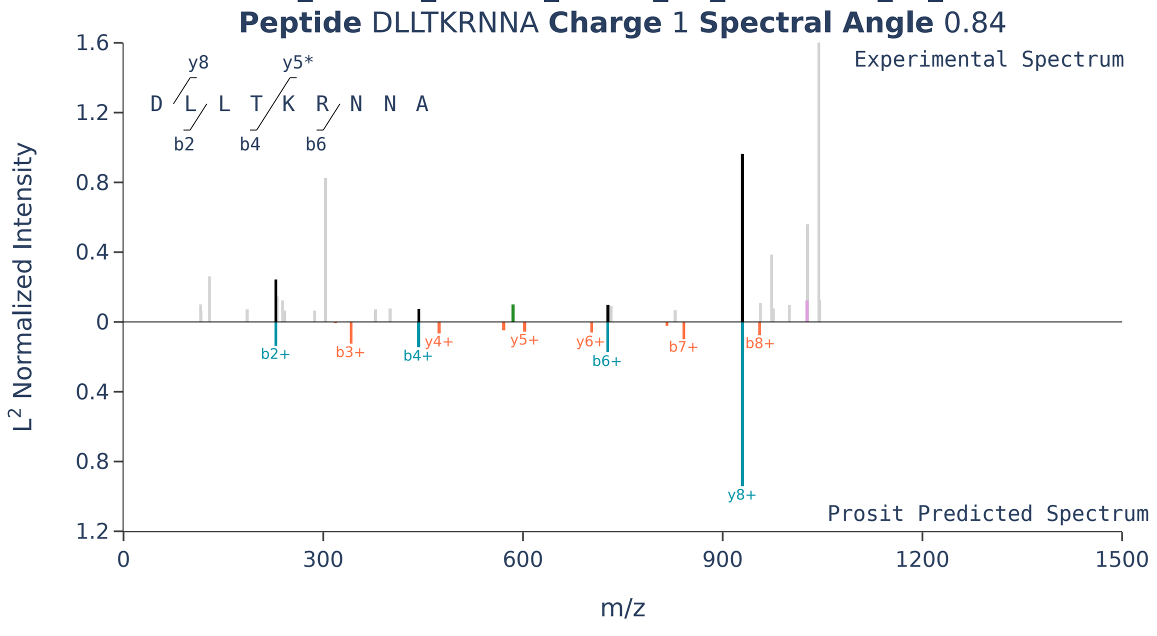


**Source** WSoh_301121_021221_HFGoe_M2_AnnexinA1_2h_R2 **Scan** 19200
**Peptide** CQAILDETKGDYEKI **Charge** 2 **Spectral Angle** 0.89

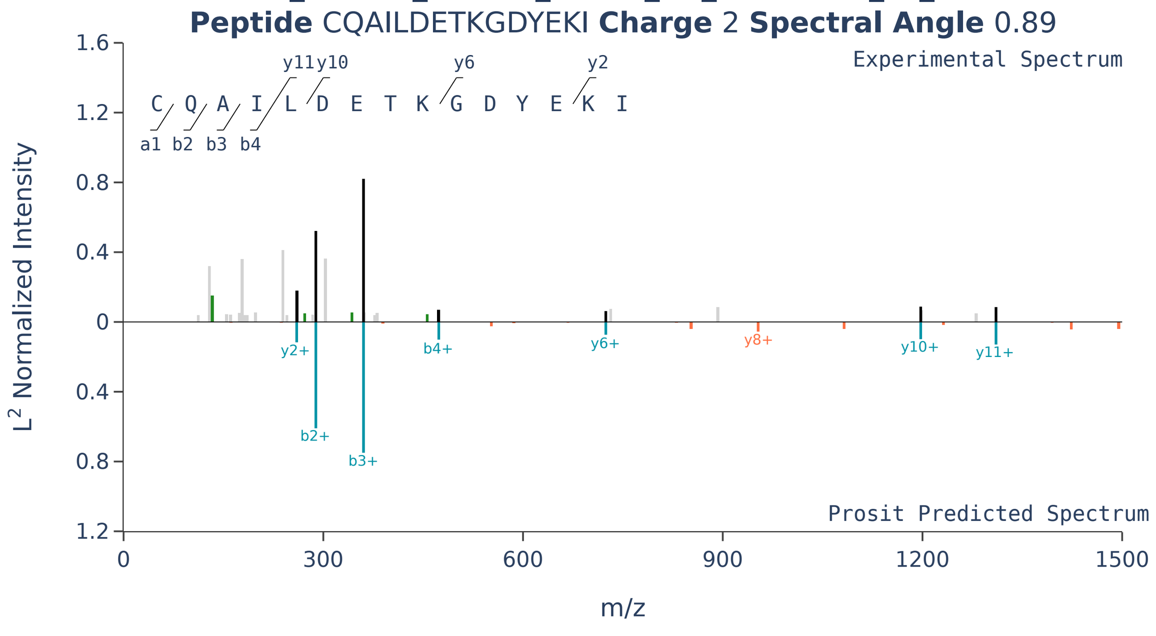


**Source** WSoh_301121_021221_HFGoe_M2_AnnexinA1_24h_R2 **Scan** 15820
**Peptide** IENEEQEYVQTVKSS **Charge** 2 **Spectral Angle** 0.85

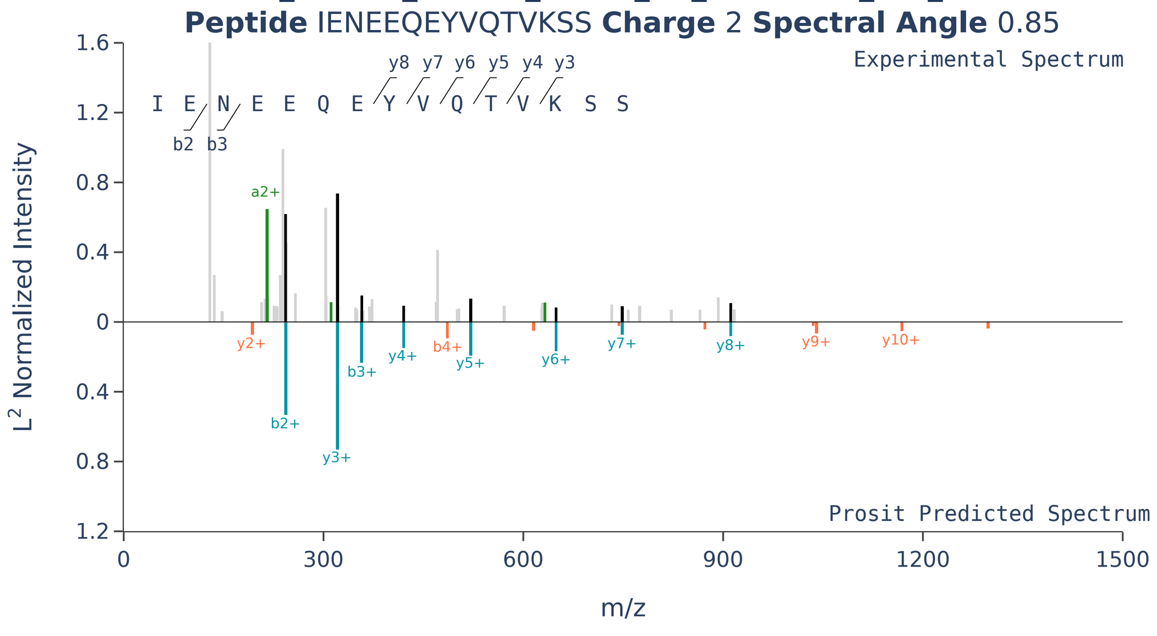

**Source** WSoh_301121_021221_HFGoe_M2_AnnexinA1_2h_R1 **Scan** 18290
**Peptide** GERRKGTDVNVFNTI **Charge** 4 **Spectral Angle** 0.72

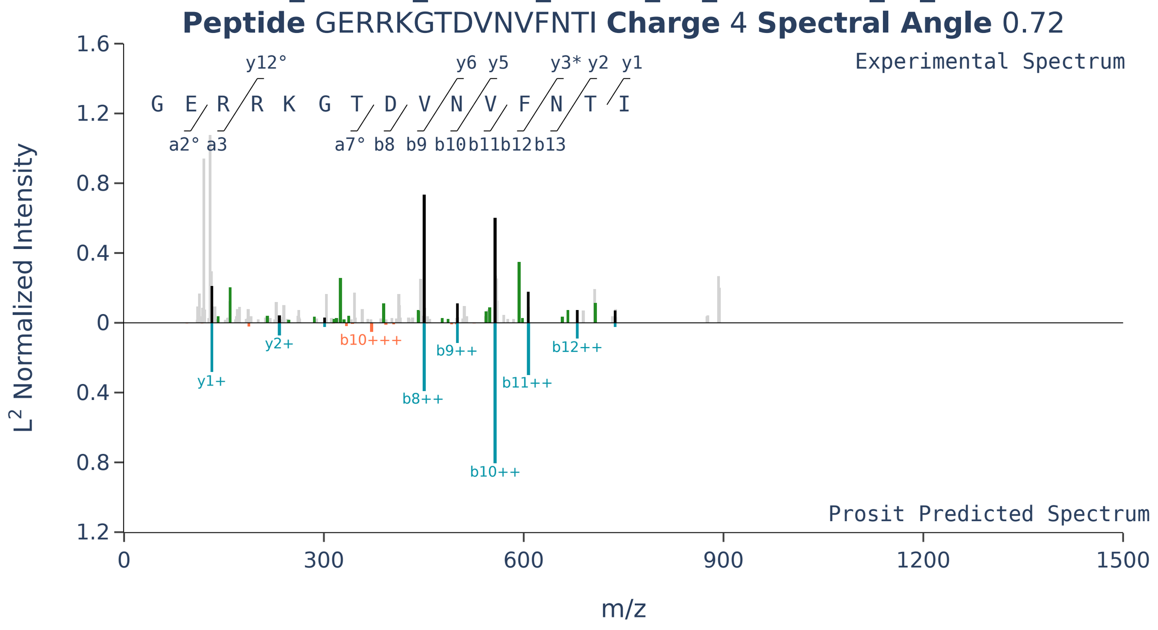


**Source** WSoh_301121_021221_HFGoe_M2_AnnexinA1_2h_R1 **Scan** 27012
**Peptide** ATIIDILTKR **Charge** 3 **Spectral Angle** 0.81

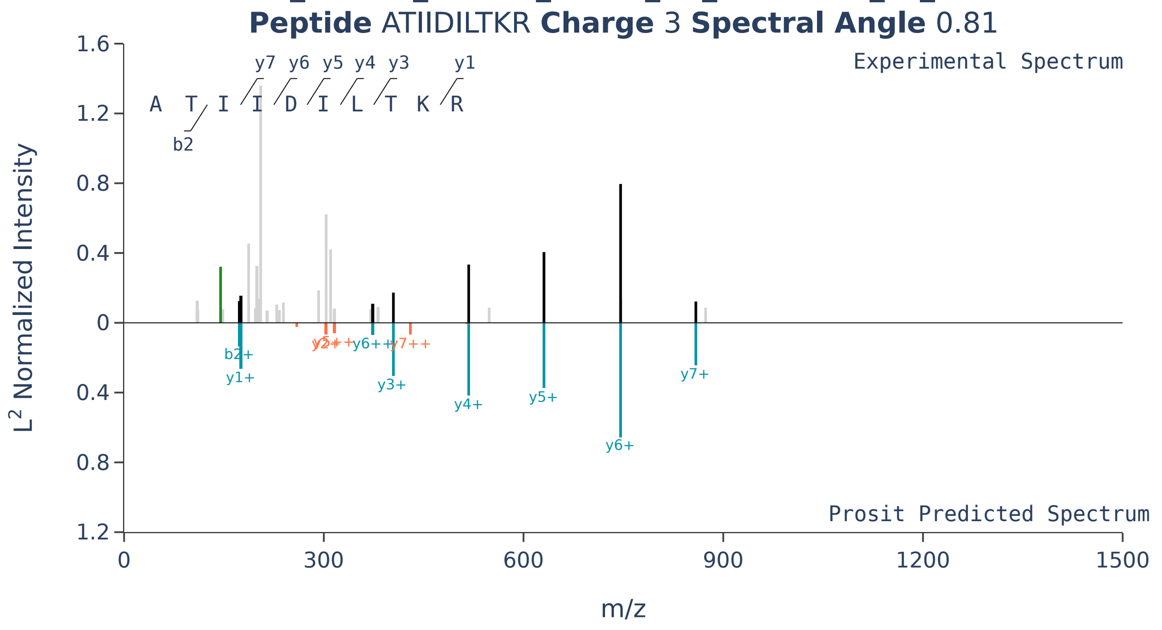


**Source** WSoh_301121_021221_HFGoe_M1_AnnexinA1_24h_R2 **Scan** 6270
**Peptide** REELKRDLAKD **Charge** 3 **Spectral Angle** 0.89

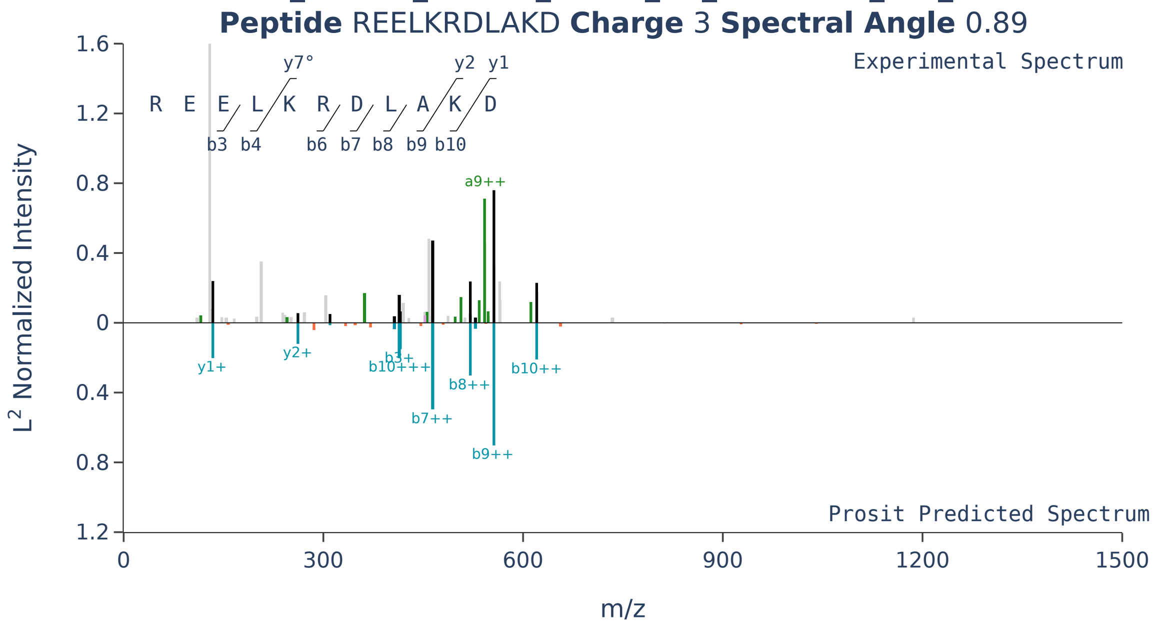


**Source** WSoh_301121_021221_HFGoe_M1_AnnexinA1_24h_R1 **Scan** 20658
**Peptide** KGGPGSAVSPYPTFNPSS **Charge** 2 **Spectral Angle** 0.89

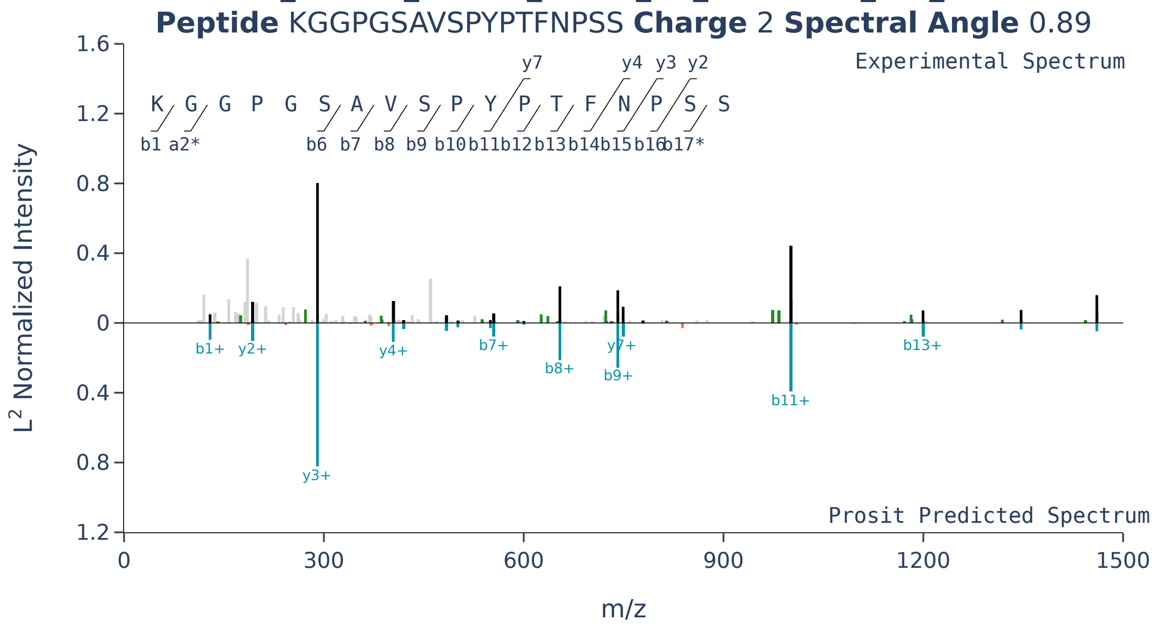


**Source** WSoh_301121_021221_HFGoe_M1_AnnexinA1_4h_R1 **Scan** 17225
**Peptide** TGKPLDETLKKALT **Charge** 3 **Spectral Angle** 0.83

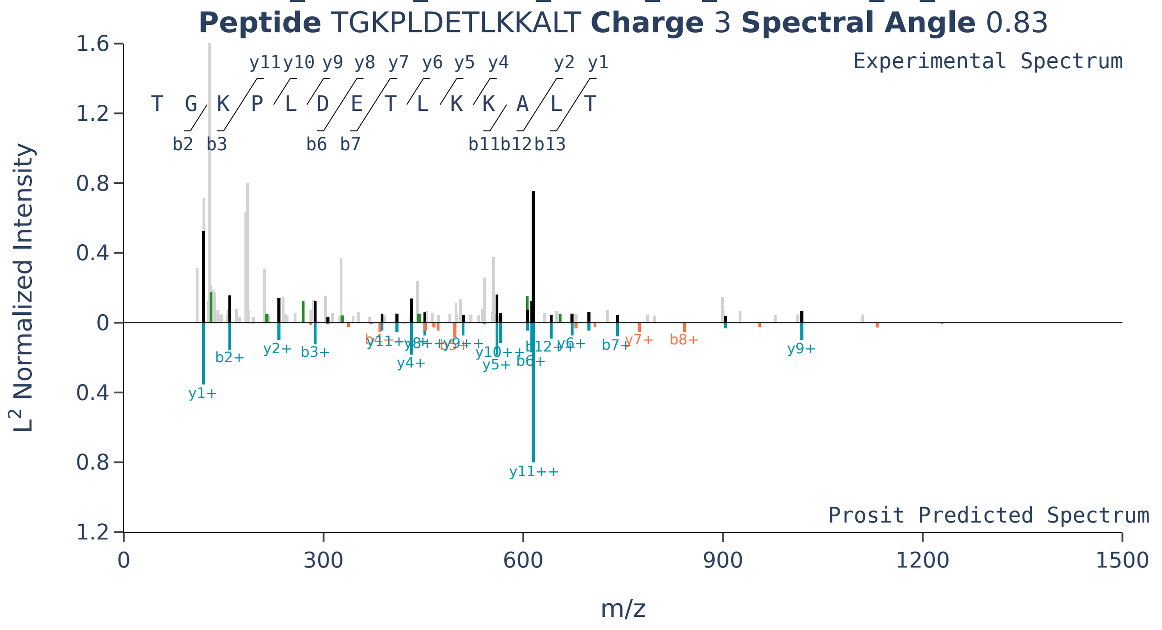


**Source** WSoh_301121_021221_HFGoe_M2_AnnexinA1_24h_R2 **Scan** 10860
**Peptide** SDARALYEAGERRKGTDV **Charge** 4 **Spectral Angle** 0.79

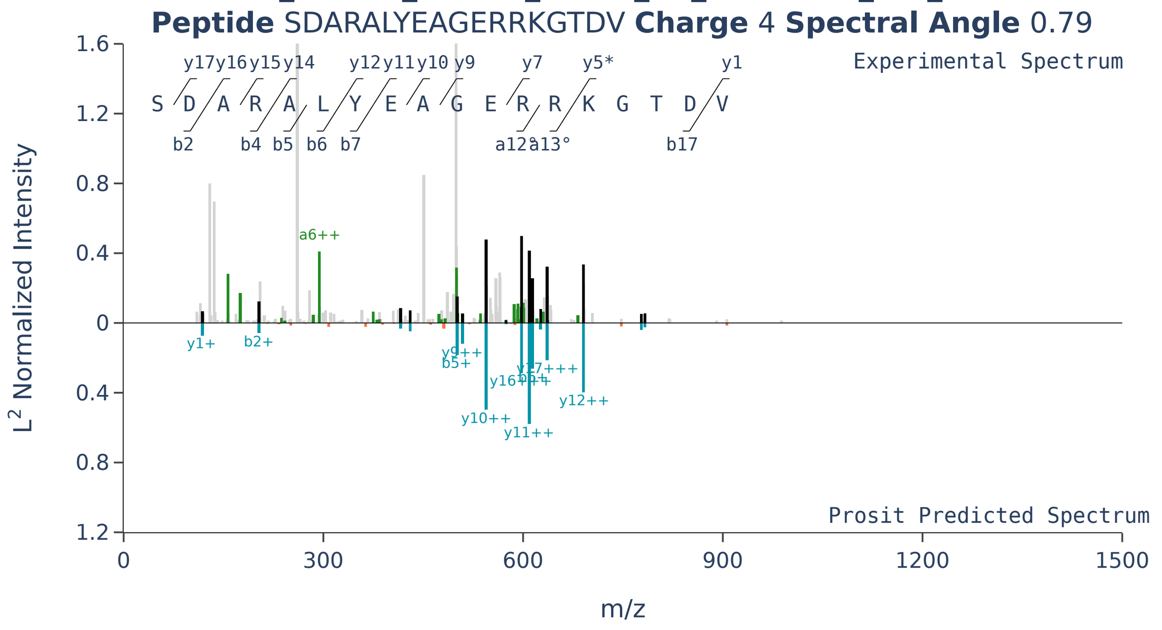


**Source** WSoh_301121_021221_HFGoe_M2_AnnexinA1_2h_R2 **Scan** 18256
**Peptide** VKSSKGGPGSAVSPYPTFNPSSD **Charge** 3 **Spectral Angle** 0.79

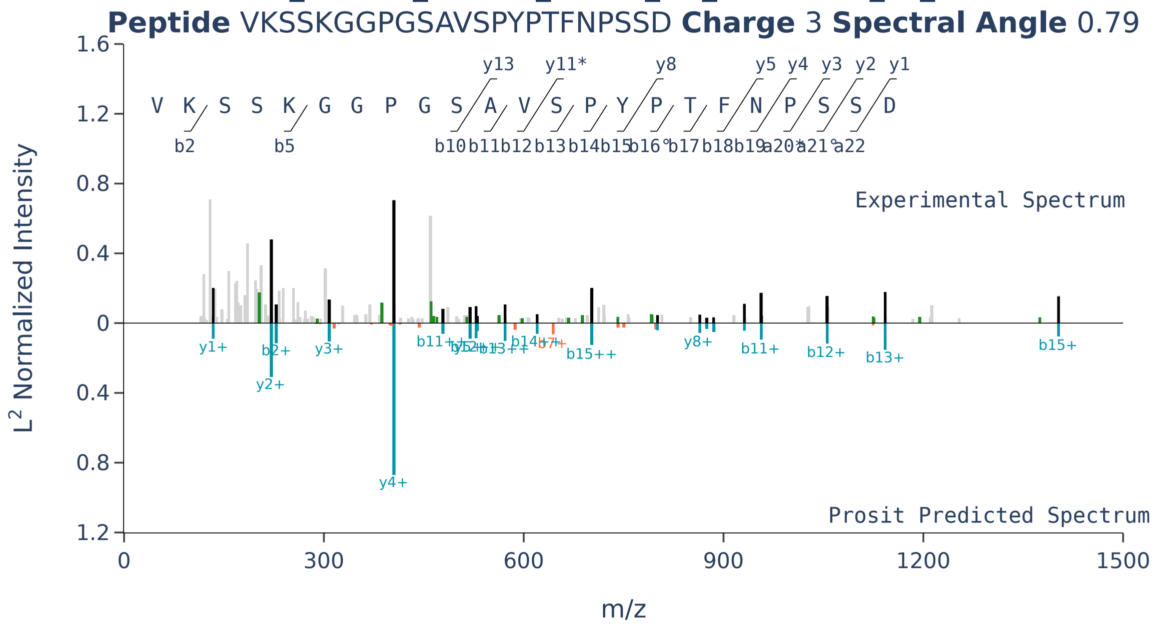


**Source** WSoh_301121_021221_HFGoe_M2_AnnexinA1_2h_R1 **Scan** 19534
**Peptide** ADELRAAMKGLGTDED **Charge** 2 **Spectral Angle** 0.77

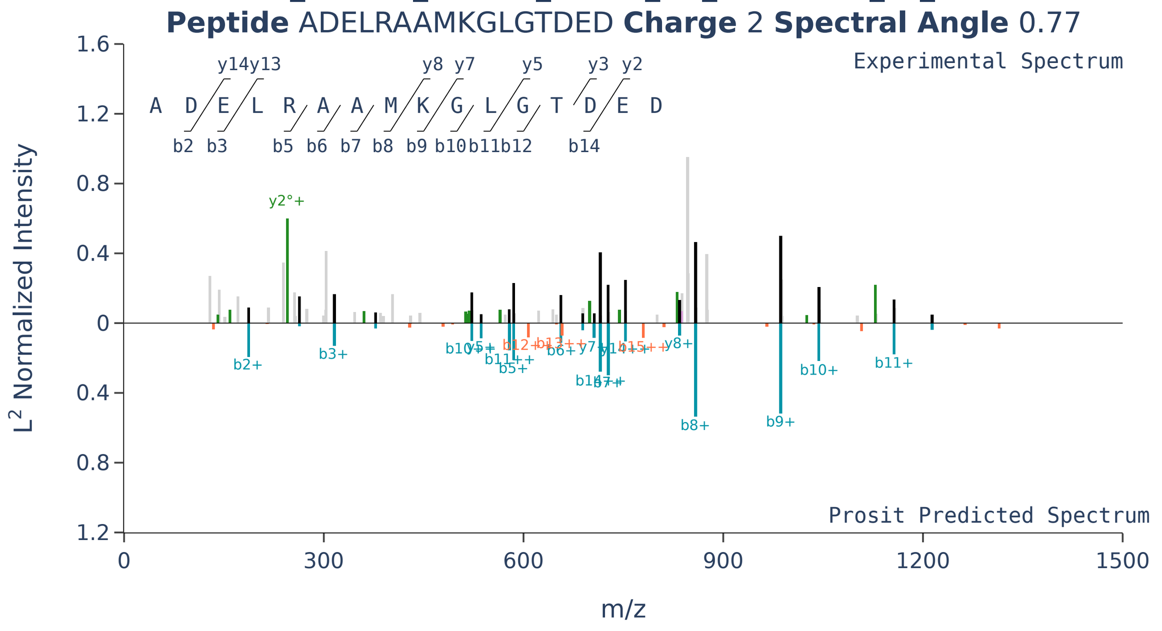


**Source** WSoh_301121_021221_HFGoe_M1_AnnexinA1_2h_R2 **Scan** 8502
**Peptide** HKAIMVKGVDEA **Charge** 3 **Spectral Angle** 0.77

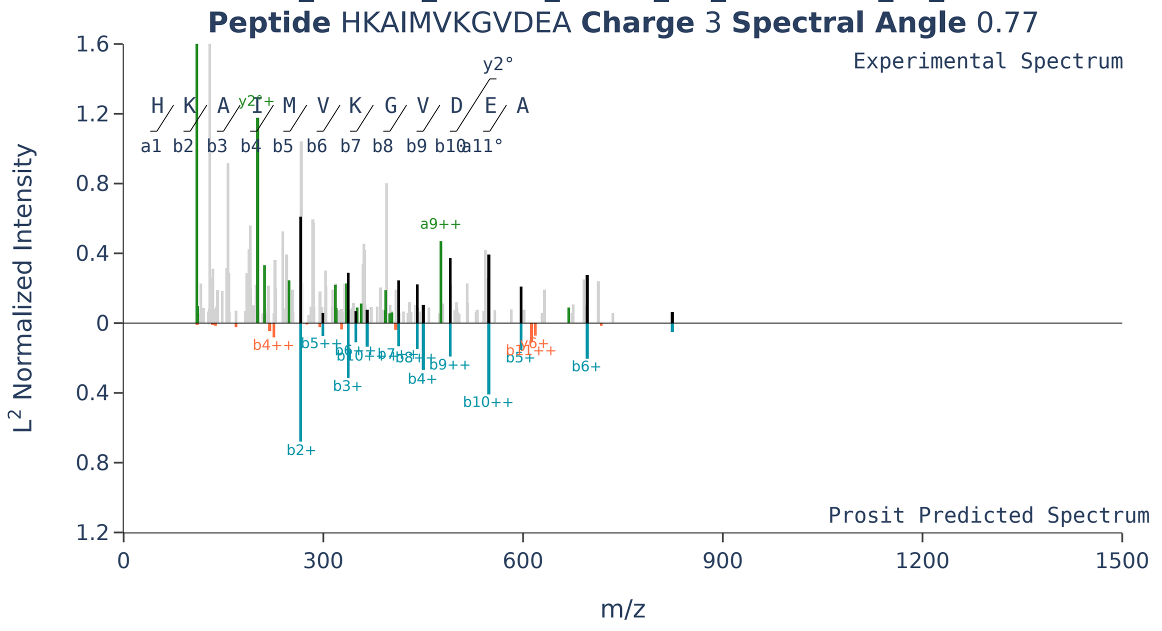


**Source** WSoh_301121_021221_HFGoe_M2_AnnexinA1_24h_R1 **Scan** 19315
**Peptide** KGLGTDEDTLIE **Charge** 2 **Spectral Angle** 0.83

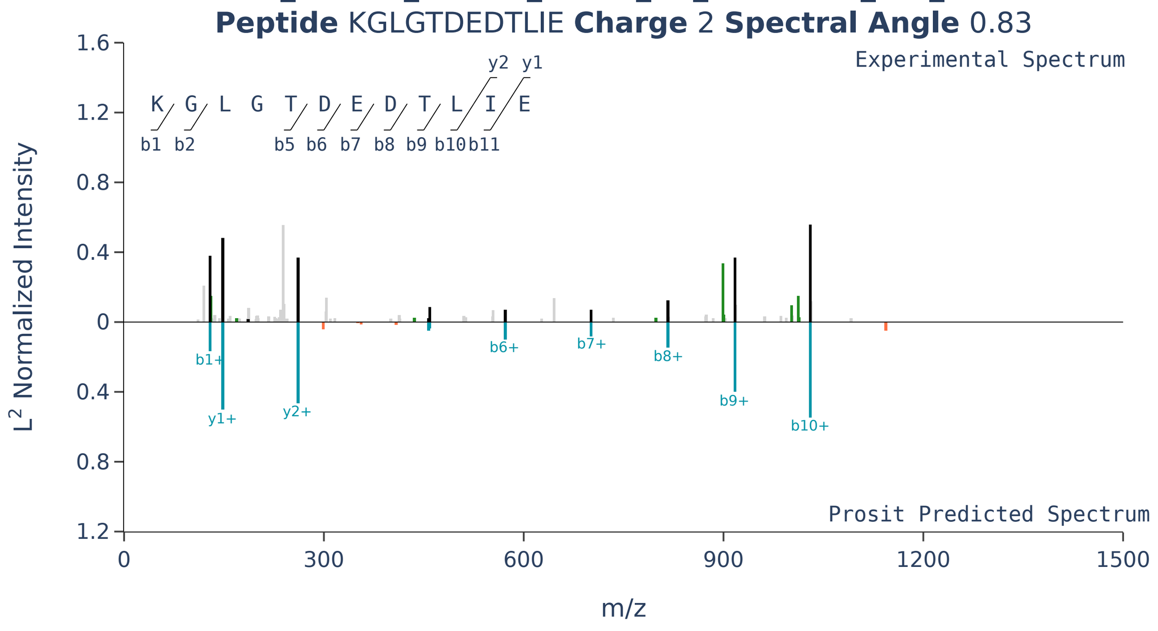


**Source** WSoh_301121_021221_HFGoe_M2_AnnexinA1_2h_R2 **Scan** 7586
**Peptide** TKYSKHDMNKVLD **Charge** 2 **Spectral Angle** 0.79

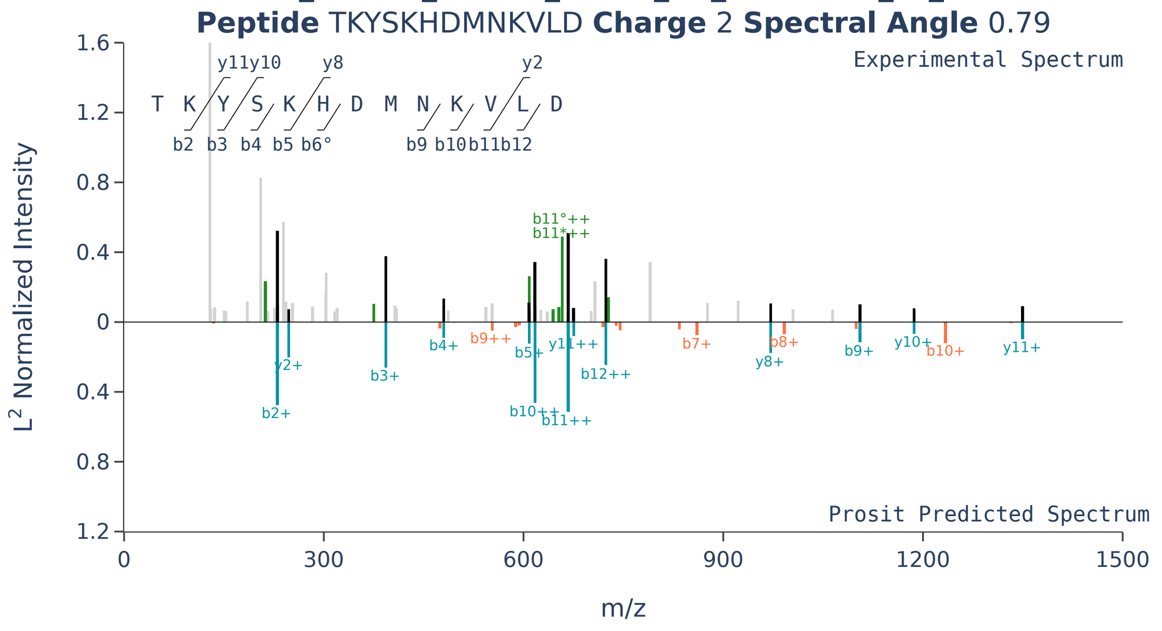


**Source** WSoh_301121_021221_HFGoe_M2_AnnexinA1_2h_R2 **Scan** 20075
**Peptide** DETKGDYEKILV **Charge** 2 **Spectral Angle** 0.79

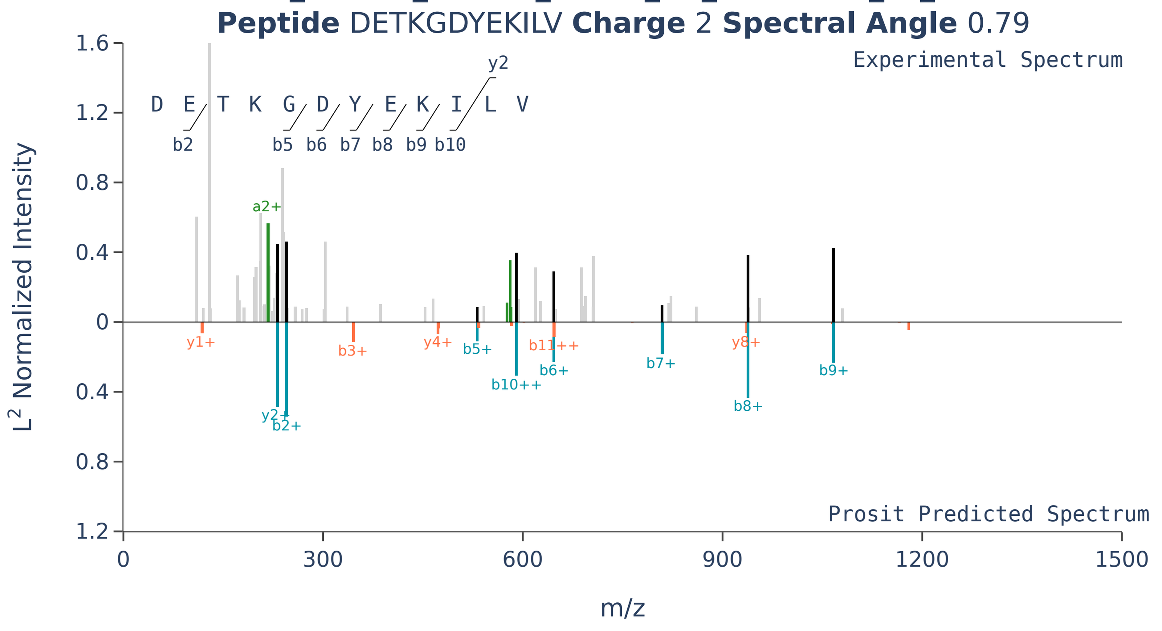


**Source** WSoh_301121_021221_HFGoe_M2_AnnexinA1_24h_R2 **Scan** 11852
**Peptide** YEAGERRKGTDVNVFN **Charge** 4 **Spectral Angle** 0.89

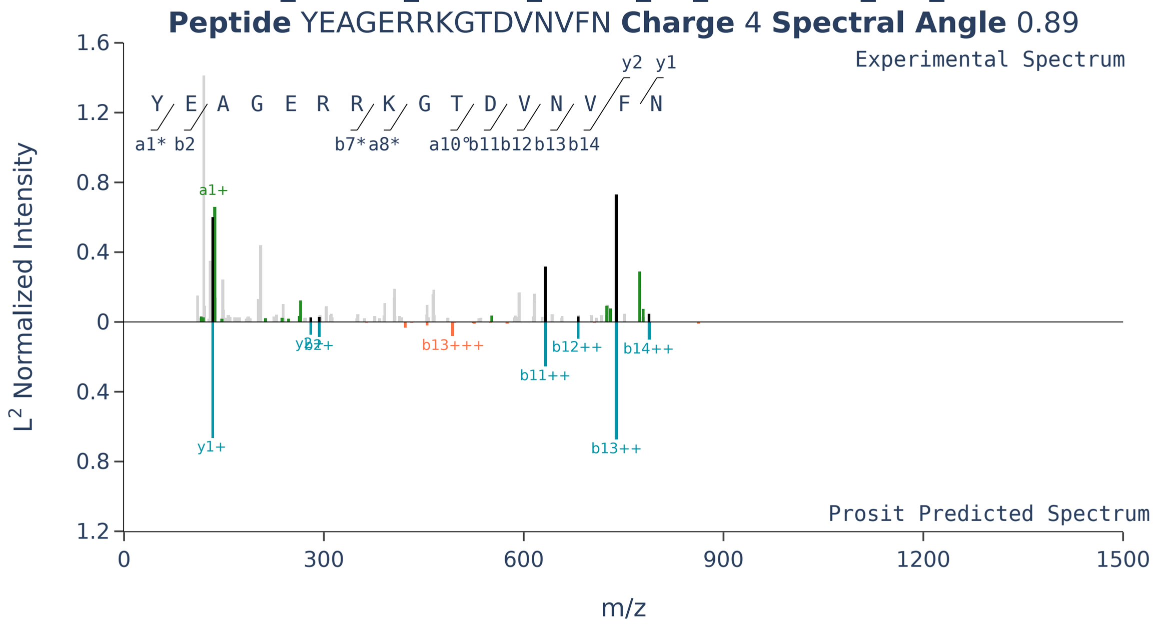


**Source** WSoh_301121_021221_HFGoe_M2_AnnexinA1_24h_R1 **Scan** 9813
**Peptide** HKALMVKGVDEA **Charge** 2 **Spectral Angle** 0.83

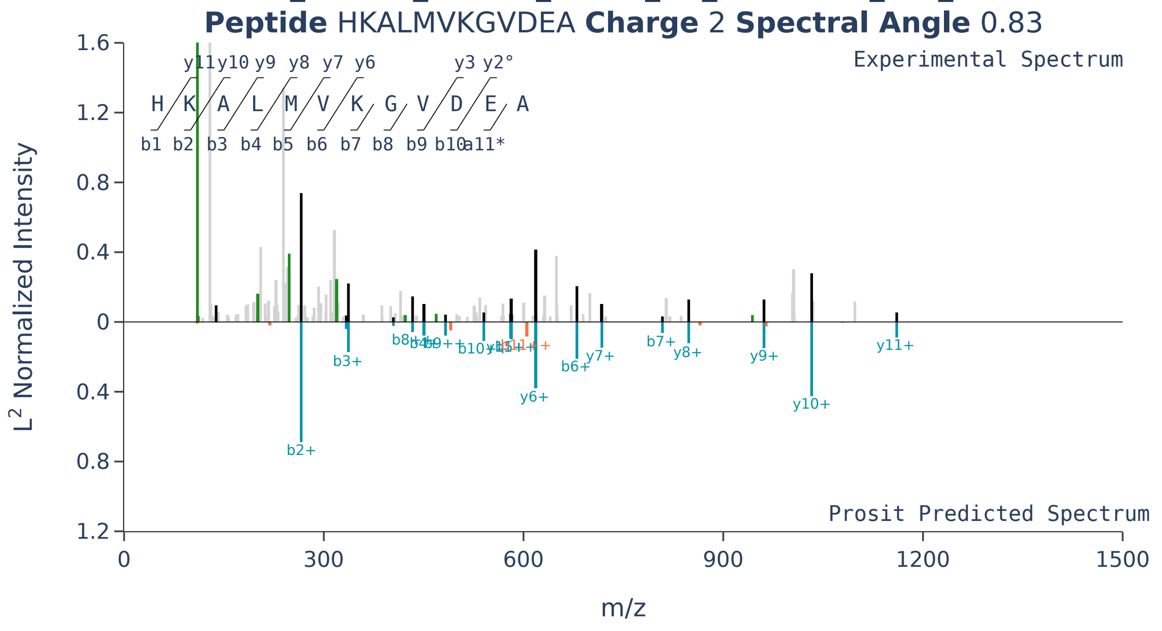


**Source** WSoh_301121_021221_HFGoe_M2_AnnexinA1_2h_R2 **Scan** 14862
**Peptide** QRQQIKAAYLQETGKPLD **Charge** 4 **Spectral Angle** 0.78

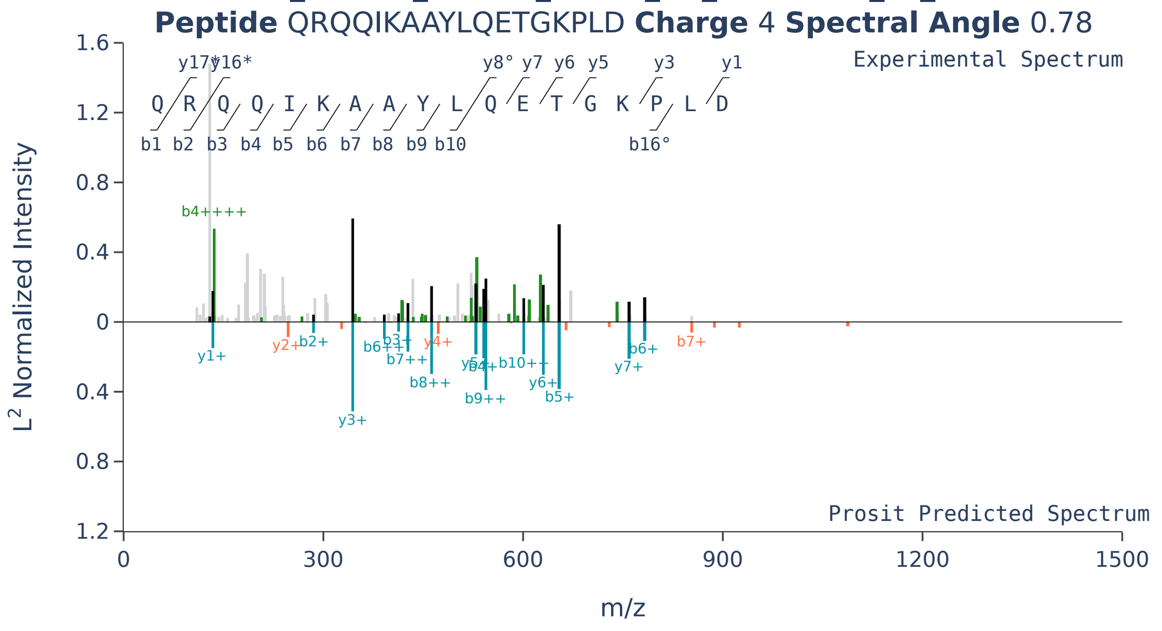

**Source** WSoh_301121_021221_HFGoe_M1_AnnexinA1_24h_R1 **Scan** 8624
**Peptide** DILTKRNNAQRQQIKA **Charge** 3 **Spectral Angle** 0.81

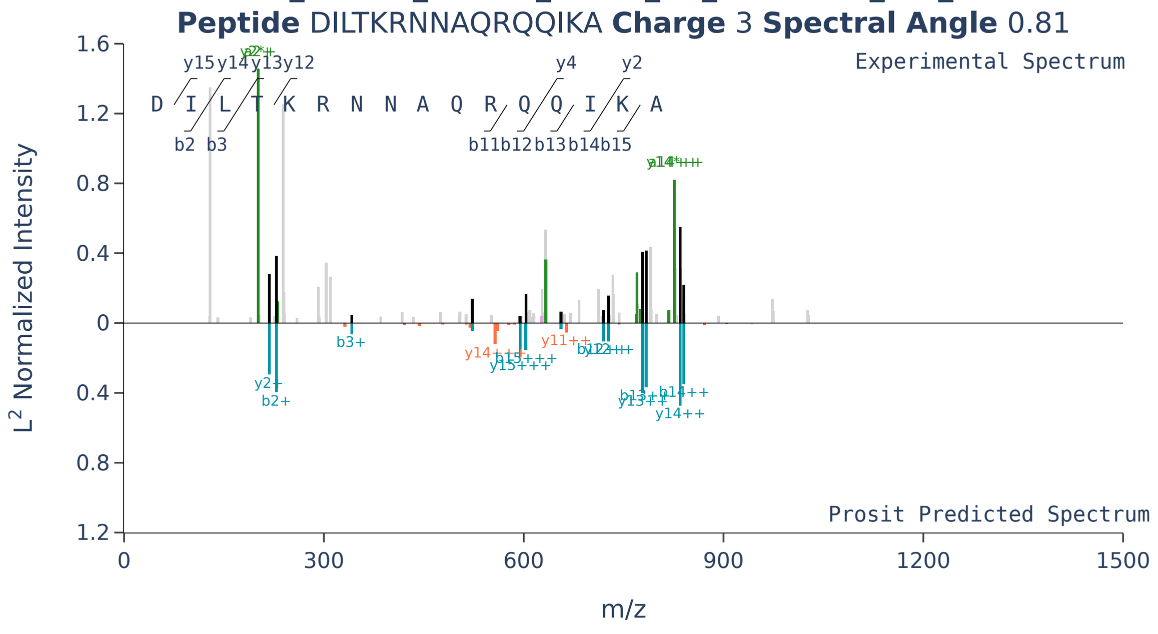


**Source** WSoh_301121_021221_HFGoe_M2_AnnexinA1_2h_R1 **Scan** 23178
**Peptide** AGERRKGTDVNVFNTILT **Charge** 3 **Spectral Angle** 0.8

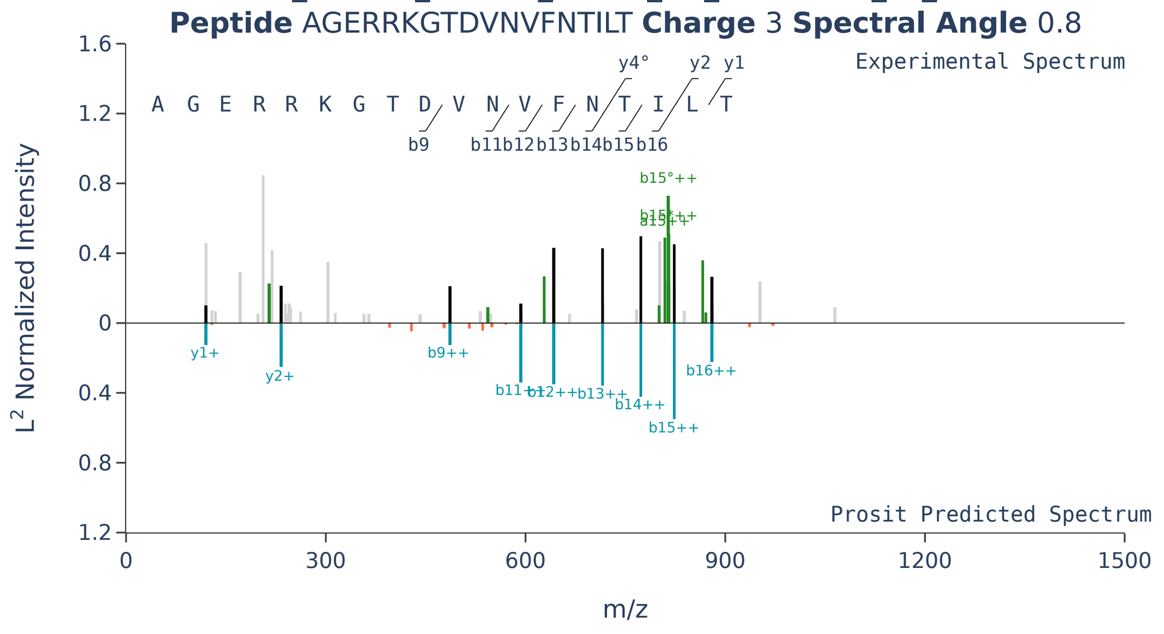


**Source** WSoh_301121_021221_HFGoe_M2_AnnexinA1_4h_R1 **Scan** 25322
**Peptide** AWFIENEEQE **Charge** 2 **Spectral Angle** 0.93

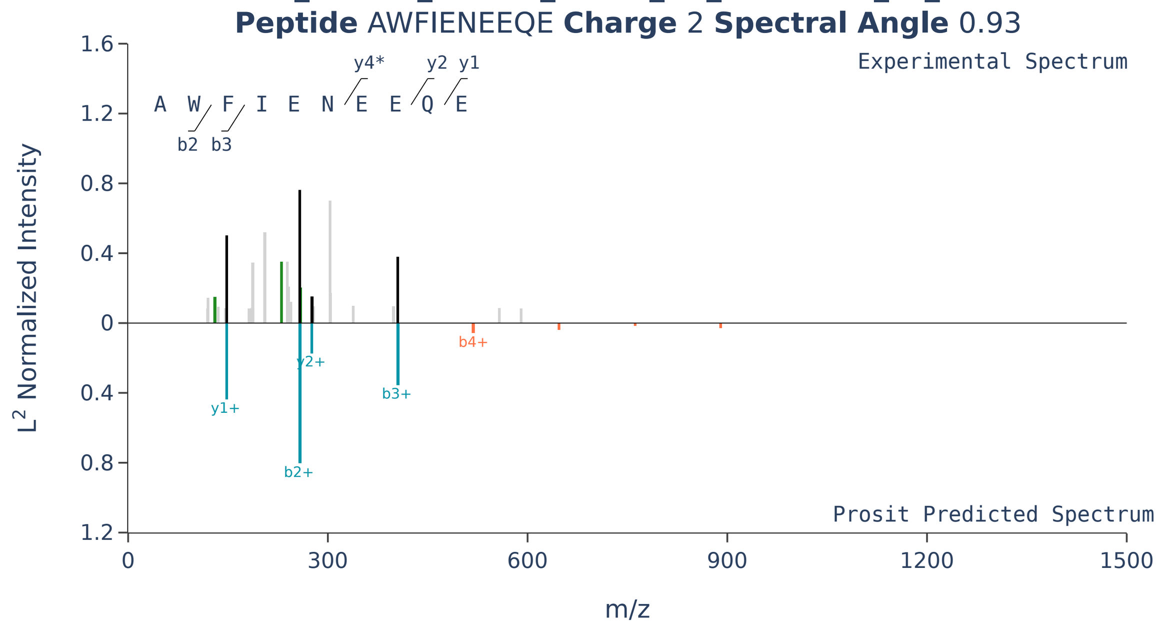


**Source** WSoh_301121_021221_HFGoe_M1_AnnexinA1_4h_R1 **Scan** 8457
**Peptide** DILTKRNNAQRQQIKAA **Charge** 3 **Spectral Angle** 0.77

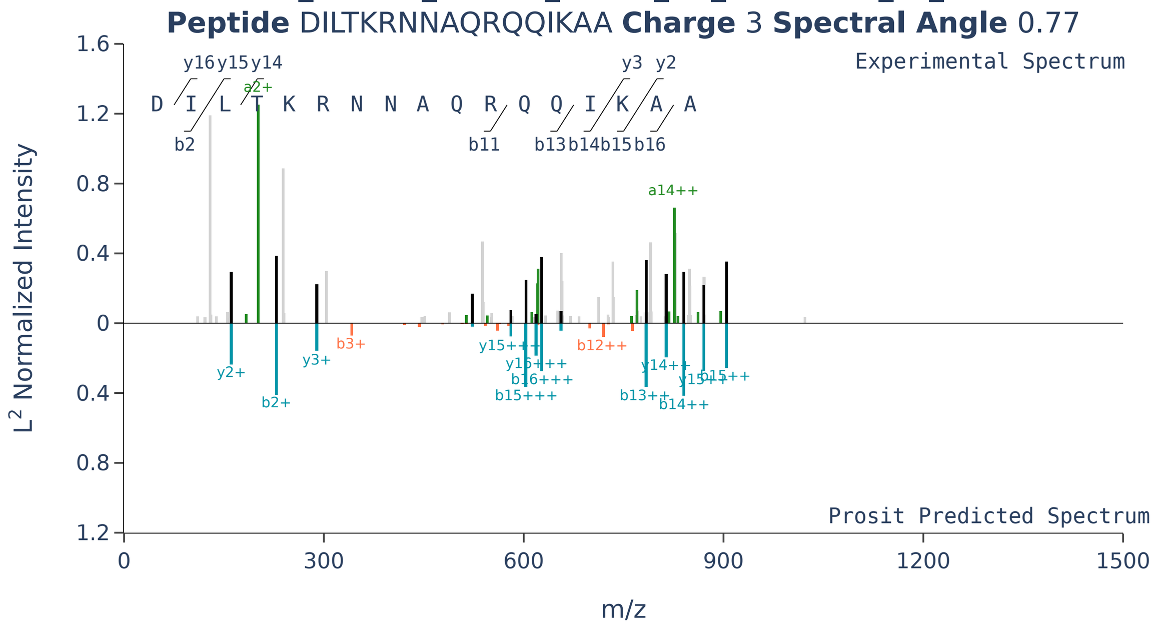


**Source** WSoh_301121_021221_HFGoe_M2_AnnexinA1_2h_R2 **Scan** 11080
**Peptide** RNNAQRQQIKAAYL **Charge** 3 **Spectral Angle** 0.76

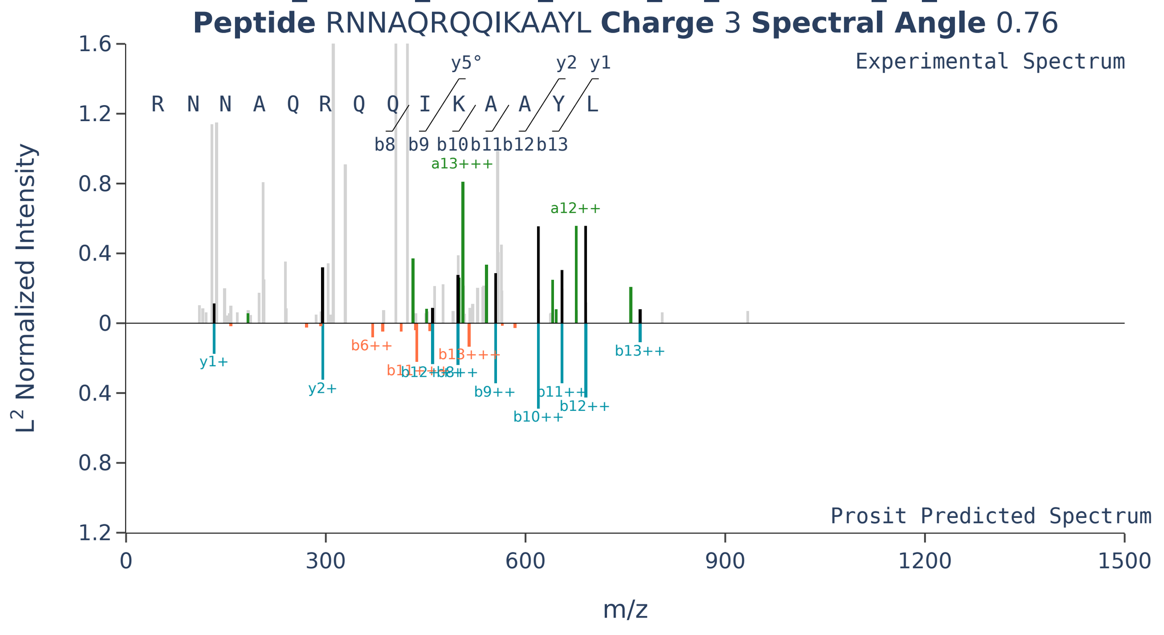


**Source** WSoh_301121_021221_HFGoe_M1_AnnexinA1_24h_R1 **Scan** 5915
**Peptide** ASRTNKEIRDINR **Charge** 4 **Spectral Angle** 0.76

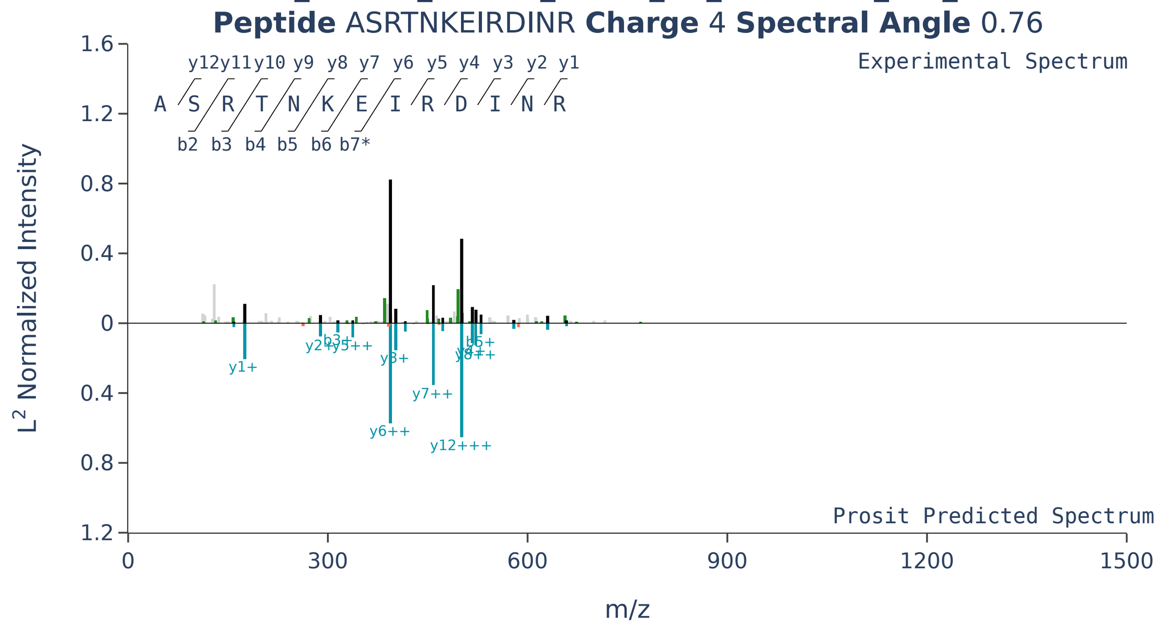


**Source** WSoh_301121_021221_HFGoe_M2_AnnexinA1_2h_R2 **Scan** 27585
**Peptide** EATIIDILTKR **Charge** 3 **Spectral Angle** 0.88

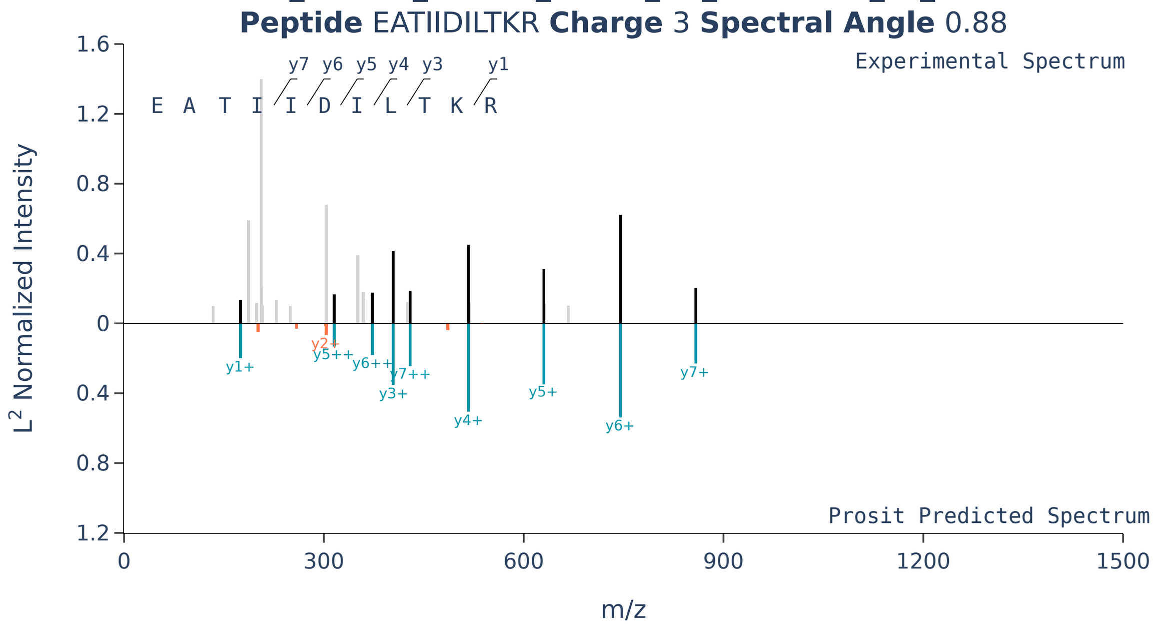


**Source** WSoh_301121_021221_HFGoe_M2_AnnexinA1_2h_R2 **Scan** 18503
**Peptide** VKCATSKPAFFAEKL **Charge** 3 **Spectral Angle** 0.8

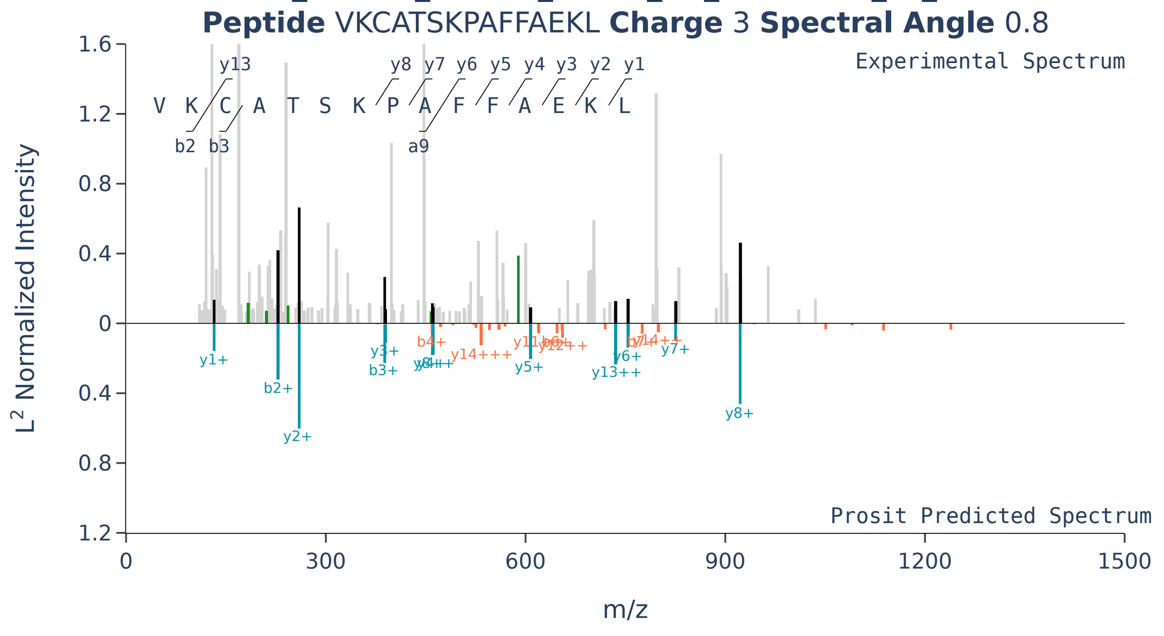


**Source** WSoh_301121_021221_HFGoe_M2_AnnexinA1_2h_R2 **Scan** 24360
**Peptide** PAQFDADELRAAM **Charge** 2 **Spectral Angle** 0.83

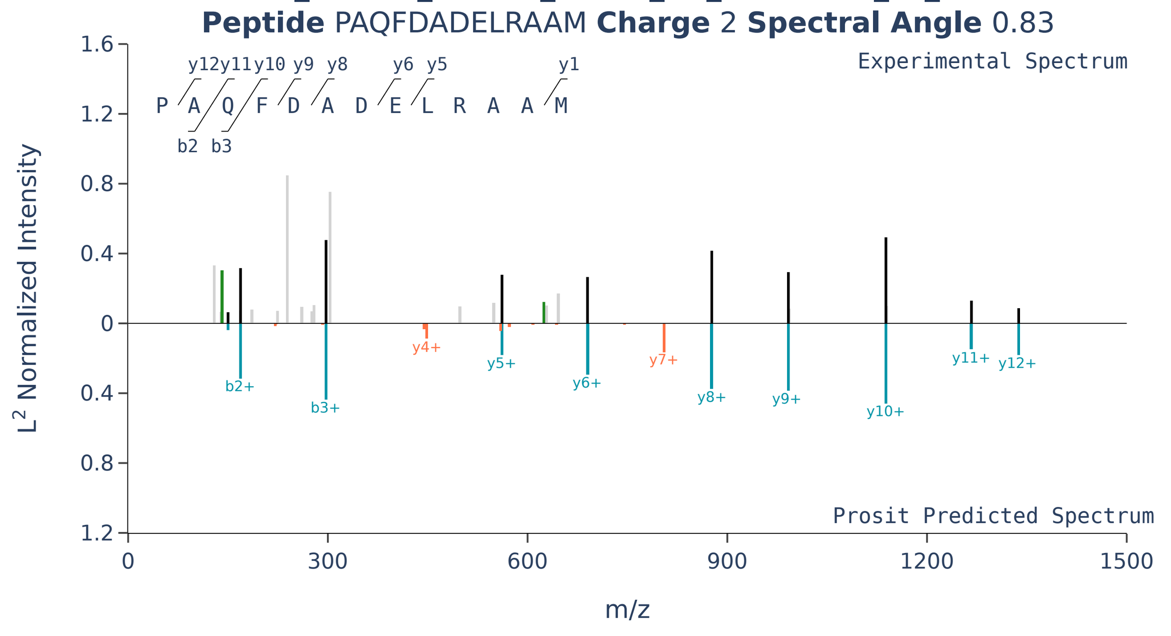


**Source** WSoh_301121_021221_HFGoe_M2_AnnexinA1_2h_R1 **Scan** 15361
**Peptide** NAQRQQIKAAYLQETGKPLD **Charge** 4 **Spectral Angle** 0.76

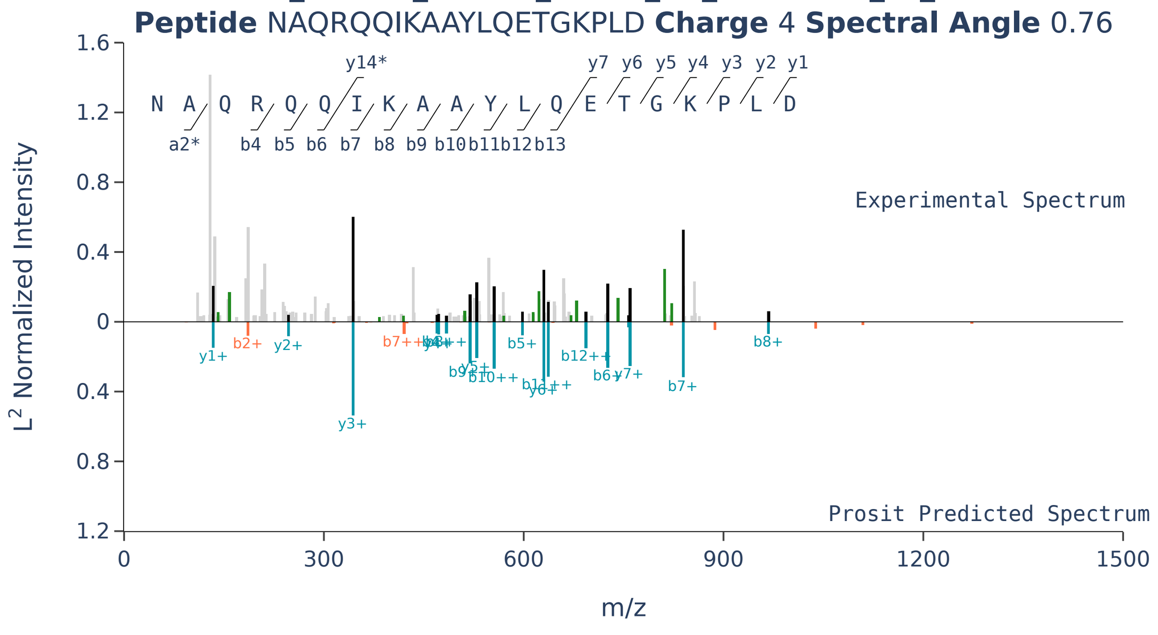


**Source** WSoh_301121_021221_HFGoe_M2_AnnexinA1_2h_R2 **Scan** 15814
**Peptide** TSKPAFFAEKLHQ **Charge** 3 **Spectral Angle** 0.76

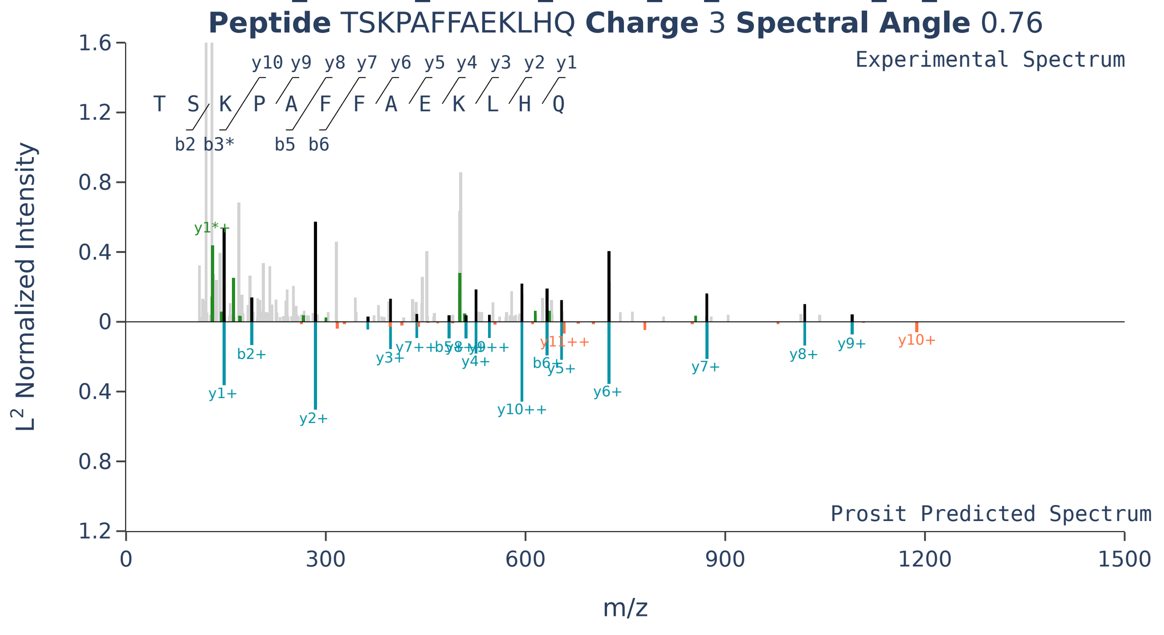


**Source** WSoh_301121_021221_HFGoe_M2_AnnexinA1_2h_R1 **Scan** 19748
**Peptide** FIENEEQEYVQTVKSS **Charge** 3 **Spectral Angle** 0.79

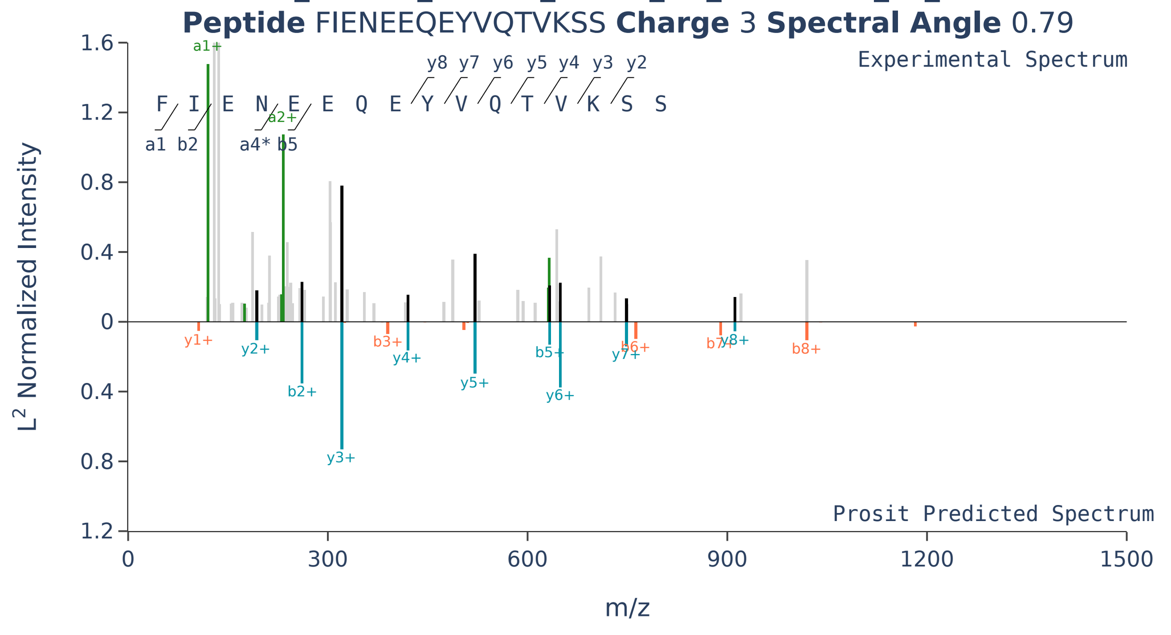


**Source** WSoh_301121_021221_HFGoe_M2_AnnexinA1_4h_R1 **Scan** 20725
**Peptide** TSGDFRNALLS **Charge** 2 **Spectral Angle** 0.78

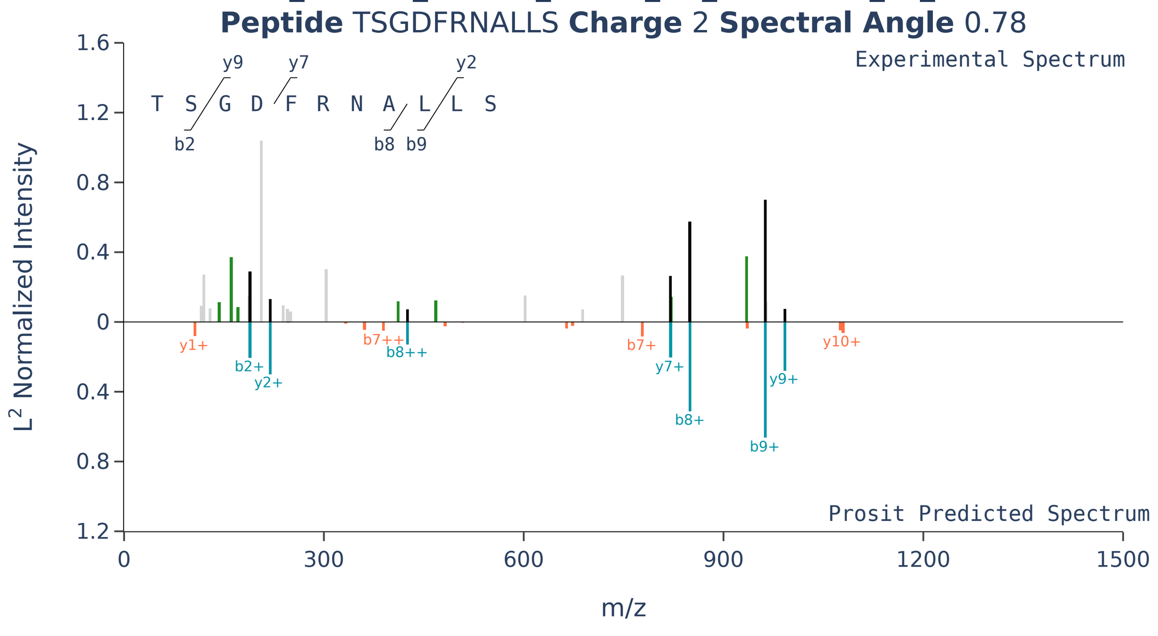


**Source** WSoh_301121_021221_HFGoe_M1_AnnexinA1_24h_R1 **Scan** 11728
**Peptide** GERRKGTDVNVFNT **Charge** 2 **Spectral Angle** 0.79

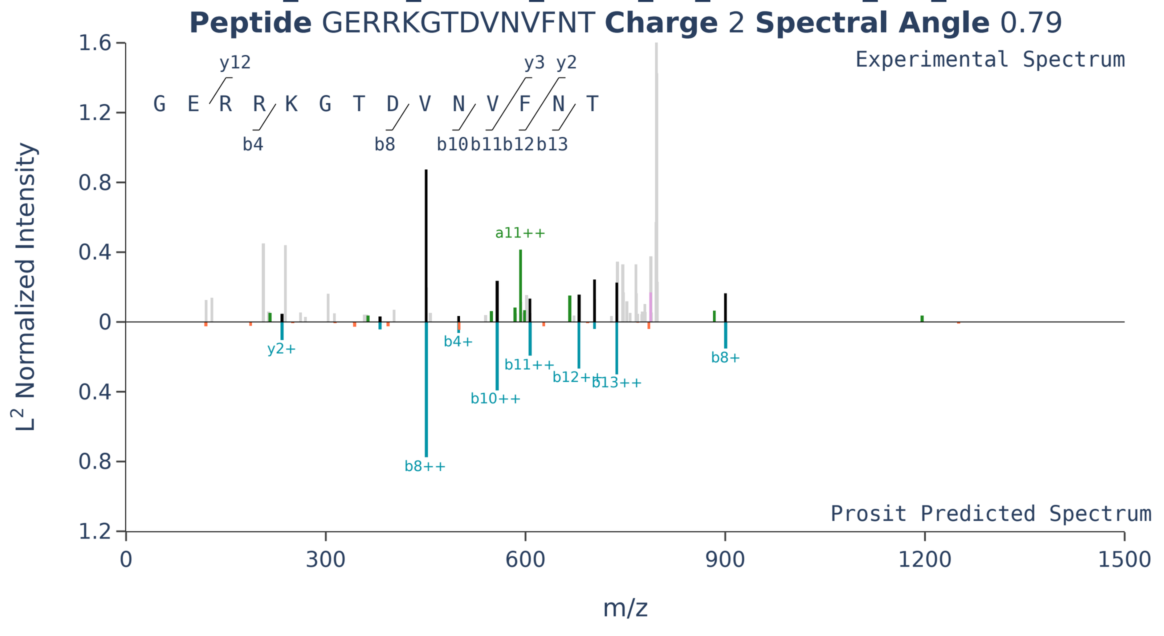


**Source** WSoh_301121_021221_HFGoe_M1_AnnexinA1_24h_R1 **Scan** 31012
**Peptide** ATIIDILT **Charge** 1 **Spectral Angle** 0.83

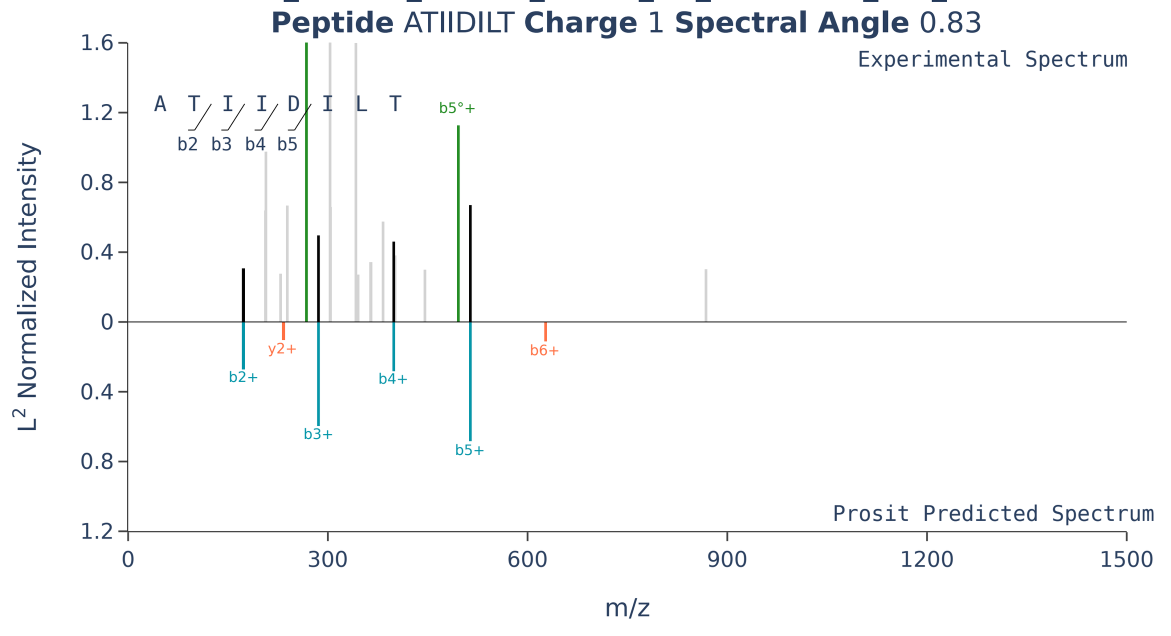

**Source** WSoh_301121_021221_HFGoe_M1_AnnexinA1_24h_R2 **Scan** 5805
**Peptide** SRTNKEIRDINR **Charge** 3 **Spectral Angle** 0.83

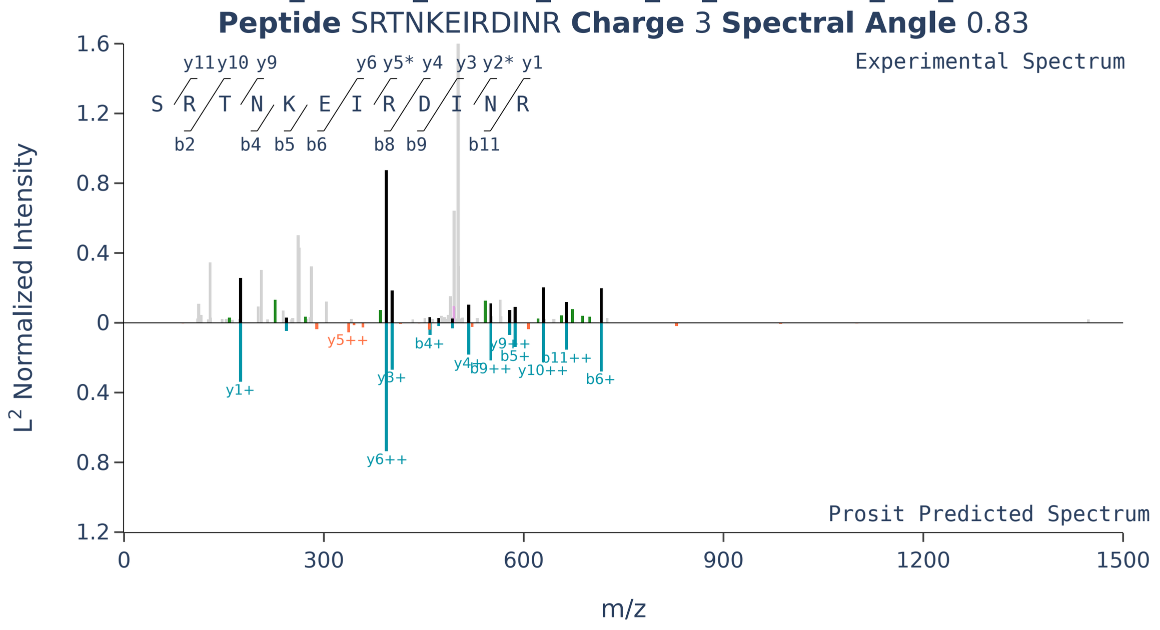


**Source** WSoh_301121_021221_HFGoe_M2_AnnexinA1_2h_R1 **Scan** 25103
**Peptide** KGDYEKILVAL **Charge** 2 **Spectral Angle** 0.58

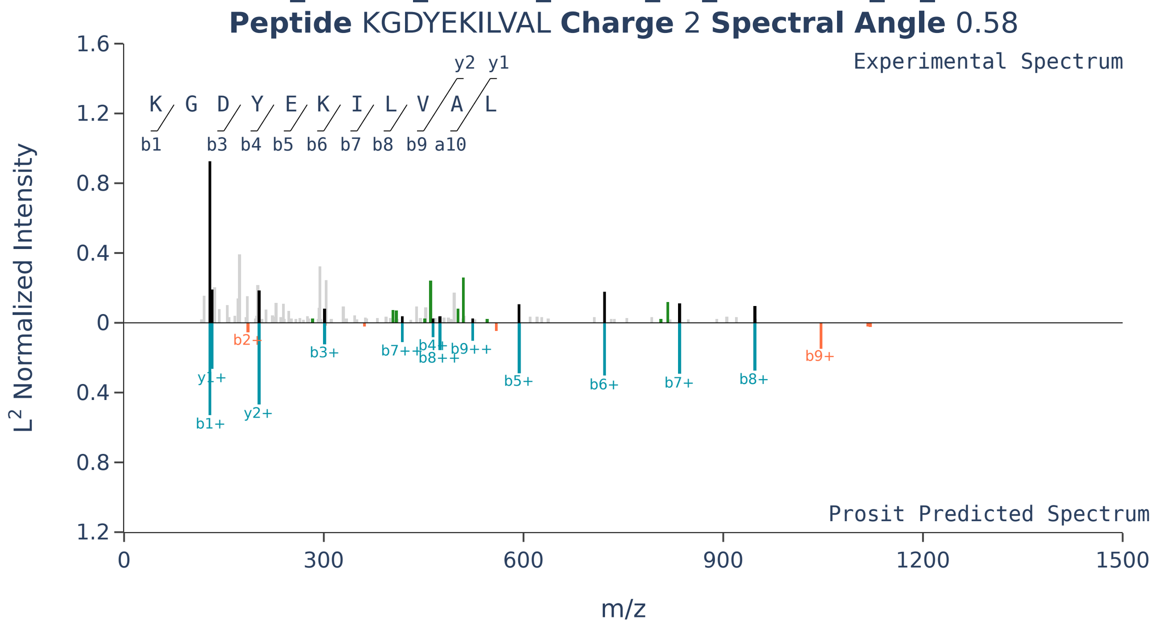


**Source** WSoh_301121_021221_HFGoe_M2_AnnexinA1_4h_R1 **Scan** 6266
**Peptide** FQKYTKYSK **Charge** 2 **Spectral Angle** 0.76

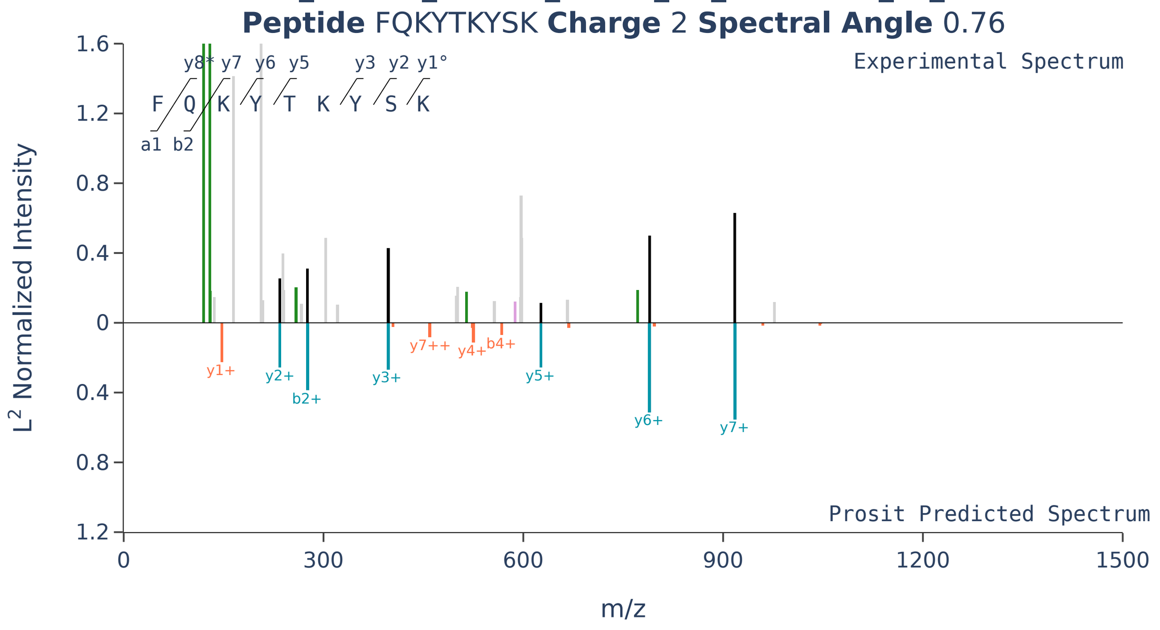


**Source** WSoh_301121_021221_HFGoe_M1_AnnexinA1_24h_R2 **Scan** 9445
**Peptide** KEIRDINRVYREE **Charge** 3 **Spectral Angle** 0.83

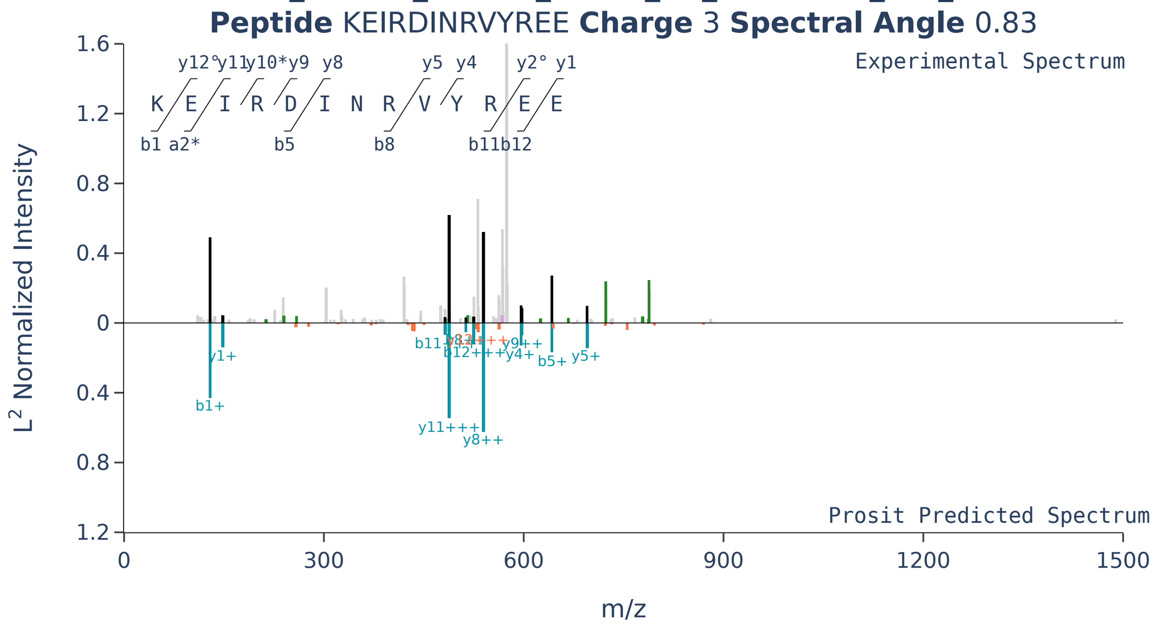


**Source** WSoh_301121_021221_HFGoe_M2_AnnexinA1_2h_R1 **Scan** 19277
**Peptide** YEAGERRKGTDVNVFNTI **Charge** 3 **Spectral Angle** 0.8

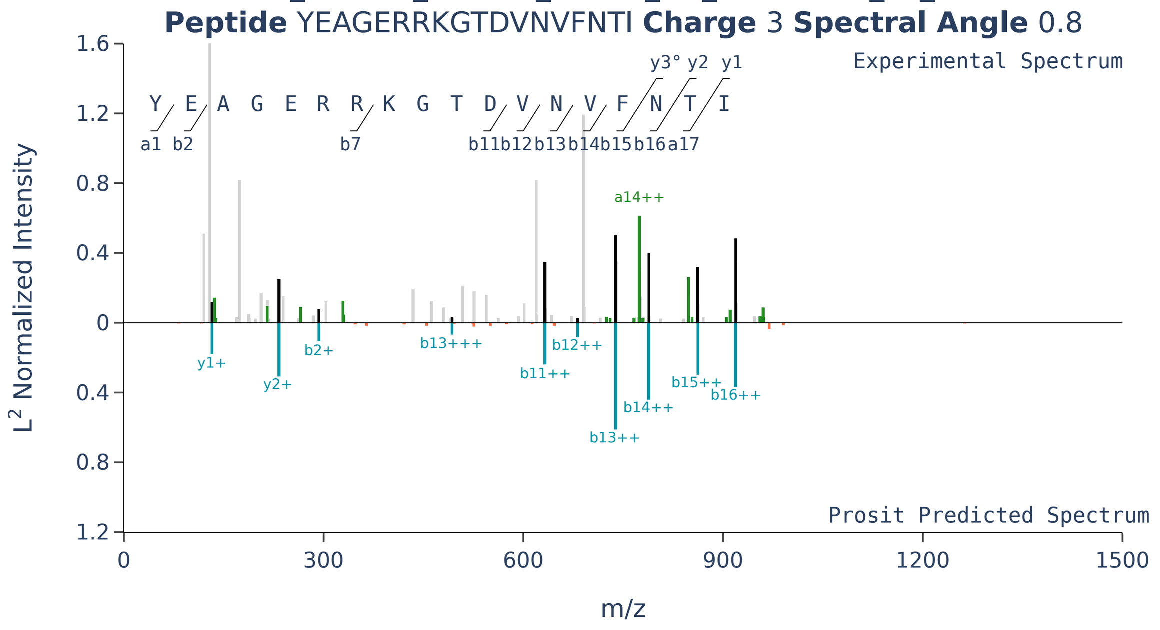


**Source** WSoh_301121_021221_HFGoe_M2_AnnexinA1_24h_R2 **Scan** 5584
**Peptide** YEAGERRKGTDV **Charge** 3 **Spectral Angle** 0.9

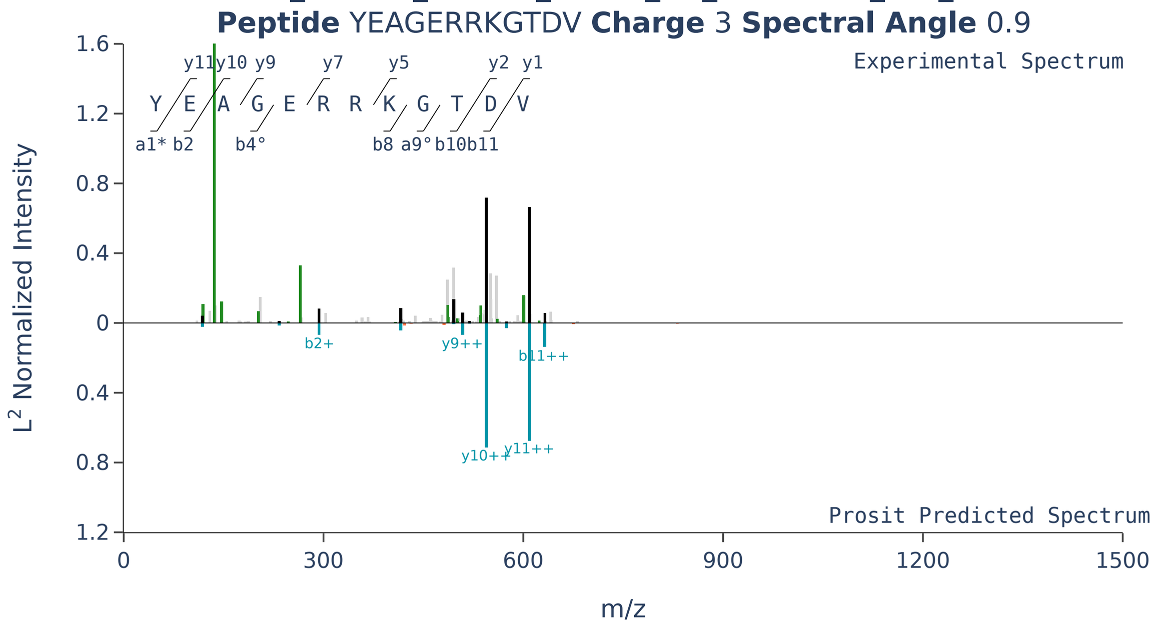


**Source** WSoh_301121_021221_HFGoe_M2_AnnexinA1_24h_R1 **Scan** 21348
**Peptide** SPYPTFNPSSD **Charge** 2 **Spectral Angle** 0.84

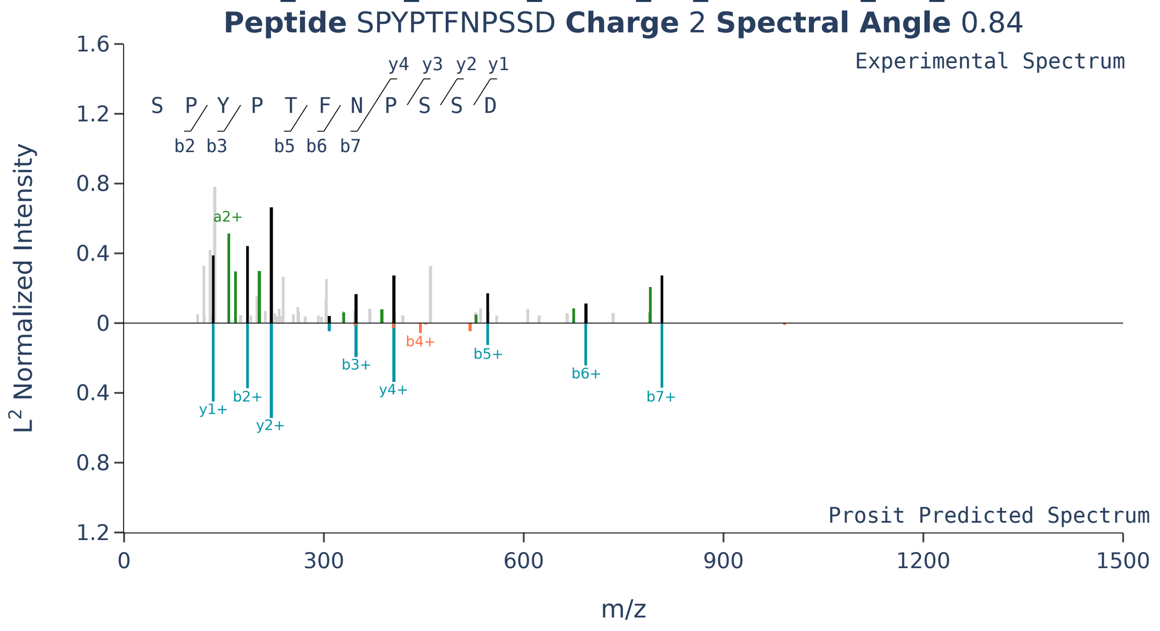


**Source** WSoh_301121_021221_HFGoe_M2_AnnexinA1_2h_R1 **Scan** 6328
**Peptide** MKGVGTRHKALIR **Charge** 3 **Spectral Angle** 0.93

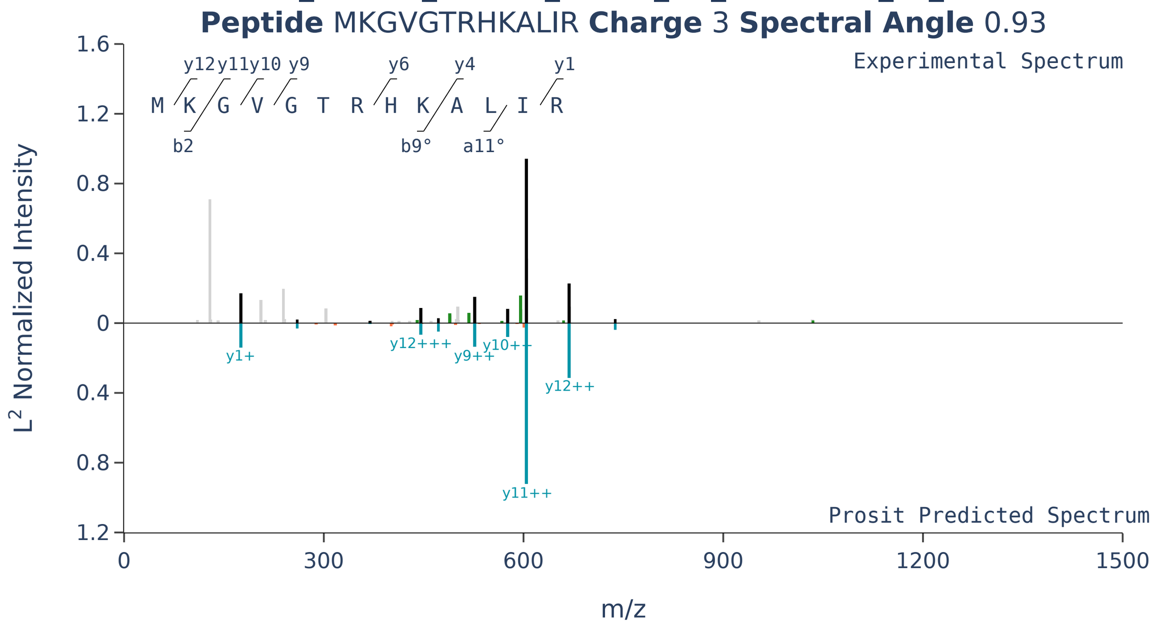


**Source** WSoh_301121_021221_HFGoe_M2_AnnexinA1_2h_R2 **Scan** 17483
**Peptide** QETGKPLDETLKKAL **Charge** 3 **Spectral Angle** 0.8

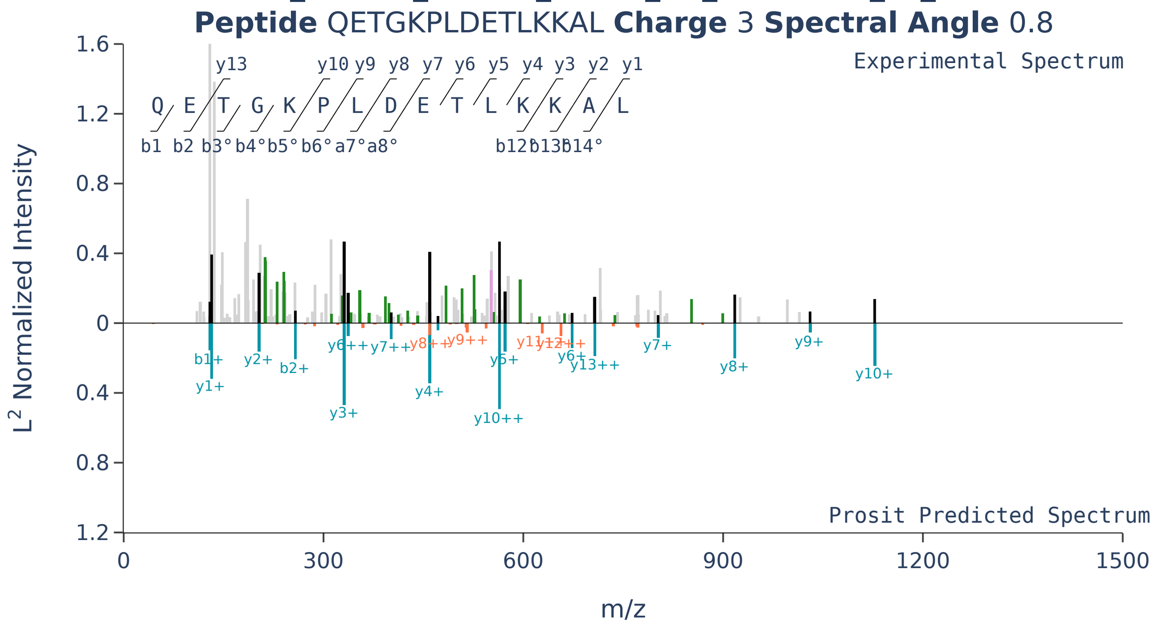


**Source** WSoh_301121_021221_HFGoe_M1_AnnexinA1_24h_R1 **Scan** 15521
**Peptide** EDFGVNE **Charge** 1 **Spectral Angle** 0.65

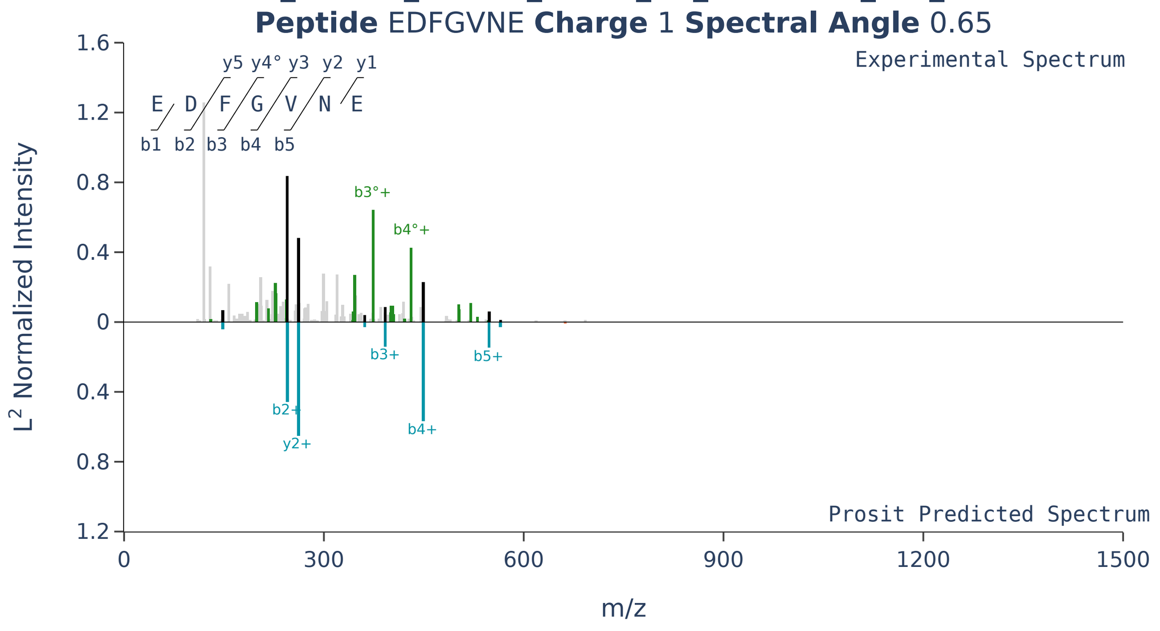


**Source** WSoh_301121_021221_HFGoe_M1_AnnexinA1_24h_R2 **Scan** 10541
**Peptide** VSRSEIDMN **Charge** 2 **Spectral Angle** 0.87

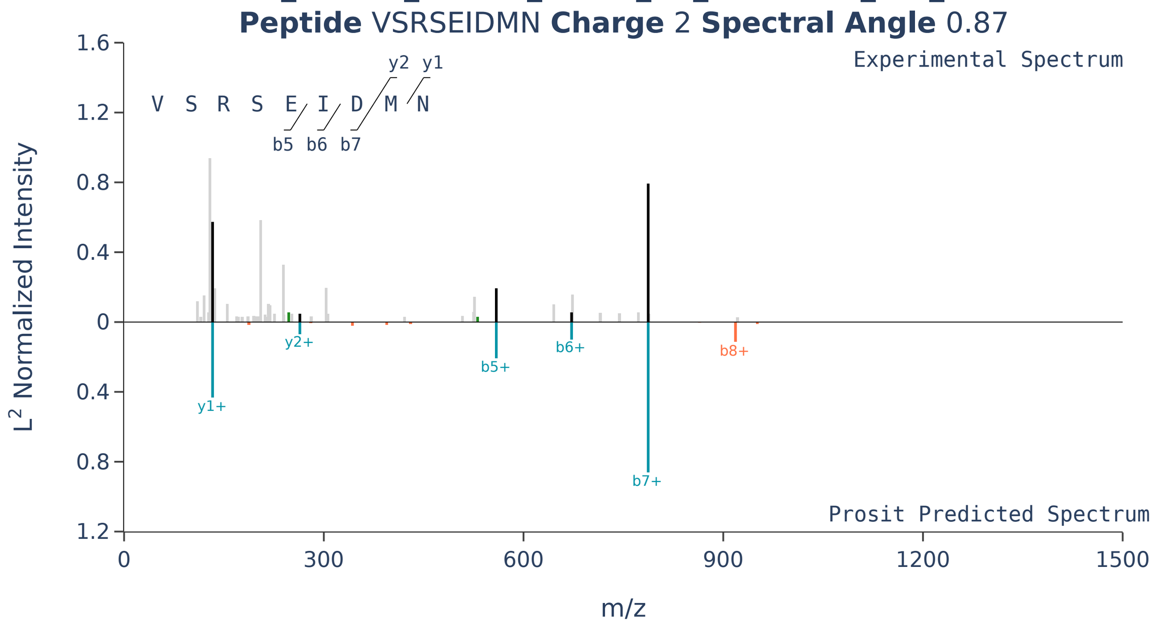


**Source** WSoh_301121_021221_HFGoe_M2_AnnexinA1_2h_R1 **Scan** 12808
**Peptide** QRQQIKAAYLQETG **Charge** 3 **Spectral Angle** 0.74

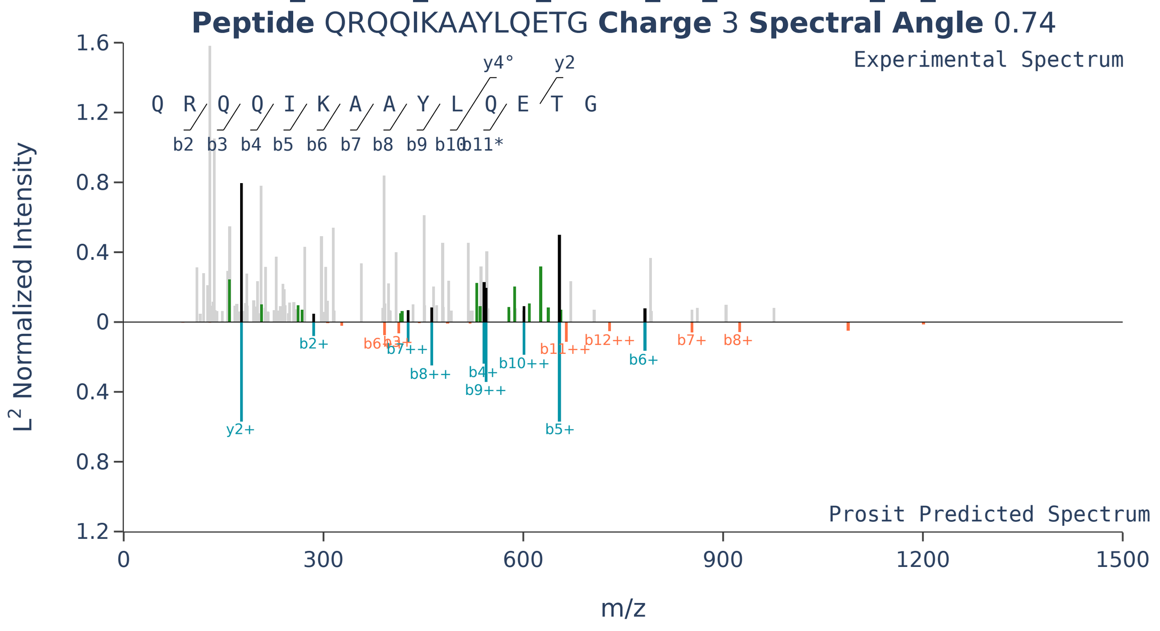


**Source** WSoh_301121_021221_HFGoe_M1_AnnexinA1_24h_R1 **Scan** 23108
**Peptide** AMKGLGTDEDTLI **Charge** 2 **Spectral Angle** 0.75

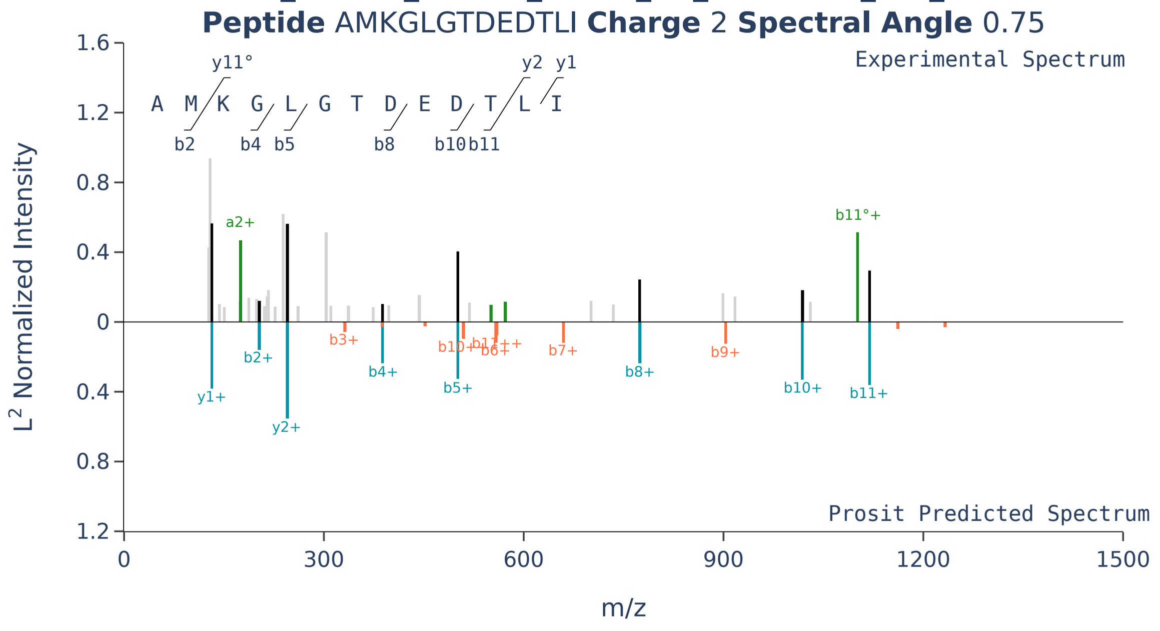


**Source** WSoh_301121_021221_HFGoe_M2_AnnexinA1_4h_R2 **Scan** 12833
**Peptide** AALHKAIMVKGVDE **Charge** 3 **Spectral Angle** 0.8

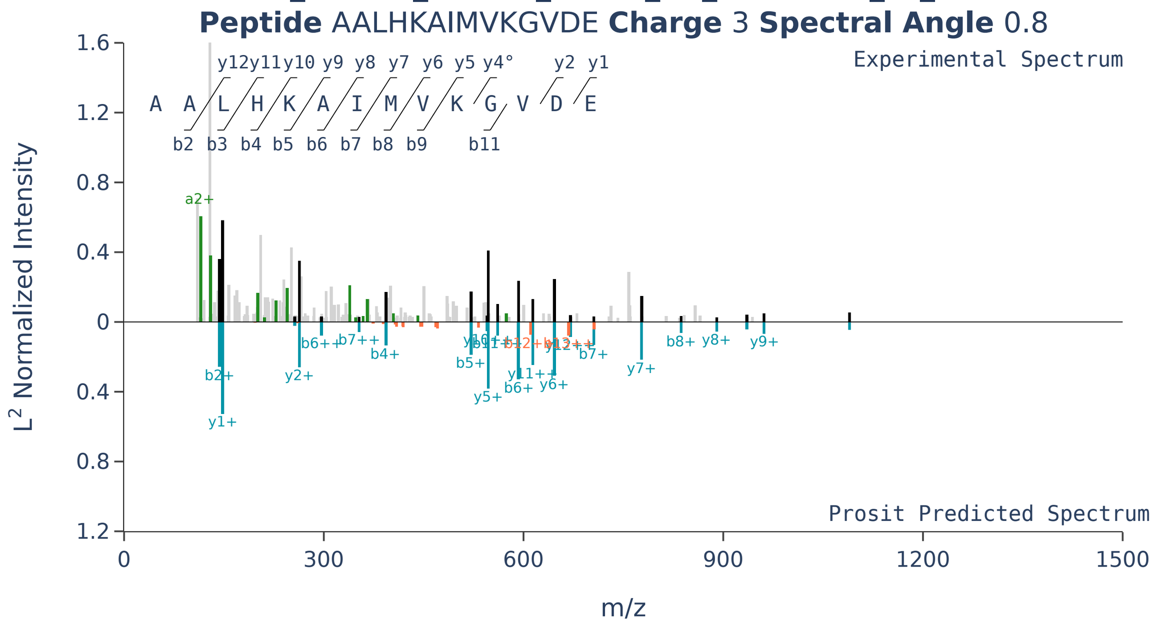


**Source** WSoh_301121_021221_HFGoe_M2_AnnexinA1_2h_R1 **Scan** 15201
**Peptide** LSLAKGDRSEDF **Charge** 3 **Spectral Angle** 0.74

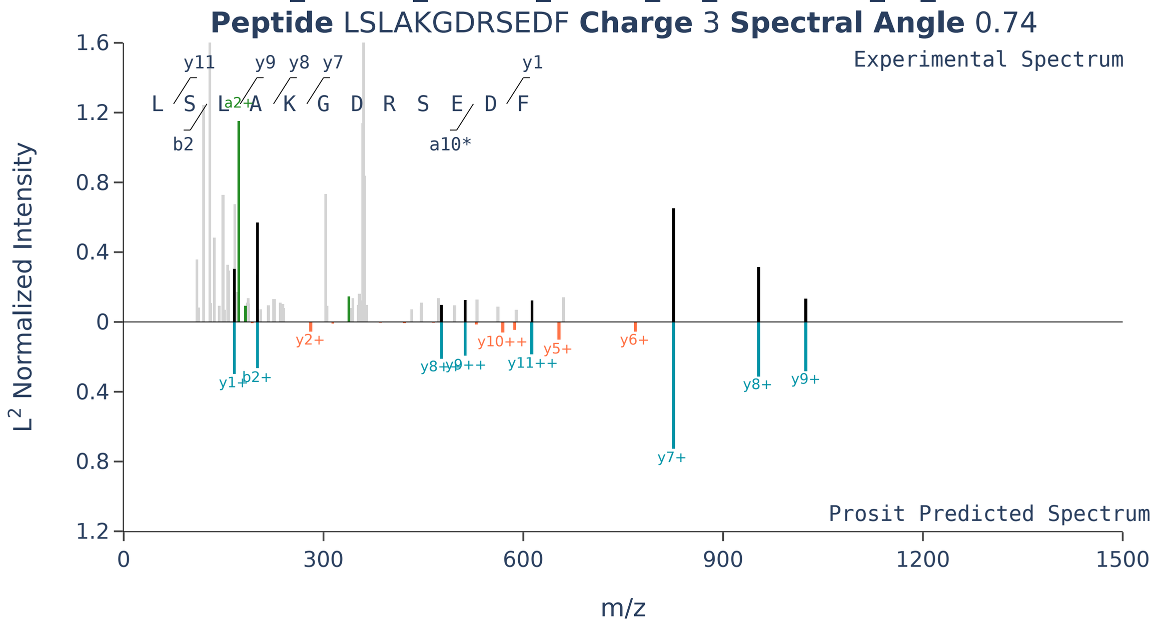

**Source** WSoh_301121_021221_HFGoe_M2_AnnexinA1_2h_R2 **Scan** 17281
**Peptide** QIKAAYLQETGKPLD **Charge** 3 **Spectral Angle** 0.82

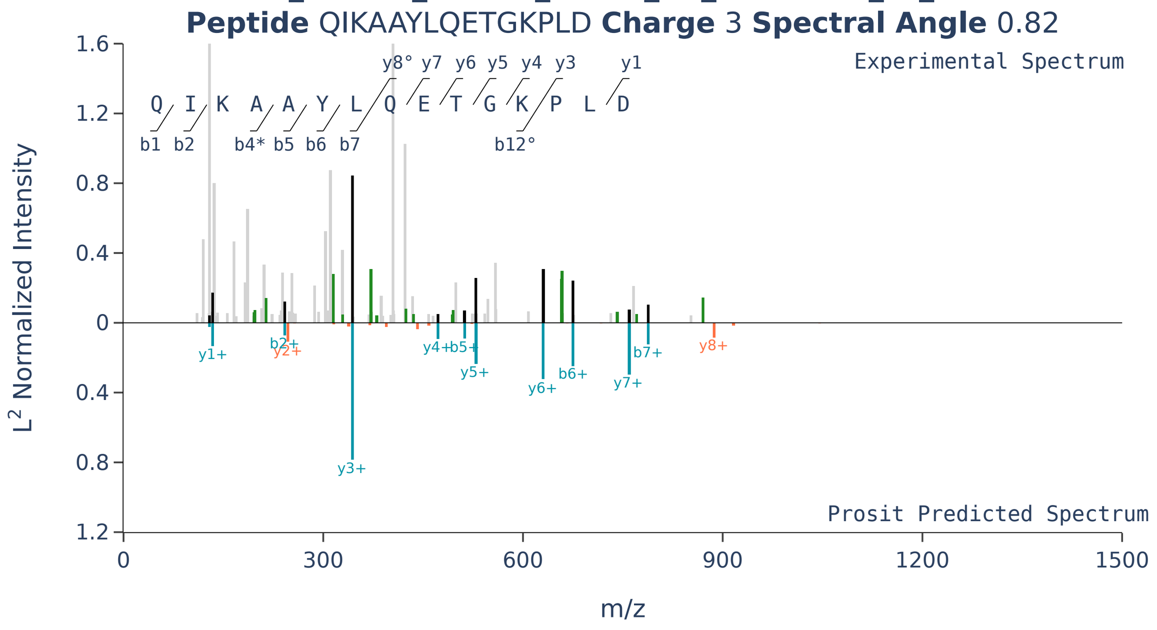


**Source** WSoh_301121_021221_HFGoe_M2_AnnexinA1_24h_R2 **Scan** 7596
**Peptide** KAAYLQETGK **Charge** 2 **Spectral Angle** 0.7

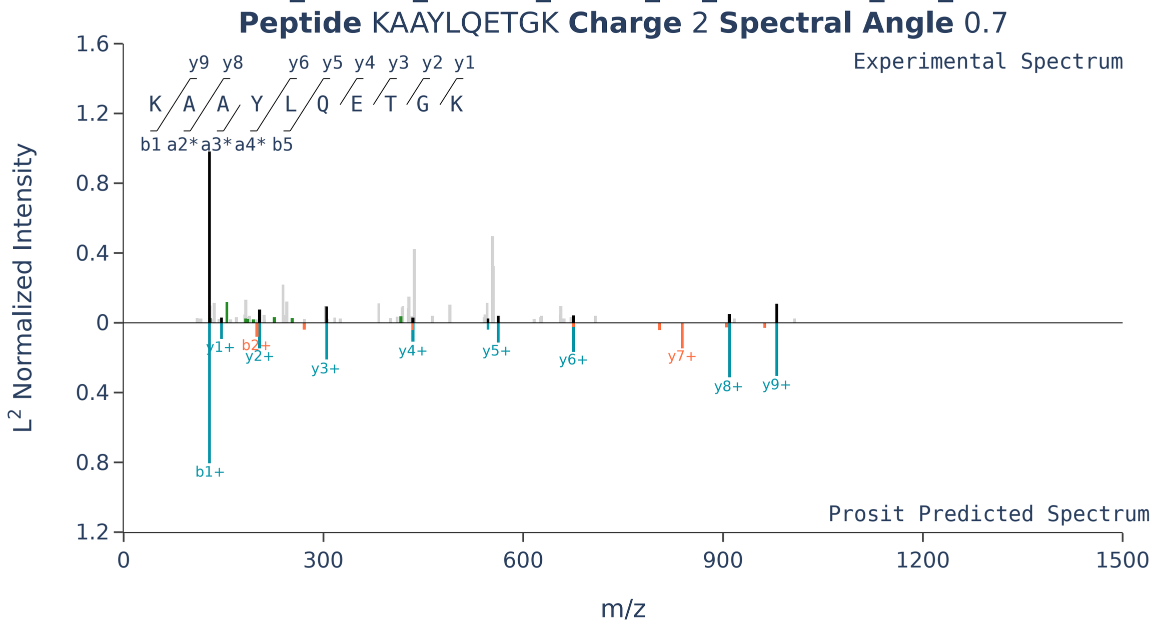


**Source** WSoh_301121_021221_HFGoe_M2_AnnexinA1_24h_R1 **Scan** 11113
**Peptide** LAKGDRSEDFGVN **Charge** 2 **Spectral Angle** 0.75

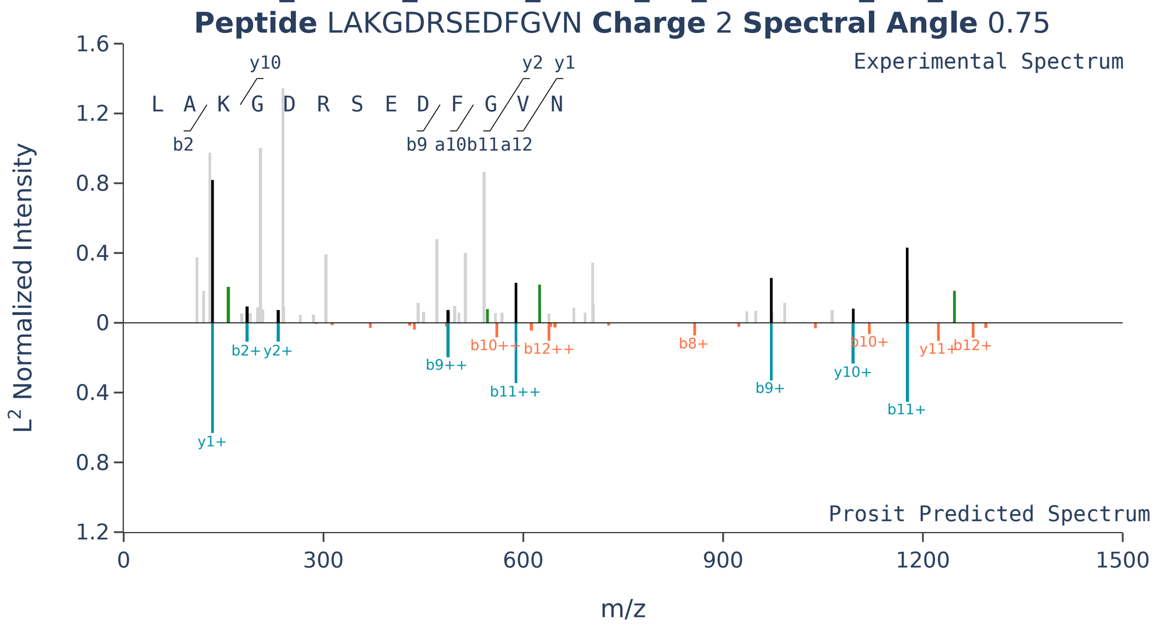


**Source** WSoh_301121_021221_HFGoe_M2_AnnexinA1_2h_R1 **Scan** 25229
**Peptide** TKGDYEKILVAL **Charge** 3 **Spectral Angle** 0.84

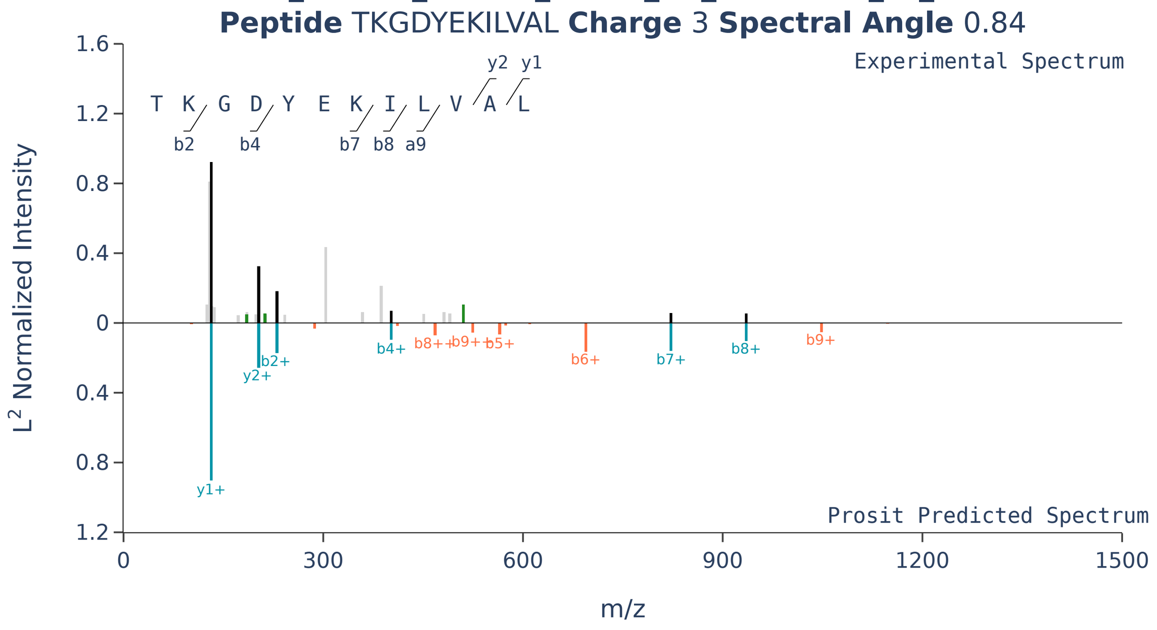


**Source** WSoh_301121_021221_HFGoe_M2_AnnexinA1_2h_R2 **Scan** 17432
**Peptide** EIRDINRVYREEL **Charge** 3 **Spectral Angle** 0.74

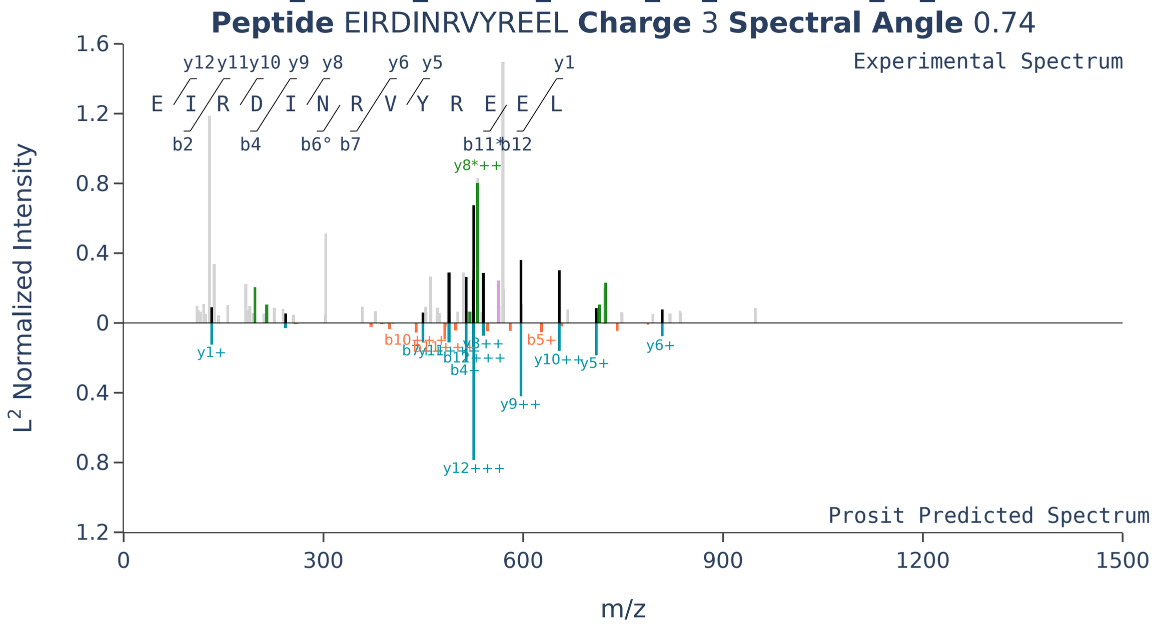


**Source** WSoh_301121_021221_HFGoe_M1_AnnexinA1_2h_R1 **Scan** 19079
**Peptide** DLKAFYQ **Charge** 2 **Spectral Angle** 0.83

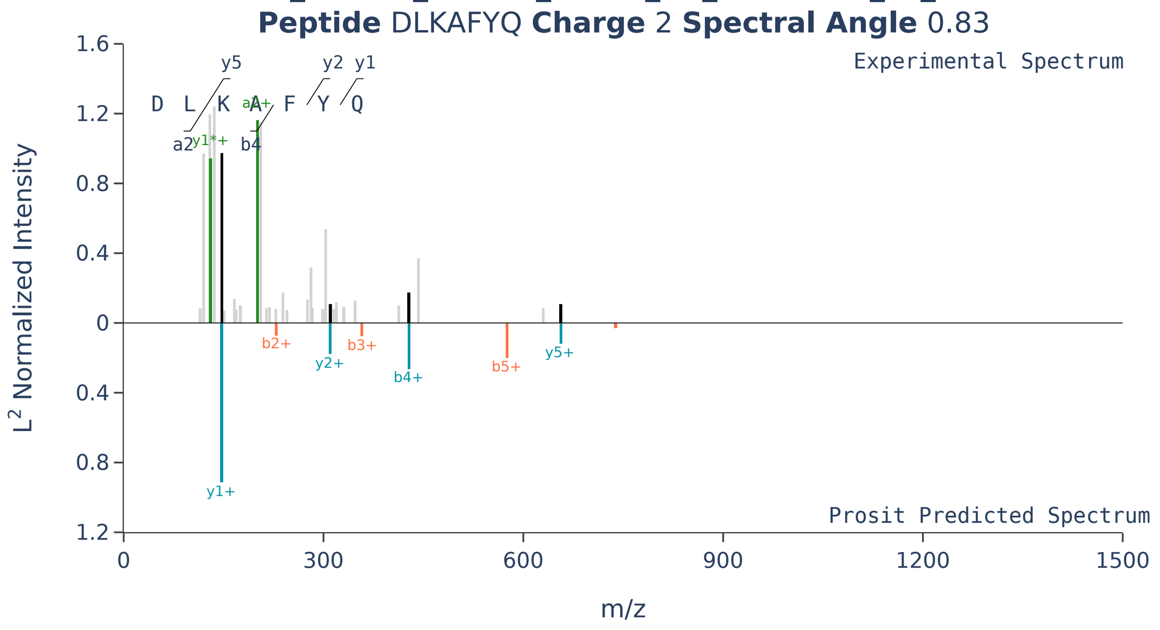


**Source** WSoh_301121_021221_HFGoe_M1_AnnexinA1_24h_R2 **Scan** 7250
**Peptide** YEAGERRKGTDVNV **Charge** 3 **Spectral Angle** 0.82

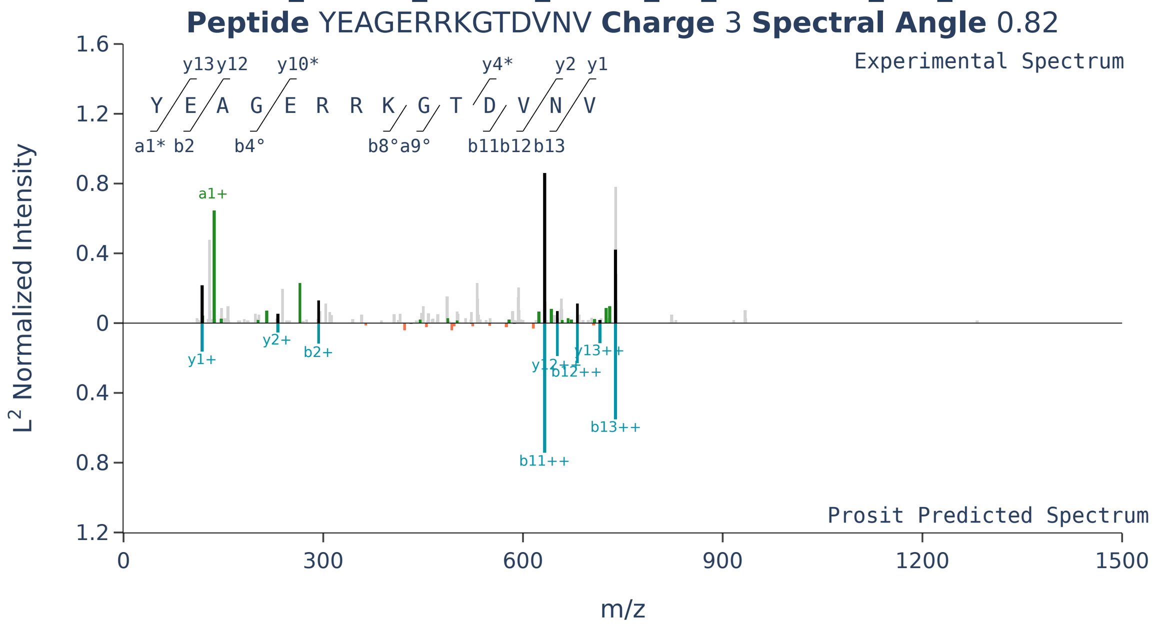


**Source** WSoh_301121_021221_HFGoe_M2_AnnexinA1_24h_R2 **Scan** 9968
**Peptide** HDMNKVL **Charge** 2 **Spectral Angle** 0.78

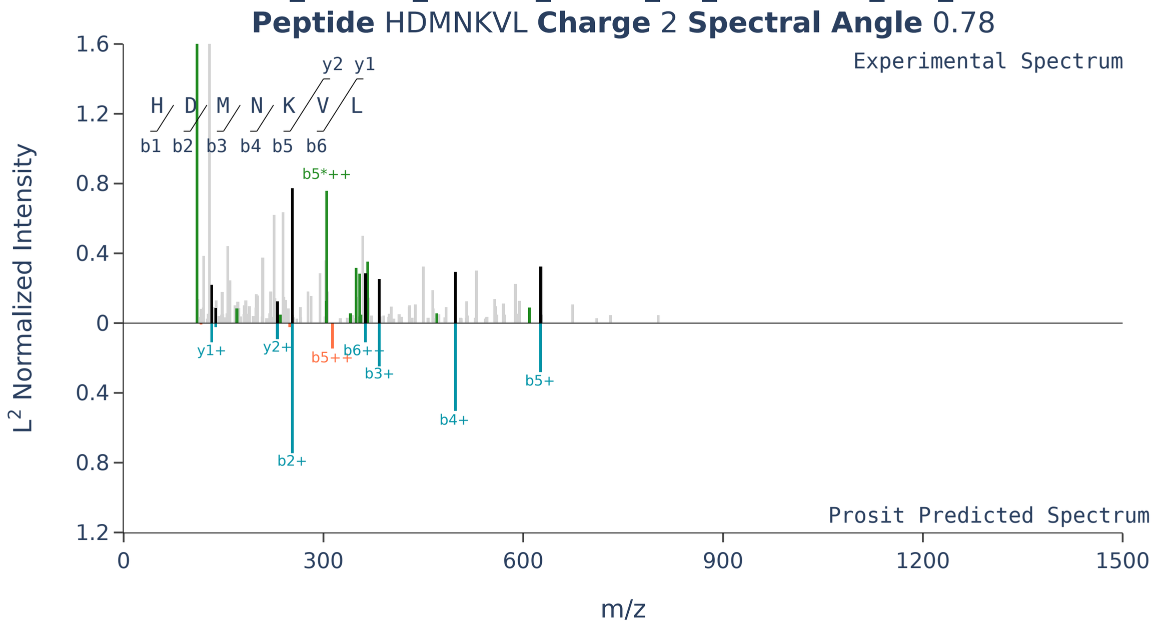


**Source** WSoh_301121_021221_HFGoe_M1_AnnexinA1_2h_R1 **Scan** 19762
**Peptide** KGGPGSAVSPYPTFN **Charge** 2 **Spectral Angle** 0.87

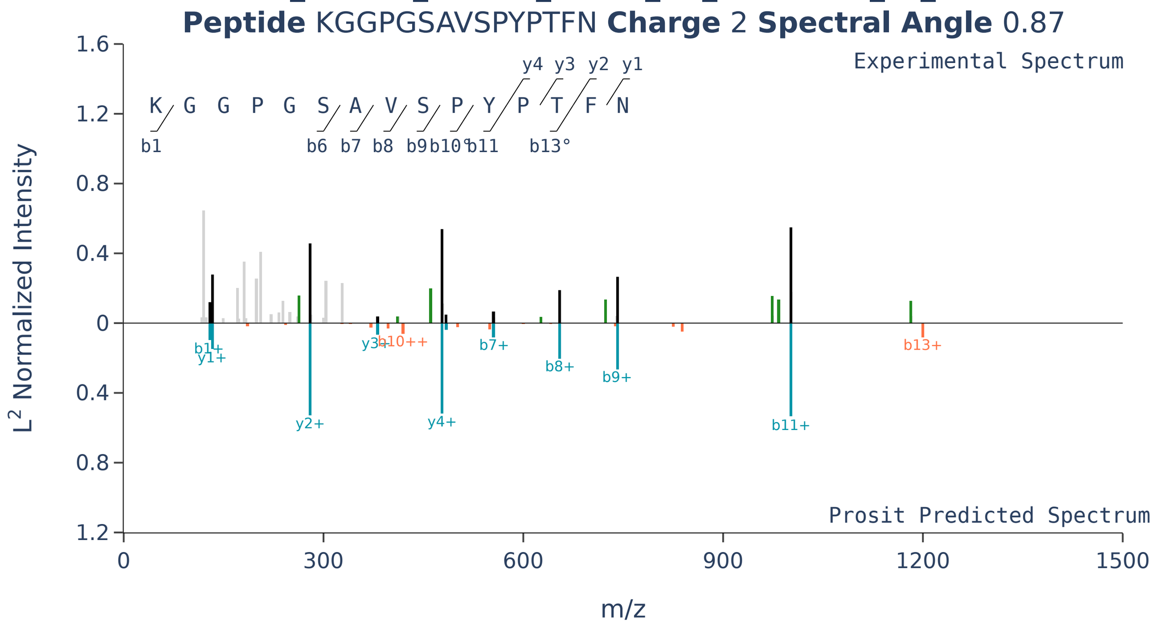


**Source** WSoh_301121_021221_HFGoe_M2_AnnexinA1_1h_R1 **Scan** 9356
**Peptide** EQEYVQTVKSSKGGPG **Charge** 3 **Spectral Angle** 0.75

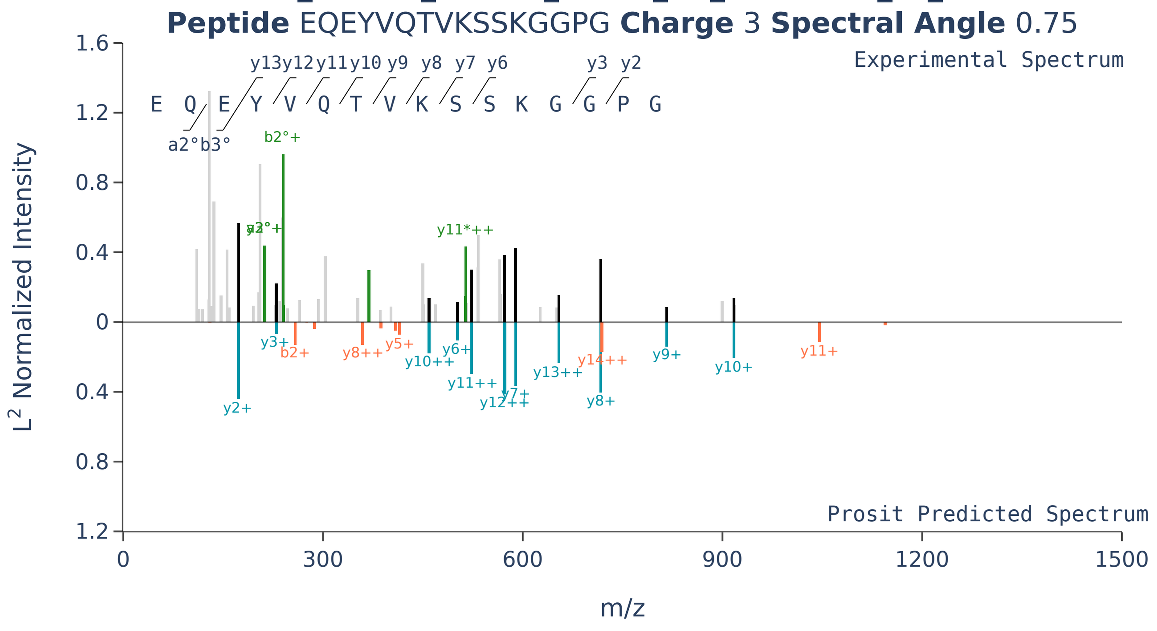


**Source** WSoh_301121_021221_HFGoe_M2_AnnexinA1_2h_R1 **Scan** 18786
**Peptide** ERRKGTDVNVFNTI **Charge** 3 **Spectral Angle** 0.75

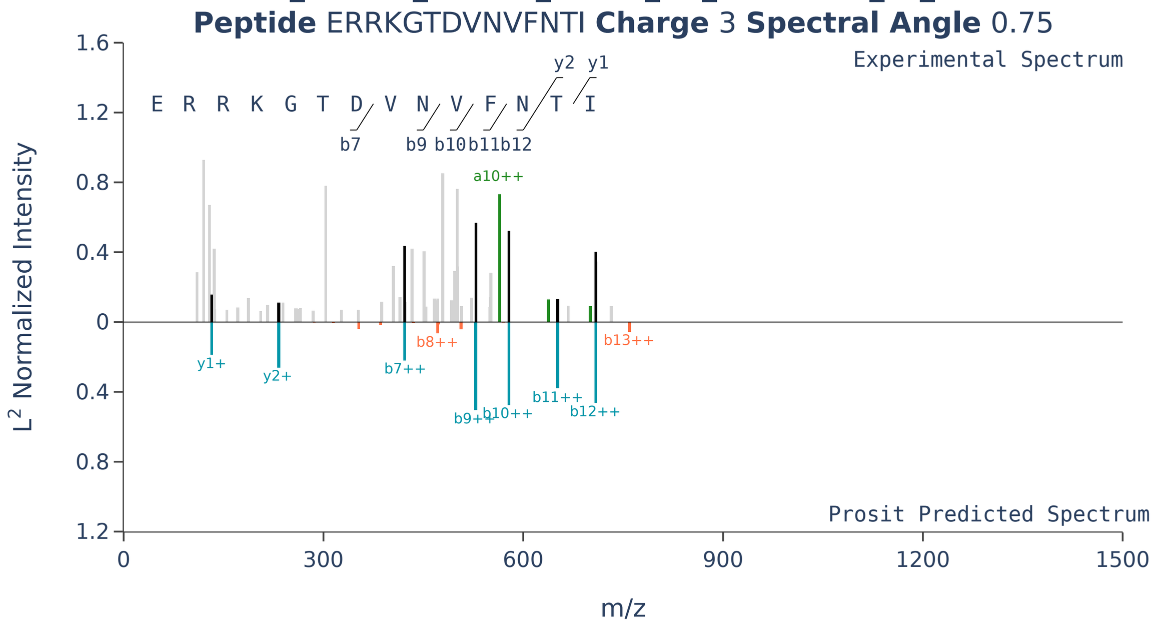


**Source** WSoh_301121_021221_HFGoe_M2_AnnexinA1_1h_R1 **Scan** 18051
**Peptide** KKALTGHLEEVVLA **Charge** 3 **Spectral Angle** 0.69

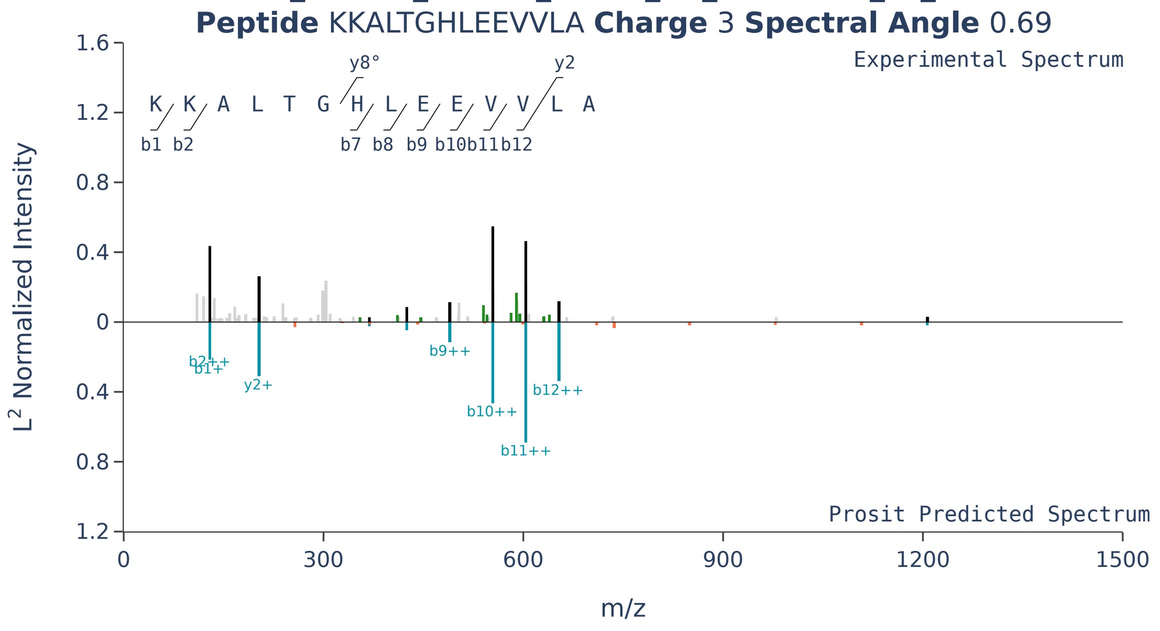


**Source** WSoh_301121_021221_HFGoe_M2_AnnexinA1_24h_R2 **Scan** 13882
**Peptide** EELKRDLAKDITSD **Charge** 2 **Spectral Angle** 0.7

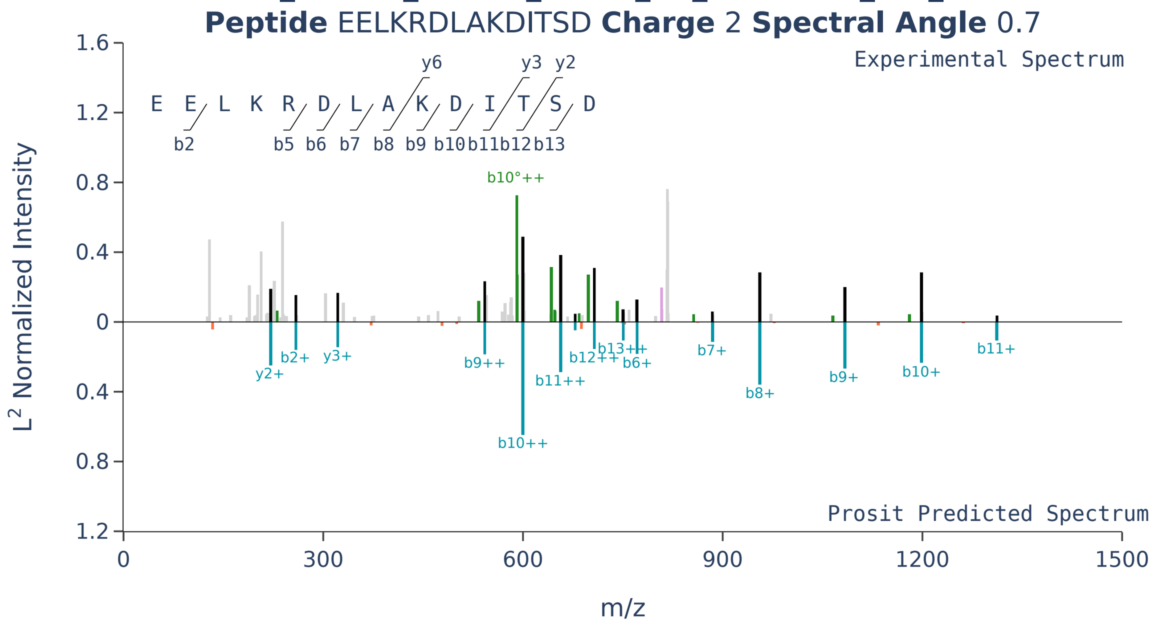


**Source** WSoh_301121_021221_HFGoe_M1_AnnexinA1_24h_R1 **Scan** 10954
**Peptide** AAMKGLGTDE **Charge** 2 **Spectral Angle** 0.79

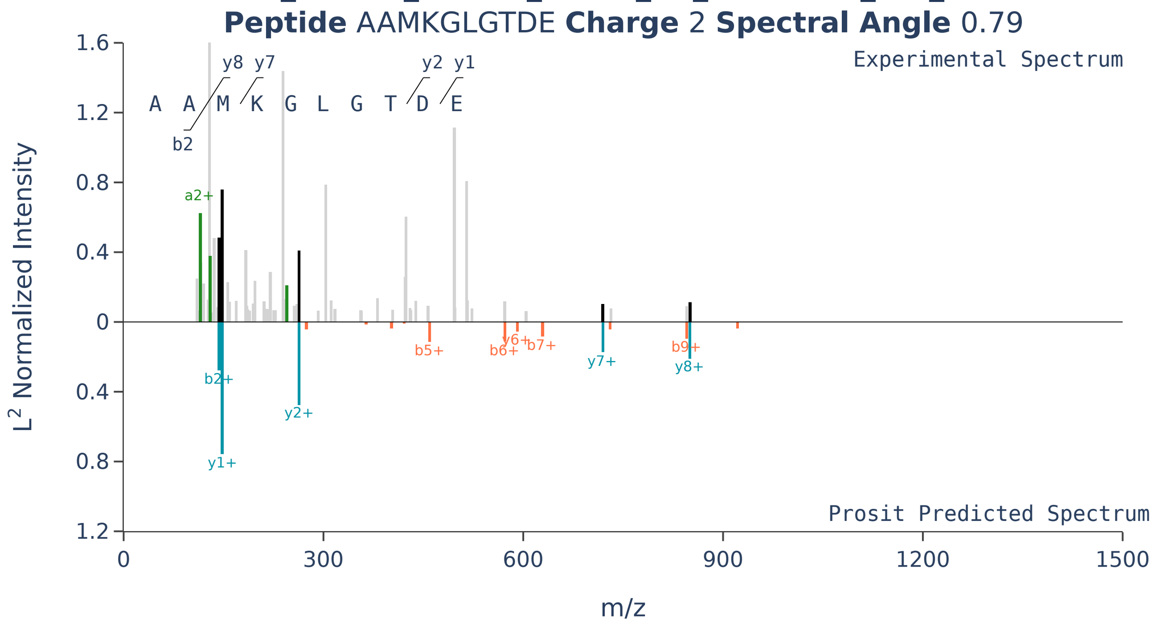


**Source** WSoh_301121_021221_HFGoe_M2_AnnexinA1_24h_R1 **Scan** 23096
**Peptide** DFGVNEDLAD **Charge** 2 **Spectral Angle** 0.8

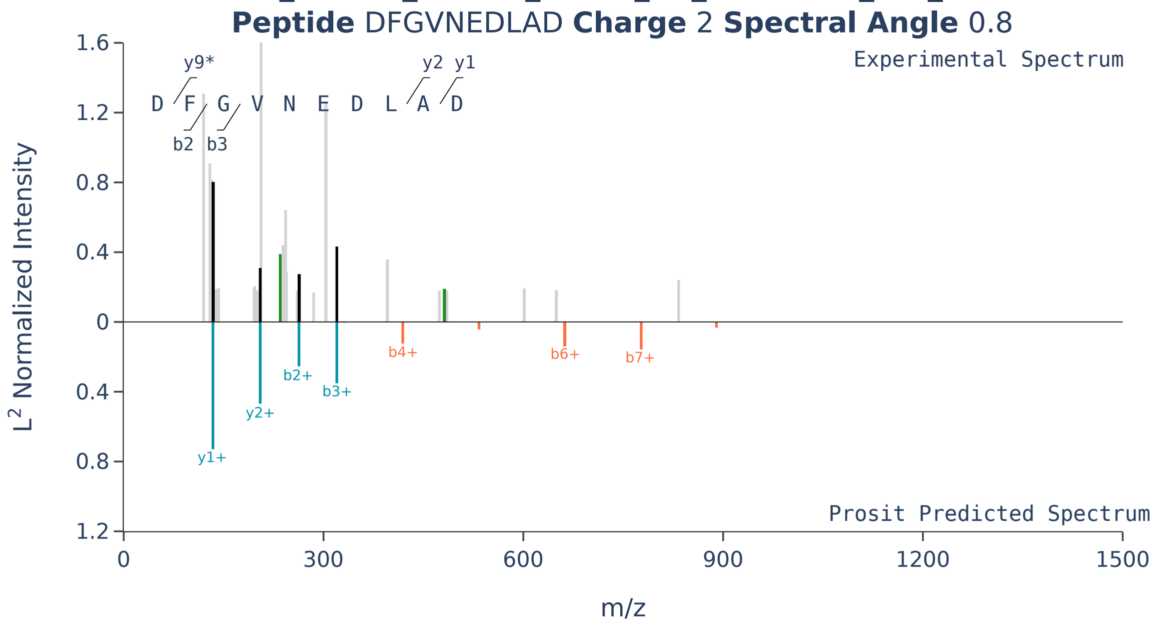

**Source** WSoh_301121_021221_HFGoe_M2_AnnexinA1_2h_R2 **Scan** 6854
**Peptide** FQKYTKYSKHDM **Charge** 3 **Spectral Angle** 0.78

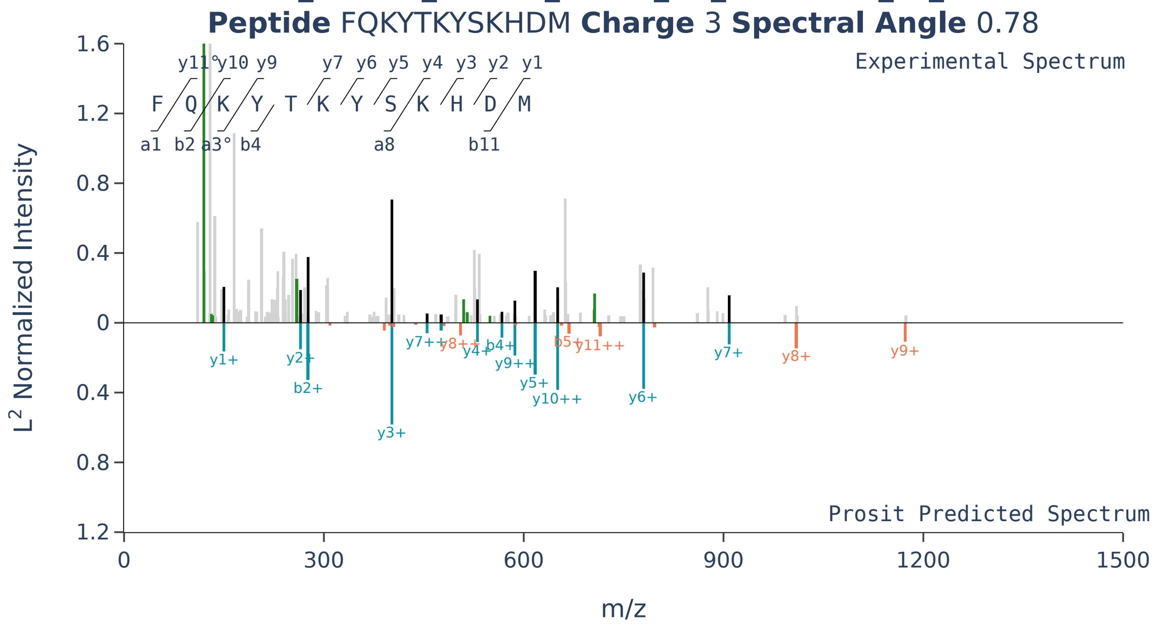


**Source** WSoh_301121_021221_HFGoe_M2_AnnexinA1_24h_R2 **Scan** 16613
**Peptide** NPSSDVAALHKAI **Charge** 3 **Spectral Angle** 0.8

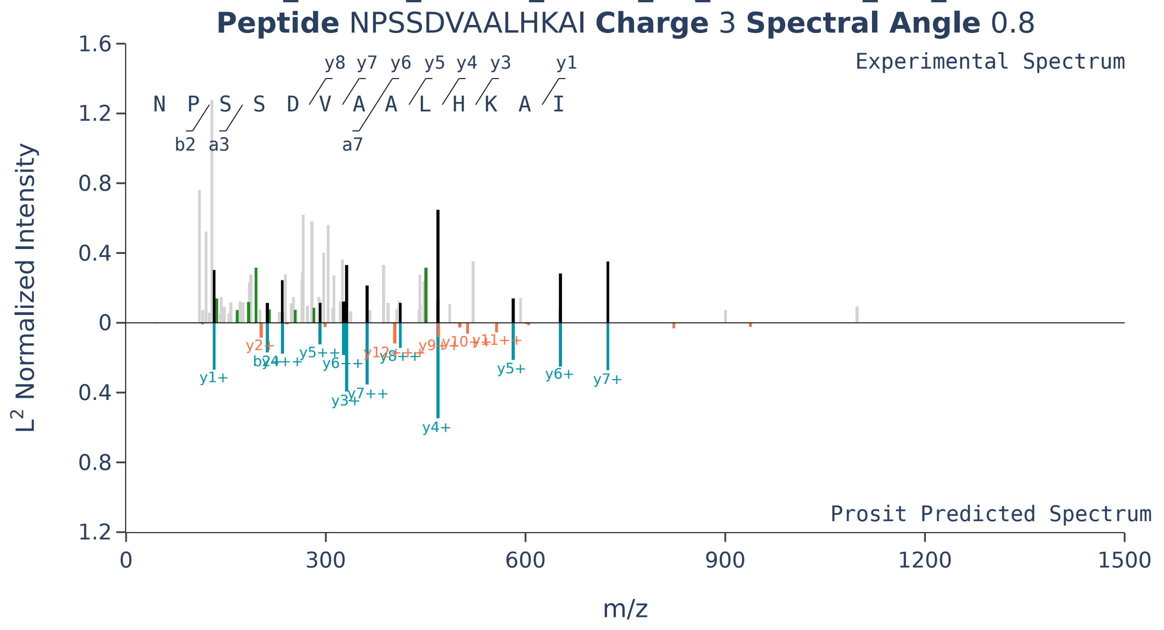


**Source** WSoh_301121_021221_HFGoe_M2_AnnexinA1_2h_R1 **Scan** 10675
**Peptide** LVALCGGNHH **Charge** 3 **Spectral Angle** 0.8

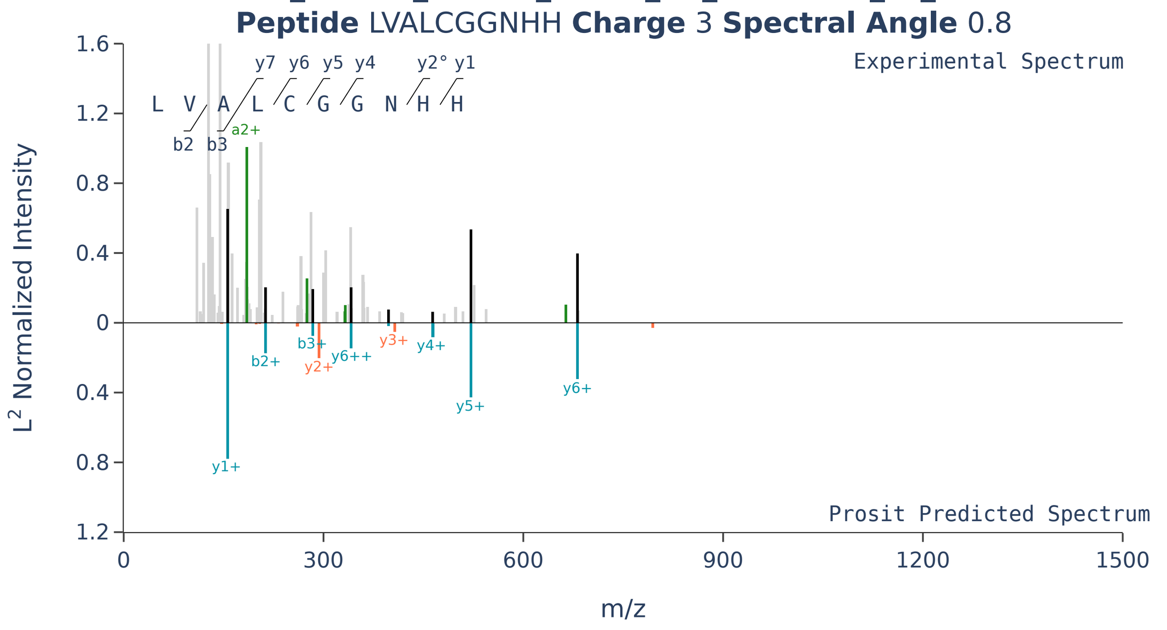


**Source** WSoh_301121_021221_HFGoe_M2_AnnexinA1_2h_R2 **Scan** 23324
**Peptide** SKGGPGSAVSPYPTFNPSSDV **Charge** 2 **Spectral Angle** 0.89

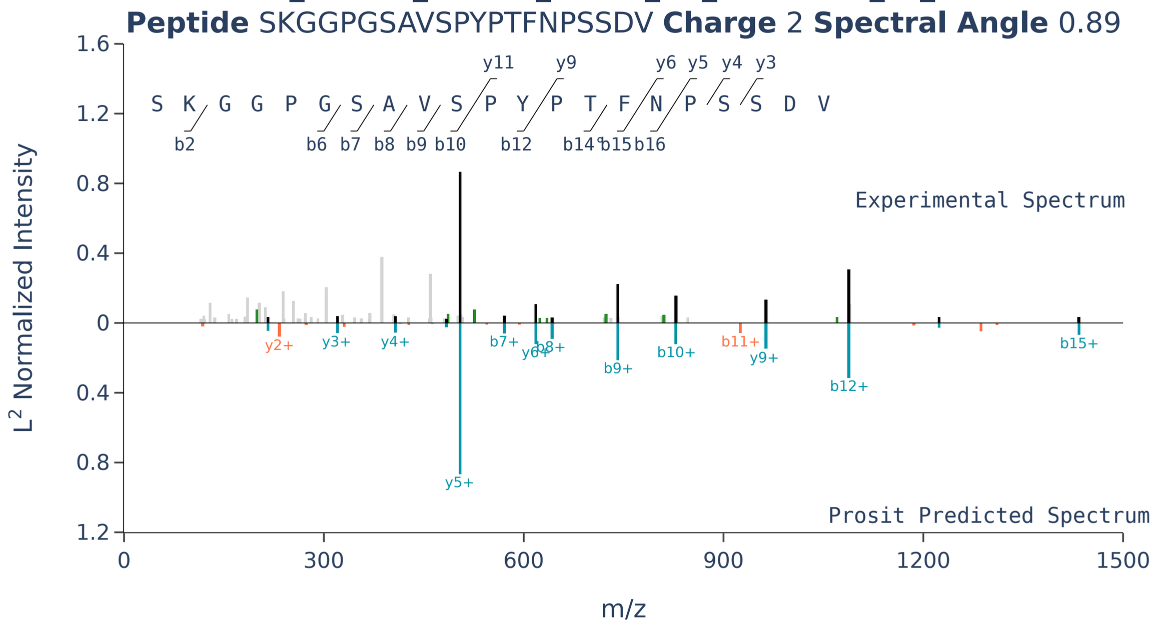


**Source** WSoh_301121_021221_HFGoe_M1_AnnexinA1_24h_R2 **Scan** 23315
**Peptide** AVSPYPTFNPSSD **Charge** 2 **Spectral Angle** 0.88

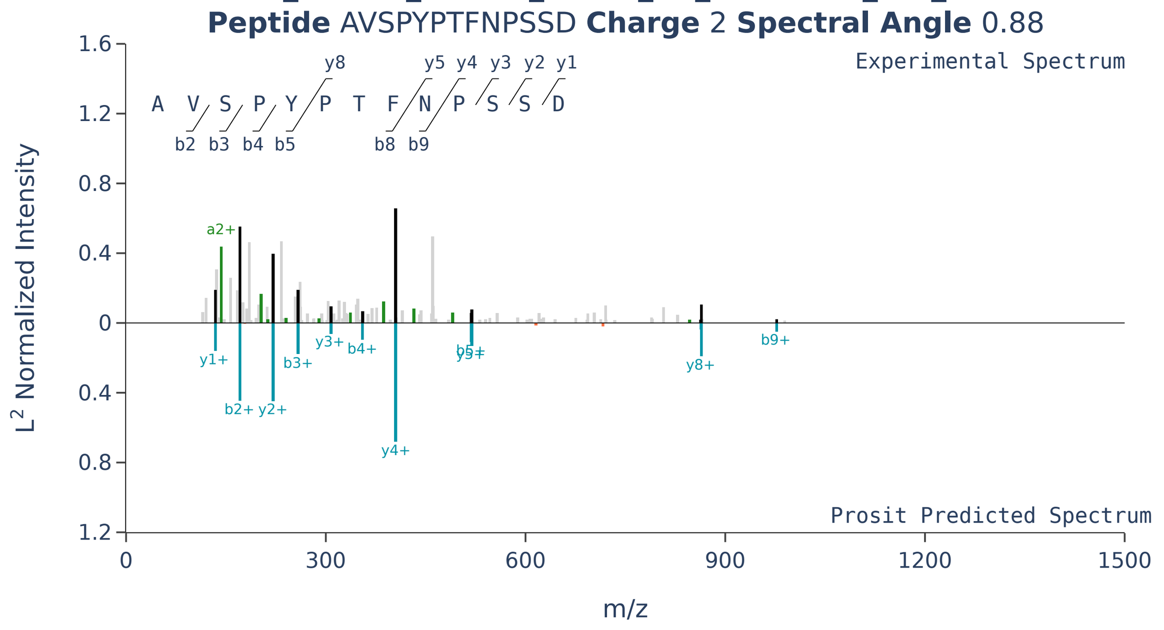


**Source** WSoh_301121_021221_HFGoe_M2_AnnexinA1_2h_R2 **Scan** 19662
**Peptide** SKGGPGSAVSPYPTFN **Charge** 2 **Spectral Angle** 0.82

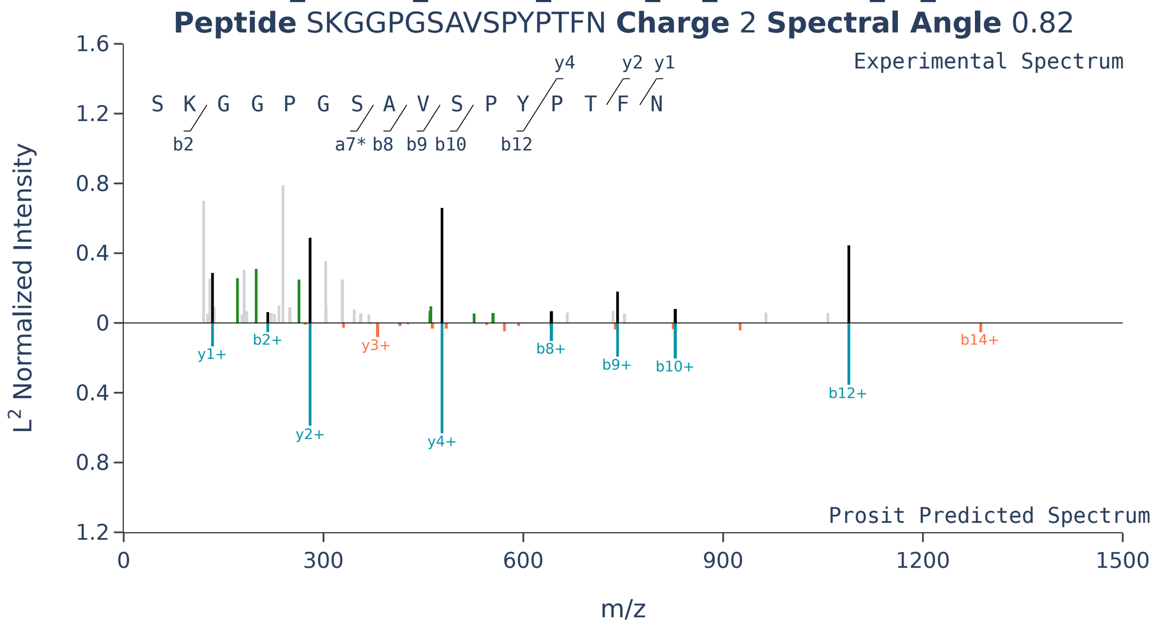


**Source** WSoh_301121_021221_HFGoe_M2_AnnexinA1_2h_R1 **Scan** 20727
**Peptide** LLSLAKGDRSEDFGVN **Charge** 3 **Spectral Angle** 0.92

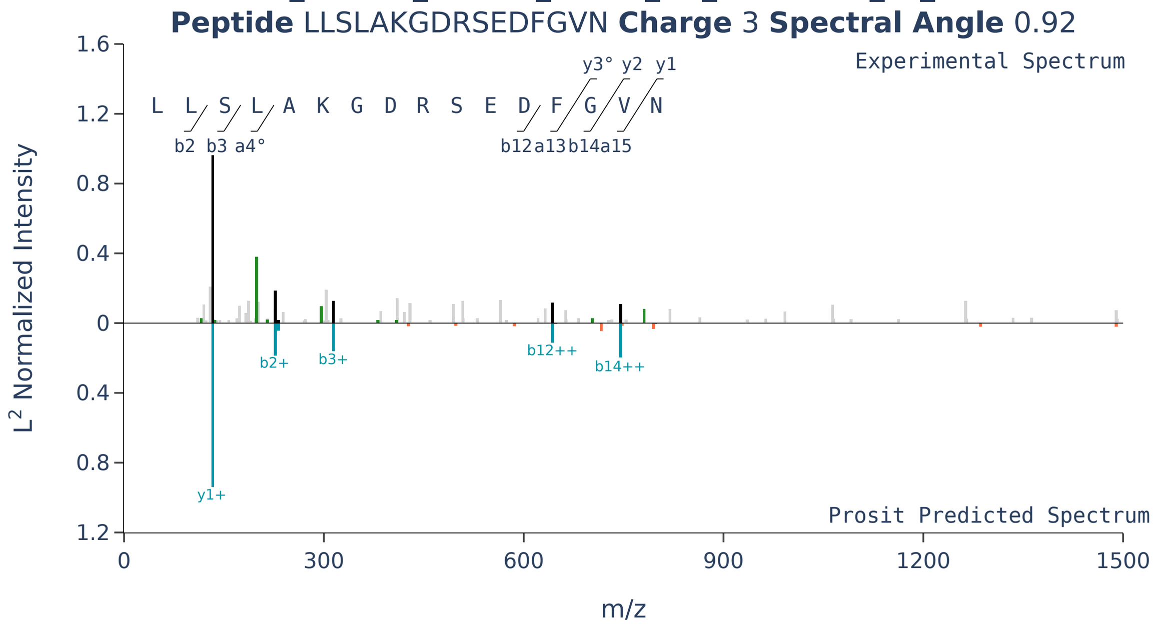


**Source** WSoh_301121_021221_HFGoe_M2_AnnexinA1_4h_R1 **Scan** 8776
**Peptide** EELKRDL **Charge** 2 **Spectral Angle** 0.7

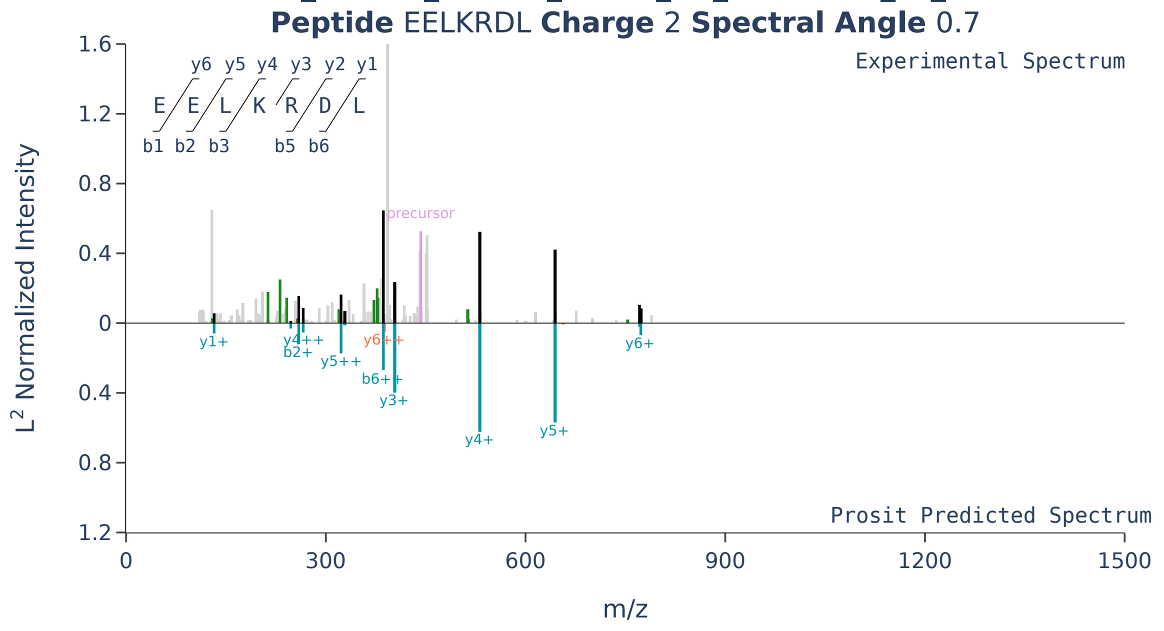


**Source** WSoh_301121_021221_HFGoe_M2_AnnexinA1_24h_R1 **Scan** 16481
**Peptide** DLELKGDIEKC **Charge** 3 **Spectral Angle** 0.76

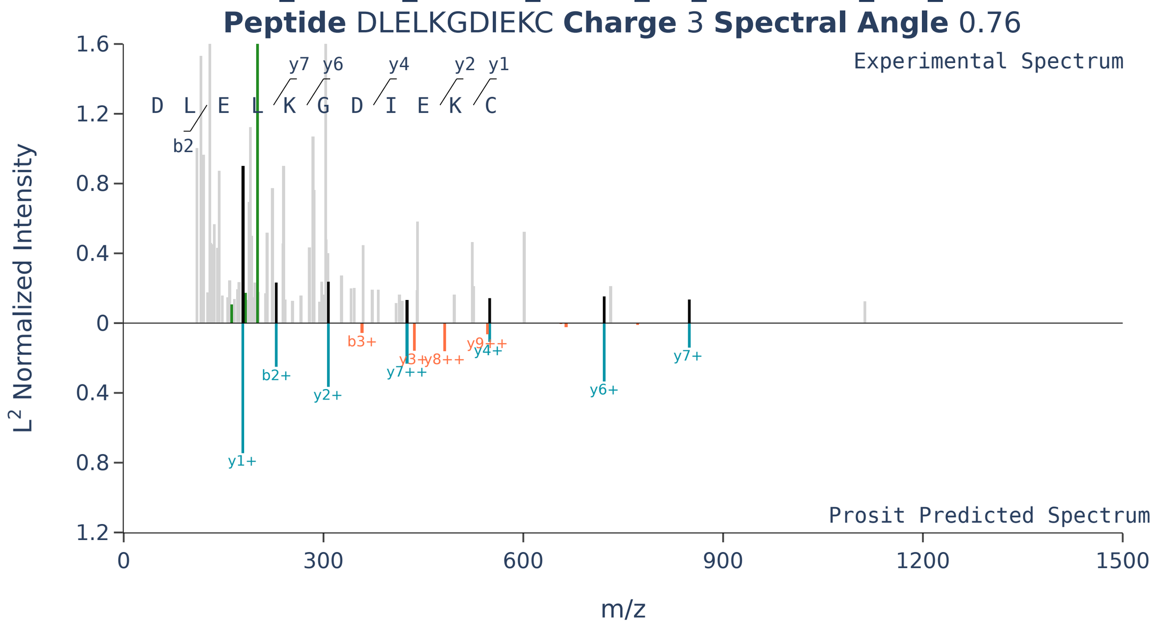


**Source** WSoh_301121_021221_HFGoe_M2_AnnexinA1_2h_R1 **Scan** 16237
**Peptide** AYLQETGKPLDE **Charge** 2 **Spectral Angle** 0.77

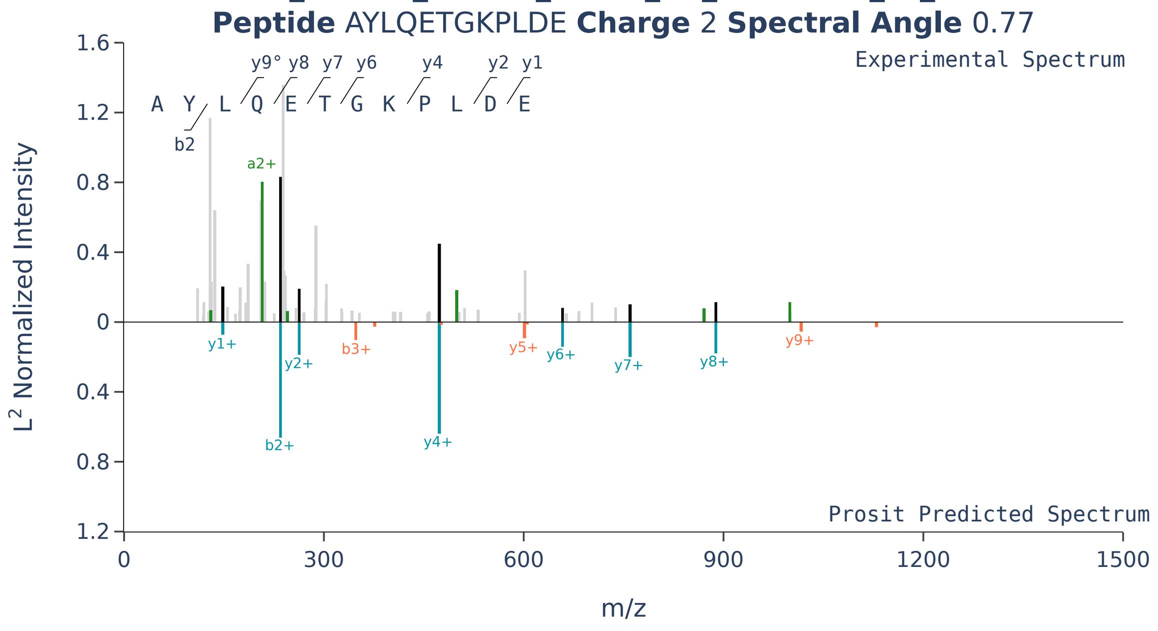


**Source** WSoh_301121_021221_HFGoe_M2_AnnexinA1_2h_R1 **Scan** 17836
**Peptide** KHDMNKVLDLELKG **Charge** 4 **Spectral Angle** 0.87

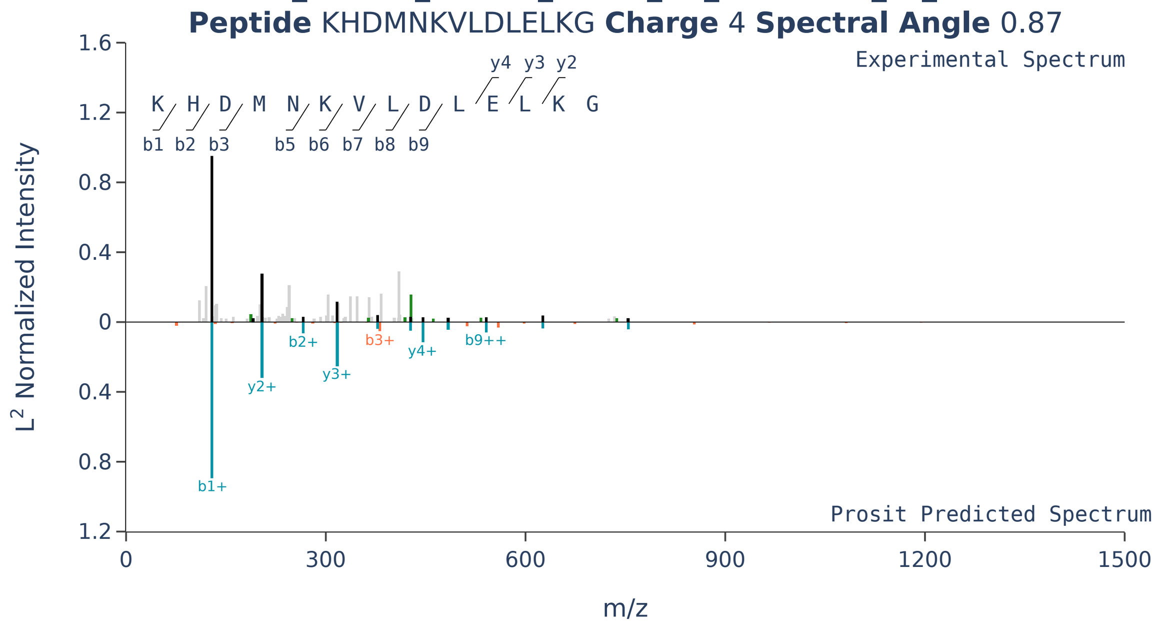


**Source** WSoh_301121_021221_HFGoe_M2_AnnexinA1_24h_R1 **Scan** 9359
**Peptide** AMKGLGT **Charge** 1 **Spectral Angle** 0.85

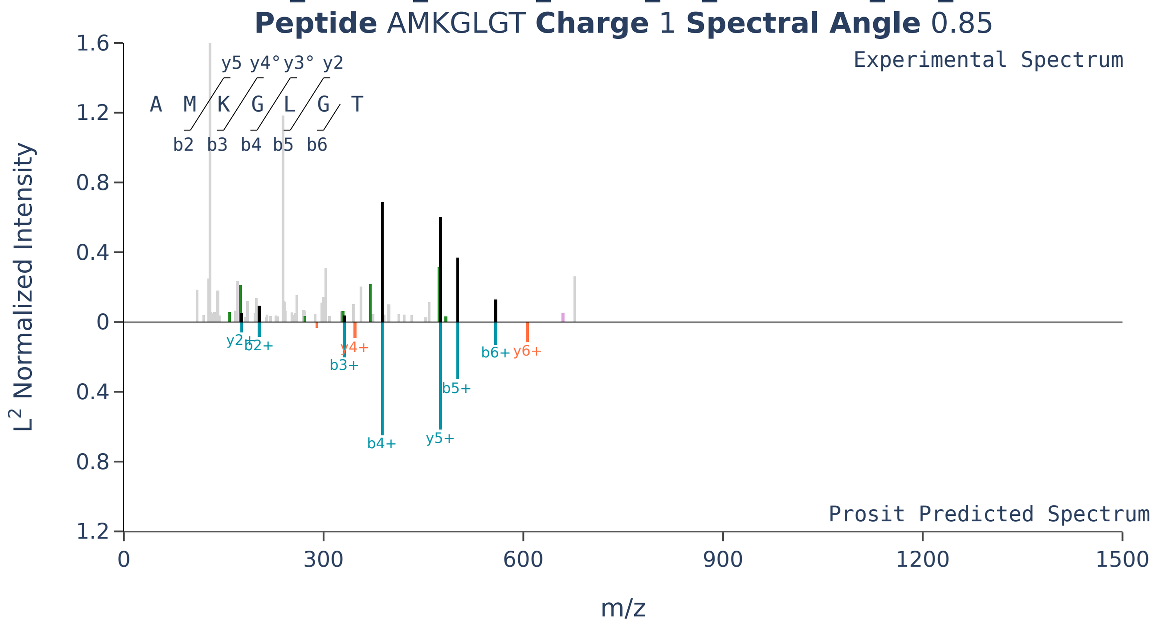


**Source** WSoh_301121_021221_HFGoe_M2_AnnexinA1_2h_R1 **Scan** 17927
**Peptide** SKHDMNKVLDLELKG **Charge** 4 **Spectral Angle** 0.82

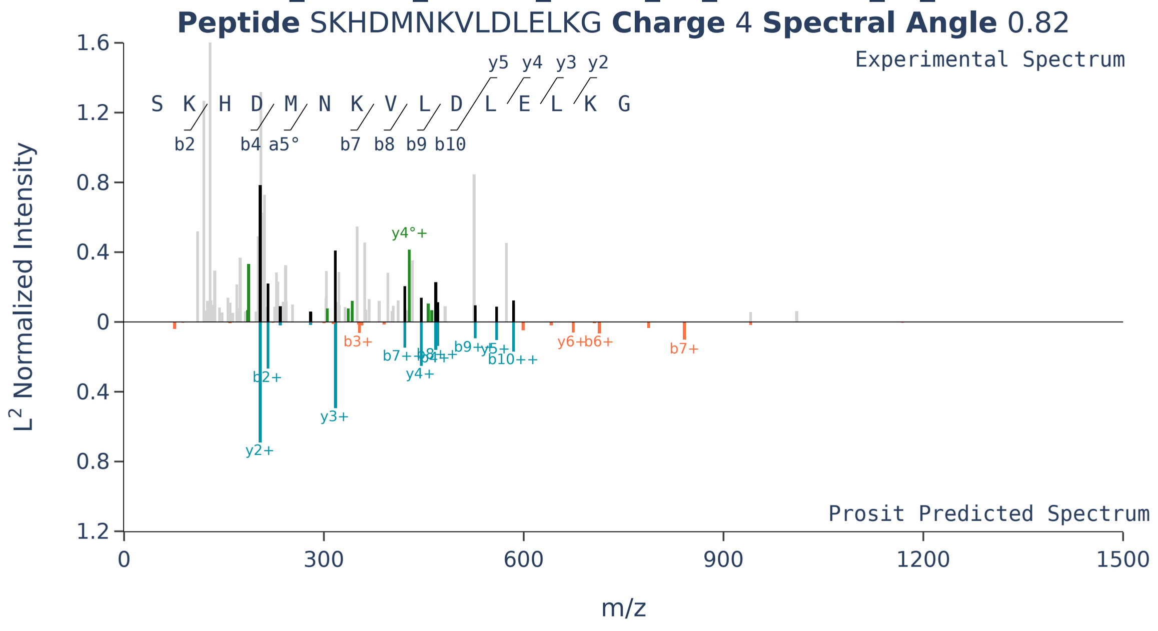


**Source** WSoh_301121_021221_HFGoe_M2_AnnexinA1_24h_R1 **Scan** 20191
**Peptide** TPAQFDADELRA **Charge** 2 **Spectral Angle** 0.81

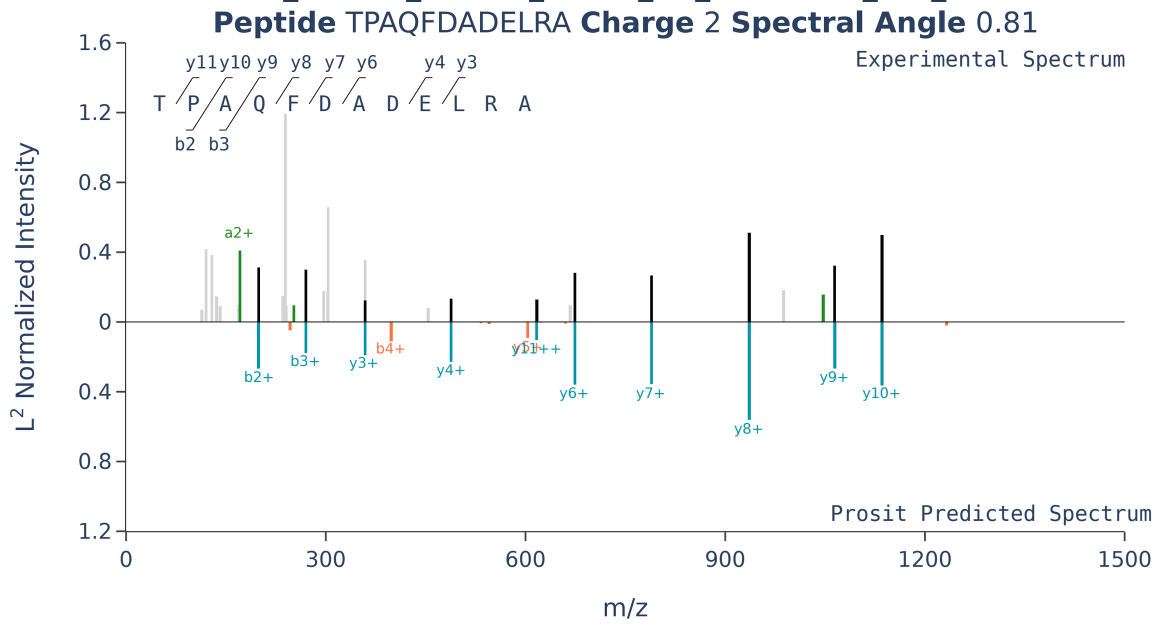


**Source** WSoh_301121_021221_HFGoe_M2_AnnexinA1_24h_R1 **Scan** 22942
**Peptide** KDITSDTSGDFRNALL **Charge** 3 **Spectral Angle** 0.65

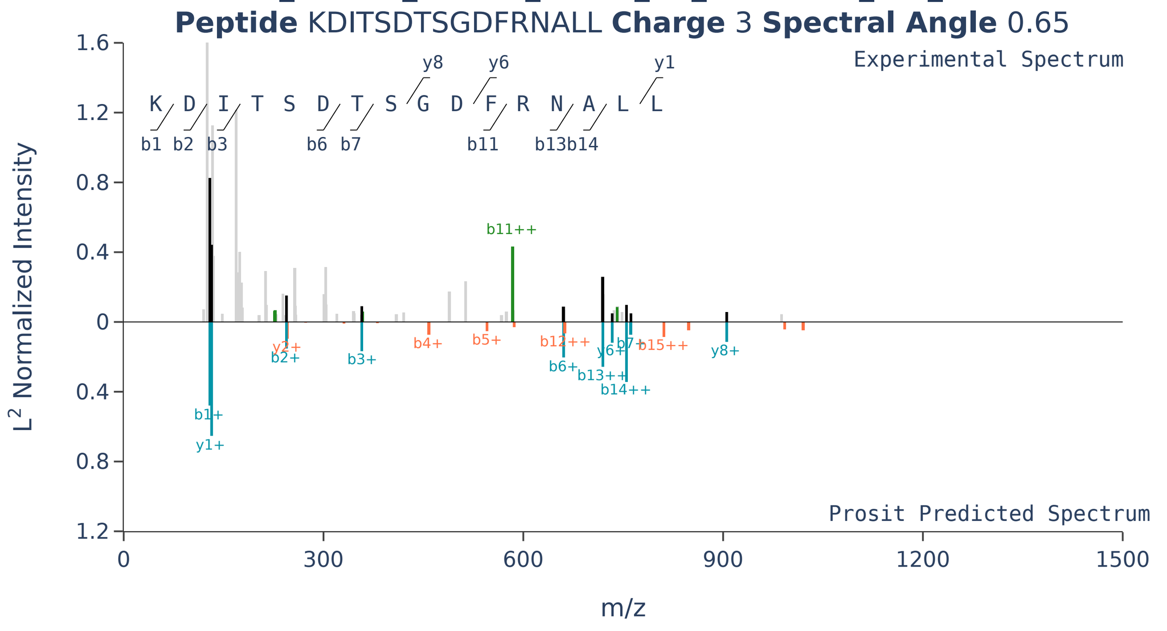

**Source** WSoh_301121_021221_HFGoe_M2_AnnexinA1_2h_R1 **Scan** 23971
**Peptide** AGERRKGTDVNVFNTILTT **Charge** 4 **Spectral Angle** 0.68

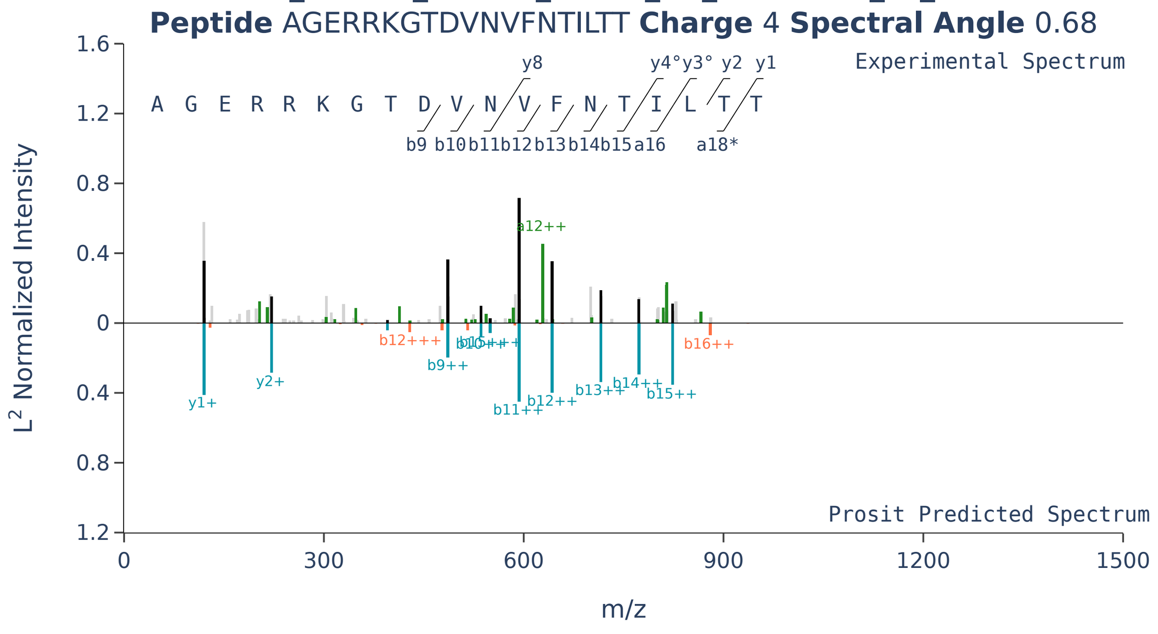


**Source** WSoh_301121_021221_HFGoe_M2_AnnexinA1_4h_R2 **Scan** 6515
**Peptide** AEKLHQAM **Charge** 2 **Spectral Angle** 0.72

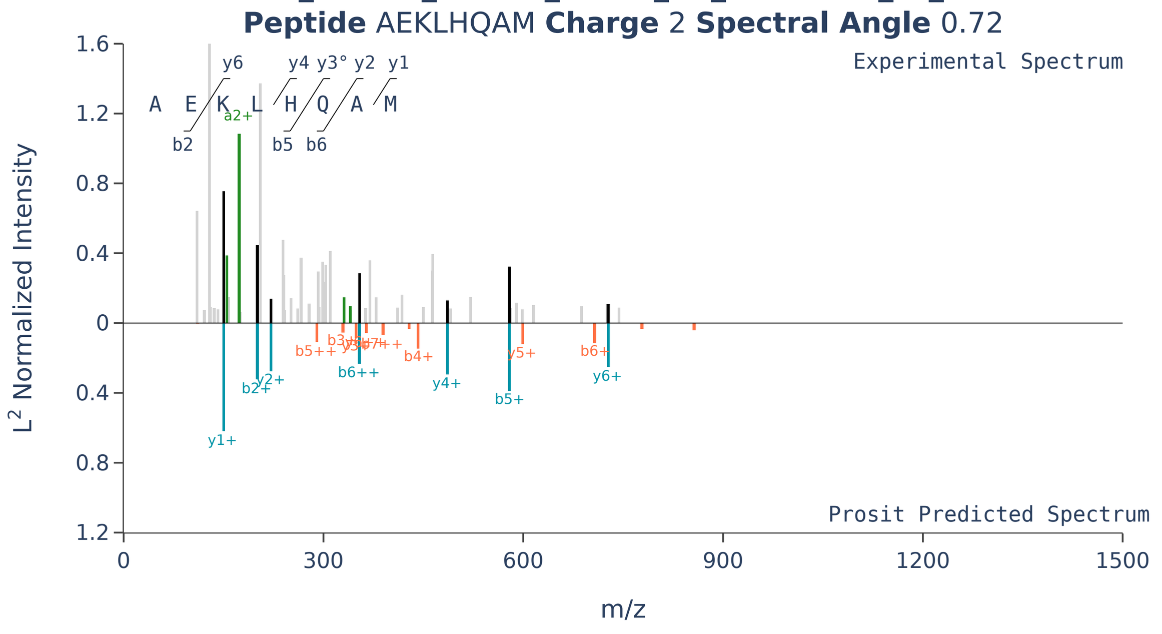


**Source** WSoh_301121_021221_HFGoe_M2_AnnexinA1_4h_R2 **Scan** 6888
**Peptide** ELKGDIEK **Charge** 2 **Spectral Angle** 0.8

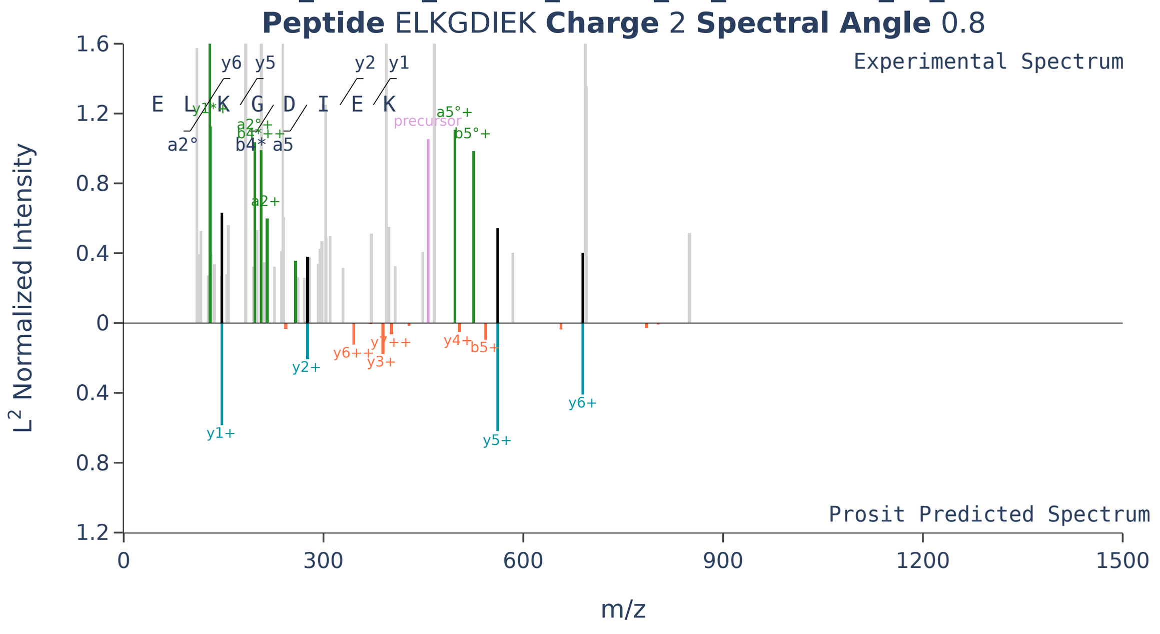


**Source** WSoh_301121_021221_HFGoe_M2_AnnexinA1_2h_R2 **Scan** 11633
**Peptide** QRQQIKAAYLQ **Charge** 3 **Spectral Angle** 0.85

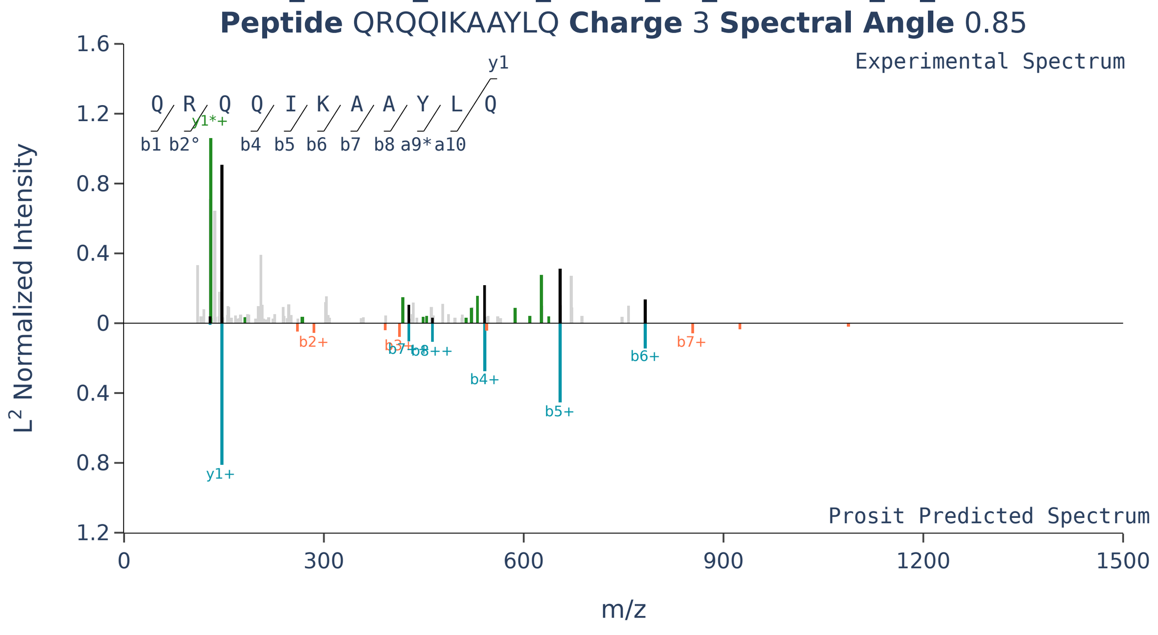


**Source** WSoh_301121_021221_HFGoe_M2_AnnexinA1_24h_R2 **Scan** 12127
**Peptide** QAILDETKGD **Charge** 2 **Spectral Angle** 0.73

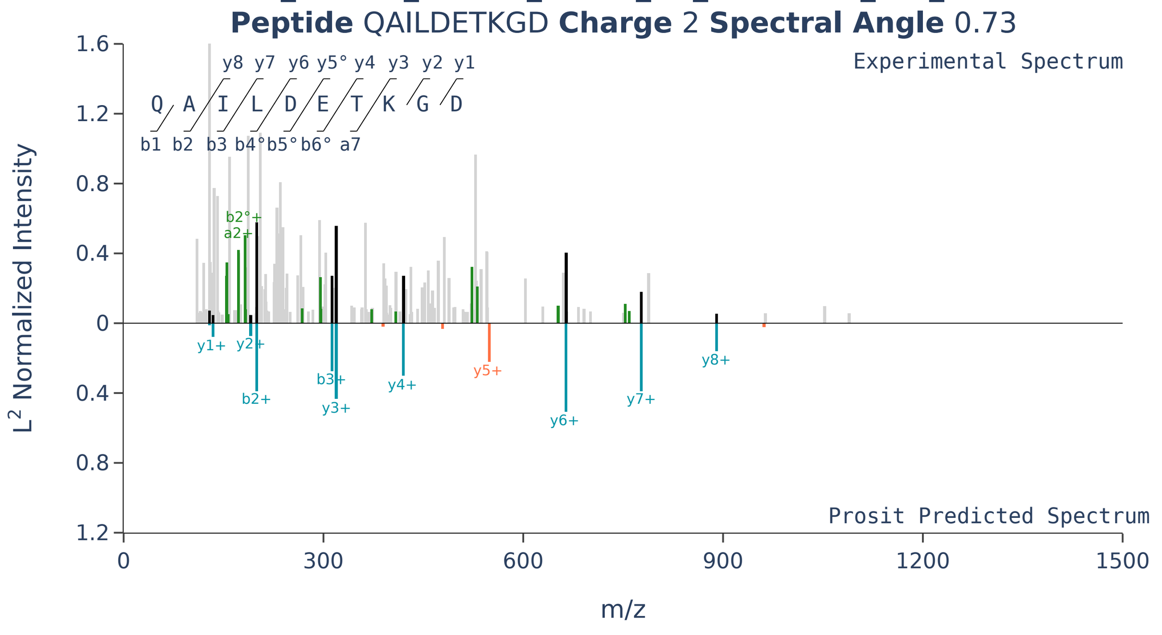


**Source** WSoh_301121_021221_HFGoe_M2_AnnexinA1_2h_R1 **Scan** 13866
**Peptide** YREELKRDLAKDITSD **Charge** 3 **Spectral Angle** 0.74

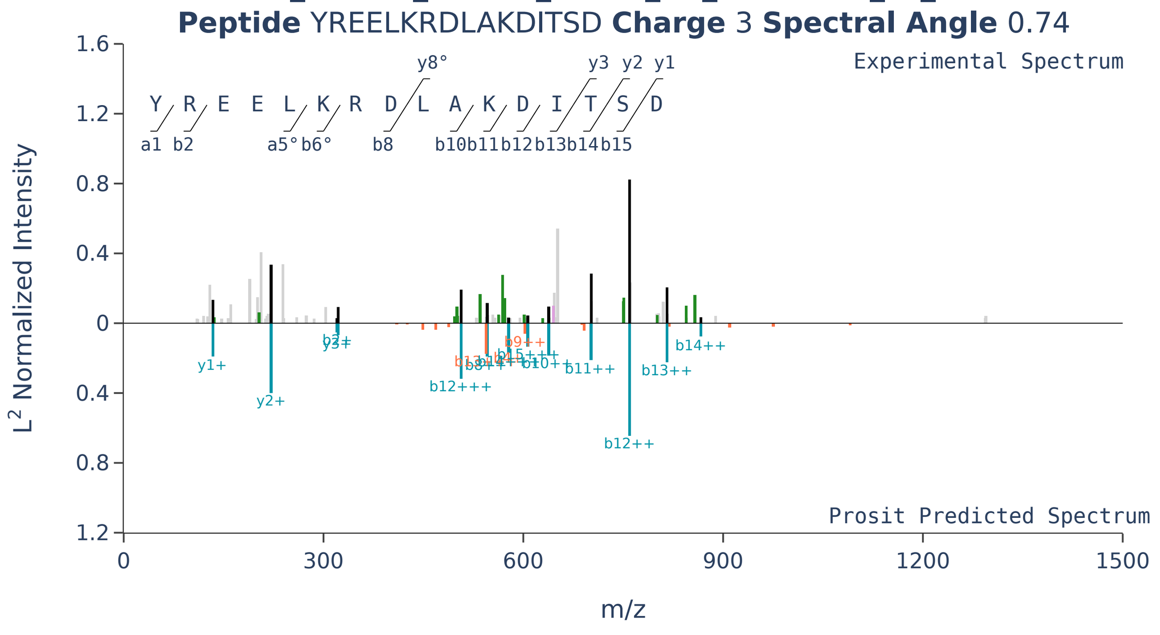


**Source** WSoh_301121_021221_HFGoe_M2_AnnexinA1_2h_R1 **Scan** 15520
**Peptide** TNKEIRDINRVYREEL **Charge** 4 **Spectral Angle** 0.84

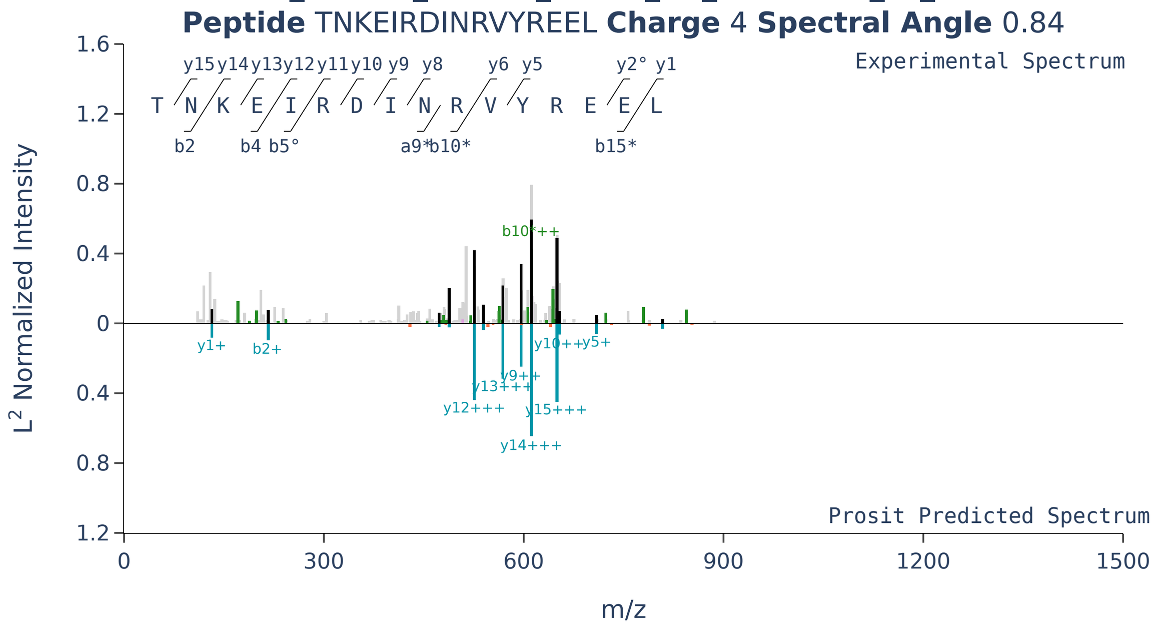


**Source** WSoh_301121_021221_HFGoe_M2_AnnexinA1_4h_R2 **Scan** 15709
**Peptide** AILDETKGDYE **Charge** 2 **Spectral Angle** 0.7

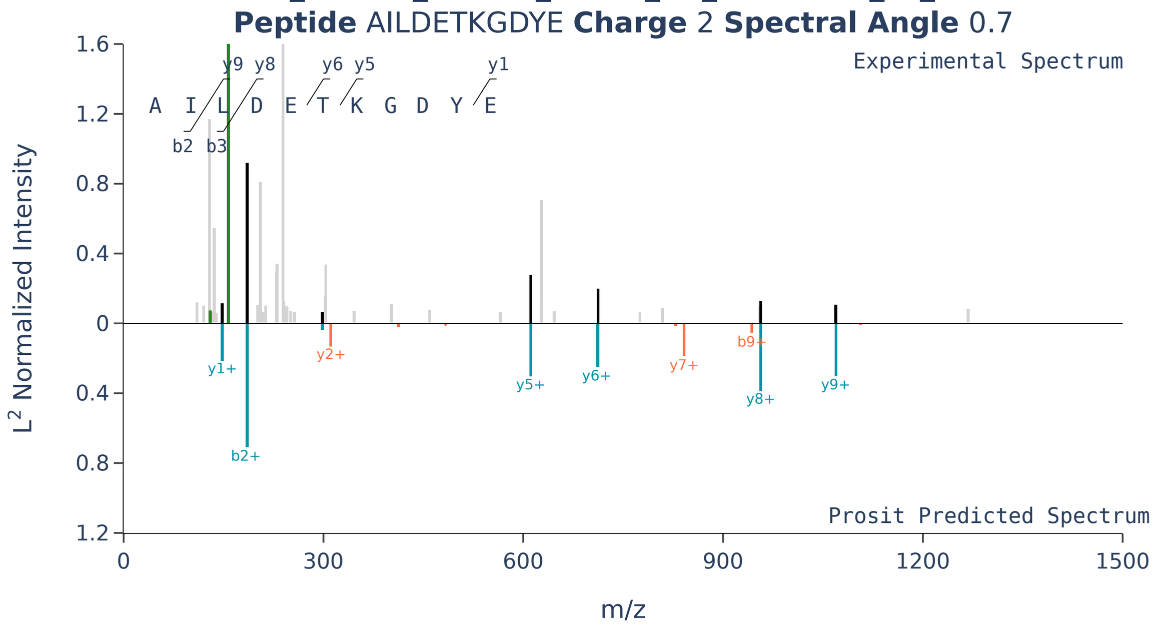


**Source** WSoh_301121_021221_HFGoe_M1_AnnexinA1_2h_R2 **Scan** 10247
**Peptide** KAAYLQETG **Charge** 2 **Spectral Angle** 0.55

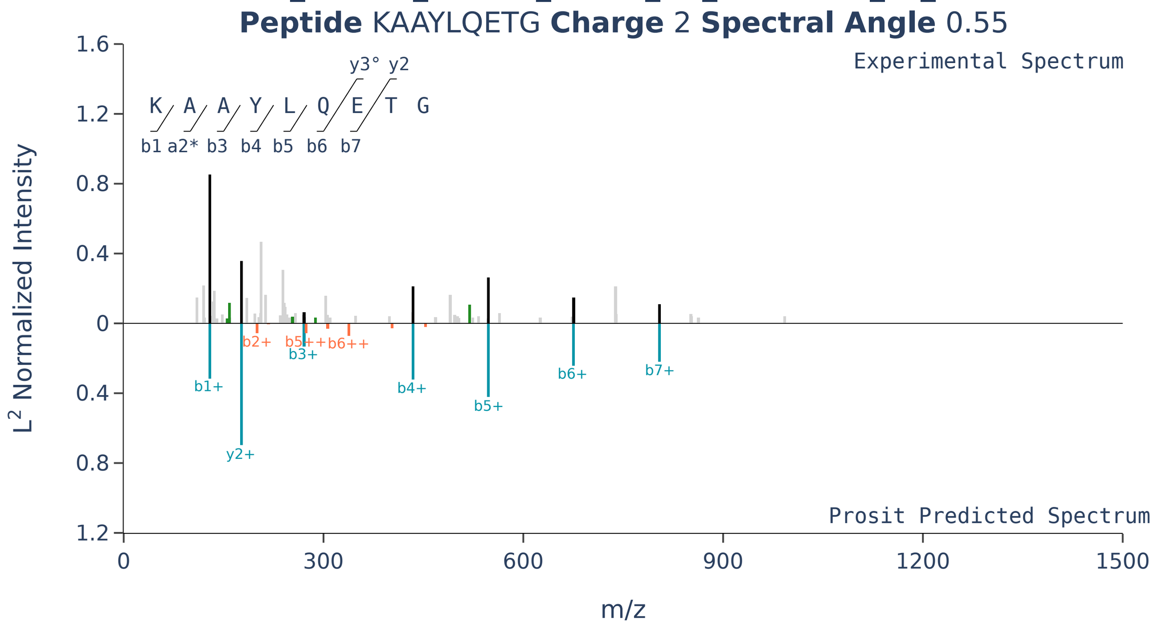


**Source** WSoh_301121_021221_HFGoe_M2_AnnexinA1_2h_R2 **Scan** 20800
**Peptide** KSSKGGPGSAVSPYPTFNPSSDV **Charge** 3 **Spectral Angle** 0.73

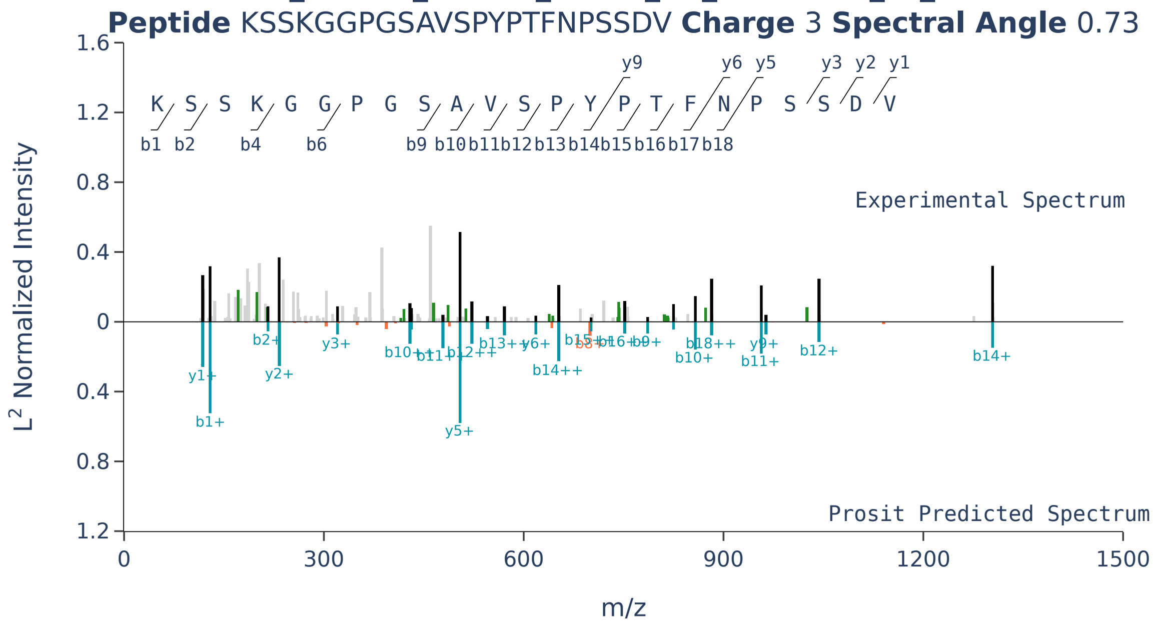


**Source** WSoh_301121_021221_HFGoe_M1_AnnexinA1_2h_R1 **Scan** 10535
**Peptide** EYVQTVKS **Charge** 1 **Spectral Angle** 0.88

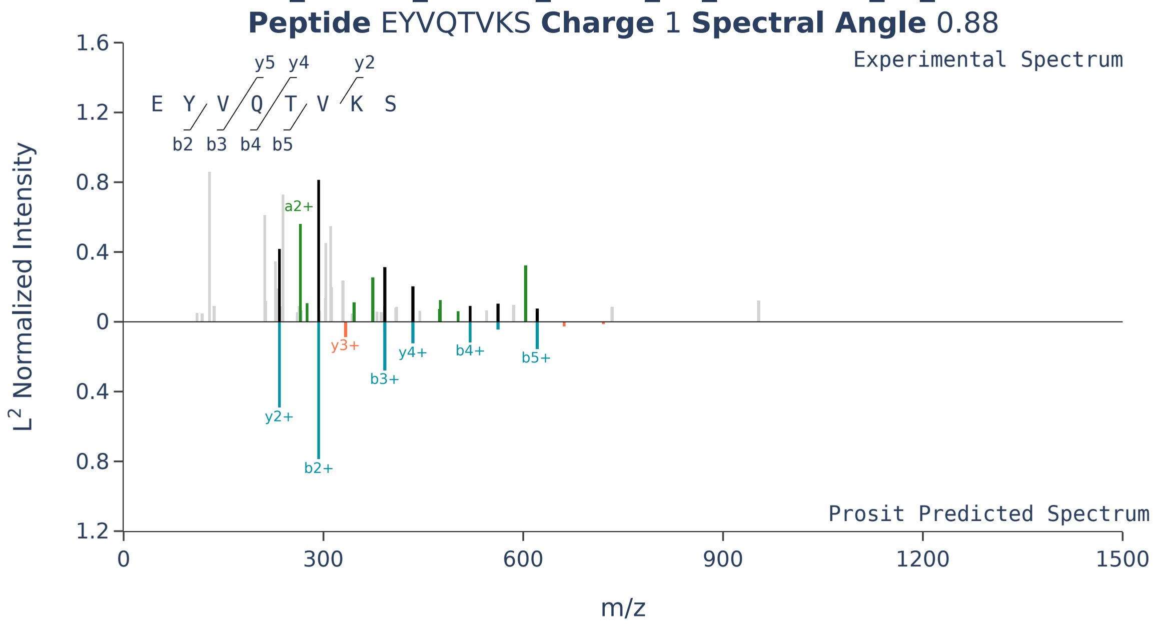


**Source** WSoh_301121_021221_HFGoe_M2_AnnexinA1_2h_R1 **Scan** 23036
**Peptide** TSDTSGDFRNALL **Charge** 2 **Spectral Angle** 0.75

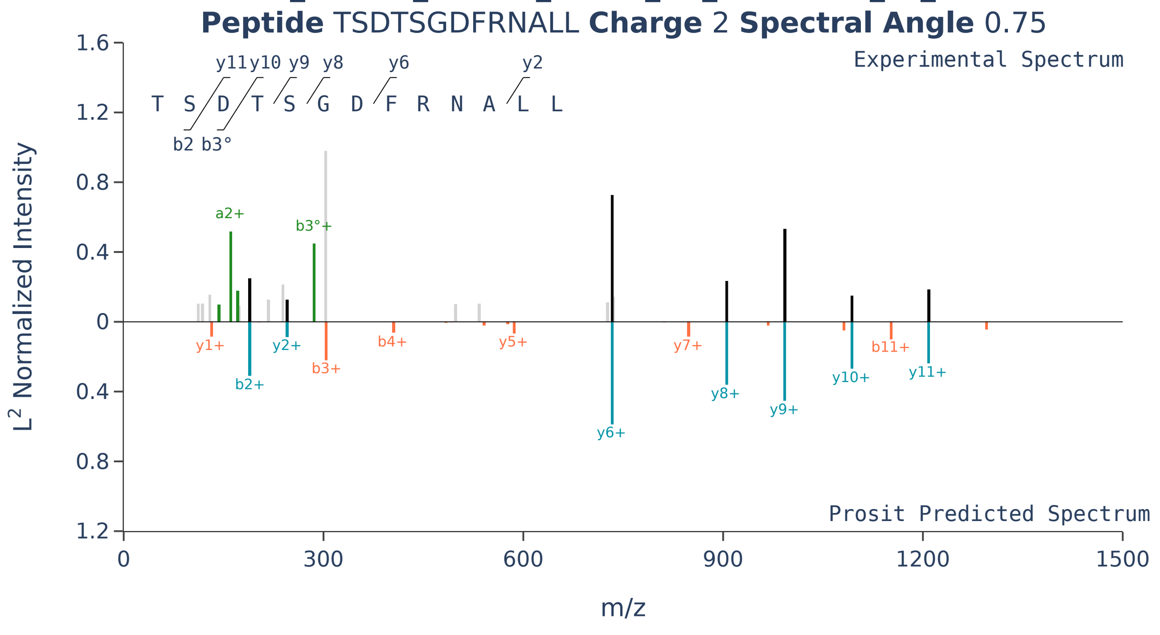


**Source** WSoh_301121_021221_HFGoe_M2_AnnexinA1_24h_R1 **Scan** 13414
**Peptide** SSKGGPGSAVSPYPT **Charge** 2 **Spectral Angle** 0.94

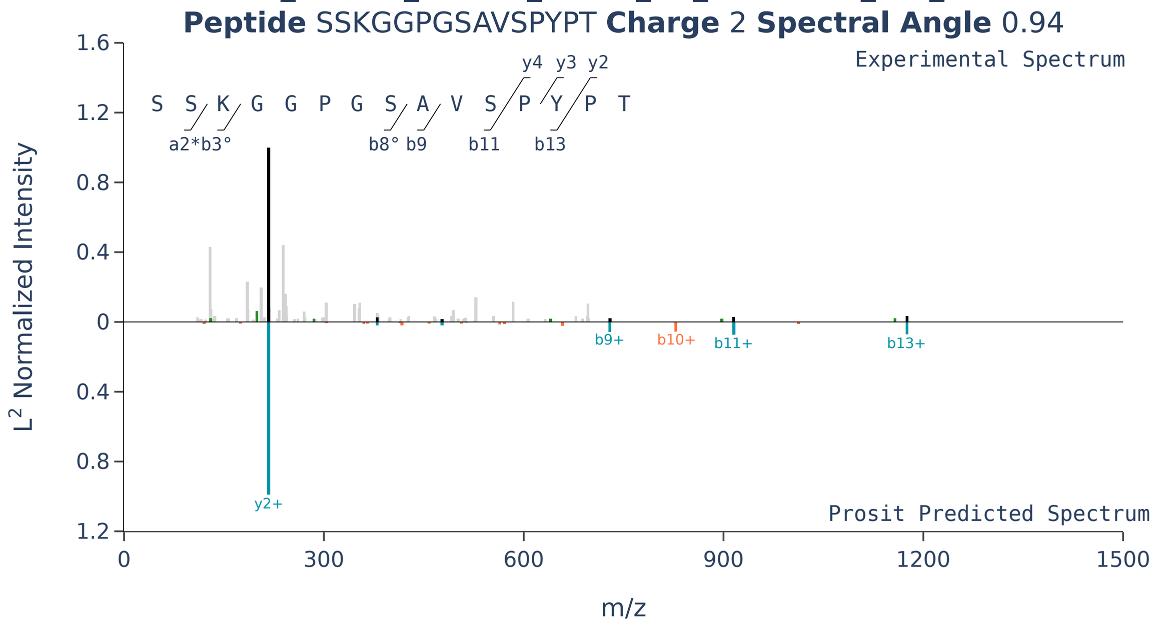


**Source** WSoh_301121_021221_HFGoe_M2_AnnexinA1_2h_R1 **Scan** 17541
**Peptide** SDARALYEAGERRKGTDVNVF **Charge** 5 **Spectral Angle** 0.82

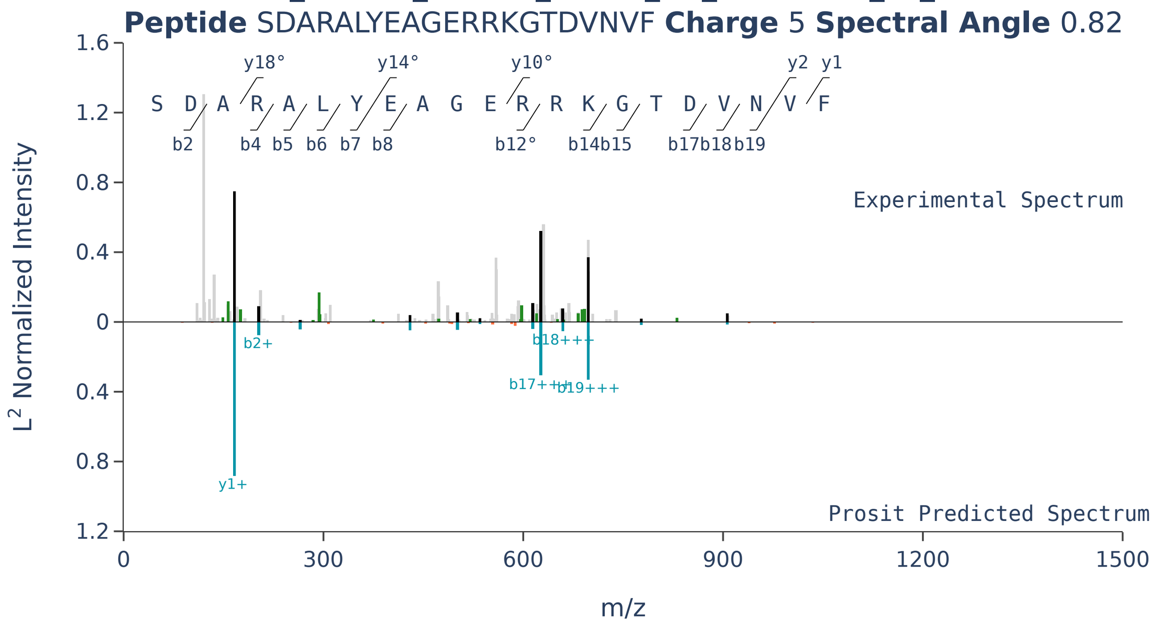


**Source** WSoh_301121_021221_HFGoe_M2_AnnexinA1_2h_R2 **Scan** 16163
**Peptide** DLADSDARALYEAGERRKG **Charge** 4 **Spectral Angle** 0.79

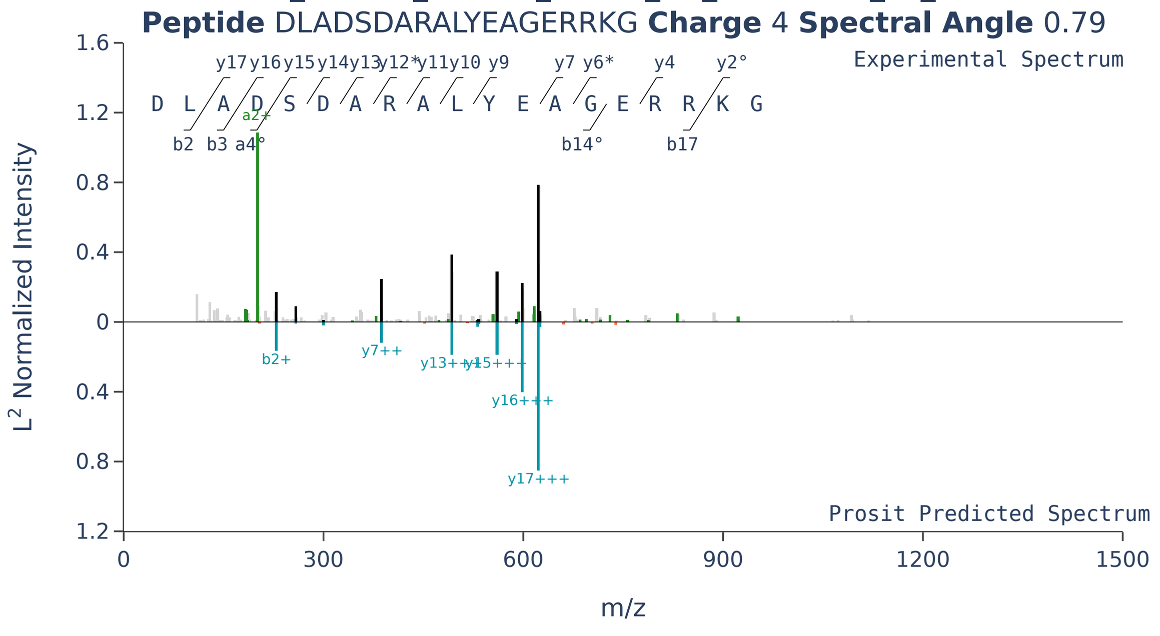

**Source** WSoh_301121_021221_HFGoe_M1_AnnexinA1_2h_R1 **Scan** 9697
**Peptide** PQLRRVFQK **Charge** 3 **Spectral Angle** 0.77

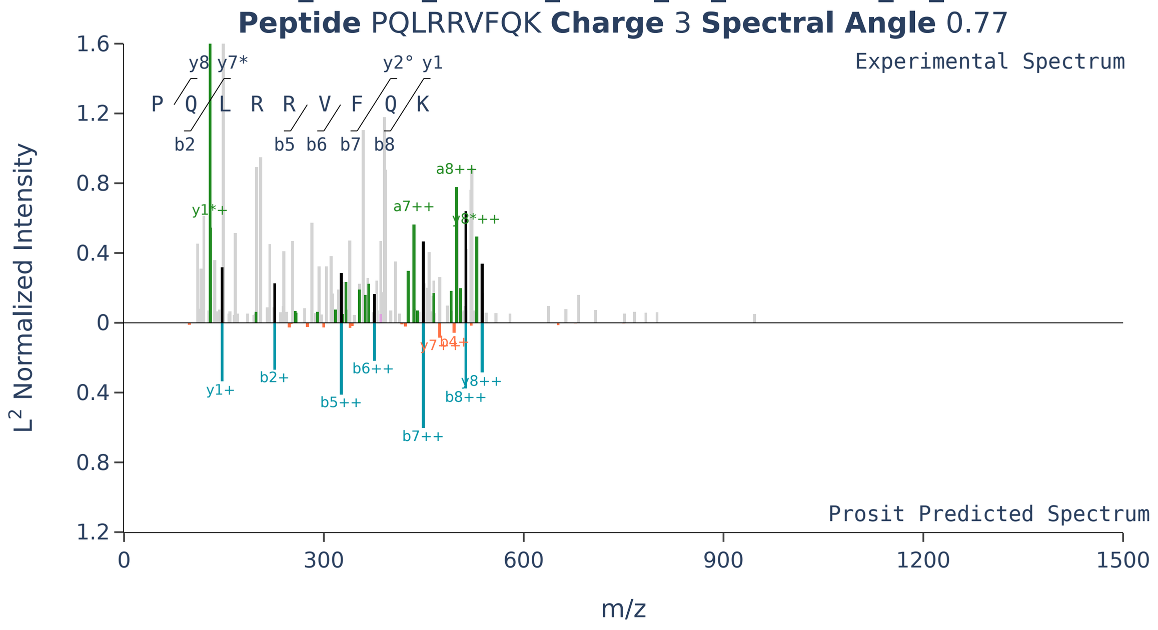


**Source** WSoh_301121_021221_HFGoe_M2_AnnexinA1_4h_R1 **Scan** 15164
**Peptide** DLEKCLTA **Charge** 1 **Spectral Angle** 0.75

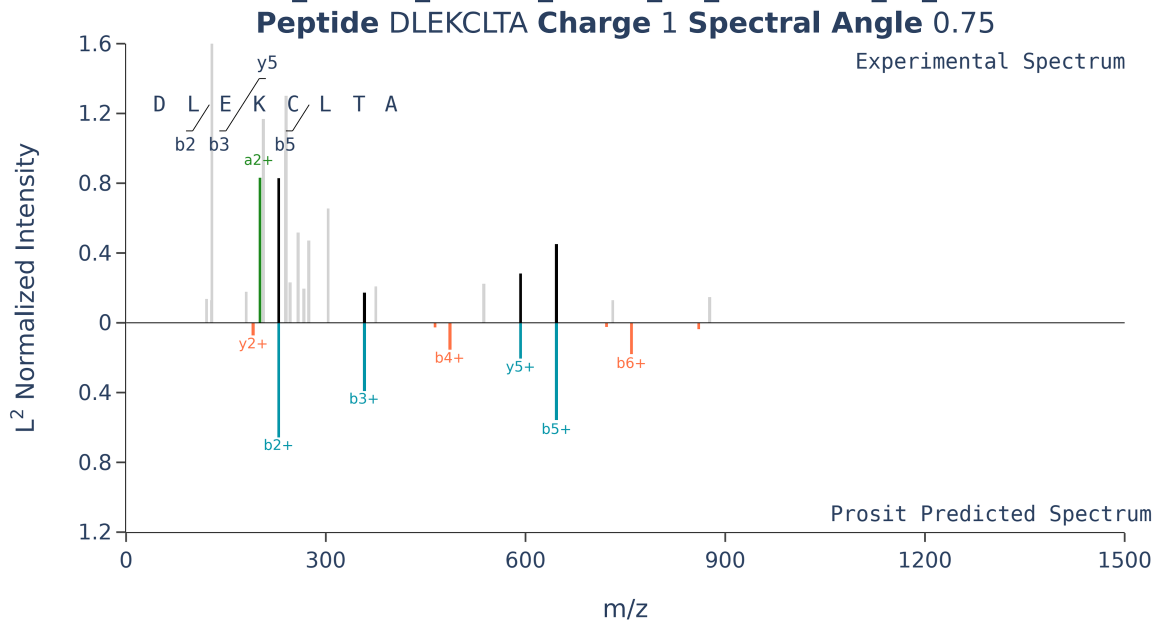


**Source** WSoh_301121_021221_HFGoe_M1_AnnexinA1_24h_R2 **Scan** 11571
**Peptide** KGDRSEDFGV **Charge** 2 **Spectral Angle** 0.48

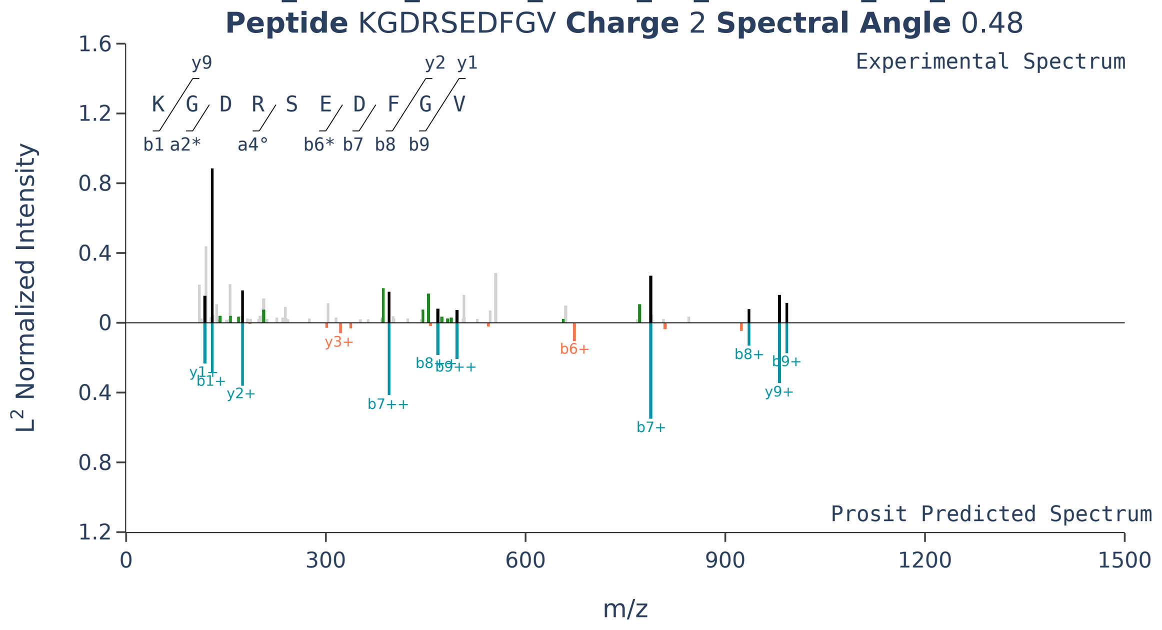


**Source** WSoh_301121_021221_HFGoe_M2_AnnexinA1_2h_R1 **Scan** 20718
**Peptide** LLSLAKGDRSEDFGVNE **Charge** 3 **Spectral Angle** 0.86

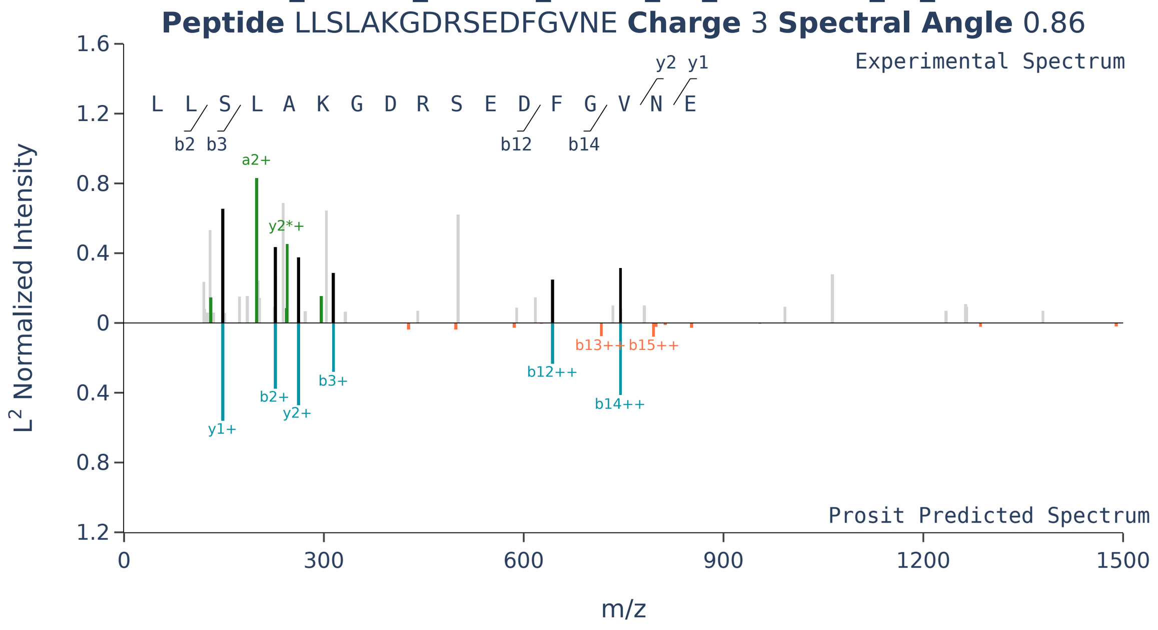


**Source** WSoh_301121_021221_HFGoe_M2_AnnexinA1_4h_R1 **Scan** 7181
**Peptide** RIMVSRSE **Charge** 2 **Spectral Angle** 0.72

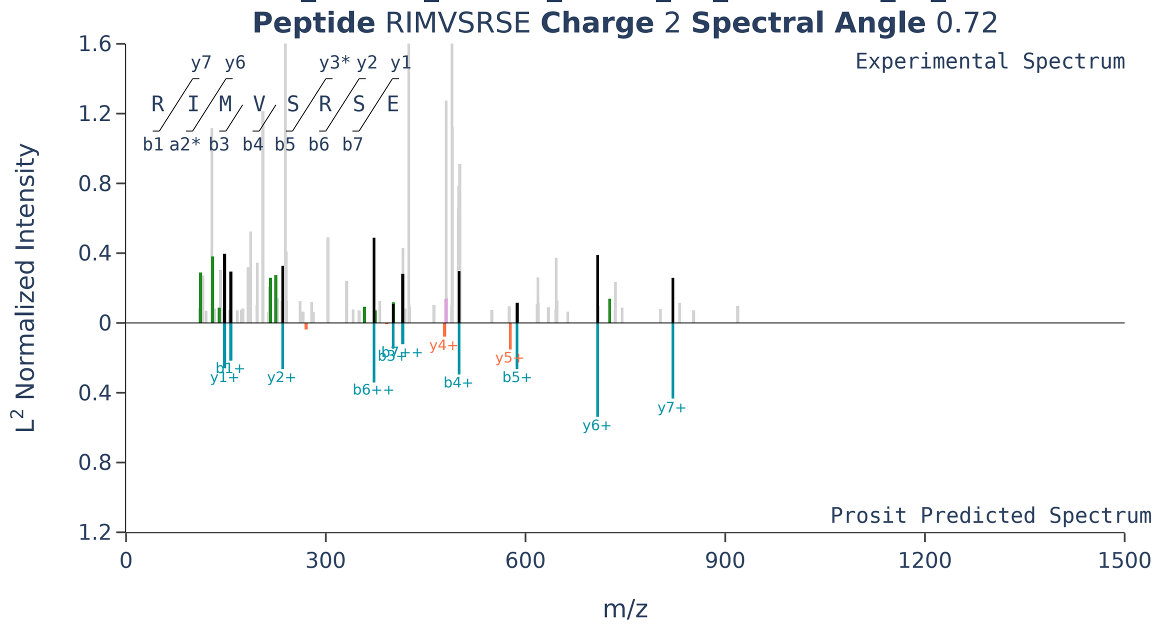


**Source** WSoh_301121_021221_HFGoe_M2_AnnexinA1_24h_R1 **Scan** 27214
**Peptide** EATIIDILTKRN **Charge** 3 **Spectral Angle** 0.85

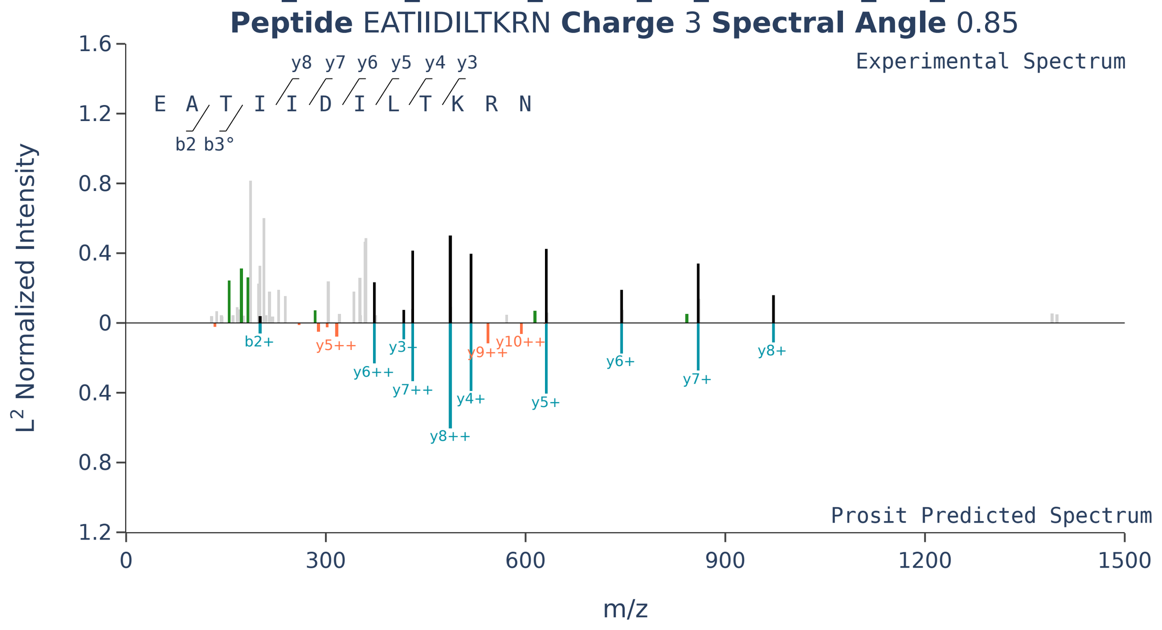


**Source** WSoh_301121_021221_HFGoe_M2_AnnexinA1_2h_R1 **Scan** 13091
**Peptide** LADSDARALYEAGERRKG **Charge** 4 **Spectral Angle** 0.78

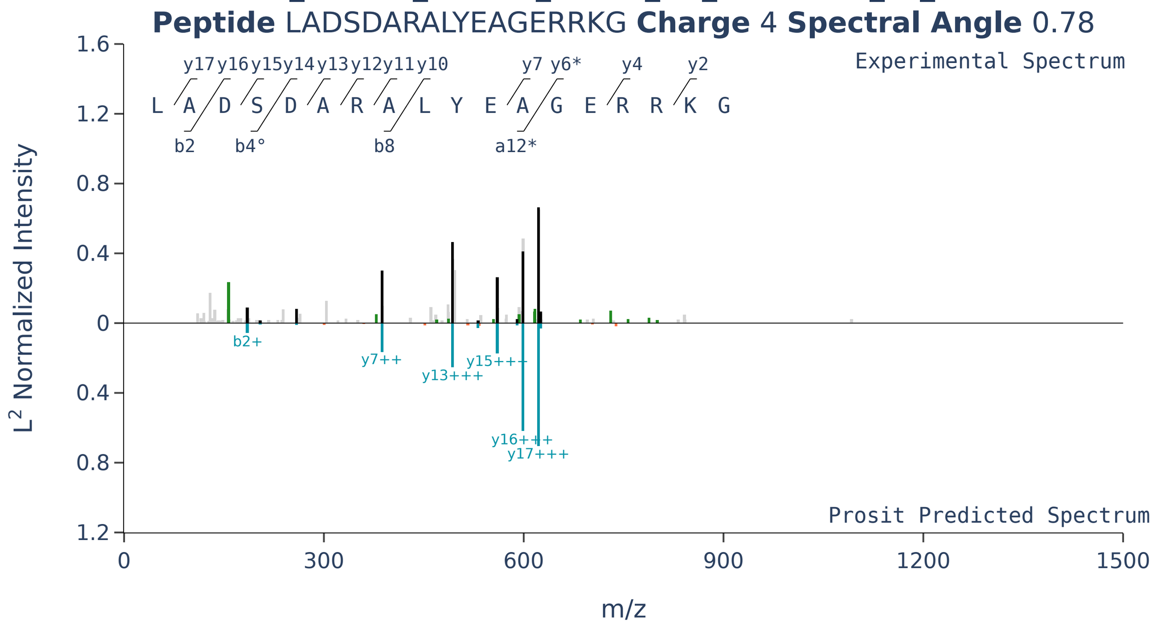


**Source** WSoh_301121_021221_HFGoe_M2_AnnexinA1_4h_R1 **Scan** 23273
**Peptide** DIEKCLTAI **Charge** 2 **Spectral Angle** 0.84

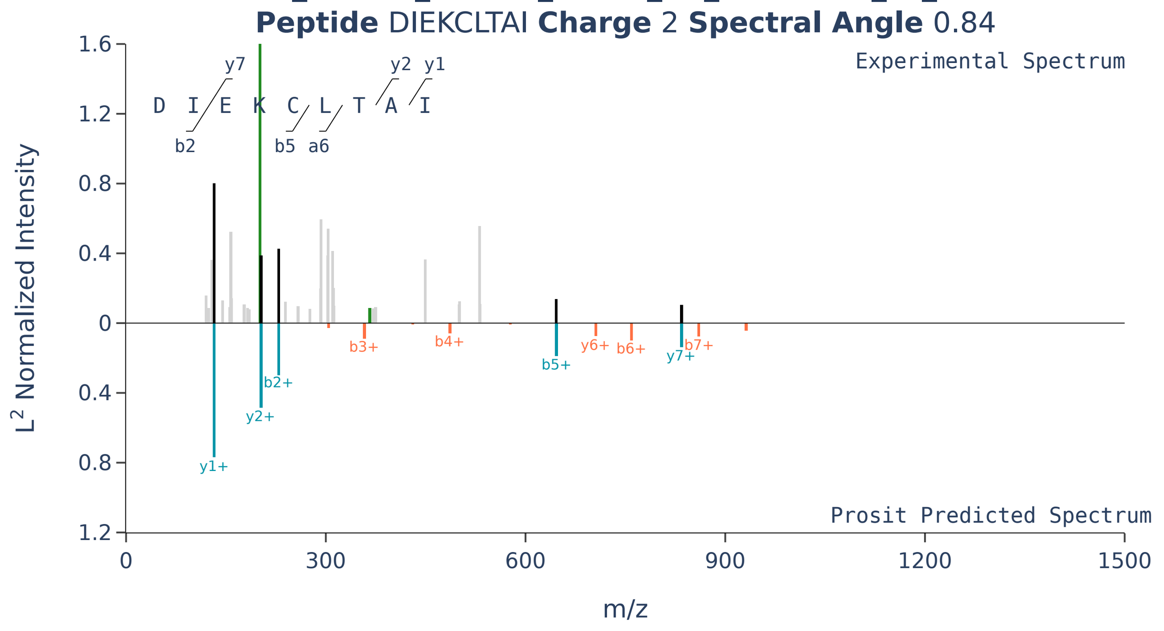


**Source** WSoh_301121_021221_HFGoe_M2_AnnexinA1_2h_R2 **Scan** 15213
**Peptide** LYEAGERRKGTDVNVF **Charge** 3 **Spectral Angle** 0.79

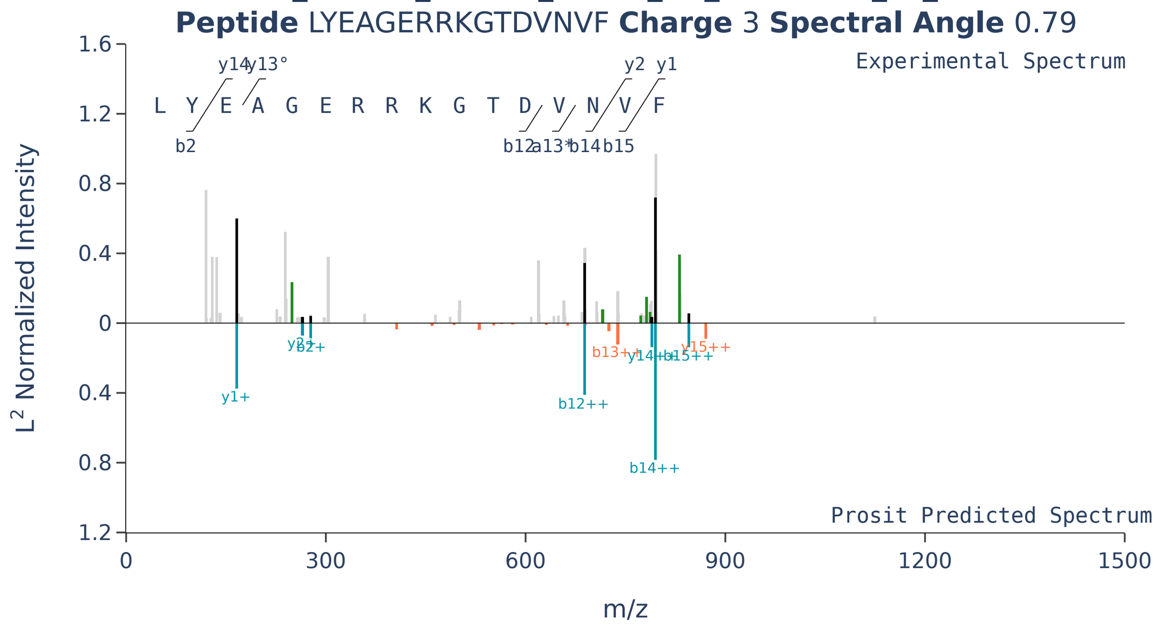


**Source** WSoh_301121_021221_HFGoe_M2_AnnexinA1_24h_R1 **Scan** 15939
**Peptide** ELKGDIEKCLT **Charge** 2 **Spectral Angle** 0.64

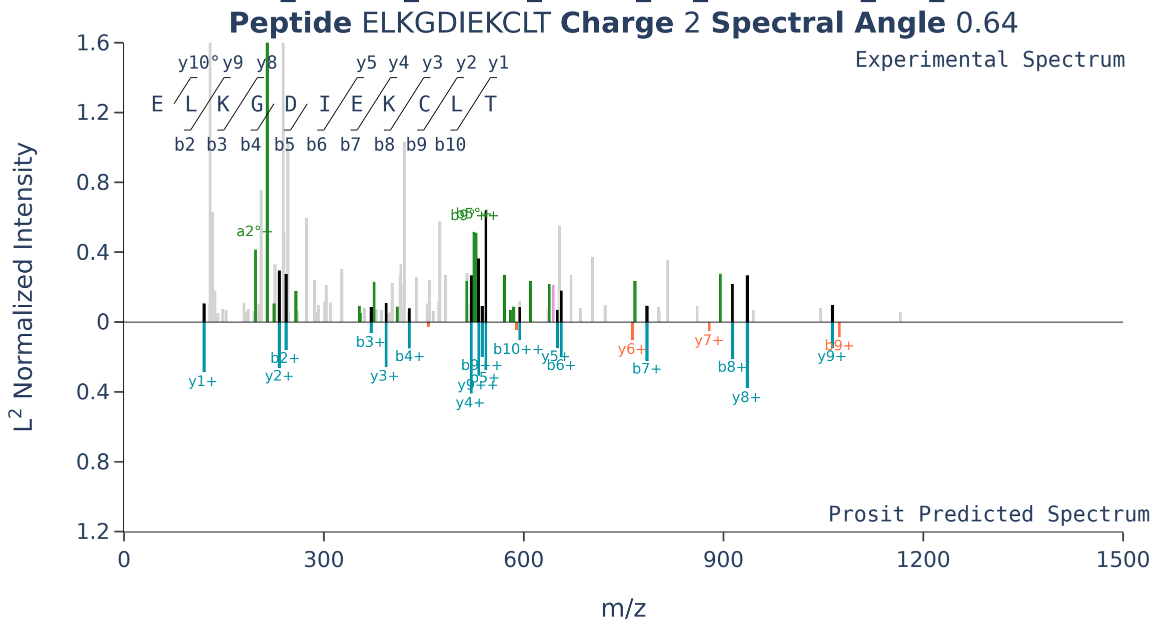


**Source** WSoh_301121_021221_HFGoe_M2_AnnexinA1_24h_R1 **Scan** 13328
**Peptide** MVSRSEIDMN **Charge** 2 **Spectral Angle** 0.78

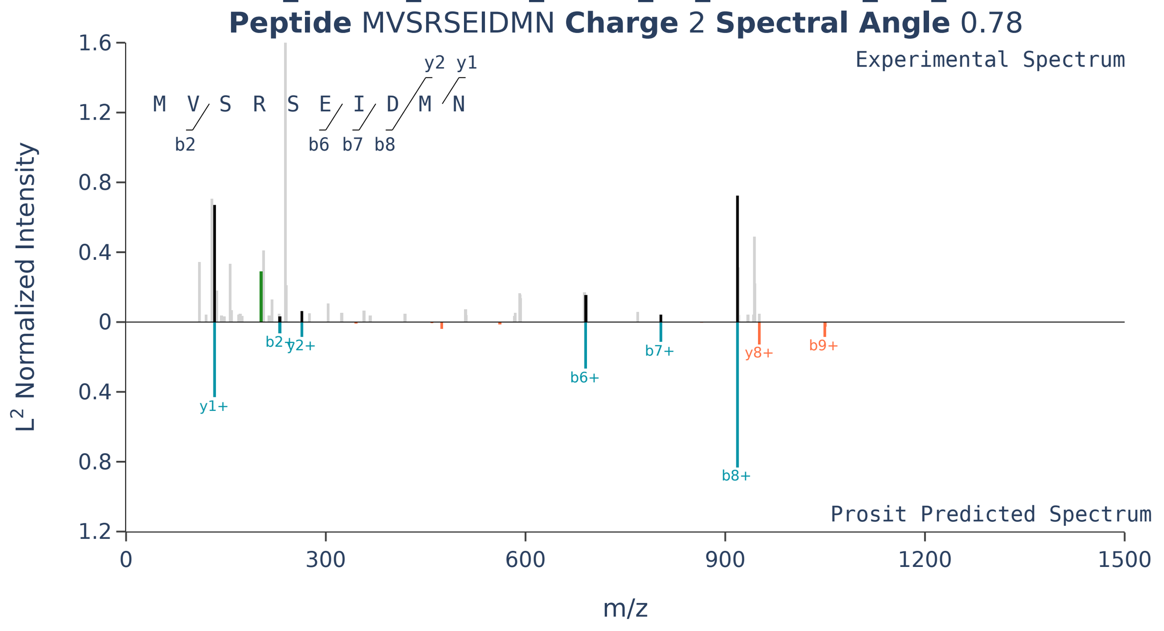


**Source** WSoh_301121_021221_HFGoe_M1_AnnexinA1_24h_R2 **Scan** 18202
**Peptide** ETKGDYEKILV **Charge** 2 **Spectral Angle** 0.73

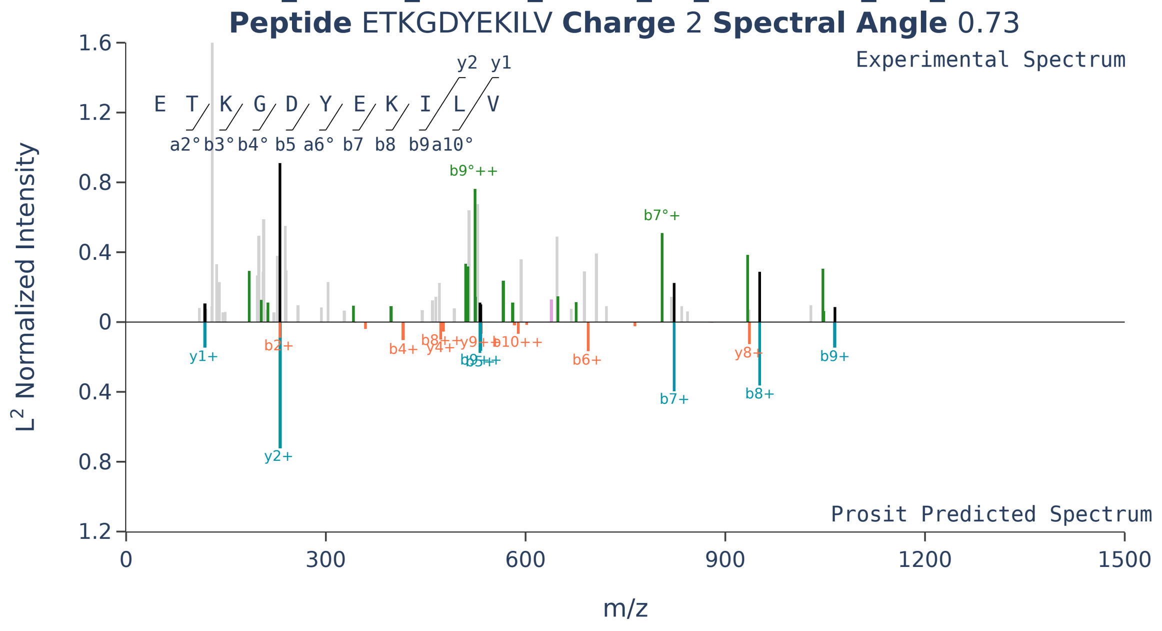


**Source** WSoh_301121_021221_HFGoe_M1_AnnexinA1_24h_R1 **Scan** 24158
**Peptide** TLIDILTKRNNA **Charge** 3 **Spectral Angle** 0.81

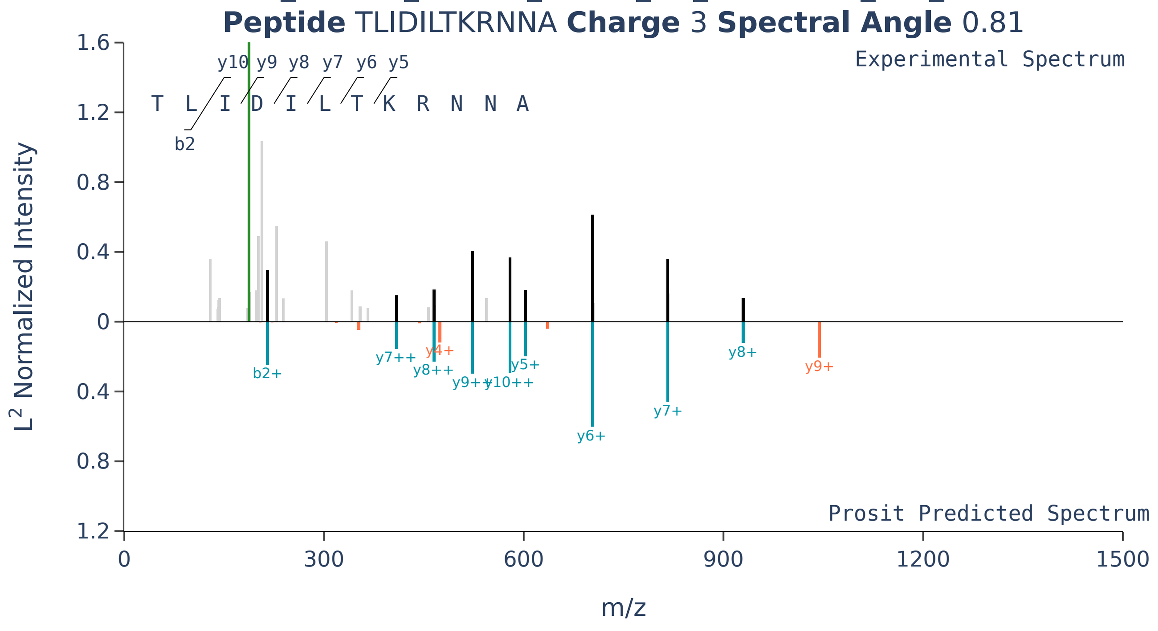


**Source** WSoh_301121_021221_HFGoe_M2_AnnexinA1_4h_R2 **Scan** 6837
**Peptide** EELKRDLAKD **Charge** 2 **Spectral Angle** 0.76

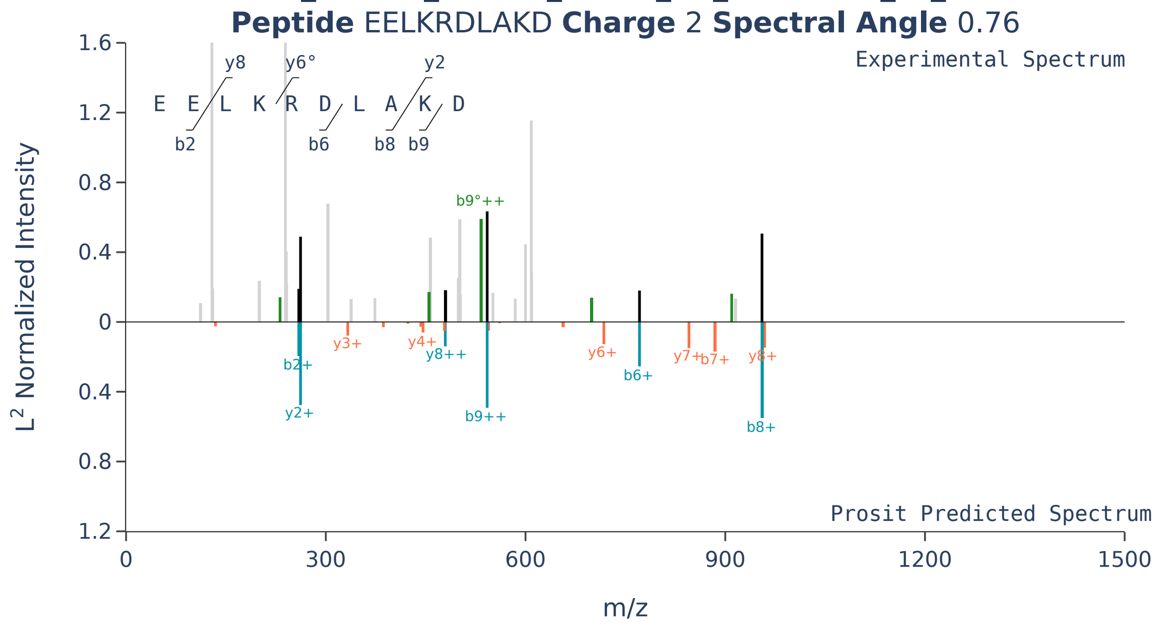


**Source** WSoh_301121_021221_HFGoe_M2_AnnexinA1_2h_R2 **Scan** 14857
**Peptide** NKEIRDINRVYREEL **Charge** 4 **Spectral Angle** 0.74

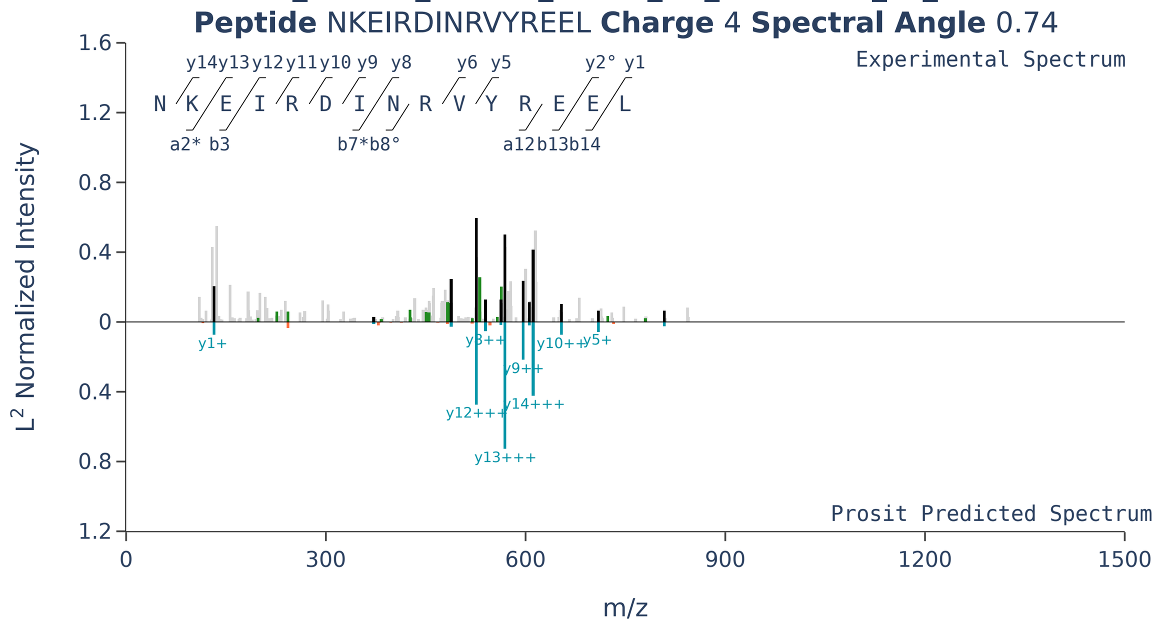

**Source** WSoh_301121_021221_HFGoe_M2_AnnexinA1_2h_R1 **Scan** 15107
**Peptide** VAALHKAIMVKGVDE **Charge** 3 **Spectral Angle** 0.75

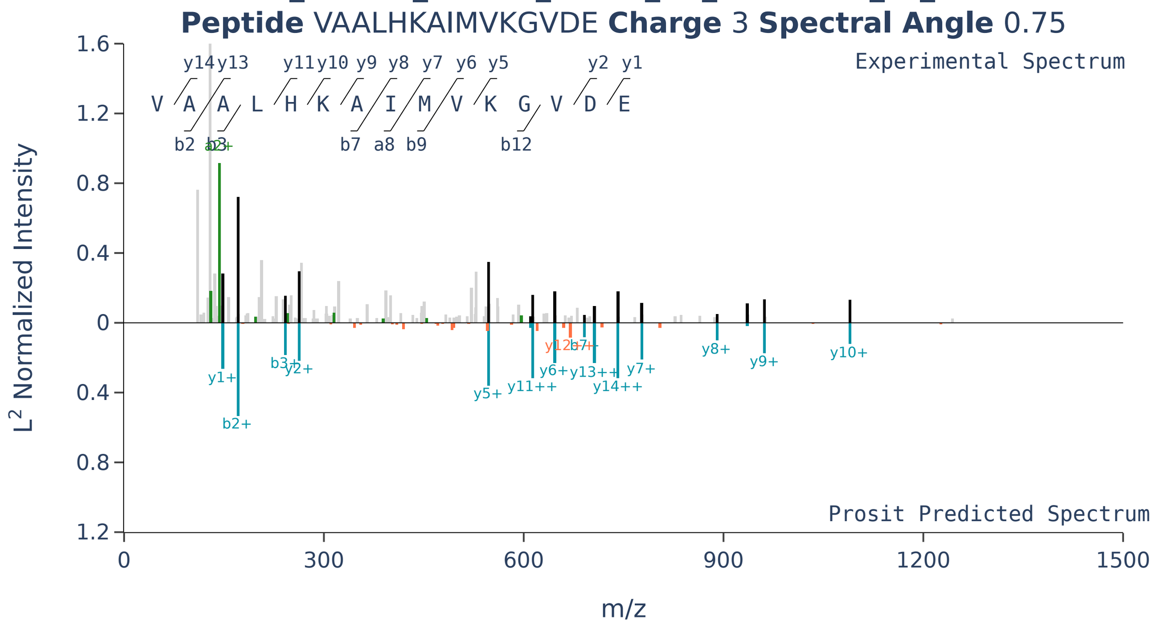


**Source** WSoh_301121_021221_HFGoe_M2_AnnexinA1_2h_R1 **Scan** 27026
**Peptide** EATIIDILTKRNNA **Charge** 2 **Spectral Angle** 0.88

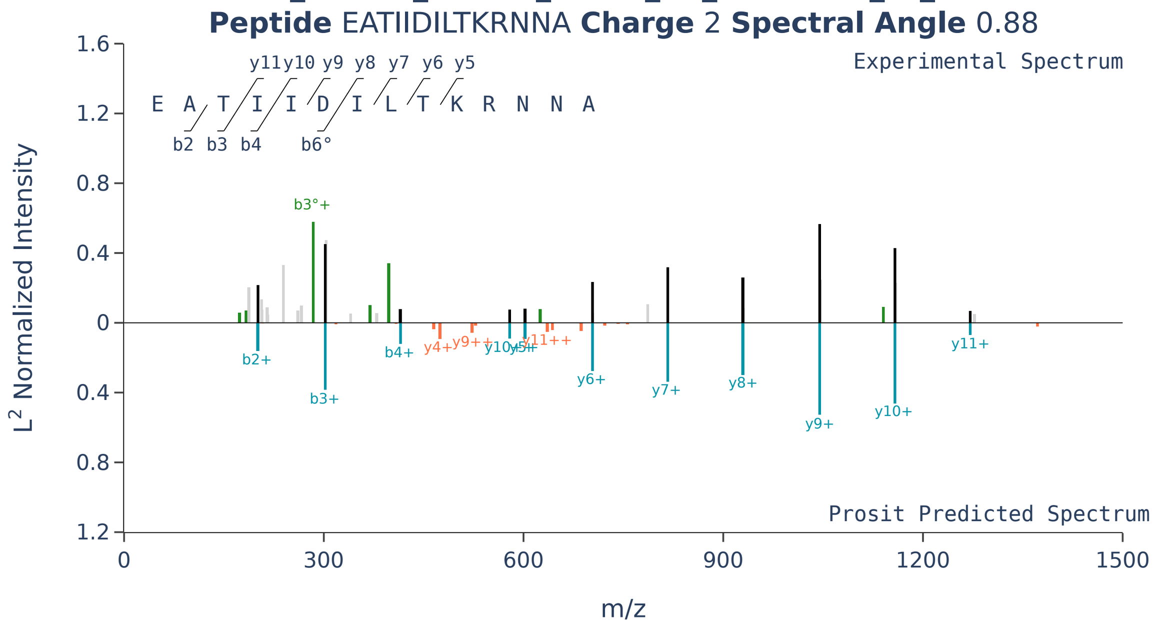


**Source** WSoh_301121_021221_HFGoe_M1_AnnexinA1_4h_R2 **Scan** 10452
**Peptide** KAAYLQET **Charge** 2 **Spectral Angle** 0.52

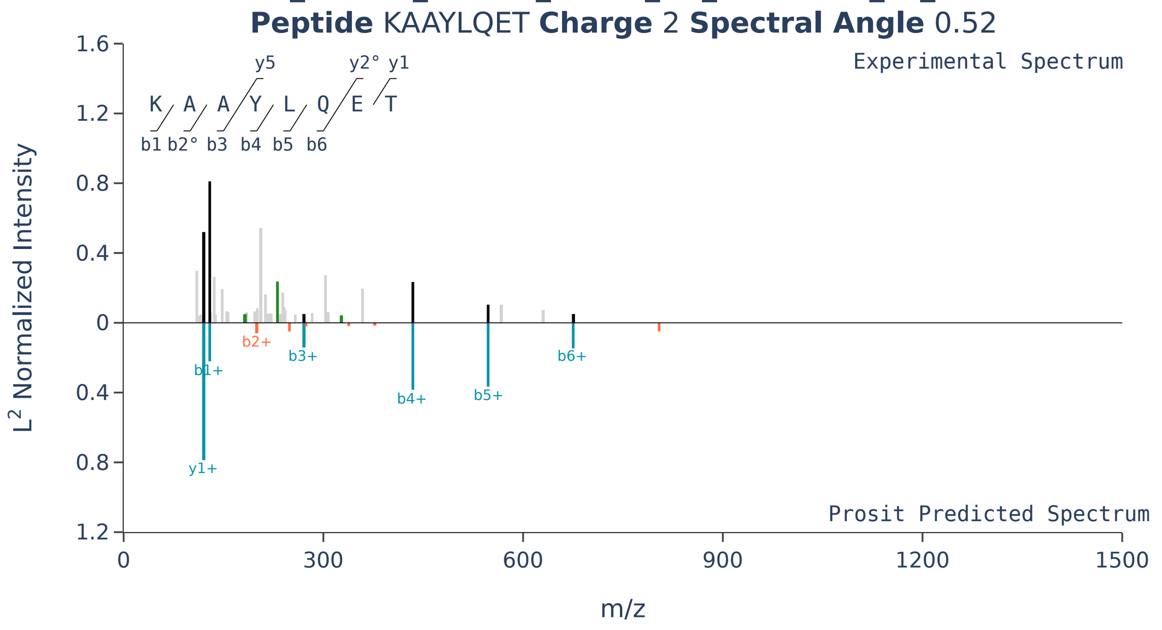


**Source** WSoh_301121_021221_HFGoe_M2_AnnexinA1_2h_R1 **Scan** 22593
**Peptide** DADELRAAMKGLGTD **Charge** 3 **Spectral Angle** 0.85

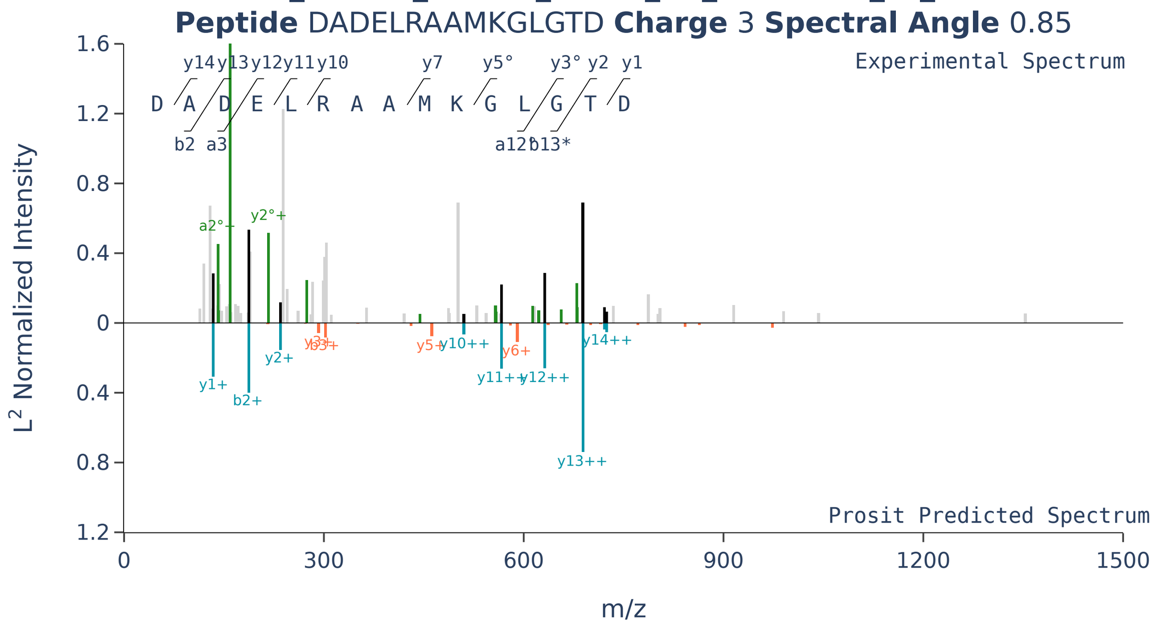


**Source** WSoh_301121_021221_HFGoe_M2_AnnexinA1_1h_R1 **Scan** 22138
**Peptide** SRSEIDMNDIKAF **Charge** 3 **Spectral Angle** 0.85

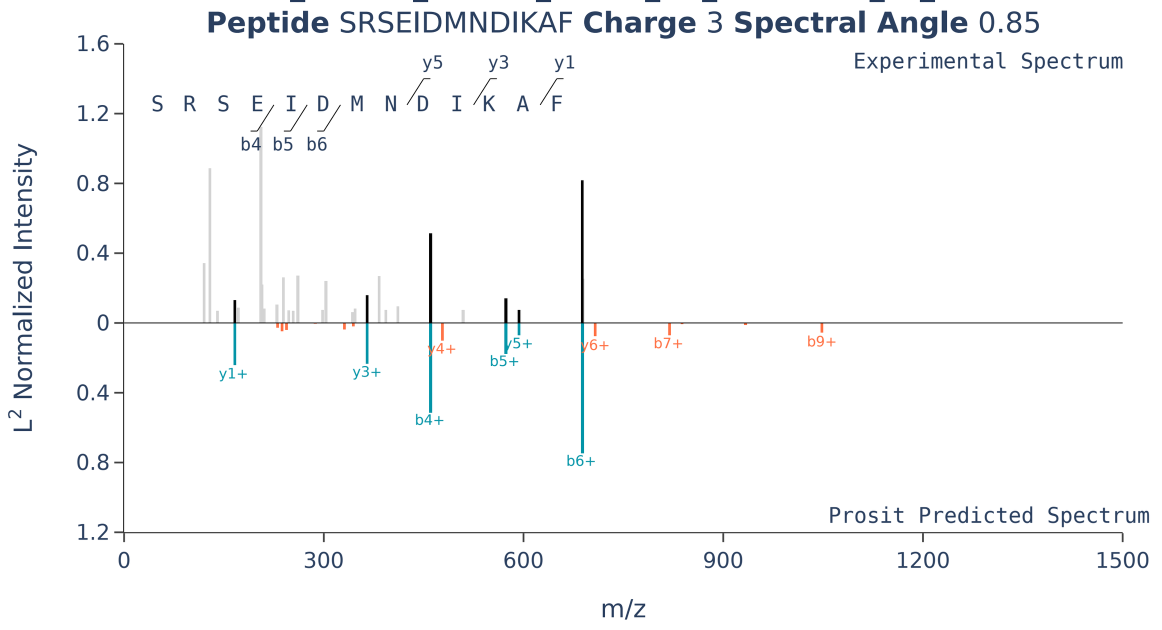


**Source** WSoh_301121_021221_HFGoe_M2_AnnexinA1_24h_R1 **Scan** 27206
**Peptide** DETKGDYEKILVAL **Charge** 3 **Spectral Angle** 0.8

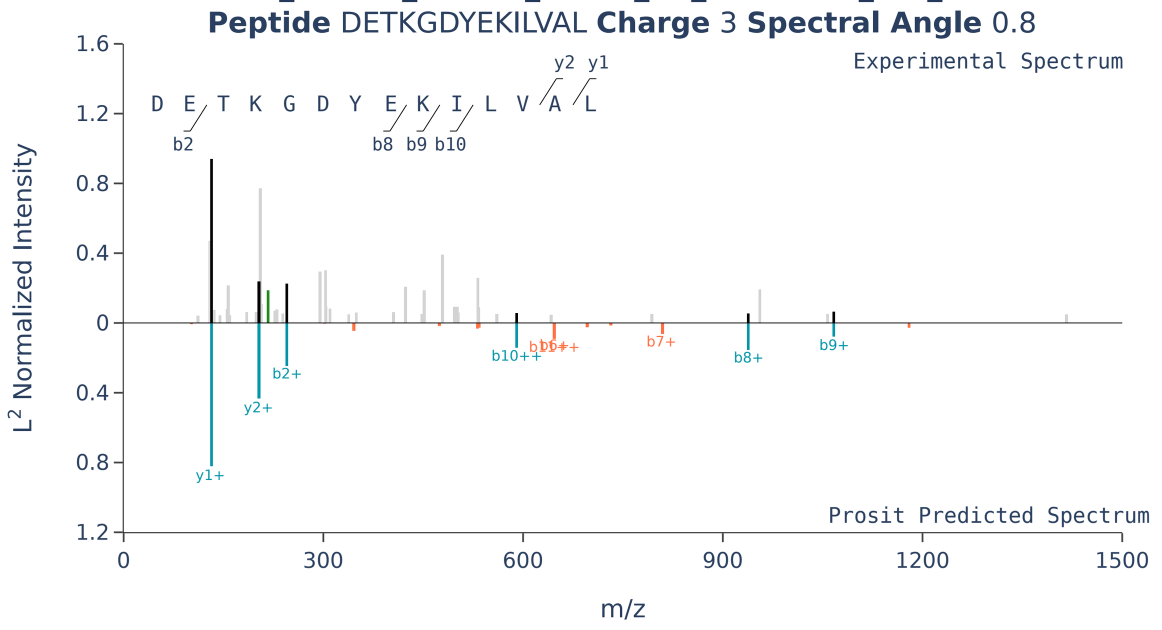


**Source** WSoh_301121_021221_HFGoe_M1_AnnexinA1_2h_R1 **Scan** 8557
**Peptide** RTNKEIRDINRVYRE **Charge** 4 **Spectral Angle** 0.78

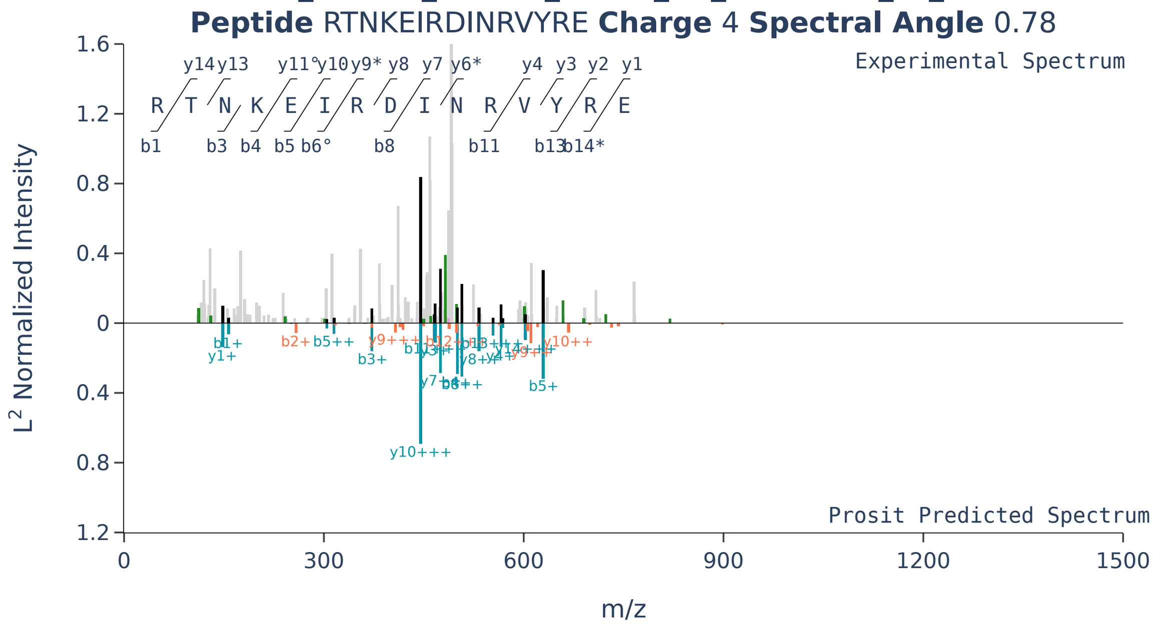


**Source** WSoh_301121_021221_HFGoe_M2_AnnexinA1_24h_R2 **Scan** 17225
**Peptide** LSLAKGDRSEDFGVN **Charge** 3 **Spectral Angle** 0.87

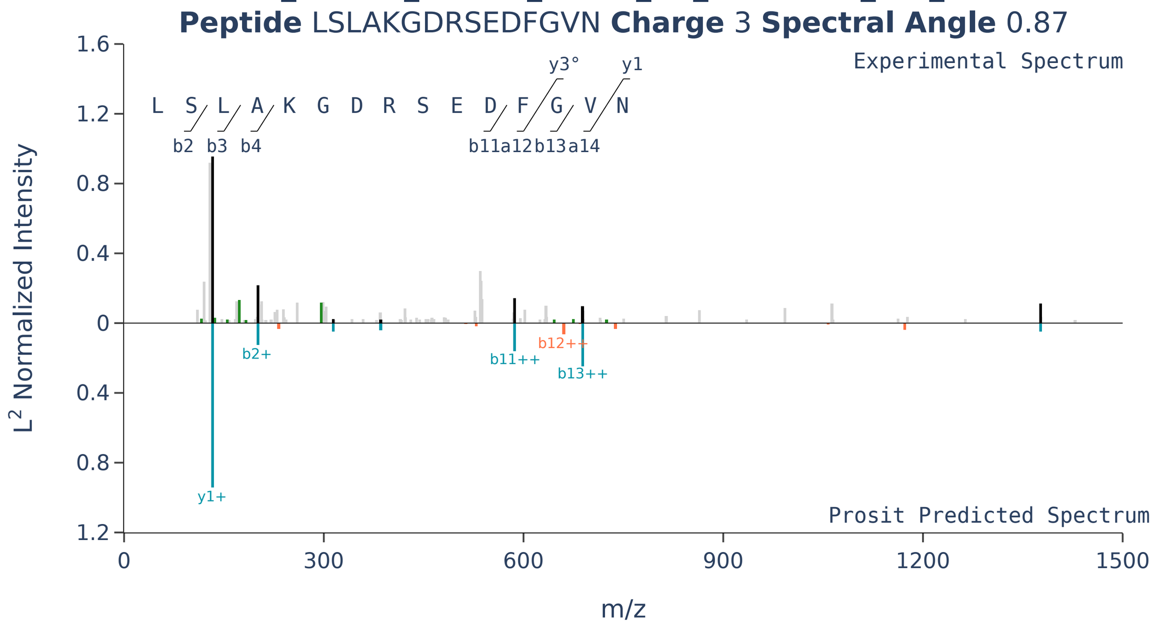


**Source** WSoh_301121_021221_HFGoe_M2_AnnexinA1_4h_R1 **Scan** 21237
**Peptide** DLADSDARALYEA **Charge** 2 **Spectral Angle** 0.81

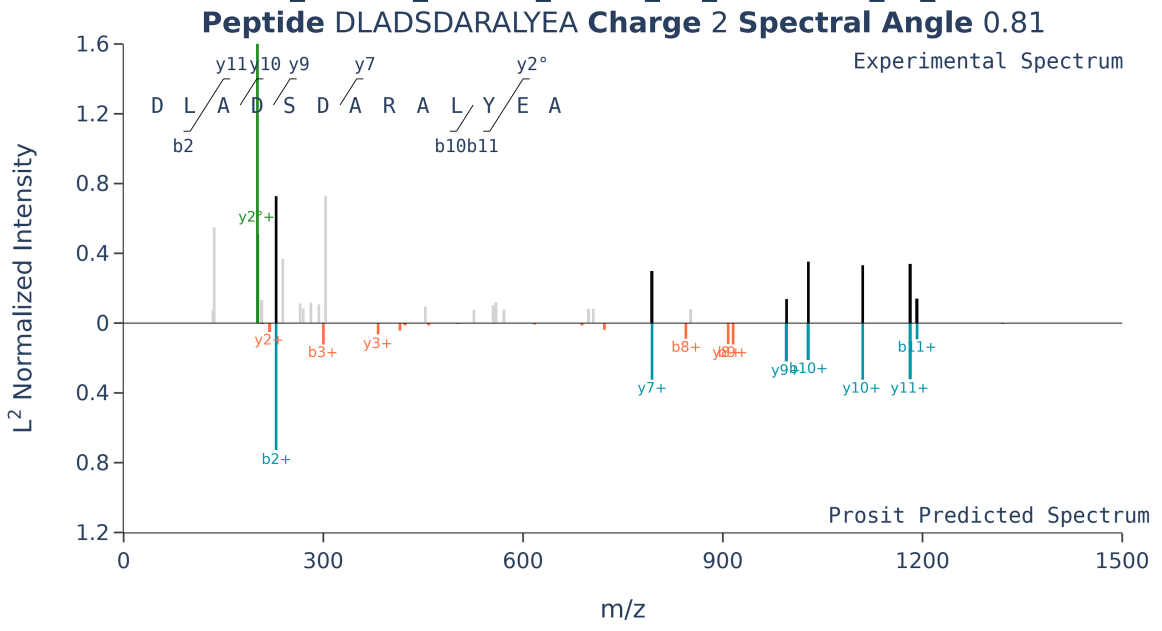


**Source** WSoh_301121_021221_HFGoe_M2_AnnexinA1_2h_R1 **Scan** 20331
**Peptide** AKGDRSEDFGVNEDLADSDARAL **Charge** 4 **Spectral Angle** 0.79

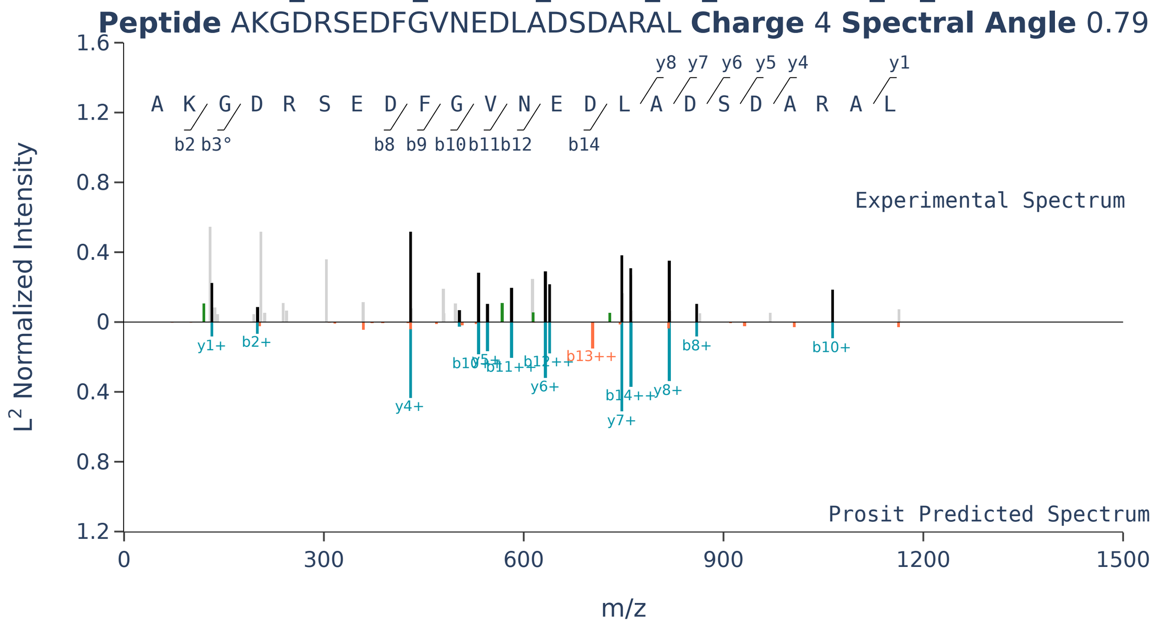


**Source** WSoh_301121_021221_HFGoe_M2_AnnexinA1_2h_R2 **Scan** 10755
**Peptide** TLKKALTGHLEE **Charge** 2 **Spectral Angle** 0.66

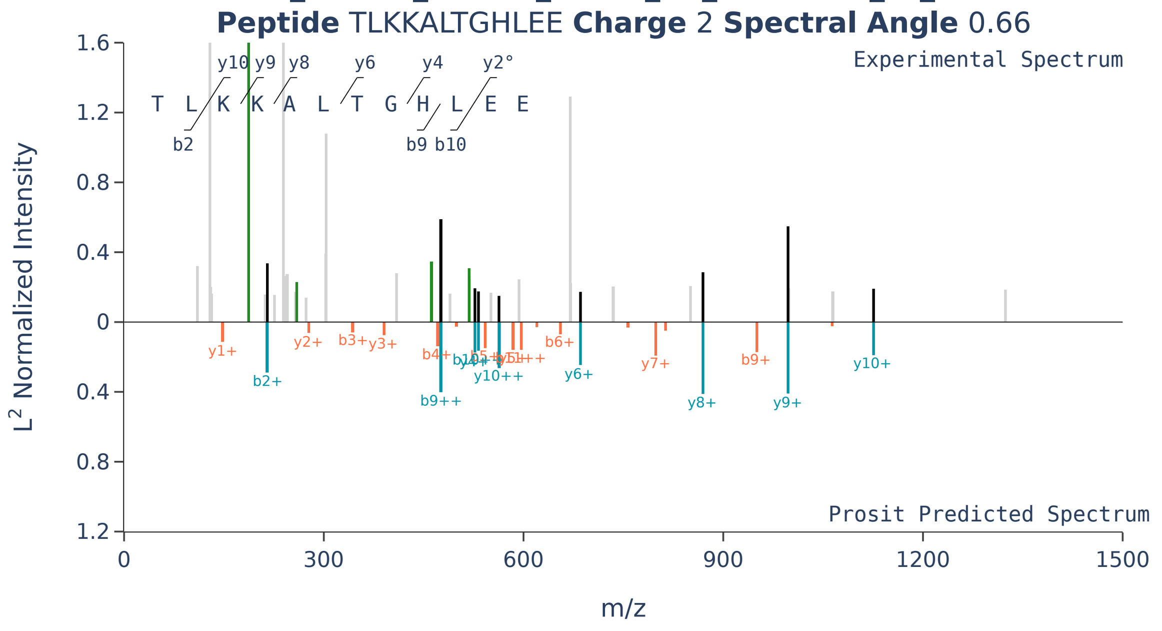


**Source** WSoh_301121_021221_HFGoe_M2_AnnexinA1_4h_R2 **Scan** 15842
**Peptide** LKTPAQFDAD **Charge** 2 **Spectral Angle** 0.82

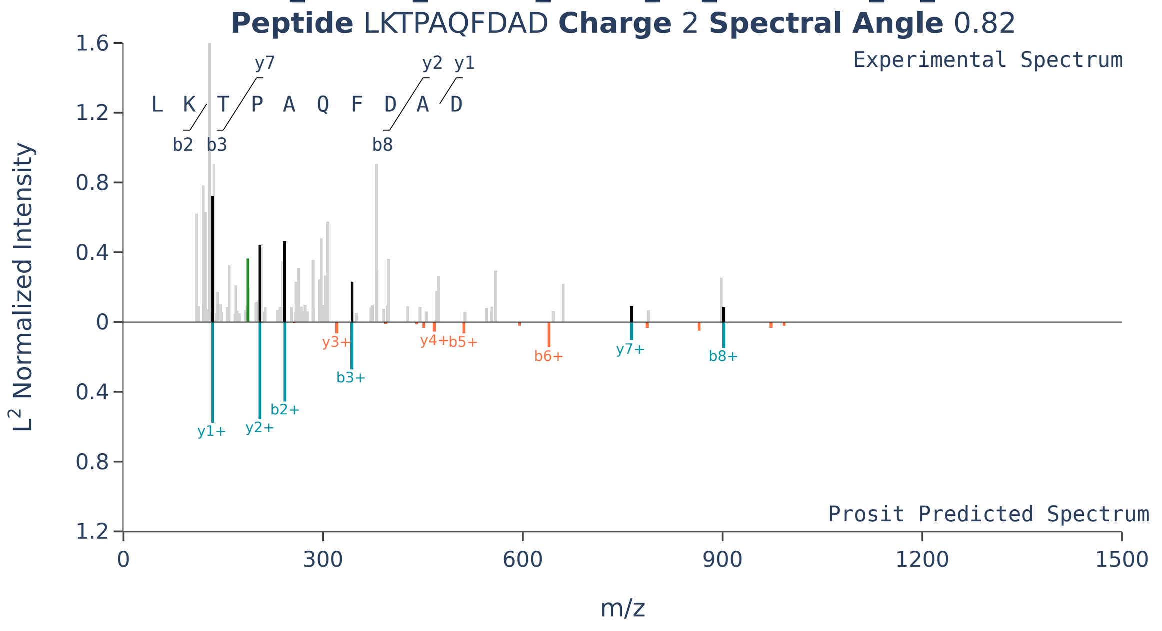


**Source** WSoh_301121_021221_HFGoe_M2_AnnexinA1_24h_R2 **Scan** 9853
**Peptide** SDARALYEAGERRKGTDVN **Charge** 4 **Spectral Angle** 0.72

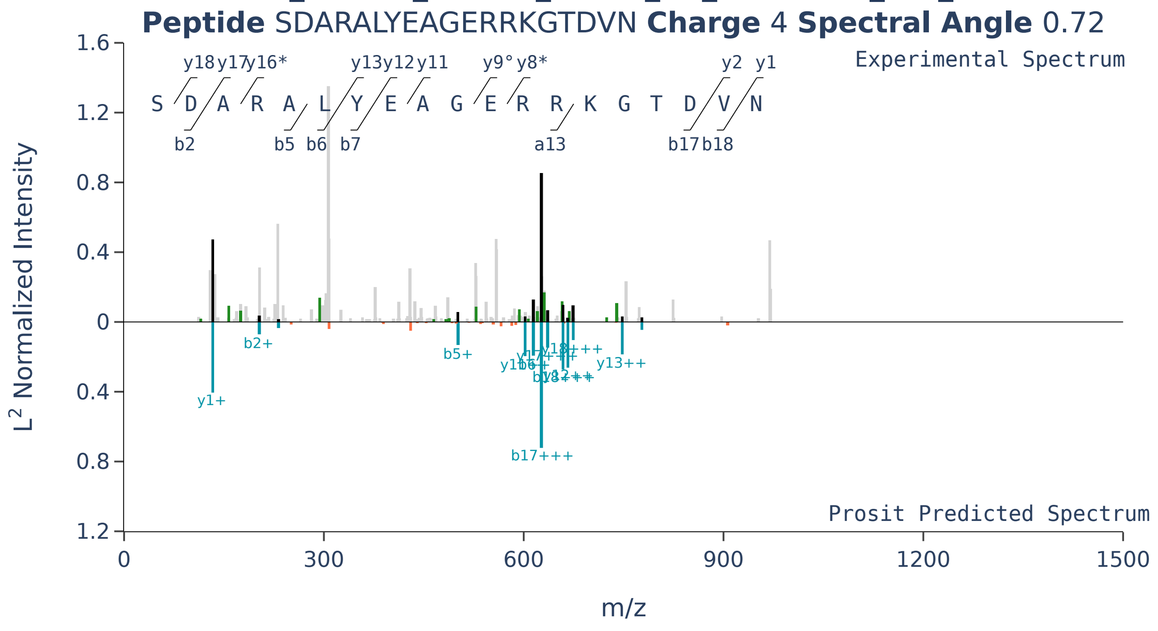


**Source** WSoh_301121_021221_HFGoe_M2_AnnexinA1_1h_R2 **Scan** 15159
**Peptide** RSYPQLRRVFQ **Charge** 3 **Spectral Angle** 0.77

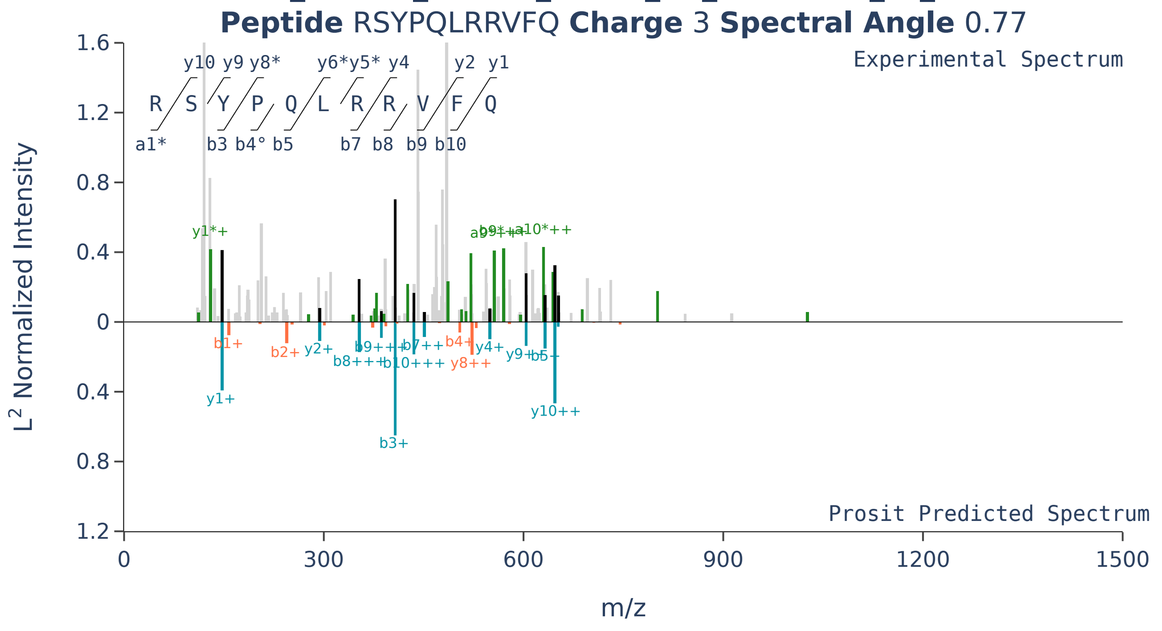


**Source** WSoh_301121_021221_HFGoe_M1_AnnexinA1_4h_R2 **Scan** 25516
**Peptide** QAWFIENEE **Charge** 2 **Spectral Angle** 0.81

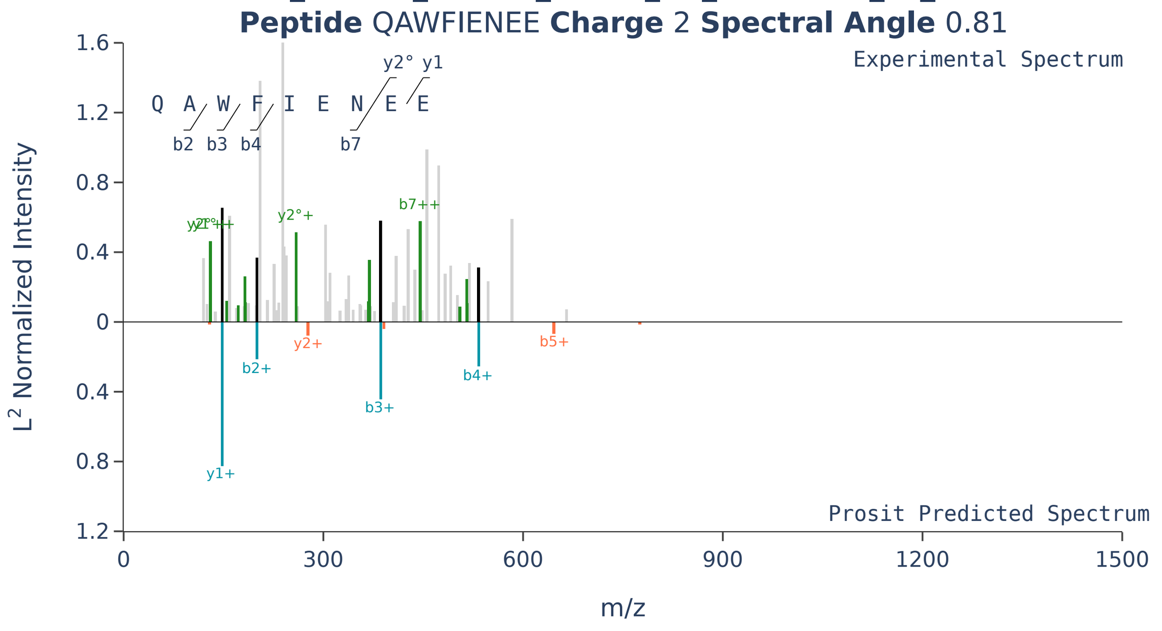

**Source** WSoh_301121_021221_HFGoe_M2_AnnexinA1_2h_R2 **Scan** 12244
**Peptide** ALHKAIMVKGVDEA **Charge** 2 **Spectral Angle** 0.77

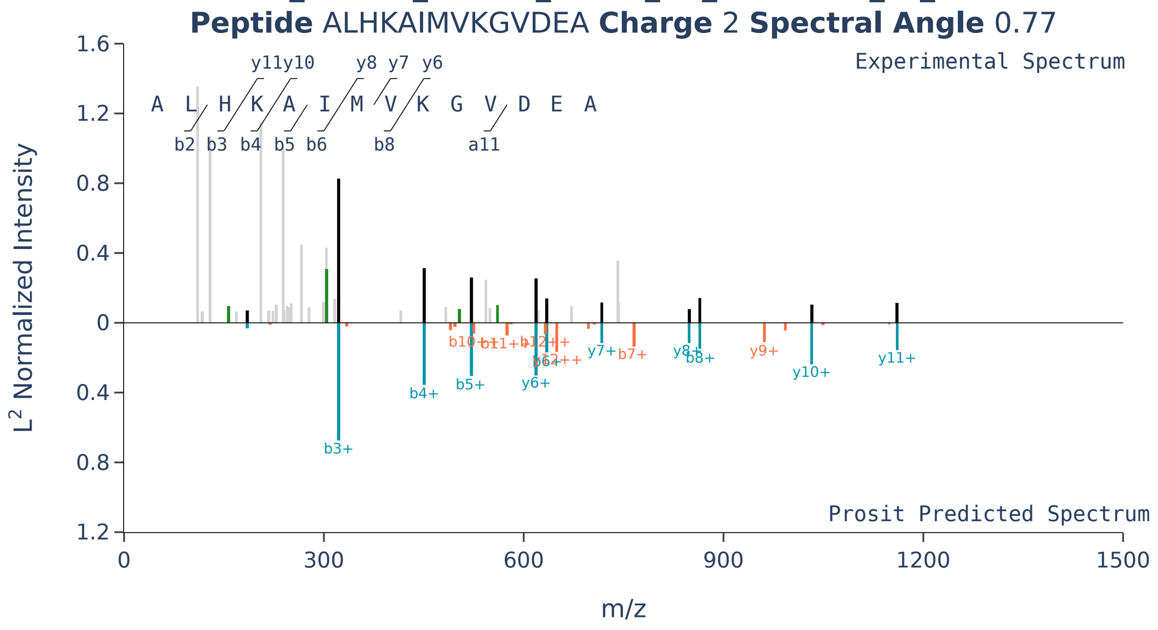


**Source** WSoh_301121_021221_HFGoe_M2_AnnexinA1_4h_R1 **Scan** 8897
**Peptide** YVQTVKSSKGGPGSAV **Charge** 2 **Spectral Angle** 0.79

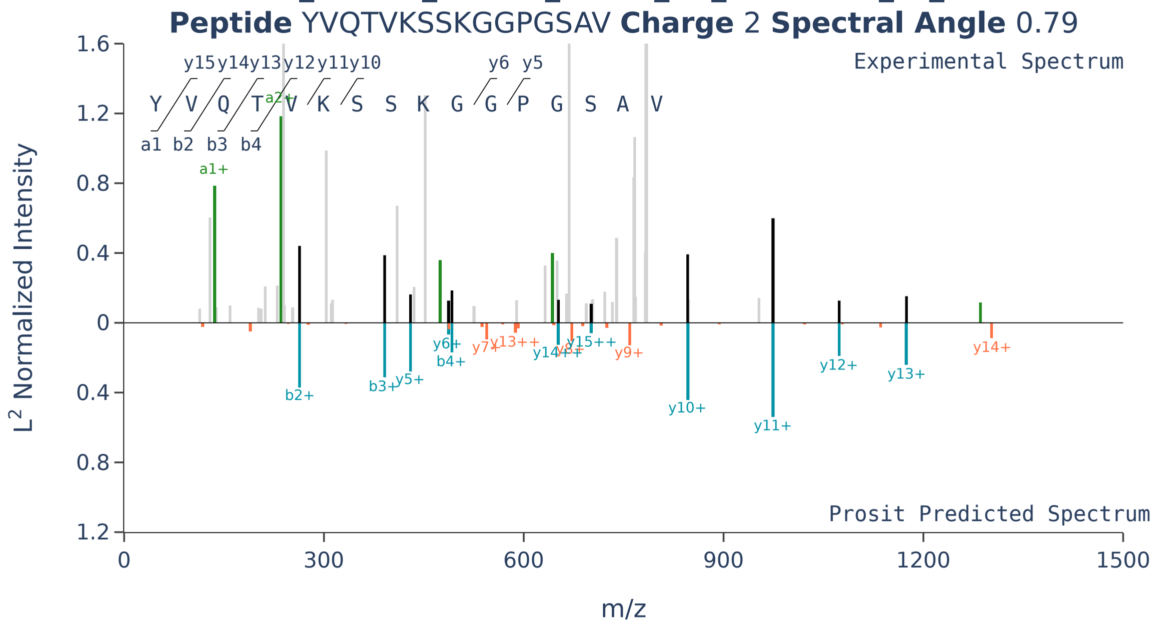


**Source** WSoh_301121_021221_HFGoe_M2_AnnexinA1_2h_R1 **Scan** 23483
**Peptide** ILDETKGDYEKILV **Charge** 2 **Spectral Angle** 0.74

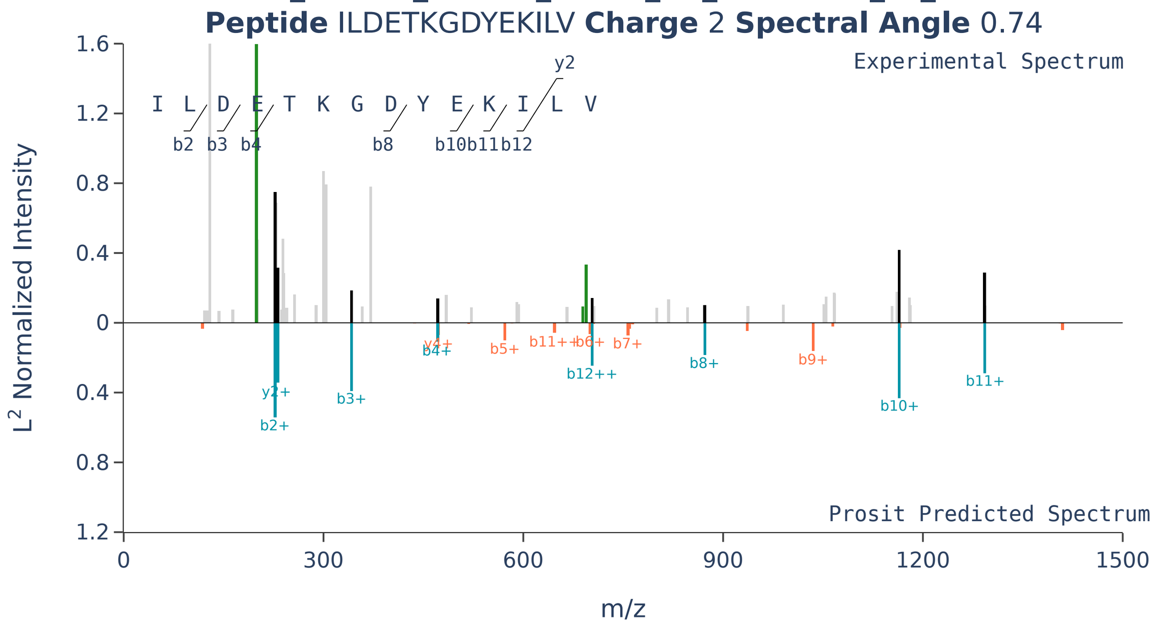


**Source** WSoh_301121_021221_HFGoe_M1_AnnexinA1_24h_R1 **Scan** 20426
**Peptide** KGDIEKCLTAIVKC **Charge** 3 **Spectral Angle** 0.89

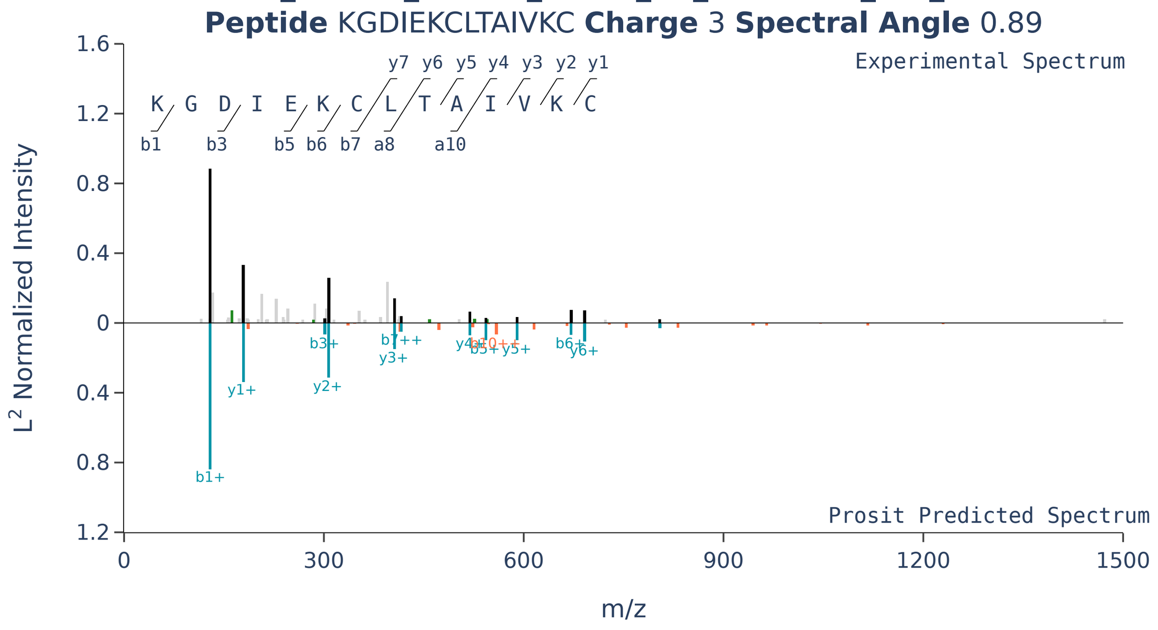


**Source** WSoh_301121_021221_HFGoe_M2_AnnexinA1_2h_R1 **Scan** 14895
**Peptide** KPAFFAEKLHQ **Charge** 3 **Spectral Angle** 0.7

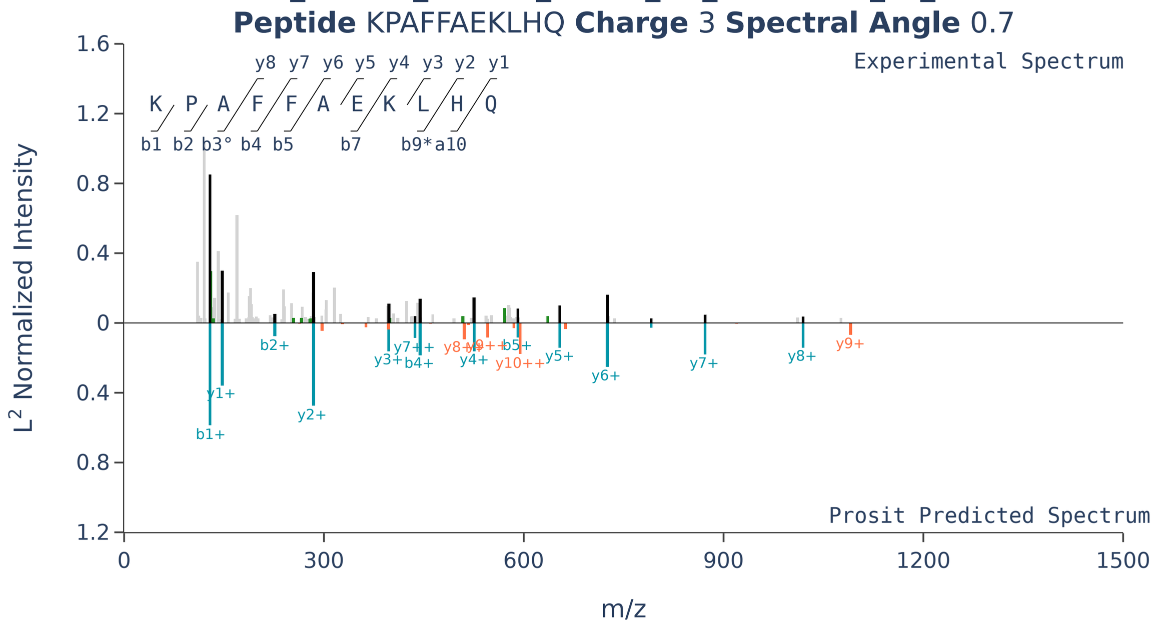


**Source** WSoh_301121_021221_HFGoe_M2_AnnexinA1_24h_R2 **Scan** 19314
**Peptide** YPTFNPSSD **Charge** 2 **Spectral Angle** 0.83

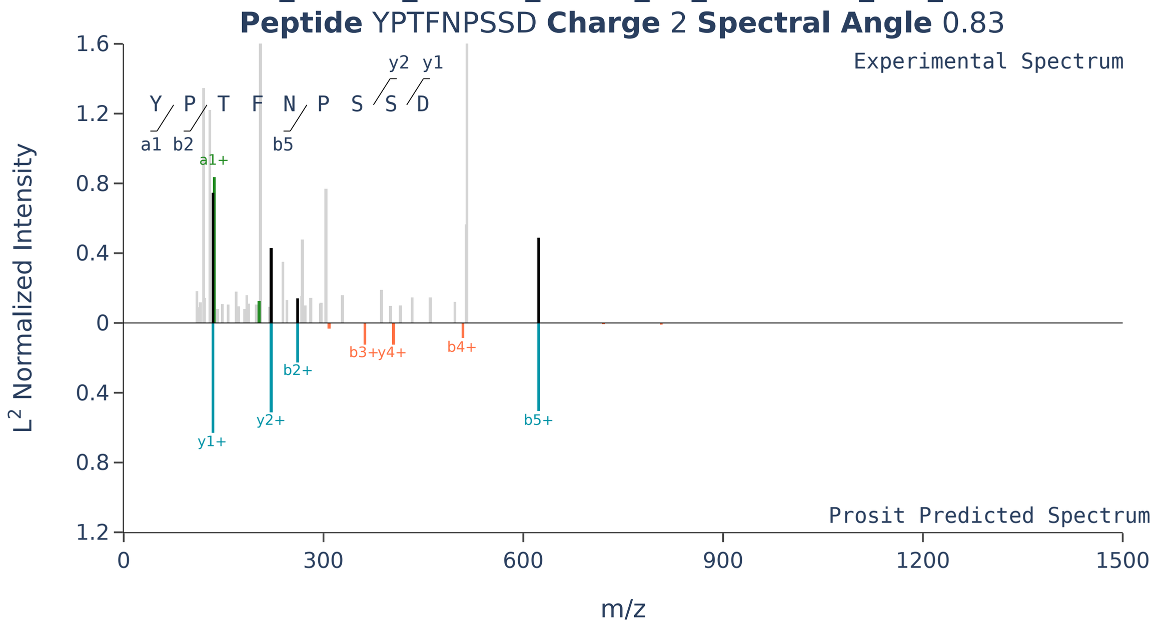


**Source** WSoh_301121_021221_HFGoe_M2_AnnexinA1_2h_R2 **Scan** 22671
**Peptide** SLCQAILDETKG **Charge** 2 **Spectral Angle** 0.74

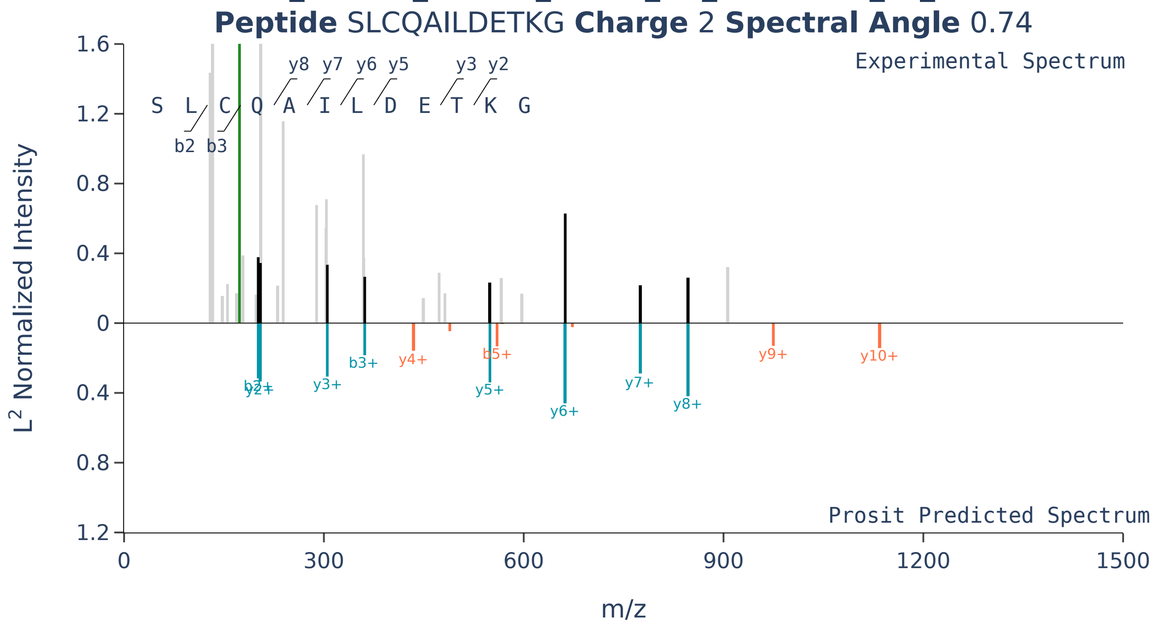


**Source** WSoh_301121_021221_HFGoe_M1_AnnexinA1_2h_R2 **Scan** 20997
**Peptide** GTDVNVFNT **Charge** 1 **Spectral Angle** 0.73

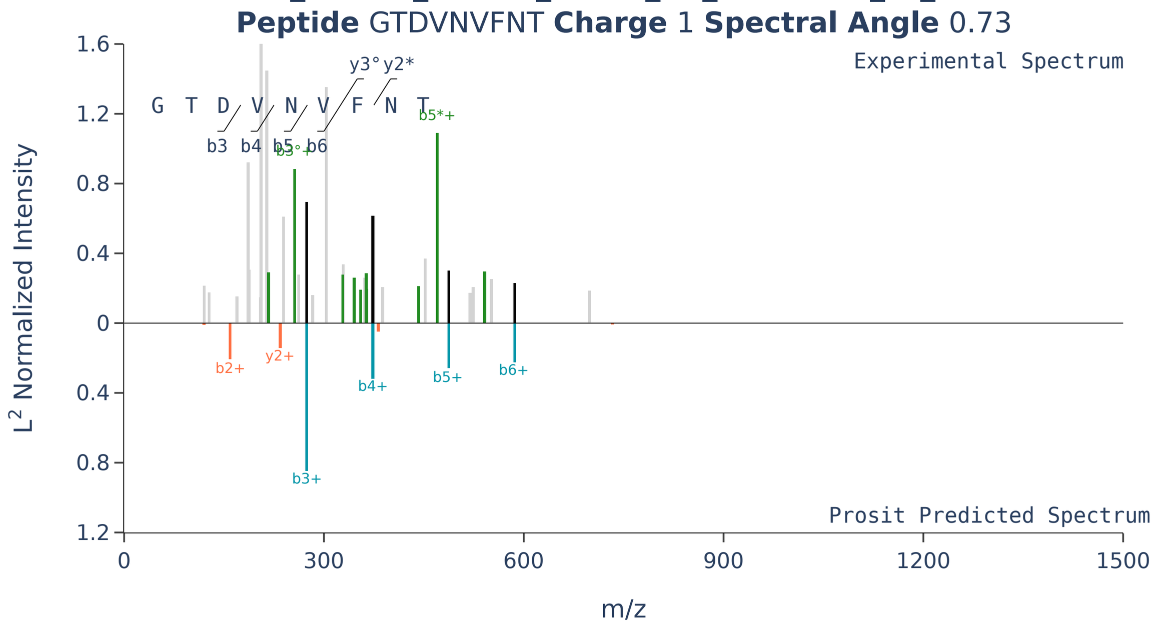


**Source** WSoh_301121_021221_HFGoe_M1_AnnexinA1_24h_R2 **Scan** 6796
**Peptide** NKEIRDIN **Charge** 2 **Spectral Angle** 0.87

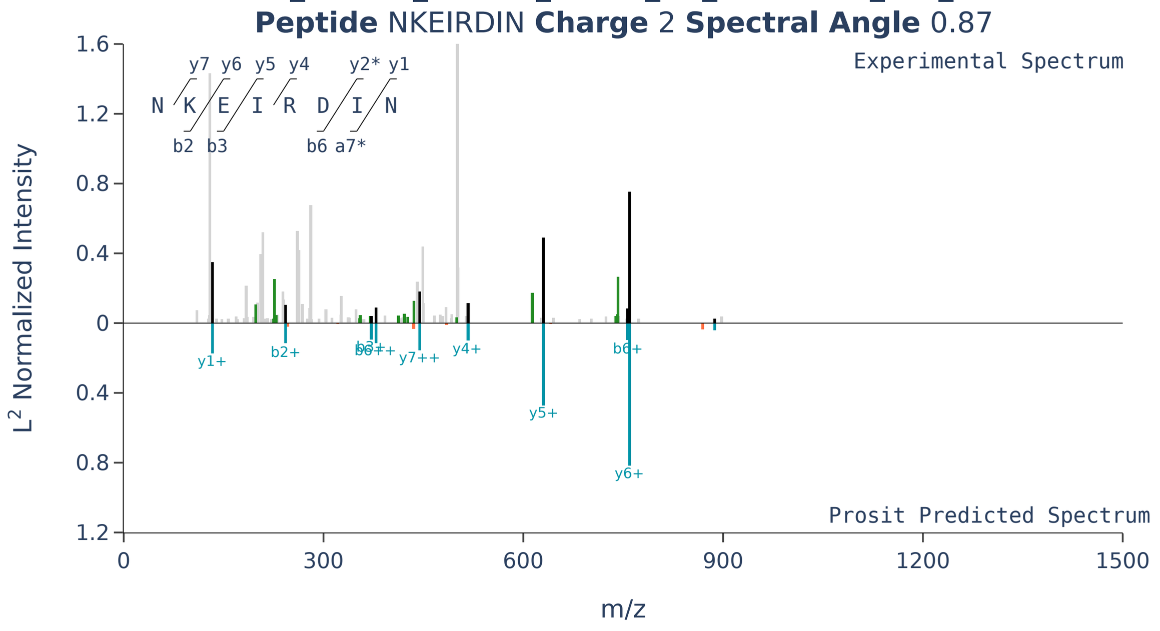


**Source** WSoh_301121_021221_HFGoe_M1_AnnexinA1_24h_R1 **Scan** 13521
**Peptide** RSEIDMNDIK **Charge** 2 **Spectral Angle** 0.86

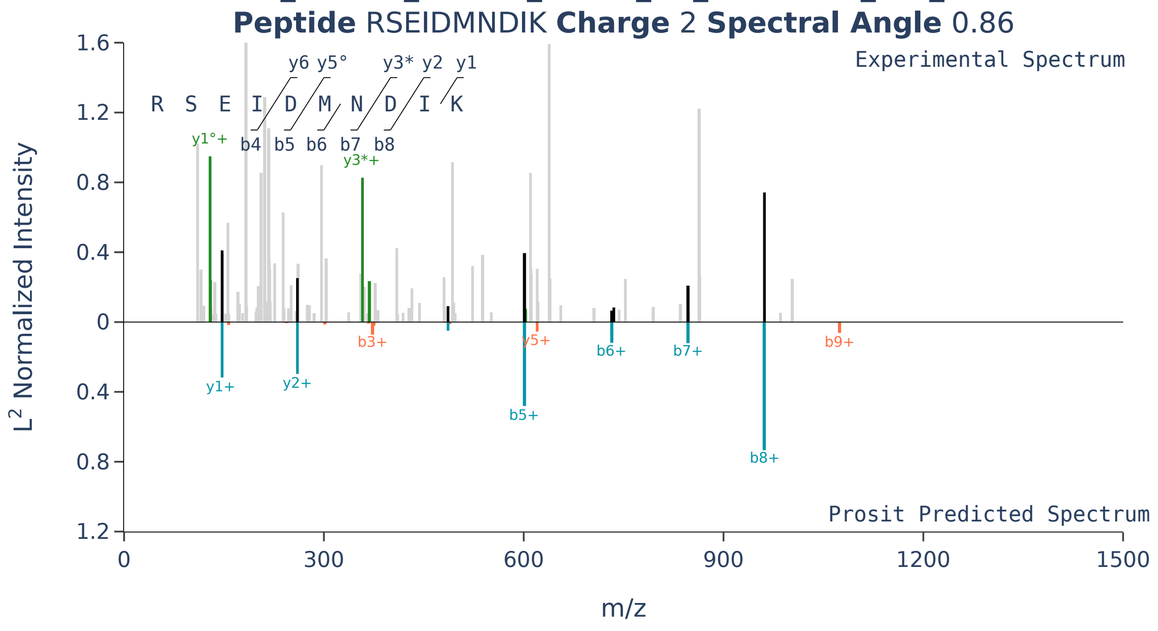


**Source** WSoh_301121_021221_HFGoe_M2_AnnexinA1_2h_R1 **Scan** 18330
**Peptide** KPLDETLKKALTG **Charge** 2 **Spectral Angle** 0.67

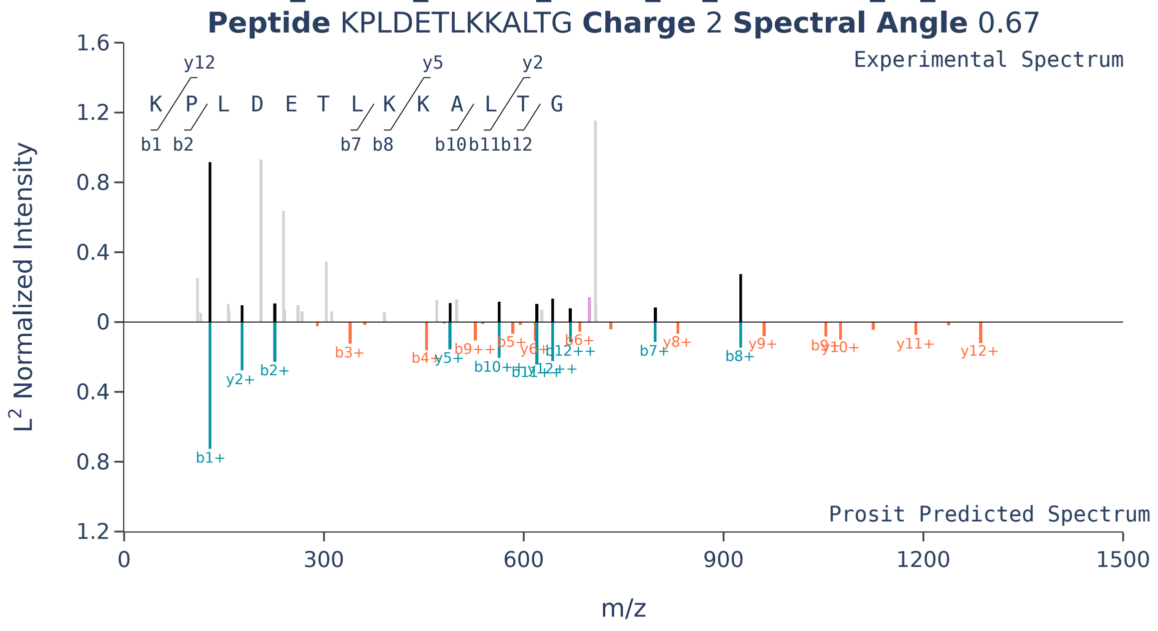


**Source** WSoh_301121_021221_HFGoe_M2_AnnexinA1_2h_R1 **Scan** 21992
**Peptide** RSEIDMNDIKAFYQK **Charge** 3 **Spectral Angle** 0.82

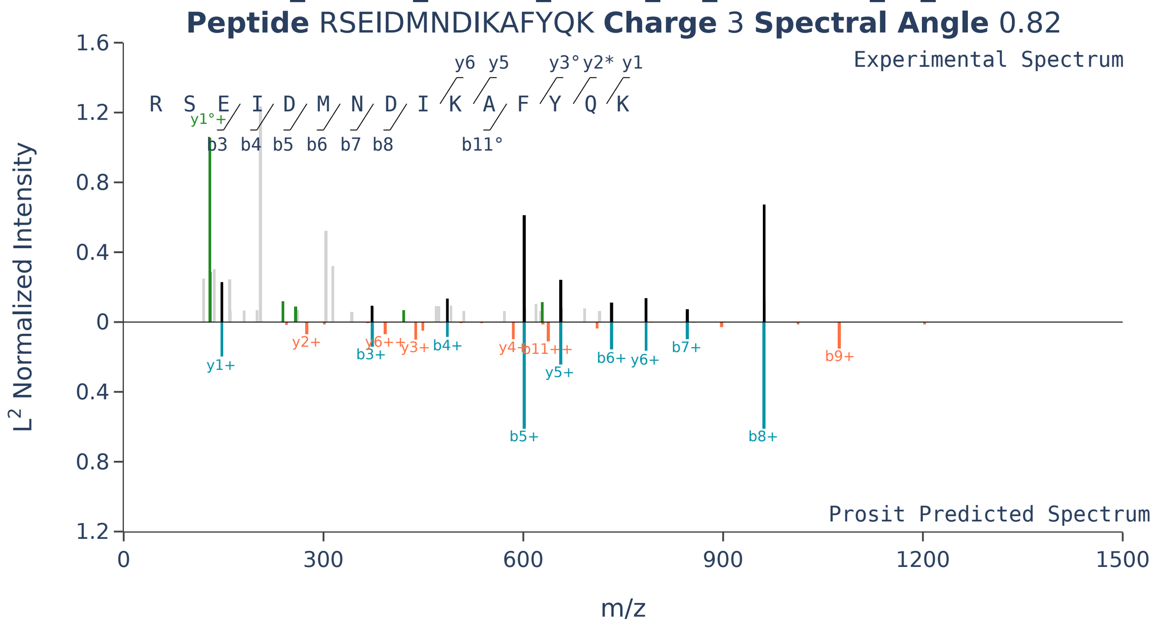


**Source** WSoh_301121_021221_HFGoe_M2_AnnexinA1_4h_R2 **Scan** 24642
**Peptide** KGVDEATIIDI **Charge** 2 **Spectral Angle** 0.76

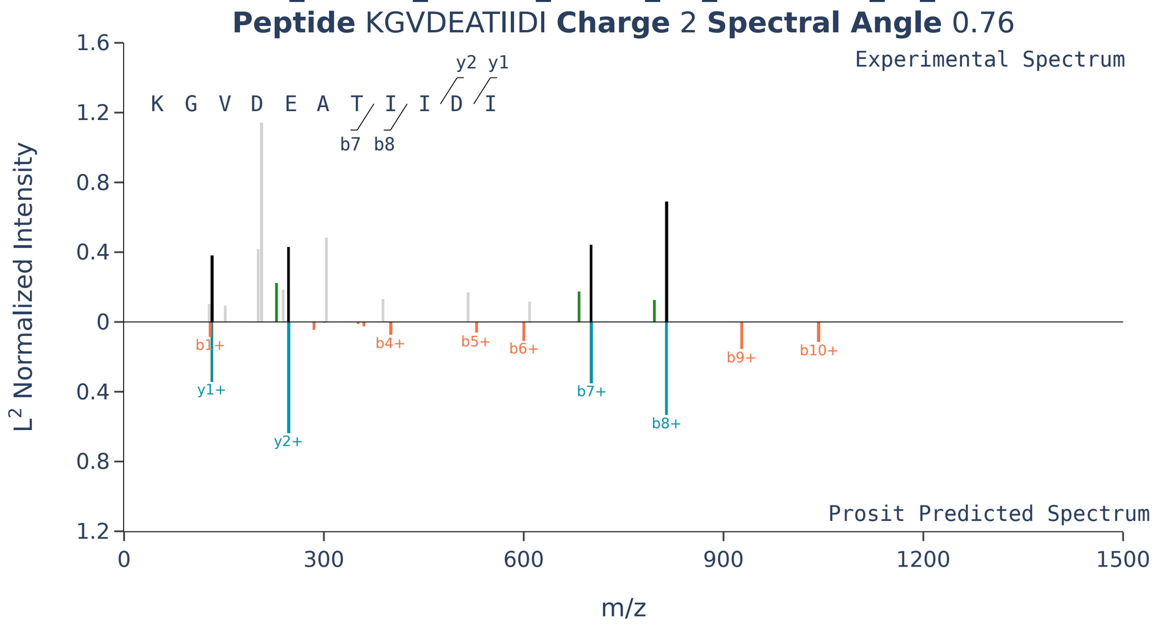


**Source** WSoh_301121_021221_HFGoe_M2_AnnexinA1_2h_R2 **Scan** 20670
**Peptide** LLSLAKGDRSEDFGVNED **Charge** 3 **Spectral Angle** 0.8

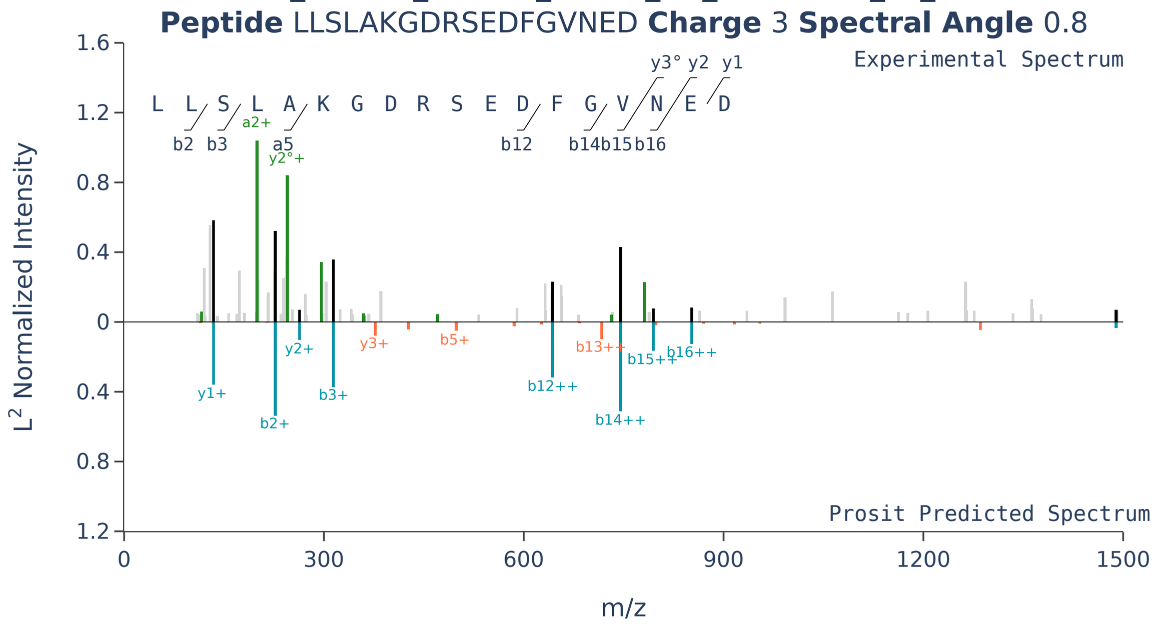


**Source** WSoh_301121_021221_HFGoe_M2_AnnexinA1_2h_R1 **Scan** 18363
**Peptide** EELKRDLAKDITSDTSGDFRNA **Charge** 3 **Spectral Angle** 0.73

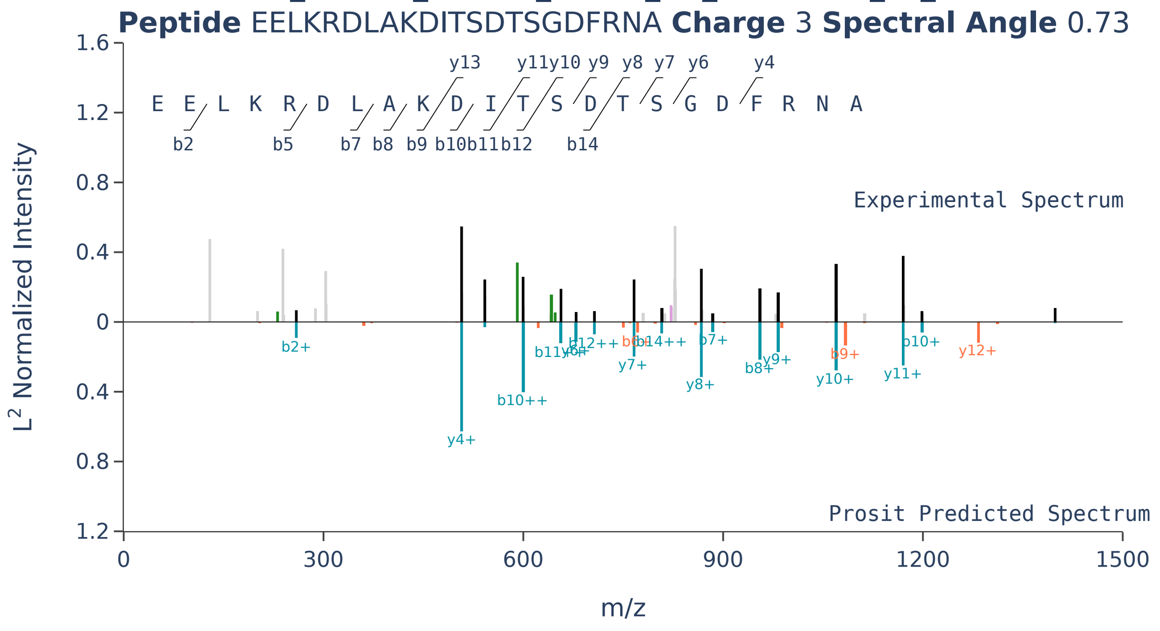

**Source** WSoh_301121_021221_HFGoe_M2_AnnexinA1_4h_R1 **Scan** 21266
**Peptide** YLQETGKPLDETL **Charge** 2 **Spectral Angle** 0.82

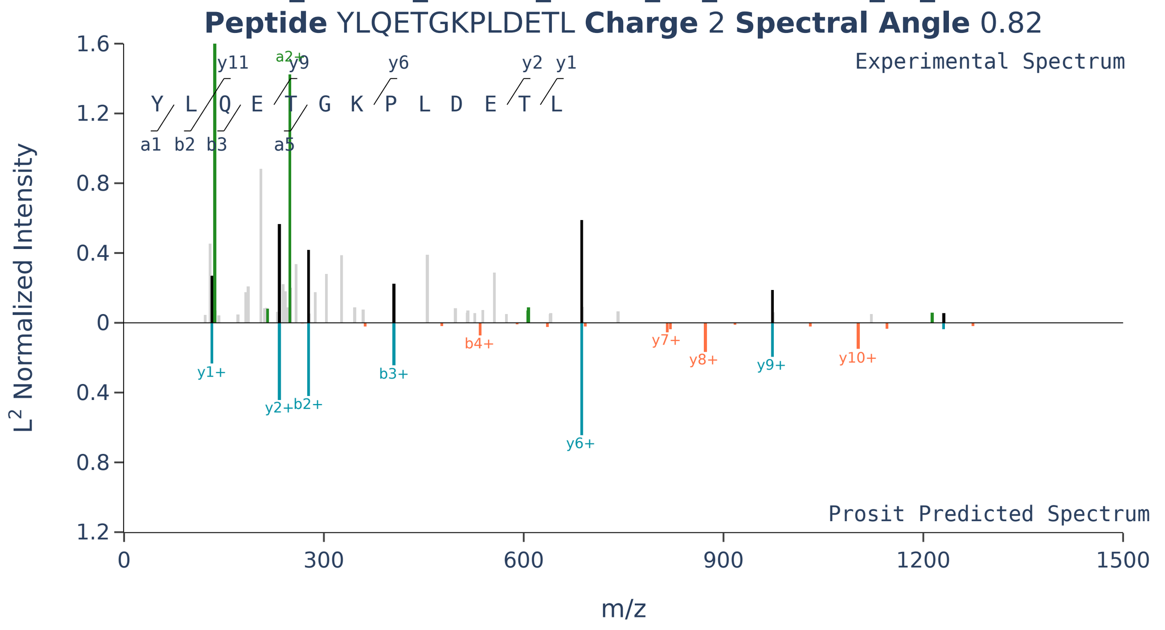


**Source** WSoh_301121_021221_HFGoe_M2_AnnexinA1_4h_R1 **Scan** 6739
**Peptide** ALHKAIMVKG **Charge** 2 **Spectral Angle** 0.76

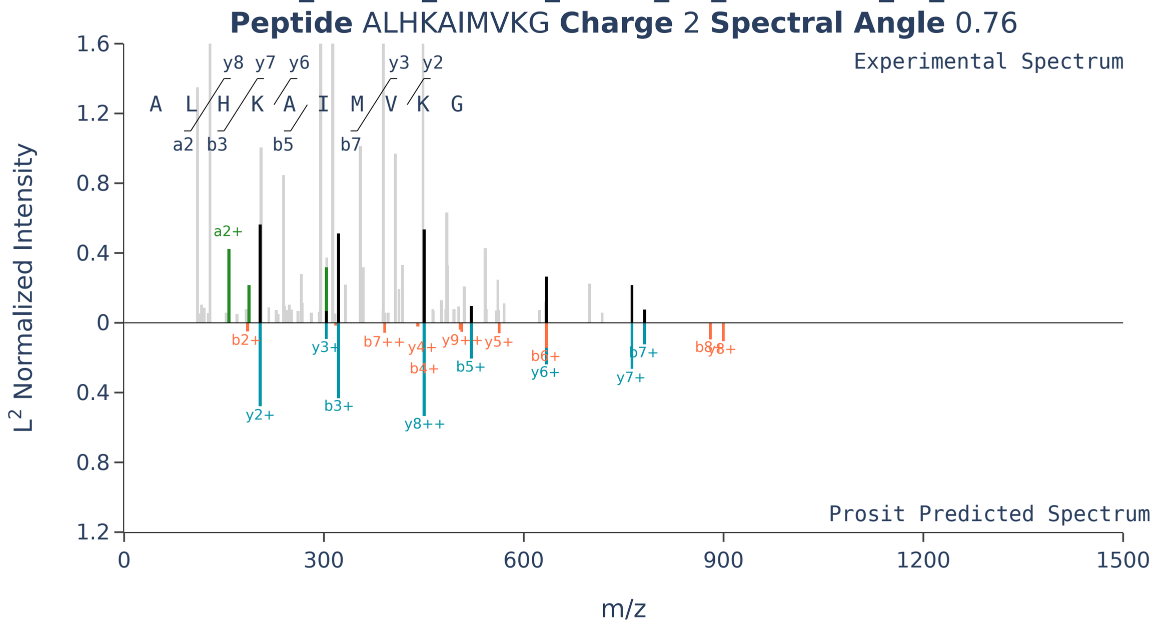


**Source** WSoh_301121_021221_HFGoe_M2_AnnexinA1_2h_R2 **Scan** 5970
**Peptide** YTKYSKHDMNKV **Charge** 3 **Spectral Angle** 0.69

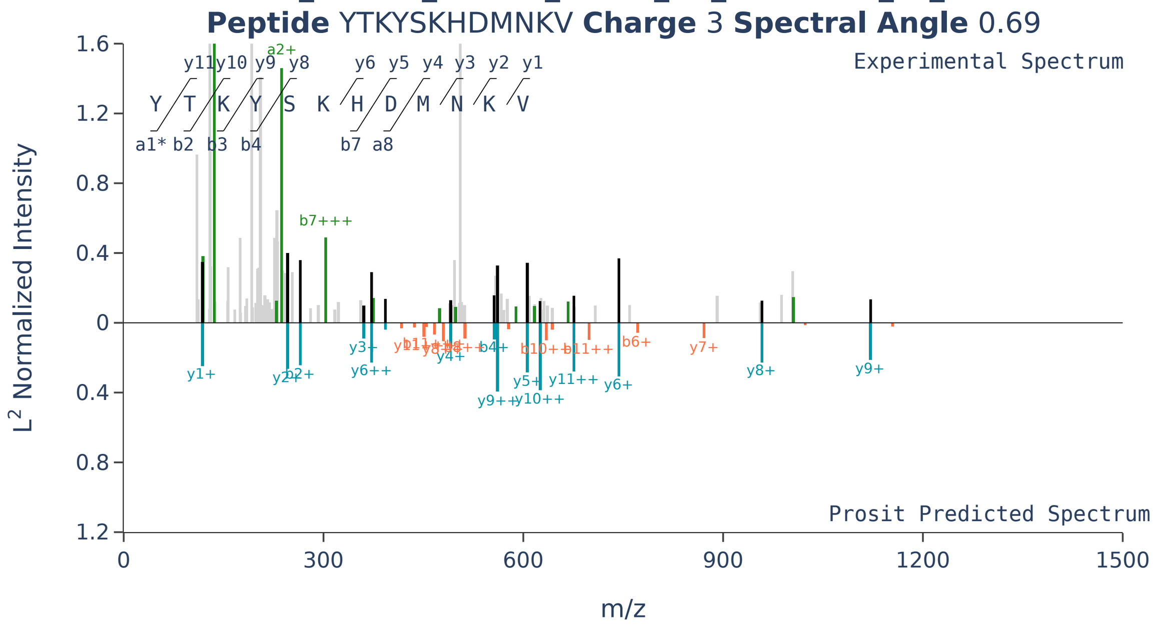


**Source** WSoh_301121_021221_HFGoe_M2_AnnexinA1_24h_R1 **Scan** 19495
**Peptide** NKVLDLELKG **Charge** 2 **Spectral Angle** 0.65

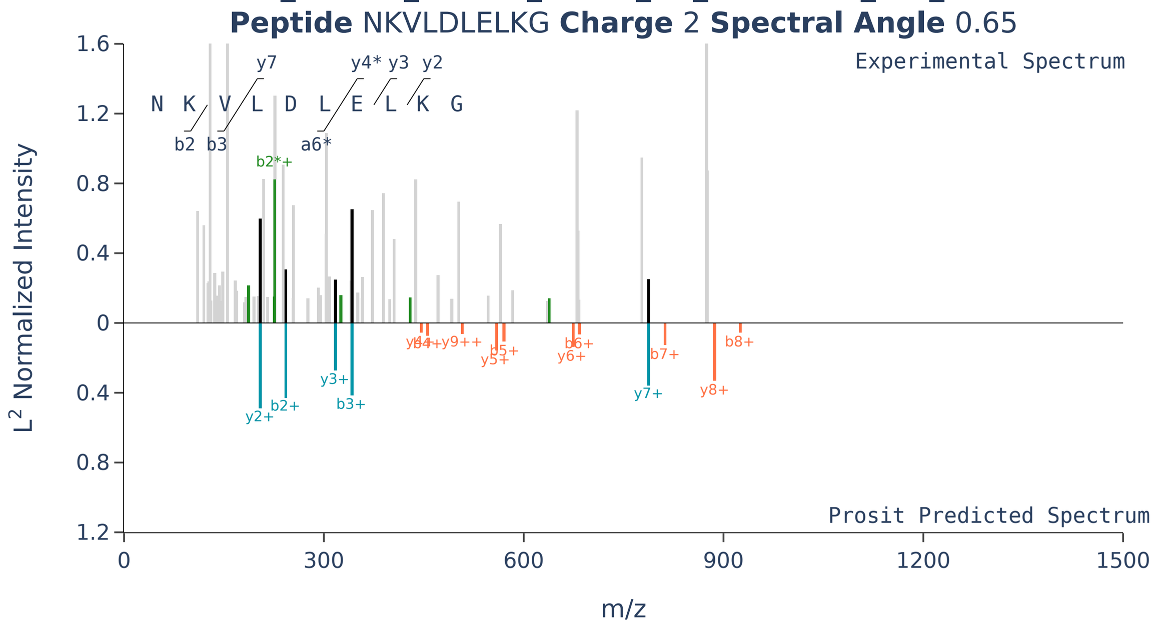


**Source** WSoh_301121_021221_HFGoe_M2_AnnexinA1_1h_R1 **Scan** 14610
**Peptide** VAALHKAIMVKGVD **Charge** 3 **Spectral Angle** 0.75

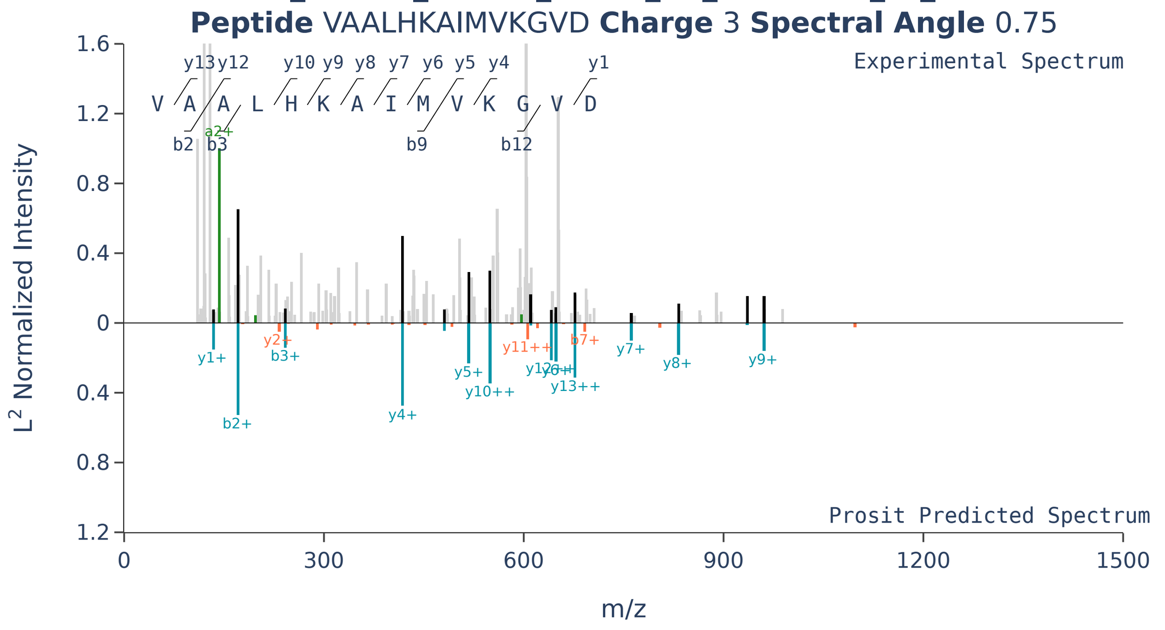


**Source** WSoh_301121_021221_HFGoe_M1_AnnexinA1_4h_R1 **Scan** 17874
**Peptide** LTTRSYPQL **Charge** 2 **Spectral Angle** 0.8

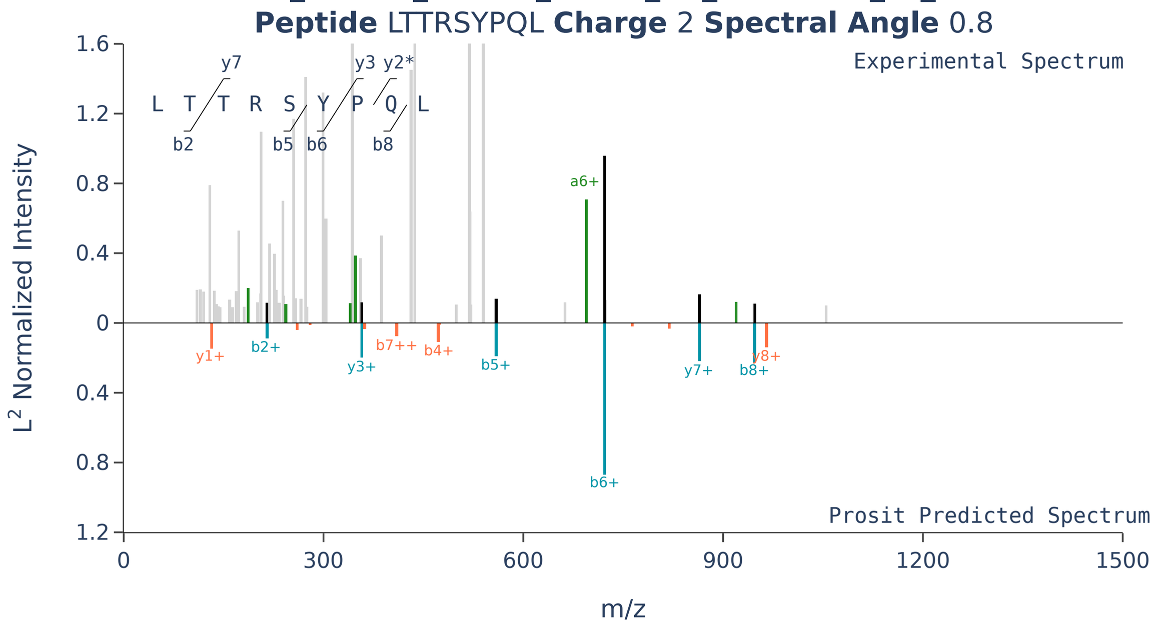


**Source** WSoh_301121_021221_HFGoe_M1_AnnexinA1_4h_R2 **Scan** 20157
**Peptide** RAAMKGLGTDEDTLL **Charge** 2 **Spectral Angle** 0.67

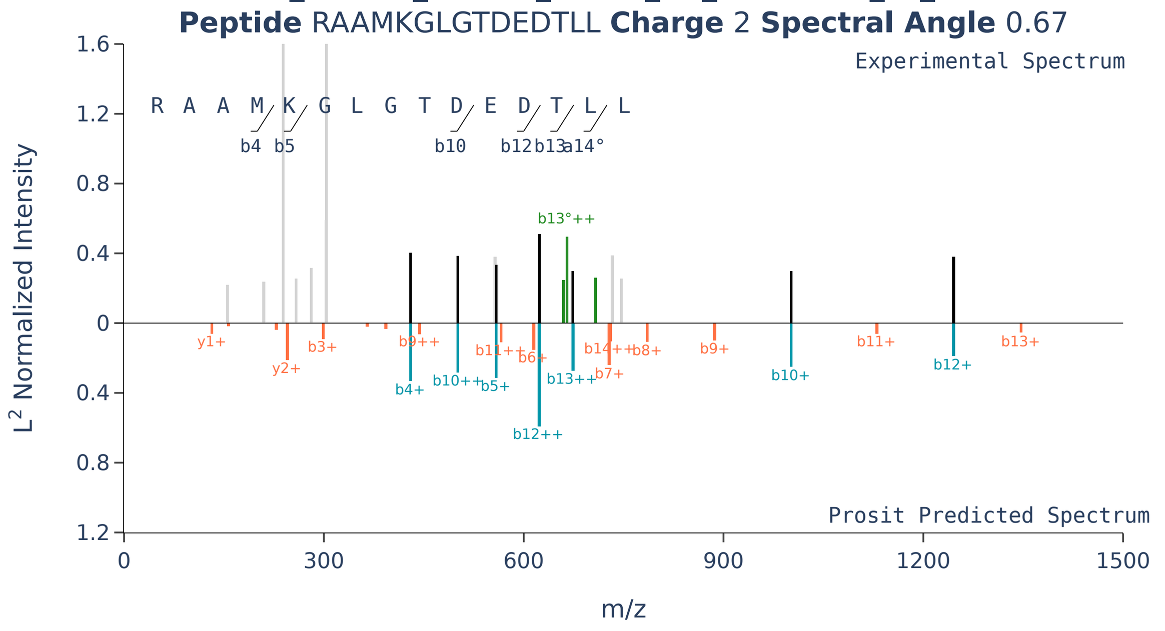


**Source** WSoh_301121_021221_HFGoe_M2_AnnexinA1_2h_R1 **Scan** 20580
**Peptide** HDMNKVLDLELKG **Charge** 3 **Spectral Angle** 0.7

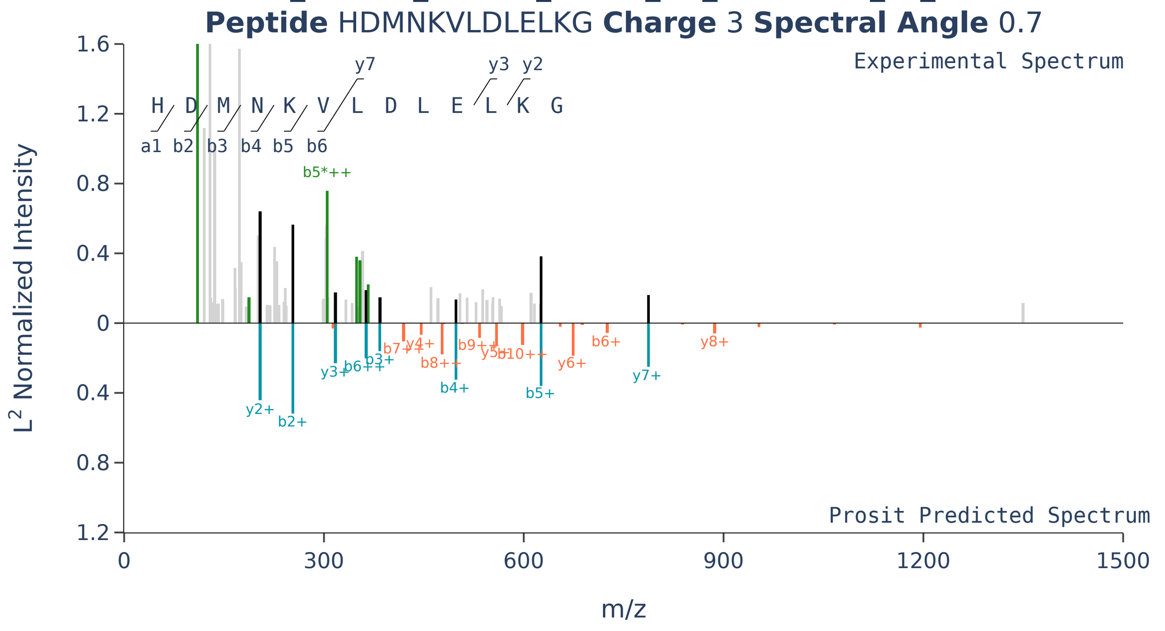


**Source** WSoh_301121_021221_HFGoe_M2_AnnexinA1_2h_R1 **Scan** 19481
**Peptide** PLDETLKKALT **Charge** 3 **Spectral Angle** 0.75

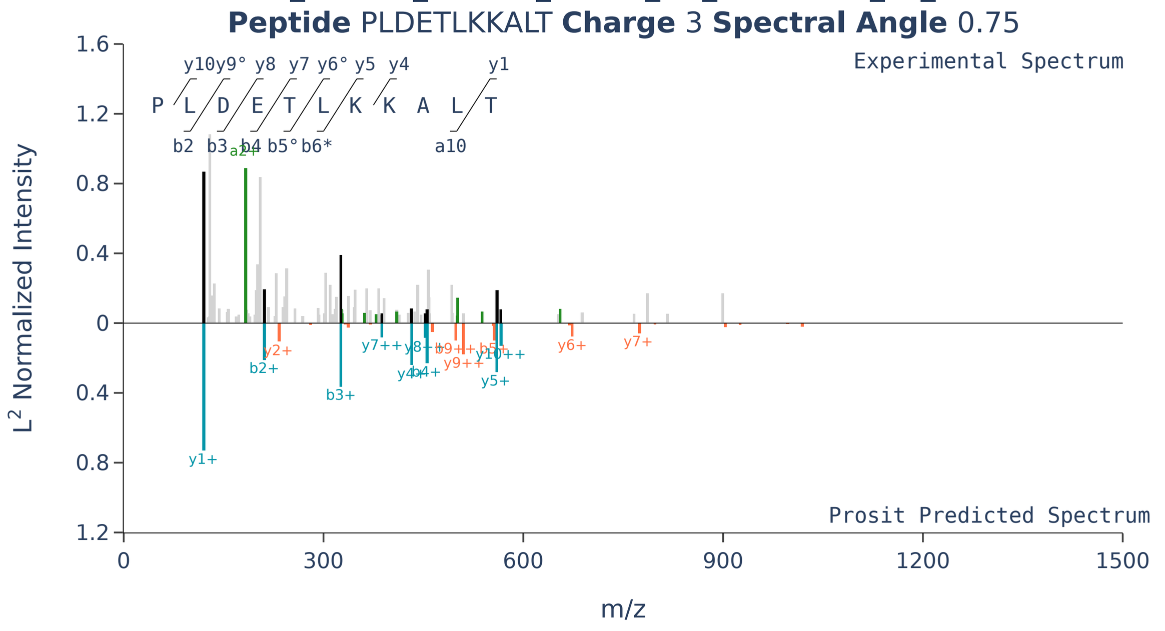


**Source** WSoh_301121_021221_HFGoe_M2_AnnexinA1_2h_R1 **Scan** 16748
**Peptide** REELKRDLAKDITSDTSGDFRNA **Charge** 4 **Spectral Angle** 0.71

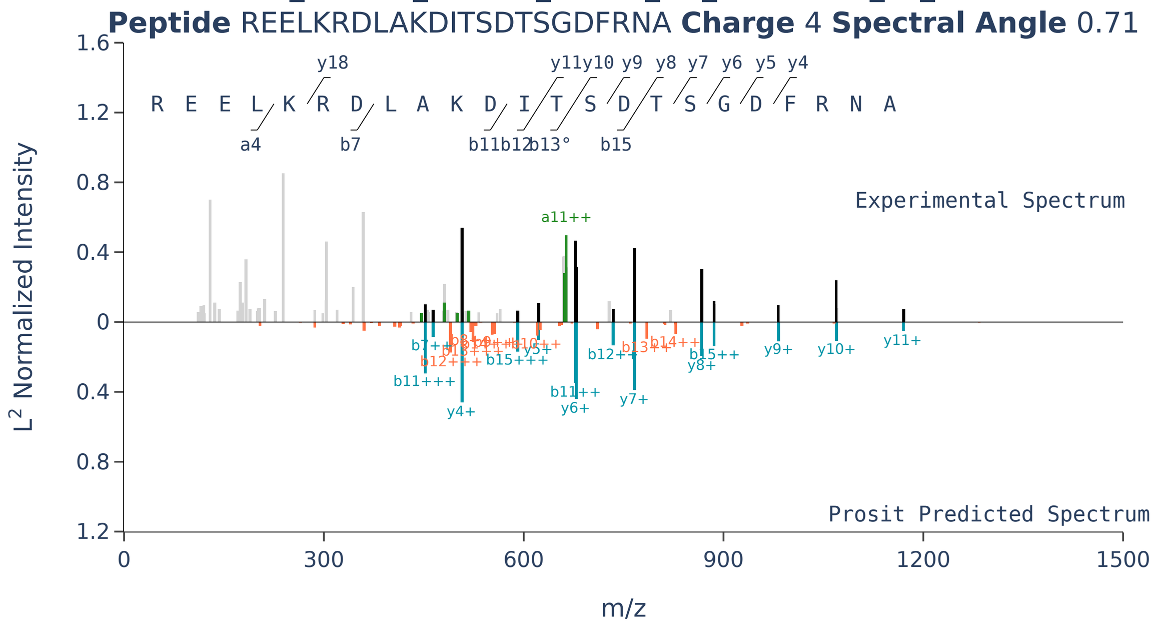


**Source** WSoh_301121_021221_HFGoe_M2_AnnexinA1_2h_R1 **Scan** 22633
**Peptide** SPYPTFNPSSDVAALHKA **Charge** 3 **Spectral Angle** 0.75

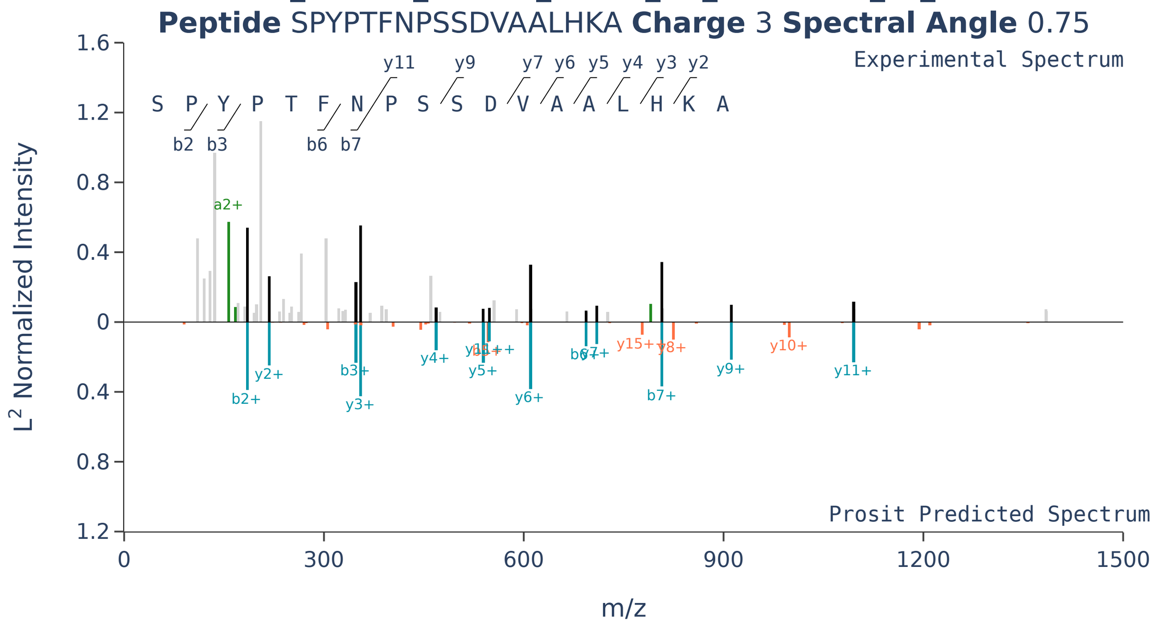


**Source** WSoh_301121_021221_HFGoe_M2_AnnexinA1_4h_R1 **Scan** 12977
**Peptide** RQQIKAAYL **Charge** 3 **Spectral Angle** 0.45

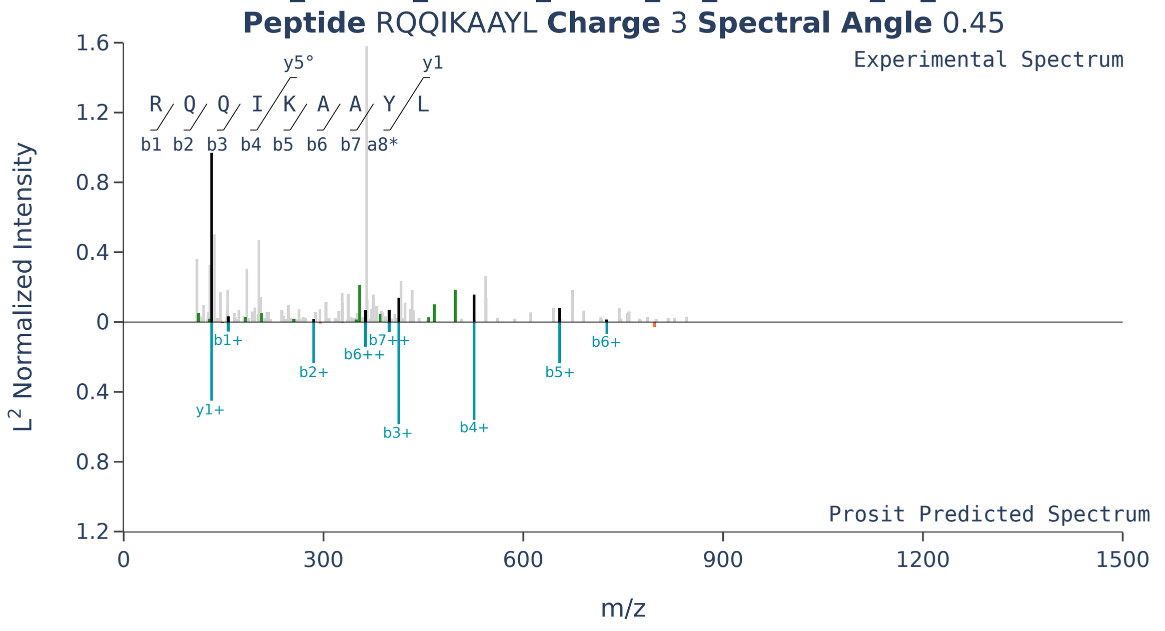


**Source** WSoh_301121_021221_HFGoe_M2_AnnexinA1_24h_R2 **Scan** 19691
**Peptide** IENEEQEYVQTV **Charge** 2 **Spectral Angle** 0.8

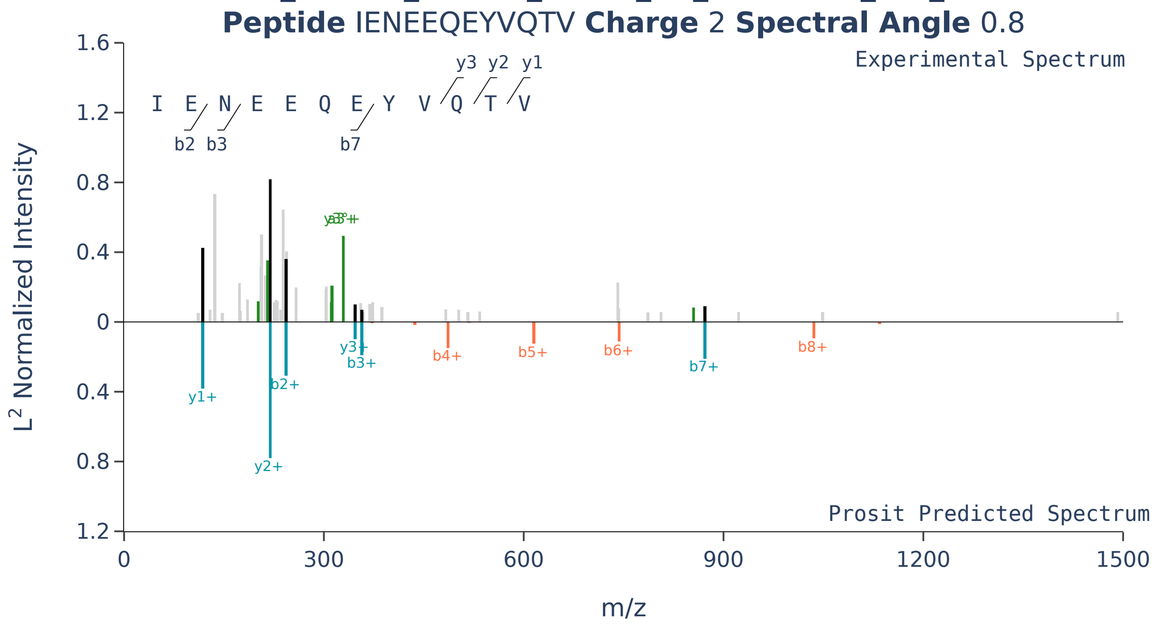


**Source** WSoh_301121_021221_HFGoe_M2_AnnexinA1_24h_R1 **Scan** 21301
**Peptide** LDETKGDYEKILVA **Charge** 2 **Spectral Angle** 0.71

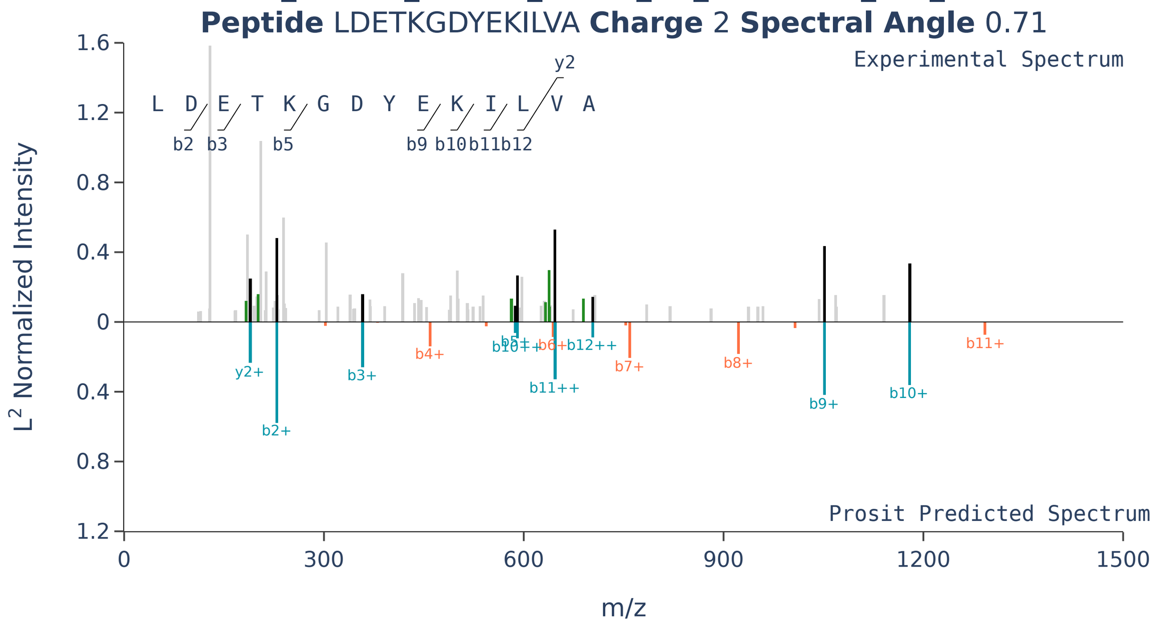


**Source** WSoh_301121_021221_HFGoe_M2_AnnexinA1_4h_R1 **Scan** 12983
**Peptide** NNAQRQQIKAAYLQE **Charge** 3 **Spectral Angle** 0.8

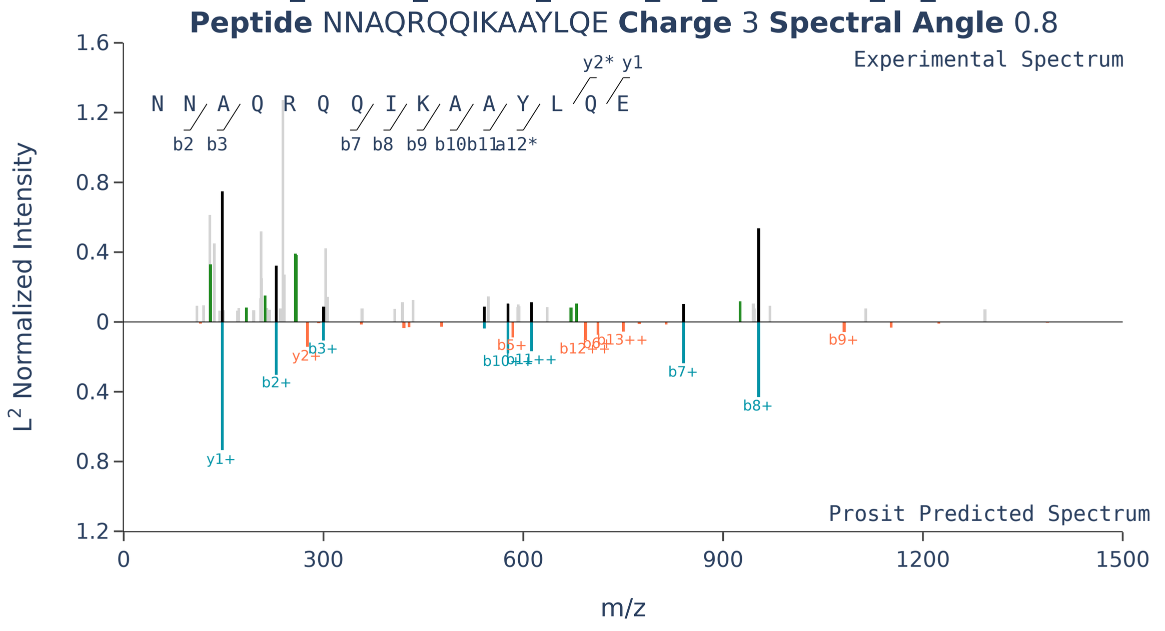

**Source** WSoh_301121_021221_HFGoe_M2_AnnexinA1_4h_R1 **Scan** 21729
**Peptide** AIMVKGVDEATI **Charge** 2 **Spectral Angle** 0.77

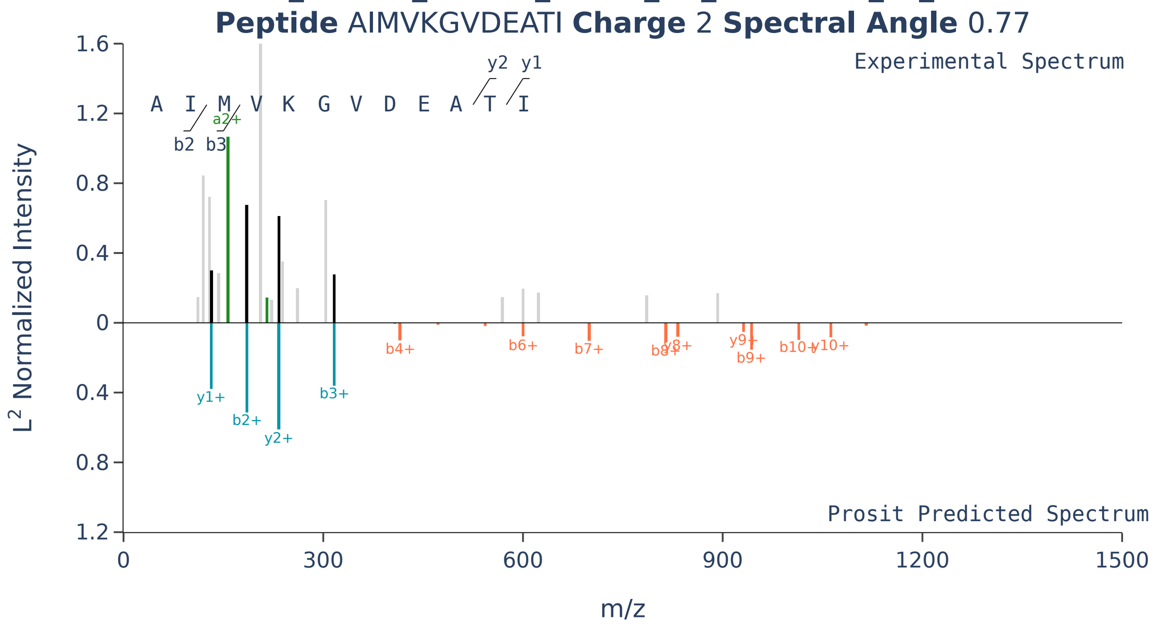


**Source** WSoh_301121_021221_HFGoe_M2_AnnexinA1_2h_R1 **Scan** 18660
**Peptide** LLSLAKGDRSEDFG **Charge** 3 **Spectral Angle** 0.66

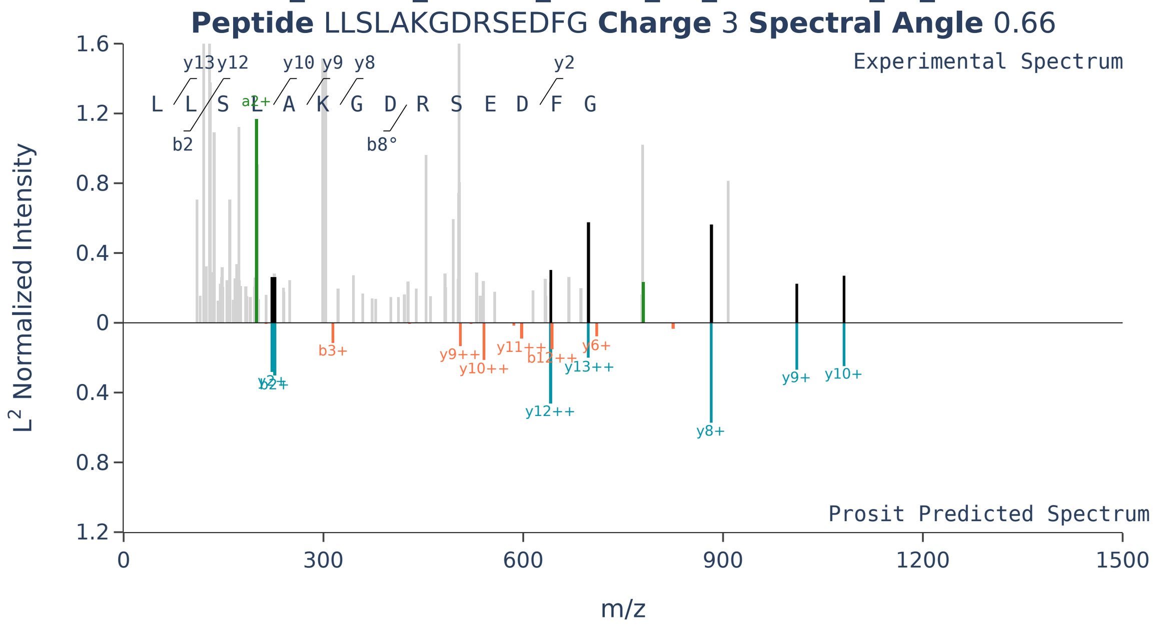


**Source** WSoh_301121_021221_HFGoe_M2_AnnexinA1_2h_R1 **Scan** 17513
**Peptide** LAKGDRSEDFGVNEDLAD **Charge** 3 **Spectral Angle** 0.7

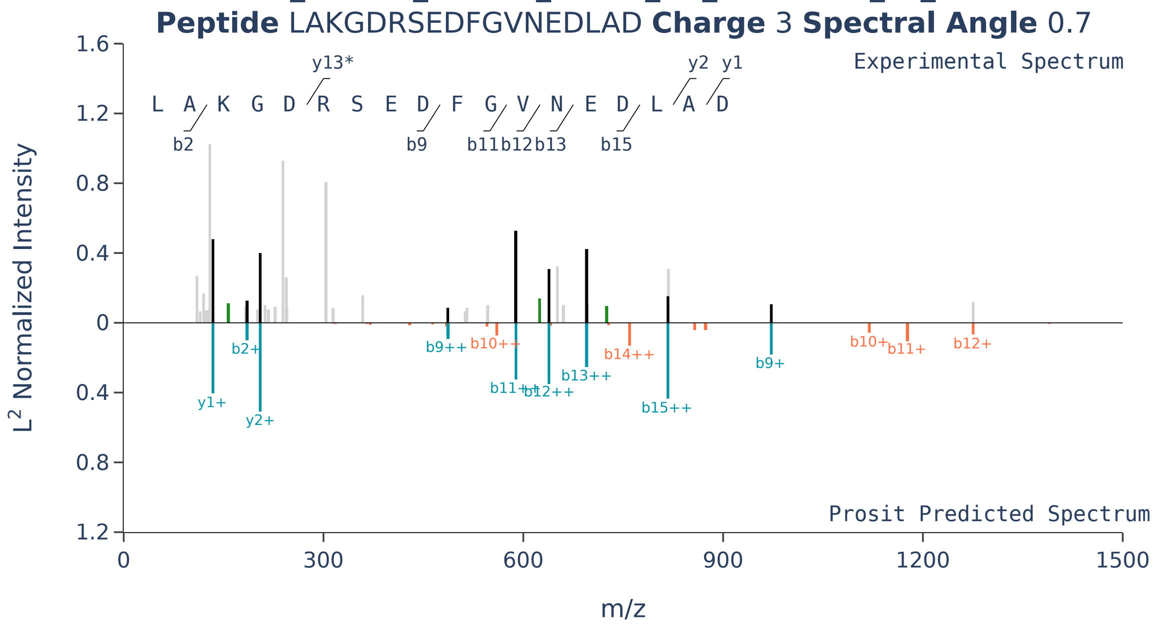


**Source** WSoh_301121_021221_HFGoe_M2_AnnexinA1_2h_R1 **Scan** 21292
**Peptide** PSSDVAALHKAIMV **Charge** 3 **Spectral Angle** 0.73

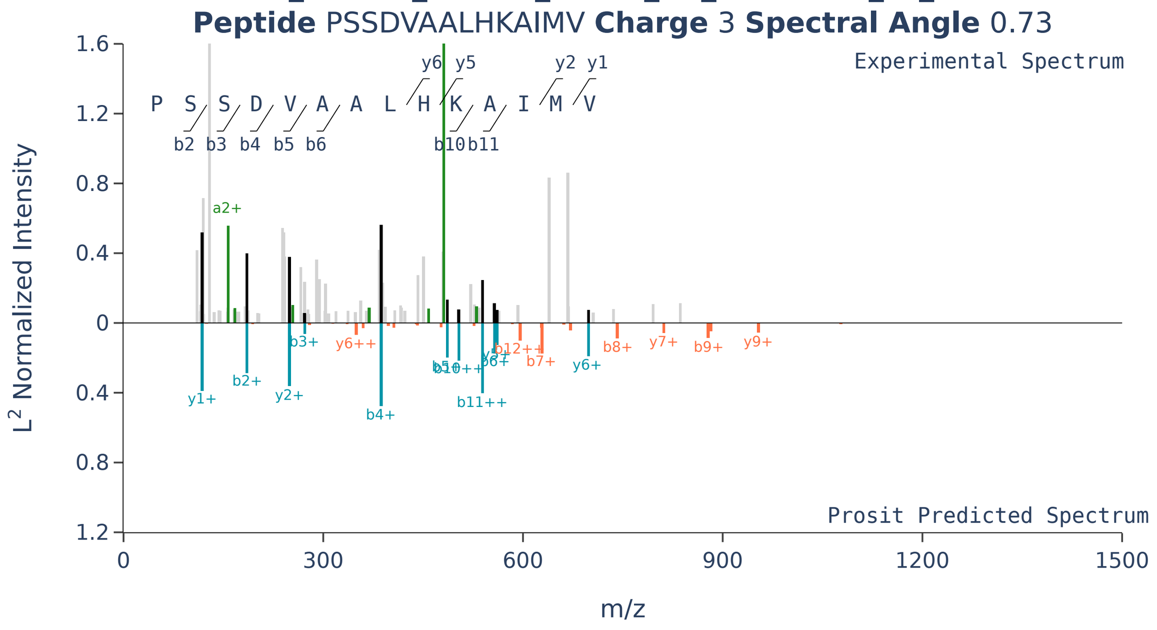


**Source** WSoh_301121_021221_HFGoe_M1_AnnexinA1_24h_R1 **Scan** 16911
**Peptide** ALTGHLEEV **Charge** 1 **Spectral Angle** 0.79

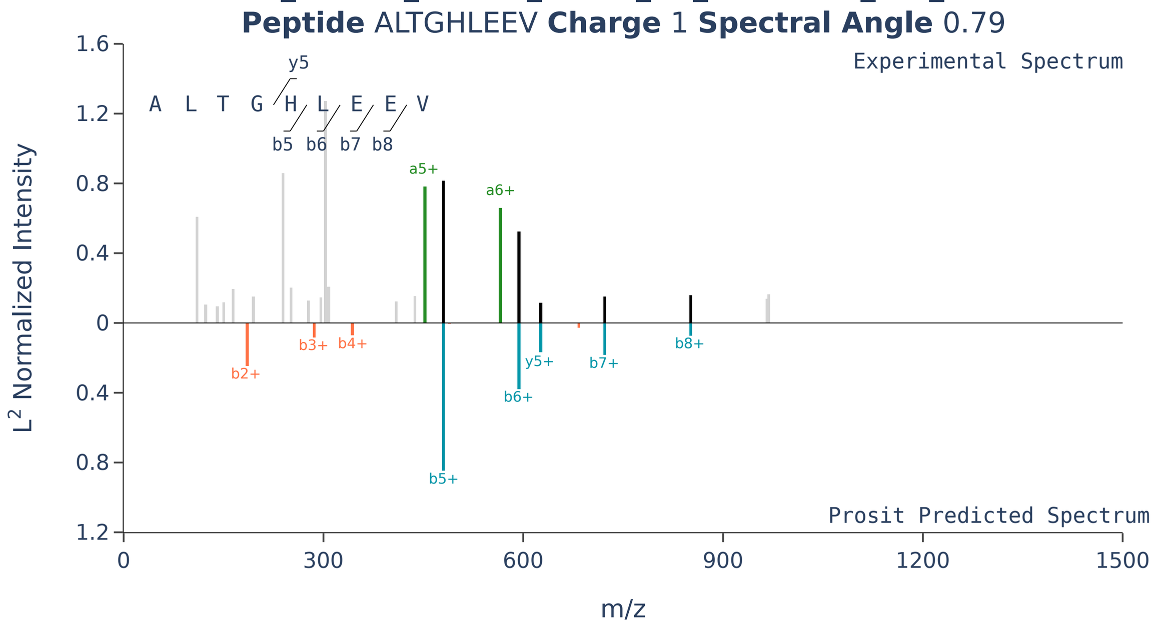


**Source** WSoh_301121_021221_HFGoe_M2_AnnexinA1_2h_R1 **Scan** 22321
**Peptide** KRDLAKDITSDTSGDFRNALL **Charge** 4 **Spectral Angle** 0.57

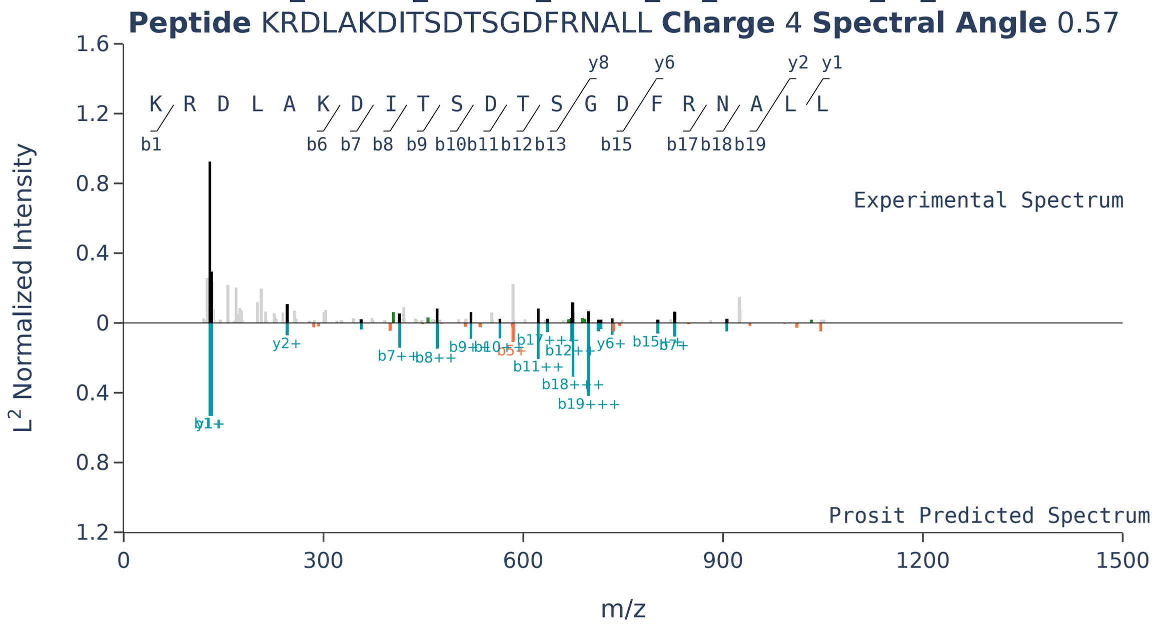


**Source** WSoh_301121_021221_HFGoe_M2_AnnexinA1_2h_R2 **Scan** 17196
**Peptide** ETLKKALTGHLEEV **Charge** 3 **Spectral Angle** 0.72

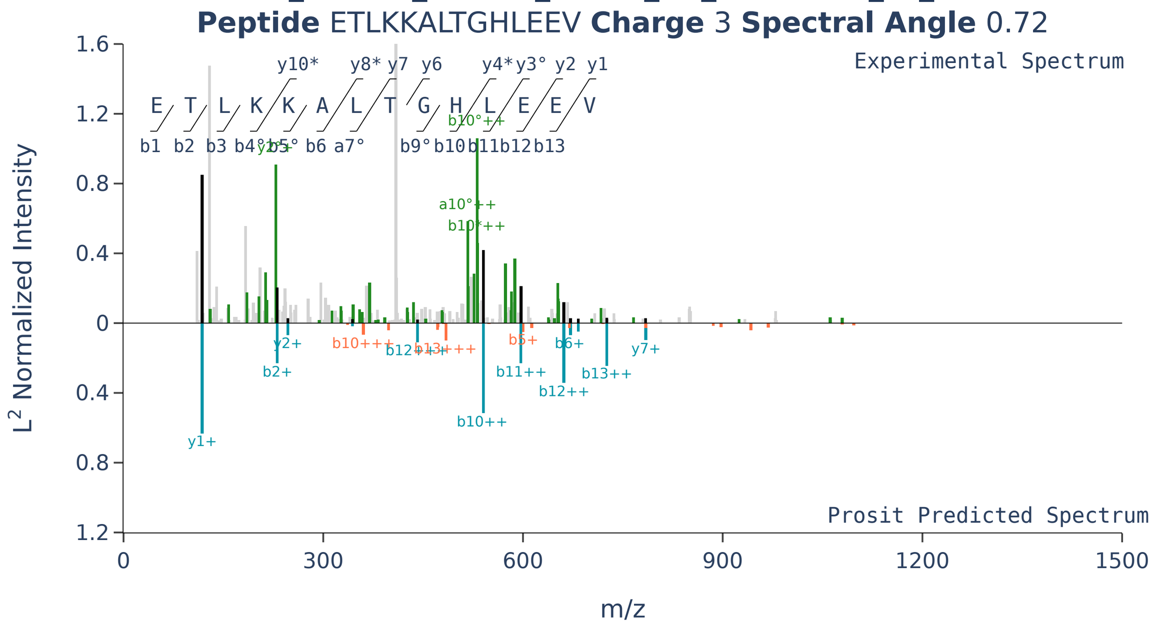


**Source** WSoh_301121_021221_HFGoe_M2_AnnexinA1_2h_R2 **Scan** 14659
**Peptide** ELRAAMKGLGTD **Charge** 3 **Spectral Angle** 0.89

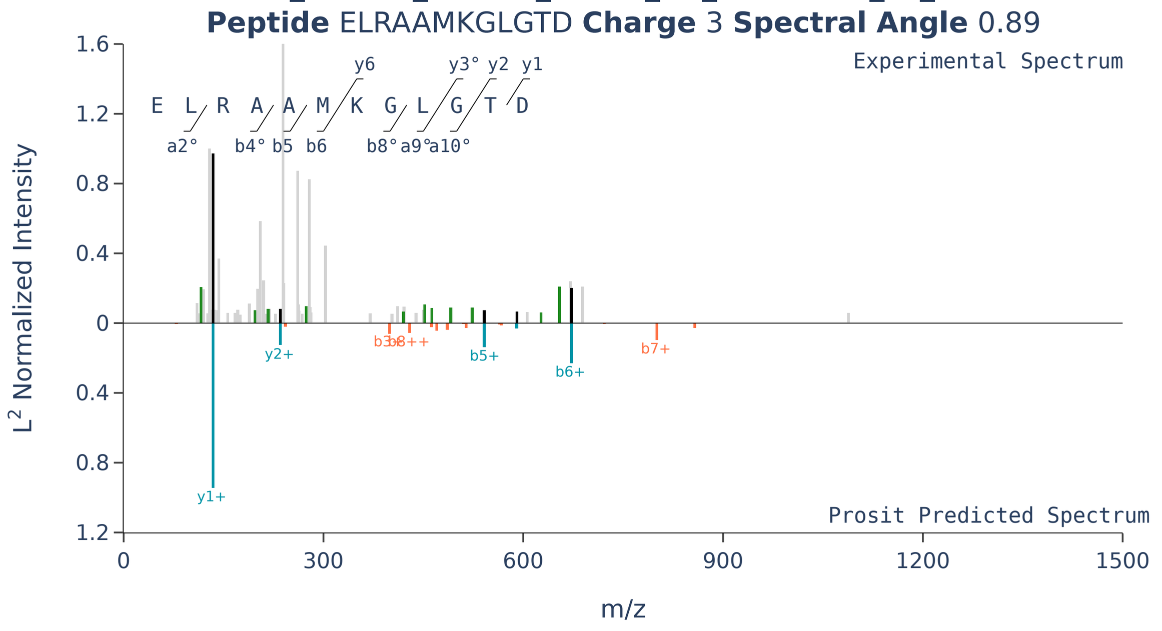


**Source** WSoh_301121_021221_HFGoe_M2_AnnexinA1_2h_R1 **Scan** 8237
**Peptide** MKGVGTRHKALL **Charge** 2 **Spectral Angle** 0.76

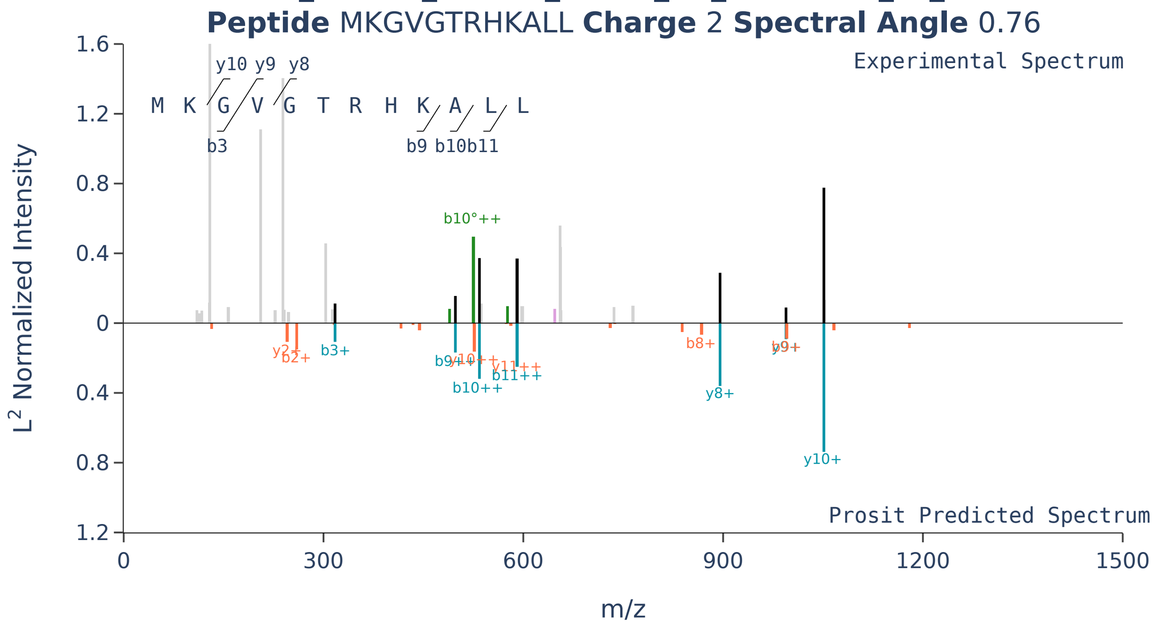


**Source** WSoh_301121_021221_HFGoe_M2_AnnexinA1_2h_R1 **Scan** 23770
**Peptide** AVSPYPTFNPSSDVAALHKA **Charge** 3 **Spectral Angle** 0.74

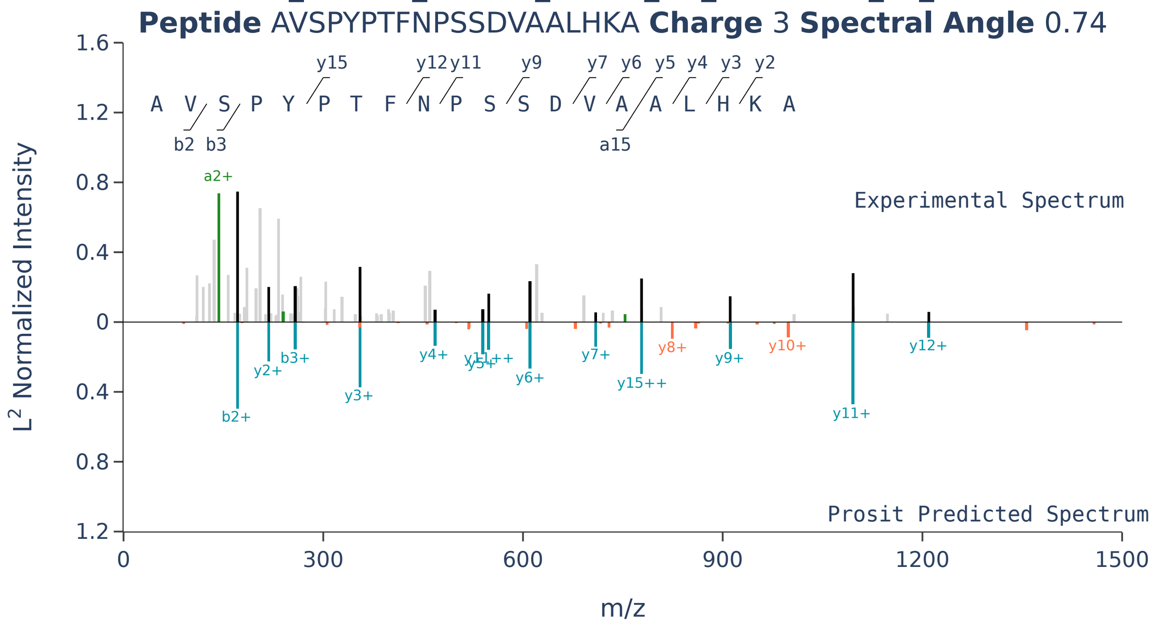


**Source** WSoh_301121_021221_HFGoe_M1_AnnexinA1_24h_R1 **Scan** 20461
**Peptide** DLAKDITSDTSGDFR **Charge** 2 **Spectral Angle** 0.69

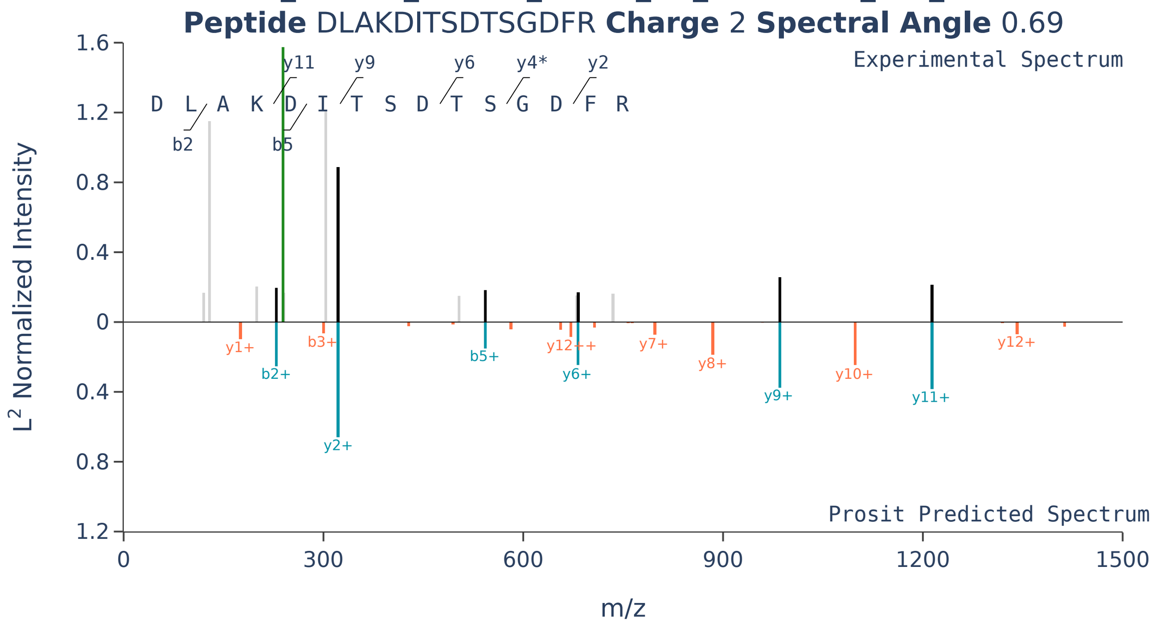


**Source** WSoh_301121_021221_HFGoe_M2_AnnexinA1_24h_R1 **Scan** 5611
**Peptide** TKYSKHDMNKV **Charge** 2 **Spectral Angle** 0.66

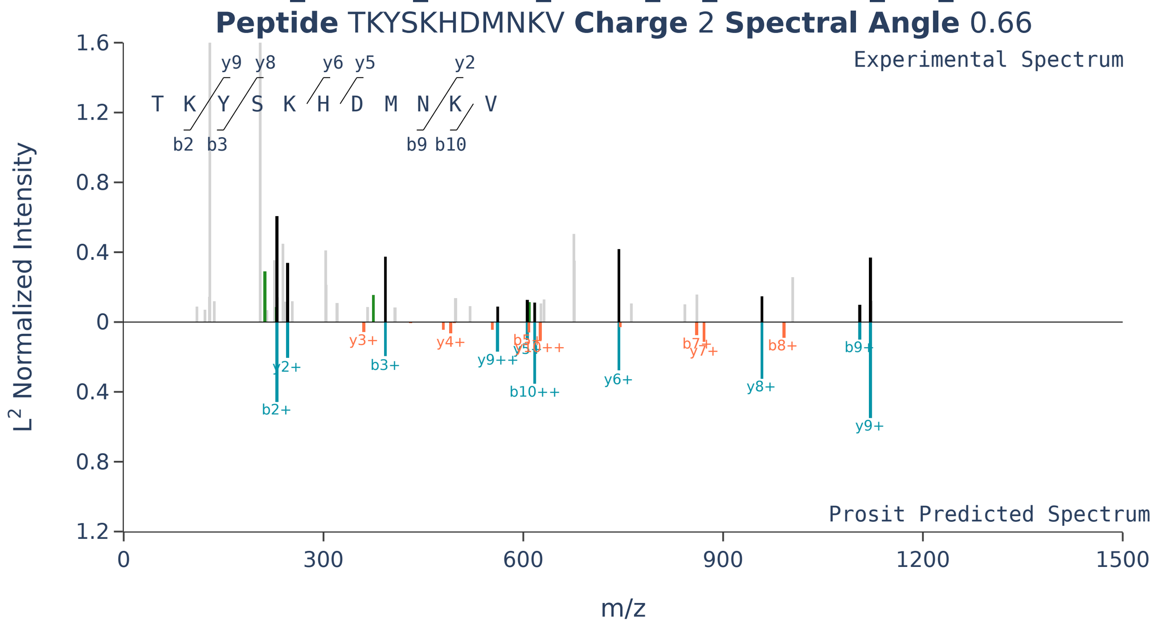


**Source** WSoh_301121_021221_HFGoe_M1_AnnexinA1_24h_R2 **Scan** 6044
**Peptide** GVGTRHKALIR **Charge** 3 **Spectral Angle** 0.84

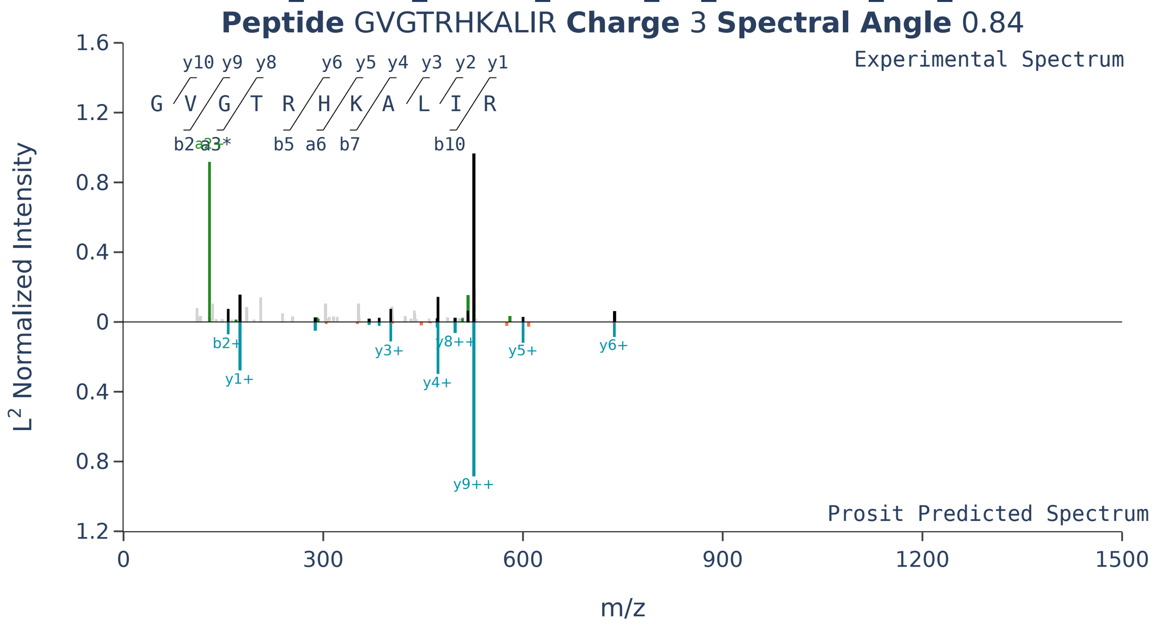


**Source** WSoh_301121_021221_HFGoe_M2_AnnexinA1_24h_R1 **Scan** 19496
**Peptide** GDRSEDFGVNEDLAD **Charge** 2 **Spectral Angle** 0.77

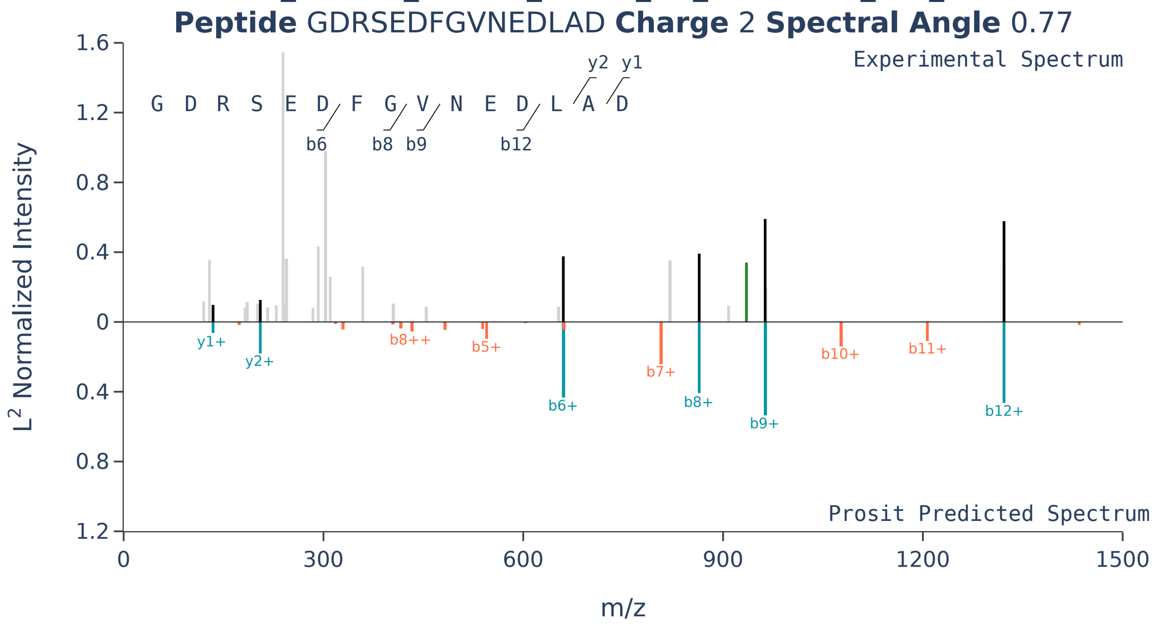


**Source** WSoh_301121_021221_HFGoe_M2_AnnexinA1_4h_R2 **Scan** 7694
**Peptide** ELRDINRV **Charge** 2 **Spectral Angle** 0.74

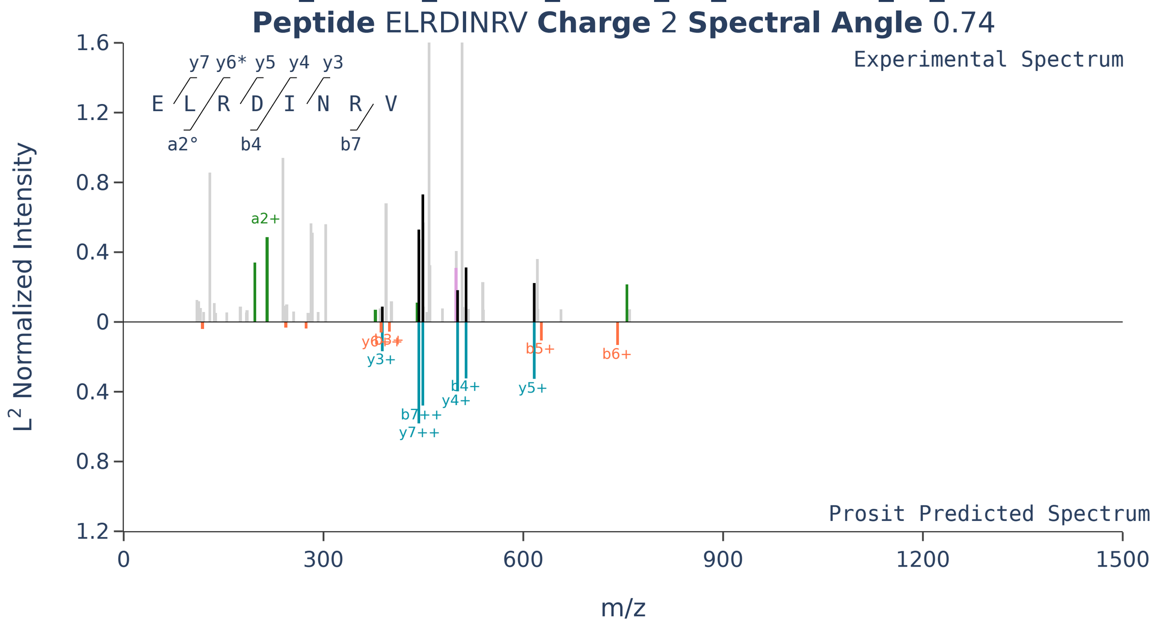

**Source** WSoh_301121_021221_HFGoe_M2_AnnexinA1_4h_R1 **Scan** 23377
**Peptide** LTGHLEEVVLA **Charge** 2 **Spectral Angle** 0.69

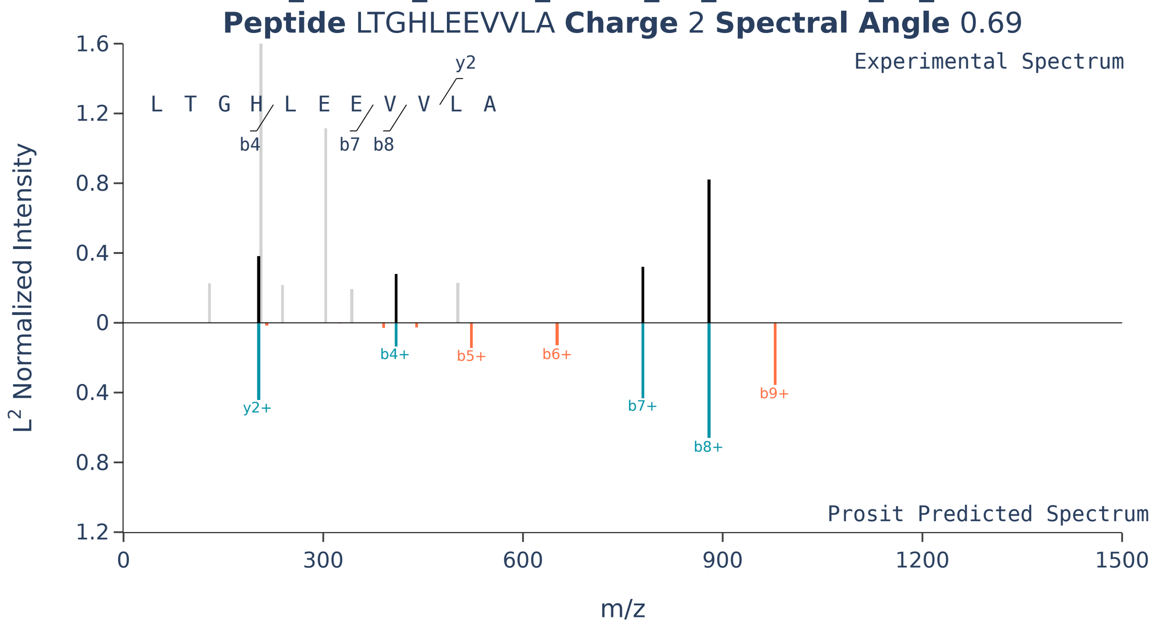


**Source** WSoh_301121_021221_HFGoe_M2_AnnexinA1_2h_R2 **Scan** 10356
**Peptide** DLLTKRNNAQRQQI **Charge** 2 **Spectral Angle** 0.85

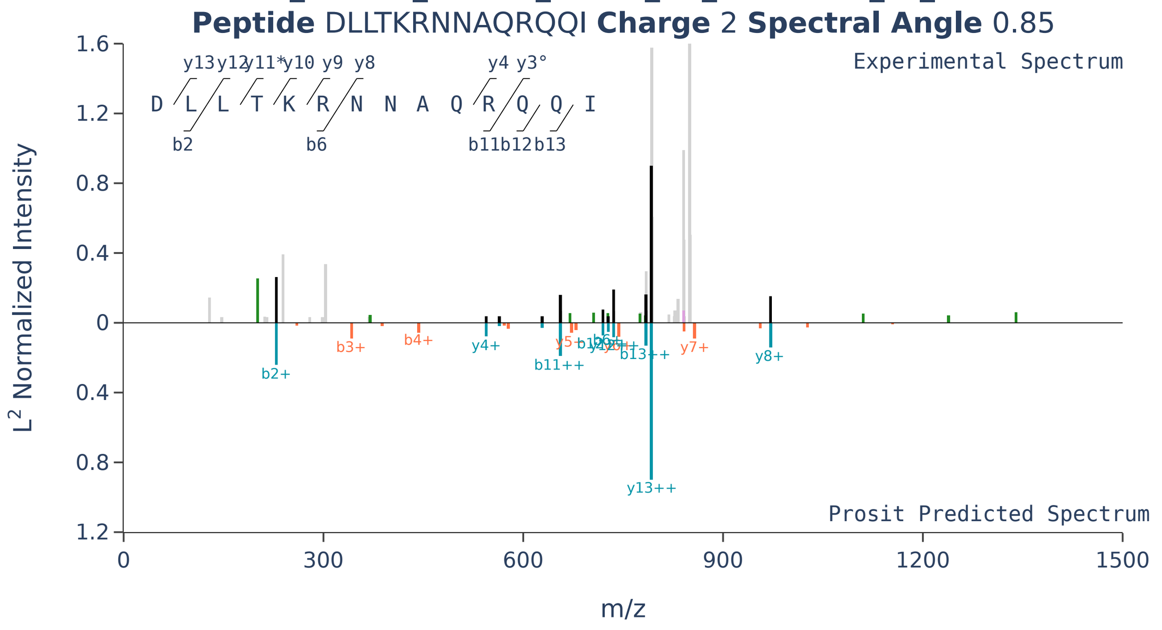


**Source** WSoh_301121_021221_HFGoe_M2_AnnexinA1_1h_R1 **Scan** 13061
**Peptide** LADSDARALYEAGERRK **Charge** 4 **Spectral Angle** 0.8

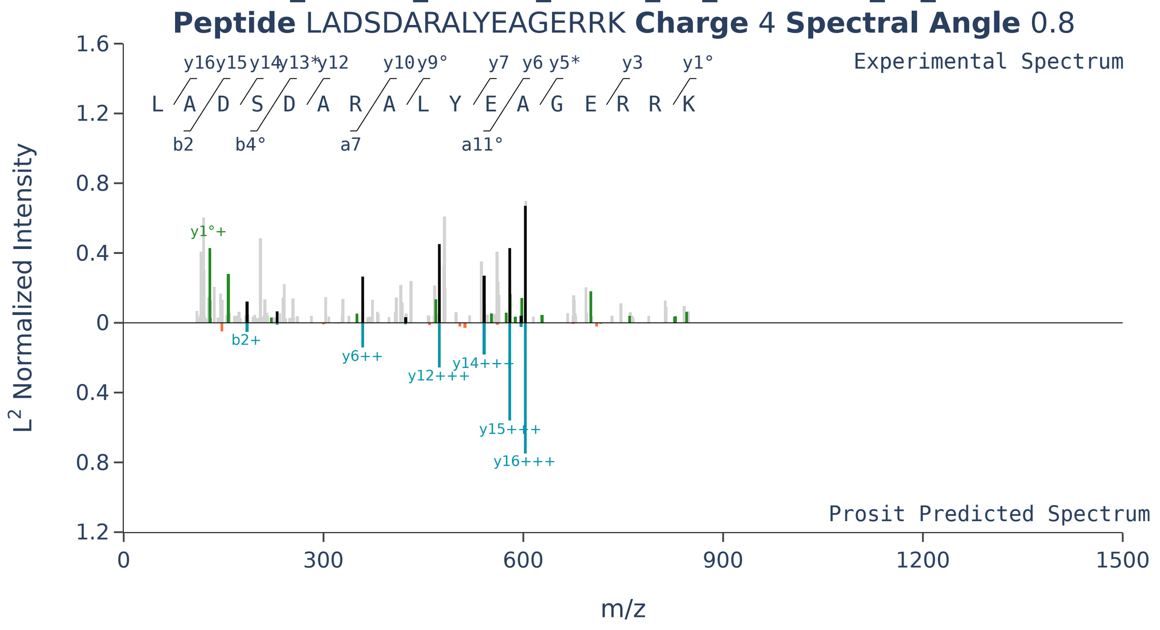


**Source** WSoh_301121_021221_HFGoe_M2_AnnexinA1_24h_R1 **Scan** 20437
**Peptide** LAKDITSDTSGDFRNAL **Charge** 3 **Spectral Angle** 0.78

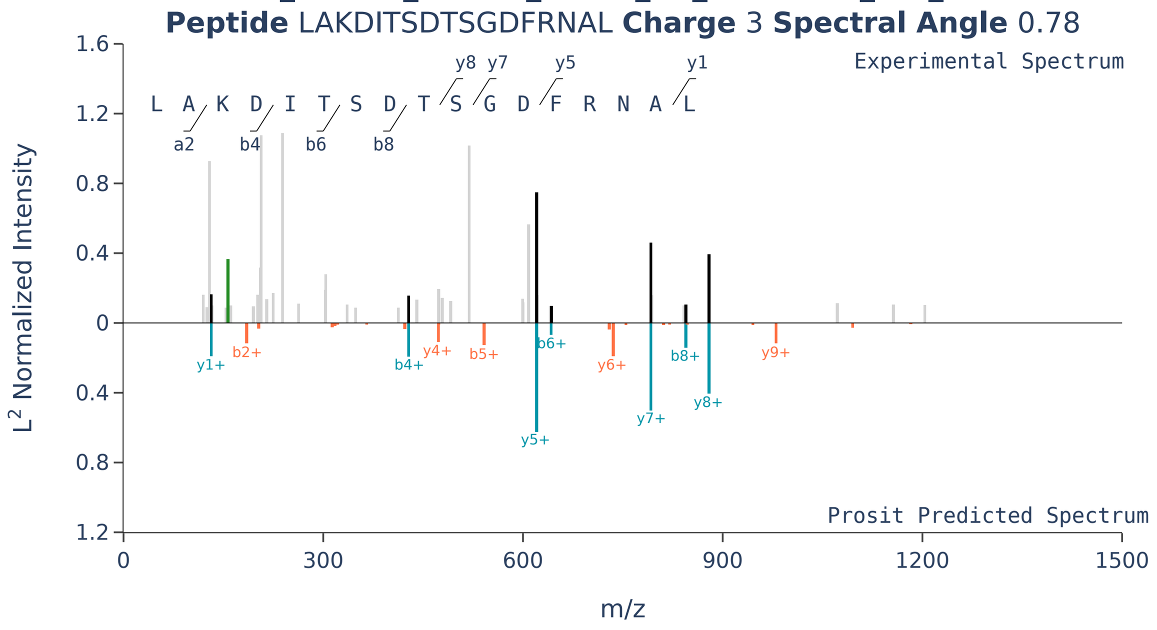


**Source** WSoh_301121_021221_HFGoe_M1_AnnexinA1_4h_R1 **Scan** 6801
**Peptide** TAIVKCATSK **Charge** 2 **Spectral Angle** 0.83

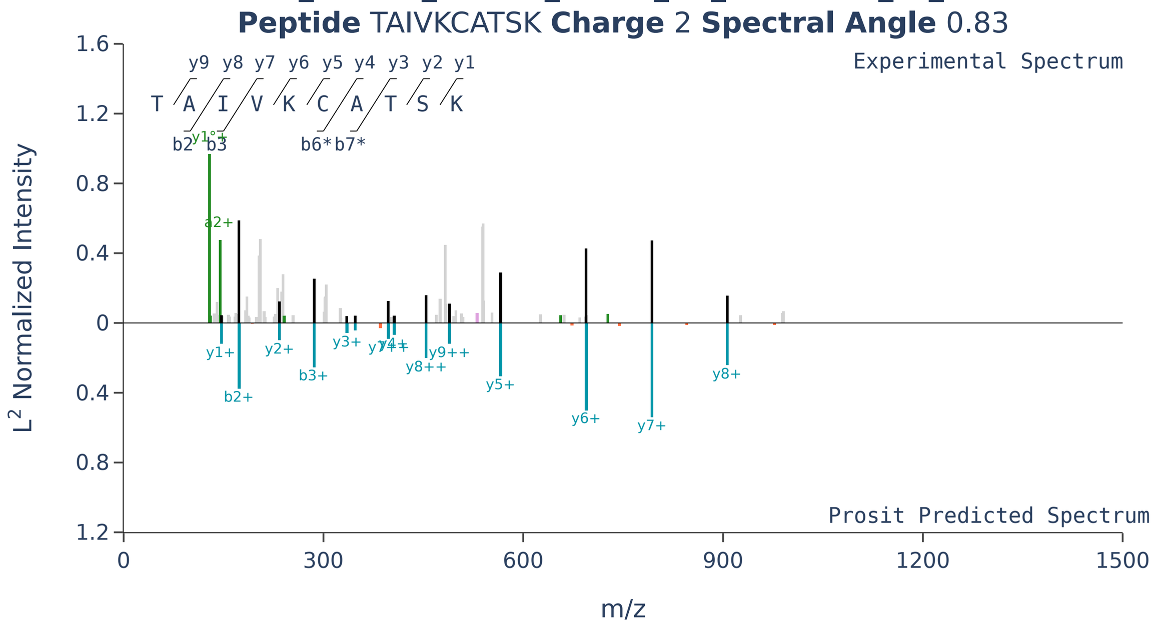


**Source** WSoh_301121_021221_HFGoe_M2_AnnexinA1_24h_R1 **Scan** 24163
**Peptide** SPYPTFNPSSDVA **Charge** 2 **Spectral Angle** 0.77

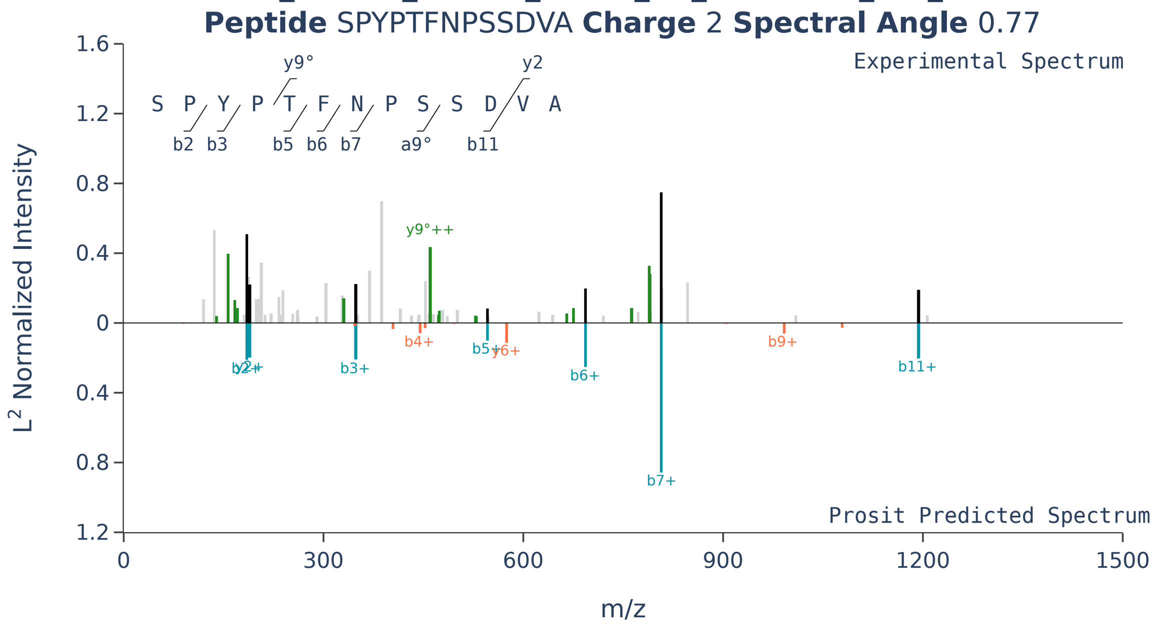


**Source** WSoh_301121_021221_HFGoe_M2_AnnexinA1_2h_R1 **Scan** 15462
**Peptide** DILTKRNNAQRQQIKAAYL **Charge** 3 **Spectral Angle** 0.68

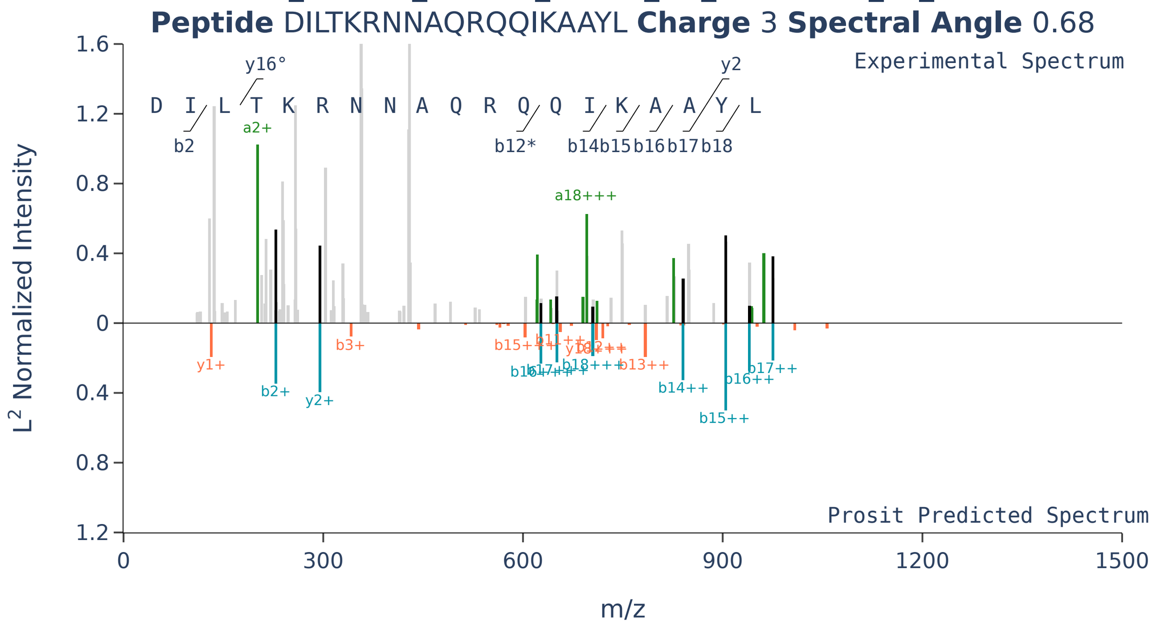


**Source** WSoh_301121_021221_HFGoe_M2_AnnexinA1_24h_R1 **Scan** 21884
**Peptide** SSKGGPGSAVSPYPTF **Charge** 2 **Spectral Angle** 0.85

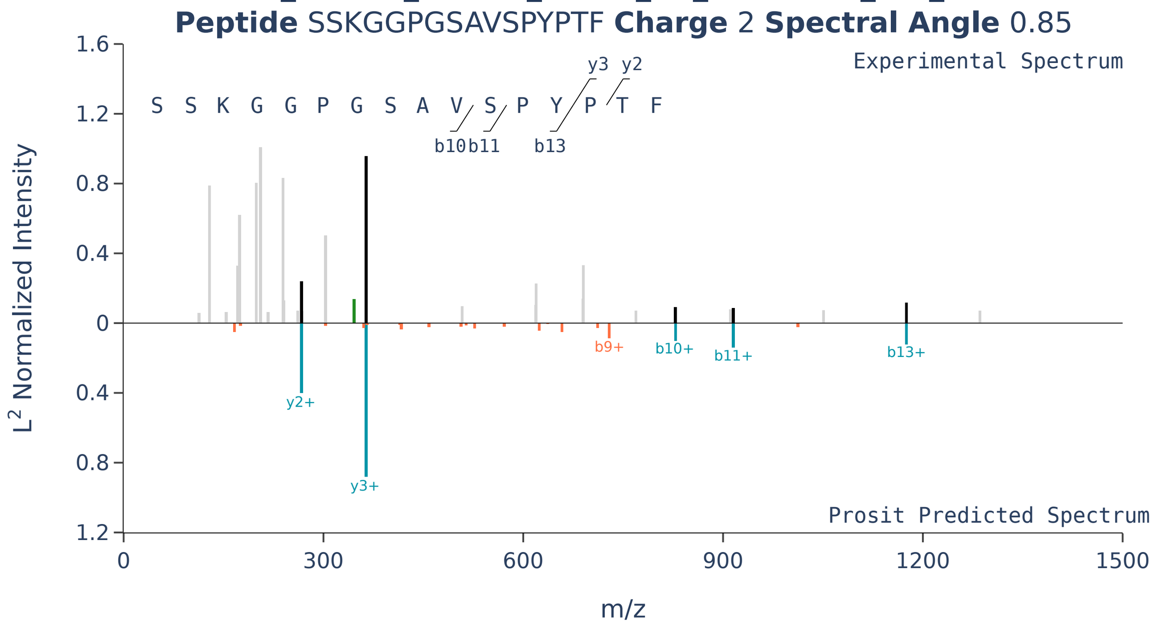


**Source** WSoh_301121_021221_HFGoe_M2_AnnexinA1_2h_R2 **Scan** 20400
**Peptide** DIKAFYQKM **Charge** 2 **Spectral Angle** 0.65

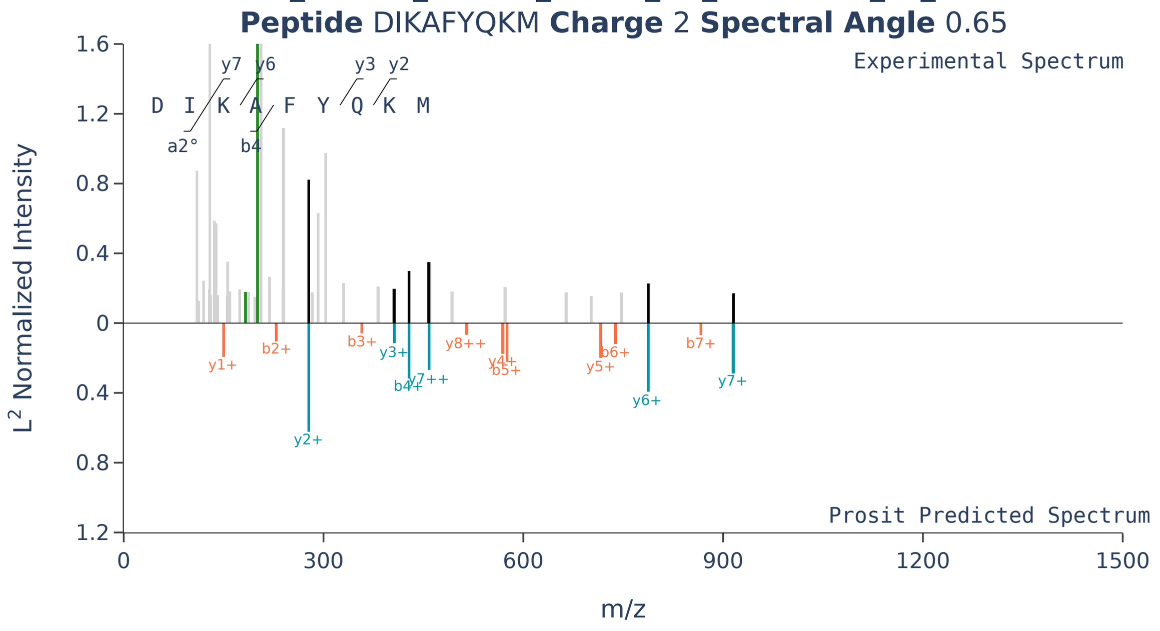


**Source** WSoh_301121_021221_HFGoe_M2_AnnexinA1_2h_R1 **Scan** 27146
**Peptide** KGGPGSAVSPYPTFNPSSDVAAL **Charge** 3 **Spectral Angle** 0.76

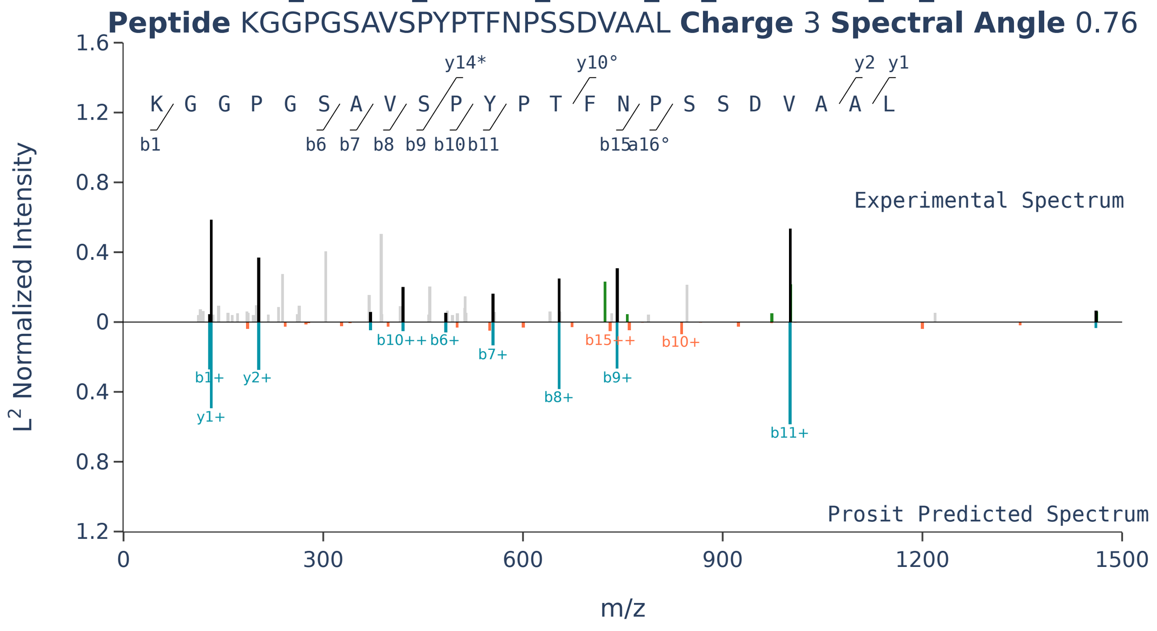


**Source** WSoh_301121_021221_HFGoe_M2_AnnexinA1_2h_R1 **Scan** 21736
**Peptide** KGGPGSAVSPYPTFNPSSDVAALHK **Charge** 4 **Spectral Angle** 0.74

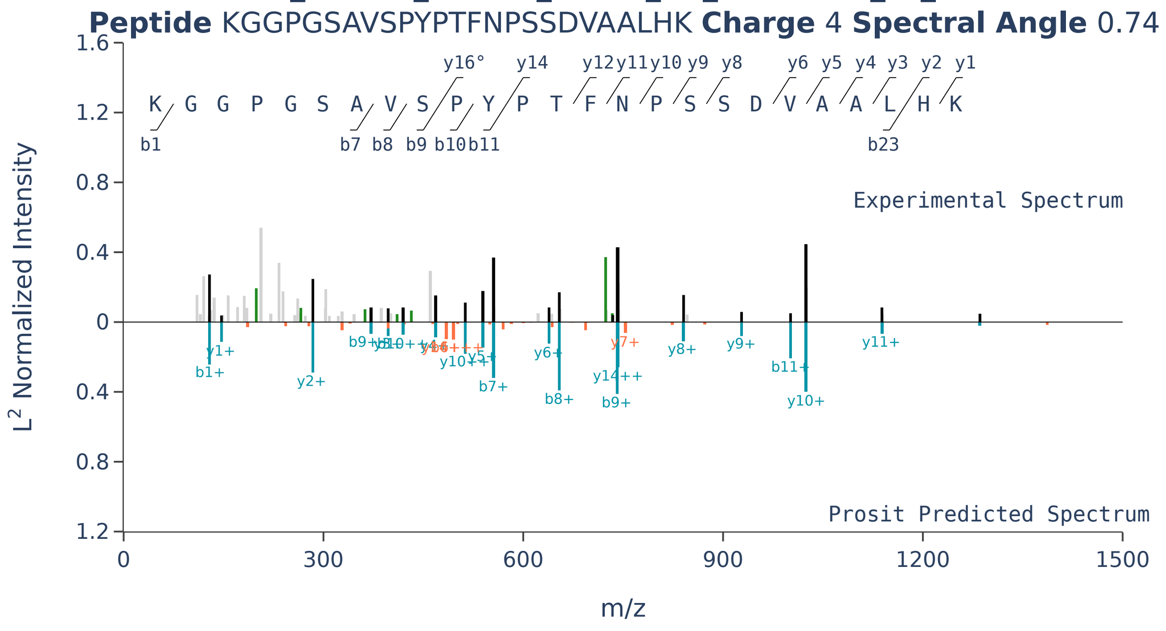


**Source** WSoh_301121_021221_HFGoe_M2_AnnexinA1_2h_R1 **Scan** 19535
**Peptide** DVAALHKAIMVKGVD **Charge** 2 **Spectral Angle** 0.78

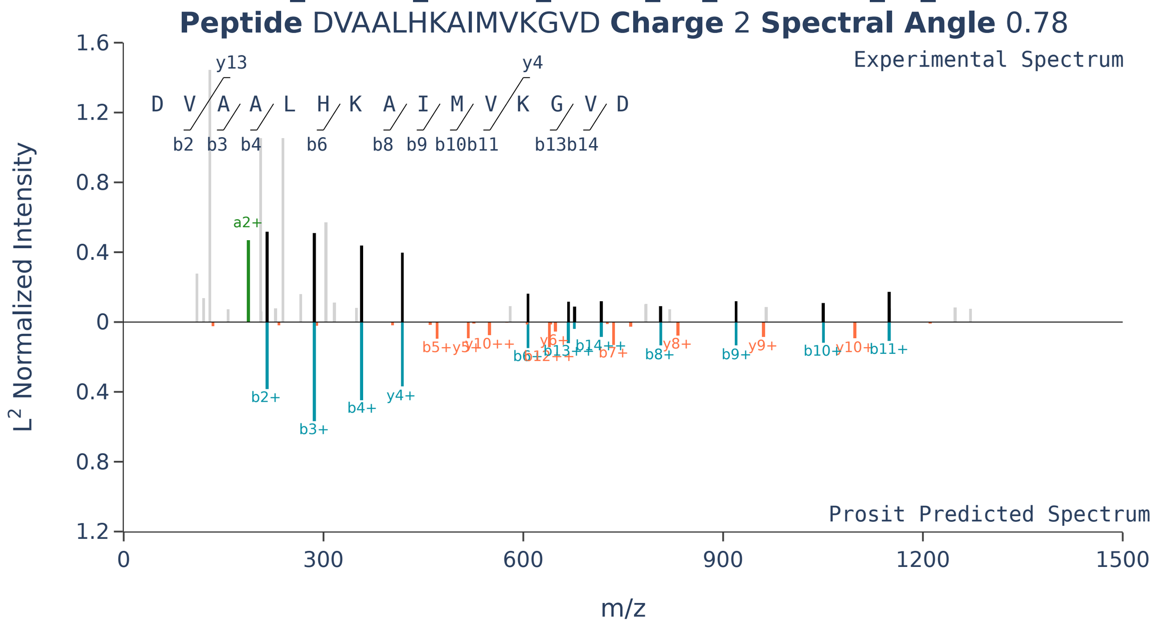


**Source** WSoh_301121_021221_HFGoe_M2_AnnexinA1_2h_R1 **Scan** 19872
**Peptide** DVAALHKAIMVKGVDE **Charge** 3 **Spectral Angle** 0.72

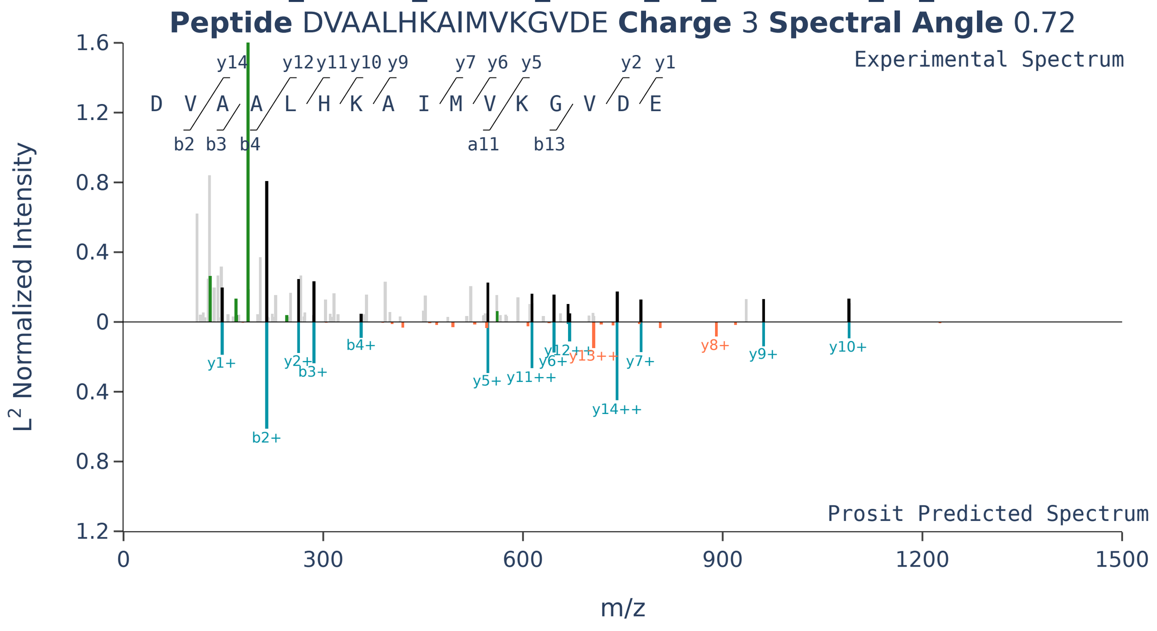


**Source** WSoh_301121_021221_HFGoe_M1_AnnexinA1_4h_R1 **Scan** 6951
**Peptide** RIMVSRS **Charge** 2 **Spectral Angle** 0.85

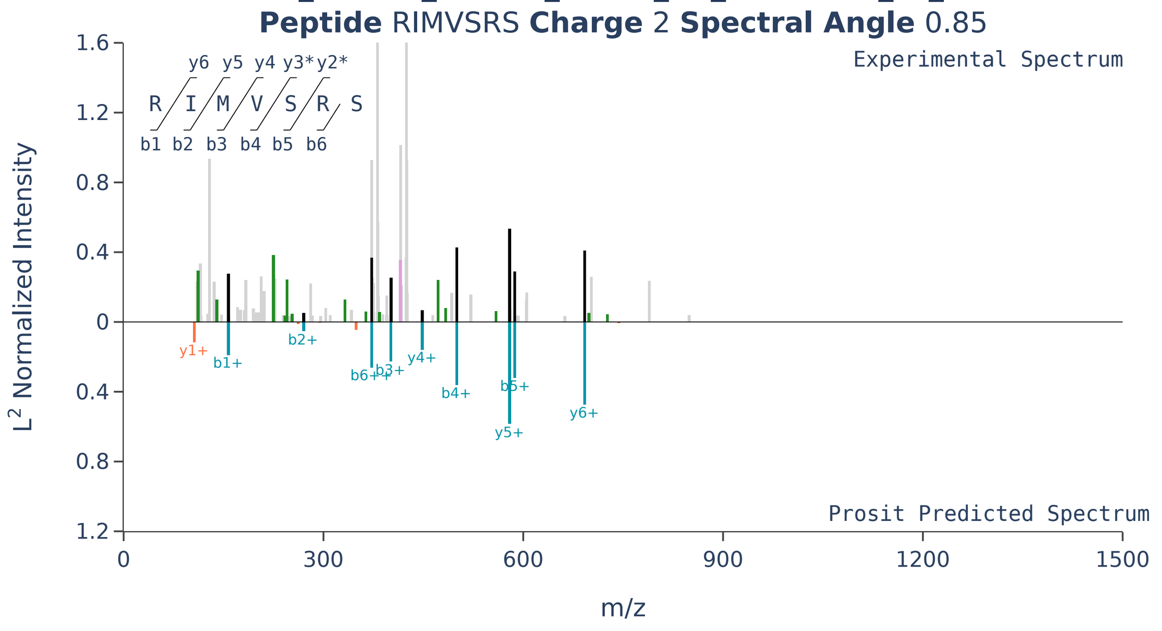


**Source** WSoh_301121_021221_HFGoe_M2_AnnexinA1_2h_R1 **Scan** 17756
**Peptide** KSSKGGPGSAVSPYPTFNPSS **Charge** 2 **Spectral Angle** 0.78

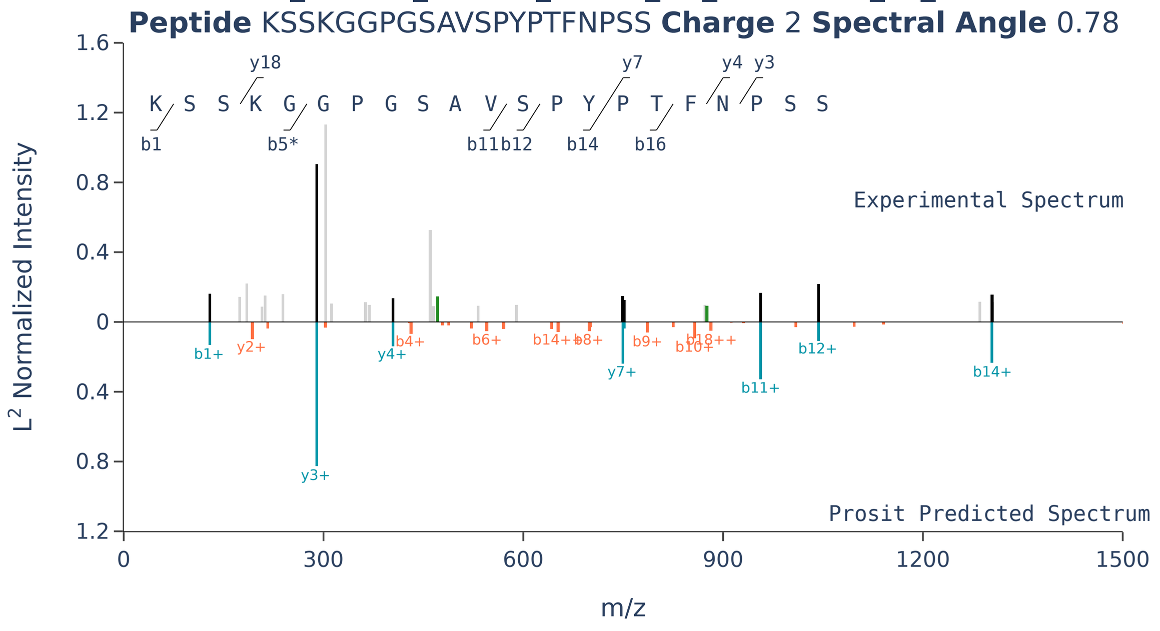

**Source** WSoh_301121_021221_HFGoe_M2_AnnexinA1_24h_R1 **Scan** 16130
**Peptide** KRDLAKDITSDTSGDF **Charge** 3 **Spectral Angle** 0.74

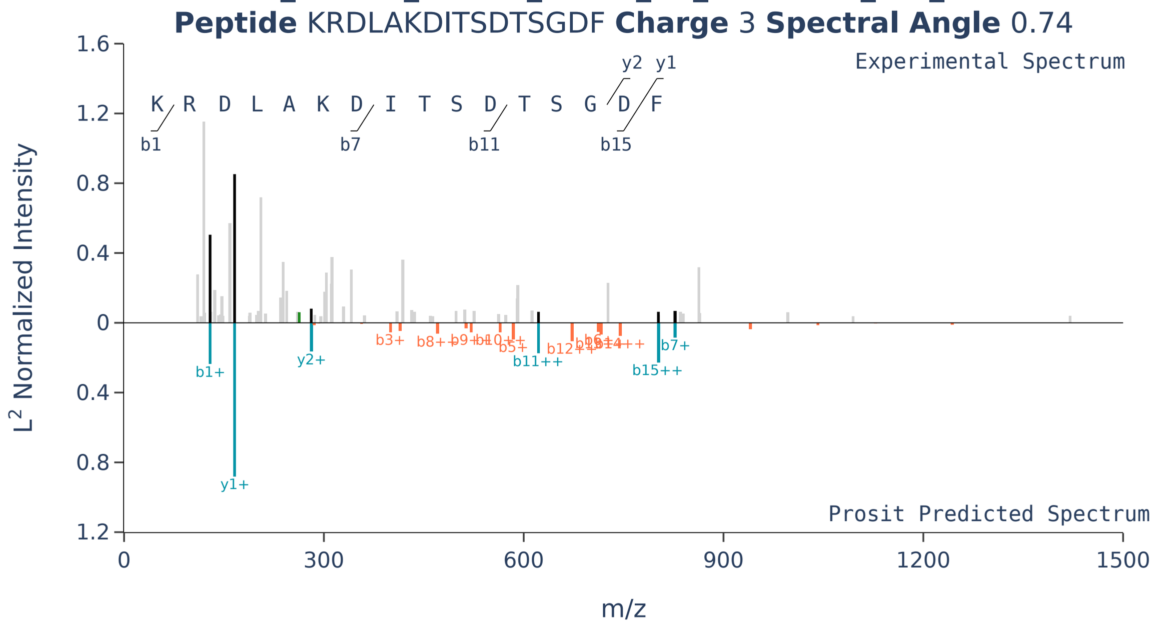


**Source** WSoh_301121_021221_HFGoe_M2_AnnexinA1_24h_R1 **Scan** 23825
**Peptide** PGSAVSPYPTFNPSSD **Charge** 2 **Spectral Angle** 0.79

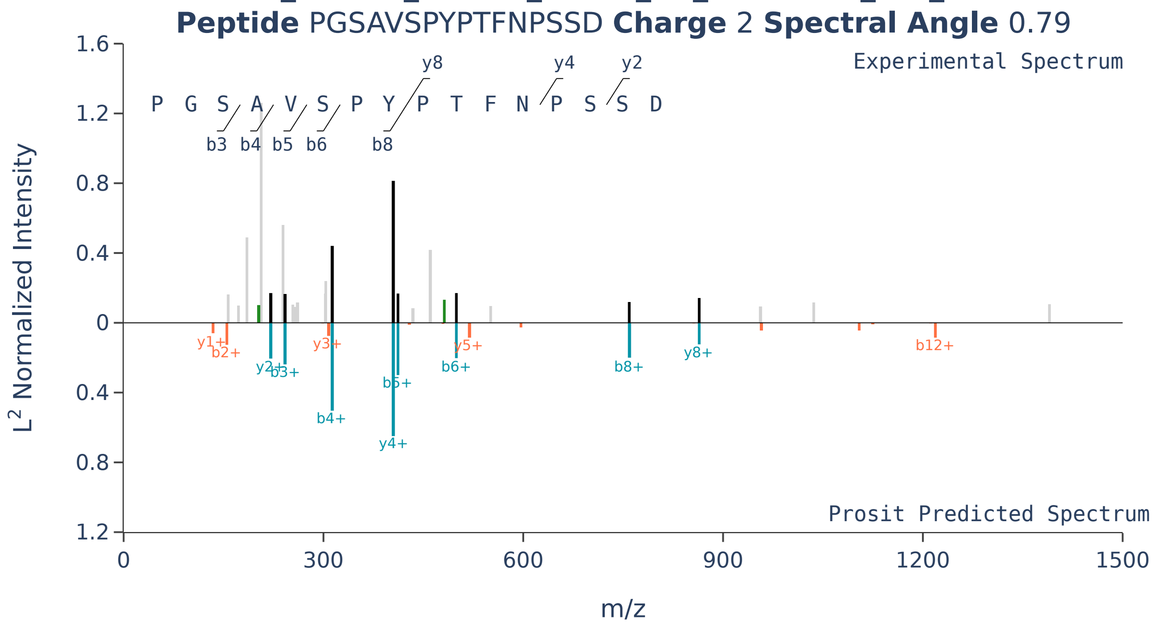


**Source** WSoh_301121_021221_HFGoe_M1_AnnexinA1_24h_R1 **Scan** 5867
**Peptide** RALYEAGERRKG **Charge** 3 **Spectral Angle** 0.69

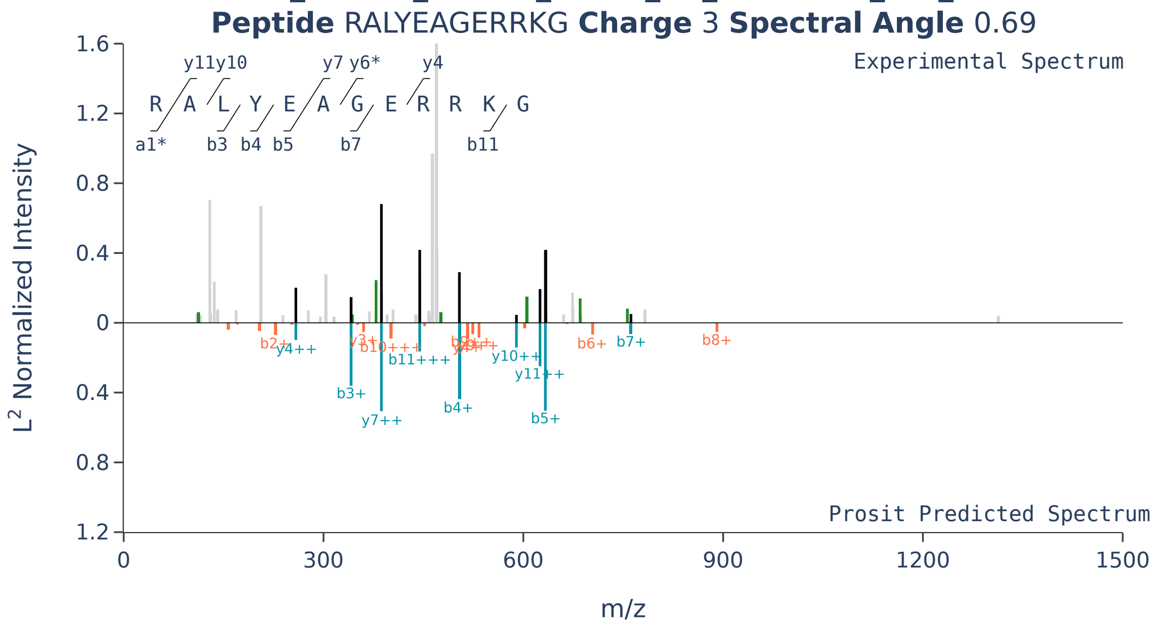


**Source** WSoh_301121_021221_HFGoe_M1_AnnexinA1_24h_R2 **Scan** 24600
**Peptide** AVSPYPTF **Charge** 1 **Spectral Angle** 0.85

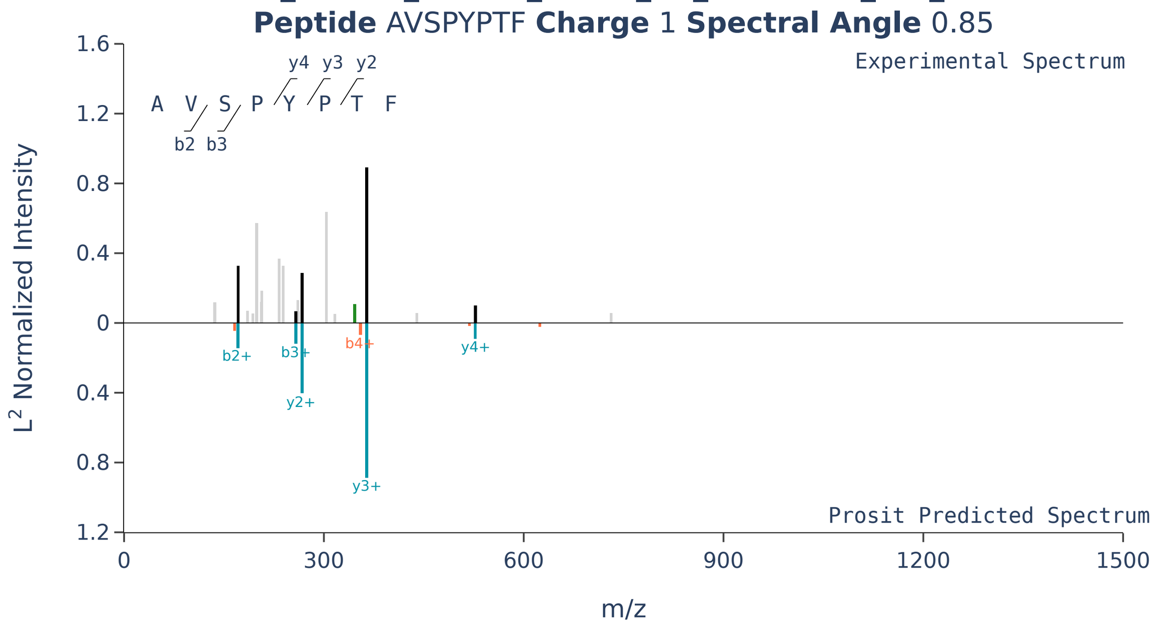


**Source** WSoh_301121_021221_HFGoe_M2_AnnexinA1_2h_R2 **Scan** 8974
**Peptide** ASRTNKEIRDINRVYRE **Charge** 5 **Spectral Angle** 0.68

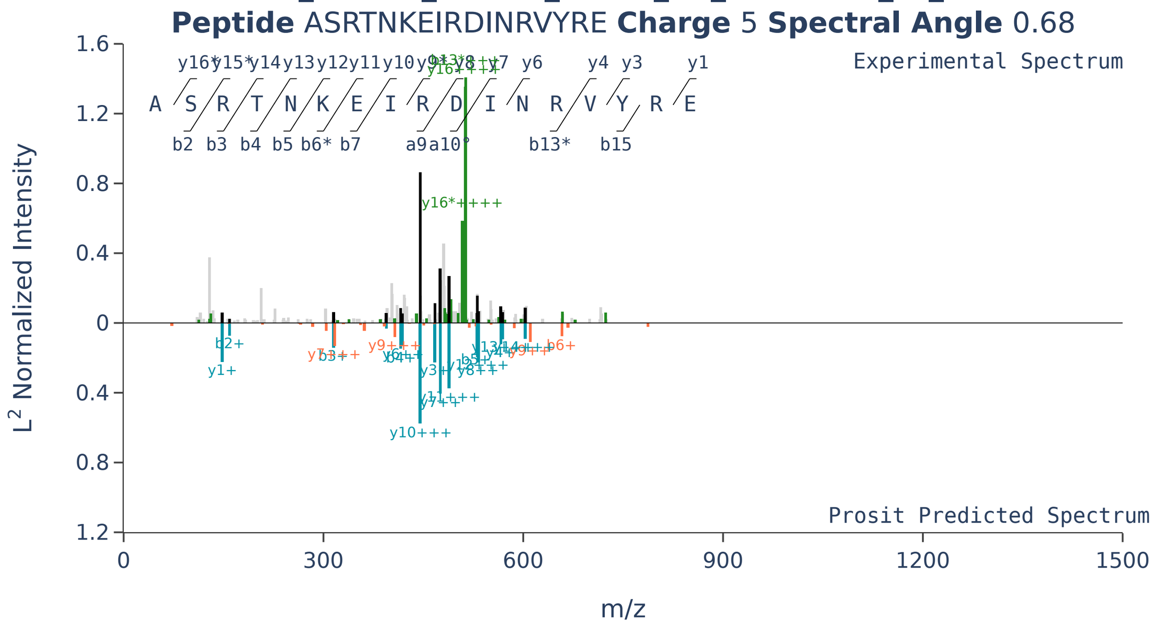


**Source** WSoh_301121_021221_HFGoe_M2_AnnexinA1_2h_R1 **Scan** 14947
**Peptide** ARALYEAGERRKGTDVNVF **Charge** 4 **Spectral Angle** 0.74

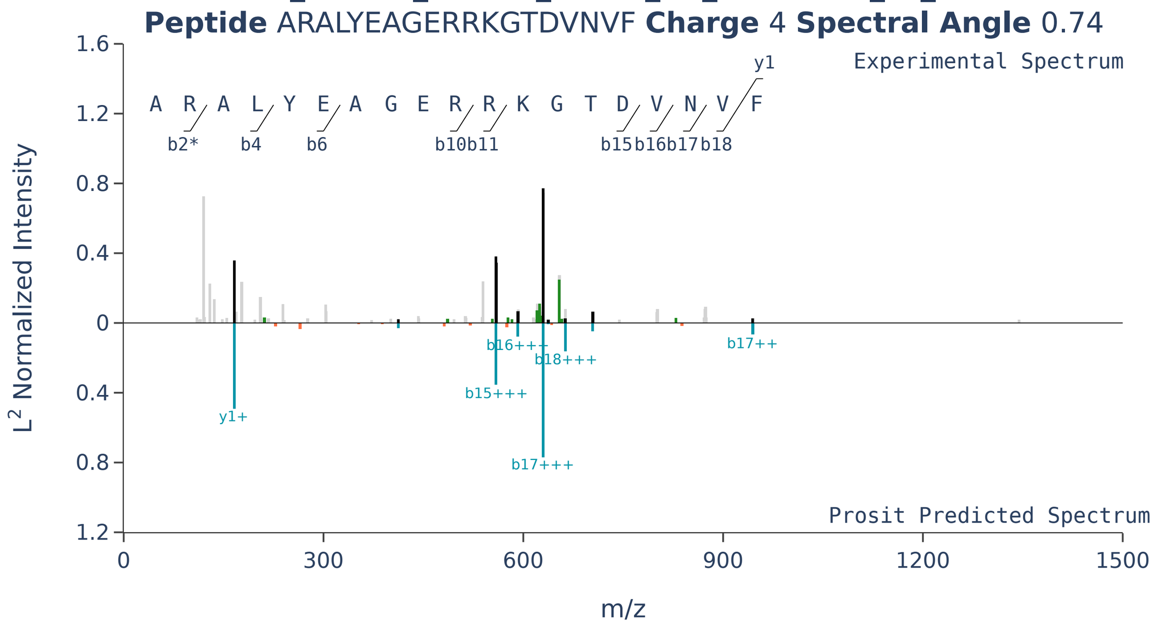


**Source** WSoh_301121_021221_HFGoe_M2_AnnexinA1_2h_R2 **Scan** 20981
**Peptide** NDIKAFYQKMY **Charge** 2 **Spectral Angle** 0.7

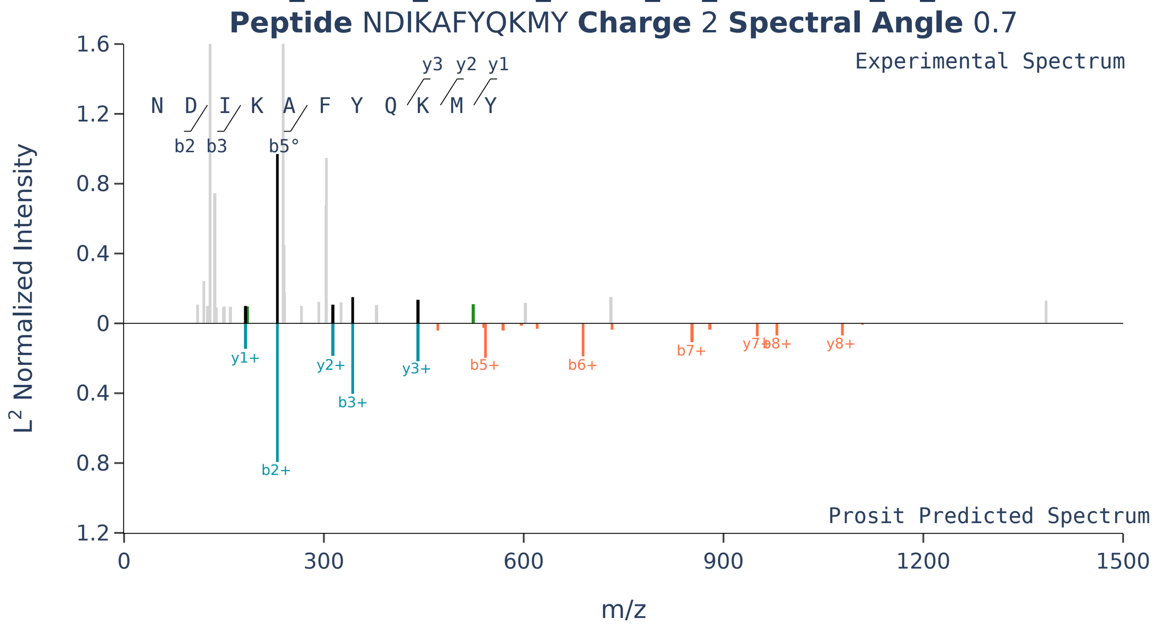


**Source** WSoh_301121_021221_HFGoe_M1_AnnexinA1_2h_R1 **Scan** 7324
**Peptide** ASRTNKEIRDL **Charge** 2 **Spectral Angle** 0.66

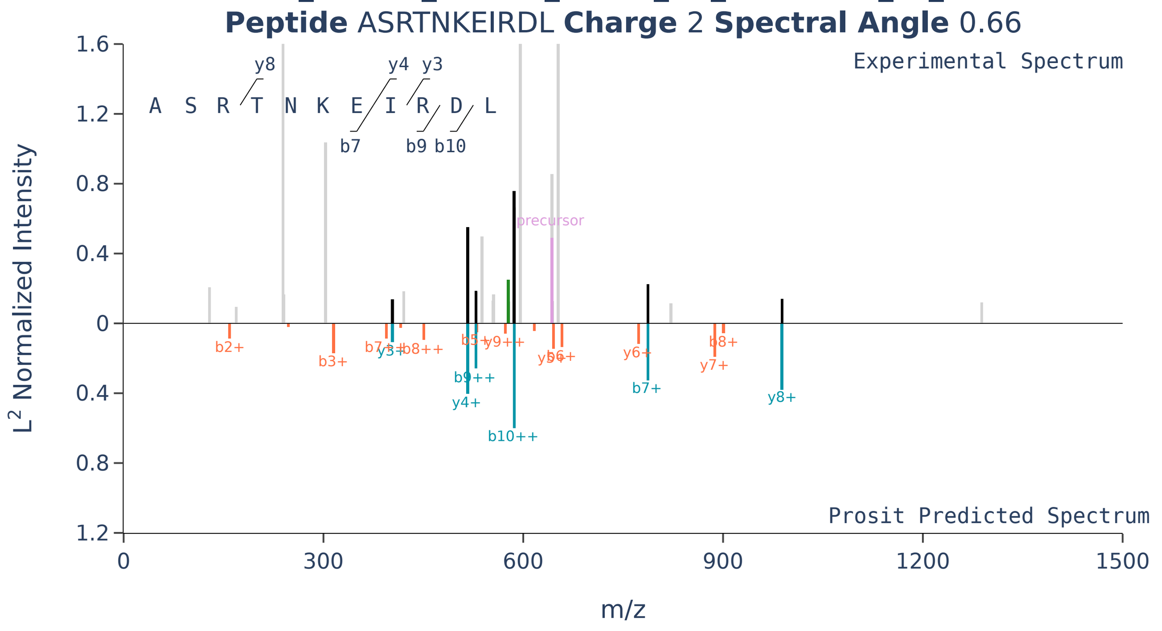


**Source** WSoh_301121_021221_HFGoe_M2_AnnexinA1_2h_R1 **Scan** 25552
**Peptide** LALLKTPAQFD **Charge** 2 **Spectral Angle** 0.79

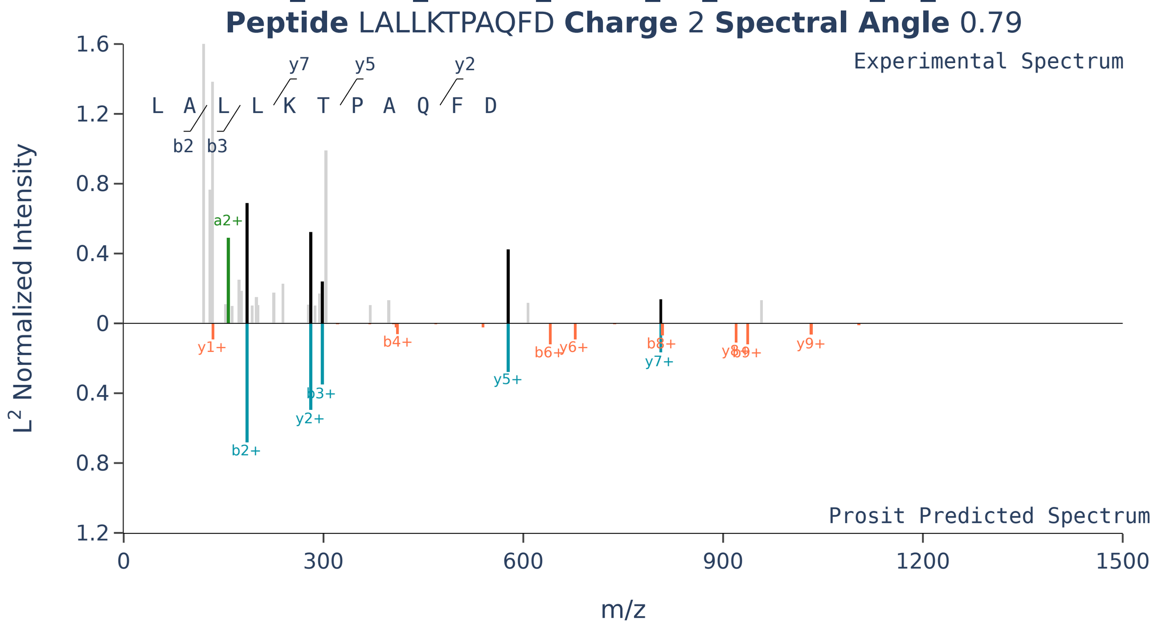


**Source** WSoh_301121_021221_HFGoe_M1_AnnexinA1_24h_R1 **Scan** 12949
**Peptide** ELKRDLAKDI **Charge** 3 **Spectral Angle** 0.72

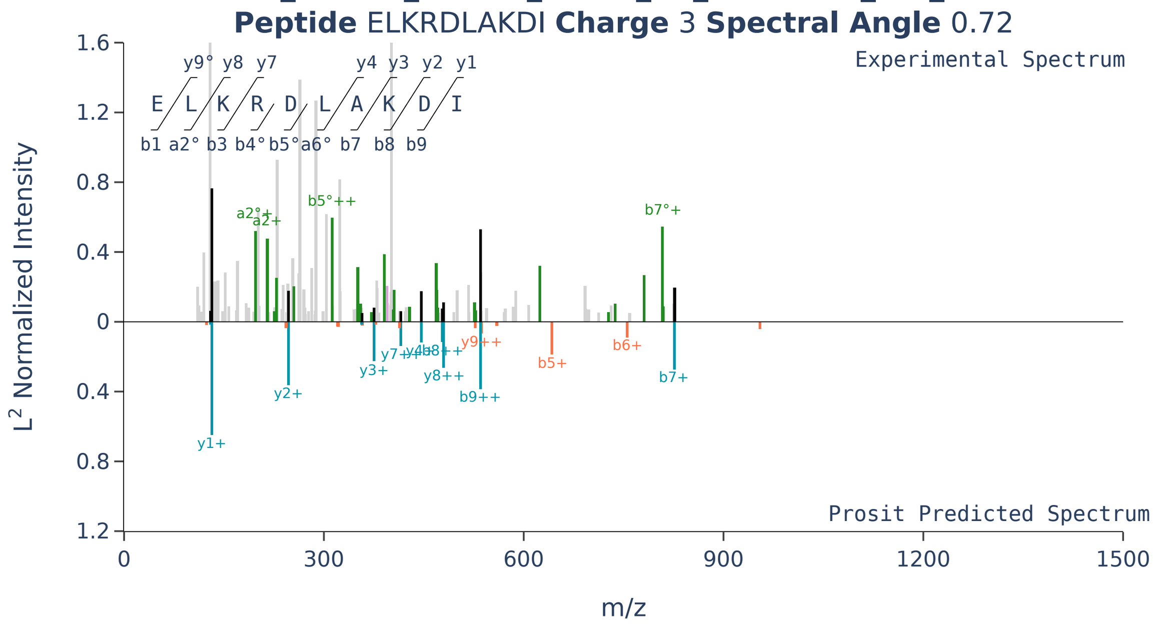


**Source** WSoh_301121_021221_HFGoe_M2_AnnexinA1_4h_R1 **Scan** 7720
**Peptide** LAKGDRSEDFG **Charge** 2 **Spectral Angle** 0.75

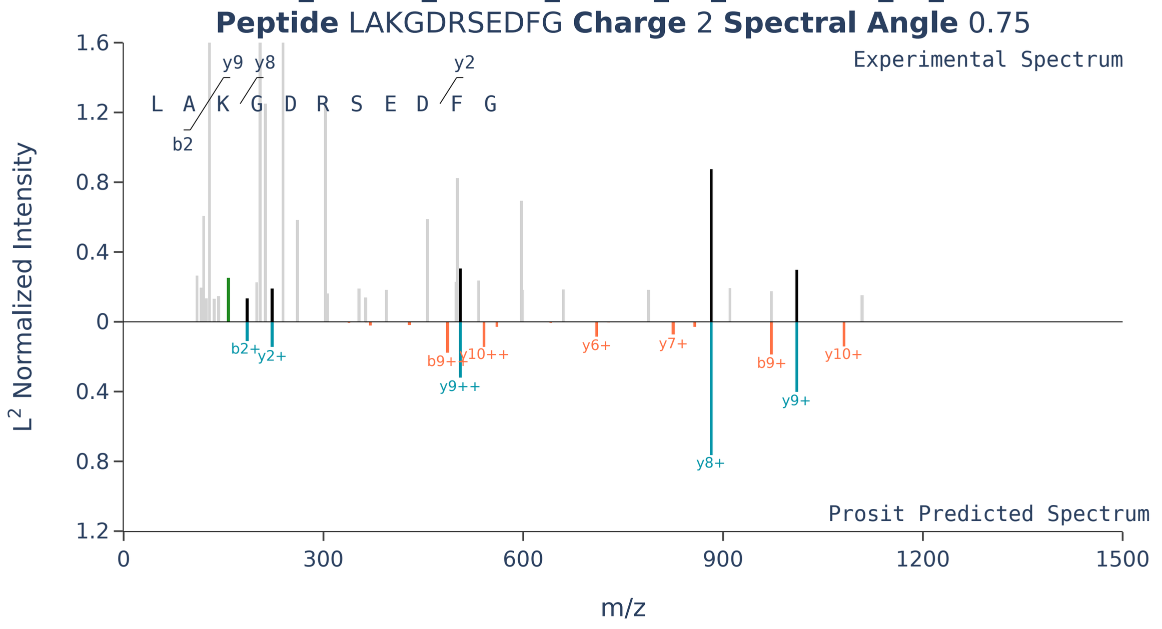


**Source** WSoh_301121_021221_HFGoe_M2_AnnexinA1_4h_R1 **Scan** 8695
**Peptide** LAKDITSDTS **Charge** 2 **Spectral Angle** 0.65

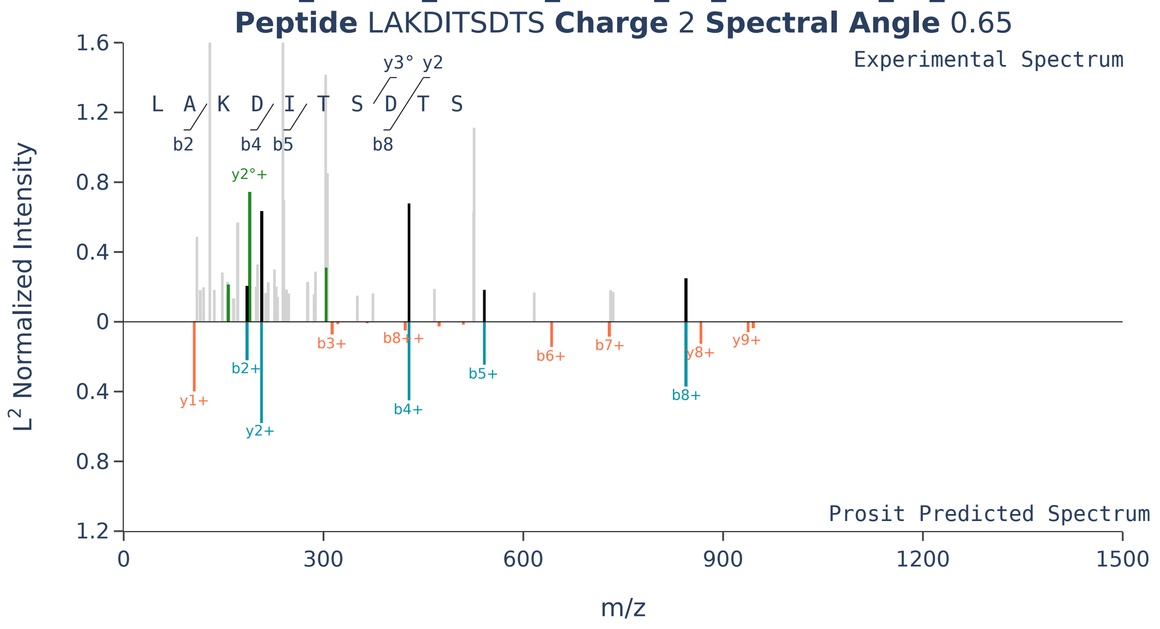


**Source** WSoh_301121_021221_HFGoe_M1_AnnexinA1_24h_R1 **Scan** 20066
**Peptide** QETGKPLDETLKKALTG **Charge** 3 **Spectral Angle** 0.77

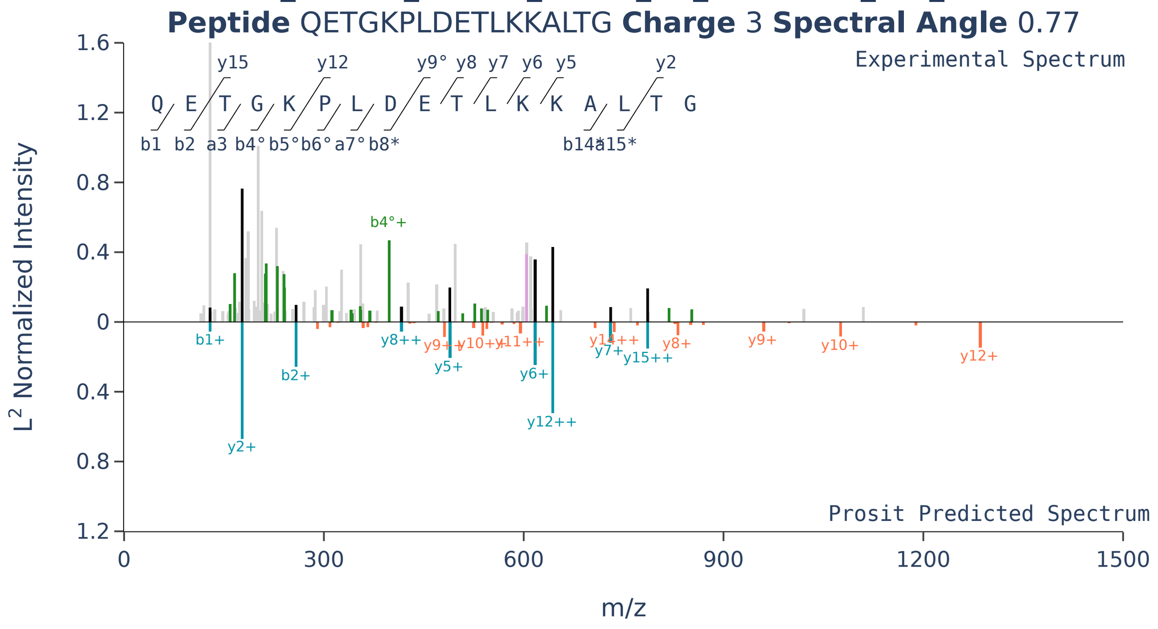


**Source** WSoh_301121_021221_HFGoe_M2_AnnexinA1_2h_R1 **Scan** 17698
**Peptide** YSKHDMNKVLDL **Charge** 3 **Spectral Angle** 0.76

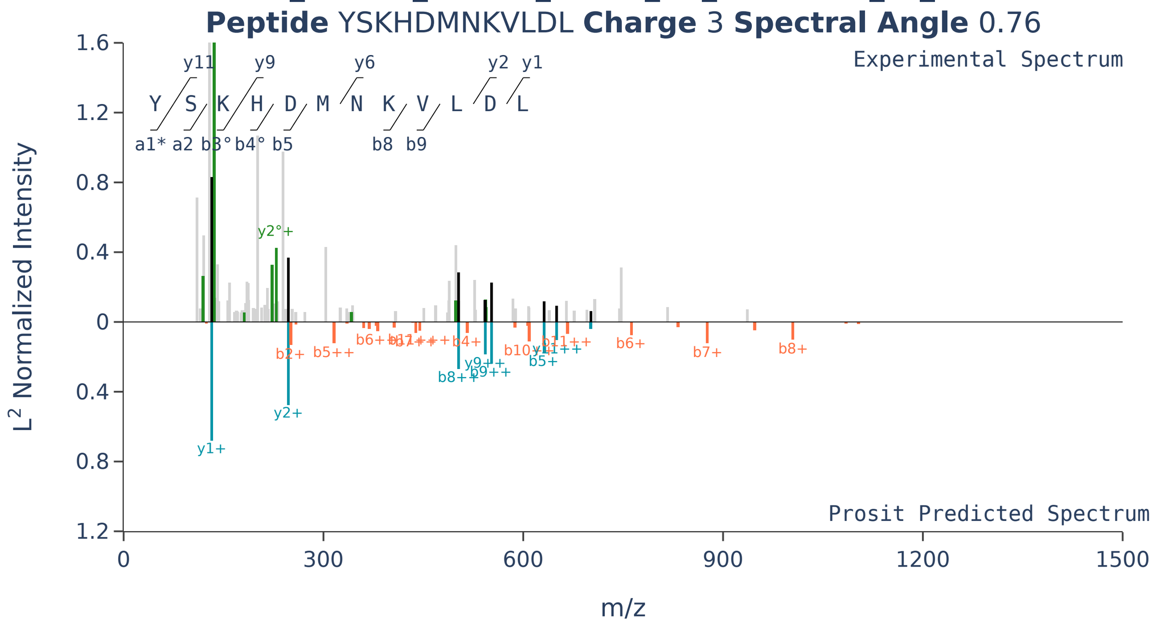


**Source** WSoh_301121_021221_HFGoe_M2_AnnexinA1_1h_R1 **Scan** 22890
**Peptide** MKGLGTDEDTLL **Charge** 2 **Spectral Angle** 0.64

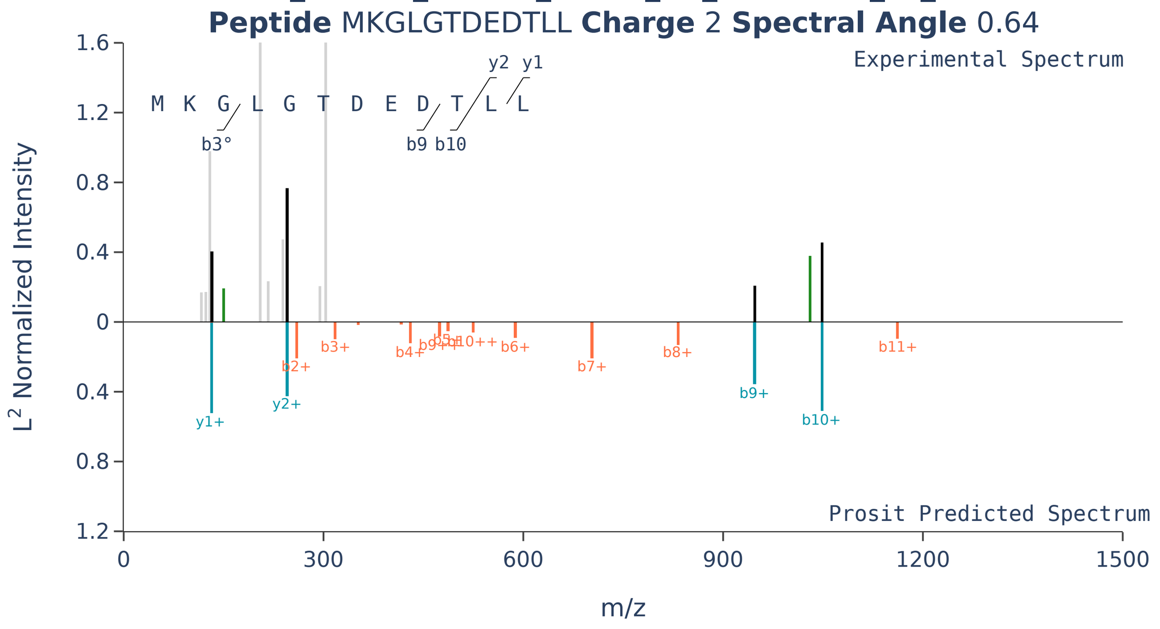

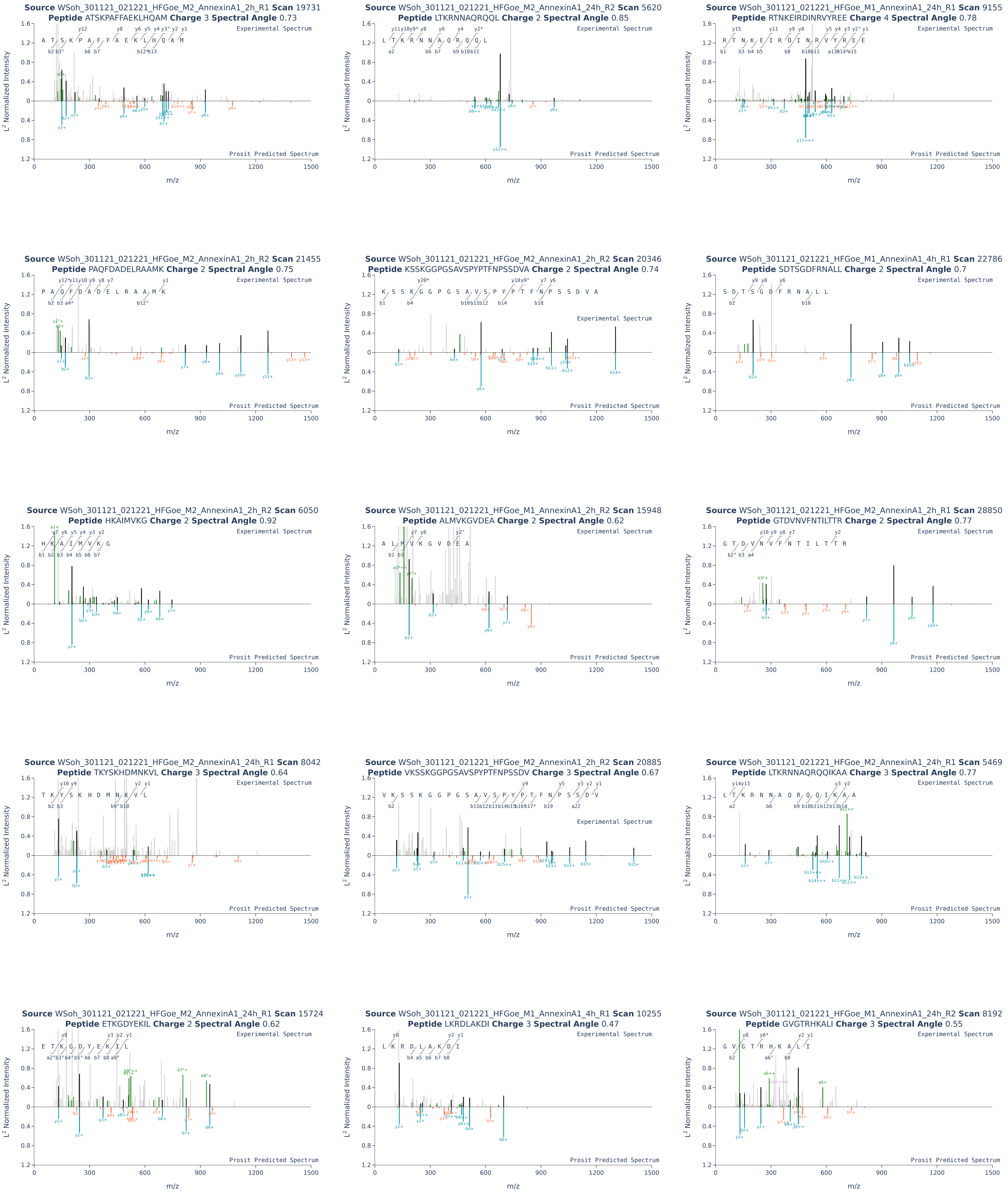
Source WSoh_301121_021221_HFGoe_M2_AnnexinA1_2h_R1 Scan 19731
Peptide ATSKPAFFAEKLHQAM Charge 3 Spectral Angle 0.73
Source WSoh_301121_021221_HFGoe_M2_AnnexinA1_24h_R2 Scan 5620
Peptide LTKRNNAQRQQL Charge 2 Spectral Angle 0.85
Source WSoh_301121_021221_HFGoe_M1_AnnexinA1_24h_R1 Scan 9155
Peptide RTNKEIRDINRVYREE Charge 4 Spectral Angle 0.78
Source WSoh_301121_021221_HFGoe_M2_AnnexinA1_2h_R2 Scan 21455
Peptide PAQFDADELRAAMK Charge 2 Spectral Angle 0.75
Source WSoh_301121_021221_HFGoe_M2_AnnexinA1_2h_R2 Scan 20346
Peptide KSSKGGPGSAVSPYPTFNPSSDVA Charge 2 Spectral Angle 0.74
Source WSoh_301121_021221_HFGoe_M1_AnnexinA1_4h_R1 Scan 22786
Peptide SDTSGDFRNALL Charge 2 Spectral Angle 0.7
Source WSoh_301121_021221_HFGoe_M2_AnnexinA1_2h_R2 Scan 6050
Peptide HKAIMVKG Charge 2 Spectral Angle 0.92
Source WSoh_301121_021221_HFGoe_M1_AnnexinA1_2h_R2 Scan 15948
Peptide ALMVKGVDEA Charge 2 Spectral Angle 0.62
Source WSoh_301121_021221_HFGoe_M2_AnnexinA1_2h_R1 Scan 28850
Peptide GTDVNVFNTILTTR Charge 2 Spectral Angle 0.77
Source WSoh_301121_021221_HFGoe_M2_AnnexinA1_24h_R1 Scan 8042
Peptide TKYSKHDMNKVL Charge 3 Spectral Angle 0.64
Source WSoh_301121_021221_HFGoe_M2_AnnexinA1_2h_R2 Scan 20885
Peptide VKSSKGGPGSAVSPYPTFNPSSDV Charge 2 Spectral Angle 0.67
Source WSoh_301121_021221_HFGoe_M2_AnnexinA1_24h_R1 Scan 5469
Peptide LTKRNNAQRQQIKAA Charge 3 Spectral Angle 0.77
Source WSoh_301121_021221_HFGoe_M2_AnnexinA1_24h_R1 Scan 15724
Peptide ETKGDYEKIL Charge 2 Spectral Angle 0.62
Source WSoh_301121_021221_HFGoe_M1_AnnexinA1_4h_R1 Scan 10255
Peptide LKRDLAKDI Charge 3 Spectral Angle 0.47
Source WSoh_301121_021221_HFGoe_M1_AnnexinA1_24h_R2 Scan 8192
Peptide GVGTRHKALI Charge 3 Spectral Angle 0.55
Experimental Spectrum
Prosit Predicted Spectrum
L² Normalized Intensity
m/z

**Source** WSoh_301121_021221_HFGoe_M2_AnnexinA1_2h_R1 **Scan** 29267
**Peptide** EATIIDILTK **Charge** 2 **Spectral Angle** 0.74

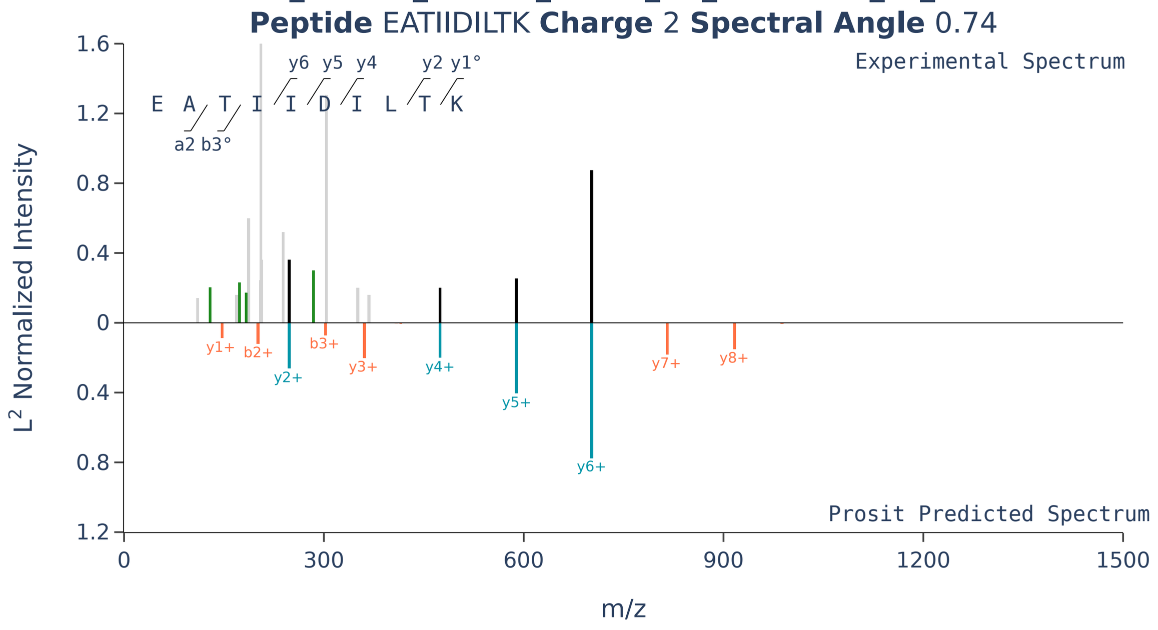


**Source** WSoh_301121_021221_HFGoe_M2_AnnexinA1_2h_R2 **Scan** 24089
**Peptide** SRSEIDMNDIKAFYQ **Charge** 3 **Spectral Angle** 0.78

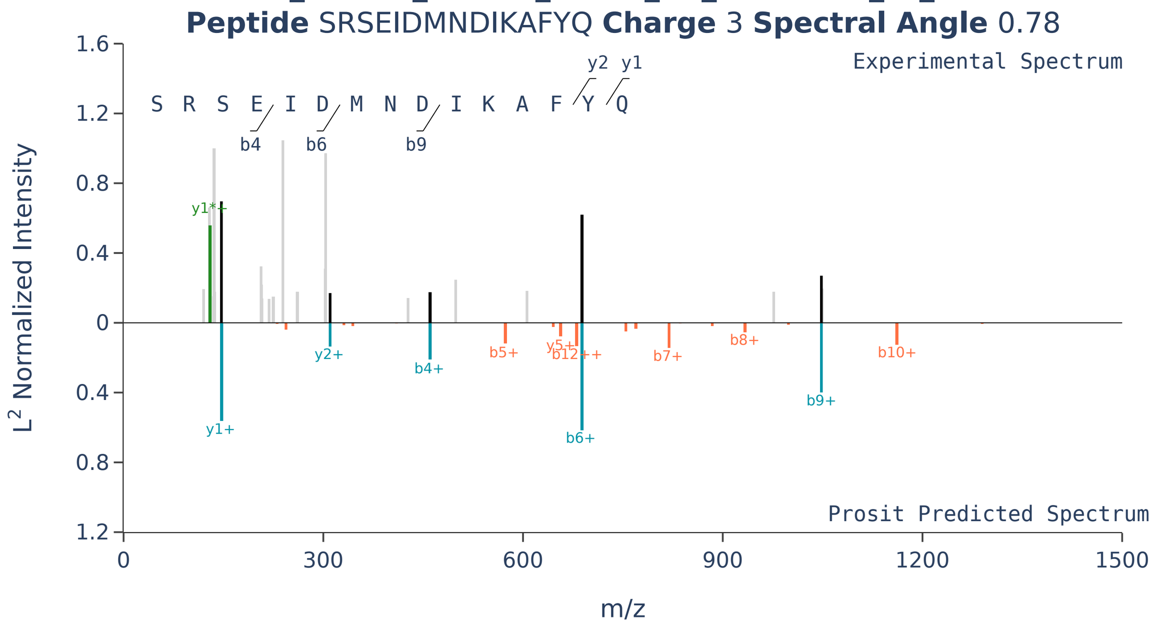


**Source** WSoh_301121_021221_HFGoe_M2_AnnexinA1_2h_R2 **Scan** 13663
**Peptide** YLQETGKPLDETLKK **Charge** 3 **Spectral Angle** 0.72

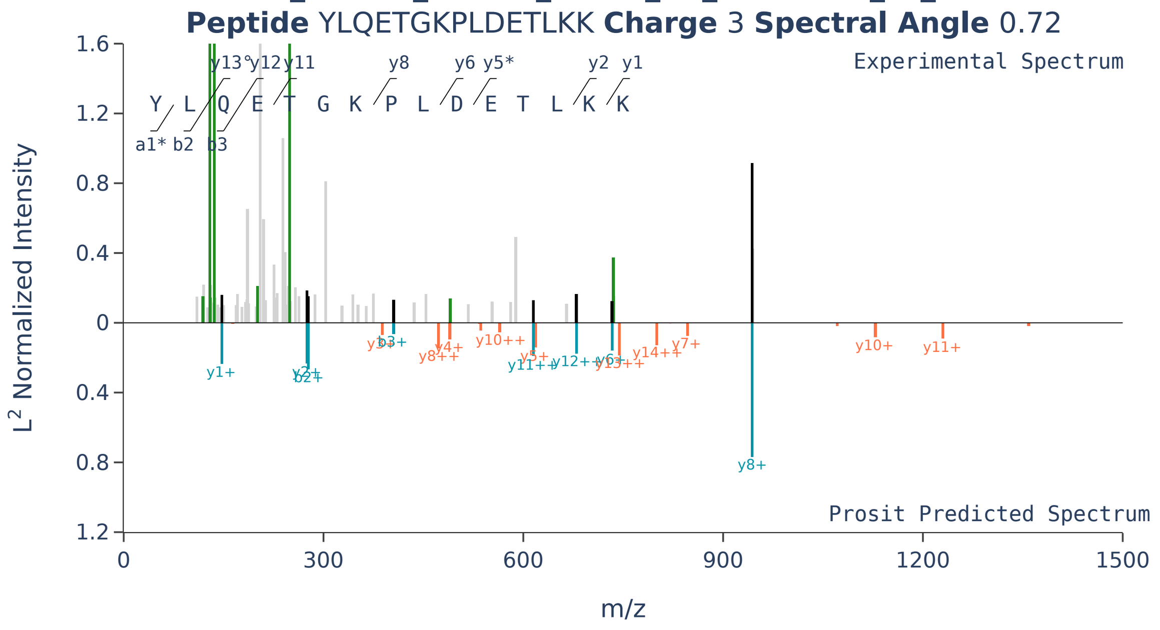


**Source** WSoh_301121_021221_HFGoe_M2_AnnexinA1_24h_R2 **Scan** 5906
**Peptide** AMKGVGTRHKAL **Charge** 3 **Spectral Angle** 0.75

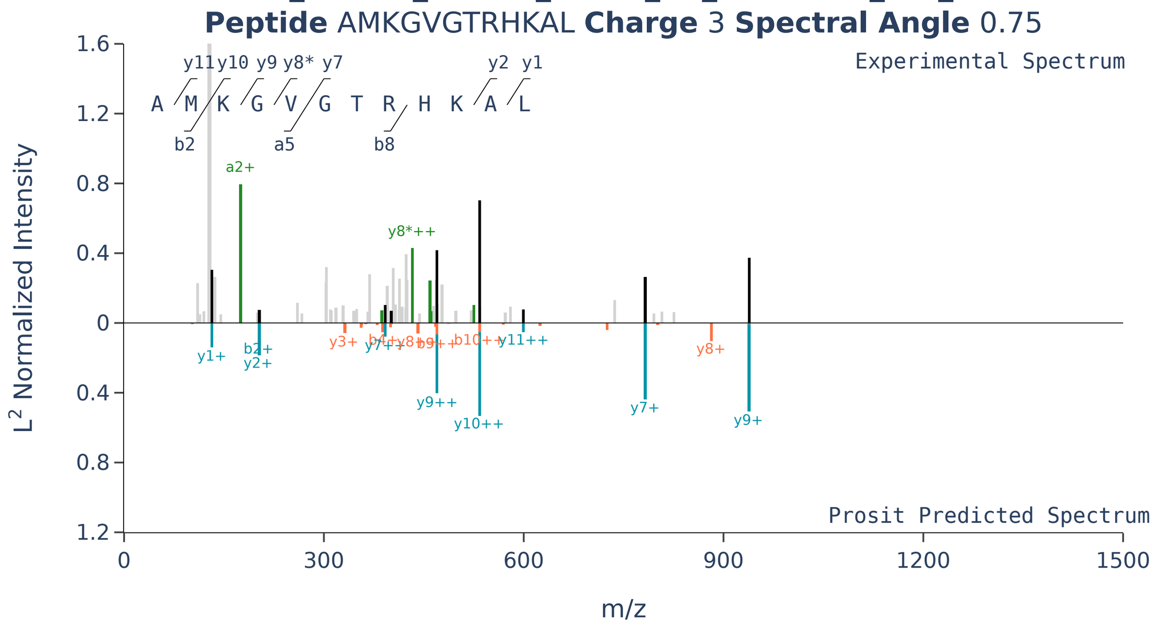


**Source** WSoh_301121_021221_HFGoe_M2_AnnexinA1_24h_R1 **Scan** 15278
**Peptide** KGTDVNVFN **Charge** 2 **Spectral Angle** 0.46

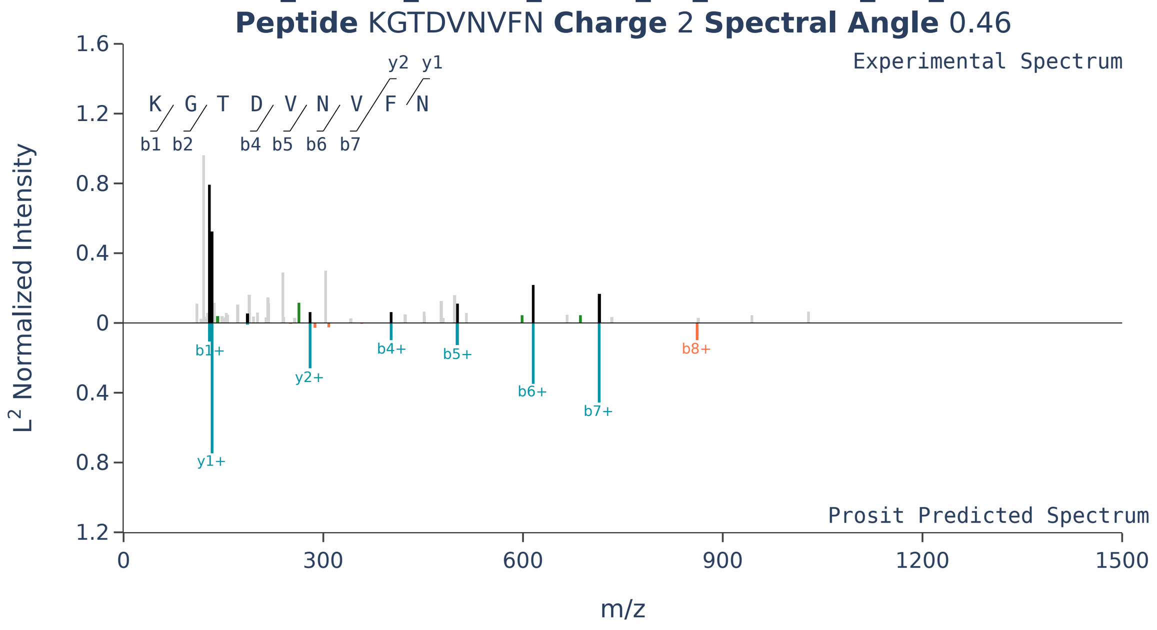


**Source** WSoh_301121_021221_HFGoe_M2_AnnexinA1_2h_R2 **Scan** 8790
**Peptide** SRTNKEIRDINRVYRE **Charge** 4 **Spectral Angle** 0.78

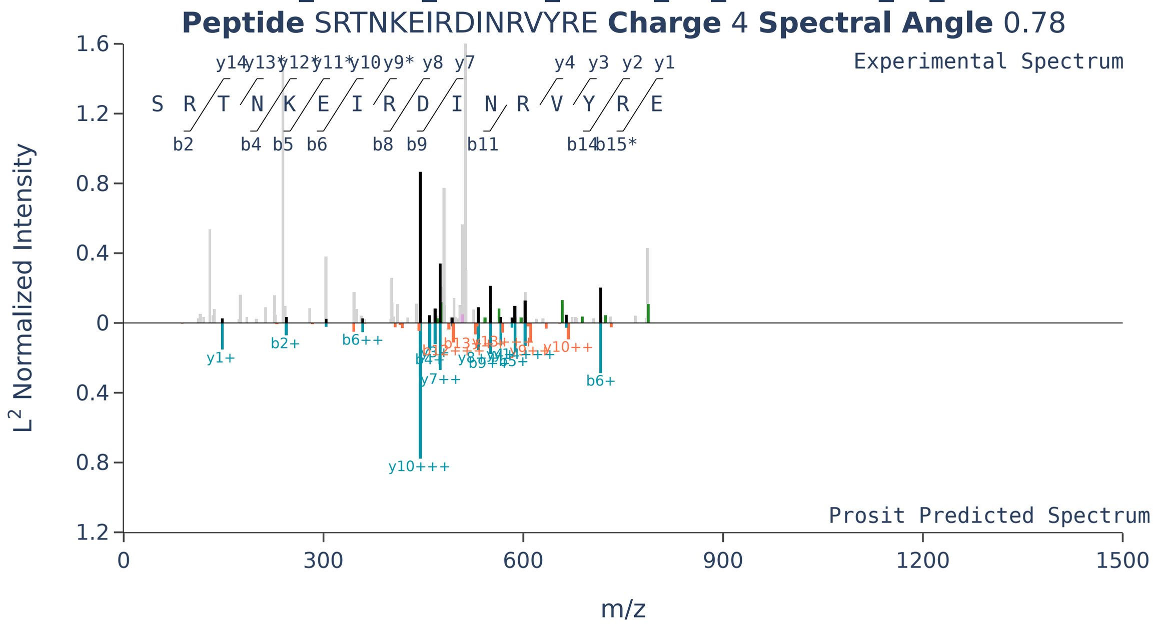


**Source** WSoh_301121_021221_HFGoe_M1_AnnexinA1_1h_R1 **Scan** 18749
**Peptide** LTSDTSGDFRNAL **Charge** 2 **Spectral Angle** 0.64

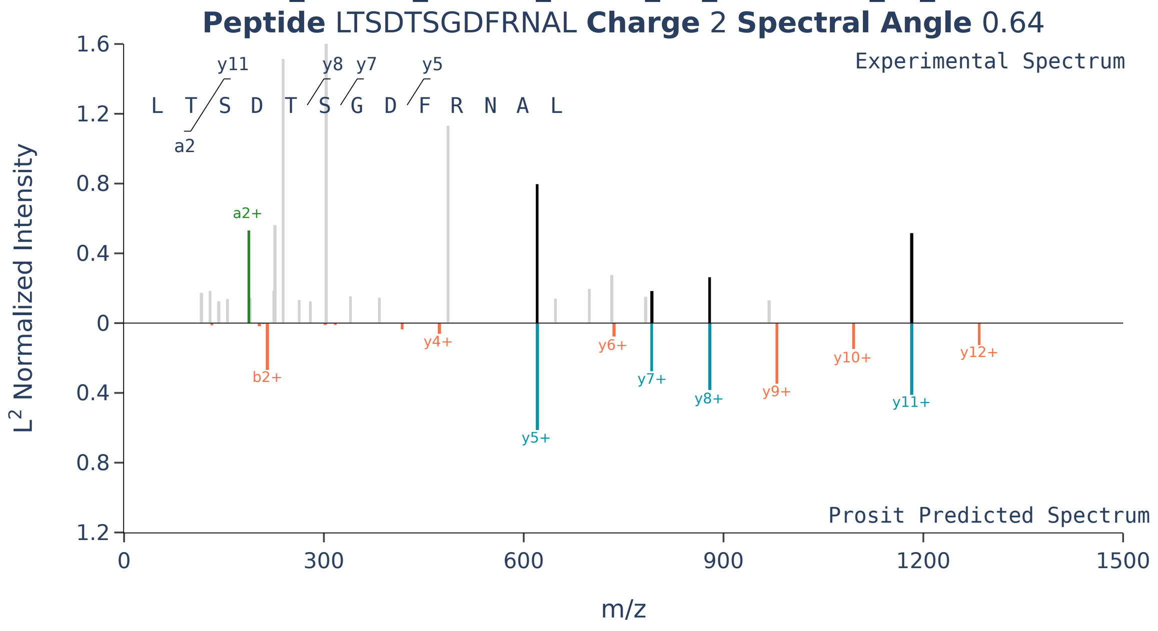


**Source** WSoh_301121_021221_HFGoe_M2_AnnexinA1_2h_R1 **Scan** 21735
**Peptide** AKDITSDTSGDFRNALLS **Charge** 3 **Spectral Angle** 0.63

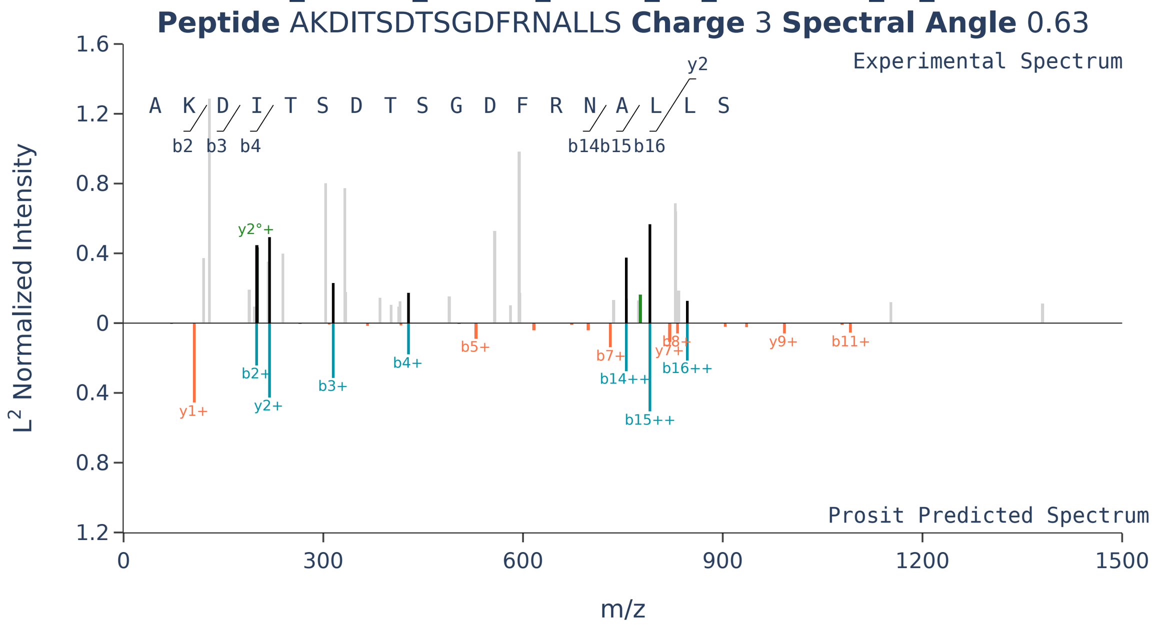


**Source** WSoh_301121_021221_HFGoe_M2_AnnexinA1_2h_R1 **Scan** 21502
**Peptide** TIIDILTKRNNAQRQQ **Charge** 3 **Spectral Angle** 0.91

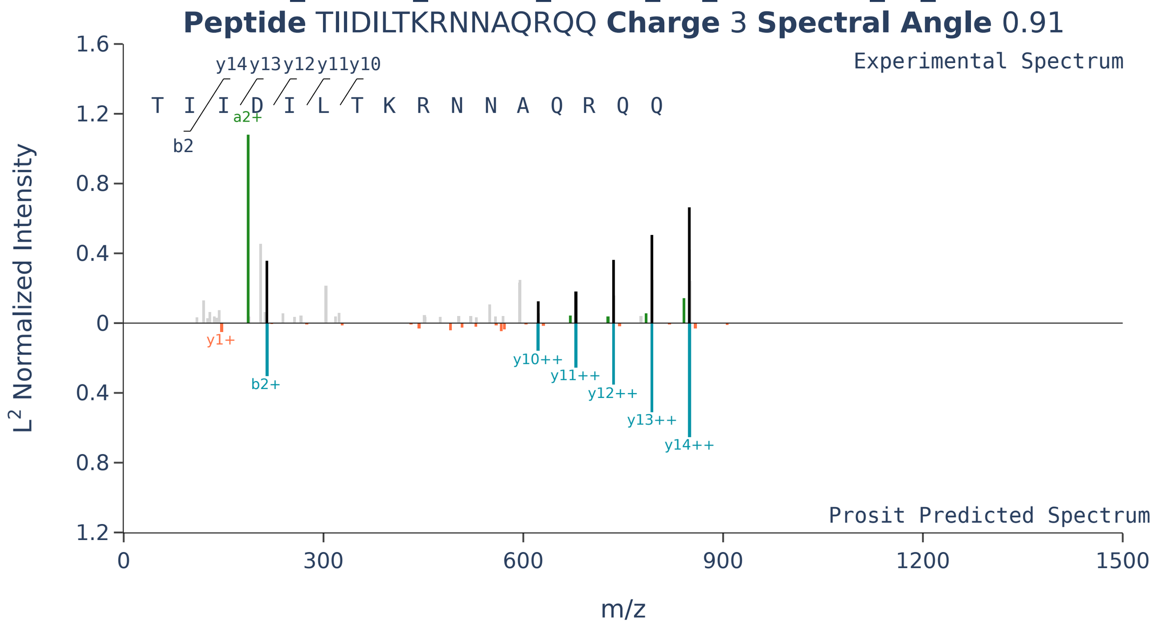


**Source** WSoh_301121_021221_HFGoe_M2_AnnexinA1_2h_R1 **Scan** 21471
**Peptide** PLDETLKKALTG **Charge** 3 **Spectral Angle** 0.74

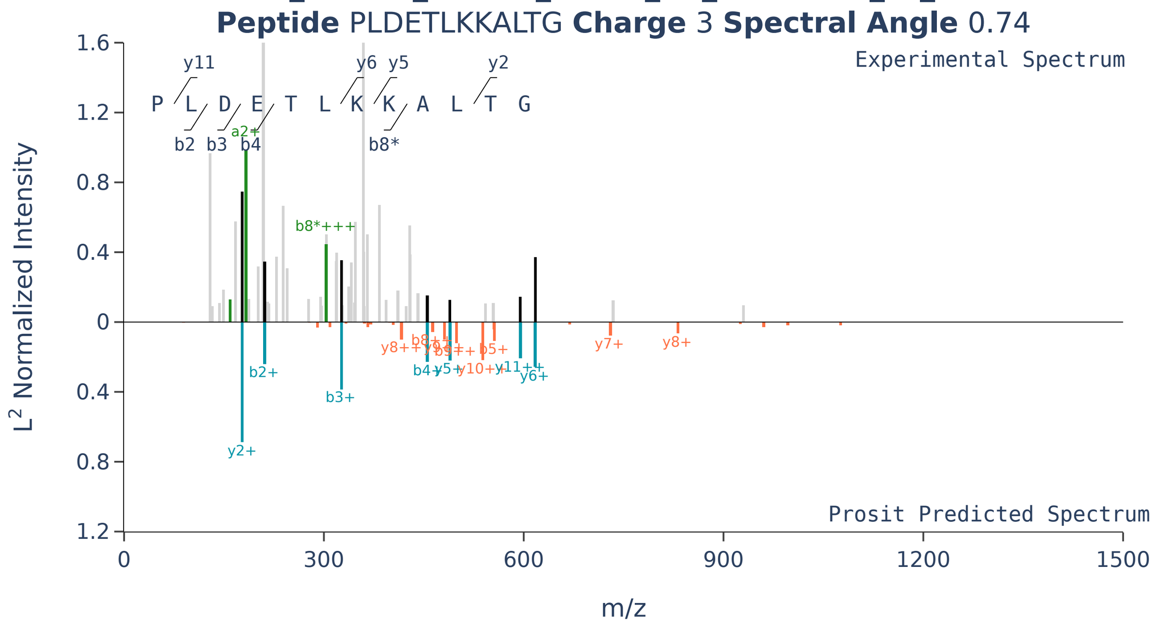


**Source** WSoh_301121_021221_HFGoe_M2_AnnexinA1_4h_R2 **Scan** 12638
**Peptide** AQRQQIKAAYLQE **Charge** 2 **Spectral Angle** 0.64

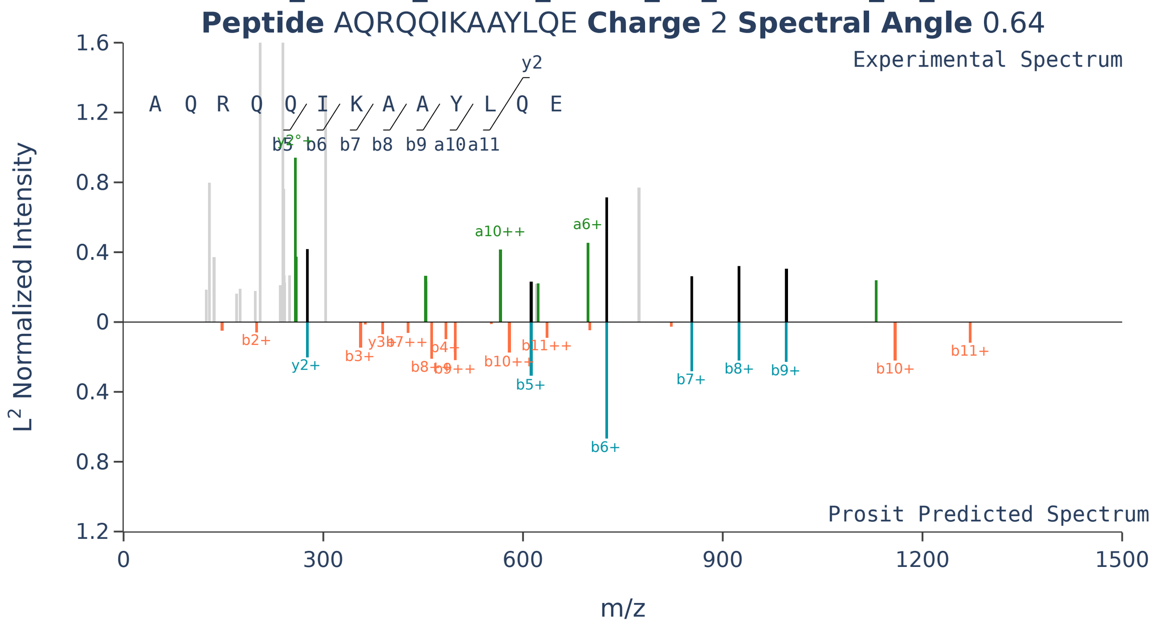


**Source** WSoh_301121_021221_HFGoe_M2_AnnexinA1_2h_R1 **Scan** 20387
**Peptide** GDRSEDFGVNEDL **Charge** 2 **Spectral Angle** 0.72

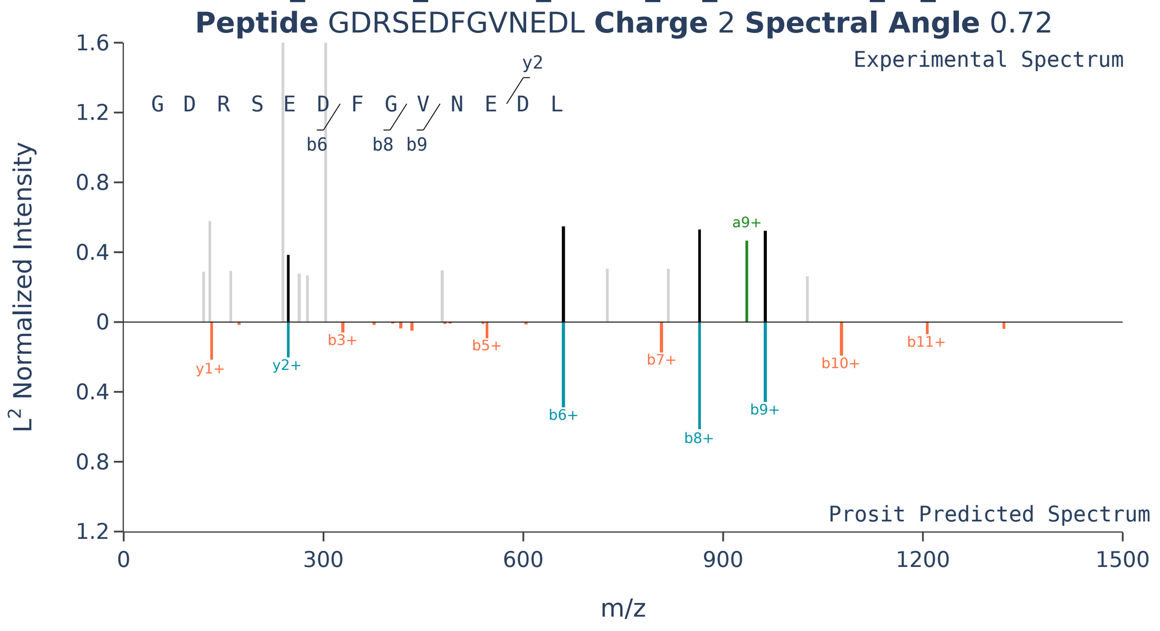


**Source** WSoh_301121_021221_HFGoe_M2_AnnexinA1_2h_R2 **Scan** 22382
**Peptide** ALLKTPAQF **Charge** 2 **Spectral Angle** 0.68

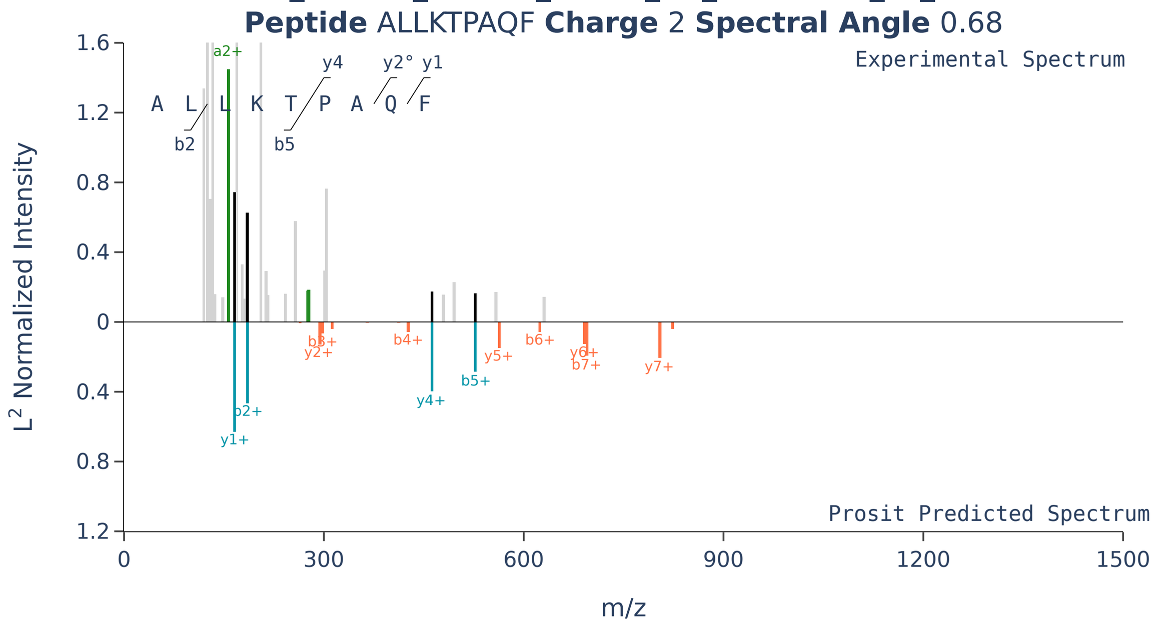


**Source** WSoh_301121_021221_HFGoe_M2_AnnexinA1_1h_R1 **Scan** 8048
**Peptide** FQKYTKYS **Charge** 3 **Spectral Angle** 0.59

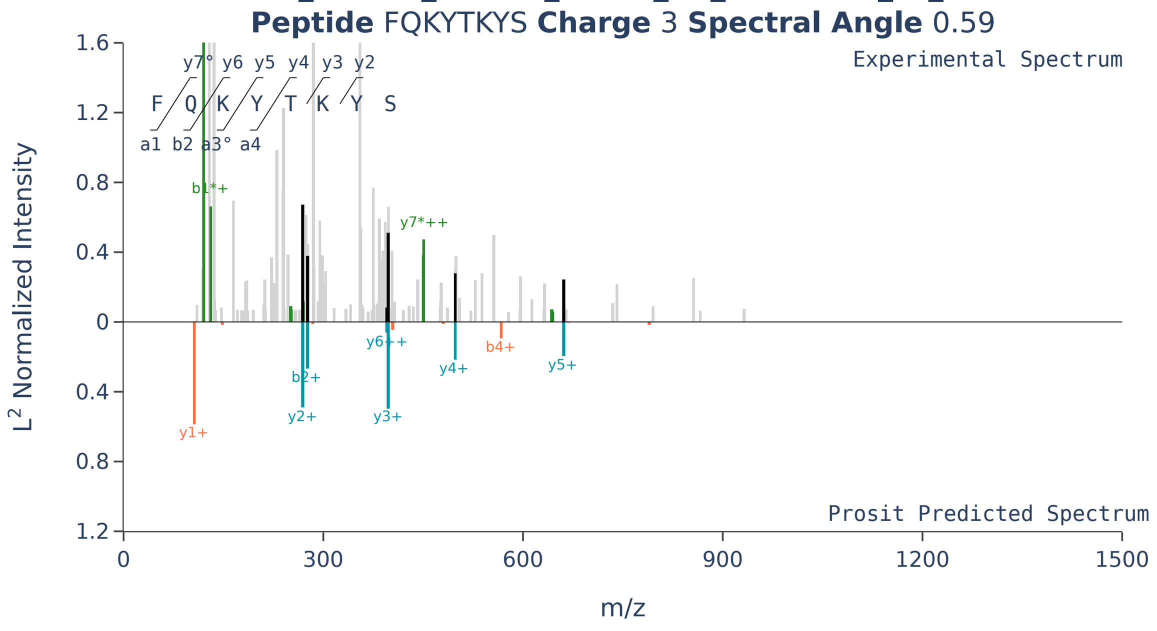


**Source** WSoh_301121_021221_HFGoe_M2_AnnexinA1_2h_R1 **Scan** 27102
**Peptide** EATIIDILTKRNN **Charge** 2 **Spectral Angle** 0.79

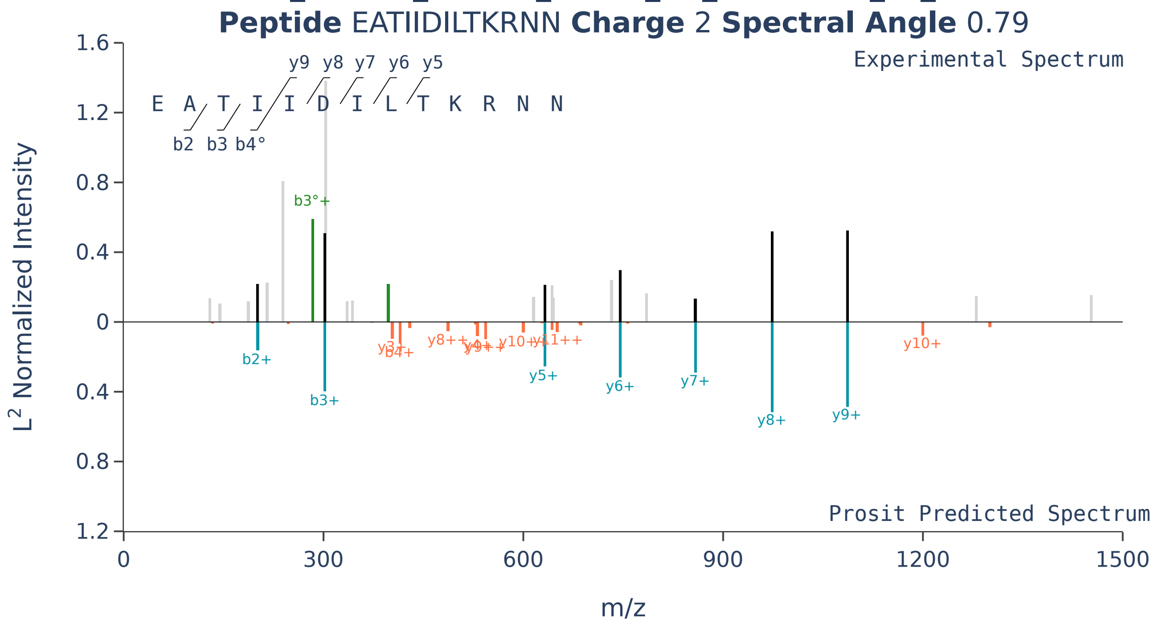

**Source** WSoh_301121_021221_HFGoe_M2_AnnexinA1_2h_R1 **Scan** 23818
**Peptide** ALLKTPAQFDADELRA **Charge** 2 **Spectral Angle** 0.82

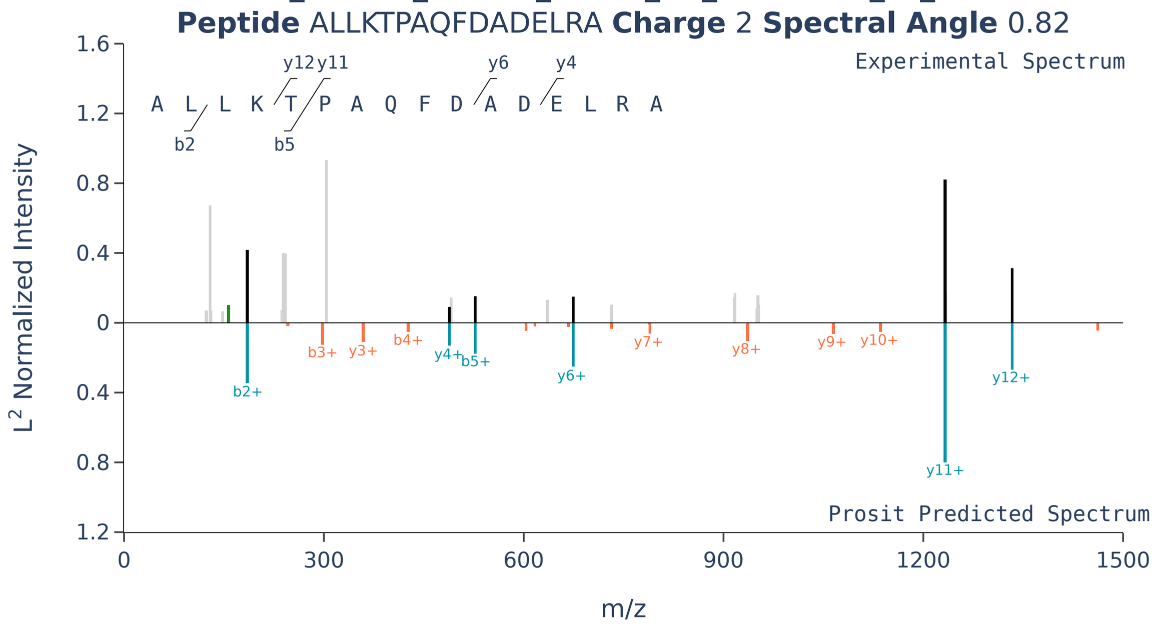


**Source** WSoh_301121_021221_HFGoe_M1_AnnexinA1_24h_R2 **Scan** 8528
**Peptide** IRDINRV **Charge** 2 **Spectral Angle** 0.42

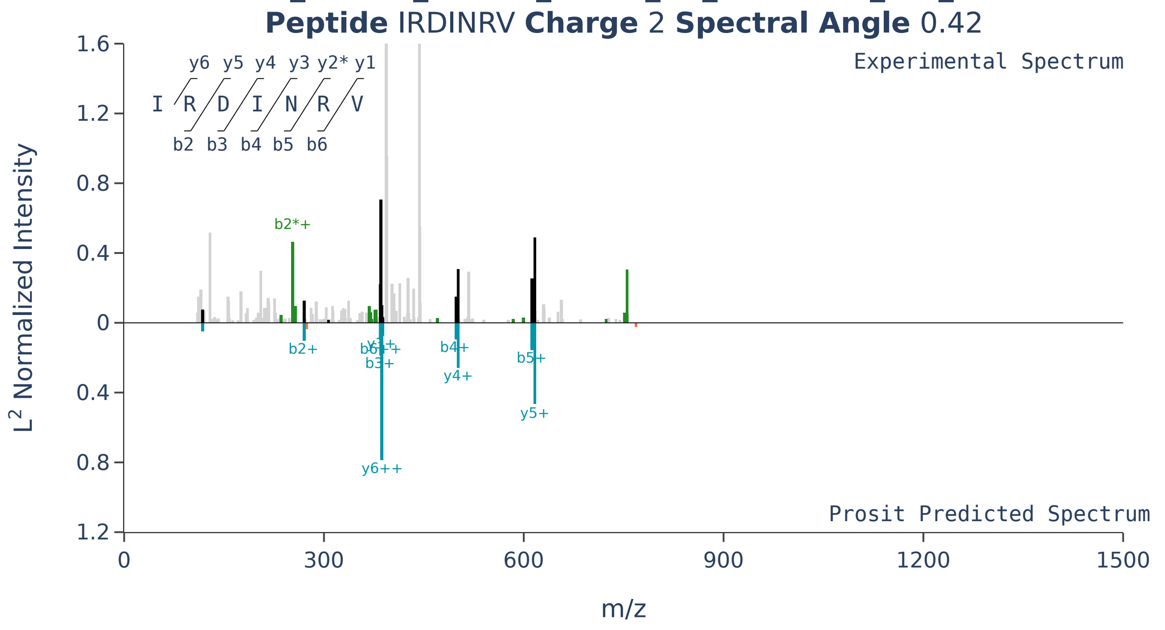


**Source** WSoh_301121_021221_HFGoe_M2_AnnexinA1_2h_R1 **Scan** 24410
**Peptide** DTSGDFRNALL **Charge** 2 **Spectral Angle** 0.65

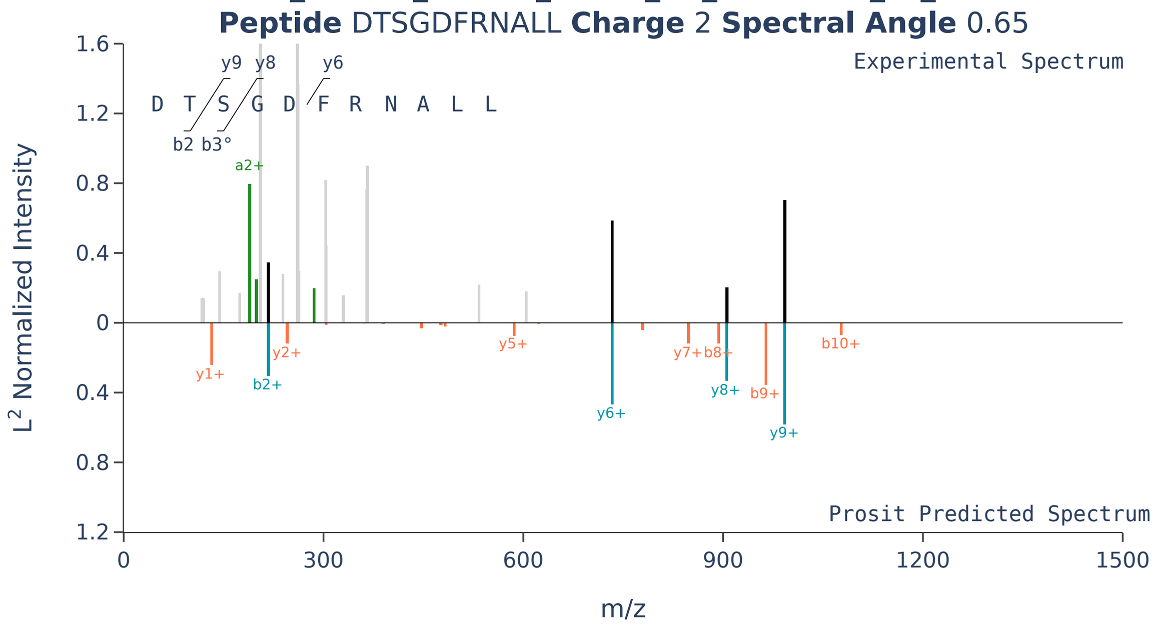


**Source** WSoh_301121_021221_HFGoe_M2_AnnexinA1_2h_R1 **Scan** 26587
**Peptide** SEIDMNDIKAFYQ **Charge** 2 **Spectral Angle** 0.81

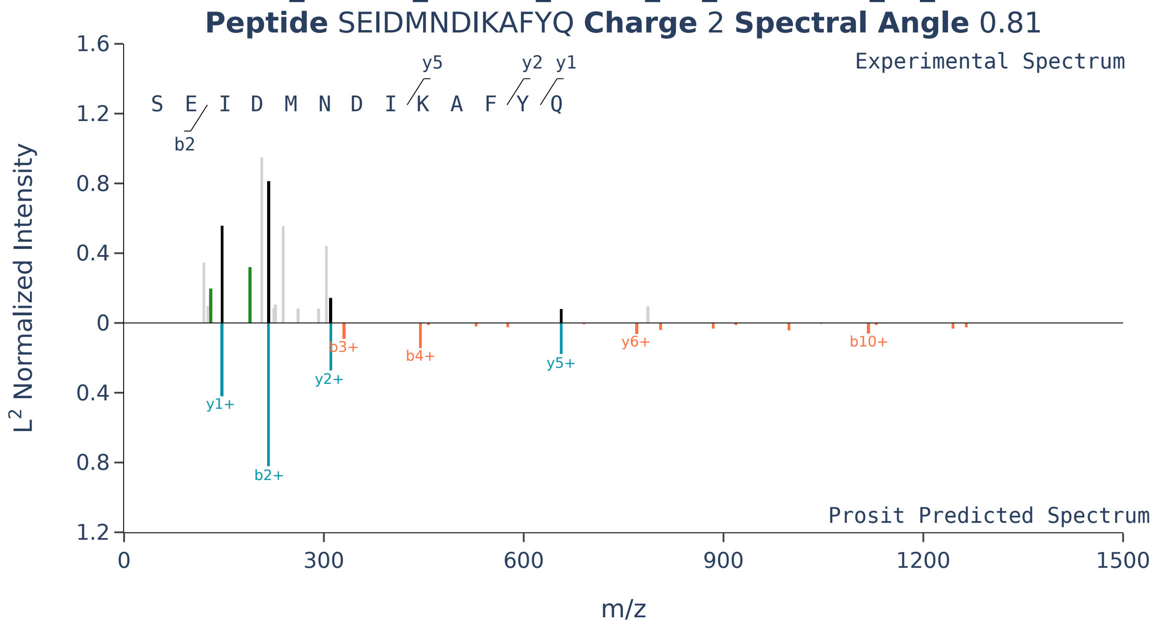


**Source** WSoh_301121_021221_HFGoe_M2_AnnexinA1_4h_R1 **Scan** 24132
**Peptide** TLIDILTKRN **Charge** 2 **Spectral Angle** 0.66

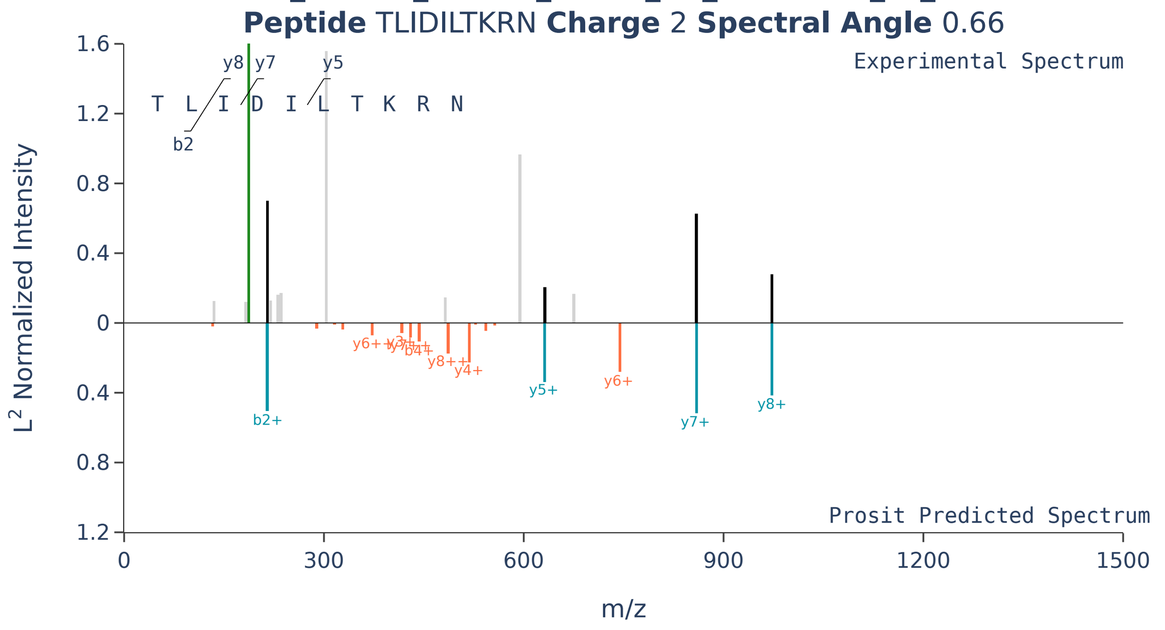


**Source** WSoh_301121_021221_HFGoe_M2_AnnexinA1_1h_R1 **Scan** 29364
**Peptide** EDTLIEILASRTN **Charge** 2 **Spectral Angle** 0.73

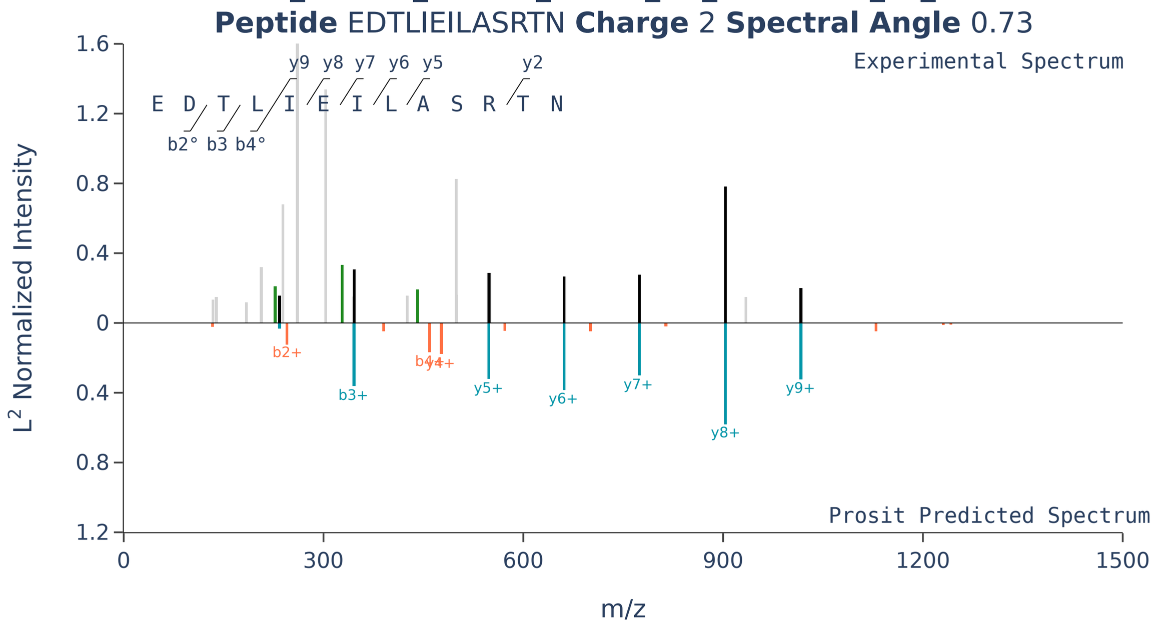


**Source** WSoh_301121_021221_HFGoe_M2_AnnexinA1_24h_R2 **Scan** 22526
**Peptide** AVSPYPTFN **Charge** 2 **Spectral Angle** 0.82

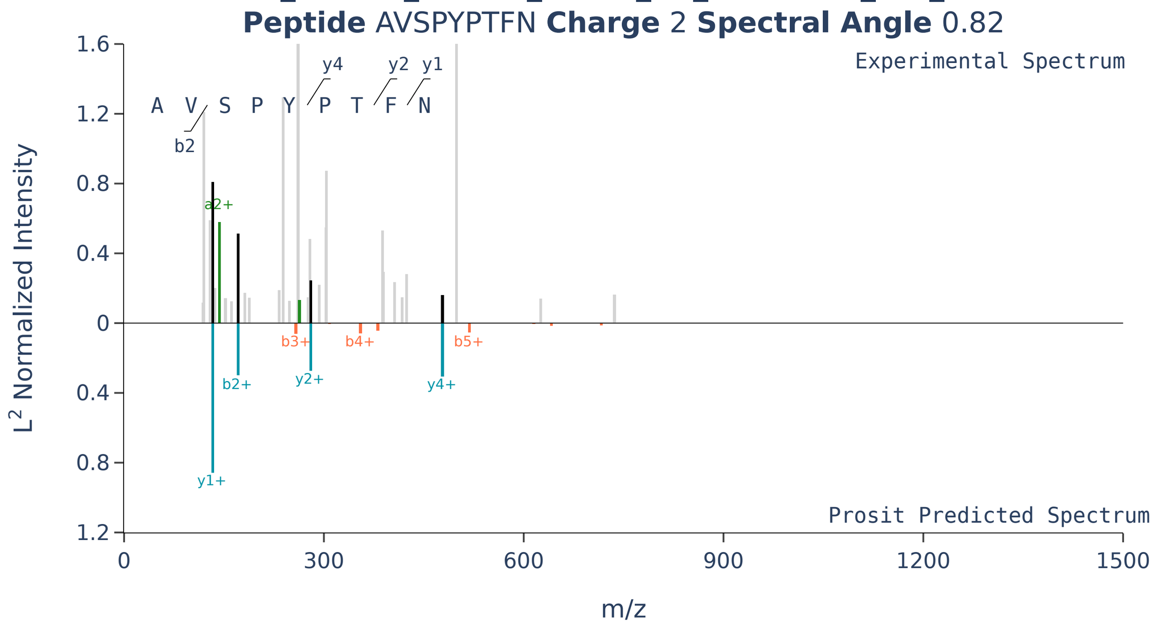


**Source** WSoh_301121_021221_HFGoe_M1_AnnexinA1_24h_R1 **Scan** 19737
**Peptide** SSKGGPGSAVSPYPTFN **Charge** 2 **Spectral Angle** 0.78

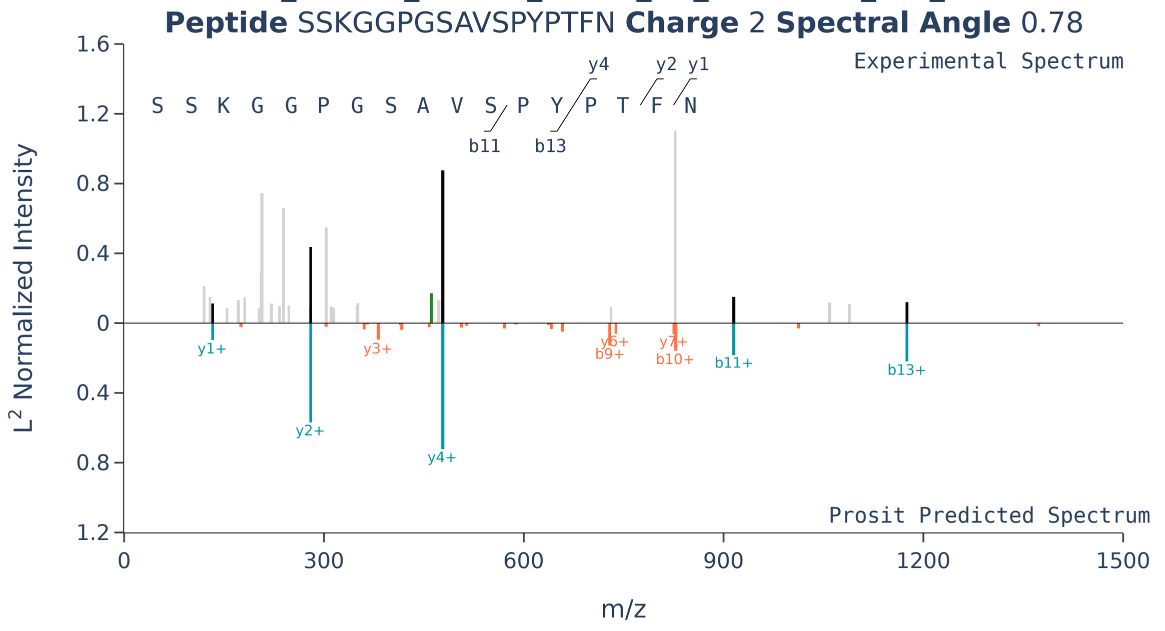


**Source** WSoh_301121_021221_HFGoe_M2_AnnexinA1_24h_R2 **Scan** 14853
**Peptide** PSSDVAALHKAI **Charge** 3 **Spectral Angle** 0.69

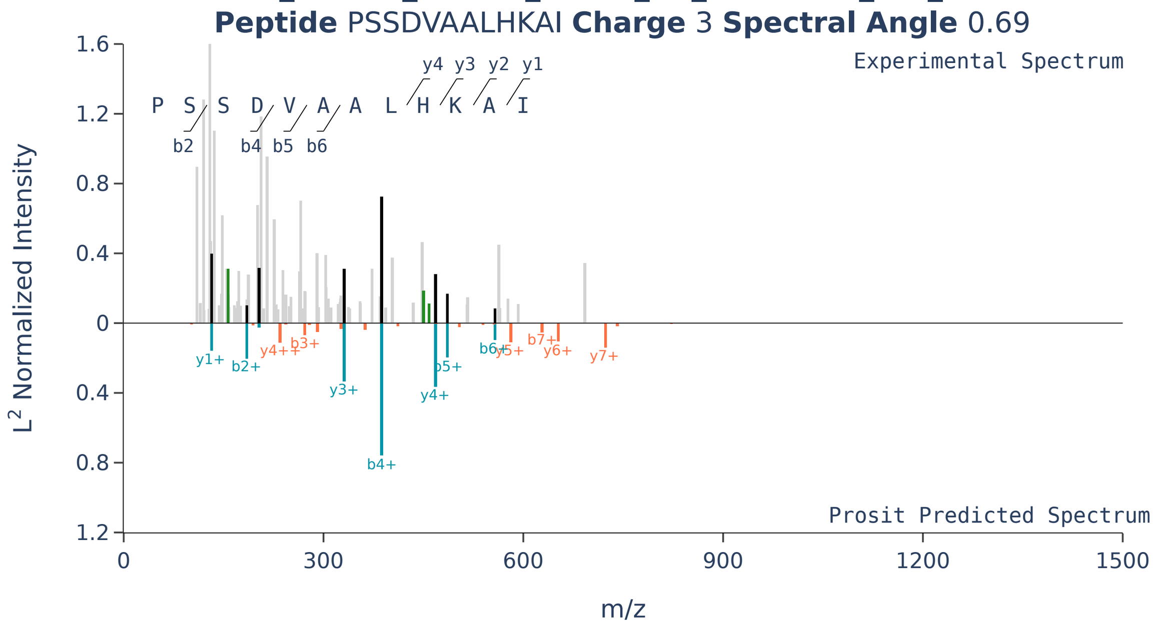


**Source** WSoh_301121_021221_HFGoe_M2_AnnexinA1_2h_R2 **Scan** 17492
**Peptide** SDARALYEAGERRKGTDVNVFNT **Charge** 4 **Spectral Angle** 0.77

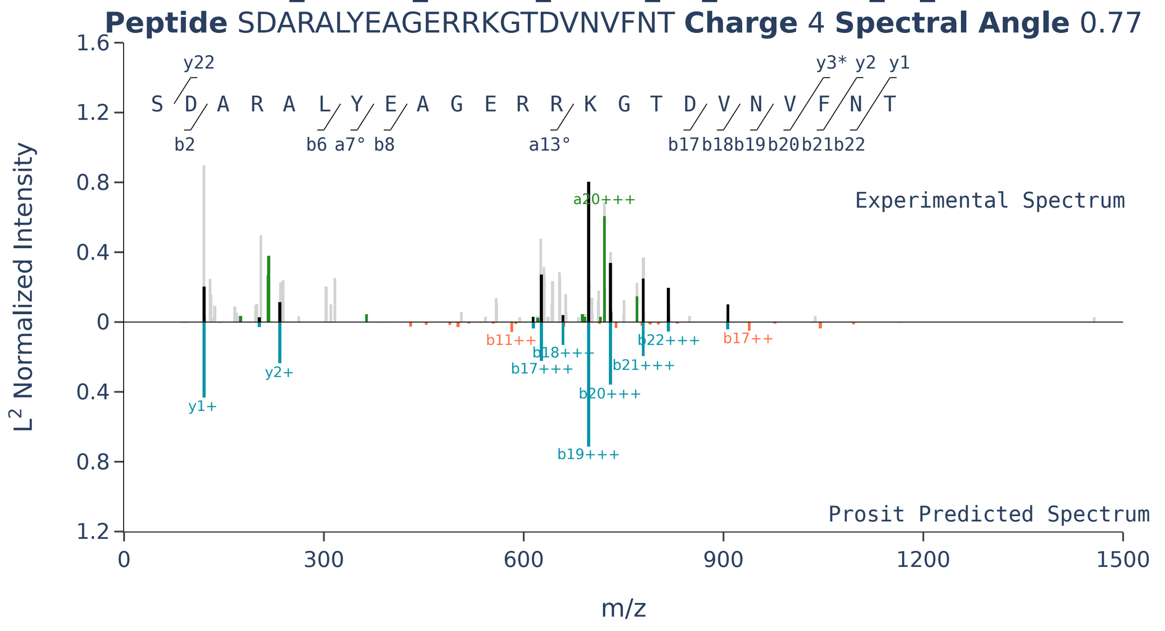


**Source** WSoh_301121_021221_HFGoe_M2_AnnexinA1_24h_R2 **Scan** 5292
**Peptide** HQAMKGVGTRH **Charge** 3 **Spectral Angle** 0.8

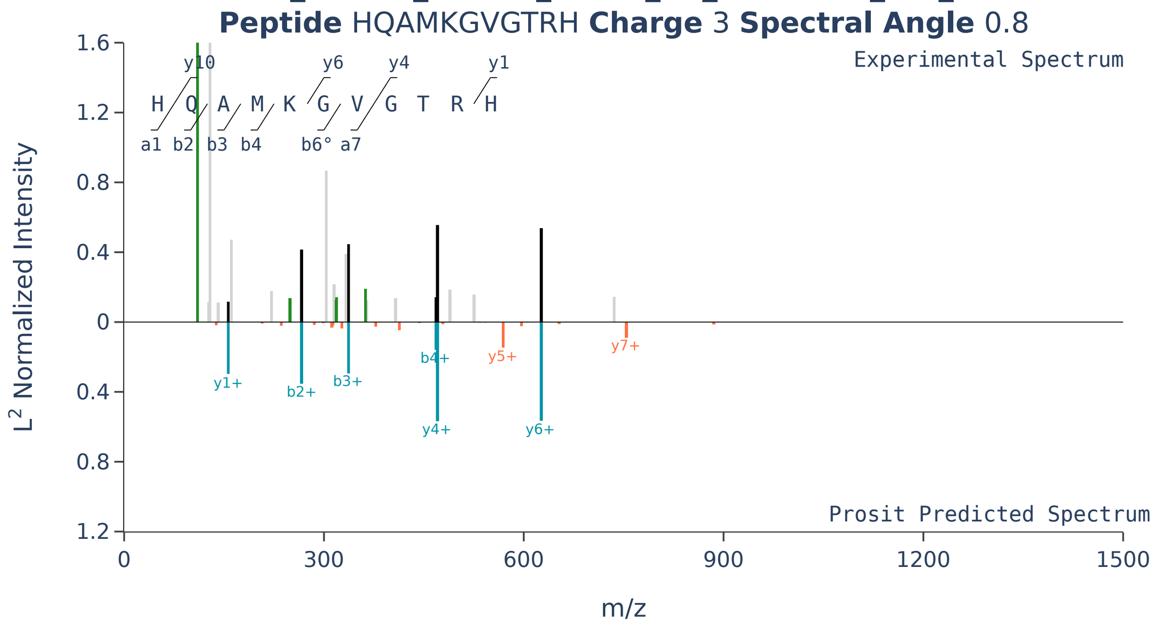


**Source** WSoh_301121_021221_HFGoe_M2_AnnexinA1_2h_R1 **Scan** 27727
**Peptide** KGVDEATIIDILTKR **Charge** 3 **Spectral Angle** 0.78

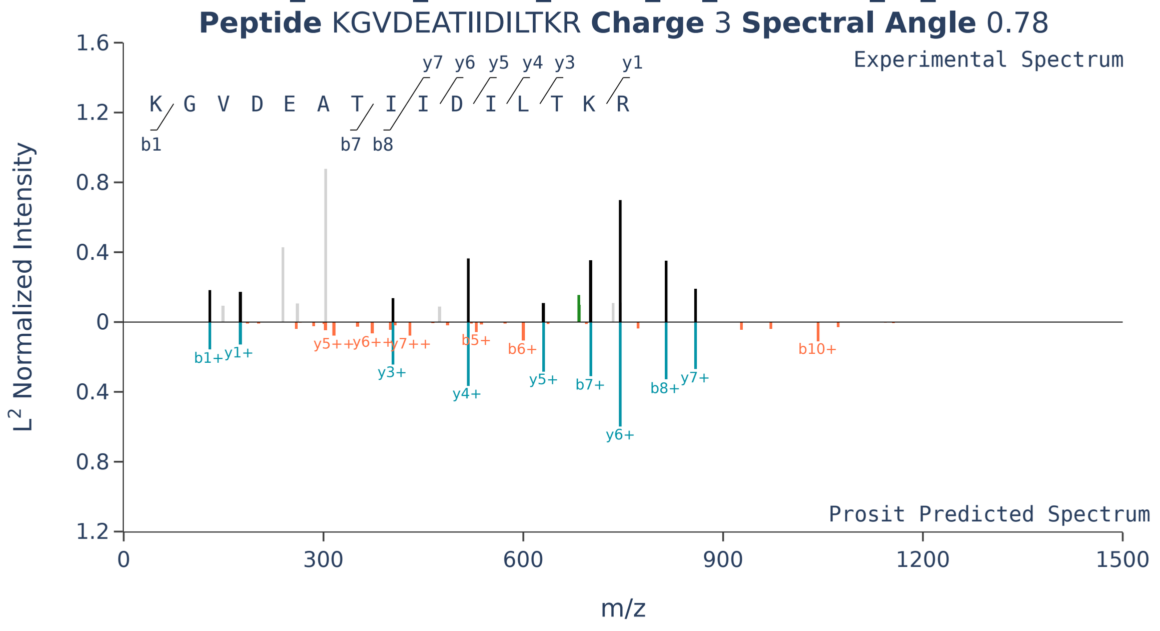


**Source** WSoh_301121_021221_HFGoe_M1_AnnexinA1_24h_R2 **Scan** 22869
**Peptide** VSPYPTFNPSSD **Charge** 2 **Spectral Angle** 0.8

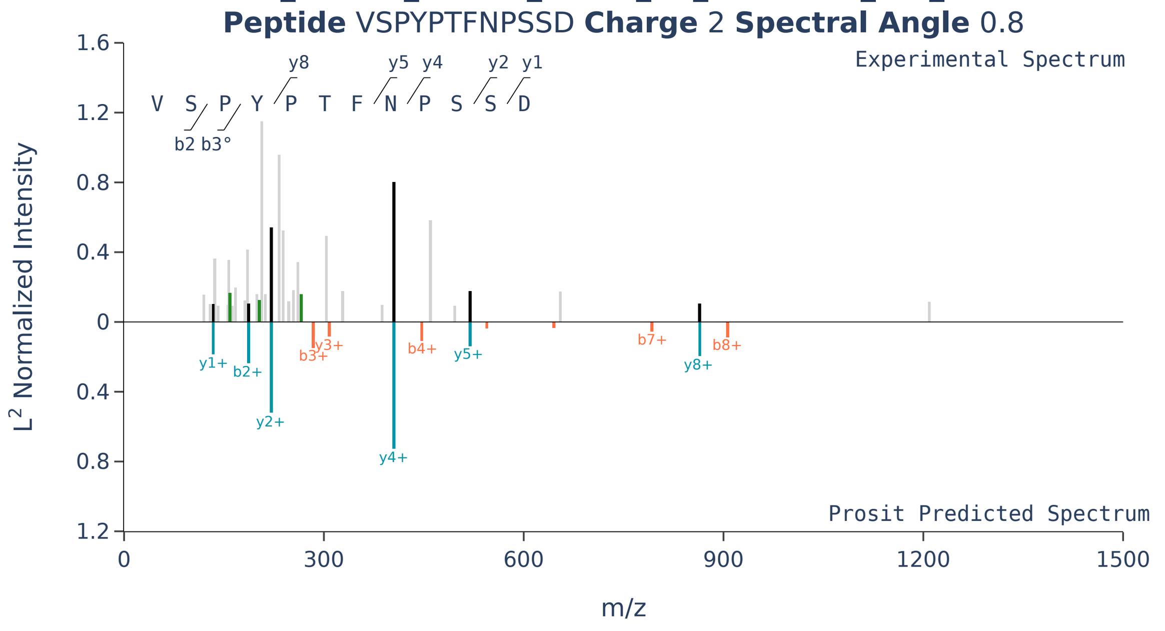


**Source** WSoh_301121_021221_HFGoe_M1_AnnexinA1_24h_R1 **Scan** 28054
**Peptide** ATIIDILTK **Charge** 2 **Spectral Angle** 0.76

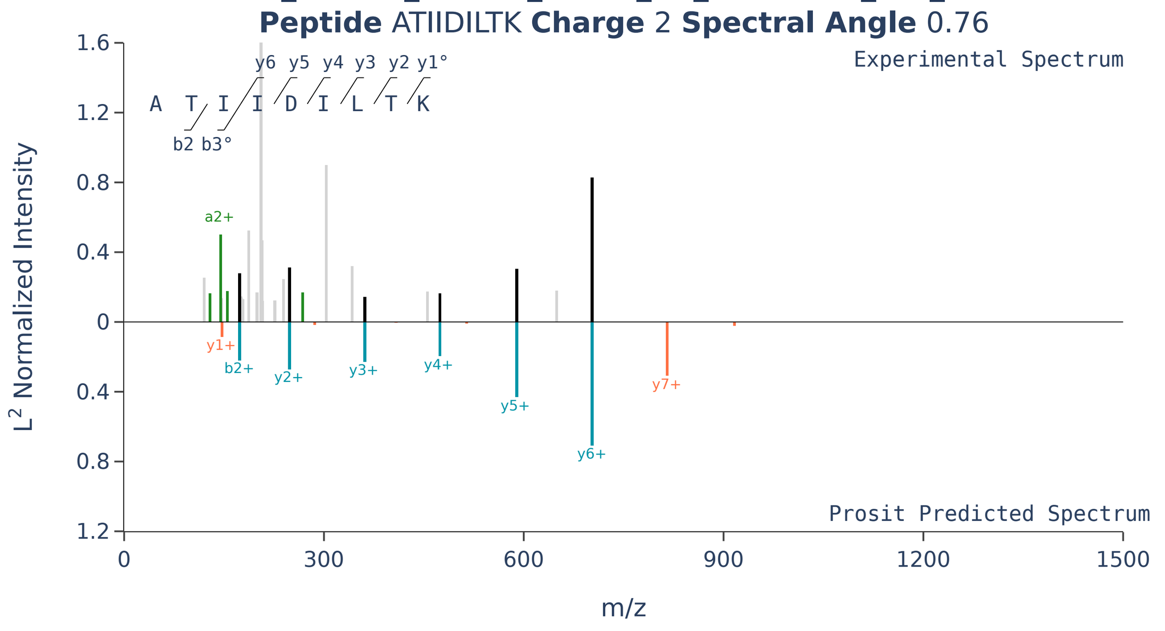


**Source** WSoh_301121_021221_HFGoe_M2_AnnexinA1_2h_R2 **Scan** 14898
**Peptide** TKYSKHDMNKVLDL **Charge** 3 **Spectral Angle** 0.7

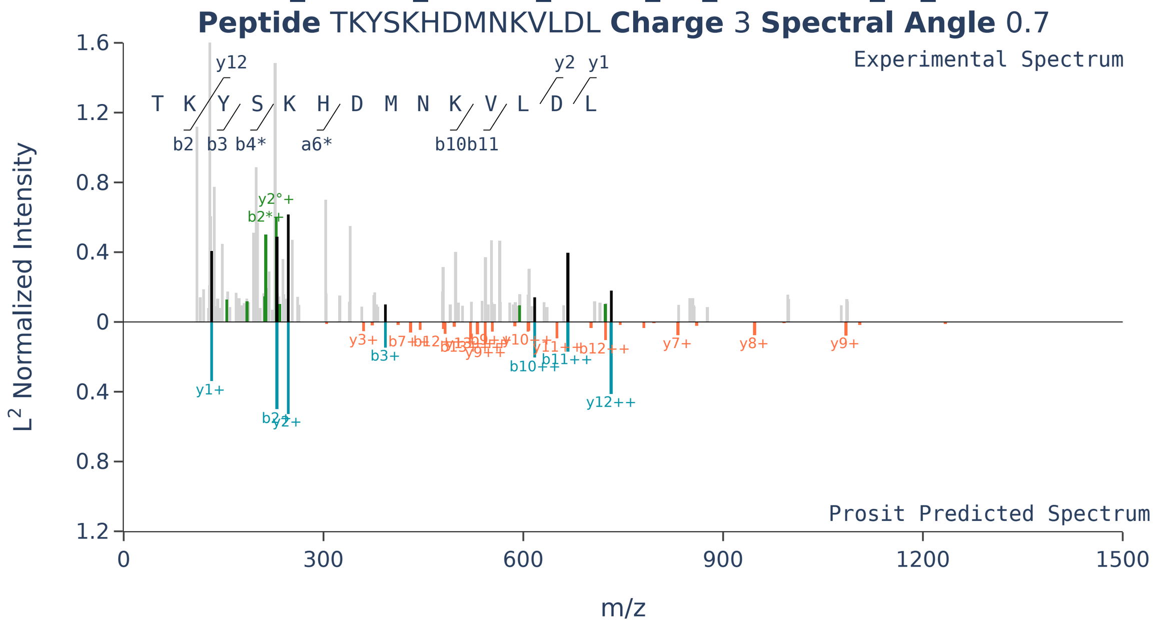

**Source** WSoh_301121_021221_HFGoe_M2_AnnexinA1_2h_R1 **Scan** 16538
**Peptide** VKCATSKPAFFAE **Charge** 2 **Spectral Angle** 0.58

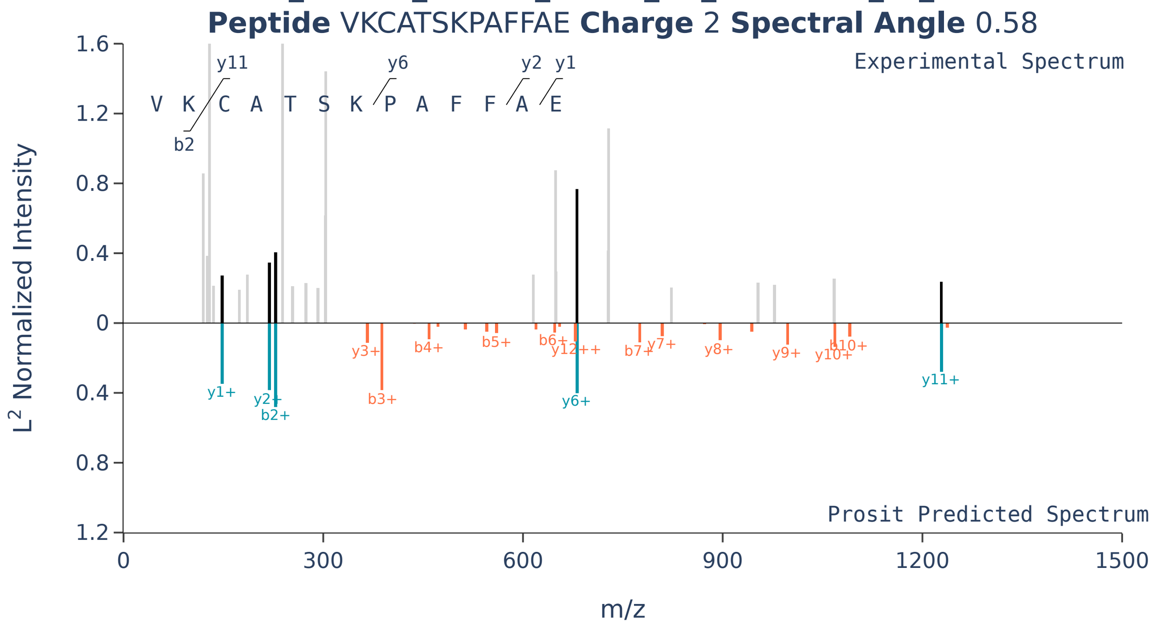


**Source** WSoh_301121_021221_HFGoe_M2_AnnexinA1_1h_R2 **Scan** 12282
**Peptide** RDLNRVYREEL **Charge** 2 **Spectral Angle** 0.62

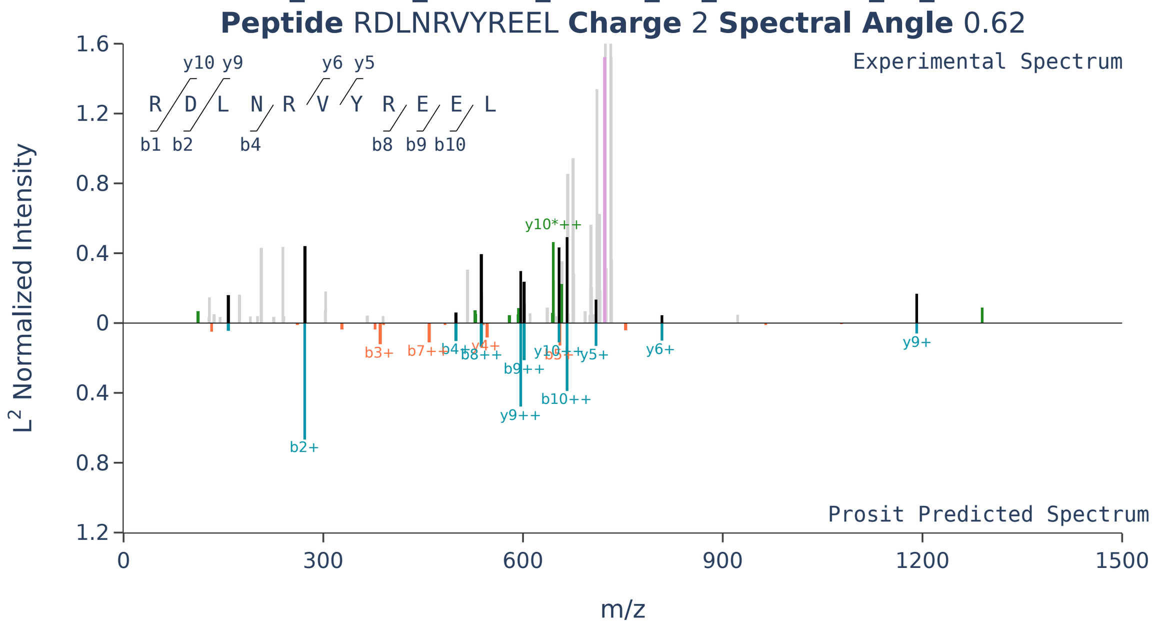


**Source** WSoh_301121_021221_HFGoe_M1_AnnexinA1_2h_R1 **Scan** 15252
**Peptide** PLDETLK **Charge** 1 **Spectral Angle** 0.67

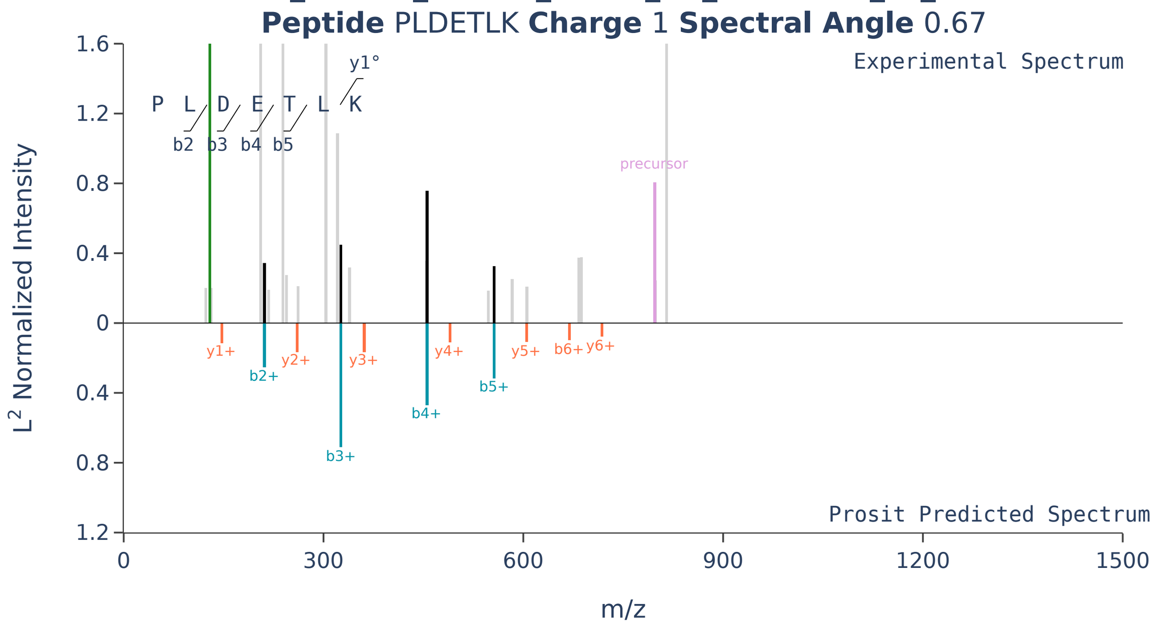


**Source** WSoh_301121_021221_HFGoe_M2_AnnexinA1_2h_R1 **Scan** 23235
**Peptide** KGGPGSAVSPYPTFNPSSDVA **Charge** 2 **Spectral Angle** 0.71

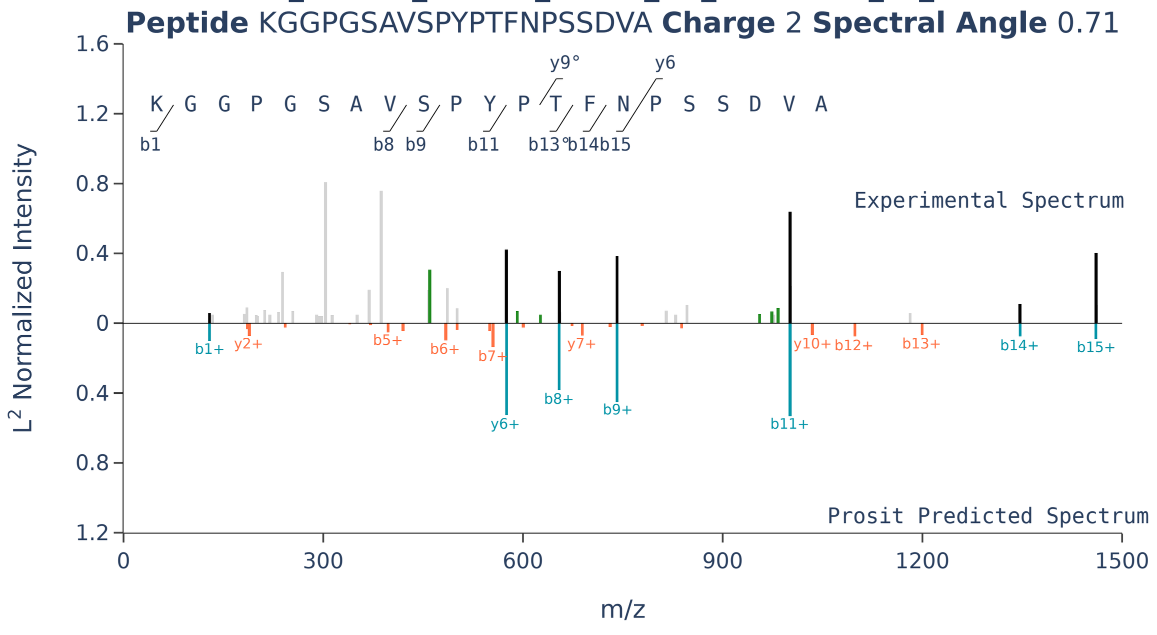


**Source** WSoh_301121_021221_HFGoe_M2_AnnexinA1_2h_R1 **Scan** 24183
**Peptide** TIIDILTKRNNAQ **Charge** 3 **Spectral Angle** 0.7

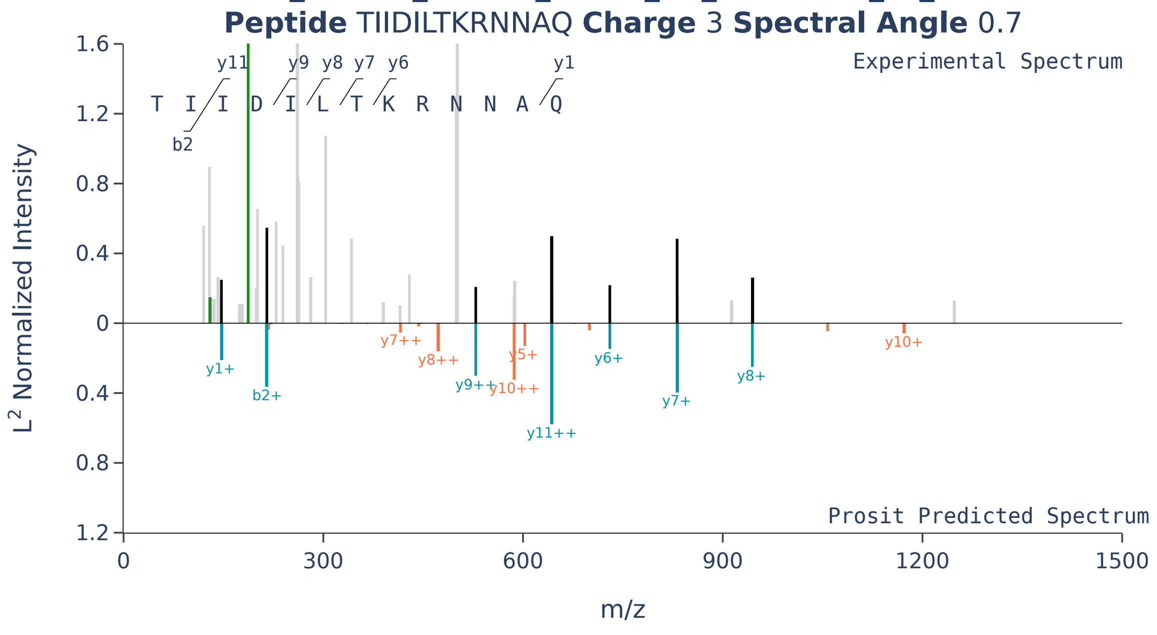


**Source** WSoh_301121_021221_HFGoe_M2_AnnexinA1_2h_R2 **Scan** 7213
**Peptide** FQKYTKYSKHDMNKV **Charge** 4 **Spectral Angle** 0.41

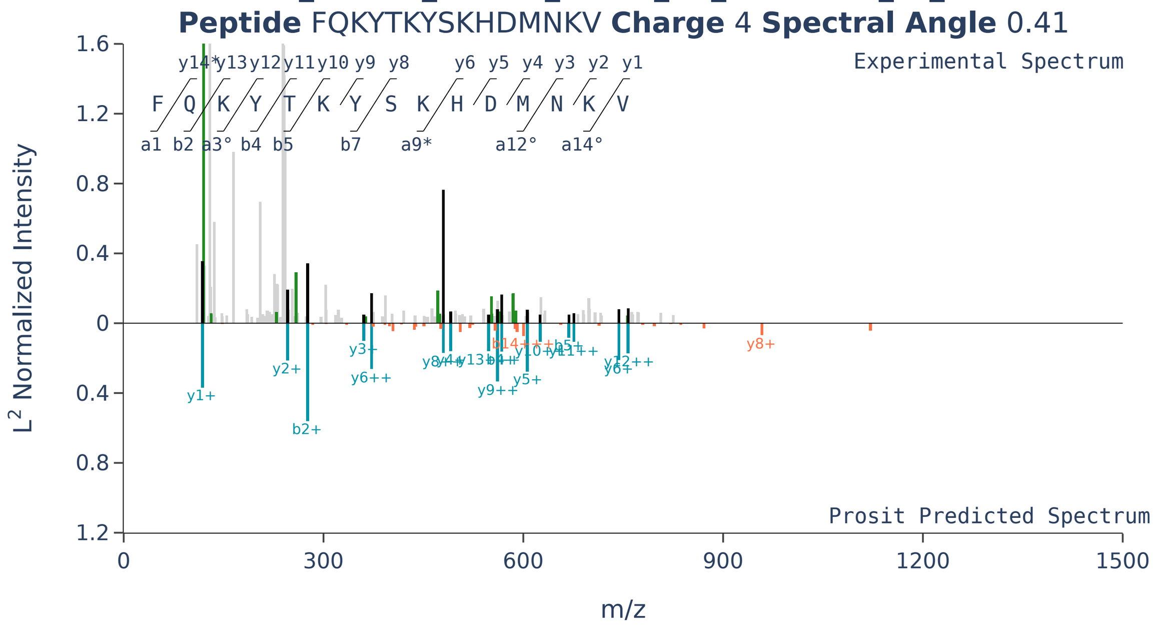


**Source** WSoh_301121_021221_HFGoe_M2_AnnexinA1_24h_R2 **Scan** 21076
**Peptide** KGLGTDEDTLL **Charge** 2 **Spectral Angle** 0.58

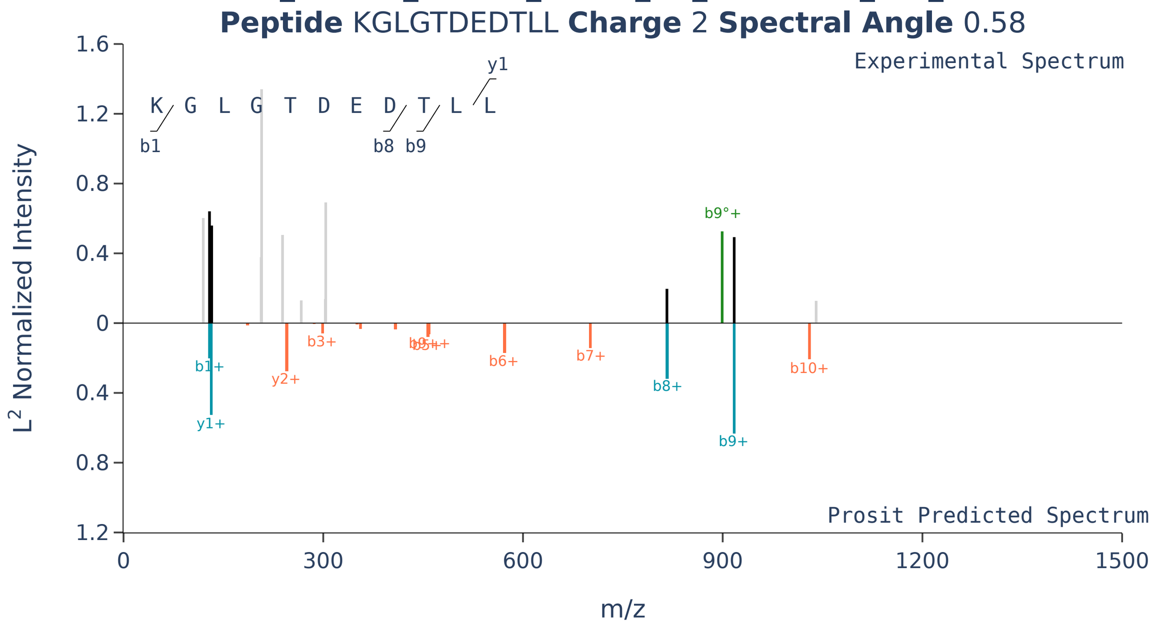


**Source** WSoh_301121_021221_HFGoe_M2_AnnexinA1_4h_R1 **Scan** 18074
**Peptide** QETGKPLDETLKKALT **Charge** 3 **Spectral Angle** 0.72

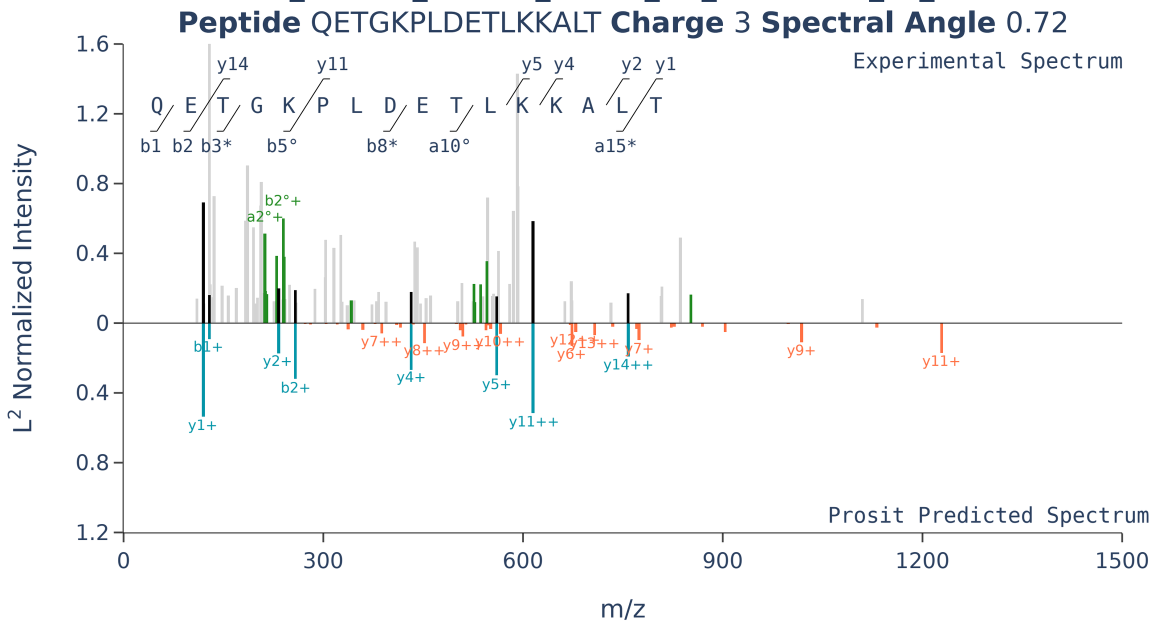


**Source** WSoh_301121_021221_HFGoe_M2_AnnexinA1_4h_R2 **Scan** 20014
**Peptide** DLADSDARALY **Charge** 2 **Spectral Angle** 0.61

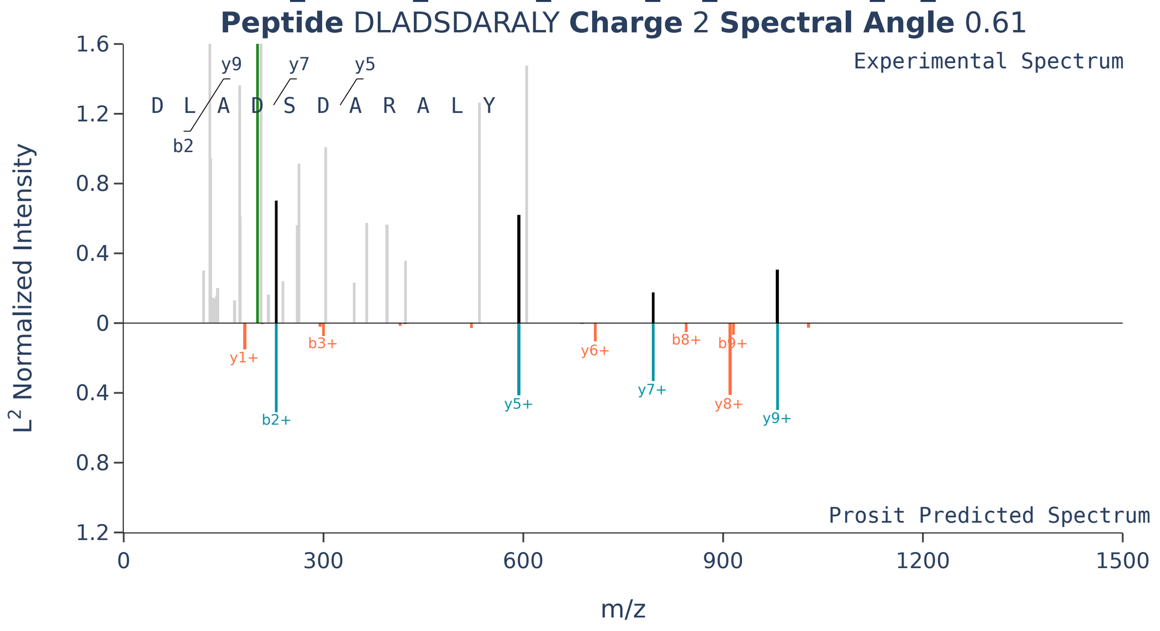


**Source** WSoh_301121_021221_HFGoe_M2_AnnexinA1_24h_R2 **Scan** 9443
**Peptide** KRDLAKDITSDTSGD **Charge** 2 **Spectral Angle** 0.58

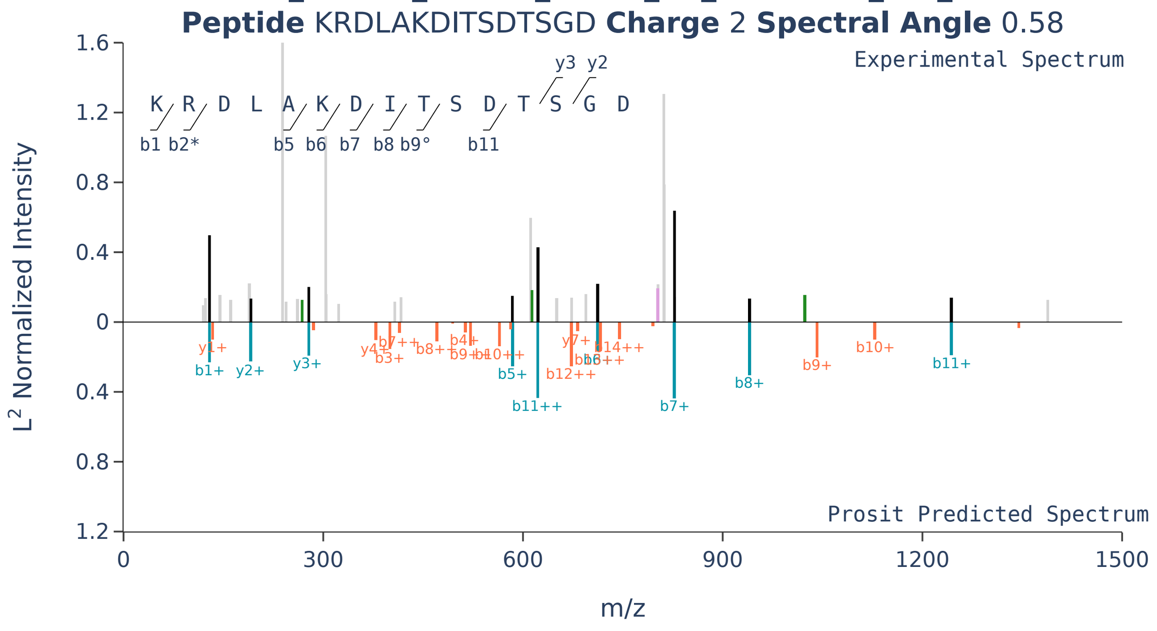


**Source** WSoh_301121_021221_HFGoe_M2_AnnexinA1_24h_R1 **Scan** 10745
**Peptide** LHKAIMVKGVD **Charge** 3 **Spectral Angle** 0.7

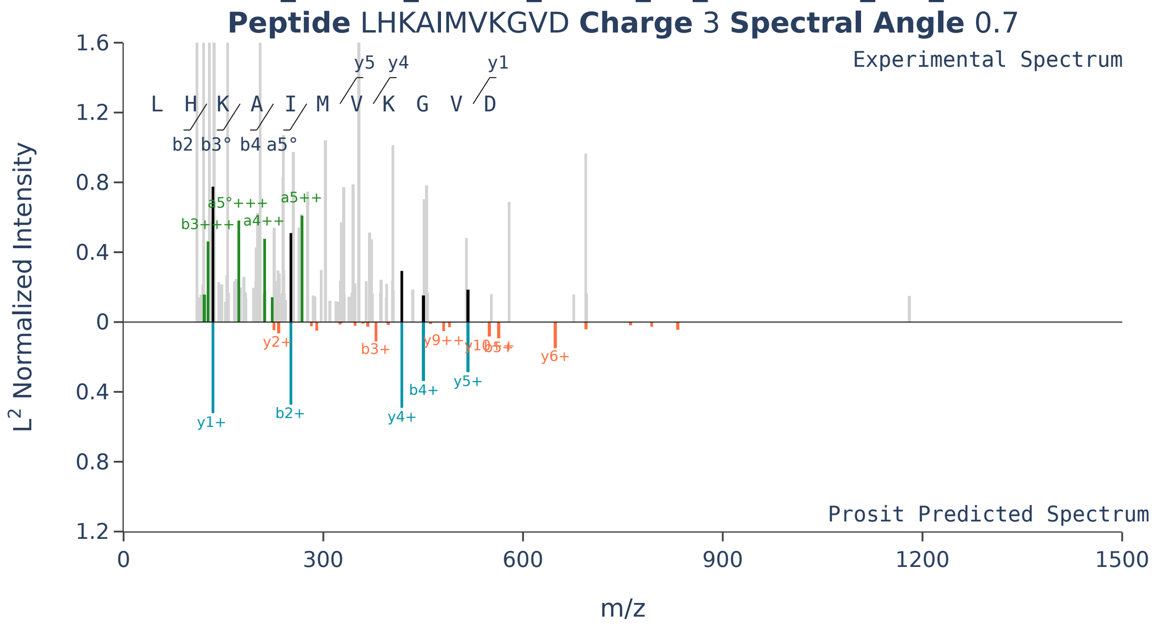


**Source** WSoh_301121_021221_HFGoe_M1_AnnexinA1_24h_R1 **Scan** 16488
**Peptide** EEQEYVQTV **Charge** 2 **Spectral Angle** 0.62

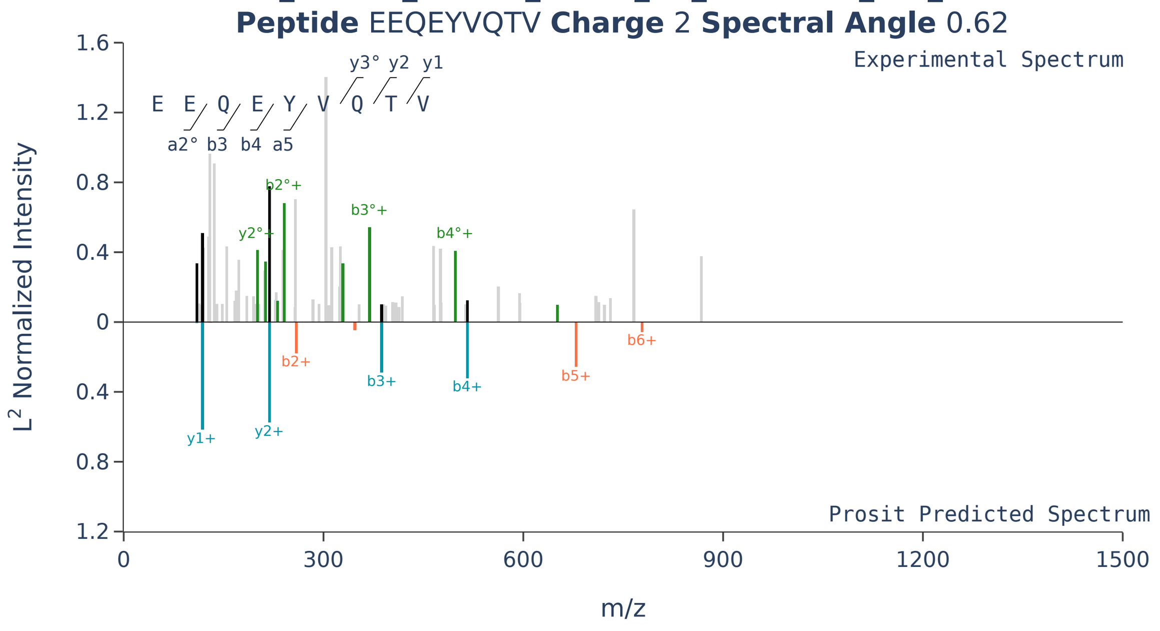


**Source** WSoh_301121_021221_HFGoe_M1_AnnexinA1_4h_R1 **Scan** 16673
**Peptide** LKTPAQFDA **Charge** 2 **Spectral Angle** 0.79

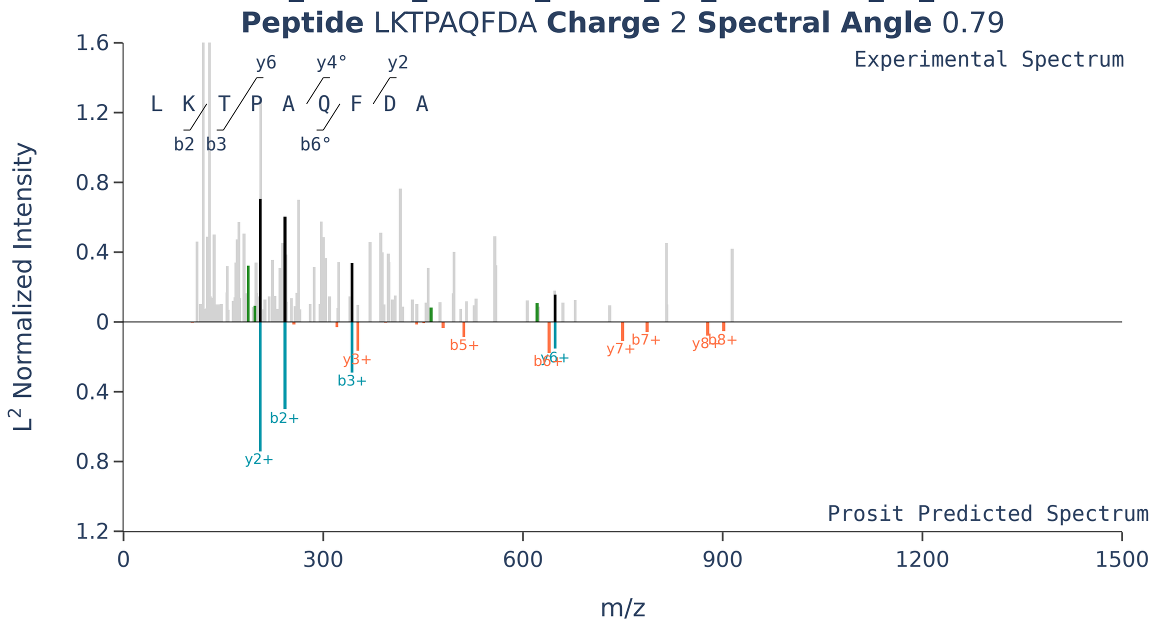


**Source** WSoh_301121_021221_HFGoe_M2_AnnexinA1_2h_R1 **Scan** 21262
**Peptide** LELKGDIEKCLTA **Charge** 3 **Spectral Angle** 0.79

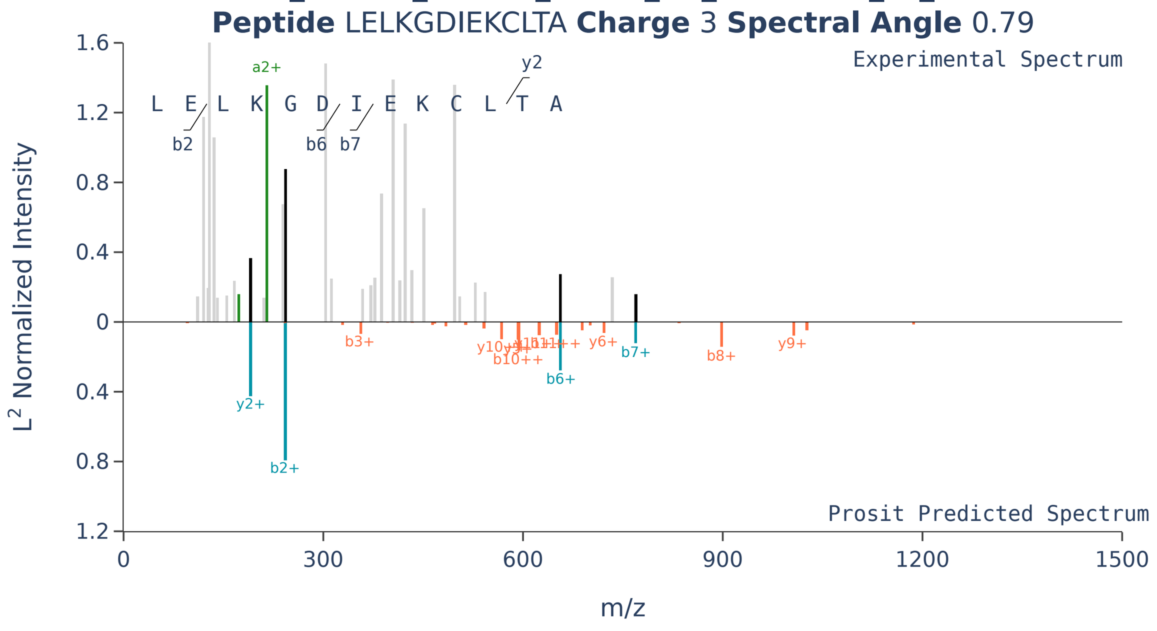


**Source** WSoh_301121_021221_HFGoe_M2_AnnexinA1_2h_R1 **Scan** 23503
**Peptide** ATIIDILTKRNNAQRQQ **Charge** 3 **Spectral Angle** 0.83

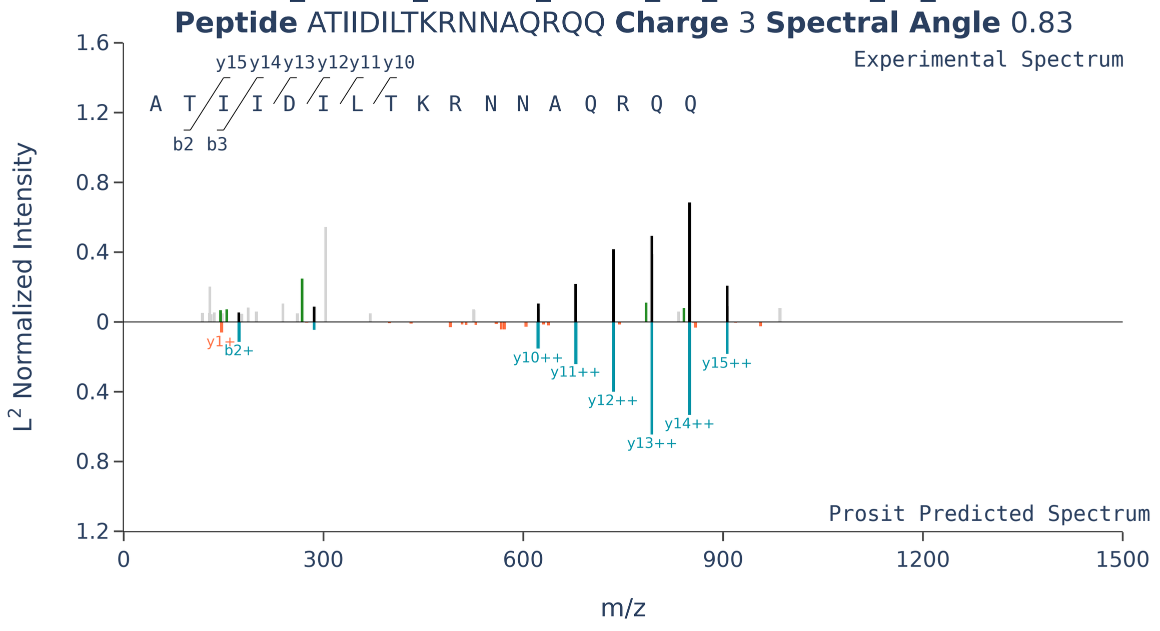

**Source** WSoh_301121_021221_HFGoe_M2_AnnexinA1_1h_R1 **Scan** 22876
**Peptide** NTILTTRSYPQL **Charge** 2 **Spectral Angle** 0.68

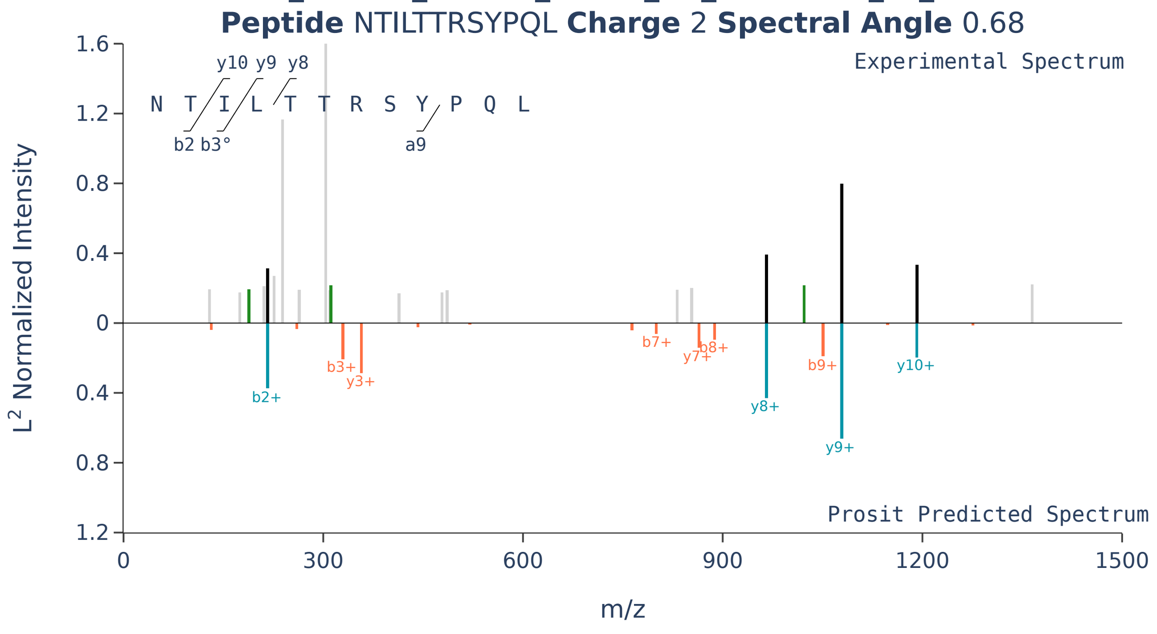


**Source** WSoh_301121_021221_HFGoe_M2_AnnexinA1_1h_R1 **Scan** 21453
**Peptide** ALLKTPAQFDAD **Charge** 2 **Spectral Angle** 0.7

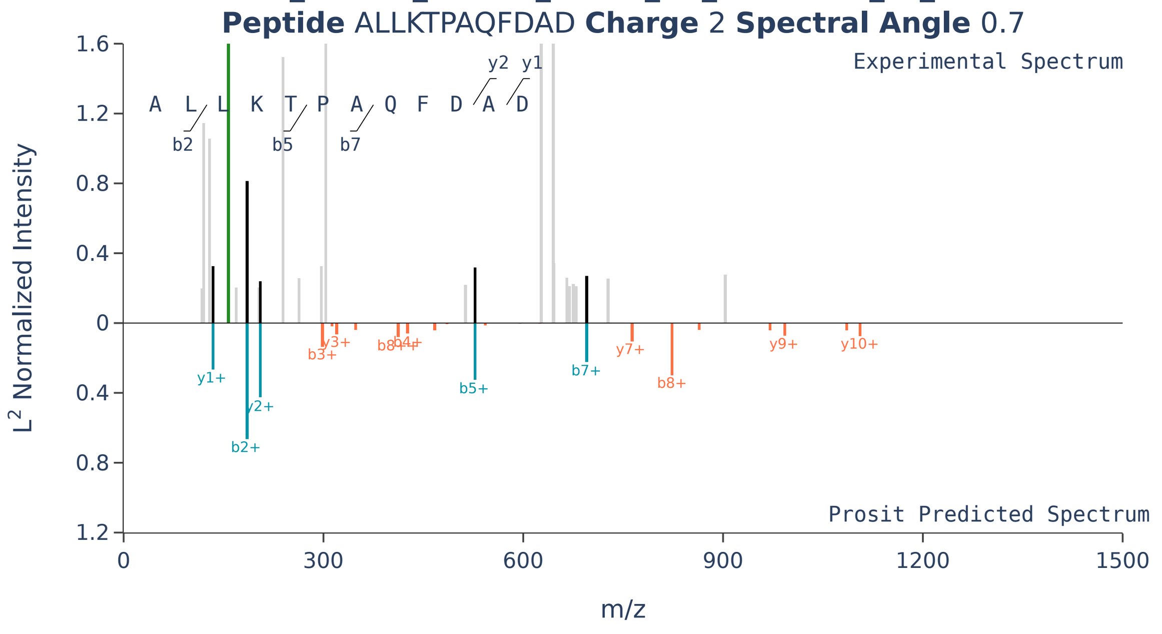


**Source** WSoh_301121_021221_HFGoe_M2_AnnexinA1_2h_R2 **Scan** 18263
**Peptide** QTVKSSKGGPGSAVSPYPTFNPSSD **Charge** 3 **Spectral Angle** 0.87

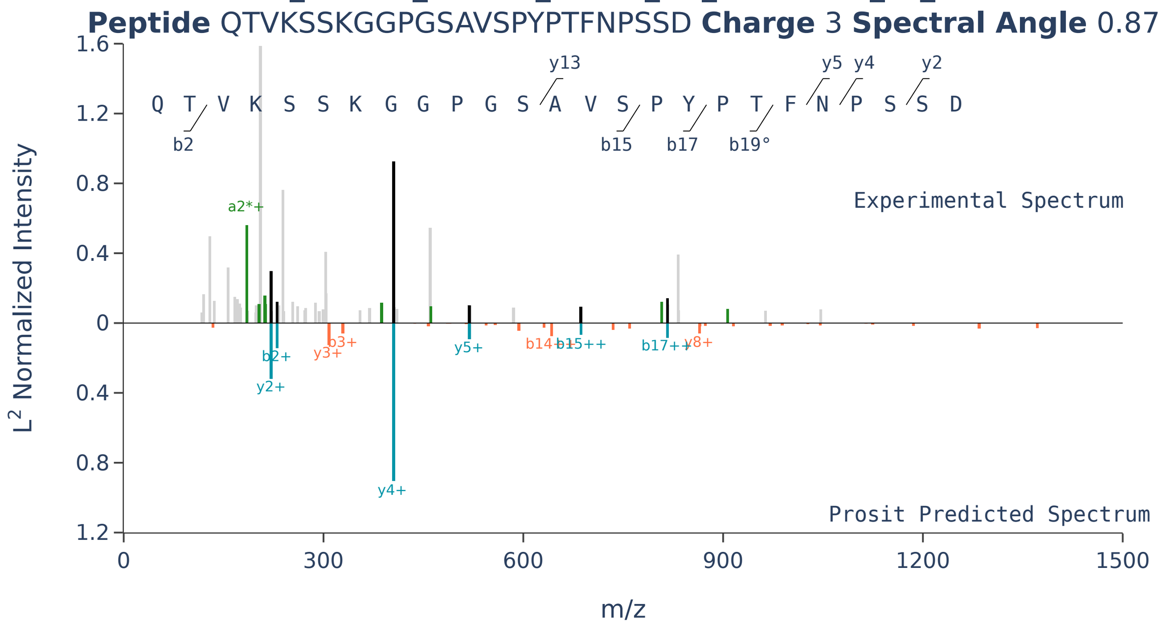


**Source** WSoh_301121_021221_HFGoe_M2_AnnexinA1_24h_R1 **Scan** 9260
**Peptide** LASRTNKEIRDINRV **Charge** 3 **Spectral Angle** 0.79

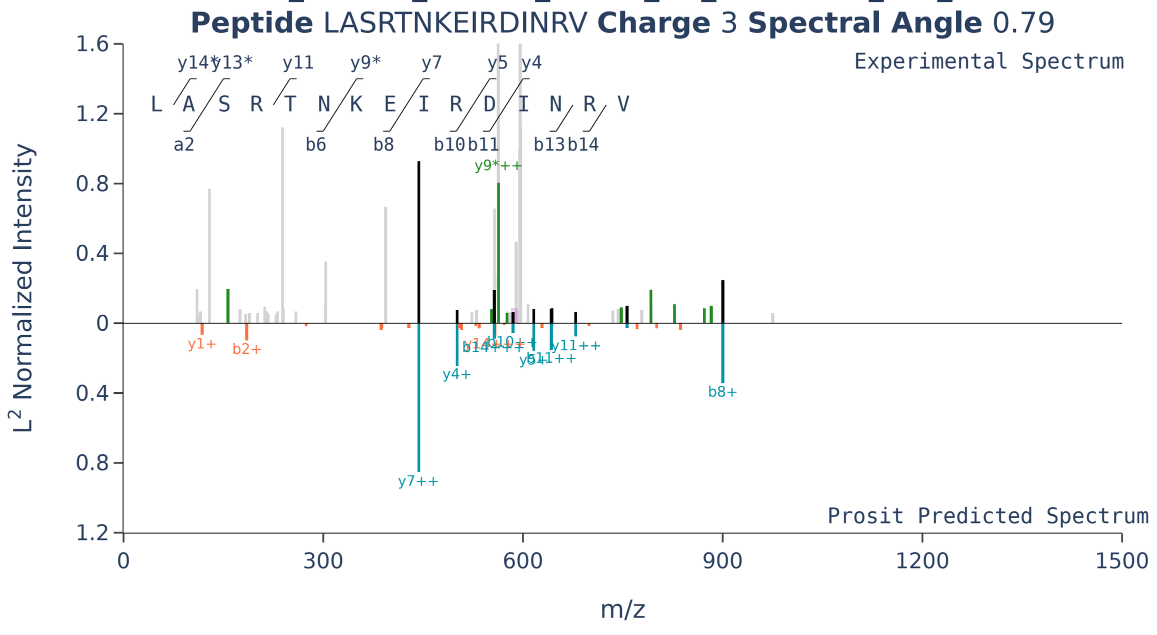


**Source** WSoh_301121_021221_HFGoe_M1_AnnexinA1_24h_R1 **Scan** 25931
**Peptide** ATIIDILTKRNN **Charge** 3 **Spectral Angle** 0.66

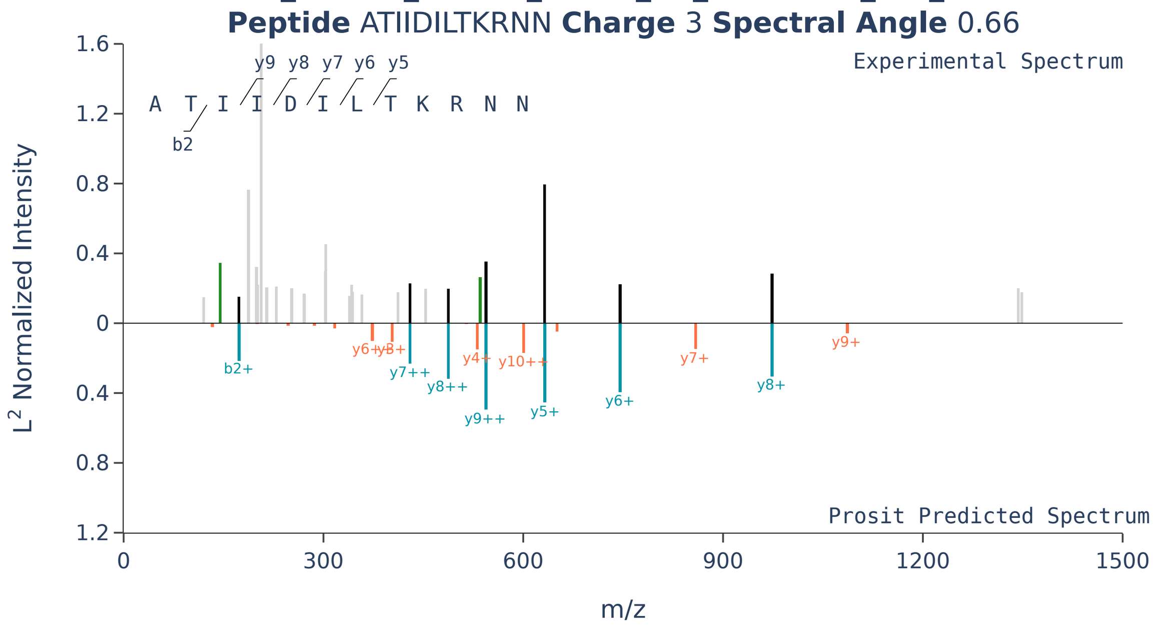


**Source** WSoh_301121_021221_HFGoe_M1_AnnexinA1_24h_R1 **Scan** 32259
**Peptide** GTDEDTLIEIL **Charge** 2 **Spectral Angle** 0.79

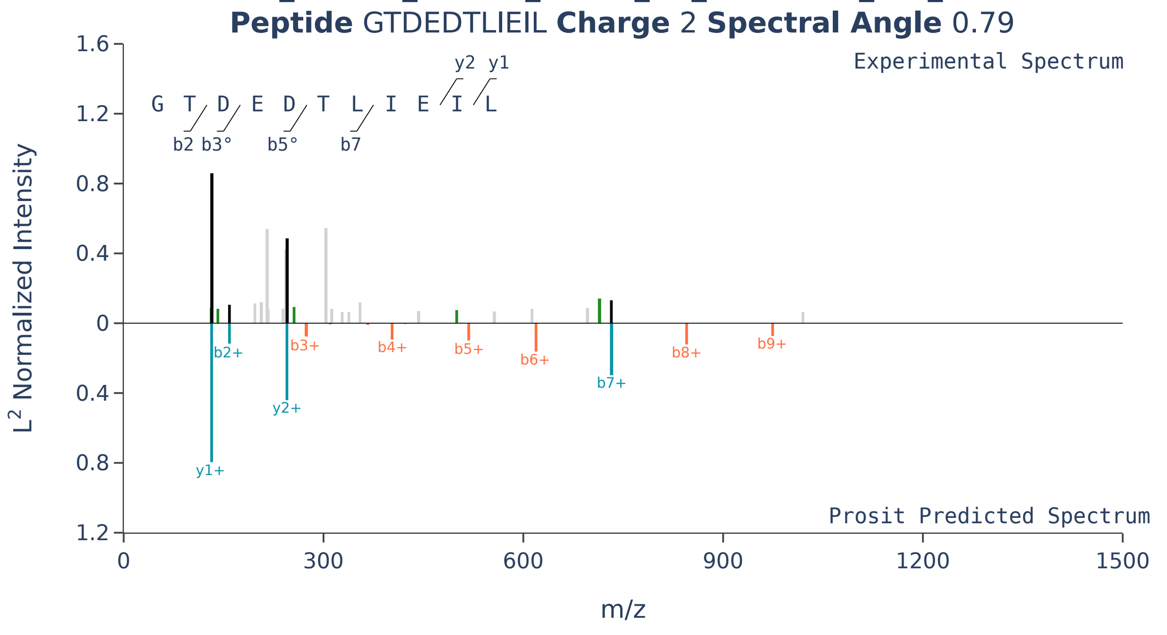


**Source** WSoh_301121_021221_HFGoe_M2_AnnexinA1_2h_R1 **Scan** 25225
**Peptide** DLAKDITSDTSGDFRNAL **Charge** 2 **Spectral Angle** 0.59

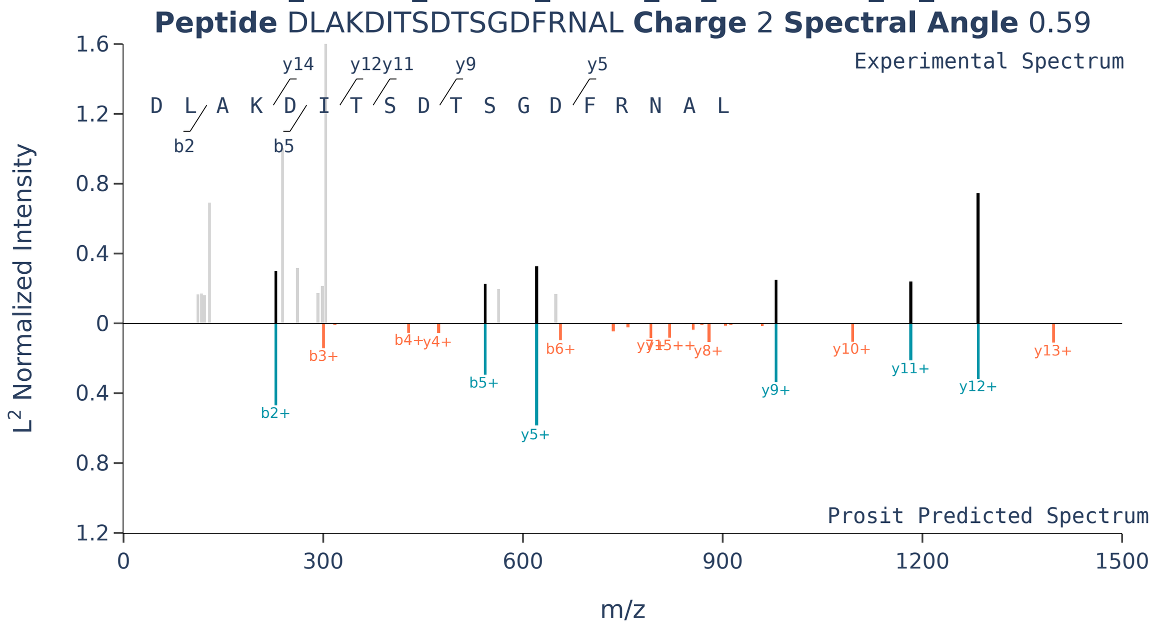


**Source** WSoh_301121_021221_HFGoe_M2_AnnexinA1_2h_R1 **Scan** 23715
**Peptide** ADELRAAMKGLGTDEDTL **Charge** 3 **Spectral Angle** 0.68

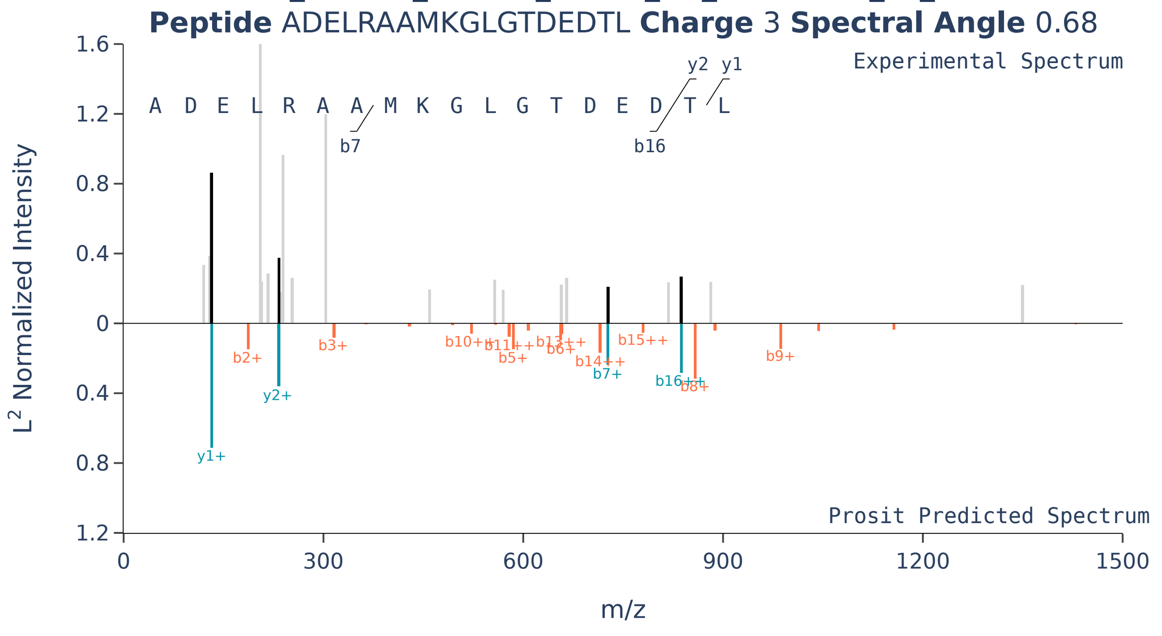


**Source** WSoh_301121_021221_HFGoe_M2_AnnexinA1_2h_R1 **Scan** 20901
**Peptide** EQEYVQTVKSSKGGPGSAVSPYPTFNPSS **Charge** 3 **Spectral Angle** 0.85

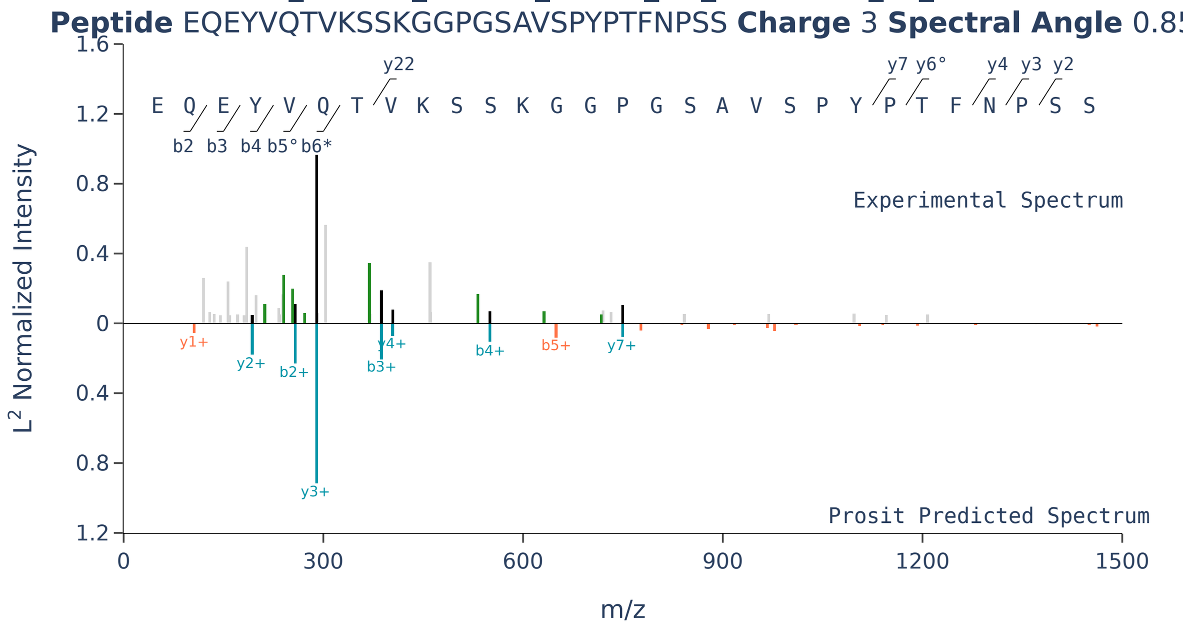


**Source** WSoh_301121_021221_HFGoe_M2_AnnexinA1_2h_R2 **Scan** 16857
**Peptide** ALYEAGERRKGTDVNVF **Charge** 3 **Spectral Angle** 0.72

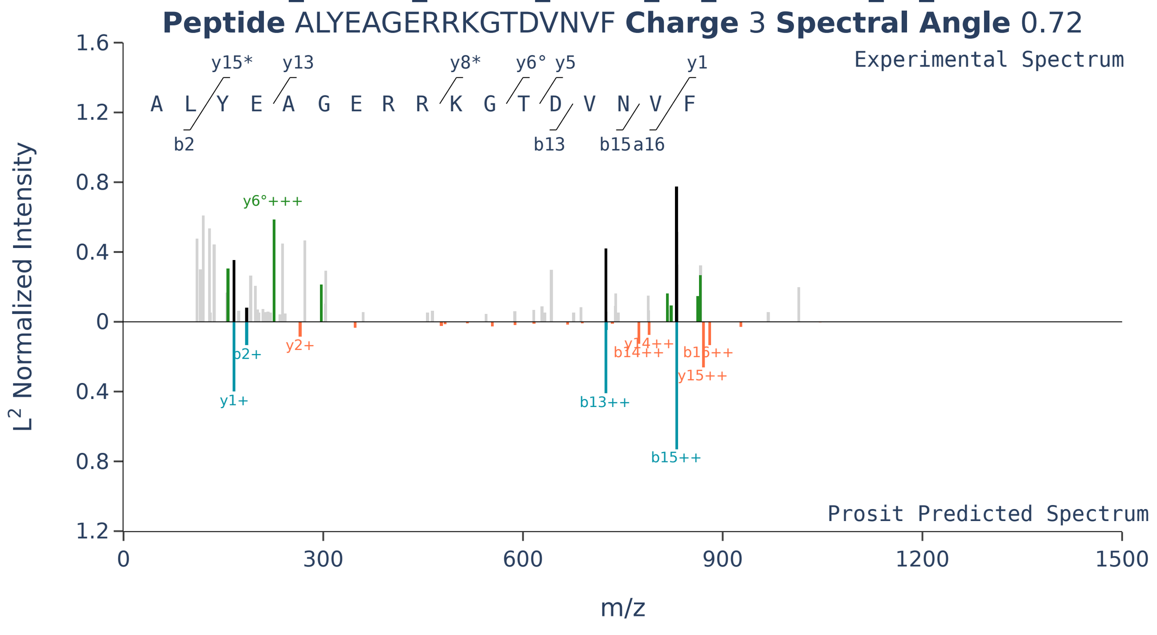


**Source** WSoh_301121_021221_HFGoe_M2_AnnexinA1_24h_R1 **Scan** 5906
**Peptide** RALYEAGERRK **Charge** 3 **Spectral Angle** 0.71

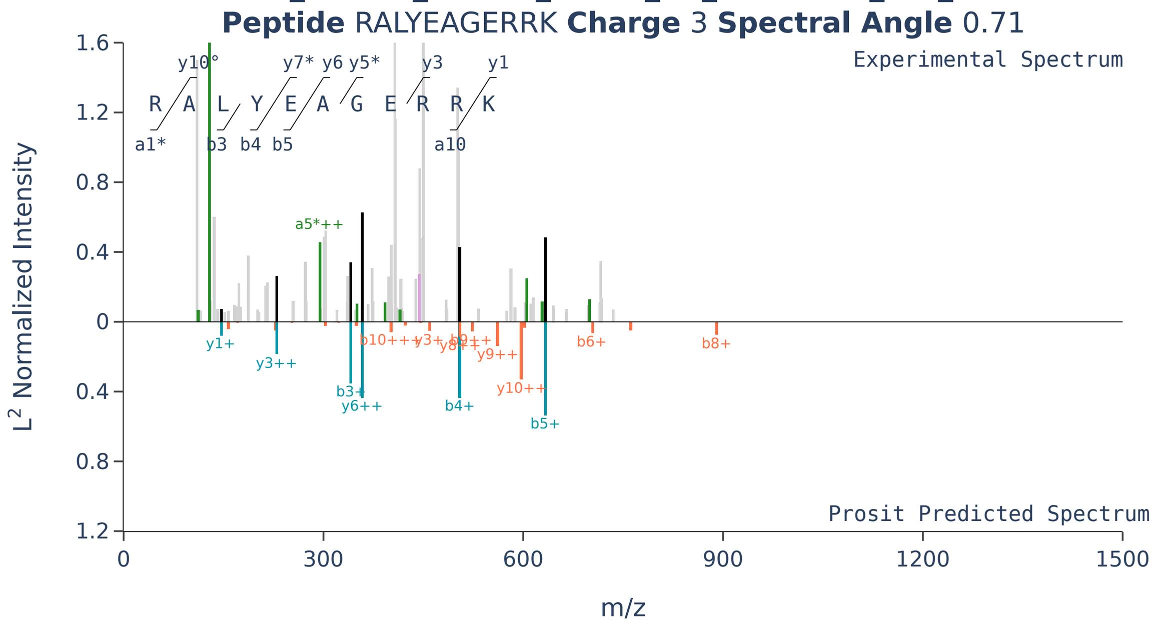


**Source** WSoh_301121_021221_HFGoe_M2_AnnexinA1_0h_R1 **Scan** 16347
**Peptide** FIENEEQEYVQTVKSSK **Charge** 3 **Spectral Angle** 0.57

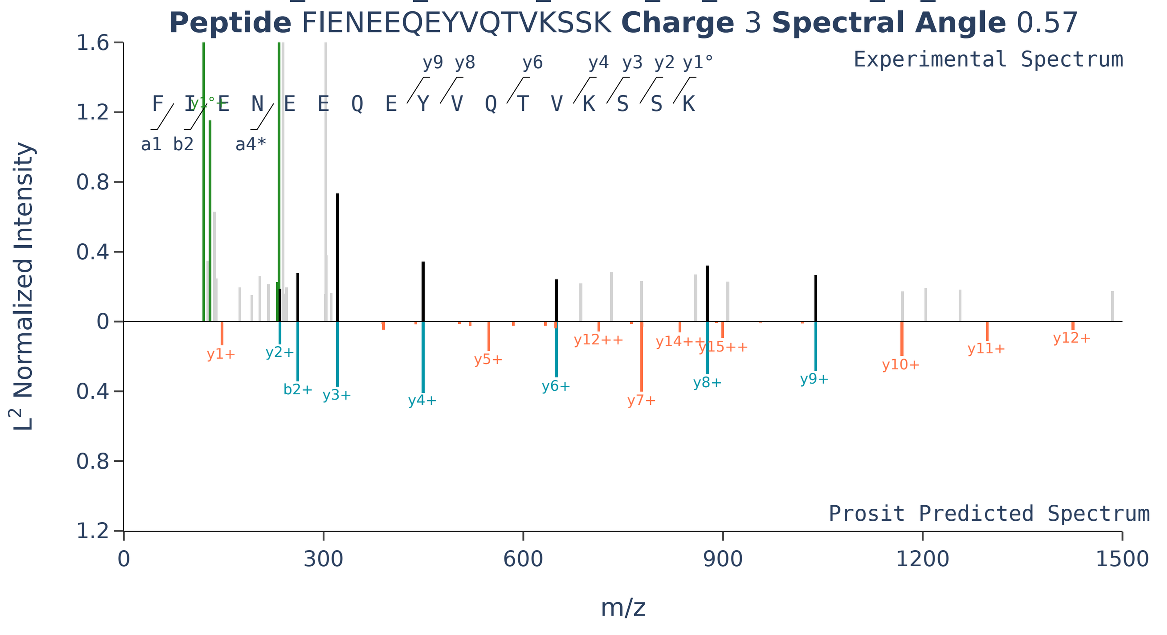


**Source** WSoh_301121_021221_HFGoe_M2_AnnexinA1_2h_R1 **Scan** 16875
**Peptide** ELKRDLAKDITSDTSGDFRN **Charge** 3 **Spectral Angle** 0.67

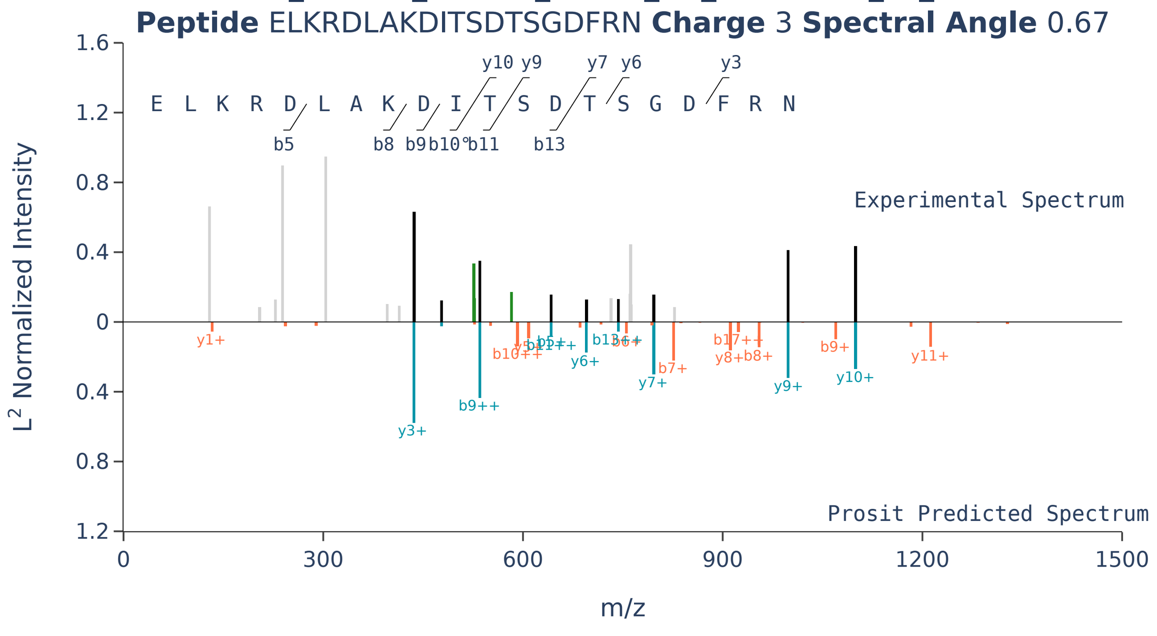


**Source** WSoh_301121_021221_HFGoe_M1_AnnexinA1_24h_R1 **Scan** 22952
**Peptide** AVSPYPTFNPSS **Charge** 2 **Spectral Angle** 0.73

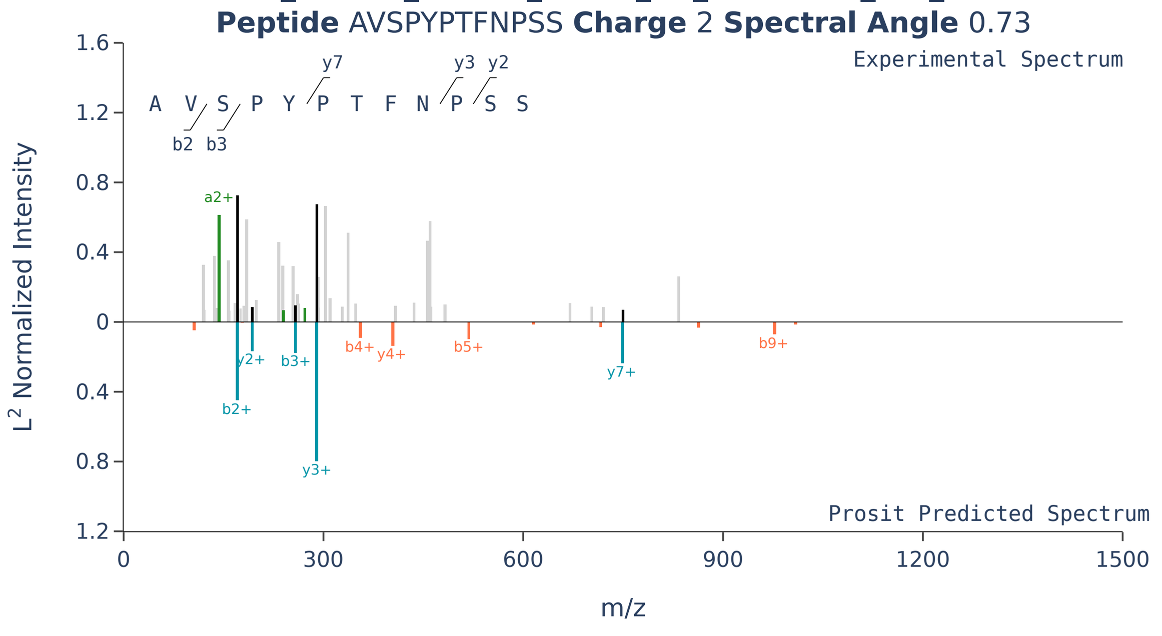


**Source** WSoh_301121_021221_HFGoe_M2_AnnexinA1_24h_R1 **Scan** 32619
**Peptide** GTDEDTLIELL **Charge** 2 **Spectral Angle** 0.79

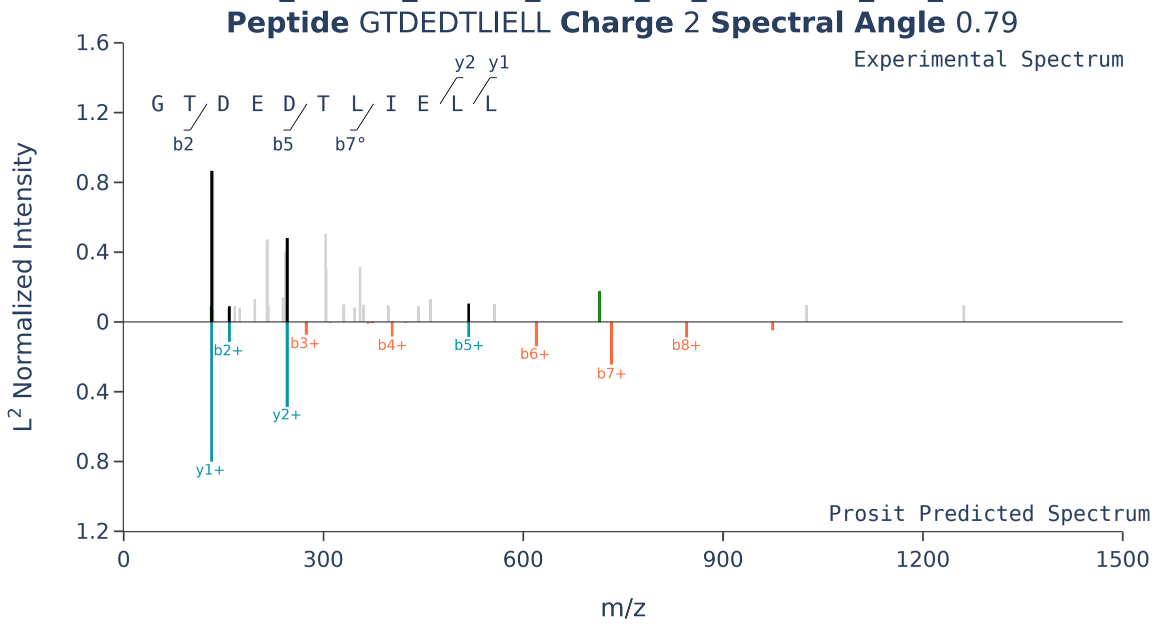

**Source** WSoh_301121_021221_HFGoe_M2_AnnexinA1_2h_R1 **Scan** 8401
**Peptide** RDINRVYREELKR **Charge** 5 **Spectral Angle** 0.62

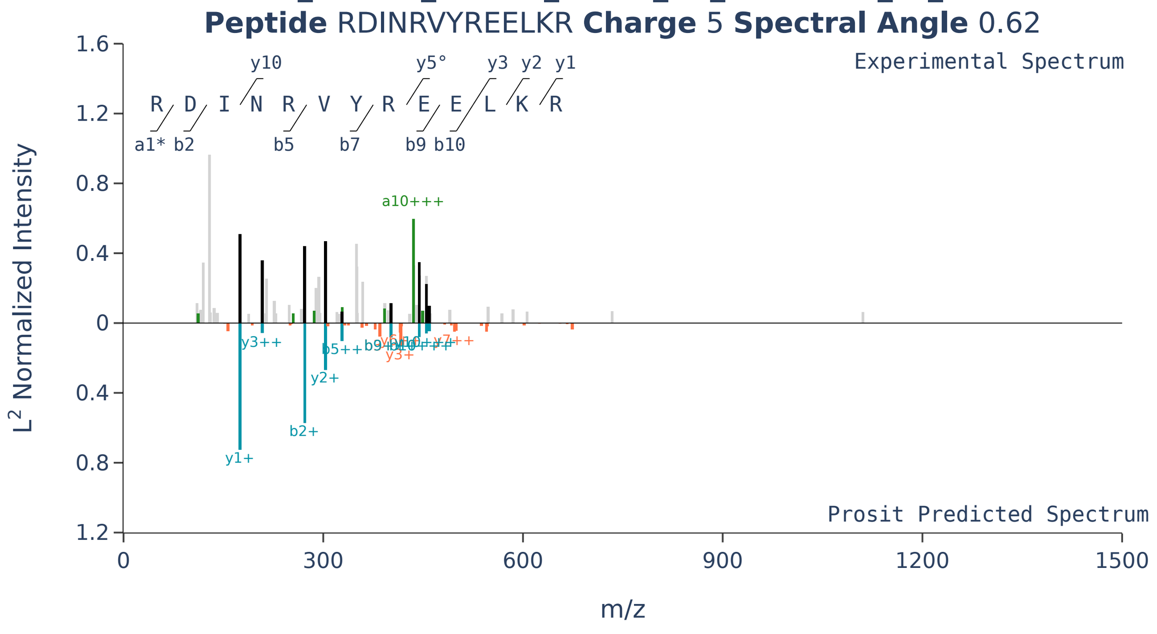


**Source** WSoh_301121_021221_HFGoe_M2_AnnexinA1_2h_R1 **Scan** 22080
**Peptide** PAFFAEKLHQAM **Charge** 3 **Spectral Angle** 0.74

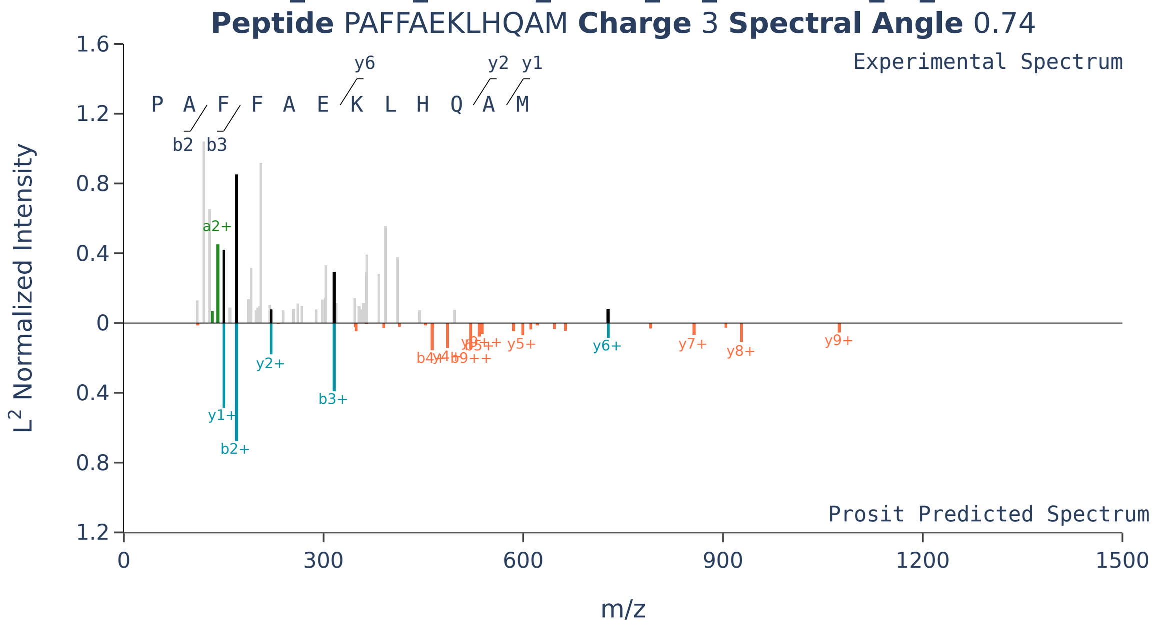


**Source** WSoh_301121_021221_HFGoe_M2_AnnexinA1_2h_R2 **Scan** 23966
**Peptide** AGERRKGTDVNVFNTIL **Charge** 4 **Spectral Angle** 0.69

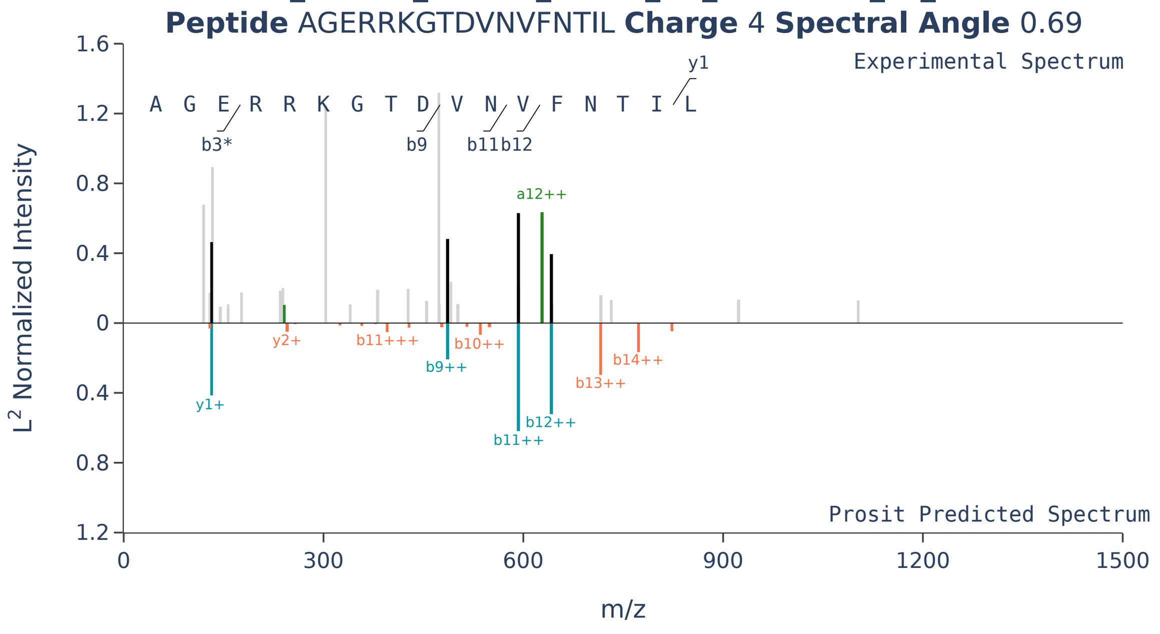


**Source** WSoh_301121_021221_HFGoe_M2_AnnexinA1_2h_R1 **Scan** 9961
**Peptide** EQEYVQTVKSSKGGPGSA **Charge** 3 **Spectral Angle** 0.6

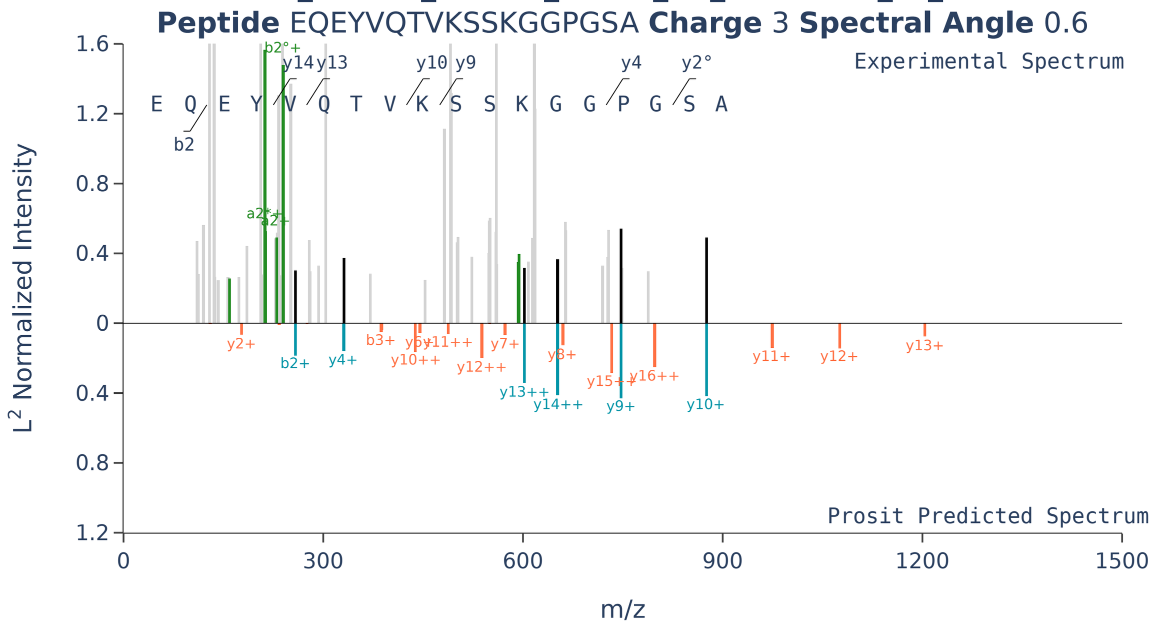


**Source** WSoh_301121_021221_HFGoe_M2_AnnexinA1_1h_R1 **Scan** 13494
**Peptide** RTNKEIRDINRVYREEL **Charge** 5 **Spectral Angle** 0.53

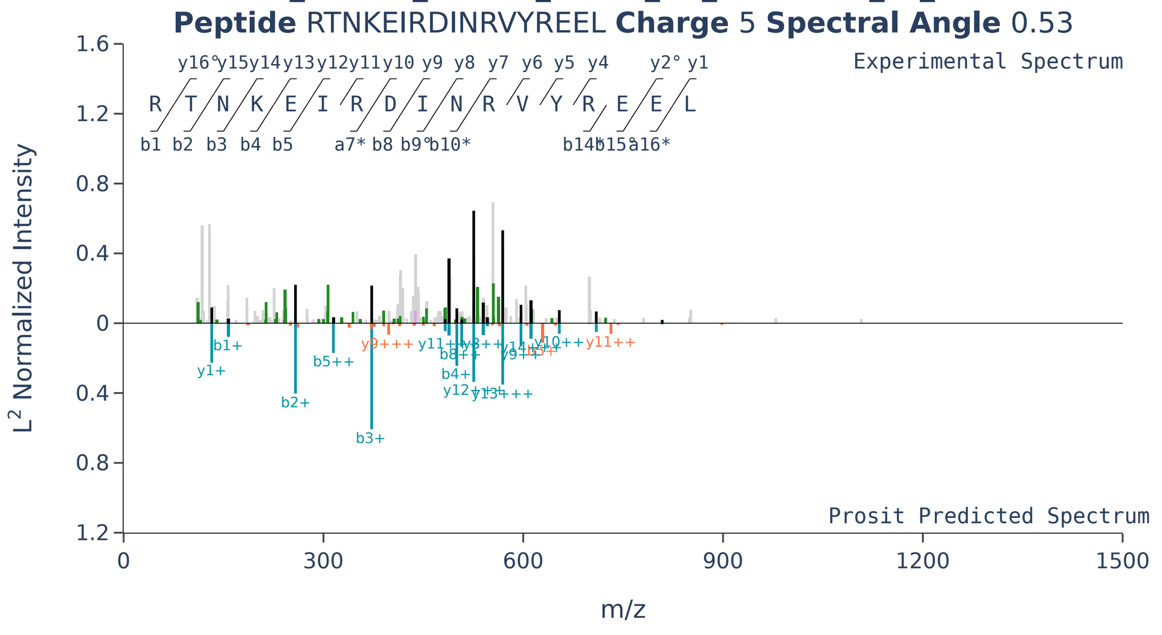


**Source** WSoh_301121_021221_HFGoe_M2_AnnexinA1_24h_R2 **Scan** 19544
**Peptide** QFDADELRA **Charge** 2 **Spectral Angle** 0.74

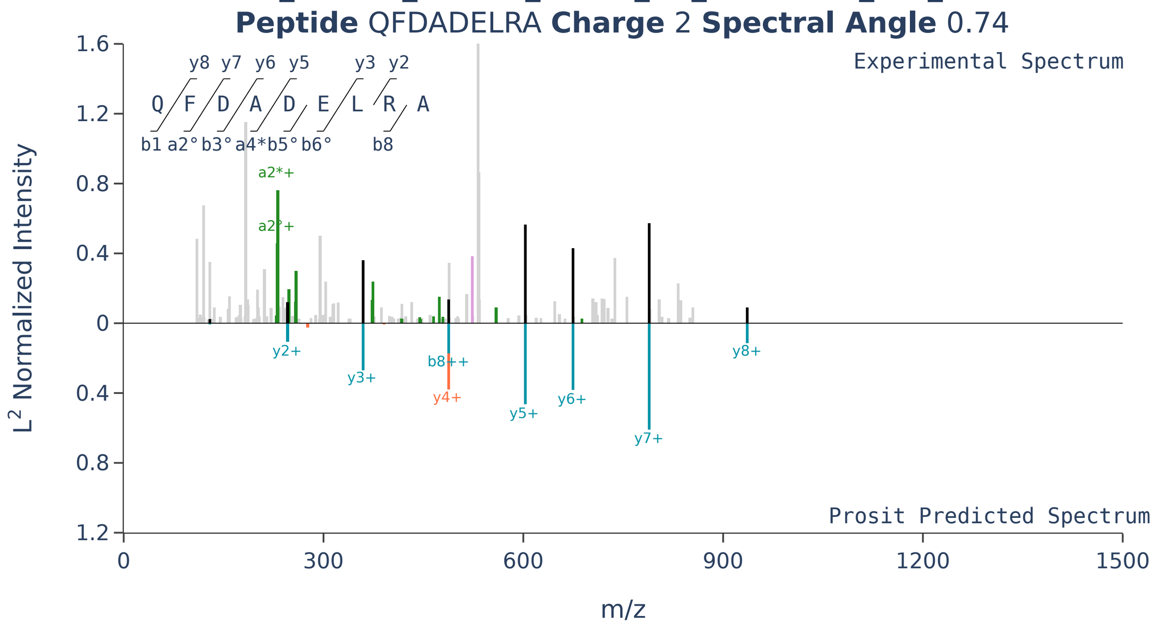


**Source** WSoh_301121_021221_HFGoe_M2_AnnexinA1_2h_R2 **Scan** 19178
**Peptide** CQAILDETKGDYEKL **Charge** 3 **Spectral Angle** 0.72

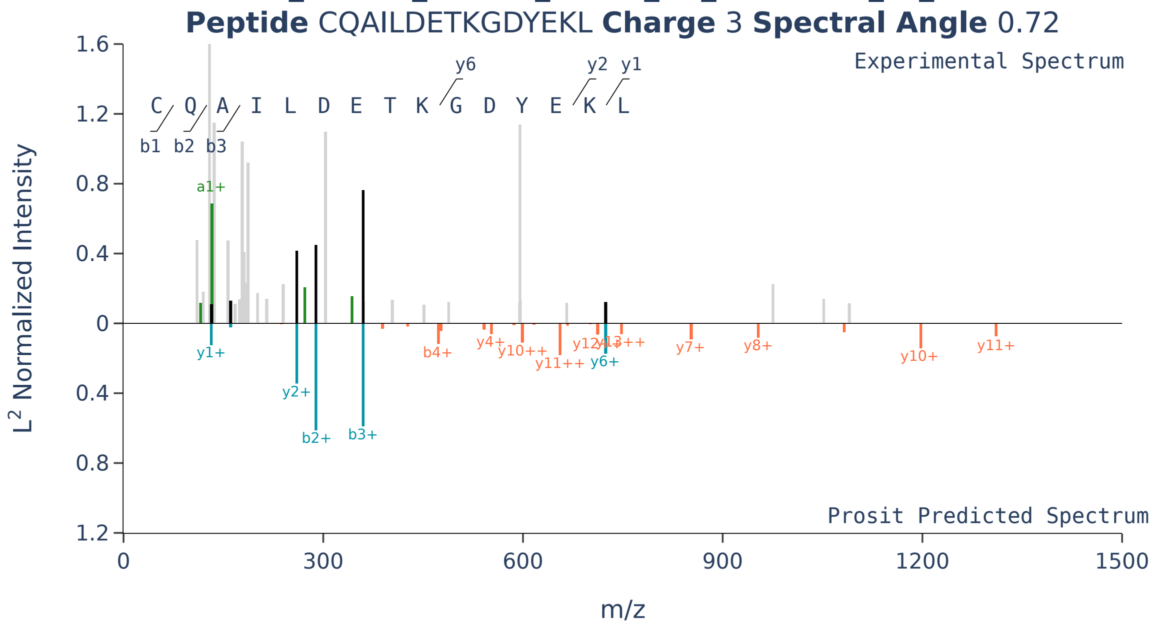


**Source** WSoh_301121_021221_HFGoe_M2_AnnexinA1_24h_R1 **Scan** 10675
**Peptide** ELRAAMKG **Charge** 2 **Spectral Angle** 0.64

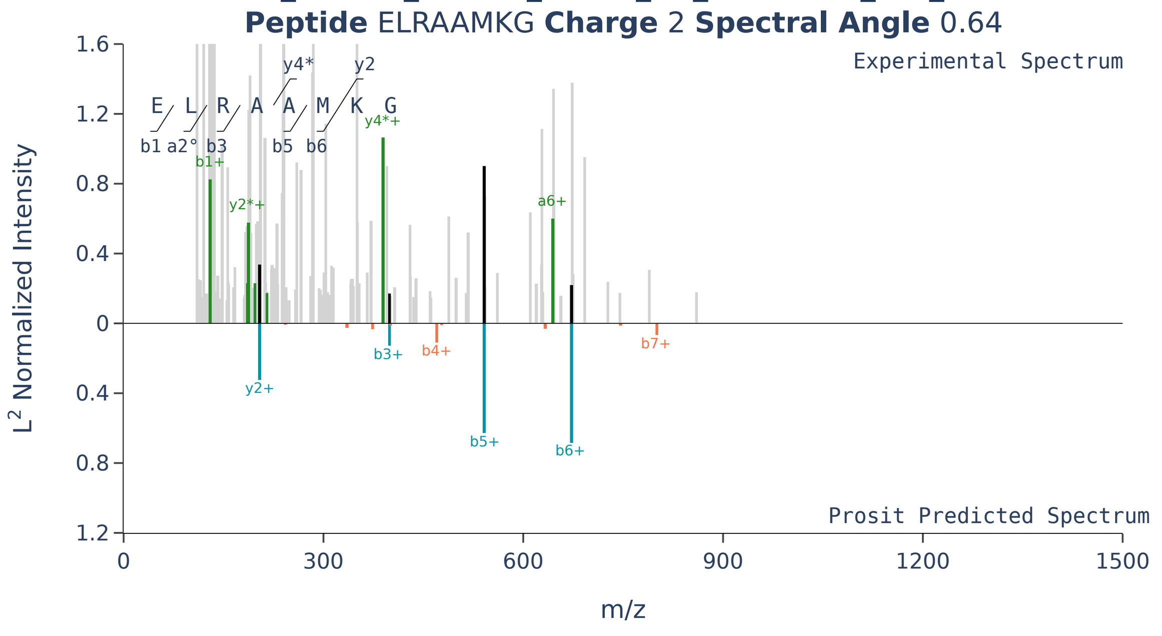


**Source** WSoh_301121_021221_HFGoe_M2_AnnexinA1_2h_R2 **Scan** 21496
**Peptide** KQAWFIENEEQE **Charge** 2 **Spectral Angle** 0.34

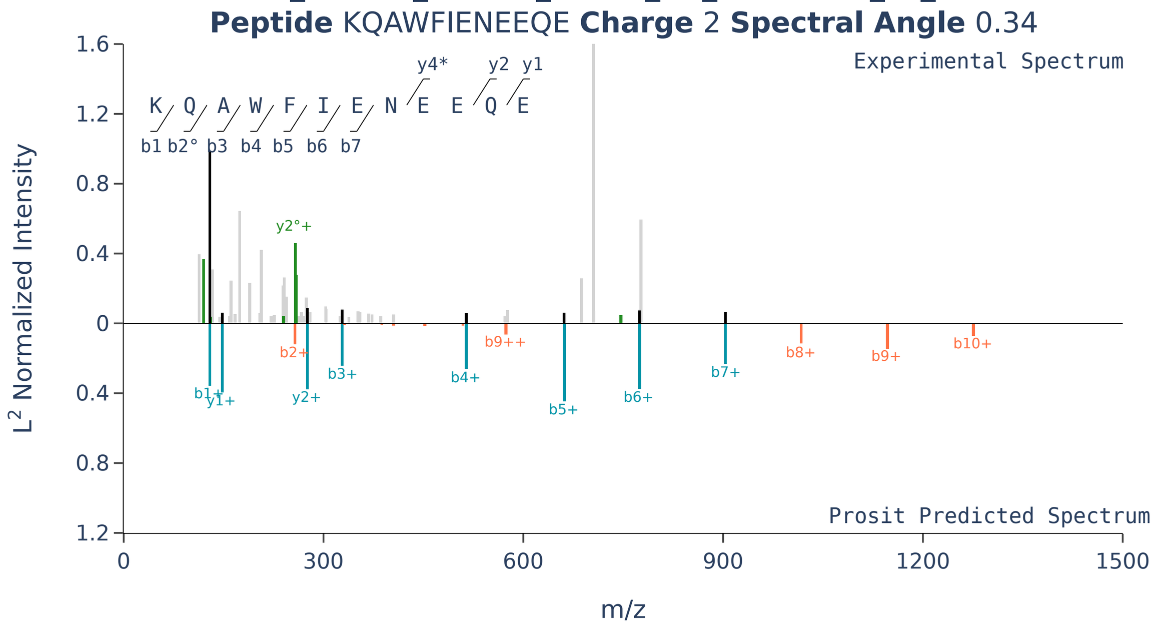


**Source** WSoh_301121_021221_HFGoe_M2_AnnexinA1_2h_R2 **Scan** 21385
**Peptide** QEYVQTVKSSKGGPGSAVSPYPTF **Charge** 3 **Spectral Angle** 0.89

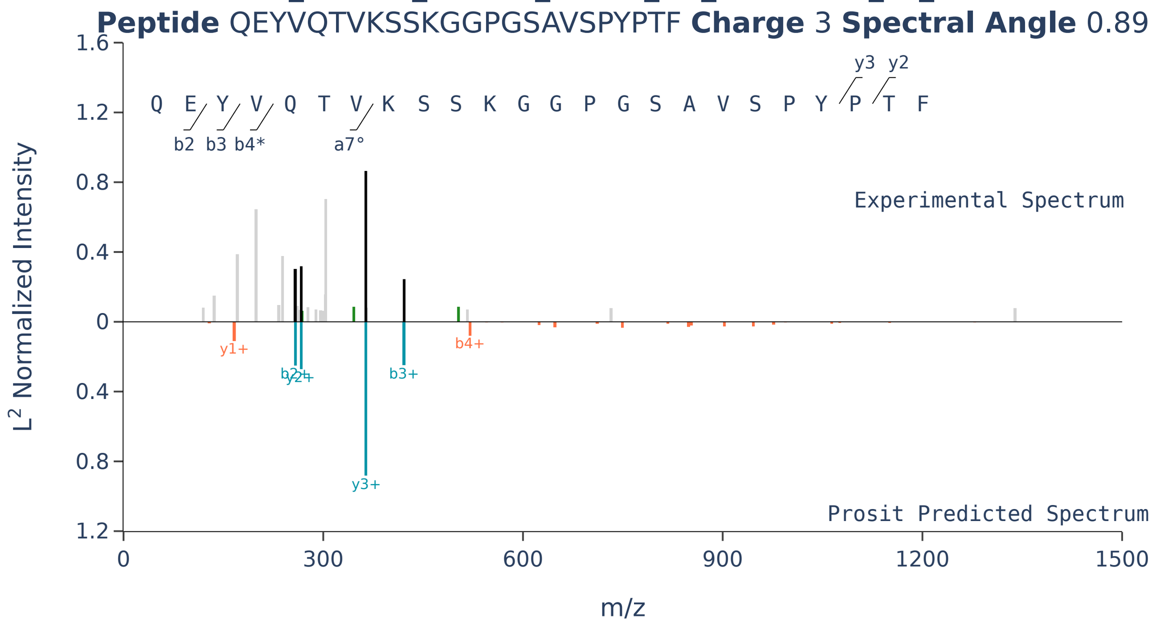


**Source** WSoh_301121_021221_HFGoe_M1_AnnexinA1_24h_R1 **Scan** 13637
**Peptide** ETGKPLD **Charge** 1 **Spectral Angle** 0.87

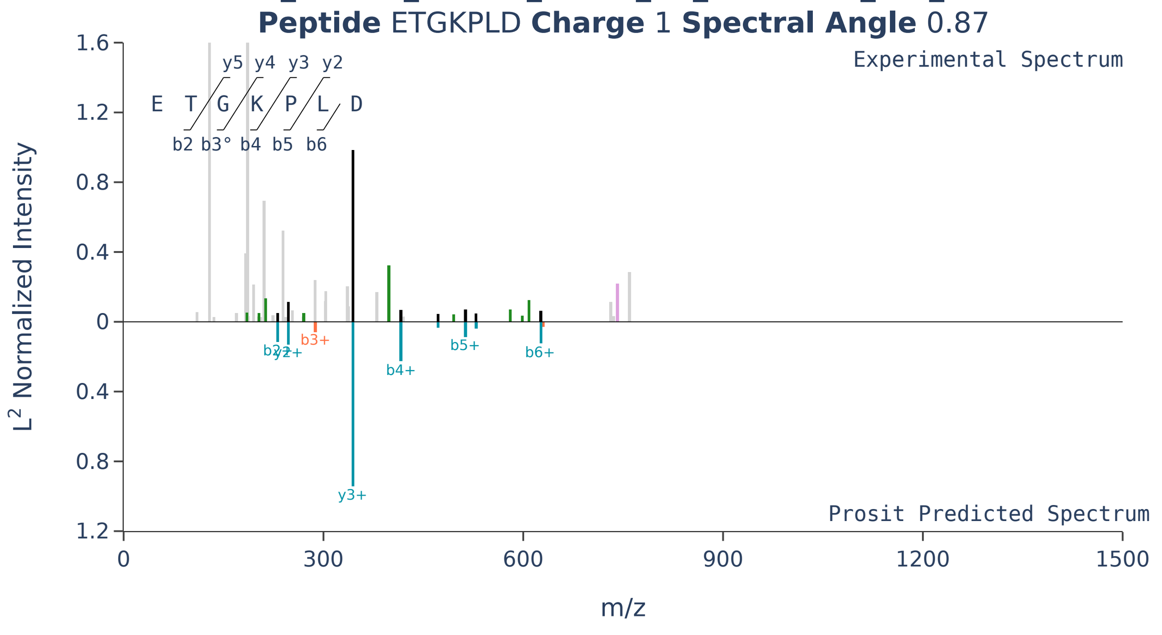


**Source** WSoh_301121_021221_HFGoe_M1_AnnexinA1_4h_R2 **Scan** 16705
**Peptide** KSSKGGPGSAVSPYPTFN **Charge** 2 **Spectral Angle** 0.69

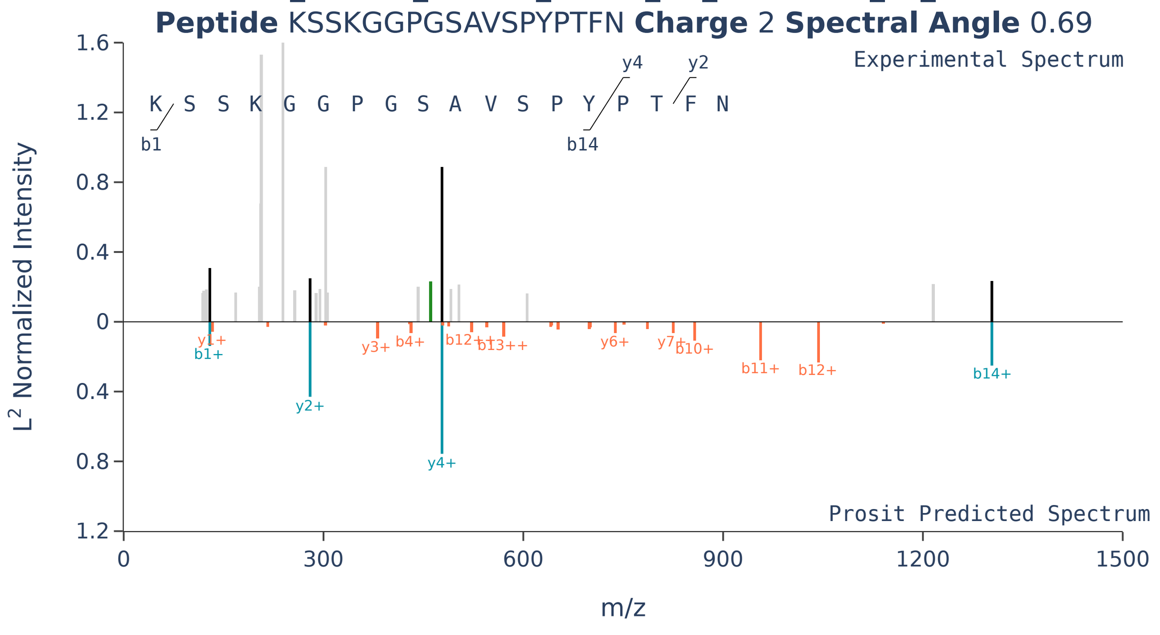


**Source** WSoh_301121_021221_HFGoe_M1_AnnexinA1_1h_R1 **Scan** 21890
**Peptide** AYLQETGKPLDETL **Charge** 2 **Spectral Angle** 0.81

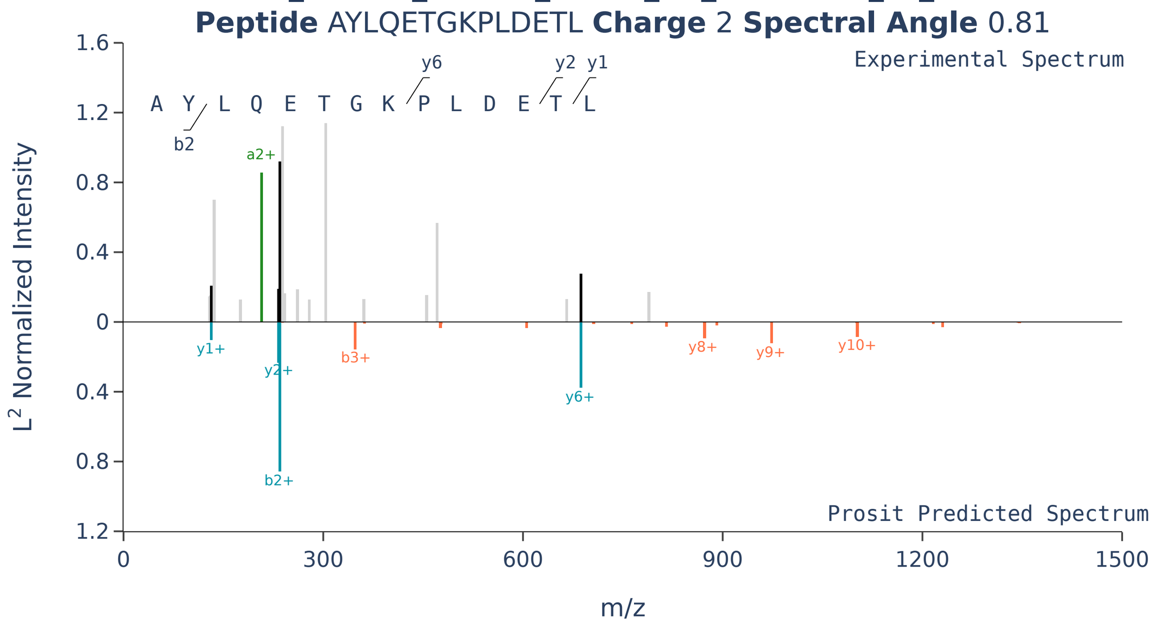


**Source** WSoh_301121_021221_HFGoe_M2_AnnexinA1_2h_R1 **Scan** 23635
**Peptide** TLIDILTKRNNAQRQQI **Charge** 3 **Spectral Angle** 0.74

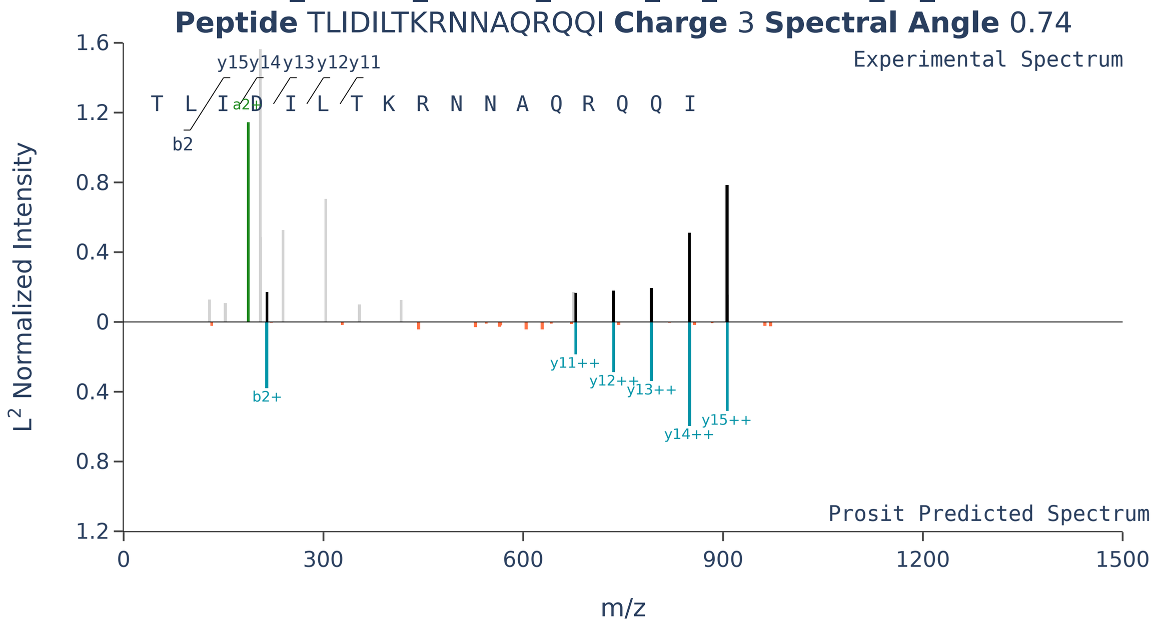


**Source** WSoh_301121_021221_HFGoe_M2_AnnexinA1_2h_R2 **Scan** 20427
**Peptide** SSKGGPGSAVSPYPTFNPSS **Charge** 2 **Spectral Angle** 0.79

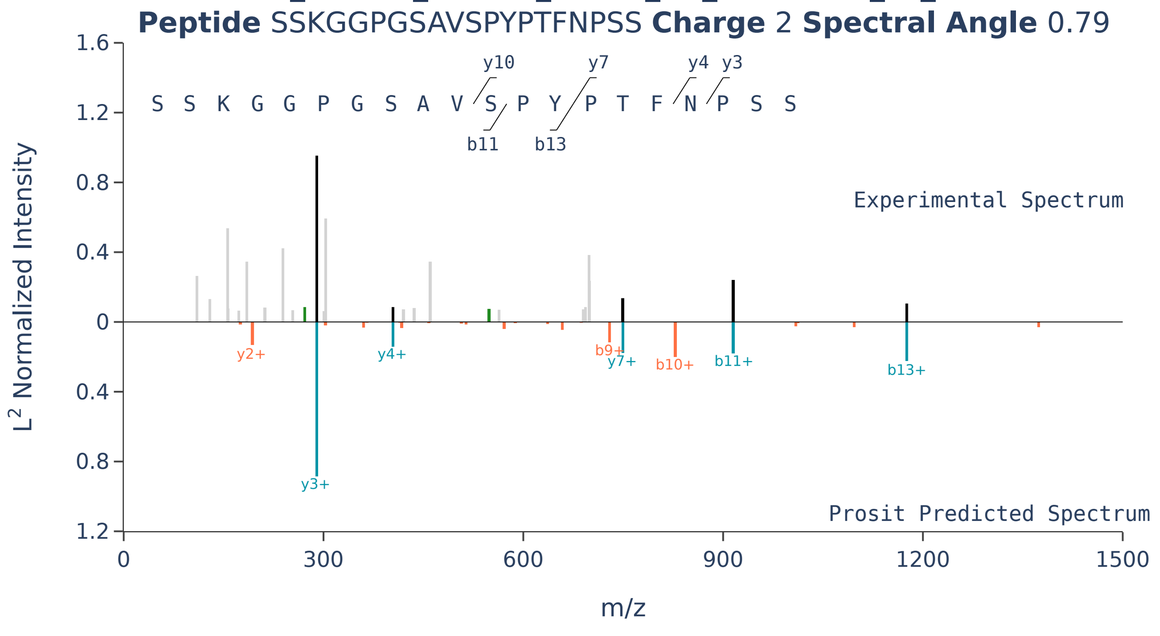

**Source** WSoh_301121_021221_HFGoe_M2_AnnexinA1_2h_R1 **Scan** 21880
**Peptide** EQEYVQTVKSSKGGPGSAVSPYPTF **Charge** 3 **Spectral Angle** 0.82

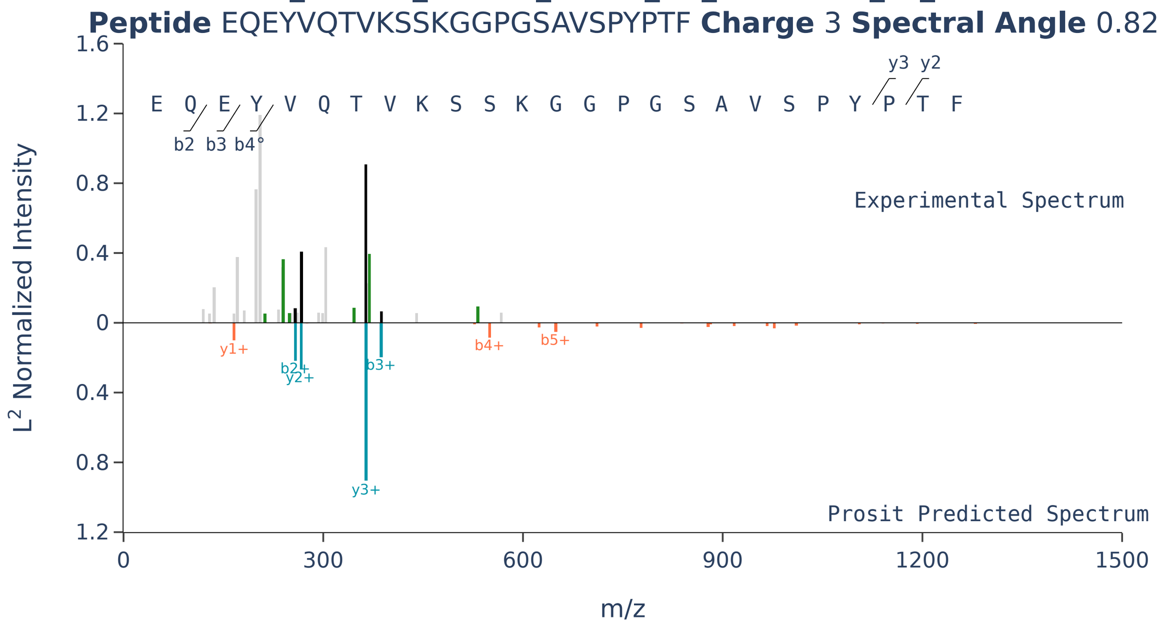


**Source** WSoh_301121_021221_HFGoe_M2_AnnexinA1_2h_R1 **Scan** 24527
**Peptide** SPYPTFNPSSDV **Charge** 2 **Spectral Angle** 0.58

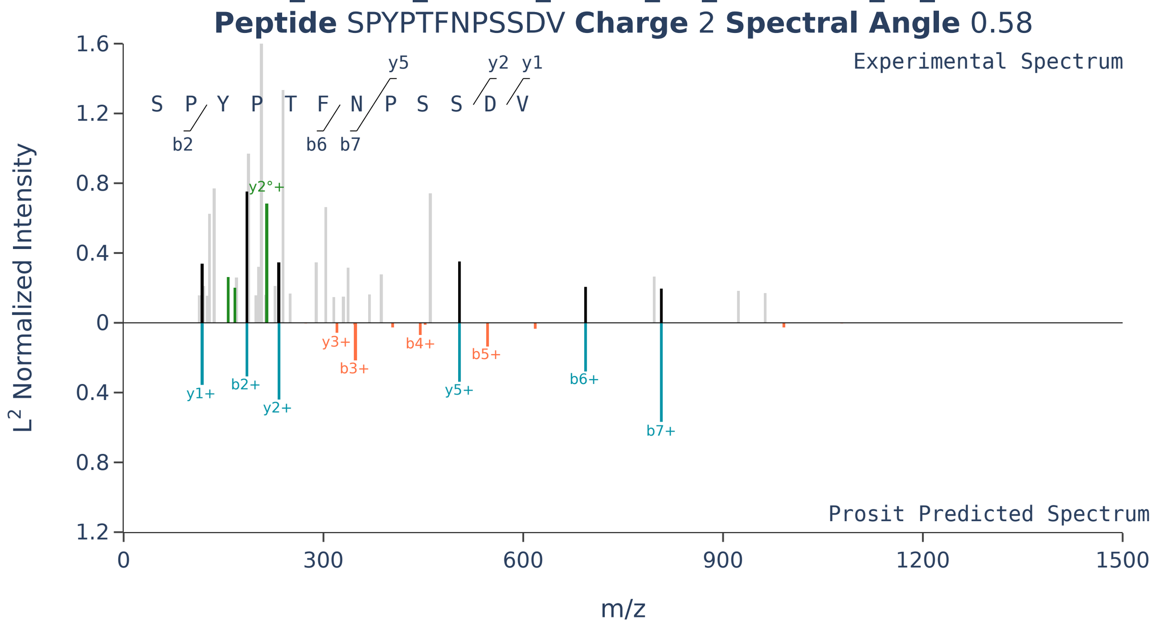


**Source** WSoh_301121_021221_HFGoe_M1_AnnexinA1_24h_R2 **Scan** 30169
**Peptide** KGLGTDEDTLIEIL **Charge** 2 **Spectral Angle** 0.66

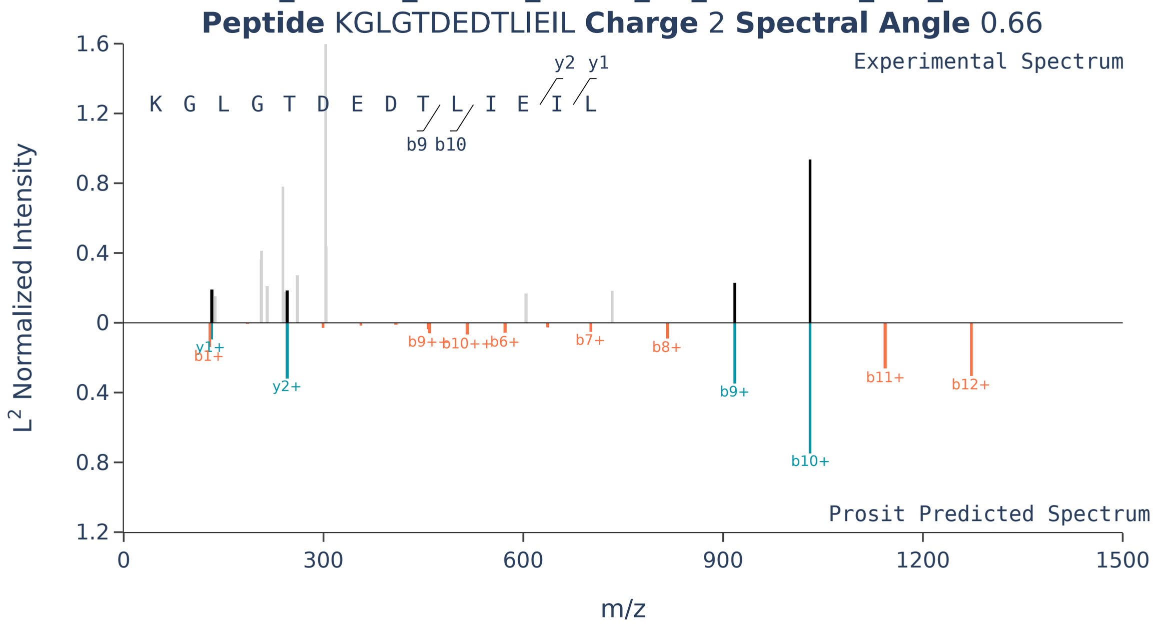


**Source** WSoh_301121_021221_HFGoe_M2_AnnexinA1_24h_R1 **Scan** 12329
**Peptide** RDLAKDITSDT **Charge** 2 **Spectral Angle** 0.63

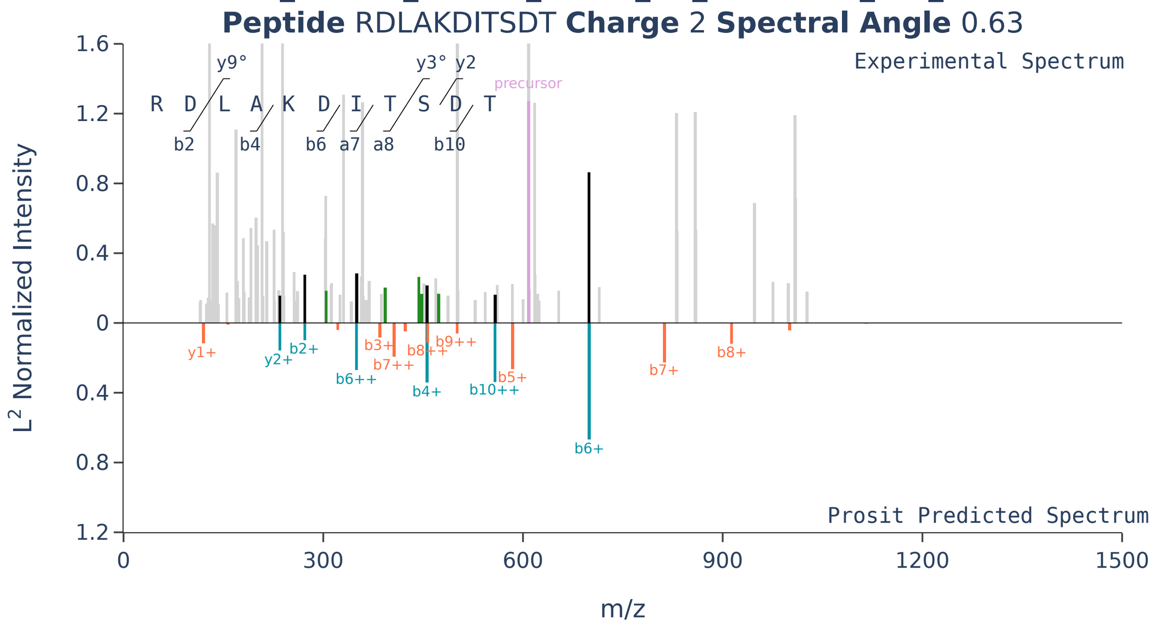


**Source** WSoh_301121_021221_HFGoe_M2_AnnexinA1_24h_R1 **Scan** 21031
**Peptide** KGGPGSAVSPYPTFNPS **Charge** 2 **Spectral Angle** 0.85

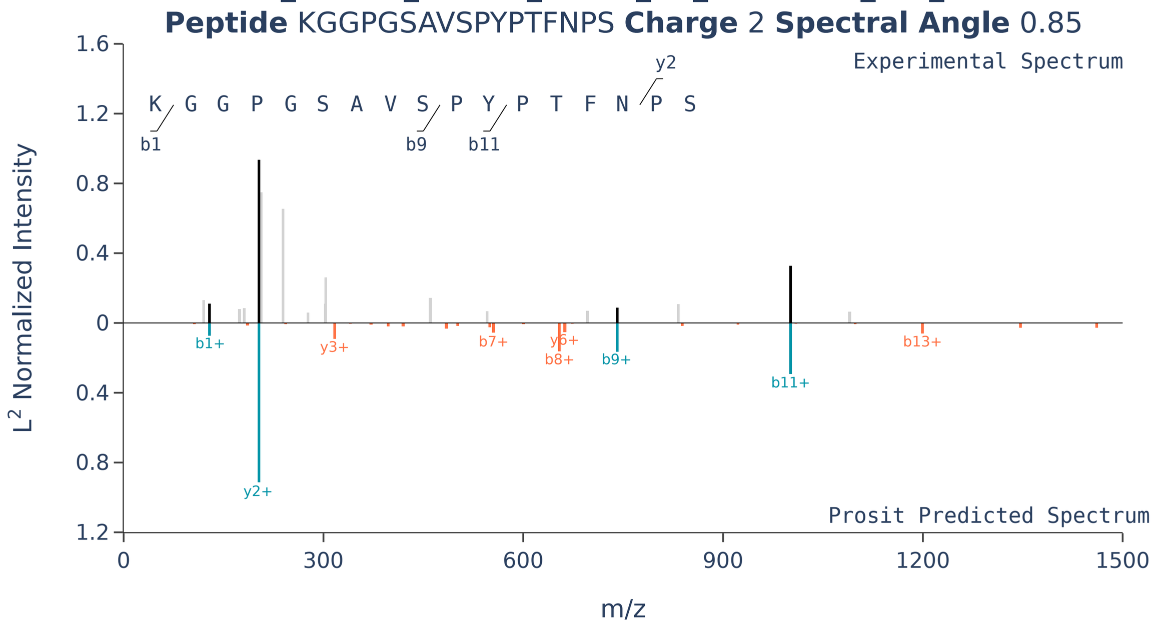


**Source** WSoh_301121_021221_HFGoe_M2_AnnexinA1_1h_R1 **Scan** 27270
**Peptide** AKDITSDTSGDFRNALLSL **Charge** 3 **Spectral Angle** 0.74

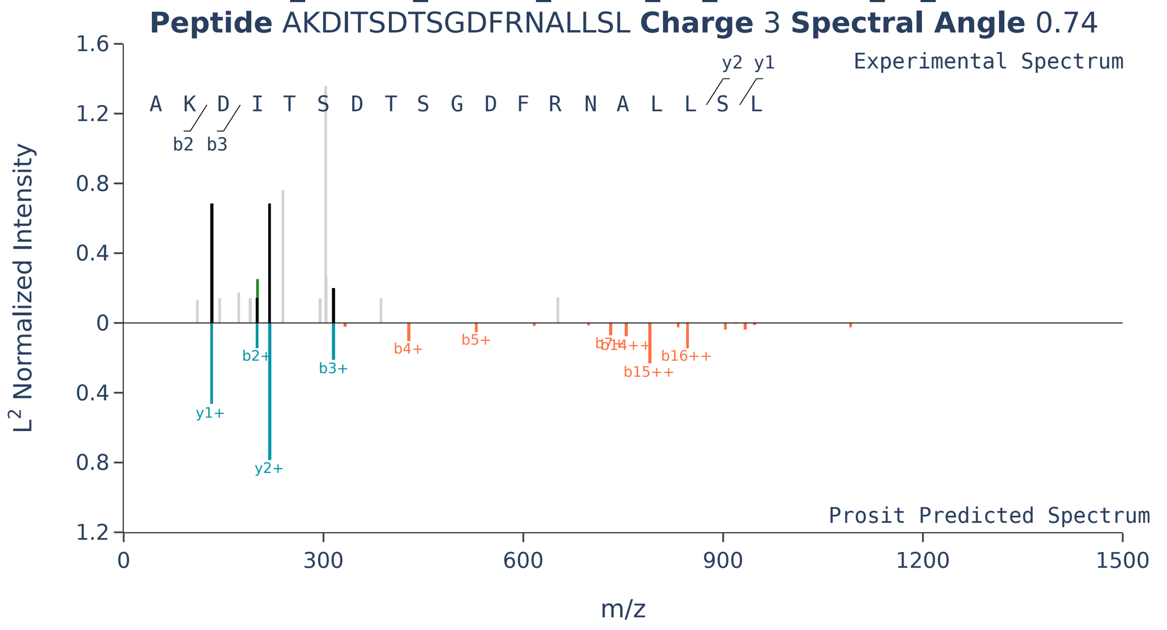


**Source** WSoh_301121_021221_HFGoe_M1_AnnexinA1_24h_R2 **Scan** 5474
**Peptide** LTKRNNAQRQQIKA **Charge** 3 **Spectral Angle** 0.69

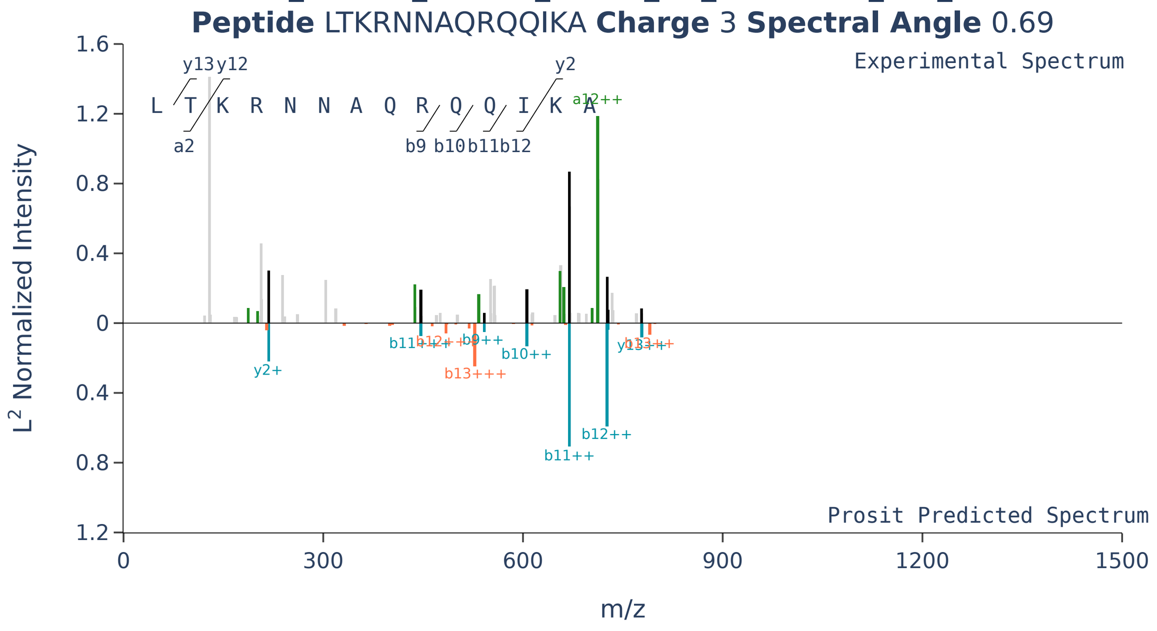


**Source** WSoh_301121_021221_HFGoe_M2_AnnexinA1_24h_R2 **Scan** 18127
**Peptide** NPSSDVAAL **Charge** 1 **Spectral Angle** 0.79

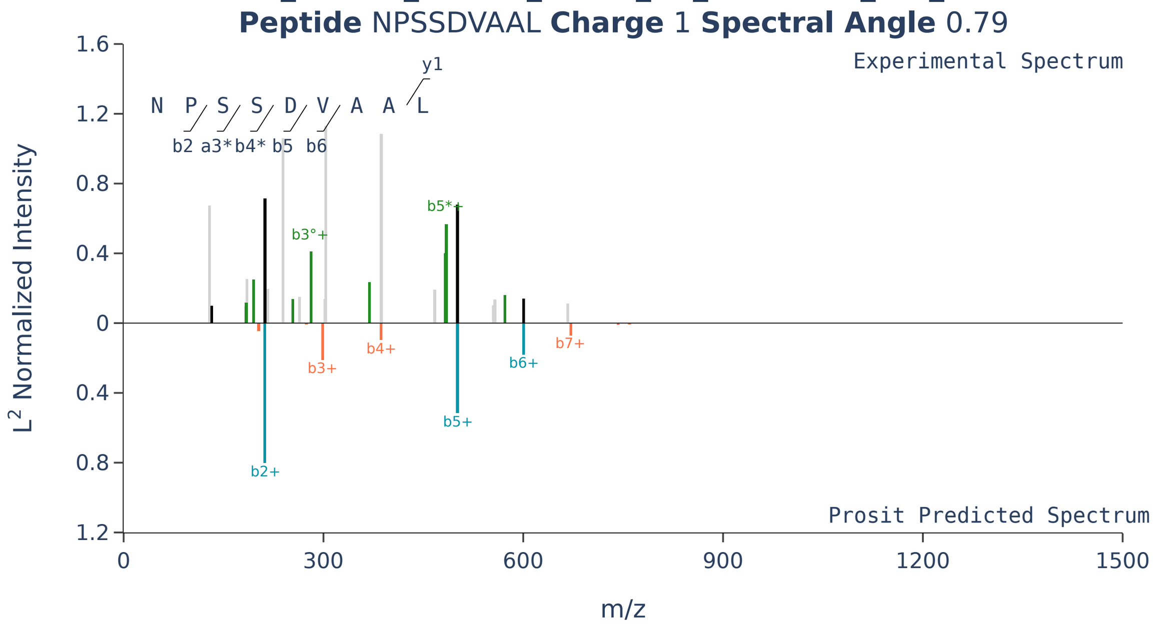


**Source** WSoh_301121_021221_HFGoe_M2_AnnexinA1_2h_R1 **Scan** 15139
**Peptide** AQRQQIKAAYLQETGKPLD **Charge** 3 **Spectral Angle** 0.54

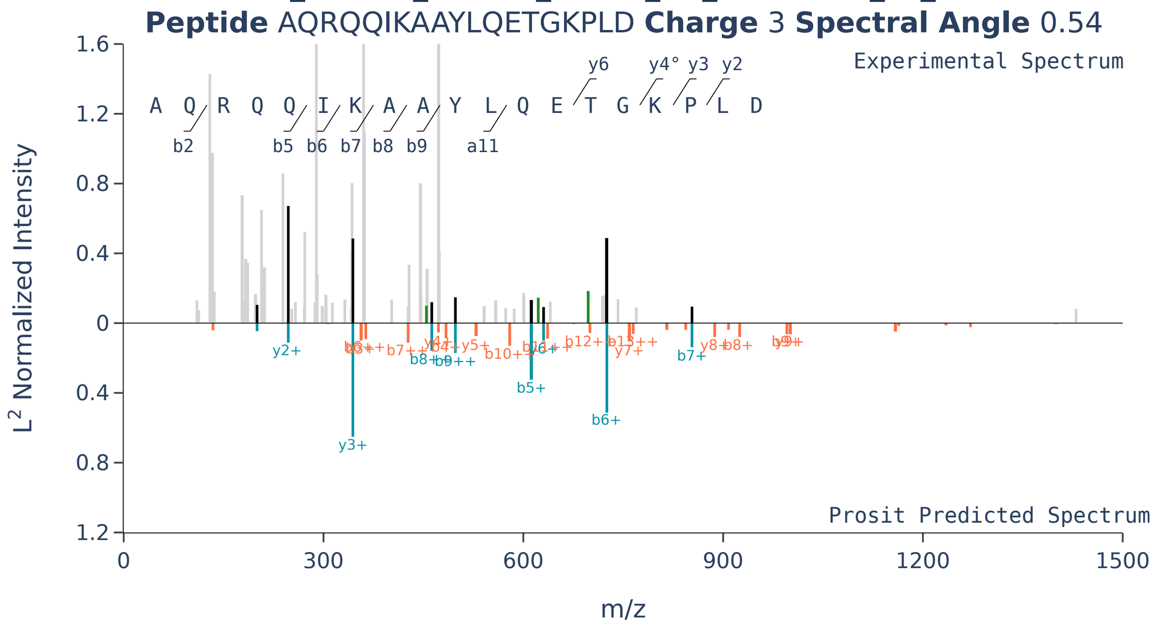


**Source** WSoh_301121_021221_HFGoe_M2_AnnexinA1_4h_R1 **Scan** 17380
**Peptide** DETKGDYEKLL **Charge** 2 **Spectral Angle** 0.66

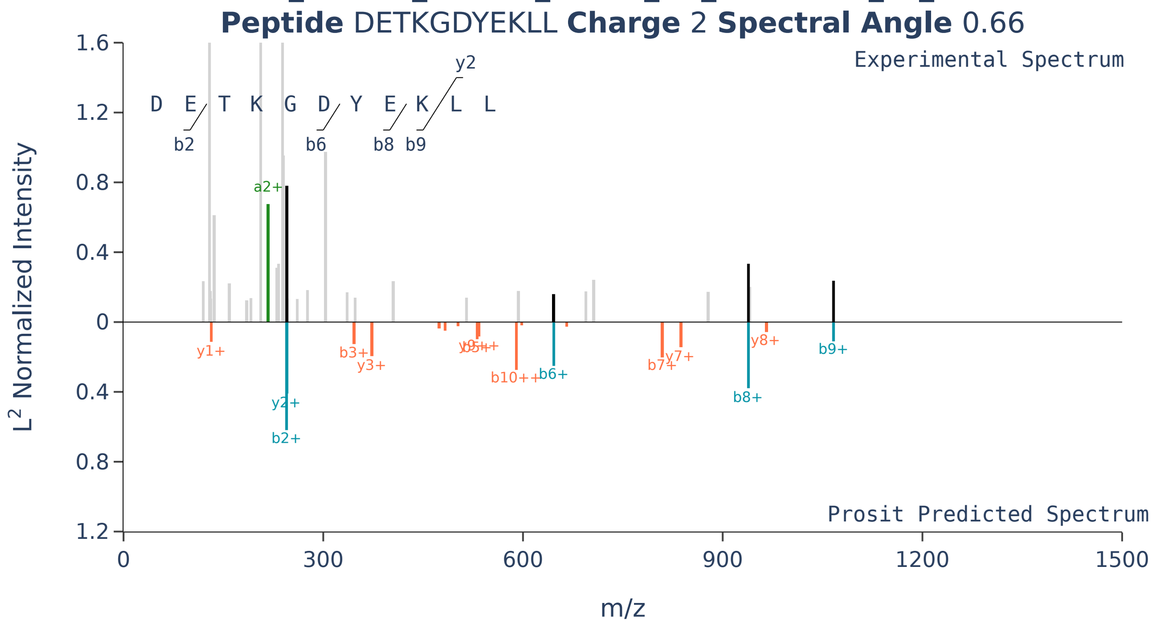


**Source** WSoh_301121_021221_HFGoe_M2_AnnexinA1_2h_R1 **Scan** 25907
**Peptide** AVSPYPTFNPSSDV **Charge** 2 **Spectral Angle** 0.7

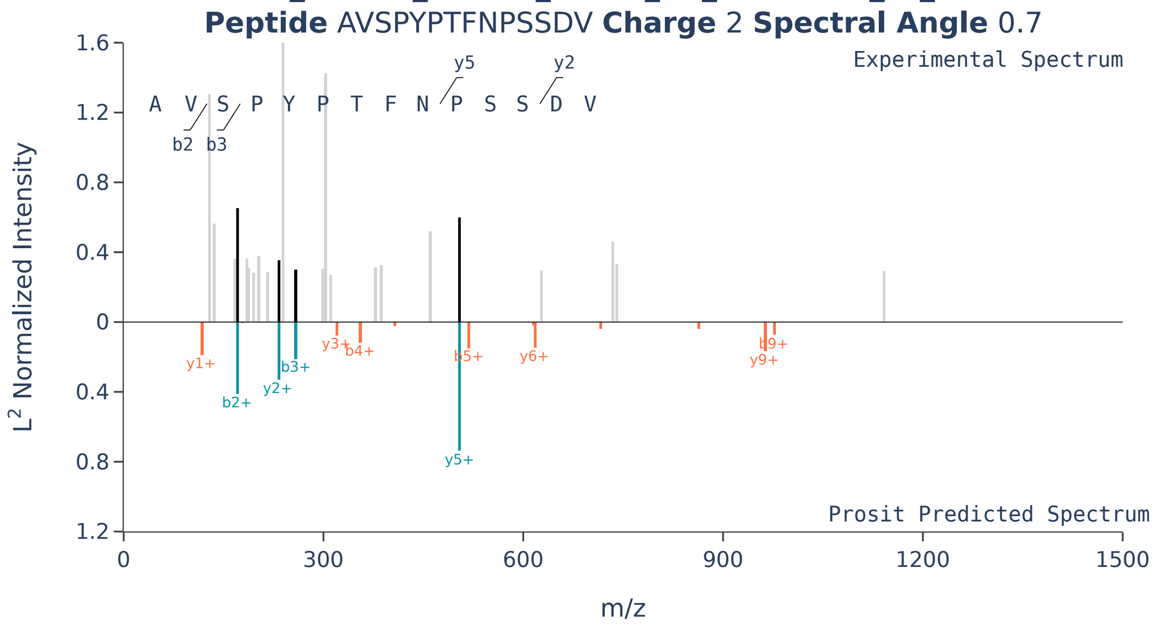


**Source** WSoh_301121_021221_HFGoe_M2_AnnexinA1_2h_R1 **Scan** 14703
**Peptide** VKCATSKPAFFAEKLHQA **Charge** 4 **Spectral Angle** 0.6

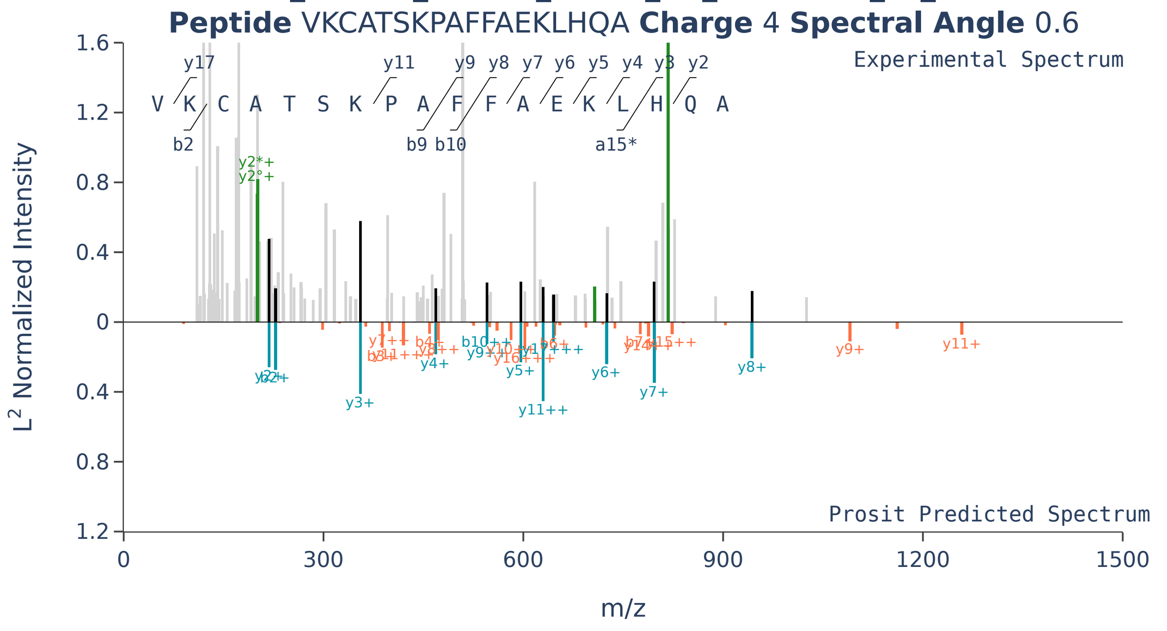


**Source** WSoh_301121_021221_HFGoe_M1_AnnexinA1_2h_R1 **Scan** 21667
**Peptide** KQAWFIEN **Charge** 2 **Spectral Angle** 0.44

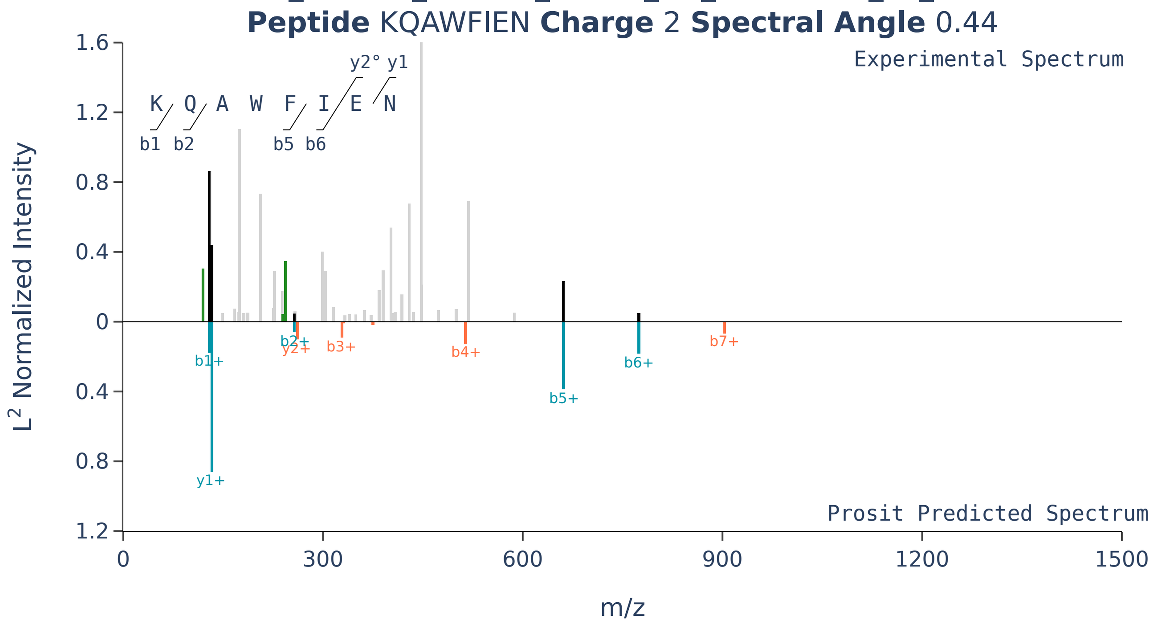


**Source** WSoh_301121_021221_HFGoe_M1_AnnexinA1_24h_R1 **Scan** 5812
**Peptide** HQAMKGVGTRHKAL **Charge** 3 **Spectral Angle** 0.71

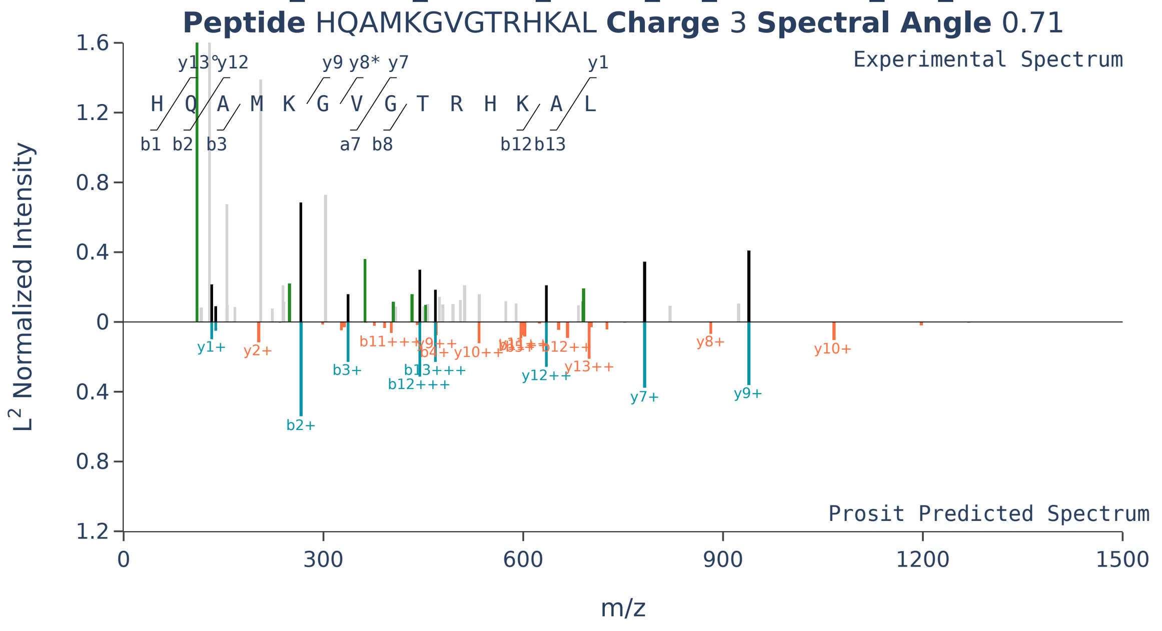


**Source** WSoh_301121_021221_HFGoe_M2_AnnexinA1_4h_R1 **Scan** 8089
**Peptide** PSSDVAALHK **Charge** 2 **Spectral Angle** 0.42

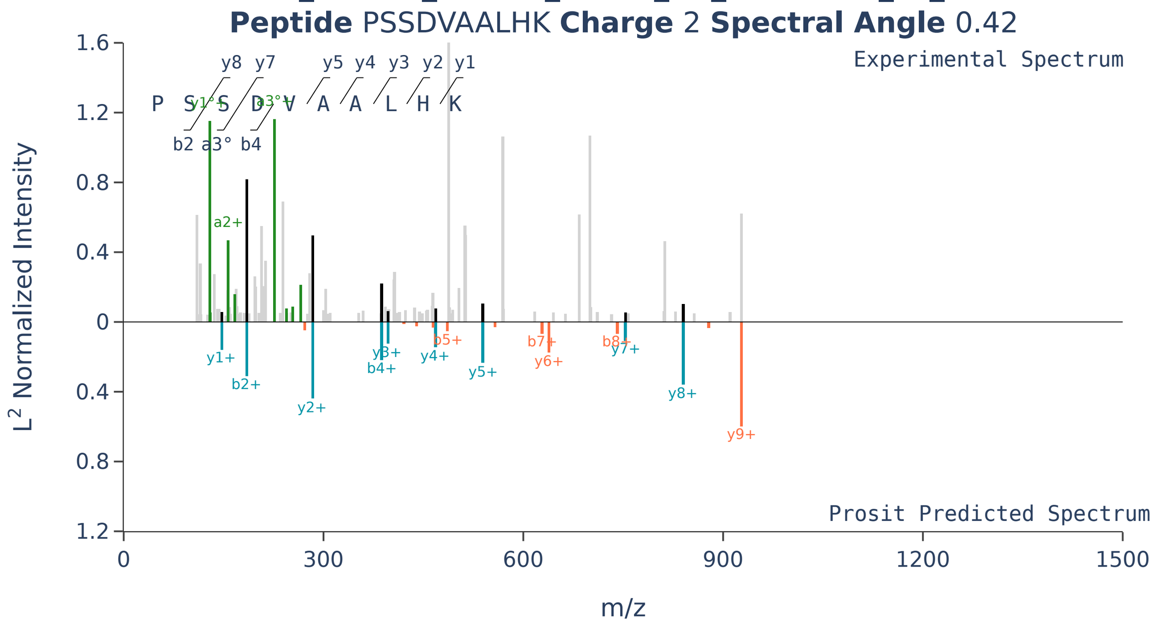

**Source** WSoh_301121_021221_HFGoe_M2_AnnexinA1_2h_R1 **Scan** 24318
**Peptide** EATIIDILTKRNNAQRQQ **Charge** 3 **Spectral Angle** 0.78

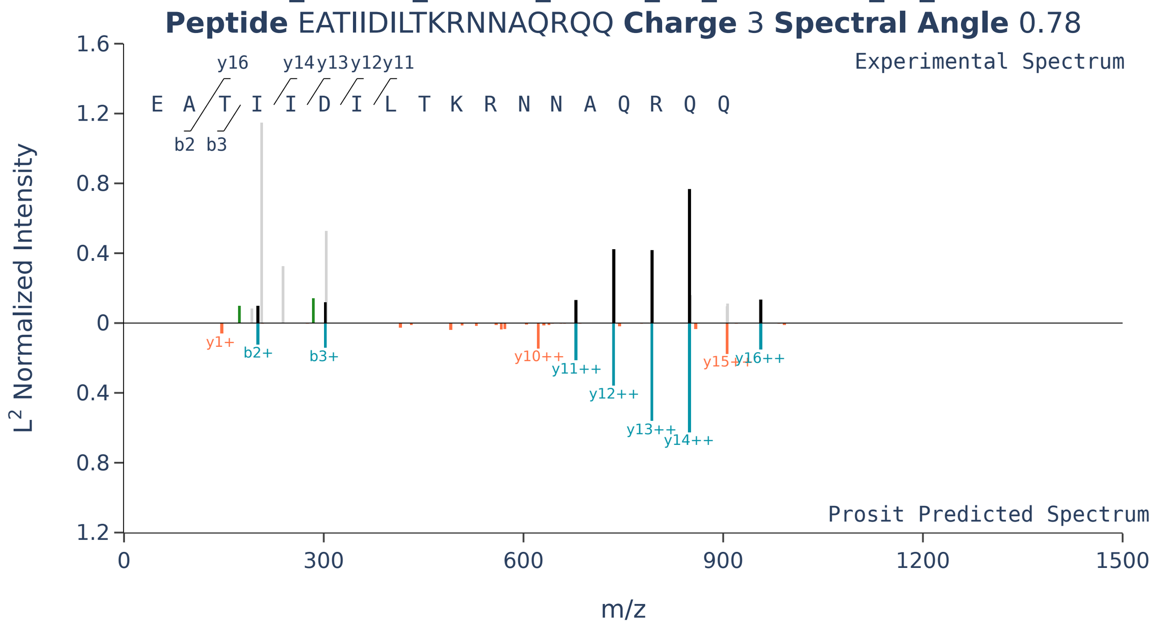


**Source** WSoh_301121_021221_HFGoe_M1_AnnexinA1_24h_R1 **Scan** 23946
**Peptide** LTSDTSGDFRNALL **Charge** 2 **Spectral Angle** 0.55

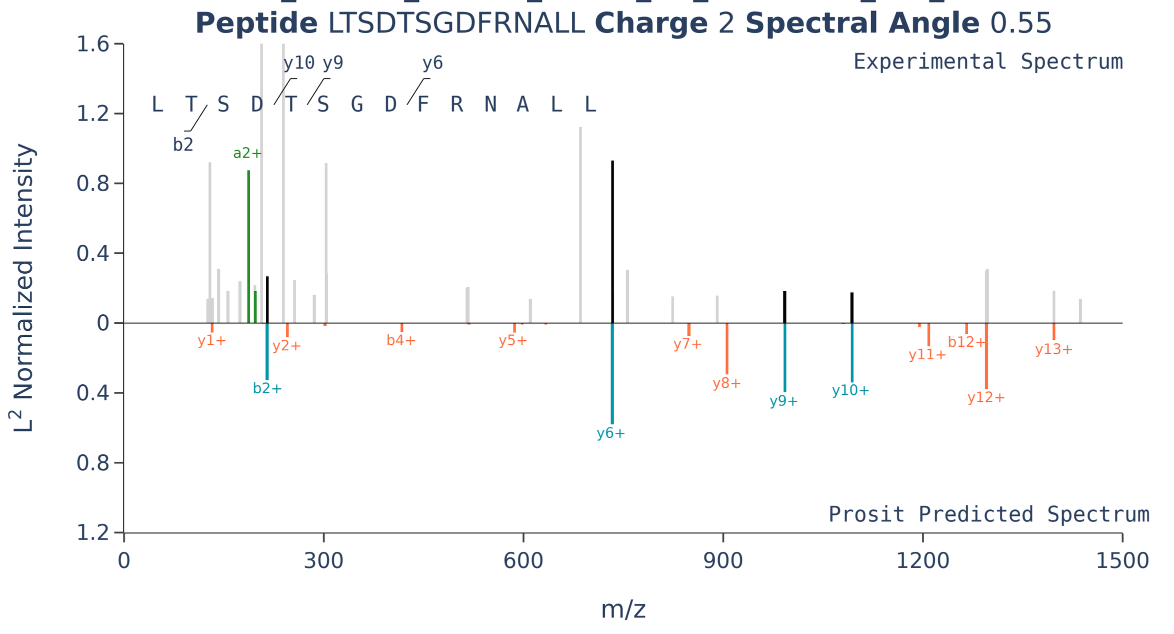


**Source** WSoh_301121_021221_HFGoe_M2_AnnexinA1_2h_R1 **Scan** 27056
**Peptide** SKGGPGSAVSPYPTFNPSSDVAAL **Charge** 3 **Spectral Angle** 0.76

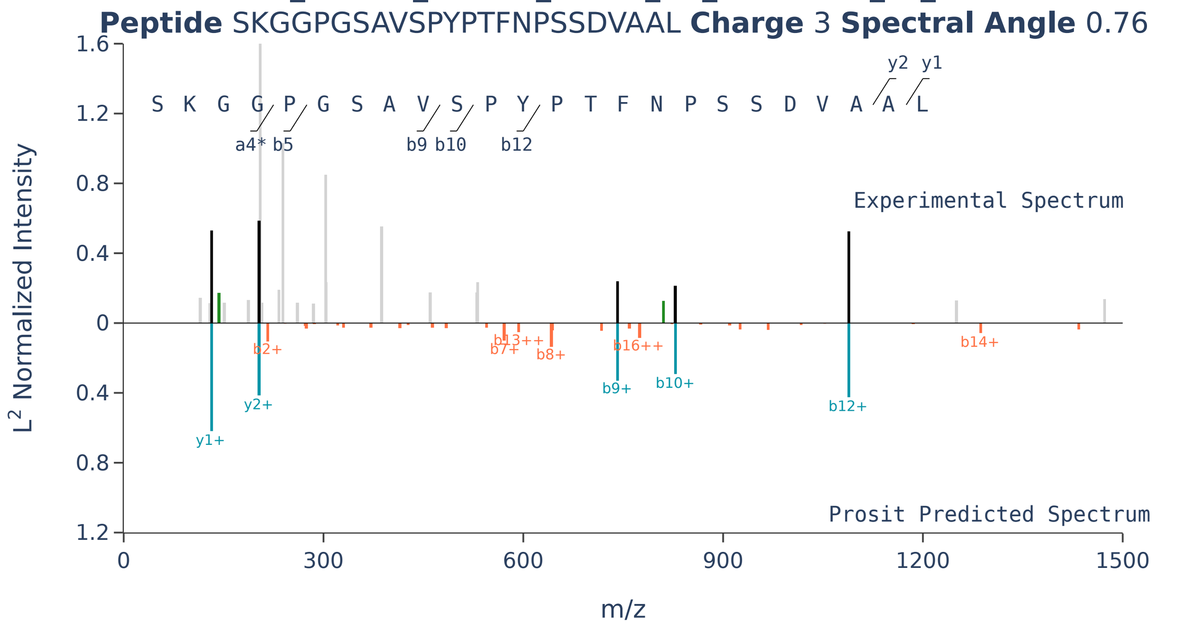


**Source** WSoh_301121_021221_HFGoe_M1_AnnexinA1_2h_R1 **Scan** 11502
**Peptide** LDLLTKRNNAQRQQIKAA **Charge** 4 **Spectral Angle** 0.4

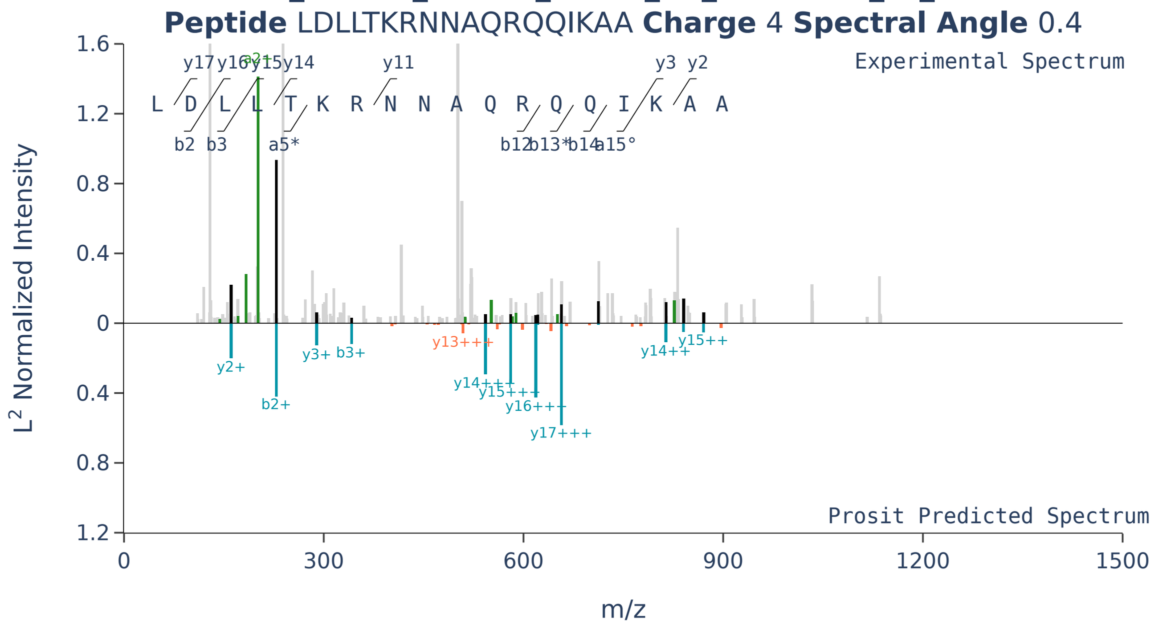


**Source** WSoh_301121_021221_HFGoe_M2_AnnexinA1_4h_R1 **Scan** 28039
**Peptide** TSGDFRNALLSL **Charge** 2 **Spectral Angle** 0.65

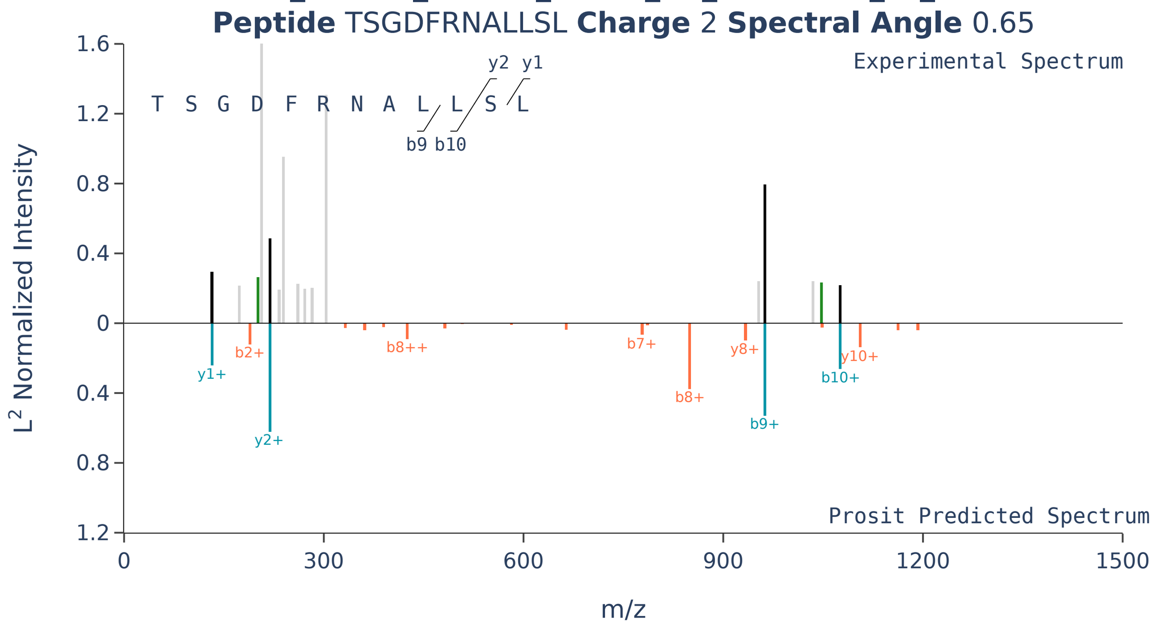


**Source** WSoh_301121_021221_HFGoe_M1_AnnexinA1_24h_R2 **Scan** 23884
**Peptide** DIEKCLTAIVKC **Charge** 3 **Spectral Angle** 0.75

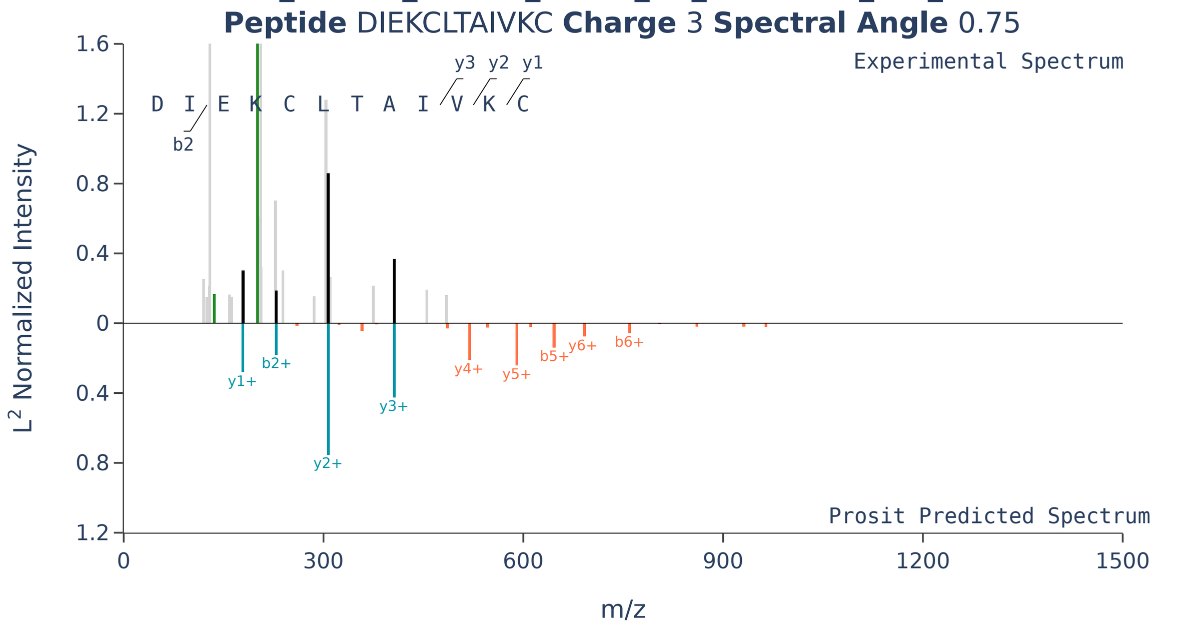


**Source** WSoh_301121_021221_HFGoe_M1_AnnexinA1_24h_R1 **Scan** 7443
**Peptide** KRDLAKDITS **Charge** 3 **Spectral Angle** 0.41

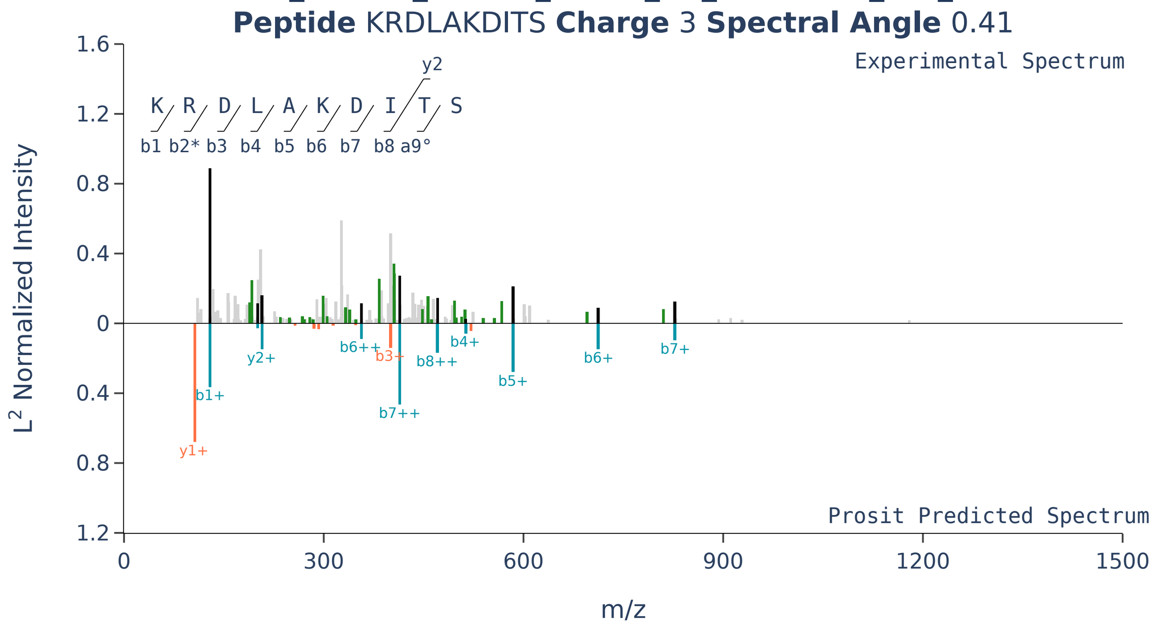


**Source** WSoh_301121_021221_HFGoe_M2_AnnexinA1_2h_R1 **Scan** 24985
**Peptide** KSSKGGPGSAVSPYPTFNPSSDVAAL **Charge** 3 **Spectral Angle** 0.62

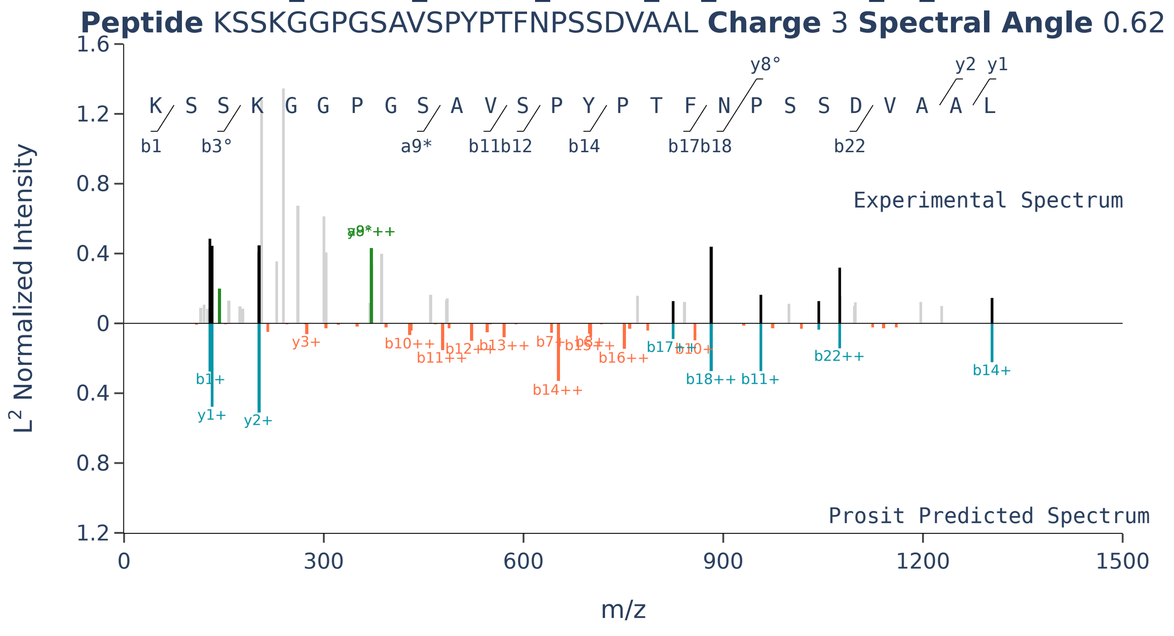


**Source** WSoh_301121_021221_HFGoe_M2_AnnexinA1_2h_R1 **Scan** 22585
**Peptide** LLSLAKGDRSEDFGV **Charge** 3 **Spectral Angle** 0.62

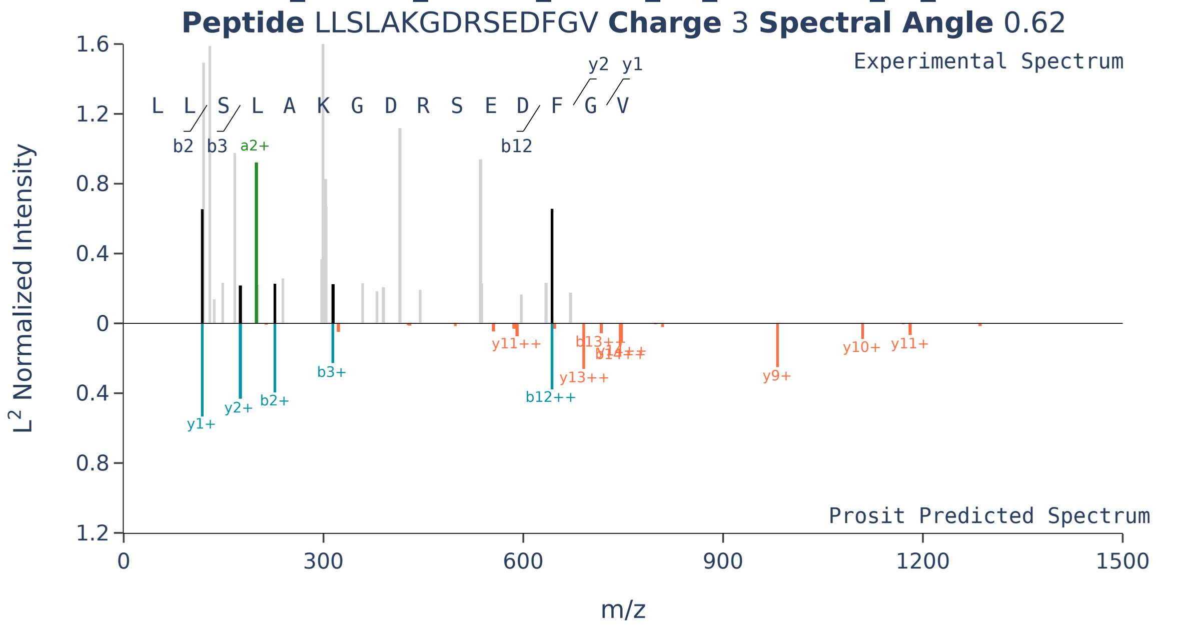


**Source** WSoh_301121_021221_HFGoe_M2_AnnexinA1_24h_R2 **Scan** 23186
**Peptide** AMKGLGTDEDTLL **Charge** 2 **Spectral Angle** 0.55

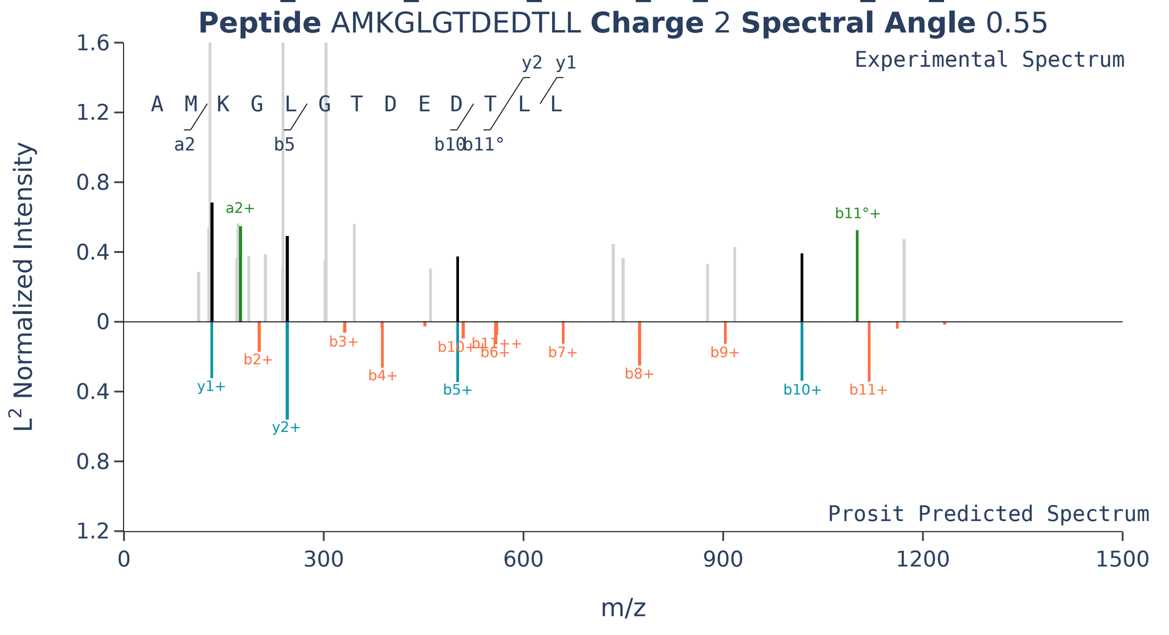


**Source** WSoh_301121_021221_HFGoe_M2_AnnexinA1_24h_R2 **Scan** 12491
**Peptide** LTTRSYPQLRRV **Charge** 3 **Spectral Angle** 0.49

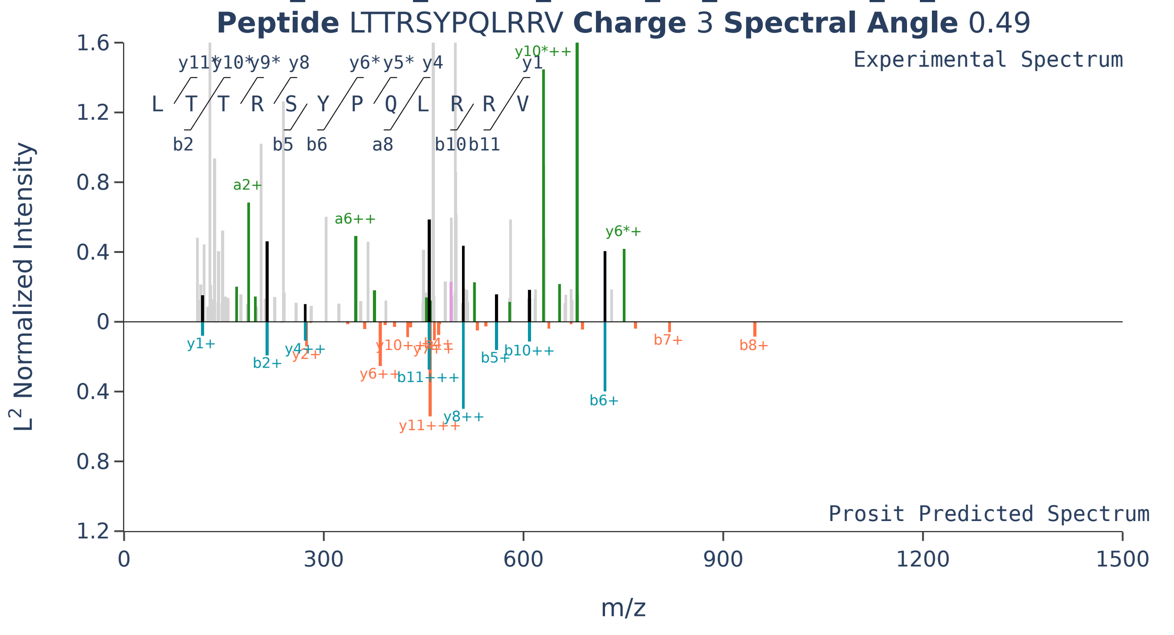


**Source** WSoh_301121_021221_HFGoe_M2_AnnexinA1_24h_R2 **Scan** 8048
**Peptide** ADELRAA **Charge** 1 **Spectral Angle** 0.83

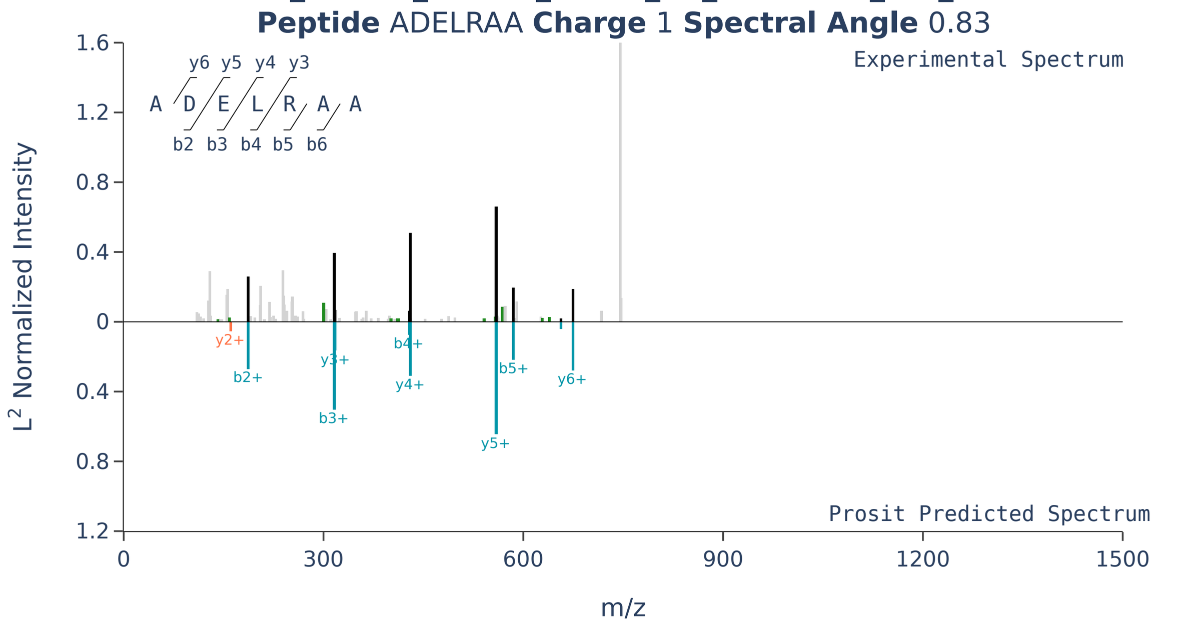


**Source** WSoh_301121_021221_HFGoe_M2_AnnexinA1_2h_R2 **Scan** 20309
**Peptide** YVQTVKSSKGGPGSAVSPYPTF **Charge** 3 **Spectral Angle** 0.76

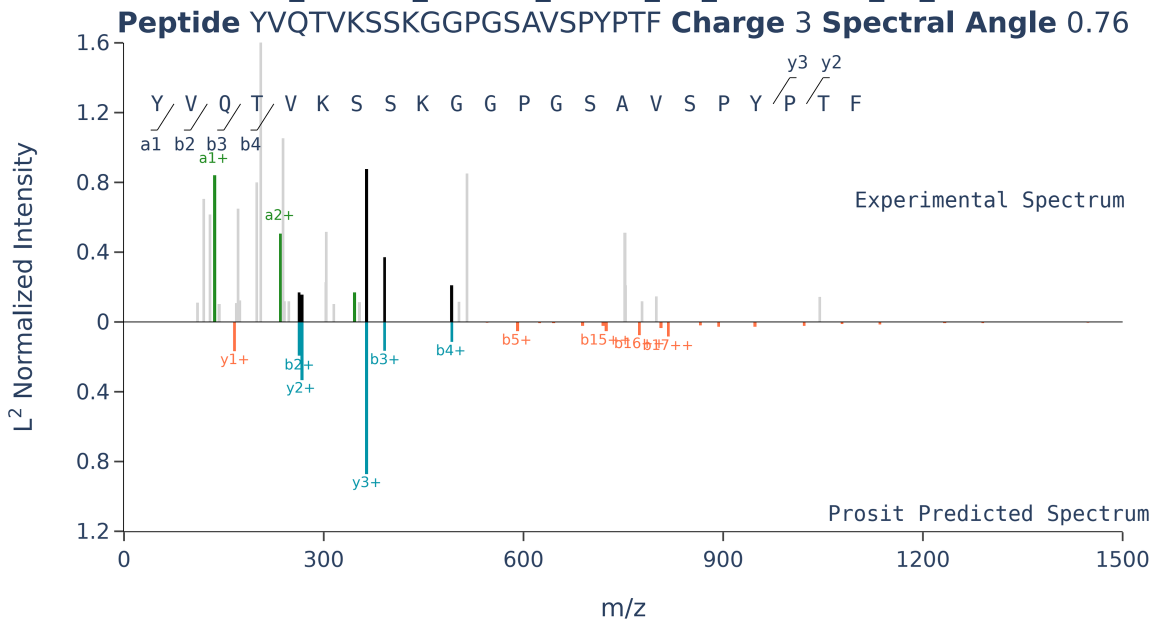


**Source** WSoh_301121_021221_HFGoe_M2_AnnexinA1_2h_R1 **Scan** 31088
**Peptide** EDTLIEILASRT **Charge** 2 **Spectral Angle** 0.63

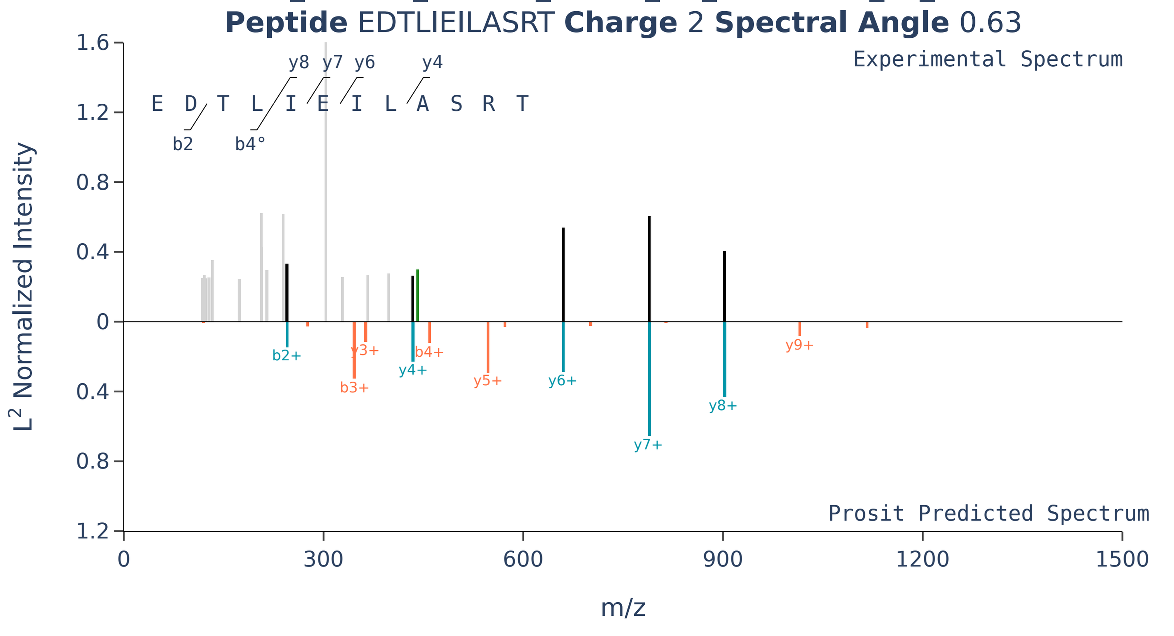


**Source** WSoh_301121_021221_HFGoe_M2_AnnexinA1_2h_R1 **Scan** 23439
**Peptide** KGGPGSAVSPYPTFNPSSDV **Charge** 2 **Spectral Angle** 0.76

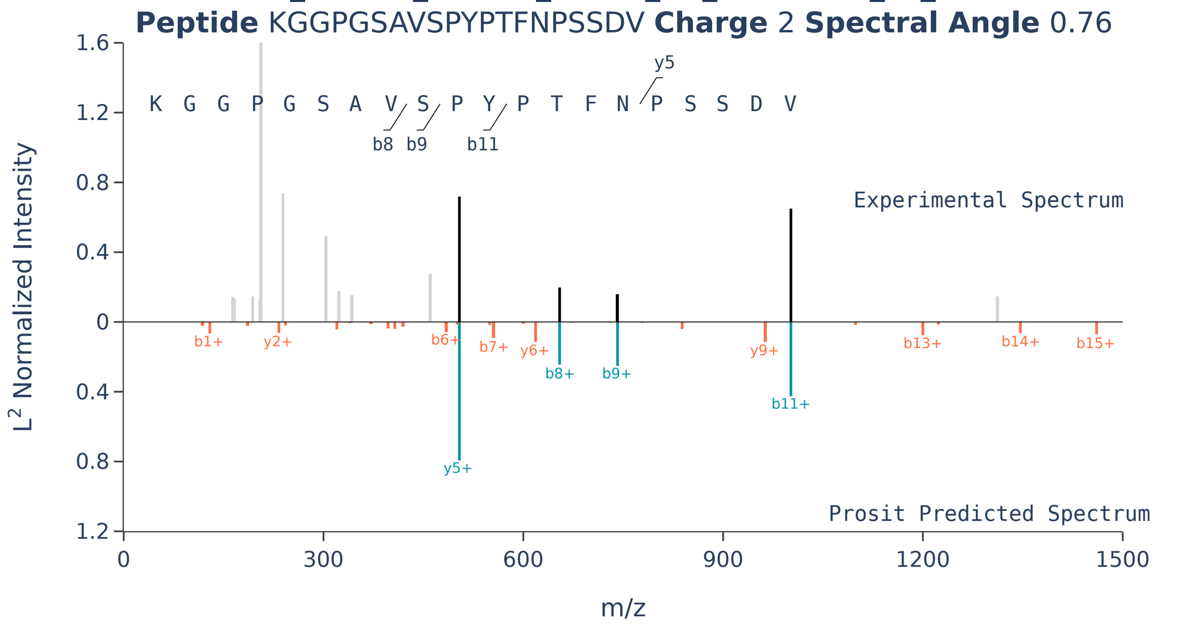

**Source** WSoh_301121_021221_HFGoe_M2_AnnexinA1_2h_R1 **Scan** 32486
**Peptide** DTLIEIL **Charge** 1 **Spectral Angle** 0.74

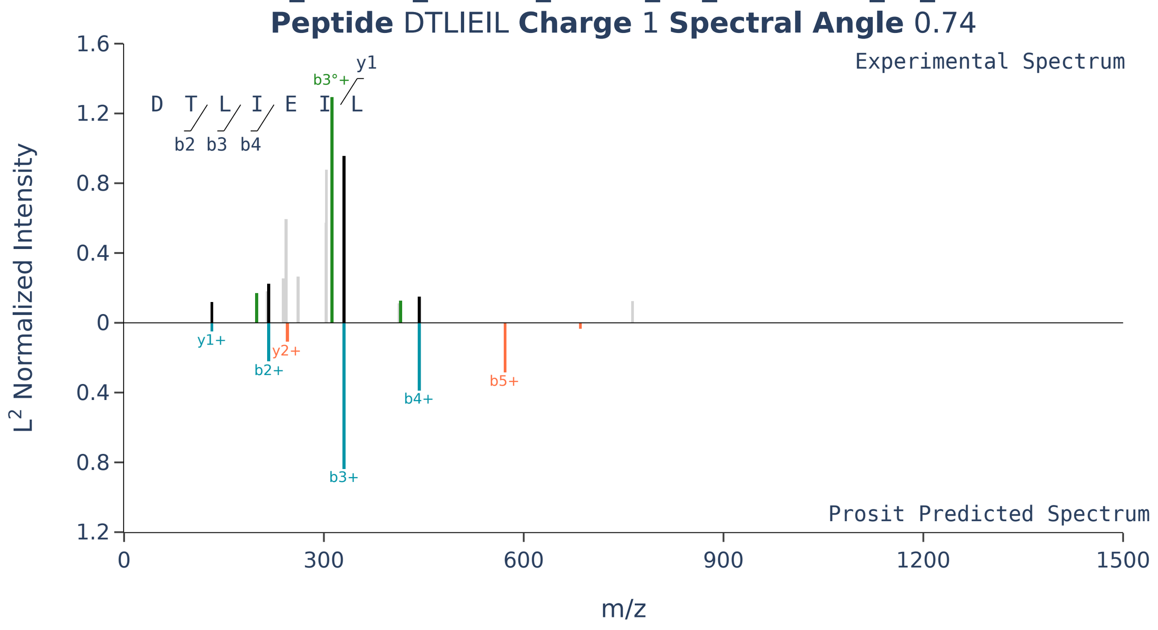


**Source** WSoh_301121_021221_HFGoe_M2_AnnexinA1_2h_R1 **Scan** 23236
**Peptide** SKGGPGSAVSPYPTFNPSSDVA **Charge** 2 **Spectral Angle** 0.71

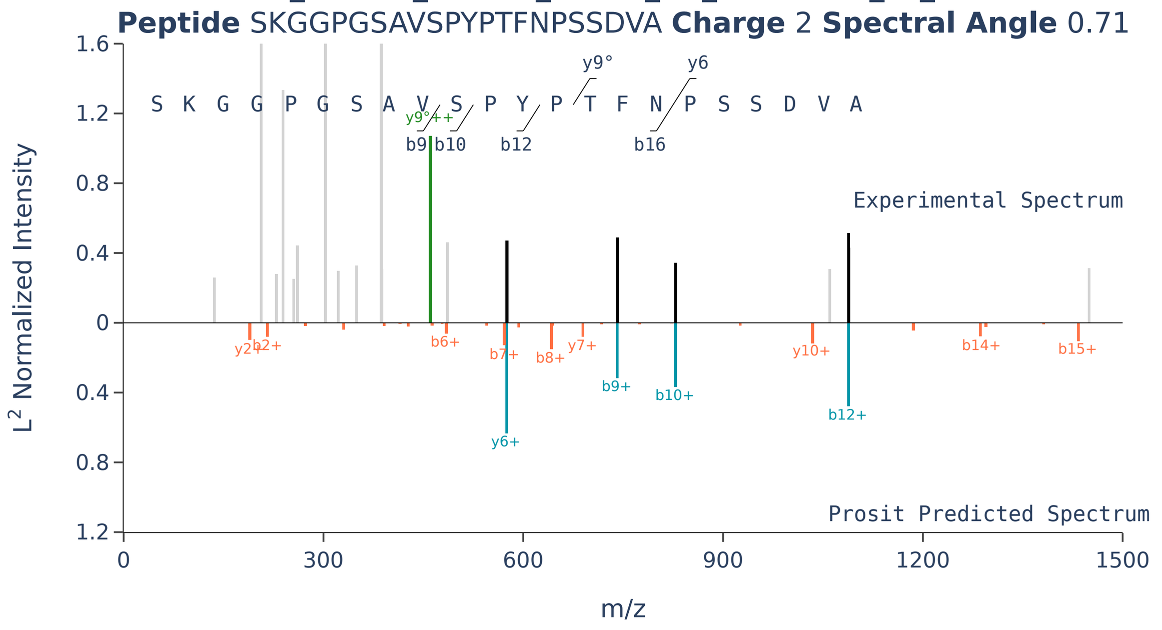


**Source** WSoh_301121_021221_HFGoe_M2_AnnexinA1_1h_R2 **Scan** 25544
**Peptide** ALLKTPAQFDADEL **Charge** 2 **Spectral Angle** 0.74

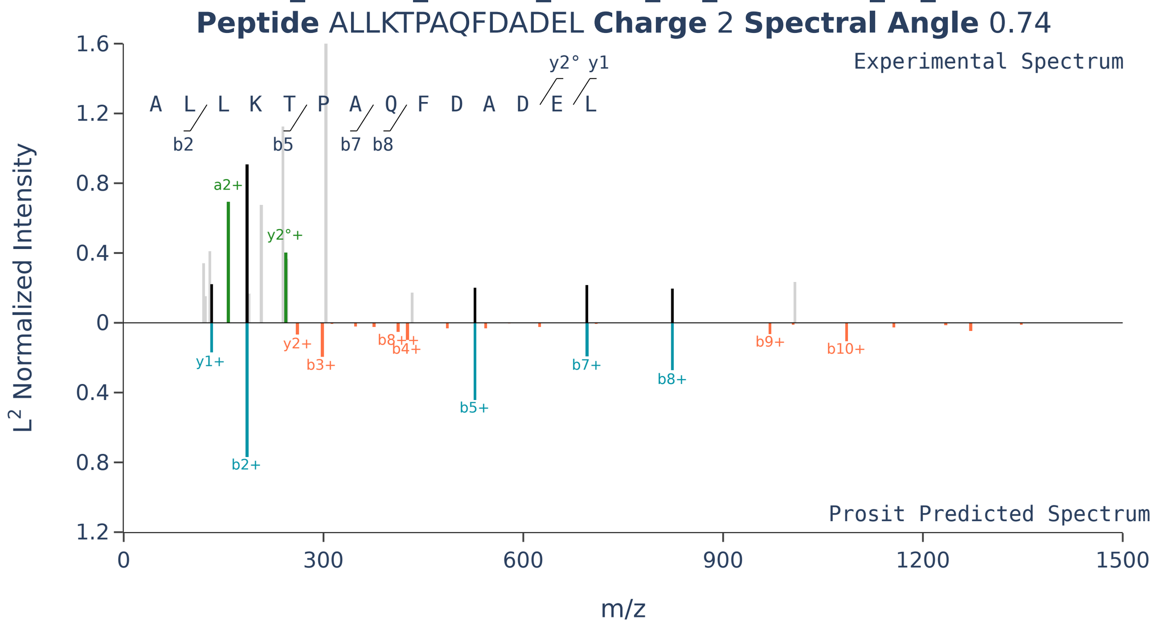


**Source** WSoh_301121_021221_HFGoe_M2_AnnexinA1_2h_R1 **Scan** 31673
**Peptide** TDVNVFNTILTT **Charge** 2 **Spectral Angle** 0.6

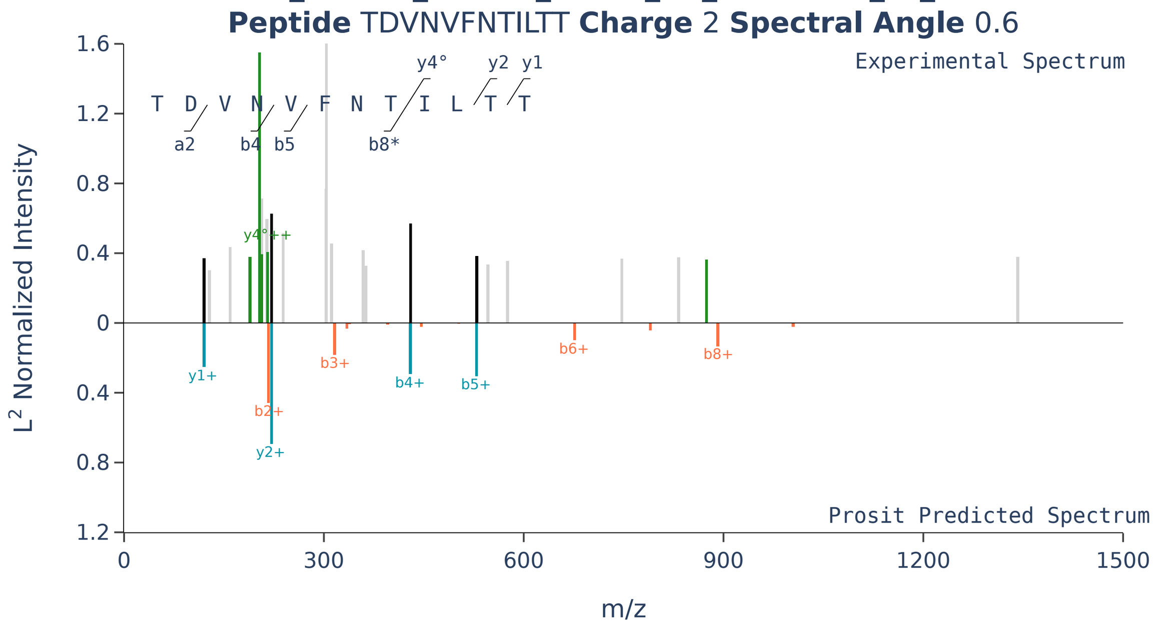


**Source** WSoh_301121_021221_HFGoe_M2_AnnexinA1_1h_R1 **Scan** 23436
**Peptide** ALLKTPAQFDADELRAA **Charge** 3 **Spectral Angle** 0.67

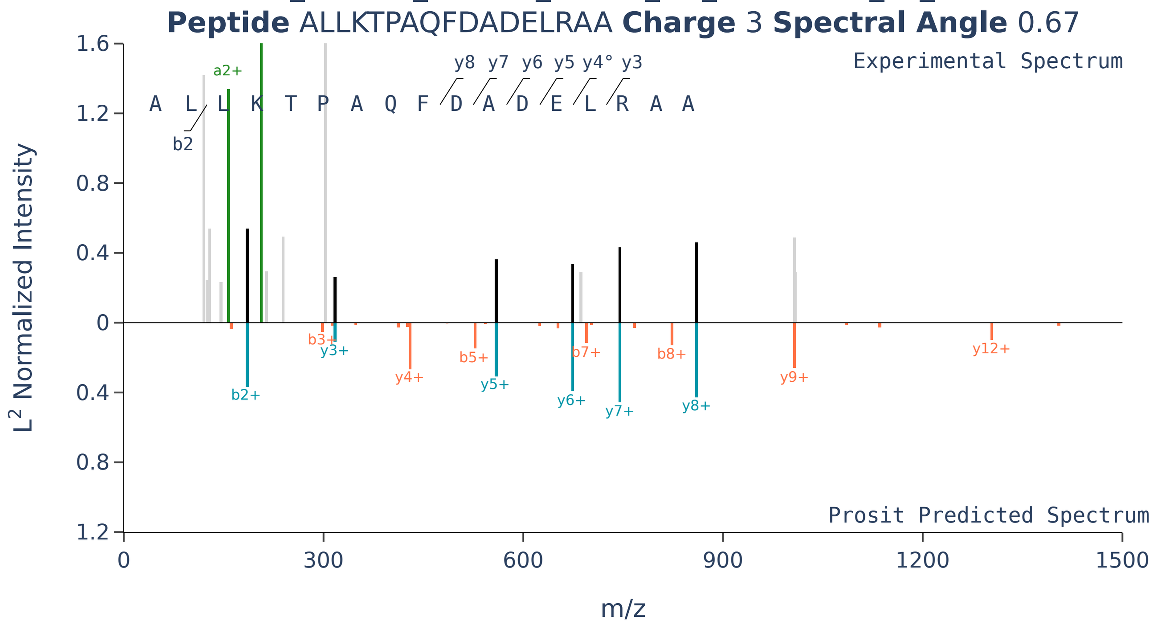


**Source** WSoh_301121_021221_HFGoe_M2_AnnexinA1_2h_R2 **Scan** 19388
**Peptide** YVQTVKSSKGGPGSAVSPYPTFNPSS **Charge** 3 **Spectral Angle** 0.86

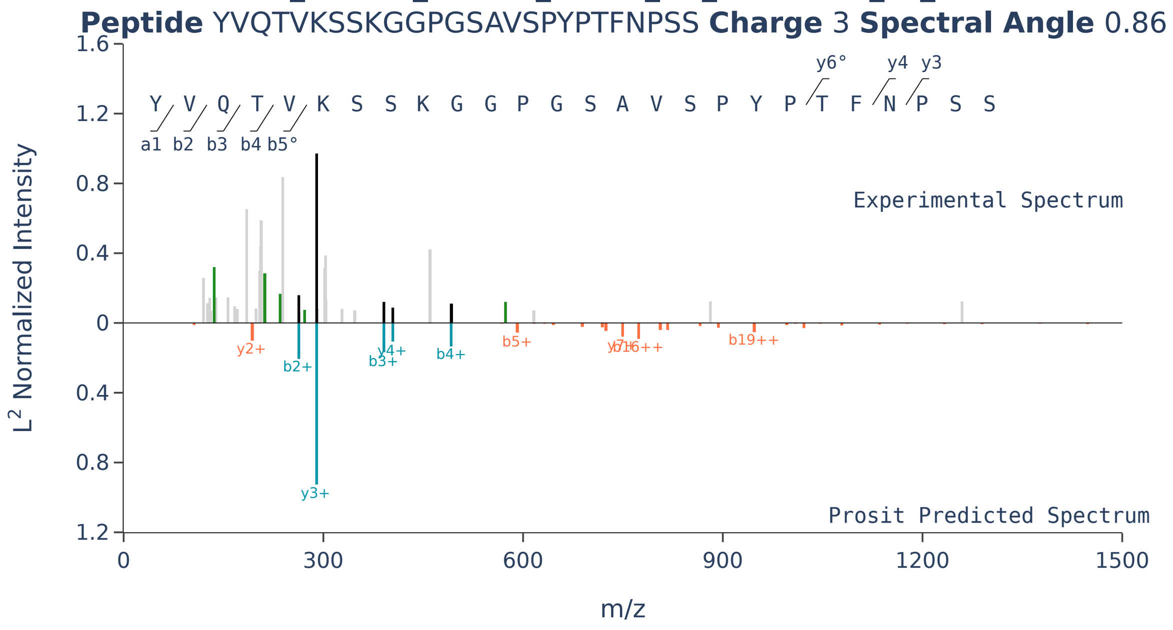


**Source** WSoh_301121_021221_HFGoe_M2_AnnexinA1_2h_R2 **Scan** 20865
**Peptide** SPYPTFNPSS **Charge** 2 **Spectral Angle** 0.56

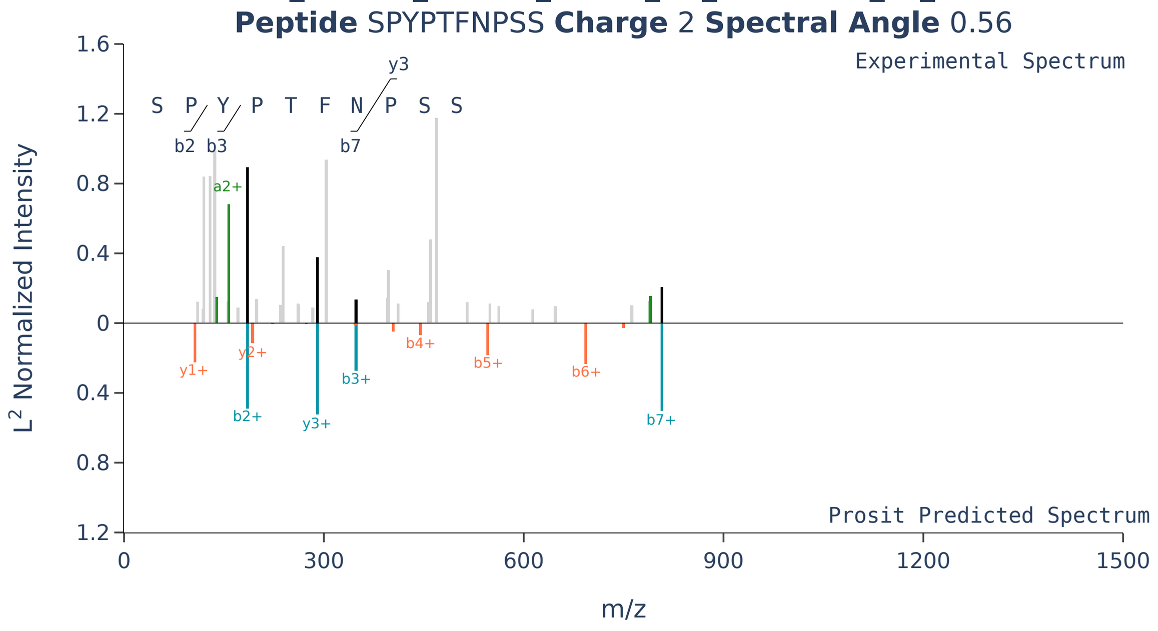


**Source** WSoh_301121_021221_HFGoe_M2_AnnexinA1_1h_R2 **Scan** 18913
**Peptide** KGLGTDEDTLLE **Charge** 2 **Spectral Angle** 0.34

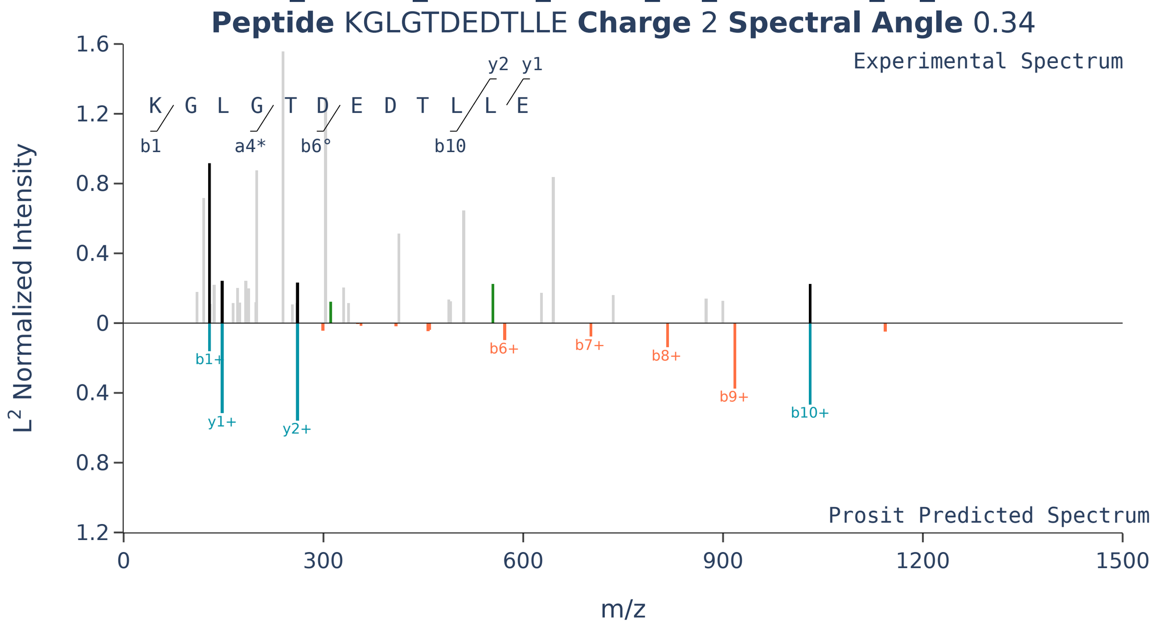


**Source** WSoh_301121_021221_HFGoe_M2_AnnexinA1_2h_R1 **Scan** 8953
**Peptide** YTKYSKHDMNKVLD **Charge** 2 **Spectral Angle** 0.49

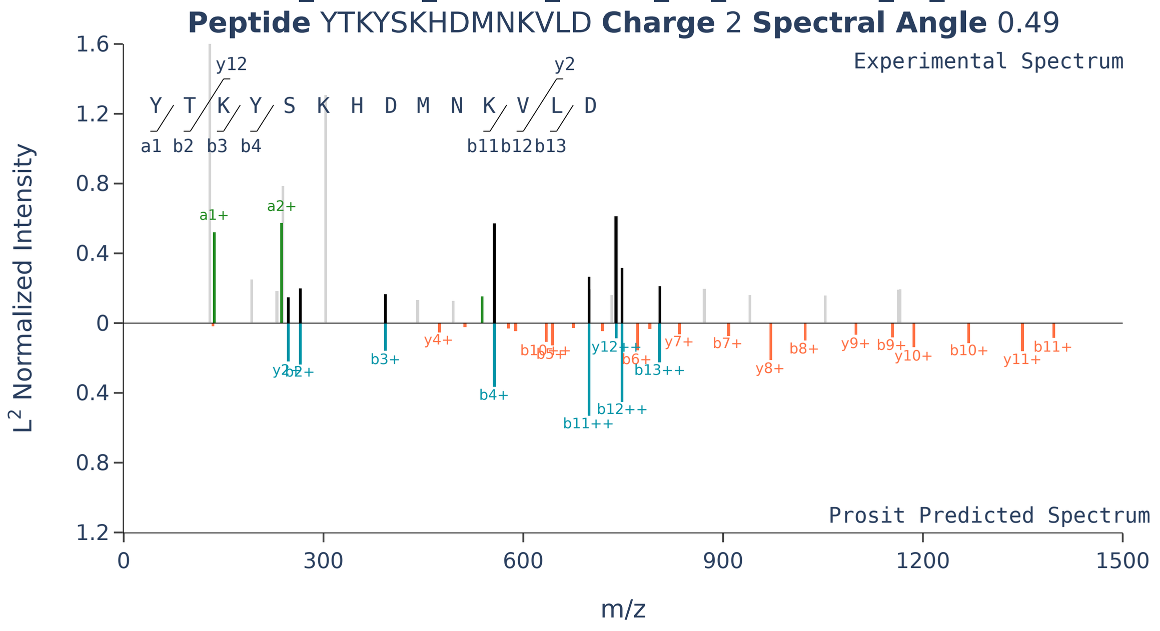


**Source** WSoh_301121_021221_HFGoe_M2_AnnexinA1_2h_R1 **Scan** 22022
**Peptide** KGGPGSAVSPYPTFNPSSDVAALHKA **Charge** 4 **Spectral Angle** 0.62

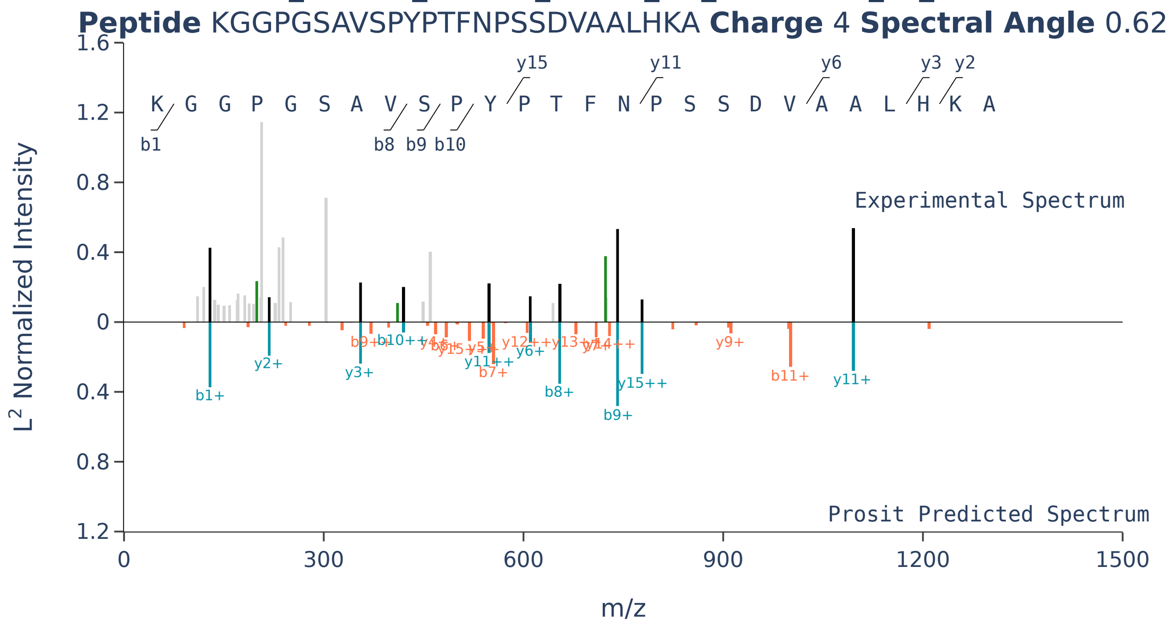


**Source** WSoh_301121_021221_HFGoe_M2_AnnexinA1_2h_R2 **Scan** 22933
**Peptide** SSKGGPGSAVSPYPTFNPSSDVA **Charge** 3 **Spectral Angle** 0.66

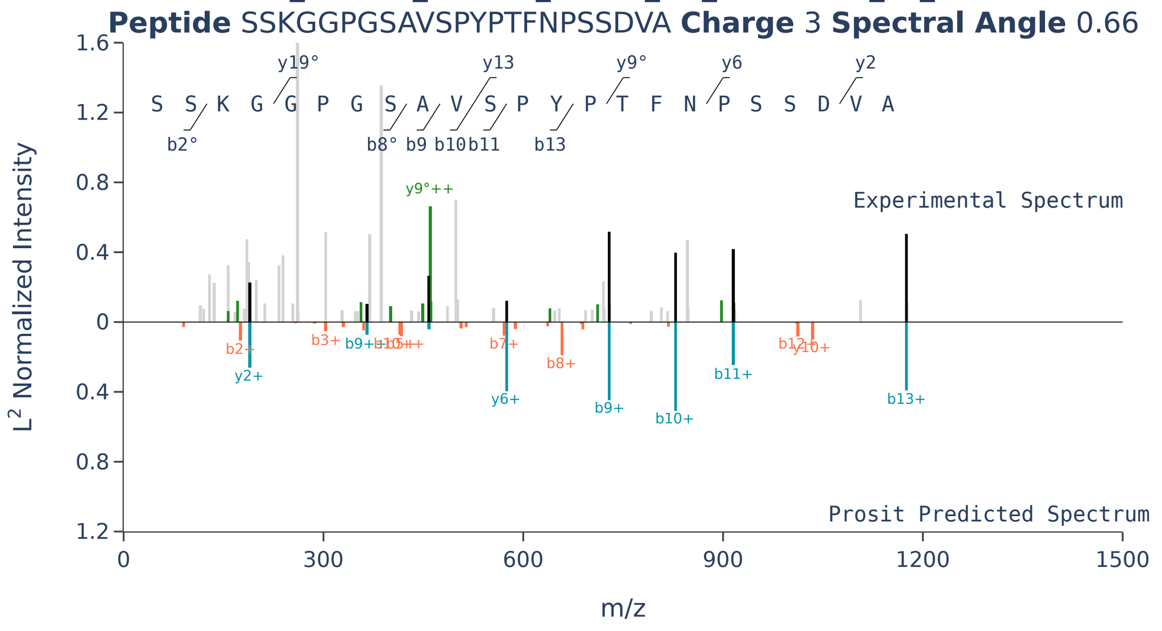


**Source** WSoh_301121_021221_HFGoe_M2_AnnexinA1_2h_R1 **Scan** 17489
**Peptide** ELRDINRVYREEL **Charge** 3 **Spectral Angle** 0.61

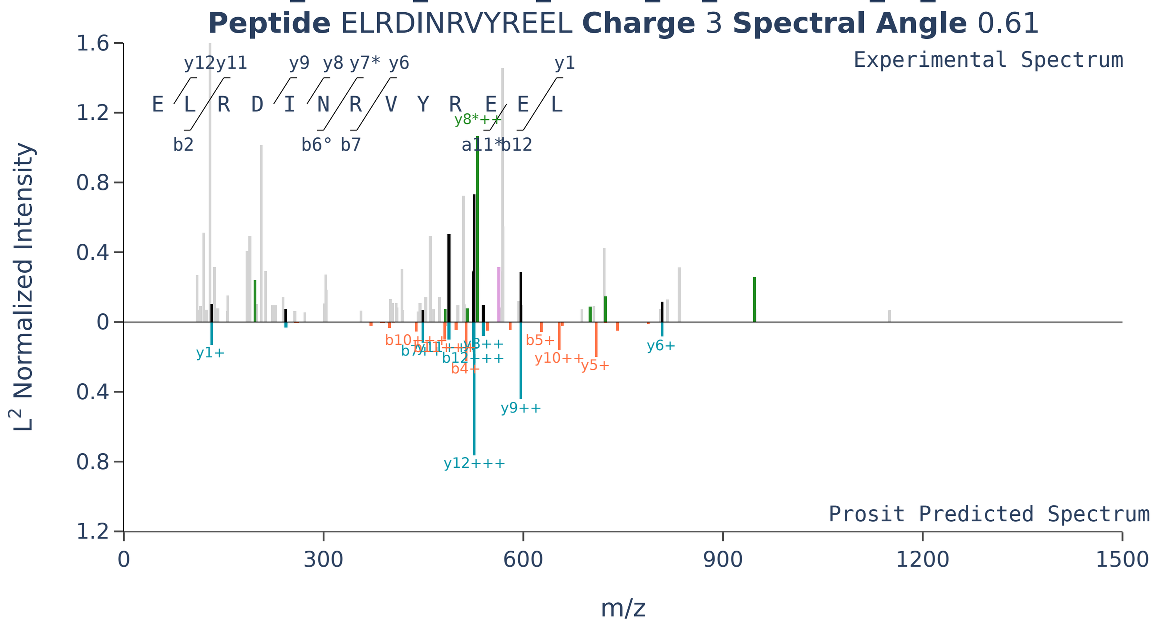


**Source** WSoh_301121_021221_HFGoe_M2_AnnexinA1_24h_R2 **Scan** 11474
**Peptide** SGDFRNA **Charge** 1 **Spectral Angle** 0.86

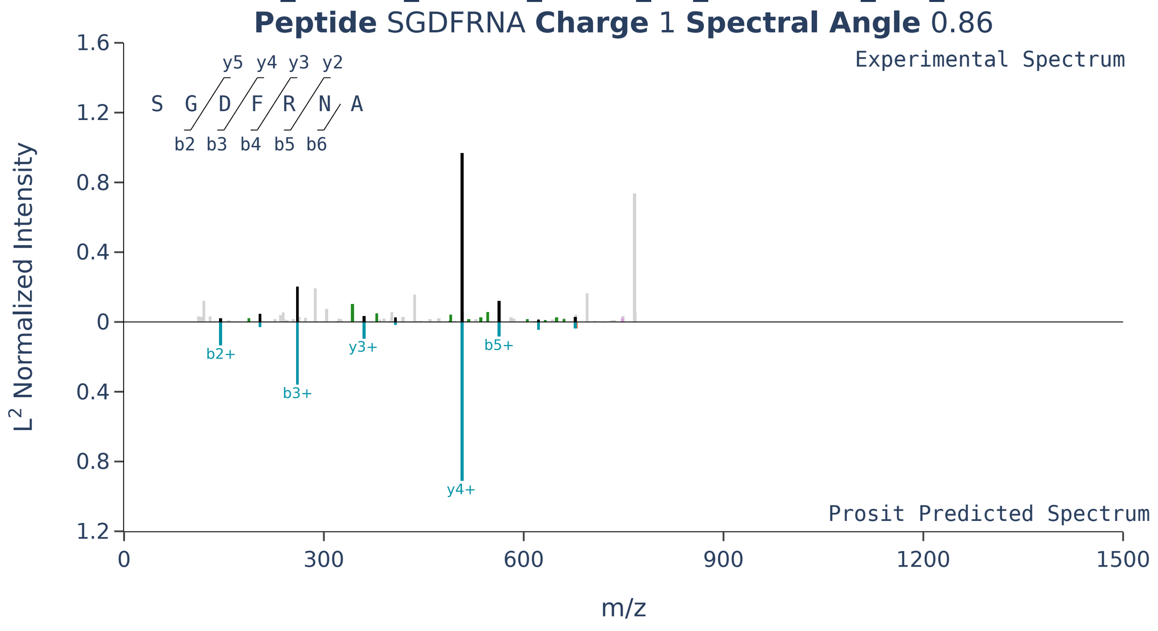


**Source** WSoh_301121_021221_HFGoe_M1_AnnexinA1_24h_R2 **Scan** 23729
**Peptide** LKQAWFIENEE **Charge** 2 **Spectral Angle** 0.63

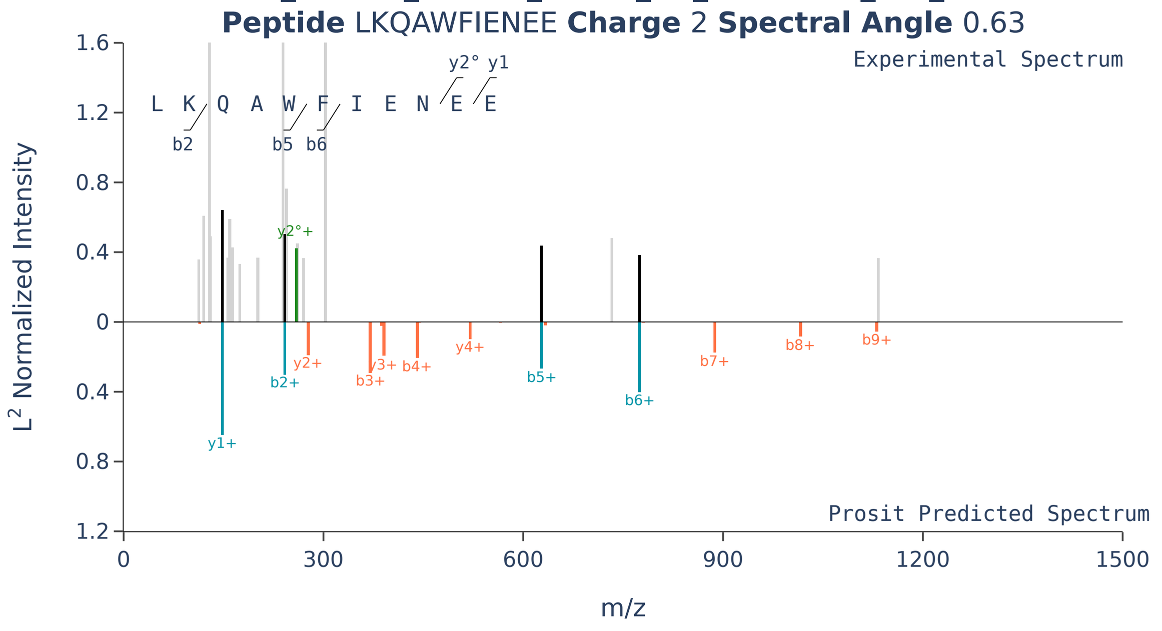


**Source** WSoh_301121_021221_HFGoe_M2_AnnexinA1_4h_R1 **Scan** 23443
**Peptide** DIEKCLTAIVK **Charge** 2 **Spectral Angle** 0.66

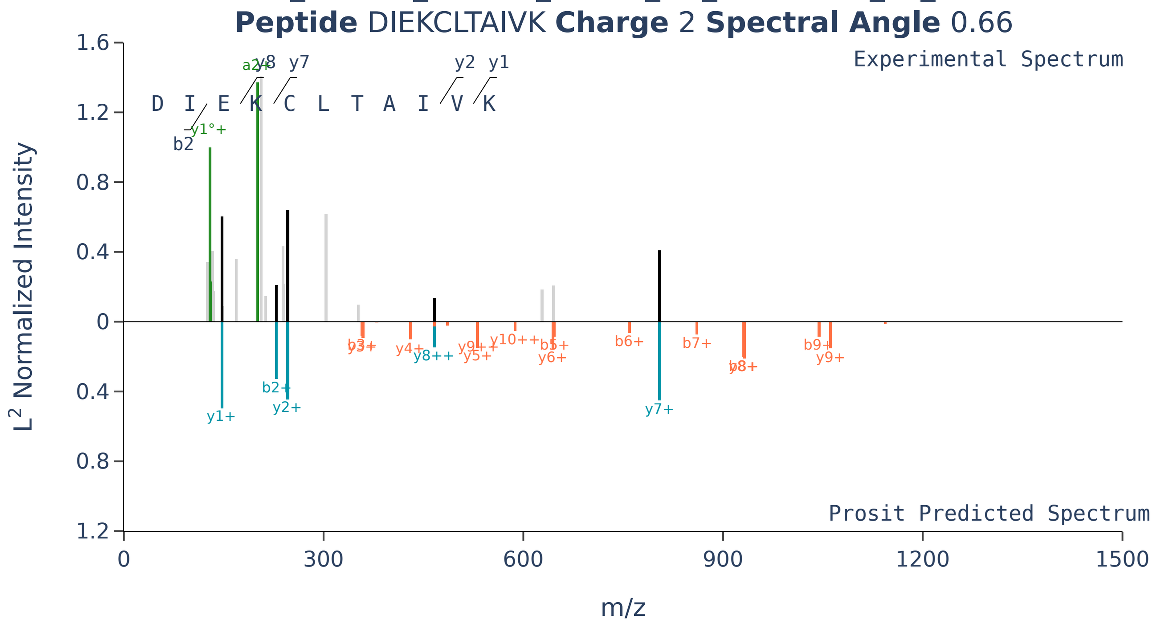

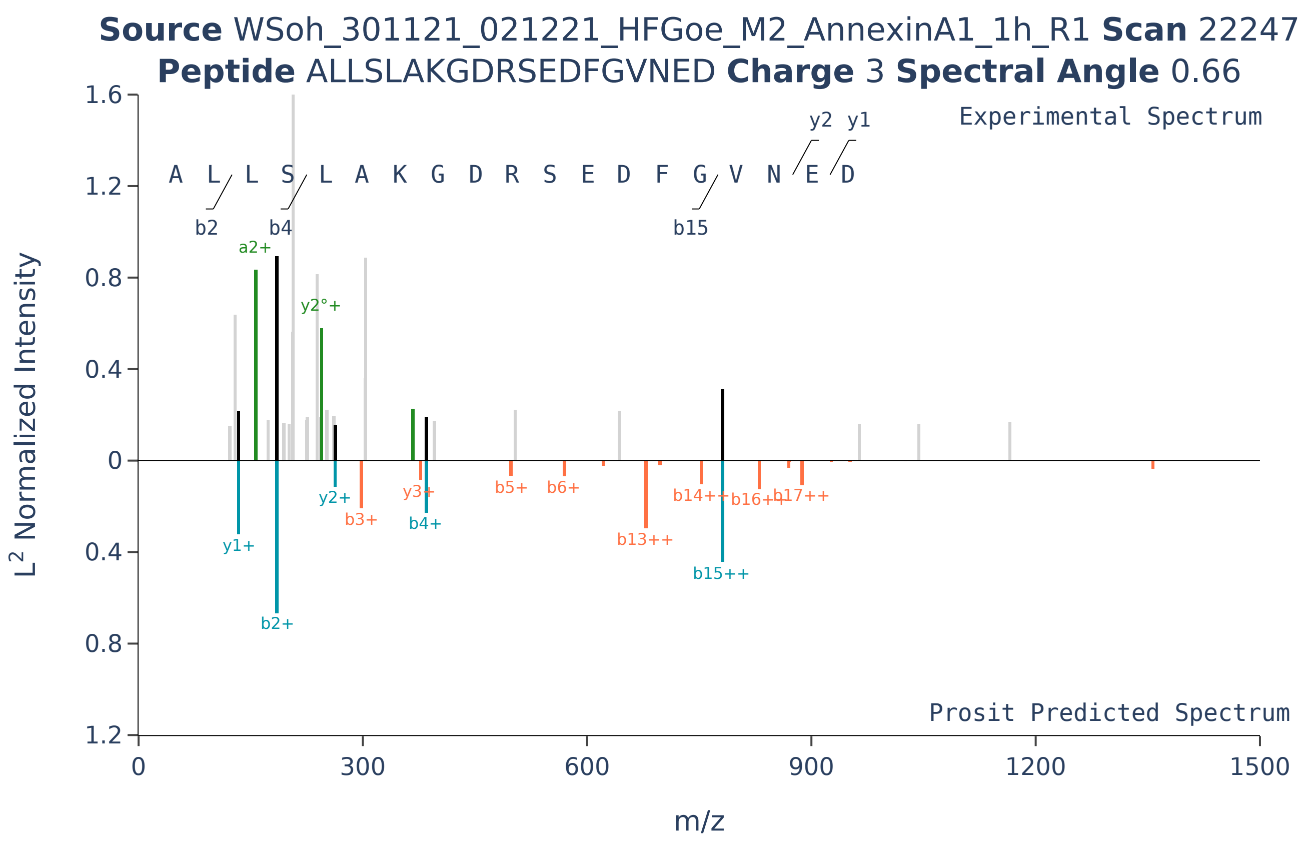
Source WSoh_301121_021221_HFGoe_M2_AnnexinA1_1h_R1 Scan 22247
Peptide ALLSLAKGDRSEDFGVNED Charge 3 Spectral Angle 0.66
Experimental Spectrum
Prosit Predicted Spectrum
L² Normalized Intensity
m/z

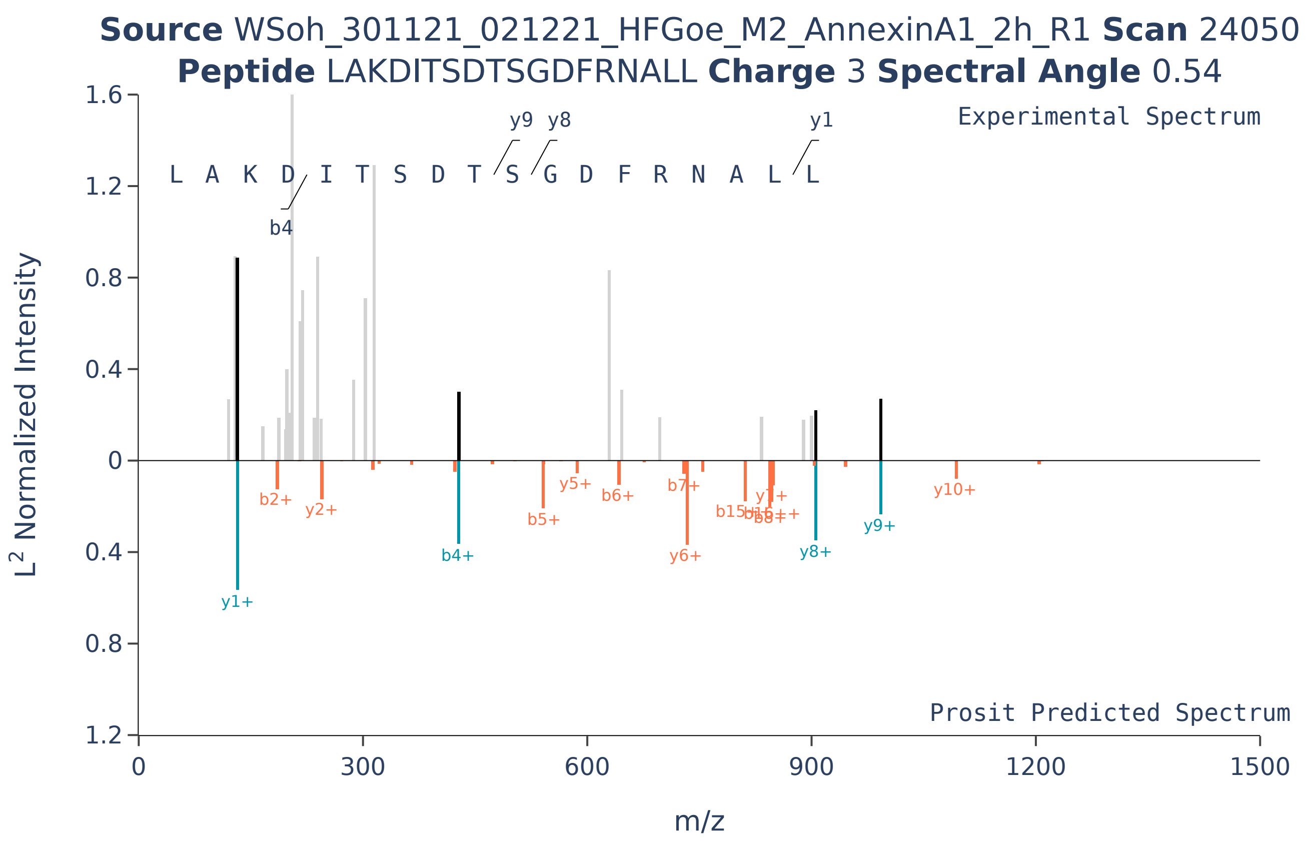
Source WSoh_301121_021221_HFGoe_M2_AnnexinA1_2h_R1 Scan 24050
Peptide LAKDITSDTSGDFRNALL Charge 3 Spectral Angle 0.54
Experimental Spectrum
Prosit Predicted Spectrum
L² Normalized Intensity
m/z

**Source** W_Soh_160821_170921_Goe_aSyn_A1_24h_R2 **Scan** 9732
**Peptide** PDNEAYEMPSE **Charge** 2 **Spectral Angle** 0.94

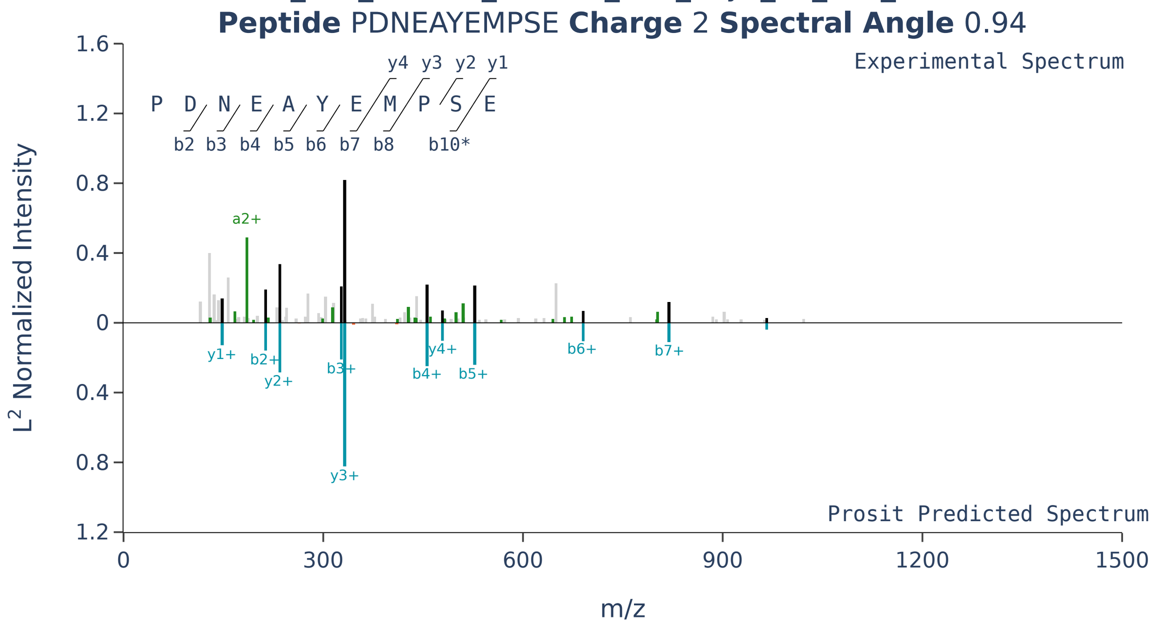


**Source** W_Soh_160821_170921_Goe_aSyn_A1_24h_R2 **Scan** 13678
**Peptide** EAYEMPSE **Charge** 2 **Spectral Angle** 0.94

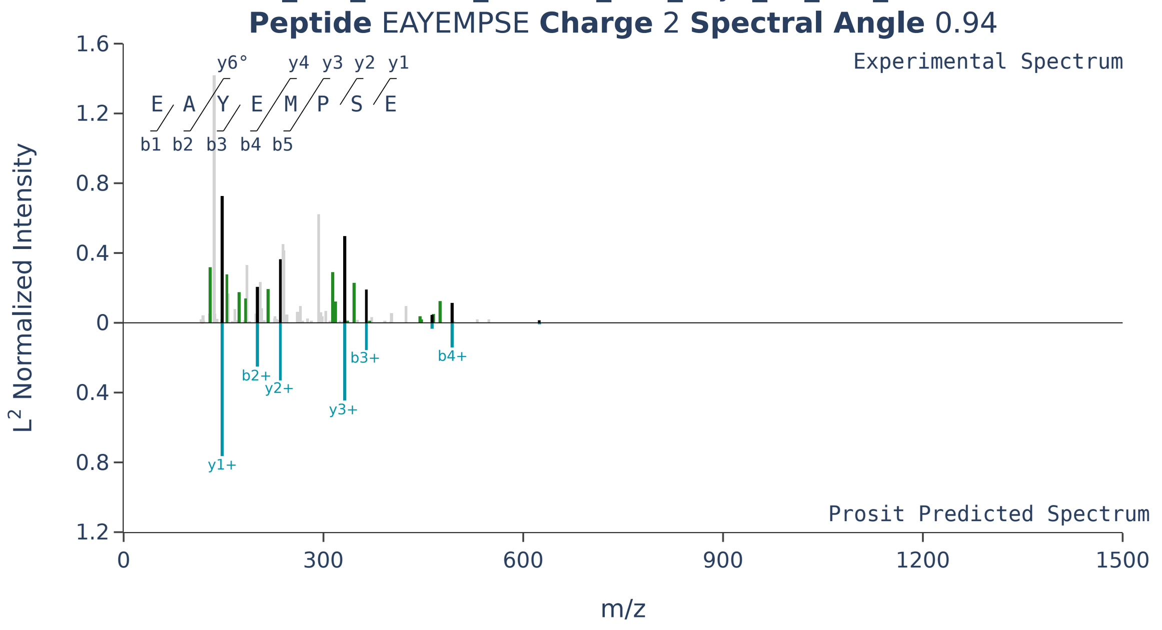


**Source** W_Soh_160821_170921_Goe_aSyn_A1_24h_R1 **Scan** 9482
**Peptide** QDYEPEA **Charge** 1 **Spectral Angle** 0.95

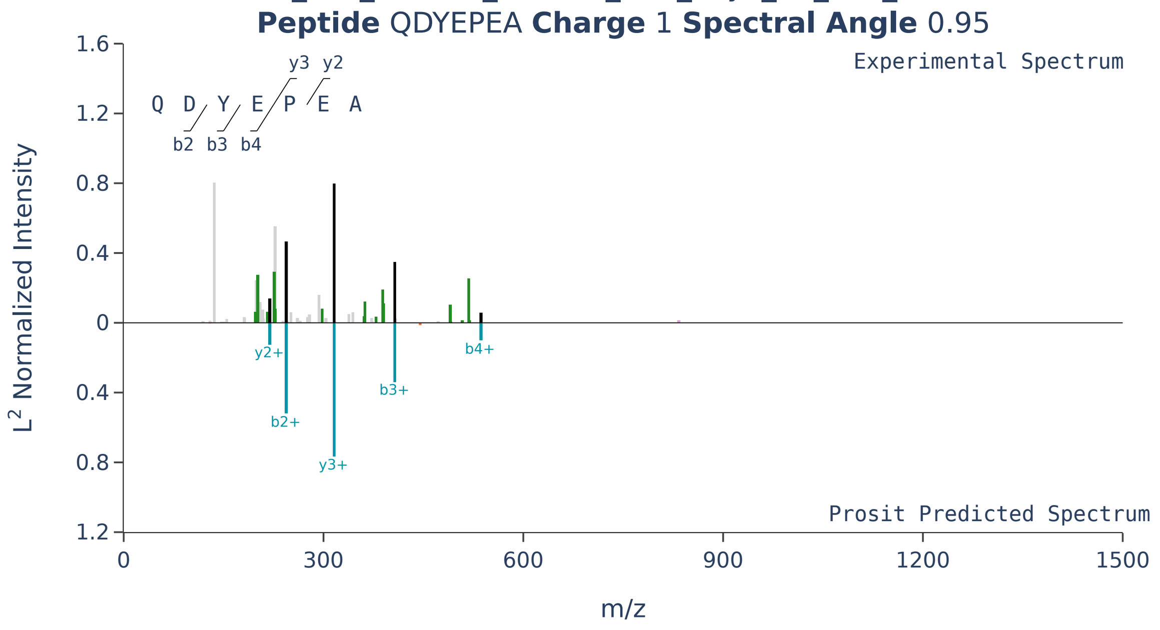


**Source** W_Soh_160821_170921_Goe_aSyn_A1_6h_R2 **Scan** 17633
**Peptide** PDNEAYEMPSEEGYQDYEPEA **Charge** 2 **Spectral Angle** 0.96

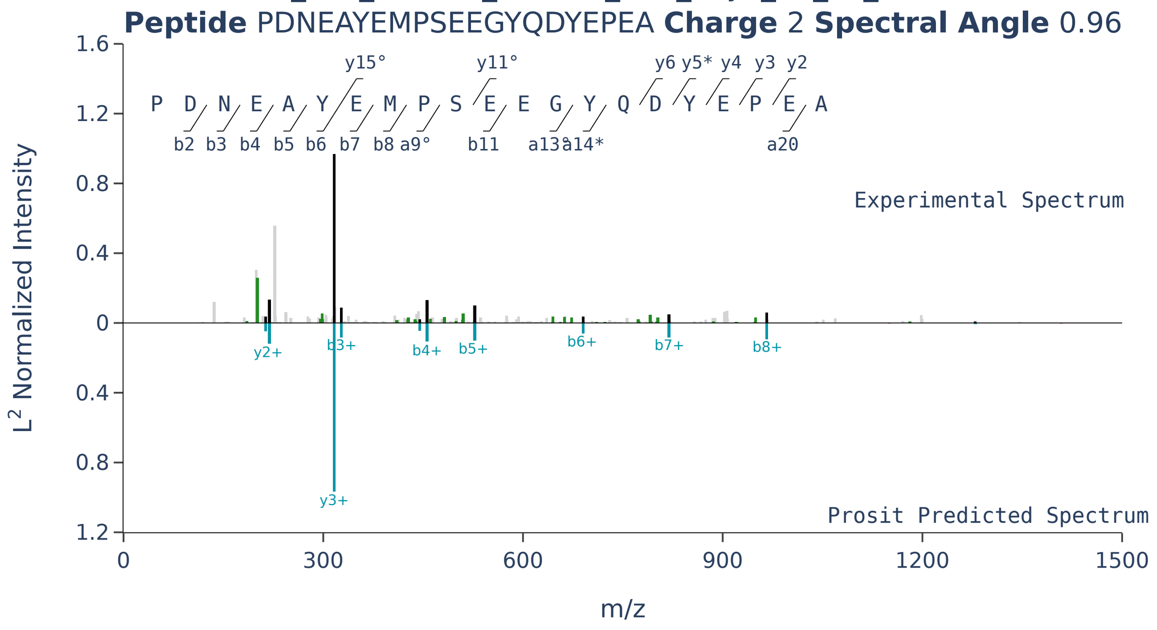


**Source** W_Soh_160821_170921_Goe_aSyn_A2_24h_R2 **Scan** 5424
**Peptide** GSKTKEGVVH **Charge** 2 **Spectral Angle** 0.94

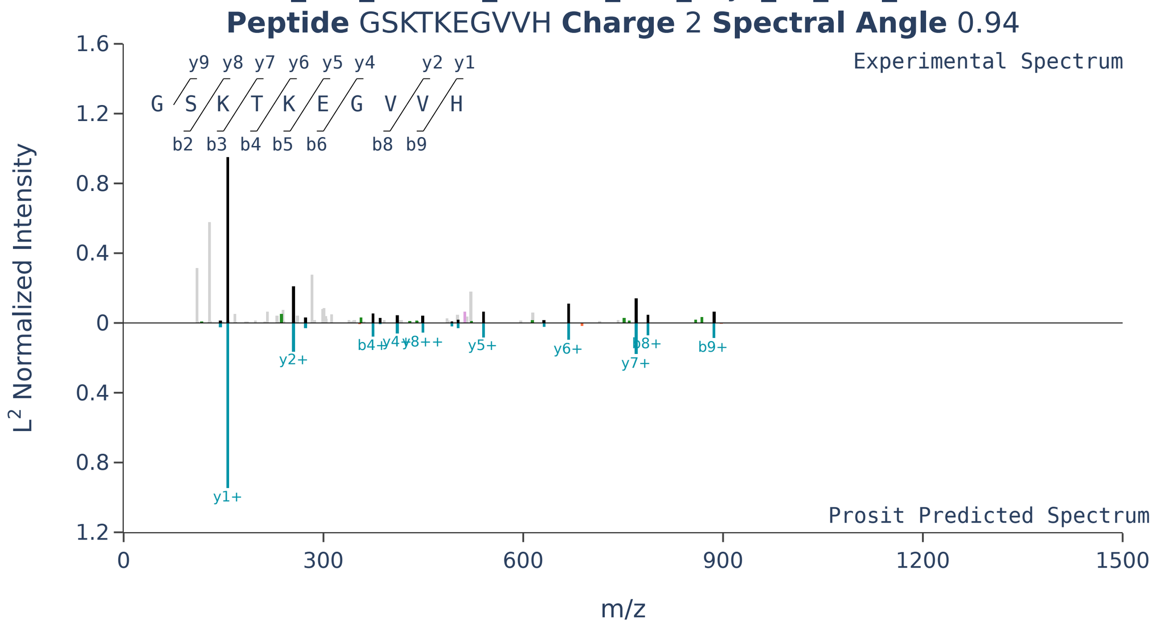


**Source** W_Soh_160821_170921_Goe_aSyn_A2_24h_R1 **Scan** 13075
**Peptide** GYQDYEPEA **Charge** 2 **Spectral Angle** 0.93

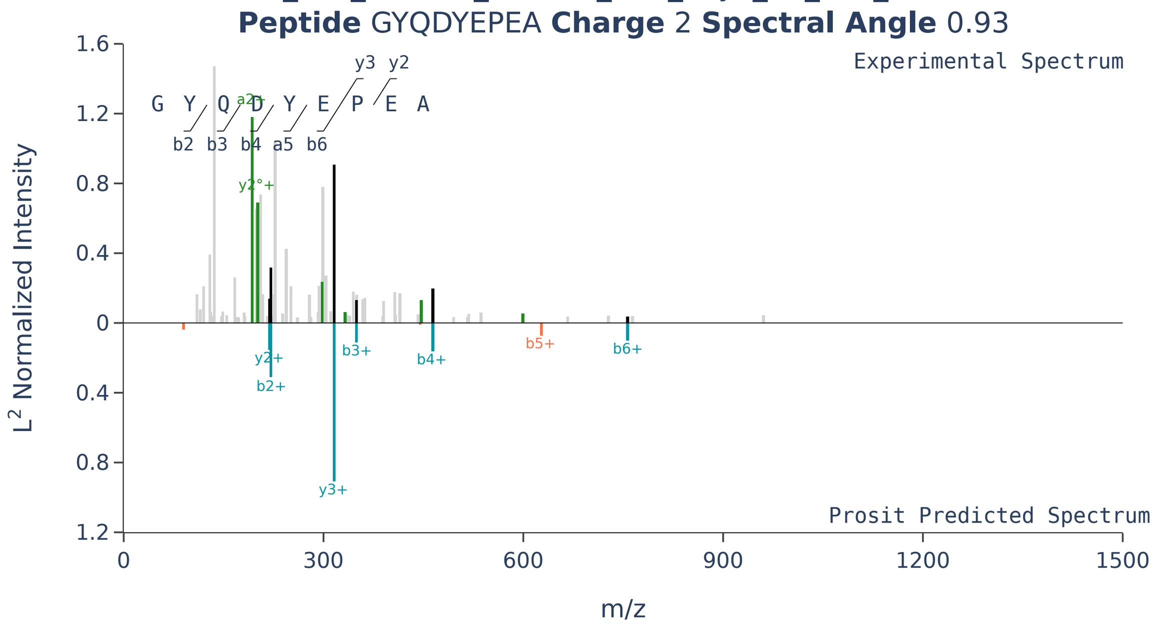


**Source** W_Soh_160821_170921_Goe_aSyn_A2_3h_R2 **Scan** 16766
**Peptide** PDNEAYEMPSEEGY **Charge** 2 **Spectral Angle** 0.9

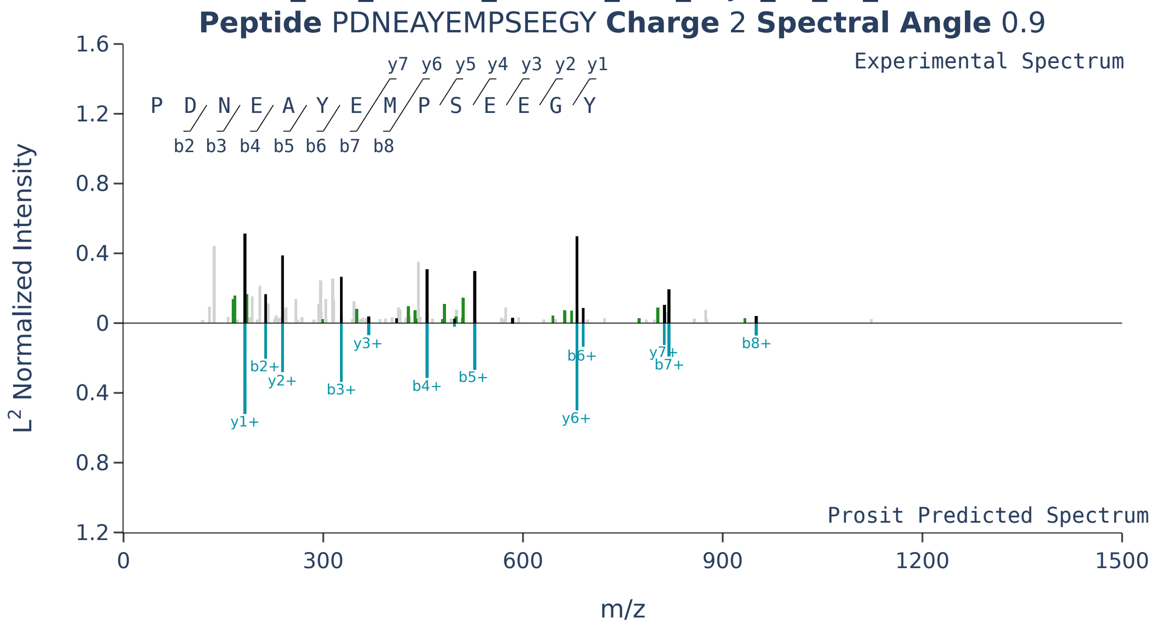


**Source** W_Soh_160821_170921_Goe_aSyn_A1_24h_R1 **Scan** 6579
**Peptide** AAATGFVKK **Charge** 2 **Spectral Angle** 0.9

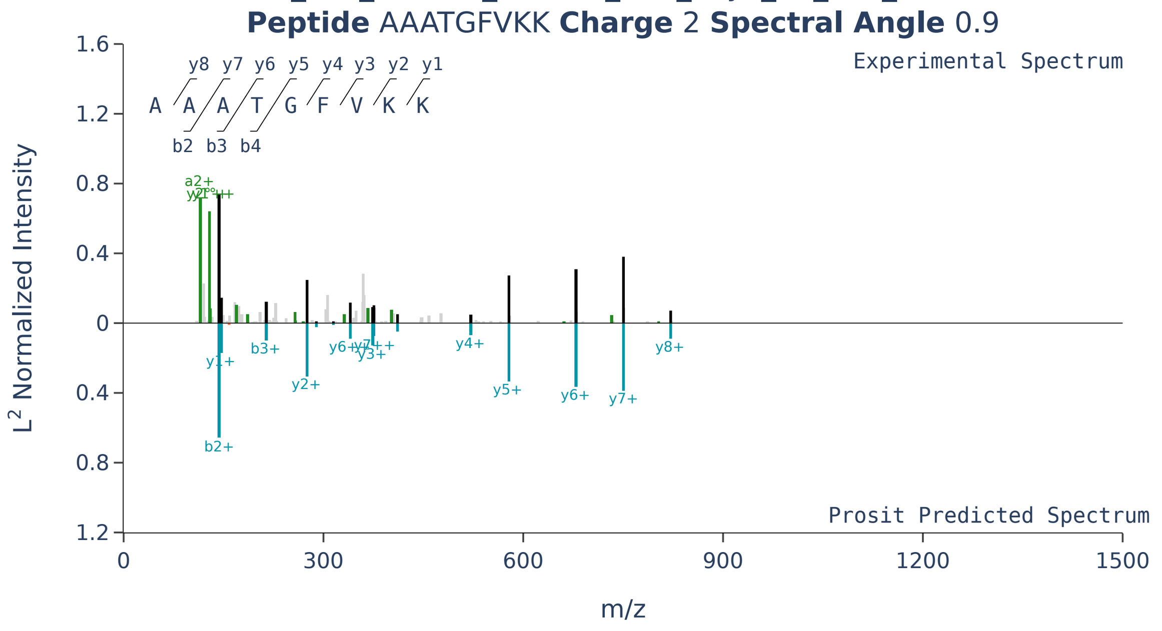


**Source** W_Soh_160821_170921_Goe_aSyn_A2_24h_R2 **Scan** 8001
**Peptide** YVGSKTKE **Charge** 2 **Spectral Angle** 0.96

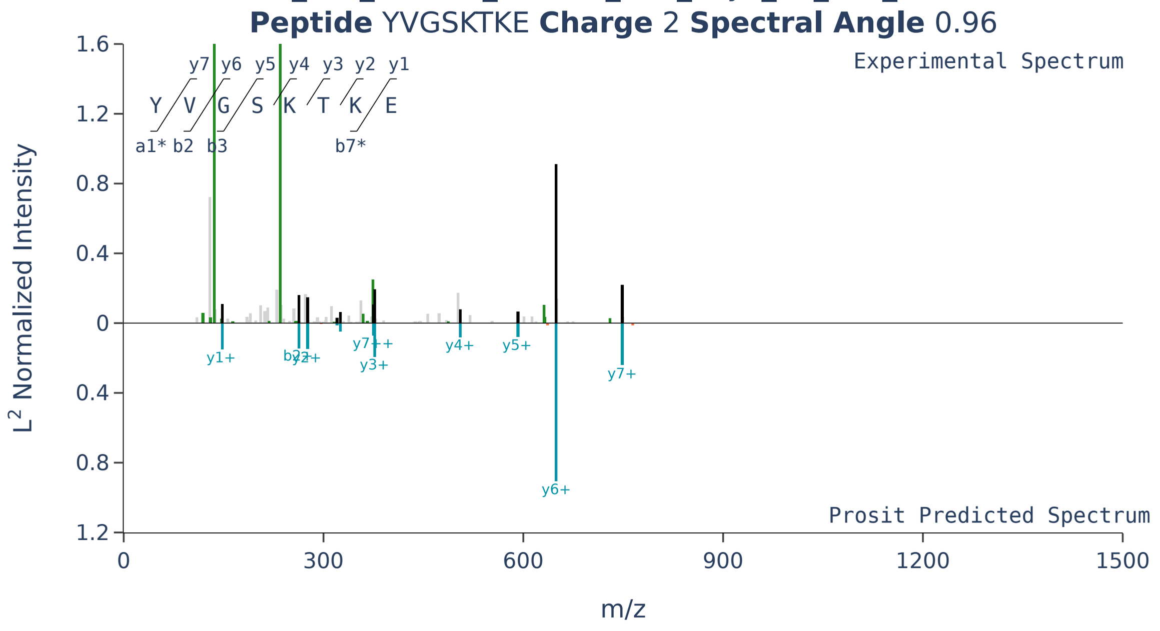


**Source** W_Soh_160821_170921_Goe_aSyn_A1_1h_R1 **Scan** 14026
**Peptide** MPSEEGYQDYEPEA **Charge** 2 **Spectral Angle** 0.95

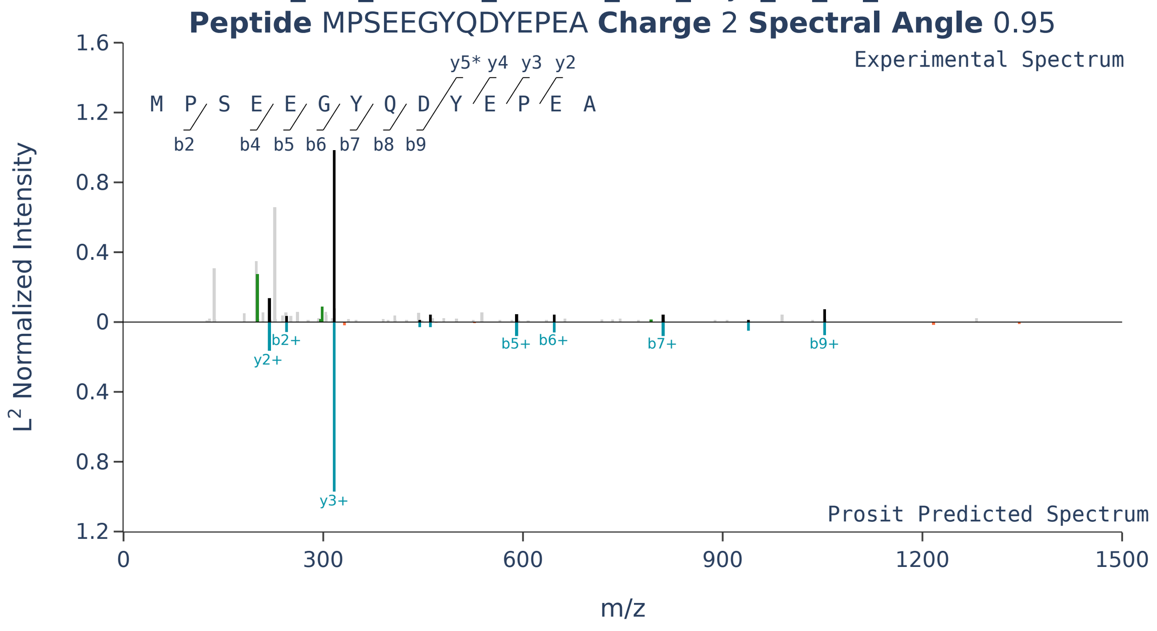


**Source** W_Soh_160821_170921_Goe_aSyn_A1_24h_R2 **Scan** 15706
**Peptide** MPVDPDNEAYE **Charge** 2 **Spectral Angle** 0.87

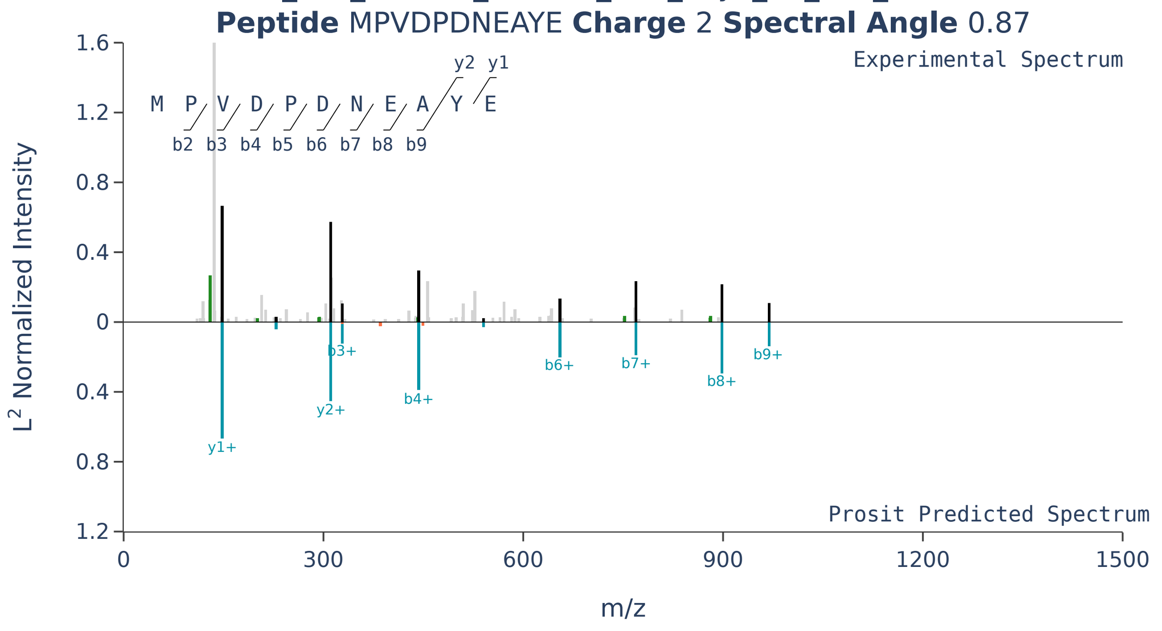


**Source** W_Soh_160821_170921_Goe_aSyn_A1_24h_R2 **Scan** 14674
**Peptide** PSEEGYQDYEPEA **Charge** 2 **Spectral Angle** 0.93

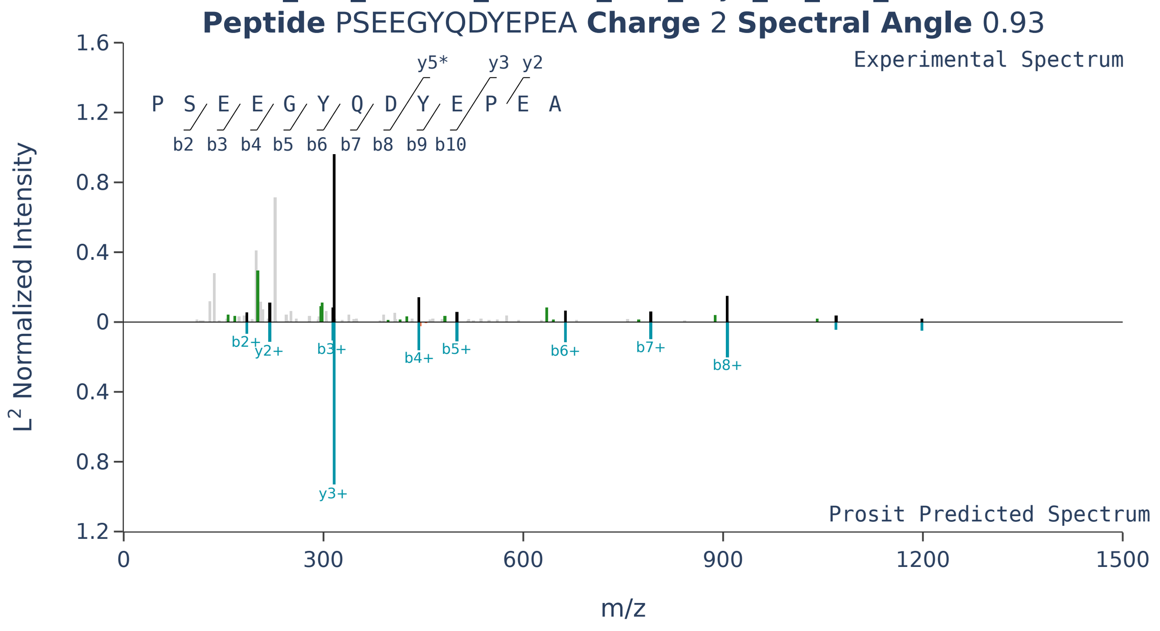


**Source** W_Soh_160821_170921_Goe_aSyn_A2_24h_R1 **Scan** 18054
**Peptide** GILEDMPVD **Charge** 1 **Spectral Angle** 0.88

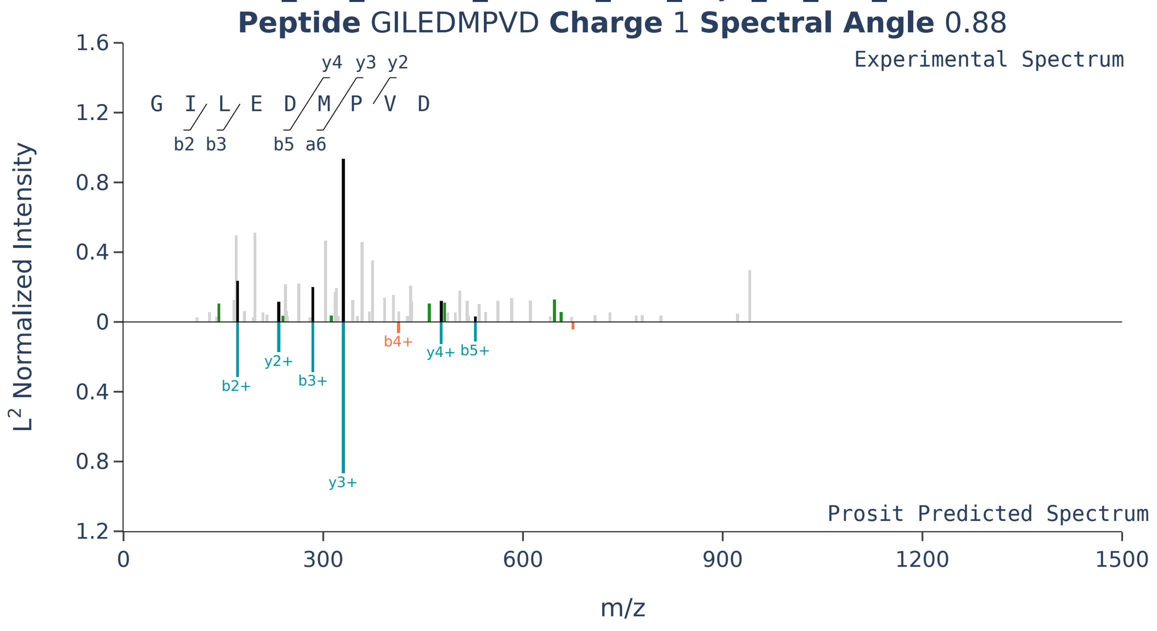


**Source** W_Soh_160821_170921_Goe_aSyn_A1_24h_R2 **Scan** 16363
**Peptide** LEDMPVD **Charge** 1 **Spectral Angle** 0.96

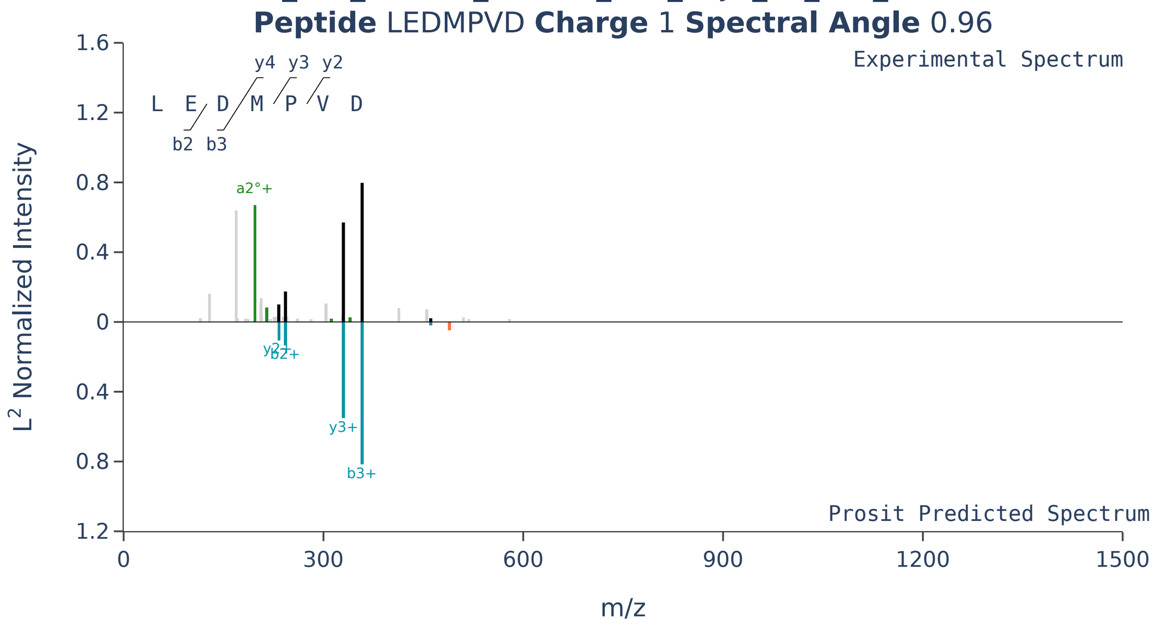


**Source** W_Soh_160821_170921_Goe_aSyn_A1_24h_R1 **Scan** 11069
**Peptide** VEGAGSIAA **Charge** 1 **Spectral Angle** 0.94

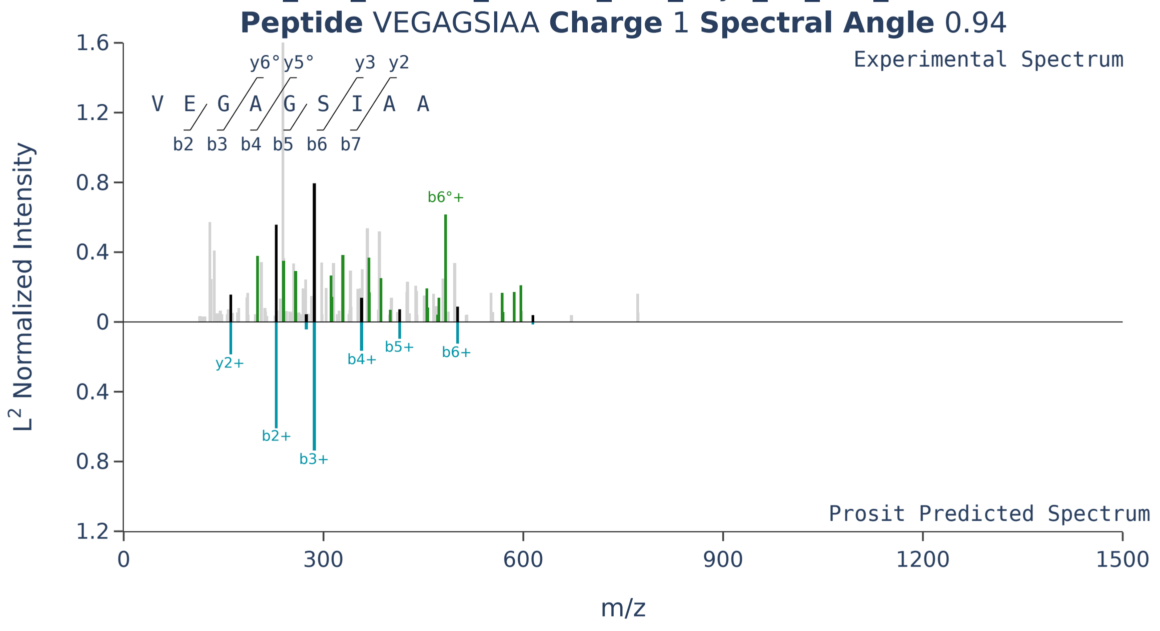

**Source** W_Soh_160821_170921_Goe_aSyn_A2_24h_R1 **Scan** 9074
**Peptide** LYVGSKTKEGVVH **Charge** 3 **Spectral Angle** 0.94

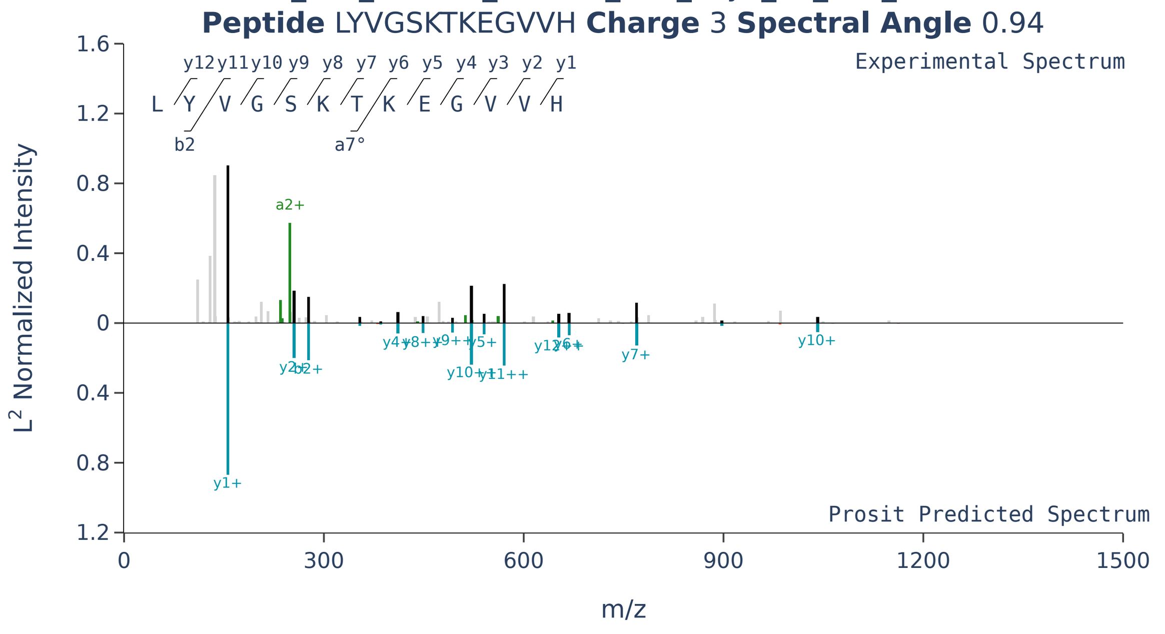


**Source** W_Soh_160821_170921_Goe_aSyn_A1_24h_R1 **Scan** 14385
**Peptide** GSIAAATGFVKKD **Charge** 2 **Spectral Angle** 0.87

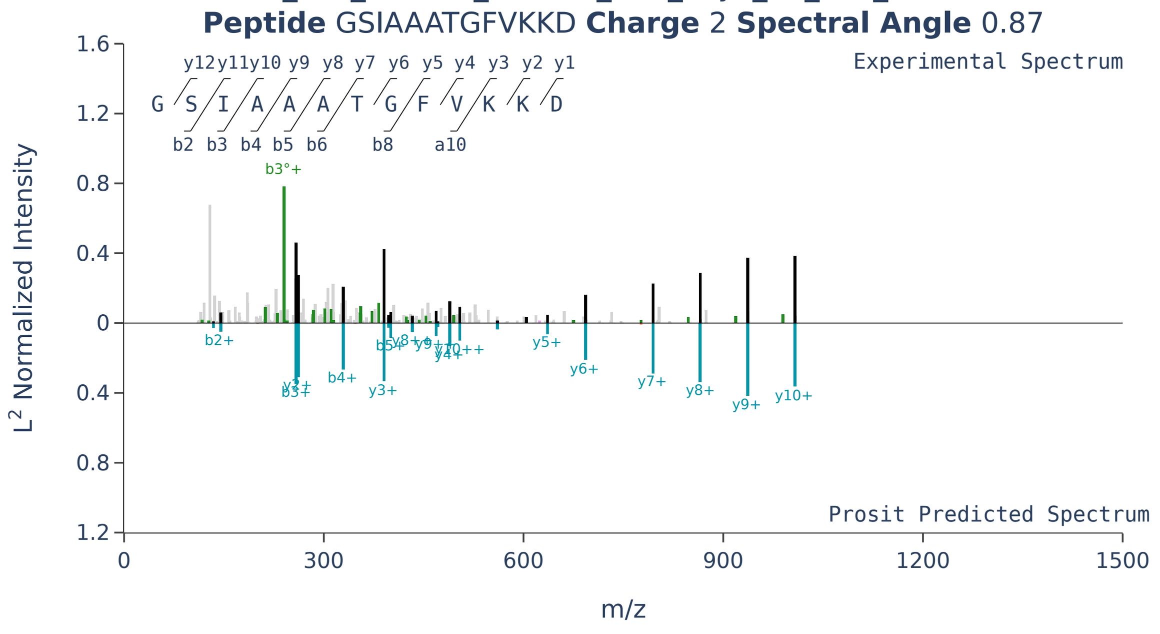


**Source** W_Soh_160821_170921_Goe_aSyn_A1_24h_R1 **Scan** 9436
**Peptide** VGGAVVTG **Charge** 1 **Spectral Angle** 0.9

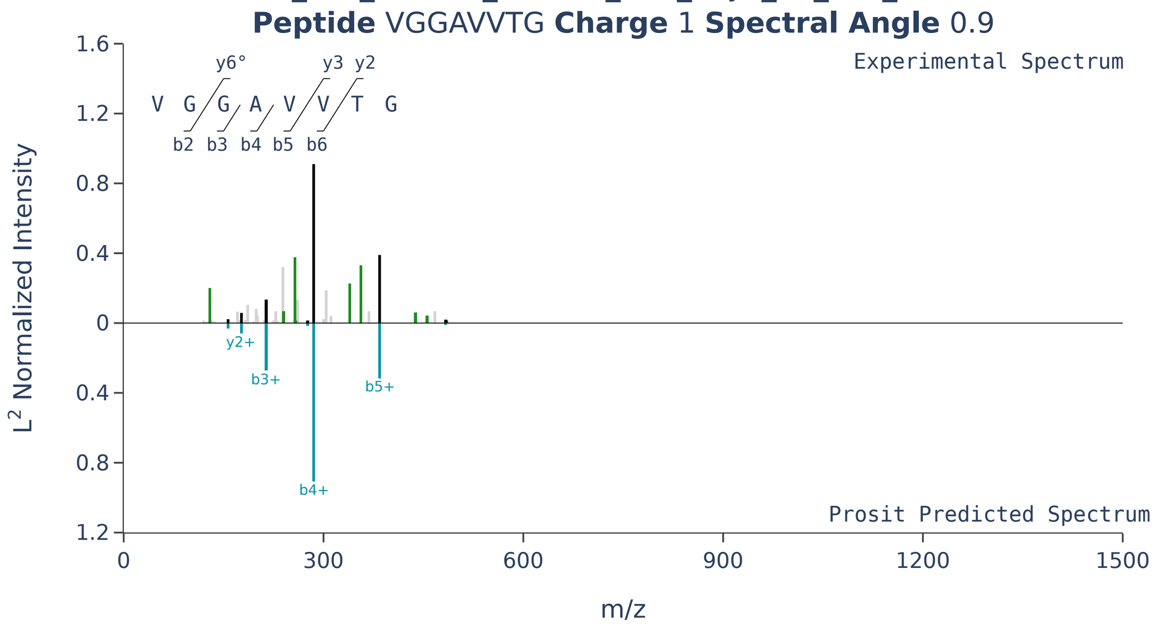


**Source** W_Soh_160821_170921_Goe_aSyn_A1_24h_R2 **Scan** 6794
**Peptide** GVTAVAQKT **Charge** 2 **Spectral Angle** 0.87

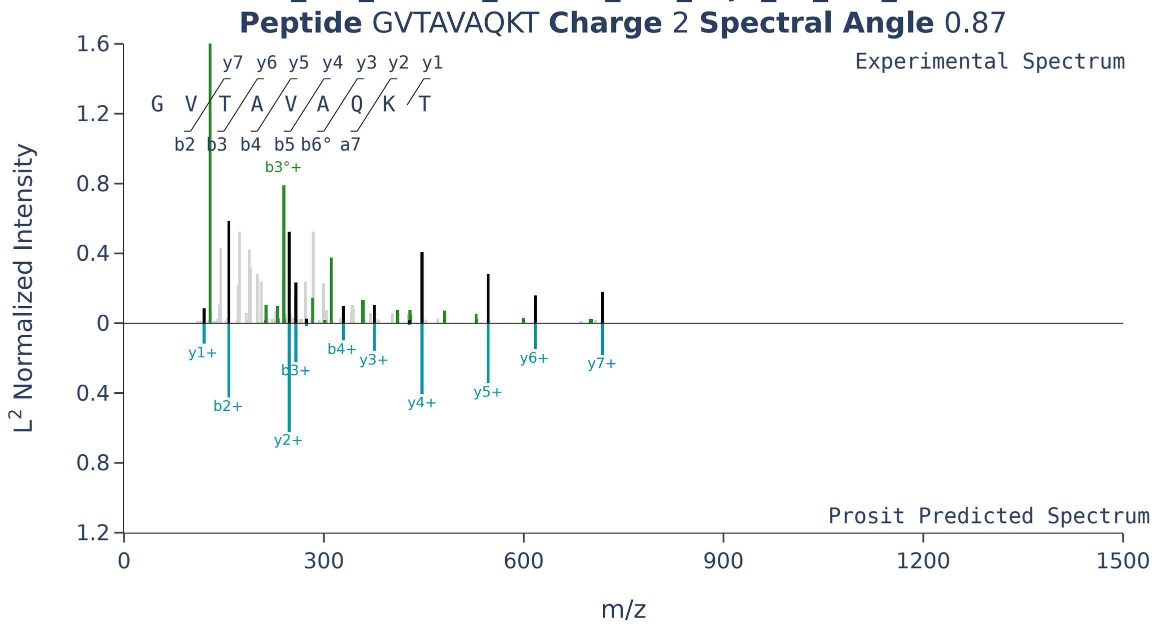


**Source** W_Soh_160821_170921_Goe_aSyn_A2_24h_R1 **Scan** 14448
**Peptide** PDNEAYEMPSEE **Charge** 1 **Spectral Angle** 0.93

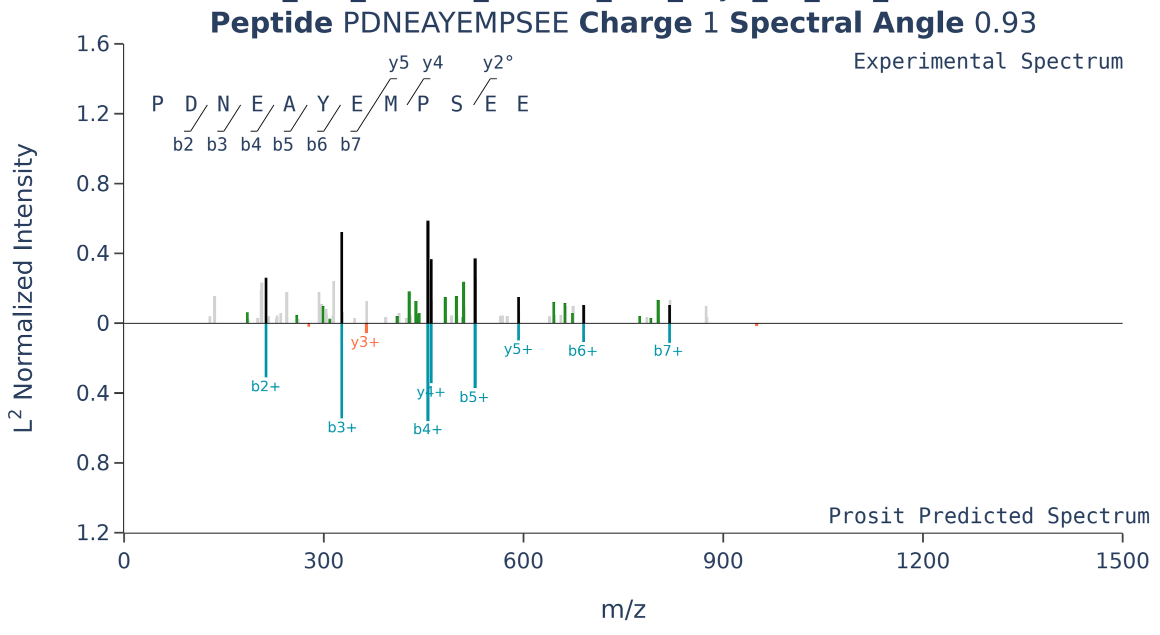


**Source** W_Soh_160821_170921_Goe_aSyn_A2_24h_R2 **Scan** 16152
**Peptide** MDVFMKG **Charge** 2 **Spectral Angle** 0.96

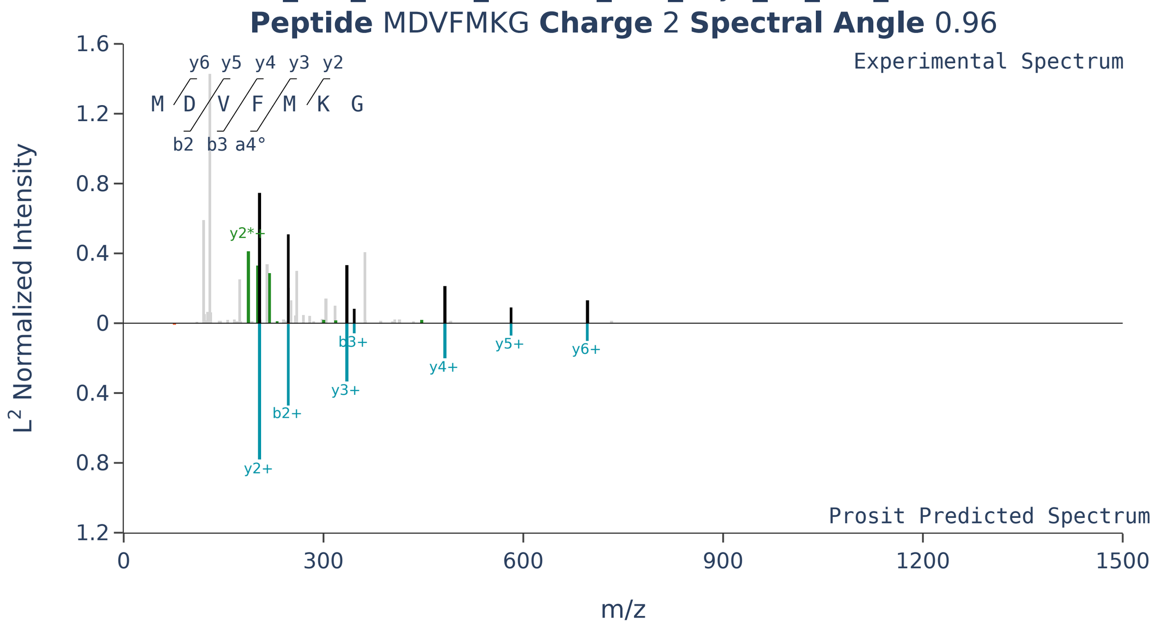


**Source** W_Soh_160821_170921_Goe_aSyn_A2_24h_R2 **Scan** 14173
**Peptide** PDNEAYEMPSEEG **Charge** 2 **Spectral Angle** 0.89

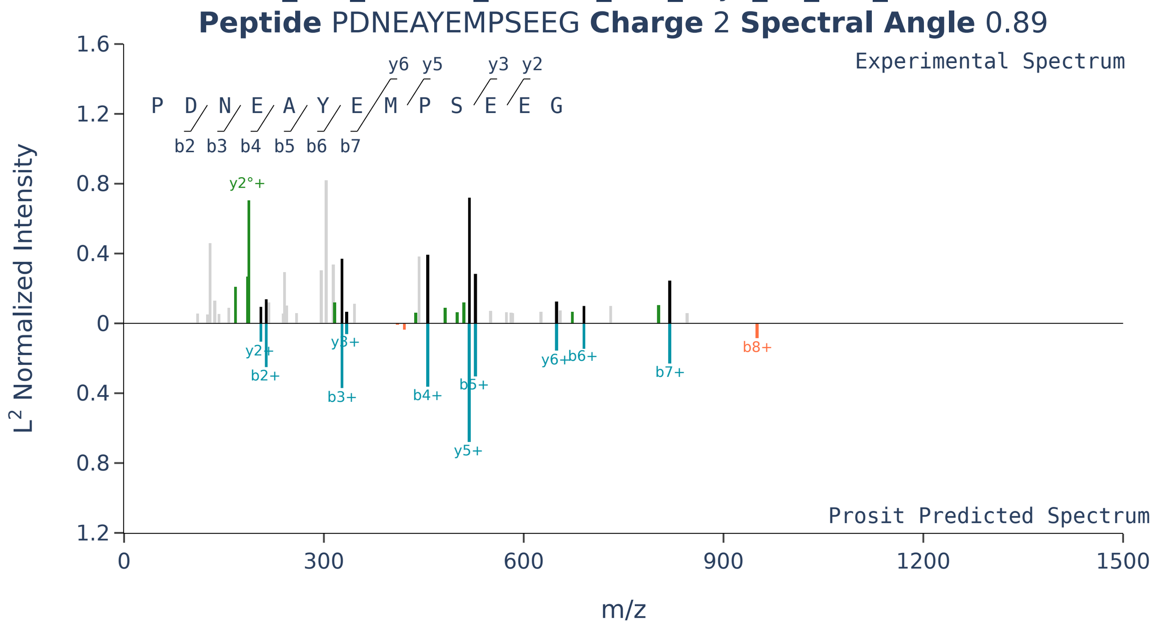


**Source** W_Soh_160821_170921_Goe_aSyn_A2_24h_R2 **Scan** 10354
**Peptide** TGFVKKDQL **Charge** 2 **Spectral Angle** 0.87

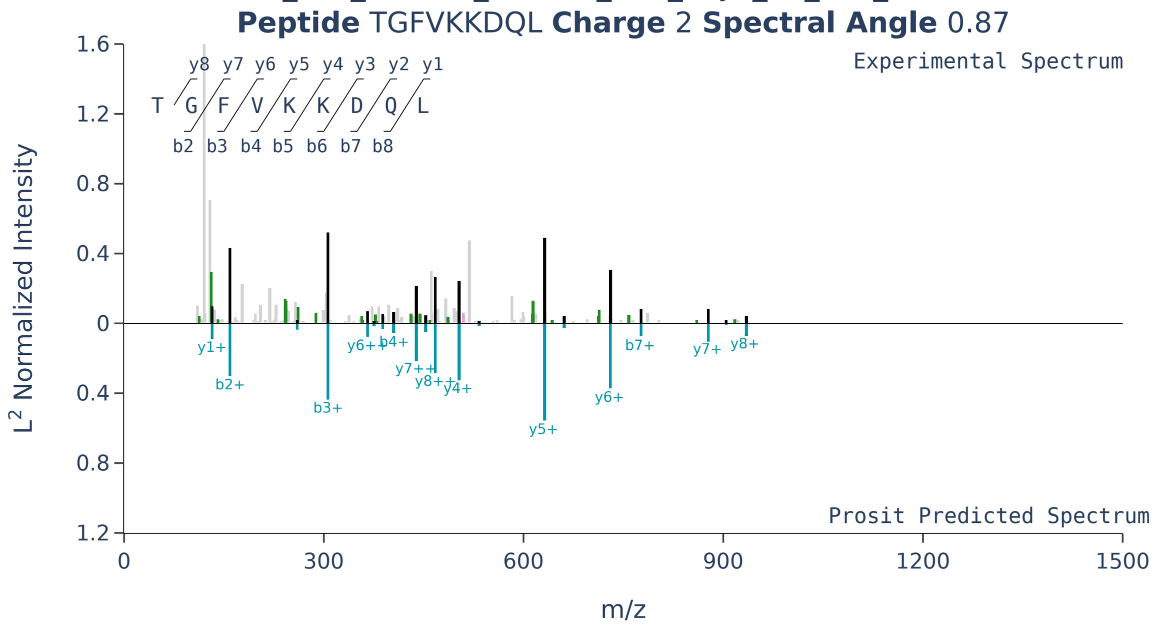


**Source** W_Soh_160821_170921_Goe_aSyn_A1_6h_R1 **Scan** 6568
**Peptide** KEGVVHGVAT **Charge** 2 **Spectral Angle** 0.89

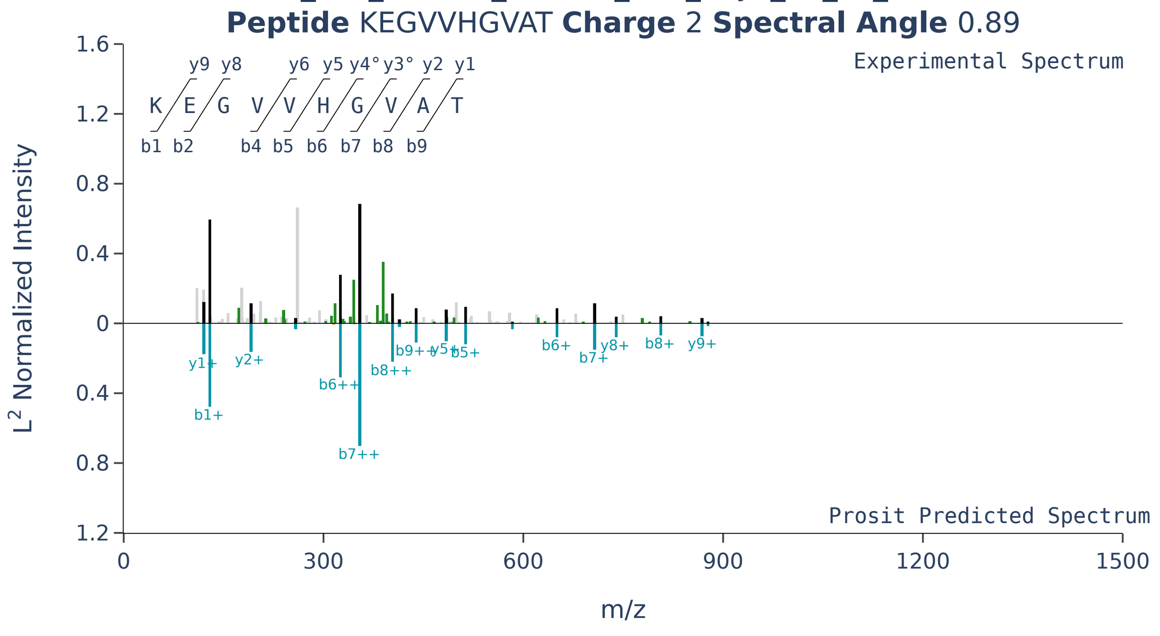


**Source** W_Soh_160821_170921_Goe_aSyn_A1_24h_R1 **Scan** 11657
**Peptide** TNVGGAVVT **Charge** 1 **Spectral Angle** 0.86

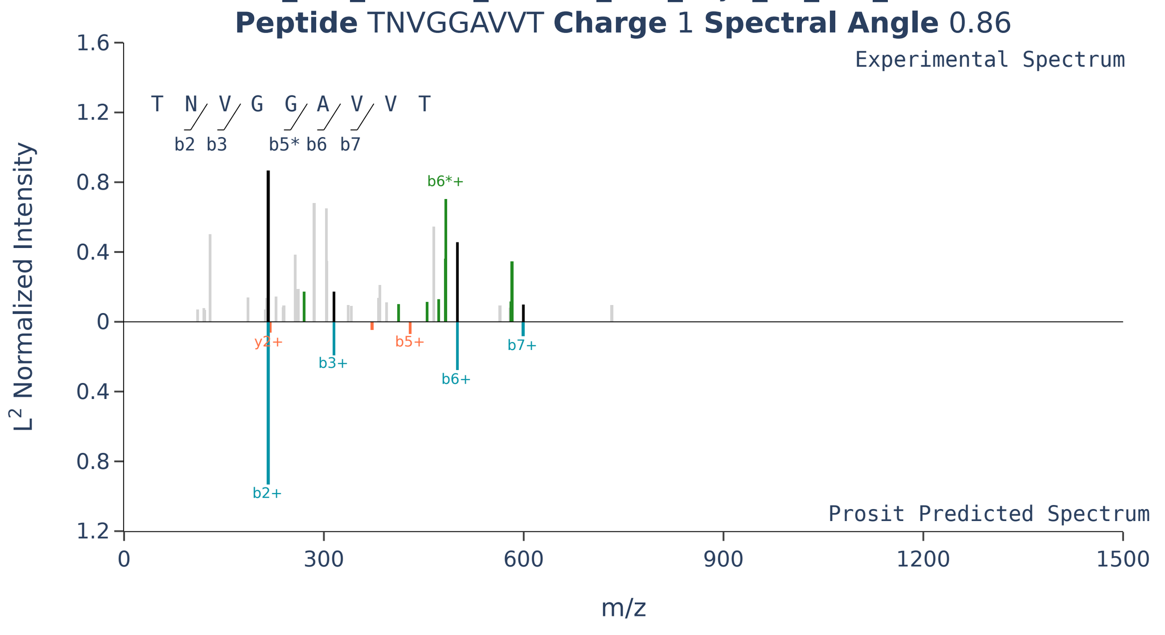


**Source** W_Soh_160821_170921_Goe_aSyn_A1_6h_R1 **Scan** 6503
**Peptide** AAATGFVKKDQ **Charge** 2 **Spectral Angle** 0.9

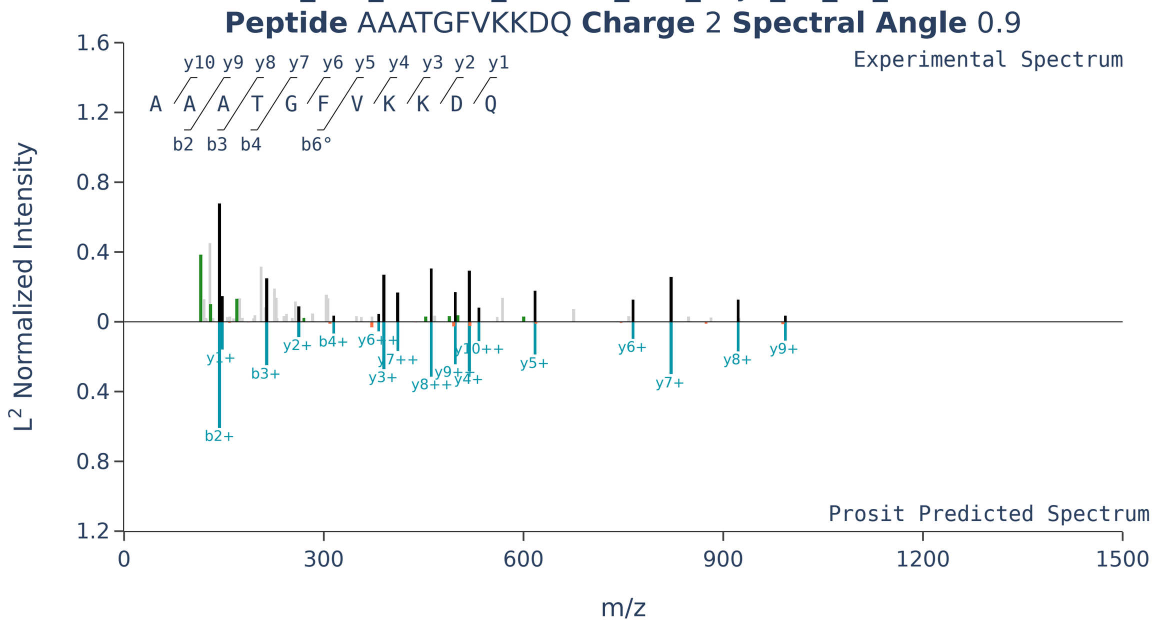


**Source** W_Soh_160821_170921_Goe_aSyn_A1_24h_R1 **Scan** 13844
**Peptide** GSIAAATGFVKK **Charge** 2 **Spectral Angle** 0.91

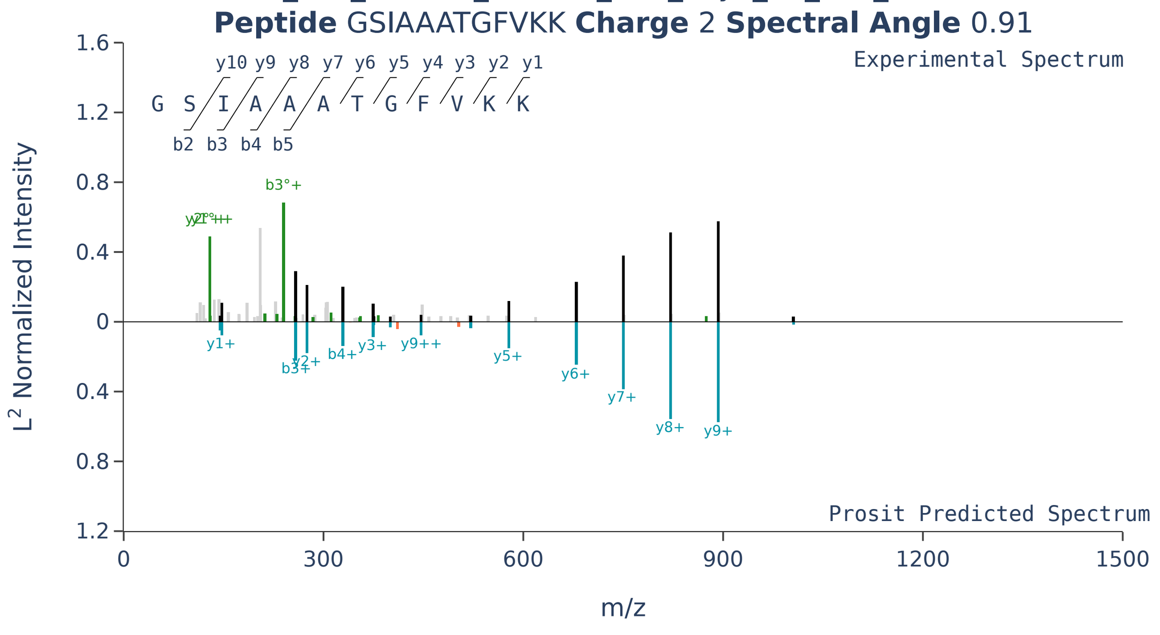


**Source** W_Soh_160821_170921_Goe_aSyn_A2_24h_R2 **Scan** 16374
**Peptide** TKEGVLYV **Charge** 2 **Spectral Angle** 0.89

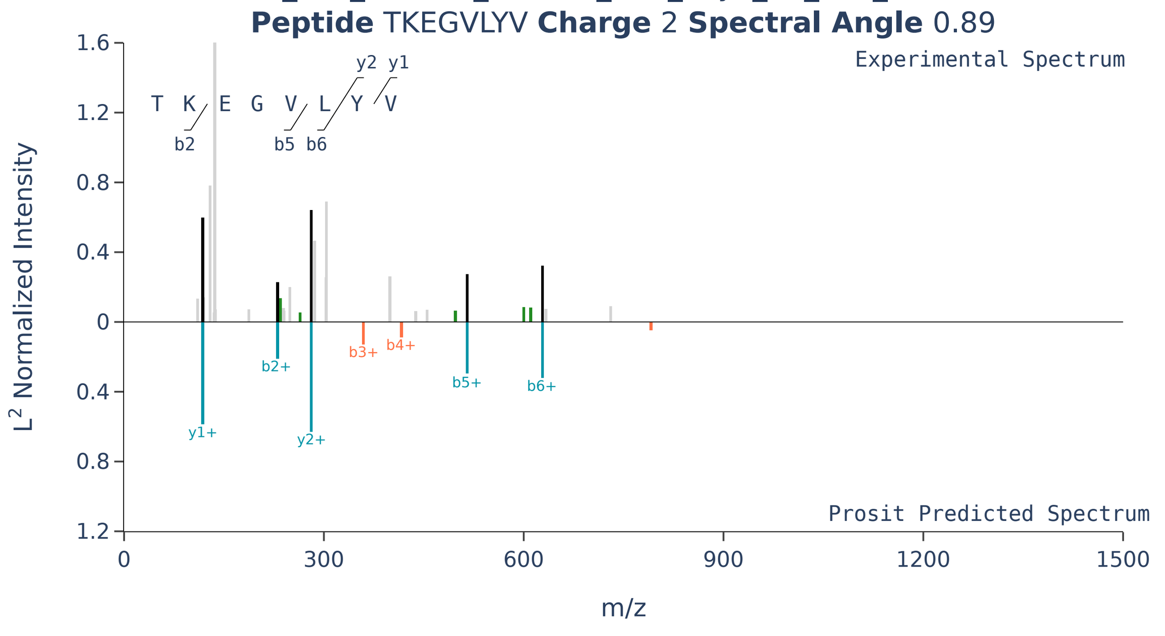


**Source** W_Soh_160821_170921_Goe_aSyn_A2_24h_R1 **Scan** 5486
**Peptide** YVGSKTKEGVVH **Charge** 2 **Spectral Angle** 0.91

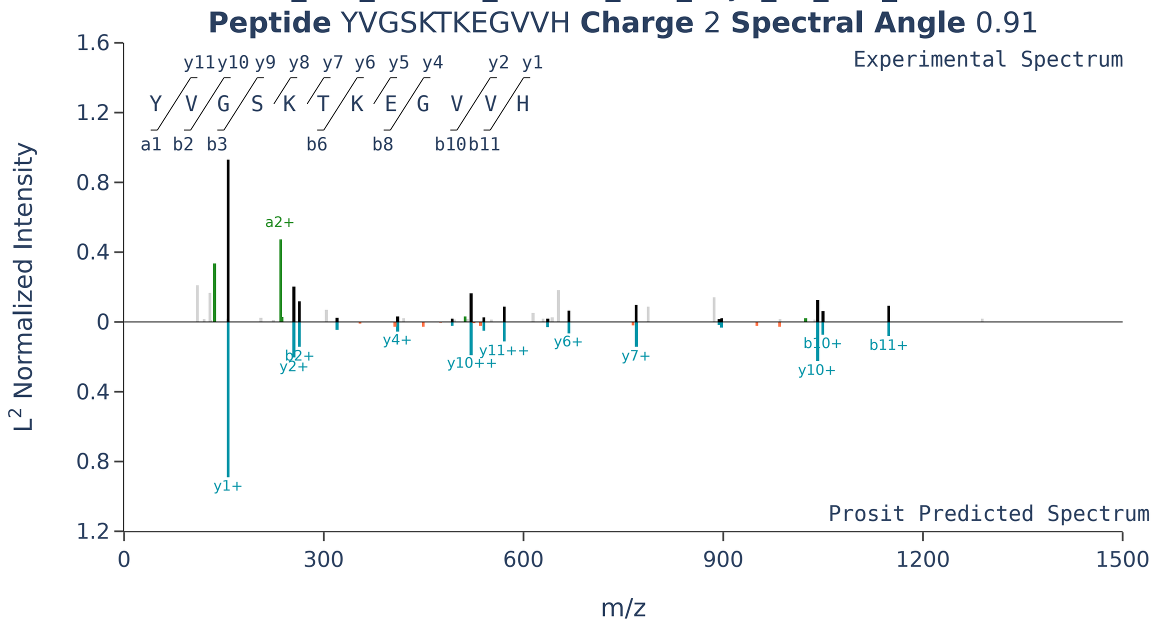


**Source** W_Soh_160821_170921_Goe_aSyn_A1_24h_R2 **Scan** 5824
**Peptide** AEKTKEQVTNVG **Charge** 2 **Spectral Angle** 0.86

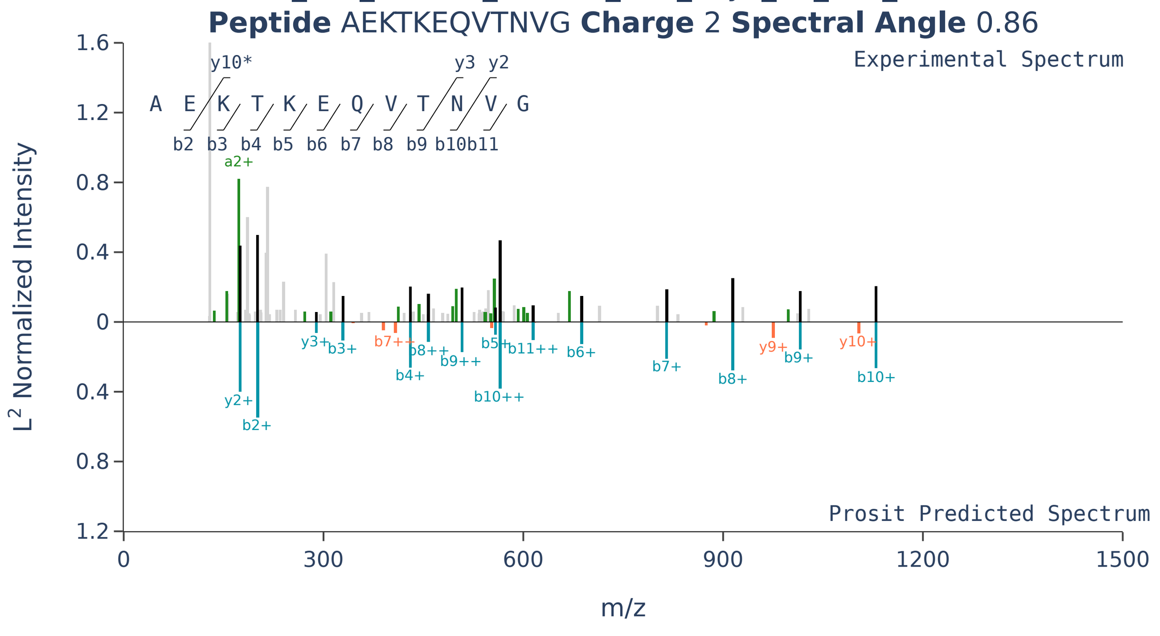

**Source** W_Soh_160821_170921_Goe_aSyn_A1_24h_R2 **Scan** 16244
**Peptide** MPVDPDNEAY **Charge** 2 **Spectral Angle** 0.88

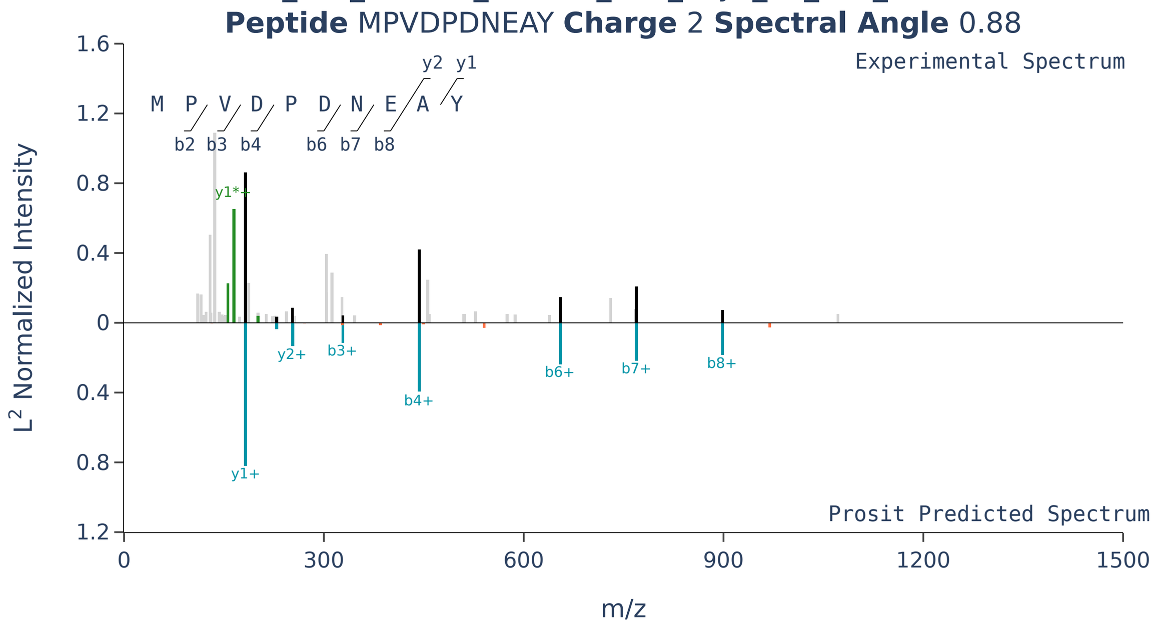


**Source** W_Soh_160821_170921_Goe_aSyn_A1_24h_R1 **Scan** 10258
**Peptide** VAQKTVEGAGSIAA **Charge** 2 **Spectral Angle** 0.92

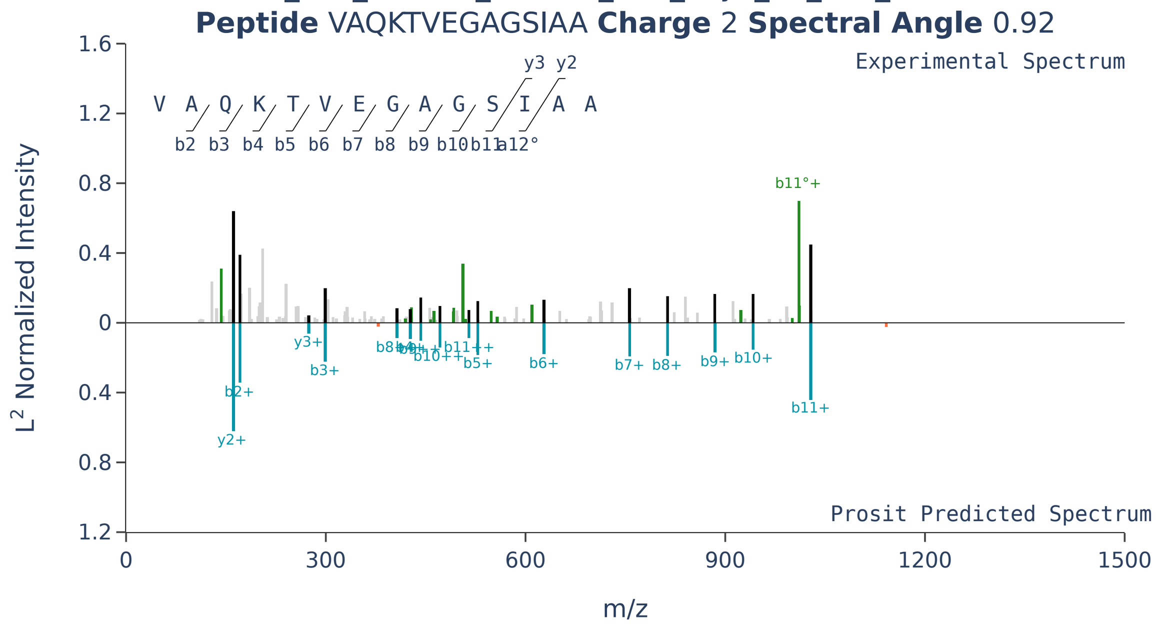


**Source** W_Soh_160821_170921_Goe_aSyn_A2_24h_R2 **Scan** 7962
**Peptide** LYVGSKTKE **Charge** 2 **Spectral Angle** 0.91

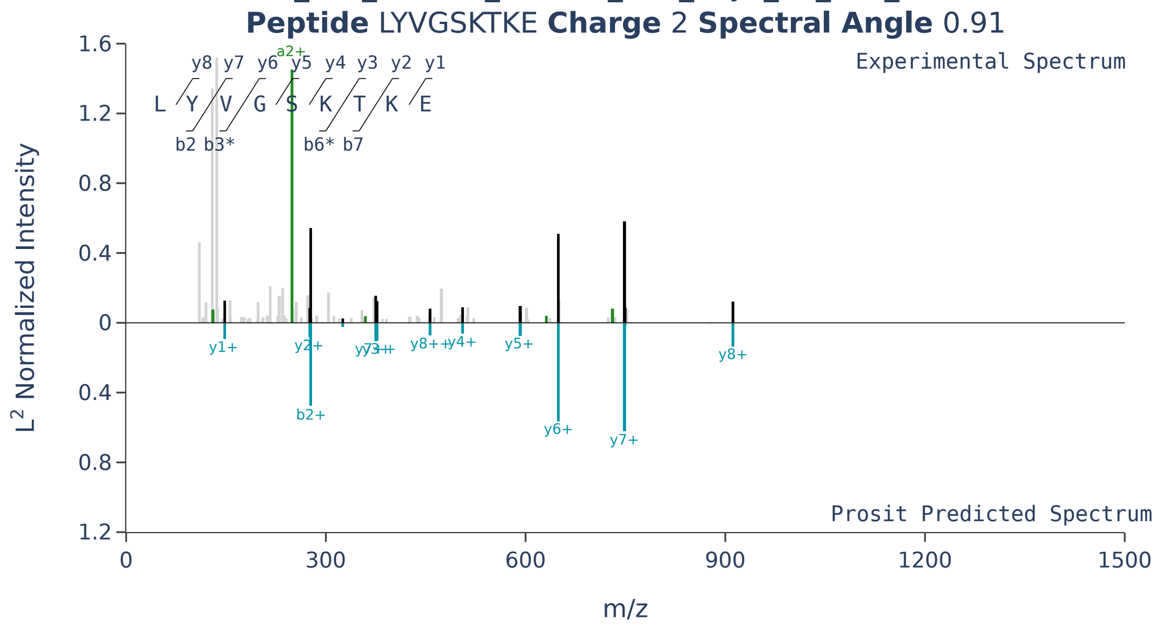


**Source** W_Soh_160821_170921_Goe_aSyn_A1_1h_R1 **Scan** 12627
**Peptide** EGYQDYEPEA **Charge** 1 **Spectral Angle** 0.85

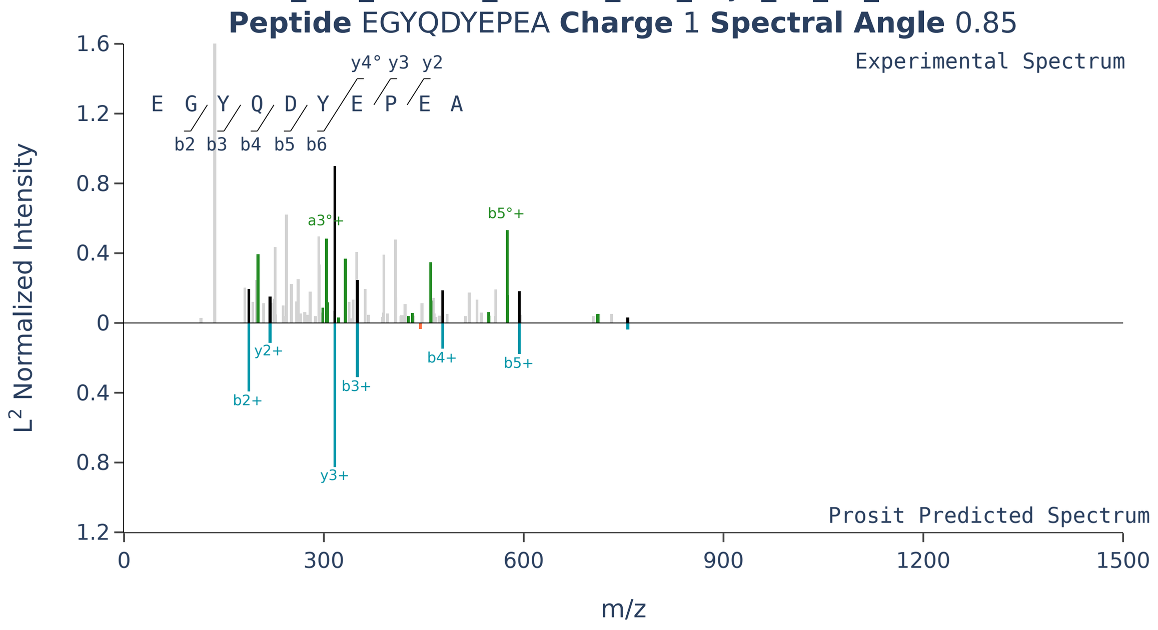


**Source** W_Soh_160821_170921_Goe_aSyn_A2_24h_R2 **Scan** 7547
**Peptide** AVAQKTVE **Charge** 1 **Spectral Angle** 0.9

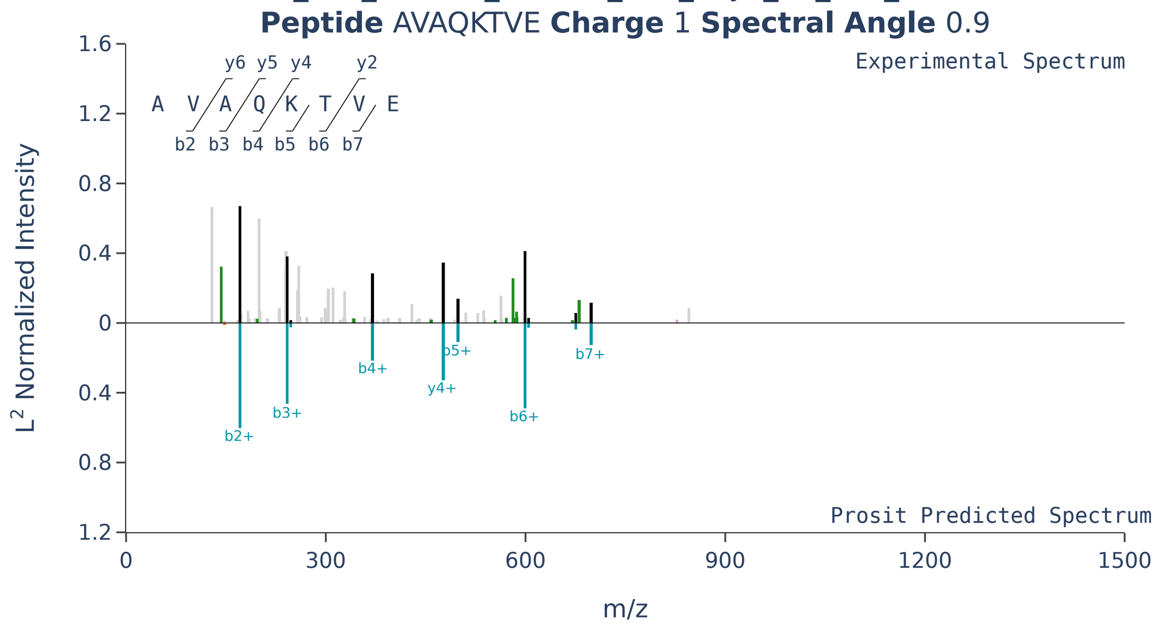


**Source** W_Soh_160821_170921_Goe_aSyn_A2_24h_R1 **Scan** 13520
**Peptide** GGAVVTGV **Charge** 1 **Spectral Angle** 0.87

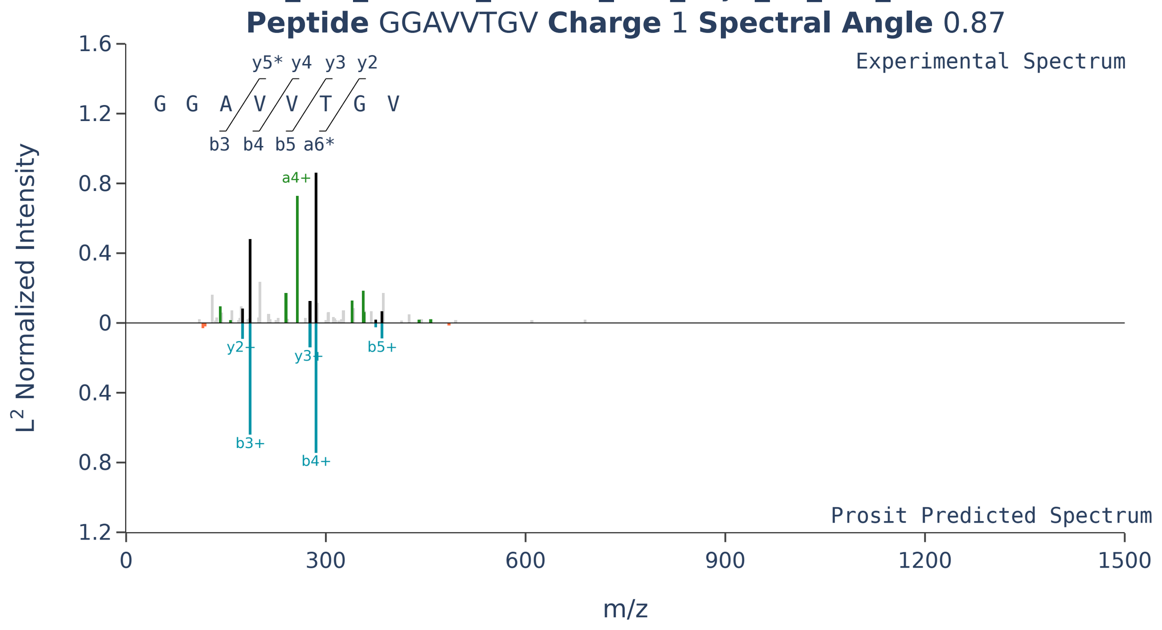


**Source** W_Soh_160821_170921_Goe_aSyn_A1_24h_R2 **Scan** 6949
**Peptide** TKEGVVHGV **Charge** 2 **Spectral Angle** 0.91

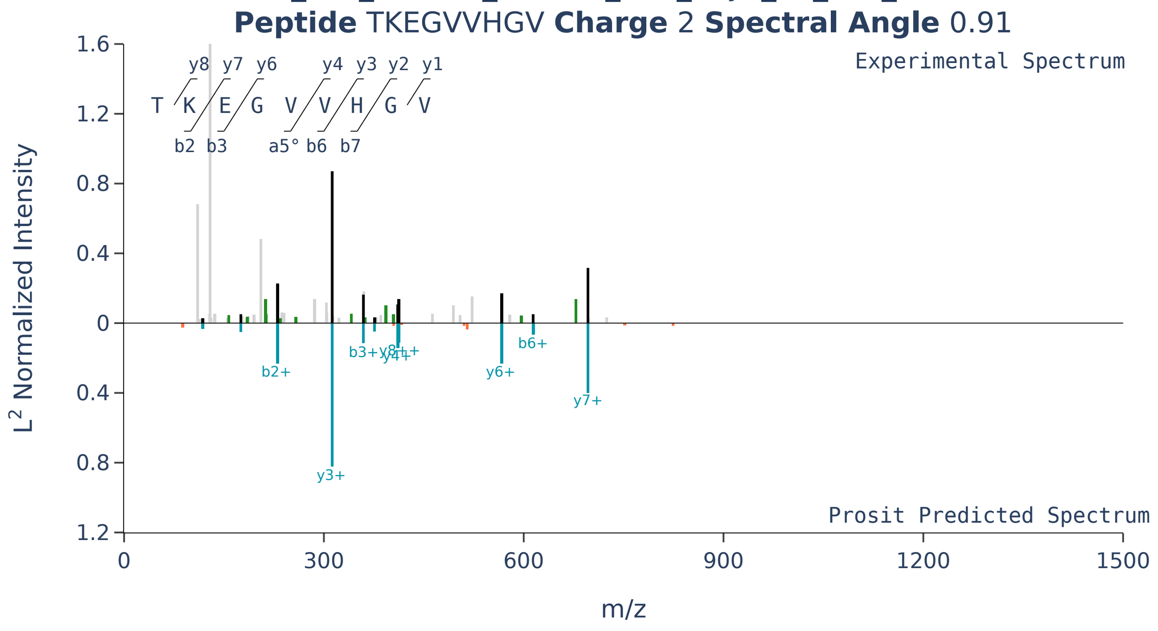


**Source** W_Soh_160821_170921_Goe_aSyn_A1_6h_R2 **Scan** 20368
**Peptide** ILEDMPVD **Charge** 1 **Spectral Angle** 0.87

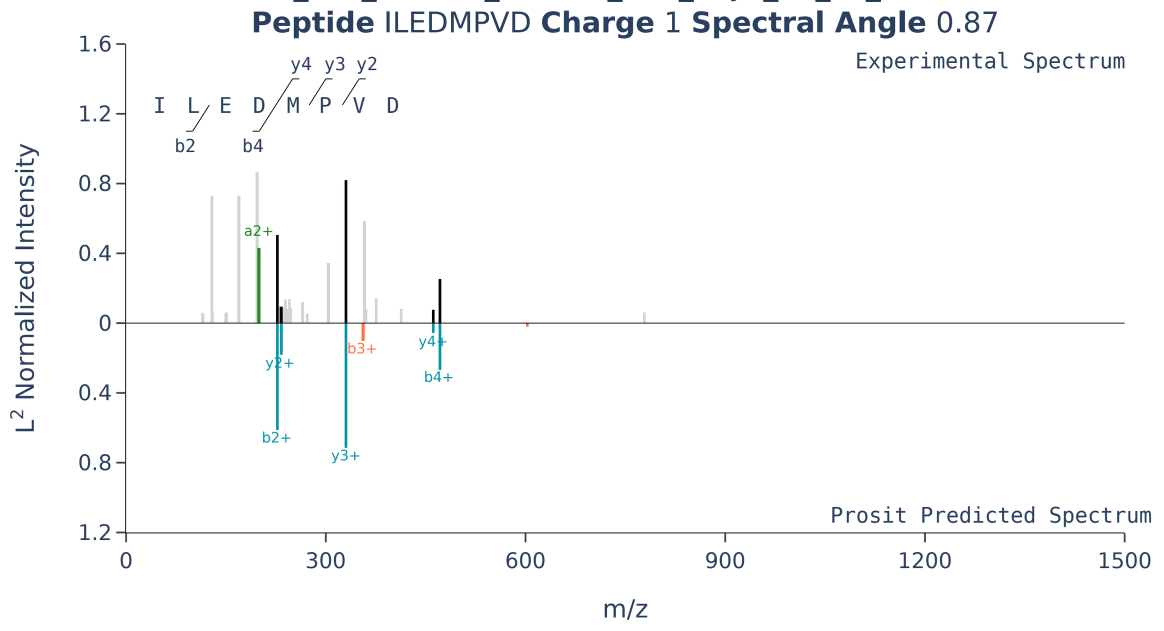


**Source** W_Soh_160821_170921_Goe_aSyn_A1_24h_R2 **Scan** 14199
**Peptide** MPSEEGYQDYE **Charge** 2 **Spectral Angle** 0.9

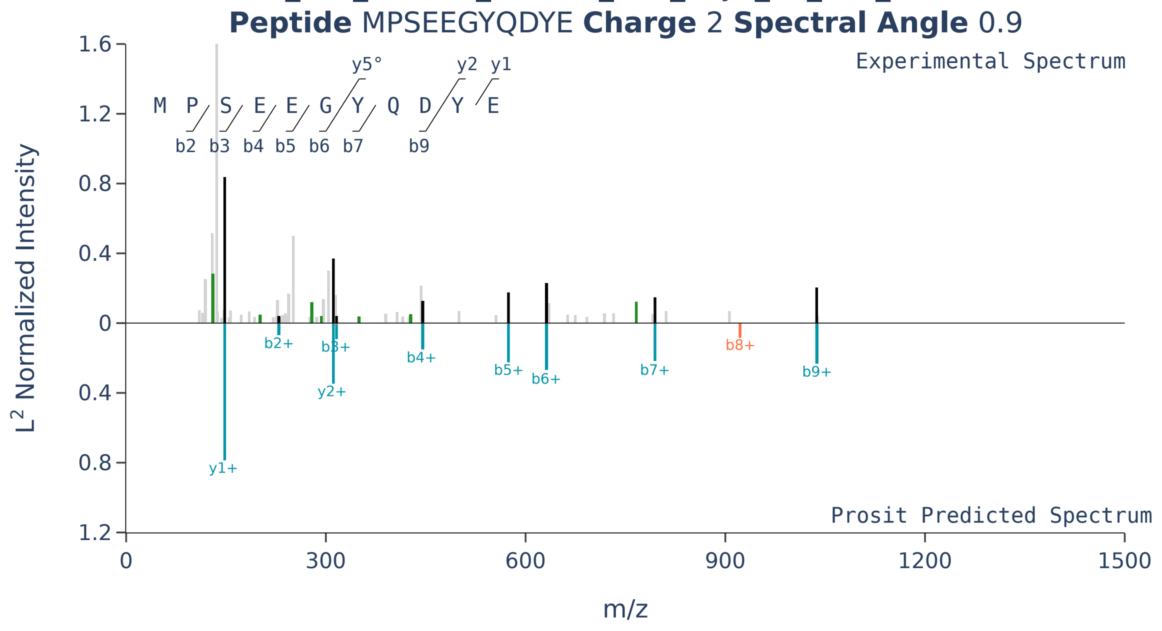


**Source** W_Soh_160821_170921_Goe_aSyn_A1_6h_R1 **Scan** 5830
**Peptide** GSKTKEGVVHGV **Charge** 2 **Spectral Angle** 0.82

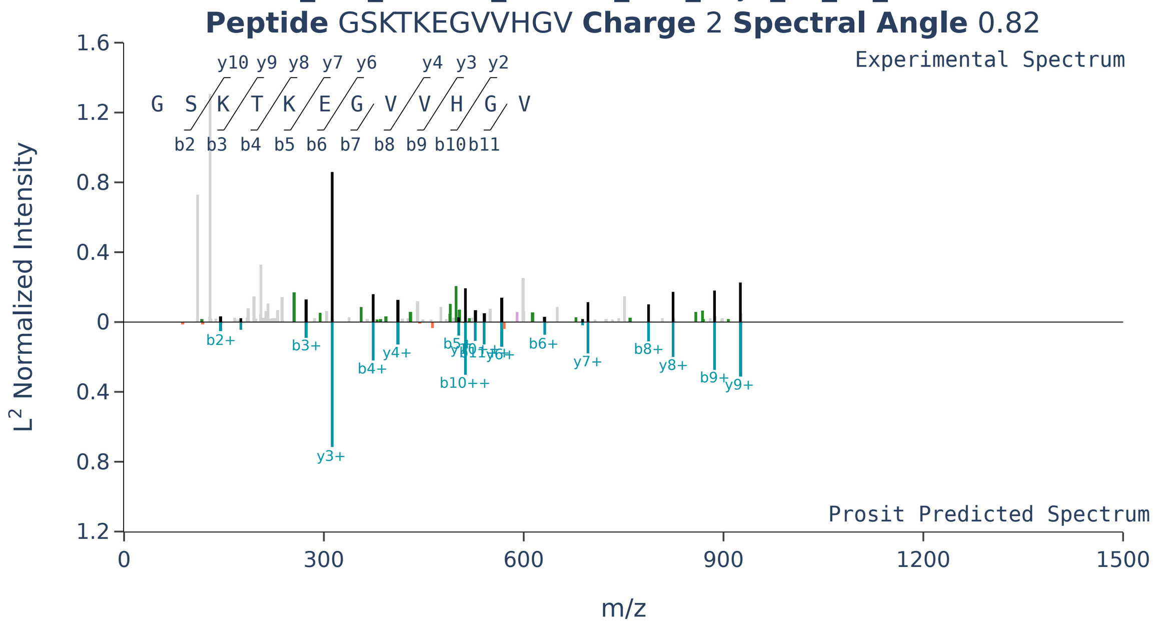


**Source** W_Soh_160821_170921_Goe_aSyn_A2_24h_R2 **Scan** 10701
**Peptide** VLYVGSKT **Charge** 2 **Spectral Angle** 0.94

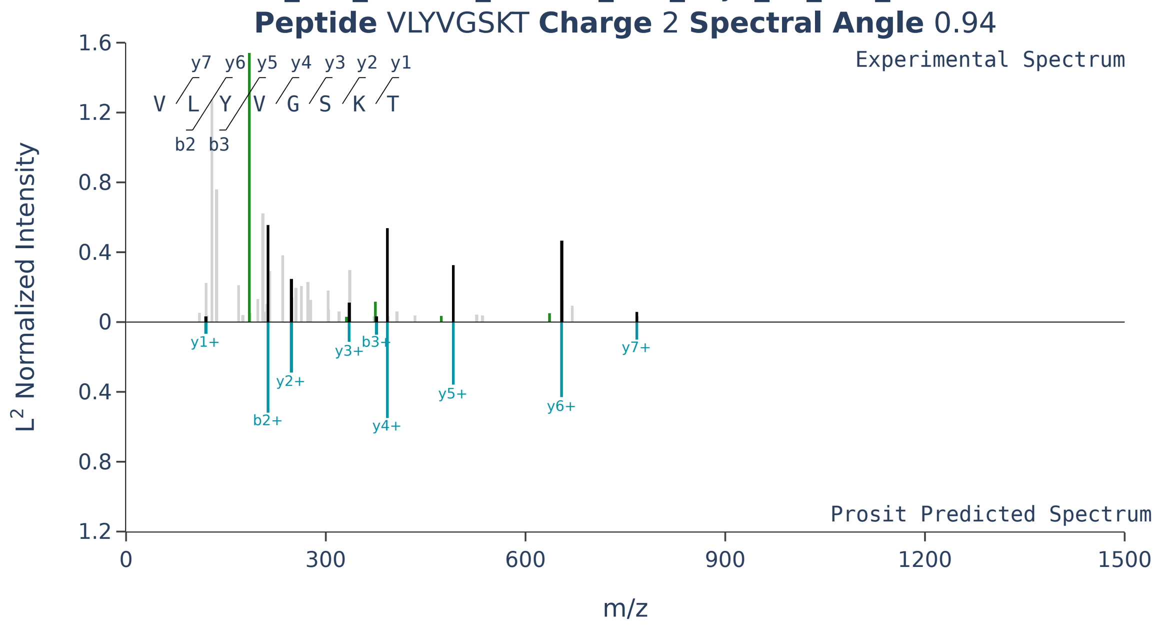


**Source** W_Soh_160821_170921_Goe_aSyn_A1_6h_R2 **Scan** 20533
**Peptide** VGGAVVTGVTAVAQKTVE **Charge** 2 **Spectral Angle** 0.92

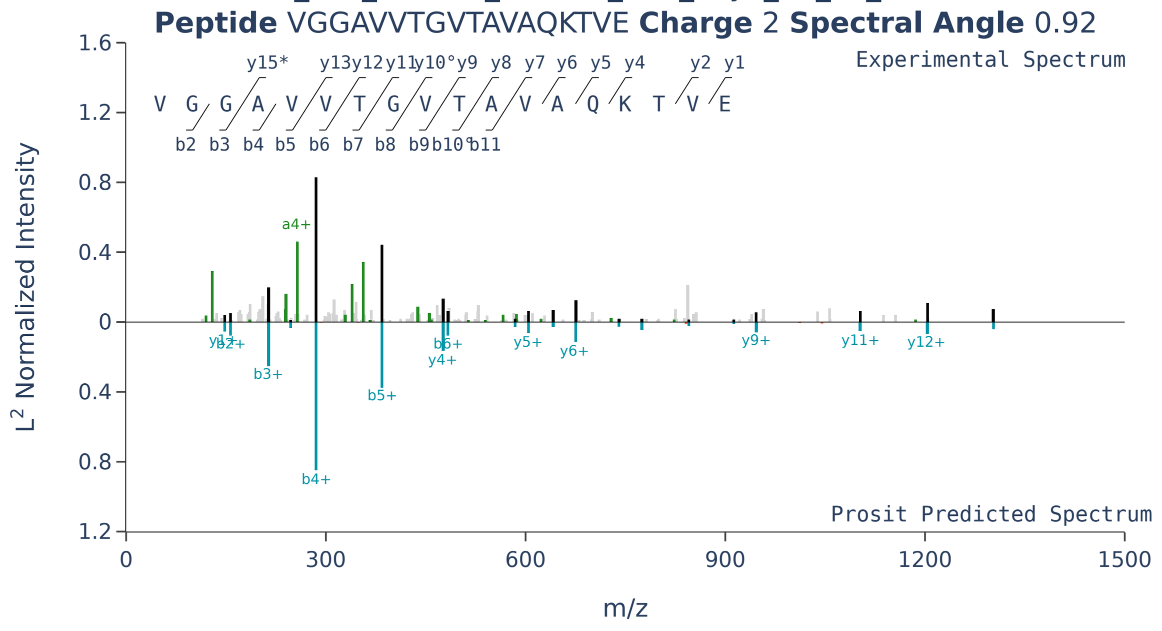


**Source** W_Soh_160821_170921_Goe_aSyn_A1_24h_R2 **Scan** 5718
**Peptide** ATVAEKTKEQVTN **Charge** 2 **Spectral Angle** 0.85

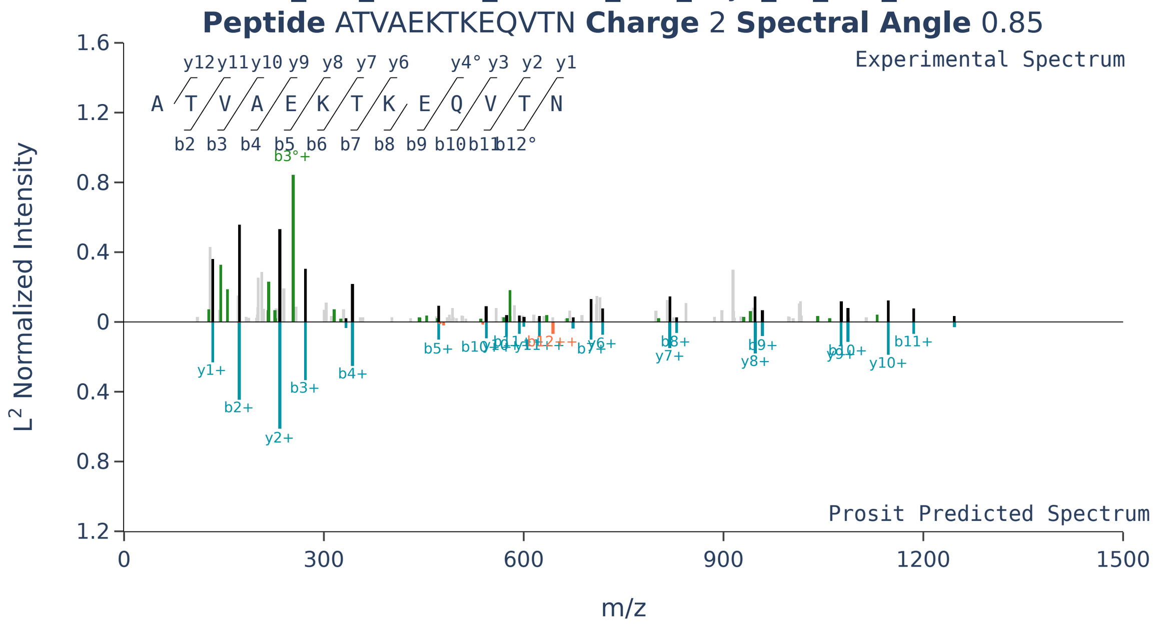


**Source** W_Soh_160821_170921_Goe_aSyn_A1_3h_R2 **Scan** 6293
**Peptide** QLGKNEEGAPQE **Charge** 1 **Spectral Angle** 0.94

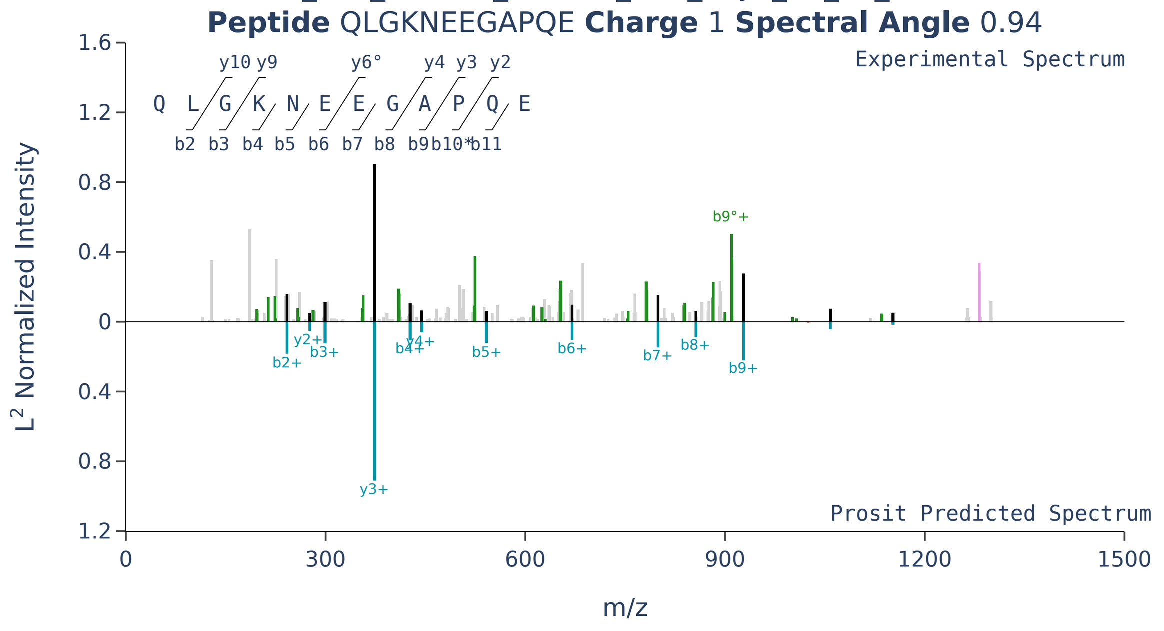


**Source** W_Soh_160821_170921_Goe_aSyn_A1_6h_R1 **Scan** 13997
**Peptide** GSIAAATGFVKKDQ **Charge** 2 **Spectral Angle** 0.85

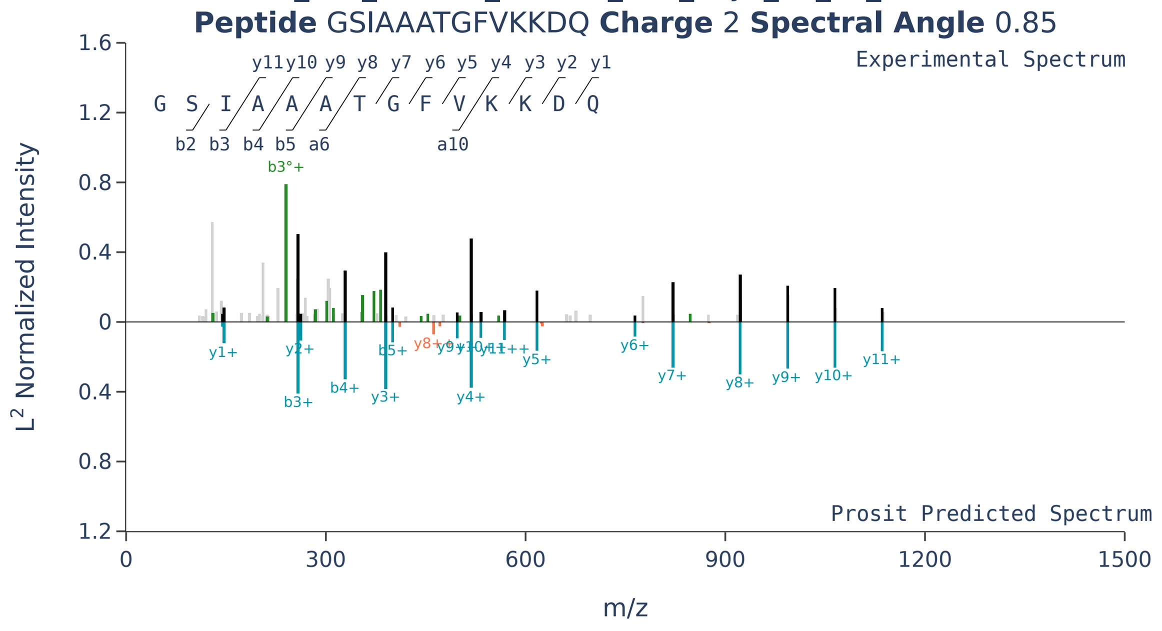

**Source** W_Soh_160821_170921_Goe_aSyn_A1_6h_R2 **Scan** 23950
**Peptide** TNVGGAVVTGVTAVAQKTVE **Charge** 2 **Spectral Angle** 0.94

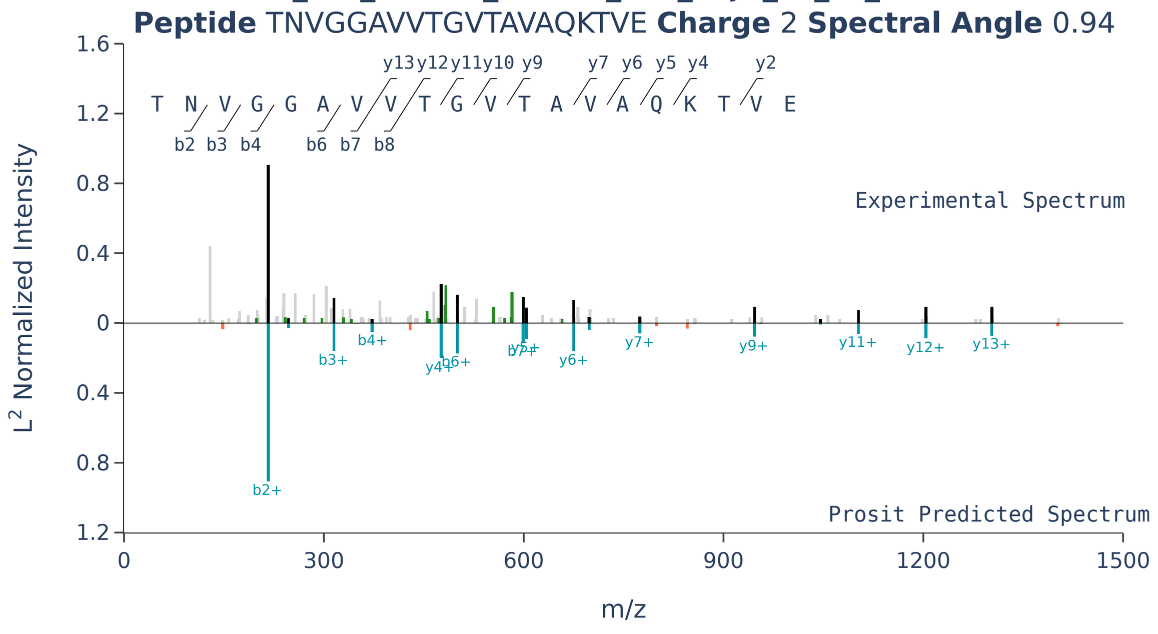


**Source** W_Soh_160821_170921_Goe_aSyn_A2_24h_R1 **Scan** 16524
**Peptide** PDNEAYEMPSEEGYQ **Charge** 2 **Spectral Angle** 0.87

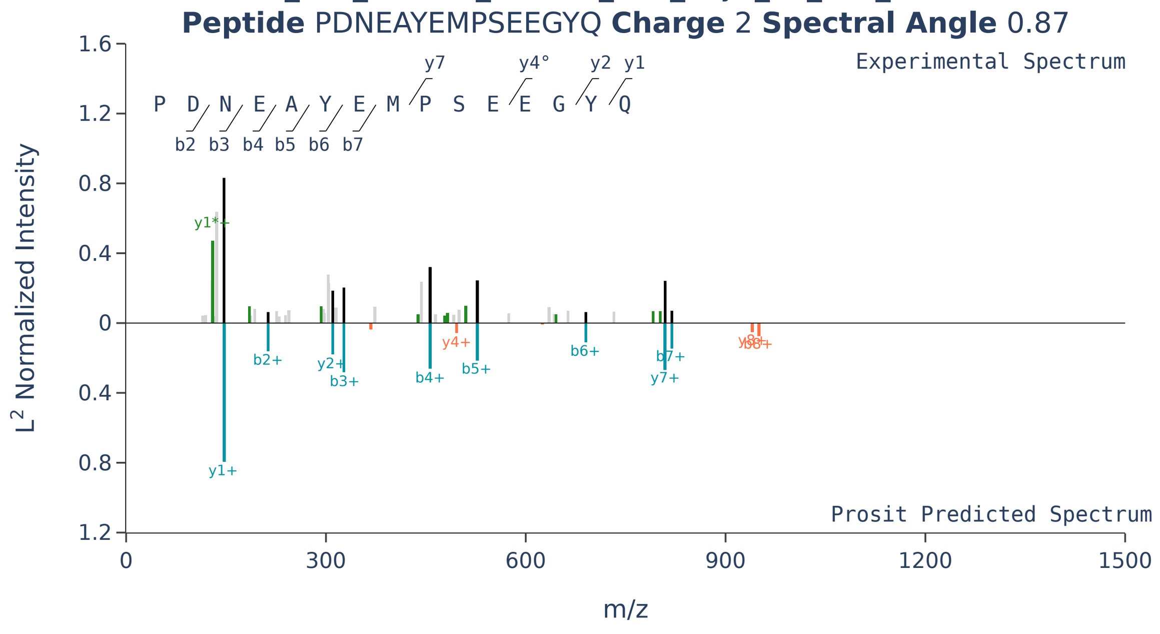


**Source** W_Soh_160821_170921_Goe_aSyn_A1_15'_R1 **Scan** 8475
**Peptide** GFVKKDQL **Charge** 2 **Spectral Angle** 0.85

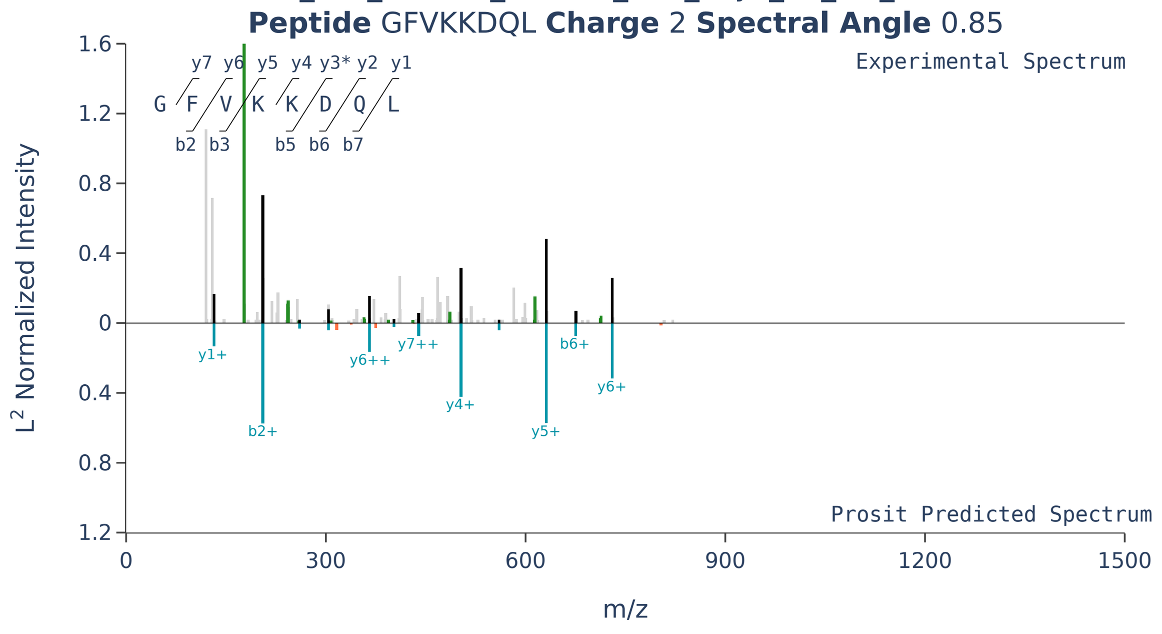


**Source** W_Soh_160821_170921_Goe_aSyn_A1_24h_R1 **Scan** 7214
**Peptide** GVATVAEKT **Charge** 1 **Spectral Angle** 0.85

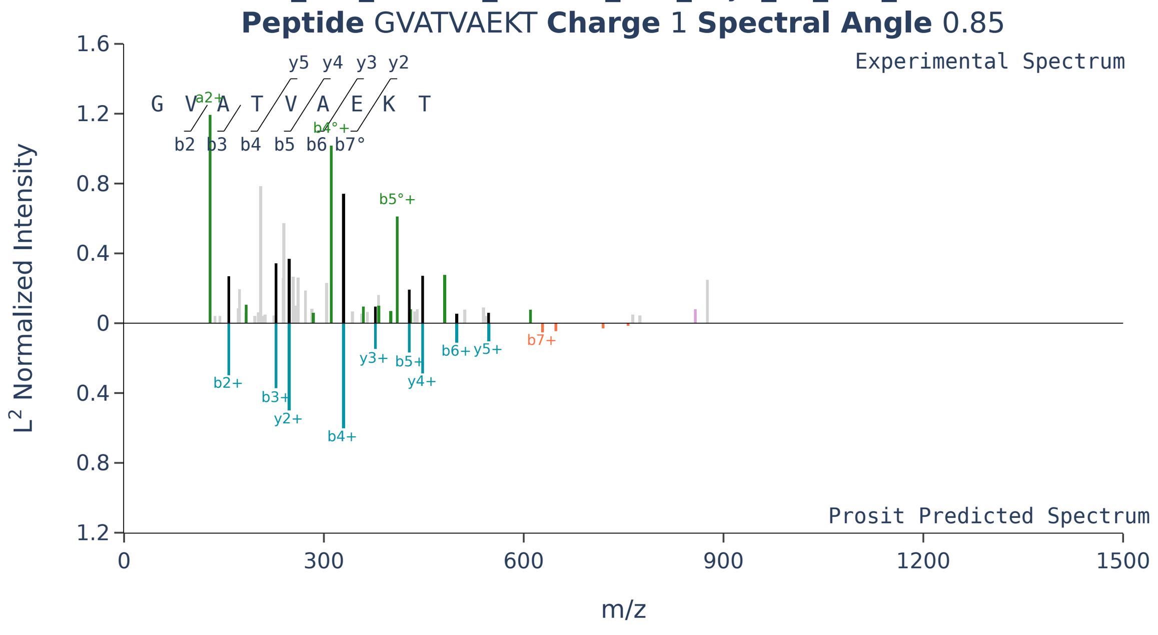


**Source** W_Soh_160821_170921_Goe_aSyn_A2_24h_R2 **Scan** 5523
**Peptide** KEGVVAAAEKTKQ **Charge** 2 **Spectral Angle** 0.88

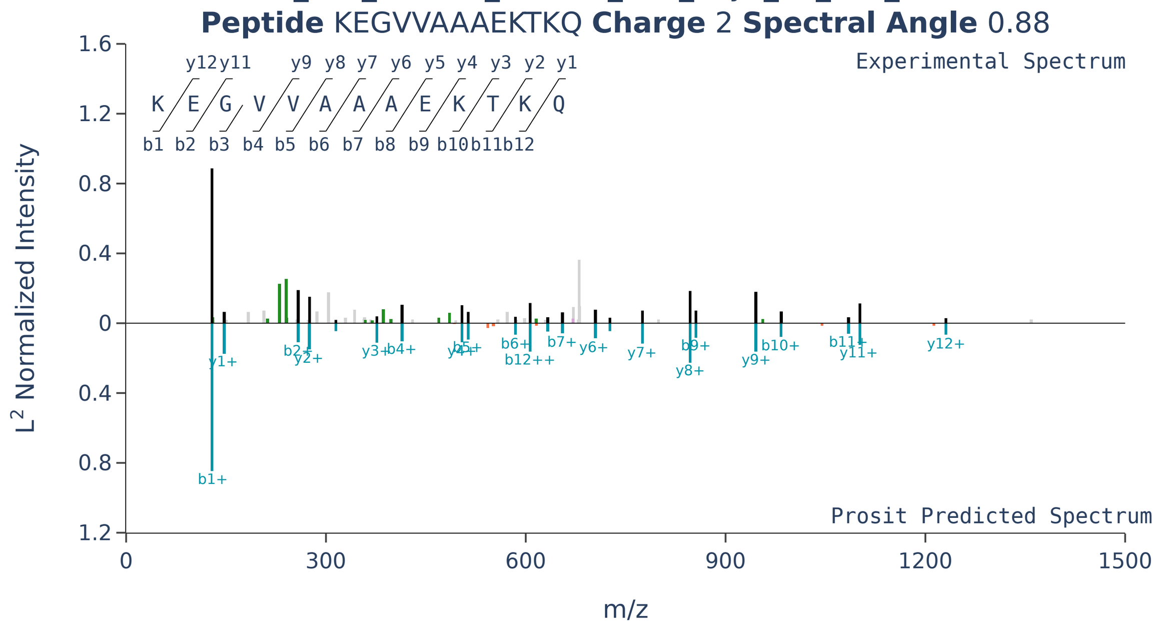


**Source** W_Soh_160821_170921_Goe_aSyn_A1_24h_R1 **Scan** 5899
**Peptide** LGKNEEGAPQE **Charge** 2 **Spectral Angle** 0.93

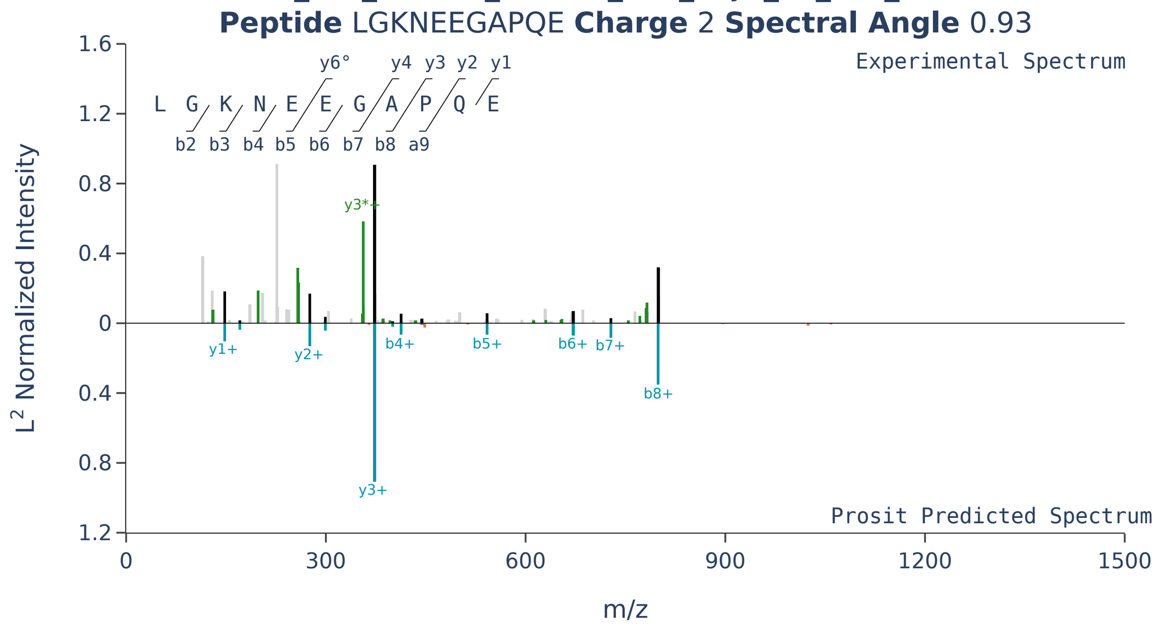


**Source** W_Soh_160821_170921_Goe_aSyn_A2_24h_R2 **Scan** 17189
**Peptide** MDVFMKGLSK **Charge** 2 **Spectral Angle** 0.88

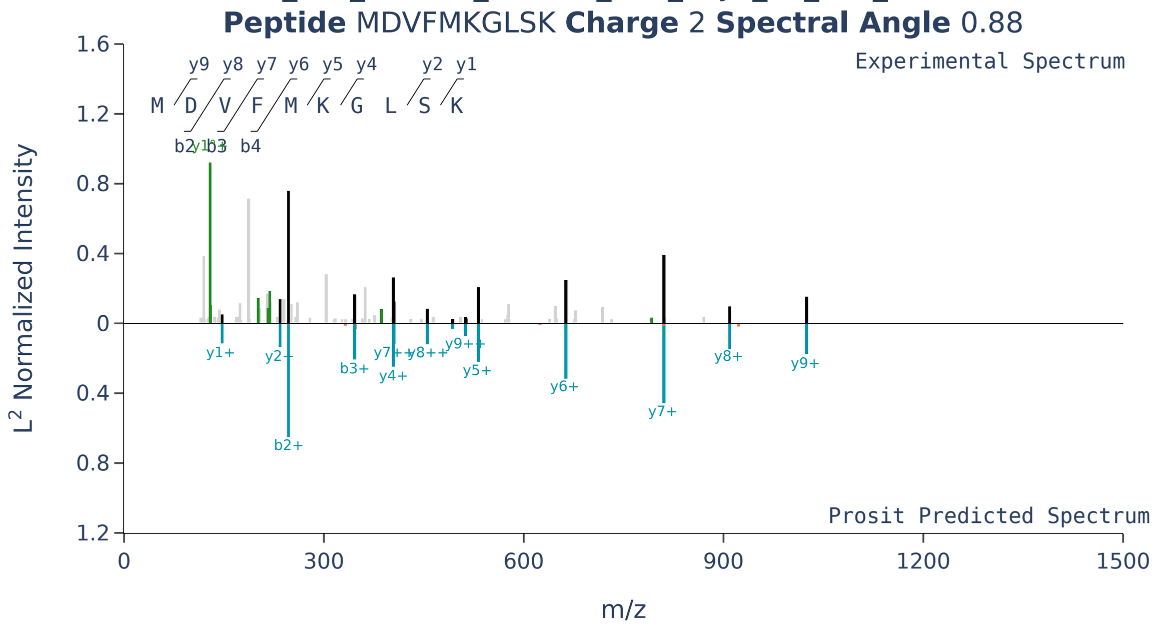


**Source** W_Soh_160821_170921_Goe_aSyn_A2_24h_R1 **Scan** 19728
**Peptide** GGAVVTGVTAVAQKTVEG **Charge** 2 **Spectral Angle** 0.92

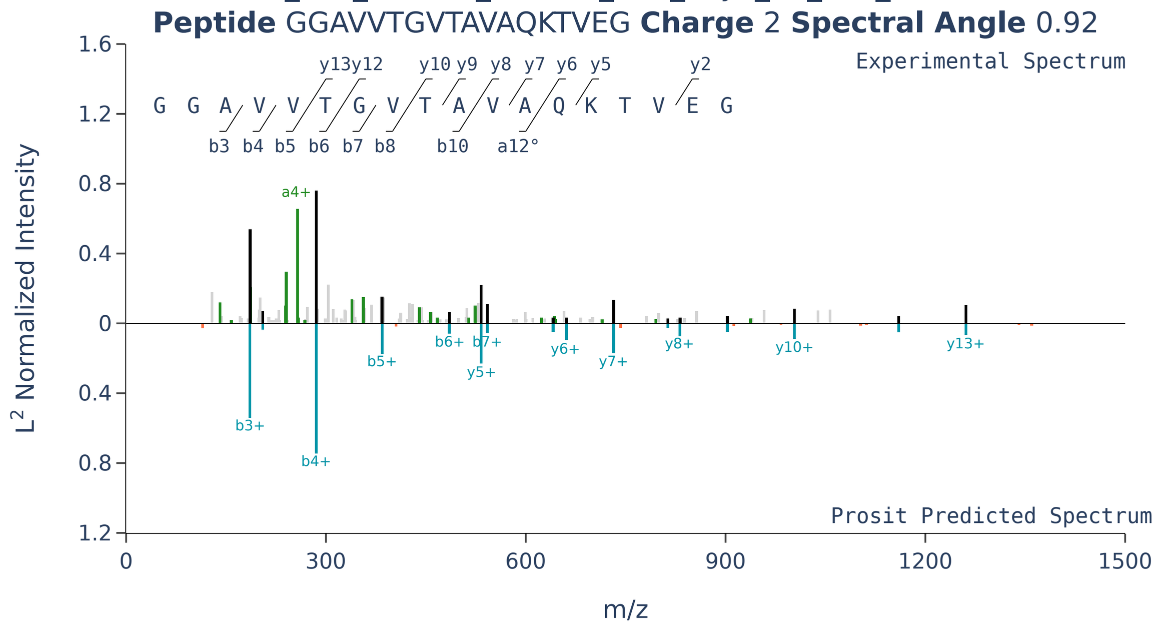


**Source** W_Soh_160821_170921_Goe_aSyn_A1_24h_R2 **Scan** 14584
**Peptide** DVFMKGLSKAKE **Charge** 3 **Spectral Angle** 0.87

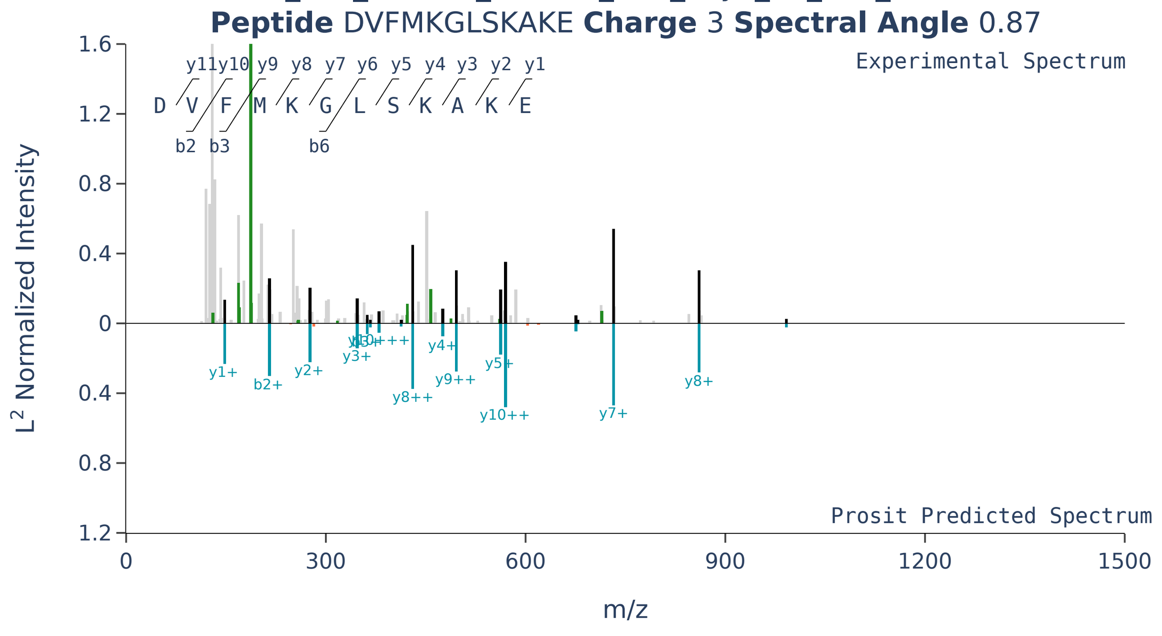


**Source** W_Soh_160821_170921_Goe_aSyn_A1_24h_R1 **Scan** 6193
**Peptide** GVATVAEKTKE **Charge** 2 **Spectral Angle** 0.86

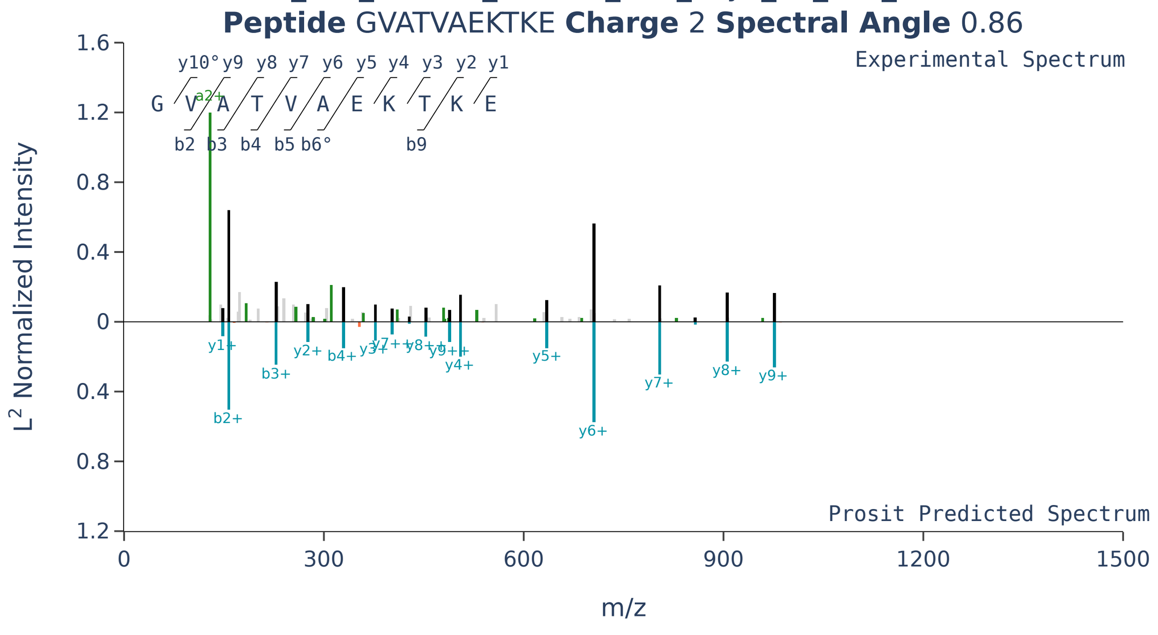


**Source** W_Soh_160821_170921_Goe_aSyn_A2_24h_R2 **Scan** 6110
**Peptide** AATGFVKKDQ **Charge** 3 **Spectral Angle** 0.86

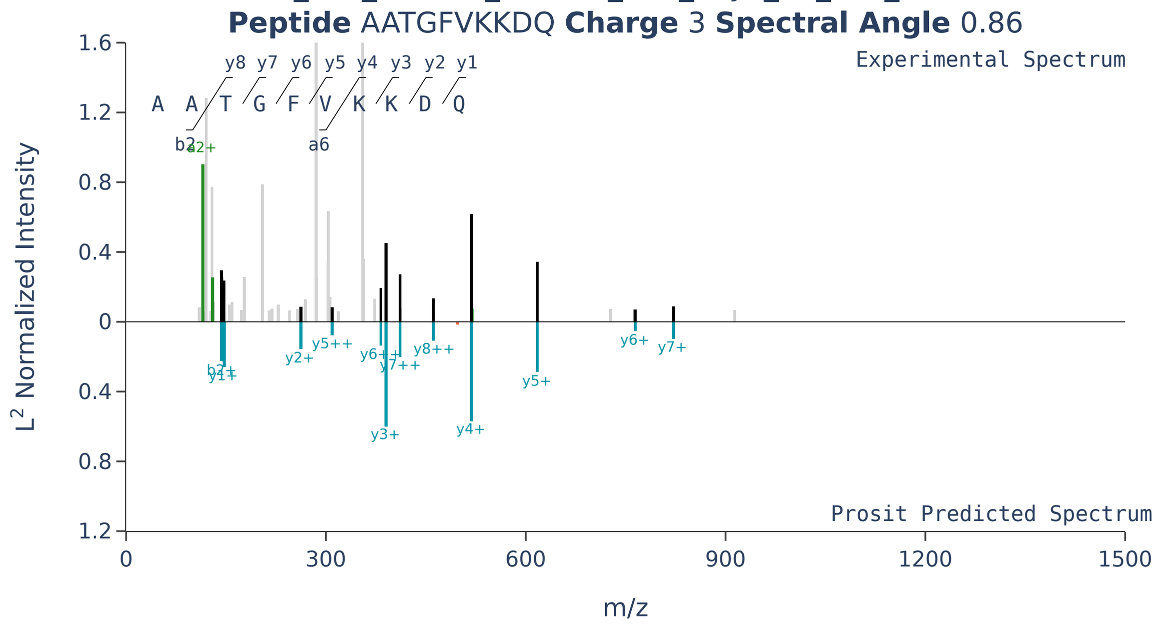


**Source** W_Soh_160821_170921_Goe_aSyn_A2_24h_R1 **Scan** 7846
**Peptide** YVGSKTKEG **Charge** 2 **Spectral Angle** 0.92

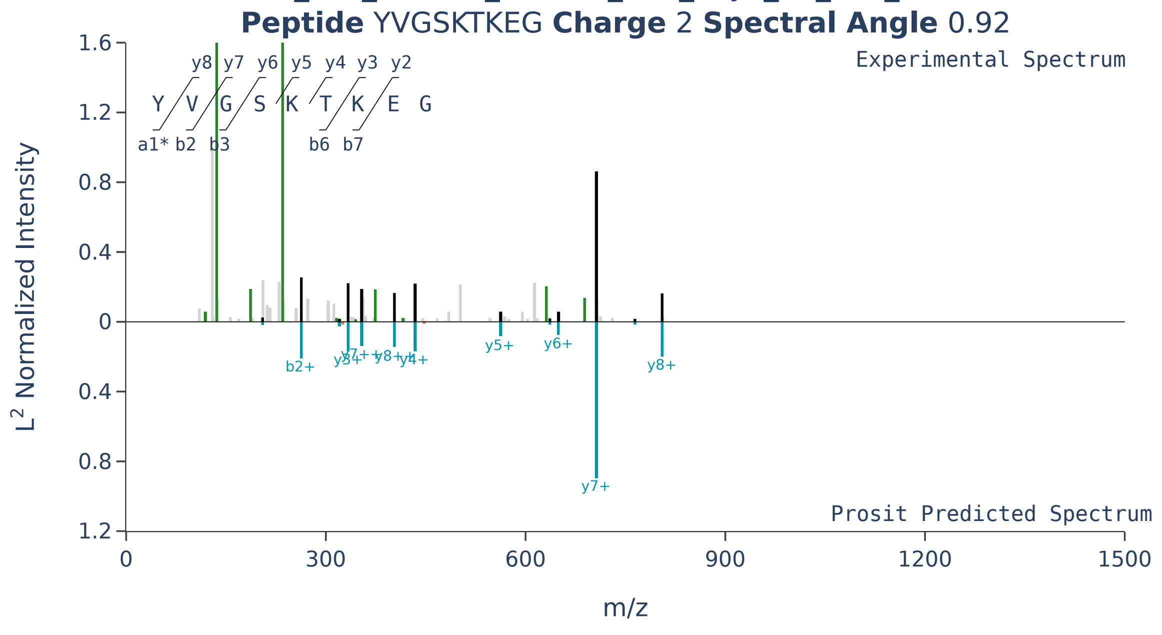


**Source** W_Soh_160821_170921_Goe_aSyn_A2_24h_R2 **Scan** 18052
**Peptide** GVTAVAQKTVEGAGSIAAA **Charge** 2 **Spectral Angle** 0.89

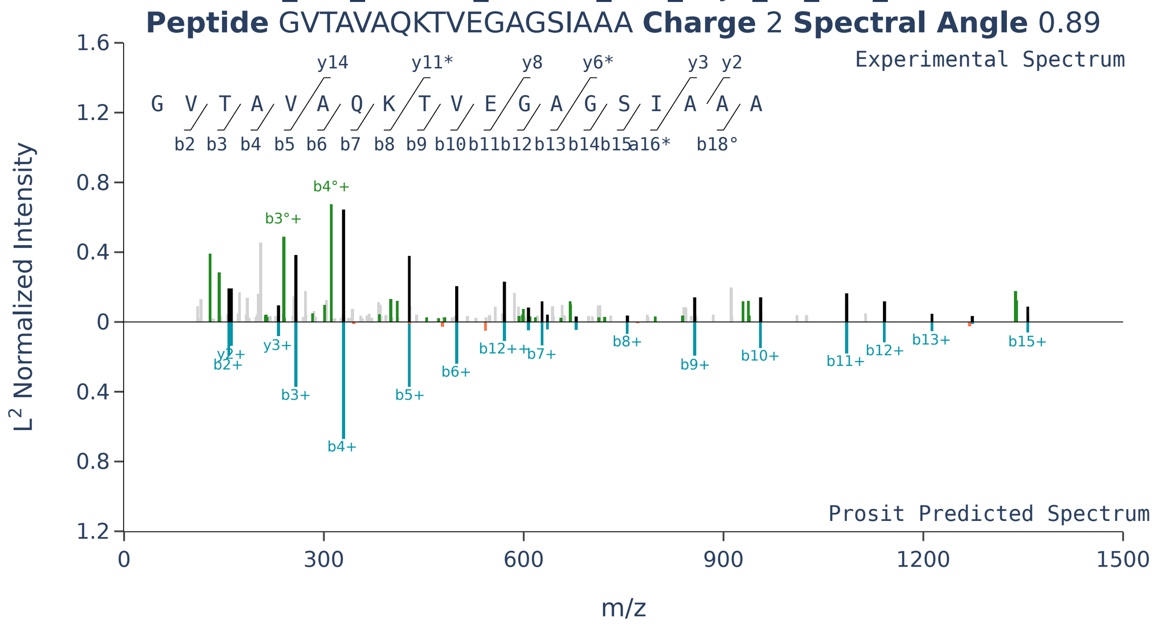


**Source** W_Soh_160821_170921_Goe_aSyn_A2_24h_R2 **Scan** 11345
**Peptide** YQDYEPEA **Charge** 2 **Spectral Angle** 0.9

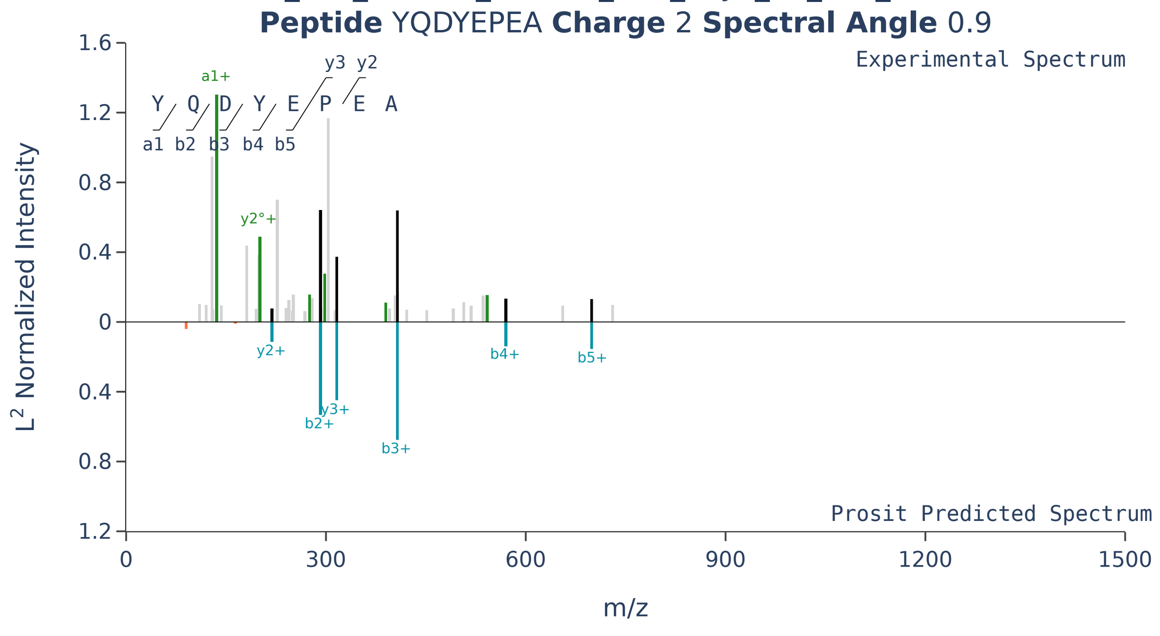


**Source** W_Soh_160821_170921_Goe_aSyn_A1_24h_R1 **Scan** 18245
**Peptide** MDVFMKGLSKA **Charge** 3 **Spectral Angle** 0.9

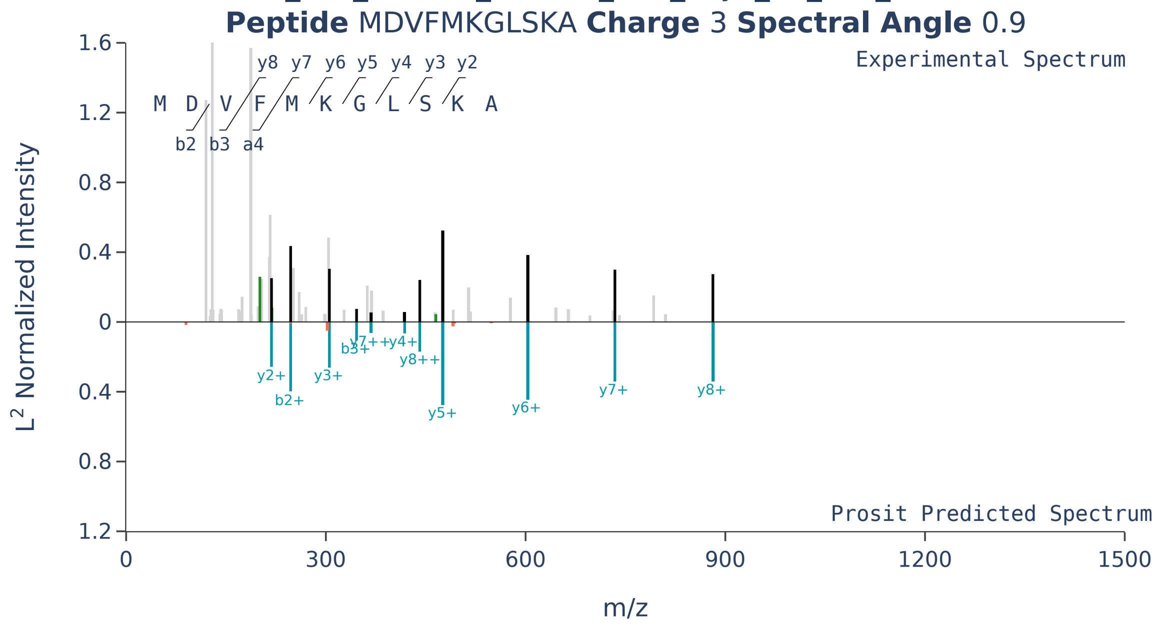

**Source** W_Soh_160821_170921_Goe_aSyn_A1_24h_R2 **Scan** 15068
**Peptide** TKEGVLYVG **Charge** 2 **Spectral Angle** 0.92

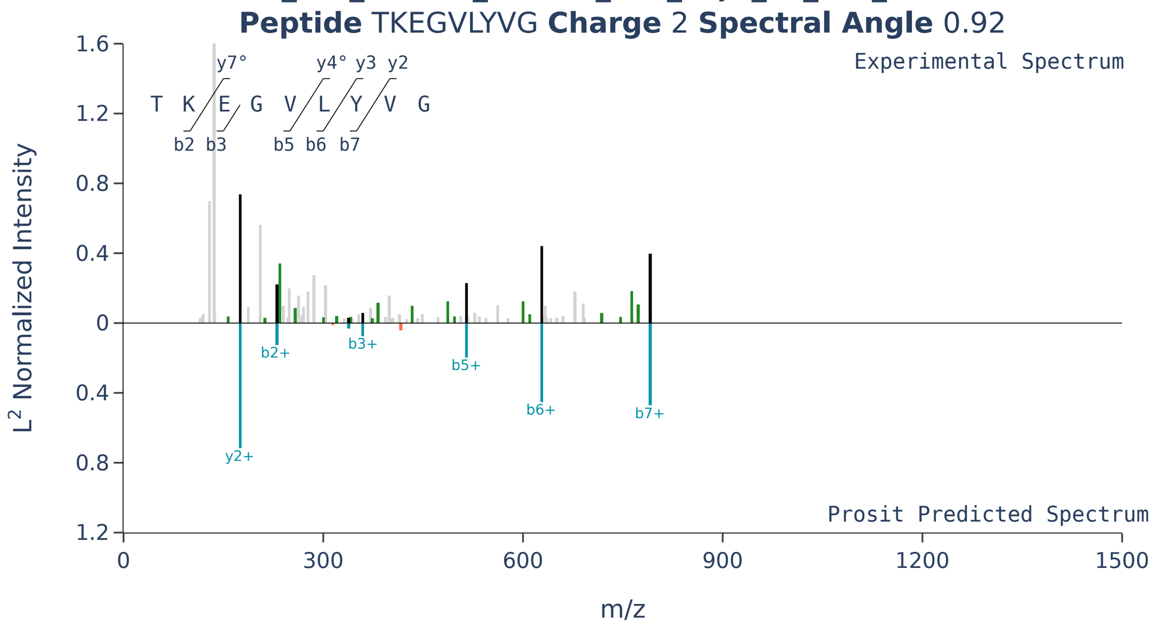


**Source** W_Soh_160821_170921_Goe_aSyn_A2_24h_R2 **Scan** 18799
**Peptide** GGAVVTGVTAVAQKTVE **Charge** 2 **Spectral Angle** 0.92

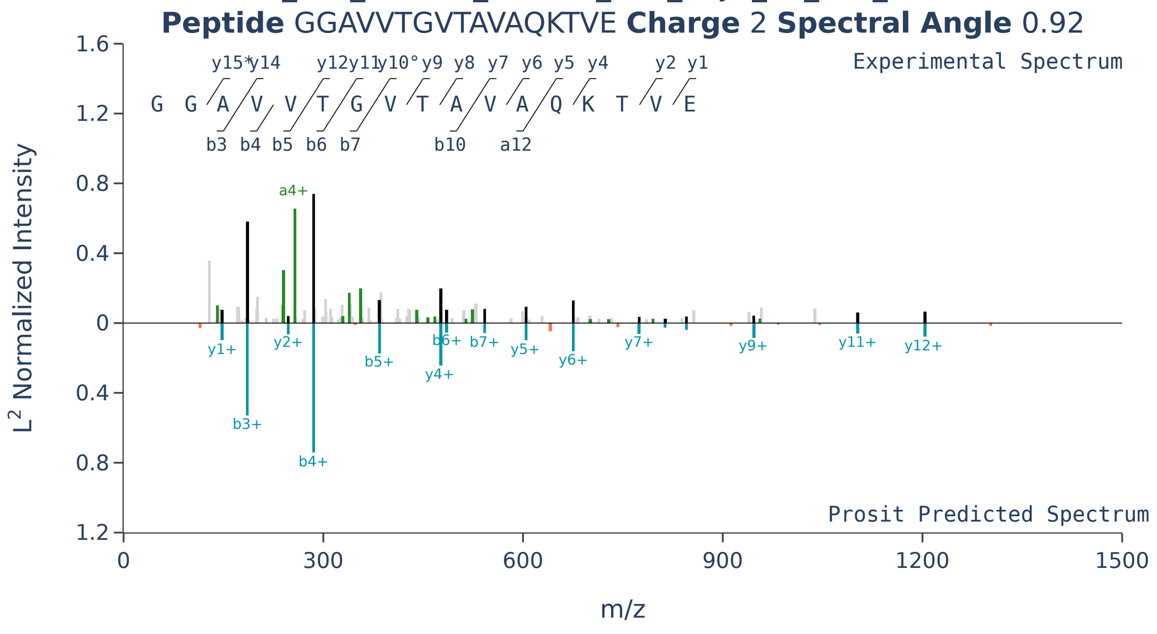


**Source** W_Soh_160821_170921_Goe_aSyn_A1_6h_R2 **Scan** 14290
**Peptide** DVFMKGLSKAKEG **Charge** 3 **Spectral Angle** 0.87

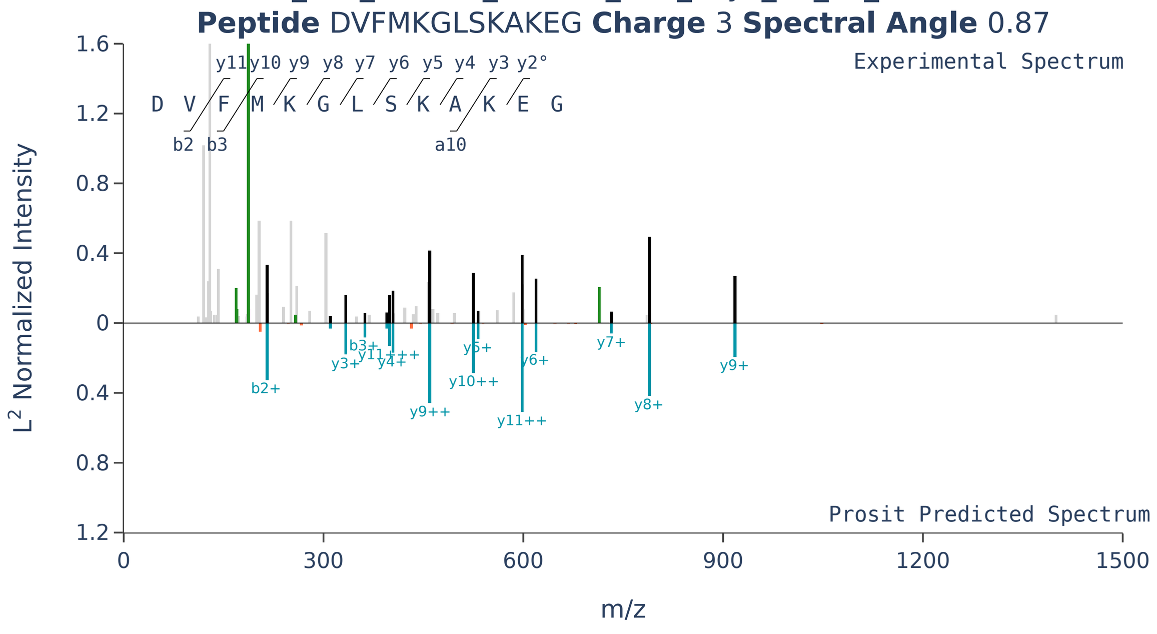


**Source** W_Soh_160821_170921_Goe_aSyn_A1_24h_R1 **Scan** 6011
**Peptide** GVATVAEKTKEQ **Charge** 3 **Spectral Angle** 0.89

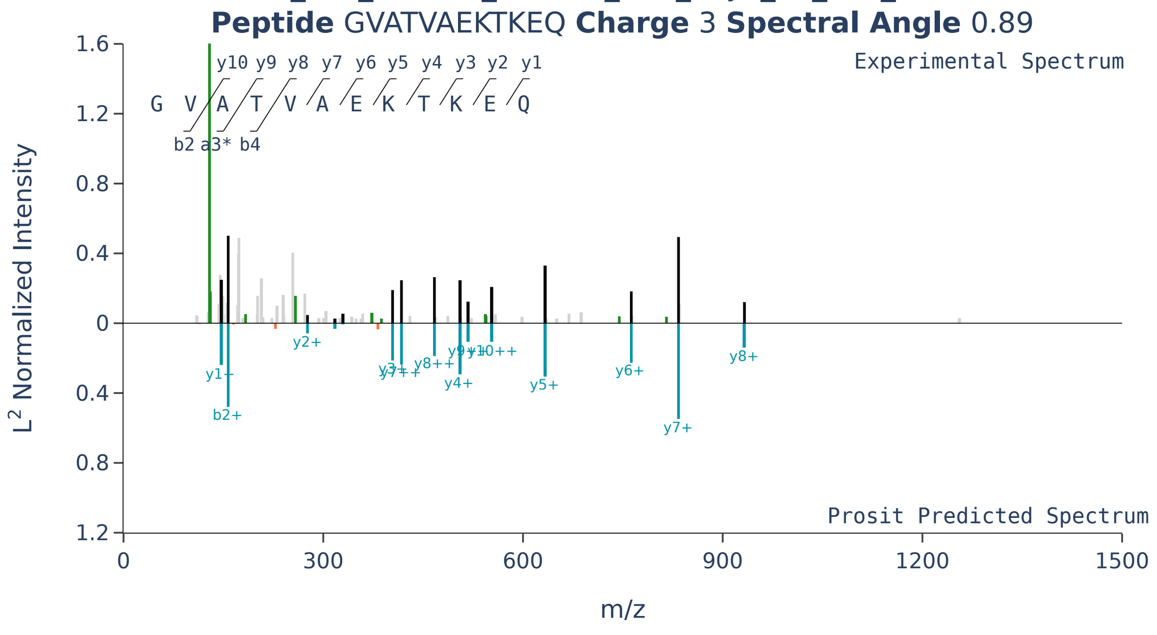


**Source** W_Soh_160821_170921_Goe_aSyn_A1_24h_R1 **Scan** 13297
**Peptide** VTNVGGAVVTG **Charge** 1 **Spectral Angle** 0.84

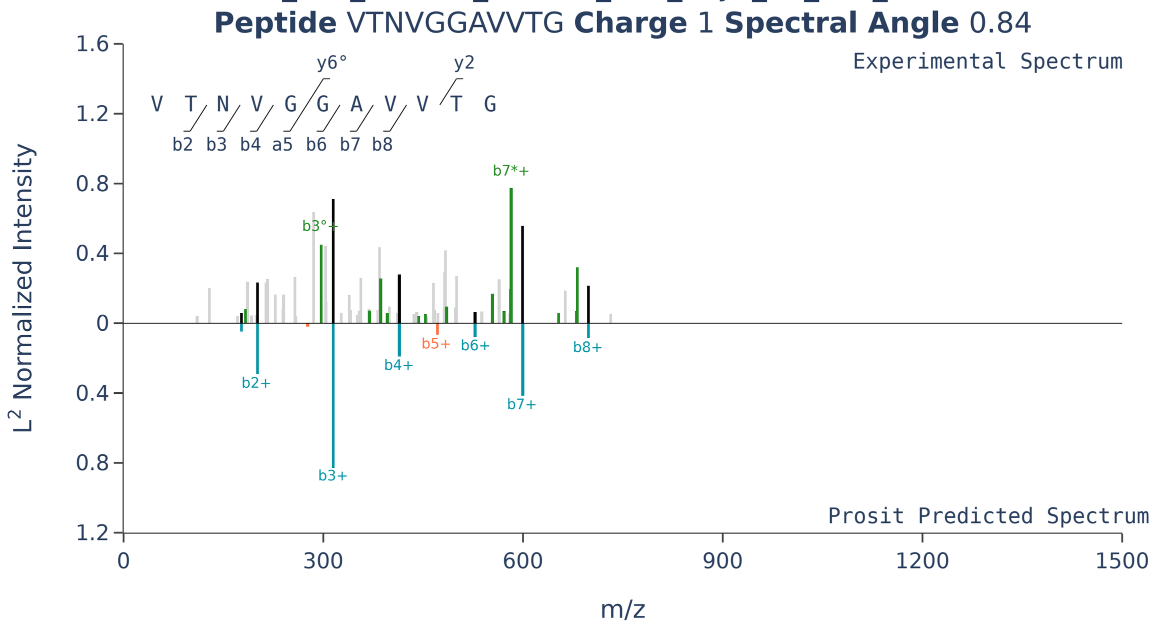


**Source** W_Soh_160821_170921_Goe_aSyn_A2_24h_R2 **Scan** 13896
**Peptide** GAGSIAAATGFVKK **Charge** 2 **Spectral Angle** 0.92

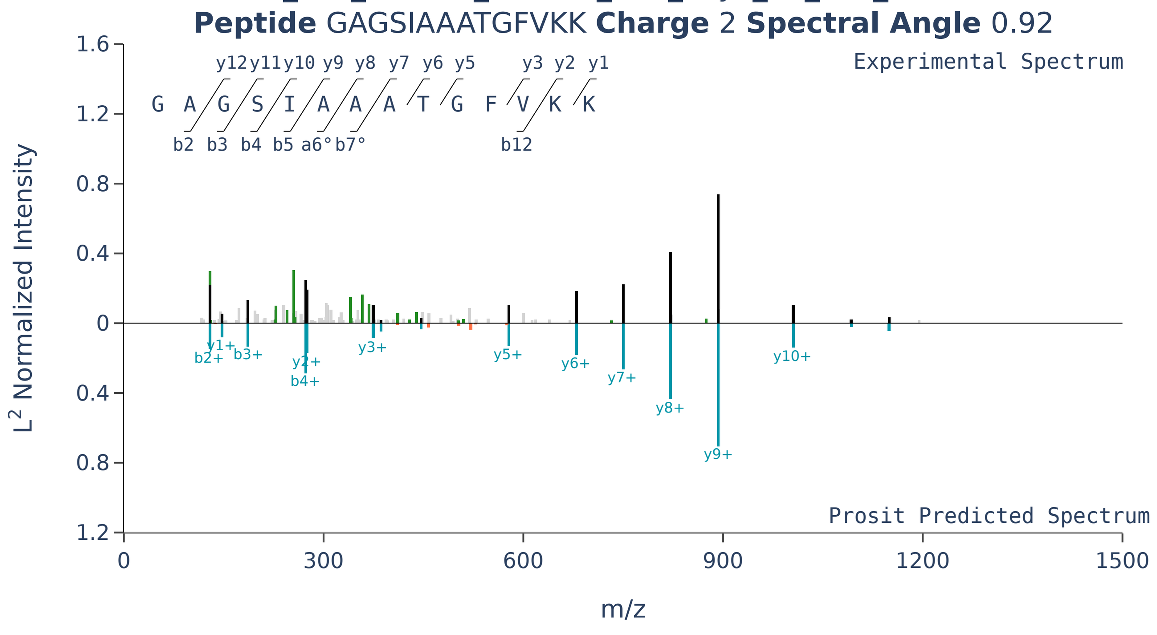


**Source** W_Soh_160821_170921_Goe_aSyn_A1_3h_R1 **Scan** 7035
**Peptide** KEGVVAAAE **Charge** 2 **Spectral Angle** 0.93

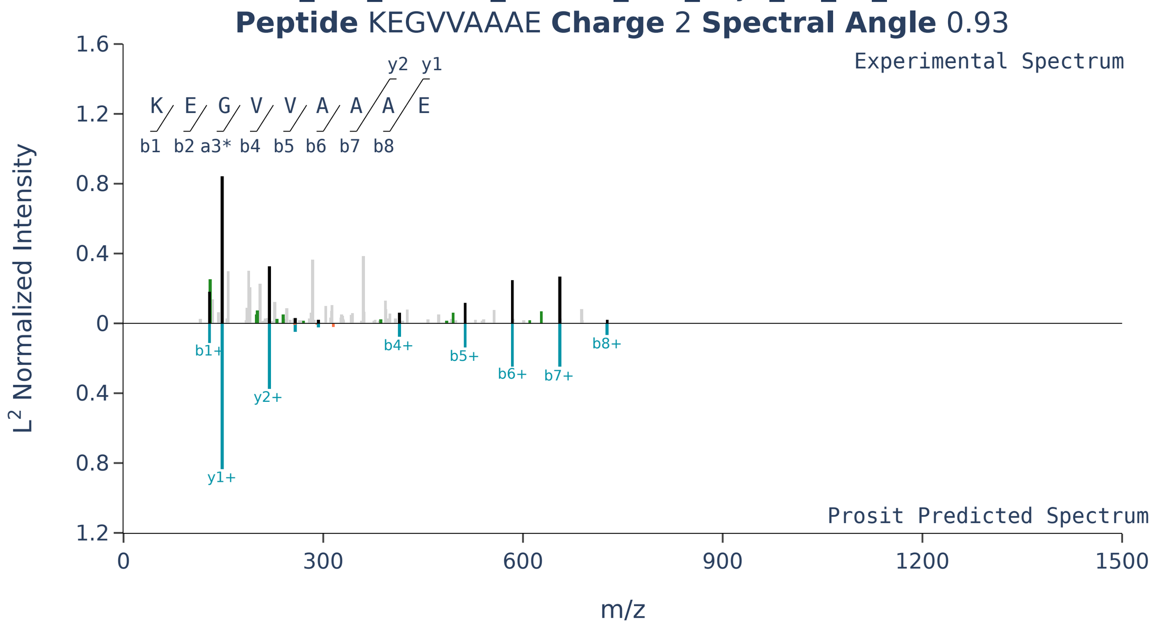


**Source** W_Soh_160821_170921_Goe_aSyn_A2_24h_R2 **Scan** 21173
**Peptide** KDQLGKNEEGAPQEGILEDMPVD **Charge** 3 **Spectral Angle** 0.83

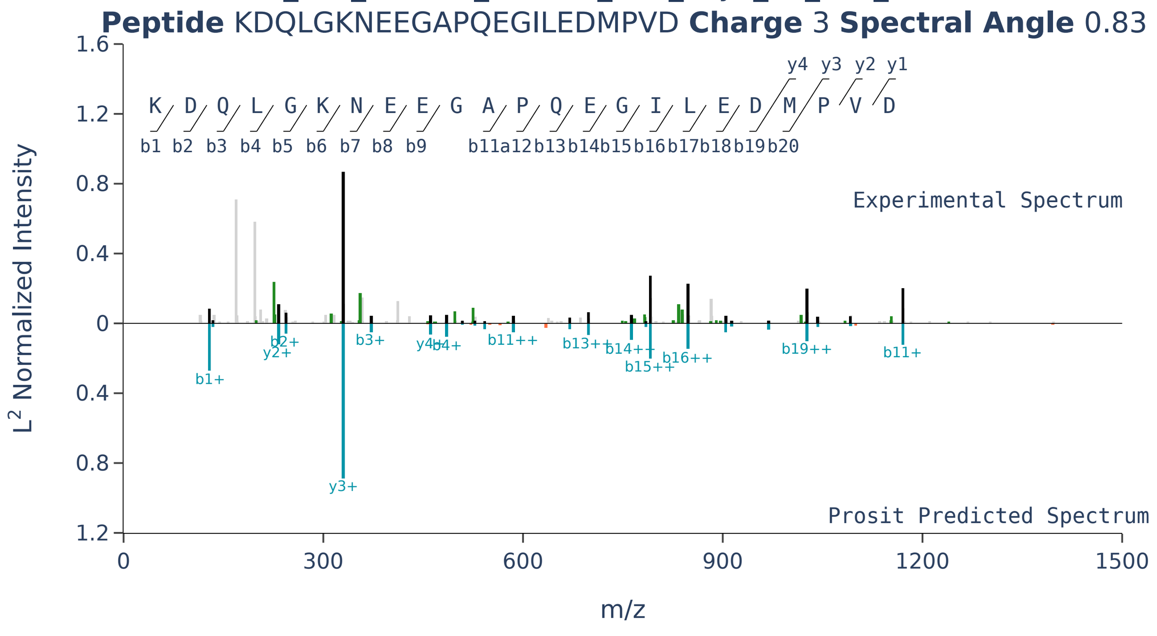


**Source** W_Soh_160821_170921_Goe_aSyn_A1_24h_R2 **Scan** 12817
**Peptide** PDNEAYEM **Charge** 2 **Spectral Angle** 0.93

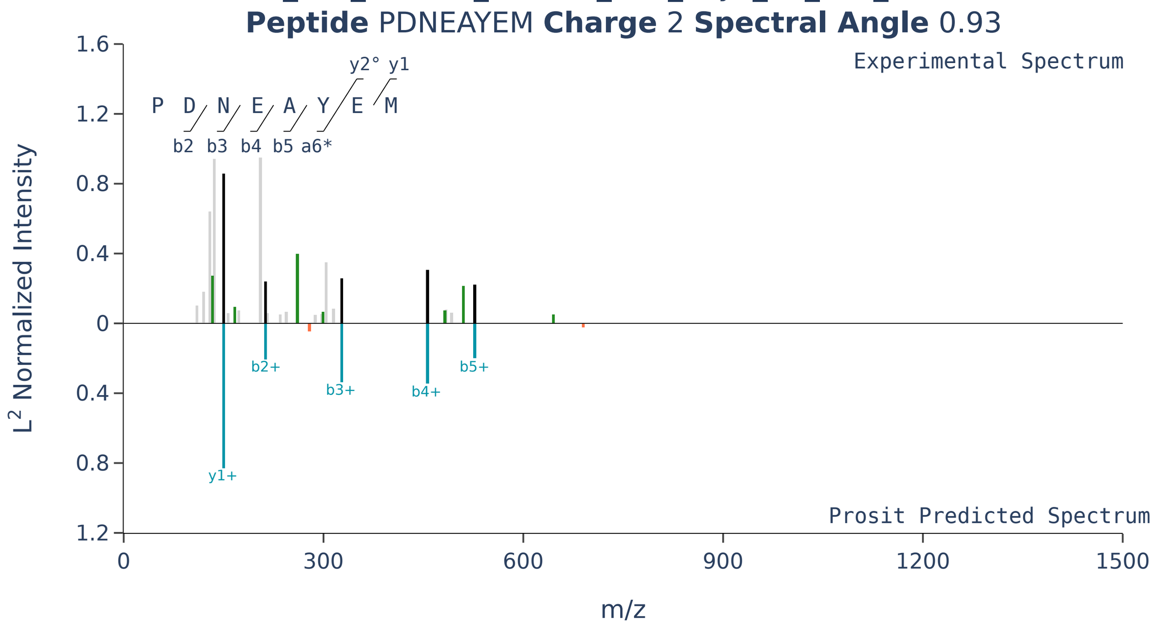


**Source** W_Soh_160821_170921_Goe_aSyn_A1_24h_R1 **Scan** 13374
**Peptide** VTNVGGAVVT **Charge** 2 **Spectral Angle** 0.86

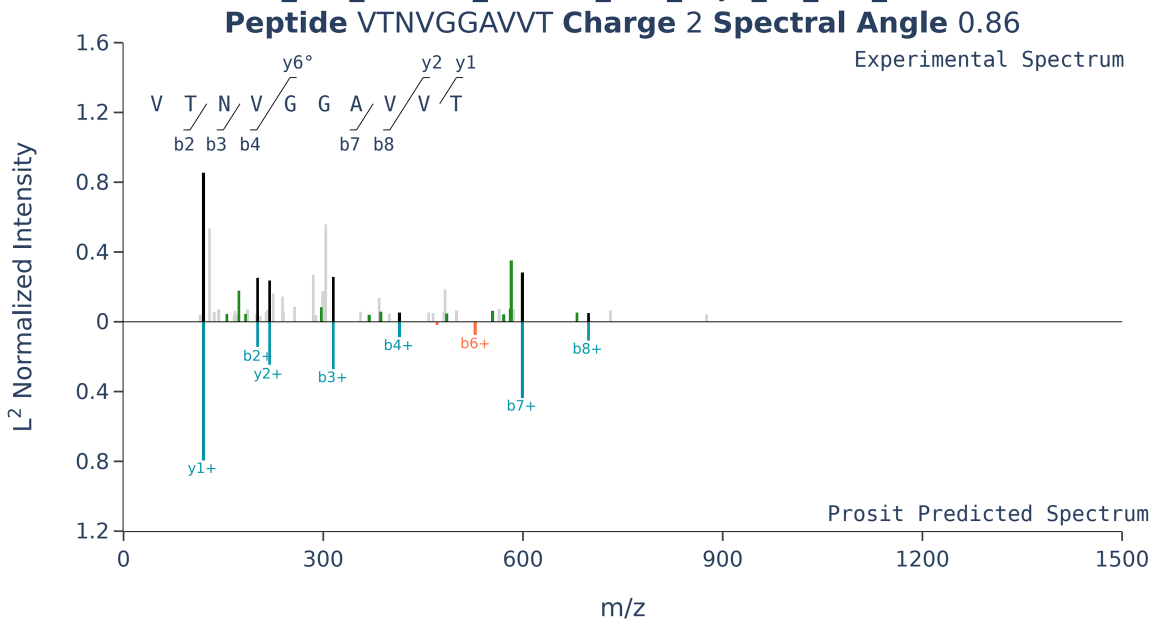


**Source** W_Soh_160821_170921_Goe_aSyn_A1_24h_R2 **Scan** 9866
**Peptide** MPVDPDN **Charge** 1 **Spectral Angle** 0.92

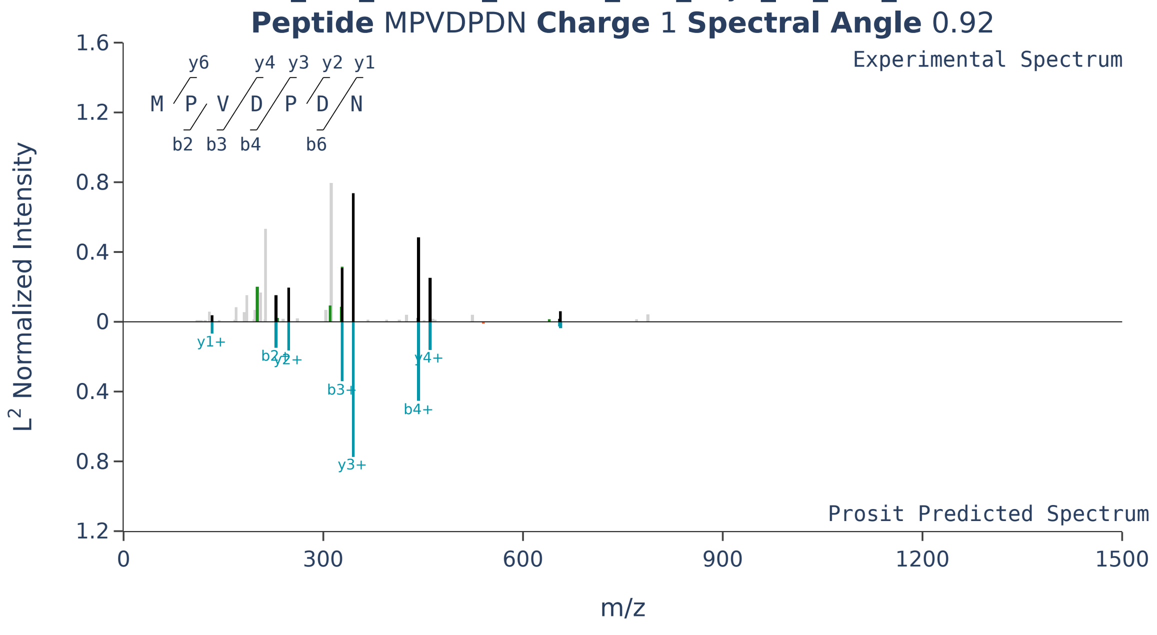


**Source** W_Soh_160821_170921_Goe_aSyn_A2_6h_R1 **Scan** 9193
**Peptide** AQKTVEGAGSIAA **Charge** 2 **Spectral Angle** 0.92

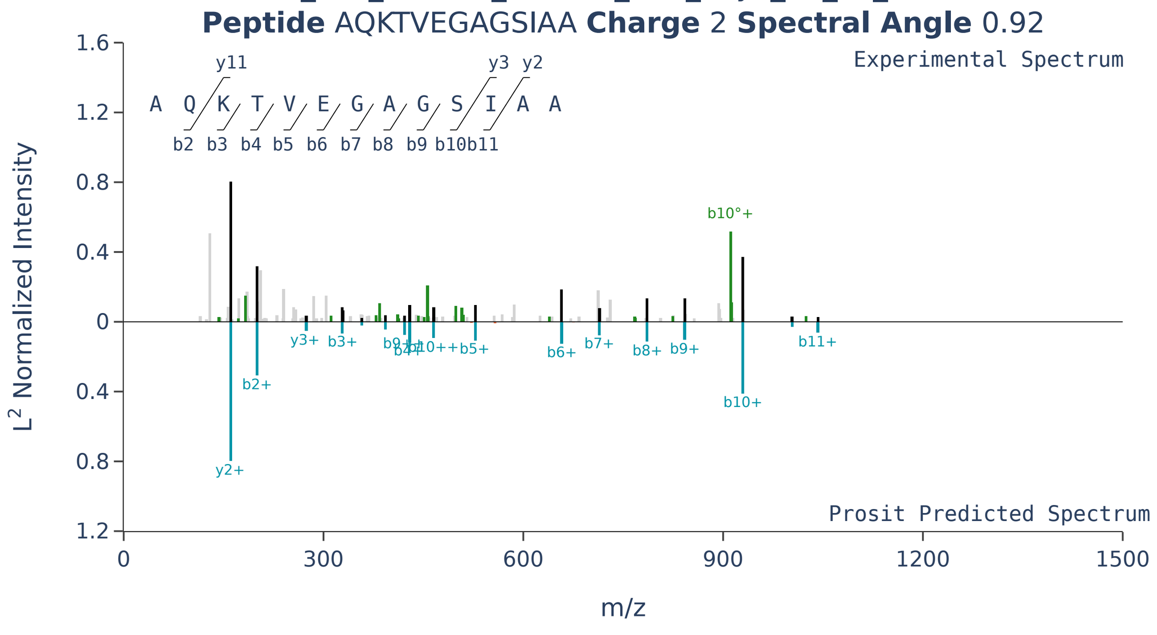


**Source** W_Soh_160821_170921_Goe_aSyn_A1_6h_R1 **Scan** 18212
**Peptide** AEKTKEQVTNVGGAVVTGVTAV **Charge** 3 **Spectral Angle** 0.89

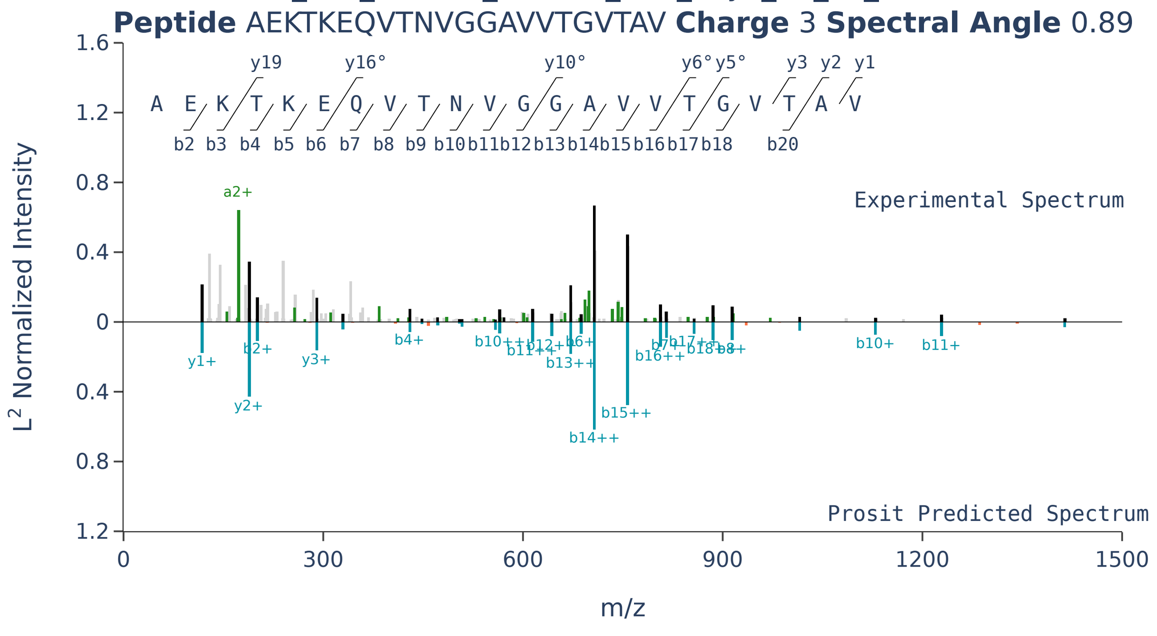


**Source** W_Soh_160821_170921_Goe_aSyn_A2_24h_R1 **Scan** 6519
**Peptide** KEGVVHGV **Charge** 2 **Spectral Angle** 0.94

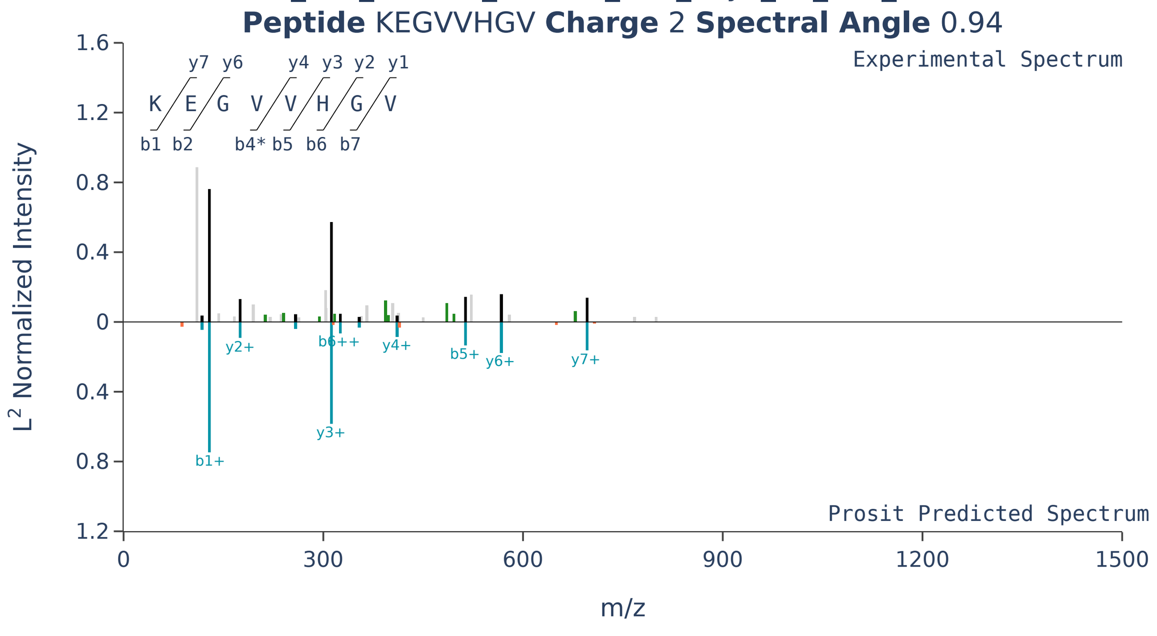


**Source** W_Soh_160821_170921_Goe_aSyn_A1_24h_R1 **Scan** 17063
**Peptide** LEDMPVDPDN **Charge** 1 **Spectral Angle** 0.89

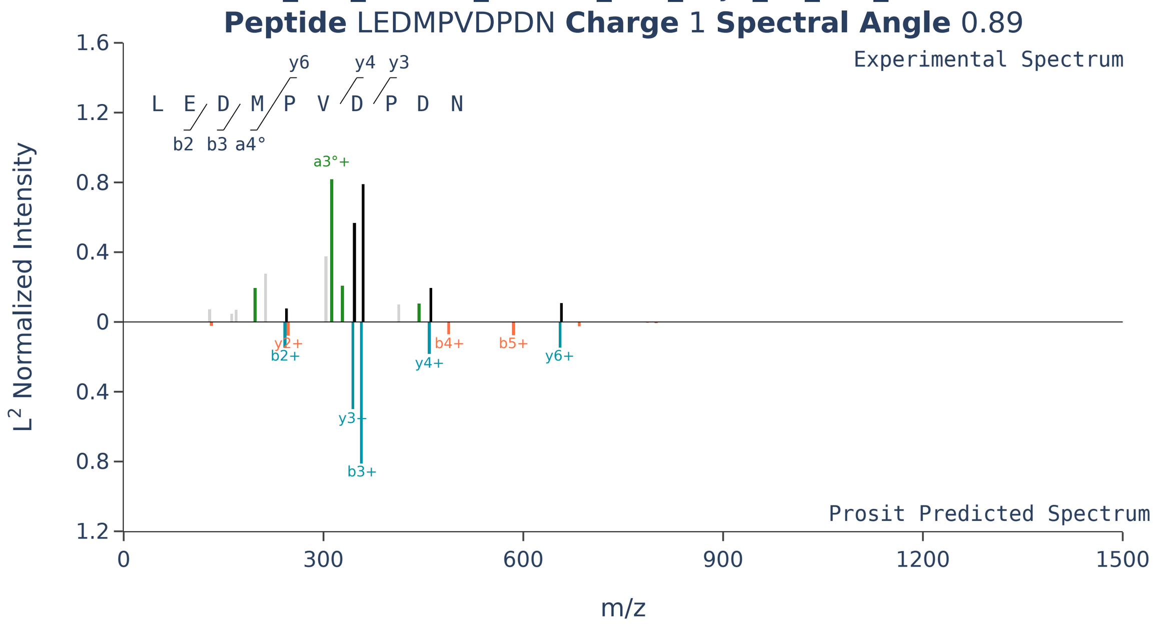

**Source** W_Soh_160821_170921_Goe_aSyn_A2_24h_R1 **Scan** 6421
**Peptide** VGSKTKEGVVHGVAT **Charge** 2 **Spectral Angle** 0.89

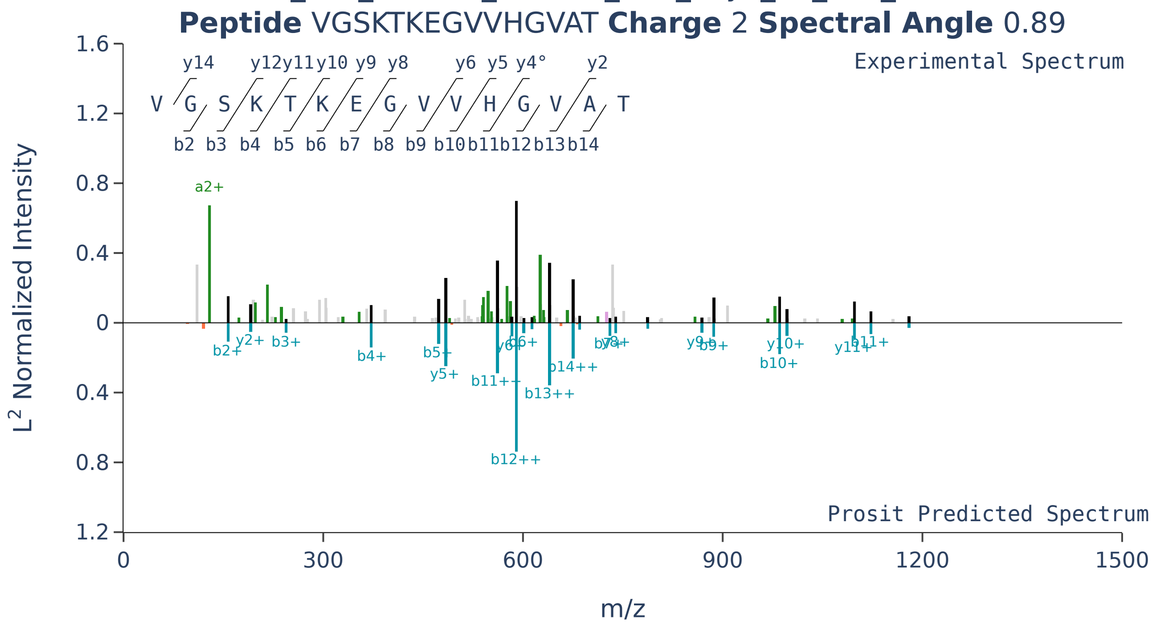


**Source** W_Soh_160821_170921_Goe_aSyn_A1_24h_R2 **Scan** 13687
**Peptide** NEAYEMPSE **Charge** 2 **Spectral Angle** 0.84

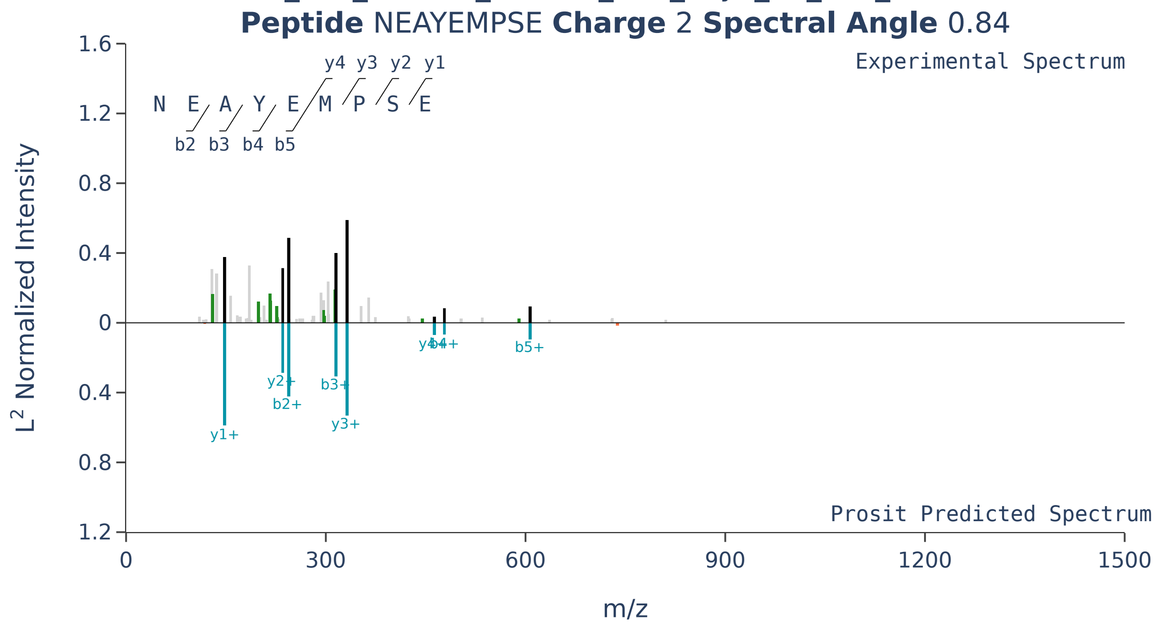


**Source** W_Soh_160821_170921_Goe_aSyn_A1_24h_R1 **Scan** 6743
**Peptide** AAATGFVKKD **Charge** 2 **Spectral Angle** 0.85

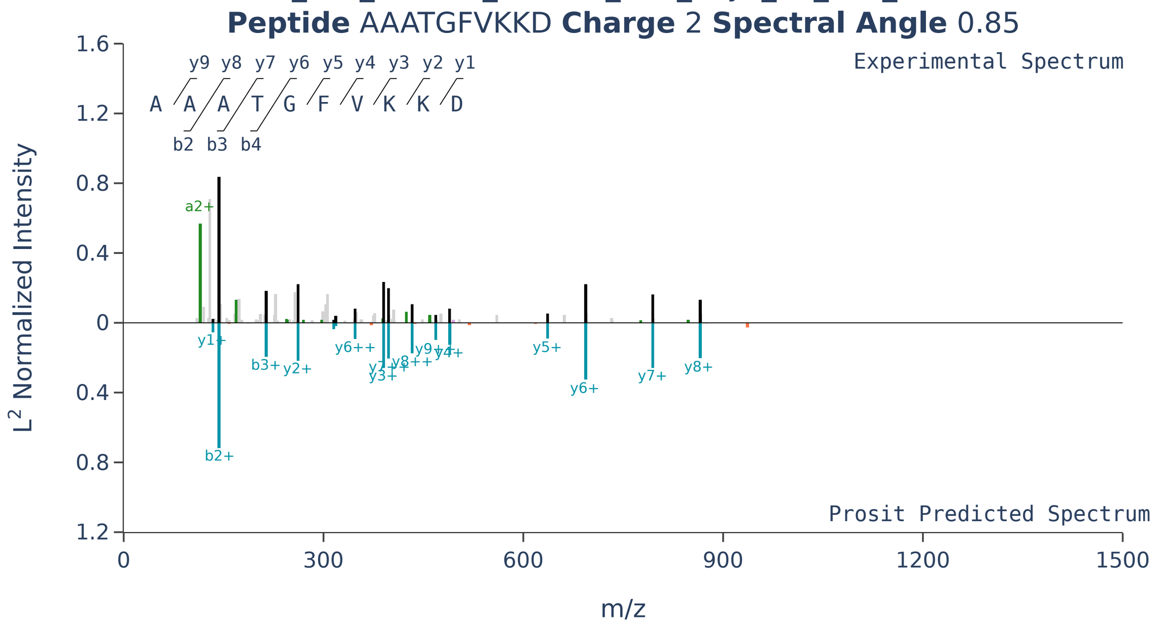


**Source** W_Soh_160821_170921_Goe_aSyn_A1_24h_R2 **Scan** 17567
**Peptide** GVTAVAQKTVEGAGSIAA **Charge** 2 **Spectral Angle** 0.9

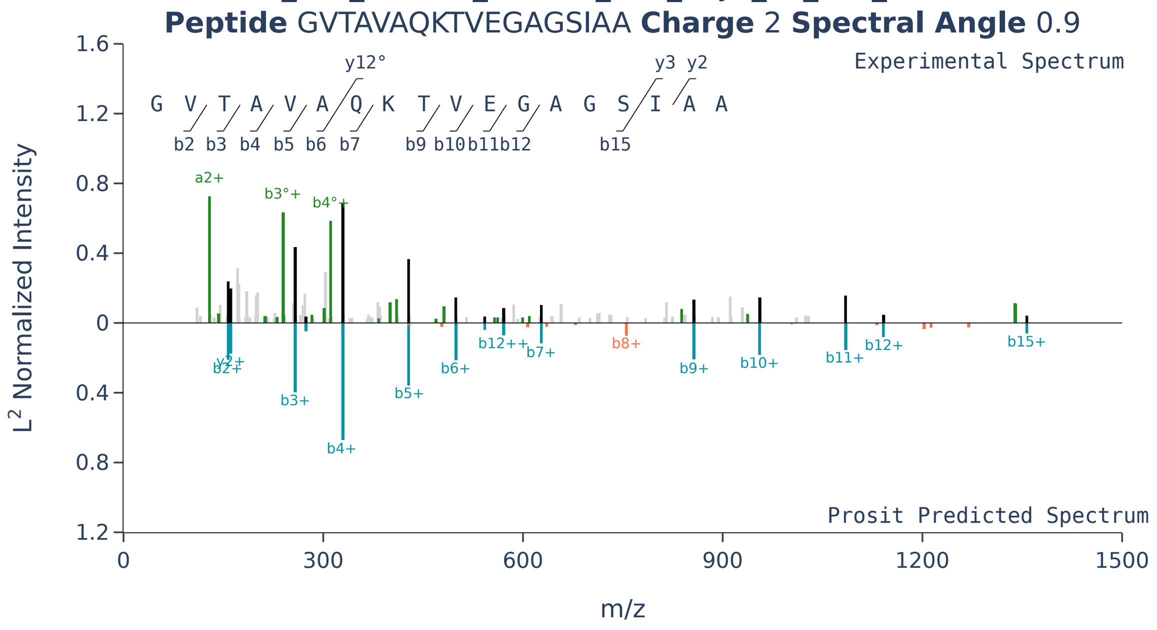


**Source** W_Soh_160821_170921_Goe_aSyn_A1_1h_R2 **Scan** 18732
**Peptide** KEQVTNVGGAVVTGVTAVA **Charge** 2 **Spectral Angle** 0.86

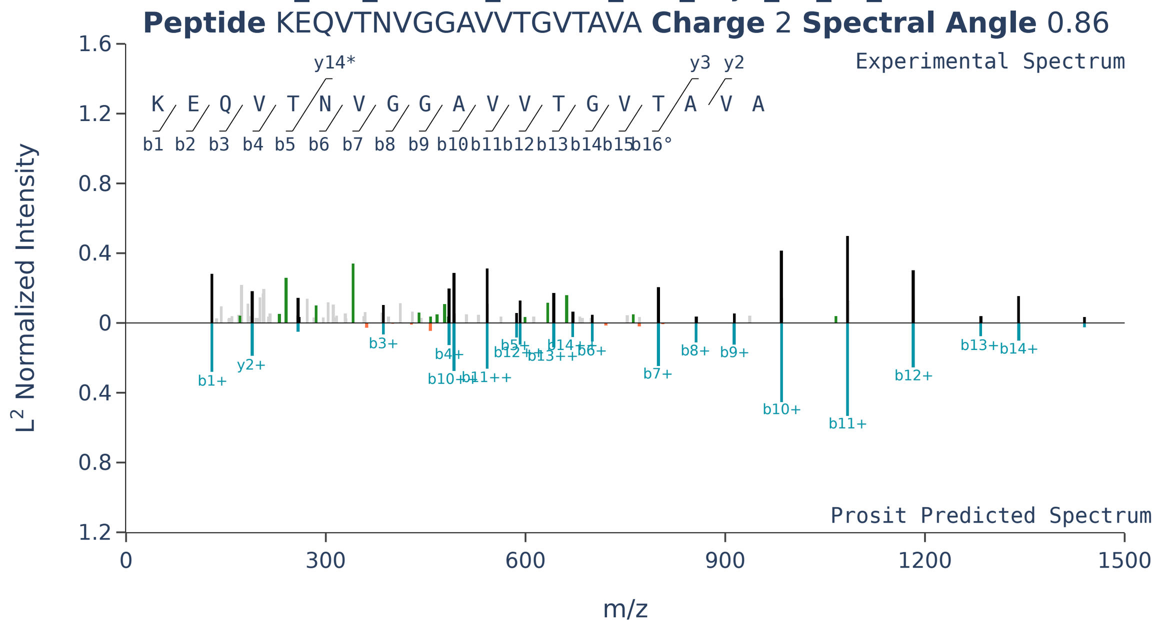


**Source** W_Soh_160821_170921_Goe_aSyn_A1_6h_R1 **Scan** 5685
**Peptide** GVVAAAEKTKQ **Charge** 3 **Spectral Angle** 0.84

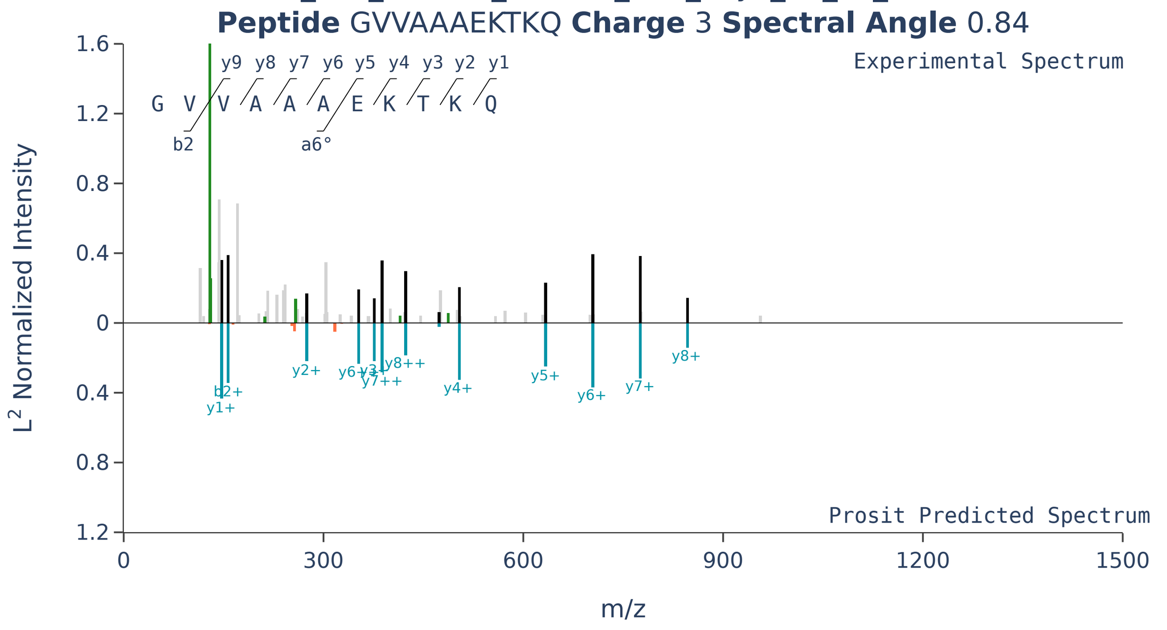


**Source** W_Soh_160821_170921_Goe_aSyn_A1_24h_R1 **Scan** 14331
**Peptide** DVFMKGLSK **Charge** 2 **Spectral Angle** 0.91

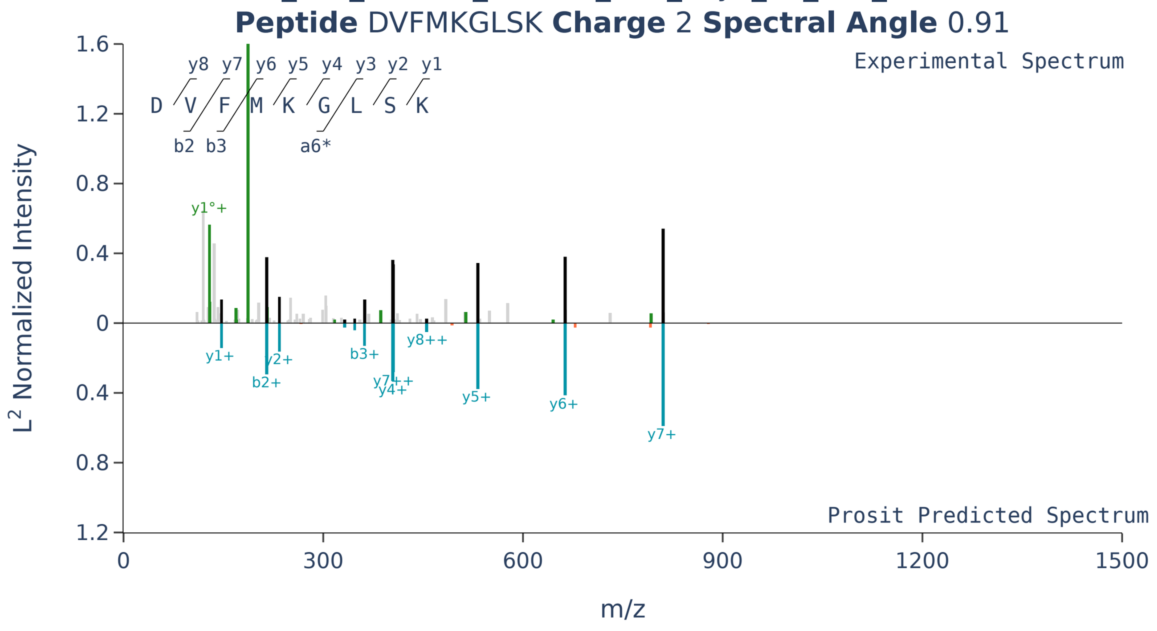


**Source** W_Soh_160821_170921_Goe_aSyn_A2_6h_R2 **Scan** 12313
**Peptide** LYVGSKTKEGVVHGV **Charge** 3 **Spectral Angle** 0.92

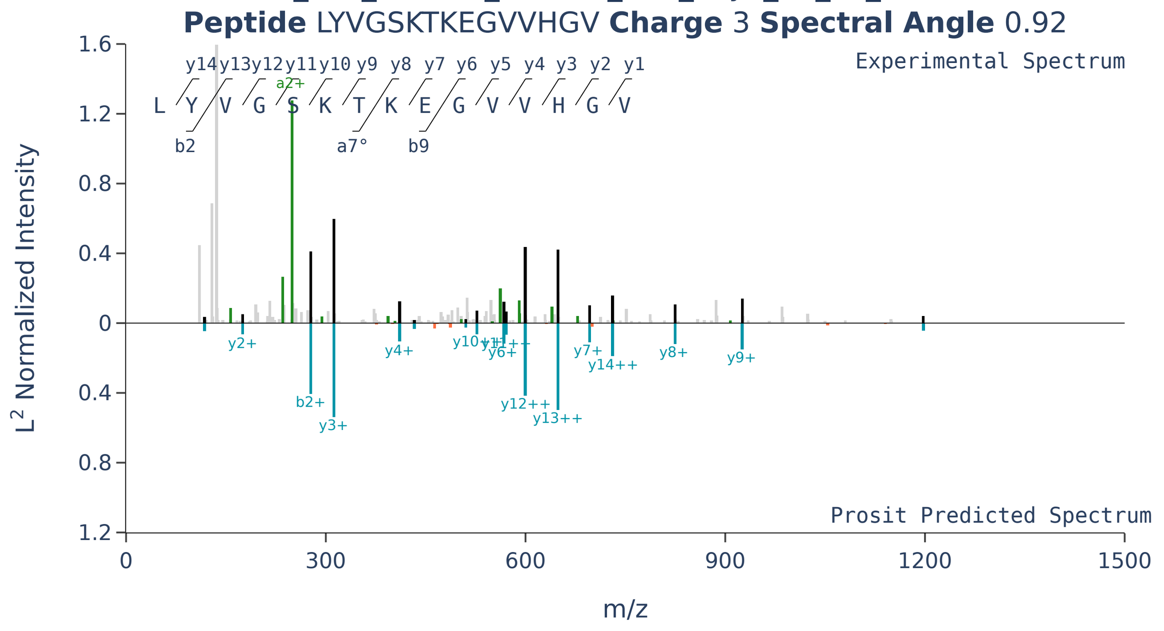


**Source** W_Soh_160821_170921_Goe_aSyn_A2_24h_R1 **Scan** 8149
**Peptide** AYEMPSE **Charge** 1 **Spectral Angle** 0.88

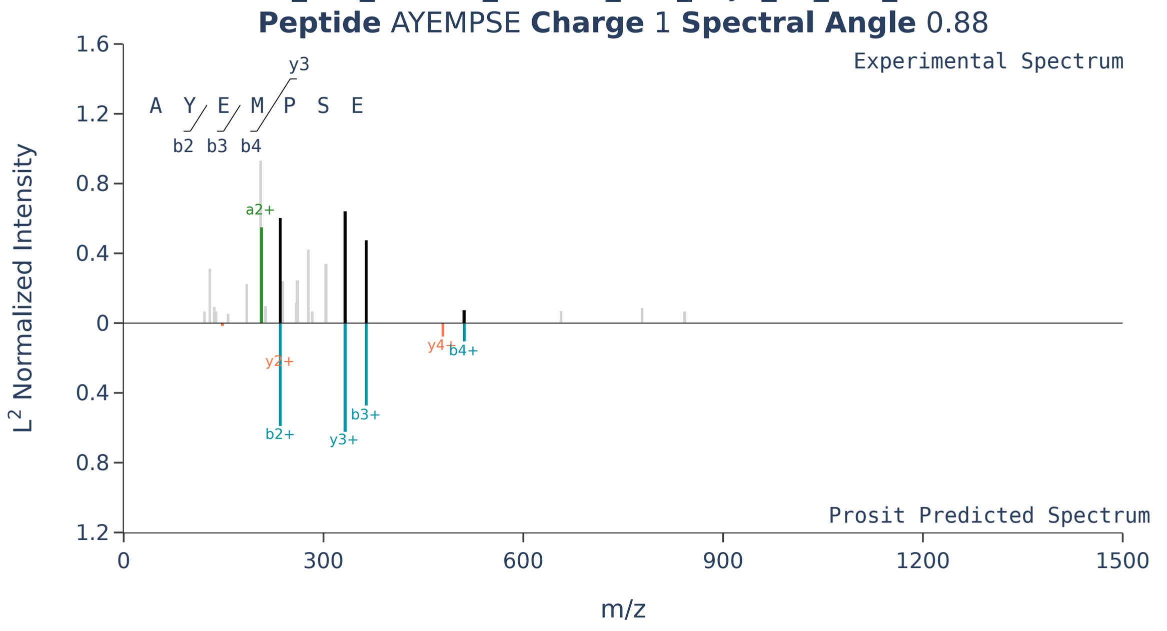


**Source** W_Soh_160821_170921_Goe_aSyn_A1_24h_R2 **Scan** 14119
**Peptide** SEEGYQDYEPEA **Charge** 2 **Spectral Angle** 0.92

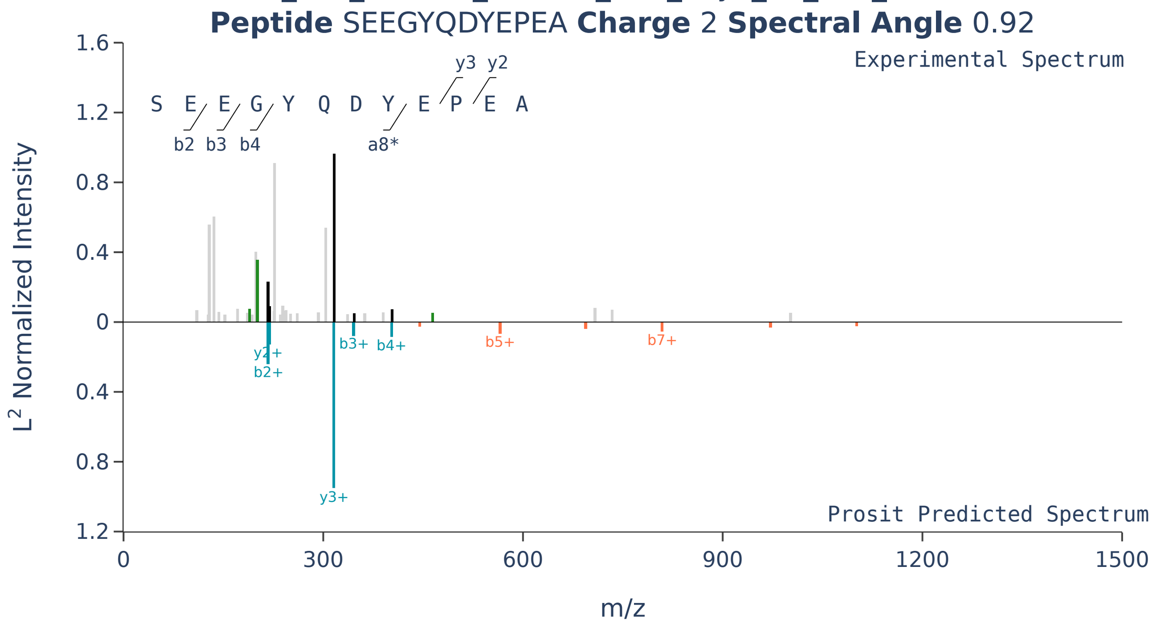


**Source** W_Soh_160821_170921_Goe_aSyn_A1_24h_R1 **Scan** 14121
**Peptide** MDVFMKGLSKAK **Charge** 3 **Spectral Angle** 0.94

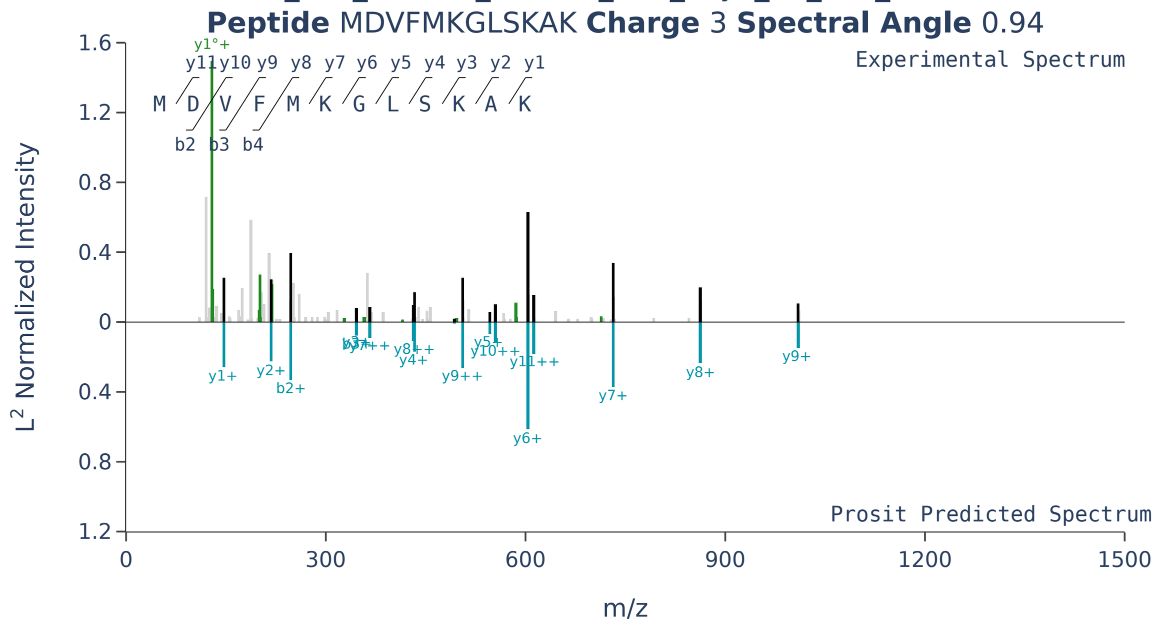


**Source** W_Soh_160821_170921_Goe_aSyn_A2_6h_R2 **Scan** 13899
**Peptide** QVTNVGGAVVT **Charge** 1 **Spectral Angle** 0.86

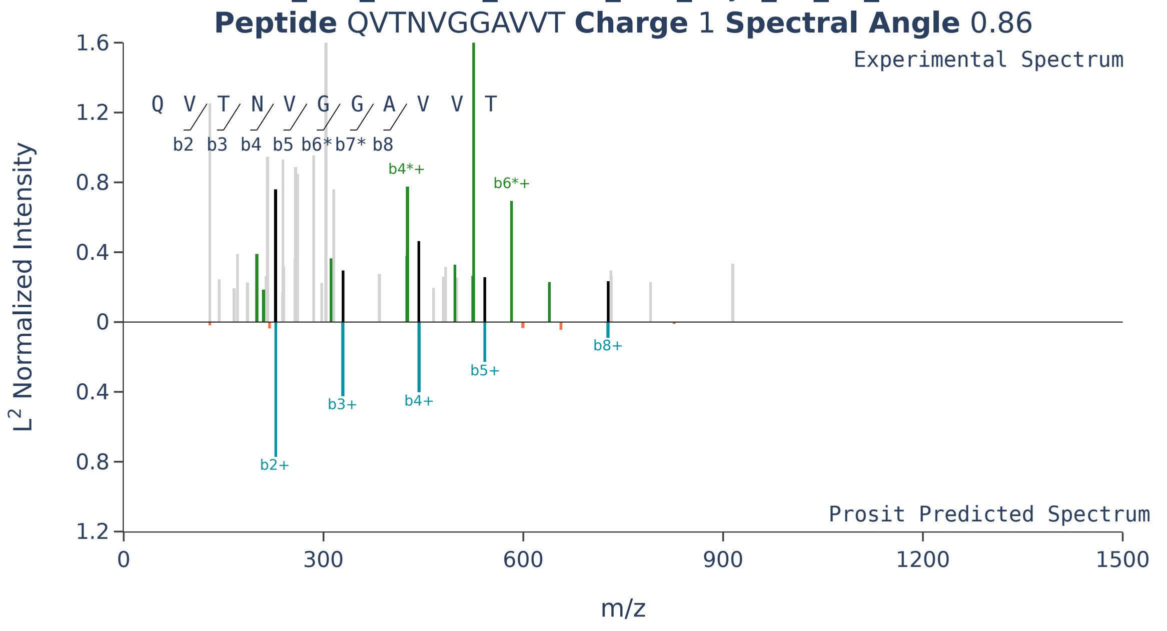


**Source** W_Soh_160821_170921_Goe_aSyn_A2_24h_R2 **Scan** 17133
**Peptide** VGGAVVTGVTAVAQKT **Charge** 2 **Spectral Angle** 0.91

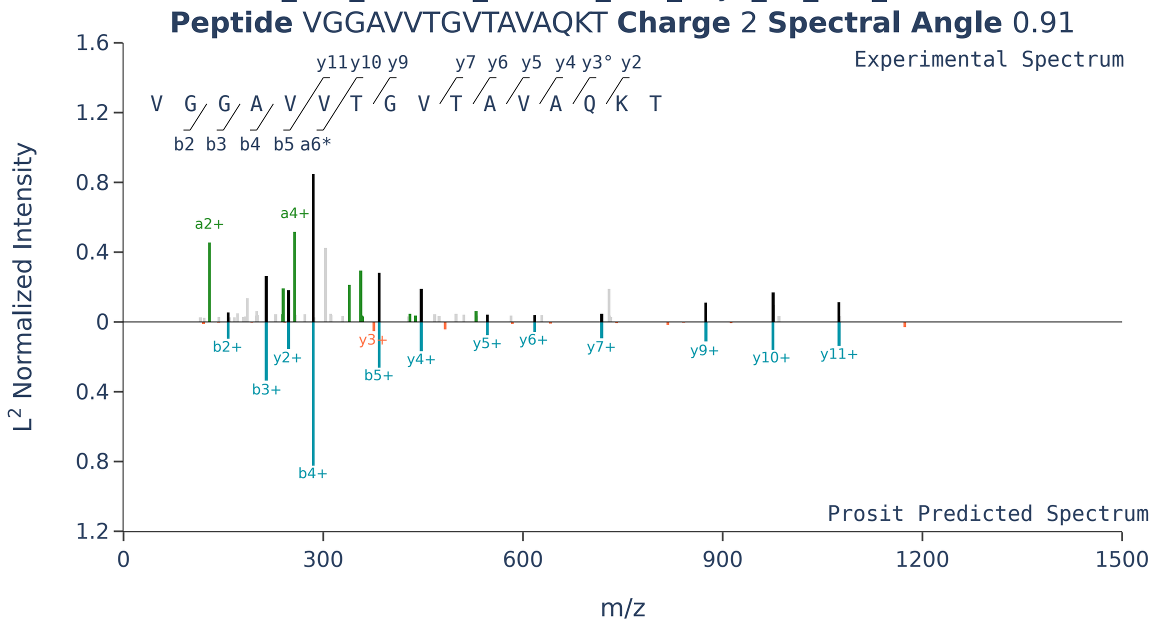


**Source** W_Soh_160821_170921_Goe_aSyn_A1_24h_R1 **Scan** 7685
**Peptide** VTAVAQKTVEG **Charge** 1 **Spectral Angle** 0.9

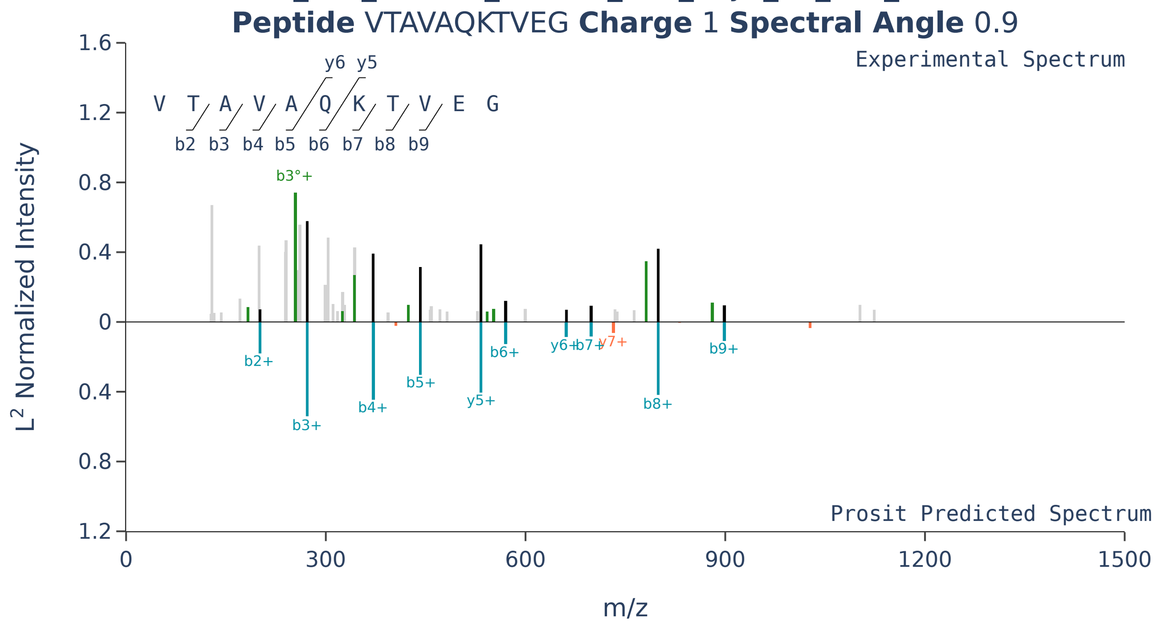


**Source** W_Soh_160821_170921_Goe_aSyn_A1_24h_R2 **Scan** 7577
**Peptide** VTAVAQKTVE **Charge** 1 **Spectral Angle** 0.9

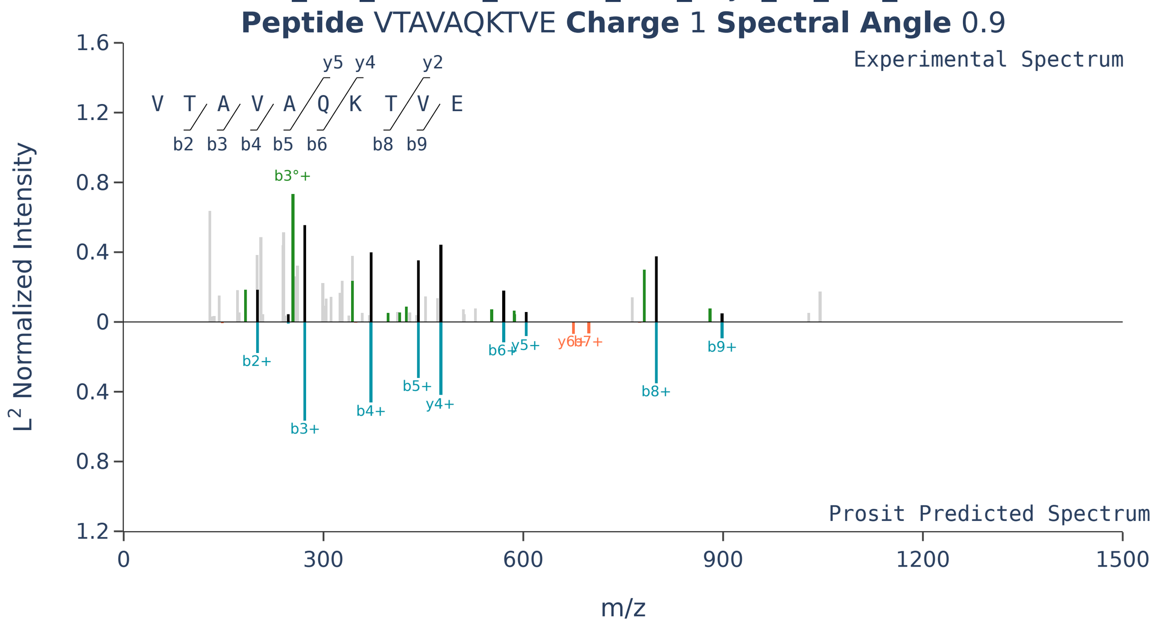

**Source** W_Soh_160821_170921_Goe_aSyn_A1_24h_R1 **Scan** 13516
**Peptide** ATVAEKTKEQVTNVGGAVVT **Charge** 2 **Spectral Angle** 0.85

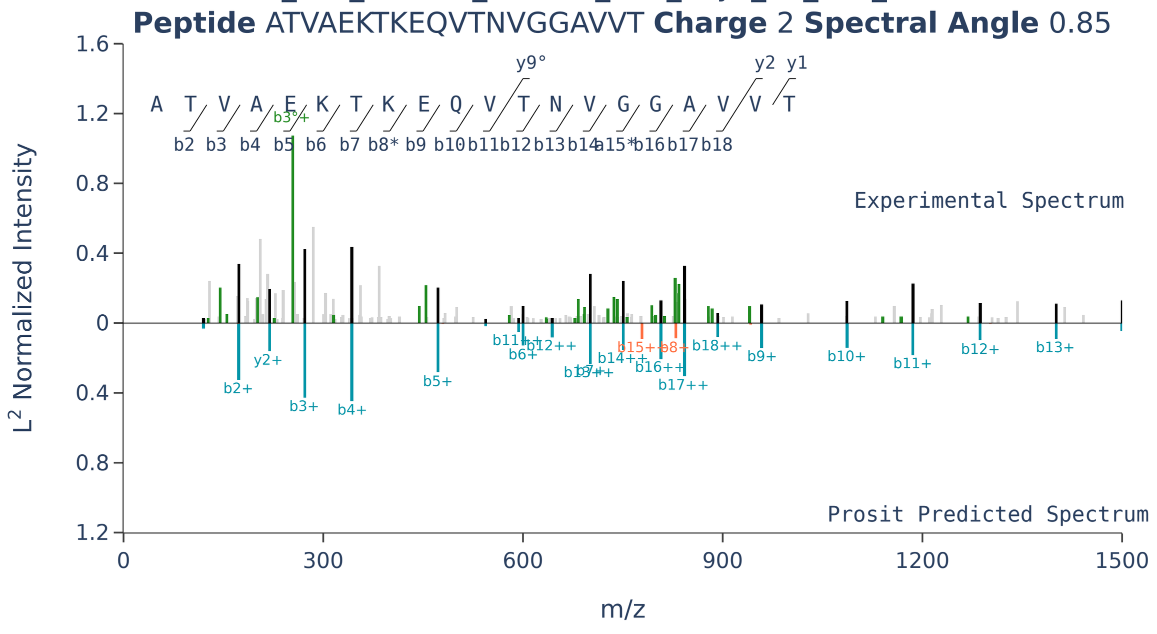


**Source** W_Soh_160821_170921_Goe_aSyn_A1_24h_R2 **Scan** 15158
**Peptide** EGAGSIAAATGFVKK **Charge** 2 **Spectral Angle** 0.88

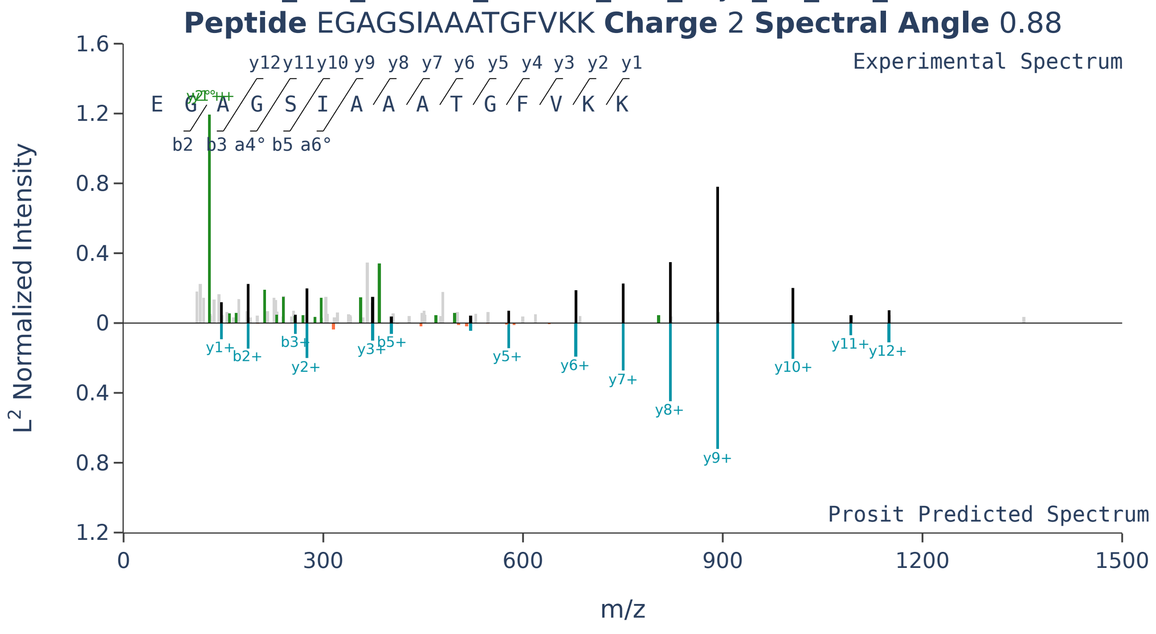


**Source** W_Soh_160821_170921_Goe_aSyn_A1_24h_R1 **Scan** 6218
**Peptide** TAVAQKTVEG **Charge** 1 **Spectral Angle** 0.86

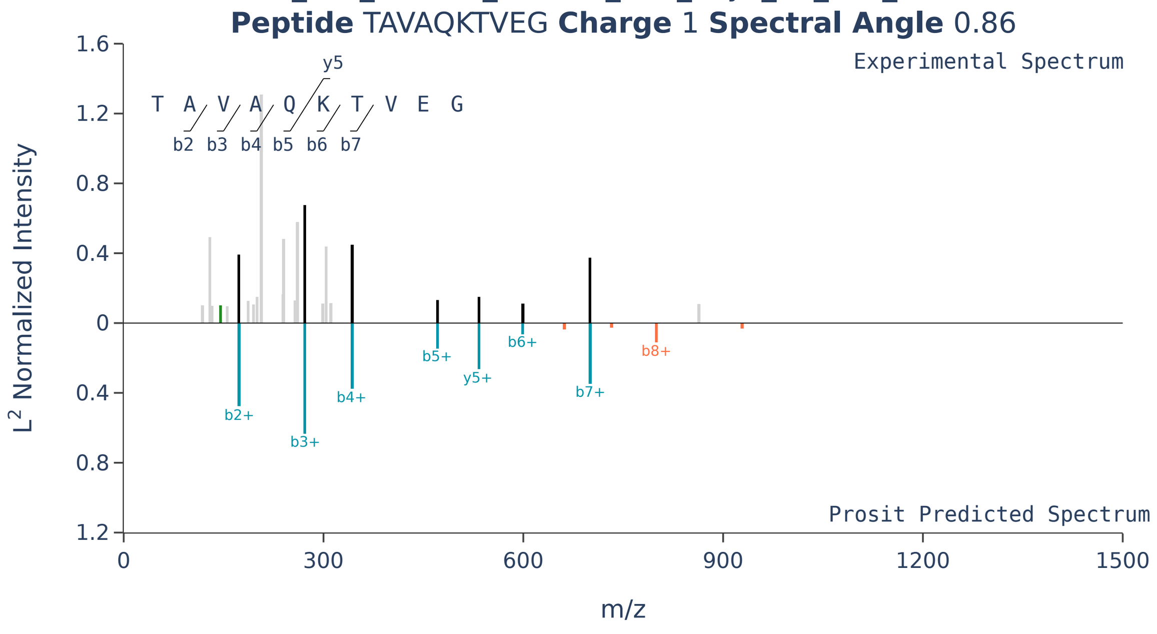


**Source** W_Soh_160821_170921_Goe_aSyn_A1_6h_R2 **Scan** 6031
**Peptide** EKTKEQVTNV **Charge** 2 **Spectral Angle** 0.88

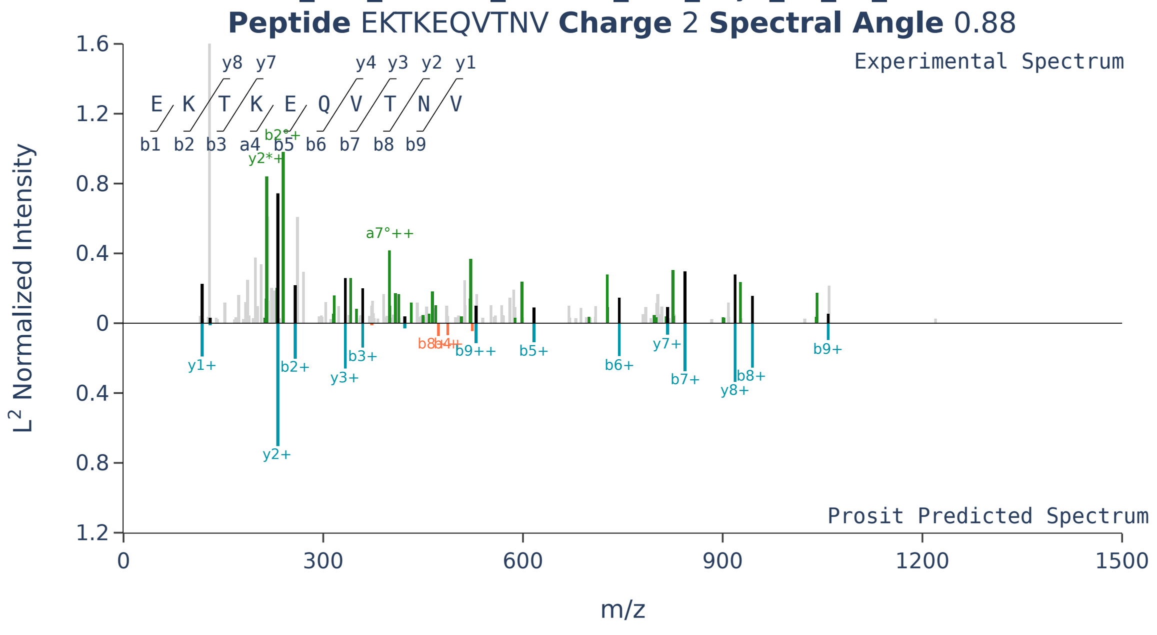


**Source** W_Soh_160821_170921_Goe_aSyn_A1_24h_R1 **Scan** 6676
**Peptide** PDNEAYE **Charge** 1 **Spectral Angle** 0.93

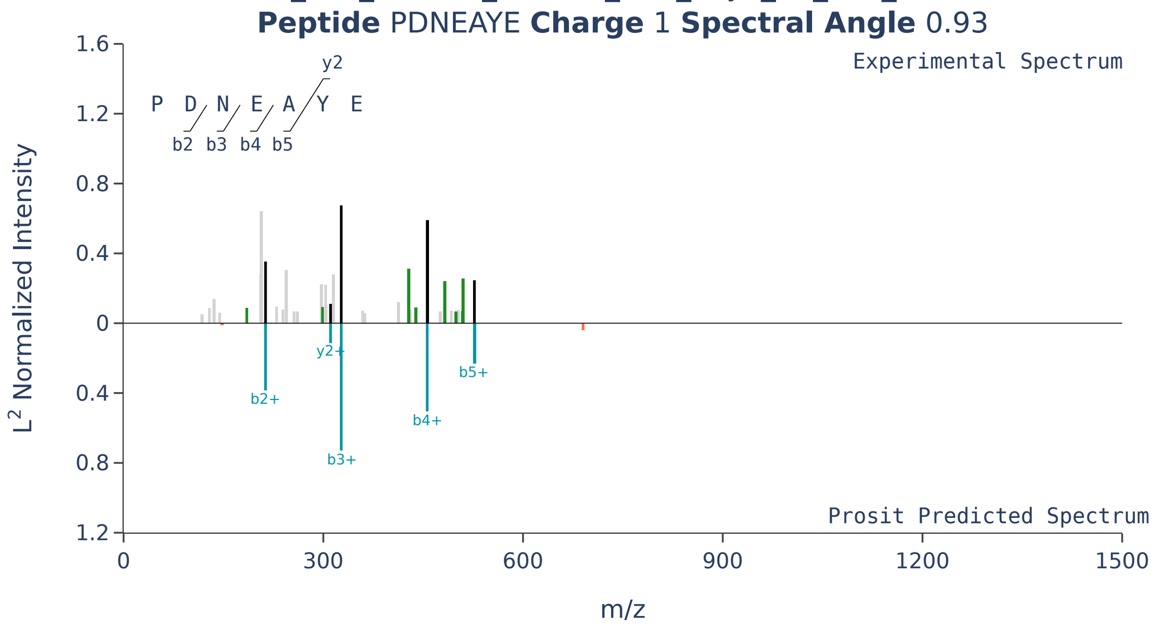


**Source** W_Soh_160821_170921_Goe_aSyn_A2_24h_R2 **Scan** 16747
**Peptide** VEGAGSIAAATGFVKKDQ **Charge** 3 **Spectral Angle** 0.86

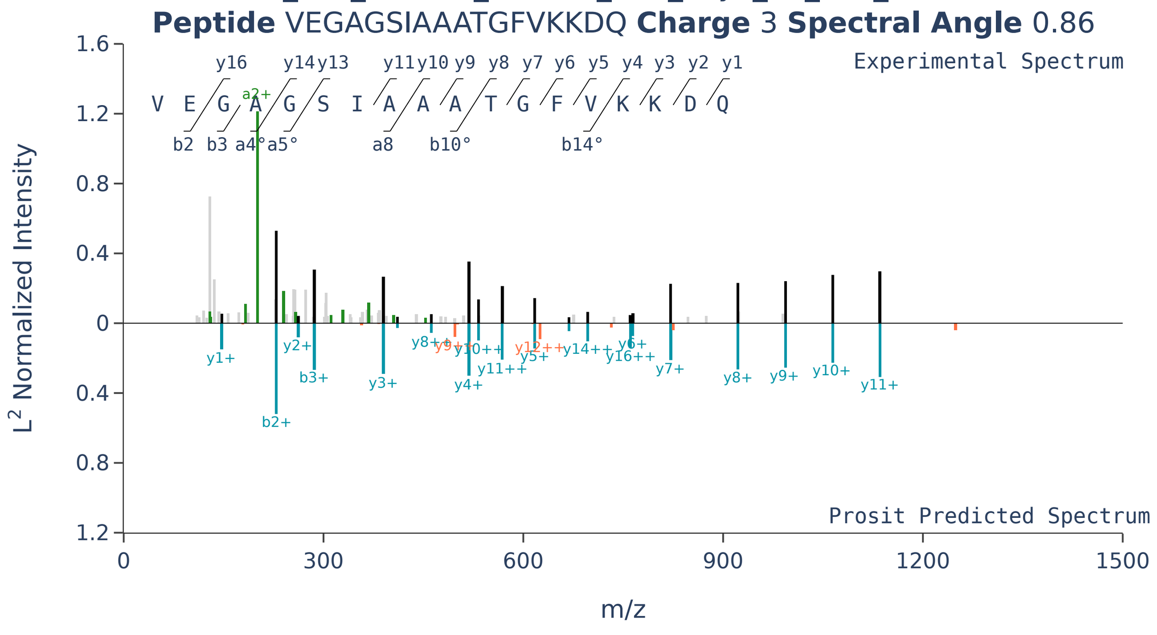


**Source** W_Soh_160821_170921_Goe_aSyn_A1_24h_R1 **Scan** 7534
**Peptide** TAVAQKTVE **Charge** 1 **Spectral Angle** 0.95

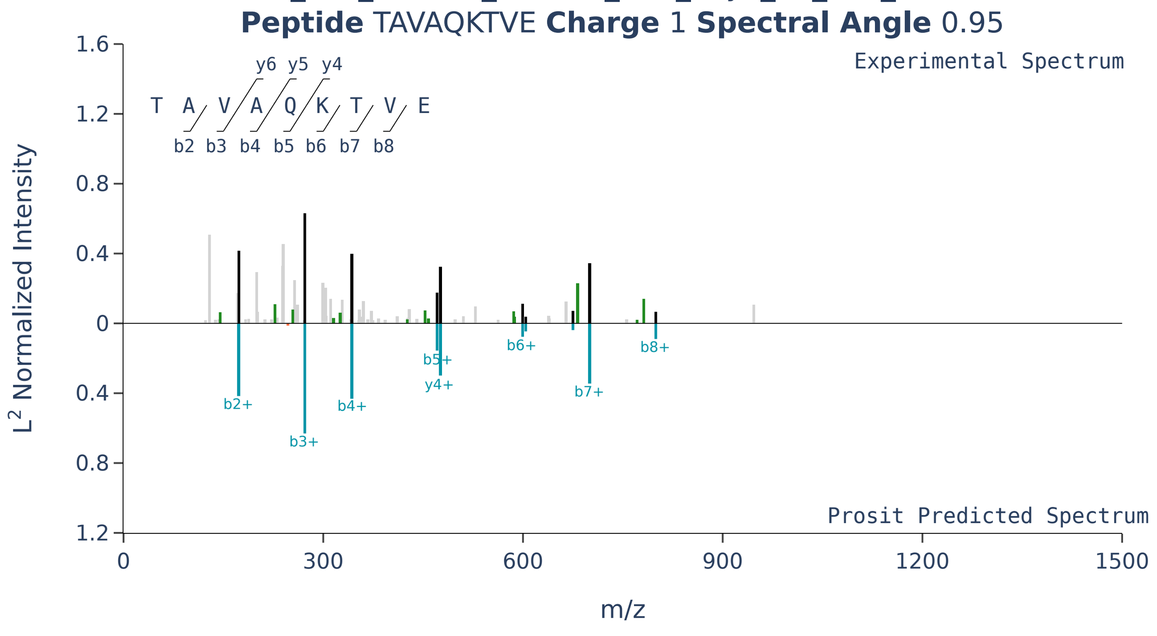


**Source** W_Soh_160821_170921_Goe_aSyn_A1_24h_R1 **Scan** 20381
**Peptide** MPVDPDNEAYEMPSE **Charge** 2 **Spectral Angle** 0.92

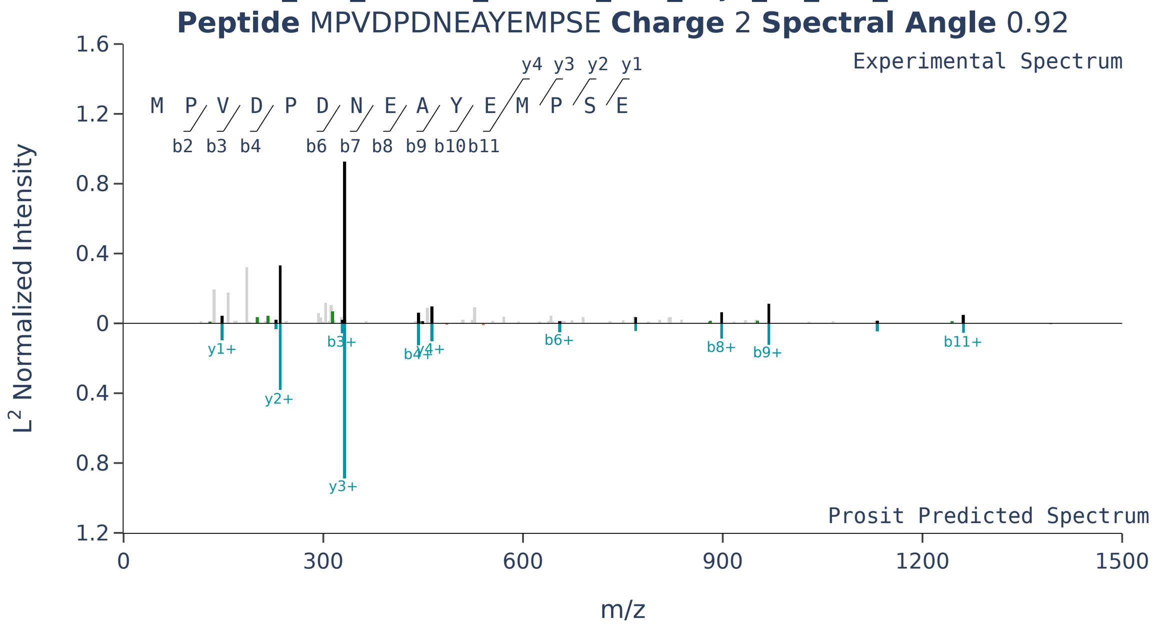


**Source** W_Soh_160821_170921_Goe_aSyn_A1_24h_R1 **Scan** 12799
**Peptide** AYEMPSEE **Charge** 2 **Spectral Angle** 0.91

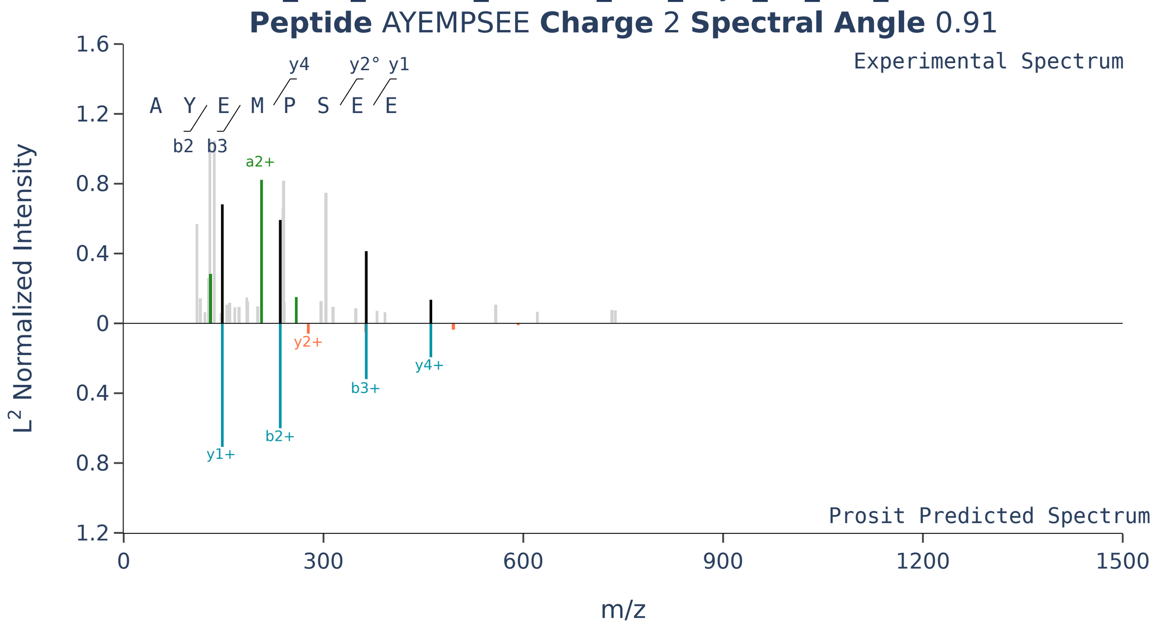


**Source** W_Soh_160821_170921_Goe_aSyn_A1_6h_R2 **Scan** 19967
**Peptide** AYEMPSEEGYQDYEPEA **Charge** 2 **Spectral Angle** 0.89

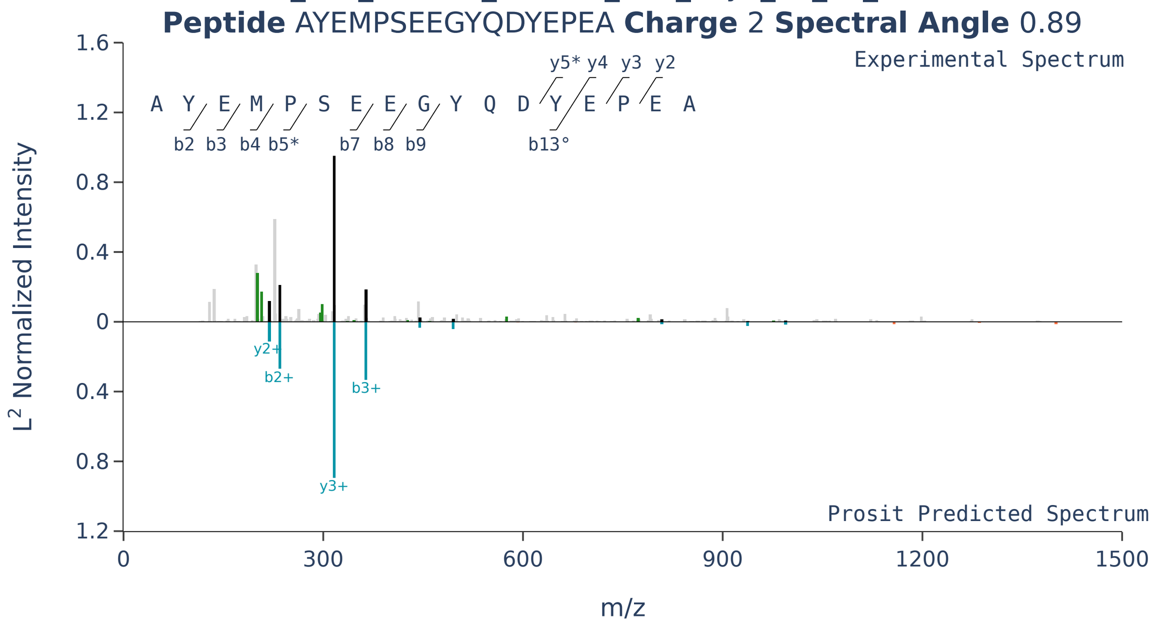


**Source** W_Soh_160821_170921_Goe_aSyn_A1_24h_R1 **Scan** 16845
**Peptide** PDNEAYEMPSEEGYQD **Charge** 2 **Spectral Angle** 0.84

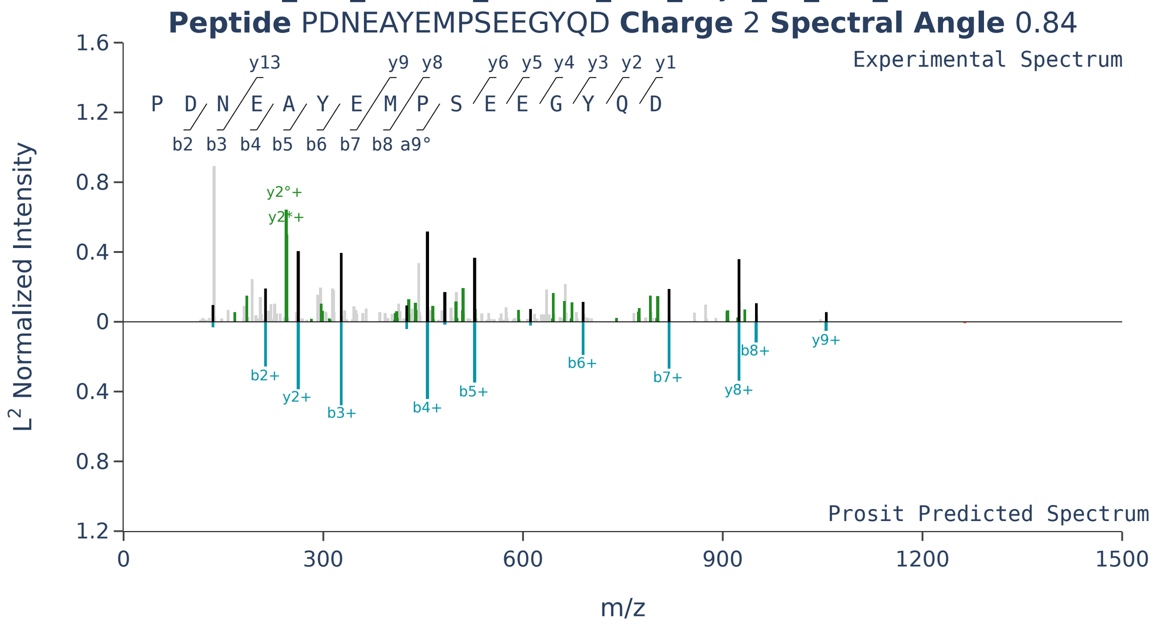


**Source** W_Soh_160821_170921_Goe_aSyn_A2_1h_R2 **Scan** 16516
**Peptide** EGAGSIAAATGFVKKDQL **Charge** 3 **Spectral Angle** 0.87

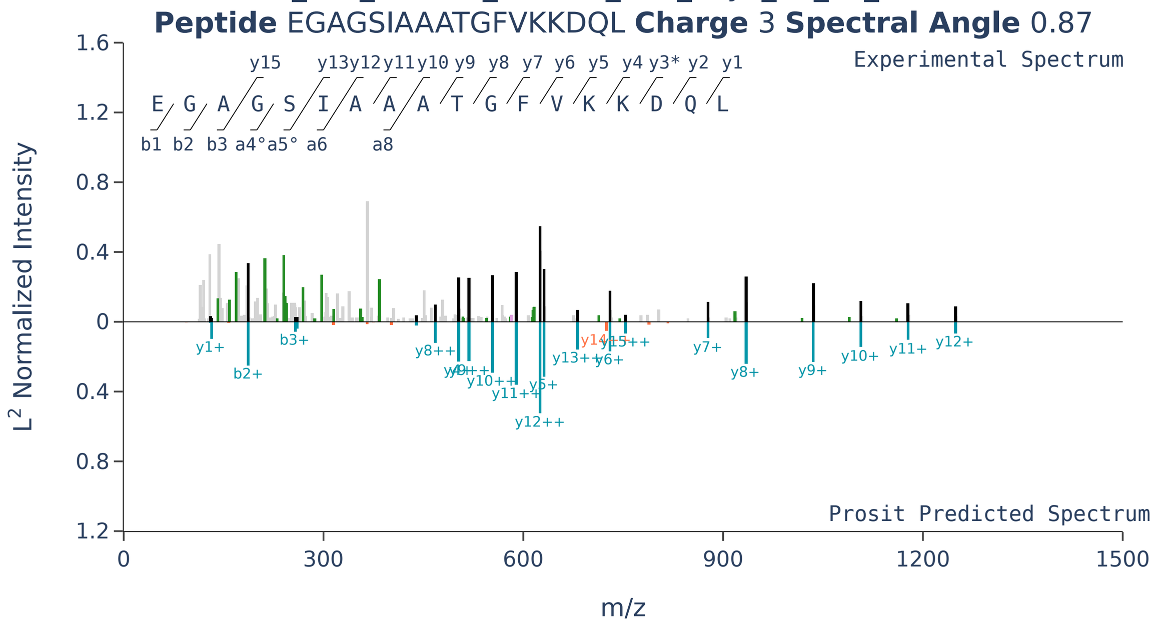


**Source** W_Soh_160821_170921_Goe_aSyn_A2_24h_R2 **Scan** 20473
**Peptide** MDVFMKGL **Charge** 2 **Spectral Angle** 0.84

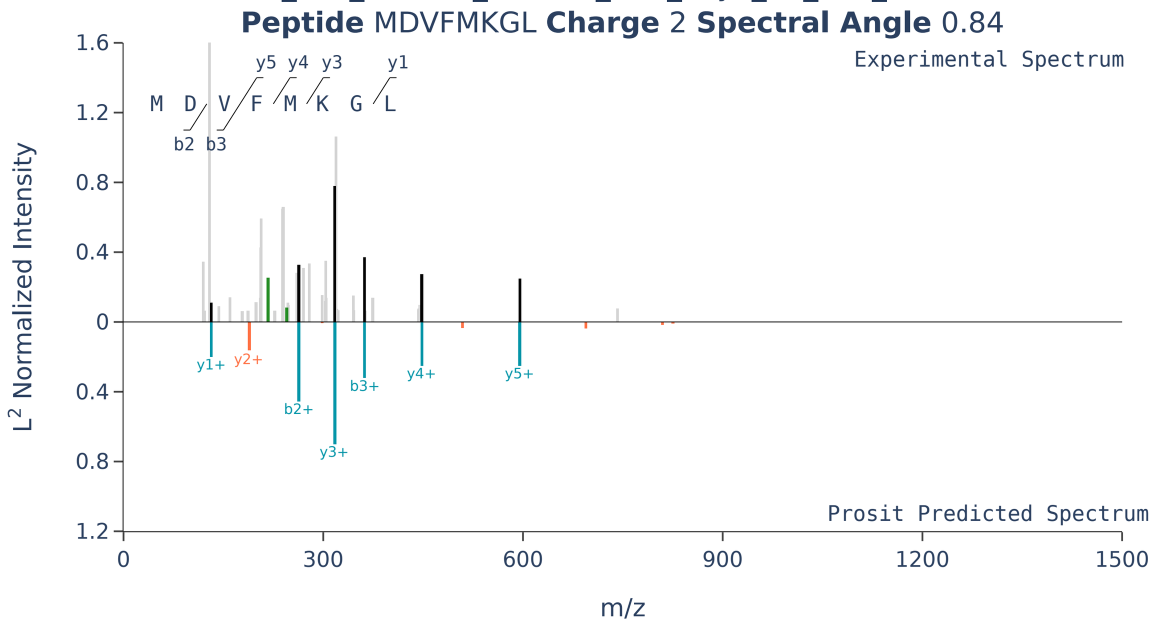


**Source** W_Soh_160821_170921_Goe_aSyn_A1_6h_R1 **Scan** 17536
**Peptide** LEDMPVDPDNE **Charge** 2 **Spectral Angle** 0.81

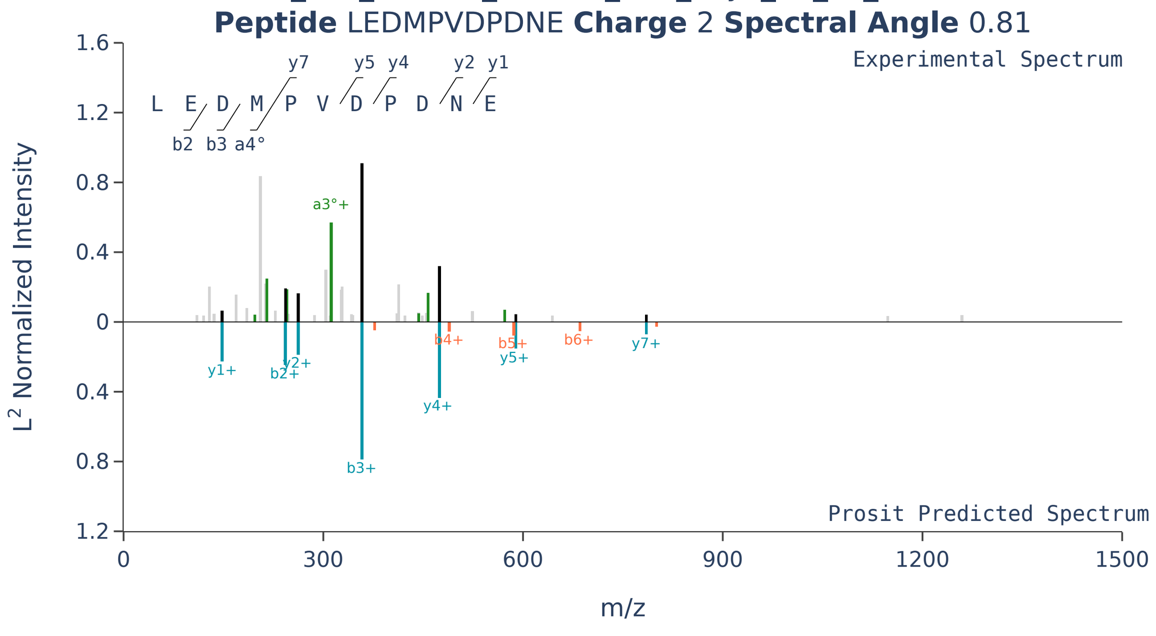


**Source** W_Soh_160821_170921_Goe_aSyn_A1_6h_R2 **Scan** 6443
**Peptide** KEGVVAAAEKT **Charge** 3 **Spectral Angle** 0.88

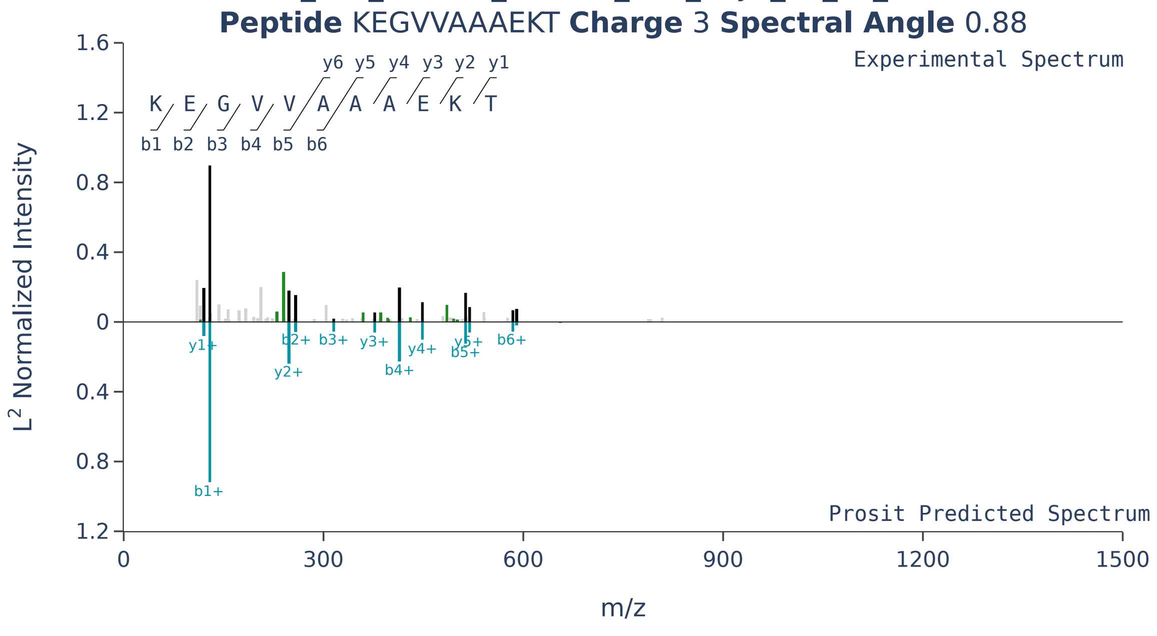

**Source** W_Soh_160821_170921_Goe_aSyn_A1_24h_R2 **Scan** 6700
**Peptide** FMKGLSKAKE **Charge** 2 **Spectral Angle** 0.88

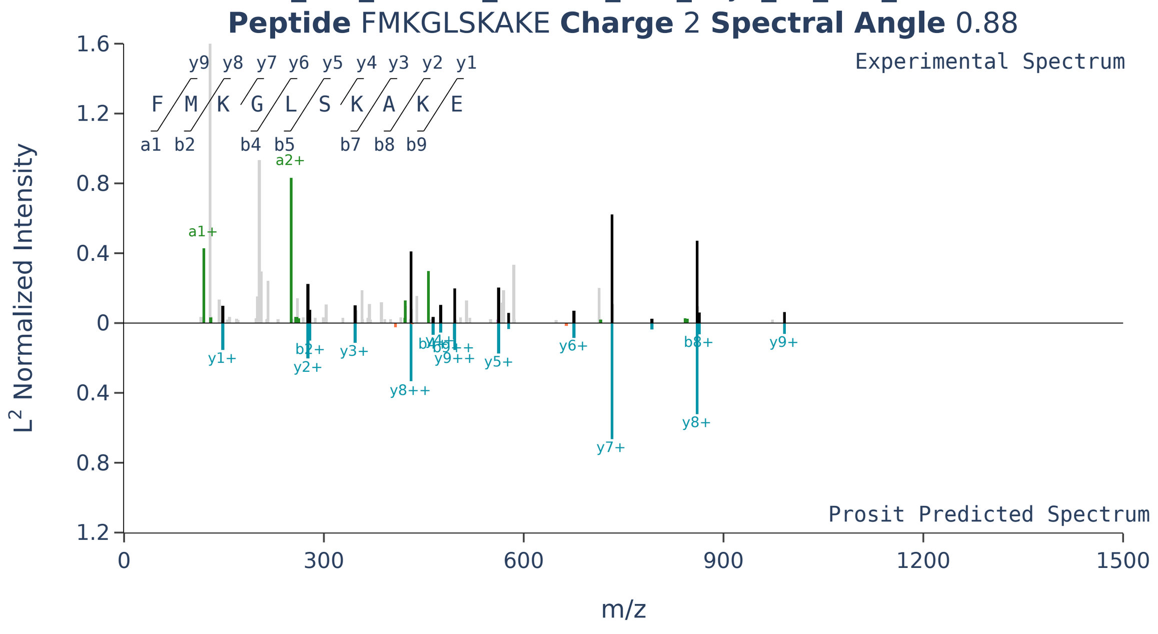


**Source** W_Soh_160821_170921_Goe_aSyn_A1_24h_R1 **Scan** 16458
**Peptide** KEQVTNVGGAVVTGV **Charge** 2 **Spectral Angle** 0.85

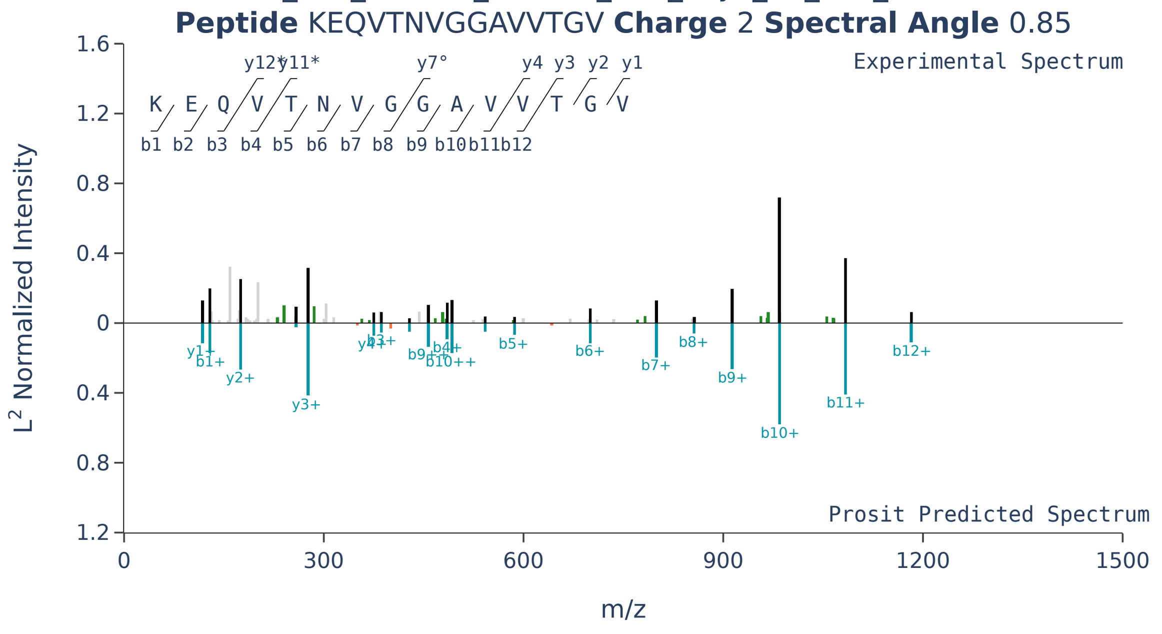


**Source** W_Soh_160821_170921_Goe_aSyn_A1_24h_R1 **Scan** 11610
**Peptide** TNVGGAVVTG **Charge** 1 **Spectral Angle** 0.76

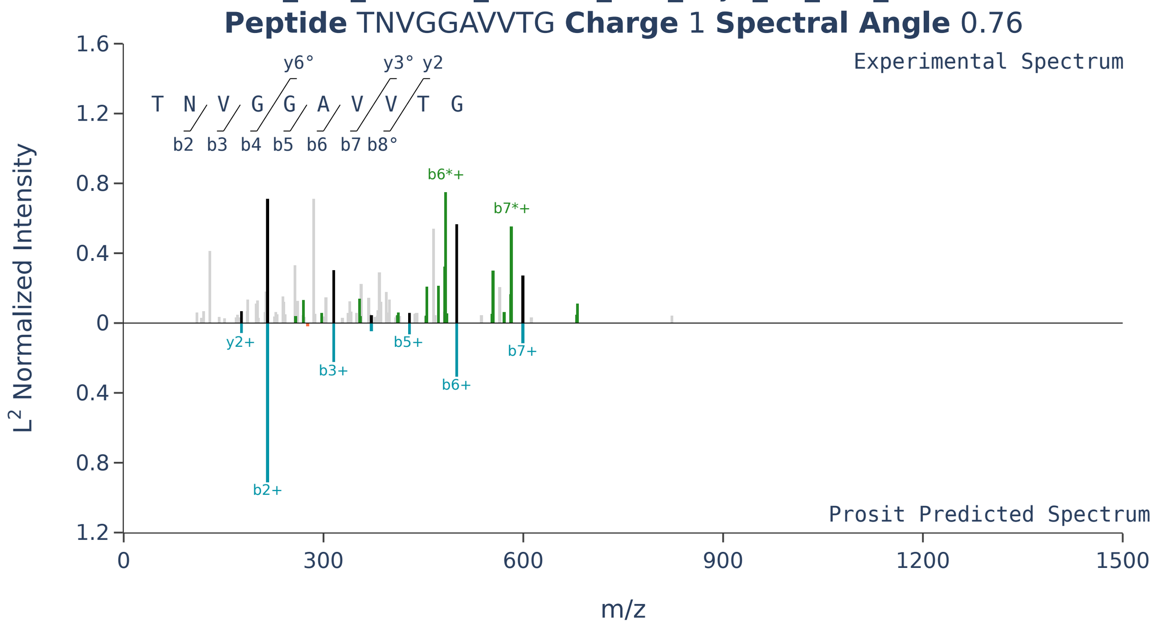


**Source** W_Soh_160821_170921_Goe_aSyn_A2_24h_R1 **Scan** 15335
**Peptide** VAEKTKEQVTNVGGAVVTGVTA **Charge** 3 **Spectral Angle** 0.87

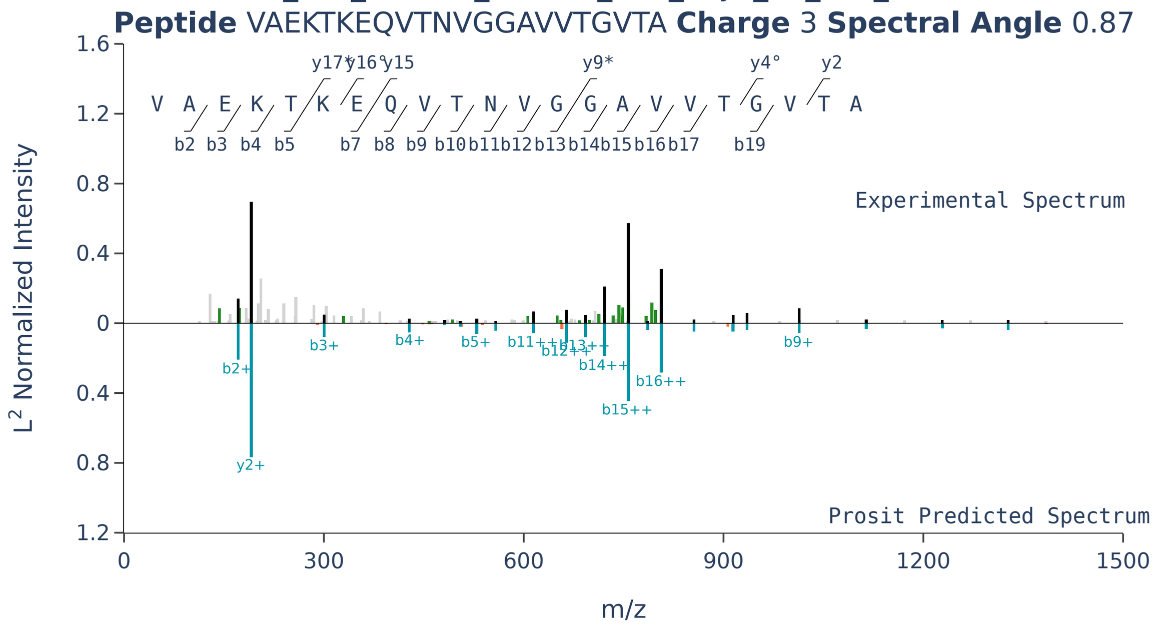


**Source** W_Soh_160821_170921_Goe_aSyn_A1_24h_R2 **Scan** 7886
**Peptide** VLYVGSKTKEG **Charge** 2 **Spectral Angle** 0.91

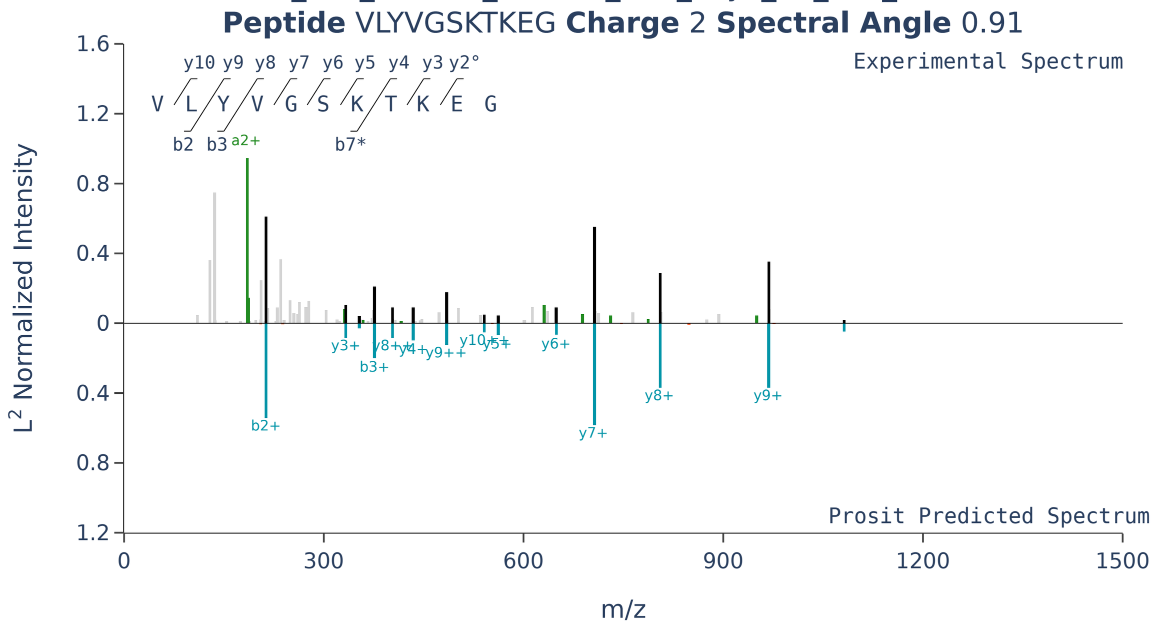


**Source** W_Soh_160821_170921_Goe_aSyn_A1_24h_R2 **Scan** 12297
**Peptide** GVLYVGSK **Charge** 2 **Spectral Angle** 0.86

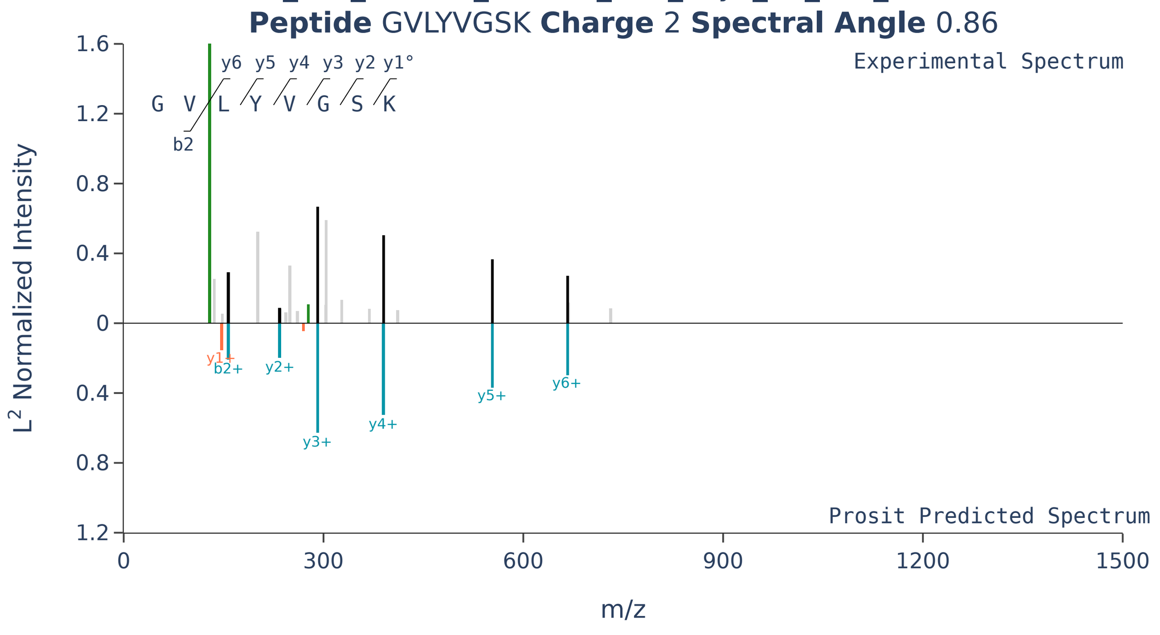


**Source** W_Soh_160821_170921_Goe_aSyn_A1_24h_R1 **Scan** 6235
**Peptide** KGLSKAKEGVVAA **Charge** 2 **Spectral Angle** 0.89

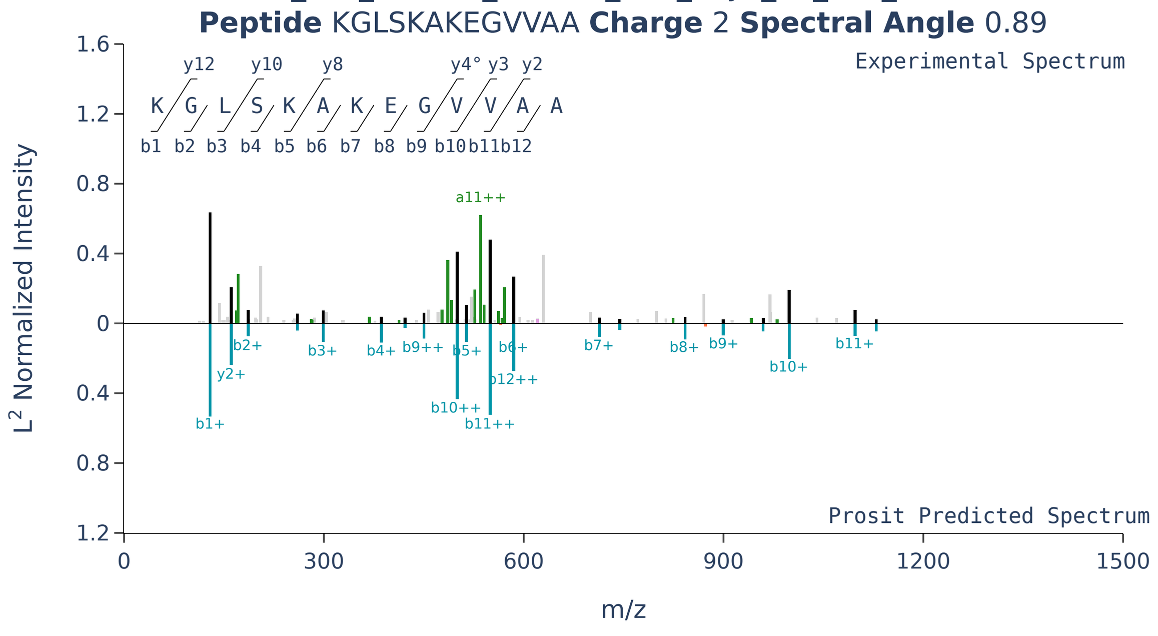


**Source** W_Soh_160821_170921_Goe_aSyn_A2_30'_R1 **Scan** 7796
**Peptide** AGKTKEGVLY **Charge** 3 **Spectral Angle** 0.92

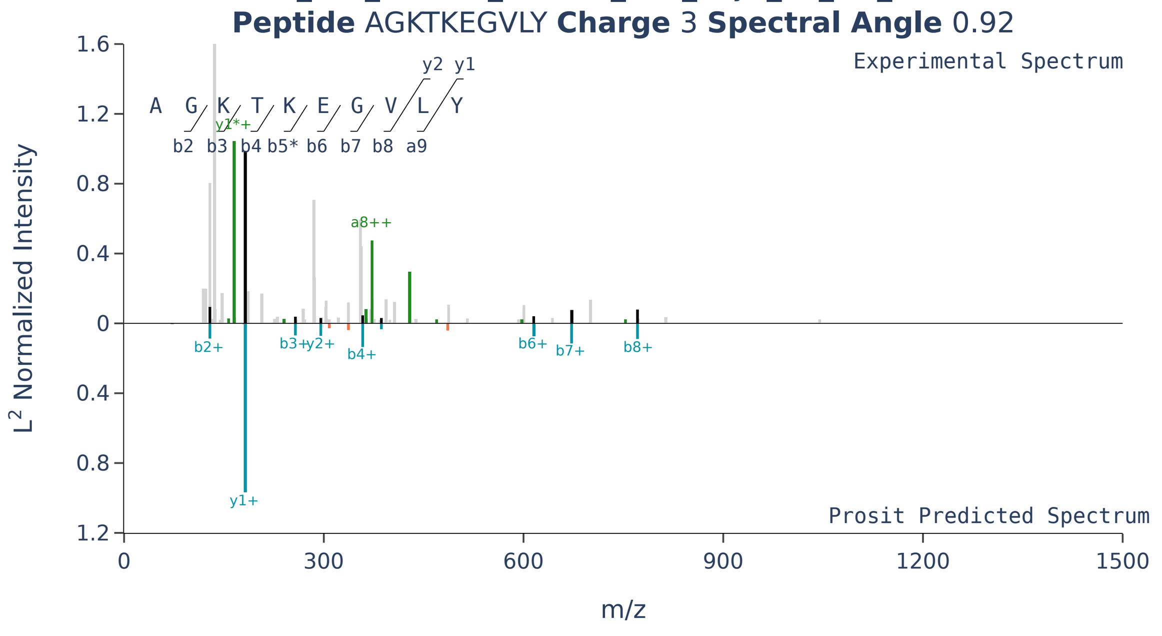


**Source** W_Soh_160821_170921_Goe_aSyn_A1_24h_R1 **Scan** 5524
**Peptide** KEGVVAAAEKTK **Charge** 3 **Spectral Angle** 0.84

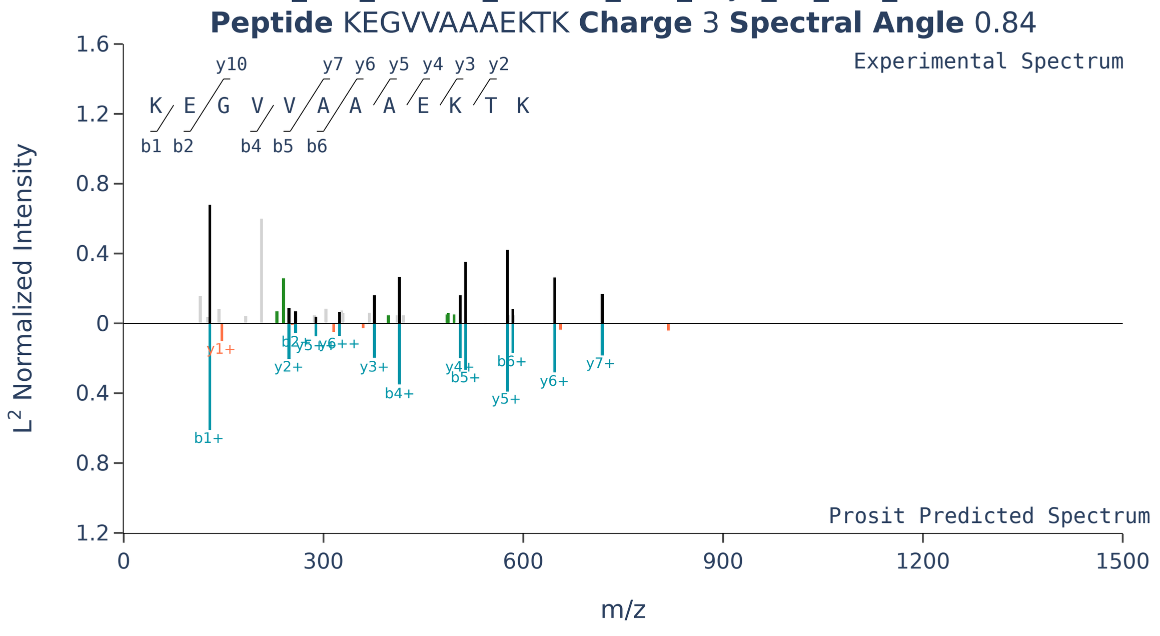


**Source** W_Soh_160821_170921_Goe_aSyn_A1_24h_R1 **Scan** 7575
**Peptide** TGVTAVAQKT **Charge** 2 **Spectral Angle** 0.84

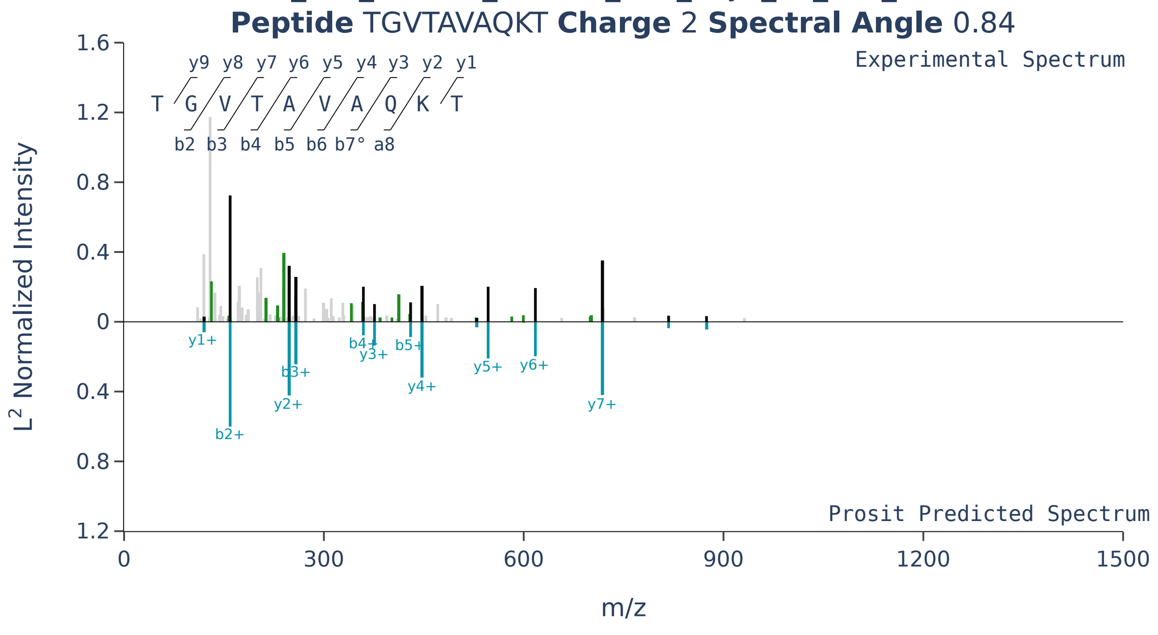


**Source** W_Soh_160821_170921_Goe_aSyn_A1_24h_R1 **Scan** 6772
**Peptide** TAVAQKT **Charge** 1 **Spectral Angle** 0.89

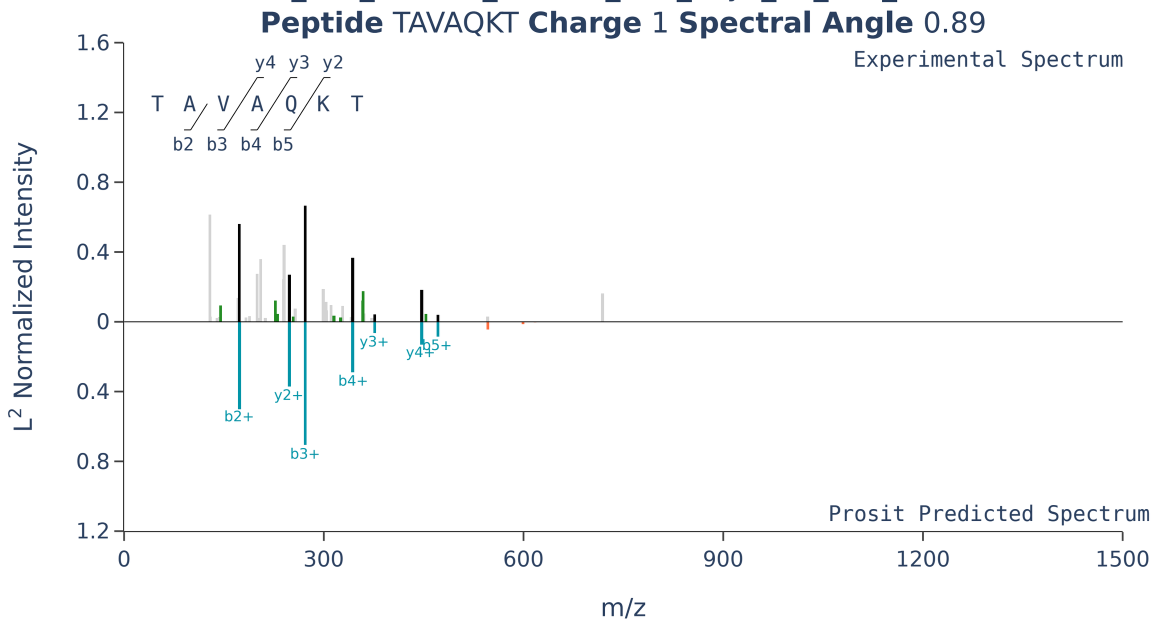


**Source** W_Soh_160821_170921_Goe_aSyn_A2_24h_R2 **Scan** 18722
**Peptide** MDVFMKGLSKAKEGVV **Charge** 2 **Spectral Angle** 0.9

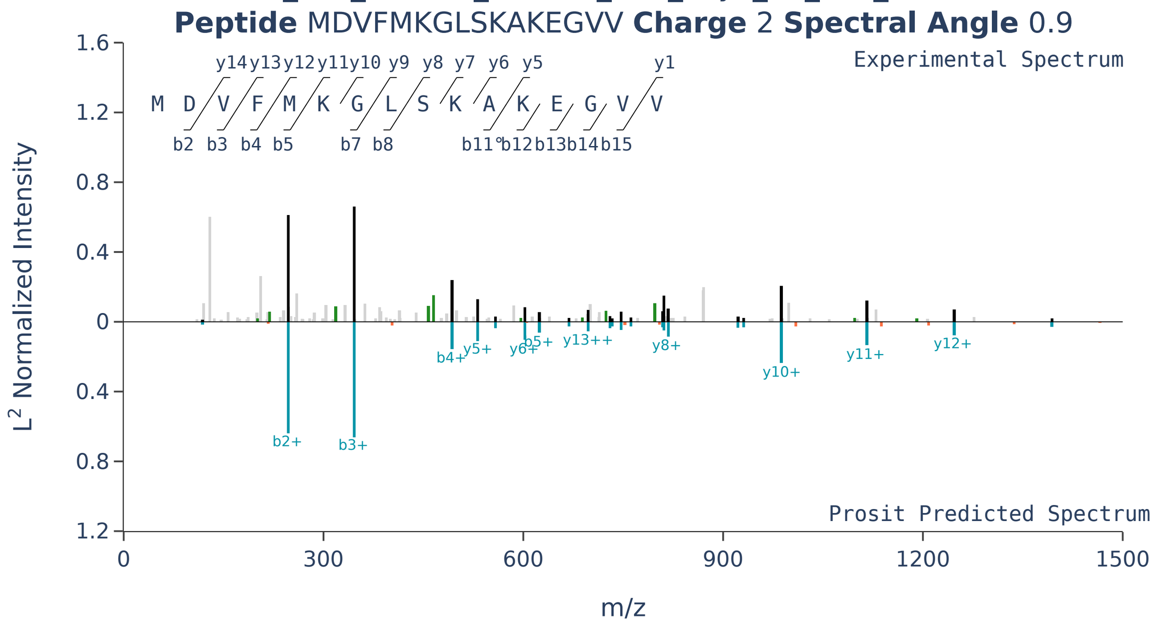


**Source** W_Soh_160821_170921_Goe_aSyn_A1_24h_R1 **Scan** 17093
**Peptide** TKEQVTNVGGAVVTGVTA **Charge** 2 **Spectral Angle** 0.82

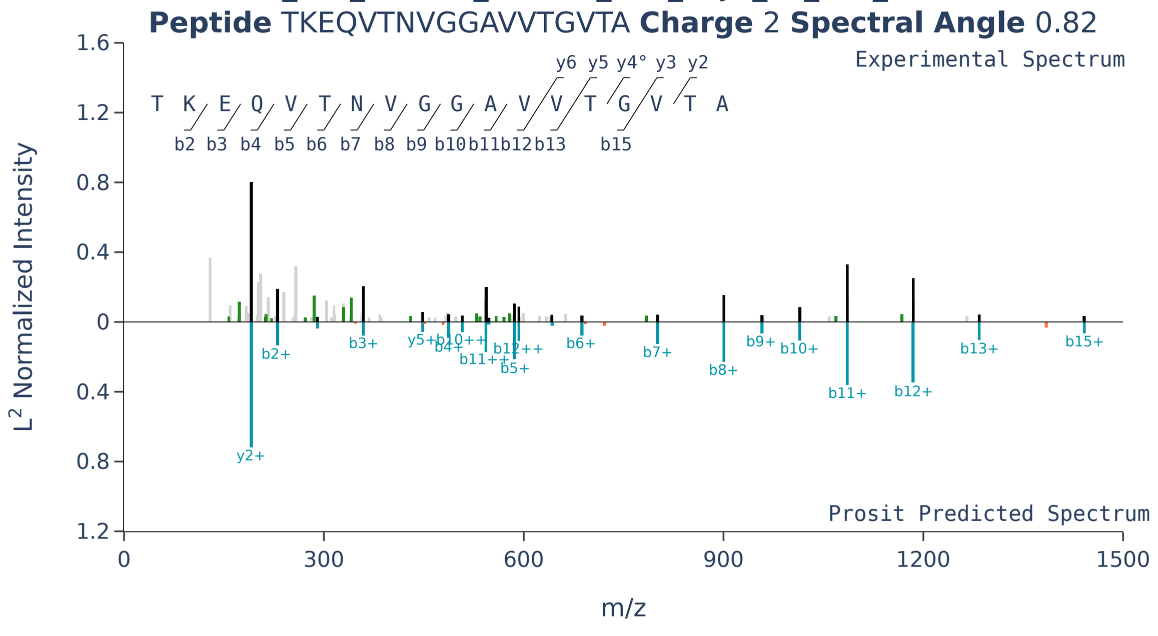


**Source** W_Soh_160821_170921_Goe_aSyn_A2_24h_R1 **Scan** 13792
**Peptide** ATVAEKTKEQVTNVGGAVVTG **Charge** 3 **Spectral Angle** 0.87

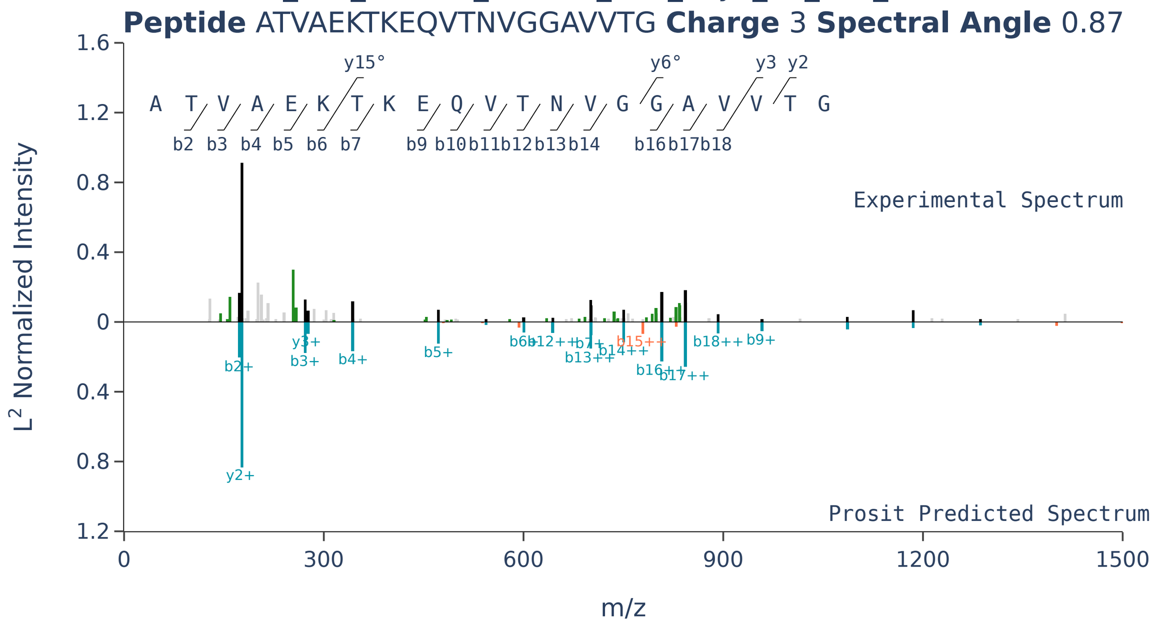


**Source** W_Soh_160821_170921_Goe_aSyn_A1_24h_R1 **Scan** 6107
**Peptide** ATVAEKTKE **Charge** 2 **Spectral Angle** 0.88

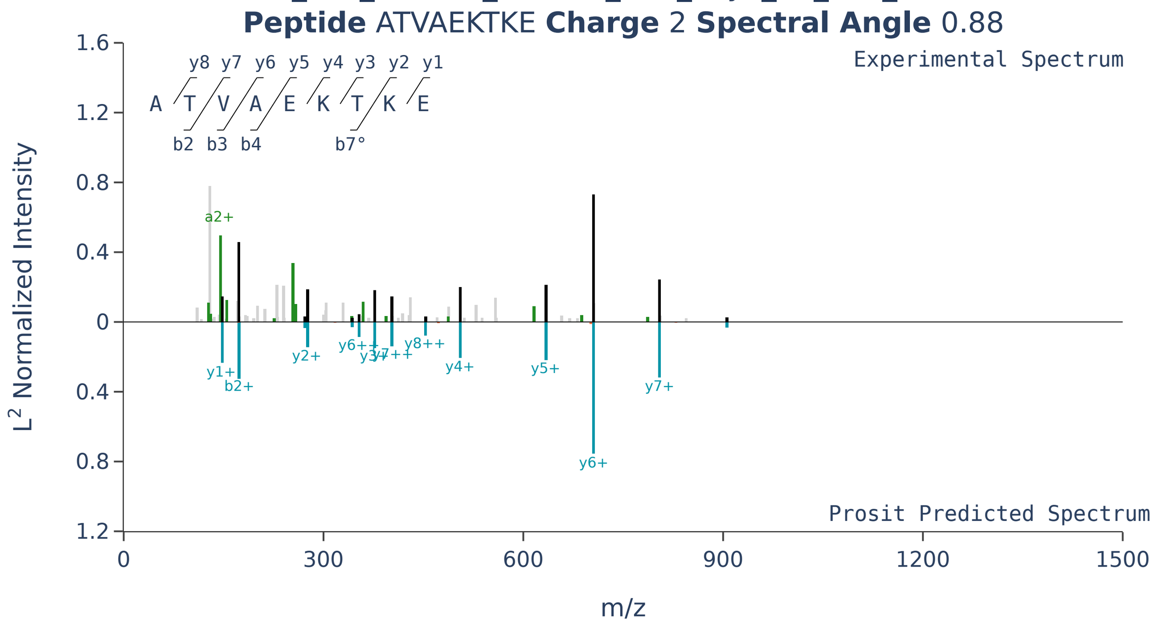

**Source** W_Soh_160821_170921_Goe_aSyn_A1_24h_R1 **Scan** 5530
**Peptide** GSKTKEGVVHG **Charge** 2 **Spectral Angle** 0.88

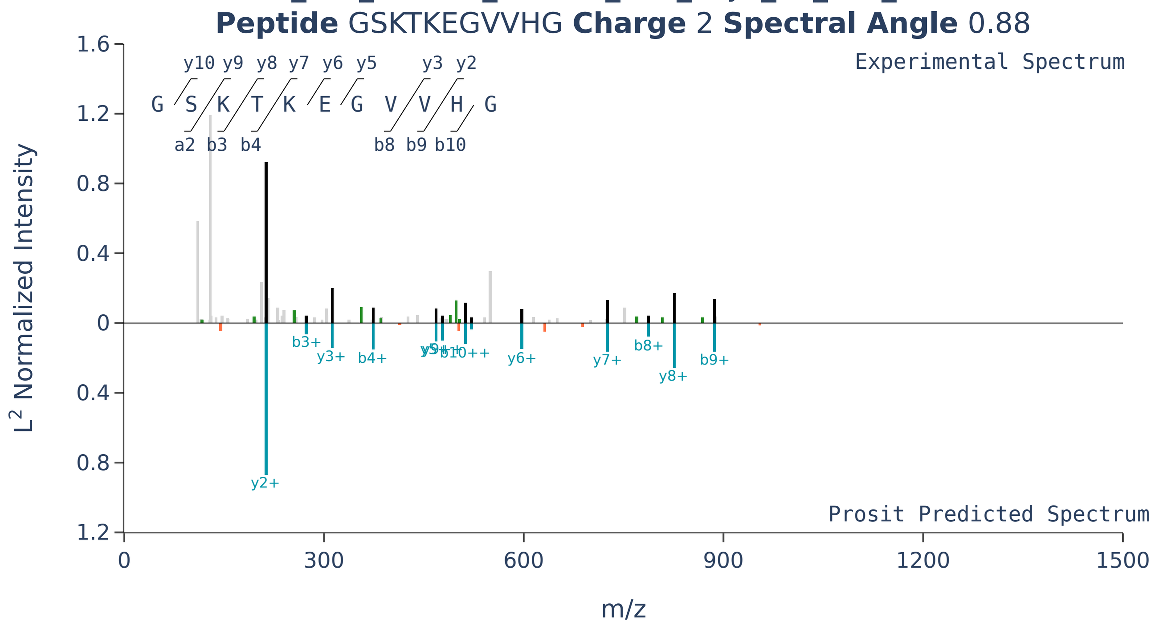


**Source** W_Soh_160821_170921_Goe_aSyn_A1_24h_R2 **Scan** 6030
**Peptide** ATVAEKTKEQV **Charge** 3 **Spectral Angle** 0.8

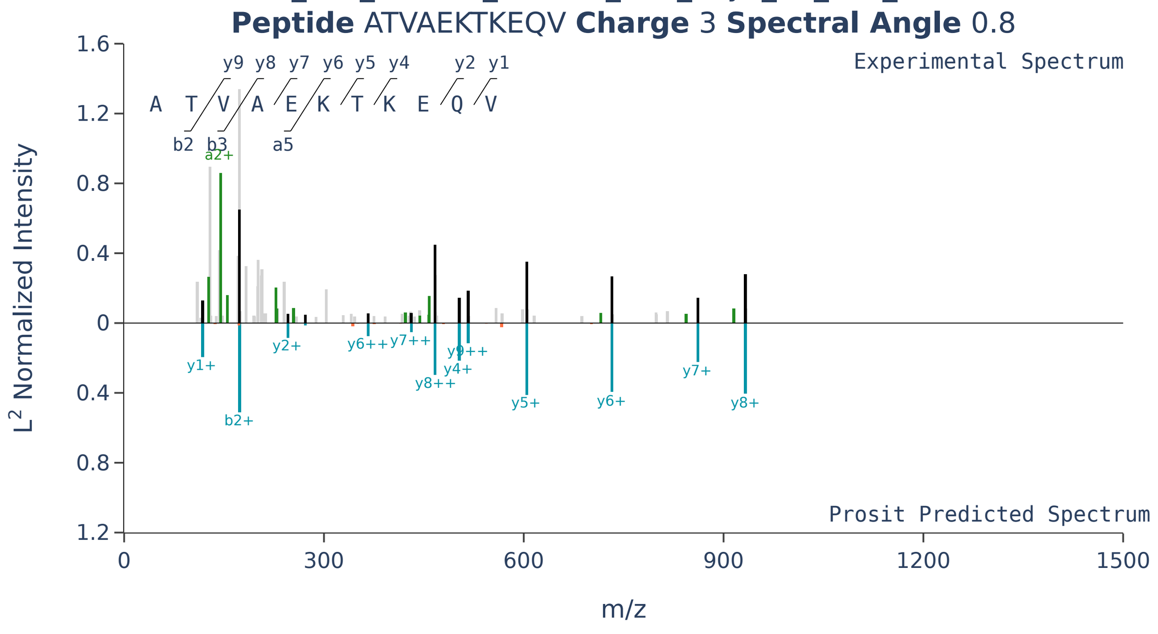


**Source** W_Soh_160821_170921_Goe_aSyn_A1_24h_R1 **Scan** 10280
**Peptide** EGVLYVGSKTKE **Charge** 3 **Spectral Angle** 0.94

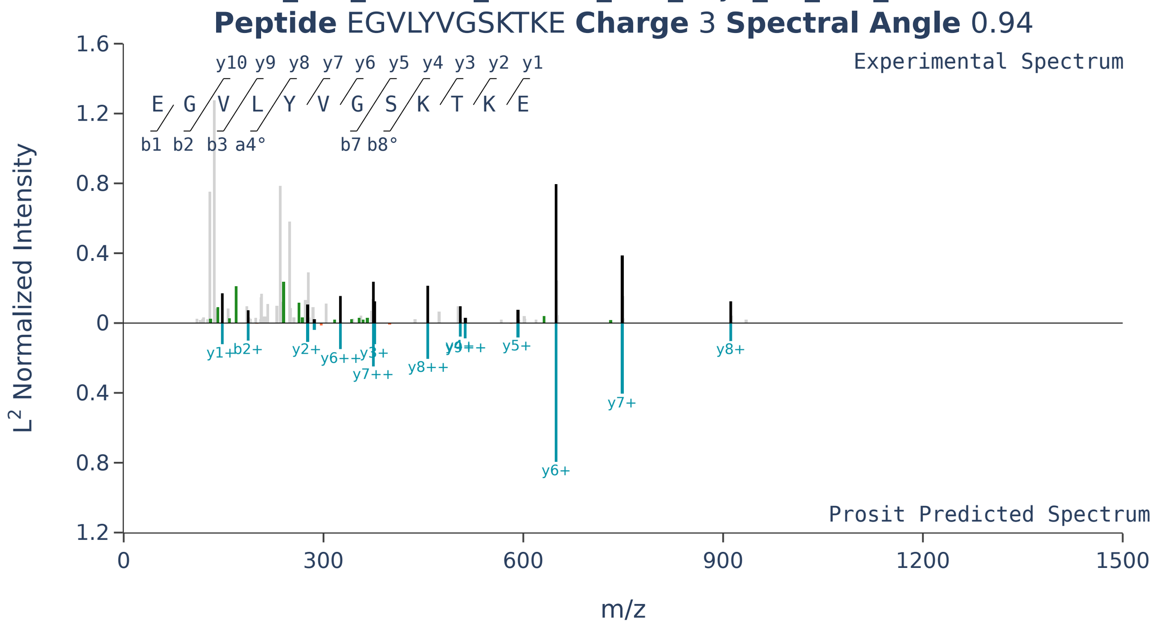


**Source** W_Soh_160821_170921_Goe_aSyn_A1_24h_R1 **Scan** 8945
**Peptide** GVLYVGSKTK **Charge** 2 **Spectral Angle** 0.87

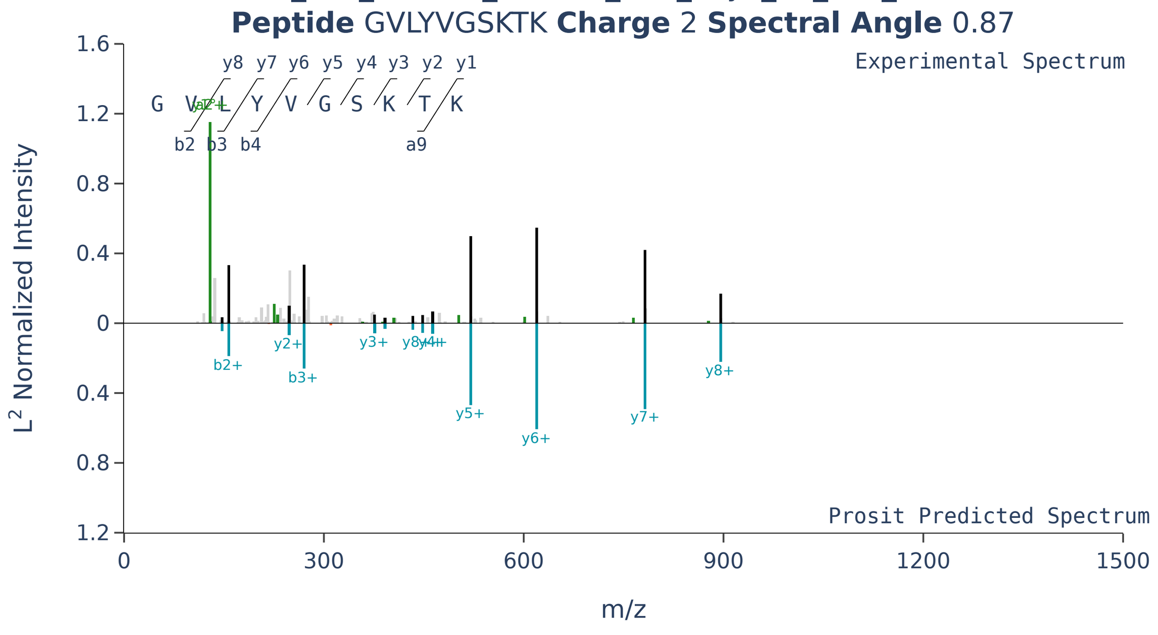


**Source** W_Soh_160821_170921_Goe_aSyn_A1_6h_R1 **Scan** 7804
**Peptide** VLYVGSKTKE **Charge** 3 **Spectral Angle** 0.91

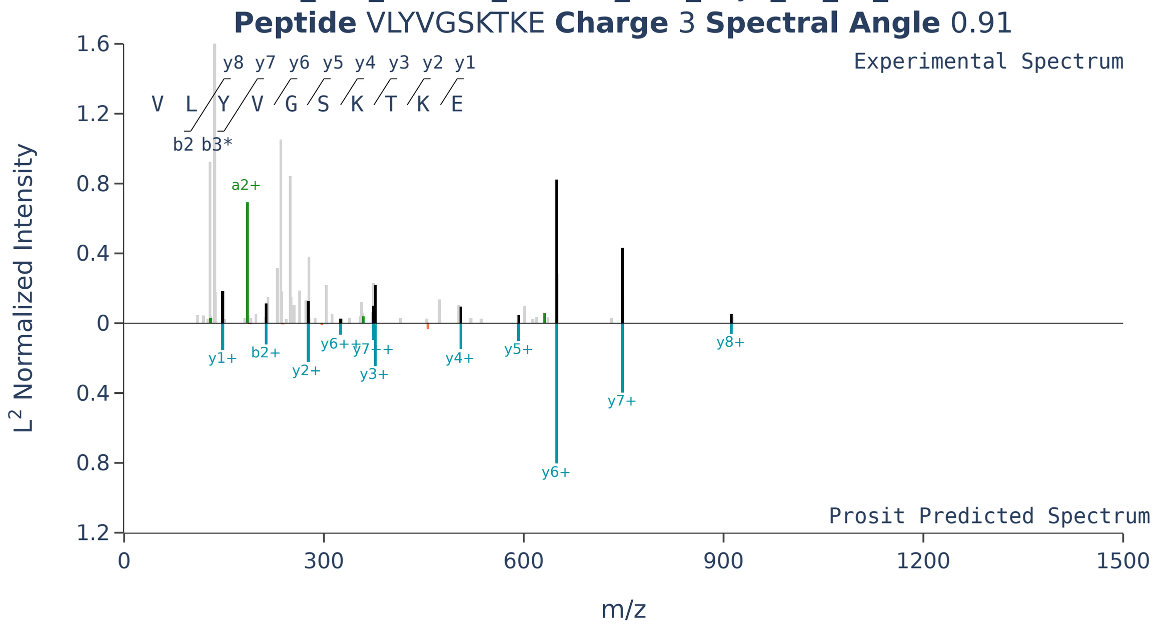


**Source** W_Soh_160821_170921_Goe_aSyn_A2_24h_R2 **Scan** 6284
**Peptide** LSKAKEGVVA **Charge** 2 **Spectral Angle** 0.83

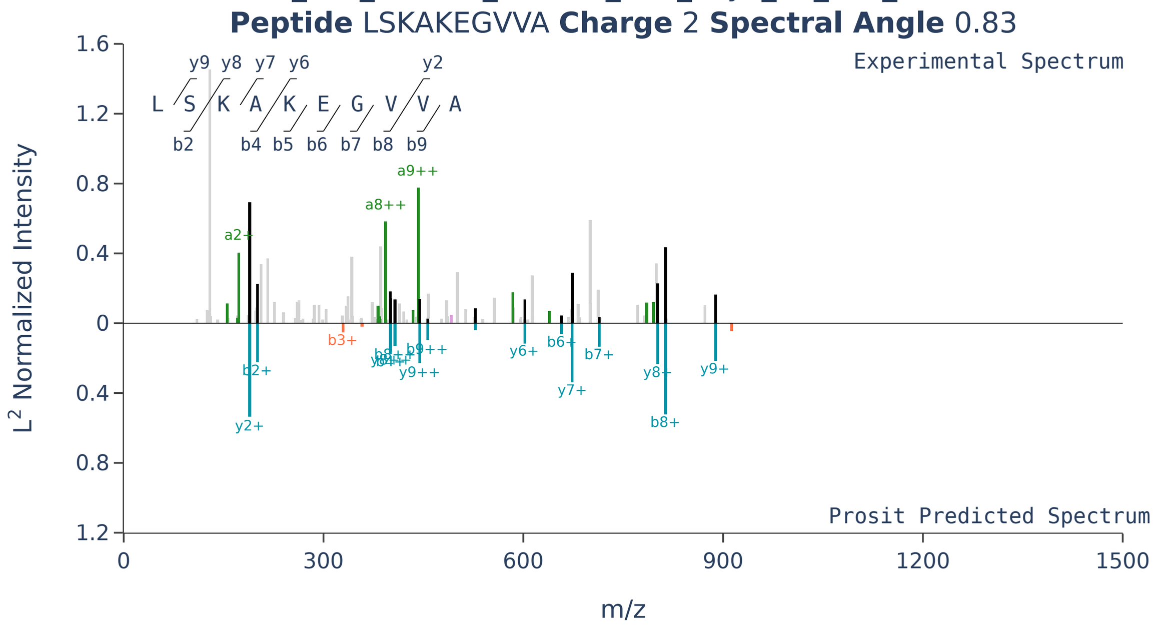


**Source** W_Soh_160821_170921_Goe_aSyn_A1_24h_R1 **Scan** 6089
**Peptide** VVAAAEKTKQGV **Charge** 2 **Spectral Angle** 0.84

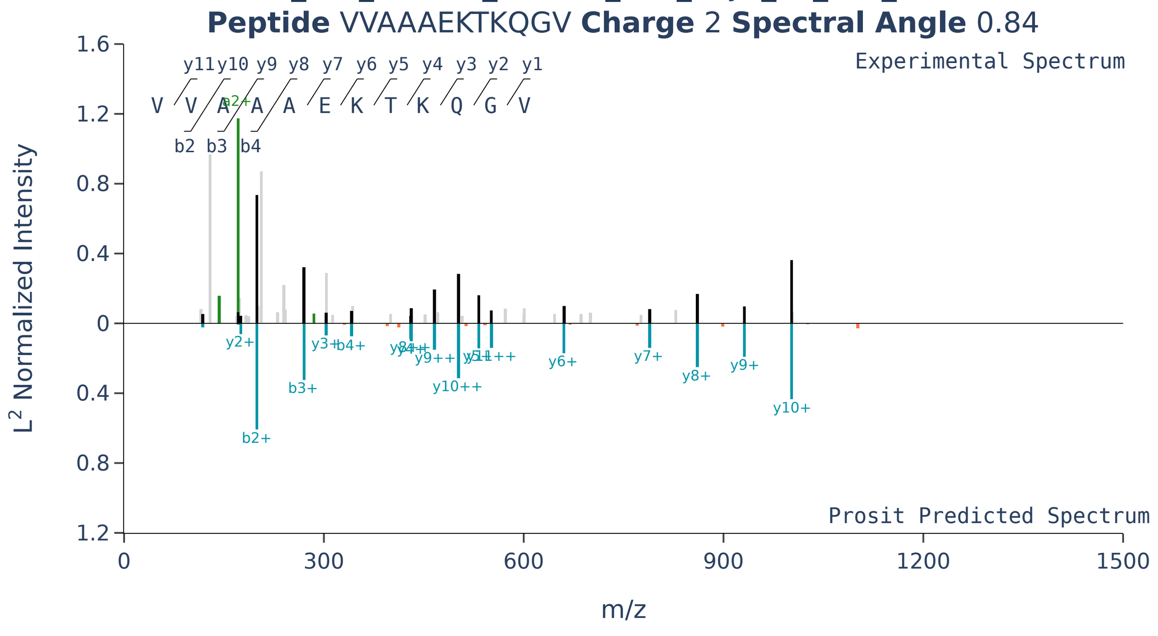


**Source** W_Soh_160821_170921_Goe_aSyn_A1_24h_R2 **Scan** 9114
**Peptide** GAGSIAAATG **Charge** 1 **Spectral Angle** 0.84

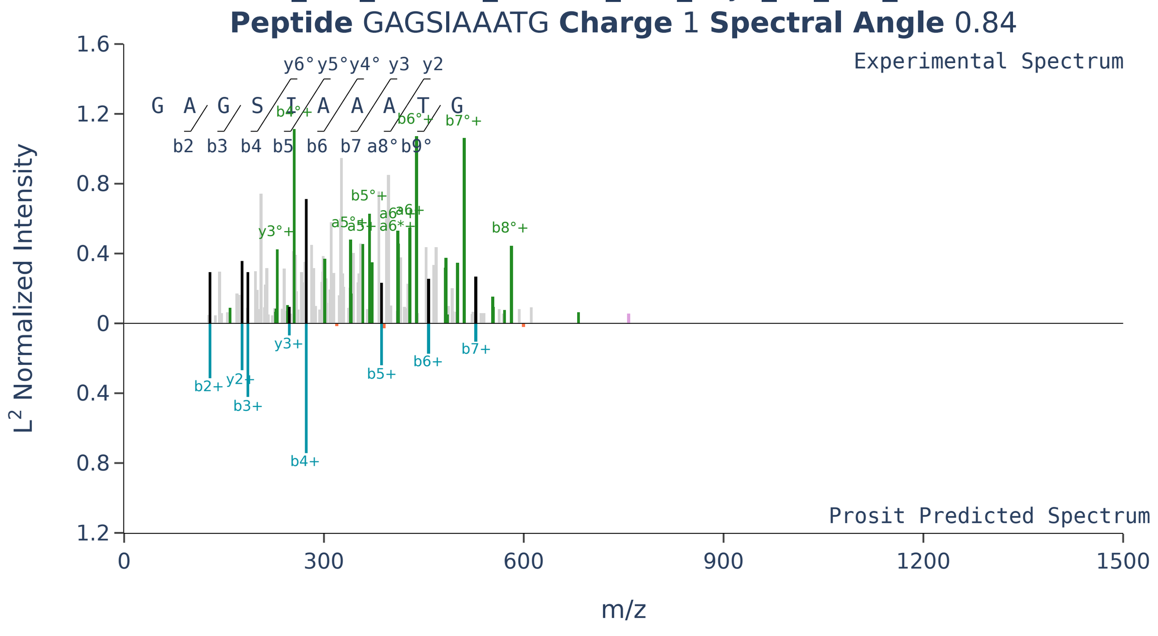


**Source** W_Soh_160821_170921_Goe_aSyn_A2_24h_R1 **Scan** 14862
**Peptide** VATVAEKTKEQVTNVGGAVVTG **Charge** 3 **Spectral Angle** 0.83

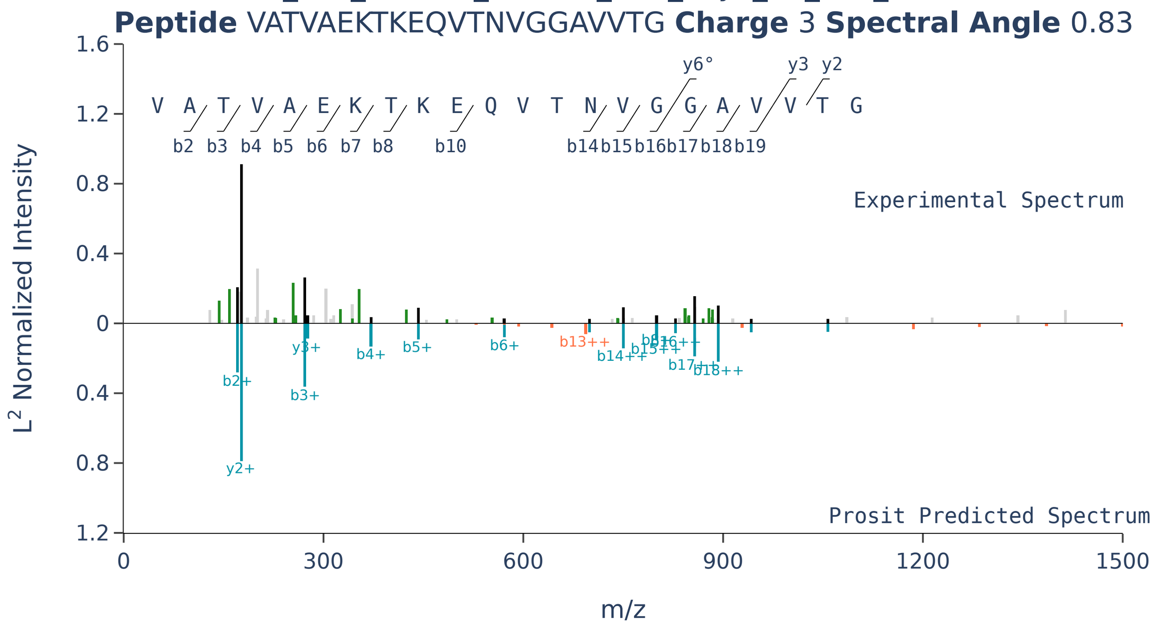


**Source** W_Soh_160821_170921_Goe_aSyn_A2_24h_R1 **Scan** 7096
**Peptide** GVVAAAEKT **Charge** 2 **Spectral Angle** 0.86

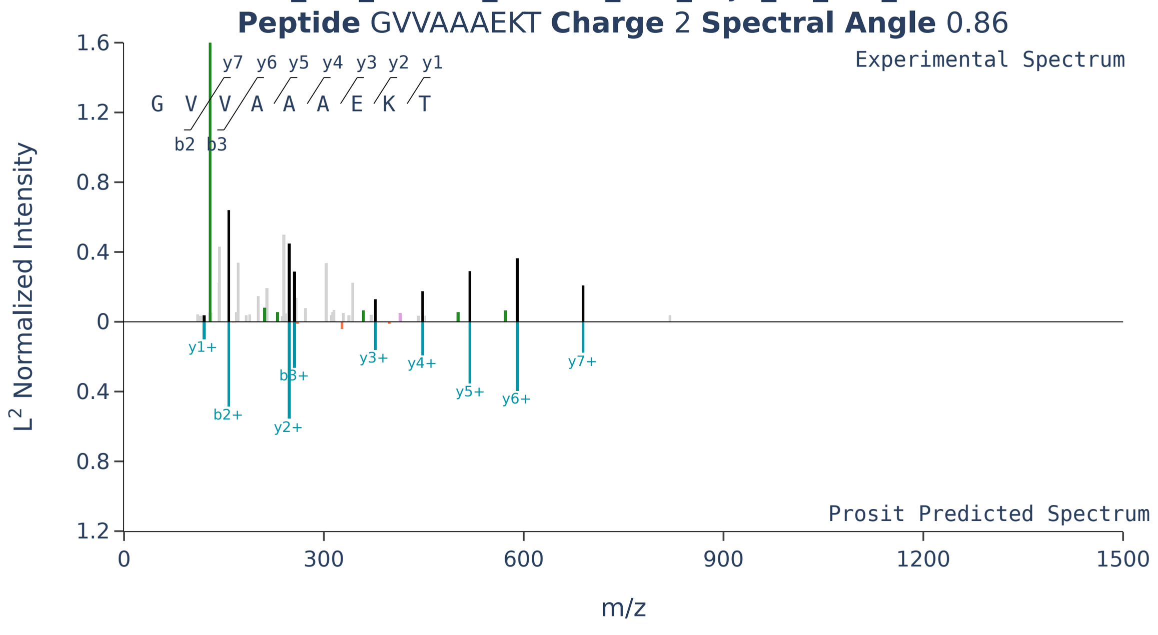


**Source** W_Soh_160821_170921_Goe_aSyn_A1_6h_R2 **Scan** 20424
**Peptide** NEAYEMPSEEGYQDYEPEA **Charge** 2 **Spectral Angle** 0.88

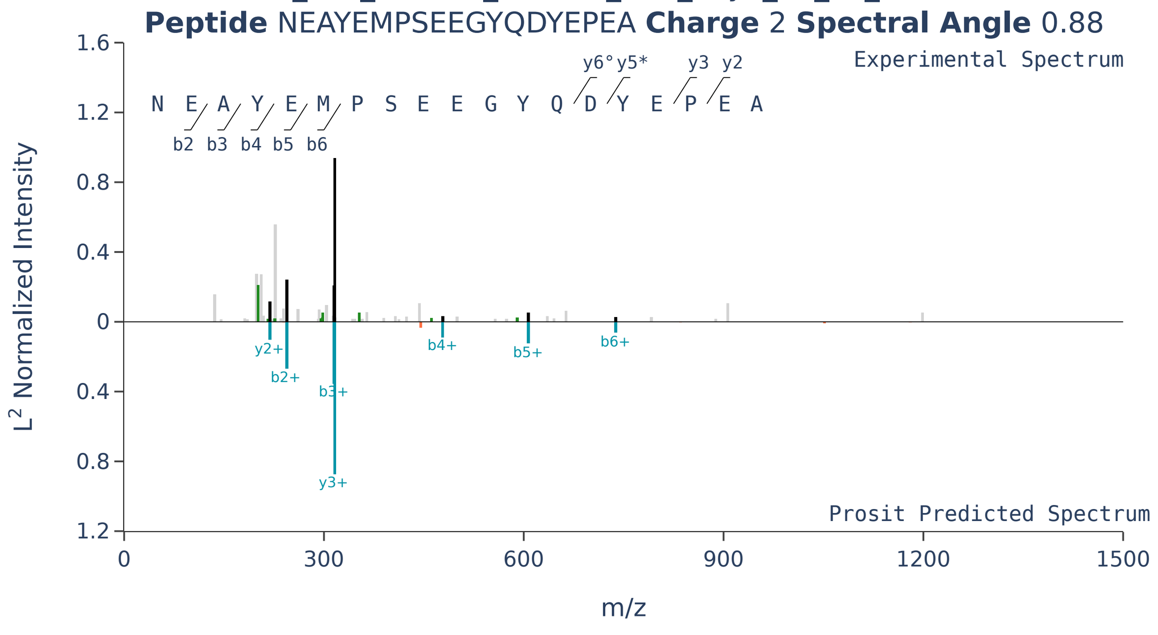


**Source** W_Soh_160821_170921_Goe_aSyn_A1_24h_R1 **Scan** 7835
**Peptide** GSKTKEGVVHGVATV **Charge** 2 **Spectral Angle** 0.89

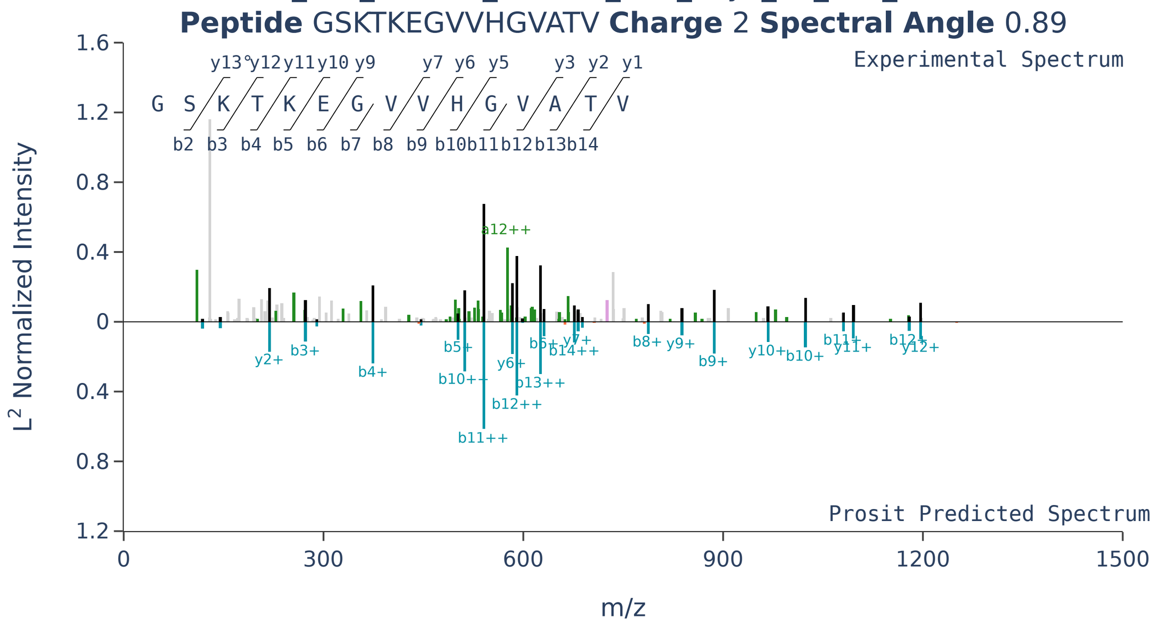


**Source** W_Soh_160821_170921_Goe_aSyn_A2_30'_R1 **Scan** 8602
**Peptide** GVTAVAQKTVE **Charge** 2 **Spectral Angle** 0.89

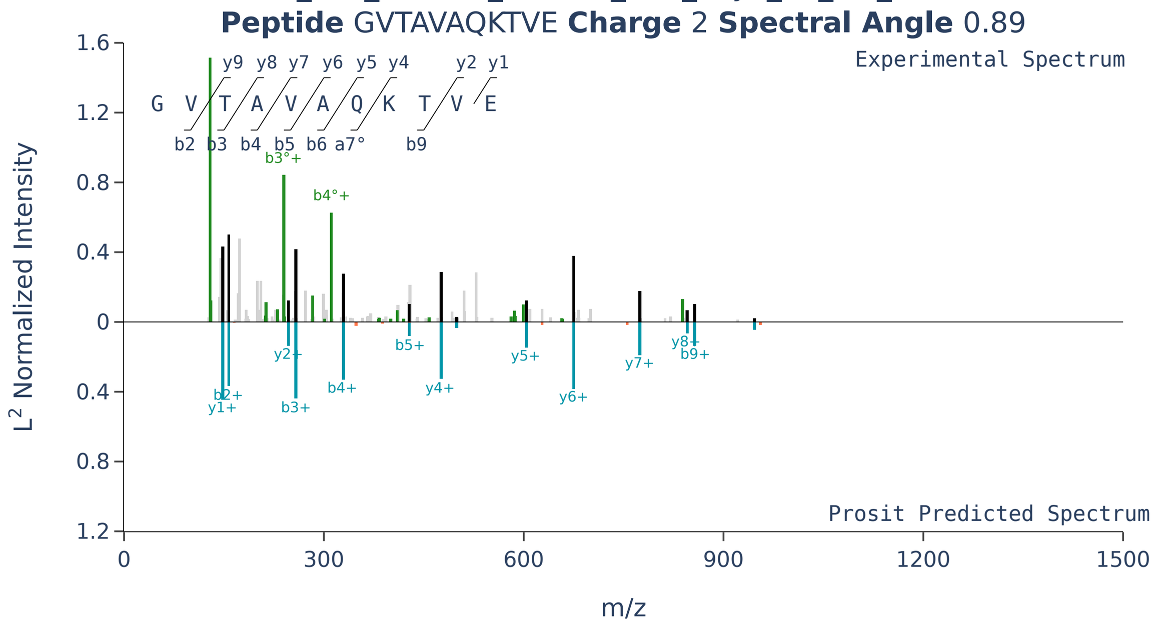


**Source** W_Soh_160821_170921_Goe_aSyn_A1_24h_R2 **Scan** 6282
**Peptide** VGSKTKEGVVHGVA **Charge** 2 **Spectral Angle** 0.82

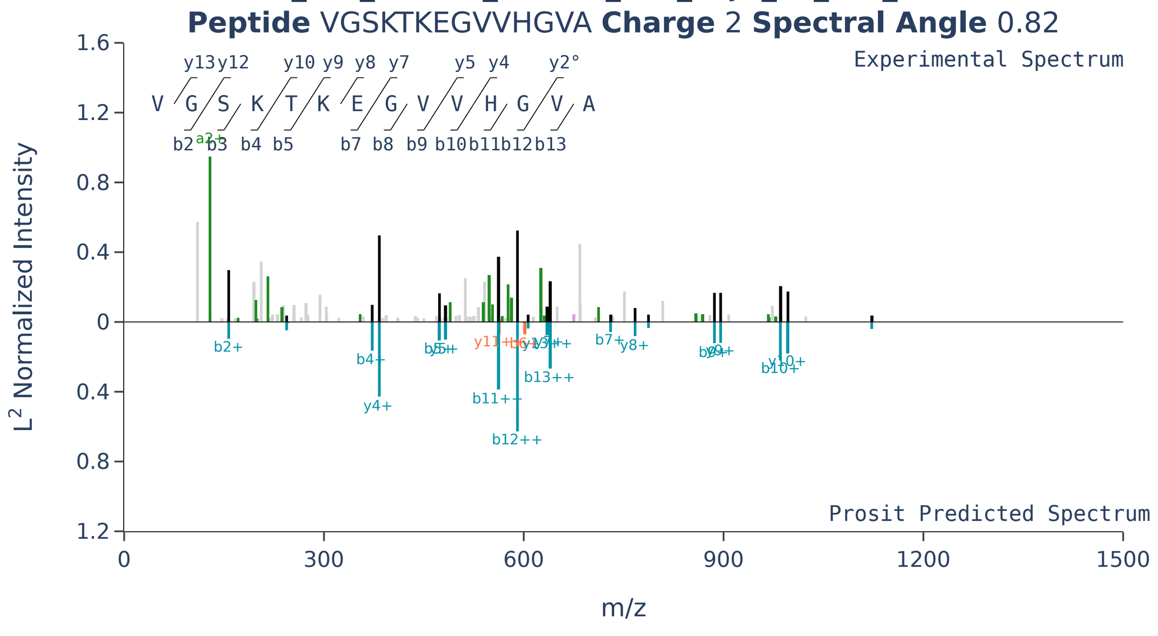


**Source** W_Soh_160821_170921_Goe_aSyn_A1_24h_R2 **Scan** 6710
**Peptide** VAEKTKEQVTNV **Charge** 1 **Spectral Angle** 0.83

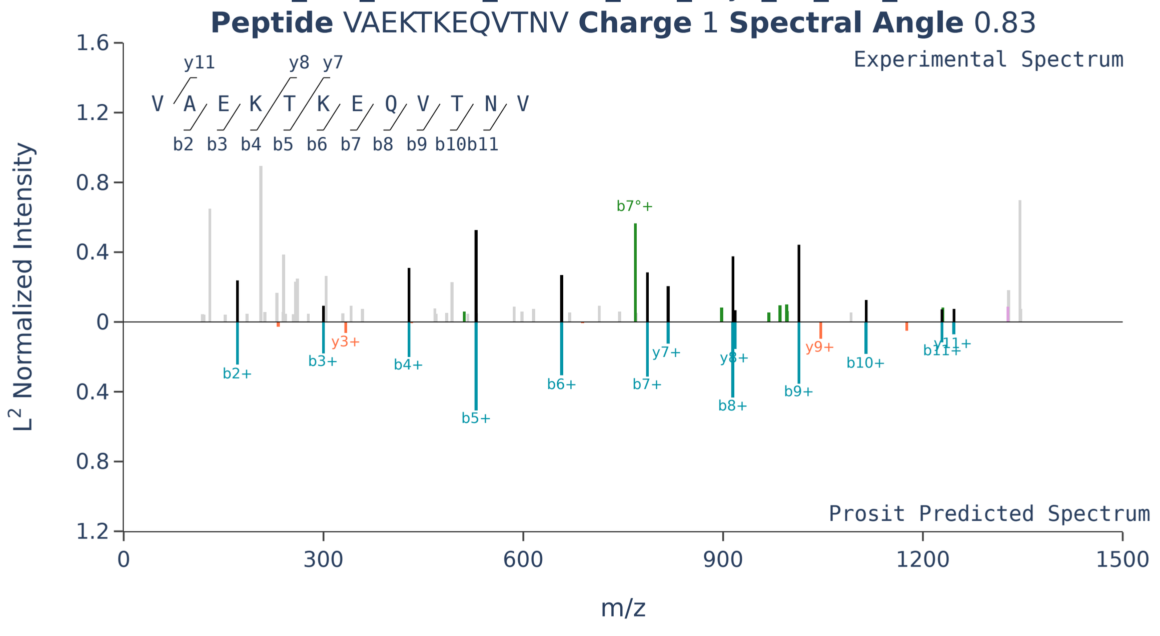

**Source** W_Soh_160821_170921_Goe_aSyn_A1_24h_R1 **Scan** 7676
**Peptide** VLYVGSKTK **Charge** 2 **Spectral Angle** 0.91

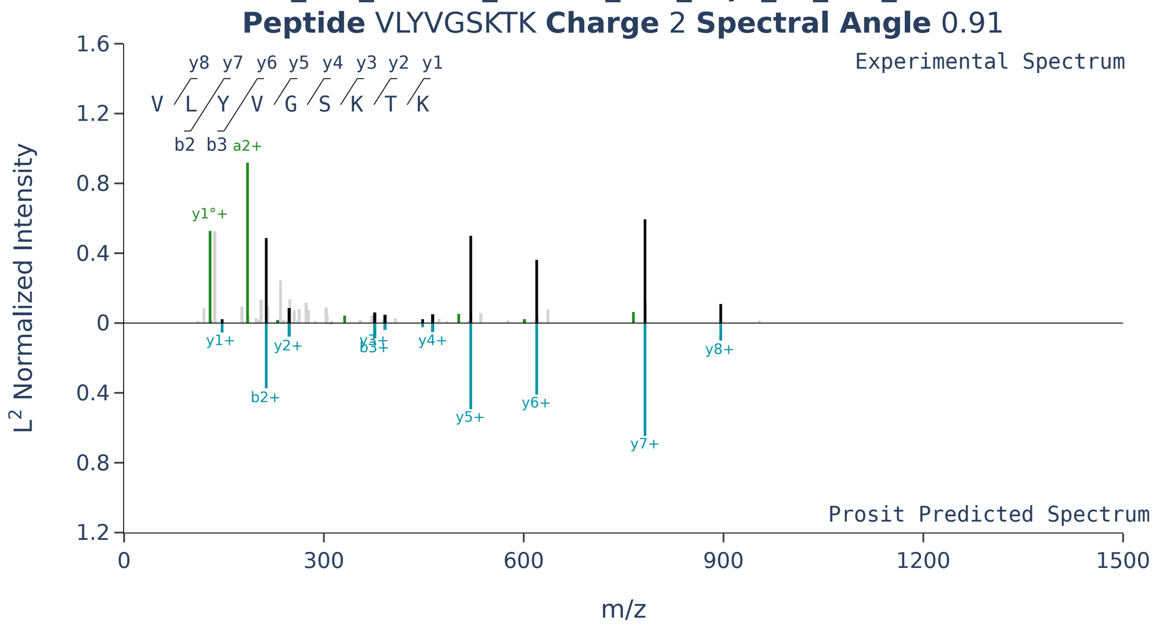


**Source** W_Soh_160821_170921_Goe_aSyn_A1_6h_R1 **Scan** 7861
**Peptide** GVVAAAEKTKQGVAE **Charge** 2 **Spectral Angle** 0.9

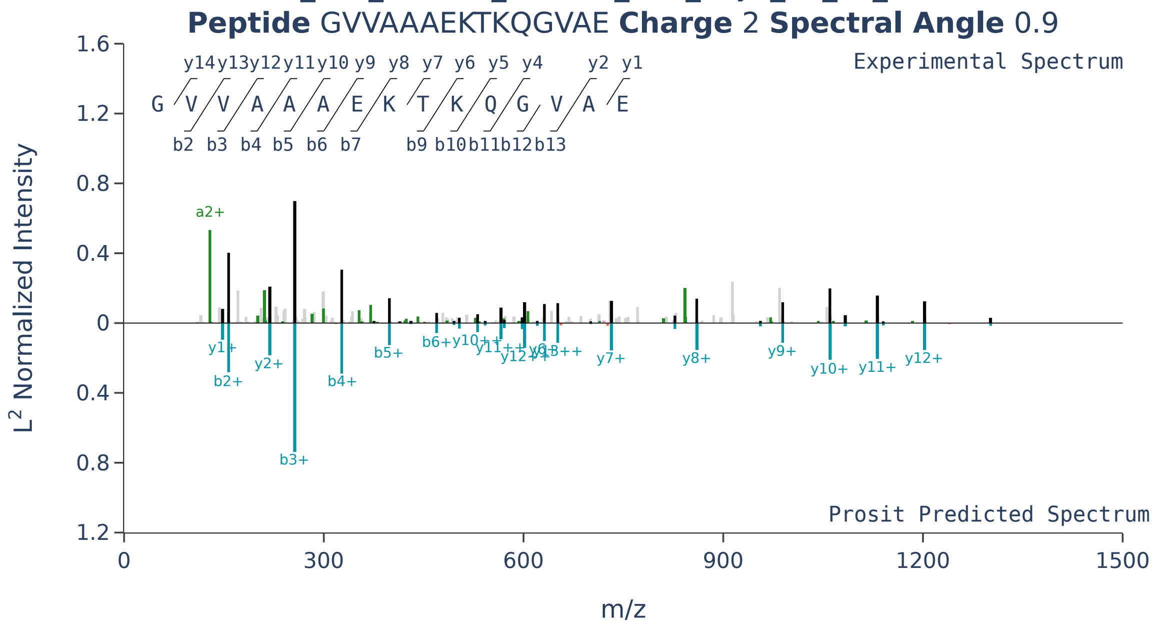


**Source** W_Soh_160821_170921_Goe_aSyn_A1_24h_R2 **Scan** 5868
**Peptide** GVVAAAEKTKQG **Charge** 2 **Spectral Angle** 0.81

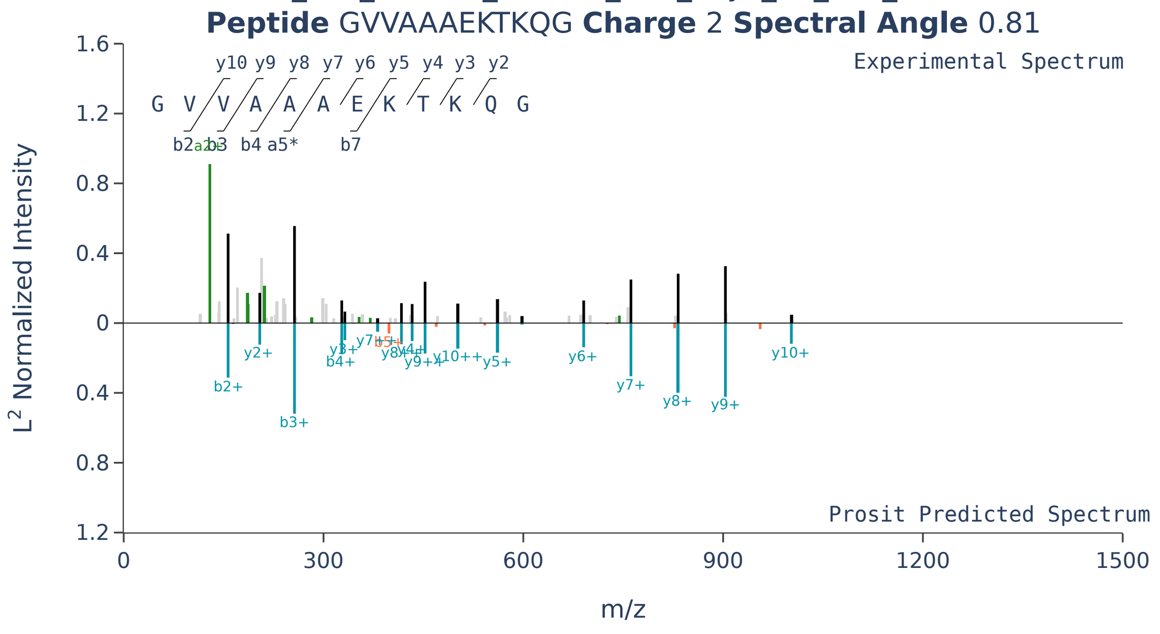


**Source** W_Soh_160821_170921_Goe_aSyn_A1_24h_R2 **Scan** 8722
**Peptide** EQVTNVGGA **Charge** 2 **Spectral Angle** 0.82

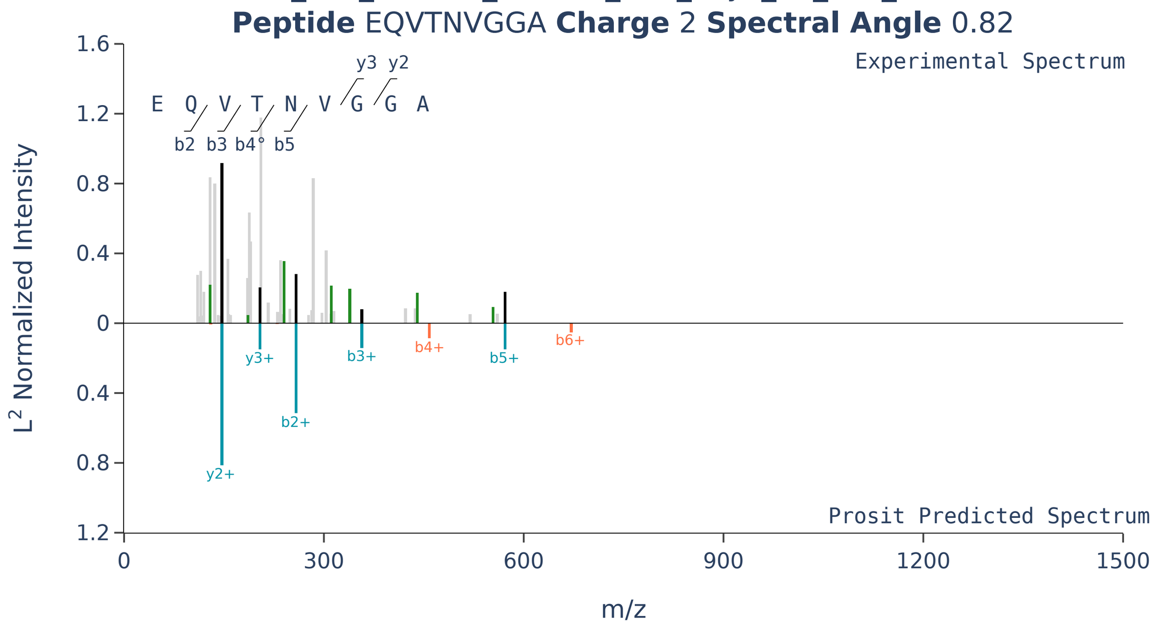


**Source** W_Soh_160821_170921_Goe_aSyn_A1_24h_R1 **Scan** 12119
**Peptide** DMPVDPDN **Charge** 1 **Spectral Angle** 0.9

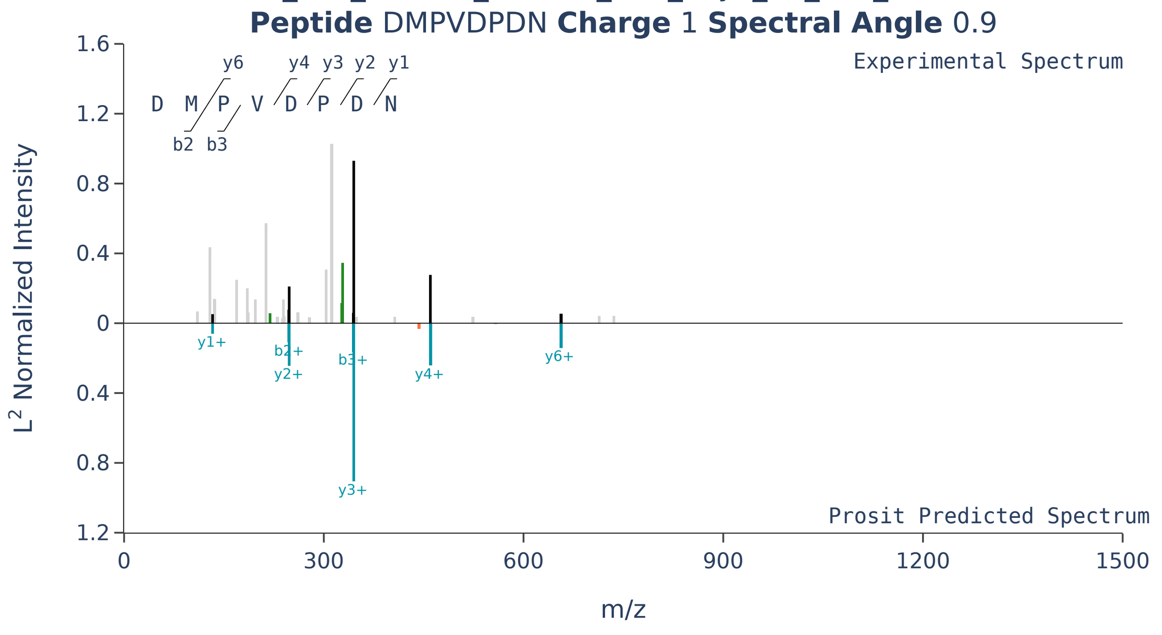


**Source** W_Soh_160821_170921_Goe_aSyn_A1_24h_R1 **Scan** 7051
**Peptide** TGFVKKDQLGKNE **Charge** 3 **Spectral Angle** 0.81

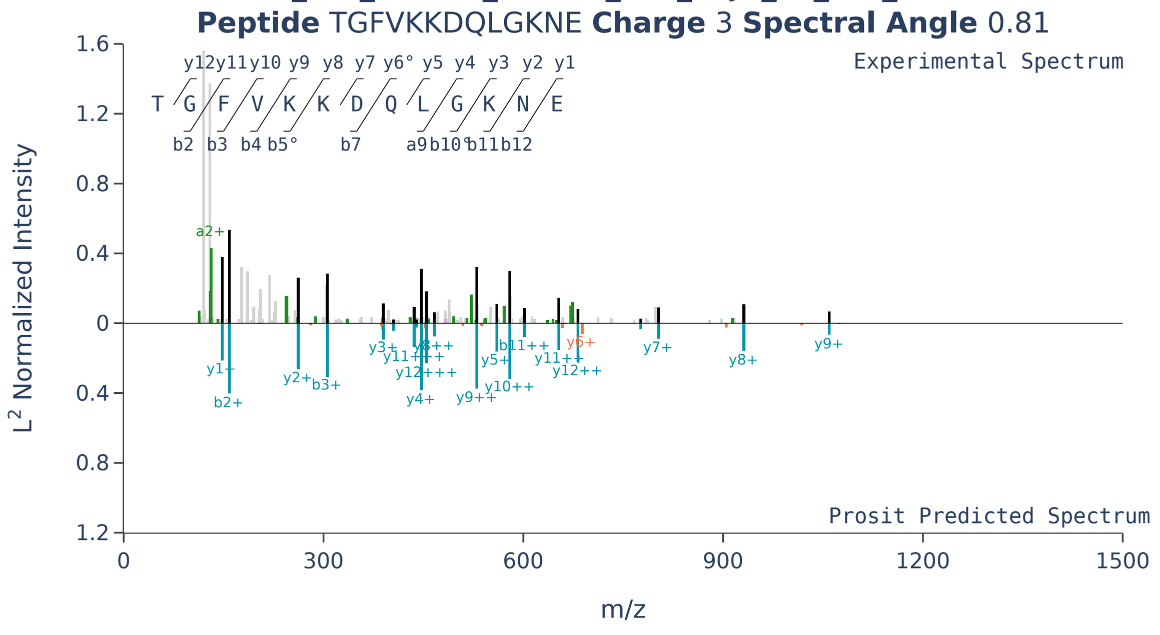


**Source** W_Soh_160821_170921_Goe_aSyn_A1_24h_R2 **Scan** 10291
**Peptide** EGVLYVGSKTKEG **Charge** 3 **Spectral Angle** 0.9

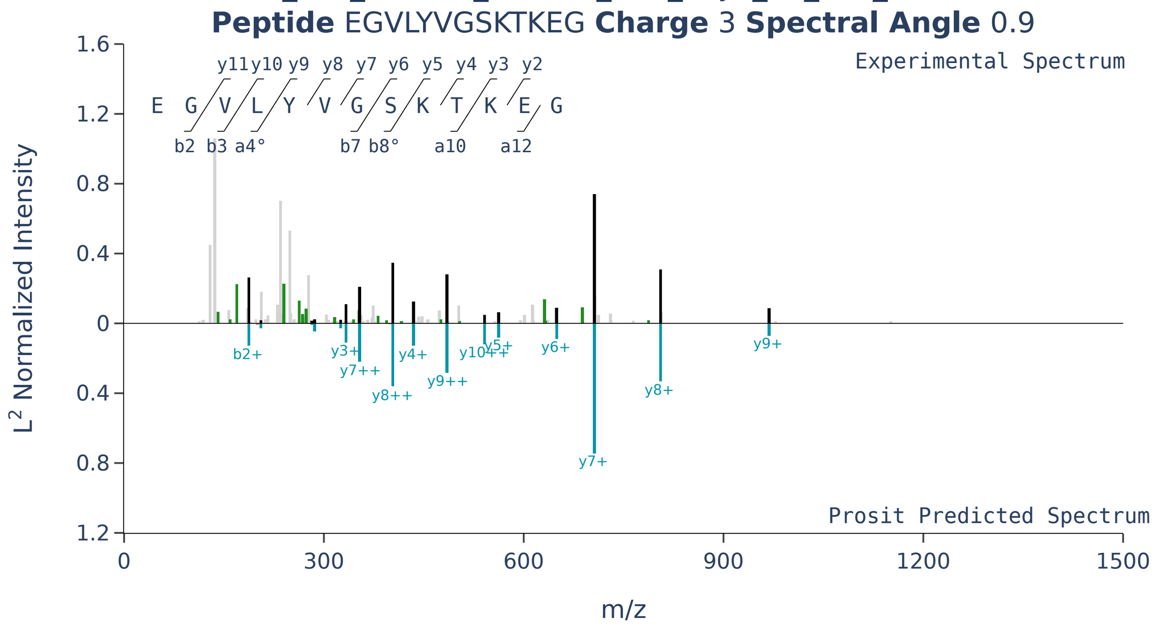


**Source** W_Soh_160821_170921_Goe_aSyn_A2_24h_R2 **Scan** 15089
**Peptide** AQKTVEGAGSIAAATGFVKKD **Charge** 2 **Spectral Angle** 0.86

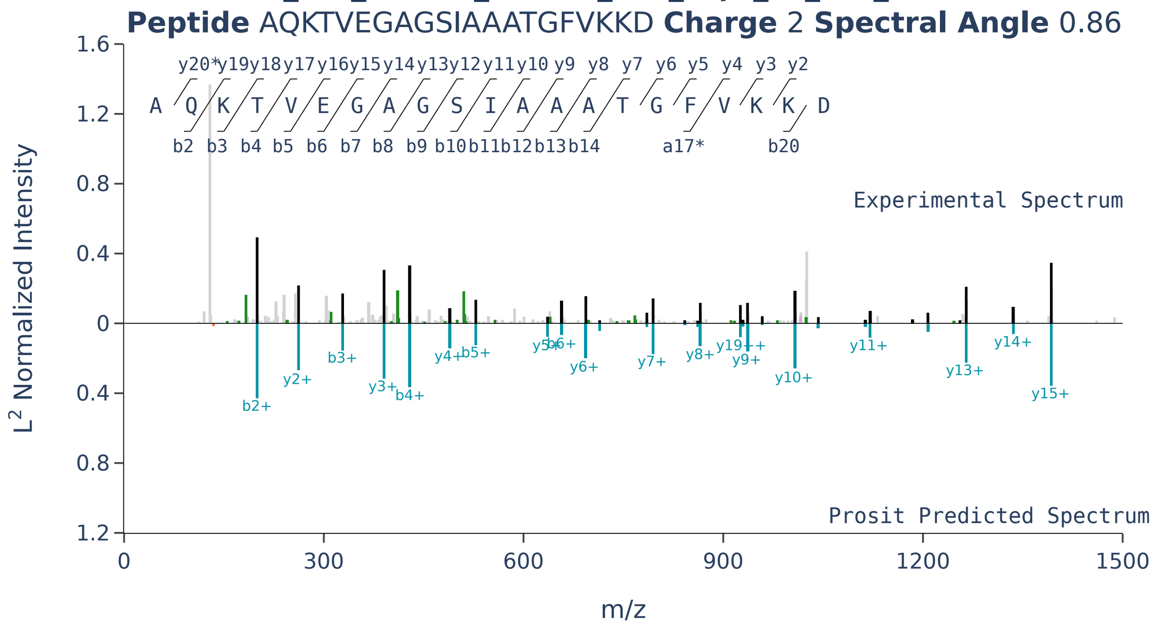


**Source** W_Soh_160821_170921_Goe_aSyn_A2_24h_R2 **Scan** 15247
**Peptide** VAQKTVEGAGSIAAATGFVKK **Charge** 3 **Spectral Angle** 0.87

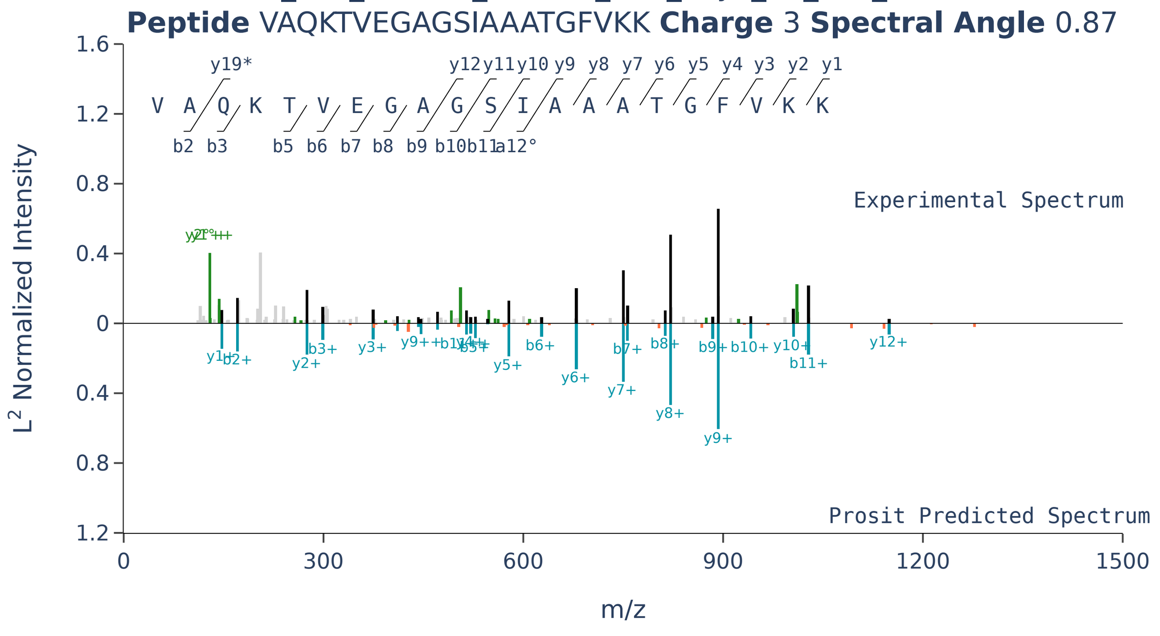


**Source** W_Soh_160821_170921_Goe_aSyn_A1_6h_R1 **Scan** 14885
**Peptide** LGKNEEGAPQEGILE **Charge** 2 **Spectral Angle** 0.92

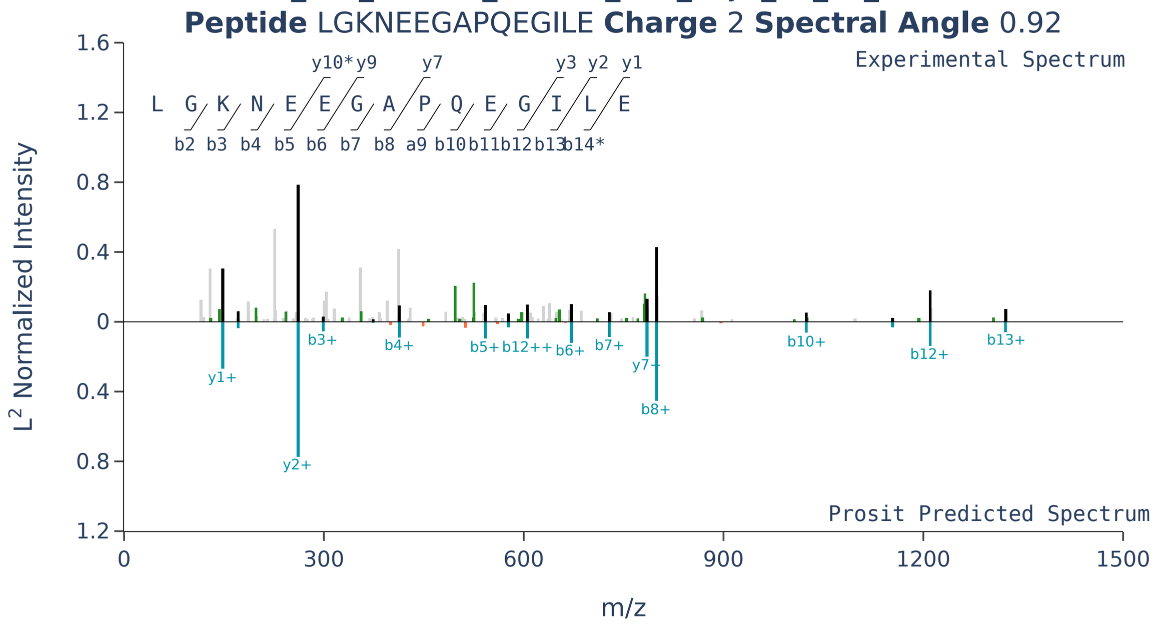


**Source** W_Soh_160821_170921_Goe_aSyn_A1_24h_R1 **Scan** 9191
**Peptide** KEGVVHGVATV **Charge** 2 **Spectral Angle** 0.91

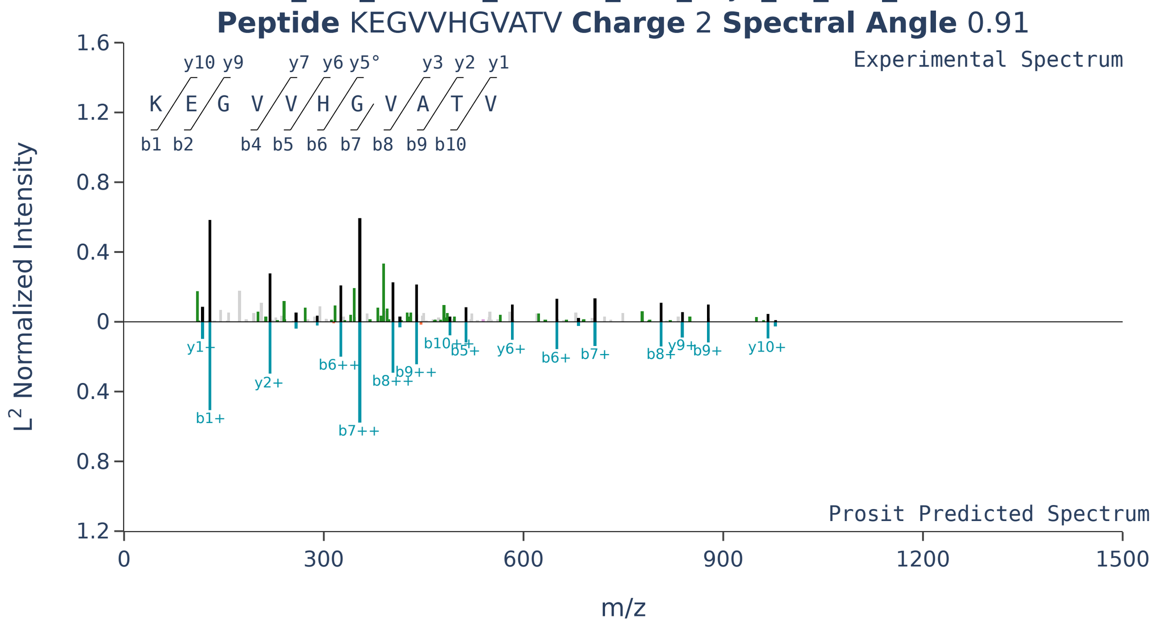


**Source** W_Soh_160821_170921_Goe_aSyn_A2_24h_R1 **Scan** 13841
**Peptide** EGVLYVGSKT **Charge** 2 **Spectral Angle** 0.9

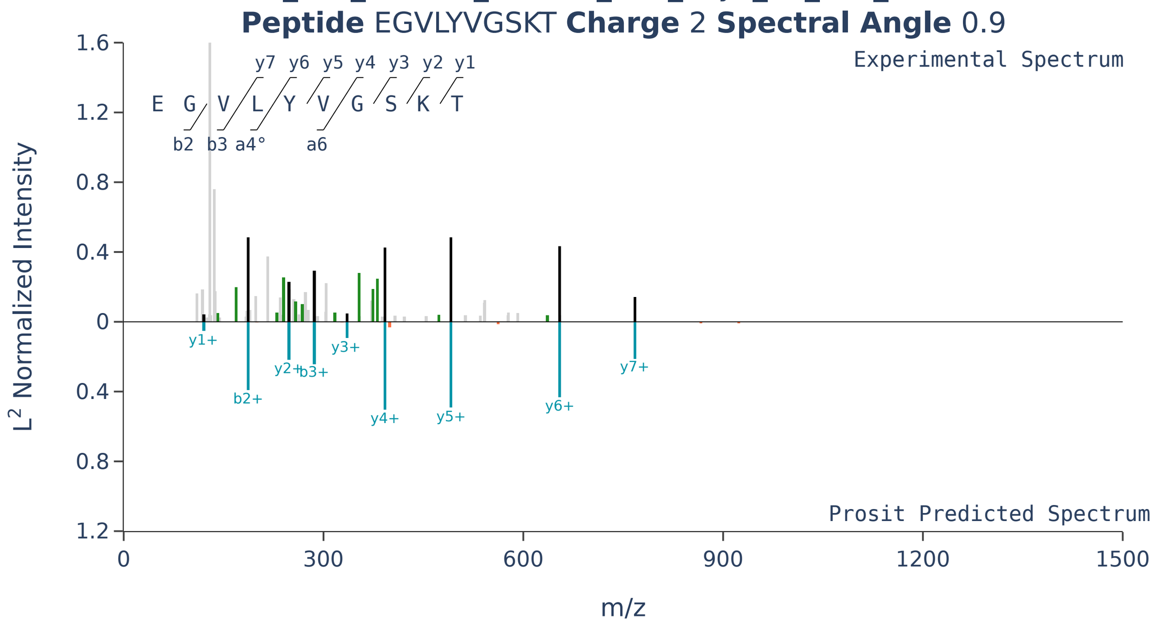


**Source** W_Soh_160821_170921_Goe_aSyn_A1_24h_R1 **Scan** 6453
**Peptide** GFVKKDQLGKNE **Charge** 2 **Spectral Angle** 0.81

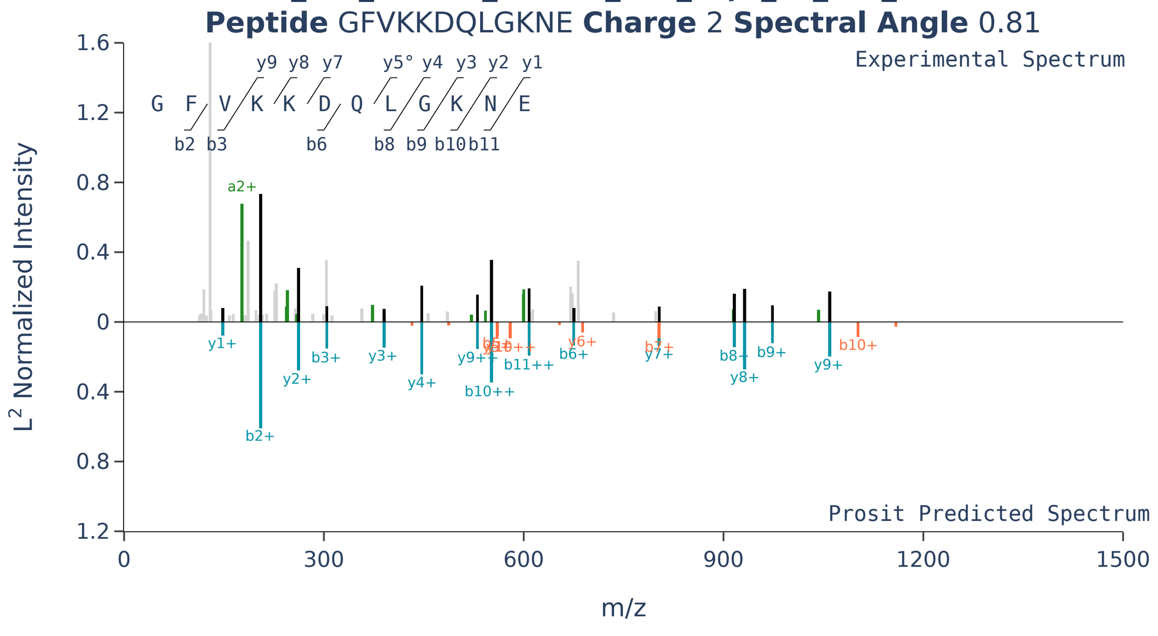


**Source** W_Soh_160821_170921_Goe_aSyn_A1_3h_R2 **Scan** 10563
**Peptide** MPVDPDNE **Charge** 1 **Spectral Angle** 0.81

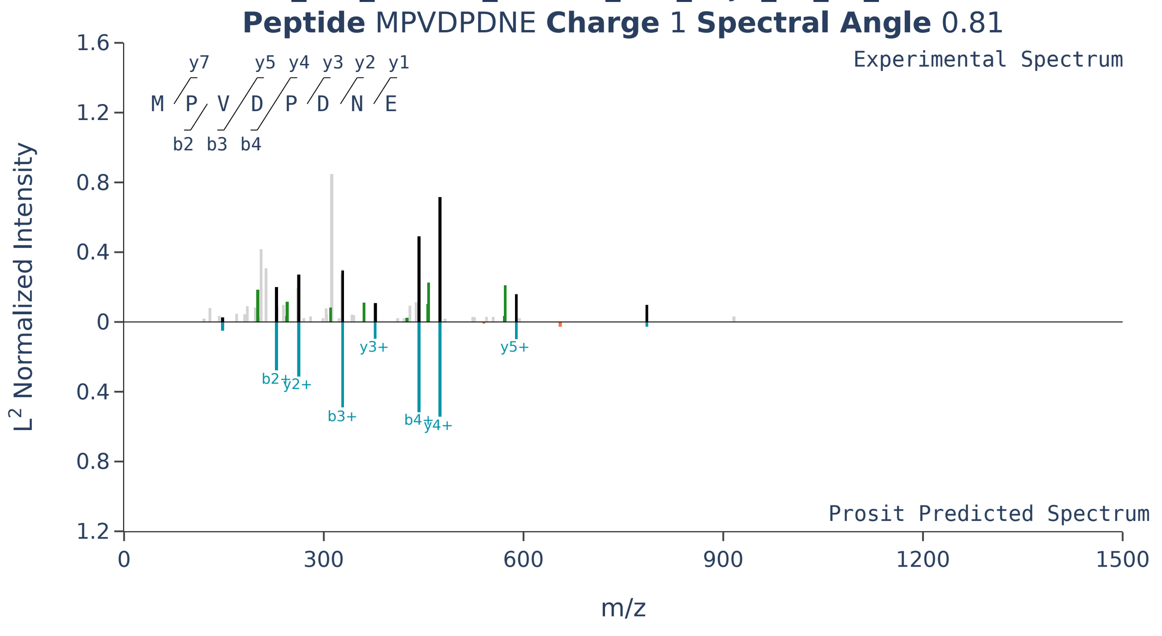


**Source** W_Soh_160821_170921_Goe_aSyn_A1_6h_R2 **Scan** 21493
**Peptide** TNVGGAVVTGVTAV **Charge** 2 **Spectral Angle** 0.88

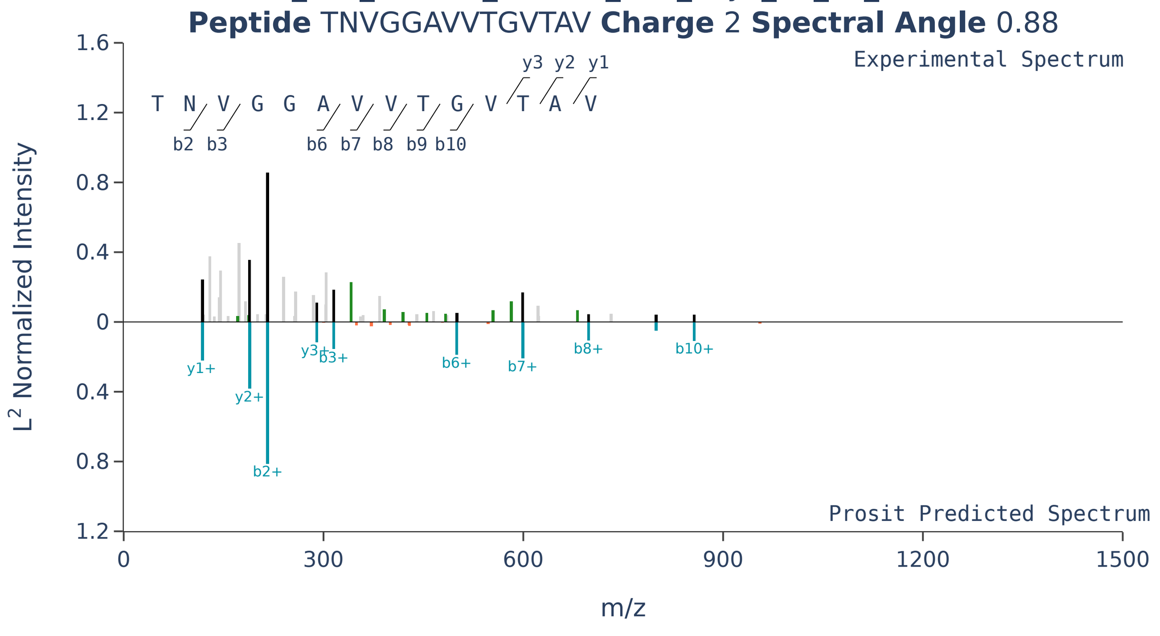

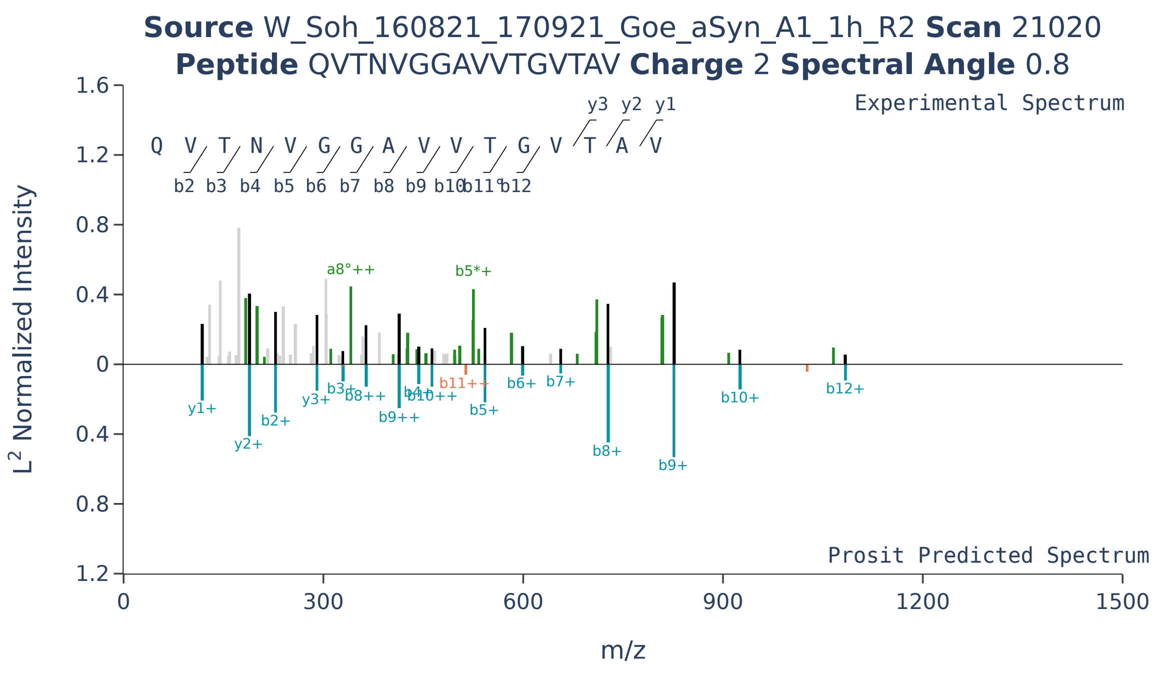
Source W_Soh_160821_170921_Goe_aSyn_A1_1h_R2 Scan 21020
Peptide QVTNVGGAVVTGVTAV Charge 2 Spectral Angle 0.8
Experimental Spectrum
Prosit Predicted Spectrum
L² Normalized Intensity
m/z

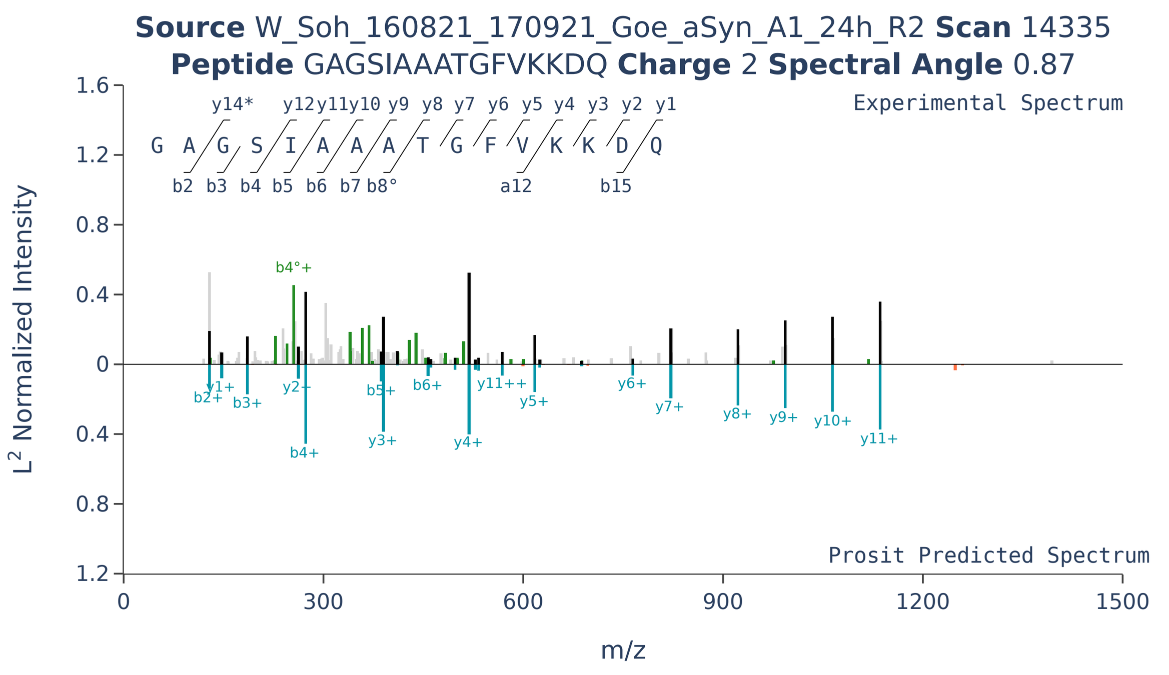
Source W_Soh_160821_170921_Goe_aSyn_A1_24h_R2 Scan 14335
Peptide GAGSIAAATGFVKKDQ Charge 2 Spectral Angle 0.87
Experimental Spectrum
Prosit Predicted Spectrum
L² Normalized Intensity
m/z

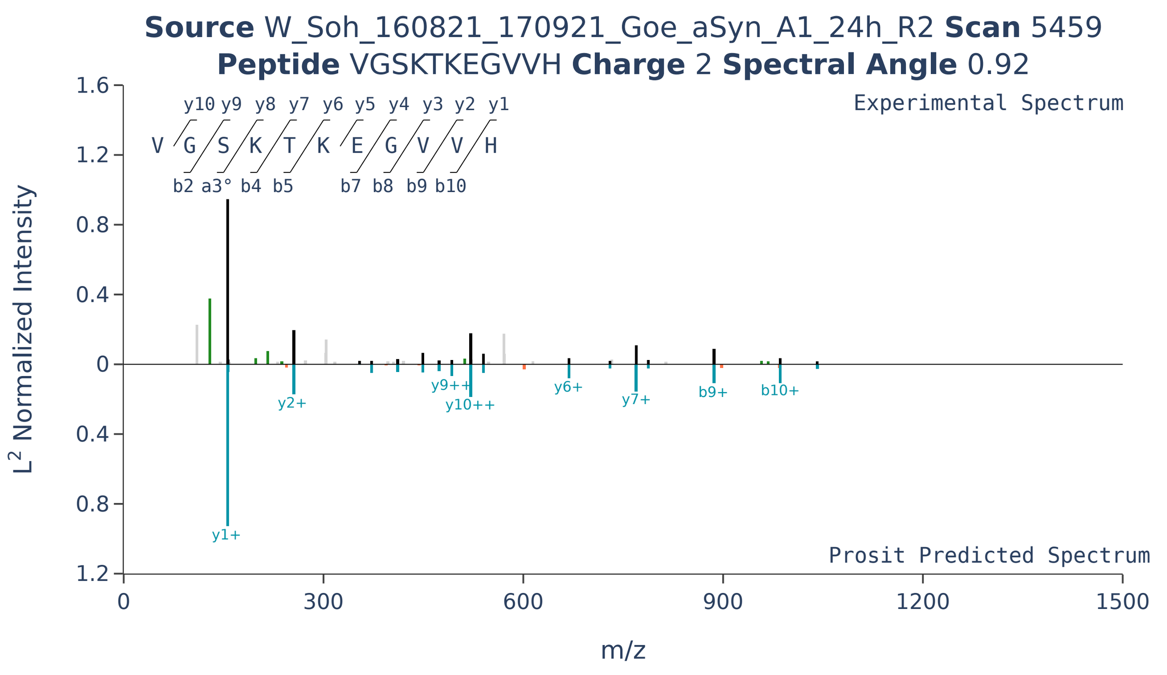
Source W_Soh_160821_170921_Goe_aSyn_A1_24h_R2 Scan 5459
Peptide VGSKTKEGVVH Charge 2 Spectral Angle 0.92
Experimental Spectrum
Prosit Predicted Spectrum
L² Normalized Intensity
m/z

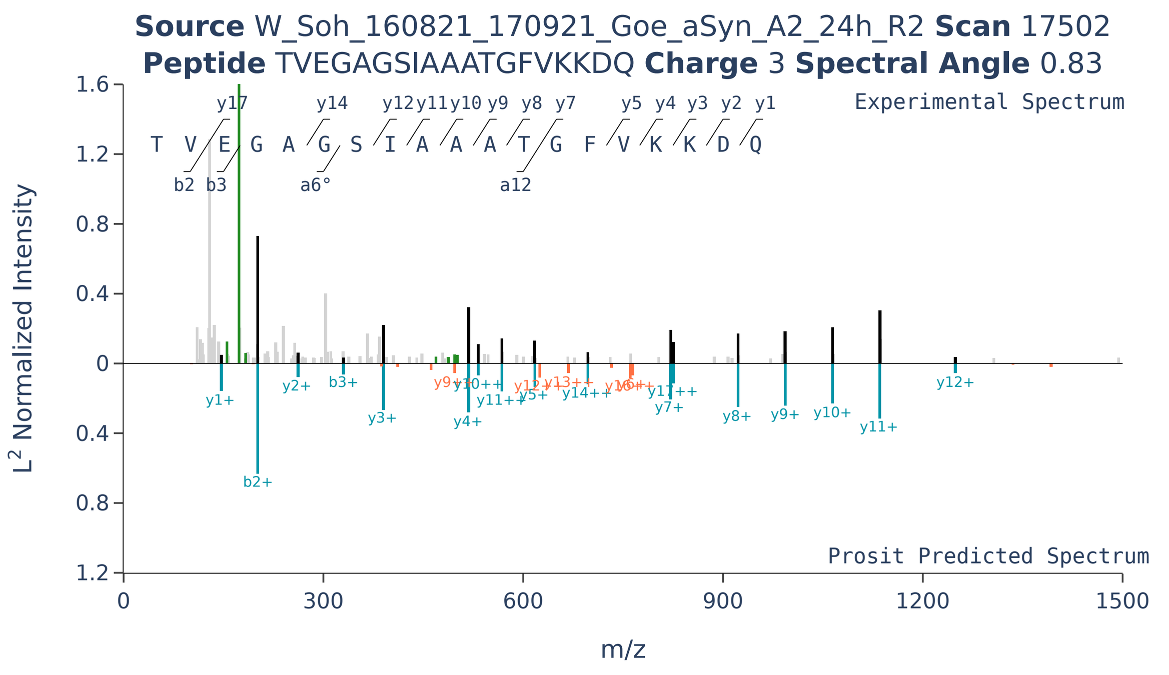
Source W_Soh_160821_170921_Goe_aSyn_A2_24h_R2 Scan 17502
Peptide TVEGAGSIAAATGFVKKDQ Charge 3 Spectral Angle 0.83
Experimental Spectrum
Prosit Predicted Spectrum
L² Normalized Intensity
m/z

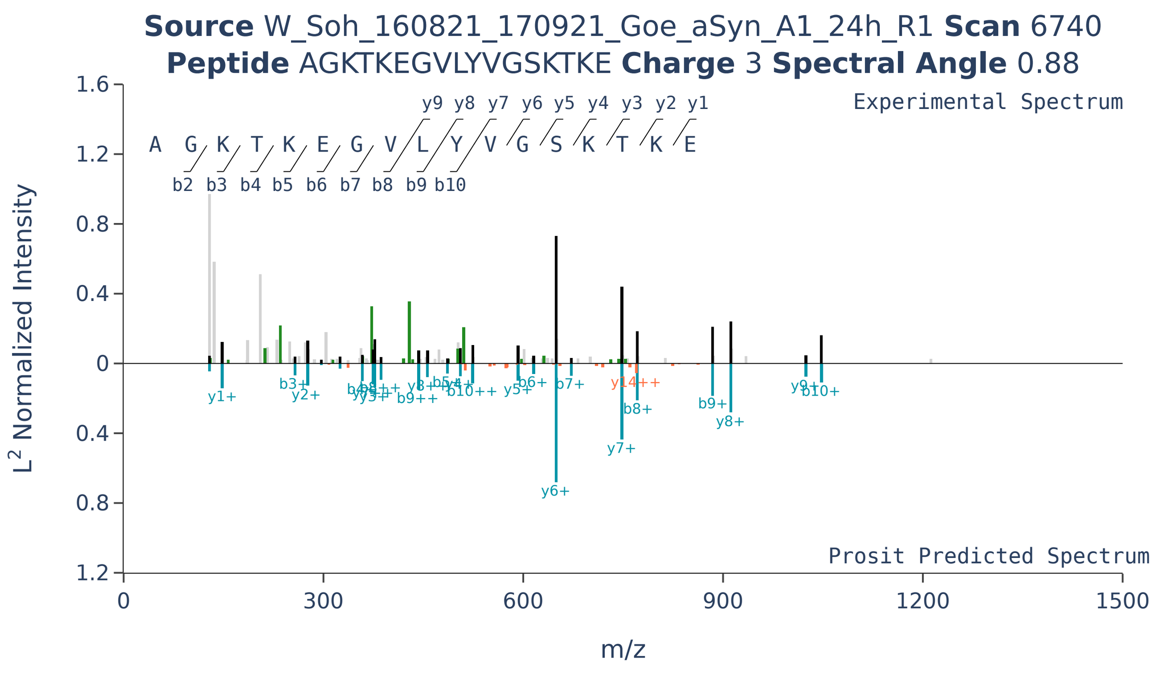
Source W_Soh_160821_170921_Goe_aSyn_A1_24h_R1 Scan 6740
Peptide AGKTKEGVLYVGSKTKE Charge 3 Spectral Angle 0.88
Experimental Spectrum
Prosit Predicted Spectrum
L² Normalized Intensity
m/z

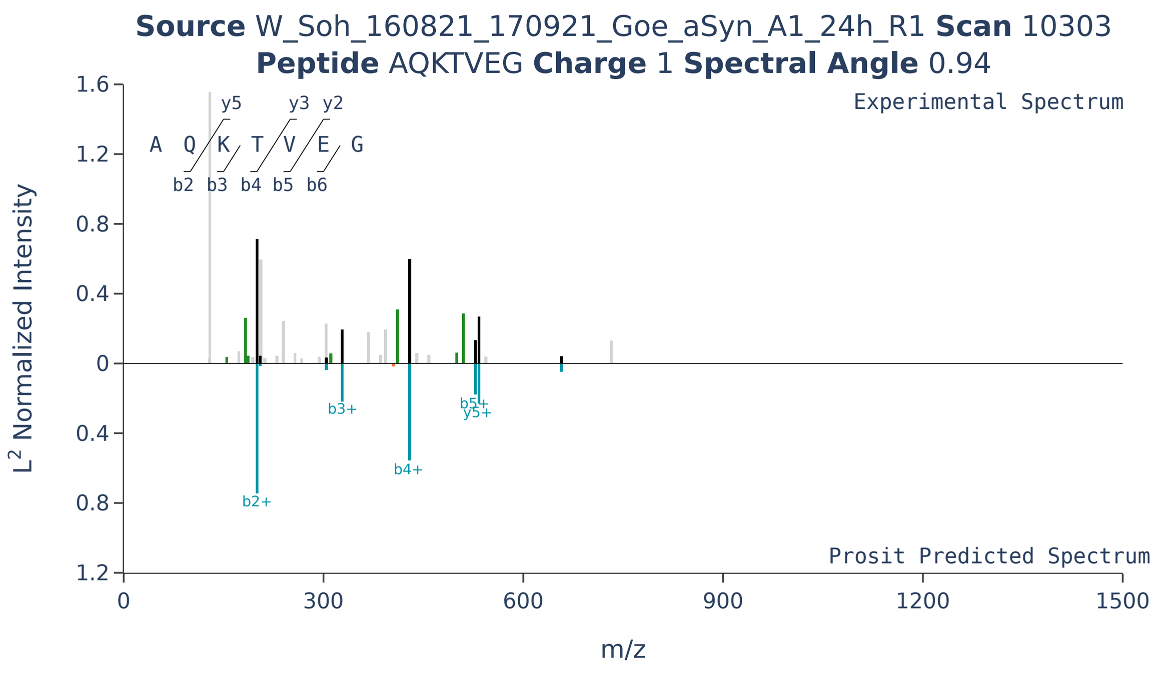
Source W_Soh_160821_170921_Goe_aSyn_A1_24h_R1 Scan 10303
Peptide AQKTVEG Charge 1 Spectral Angle 0.94
Experimental Spectrum
Prosit Predicted Spectrum
L² Normalized Intensity
m/z

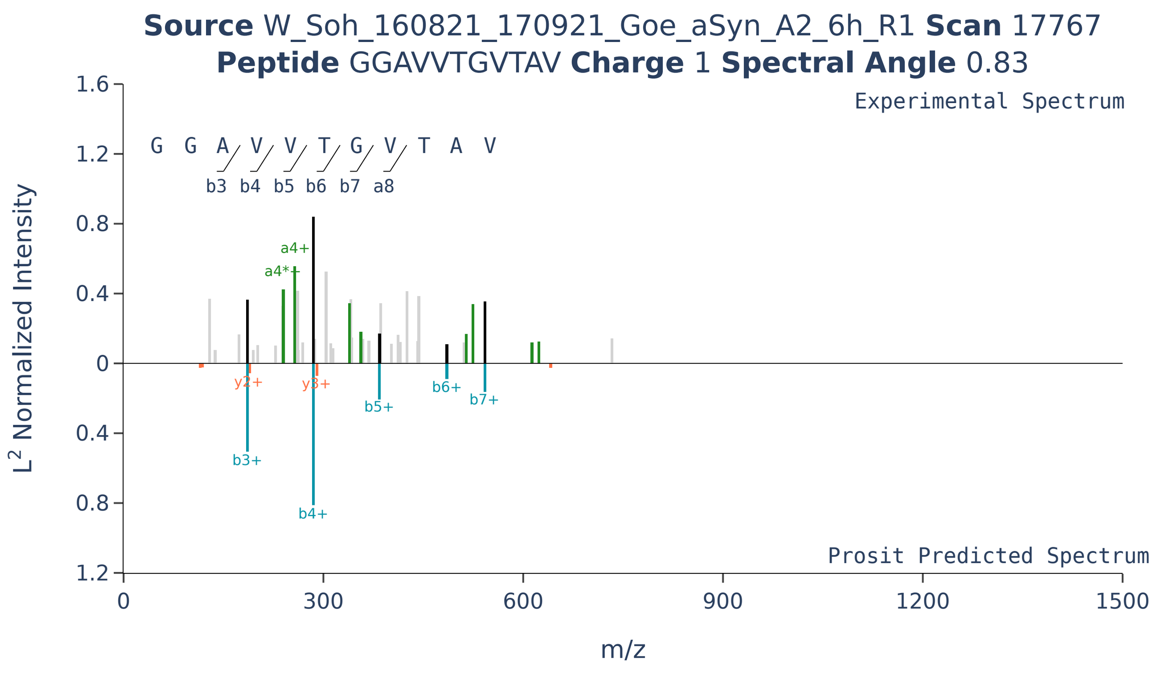
Source W_Soh_160821_170921_Goe_aSyn_A2_6h_R1 Scan 17767
Peptide GGAVVTGVTAV Charge 1 Spectral Angle 0.83
Experimental Spectrum
Prosit Predicted Spectrum
L² Normalized Intensity
m/z

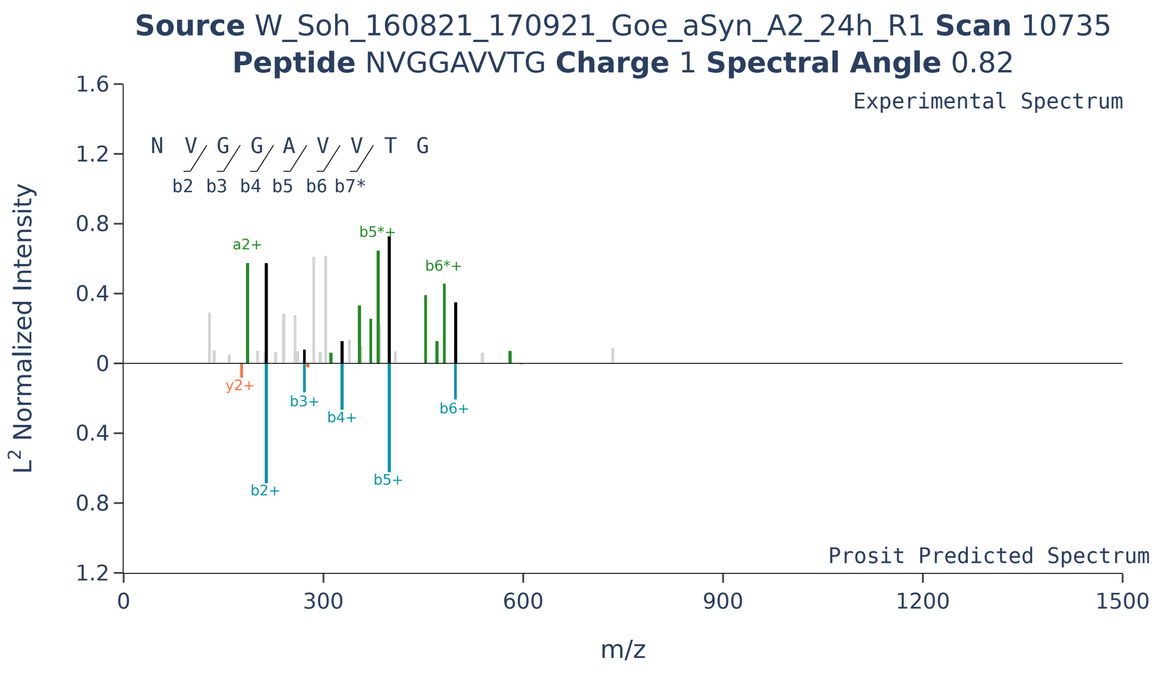
Source W_Soh_160821_170921_Goe_aSyn_A2_24h_R1 Scan 10735
Peptide NVGGAVVTG Charge 1 Spectral Angle 0.82
Experimental Spectrum
Prosit Predicted Spectrum
L² Normalized Intensity
m/z

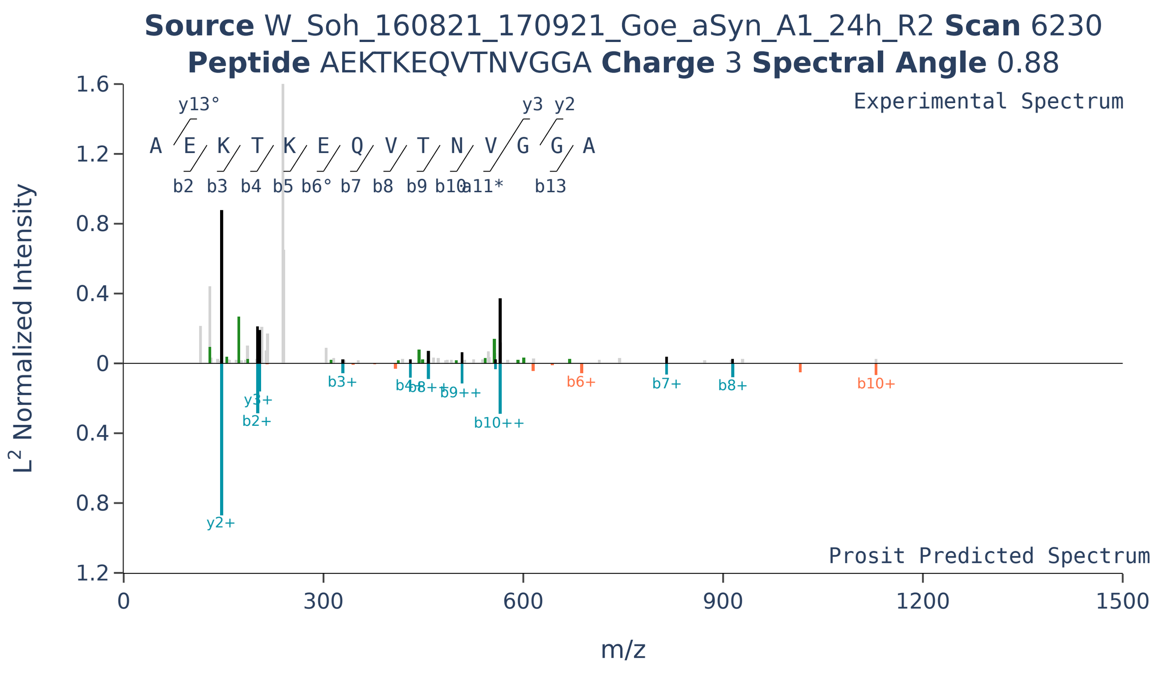
Source W_Soh_160821_170921_Goe_aSyn_A1_24h_R2 Scan 6230
Peptide AEKTKEQVTNVGGA Charge 3 Spectral Angle 0.88
Experimental Spectrum
Prosit Predicted Spectrum
L² Normalized Intensity
m/z

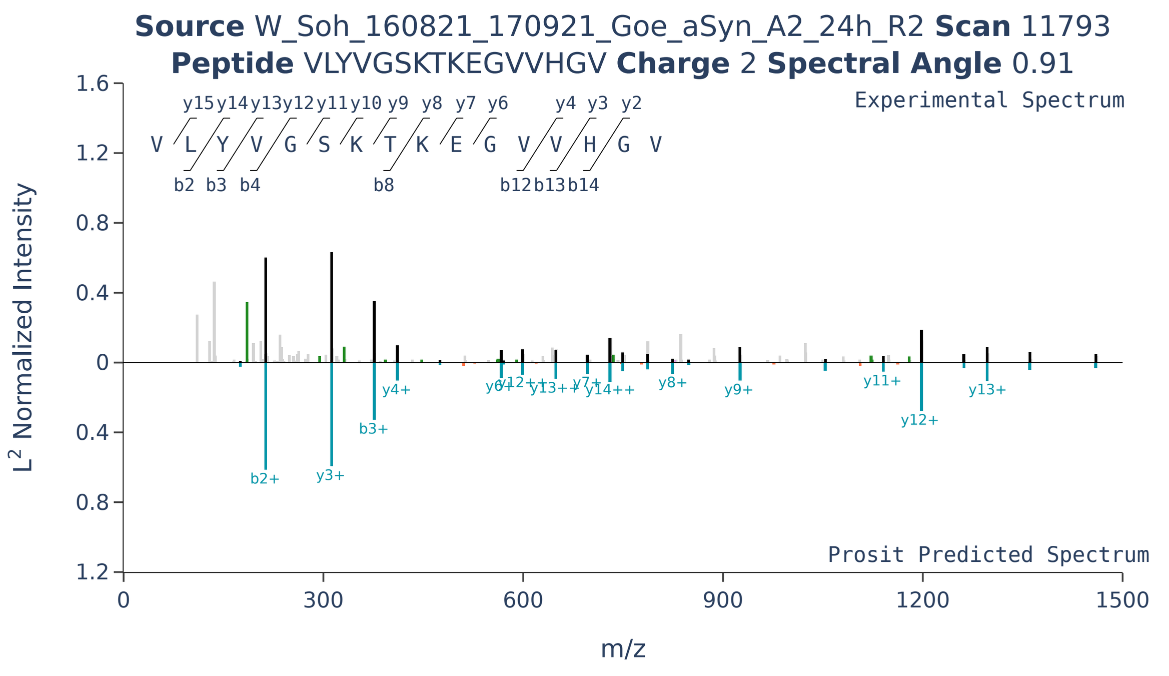
Source W_Soh_160821_170921_Goe_aSyn_A2_24h_R2 Scan 11793
Peptide VLYVGSKTKEGVVHGV Charge 2 Spectral Angle 0.91
Experimental Spectrum
Prosit Predicted Spectrum
L² Normalized Intensity
m/z

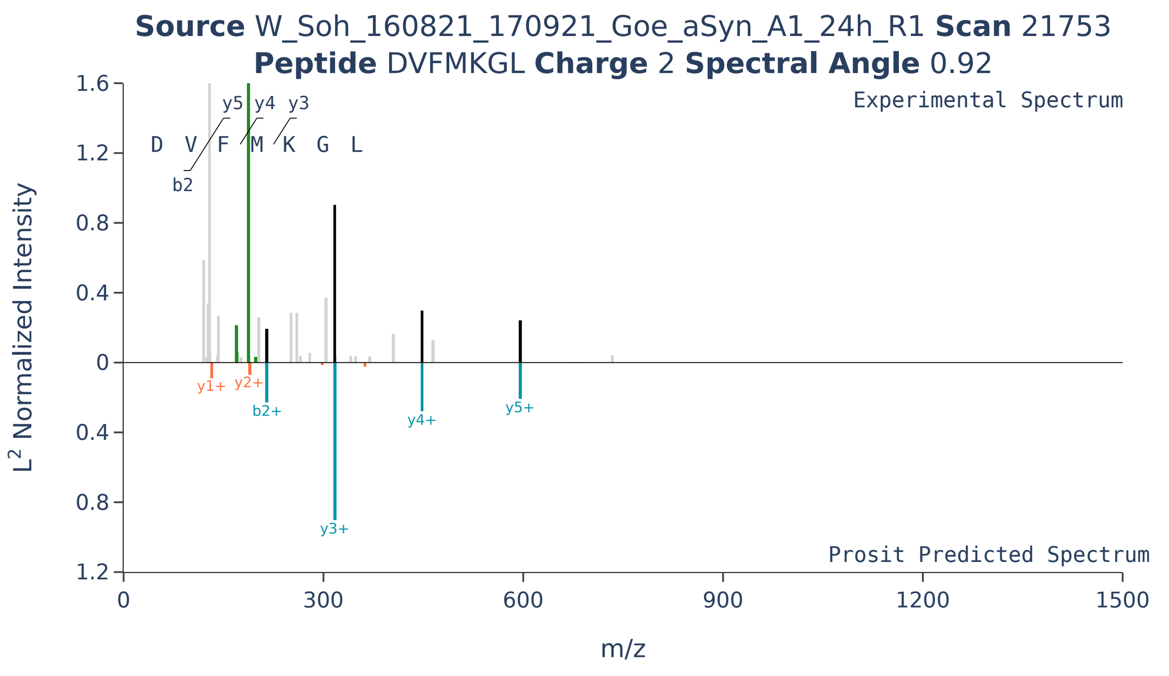
Source W_Soh_160821_170921_Goe_aSyn_A1_24h_R1 Scan 21753
Peptide DVFMKGL Charge 2 Spectral Angle 0.92
Experimental Spectrum
Prosit Predicted Spectrum
L² Normalized Intensity
m/z

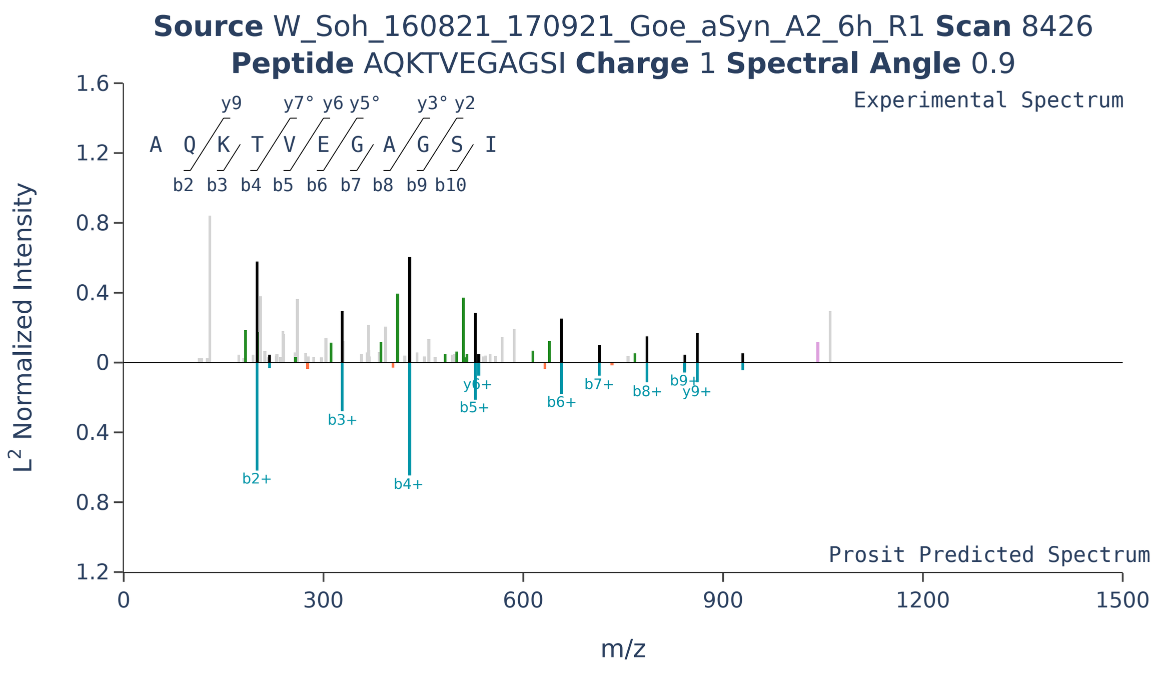
Source W_Soh_160821_170921_Goe_aSyn_A2_6h_R1 Scan 8426
Peptide AQKTVEGAGSI Charge 1 Spectral Angle 0.9
Experimental Spectrum
Prosit Predicted Spectrum
L² Normalized Intensity
m/z

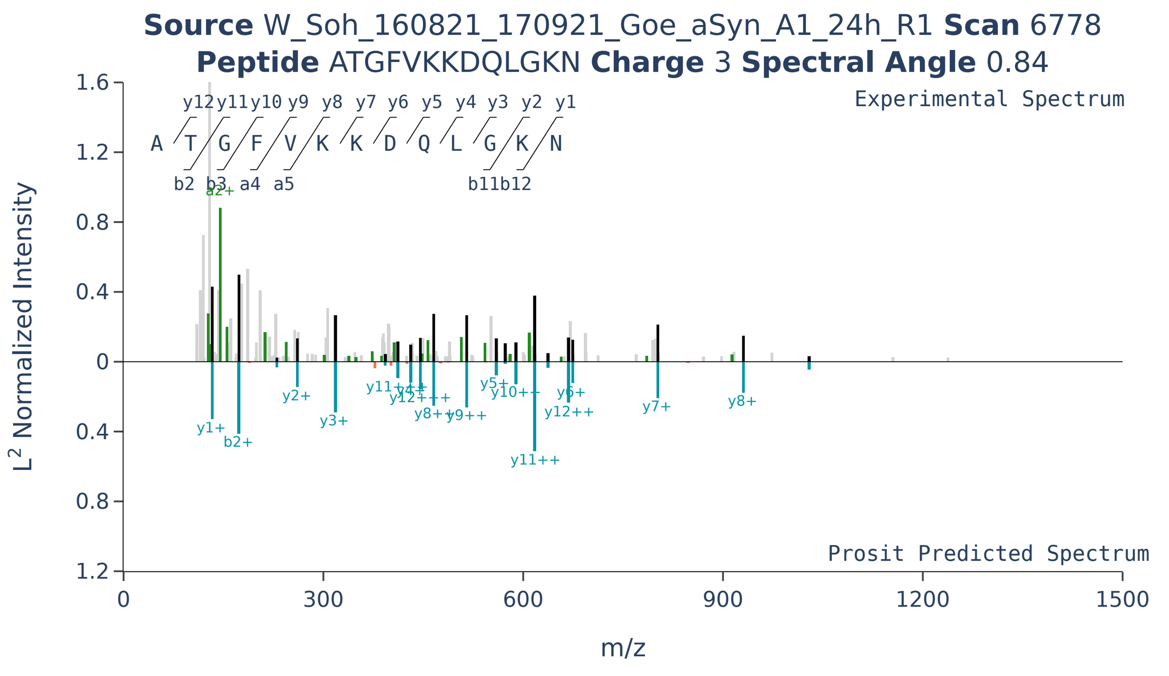
Source W_Soh_160821_170921_Goe_aSyn_A1_24h_R1 Scan 6778
Peptide ATGFVKKDQLGKN Charge 3 Spectral Angle 0.84
Experimental Spectrum
Prosit Predicted Spectrum
L² Normalized Intensity
m/z

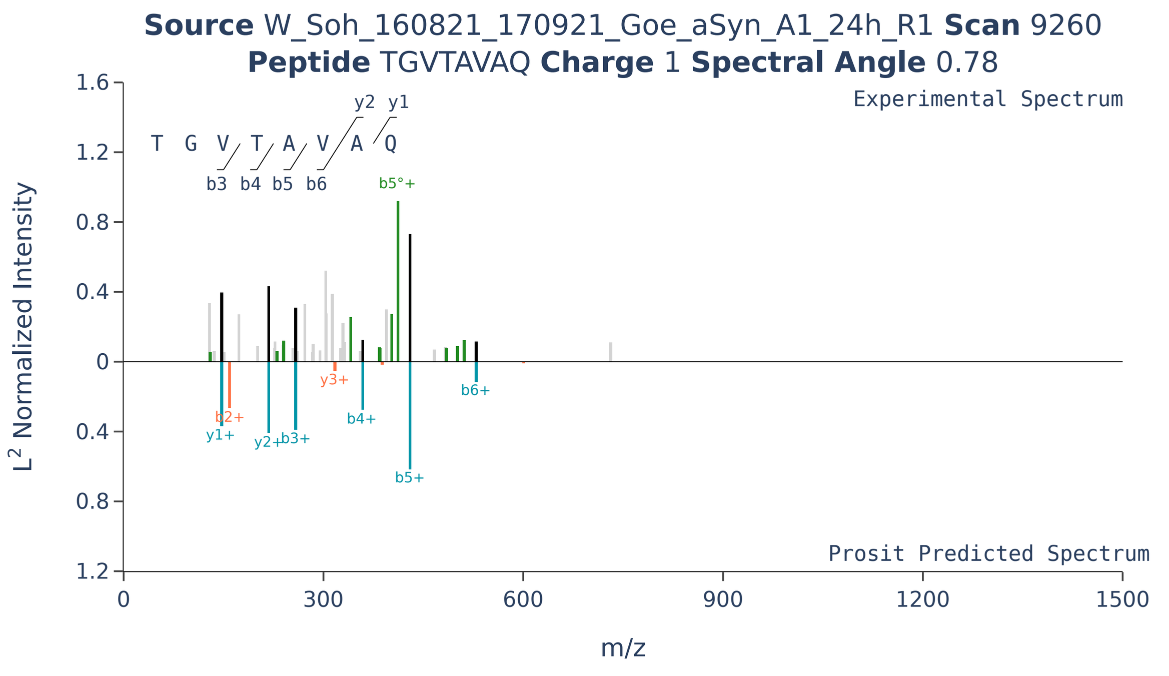
Source W_Soh_160821_170921_Goe_aSyn_A1_24h_R1 Scan 9260
Peptide TGVTAVAQ Charge 1 Spectral Angle 0.78
Experimental Spectrum
Prosit Predicted Spectrum
L² Normalized Intensity
m/z

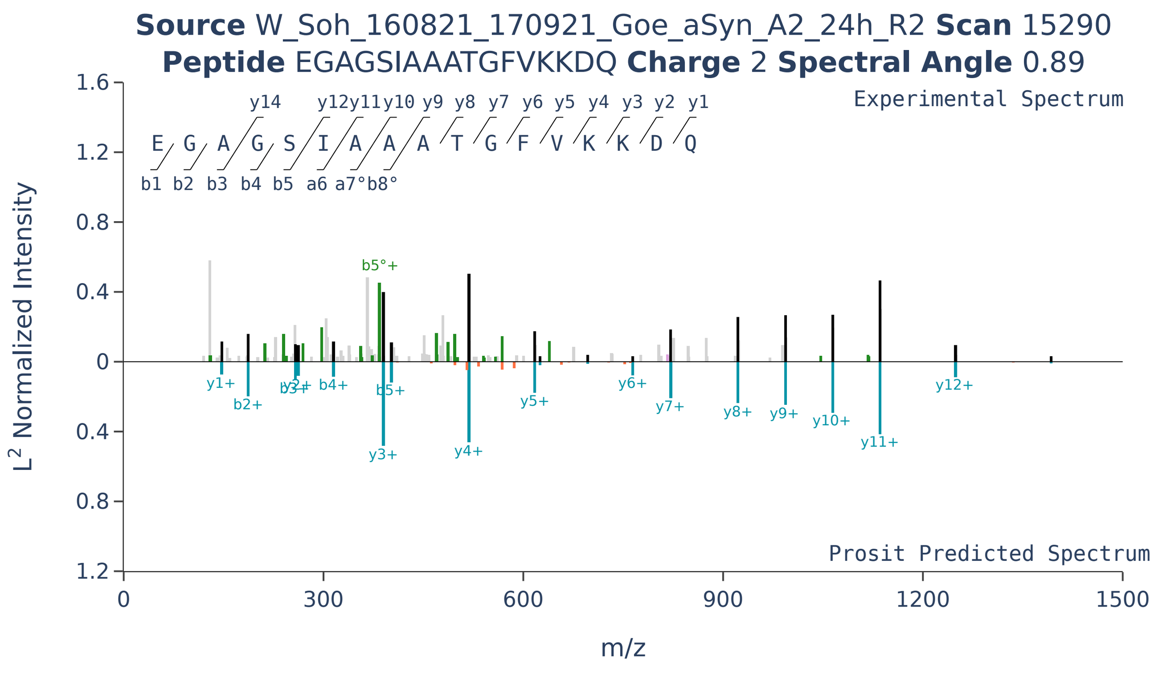
Source W_Soh_160821_170921_Goe_aSyn_A2_24h_R2 Scan 15290
Peptide EGAGSIAAATGFVKKDQ Charge 2 Spectral Angle 0.89
Experimental Spectrum
Prosit Predicted Spectrum
L² Normalized Intensity
m/z

**Source** W_Soh_160821_170921_Goe_aSyn_A1_6h_R2 **Scan** 12634
**Peptide** GVLYVGSKT **Charge** 2 **Spectral Angle** 0.88

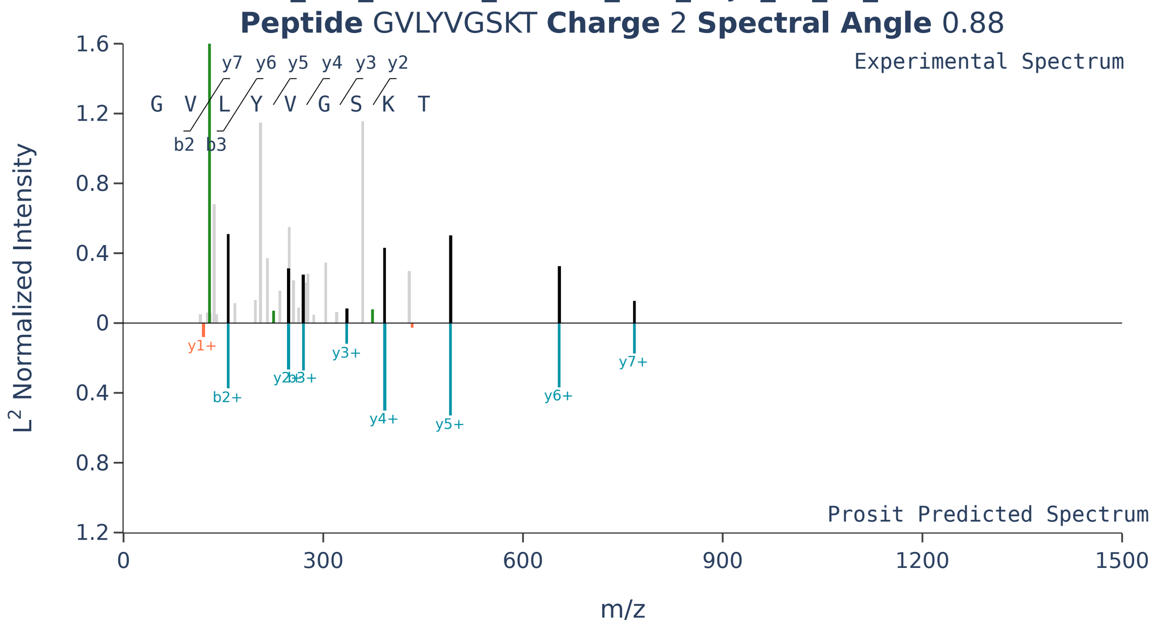


**Source** W_Soh_160821_170921_Goe_aSyn_A2_24h_R2 **Scan** 13758
**Peptide** GGAVVTGVTA **Charge** 1 **Spectral Angle** 0.89

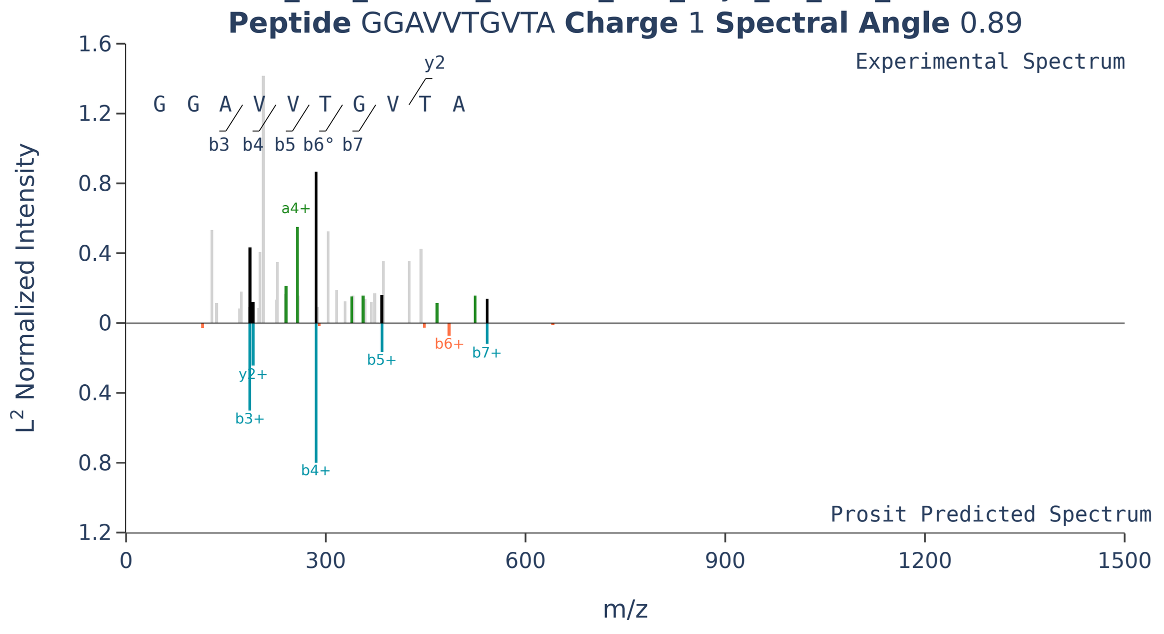


**Source** W_Soh_160821_170921_Goe_aSyn_A2_24h_R2 **Scan** 17541
**Peptide** AQKTVEGAGSIAAATGFVK **Charge** 2 **Spectral Angle** 0.78

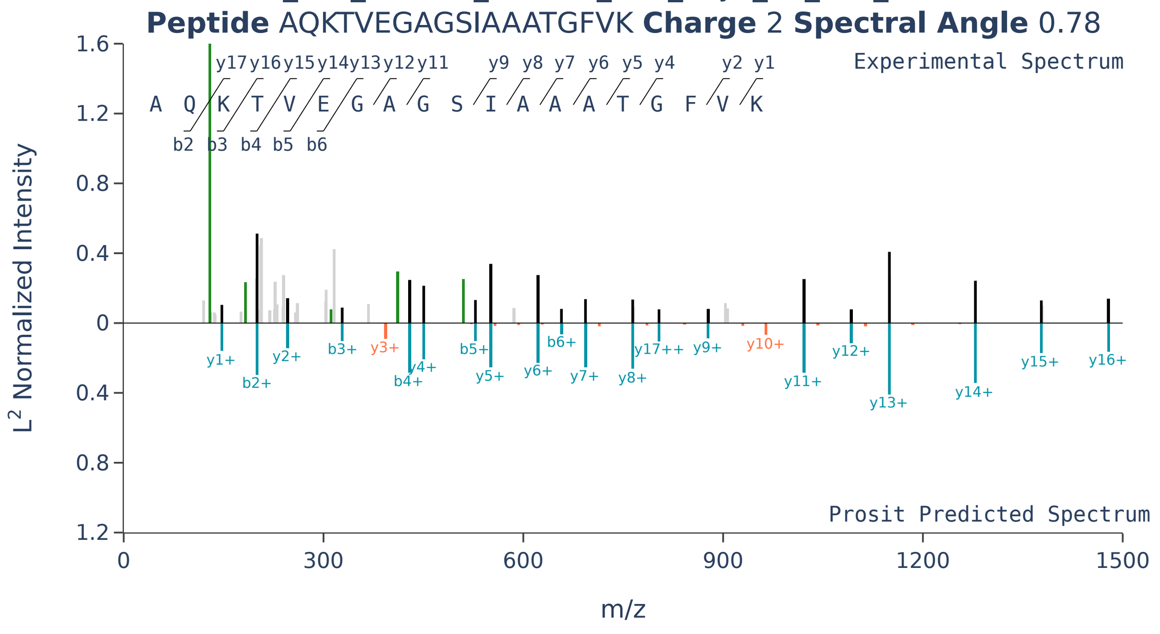


**Source** W_Soh_160821_170921_Goe_aSyn_A1_24h_R1 **Scan** 10940
**Peptide** NVGGAVVT **Charge** 1 **Spectral Angle** 0.76

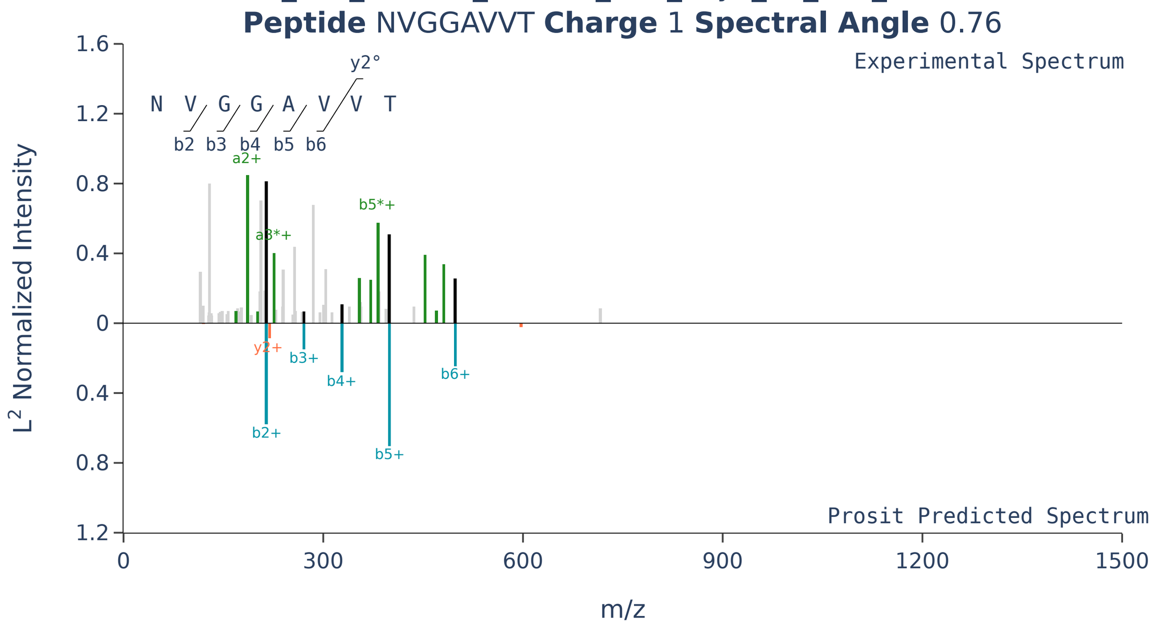


**Source** W_Soh_160821_170921_Goe_aSyn_A2_24h_R2 **Scan** 15033
**Peptide** AQKTVEGAGSIAAATGFVKKDQ **Charge** 3 **Spectral Angle** 0.84

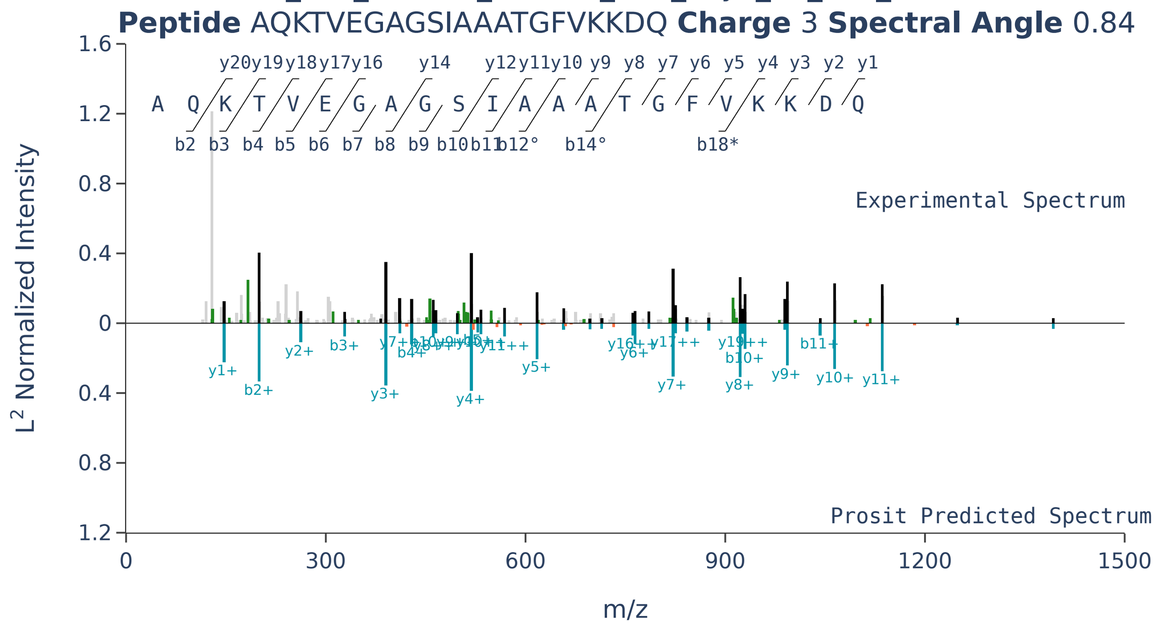


**Source** W_Soh_160821_170921_Goe_aSyn_A2_24h_R1 **Scan** 13431
**Peptide** AGSIAAATGFVKKD **Charge** 2 **Spectral Angle** 0.91

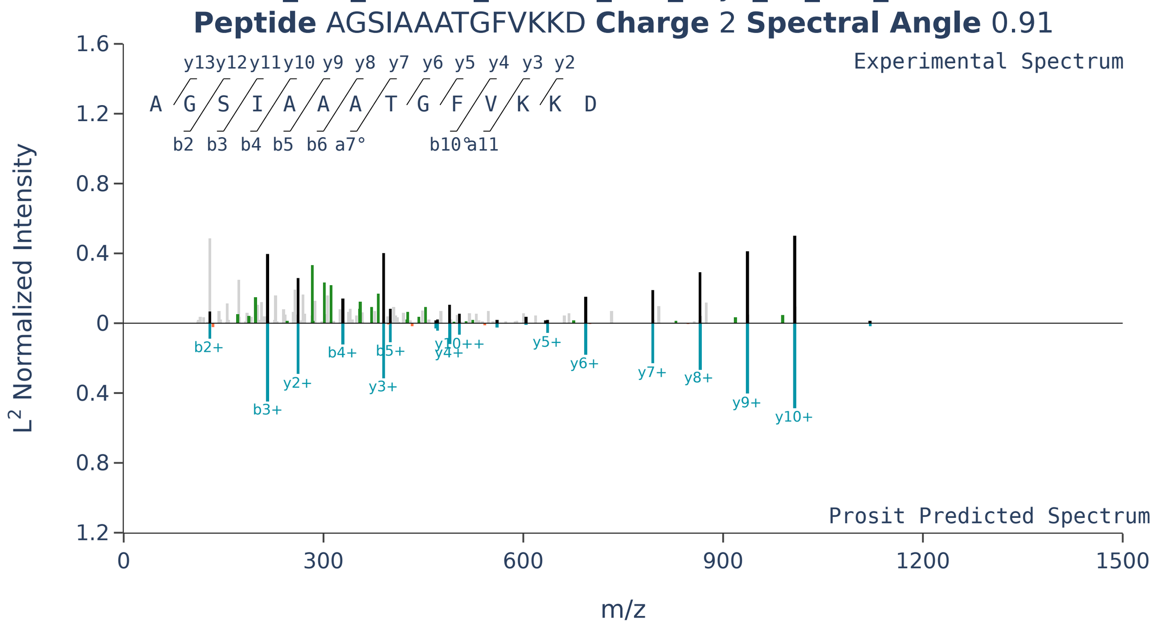


**Source** W_Soh_160821_170921_Goe_aSyn_A1_24h_R1 **Scan** 15367
**Peptide** DVFMKGLSKA **Charge** 3 **Spectral Angle** 0.86

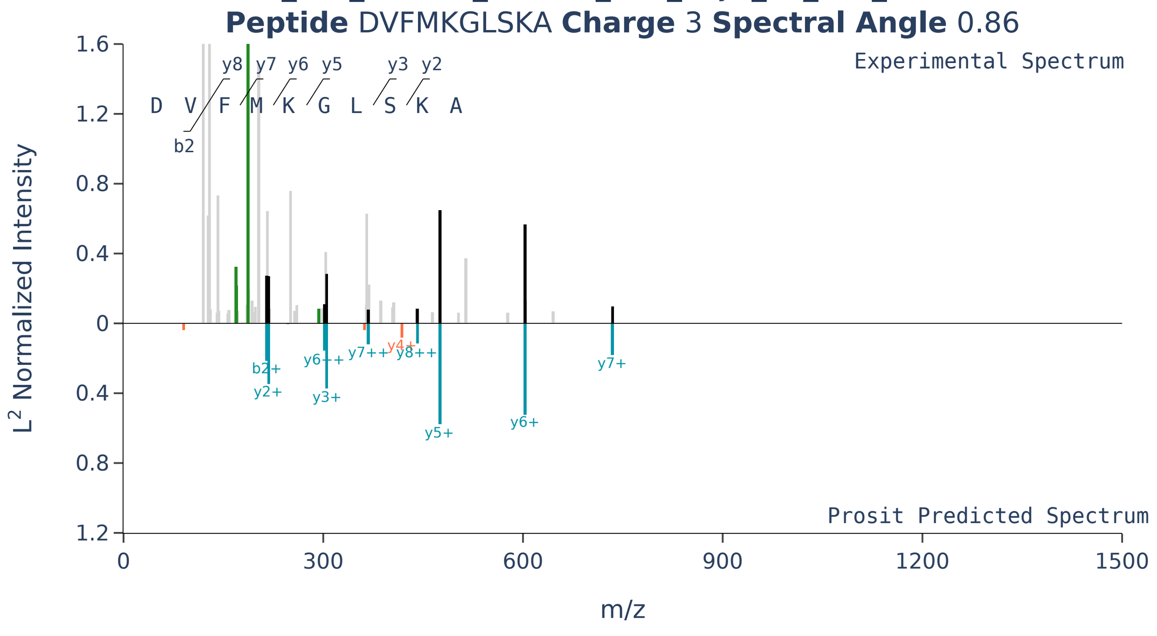


**Source** W_Soh_160821_170921_Goe_aSyn_A2_24h_R1 **Scan** 14649
**Peptide** AQKTVEGAGSIAAATGFVKK **Charge** 3 **Spectral Angle** 0.91

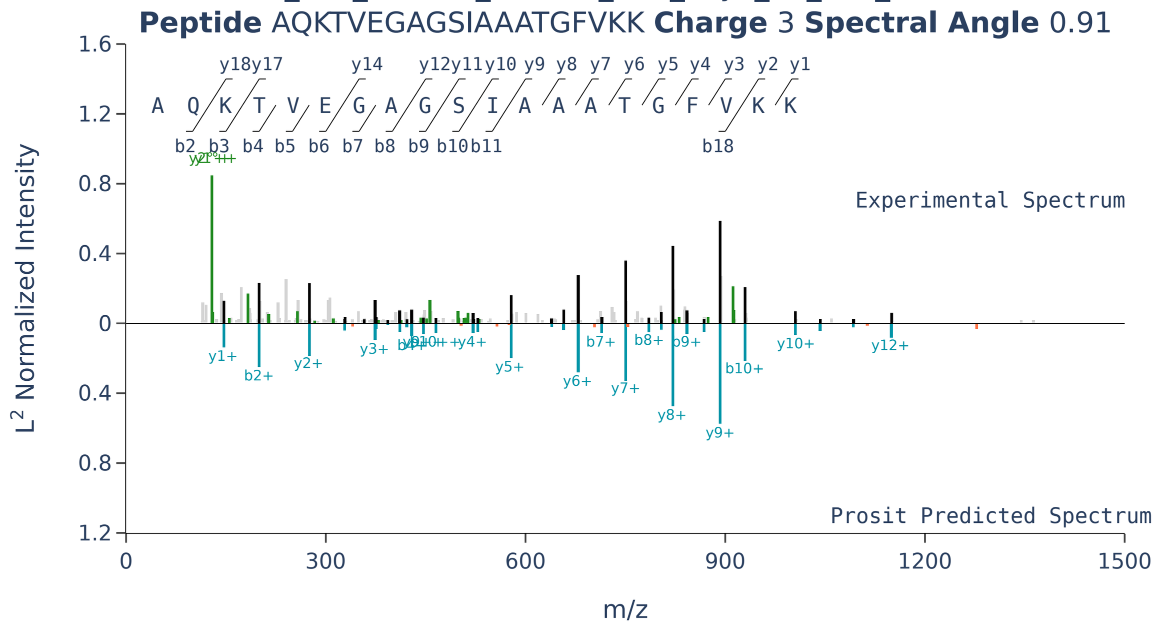


**Source** W_Soh_160821_170921_Goe_aSyn_A2_24h_R1 **Scan** 7551
**Peptide** GLSKAKEGVVAA **Charge** 1 **Spectral Angle** 0.87

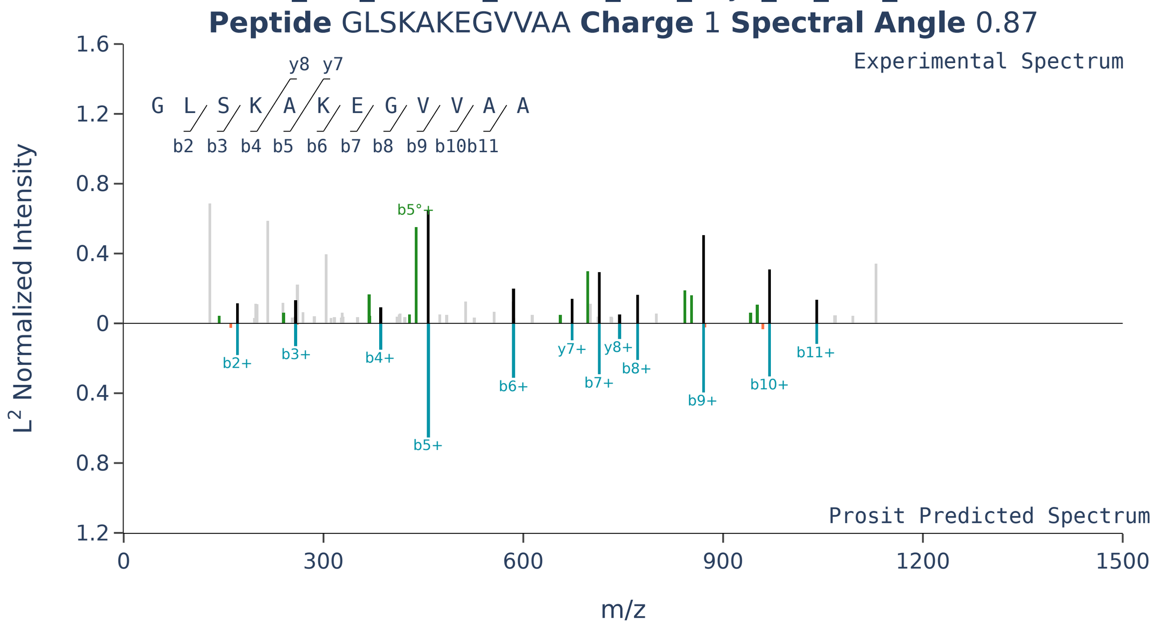


**Source** W_Soh_160821_170921_Goe_aSyn_A1_6h_R2 **Scan** 14053
**Peptide** EEGYQDYEPEA **Charge** 2 **Spectral Angle** 0.9

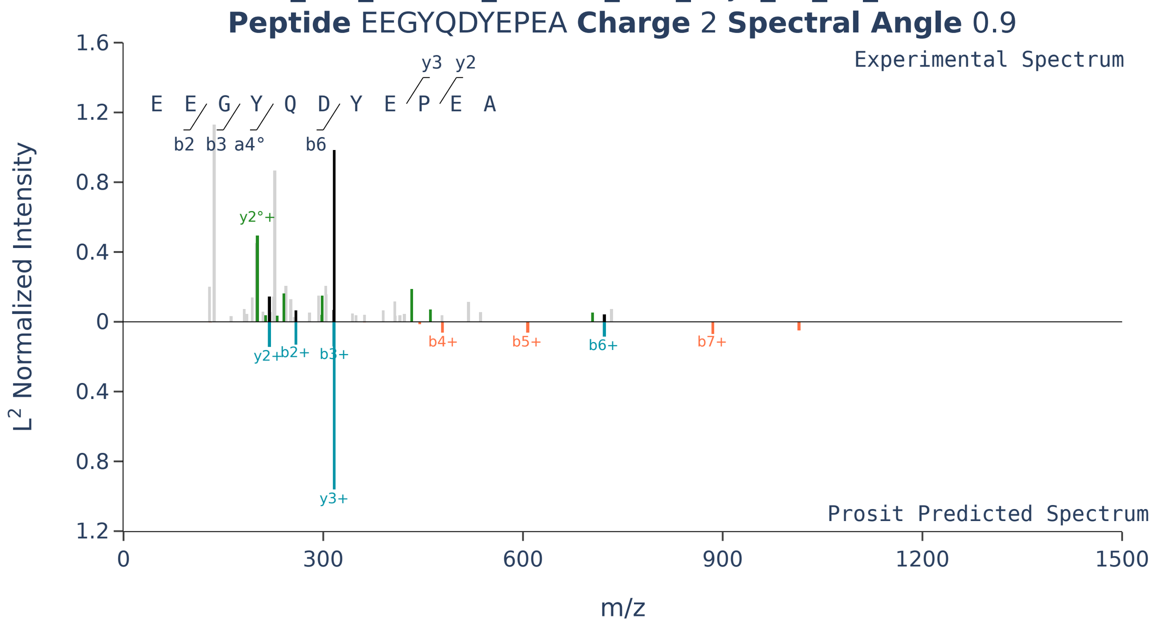


**Source** W_Soh_160821_170921_Goe_aSyn_A1_24h_R1 **Scan** 6719
**Peptide** EGVVAAAEKTKQG **Charge** 2 **Spectral Angle** 0.85

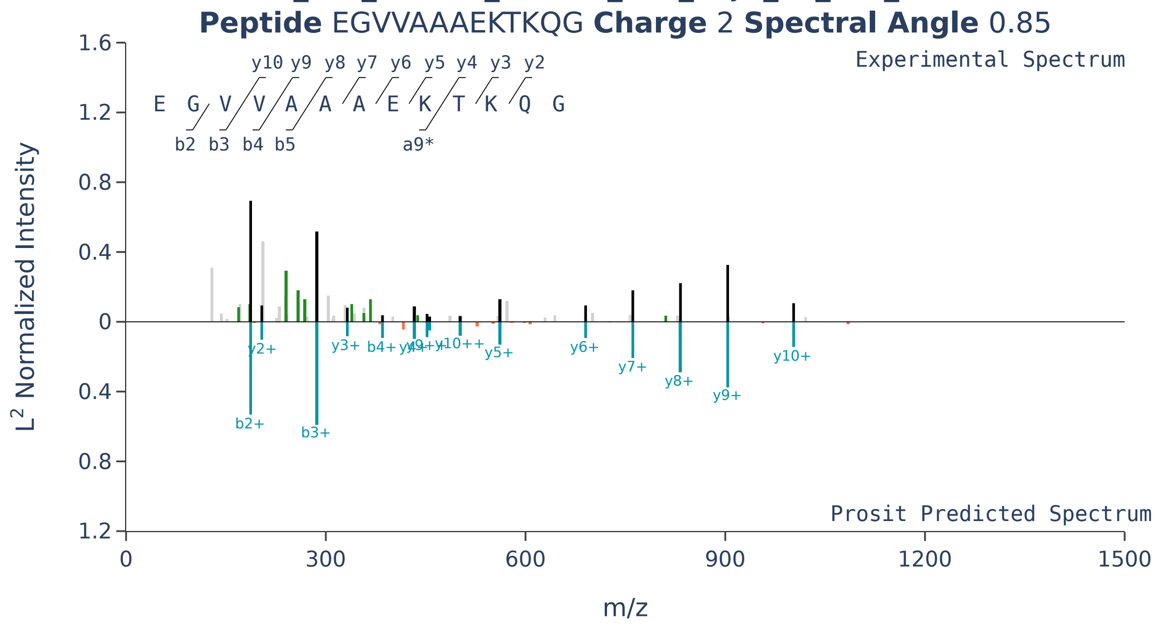


**Source** W_Soh_160821_170921_Goe_aSyn_A1_24h_R2 **Scan** 17730
**Peptide** TNVGGAVVTGV **Charge** 2 **Spectral Angle** 0.85

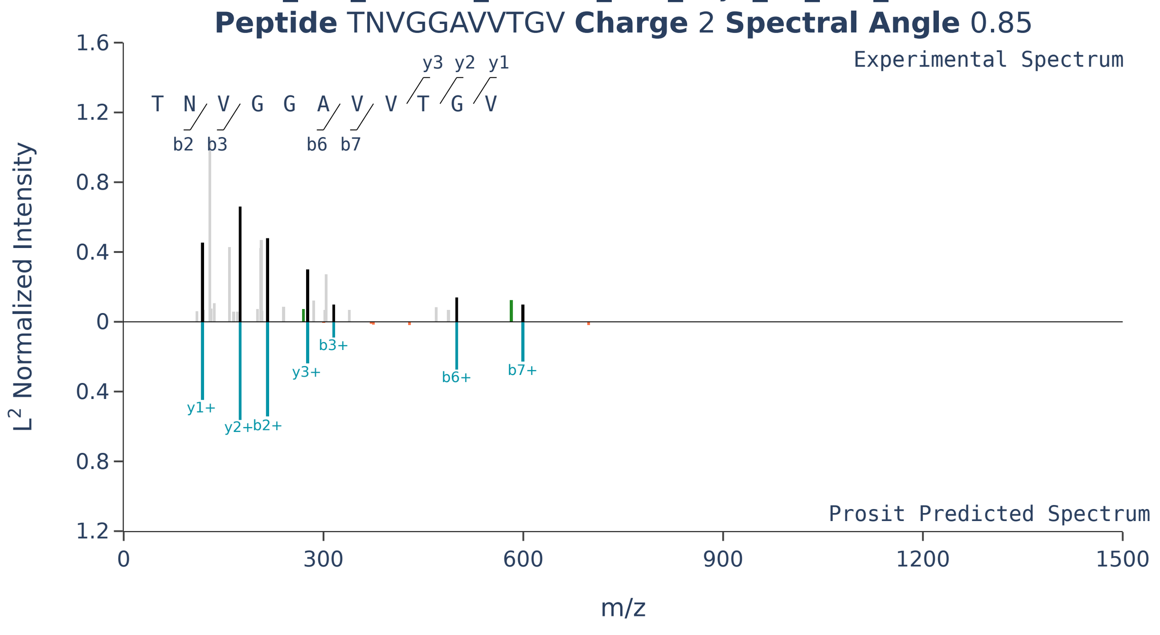


**Source** W_Soh_160821_170921_Goe_aSyn_A1_6h_R1 **Scan** 5475
**Peptide** KEGVVAAAEKTKQG **Charge** 3 **Spectral Angle** 0.82

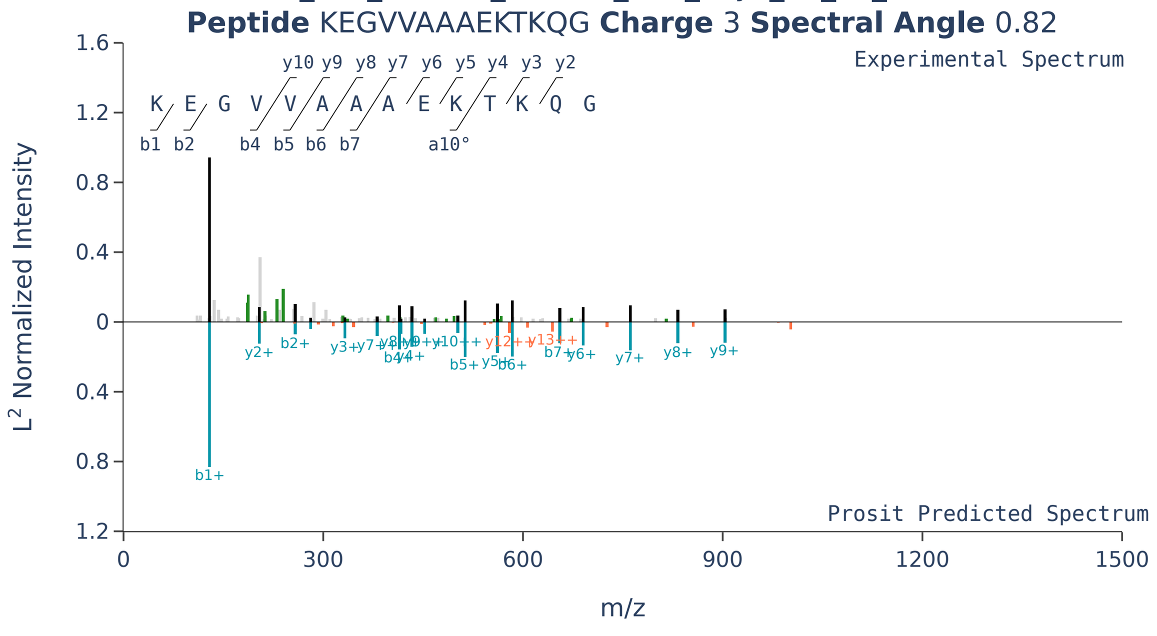


**Source** W_Soh_160821_170921_Goe_aSyn_A2_24h_R1 **Scan** 13392
**Peptide** SIAAATGFVKKD **Charge** 2 **Spectral Angle** 0.83

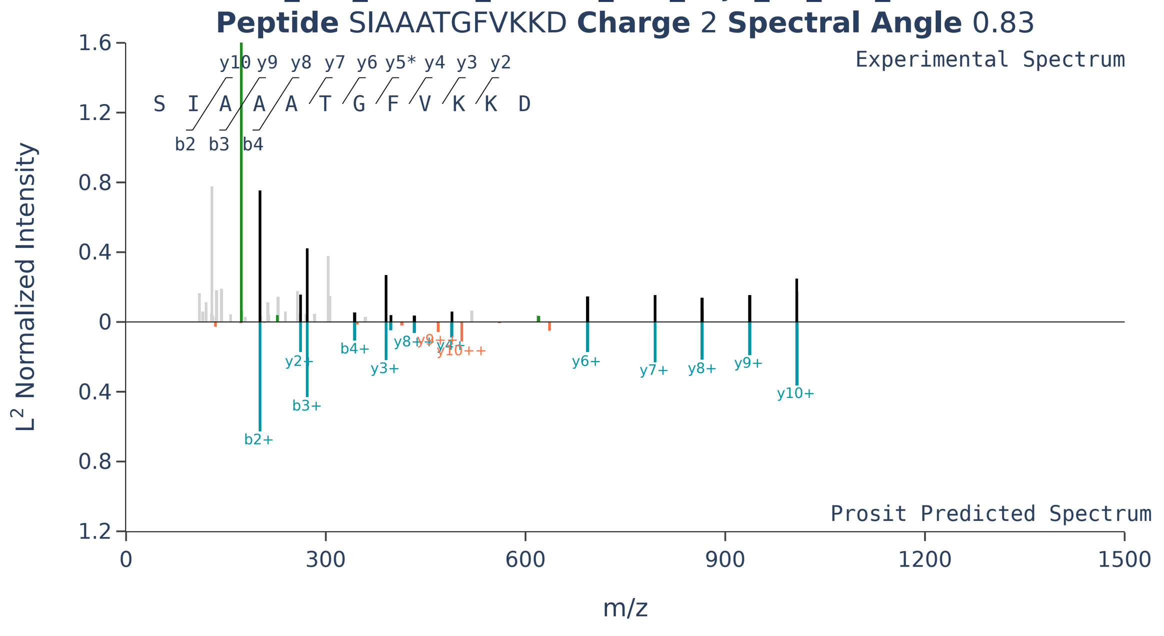


**Source** W_Soh_160821_170921_Goe_aSyn_A1_24h_R2 **Scan** 7101
**Peptide** GLSKAKEGVVA **Charge** 1 **Spectral Angle** 0.86

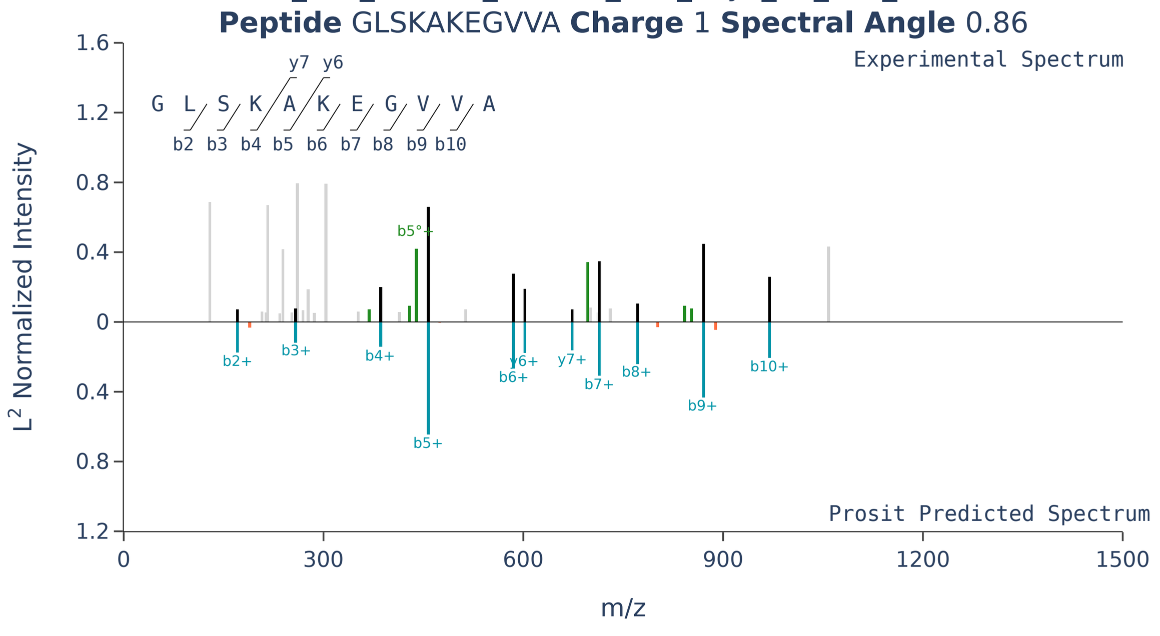

**Source** W_Soh_160821_170921_Goe_aSyn_A1_24h_R2 **Scan** 13102
**Peptide** TAVAQKTVEGAGSIAAA **Charge** 2 **Spectral Angle** 0.89

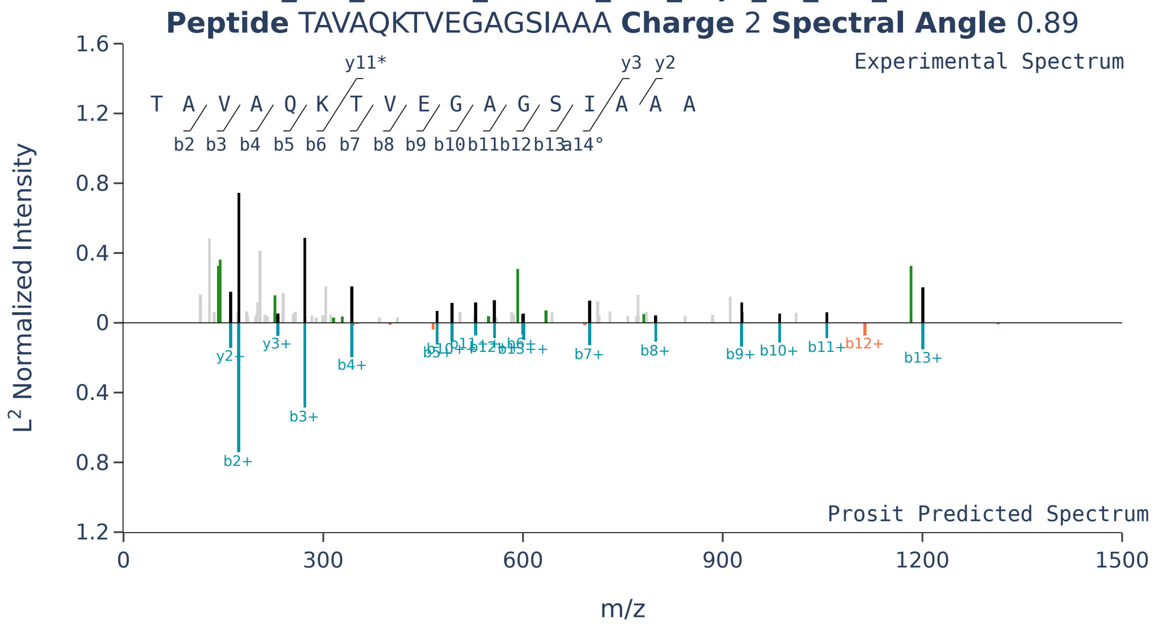


**Source** W_Soh_160821_170921_Goe_aSyn_A1_24h_R2 **Scan** 7341
**Peptide** MPSEEGYQD **Charge** 1 **Spectral Angle** 0.85

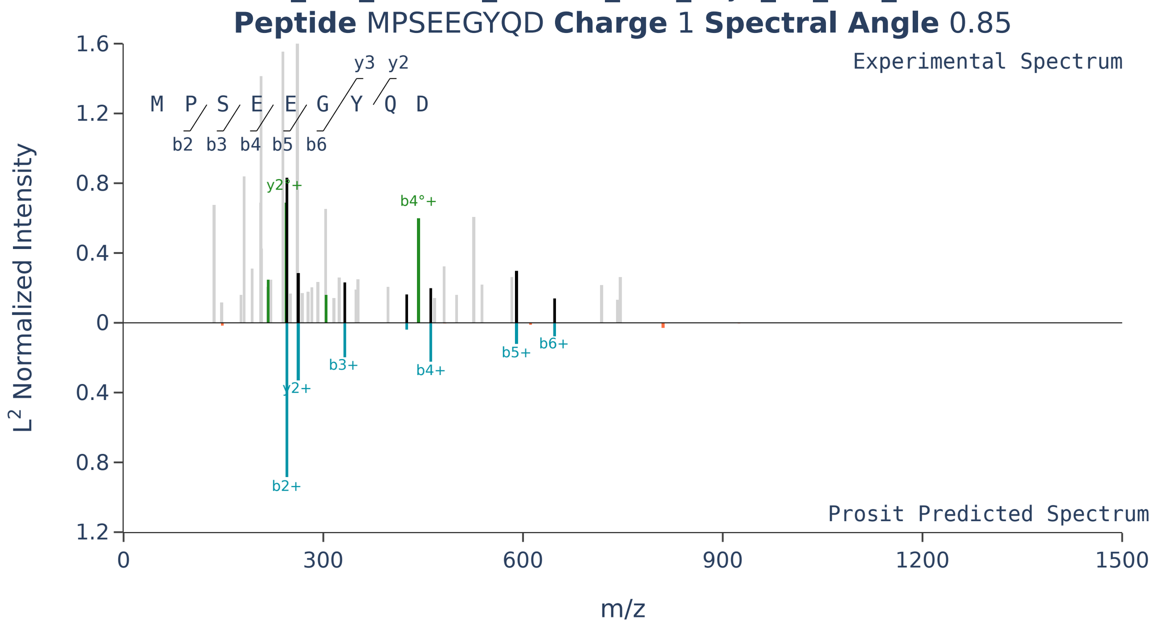


**Source** W_Soh_160821_170921_Goe_aSyn_A2_24h_R1 **Scan** 7191
**Peptide** TGFVKKDQLGKNEE **Charge** 3 **Spectral Angle** 0.84

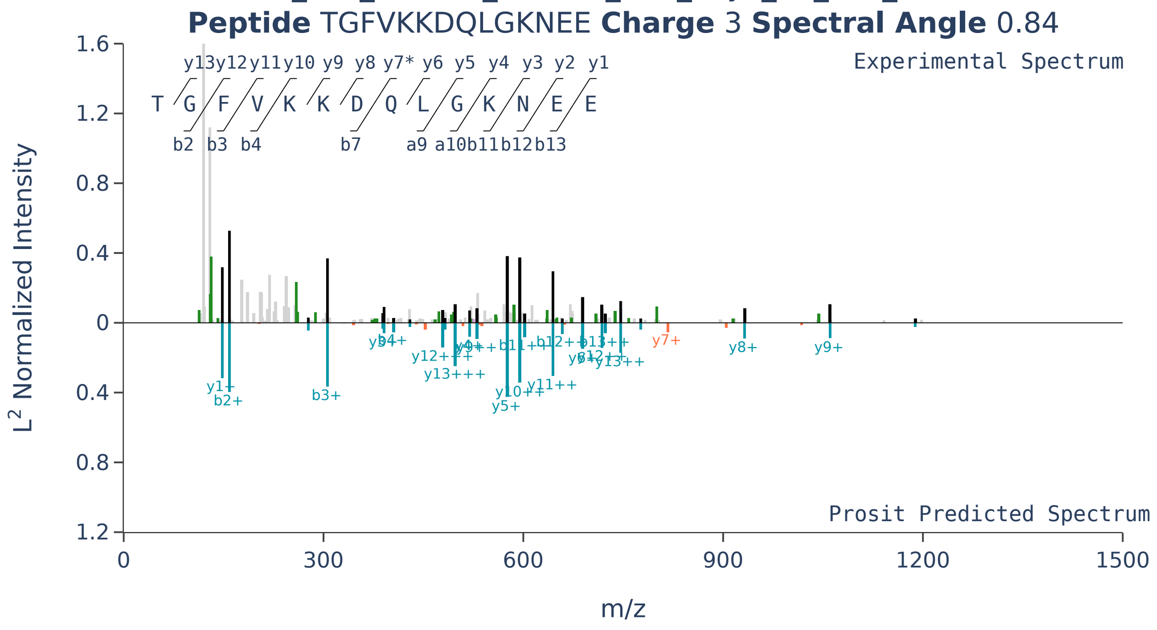


**Source** W_Soh_160821_170921_Goe_aSyn_A1_24h_R2 **Scan** 12328
**Peptide** YEMPSEE **Charge** 1 **Spectral Angle** 0.8

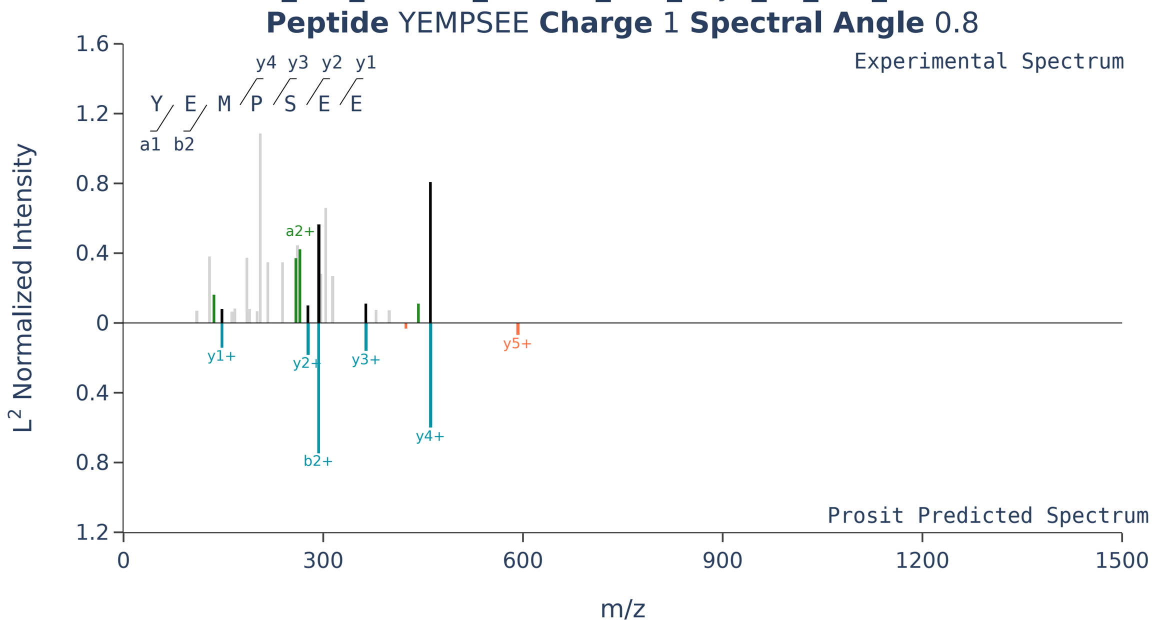


**Source** W_Soh_160821_170921_Goe_aSyn_A1_3h_R2 **Scan** 8482
**Peptide** KTVEGAGSIAA **Charge** 2 **Spectral Angle** 0.87

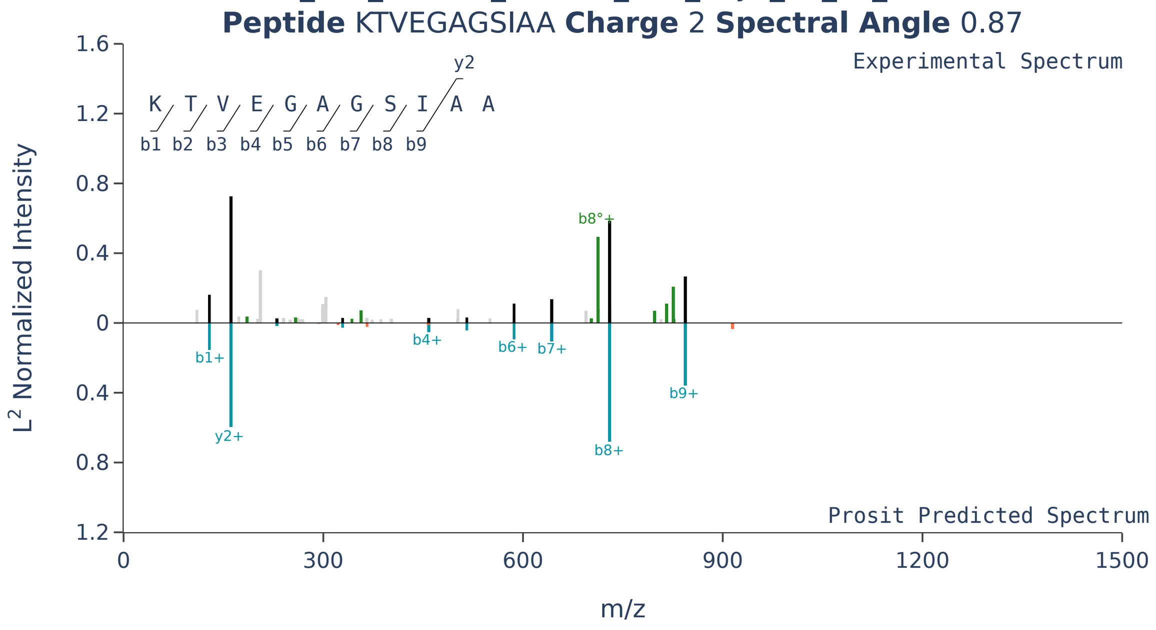


**Source** W_Soh_160821_170921_Goe_aSyn_A2_3h_R2 **Scan** 5944
**Peptide** ATVAEKTKEQ **Charge** 2 **Spectral Angle** 0.79

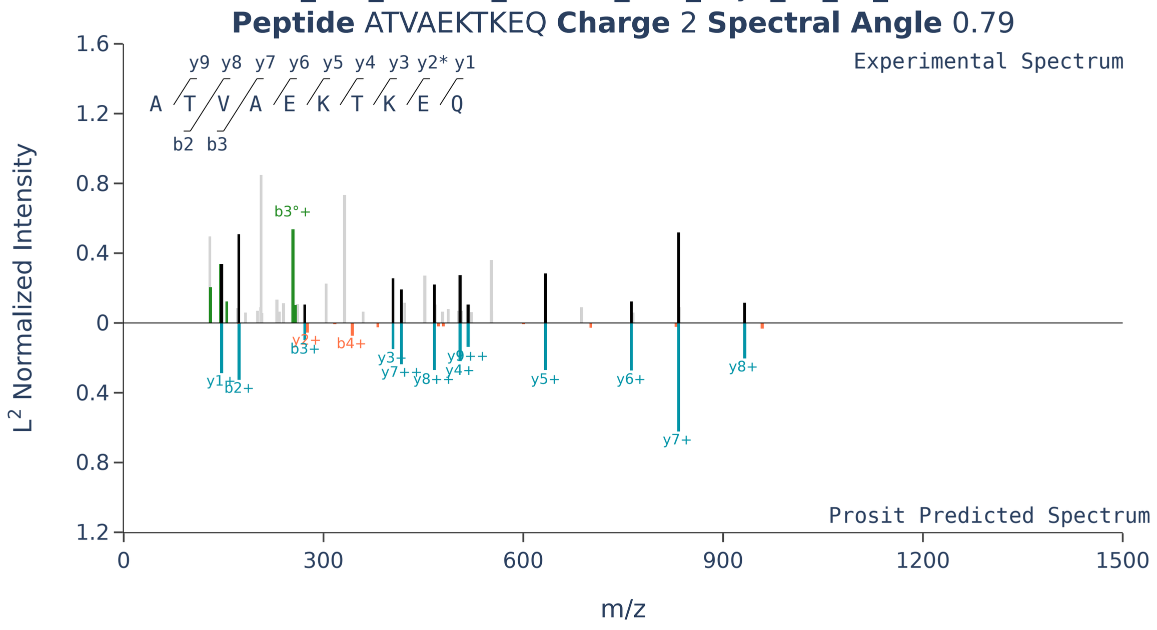


**Source** W_Soh_160821_170921_Goe_aSyn_A1_24h_R2 **Scan** 6115
**Peptide** AEKTKEQVTNV **Charge** 1 **Spectral Angle** 0.8

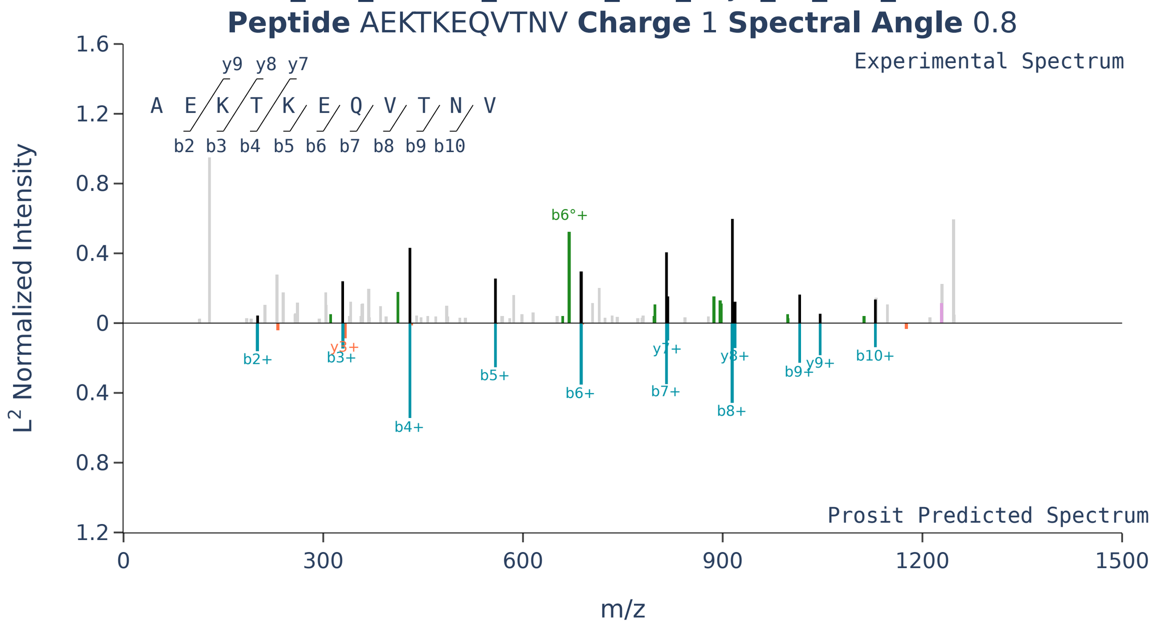


**Source** W_Soh_160821_170921_Goe_aSyn_A1_24h_R2 **Scan** 18266
**Peptide** VAQKTVEGAGSIAAATGFVK **Charge** 3 **Spectral Angle** 0.86

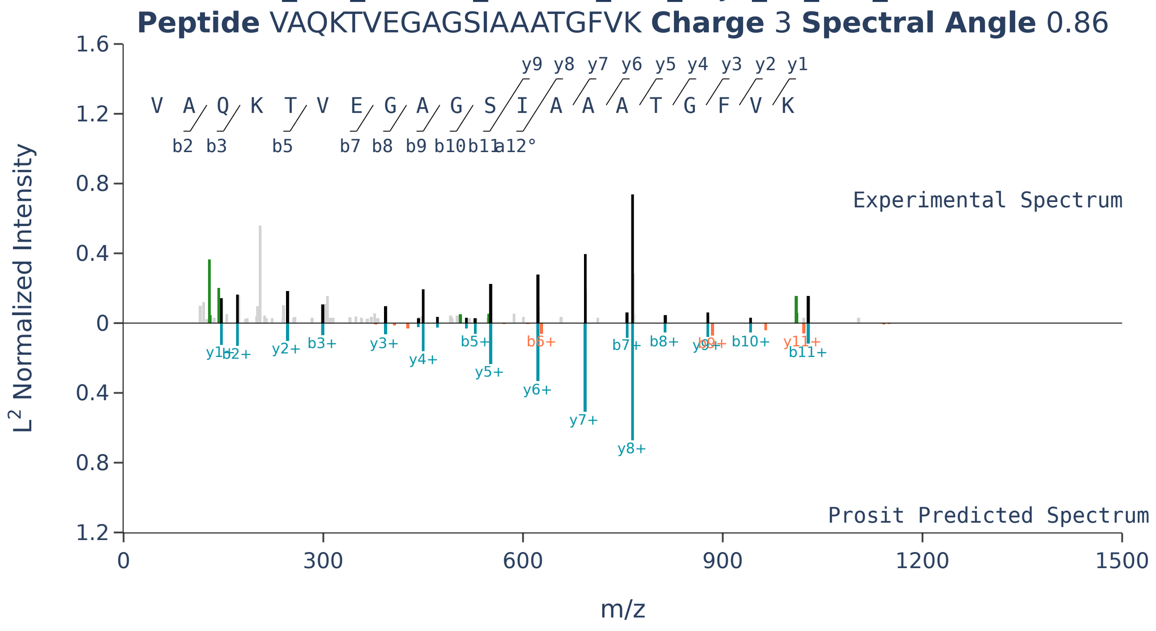


**Source** W_Soh_160821_170921_Goe_aSyn_A1_3h_R1 **Scan** 16345
**Peptide** ATVAEKTKEQVTNVGGAVVTGV **Charge** 3 **Spectral Angle** 0.88

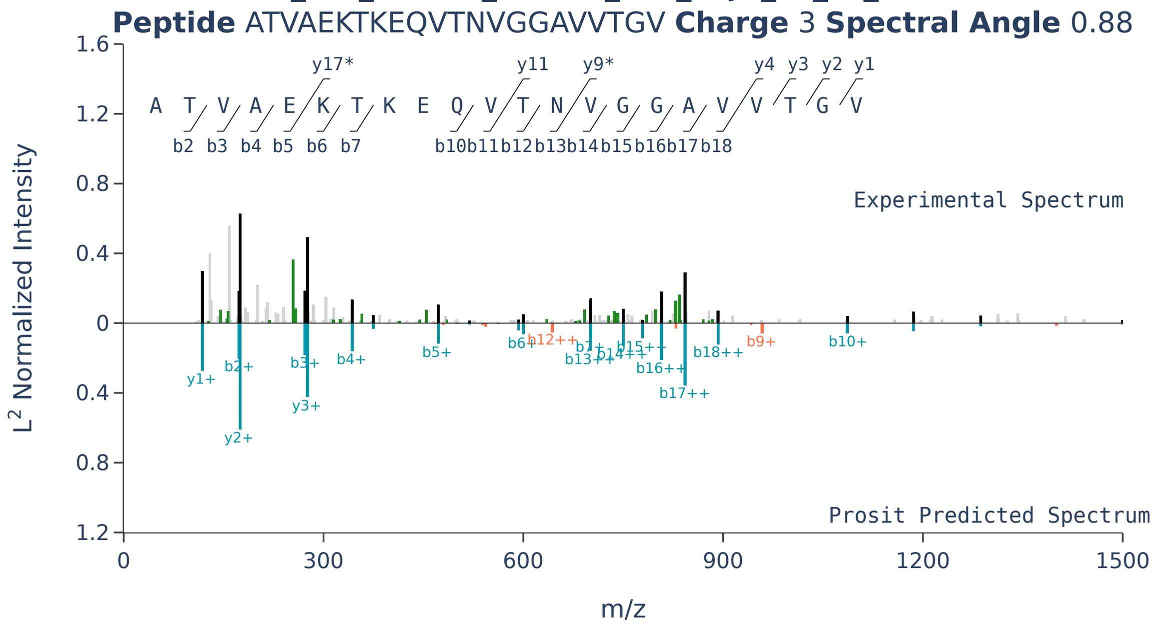


**Source** W_Soh_160821_170921_Goe_aSyn_A1_24h_R1 **Scan** 7183
**Peptide** YVGSKTKEGVVHGV **Charge** 2 **Spectral Angle** 0.85

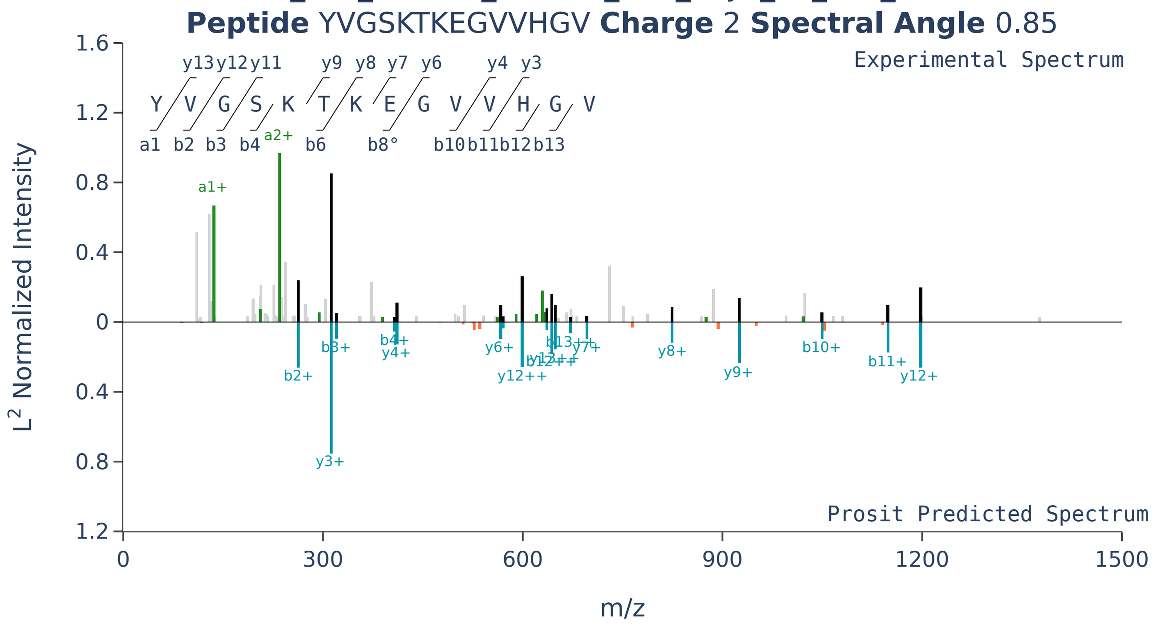


**Source** W_Soh_160821_170921_Goe_aSyn_A1_24h_R1 **Scan** 6314
**Peptide** LYVGSKTKEG **Charge** 2 **Spectral Angle** 0.88

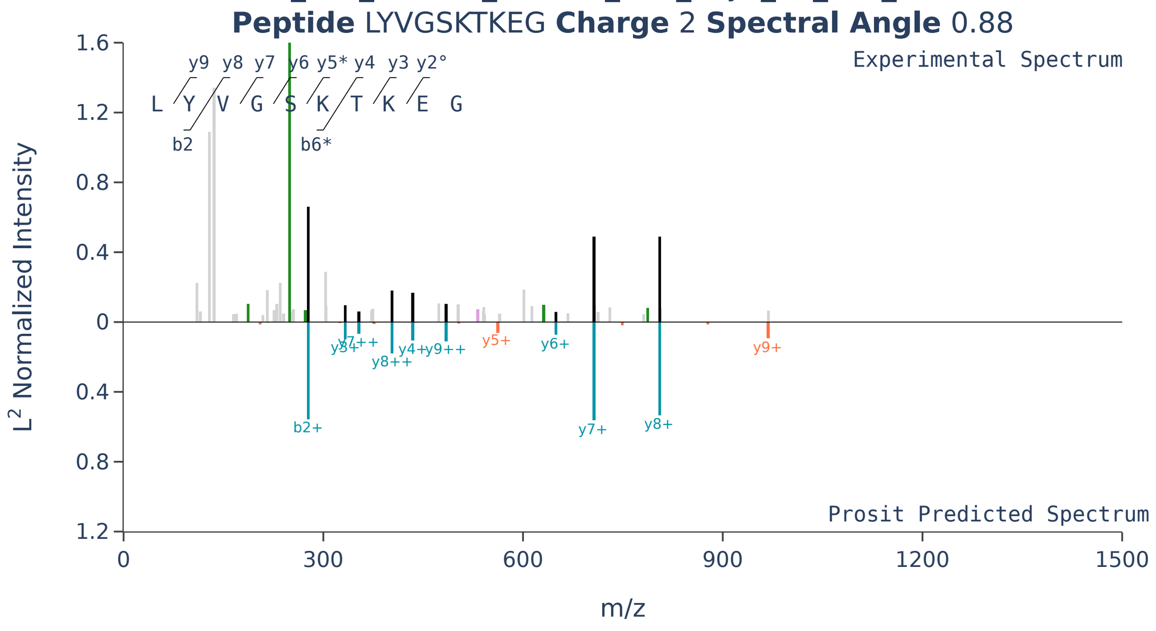


**Source** W_Soh_160821_170921_Goe_aSyn_A2_24h_R1 **Scan** 8064
**Peptide** GSKTKEGVVHGVATVA **Charge** 3 **Spectral Angle** 0.86

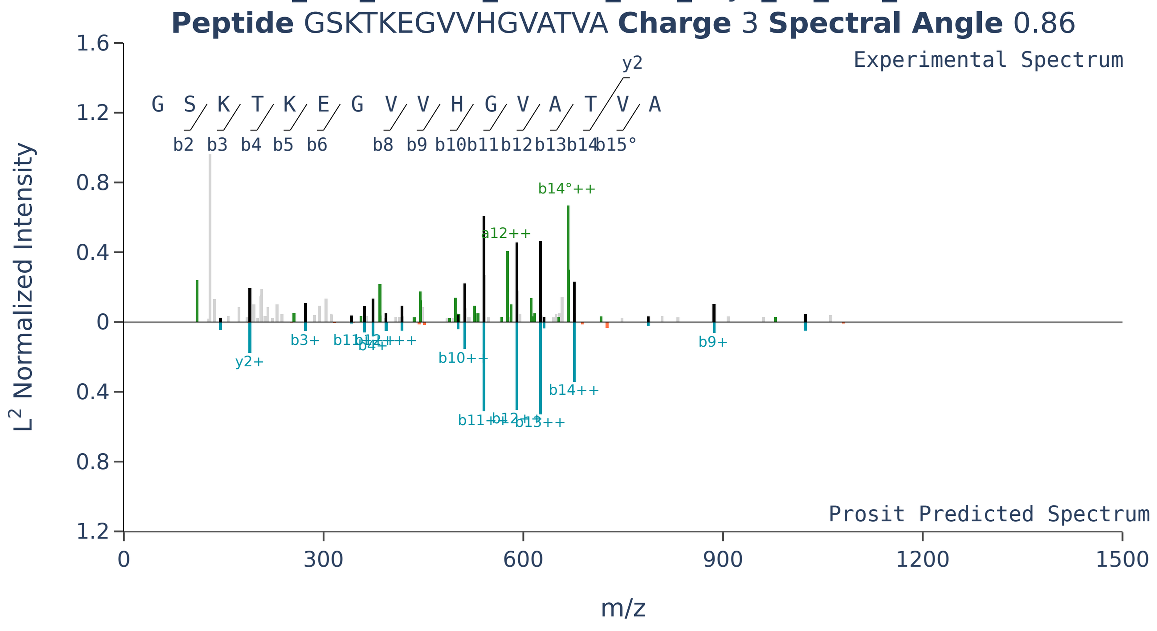


**Source** W_Soh_160821_170921_Goe_aSyn_A2_6h_R2 **Scan** 17913
**Peptide** VAEKTKEQVTNVGGAVVTGVTAV **Charge** 3 **Spectral Angle** 0.86

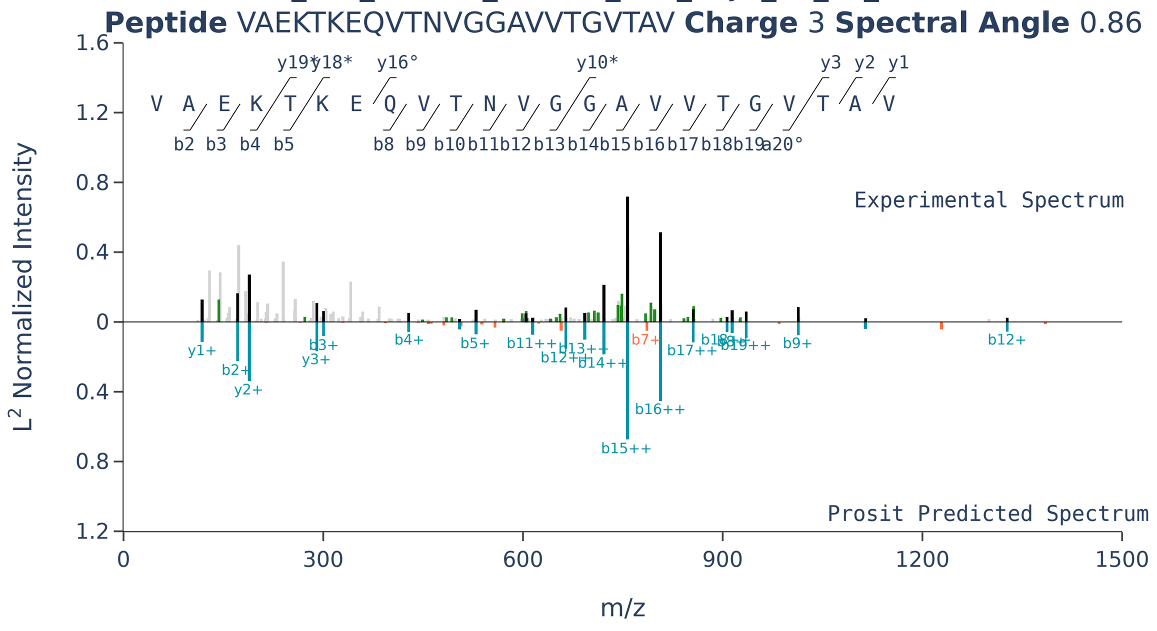


**Source** W_Soh_160821_170921_Goe_aSyn_A1_6h_R2 **Scan** 24880
**Peptide** GILEDMPVPDPN **Charge** 2 **Spectral Angle** 0.89

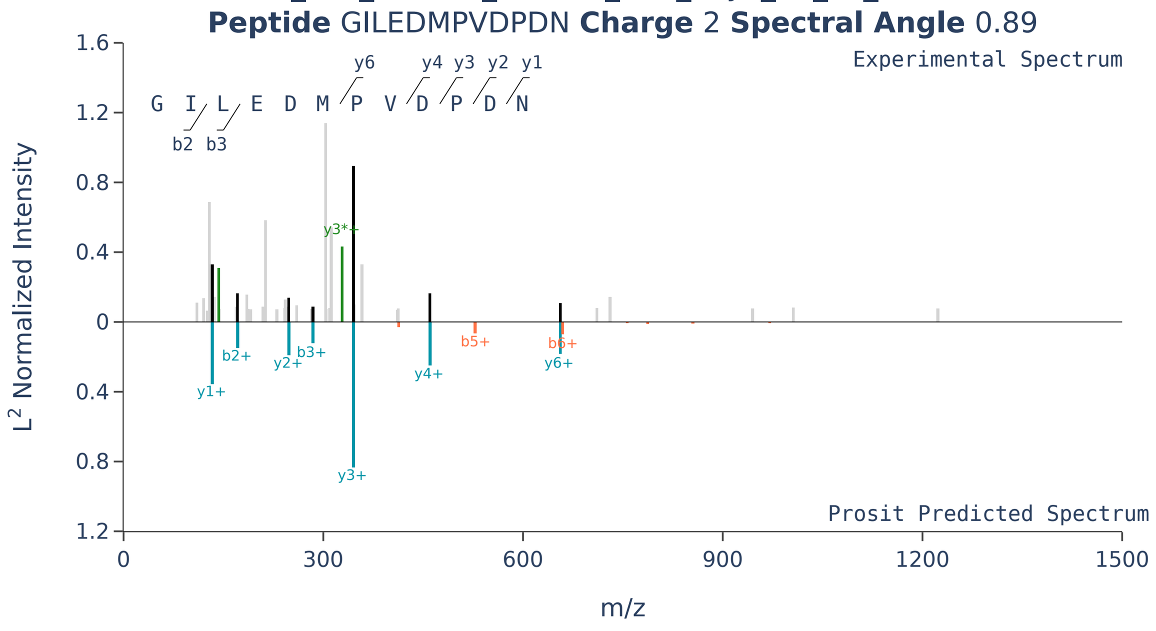


**Source** W_Soh_160821_170921_Goe_aSyn_A2_24h_R2 **Scan** 8683
**Peptide** AQKTVEGAGSIA **Charge** 2 **Spectral Angle** 0.87

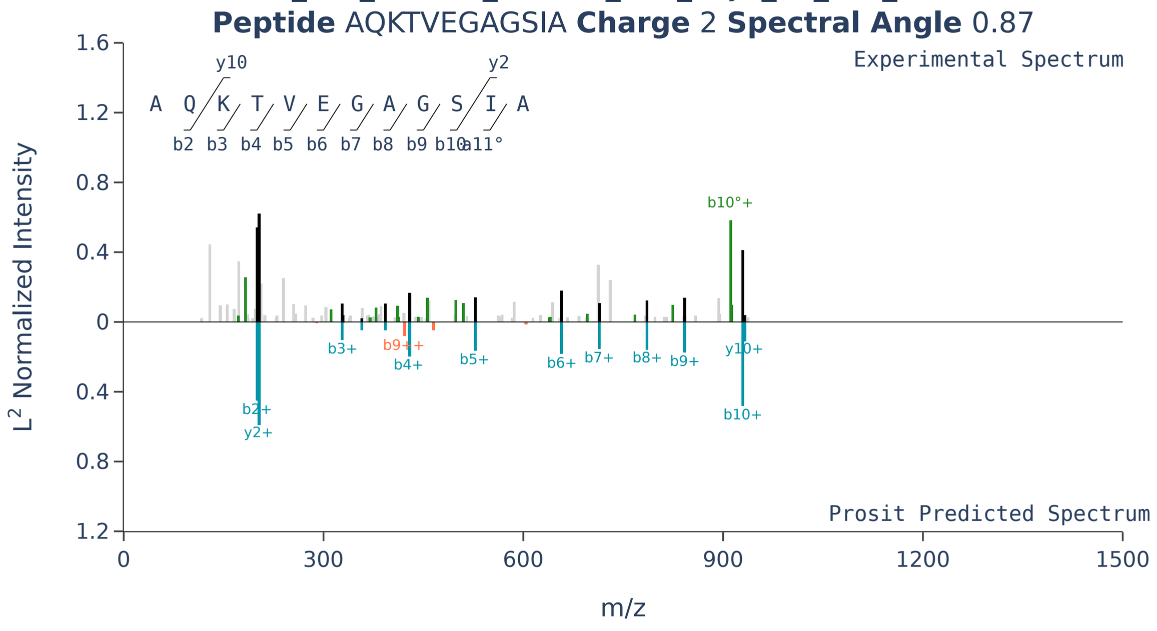

**Source** W_Soh_160821_170921_Goe_aSyn_A1_30'_R2 **Scan** 5843
**Peptide** SKTKEGVVHGVAT **Charge** 3 **Spectral Angle** 0.87

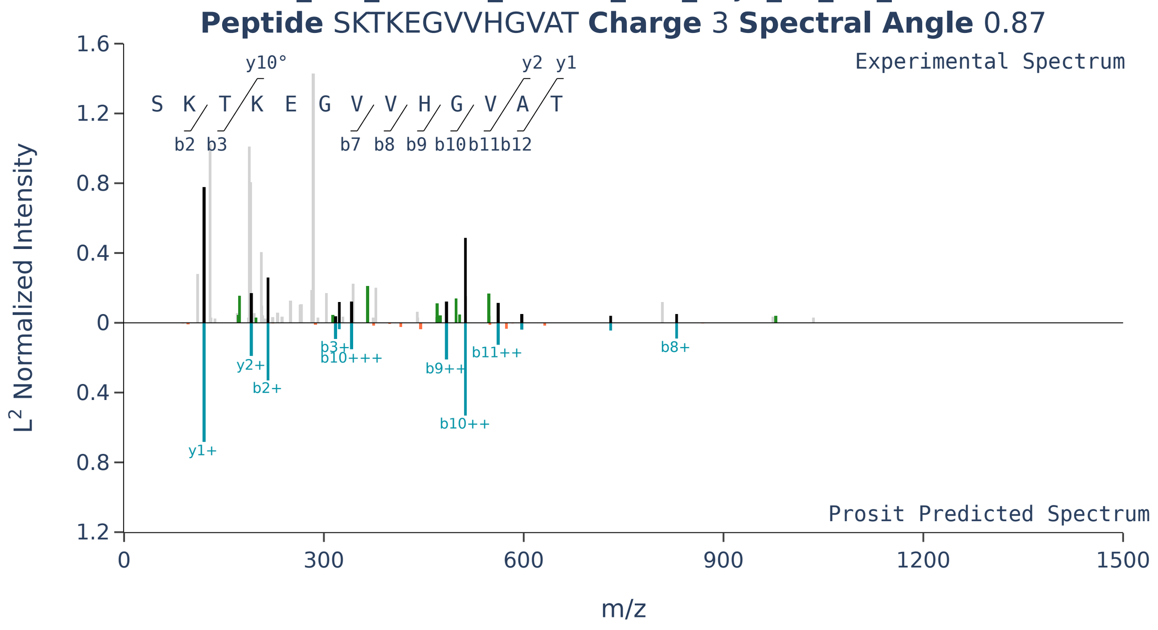


**Source** W_Soh_160821_170921_Goe_aSyn_A1_6h_R1 **Scan** 6183
**Peptide** AATGFVKKD **Charge** 2 **Spectral Angle** 0.82

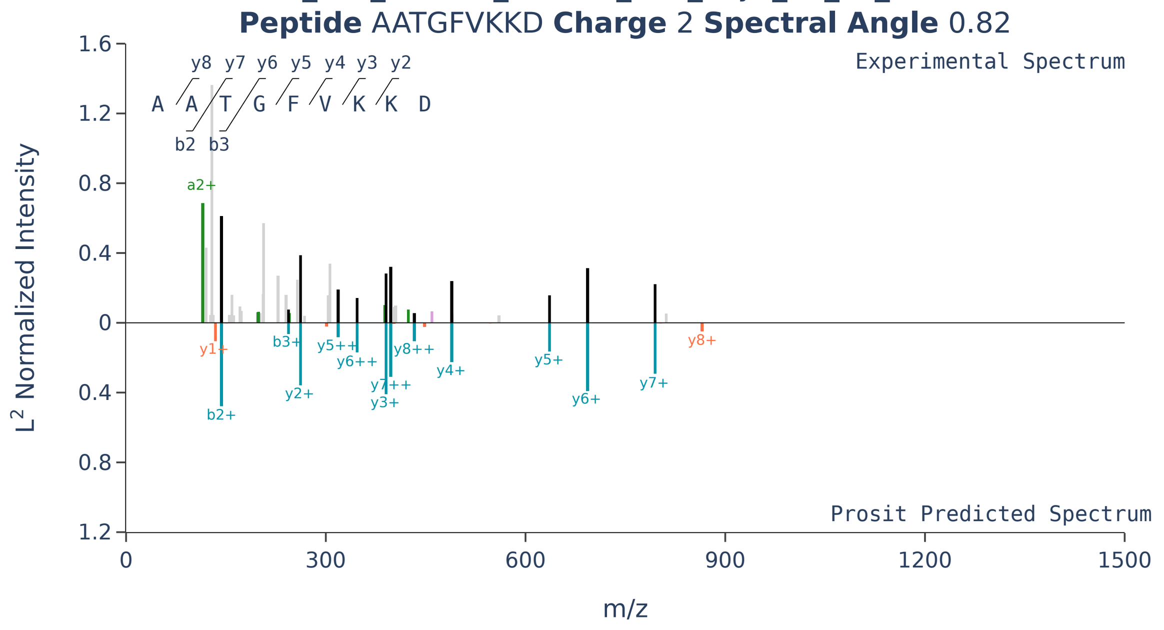


**Source** W_Soh_160821_170921_Goe_aSyn_A1_24h_R2 **Scan** 17114
**Peptide** AYEMPSEEGY **Charge** 2 **Spectral Angle** 0.84

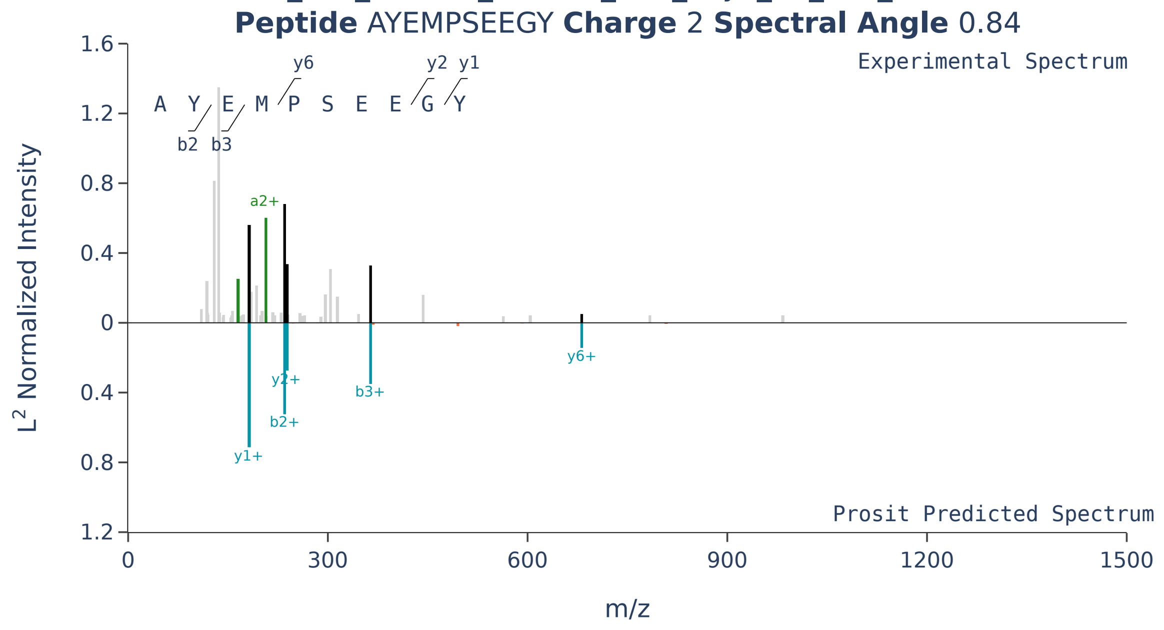


**Source** W_Soh_160821_170921_Goe_aSyn_A1_24h_R1 **Scan** 5977
**Peptide** KTKEGVVHGVAT **Charge** 2 **Spectral Angle** 0.86

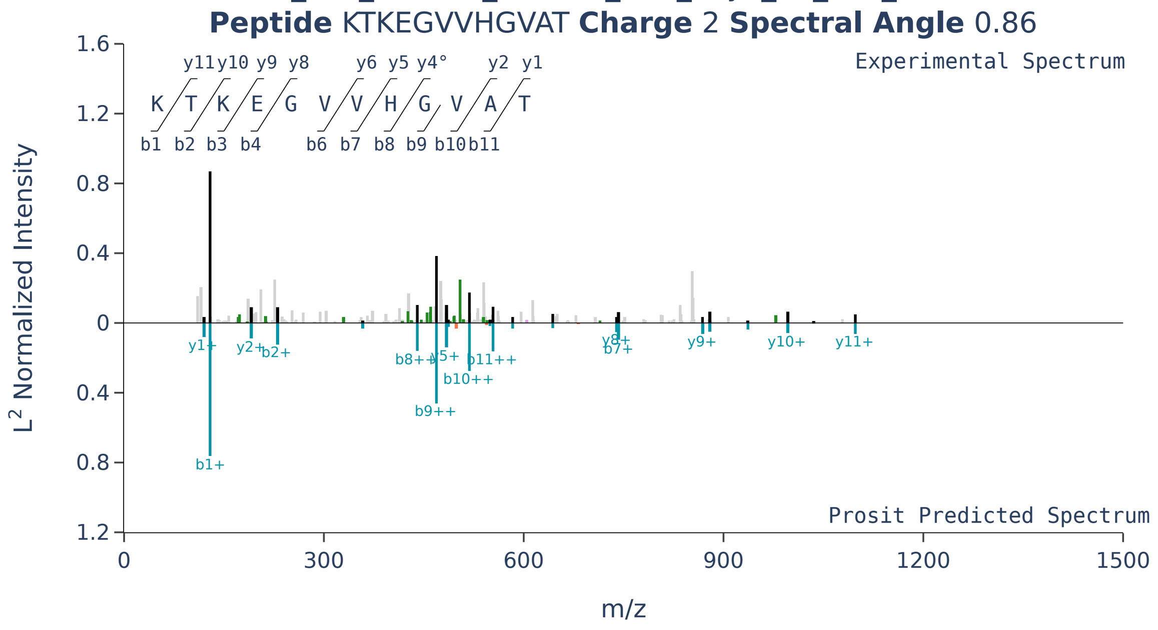


**Source** W_Soh_160821_170921_Goe_aSyn_A1_24h_R2 **Scan** 14299
**Peptide** VTAVAQKTVEGAGSIAA **Charge** 2 **Spectral Angle** 0.81

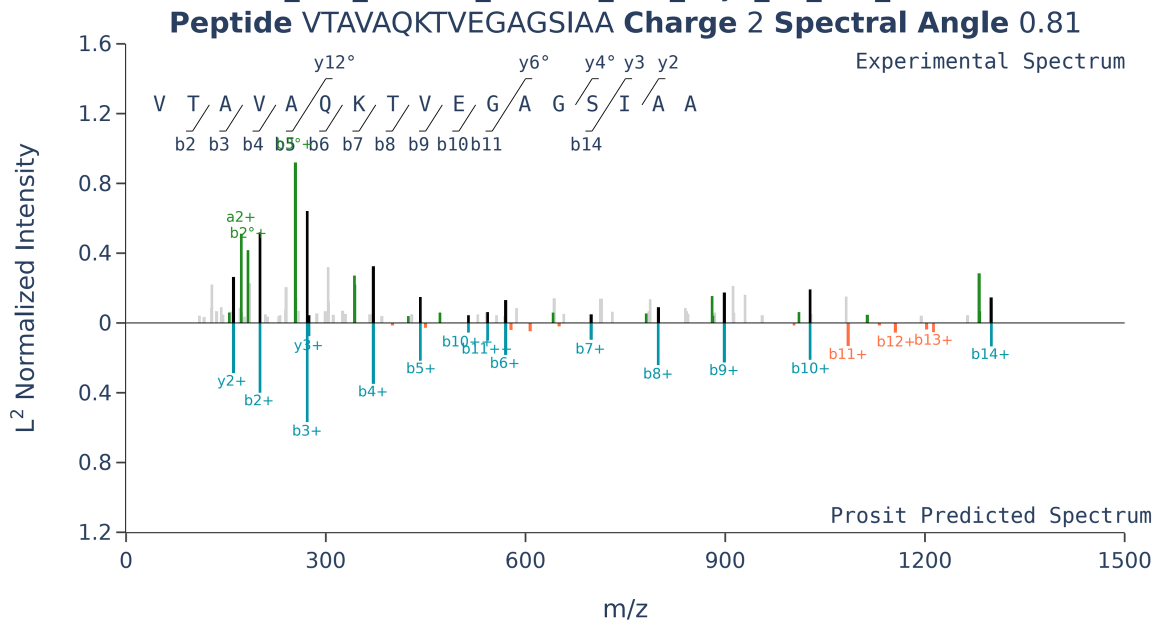


**Source** W_Soh_160821_170921_Goe_aSyn_A2_24h_R2 **Scan** 14589
**Peptide** GAGSIAAATGFVKKDQLGKNE **Charge** 3 **Spectral Angle** 0.84

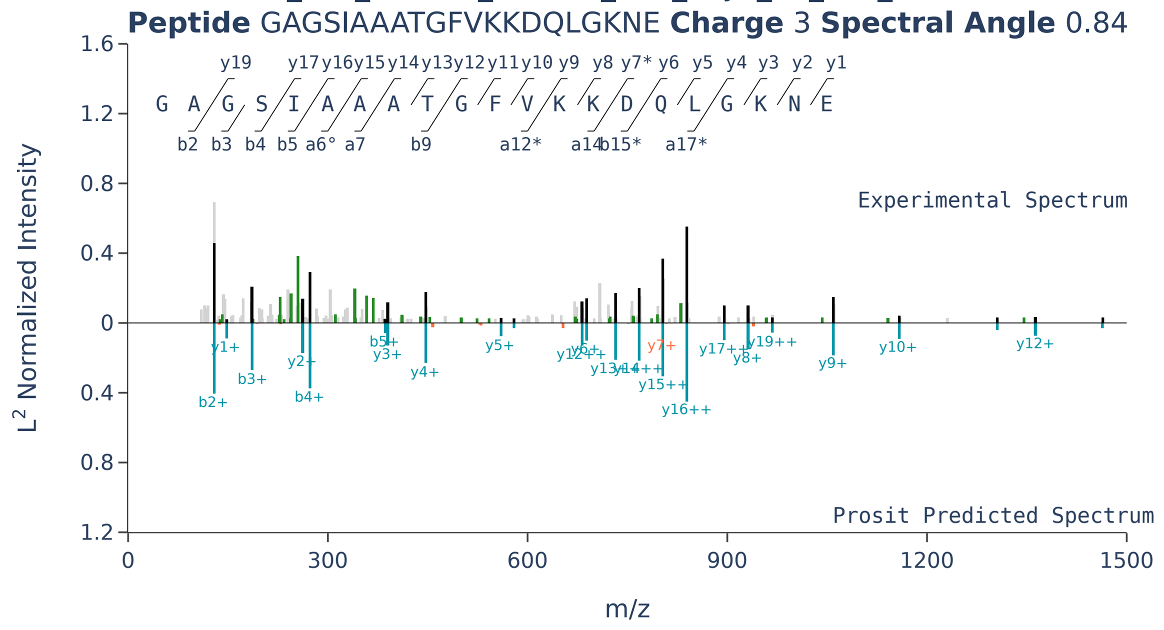


**Source** W_Soh_160821_170921_Goe_aSyn_A1_6h_R1 **Scan** 24061
**Peptide** EQVTNVGGAVVTGVTAV **Charge** 2 **Spectral Angle** 0.86

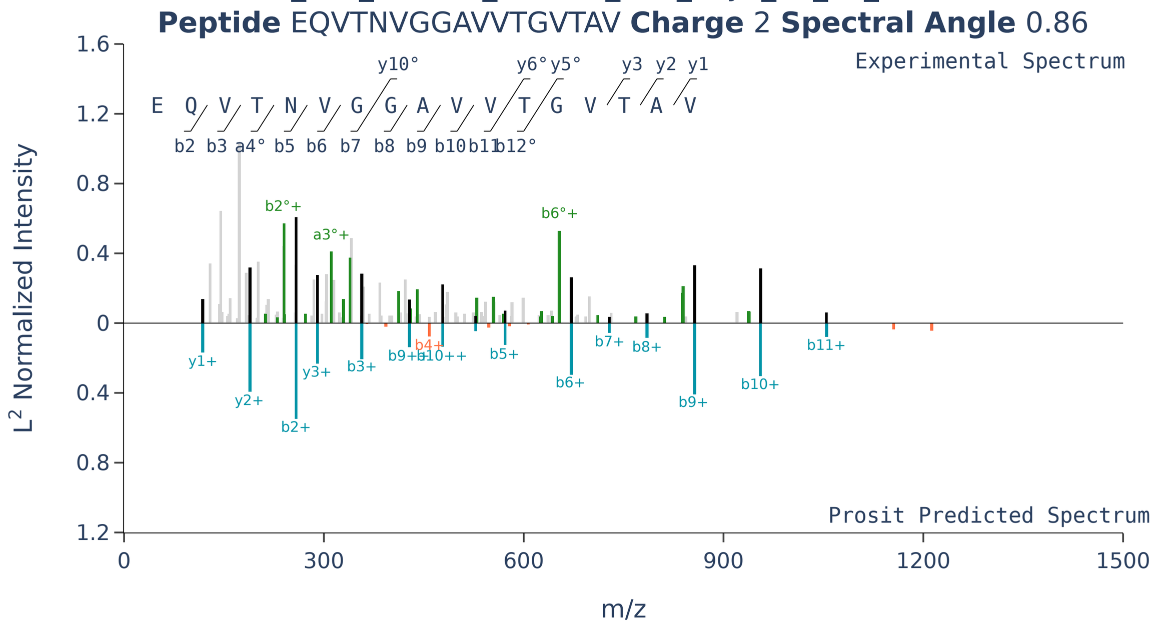


**Source** W_Soh_160821_170921_Goe_aSyn_A1_24h_R1 **Scan** 10694
**Peptide** TGVTAVAQKTVE **Charge** 2 **Spectral Angle** 0.92

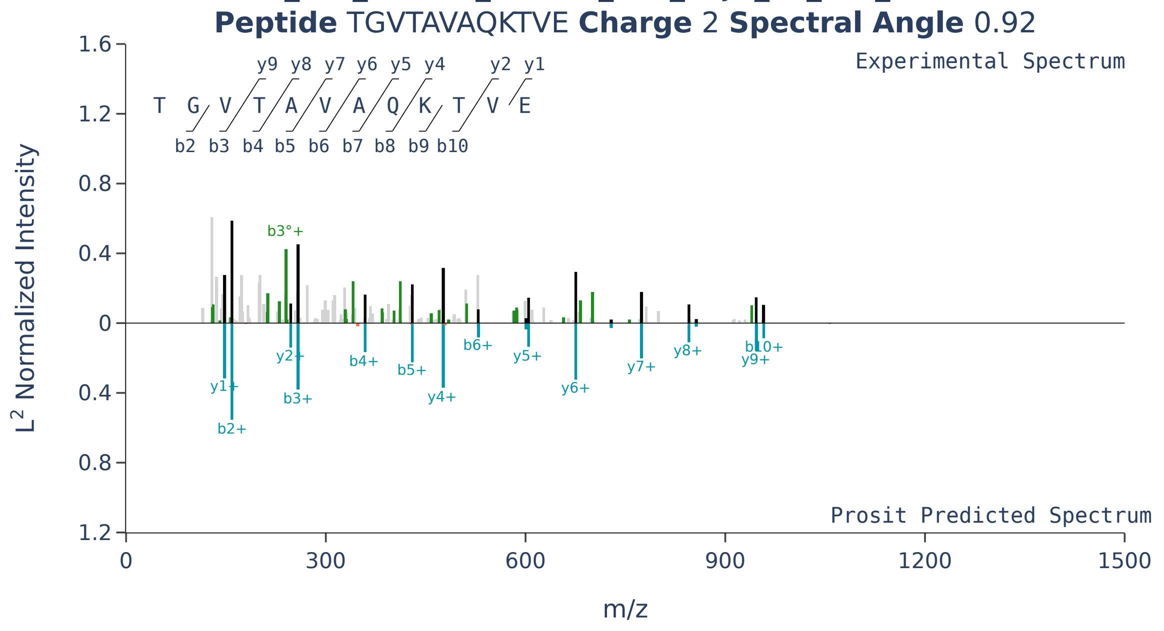


**Source** W_Soh_160821_170921_Goe_aSyn_A2_24h_R1 **Scan** 9975
**Peptide** AQKTVEGAGSIAAA **Charge** 2 **Spectral Angle** 0.86

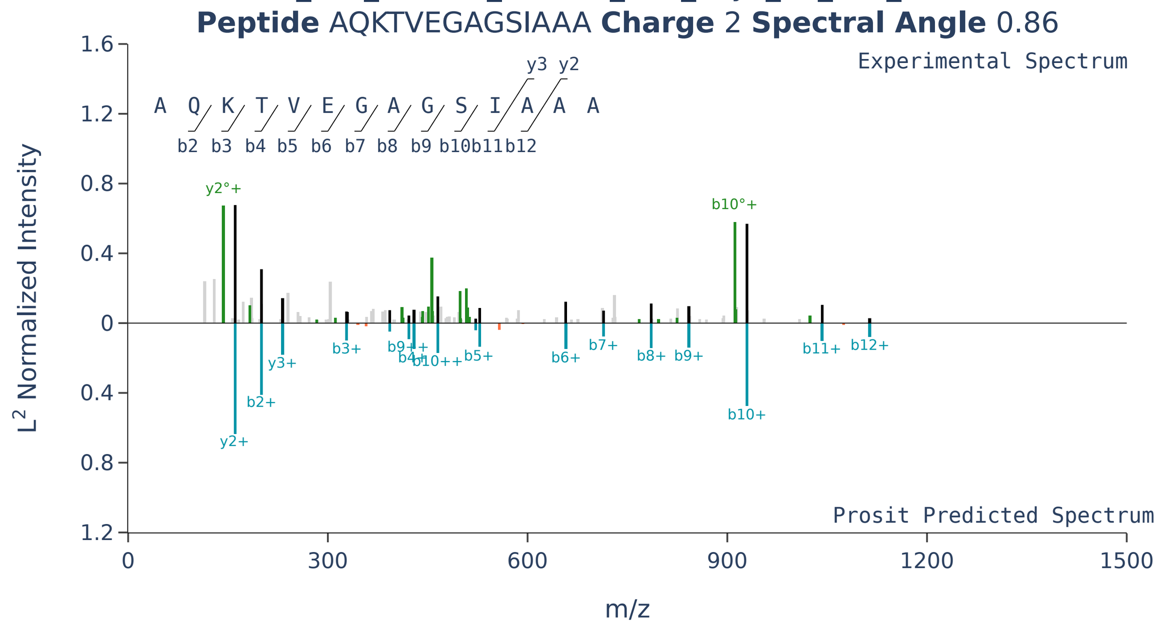


**Source** W_Soh_160821_170921_Goe_aSyn_A1_6h_R1 **Scan** 5711
**Peptide** KTKEGVVHGV **Charge** 2 **Spectral Angle** 0.82

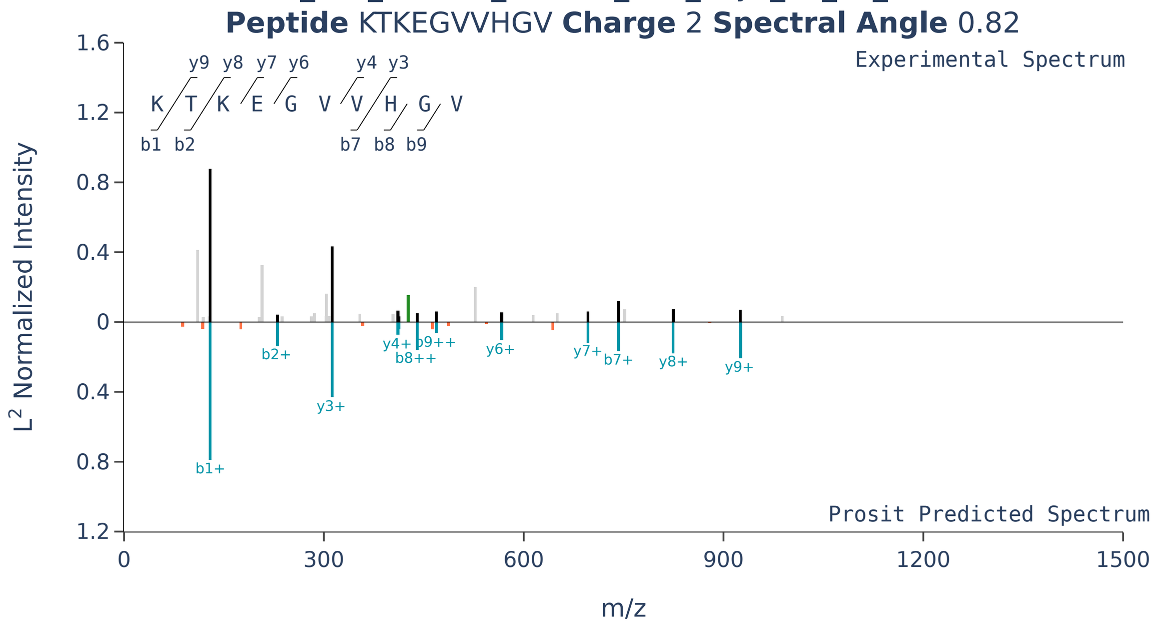


**Source** W_Soh_160821_170921_Goe_aSyn_A1_24h_R2 **Scan** 6211
**Peptide** MKGLSKAKEGVVA **Charge** 3 **Spectral Angle** 0.9

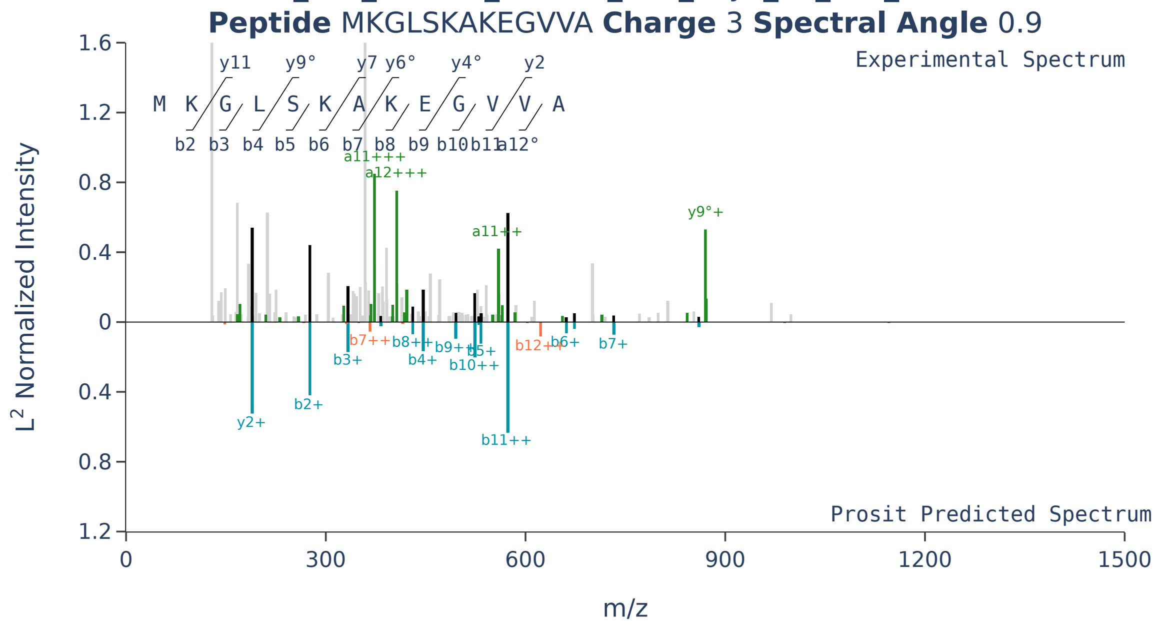


**Source** W_Soh_160821_170921_Goe_aSyn_A1_24h_R2 **Scan** 17384
**Peptide** AEKTKEQVTNVGGAVVTGVTAVAQK **Charge** 3 **Spectral Angle** 0.84

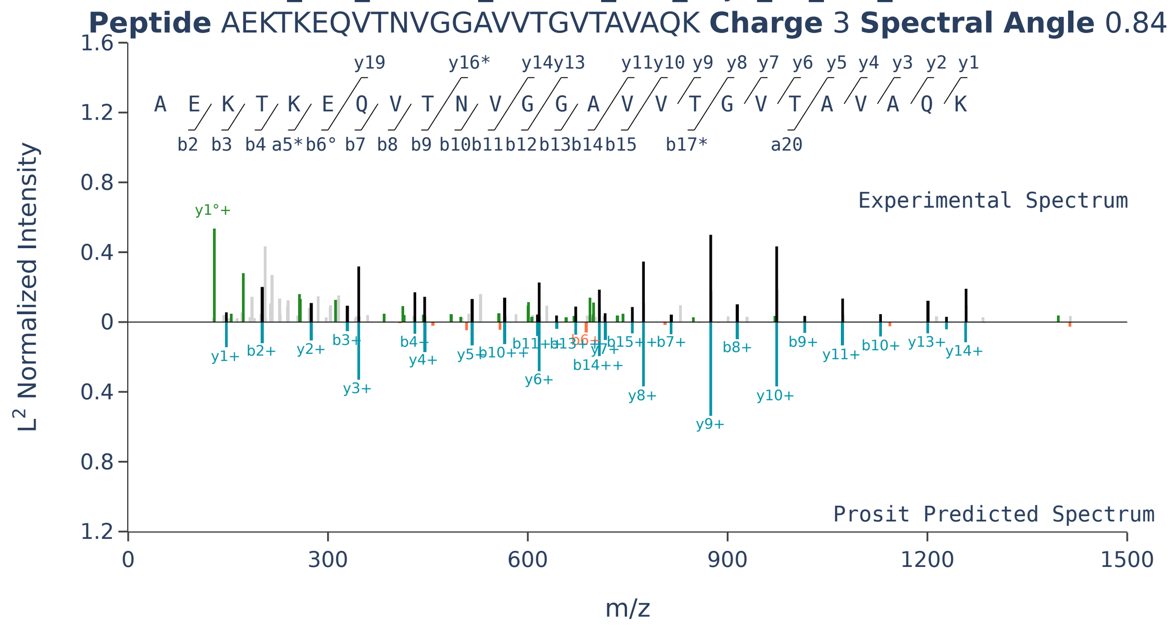


**Source** W_Soh_160821_170921_Goe_aSyn_A1_6h_R2 **Scan** 24629
**Peptide** GGAVVTGVTAVAQKTVEGAGSI **Charge** 2 **Spectral Angle** 0.87

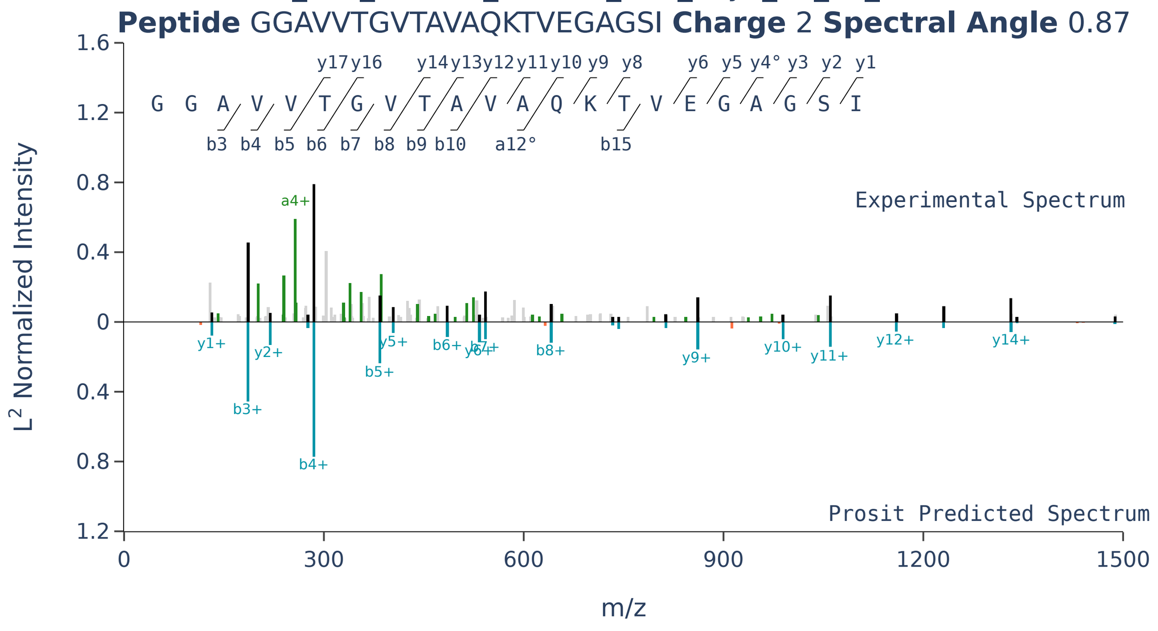


**Source** W_Soh_160821_170921_Goe_aSyn_A1_6h_R1 **Scan** 11166
**Peptide** EGVLYVGSKTKEGVVH **Charge** 4 **Spectral Angle** 0.95

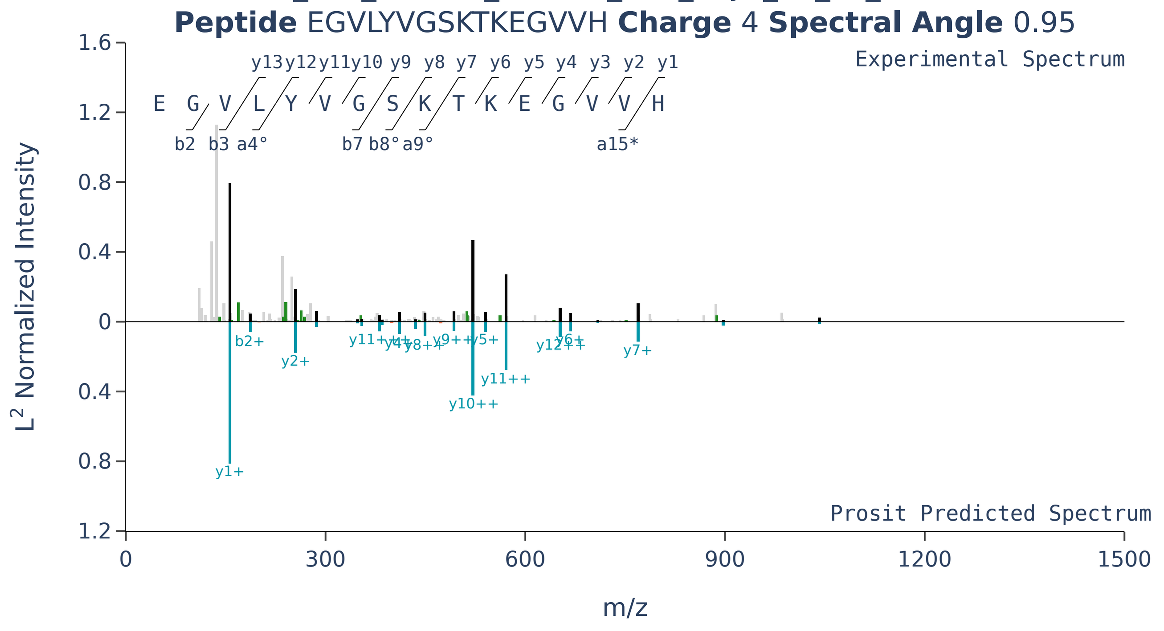


**Source** W_Soh_160821_170921_Goe_aSyn_A2_6h_R2 **Scan** 9275
**Peptide** FVKKDQL **Charge** 2 **Spectral Angle** 0.87

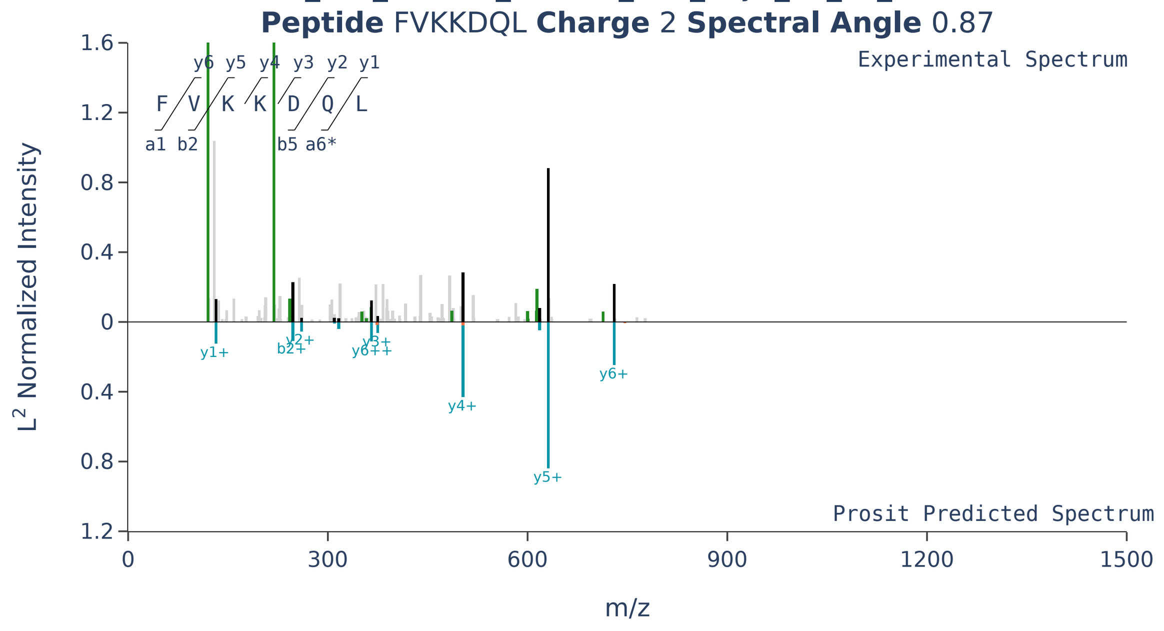

**Source** W_Soh_160821_170921_Goe_aSyn_A1_24h_R2 **Scan** 14623
**Peptide** VATVAEKTKEQVTNVGGAVVT **Charge** 2 **Spectral Angle** 0.81

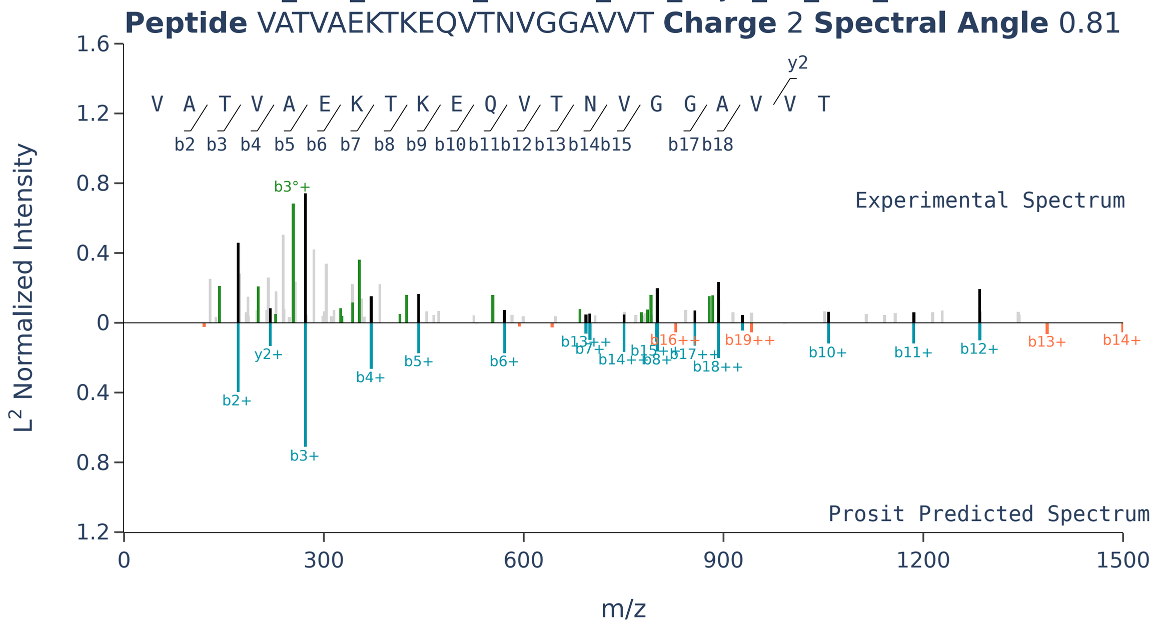


**Source** W_Soh_160821_170921_Goe_aSyn_A2_24h_R1 **Scan** 9229
**Peptide** GVLYVGSKTKE **Charge** 1 **Spectral Angle** 0.87

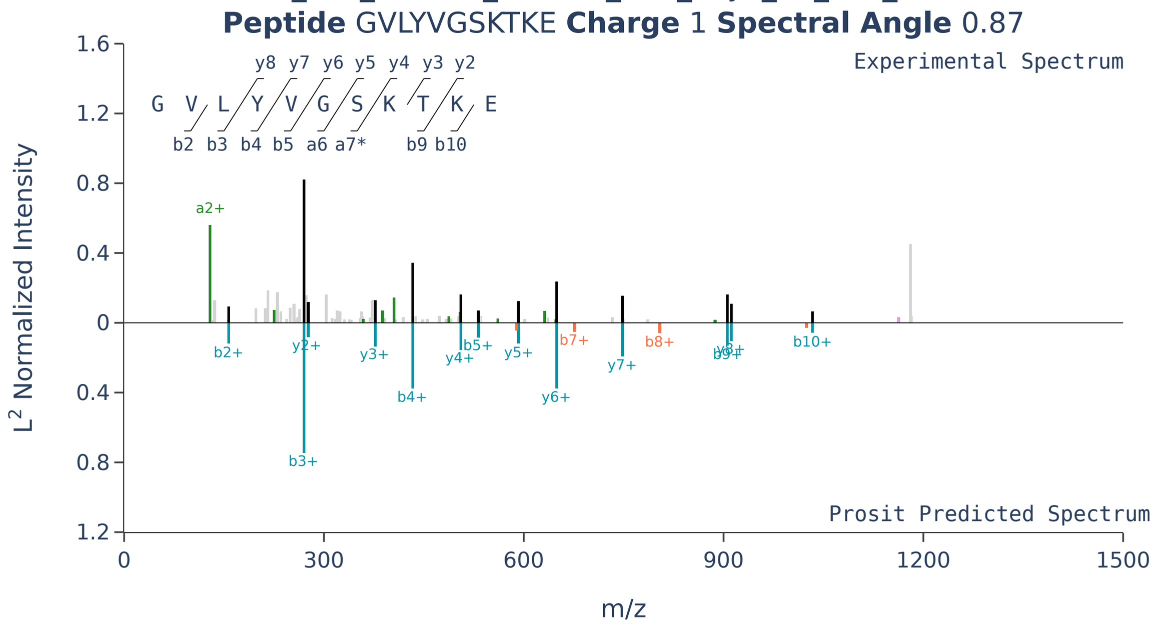


**Source** W_Soh_160821_170921_Goe_aSyn_A2_24h_R2 **Scan** 9523
**Peptide** ATGFVKKDQL **Charge** 1 **Spectral Angle** 0.86

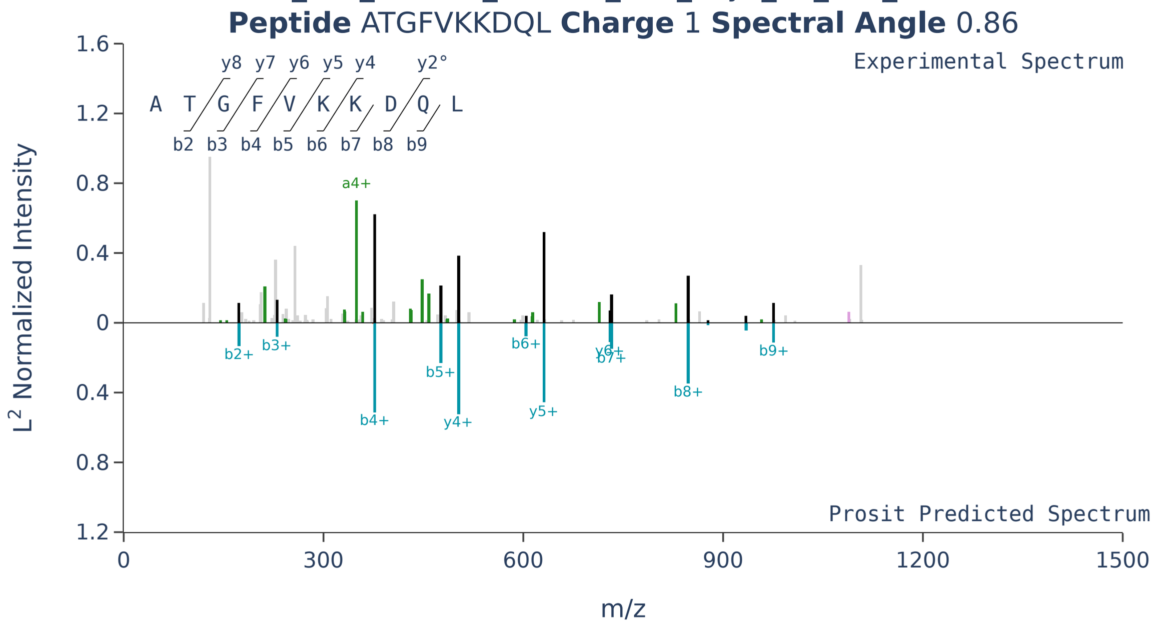


**Source** W_Soh_160821_170921_Goe_aSyn_A2_24h_R1 **Scan** 6616
**Peptide** TGFVKKDQLGKN **Charge** 3 **Spectral Angle** 0.87

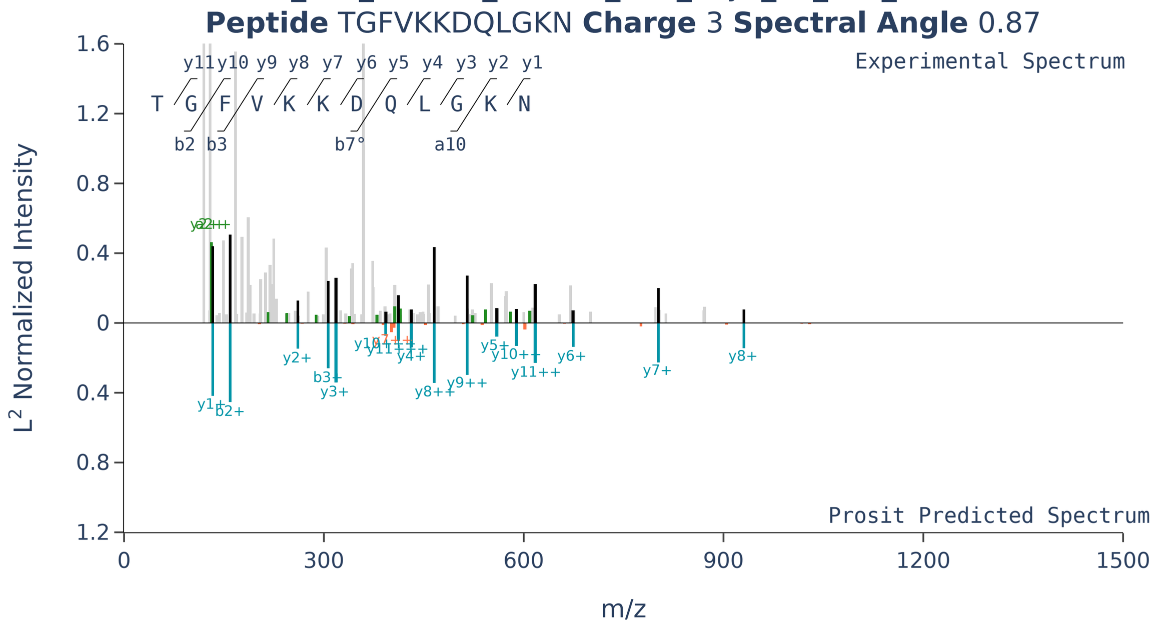


**Source** W_Soh_160821_170921_Goe_aSyn_A1_24h_R2 **Scan** 11361
**Peptide** GKTKEGVLYVG **Charge** 2 **Spectral Angle** 0.9

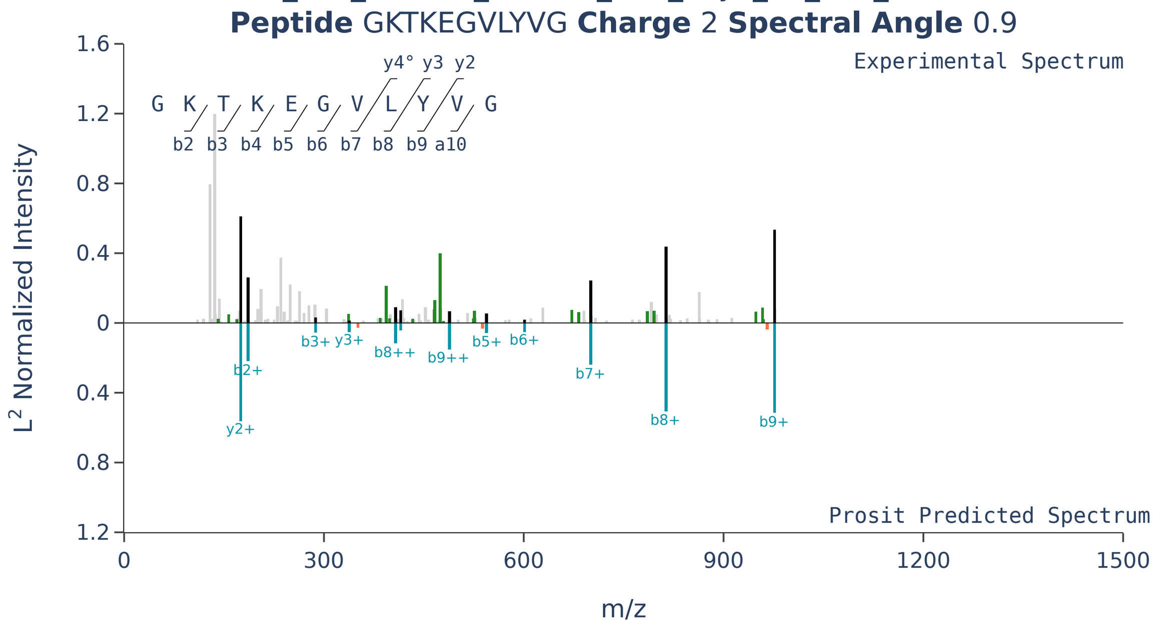


**Source** W_Soh_160821_170921_Goe_aSyn_A2_24h_R2 **Scan** 12263
**Peptide** TKEQVTNVGGAVVT **Charge** 2 **Spectral Angle** 0.84

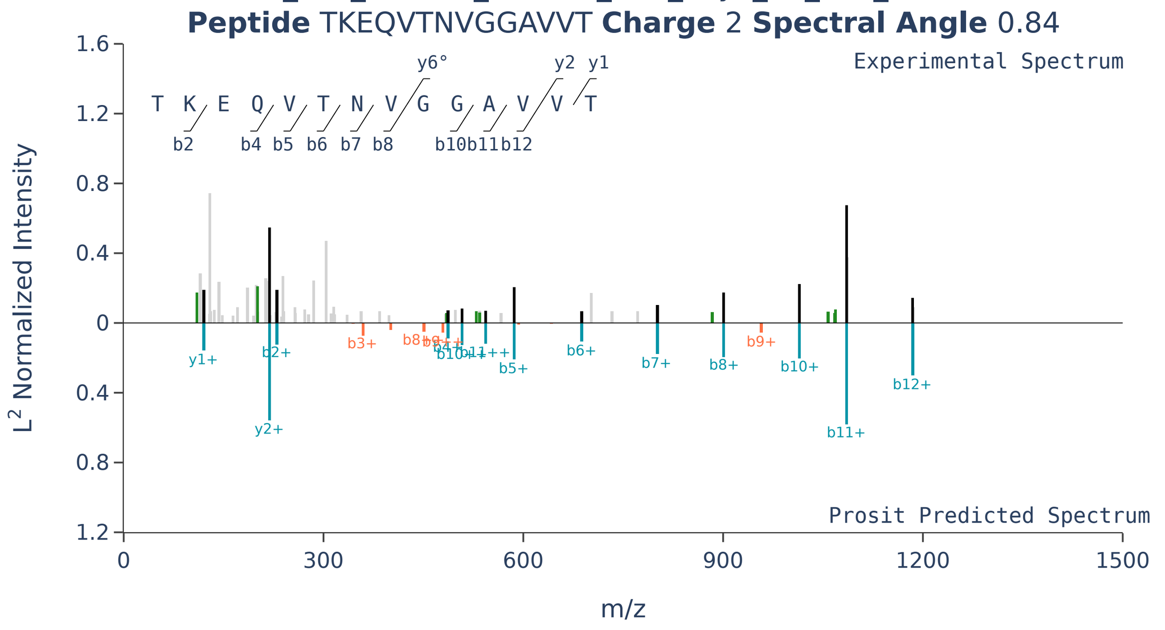


**Source** W_Soh_160821_170921_Goe_aSyn_A1_6h_R1 **Scan** 11109
**Peptide** AGKTKEGVLYVG **Charge** 1 **Spectral Angle** 0.87

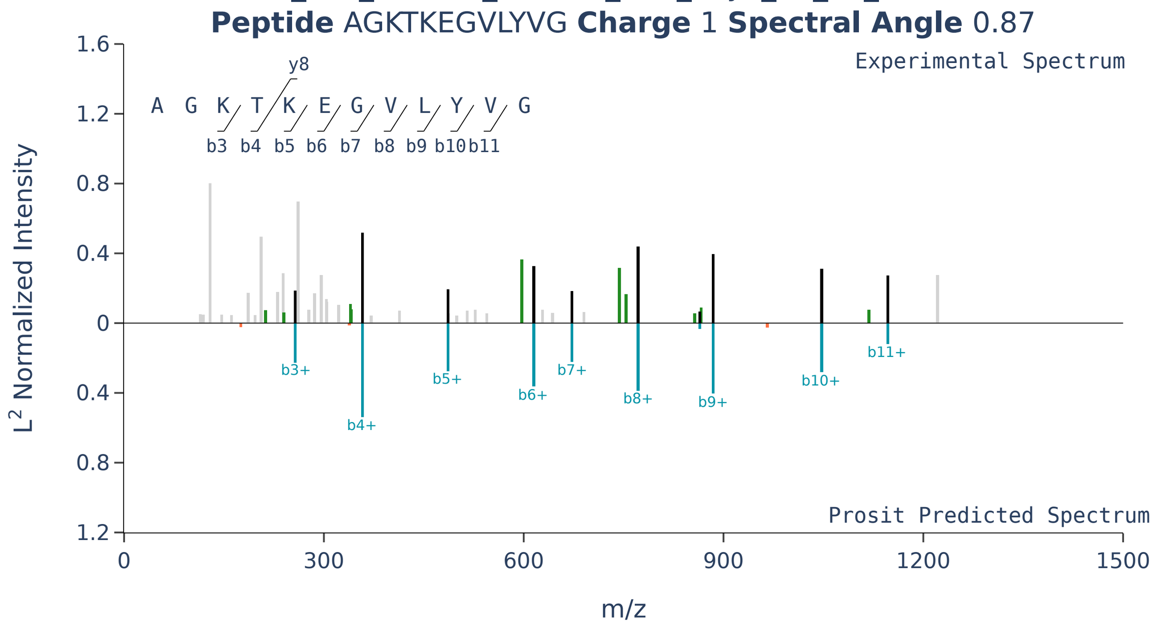


**Source** W_Soh_160821_170921_Goe_aSyn_A1_6h_R1 **Scan** 6997
**Peptide** DQLGKNEEGAPQE **Charge** 1 **Spectral Angle** 0.81

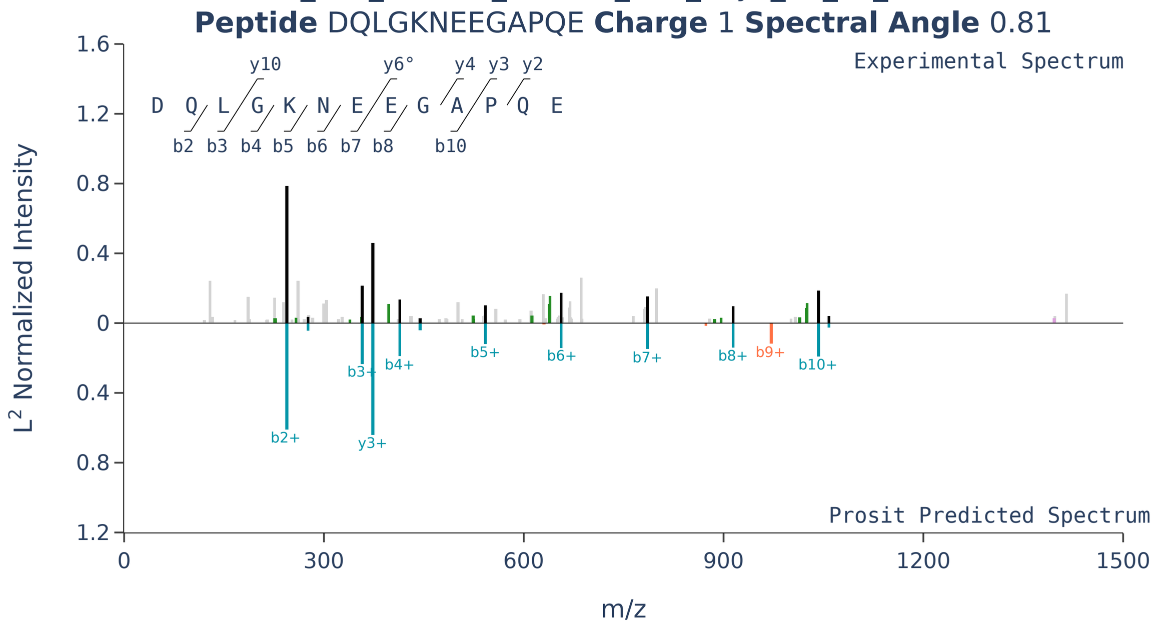


**Source** W_Soh_160821_170921_Goe_aSyn_A1_24h_R2 **Scan** 8266
**Peptide** GKTKEGVLYVGSKT **Charge** 3 **Spectral Angle** 0.91

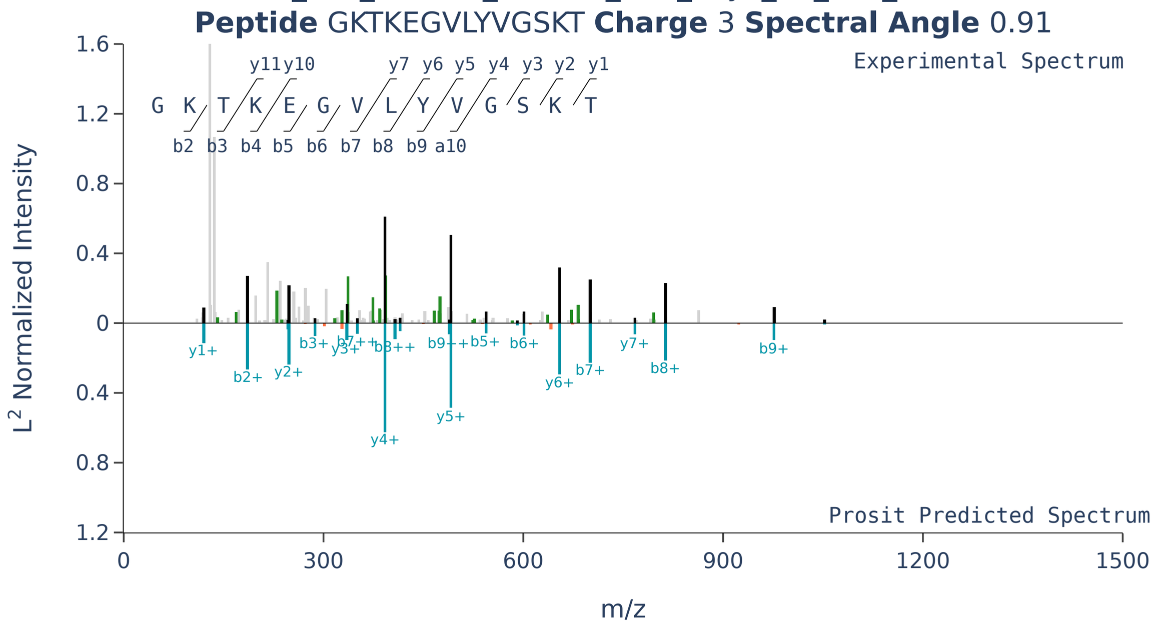


**Source** W_Soh_160821_170921_Goe_aSyn_A1_24h_R1 **Scan** 10434
**Peptide** TKEGVLYVGSKT **Charge** 3 **Spectral Angle** 0.85

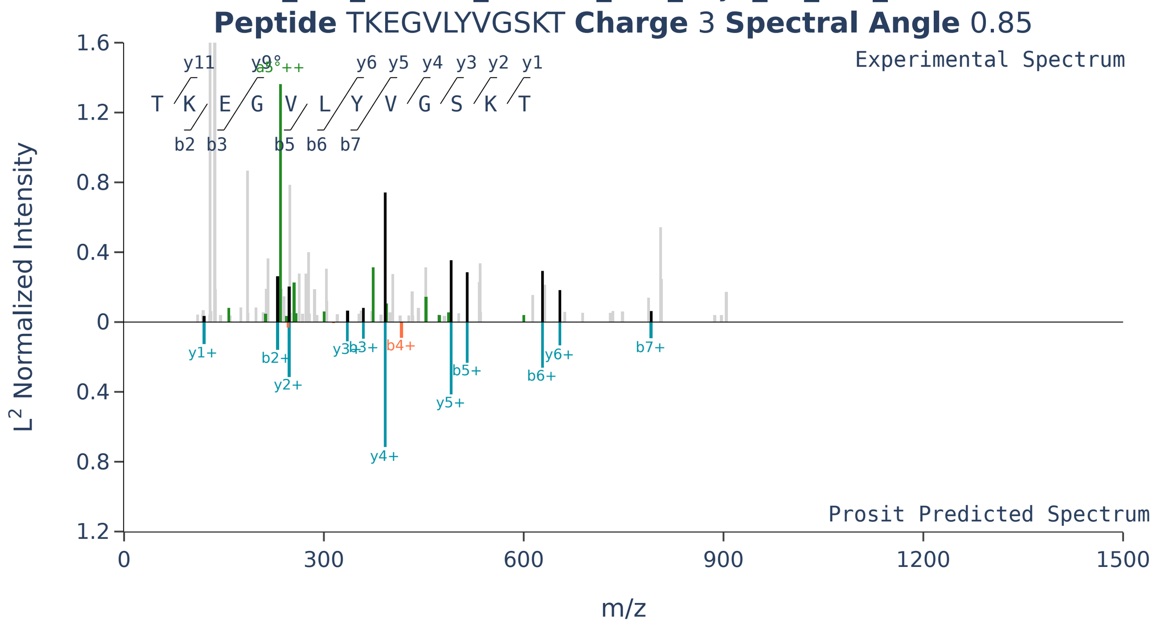


**Source** W_Soh_160821_170921_Goe_aSyn_A2_24h_R1 **Scan** 15994
**Peptide** VGGAVVTGVTA **Charge** 1 **Spectral Angle** 0.84

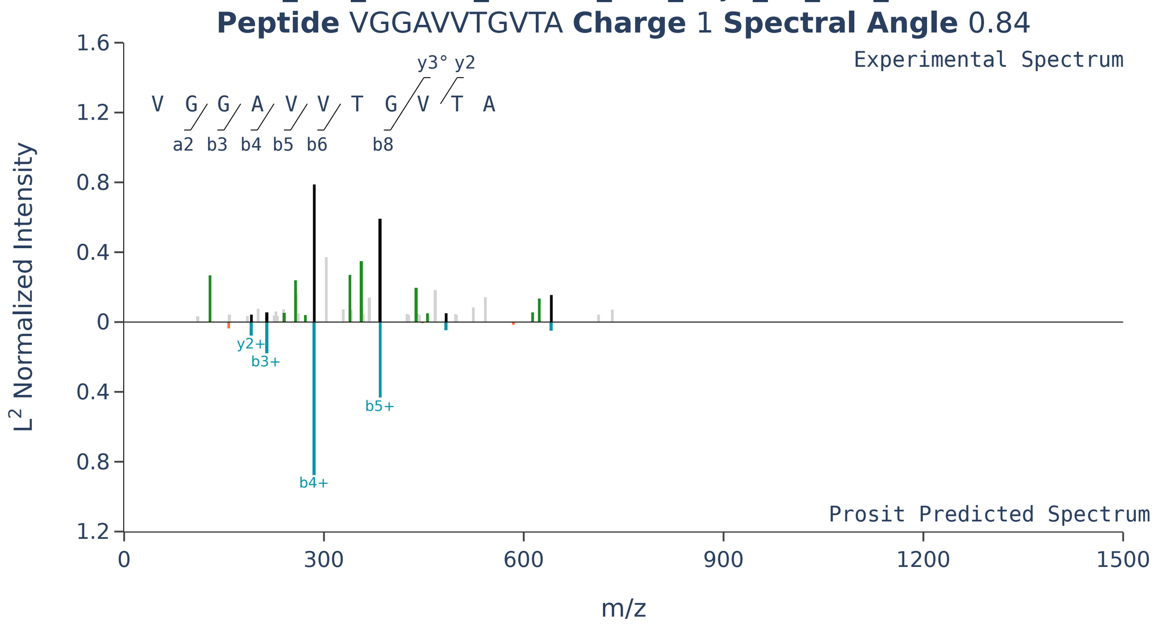


**Source** W_Soh_160821_170921_Goe_aSyn_A1_6h_R2 **Scan** 18779
**Peptide** VTNVGGAVVTGVTA **Charge** 2 **Spectral Angle** 0.9

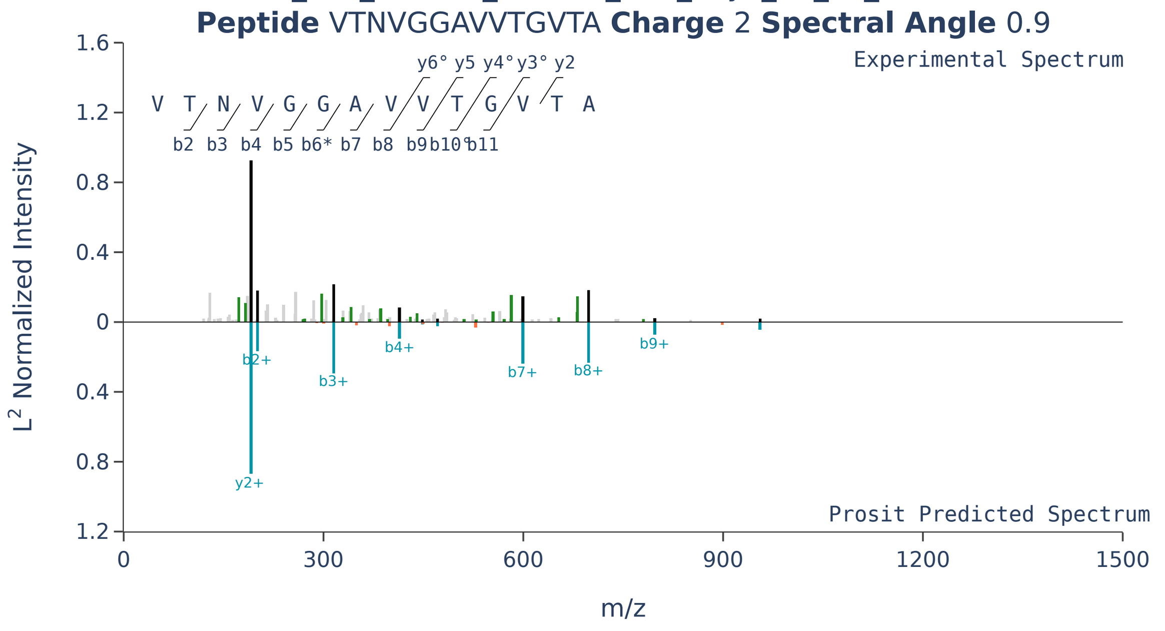


**Source** W_Soh_160821_170921_Goe_aSyn_A1_24h_R1 **Scan** 11081
**Peptide** AGKTKEGVLYVGS **Charge** 1 **Spectral Angle** 0.84

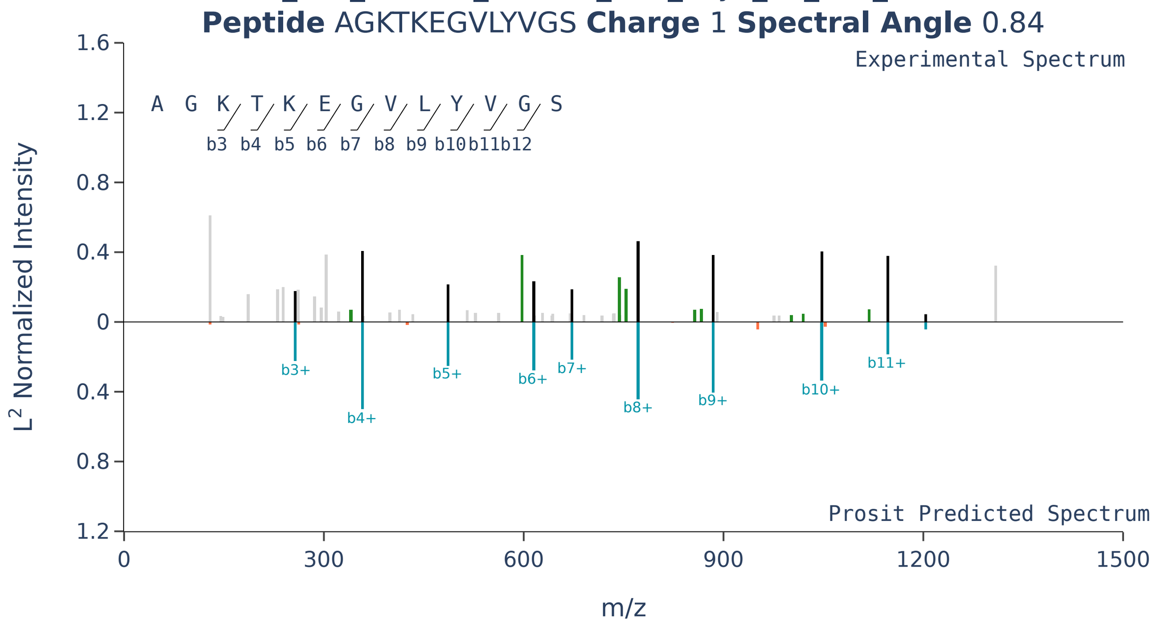


**Source** W_Soh_160821_170921_Goe_aSyn_A1_24h_R2 **Scan** 14268
**Peptide** KTVEGAGSIAAATGFVKK **Charge** 4 **Spectral Angle** 0.85

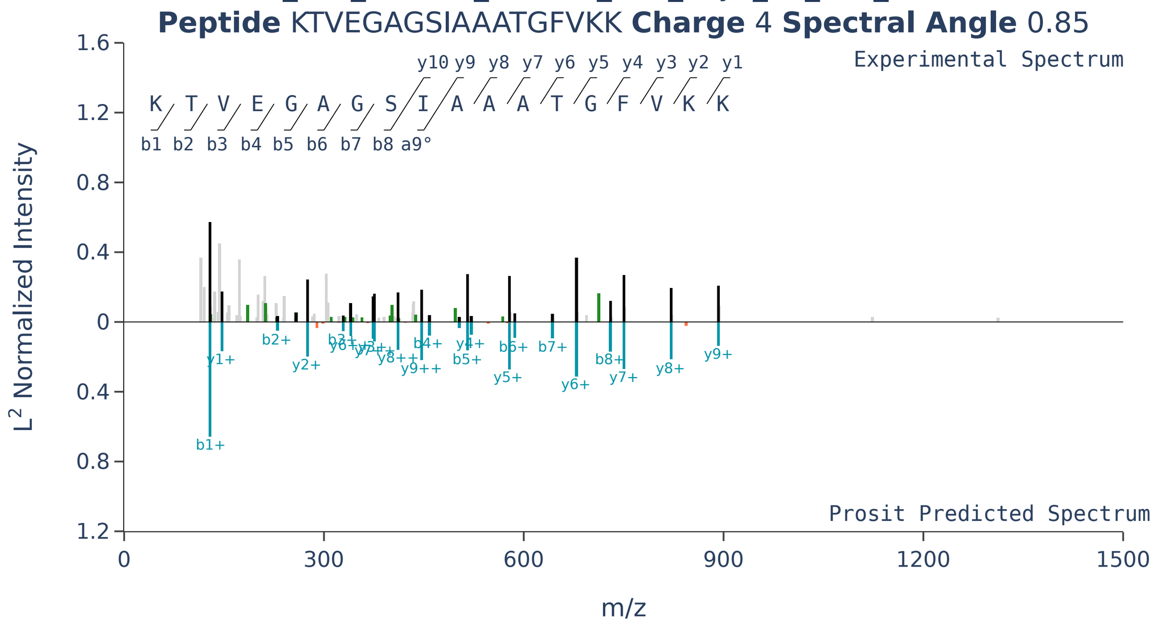


**Source** W_Soh_160821_170921_Goe_aSyn_A2_24h_R1 **Scan** 16688
**Peptide** KEQVTNVGGAVVTGVTA **Charge** 2 **Spectral Angle** 0.79

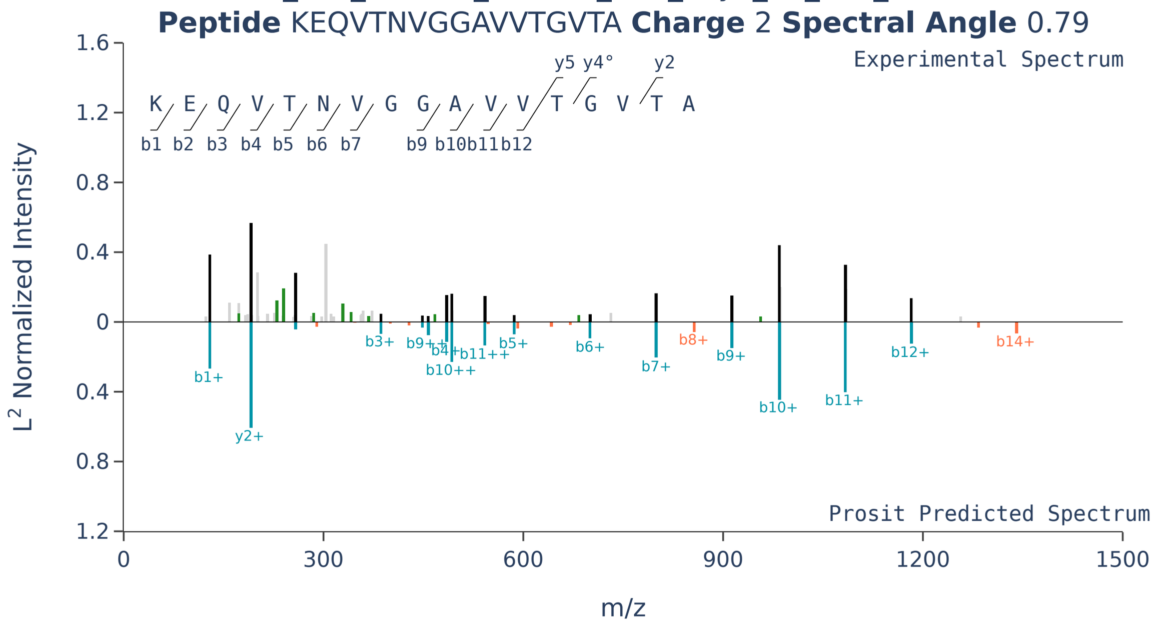

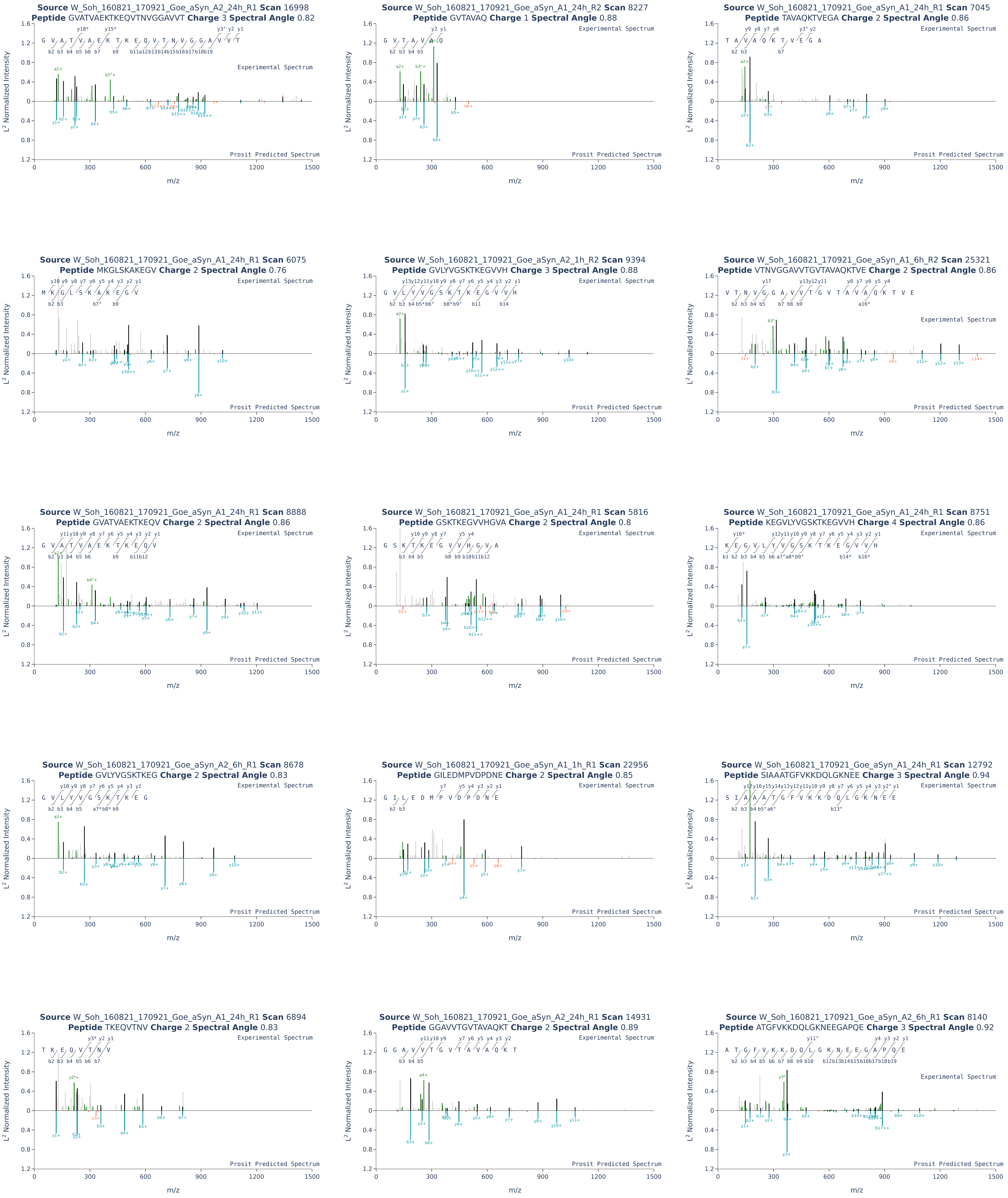
Source W_Soh_160821_170921_Goe_aSyn_A2_24h_R1 Scan 16998
Peptide GVATVAEKTKEQVTNVGGAVVT Charge 3 Spectral Angle 0.82
Source W_Soh_160821_170921_Goe_aSyn_A1_24h_R2 Scan 8227
Peptide GVTAVAQ Charge 1 Spectral Angle 0.88
Source W_Soh_160821_170921_Goe_aSyn_A1_24h_R1 Scan 7045
Peptide TAVAQKTVEGA Charge 2 Spectral Angle 0.86
Source W_Soh_160821_170921_Goe_aSyn_A1_24h_R1 Scan 6075
Peptide MKGLSKAKEGV Charge 2 Spectral Angle 0.76
Source W_Soh_160821_170921_Goe_aSyn_A2_1h_R2 Scan 9394
Peptide GVLYVGSKTKEGVVH Charge 3 Spectral Angle 0.88
Source W_Soh_160821_170921_Goe_aSyn_A1_6h_R2 Scan 25321
Peptide VTNVGGAVVTGVTAVAQKTVE Charge 2 Spectral Angle 0.86
Source W_Soh_160821_170921_Goe_aSyn_A1_24h_R1 Scan 8888
Peptide GVATVAEKTKEQV Charge 2 Spectral Angle 0.86
Source W_Soh_160821_170921_Goe_aSyn_A1_24h_R1 Scan 5816
Peptide GSKTKEGVVHGVA Charge 2 Spectral Angle 0.8
Source W_Soh_160821_170921_Goe_aSyn_A1_24h_R1 Scan 8751
Peptide KEGVLYVGSKTKEGVVH Charge 4 Spectral Angle 0.86
Source W_Soh_160821_170921_Goe_aSyn_A2_6h_R1 Scan 8678
Peptide GVLYVGSKTKEG Charge 2 Spectral Angle 0.83
Source W_Soh_160821_170921_Goe_aSyn_A1_1h_R1 Scan 22956
Peptide GILEDMPVDPDNE Charge 2 Spectral Angle 0.85
Source W_Soh_160821_170921_Goe_aSyn_A1_24h_R1 Scan 12792
Peptide SIAAATGFVKKDQLGKNEE Charge 3 Spectral Angle 0.94
Source W_Soh_160821_170921_Goe_aSyn_A1_24h_R1 Scan 6894
Peptide TKEQVTNV Charge 2 Spectral Angle 0.83
Source W_Soh_160821_170921_Goe_aSyn_A2_24h_R1 Scan 14931
Peptide GGAVVTGVTAVAQKT Charge 2 Spectral Angle 0.89
Source W_Soh_160821_170921_Goe_aSyn_A2_6h_R1 Scan 8140
Peptide ATGFVKKDQLGKNEEGAPQE Charge 3 Spectral Angle 0.92
Experimental Spectrum
Prosit Predicted Spectrum
L² Normalized Intensity
m/z

**Source** W_Soh_160821_170921_Goe_aSyn_A2_1h_R1 **Scan** 8703
**Peptide** VAQKTVEGAGSIA **Charge** 2 **Spectral Angle** 0.84

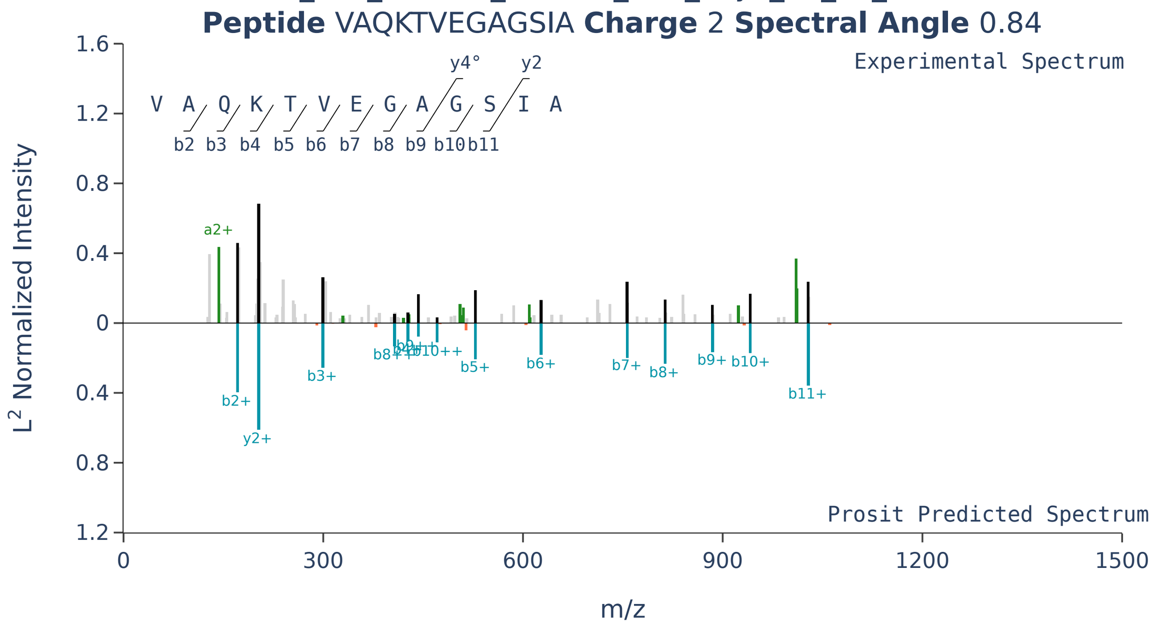


**Source** W_Soh_160821_170921_Goe_aSyn_A2_24h_R1 **Scan** 9207
**Peptide** TKEGVLYVGSKTKEGVVH **Charge** 4 **Spectral Angle** 0.91

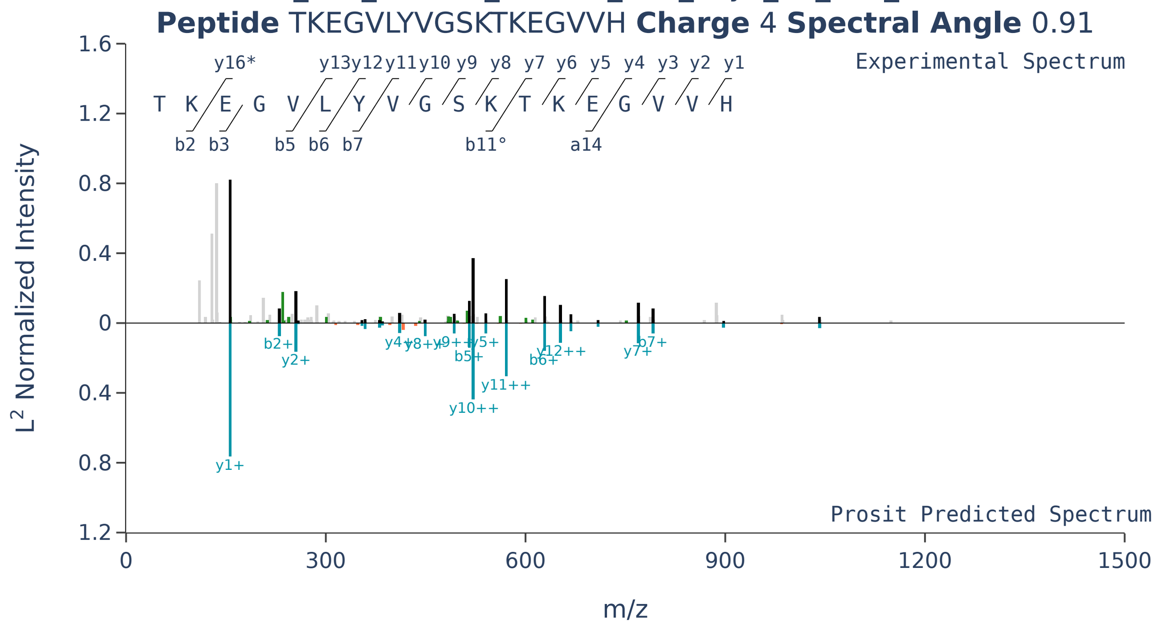


**Source** W_Soh_160821_170921_Goe_aSyn_A1_24h_R2 **Scan** 5657
**Peptide** ATGFVKKDQ **Charge** 2 **Spectral Angle** 0.88

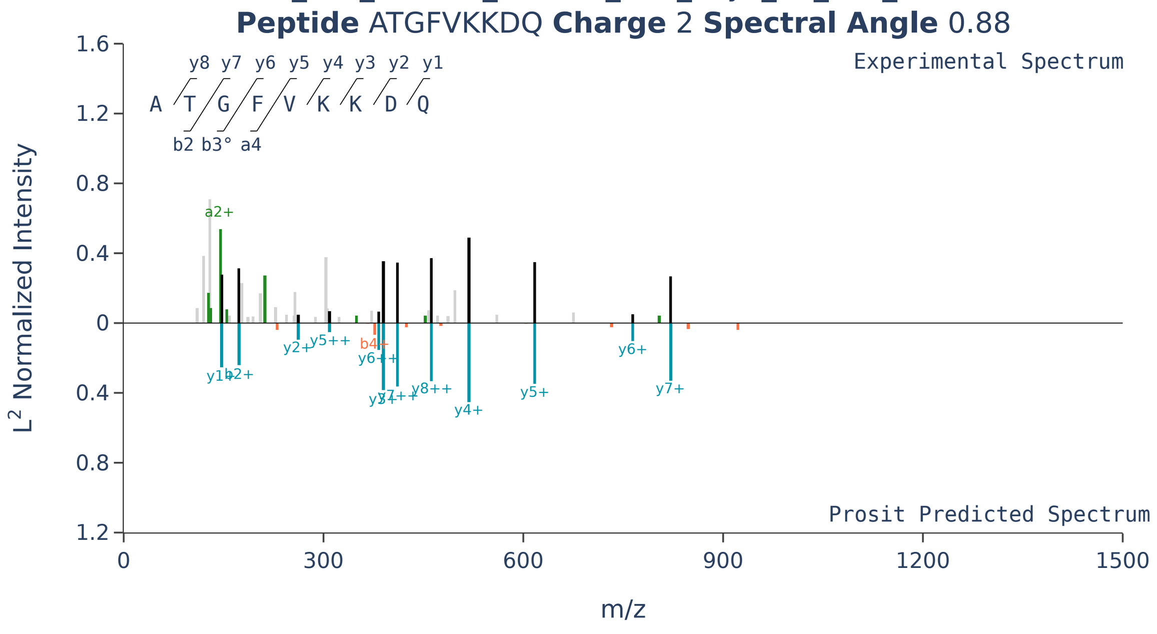


**Source** W_Soh_160821_170921_Goe_aSyn_A1_24h_R2 **Scan** 13010
**Peptide** AGSIAAATGFVKK **Charge** 3 **Spectral Angle** 0.87

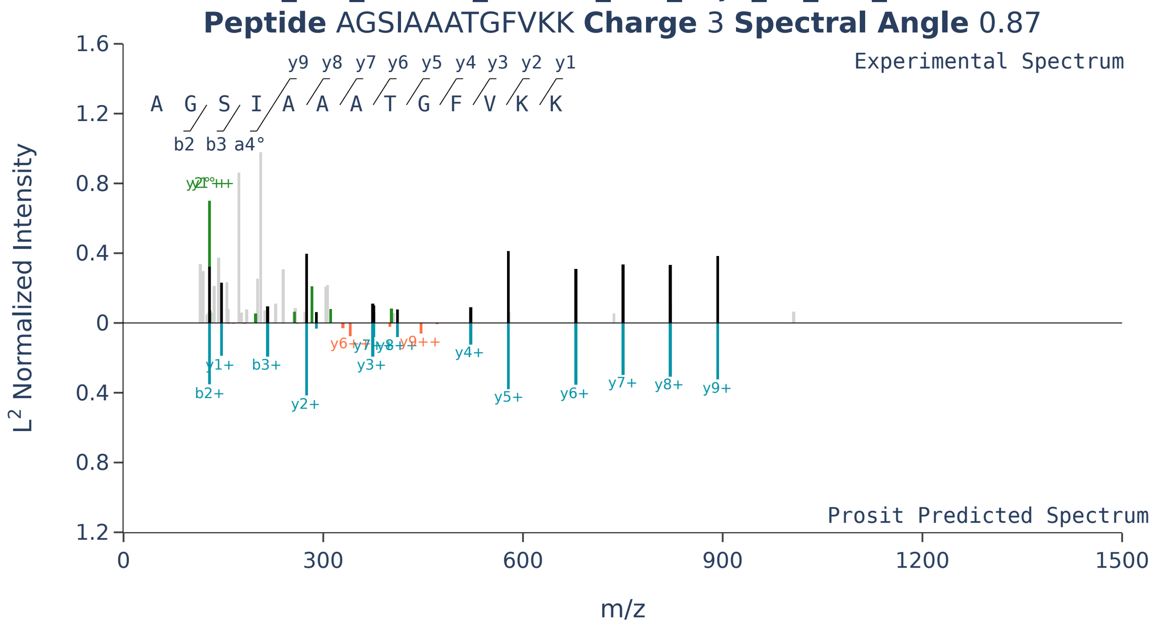


**Source** W_Soh_160821_170921_Goe_aSyn_A1_24h_R1 **Scan** 15081
**Peptide** VVTGVTAVAQKTVE **Charge** 2 **Spectral Angle** 0.89

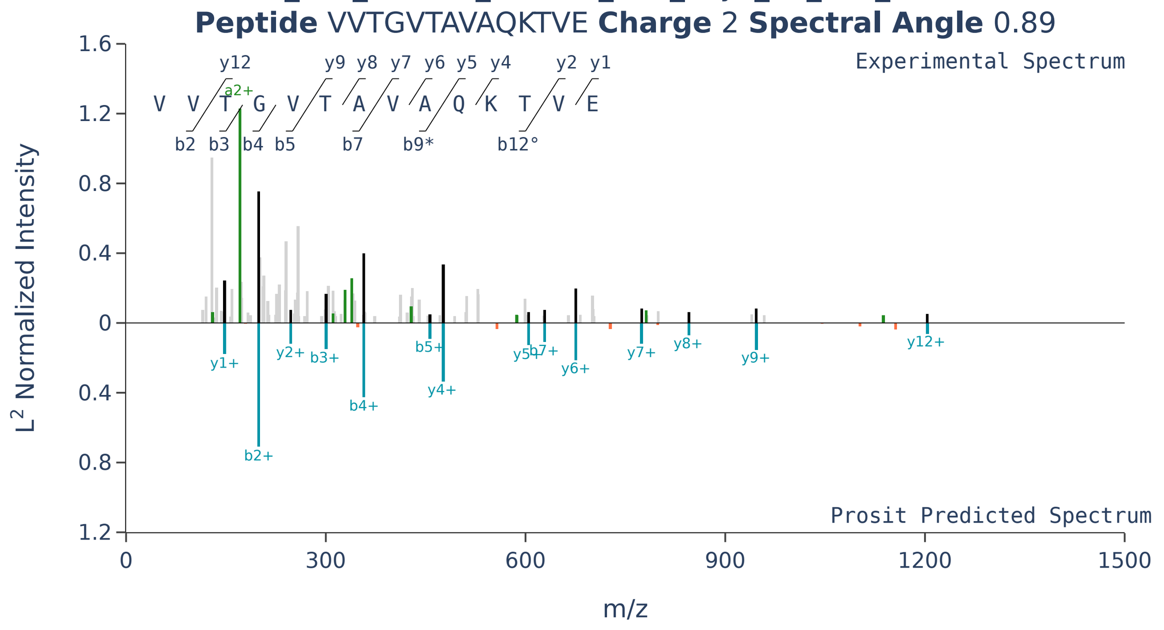


**Source** W_Soh_160821_170921_Goe_aSyn_A1_24h_R1 **Scan** 9285
**Peptide** KEGVVHGVATVA **Charge** 3 **Spectral Angle** 0.86

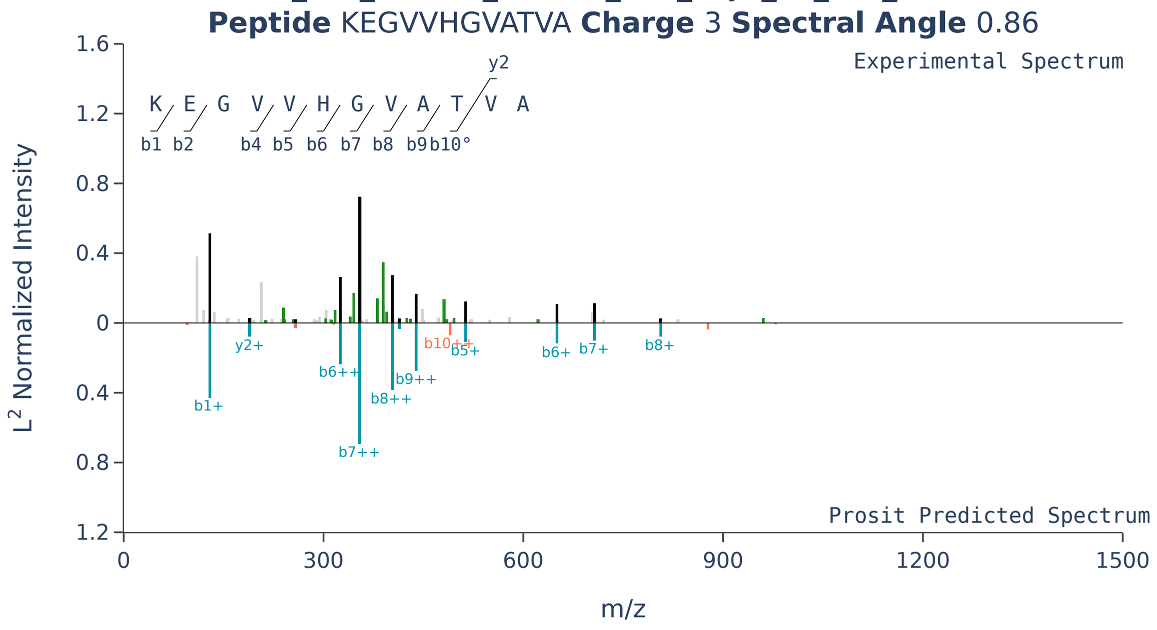


**Source** W_Soh_160821_170921_Goe_aSyn_A2_6h_R1 **Scan** 8783
**Peptide** VLYVGSKTKEGVVH **Charge** 2 **Spectral Angle** 0.84

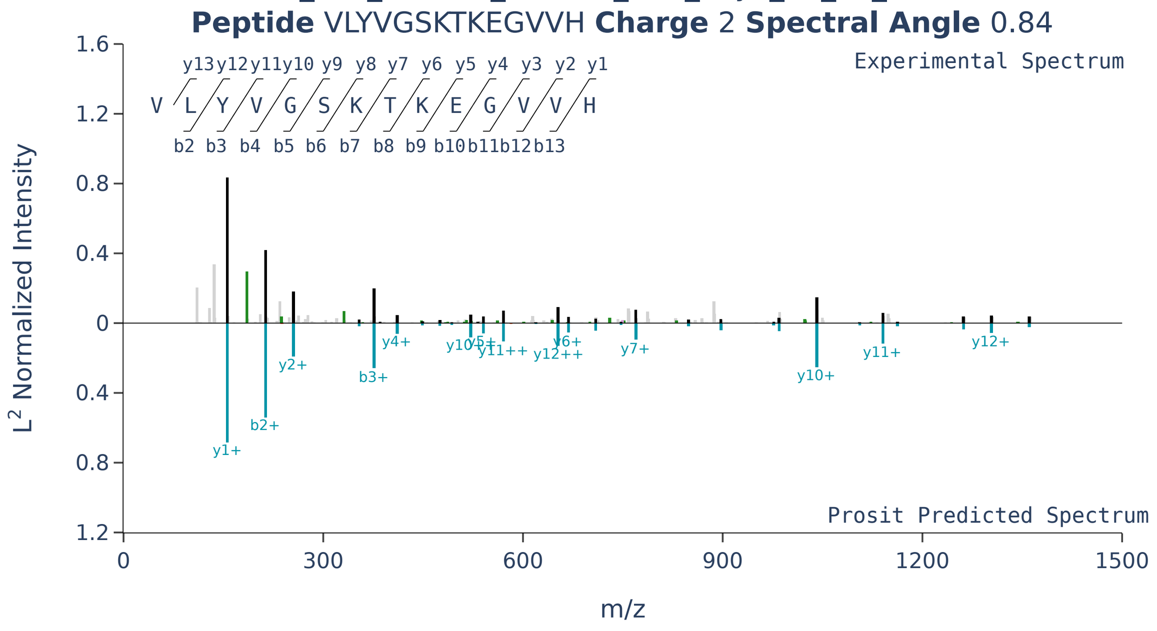


**Source** W_Soh_160821_170921_Goe_aSyn_A2_3h_R2 **Scan** 9699
**Peptide** LYVGSKTKEGVVHG **Charge** 3 **Spectral Angle** 0.86

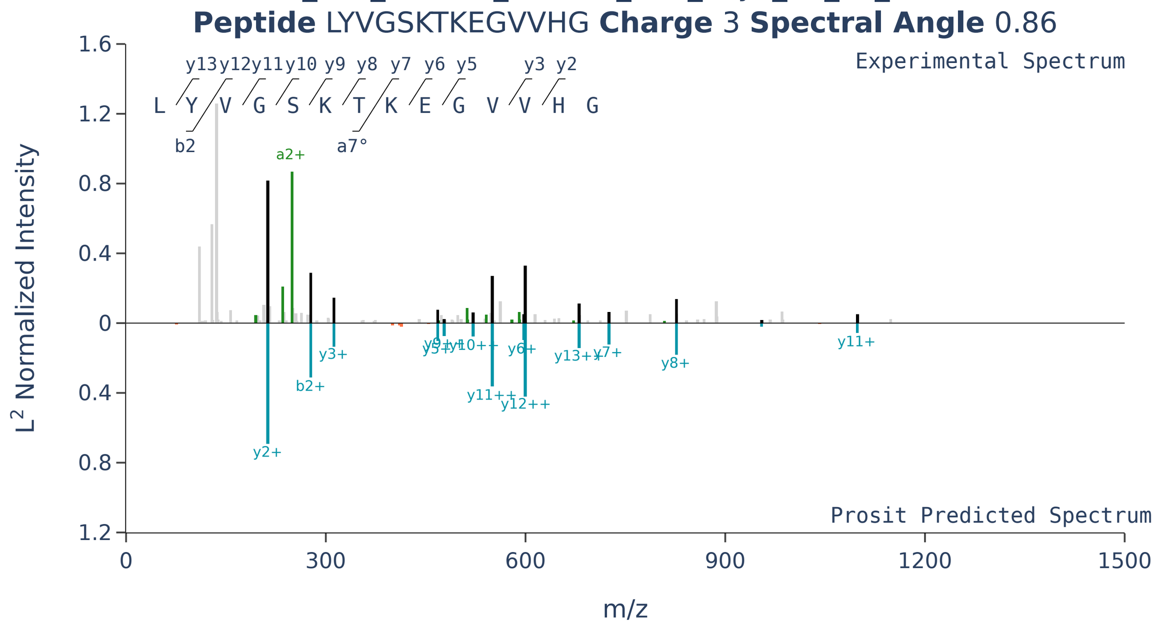


**Source** W_Soh_160821_170921_Goe_aSyn_A1_24h_R1 **Scan** 9248
**Peptide** MPSEEGYQ **Charge** 1 **Spectral Angle** 0.85

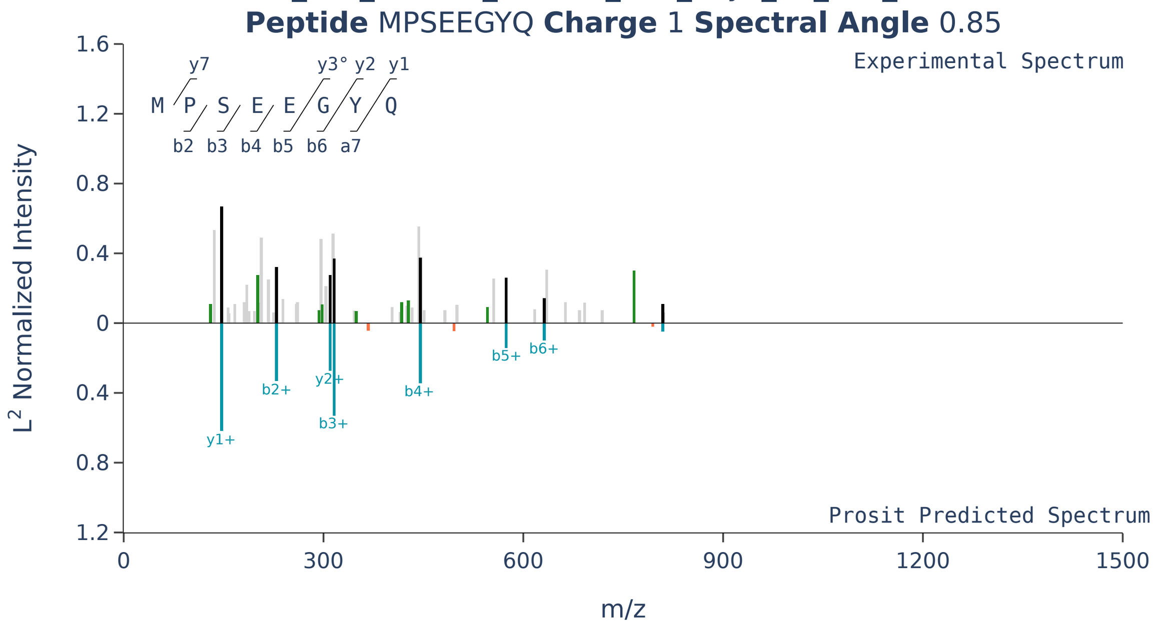


**Source** W_Soh_160821_170921_Goe_aSyn_A2_24h_R1 **Scan** 17550
**Peptide** TAVAQKTVEGAGSIAAATGFVKKDQ **Charge** 3 **Spectral Angle** 0.81

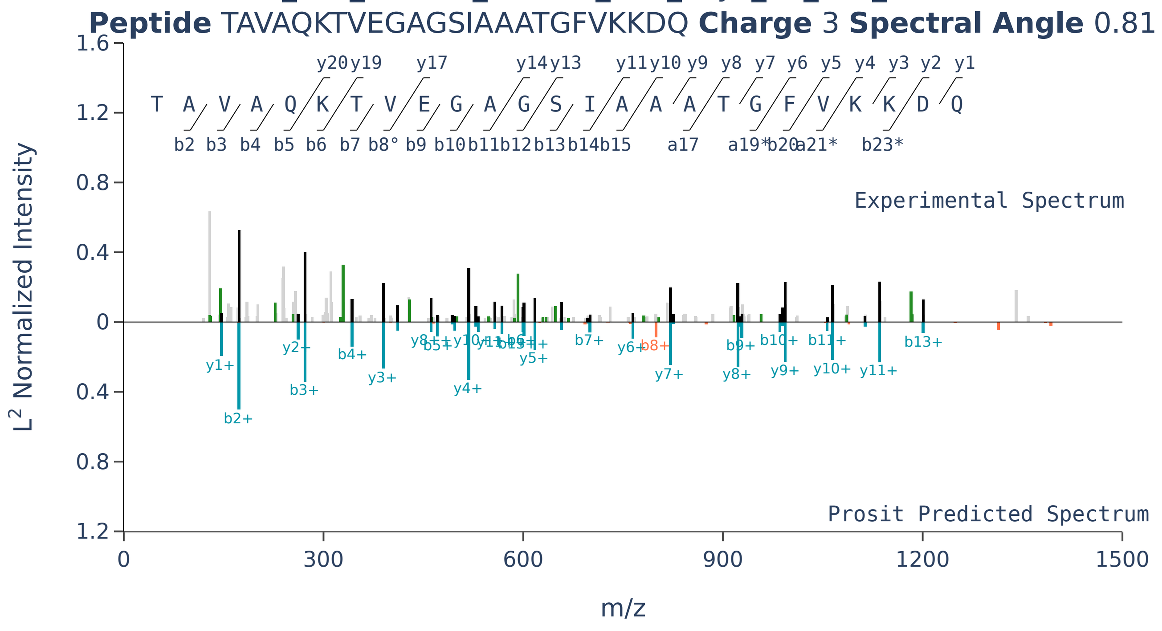


**Source** W_Soh_160821_170921_Goe_aSyn_A1_24h_R2 **Scan** 14527
**Peptide** EKTKEQVTNVGGAVVTGV **Charge** 2 **Spectral Angle** 0.85

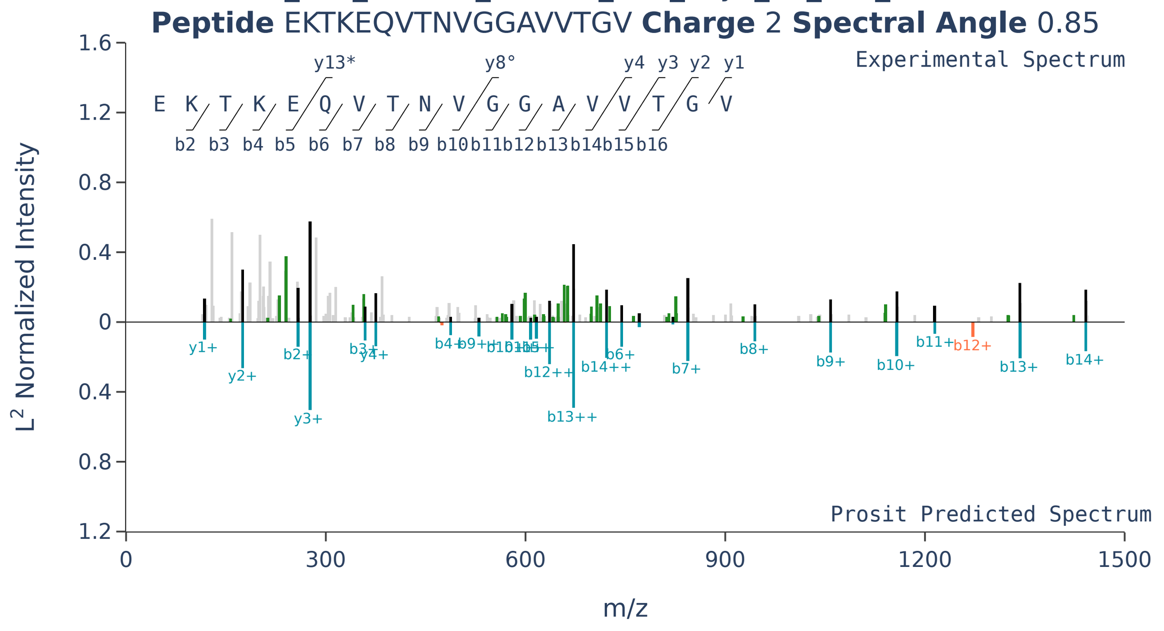


**Source** W_Soh_160821_170921_Goe_aSyn_A2_6h_R2 **Scan** 11845
**Peptide** AGKTKEGVLYV **Charge** 1 **Spectral Angle** 0.89

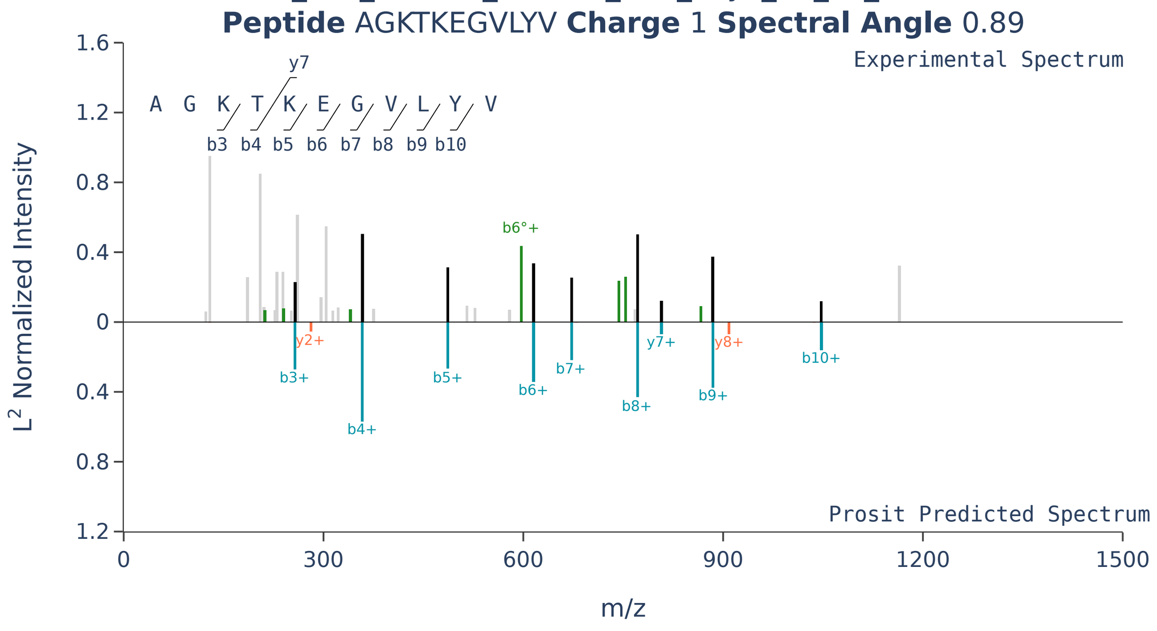


**Source** W_Soh_160821_170921_Goe_aSyn_A1_24h_R2 **Scan** 10402
**Peptide** GVTAVAQKTVEG **Charge** 1 **Spectral Angle** 0.89

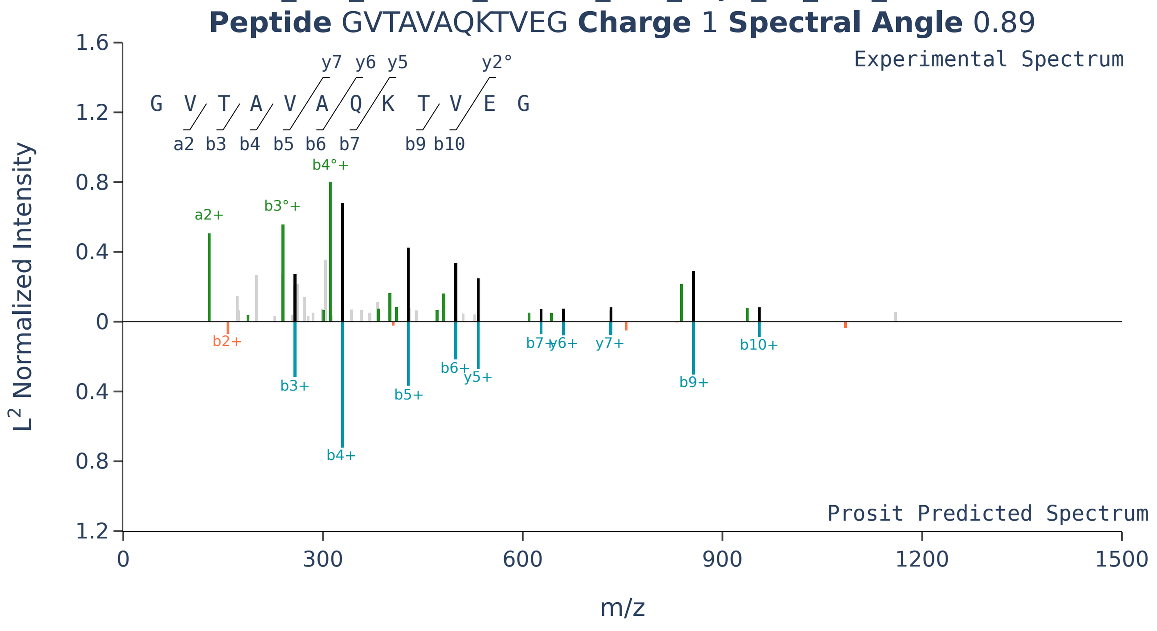


**Source** W_Soh_160821_170921_Goe_aSyn_A1_24h_R1 **Scan** 6681
**Peptide** LSKAKEGVVAA **Charge** 2 **Spectral Angle** 0.78

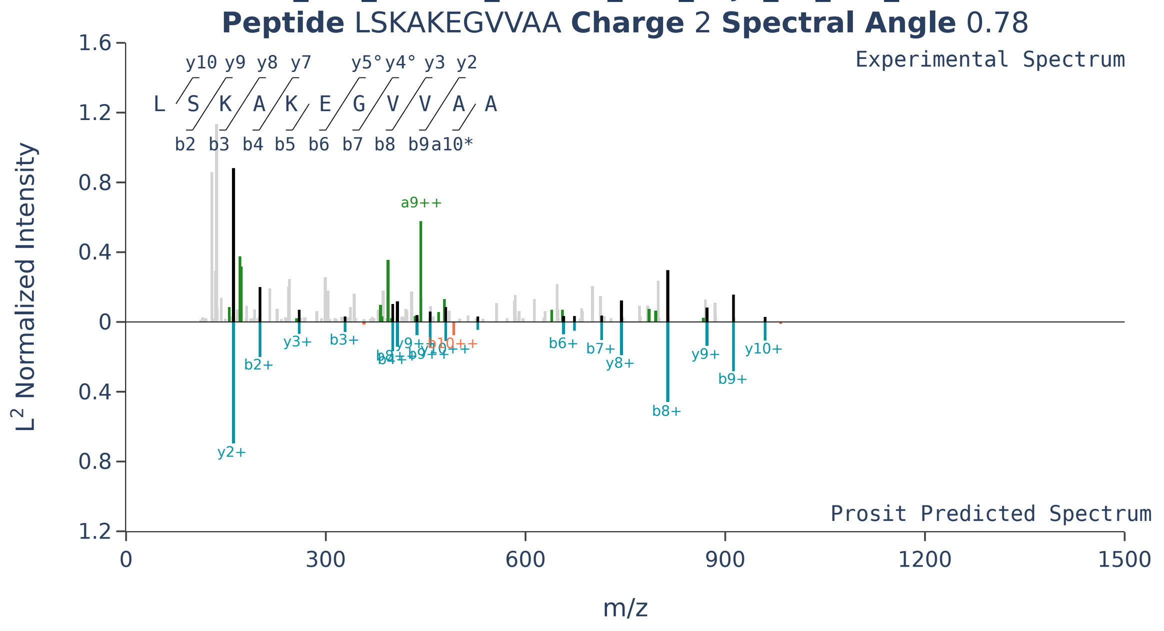


**Source** W_Soh_160821_170921_Goe_aSyn_A1_24h_R1 **Scan** 15534
**Peptide** GVAEAAGKTKEGVLYV **Charge** 2 **Spectral Angle** 0.92

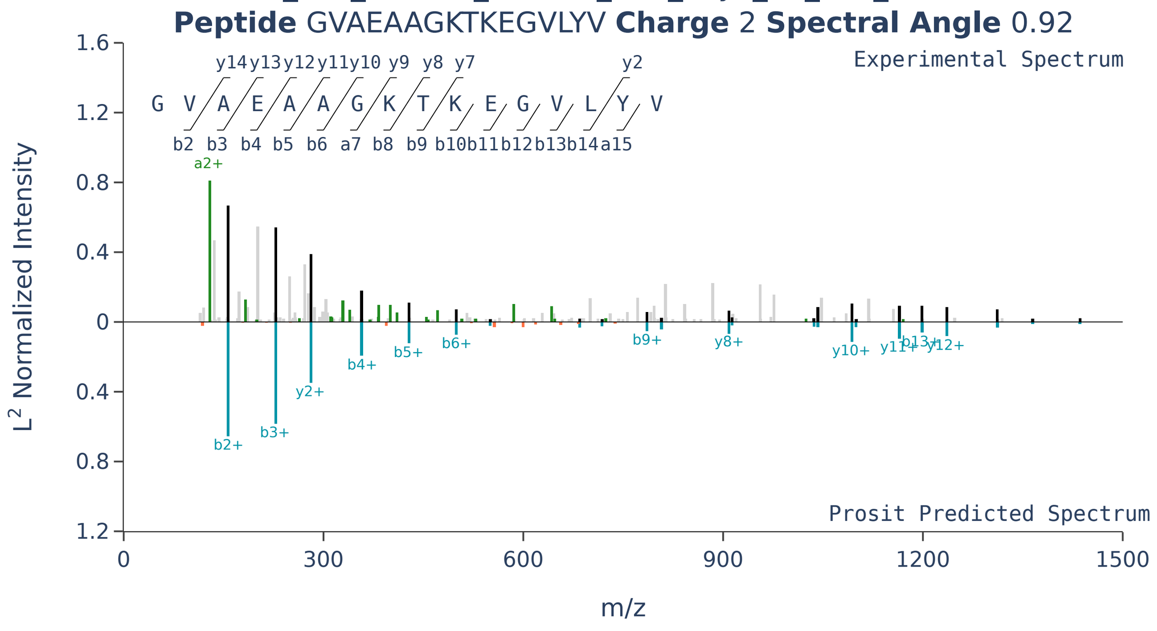

**Source** W_Soh_160821_170921_Goe_aSyn_A1_24h_R1 **Scan** 14909
**Peptide** VTAVAQKTVEGAGSIAAA **Charge** 2 **Spectral Angle** 0.83

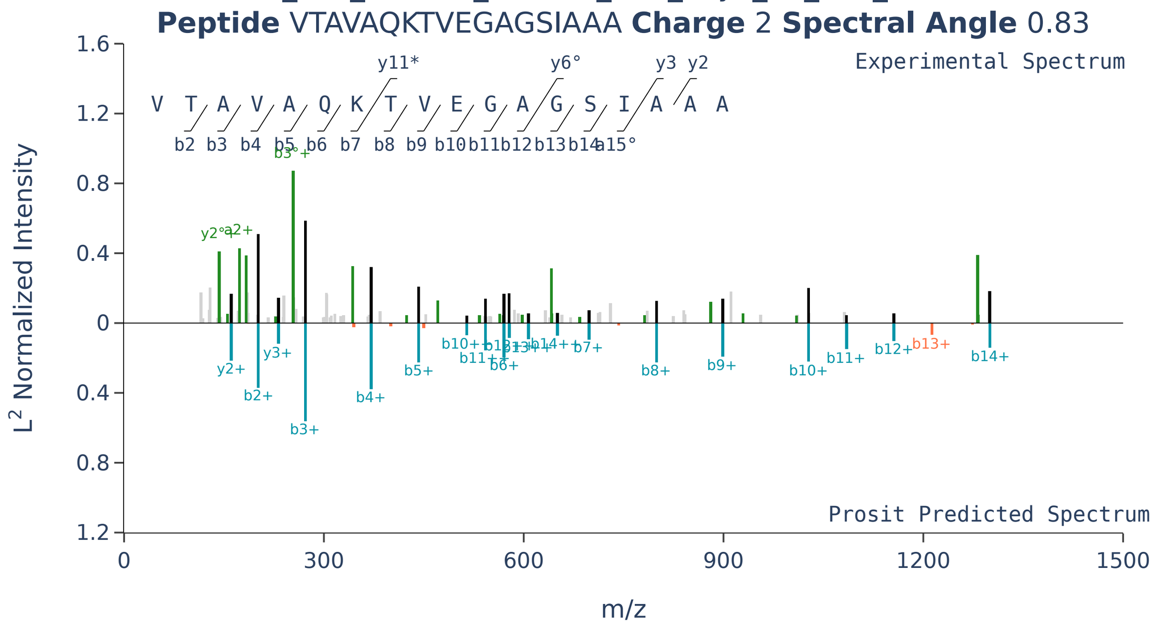


**Source** W_Soh_160821_170921_Goe_aSyn_A2_24h_R2 **Scan** 12713
**Peptide** EDMPVDPDN **Charge** 2 **Spectral Angle** 0.88

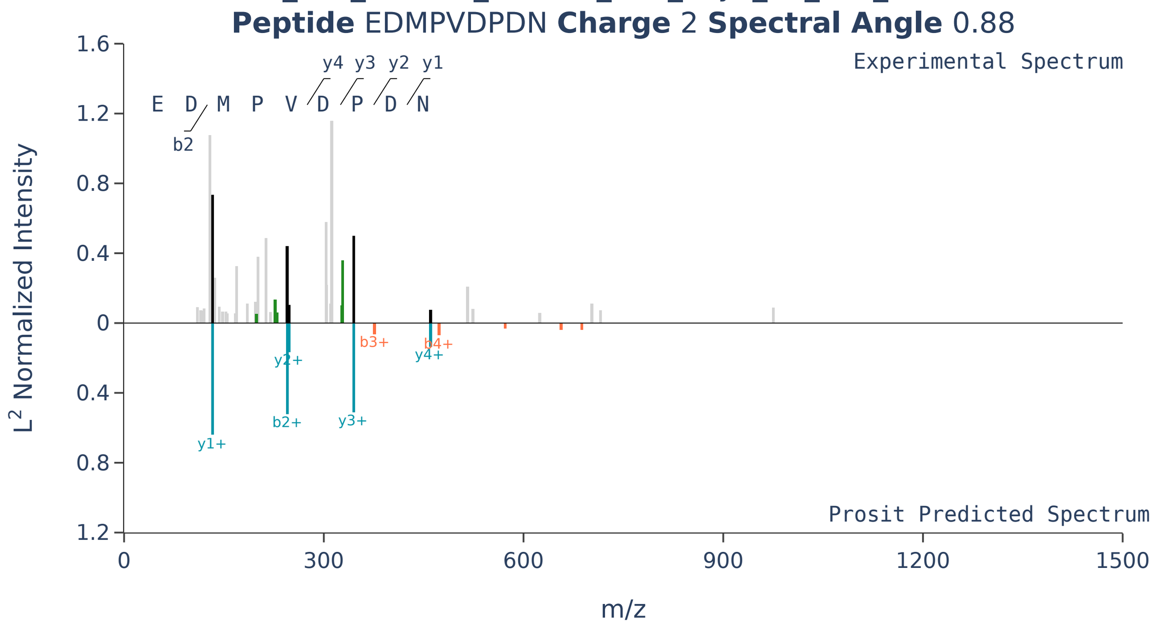


**Source** W_Soh_160821_170921_Goe_aSyn_A2_24h_R2 **Scan** 15700
**Peptide** VAQKTVEGAGSIAAATGFVKKD **Charge** 2 **Spectral Angle** 0.79

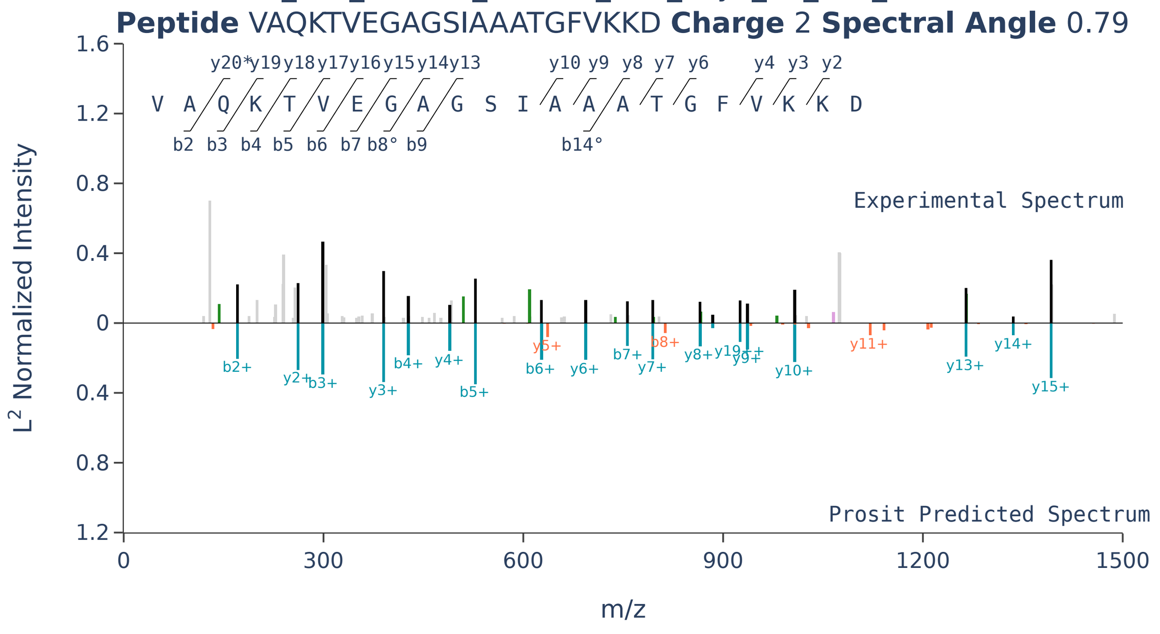


**Source** W_Soh_160821_170921_Goe_aSyn_A1_24h_R1 **Scan** 7843
**Peptide** KTKEGVVHGVATVA **Charge** 2 **Spectral Angle** 0.81

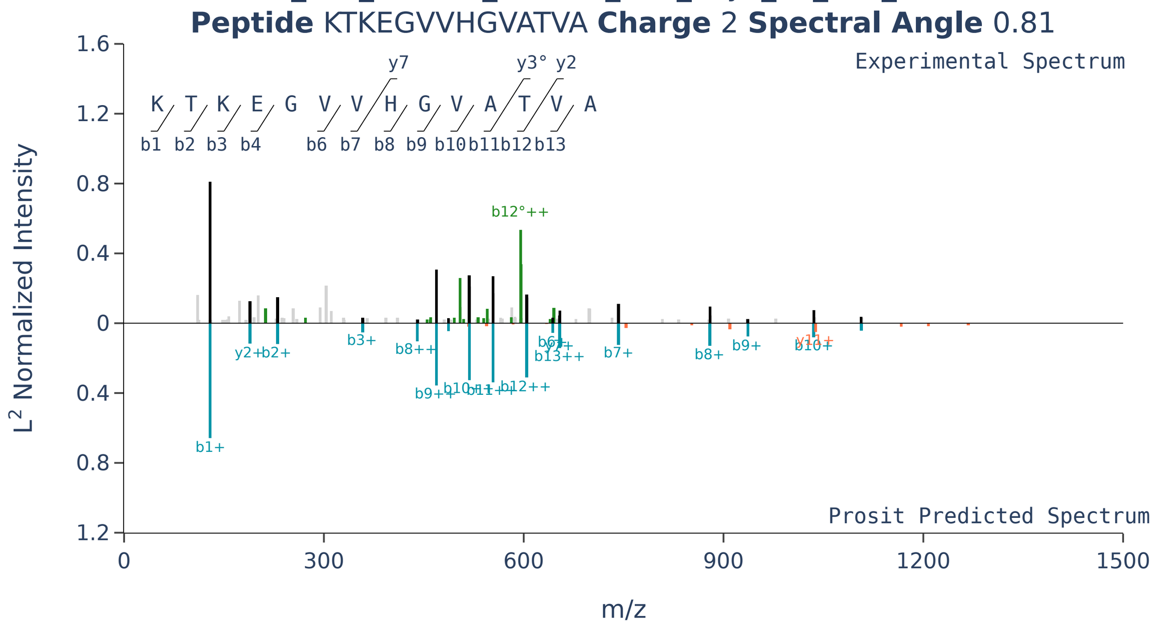


**Source** W_Soh_160821_170921_Goe_aSyn_A2_1h_R1 **Scan** 19137
**Peptide** AEKTKEQVTNVGGAVVTGVTAVAQKTVE **Charge** 4 **Spectral Angle** 0.81

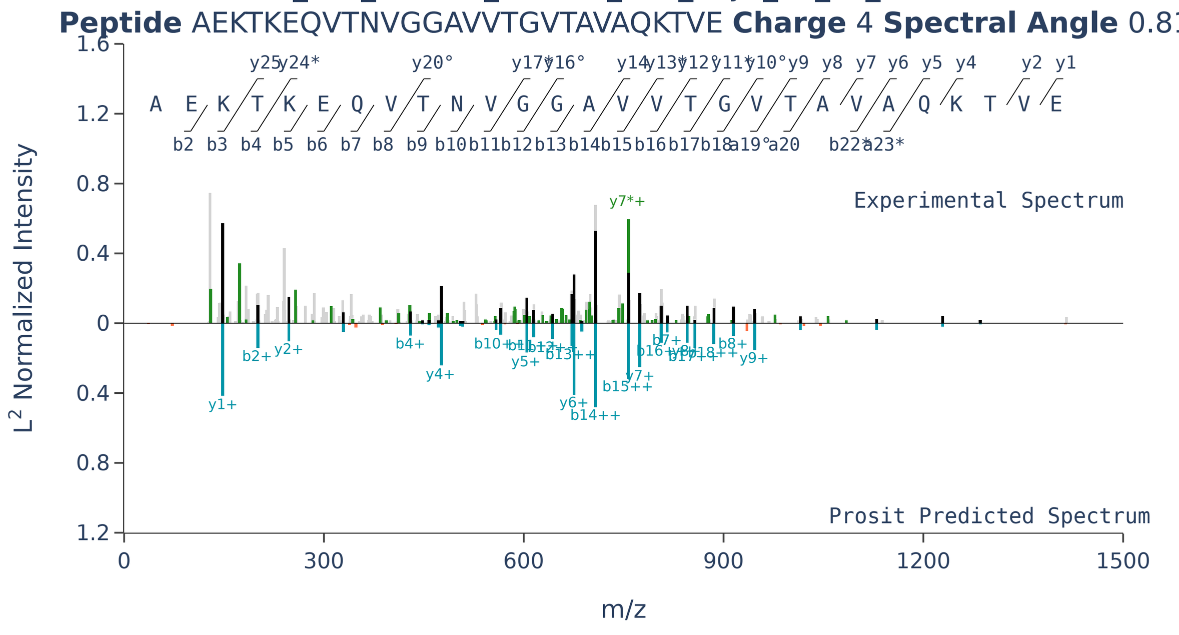


**Source** W_Soh_160821_170921_Goe_aSyn_A1_24h_R2 **Scan** 8468
**Peptide** FMKGLSKA **Charge** 2 **Spectral Angle** 0.89

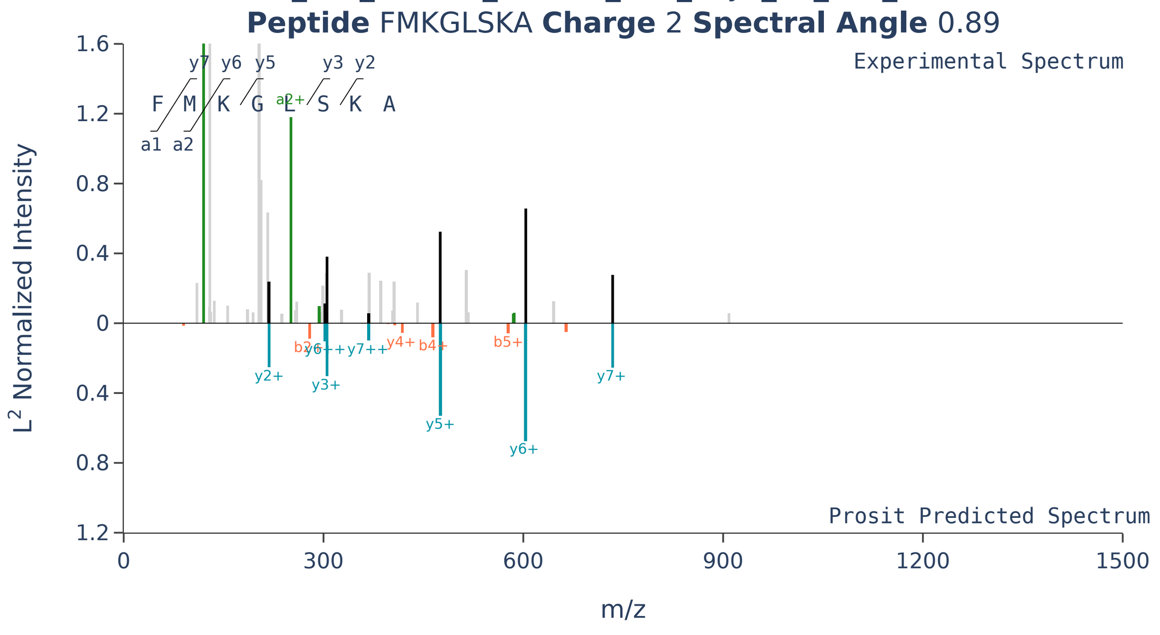


**Source** W_Soh_160821_170921_Goe_aSyn_A1_24h_R2 **Scan** 13028
**Peptide** GVATVAEKTKEQVTNV **Charge** 2 **Spectral Angle** 0.91

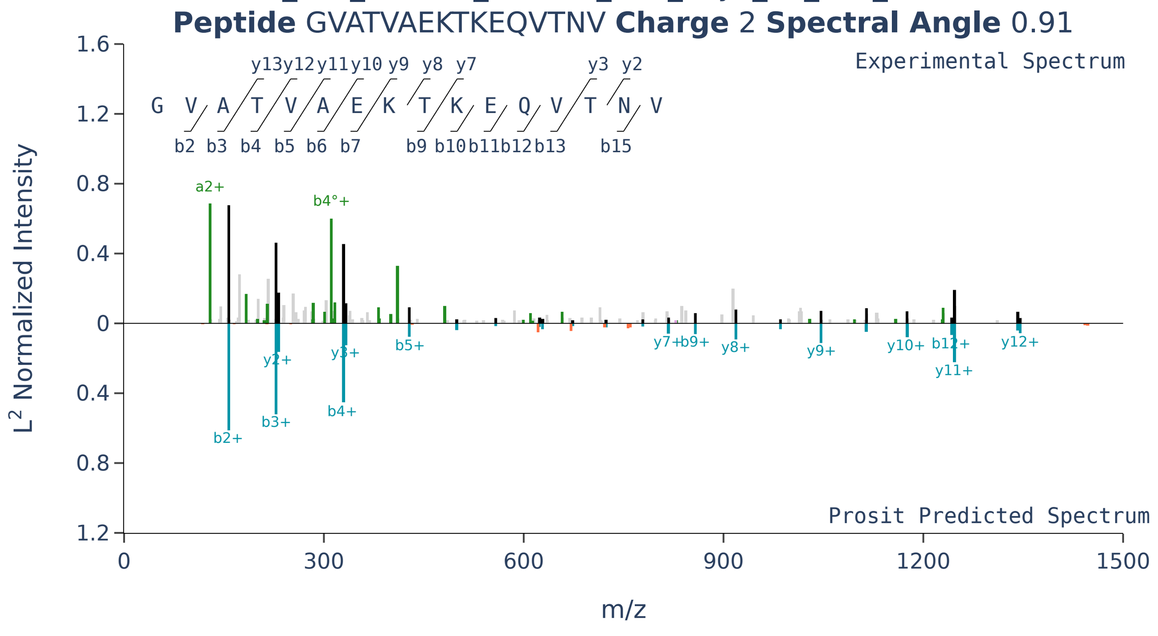


**Source** W_Soh_160821_170921_Goe_aSyn_A1_24h_R1 **Scan** 7110
**Peptide** ATGFVKKDQLGKNEE **Charge** 2 **Spectral Angle** 0.81

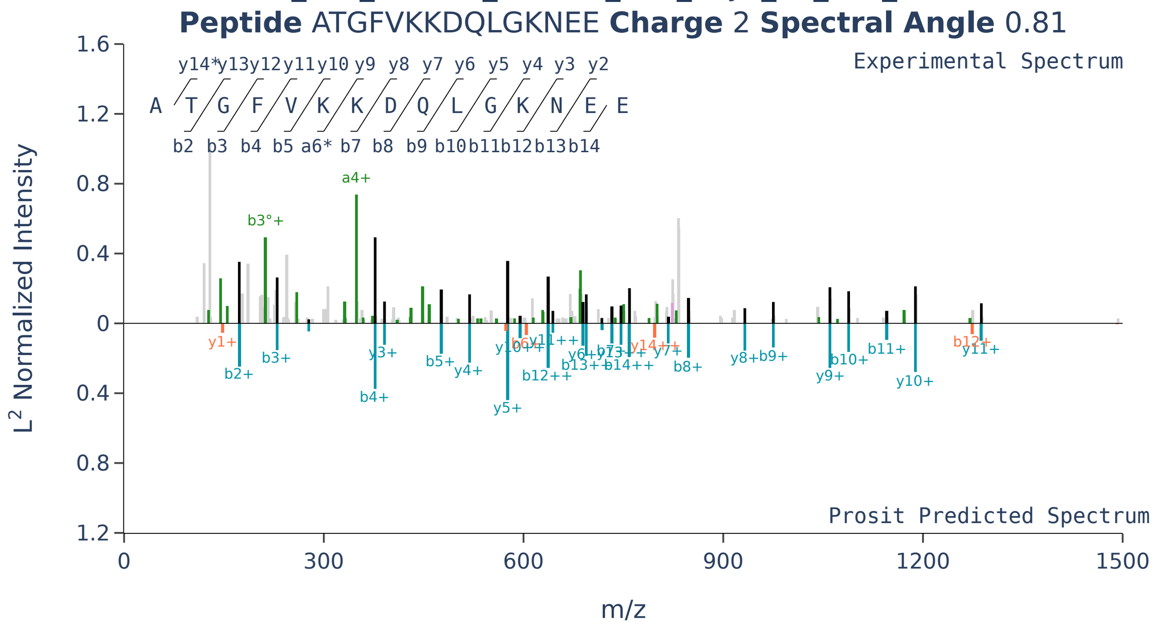


**Source** W_Soh_160821_170921_Goe_aSyn_A2_24h_R1 **Scan** 9303
**Peptide** KTVEGAGSIAAA **Charge** 2 **Spectral Angle** 0.85

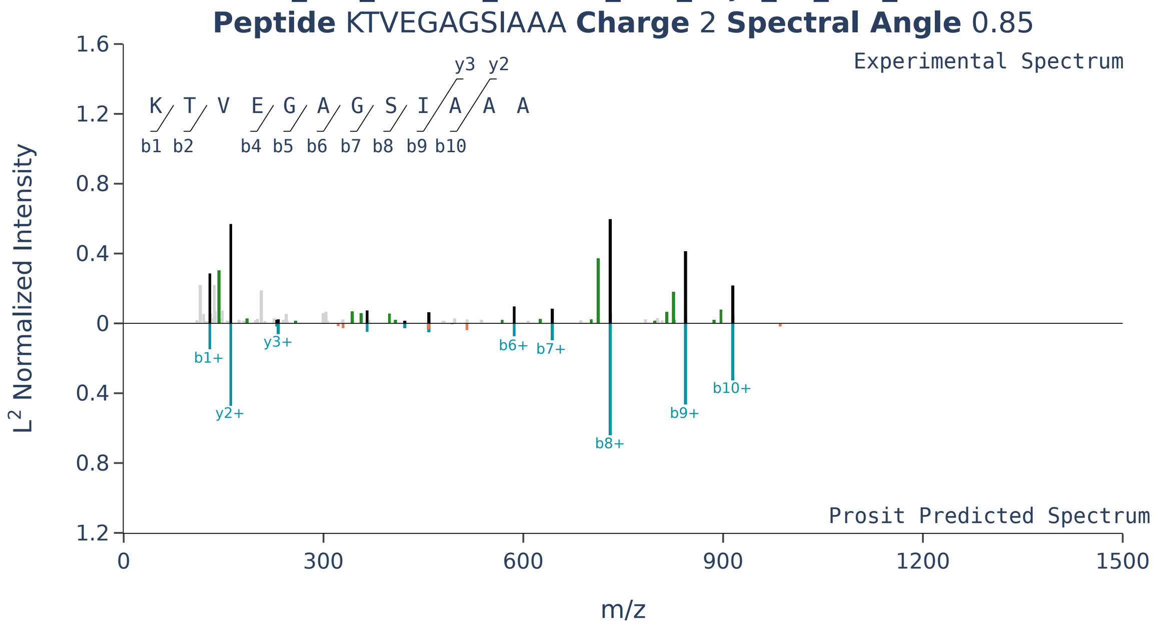


**Source** W_Soh_160821_170921_Goe_aSyn_A1_6h_R1 **Scan** 6803
**Peptide** VATVAEKTKEQVT **Charge** 3 **Spectral Angle** 0.84

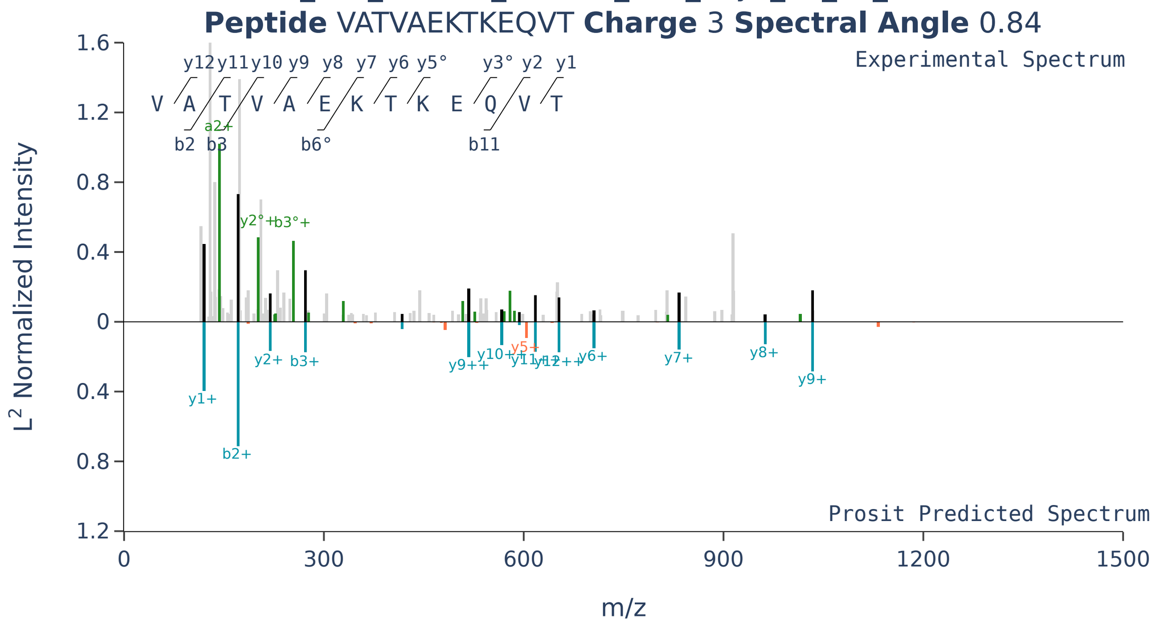


**Source** W_Soh_160821_170921_Goe_aSyn_A1_30'_R2 **Scan** 5799
**Peptide** KGLSKAKEGVVA **Charge** 2 **Spectral Angle** 0.86

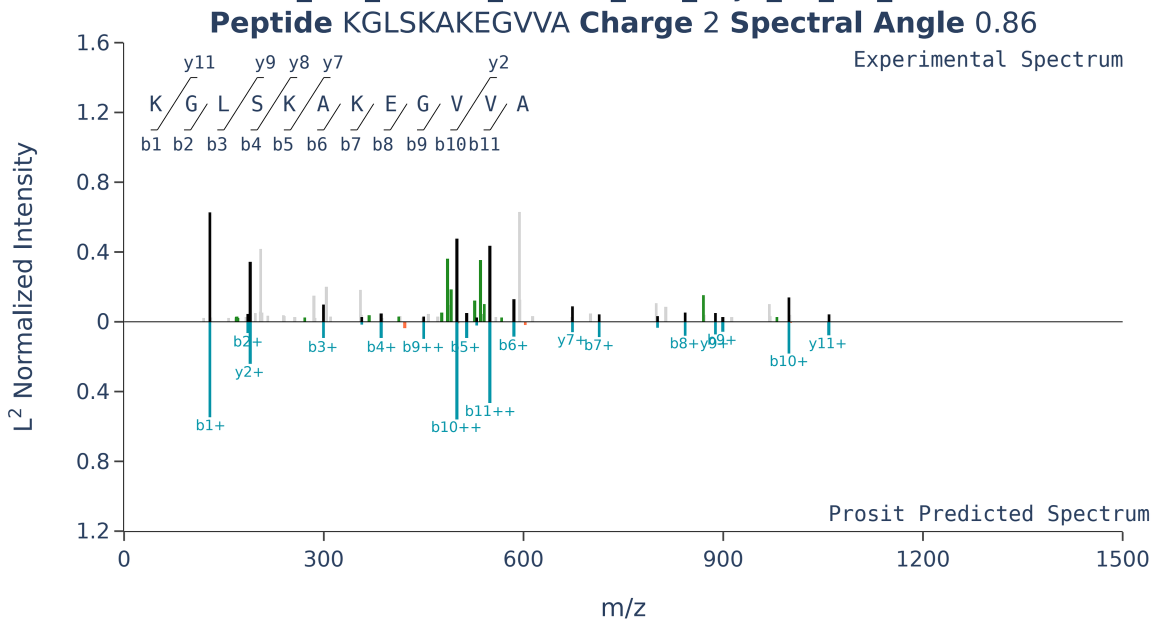


**Source** W_Soh_160821_170921_Goe_aSyn_A1_24h_R2 **Scan** 12363
**Peptide** GKTKEGVLYV **Charge** 2 **Spectral Angle** 0.89

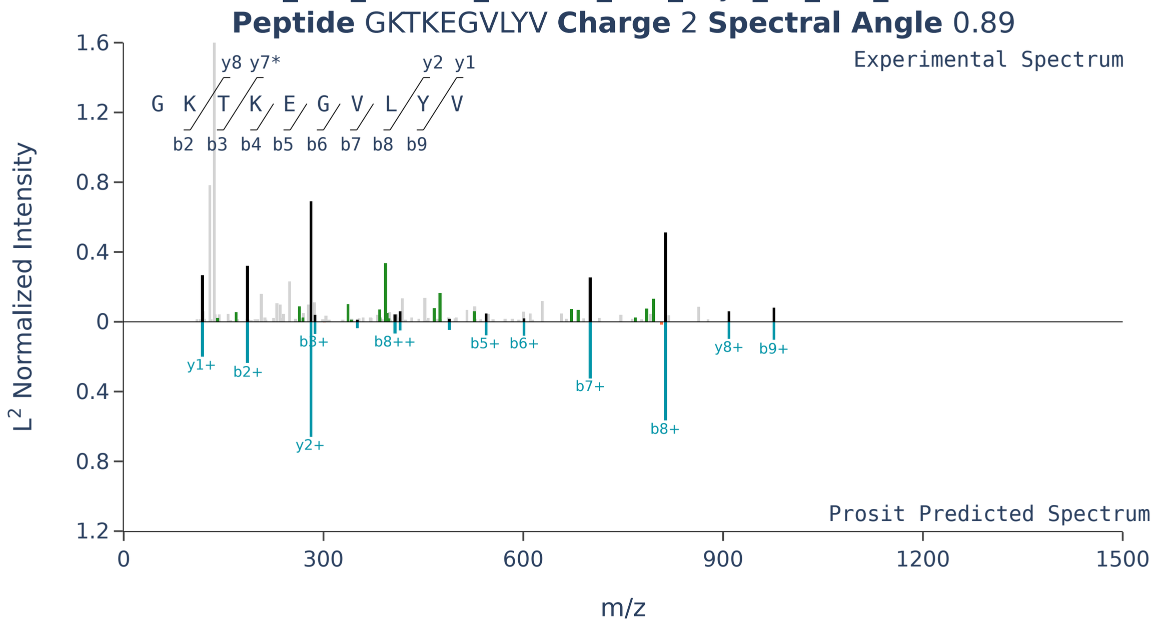


**Source** W_Soh_160821_170921_Goe_aSyn_A1_24h_R2 **Scan** 17969
**Peptide** GVLYVGS **Charge** 1 **Spectral Angle** 0.86

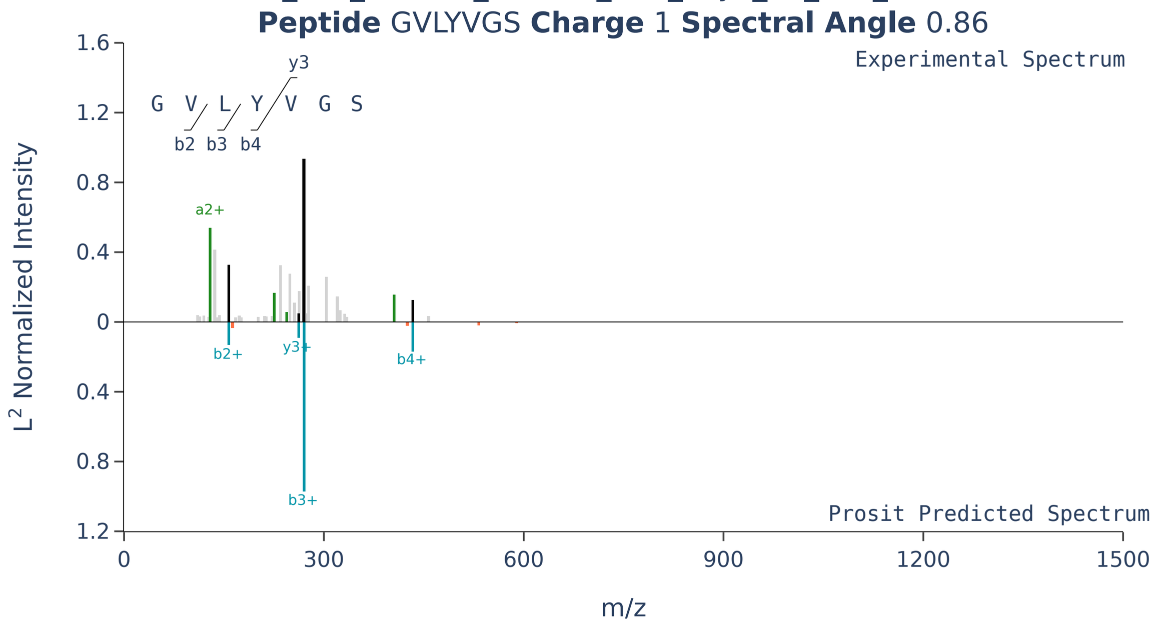


**Source** W_Soh_160821_170921_Goe_aSyn_A2_24h_R2 **Scan** 14600
**Peptide** GAGSIAAATGFVKKD **Charge** 3 **Spectral Angle** 0.82

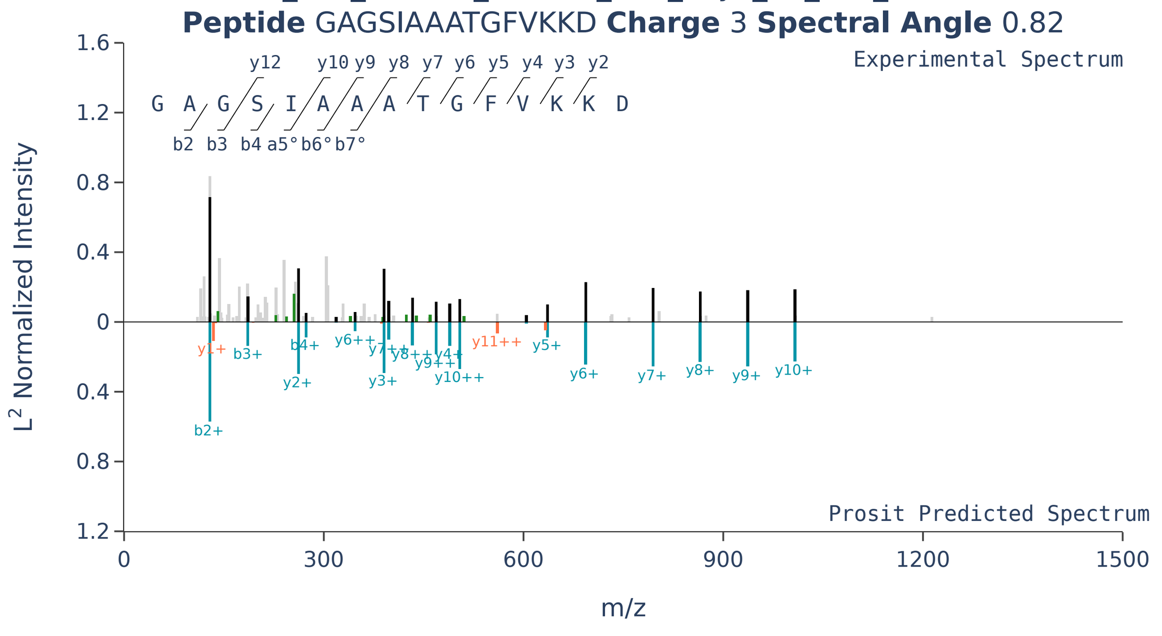


**Source** W_Soh_160821_170921_Goe_aSyn_A2_6h_R1 **Scan** 7963
**Peptide** AGKTKEGVLYVGSKT **Charge** 3 **Spectral Angle** 0.89

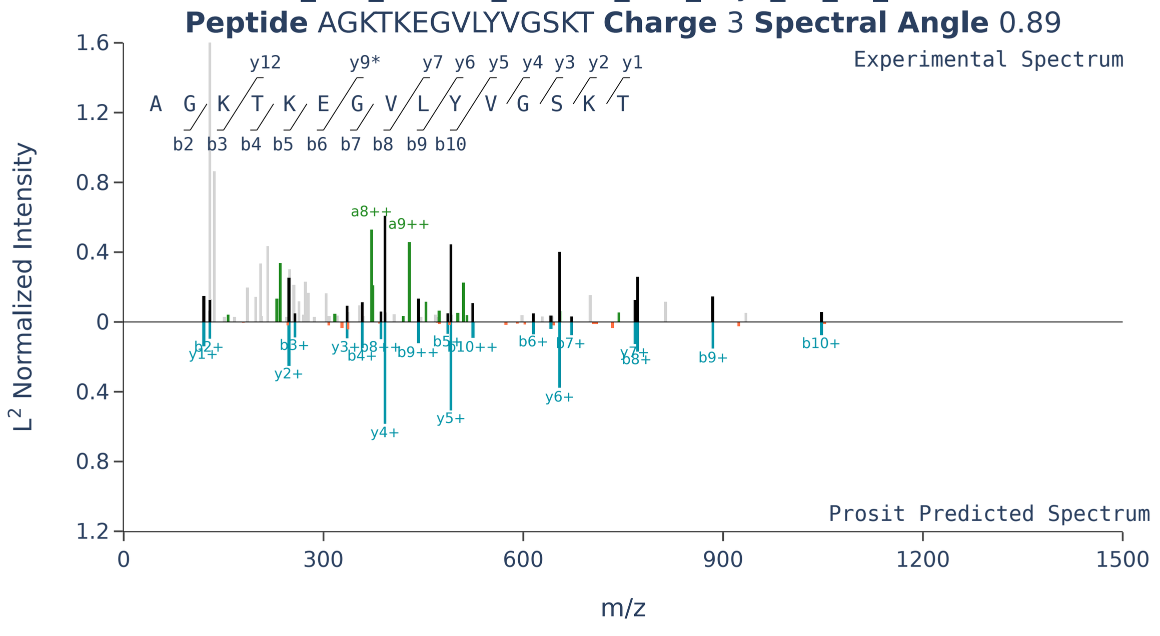

**Source** W_Soh_160821_170921_Goe_aSyn_A1_24h_R1 **Scan** 6542
**Peptide** AGKTKEGVLYVGSKTK **Charge** 4 **Spectral Angle** 0.87

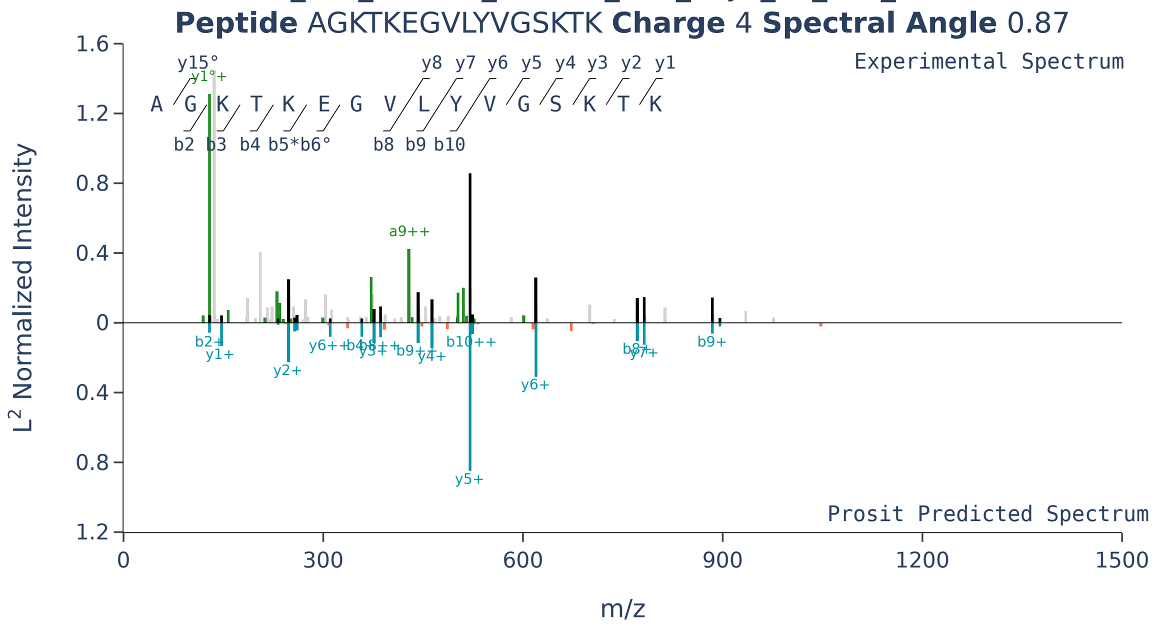


**Source** W_Soh_160821_170921_Goe_aSyn_A2_24h_R1 **Scan** 8066
**Peptide** KTVEGAGSIA **Charge** 2 **Spectral Angle** 0.86

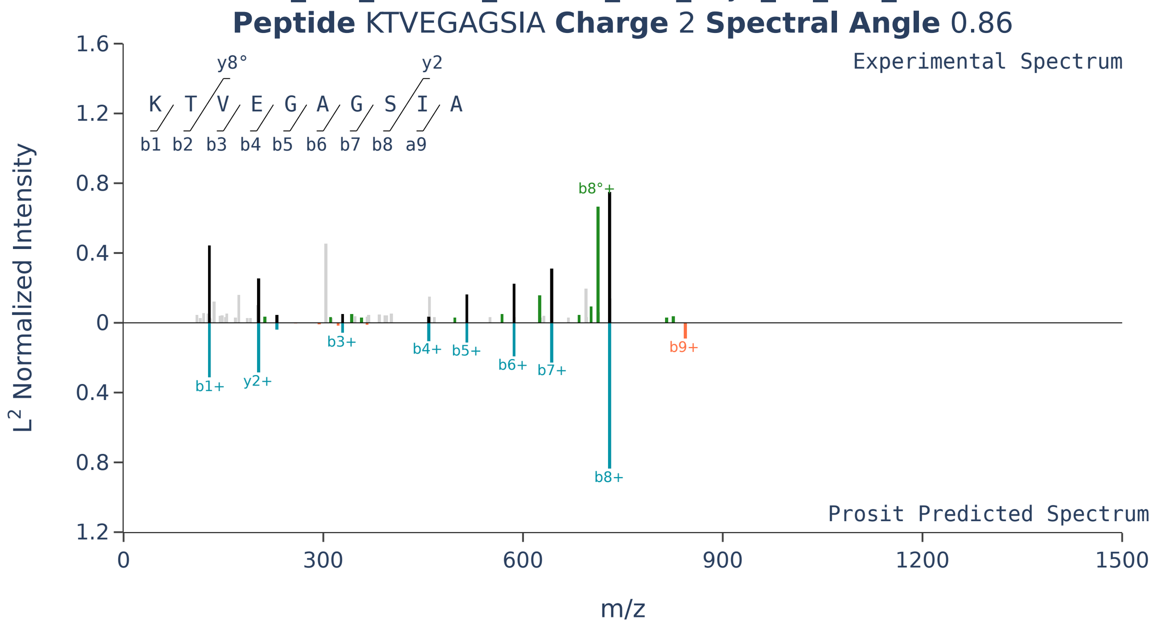


**Source** W_Soh_160821_170921_Goe_aSyn_A1_24h_R1 **Scan** 14866
**Peptide** QKTVEGAGSIAAATGFVKKDQ **Charge** 3 **Spectral Angle** 0.85

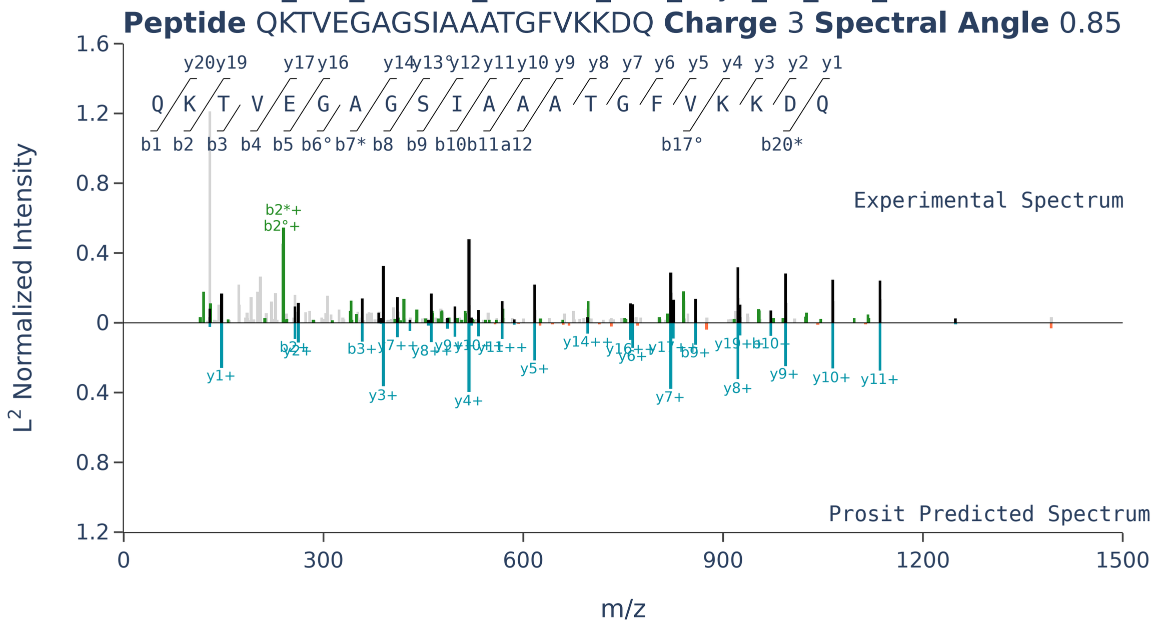


**Source** W_Soh_160821_170921_Goe_aSyn_A2_24h_R1 **Scan** 6765
**Peptide** VVHGVATVAEKTKEQ **Charge** 3 **Spectral Angle** 0.88

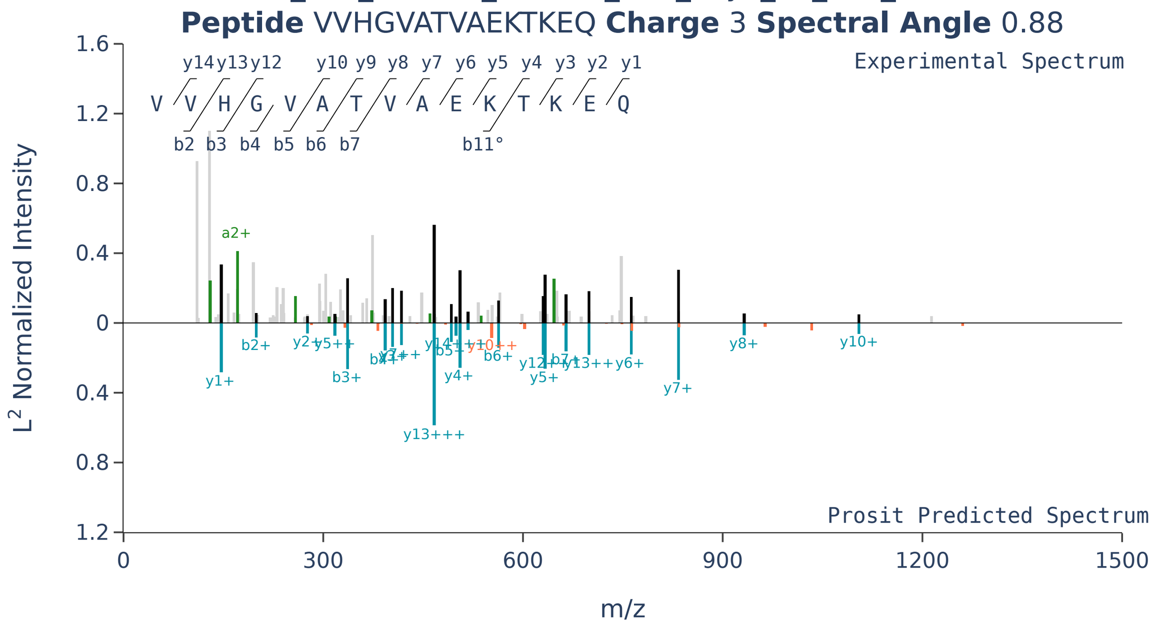


**Source** W_Soh_160821_170921_Goe_aSyn_A2_24h_R2 **Scan** 14579
**Peptide** KTVEGAGSIAAATGFVKKD **Charge** 3 **Spectral Angle** 0.87

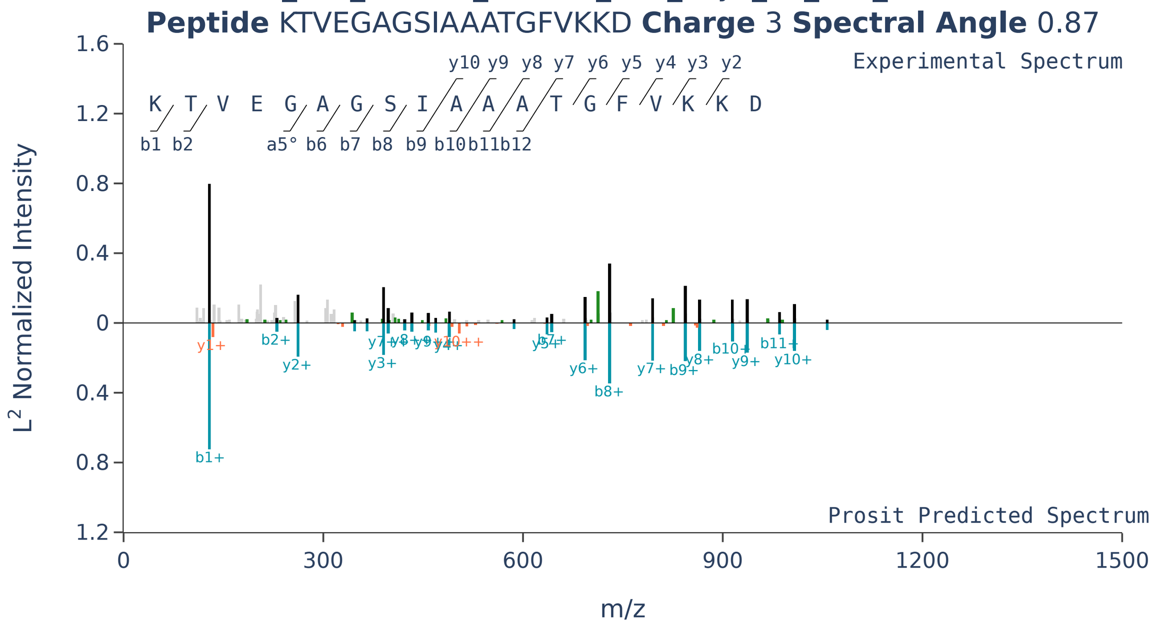


**Source** W_Soh_160821_170921_Goe_aSyn_A1_3h_R2 **Scan** 12490
**Peptide** MDVFMKGLSKAKE **Charge** 3 **Spectral Angle** 0.78

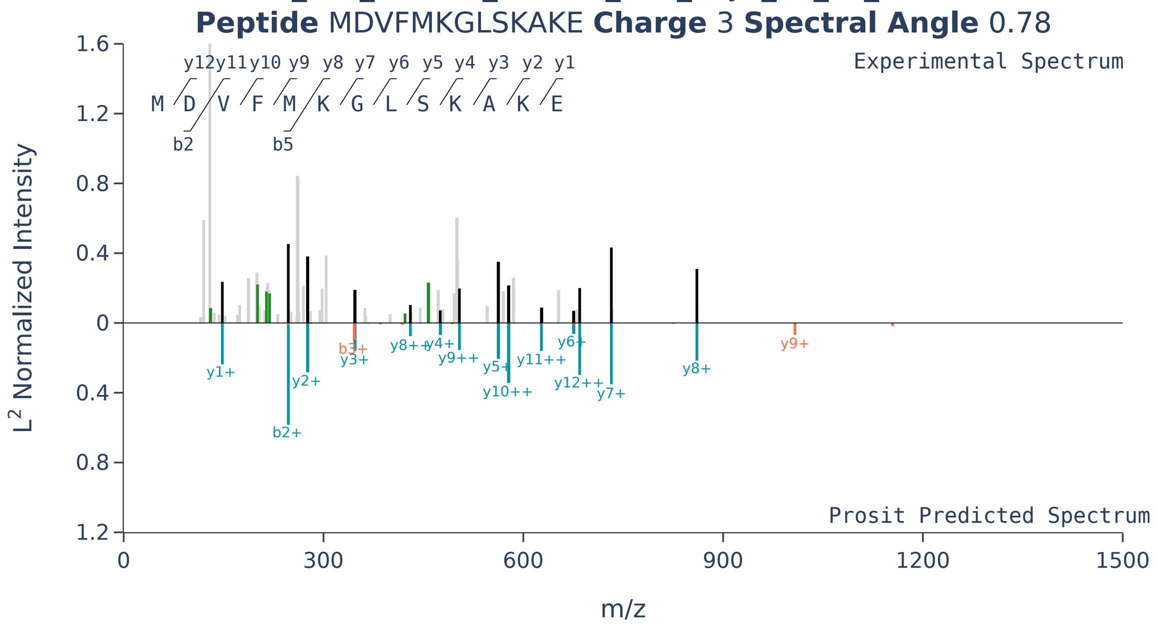


**Source** W_Soh_160821_170921_Goe_aSyn_A1_24h_R2 **Scan** 9596
**Peptide** EGAGSIAAA **Charge** 1 **Spectral Angle** 0.81

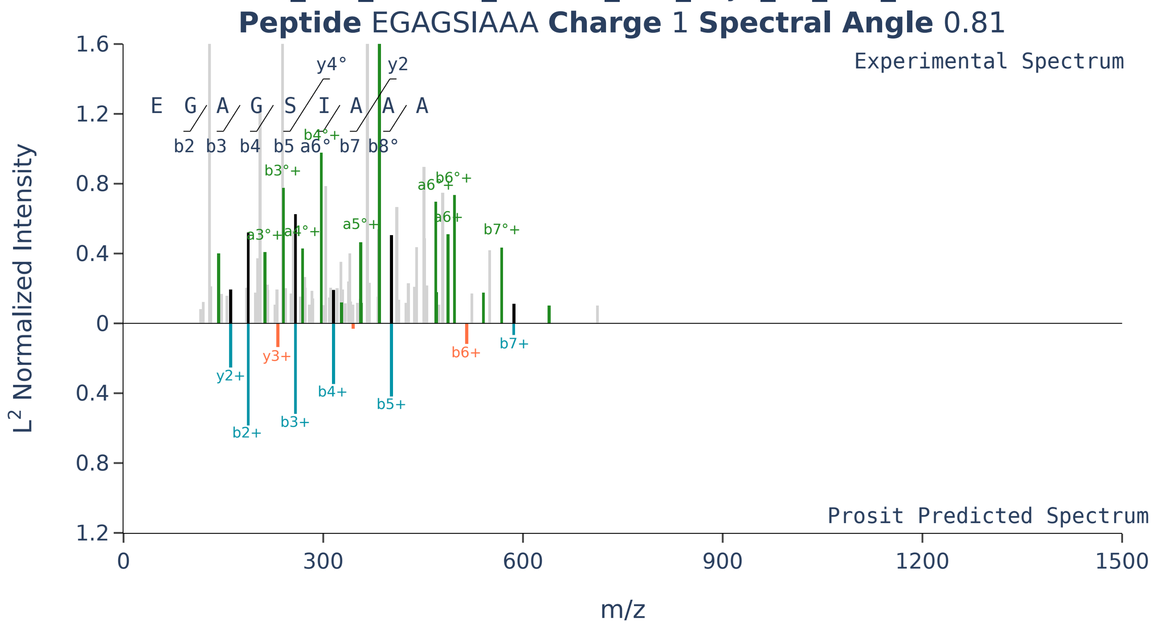


**Source** W_Soh_160821_170921_Goe_aSyn_A2_6h_R1 **Scan** 19566
**Peptide** GVATVAEKTKEQVTNVGGAVVTGV **Charge** 3 **Spectral Angle** 0.88

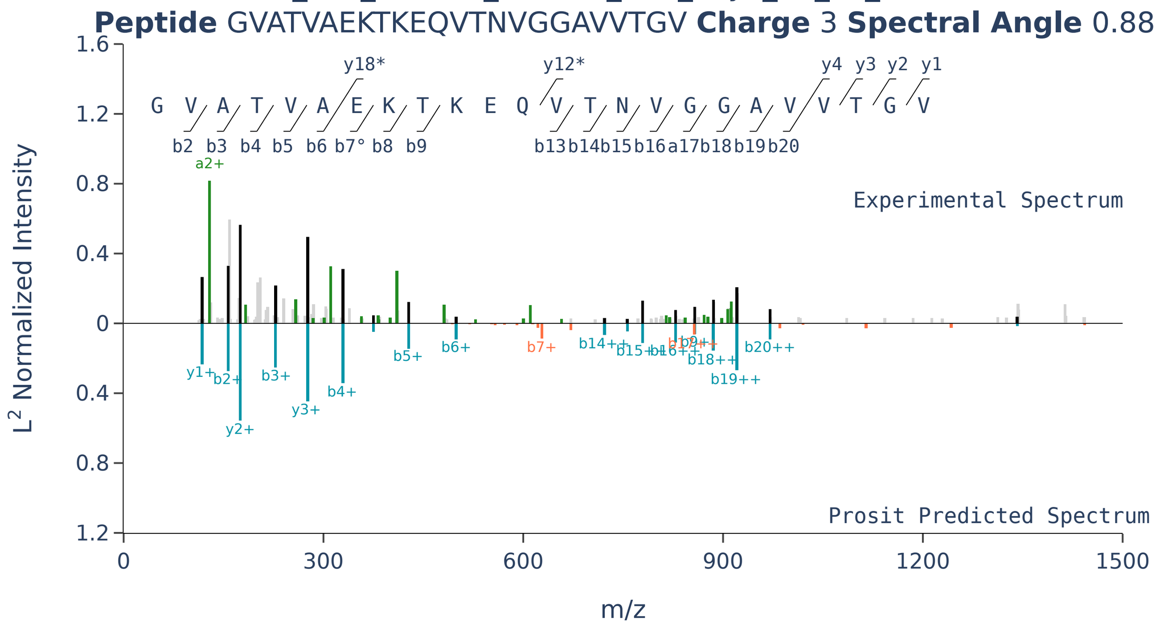


**Source** W_Soh_160821_170921_Goe_aSyn_A1_24h_R2 **Scan** 6852
**Peptide** VVAAAEKTKQGVAEA **Charge** 2 **Spectral Angle** 0.9

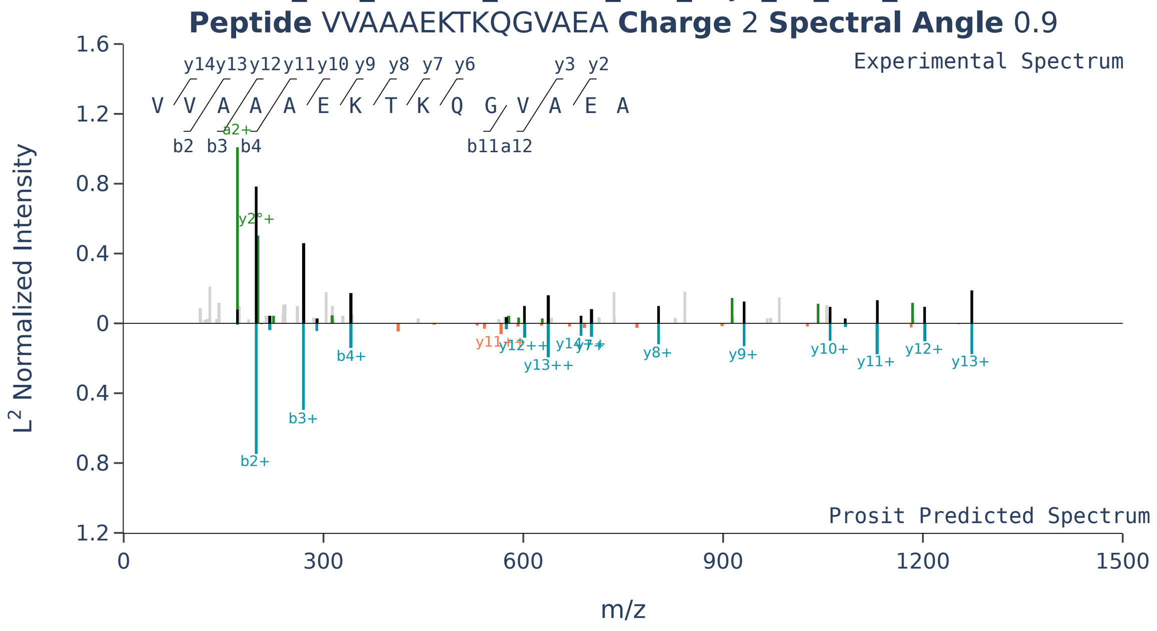


**Source** W_Soh_160821_170921_Goe_aSyn_A1_24h_R1 **Scan** 11651
**Peptide** TKEGVLY **Charge** 2 **Spectral Angle** 0.91

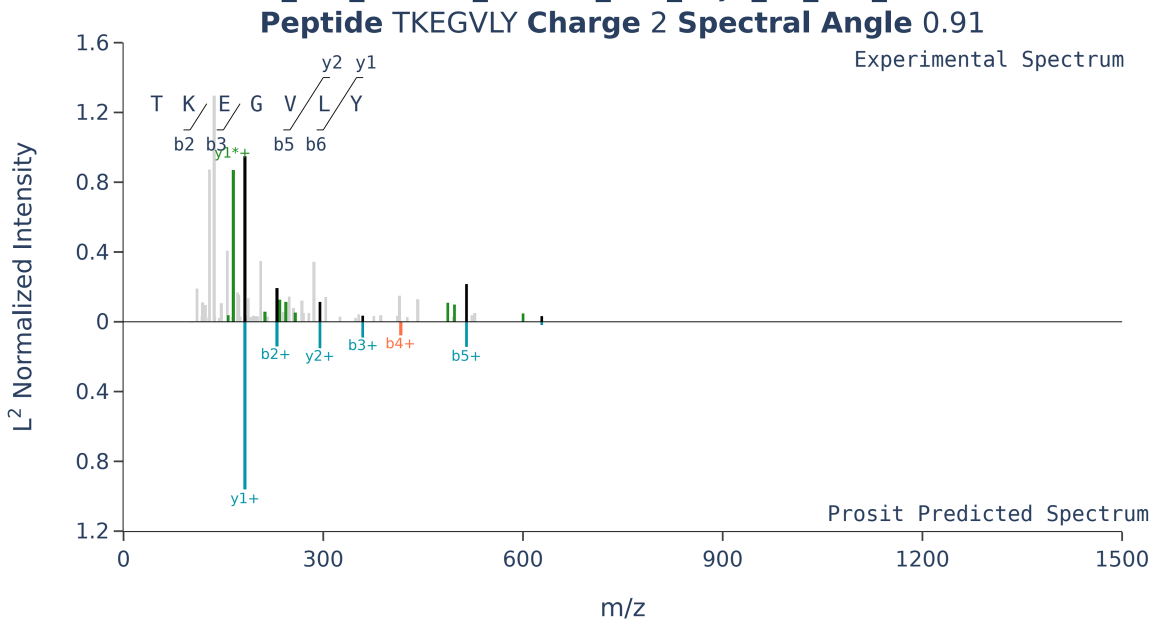


**Source** W_Soh_160821_170921_Goe_aSyn_A1_6h_R1 **Scan** 21566
**Peptide** EDMPVDPDNEAYEMPSE **Charge** 2 **Spectral Angle** 0.95

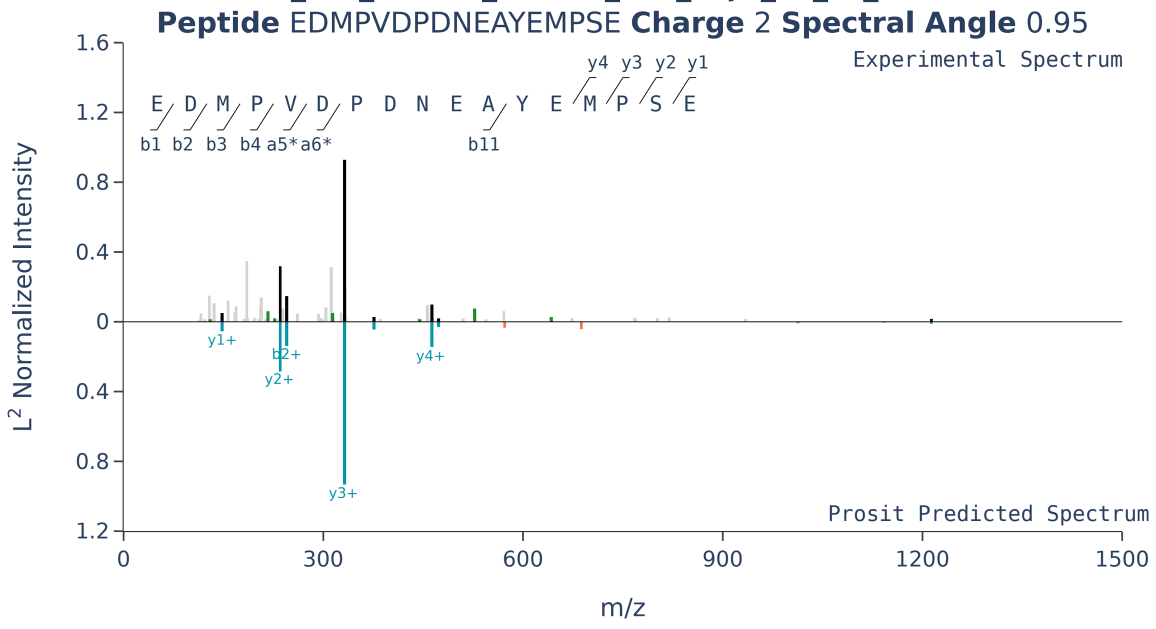


**Source** W_Soh_160821_170921_Goe_aSyn_A1_24h_R2 **Scan** 13499
**Peptide** DMPVDPDNE **Charge** 1 **Spectral Angle** 0.82

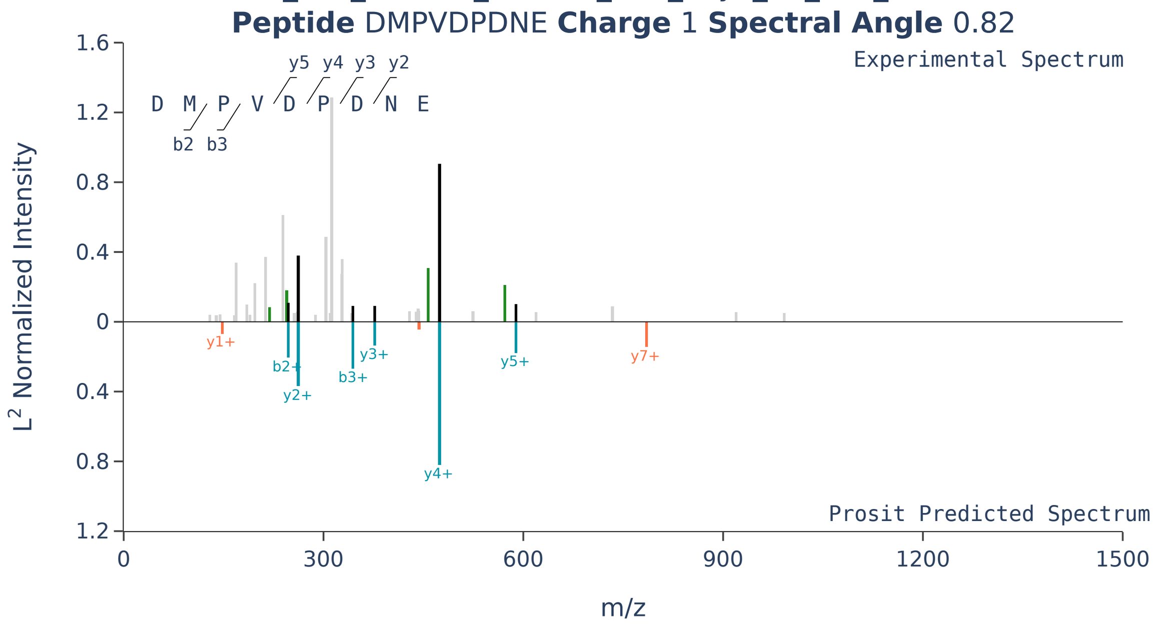


**Source** W_Soh_160821_170921_Goe_aSyn_A1_24h_R1 **Scan** 11039
**Peptide** AAATGFVKKDQL **Charge** 2 **Spectral Angle** 0.86

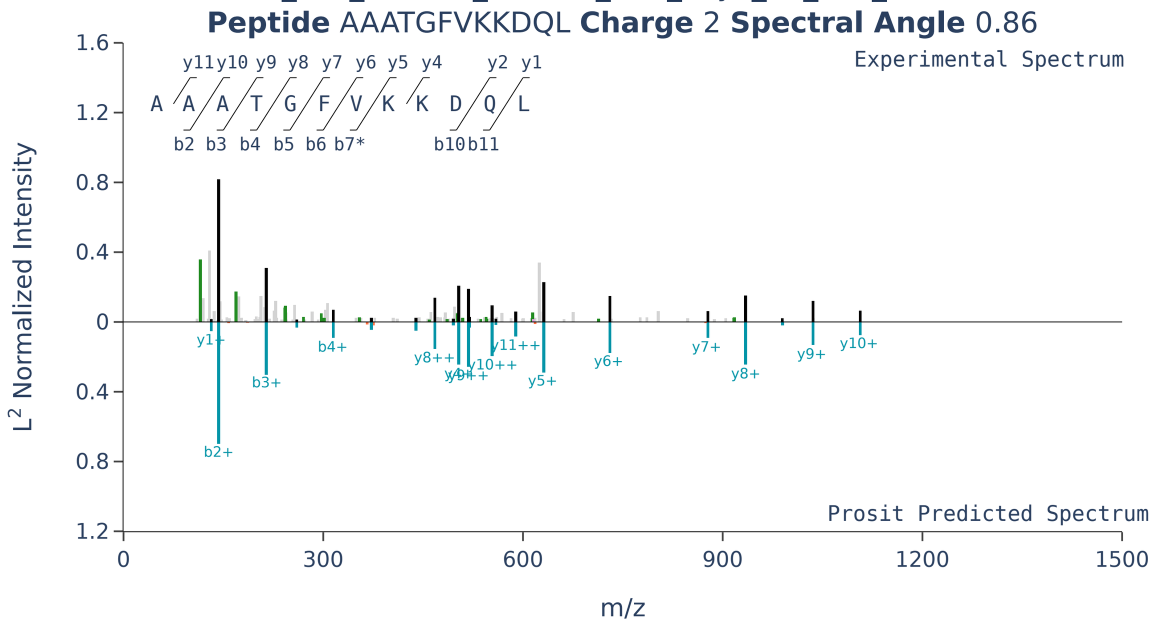


**Source** W_Soh_160821_170921_Goe_aSyn_A2_6h_R1 **Scan** 14060
**Peptide** AEKTKEQVTNVGGAVVTGVTA **Charge** 3 **Spectral Angle** 0.92

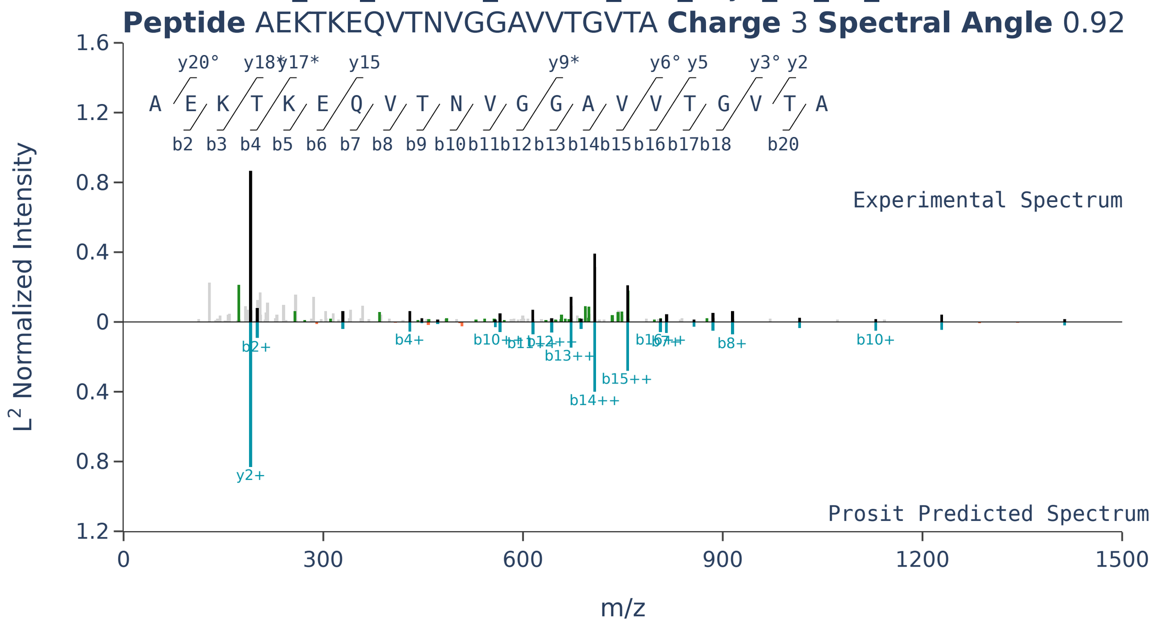


**Source** W_Soh_160821_170921_Goe_aSyn_A1_24h_R1 **Scan** 13618
**Peptide** GVLYVGSKTKEGVVHGVATVAEK **Charge** 4 **Spectral Angle** 0.82

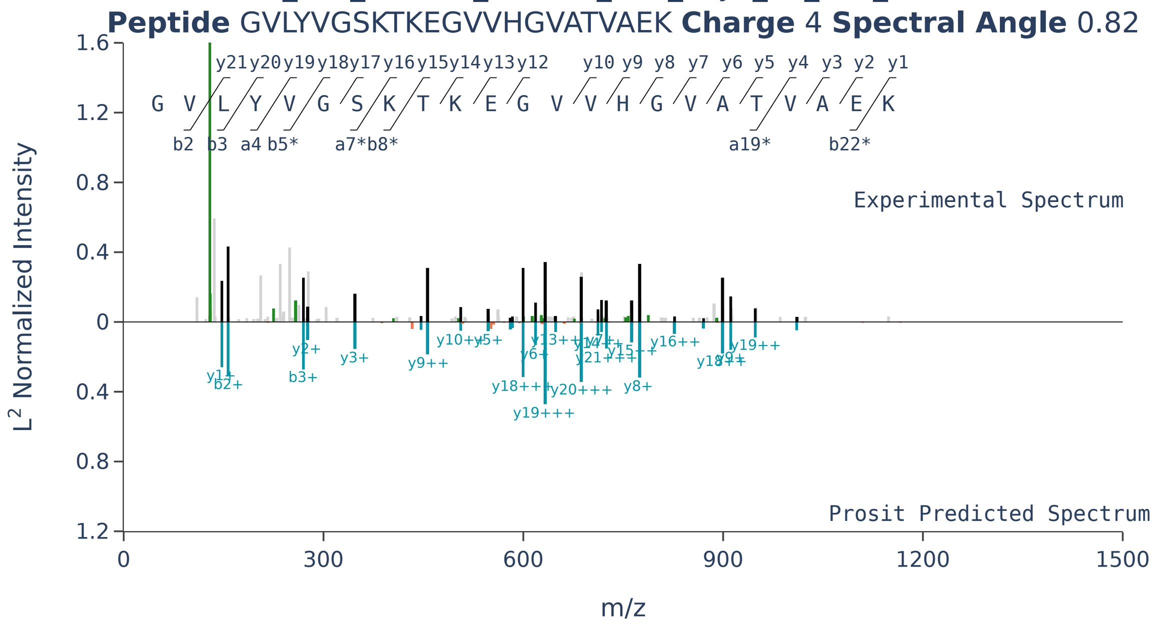

**Source** W_Soh_160821_170921_Goe_aSyn_A1_24h_R2 **Scan** 6278
**Peptide** MKGLSKAKEGVV **Charge** 2 **Spectral Angle** 0.81

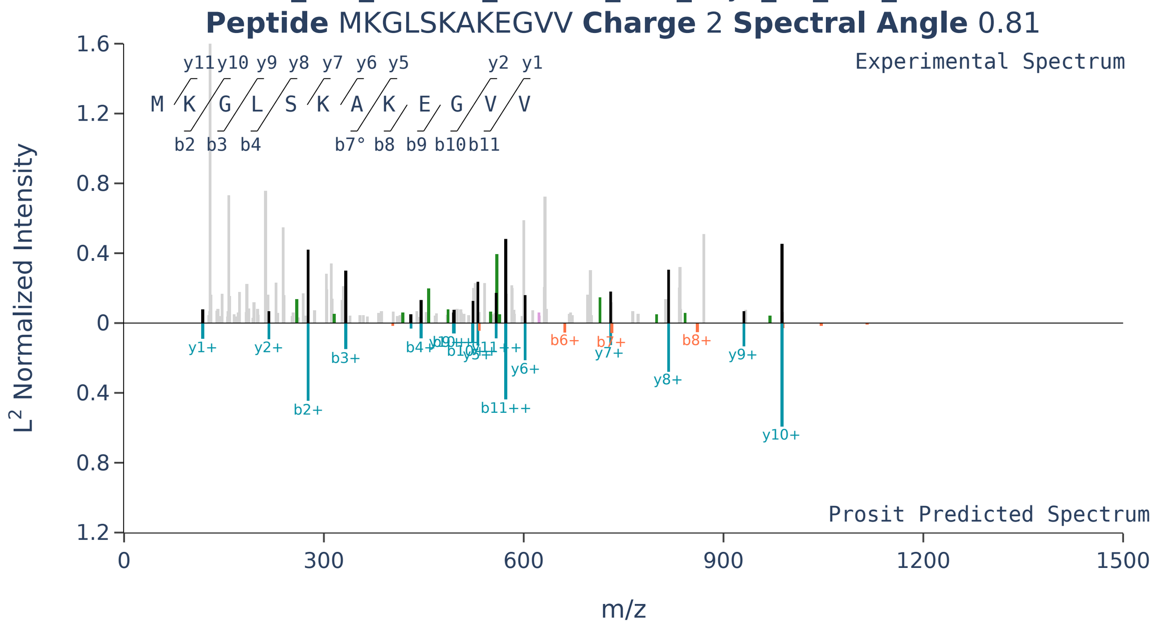


**Source** W_Soh_160821_170921_Goe_aSyn_A2_24h_R2 **Scan** 17921
**Peptide** DMPVDPDNEAYE **Charge** 2 **Spectral Angle** 0.92

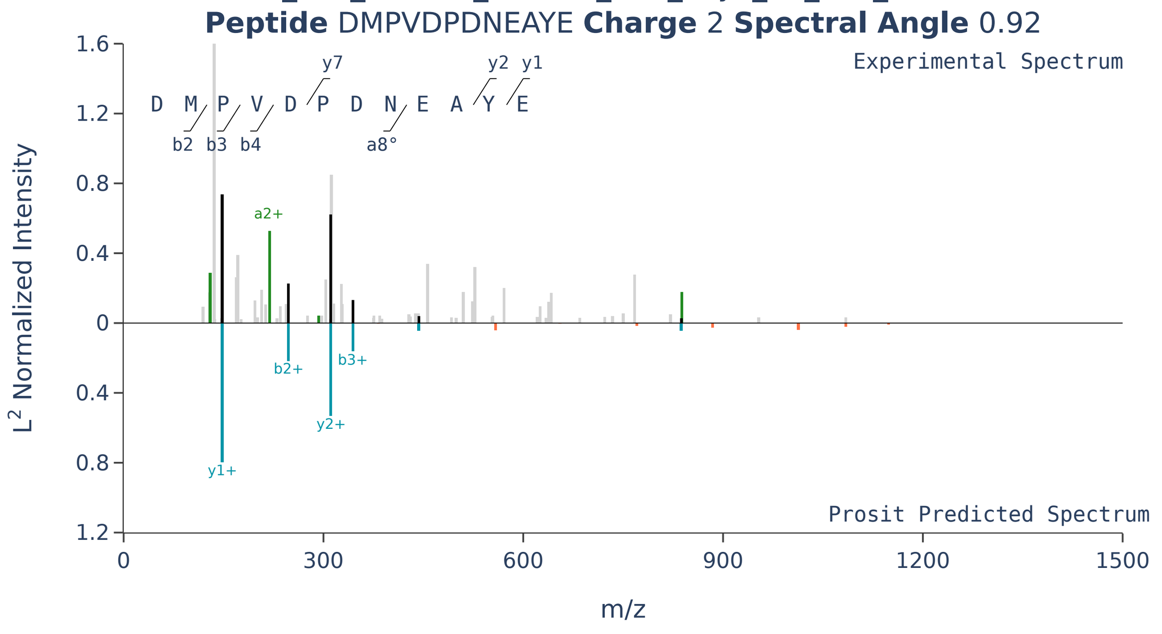


**Source** W_Soh_160821_170921_Goe_aSyn_A2_24h_R2 **Scan** 8761
**Peptide** AQKTVEGAGSL **Charge** 1 **Spectral Angle** 0.88

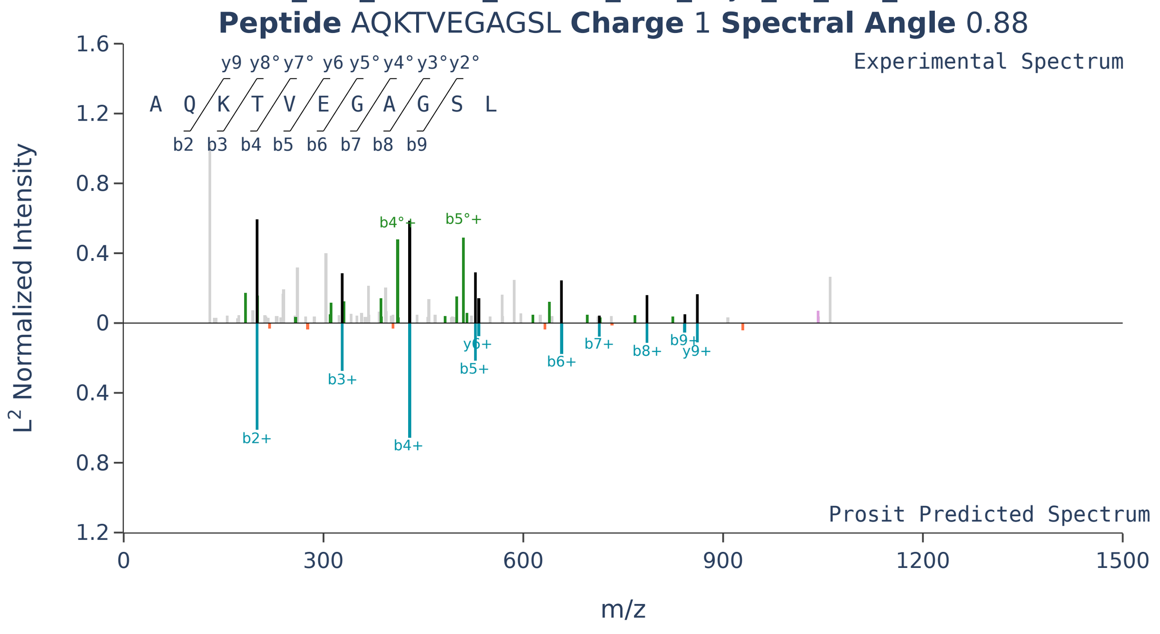


**Source** W_Soh_160821_170921_Goe_aSyn_A2_24h_R2 **Scan** 18082
**Peptide** AQKTVEGAGSIAAATGFVKKDQL **Charge** 3 **Spectral Angle** 0.84

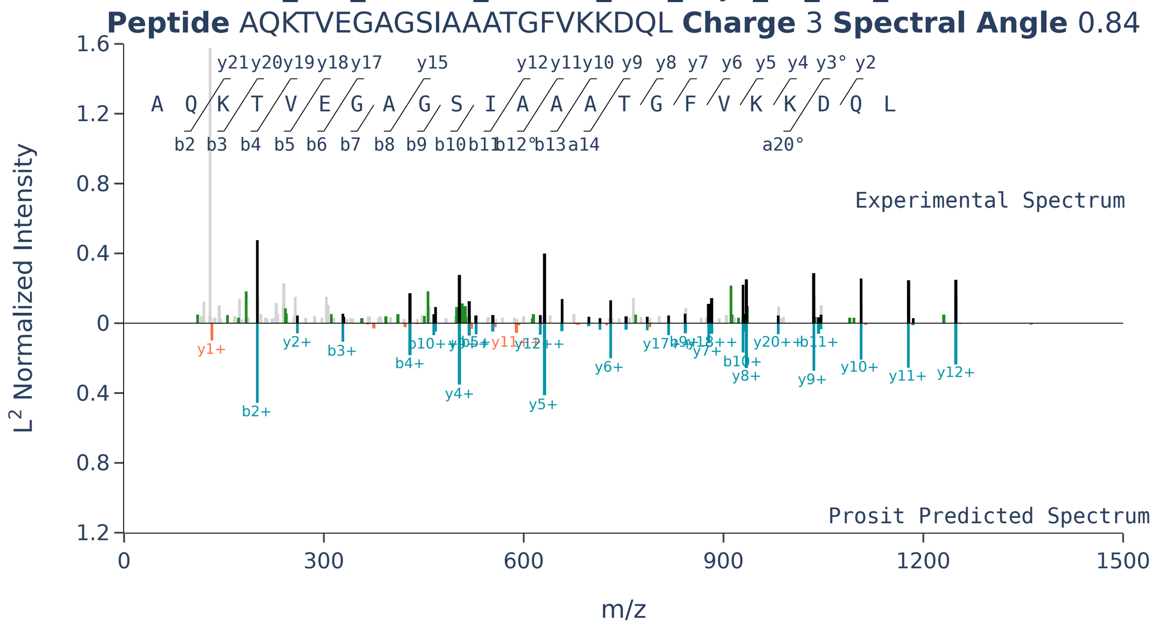


**Source** W_Soh_160821_170921_Goe_aSyn_A1_6h_R1 **Scan** 12281
**Peptide** GGAVVTGVT **Charge** 1 **Spectral Angle** 0.84

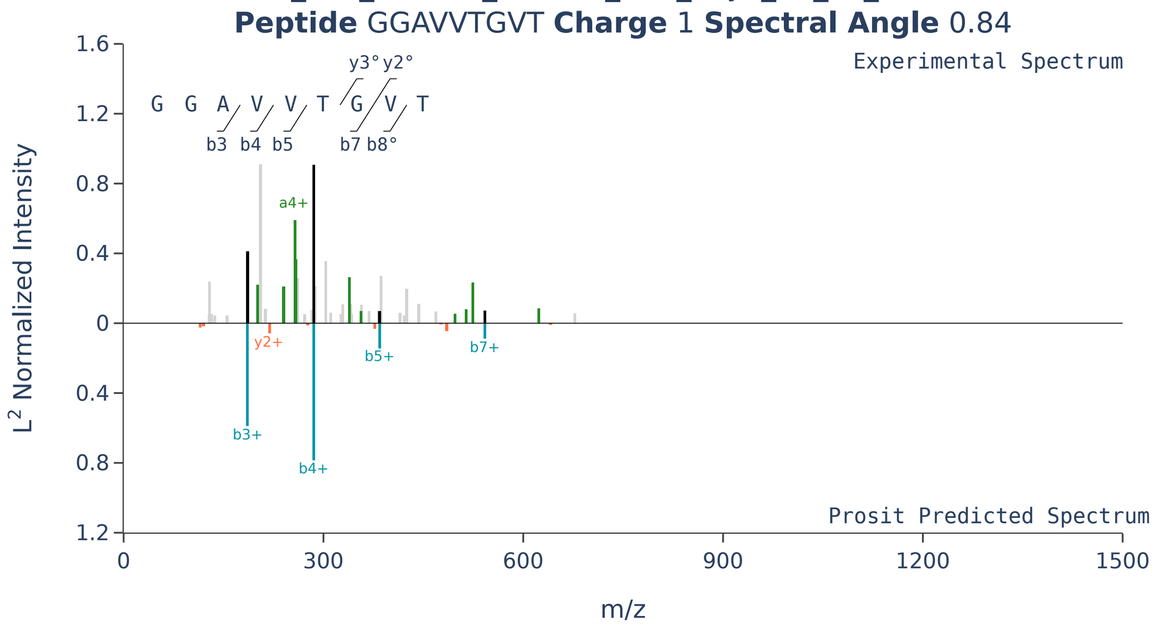


**Source** W_Soh_160821_170921_Goe_aSyn_A1_6h_R2 **Scan** 21962
**Peptide** DMPVDPDNEAYEMPSE **Charge** 2 **Spectral Angle** 0.92

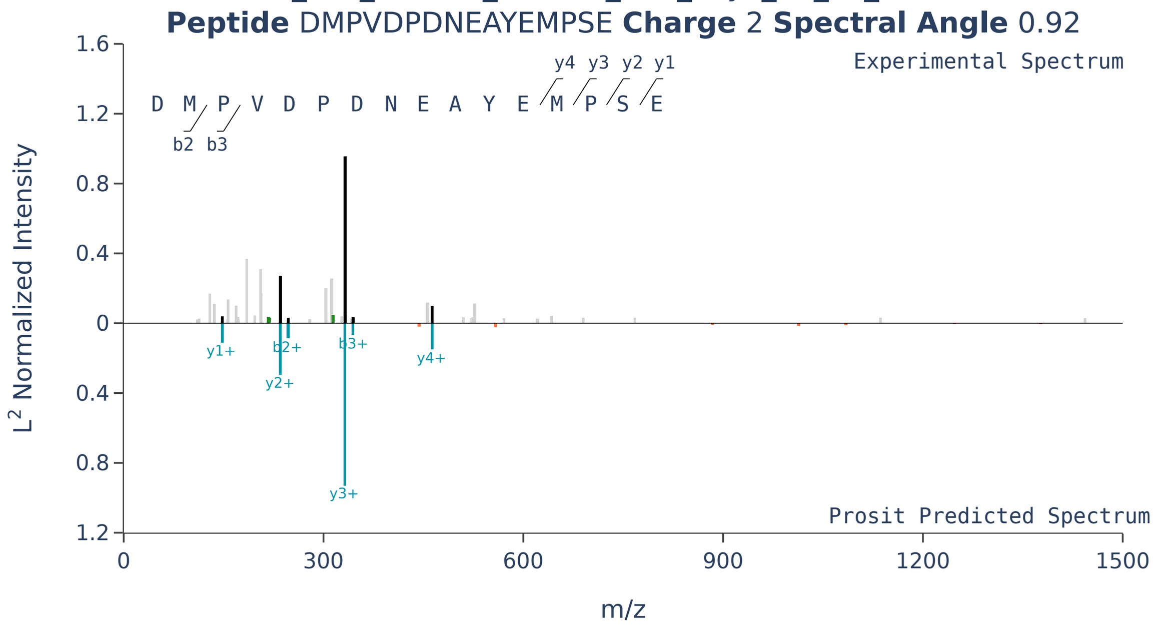


**Source** W_Soh_160821_170921_Goe_aSyn_A1_24h_R2 **Scan** 15355
**Peptide** AYEMPSEEGYQD **Charge** 2 **Spectral Angle** 0.95

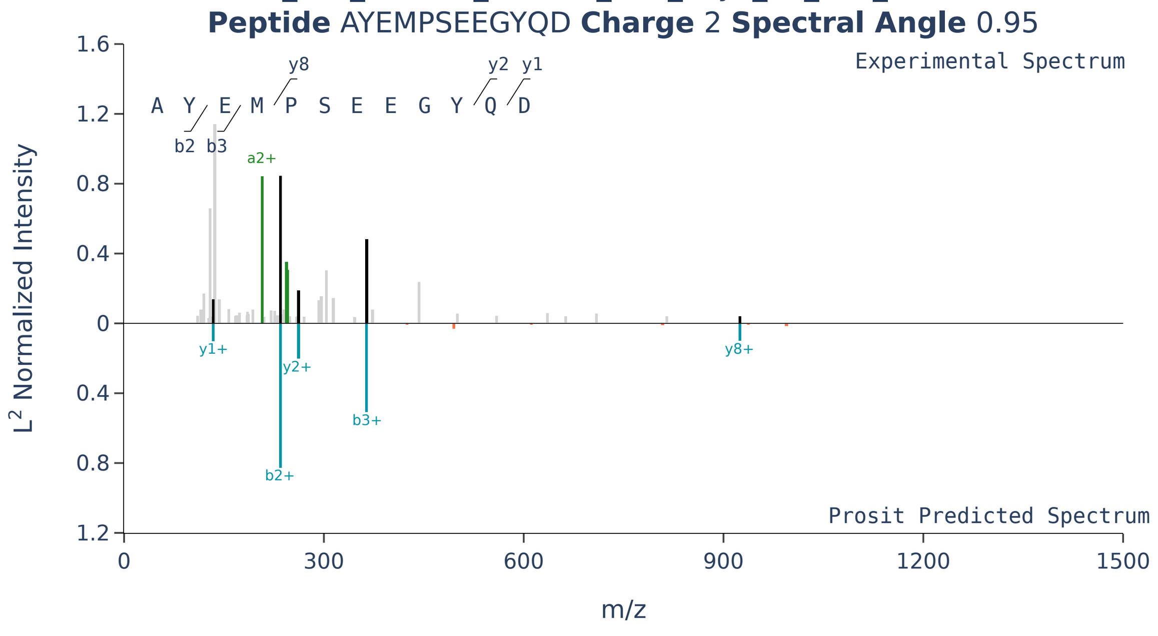


**Source** W_Soh_160821_170921_Goe_aSyn_A1_24h_R2 **Scan** 9295
**Peptide** VLYVGSKTKEGVVHG **Charge** 2 **Spectral Angle** 0.89

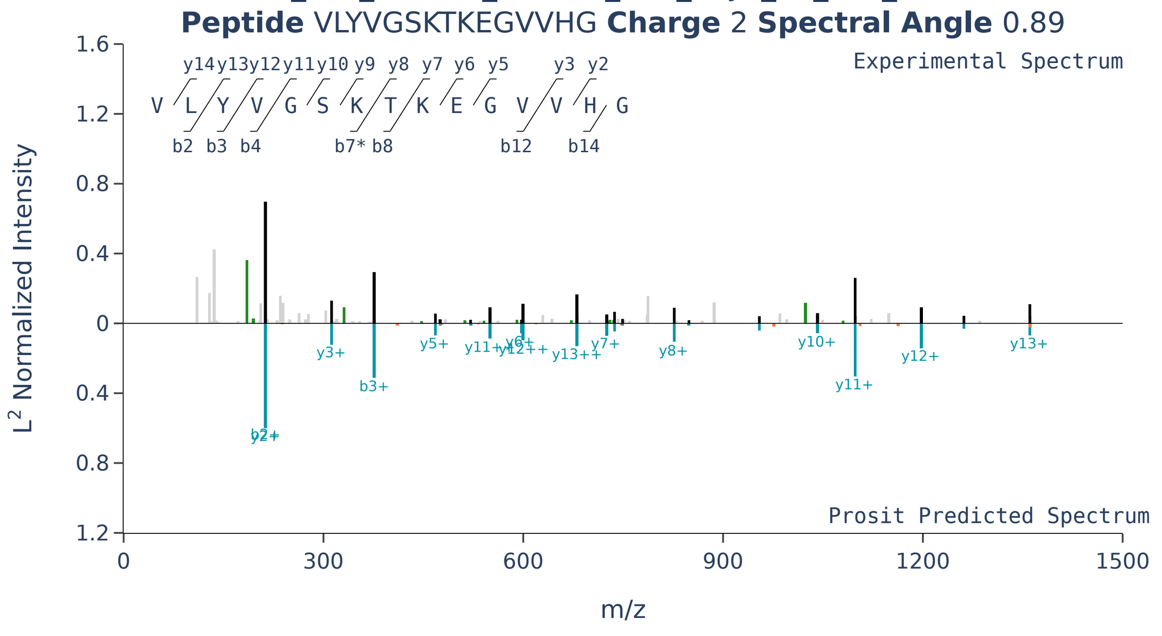


**Source** W_Soh_160821_170921_Goe_aSyn_A1_24h_R1 **Scan** 6347
**Peptide** LSKAKEGVV **Charge** 2 **Spectral Angle** 0.83

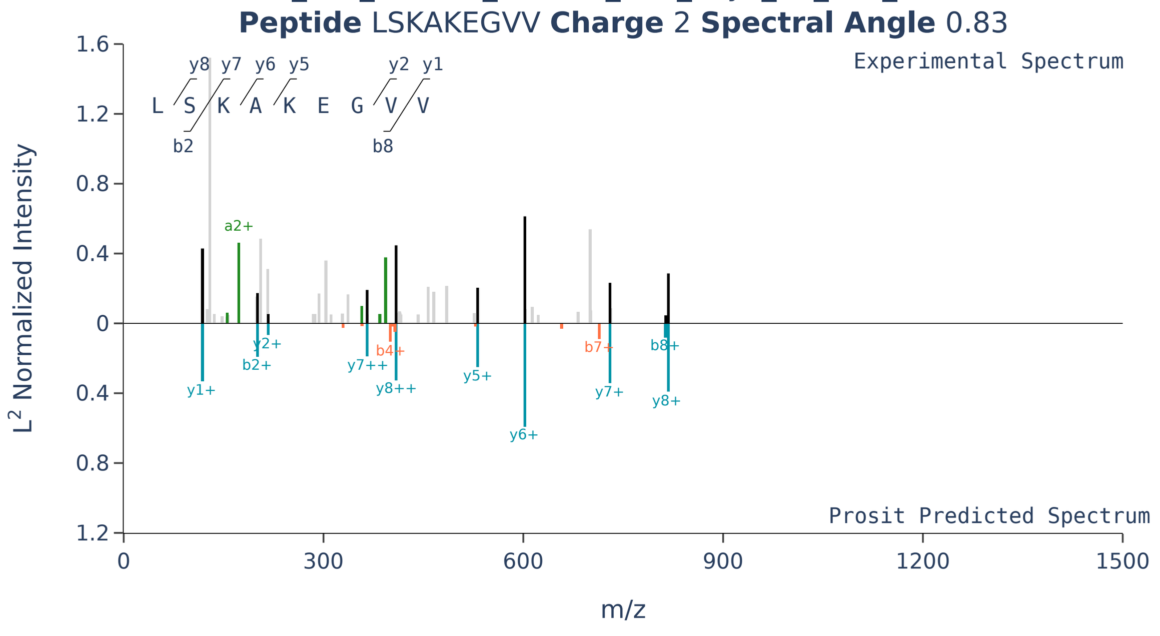


**Source** W_Soh_160821_170921_Goe_aSyn_A2_3h_R2 **Scan** 16353
**Peptide** GVATVAEKTKEQVTNVGGAVV **Charge** 3 **Spectral Angle** 0.81

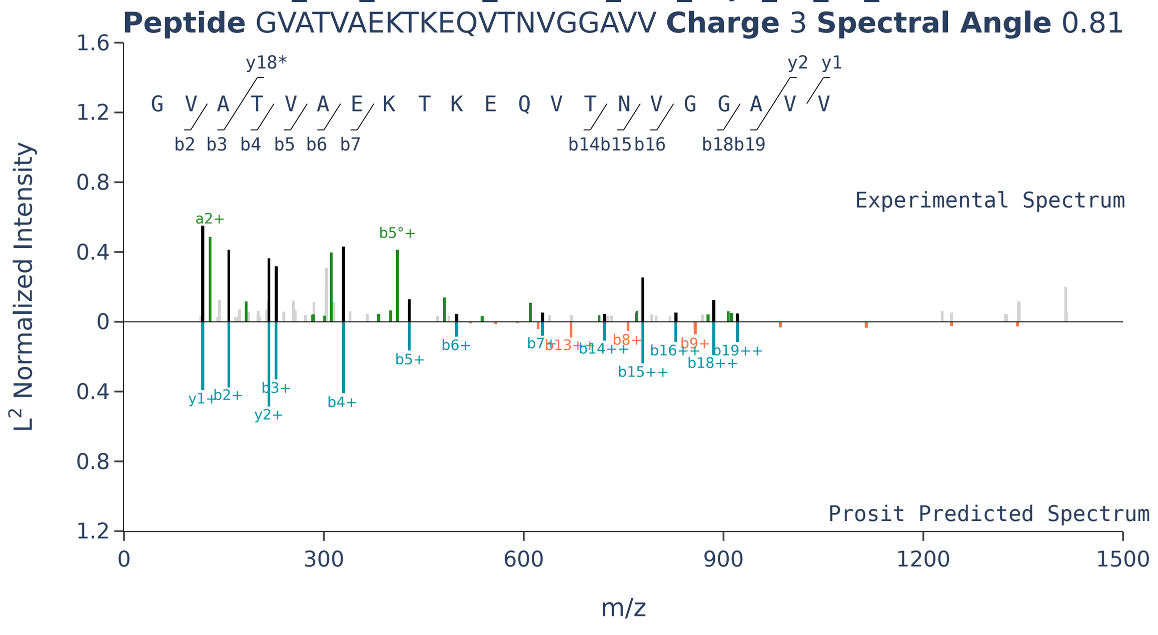


**Source** W_Soh_160821_170921_Goe_aSyn_A2_24h_R1 **Scan** 8278
**Peptide** TGFVKKDQLGKNEEGAPQE **Charge** 3 **Spectral Angle** 0.89

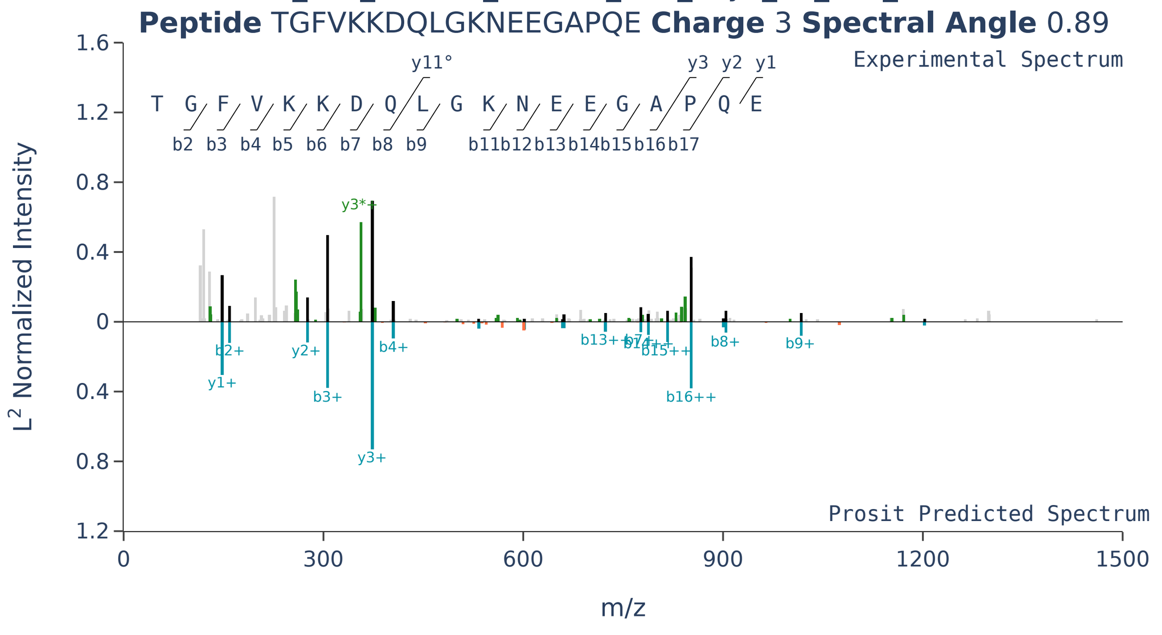


**Source** W_Soh_160821_170921_Goe_aSyn_A1_6h_R1 **Scan** 21388
**Peptide** GILEDMPVDPDNEAYE **Charge** 2 **Spectral Angle** 0.85

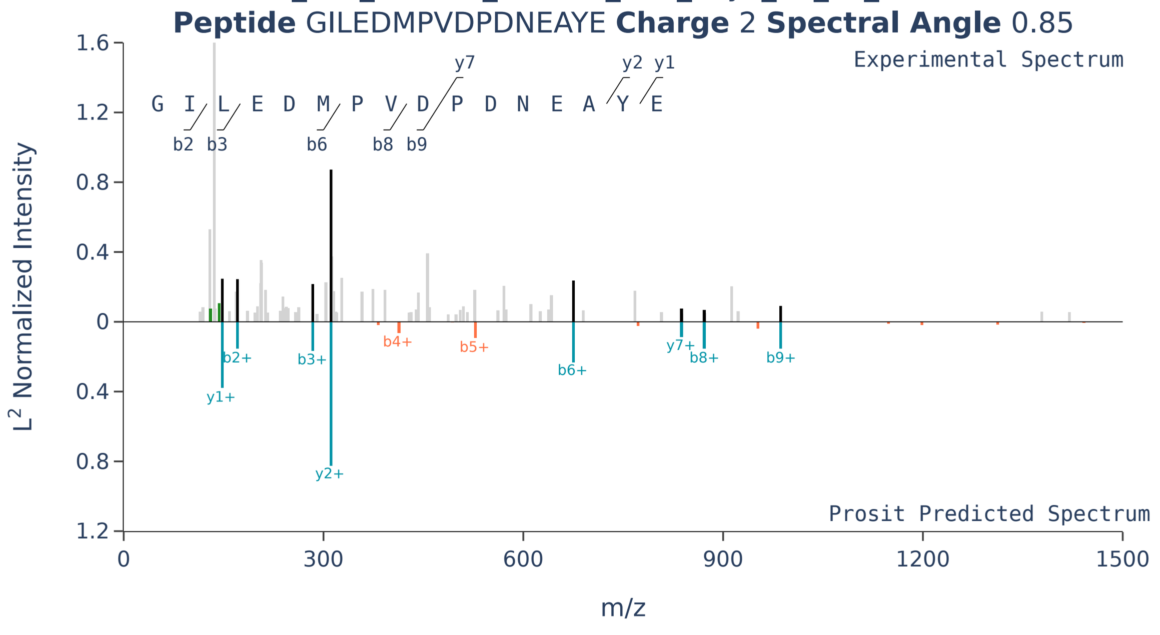


**Source** W_Soh_160821_170921_Goe_aSyn_A1_6h_R1 **Scan** 7377
**Peptide** KEGVVAAAEKTKQGVAE **Charge** 4 **Spectral Angle** 0.85

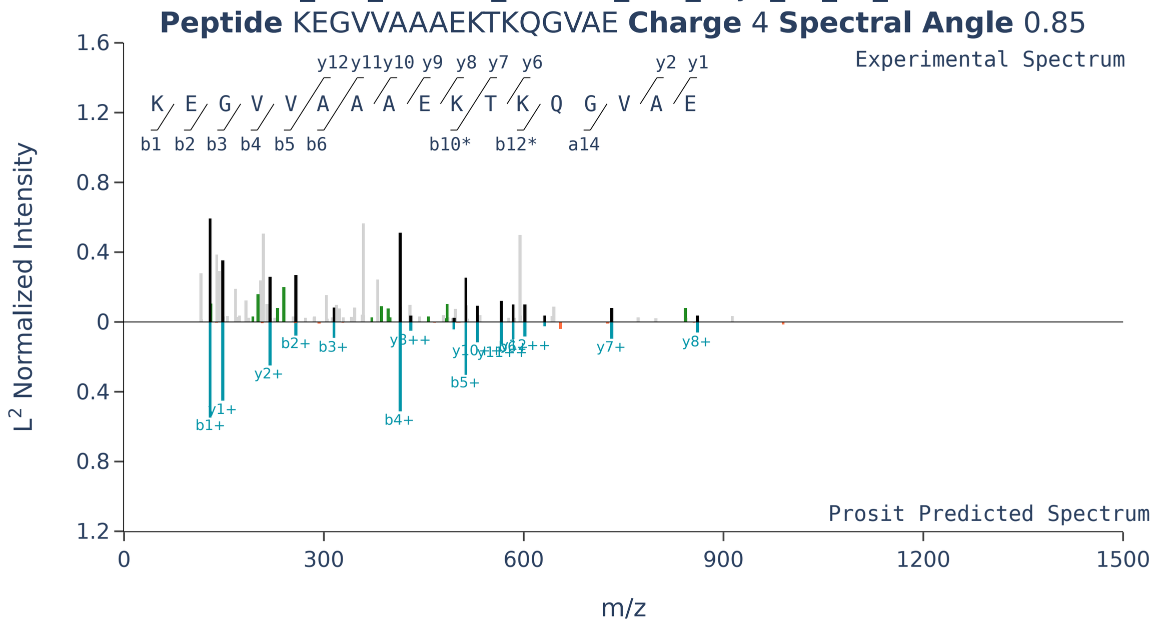


**Source** W_Soh_160821_170921_Goe_aSyn_A2_24h_R1 **Scan** 6552
**Peptide** FMKGLSKAKEG **Charge** 3 **Spectral Angle** 0.79

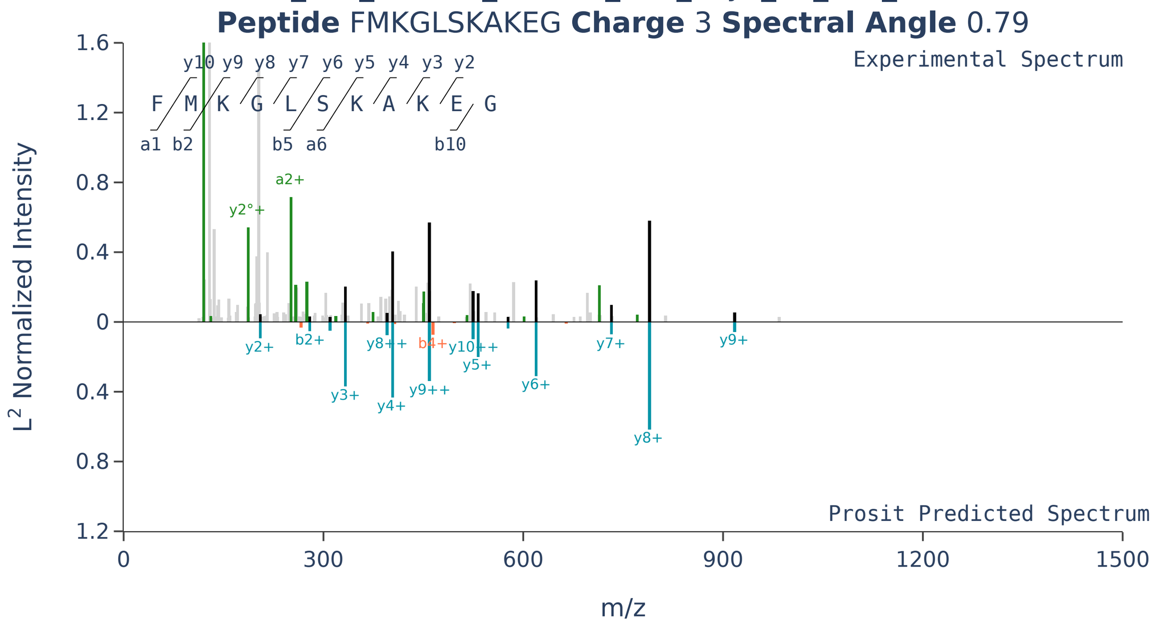


**Source** W_Soh_160821_170921_Goe_aSyn_A1_6h_R1 **Scan** 17966
**Peptide** EKTKEQVTNVGGAVVTGVTAV **Charge** 3 **Spectral Angle** 0.84

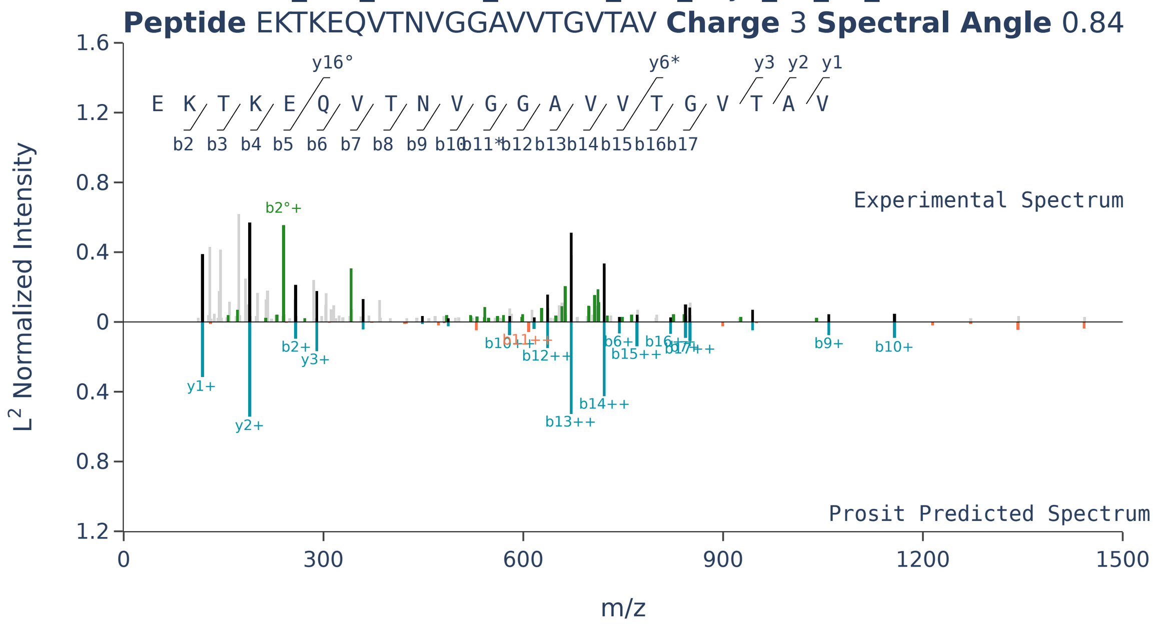

**Source** W_Soh_160821_170921_Goe_aSyn_A1_24h_R2 **Scan** 15495
**Peptide** GVLYVGSKTKEGVVHGVATVAE **Charge** 3 **Spectral Angle** 0.85

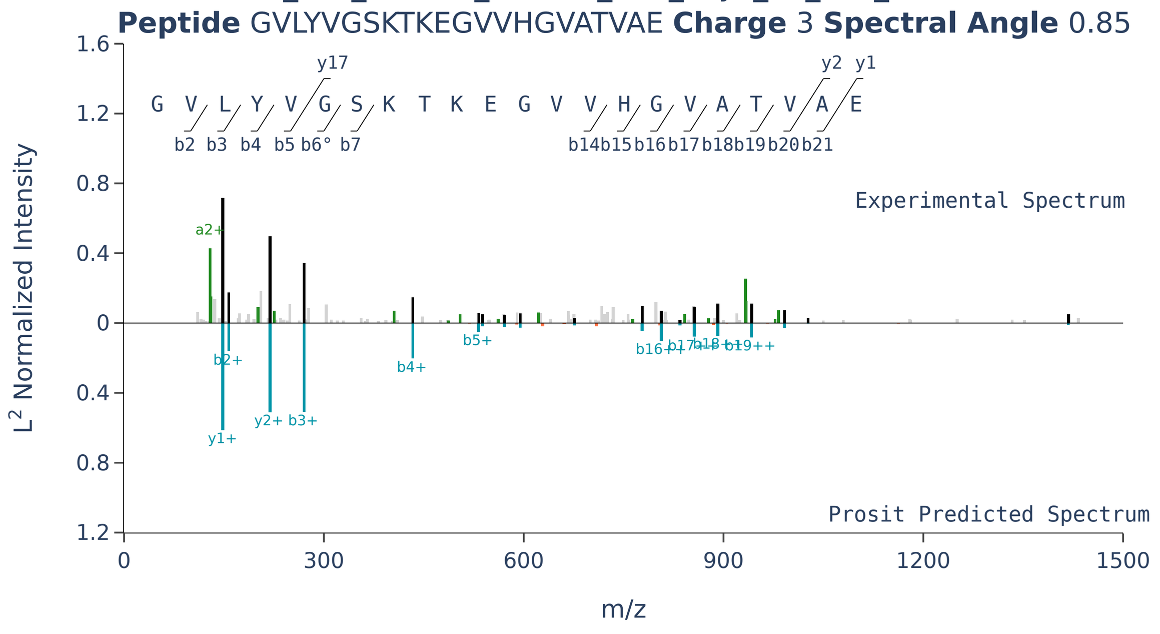


**Source** W_Soh_160821_170921_Goe_aSyn_A1_6h_R2 **Scan** 8318
**Peptide** KEGVVHGVATVAEKT **Charge** 3 **Spectral Angle** 0.92

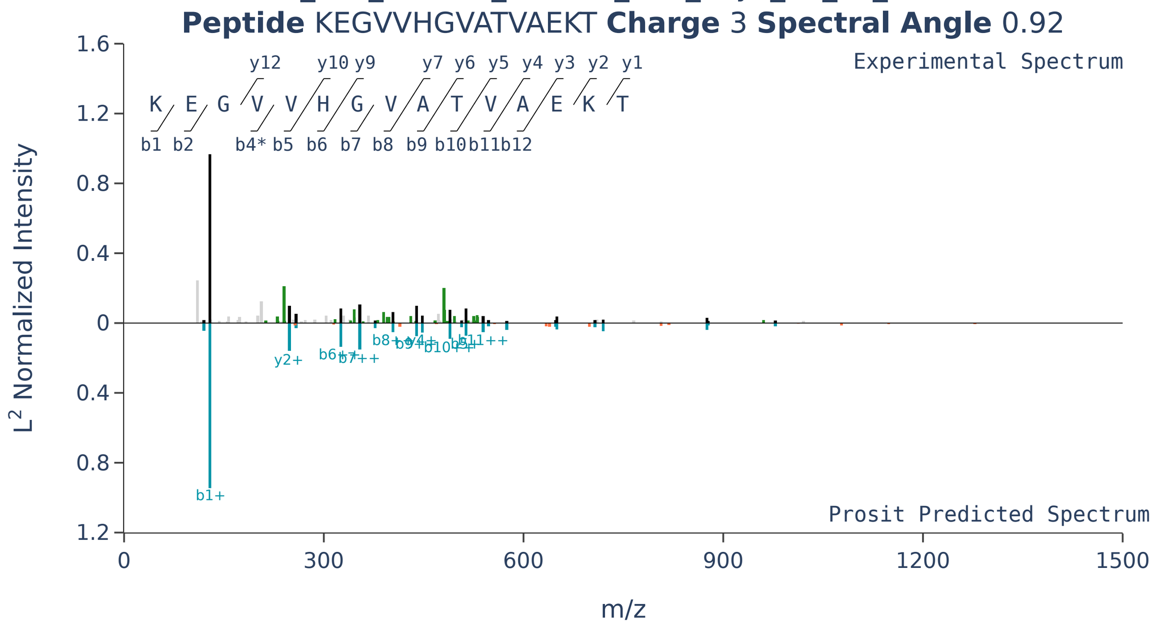


**Source** W_Soh_160821_170921_Goe_aSyn_A2_24h_R2 **Scan** 15592
**Peptide** VAQKTVEGAGSIAAATGFVKKDQ **Charge** 3 **Spectral Angle** 0.81

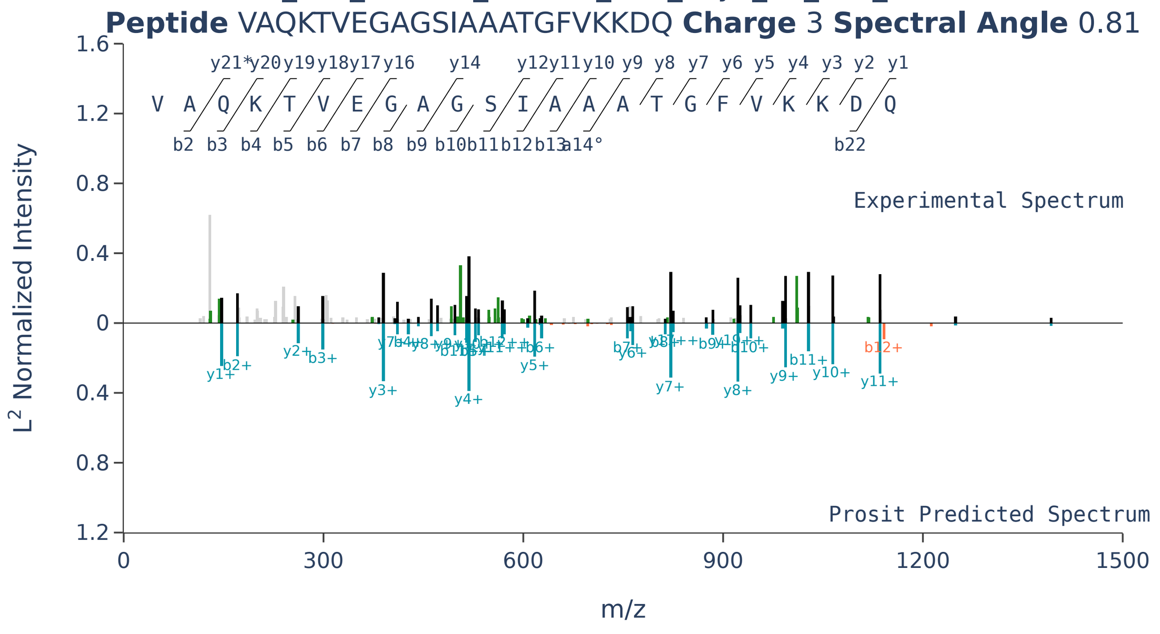


**Source** W_Soh_160821_170921_Goe_aSyn_A2_24h_R1 **Scan** 7804
**Peptide** AGKTKEGVLYVGSKTKEGVVH **Charge** 5 **Spectral Angle** 0.92

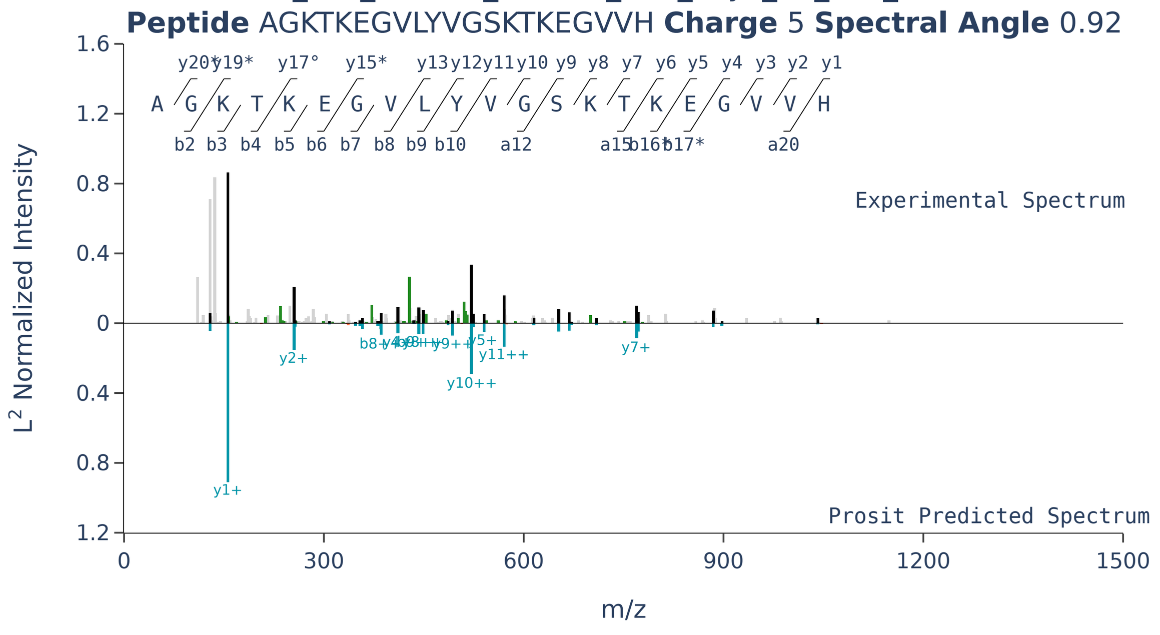


**Source** W_Soh_160821_170921_Goe_aSyn_A1_6h_R2 **Scan** 7152
**Peptide** GFVKKDQLGKNEE **Charge** 3 **Spectral Angle** 0.81

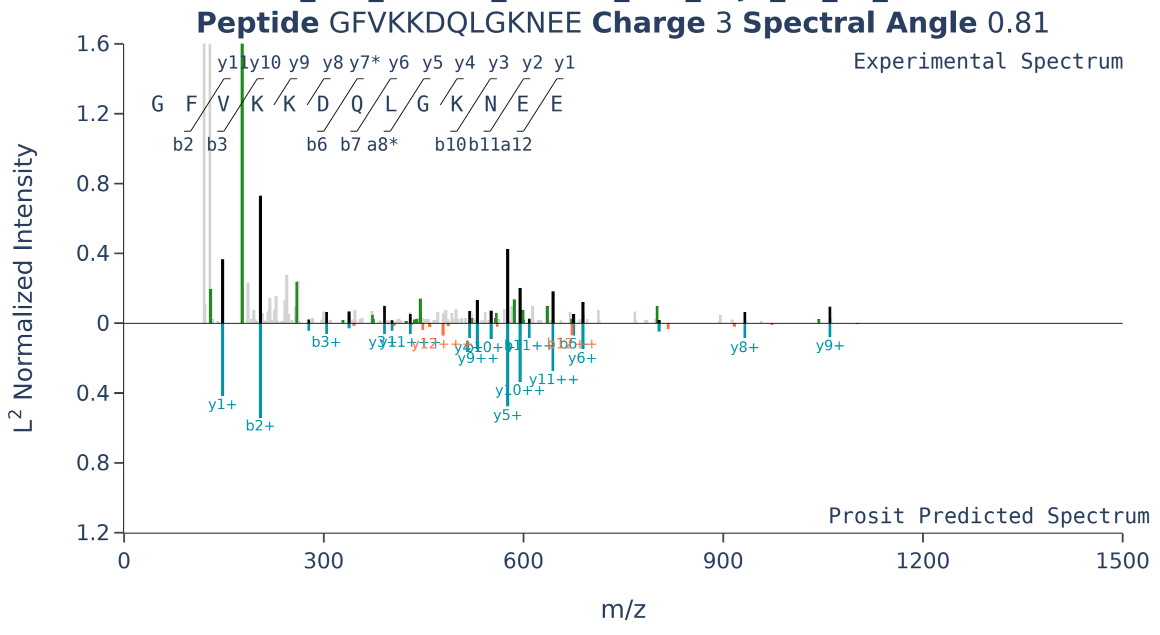


**Source** W_Soh_160821_170921_Goe_aSyn_A2_24h_R2 **Scan** 15731
**Peptide** EGAGSIAAATGFVKKD **Charge** 3 **Spectral Angle** 0.77

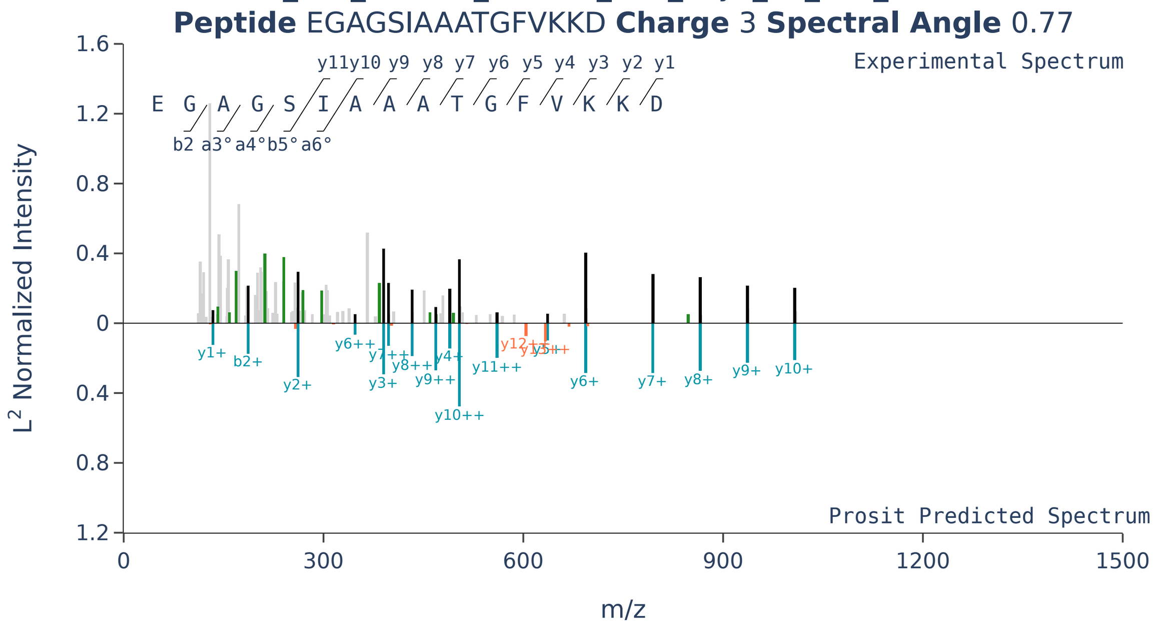


**Source** W_Soh_160821_170921_Goe_aSyn_A2_24h_R1 **Scan** 5848
**Peptide** AKEGVVAAAEKTKQ **Charge** 3 **Spectral Angle** 0.77

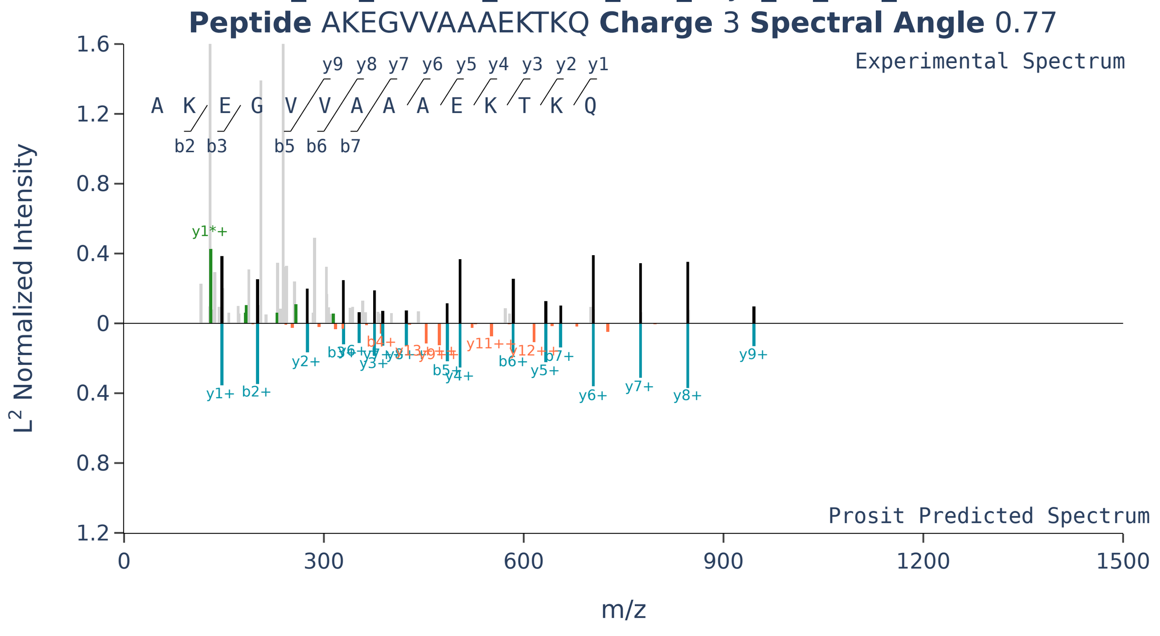


**Source** W_Soh_160821_170921_Goe_aSyn_A2_24h_R2 **Scan** 11896
**Peptide** MPVDPDNEA **Charge** 2 **Spectral Angle** 0.88

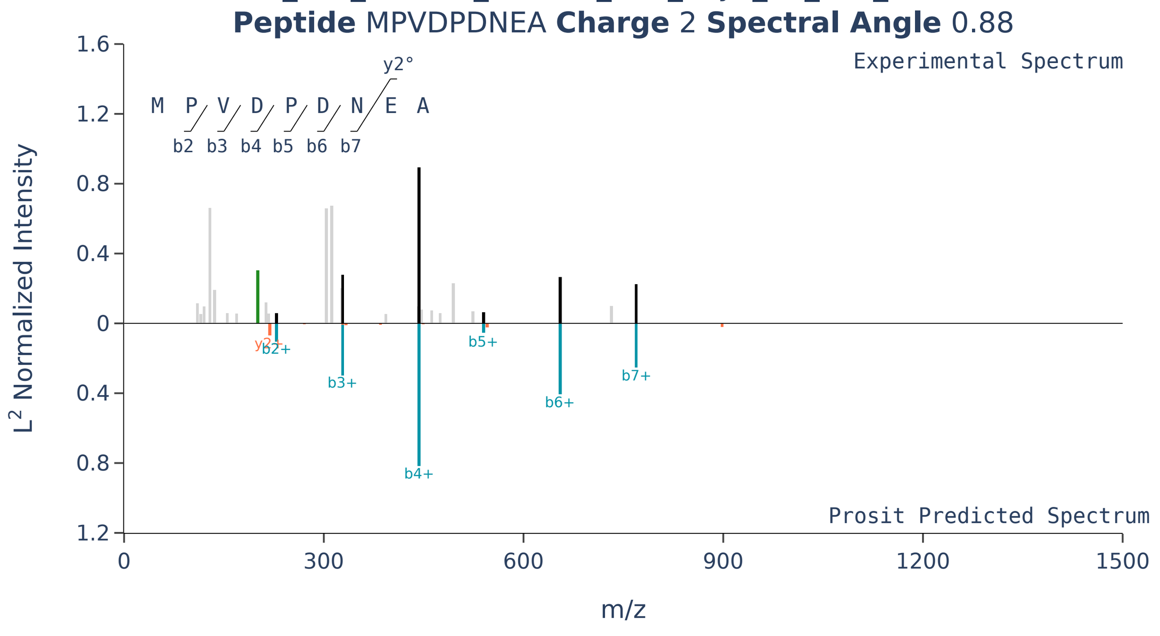


**Source** W_Soh_160821_170921_Goe_aSyn_A2_24h_R1 **Scan** 14424
**Peptide** MDVFMKGLSKAKEG **Charge** 2 **Spectral Angle** 0.9

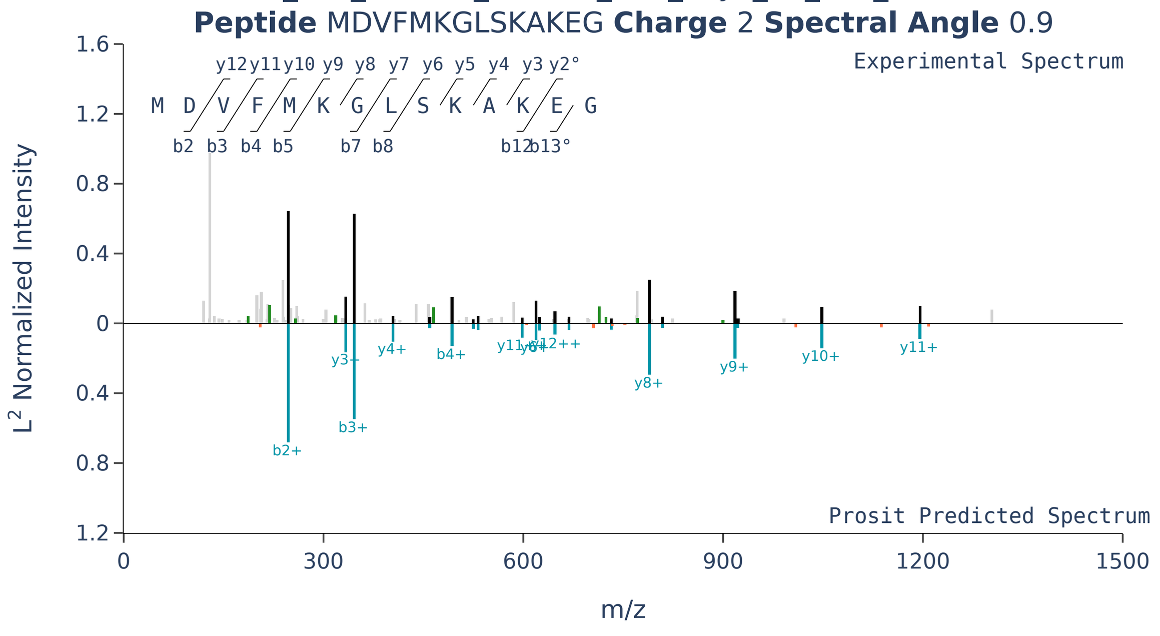


**Source** W_Soh_160821_170921_Goe_aSyn_A2_24h_R2 **Scan** 16703
**Peptide** GSIAAATGFVKKDQL **Charge** 2 **Spectral Angle** 0.9

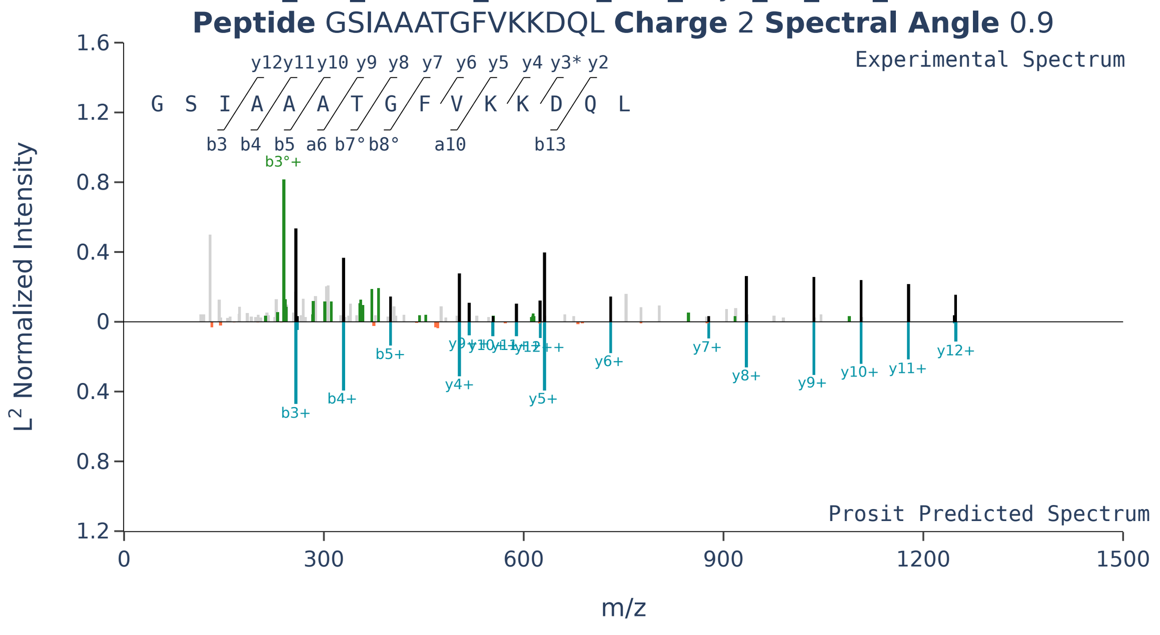


**Source** W_Soh_160821_170921_Goe_aSyn_A2_24h_R2 **Scan** 6749
**Peptide** GKTKEGVLYVGSKTKE **Charge** 4 **Spectral Angle** 0.9

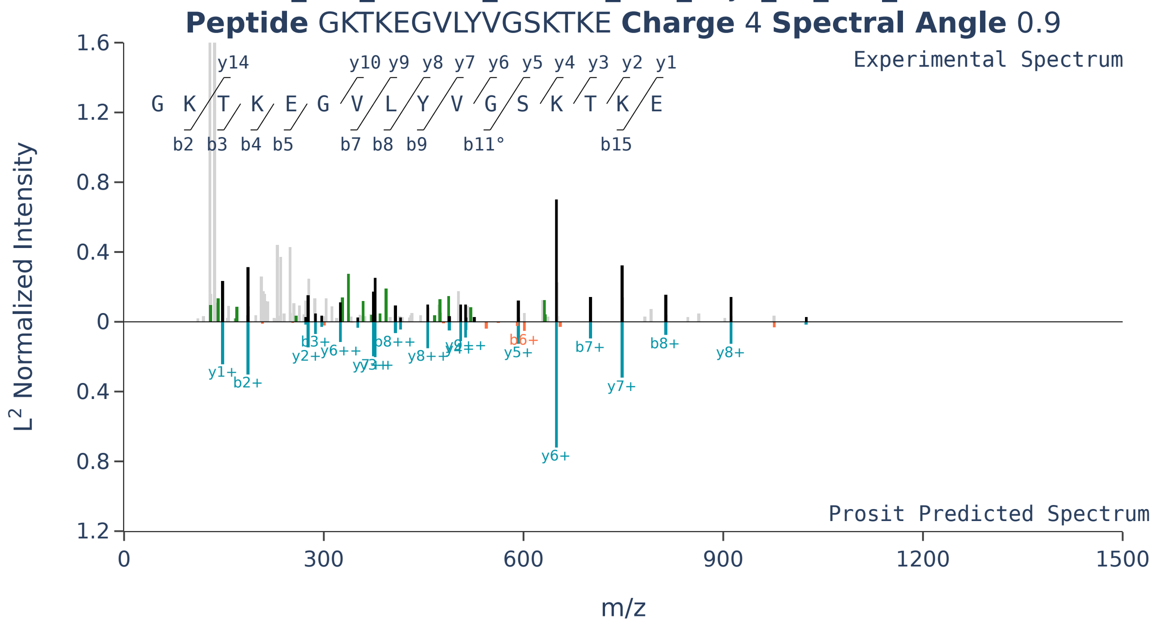


**Source** W_Soh_160821_170921_Goe_aSyn_A1_24h_R1 **Scan** 14512
**Peptide** QKTVEGAGSIAAATGFVKK **Charge** 3 **Spectral Angle** 0.89

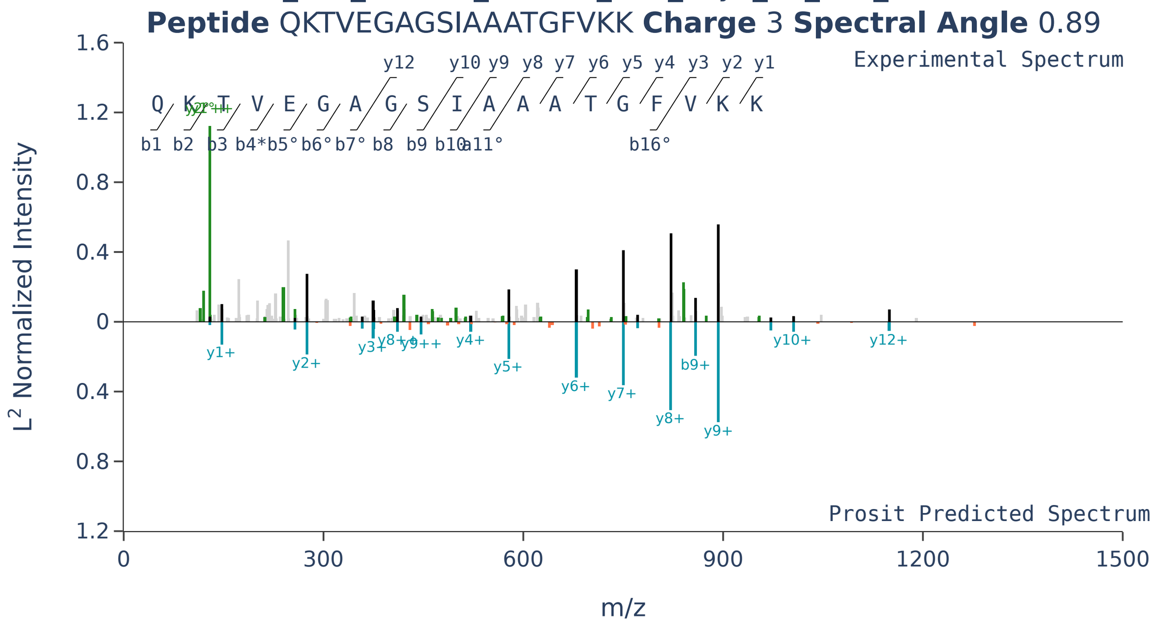


**Source** W_Soh_160821_170921_Goe_aSyn_A1_24h_R1 **Scan** 7082
**Peptide** VATVAEKTKEQV **Charge** 3 **Spectral Angle** 0.81

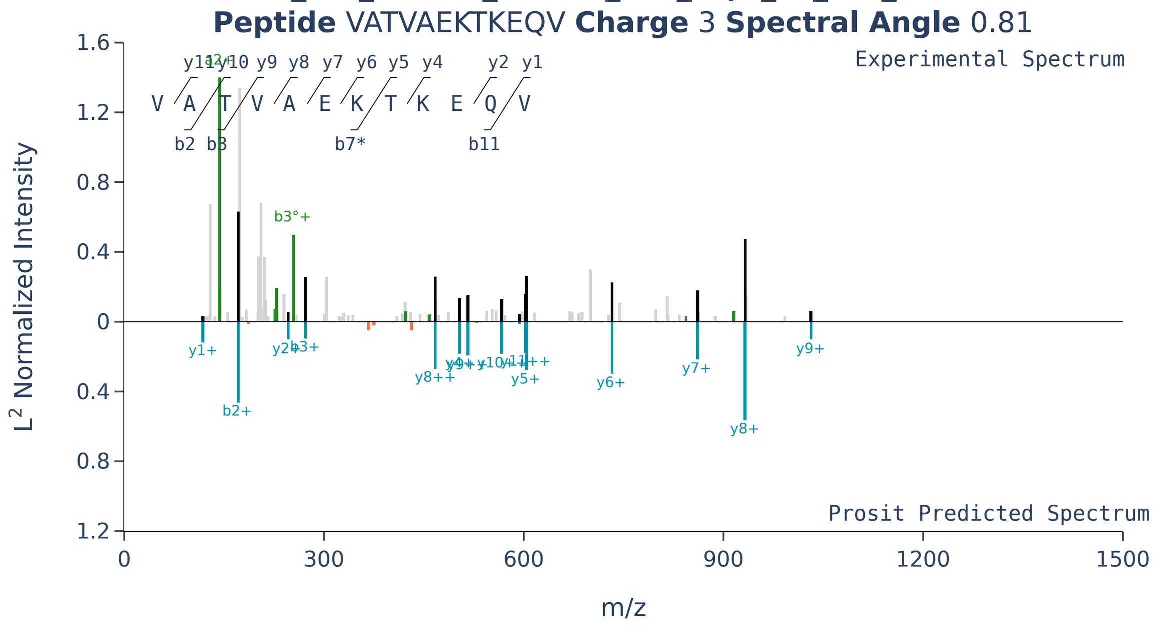


**Source** W_Soh_160821_170921_Goe_aSyn_A1_6h_R1 **Scan** 14025
**Peptide** AEKTKEQVTNVGGAVVTGV **Charge** 3 **Spectral Angle** 0.84

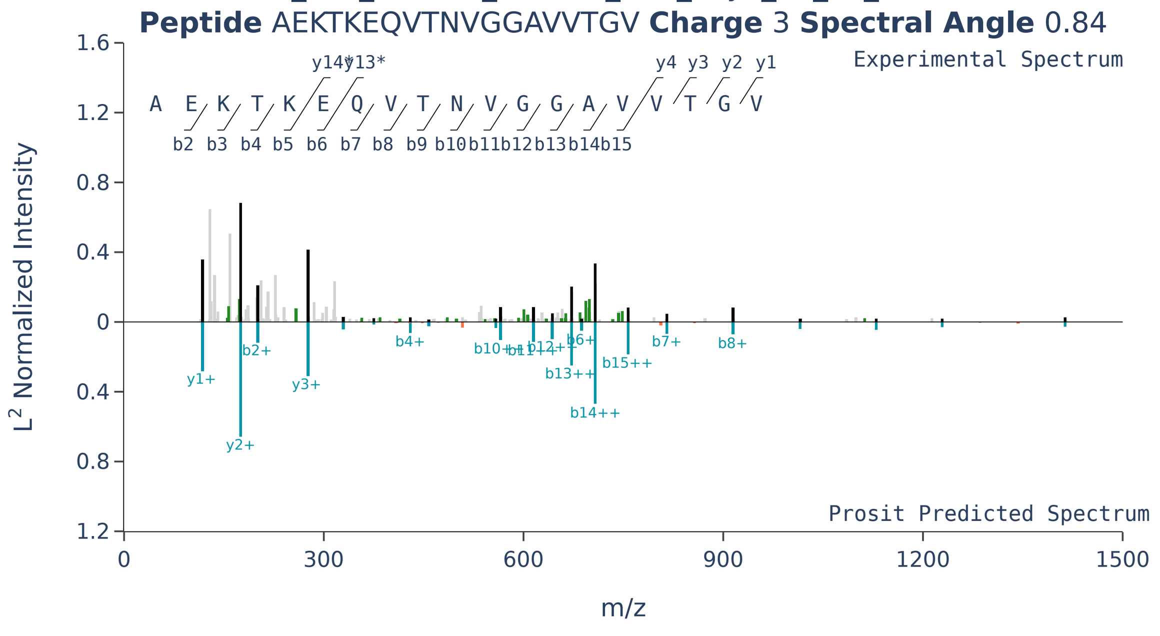


**Source** W_Soh_160821_170921_Goe_aSyn_A1_24h_R2 **Scan** 8325
**Peptide** VFMKGLSKAKE **Charge** 3 **Spectral Angle** 0.87

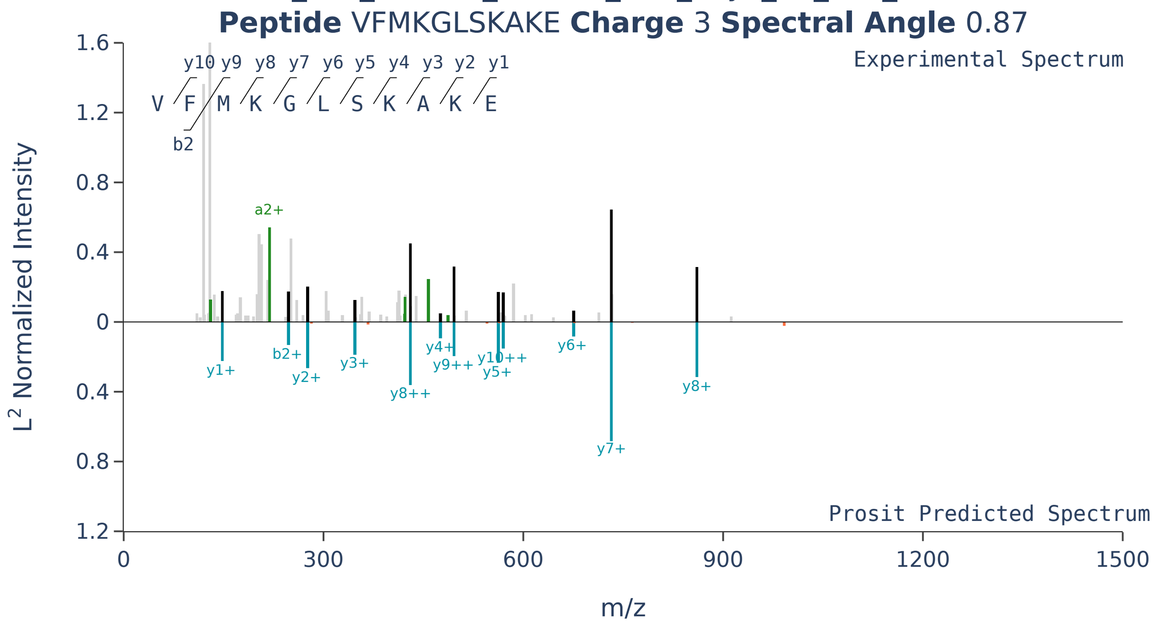

**Source** W_Soh_160821_170921_Goe_aSyn_A2_6h_R2 **Scan** 8548
**Peptide** EGAGSIAA **Charge** 1 **Spectral Angle** 0.9

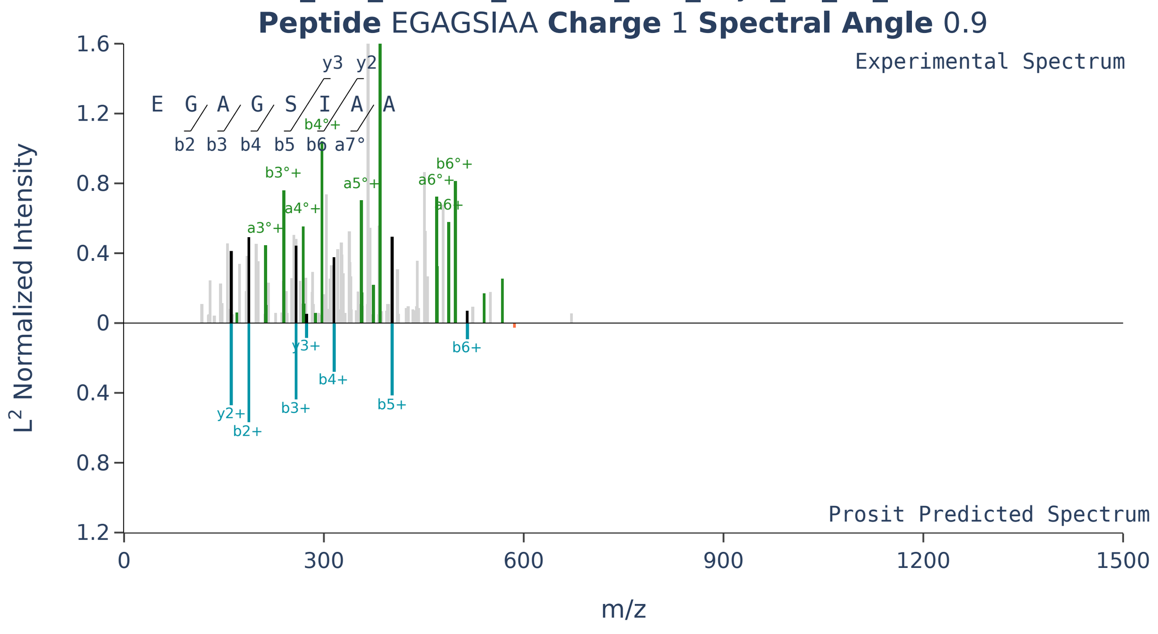


**Source** W_Soh_160821_170921_Goe_aSyn_A1_24h_R1 **Scan** 23935
**Peptide** GVTAVAQKTVEGAGSIAAATGFVKKDQL **Charge** 4 **Spectral Angle** 0.8

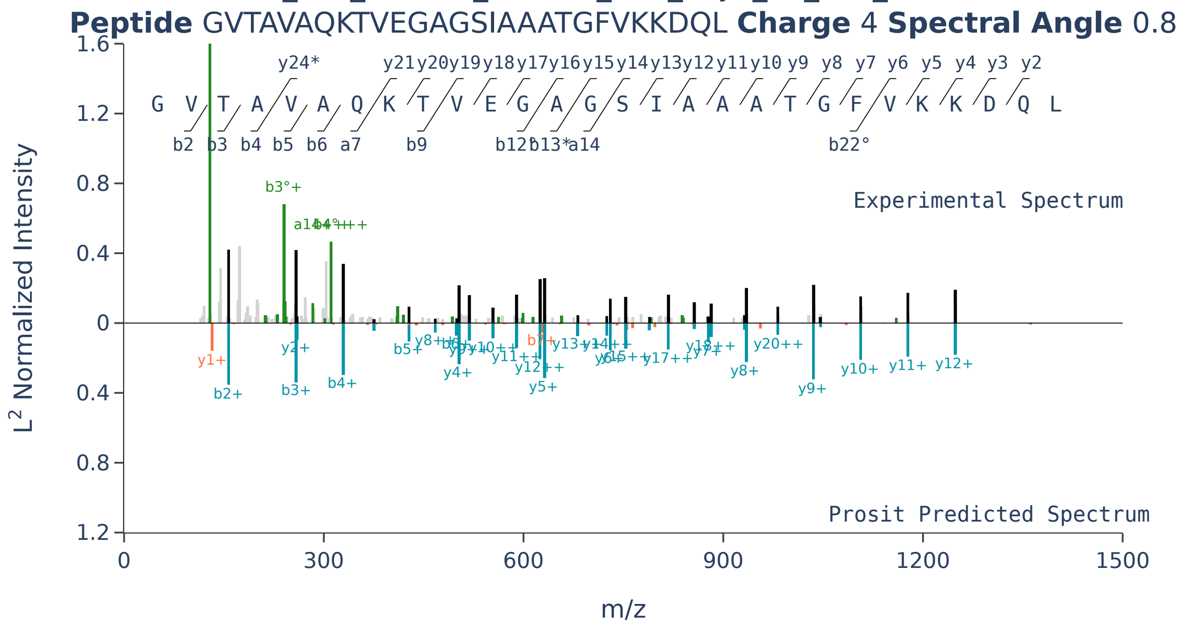


**Source** W_Soh_160821_170921_Goe_aSyn_A1_6h_R1 **Scan** 11540
**Peptide** VLYVGSKTKEGVVHGVAT **Charge** 3 **Spectral Angle** 0.92

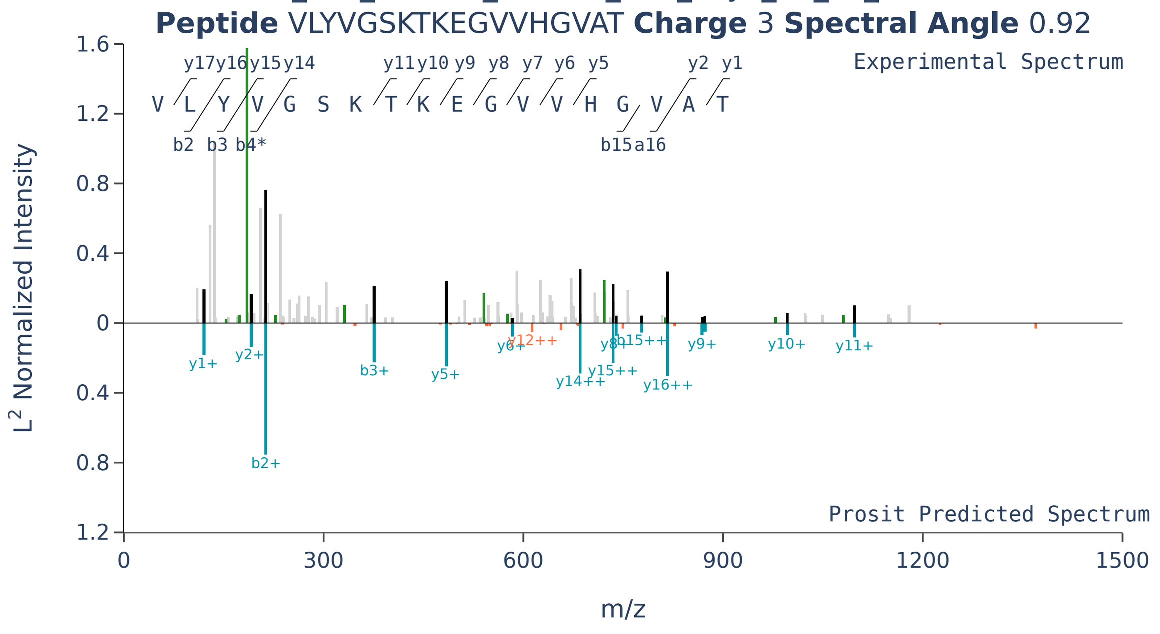


**Source** W_Soh_160821_170921_Goe_aSyn_A1_24h_R2 **Scan** 7813
**Peptide** GKTKEGVLYVGSKTKEGVVH **Charge** 3 **Spectral Angle** 0.93

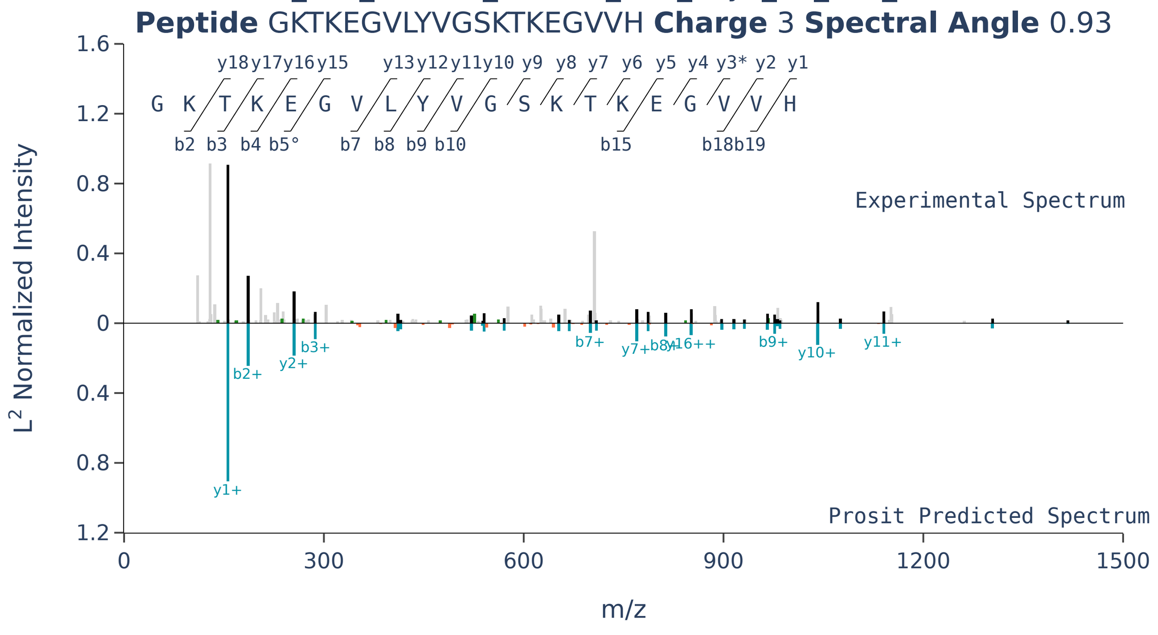


**Source** W_Soh_160821_170921_Goe_aSyn_A2_24h_R2 **Scan** 17964
**Peptide** QKTVEGAGSIAAATGFVKKDQL **Charge** 3 **Spectral Angle** 0.84

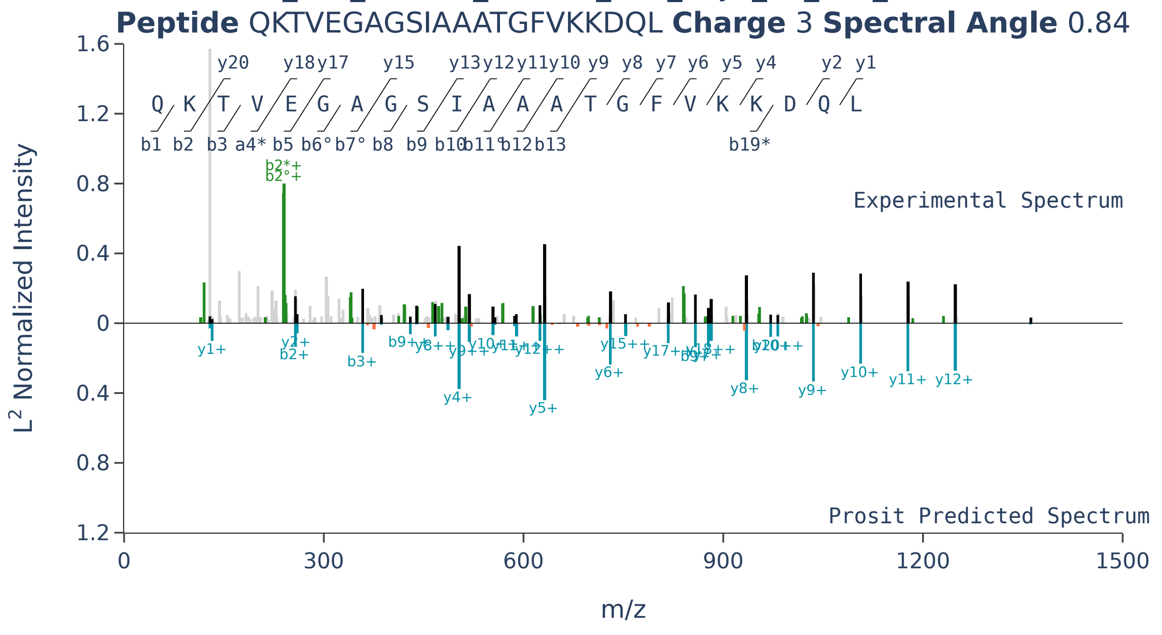


**Source** W_Soh_160821_170921_Goe_aSyn_A2_24h_R2 **Scan** 7272
**Peptide** KEGVVAAAEKTKQGV **Charge** 3 **Spectral Angle** 0.86

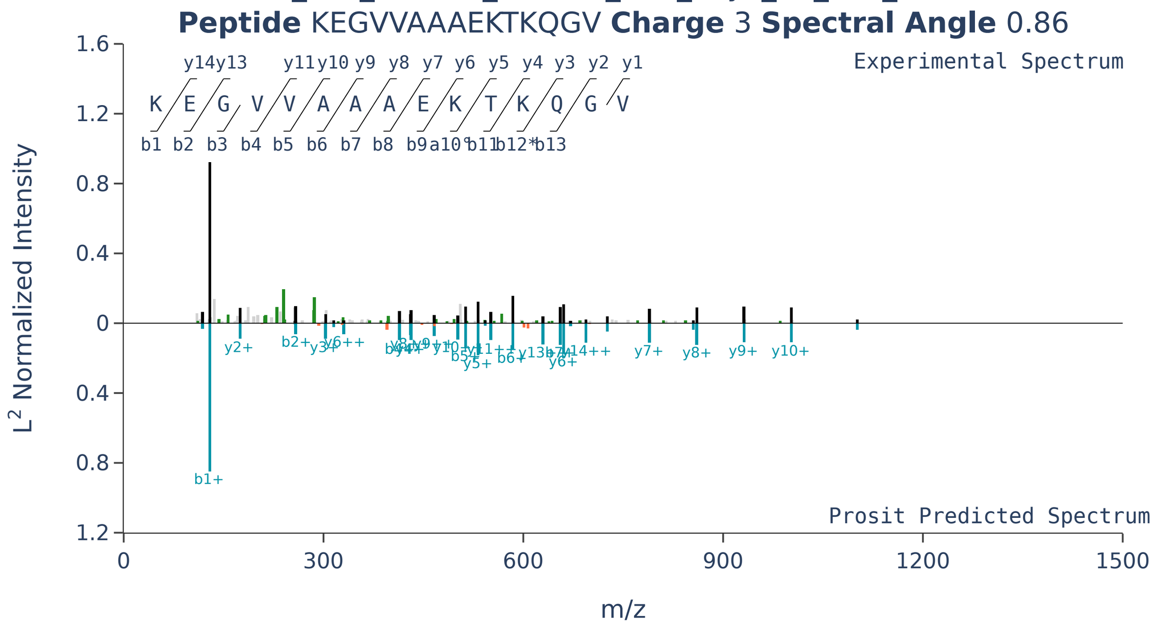


**Source** W_Soh_160821_170921_Goe_aSyn_A2_6h_R2 **Scan** 17050
**Peptide** TNVGGAVVTGVTA **Charge** 2 **Spectral Angle** 0.91

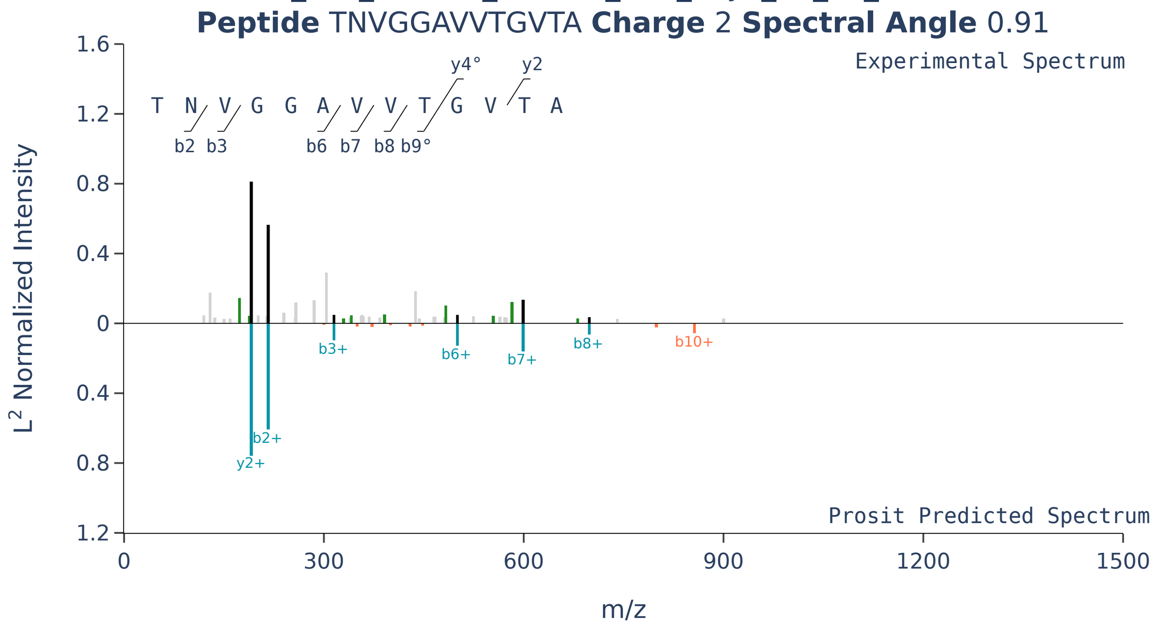


**Source** W_Soh_160821_170921_Goe_aSyn_A1_24h_R2 **Scan** 13891
**Peptide** AGSIAAATGFVKKDQLGKNE **Charge** 3 **Spectral Angle** 0.86

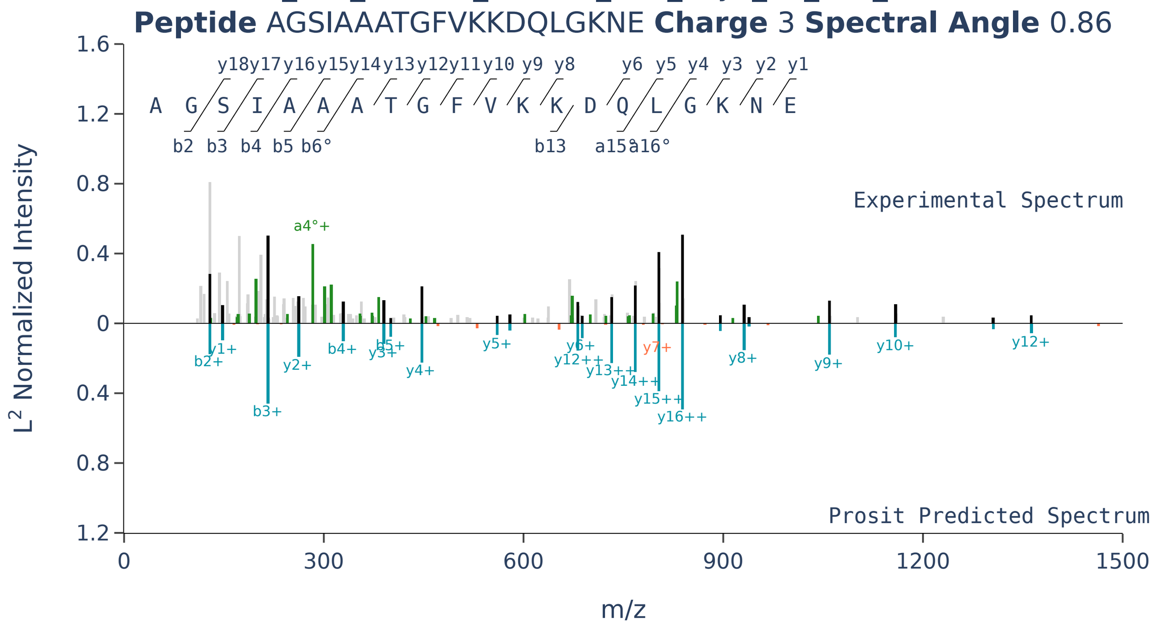


**Source** W_Soh_160821_170921_Goe_aSyn_A2_24h_R1 **Scan** 22757
**Peptide** KNEEGAPQEGILEDMPVD **Charge** 2 **Spectral Angle** 0.92

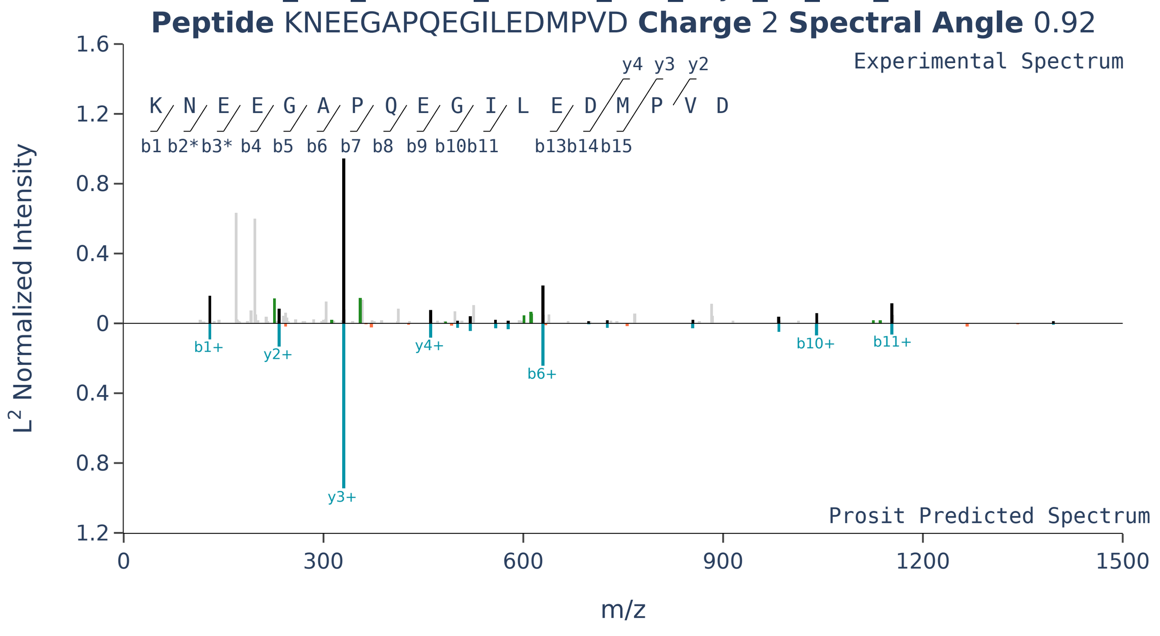


**Source** W_Soh_160821_170921_Goe_aSyn_A1_24h_R1 **Scan** 20653
**Peptide** LEDMPVDPDNEAYE **Charge** 2 **Spectral Angle** 0.87

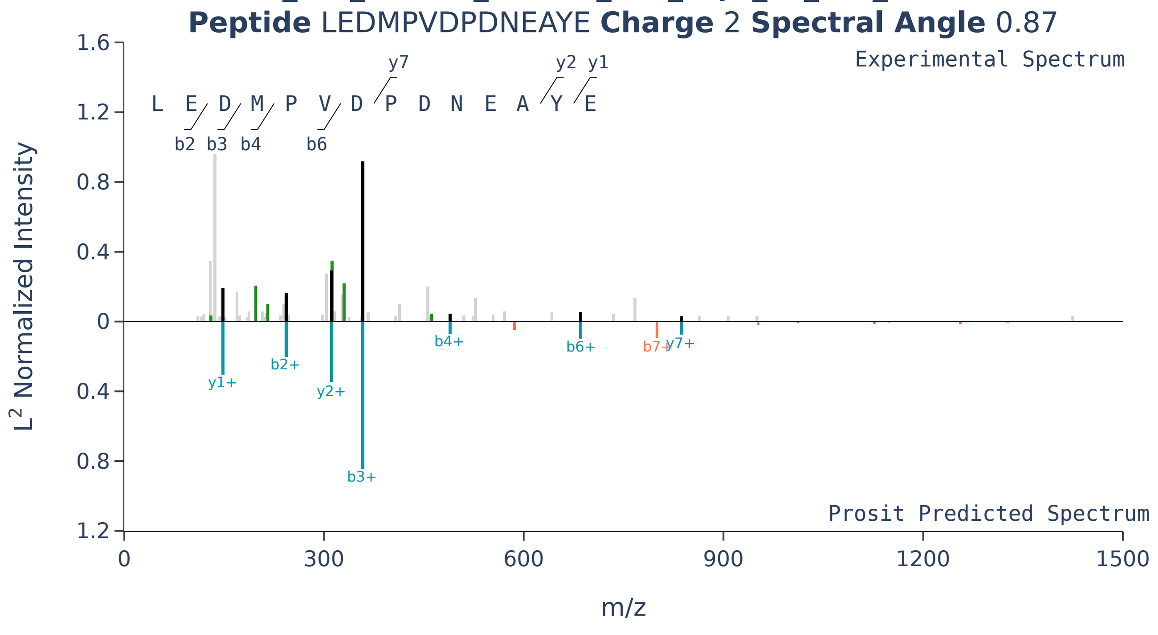


**Source** W_Soh_160821_170921_Goe_aSyn_A1_24h_R1 **Scan** 9525
**Peptide** IAAATGFVKKDQ **Charge** 3 **Spectral Angle** 0.84

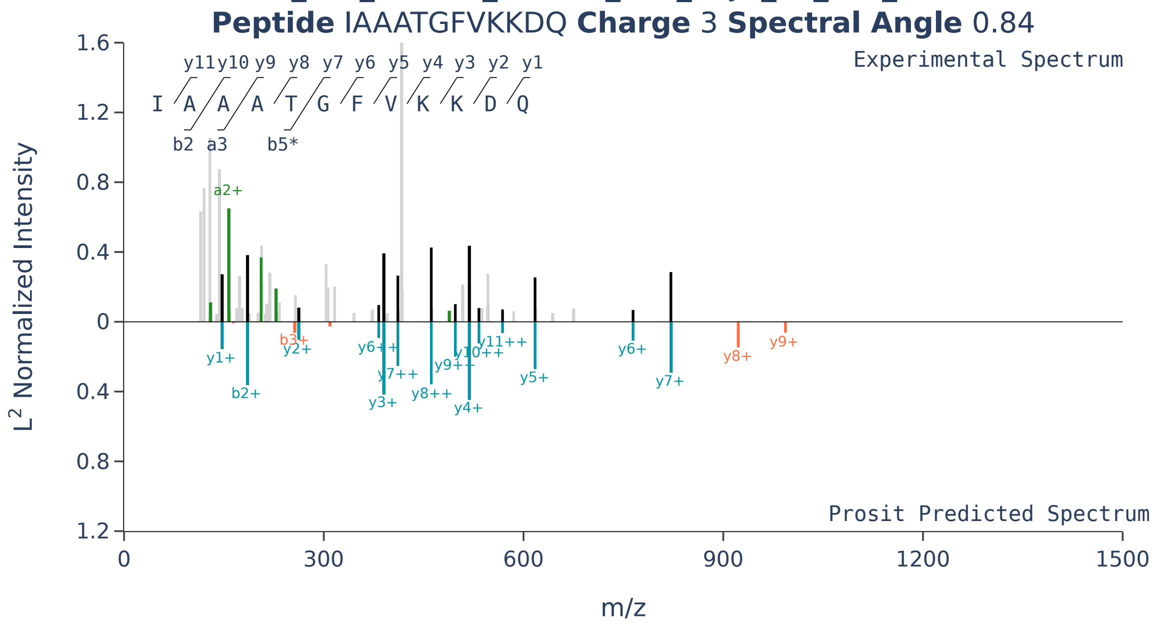


**Source** W_Soh_160821_170921_Goe_aSyn_A1_24h_R1 **Scan** 9348
**Peptide** TKEGVLYVGSKTKEGVVHG **Charge** 4 **Spectral Angle** 0.85

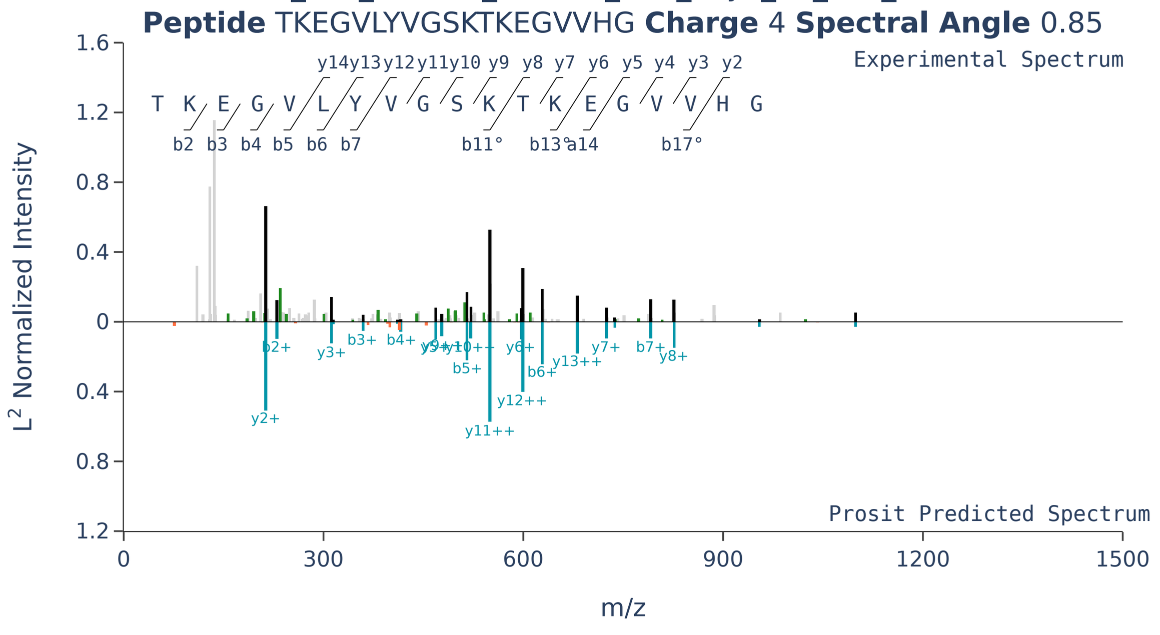


**Source** W_Soh_160821_170921_Goe_aSyn_A2_24h_R1 **Scan** 7145
**Peptide** SKAKEGVVAAAEKTKQGVAE **Charge** 3 **Spectral Angle** 0.79

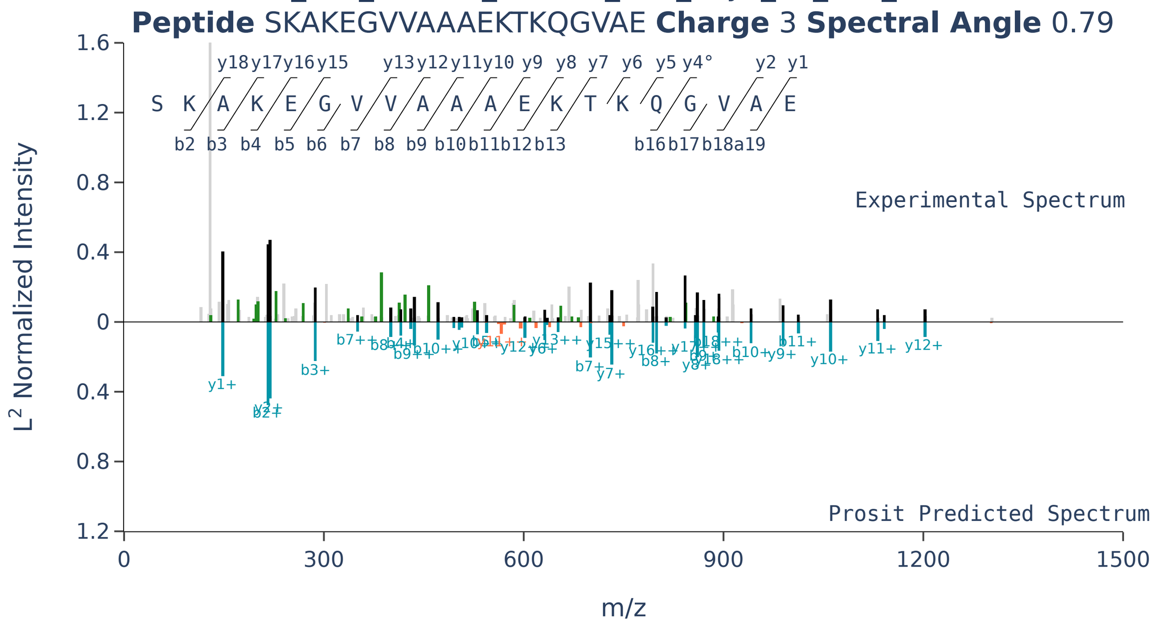


**Source** W_Soh_160821_170921_Goe_aSyn_A1_24h_R1 **Scan** 5773
**Peptide** SKTKEGVVHGVA **Charge** 2 **Spectral Angle** 0.79

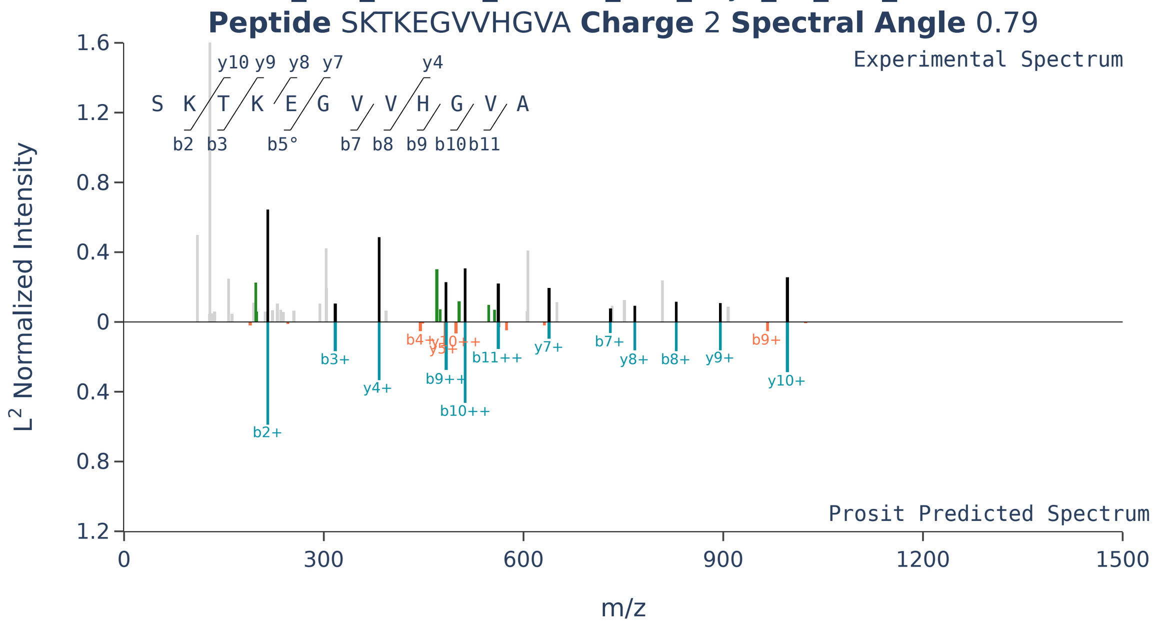


**Source** W_Soh_160821_170921_Goe_aSyn_A2_24h_R2 **Scan** 18437
**Peptide** GVVHGVATVAEKTKEQVTNVGGAVVTG **Charge** 4 **Spectral Angle** 0.85

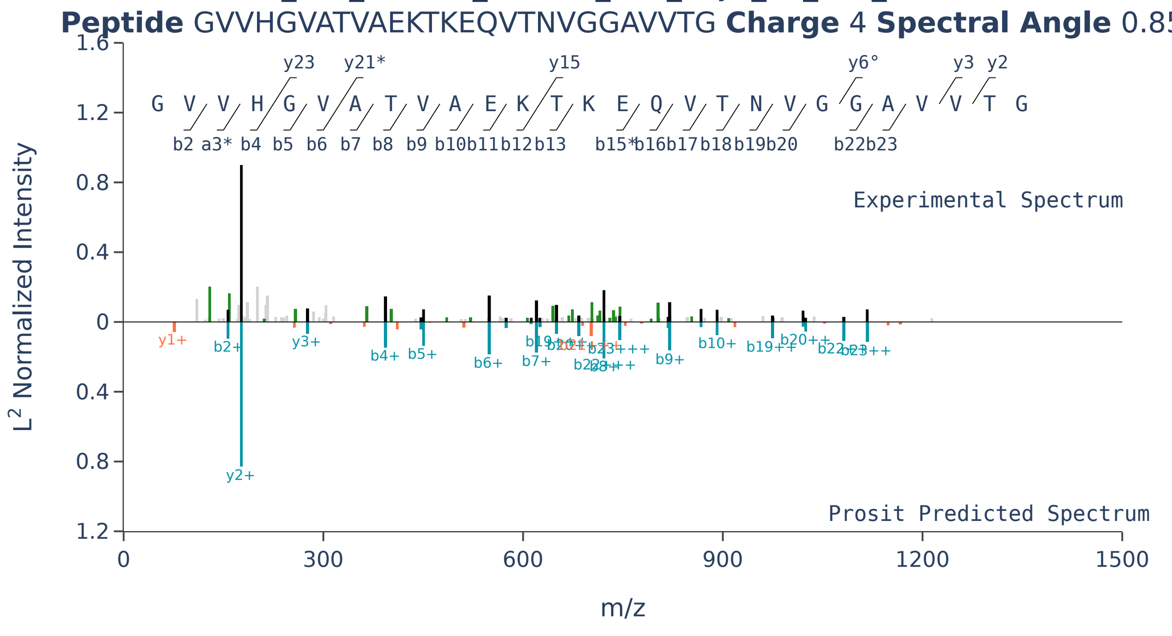

**Source** W_Soh_160821_170921_Goe_aSyn_A2_30'_R1 **Scan** 9130
**Peptide** AATGFVKKDQL **Charge** 3 **Spectral Angle** 0.84

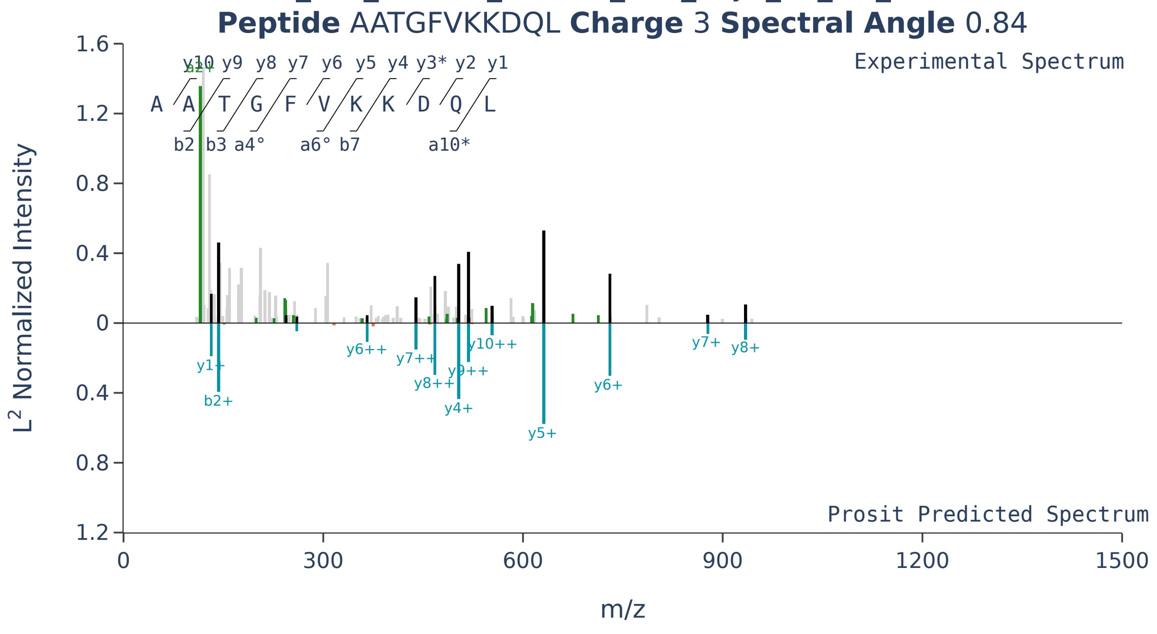


**Source** W_Soh_160821_170921_Goe_aSyn_A1_6h_R2 **Scan** 8063
**Peptide** AAATGFVKKDQLGKNE **Charge** 3 **Spectral Angle** 0.86

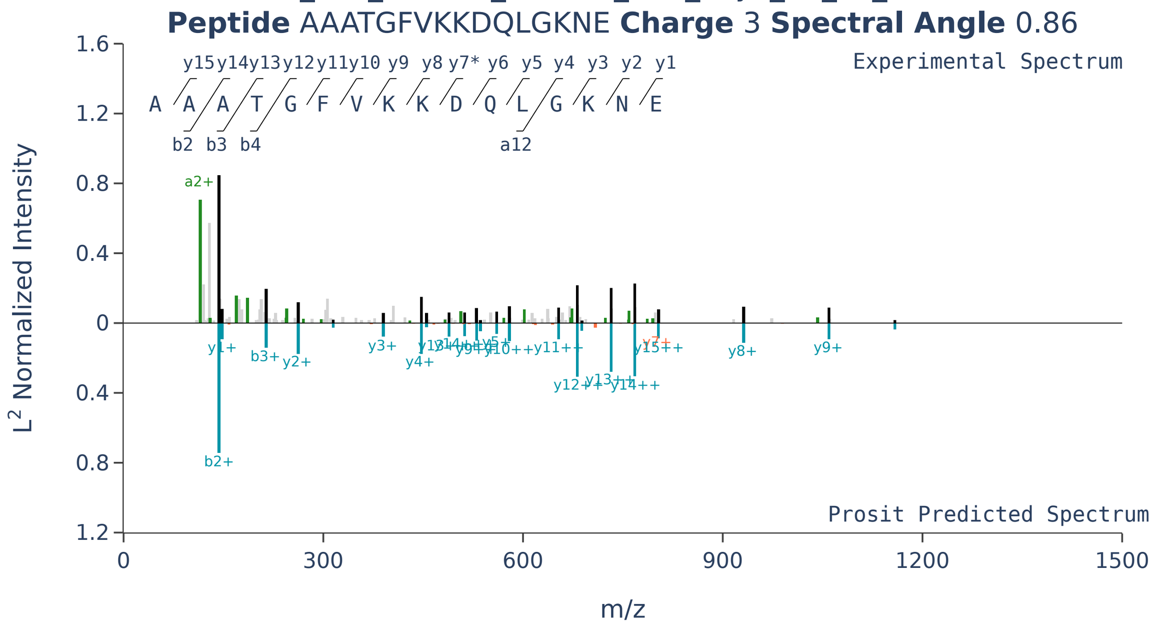


**Source** W_Soh_160821_170921_Goe_aSyn_A2_1h_R1 **Scan** 14933
**Peptide** GVATVAEKTKEQVTNVGGAVVTG **Charge** 3 **Spectral Angle** 0.82

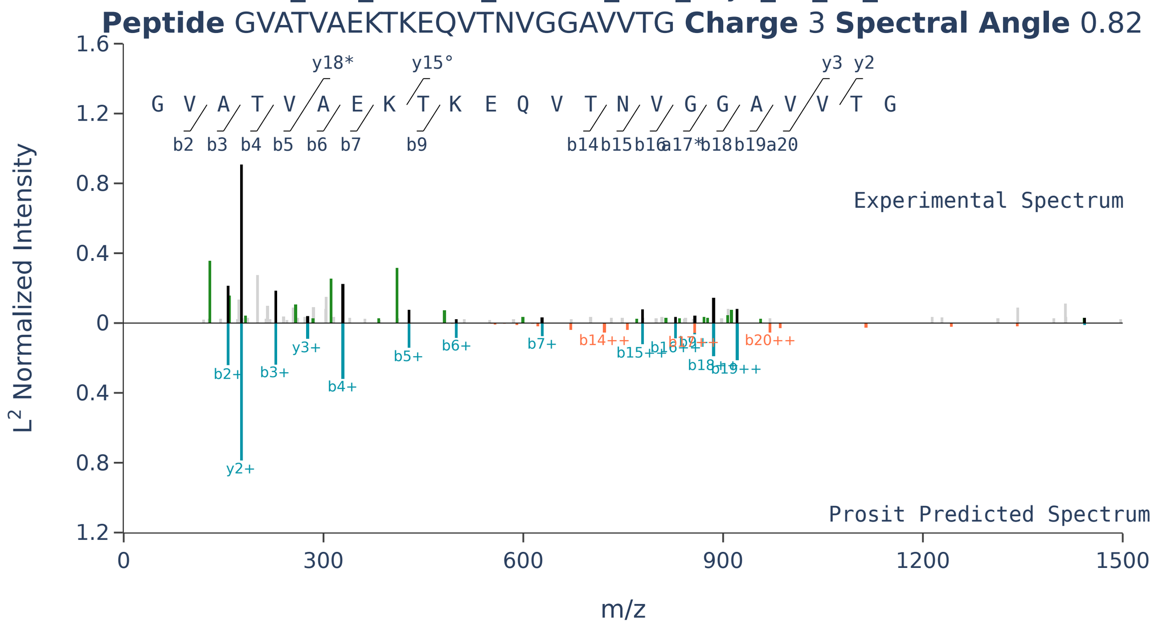


**Source** W_Soh_160821_170921_Goe_aSyn_A1_6h_R2 **Scan** 19339
**Peptide** AQKTVEGAGSIAAATGF **Charge** 2 **Spectral Angle** 0.85

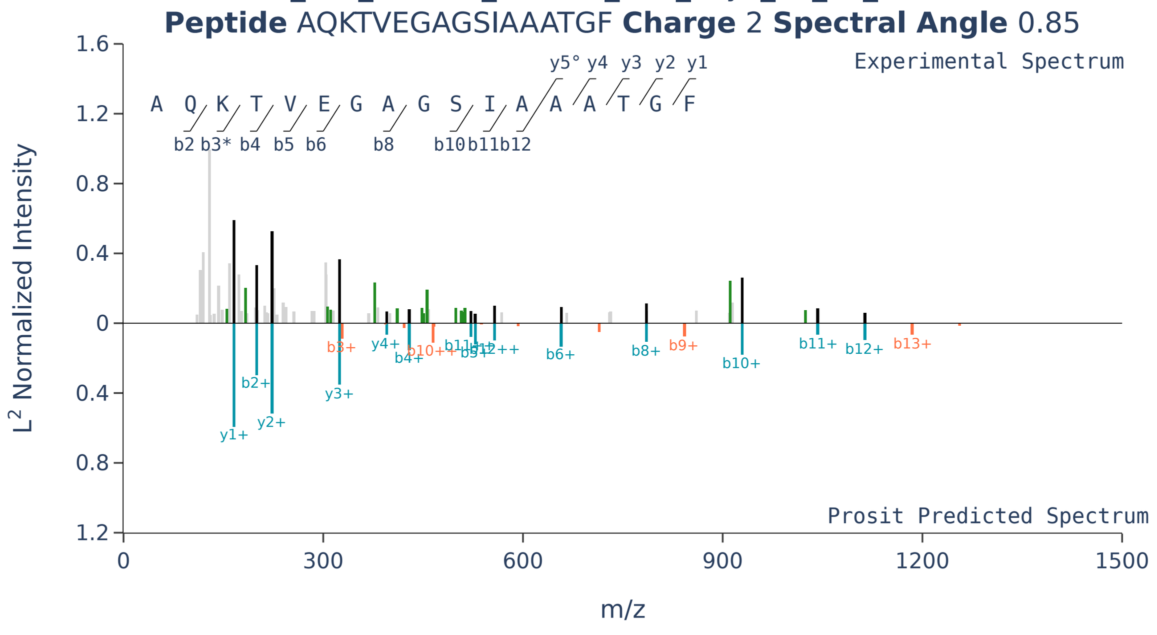


**Source** W_Soh_160821_170921_Goe_aSyn_A1_3h_R2 **Scan** 21483
**Peptide** EKTKEQVTNVGGAVVTGVTAVAQKTVE **Charge** 3 **Spectral Angle** 0.81

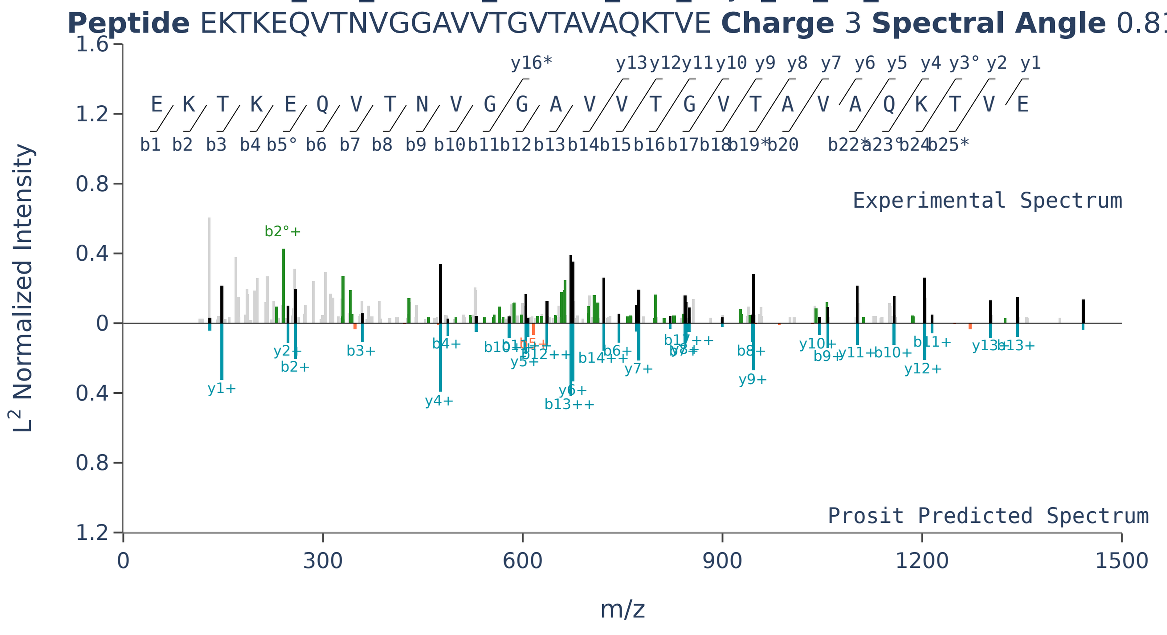


**Source** W_Soh_160821_170921_Goe_aSyn_A1_3h_R2 **Scan** 5919
**Peptide** GSKTKEGVVHGVAT **Charge** 3 **Spectral Angle** 0.84

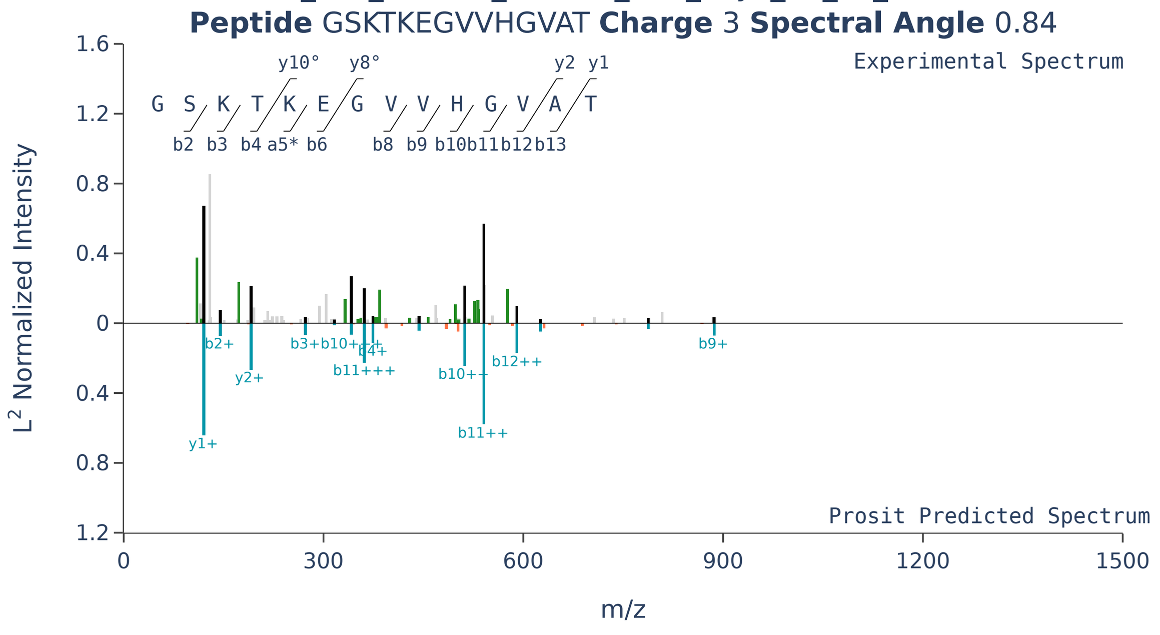


**Source** W_Soh_160821_170921_Goe_aSyn_A2_6h_R1 **Scan** 13333
**Peptide** PDNEAYEMPS **Charge** 2 **Spectral Angle** 0.92

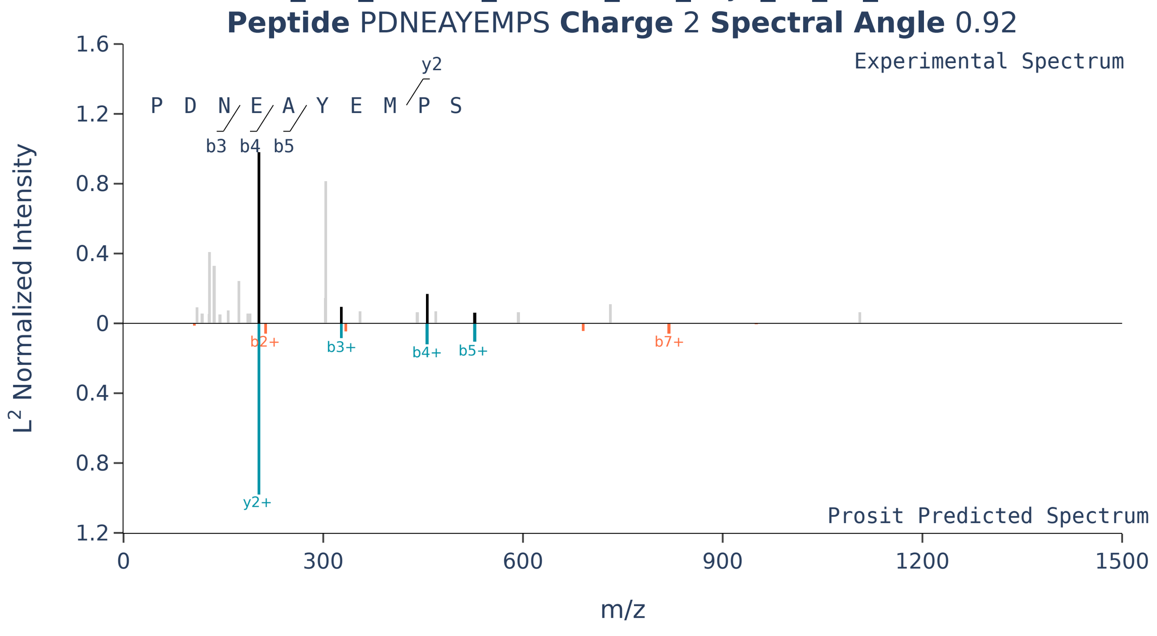


**Source** W_Soh_160821_170921_Goe_aSyn_A1_24h_R1 **Scan** 9797
**Peptide** GVTAVAQKTV **Charge** 2 **Spectral Angle** 0.81

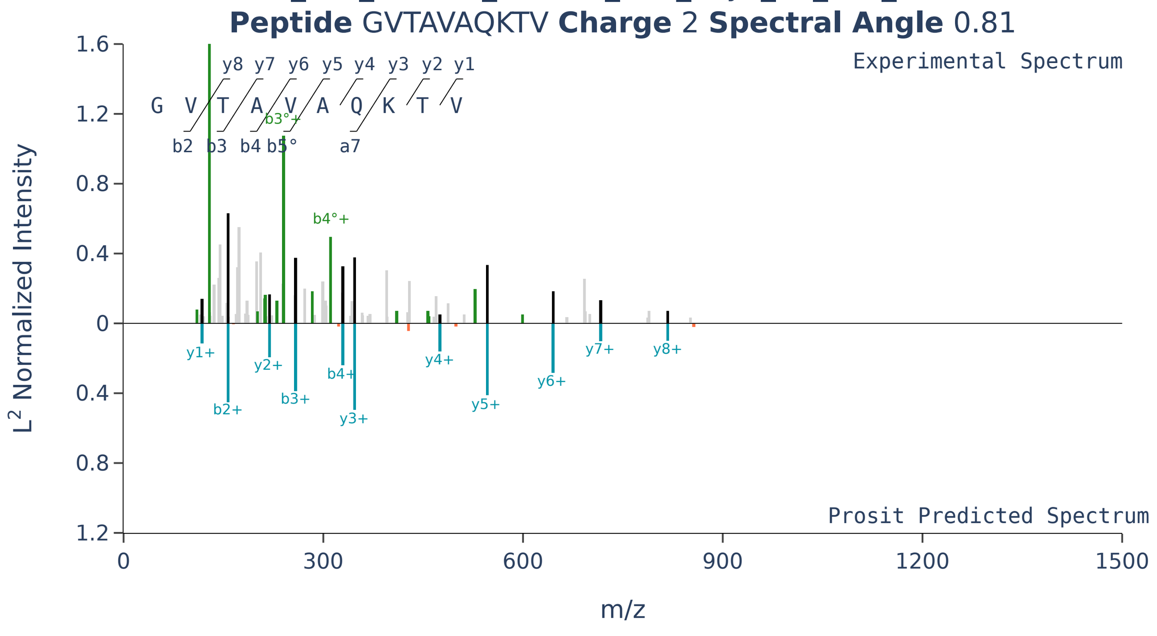


**Source** W_Soh_160821_170921_Goe_aSyn_A1_6h_R2 **Scan** 6176
**Peptide** TVAEKTKE **Charge** 2 **Spectral Angle** 0.91

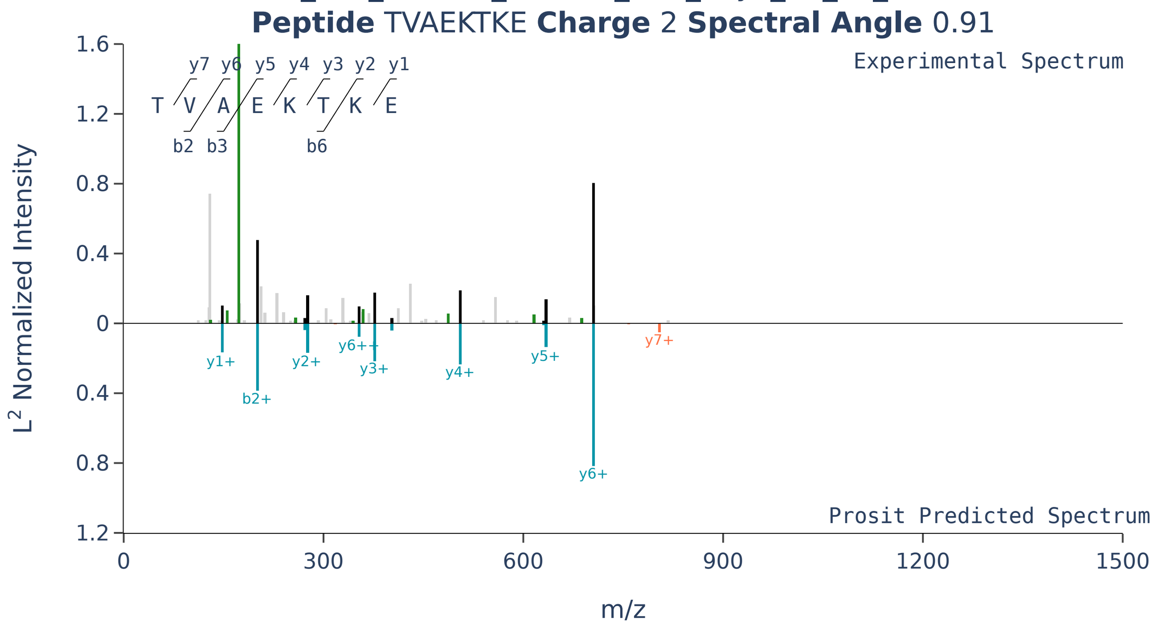


**Source** W_Soh_160821_170921_Goe_aSyn_A2_1h_R1 **Scan** 12839
**Peptide** EKTKEQVTNVGGAVVTGVTA **Charge** 3 **Spectral Angle** 0.85

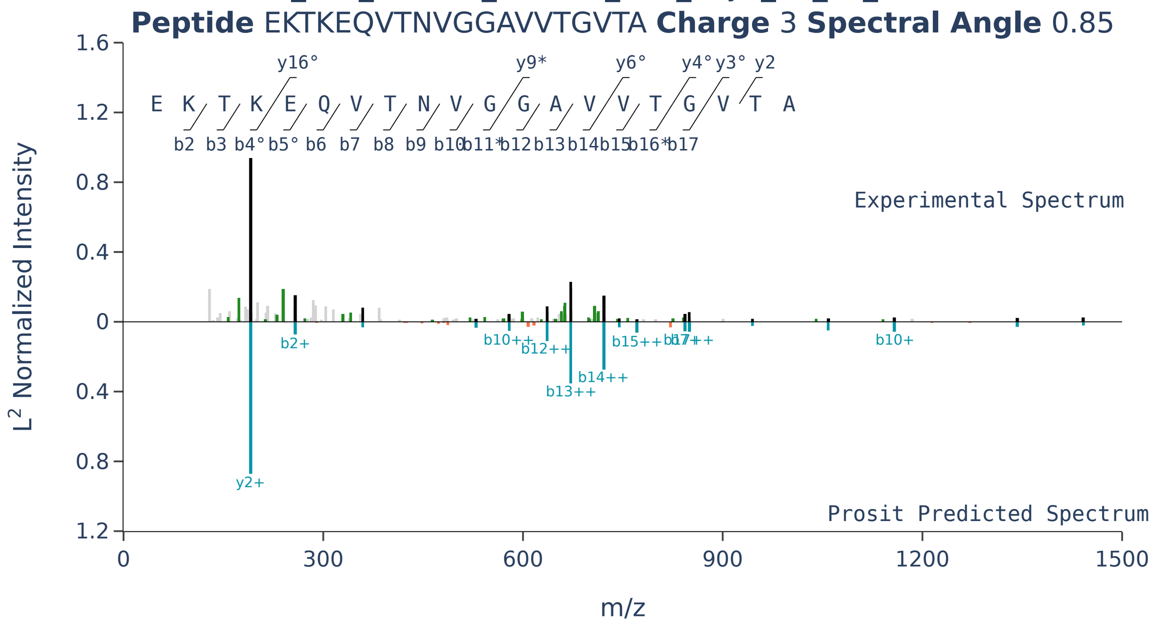


**Source** W_Soh_160821_170921_Goe_aSyn_A2_24h_R1 **Scan** 11410
**Peptide** AAGKTKEGVLYVG **Charge** 3 **Spectral Angle** 0.77

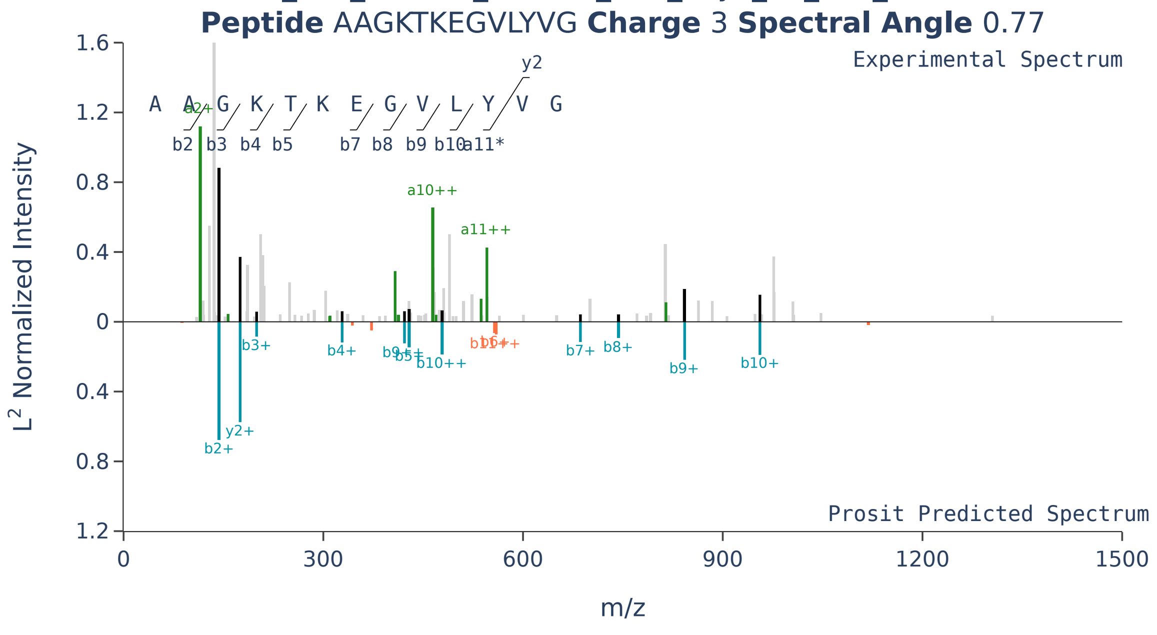


**Source** W_Soh_160821_170921_Goe_aSyn_A2_24h_R1 **Scan** 21851
**Peptide** MDVFMKGLS **Charge** 2 **Spectral Angle** 0.85

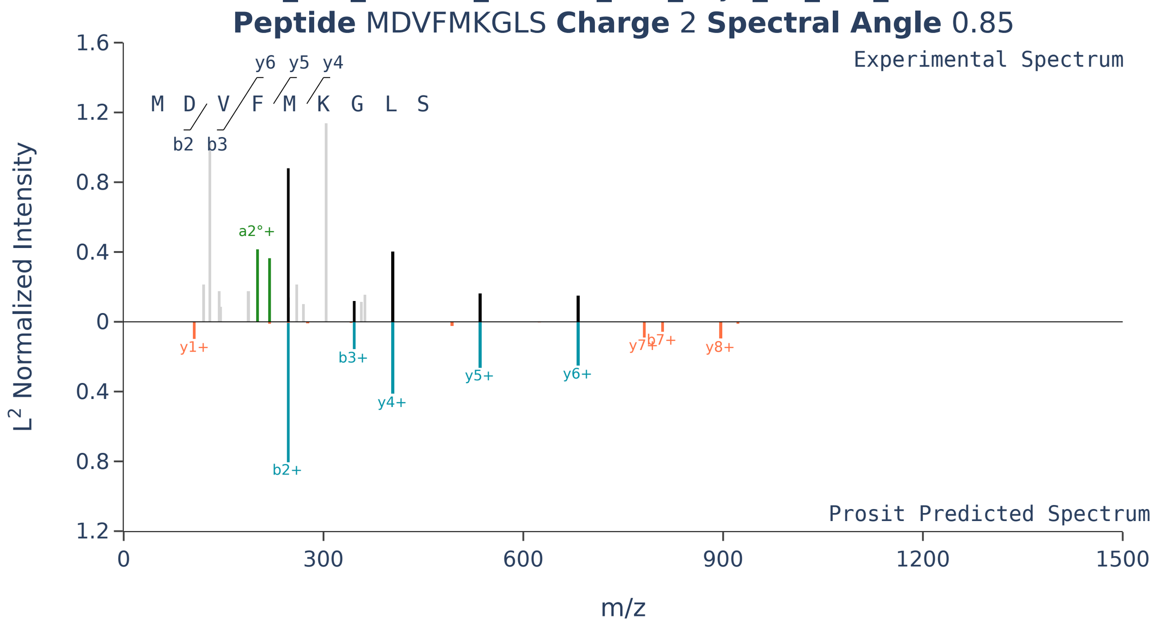


**Source** W_Soh_160821_170921_Goe_aSyn_A2_24h_R1 **Scan** 16661
**Peptide** DVFMKGLSKAKEGVVA **Charge** 2 **Spectral Angle** 0.88

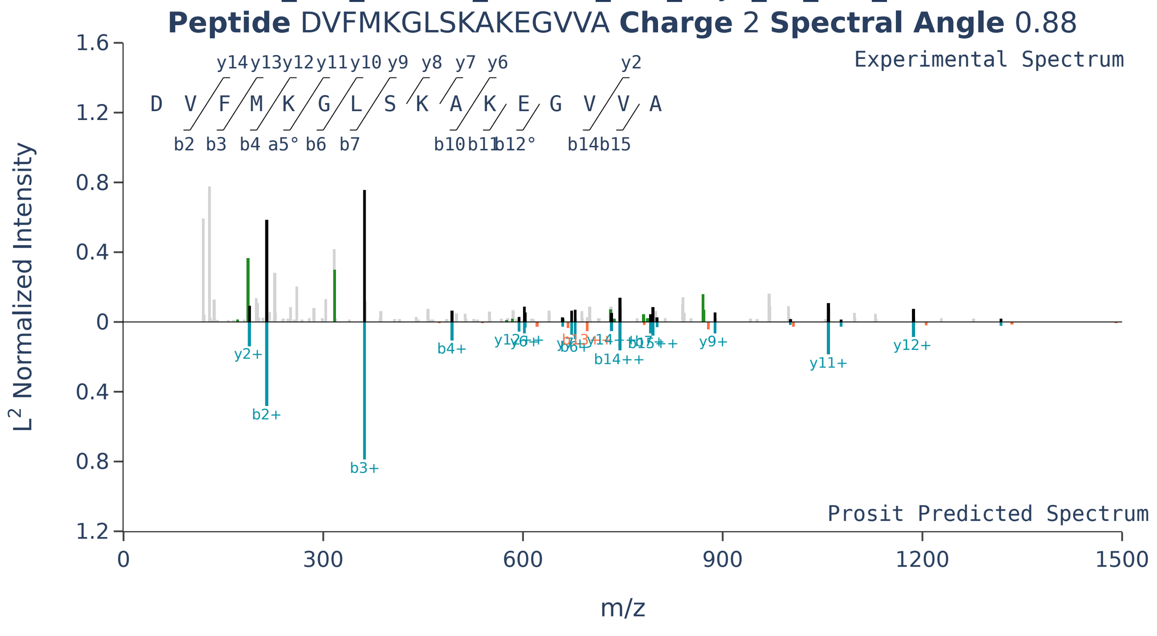


**Source** W_Soh_160821_170921_Goe_aSyn_A1_24h_R2 **Scan** 8384
**Peptide** GSKTKEGVVHGVATVAE **Charge** 3 **Spectral Angle** 0.85

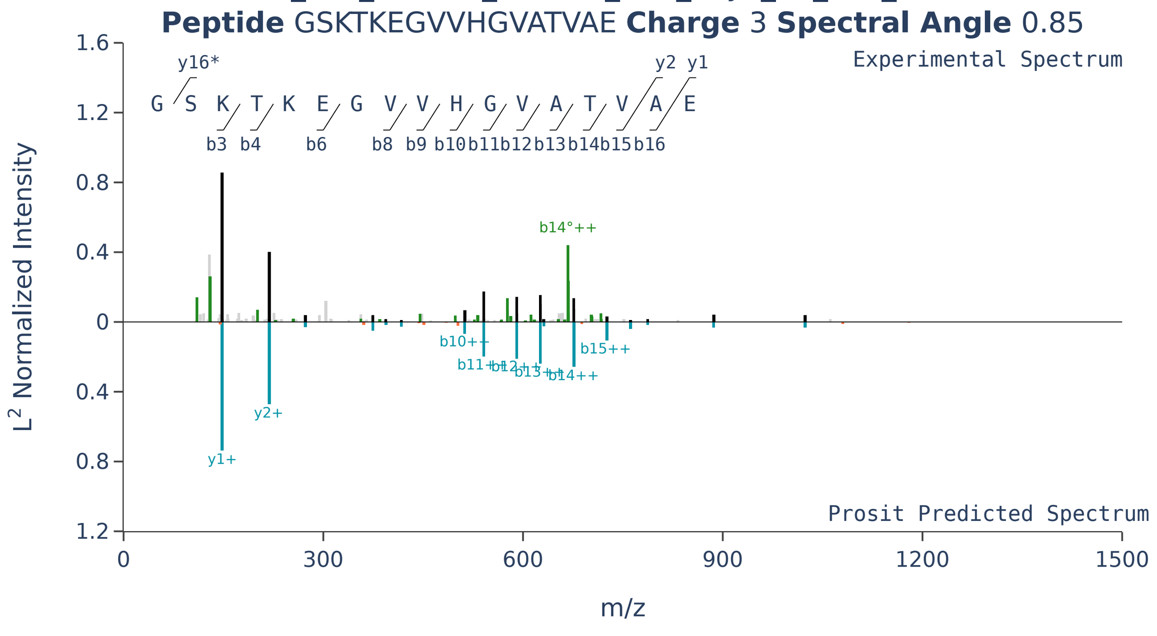


**Source** W_Soh_160821_170921_Goe_aSyn_A1_6h_R1 **Scan** 13144
**Peptide** TKEQVTNVGGAVV **Charge** 2 **Spectral Angle** 0.81

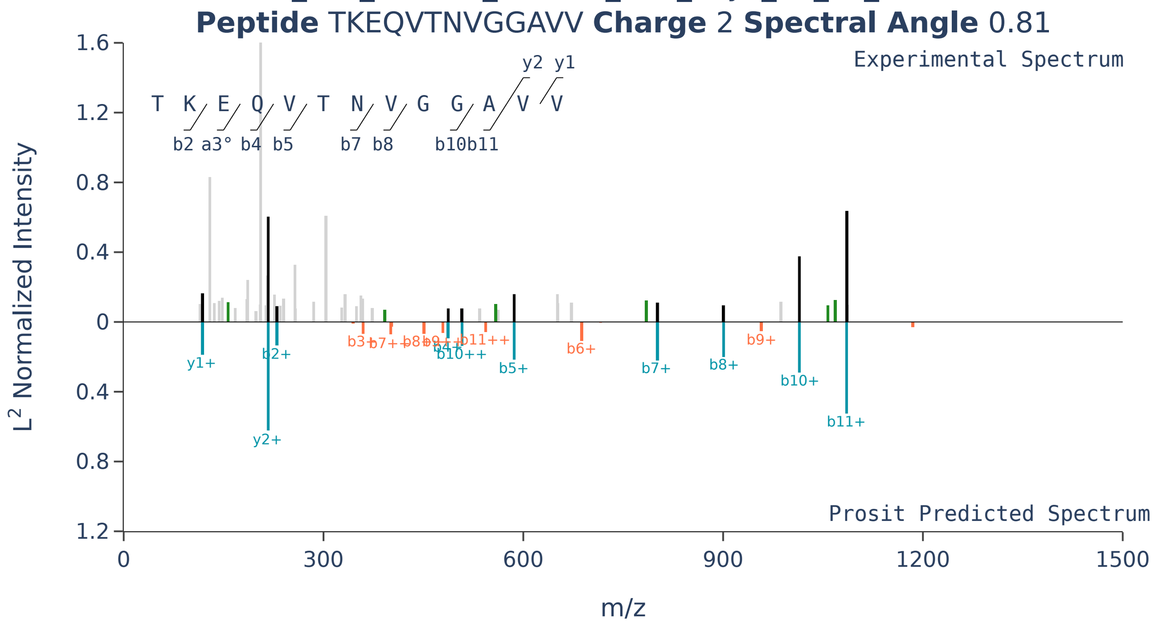

**Source** W_Soh_160821_170921_Goe_aSyn_A1_24h_R2 **Scan** 7718
**Peptide** GFVKKDQLGKNEEGAPQE **Charge** 3 **Spectral Angle** 0.86

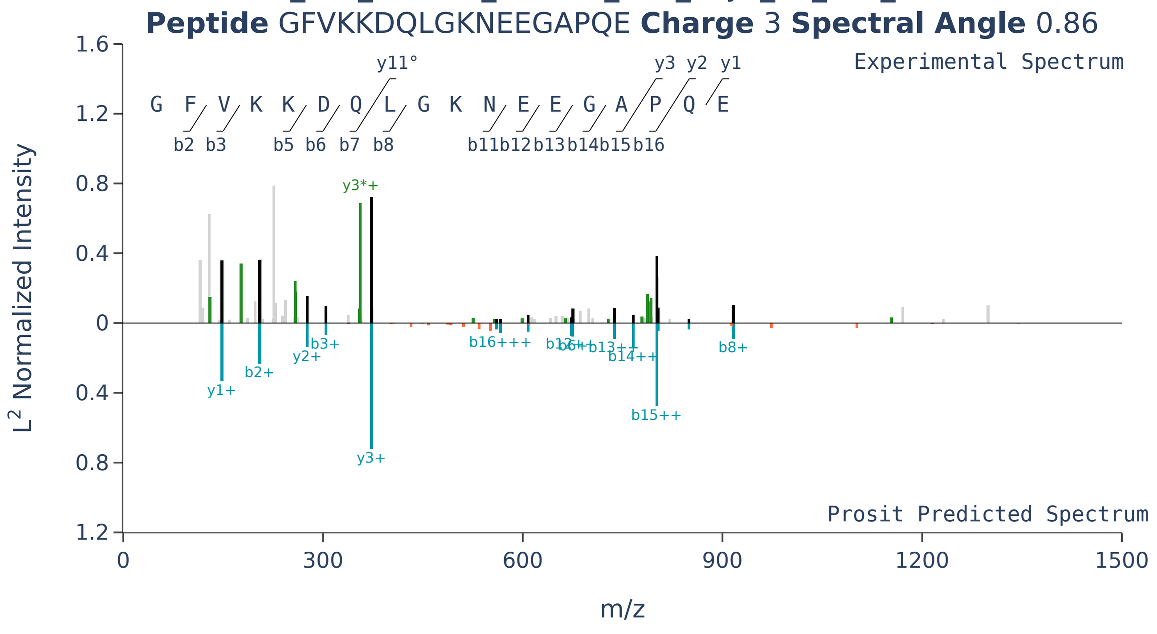


**Source** W_Soh_160821_170921_Goe_aSyn_A1_24h_R1 **Scan** 8840
**Peptide** AATGFVKKDQLGKNEEGAPQE **Charge** 3 **Spectral Angle** 0.92

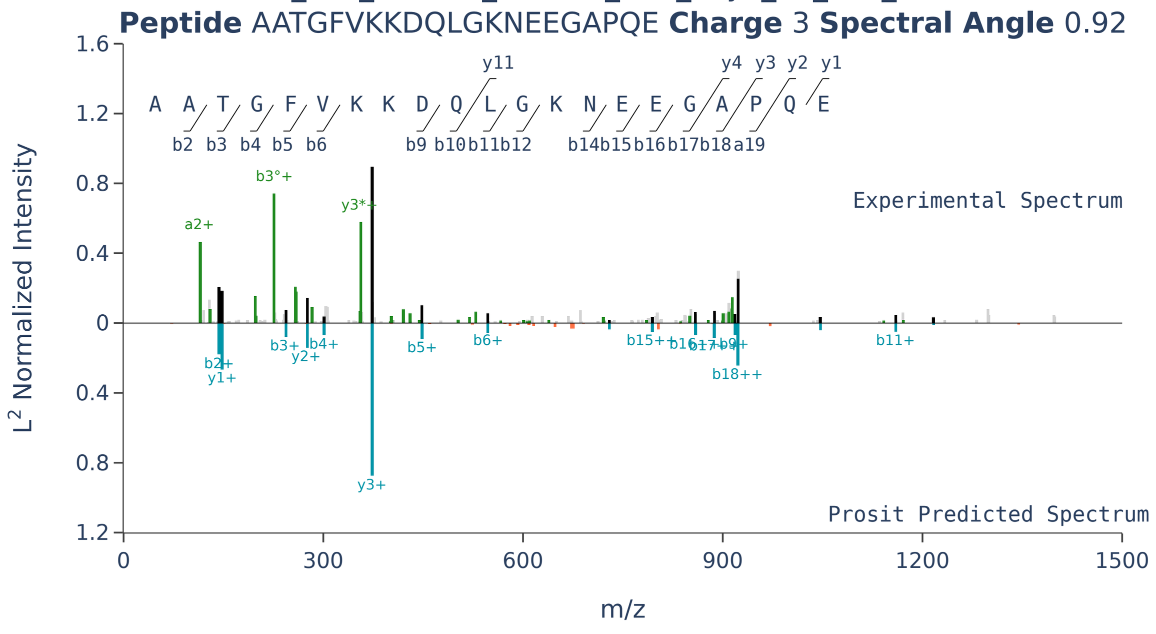


**Source** W_Soh_160821_170921_Goe_aSyn_A2_24h_R1 **Scan** 8859
**Peptide** ATGFVKKDQLG **Charge** 3 **Spectral Angle** 0.84

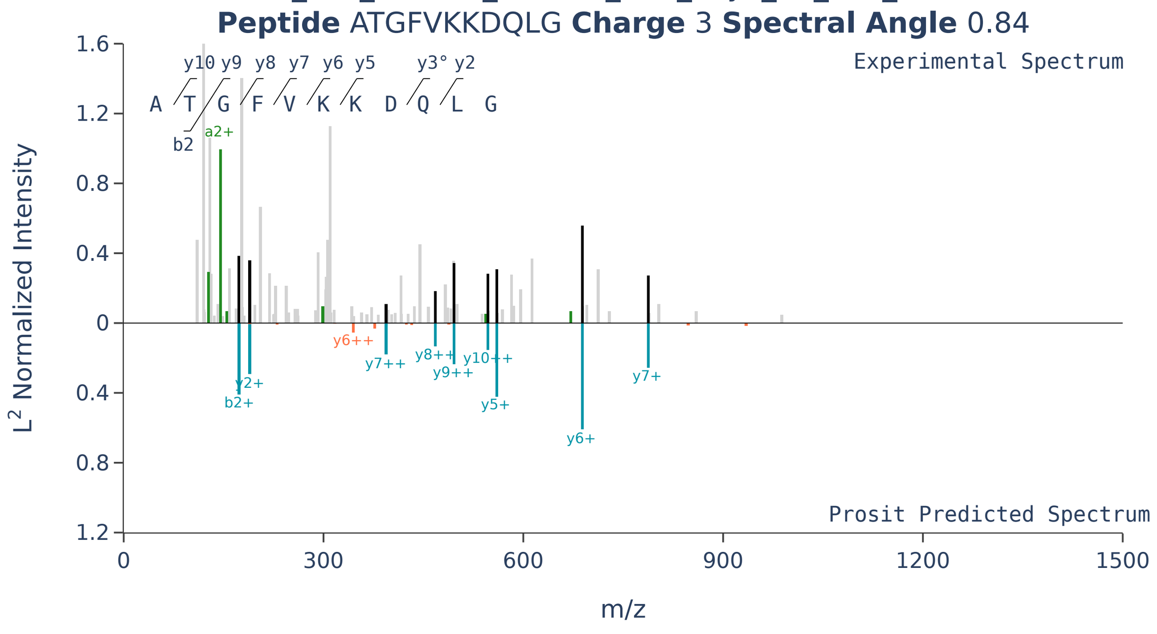


**Source** W_Soh_160821_170921_Goe_aSyn_A2_24h_R2 **Scan** 12073
**Peptide** GVAEAAGKTKEGVLYVGSKT **Charge** 3 **Spectral Angle** 0.89

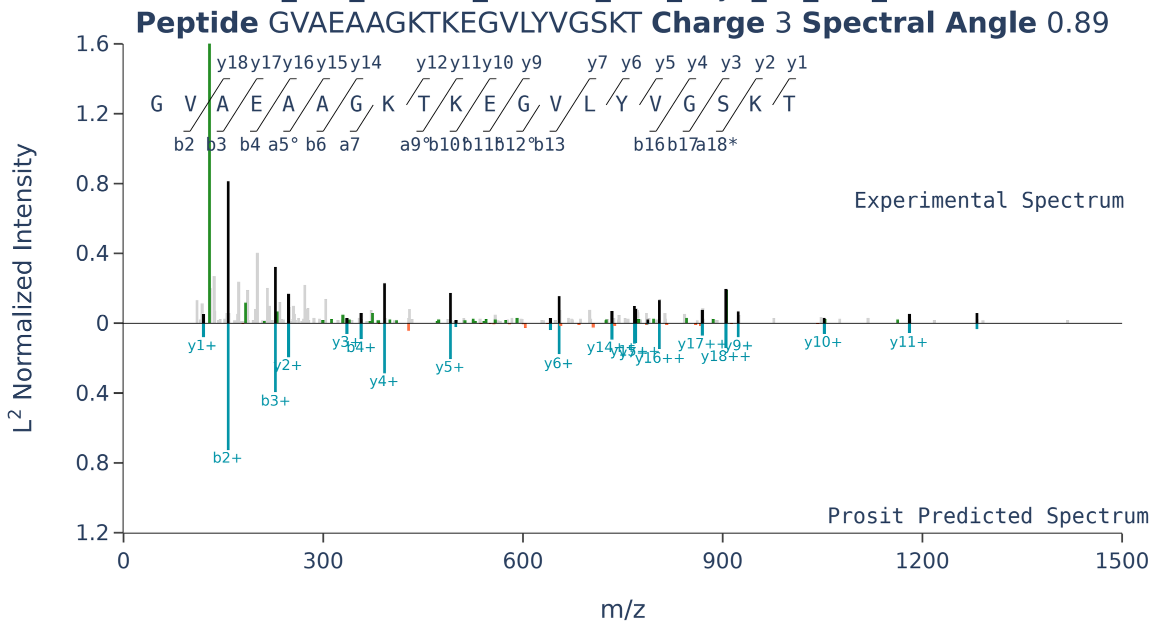


**Source** W_Soh_160821_170921_Goe_aSyn_A1_24h_R2 **Scan** 5909
**Peptide** SKTKEGVVHGV **Charge** 2 **Spectral Angle** 0.77

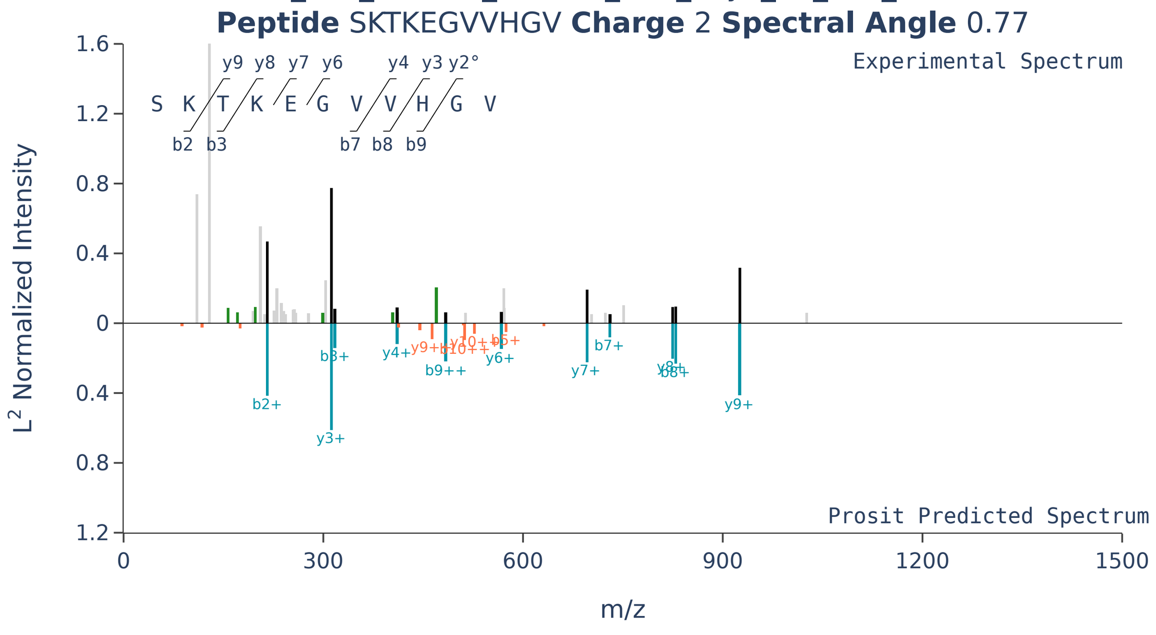


**Source** W_Soh_160821_170921_Goe_aSyn_A1_24h_R1 **Scan** 17030
**Peptide** VEGAGSIAAATGFVKKD **Charge** 2 **Spectral Angle** 0.82

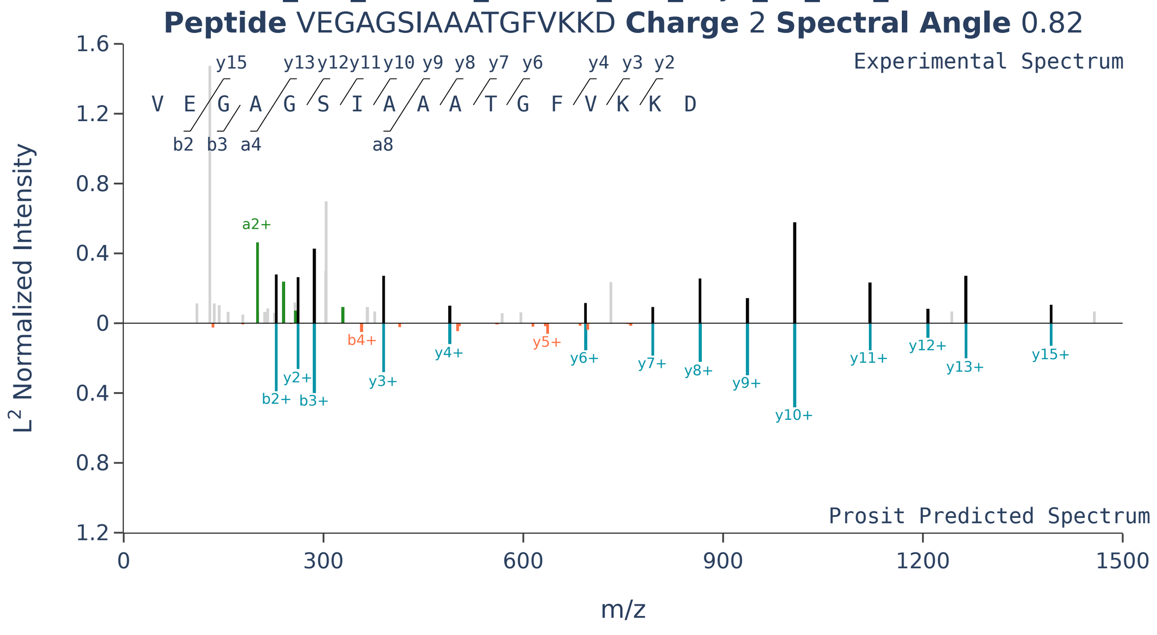


**Source** W_Soh_160821_170921_Goe_aSyn_A1_24h_R2 **Scan** 5998
**Peptide** SKAKEGVVAAAEKT **Charge** 3 **Spectral Angle** 0.86

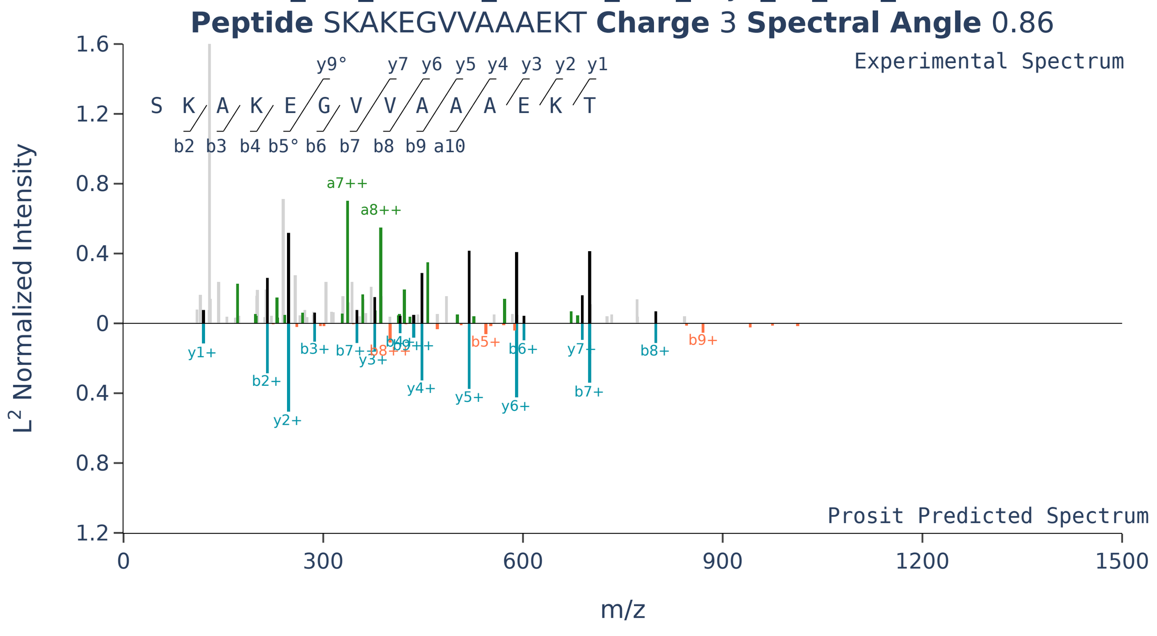


**Source** W_Soh_160821_170921_Goe_aSyn_A1_24h_R2 **Scan** 14735
**Peptide** GAGSIAAATGFVKKDQLGKNEE **Charge** 3 **Spectral Angle** 0.79

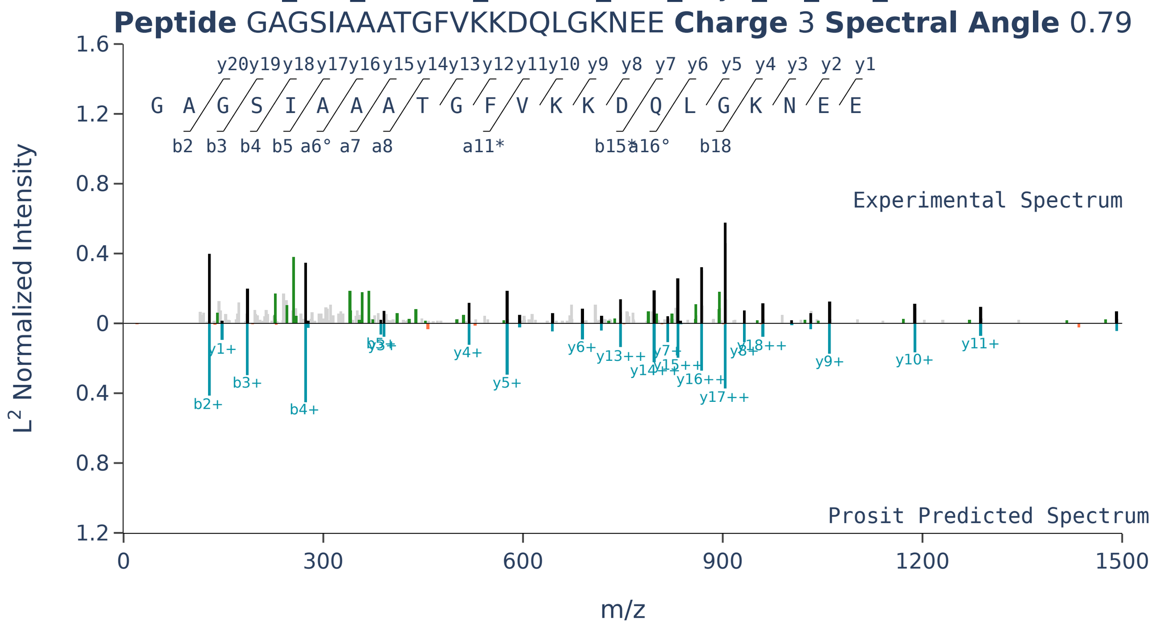


**Source** W_Soh_160821_170921_Goe_aSyn_A2_24h_R2 **Scan** 8504
**Peptide** GKTKEGVLY **Charge** 2 **Spectral Angle** 0.87

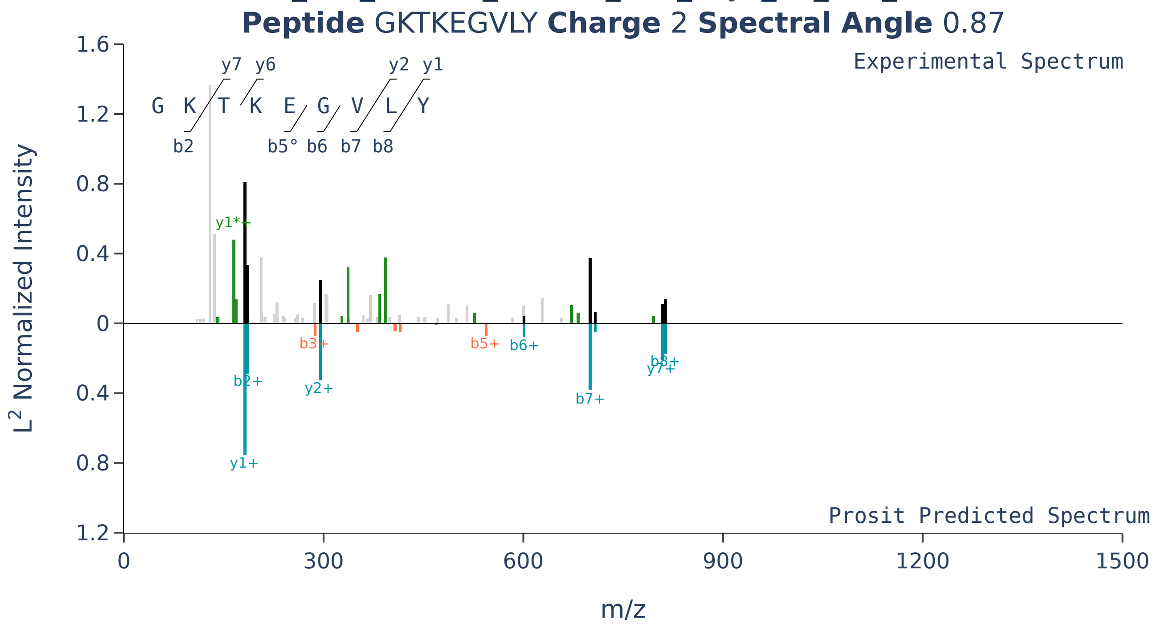


**Source** W_Soh_160821_170921_Goe_aSyn_A1_24h_R1 **Scan** 14804
**Peptide** GVLYVGSKTKEGVVHGVATVAEKT **Charge** 3 **Spectral Angle** 0.87

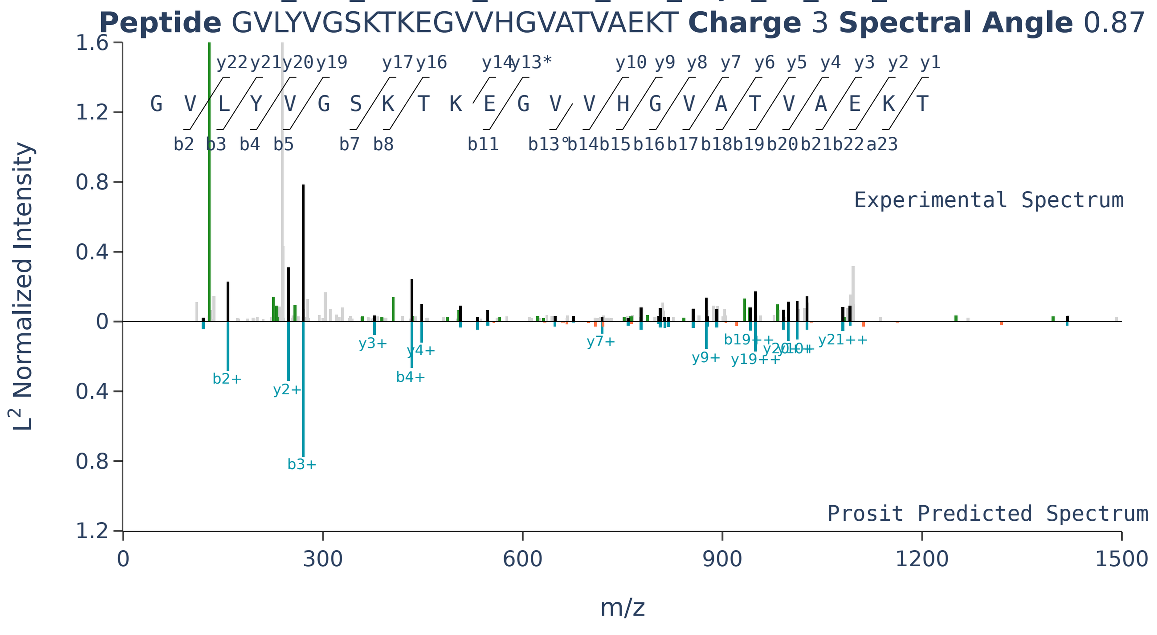


**Source** W_Soh_160821_170921_Goe_aSyn_A1_6h_R1 **Scan** 12904
**Peptide** AGSIAAATGFVKKDQ **Charge** 3 **Spectral Angle** 0.81

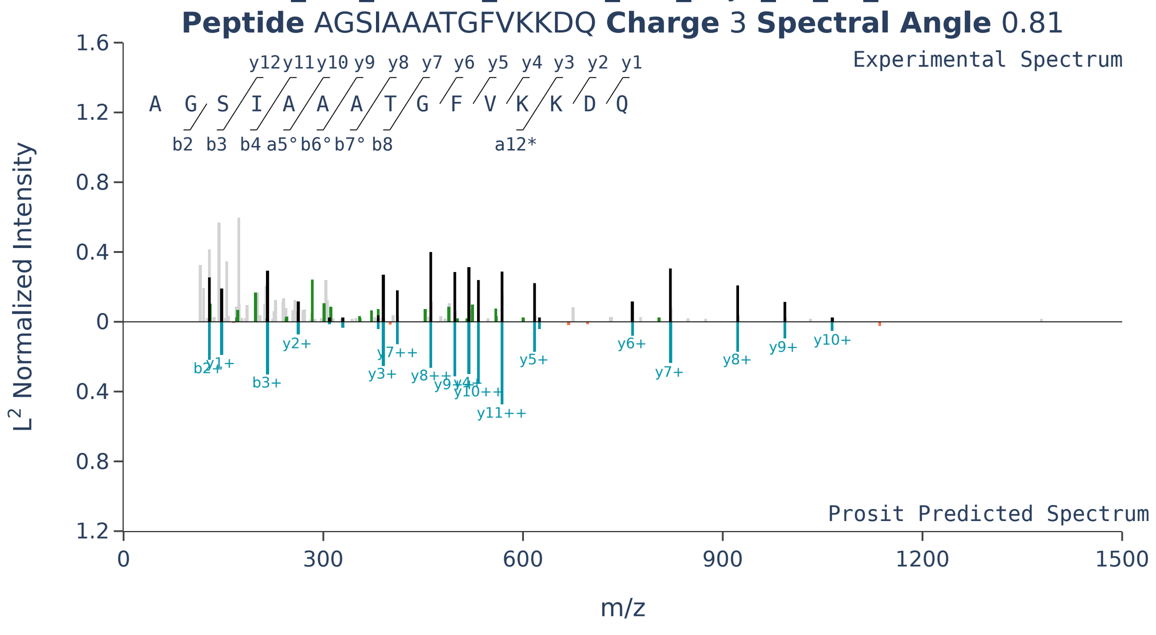


**Source** W_Soh_160821_170921_Goe_aSyn_A2_6h_R1 **Scan** 16571
**Peptide** AGSIAAATGFVKKDQL **Charge** 3 **Spectral Angle** 0.83

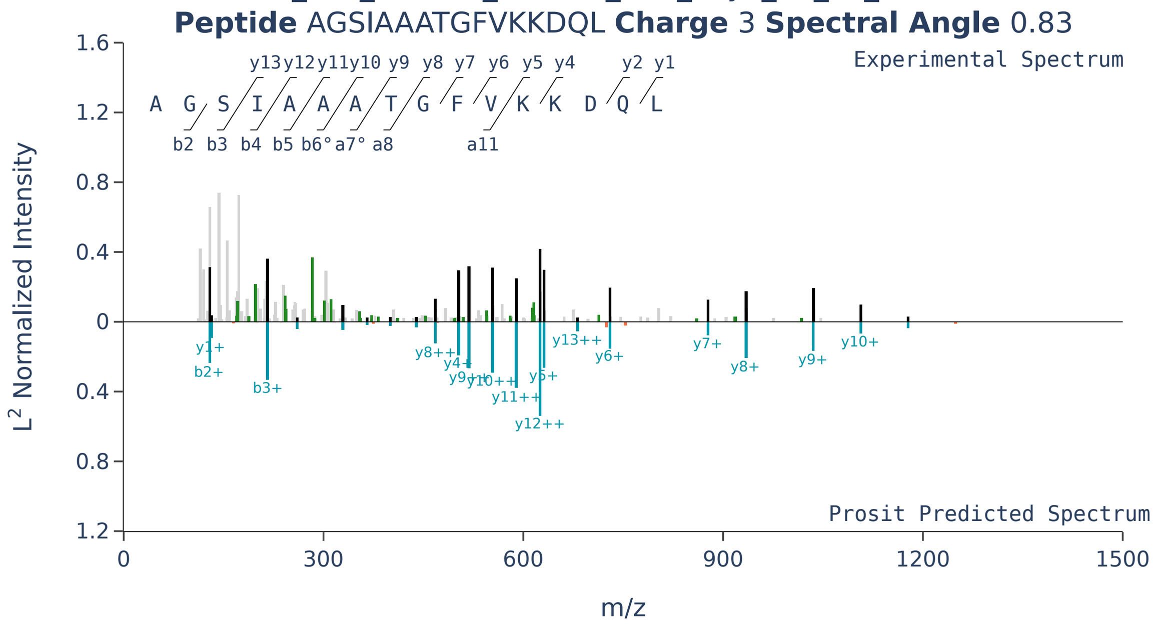


**Source** W_Soh_160821_170921_Goe_aSyn_A2_24h_R1 **Scan** 14103
**Peptide** GVLYVGSKTKEGVV **Charge** 2 **Spectral Angle** 0.86

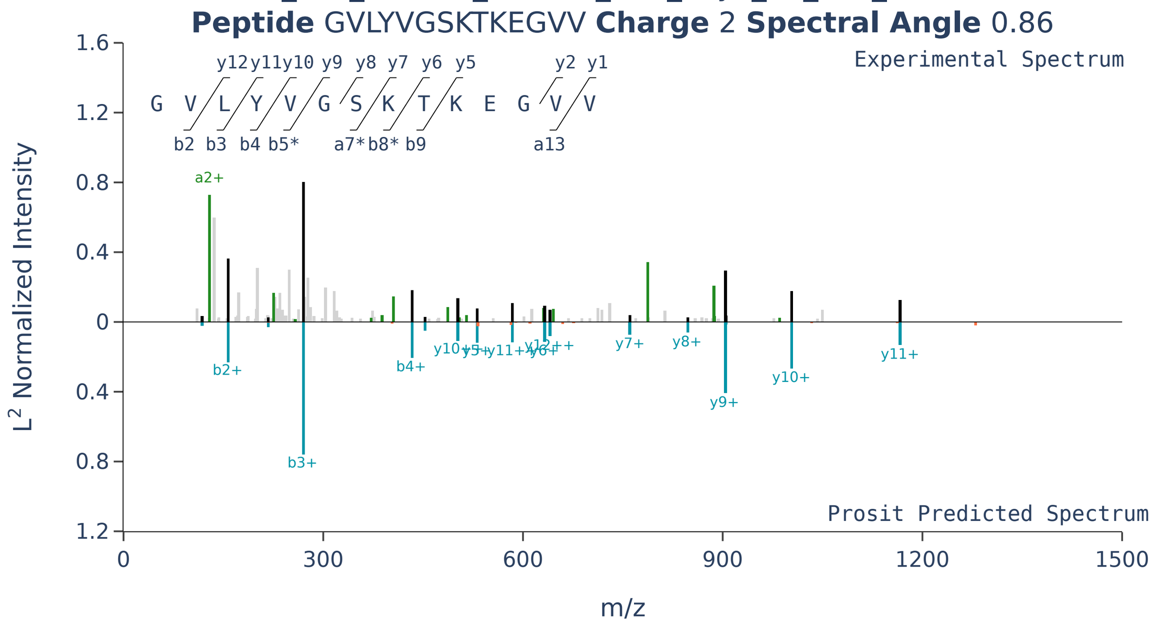


**Source** W_Soh_160821_170921_Goe_aSyn_A1_24h_R2 **Scan** 7655
**Peptide** AATGFVKKDQLGKNE **Charge** 3 **Spectral Angle** 0.82

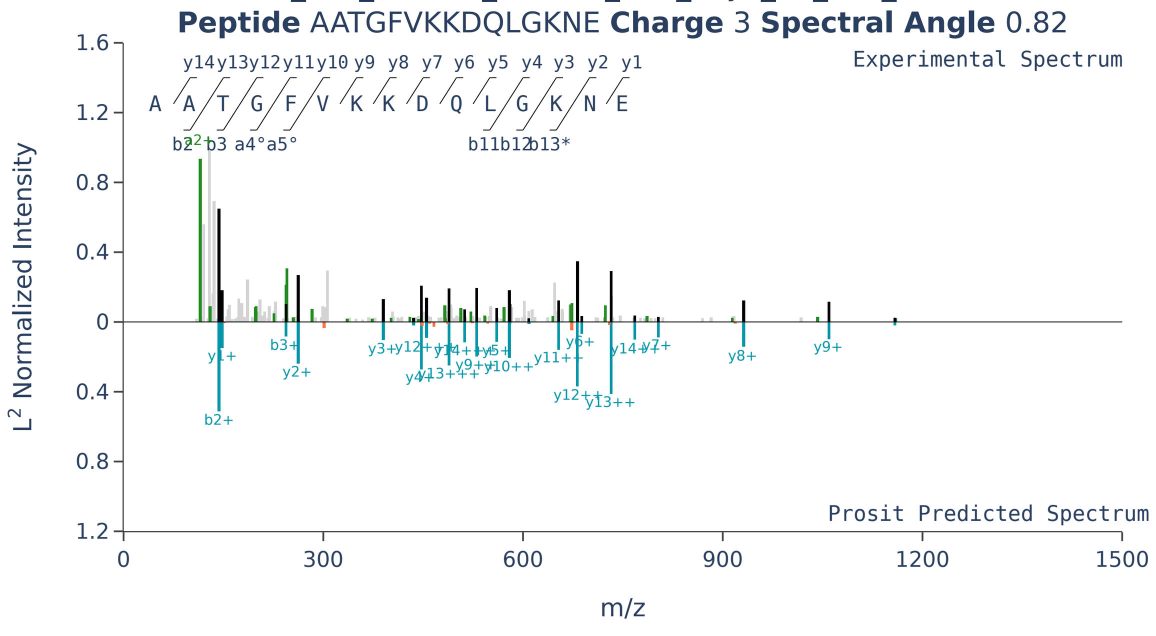


**Source** W_Soh_160821_170921_Goe_aSyn_A1_24h_R2 **Scan** 6352
**Peptide** QLGKNEEGAPQEG **Charge** 2 **Spectral Angle** 0.94

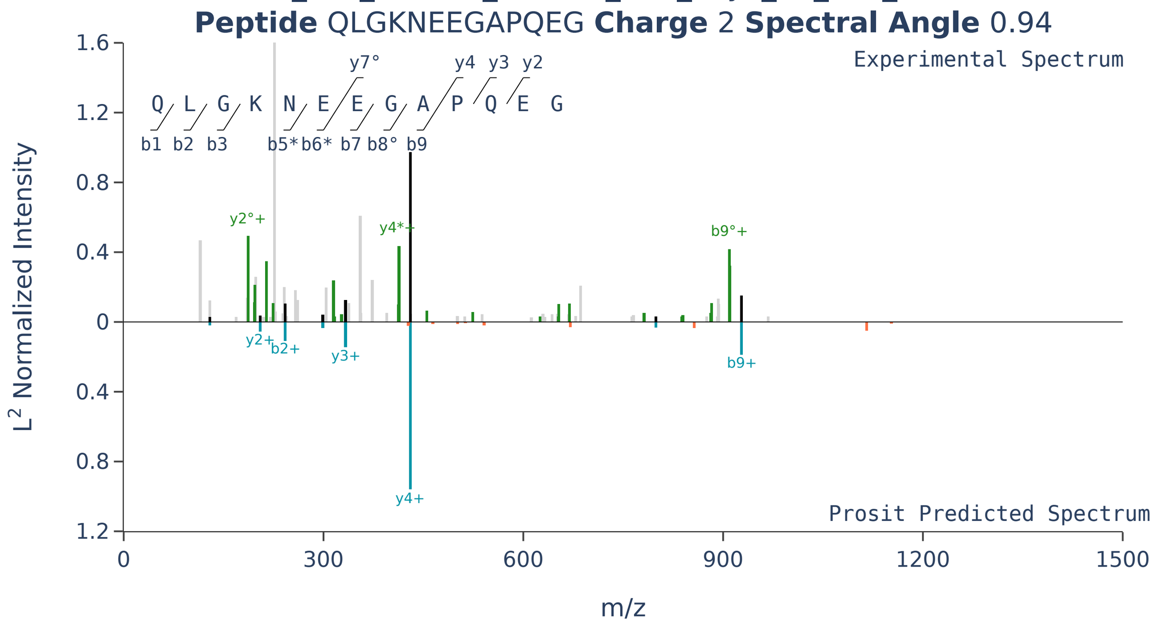

**Source** W_Soh_160821_170921_Goe_aSyn_A2_6h_R1 **Scan** 21183
**Peptide** VGGAVVTGVTAVAQKTVEG **Charge** 3 **Spectral Angle** 0.83

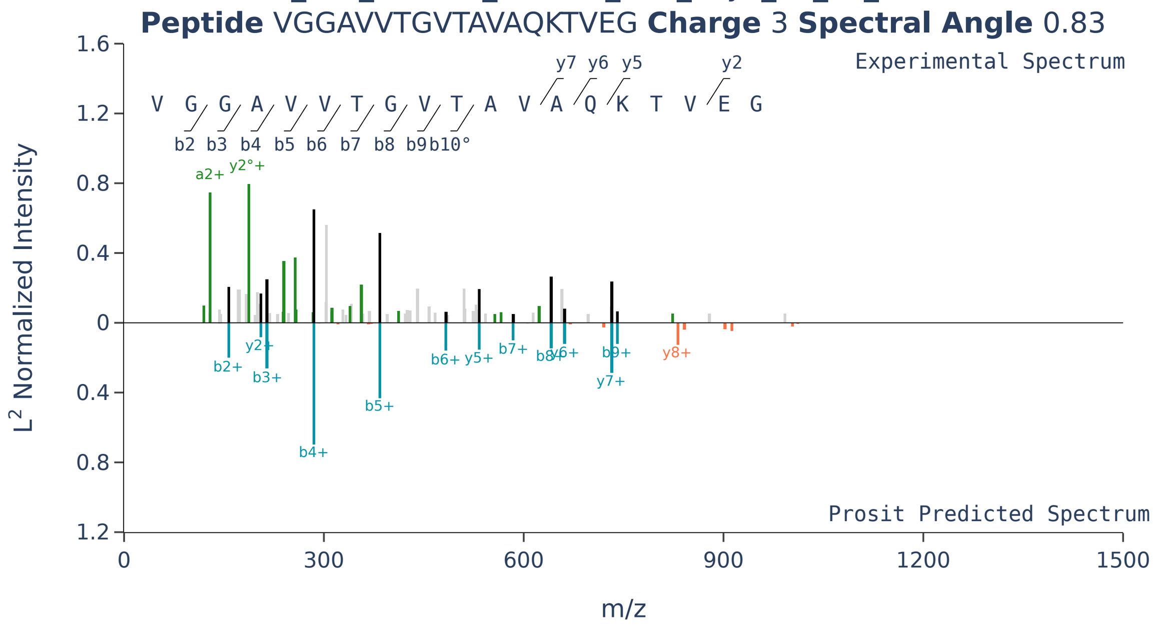


**Source** W_Soh_160821_170921_Goe_aSyn_A2_24h_R2 **Scan** 14576
**Peptide** ATGFVKKDQLGKNEEGAPQEGILED **Charge** 3 **Spectral Angle** 0.83

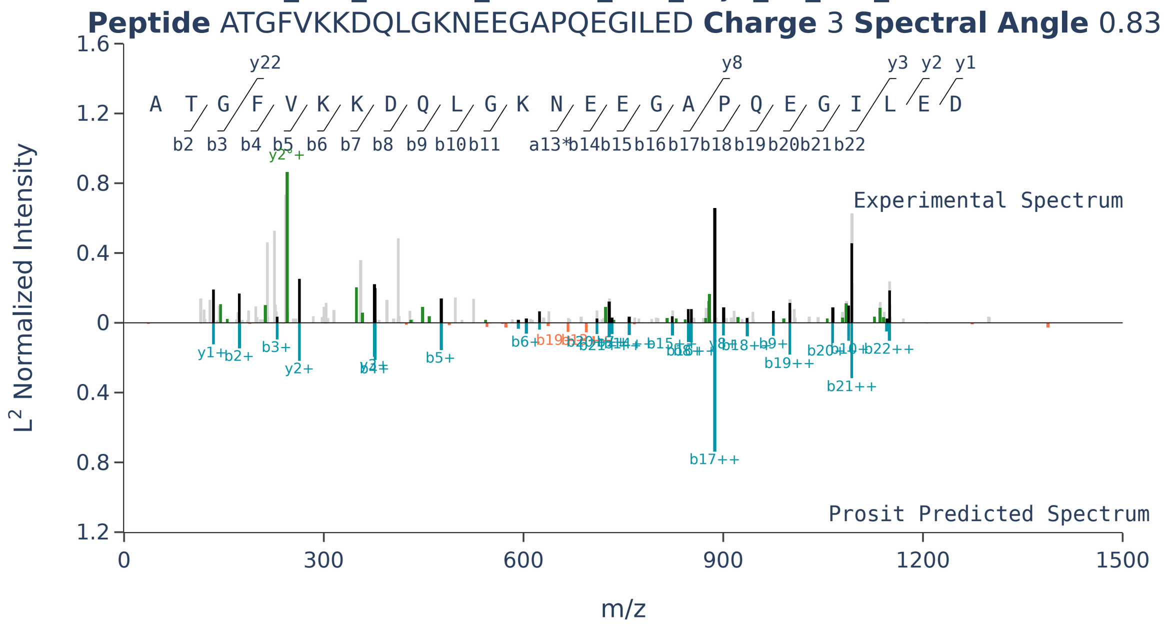


**Source** W_Soh_160821_170921_Goe_aSyn_A2_24h_R2 **Scan** 12793
**Peptide** AGKTKEGVLYVGSKTKEGVVHGVATVAE **Charge** 4 **Spectral Angle** 0.87

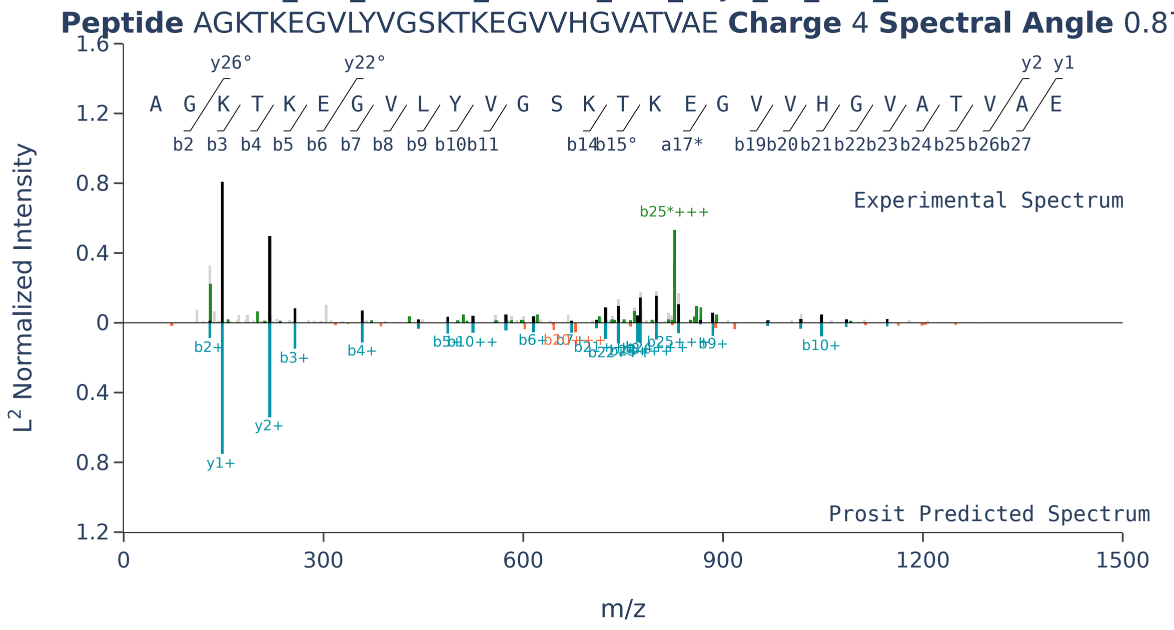


**Source** W_Soh_160821_170921_Goe_aSyn_A1_24h_R1 **Scan** 13975
**Peptide** EGVLYVGSKTKEGVVHGV **Charge** 4 **Spectral Angle** 0.88

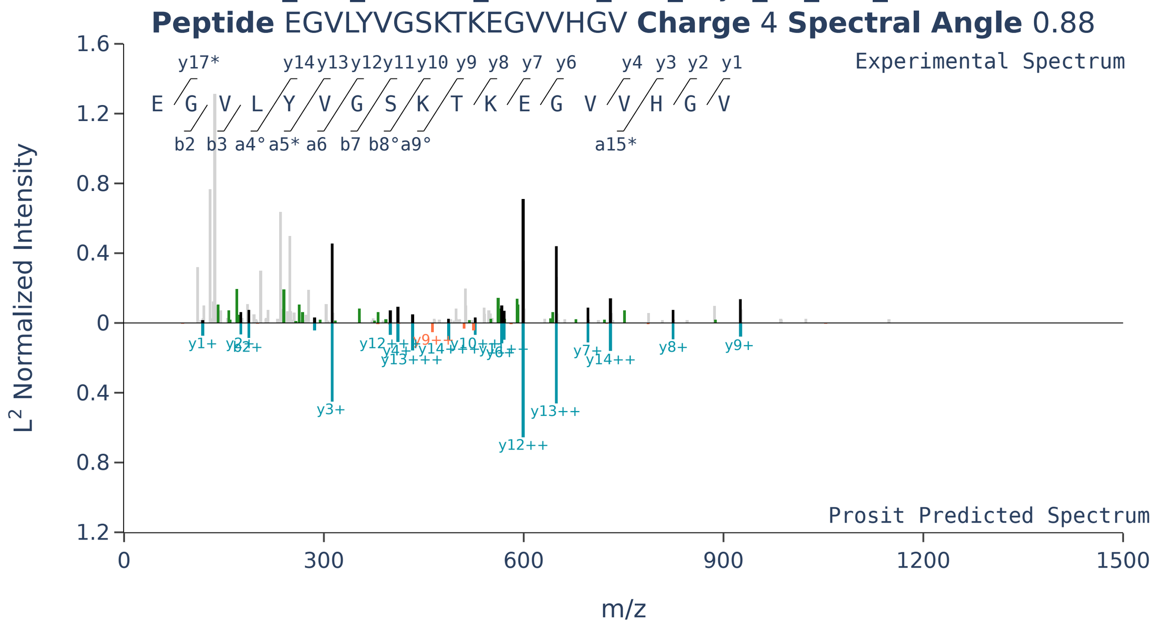


**Source** W_Soh_160821_170921_Goe_aSyn_A2_24h_R2 **Scan** 13168
**Peptide** GKNEEGAPQEGILE **Charge** 2 **Spectral Angle** 0.89

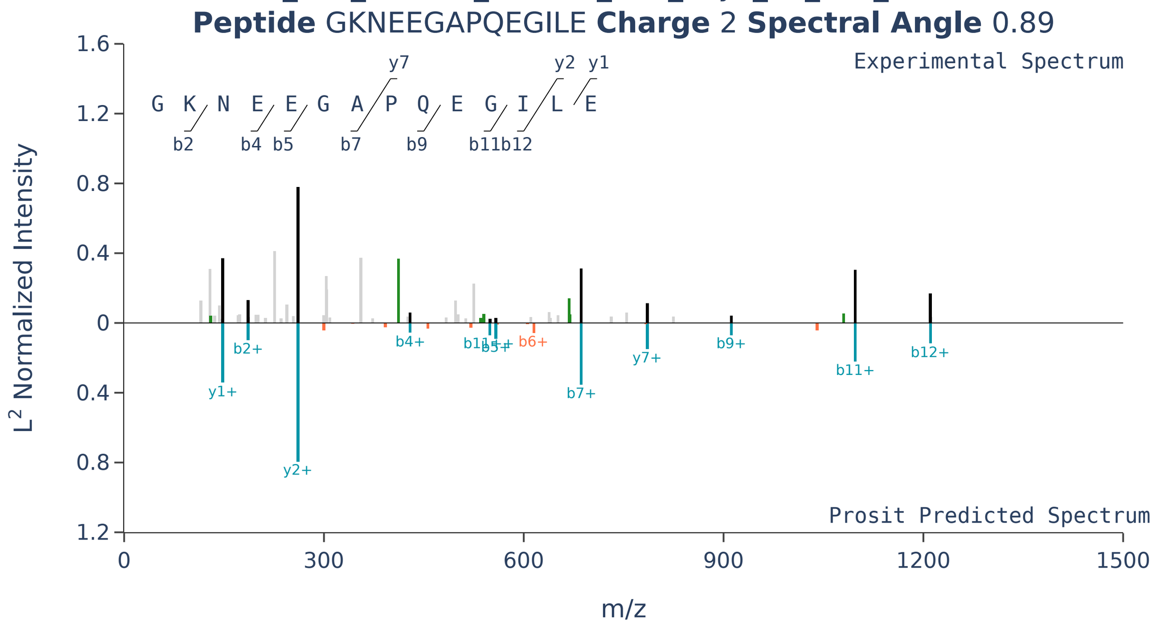


**Source** W_Soh_160821_170921_Goe_aSyn_A1_24h_R2 **Scan** 17818
**Peptide** TAVAQKTVEGAGSIAAATGFVKKD **Charge** 3 **Spectral Angle** 0.84

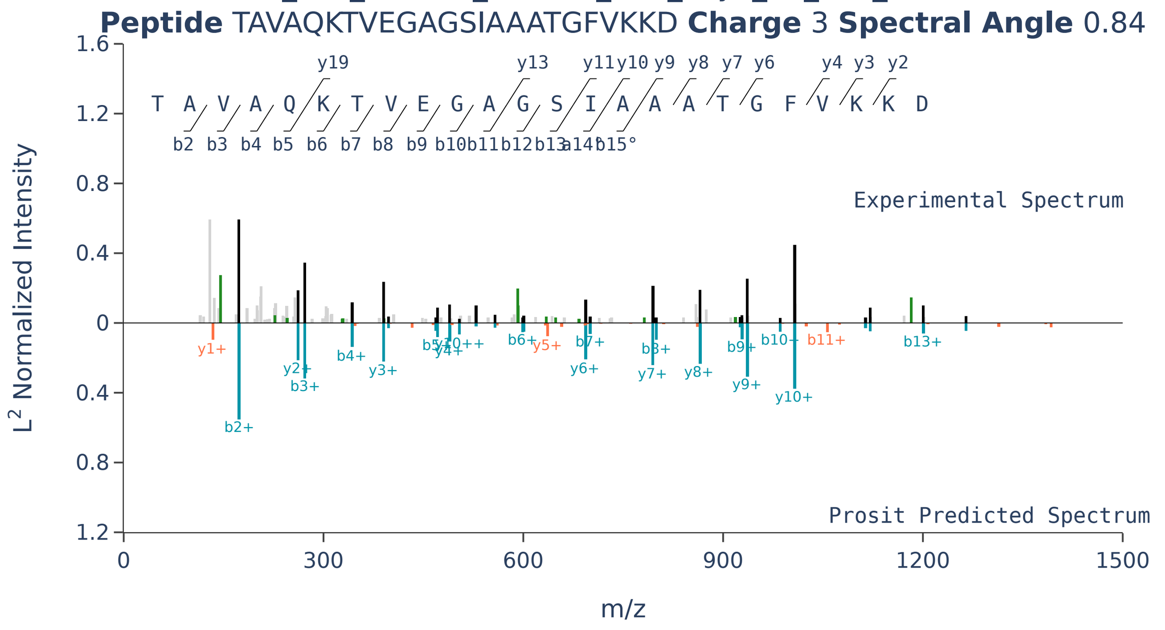


**Source** W_Soh_160821_170921_Goe_aSyn_A2_6h_R1 **Scan** 11046
**Peptide** ATGFVKKDQLGKNEEGAPQEGI **Charge** 3 **Spectral Angle** 0.87

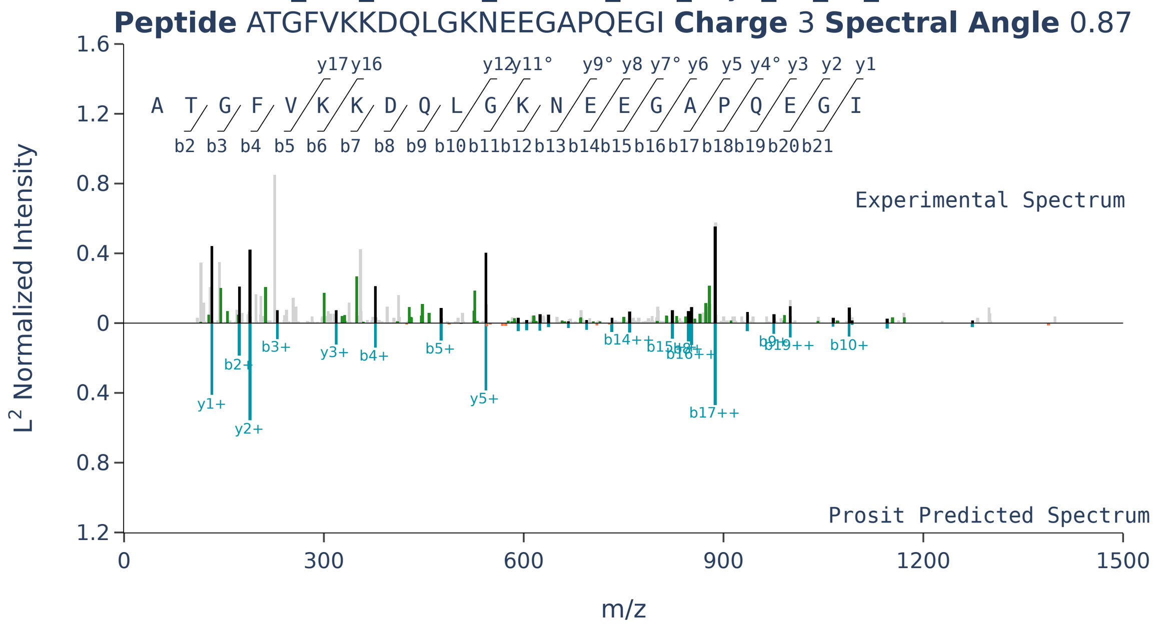


**Source** W_Soh_160821_170921_Goe_aSyn_A1_24h_R2 **Scan** 17062
**Peptide** DQLGKNEEGAPQEGILE **Charge** 2 **Spectral Angle** 0.88

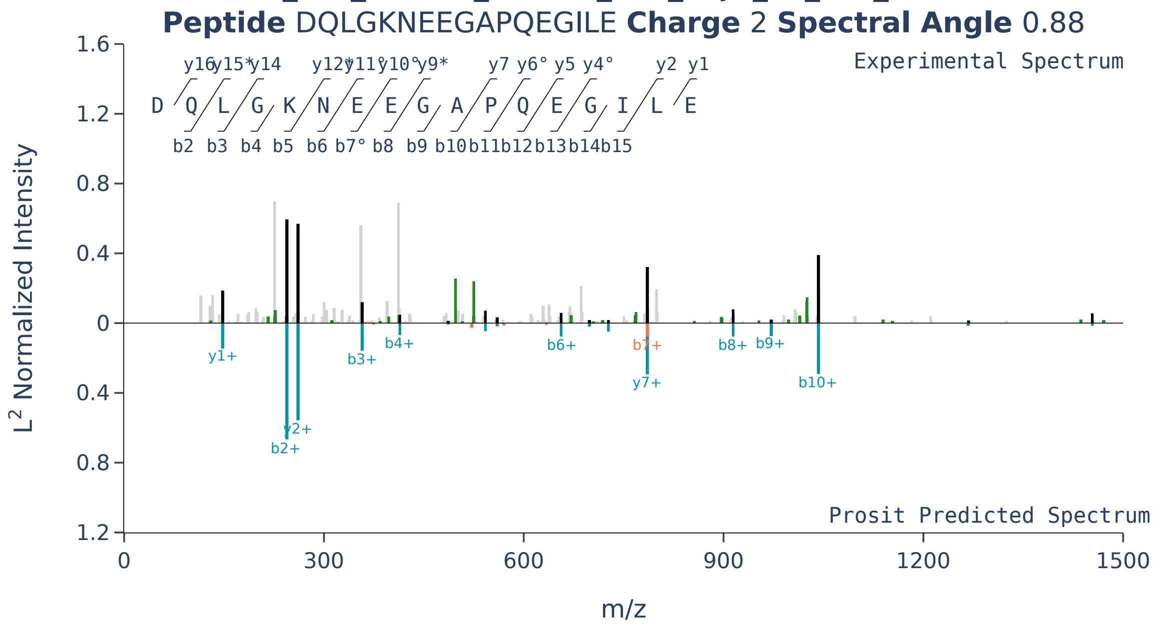


**Source** W_Soh_160821_170921_Goe_aSyn_A1_24h_R2 **Scan** 9794
**Peptide** TKEGVVHGVATV **Charge** 2 **Spectral Angle** 0.8

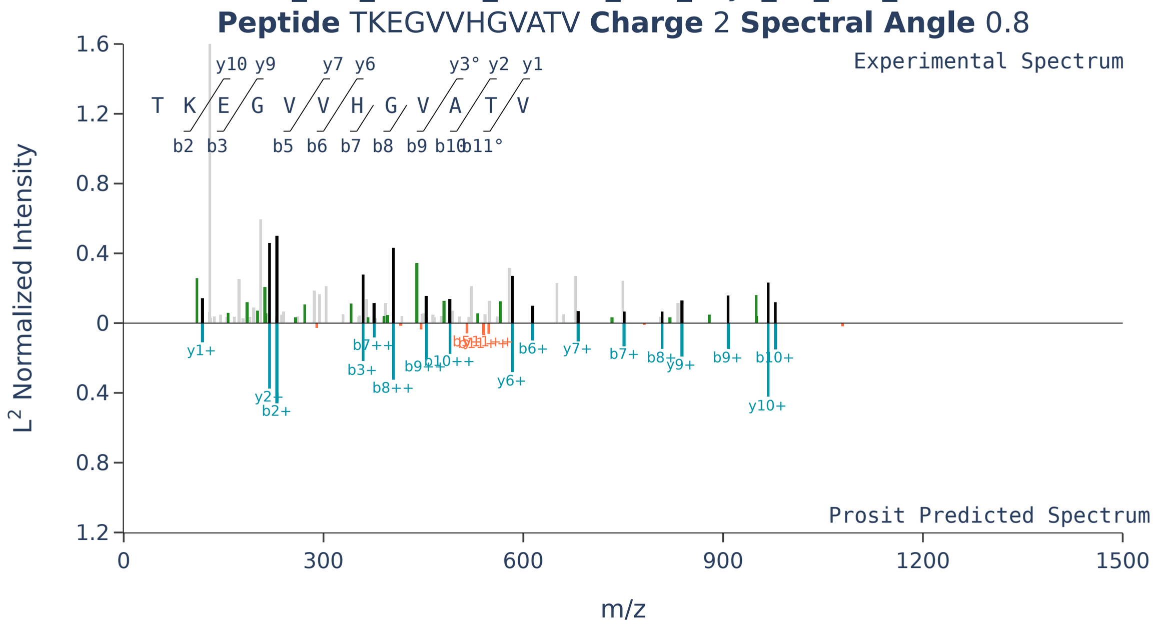


**Source** W_Soh_160821_170921_Goe_aSyn_A1_24h_R1 **Scan** 6720
**Peptide** KTKEGVVHGVATVAEK **Charge** 3 **Spectral Angle** 0.76

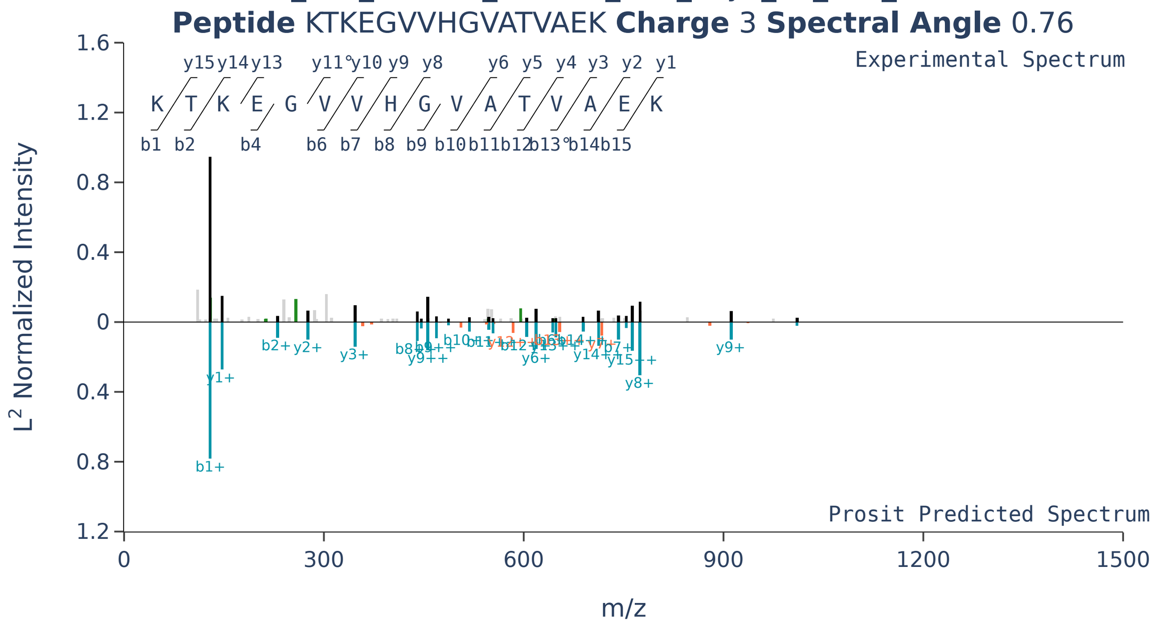


**Source** W_Soh_160821_170921_Goe_aSyn_A2_24h_R2 **Scan** 18010
**Peptide** GLLEDMPVD **Charge** 1 **Spectral Angle** 0.85

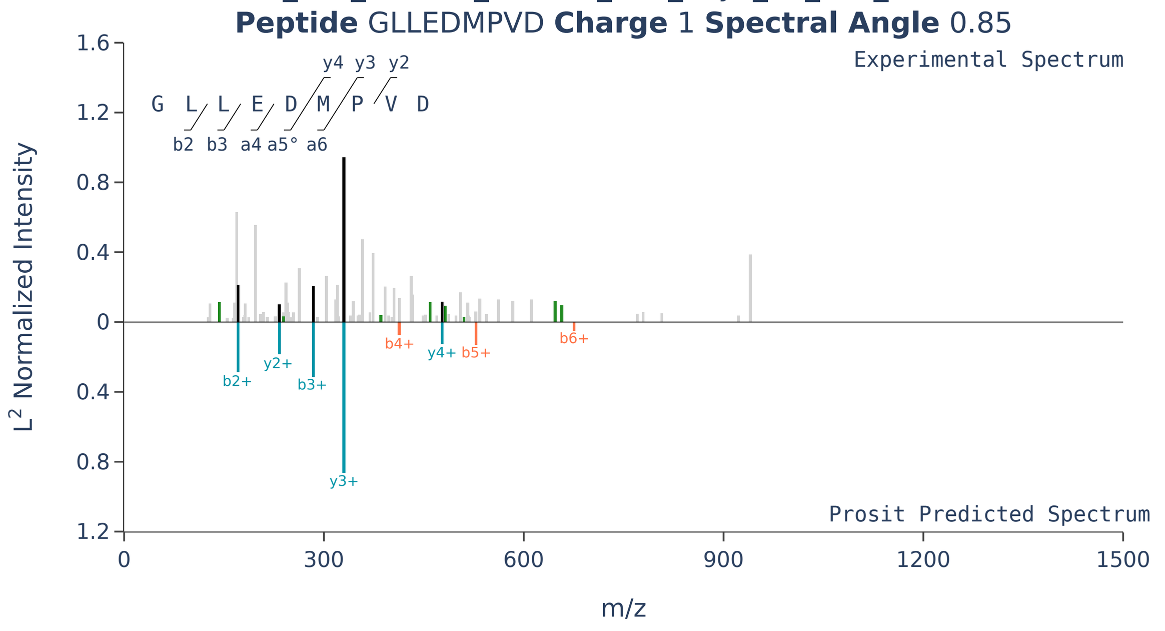


**Source** W_Soh_160821_170921_Goe_aSyn_A1_24h_R2 **Scan** 7921
**Peptide** GVVAAAEKTKQGV **Charge** 2 **Spectral Angle** 0.81

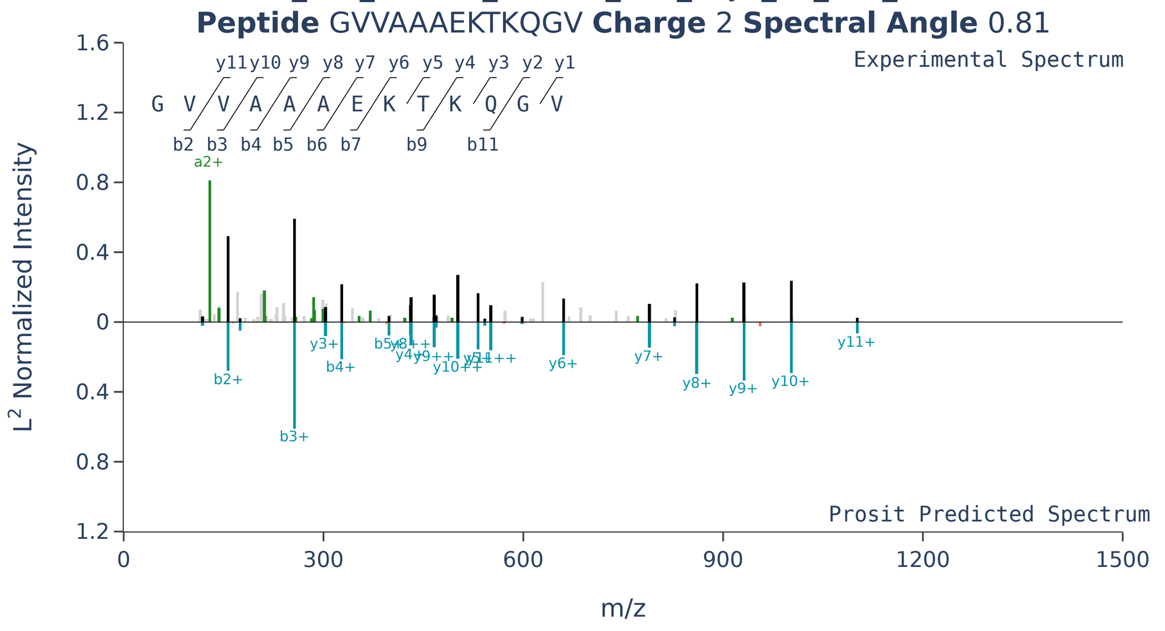


**Source** W_Soh_160821_170921_Goe_aSyn_A1_6h_R2 **Scan** 19985
**Peptide** KEQVTNVGGAVVTGVTAV **Charge** 3 **Spectral Angle** 0.69

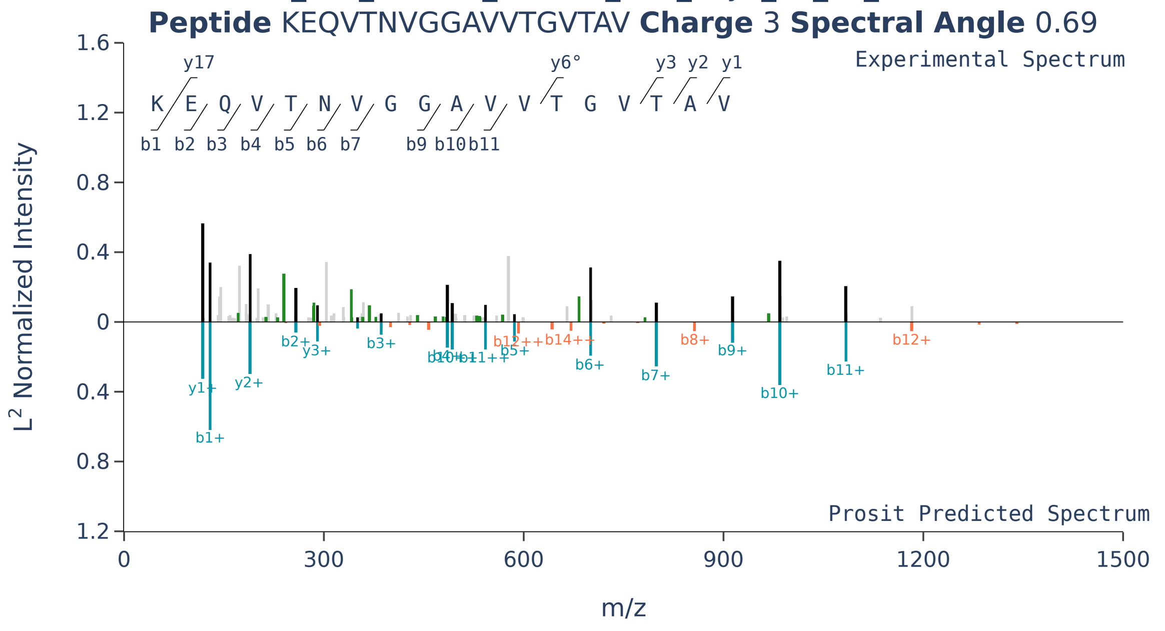


**Source** W_Soh_160821_170921_Goe_aSyn_A1_6h_R2 **Scan** 14364
**Peptide** KTVEGAGSIAAATGFVKKDQ **Charge** 3 **Spectral Angle** 0.82

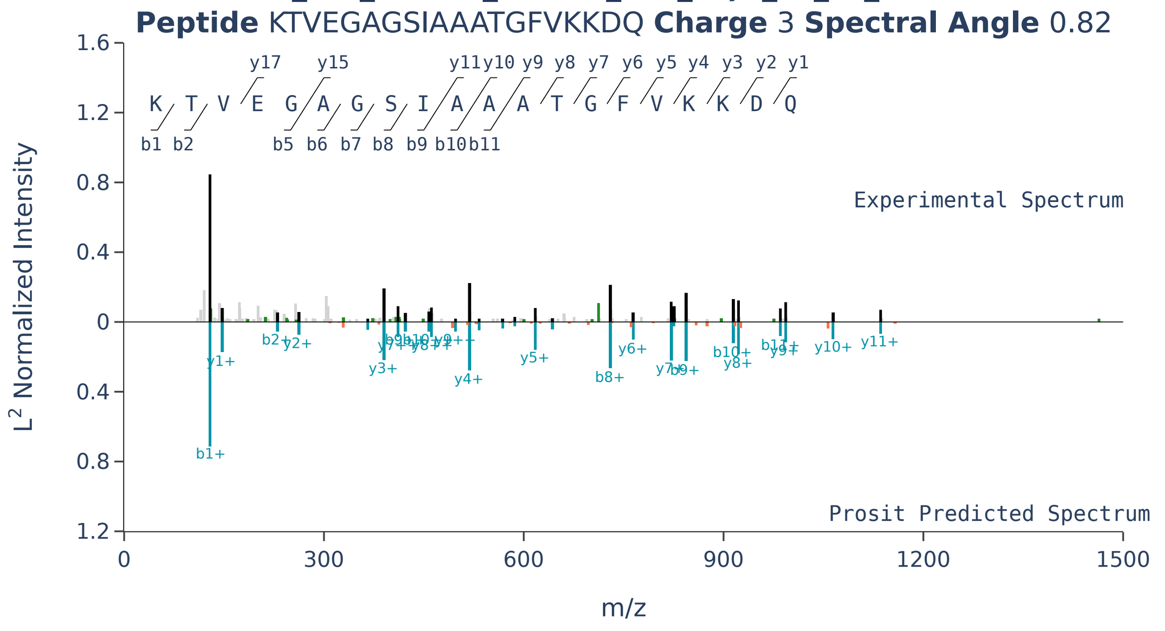


**Source** W_Soh_160821_170921_Goe_aSyn_A1_6h_R1 **Scan** 19366
**Peptide** TVAEKTKEQVTNVGGAVVTGVTAV **Charge** 3 **Spectral Angle** 0.83

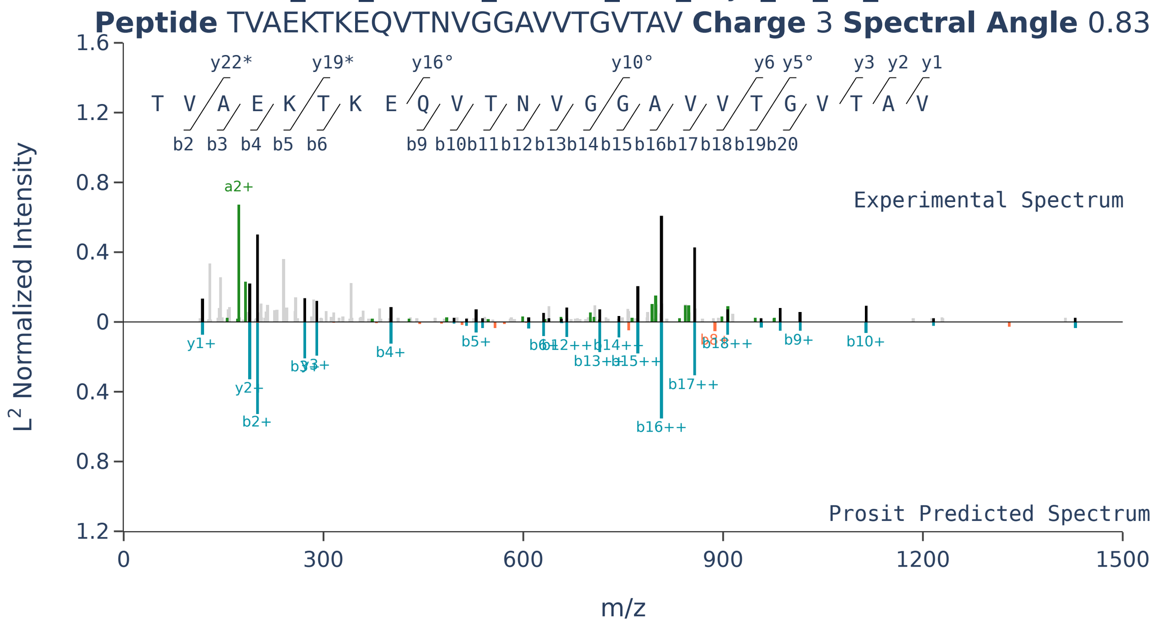

**Source** W_Soh_160821_170921_Goe_aSyn_A1_6h_R2 **Scan** 9947
**Peptide** KEGVLYVGSKT **Charge** 3 **Spectral Angle** 0.87

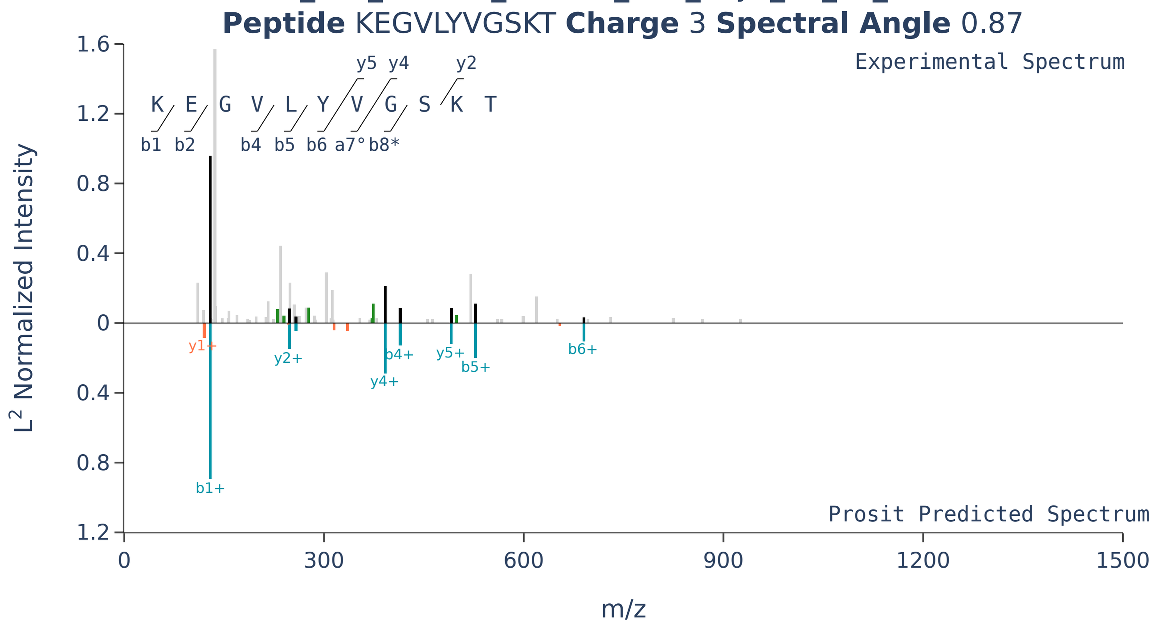


**Source** W_Soh_160821_170921_Goe_aSyn_A2_24h_R2 **Scan** 14889
**Peptide** AATGFVKKDQLGKNEEGAPQEGILE **Charge** 3 **Spectral Angle** 0.9

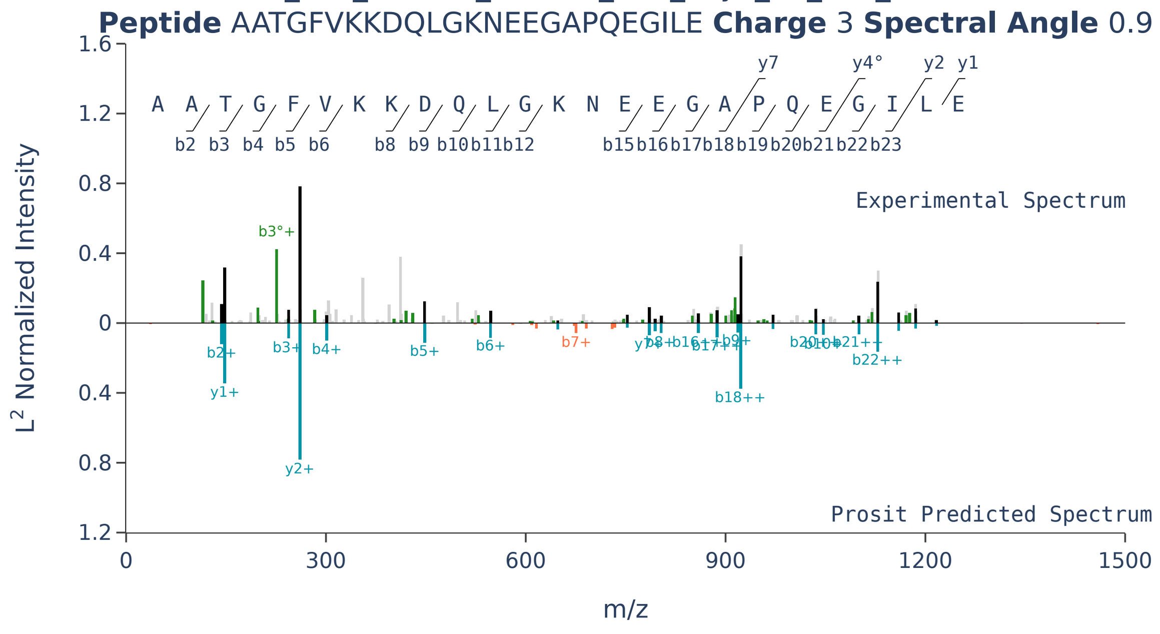


**Source** W_Soh_160821_170921_Goe_aSyn_A1_24h_R1 **Scan** 22317
**Peptide** AEKTKEQVTNVGGAVVTGVTAVAQKTV **Charge** 3 **Spectral Angle** 0.8

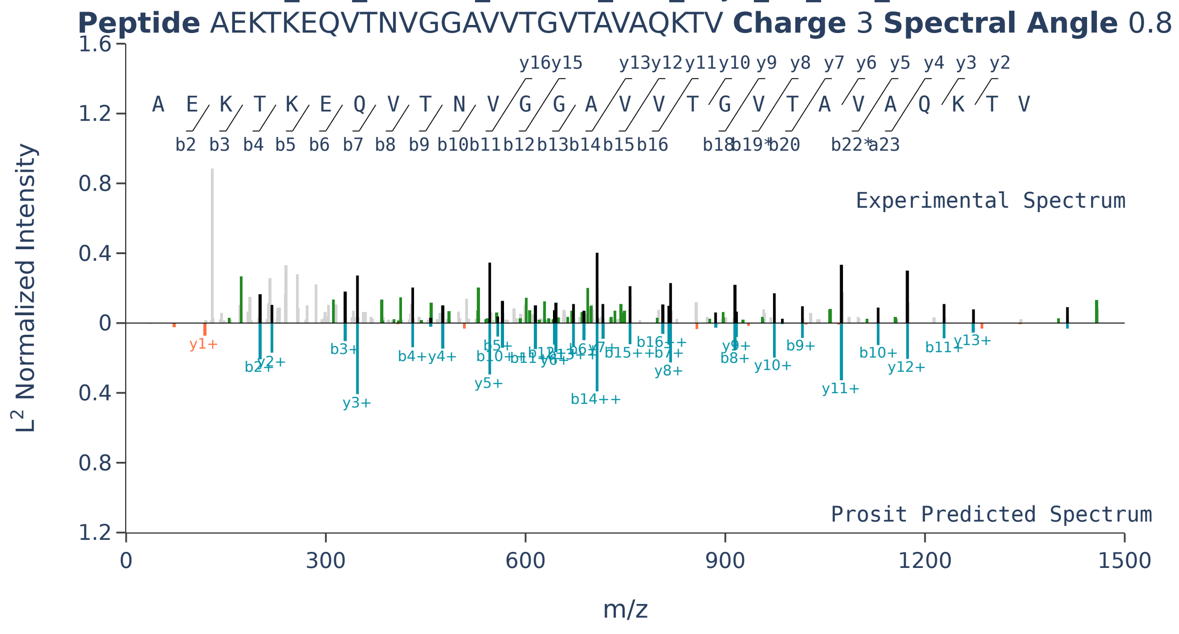


**Source** W_Soh_160821_170921_Goe_aSyn_A1_6h_R1 **Scan** 10784
**Peptide** AEKTKEQVTNVGGAVV **Charge** 3 **Spectral Angle** 0.82

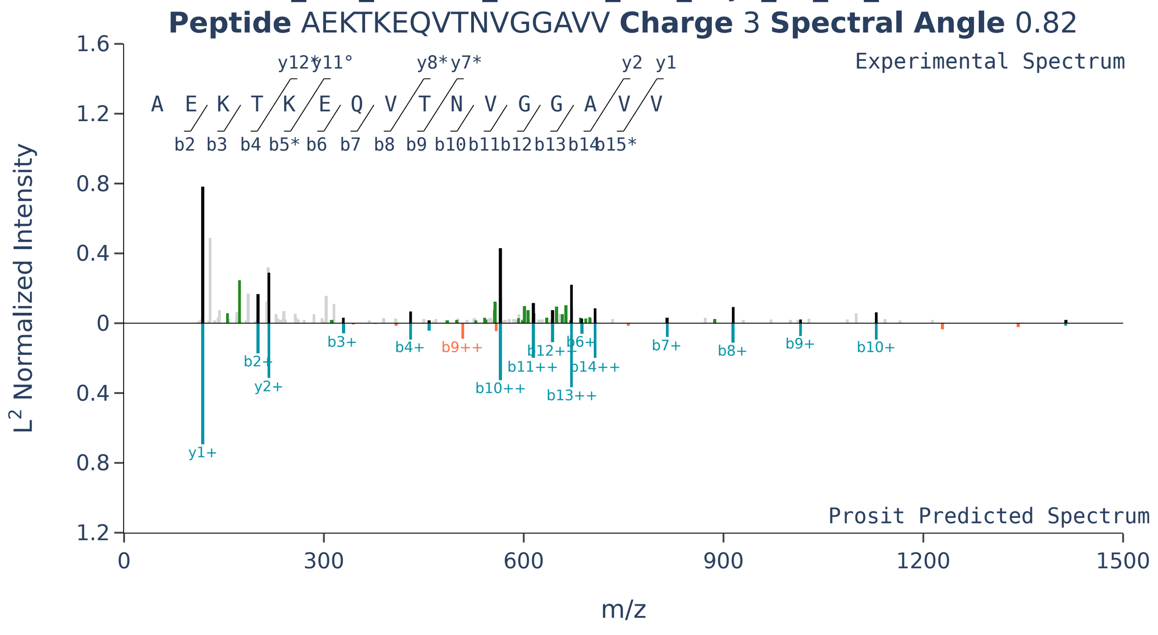


**Source** W_Soh_160821_170921_Goe_aSyn_A1_6h_R2 **Scan** 17782
**Peptide** VATVAEKTKEQVTNVGGAVVTGVT **Charge** 3 **Spectral Angle** 0.81

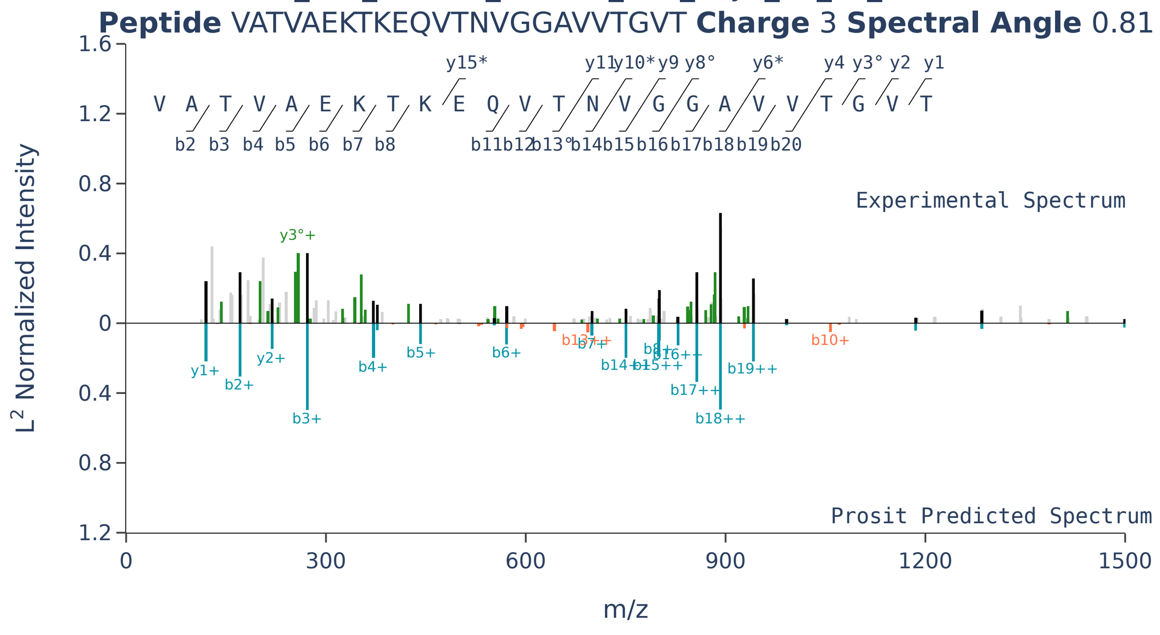


**Source** W_Soh_160821_170921_Goe_aSyn_A2_6h_R1 **Scan** 17361
**Peptide** GAGSIAAATGFVKKDQL **Charge** 3 **Spectral Angle** 0.77

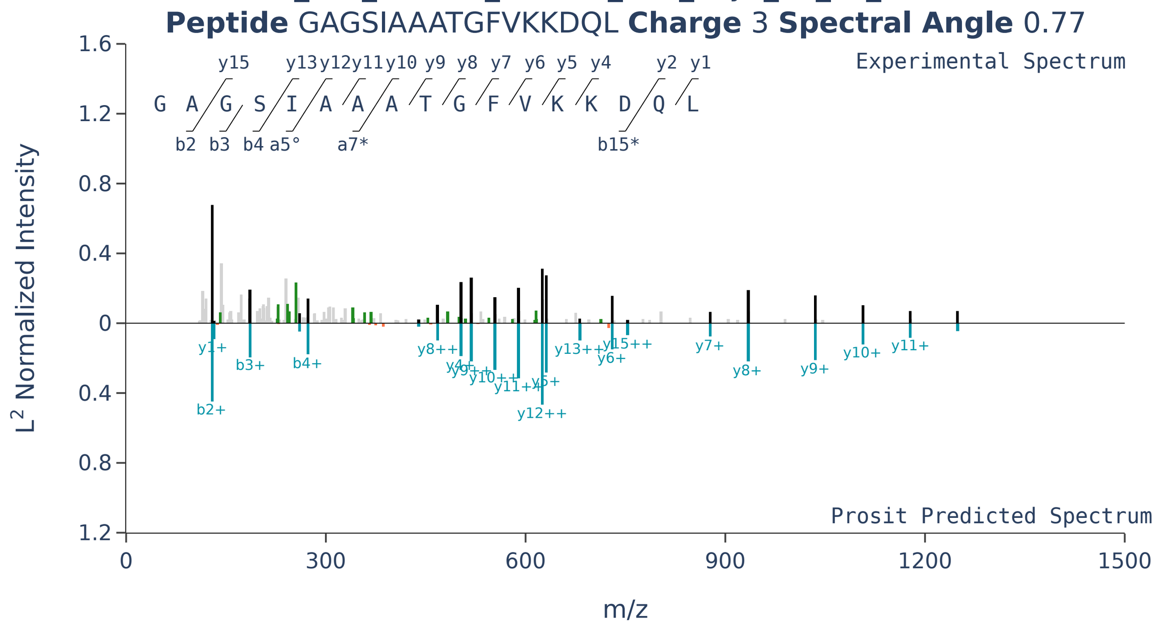


**Source** W_Soh_160821_170921_Goe_aSyn_A1_24h_R2 **Scan** 9172
**Peptide** KEGVVAAAEKTKQGVAEAAGK **Charge** 3 **Spectral Angle** 0.93

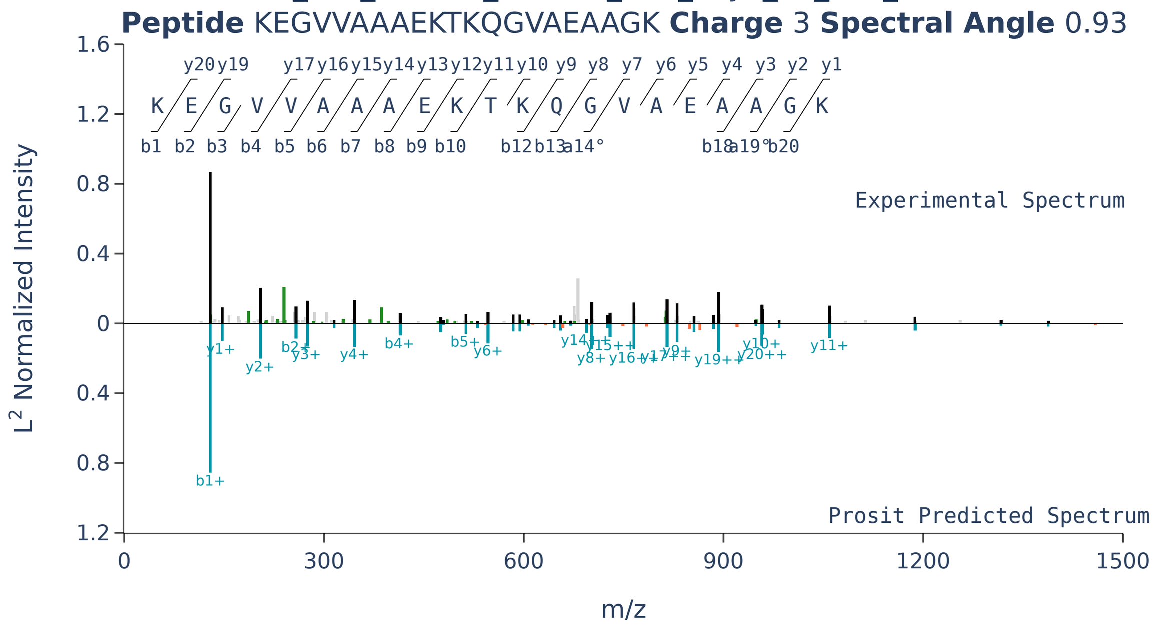


**Source** W_Soh_160821_170921_Goe_aSyn_A1_24h_R1 **Scan** 16654
**Peptide** TNVGGAVVTGVT **Charge** 2 **Spectral Angle** 0.89

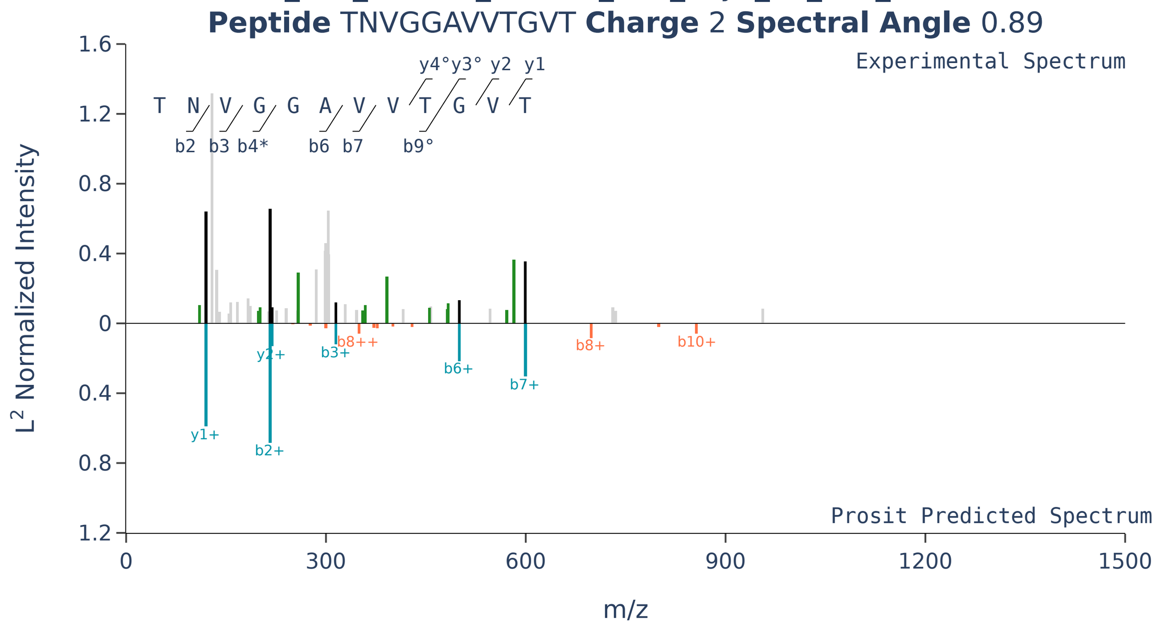


**Source** W_Soh_160821_170921_Goe_aSyn_A1_3h_R1 **Scan** 9837
**Peptide** GVLYVGSKTKEGVVHG **Charge** 3 **Spectral Angle** 0.83

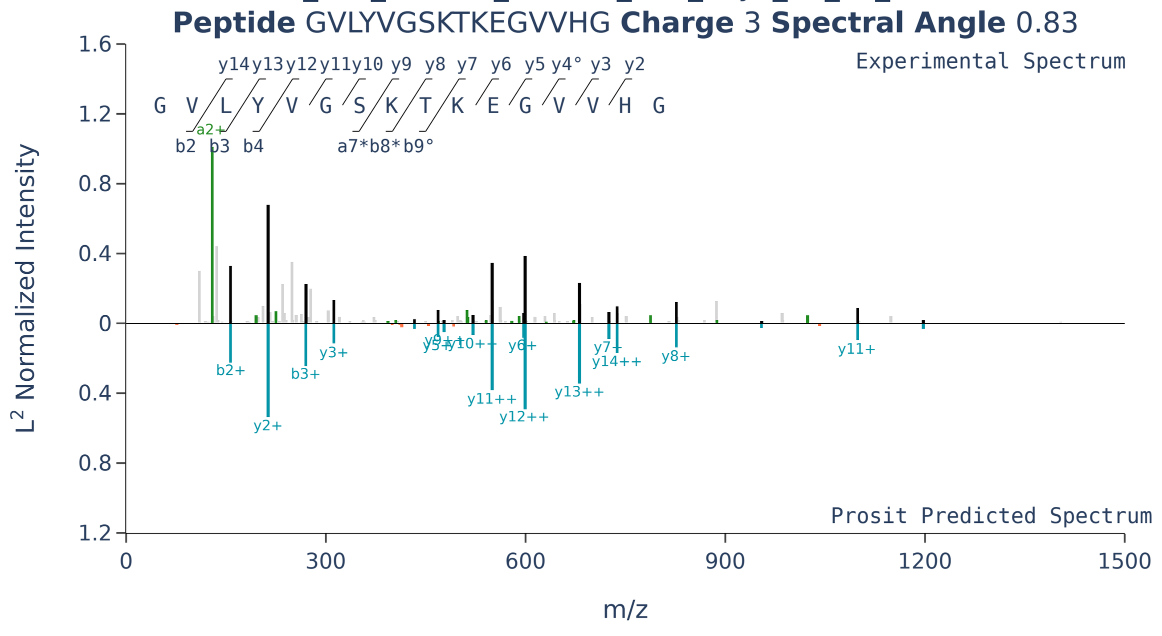


**Source** W_Soh_160821_170921_Goe_aSyn_A1_3h_R1 **Scan** 22584
**Peptide** GVATVAEKTKEQVTNVGGAVVTGVTAVAQK **Charge** 4 **Spectral Angle** 0.83

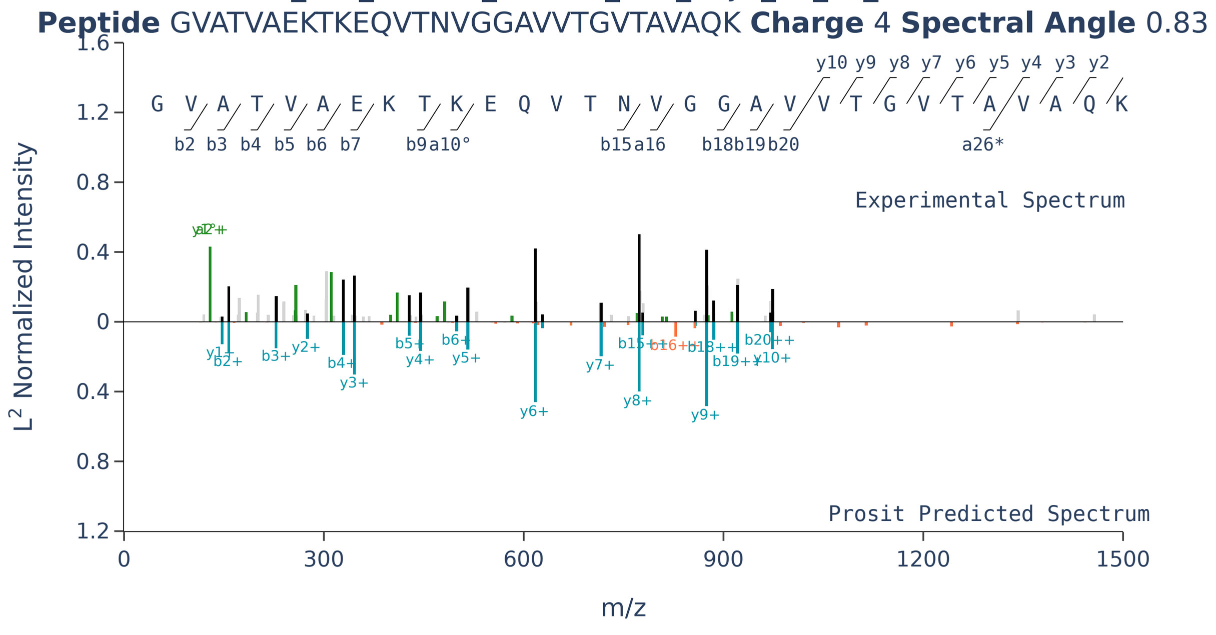


**Source** W_Soh_160821_170921_Goe_aSyn_A1_24h_R1 **Scan** 11480
**Peptide** TGVTAVAQKTVEG **Charge** 2 **Spectral Angle** 0.89

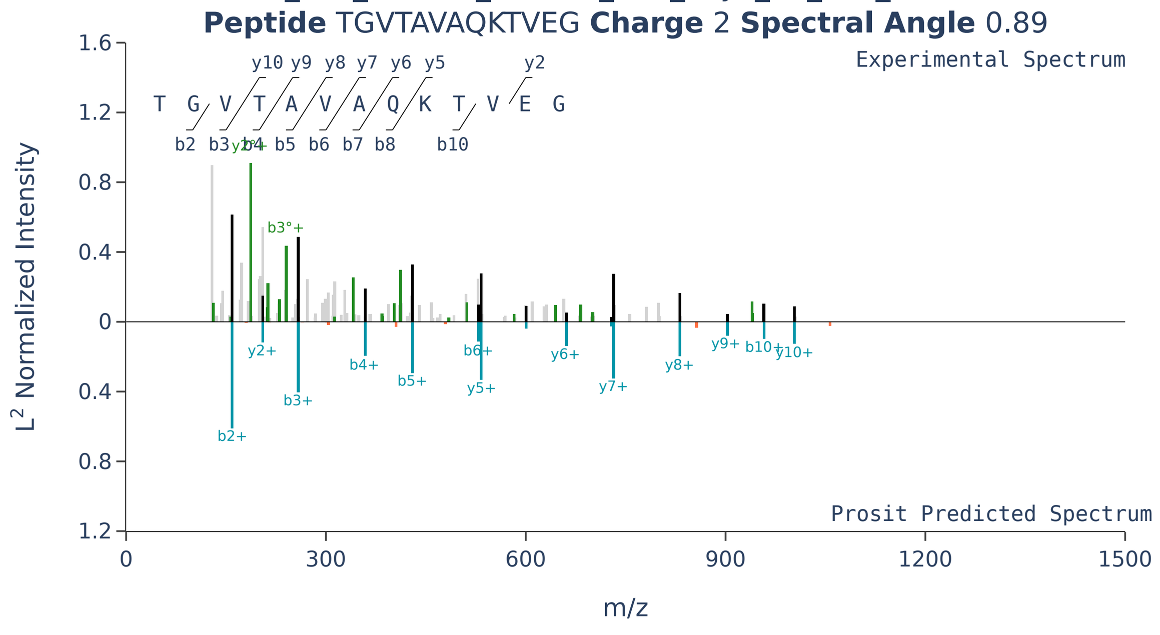


**Source** W_Soh_160821_170921_Goe_aSyn_A1_24h_R1 **Scan** 17601
**Peptide** EDMPVDPDNEAYE **Charge** 2 **Spectral Angle** 0.84

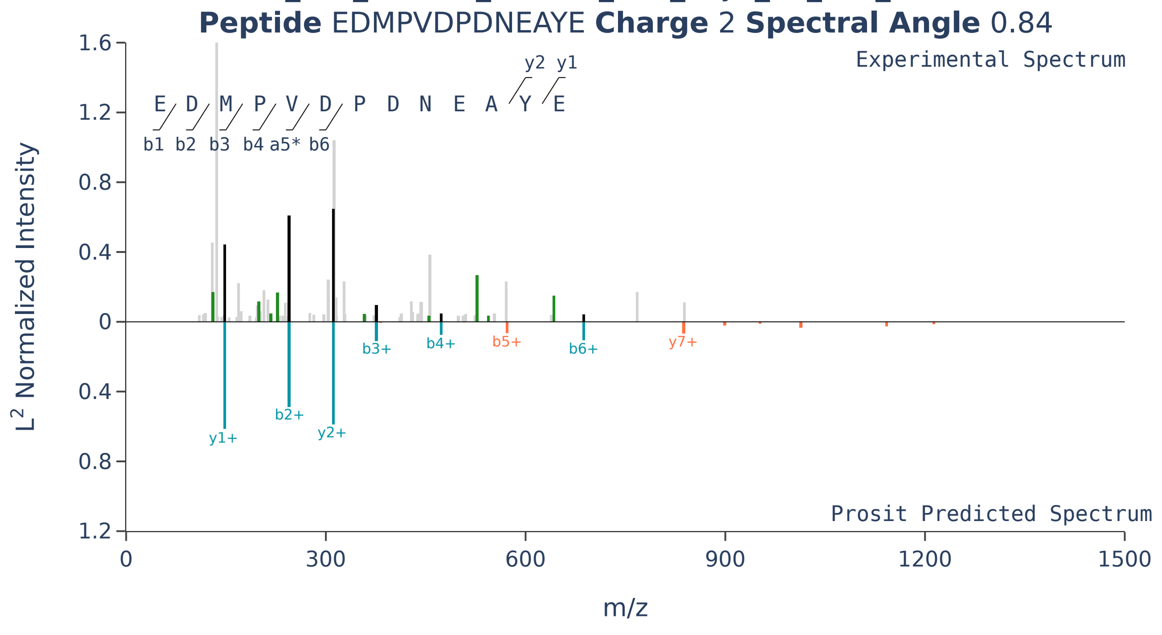


**Source** W_Soh_160821_170921_Goe_aSyn_A1_6h_R2 **Scan** 9280
**Peptide** QKTVEGAGSIAA **Charge** 2 **Spectral Angle** 0.89

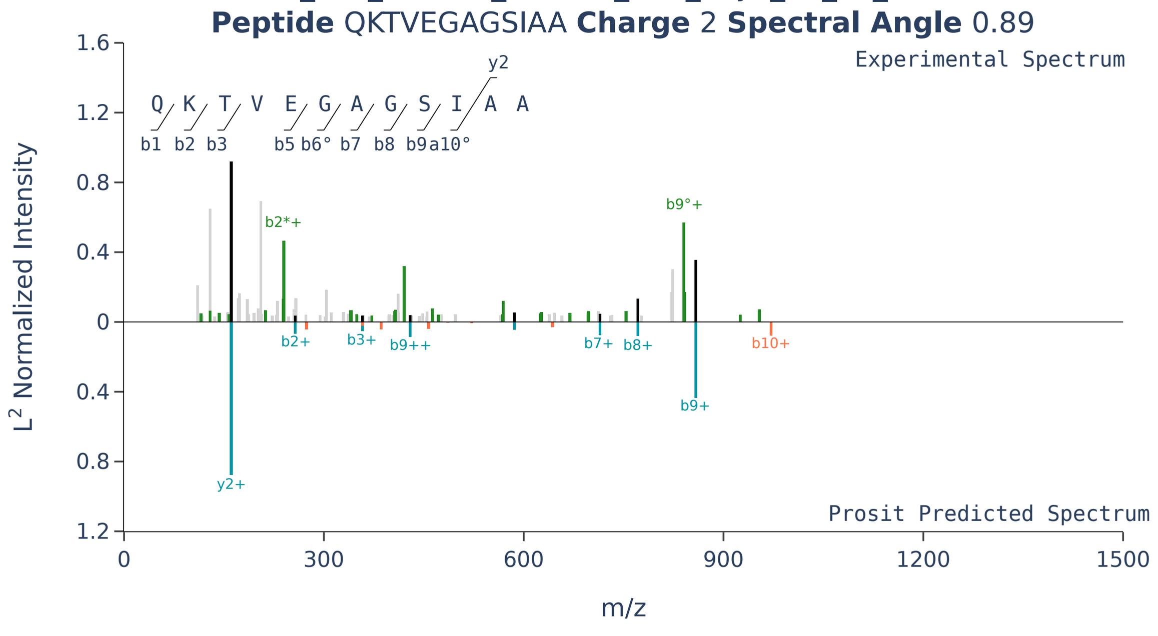


**Source** W_Soh_160821_170921_Goe_aSyn_A1_24h_R2 **Scan** 17544
**Peptide** KTKEQVTNVGGAVVTGVTAV **Charge** 3 **Spectral Angle** 0.82

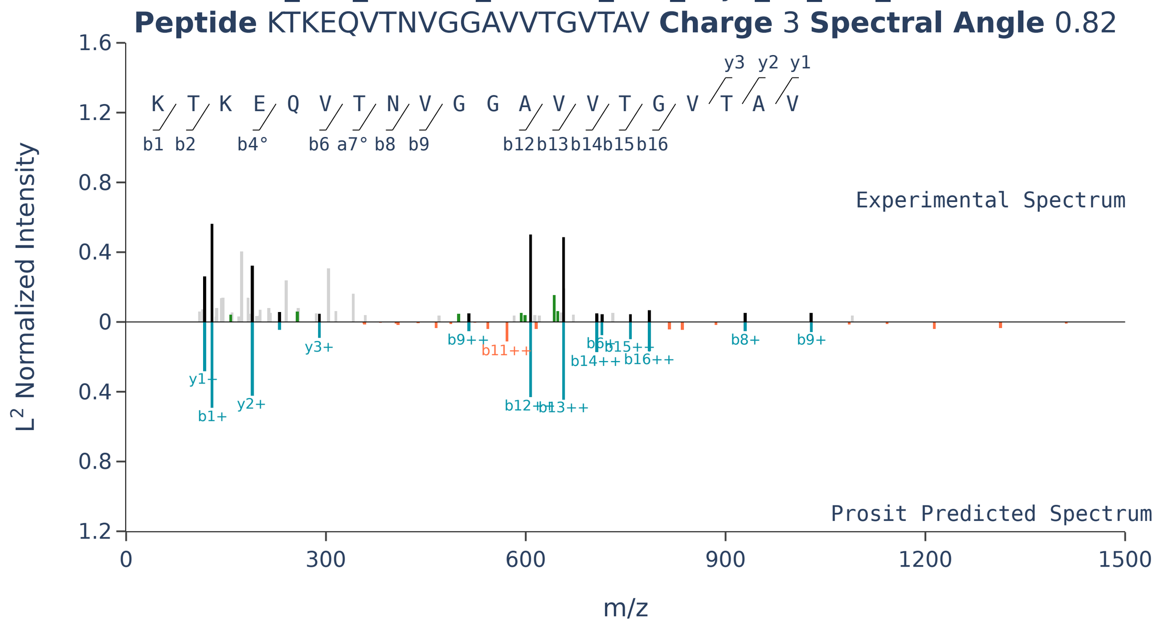


**Source** W_Soh_160821_170921_Goe_aSyn_A2_24h_R2 **Scan** 17171
**Peptide** GGAVVTGVTAVAQ **Charge** 2 **Spectral Angle** 0.87

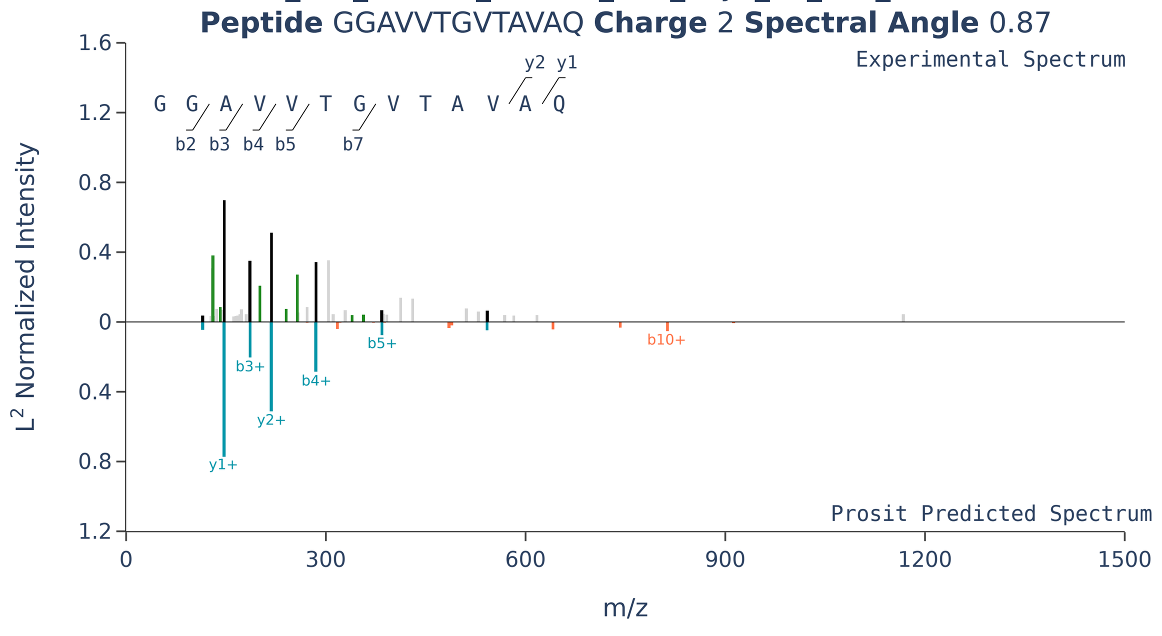

**Source** W_Soh_160821_170921_Goe_aSyn_A2_24h_R1 **Scan** 13709
**Peptide** NEAYEMPSEE **Charge** 2 **Spectral Angle** 0.81

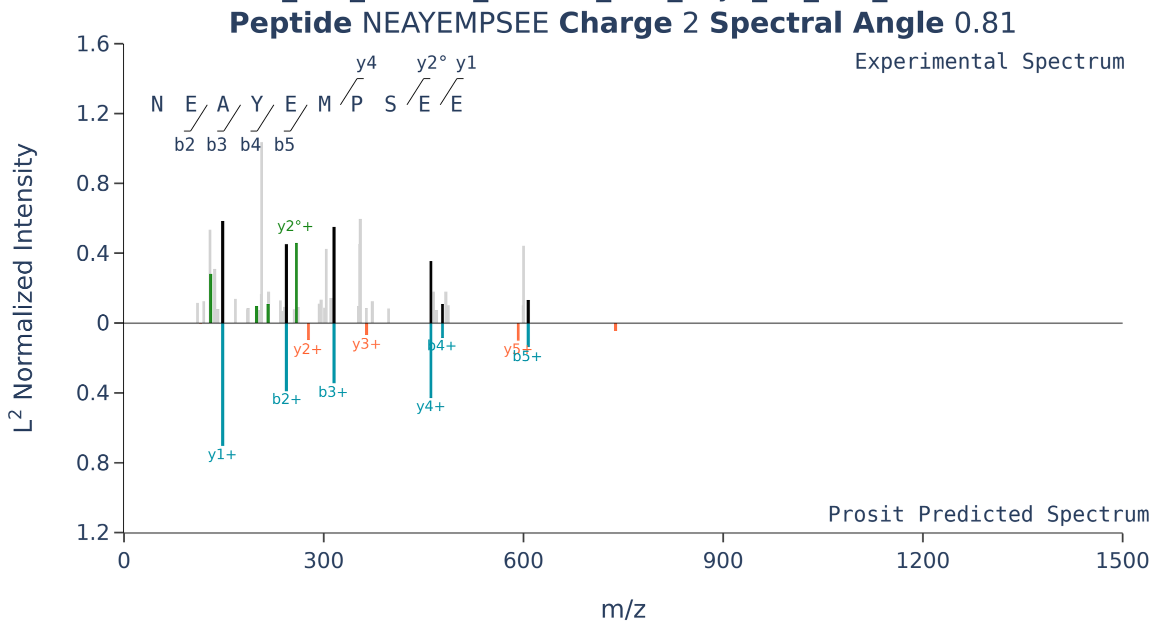


**Source** W_Soh_160821_170921_Goe_aSyn_A1_24h_R2 **Scan** 11156
**Peptide** VAEKTKEQVTNVGGAVVTG **Charge** 3 **Spectral Angle** 0.85

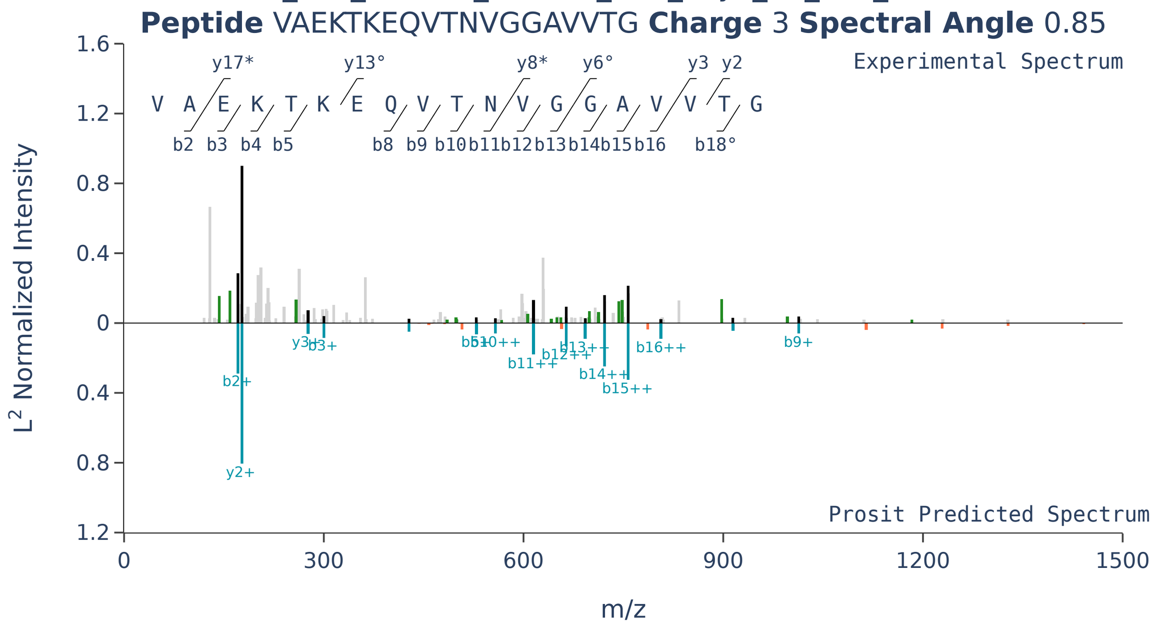


**Source** W_Soh_160821_170921_Goe_aSyn_A1_24h_R2 **Scan** 23416
**Peptide** NVGGAVVTGVTAVAQKTVE **Charge** 2 **Spectral Angle** 0.83

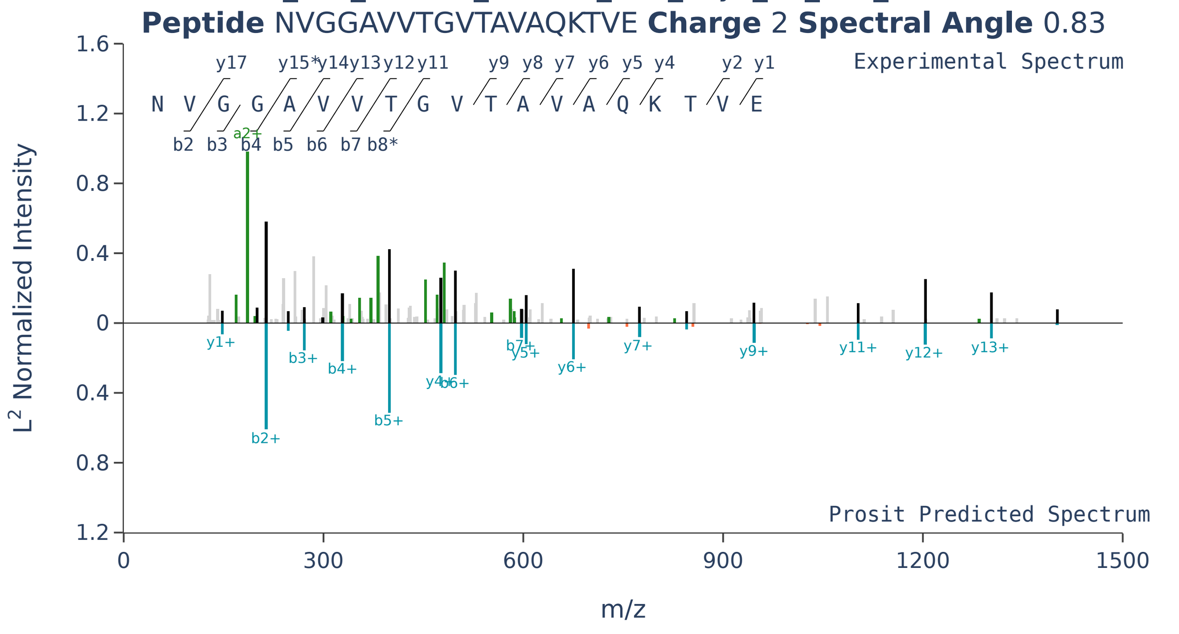


**Source** W_Soh_160821_170921_Goe_aSyn_A2_24h_R1 **Scan** 20150
**Peptide** VTNVGGAVVTGVTAVAQK **Charge** 2 **Spectral Angle** 0.76

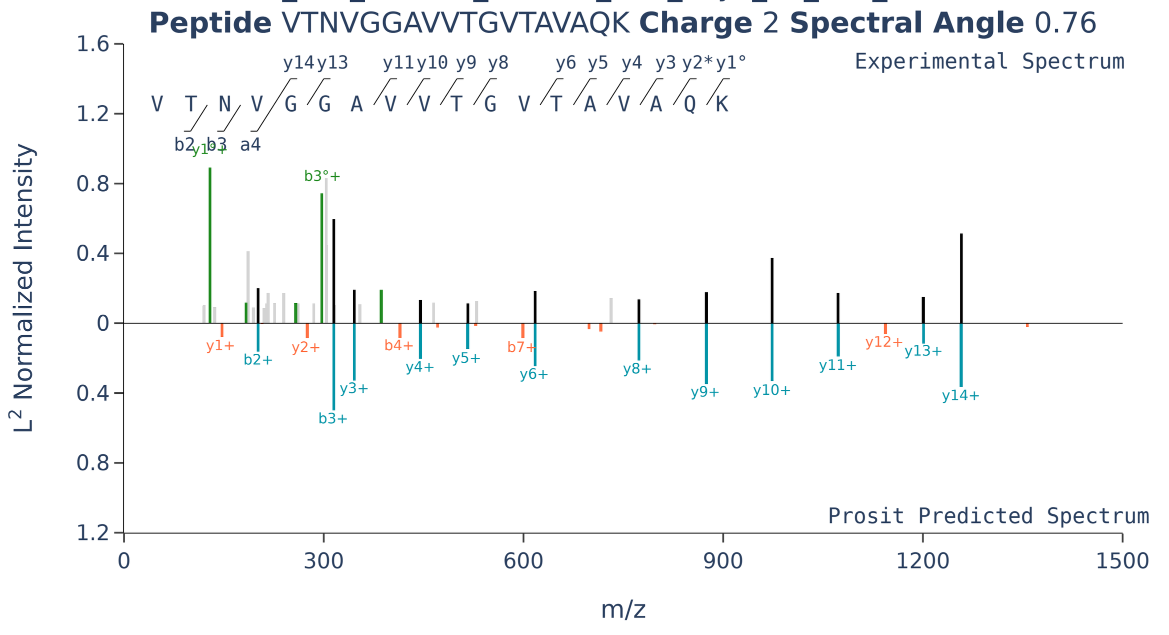


**Source** W_Soh_160821_170921_Goe_aSyn_A1_6h_R2 **Scan** 16479
**Peptide** TVAEKTKEQVTNVGGAVVTGVTA **Charge** 3 **Spectral Angle** 0.89

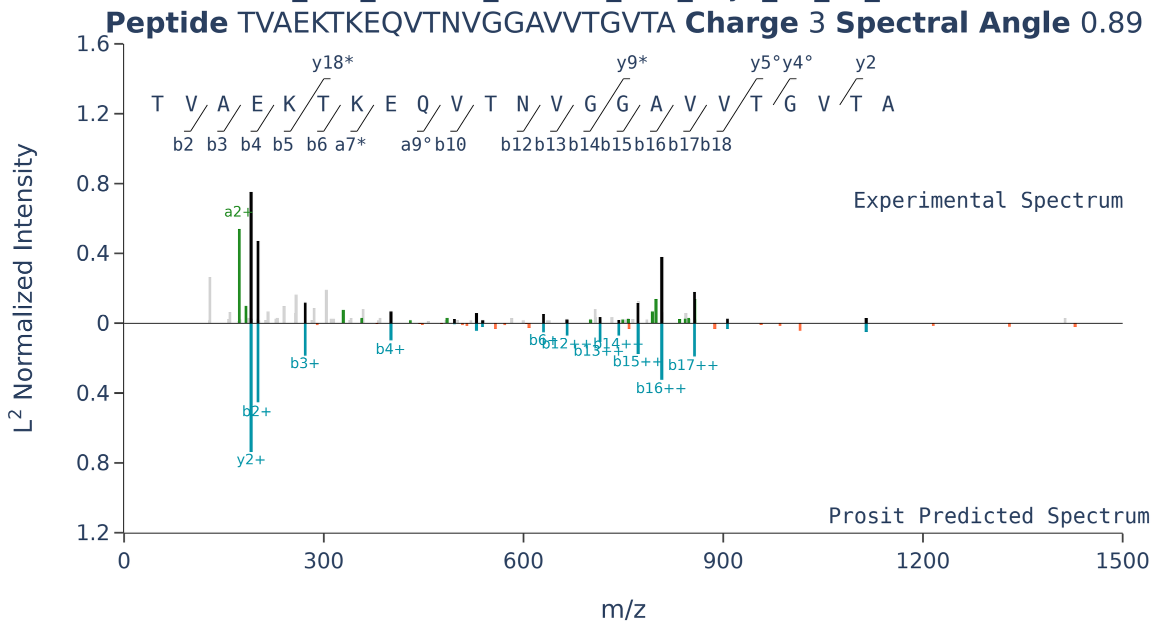


**Source** W_Soh_160821_170921_Goe_aSyn_A1_24h_R1 **Scan** 8152
**Peptide** VFMKGLSKAKEG **Charge** 3 **Spectral Angle** 0.79

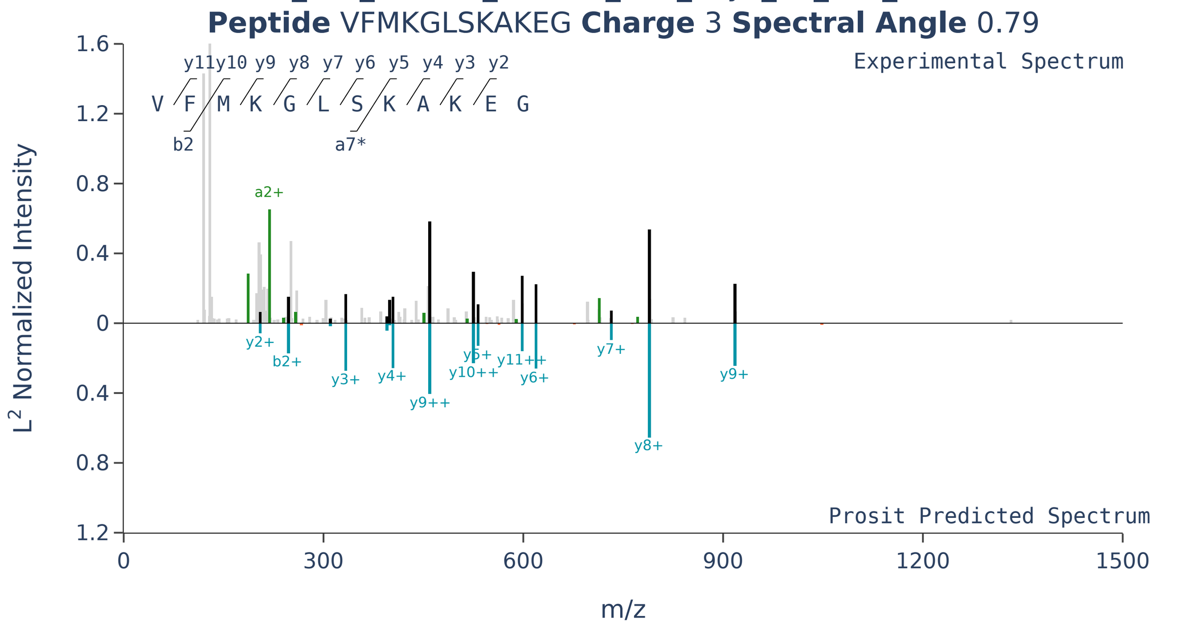


**Source** W_Soh_160821_170921_Goe_aSyn_A1_6h_R2 **Scan** 6233
**Peptide** VGSKTKEGVVHGV **Charge** 2 **Spectral Angle** 0.77

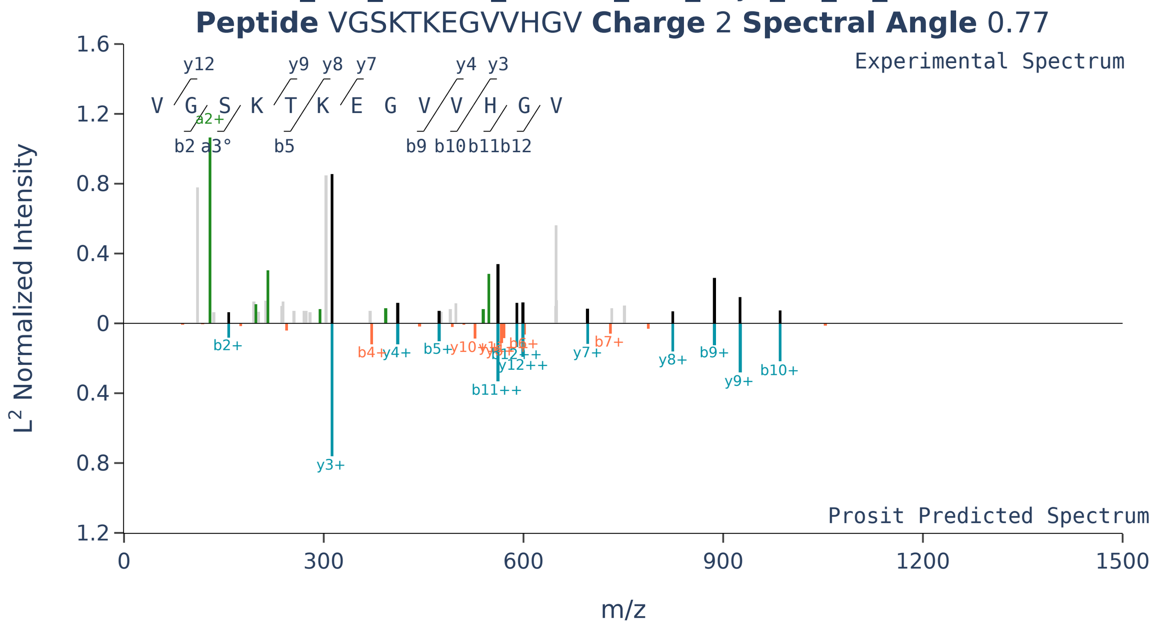


**Source** W_Soh_160821_170921_Goe_aSyn_A1_24h_R1 **Scan** 12769
**Peptide** VLYVGSKTKEGVV **Charge** 3 **Spectral Angle** 0.86

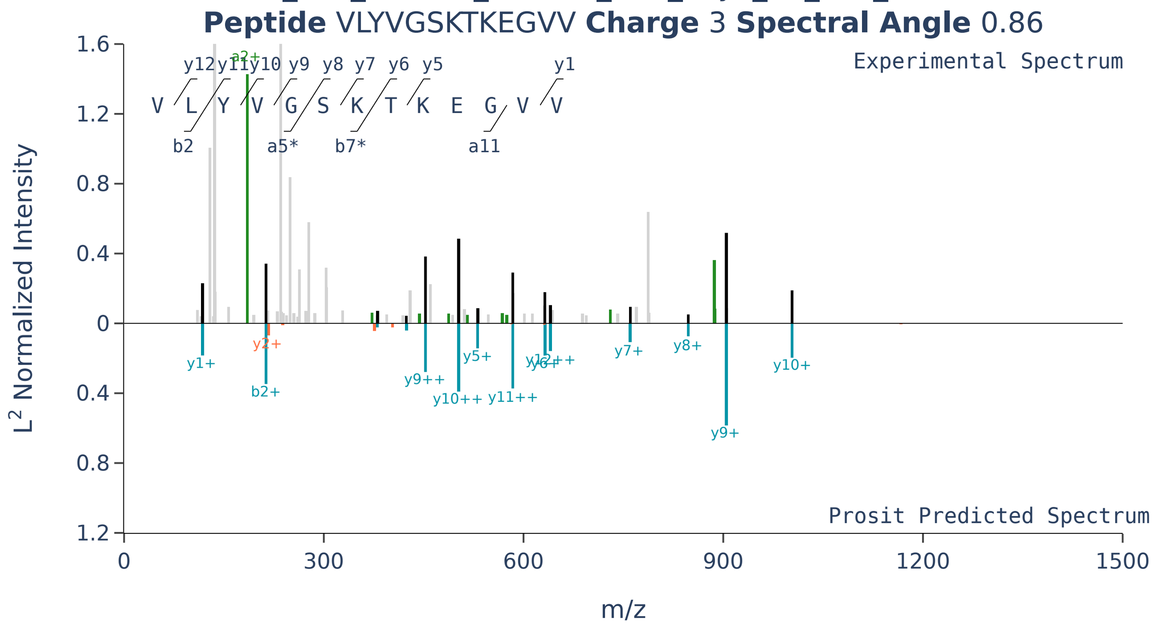


**Source** W_Soh_160821_170921_Goe_aSyn_A1_24h_R1 **Scan** 19021
**Peptide** AEKTKEQVTNVGGAVVTGVTAVAQKT **Charge** 4 **Spectral Angle** 0.8

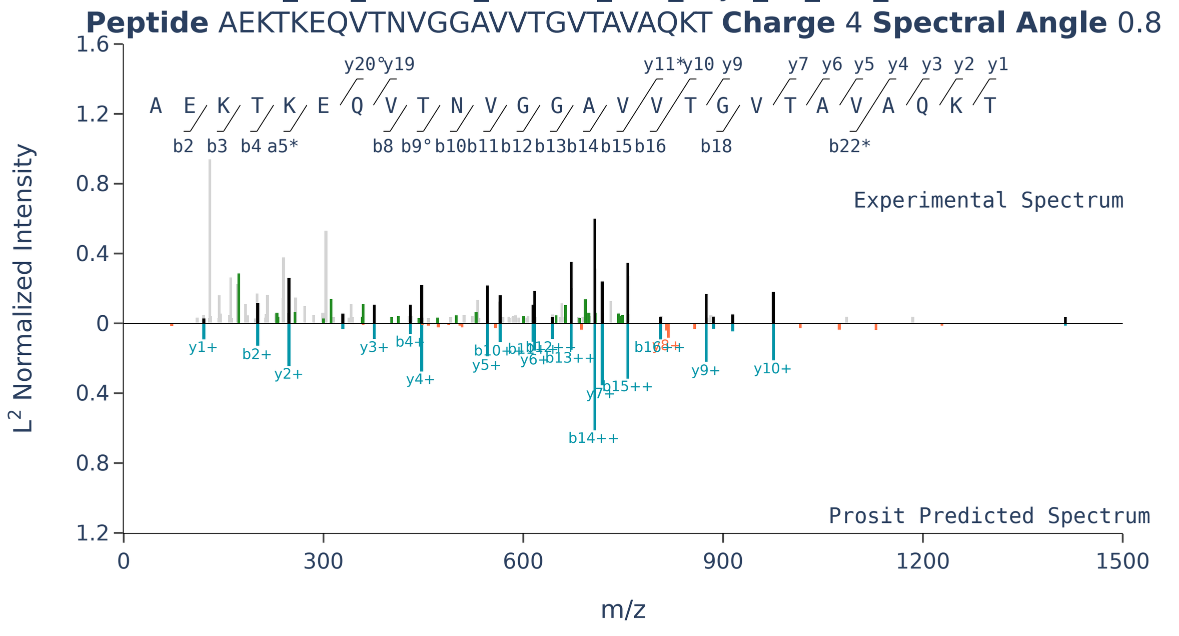


**Source** W_Soh_160821_170921_Goe_aSyn_A2_24h_R1 **Scan** 15066
**Peptide** ATGFVKKDQLGKNEEGAPQEGIL **Charge** 3 **Spectral Angle** 0.88

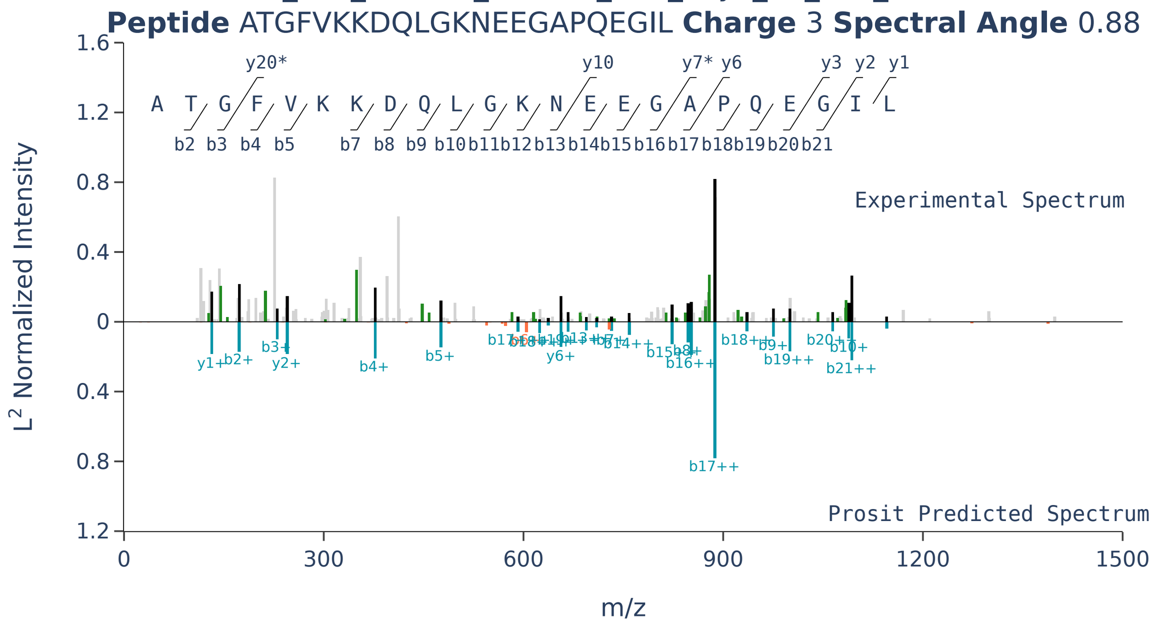


**Source** W_Soh_160821_170921_Goe_aSyn_A2_24h_R1 **Scan** 5550
**Peptide** FVKKDQLGKNE **Charge** 2 **Spectral Angle** 0.74

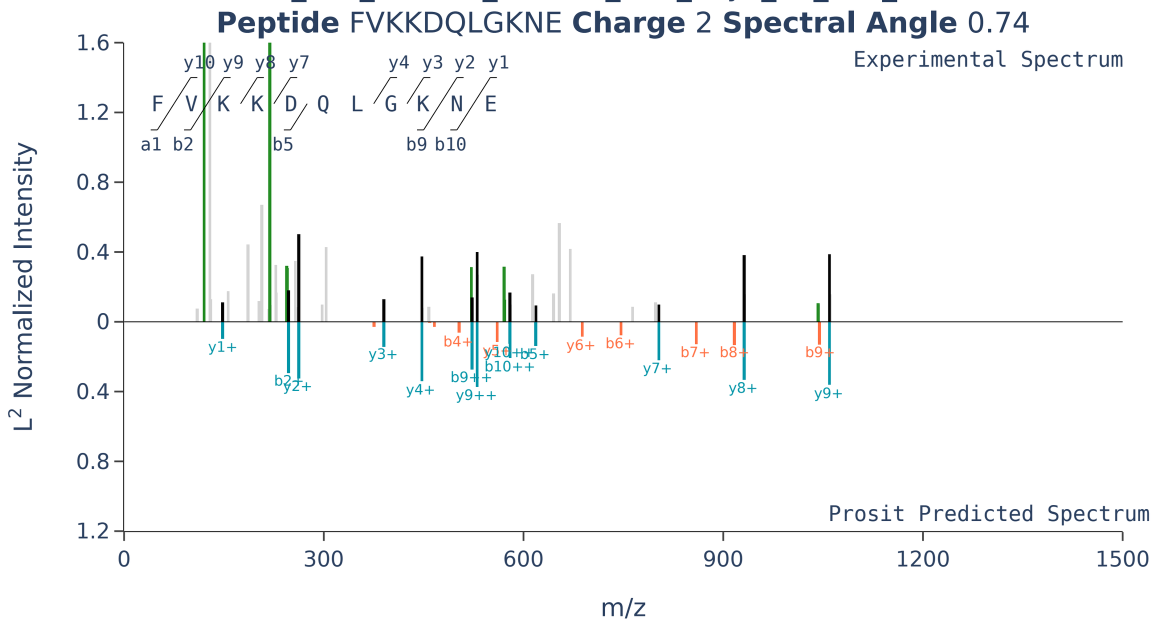


**Source** W_Soh_160821_170921_Goe_aSyn_A2_24h_R2 **Scan** 14373
**Peptide** ATGFVKKDQLGKNEEGAPQEGILE **Charge** 3 **Spectral Angle** 0.89

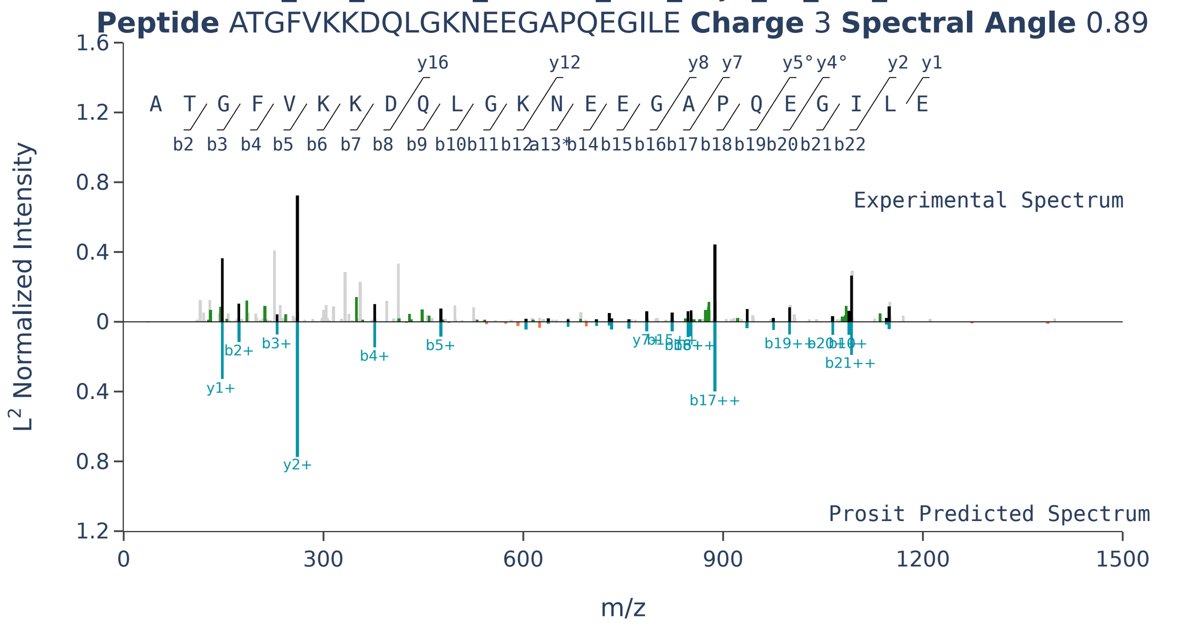


**Source** W_Soh_160821_170921_Goe_aSyn_A2_24h_R1 **Scan** 10958
**Peptide** VAQKTVEGAGSIAAA **Charge** 1 **Spectral Angle** 0.78

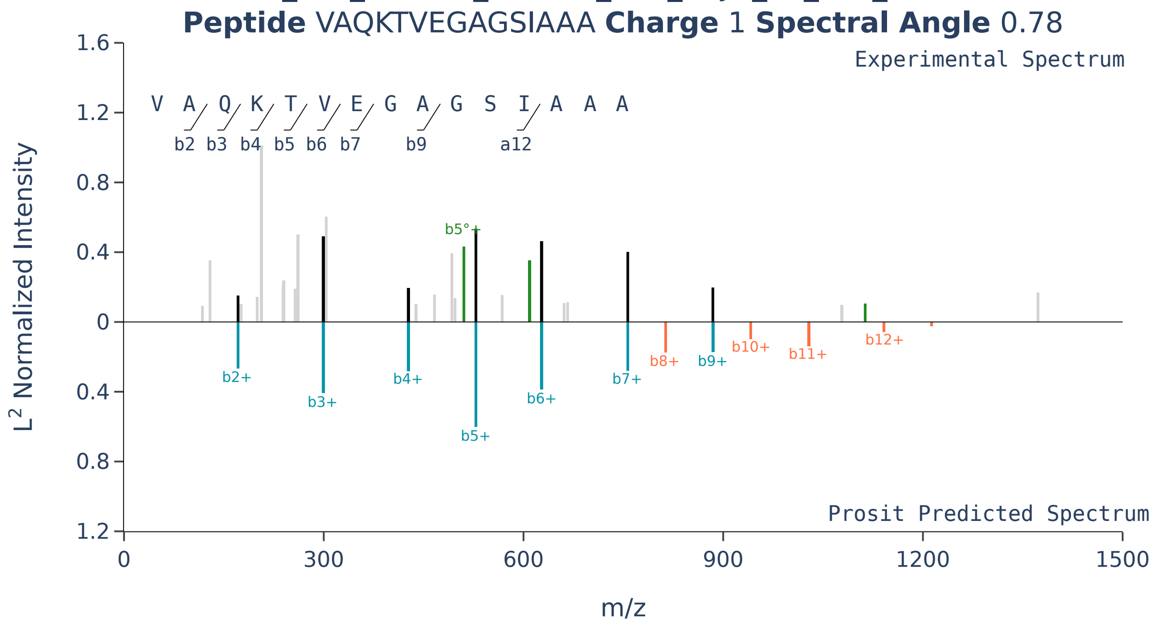


**Source** W_Soh_160821_170921_Goe_aSyn_A1_24h_R1 **Scan** 14551
**Peptide** GAGSIAAATGFVKKDQLGKN **Charge** 3 **Spectral Angle** 0.8

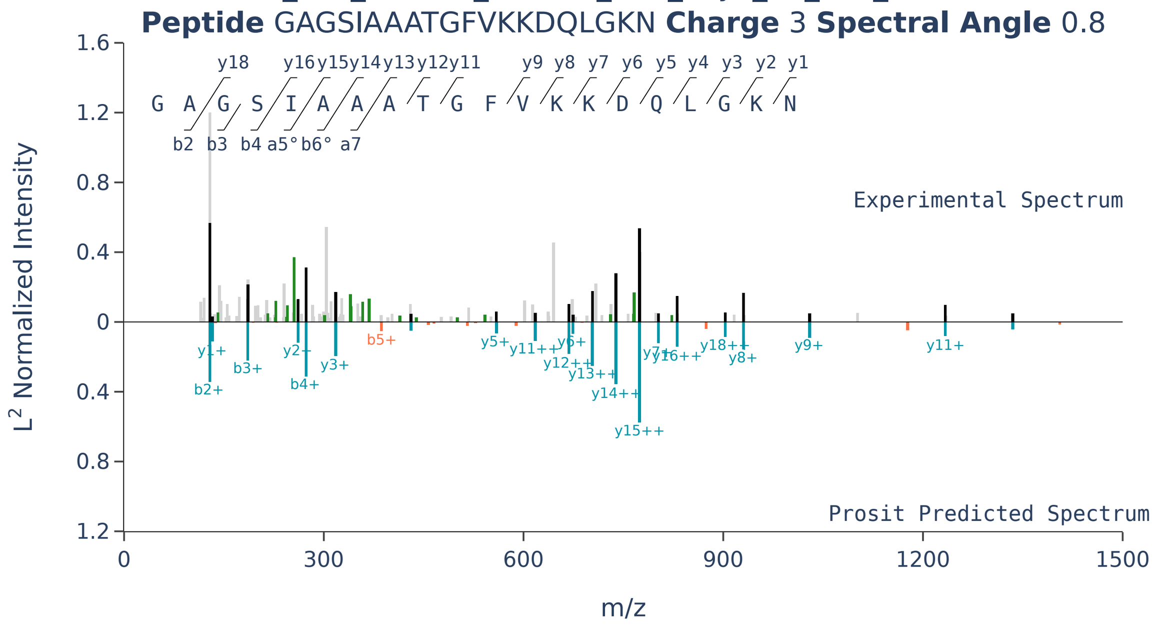


**Source** W_Soh_160821_170921_Goe_aSyn_A2_24h_R1 **Scan** 14364
**Peptide** VLYVGSKTKEGVVHGVATVAE **Charge** 3 **Spectral Angle** 0.84

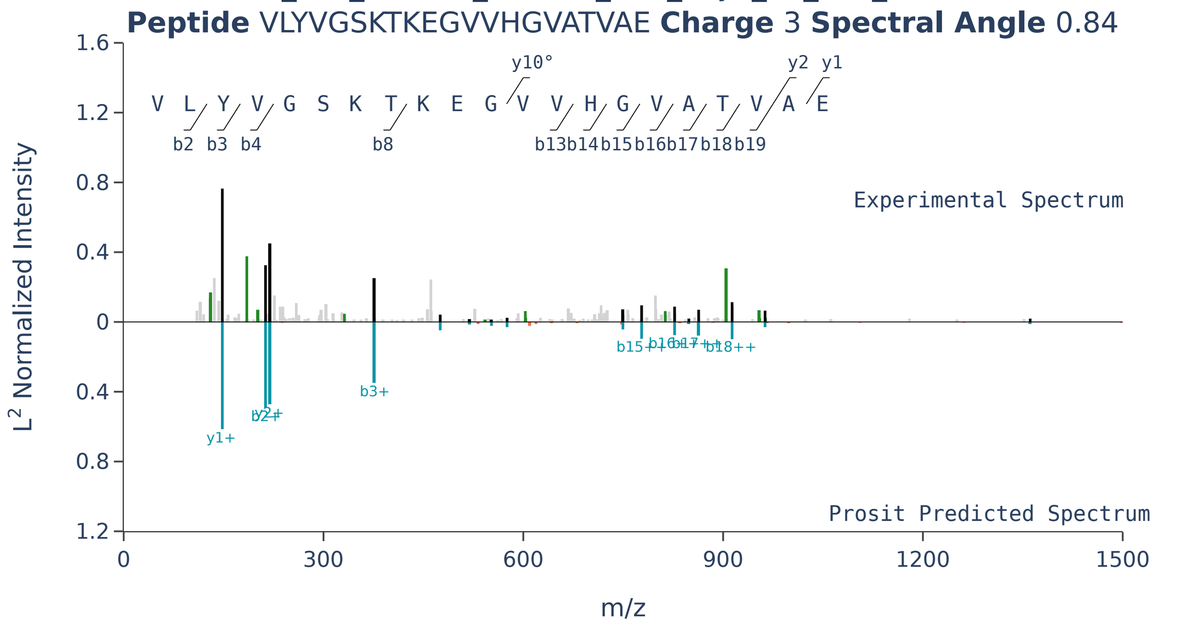

**Source** W_Soh_160821_170921_Goe_aSyn_A1_24h_R2 **Scan** 7647
**Peptide** VAQKTVE **Charge** 1 **Spectral Angle** 0.86

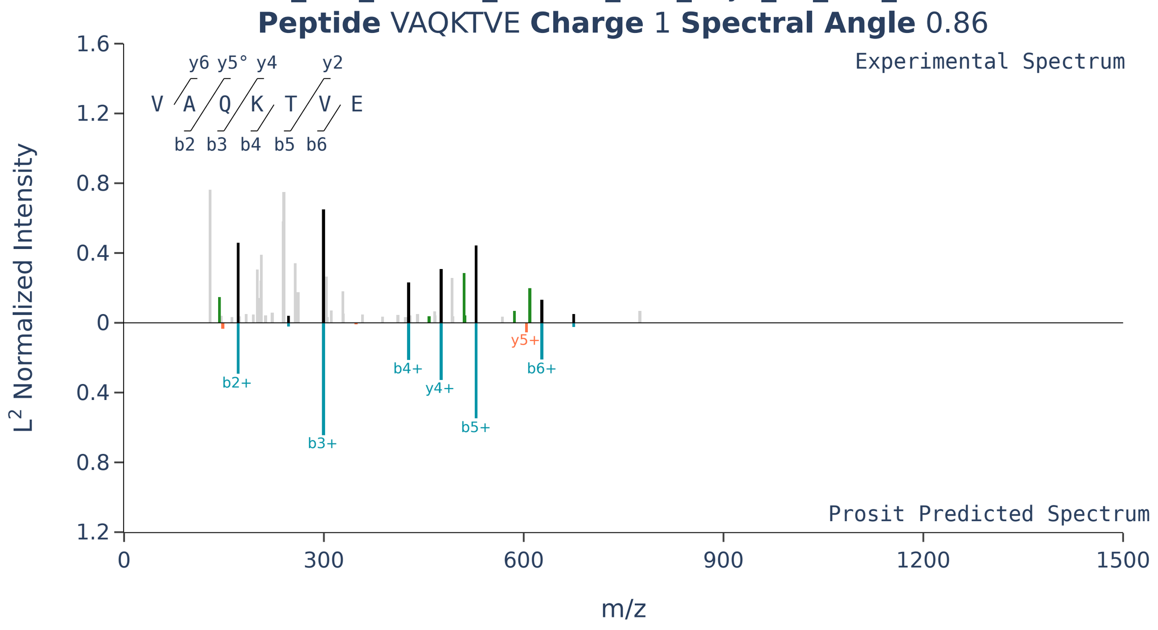


**Source** W_Soh_160821_170921_Goe_aSyn_A2_24h_R1 **Scan** 9176
**Peptide** GVATVAEKTKEQVT **Charge** 2 **Spectral Angle** 0.85

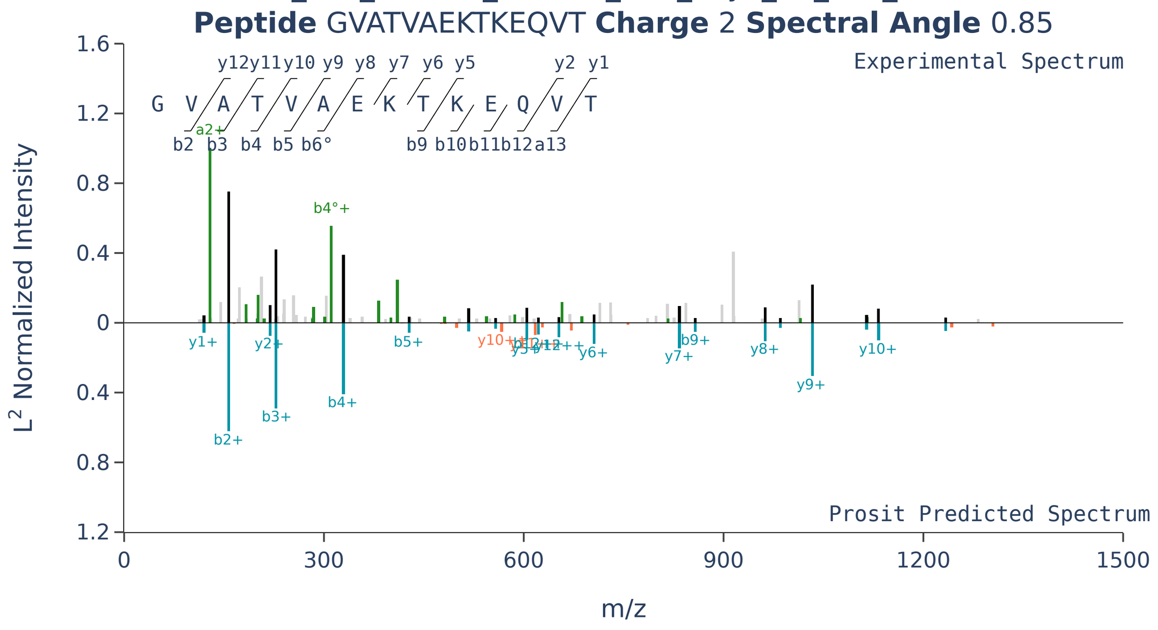


**Source** W_Soh_160821_170921_Goe_aSyn_A1_24h_R1 **Scan** 20857
**Peptide** EAYEMPSEEGYQDYEPEA **Charge** 2 **Spectral Angle** 0.84

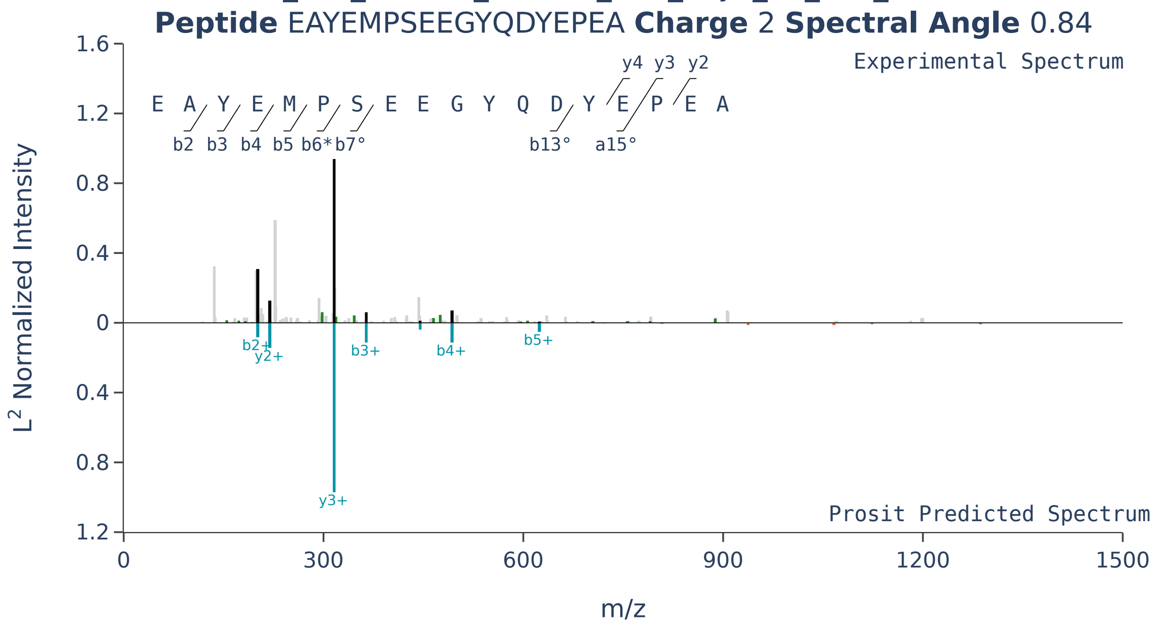


**Source** W_Soh_160821_170921_Goe_aSyn_A1_24h_R2 **Scan** 9495
**Peptide** KEGVVAAAEKTKQGVAEAAGKTKE **Charge** 3 **Spectral Angle** 0.9

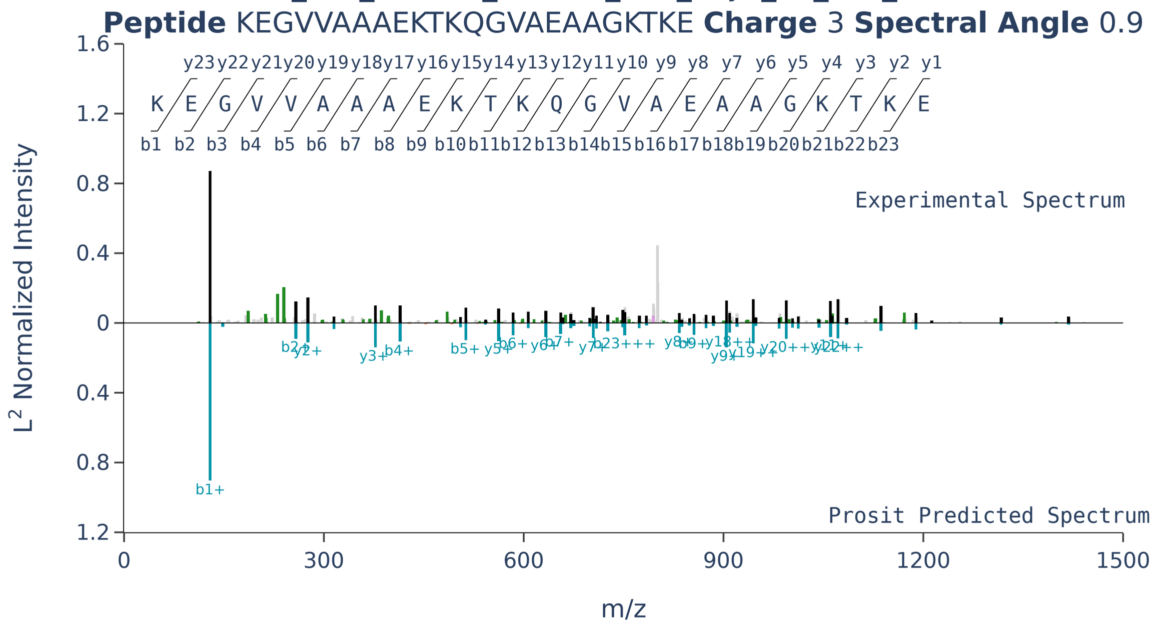


**Source** W_Soh_160821_170921_Goe_aSyn_A2_24h_R1 **Scan** 7847
**Peptide** GSKTKEGVVHGVATVAEKT **Charge** 4 **Spectral Angle** 0.8

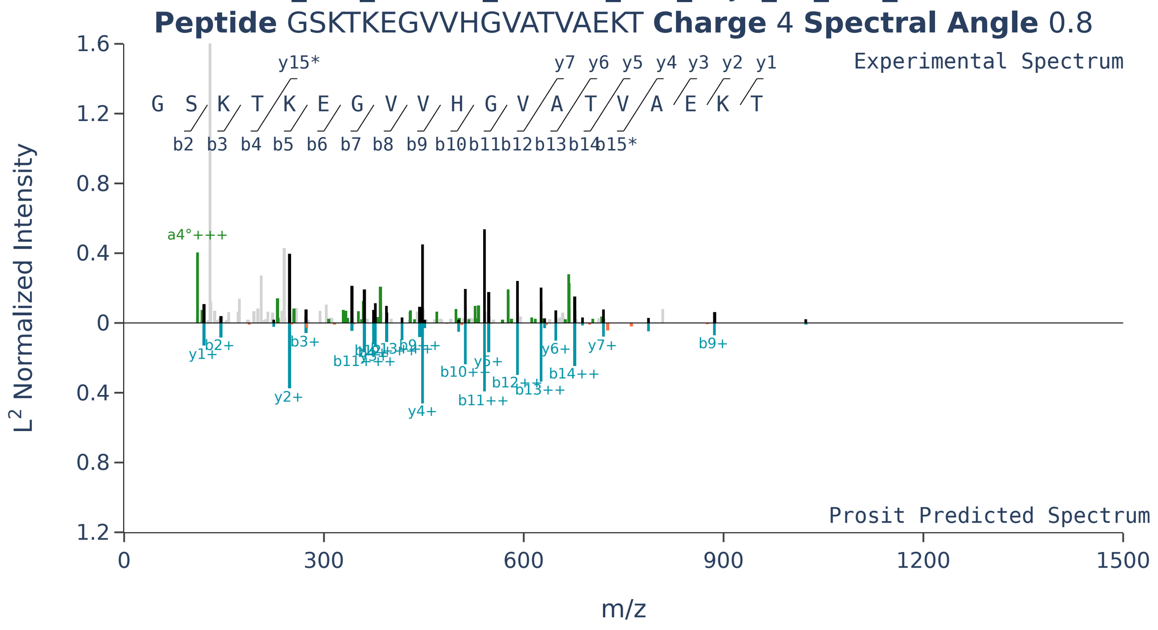


**Source** W_Soh_160821_170921_Goe_aSyn_A2_24h_R2 **Scan** 7829
**Peptide** VTAVAQKTV **Charge** 2 **Spectral Angle** 0.84

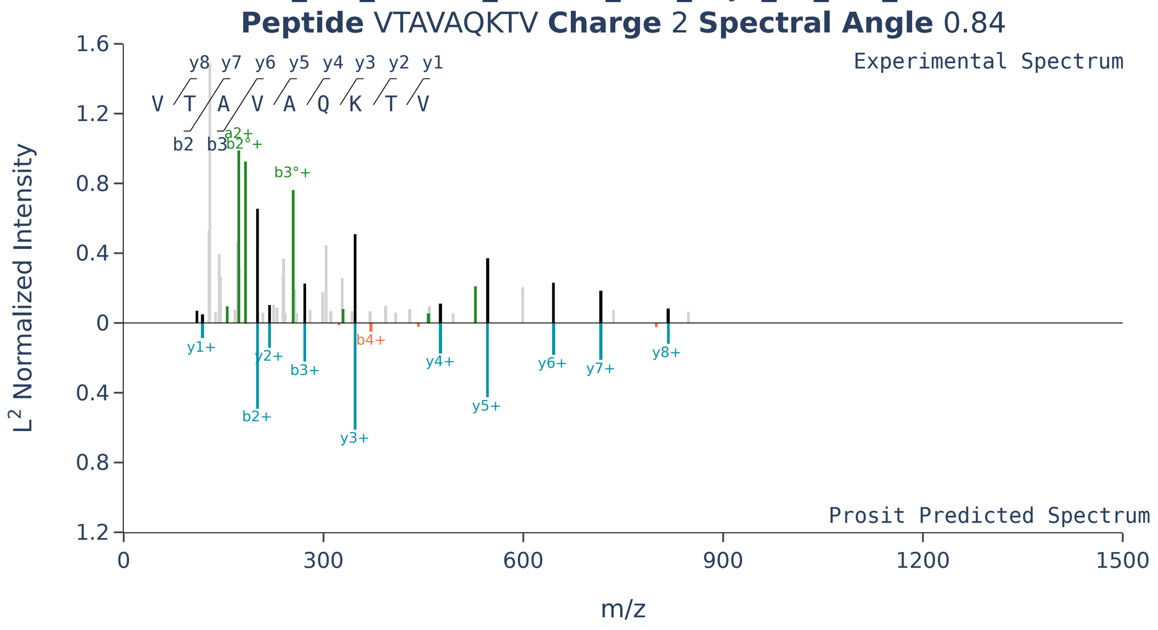


**Source** W_Soh_160821_170921_Goe_aSyn_A1_6h_R1 **Scan** 17671
**Peptide** KTVEGAGSIAAATGFVKKDQL **Charge** 3 **Spectral Angle** 0.86

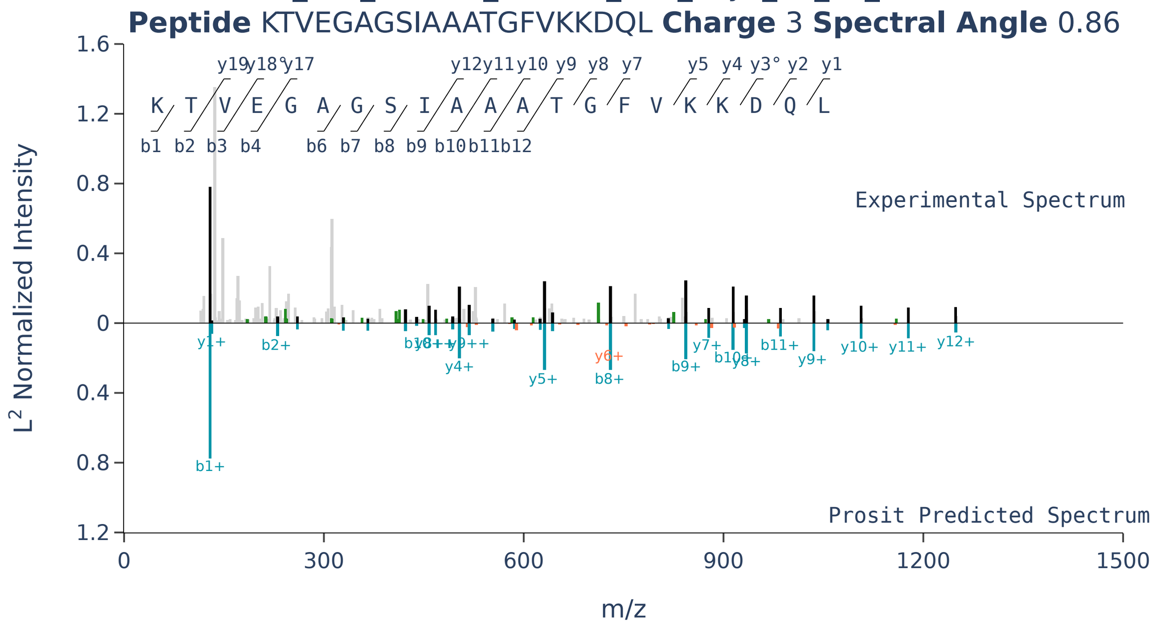


**Source** W_Soh_160821_170921_Goe_aSyn_A1_6h_R2 **Scan** 18382
**Peptide** GGAVVTGVTAVA **Charge** 1 **Spectral Angle** 0.84

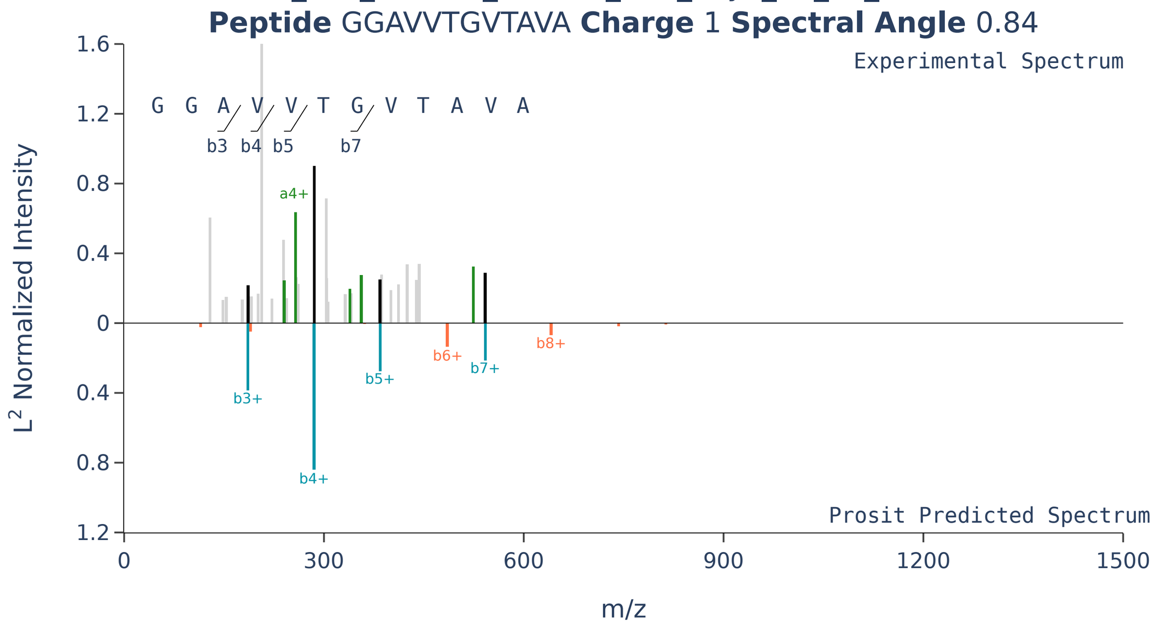


**Source** W_Soh_160821_170921_Goe_aSyn_A1_24h_R2 **Scan** 17321
**Peptide** DVFMKGLSKAKEGVVAA **Charge** 2 **Spectral Angle** 0.92

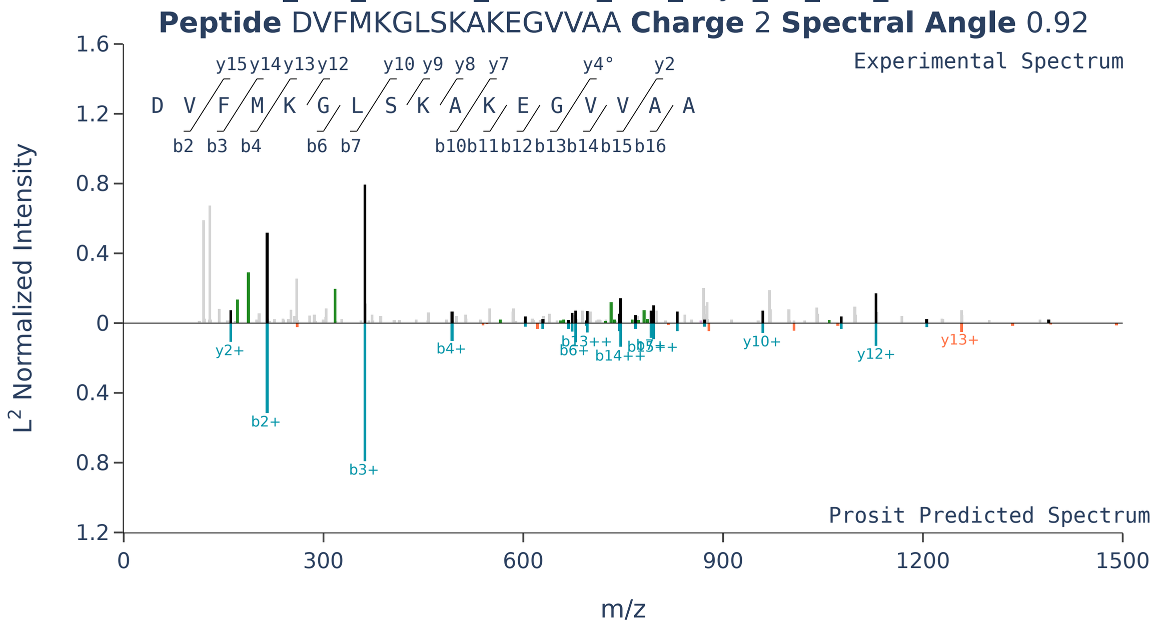


**Source** W_Soh_160821_170921_Goe_aSyn_A2_24h_R2 **Scan** 19912
**Peptide** MDVFMKGLSKAKEGVVAAAEKT **Charge** 3 **Spectral Angle** 0.9

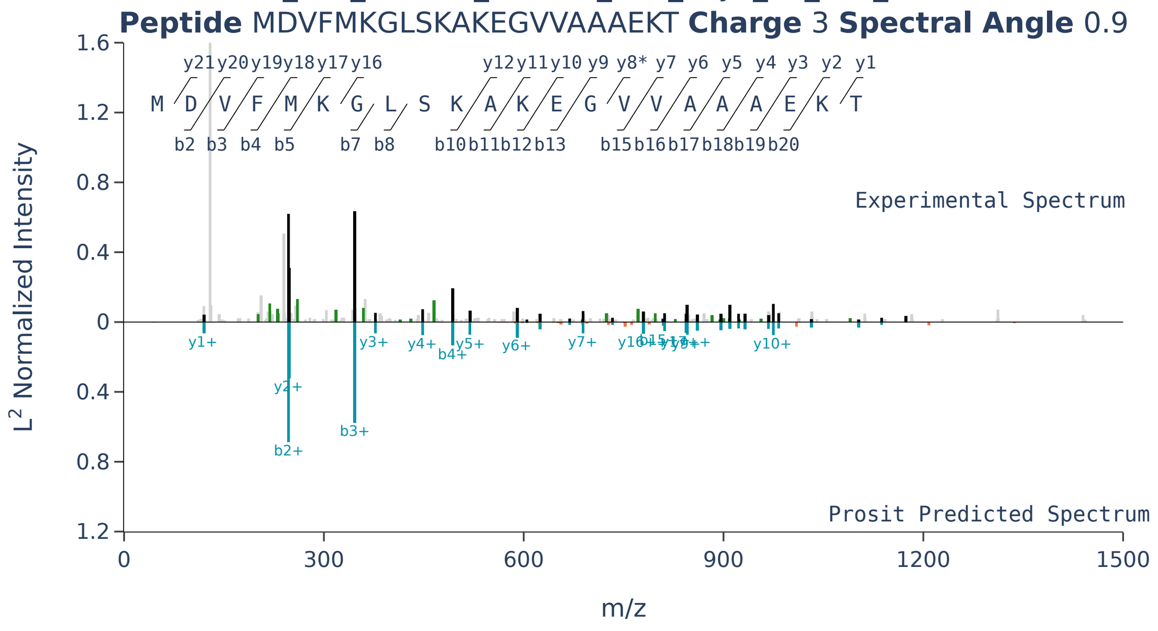


**Source** W_Soh_160821_170921_Goe_aSyn_A1_24h_R1 **Scan** 12355
**Peptide** TAVAQKTVEGAGSIAA **Charge** 2 **Spectral Angle** 0.85

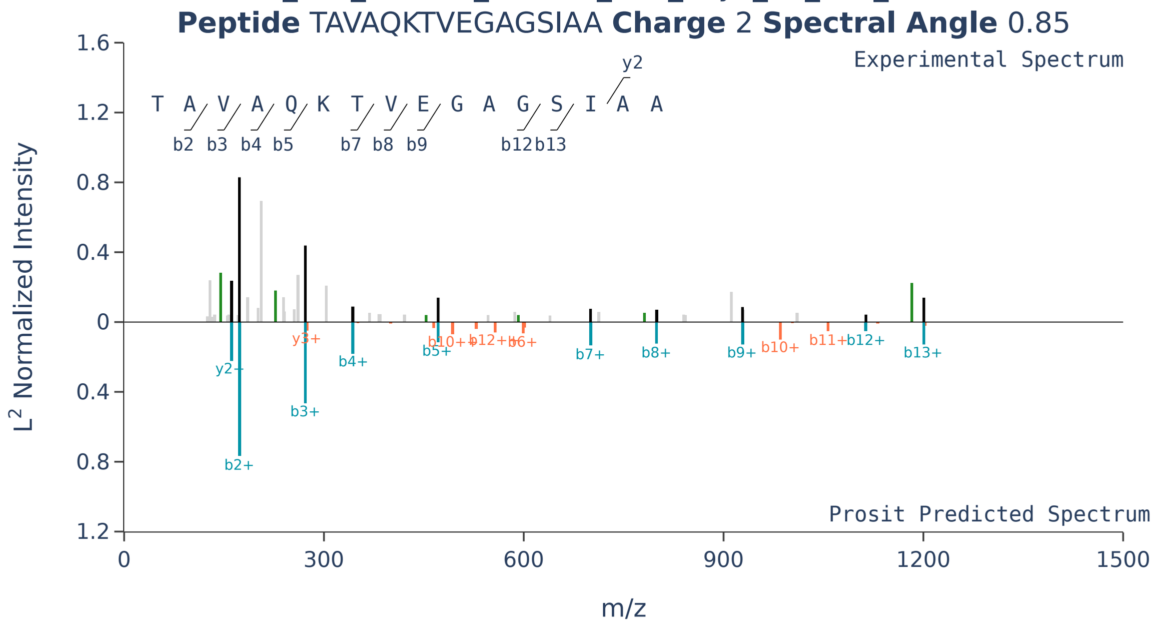


**Source** W_Soh_160821_170921_Goe_aSyn_A1_24h_R2 **Scan** 7749
**Peptide** AATGFVKKDQLGKNEE **Charge** 4 **Spectral Angle** 0.86

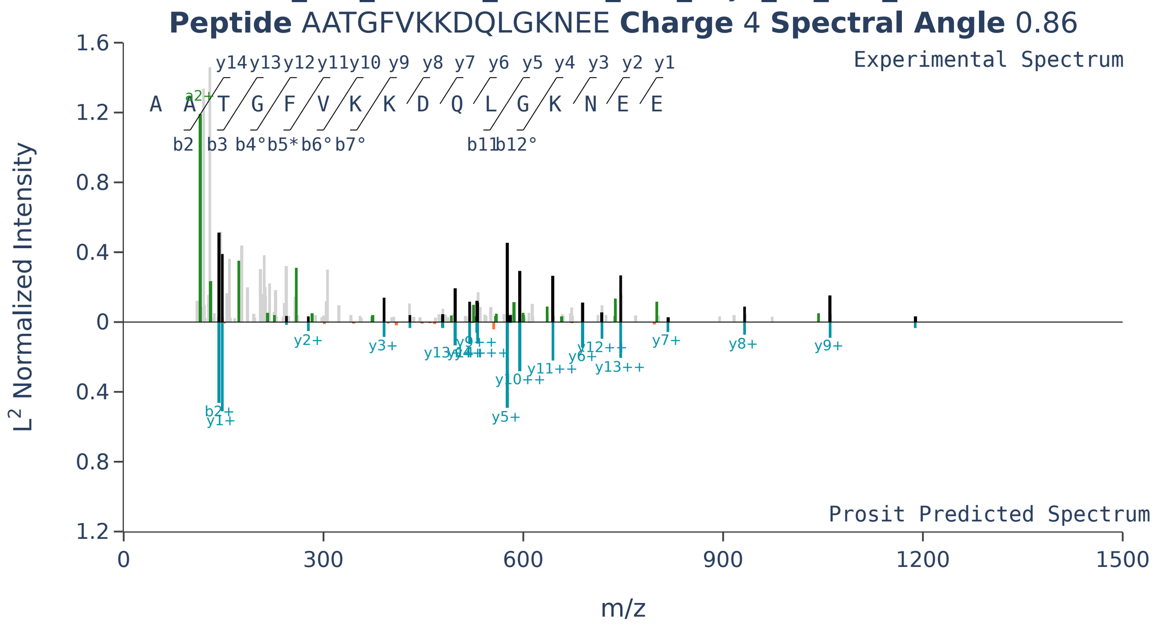


**Source** W_Soh_160821_170921_Goe_aSyn_A1_24h_R2 **Scan** 6495
**Peptide** SKAKEGVVAAAE **Charge** 2 **Spectral Angle** 0.82

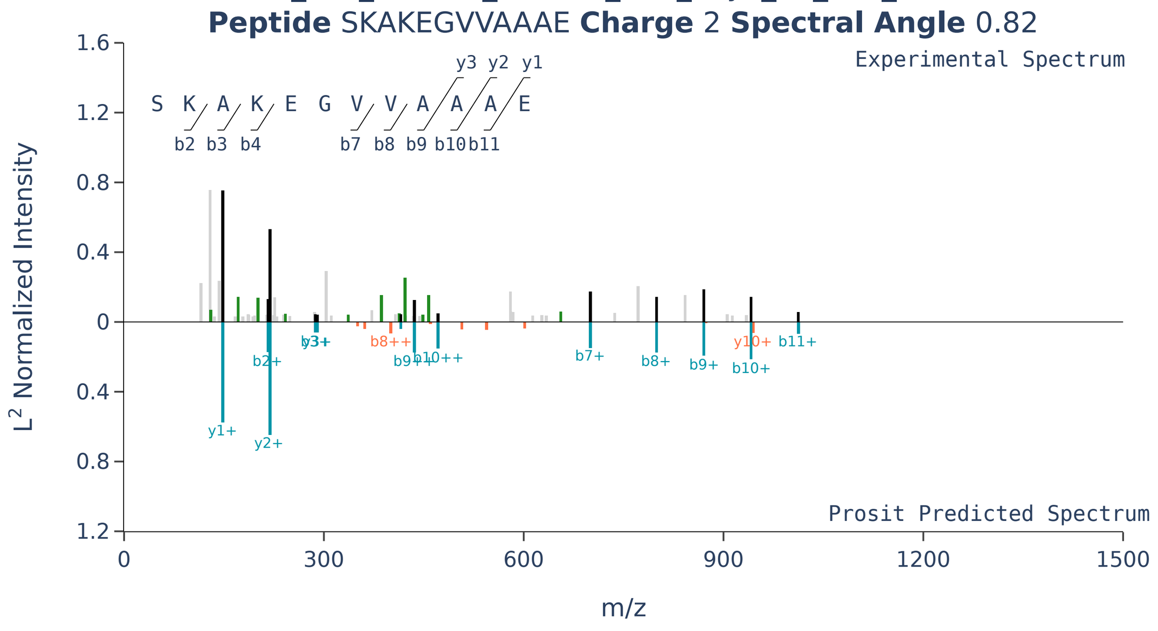


**Source** W_Soh_160821_170921_Goe_aSyn_A1_24h_R1 **Scan** 10978
**Peptide** VFMKGLSKA **Charge** 2 **Spectral Angle** 0.85

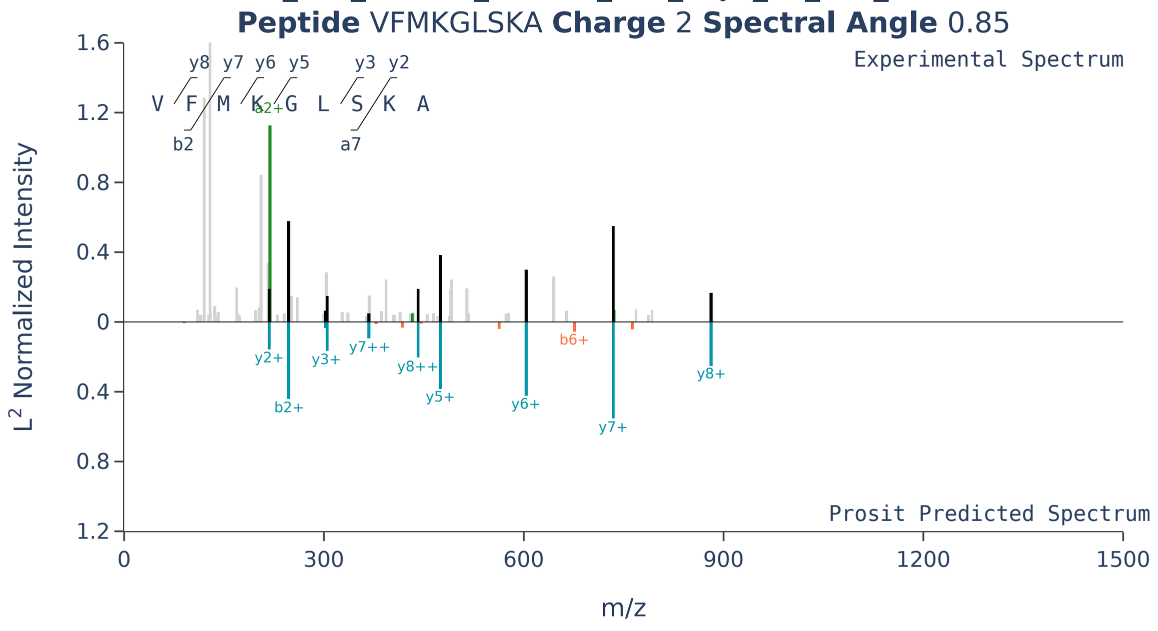


**Source** W_Soh_160821_170921_Goe_aSyn_A1_24h_R2 **Scan** 14406
**Peptide** GKNEEGAPQEGIL **Charge** 2 **Spectral Angle** 0.84

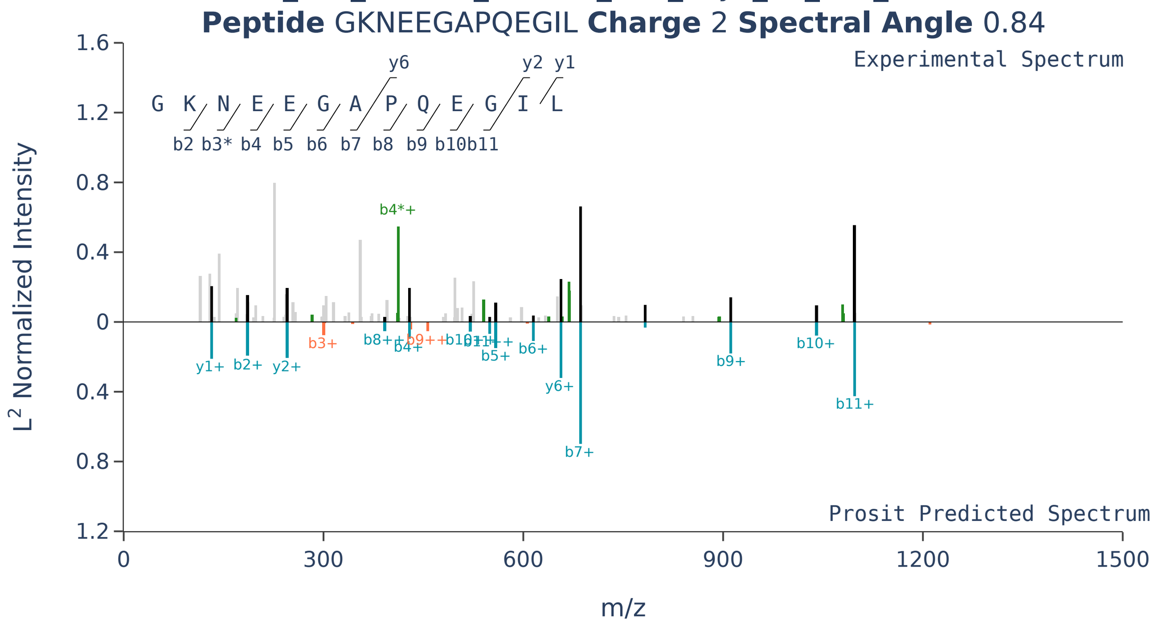

**Source** W_Soh_160821_170921_Goe_aSyn_A1_24h_R2 **Scan** 6871
**Peptide** AKEGVVAAAEKT **Charge** 2 **Spectral Angle** 0.8

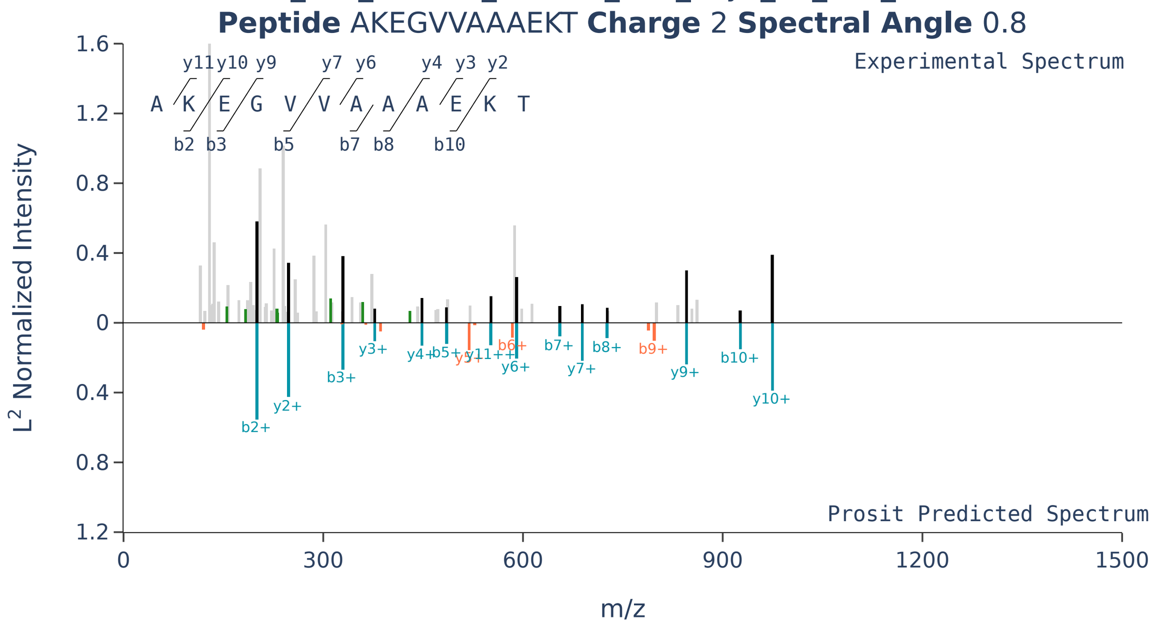


**Source** W_Soh_160821_170921_Goe_aSyn_A1_24h_R1 **Scan** 7120
**Peptide** VVAAAEKTKQGVAEAAGKTKE **Charge** 3 **Spectral Angle** 0.87

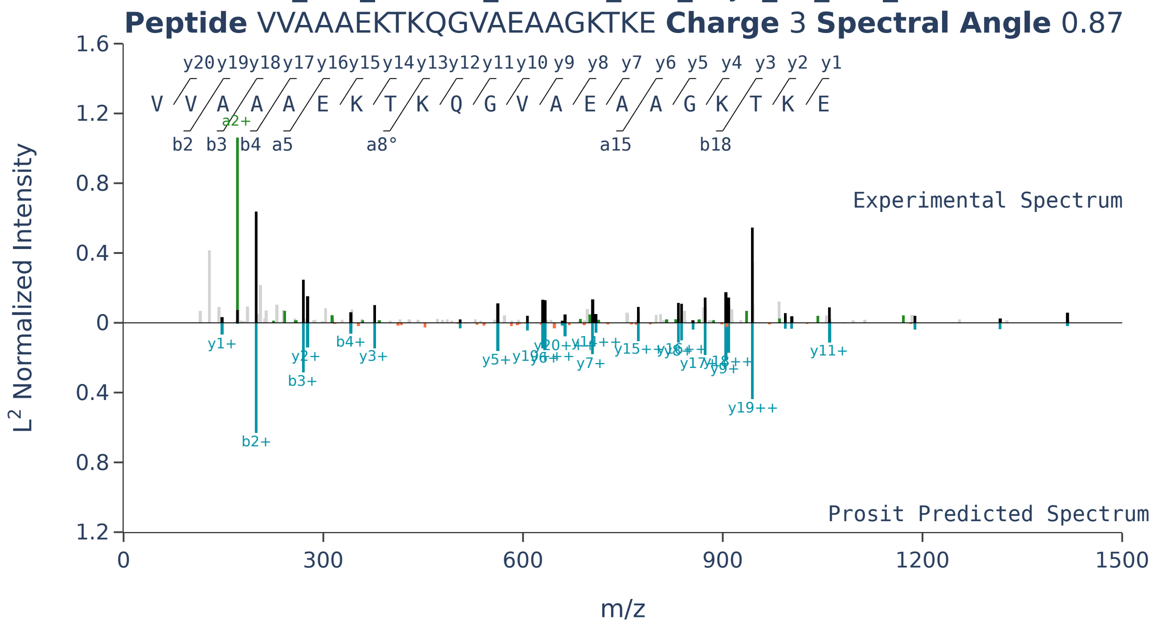


**Source** W_Soh_160821_170921_Goe_aSyn_A1_6h_R2 **Scan** 12763
**Peptide** KEQVTNVGGAVV **Charge** 2 **Spectral Angle** 0.81

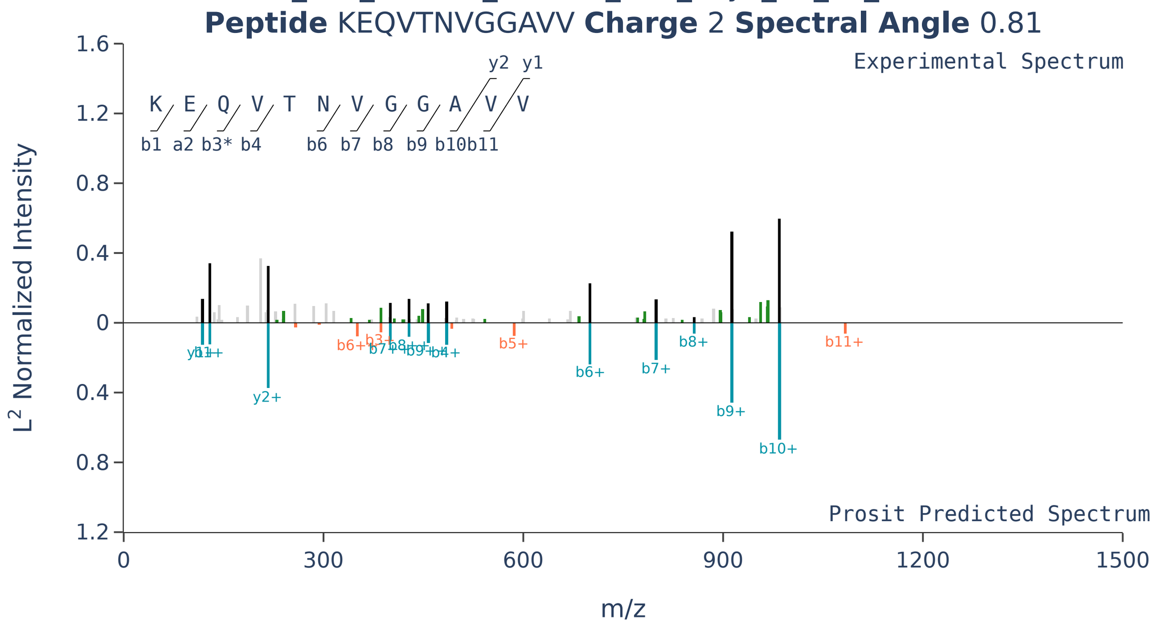


**Source** W_Soh_160821_170921_Goe_aSyn_A1_24h_R1 **Scan** 7968
**Peptide** AGKTKEGVLYVGSK **Charge** 2 **Spectral Angle** 0.82

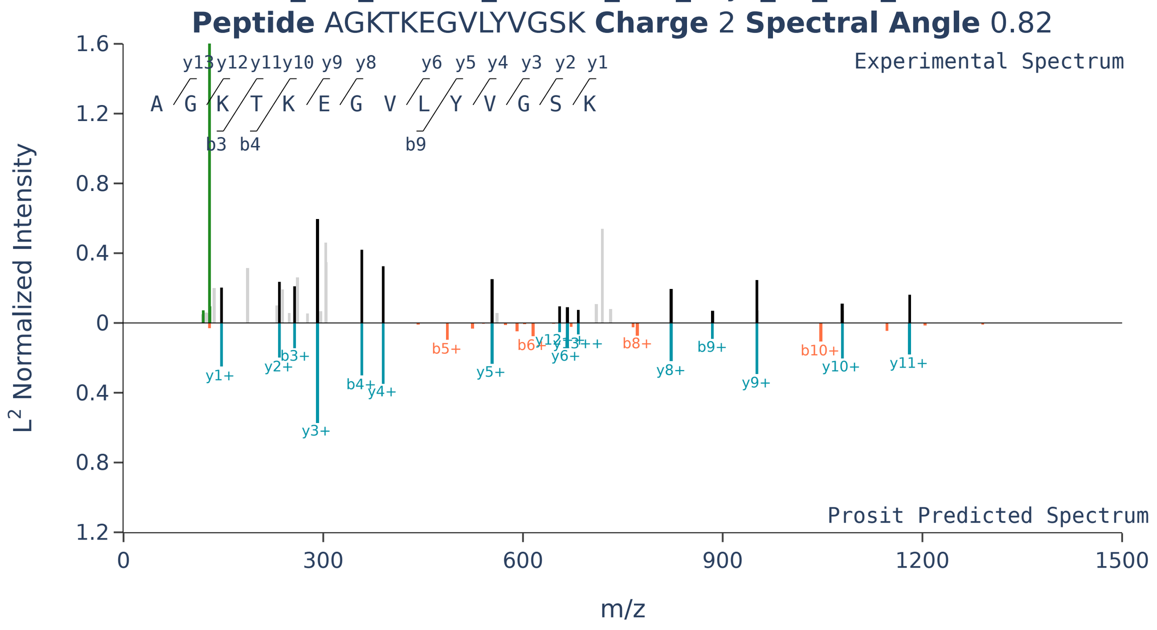


**Source** W_Soh_160821_170921_Goe_aSyn_A1_6h_R1 **Scan** 14105
**Peptide** ATVAEKTKEQVTNVGGAVV **Charge** 2 **Spectral Angle** 0.77

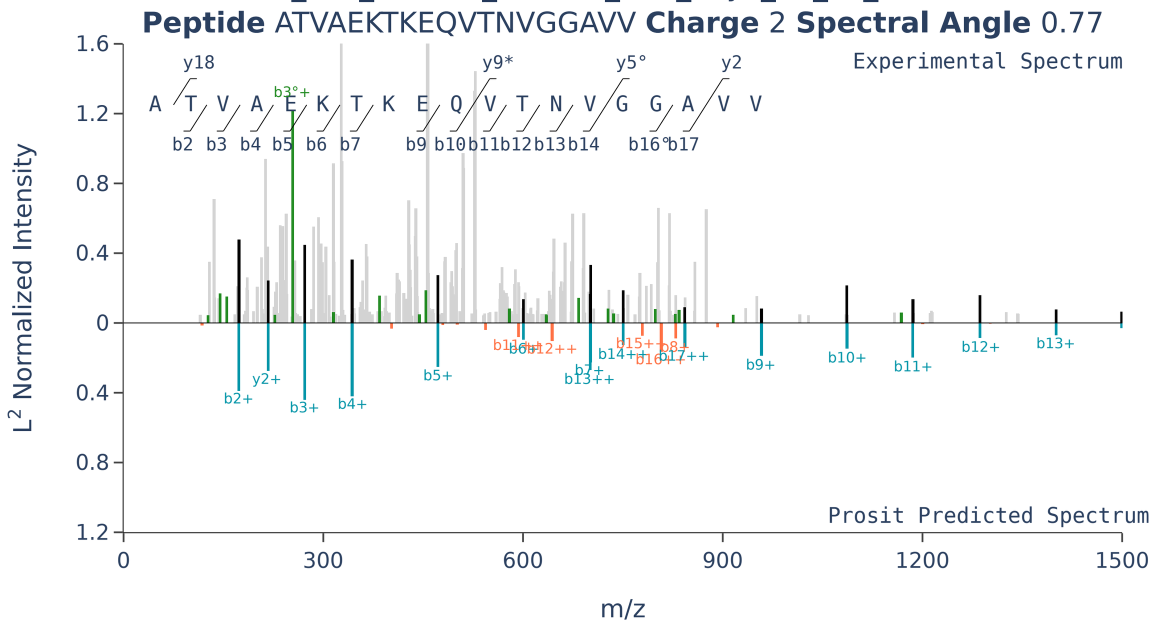


**Source** W_Soh_160821_170921_Goe_aSyn_A2_24h_R1 **Scan** 13176
**Peptide** KTKEGVVHGVATVAEKTKEQVTNV **Charge** 4 **Spectral Angle** 0.79

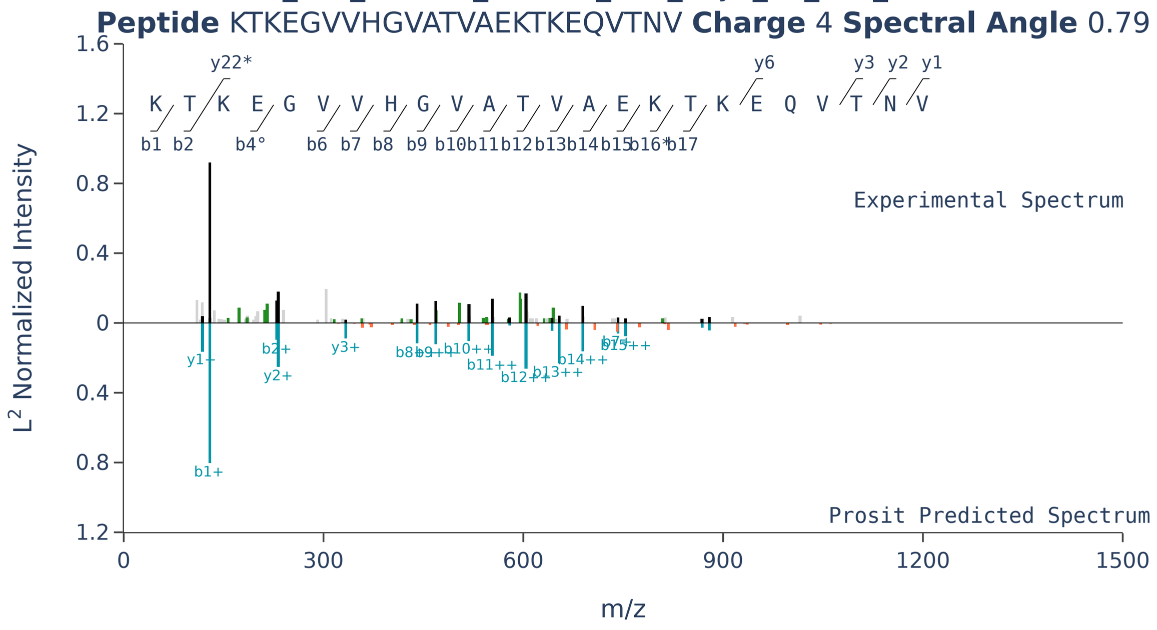


**Source** W_Soh_160821_170921_Goe_aSyn_A1_6h_R1 **Scan** 24697
**Peptide** GGAVVTGVTAVAQKTVEGAGSL **Charge** 2 **Spectral Angle** 0.84

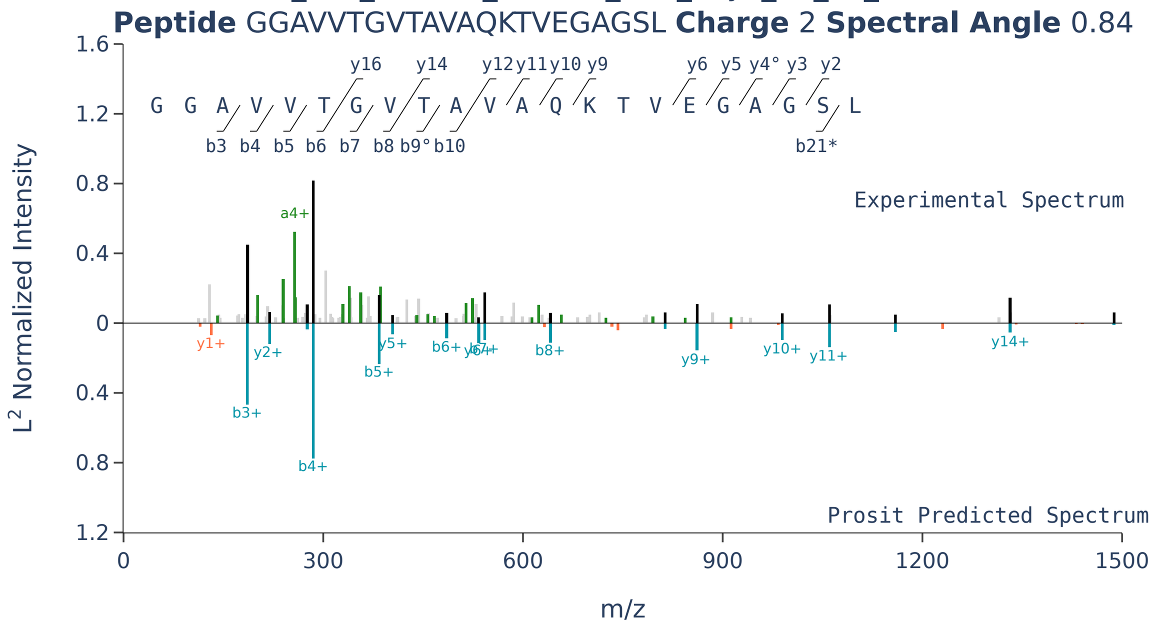


**Source** W_Soh_160821_170921_Goe_aSyn_A1_24h_R1 **Scan** 6624
**Peptide** EGVVAAAEKTKQ **Charge** 2 **Spectral Angle** 0.85

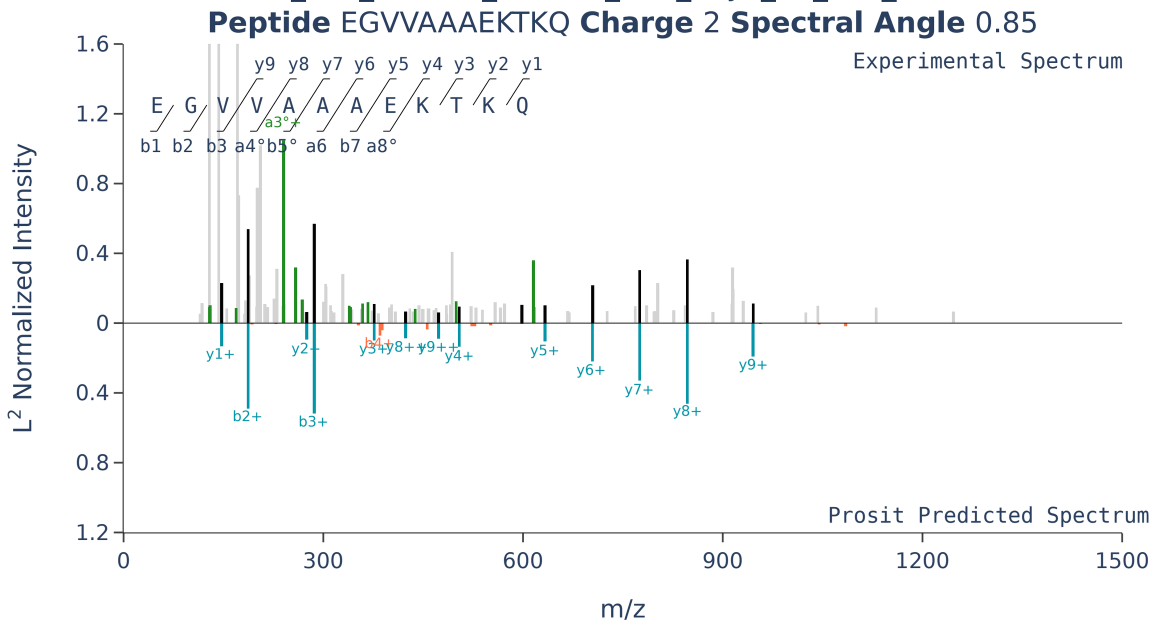


**Source** W_Soh_160821_170921_Goe_aSyn_A1_24h_R1 **Scan** 12839
**Peptide** GVLYVGSKTKEGVVHGV **Charge** 4 **Spectral Angle** 0.85

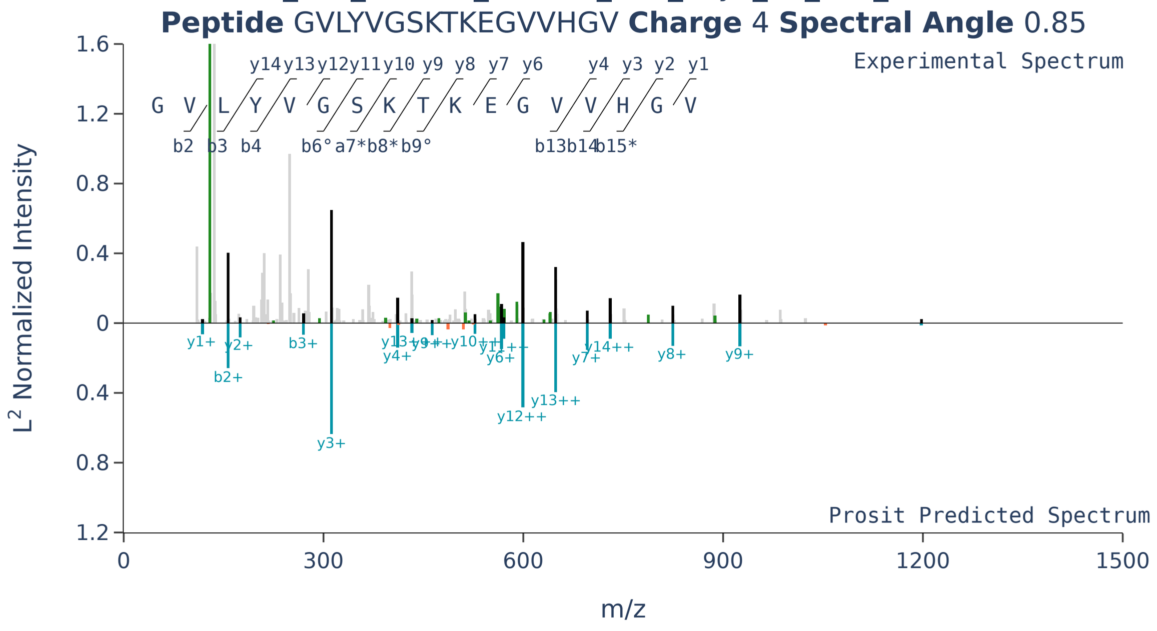


**Source** W_Soh_160821_170921_Goe_aSyn_A1_24h_R1 **Scan** 10391
**Peptide** VLYVGSKTKEGV **Charge** 3 **Spectral Angle** 0.87

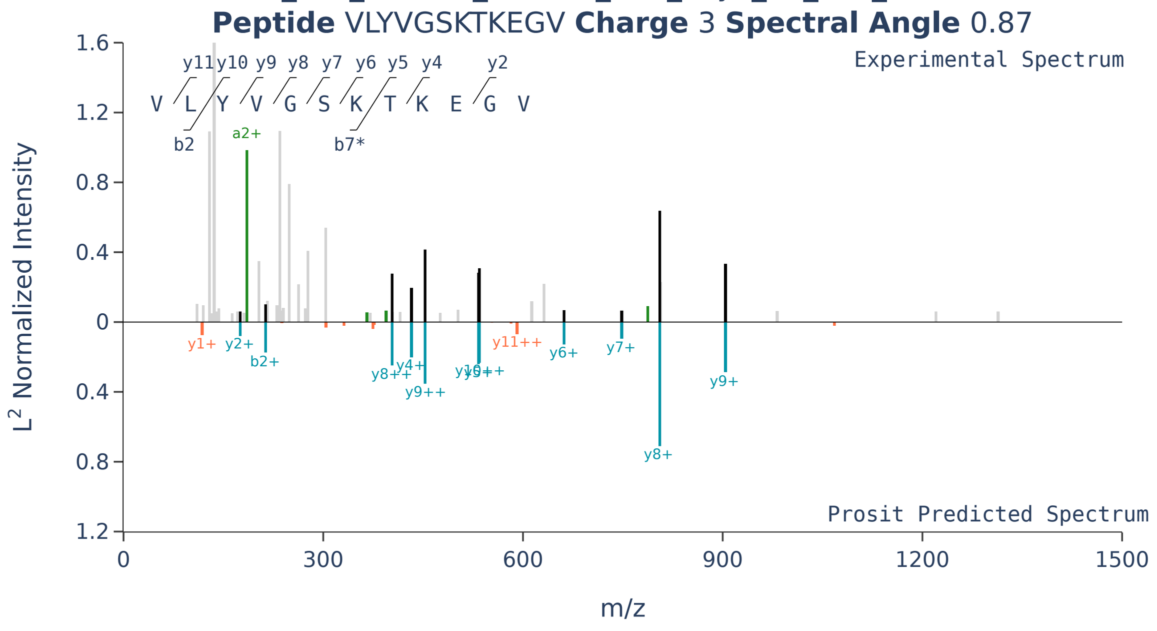


**Source** W_Soh_160821_170921_Goe_aSyn_A1_24h_R2 **Scan** 16139
**Peptide** TVAEKTKEQVTNVGGAVVTGV **Charge** 3 **Spectral Angle** 0.83

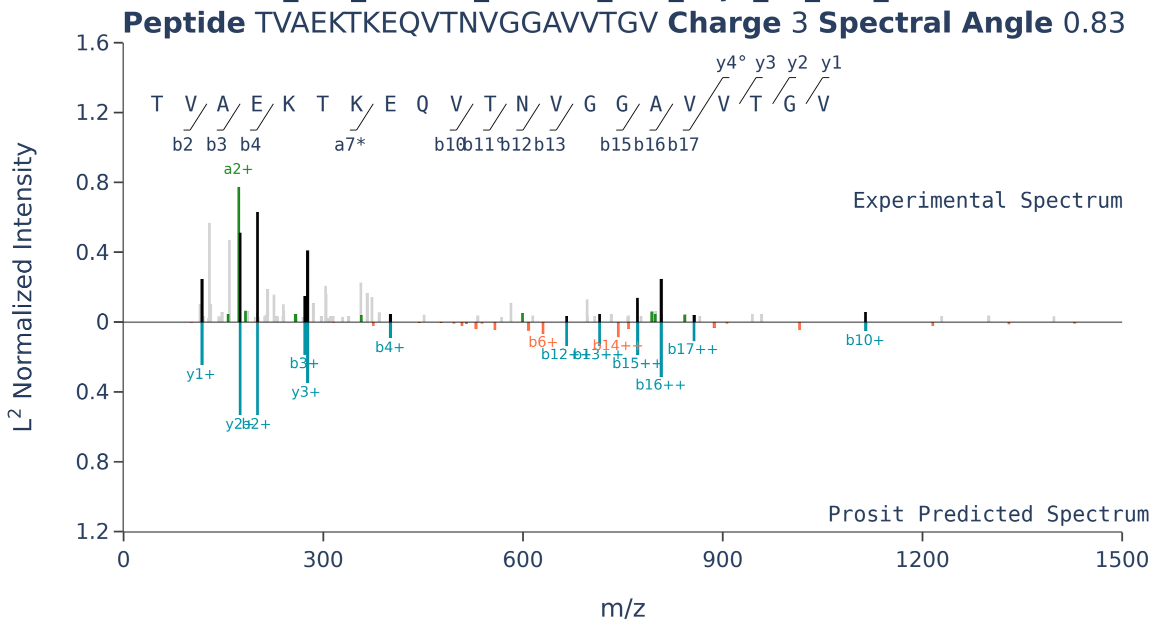


**Source** W_Soh_160821_170921_Goe_aSyn_A1_24h_R2 **Scan** 9393
**Peptide** AEAAGKTKEGVLY **Charge** 2 **Spectral Angle** 0.93

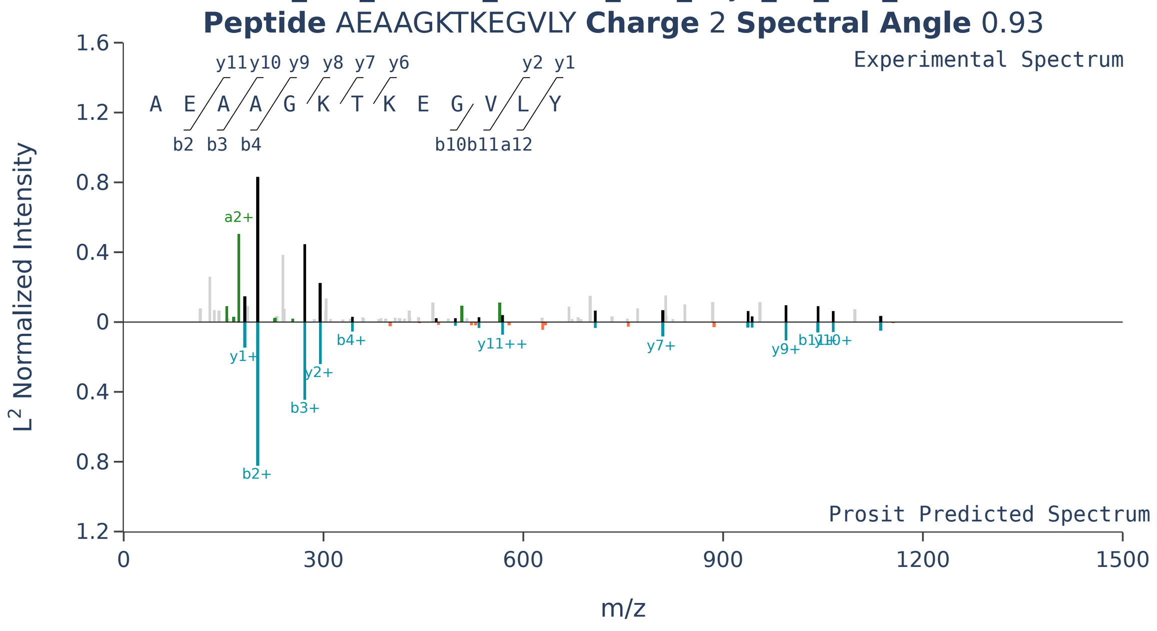


**Source** W_Soh_160821_170921_Goe_aSyn_A2_24h_R1 **Scan** 18448
**Peptide** MDVFMKGLSKAKEGVVA **Charge** 3 **Spectral Angle** 0.87

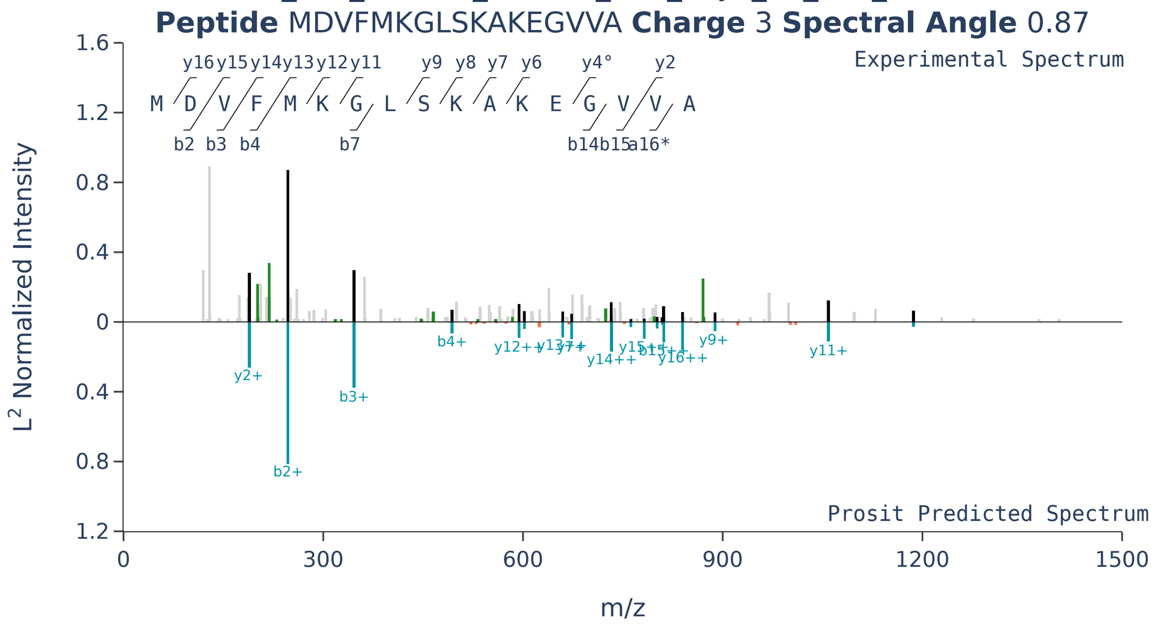


**Source** W_Soh_160821_170921_Goe_aSyn_A2_24h_R2 **Scan** 14599
**Peptide** AGSIAAATGFVKKDQLGKNEEGAPQE **Charge** 3 **Spectral Angle** 0.89

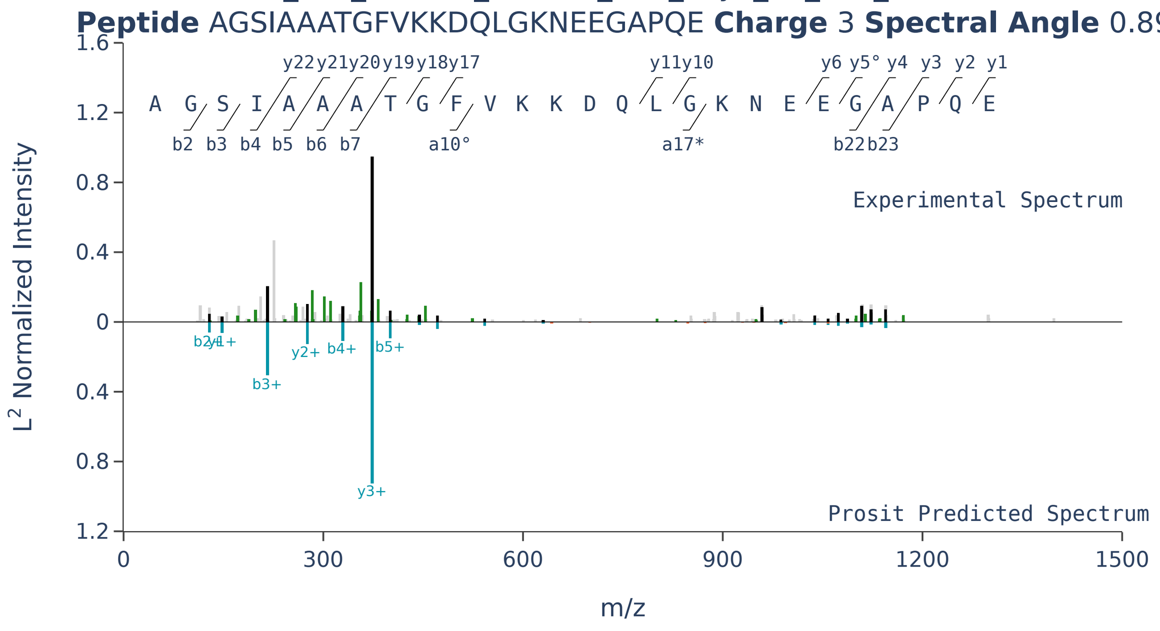


**Source** W_Soh_160821_170921_Goe_aSyn_A1_24h_R1 **Scan** 7920
**Peptide** SKTKEGVVHGVATVA **Charge** 3 **Spectral Angle** 0.83

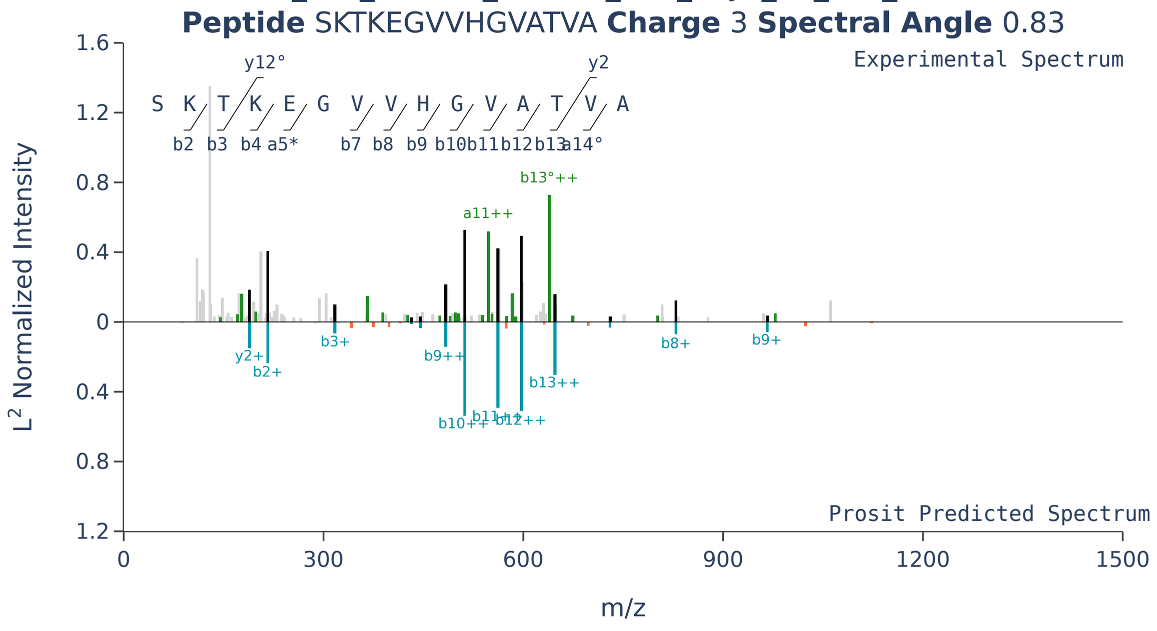

**Source** W_Soh_160821_170921_Goe_aSyn_A1_24h_R2 **Scan** 7222
**Peptide** SKAKEGVV **Charge** 2 **Spectral Angle** 0.87

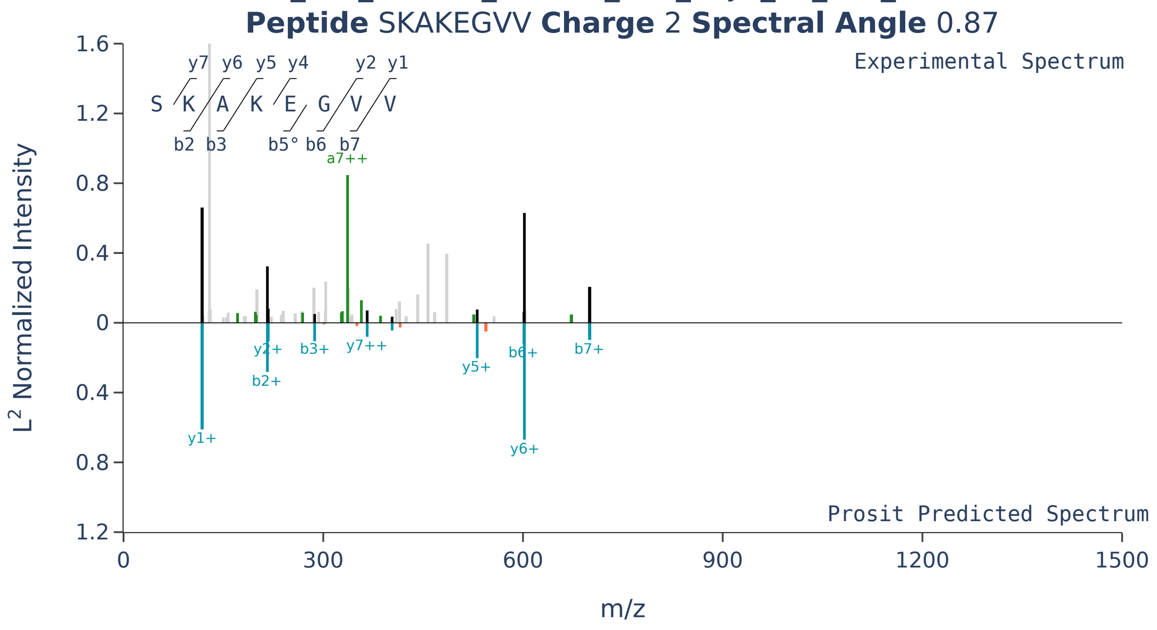


**Source** W_Soh_160821_170921_Goe_aSyn_A1_24h_R1 **Scan** 22162
**Peptide** VTNVGGAVVTGVTAVAQKT **Charge** 2 **Spectral Angle** 0.81

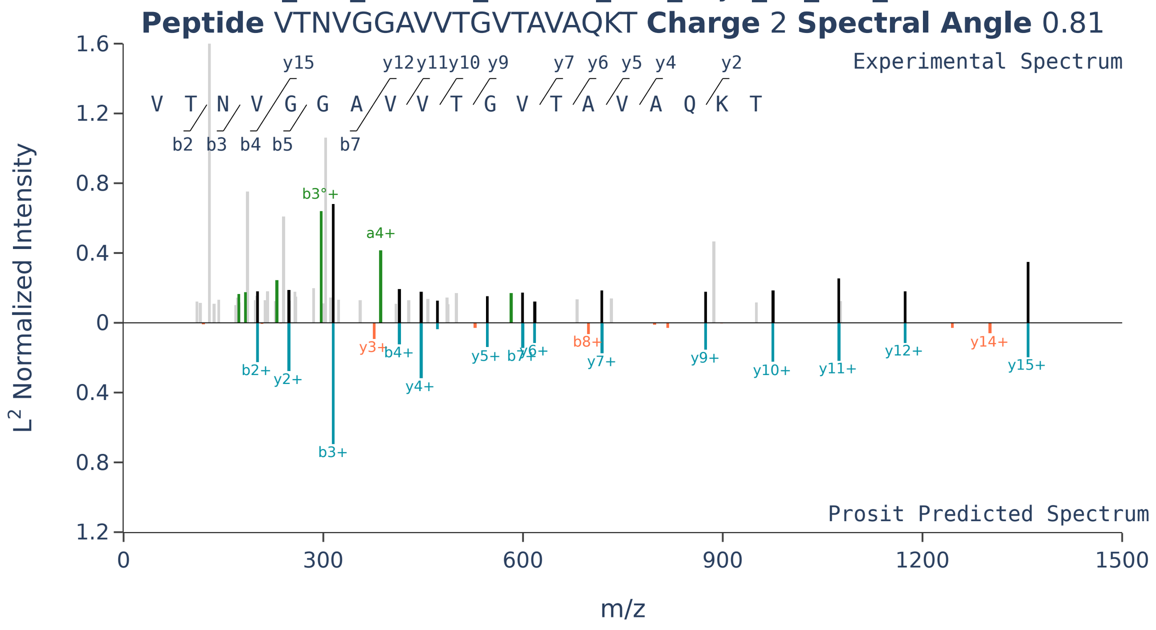


**Source** W_Soh_160821_170921_Goe_aSyn_A1_24h_R1 **Scan** 7130
**Peptide** YVGSKTKEGVVHGVA **Charge** 3 **Spectral Angle** 0.77

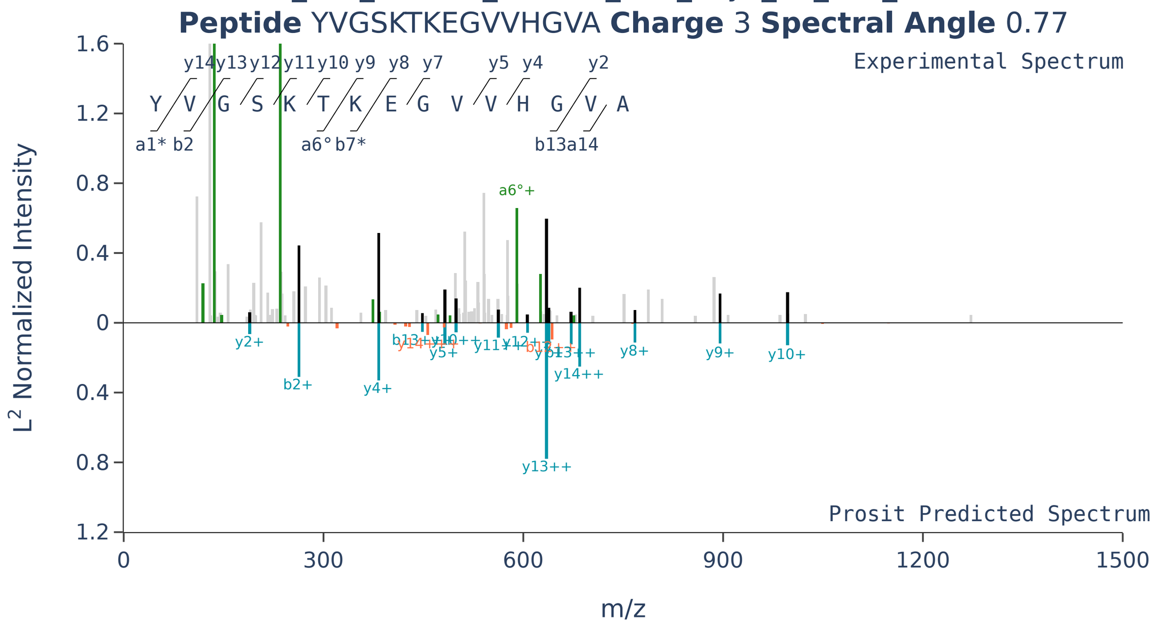


**Source** W_Soh_160821_170921_Goe_aSyn_A1_24h_R1 **Scan** 6673
**Peptide** AGKTKEGVLYVGSKTKEG **Charge** 3 **Spectral Angle** 0.86

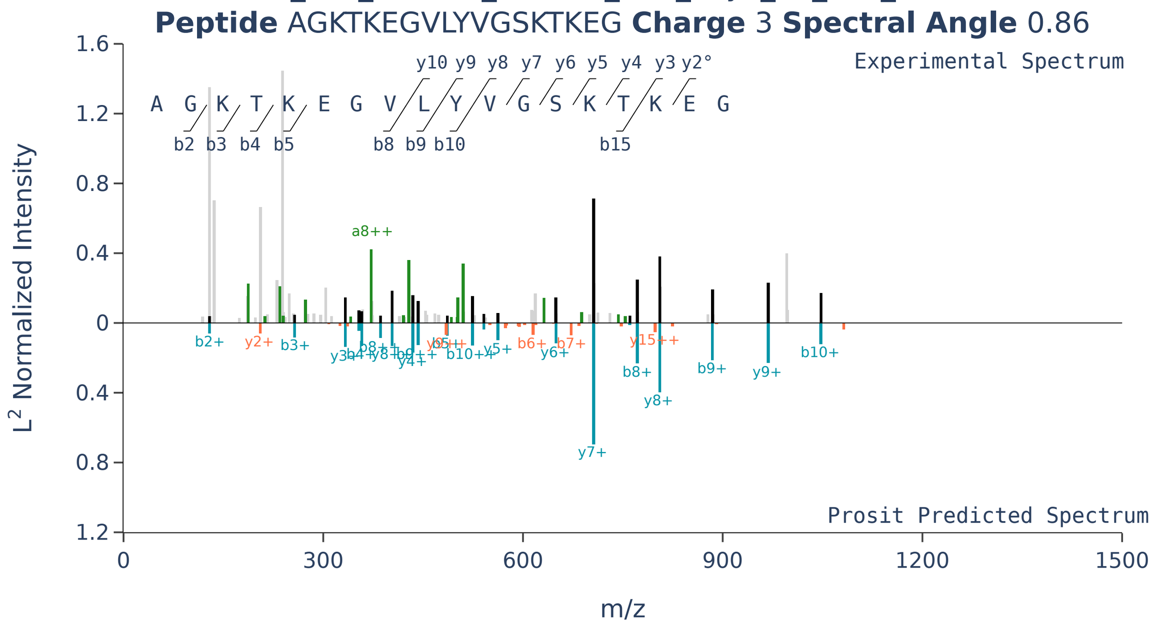


**Source** W_Soh_160821_170921_Goe_aSyn_A1_24h_R2 **Scan** 22013
**Peptide** GVTAVAQKTVEGAGSIAAATGFVKKDQ **Charge** 3 **Spectral Angle** 0.81

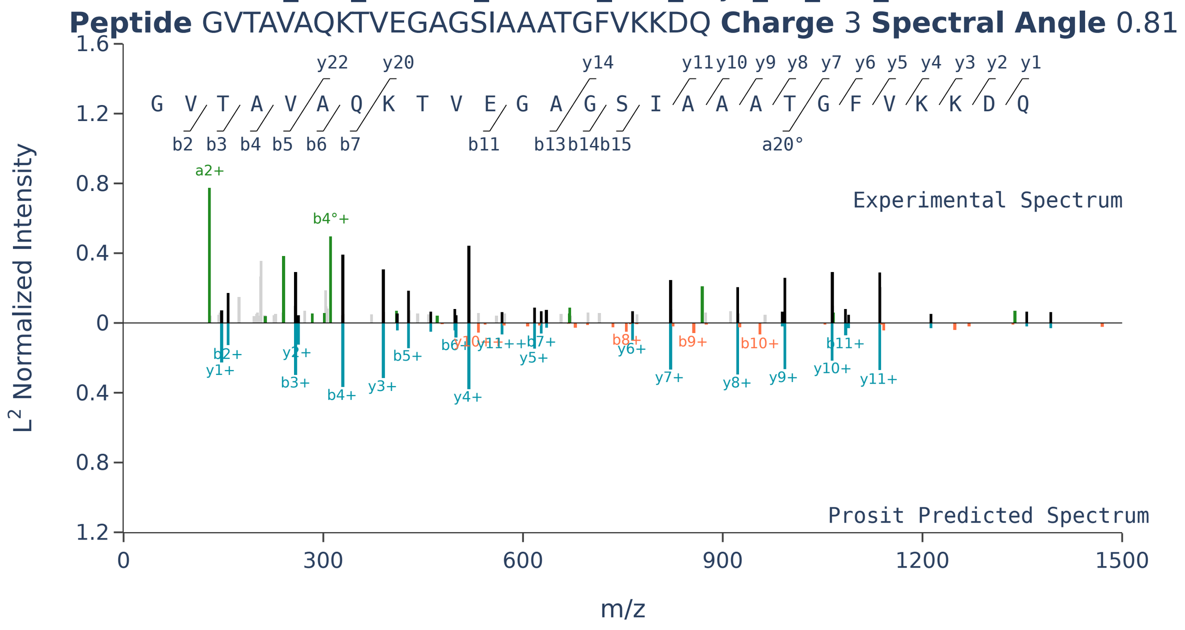


**Source** W_Soh_160821_170921_Goe_aSyn_A2_24h_R1 **Scan** 7478
**Peptide** KTKEGVVHGVATVAEKT **Charge** 4 **Spectral Angle** 0.84

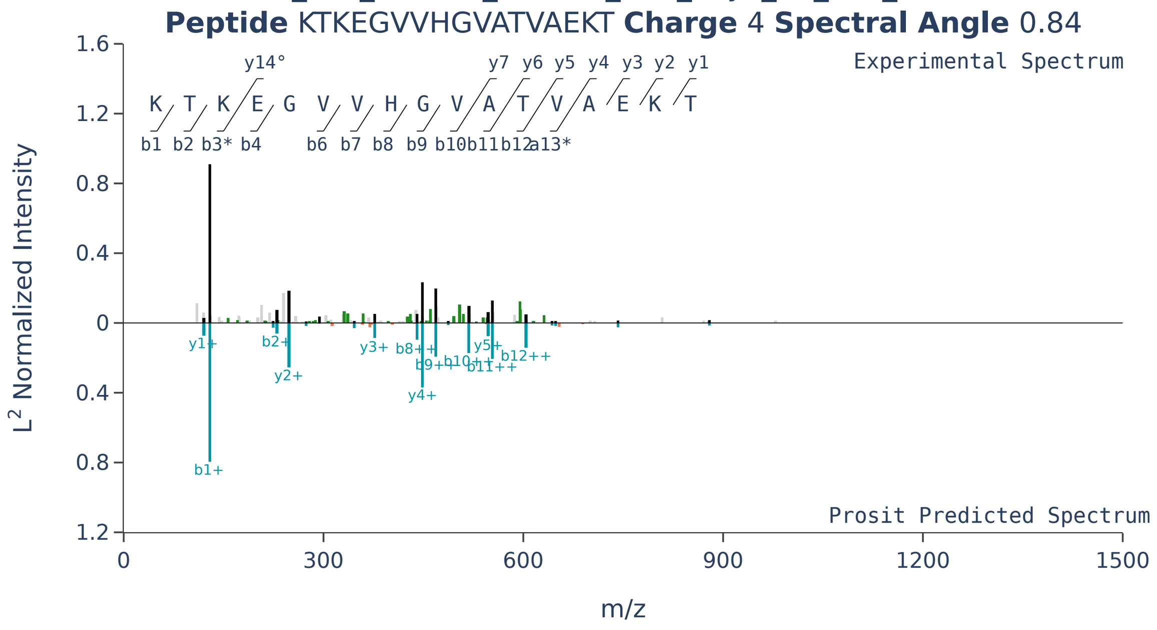


**Source** W_Soh_160821_170921_Goe_aSyn_A1_3h_R1 **Scan** 9012
**Peptide** KEGVVAAAEKTKQGVAEAAGKTKEG **Charge** 5 **Spectral Angle** 0.87

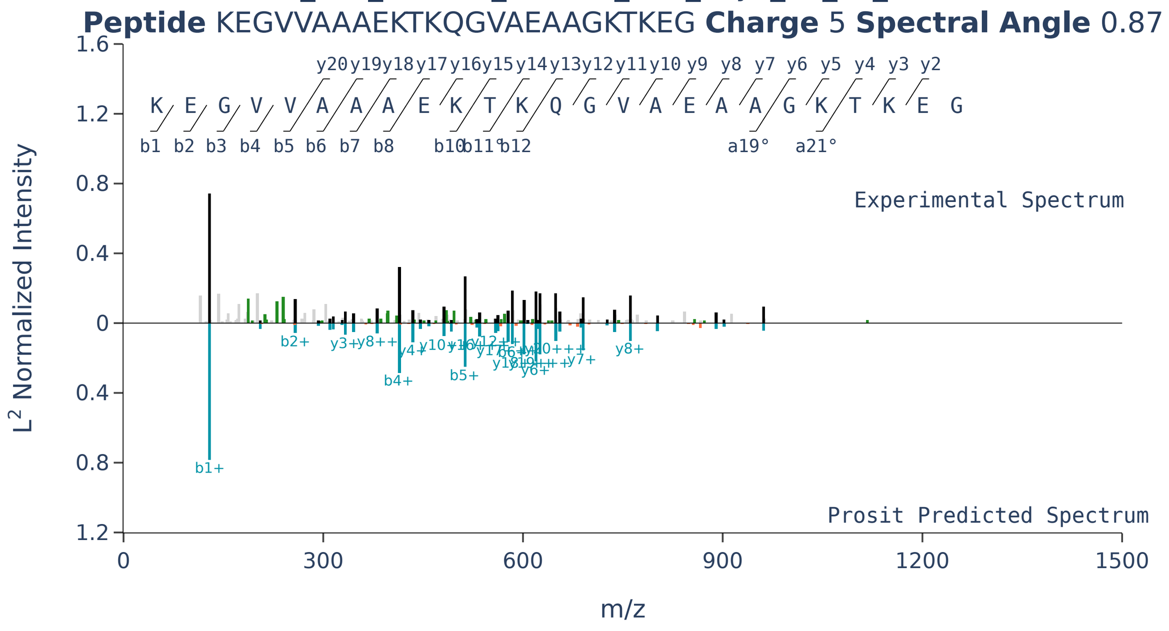


**Source** W_Soh_160821_170921_Goe_aSyn_A1_24h_R2 **Scan** 20252
**Peptide** TAVAQKTVEGAGSIAAATGFVKKDQL **Charge** 3 **Spectral Angle** 0.84

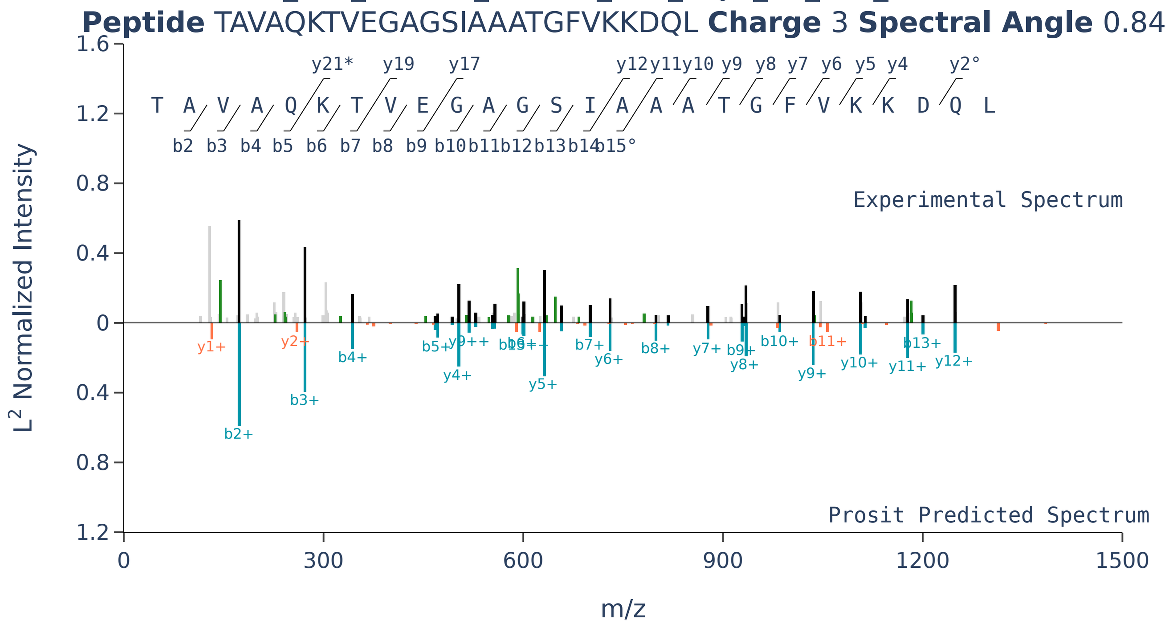


**Source** W_Soh_160821_170921_Goe_aSyn_A2_24h_R2 **Scan** 8685
**Peptide** AAGKTKEGVLY **Charge** 2 **Spectral Angle** 0.75

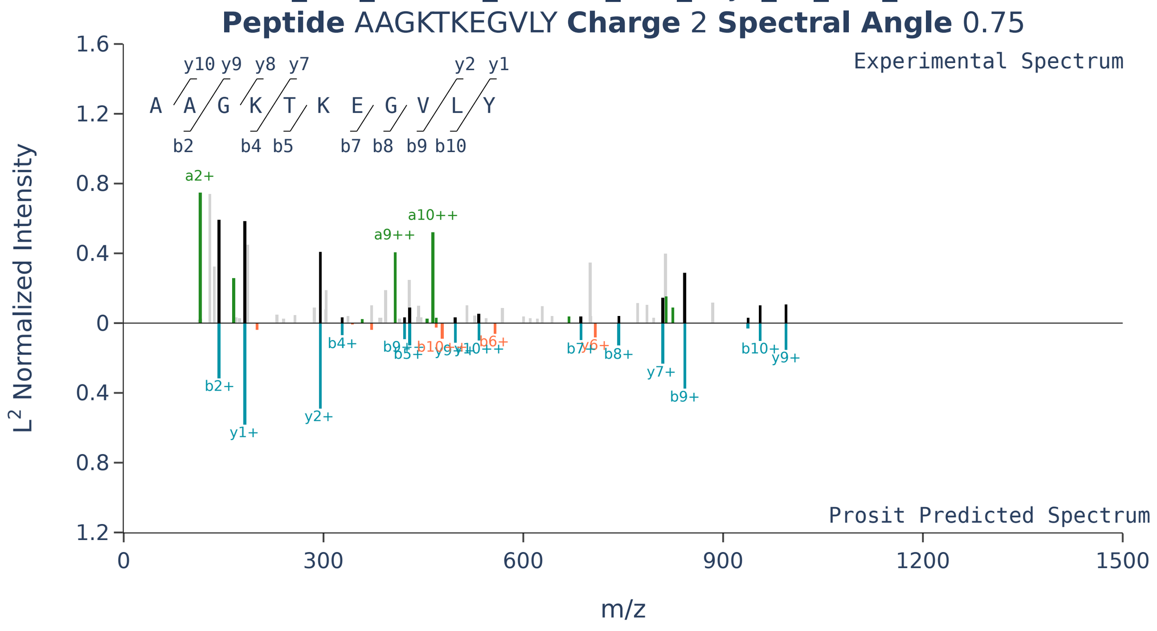


**Source** W_Soh_160821_170921_Goe_aSyn_A2_6h_R1 **Scan** 7281
**Peptide** KEGVVHGVATVAEKTKE **Charge** 4 **Spectral Angle** 0.87

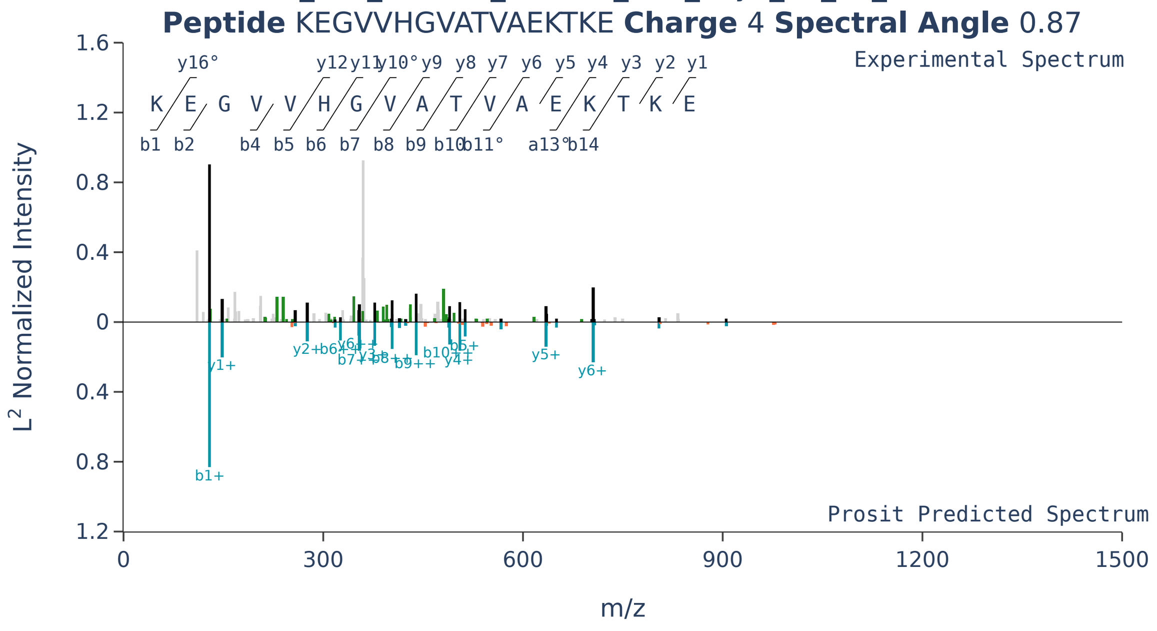


**Source** W_Soh_160821_170921_Goe_aSyn_A1_24h_R2 **Scan** 6846
**Peptide** KGLSKAKEGVVAAAEKT **Charge** 3 **Spectral Angle** 0.91

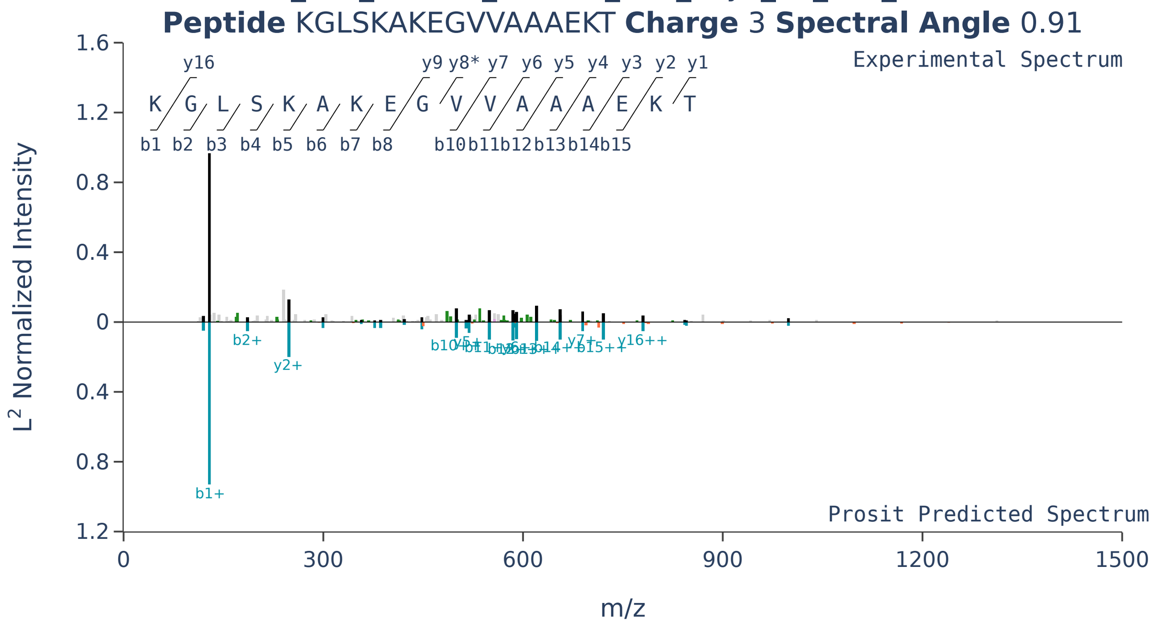


**Source** W_Soh_160821_170921_Goe_aSyn_A2_24h_R2 **Scan** 9781
**Peptide** KTKEQVTNVGGAVVTG **Charge** 3 **Spectral Angle** 0.8

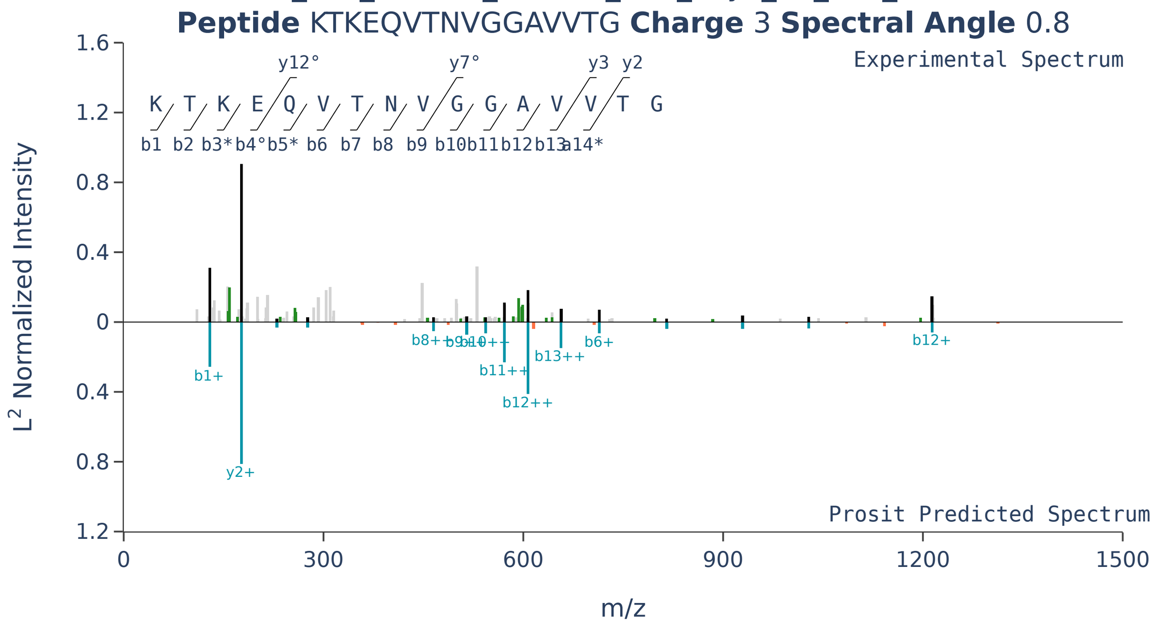


**Source** W_Soh_160821_170921_Goe_aSyn_A1_24h_R1 **Scan** 15749
**Peptide** EGAGSIAAATGFVKKDQLGKNE **Charge** 3 **Spectral Angle** 0.79

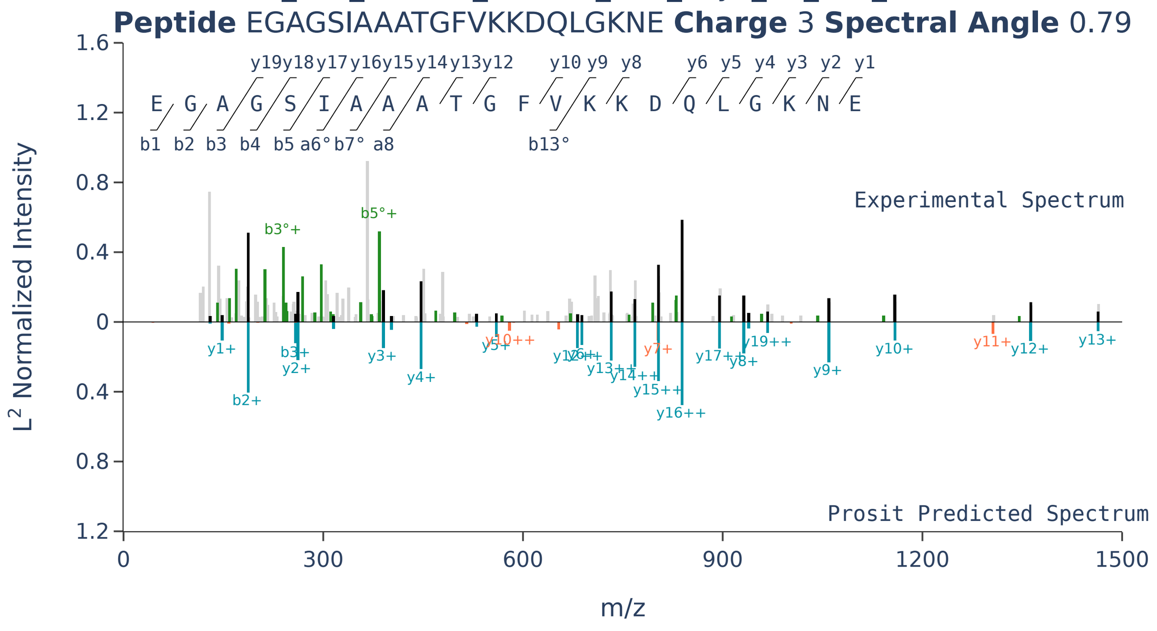


**Source** W_Soh_160821_170921_Goe_aSyn_A1_24h_R1 **Scan** 19312
**Peptide** VGGAVVTGVTAVAQ **Charge** 2 **Spectral Angle** 0.9

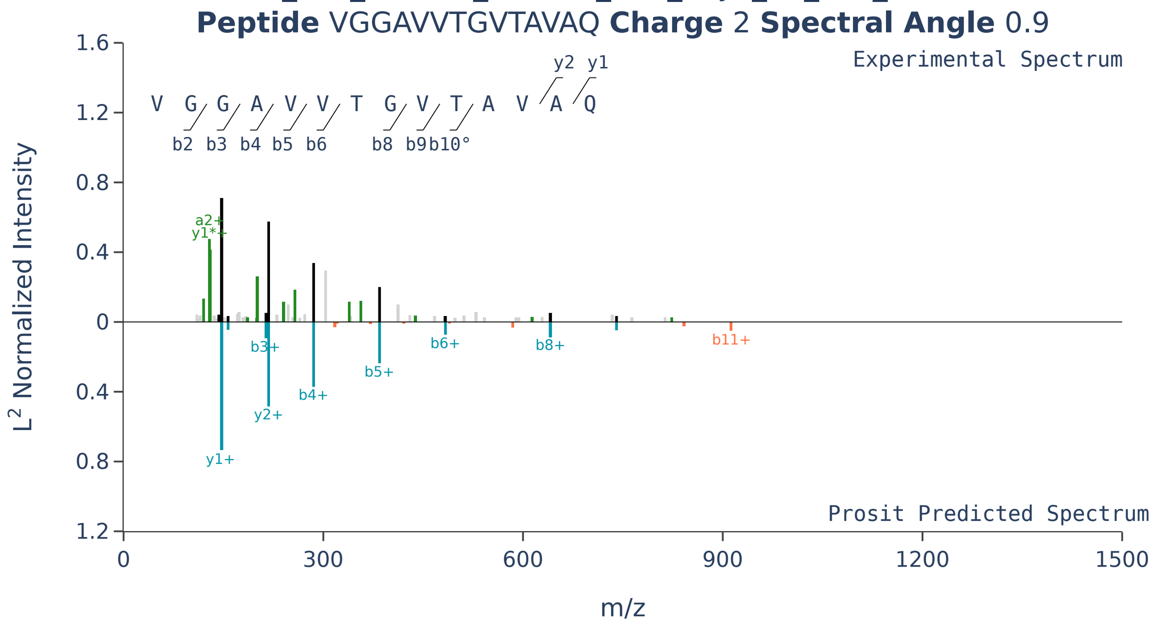


**Source** W_Soh_160821_170921_Goe_aSyn_A2_24h_R2 **Scan** 10706
**Peptide** AQKTVEGAGSIAAATG **Charge** 2 **Spectral Angle** 0.85

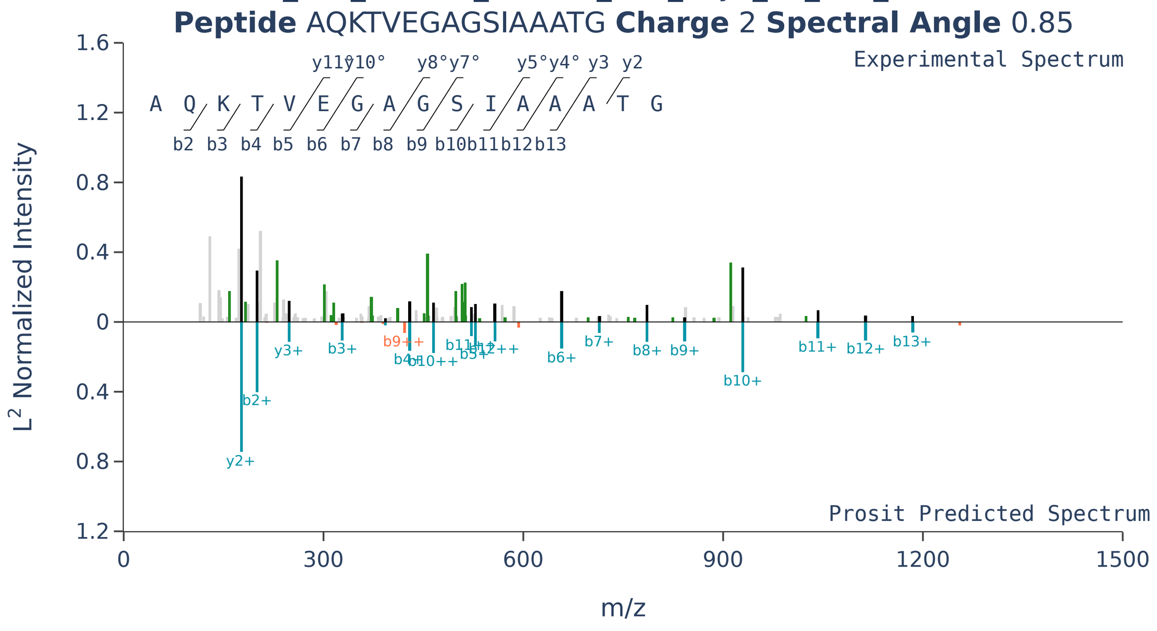

**Source** W_Soh_160821_170921_Goe_aSyn_A2_24h_R2 **Scan** 7715
**Peptide** KTKEGVVHGVATV **Charge** 2 **Spectral Angle** 0.91

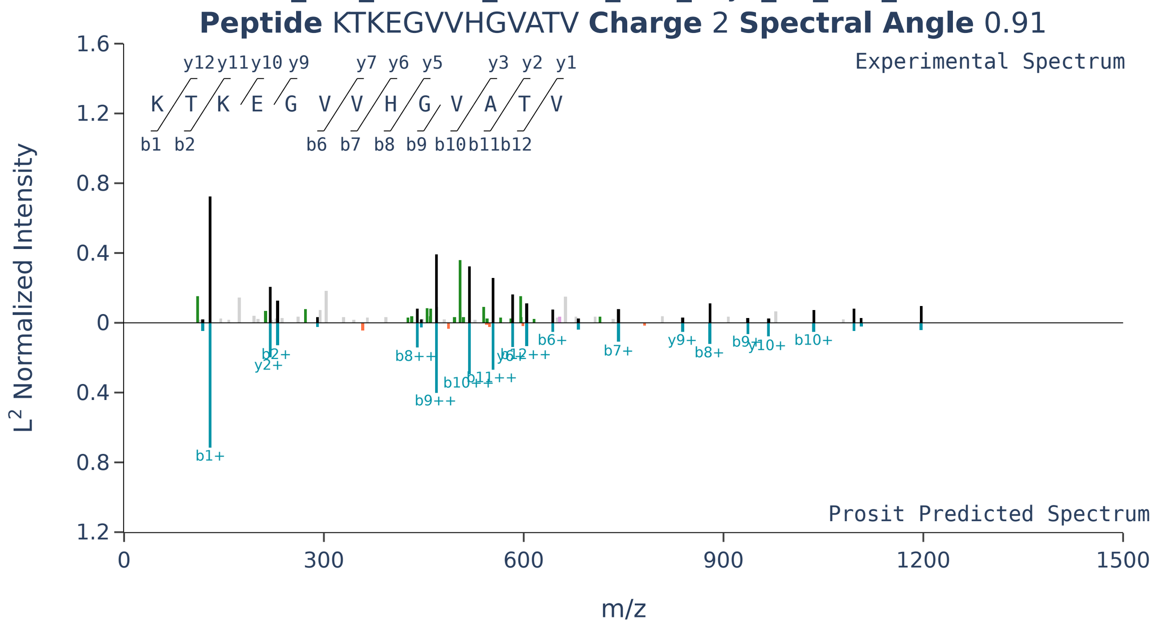


**Source** W_Soh_160821_170921_Goe_aSyn_A2_24h_R2 **Scan** 10104
**Peptide** EGVLYVGSKTK **Charge** 2 **Spectral Angle** 0.86

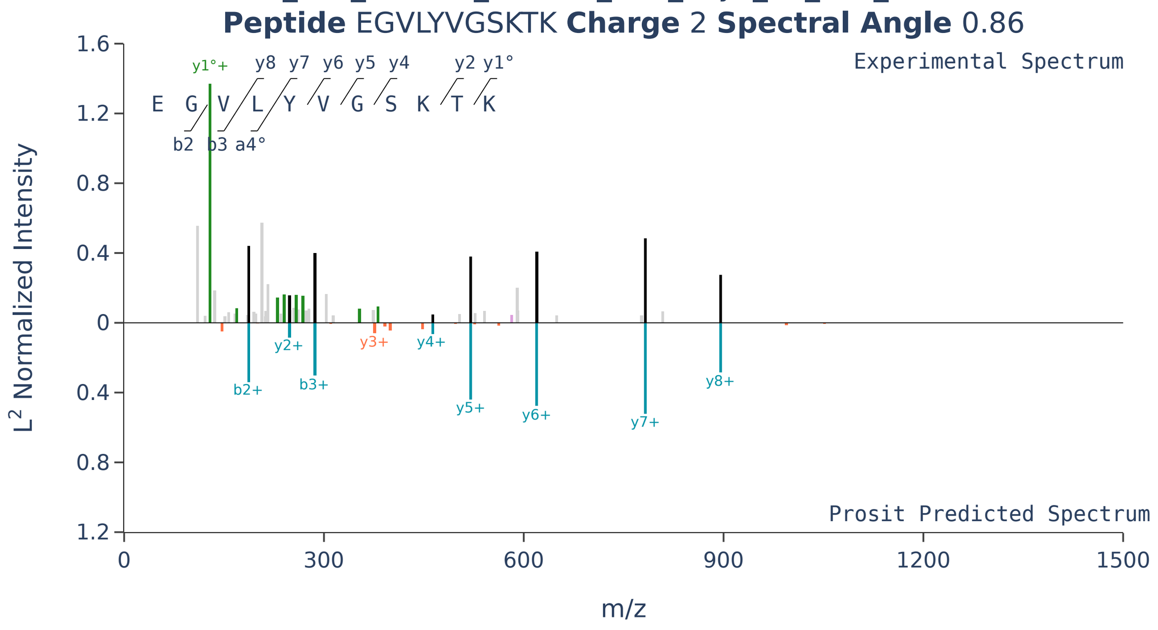


**Source** W_Soh_160821_170921_Goe_aSyn_A1_24h_R1 **Scan** 11327
**Peptide** TGFVKKDQLGKNEEGAPQEGI **Charge** 3 **Spectral Angle** 0.87

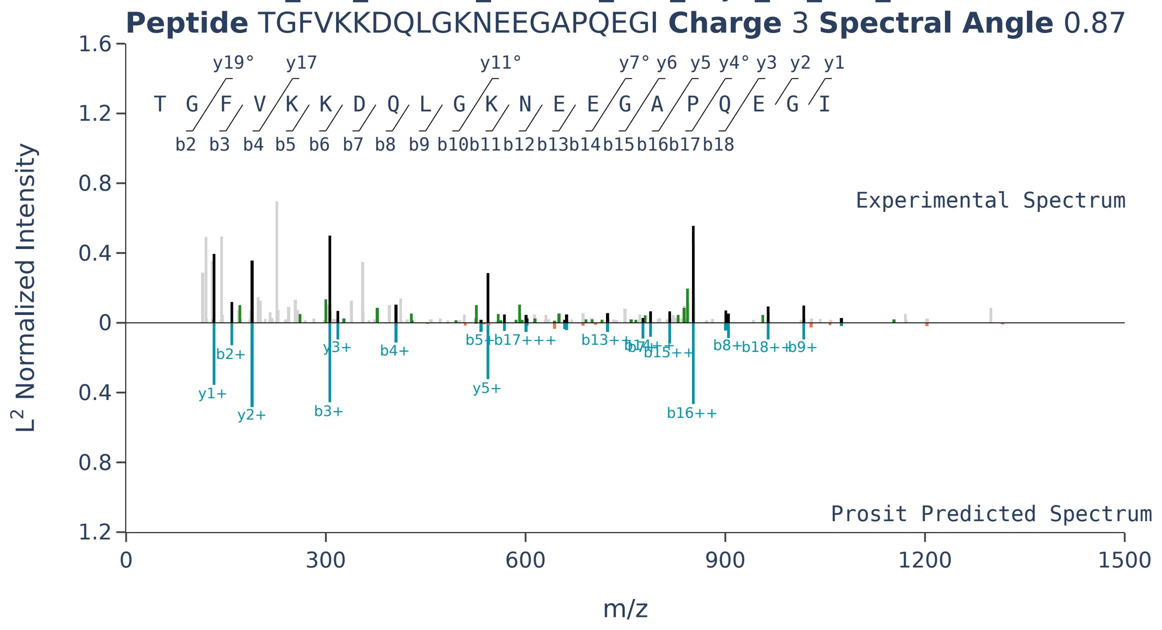


**Source** W_Soh_160821_170921_Goe_aSyn_A1_24h_R1 **Scan** 13558
**Peptide** GGAVVTGVTAVAQK **Charge** 2 **Spectral Angle** 0.84

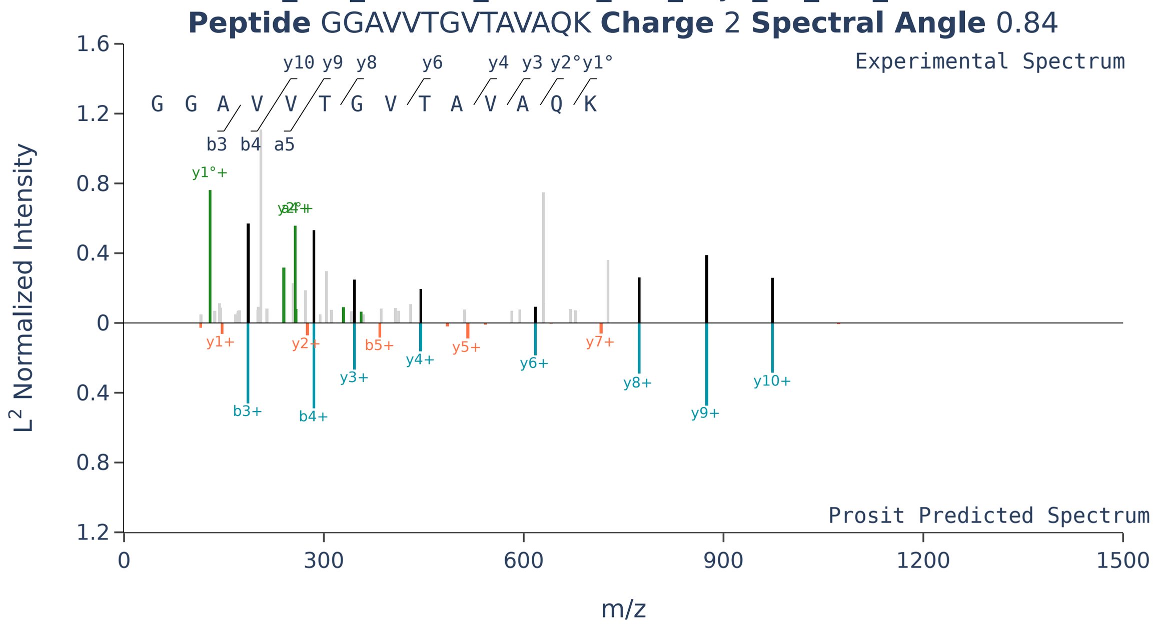


**Source** W_Soh_160821_170921_Goe_aSyn_A1_24h_R1 **Scan** 7105
**Peptide** GLSKAKEGVV **Charge** 2 **Spectral Angle** 0.79

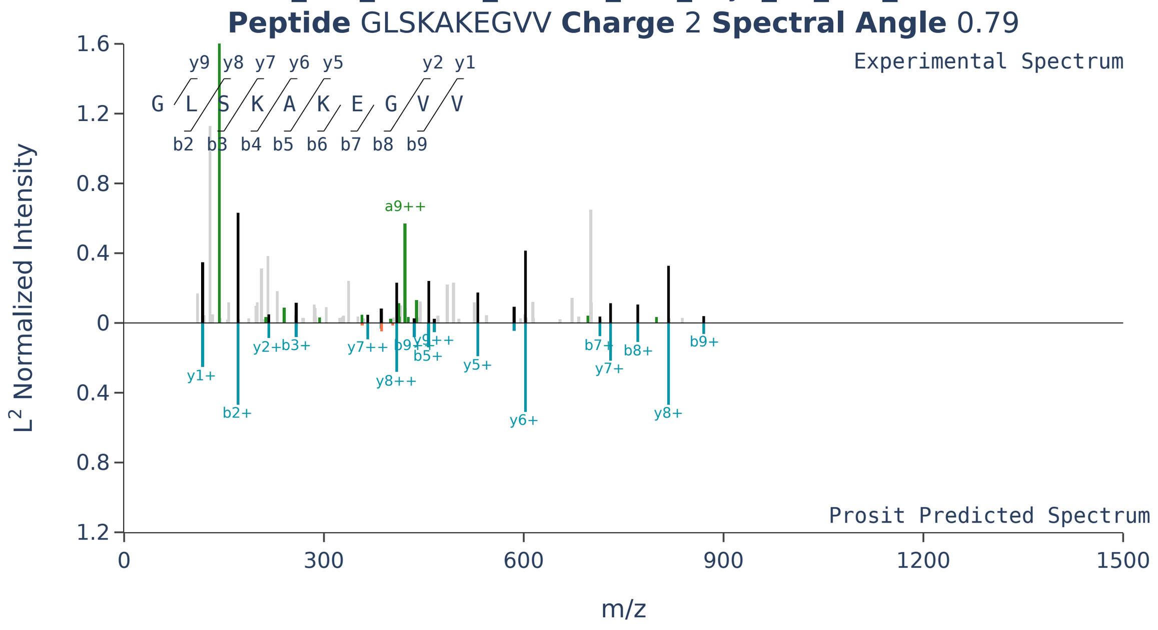


**Source** W_Soh_160821_170921_Goe_aSyn_A2_24h_R1 **Scan** 18847
**Peptide** EGAGSIAAATGFVK **Charge** 2 **Spectral Angle** 0.75

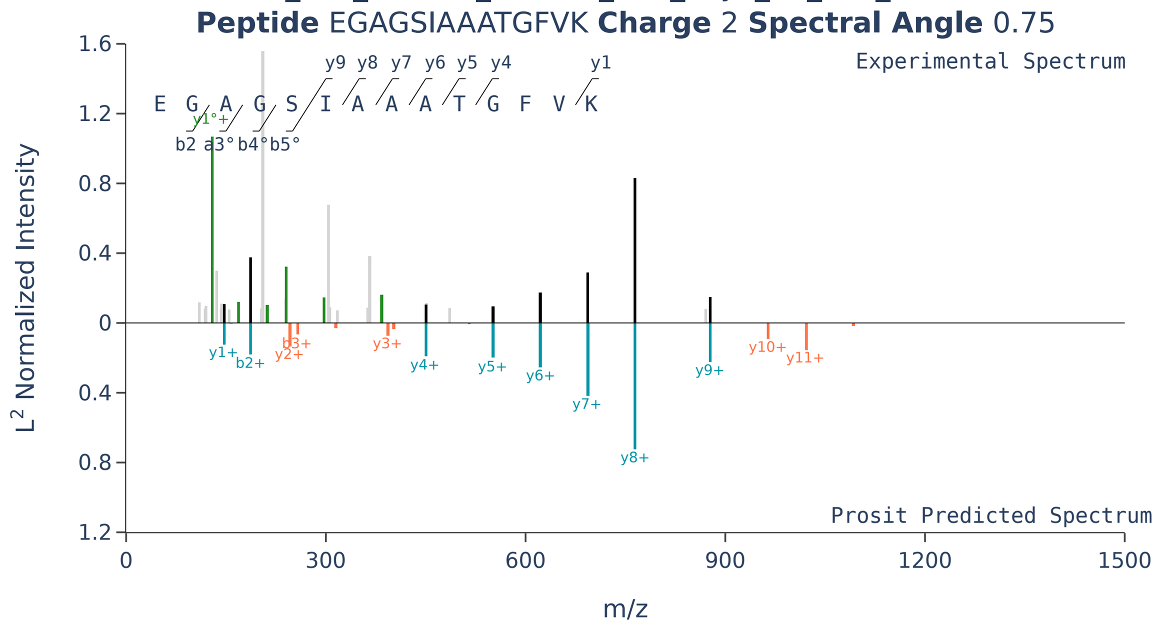


**Source** W_Soh_160821_170921_Goe_aSyn_A1_24h_R1 **Scan** 15038
**Peptide** KTVEGAGSIAAATGFVKKDQLGKNE **Charge** 4 **Spectral Angle** 0.86

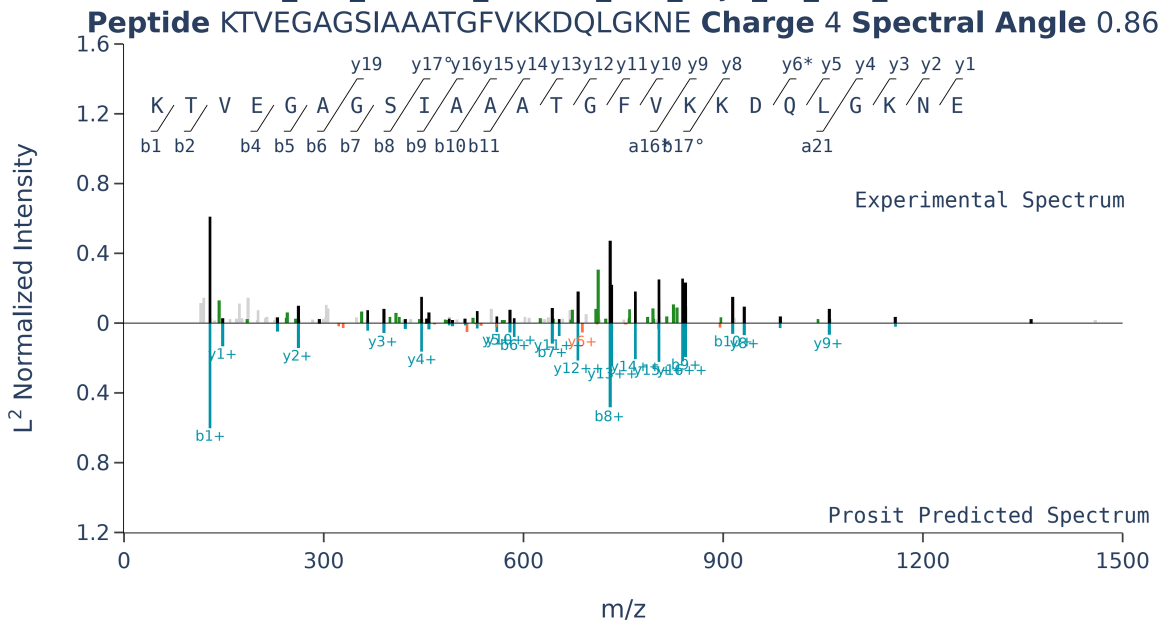


**Source** W_Soh_160821_170921_Goe_aSyn_A1_24h_R1 **Scan** 7932
**Peptide** AAGKTKEGVLYVGSKTKEGVVH **Charge** 3 **Spectral Angle** 0.91

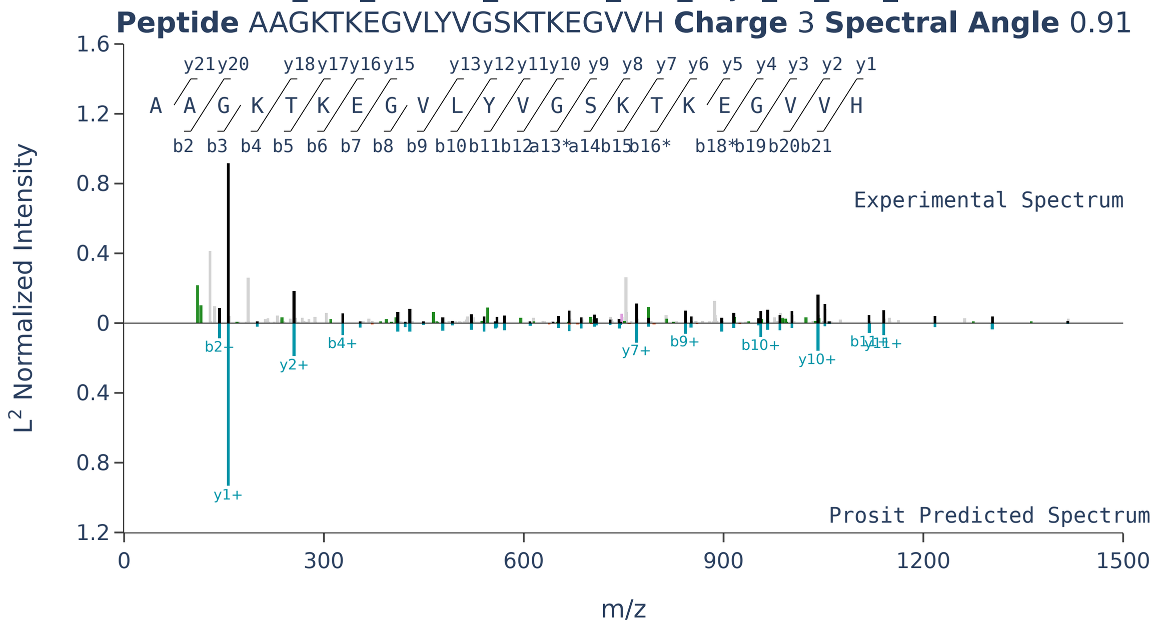


**Source** W_Soh_160821_170921_Goe_aSyn_A2_24h_R1 **Scan** 15211
**Peptide** GSIAAATGFVKKDQLGKNEEGAPQE **Charge** 3 **Spectral Angle** 0.88

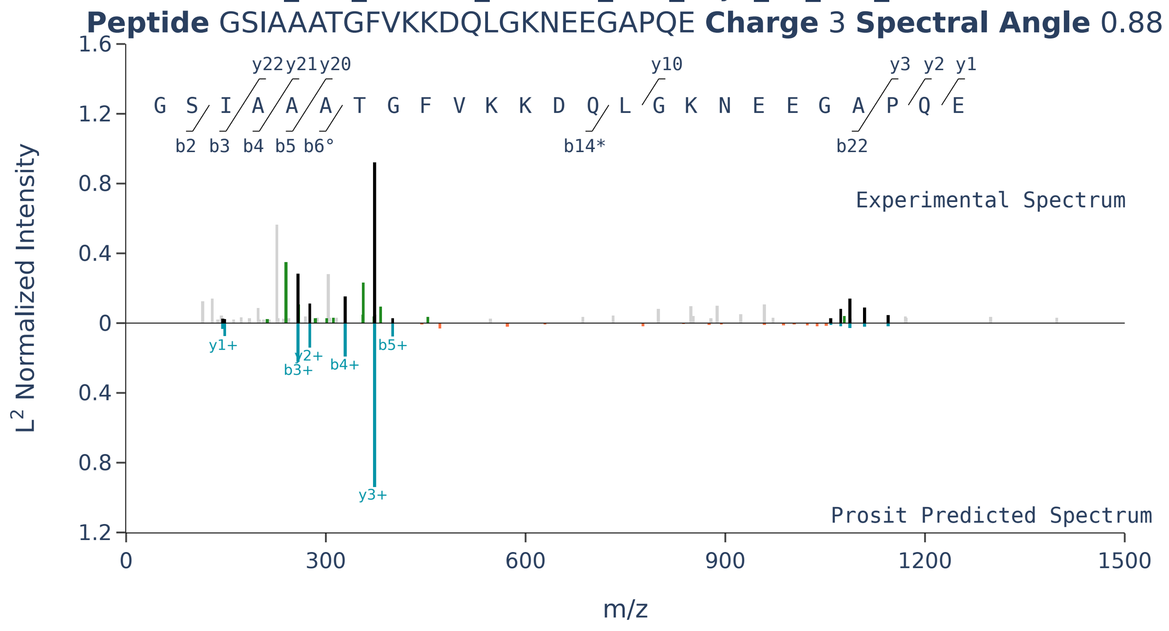


**Source** W_Soh_160821_170921_Goe_aSyn_A1_6h_R2 **Scan** 5803
**Peptide** KDQLGKNEEGAPQE **Charge** 3 **Spectral Angle** 0.7

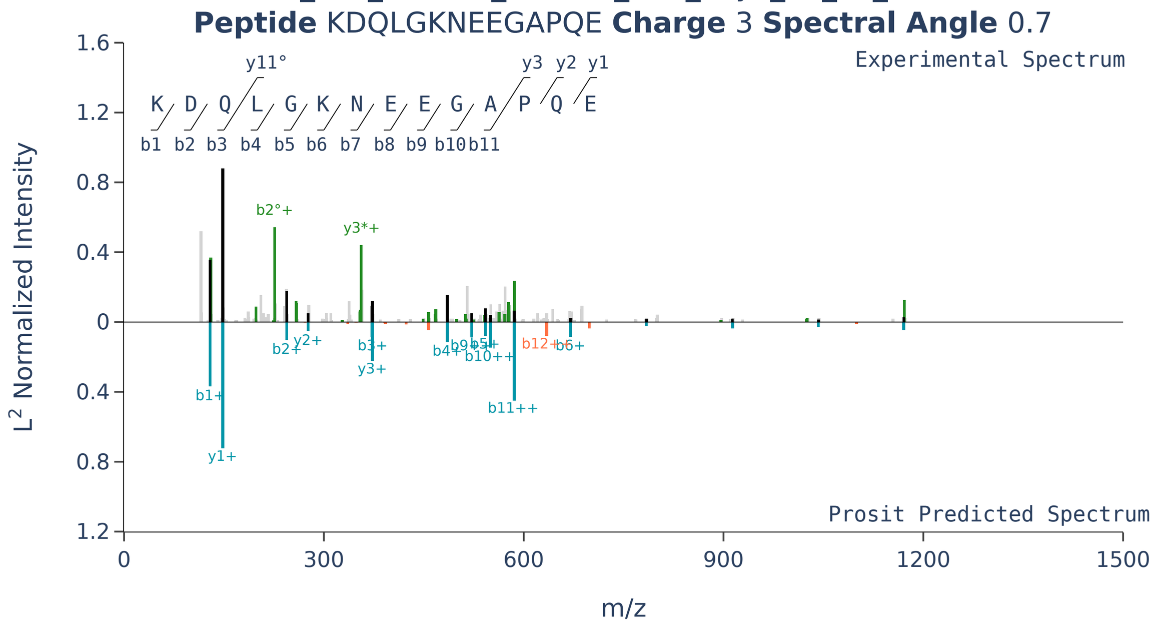


**Source** W_Soh_160821_170921_Goe_aSyn_A2_3h_R2 **Scan** 17714
**Peptide** EKTKEQVTNVGGAVVTGVTAVA **Charge** 3 **Spectral Angle** 0.84

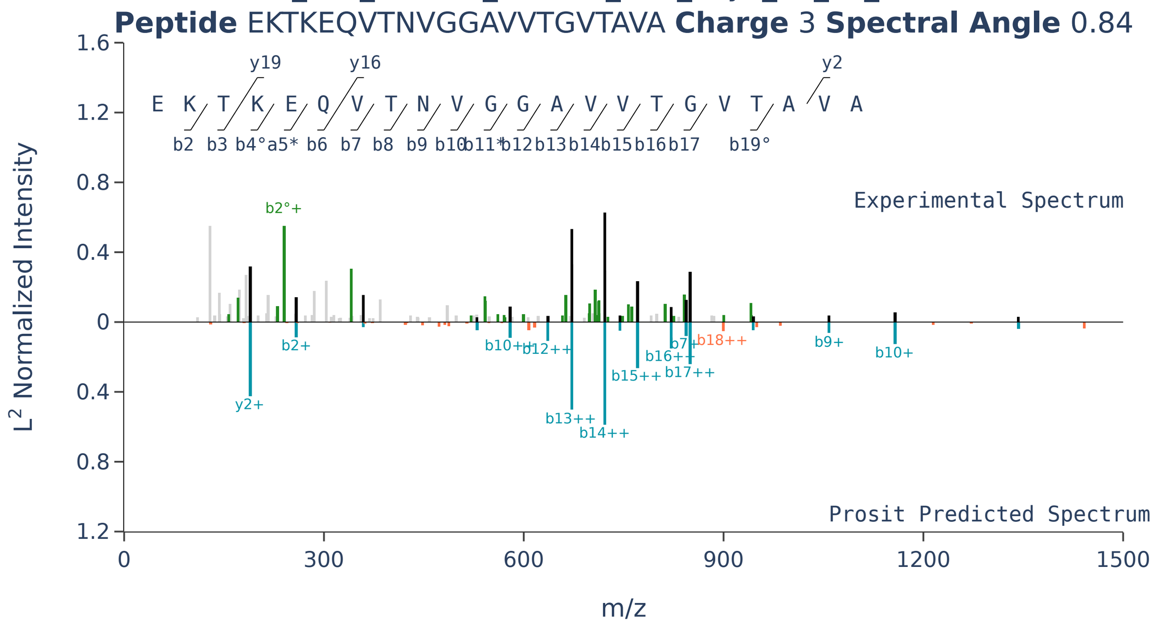


**Source** W_Soh_160821_170921_Goe_aSyn_A1_24h_R1 **Scan** 14364
**Peptide** GVAEAAGKTKEGVLYVG **Charge** 2 **Spectral Angle** 0.87

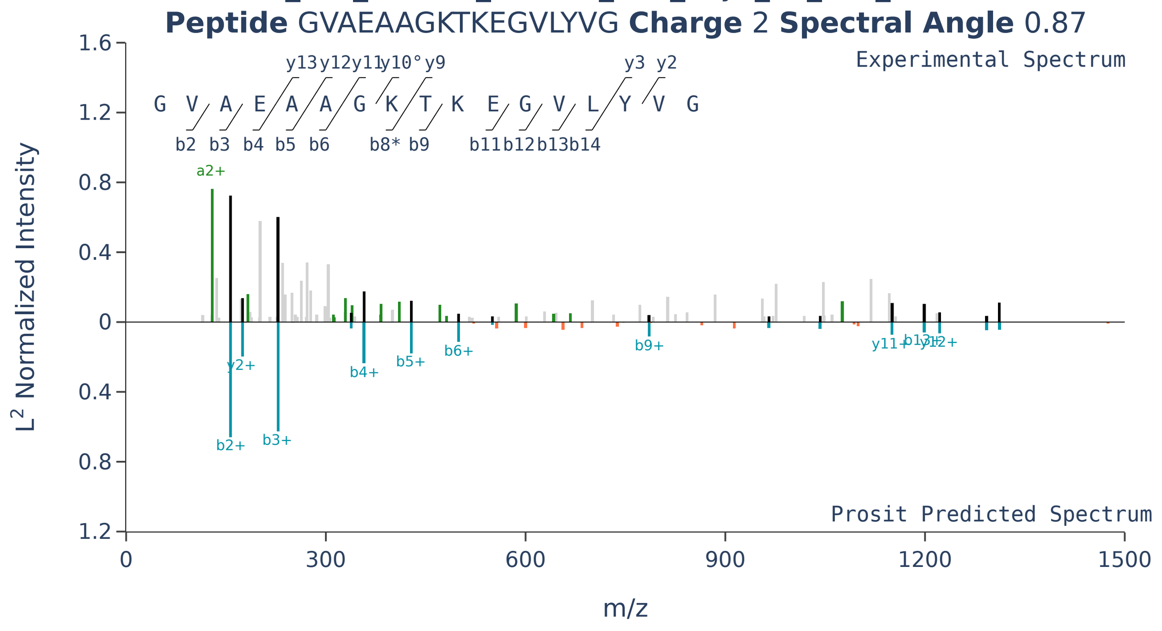


**Source** W_Soh_160821_170921_Goe_aSyn_A1_24h_R2 **Scan** 12374
**Peptide** TVAEKTKEQVTNVGGAVVT **Charge** 3 **Spectral Angle** 0.81

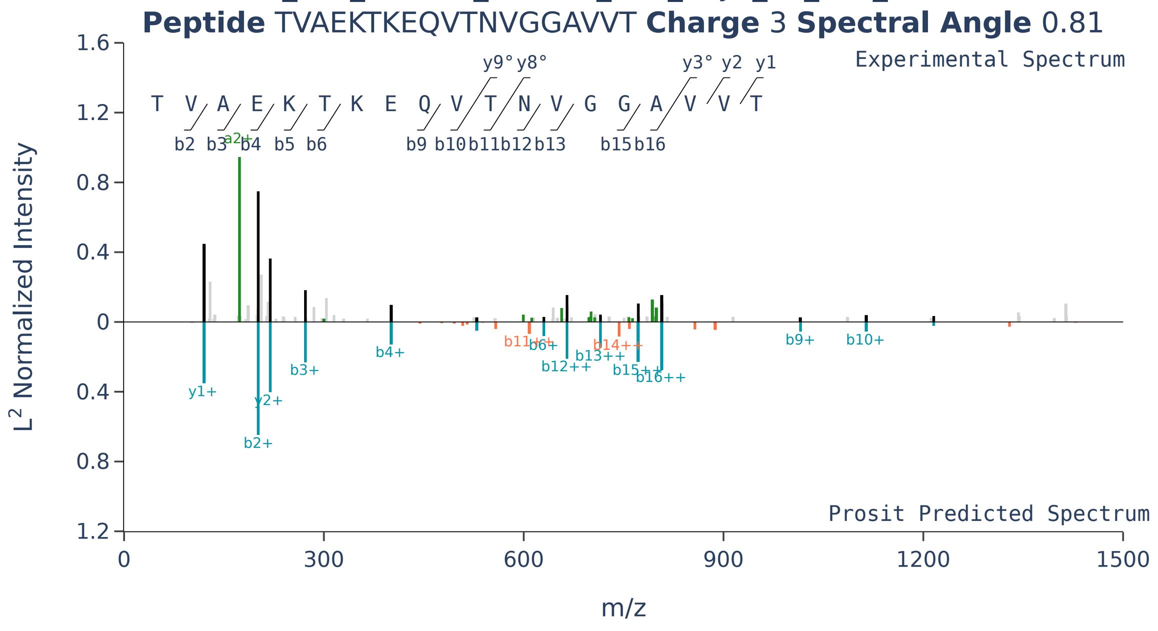


**Source** W_Soh_160821_170921_Goe_aSyn_A1_24h_R2 **Scan** 9544
**Peptide** SKAKEGVVAAAEKTKQGVAEAAGKTKE **Charge** 6 **Spectral Angle** 0.79

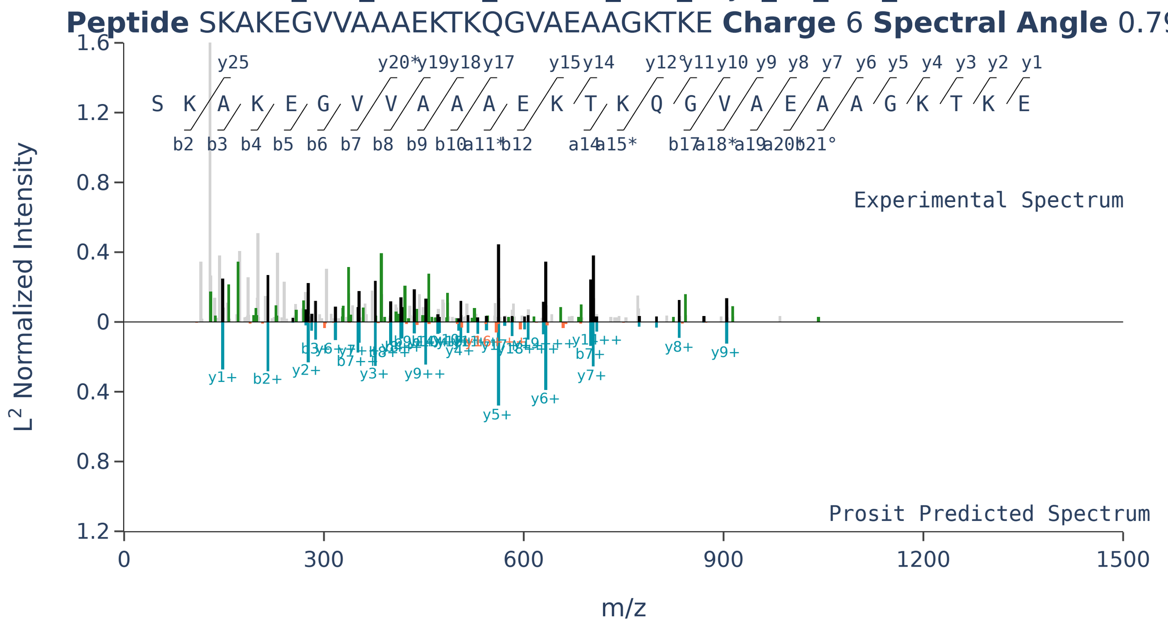


**Source** W_Soh_160821_170921_Goe_aSyn_A1_24h_R2 **Scan** 15042
**Peptide** QKTVEGAGSIAAATGFVKKD **Charge** 2 **Spectral Angle** 0.82

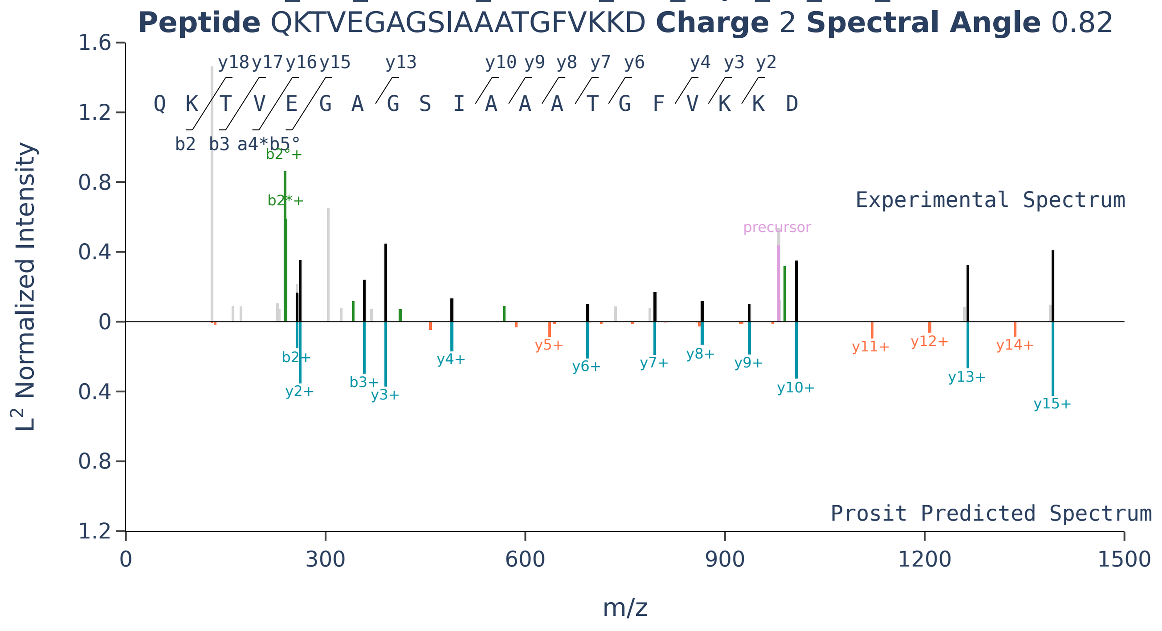

**Source** W_Soh_160821_170921_Goe_aSyn_A1_24h_R2 **Scan** 14441
**Peptide** VGSKTKEGVVHGVATVAEKTKEQVTNV **Charge** 4 **Spectral Angle** 0.76

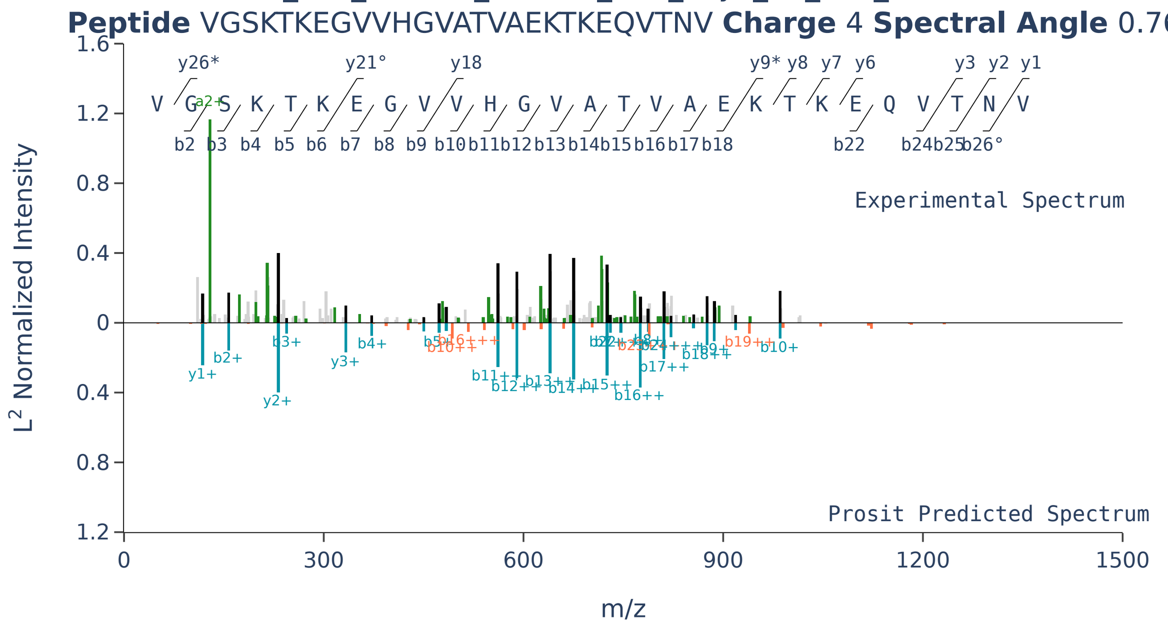


**Source** W_Soh_160821_170921_Goe_aSyn_A2_24h_R2 **Scan** 13288
**Peptide** GSIAAATGFVKKDQLGKNE **Charge** 3 **Spectral Angle** 0.84

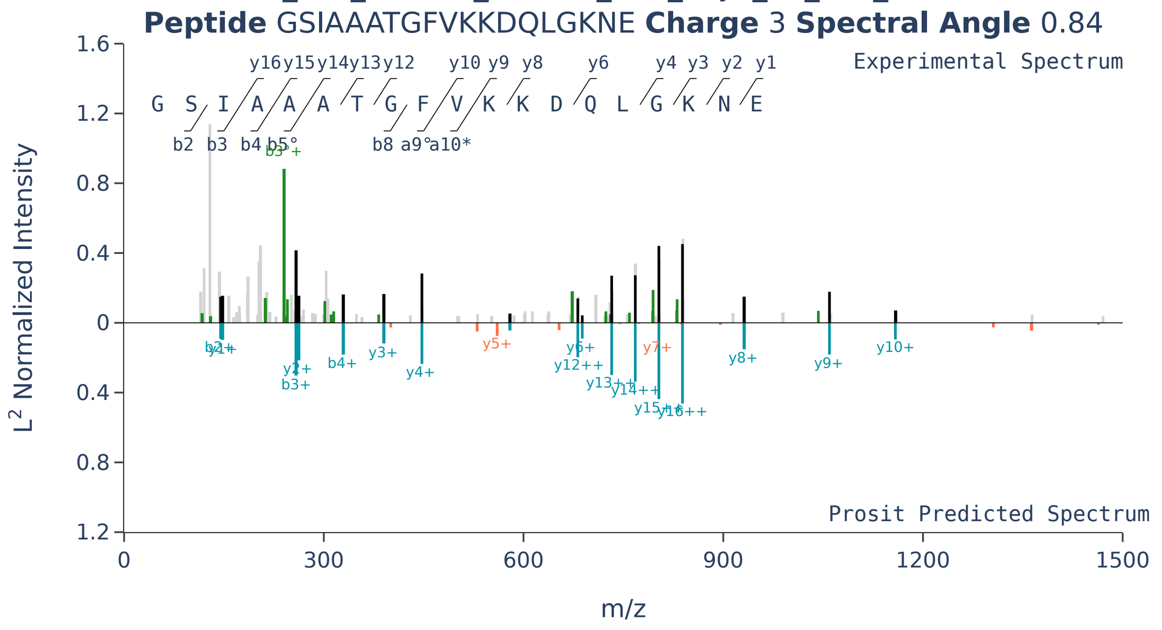


**Source** W_Soh_160821_170921_Goe_aSyn_A1_6h_R2 **Scan** 22616
**Peptide** VTNVGGAVVTGVTAVAQ **Charge** 2 **Spectral Angle** 0.9

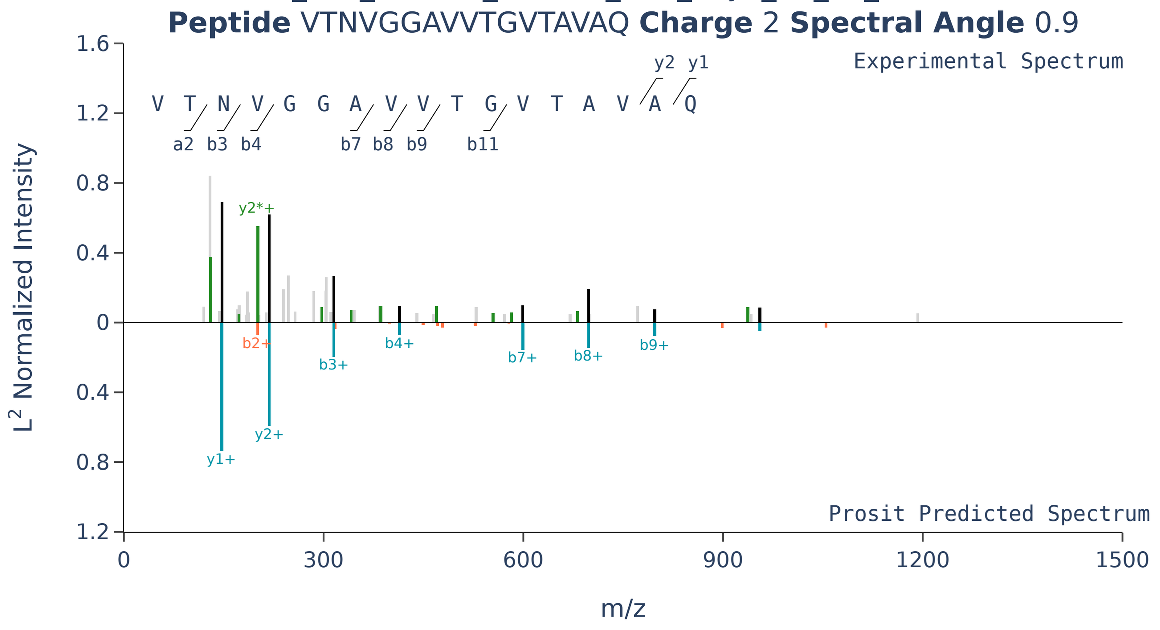


**Source** W_Soh_160821_170921_Goe_aSyn_A1_24h_R1 **Scan** 6474
**Peptide** KGLSKAKEGVVAAAEKTKQG **Charge** 3 **Spectral Angle** 0.85

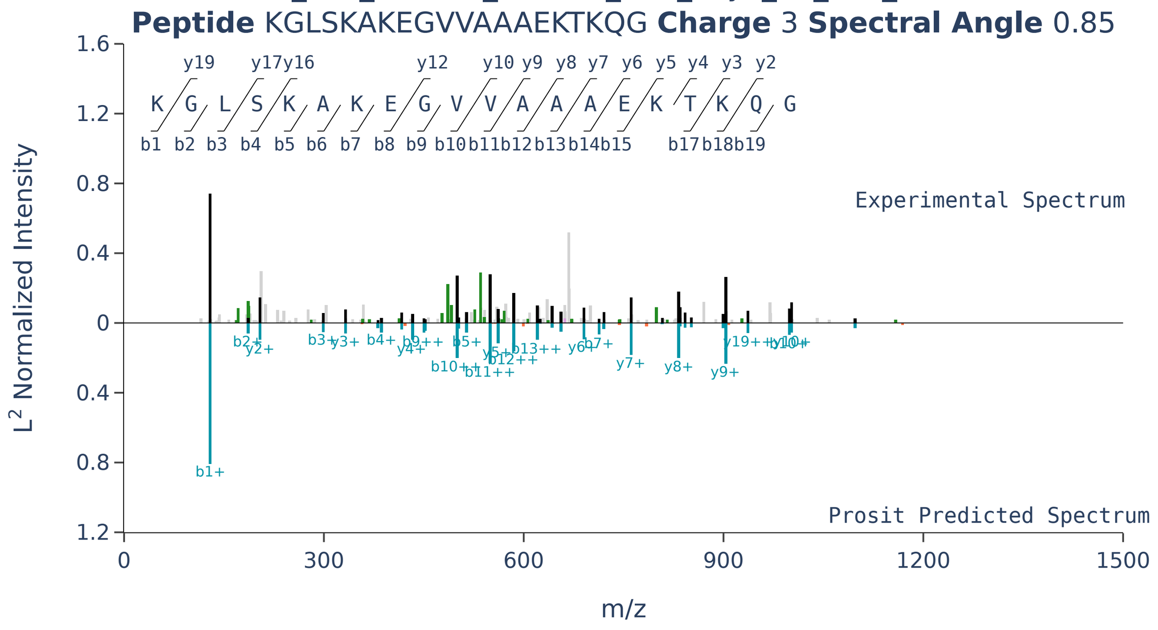


**Source** W_Soh_160821_170921_Goe_aSyn_A1_24h_R2 **Scan** 5696
**Peptide** FVKKDQLGKNEE **Charge** 3 **Spectral Angle** 0.78

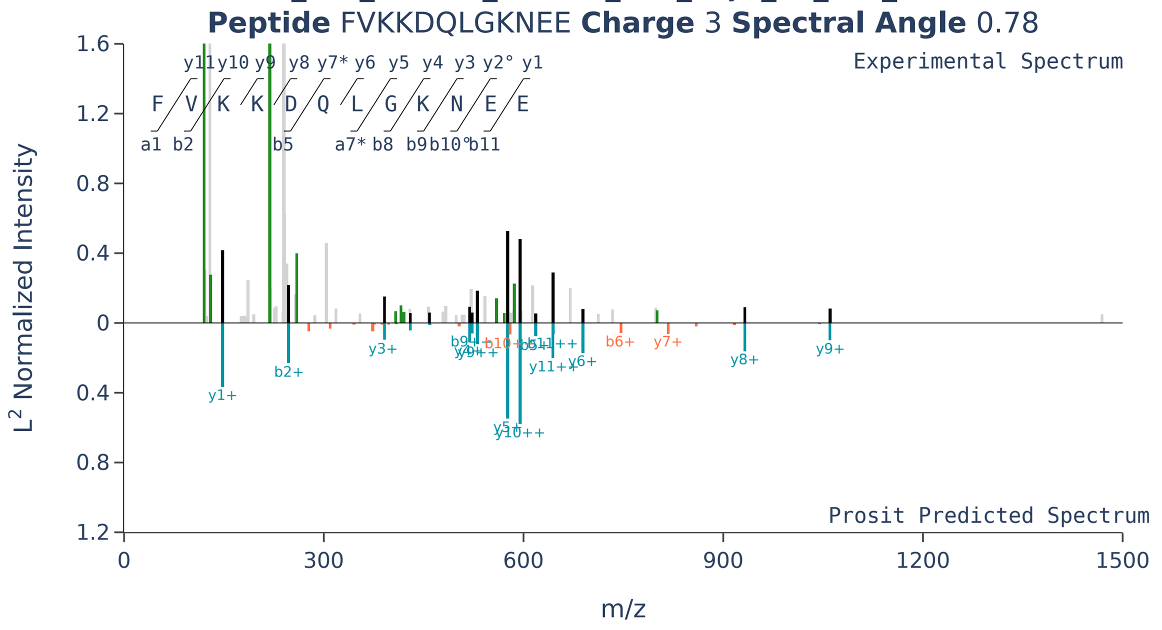


**Source** W_Soh_160821_170921_Goe_aSyn_A1_24h_R1 **Scan** 14051
**Peptide** GFVKKDQLGKNEEGAPQEGILE **Charge** 3 **Spectral Angle** 0.9

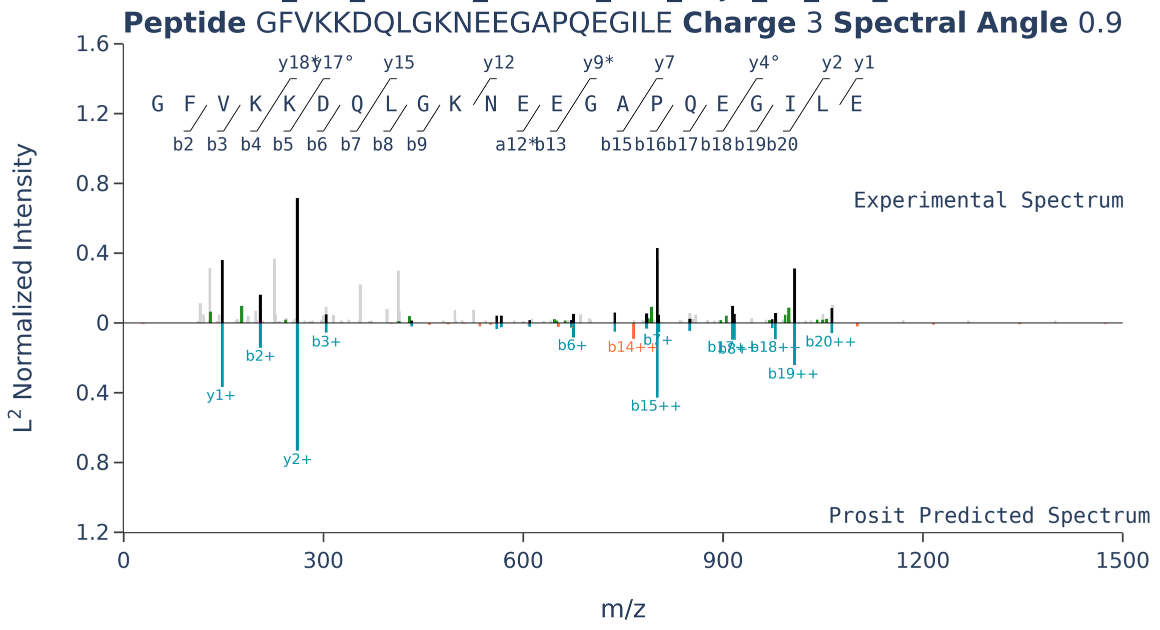


**Source** W_Soh_160821_170921_Goe_aSyn_A1_24h_R2 **Scan** 12660
**Peptide** VLYVGSKTKEGVVHGVATVAEK **Charge** 4 **Spectral Angle** 0.81

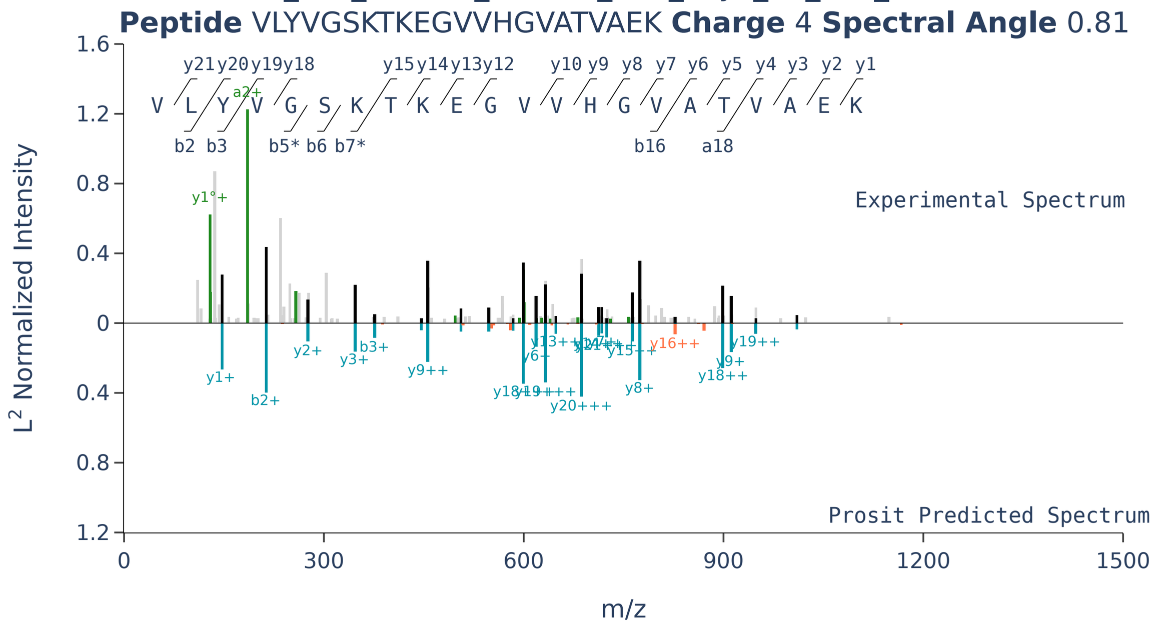


**Source** W_Soh_160821_170921_Goe_aSyn_A1_24h_R2 **Scan** 8650
**Peptide** TGFVKKDQLG **Charge** 3 **Spectral Angle** 0.74

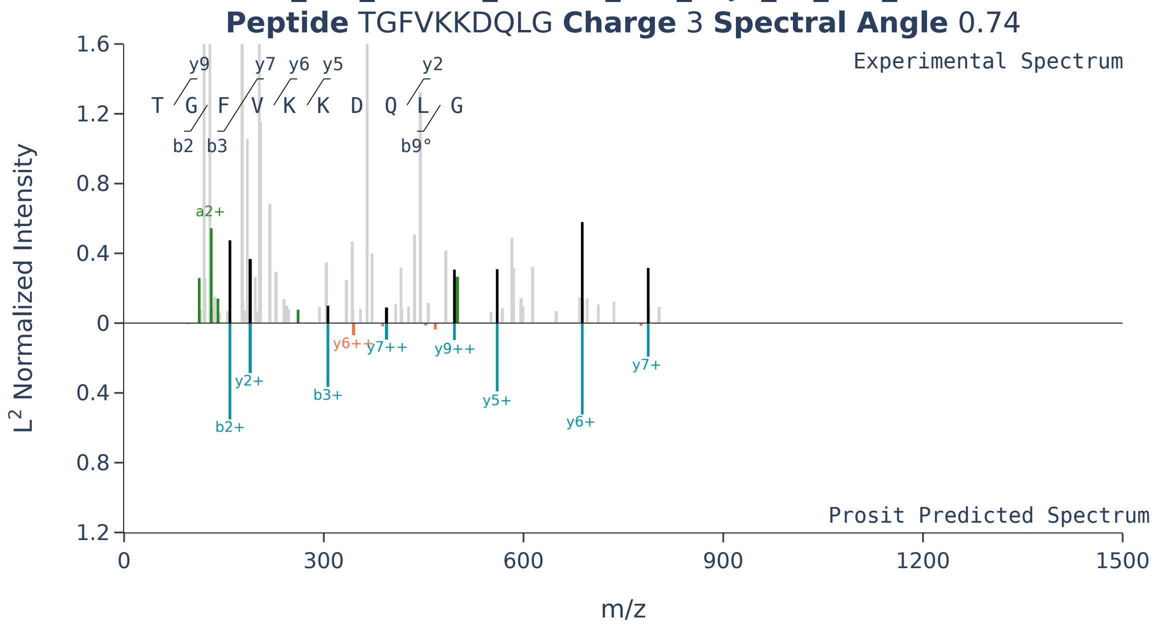


**Source** W_Soh_160821_170921_Goe_aSyn_A1_6h_R1 **Scan** 9992
**Peptide** AAGKTKEGVLYVGSKTKEGVVHGV **Charge** 4 **Spectral Angle** 0.87

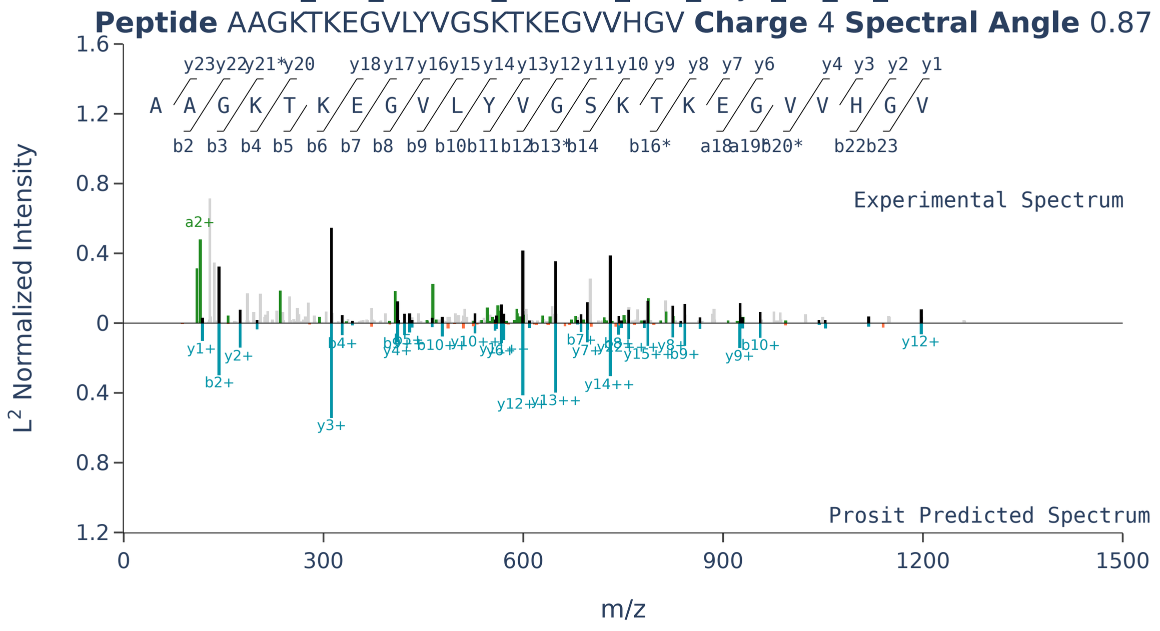


**Source** W_Soh_160821_170921_Goe_aSyn_A1_24h_R2 **Scan** 13788
**Peptide** VLYVGSKTKEGVVHGVATVAEKT **Charge** 4 **Spectral Angle** 0.84

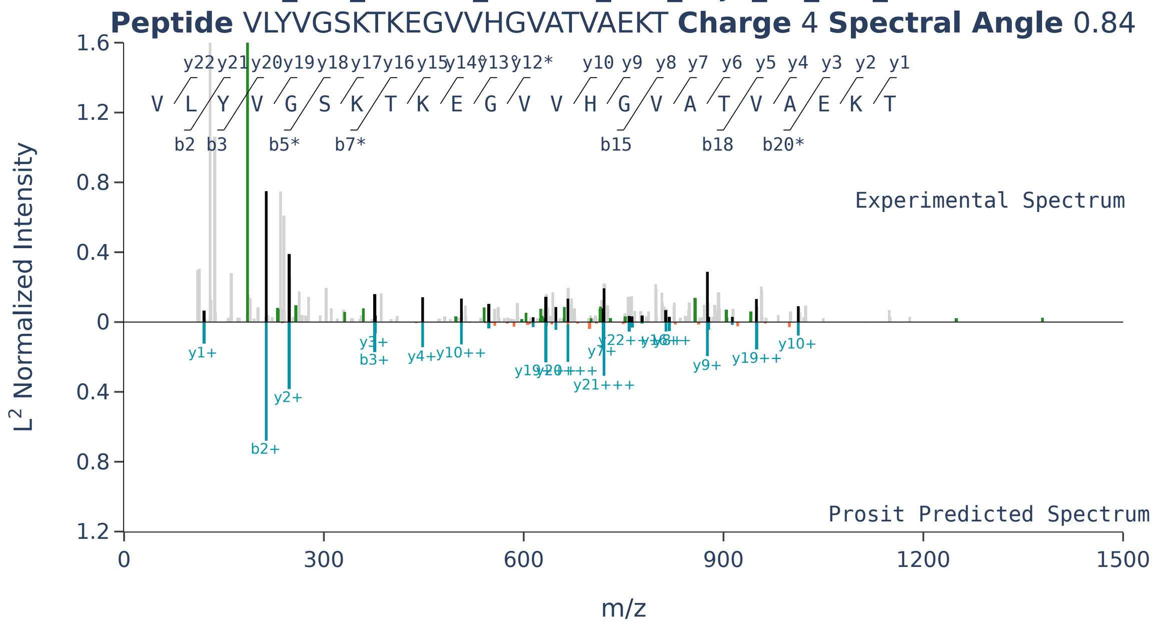


**Source** W_Soh_160821_170921_Goe_aSyn_A2_24h_R2 **Scan** 10287
**Peptide** AEKTKEQVTNVGGAVVTG **Charge** 3 **Spectral Angle** 0.88

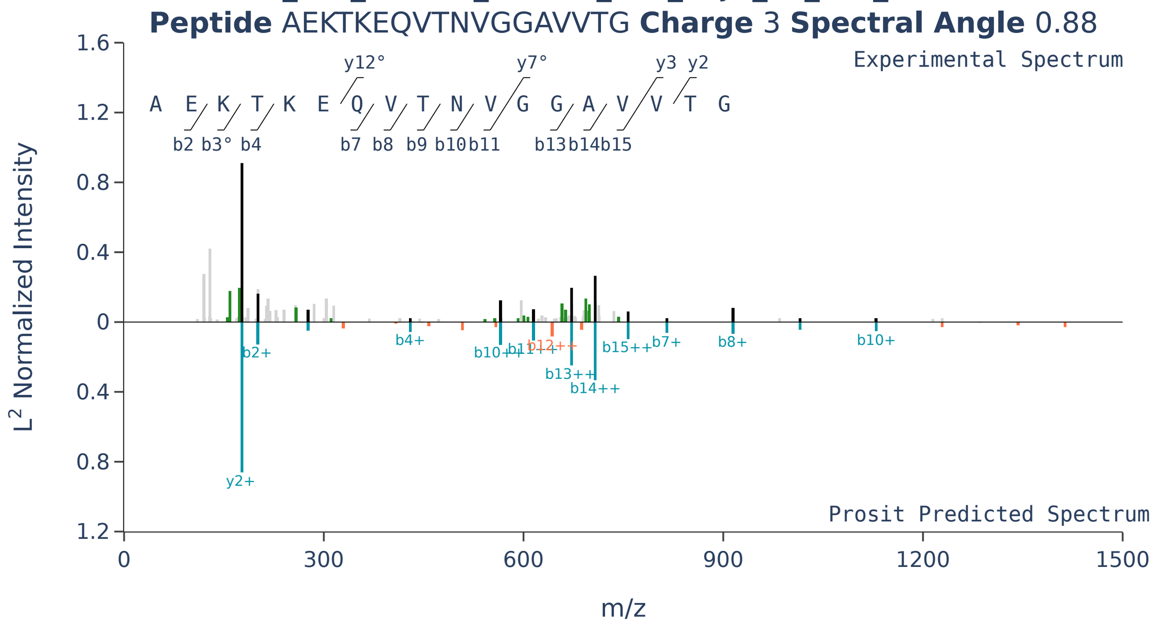


**Source** W_Soh_160821_170921_Goe_aSyn_A1_24h_R1 **Scan** 14550
**Peptide** VAEKTKEQVTNVGGAVVTGVT **Charge** 3 **Spectral Angle** 0.87

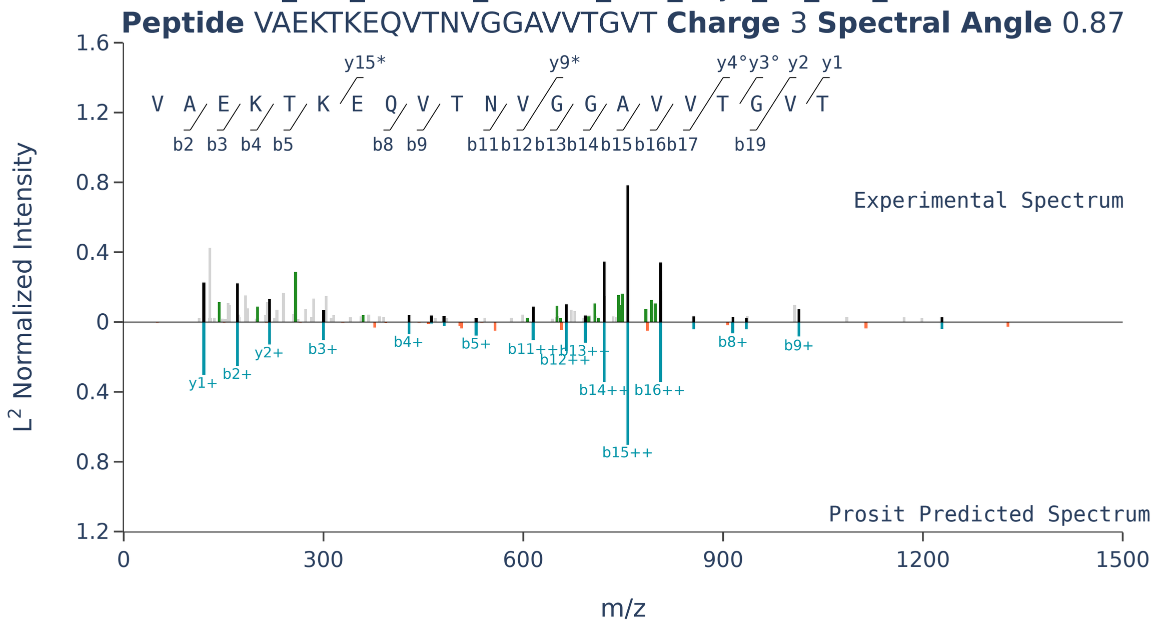


**Source** W_Soh_160821_170921_Goe_aSyn_A2_24h_R1 **Scan** 17727
**Peptide** TVEGAGSIAAATGFVKKD **Charge** 3 **Spectral Angle** 0.81

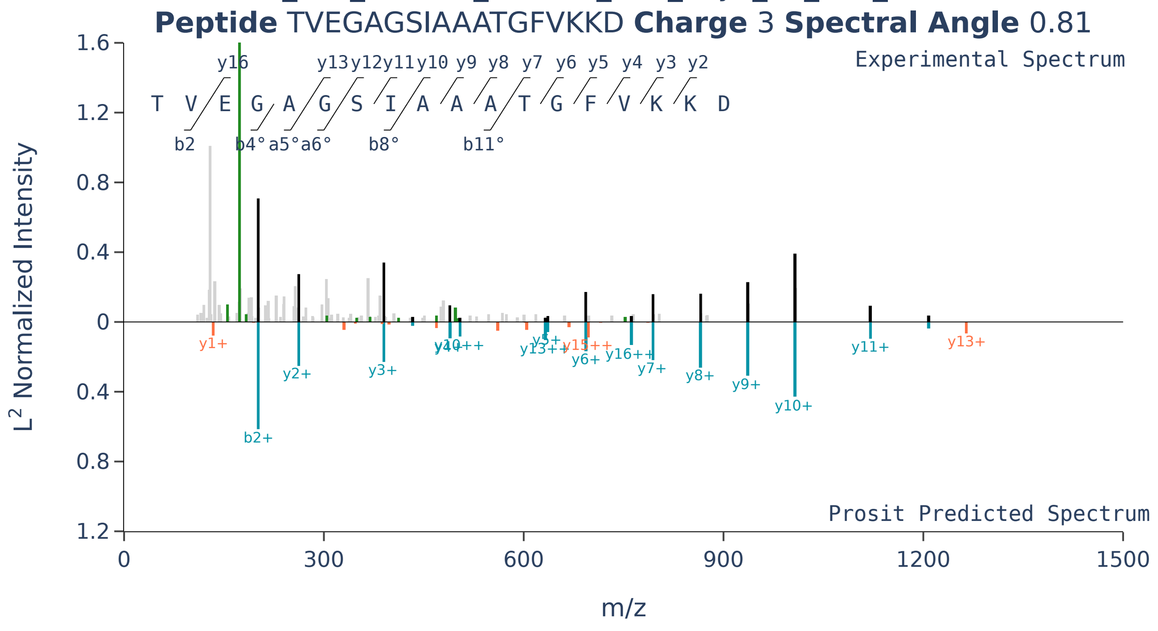


**Source** W_Soh_160821_170921_Goe_aSyn_A1_24h_R2 **Scan** 13904
**Peptide** EGVLYVGSKTKEGVVHGVAT **Charge** 3 **Spectral Angle** 0.88

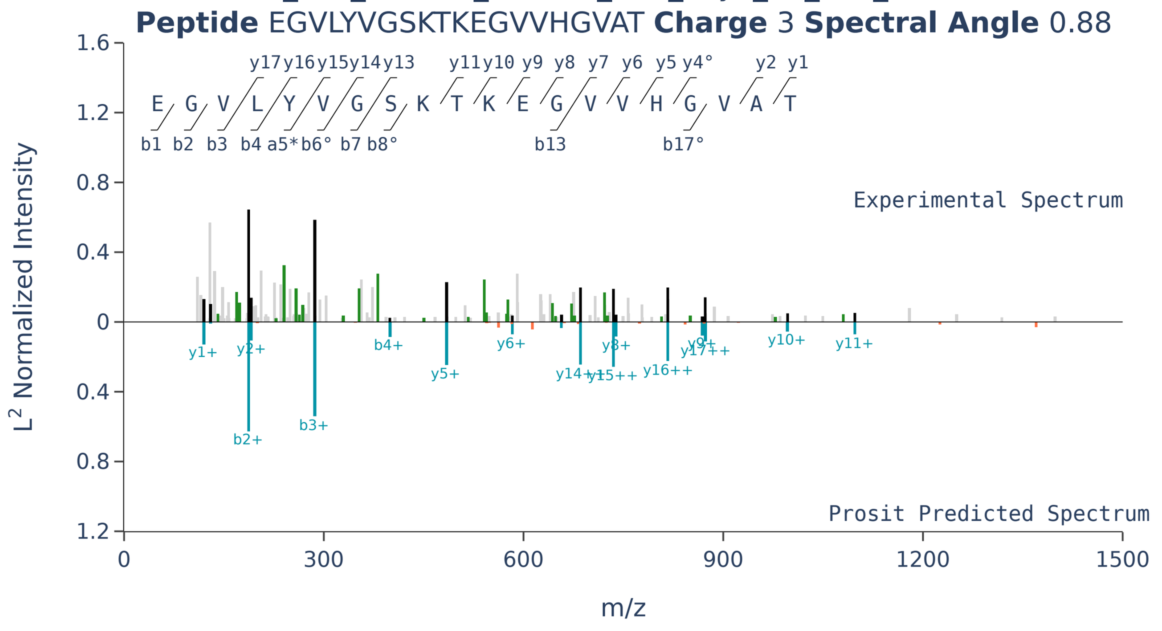


**Source** W_Soh_160821_170921_Goe_aSyn_A1_6h_R2 **Scan** 29102
**Peptide** GILEDMPVDPDNEAYEMPSE **Charge** 2 **Spectral Angle** 0.92

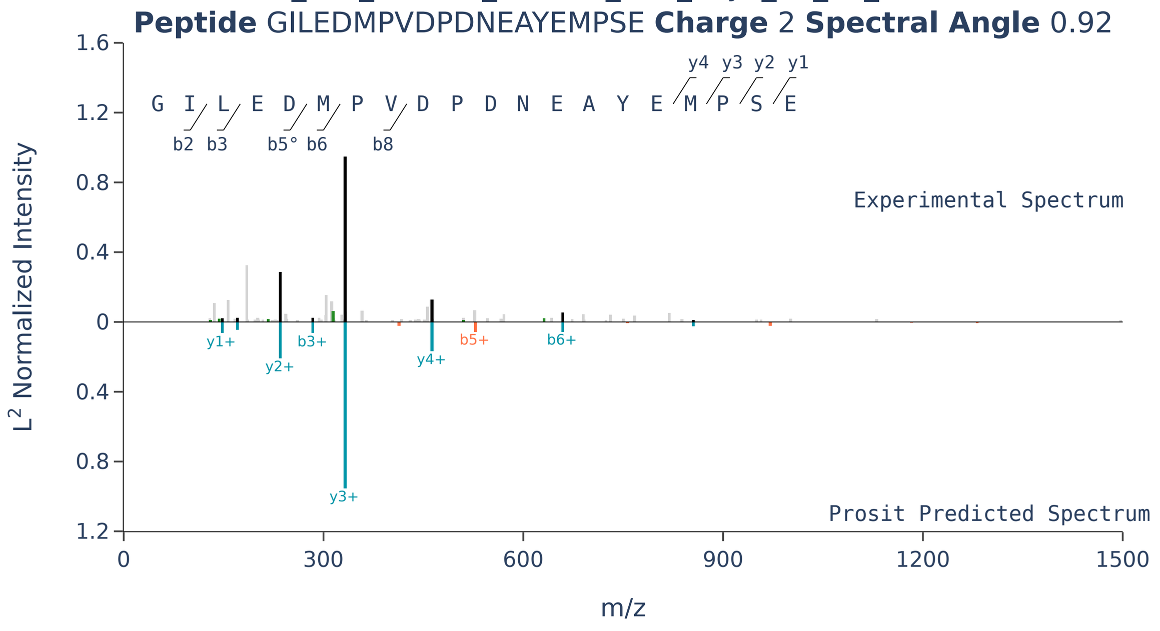

**Source** W_Soh_160821_170921_Goe_aSyn_A1_24h_R2 **Scan** 8703
**Peptide** GAGSIAAA **Charge** 1 **Spectral Angle** 0.9

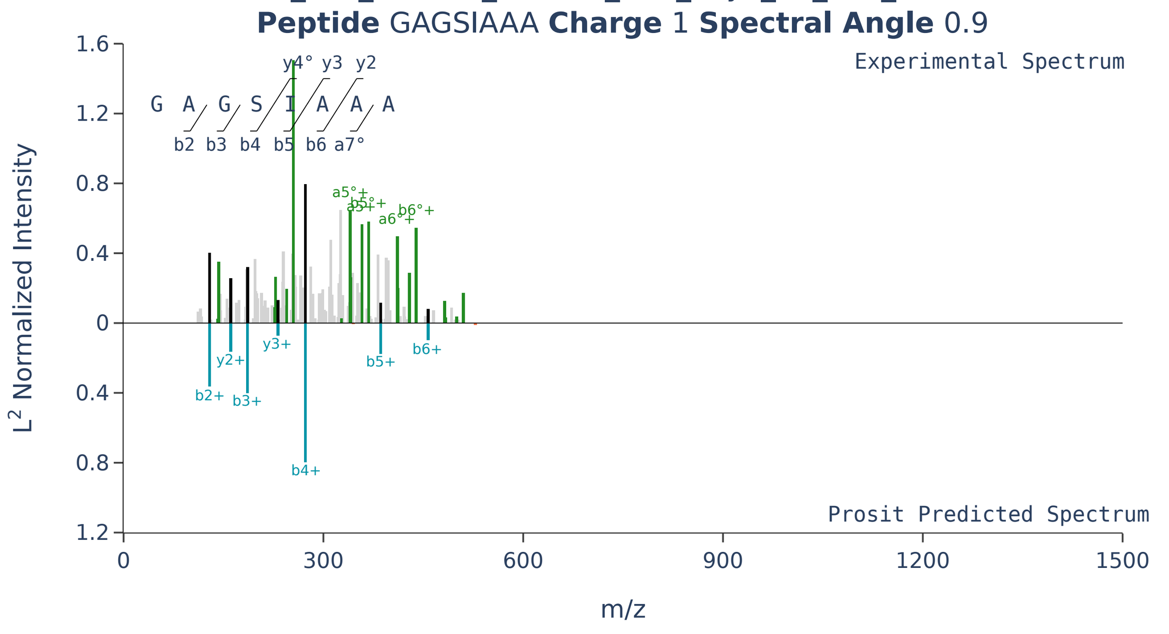


**Source** W_Soh_160821_170921_Goe_aSyn_A2_24h_R2 **Scan** 7588
**Peptide** KAKEGVVAAAEKTKQGVAEA **Charge** 3 **Spectral Angle** 0.9

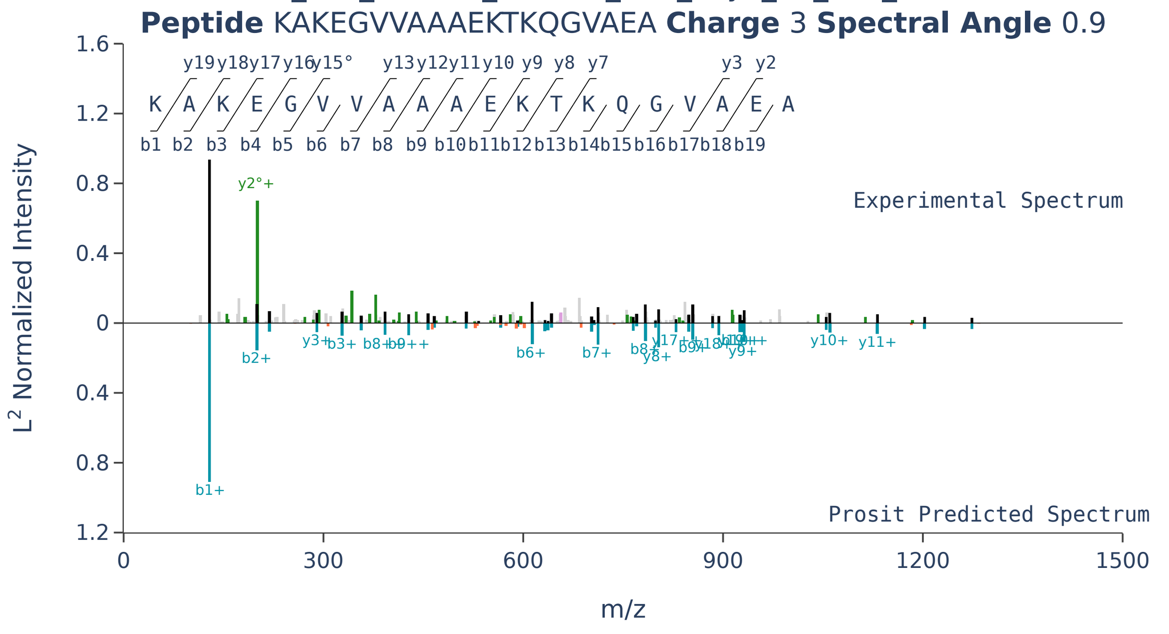


**Source** W_Soh_160821_170921_Goe_aSyn_A1_6h_R2 **Scan** 8269
**Peptide** KEGVVAAAEKTKQGVAEA **Charge** 2 **Spectral Angle** 0.81

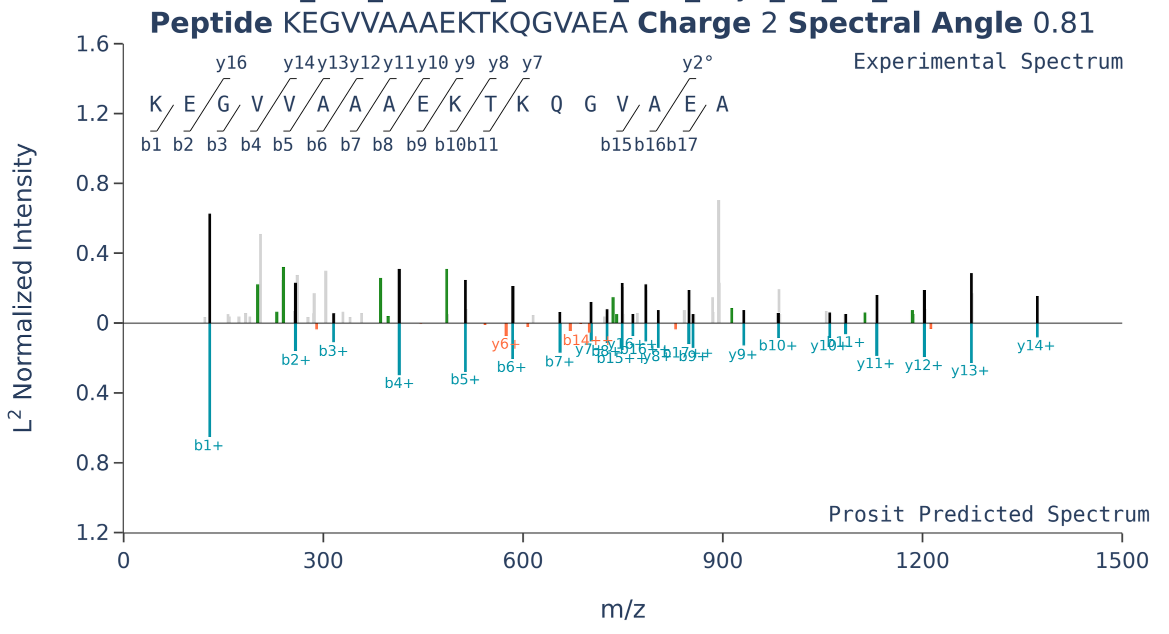


**Source** W_Soh_160821_170921_Goe_aSyn_A1_24h_R2 **Scan** 8821
**Peptide** KAKEGVVAAAEKTKQGVAEAAGKTKE **Charge** 5 **Spectral Angle** 0.88

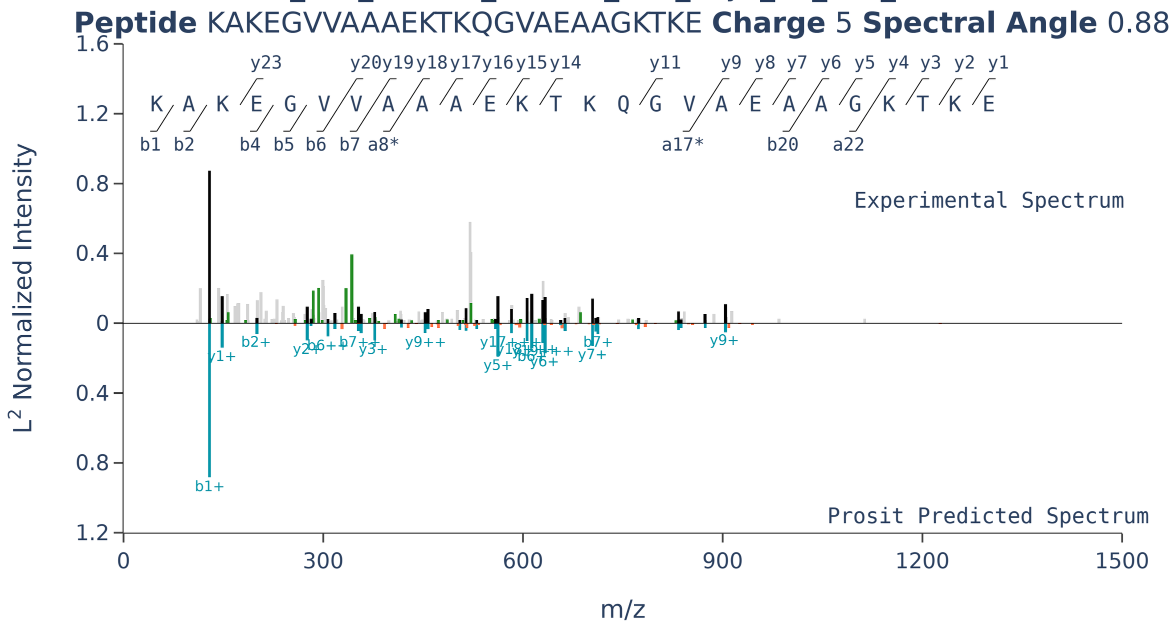


**Source** W_Soh_160821_170921_Goe_aSyn_A1_6h_R1 **Scan** 18245
**Peptide** KTKEQVTNVGGAVVTGVTAVA **Charge** 3 **Spectral Angle** 0.87

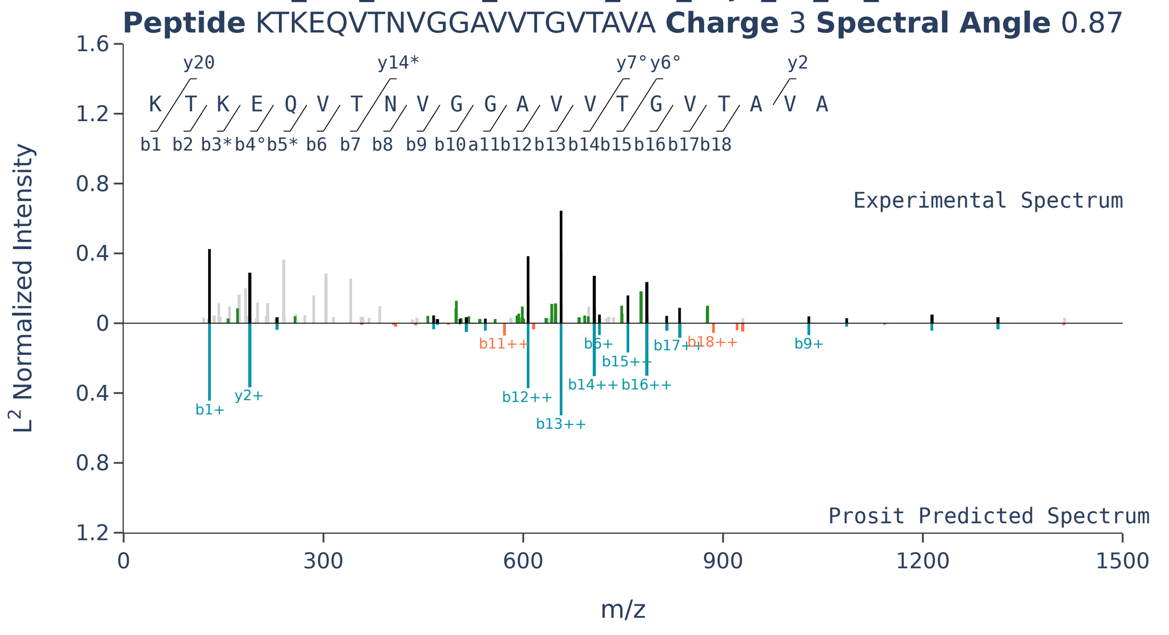


**Source** W_Soh_160821_170921_Goe_aSyn_A1_24h_R2 **Scan** 15309
**Peptide** GVLYVGSKTKEGVVHGVATVA **Charge** 3 **Spectral Angle** 0.85

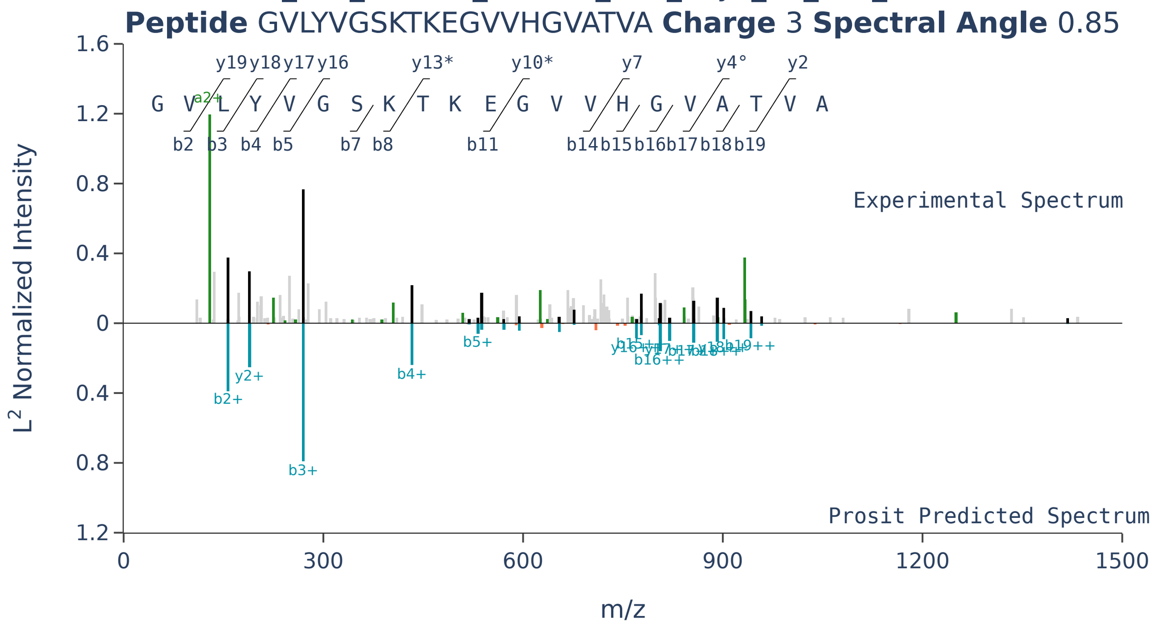


**Source** W_Soh_160821_170921_Goe_aSyn_A1_24h_R2 **Scan** 15193
**Peptide** GVLYVGSKTKEGVVHGVATV **Charge** 2 **Spectral Angle** 0.89

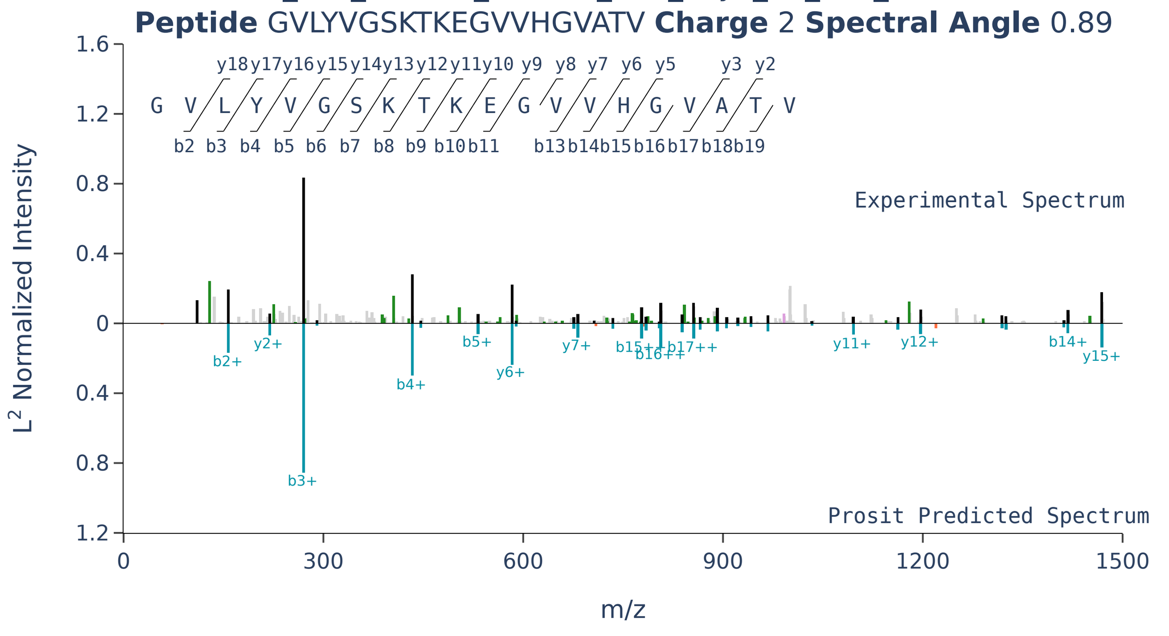


**Source** W_Soh_160821_170921_Goe_aSyn_A1_24h_R2 **Scan** 17128
**Peptide** MDVFMKGLSKAKEGV **Charge** 3 **Spectral Angle** 0.83

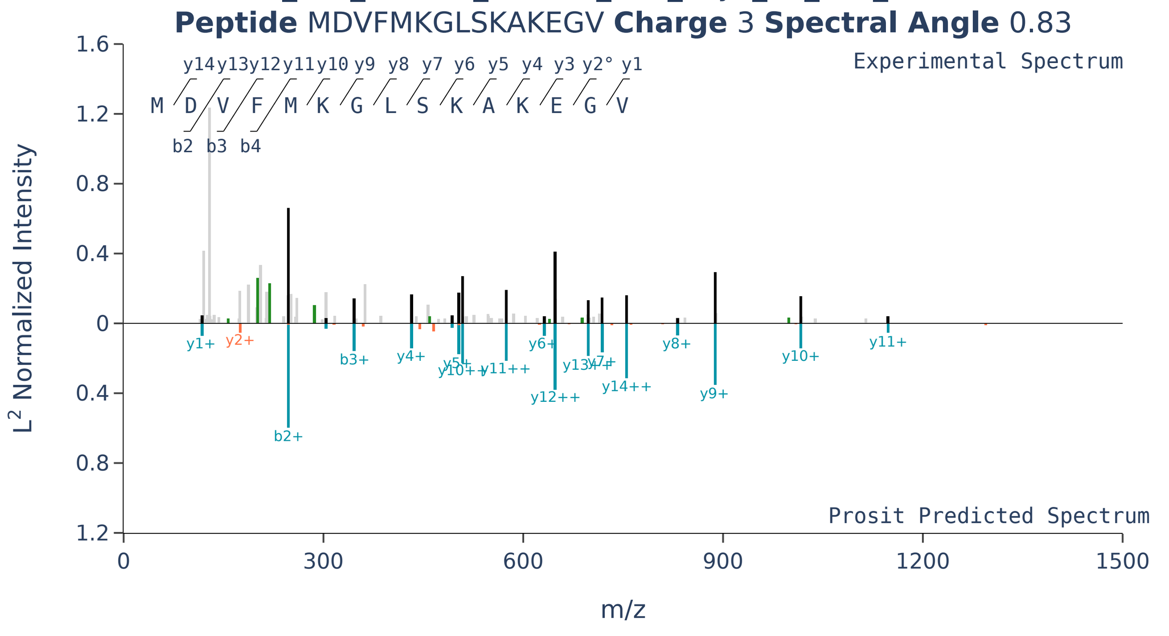


**Source** W_Soh_160821_170921_Goe_aSyn_A1_24h_R2 **Scan** 21004
**Peptide** TVEGAGSIAAATGFVKKDQL **Charge** 3 **Spectral Angle** 0.79

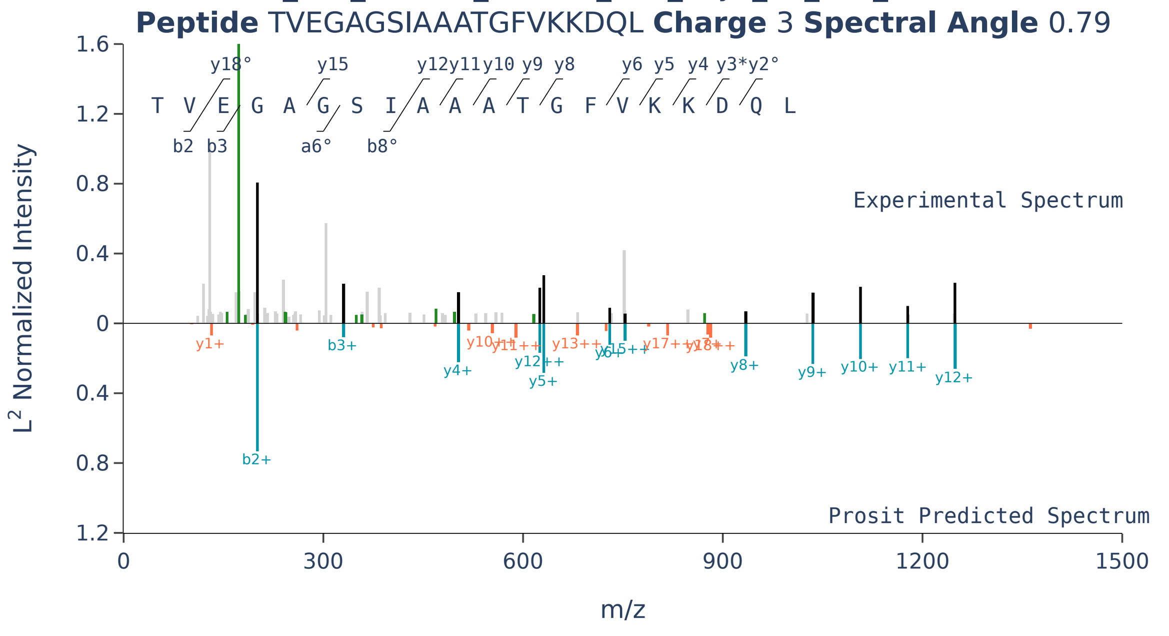


**Source** W_Soh_160821_170921_Goe_aSyn_A1_6h_R2 **Scan** 23190
**Peptide** QLGKNEEGAPQEGILEDMPVD **Charge** 3 **Spectral Angle** 0.93

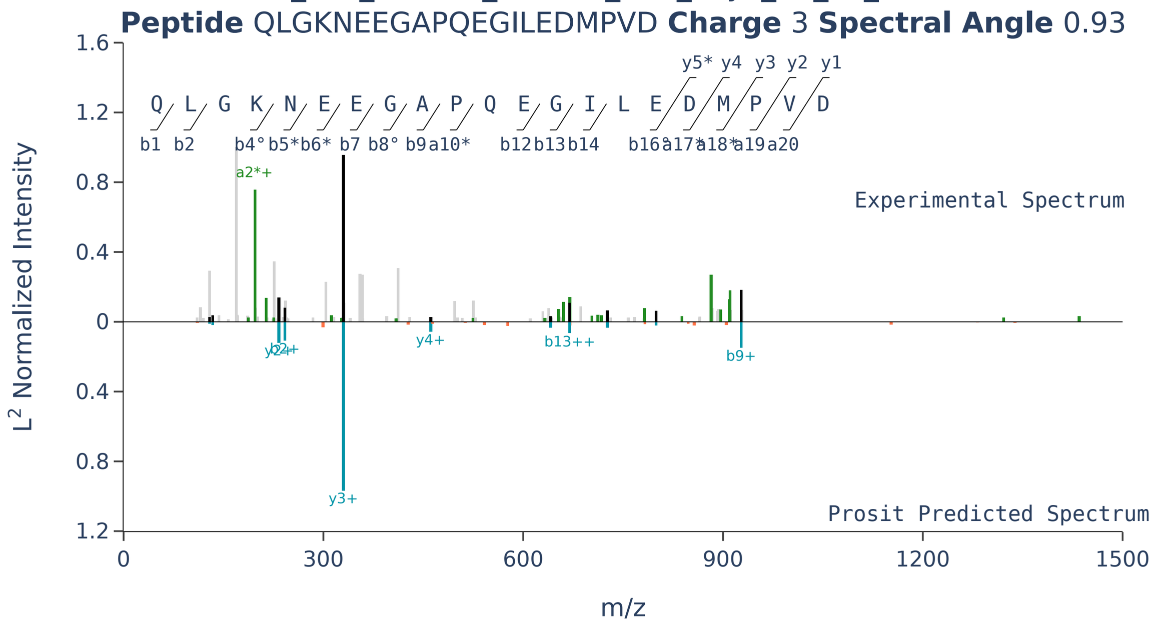


**Source** W_Soh_160821_170921_Goe_aSyn_A2_24h_R1 **Scan** 6867
**Peptide** AAGKTKEGVLYVGSKTKE **Charge** 4 **Spectral Angle** 0.87

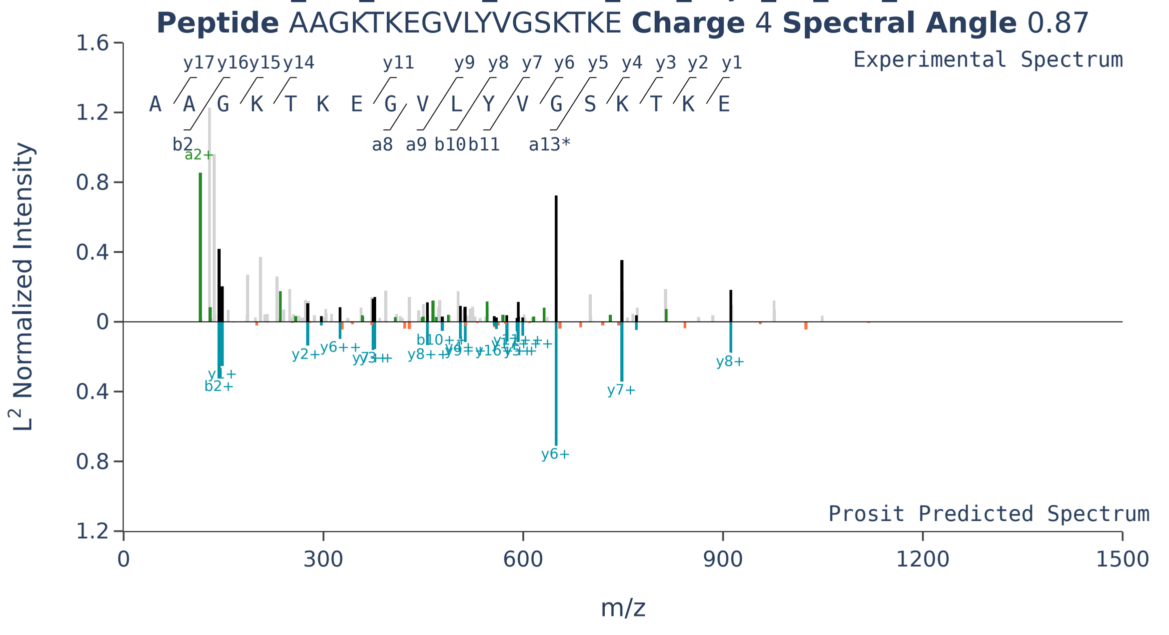


**Source** W_Soh_160821_170921_Goe_aSyn_A1_24h_R2 **Scan** 17214
**Peptide** GVTAVAQKTVEGAGSIA **Charge** 2 **Spectral Angle** 0.85

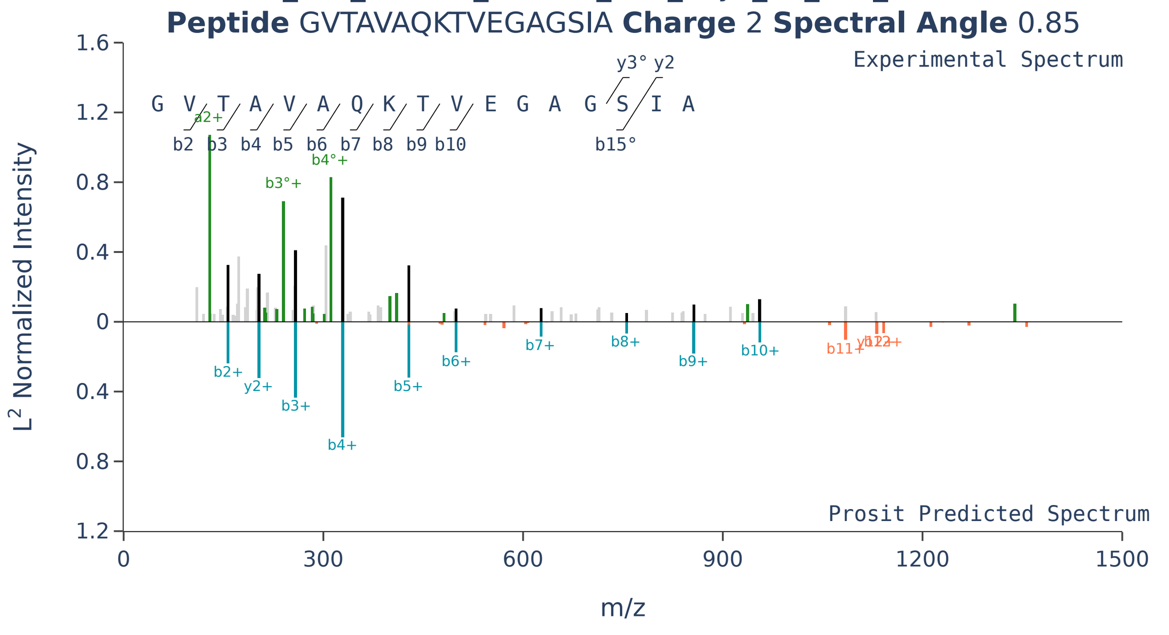


**Source** W_Soh_160821_170921_Goe_aSyn_A1_24h_R1 **Scan** 14285
**Peptide** KTKEQVTNVGGAVVTGVTA **Charge** 3 **Spectral Angle** 0.86

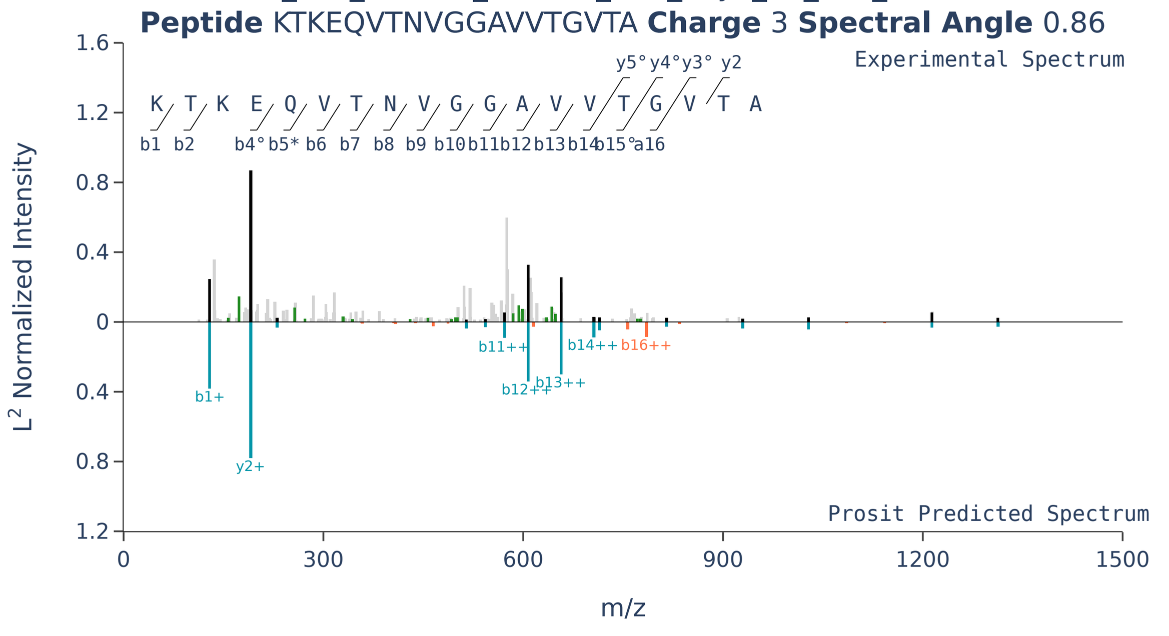


**Source** W_Soh_160821_170921_Goe_aSyn_A2_24h_R2 **Scan** 16845
**Peptide** AGSIAAATGFVK **Charge** 2 **Spectral Angle** 0.83

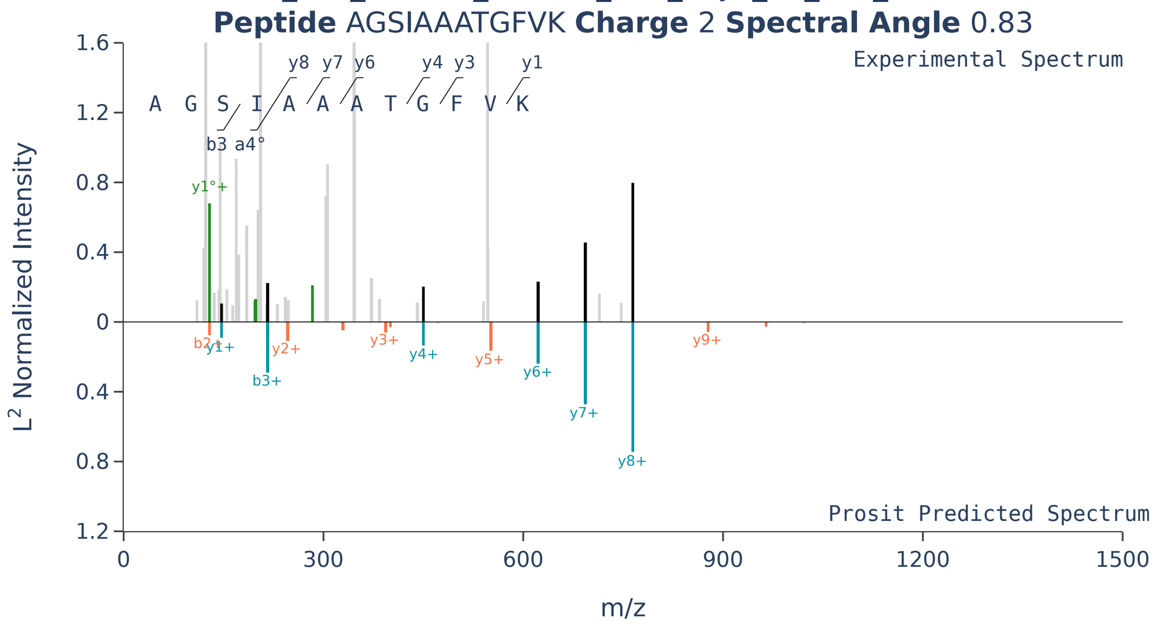


**Source** W_Soh_160821_170921_Goe_aSyn_A1_24h_R2 **Scan** 8502
**Peptide** GKNEEGAPQEGI **Charge** 2 **Spectral Angle** 0.86

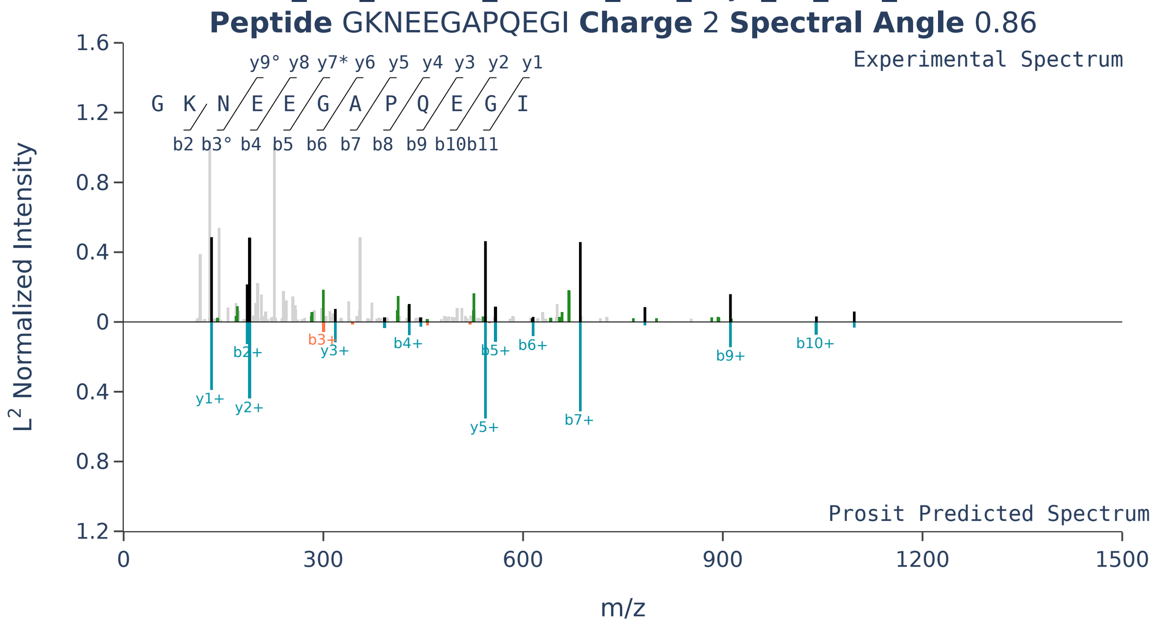

**Source** W_Soh_160821_170921_Goe_aSyn_A1_24h_R2 **Scan** 19145
**Peptide** GVTAVAQKTVEGAGSIAAAT **Charge** 2 **Spectral Angle** 0.81

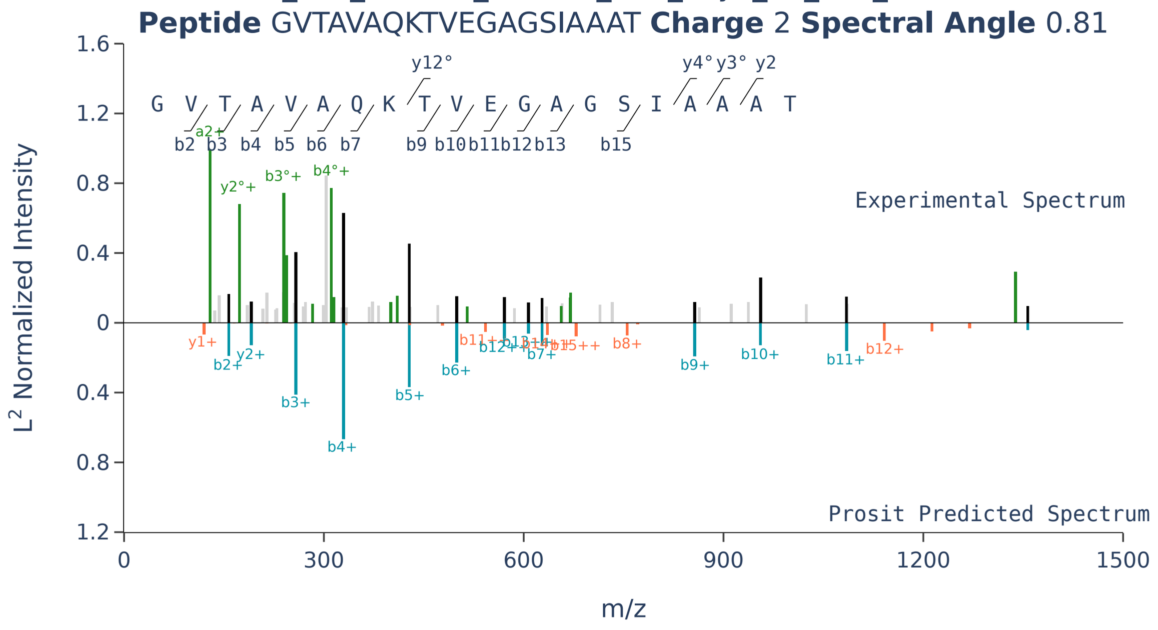


**Source** W_Soh_160821_170921_Goe_aSyn_A1_6h_R2 **Scan** 19435
**Peptide** QVTNVGGAVVTGVTA **Charge** 2 **Spectral Angle** 0.87

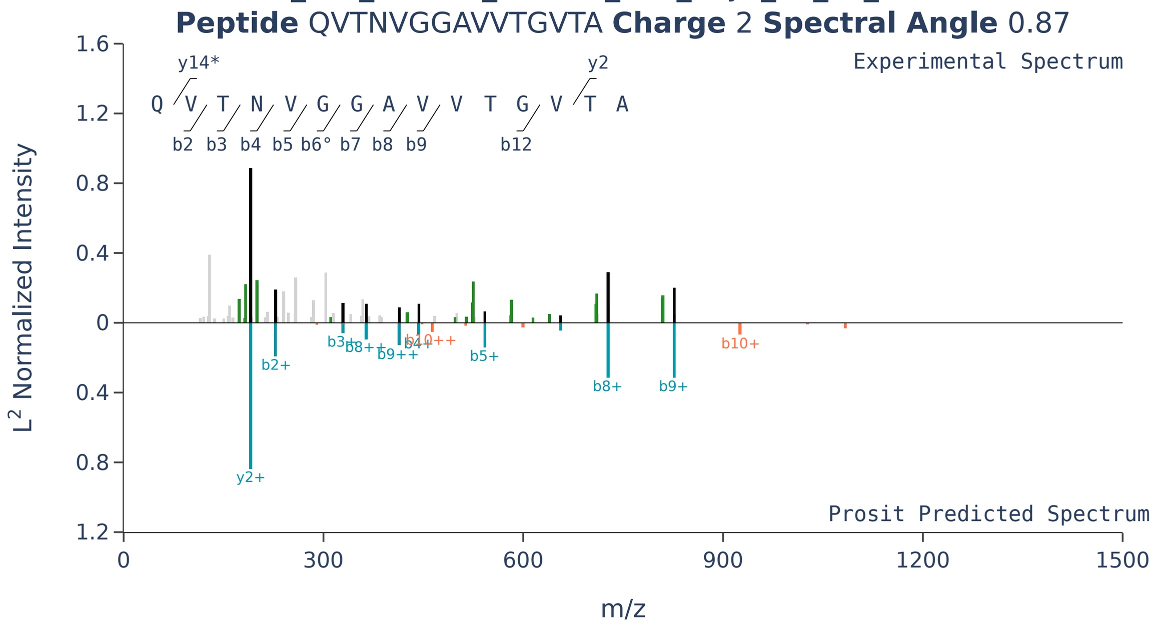


**Source** W_Soh_160821_170921_Goe_aSyn_A1_30'_R1 **Scan** 5371
**Peptide** YVGSKTKEGVVHG **Charge** 2 **Spectral Angle** 0.79

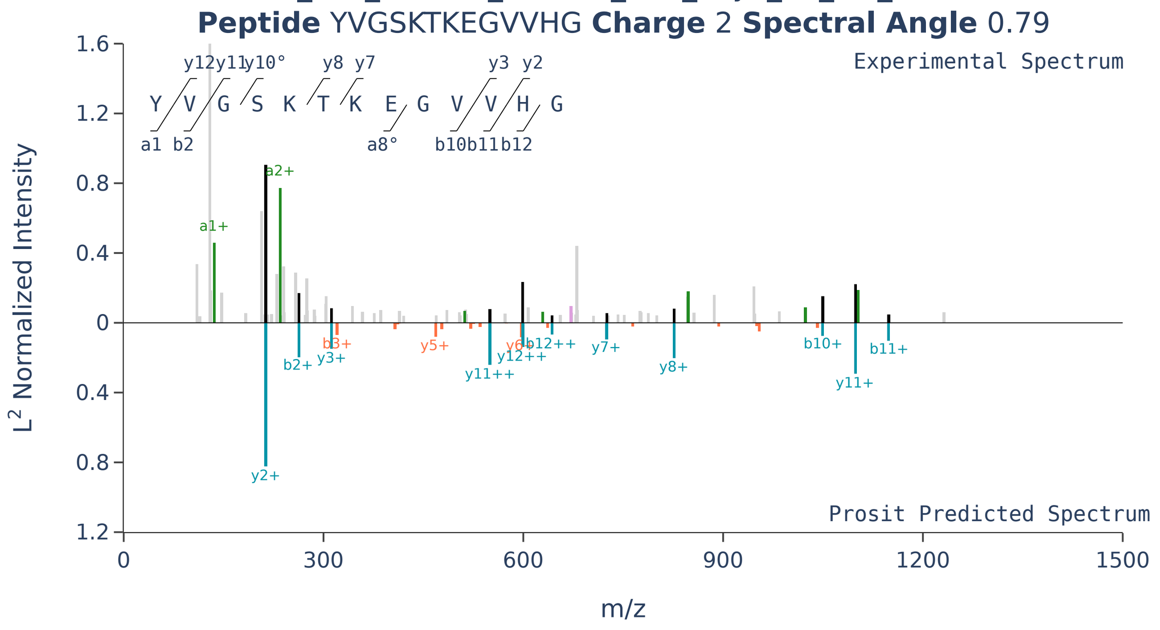


**Source** W_Soh_160821_170921_Goe_aSyn_A1_3h_R1 **Scan** 17708
**Peptide** VATVAEKTKEQVTNVGGAVVTGVTA **Charge** 3 **Spectral Angle** 0.86

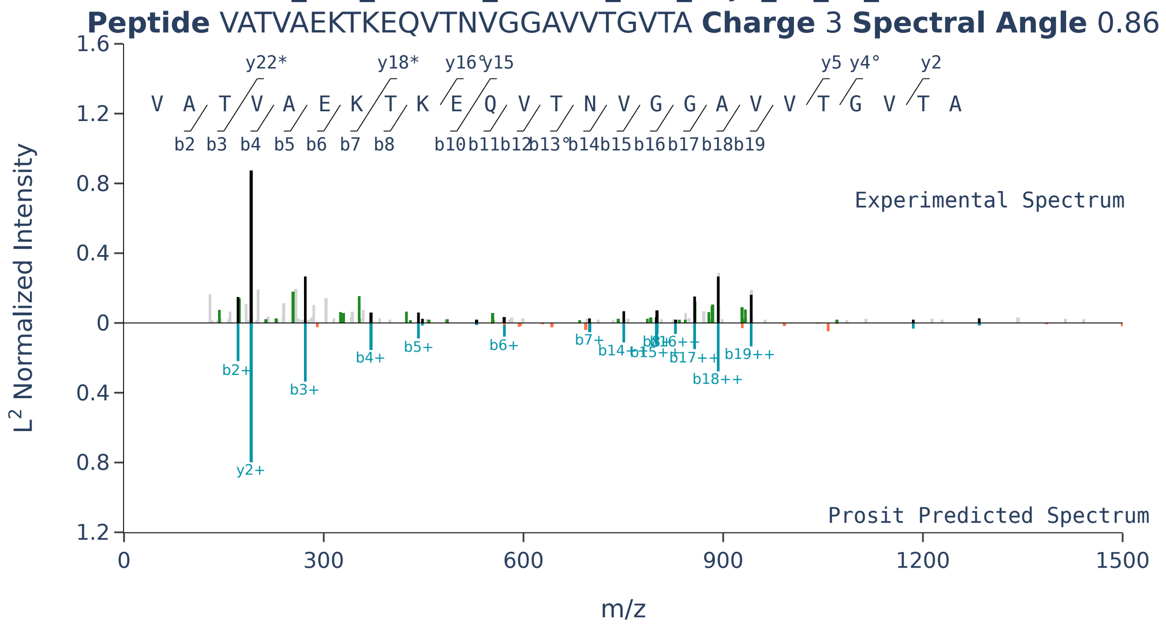


**Source** W_Soh_160821_170921_Goe_aSyn_A1_24h_R2 **Scan** 13856
**Peptide** EDMPVDPDNE **Charge** 2 **Spectral Angle** 0.8

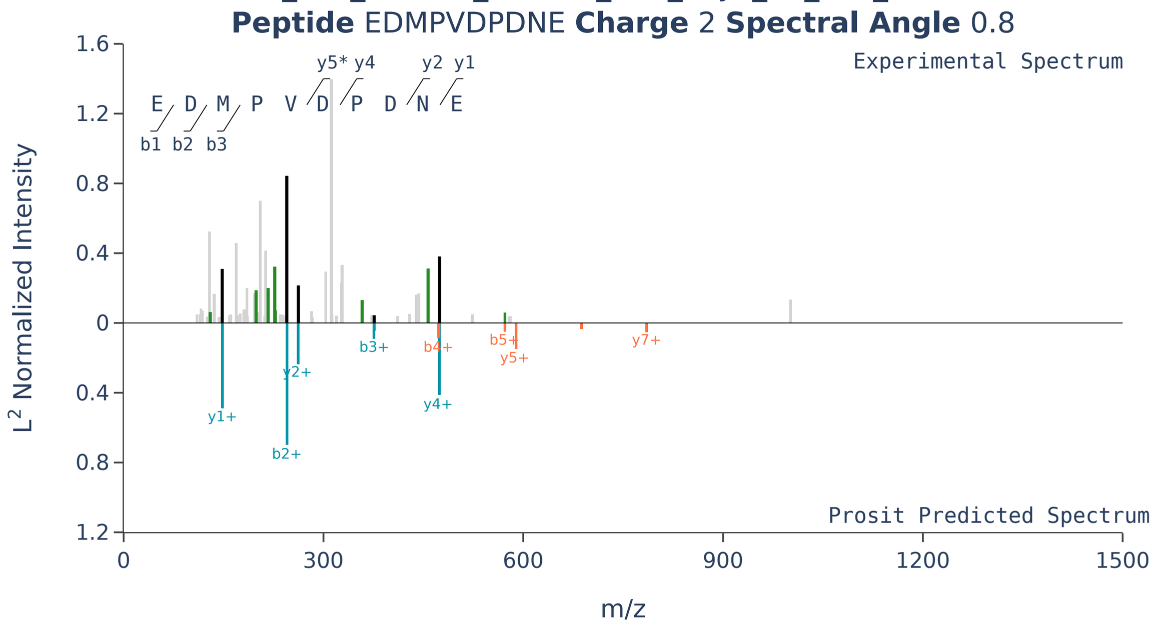


**Source** W_Soh_160821_170921_Goe_aSyn_A1_24h_R1 **Scan** 12551
**Peptide** AYEMPSEEG **Charge** 1 **Spectral Angle** 0.73

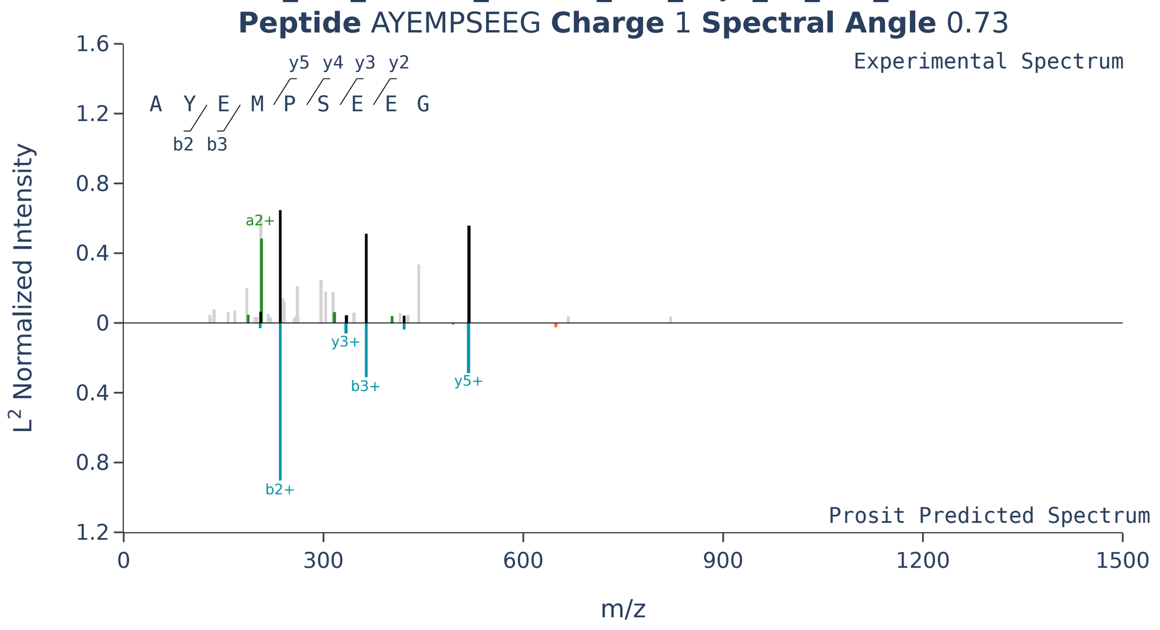


**Source** W_Soh_160821_170921_Goe_aSyn_A1_24h_R1 **Scan** 5520
**Peptide** AAAEKTKQ **Charge** 2 **Spectral Angle** 0.79

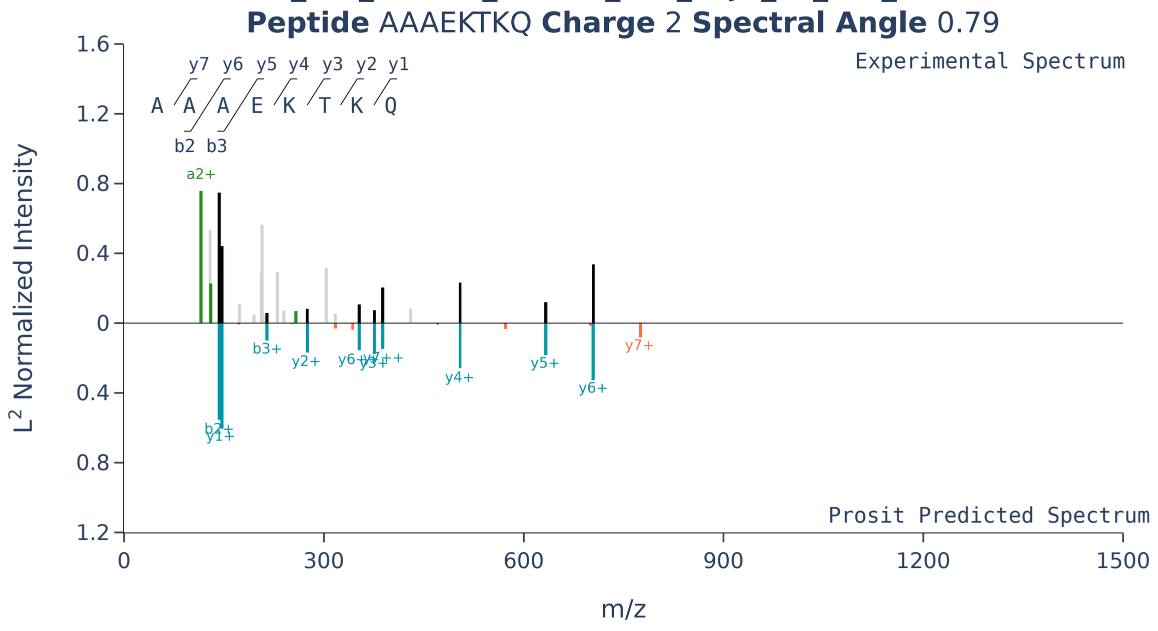


**Source** W_Soh_160821_170921_Goe_aSyn_A2_6h_R2 **Scan** 22736
**Peptide** LGKNEEGAPQEGILEDMPVD **Charge** 2 **Spectral Angle** 0.94

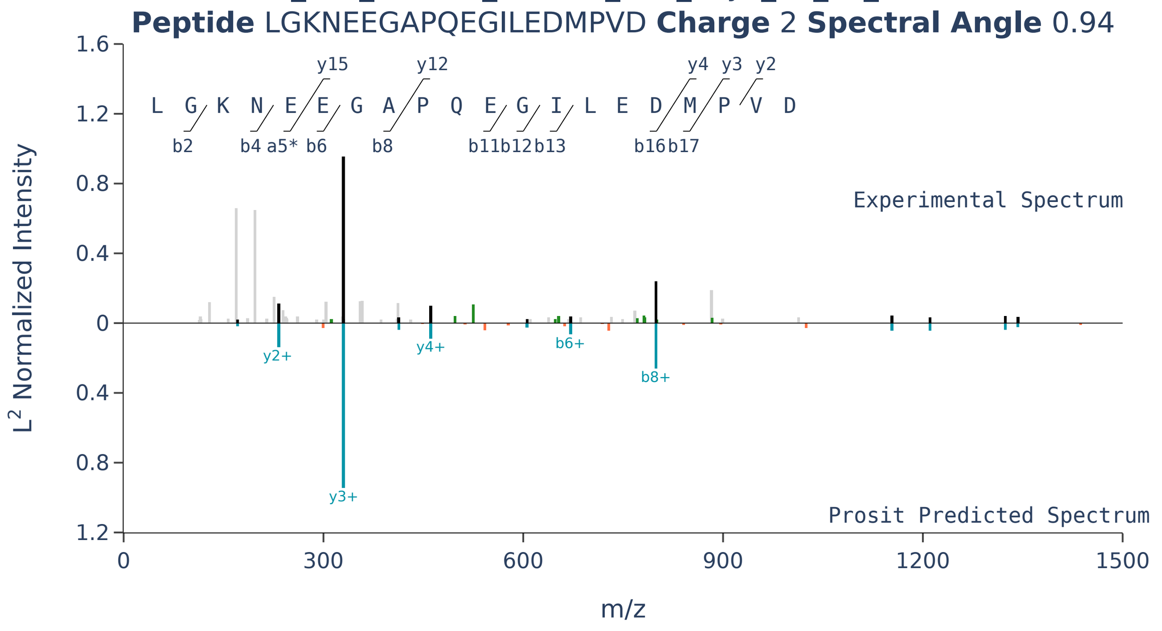


**Source** W_Soh_160821_170921_Goe_aSyn_A1_24h_R2 **Scan** 14305
**Peptide** IAAATGFVKKDQL **Charge** 3 **Spectral Angle** 0.82

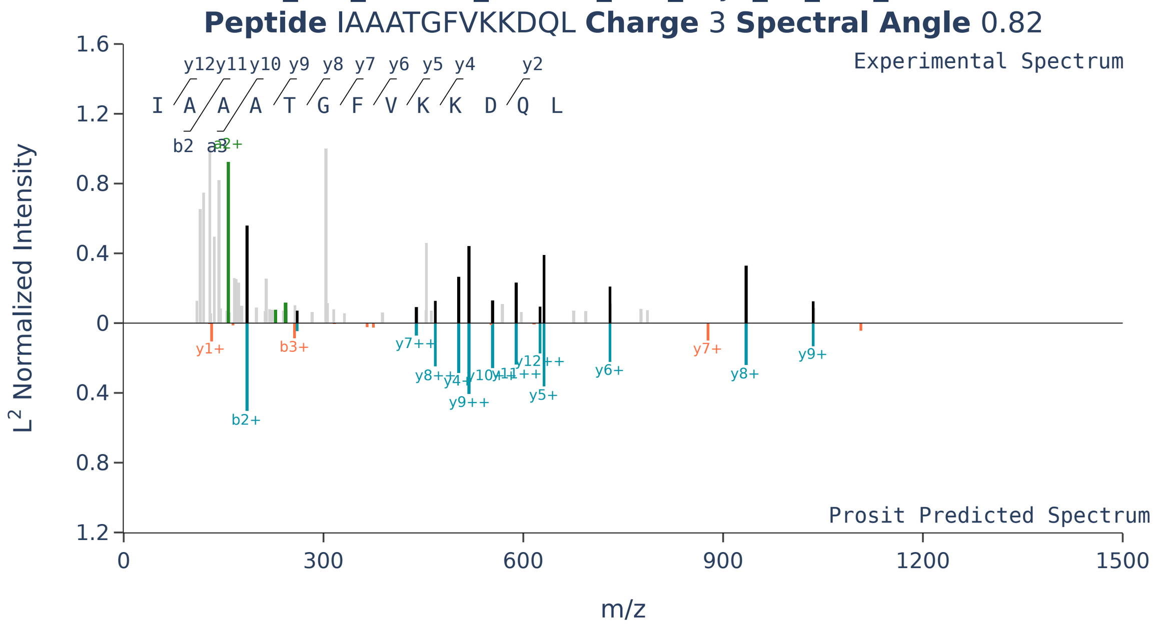


**Source** W_Soh_160821_170921_Goe_aSyn_A2_24h_R2 **Scan** 7566
**Peptide** KEGVVHGVATVAEKTKEQ **Charge** 4 **Spectral Angle** 0.9

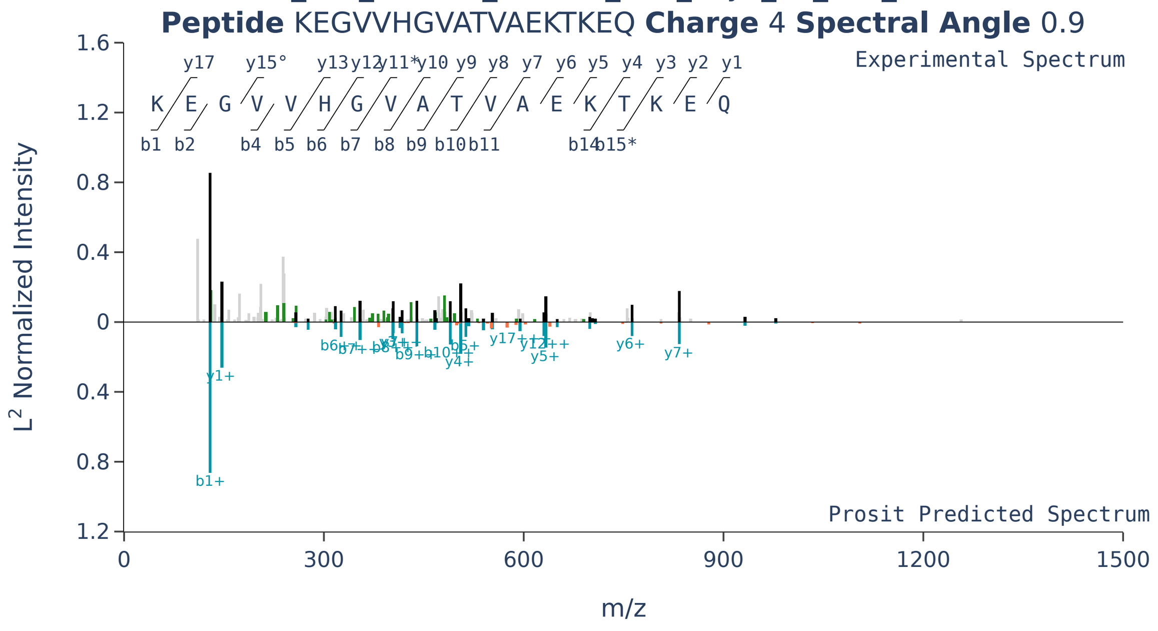


**Source** W_Soh_160821_170921_Goe_aSyn_A1_24h_R1 **Scan** 15442
**Peptide** AQKTVEGAGSIAAATGFVKKDQLGKNE **Charge** 5 **Spectral Angle** 0.77

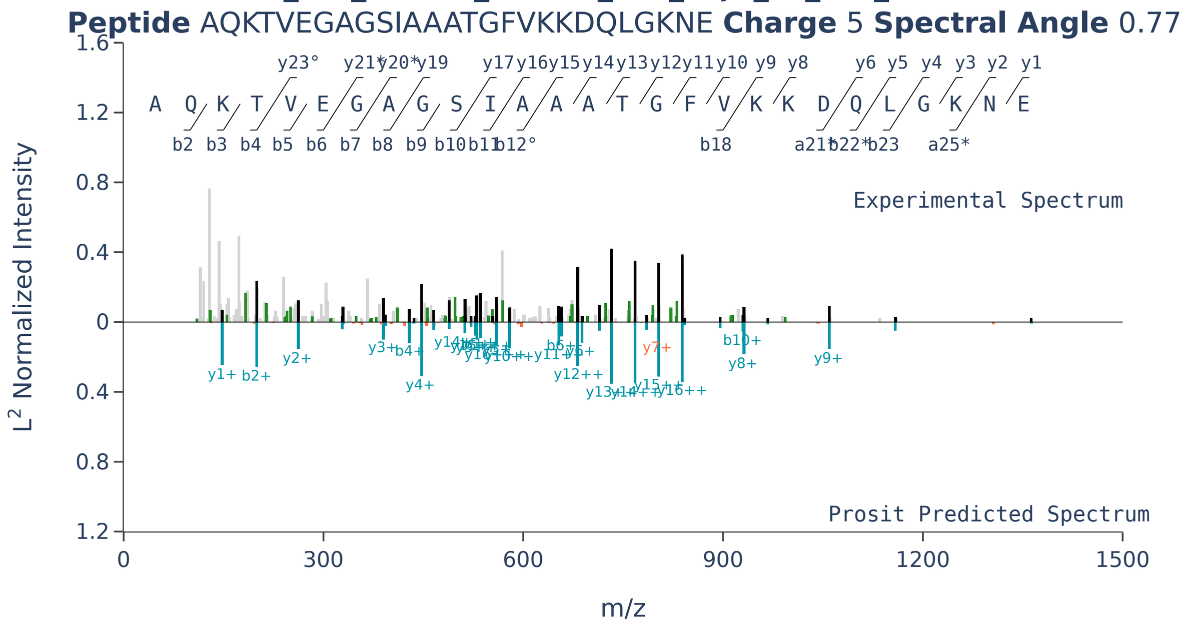


**Source** W_Soh_160821_170921_Goe_aSyn_A2_24h_R2 **Scan** 15404
**Peptide** EQVTNVGGAVVTG **Charge** 2 **Spectral Angle** 0.82

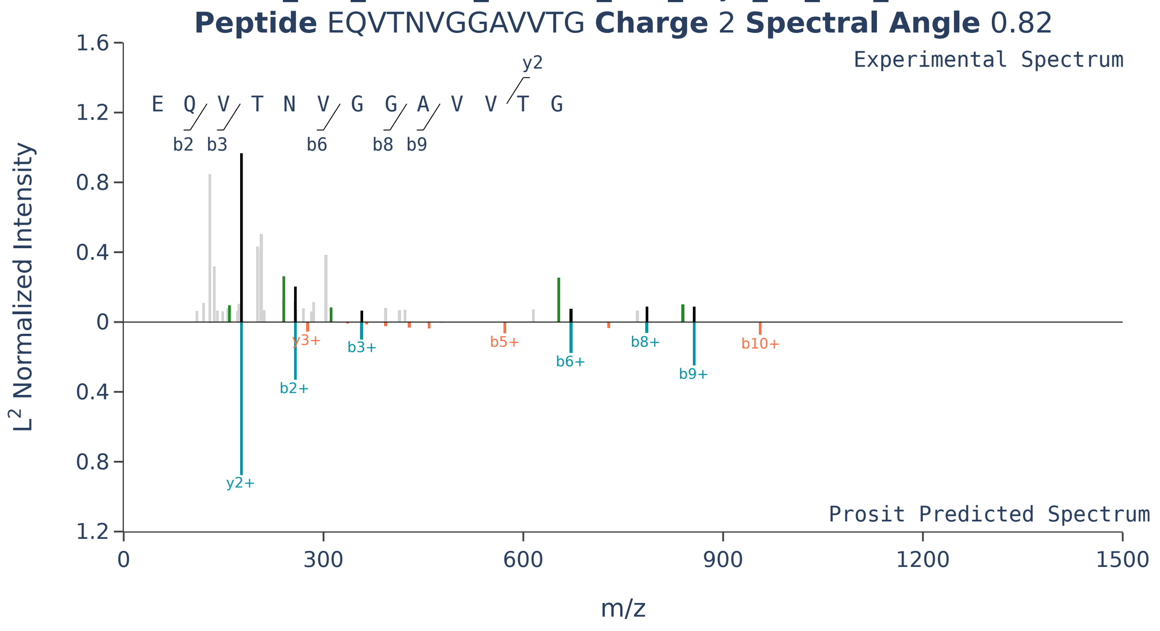


**Source** W_Soh_160821_170921_Goe_aSyn_A1_24h_R1 **Scan** 14782
**Peptide** VAEKTKEQVTNVGGAVVTGV **Charge** 3 **Spectral Angle** 0.8

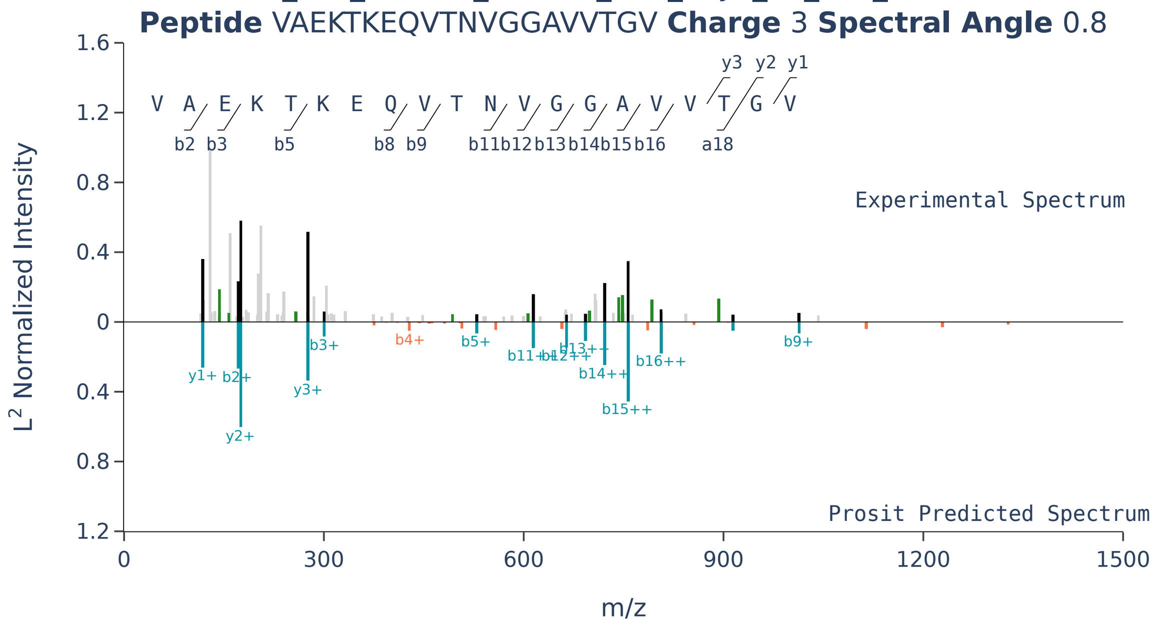


**Source** W_Soh_160821_170921_Goe_aSyn_A1_24h_R2 **Scan** 17386
**Peptide** TVEGAGSIAAATGFVKK **Charge** 3 **Spectral Angle** 0.83

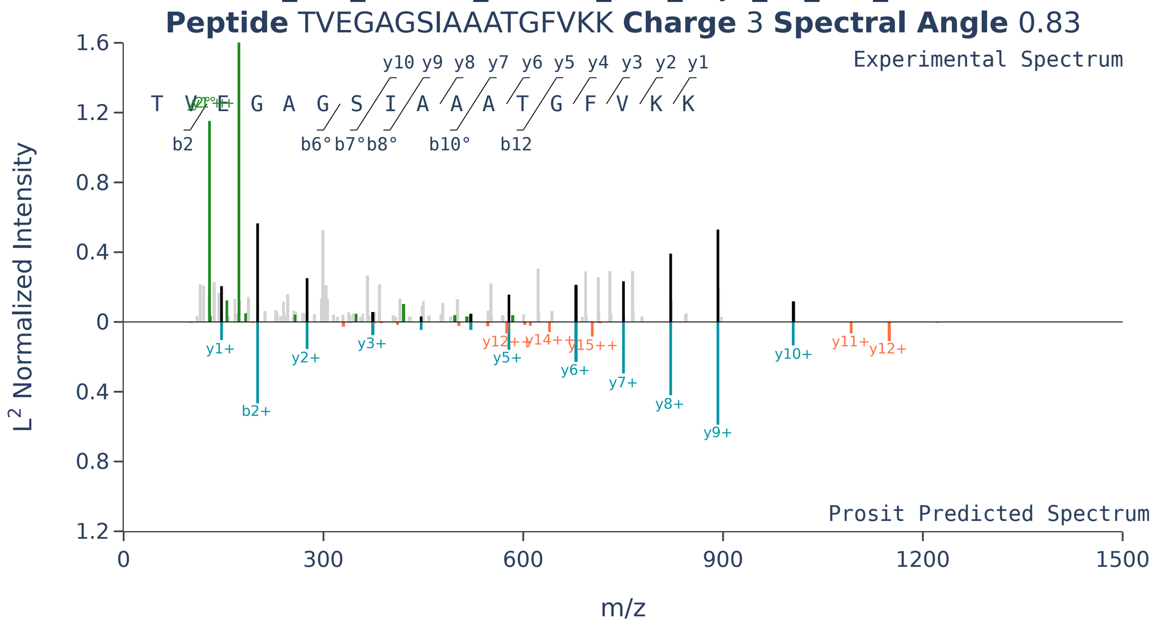


**Source** W_Soh_160821_170921_Goe_aSyn_A2_24h_R2 **Scan** 13723
**Peptide** GSKTKEGVVHGVATVAEKTKEQVTNV **Charge** 4 **Spectral Angle** 0.83

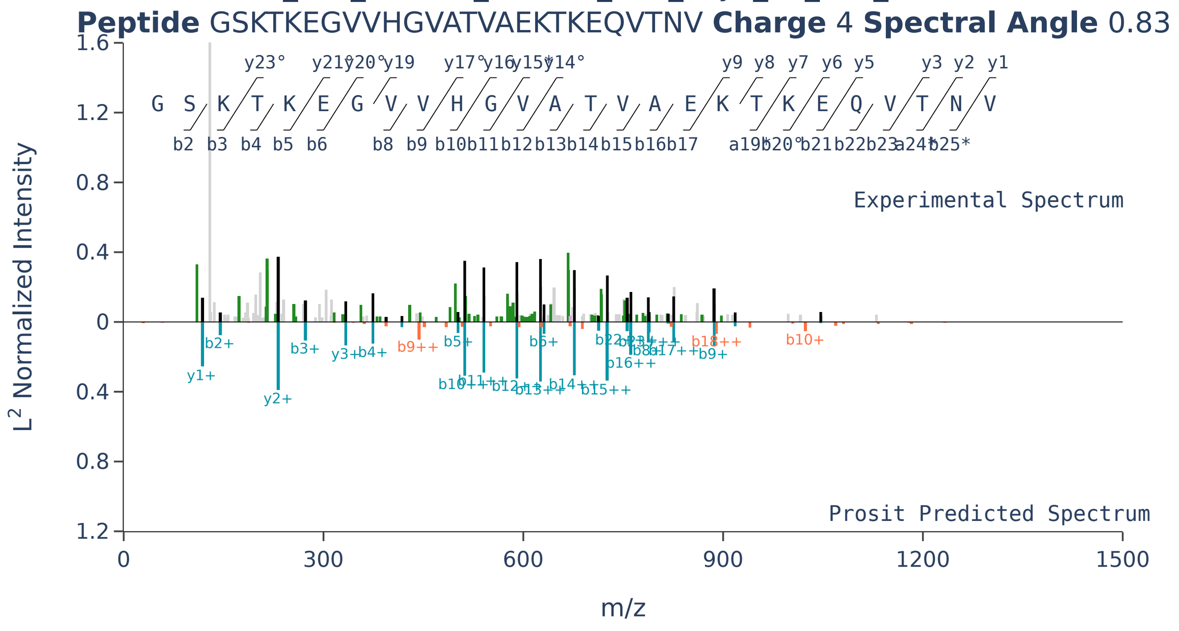

**Source** W_Soh_160821_170921_Goe_aSyn_A1_24h_R1 **Scan** 7922
**Peptide** GKTKEGVLYVGSK **Charge** 3 **Spectral Angle** 0.89

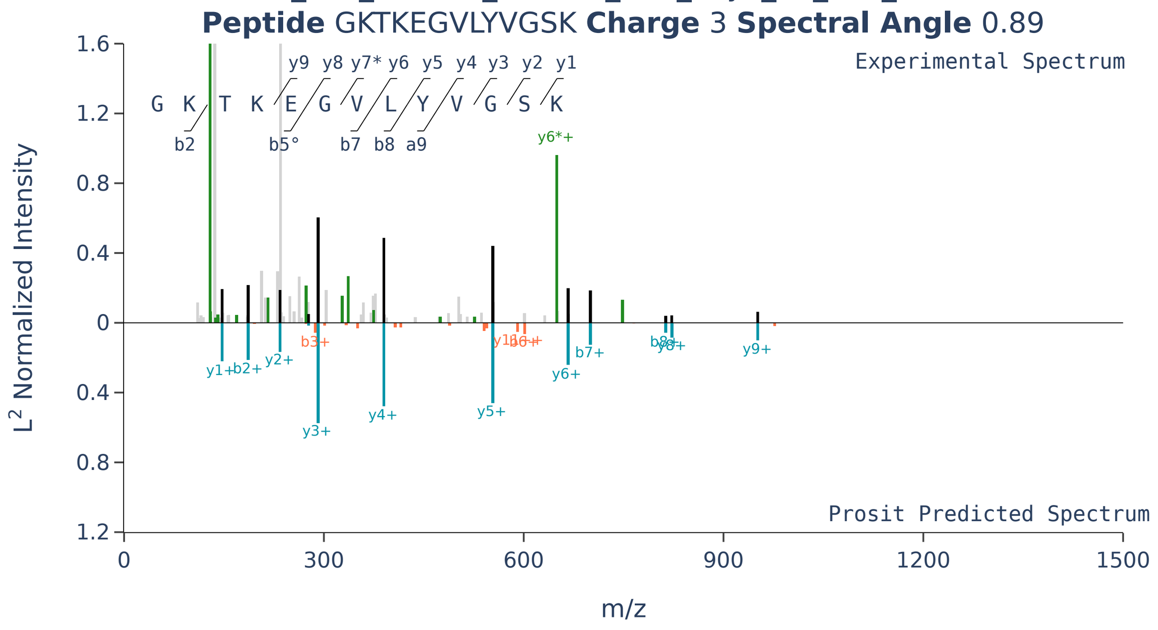


**Source** W_Soh_160821_170921_Goe_aSyn_A2_24h_R1 **Scan** 17484
**Peptide** KEGVVHGVATVAEKTKEQVTNVGGAVVT **Charge** 4 **Spectral Angle** 0.84

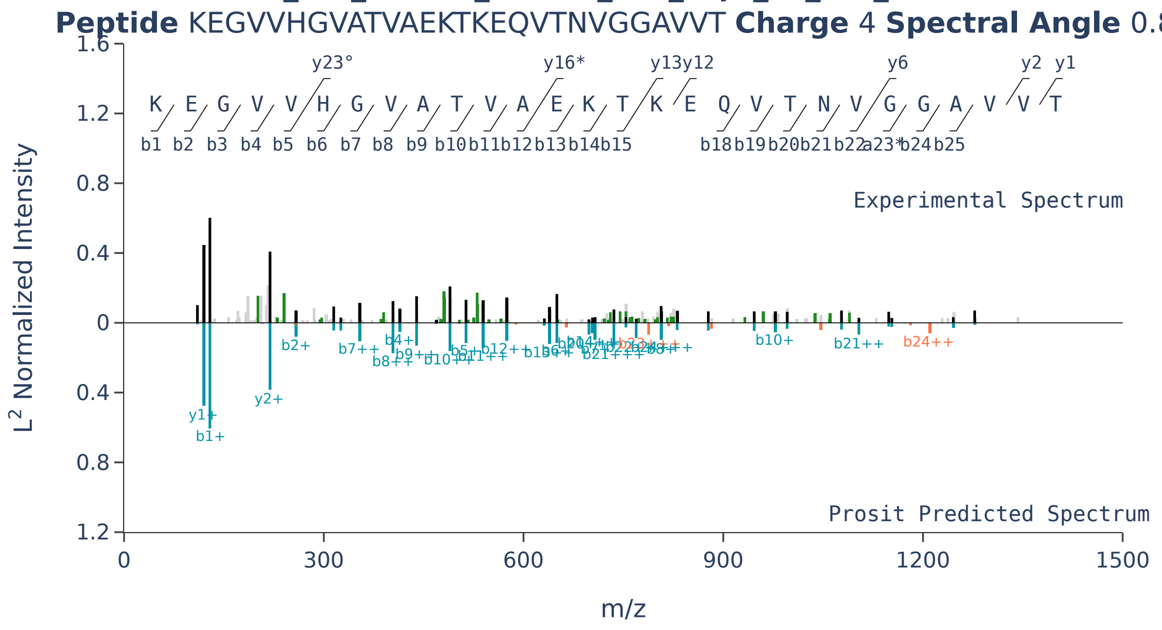


**Source** W_Soh_160821_170921_Goe_aSyn_A1_24h_R1 **Scan** 14312
**Peptide** TGFVKKDQLGKNEEGAPQEGILE **Charge** 3 **Spectral Angle** 0.89

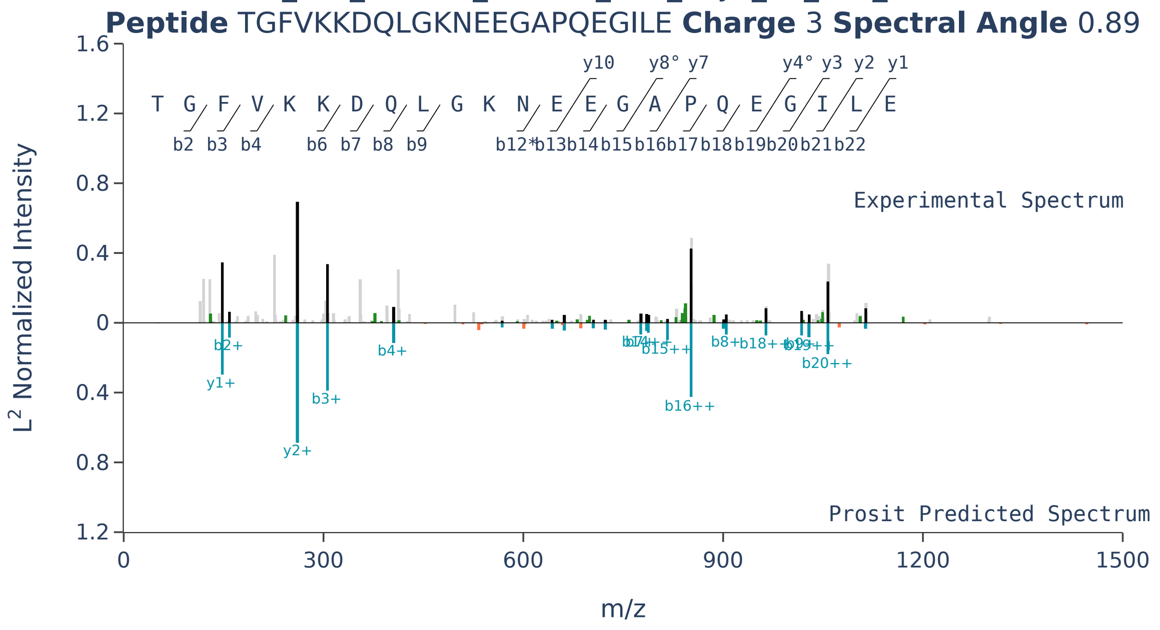


**Source** W_Soh_160821_170921_Goe_aSyn_A2_24h_R1 **Scan** 8171
**Peptide** AGKTKEGVLYVGSKTKEGVVHG **Charge** 4 **Spectral Angle** 0.84

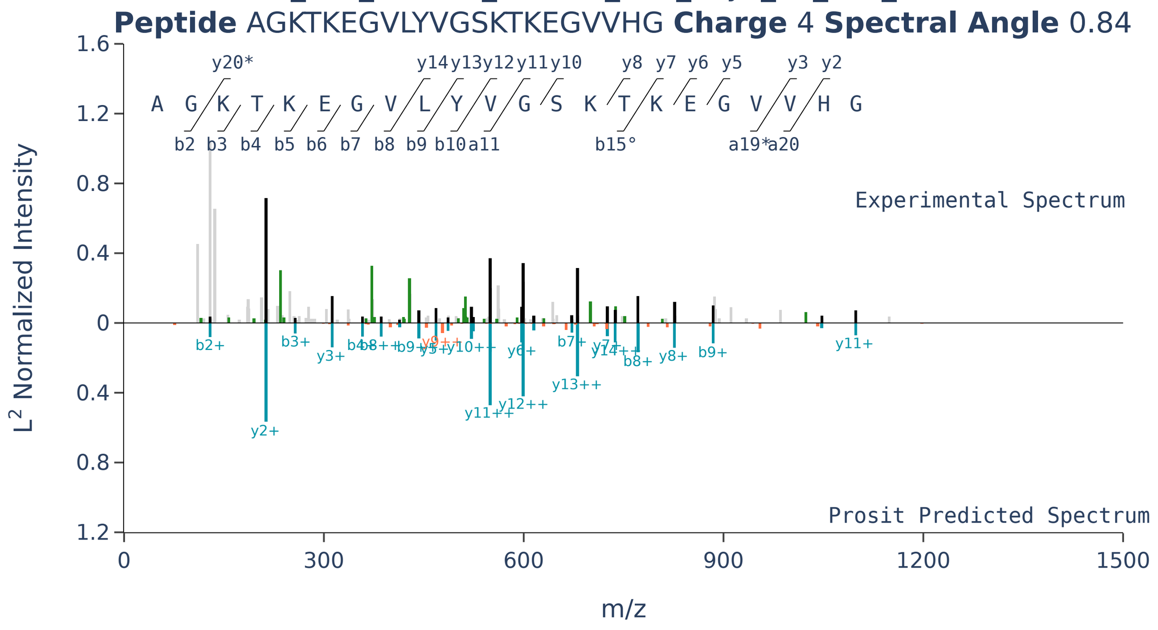


**Source** W_Soh_160821_170921_Goe_aSyn_A1_24h_R1 **Scan** 11001
**Peptide** QLGKNEEGAPQEGI **Charge** 2 **Spectral Angle** 0.9

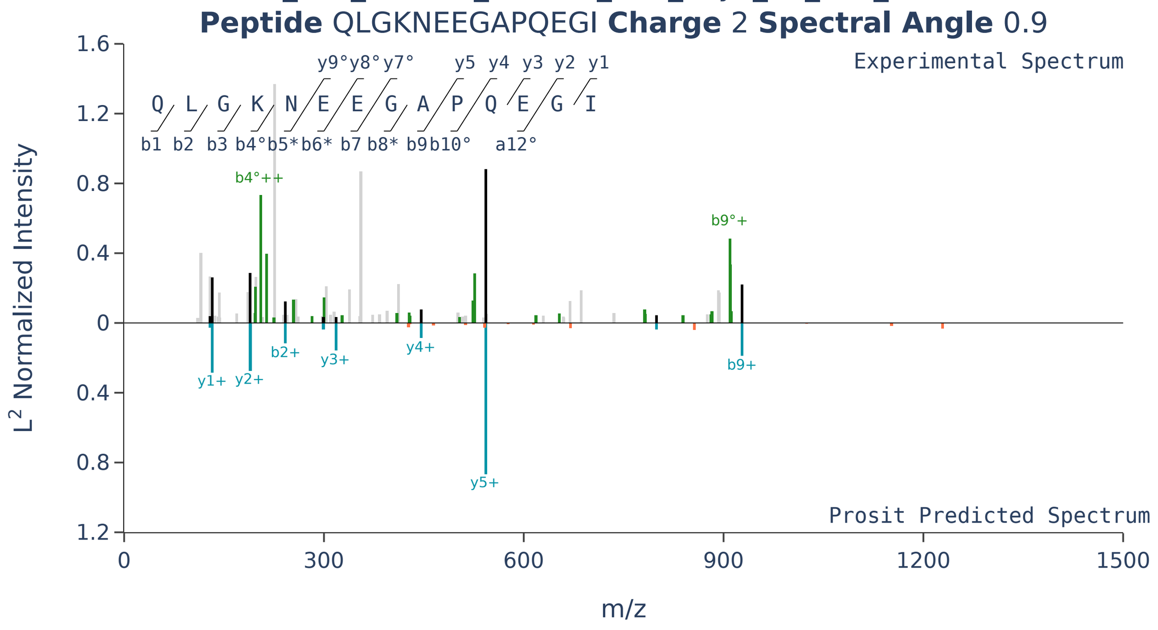


**Source** W_Soh_160821_170921_Goe_aSyn_A1_24h_R1 **Scan** 7018
**Peptide** KTKEGVVHGVATVAEKTKEQ **Charge** 5 **Spectral Angle** 0.86

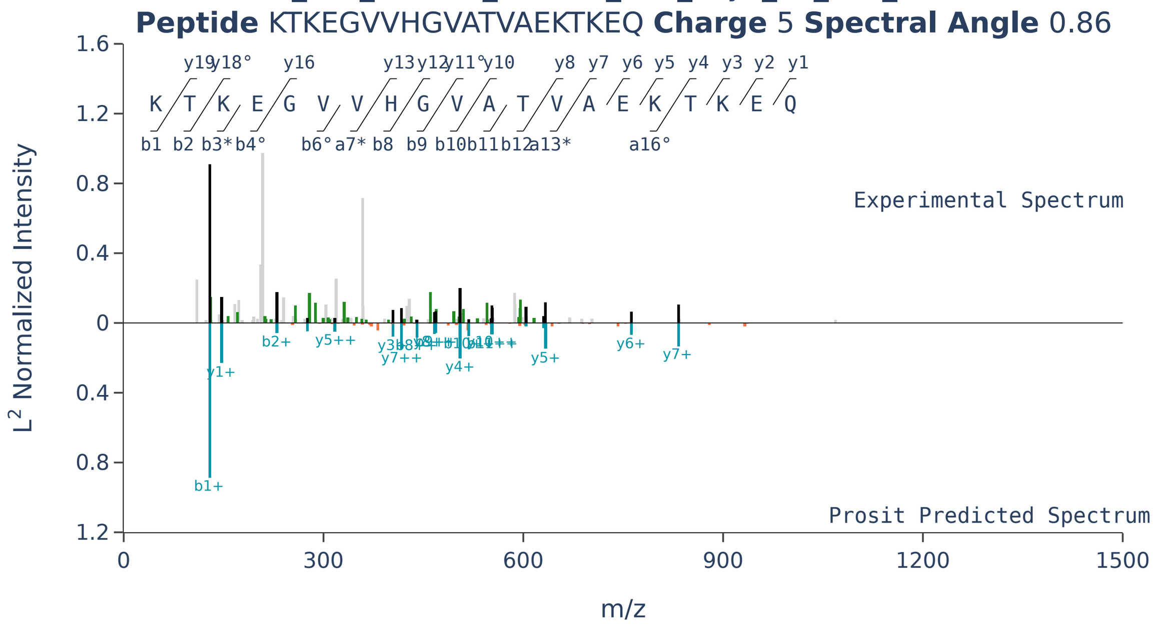


**Source** W_Soh_160821_170921_Goe_aSyn_A2_24h_R1 **Scan** 16973
**Peptide** AVVTGVTAVAQKTVE **Charge** 2 **Spectral Angle** 0.85

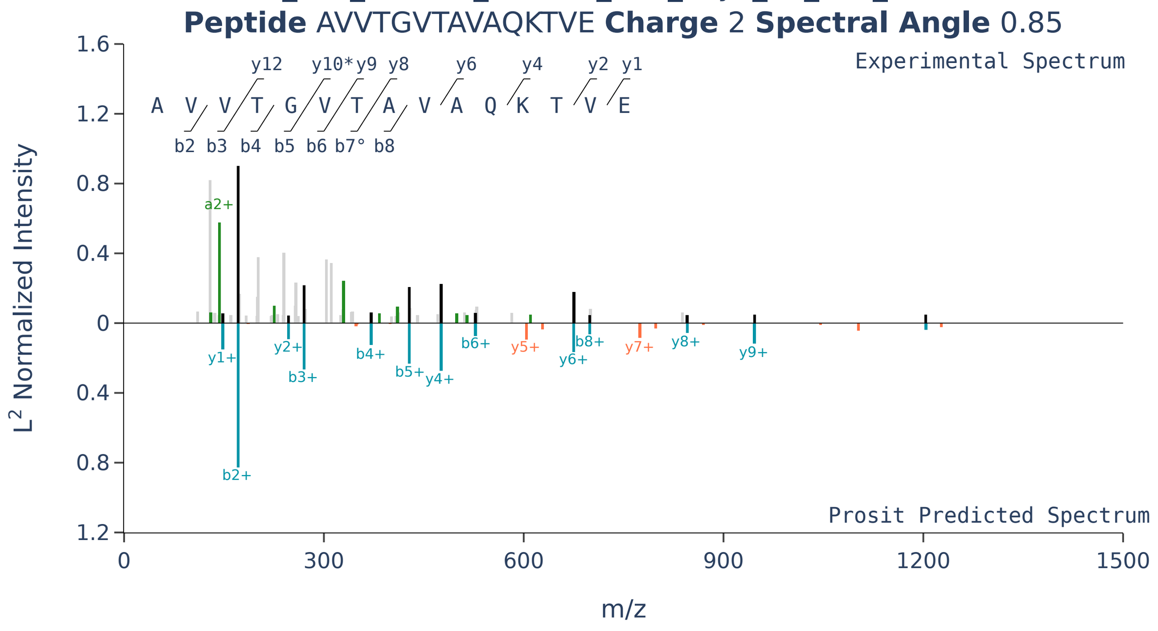


**Source** W_Soh_160821_170921_Goe_aSyn_A1_6h_R1 **Scan** 15134
**Peptide** AAATGFVKKDQLGKNEEGAPQEGILED **Charge** 4 **Spectral Angle** 0.81

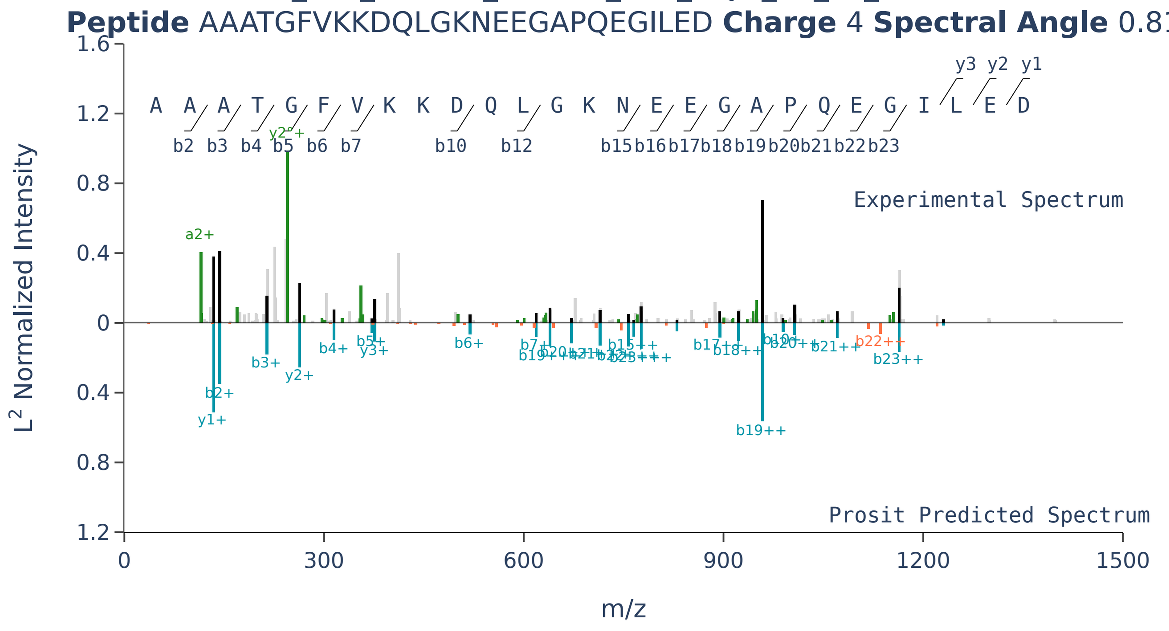


**Source** W_Soh_160821_170921_Goe_aSyn_A2_24h_R2 **Scan** 7569
**Peptide** TVAEKTKEQVTNV **Charge** 2 **Spectral Angle** 0.82

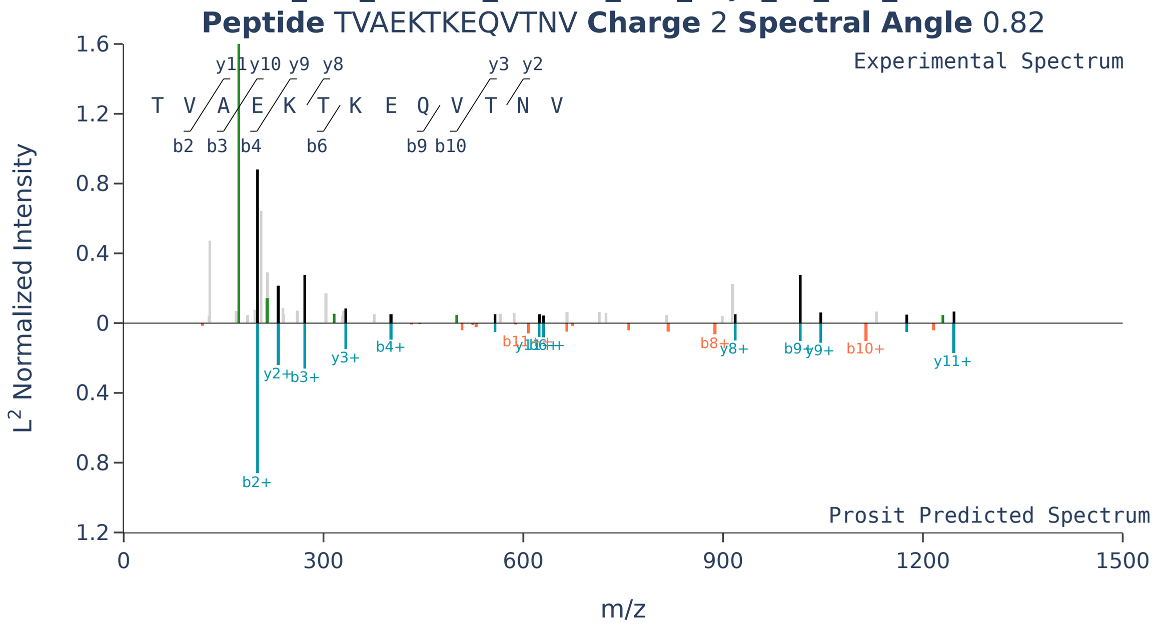


**Source** W_Soh_160821_170921_Goe_aSyn_A1_24h_R1 **Scan** 14272
**Peptide** KEGVVHGVATVAEKTKEQVTNV **Charge** 3 **Spectral Angle** 0.76

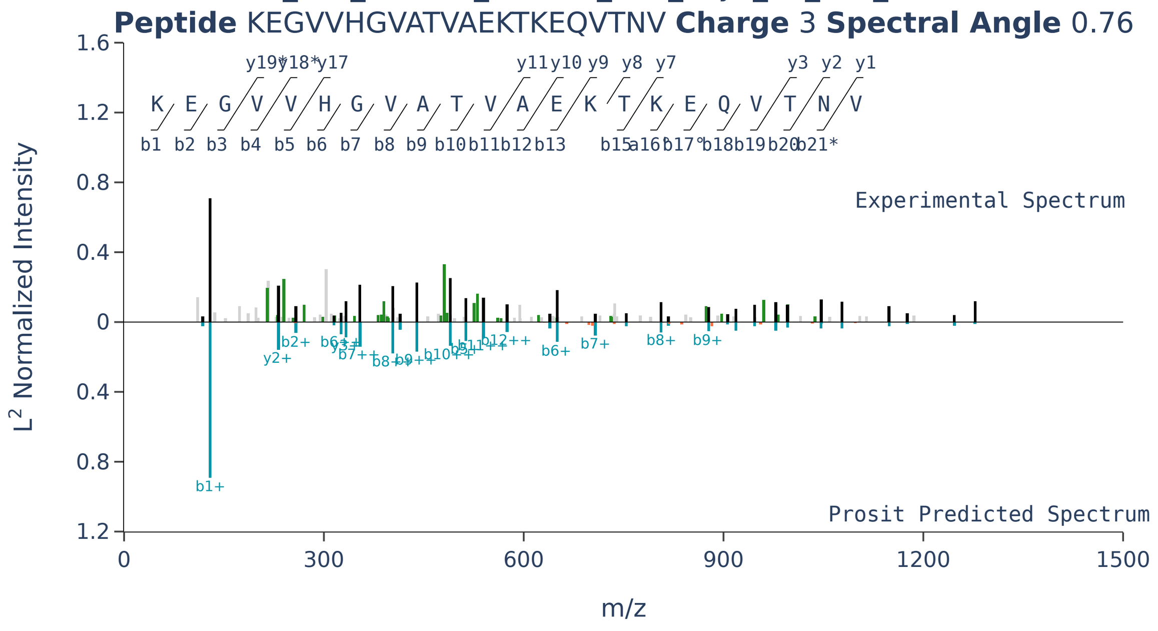


**Source** W_Soh_160821_170921_Goe_aSyn_A1_24h_R2 **Scan** 10339
**Peptide** VLYVGSK **Charge** 2 **Spectral Angle** 0.84

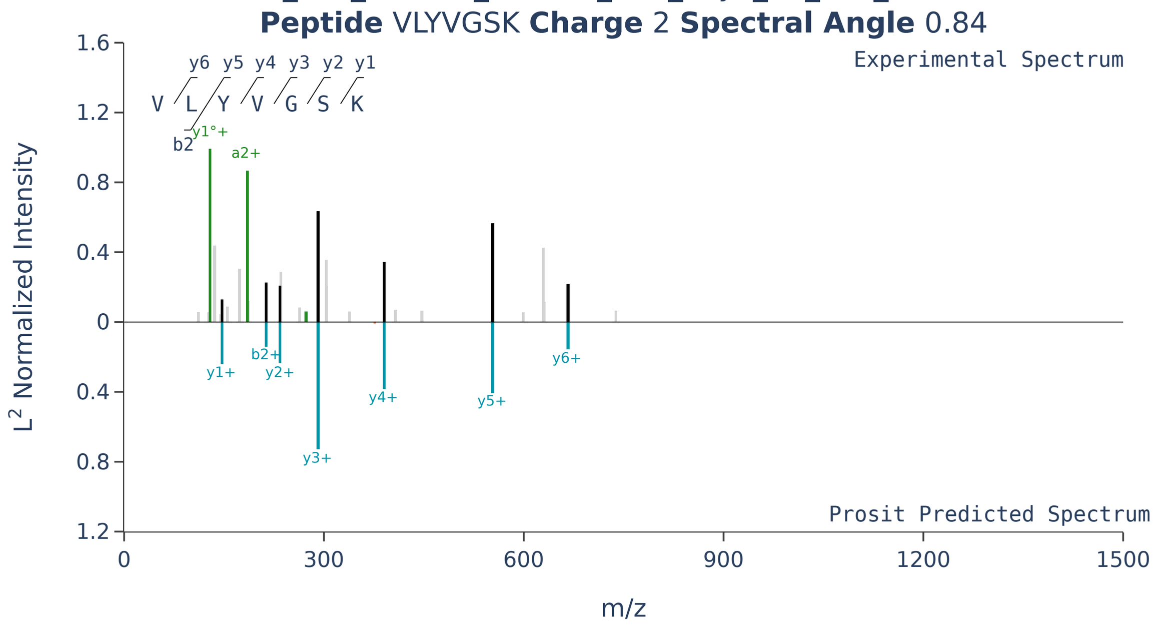


**Source** W_Soh_160821_170921_Goe_aSyn_A1_24h_R2 **Scan** 7763
**Peptide** SKTKEGVVHGVATVAEKT **Charge** 3 **Spectral Angle** 0.82

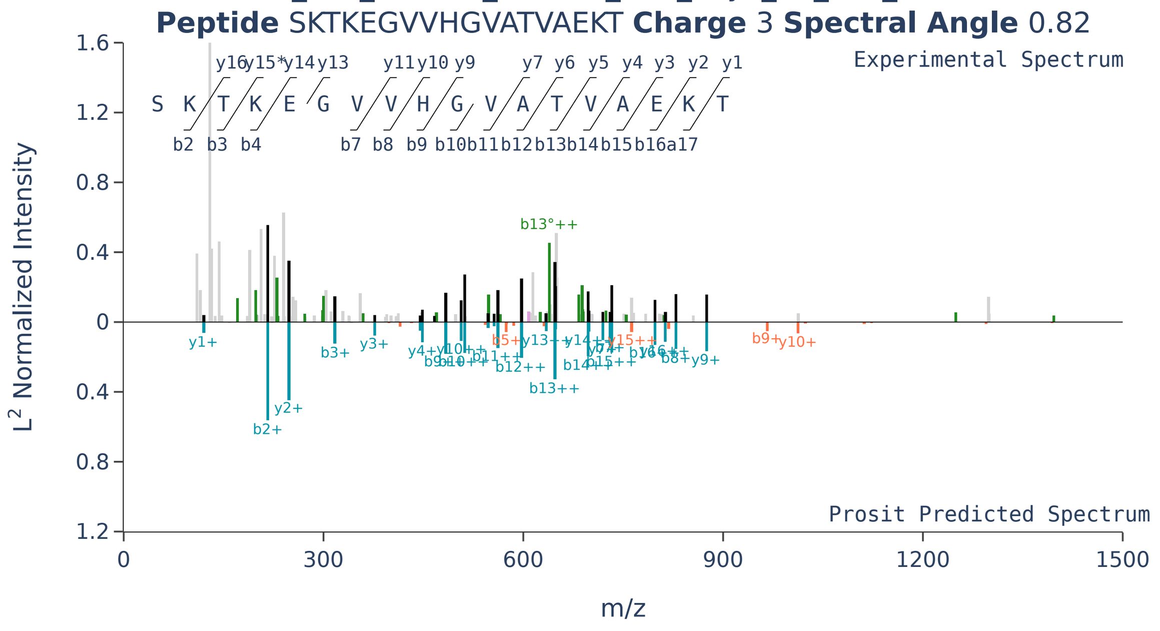


**Source** W_Soh_160821_170921_Goe_aSyn_A2_24h_R2 **Scan** 5057
**Peptide** GVAEAAGKTKE **Charge** 2 **Spectral Angle** 0.8

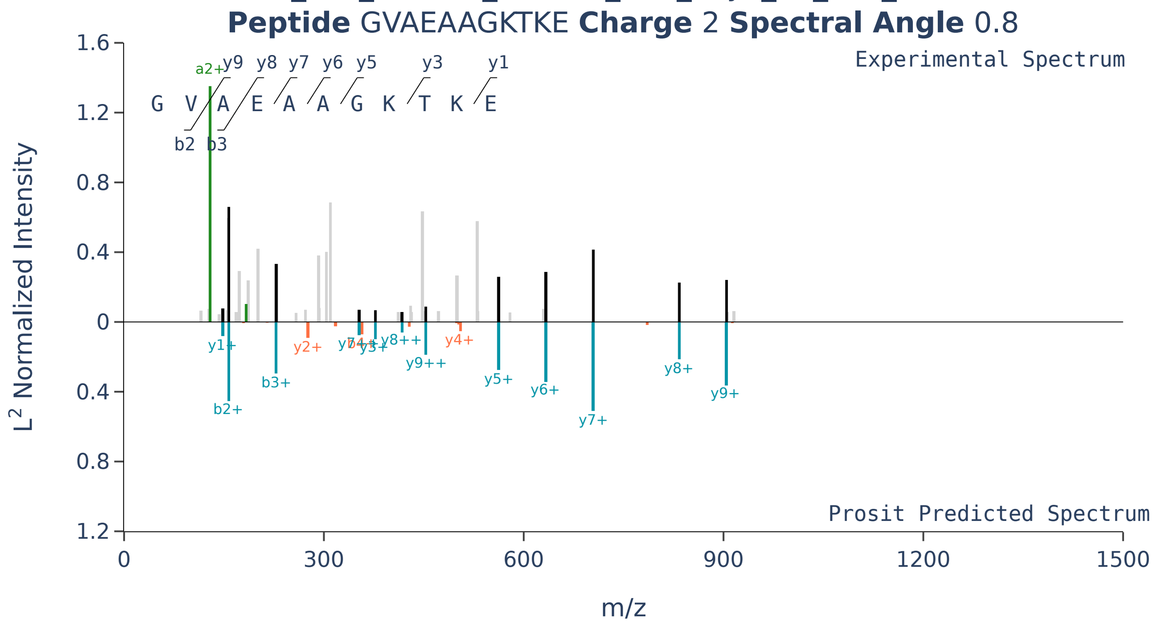


**Source** W_Soh_160821_170921_Goe_aSyn_A1_24h_R1 **Scan** 5378
**Peptide** VKKDQLGKNEEGAPQE **Charge** 2 **Spectral Angle** 0.85

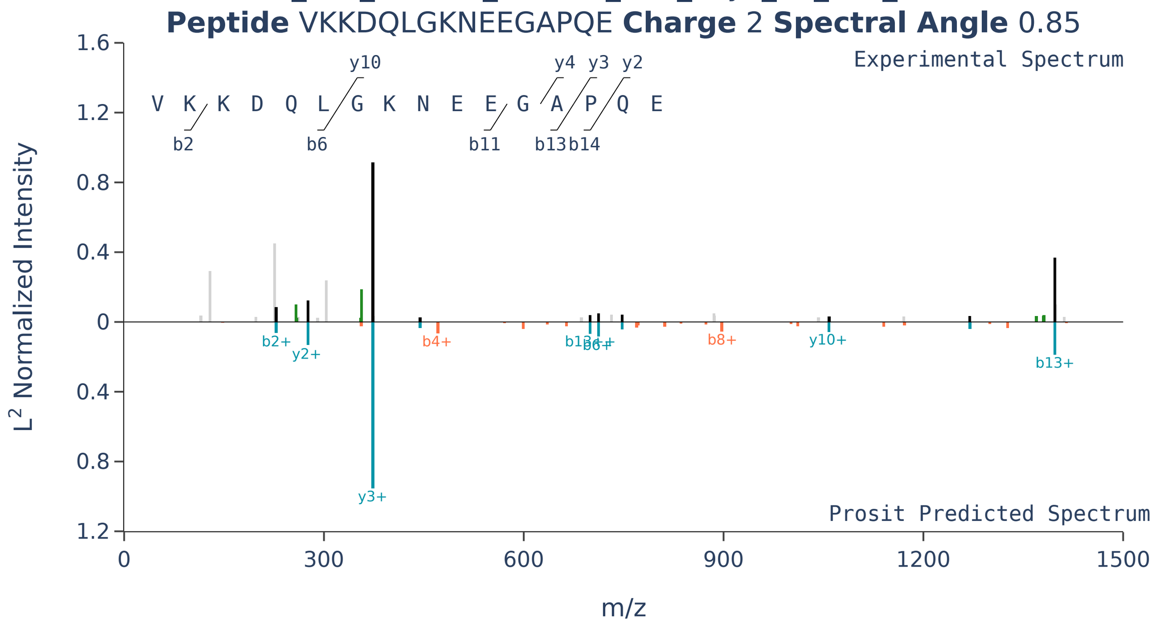


**Source** W_Soh_160821_170921_Goe_aSyn_A2_24h_R2 **Scan** 13192
**Peptide** KDQLGKNEEGAPQEGILE **Charge** 3 **Spectral Angle** 0.86

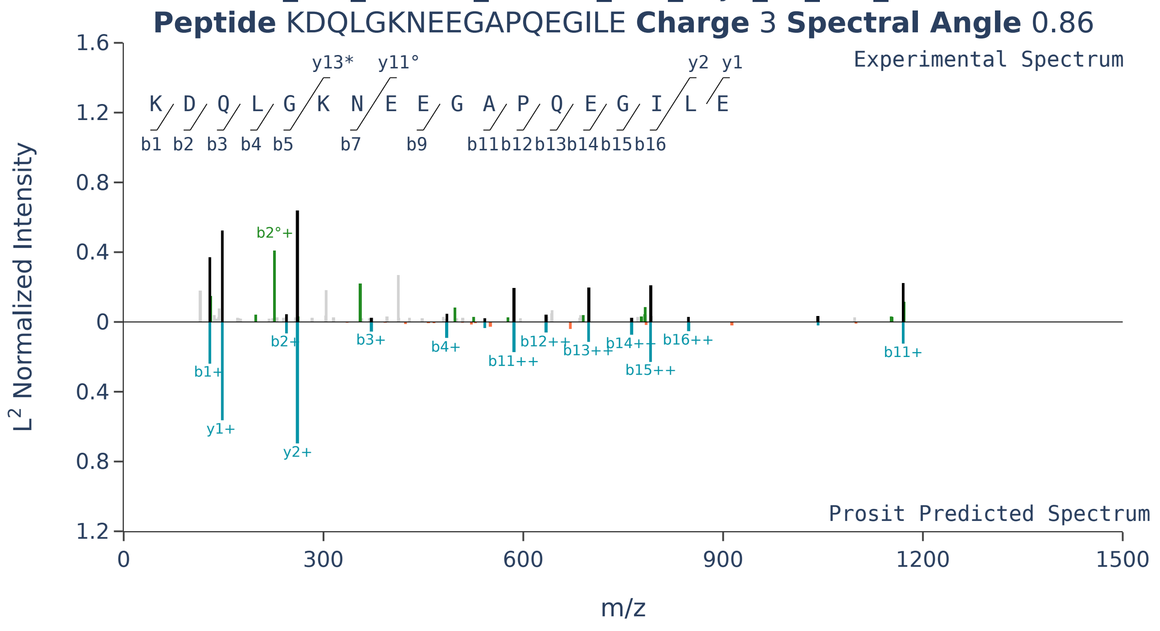

**Source** W_Soh_160821_170921_Goe_aSyn_A1_24h_R2 **Scan** 10203
**Peptide** KTVEGAGSIAAATG **Charge** 2 **Spectral Angle** 0.82

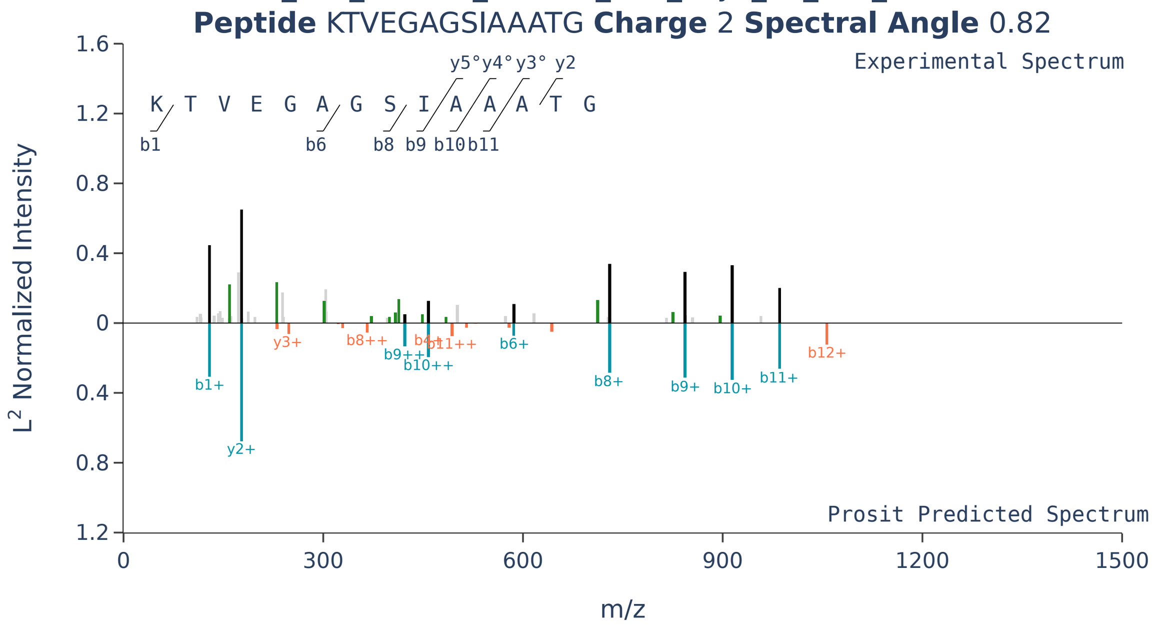


**Source** W_Soh_160821_170921_Goe_aSyn_A1_24h_R1 **Scan** 12064
**Peptide** YEMPSEEG **Charge** 1 **Spectral Angle** 0.74

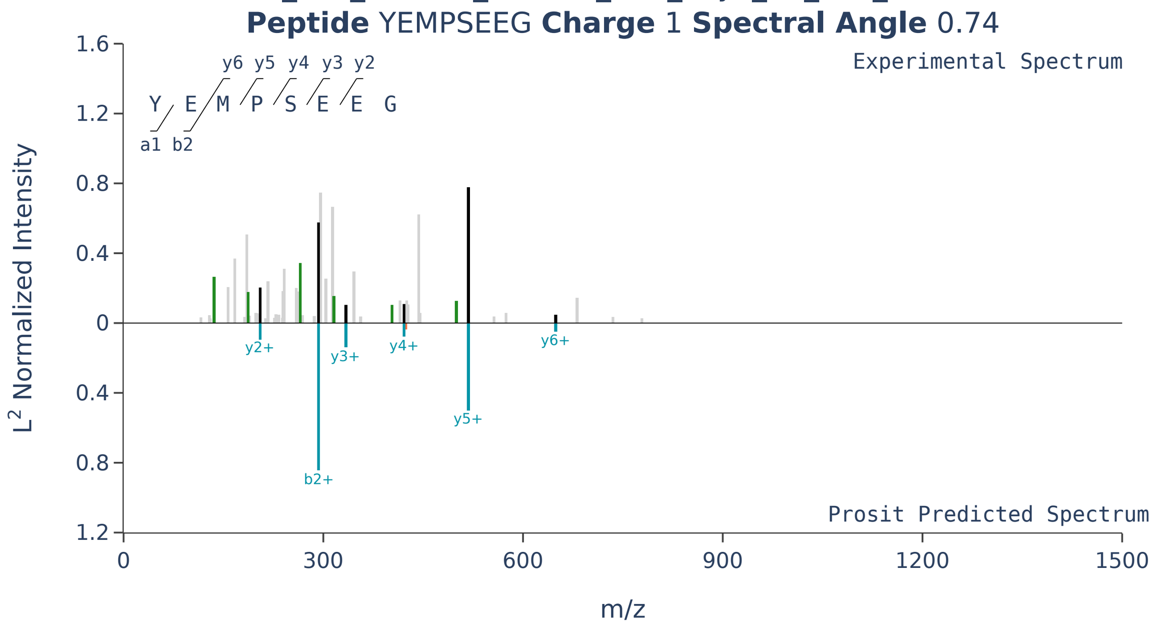


**Source** W_Soh_160821_170921_Goe_aSyn_A2_6h_R1 **Scan** 18158
**Peptide** GVVHGVATVAEKTKEQVTNVGGAVV **Charge** 3 **Spectral Angle** 0.77

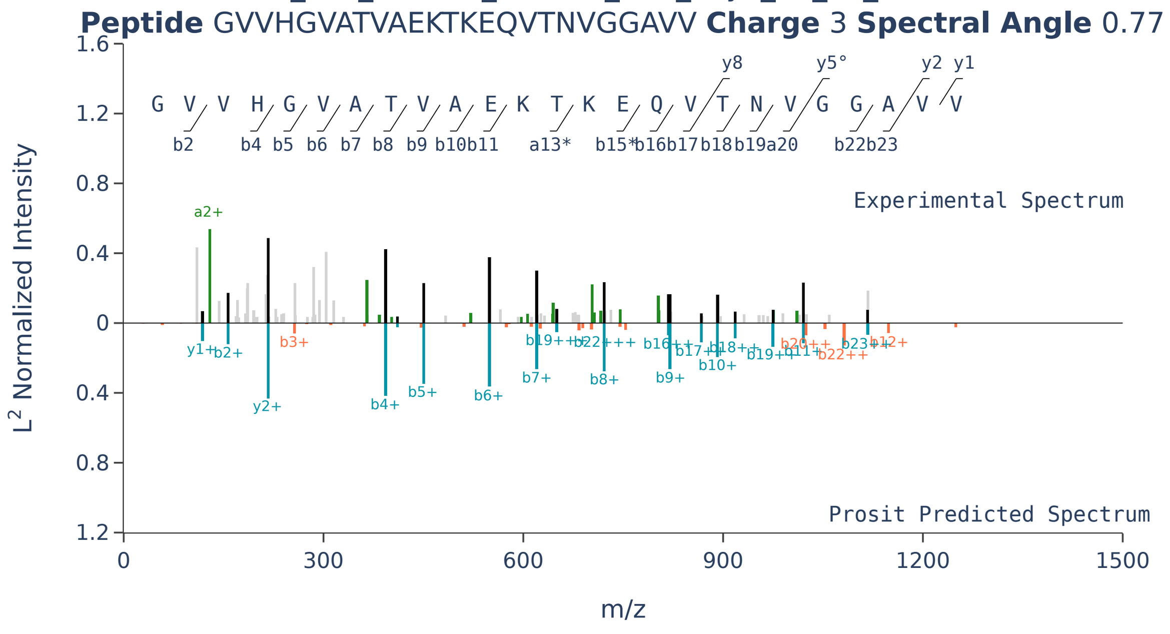


**Source** W_Soh_160821_170921_Goe_aSyn_A1_24h_R2 **Scan** 8046
**Peptide** AKEGVVAAAEKTKQGVAE **Charge** 3 **Spectral Angle** 0.76

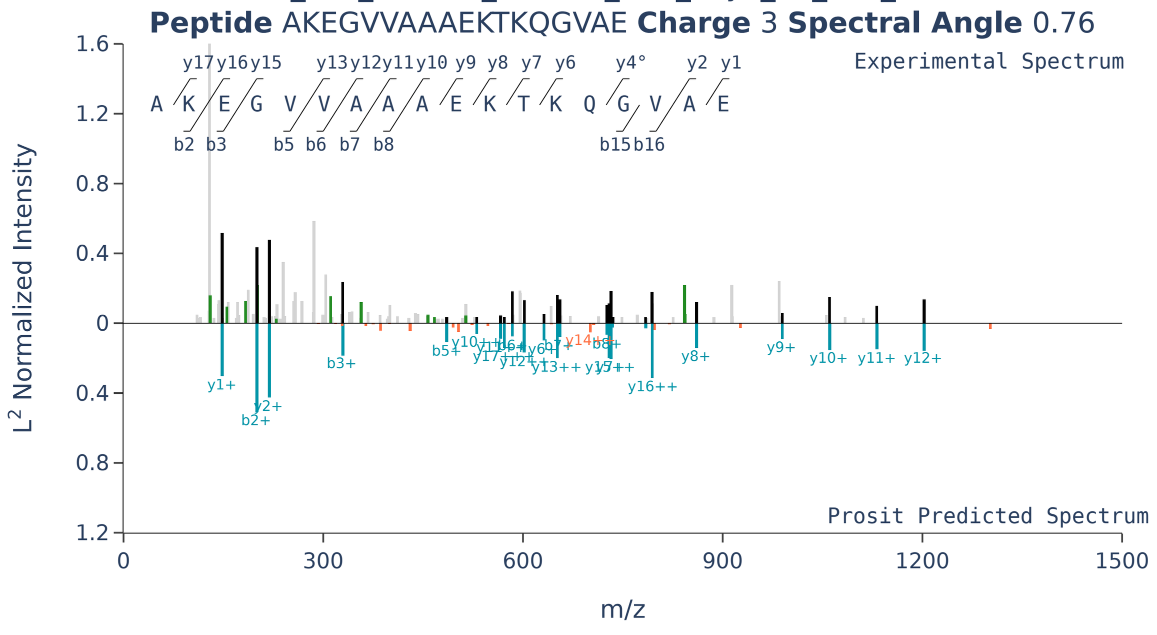


**Source** W_Soh_160821_170921_Goe_aSyn_A2_24h_R2 **Scan** 20412
**Peptide** VGGAVVTGVTAV **Charge** 1 **Spectral Angle** 0.76

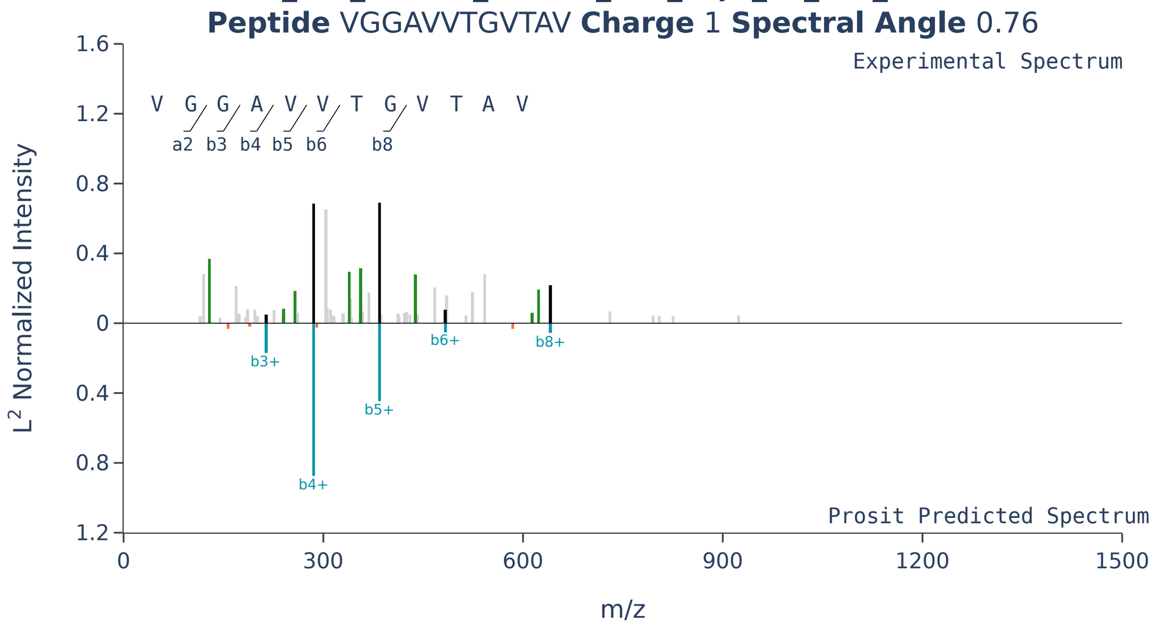


**Source** W_Soh_160821_170921_Goe_aSyn_A1_24h_R2 **Scan** 7301
**Peptide** SKTKEGVVHGVATVAEKTKE **Charge** 4 **Spectral Angle** 0.8

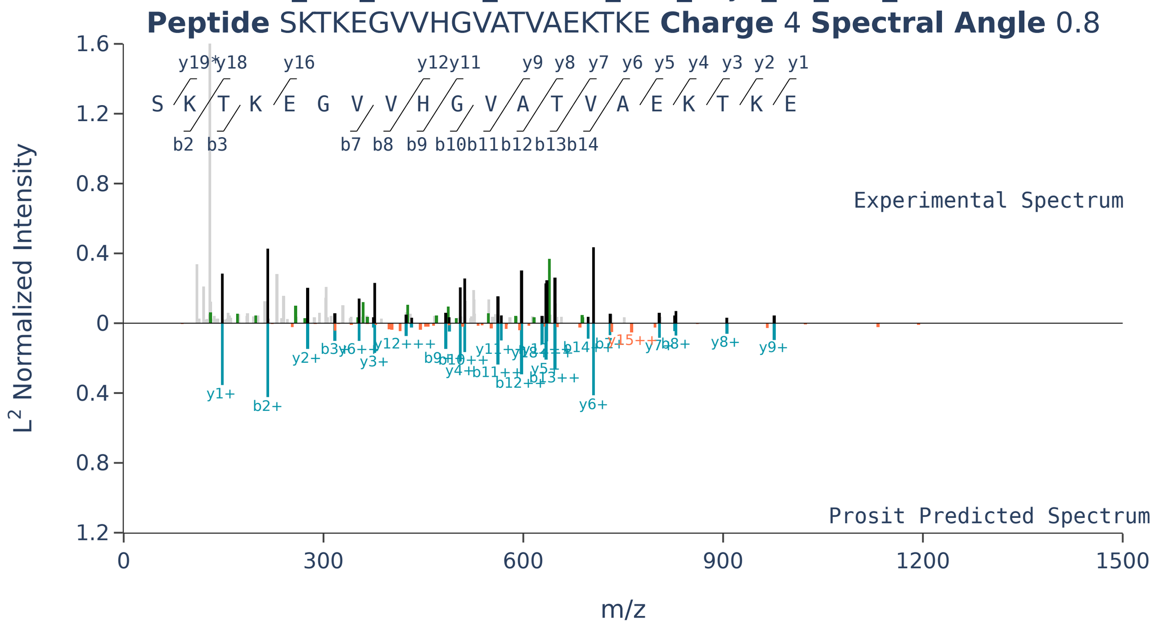


**Source** W_Soh_160821_170921_Goe_aSyn_A2_24h_R2 **Scan** 16057
**Peptide** EGVLYVGSKTKEGVVHGVATV **Charge** 3 **Spectral Angle** 0.87

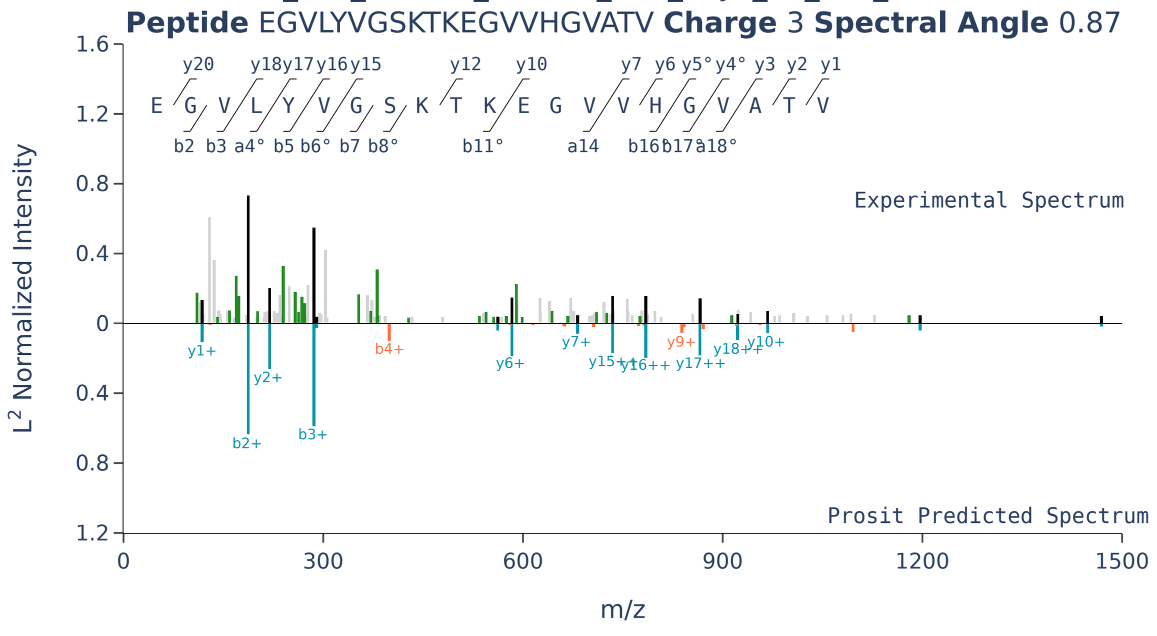


**Source** W_Soh_160821_170921_Goe_aSyn_A2_6h_R1 **Scan** 15938
**Peptide** GVLYVGSKTKEGVVHGVATVAEKTKEQV **Charge** 4 **Spectral Angle** 0.84

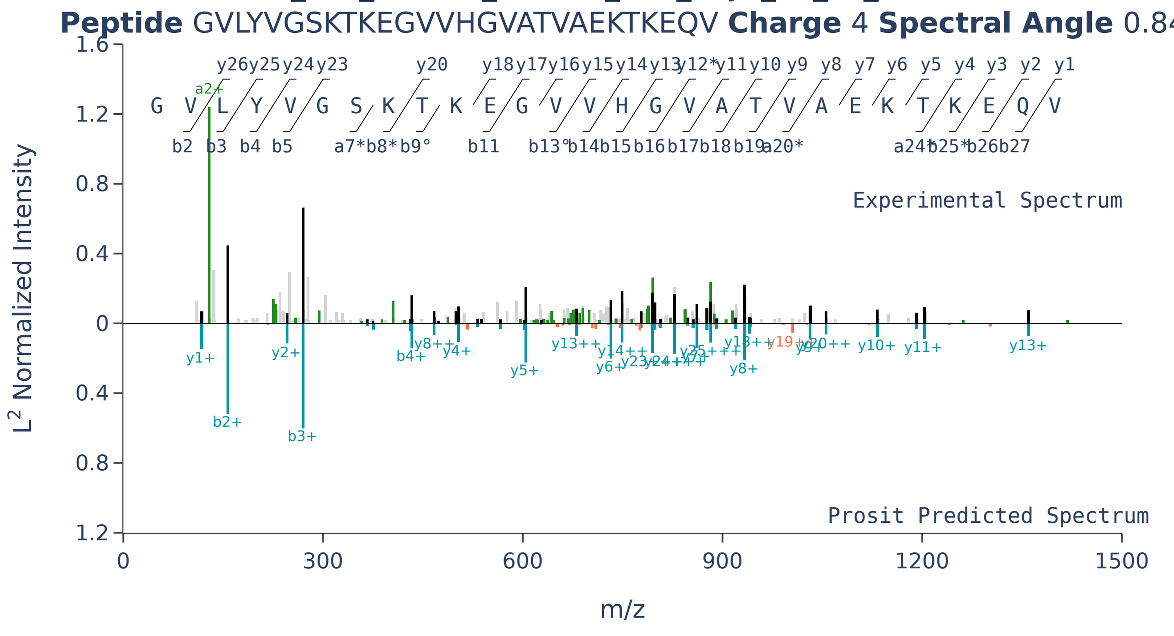


**Source** W_Soh_160821_170921_Goe_aSyn_A2_24h_R2 **Scan** 12345
**Peptide** TKEQVTNVGGAVVTG **Charge** 2 **Spectral Angle** 0.81

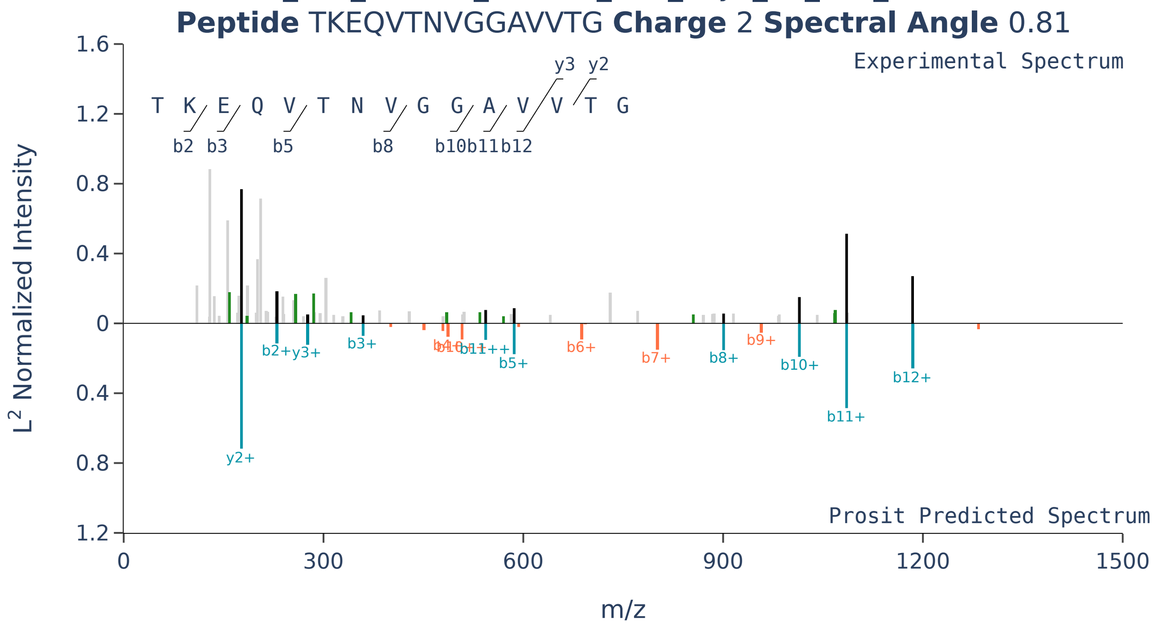


**Source** W_Soh_160821_170921_Goe_aSyn_A1_24h_R2 **Scan** 18161
**Peptide** VATVAEKTKEQVTNVGGAVVTGV **Charge** 3 **Spectral Angle** 0.81

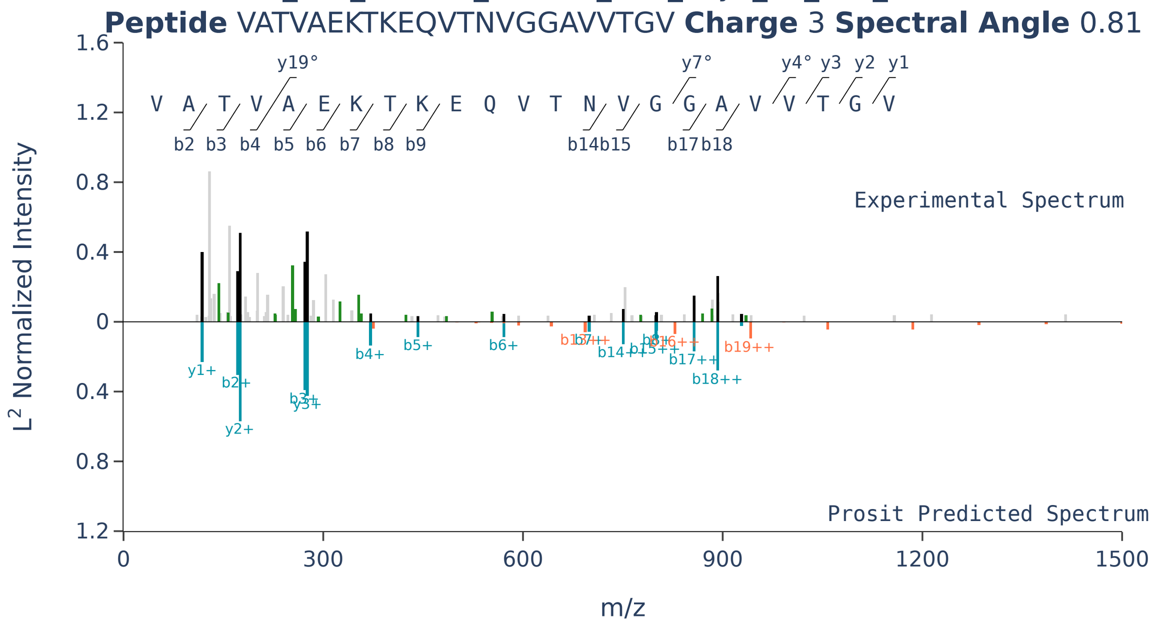


**Source** W_Soh_160821_170921_Goe_aSyn_A1_24h_R1 **Scan** 22227
**Peptide** GVTAVAQKTVEGAGSIAAATGFVKKD **Charge** 3 **Spectral Angle** 0.81

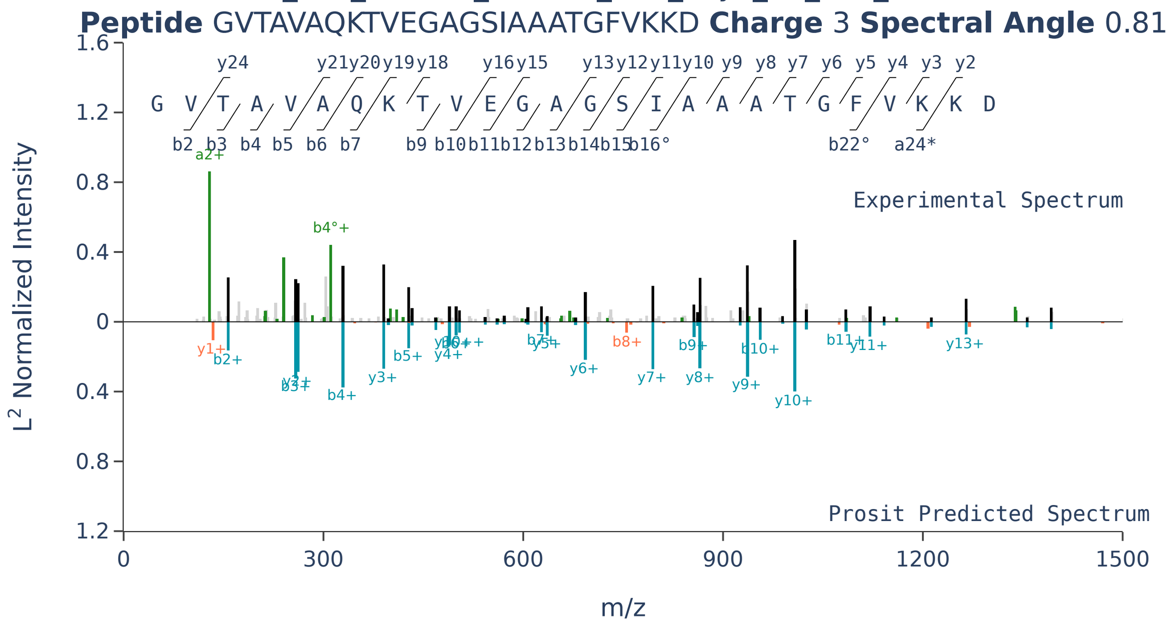


**Source** W_Soh_160821_170921_Goe_aSyn_A1_24h_R2 **Scan** 21647
**Peptide** KTKEQVTNVGGAVVTGVTAVAQKTVE **Charge** 4 **Spectral Angle** 0.83

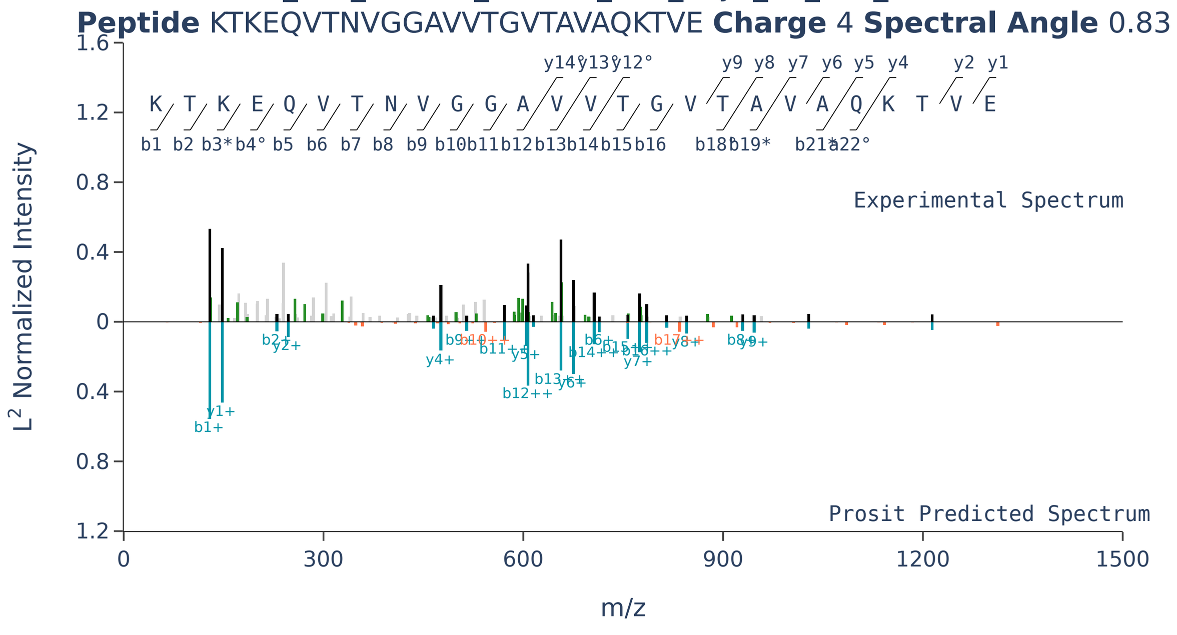


**Source** W_Soh_160821_170921_Goe_aSyn_A1_3h_R2 **Scan** 8299
**Peptide** VGSKTKEGVVHGVATVA **Charge** 3 **Spectral Angle** 0.8

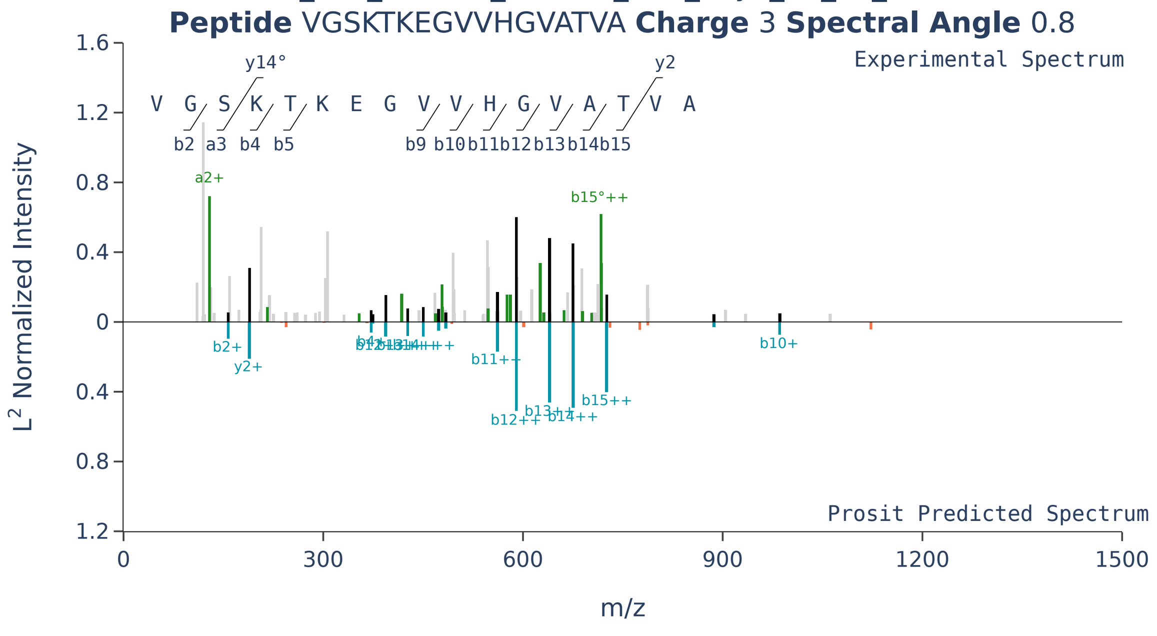


**Source** W_Soh_160821_170921_Goe_aSyn_A1_6h_R2 **Scan** 23922
**Peptide** TKEQVTNVGGAVVTGVTAVAQKTVE **Charge** 3 **Spectral Angle** 0.75

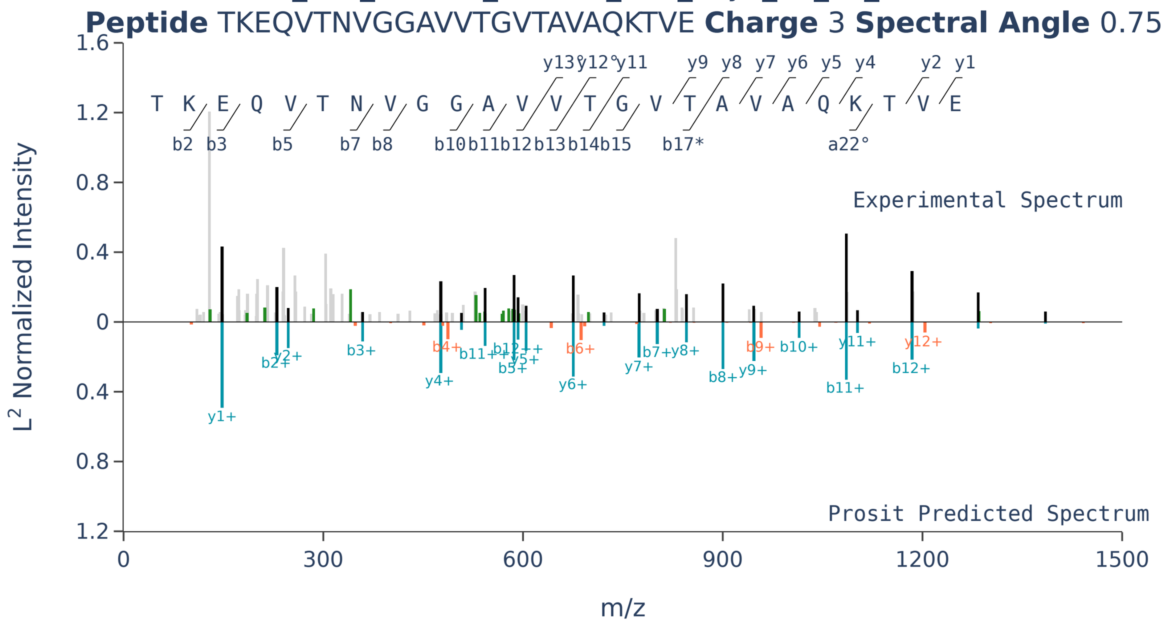


**Source** W_Soh_160821_170921_Goe_aSyn_A1_3h_R2 **Scan** 18706
**Peptide** QKTVEGAGSIAAATGF **Charge** 2 **Spectral Angle** 0.87

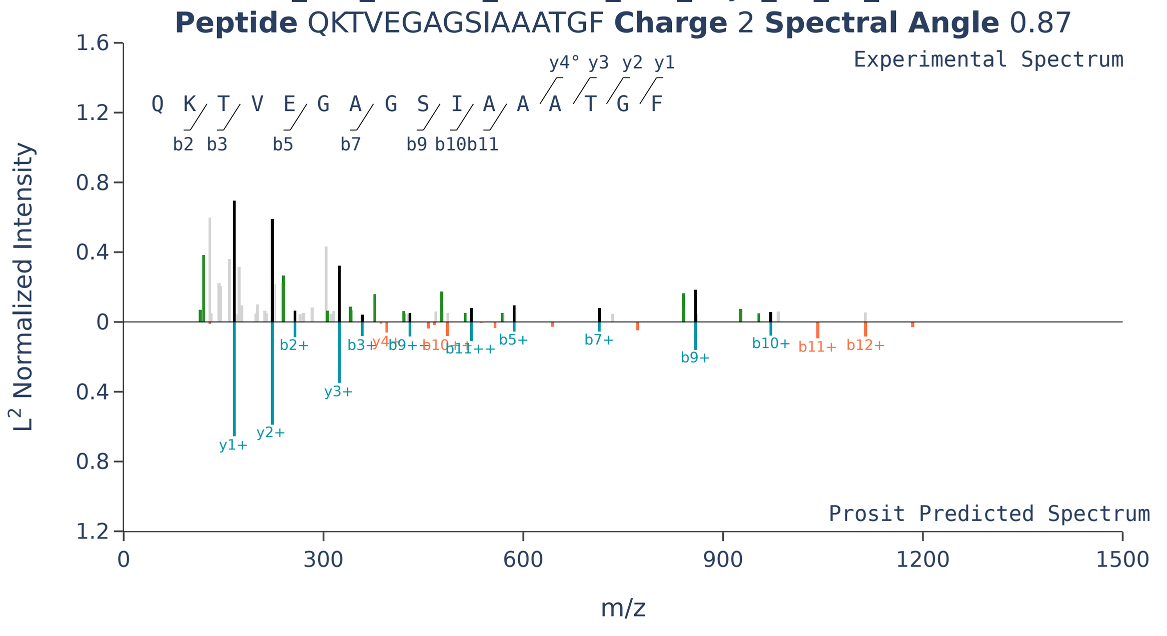

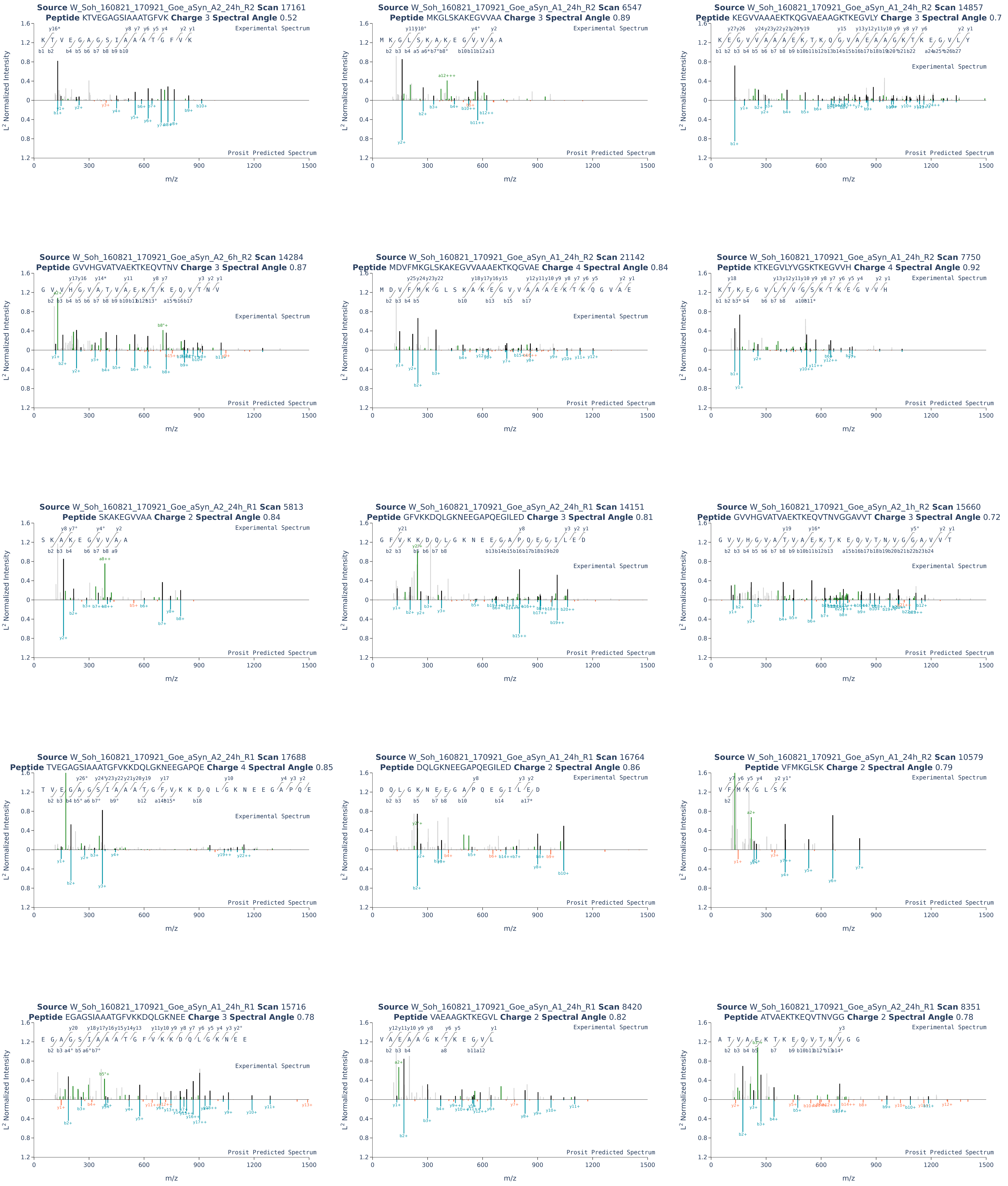

Source W_Soh_160821_170921_Goe_aSyn_A2_24h_R2 Scan 17161
Peptide KTVEGAGSIAAATGFVK Charge 3 Spectral Angle 0.52
Source W_Soh_160821_170921_Goe_aSyn_A1_24h_R2 Scan 6547
Peptide MKGLSKAKEGVVAA Charge 3 Spectral Angle 0.89
Source W_Soh_160821_170921_Goe_aSyn_A1_24h_R2 Scan 14857
Peptide KEGVVAAAEKTKQGVAEAAGKTKEGVLY Charge 3 Spectral Angle 0.7
Source W_Soh_160821_170921_Goe_aSyn_A2_6h_R2 Scan 14284
Peptide GVVHGVATVAEKTKEQVTNV Charge 3 Spectral Angle 0.87
Source W_Soh_160821_170921_Goe_aSyn_A1_24h_R2 Scan 21142
Peptide MDVFMKGLSKAKEGVVAAAEKTKQGVAE Charge 4 Spectral Angle 0.84
Source W_Soh_160821_170921_Goe_aSyn_A1_24h_R2 Scan 7750
Peptide KTKEGVLYVGSKTKEGVVH Charge 4 Spectral Angle 0.92
Source W_Soh_160821_170921_Goe_aSyn_A2_24h_R1 Scan 5813
Peptide SKAKEGVVAA Charge 2 Spectral Angle 0.84
Source W_Soh_160821_170921_Goe_aSyn_A2_24h_R1 Scan 14151
Peptide GFVKKDQLGKNEEGAPQEGILED Charge 3 Spectral Angle 0.81
Source W_Soh_160821_170921_Goe_aSyn_A2_1h_R2 Scan 15660
Peptide GVVHGVATVAEKTKEQVTNVGGAVVT Charge 3 Spectral Angle 0.72
Source W_Soh_160821_170921_Goe_aSyn_A2_24h_R1 Scan 17688
Peptide TVEGAGSIAAATGFVKKDQLGKNEEGAPQE Charge 4 Spectral Angle 0.85
Source W_Soh_160821_170921_Goe_aSyn_A1_24h_R1 Scan 16764
Peptide DQLGKNEEGAPQEGILED Charge 2 Spectral Angle 0.86
Source W_Soh_160821_170921_Goe_aSyn_A1_24h_R2 Scan 10579
Peptide VFMKGLSK Charge 2 Spectral Angle 0.79
Source W_Soh_160821_170921_Goe_aSyn_A1_24h_R1 Scan 15716
Peptide EGAGSIAAATGFVKKDQLGKNEE Charge 3 Spectral Angle 0.78
Source W_Soh_160821_170921_Goe_aSyn_A1_24h_R1 Scan 8420
Peptide VAEAAGKTKEGVL Charge 2 Spectral Angle 0.82
Source W_Soh_160821_170921_Goe_aSyn_A2_24h_R1 Scan 8351
Peptide ATVAEKTKEQVTNVGG Charge 2 Spectral Angle 0.78
Experimental Spectrum
Prosit Predicted Spectrum
L² Normalized Intensity
m/z

**Source** W_Soh_160821_170921_Goe_aSyn_A2_24h_R2 **Scan** 12479
**Peptide** TVAEKTKEQVTNVGGAVVTG **Charge** 3 **Spectral Angle** 0.83

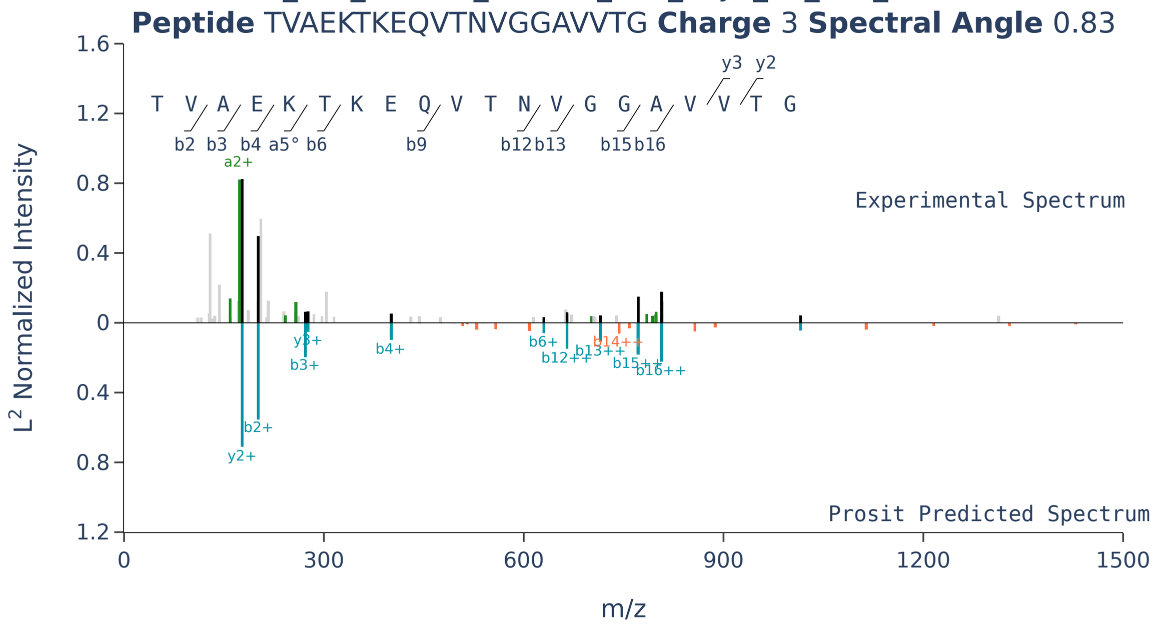


**Source** W_Soh_160821_170921_Goe_aSyn_A1_6h_R1 **Scan** 6336
**Peptide** KEGVVHGVA **Charge** 2 **Spectral Angle** 0.66

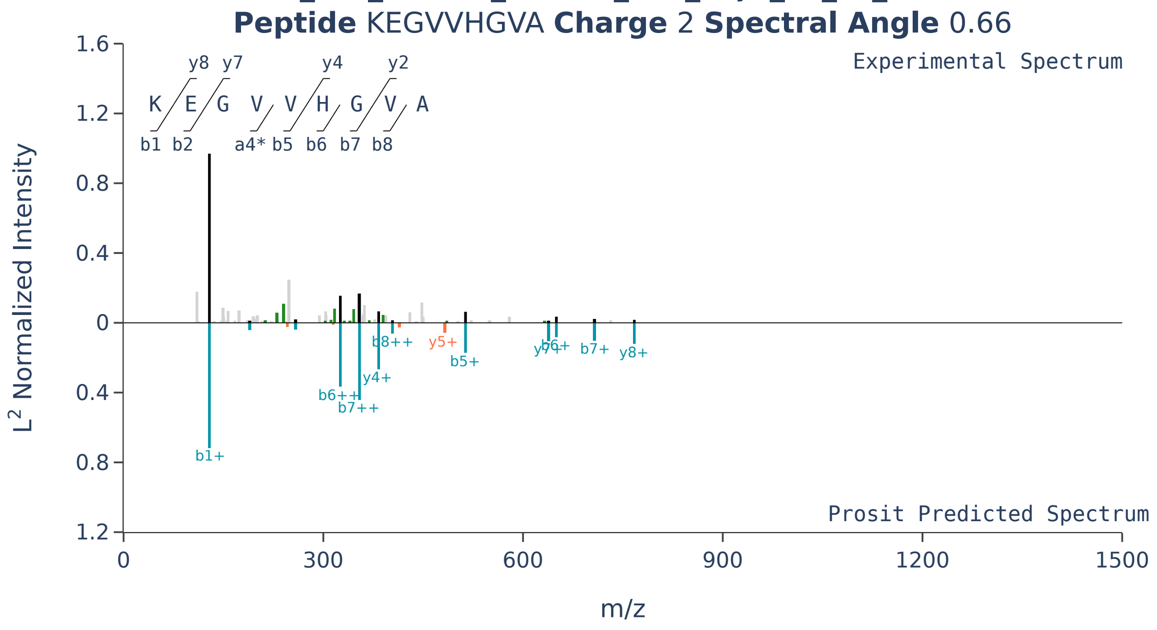


**Source** W_Soh_160821_170921_Goe_aSyn_A1_24h_R2 **Scan** 17931
**Peptide** KEGVVHGVATVAEKTKEQVTNVGGAVVTG **Charge** 4 **Spectral Angle** 0.82

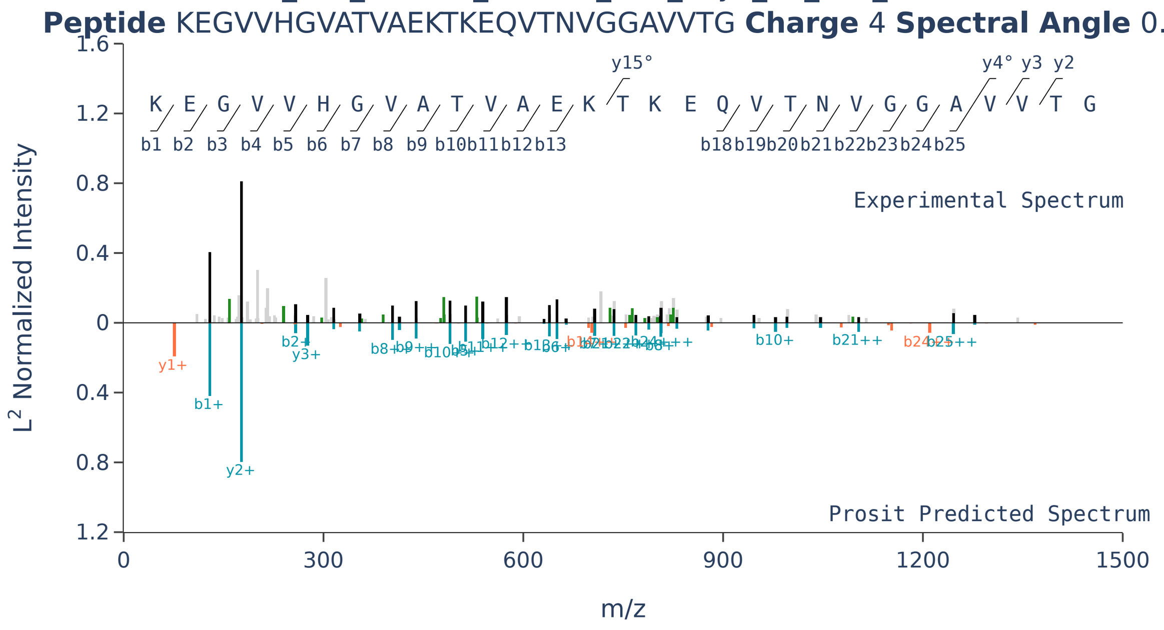


**Source** W_Soh_160821_170921_Goe_aSyn_A2_6h_R2 **Scan** 17218
**Peptide** ATVAEKTKEQVTNVGGAVVTGVTA **Charge** 3 **Spectral Angle** 0.87

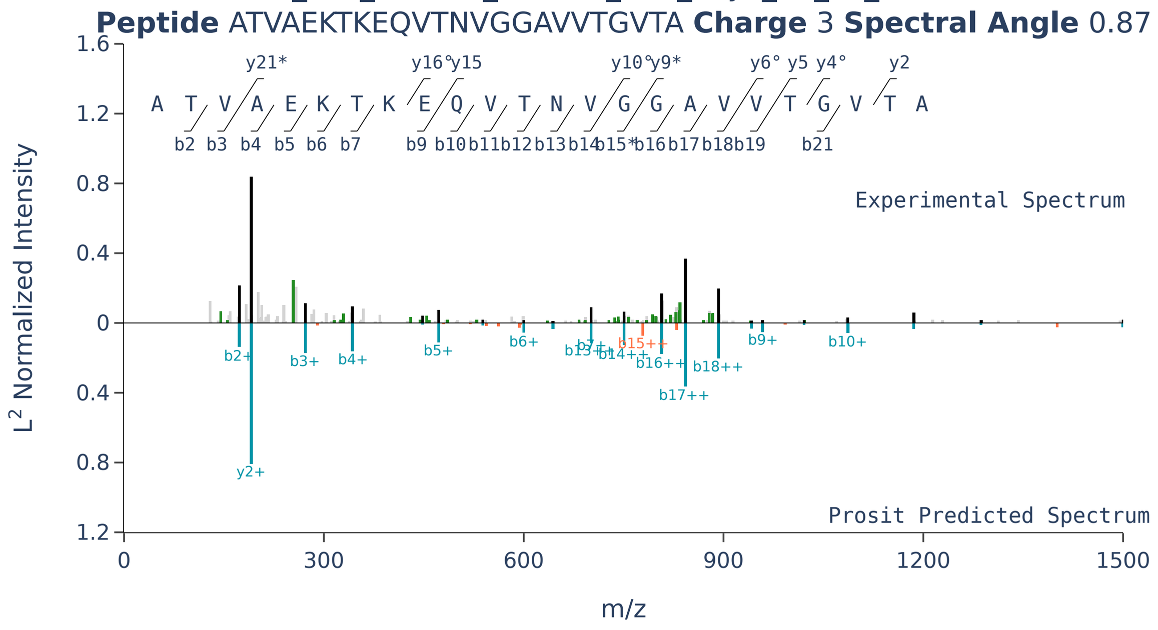


**Source** W_Soh_160821_170921_Goe_aSyn_A1_24h_R1 **Scan** 8918
**Peptide** SKAKEGVVAAAEKTKQGVAEAAGK **Charge** 4 **Spectral Angle** 0.83

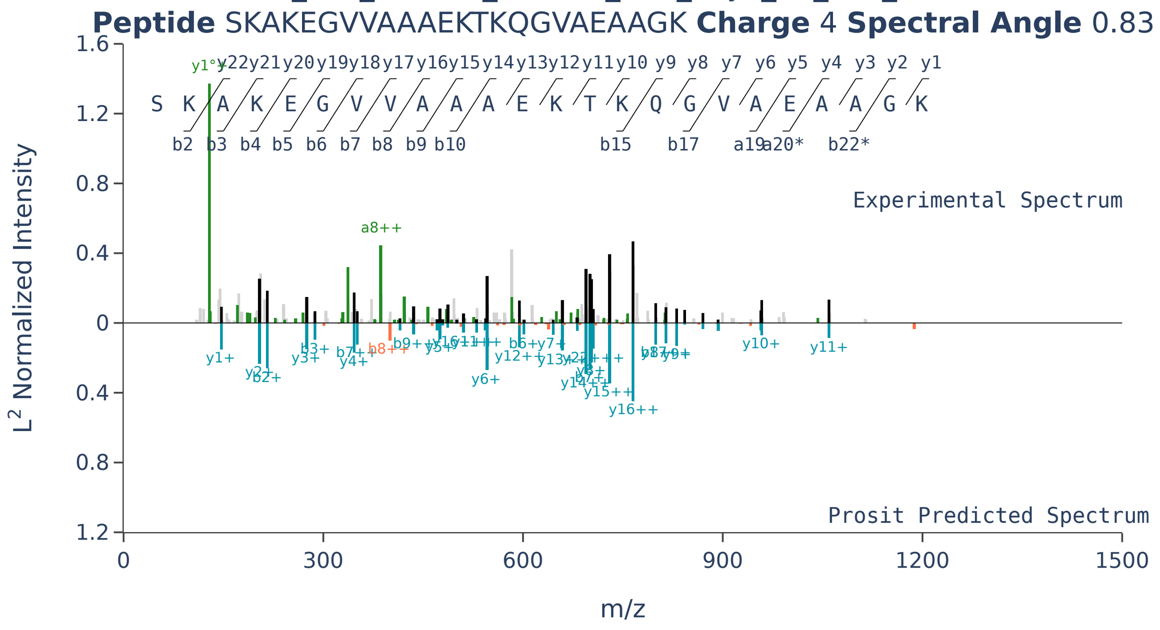


**Source** W_Soh_160821_170921_Goe_aSyn_A2_24h_R1 **Scan** 9878
**Peptide** VAEAAGKTKEGVLYVGSKTKEGVVH **Charge** 5 **Spectral Angle** 0.84

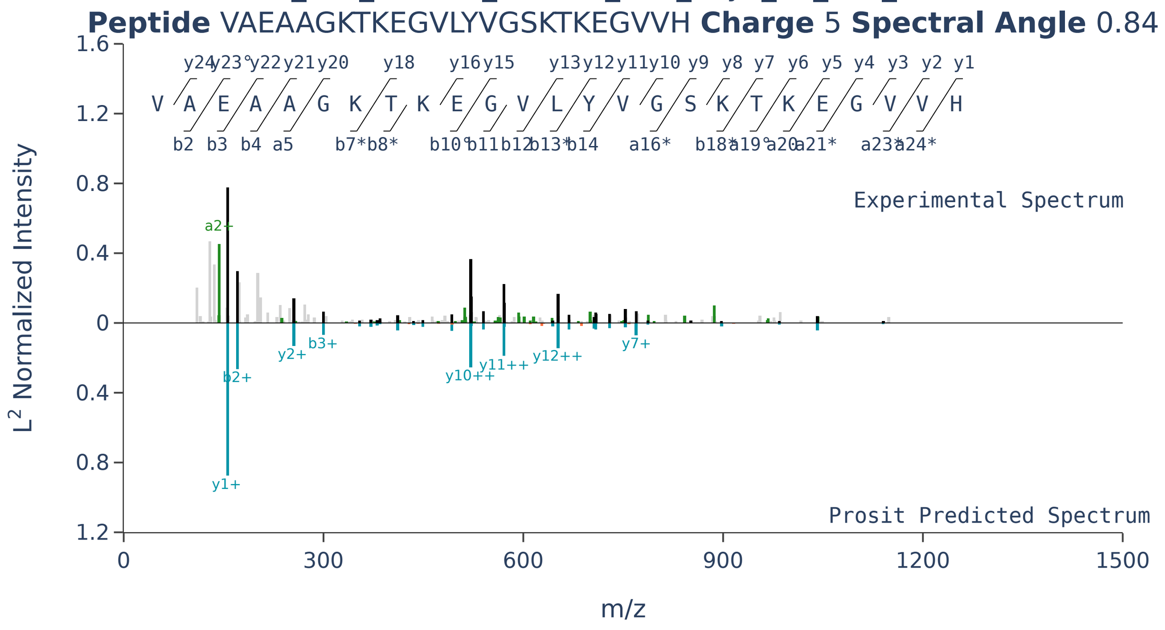


**Source** W_Soh_160821_170921_Goe_aSyn_A1_24h_R1 **Scan** 9662
**Peptide** KTKEQVTNVGGAVVT **Charge** 2 **Spectral Angle** 0.78

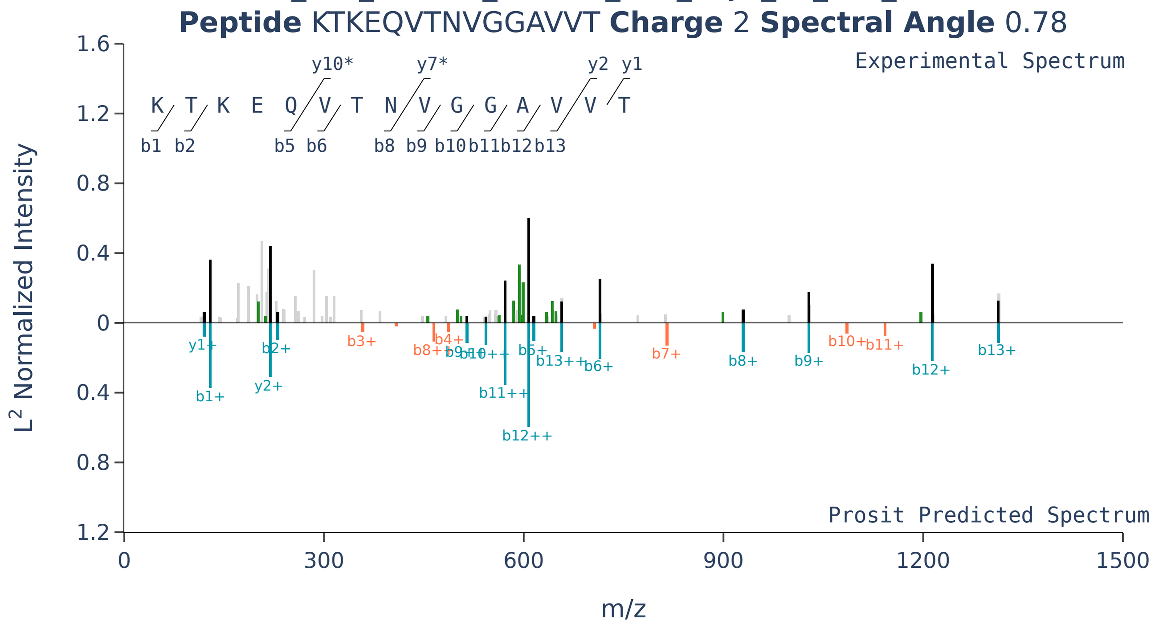


**Source** W_Soh_160821_170921_Goe_aSyn_A1_6h_R2 **Scan** 17245
**Peptide** EMPSEEGYQDYEPEA **Charge** 2 **Spectral Angle** 0.94

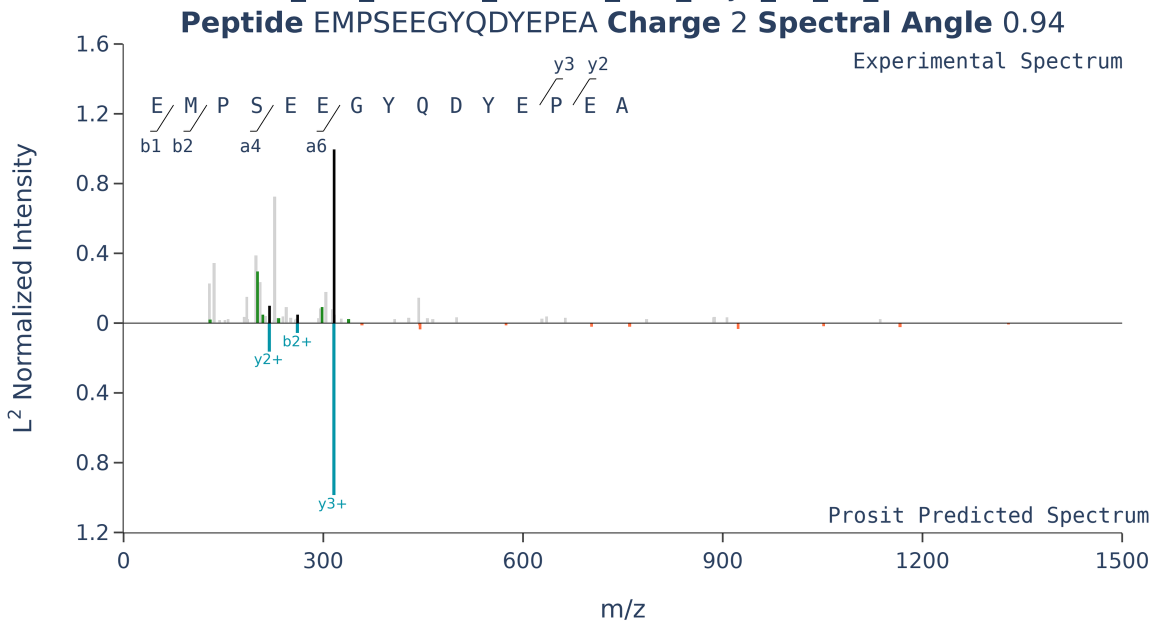


**Source** W_Soh_160821_170921_Goe_aSyn_A1_15'_R2 **Scan** 18475
**Peptide** VTNVGGAVVTGVTAV **Charge** 2 **Spectral Angle** 0.68

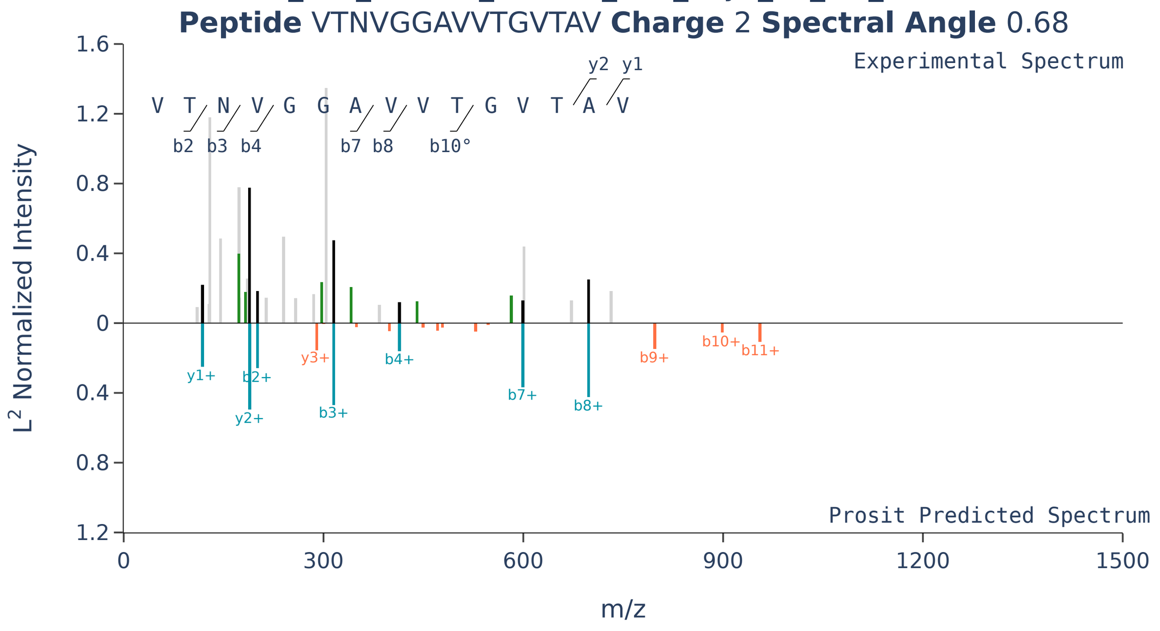


**Source** W_Soh_160821_170921_Goe_aSyn_A2_3h_R2 **Scan** 9131
**Peptide** QKTVEGAGSIAAA **Charge** 2 **Spectral Angle** 0.9

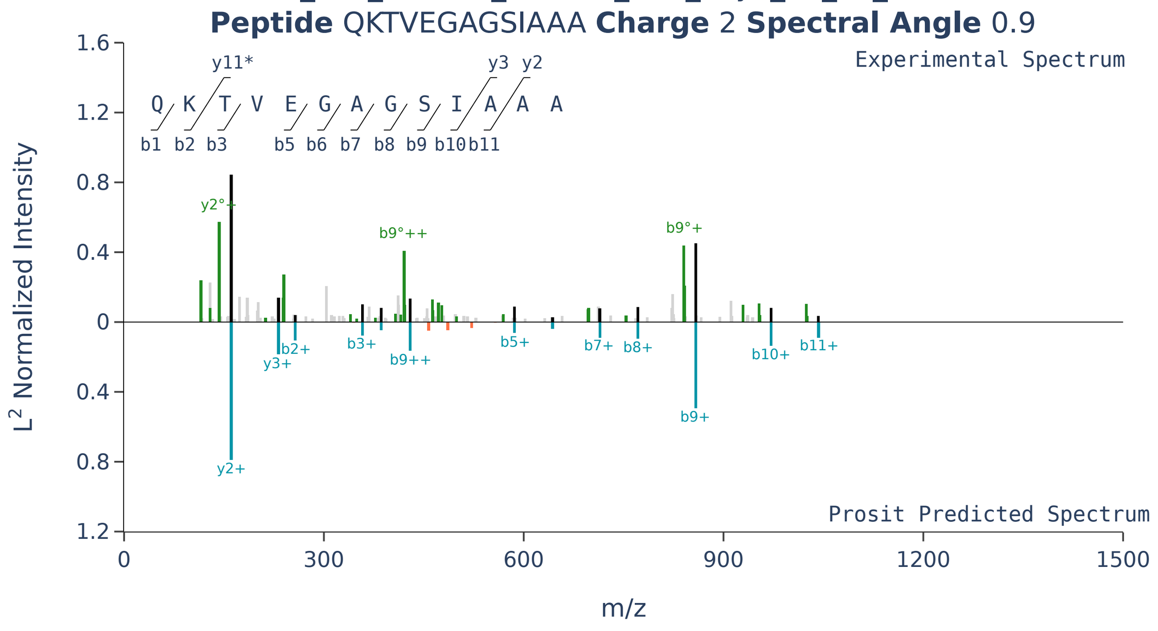


**Source** W_Soh_160821_170921_Goe_aSyn_A1_24h_R2 **Scan** 8195
**Peptide** AAGKTKEGVLYVGSKTKEGVVHG **Charge** 5 **Spectral Angle** 0.89

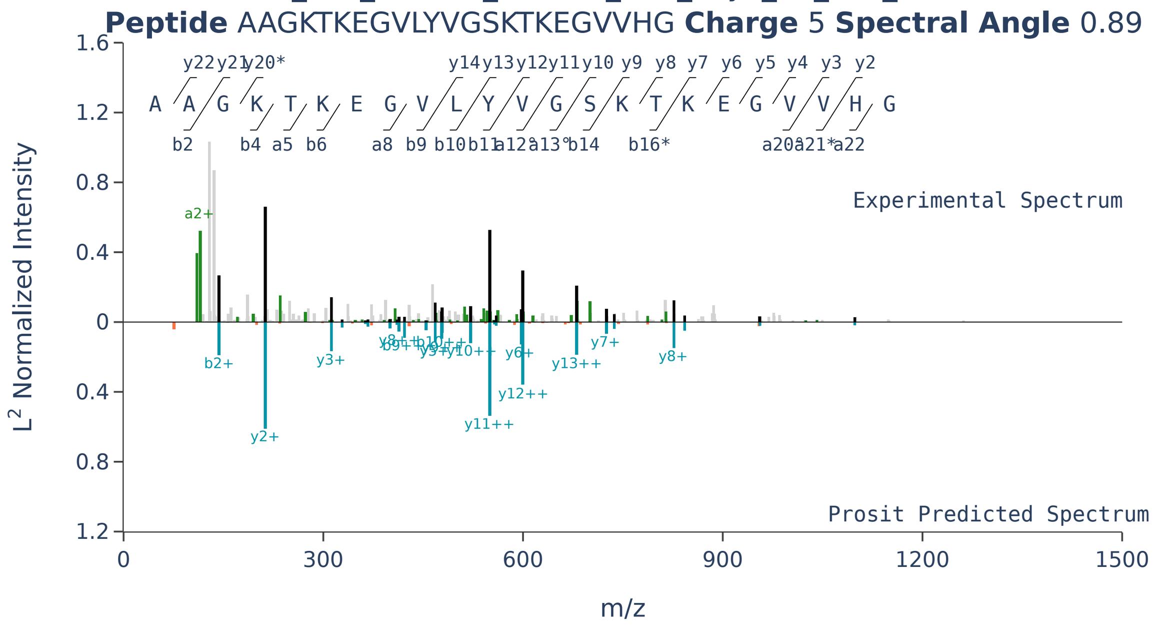


**Source** W_Soh_160821_170921_Goe_aSyn_A1_24h_R1 **Scan** 6500
**Peptide** GKTKEGVLYVGSKTK **Charge** 3 **Spectral Angle** 0.83

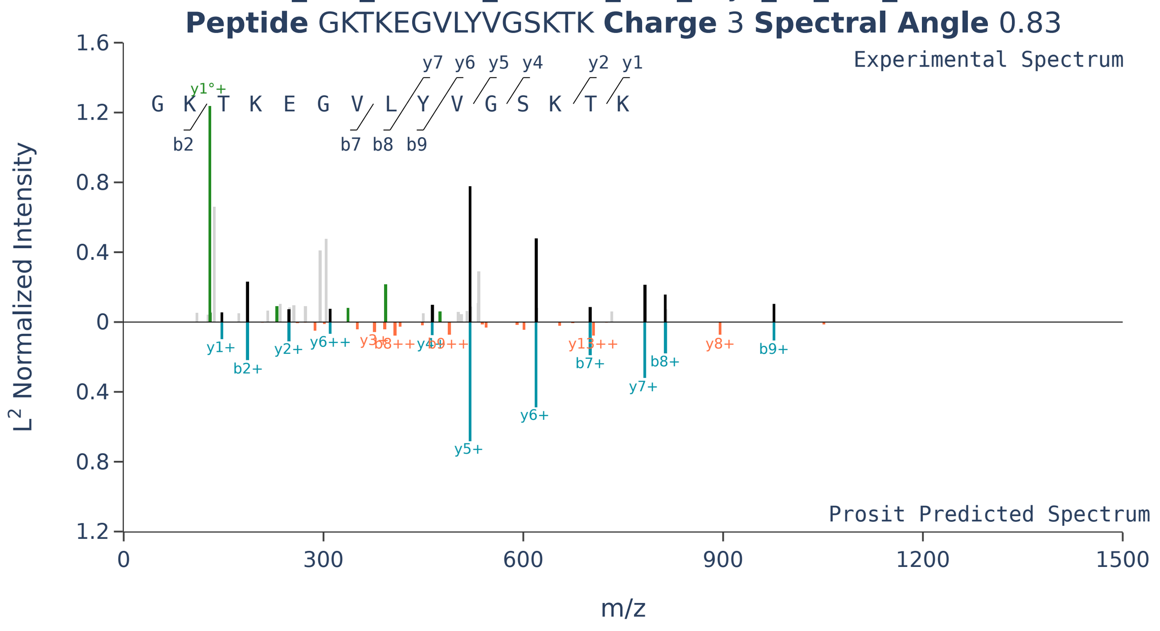


**Source** W_Soh_160821_170921_Goe_aSyn_A2_24h_R1 **Scan** 6580
**Peptide** VATVAEKTKEQVTN **Charge** 2 **Spectral Angle** 0.87

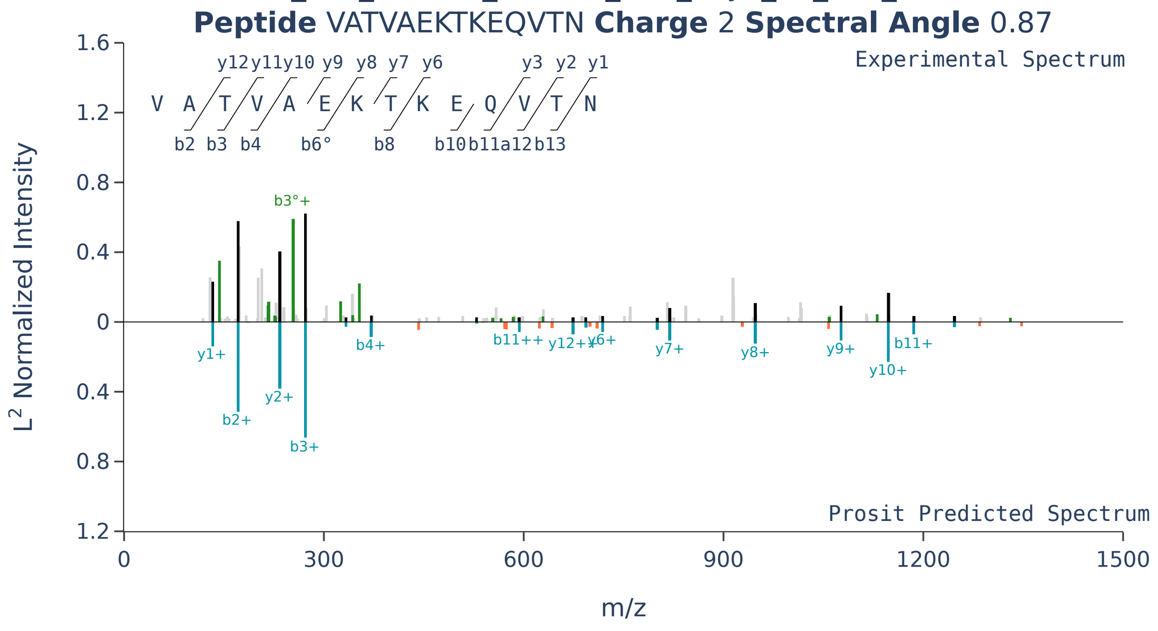


**Source** W_Soh_160821_170921_Goe_aSyn_A1_24h_R2 **Scan** 10453
**Peptide** KTKEGVVHGVATVAEKTKEQVTN **Charge** 3 **Spectral Angle** 0.82

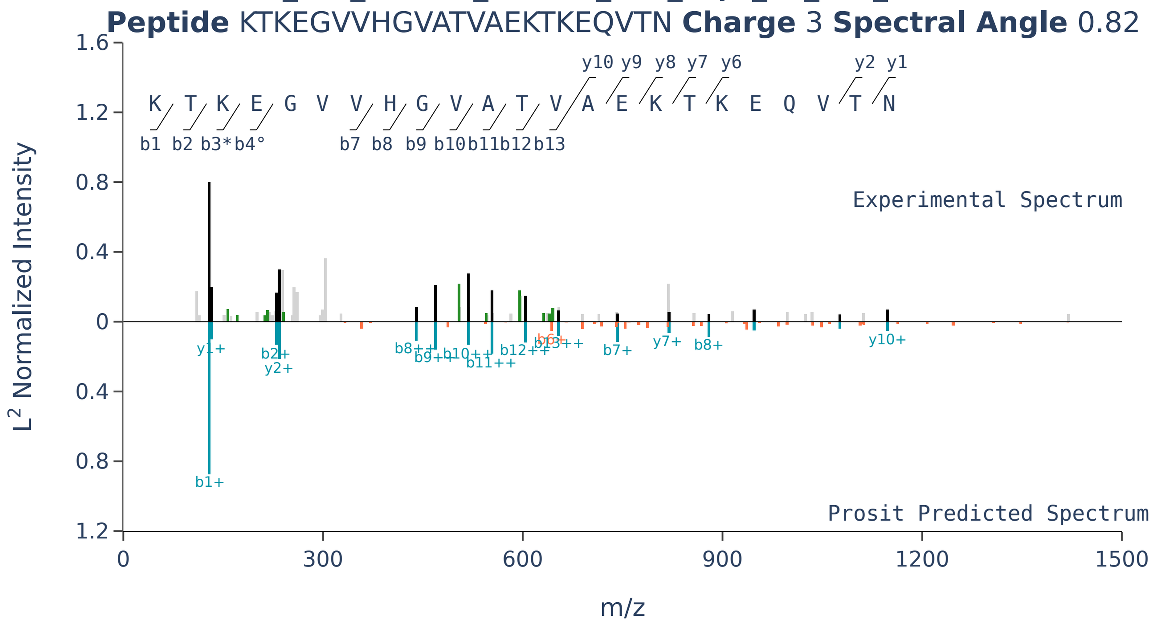


**Source** W_Soh_160821_170921_Goe_aSyn_A1_24h_R2 **Scan** 10604
**Peptide** MKGLSKAKEGVVAAAEKTKQGVAE **Charge** 4 **Spectral Angle** 0.74

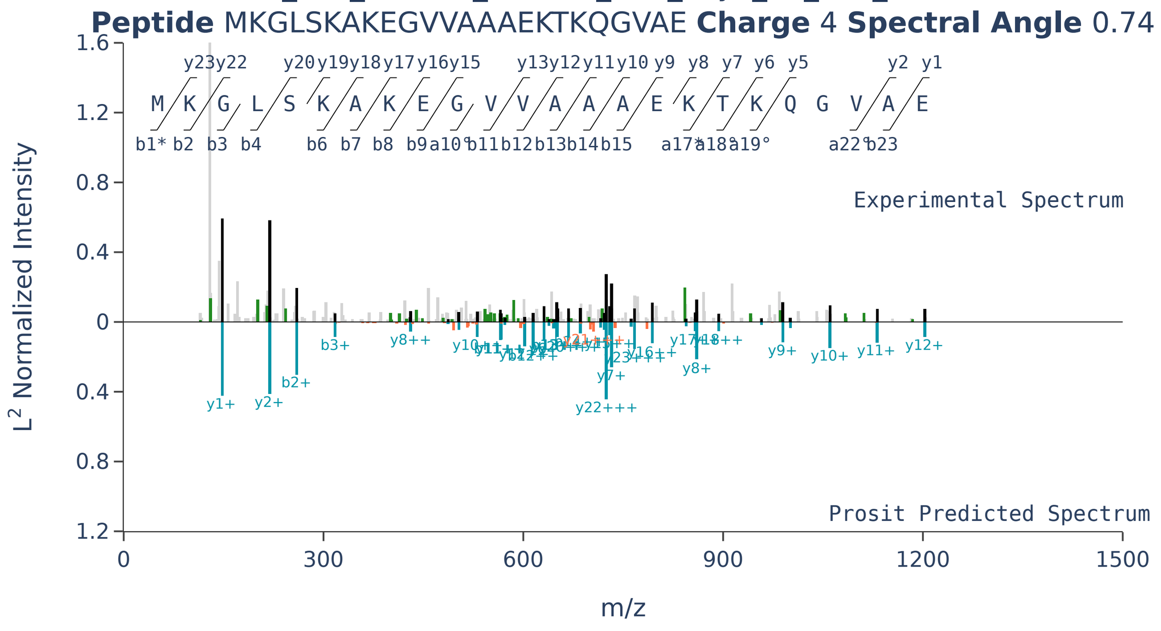

**Source** W_Soh_160821_170921_Goe_aSyn_A2_24h_R2 **Scan** 7109
**Peptide** GLSKAKEGVVAAAEK **Charge** 3 **Spectral Angle** 0.81

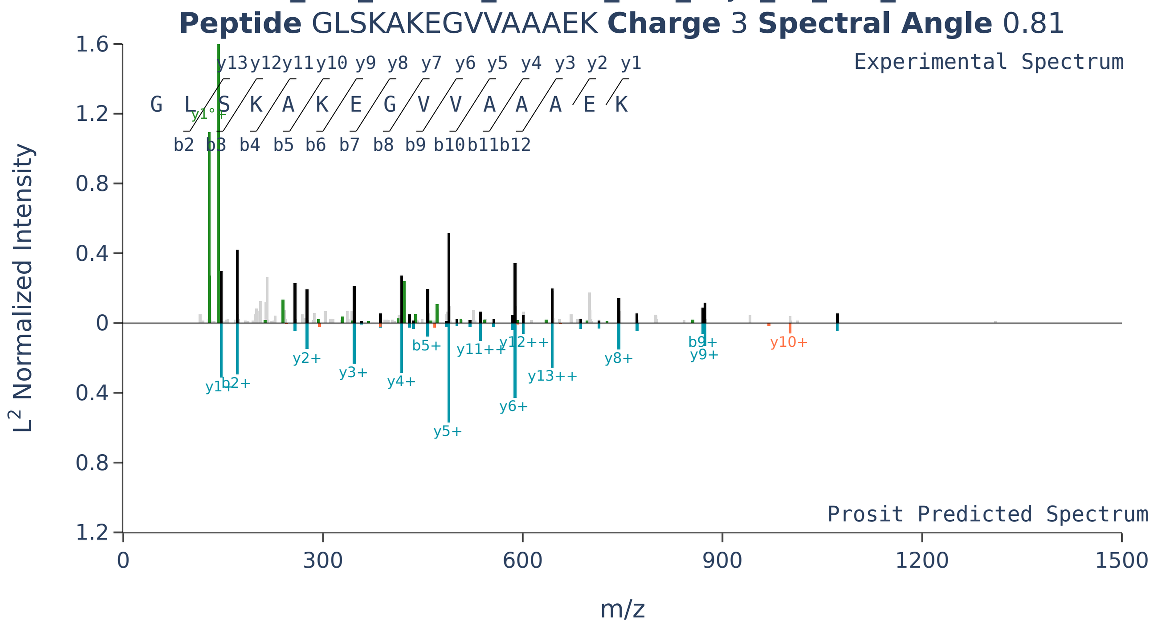


**Source** W_Soh_160821_170921_Goe_aSyn_A2_6h_R1 **Scan** 20901
**Peptide** GLLEDMPVDPDNEAYE **Charge** 2 **Spectral Angle** 0.84

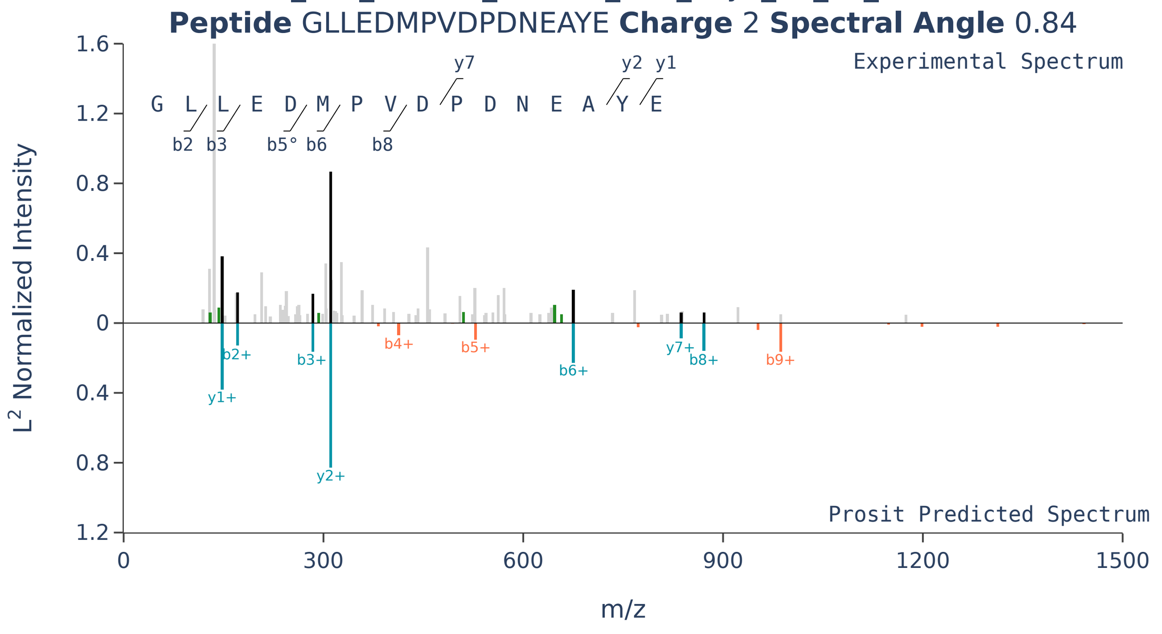


**Source** W_Soh_160821_170921_Goe_aSyn_A1_3h_R1 **Scan** 19613
**Peptide** TKEQVTNVGGAVVTGVTAV **Charge** 3 **Spectral Angle** 0.72

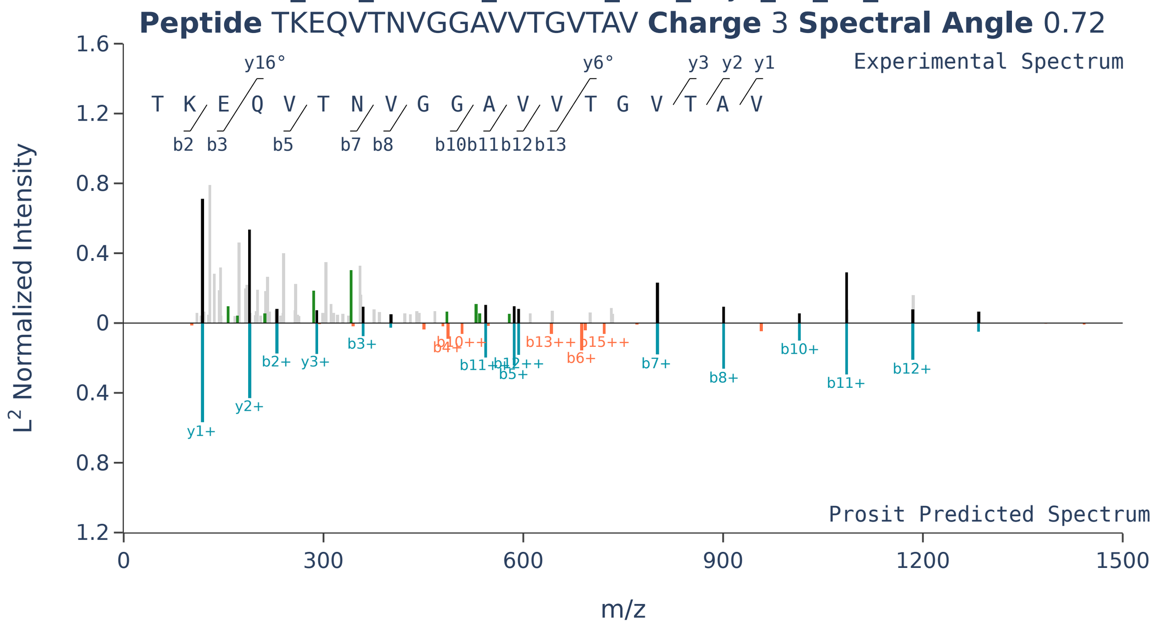


**Source** W_Soh_160821_170921_Goe_aSyn_A2_24h_R1 **Scan** 21498
**Peptide** TNVGGAVVTGVTAVAQ **Charge** 2 **Spectral Angle** 0.89

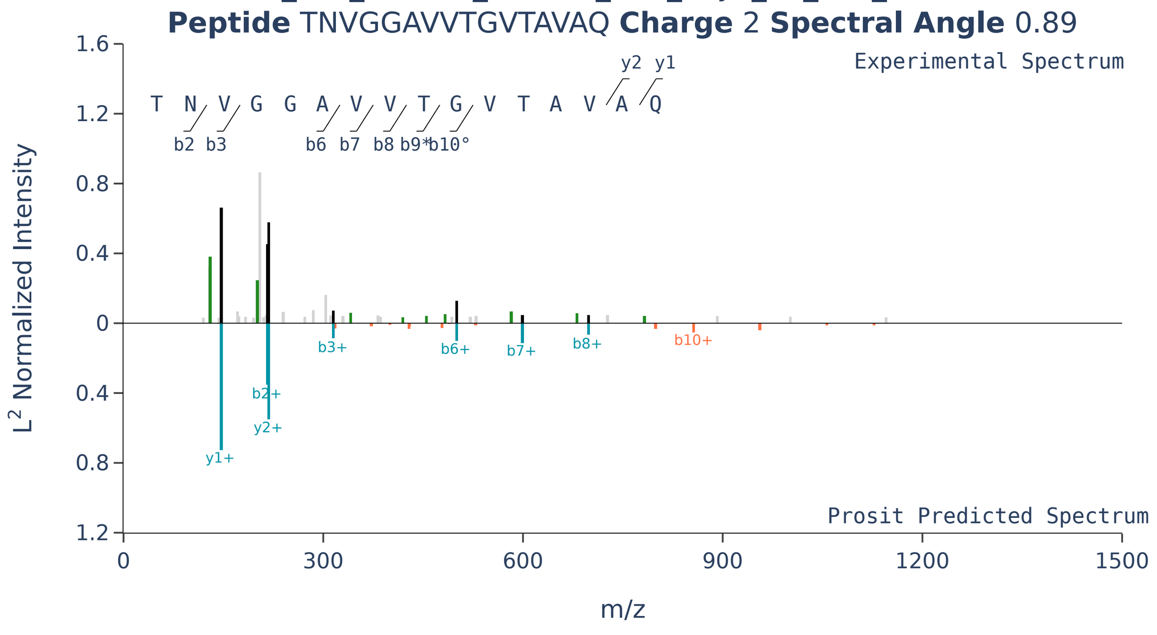


**Source** W_Soh_160821_170921_Goe_aSyn_A1_24h_R1 **Scan** 15434
**Peptide** VATVAEKTKEQVTNVGGAVV **Charge** 2 **Spectral Angle** 0.81

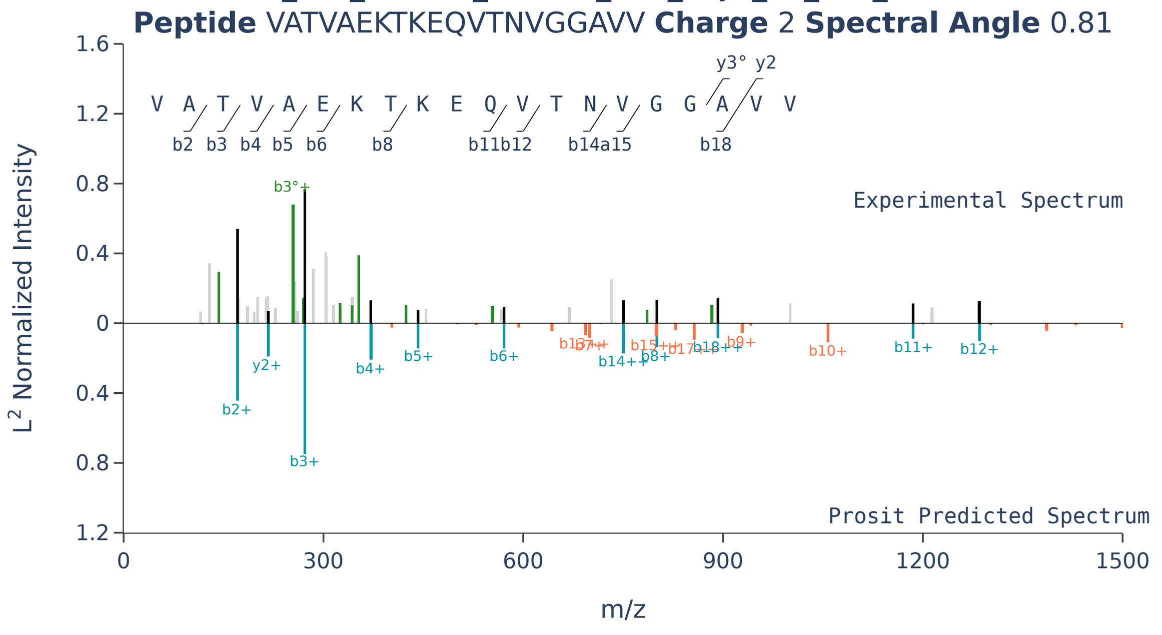


**Source** W_Soh_160821_170921_Goe_aSyn_A1_24h_R2 **Scan** 10180
**Peptide** KQGVAEAAGKTKEGVLYVGSKT **Charge** 4 **Spectral Angle** 0.9

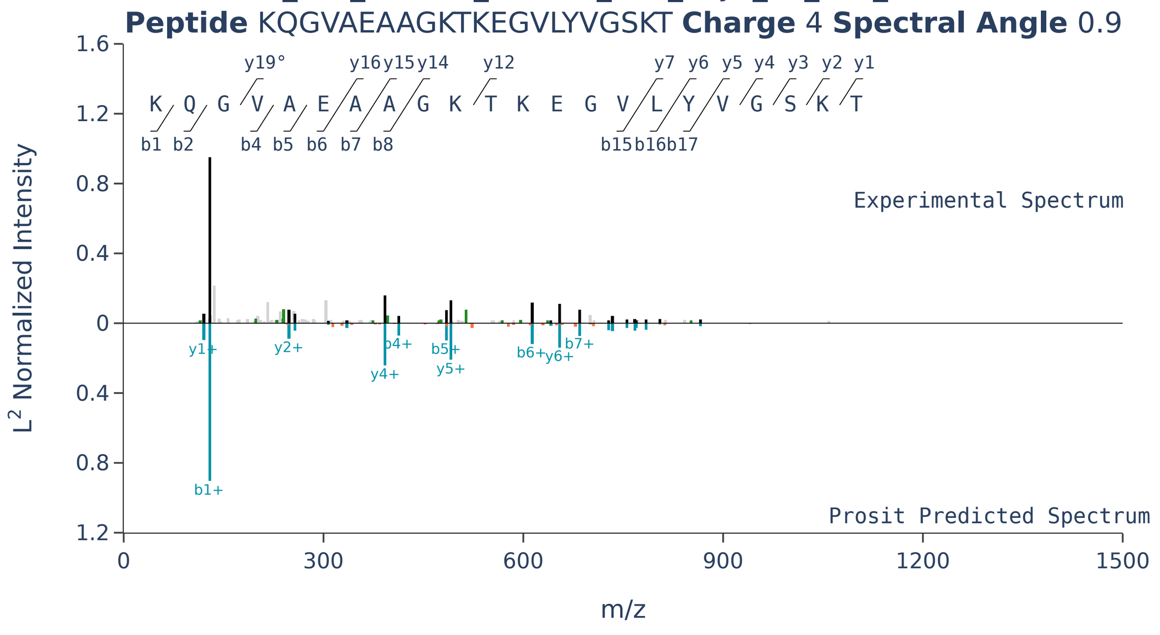


**Source** W_Soh_160821_170921_Goe_aSyn_A2_6h_R2 **Scan** 12066
**Peptide** GVAEAAGKTKEGVLYVGSKTKEGVVHG **Charge** 4 **Spectral Angle** 0.84

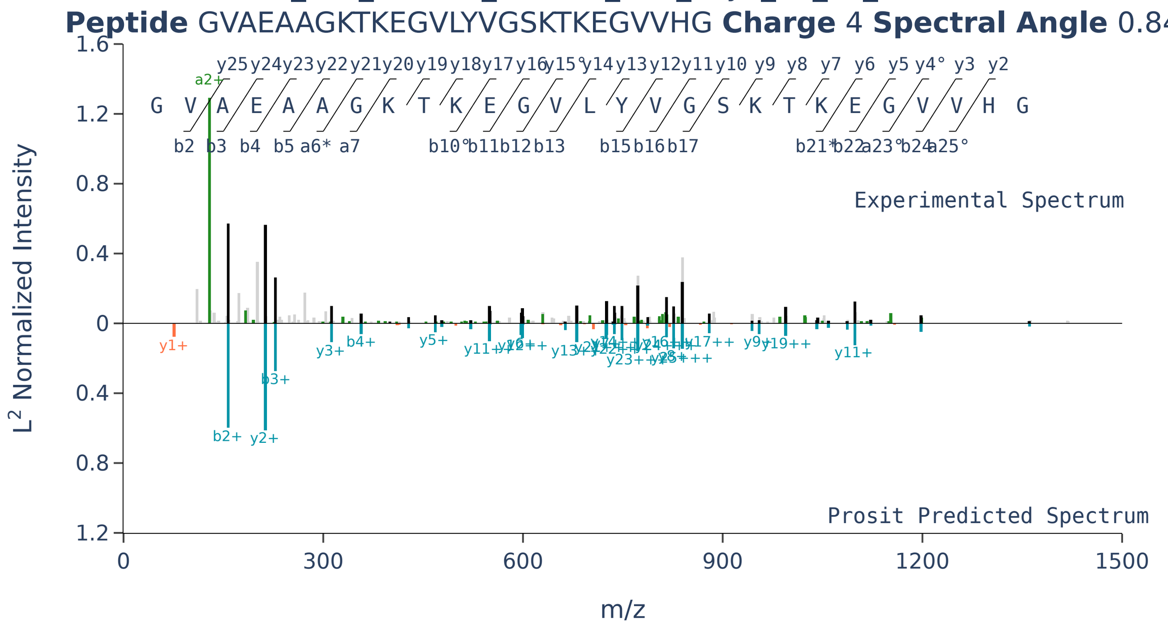


**Source** W_Soh_160821_170921_Goe_aSyn_A1_24h_R1 **Scan** 13872
**Peptide** AEKTKEQVTNVGGAVVTGVT **Charge** 3 **Spectral Angle** 0.88

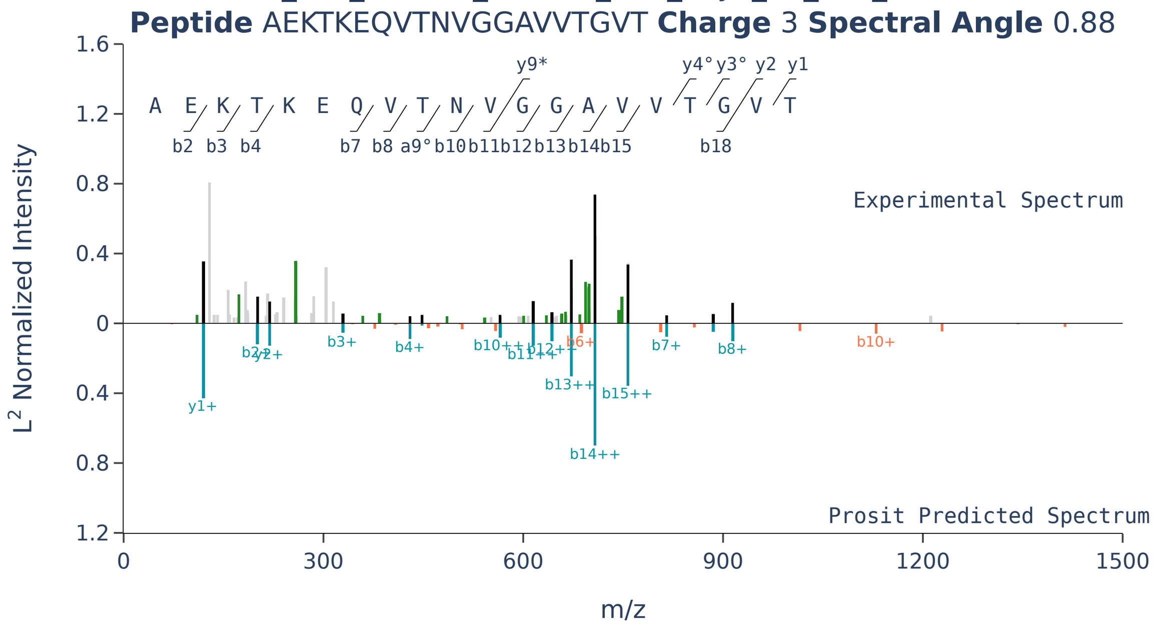


**Source** W_Soh_160821_170921_Goe_aSyn_A1_24h_R2 **Scan** 10152
**Peptide** YVGSKTKEGVVHGVATVAE **Charge** 3 **Spectral Angle** 0.88

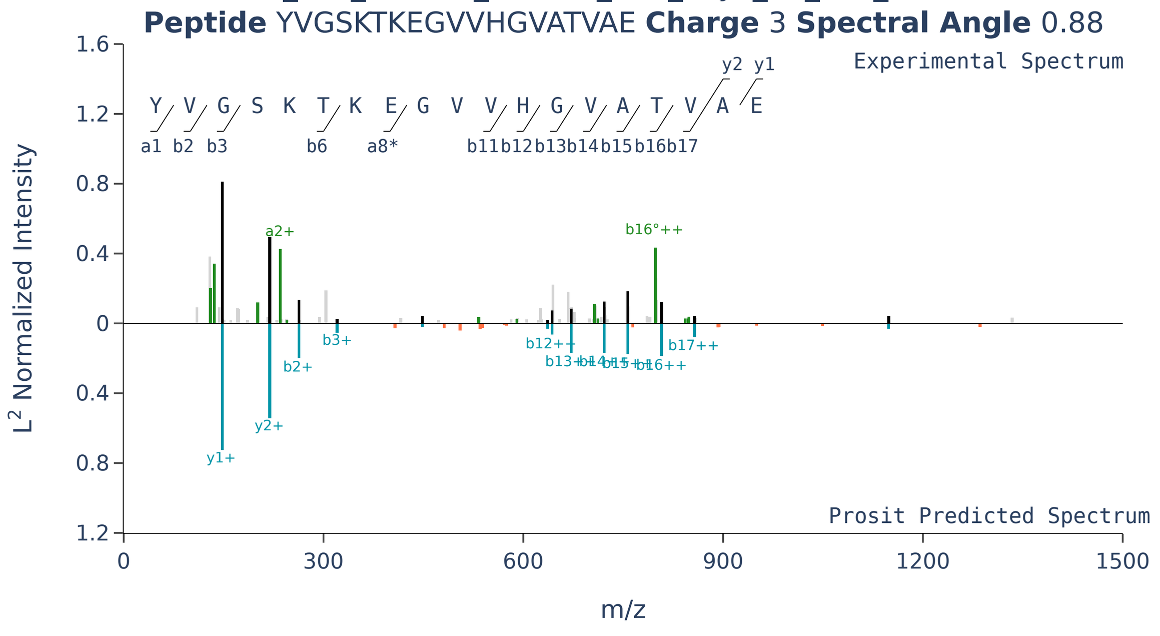


**Source** W_Soh_160821_170921_Goe_aSyn_A1_24h_R2 **Scan** 6801
**Peptide** TGFVKKDQLGK **Charge** 3 **Spectral Angle** 0.73

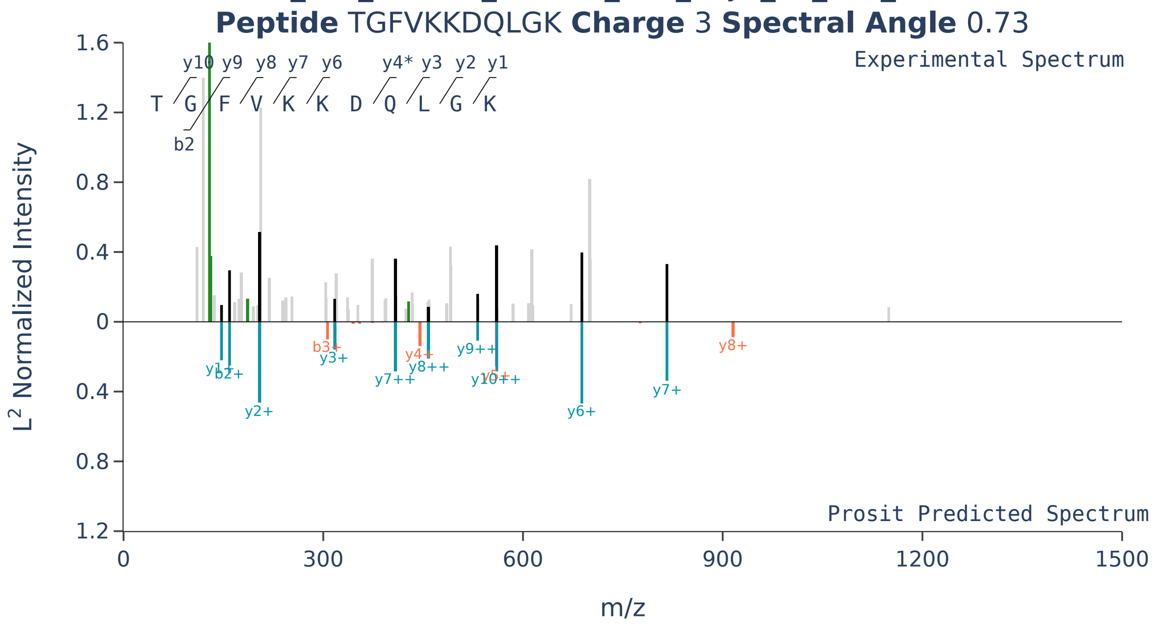


**Source** W_Soh_160821_170921_Goe_aSyn_A1_24h_R2 **Scan** 7403
**Peptide** KEGVVAAAEKTKQGVA **Charge** 3 **Spectral Angle** 0.8

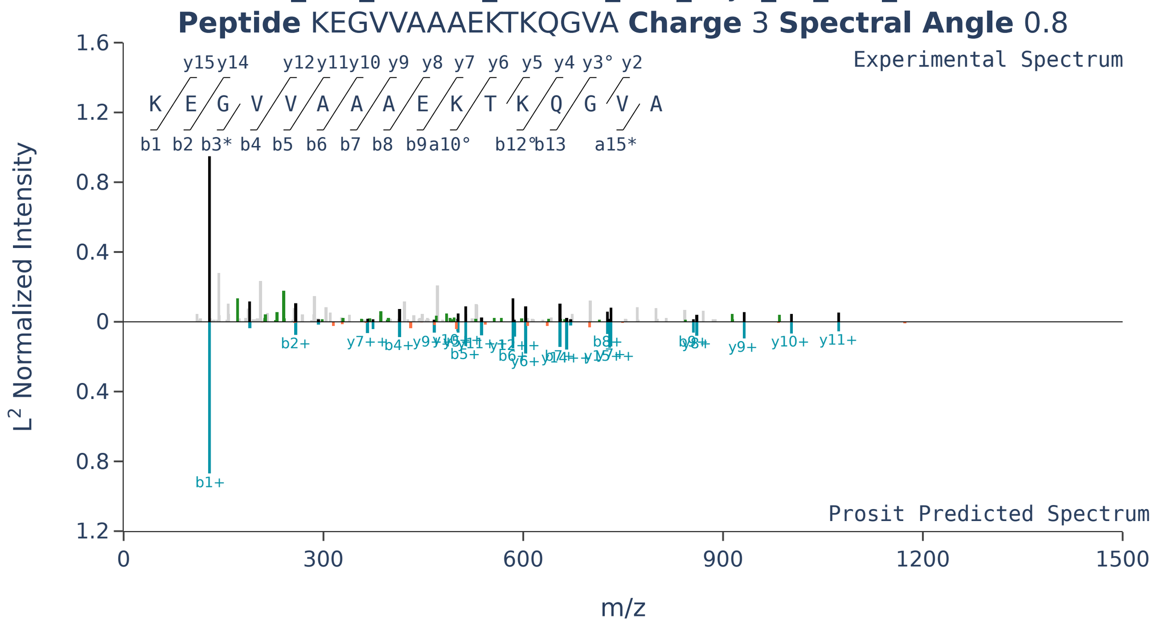


**Source** W_Soh_160821_170921_Goe_aSyn_A2_6h_R1 **Scan** 17036
**Peptide** HGVATVAEKTKEQVTNVGGAVVTGV **Charge** 3 **Spectral Angle** 0.74

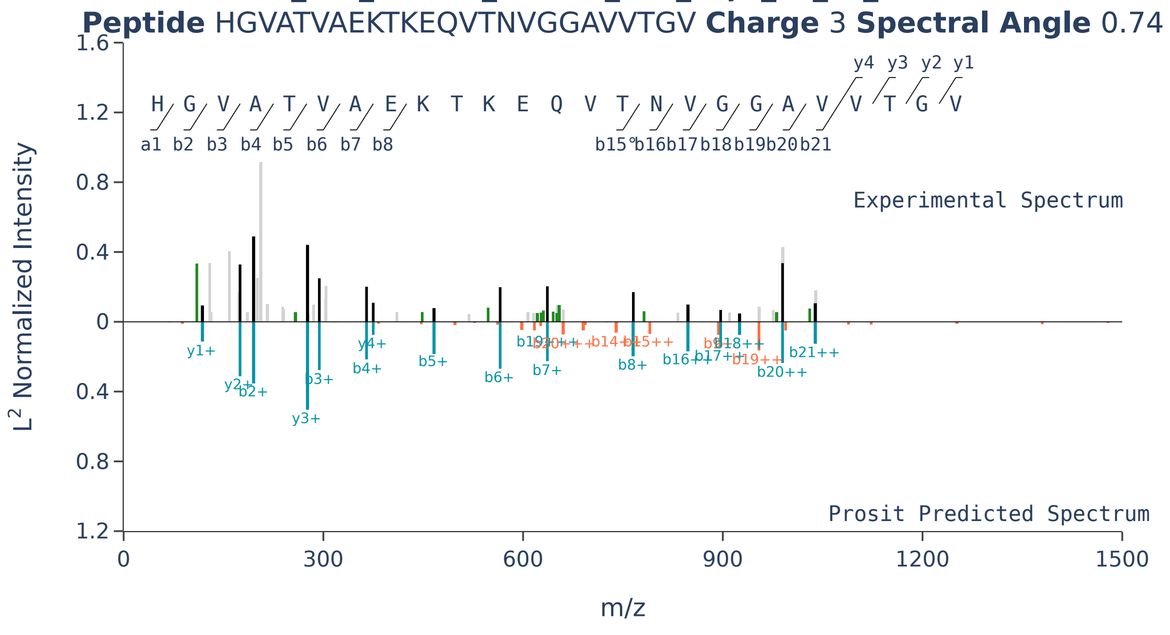


**Source** W_Soh_160821_170921_Goe_aSyn_A1_24h_R1 **Scan** 7056
**Peptide** YVGSKTKEGVV **Charge** 3 **Spectral Angle** 0.86

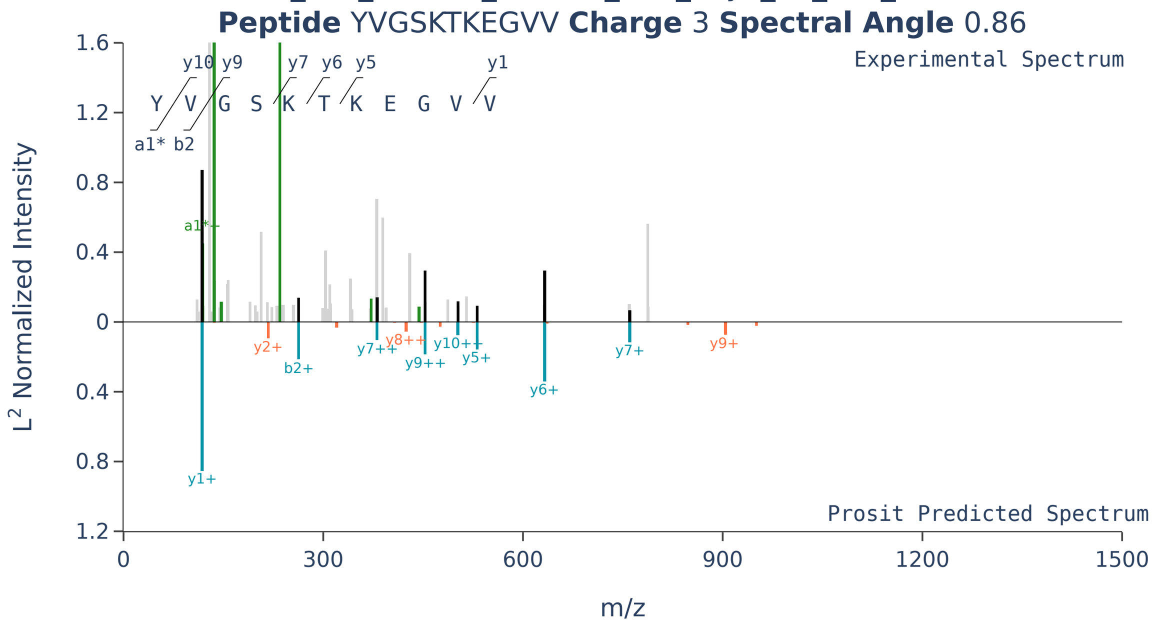


**Source** W_Soh_160821_170921_Goe_aSyn_A1_24h_R2 **Scan** 19156
**Peptide** MDVFMKGLSKAKEGVVAAAEKTKQG **Charge** 4 **Spectral Angle** 0.88

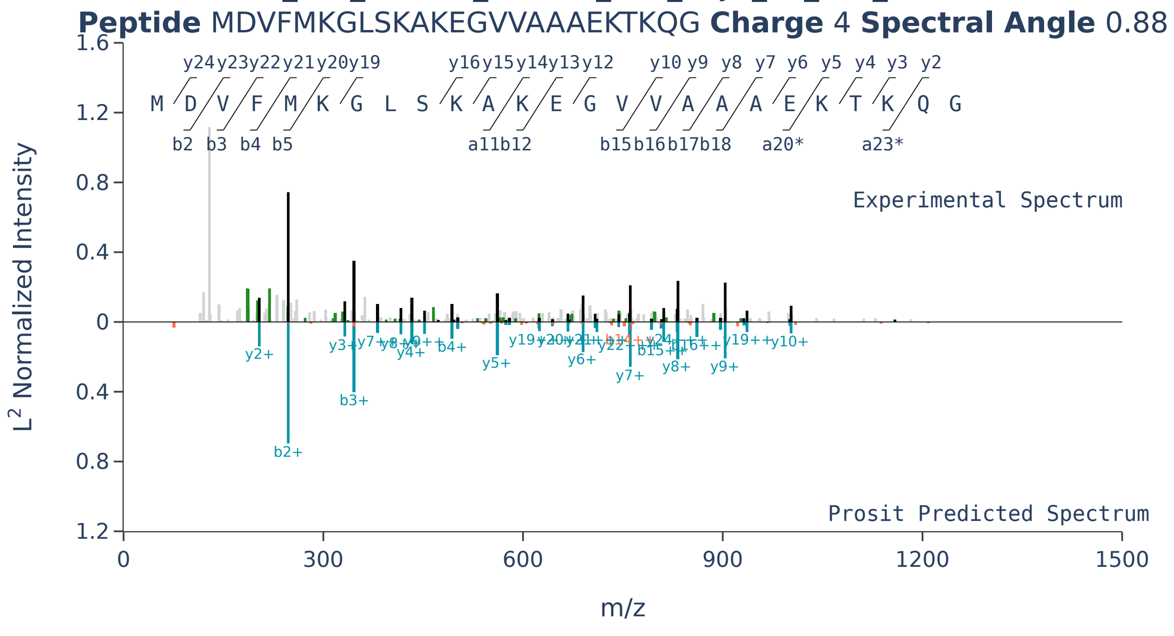


**Source** W_Soh_160821_170921_Goe_aSyn_A1_24h_R1 **Scan** 11163
**Peptide** AEKTKQGVAEAAGKTKEGVLYV **Charge** 3 **Spectral Angle** 0.82

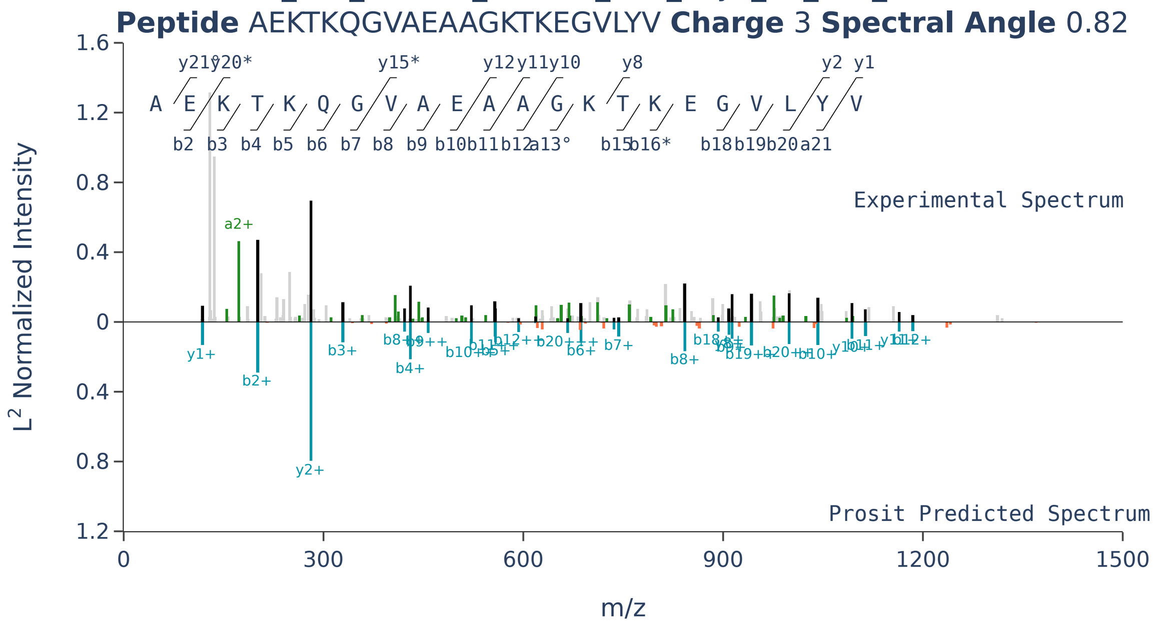

**Source** W_Soh_160821_170921_Goe_aSyn_A1_24h_R1 **Scan** 18124
**Peptide** TGVTAVAQKTVEGAGSI **Charge** 2 **Spectral Angle** 0.79

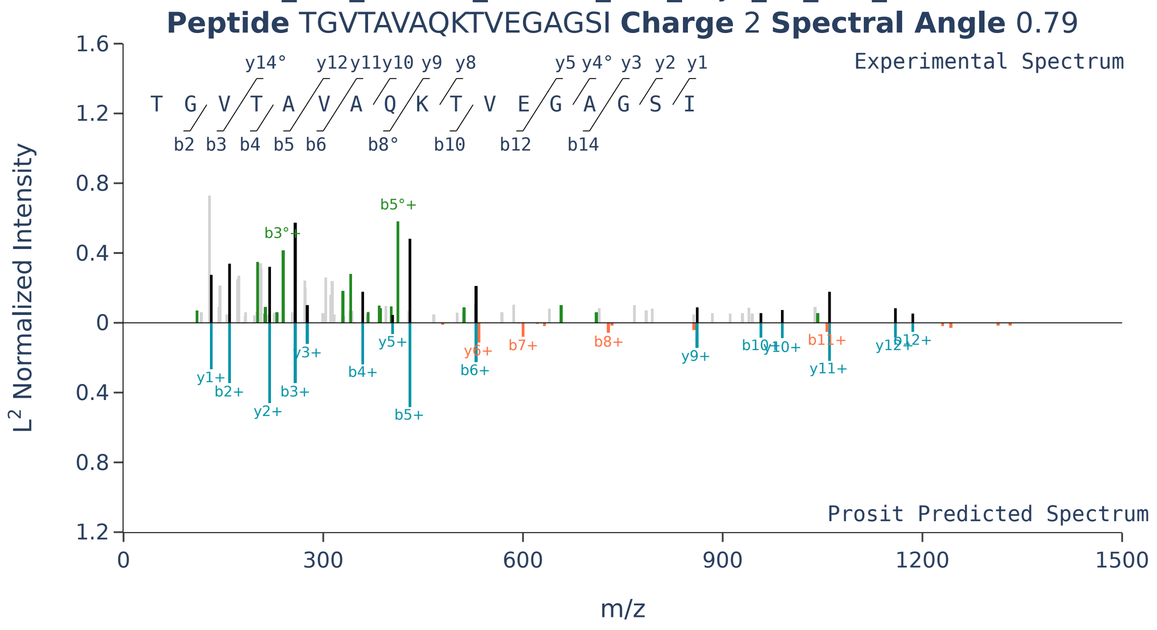


**Source** W_Soh_160821_170921_Goe_aSyn_A1_6h_R2 **Scan** 18715
**Peptide** AEKTKEQVTNVGGAVVTGVTAVA **Charge** 3 **Spectral Angle** 0.84

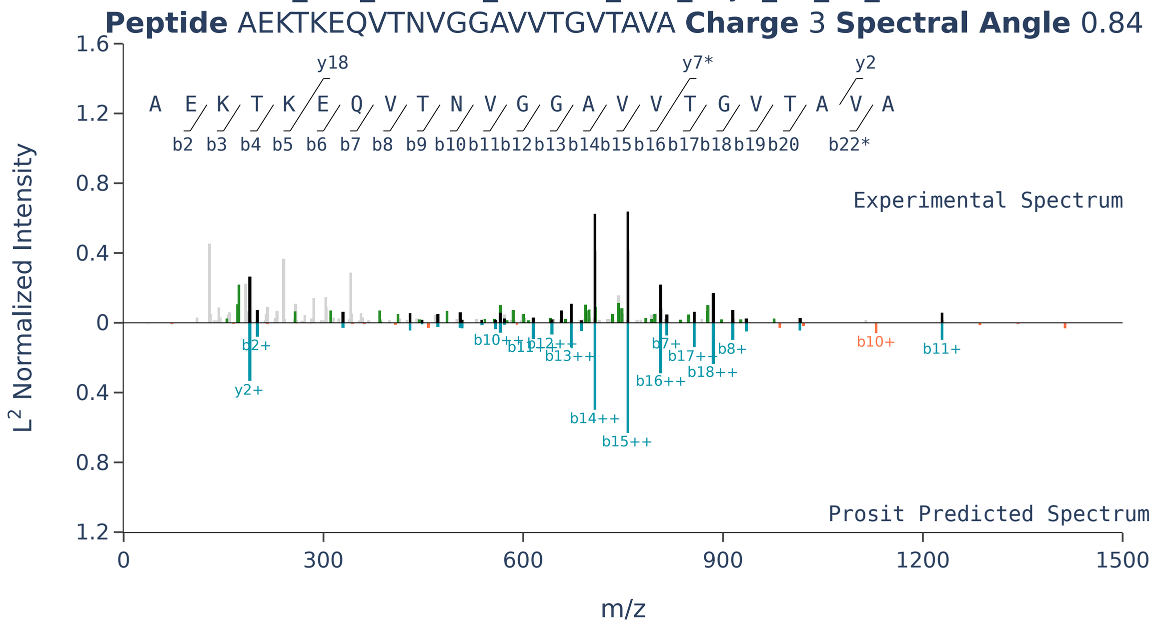


**Source** W_Soh_160821_170921_Goe_aSyn_A1_6h_R1 **Scan** 26023
**Peptide** QVTNVGGAVVTGVTAVAQKTVE **Charge** 2 **Spectral Angle** 0.79

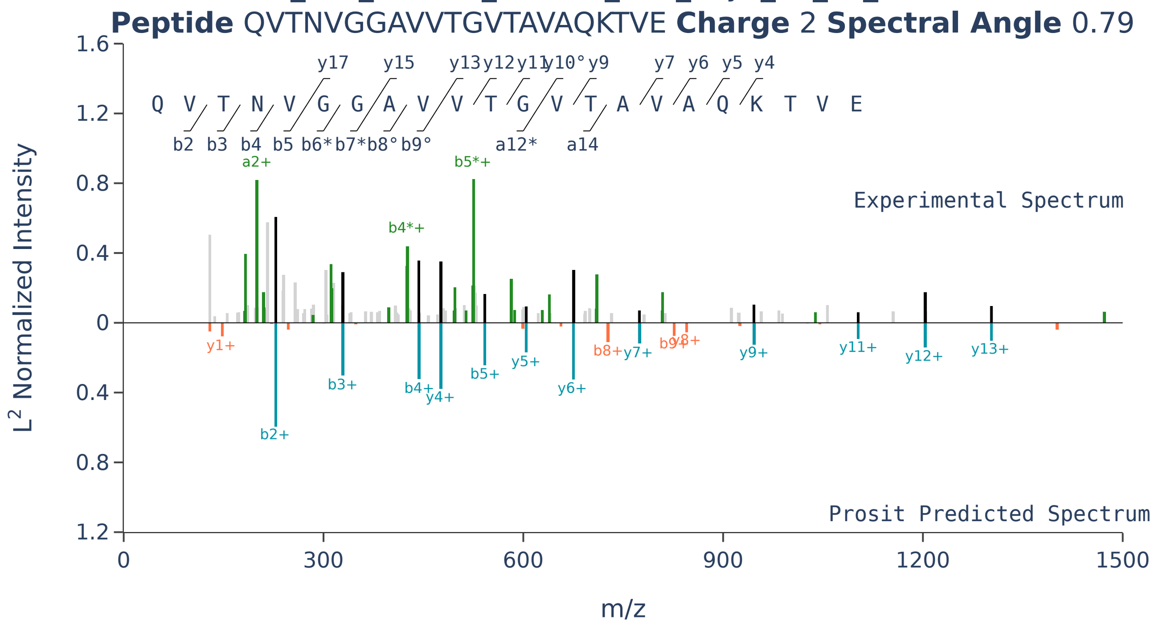


**Source** W_Soh_160821_170921_Goe_aSyn_A2_24h_R2 **Scan** 10037
**Peptide** AGKTKEGVLYVGSKTKEGVVHGV **Charge** 4 **Spectral Angle** 0.84

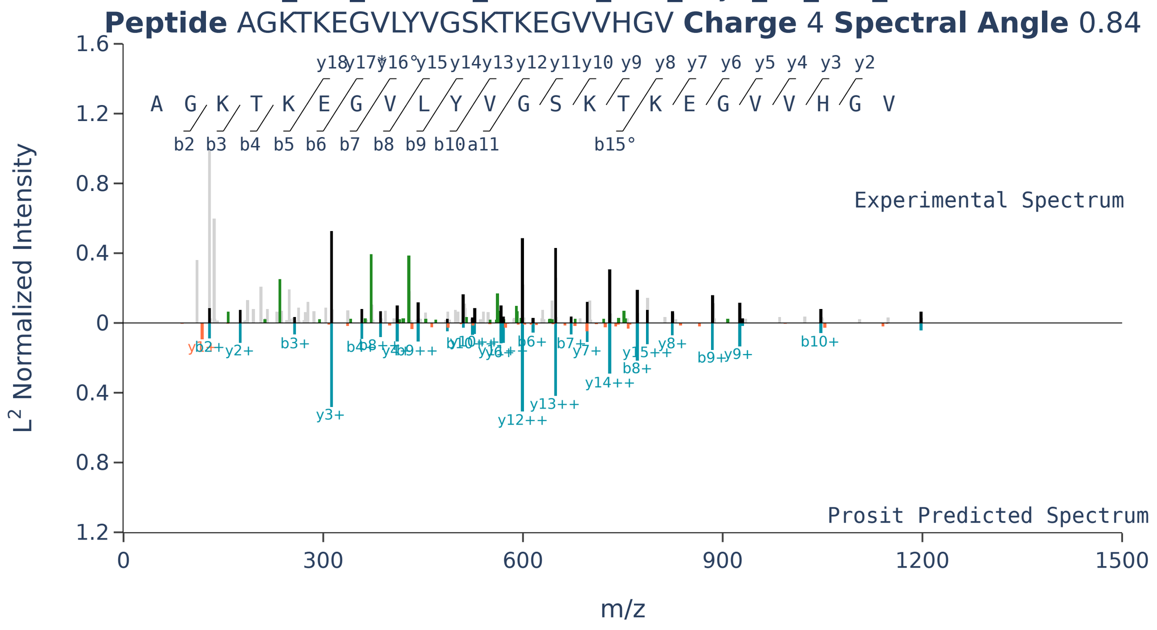


**Source** W_Soh_160821_170921_Goe_aSyn_A2_6h_R1 **Scan** 19737
**Peptide** TGFVKKDQLGKNEEGAPQEGILEDMPVD **Charge** 4 **Spectral Angle** 0.81

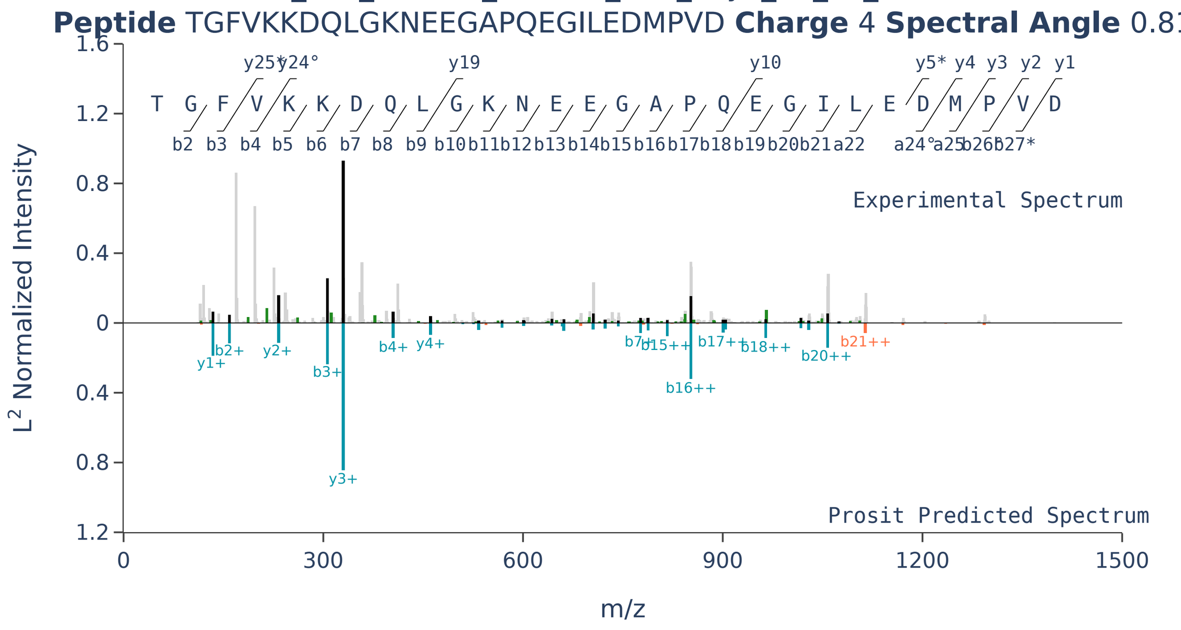


**Source** W_Soh_160821_170921_Goe_aSyn_A1_6h_R1 **Scan** 8142
**Peptide** AAATGFVKKDQLGKNEE **Charge** 2 **Spectral Angle** 0.78

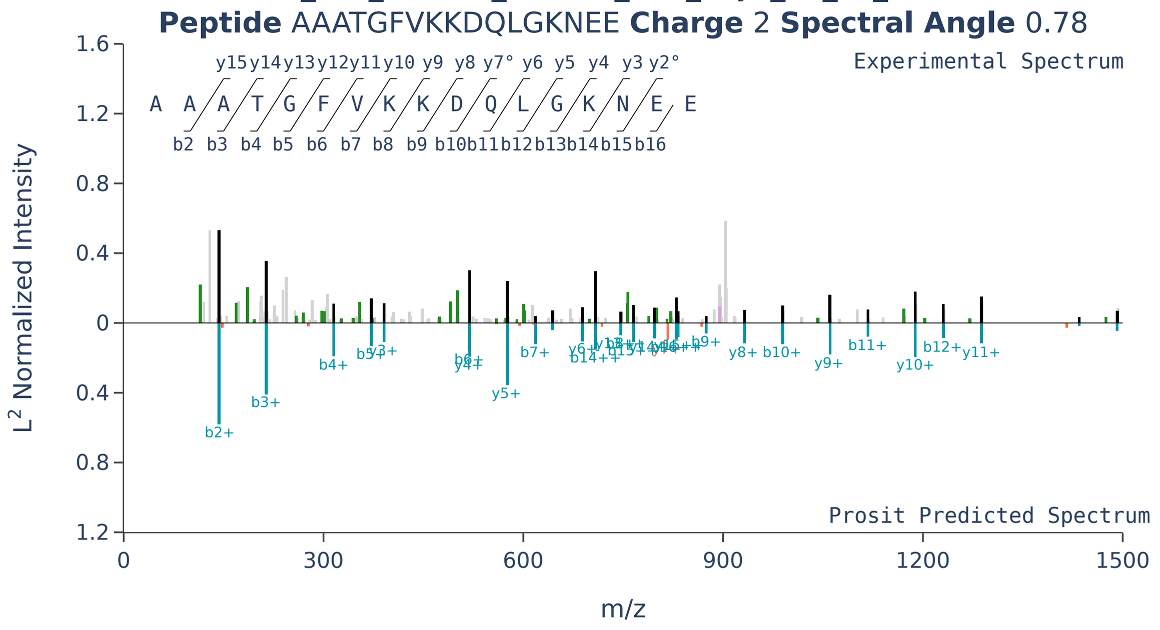


**Source** W_Soh_160821_170921_Goe_aSyn_A2_24h_R1 **Scan** 10767
**Peptide** MPSEEGY **Charge** 1 **Spectral Angle** 0.81

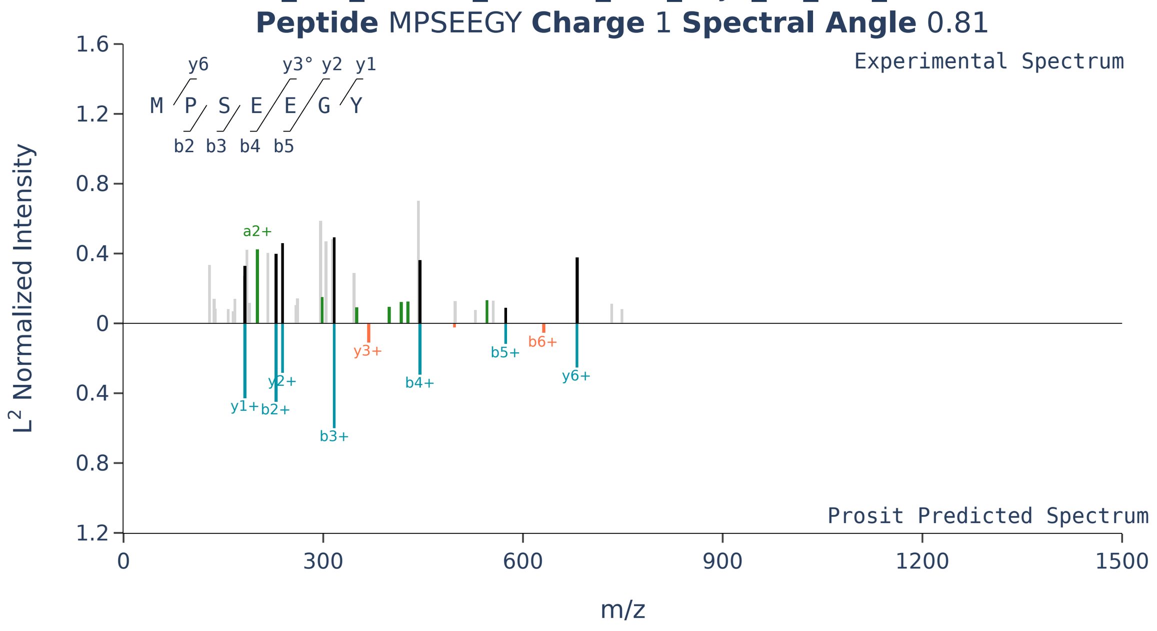


**Source** W_Soh_160821_170921_Goe_aSyn_A2_24h_R1 **Scan** 8134
**Peptide** VAEAAGKTKEGVLYVGSKTK **Charge** 3 **Spectral Angle** 0.87

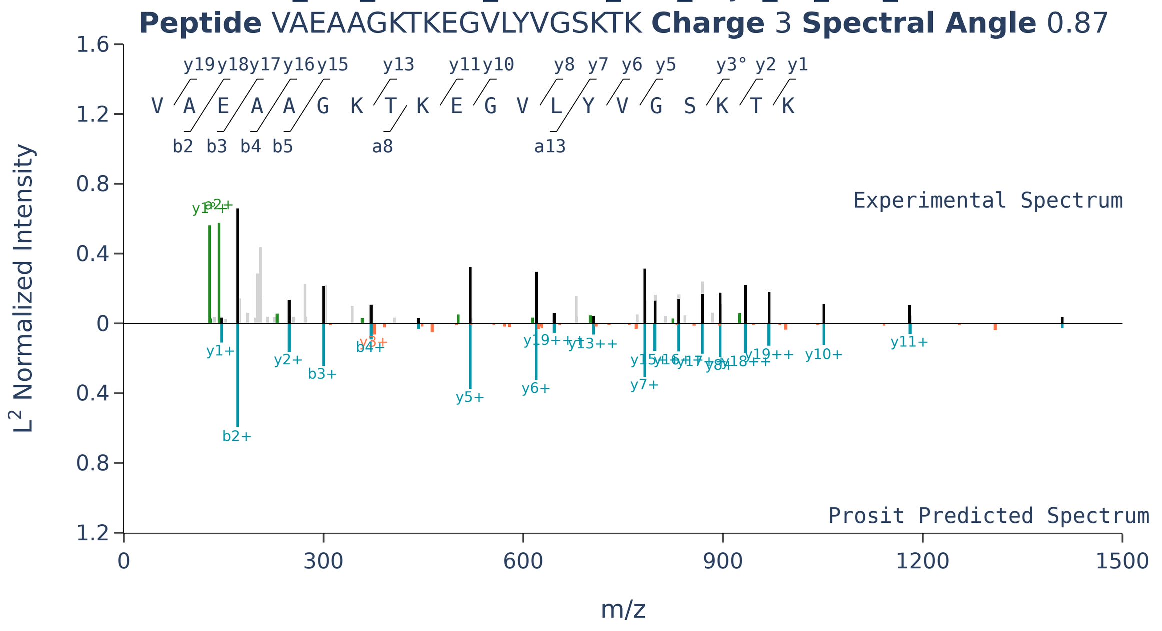


**Source** W_Soh_160821_170921_Goe_aSyn_A1_6h_R1 **Scan** 15087
**Peptide** AAATGFVKKDQLGKNEEGAPQEGILE **Charge** 3 **Spectral Angle** 0.81

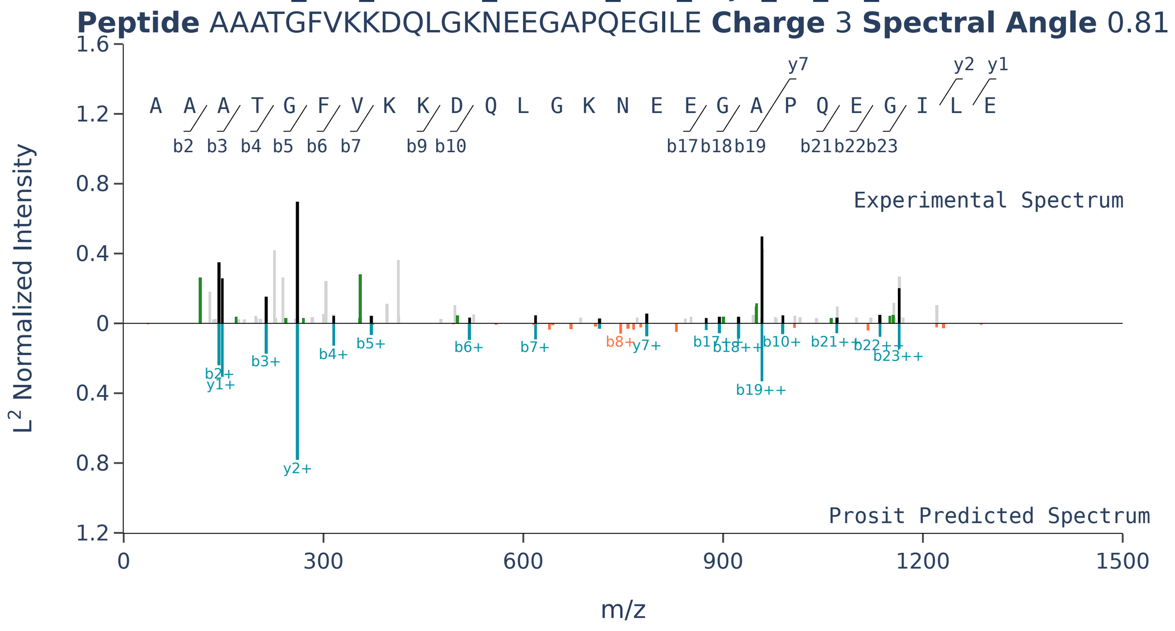


**Source** W_Soh_160821_170921_Goe_aSyn_A1_24h_R2 **Scan** 15705
**Peptide** GAGSIAAATGFVKKDQLGKNEEGAPQE **Charge** 3 **Spectral Angle** 0.88

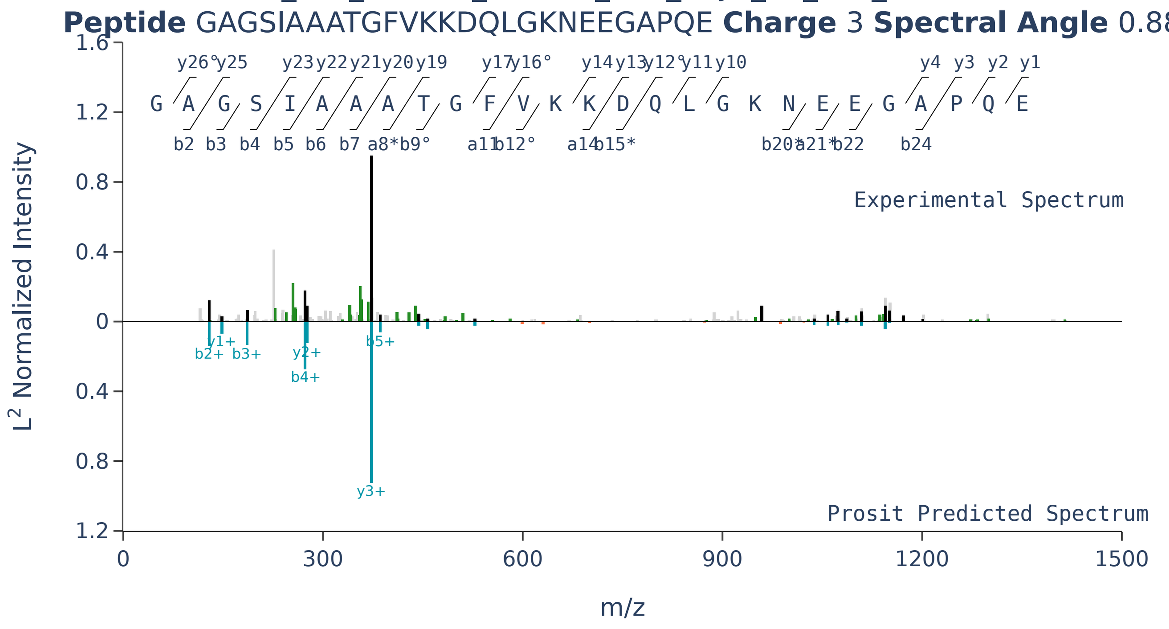


**Source** W_Soh_160821_170921_Goe_aSyn_A2_24h_R2 **Scan** 10173
**Peptide** AAGKTKEGVLYVGSKTKEGVVHGVAT **Charge** 4 **Spectral Angle** 0.77

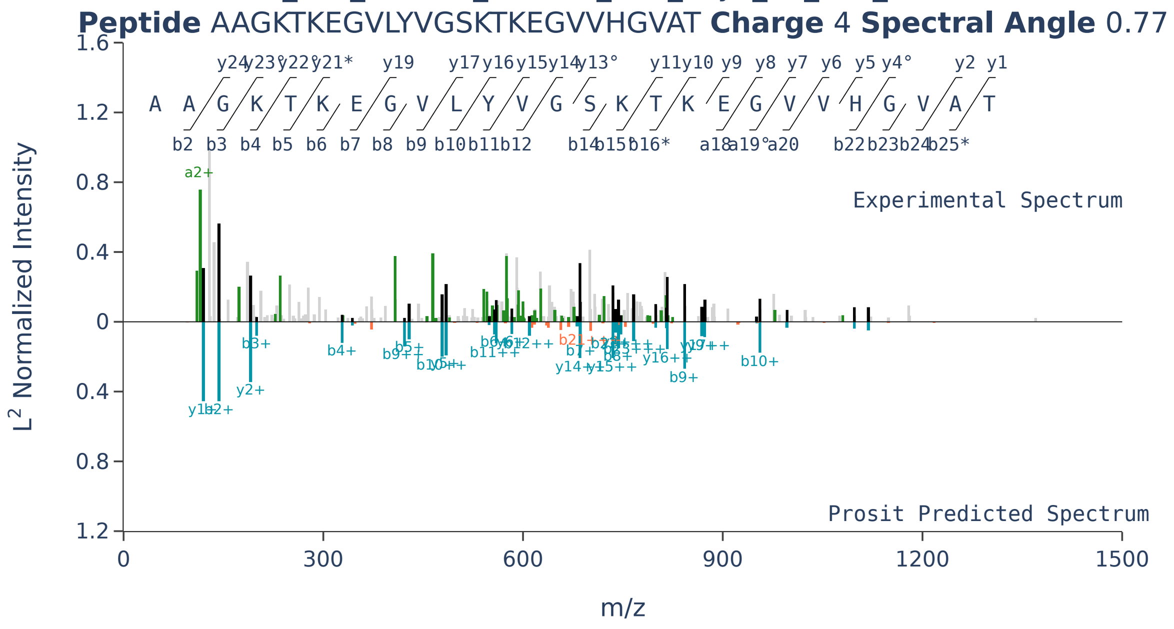


**Source** W_Soh_160821_170921_Goe_aSyn_A1_24h_R1 **Scan** 8106
**Peptide** MKGLSKAKEGVVAAAEKTKQG **Charge** 3 **Spectral Angle** 0.82

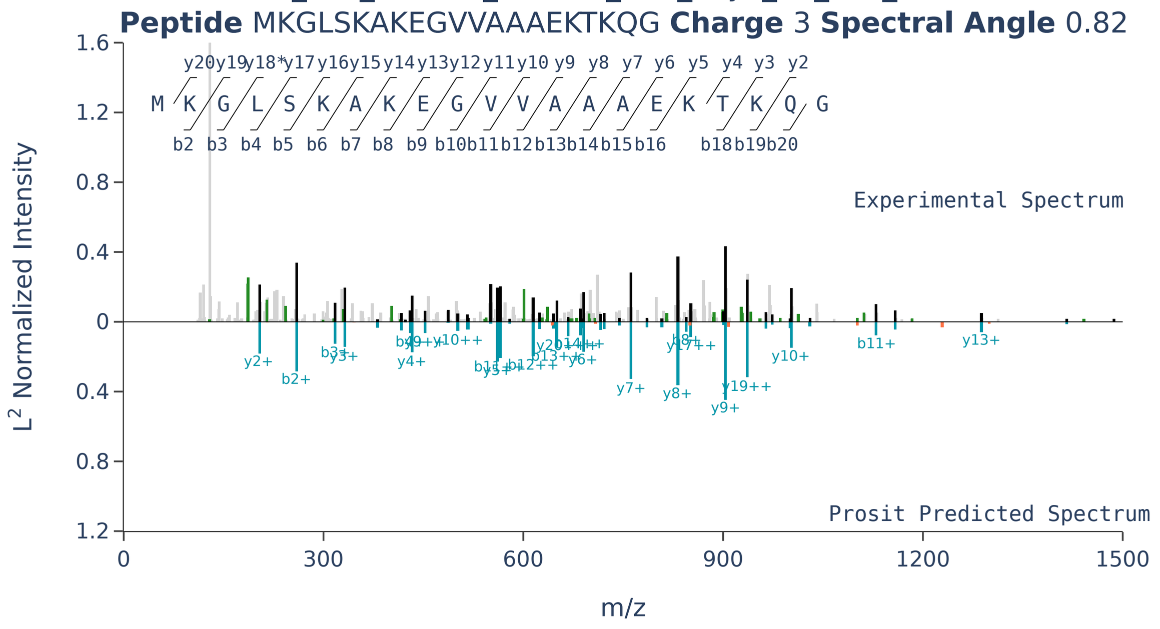


**Source** W_Soh_160821_170921_Goe_aSyn_A2_6h_R1 **Scan** 12314
**Peptide** VLYVGSKTKEGVVHGVATVAEKTKE **Charge** 4 **Spectral Angle** 0.83

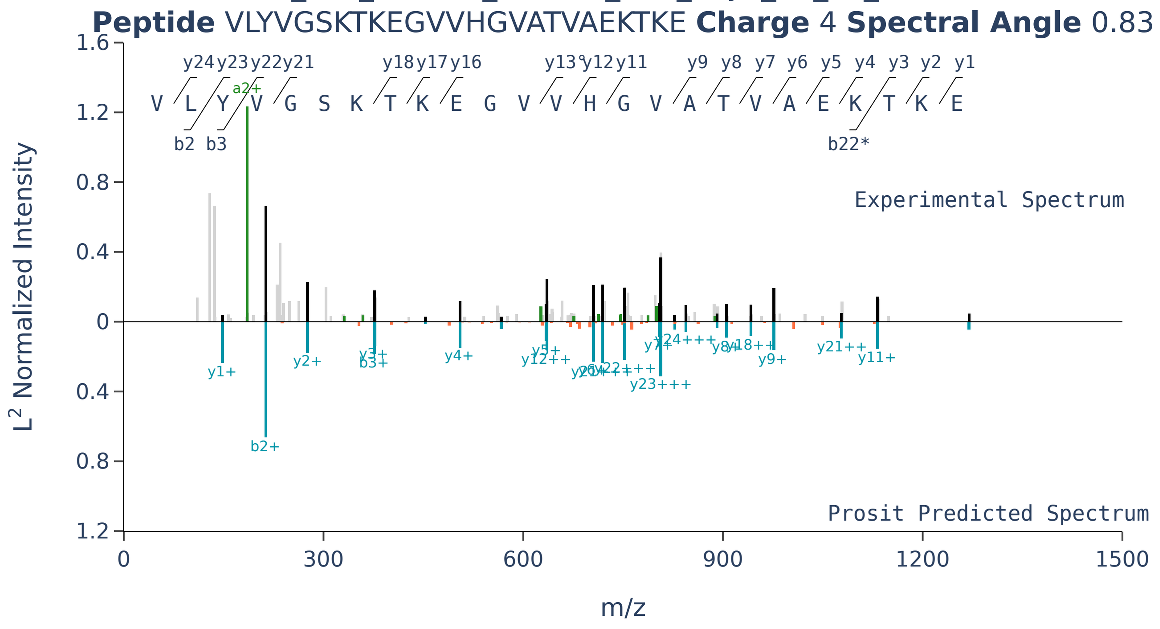


**Source** W_Soh_160821_170921_Goe_aSyn_A2_24h_R2 **Scan** 13574
**Peptide** GKNEEGAPQEGILED **Charge** 2 **Spectral Angle** 0.79

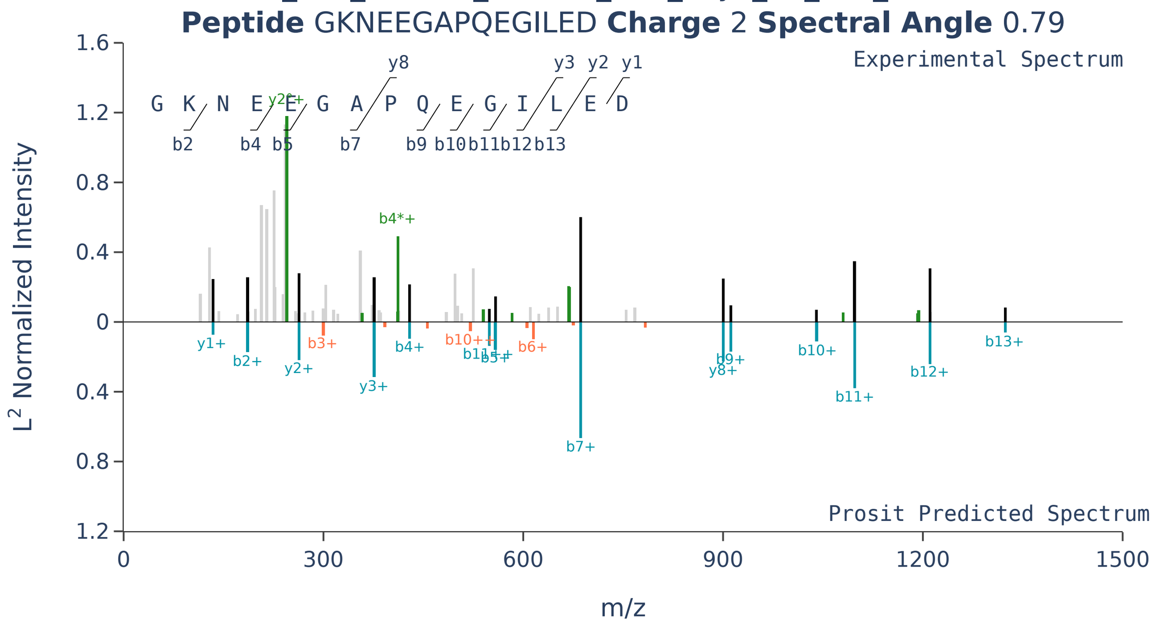


**Source** W_Soh_160821_170921_Goe_aSyn_A2_6h_R2 **Scan** 20223
**Peptide** GVATVAEKTKEQVTNVGGAVVTGVTA **Charge** 3 **Spectral Angle** 0.85

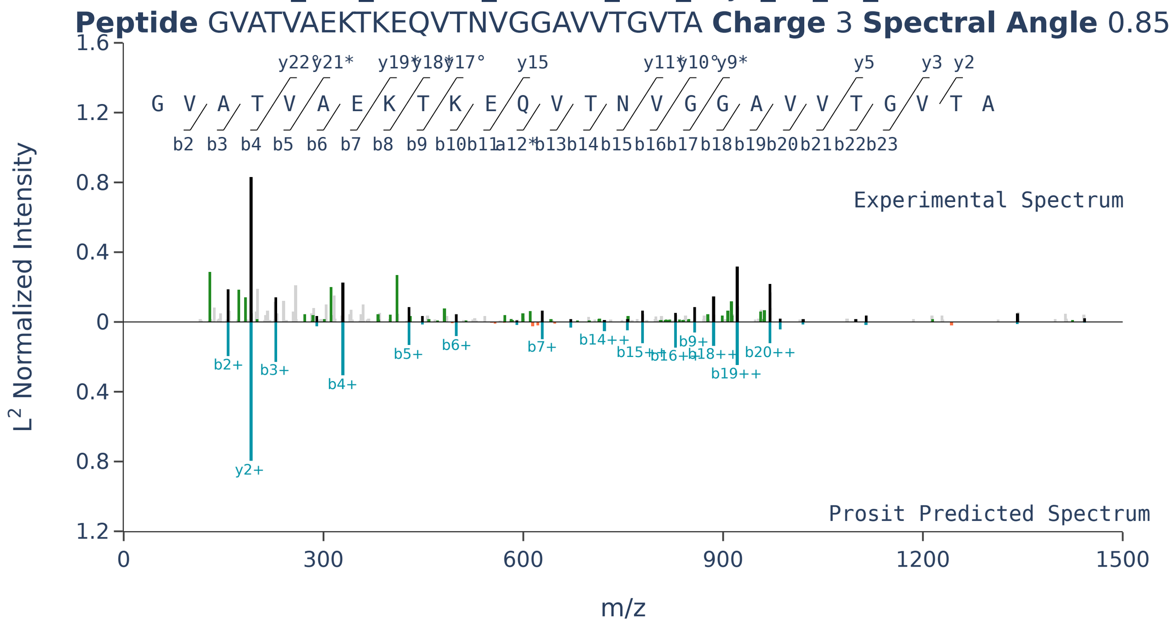

**Source** W_Soh_160821_170921_Goe_aSyn_A2_24h_R2 **Scan** 15391
**Peptide** QLGKNEEGAPQEGILED **Charge** 2 **Spectral Angle** 0.81

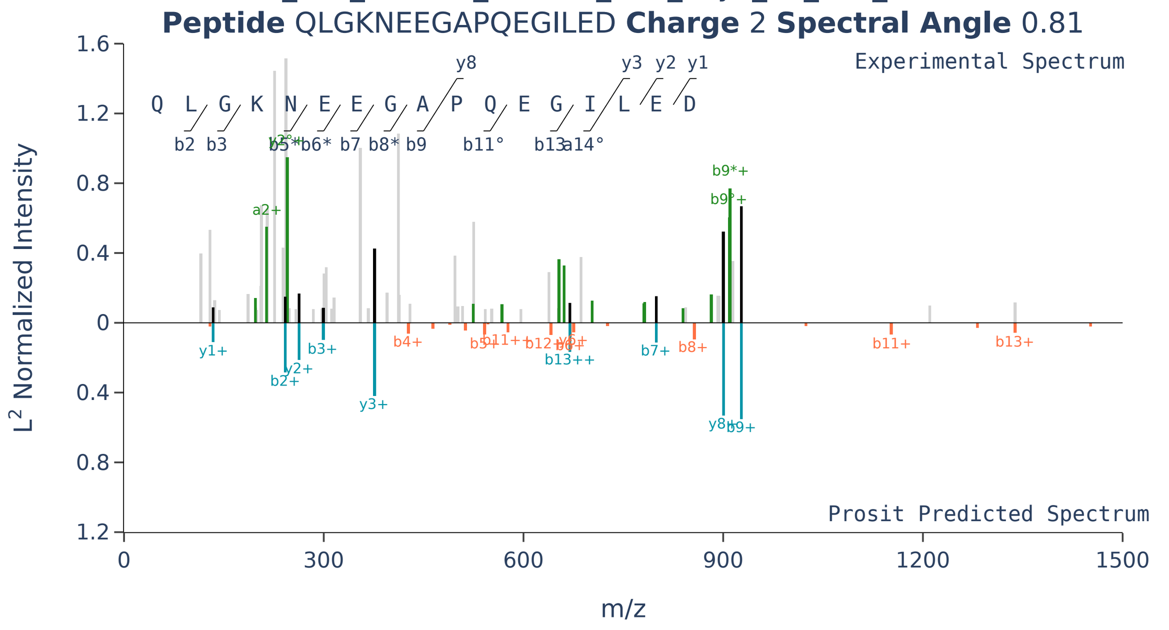


**Source** W_Soh_160821_170921_Goe_aSyn_A1_24h_R2 **Scan** 7950
**Peptide** SKTKEGVVHGVATV **Charge** 1 **Spectral Angle** 0.78

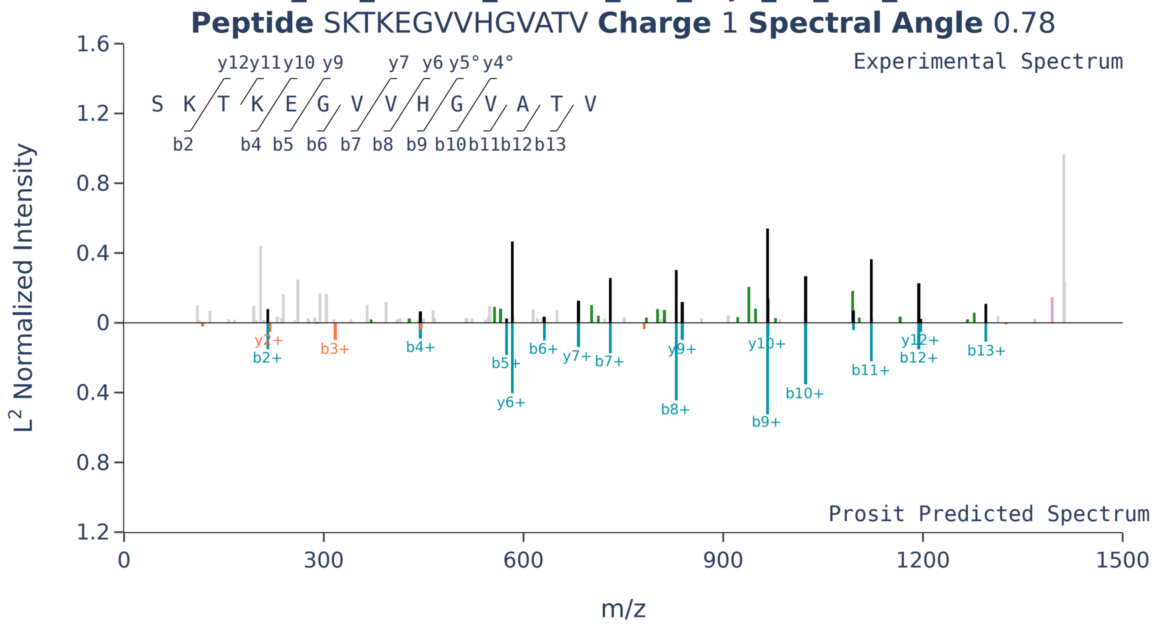


**Source** W_Soh_160821_170921_Goe_aSyn_A1_6h_R2 **Scan** 11093
**Peptide** EKTKEQVTNVGGAVV **Charge** 3 **Spectral Angle** 0.79

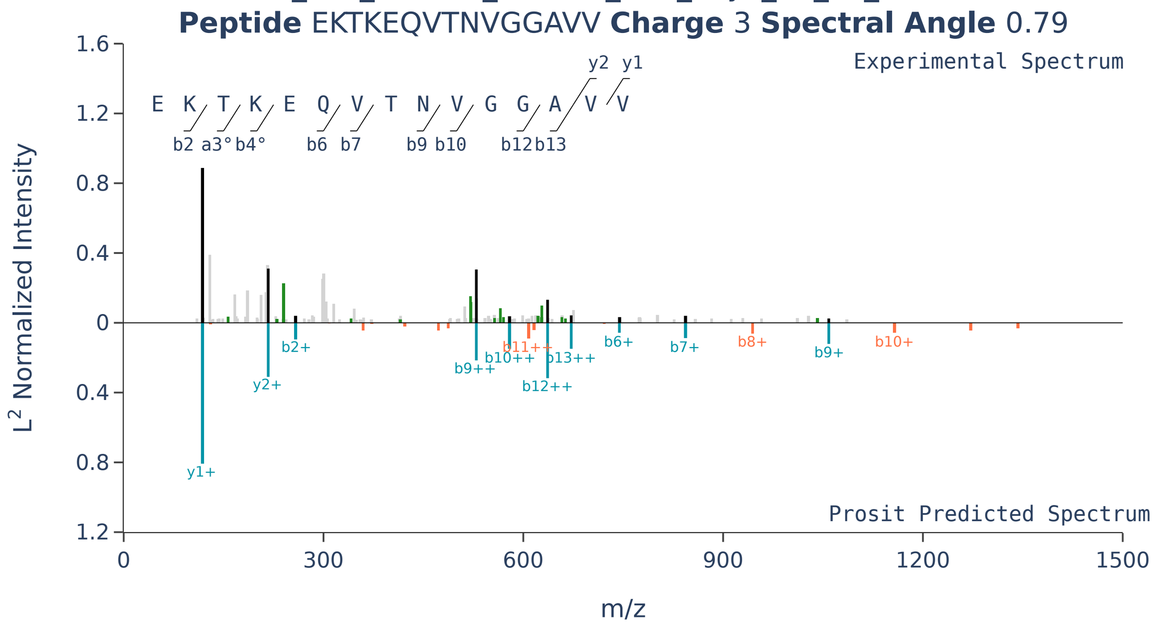


**Source** W_Soh_160821_170921_Goe_aSyn_A2_24h_R2 **Scan** 18567
**Peptide** MDVFMKGLSKAKEGVVAAAEK **Charge** 4 **Spectral Angle** 0.79

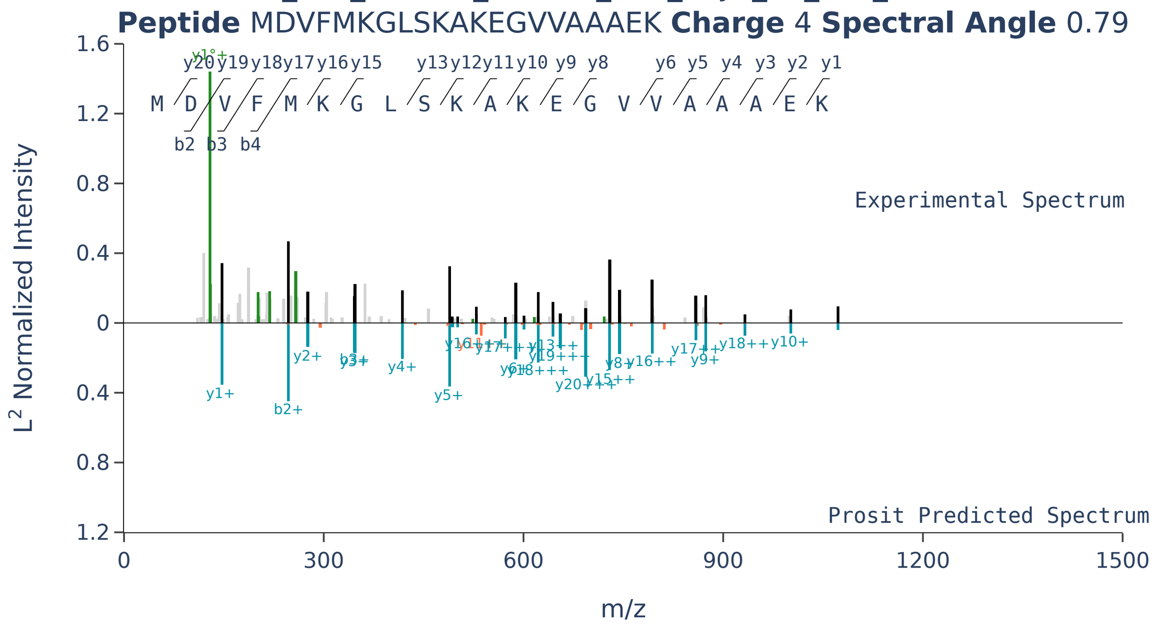


**Source** W_Soh_160821_170921_Goe_aSyn_A1_24h_R2 **Scan** 6281
**Peptide** AAAEKTKQGVAE **Charge** 2 **Spectral Angle** 0.79

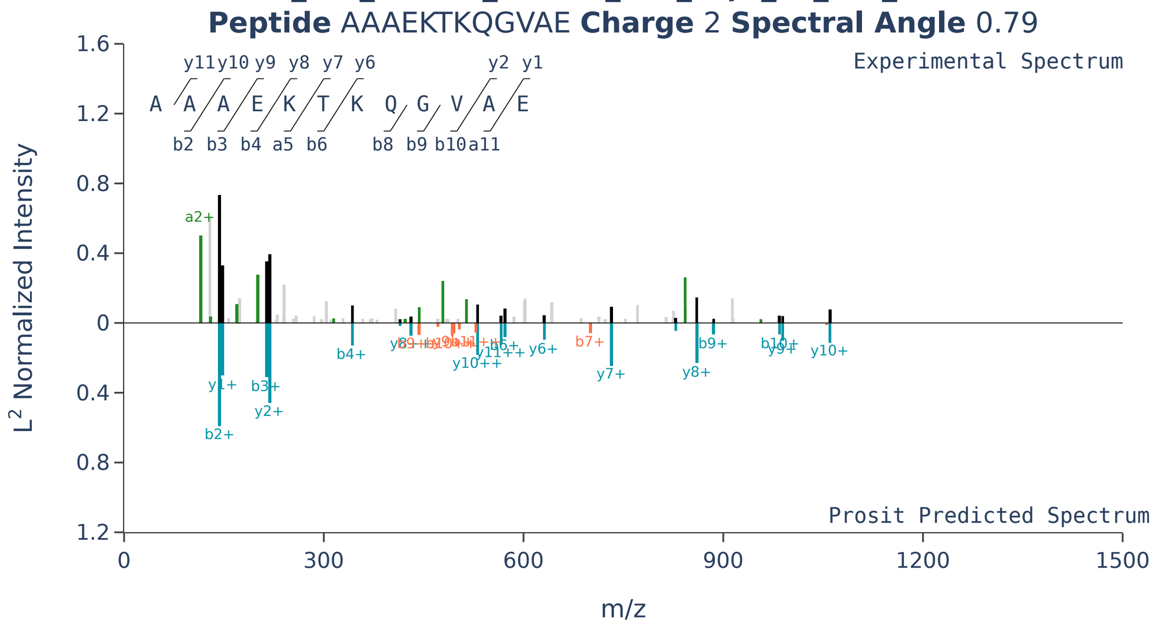


**Source** W_Soh_160821_170921_Goe_aSyn_A1_3h_R1 **Scan** 5085
**Peptide** KKDQLGKNEEGAPQE **Charge** 2 **Spectral Angle** 0.8

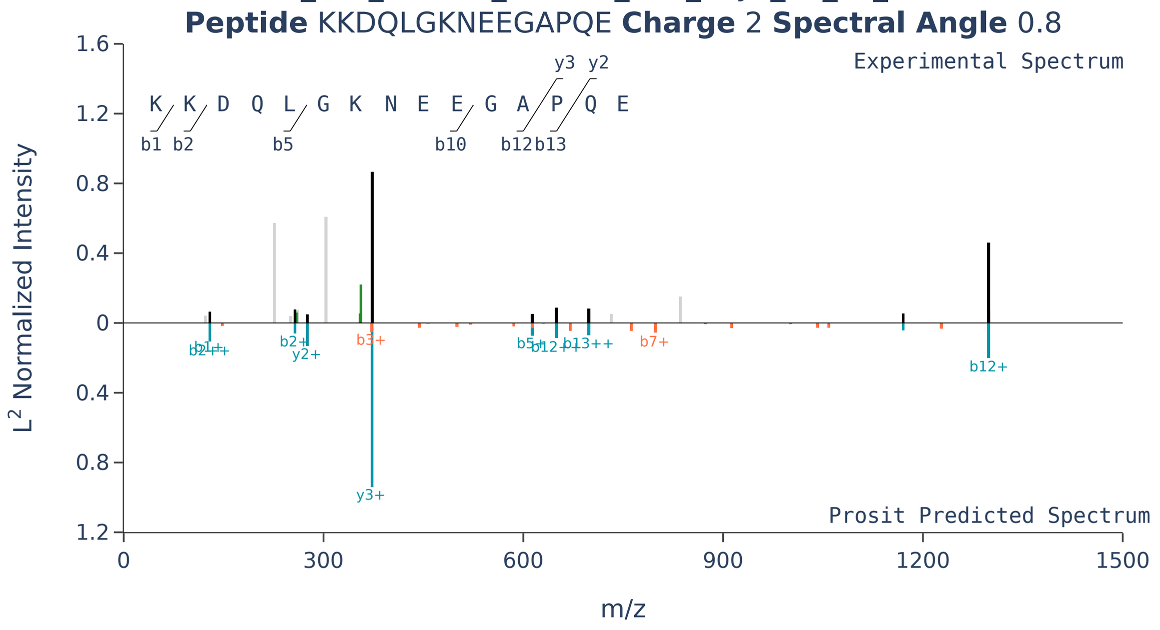


**Source** W_Soh_160821_170921_Goe_aSyn_A2_24h_R1 **Scan** 15839
**Peptide** VAQKTVEGAGSIAAATGFVKKDQLGKNEE **Charge** 5 **Spectral Angle** 0.69

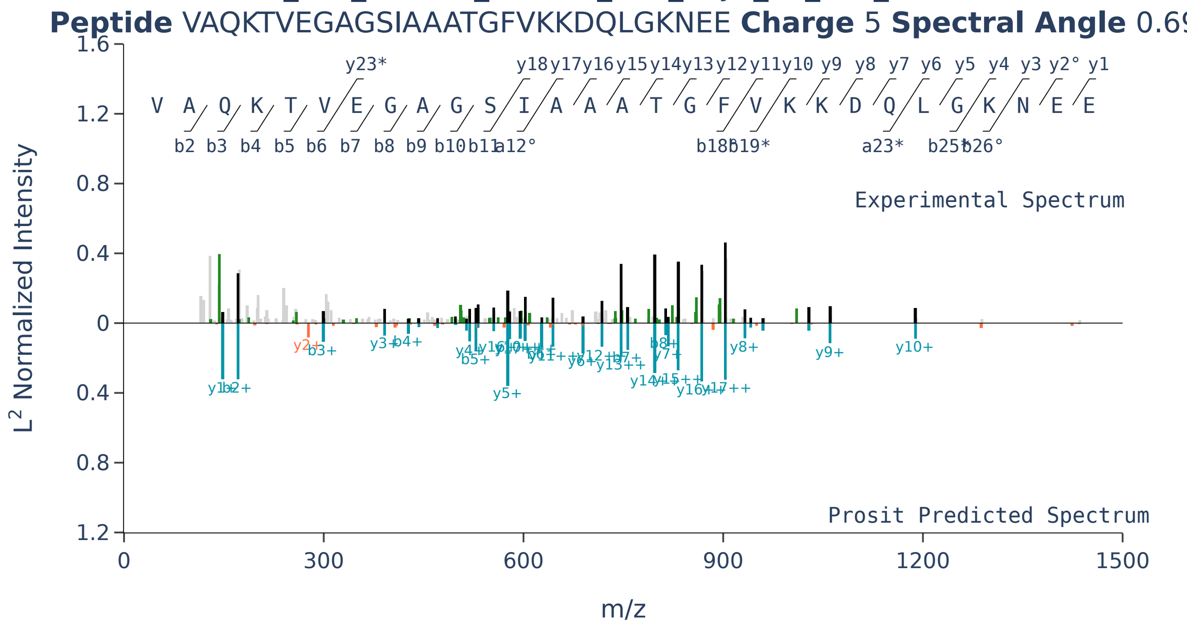


**Source** W_Soh_160821_170921_Goe_aSyn_A1_24h_R1 **Scan** 7485
**Peptide** KEGVVHGVATVAEK **Charge** 3 **Spectral Angle** 0.71

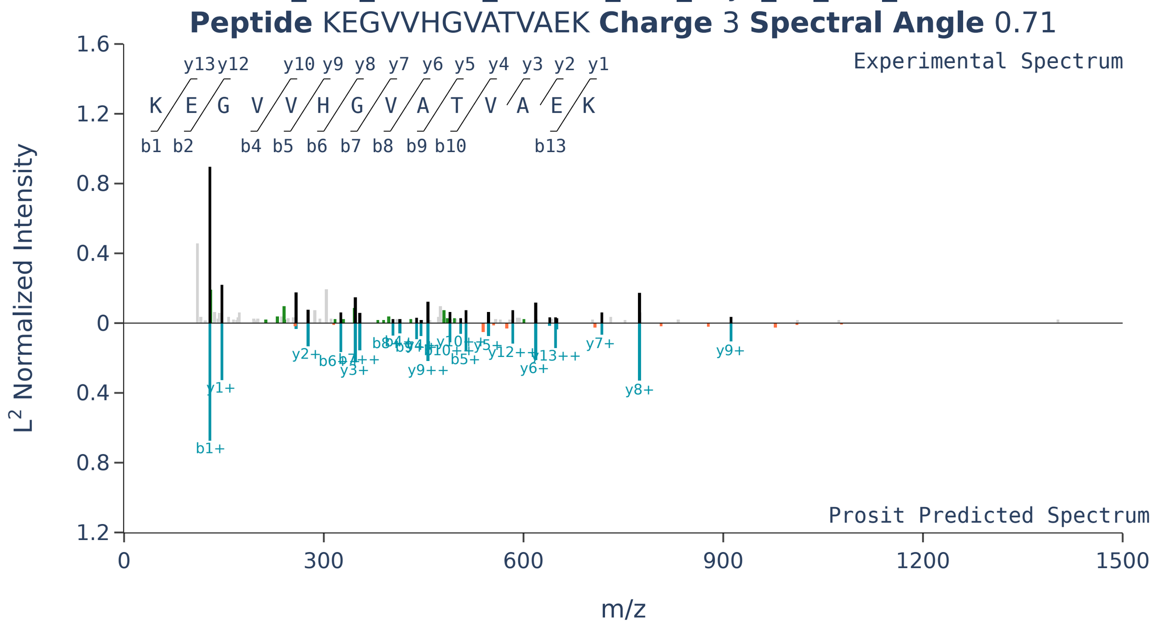


**Source** W_Soh_160821_170921_Goe_aSyn_A1_24h_R1 **Scan** 11667
**Peptide** TKEGVLYVGSKTKEGVVHGV **Charge** 4 **Spectral Angle** 0.85

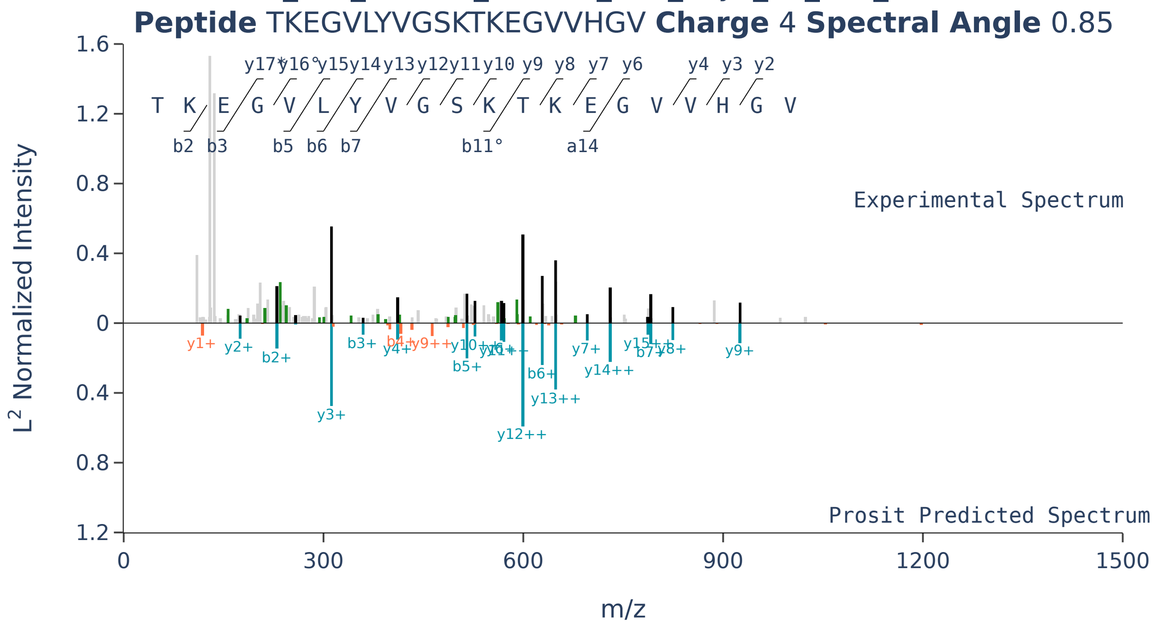


**Source** W_Soh_160821_170921_Goe_aSyn_A1_24h_R1 **Scan** 14968
**Peptide** YEMPSEEGYQD **Charge** 2 **Spectral Angle** 0.87

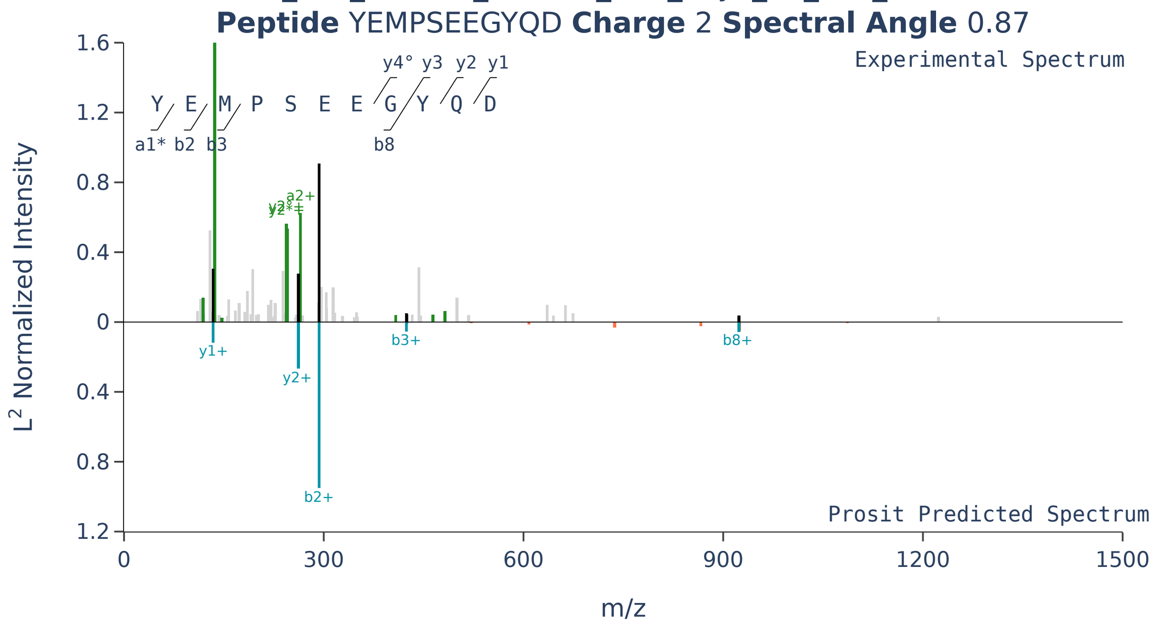


**Source** W_Soh_160821_170921_Goe_aSyn_A1_24h_R2 **Scan** 6339
**Peptide** LSKAKEGVVAAAEKTKQG **Charge** 4 **Spectral Angle** 0.77

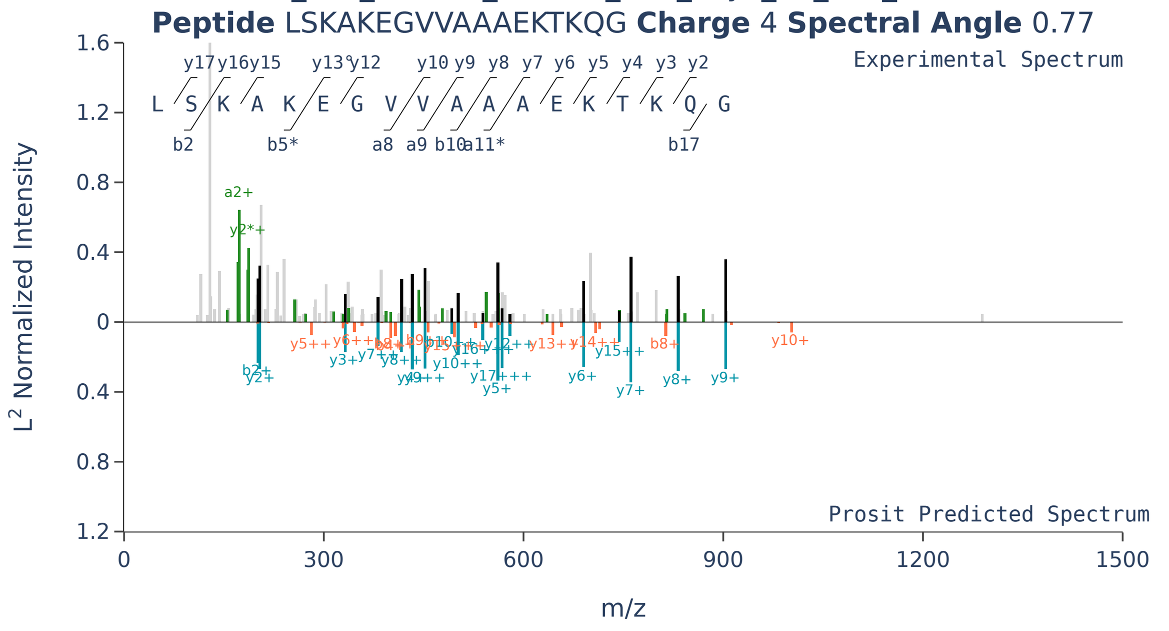


**Source** W_Soh_160821_170921_Goe_aSyn_A1_6h_R2 **Scan** 18397
**Peptide** AEKTKEQVTNVGGAVVTGVTAVAQ **Charge** 3 **Spectral Angle** 0.84

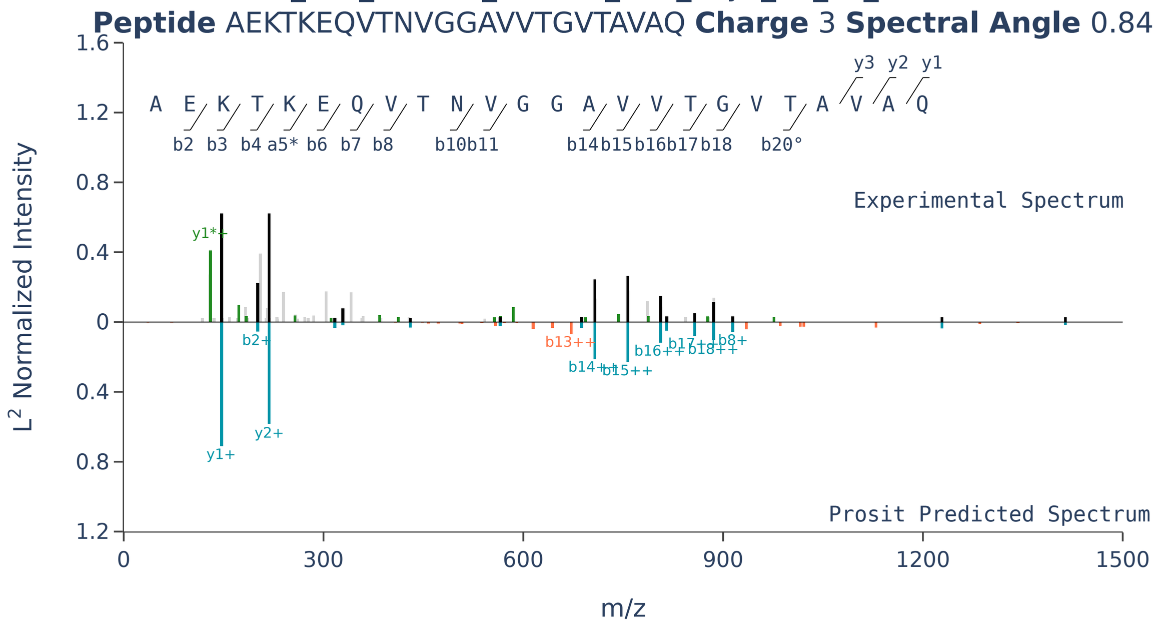


**Source** W_Soh_160821_170921_Goe_aSyn_A1_3h_R1 **Scan** 13555
**Peptide** TGFVKKDQLGKNEEGAPQEGILED **Charge** 4 **Spectral Angle** 0.85

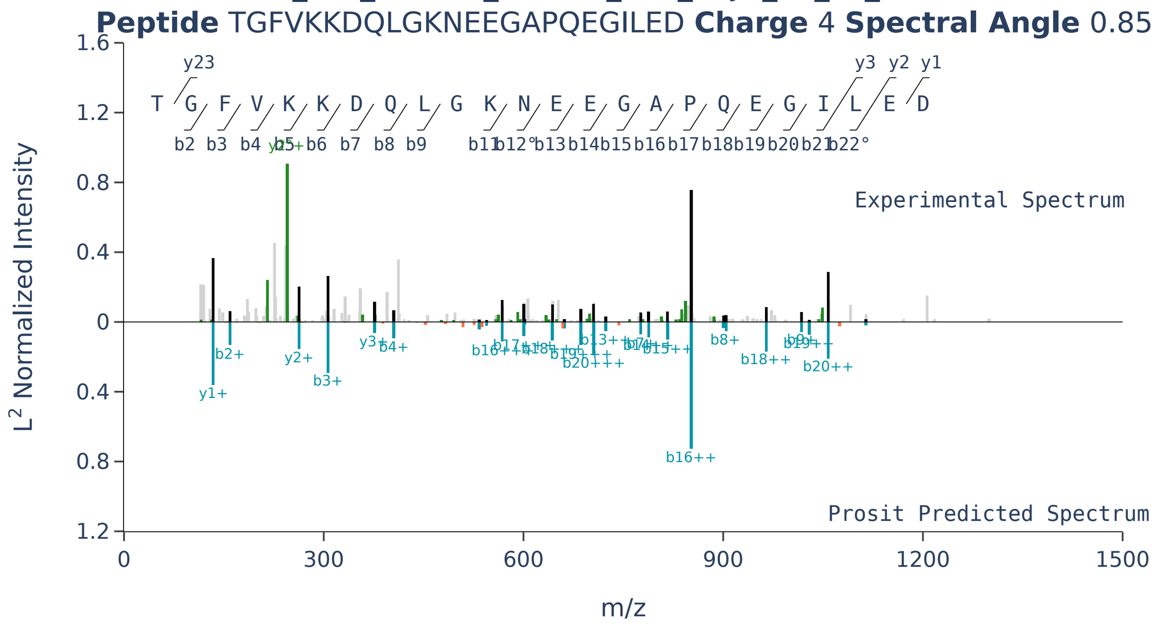


**Source** W_Soh_160821_170921_Goe_aSyn_A2_24h_R2 **Scan** 6988
**Peptide** ATGFVKKDQLGKNE **Charge** 3 **Spectral Angle** 0.76

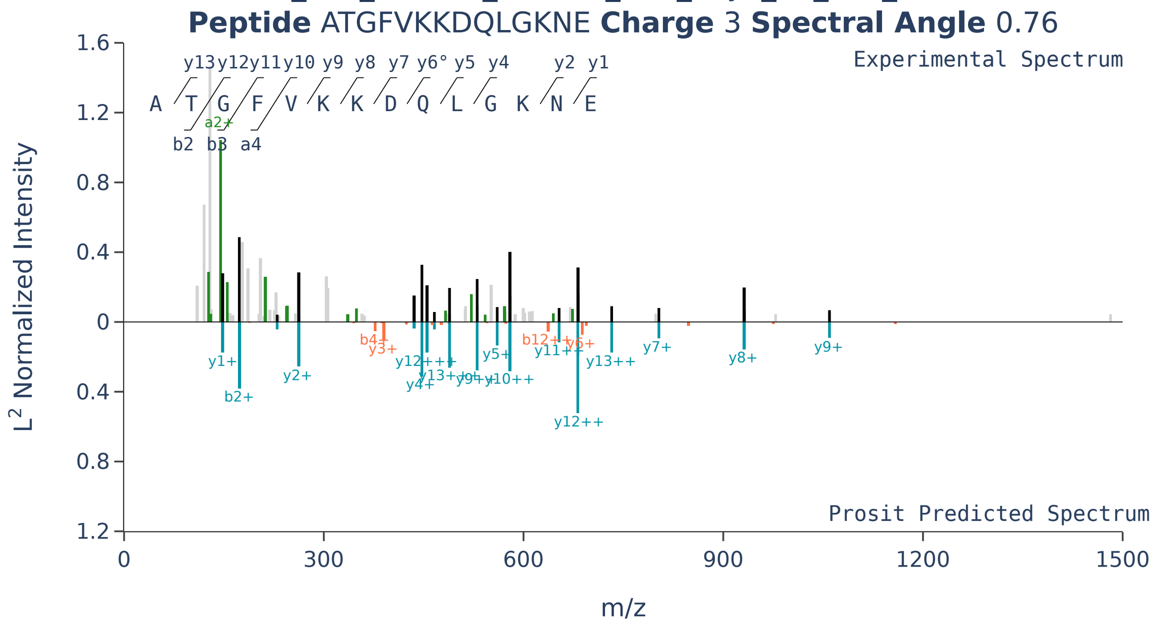


**Source** W_Soh_160821_170921_Goe_aSyn_A1_24h_R2 **Scan** 17530
**Peptide** GAGSIAAATGFVKKDQLG **Charge** 3 **Spectral Angle** 0.7

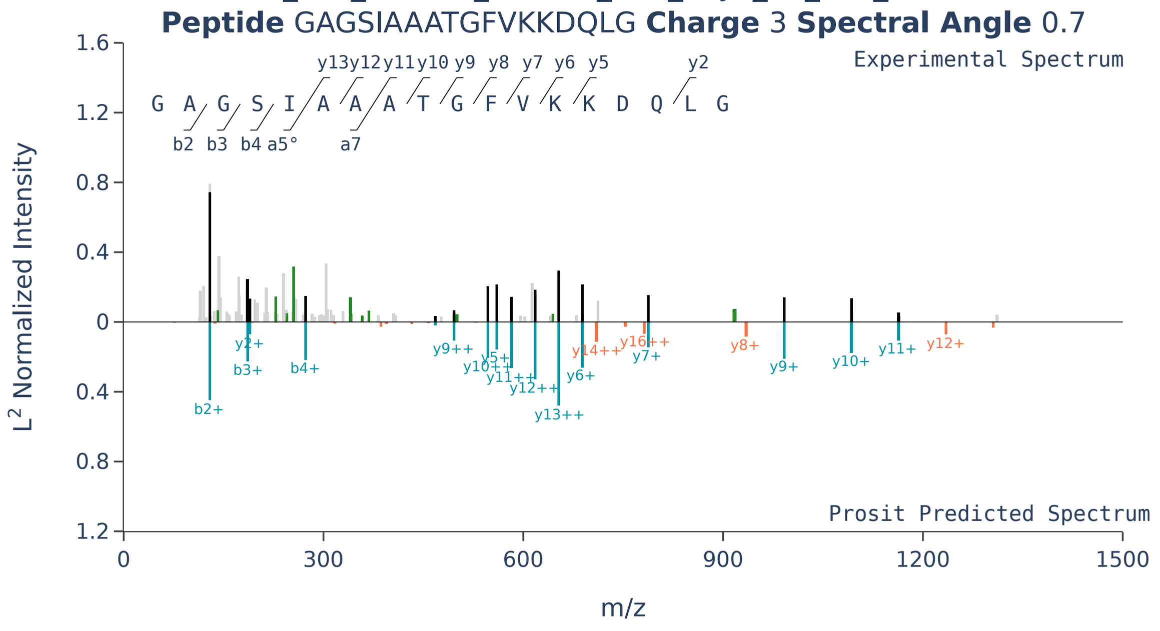

**Source** W_Soh_160821_170921_Goe_aSyn_A1_24h_R2 **Scan** 5958
**Peptide** AKEGVVAAAEKTKQG **Charge** 3 **Spectral Angle** 0.7

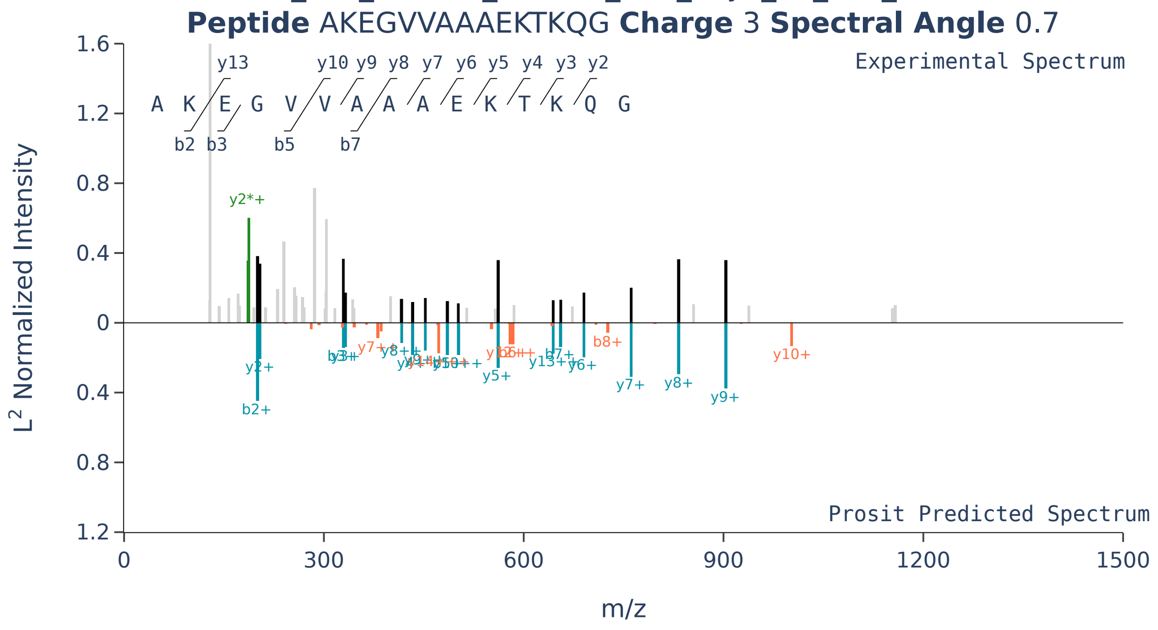


**Source** W_Soh_160821_170921_Goe_aSyn_A2_24h_R2 **Scan** 16697
**Peptide** MDVFMKGLSKAKEGVVAA **Charge** 2 **Spectral Angle** 0.85

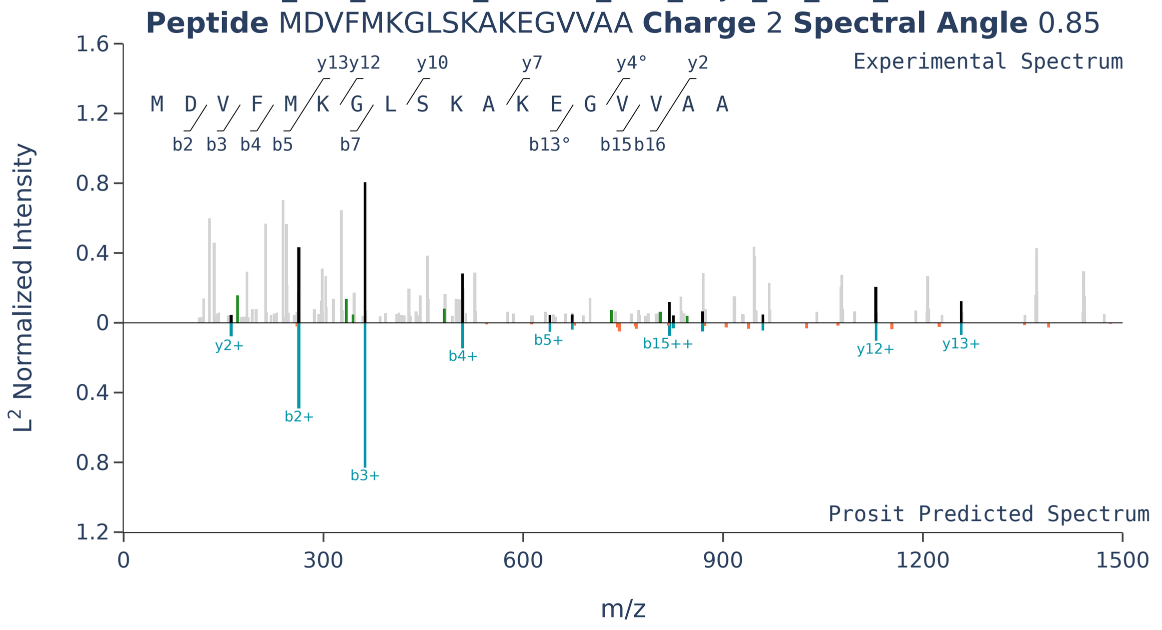


**Source** W_Soh_160821_170921_Goe_aSyn_A2_24h_R2 **Scan** 15382
**Peptide** AQKTVEGAGSIAAATGFVKKDQLGKNEE **Charge** 4 **Spectral Angle** 0.69

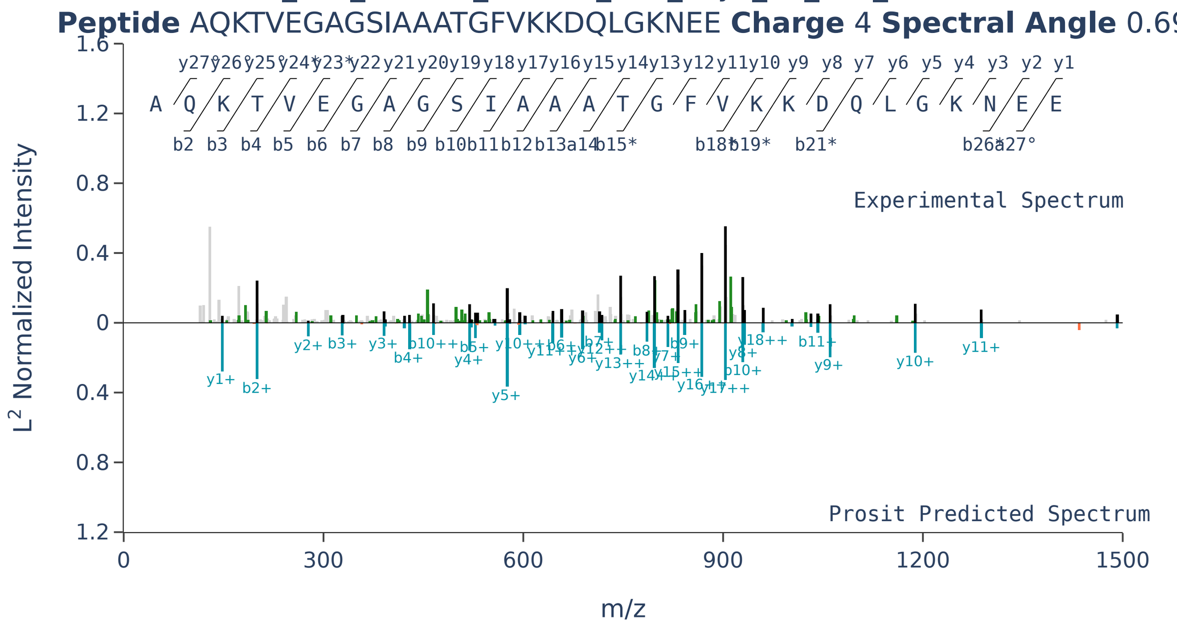


**Source** W_Soh_160821_170921_Goe_aSyn_A1_24h_R1 **Scan** 5036
**Peptide** AAAEKTKQGVAEAAGKTKE **Charge** 4 **Spectral Angle** 0.72

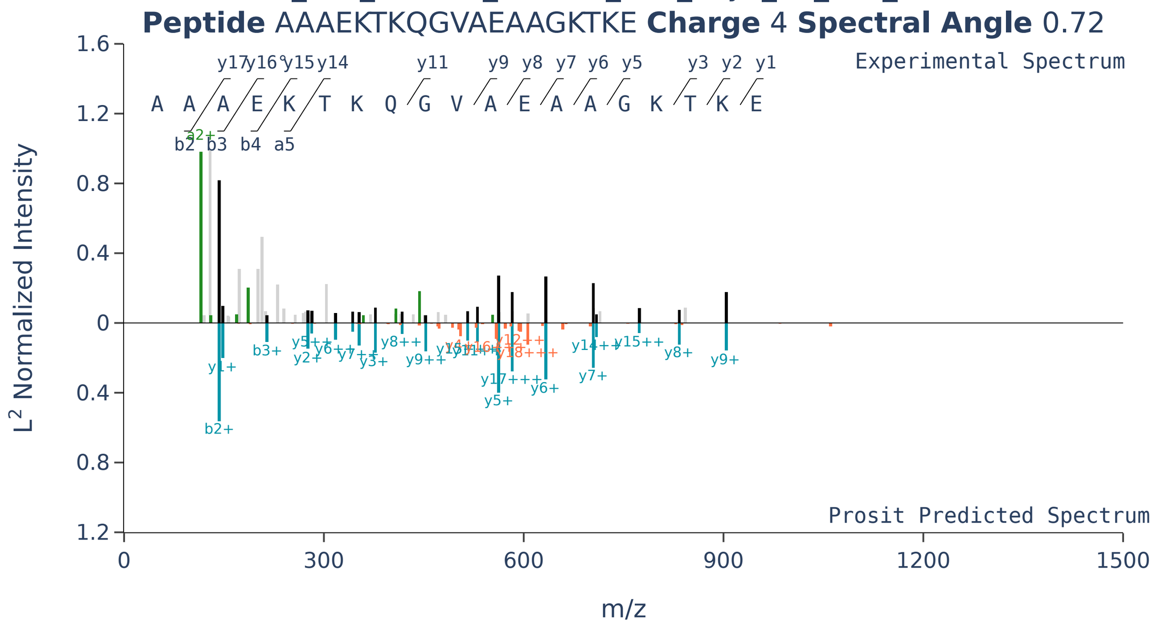


**Source** W_Soh_160821_170921_Goe_aSyn_A2_24h_R2 **Scan** 15034
**Peptide** KTVEGAGSIAAATGFVKKDQLGKNEE **Charge** 4 **Spectral Angle** 0.76

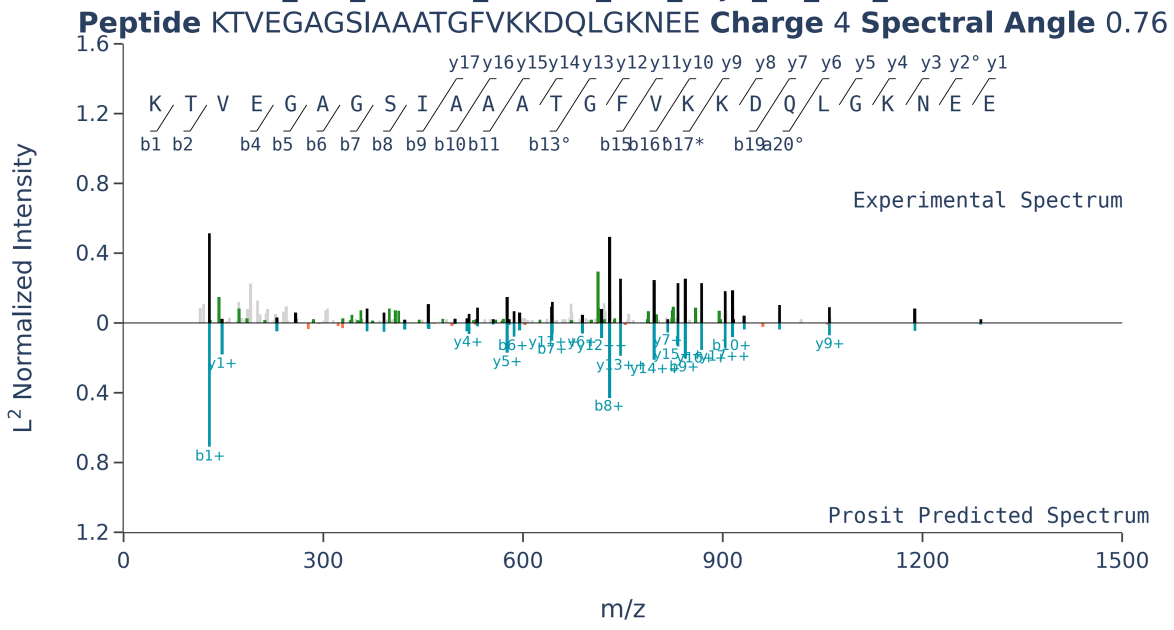


**Source** W_Soh_160821_170921_Goe_aSyn_A1_24h_R1 **Scan** 8680
**Peptide** EAAGKTKEGVLYVGSKTKEGVVH **Charge** 4 **Spectral Angle** 0.92

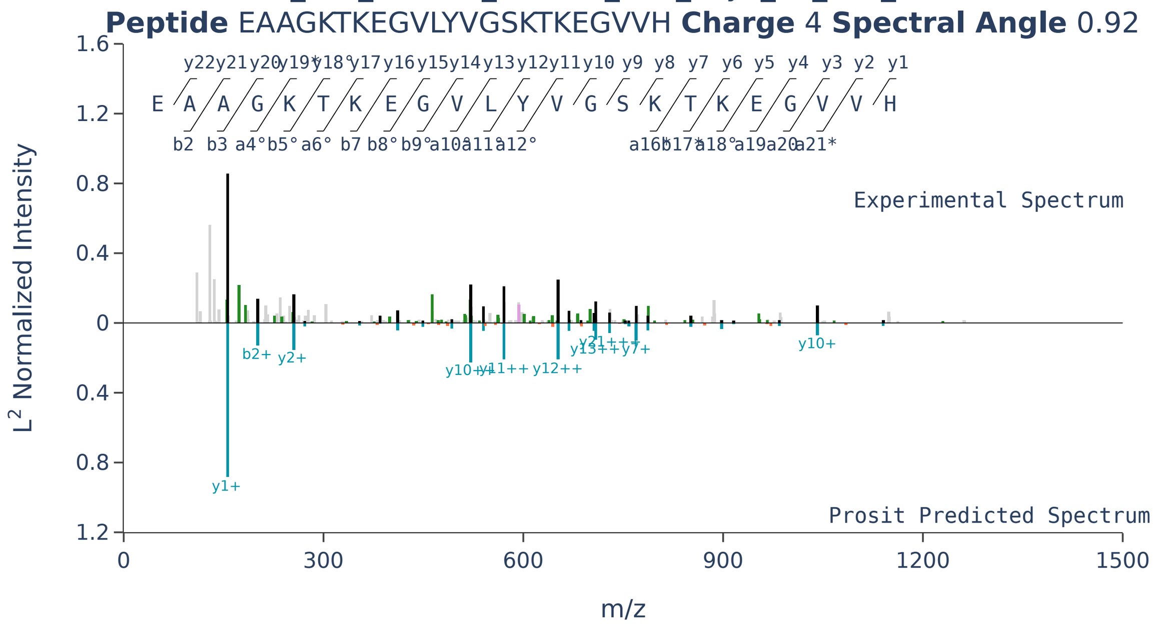


**Source** W_Soh_160821_170921_Goe_aSyn_A1_24h_R2 **Scan** 17651
**Peptide** TVEGAGSIAAATGFVKKDQLGKNEE **Charge** 4 **Spectral Angle** 0.82

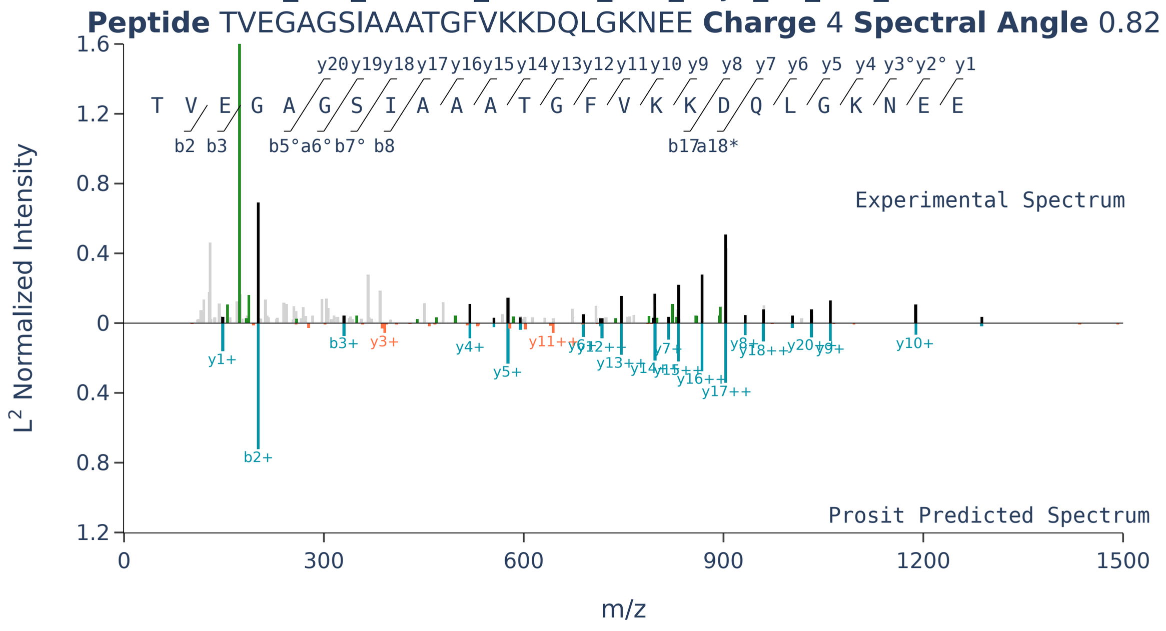


**Source** W_Soh_160821_170921_Goe_aSyn_A1_24h_R1 **Scan** 20396
**Peptide** GVATVAEKTKEQVTNVGGAVVTGVT **Charge** 3 **Spectral Angle** 0.78

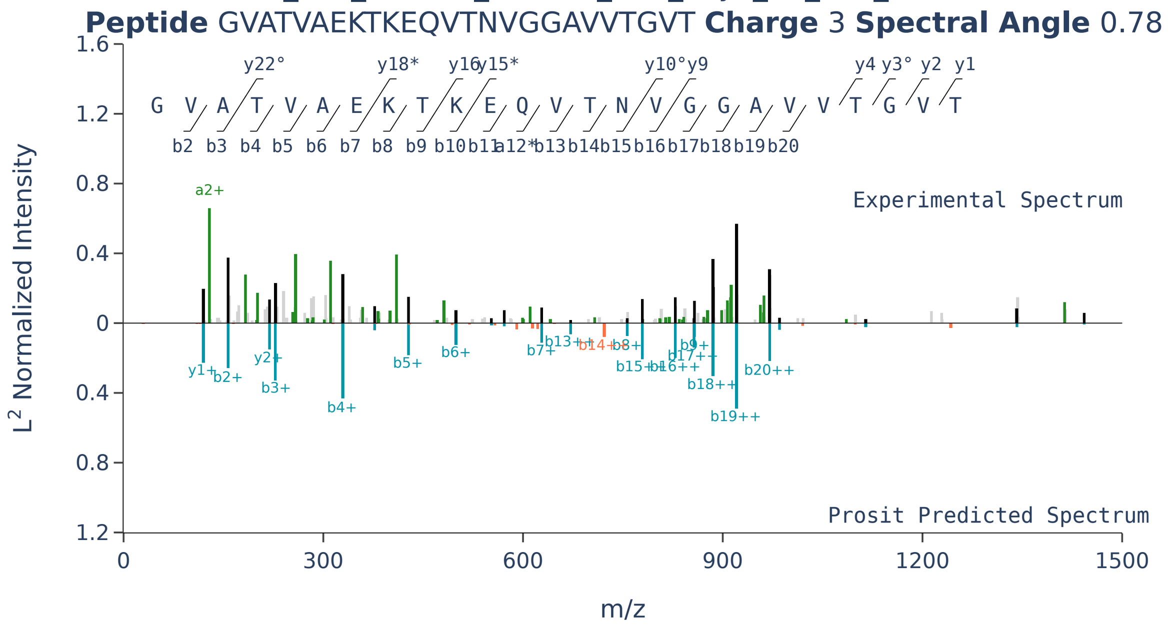


**Source** W_Soh_160821_170921_Goe_aSyn_A1_24h_R2 **Scan** 6886
**Peptide** KAKEGVVAAAEKTKQGVAE **Charge** 3 **Spectral Angle** 0.83

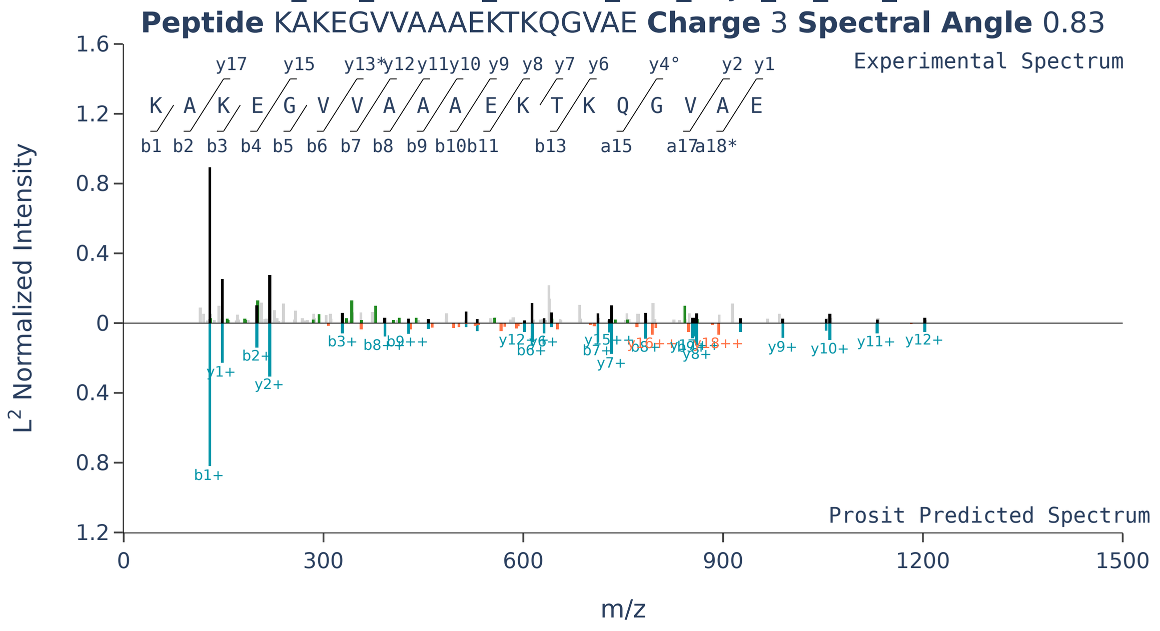


**Source** W_Soh_160821_170921_Goe_aSyn_A2_24h_R1 **Scan** 12382
**Peptide** GVAEAAGKTKEGVLYVGSKTKEGVVH **Charge** 4 **Spectral Angle** 0.82

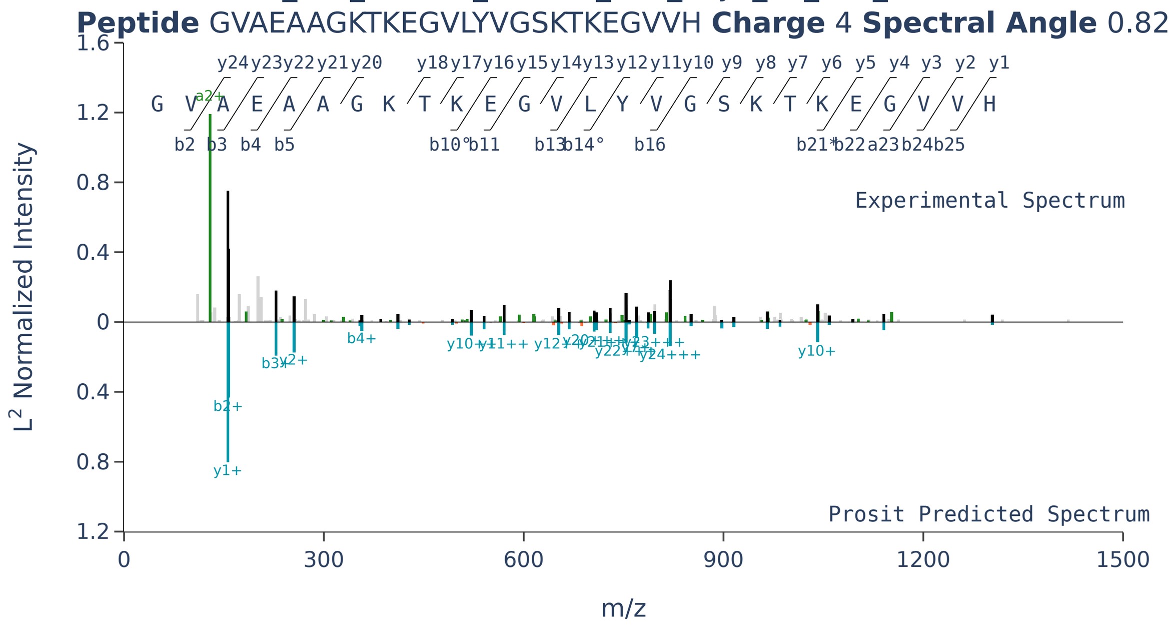


**Source** W_Soh_160821_170921_Goe_aSyn_A2_24h_R1 **Scan** 13859
**Peptide** AGSIAAATGFVKKDQLGKNEE **Charge** 3 **Spectral Angle** 0.79

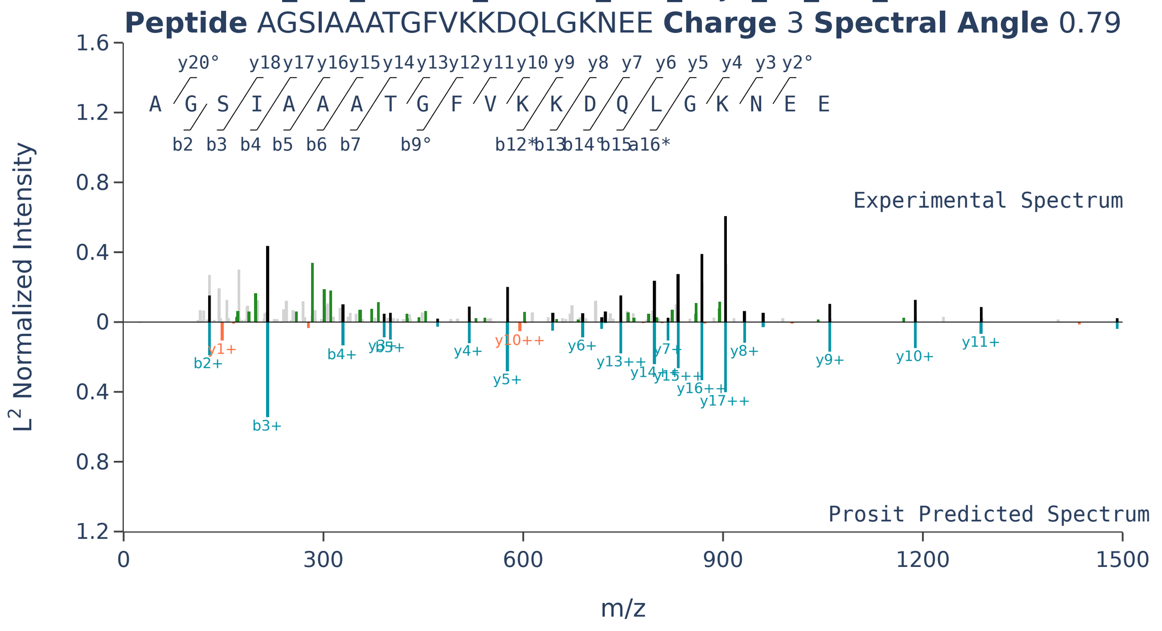


**Source** W_Soh_160821_170921_Goe_aSyn_A1_1h_R2 **Scan** 18527
**Peptide** MDVFMKGLSKAKEGVVAAAEKTKQGV **Charge** 4 **Spectral Angle** 0.86

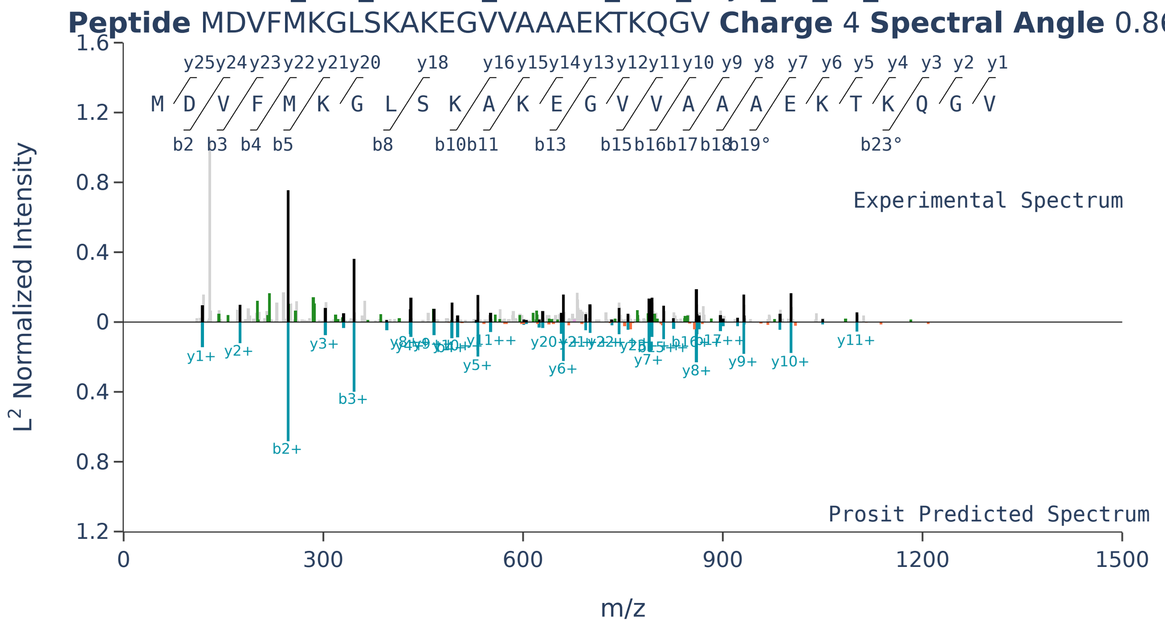


**Source** W_Soh_160821_170921_Goe_aSyn_A1_24h_R1 **Scan** 6085
**Peptide** LSKAKEGVVAAAEKTKQ **Charge** 4 **Spectral Angle** 0.8

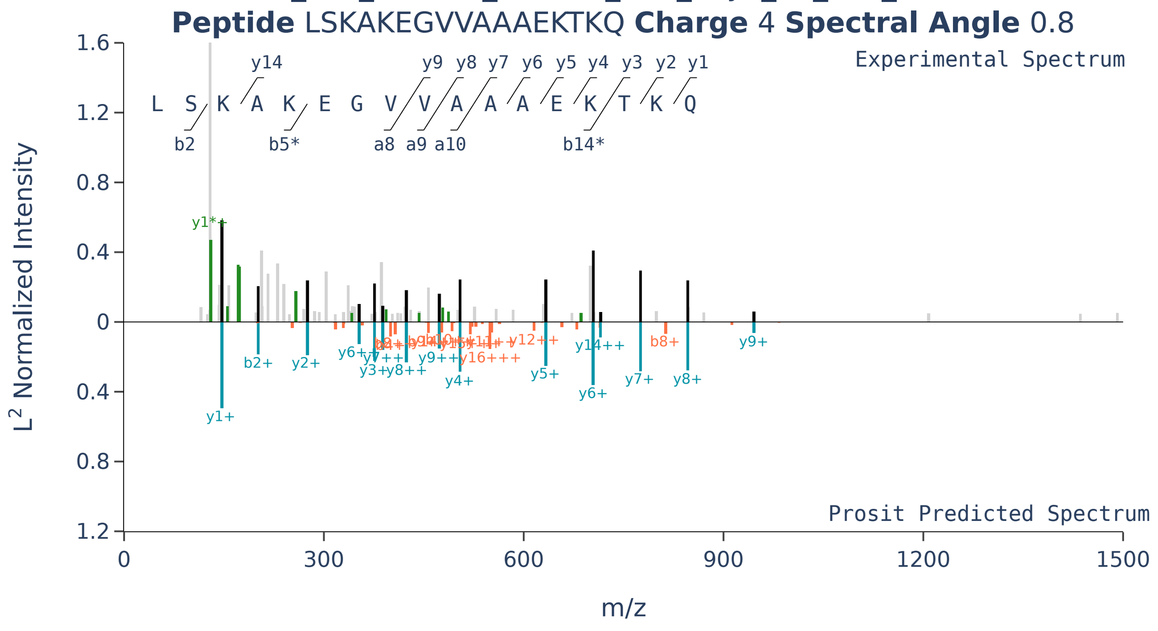


**Source** W_Soh_160821_170921_Goe_aSyn_A1_24h_R1 **Scan** 22826
**Peptide** TGVTAVAQKTVEGAGSIAAATGFVKK **Charge** 3 **Spectral Angle** 0.78

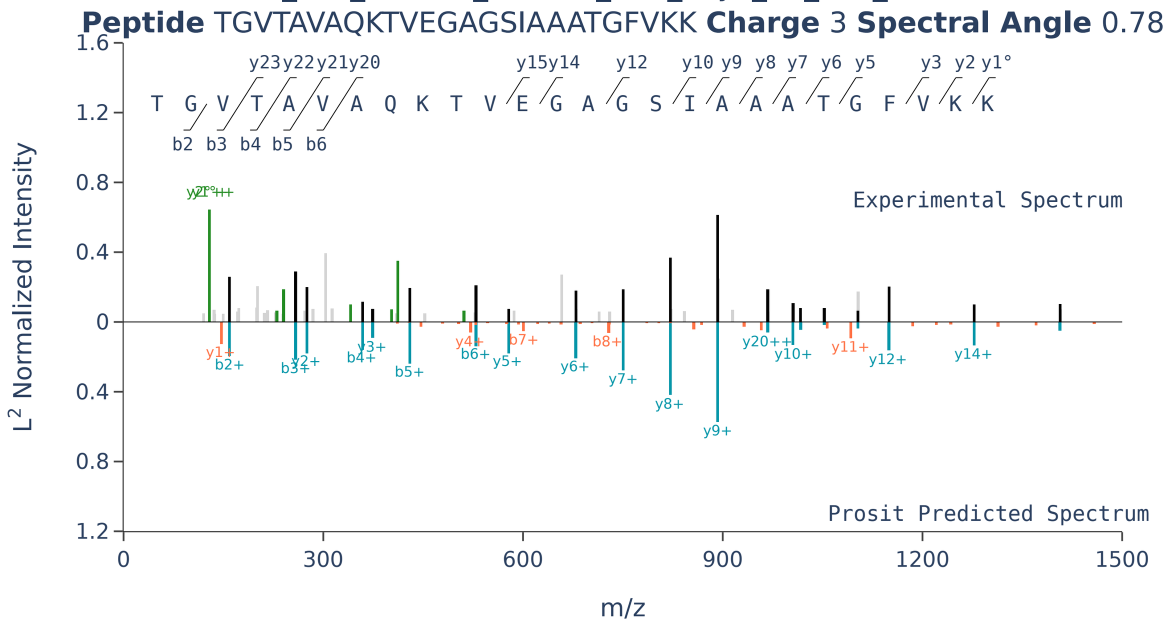


**Source** W_Soh_160821_170921_Goe_aSyn_A1_6h_R1 **Scan** 24565
**Peptide** DQLGKNEEGAPQEGILEDMPVD **Charge** 3 **Spectral Angle** 0.9

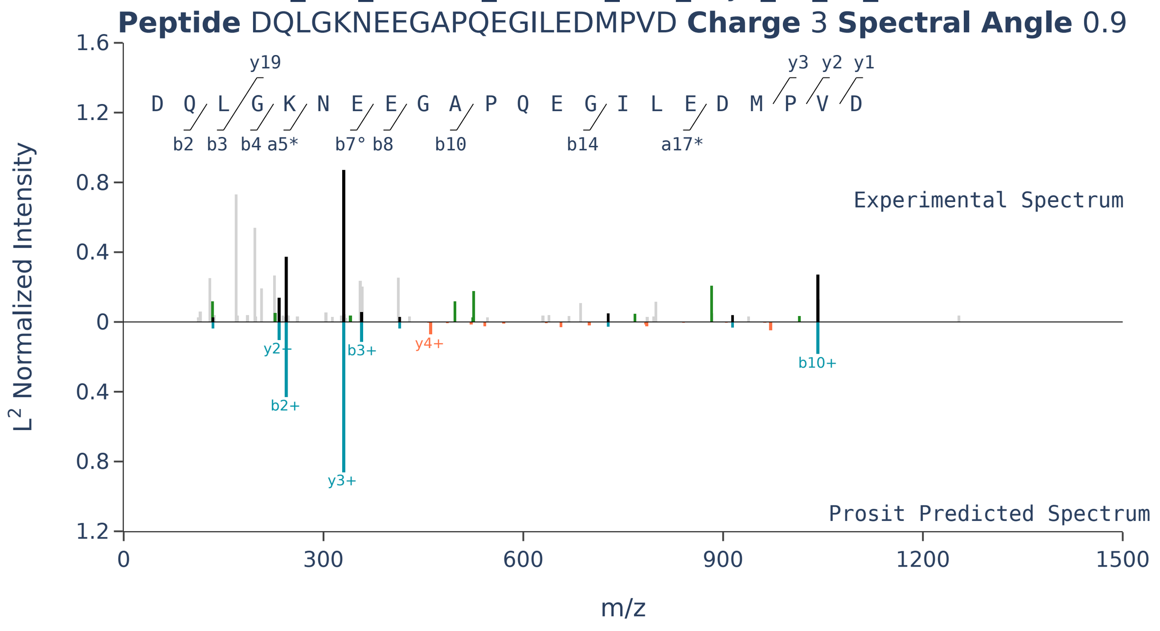

**Source** W_Soh_160821_170921_Goe_aSyn_A2_24h_R1 **Scan** 16474
**Peptide** VEGAGSIAAATGFVKK **Charge** 3 **Spectral Angle** 0.8

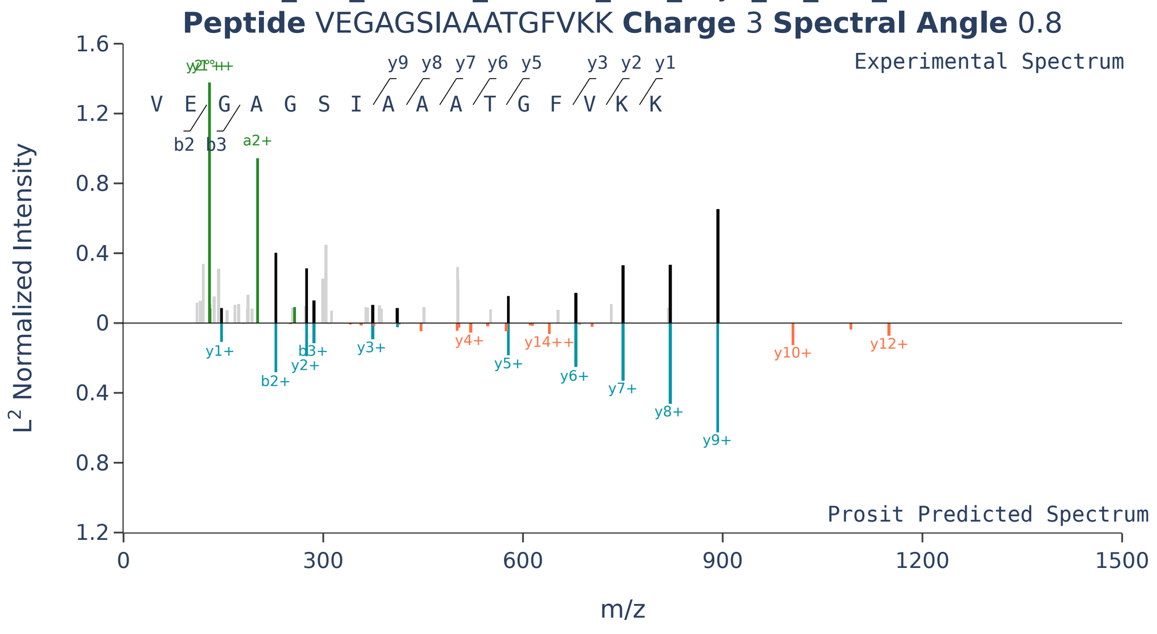


**Source** W_Soh_160821_170921_Goe_aSyn_A1_3h_R2 **Scan** 11165
**Peptide** MKGLSKAKEGVVAAAEKTKQGVAEA **Charge** 4 **Spectral Angle** 0.8

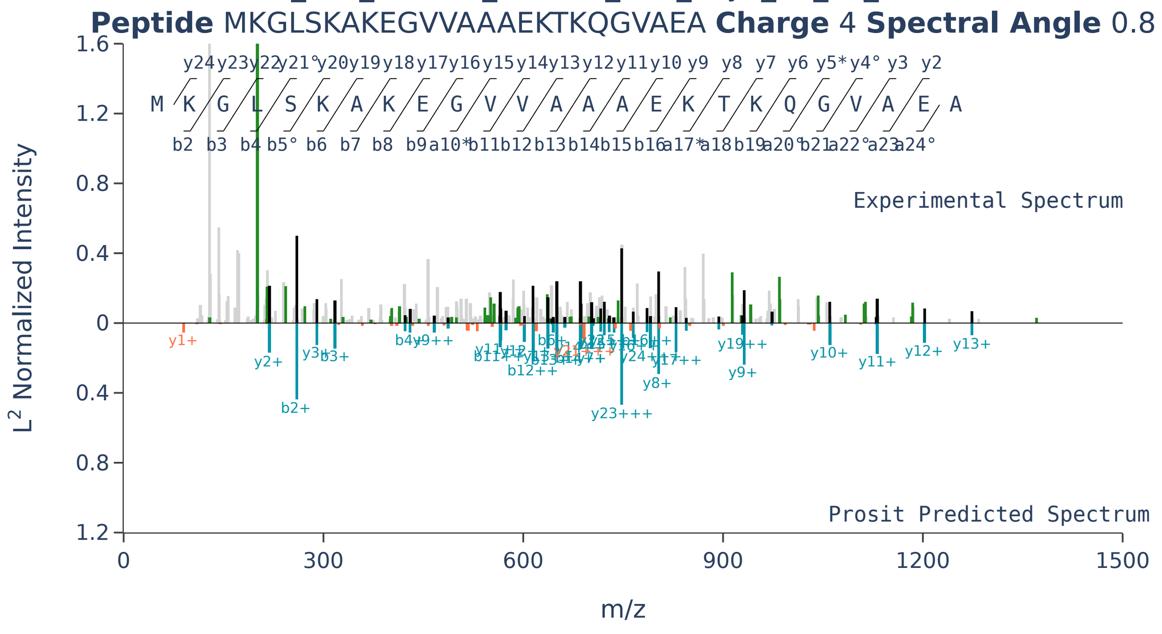


**Source** W_Soh_160821_170921_Goe_aSyn_A1_24h_R1 **Scan** 8889
**Peptide** GVVAAAEKTKQGVAEA **Charge** 2 **Spectral Angle** 0.83

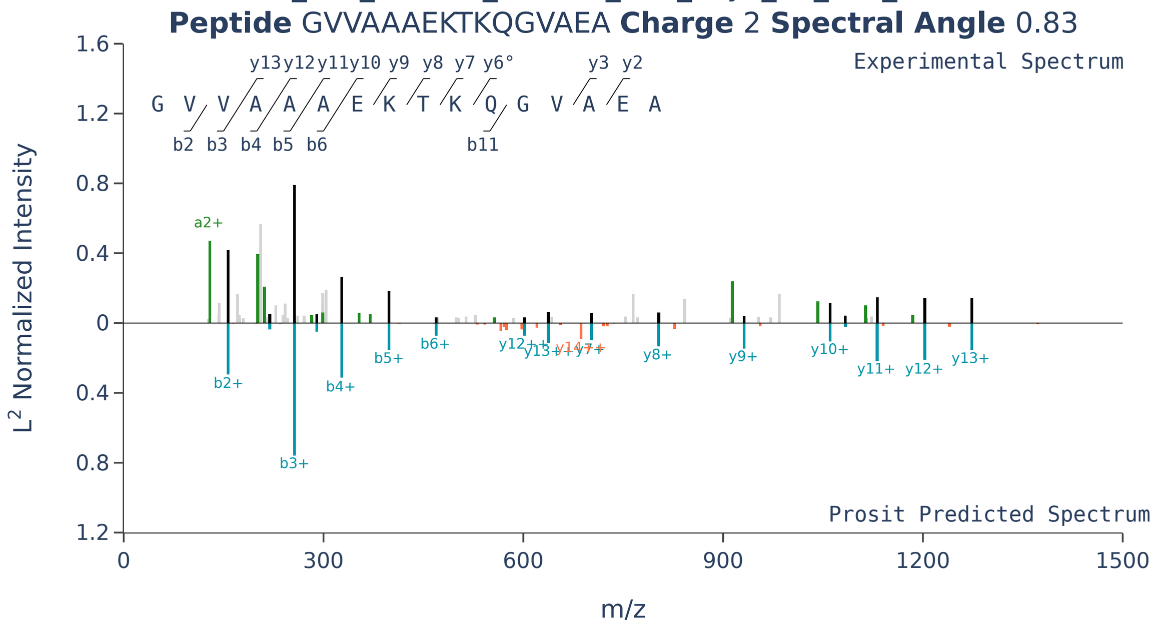


**Source** W_Soh_160821_170921_Goe_aSyn_A1_24h_R2 **Scan** 13054
**Peptide** VAEAAGKTKEGVLYVG **Charge** 2 **Spectral Angle** 0.89

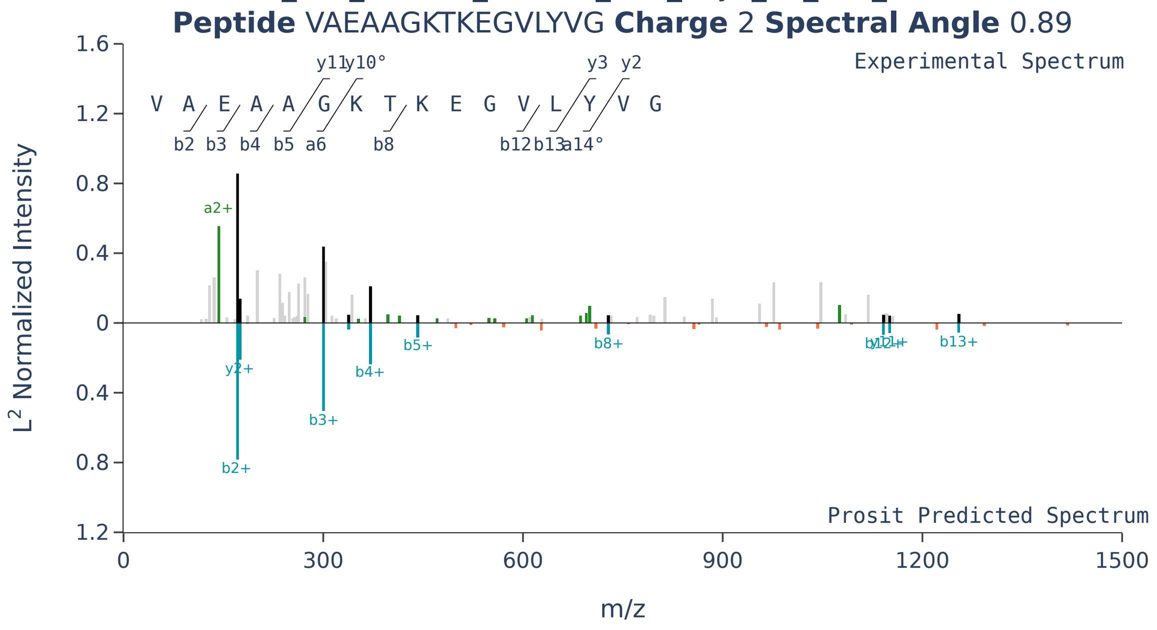


**Source** W_Soh_160821_170921_Goe_aSyn_A2_24h_R1 **Scan** 11068
**Peptide** GFVKKDQLGKNEEGAPQEGI **Charge** 3 **Spectral Angle** 0.85

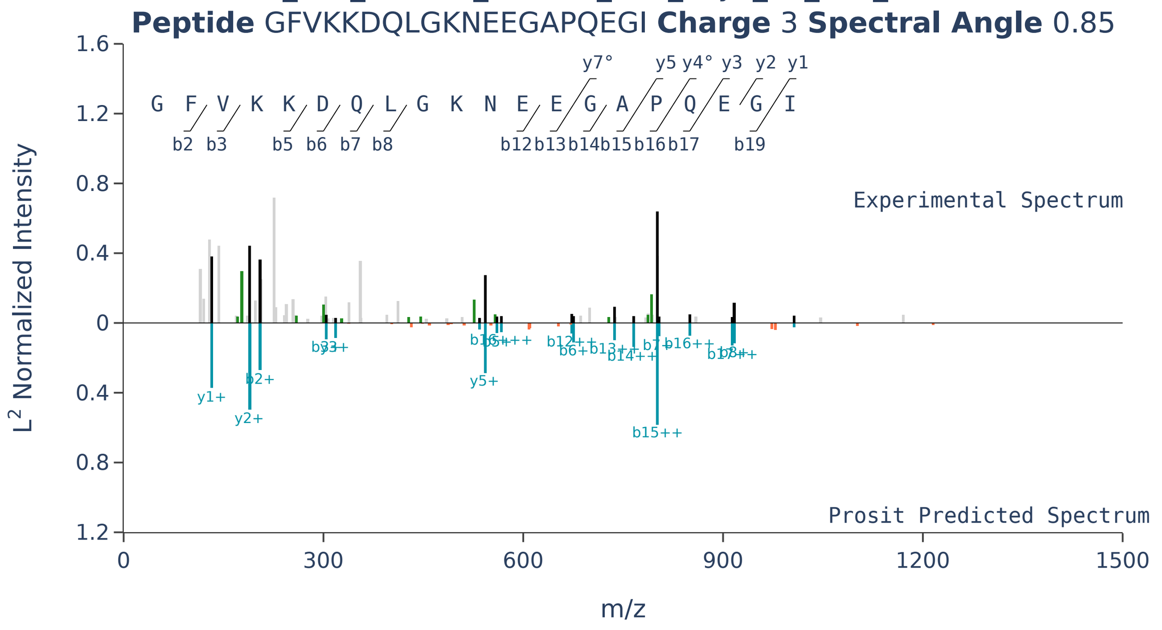


**Source** W_Soh_160821_170921_Goe_aSyn_A1_3h_R1 **Scan** 21689
**Peptide** VAEKTKEQVTNVGGAVVTGVTAVAQKTVE **Charge** 3 **Spectral Angle** 0.68

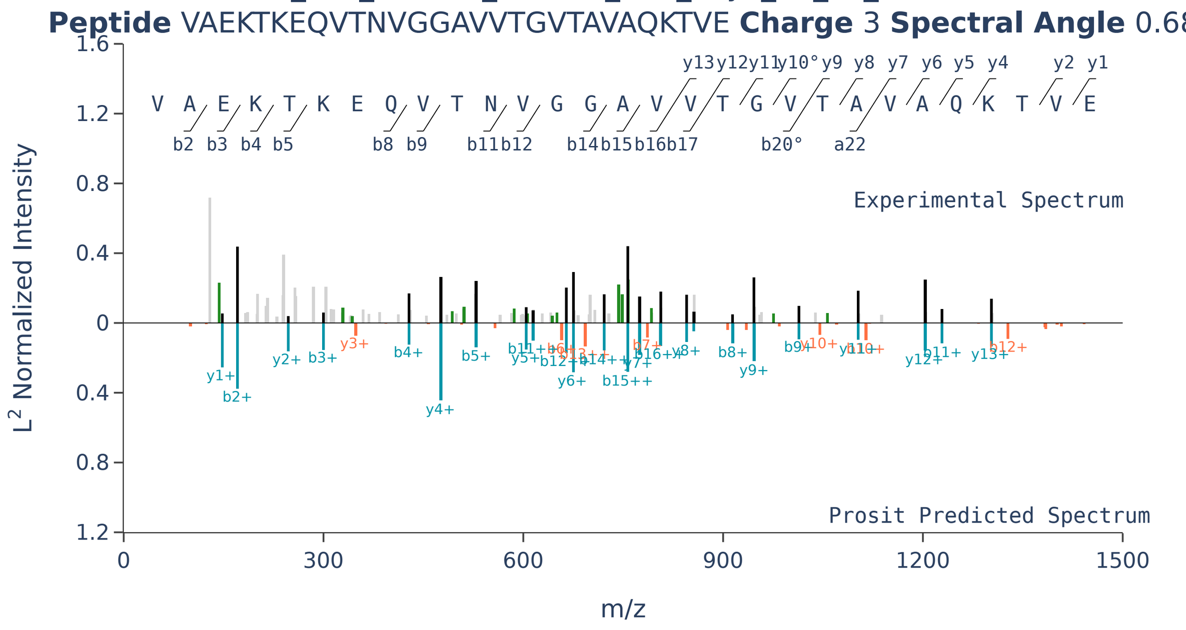


**Source** W_Soh_160821_170921_Goe_aSyn_A1_24h_R2 **Scan** 19321
**Peptide** VTAVAQKTVEGAGSIAAATGFVKKD **Charge** 4 **Spectral Angle** 0.8

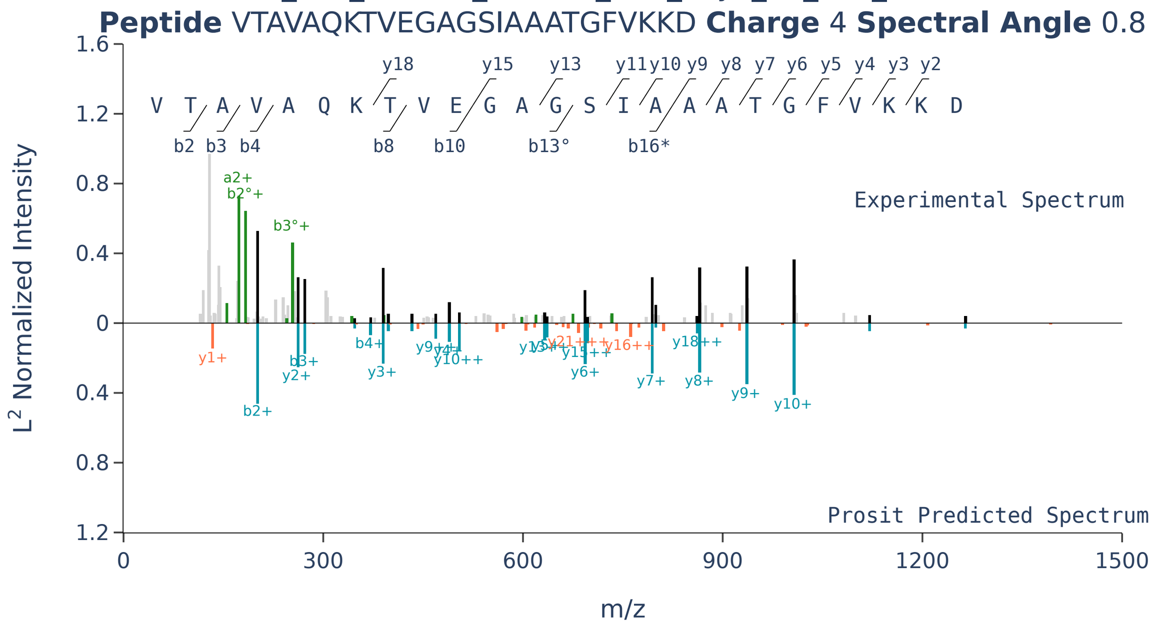


**Source** W_Soh_160821_170921_Goe_aSyn_A2_6h_R1 **Scan** 17867
**Peptide** VAQKTVEGAGSIAAATGFVKKDQL **Charge** 4 **Spectral Angle** 0.79

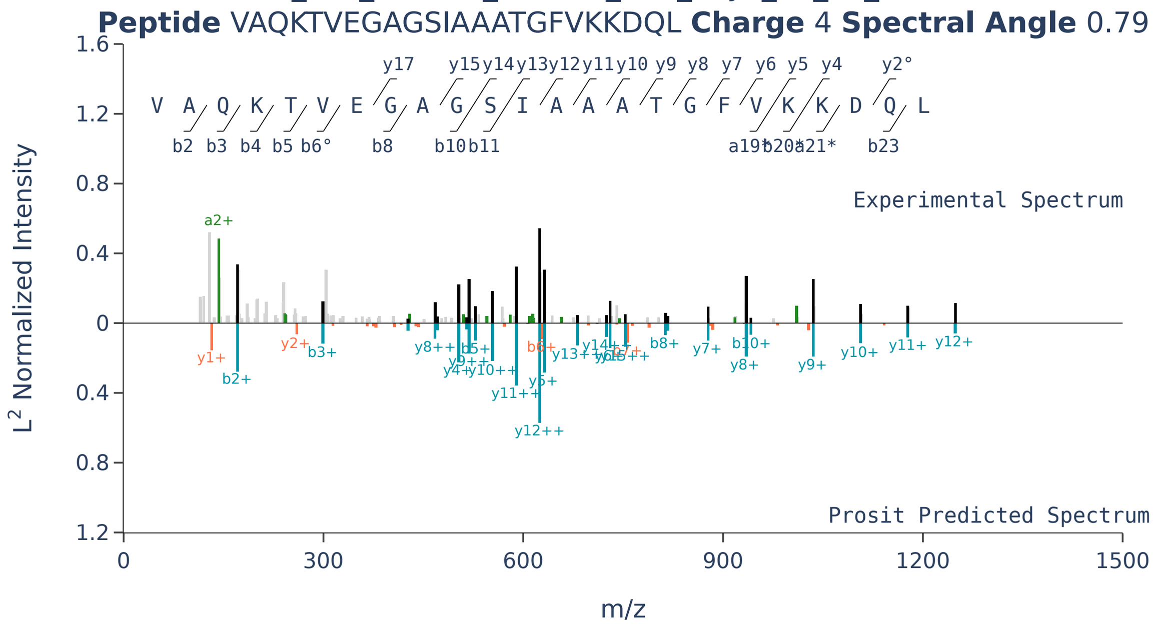


**Source** W_Soh_160821_170921_Goe_aSyn_A1_24h_R1 **Scan** 19075
**Peptide** VTAVAQKTVEGAGSIAAATGFVKKDQ **Charge** 3 **Spectral Angle** 0.77

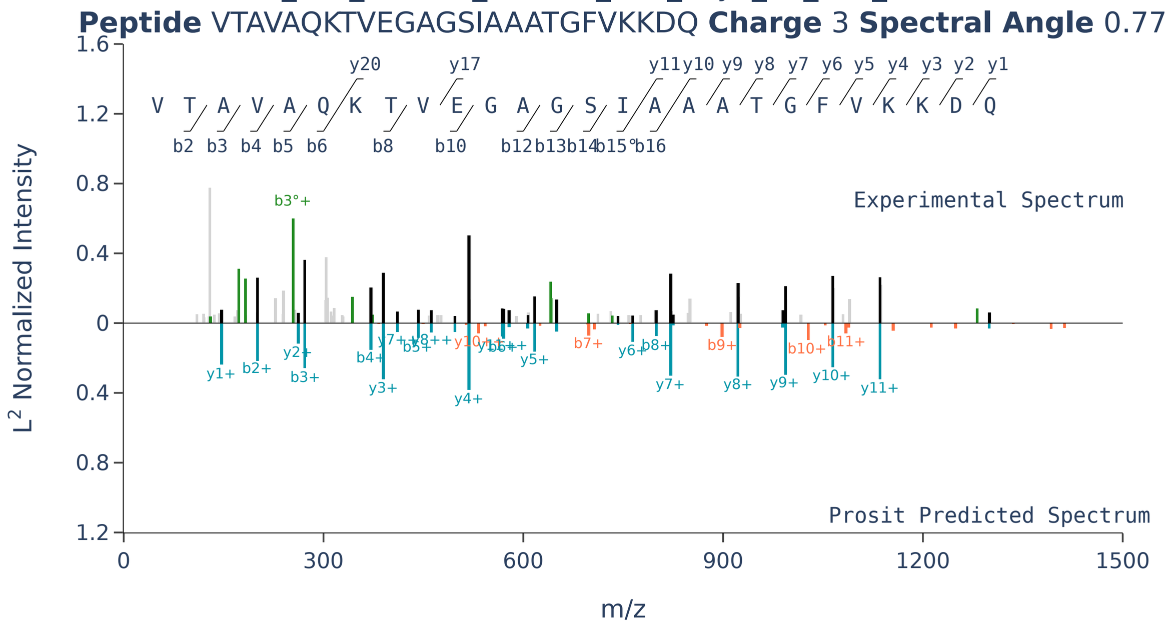


**Source** W_Soh_160821_170921_Goe_aSyn_A1_24h_R2 **Scan** 10968
**Peptide** EGAPQEGI **Charge** 2 **Spectral Angle** 0.78

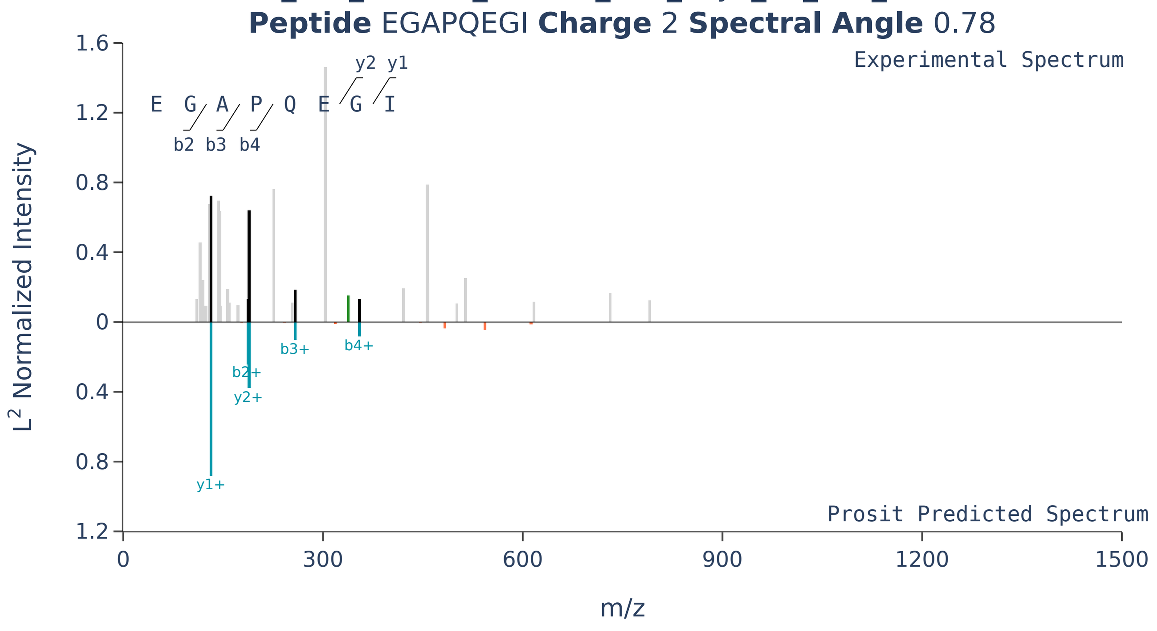


**Source** W_Soh_160821_170921_Goe_aSyn_A1_3h_R1 **Scan** 9838
**Peptide** EKTKEQVTNVGGAVVT **Charge** 3 **Spectral Angle** 0.79

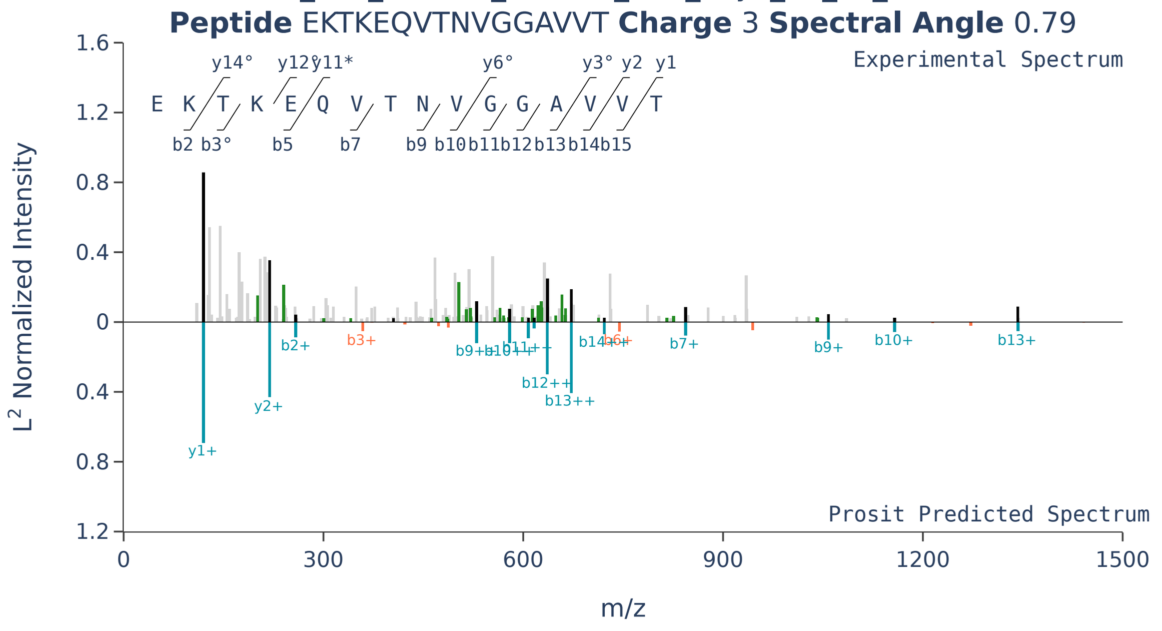


**Source** W_Soh_160821_170921_Goe_aSyn_A1_24h_R2 **Scan** 15477
**Peptide** QKTVEGAGSIAAATGFVKKDQLGKNE **Charge** 4 **Spectral Angle** 0.74

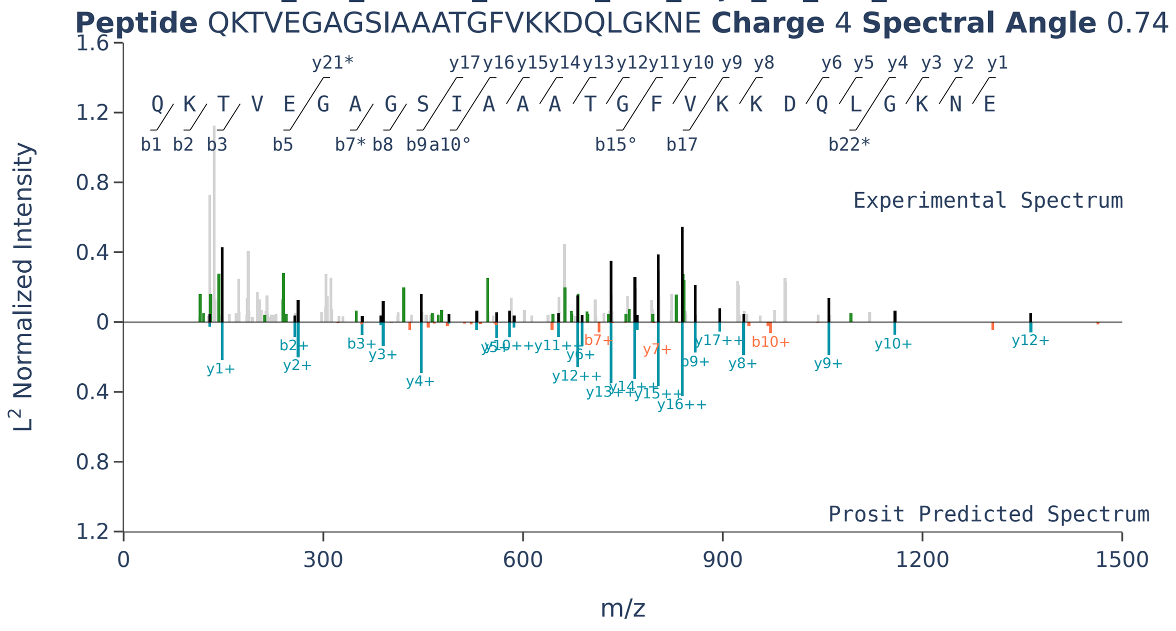


**Source** W_Soh_160821_170921_Goe_aSyn_A2_6h_R1 **Scan** 5294
**Peptide** HGVATVAEKTKE **Charge** 3 **Spectral Angle** 0.72

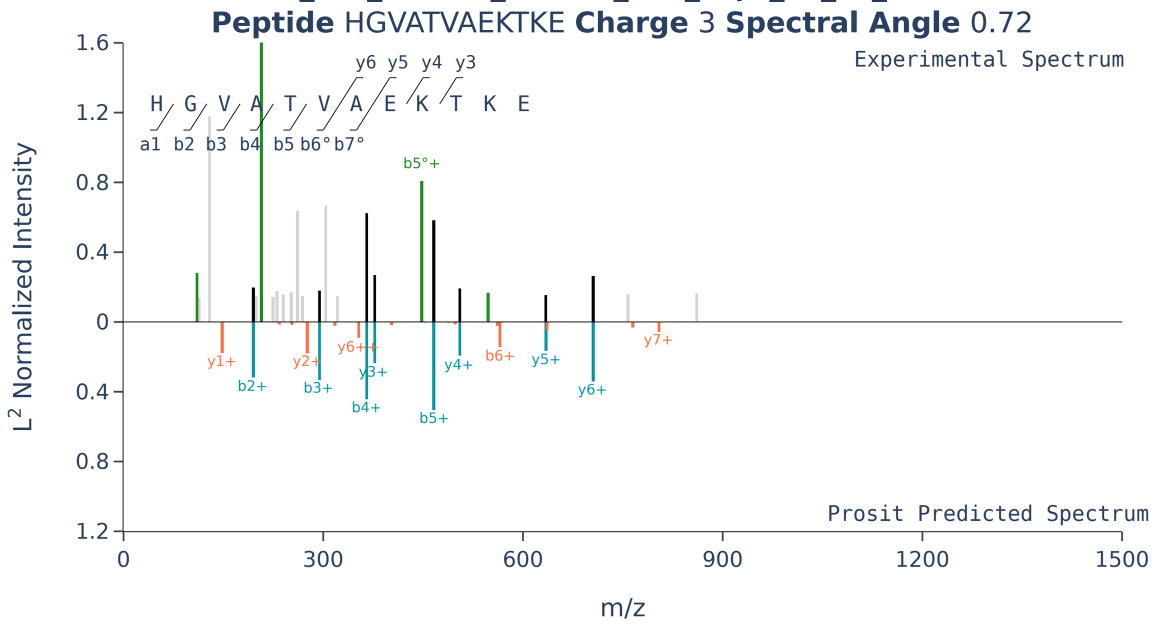


**Source** W_Soh_160821_170921_Goe_aSyn_A1_24h_R2 **Scan** 20284
**Peptide** MPVDPDNEAYEMPSEE **Charge** 2 **Spectral Angle** 0.82

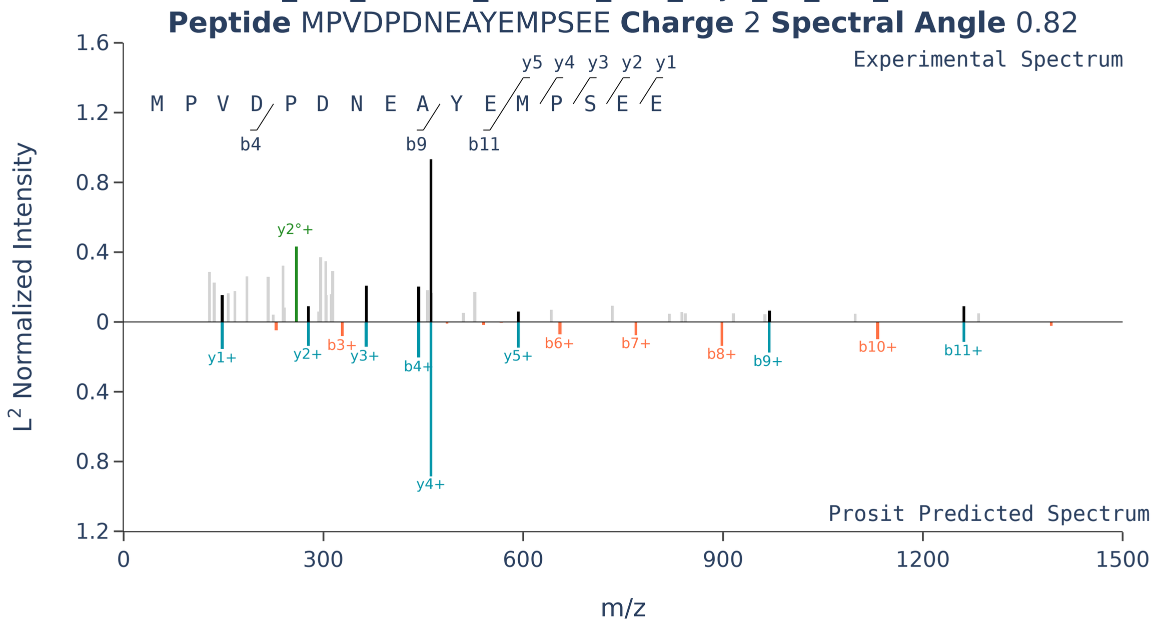


**Source** W_Soh_160821_170921_Goe_aSyn_A2_6h_R1 **Scan** 14716
**Peptide** QLGKNEEGAPQEGILE **Charge** 2 **Spectral Angle** 0.89

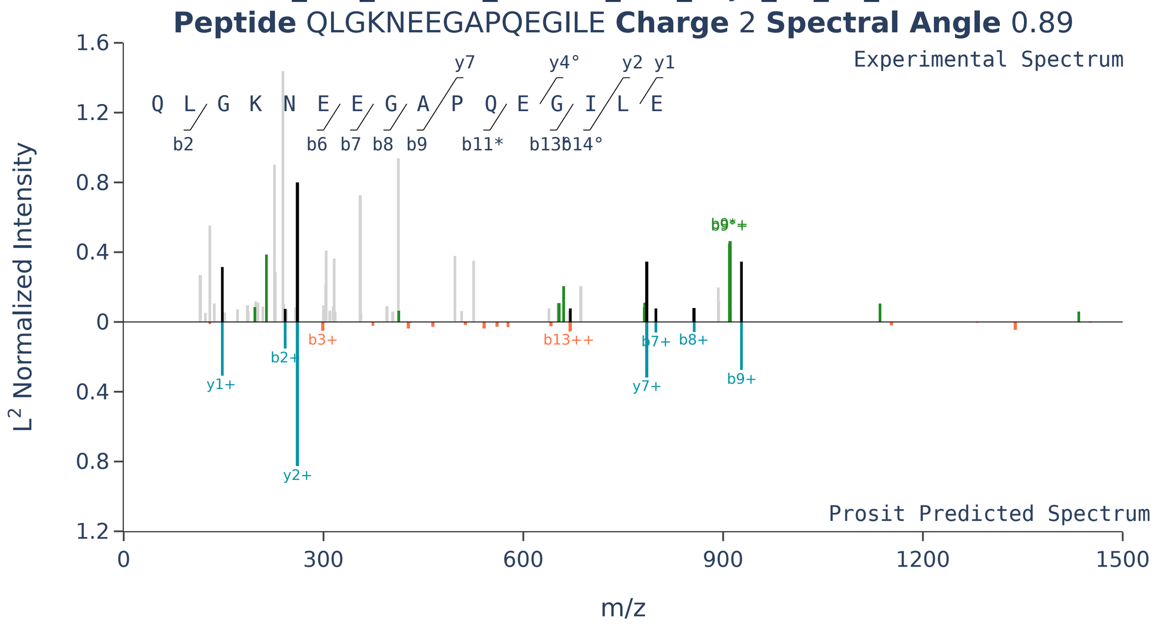

**Source** W_Soh_160821_170921_Goe_aSyn_A2_24h_R2 **Scan** 14519
**Peptide** HGVATVAEKTKEQVTNVGGAVVT **Charge** 3 **Spectral Angle** 0.69

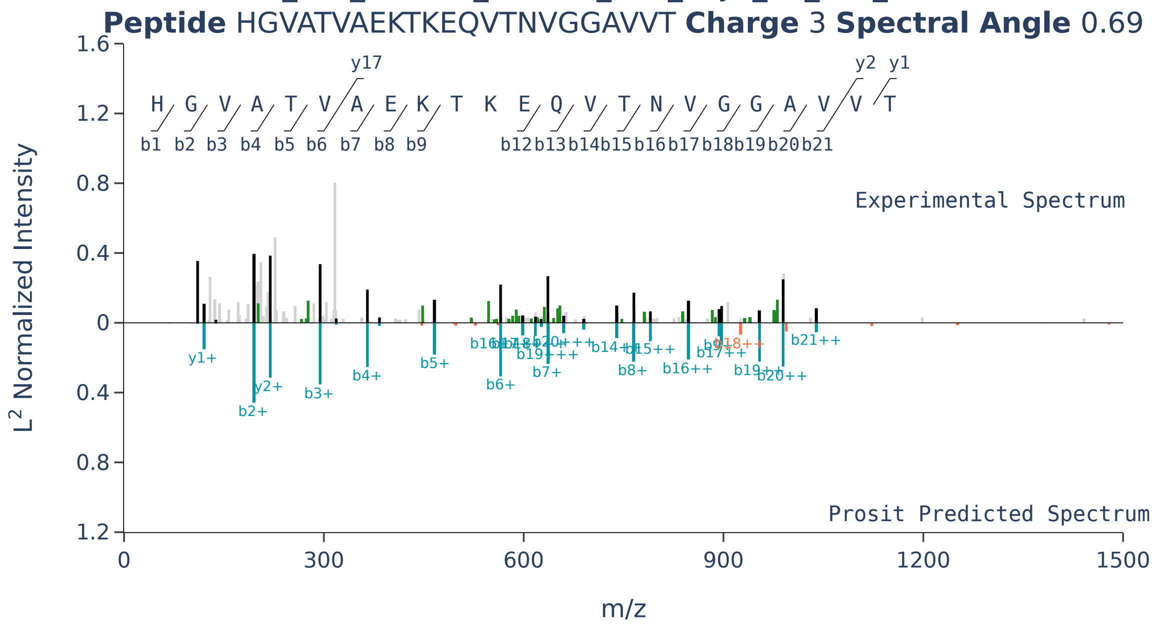


**Source** W_Soh_160821_170921_Goe_aSyn_A1_6h_R2 **Scan** 19983
**Peptide** VAQKTVEGAGSIAAATGF **Charge** 2 **Spectral Angle** 0.7

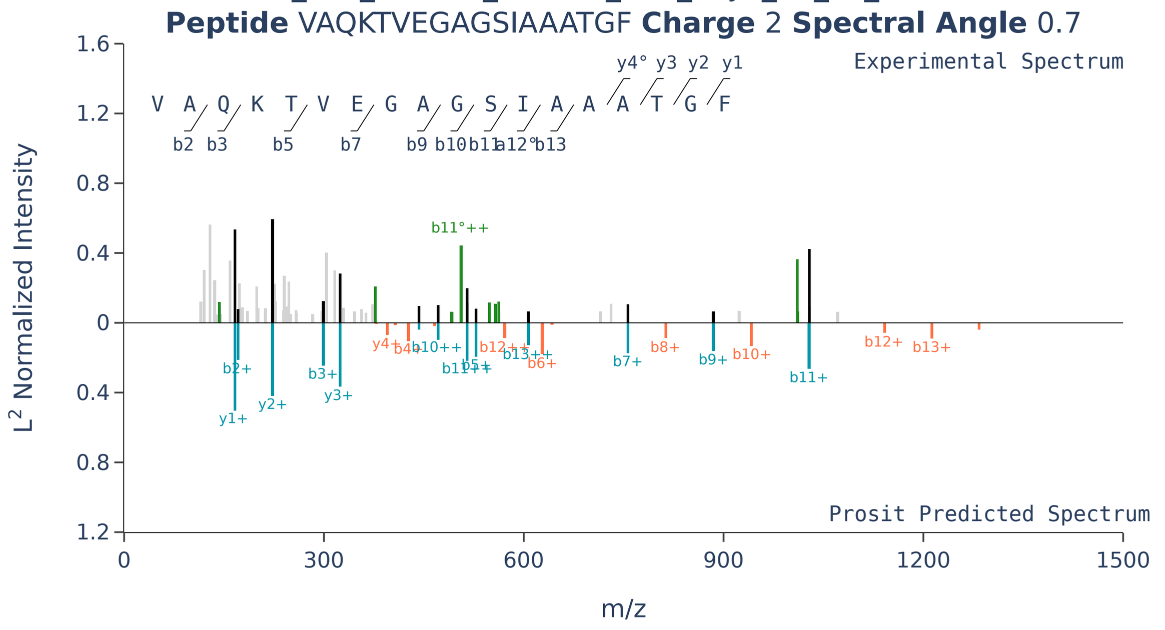


**Source** W_Soh_160821_170921_Goe_aSyn_A1_24h_R2 **Scan** 9236
**Peptide** AEKTKQGVAEAAGKTKEGVLYVGSKTKE **Charge** 4 **Spectral Angle** 0.75

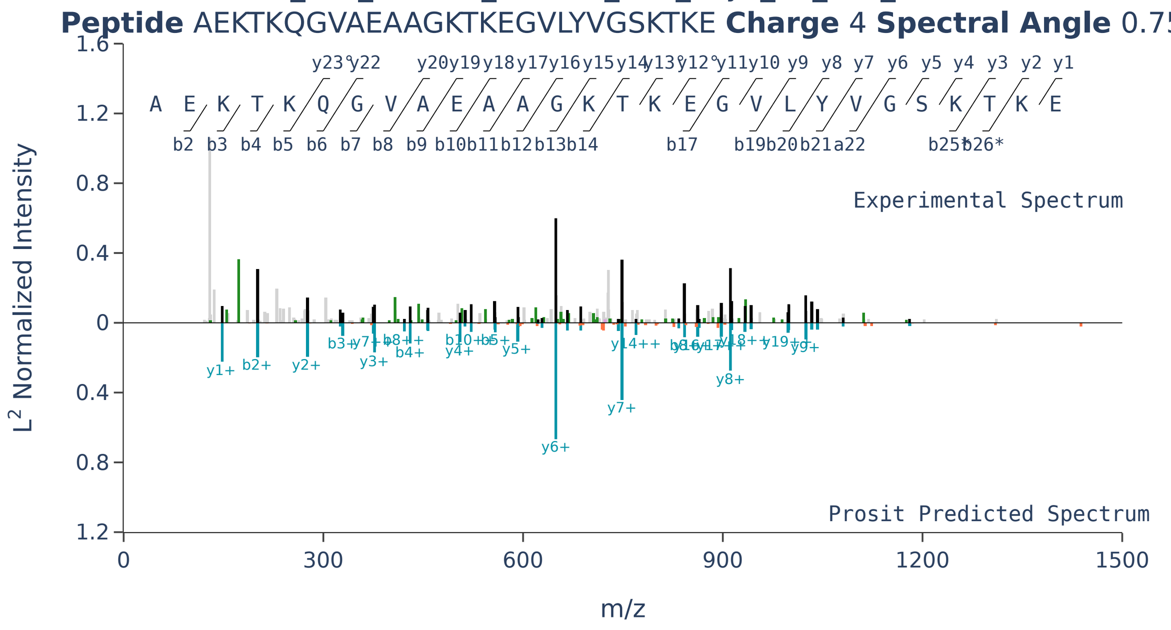


**Source** W_Soh_160821_170921_Goe_aSyn_A1_24h_R1 **Scan** 14077
**Peptide** VLYVGSKTKEGVVHGVATV **Charge** 2 **Spectral Angle** 0.88

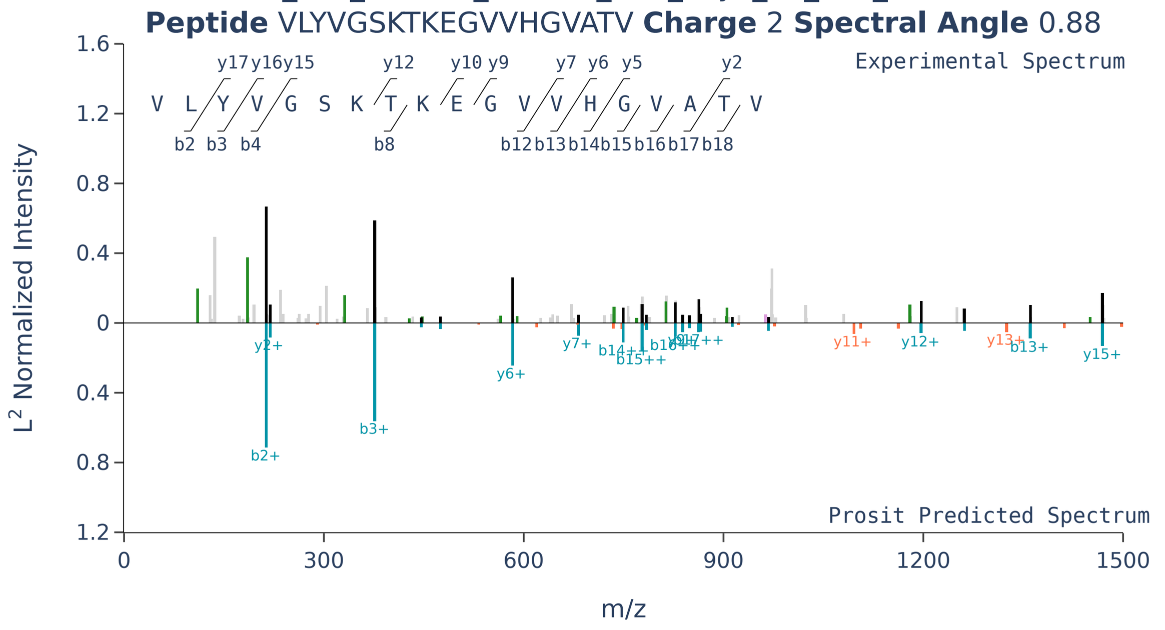


**Source** W_Soh_160821_170921_Goe_aSyn_A2_24h_R2 **Scan** 12952
**Peptide** VTGVTAVAQKTVE **Charge** 2 **Spectral Angle** 0.79

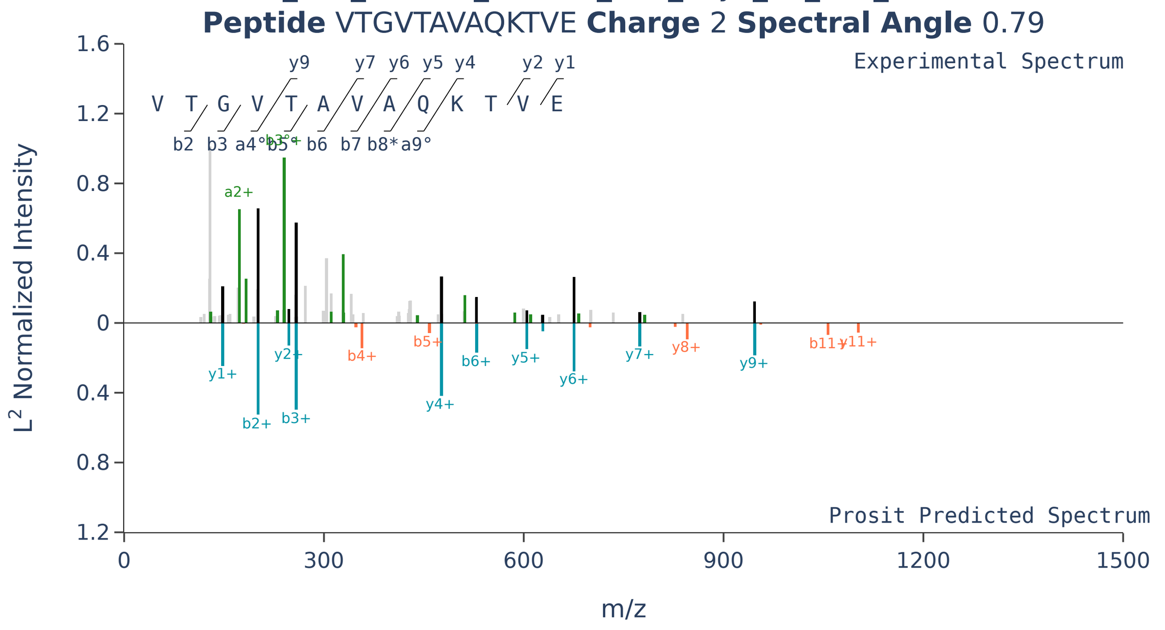


**Source** W_Soh_160821_170921_Goe_aSyn_A1_24h_R1 **Scan** 25543
**Peptide** TNVGGAVVTGVTAVAQKTVEG **Charge** 2 **Spectral Angle** 0.88

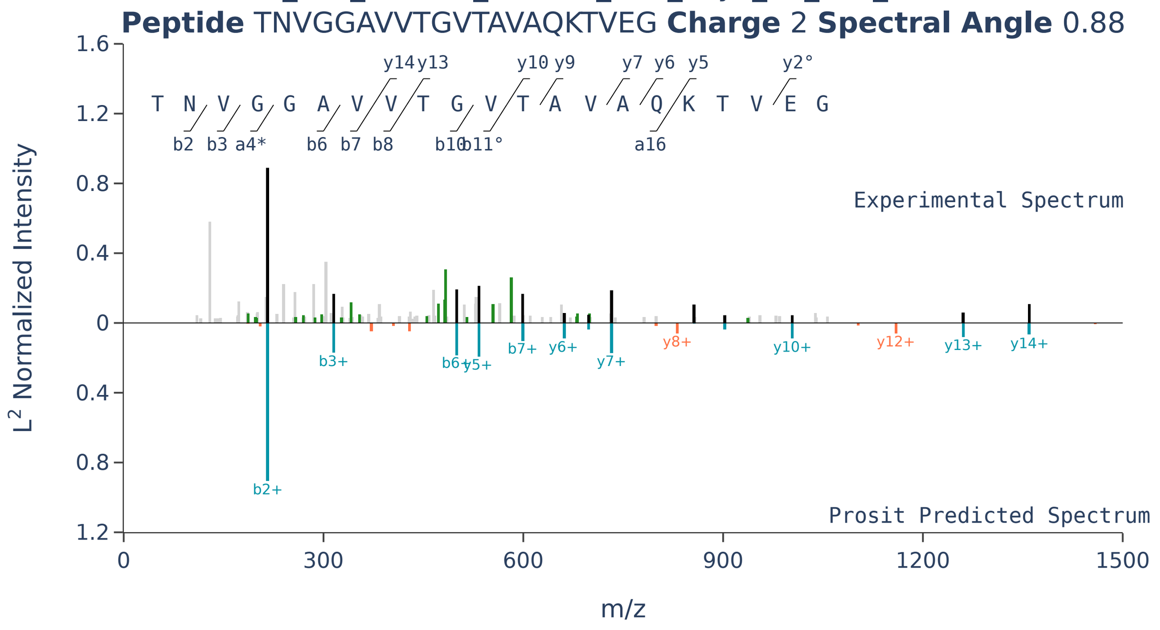


**Source** W_Soh_160821_170921_Goe_aSyn_A1_24h_R1 **Scan** 20344
**Peptide** VEGAGSIAAATGFVKKDQL **Charge** 3 **Spectral Angle** 0.77

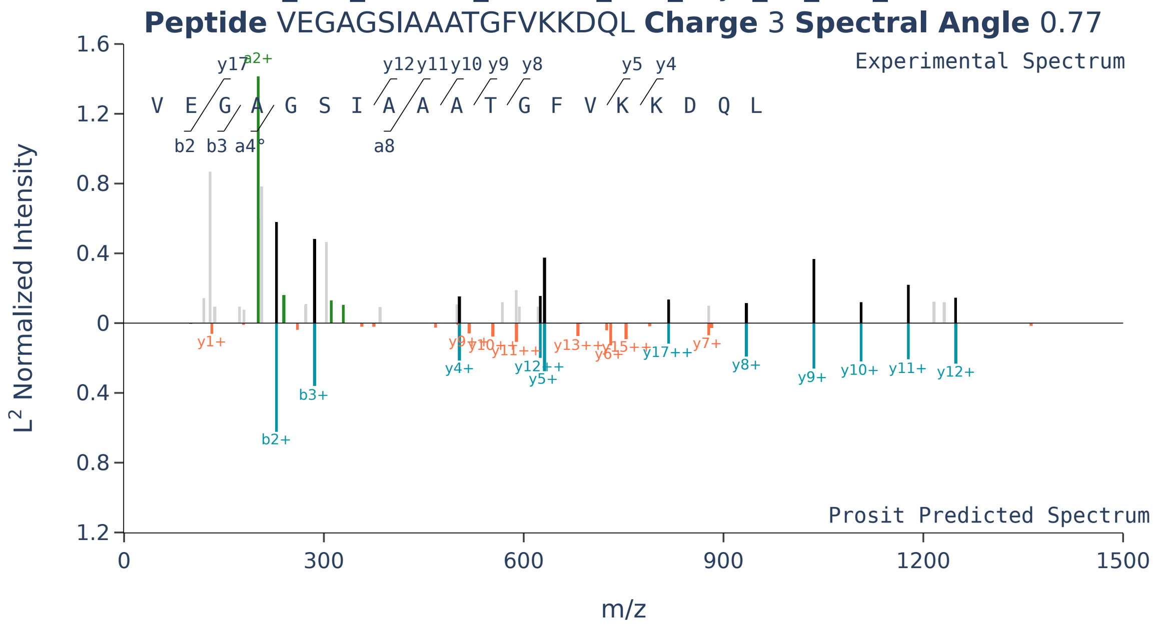


**Source** W_Soh_160821_170921_Goe_aSyn_A1_24h_R2 **Scan** 13632
**Peptide** SKTKEGVVHGVATVAEKTKEQVTNV **Charge** 4 **Spectral Angle** 0.82

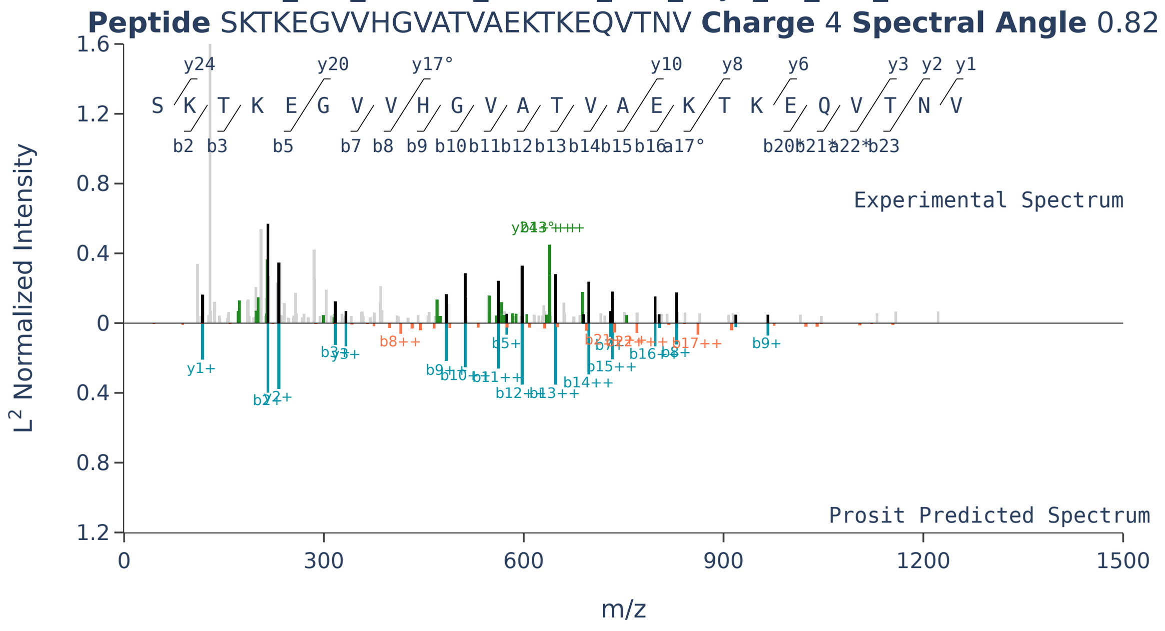


**Source** W_Soh_160821_170921_Goe_aSyn_A1_24h_R1 **Scan** 17191
**Peptide** VEGAGSIAAATGFVKKDQLGKNEEGAPQE **Charge** 4 **Spectral Angle** 0.87

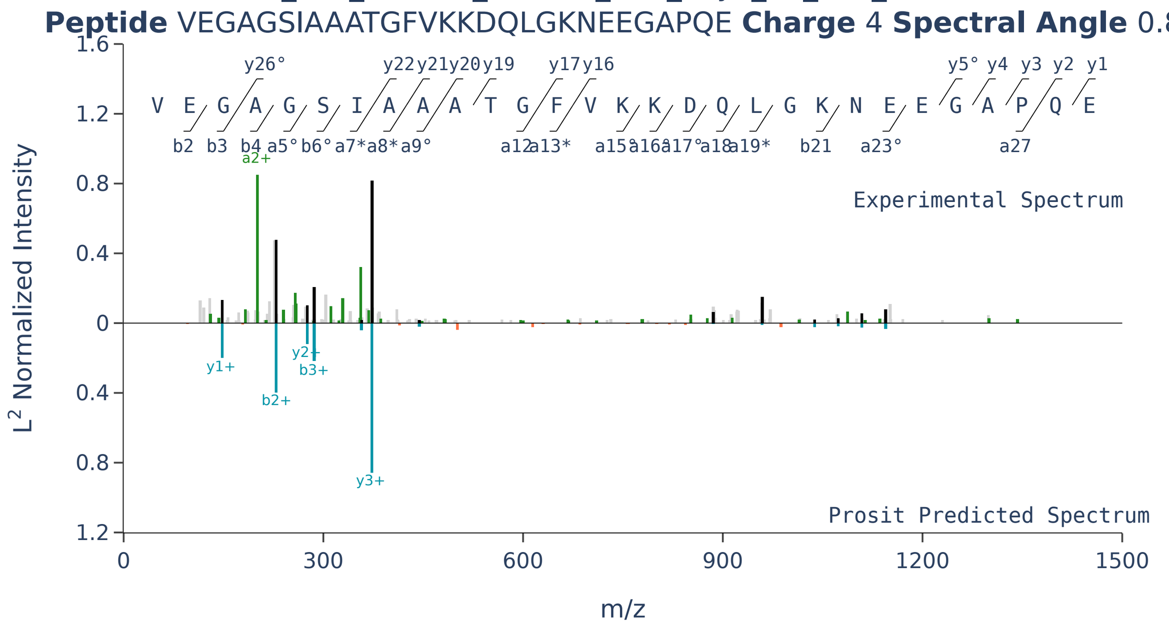


**Source** W_Soh_160821_170921_Goe_aSyn_A1_6h_R2 **Scan** 11400
**Peptide** EGVLYVGSKTKEGVVHG **Charge** 4 **Spectral Angle** 0.87

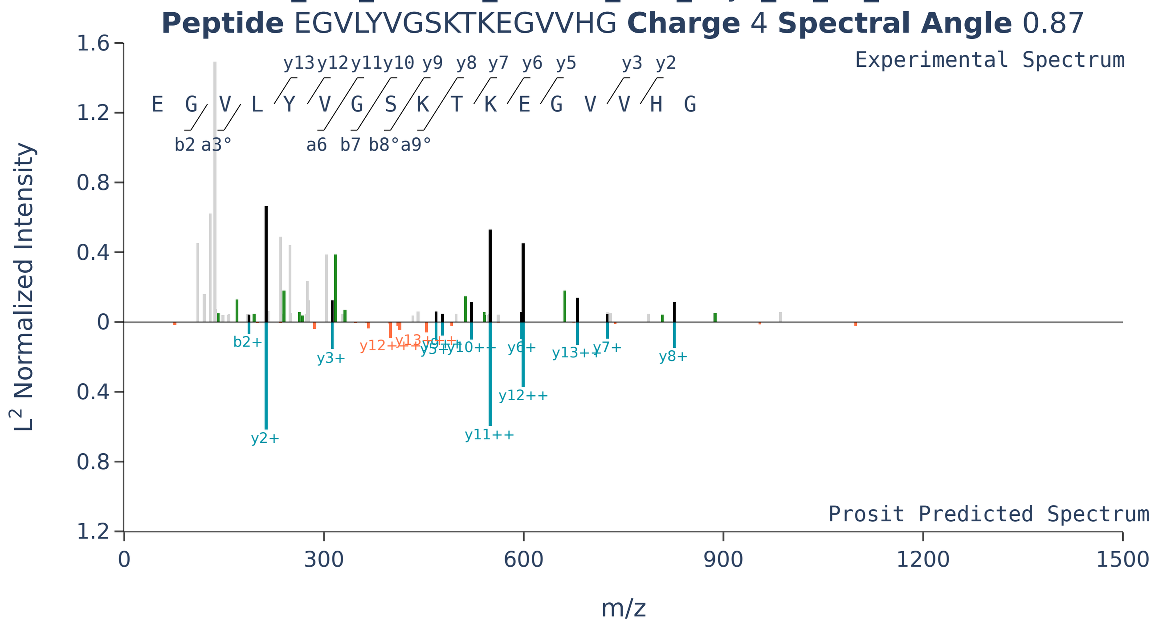


**Source** W_Soh_160821_170921_Goe_aSyn_A2_24h_R2 **Scan** 9905
**Peptide** GKTKEGVLYVGSKTKEGVVHGV **Charge** 5 **Spectral Angle** 0.81

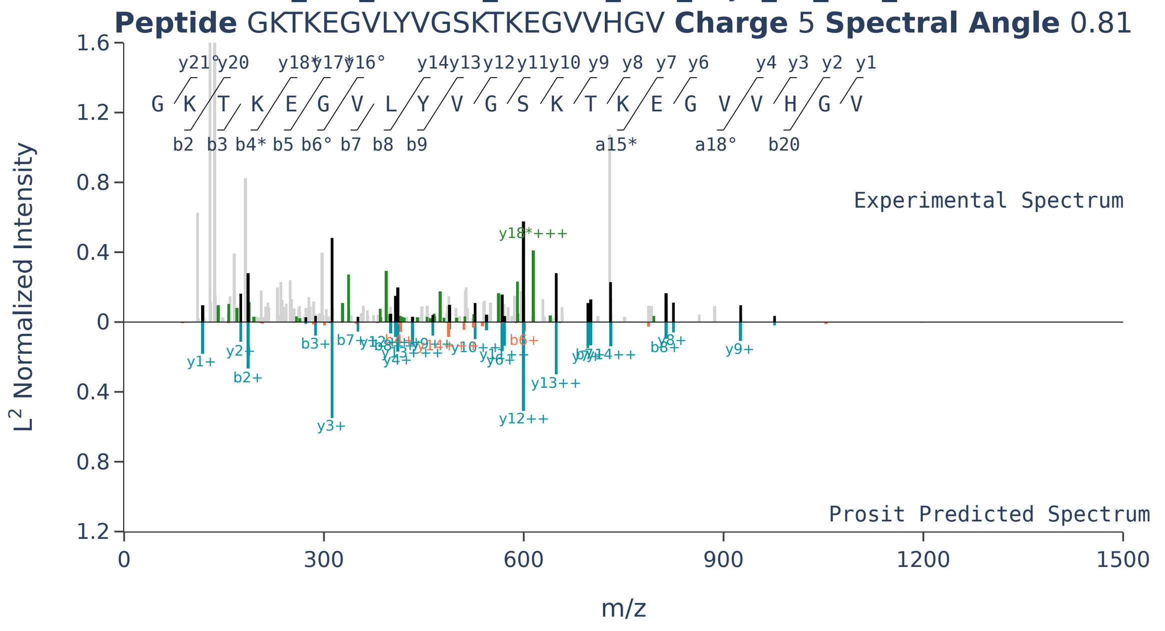


**Source** W_Soh_160821_170921_Goe_aSyn_A2_24h_R2 **Scan** 16857
**Peptide** EGAPQEGILE **Charge** 2 **Spectral Angle** 0.78

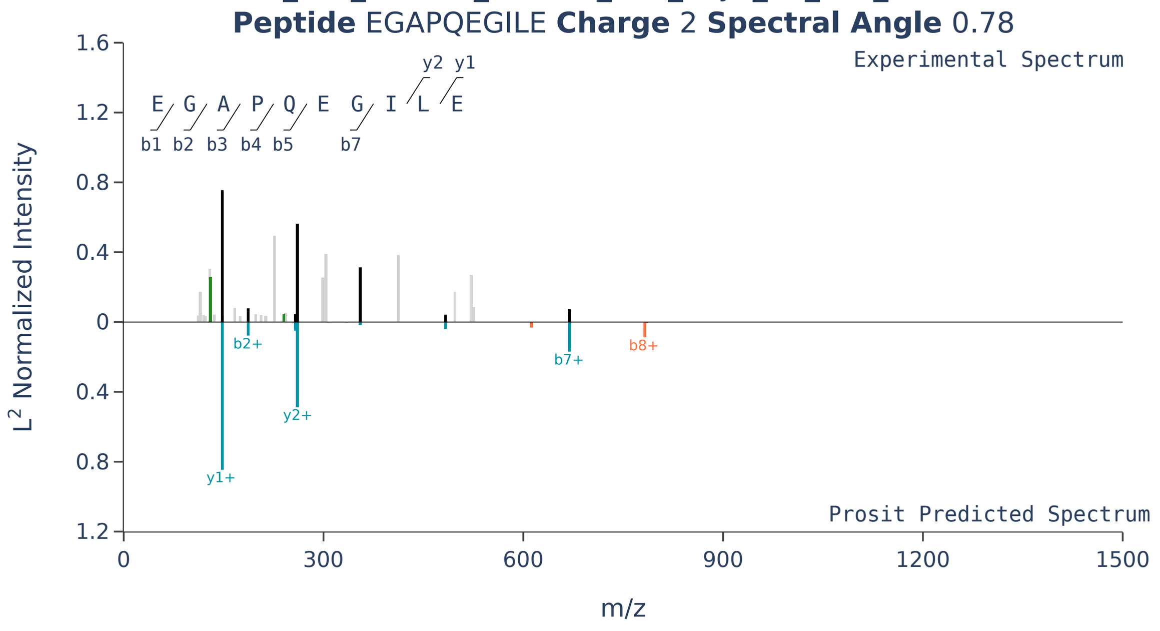


**Source** W_Soh_160821_170921_Goe_aSyn_A2_24h_R2 **Scan** 16538
**Peptide** AVAQKTVEGAGSIAAATGFVKKD **Charge** 3 **Spectral Angle** 0.77

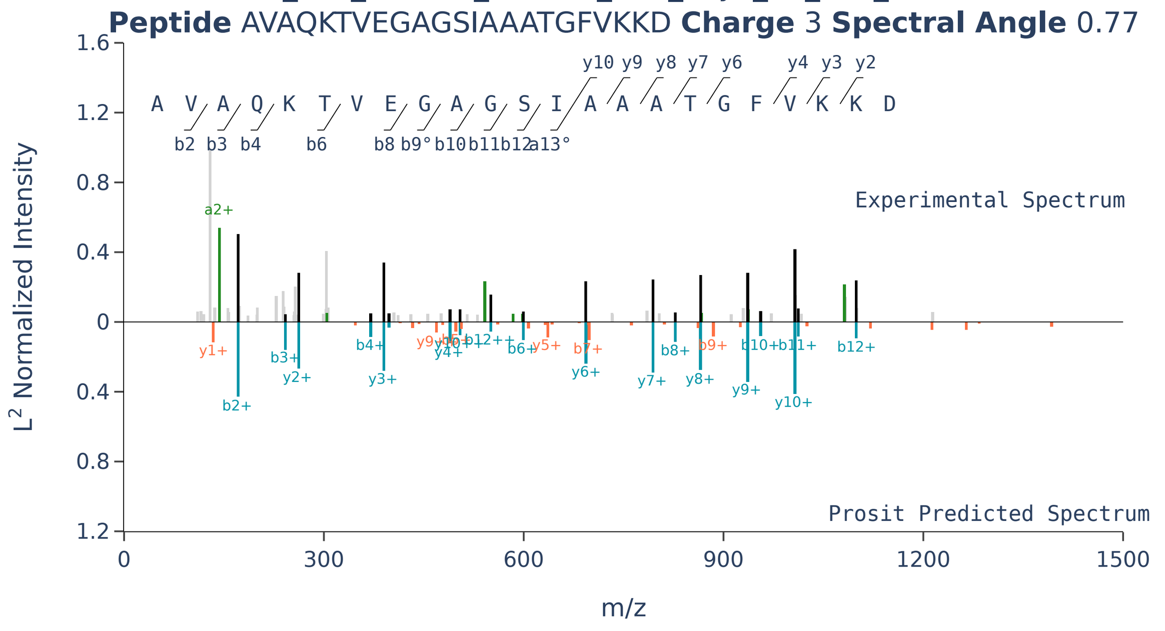


**Source** W_Soh_160821_170921_Goe_aSyn_A1_24h_R2 **Scan** 5185
**Peptide** QGVAEAAGKTKE **Charge** 3 **Spectral Angle** 0.73

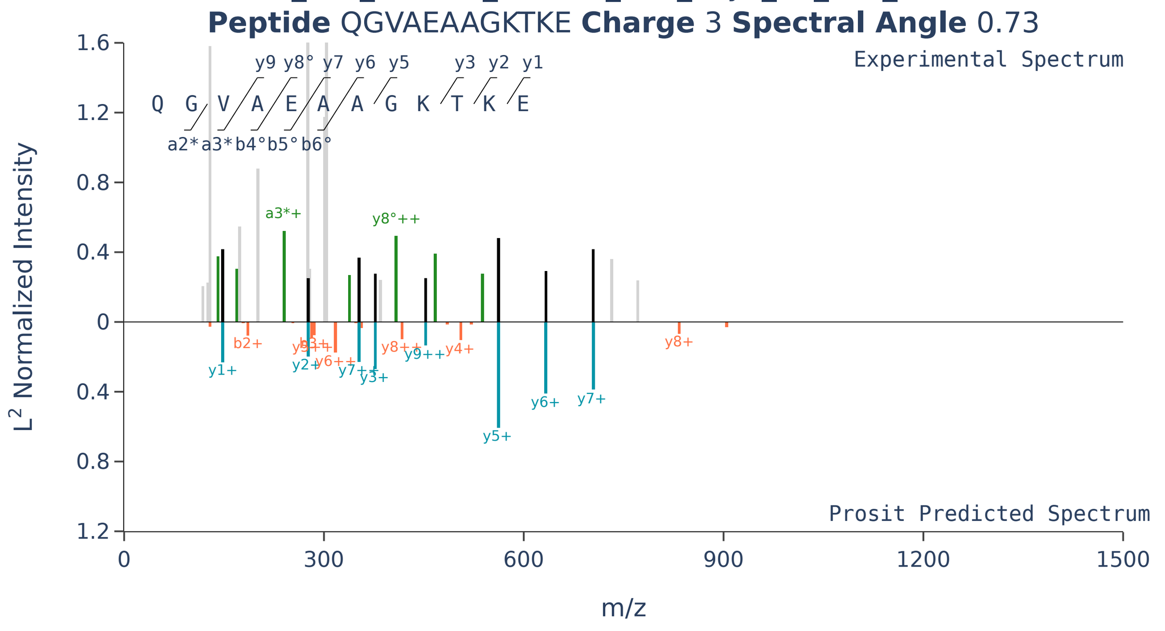


**Source** W_Soh_160821_170921_Goe_aSyn_A1_24h_R2 **Scan** 5496
**Peptide** VAEAAGKTKEGV **Charge** 2 **Spectral Angle** 0.72

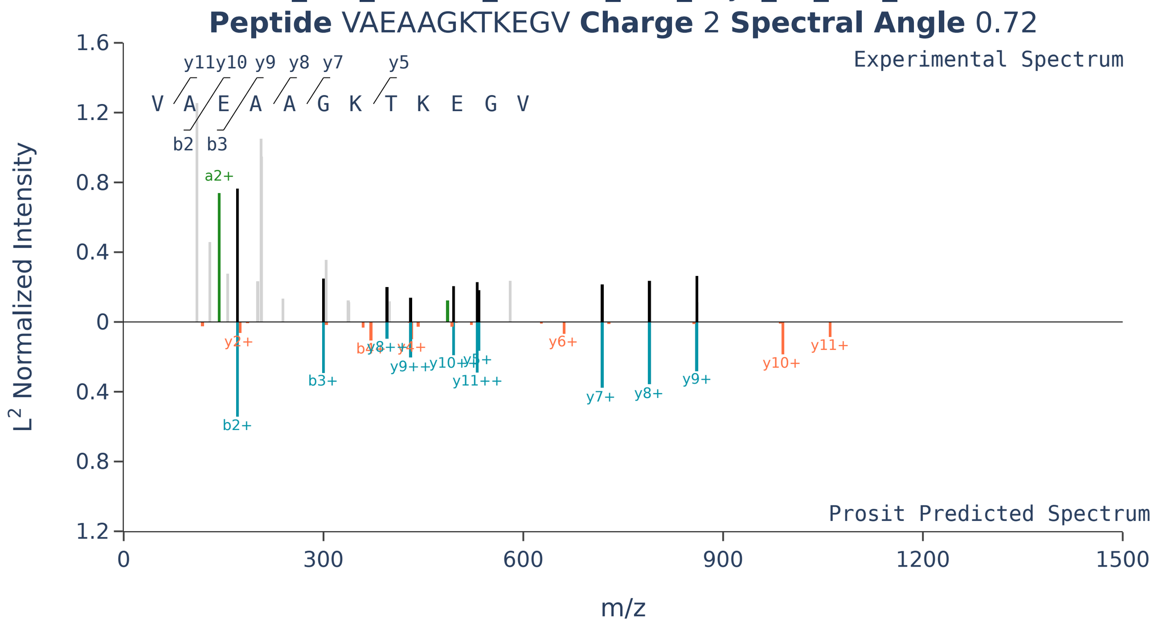

**Source** W_Soh_160821_170921_Goe_aSyn_A1_6h_R1 **Scan** 22095
**Peptide** TNVGGAVVTGVTAVA **Charge** 2 **Spectral Angle** 0.8

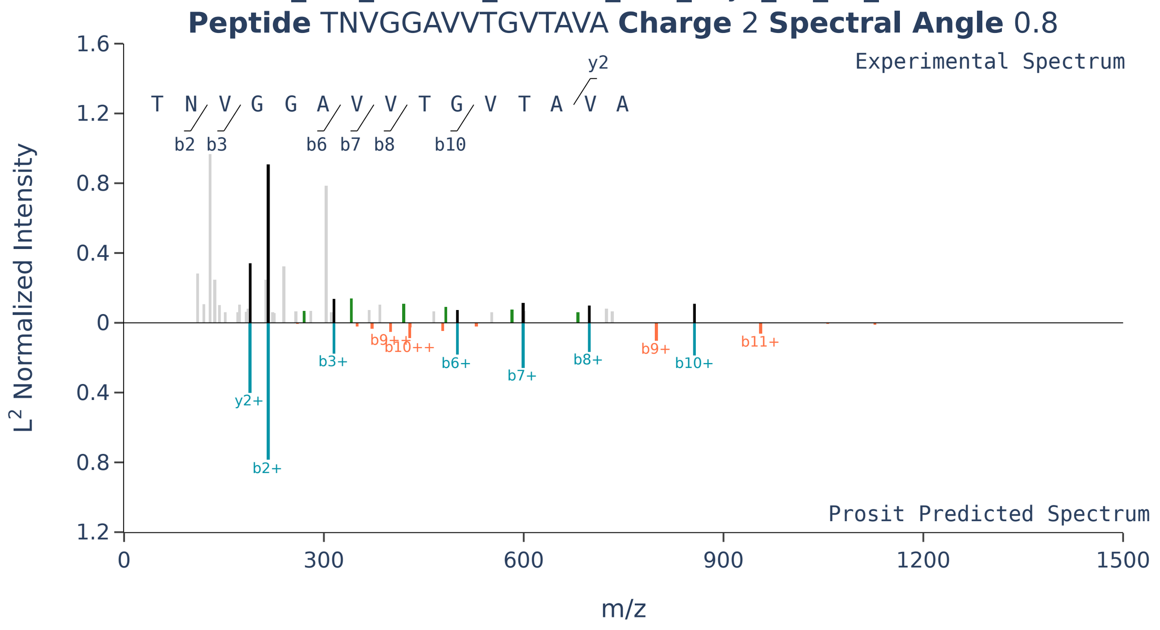


**Source** W_Soh_160821_170921_Goe_aSyn_A1_24h_R2 **Scan** 17322
**Peptide** PVDPDNEAYEMPSE **Charge** 2 **Spectral Angle** 0.86

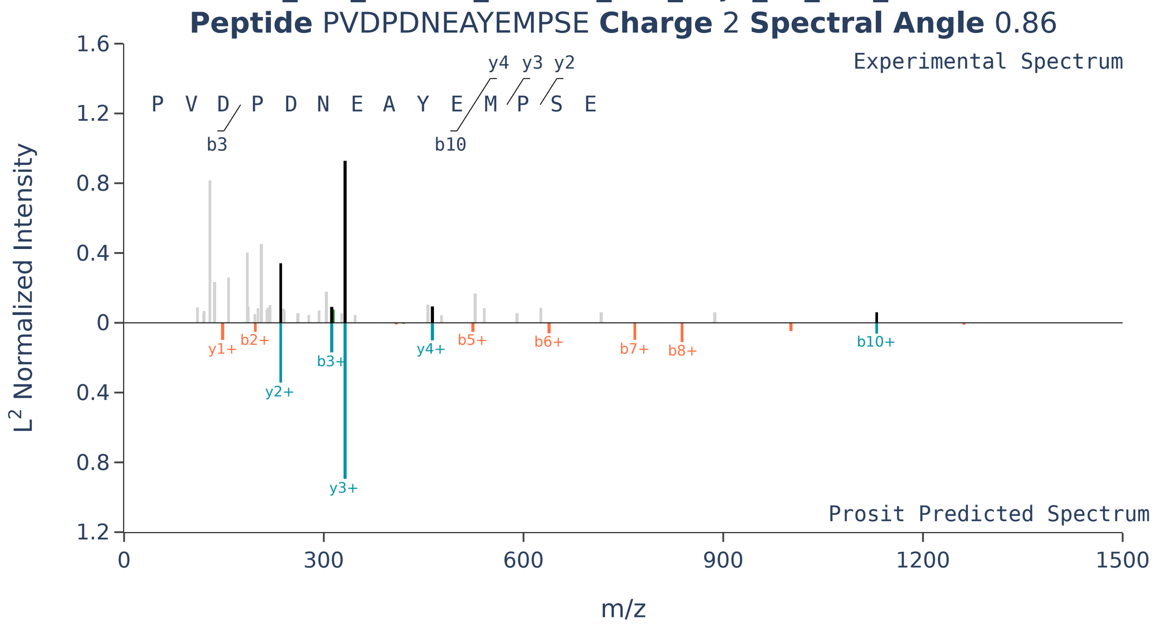


**Source** W_Soh_160821_170921_Goe_aSyn_A1_24h_R1 **Scan** 22413
**Peptide** VAQKTVEGAGSIAAATGFV **Charge** 2 **Spectral Angle** 0.71

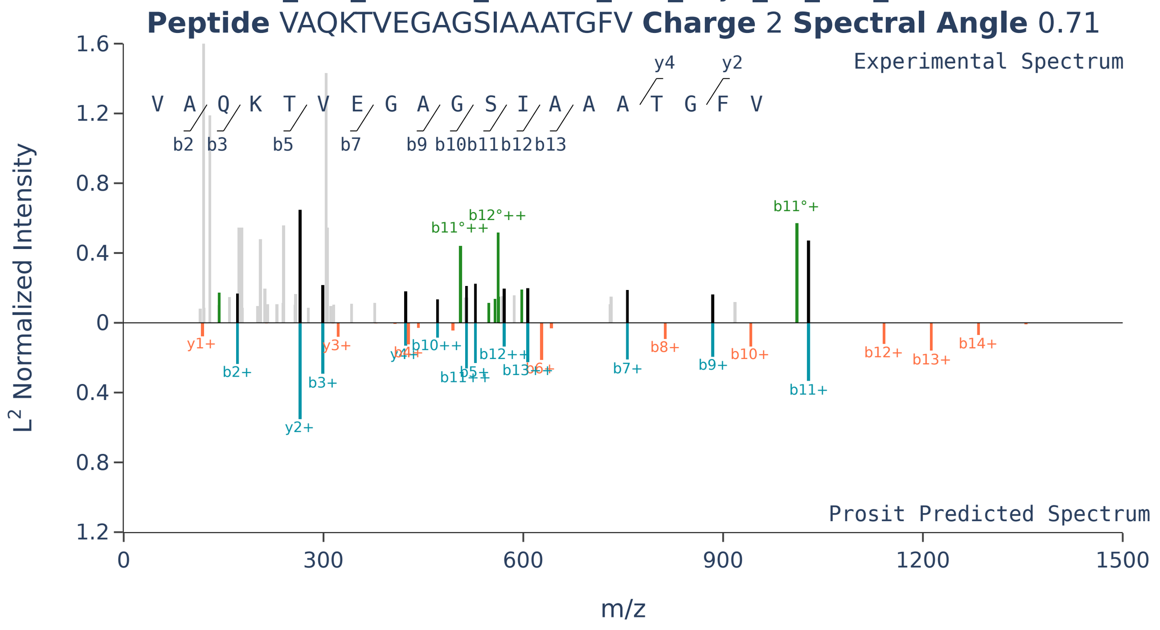


**Source** W_Soh_160821_170921_Goe_aSyn_A1_24h_R2 **Scan** 8182
**Peptide** GVVHGVATVAEKTKEQ **Charge** 4 **Spectral Angle** 0.78

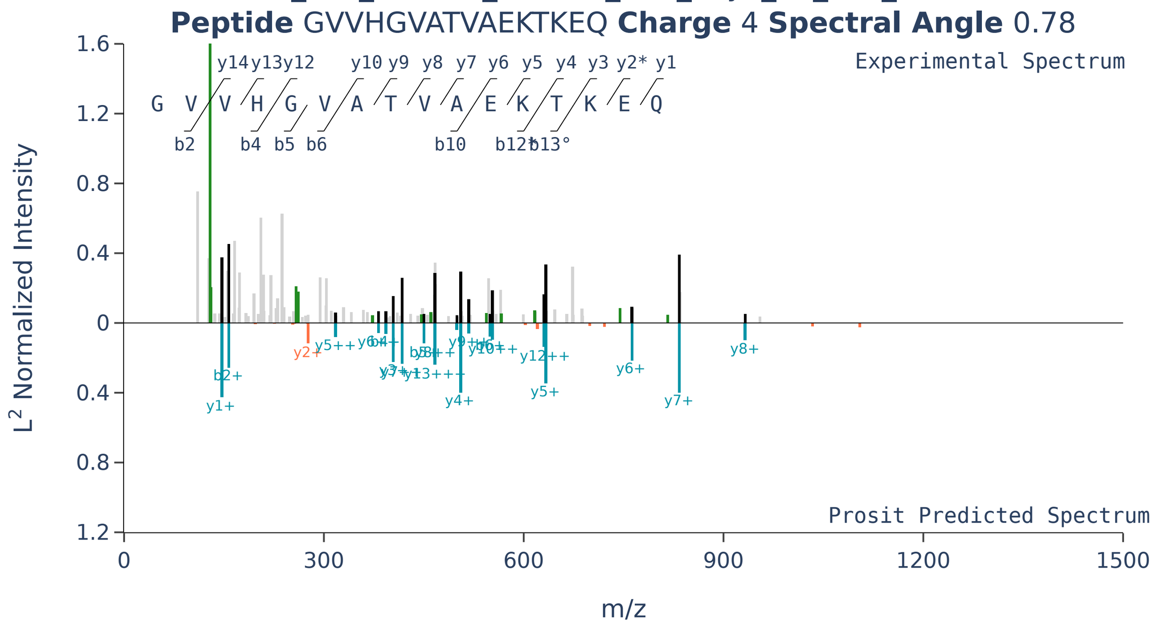


**Source** W_Soh_160821_170921_Goe_aSyn_A2_24h_R1 **Scan** 12416
**Peptide** AAGKTKEGVLYV **Charge** 3 **Spectral Angle** 0.86

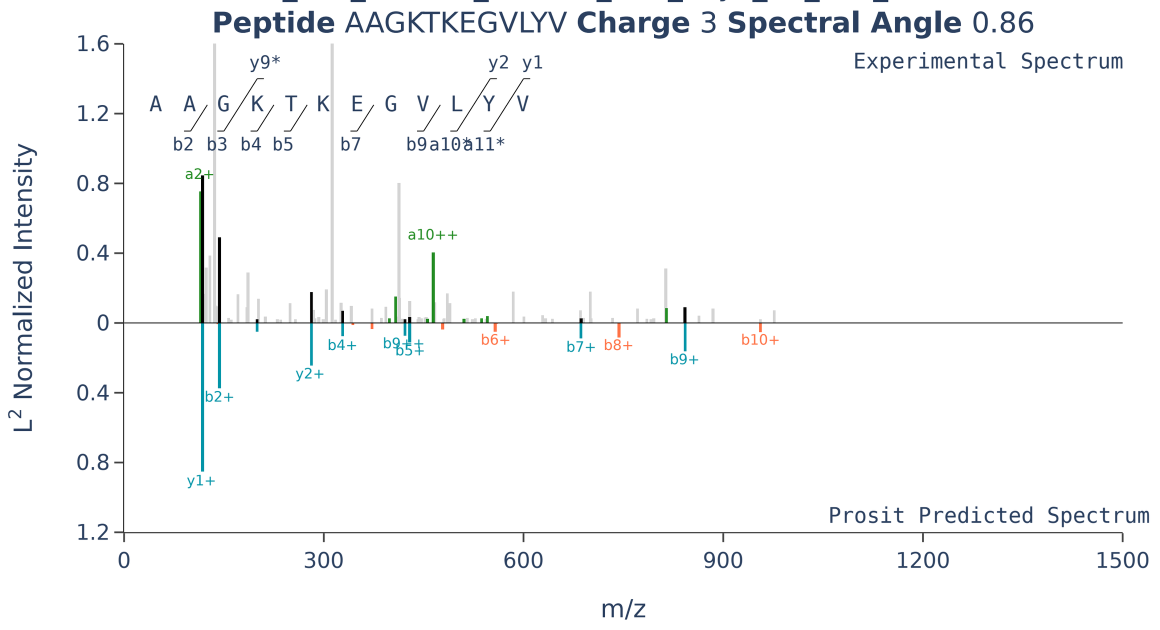


**Source** W_Soh_160821_170921_Goe_aSyn_A1_24h_R1 **Scan** 19212
**Peptide** AGSIAAATGFVKKDQLGKNEEGAPQEGILE **Charge** 3 **Spectral Angle** 0.7

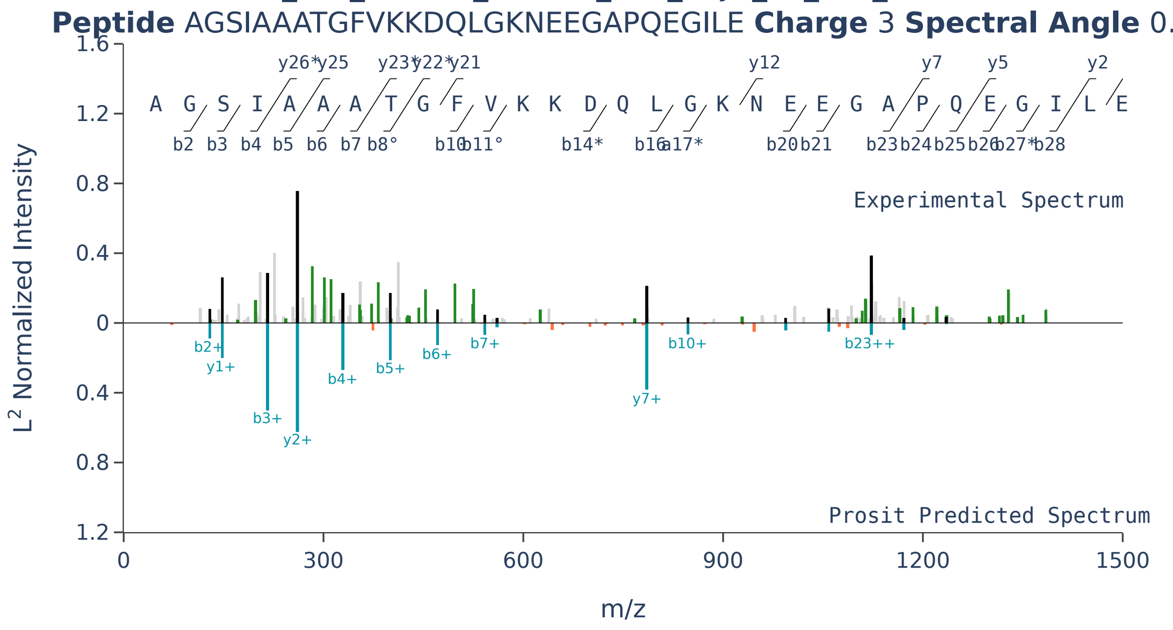


**Source** W_Soh_160821_170921_Goe_aSyn_A2_6h_R2 **Scan** 18285
**Peptide** KKDQLGKNEEGAPQEGILEDMPVD **Charge** 3 **Spectral Angle** 0.88

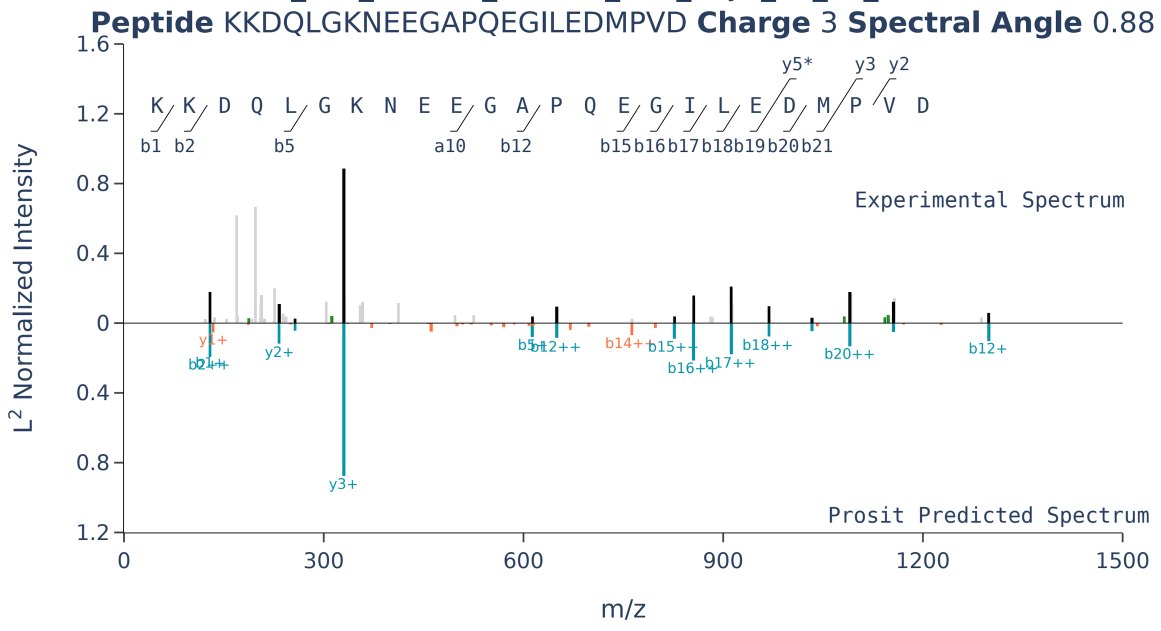


**Source** W_Soh_160821_170921_Goe_aSyn_A1_6h_R1 **Scan** 14046
**Peptide** VLYVGSKTKEGVVHGVATVA **Charge** 2 **Spectral Angle** 0.85

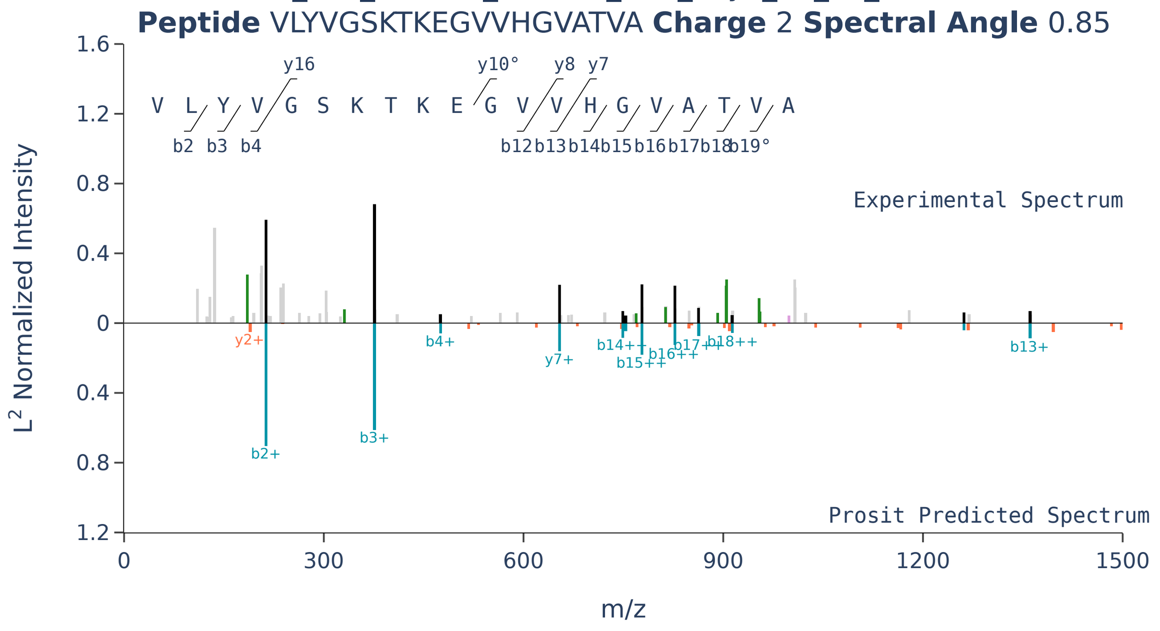


**Source** W_Soh_160821_170921_Goe_aSyn_A2_24h_R1 **Scan** 8490
**Peptide** VGSKTKEGVVHGVATVAEKT **Charge** 3 **Spectral Angle** 0.78

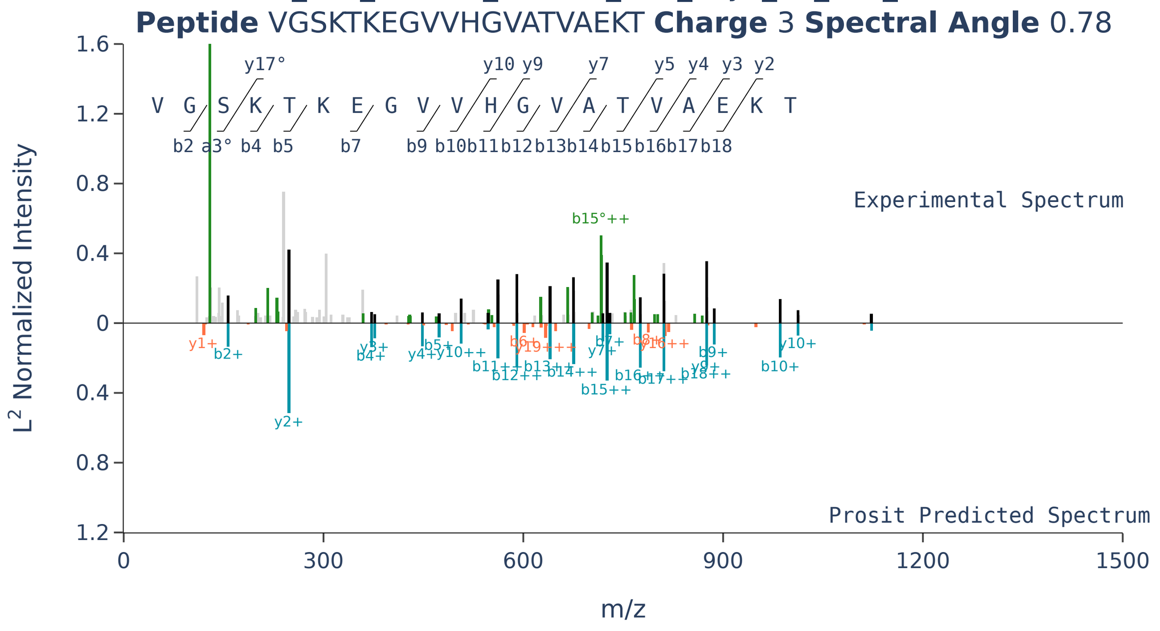


**Source** W_Soh_160821_170921_Goe_aSyn_A2_6h_R1 **Scan** 15125
**Peptide** KEQVTNVGGAVVTGVT **Charge** 2 **Spectral Angle** 0.79

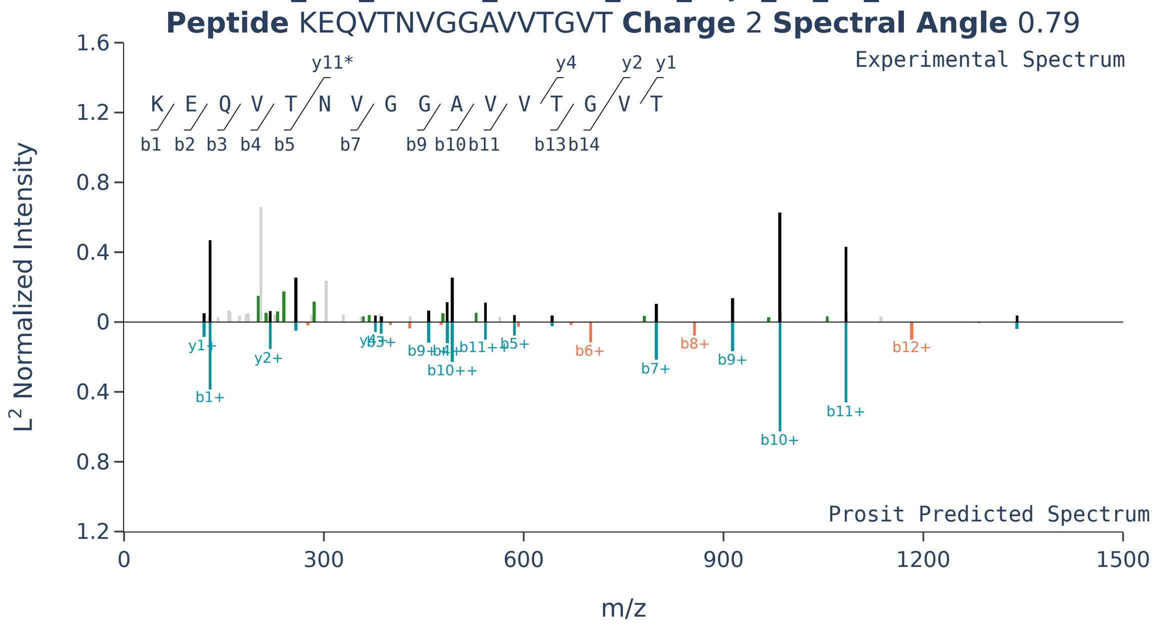


**Source** W_Soh_160821_170921_Goe_aSyn_A2_24h_R1 **Scan** 10091
**Peptide** KTVEGAGSIAAAT **Charge** 2 **Spectral Angle** 0.67

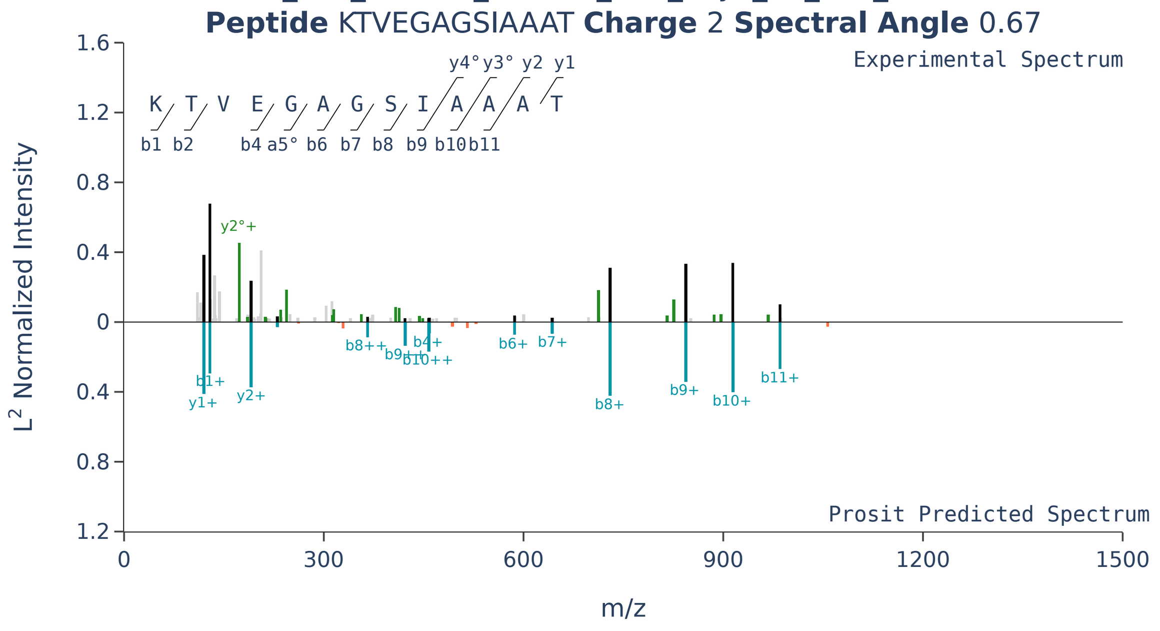


**Source** W_Soh_160821_170921_Goe_aSyn_A1_6h_R2 **Scan** 24279
**Peptide** GKNEEGAPQEGILEDMPVDPDNEAYE **Charge** 3 **Spectral Angle** 0.83

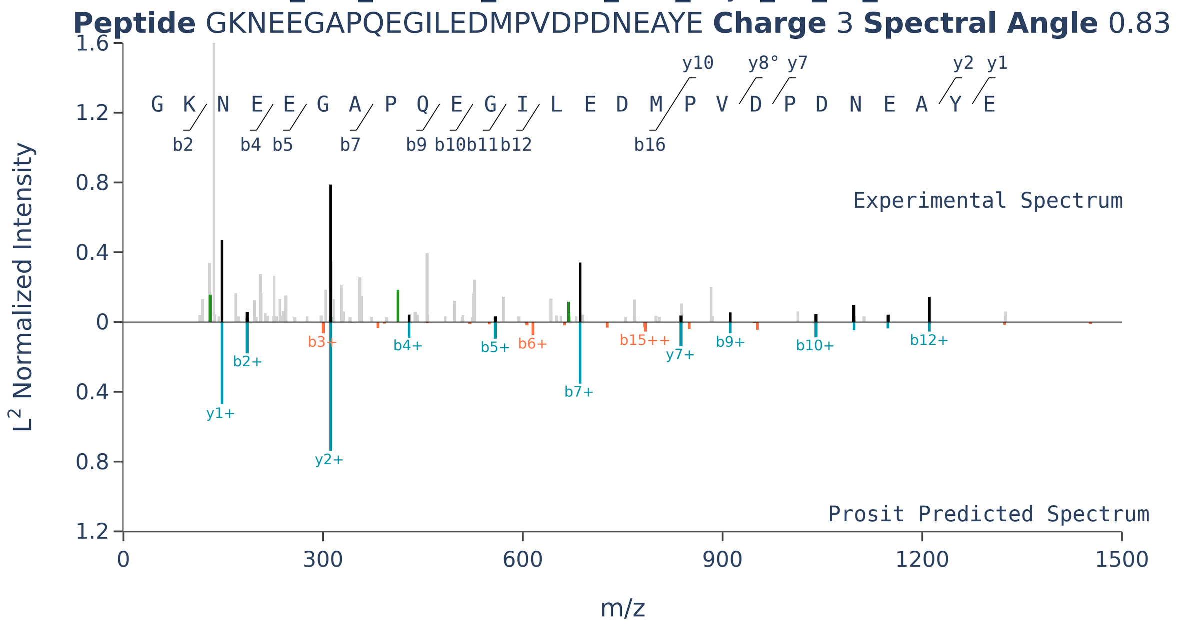


**Source** W_Soh_160821_170921_Goe_aSyn_A1_6h_R1 **Scan** 19750
**Peptide** MDVFMKGLSKAKEGVVAAAE **Charge** 3 **Spectral Angle** 0.9

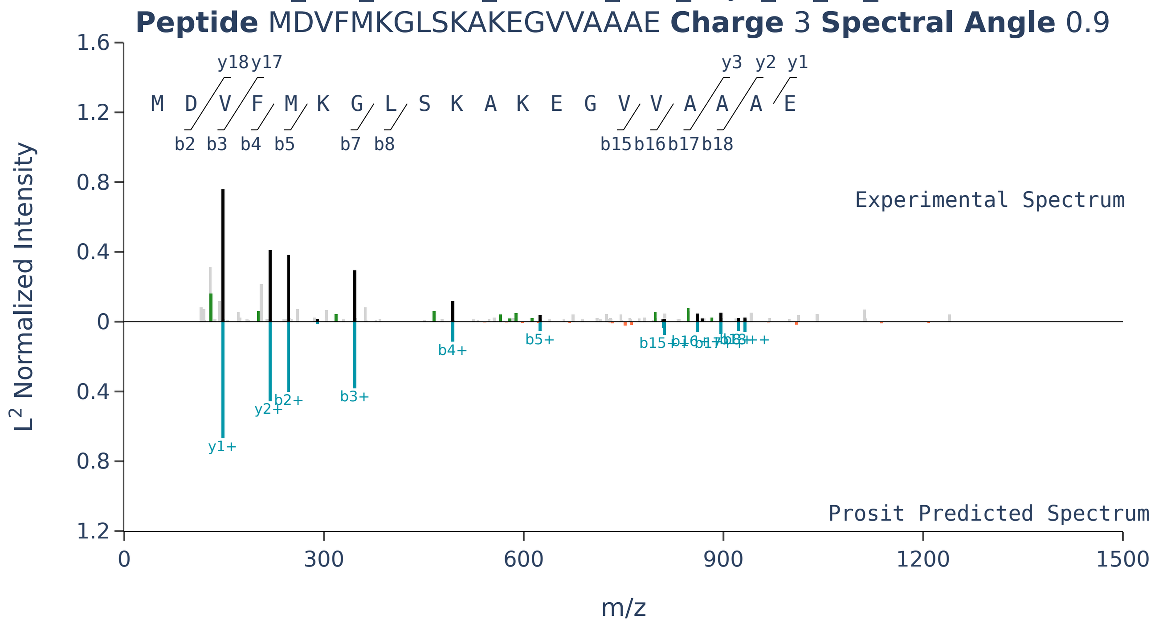


**Source** W_Soh_160821_170921_Goe_aSyn_A1_24h_R2 **Scan** 25839
**Peptide** VTNVGGAVVTGVTAVAQKTV **Charge** 2 **Spectral Angle** 0.8

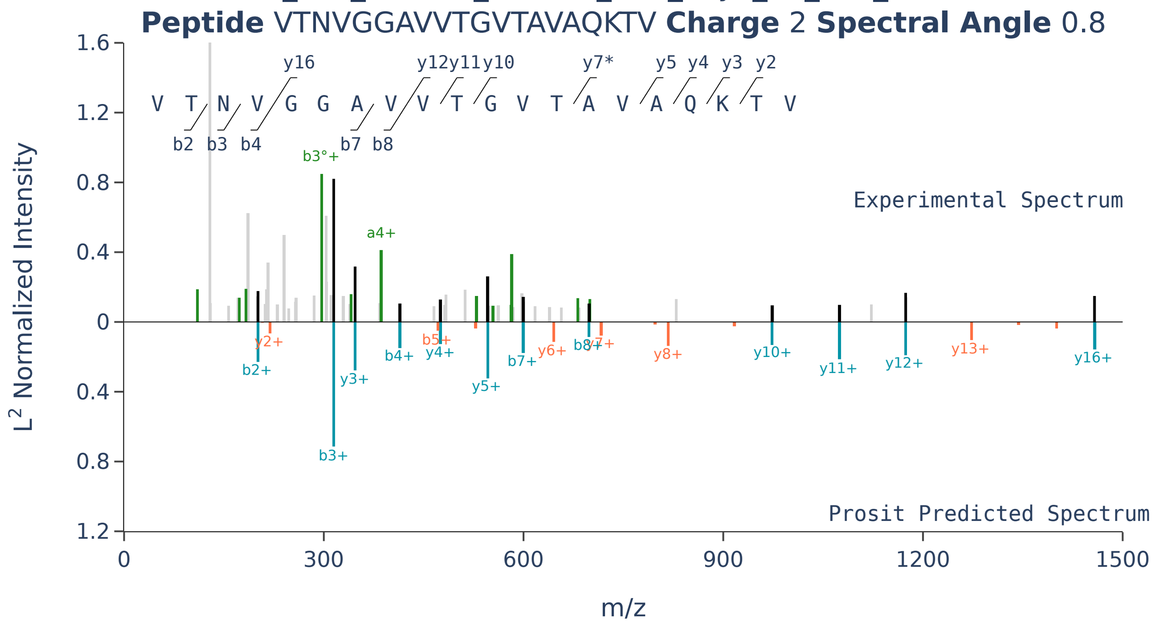


**Source** W_Soh_160821_170921_Goe_aSyn_A2_24h_R1 **Scan** 9535
**Peptide** GVVAAAEKTKQGVAEAAGKTKE **Charge** 3 **Spectral Angle** 0.85

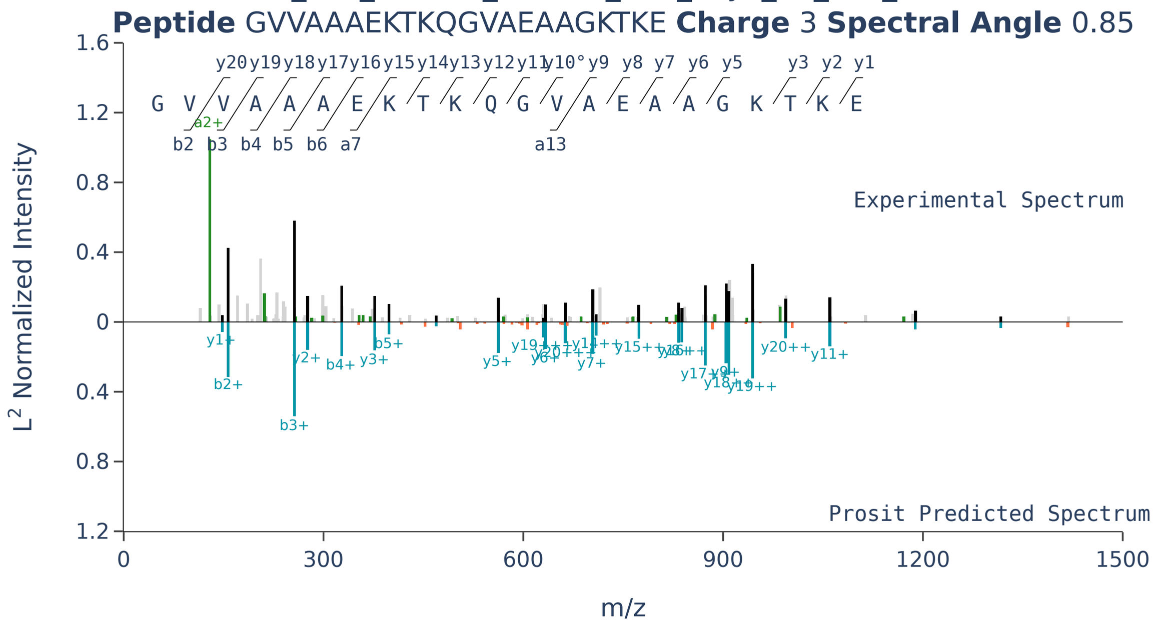

**Source** W_Soh_160821_170921_Goe_aSyn_A1_24h_R2 **Scan** 13120
**Peptide** VLYVGSKTKEGVVHGVATVAEKTKEQ **Charge** 3 **Spectral Angle** 0.78

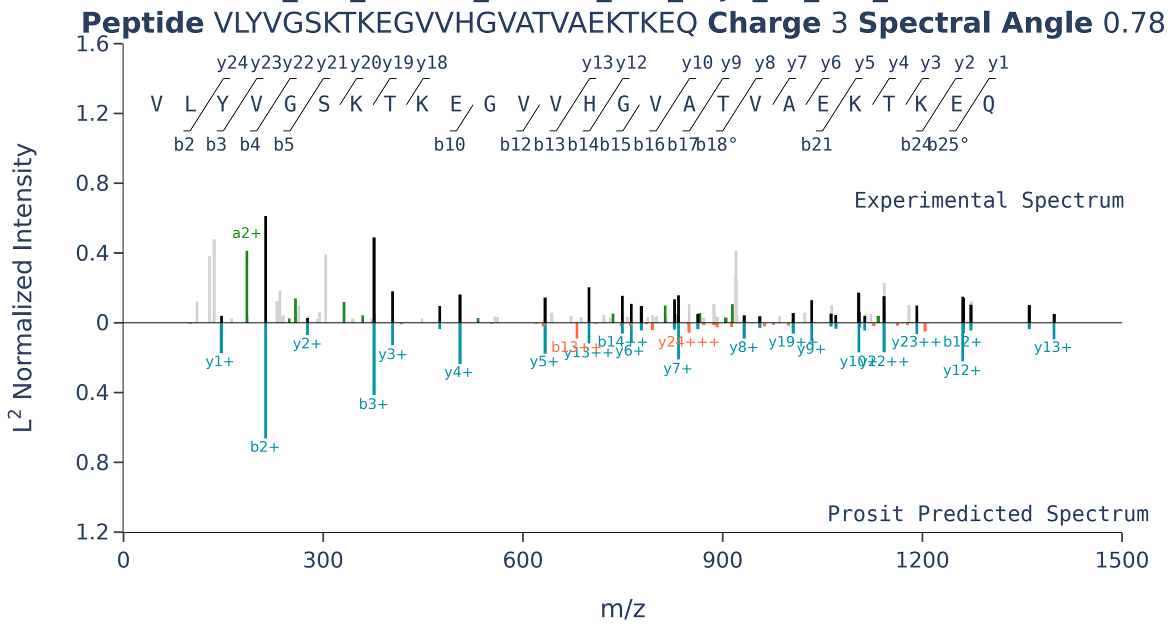


**Source** W_Soh_160821_170921_Goe_aSyn_A2_6h_R1 **Scan** 15717
**Peptide** KEGVVAAAEKTKQGVAEAAGKTKEGVLYV **Charge** 5 **Spectral Angle** 0.89

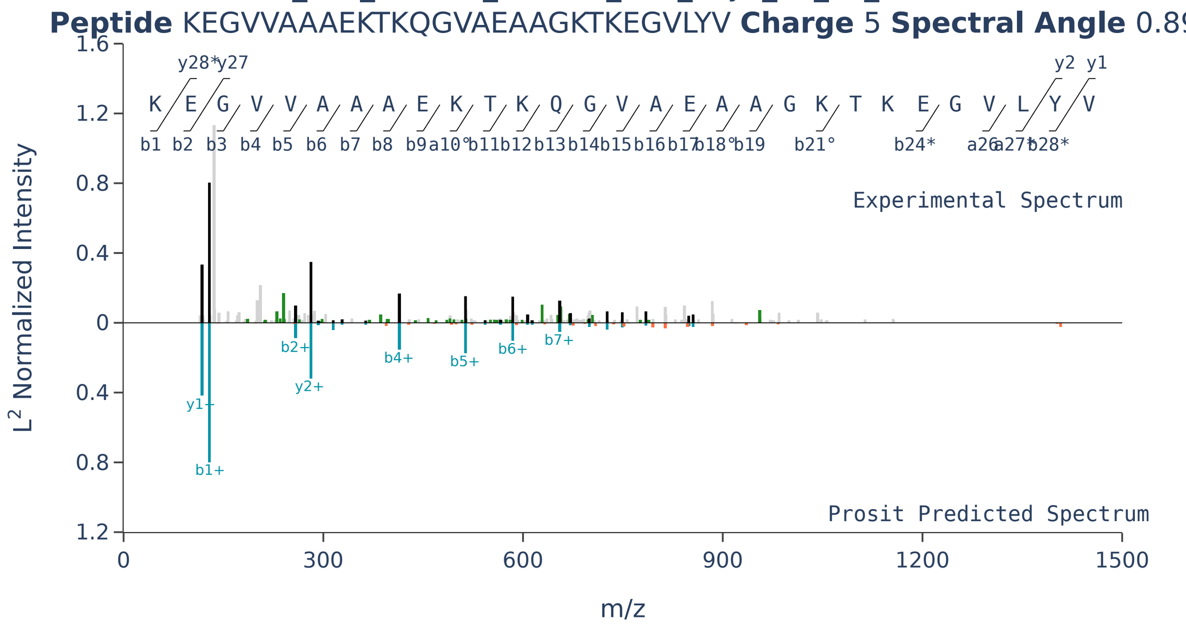


**Source** W_Soh_160821_170921_Goe_aSyn_A1_6h_R1 **Scan** 16720
**Peptide** DVFMKGLSKAKEGVV **Charge** 3 **Spectral Angle** 0.81

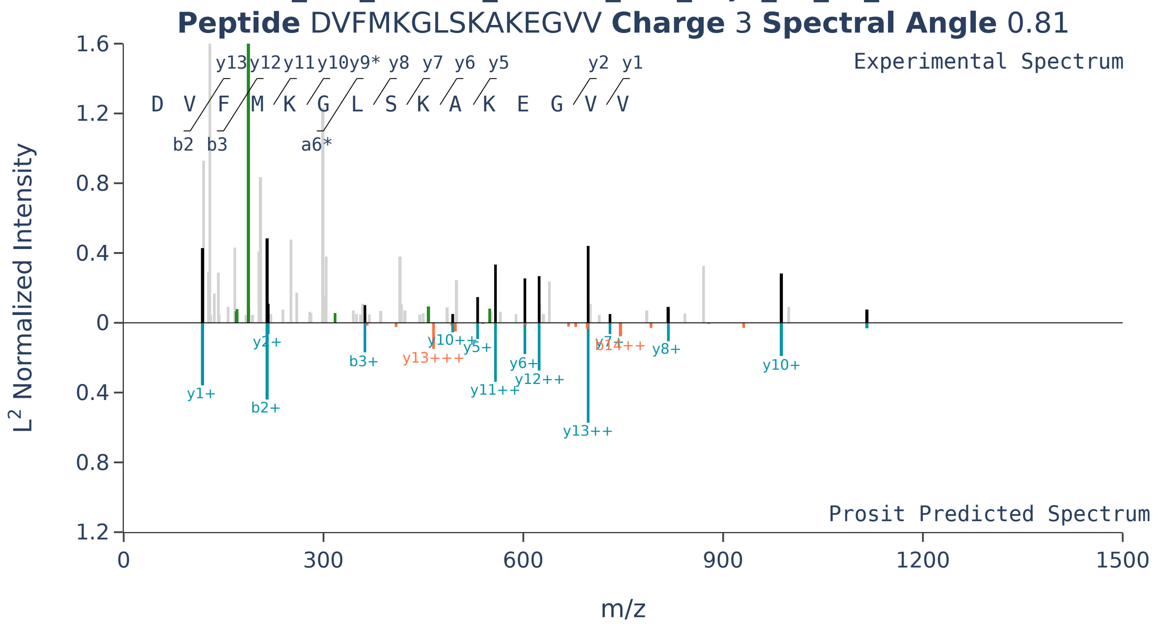


**Source** W_Soh_160821_170921_Goe_aSyn_A1_1h_R2 **Scan** 20716
**Peptide** GVATVAEKTKEQVTNVGGAVVTGVTAV **Charge** 3 **Spectral Angle** 0.69

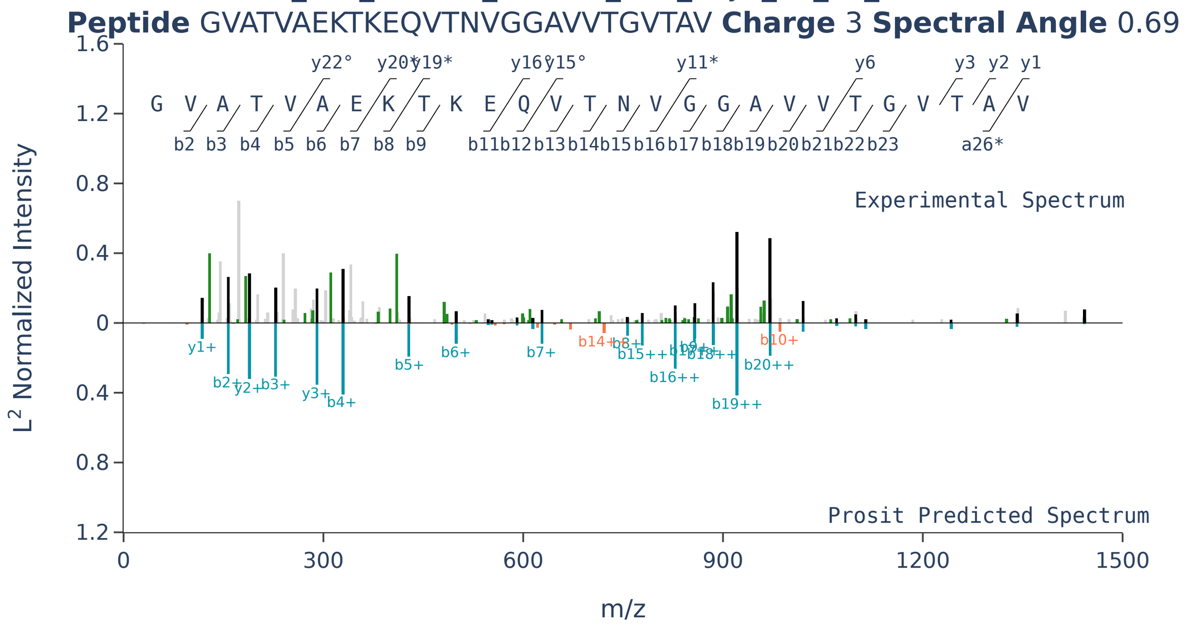


**Source** W_Soh_160821_170921_Goe_aSyn_A2_24h_R2 **Scan** 12709
**Peptide** VVHGVATVAEKTKEQVTNV **Charge** 3 **Spectral Angle** 0.83

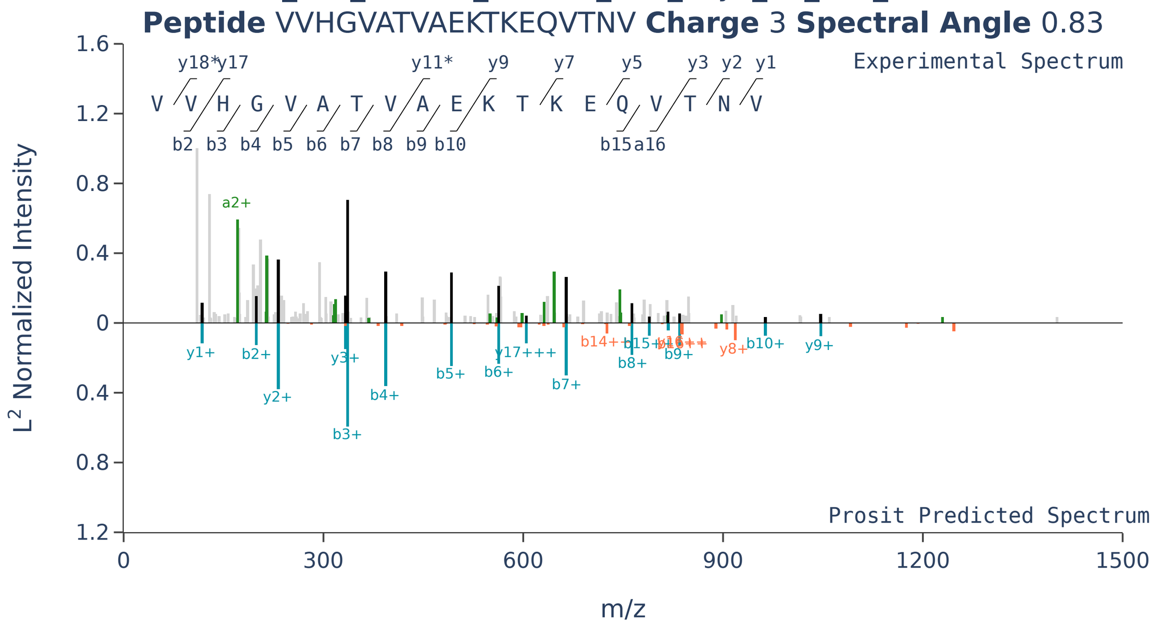


**Source** W_Soh_160821_170921_Goe_aSyn_A2_1h_R1 **Scan** 9150
**Peptide** AGKTKEGVLYVGSKTKEGVVHGVAT **Charge** 5 **Spectral Angle** 0.85

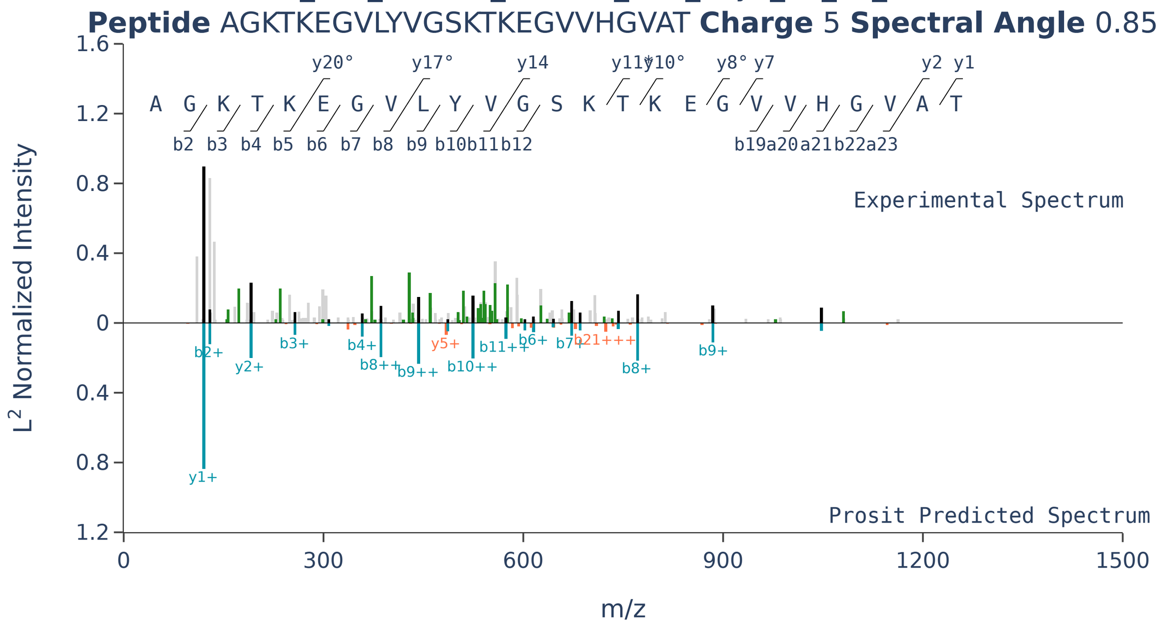


**Source** W_Soh_160821_170921_Goe_aSyn_A1_24h_R1 **Scan** 7224
**Peptide** GLSKAKEGVVAAAEKTKQ **Charge** 3 **Spectral Angle** 0.77

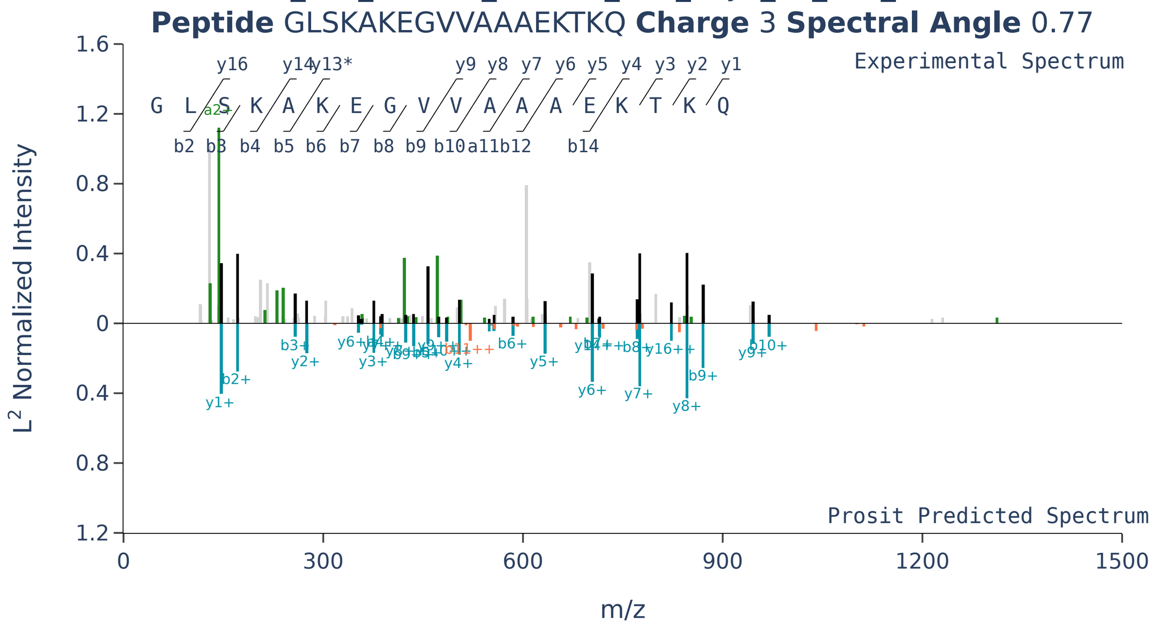


**Source** W_Soh_160821_170921_Goe_aSyn_A2_24h_R2 **Scan** 5980
**Peptide** HGVATVAEKT **Charge** 2 **Spectral Angle** 0.74

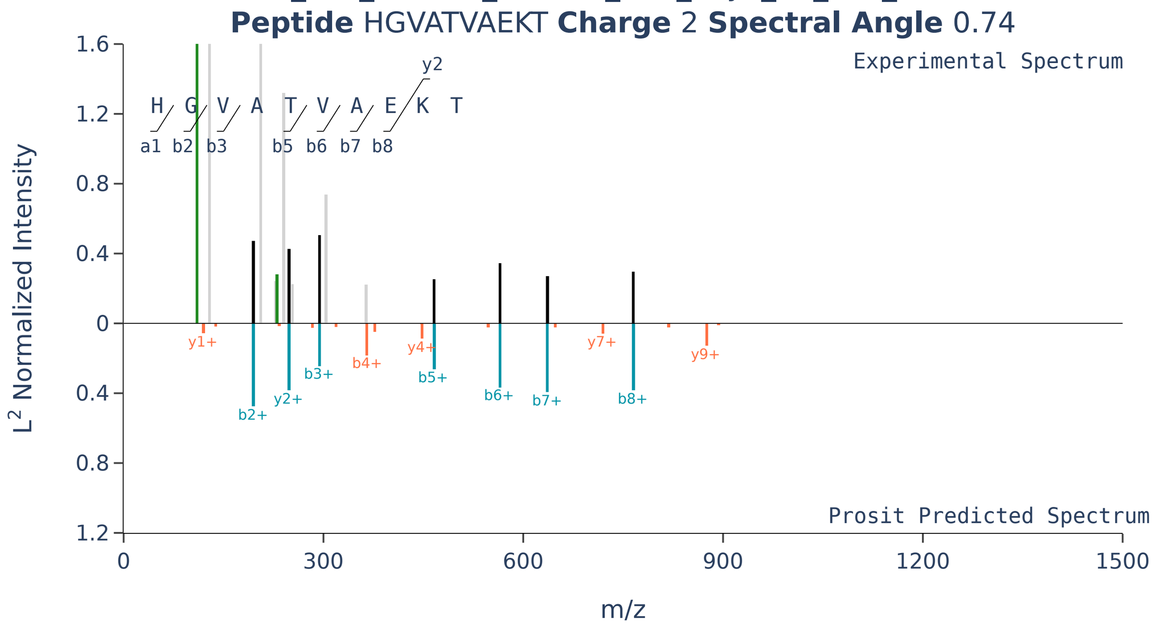


**Source** W_Soh_160821_170921_Goe_aSyn_A1_3h_R1 **Scan** 20368
**Peptide** NVGGAVVTGVTAV **Charge** 2 **Spectral Angle** 0.78

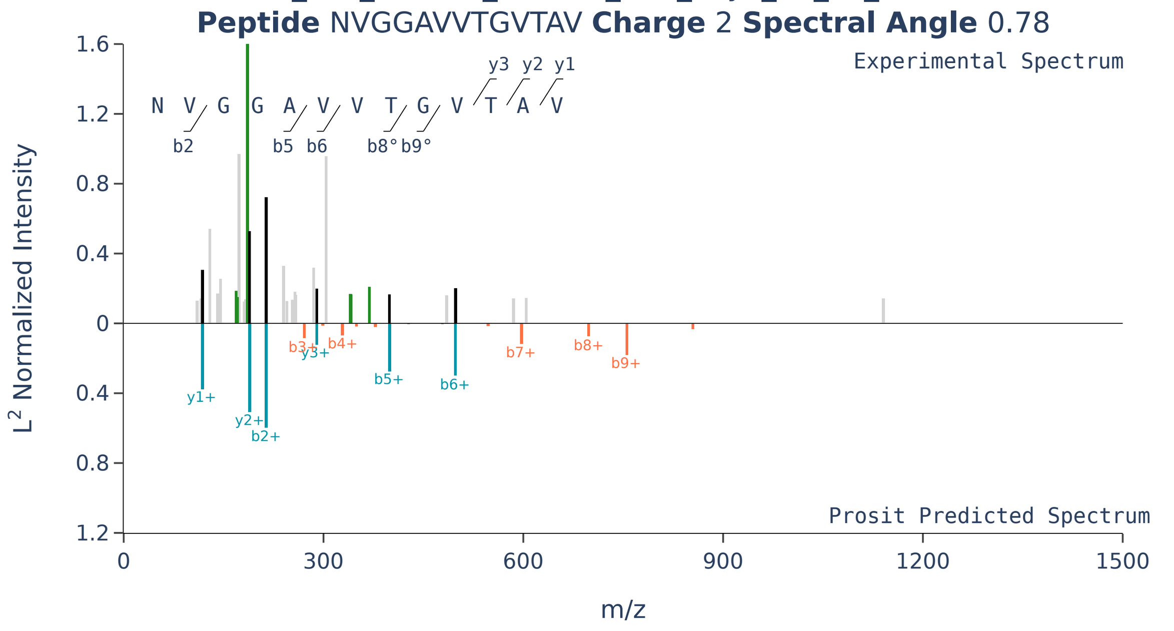


**Source** W_Soh_160821_170921_Goe_aSyn_A1_24h_R1 **Scan** 6217
**Peptide** VAEKTKEQVTNVGG **Charge** 3 **Spectral Angle** 0.83

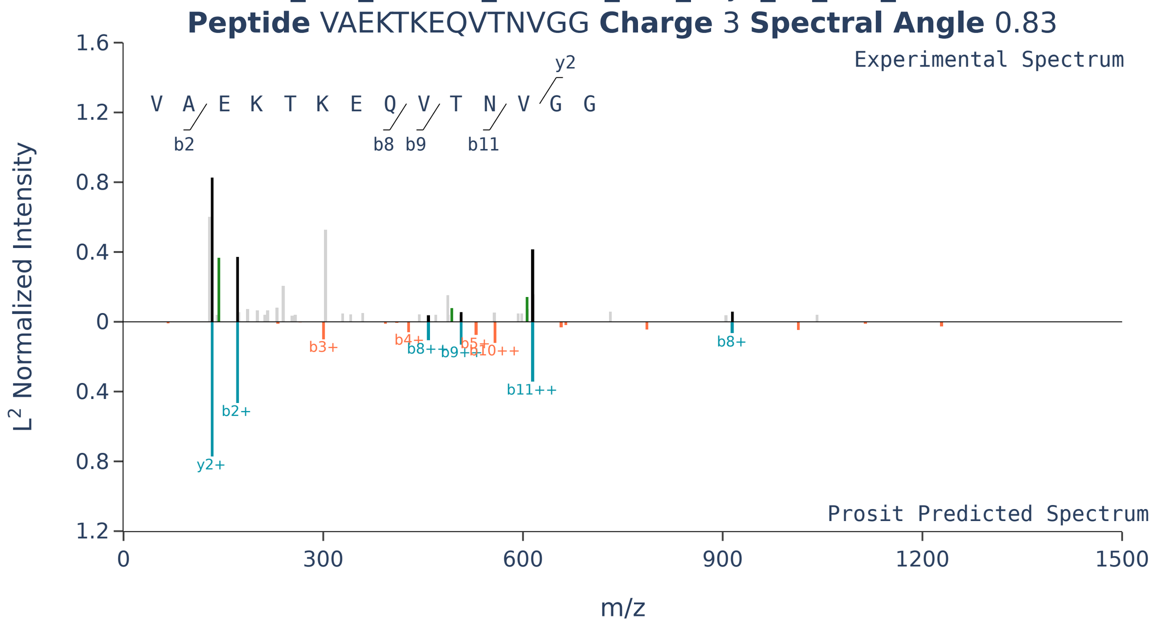


**Source** W_Soh_160821_170921_Goe_aSyn_A2_24h_R1 **Scan** 11675
**Peptide** VAQKTVEGAGSIAAATG **Charge** 2 **Spectral Angle** 0.75

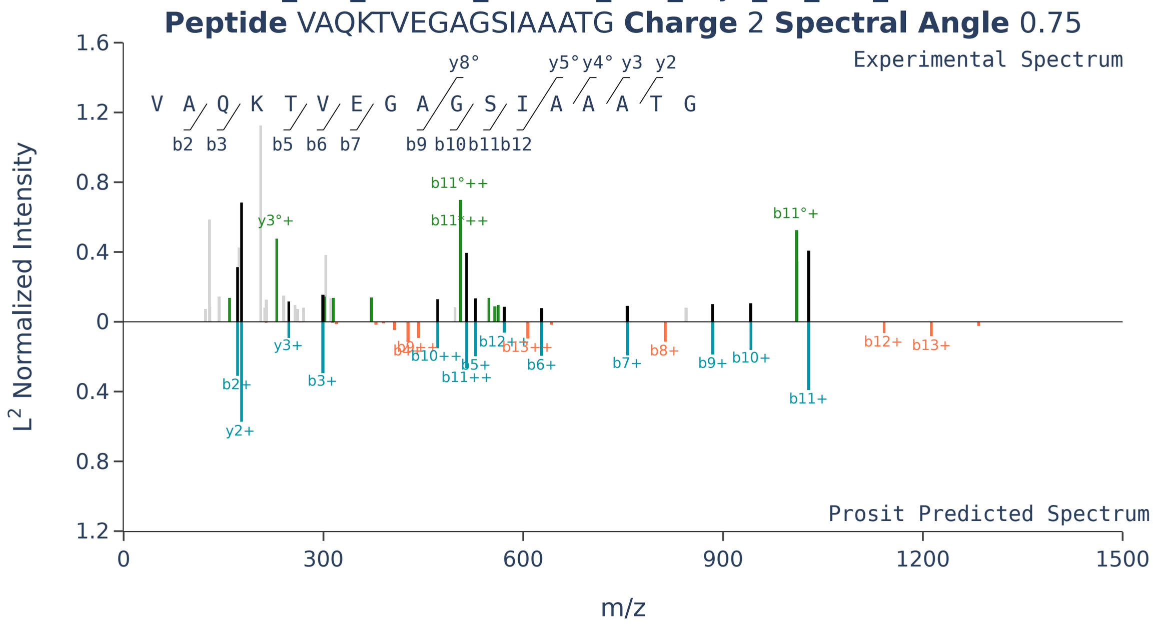


**Source** W_Soh_160821_170921_Goe_aSyn_A2_24h_R2 **Scan** 7500
**Peptide** QVTNVGGA **Charge** 1 **Spectral Angle** 0.68

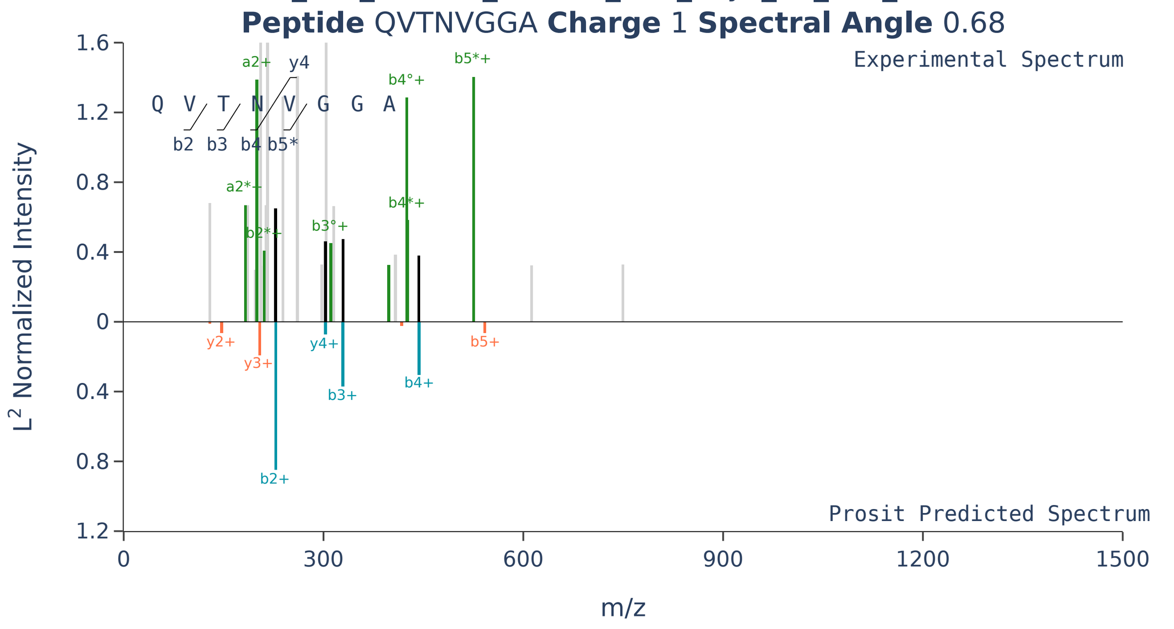


**Source** W_Soh_160821_170921_Goe_aSyn_A1_6h_R1 **Scan** 20424
**Peptide** ATVAEKTKEQVTNVGGAVVTGVTAV **Charge** 3 **Spectral Angle** 0.67

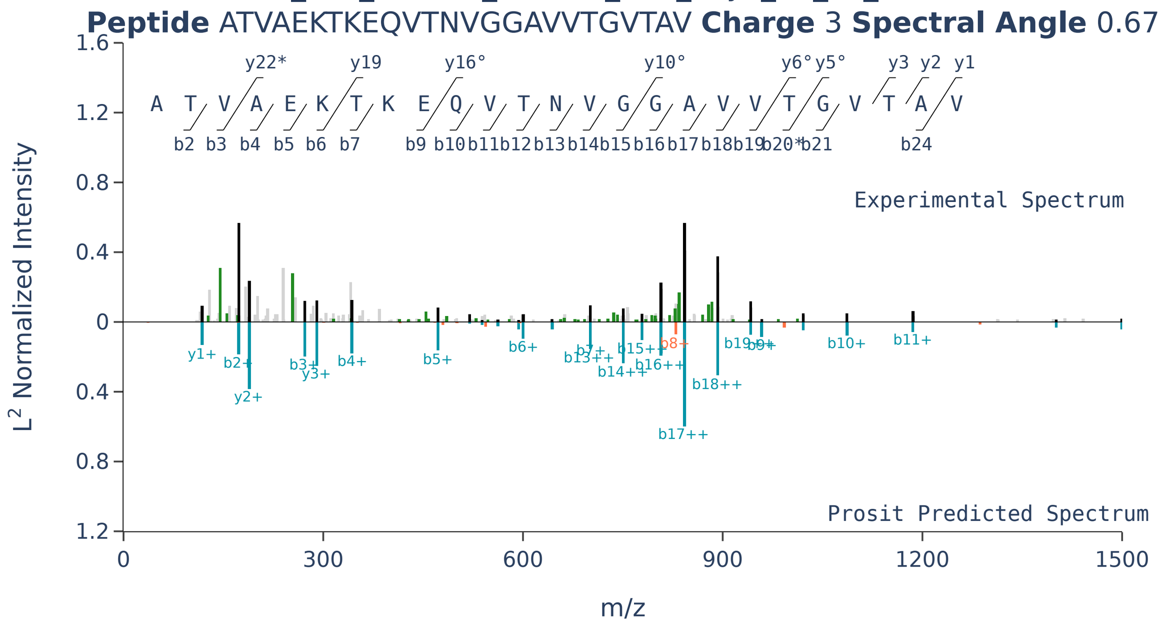


**Source** W_Soh_160821_170921_Goe_aSyn_A1_24h_R2 **Scan** 11046
**Peptide** VAEKTKEQVTNVGGAVVT **Charge** 2 **Spectral Angle** 0.75

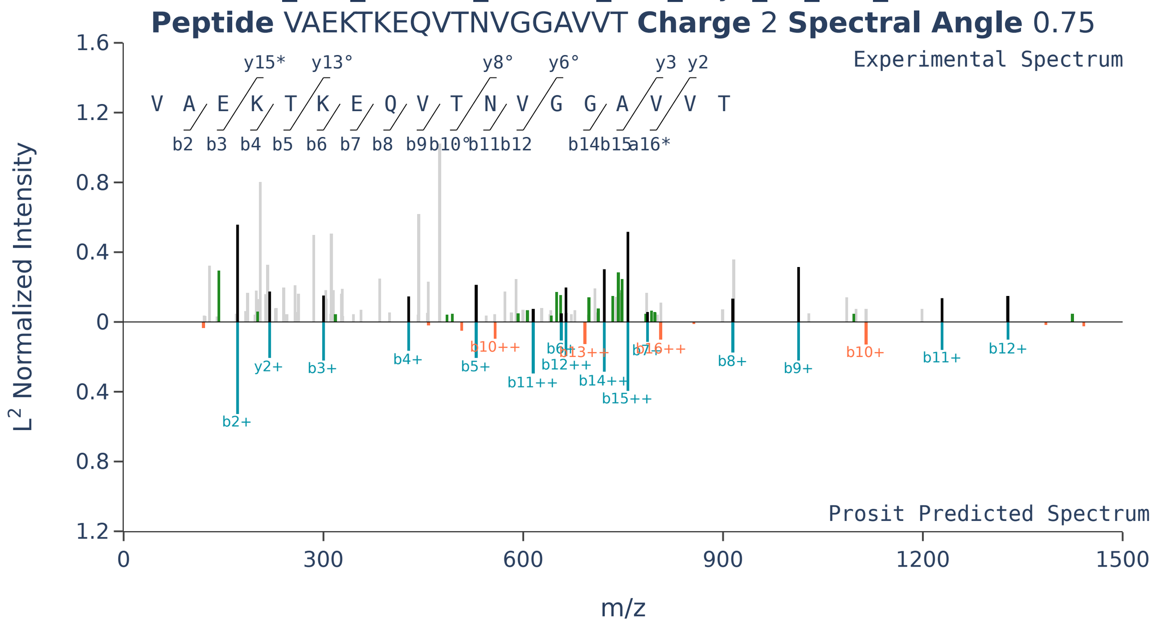


**Source** W_Soh_160821_170921_Goe_aSyn_A1_24h_R2 **Scan** 10854
**Peptide** KEGVVHGVATVAEKTKEQV **Charge** 3 **Spectral Angle** 0.86

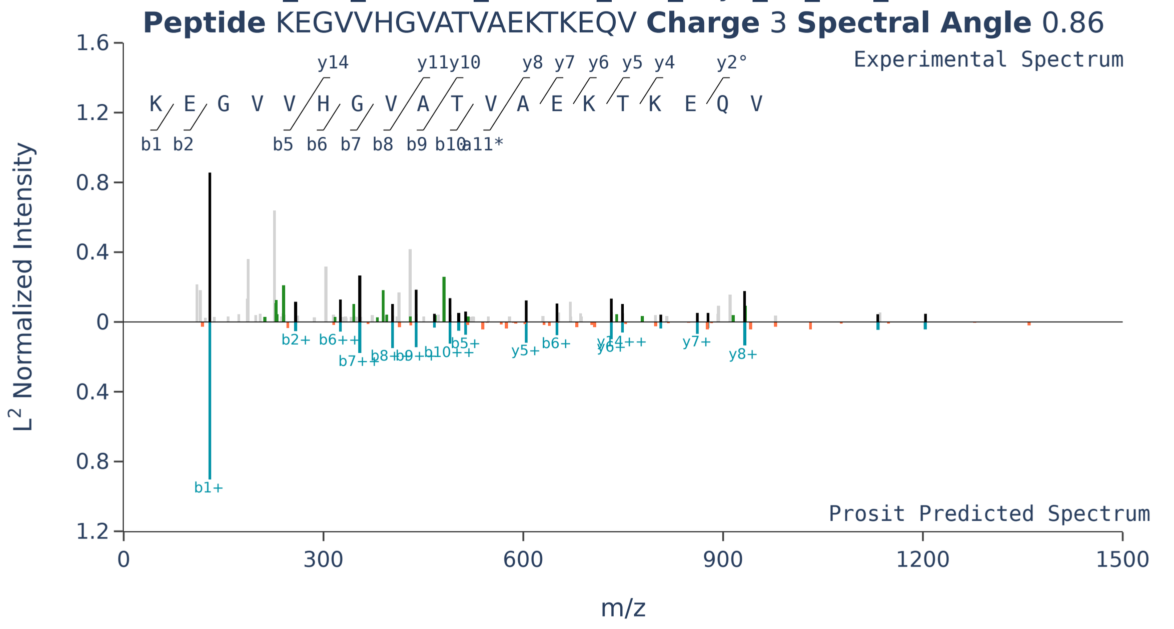

**Source** W_Soh_160821_170921_Goe_aSyn_A2_24h_R1 **Scan** 10059
**Peptide** GVVAAAEKTKQGVAEAA **Charge** 2 **Spectral Angle** 0.84

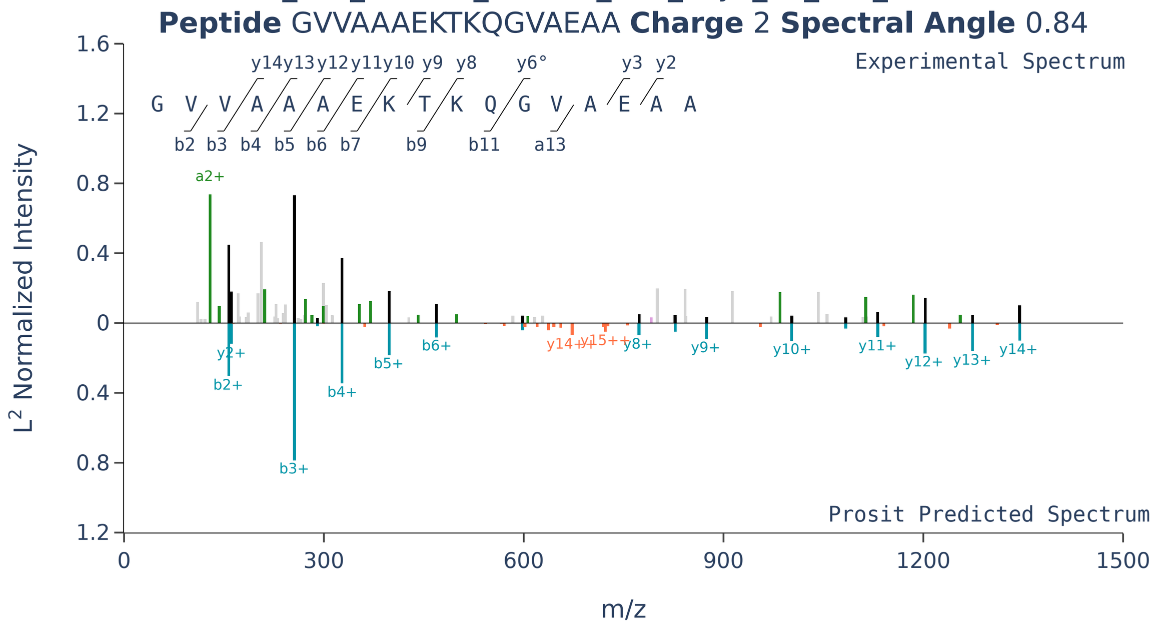

**Source** W_Soh_160821_170921_Goe_aSyn_A2_6h_R1 **Scan** 18661
**Peptide** VAEKTKEQVTNVGGAVVTGVTAVA **Charge** 3 **Spectral Angle** 0.82

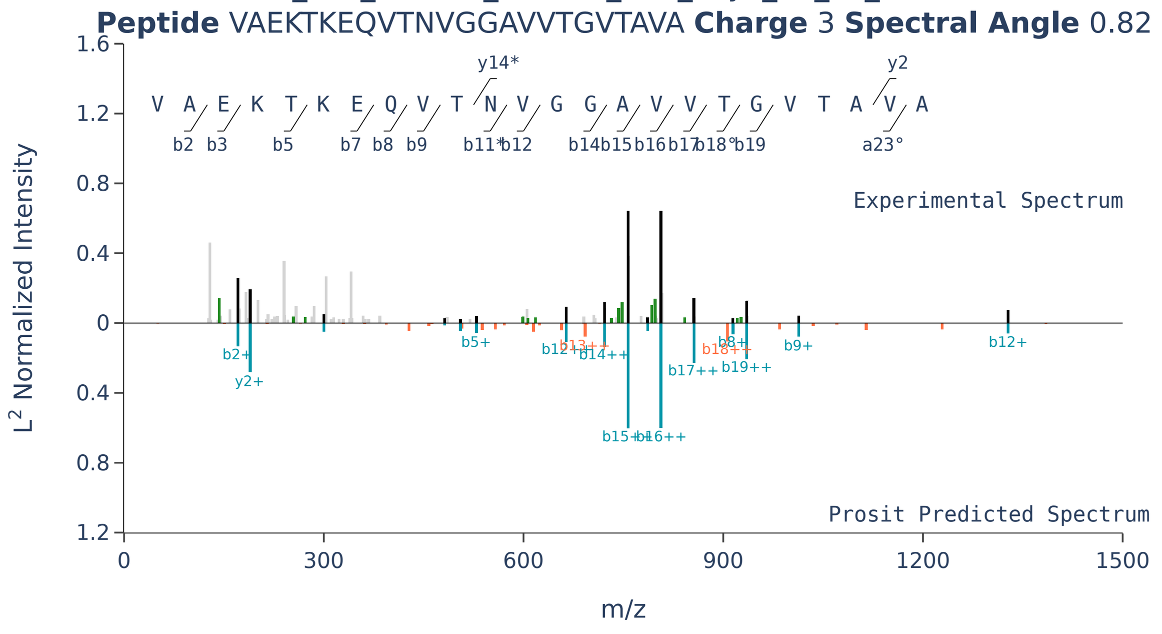

**Source** W_Soh_160821_170921_Goe_aSyn_A1_24h_R1 **Scan** 10368
**Peptide** EMPSEEGYQD **Charge** 2 **Spectral Angle** 0.87

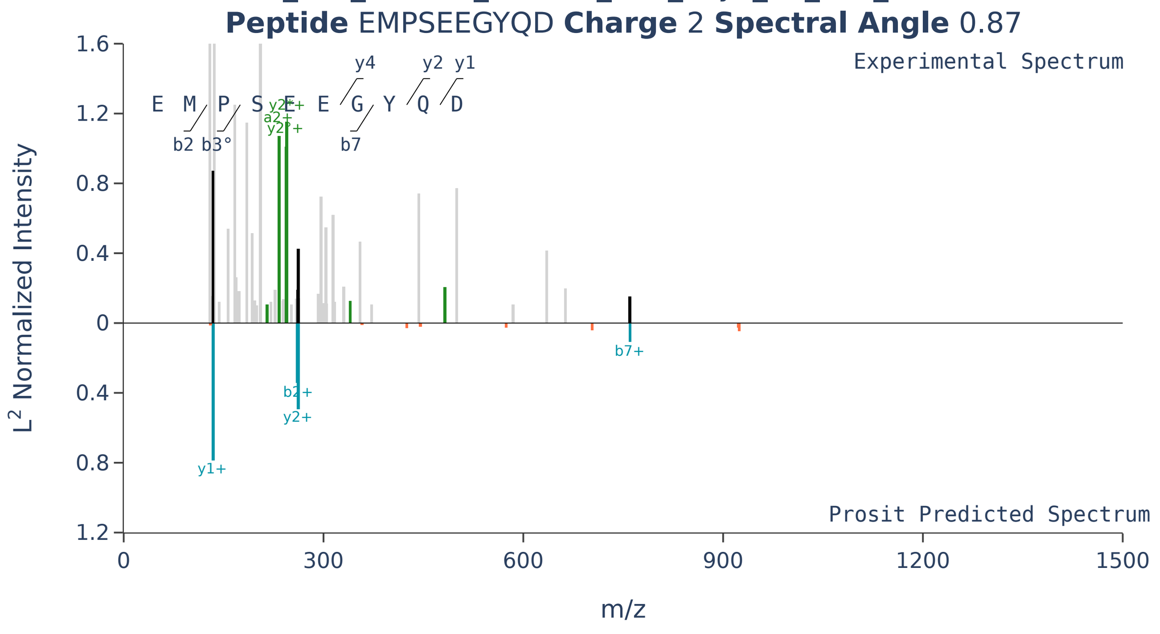

**Source** W_Soh_160821_170921_Goe_aSyn_A1_24h_R1 **Scan** 6678
**Peptide** AAGKTKEGVLYVGSKTK **Charge** 4 **Spectral Angle** 0.86

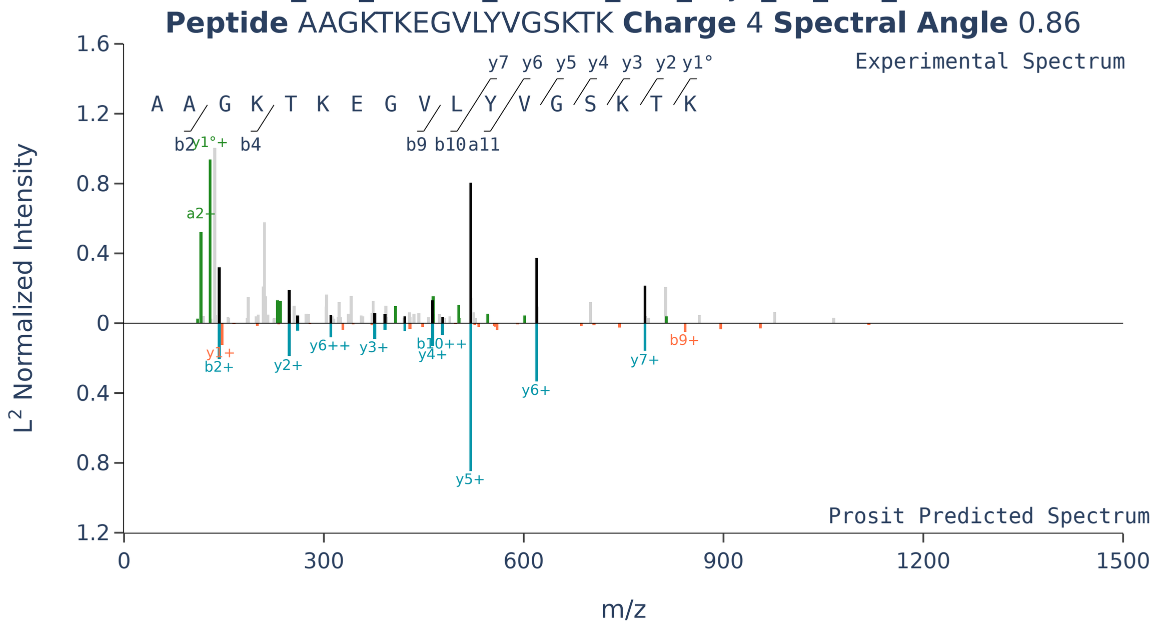

**Source** W_Soh_160821_170921_Goe_aSyn_A2_24h_R1 **Scan** 18105
**Peptide** KEGVVHGVATVAEKTKEQVTNVGGAVV **Charge** 4 **Spectral Angle** 0.75

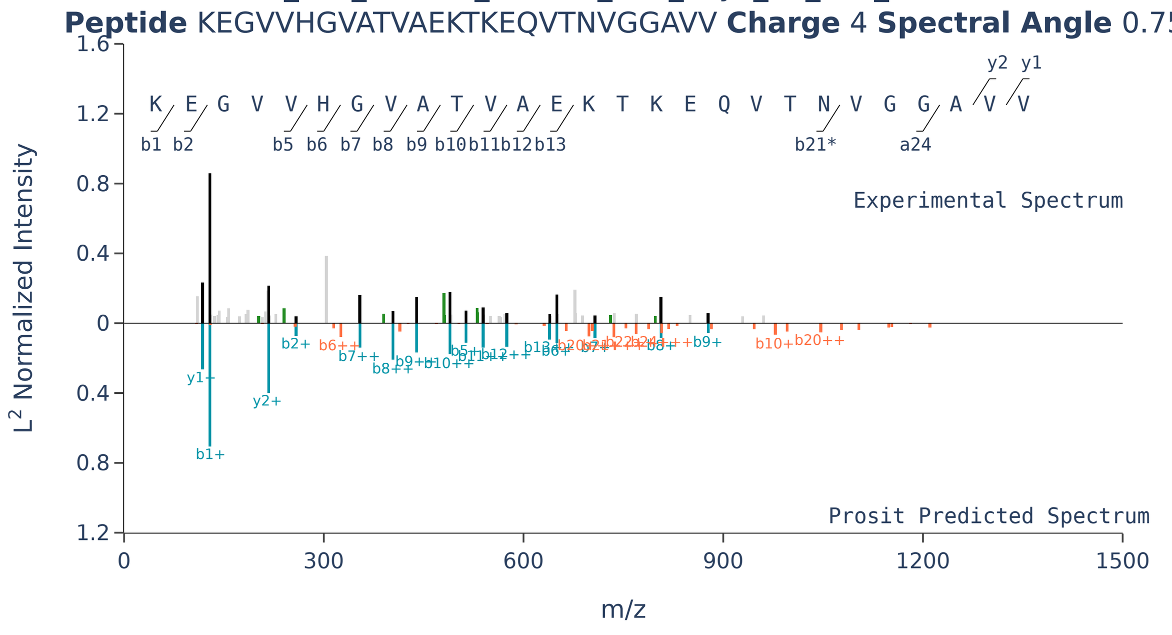

**Source** W_Soh_160821_170921_Goe_aSyn_A1_24h_R2 **Scan** 20519
**Peptide** GFVKKDQLGKNEEGAPQEGILEDMPVD **Charge** 3 **Spectral Angle** 0.9

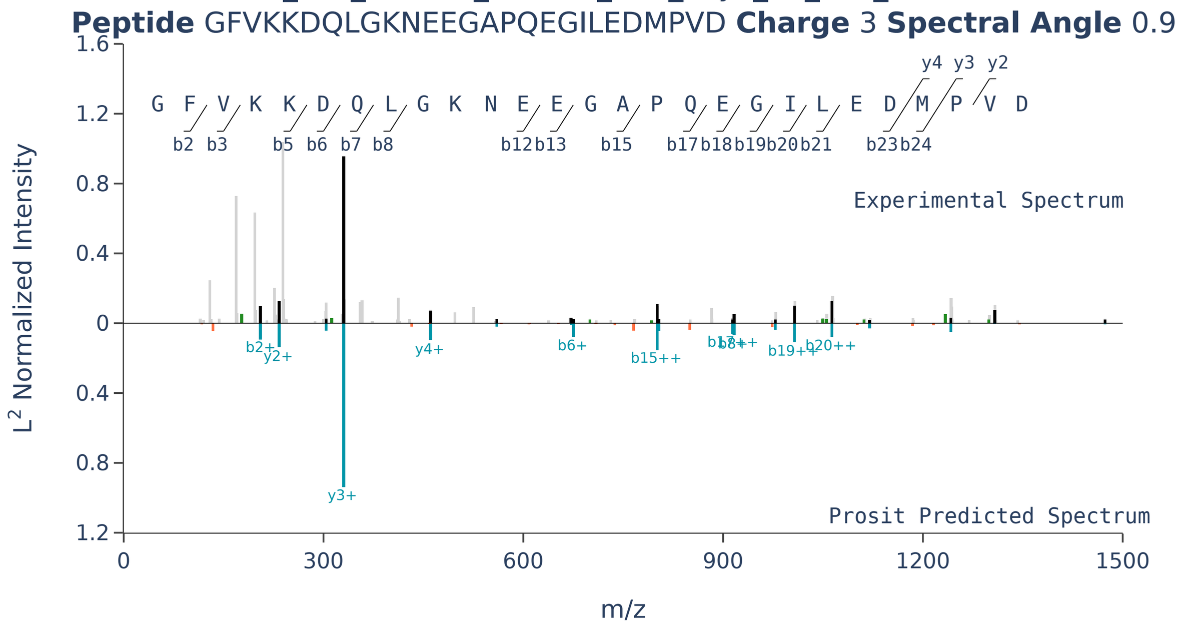

**Source** W_Soh_160821_170921_Goe_aSyn_A2_24h_R2 **Scan** 20129
**Peptide** YEMPSEEGYQDYEPEA **Charge** 2 **Spectral Angle** 0.91

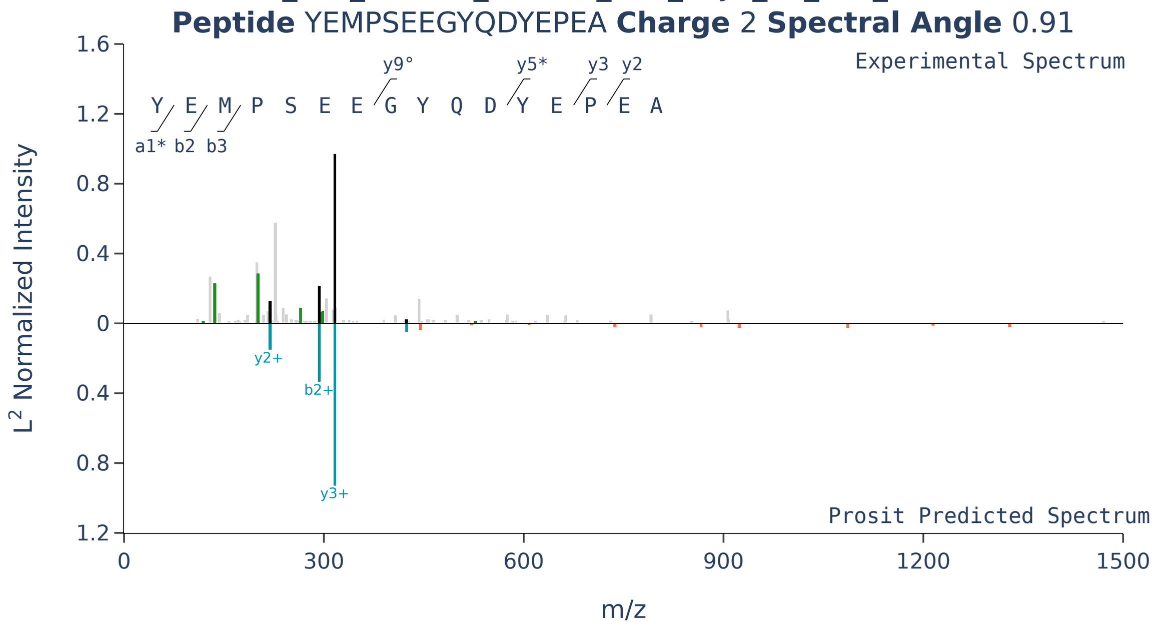

**Source** W_Soh_160821_170921_Goe_aSyn_A1_24h_R2 **Scan** 8707
**Peptide** QKTVEGAGSI **Charge** 1 **Spectral Angle** 0.8

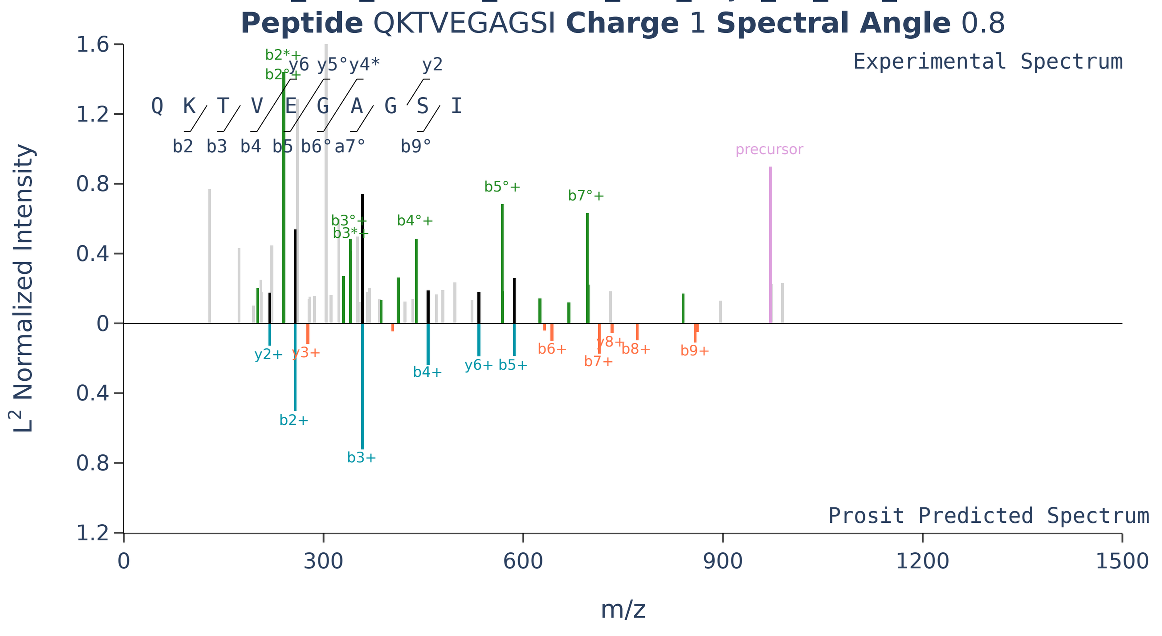

**Source** W_Soh_160821_170921_Goe_aSyn_A1_6h_R1 **Scan** 17218
**Peptide** QKTVEGAGSIAAATGFVK **Charge** 3 **Spectral Angle** 0.88

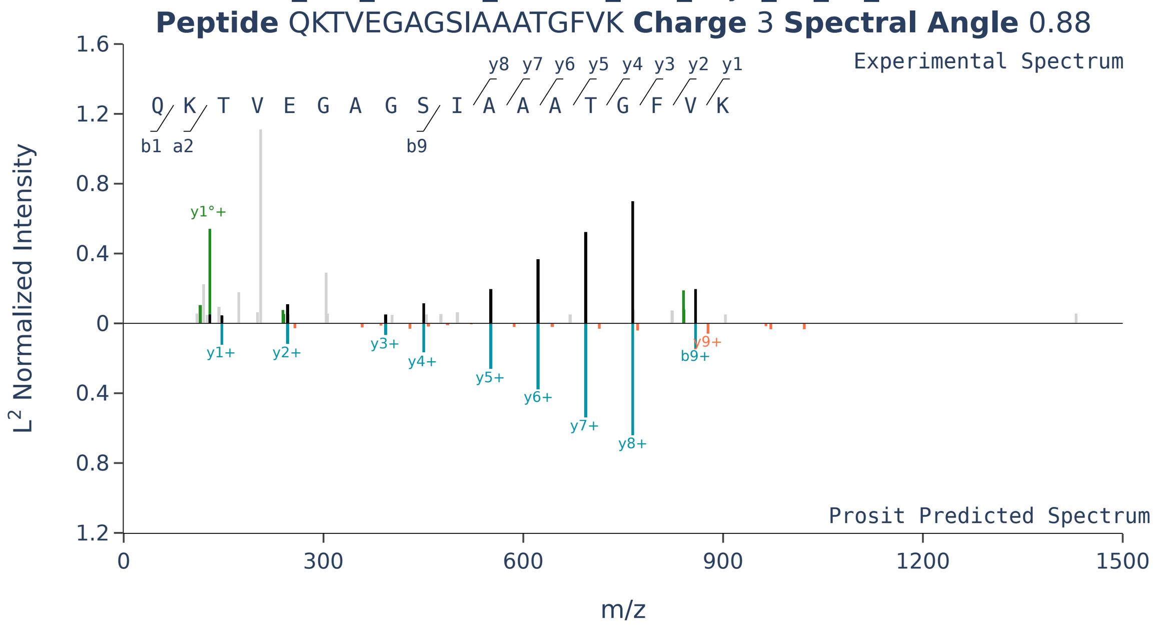

**Source** W_Soh_160821_170921_Goe_aSyn_A2_24h_R1 **Scan** 11821
**Peptide** AGKTKEGVLYVGSKTKEGVVHGVATVAEK **Charge** 5 **Spectral Angle** 0.65

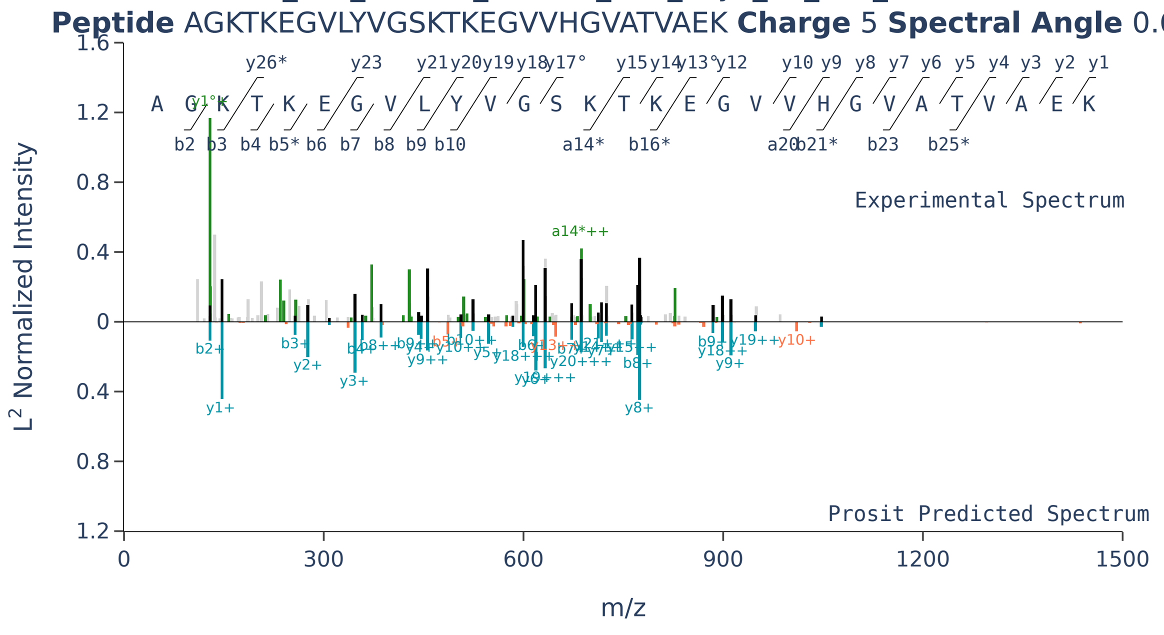

**Source** W_Soh_160821_170921_Goe_aSyn_A1_24h_R2 **Scan** 7290
**Peptide** VVAAAEKTKQGVAEAAGKTKEG **Charge** 3 **Spectral Angle** 0.88

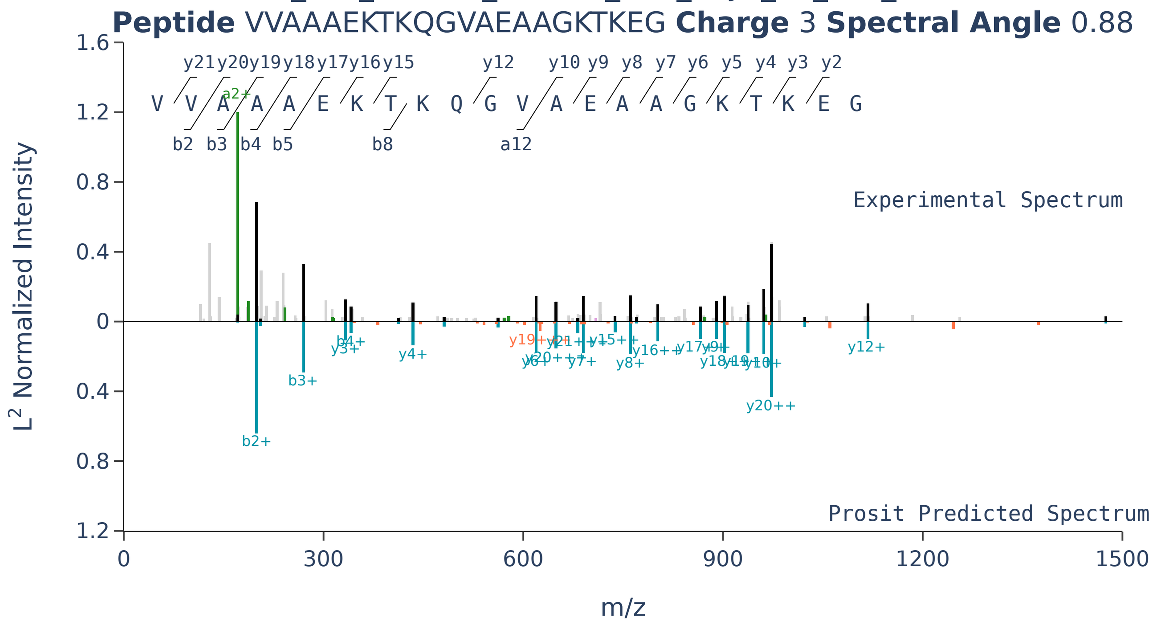

**Source** W_Soh_160821_170921_Goe_aSyn_A1_24h_R2 **Scan** 5669
**Peptide** AAEKTKQG **Charge** 2 **Spectral Angle** 0.75

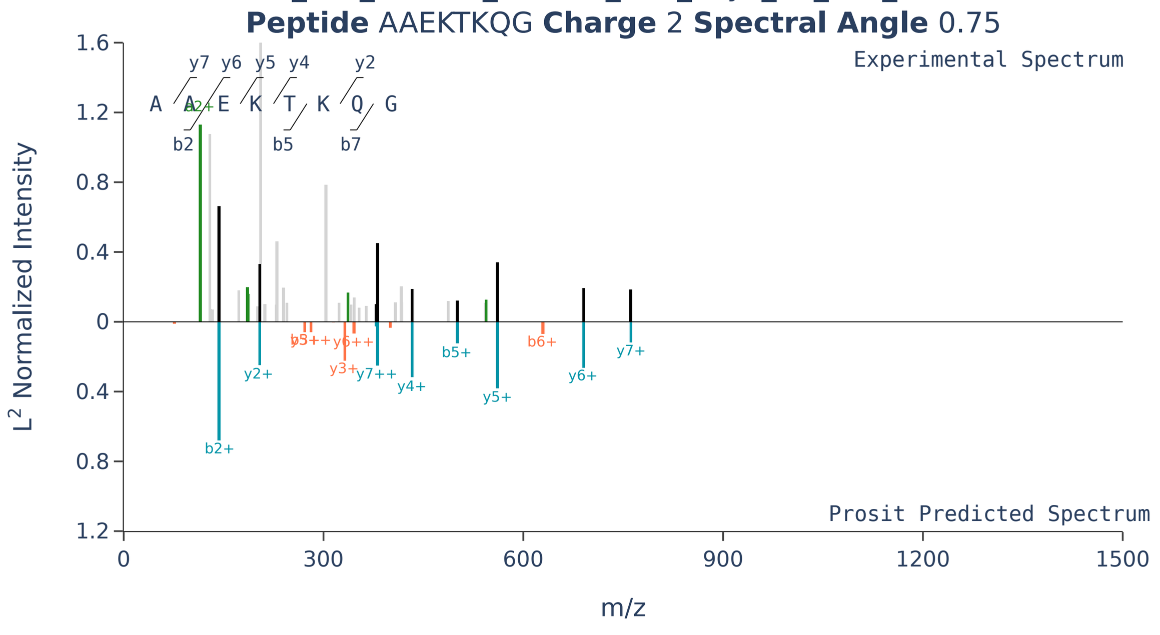

**Source** W_Soh_160821_170921_Goe_aSyn_A2_24h_R2 **Scan** 11641
**Peptide** VAEKTKEQVTNVGGAVV **Charge** 3 **Spectral Angle** 0.76

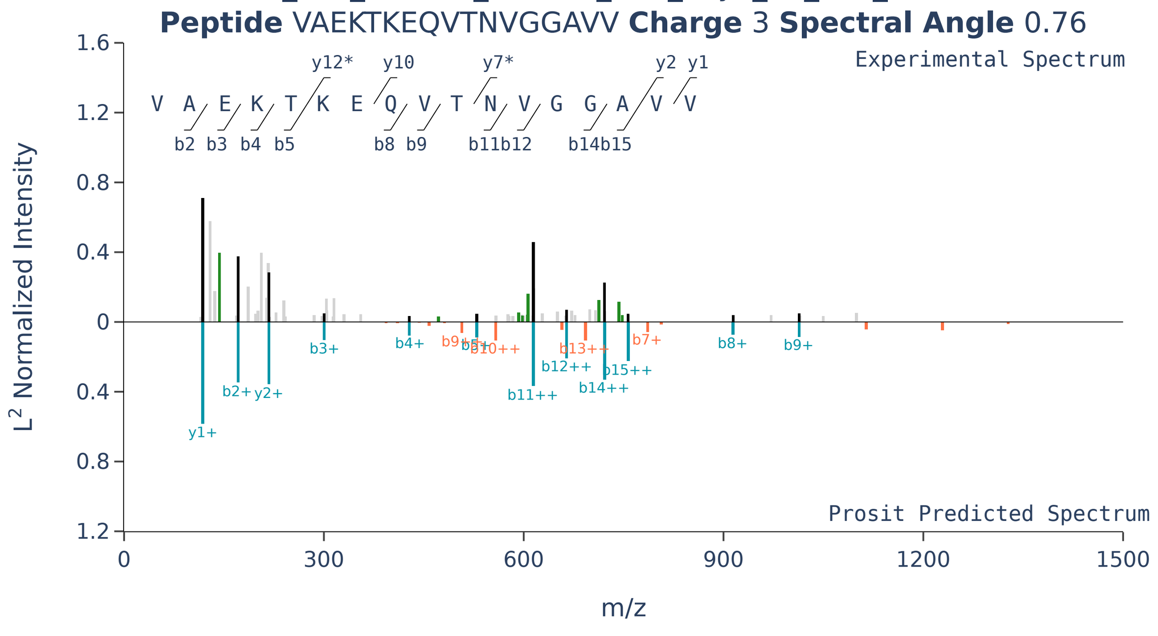

**Source** W_Soh_160821_170921_Goe_aSyn_A1_6h_R1 **Scan** 9919
**Peptide** VAEAAGKTKEGVLYVGSKTKEGVVHG **Charge** 4 **Spectral Angle** 0.82

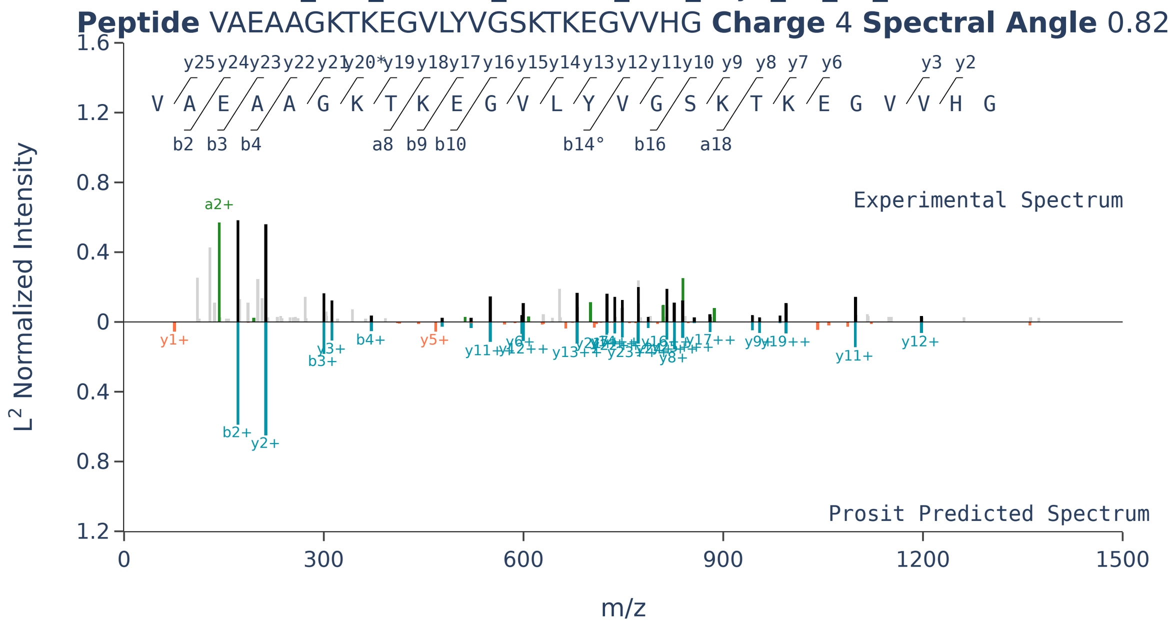

**Source** W_Soh_160821_170921_Goe_aSyn_A1_24h_R1 **Scan** 5230
**Peptide** KAKEGVVAAAEKTKQG **Charge** 3 **Spectral Angle** 0.78

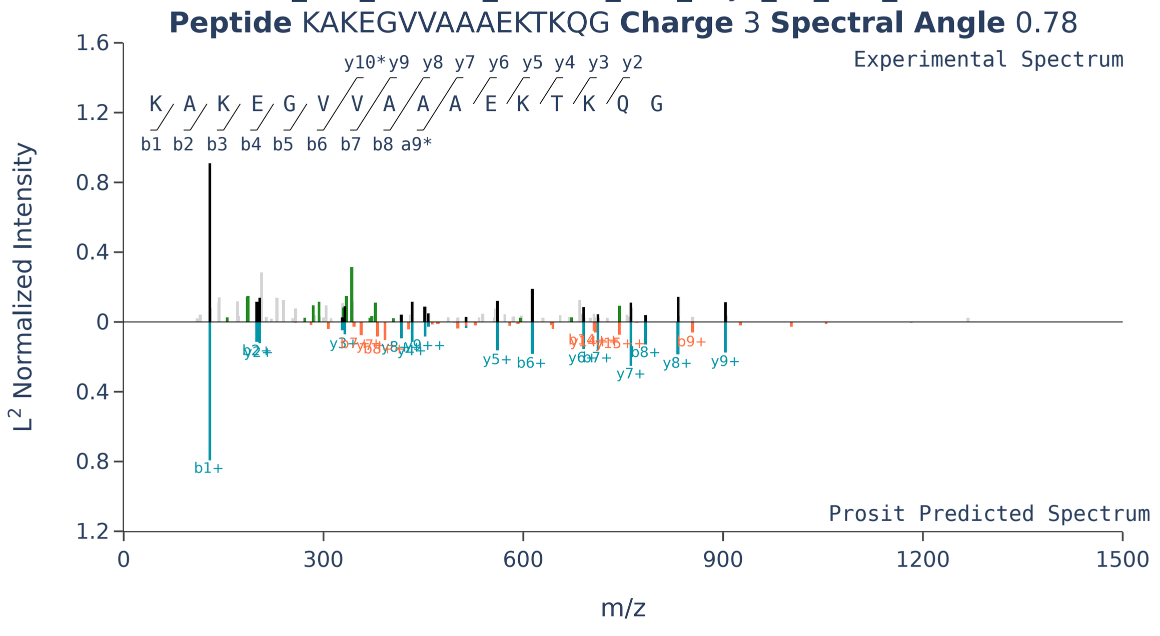

**Source** W_Soh_160821_170921_Goe_aSyn_A1_24h_R2 **Scan** 13511
**Peptide** FVKKDQLGKNEEGAPQEGIL **Charge** 3 **Spectral Angle** 0.84

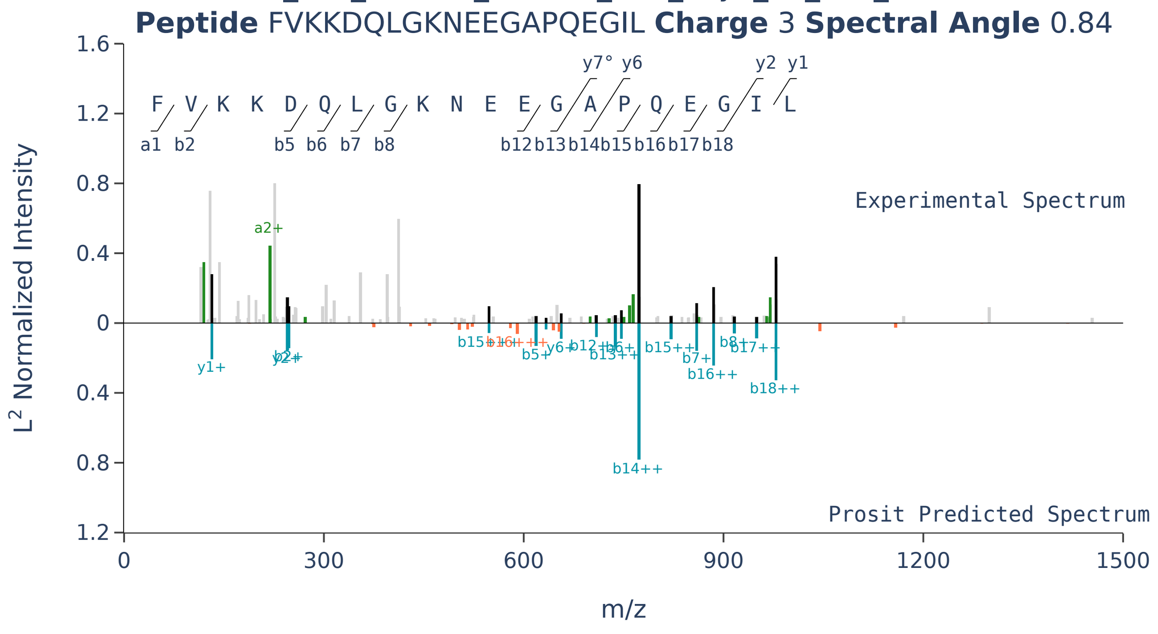


**Source** W_Soh_160821_170921_Goe_aSyn_A1_24h_R1 **Scan** 8813
**Peptide** VGSKTKEGVVHGVATVAE **Charge** 3 **Spectral Angle** 0.86

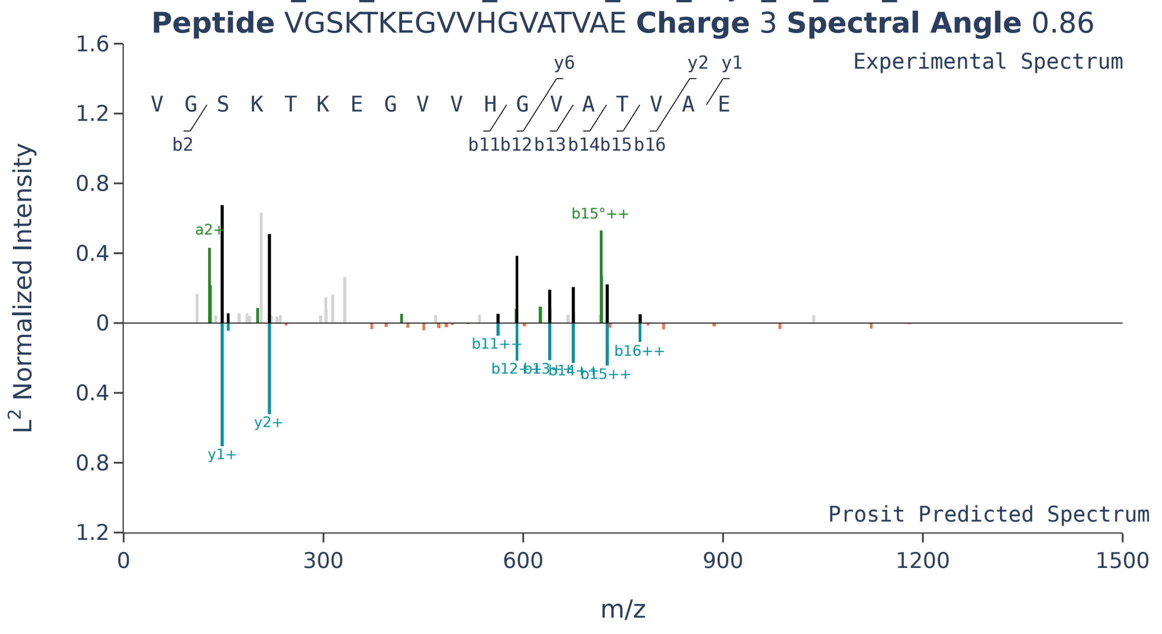


**Source** W_Soh_160821_170921_Goe_aSyn_A1_24h_R2 **Scan** 8666
**Peptide** MKGLSKAKEGVVAAAE **Charge** 3 **Spectral Angle** 0.89

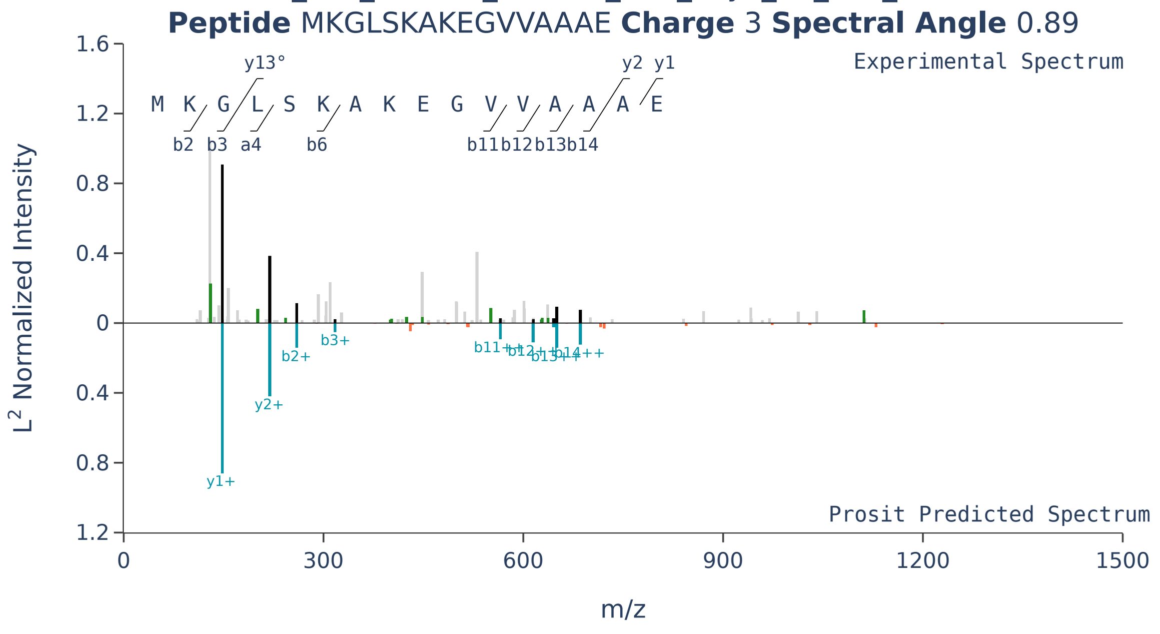


**Source** W_Soh_160821_170921_Goe_aSyn_A1_24h_R1 **Scan** 7655
**Peptide** KEGVLYVGSKTKE **Charge** 3 **Spectral Angle** 0.7

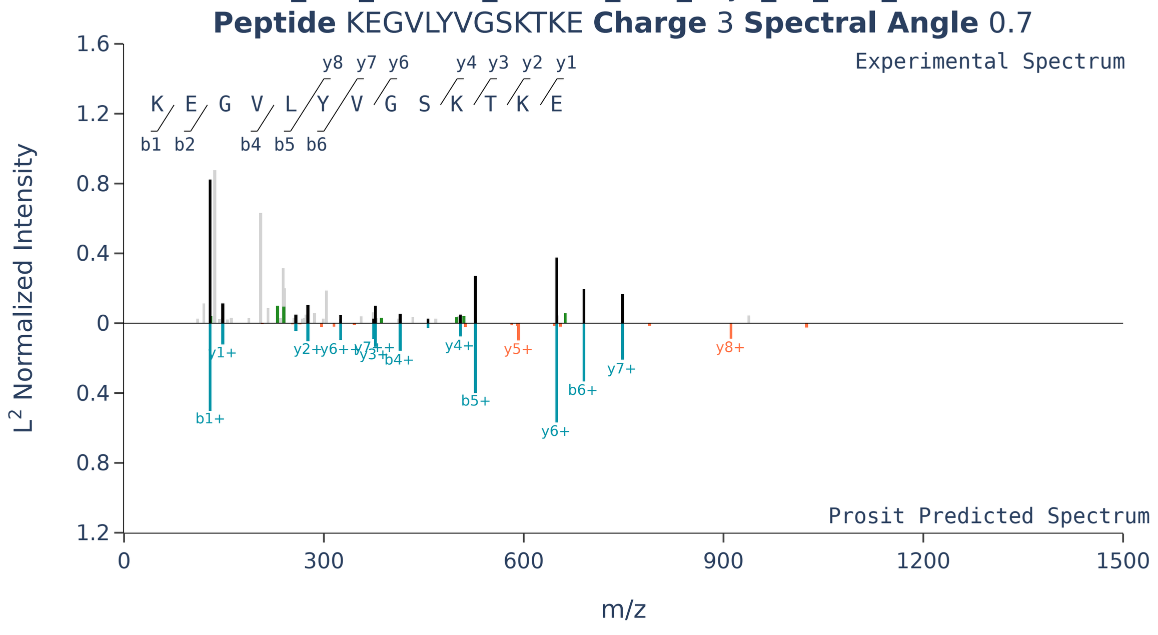


**Source** W_Soh_160821_170921_Goe_aSyn_A1_24h_R2 **Scan** 8170
**Peptide** AAGKTKEGVLYVGSK **Charge** 3 **Spectral Angle** 0.82

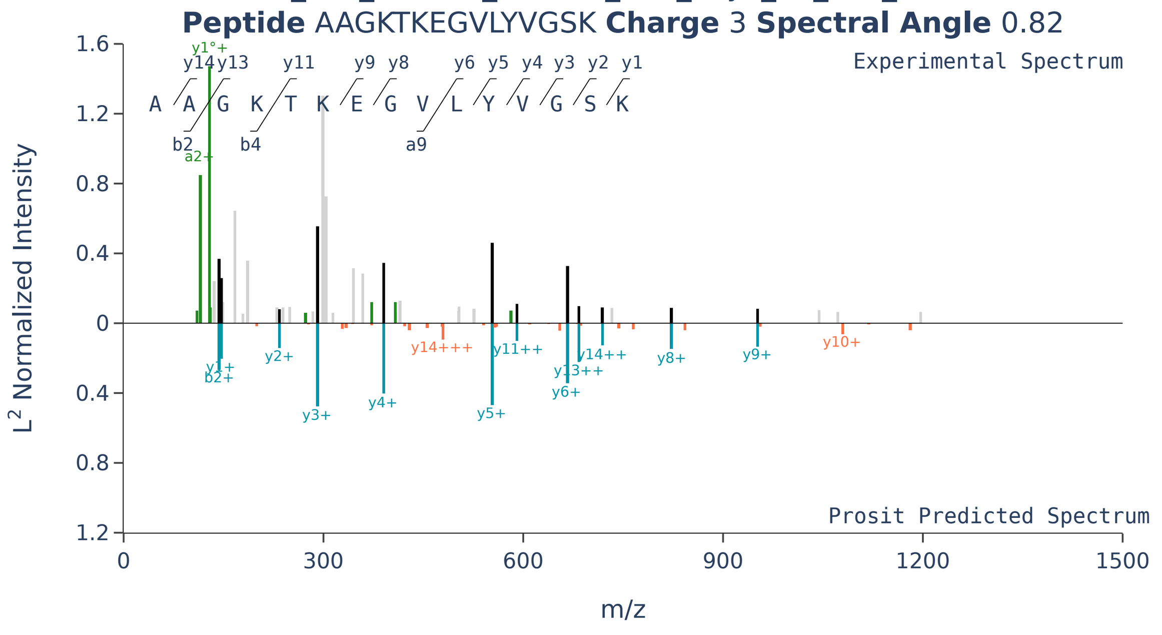


**Source** W_Soh_160821_170921_Goe_aSyn_A1_24h_R2 **Scan** 13802
**Peptide** KTKEQVTNVGGAVVTGV **Charge** 2 **Spectral Angle** 0.77

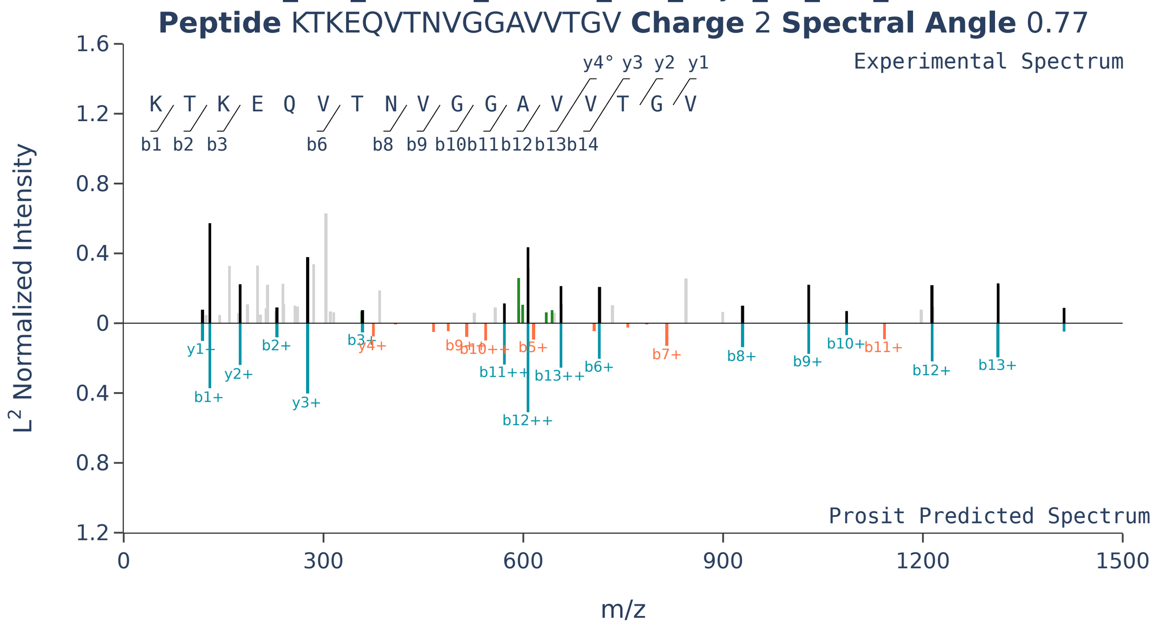


**Source** W_Soh_160821_170921_Goe_aSyn_A1_24h_R1 **Scan** 18825
**Peptide** QVTNVGGAVVTGVT **Charge** 2 **Spectral Angle** 0.79

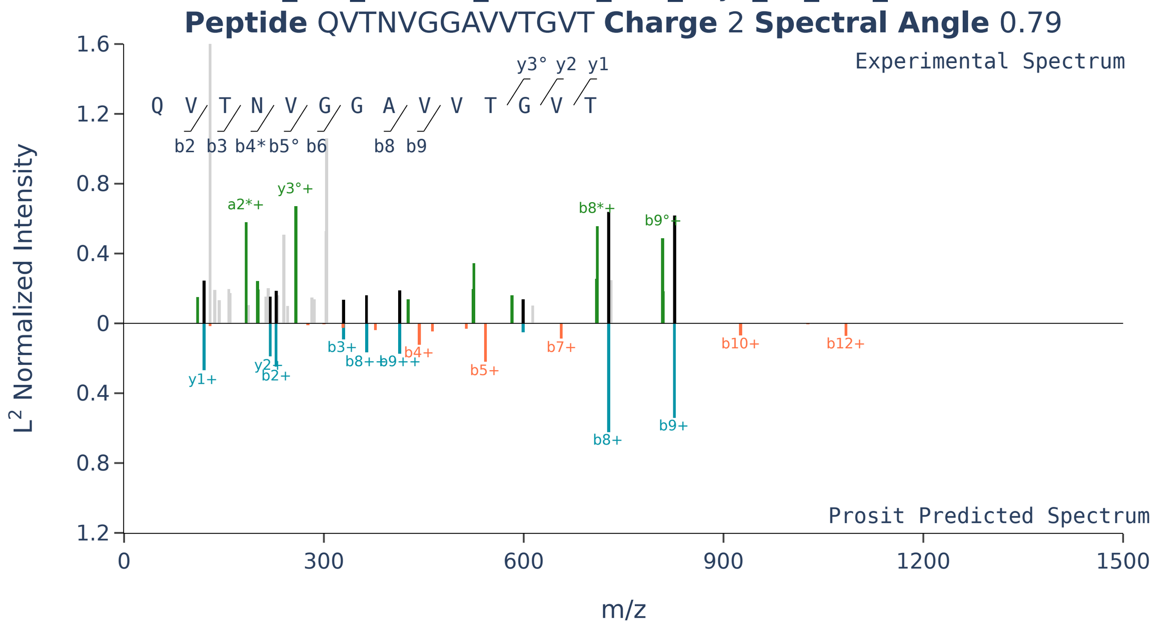


**Source** W_Soh_160821_170921_Goe_aSyn_A1_24h_R1 **Scan** 8990
**Peptide** SKAKEGVVAAAEKTKQGVAEAA **Charge** 3 **Spectral Angle** 0.77

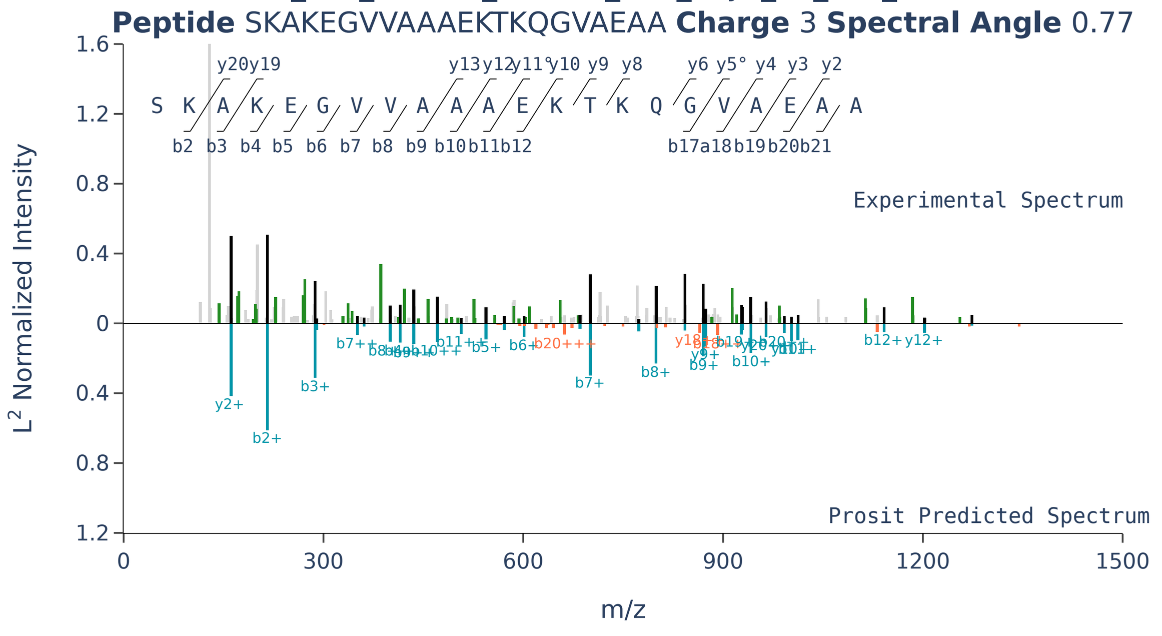


**Source** W_Soh_160821_170921_Goe_aSyn_A1_24h_R2 **Scan** 18155
**Peptide** AVVTGVTAVAQKTVEG **Charge** 2 **Spectral Angle** 0.82

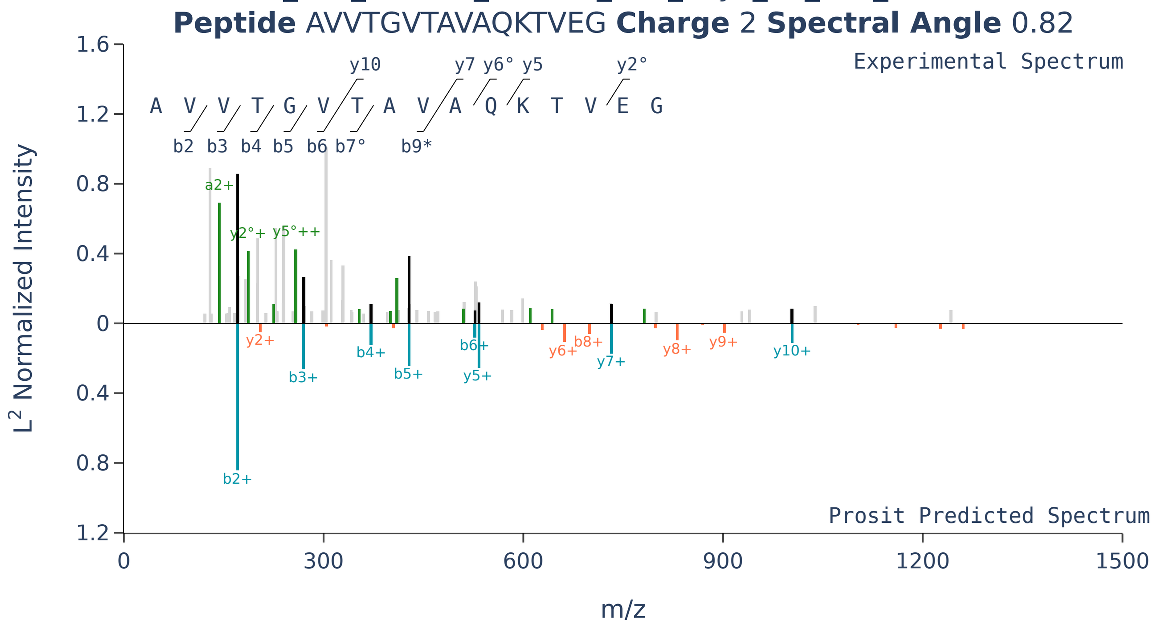


**Source** W_Soh_160821_170921_Goe_aSyn_A1_24h_R2 **Scan** 21902
**Peptide** GVTAVAQKTVEGAGSIAAATGFVKK **Charge** 3 **Spectral Angle** 0.78

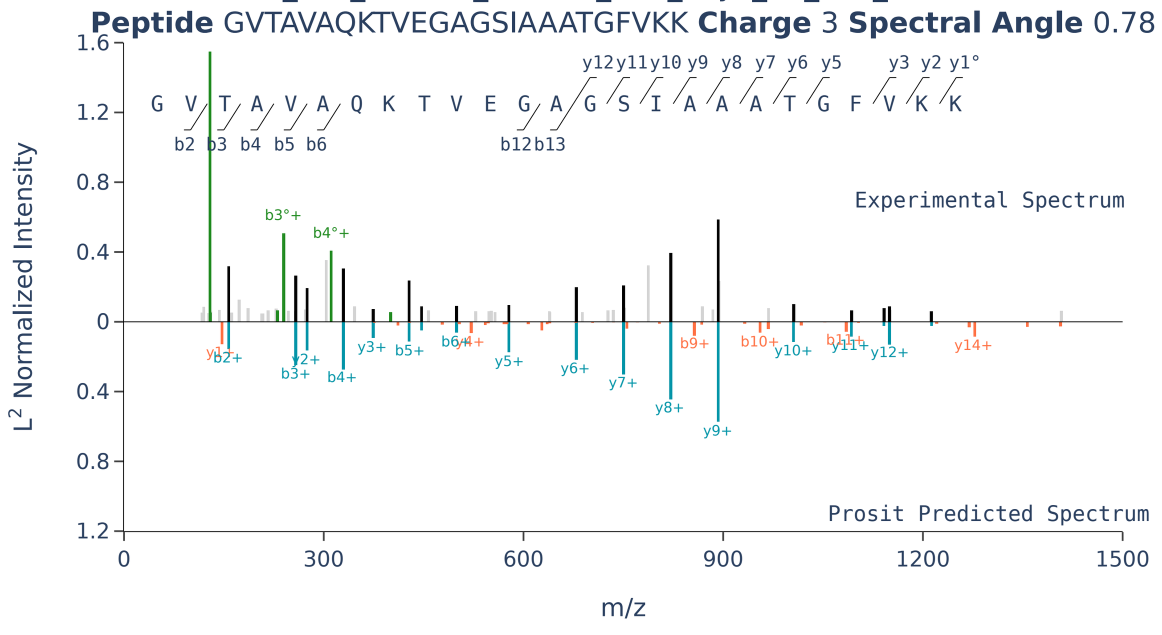


**Source** W_Soh_160821_170921_Goe_aSyn_A1_24h_R2 **Scan** 6897
**Peptide** VVAAAEKTKQGVAEAAGK **Charge** 3 **Spectral Angle** 0.75

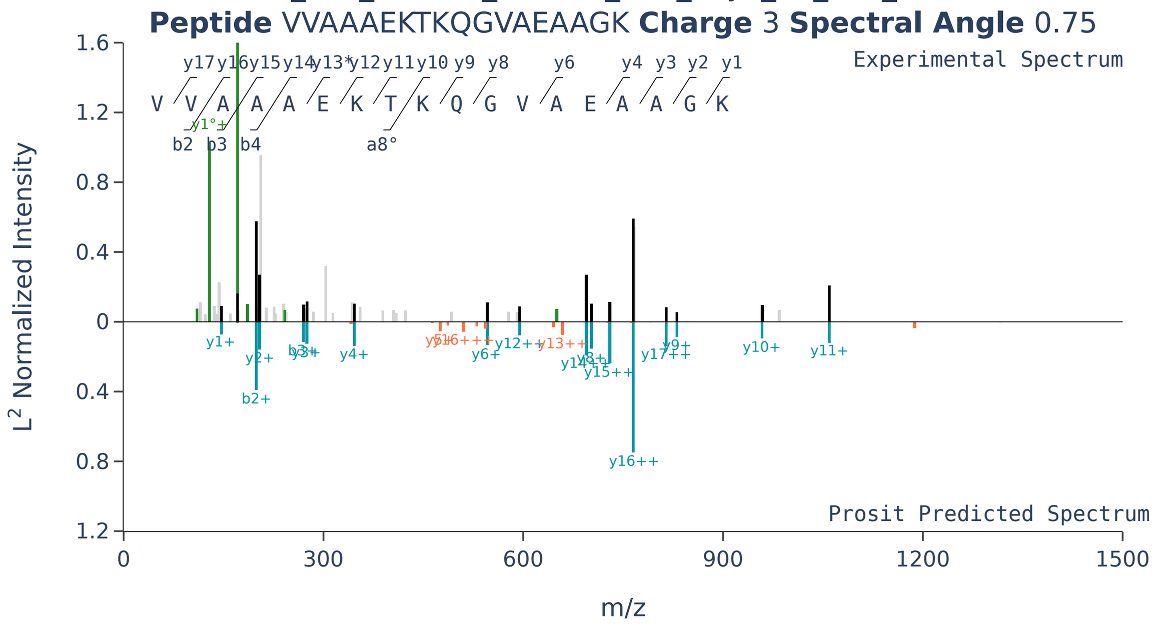


**Source** W_Soh_160821_170921_Goe_aSyn_A2_24h_R1 **Scan** 6808
**Peptide** LSKAKEGVVAAAEKT **Charge** 3 **Spectral Angle** 0.73

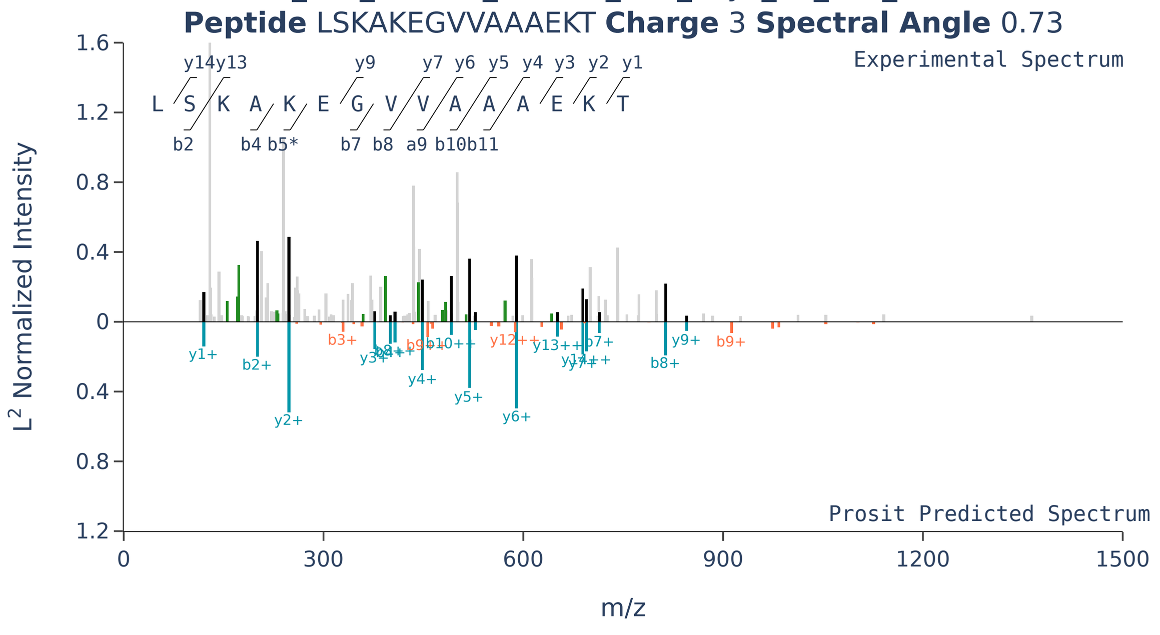


**Source** W_Soh_160821_170921_Goe_aSyn_A1_24h_R1 **Scan** 11995
**Peptide** VAEAAGKTKEGVLYVGSKTKEGVVHGV **Charge** 4 **Spectral Angle** 0.83

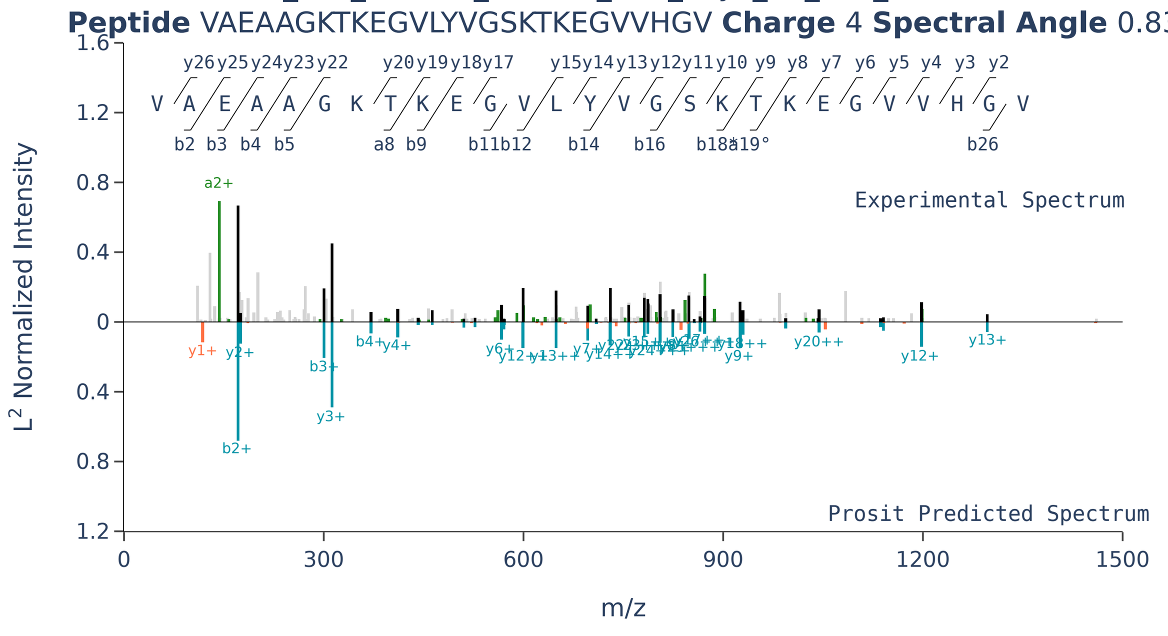


**Source** W_Soh_160821_170921_Goe_aSyn_A1_24h_R2 **Scan** 20801
**Peptide** TNVGGAVVTGVTAVAQKT **Charge** 2 **Spectral Angle** 0.85

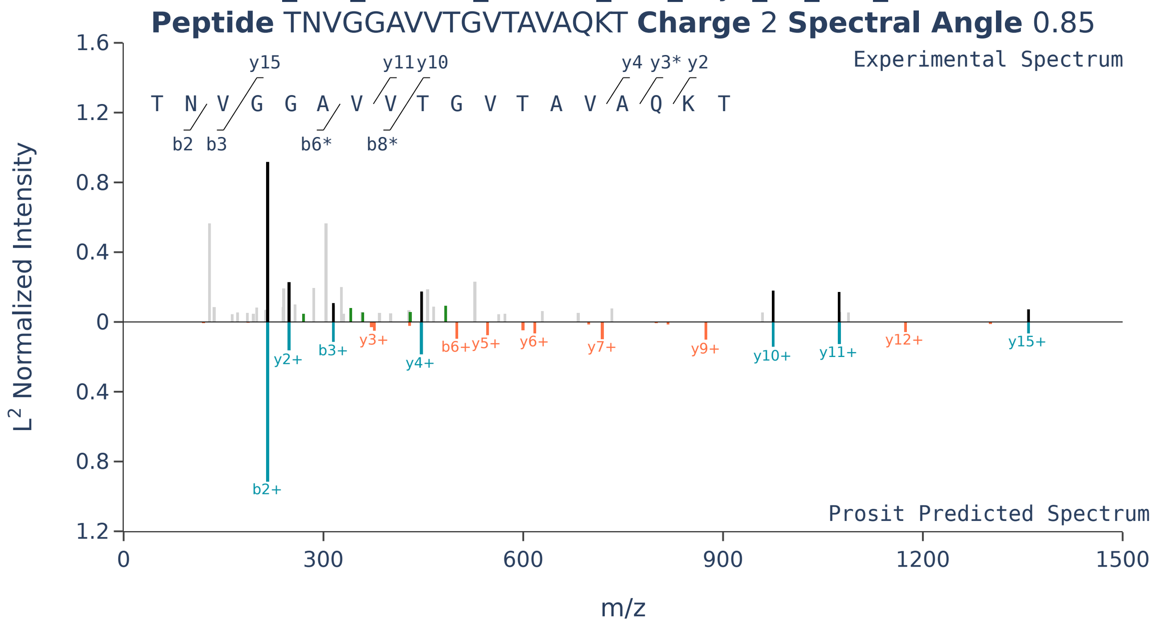


**Source** W_Soh_160821_170921_Goe_aSyn_A2_24h_R2 **Scan** 17484
**Peptide** TVEGAGSIAAATGFVKKDQLGKNE **Charge** 3 **Spectral Angle** 0.76

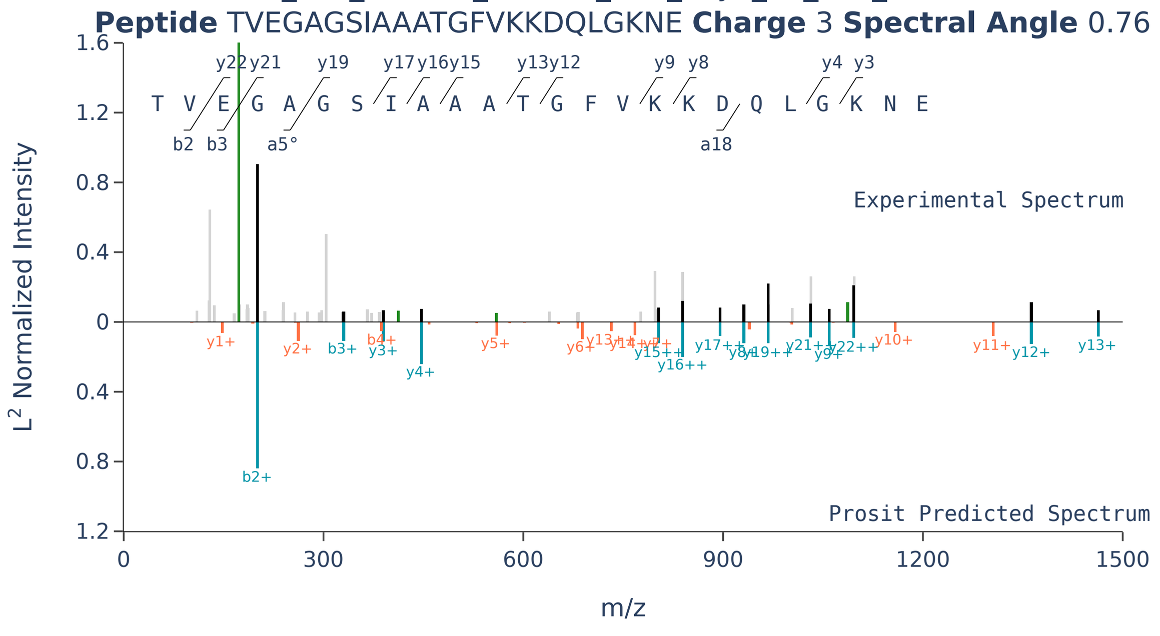

**Source** W_Soh_160821_170921_Goe_aSyn_A2_24h_R2 **Scan** 7039
**Peptide** TKEGVVHGVAT **Charge** 2 **Spectral Angle** 0.7

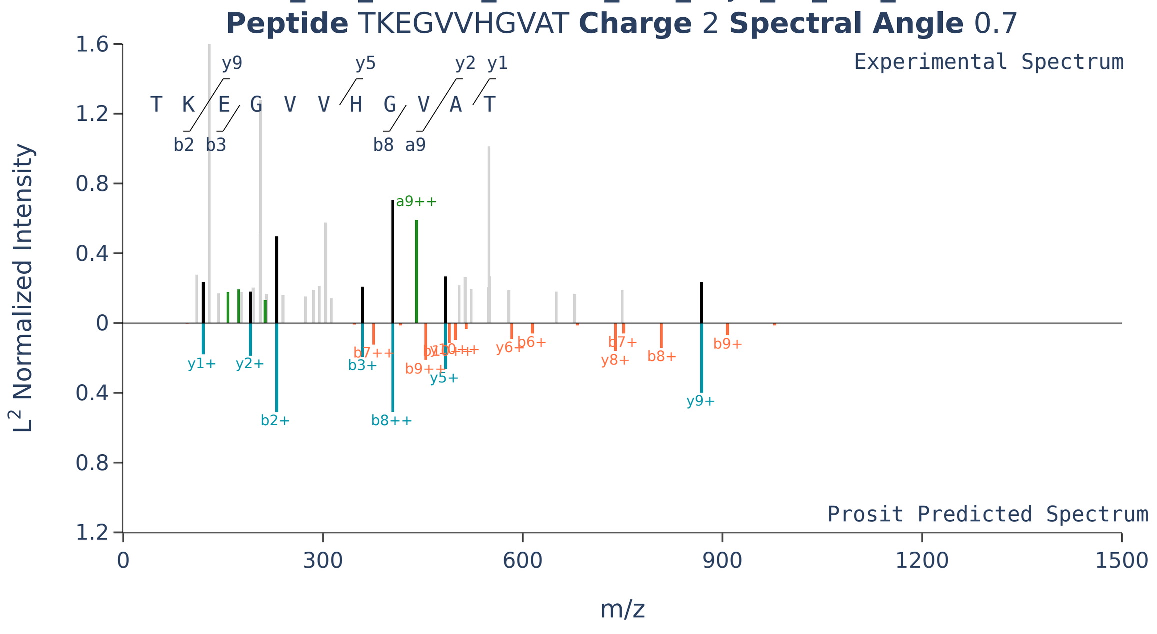


**Source** W_Soh_160821_170921_Goe_aSyn_A1_24h_R1 **Scan** 10342
**Peptide** VAEAAGKTKEGVLY **Charge** 2 **Spectral Angle** 0.87

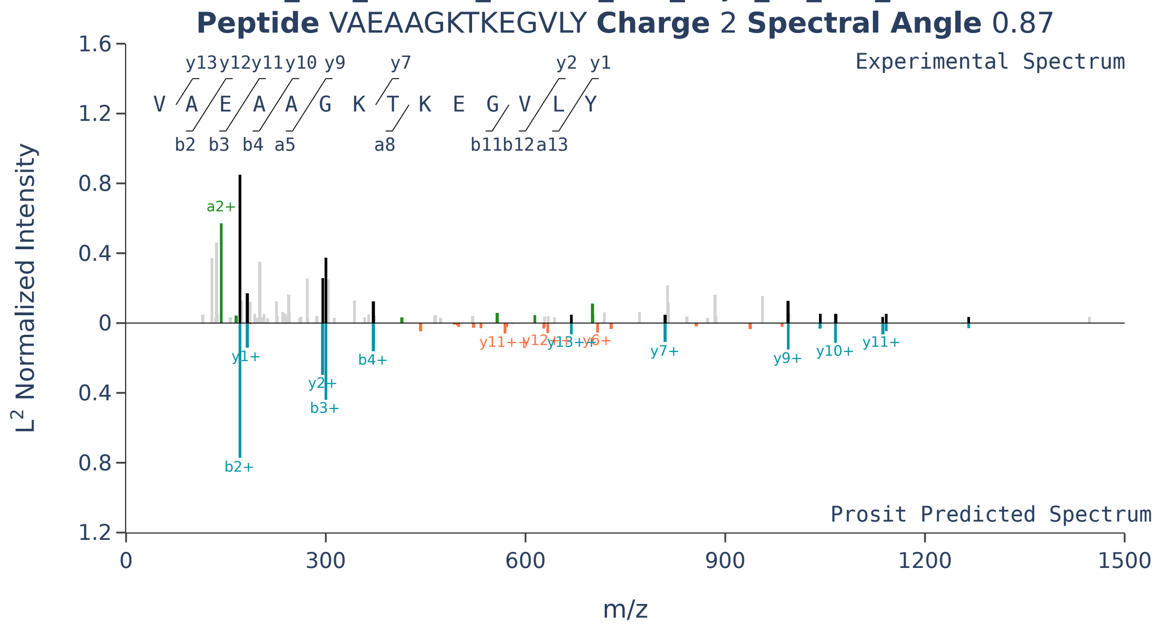


**Source** W_Soh_160821_170921_Goe_aSyn_A2_24h_R1 **Scan** 15932
**Peptide** EAYEMPSEEGYQD **Charge** 2 **Spectral Angle** 0.81

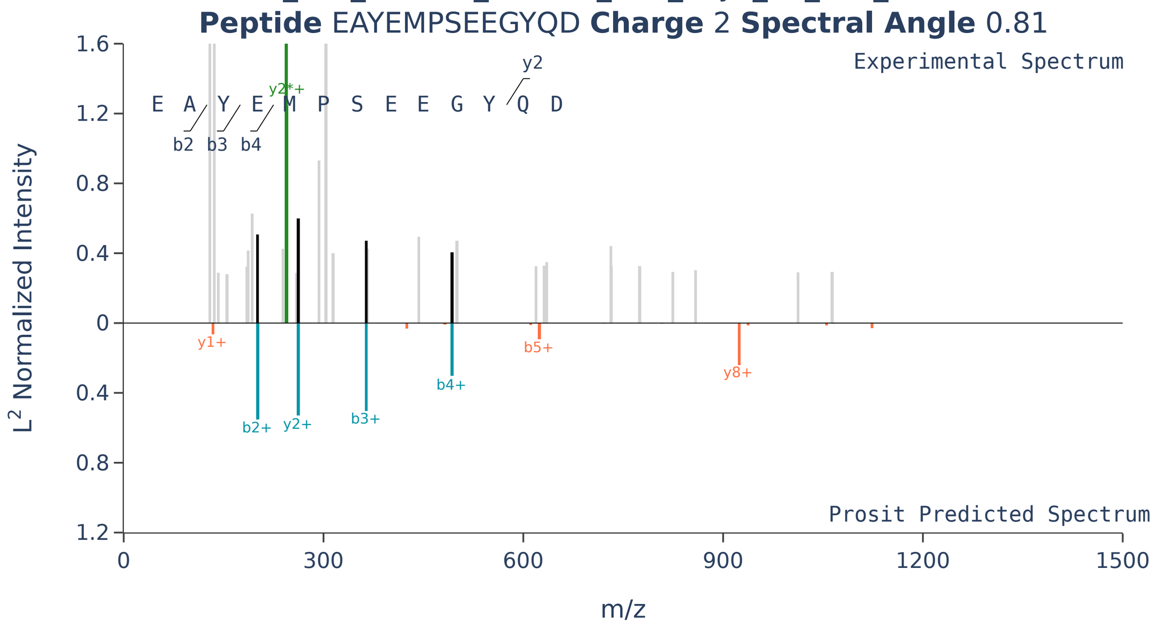


**Source** W_Soh_160821_170921_Goe_aSyn_A2_24h_R2 **Scan** 7855
**Peptide** MKGLSKAKEGVVAAAEKTKQ **Charge** 4 **Spectral Angle** 0.79

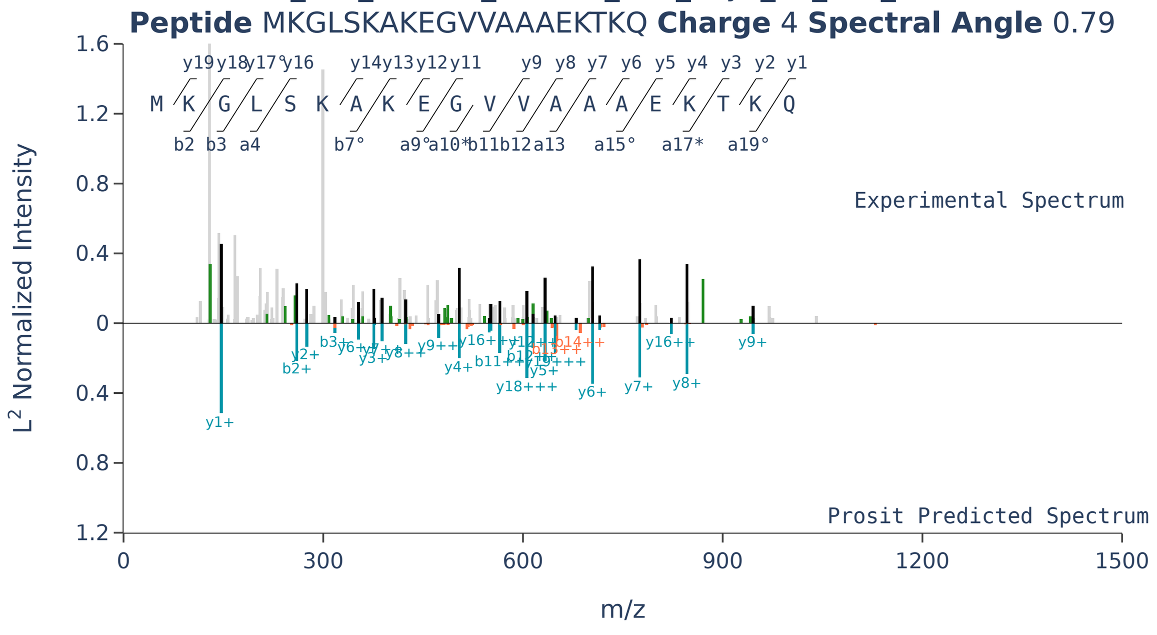


**Source** W_Soh_160821_170921_Goe_aSyn_A2_24h_R1 **Scan** 8392
**Peptide** VAEAAGKTKEGVLYVGSKTKE **Charge** 3 **Spectral Angle** 0.87

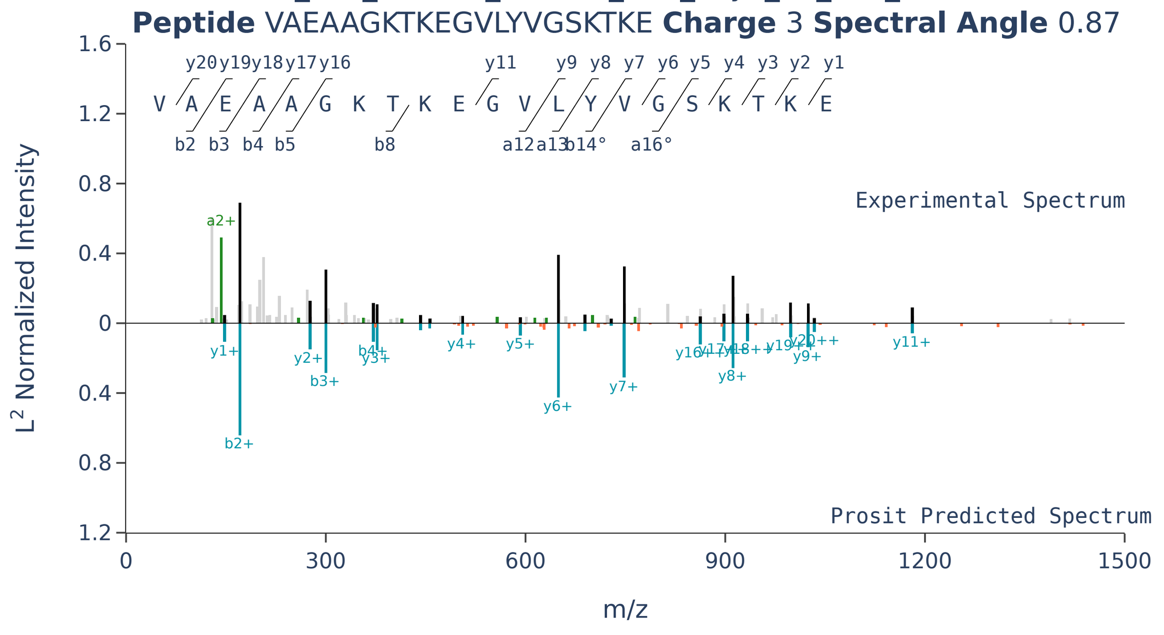


**Source** W_Soh_160821_170921_Goe_aSyn_A2_24h_R2 **Scan** 8980
**Peptide** GVATVAE **Charge** 1 **Spectral Angle** 0.77

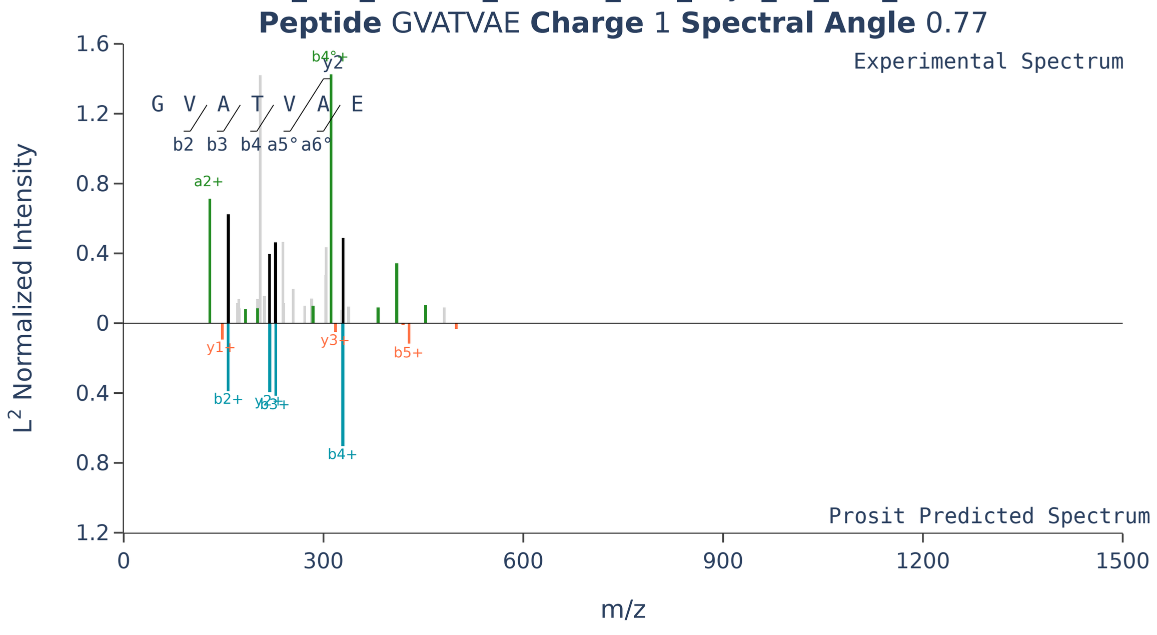


**Source** W_Soh_160821_170921_Goe_aSyn_A1_6h_R1 **Scan** 15755
**Peptide** KEGVVAAAEKTKQGVAEAAGKTKEGVLYVG **Charge** 4 **Spectral Angle** 0.77

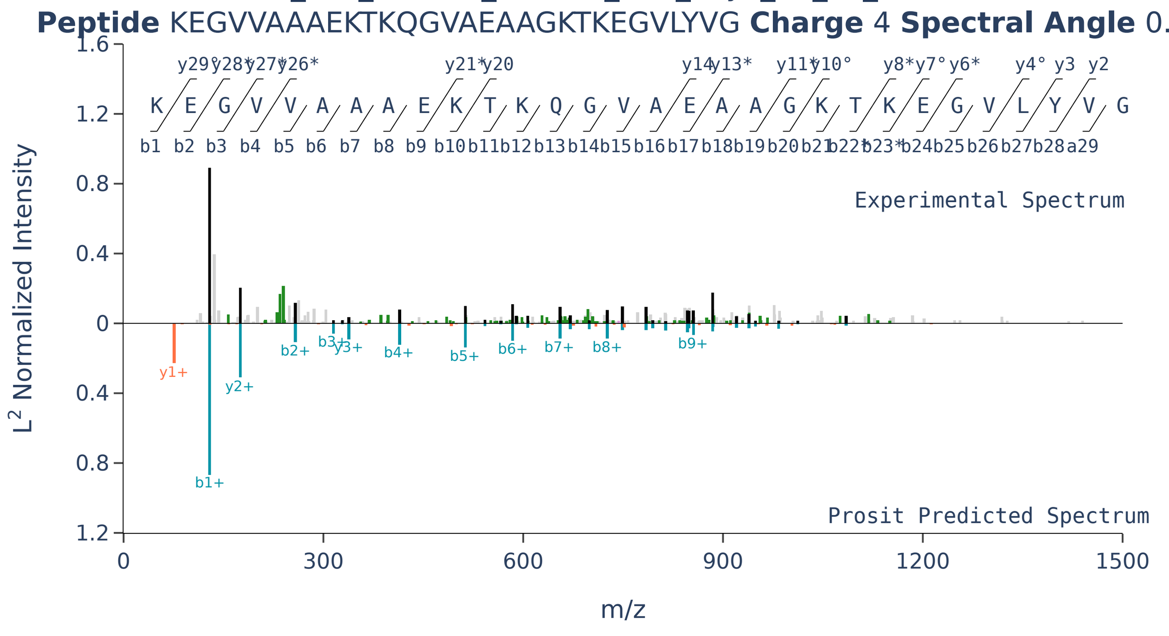


**Source** W_Soh_160821_170921_Goe_aSyn_A2_6h_R1 **Scan** 13608
**Peptide** GVAEAAGKTKEGVLYVGSKTKEGVVHGV **Charge** 4 **Spectral Angle** 0.8

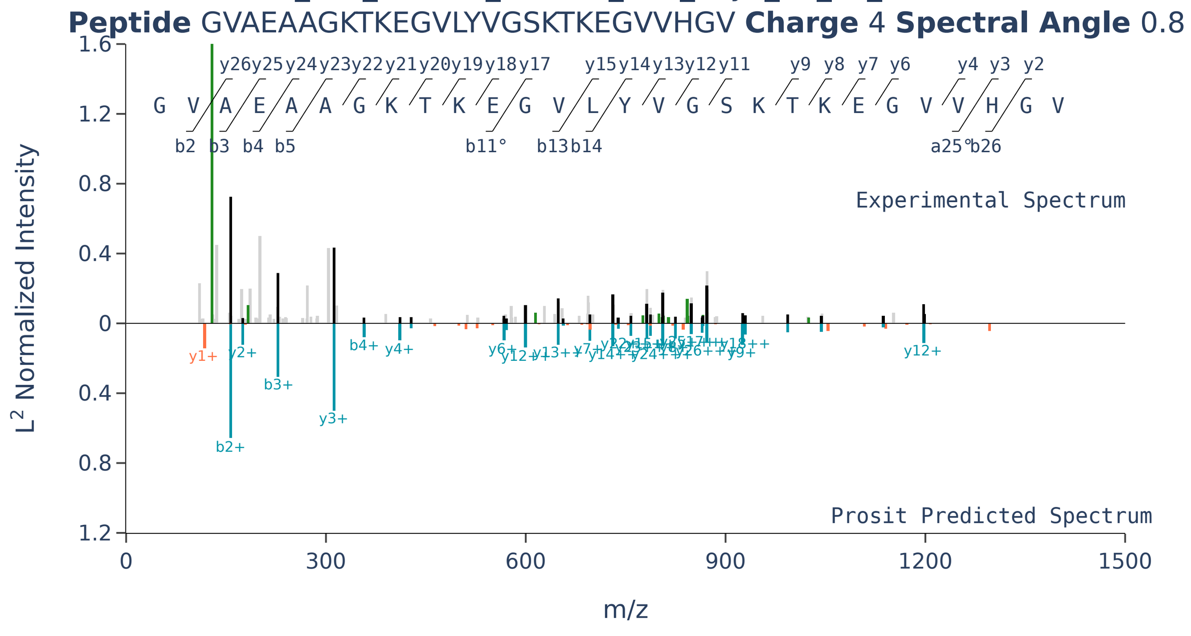


**Source** W_Soh_160821_170921_Goe_aSyn_A1_3h_R1 **Scan** 19963
**Peptide** GVVHGVATVAEKTKEQVTNVGGAVVTGV **Charge** 3 **Spectral Angle** 0.74

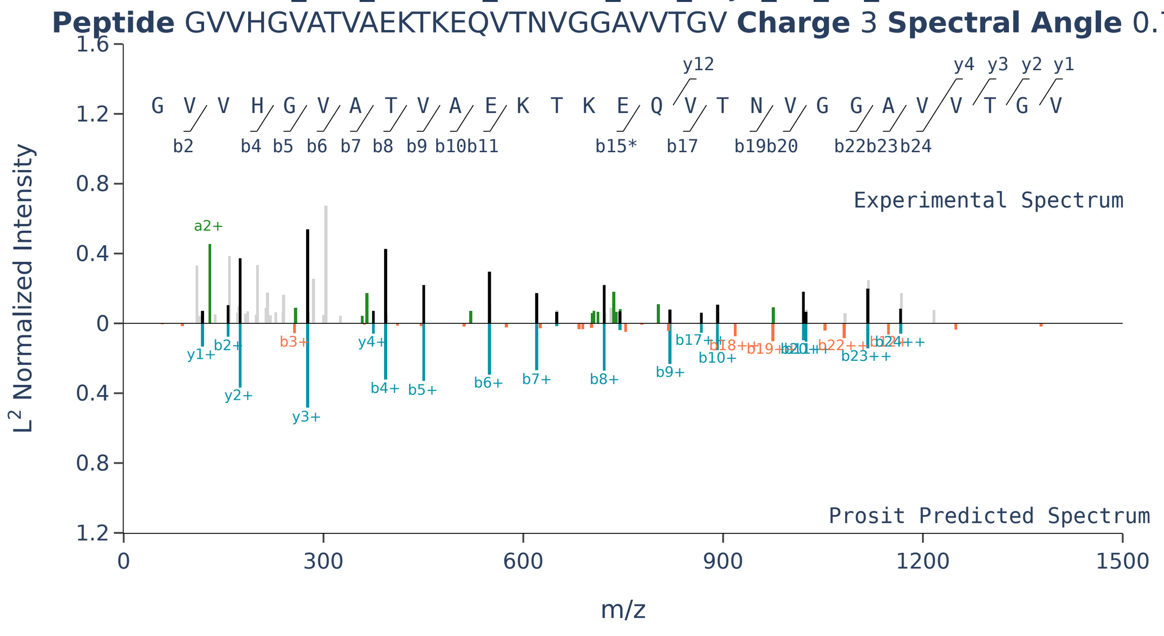


**Source** W_Soh_160821_170921_Goe_aSyn_A1_6h_R2 **Scan** 23311
**Peptide** VTNVGGAVVTGVTAVA **Charge** 2 **Spectral Angle** 0.75

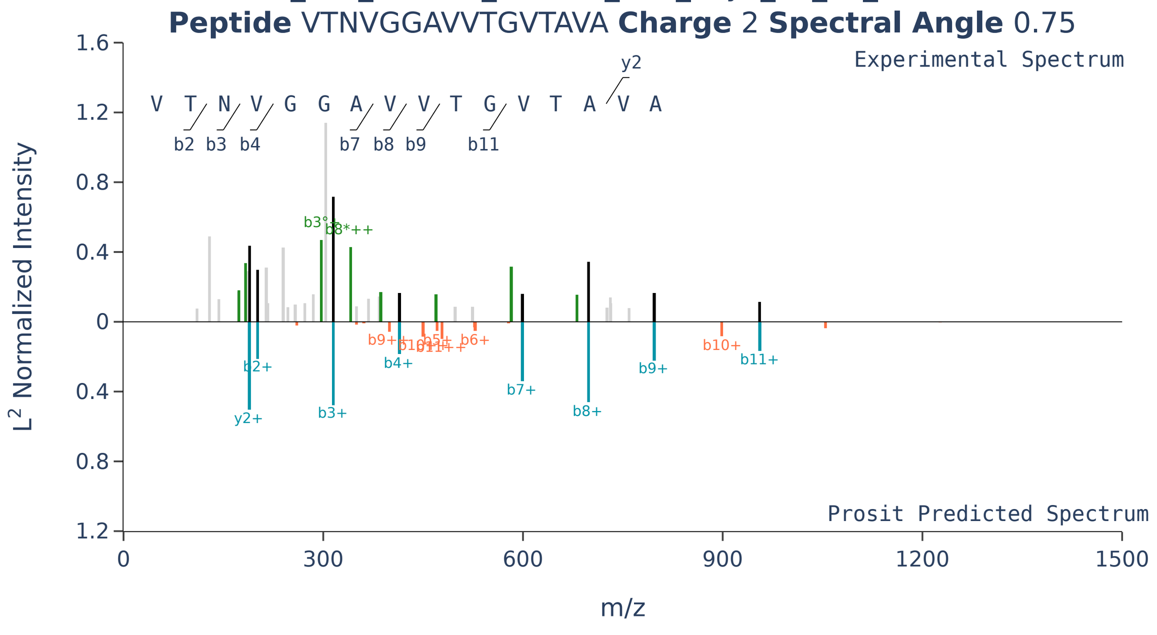


**Source** W_Soh_160821_170921_Goe_aSyn_A2_24h_R2 **Scan** 8992
**Peptide** GVATVAEKTKEQVTN **Charge** 3 **Spectral Angle** 0.82

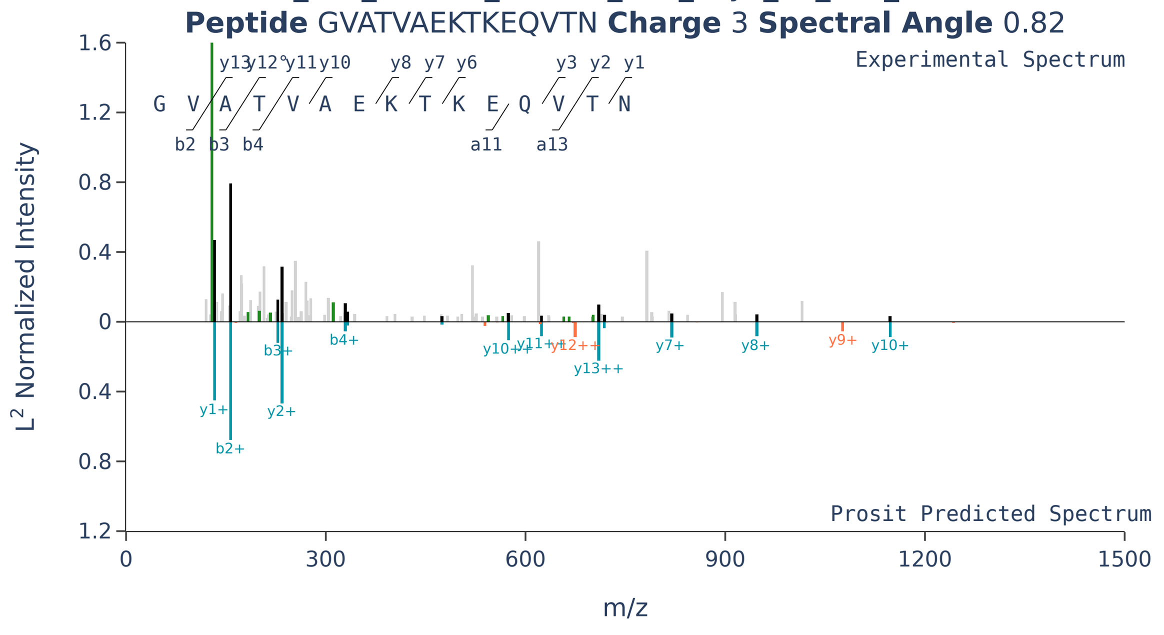


**Source** W_Soh_160821_170921_Goe_aSyn_A2_24h_R2 **Scan** 7044
**Peptide** TVAEKTKEQV **Charge** 2 **Spectral Angle** 0.78

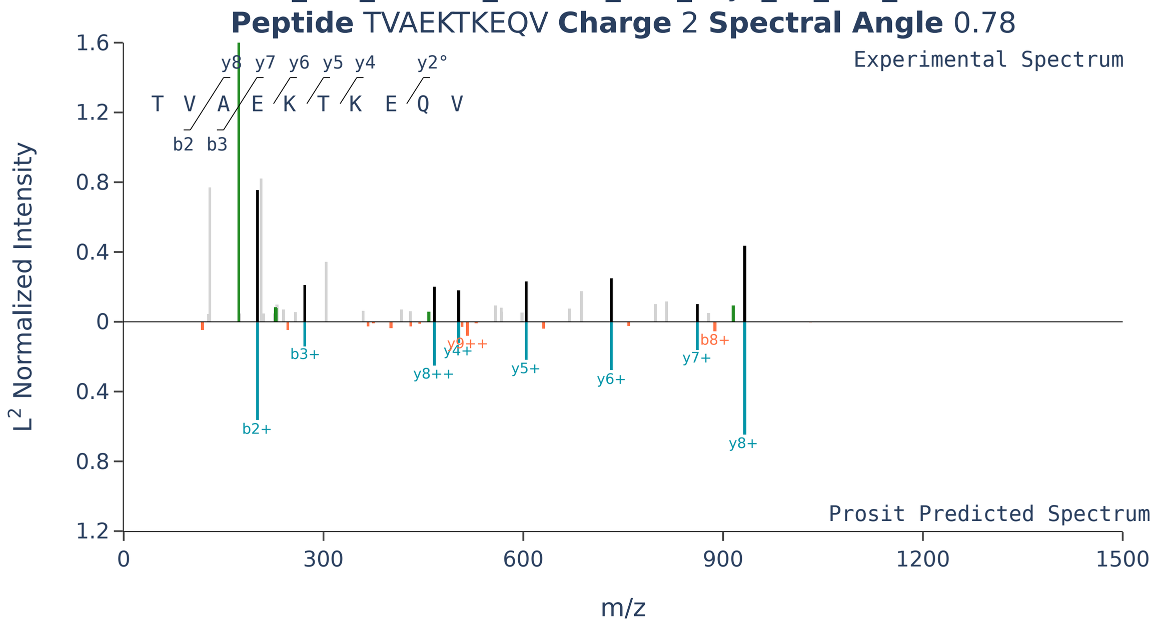


**Source** W_Soh_160821_170921_Goe_aSyn_A1_24h_R2 **Scan** 9812
**Peptide** VAQKTVEGAGSI **Charge** 1 **Spectral Angle** 0.79

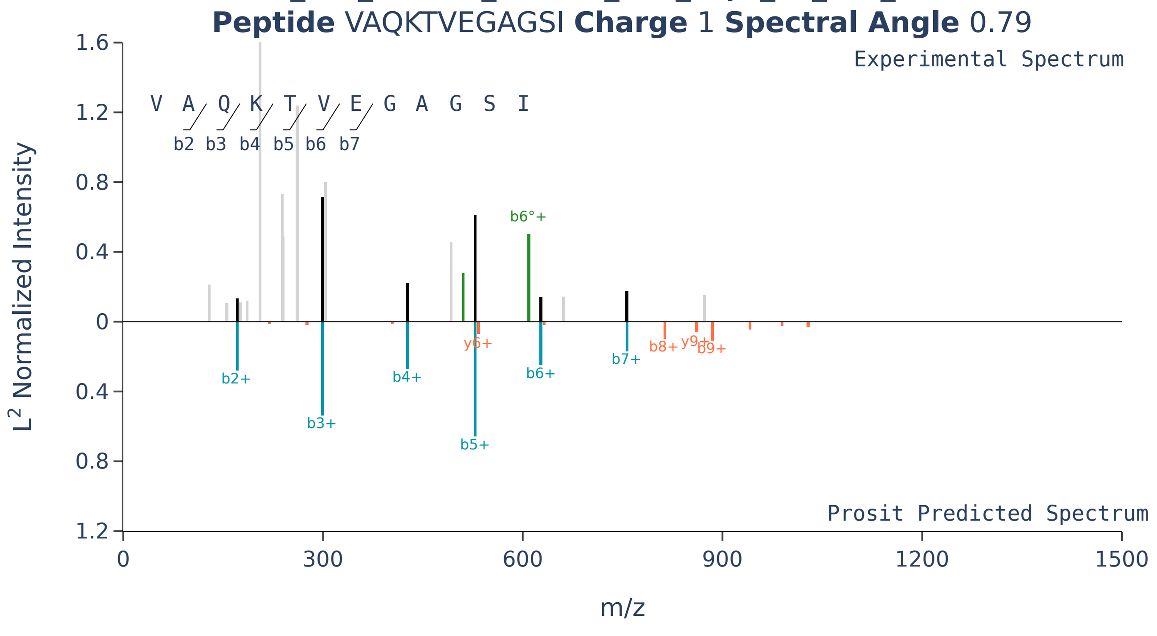


**Source** W_Soh_160821_170921_Goe_aSyn_A2_24h_R2 **Scan** 14602
**Peptide** GFVKKDQLGKNEEGAPQEGIL **Charge** 3 **Spectral Angle** 0.8

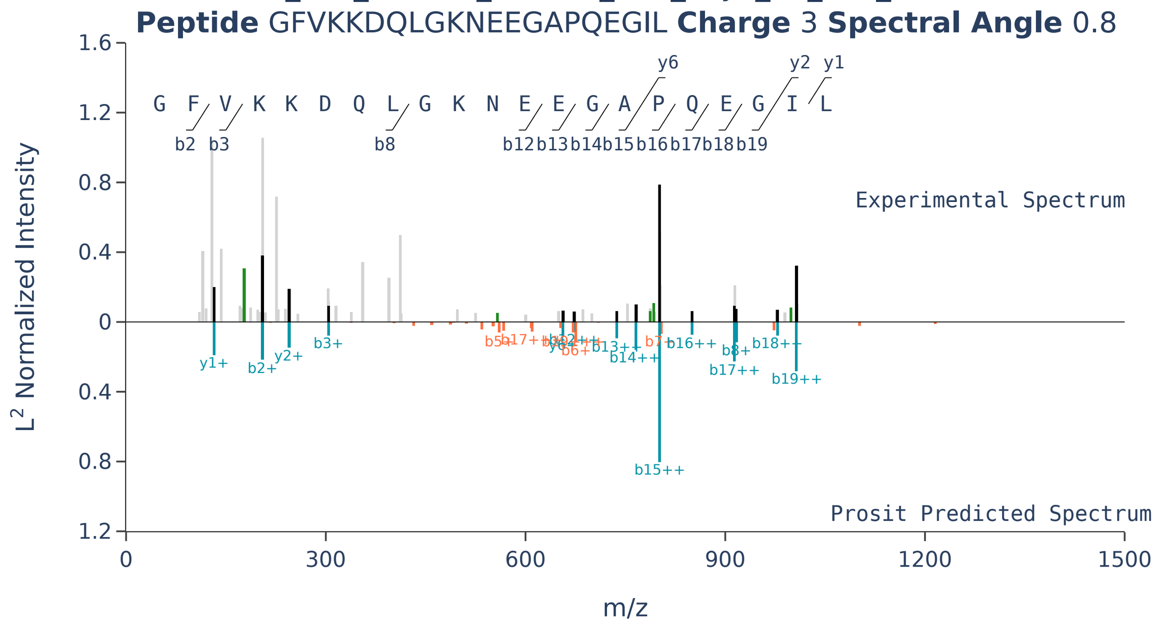


**Source** W_Soh_160821_170921_Goe_aSyn_A2_24h_R2 **Scan** 8944
**Peptide** KEGVLYVGSKTKEGVVHG **Charge** 3 **Spectral Angle** 0.87

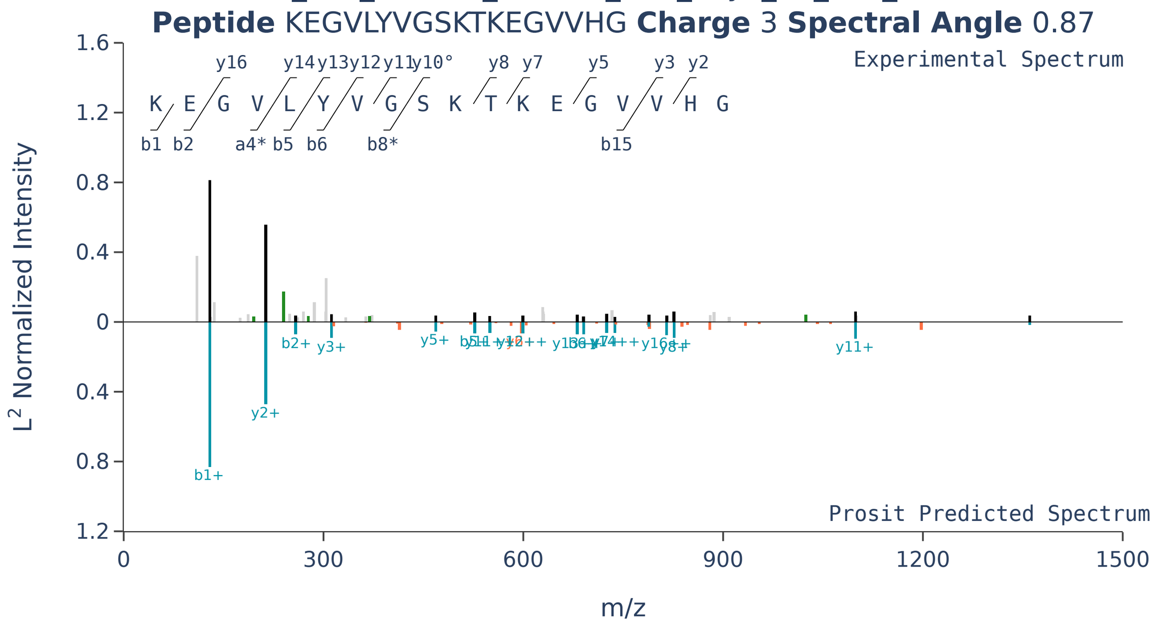

**Source** W_Soh_160821_170921_Goe_aSyn_A1_24h_R1 **Scan** 8327
**Peptide** AGKTKEGVLYVGSKTKEGV **Charge** 4 **Spectral Angle** 0.8

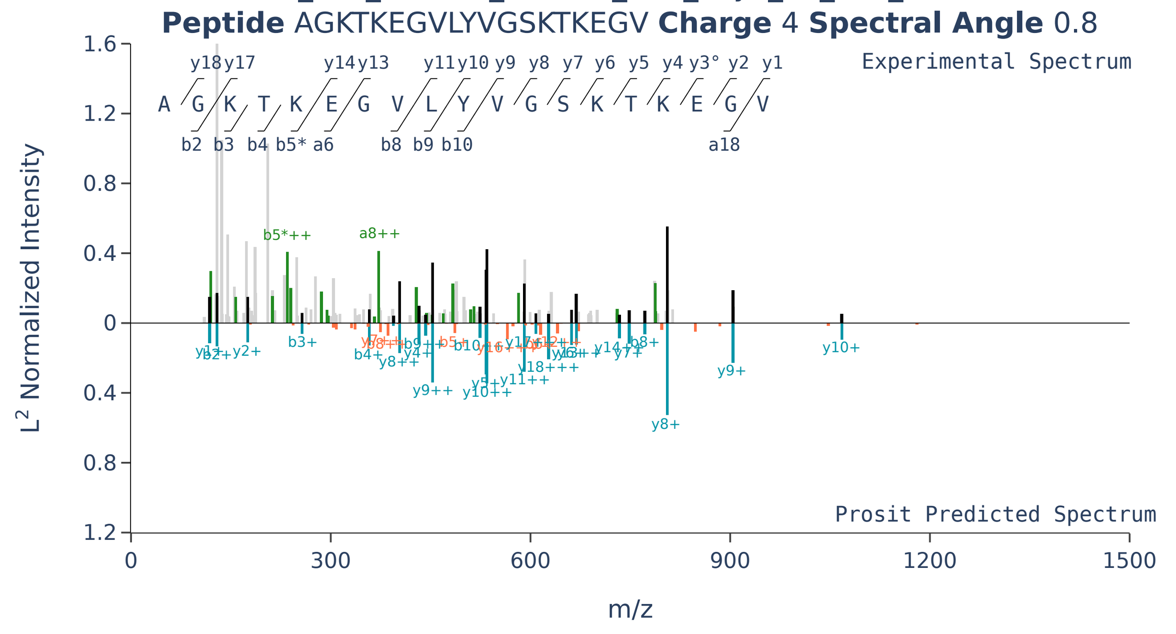


**Source** W_Soh_160821_170921_Goe_aSyn_A2_6h_R2 **Scan** 21150
**Peptide** AQKTVEGAGSIAAATGFV **Charge** 2 **Spectral Angle** 0.73

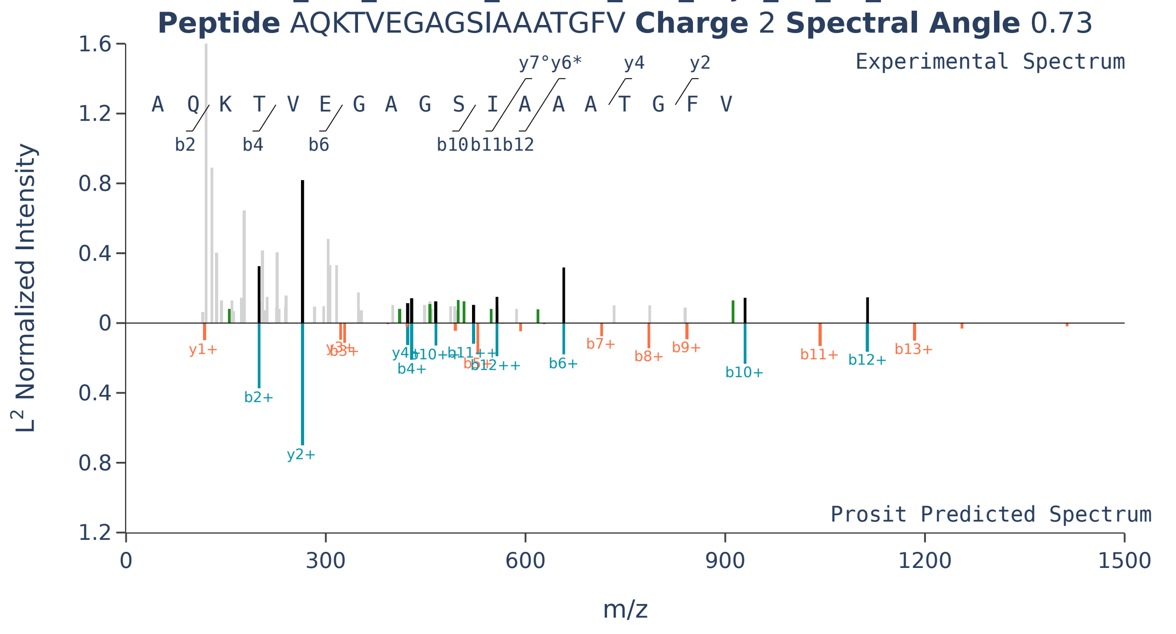


**Source** W_Soh_160821_170921_Goe_aSyn_A1_24h_R1 **Scan** 15917
**Peptide** TVAEKTKEQVTNVGGAVVTGVT **Charge** 3 **Spectral Angle** 0.8

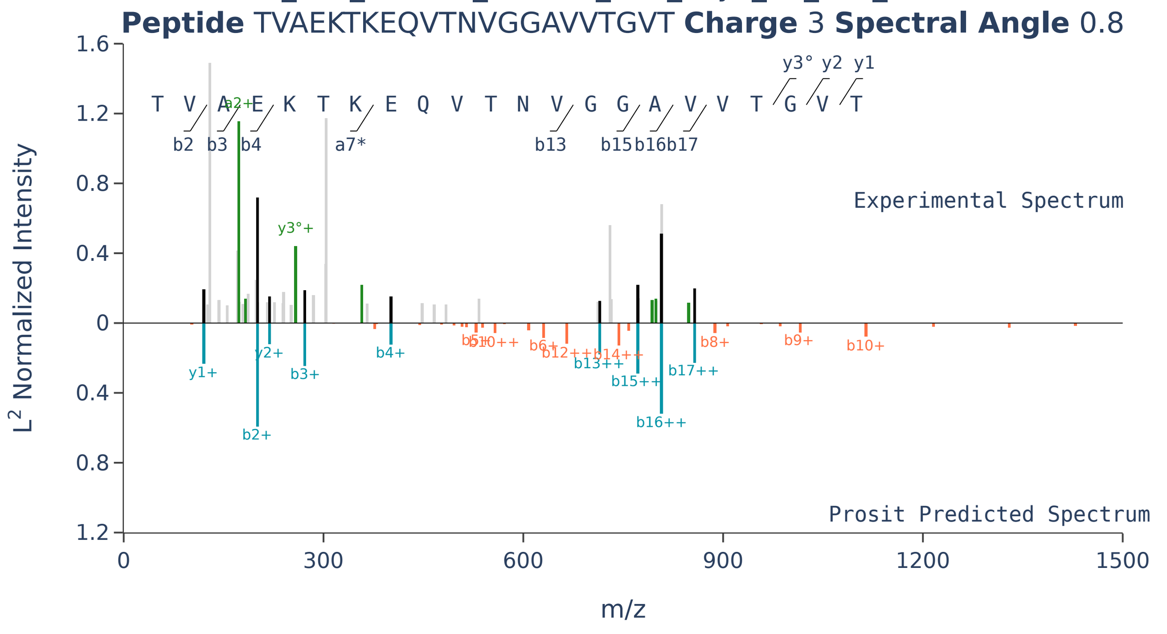


**Source** W_Soh_160821_170921_Goe_aSyn_A2_24h_R2 **Scan** 18673
**Peptide** MDVFMKGLSKAKEGVVAAAEKTKQ **Charge** 3 **Spectral Angle** 0.83

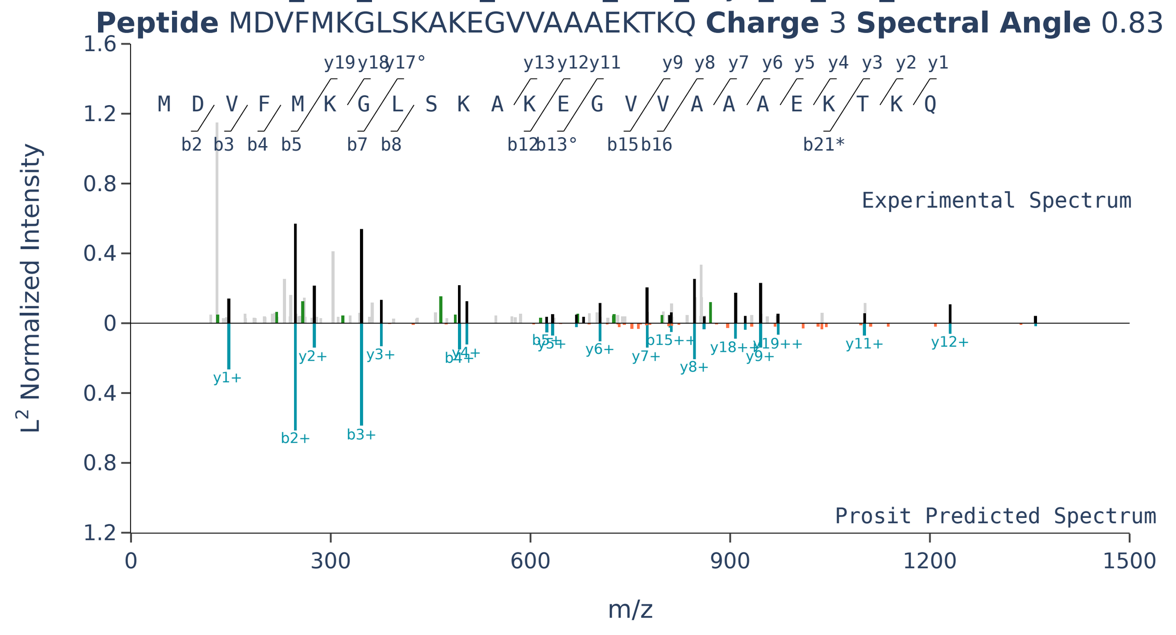


**Source** W_Soh_160821_170921_Goe_aSyn_A2_3h_R2 **Scan** 16833
**Peptide** DQLGKNEEGAPQEGIL **Charge** 2 **Spectral Angle** 0.86

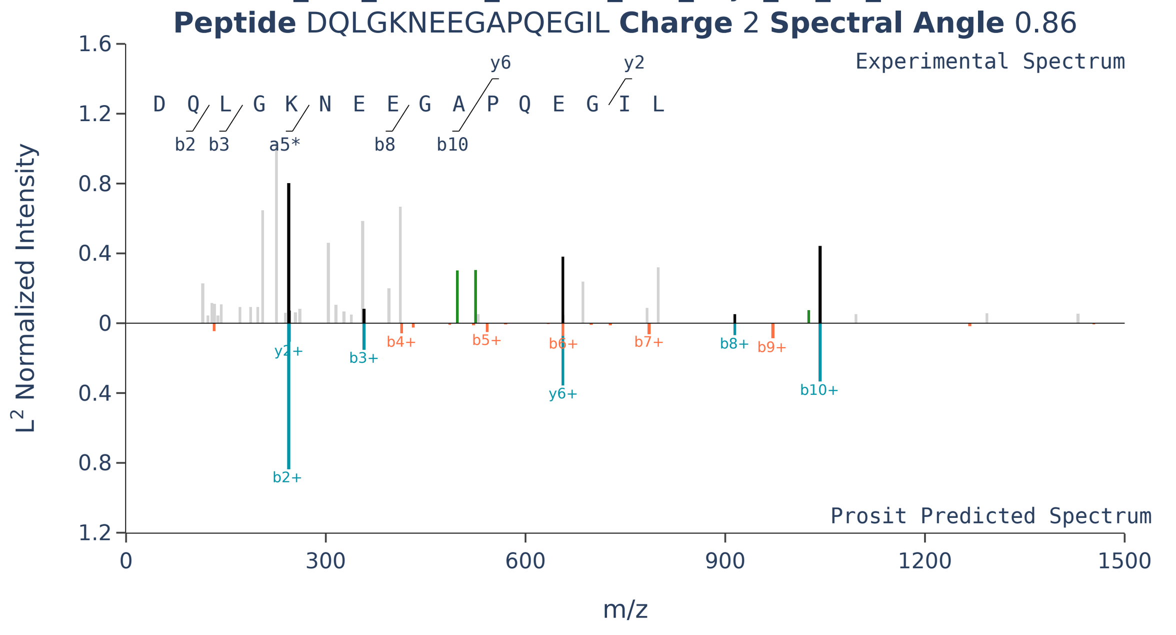


**Source** W_Soh_160821_170921_Goe_aSyn_A1_24h_R2 **Scan** 12870
**Peptide** SIAAATGFVKKDQLGKNE **Charge** 3 **Spectral Angle** 0.83

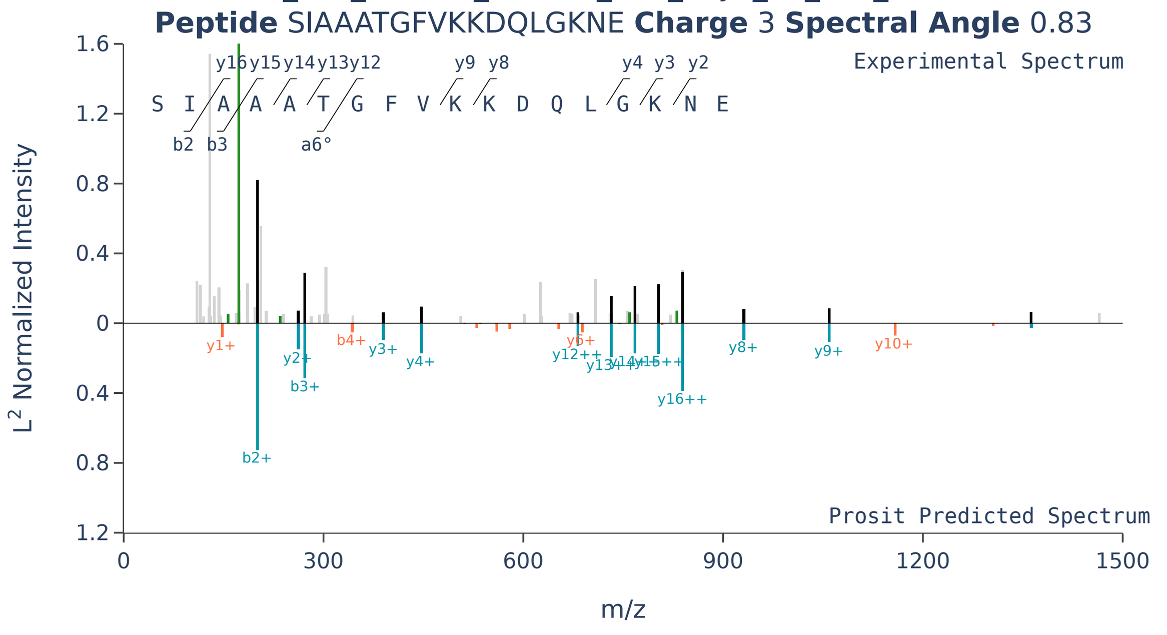


**Source** W_Soh_160821_170921_Goe_aSyn_A1_24h_R2 **Scan** 5055
**Peptide** GVAEAAGKTKEG **Charge** 2 **Spectral Angle** 0.68

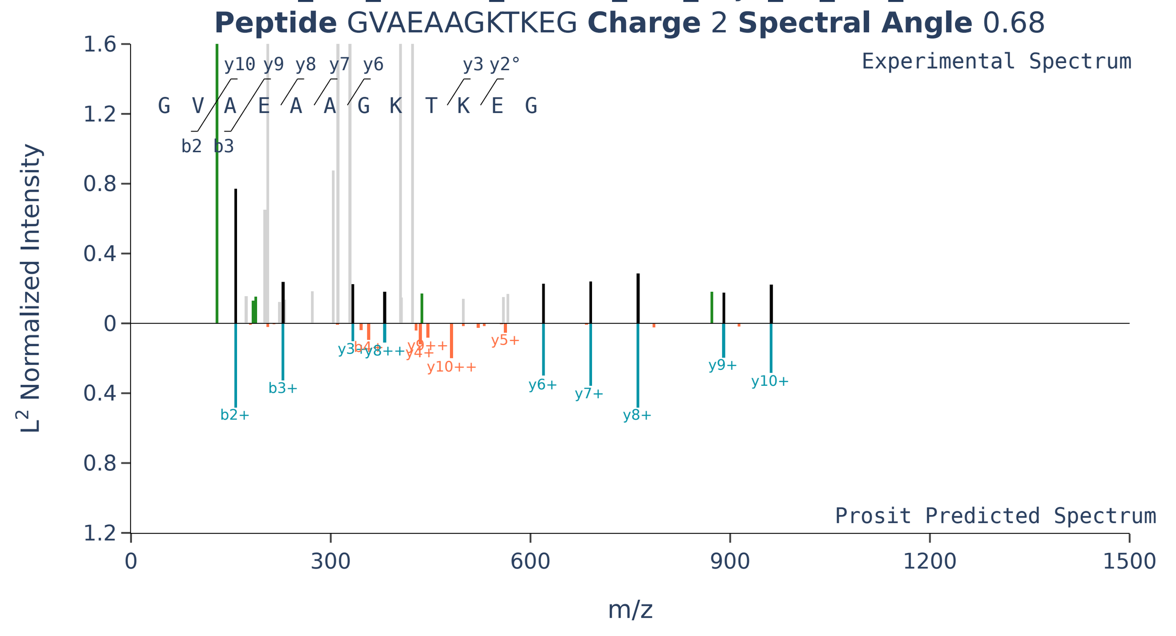


**Source** W_Soh_160821_170921_Goe_aSyn_A2_24h_R2 **Scan** 6908
**Peptide** SKTKEGVVHGVATVAEK **Charge** 4 **Spectral Angle** 0.75

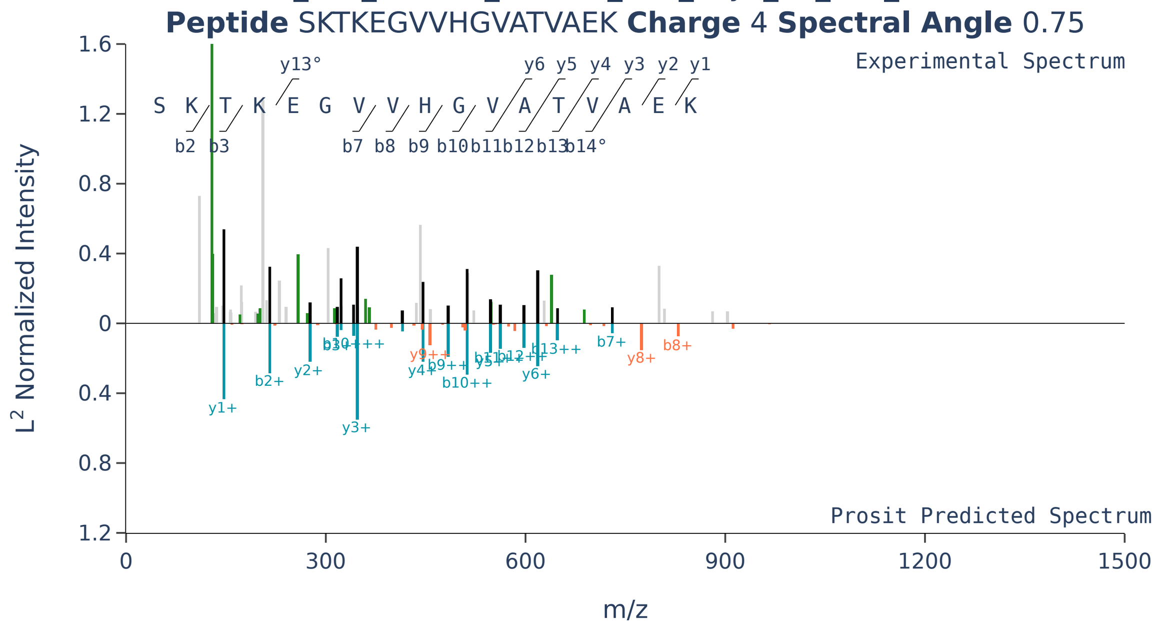


**Source** W_Soh_160821_170921_Goe_aSyn_A1_24h_R2 **Scan** 14065
**Peptide** KEGVVAAAEKTKQGVAEAAGKTKEGVL **Charge** 4 **Spectral Angle** 0.87

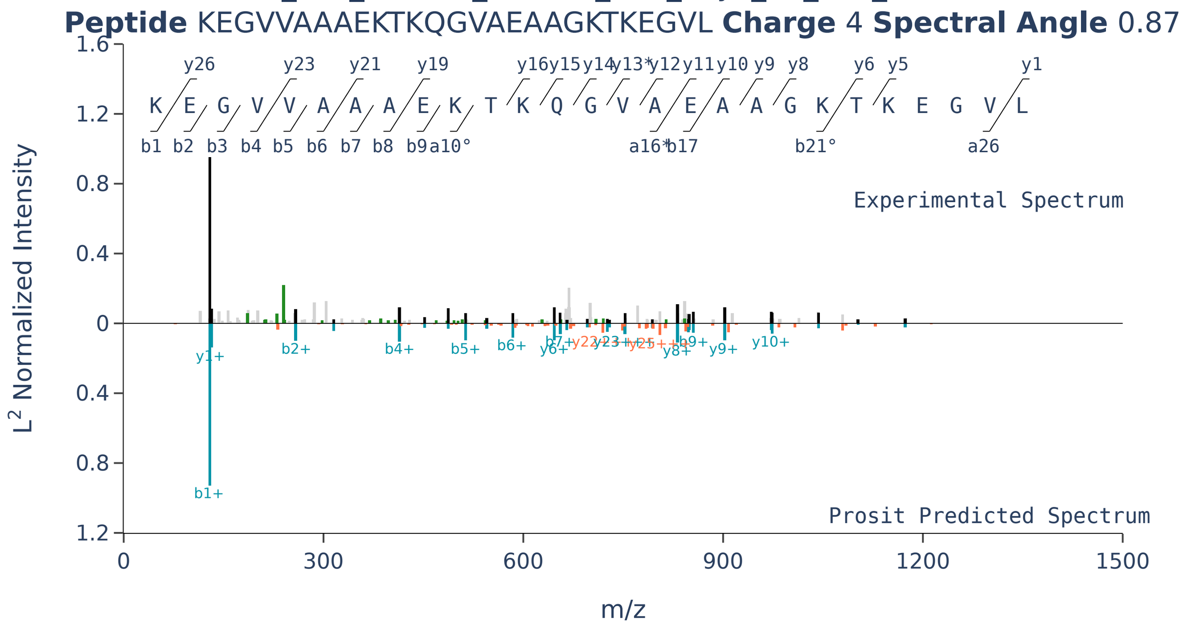


**Source** W_Soh_160821_170921_Goe_aSyn_A2_6h_R2 **Scan** 14098
**Peptide** IAAATGFVKKDQLGKNE **Charge** 3 **Spectral Angle** 0.86

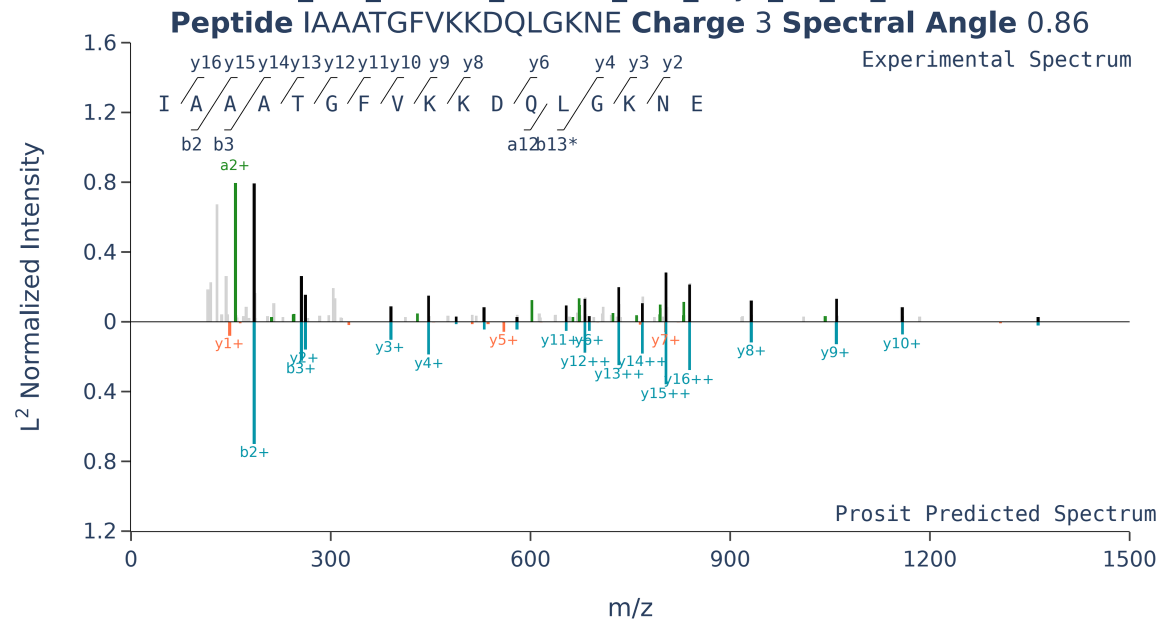


**Source** W_Soh_160821_170921_Goe_aSyn_A2_24h_R2 **Scan** 16150
**Peptide** EGAGSIAAATGFVKKDQLGKNEEGAPQE **Charge** 3 **Spectral Angle** 0.87

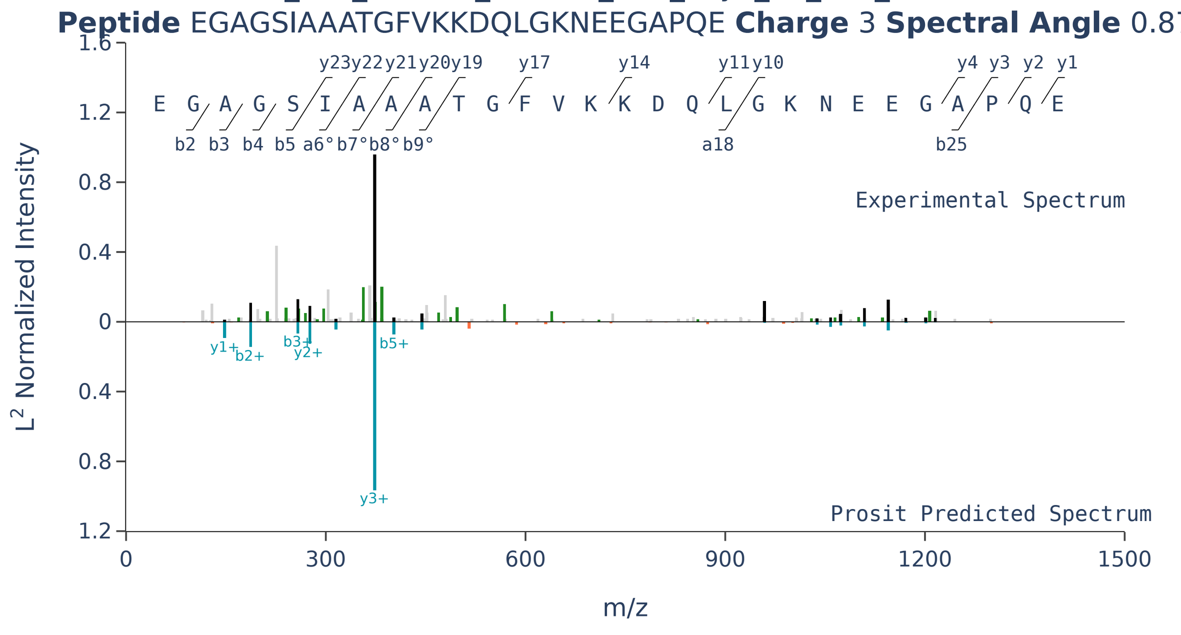


**Source** W_Soh_160821_170921_Goe_aSyn_A1_24h_R1 **Scan** 10442
**Peptide** GSKTKEGVVHGVATVAEKTKEQV **Charge** 4 **Spectral Angle** 0.73

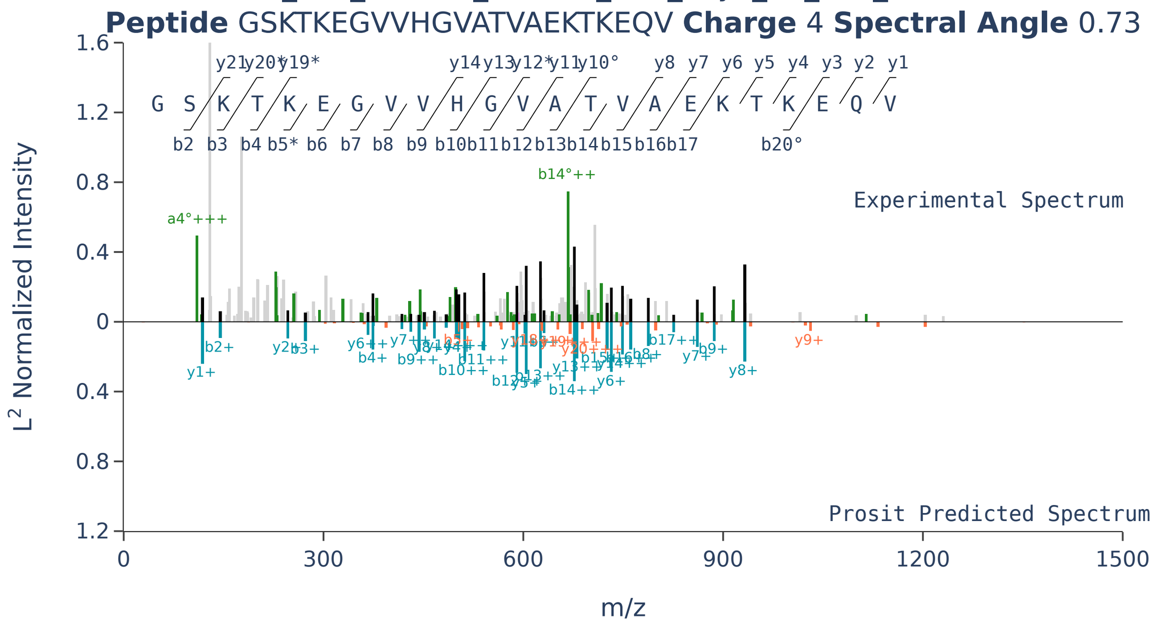


**Source** W_Soh_160821_170921_Goe_aSyn_A1_24h_R1 **Scan** 7188
**Peptide** MKGLSKAKEGVVAAAEKT **Charge** 4 **Spectral Angle** 0.86

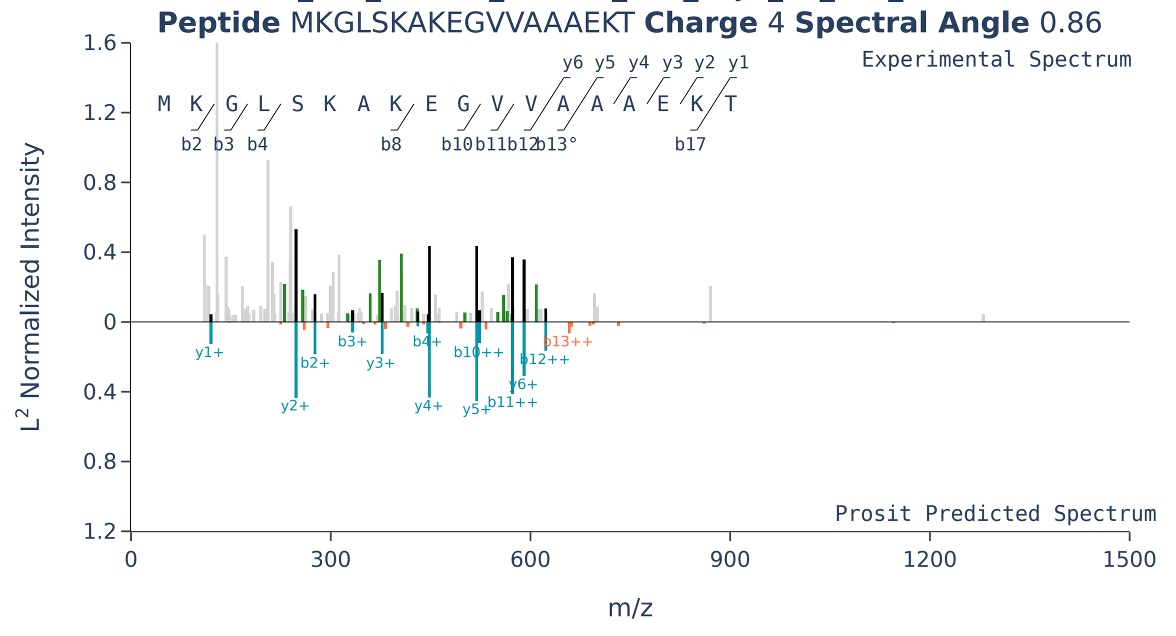


**Source** W_Soh_160821_170921_Goe_aSyn_A1_24h_R1 **Scan** 7311
**Peptide** SKTKEGVVHGVATVAEKTKEQ **Charge** 5 **Spectral Angle** 0.69

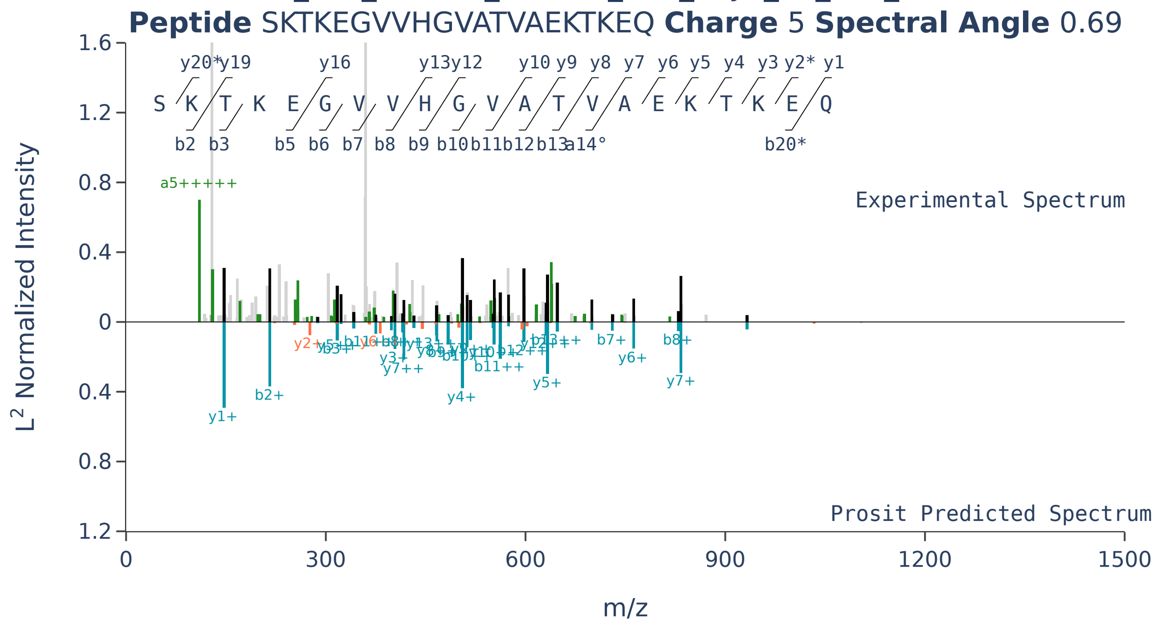


**Source** W_Soh_160821_170921_Goe_aSyn_A1_24h_R1 **Scan** 6476
**Peptide** GVAEAAGKTKEGV **Charge** 2 **Spectral Angle** 0.73

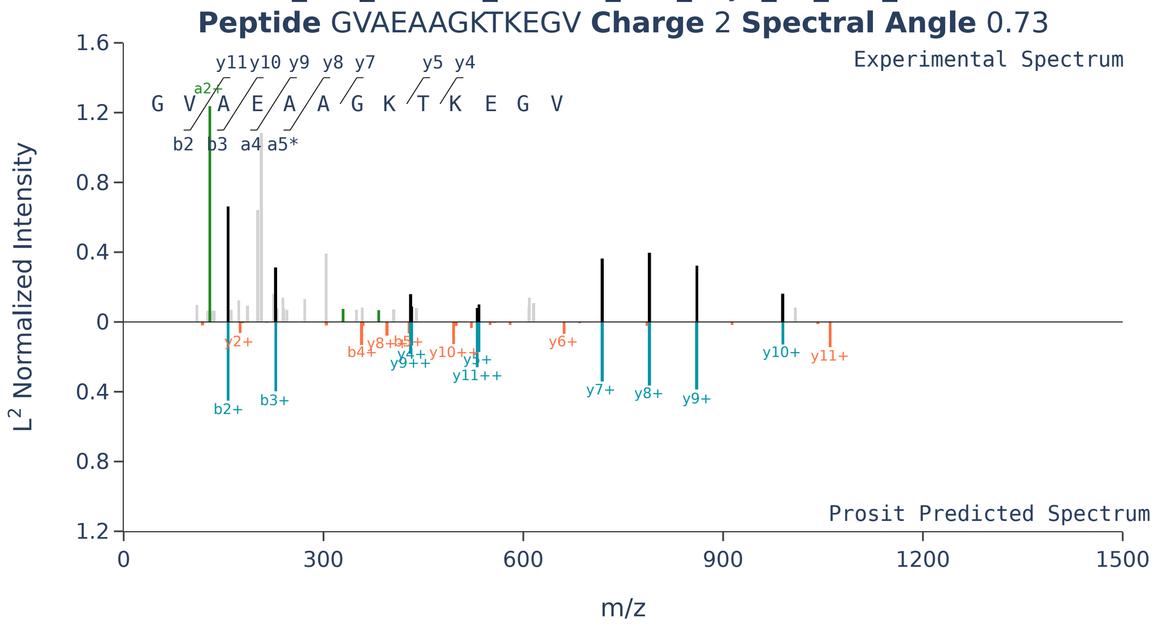

**Source** W_Soh_160821_170921_Goe_aSyn_A1_24h_R1 **Scan** 5069
**Peptide** AAAEKTKQGVAEAAGKTKEG **Charge** 4 **Spectral Angle** 0.75

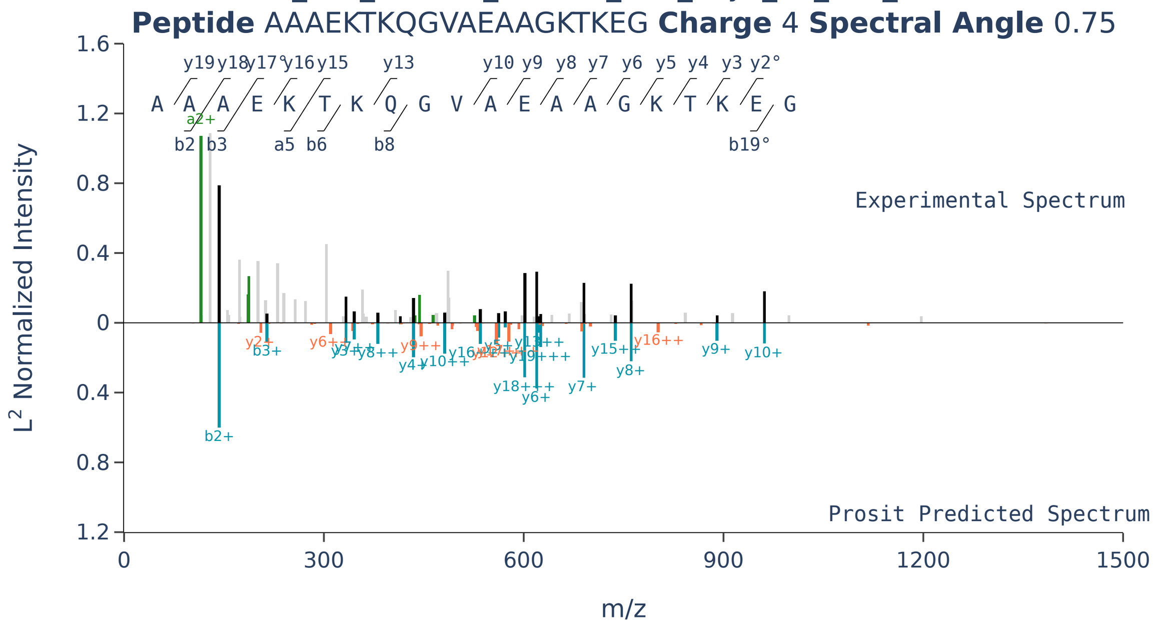


**Source** W_Soh_160821_170921_Goe_aSyn_A1_24h_R2 **Scan** 16381
**Peptide** SIAAATGFVKKDQL **Charge** 2 **Spectral Angle** 0.82

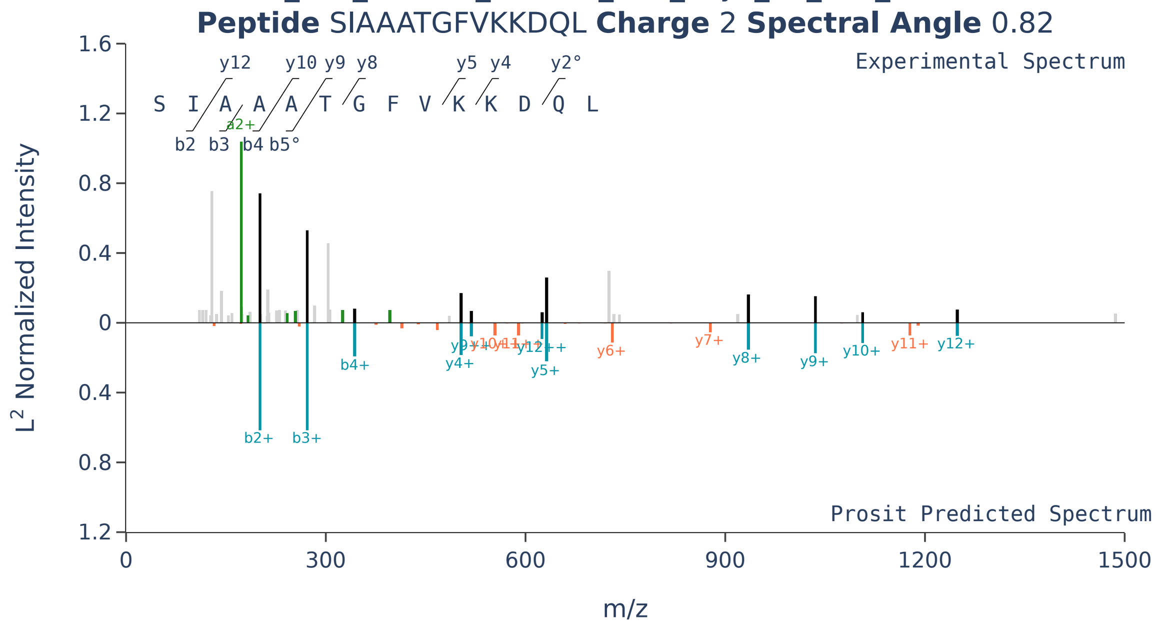


**Source** W_Soh_160821_170921_Goe_aSyn_A1_24h_R1 **Scan** 8101
**Peptide** GVVHGVATVAEKTKE **Charge** 4 **Spectral Angle** 0.78

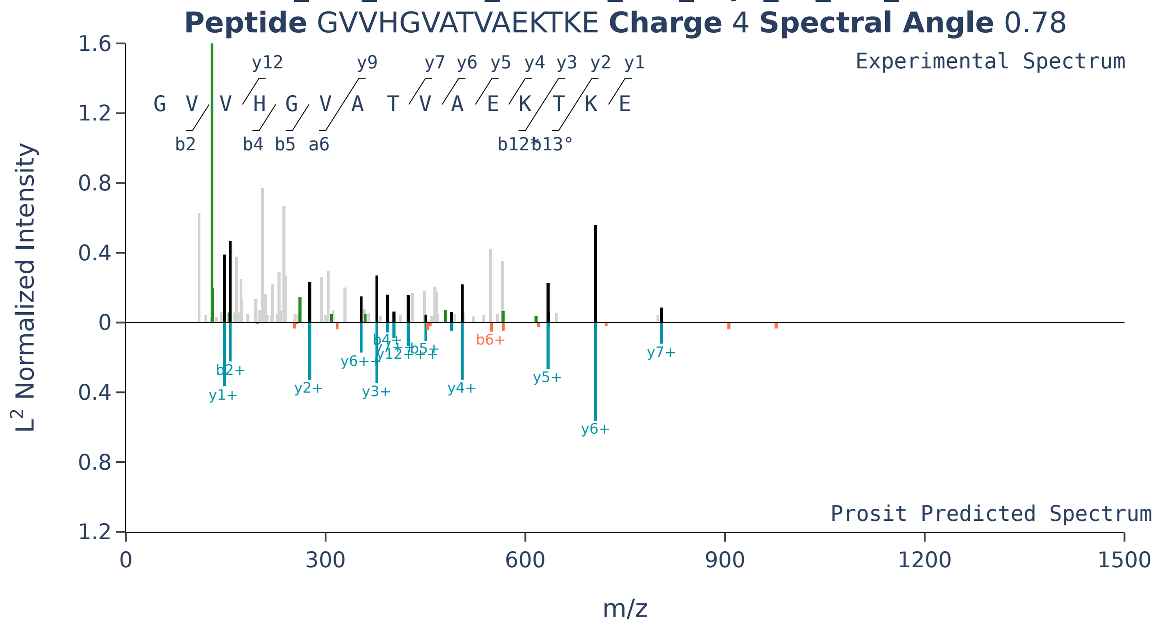


**Source** W_Soh_160821_170921_Goe_aSyn_A1_24h_R2 **Scan** 5260
**Peptide** VATVAEKTKE **Charge** 2 **Spectral Angle** 0.71

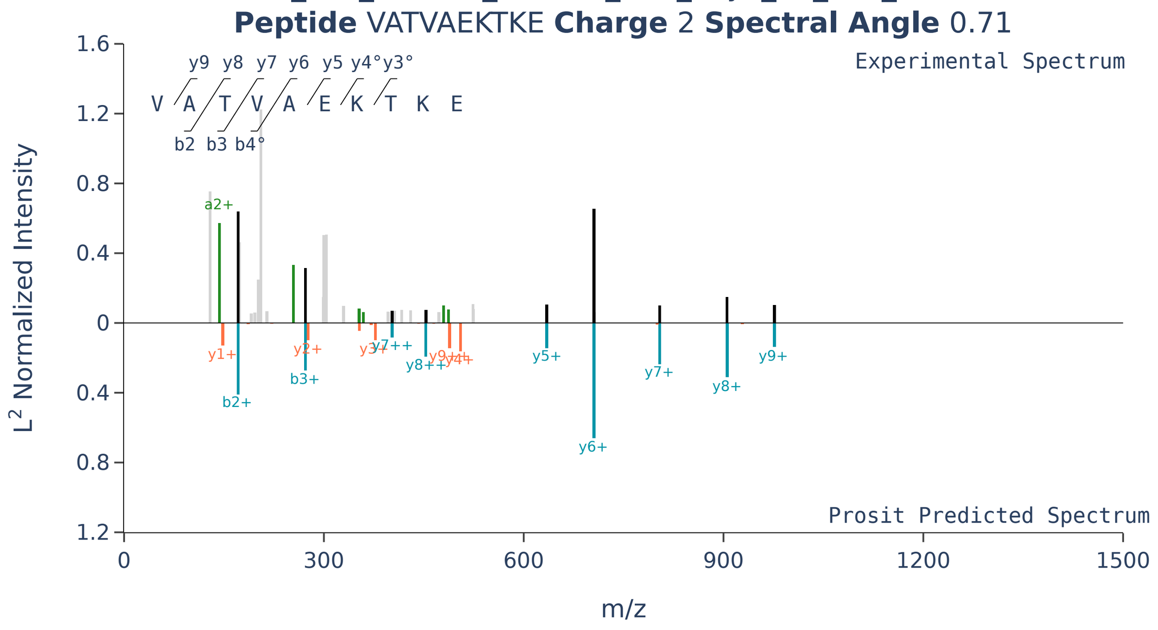


**Source** W_Soh_160821_170921_Goe_aSyn_A1_24h_R1 **Scan** 15012
**Peptide** DVFMKGLSKAKEGV **Charge** 3 **Spectral Angle** 0.77

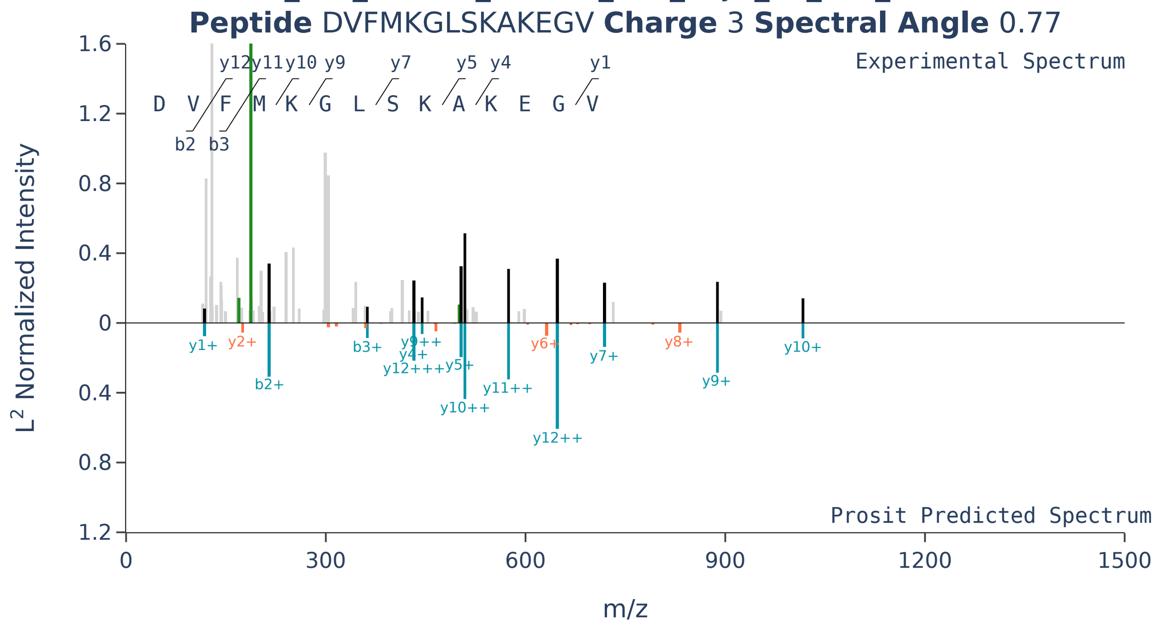


**Source** W_Soh_160821_170921_Goe_aSyn_A2_24h_R2 **Scan** 9430
**Peptide** KEGVVAAAEKTKQGVAEAA **Charge** 3 **Spectral Angle** 0.91

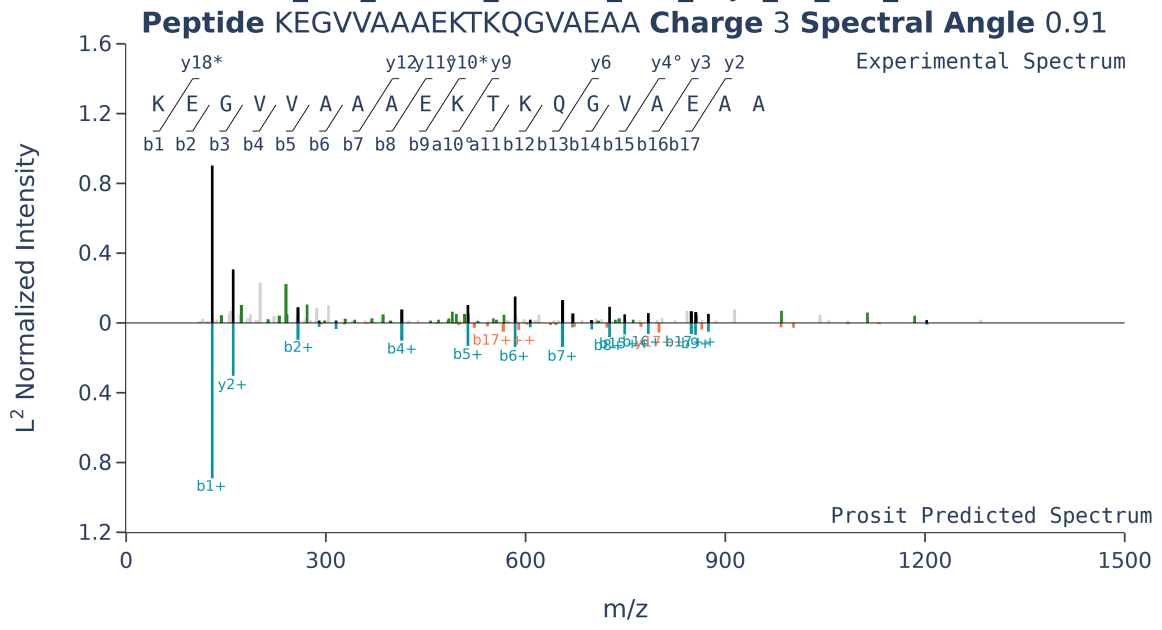


**Source** W_Soh_160821_170921_Goe_aSyn_A2_24h_R2 **Scan** 14039
**Peptide** GVLYVGSKTKEGVVHGVATVAEKTKEQ **Charge** 5 **Spectral Angle** 0.7

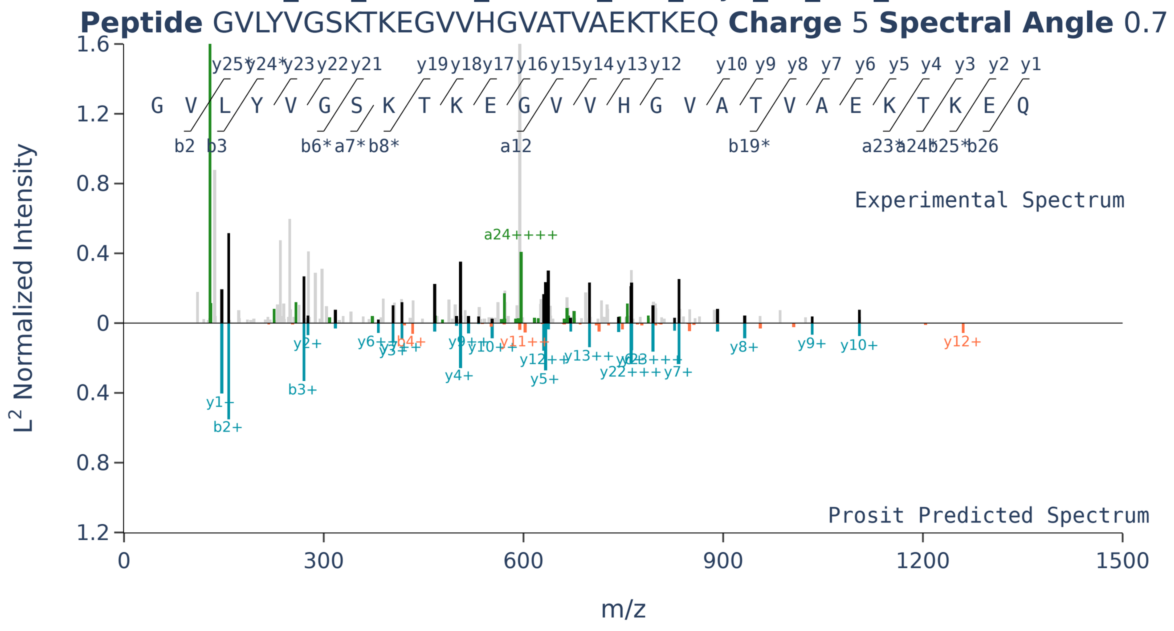


**Source** W_Soh_160821_170921_Goe_aSyn_A2_24h_R2 **Scan** 9105
**Peptide** GAGSIAAAT **Charge** 1 **Spectral Angle** 0.71

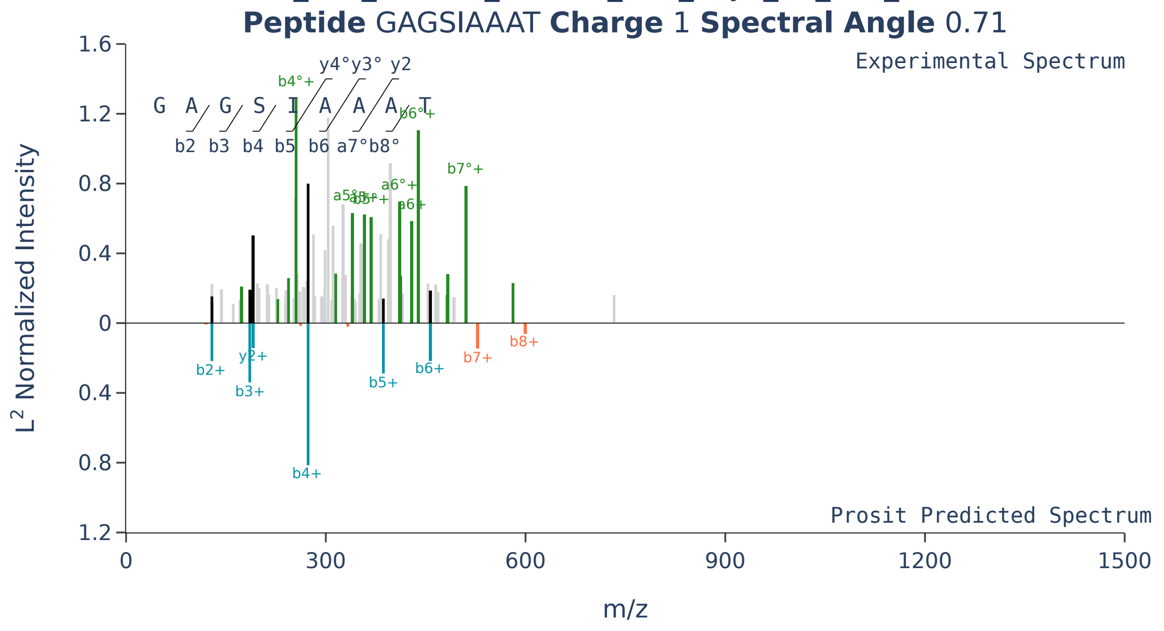


**Source** W_Soh_160821_170921_Goe_aSyn_A2_24h_R1 **Scan** 11087
**Peptide** AAAEKTKQGVAEAAGKTKEGVLYVGSKT **Charge** 5 **Spectral Angle** 0.79

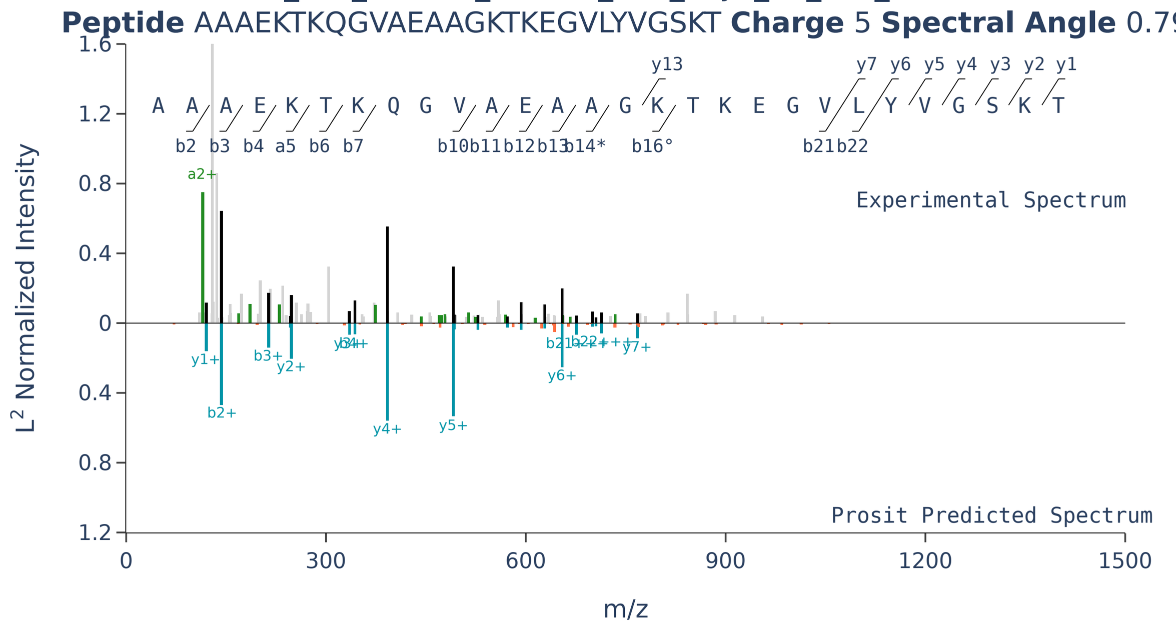


**Source** W_Soh_160821_170921_Goe_aSyn_A2_24h_R1 **Scan** 7334
**Peptide** YVGSKTKEGVVHGVAT **Charge** 2 **Spectral Angle** 0.74

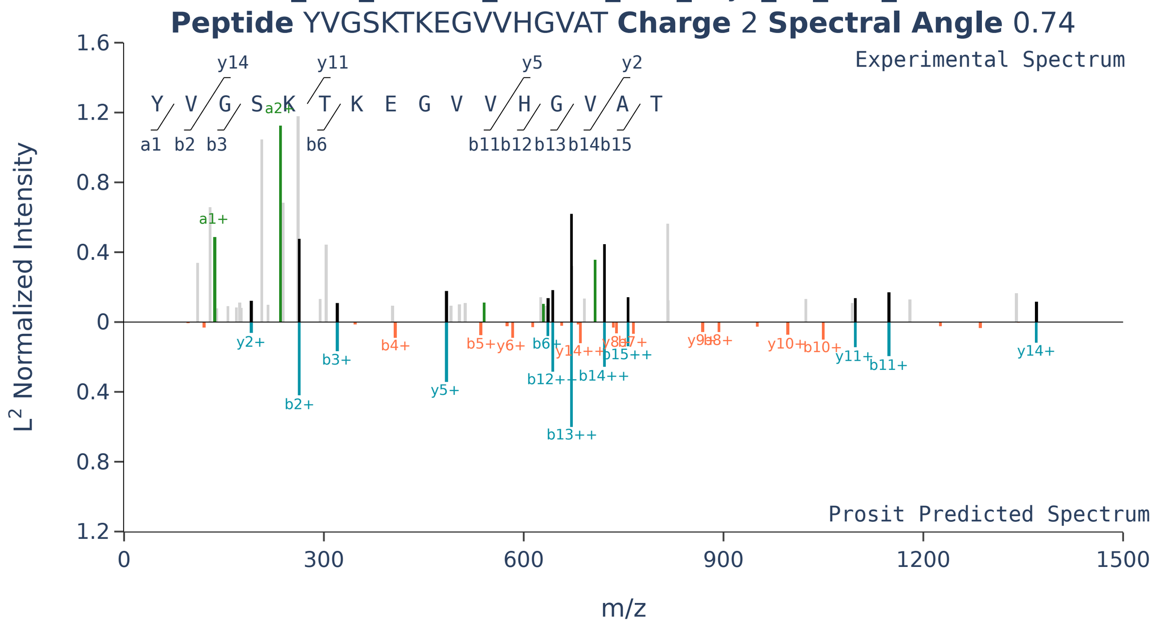


**Source** W_Soh_160821_170921_Goe_aSyn_A1_3h_R2 **Scan** 9259
**Peptide** KEGVVHGVATVAE **Charge** 2 **Spectral Angle** 0.74

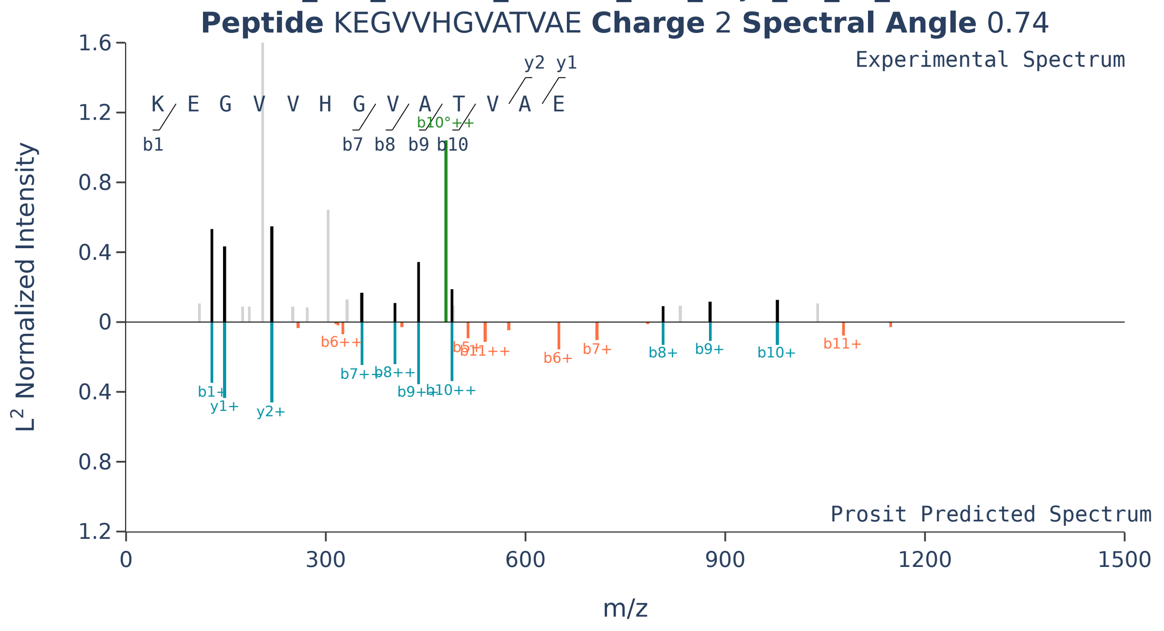


**Source** W_Soh_160821_170921_Goe_aSyn_A2_24h_R1 **Scan** 14138
**Peptide** LYVGSKTKEGVVHGVATV **Charge** 3 **Spectral Angle** 0.82

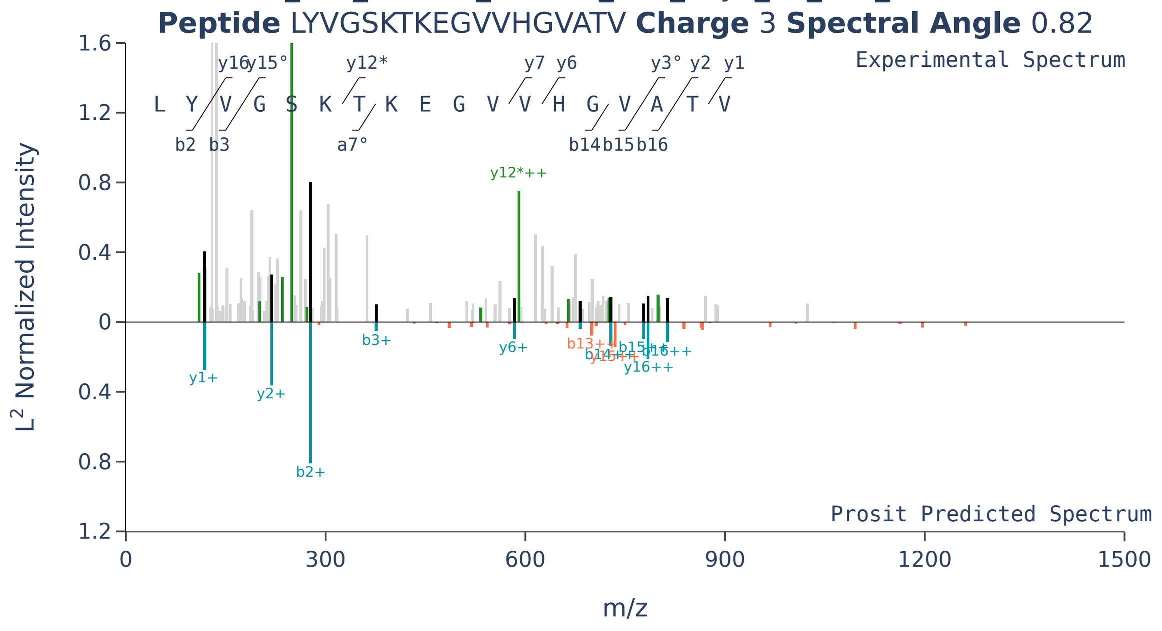


**Source** W_Soh_160821_170921_Goe_aSyn_A1_24h_R2 **Scan** 18681
**Peptide** DVFMKGLSKAKEGVVAAAEKT **Charge** 3 **Spectral Angle** 0.87

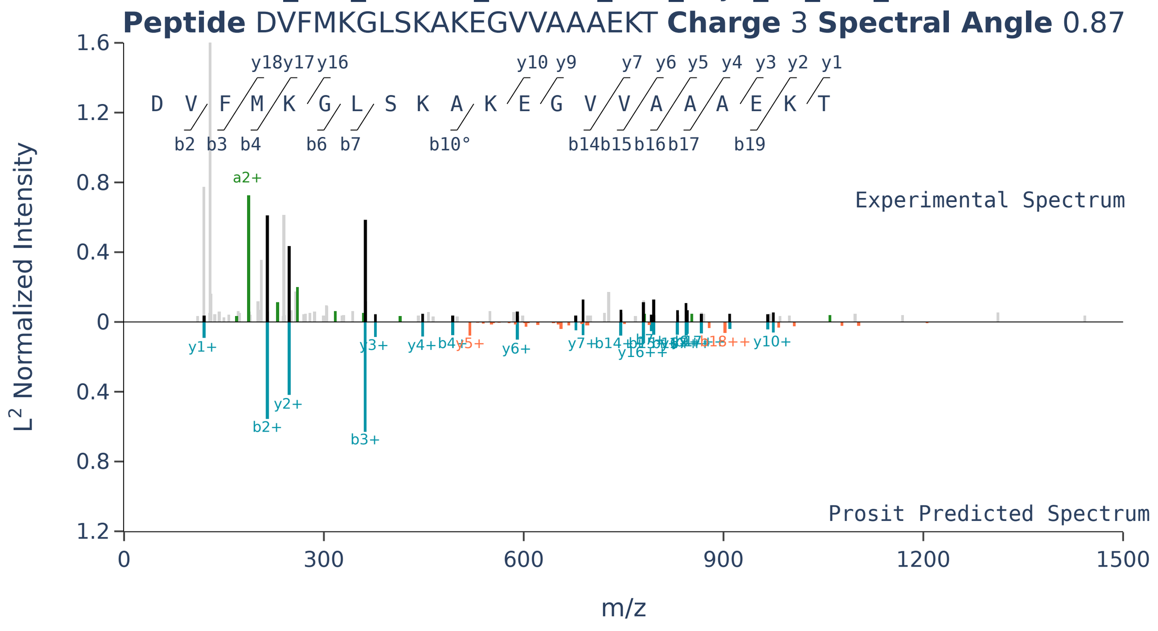


**Source** W_Soh_160821_170921_Goe_aSyn_A1_3h_R2 **Scan** 9902
**Peptide** AAEKTKQGVAEAAGKTKEGVLYVGSKT **Charge** 5 **Spectral Angle** 0.81

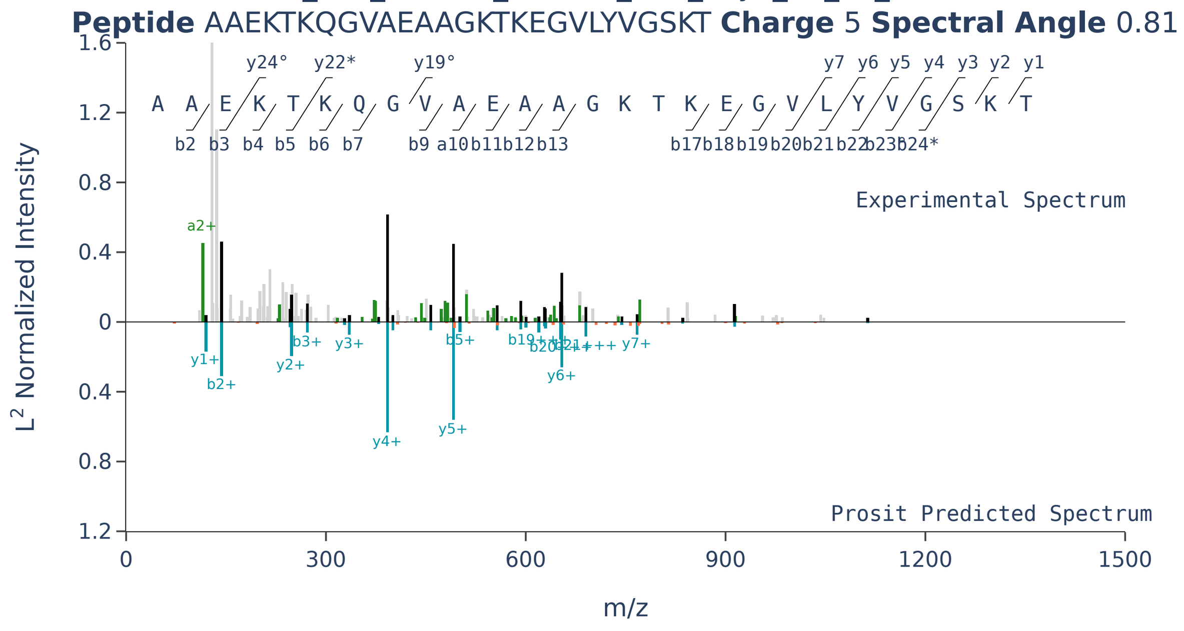


**Source** W_Soh_160821_170921_Goe_aSyn_A1_24h_R2 **Scan** 17711
**Peptide** DVFMKGLSKAKEGVVAAAEKTKQG **Charge** 4 **Spectral Angle** 0.81

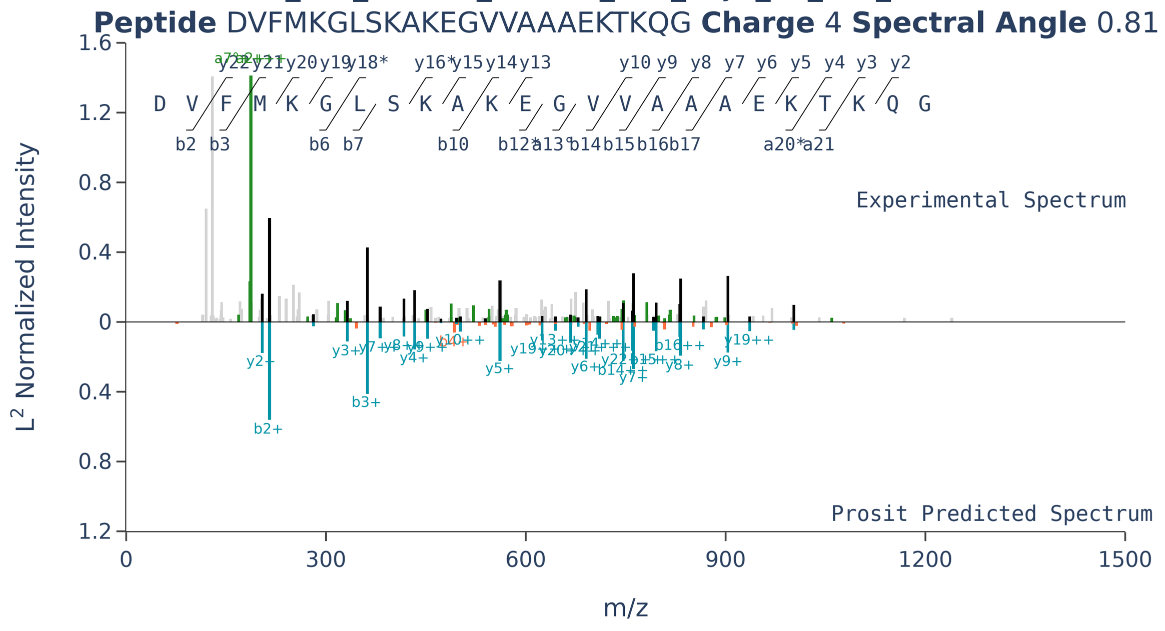

**Source** W_Soh_160821_170921_Goe_aSyn_A1_24h_R1 **Scan** 14483
**Peptide** TKEGVLYVGS **Charge** 2 **Spectral Angle** 0.71

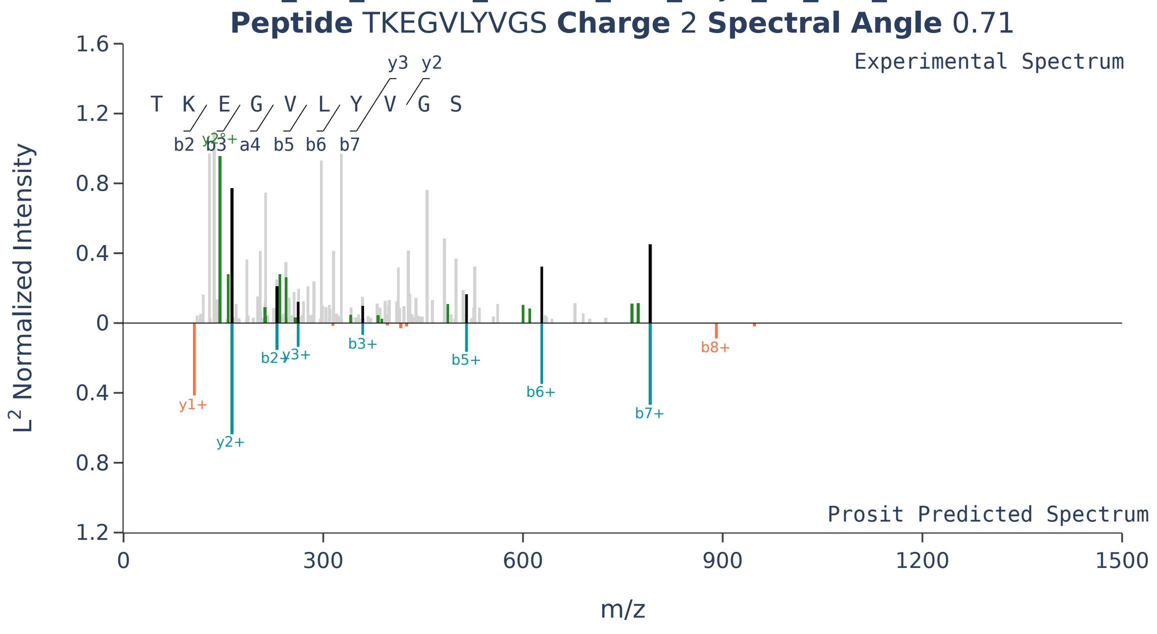

**Source** W_Soh_160821_170921_Goe_aSyn_A1_6h_R2 **Scan** 9395
**Peptide** SKAKEGVVAAAEKTKQGVAEAAGKTKEG **Charge** 5 **Spectral Angle** 0.76

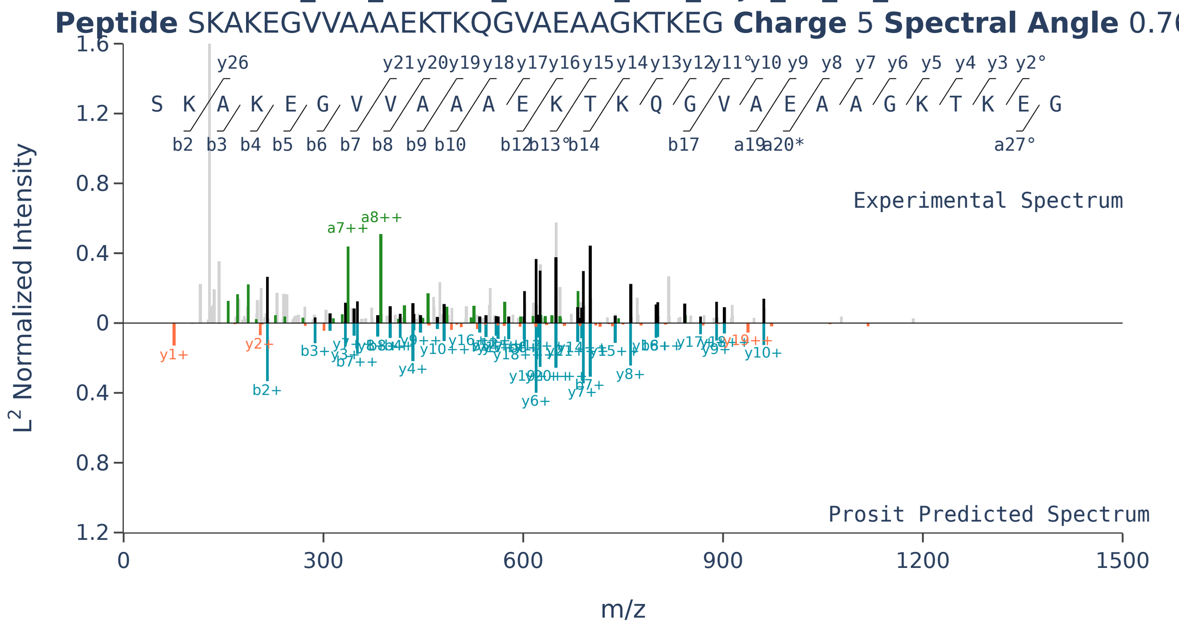

**Source** W_Soh_160821_170921_Goe_aSyn_A1_6h_R2 **Scan** 10107
**Peptide** AEKTKEQVTNVGGAVVT **Charge** 3 **Spectral Angle** 0.78

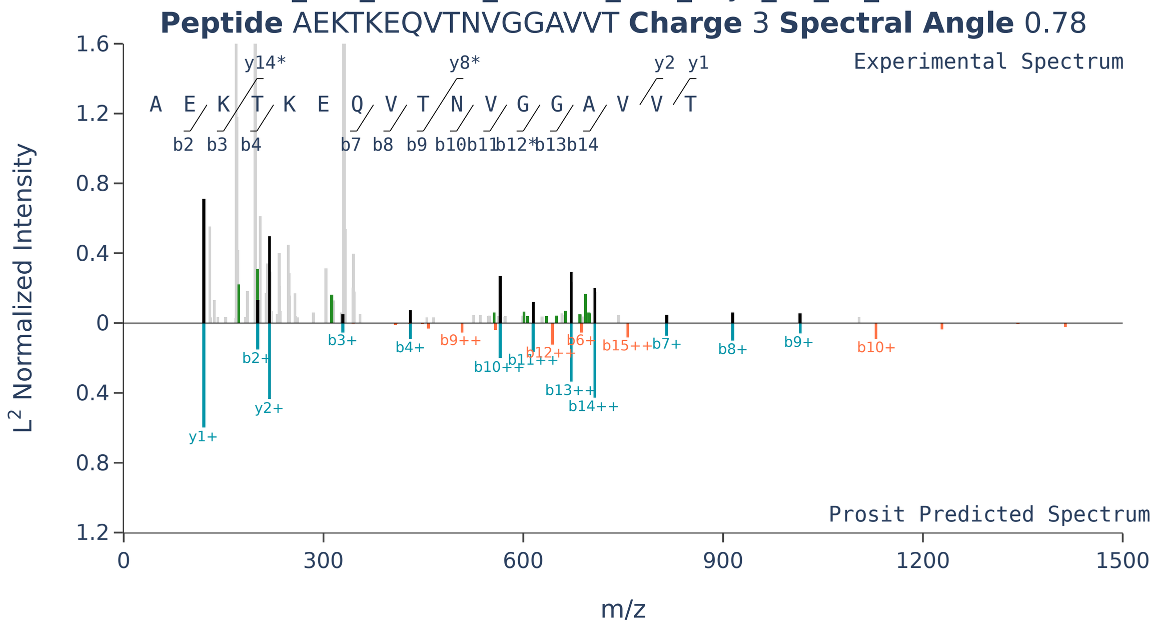

**Source** W_Soh_160821_170921_Goe_aSyn_A2_24h_R1 **Scan** 11212
**Peptide** KKDQLGKNEEGAPQEGILED **Charge** 3 **Spectral Angle** 0.83

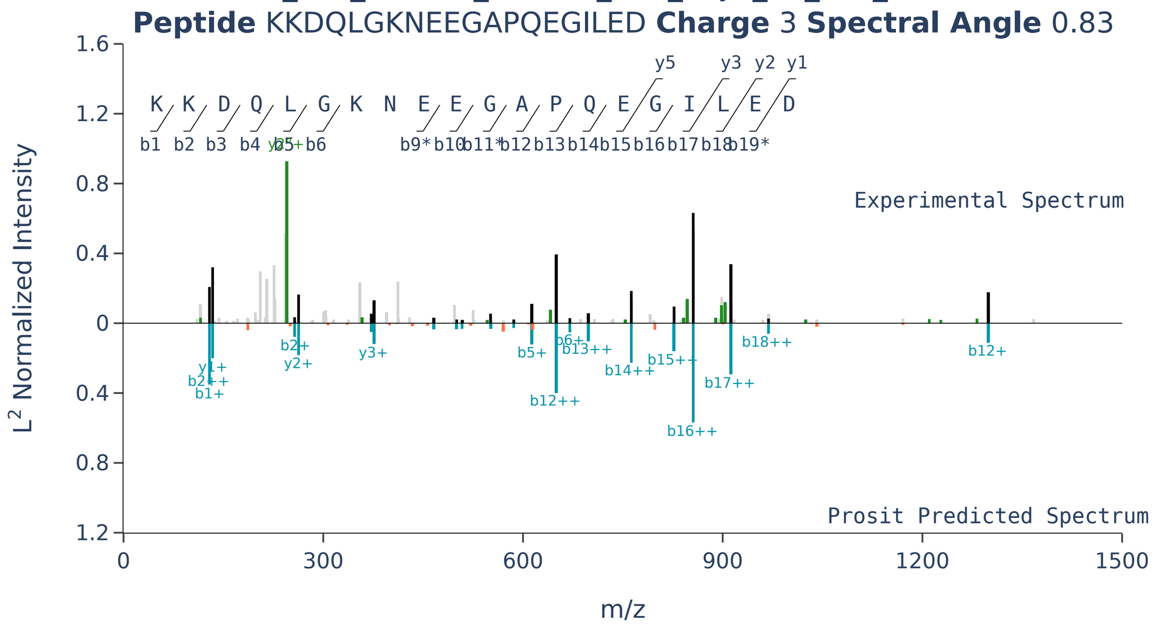

**Source** W_Soh_160821_170921_Goe_aSyn_A2_24h_R2 **Scan** 11594
**Peptide** VGSKTKEGVVHGVATVAEKTKEQVTN **Charge** 4 **Spectral Angle** 0.78

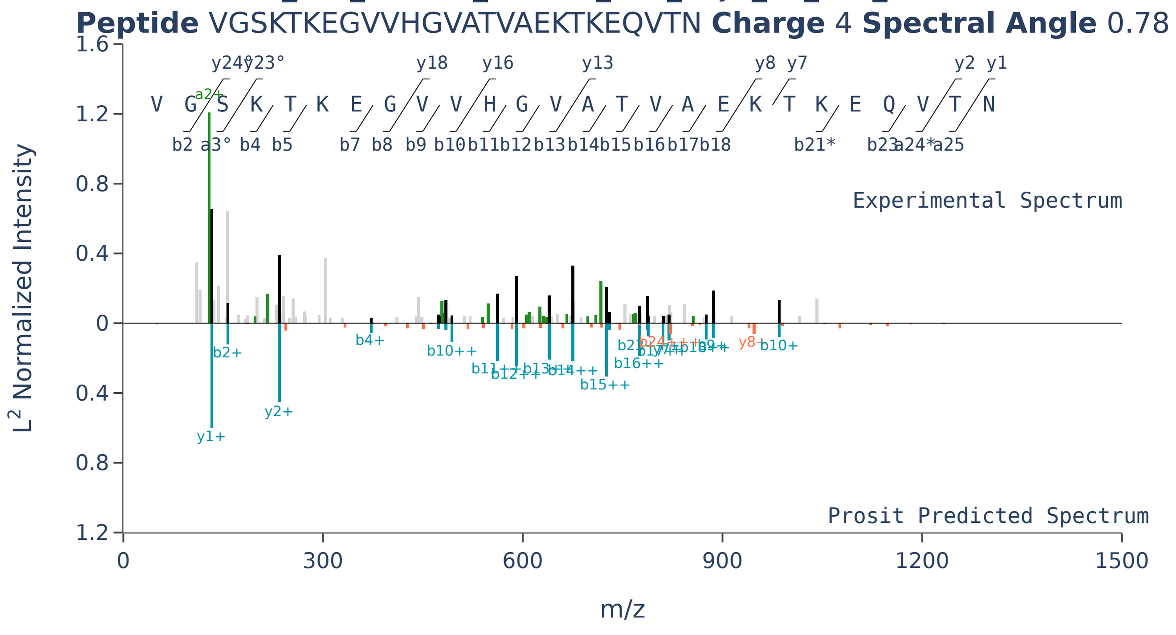

**Source** W_Soh_160821_170921_Goe_aSyn_A1_6h_R1 **Scan** 22291
**Peptide** GKNEEGAPQEGILEDMPVD **Charge** 2 **Spectral Angle** 0.9

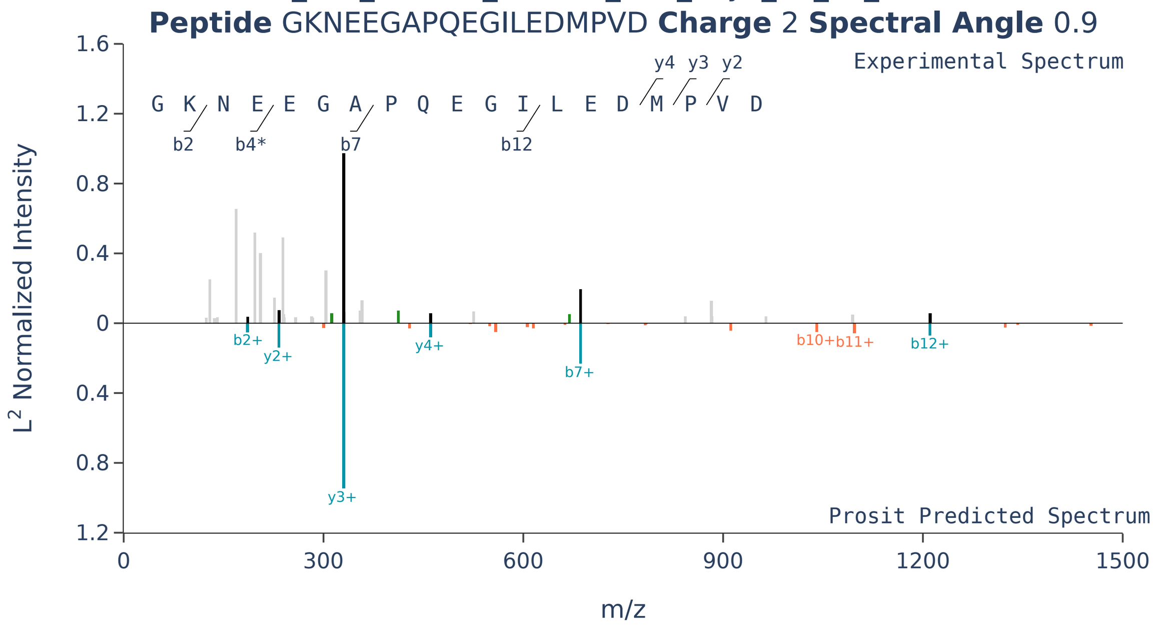

**Source** W_Soh_160821_170921_Goe_aSyn_A1_24h_R1 **Scan** 13991
**Peptide** GVLYVGSKTKEGVVHGVATVAEKTKE **Charge** 3 **Spectral Angle** 0.8

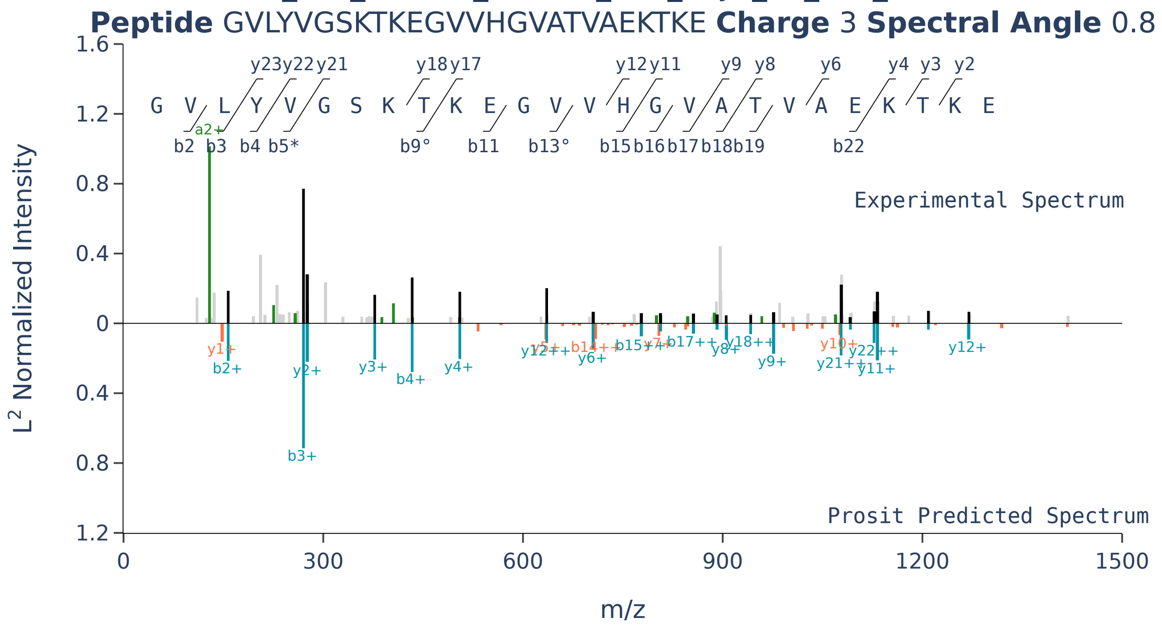

**Source** W_Soh_160821_170921_Goe_aSyn_A1_6h_R2 **Scan** 11534
**Peptide** GVVHGVATV **Charge** 2 **Spectral Angle** 0.76

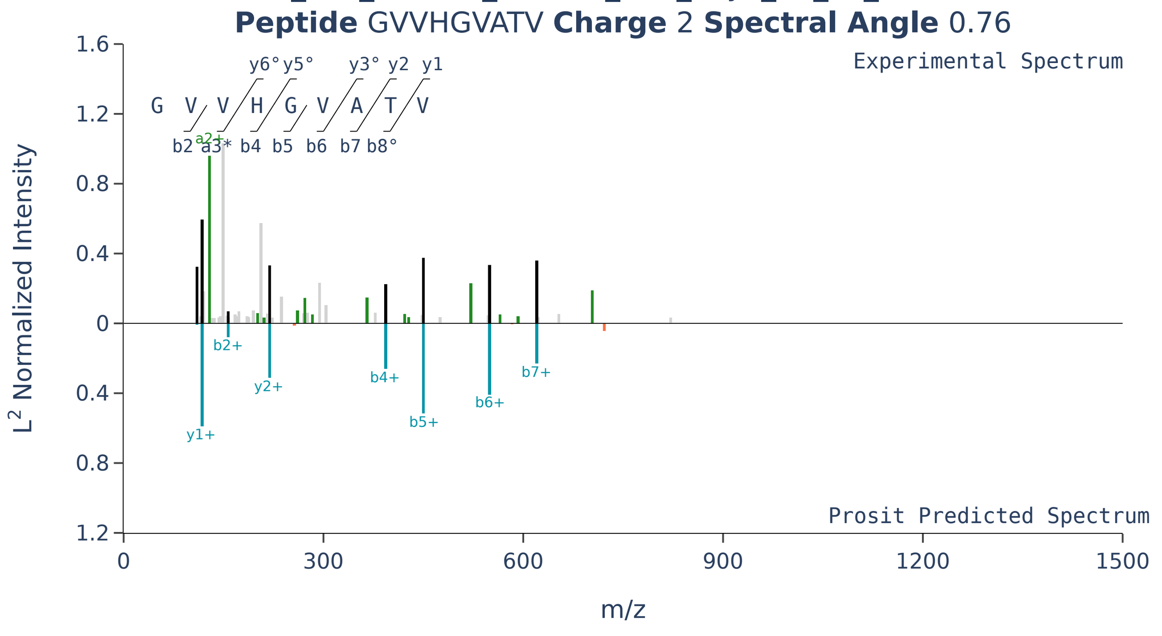

**Source** W_Soh_160821_170921_Goe_aSyn_A2_24h_R2 **Scan** 17880
**Peptide** VAEKTKEQVTNVGGAVVTGVTAVAQK **Charge** 4 **Spectral Angle** 0.78

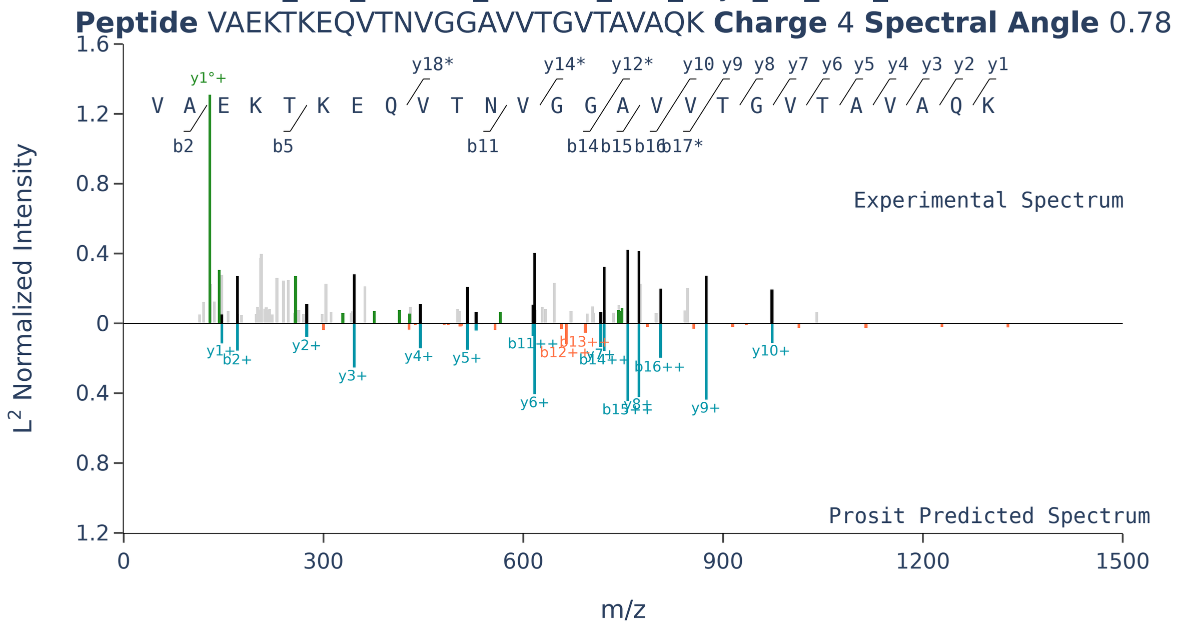

**Source** W_Soh_160821_170921_Goe_aSyn_A2_24h_R2 **Scan** 5494
**Peptide** SKAKEGVVAAAEKTKQG **Charge** 3 **Spectral Angle** 0.69

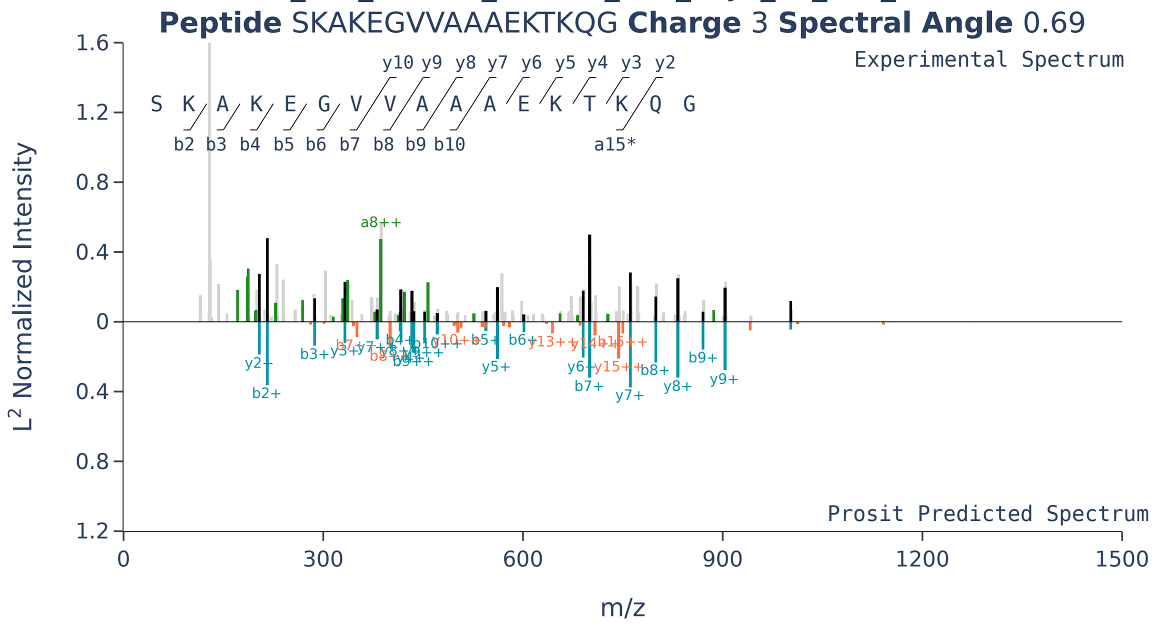

**Source** W_Soh_160821_170921_Goe_aSyn_A1_24h_R2 **Scan** 7461
**Peptide** GSKTKEGVVHGVATVAEKTKE **Charge** 5 **Spectral Angle** 0.73

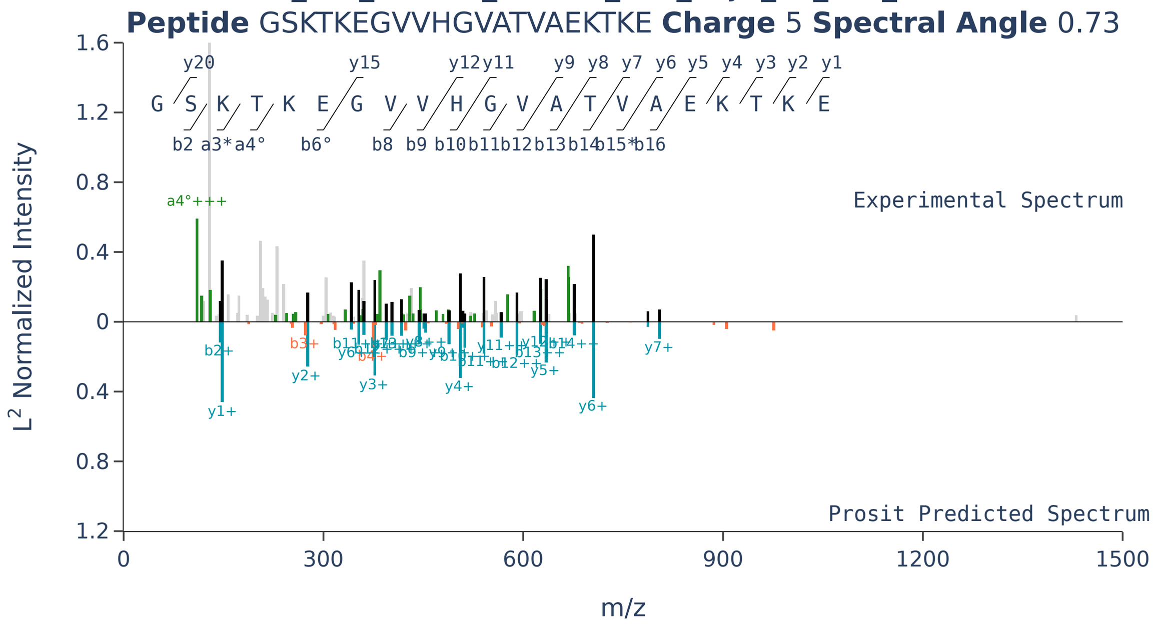

**Source** W_Soh_160821_170921_Goe_aSyn_A1_24h_R1 **Scan** 7451
**Peptide** GLSKAKEGVVAAAEKTKQG **Charge** 3 **Spectral Angle** 0.75

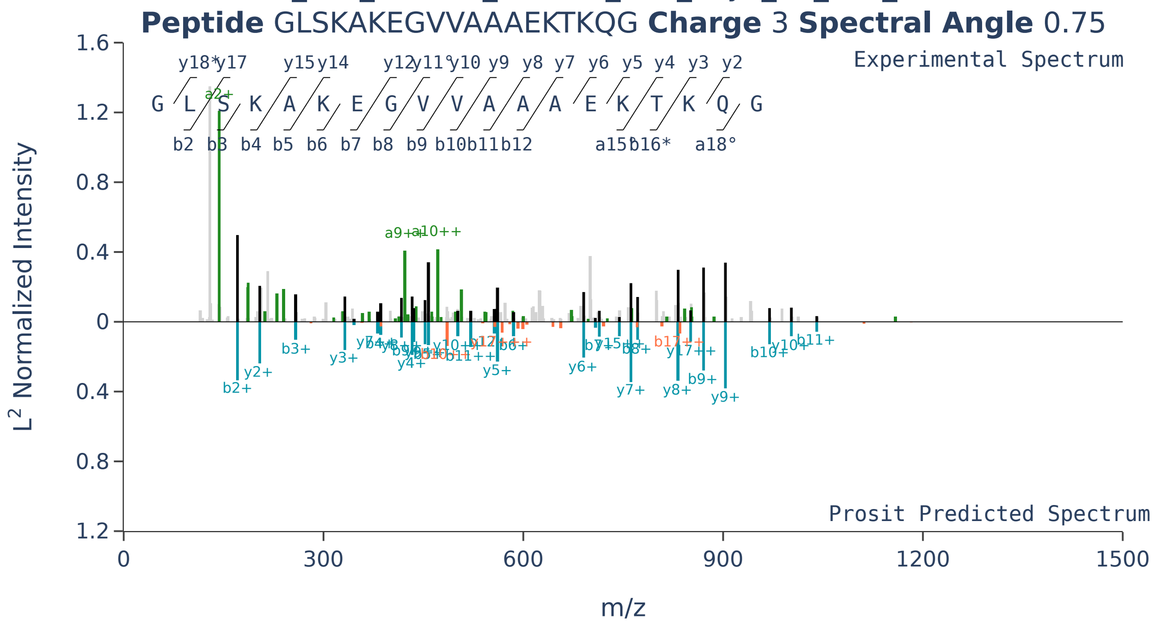

**Source** W_Soh_160821_170921_Goe_aSyn_A2_3h_R2 **Scan** 10707
**Peptide** ATGFVKKDQLGKNEEGAPQEGL **Charge** 4 **Spectral Angle** 0.85

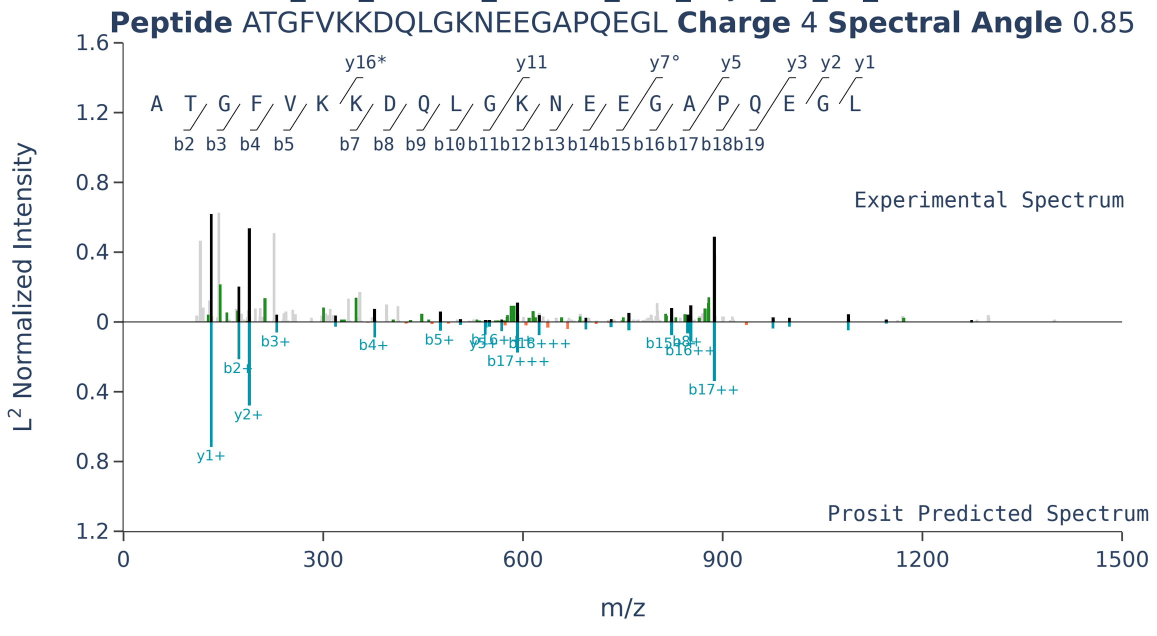

**Source** W_Soh_160821_170921_Goe_aSyn_A1_3h_R2 **Scan** 5142
**Peptide** QGVAEAAGKTKEG **Charge** 2 **Spectral Angle** 0.67

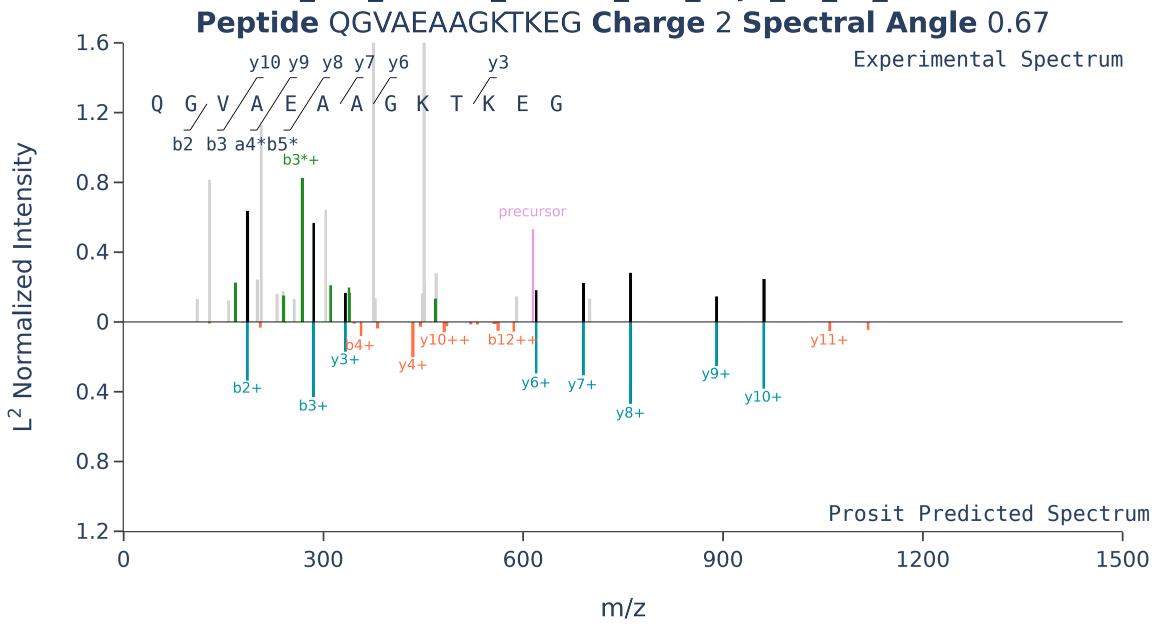

**Source** W_Soh_160821_170921_Goe_aSyn_A2_6h_R2 **Scan** 14330
**Peptide** AATGFVKKDQLGKNEEGAPQEGILED **Charge** 4 **Spectral Angle** 0.8

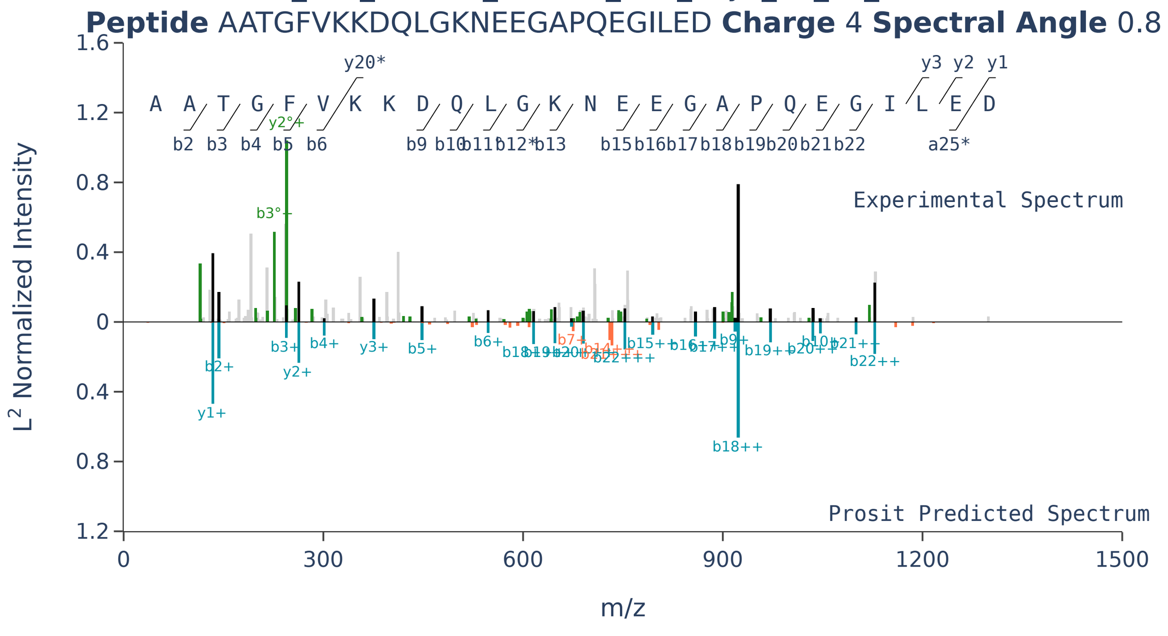

**Source** W_Soh_160821_170921_Goe_aSyn_A1_6h_R2 **Scan** 11125
**Peptide** KEGVLYVGSKTKEGVVHGV **Charge** 4 **Spectral Angle** 0.69

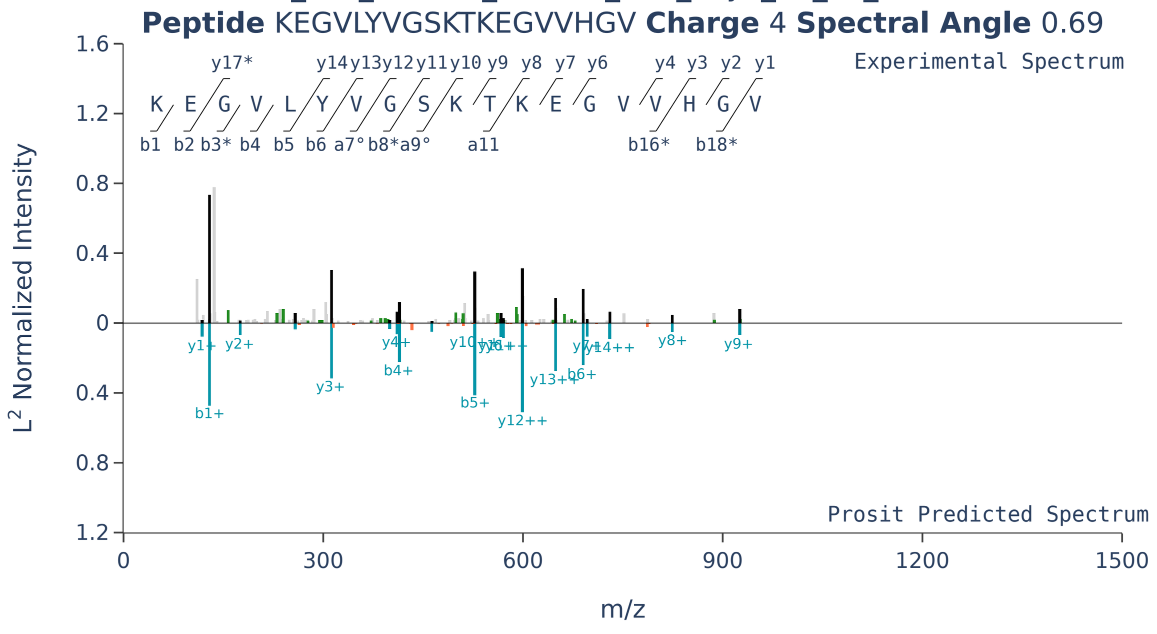


**Source** W_Soh_160821_170921_Goe_aSyn_A1_3h_R1 **Scan** 20348
**Peptide** VATVAEKTKEQVTNVGGAVVTGVTAV **Charge** 3 **Spectral Angle** 0.77

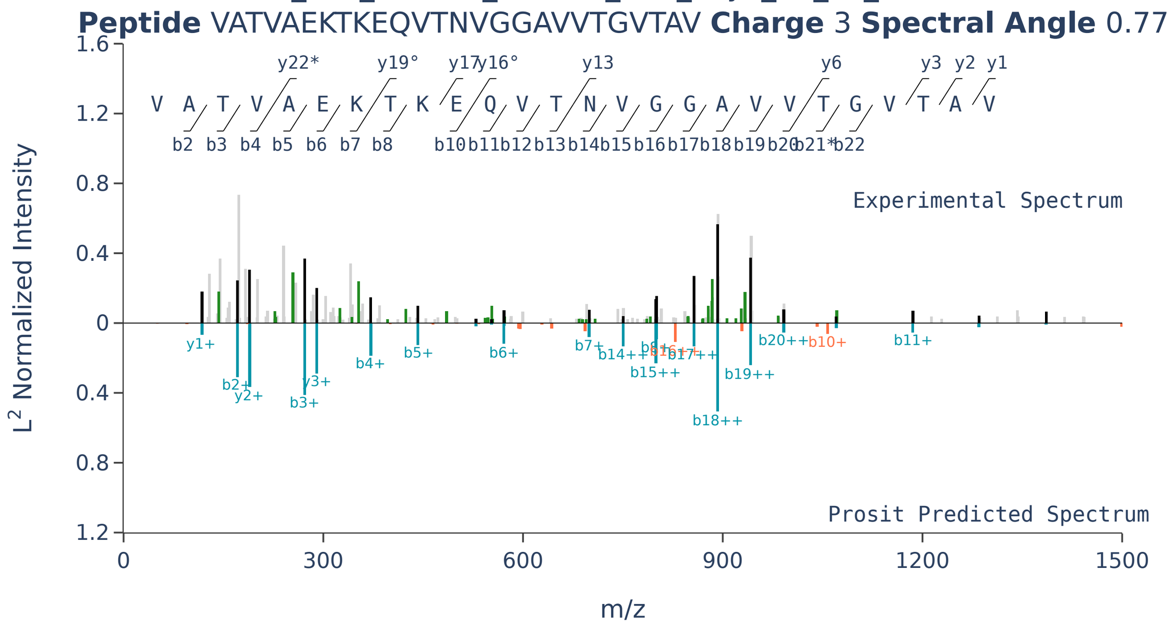


**Source** W_Soh_160821_170921_Goe_aSyn_A2_24h_R2 **Scan** 8999
**Peptide** AKEGVVAAAEKTKQGVAEA **Charge** 3 **Spectral Angle** 0.72

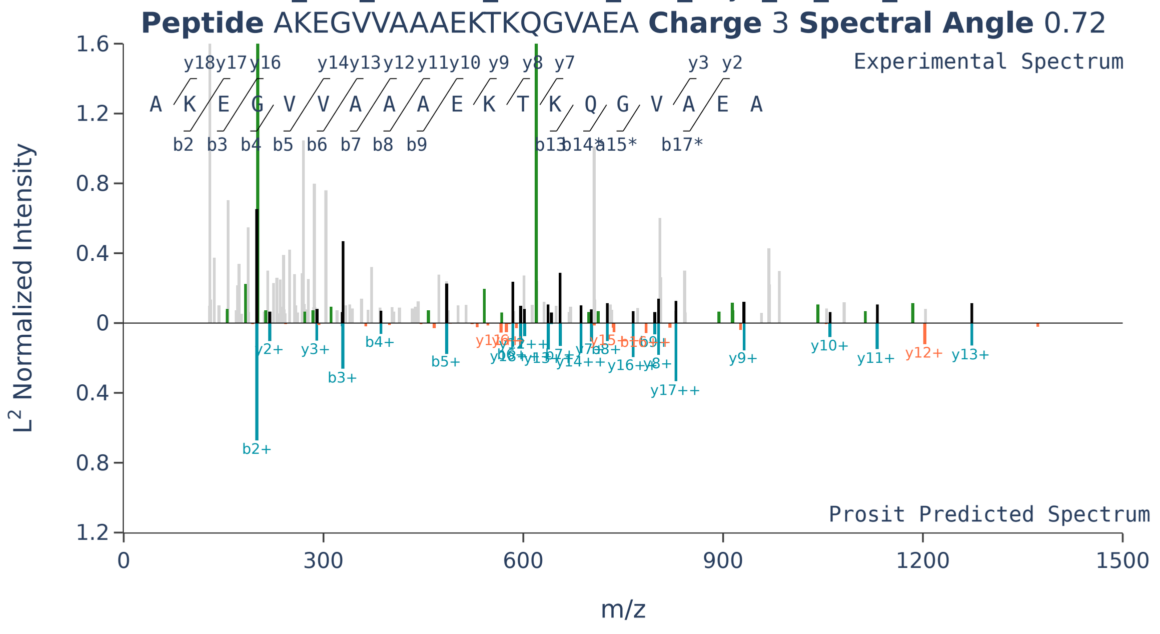


**Source** W_Soh_160821_170921_Goe_aSyn_A1_24h_R1 **Scan** 9624
**Peptide** GVVAAAEKTKQGVAEAAGKTKEG **Charge** 3 **Spectral Angle** 0.84

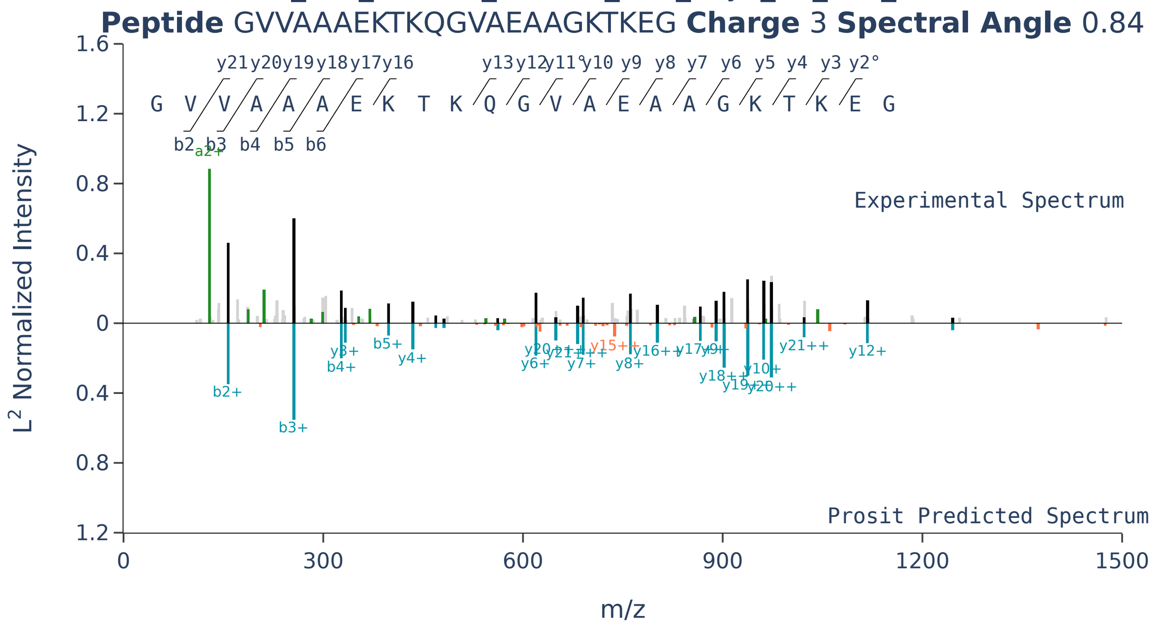


**Source** W_Soh_160821_170921_Goe_aSyn_A1_24h_R2 **Scan** 10135
**Peptide** AKEGVVAAAEKTKQGVAEAAGKTKE **Charge** 5 **Spectral Angle** 0.78

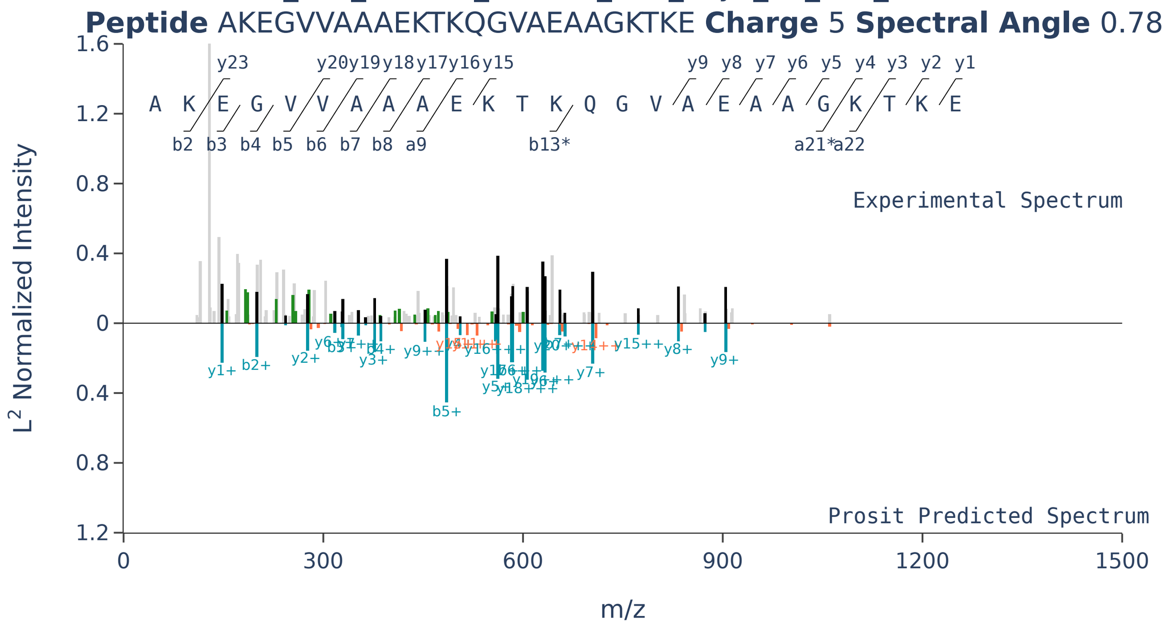


**Source** W_Soh_160821_170921_Goe_aSyn_A1_24h_R2 **Scan** 9617
**Peptide** KGLSKAKEGVVAAAEKTKQGVAEA **Charge** 3 **Spectral Angle** 0.75

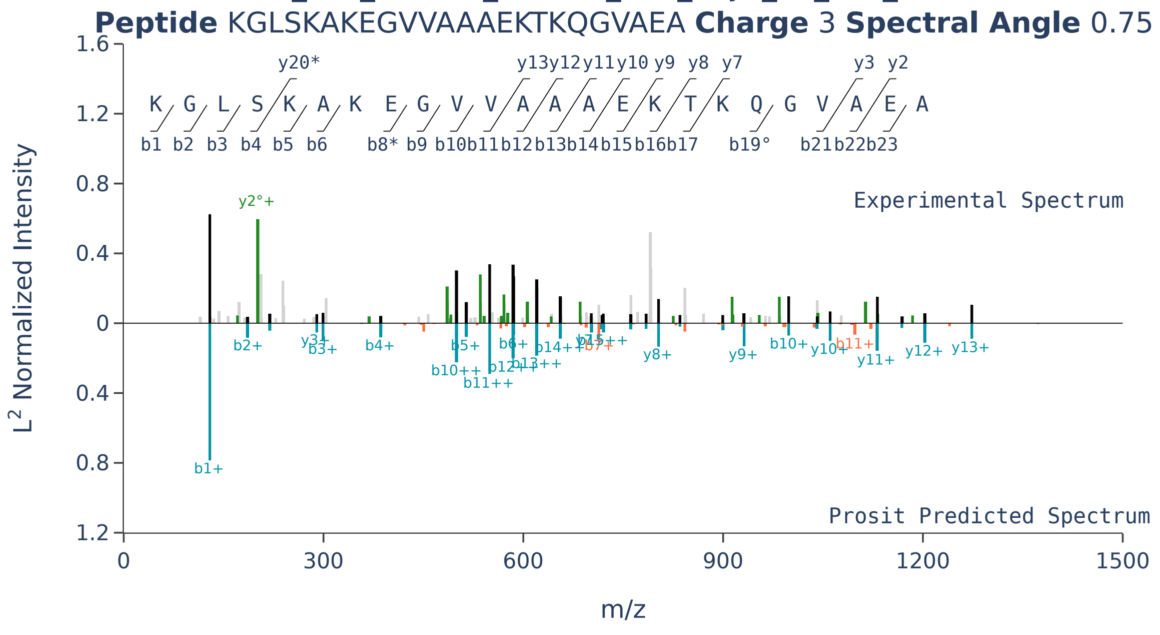


**Source** W_Soh_160821_170921_Goe_aSyn_A1_3h_R2 **Scan** 4930
**Peptide** AEKTKQGVAEAAGKTKE **Charge** 4 **Spectral Angle** 0.72

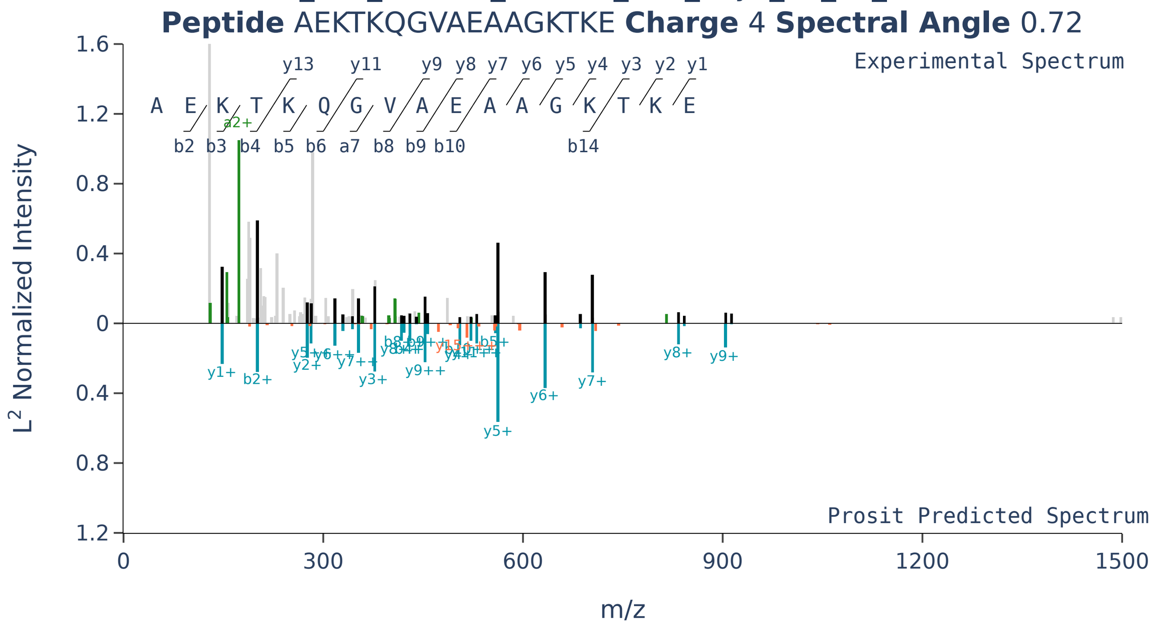


**Source** W_Soh_160821_170921_Goe_aSyn_A2_6h_R1 **Scan** 18419
**Peptide** VAEKTKEQVTNVGGAVVTGVTAVAQ **Charge** 3 **Spectral Angle** 0.86

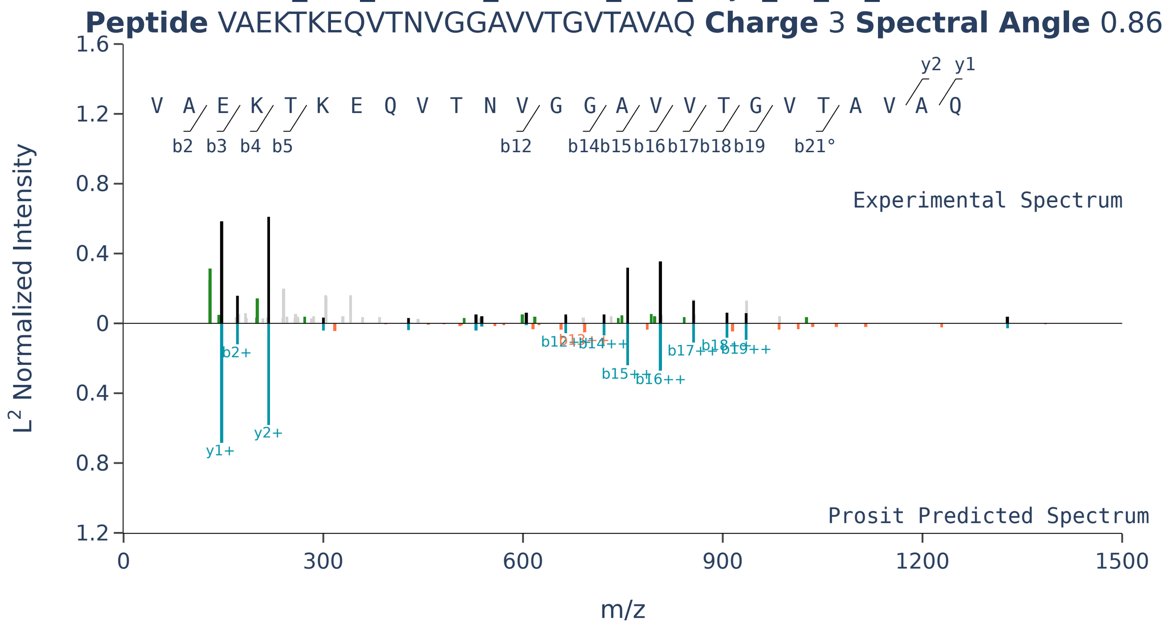


**Source** W_Soh_160821_170921_Goe_aSyn_A2_24h_R1 **Scan** 9026
**Peptide** AAEKTKQGVAEAAGKTKEGVLY **Charge** 3 **Spectral Angle** 0.78

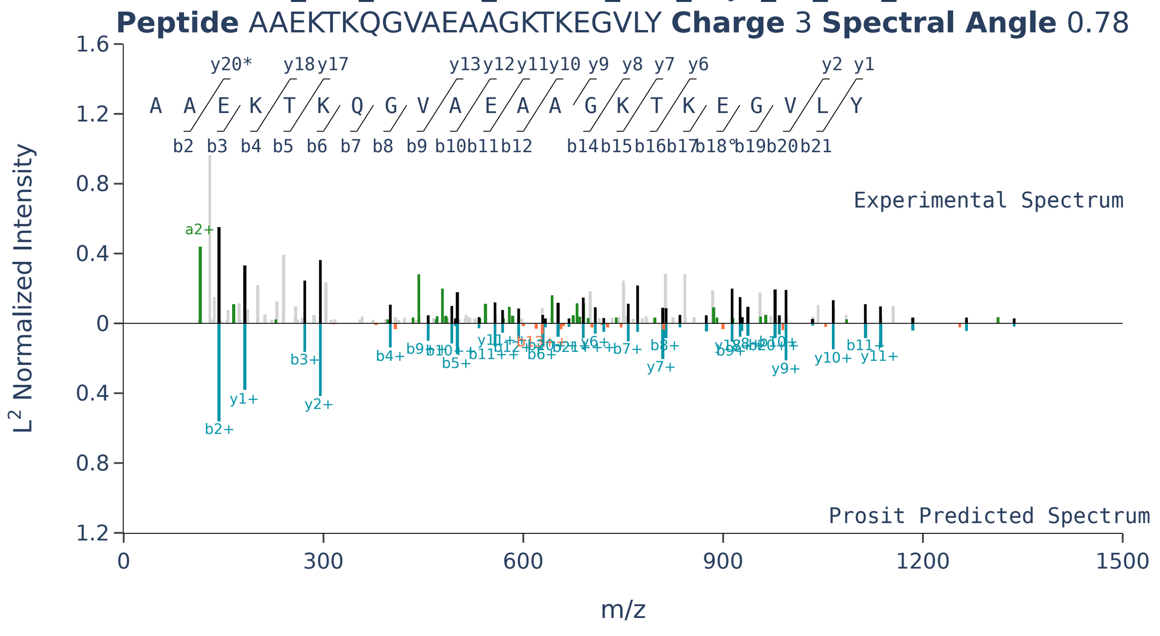


**Source** W_Soh_160821_170921_Goe_aSyn_A1_6h_R2 **Scan** 28910
**Peptide** GLLEDMPVDPDNEAYEMPSE **Charge** 3 **Spectral Angle** 0.84

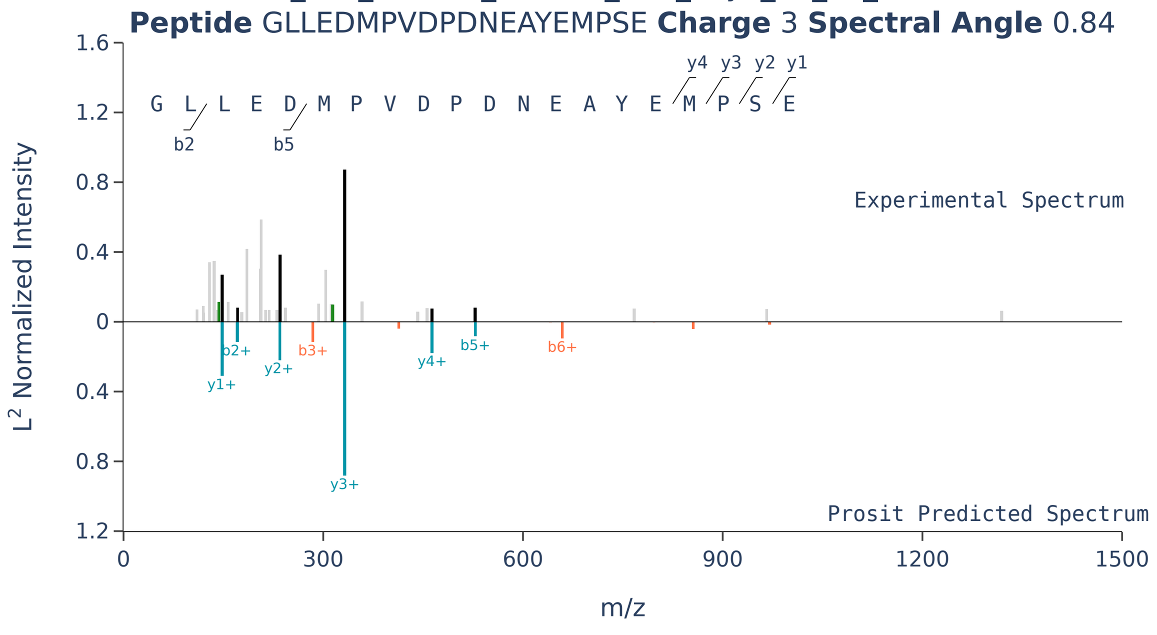


**Source** W_Soh_160821_170921_Goe_aSyn_A2_24h_R2 **Scan** 10627
**Peptide** PVDPDNEAYE **Charge** 2 **Spectral Angle** 0.75

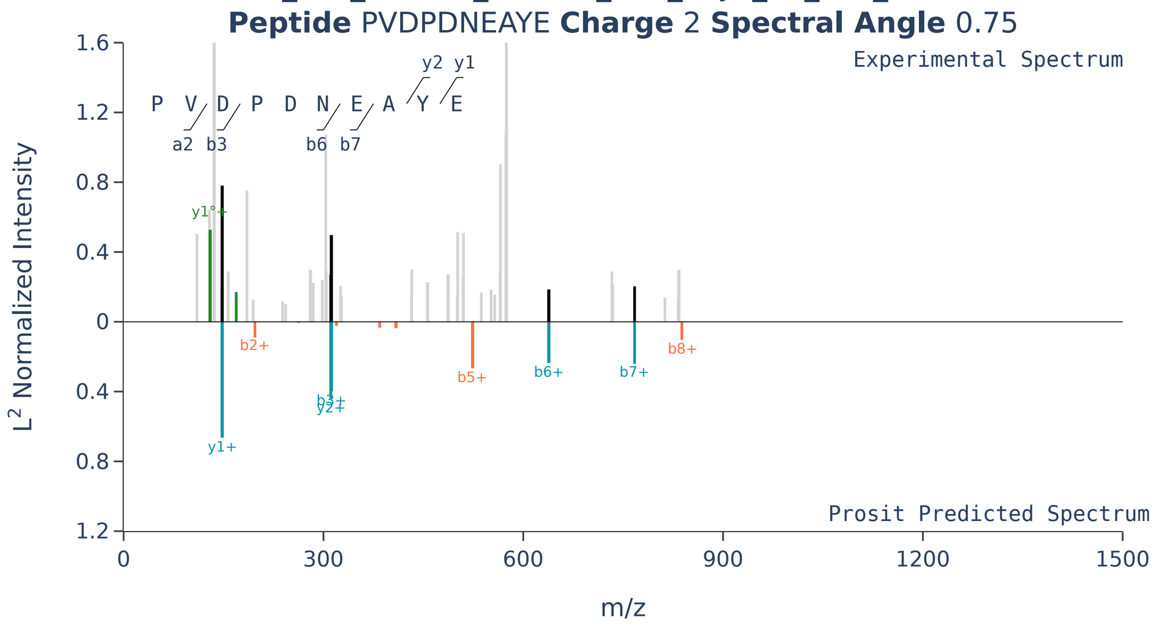


**Source** W_Soh_160821_170921_Goe_aSyn_A1_3h_R1 **Scan** 20390
**Peptide** MPVDPDNEAYEMPSEEGYQDYEPEA **Charge** 2 **Spectral Angle** 0.87

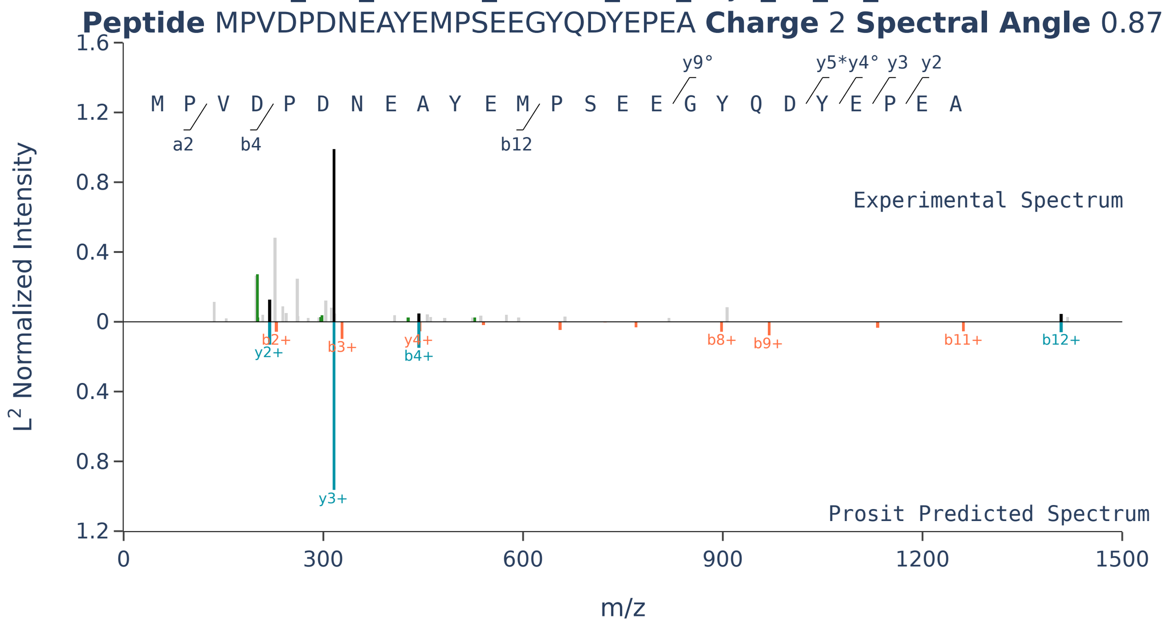


**Source** W_Soh_160821_170921_Goe_aSyn_A1_24h_R1 **Scan** 10256
**Peptide** SKTKEGVVHGVATVAEKTKEQV **Charge** 4 **Spectral Angle** 0.75

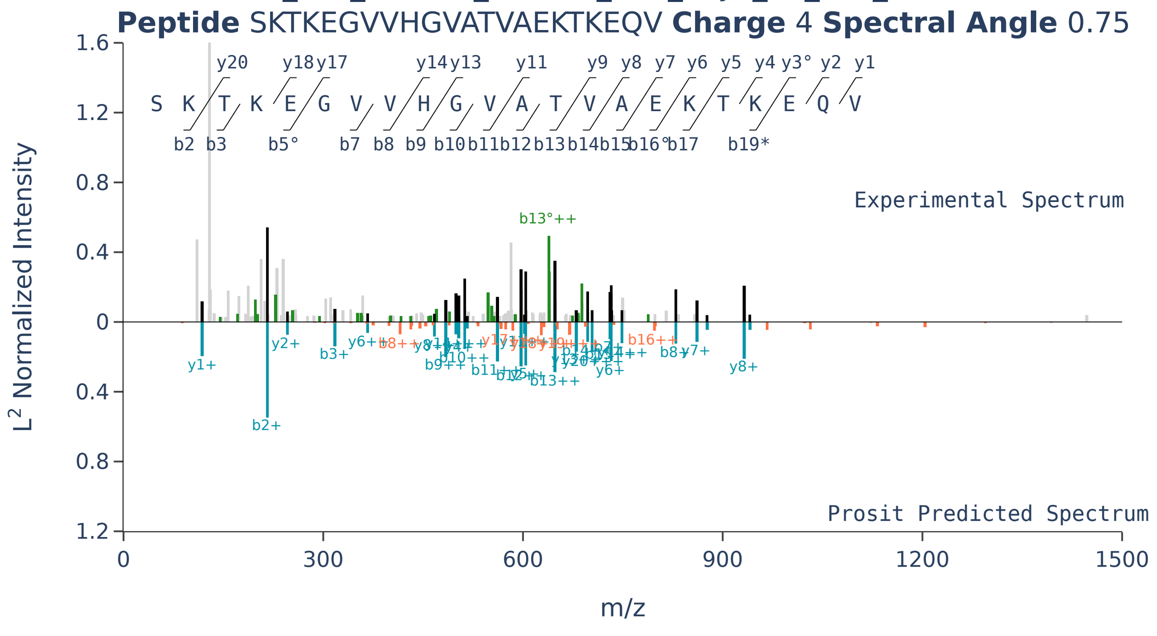


**Source** W_Soh_160821_170921_Goe_aSyn_A2_24h_R1 **Scan** 11073
**Peptide** GKTKEGVLYVGS **Charge** 2 **Spectral Angle** 0.68

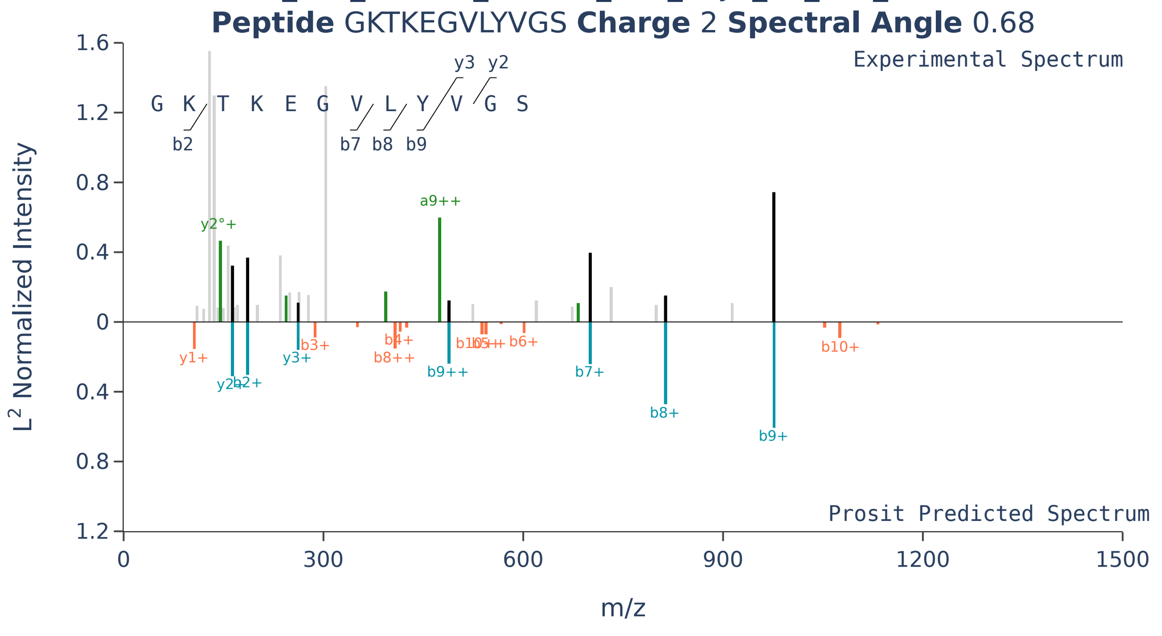


**Source** W_Soh_160821_170921_Goe_aSyn_A1_24h_R2 **Scan** 24761
**Peptide** GLLEDMP **Charge** 1 **Spectral Angle** 0.67

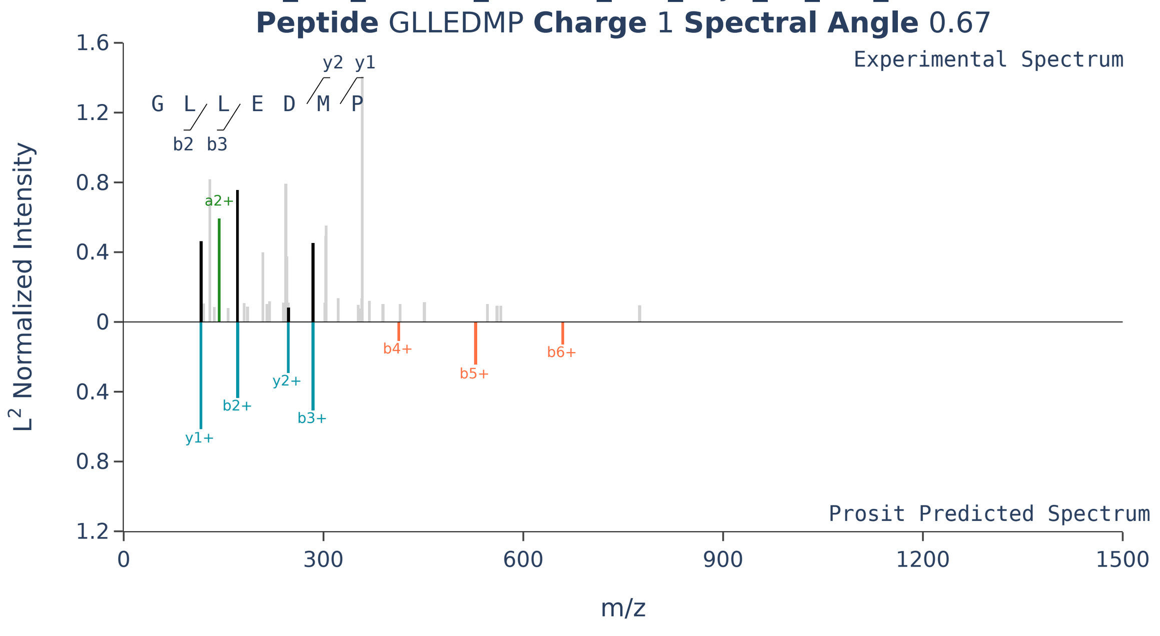

**Source** W_Soh_160821_170921_Goe_aSyn_A1_24h_R1 **Scan** 18597
**Peptide** TGVTAVAQKTVEGAGSIAA **Charge** 2 **Spectral Angle** 0.75

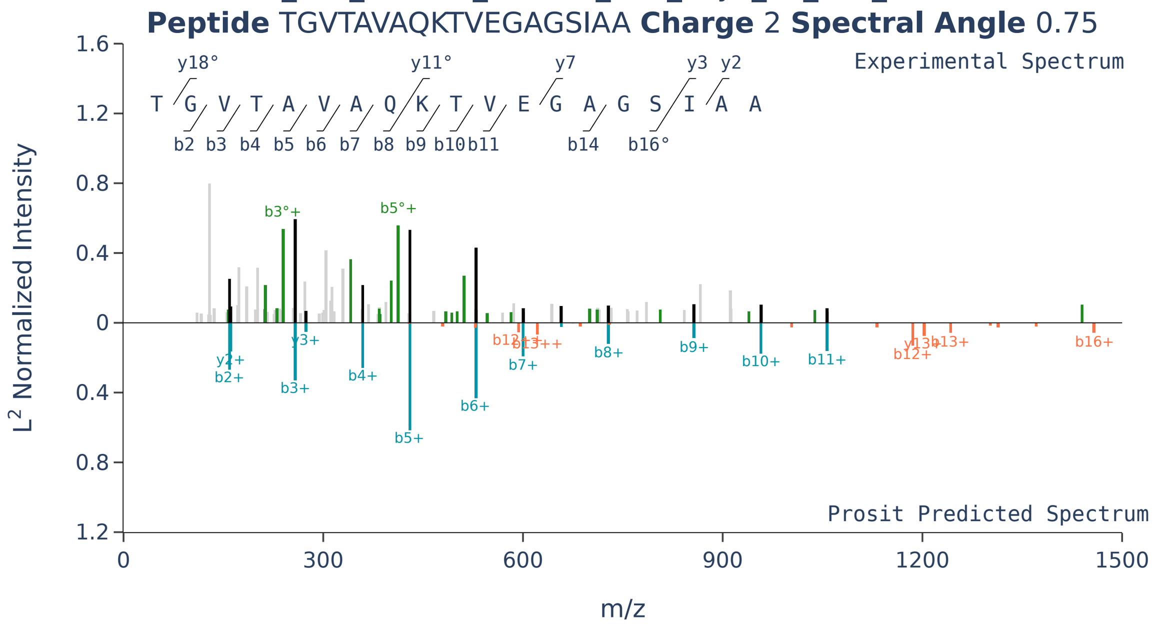


**Source** W_Soh_160821_170921_Goe_aSyn_A1_24h_R2 **Scan** 8979
**Peptide** EAAGKTKEGVLYVGSKTKEGVVHG **Charge** 4 **Spectral Angle** 0.87

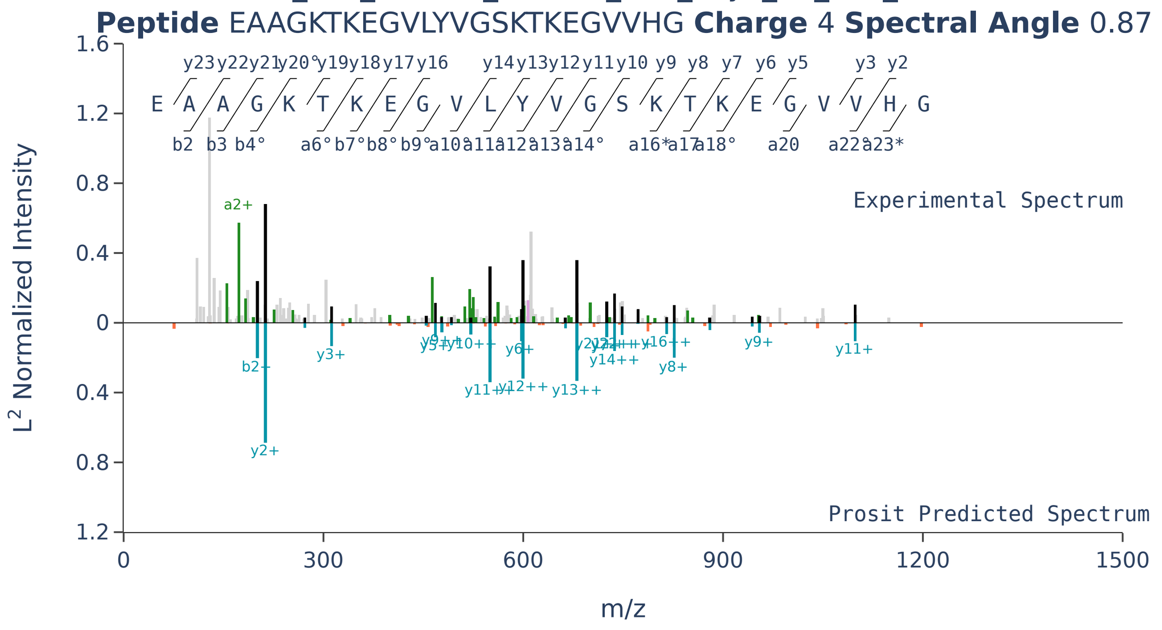


**Source** W_Soh_160821_170921_Goe_aSyn_A2_24h_R2 **Scan** 9573
**Peptide** AAEKTKQGVAEAAGKTKEGVLYVGSKTKE **Charge** 5 **Spectral Angle** 0.79

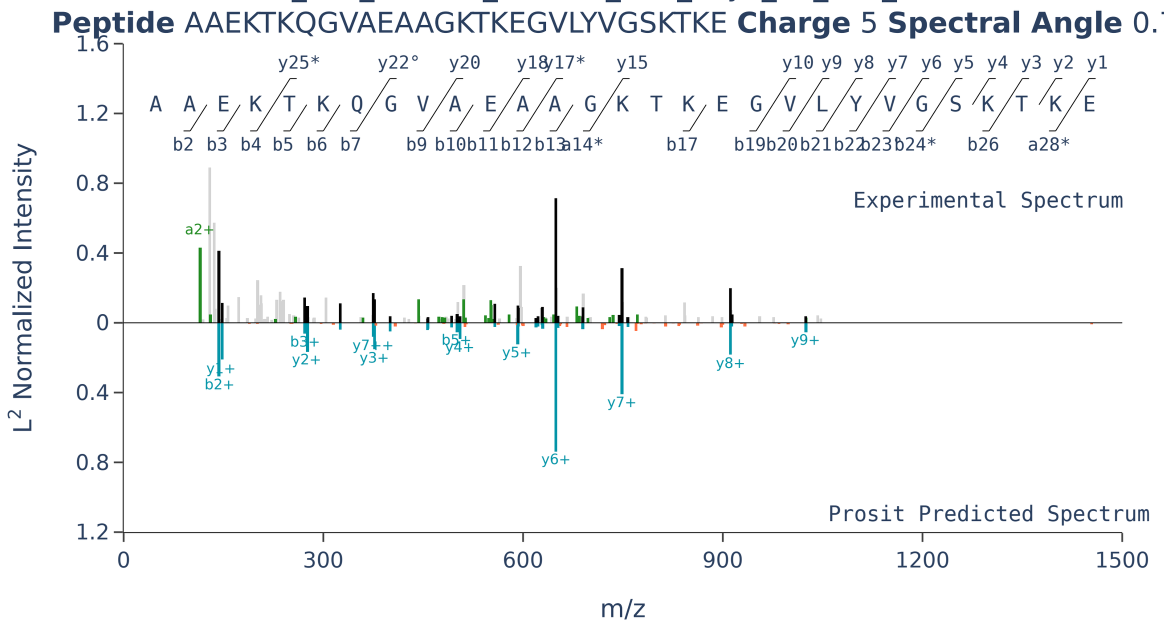


**Source** W_Soh_160821_170921_Goe_aSyn_A2_24h_R1 **Scan** 15893
**Peptide** AAATGFVKKDQLGKNEEGAPQEGIL **Charge** 3 **Spectral Angle** 0.78

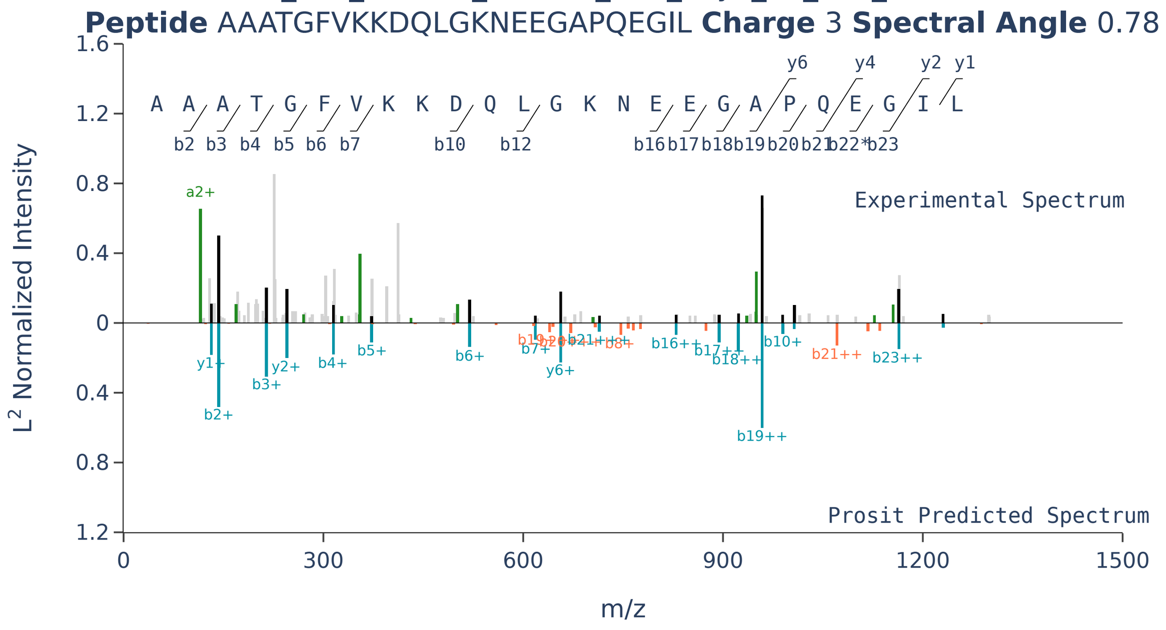


**Source** W_Soh_160821_170921_Goe_aSyn_A2_24h_R2 **Scan** 24412
**Peptide** AEKTKEQVTNVGGAVVTGVTAVAQKTVEGA **Charge** 4 **Spectral Angle** 0.71

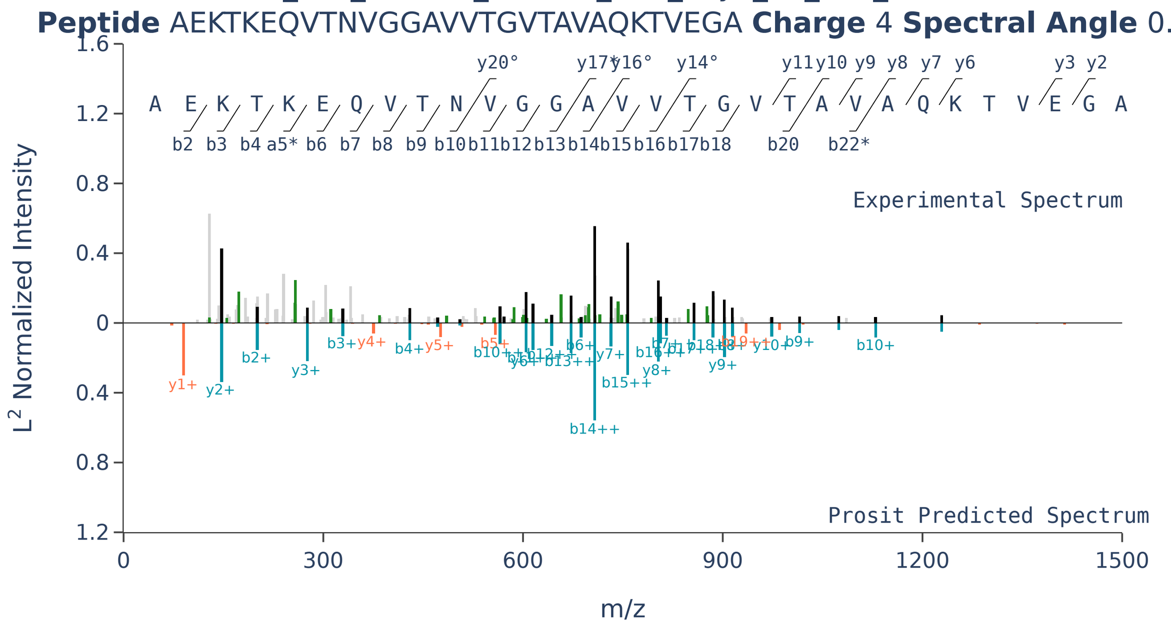


**Source** W_Soh_160821_170921_Goe_aSyn_A1_24h_R2 **Scan** 12744
**Peptide** GVATVAEKTKEQVTNVGG **Charge** 3 **Spectral Angle** 0.83

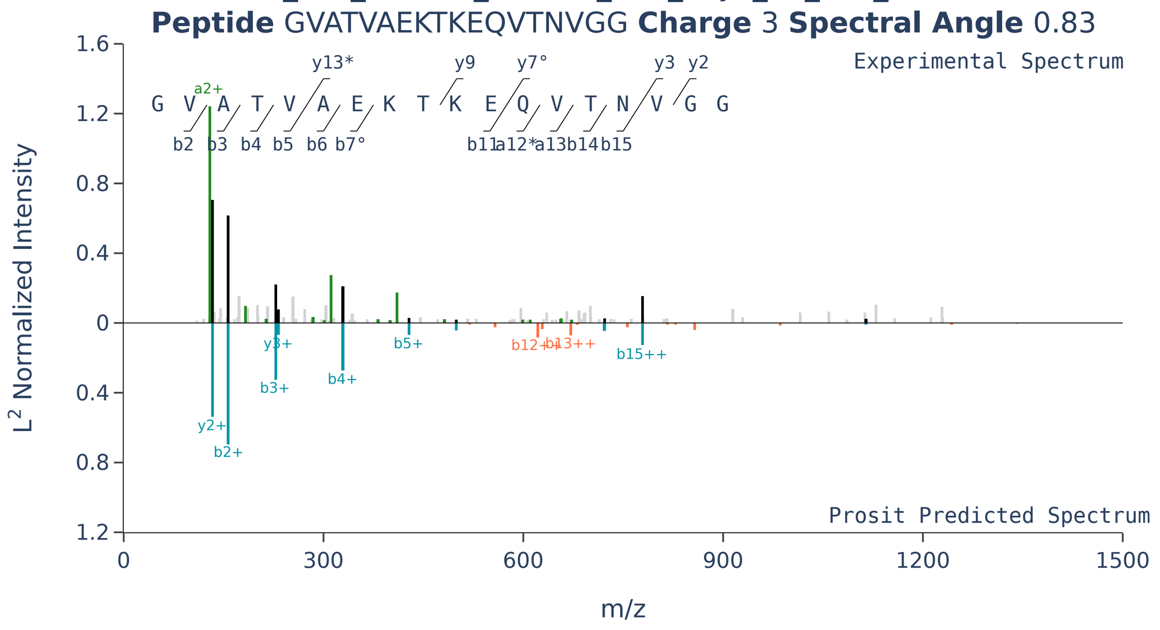


**Source** W_Soh_160821_170921_Goe_aSyn_A2_24h_R2 **Scan** 6864
**Peptide** VVHGVATVAEKTKE **Charge** 4 **Spectral Angle** 0.7

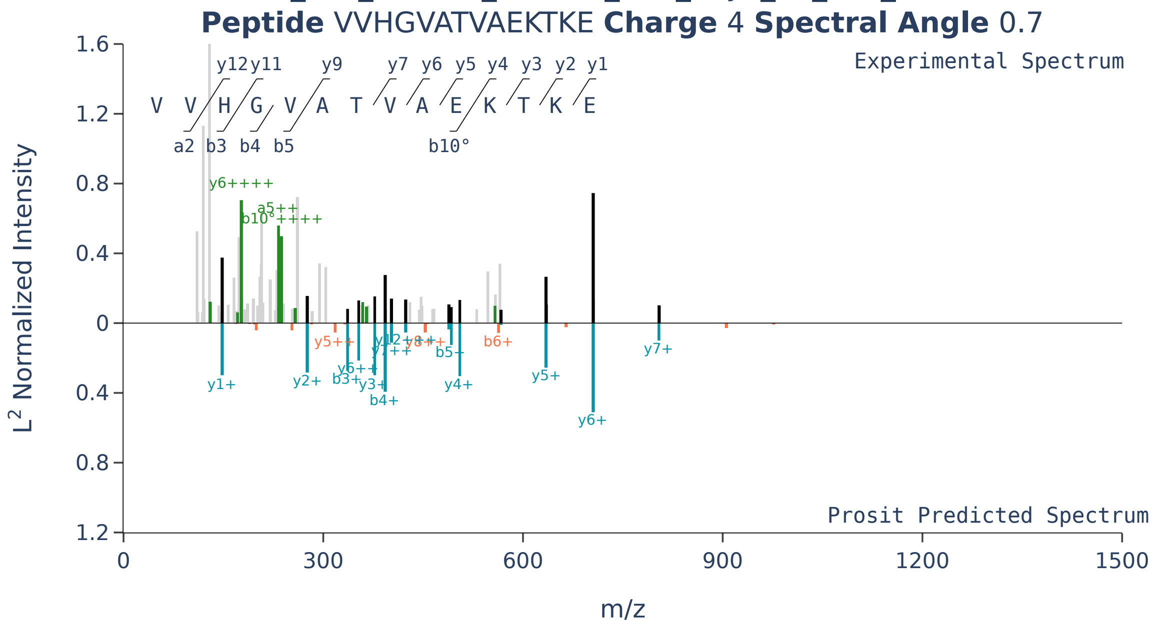


**Source** W_Soh_160821_170921_Goe_aSyn_A2_24h_R1 **Scan** 12390
**Peptide** DQLGKNEEGAPQEGI **Charge** 2 **Spectral Angle** 0.86

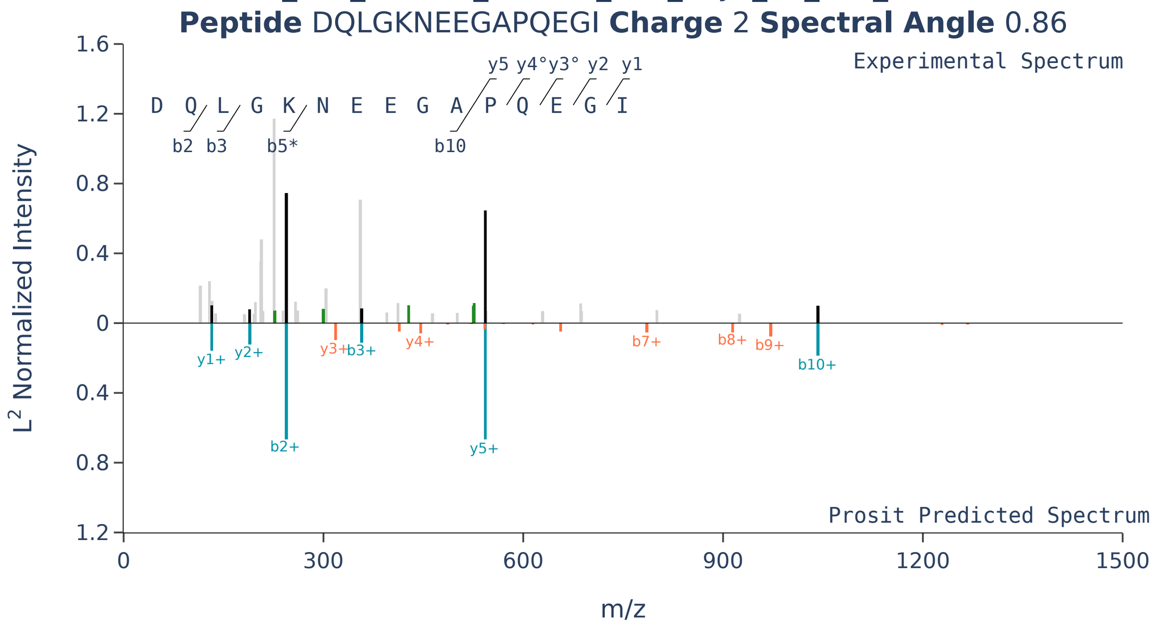


**Source** W_Soh_160821_170921_Goe_aSyn_A2_24h_R1 **Scan** 8010
**Peptide** VAAAEKTKQGVAE **Charge** 2 **Spectral Angle** 0.86

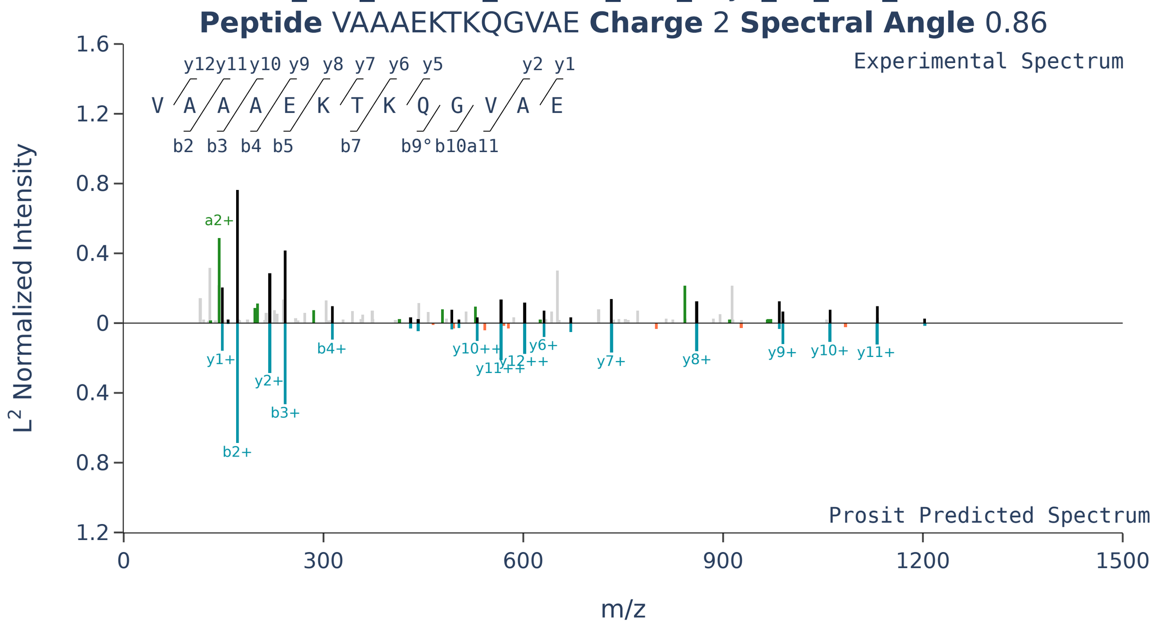


**Source** W_Soh_160821_170921_Goe_aSyn_A1_3h_R1 **Scan** 8068
**Peptide** QKTVEGAGSIA **Charge** 2 **Spectral Angle** 0.82

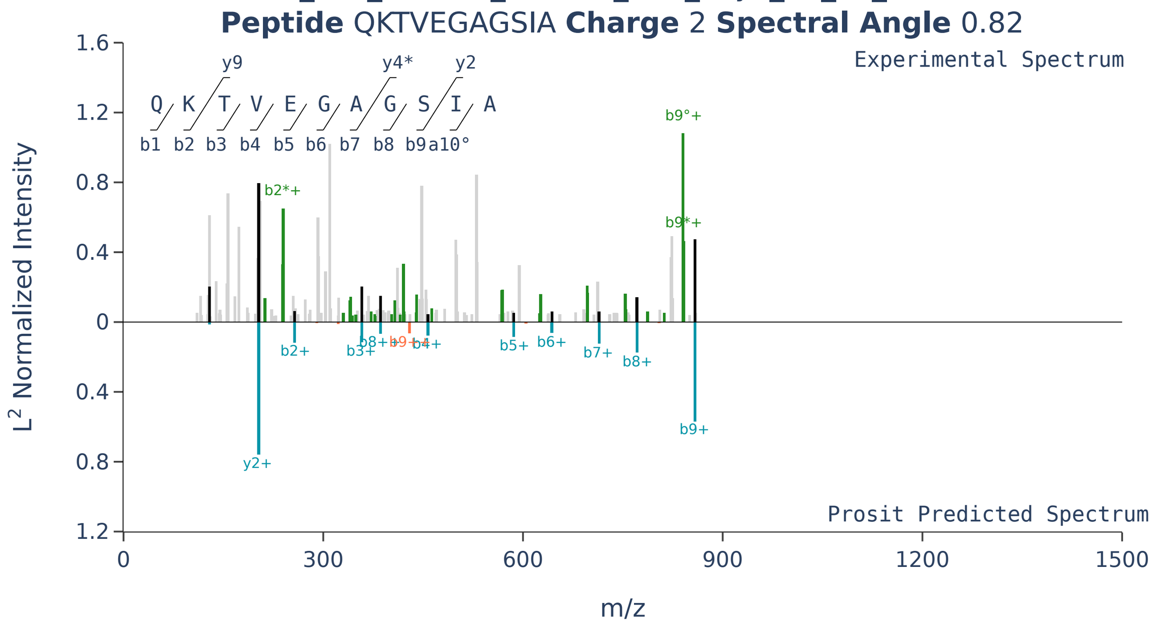


**Source** W_Soh_160821_170921_Goe_aSyn_A1_24h_R1 **Scan** 8971
**Peptide** LYVGSKTK **Charge** 2 **Spectral Angle** 0.93

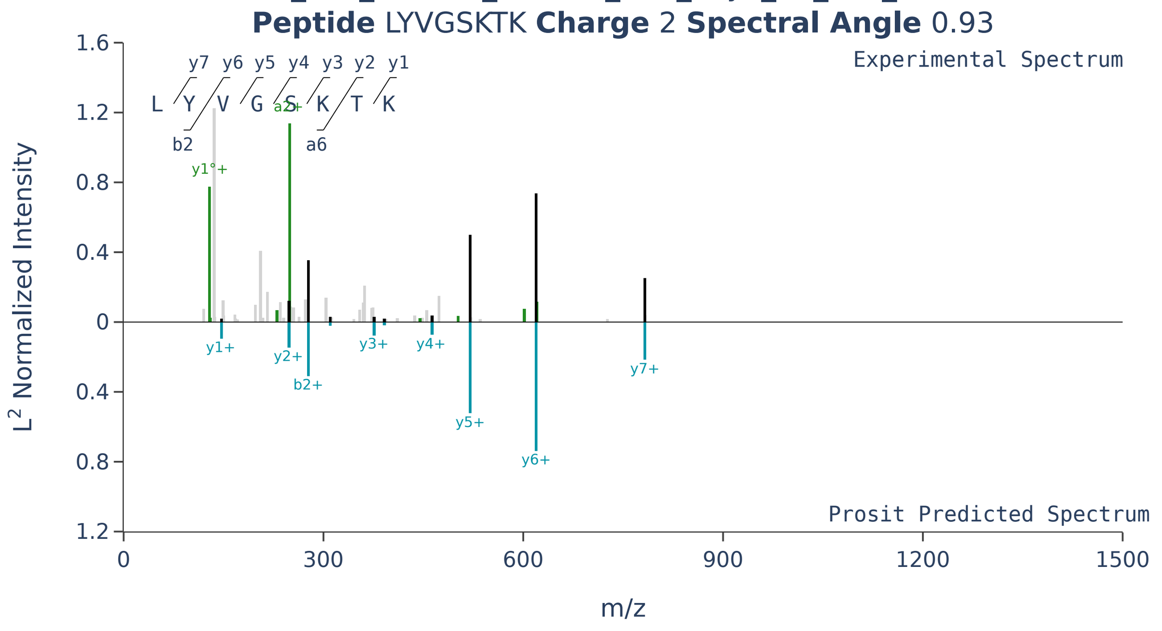


**Source** W_Soh_160821_170921_Goe_aSyn_A1_24h_R2 **Scan** 15972
**Peptide** VAQKTVEGAGSIAAATGFVKKDQLGKNE **Charge** 5 **Spectral Angle** 0.72

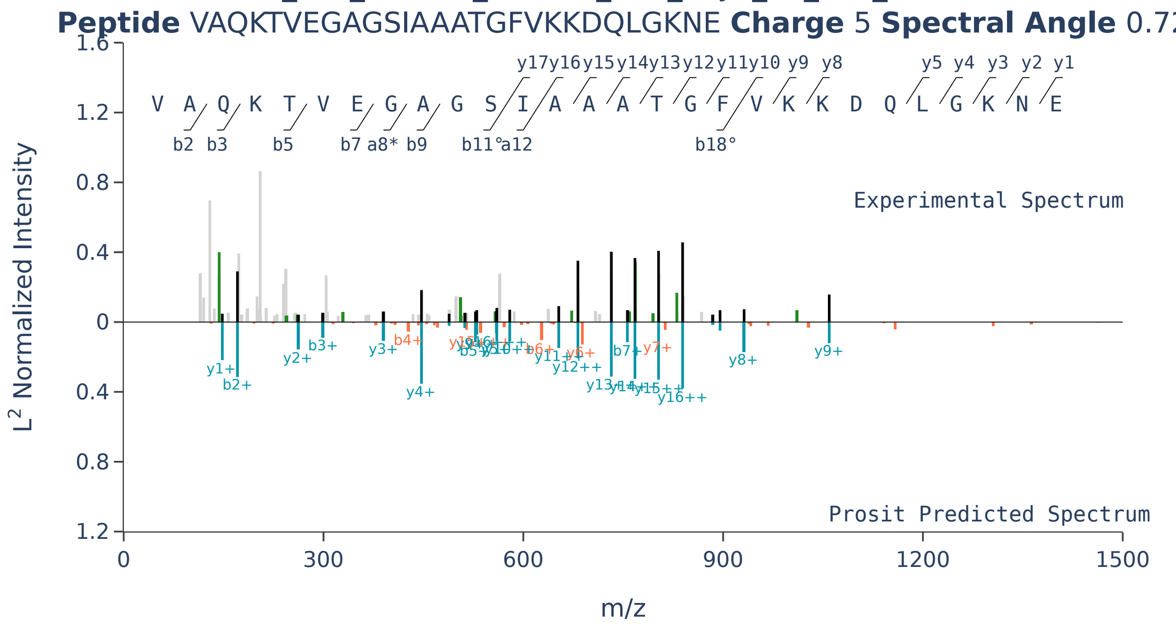


**Source** W_Soh_160821_170921_Goe_aSyn_A2_6h_R2 **Scan** 17401
**Peptide** GAVVTGVTAVAQKTVE **Charge** 3 **Spectral Angle** 0.8

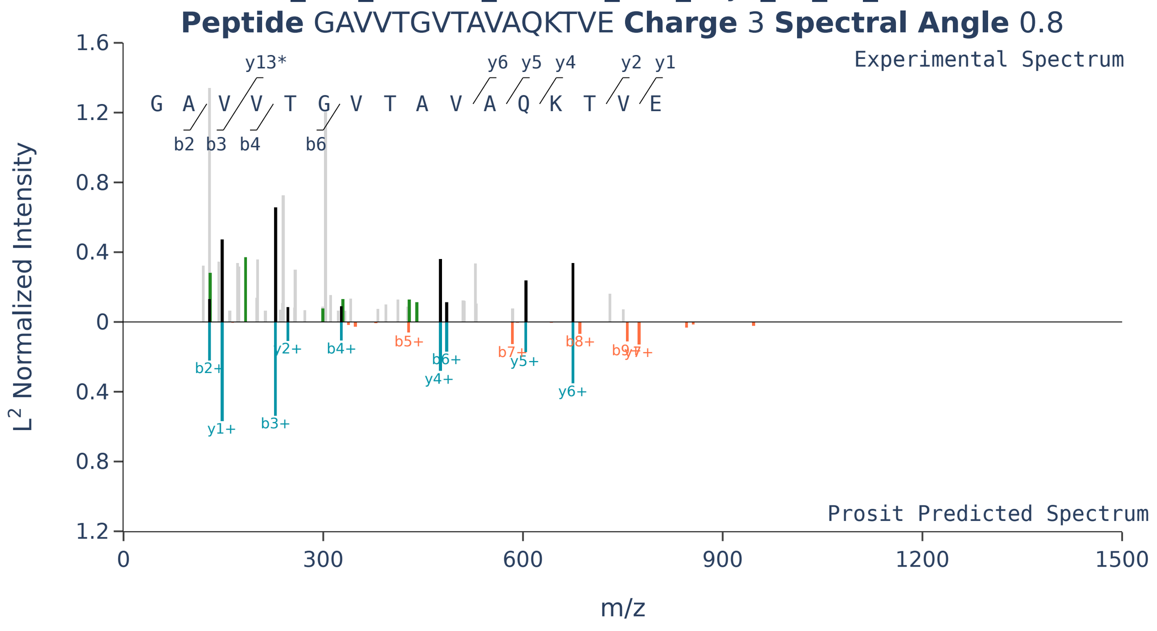


**Source** W_Soh_160821_170921_Goe_aSyn_A1_24h_R2 **Scan** 8271
**Peptide** KTVEGAGSI **Charge** 1 **Spectral Angle** 0.74

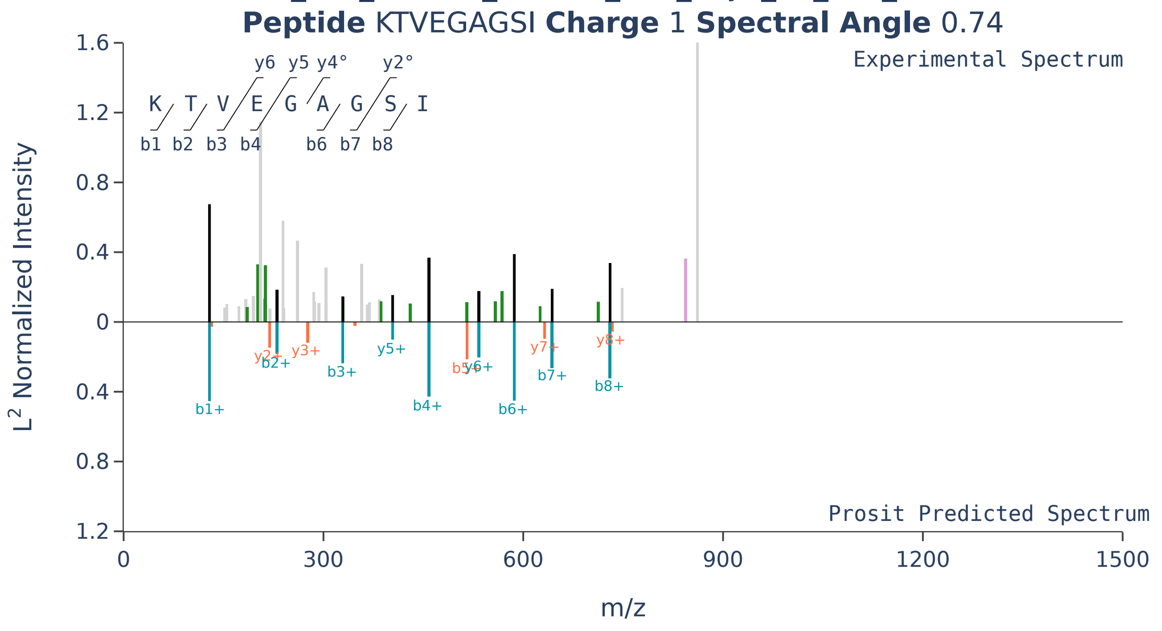


**Source** W_Soh_160821_170921_Goe_aSyn_A2_6h_R1 **Scan** 12720
**Peptide** GSIAAATGFVKKDQLGKNEE **Charge** 3 **Spectral Angle** 0.8

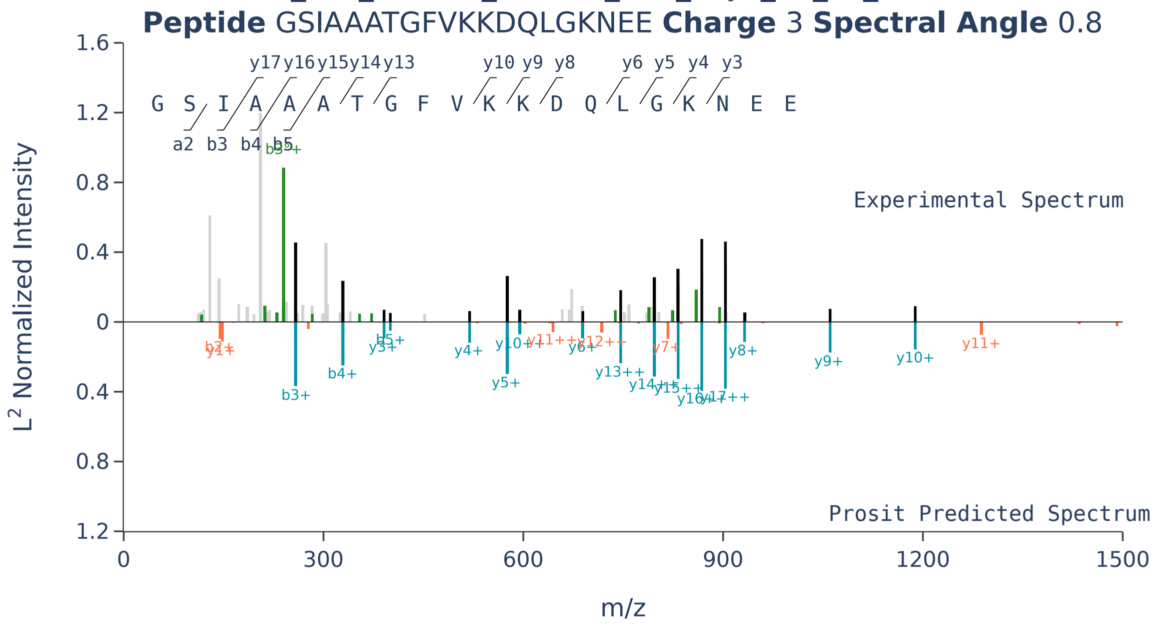

**Source** W_Soh_160821_170921_Goe_aSyn_A2_24h_R1 **Scan** 20736
**Peptide** NVGGAVVTGVTAVAQ **Charge** 2 **Spectral Angle** 0.83

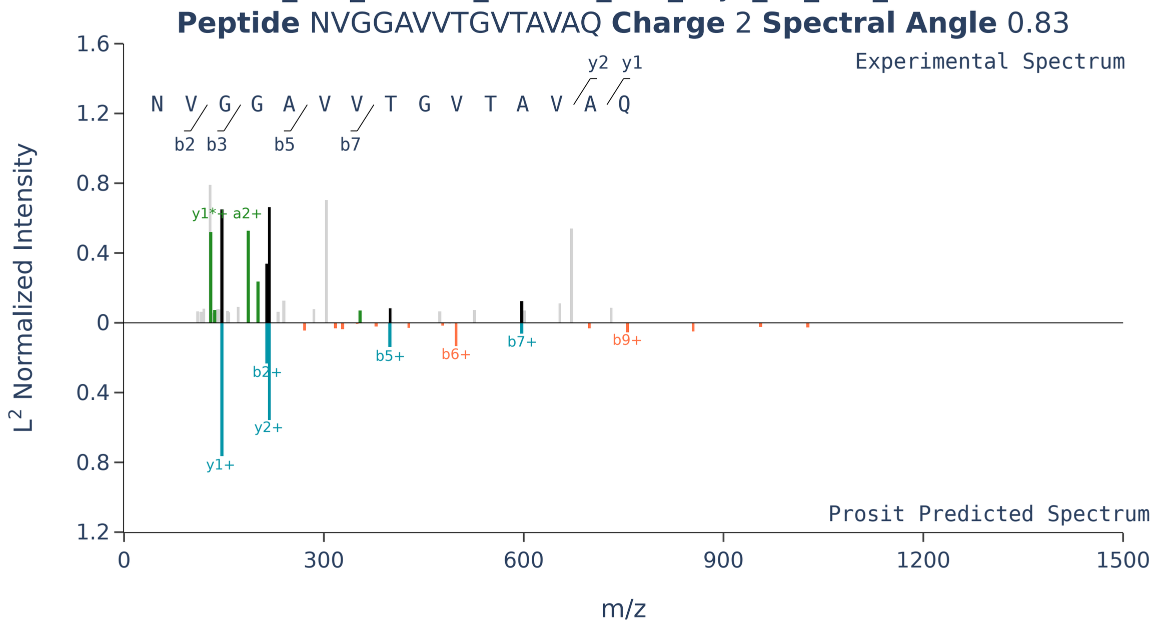


**Source** W_Soh_160821_170921_Goe_aSyn_A1_24h_R2 **Scan** 19752
**Peptide** MDVFMKGLSKAKEGVVAAA **Charge** 3 **Spectral Angle** 0.89

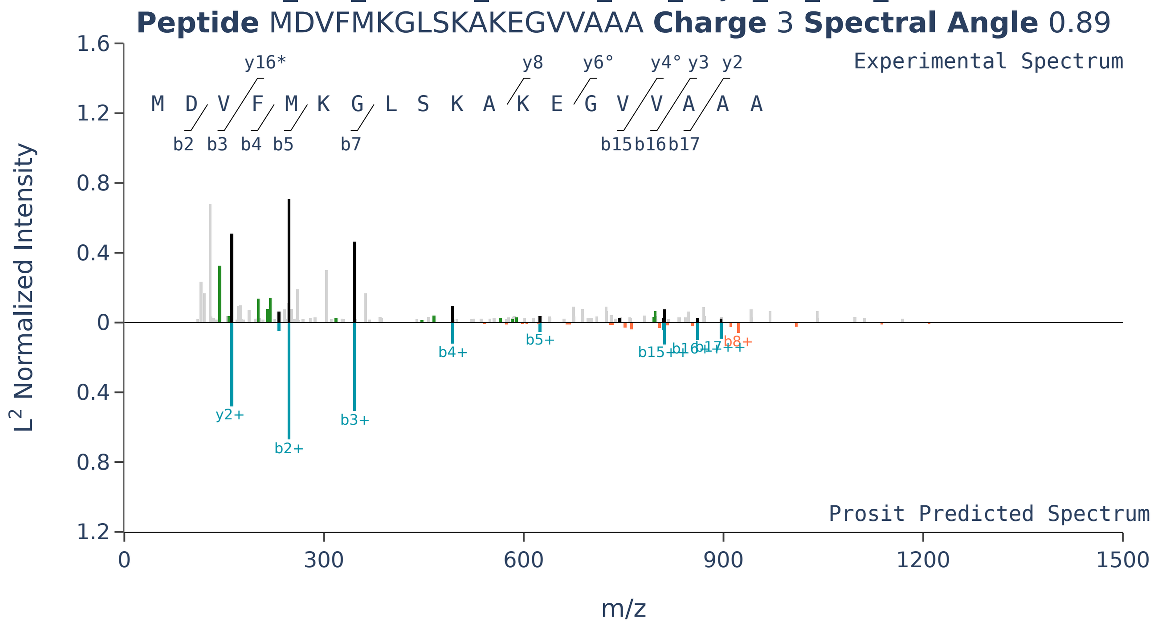


**Source** W_Soh_160821_170921_Goe_aSyn_A1_24h_R2 **Scan** 11050
**Peptide** GSKTKEGVVHGVATVAEKTKEQVTN **Charge** 5 **Spectral Angle** 0.81

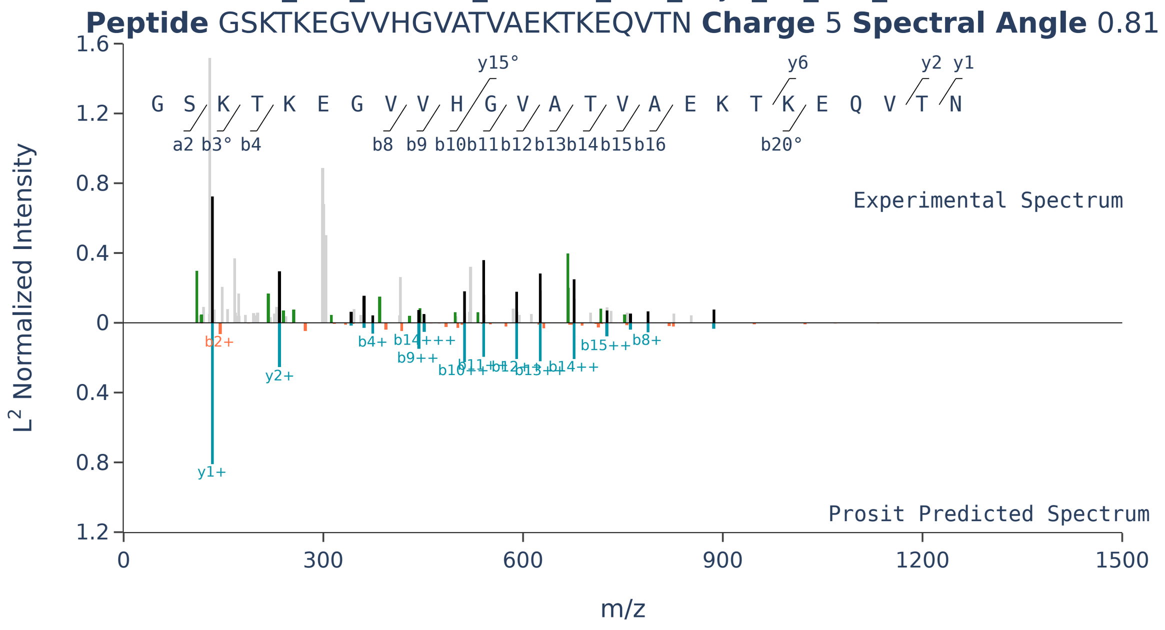


**Source** W_Soh_160821_170921_Goe_aSyn_A2_24h_R1 **Scan** 20609
**Peptide** KEQVTNVGGAVVTGVTAVAQ **Charge** 2 **Spectral Angle** 0.68

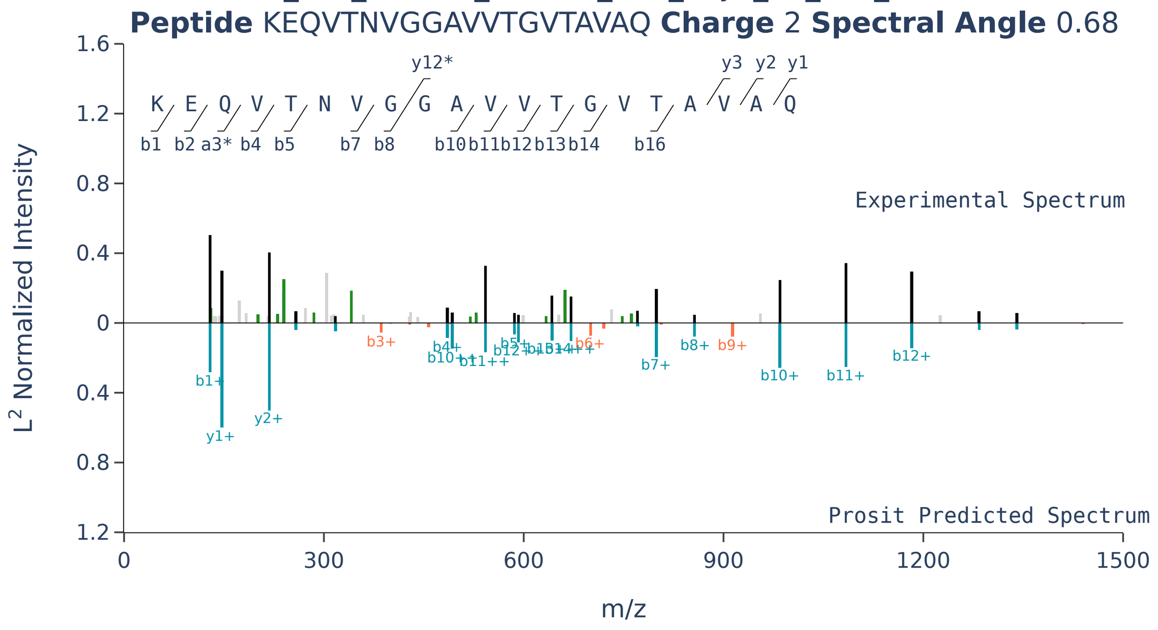


**Source** W_Soh_160821_170921_Goe_aSyn_A1_3h_R1 **Scan** 16196
**Peptide** ATVAEKTKEQVTNVGGAVVTGVT **Charge** 3 **Spectral Angle** 0.7

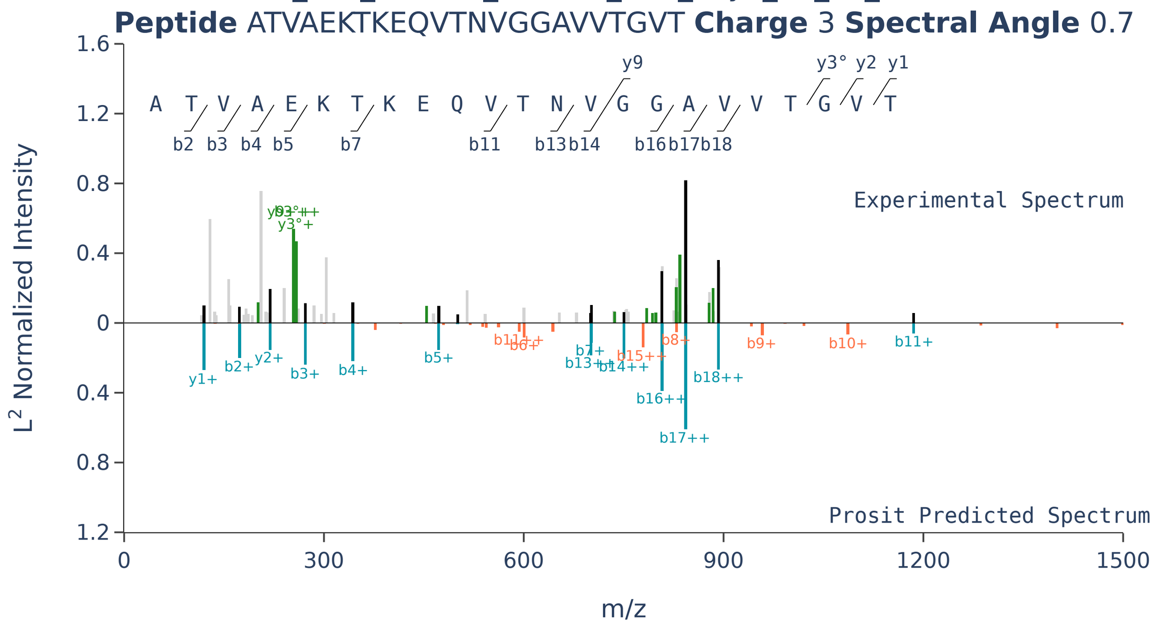


**Source** W_Soh_160821_170921_Goe_aSyn_A1_24h_R2 **Scan** 8187
**Peptide** TKEGVLYVGSKTKE **Charge** 4 **Spectral Angle** 0.77

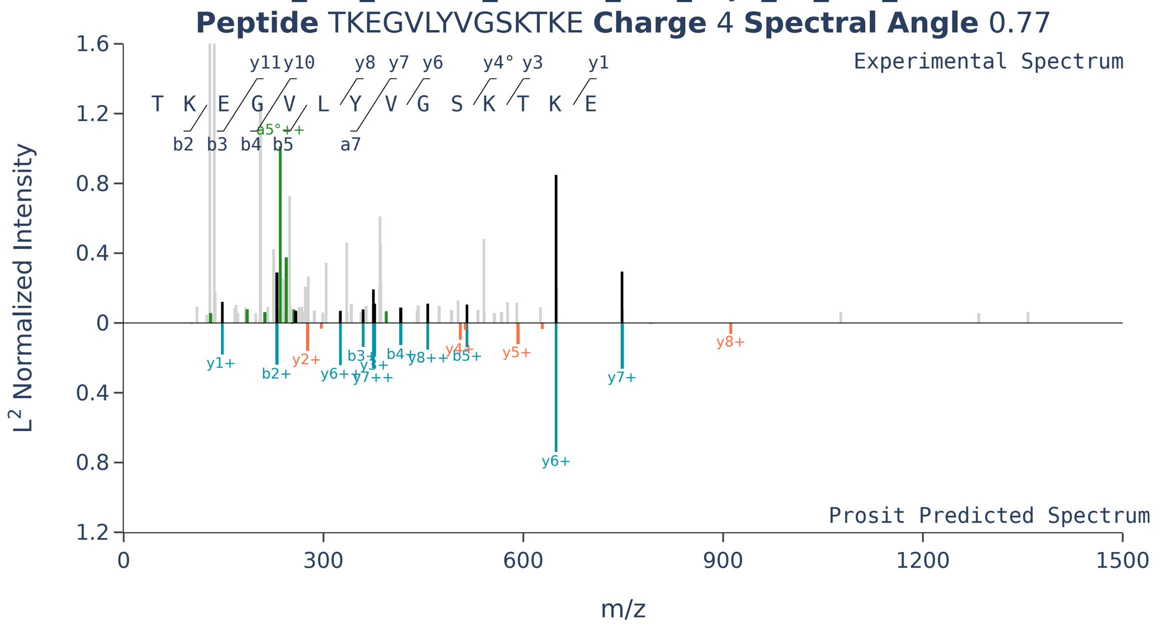


**Source** W_Soh_160821_170921_Goe_aSyn_A2_24h_R1 **Scan** 5157
**Peptide** VATVAEKTKEQ **Charge** 2 **Spectral Angle** 0.71

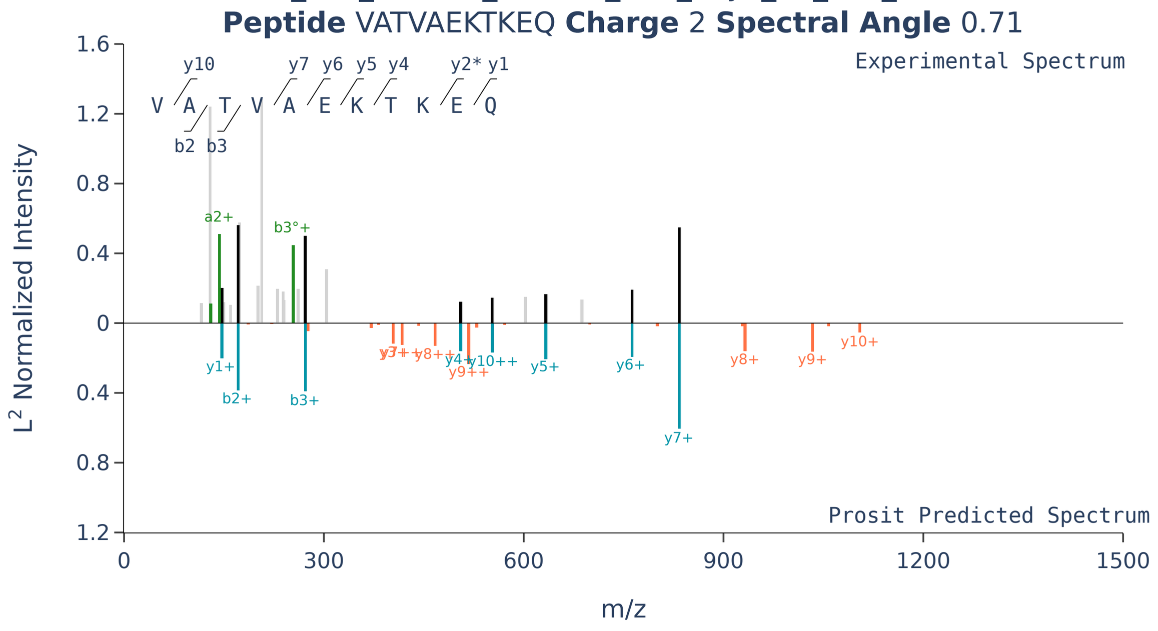


**Source** W_Soh_160821_170921_Goe_aSyn_A1_6h_R2 **Scan** 16114
**Peptide** PDNEAYEMP **Charge** 2 **Spectral Angle** 0.85

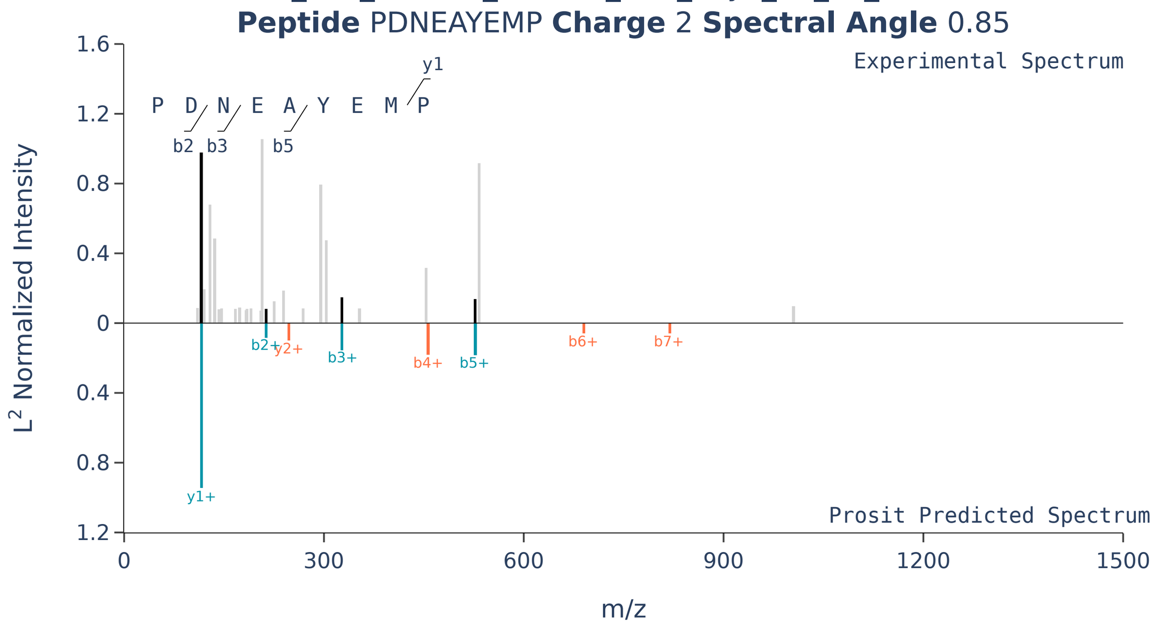


**Source** W_Soh_160821_170921_Goe_aSyn_A1_6h_R2 **Scan** 23544
**Peptide** LEDMPVDPDNEAYEMPSE **Charge** 2 **Spectral Angle** 0.89

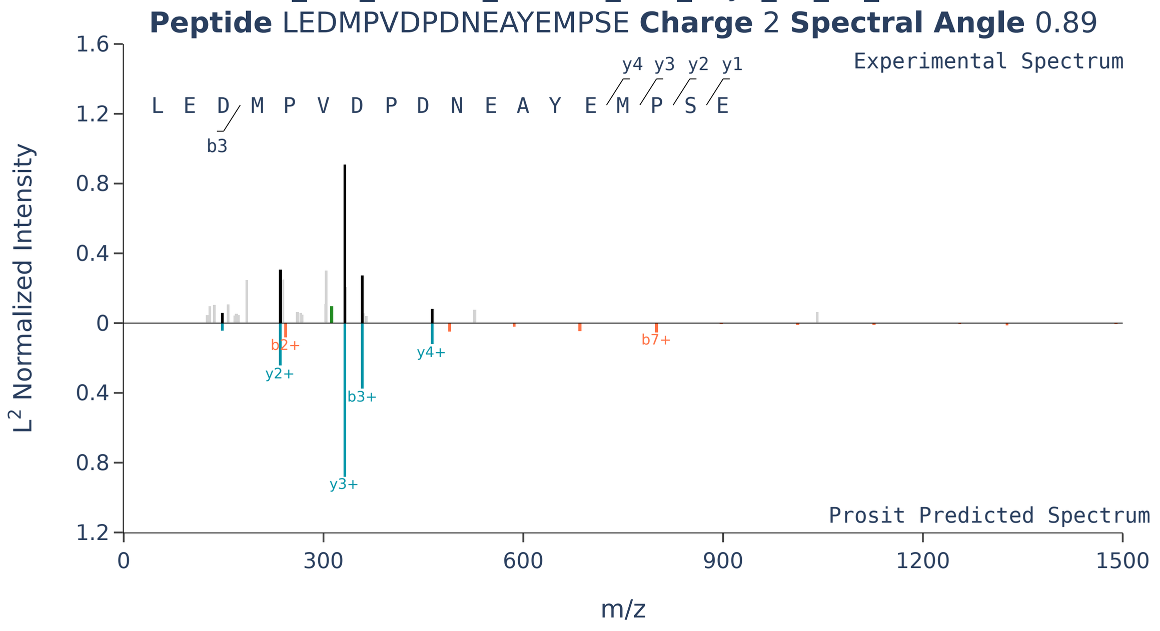


**Source** W_Soh_160821_170921_Goe_aSyn_A2_24h_R2 **Scan** 18713
**Peptide** GGAVVTGVTAVAQKTV **Charge** 3 **Spectral Angle** 0.7

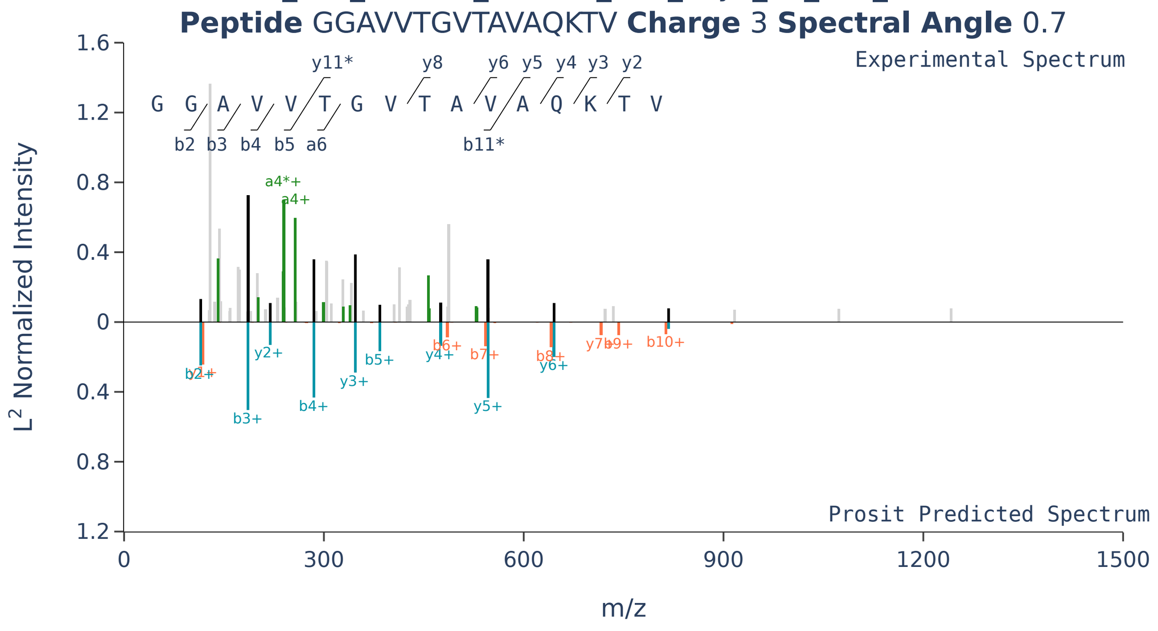


**Source** W_Soh_160821_170921_Goe_aSyn_A1_6h_R2 **Scan** 20008
**Peptide** GKNEEGAPQEGILEDM **Charge** 2 **Spectral Angle** 0.71

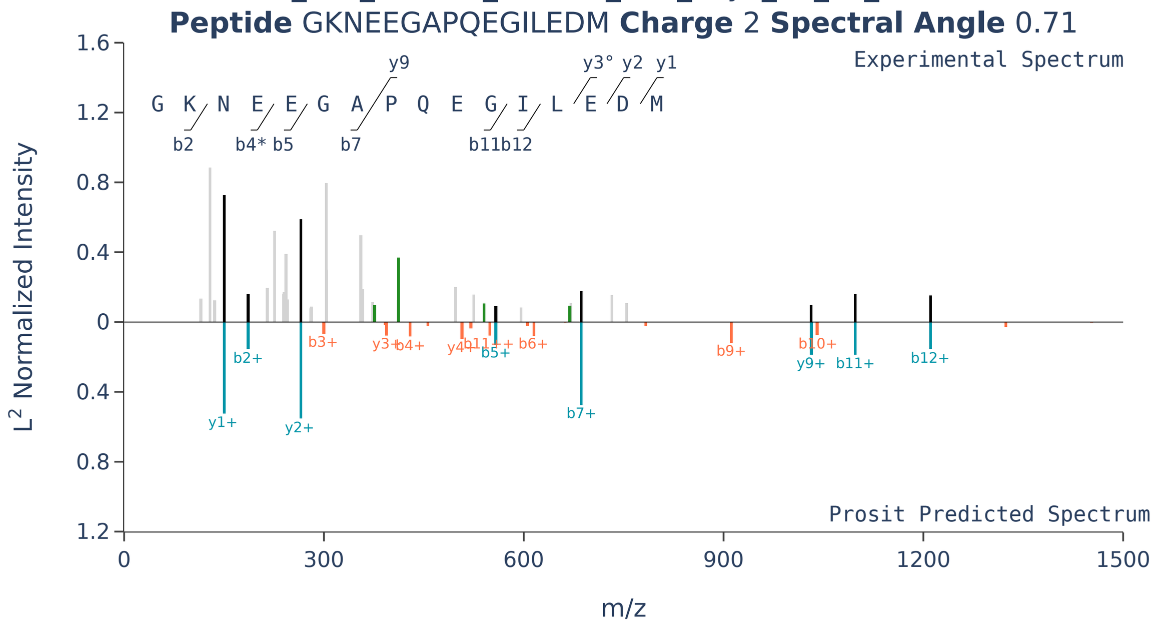


**Source** W_Soh_160821_170921_Goe_aSyn_A2_24h_R2 **Scan** 6592
**Peptide** FVKKDQLGKNEEGAPQE **Charge** 2 **Spectral Angle** 0.88

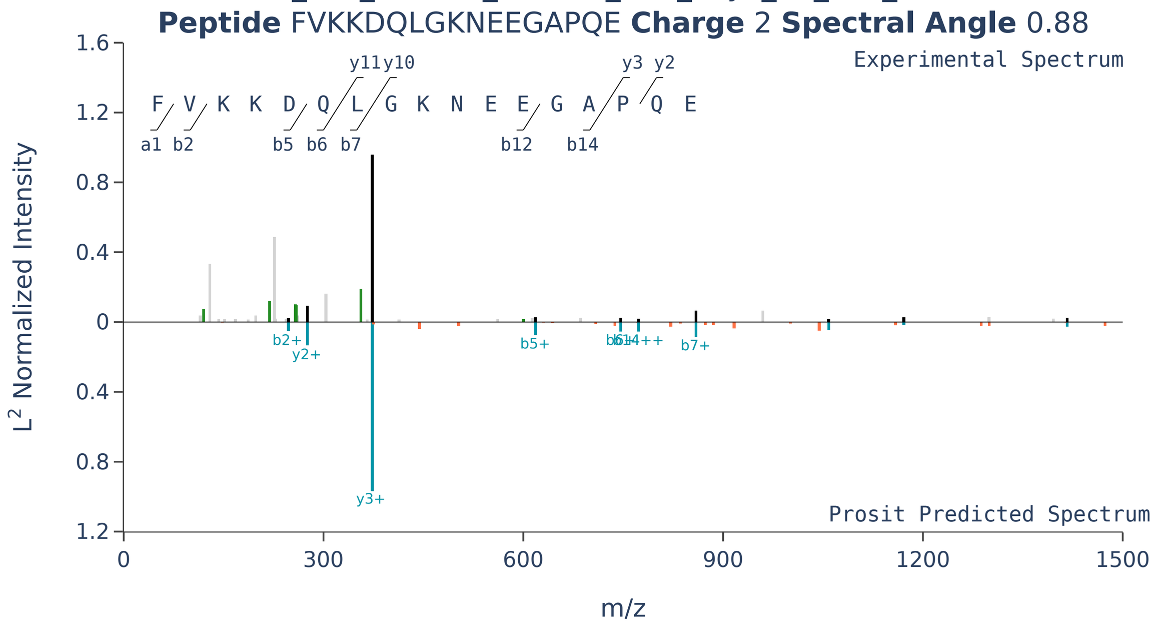


**Source** W_Soh_160821_170921_Goe_aSyn_A1_24h_R2 **Scan** 15122
**Peptide** TGFVKKDQLGKNEEGAPQEGIL **Charge** 2 **Spectral Angle** 0.75

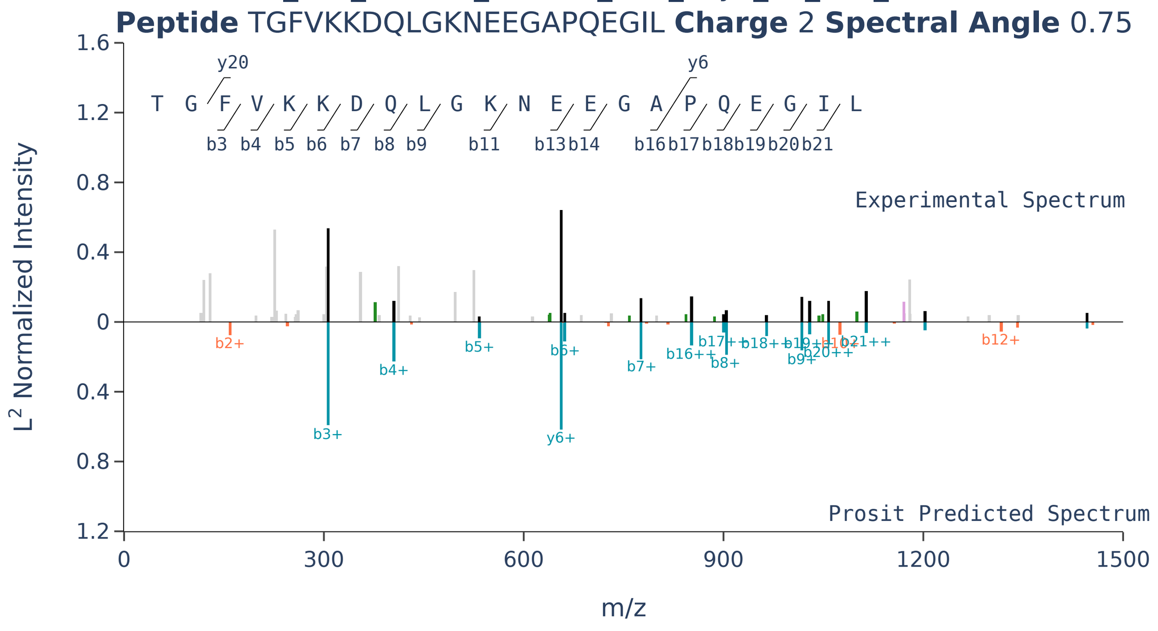


**Source** W_Soh_160821_170921_Goe_aSyn_A2_24h_R1 **Scan** 14101
**Peptide** KDQLGKNEEGAPQEGIL **Charge** 3 **Spectral Angle** 0.75

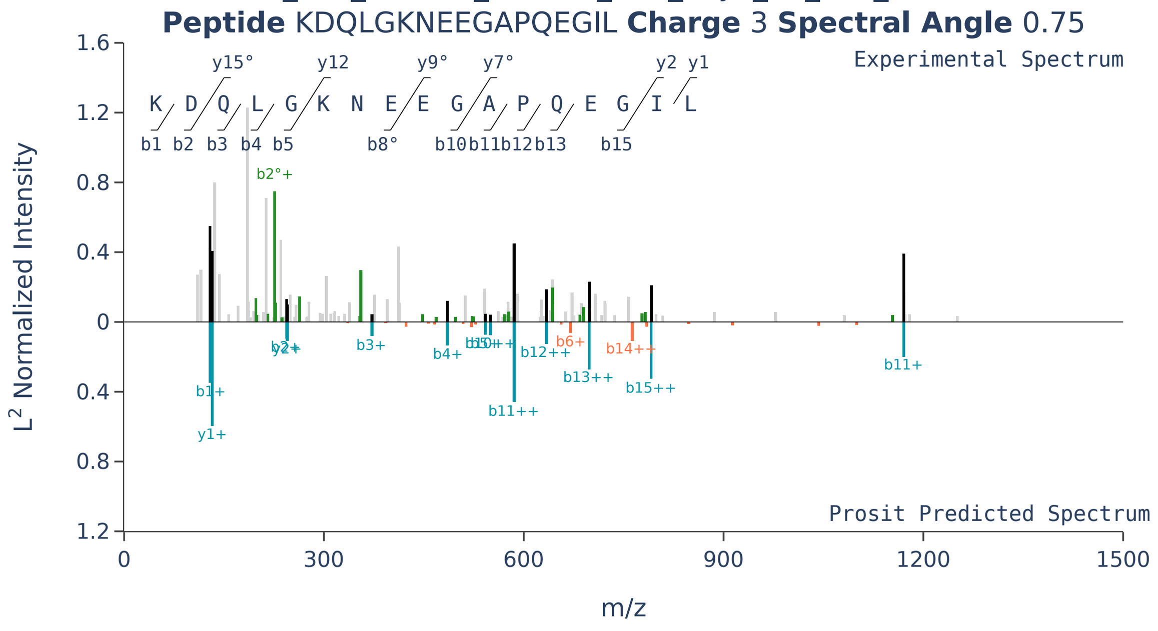


**Source** W_Soh_160821_170921_Goe_aSyn_A2_6h_R2 **Scan** 19505
**Peptide** EGAPQEGILEDMPVD **Charge** 2 **Spectral Angle** 0.68

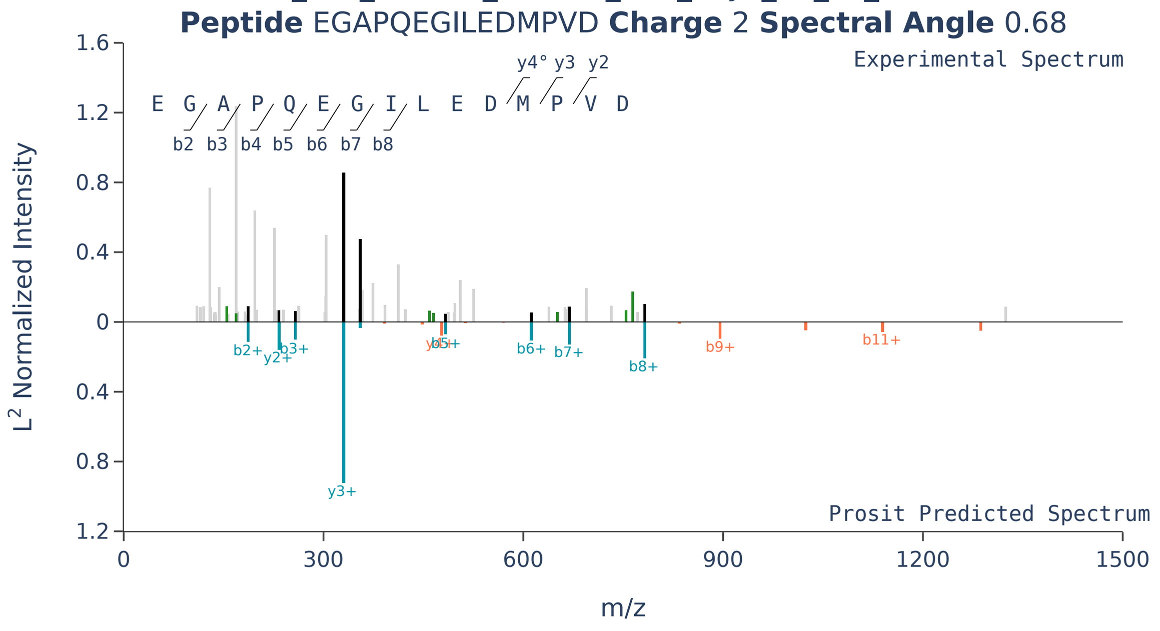

**Source** W_Soh_160821_170921_Goe_aSyn_A1_24h_R1 **Scan** 12471
**Peptide** GLSKAKEGVVAAAEKTKQGVAEAAGKTKE **Charge** 5 **Spectral Angle** 0.74

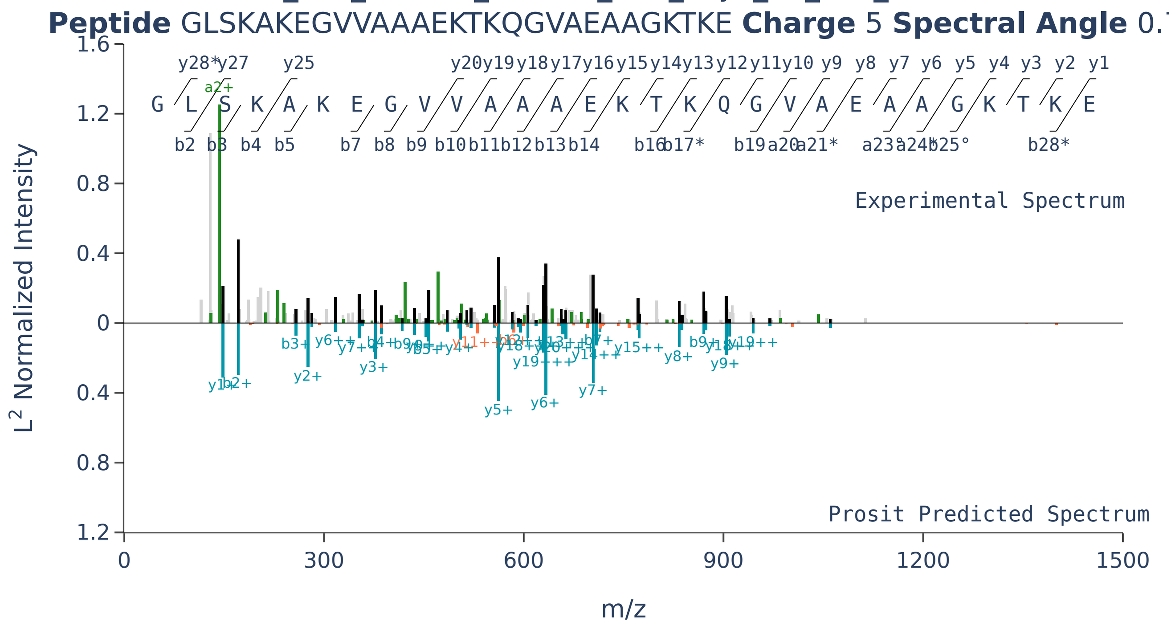


**Source** W_Soh_160821_170921_Goe_aSyn_A2_24h_R2 **Scan** 13132
**Peptide** GVATVAEKTKEQVTNVGGA **Charge** 3 **Spectral Angle** 0.85

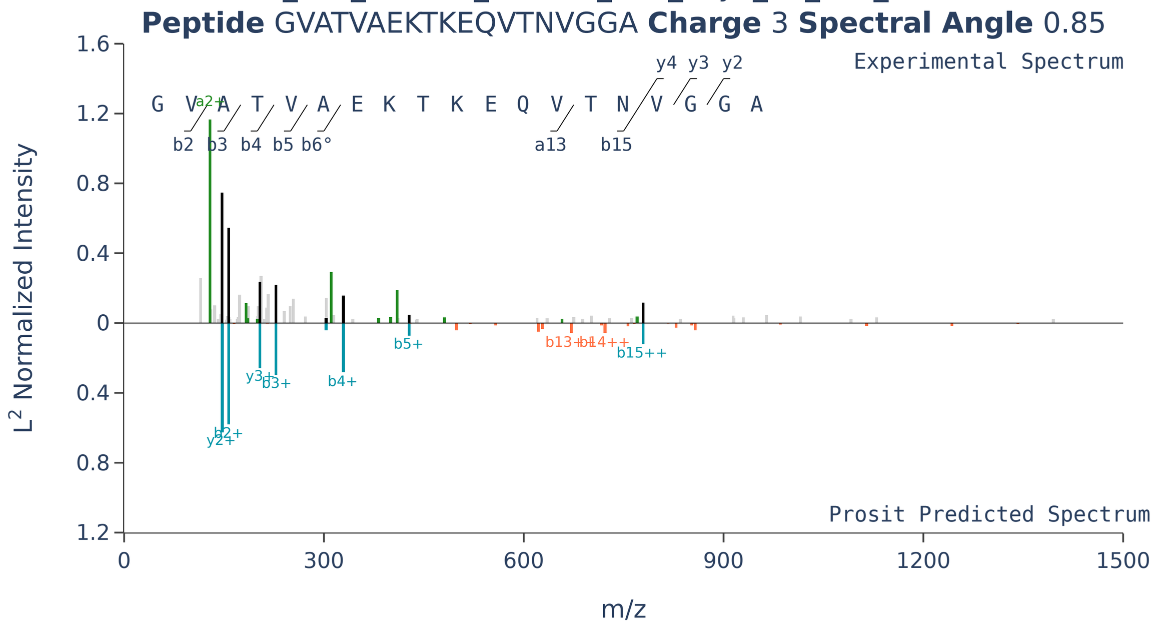


**Source** W_Soh_160821_170921_Goe_aSyn_A1_24h_R2 **Scan** 8318
**Peptide** SKTKEGVVHGVATVAE **Charge** 2 **Spectral Angle** 0.75

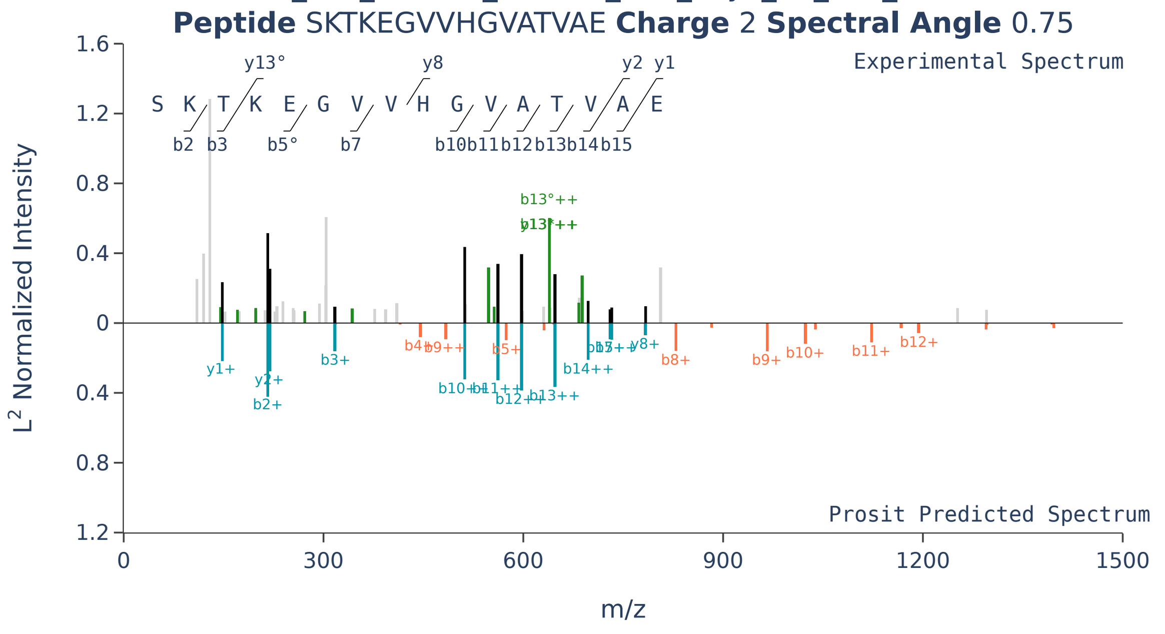


**Source** W_Soh_160821_170921_Goe_aSyn_A2_24h_R2 **Scan** 8624
**Peptide** KTKEGVLY **Charge** 2 **Spectral Angle** 0.61

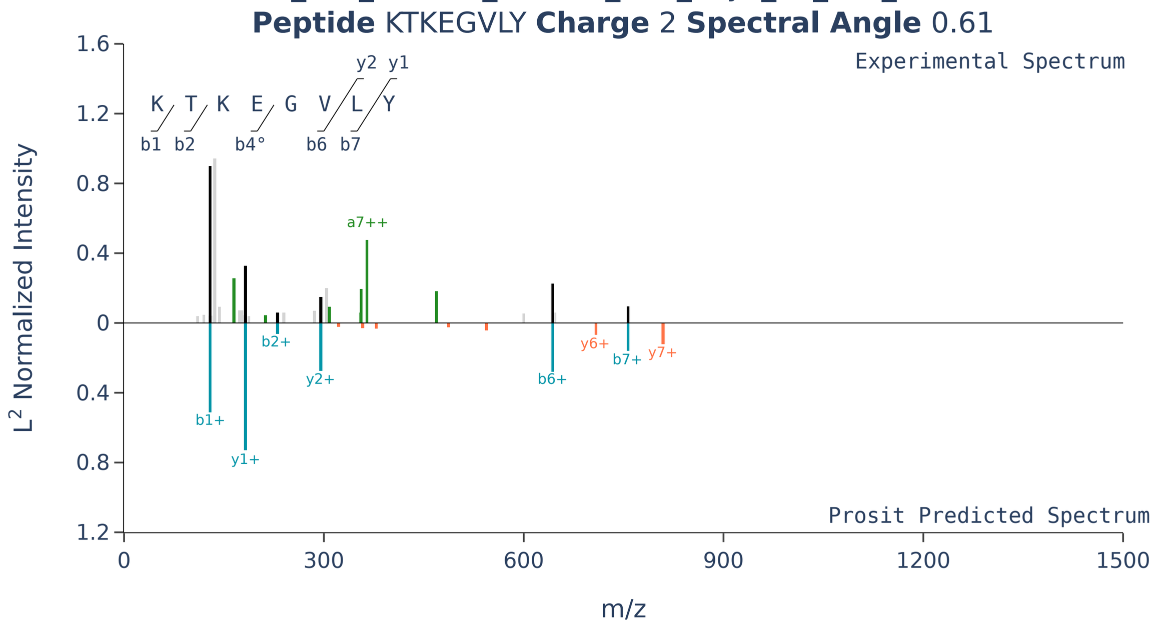


**Source** W_Soh_160821_170921_Goe_aSyn_A1_6h_R2 **Scan** 19459
**Peptide** QVTNVGGAVVTGV **Charge** 2 **Spectral Angle** 0.71

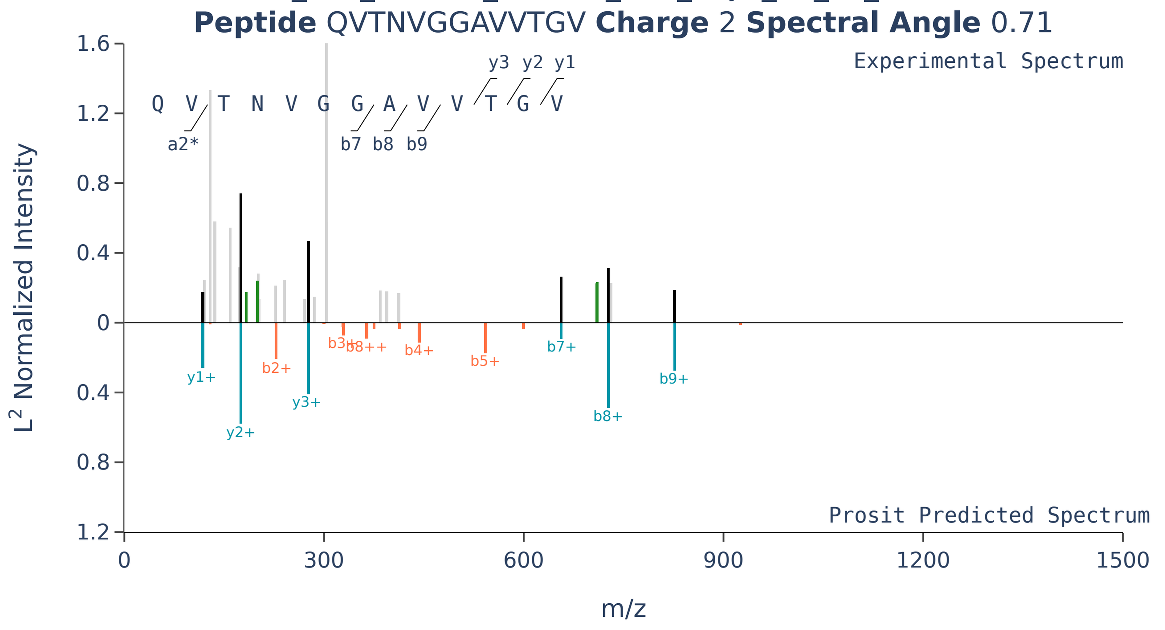


**Source** W_Soh_160821_170921_Goe_aSyn_A1_24h_R2 **Scan** 17435
**Peptide** TAVAQKTVEGAGSIAAATGFVKK **Charge** 3 **Spectral Angle** 0.76

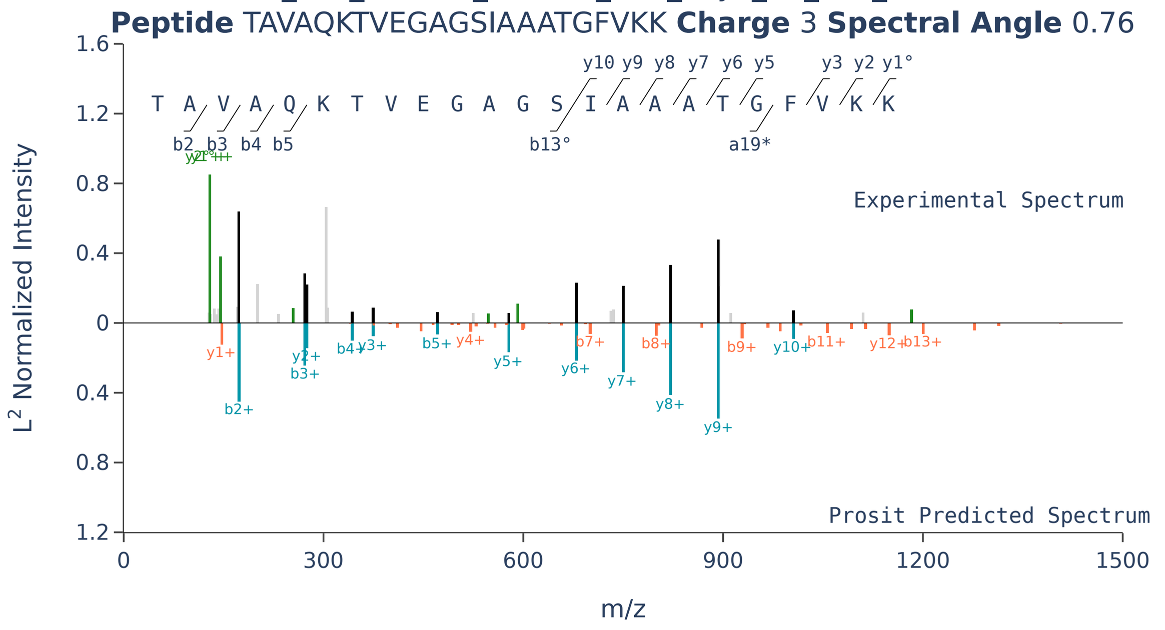


**Source** W_Soh_160821_170921_Goe_aSyn_A1_6h_R1 **Scan** 6831
**Peptide** KTKEGVVHGVATVAEKTKE **Charge** 4 **Spectral Angle** 0.81

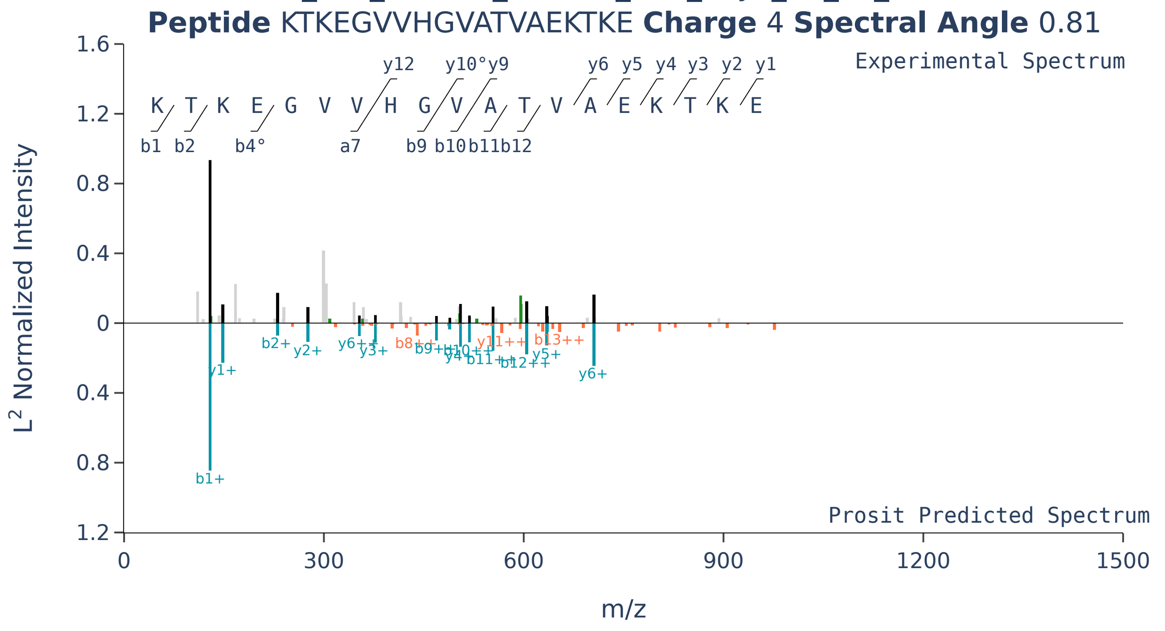


**Source** W_Soh_160821_170921_Goe_aSyn_A1_24h_R1 **Scan** 17475
**Peptide** EKTKEQVTNVGGAVVTGVTAVAQK **Charge** 4 **Spectral Angle** 0.74

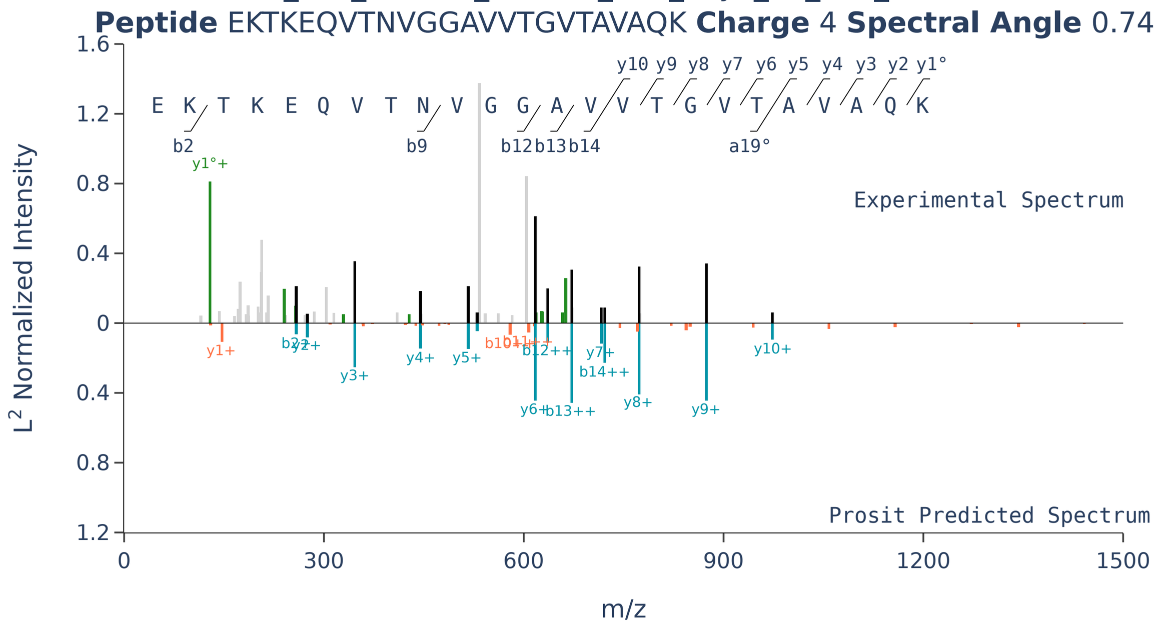


**Source** W_Soh_160821_170921_Goe_aSyn_A1_24h_R1 **Scan** 9812
**Peptide** AEKTKQGVAEAAGKTKEGVLYVGSKT **Charge** 5 **Spectral Angle** 0.78

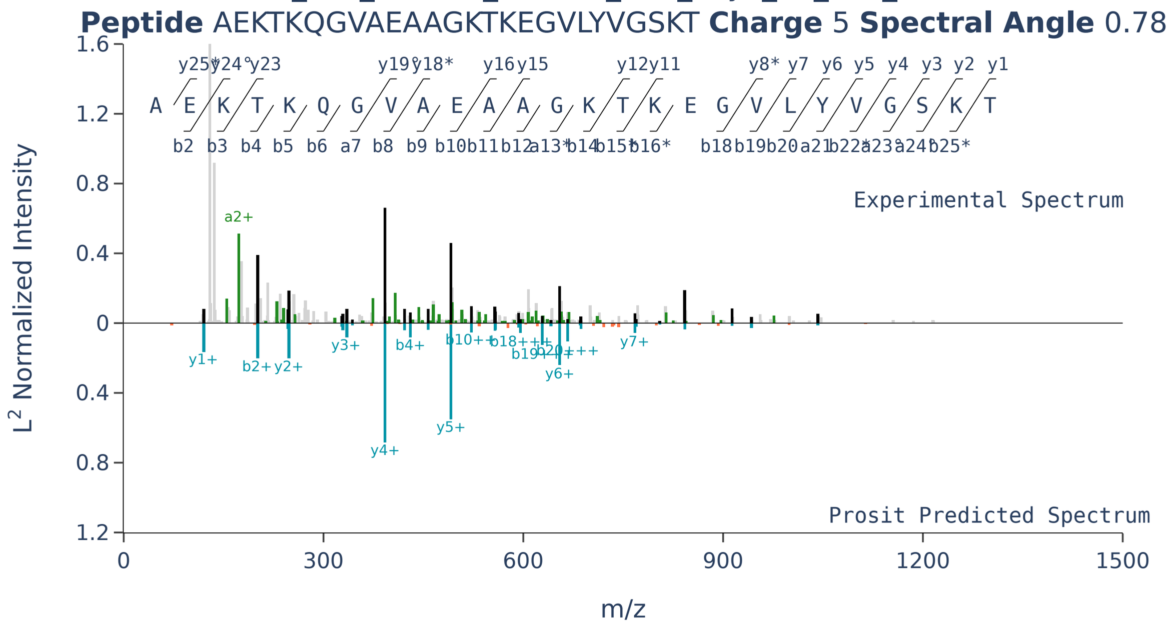


**Source** W_Soh_160821_170921_Goe_aSyn_A2_24h_R2 **Scan** 16860
**Peptide** VEGAGSIAAATGFVKKDQLGKNEE **Charge** 4 **Spectral Angle** 0.73

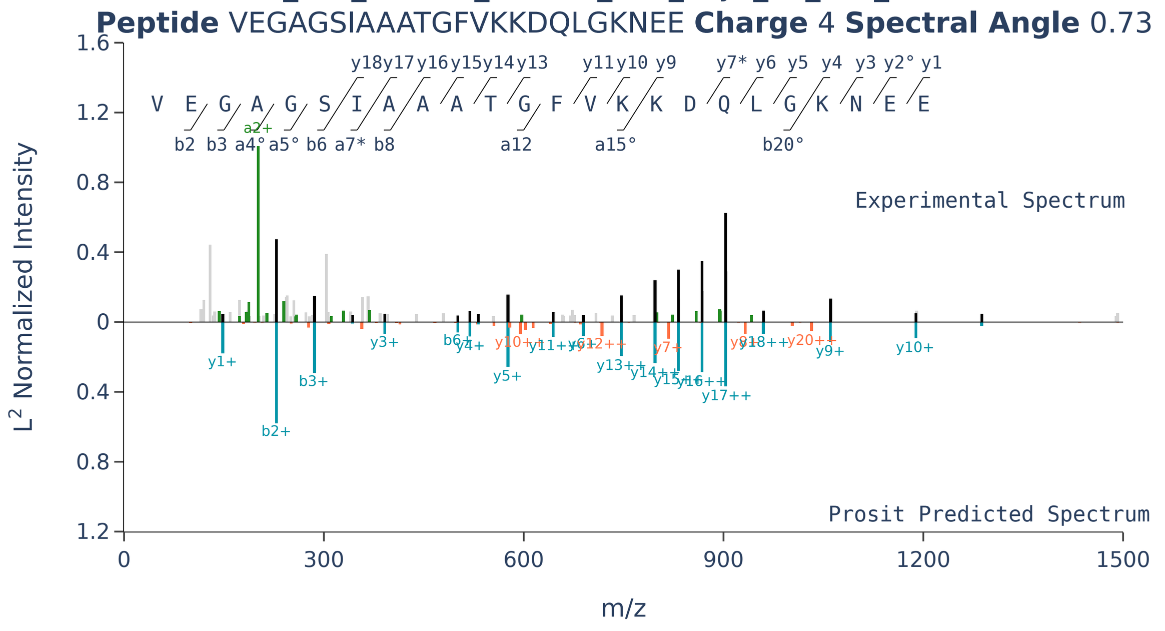


**Source** W_Soh_160821_170921_Goe_aSyn_A1_3h_R2 **Scan** 11417
**Peptide** KEQVTNVGGAVVTG **Charge** 2 **Spectral Angle** 0.76

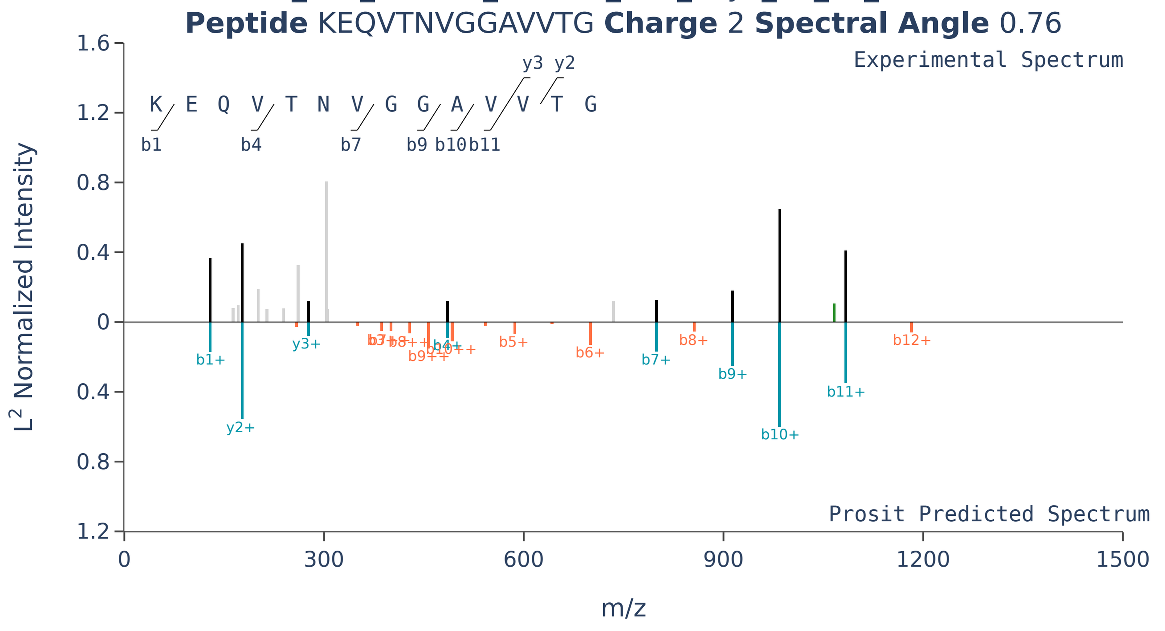


**Source** W_Soh_160821_170921_Goe_aSyn_A1_6h_R1 **Scan** 24784
**Peptide** GGAVVTGVTAVAQKTVEGAGSIA **Charge** 2 **Spectral Angle** 0.81

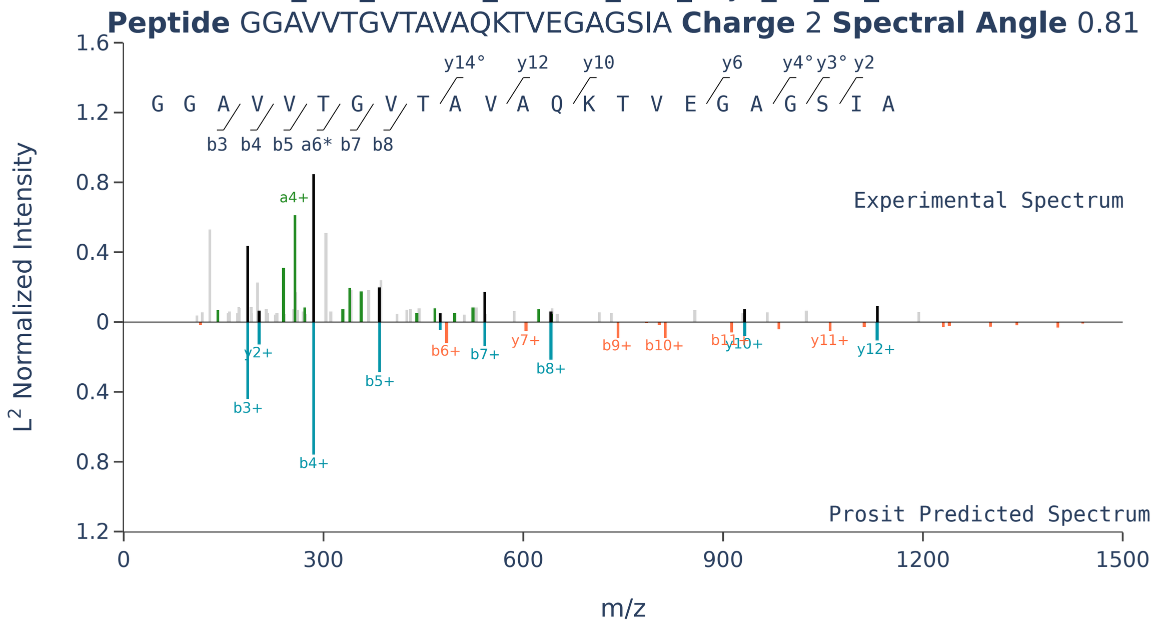


**Source** W_Soh_160821_170921_Goe_aSyn_A2_24h_R1 **Scan** 6524
**Peptide** FVKKDQLGKNEEGAPQEG **Charge** 3 **Spectral Angle** 0.78

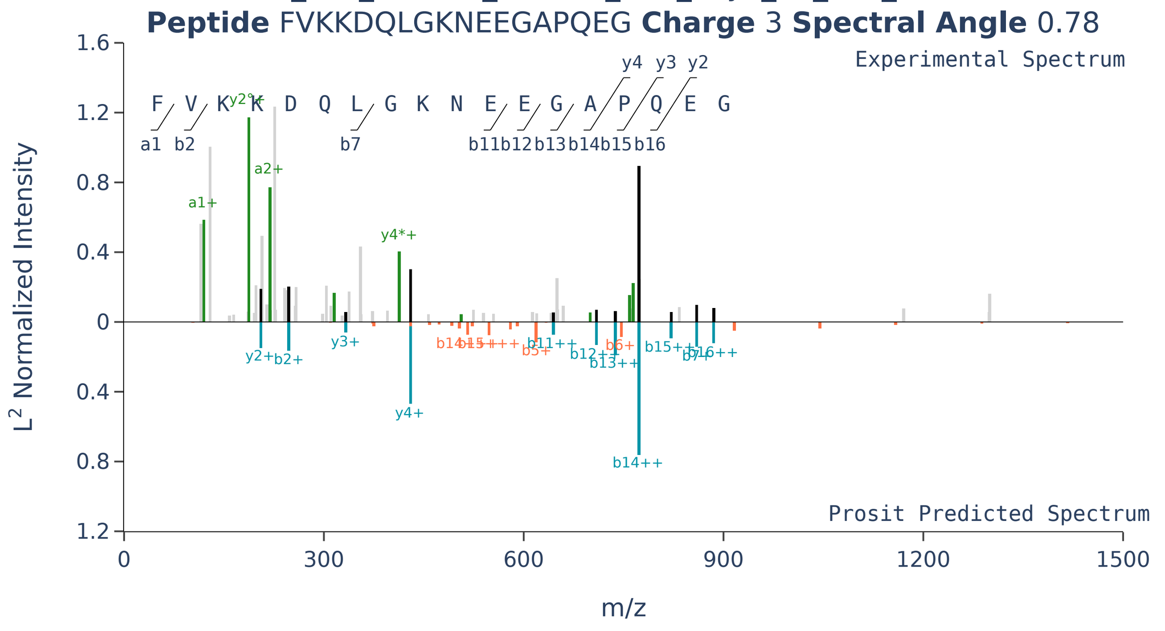


**Source** W_Soh_160821_170921_Goe_aSyn_A1_24h_R2 **Scan** 16807
**Peptide** TKEQVTNVGGAVVTGV **Charge** 2 **Spectral Angle** 0.68

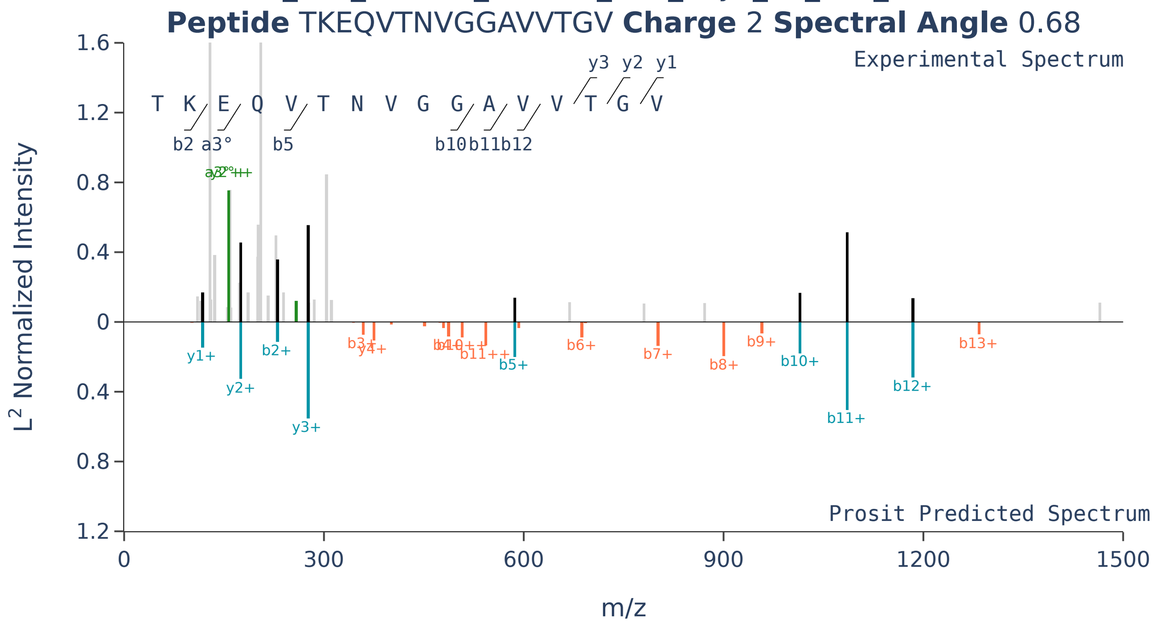


**Source** W_Soh_160821_170921_Goe_aSyn_A1_6h_R1 **Scan** 19486
**Peptide** FVKKDQLGKNEEGAPQEGILEDMPVD **Charge** 4 **Spectral Angle** 0.8

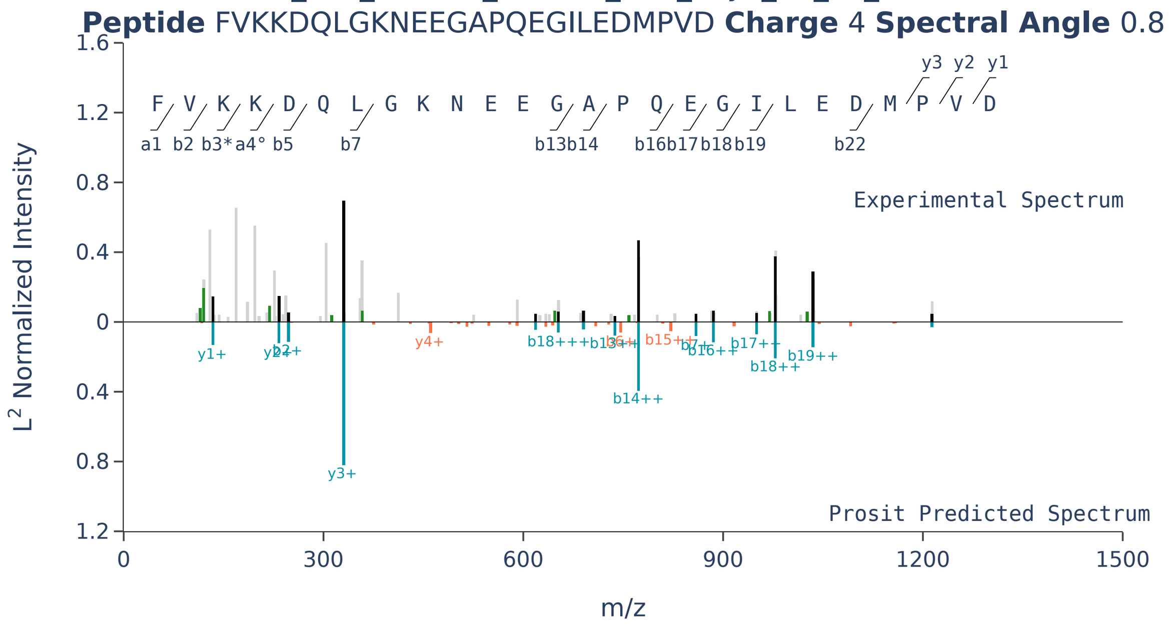

**Source** W_Soh_160821_170921_Goe_aSyn_A1_24h_R1 **Scan** 5204
**Peptide** HGVATVAEKTKEQ **Charge** 3 **Spectral Angle** 0.66

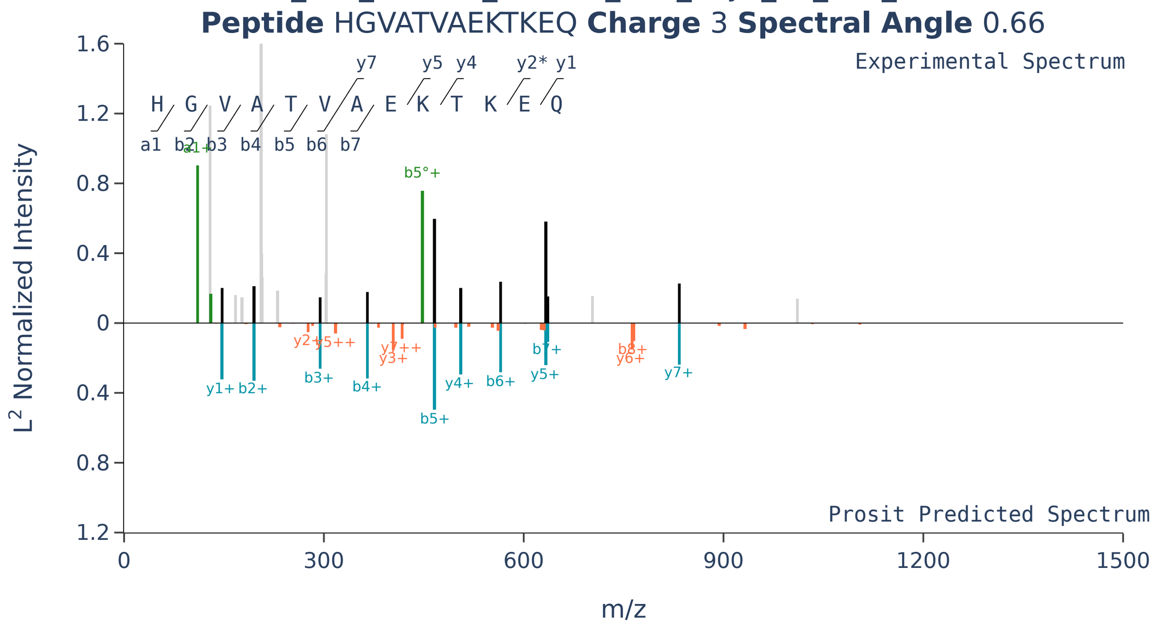


**Source** W_Soh_160821_170921_Goe_aSyn_A1_24h_R1 **Scan** 22256
**Peptide** VVHGVATVAEKTKEQVTNVGGAVVTGVTAV **Charge** 3 **Spectral Angle** 0.69

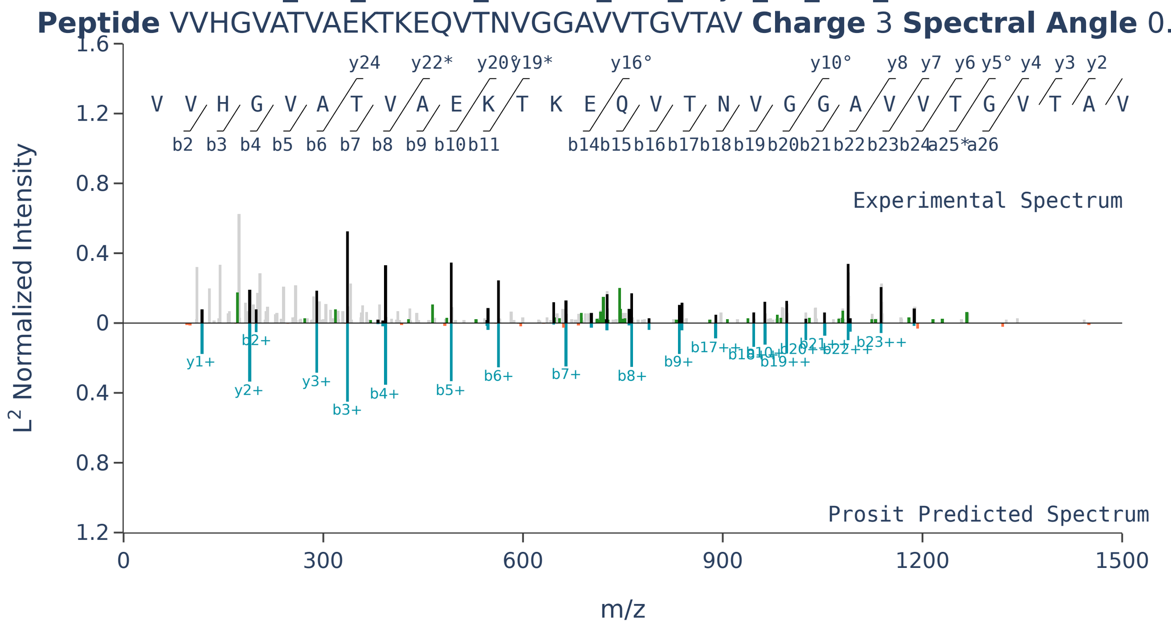


**Source** W_Soh_160821_170921_Goe_aSyn_A2_24h_R1 **Scan** 11401
**Peptide** KQGVAEAAGKTKEGVLYVGS **Charge** 3 **Spectral Angle** 0.82

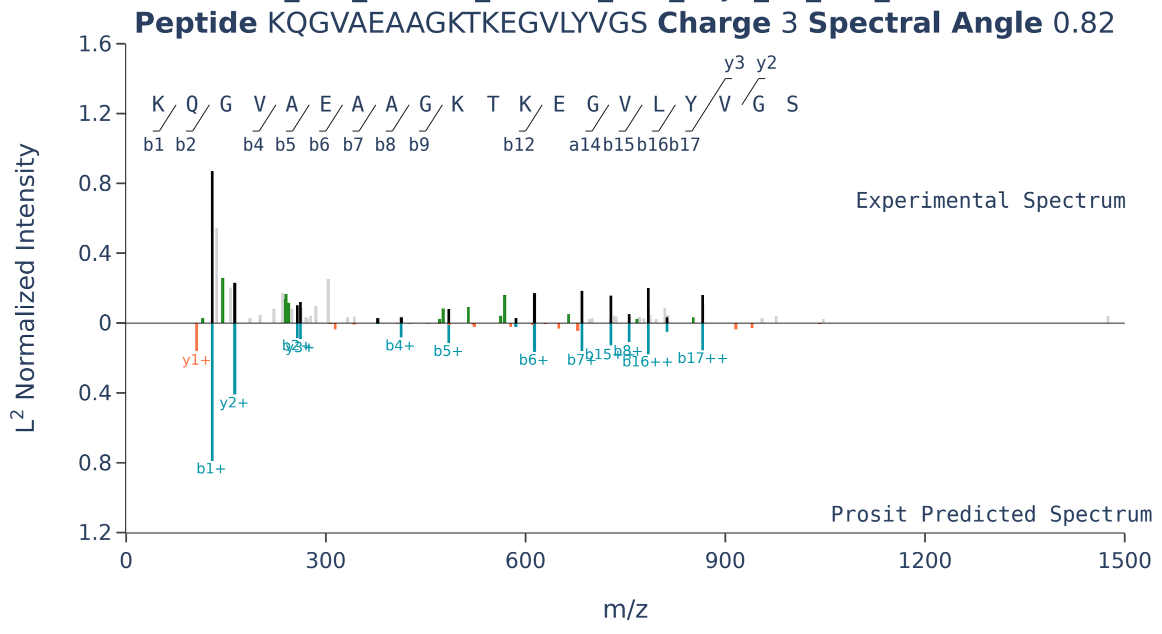


**Source** W_Soh_160821_170921_Goe_aSyn_A1_24h_R1 **Scan** 10748
**Peptide** KQGVAEAAGKTKEGVLYVGSKTKEGVVH **Charge** 5 **Spectral Angle** 0.79

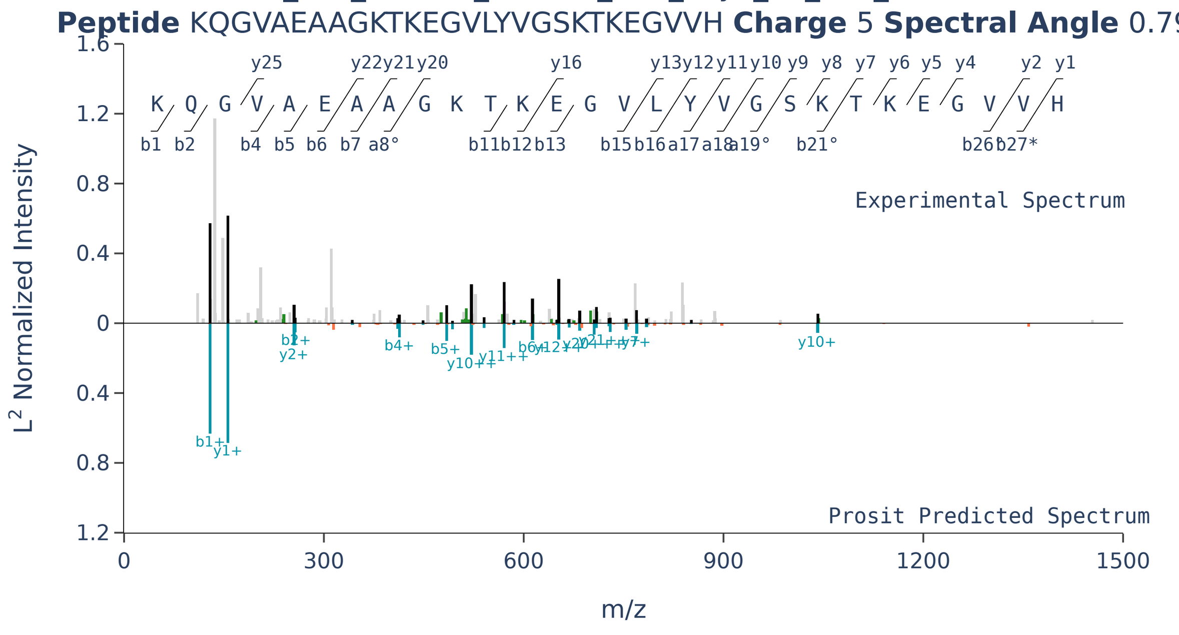


**Source** W_Soh_160821_170921_Goe_aSyn_A2_24h_R1 **Scan** 8473
**Peptide** KGLSKAKEGVVAAAEKTKQGVAE **Charge** 4 **Spectral Angle** 0.84

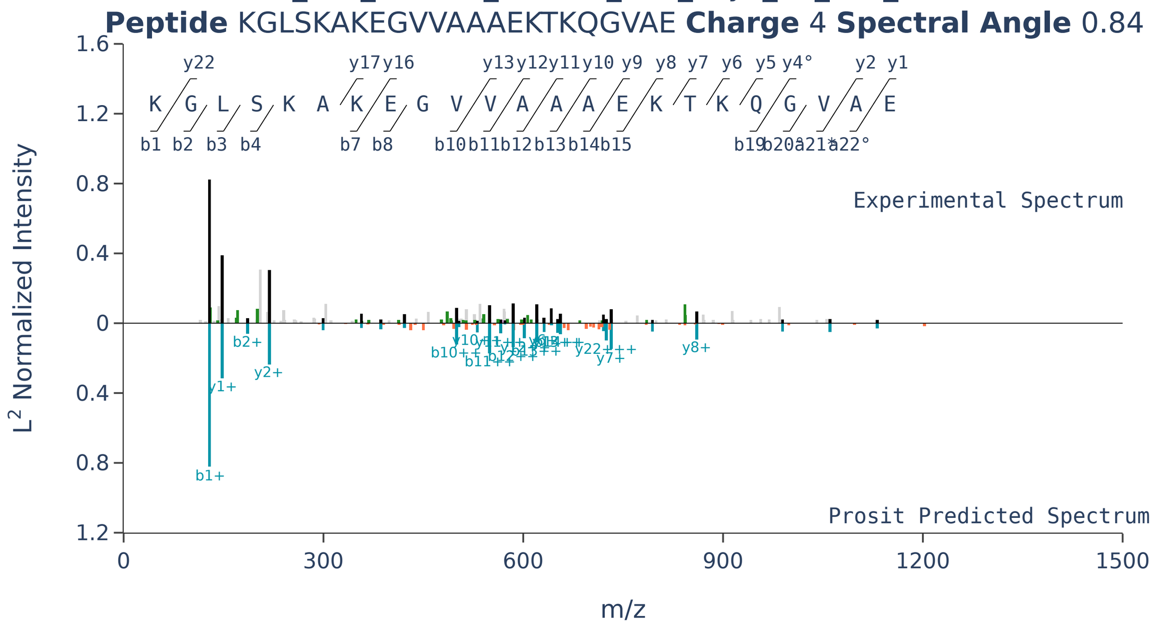


**Source** W_Soh_160821_170921_Goe_aSyn_A1_24h_R2 **Scan** 10448
**Peptide** KTKEGVVHGVATVAEKTKEQVT **Charge** 4 **Spectral Angle** 0.81

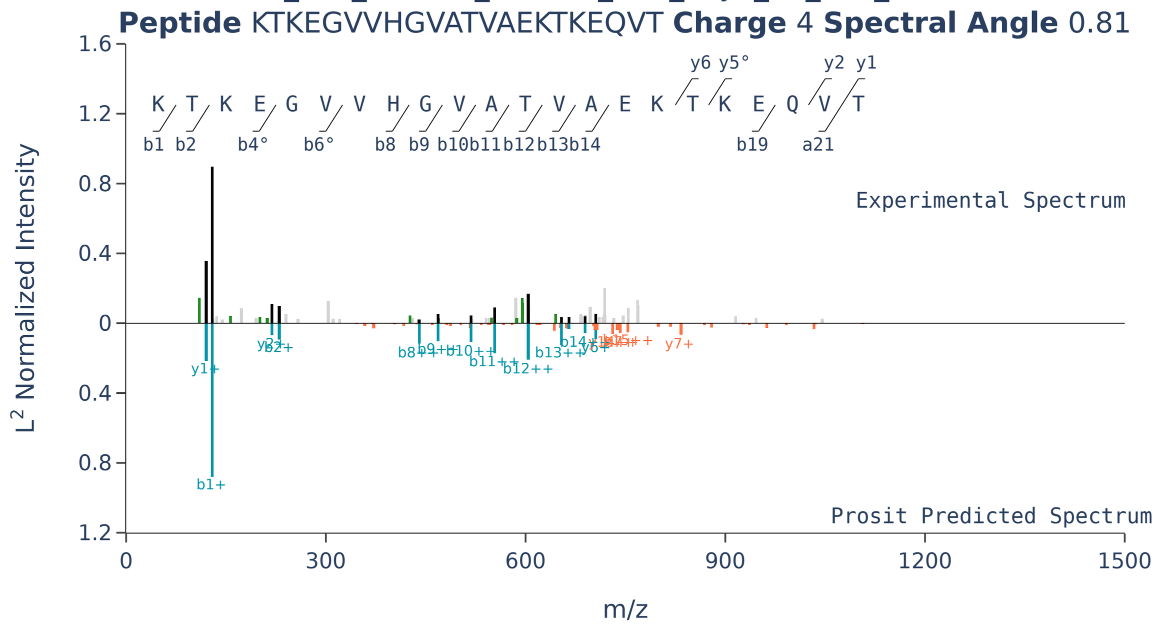


**Source** W_Soh_160821_170921_Goe_aSyn_A1_24h_R1 **Scan** 9406
**Peptide** AAATGFVKKDQLGKNEEGAPQE **Charge** 2 **Spectral Angle** 0.86

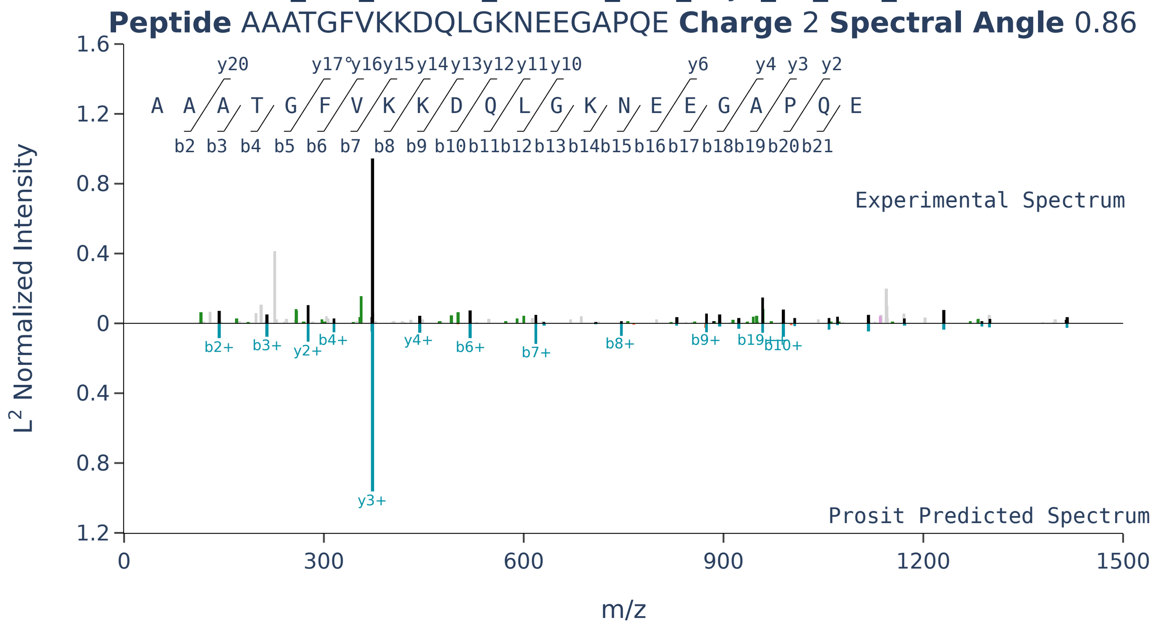


**Source** W_Soh_160821_170921_Goe_aSyn_A2_1h_R1 **Scan** 10921
**Peptide** AGKTKEGVLYVGSKTKEGVVHGVATVA **Charge** 4 **Spectral Angle** 0.66

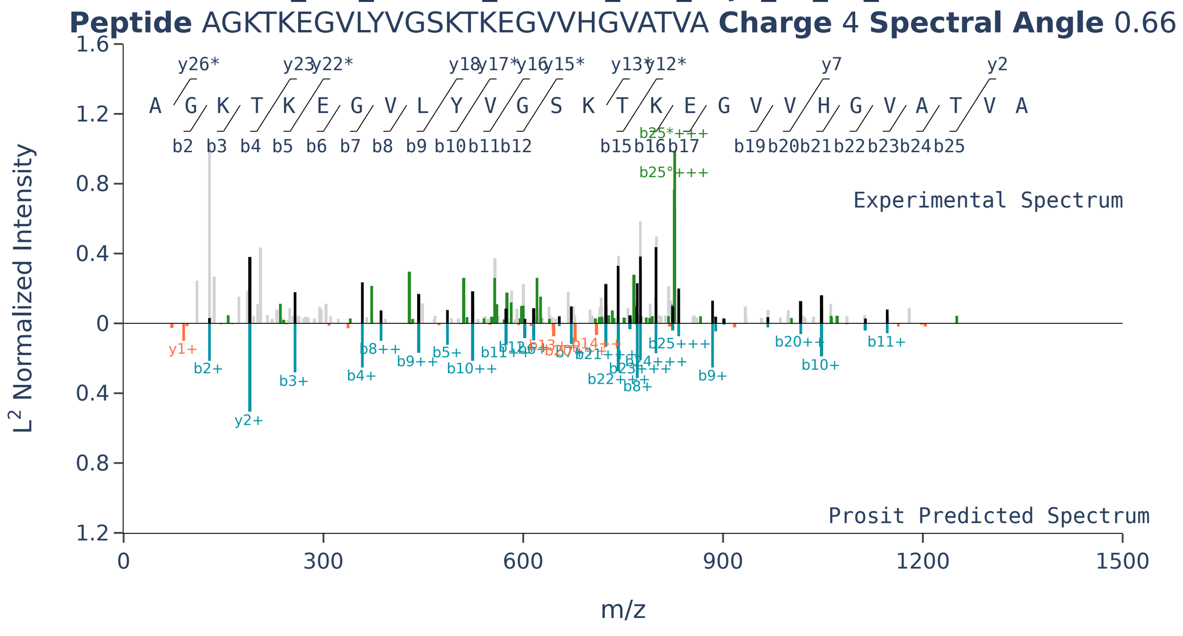


**Source** W_Soh_160821_170921_Goe_aSyn_A2_30'_R2 **Scan** 10377
**Peptide** KEQVTNVGGAVVT **Charge** 2 **Spectral Angle** 0.71

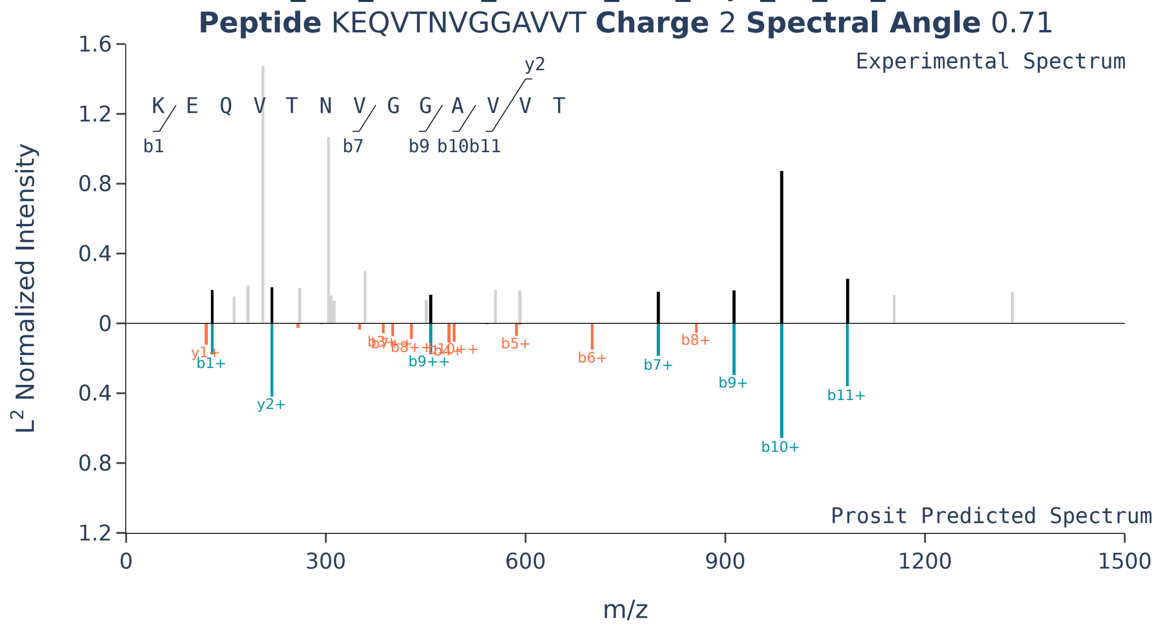


**Source** W_Soh_160821_170921_Goe_aSyn_A1_24h_R2 **Scan** 12222
**Peptide** AGKTKEGVLYVGSKTKEGVVHGVATV **Charge** 2 **Spectral Angle** 0.7

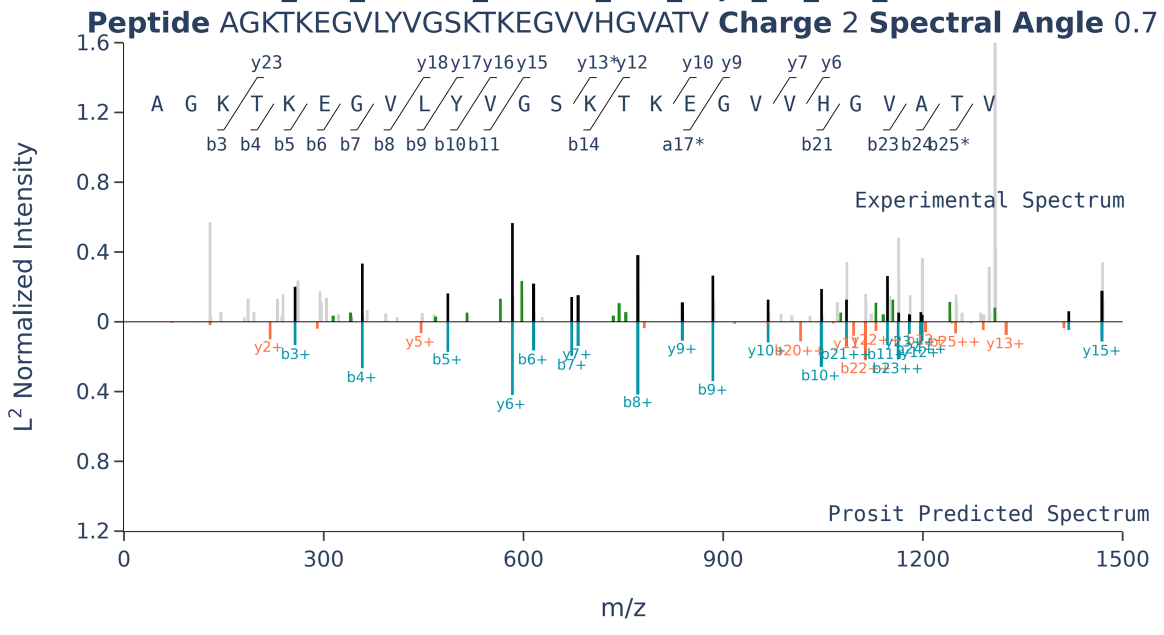


**Source** W_Soh_160821_170921_Goe_aSyn_A1_30'_R2 **Scan** 7767
**Peptide** GKNEEGAPQEGL **Charge** 2 **Spectral Angle** 0.83

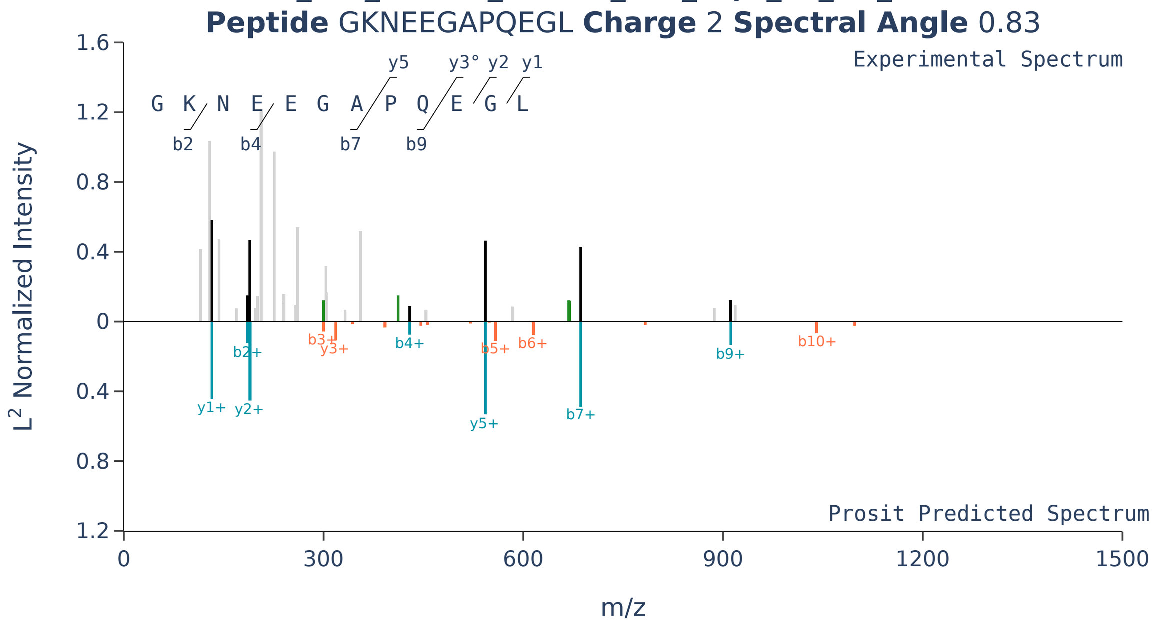


**Source** W_Soh_160821_170921_Goe_aSyn_A1_24h_R2 **Scan** 8954
**Peptide** AEAAGKTKEGVLYVGSKTKEGVVH **Charge** 3 **Spectral Angle** 0.9

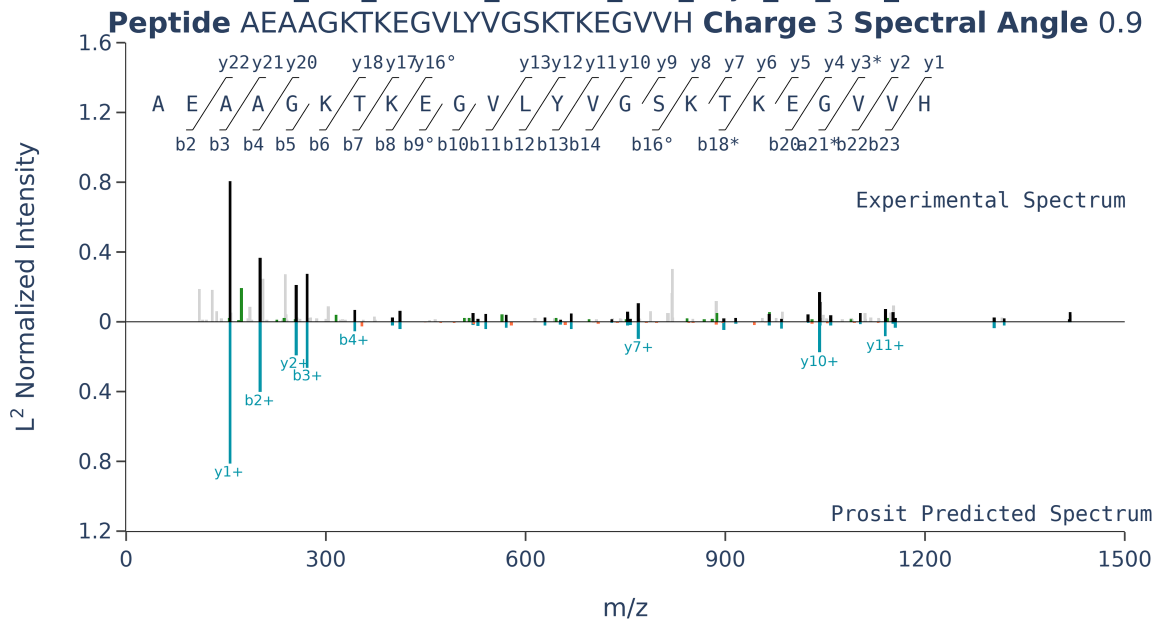


**Source** W_Soh_160821_170921_Goe_aSyn_A2_24h_R1 **Scan** 10285
**Peptide** MKGLSKAKEGVVAAAEKTKQGV **Charge** 4 **Spectral Angle** 0.74

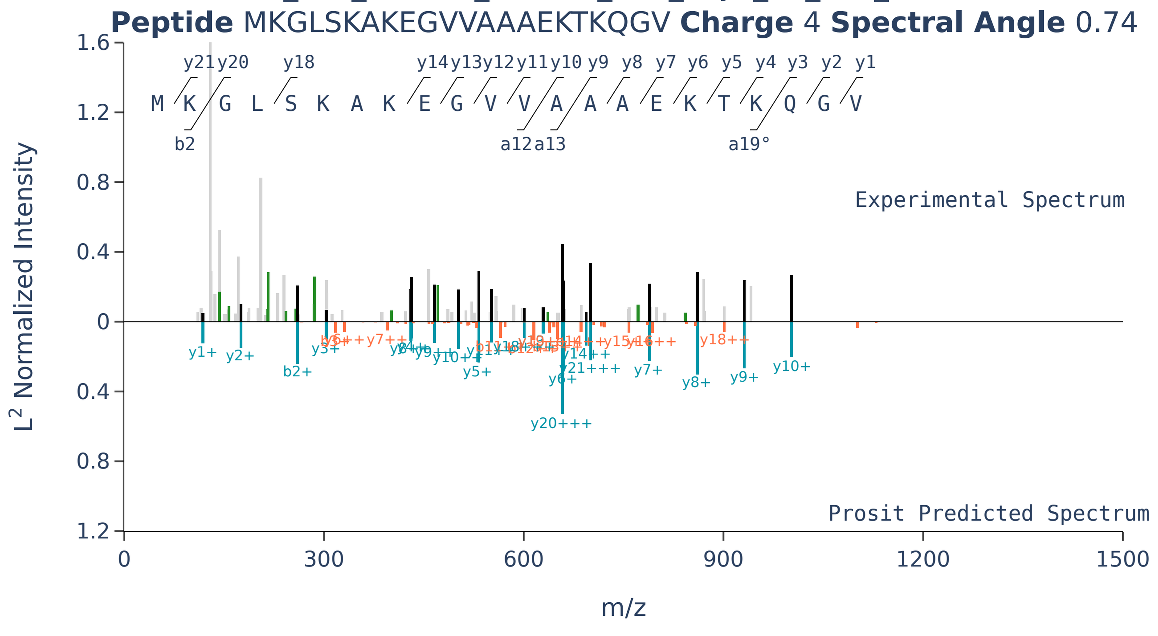


**Source** W_Soh_160821_170921_Goe_aSyn_A1_24h_R1 **Scan** 9489
**Peptide** YVGSKTKEGVVHGVATV **Charge** 3 **Spectral Angle** 0.83

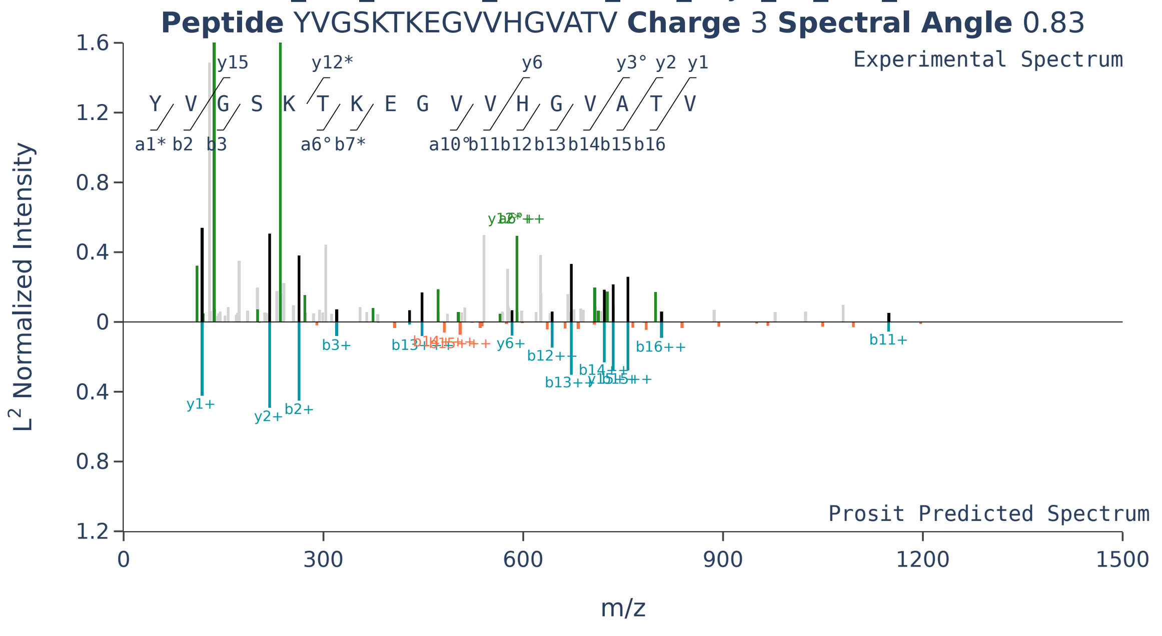


**Source** W_Soh_160821_170921_Goe_aSyn_A1_24h_R1 **Scan** 9993
**Peptide** VAEAAGKTKEGVLYVGSKT **Charge** 4 **Spectral Angle** 0.87

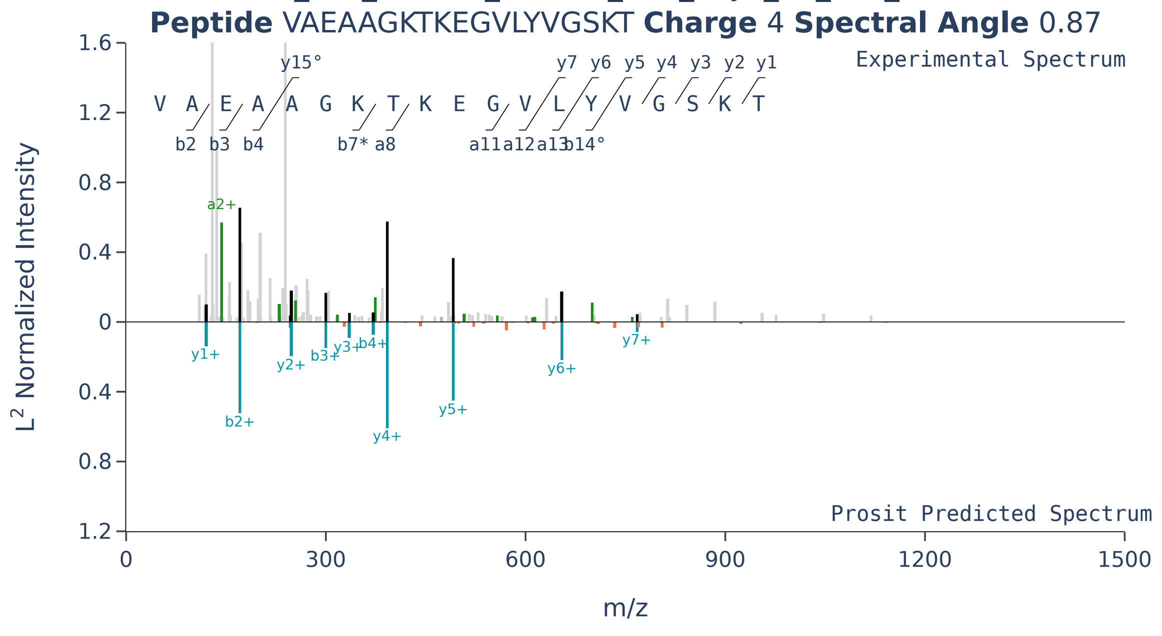

**Source** W_Soh_160821_170921_Goe_aSyn_A2_24h_R1 **Scan** 18610
**Peptide** MDVFMKGLSKAKEGVVAAAEKTK **Charge** 4 **Spectral Angle** 0.8

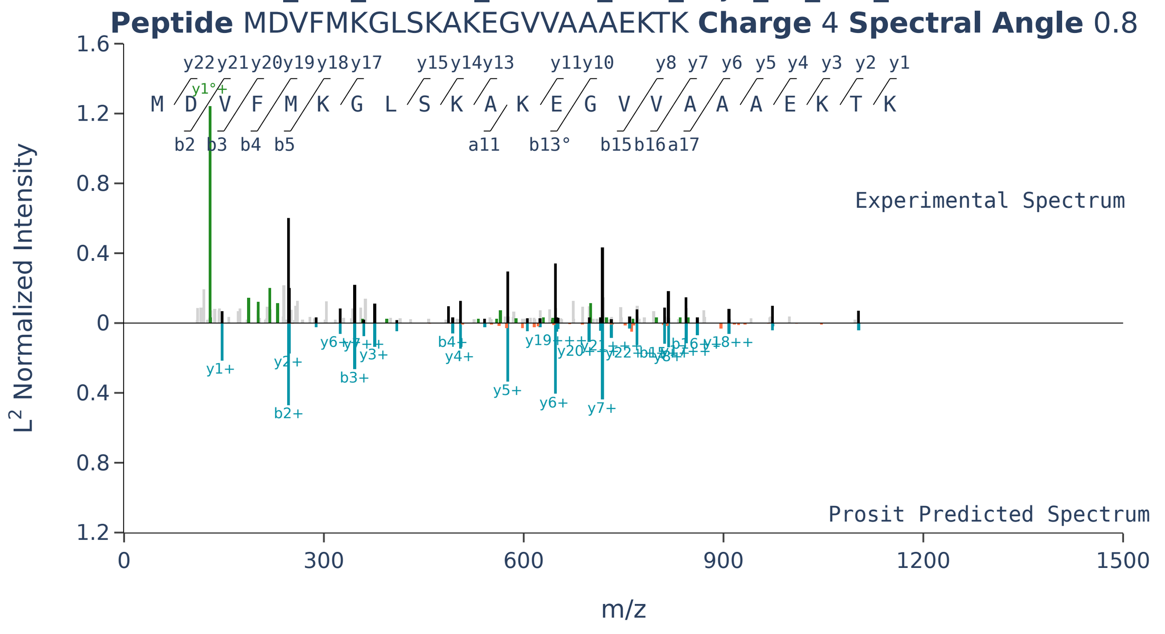


**Source** W_Soh_160821_170921_Goe_aSyn_A1_24h_R2 **Scan** 9445
**Peptide** KDQLGKNEEGAPQEGI **Charge** 2 **Spectral Angle** 0.78

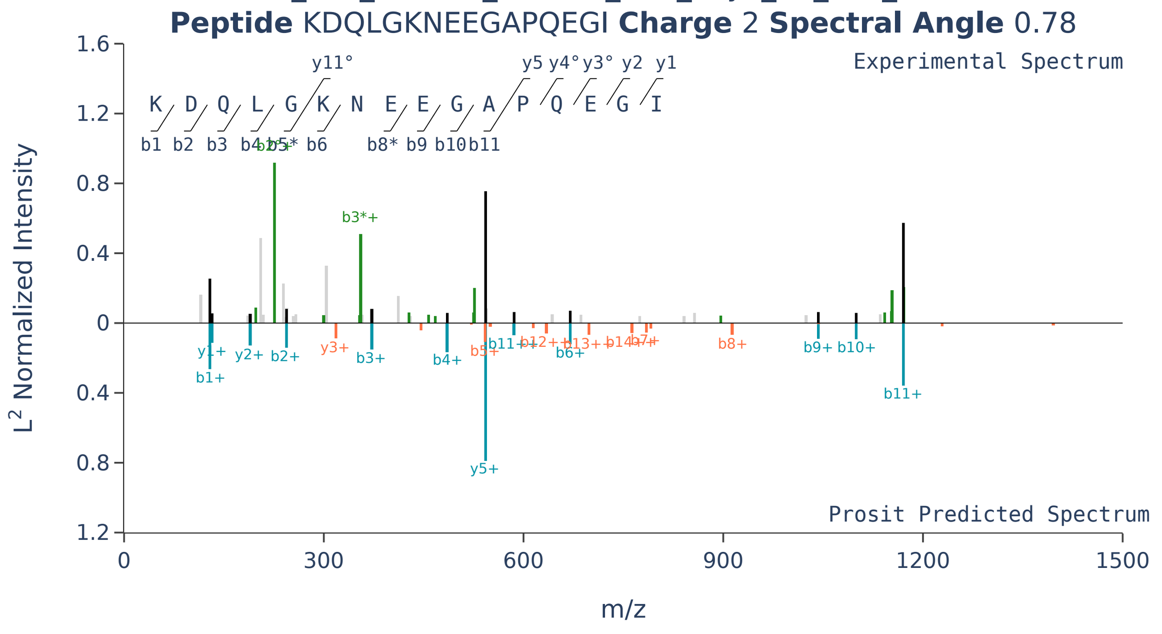


**Source** W_Soh_160821_170921_Goe_aSyn_A1_24h_R1 **Scan** 27665
**Peptide** VGGAVVTGVTAVAQKTVEGAGSIAAA **Charge** 3 **Spectral Angle** 0.84

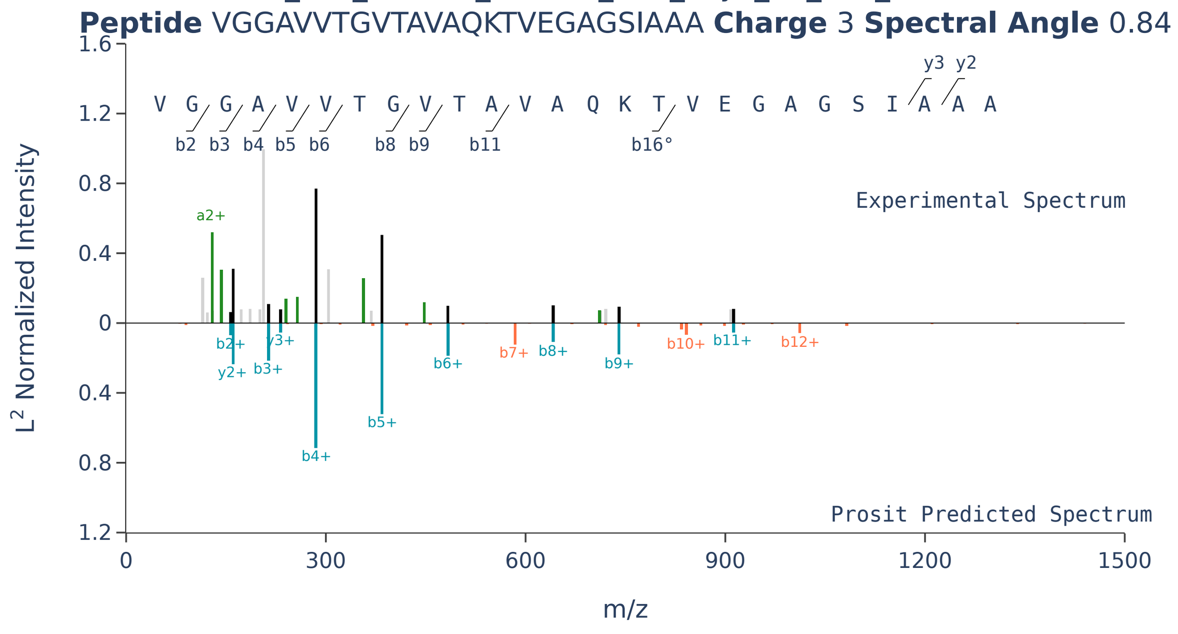


**Source** W_Soh_160821_170921_Goe_aSyn_A1_24h_R2 **Scan** 8929
**Peptide** KAKEGVVAAAEKTKQGVAEAAGKTKEG **Charge** 4 **Spectral Angle** 0.85

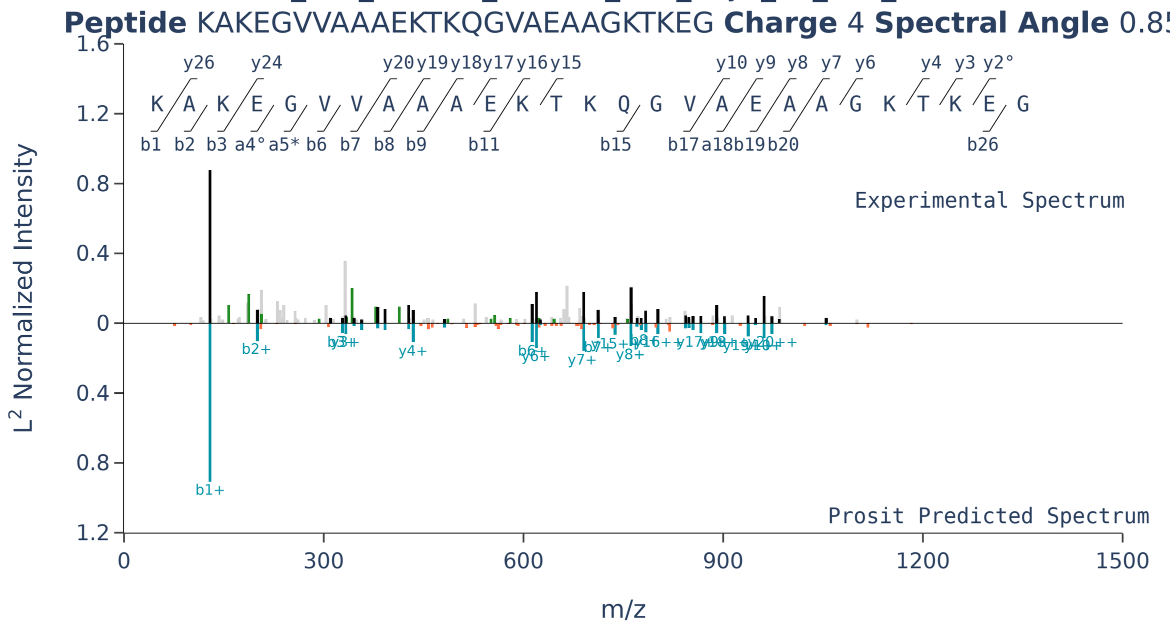


**Source** W_Soh_160821_170921_Goe_aSyn_A1_6h_R1 **Scan** 5575
**Peptide** ATGFVKK **Charge** 2 **Spectral Angle** 0.72

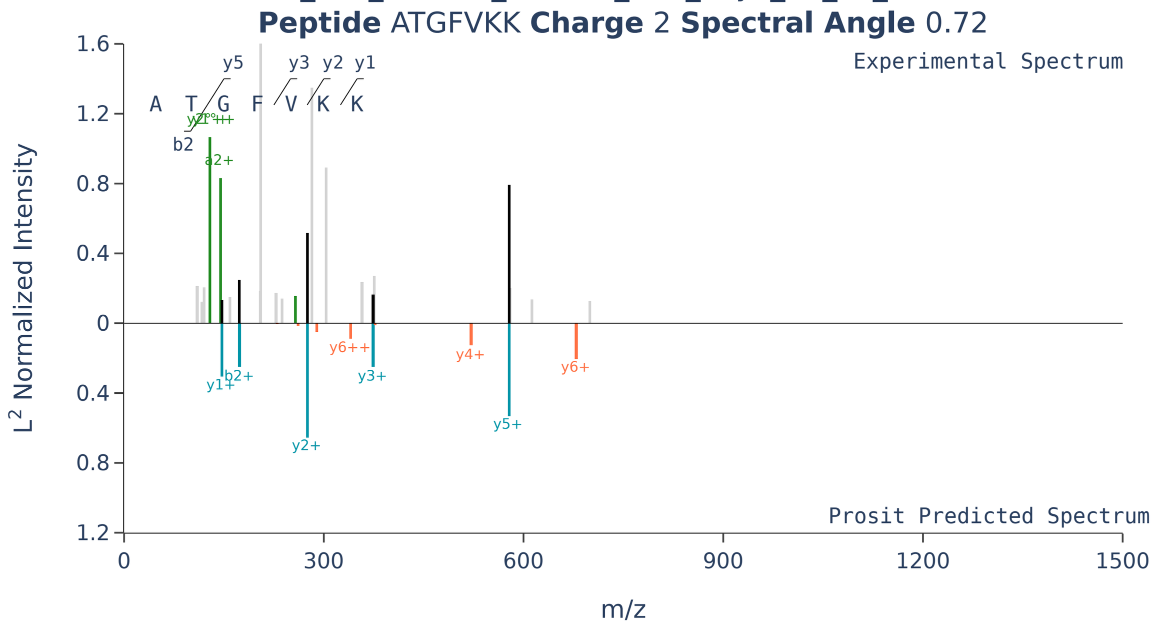


**Source** W_Soh_160821_170921_Goe_aSyn_A2_6h_R1 **Scan** 12141
**Peptide** GVLYVGSKTKEGVVHGVAT **Charge** 2 **Spectral Angle** 0.82

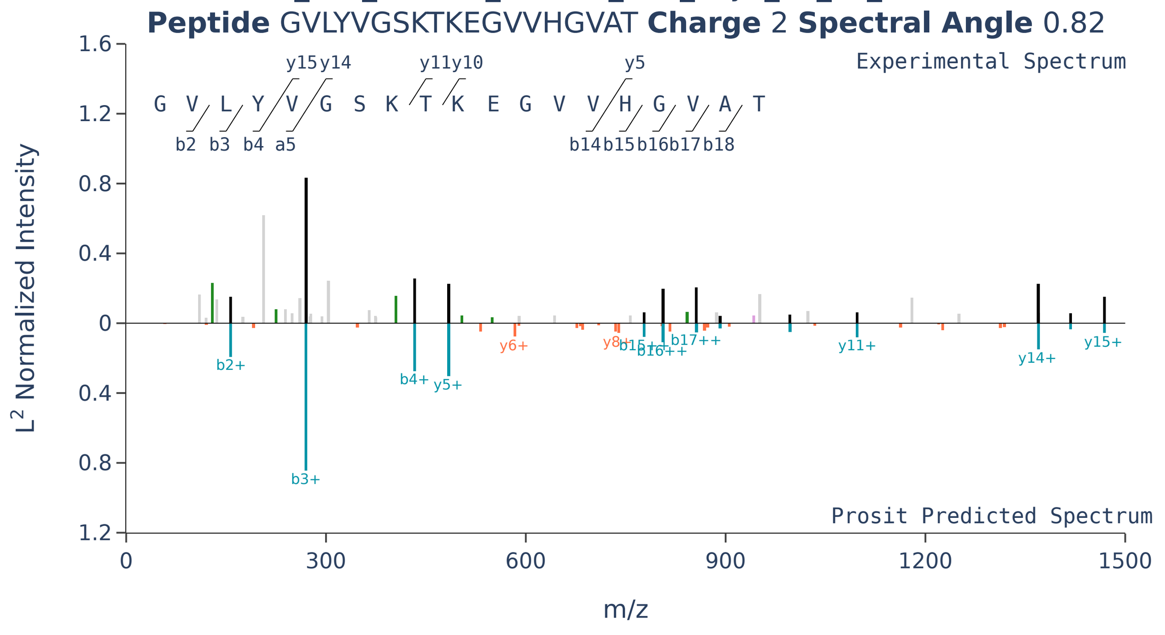


**Source** W_Soh_160821_170921_Goe_aSyn_A1_24h_R1 **Scan** 23116
**Peptide** TGVTAVAQKTVEGAGSIAAATGFVKKD **Charge** 4 **Spectral Angle** 0.71

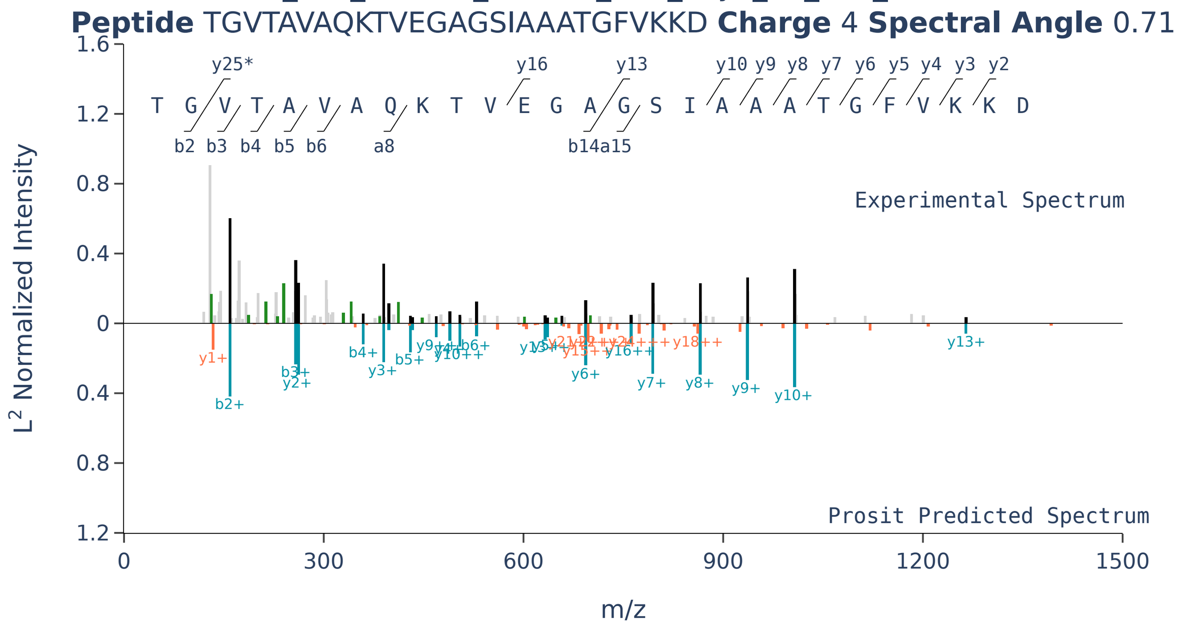


**Source** W_Soh_160821_170921_Goe_aSyn_A1_24h_R1 **Scan** 5421
**Peptide** TGFVKKDQ **Charge** 2 **Spectral Angle** 0.8

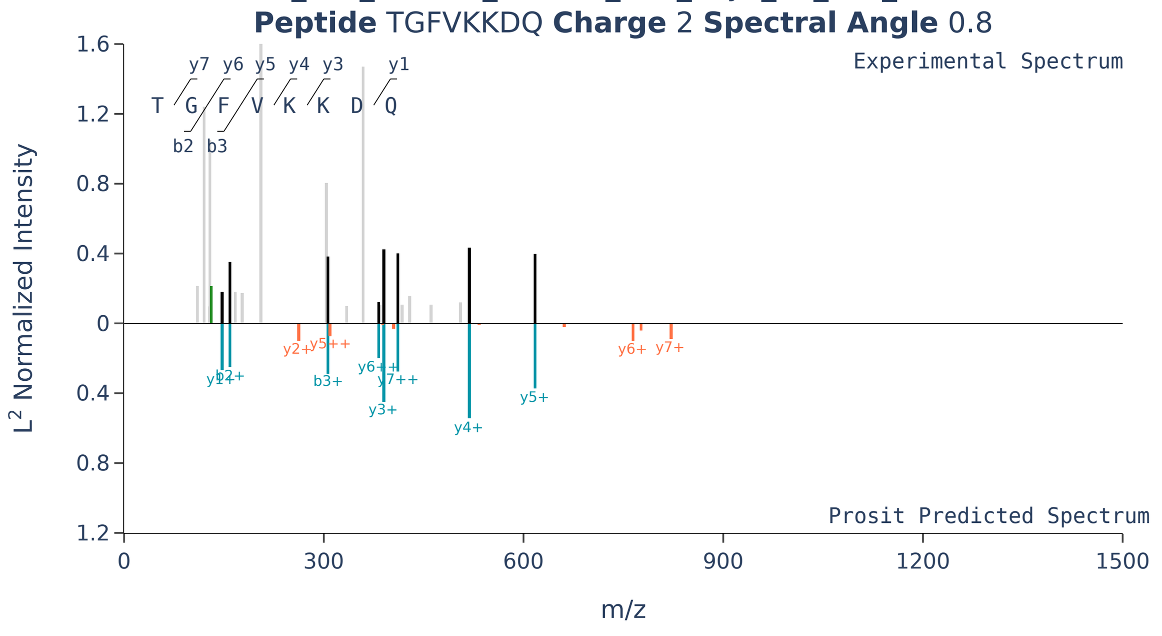


**Source** W_Soh_160821_170921_Goe_aSyn_A1_24h_R1 **Scan** 10052
**Peptide** KEGVVAAAEKTKQGVAEAAGKT **Charge** 3 **Spectral Angle** 0.9

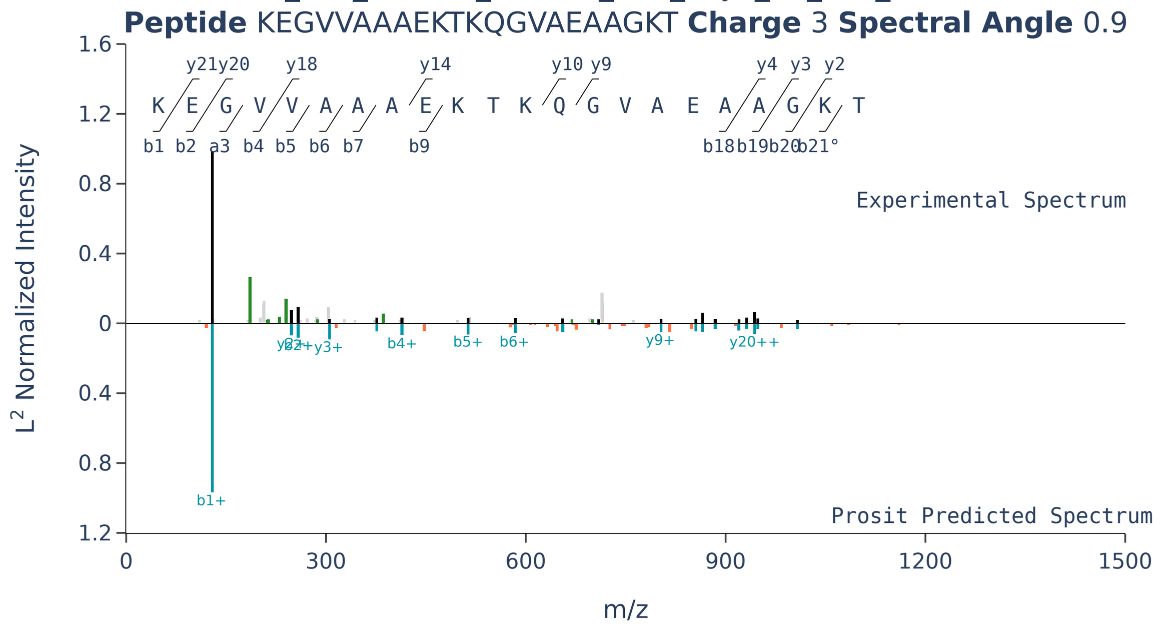


**Source** W_Soh_160821_170921_Goe_aSyn_A1_24h_R1 **Scan** 15619
**Peptide** QGVAEAAGKTKEGVLYV **Charge** 2 **Spectral Angle** 0.87

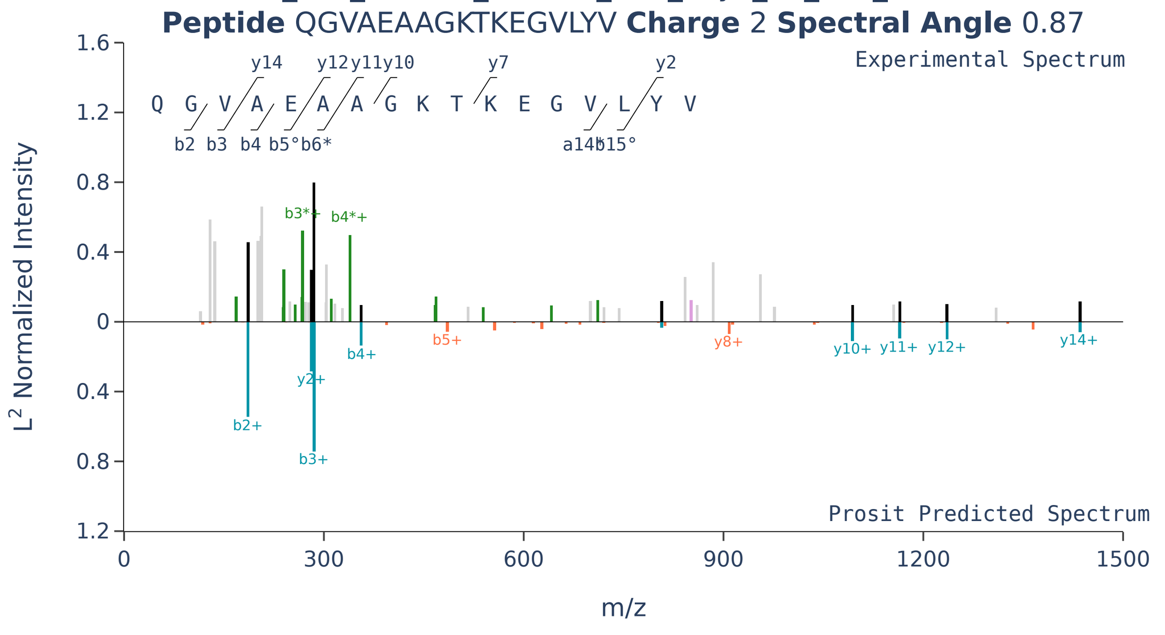


**Source** W_Soh_160821_170921_Goe_aSyn_A2_24h_R2 **Scan** 8864
**Peptide** EGVVHGVAT **Charge** 2 **Spectral Angle** 0.85

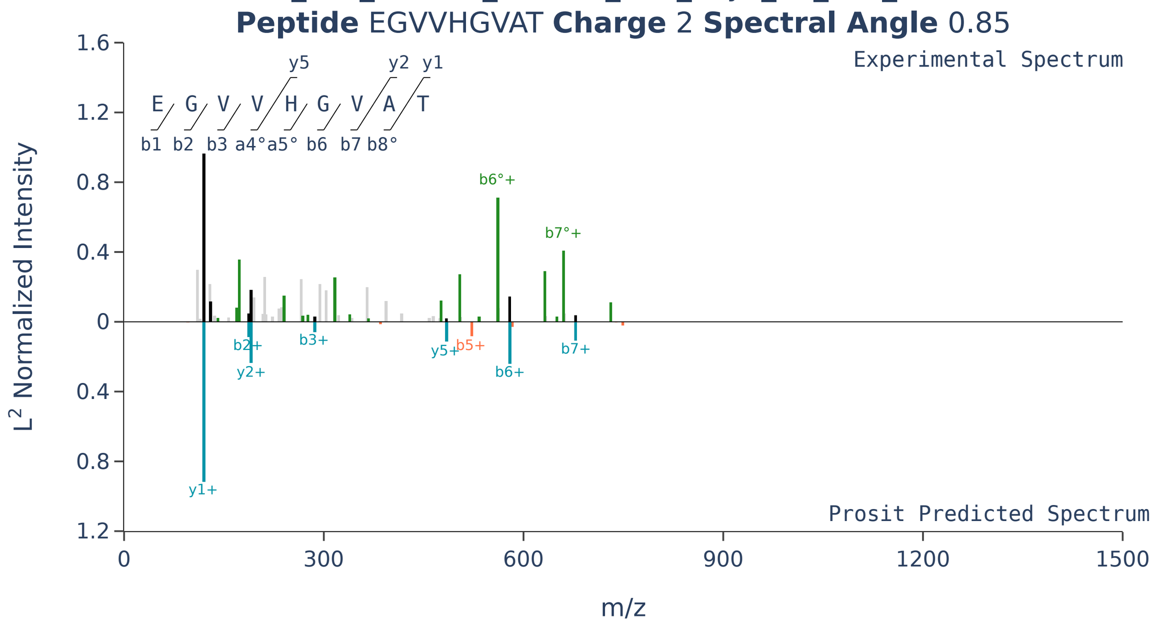


**Source** W_Soh_160821_170921_Goe_aSyn_A2_24h_R1 **Scan** 7196
**Peptide** GLSKAKEGVVAAAEKTK **Charge** 3 **Spectral Angle** 0.79

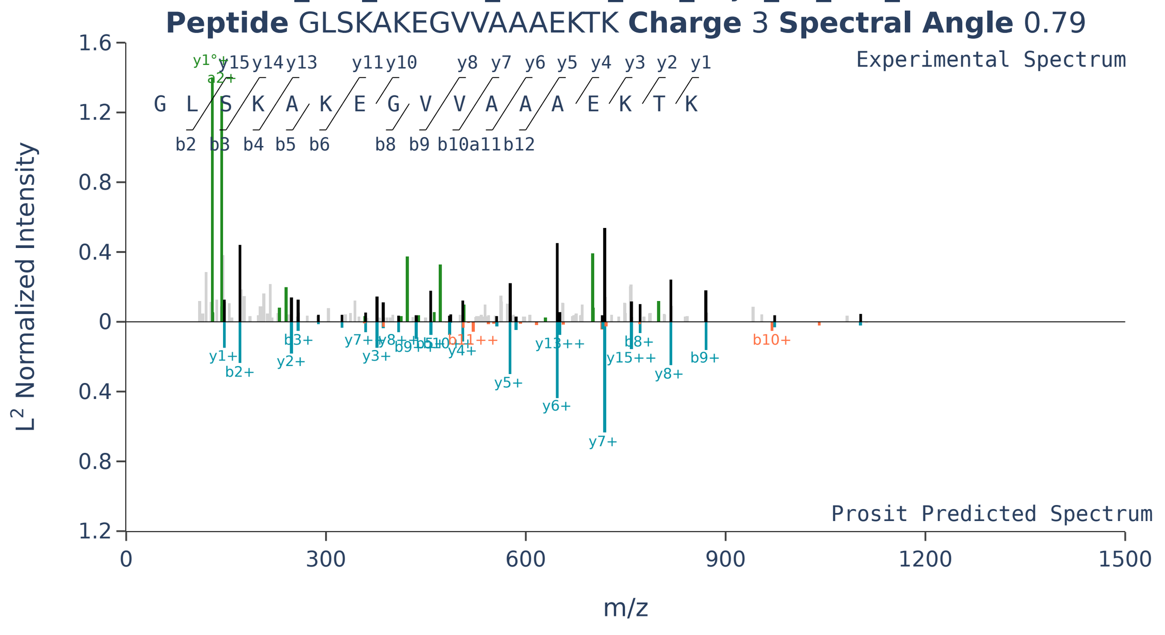


**Source** W_Soh_160821_170921_Goe_aSyn_A1_24h_R1 **Scan** 17032
**Peptide** GVVAAAEKTKQGVAEAAGKTKEGVLYV **Charge** 3 **Spectral Angle** 0.86

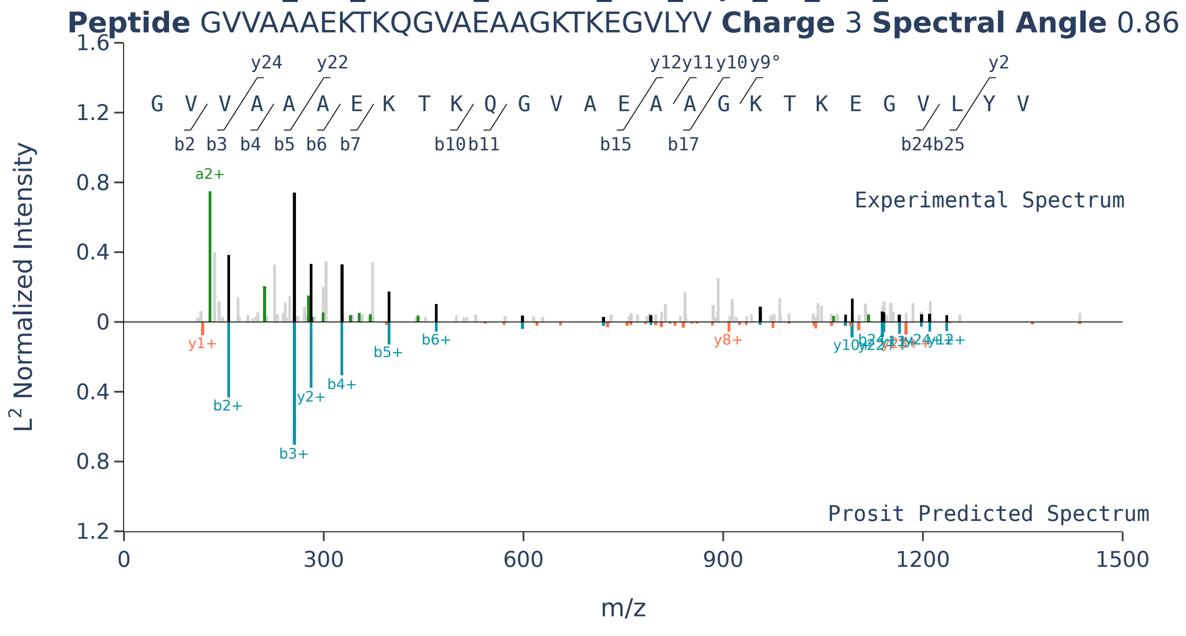


**Source** W_Soh_160821_170921_Goe_aSyn_A2_24h_R1 **Scan** 21741
**Peptide** QKTVEGAGSIAAATGFV **Charge** 2 **Spectral Angle** 0.76

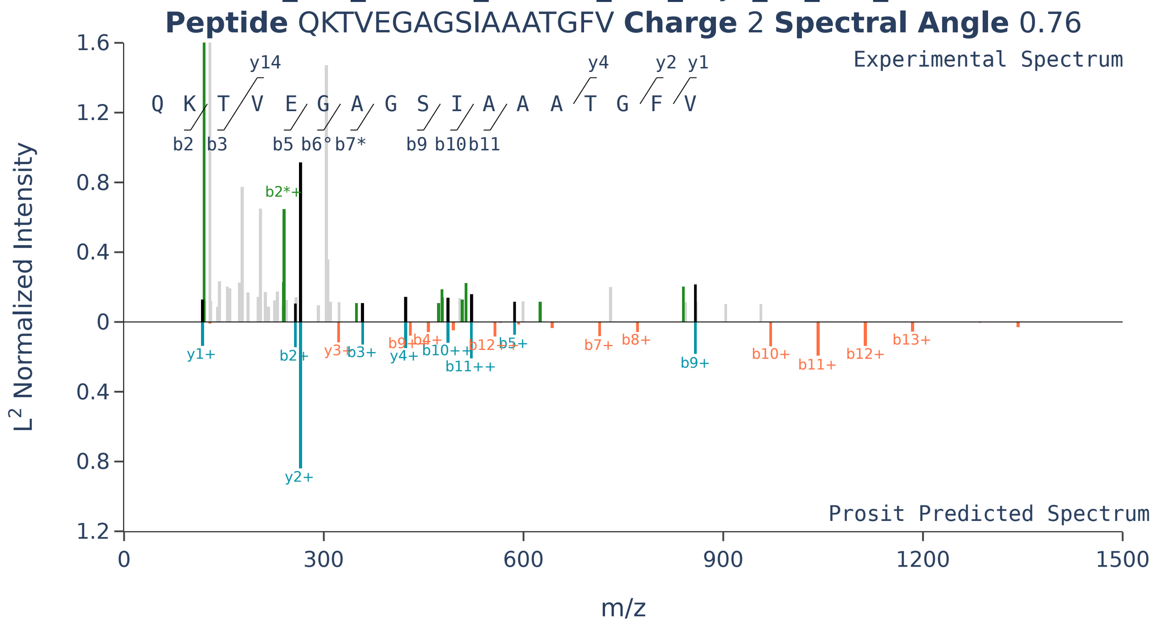


**Source** W_Soh_160821_170921_Goe_aSyn_A1_24h_R2 **Scan** 18319
**Peptide** TGVTAVAQKTVEGAGSIA **Charge** 2 **Spectral Angle** 0.72

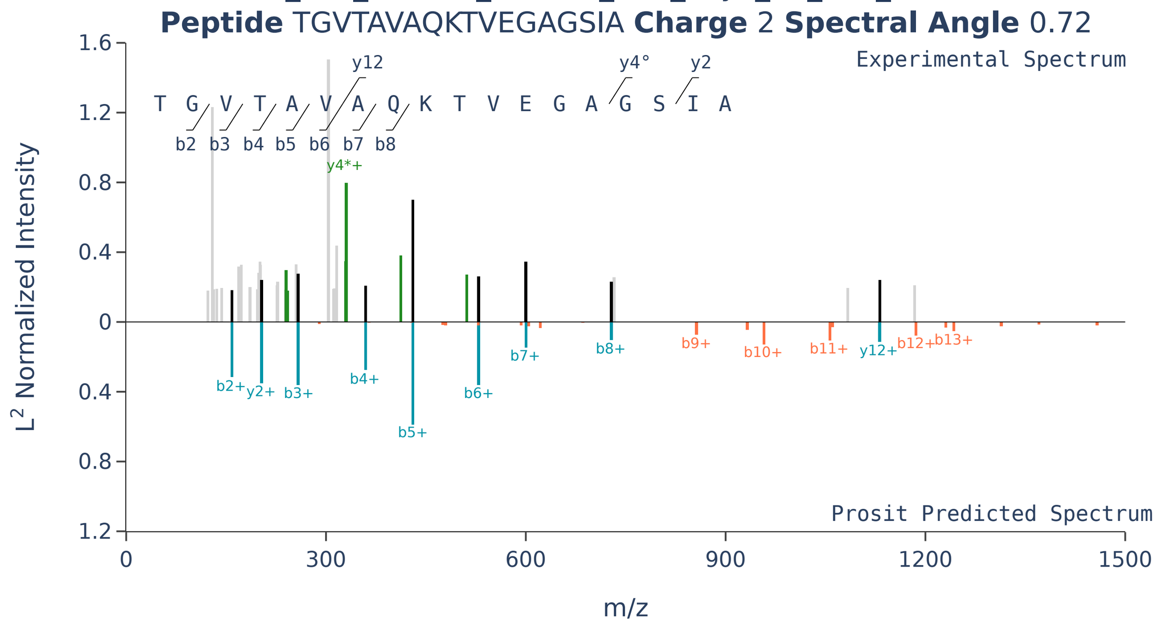

**Source** W_Soh_160821_170921_Goe_aSyn_A2_24h_R2 **Scan** 20711
**Peptide** MDVFMKGLSKAKEGVVAAAEKTKQGVA **Charge** 3 **Spectral Angle** 0.78

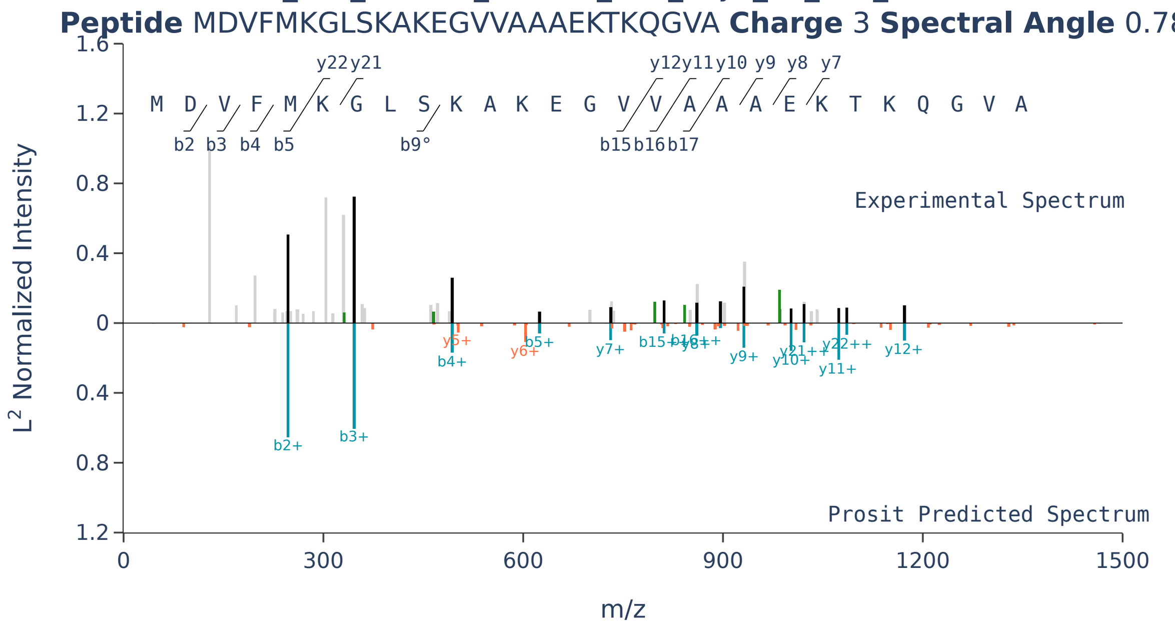


**Source** W_Soh_160821_170921_Goe_aSyn_A1_3h_R2 **Scan** 4965
**Peptide** AAEKTKQGVAEAAGKTKE **Charge** 2 **Spectral Angle** 0.66

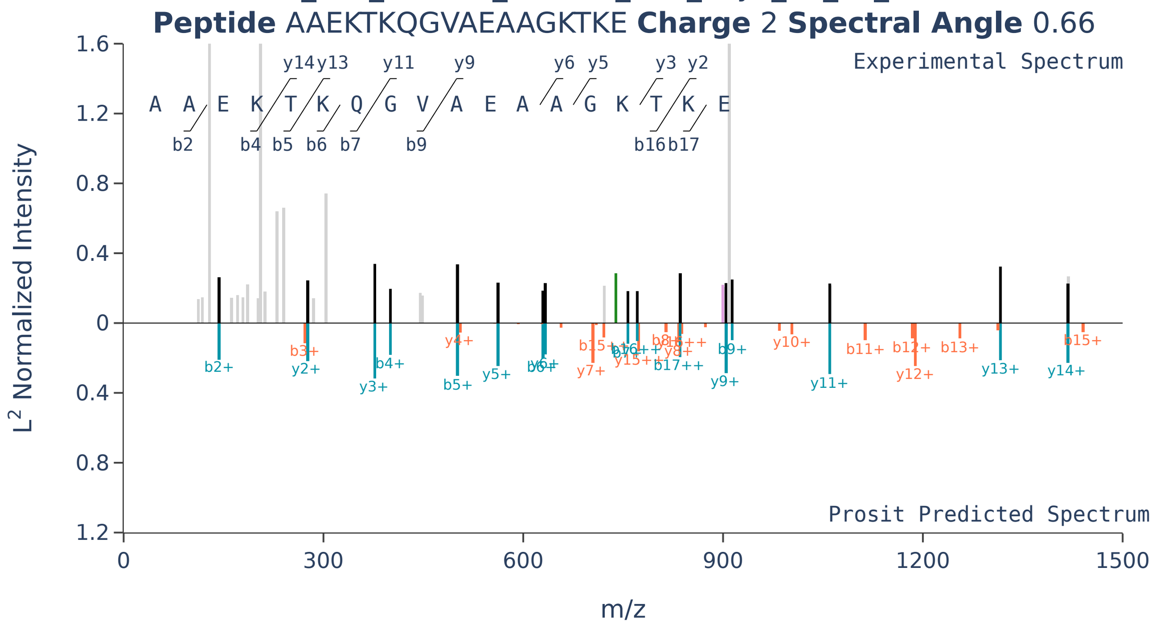


**Source** W_Soh_160821_170921_Goe_aSyn_A2_24h_R2 **Scan** 13196
**Peptide** SIAAATGFVKKDQ **Charge** 2 **Spectral Angle** 0.74

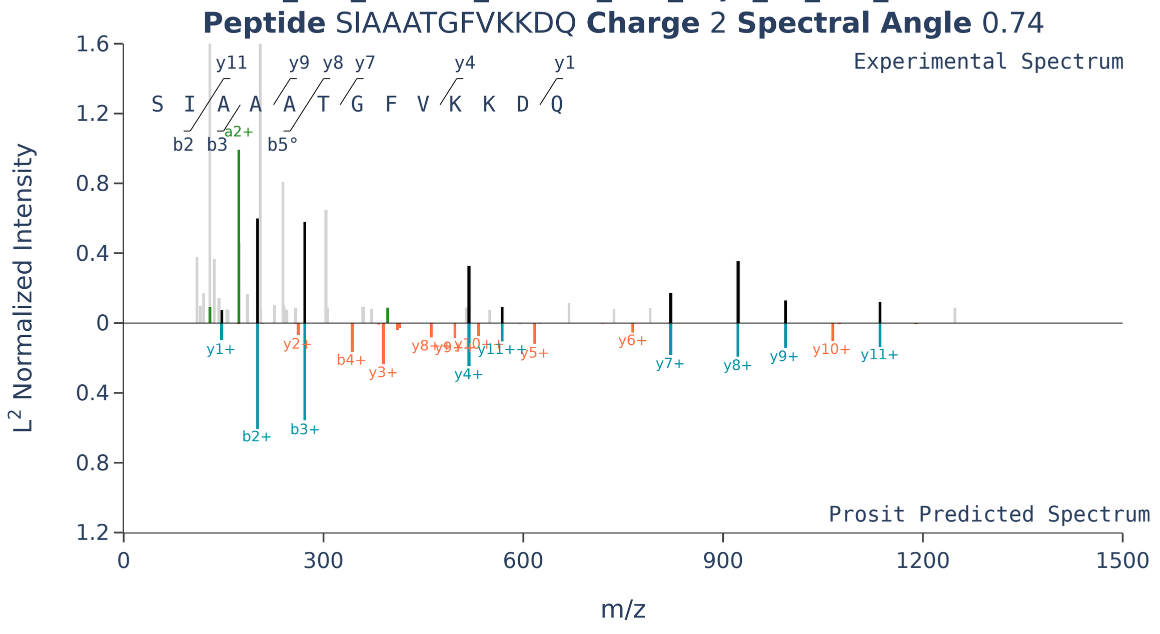


**Source** W_Soh_160821_170921_Goe_aSyn_A1_24h_R2 **Scan** 6906
**Peptide** AAGKTKEGVLYVGSKTKEG **Charge** 3 **Spectral Angle** 0.77

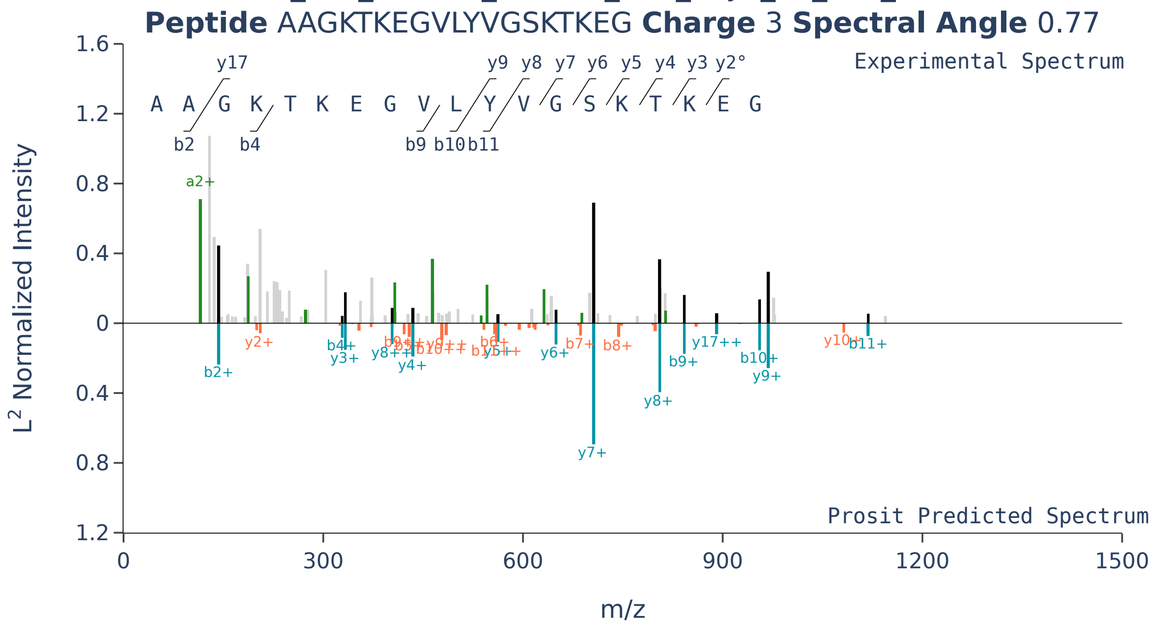


**Source** W_Soh_160821_170921_Goe_aSyn_A2_24h_R2 **Scan** 9992
**Peptide** AGKTKEGVLYVGSKTKEGVV **Charge** 3 **Spectral Angle** 0.76

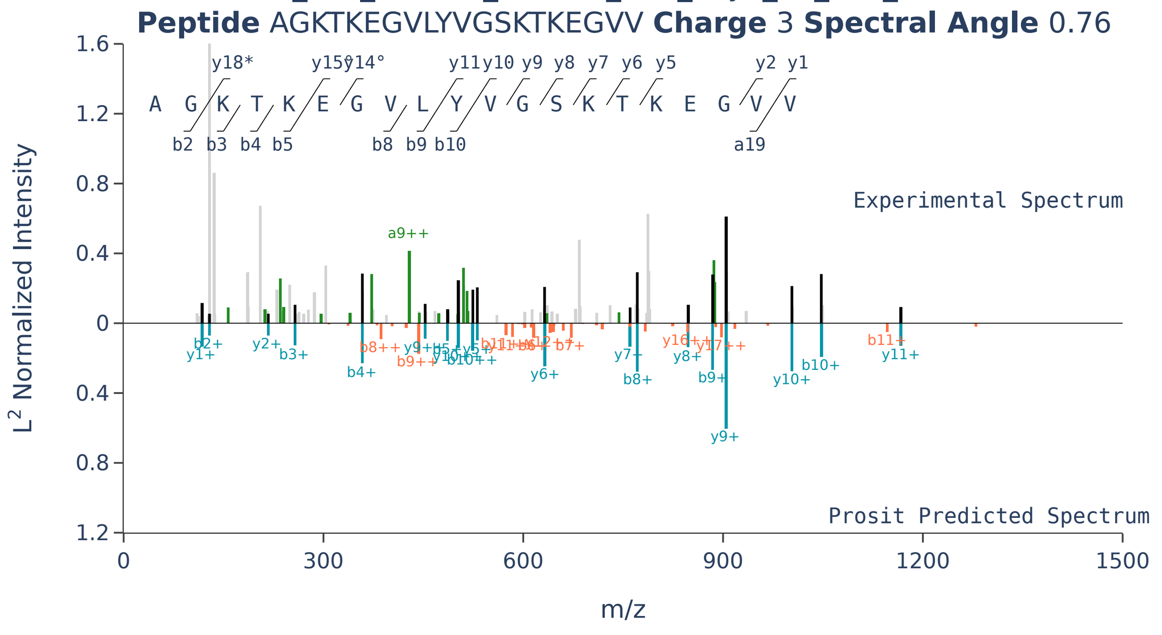


**Source** W_Soh_160821_170921_Goe_aSyn_A2_24h_R2 **Scan** 9991
**Peptide** GLSKAKEGVVAAAEKTKQGVAE **Charge** 3 **Spectral Angle** 0.71

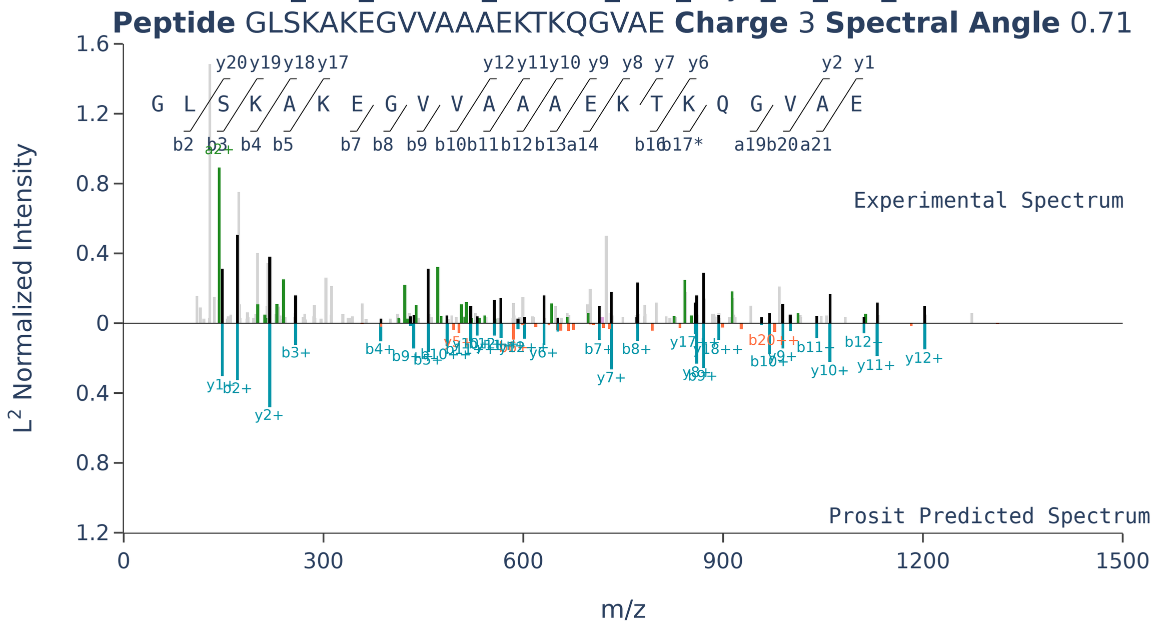


**Source** W_Soh_160821_170921_Goe_aSyn_A1_24h_R1 **Scan** 4998
**Peptide** VGSKTKEGVVHG **Charge** 3 **Spectral Angle** 0.85

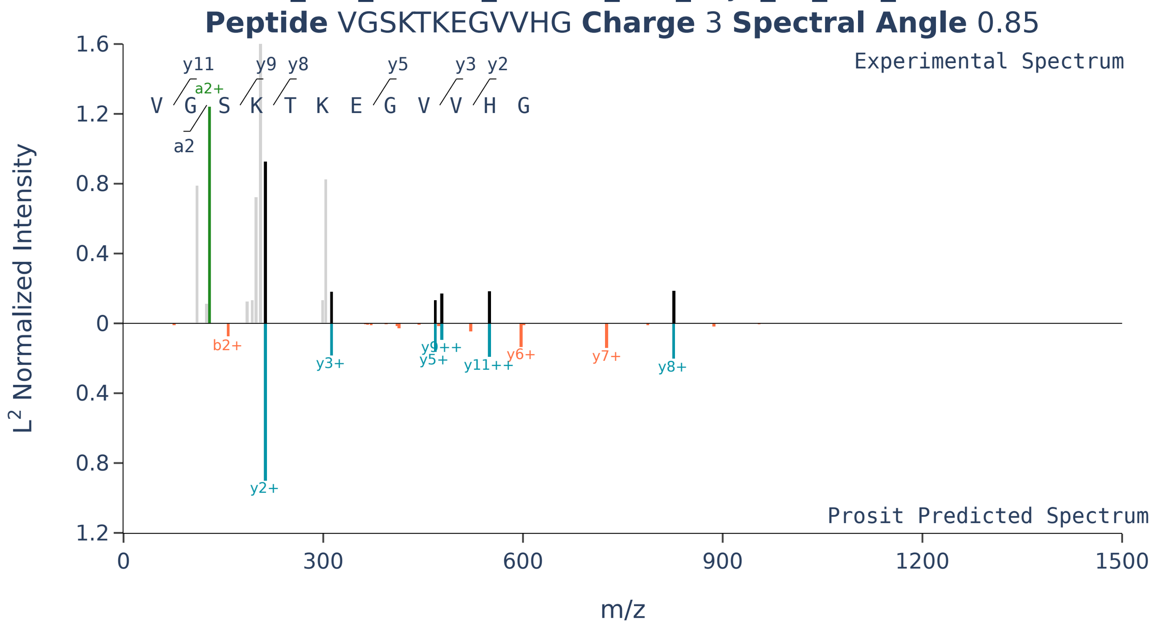


**Source** W_Soh_160821_170921_Goe_aSyn_A1_24h_R2 **Scan** 5021
**Peptide** AAAEKTKQGVAEAAGK **Charge** 2 **Spectral Angle** 0.69

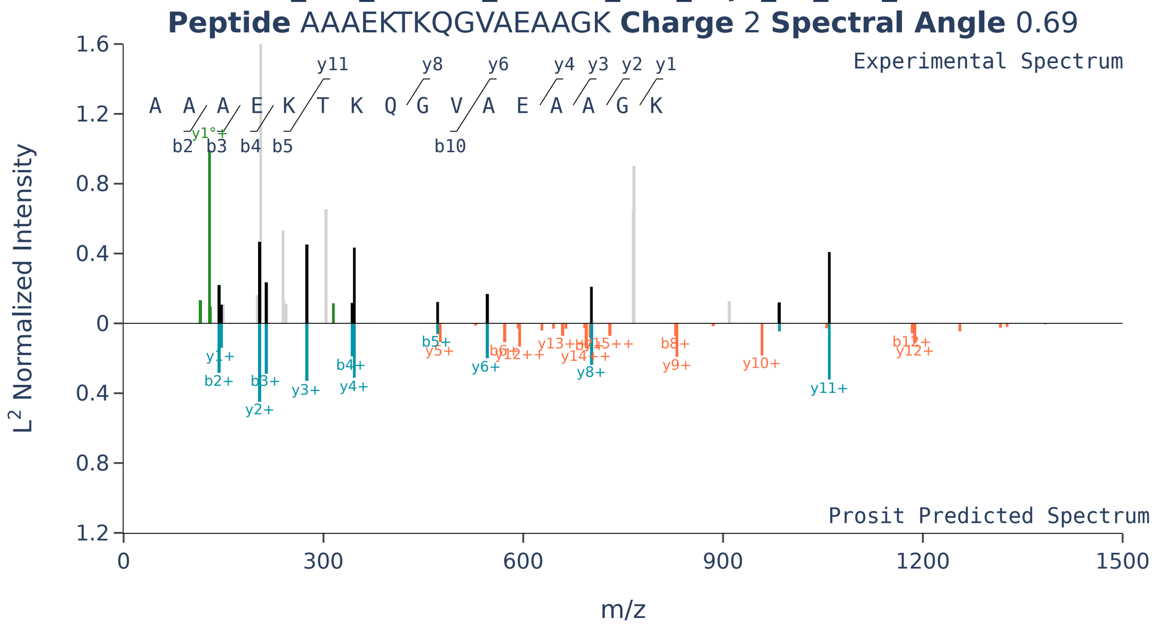


**Source** W_Soh_160821_170921_Goe_aSyn_A1_24h_R1 **Scan** 8991
**Peptide** AEAAGKTKEGVLYVGSKTKEGVVHG **Charge** 4 **Spectral Angle** 0.85

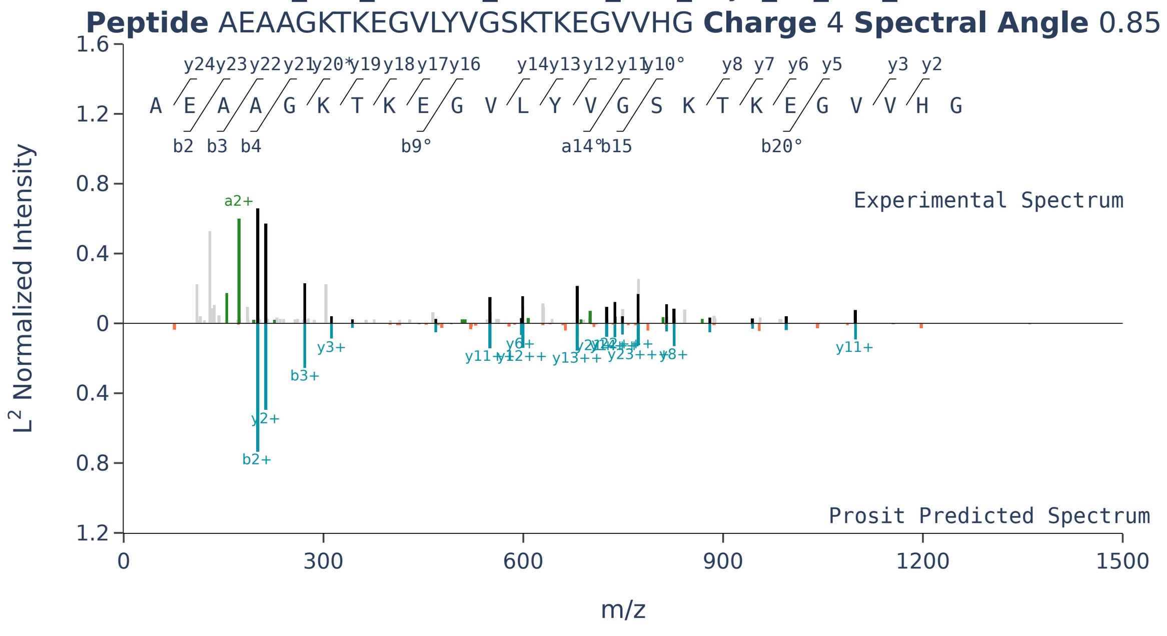


**Source** W_Soh_160821_170921_Goe_aSyn_A2_24h_R1 **Scan** 5601
**Peptide** TGFVKKD **Charge** 2 **Spectral Angle** 0.74

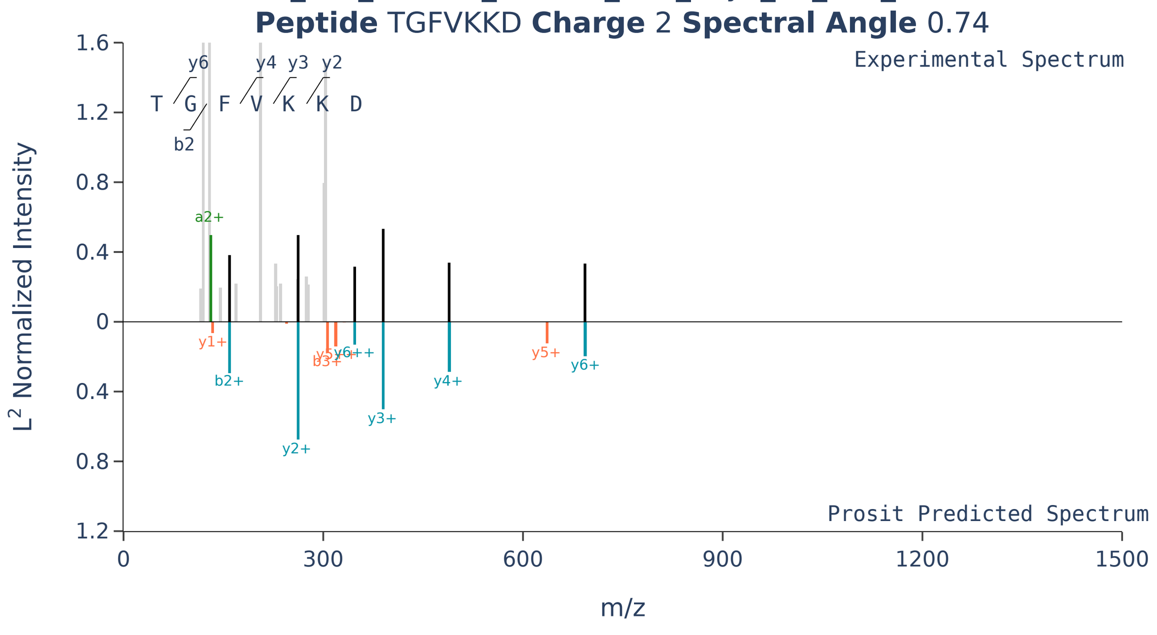


**Source** W_Soh_160821_170921_Goe_aSyn_A1_24h_R2 **Scan** 7974
**Peptide** GKTKEGVLYVGSKTKEGVVHG **Charge** 5 **Spectral Angle** 0.77

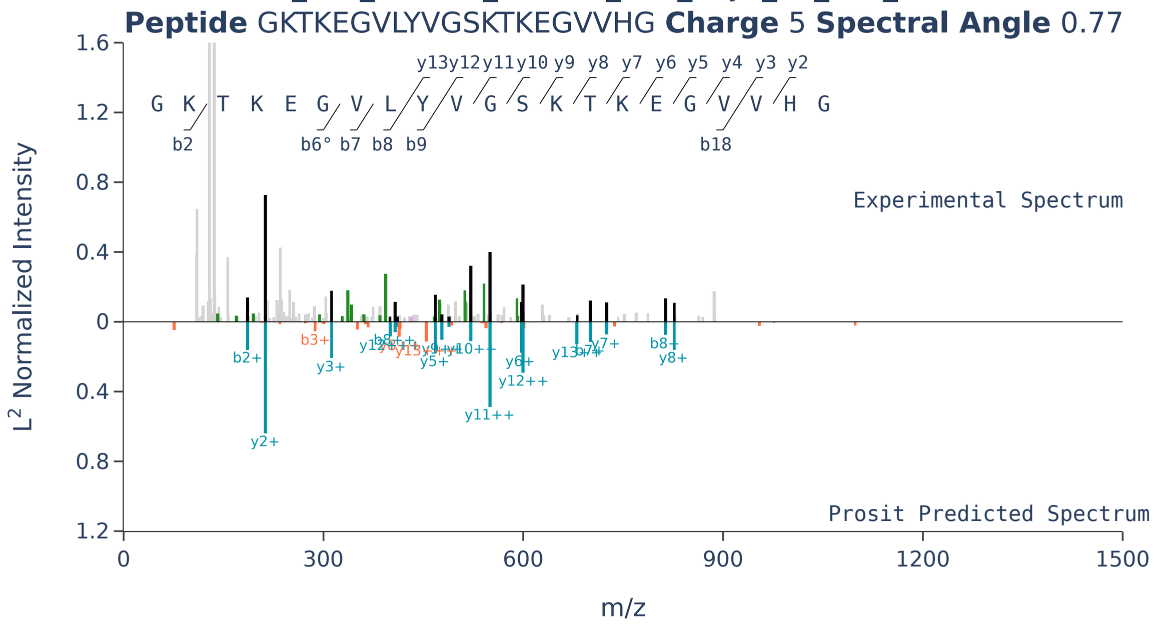


**Source** W_Soh_160821_170921_Goe_aSyn_A1_24h_R2 **Scan** 11867
**Peptide** IAAATGFVKKDQLGKNEEGAPQE **Charge** 3 **Spectral Angle** 0.92

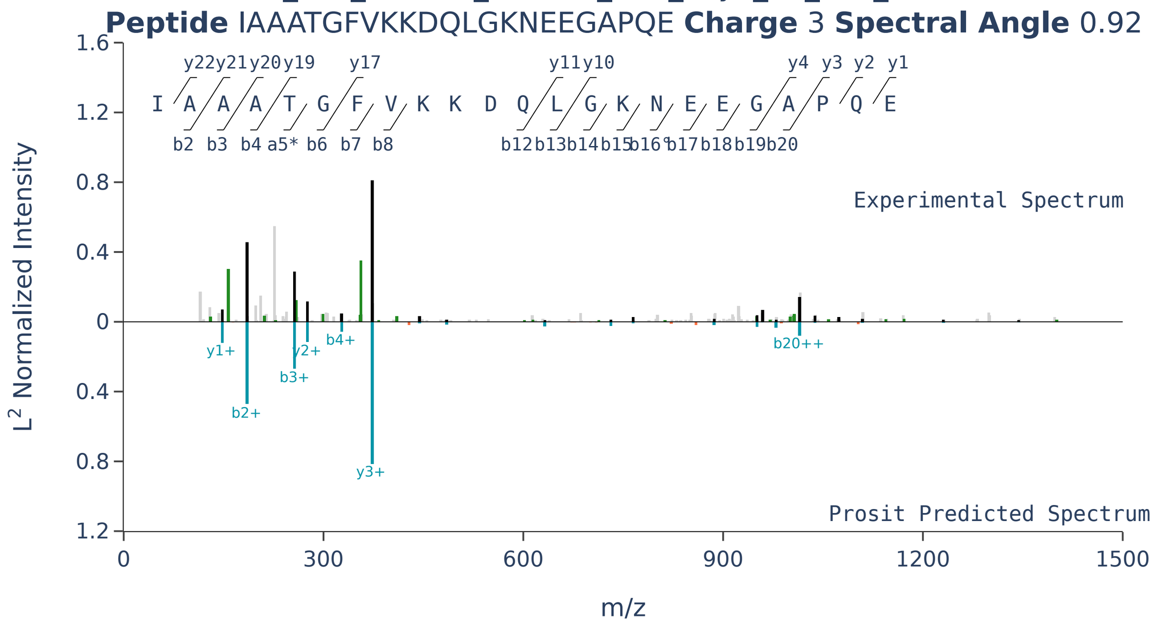


**Source** W_Soh_160821_170921_Goe_aSyn_A2_6h_R1 **Scan** 8131
**Peptide** VGSKTKEGVVHGVATV **Charge** 3 **Spectral Angle** 0.76

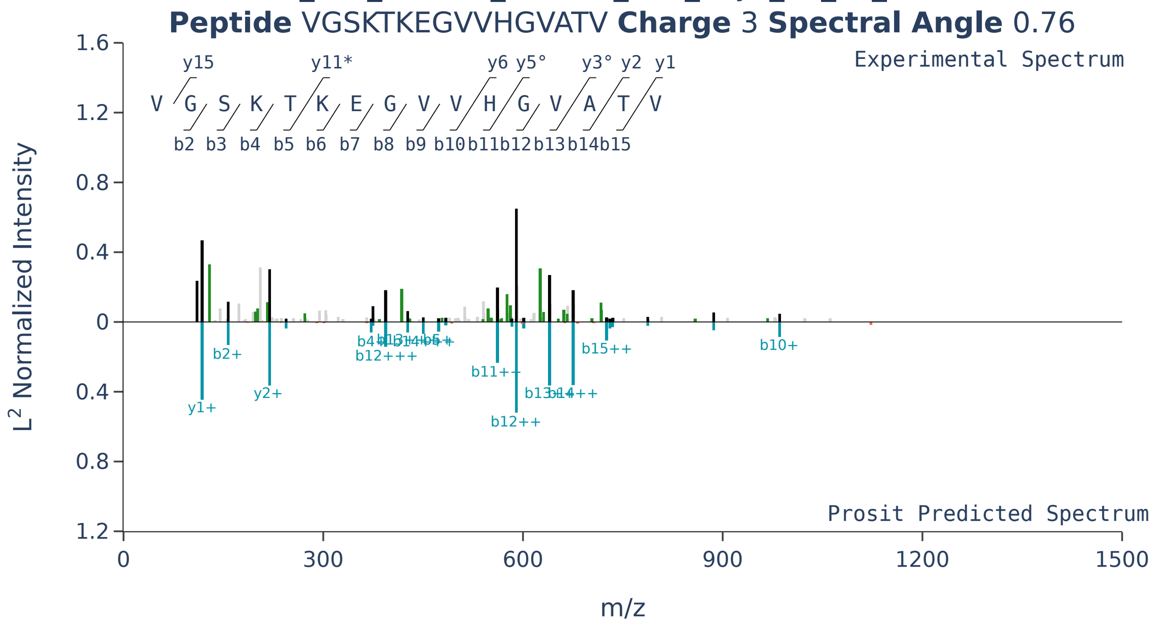


**Source** W_Soh_160821_170921_Goe_aSyn_A1_24h_R2 **Scan** 9339
**Peptide** AEKTKQGVAEAAGKTKEGVLYVGSKTKEG **Charge** 4 **Spectral Angle** 0.74

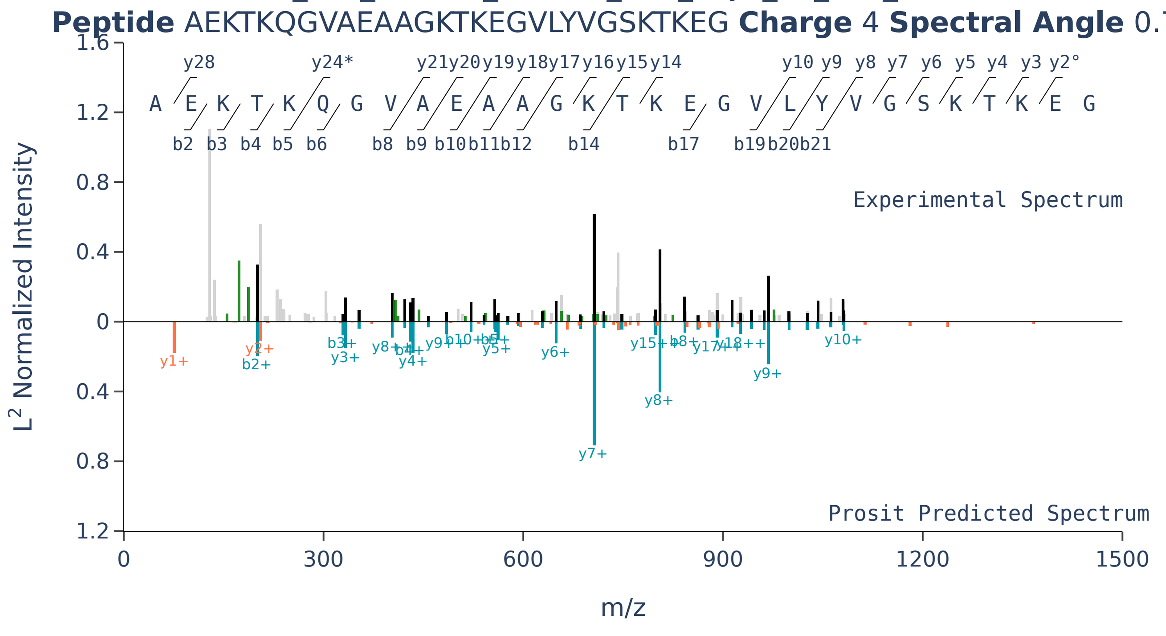


**Source** W_Soh_160821_170921_Goe_aSyn_A2_24h_R1 **Scan** 12604
**Peptide** QGVAEAAGKTKEGVLYVGSKTKEGVVH **Charge** 5 **Spectral Angle** 0.73

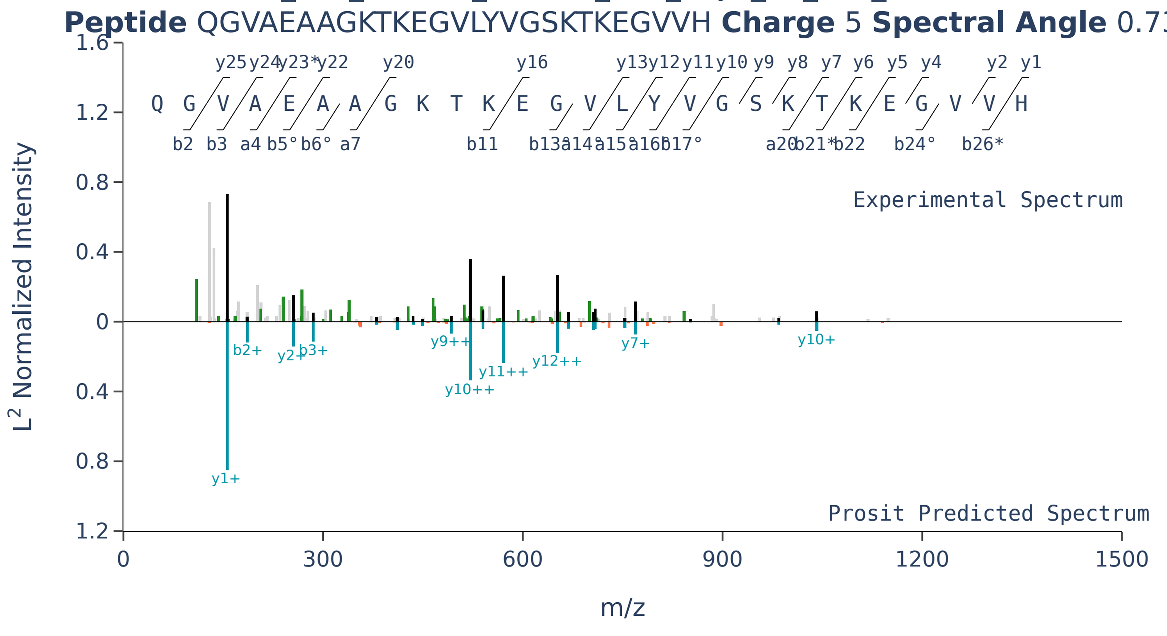

**Source** W_Soh_160821_170921_Goe_aSyn_A2_24h_R2 **Scan** 11596
**Peptide** KQGVAEAAGKTKEGVLYVG **Charge** 4 **Spectral Angle** 0.8

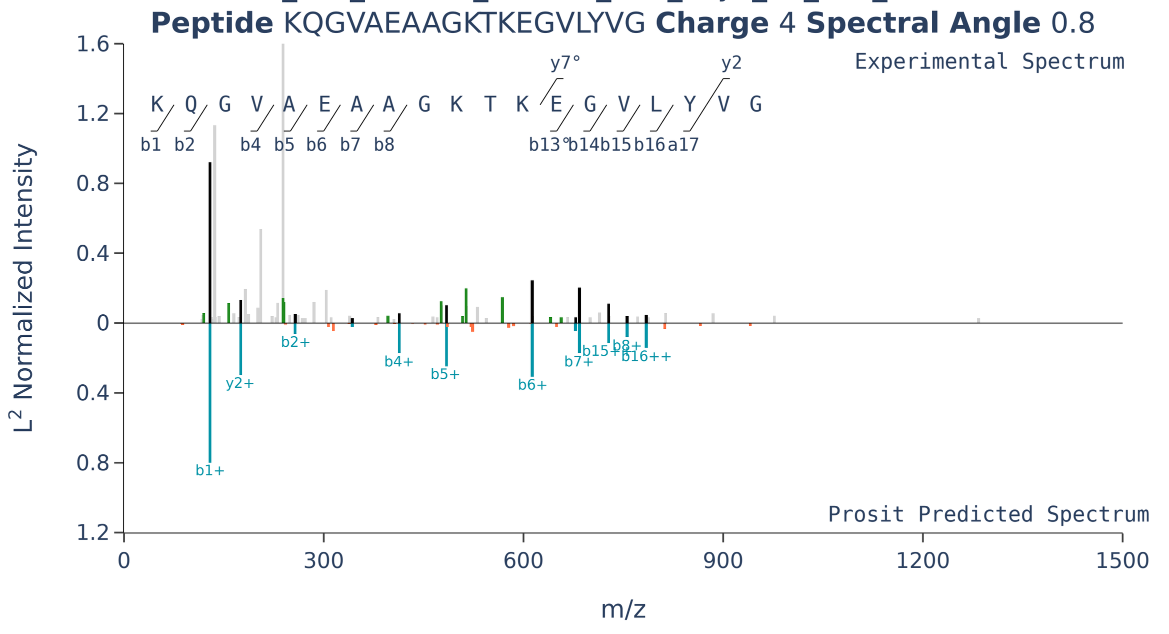


**Source** W_Soh_160821_170921_Goe_aSyn_A1_24h_R1 **Scan** 18516
**Peptide** HGVATVAEKTKEQVTNVGGAVVTGVTA **Charge** 3 **Spectral Angle** 0.69

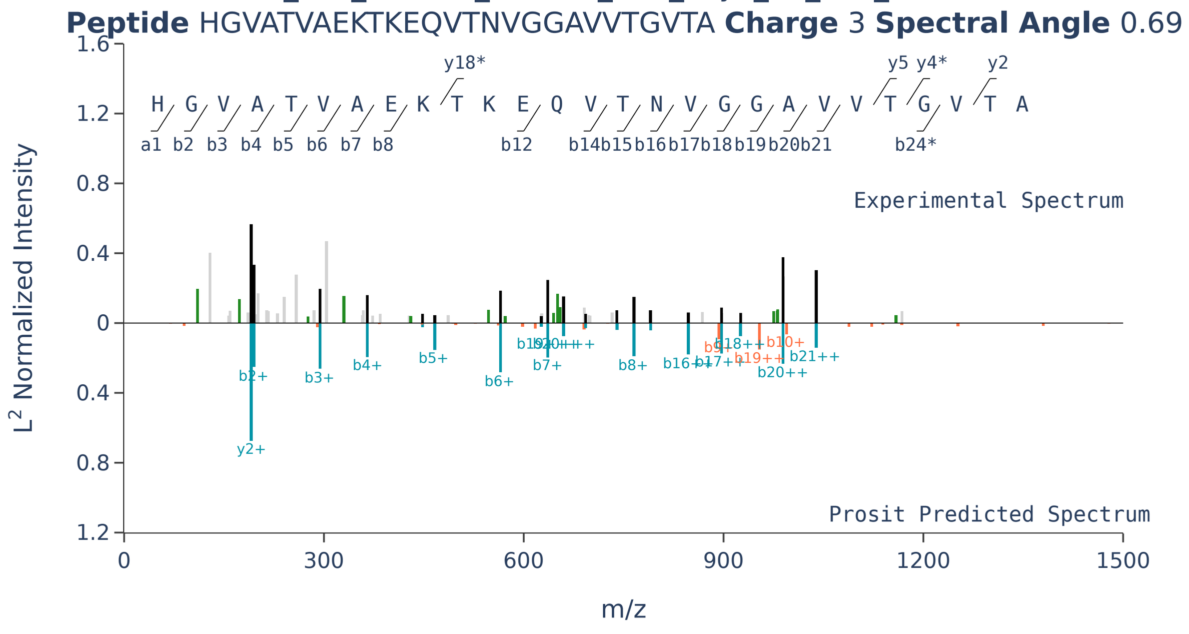


**Source** W_Soh_160821_170921_Goe_aSyn_A1_6h_R1 **Scan** 26523
**Peptide** VTNVGGAVVTGVTAVAQKTVEG **Charge** 2 **Spectral Angle** 0.76

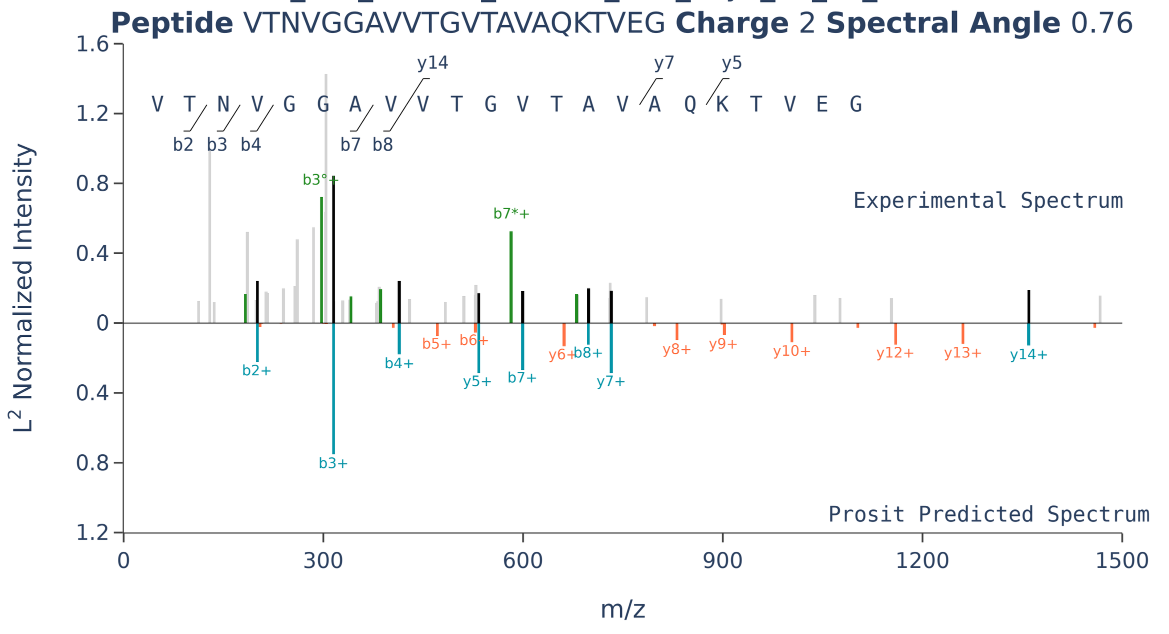


**Source** W_Soh_160821_170921_Goe_aSyn_A1_24h_R2 **Scan** 10296
**Peptide** GVAEAAGKTKEGVL **Charge** 2 **Spectral Angle** 0.77

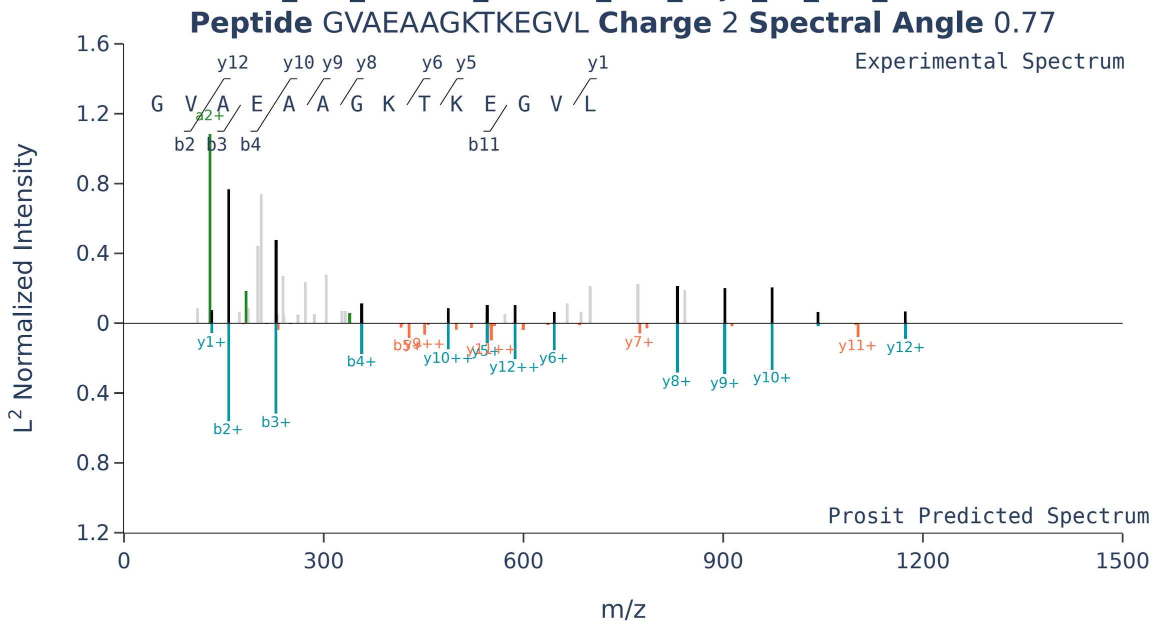


**Source** W_Soh_160821_170921_Goe_aSyn_A2_3h_R1 **Scan** 21686
**Peptide** AEKTKEQVTNVGGAVVTGVTAVAQKTVEG **Charge** 3 **Spectral Angle** 0.64

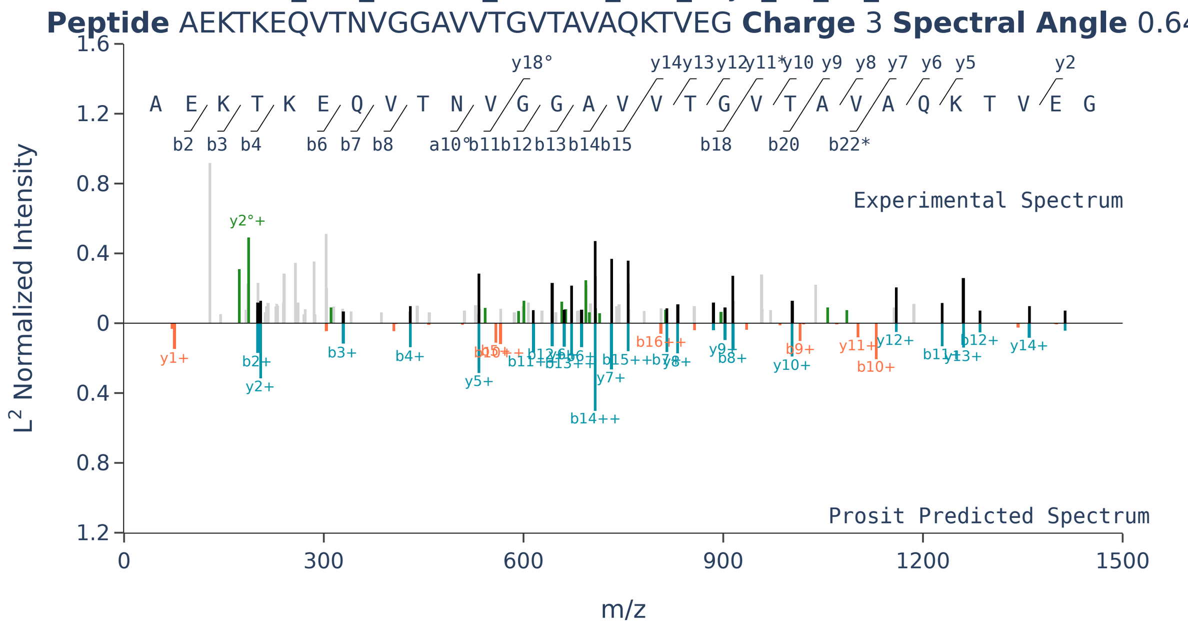


**Source** W_Soh_160821_170921_Goe_aSyn_A2_24h_R2 **Scan** 21713
**Peptide** MDVFMKGLSKAKEGVVAAAEKTKQGVAEA **Charge** 4 **Spectral Angle** 0.77

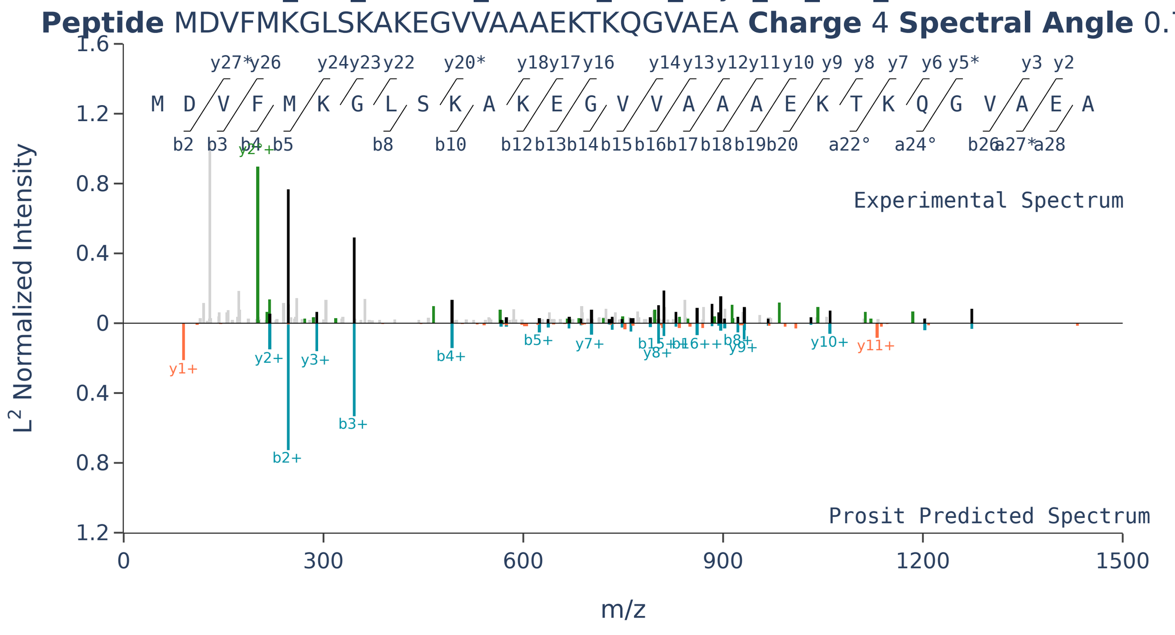


**Source** W_Soh_160821_170921_Goe_aSyn_A2_24h_R1 **Scan** 11574
**Peptide** VLYVGSKTKEGVVHGVA **Charge** 3 **Spectral Angle** 0.83

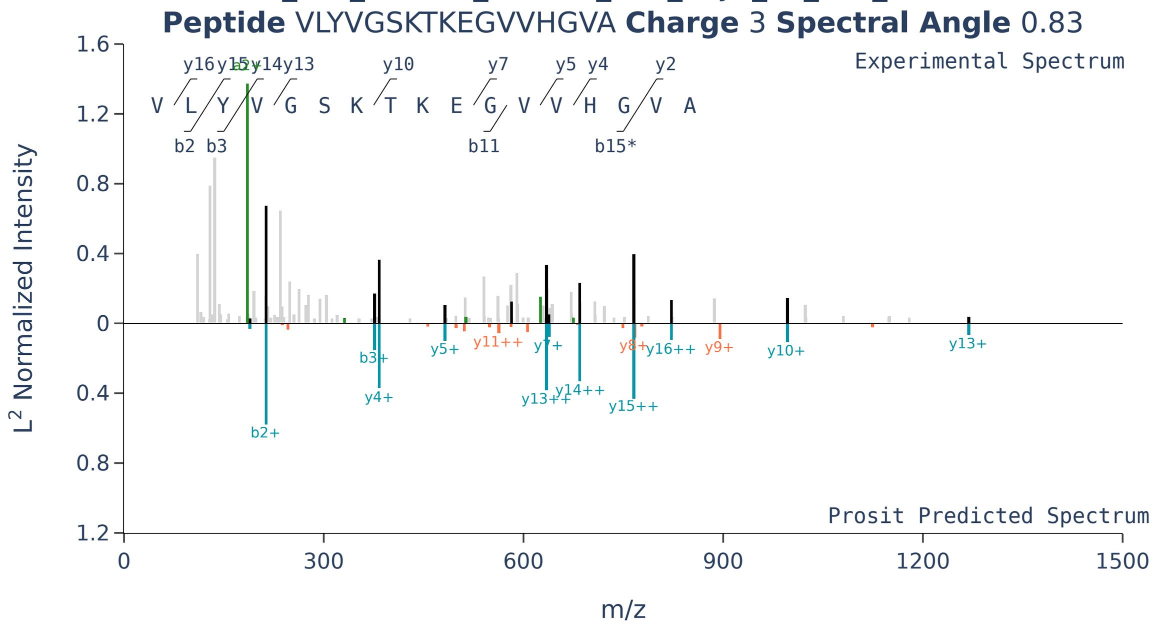


**Source** W_Soh_160821_170921_Goe_aSyn_A2_6h_R2 **Scan** 16661
**Peptide** DVFMKGLSKAKEGVVAAAEKTKQ **Charge** 4 **Spectral Angle** 0.75

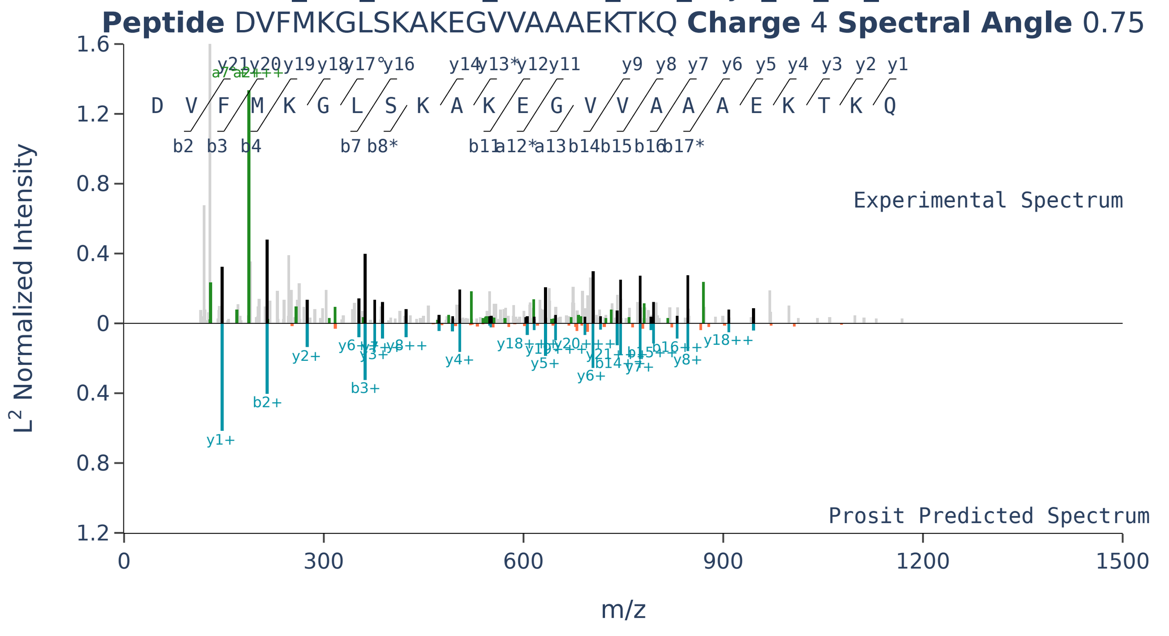


**Source** W_Soh_160821_170921_Goe_aSyn_A2_24h_R1 **Scan** 7920
**Peptide** TGFVKKDQLGKNEEGAPQEG **Charge** 3 **Spectral Angle** 0.82

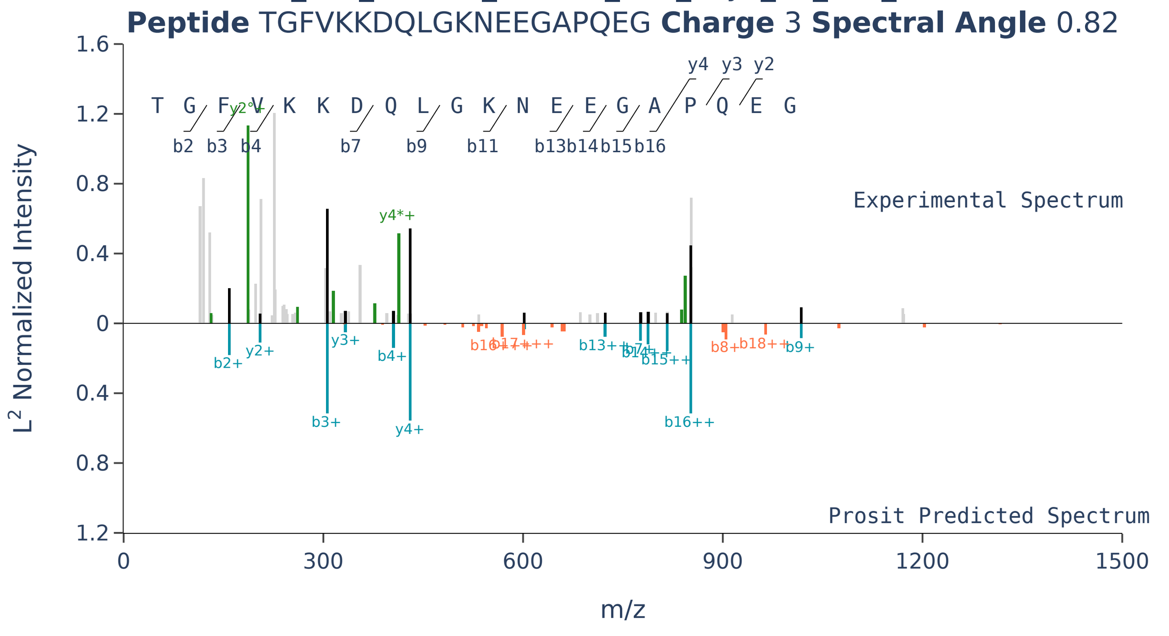


**Source** W_Soh_160821_170921_Goe_aSyn_A2_24h_R1 **Scan** 12896
**Peptide** EGVVHGVATV **Charge** 2 **Spectral Angle** 0.78

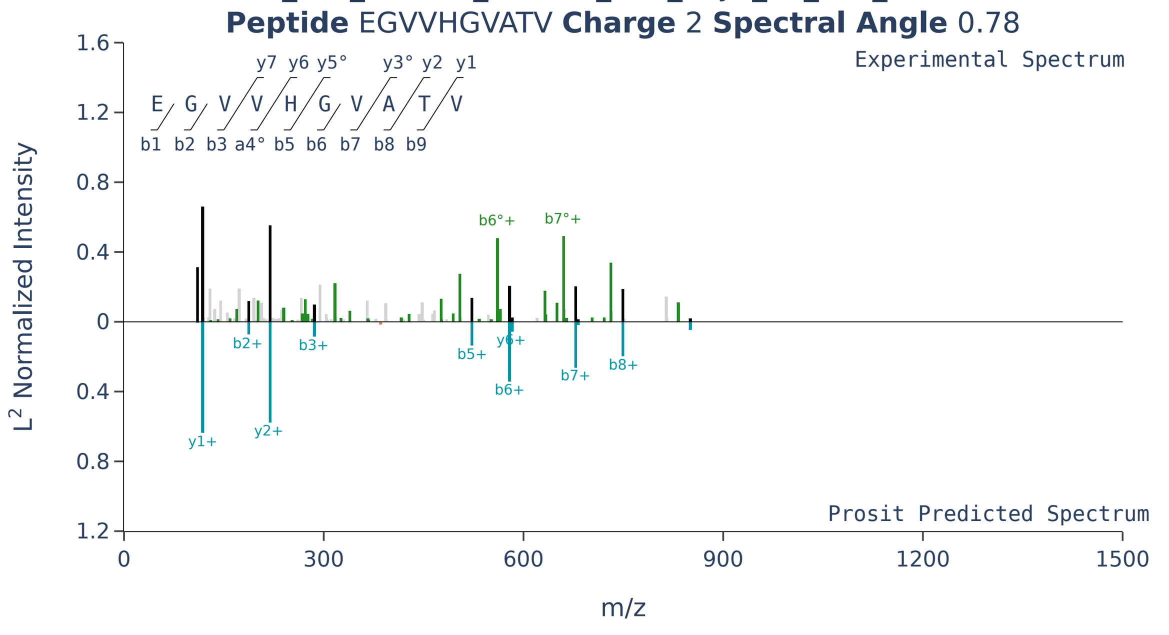


**Source** W_Soh_160821_170921_Goe_aSyn_A1_6h_R1 **Scan** 9777
**Peptide** VATVAEKTKEQVTNV **Charge** 2 **Spectral Angle** 0.84

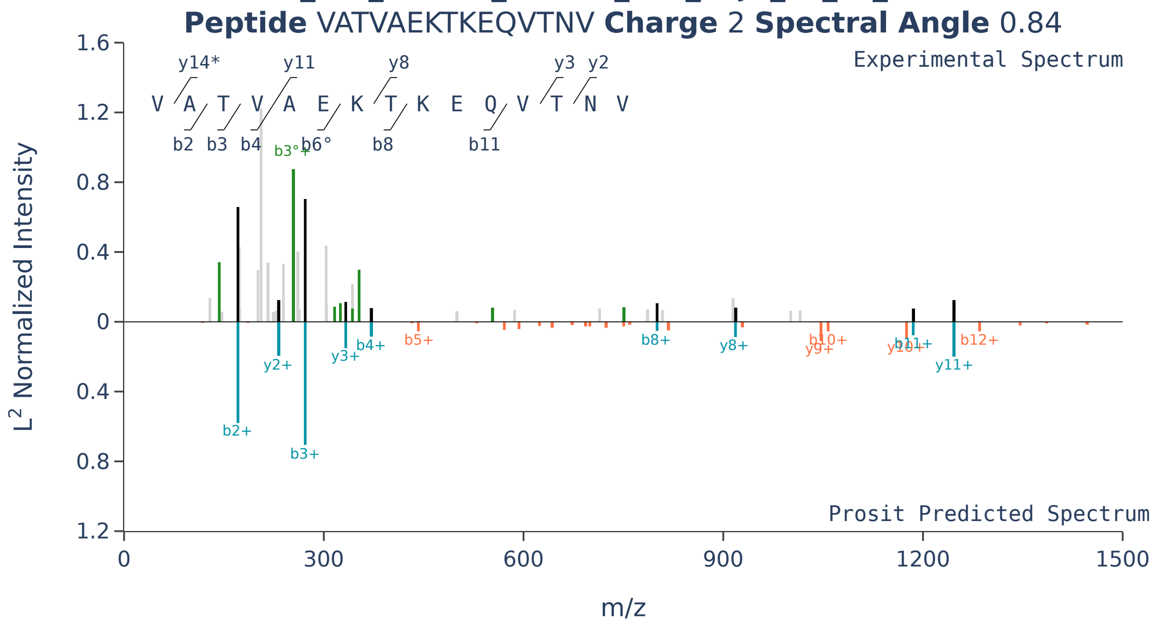


**Source** W_Soh_160821_170921_Goe_aSyn_A1_24h_R1 **Scan** 15253
**Peptide** HGVATVAEKTKEQVTNVGGAVV **Charge** 3 **Spectral Angle** 0.66

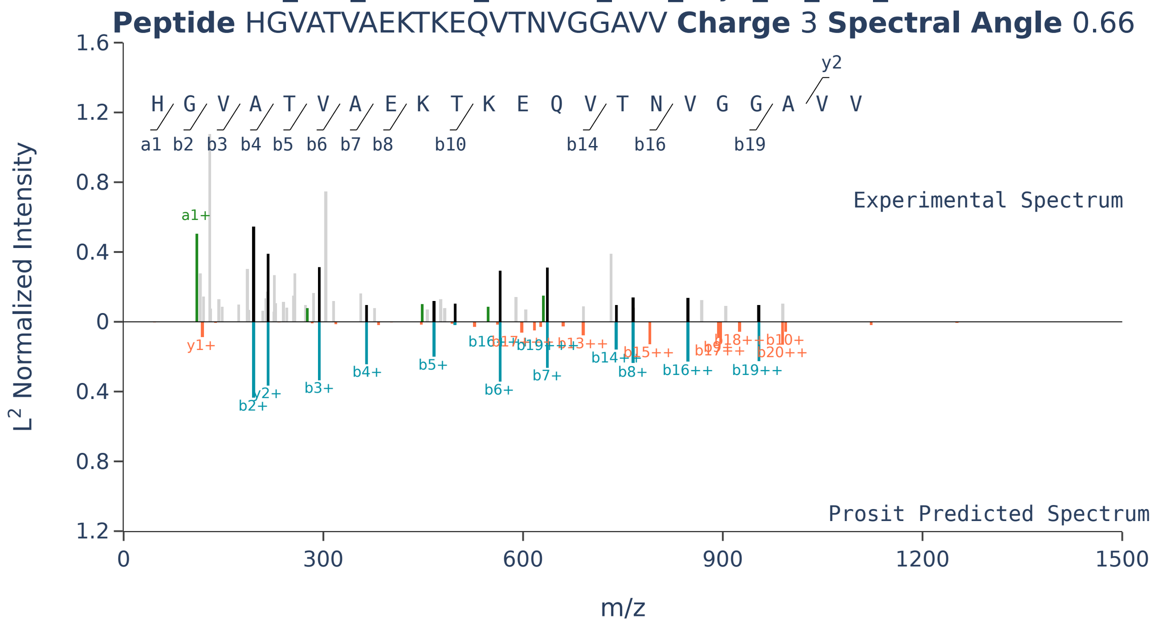


**Source** W_Soh_160821_170921_Goe_aSyn_A1_6h_R2 **Scan** 12785
**Peptide** ATVAEKTKEQVTNV **Charge** 2 **Spectral Angle** 0.81

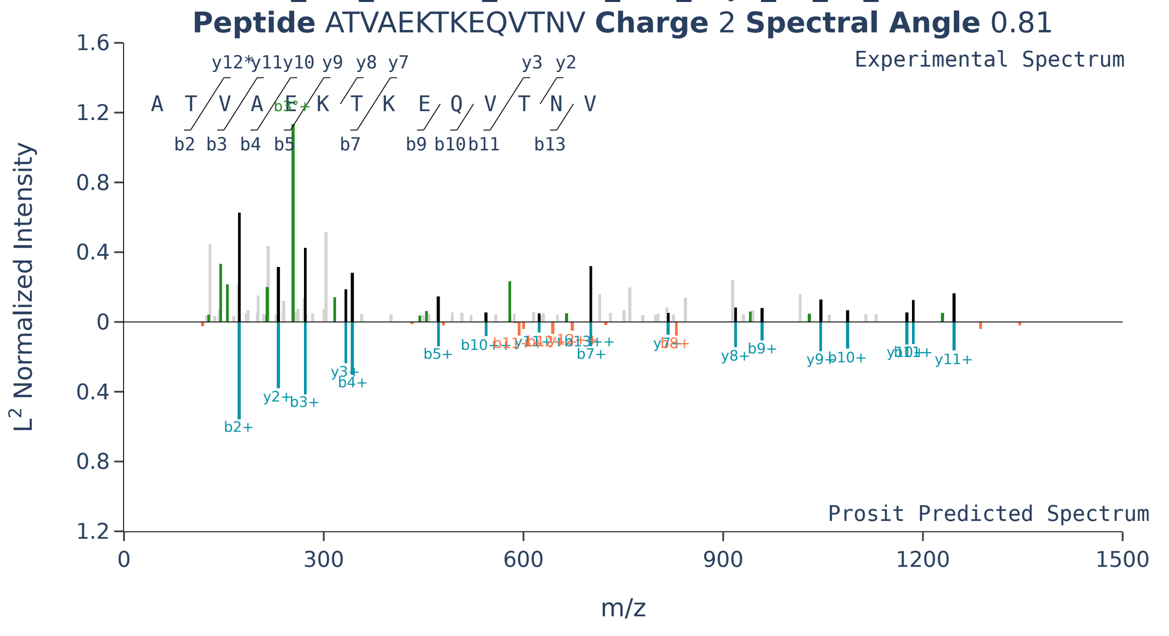


**Source** W_Soh_160821_170921_Goe_aSyn_A1_24h_R1 **Scan** 12575
**Peptide** AAATGFVKKDQLGKNEEGAPQEGI **Charge** 4 **Spectral Angle** 0.84

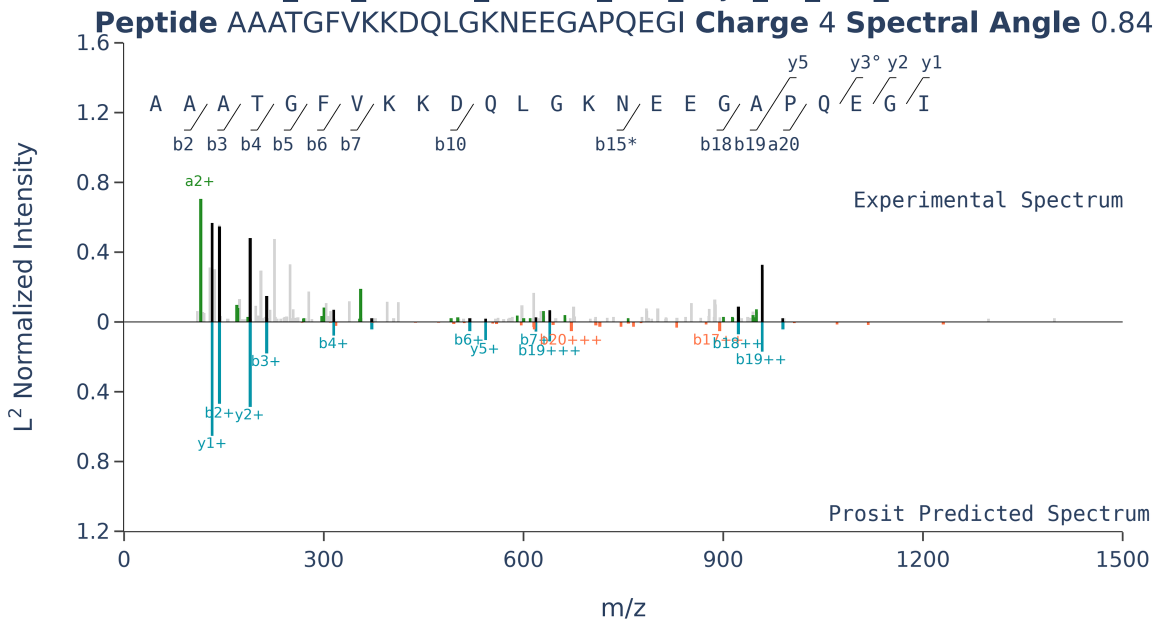


**Source** W_Soh_160821_170921_Goe_aSyn_A2_24h_R1 **Scan** 8769
**Peptide** VTAVAQKTVEGA **Charge** 2 **Spectral Angle** 0.73

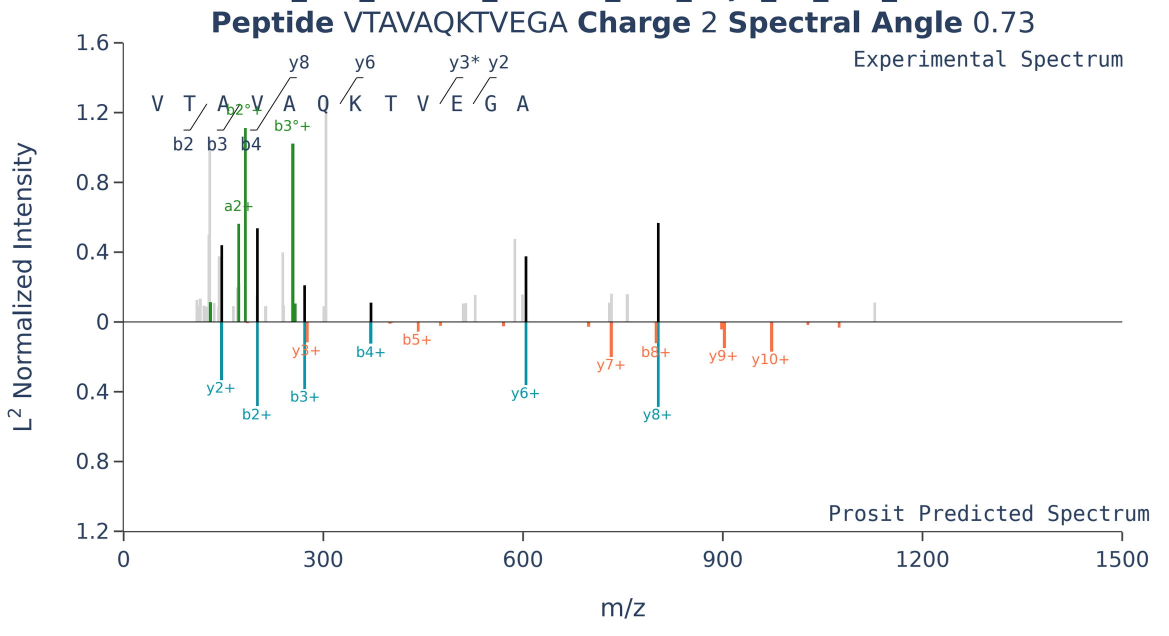

**Source** W_Soh_160821_170921_Goe_aSyn_A2_24h_R1 **Scan** 10864
**Peptide** AAEKTKQGVAEAAGKTKEGVLYVG **Charge** 3 **Spectral Angle** 0.75

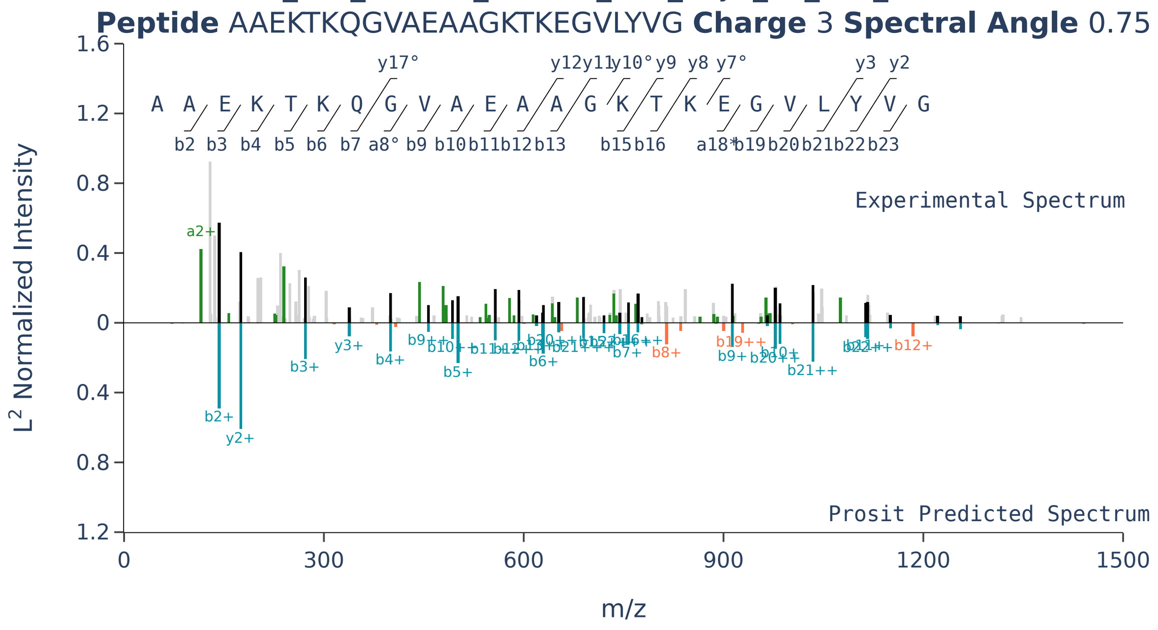


**Source** W_Soh_160821_170921_Goe_aSyn_A2_24h_R2 **Scan** 15535
**Peptide** AATGFVKKDQLGKNEEGAPQEGIL **Charge** 4 **Spectral Angle** 0.69

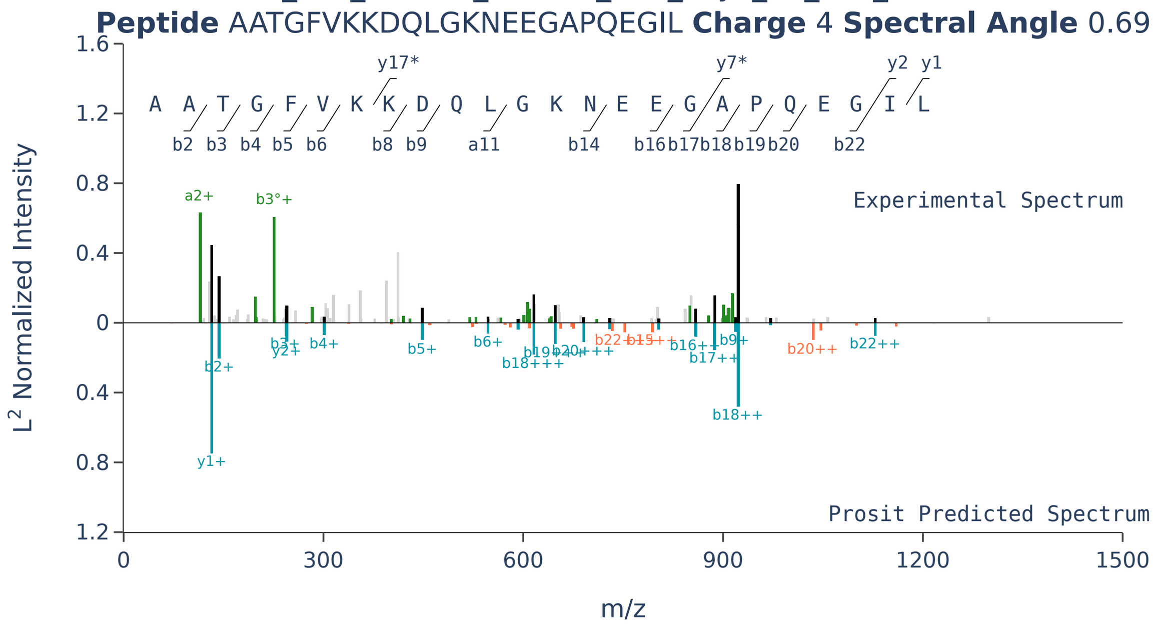


**Source** W_Soh_160821_170921_Goe_aSyn_A2_6h_R1 **Scan** 5049
**Peptide** AAEKTKQGVAEAAGKT **Charge** 3 **Spectral Angle** 0.67

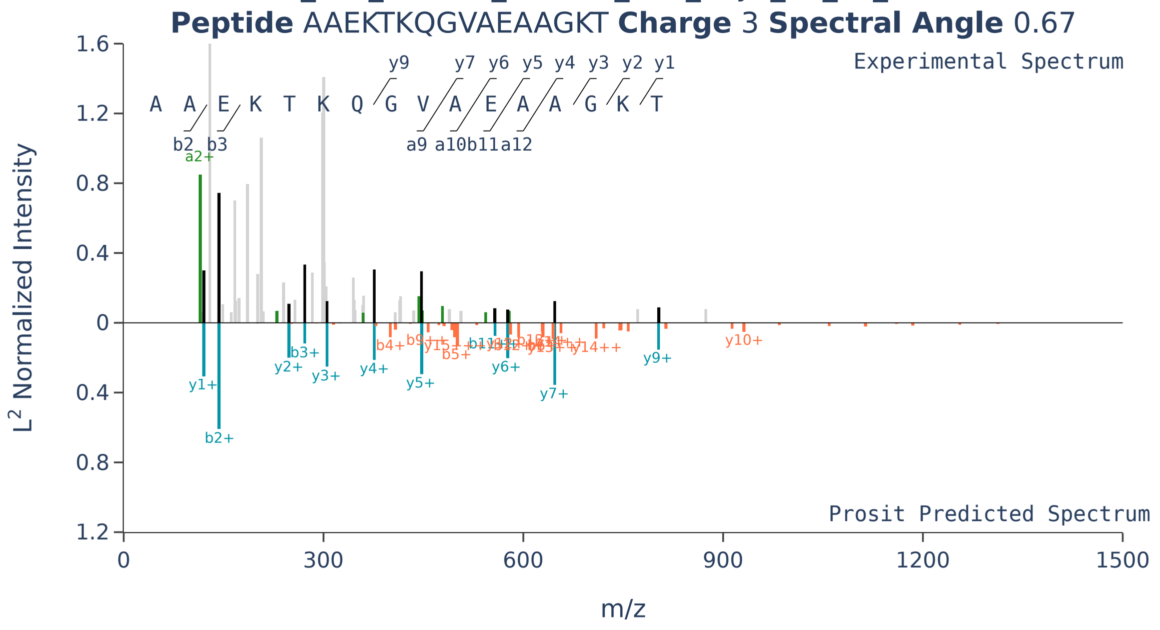


**Source** W_Soh_160821_170921_Goe_aSyn_A1_24h_R2 **Scan** 17762
**Peptide** AQKTVEGAGSIAAATGFVKKDQLG **Charge** 4 **Spectral Angle** 0.76

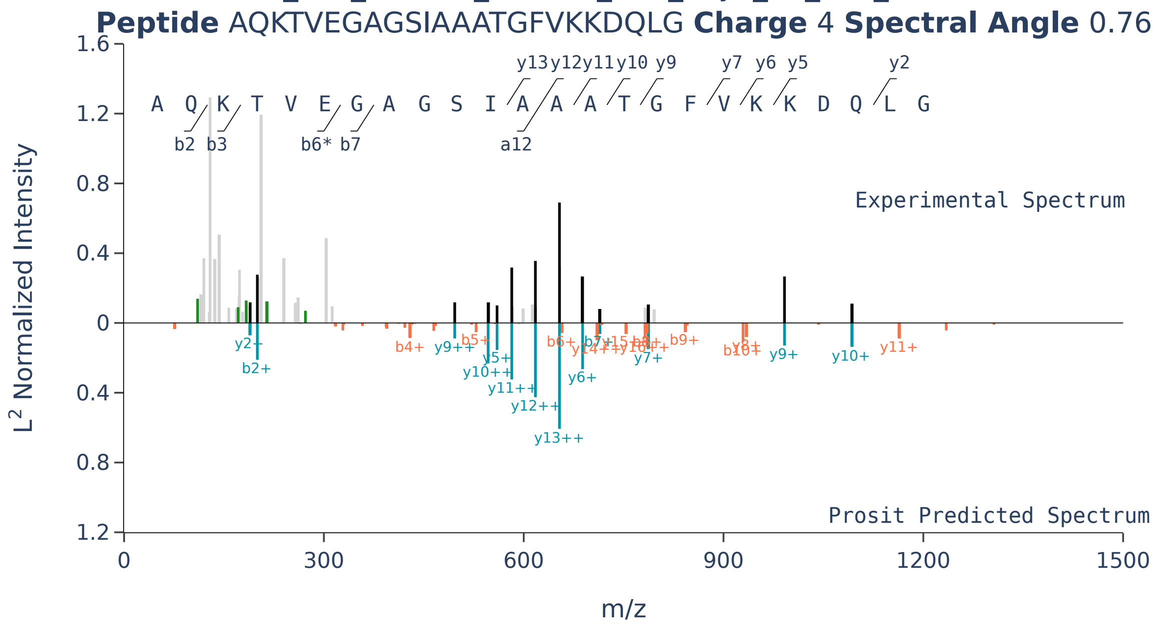


**Source** W_Soh_160821_170921_Goe_aSyn_A1_24h_R2 **Scan** 10895
**Peptide** AQKTVEGAGSIAAAT **Charge** 2 **Spectral Angle** 0.75

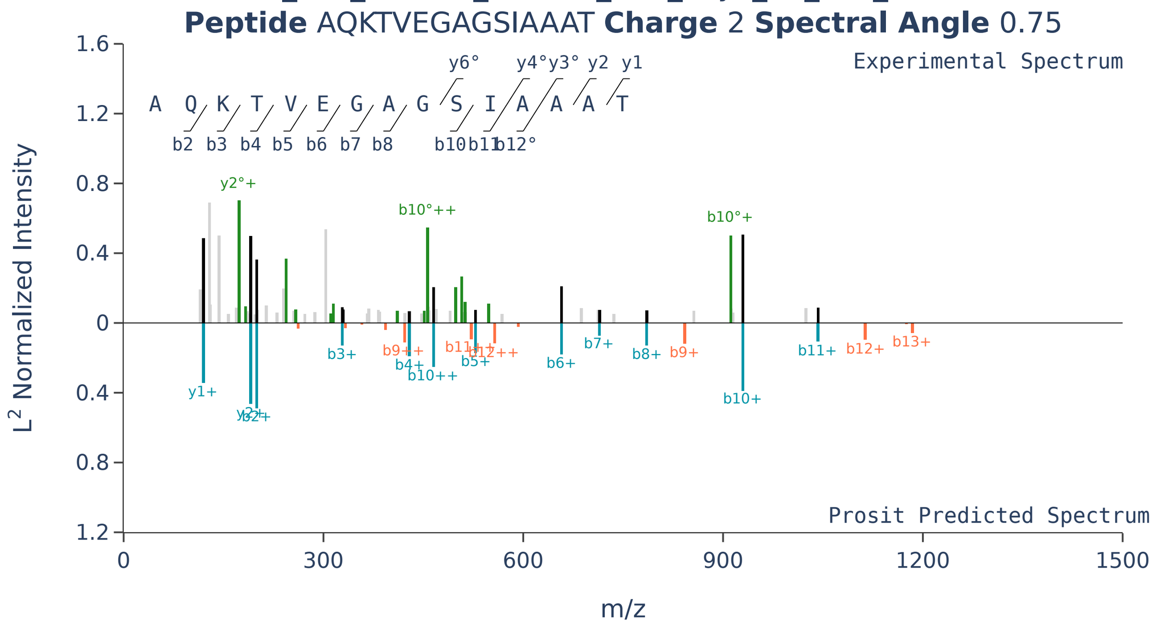


**Source** W_Soh_160821_170921_Goe_aSyn_A1_24h_R2 **Scan** 9276
**Peptide** AAEKTKQGVAEAAGKTKEGVLYVGSKTK **Charge** 5 **Spectral Angle** 0.79

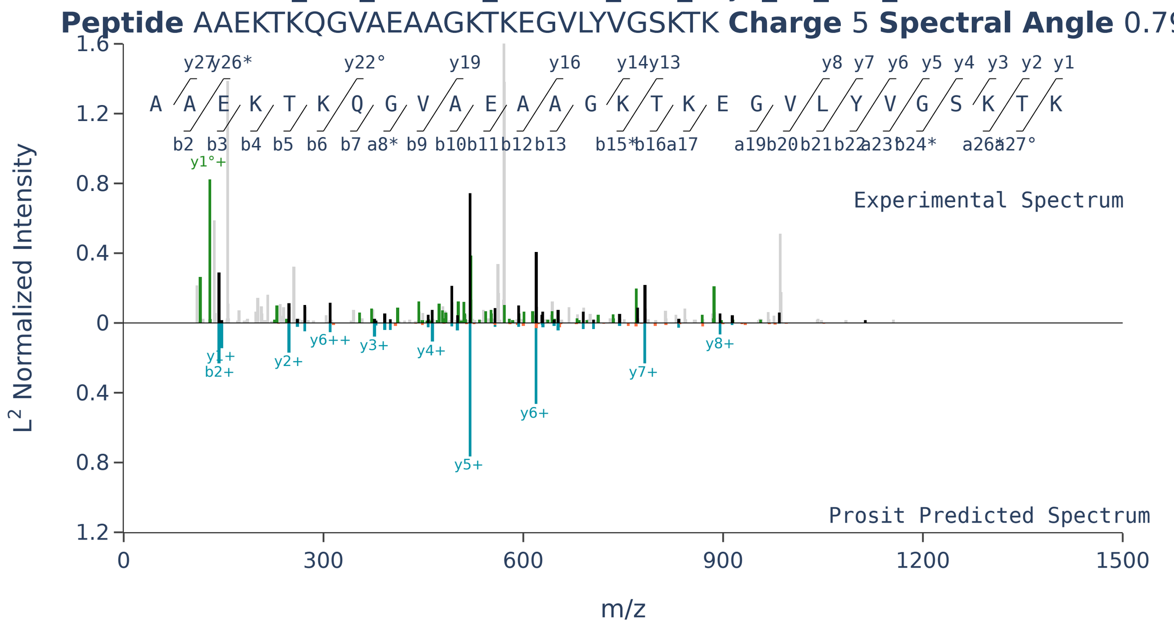


**Source** W_Soh_160821_170921_Goe_aSyn_A1_24h_R1 **Scan** 9796
**Peptide** AGKTKEGVLYVGSKTKEGVVHGVA **Charge** 4 **Spectral Angle** 0.78

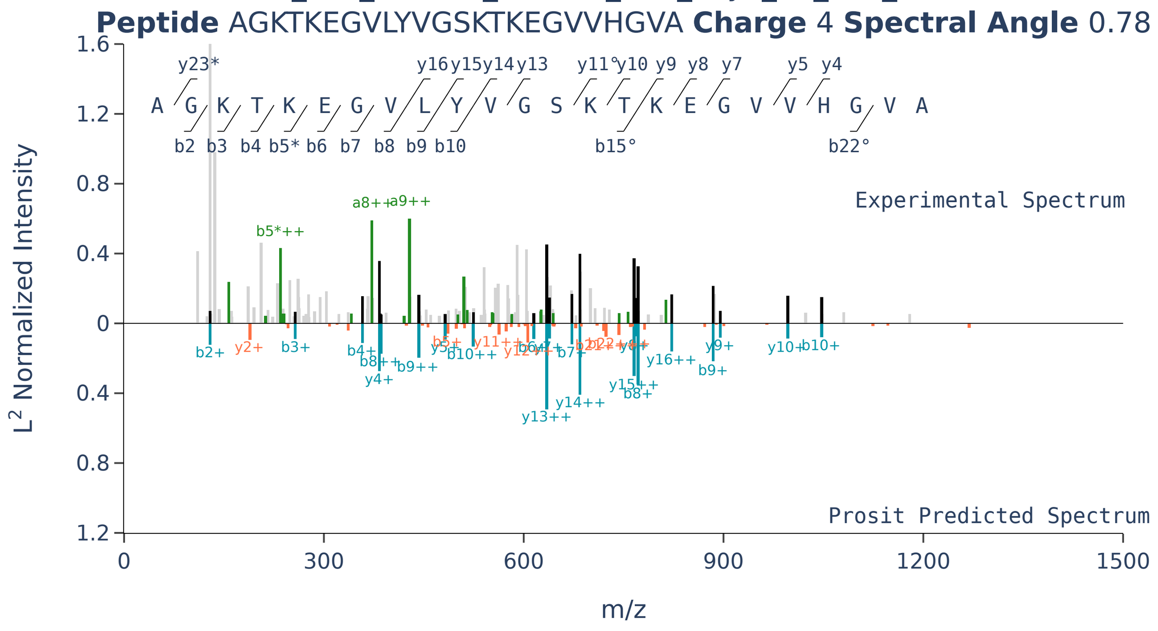


**Source** W_Soh_160821_170921_Goe_aSyn_A1_24h_R2 **Scan** 9446
**Peptide** GVVAAAEKTKQGVAEAAGK **Charge** 3 **Spectral Angle** 0.7

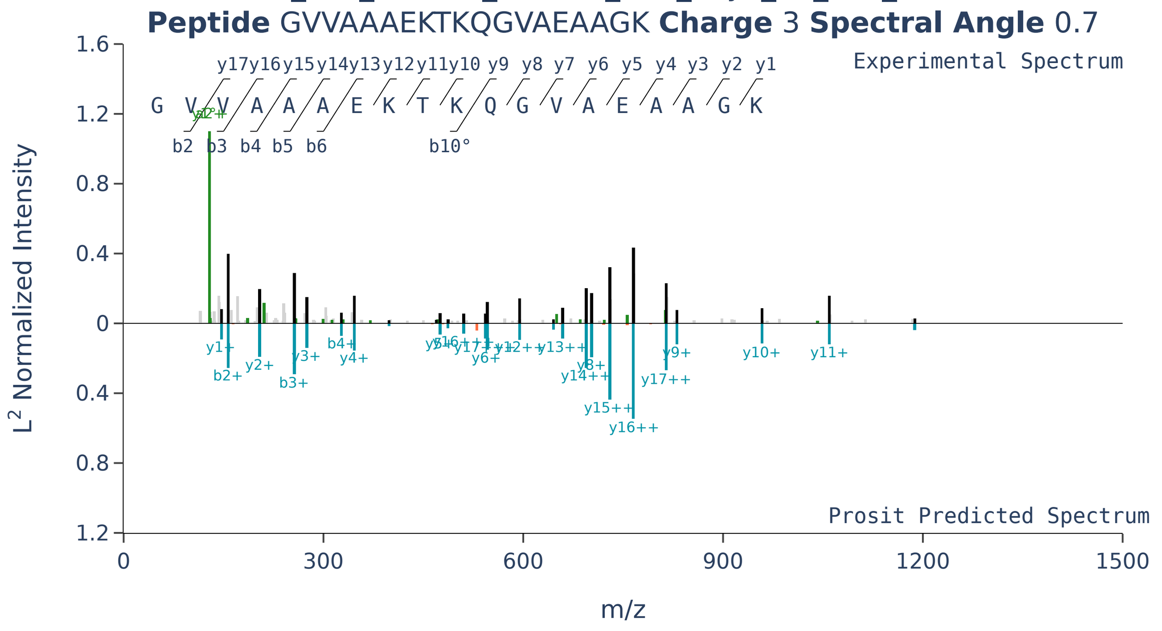


**Source** W_Soh_160821_170921_Goe_aSyn_A2_24h_R2 **Scan** 11102
**Peptide** AEAAGKTKEGVLYVGSKTKEGVVHGVAT **Charge** 4 **Spectral Angle** 0.89

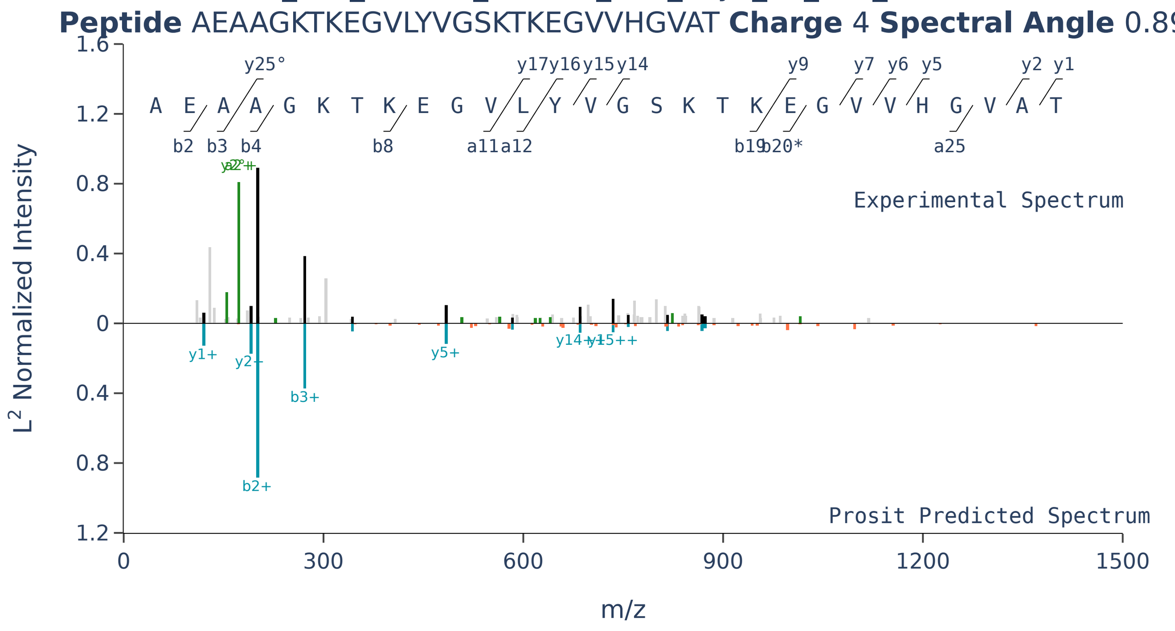


**Source** W_Soh_160821_170921_Goe_aSyn_A2_6h_R1 **Scan** 9859
**Peptide** HGVATVAEKTKEQVTNV **Charge** 3 **Spectral Angle** 0.76

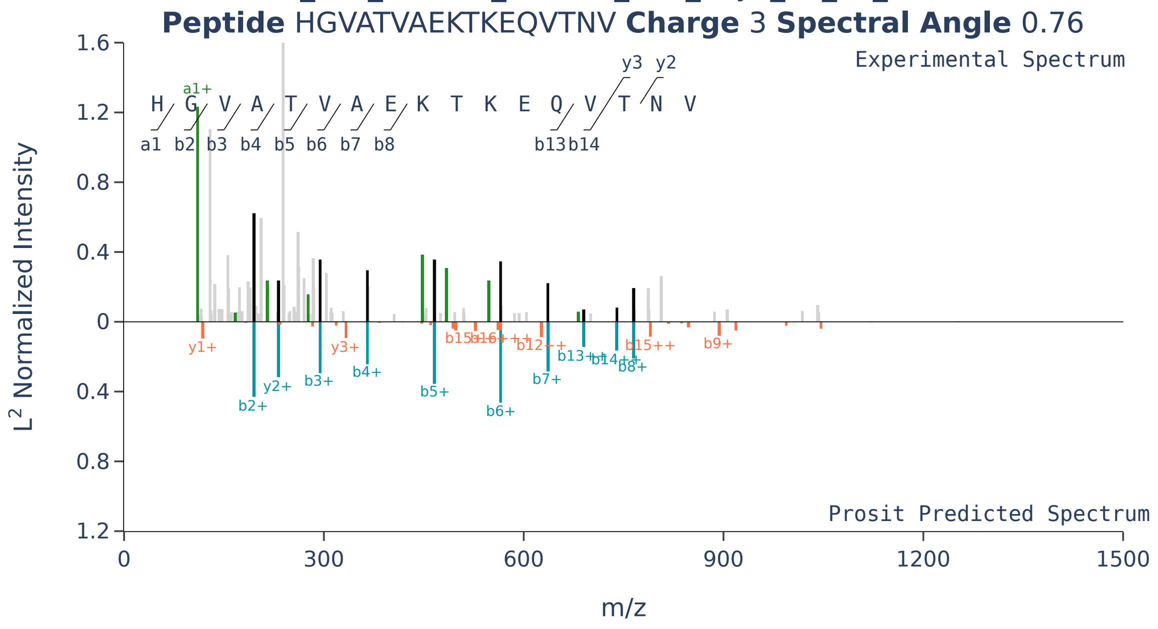


**Source** W_Soh_160821_170921_Goe_aSyn_A1_24h_R2 **Scan** 18122
**Peptide** VTNVGGAVVTGVT **Charge** 2 **Spectral Angle** 0.63

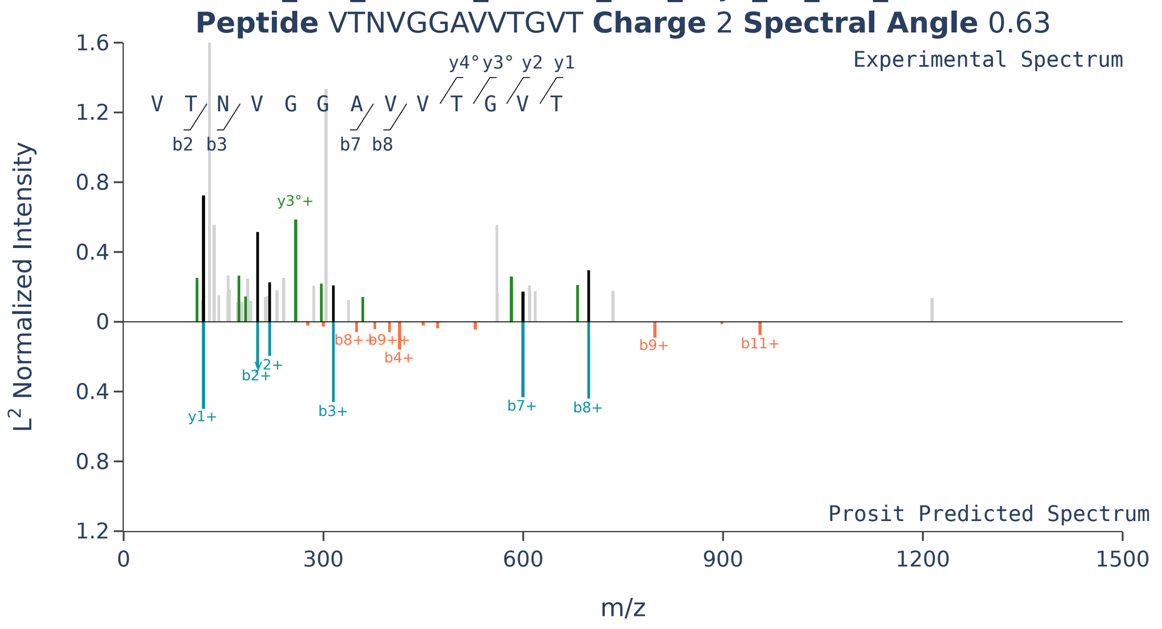


**Source** W_Soh_160821_170921_Goe_aSyn_A2_24h_R2 **Scan** 19121
**Peptide** GAVVTGVTAVAQKTVEG **Charge** 2 **Spectral Angle** 0.83

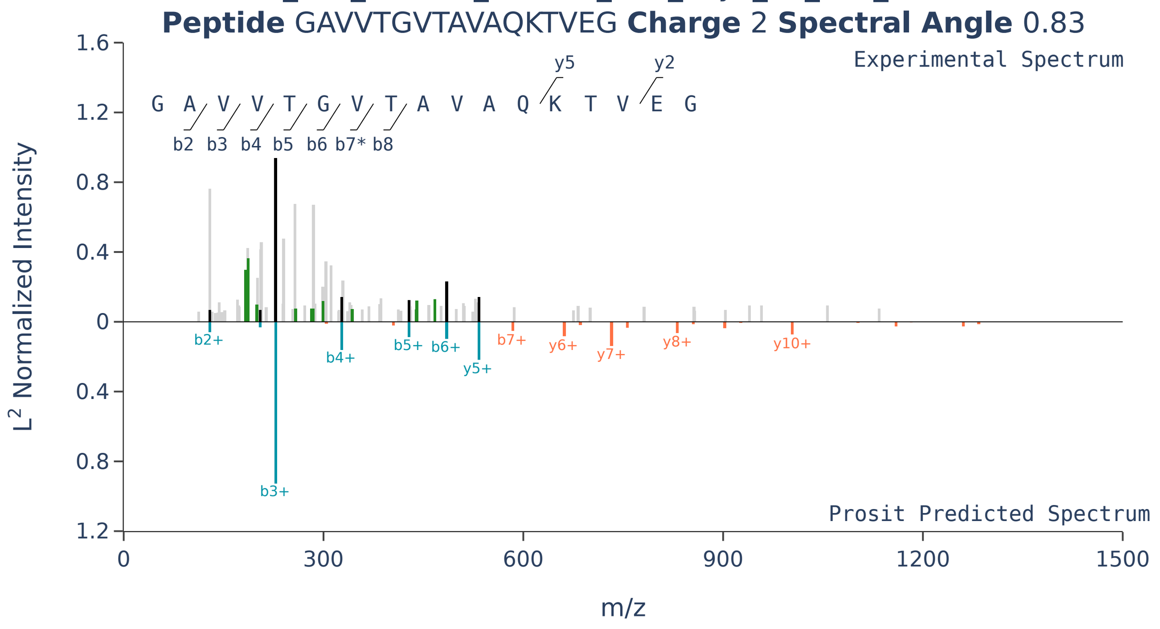


**Source** W_Soh_160821_170921_Goe_aSyn_A1_6h_R2 **Scan** 22215
**Peptide** VATVAEKTKEQVTNVGGAVVTGVTAVA **Charge** 3 **Spectral Angle** 0.76

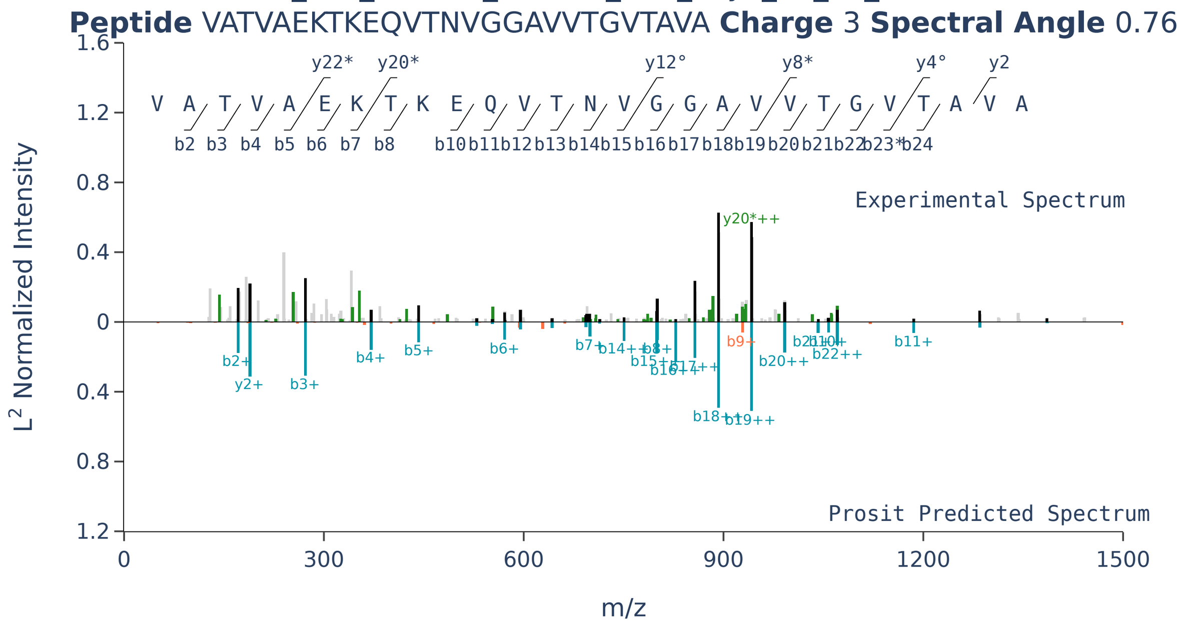


**Source** W_Soh_160821_170921_Goe_aSyn_A2_6h_R1 **Scan** 6800
**Peptide** GSKTKEGVVHGVATVAEK **Charge** 4 **Spectral Angle** 0.71

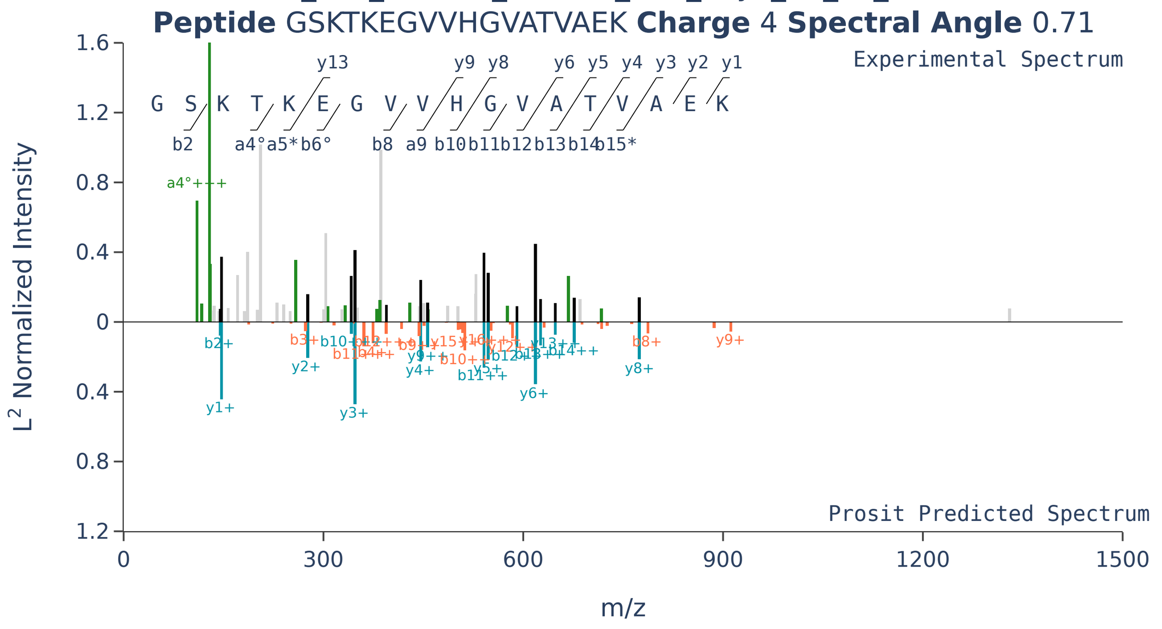


**Source** W_Soh_160821_170921_Goe_aSyn_A1_24h_R1 **Scan** 13449
**Peptide** KNEEGAPQEGILED **Charge** 2 **Spectral Angle** 0.71

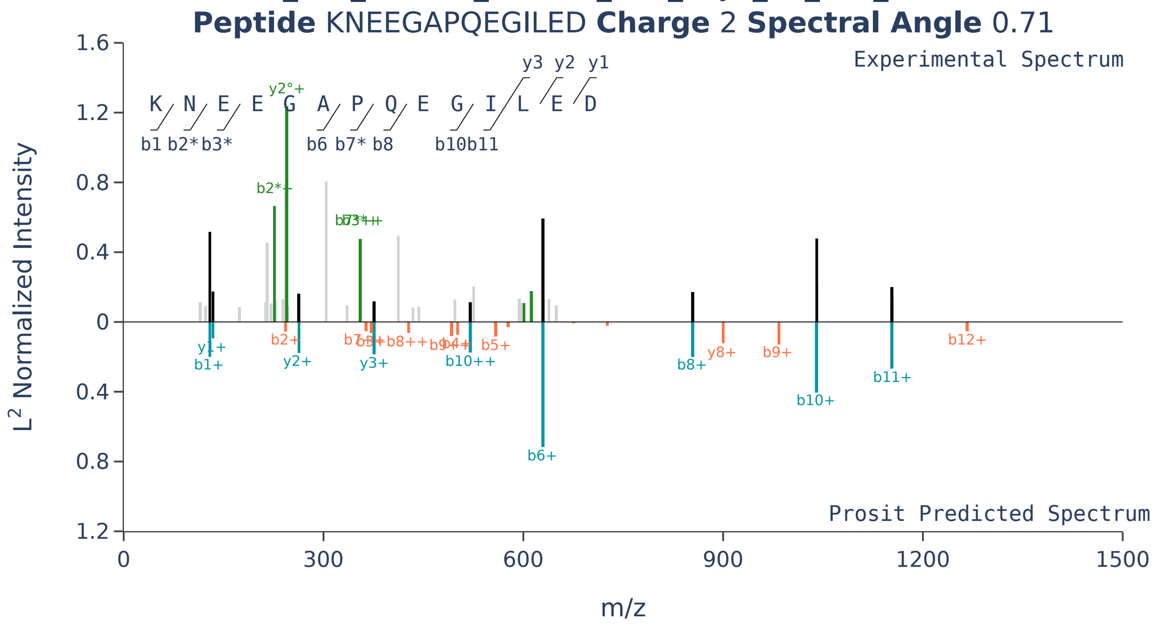

**Source** W_Soh_160821_170921_Goe_aSyn_A1_24h_R2 **Scan** 14103
**Peptide** LYVGSKTKEGVVHGVATVAEKTKE **Charge** 5 **Spectral Angle** 0.79

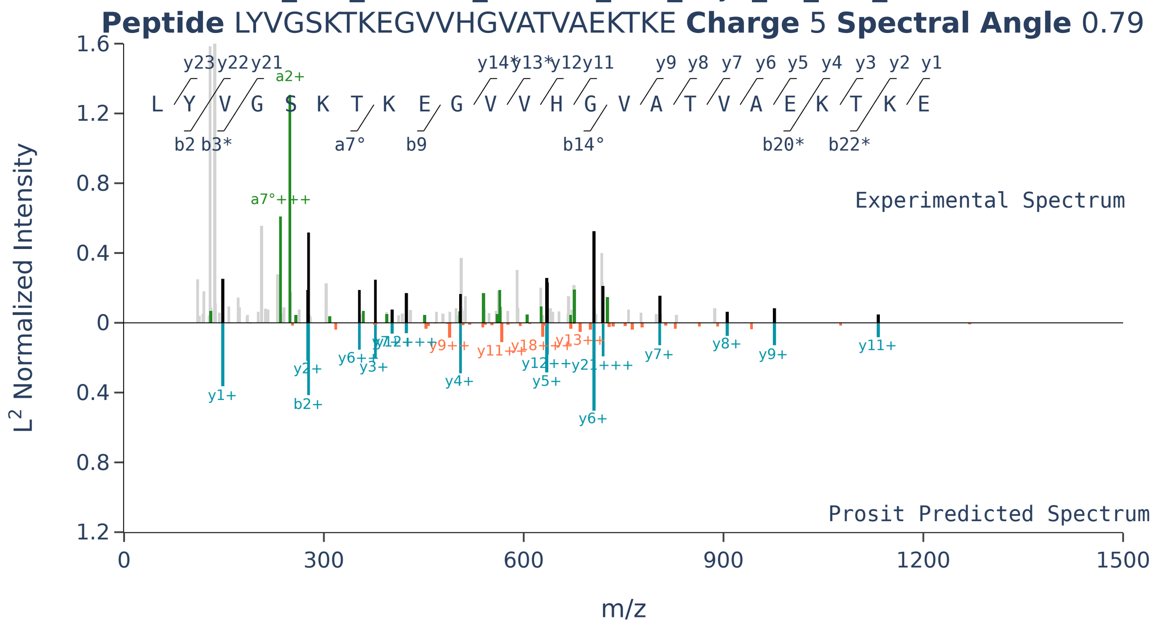


**Source** W_Soh_160821_170921_Goe_aSyn_A1_6h_R2 **Scan** 21311
**Peptide** ATVAEKTKEQVTNVGGAVVTGVTAVAQ **Charge** 3 **Spectral Angle** 0.85

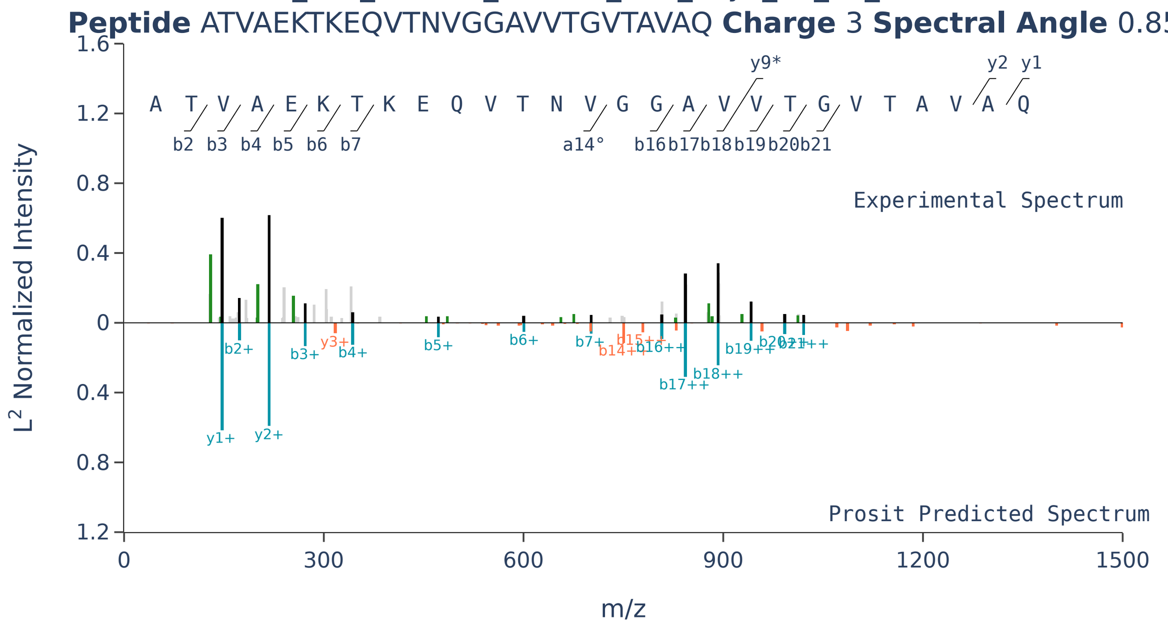


**Source** W_Soh_160821_170921_Goe_aSyn_A1_24h_R1 **Scan** 14034
**Peptide** EKTKEQVTNVGGAVVTGVT **Charge** 3 **Spectral Angle** 0.8

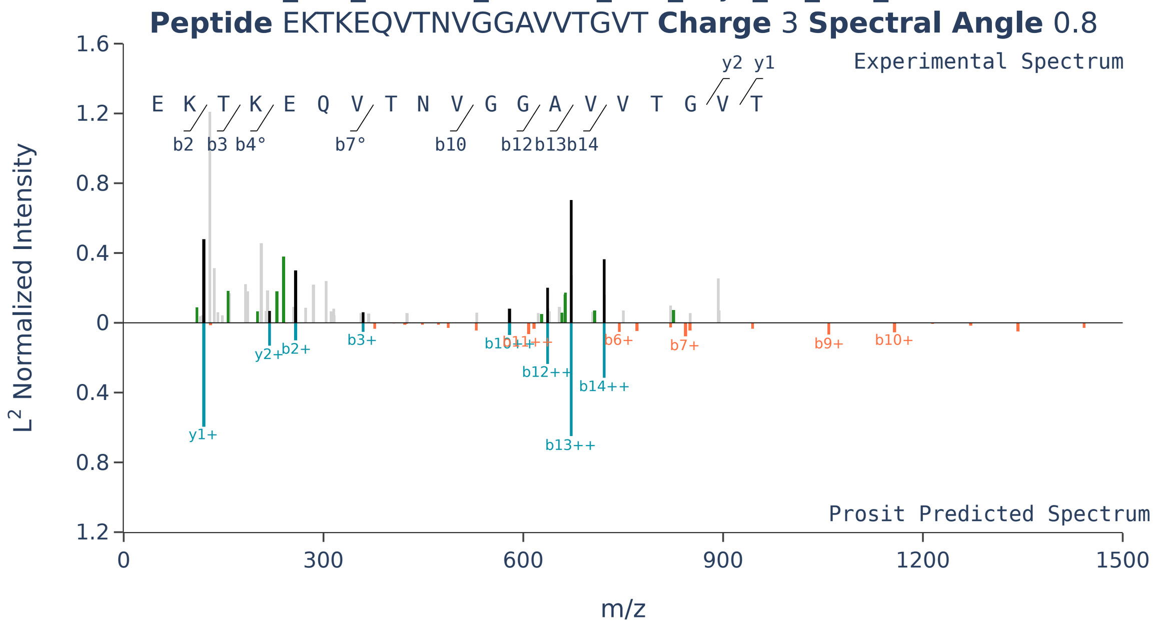


**Source** W_Soh_160821_170921_Goe_aSyn_A1_24h_R1 **Scan** 13367
**Peptide** SIAAATGFVKKDQLGKNEEGAPQE **Charge** 3 **Spectral Angle** 0.9

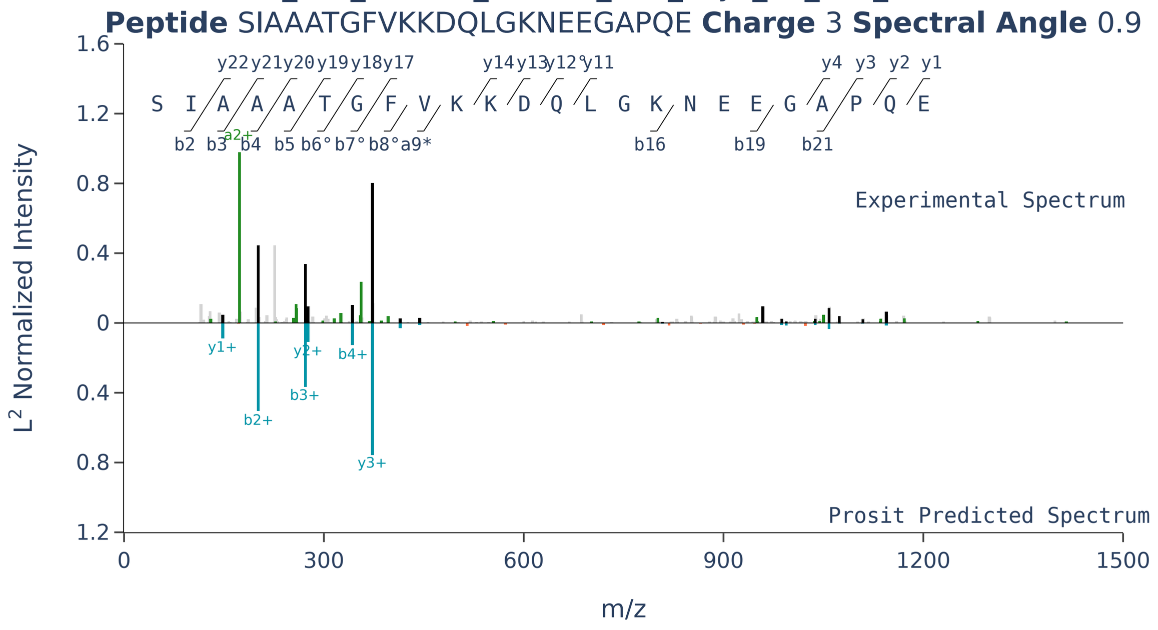


**Source** W_Soh_160821_170921_Goe_aSyn_A2_24h_R2 **Scan** 13981
**Peptide** GVAEAAGKTKEGVLYVGS **Charge** 2 **Spectral Angle** 0.78

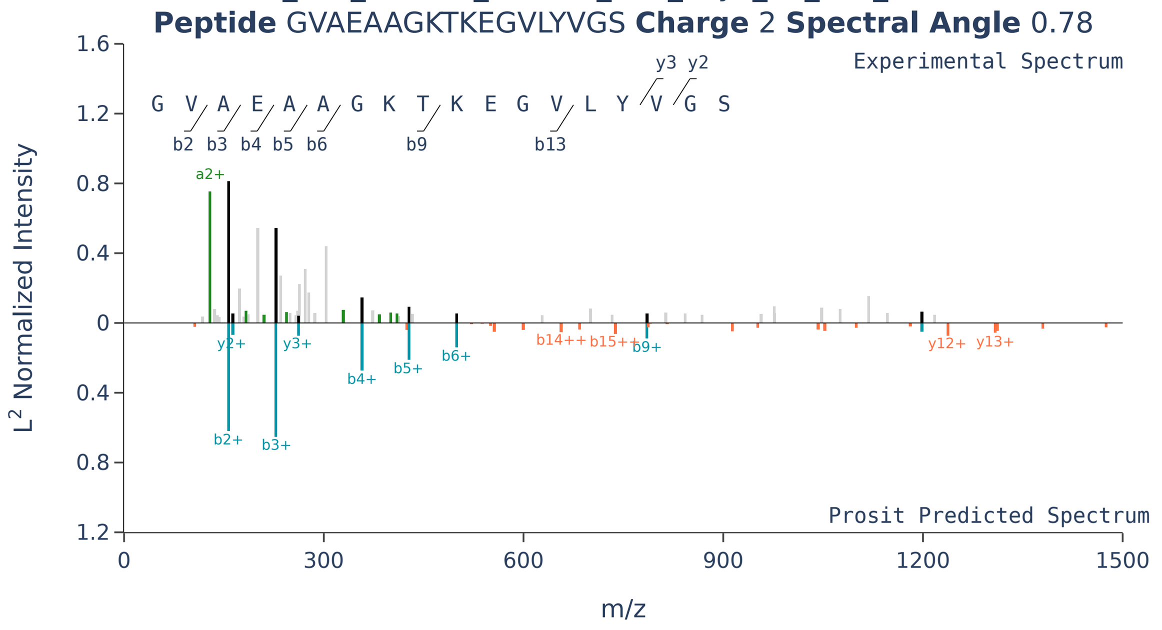


**Source** W_Soh_160821_170921_Goe_aSyn_A2_6h_R1 **Scan** 19135
**Peptide** AGSIAAATGFVKKDQLGKNEEGAPQEGIL **Charge** 4 **Spectral Angle** 0.84

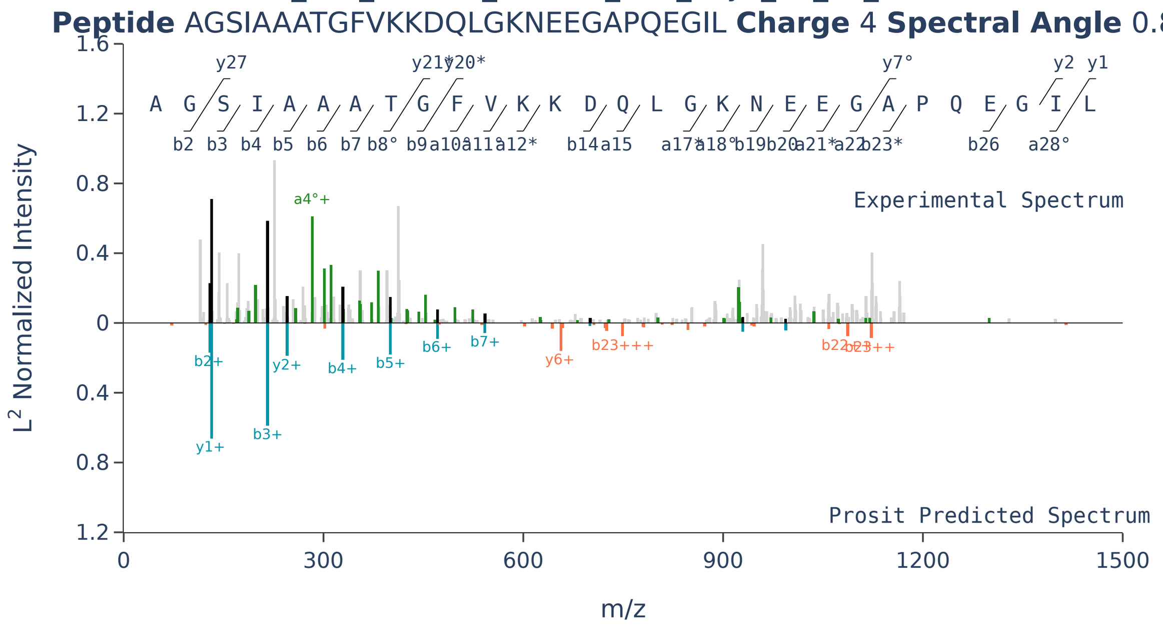


**Source** W_Soh_160821_170921_Goe_aSyn_A2_24h_R1 **Scan** 6679
**Peptide** KEQVTNVGGA **Charge** 2 **Spectral Angle** 0.56

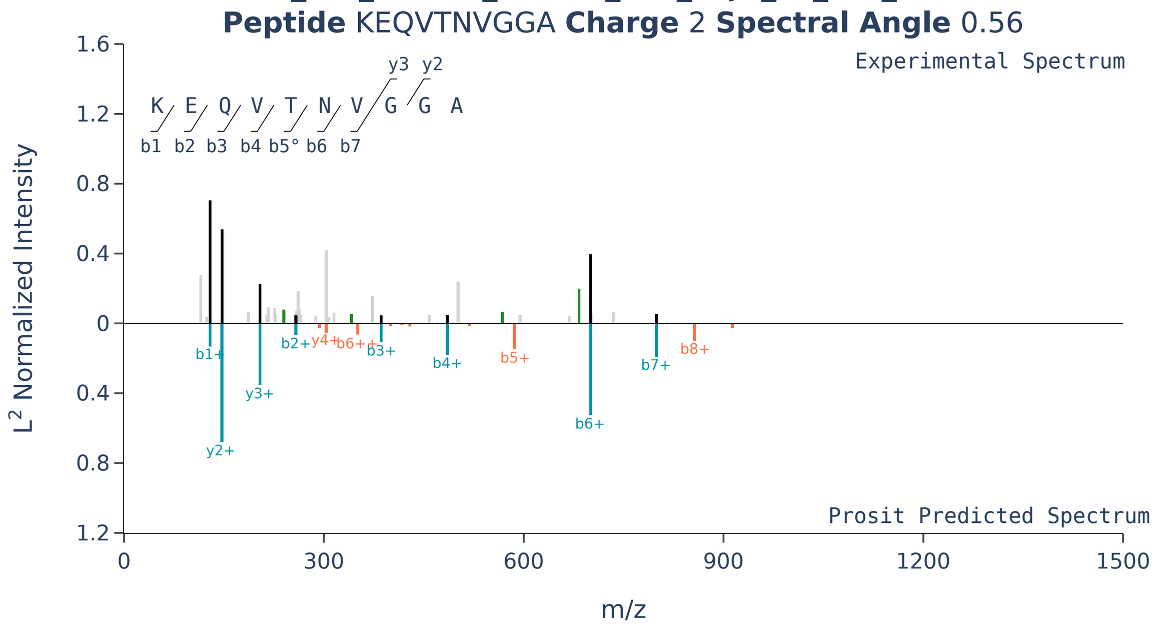


**Source** W_Soh_160821_170921_Goe_aSyn_A1_24h_R1 **Scan** 17000
**Peptide** VEGAGSIAAATGFVKKDQLGKNE **Charge** 3 **Spectral Angle** 0.77

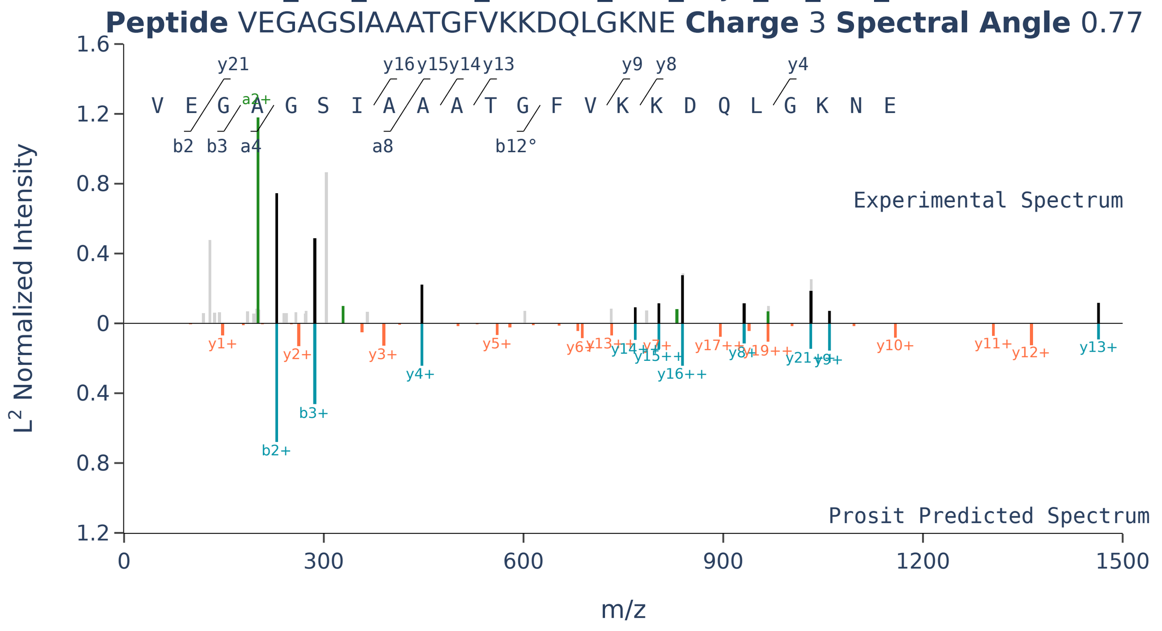


**Source** W_Soh_160821_170921_Goe_aSyn_A1_24h_R2 **Scan** 14960
**Peptide** HGVATVAEKTKEQVTNVGGAVVTG **Charge** 3 **Spectral Angle** 0.69

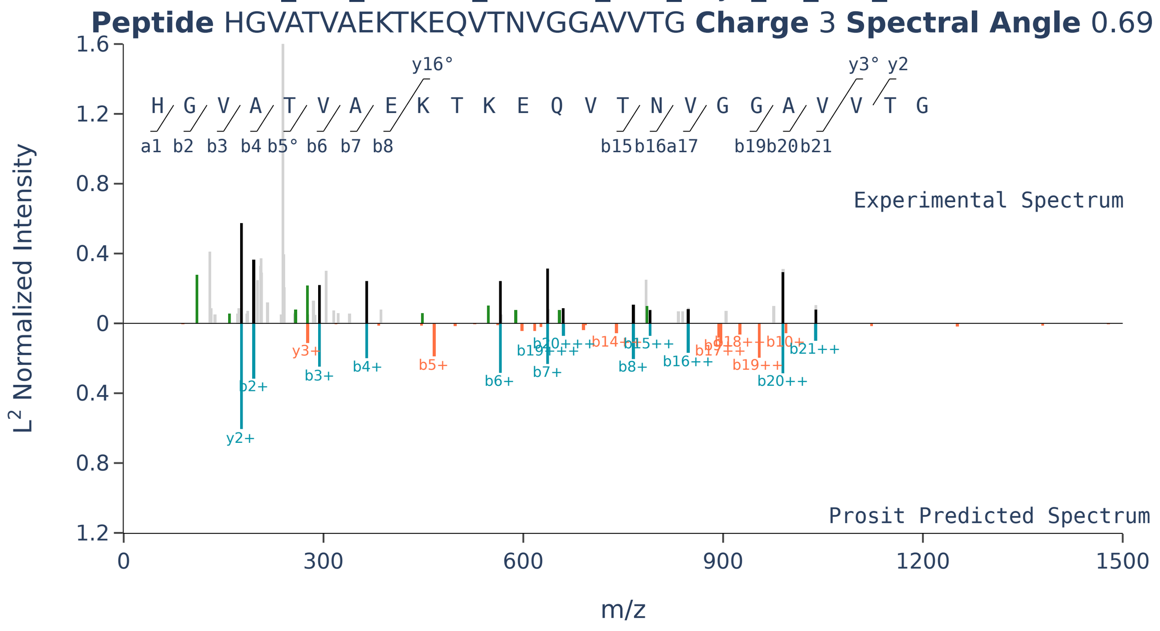


**Source** W_Soh_160821_170921_Goe_aSyn_A1_24h_R1 **Scan** 21641
**Peptide** VATVAEKTKEQVTNVGGAVVTGVTAVAQK **Charge** 4 **Spectral Angle** 0.79

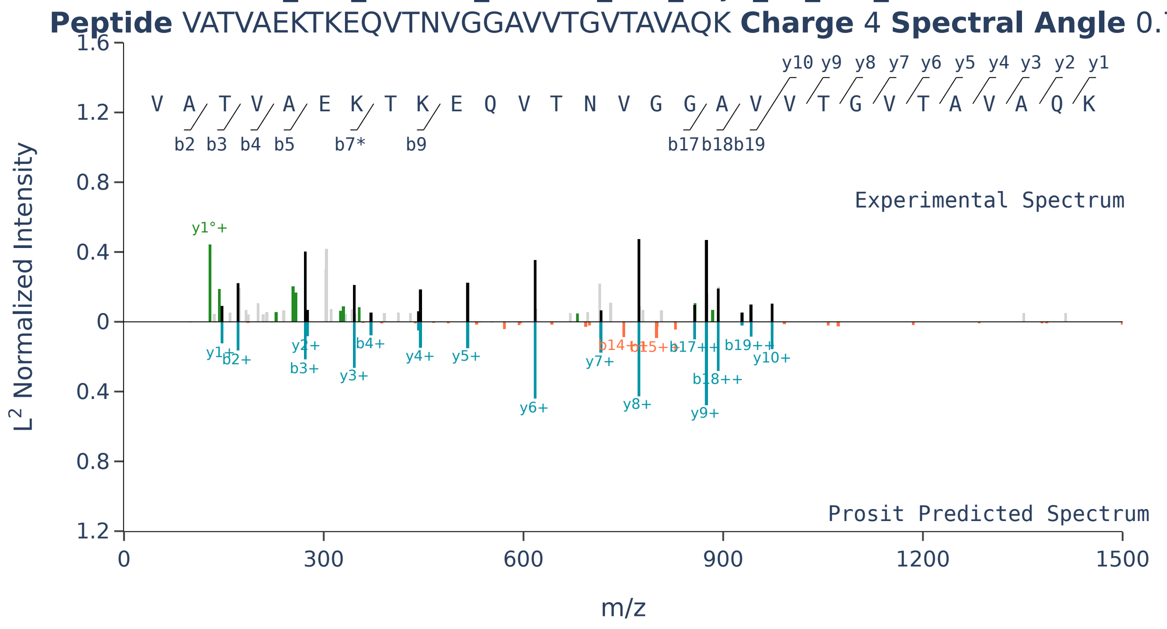


**Source** W_Soh_160821_170921_Goe_aSyn_A2_6h_R1 **Scan** 8101
**Peptide** AEKTKEQVTNVGGAV **Charge** 3 **Spectral Angle** 0.86

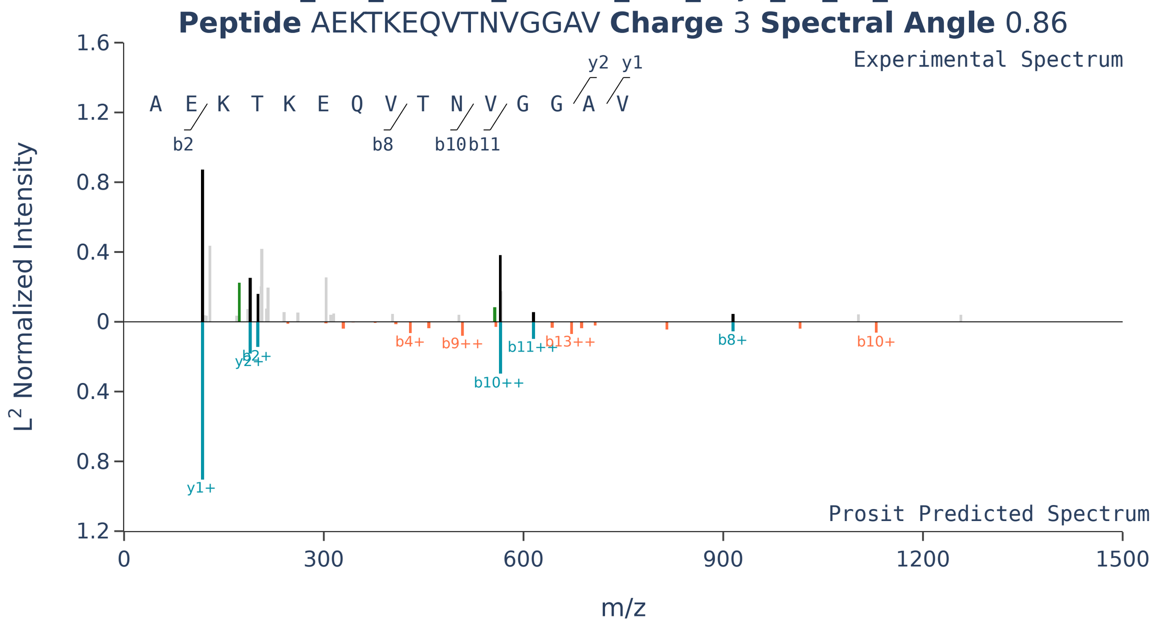


**Source** W_Soh_160821_170921_Goe_aSyn_A1_24h_R1 **Scan** 8976
**Peptide** KEGVVAAAEKTKQGVAEAAGKTK **Charge** 4 **Spectral Angle** 0.88

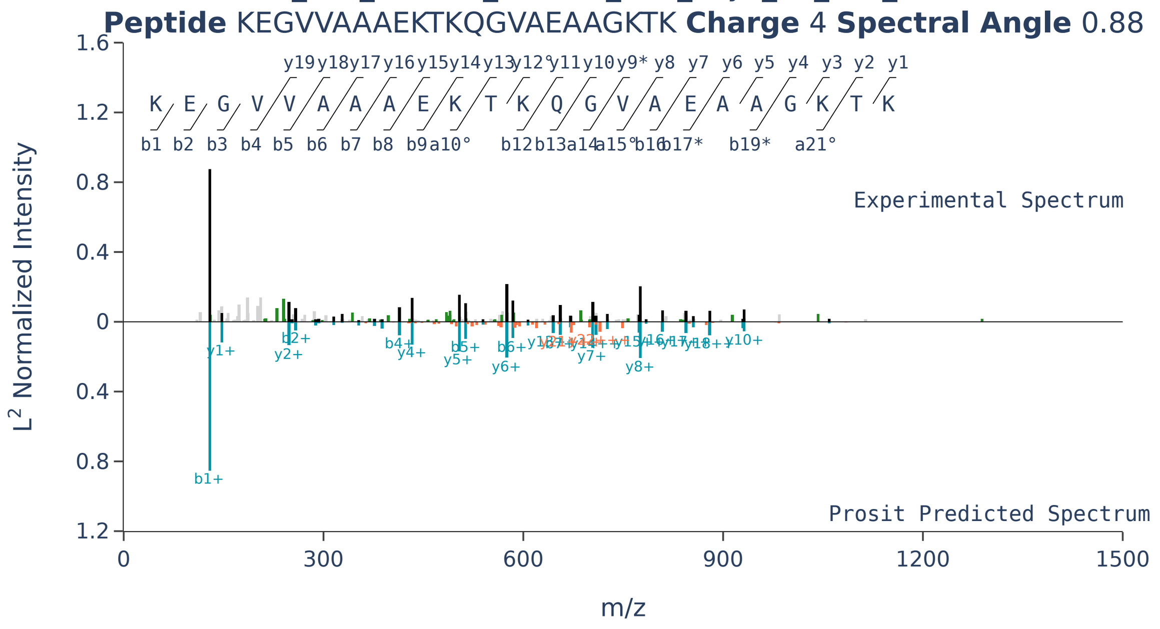


**Source** W_Soh_160821_170921_Goe_aSyn_A2_24h_R2 **Scan** 21282
**Peptide** GGAVVTGVTAVAQKTVEGA **Charge** 2 **Spectral Angle** 0.82

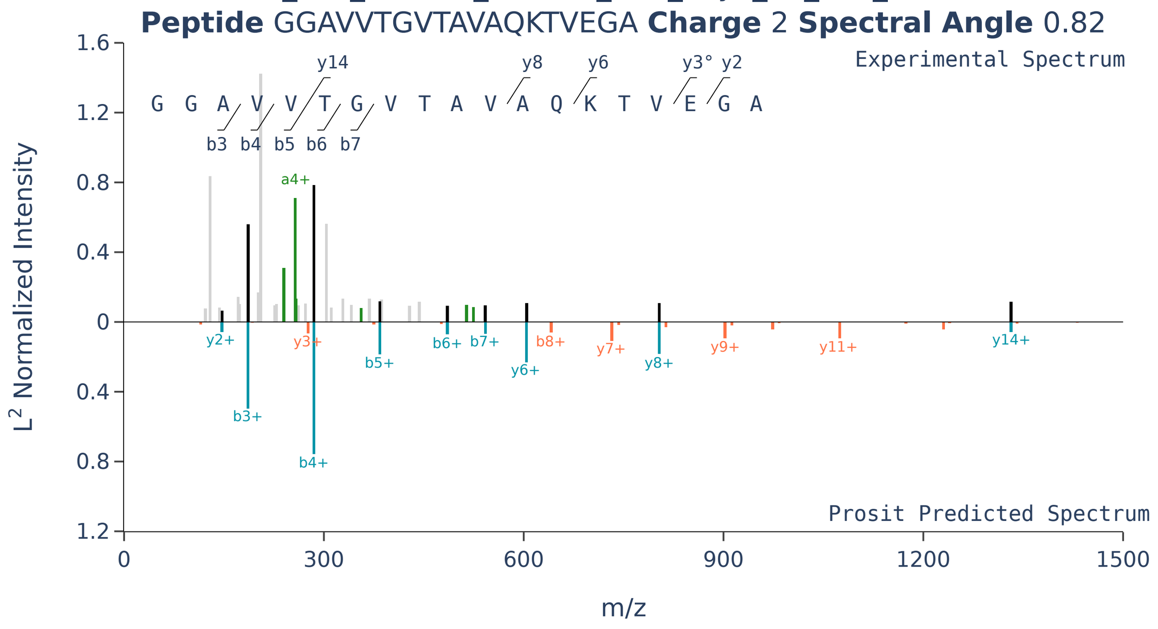


**Source** W_Soh_160821_170921_Goe_aSyn_A1_6h_R1 **Scan** 16317
**Peptide** QLGKNEEGAPQEGIL **Charge** 2 **Spectral Angle** 0.79

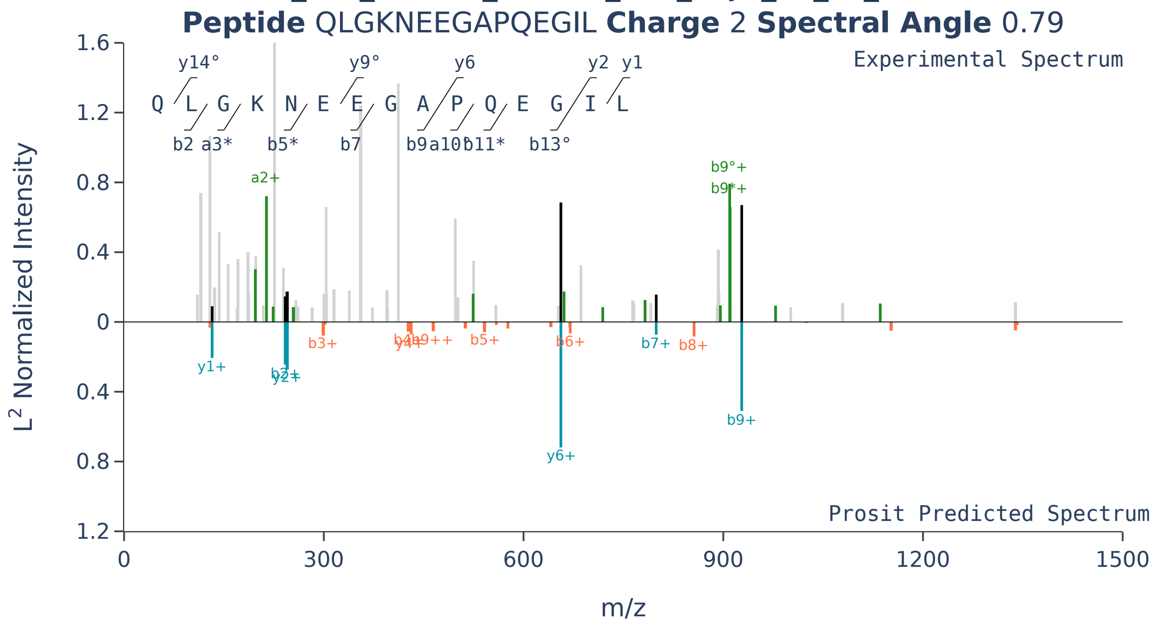


**Source** W_Soh_160821_170921_Goe_aSyn_A1_24h_R2 **Scan** 10514
**Peptide** KTKEQVTNVGGAVV **Charge** 2 **Spectral Angle** 0.63

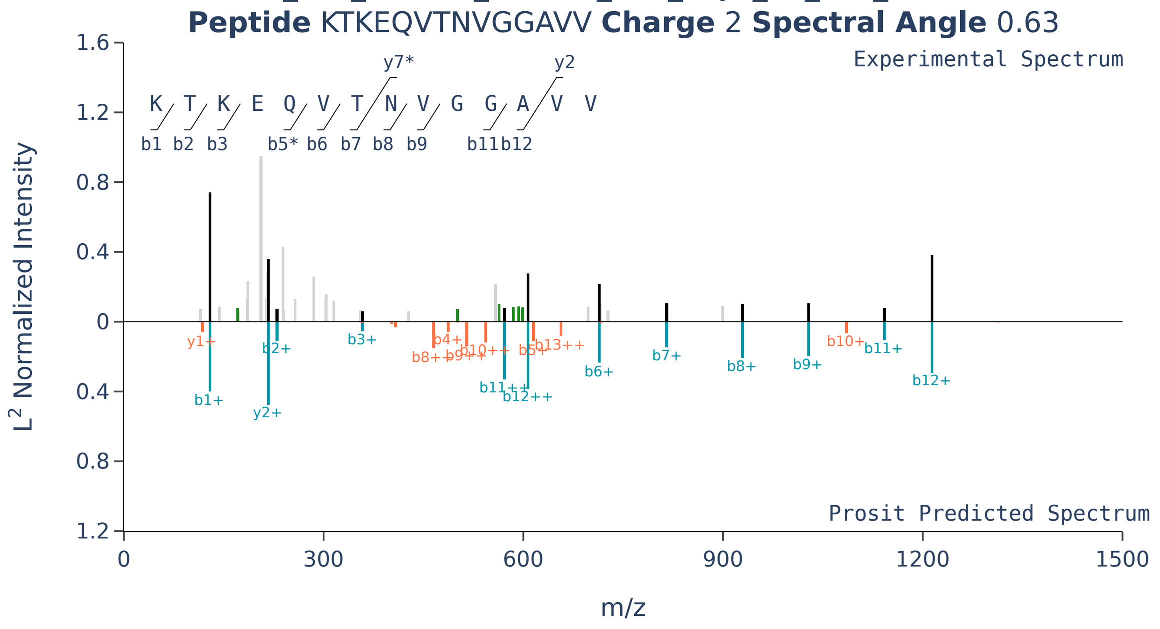

**Source** W_Soh_160821_170921_Goe_aSyn_A1_6h_R1 **Scan** 26042
**Peptide** GKNEEGAPQEGILEDMPVDPDNEAYEMPSE **Charge** 3 **Spectral Angle** 0.84

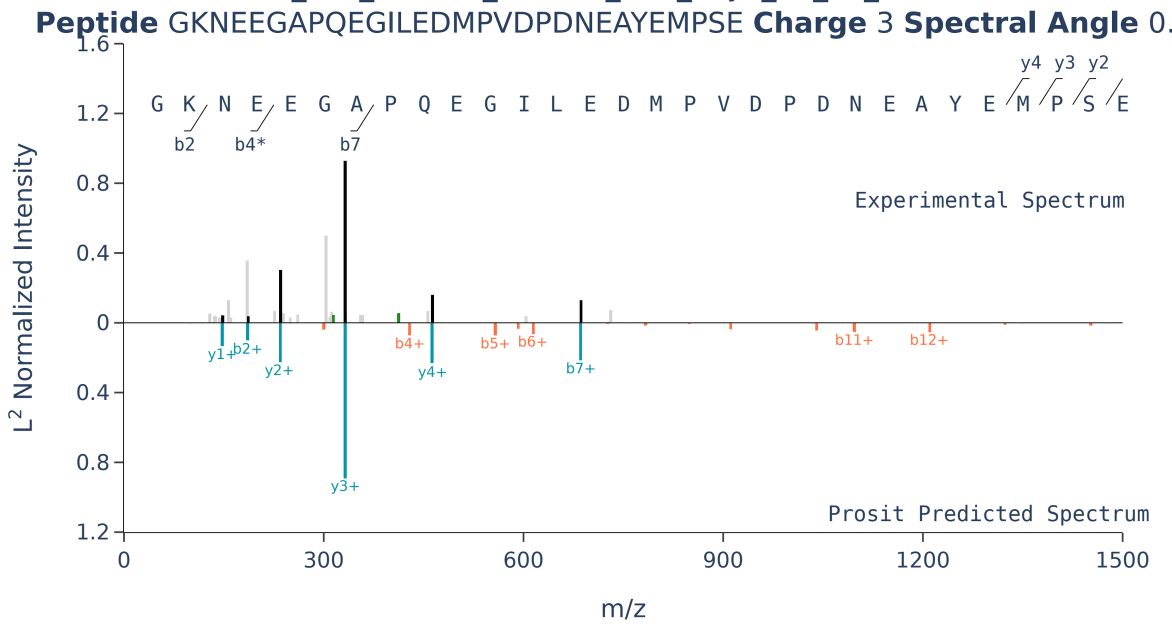


**Source** W_Soh_160821_170921_Goe_aSyn_A2_24h_R2 **Scan** 8467
**Peptide** GLSKAKEGVVAAAE **Charge** 3 **Spectral Angle** 0.85

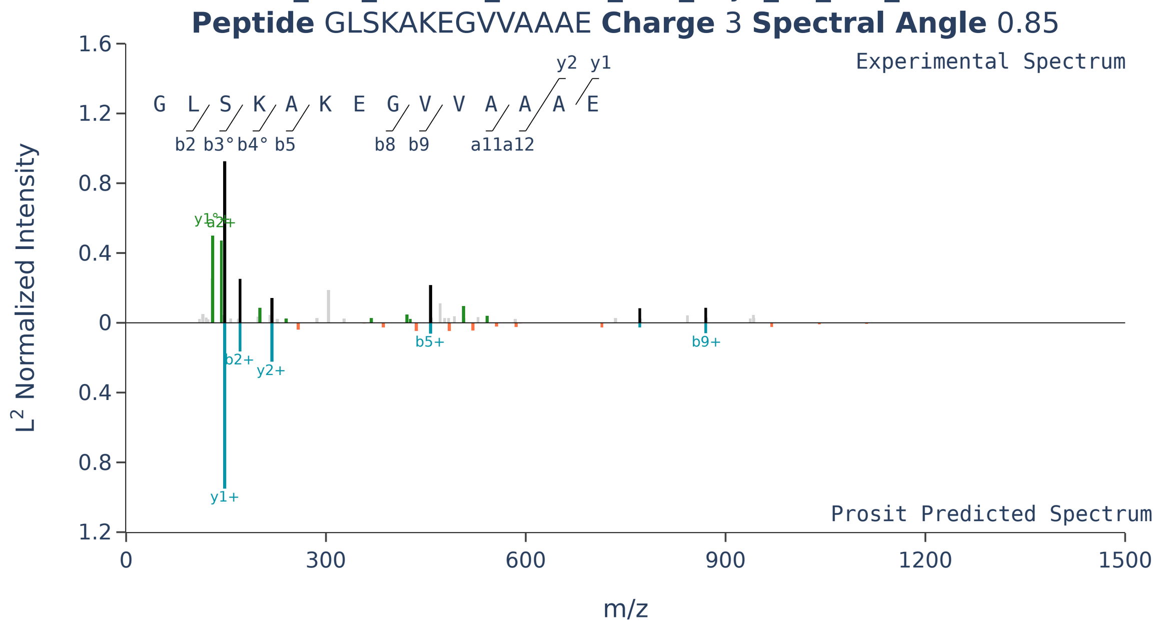


**Source** W_Soh_160821_170921_Goe_aSyn_A1_24h_R2 **Scan** 12154
**Peptide** GVAEAAGKTKEGVLY **Charge** 3 **Spectral Angle** 0.8

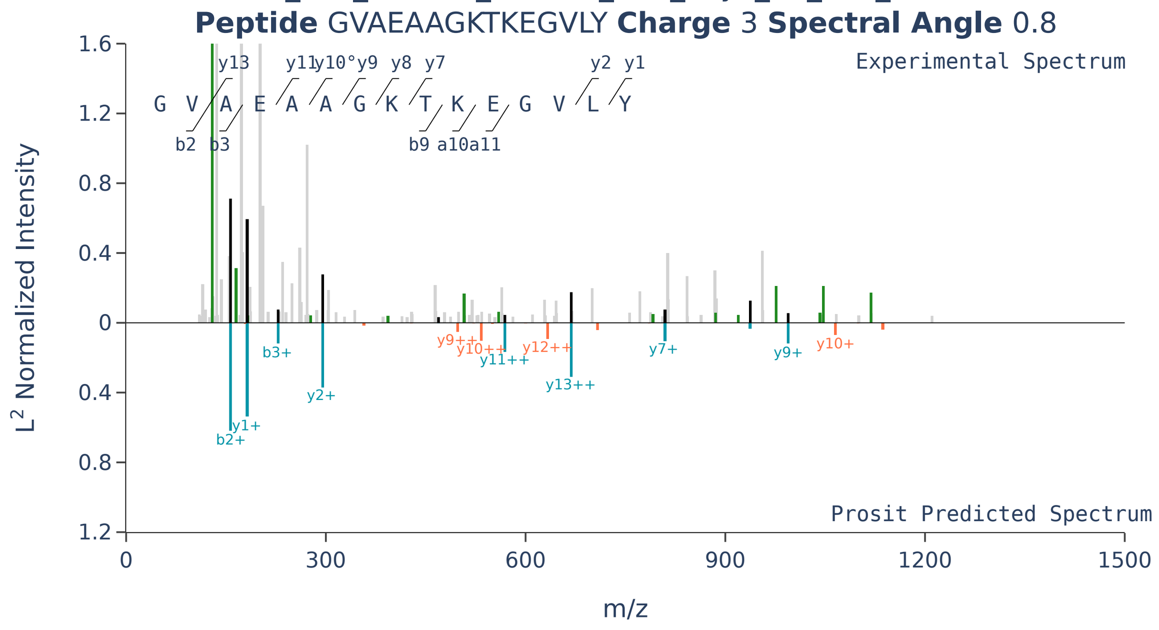


**Source** W_Soh_160821_170921_Goe_aSyn_A1_24h_R2 **Scan** 12199
**Peptide** AATGFVKKDQLGKNEEGAPQEGI **Charge** 3 **Spectral Angle** 0.67

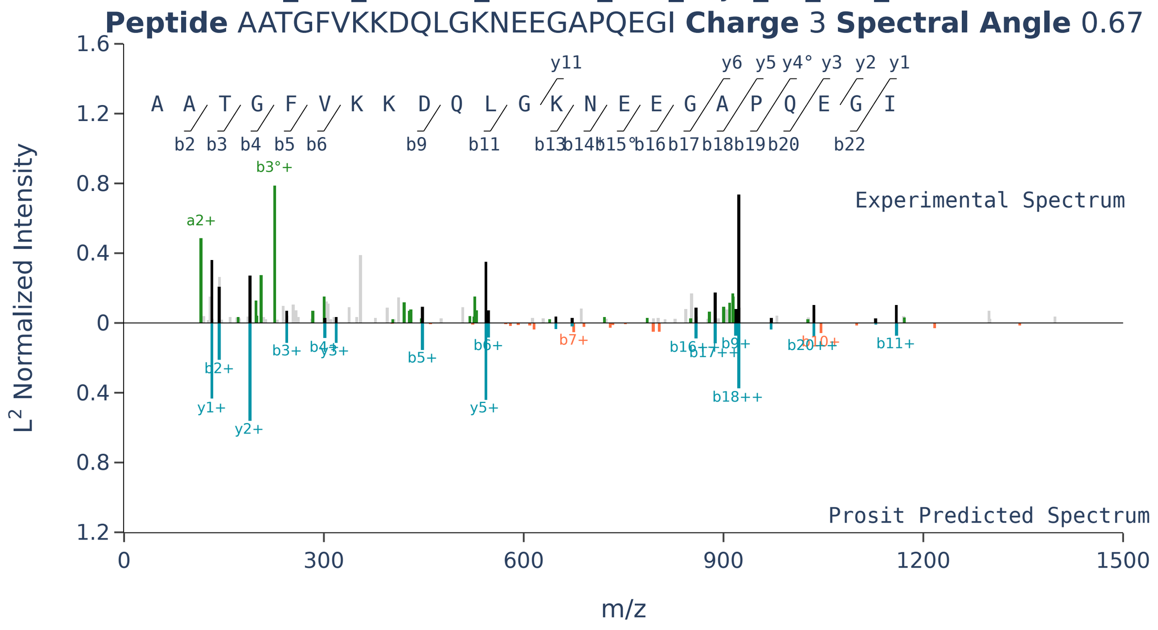


**Source** W_Soh_160821_170921_Goe_aSyn_A1_3h_R1 **Scan** 15248
**Peptide** GAPQEGILED **Charge** 2 **Spectral Angle** 0.6

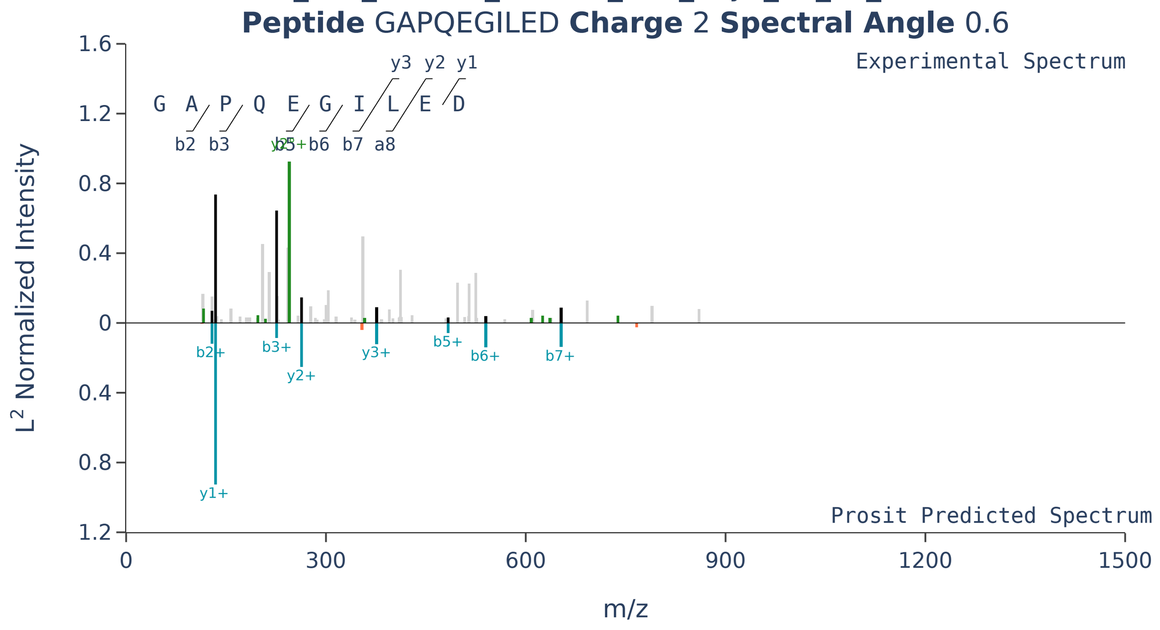


**Source** W_Soh_160821_170921_Goe_aSyn_A2_24h_R2 **Scan** 9237
**Peptide** YVGSKTKEGVVHGVATVAEKTKE **Charge** 4 **Spectral Angle** 0.73

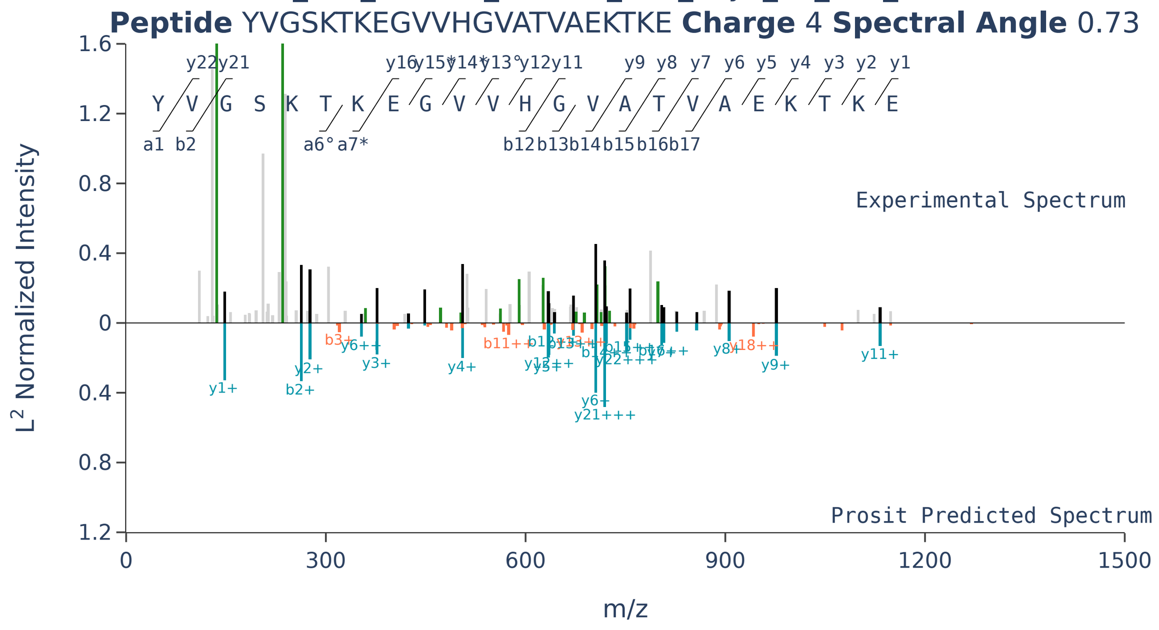


**Source** W_Soh_160821_170921_Goe_aSyn_A2_24h_R1 **Scan** 12898
**Peptide** AAGKTKEGVLYVGSKTKEGVVHGVATVAE **Charge** 4 **Spectral Angle** 0.78

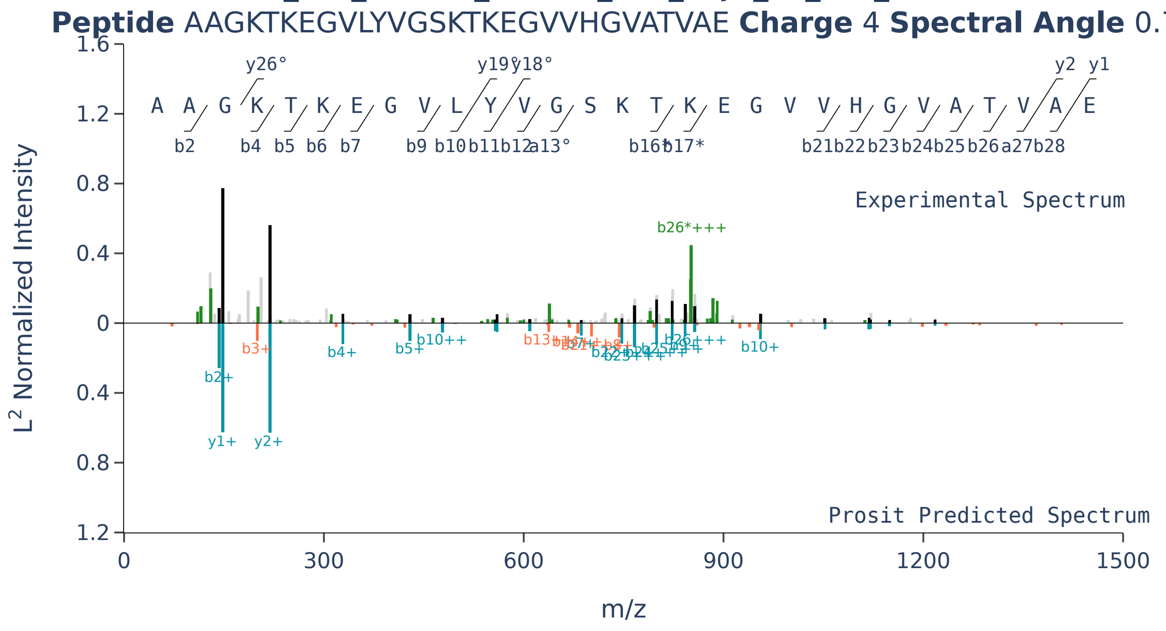


**Source** W_Soh_160821_170921_Goe_aSyn_A2_24h_R1 **Scan** 15411
**Peptide** EQVTNVGGAVVT **Charge** 2 **Spectral Angle** 0.59

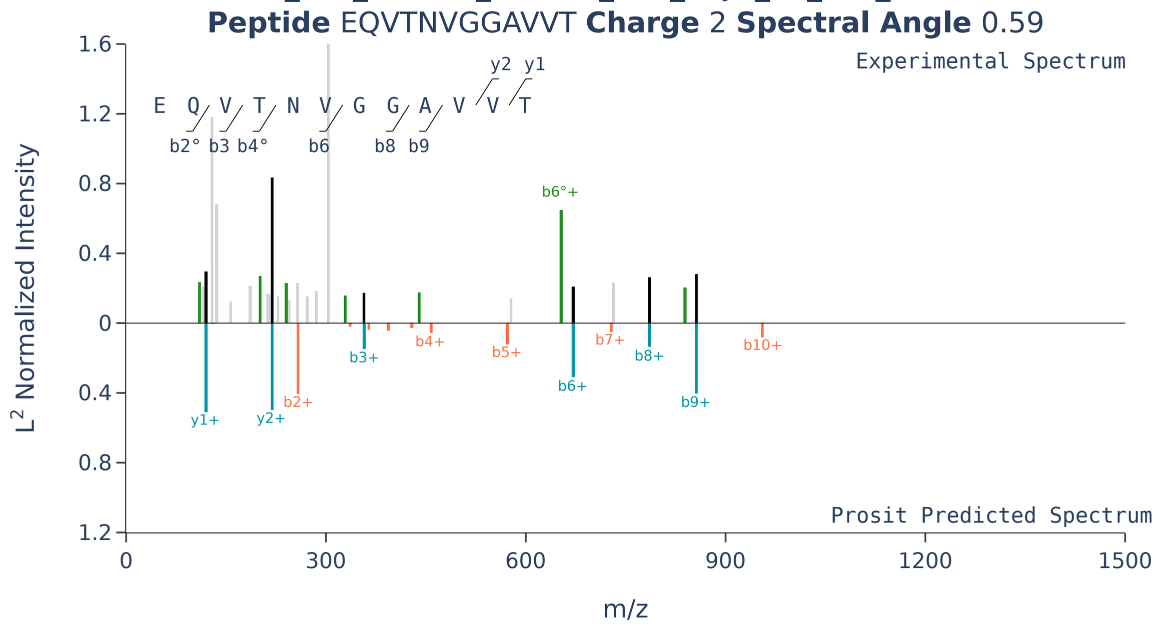


**Source** W_Soh_160821_170921_Goe_aSyn_A1_3h_R2 **Scan** 23293
**Peptide** GVATVAEKTKEQVTNVGGAVVTGVTAVAQ **Charge** 3 **Spectral Angle** 0.73

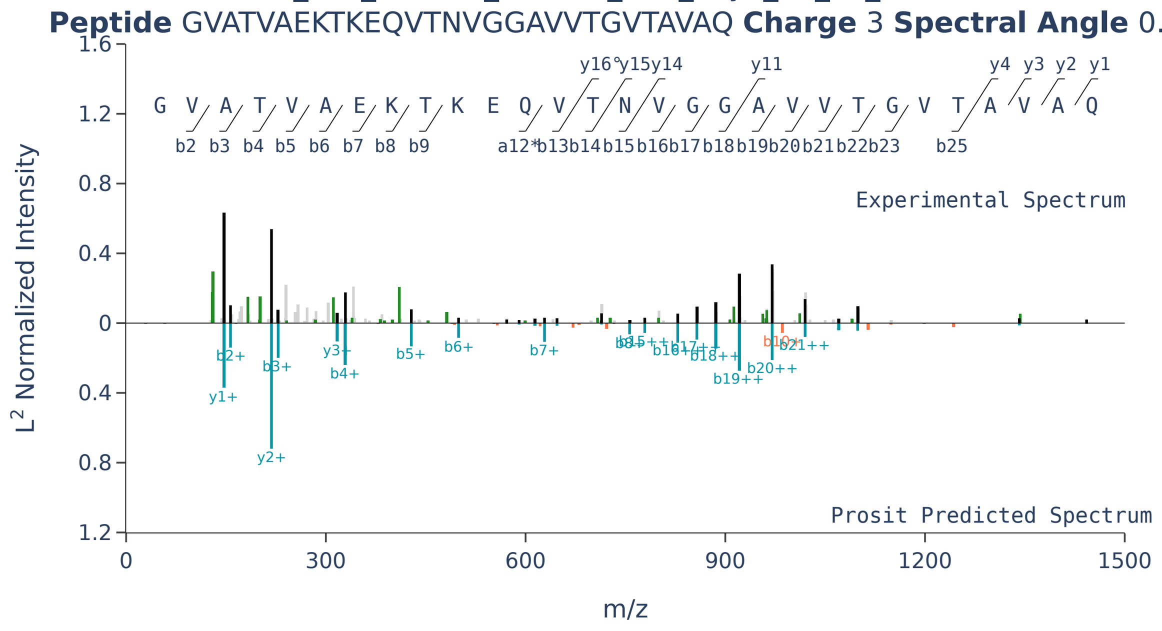


**Source** W_Soh_160821_170921_Goe_aSyn_A2_6h_R2 **Scan** 18340
**Peptide** VKKDQLGKNEEGAPQEGILEDMPVD **Charge** 3 **Spectral Angle** 0.85

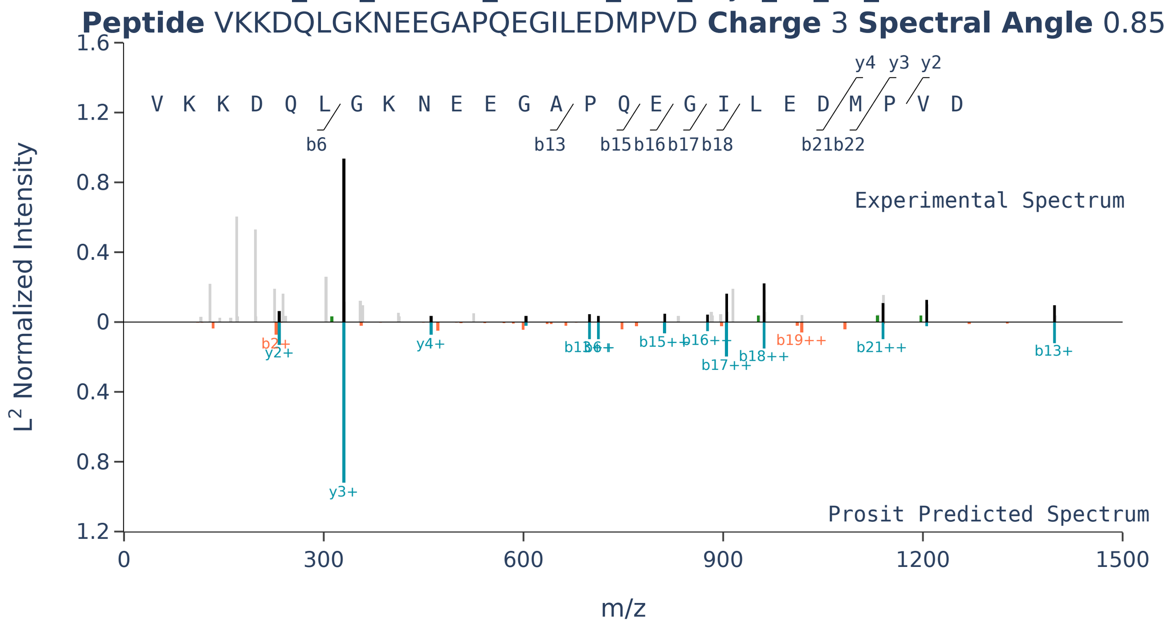


**Source** W_Soh_160821_170921_Goe_aSyn_A2_24h_R2 **Scan** 9210
**Peptide** AAATGFVK **Charge** 2 **Spectral Angle** 0.67

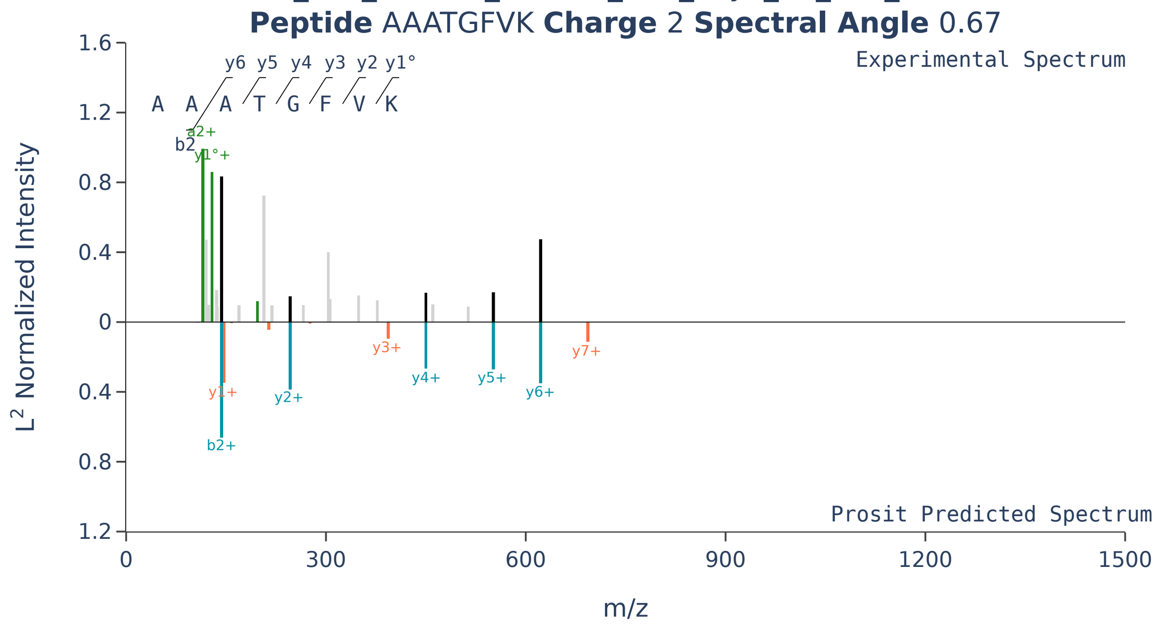


**Source** W_Soh_160821_170921_Goe_aSyn_A1_24h_R1 **Scan** 8707
**Peptide** AEKTKQGVAEAAGKTKEGVLYVGSKTK **Charge** 6 **Spectral Angle** 0.76

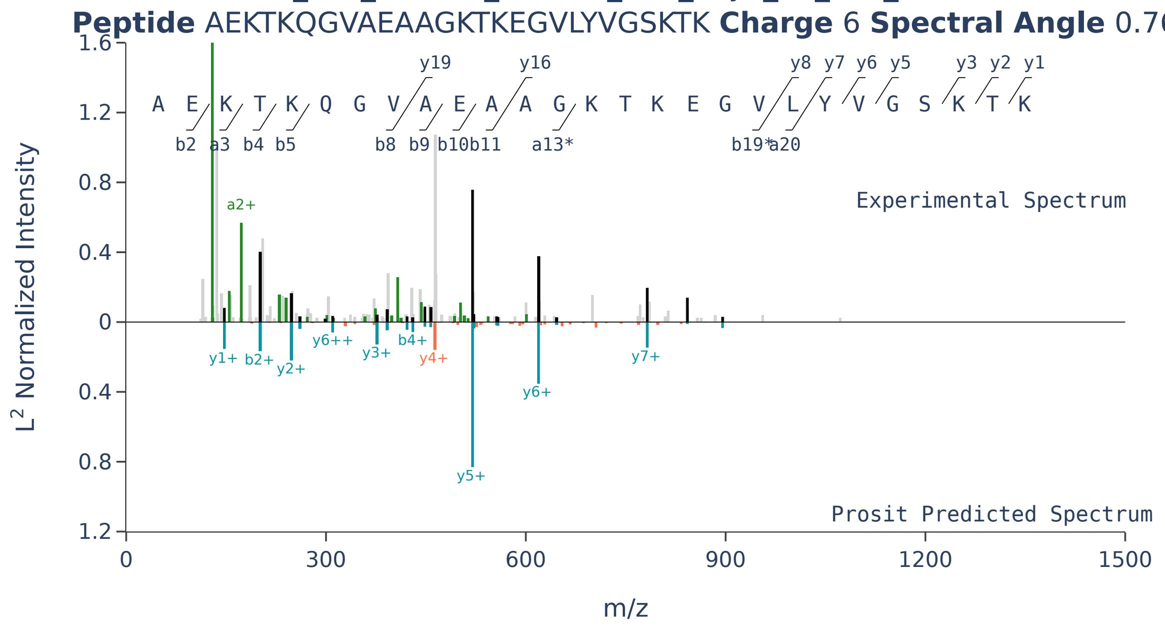


**Source** W_Soh_160821_170921_Goe_aSyn_A1_24h_R1 **Scan** 24278
**Peptide** QVTNVGGAVVTGVTAVA **Charge** 2 **Spectral Angle** 0.71

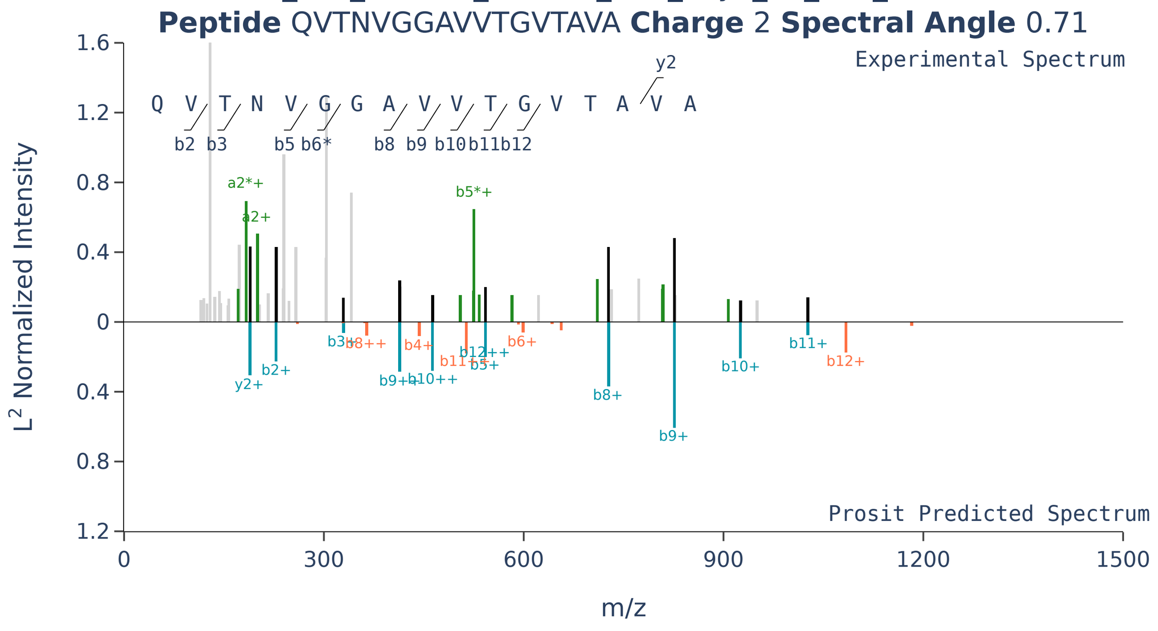


**Source** W_Soh_160821_170921_Goe_aSyn_A1_24h_R2 **Scan** 12442
**Peptide** QGVAEAAGKTKEGVLY **Charge** 3 **Spectral Angle** 0.78

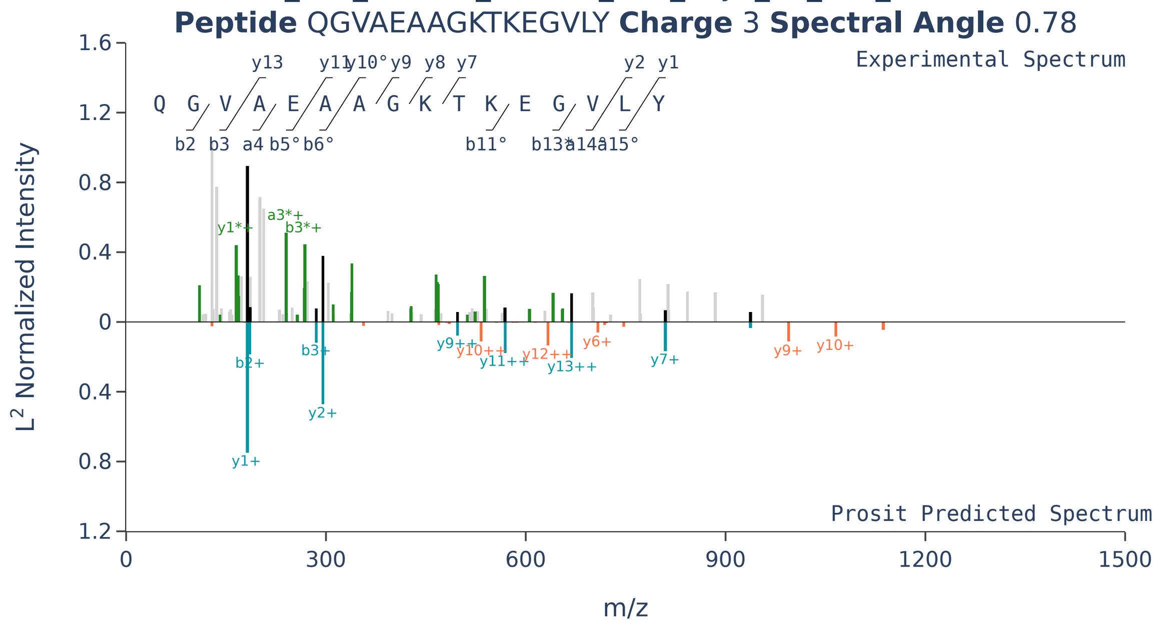


**Source** W_Soh_160821_170921_Goe_aSyn_A1_24h_R1 **Scan** 15040
**Peptide** GAGSIAAATGFVKKDQLGKNEEGA **Charge** 3 **Spectral Angle** 0.66

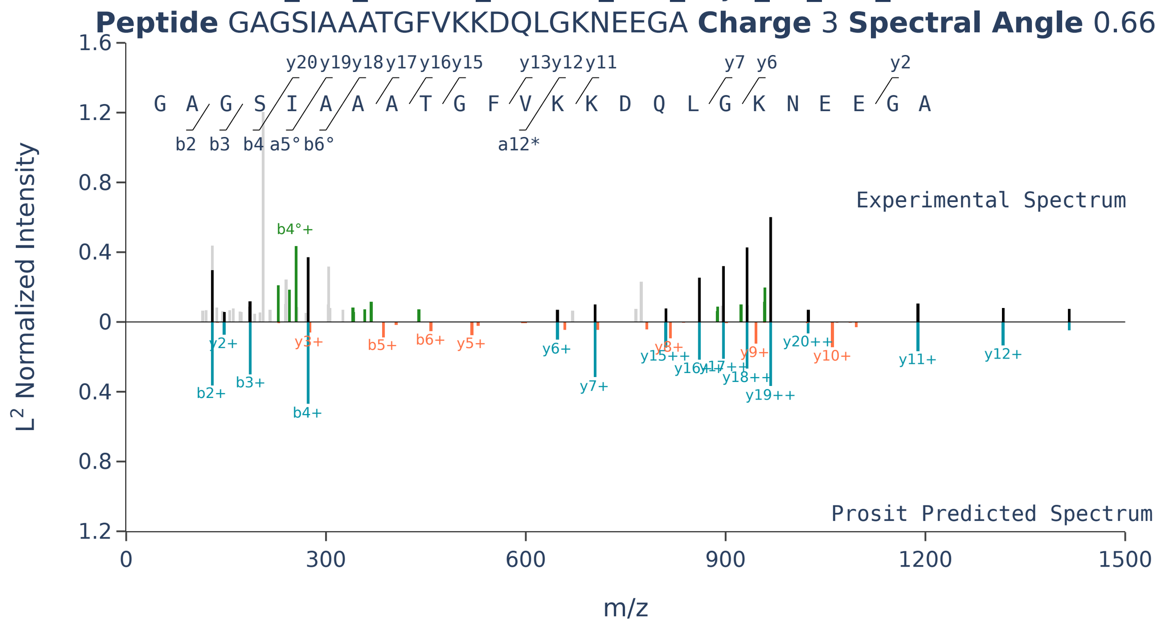

**Source** W_Soh_160821_170921_Goe_aSyn_A2_6h_R2 **Scan** 26250
**Peptide** VGGAVVTGVTAVAQKTVEGAGSIAA **Charge** 3 **Spectral Angle** 0.78

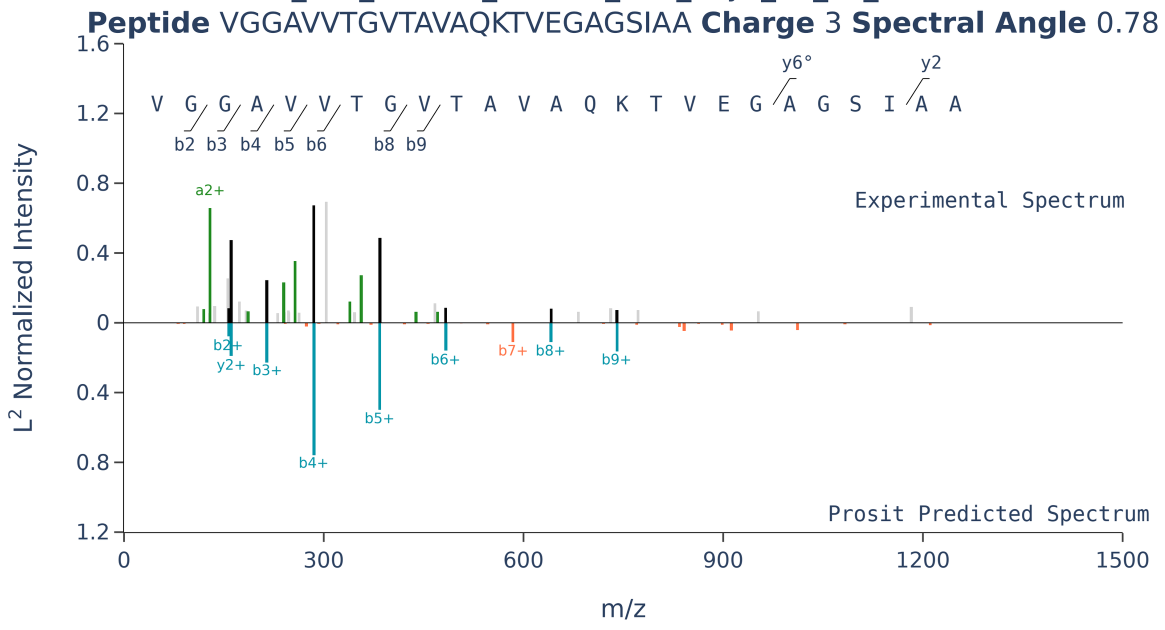


**Source** W_Soh_160821_170921_Goe_aSyn_A1_24h_R2 **Scan** 13468
**Peptide** KTKEQVTNVGGAVVTGVT **Charge** 2 **Spectral Angle** 0.68

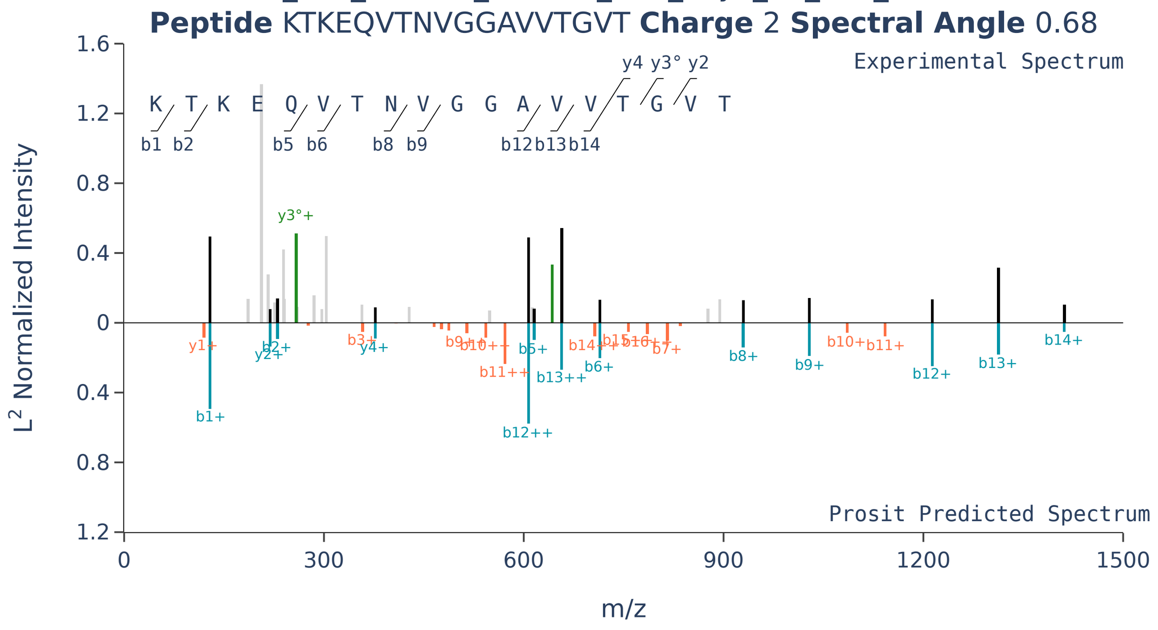


**Source** W_Soh_160821_170921_Goe_aSyn_A1_24h_R2 **Scan** 13937
**Peptide** TKEGVLYVGSKTKEGVVHGVATV **Charge** 3 **Spectral Angle** 0.81

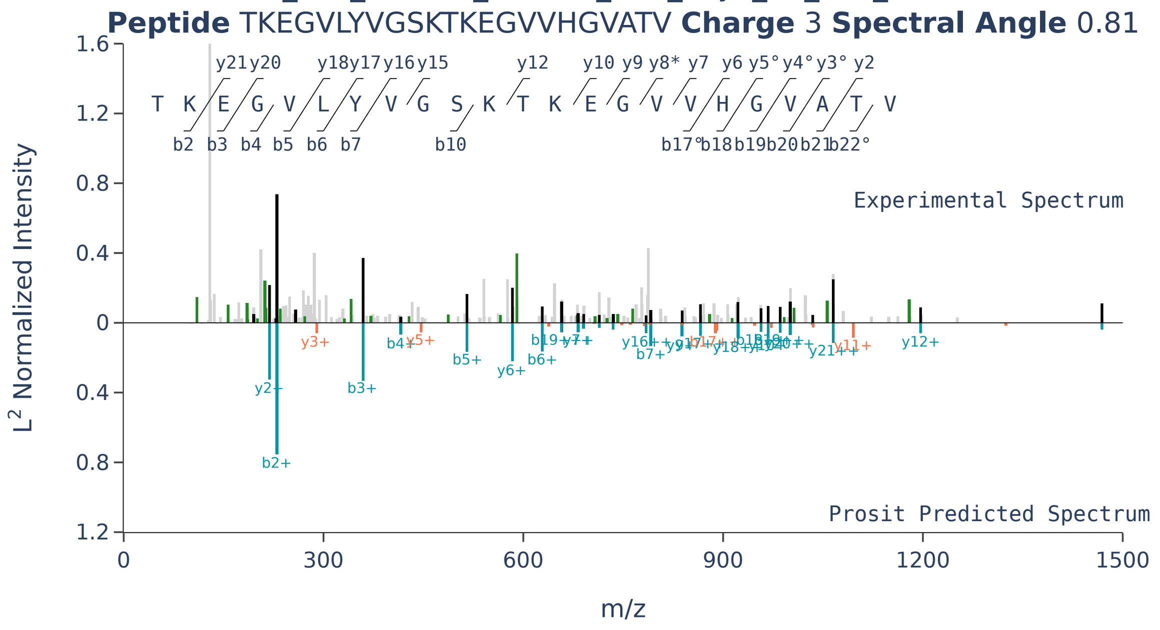


**Source** W_Soh_160821_170921_Goe_aSyn_A1_24h_R1 **Scan** 12428
**Peptide** GVATVAEKTKEQVTNVG **Charge** 3 **Spectral Angle** 0.89

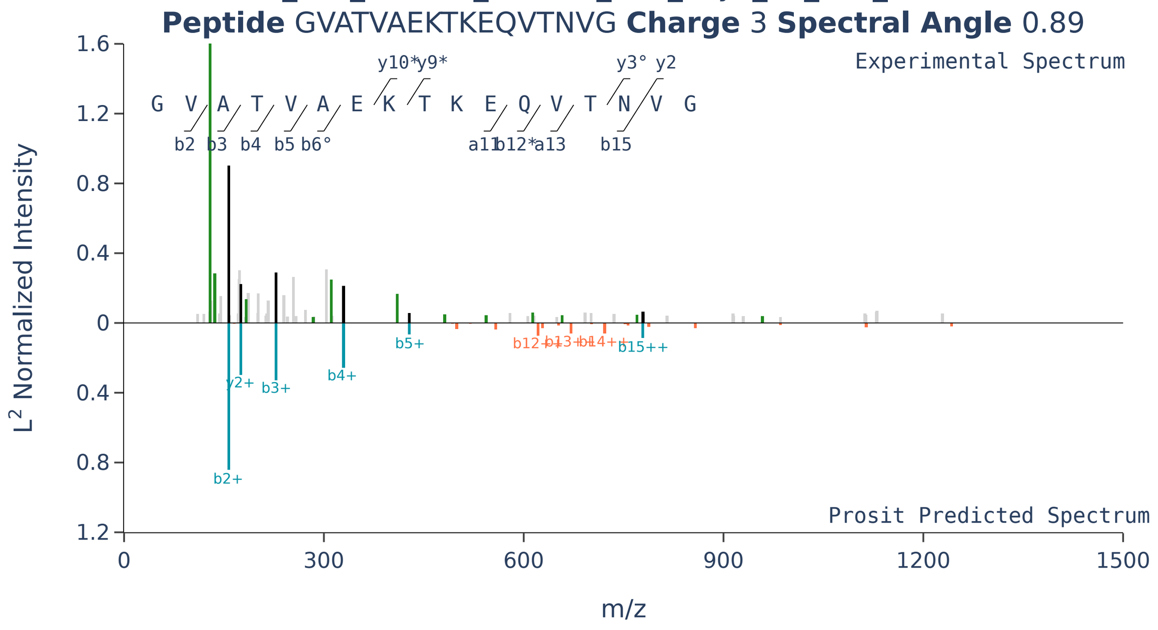


**Source** W_Soh_160821_170921_Goe_aSyn_A1_24h_R1 **Scan** 11270
**Peptide** TKQGVAEAAGKTKEGVLYVGSKTKEGVVH **Charge** 5 **Spectral Angle** 0.77

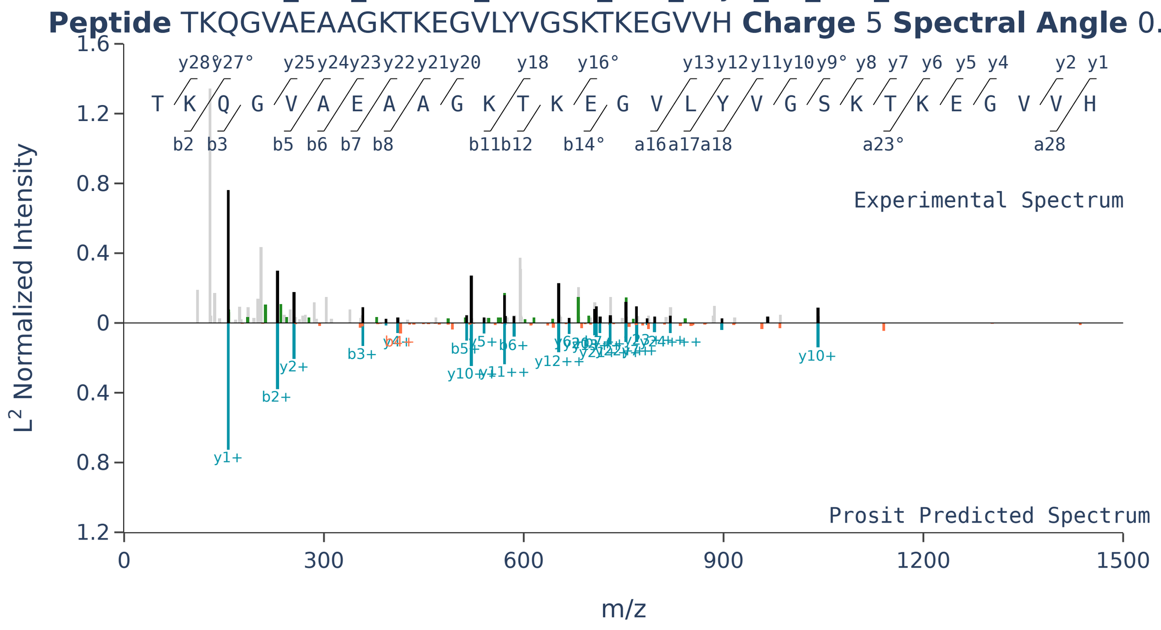


**Source** W_Soh_160821_170921_Goe_aSyn_A2_24h_R2 **Scan** 13130
**Peptide** AEAAGKTKEGVLYV **Charge** 3 **Spectral Angle** 0.91

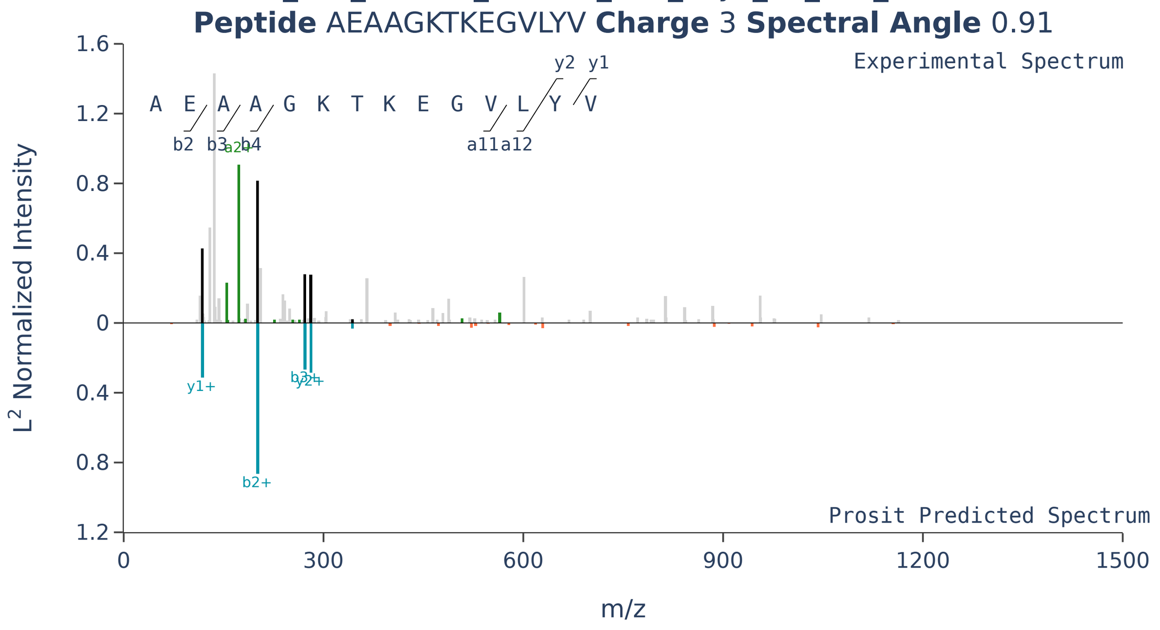


**Source** W_Soh_160821_170921_Goe_aSyn_A1_24h_R1 **Scan** 13160
**Peptide** TVAEKTKEQVTNVGGAVV **Charge** 3 **Spectral Angle** 0.74

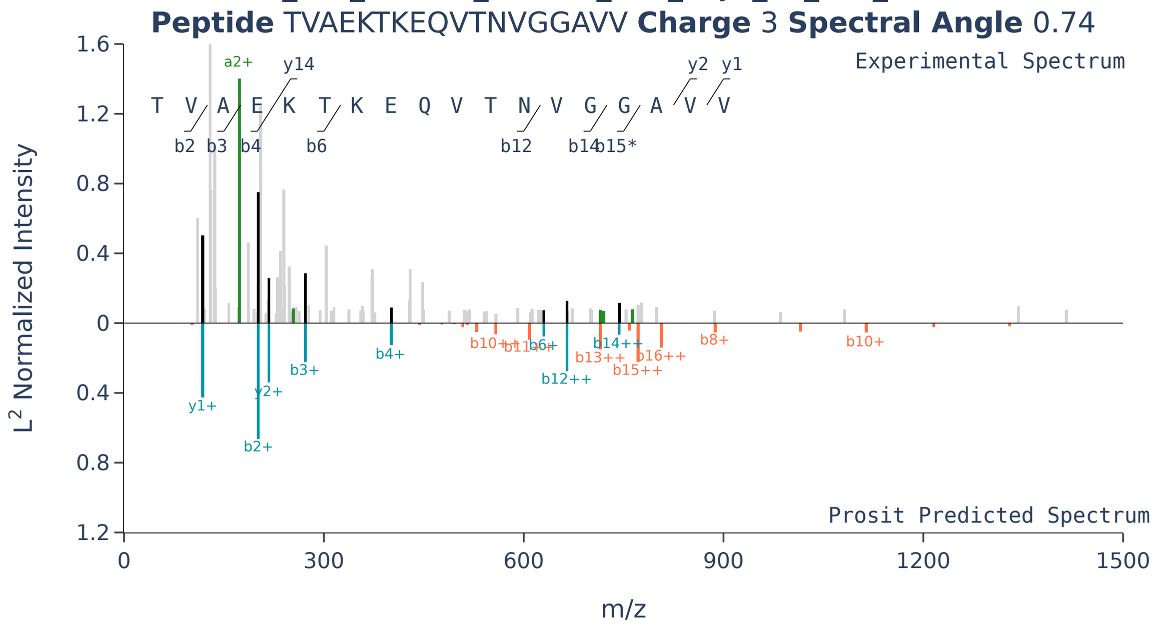


**Source** W_Soh_160821_170921_Goe_aSyn_A2_24h_R2 **Scan** 18489
**Peptide** SIAAATGFVKKDQLGKNEEGAPQEGILED **Charge** 3 **Spectral Angle** 0.74

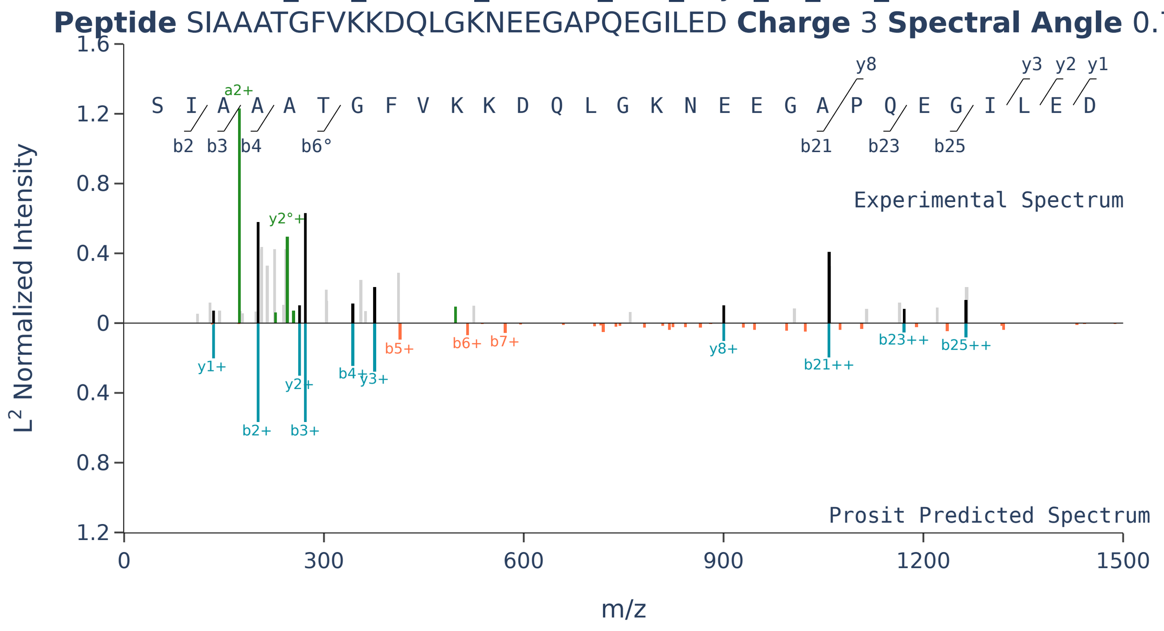


**Source** W_Soh_160821_170921_Goe_aSyn_A2_6h_R1 **Scan** 17187
**Peptide** GAGSIAAATGFVKKDQLGKNEEGAPQEGI **Charge** 3 **Spectral Angle** 0.81

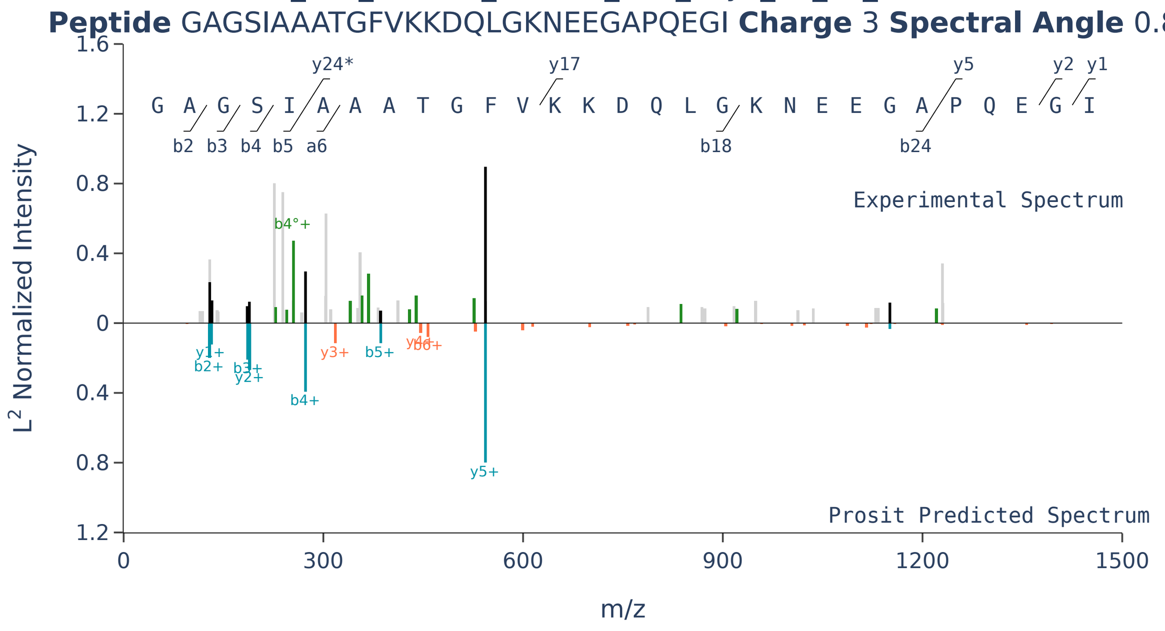


**Source** W_Soh_160821_170921_Goe_aSyn_A1_24h_R2 **Scan** 6340
**Peptide** VAEKTKEQVTNVG **Charge** 3 **Spectral Angle** 0.75

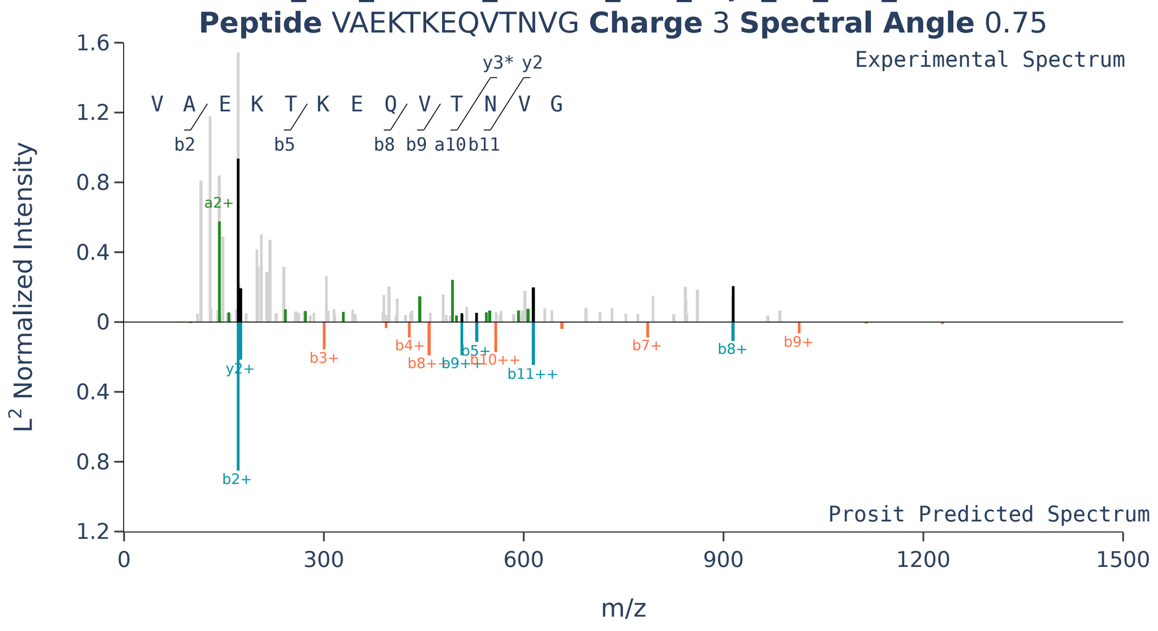


**Source** W_Soh_160821_170921_Goe_aSyn_A2_24h_R2 **Scan** 12622
**Peptide** GVLYVGSKTKEGVVHGVA **Charge** 2 **Spectral Angle** 0.82

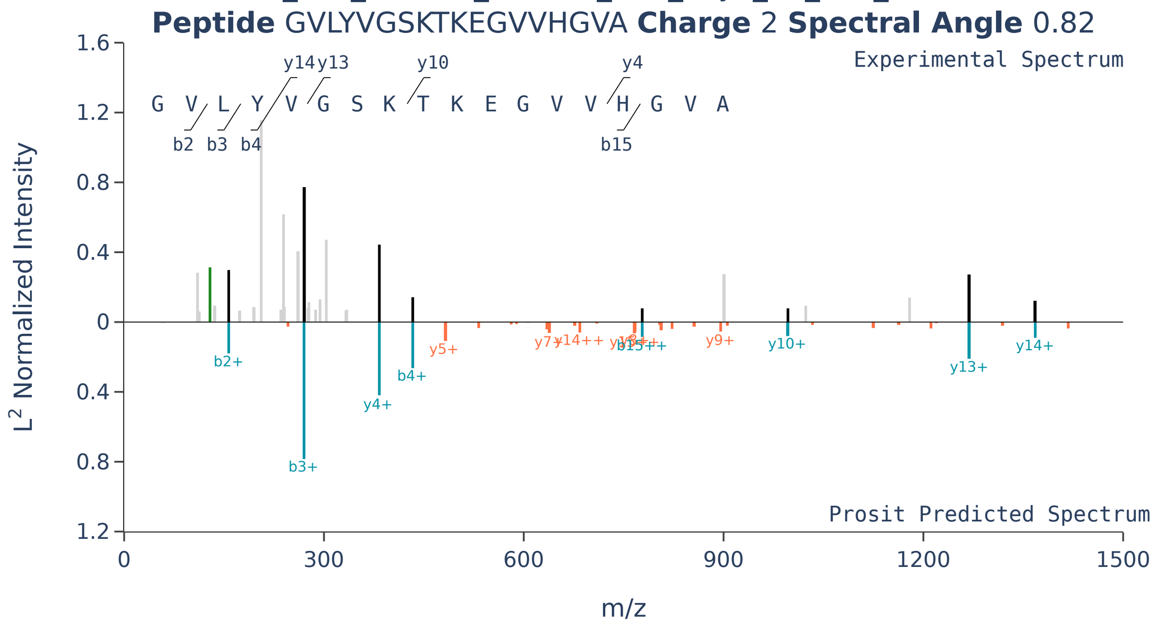


**Source** W_Soh_160821_170921_Goe_aSyn_A1_24h_R2 **Scan** 16078
**Peptide** VLYVGSKTKEGVVHGVATVAEKTKEQVTN **Charge** 4 **Spectral Angle** 0.78

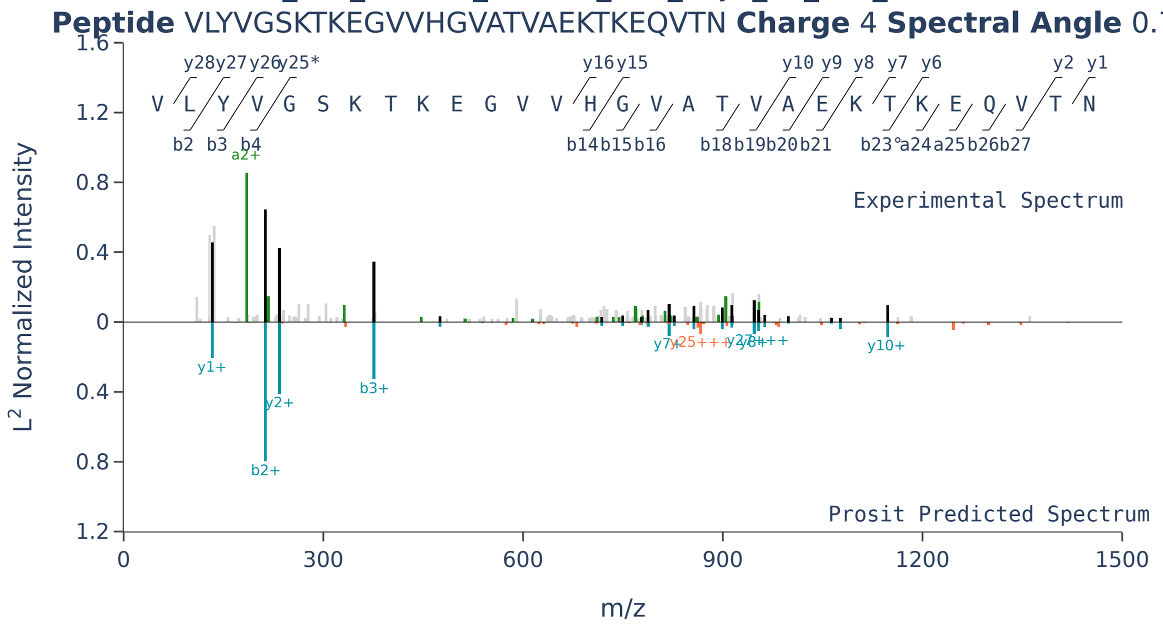


**Source** W_Soh_160821_170921_Goe_aSyn_A1_24h_R1 **Scan** 6936
**Peptide** ATGFVKKDQLGK **Charge** 2 **Spectral Angle** 0.66

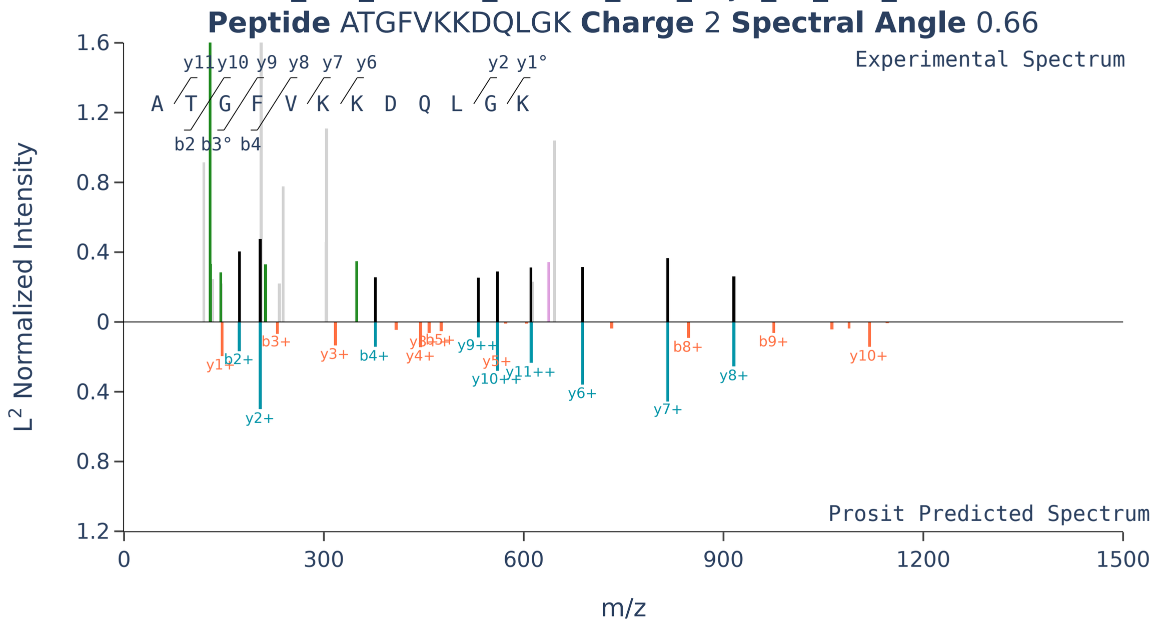


**Source** W_Soh_160821_170921_Goe_aSyn_A1_24h_R1 **Scan** 6544
**Peptide** TAVAQKTVEGAGS **Charge** 2 **Spectral Angle** 0.83

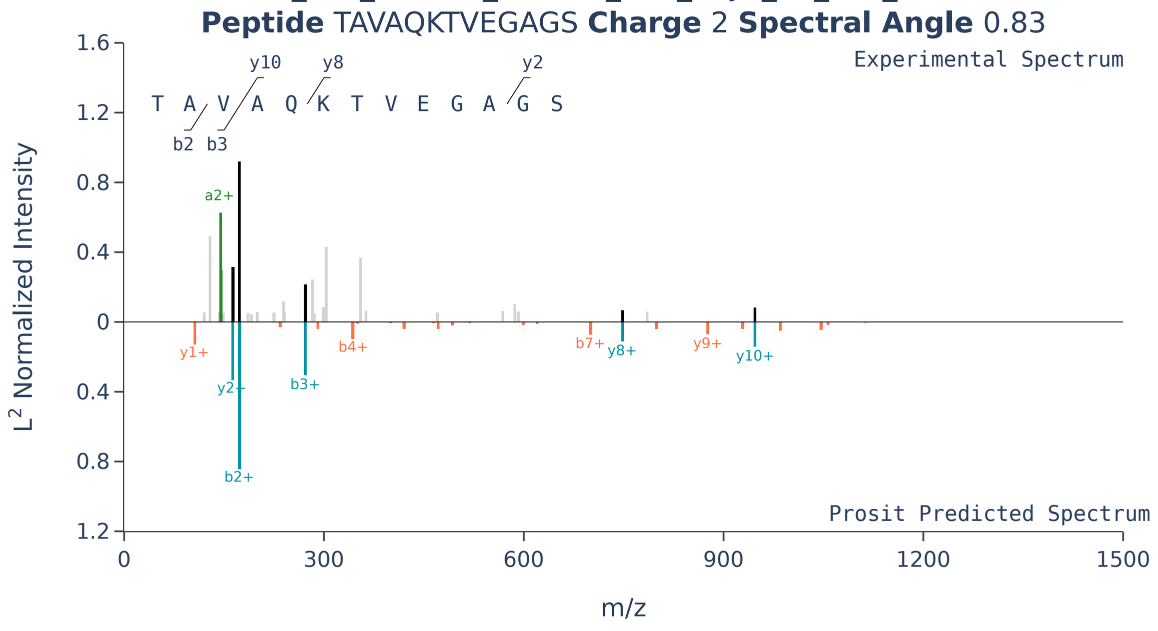


**Source** W_Soh_160821_170921_Goe_aSyn_A1_24h_R2 **Scan** 16923
**Peptide** EGVVHGVATVAEKTKEQVTNV **Charge** 3 **Spectral Angle** 0.7

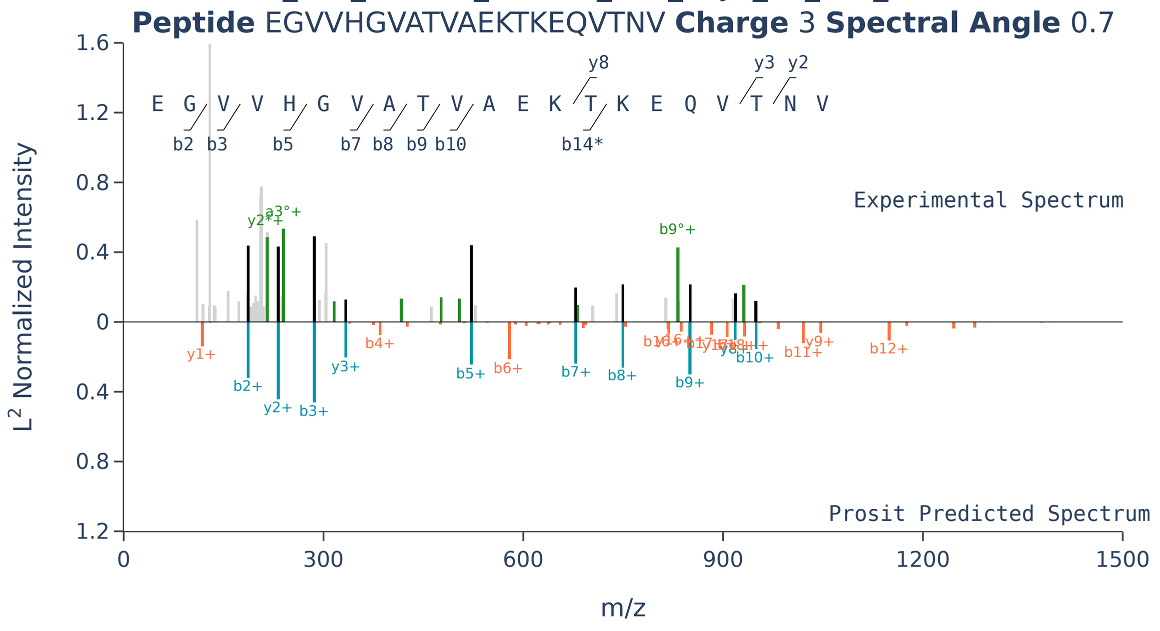

**Source** W_Soh_160821_170921_Goe_aSyn_A2_6h_R2 **Scan** 19698
**Peptide** ATGFVKKDQLGKNEEGAPQEGILEDMPVD **Charge** 3 **Spectral Angle** 0.85

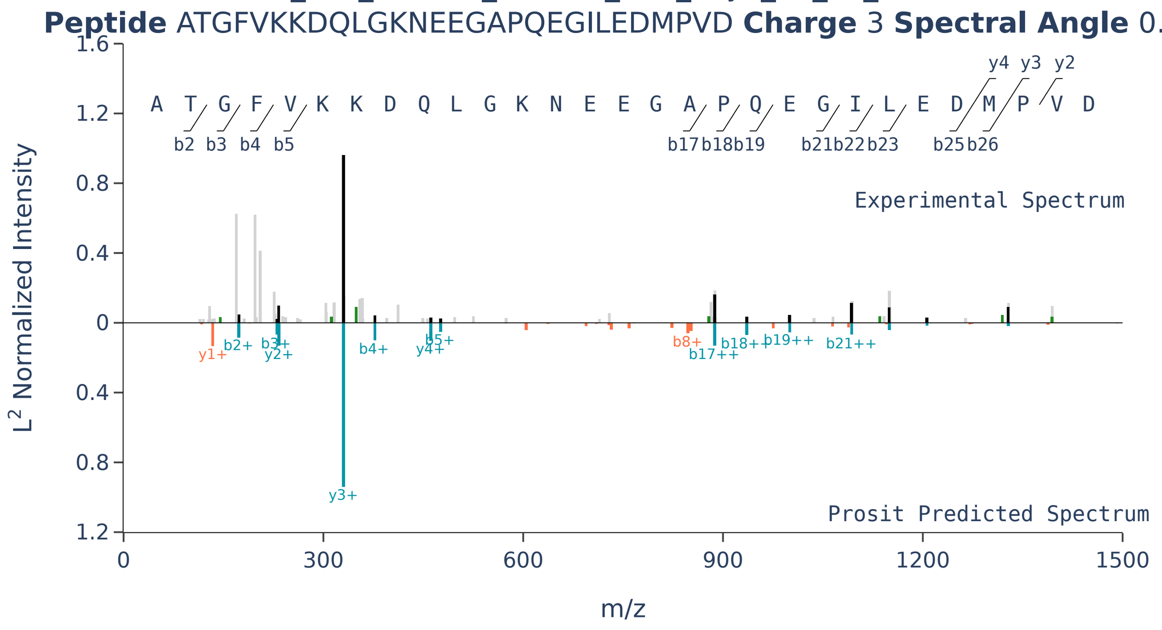


**Source** W_Soh_160821_170921_Goe_aSyn_A1_24h_R1 **Scan** 31764
**Peptide** VTNVGGAVVTGVTAVAQKTVEGAGSIAAA **Charge** 3 **Spectral Angle** 0.68

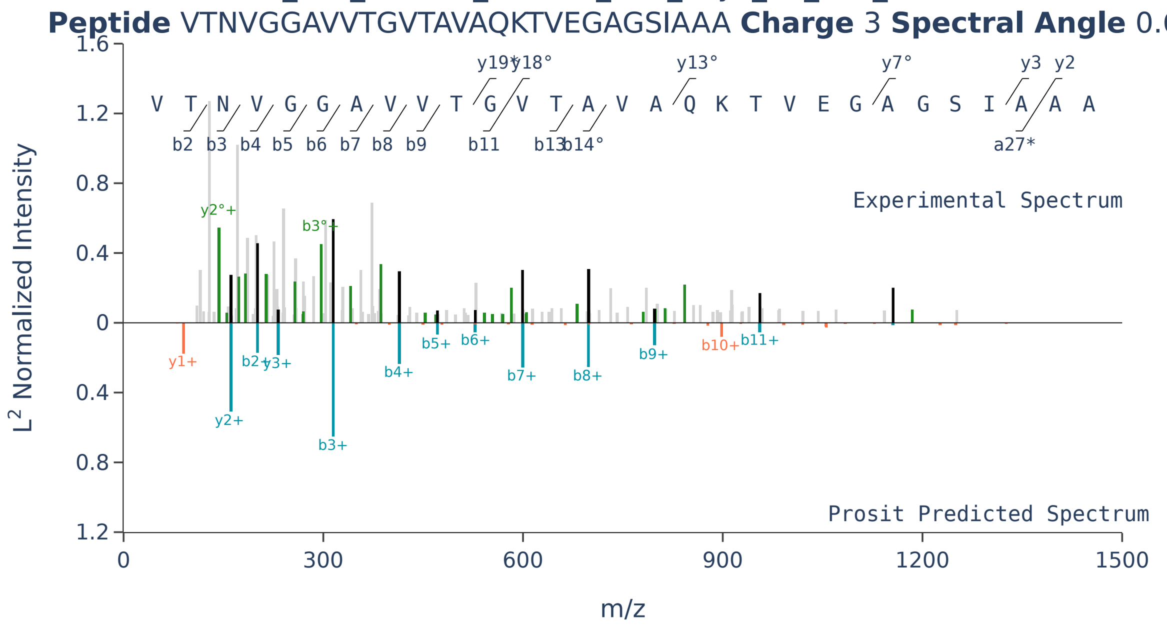


**Source** W_Soh_160821_170921_Goe_aSyn_A1_24h_R1 **Scan** 11299
**Peptide** AAAEKTKQGVAEAAGKTKEGVLYVG **Charge** 4 **Spectral Angle** 0.83

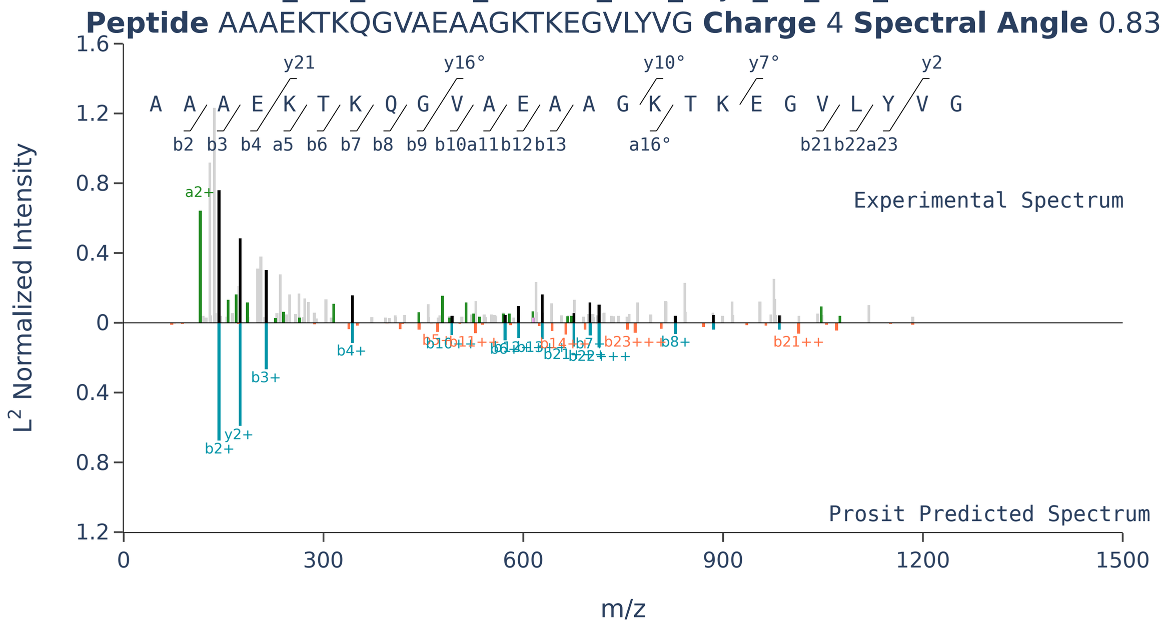


**Source** W_Soh_160821_170921_Goe_aSyn_A1_24h_R1 **Scan** 9761
**Peptide** KTKEGVVHGVATVAEKTKEQV **Charge** 3 **Spectral Angle** 0.81

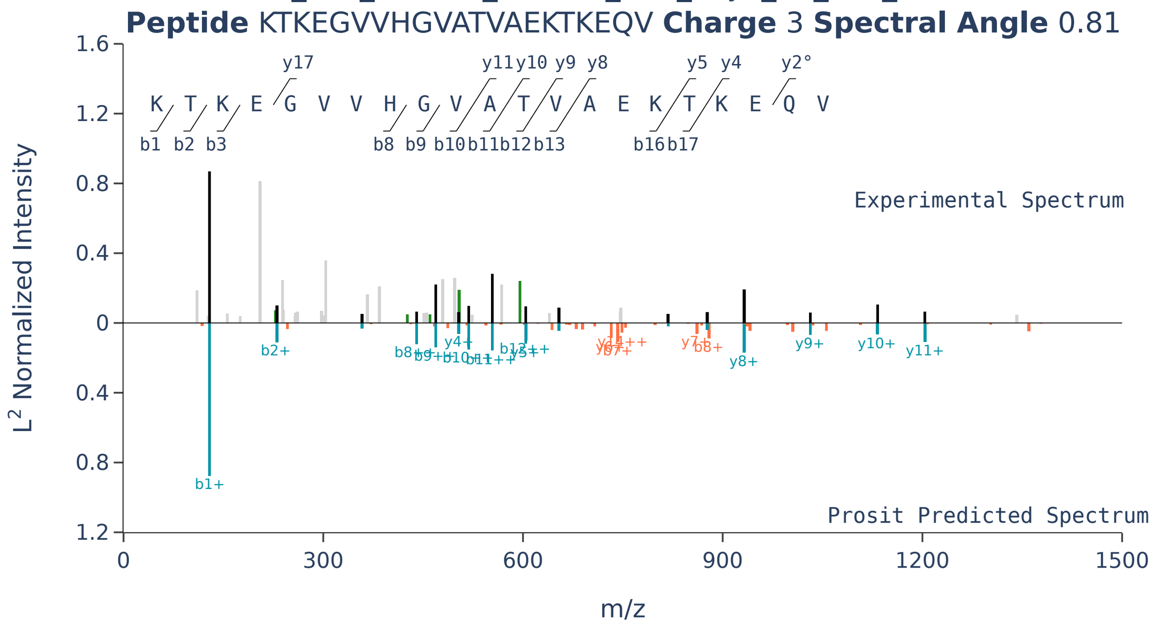


**Source** W_Soh_160821_170921_Goe_aSyn_A2_24h_R2 **Scan** 11179
**Peptide** KEGVVHGVATVAEKTKEQVTN **Charge** 3 **Spectral Angle** 0.81

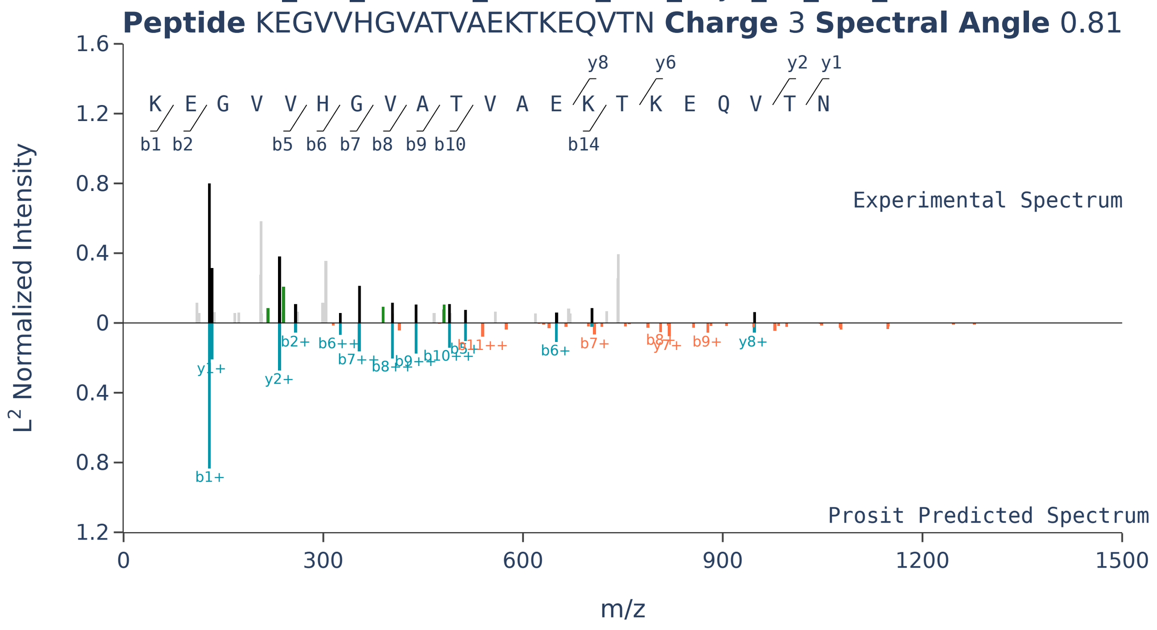


**Source** W_Soh_160821_170921_Goe_aSyn_A2_24h_R1 **Scan** 26177
**Peptide** KEQVTNVGGAVVTGVTAVAQKTVEGA **Charge** 3 **Spectral Angle** 0.73

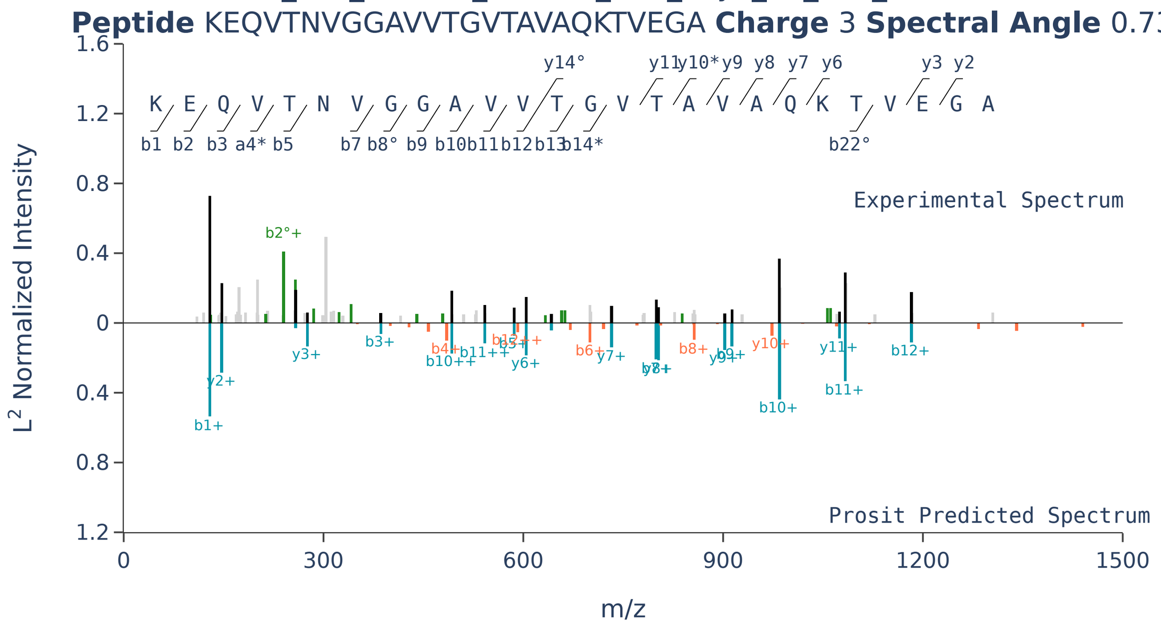


**Source** W_Soh_160821_170921_Goe_aSyn_A2_24h_R1 **Scan** 12771
**Peptide** QGVAEAAGKTKEGVLYVGSKTKEGVVHG **Charge** 5 **Spectral Angle** 0.75

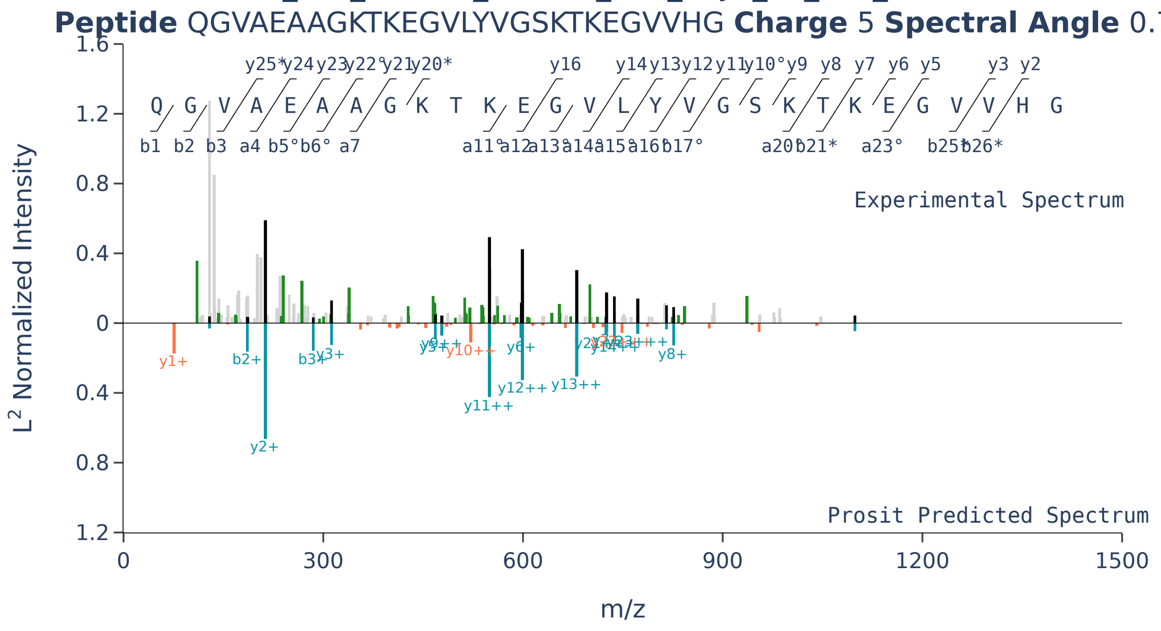


**Source** W_Soh_160821_170921_Goe_aSyn_A2_24h_R1 **Scan** 12793
**Peptide** KQGVAEAAGKTKEGVLYVGSKTKEGVVHGV **Charge** 6 **Spectral Angle** 0.77

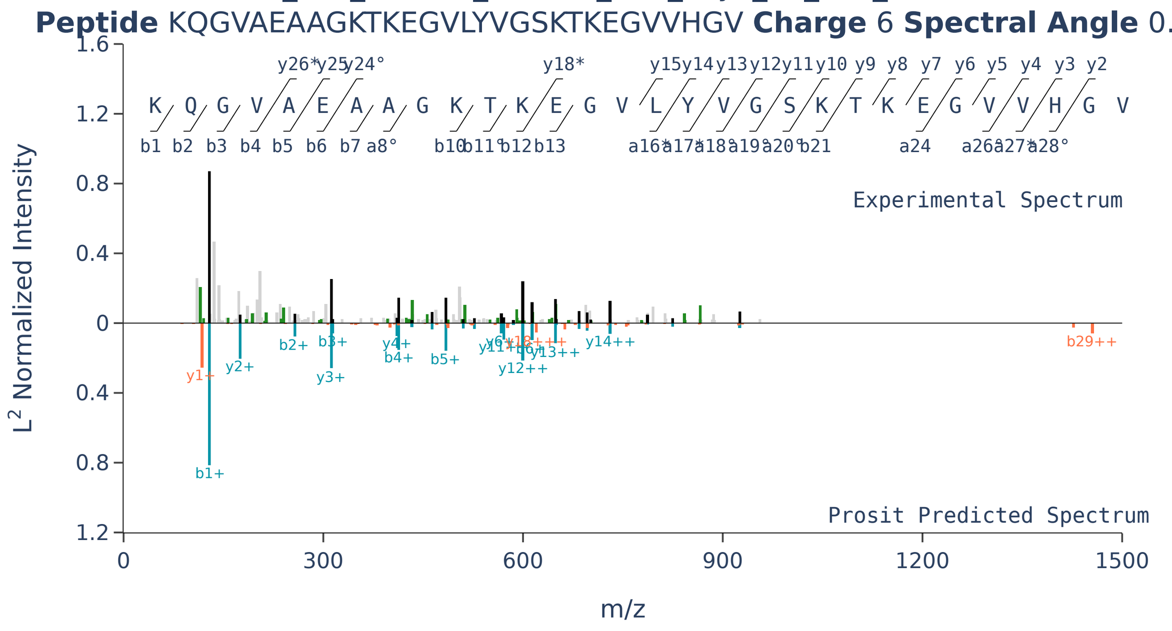


**Source** W_Soh_160821_170921_Goe_aSyn_A2_24h_R1 **Scan** 13595
**Peptide** EAYEMPSEE **Charge** 1 **Spectral Angle** 0.6

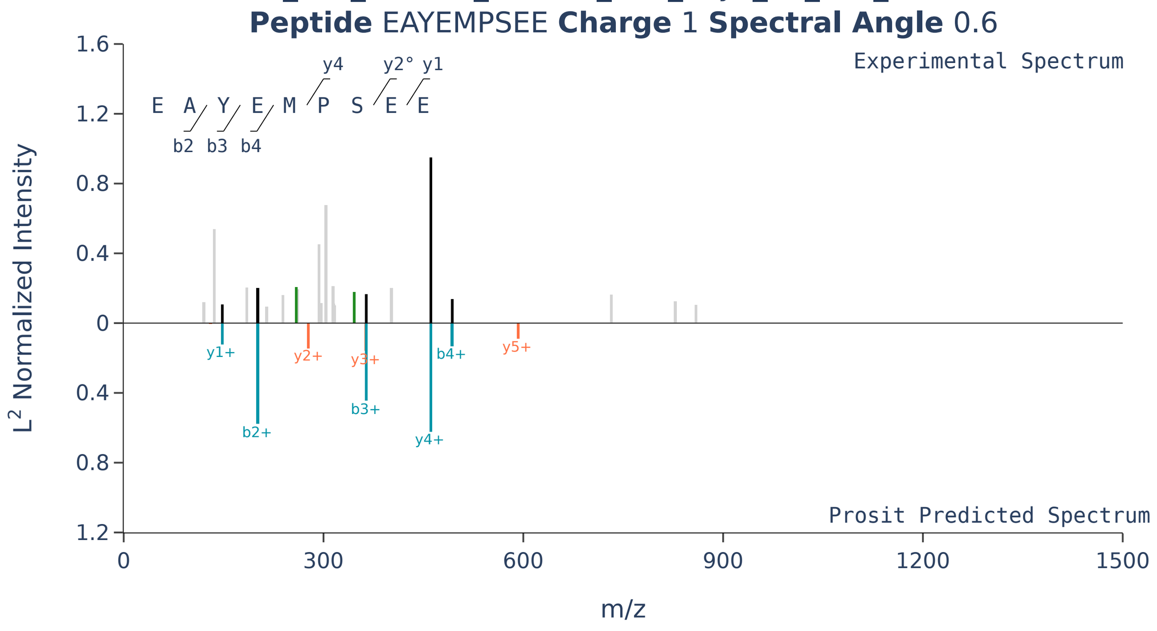


**Source** W_Soh_160821_170921_Goe_aSyn_A1_24h_R2 **Scan** 11561
**Peptide** AAEKTKQGVAEAAGKTKEGVLYV **Charge** 2 **Spectral Angle** 0.65

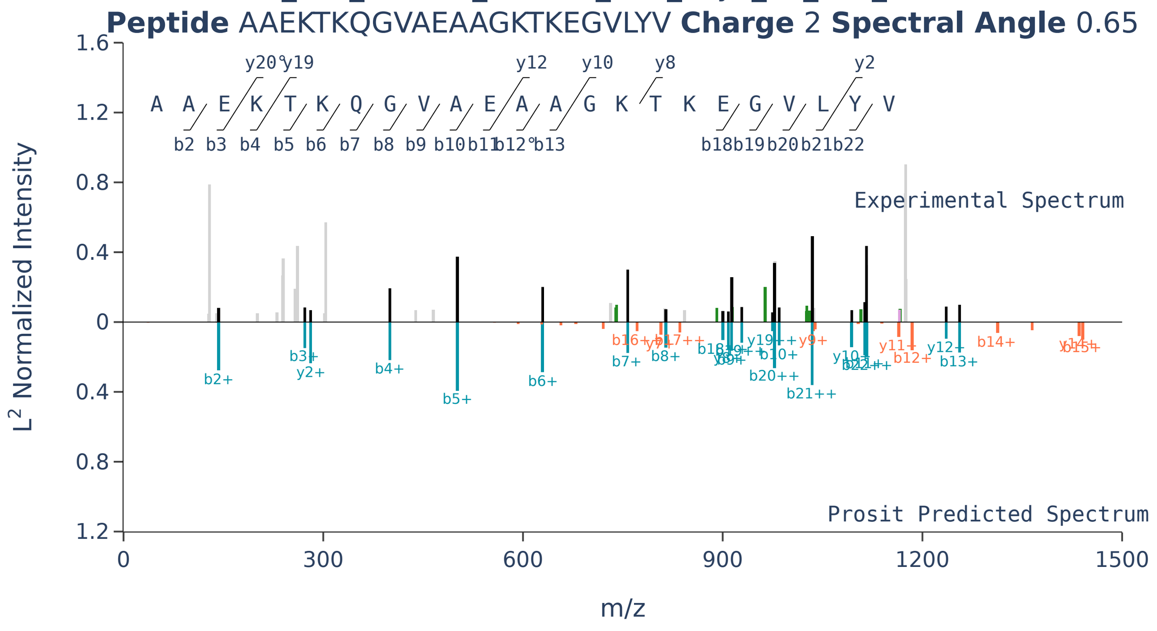


**Source** W_Soh_160821_170921_Goe_aSyn_A2_24h_R1 **Scan** 11933
**Peptide** ATVAEKTKEQVTNVGGAV **Charge** 3 **Spectral Angle** 0.81

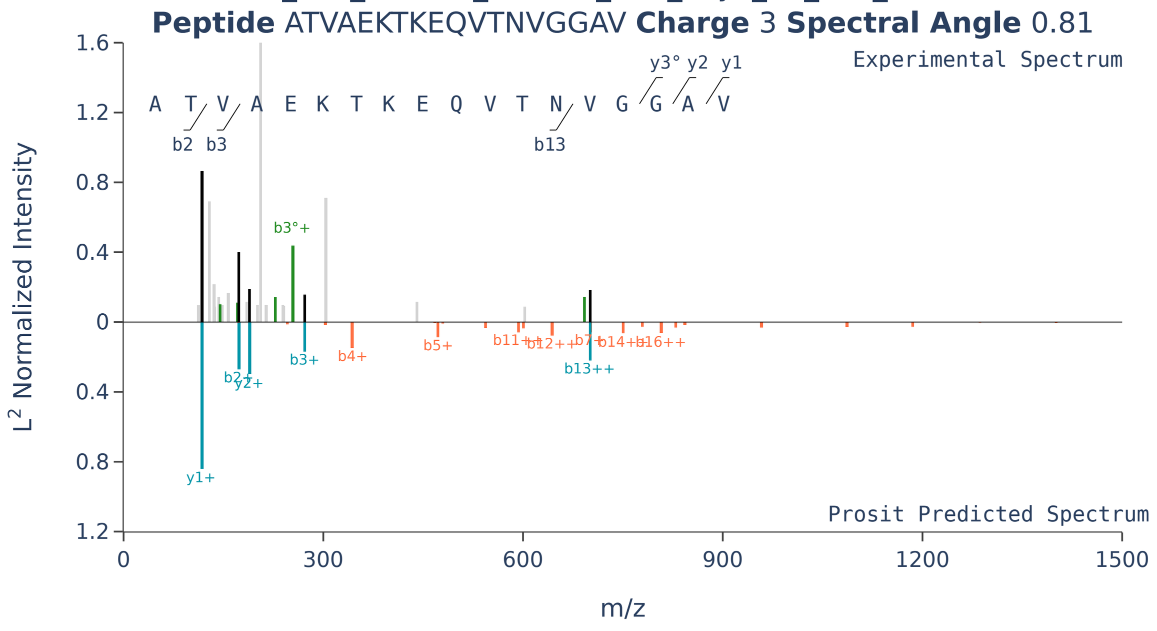


**Source** W_Soh_160821_170921_Goe_aSyn_A1_6h_R2 **Scan** 10285
**Peptide** LGKNEEGAPQEGI **Charge** 2 **Spectral Angle** 0.8

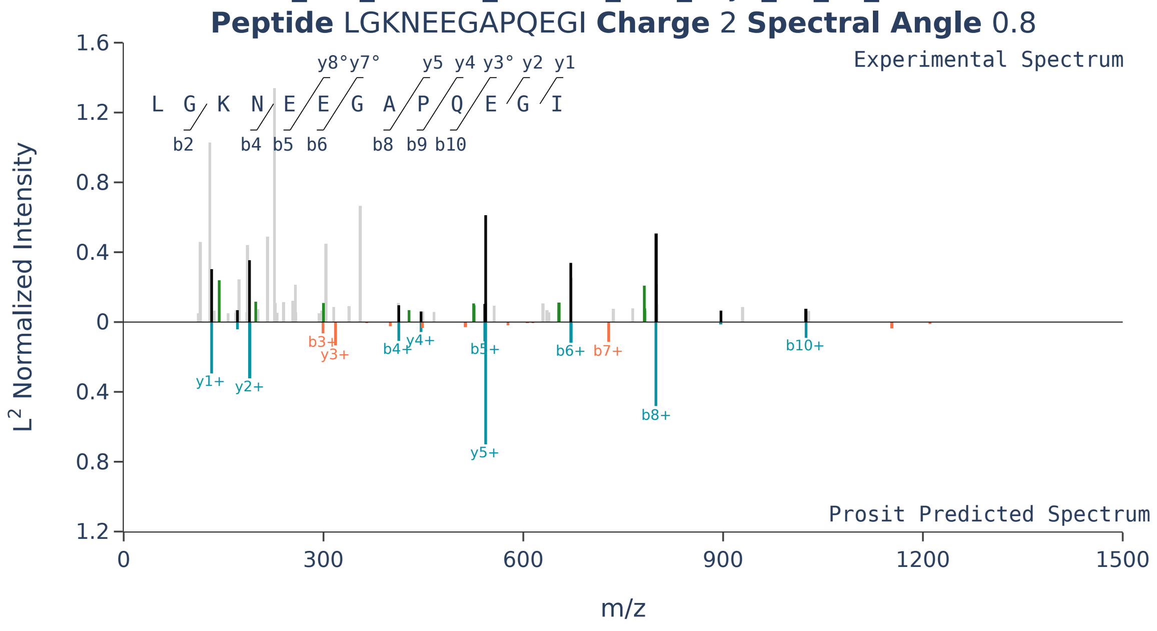


**Source** W_Soh_160821_170921_Goe_aSyn_A2_24h_R1 **Scan** 21416
**Peptide** GVVHGVATVAEKTKEQVTNVGGAVVTGVTA **Charge** 4 **Spectral Angle** 0.65

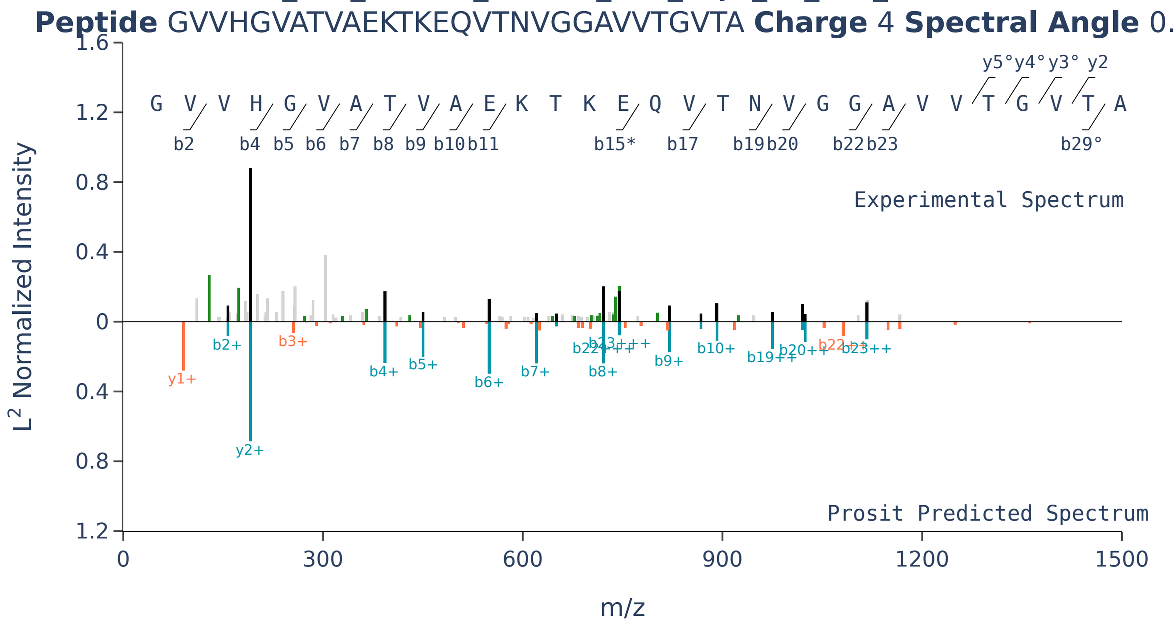


**Source** W_Soh_160821_170921_Goe_aSyn_A2_24h_R1 **Scan** 6117
**Peptide** VVAAAEKTKQGVAE **Charge** 2 **Spectral Angle** 0.75

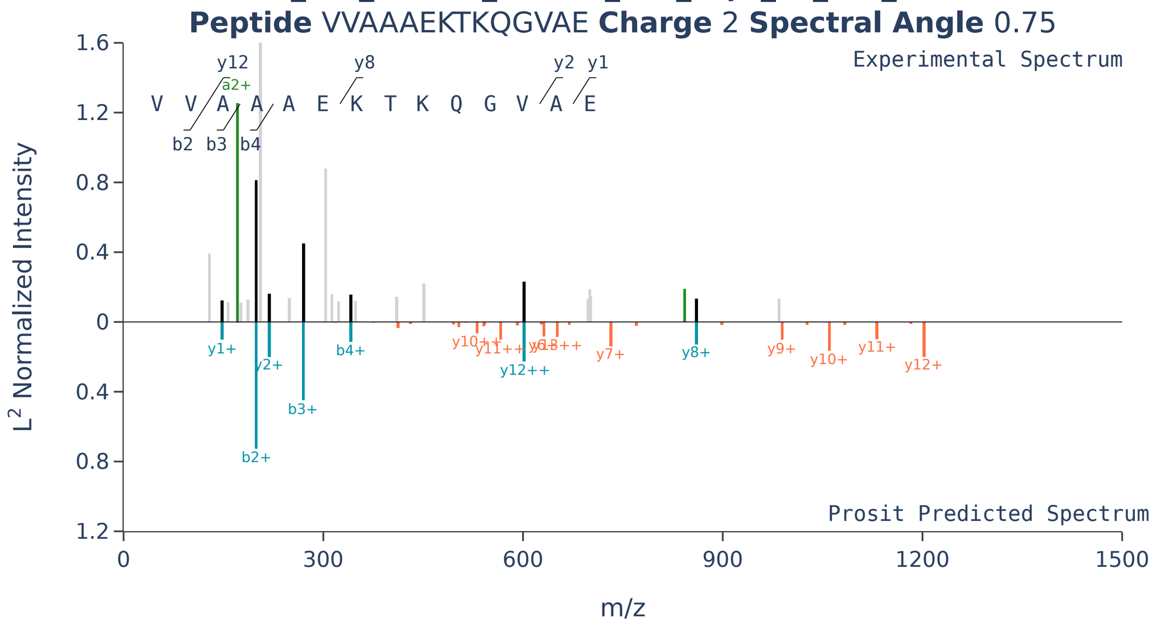


**Source** W_Soh_160821_170921_Goe_aSyn_A1_24h_R2 **Scan** 12472
**Peptide** KQGVAEAAGKTKEGVLYV **Charge** 4 **Spectral Angle** 0.86

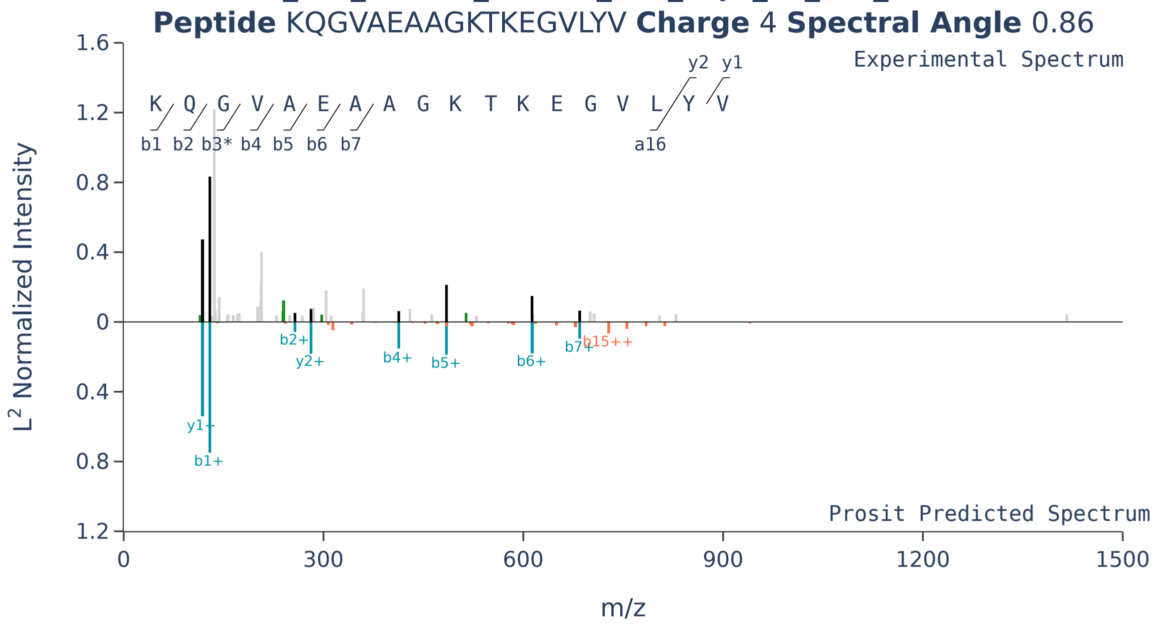

**Source** W_Soh_160821_170921_Goe_aSyn_A1_6h_R2 **Scan** 23615
**Peptide** ILEDMPVDPDNEAYE **Charge** 2 **Spectral Angle** 0.72

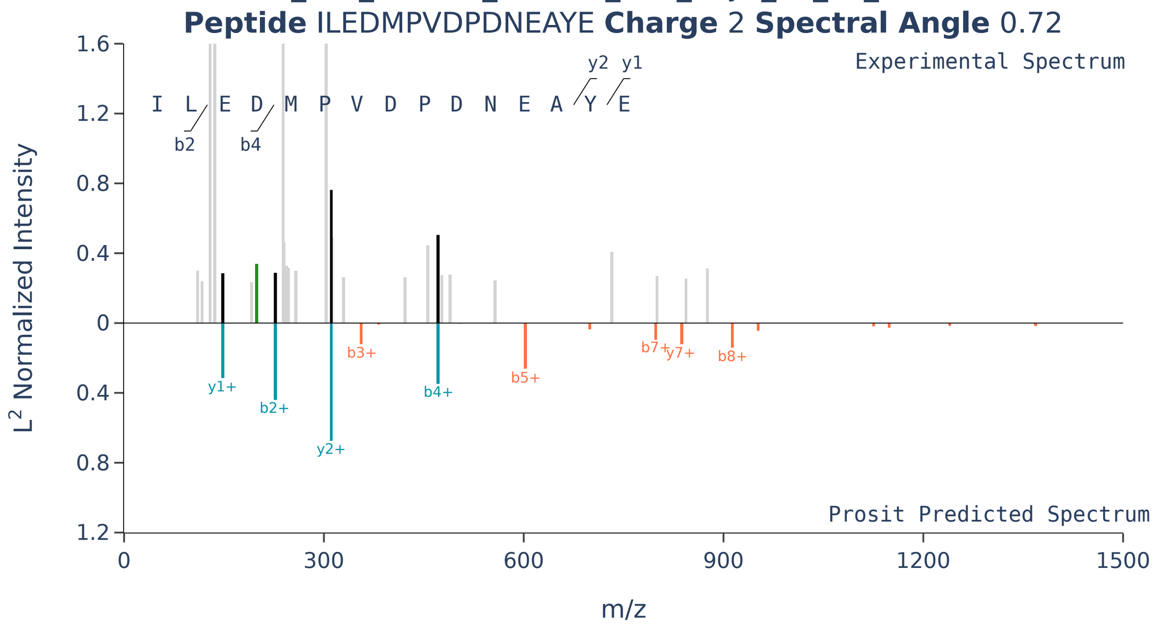


**Source** W_Soh_160821_170921_Goe_aSyn_A1_6h_R2 **Scan** 14835
**Peptide** LGKNEEGAPQEGILED **Charge** 2 **Spectral Angle** 0.75

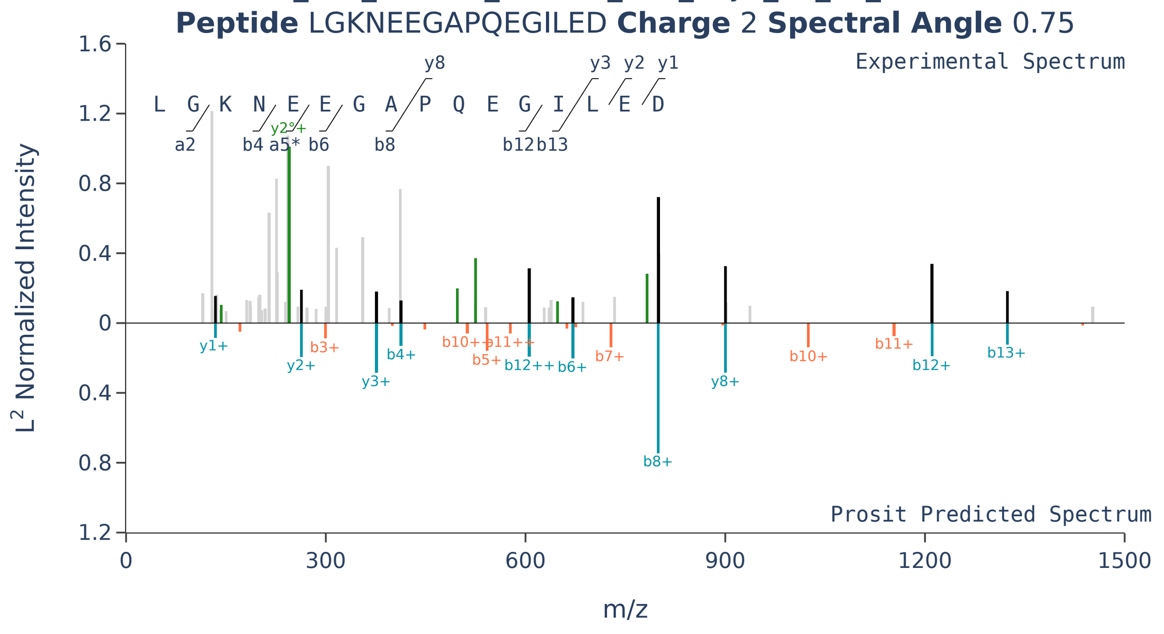


**Source** W_Soh_160821_170921_Goe_aSyn_A2_24h_R1 **Scan** 6950
**Peptide** DQLGKNEEGAPQEG **Charge** 2 **Spectral Angle** 0.84

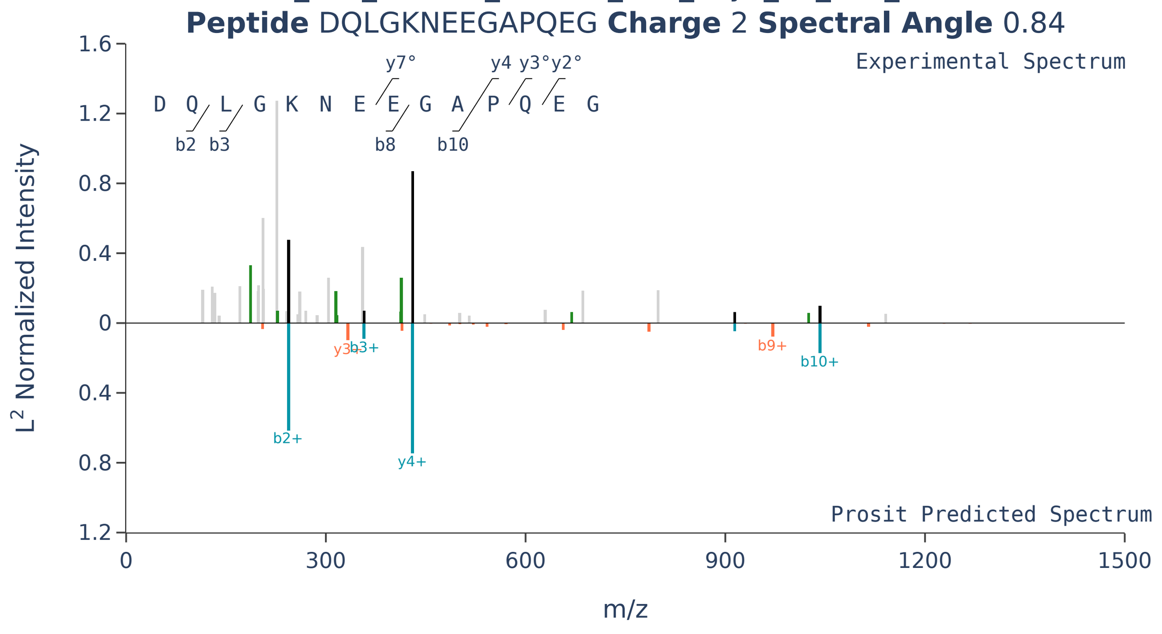


**Source** W_Soh_160821_170921_Goe_aSyn_A1_24h_R1 **Scan** 12372
**Peptide** AAGKTKEGVLYVGSKTKEGVVHGVATV **Charge** 4 **Spectral Angle** 0.64

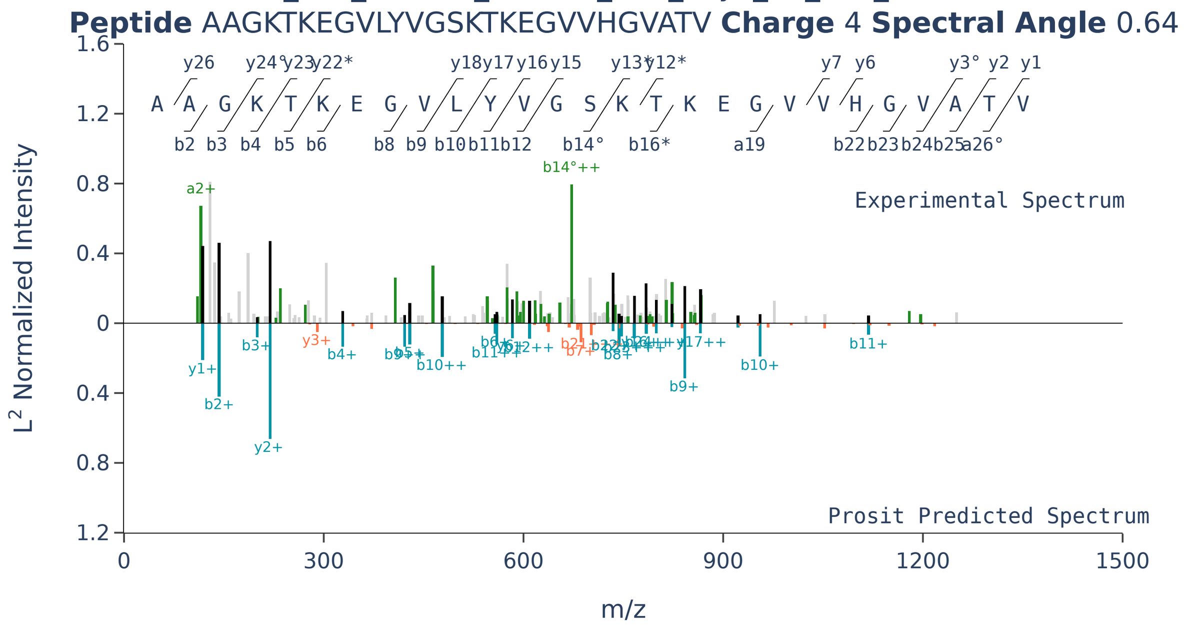


**Source** W_Soh_160821_170921_Goe_aSyn_A2_24h_R1 **Scan** 25282
**Peptide** GGAVVTGVTAVAQKTVEGAGSIAA **Charge** 3 **Spectral Angle** 0.71

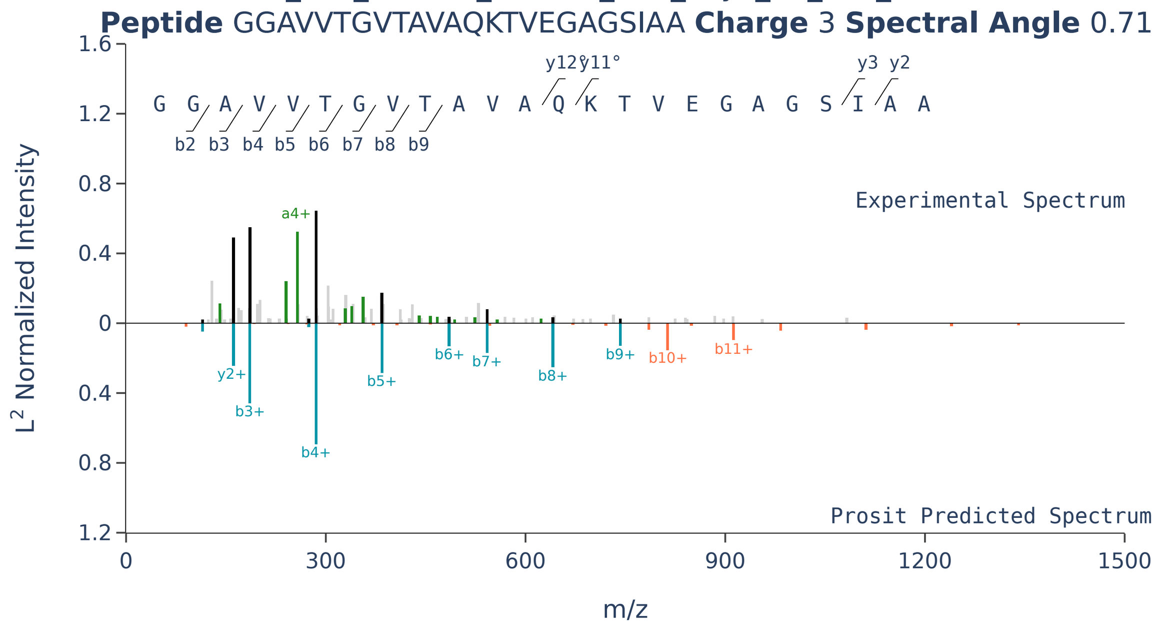


**Source** W_Soh_160821_170921_Goe_aSyn_A1_24h_R1 **Scan** 7907
**Peptide** LSKAKEGVVAAAEKTKQGV **Charge** 4 **Spectral Angle** 0.65

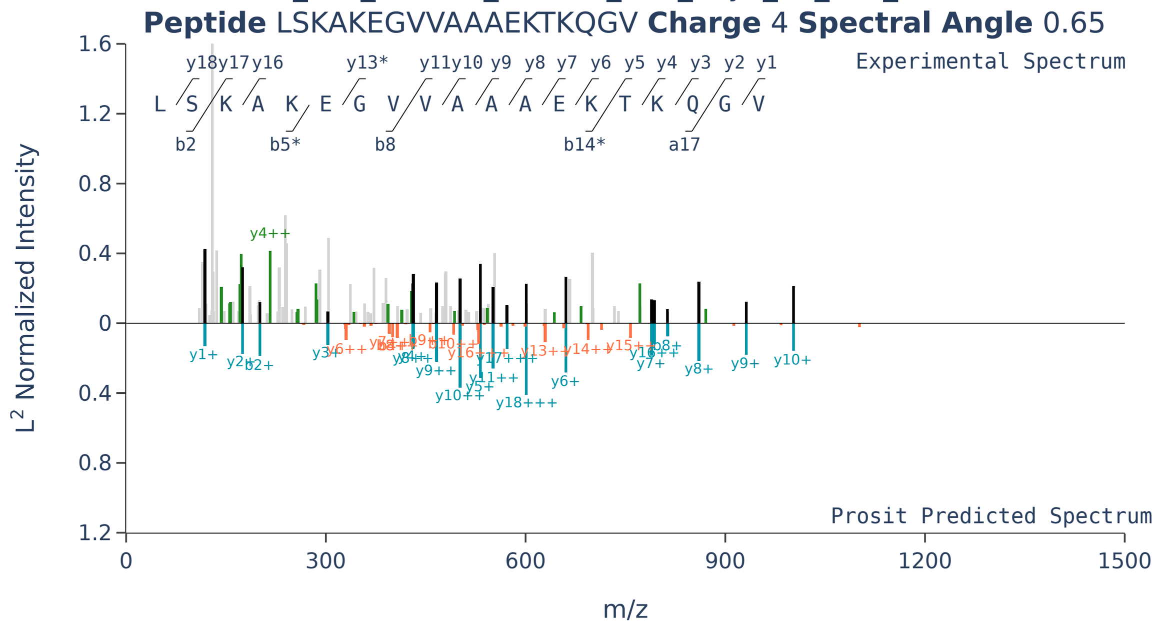


**Source** W_Soh_160821_170921_Goe_aSyn_A2_24h_R1 **Scan** 15605
**Peptide** APQEGILED **Charge** 2 **Spectral Angle** 0.8

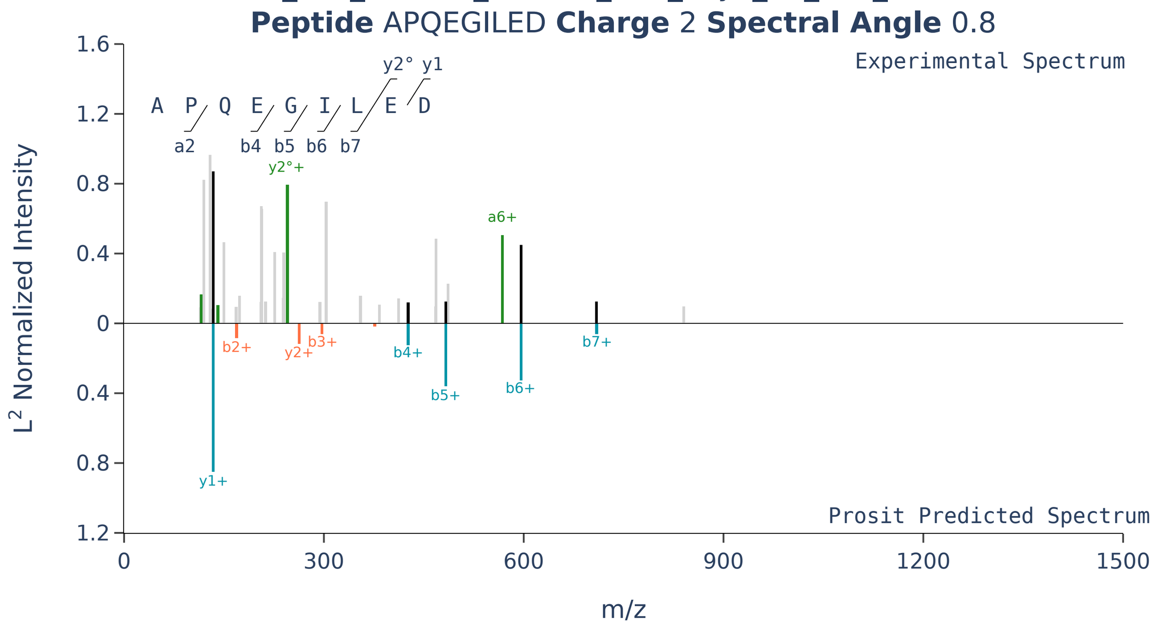


**Source** W_Soh_160821_170921_Goe_aSyn_A2_24h_R1 **Scan** 26735
**Peptide** VGGAVVTGVTAVAQKTVEGAGSI **Charge** 3 **Spectral Angle** 0.83

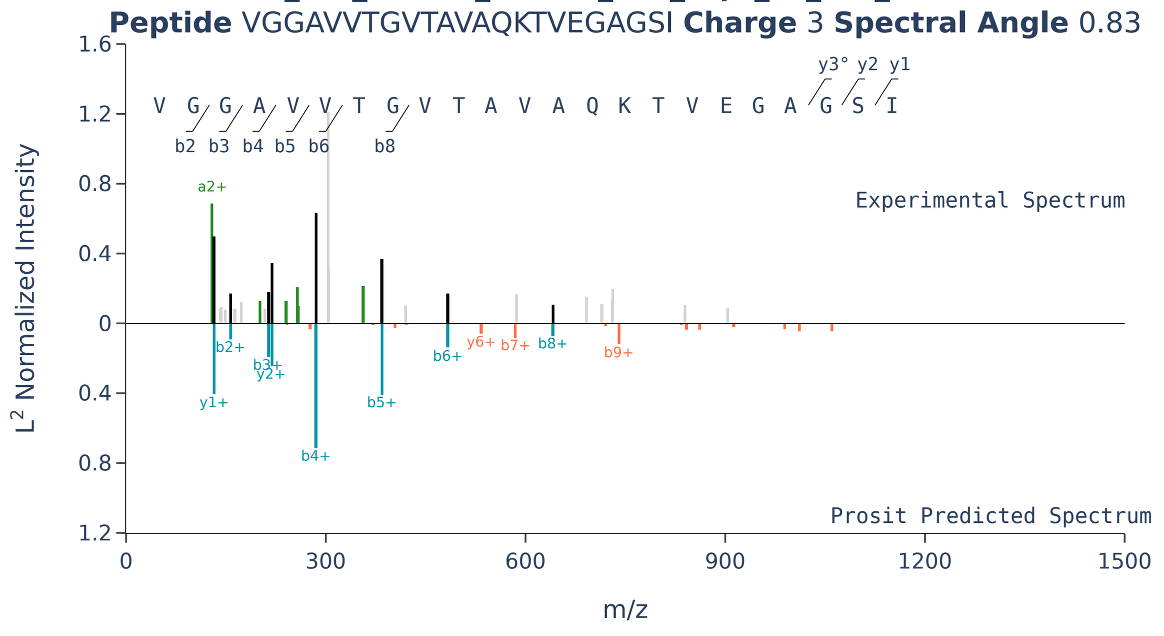


**Source** W_Soh_160821_170921_Goe_aSyn_A1_24h_R1 **Scan** 19641
**Peptide** DVFMKGLSKAKEGVVAAAEKTKQGV **Charge** 4 **Spectral Angle** 0.74

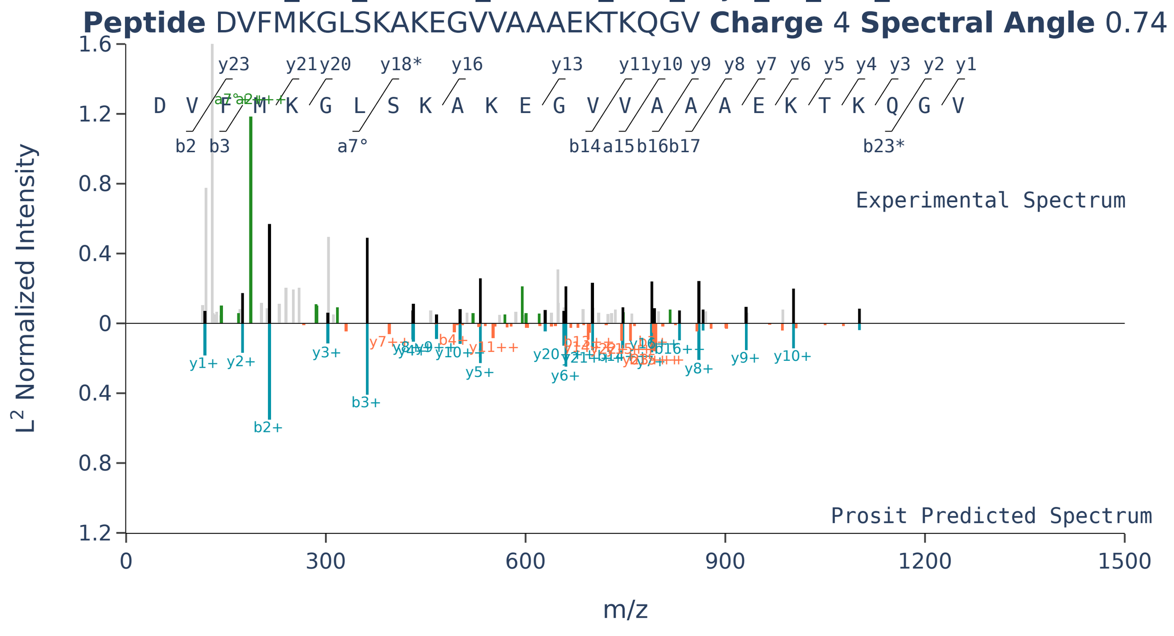


**Source** W_Soh_160821_170921_Goe_aSyn_A2_24h_R2 **Scan** 20306
**Peptide** DVFMKGLSKAKEGVVAAAEKTKQGVAEA **Charge** 3 **Spectral Angle** 0.74

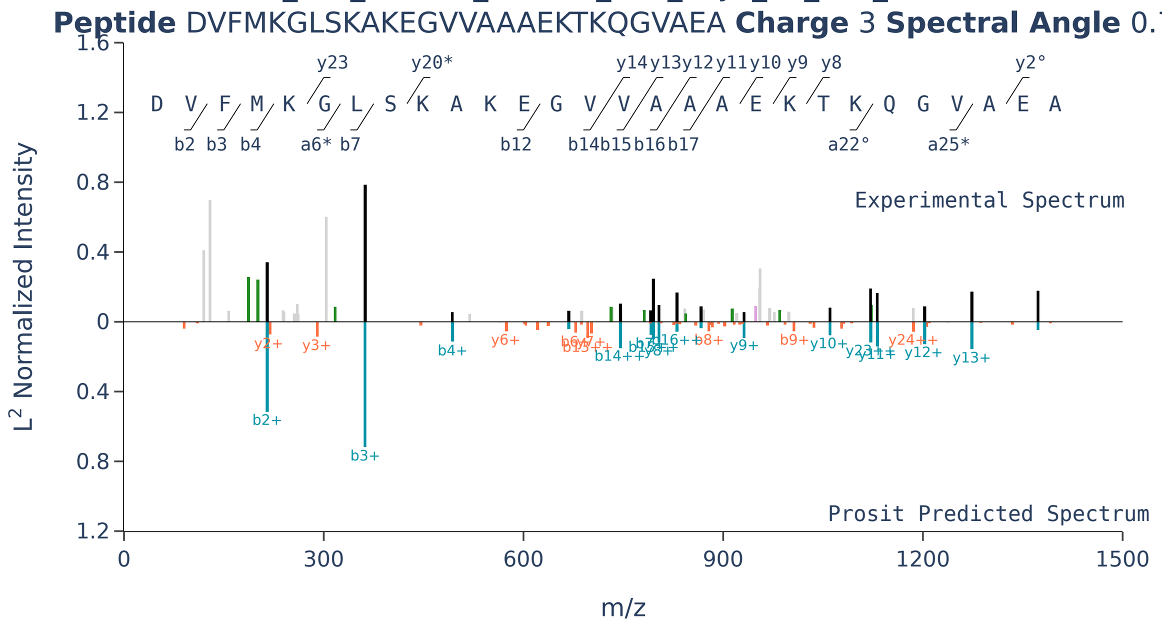


**Source** W_Soh_160821_170921_Goe_aSyn_A1_24h_R2 **Scan** 11516
**Peptide** TGFVKKDQLGKNEEGAPQEGL **Charge** 2 **Spectral Angle** 0.85

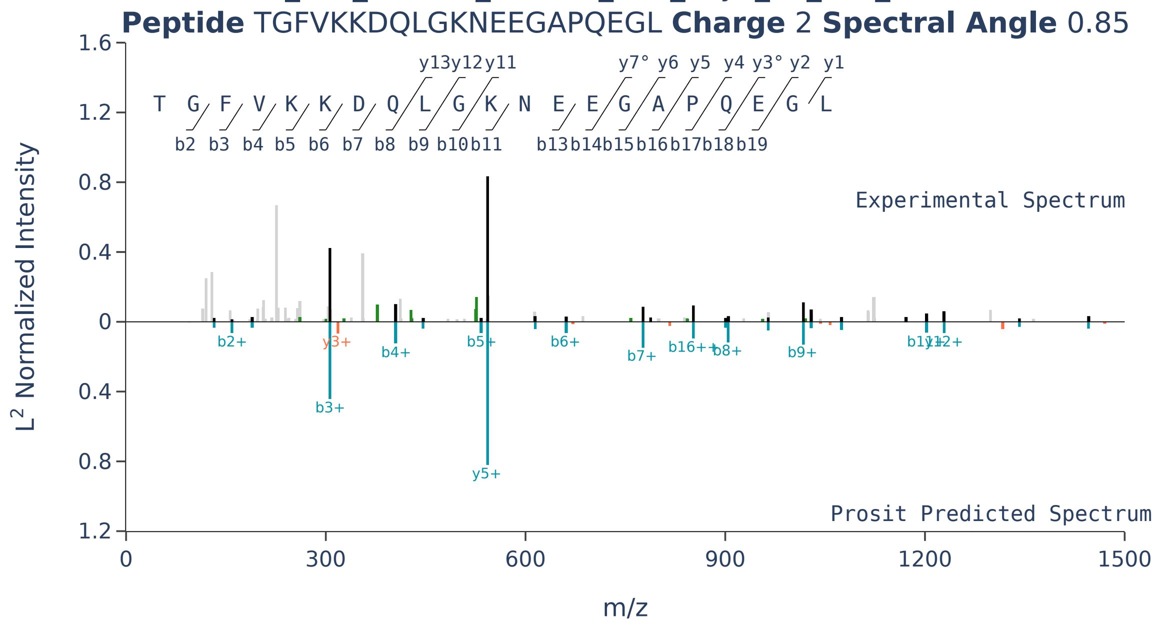


**Source** W_Soh_160821_170921_Goe_aSyn_A1_24h_R1 **Scan** 8081
**Peptide** GLSKAKEGVVAAAEKT **Charge** 4 **Spectral Angle** 0.72

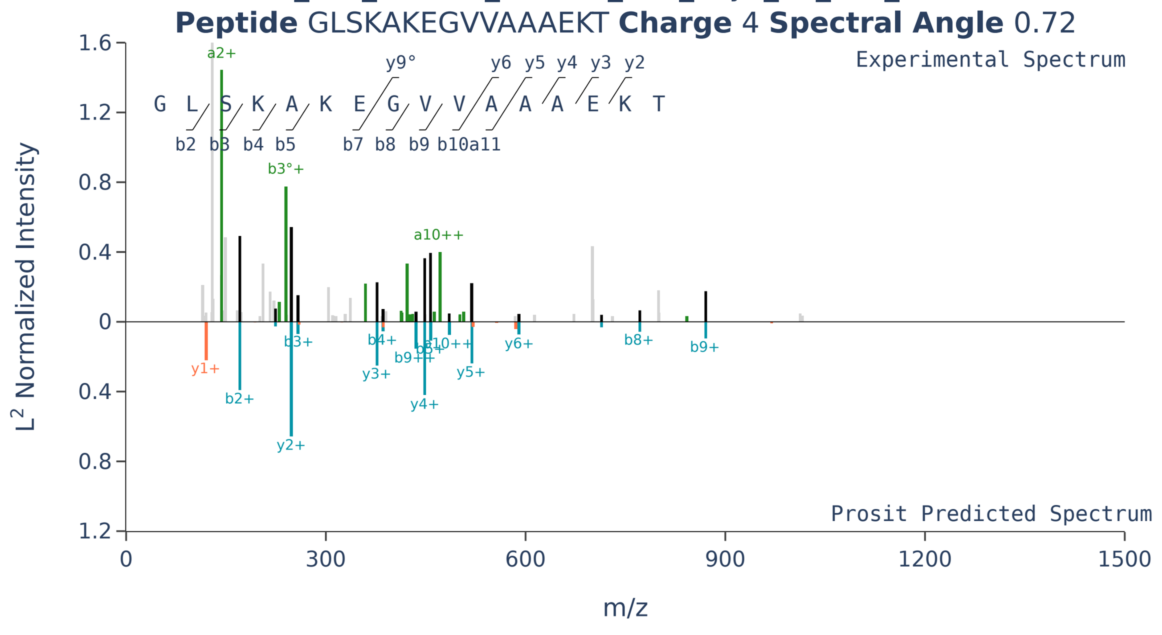


**Source** W_Soh_160821_170921_Goe_aSyn_A2_24h_R1 **Scan** 10525
**Peptide** AEKTKQGVAEAAGKTKEGVLYVG **Charge** 3 **Spectral Angle** 0.67

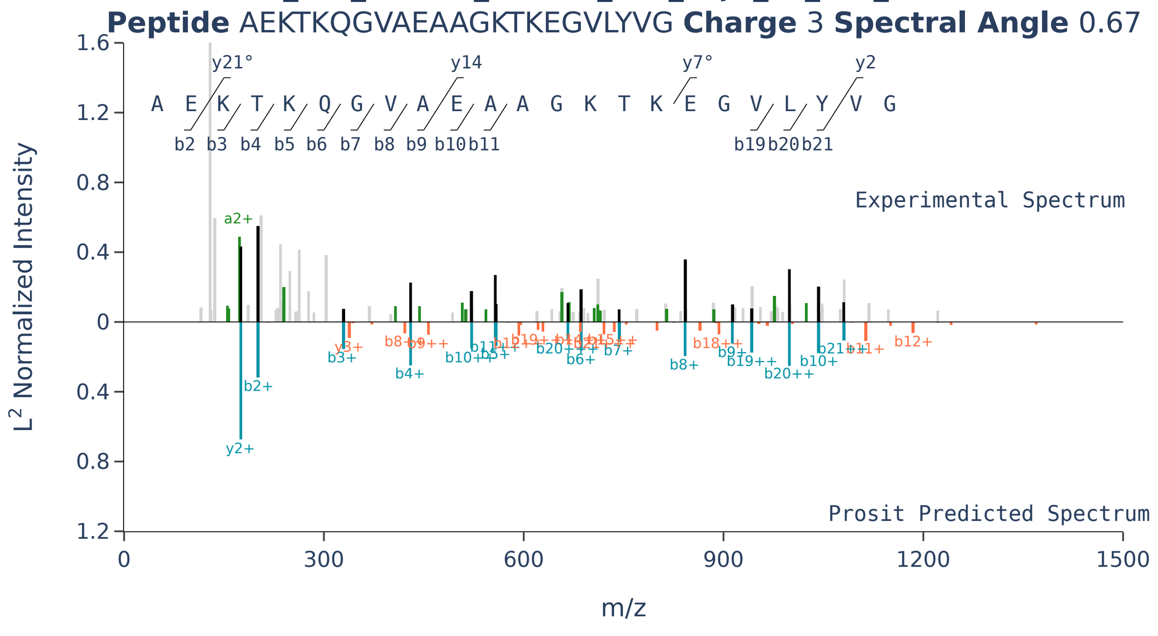


**Source** W_Soh_160821_170921_Goe_aSyn_A1_24h_R2 **Scan** 13068
**Peptide** FVKKDQLGKNEEGAPQEGILED **Charge** 2 **Spectral Angle** 0.79

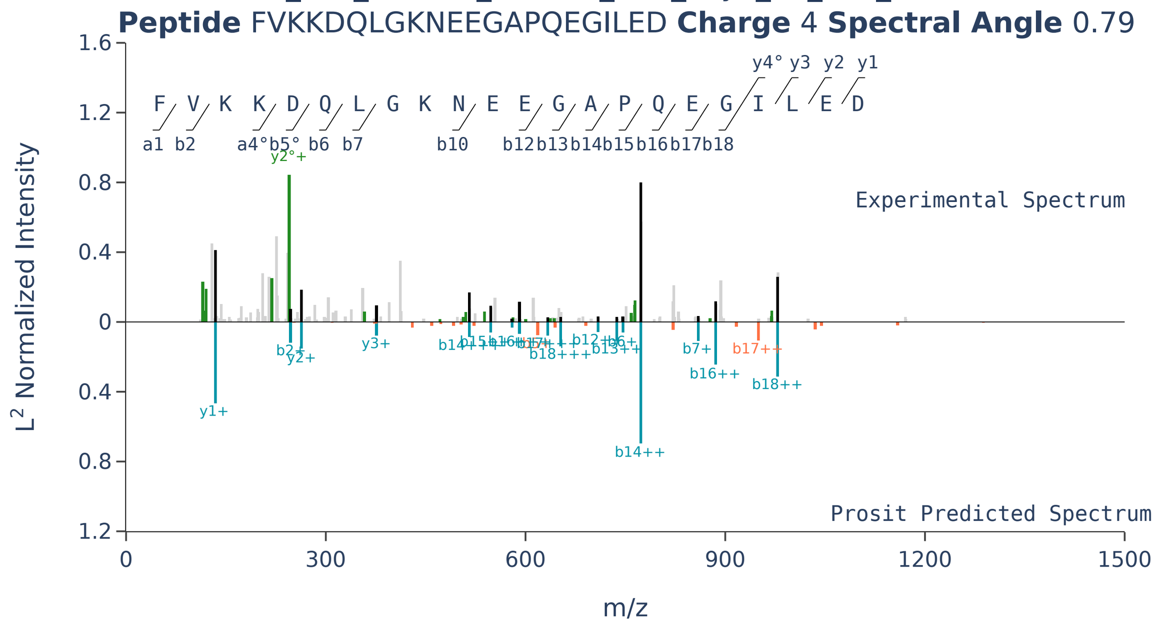


**Source** W_Soh_160821_170921_Goe_aSyn_A1_1h_R2 **Scan** 18525
**Peptide** HGVATVAEKTKEQVTNVGGAVVTGVTAV **Charge** 3 **Spectral Angle** 0.65

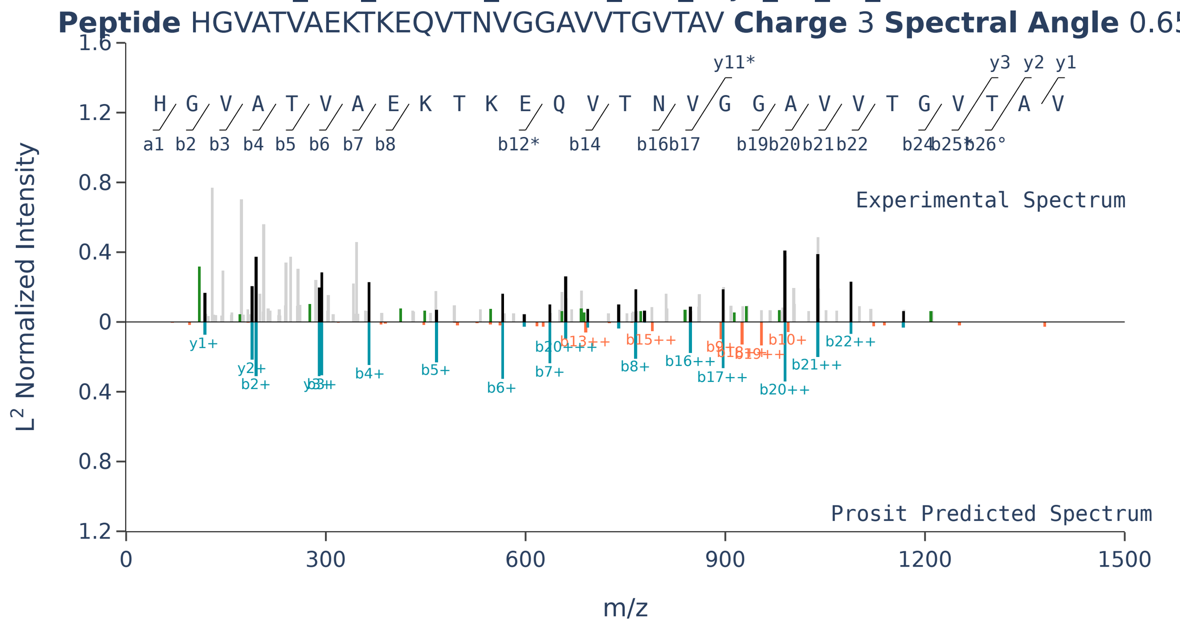

**Source** W_Soh_160821_170921_Goe_aSyn_A1_24h_R2 **Scan** 13803
**Peptide** VTAVAQKTVEGAGSIA **Charge** 2 **Spectral Angle** 0.72

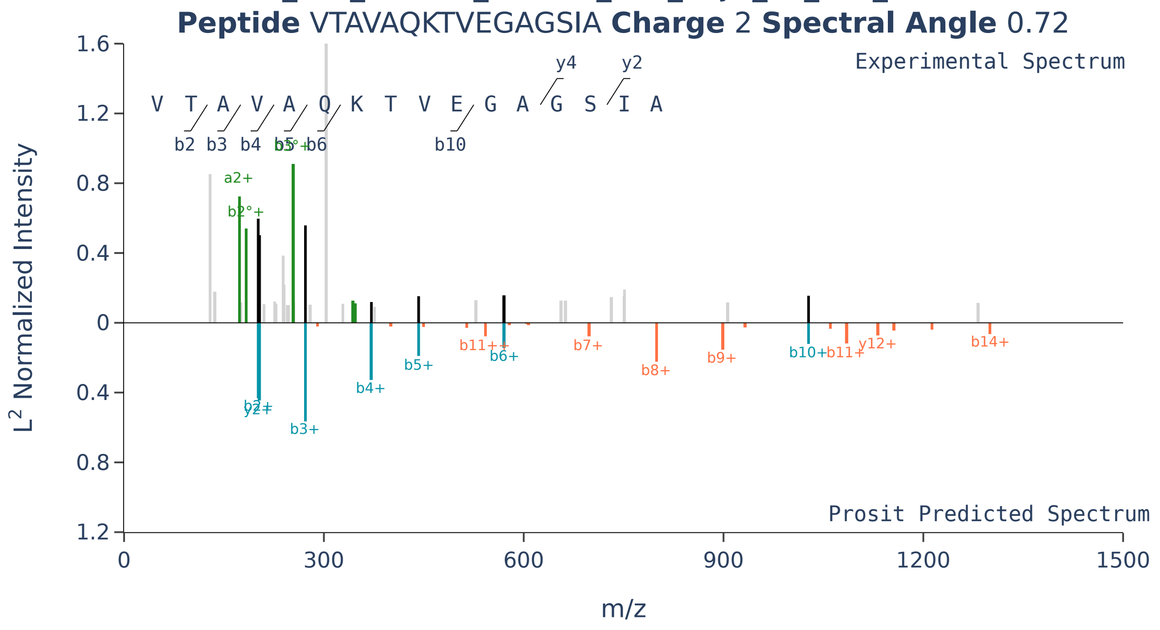


**Source** W_Soh_160821_170921_Goe_aSyn_A1_30'_R2 **Scan** 19820
**Peptide** KEQVTNVGGAVVTGVTAVAQKTVE **Charge** 3 **Spectral Angle** 0.71

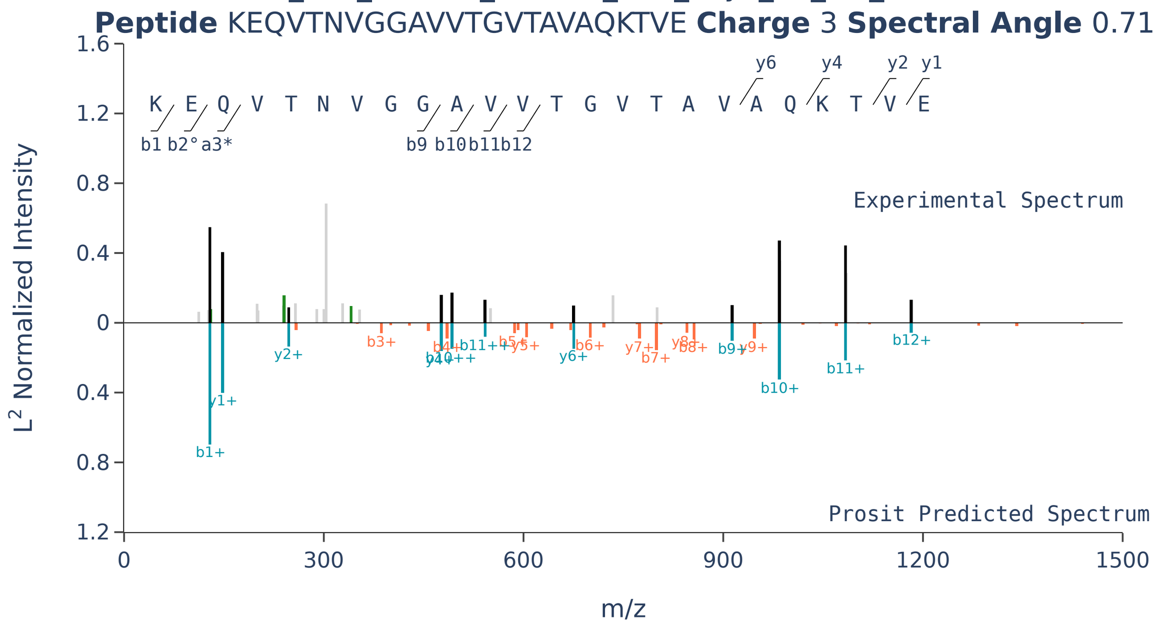


**Source** W_Soh_160821_170921_Goe_aSyn_A1_24h_R2 **Scan** 4986
**Peptide** AAEKTKQGVAEAAGKTKEG **Charge** 5 **Spectral Angle** 0.6

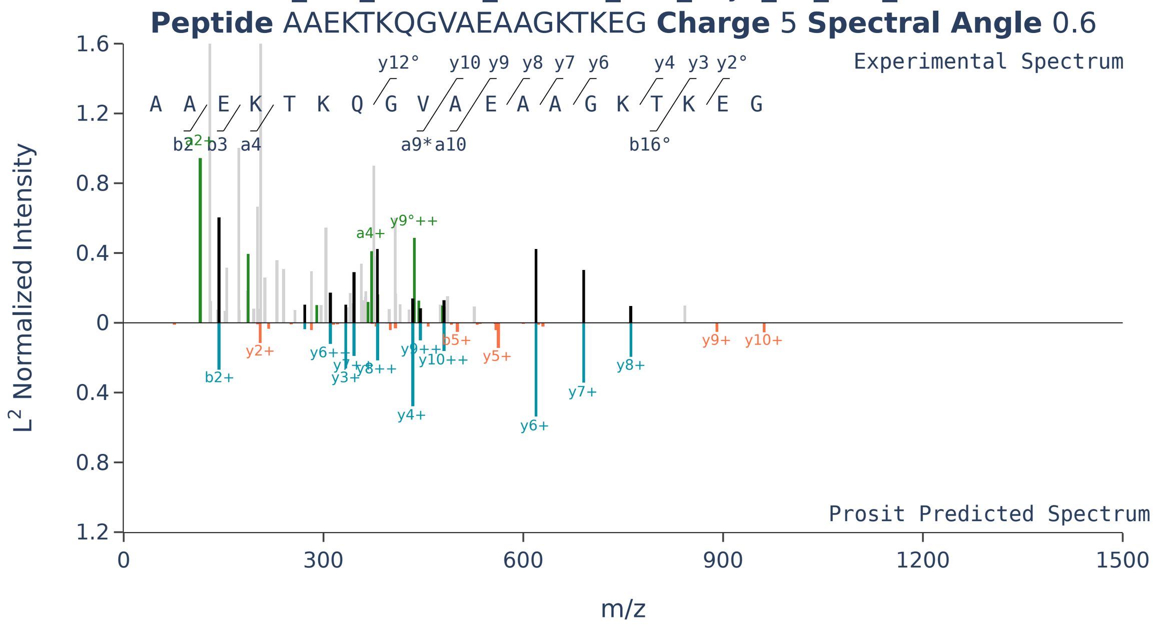


**Source** W_Soh_160821_170921_Goe_aSyn_A1_24h_R1 **Scan** 14590
**Peptide** QGVAEAAGKTKEGVLYVGSKTKEGVVHGV **Charge** 5 **Spectral Angle** 0.71

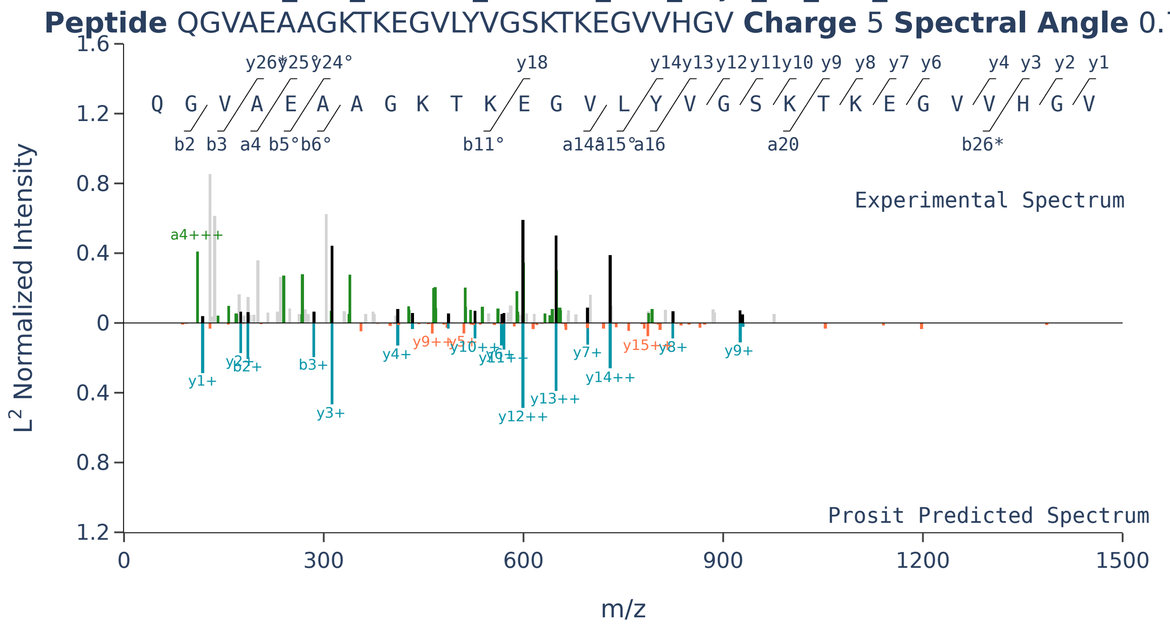


**Source** W_Soh_160821_170921_Goe_aSyn_A1_24h_R1 **Scan** 11422
**Peptide** VKKDQLGKNEEGAPQEGILED **Charge** 3 **Spectral Angle** 0.79

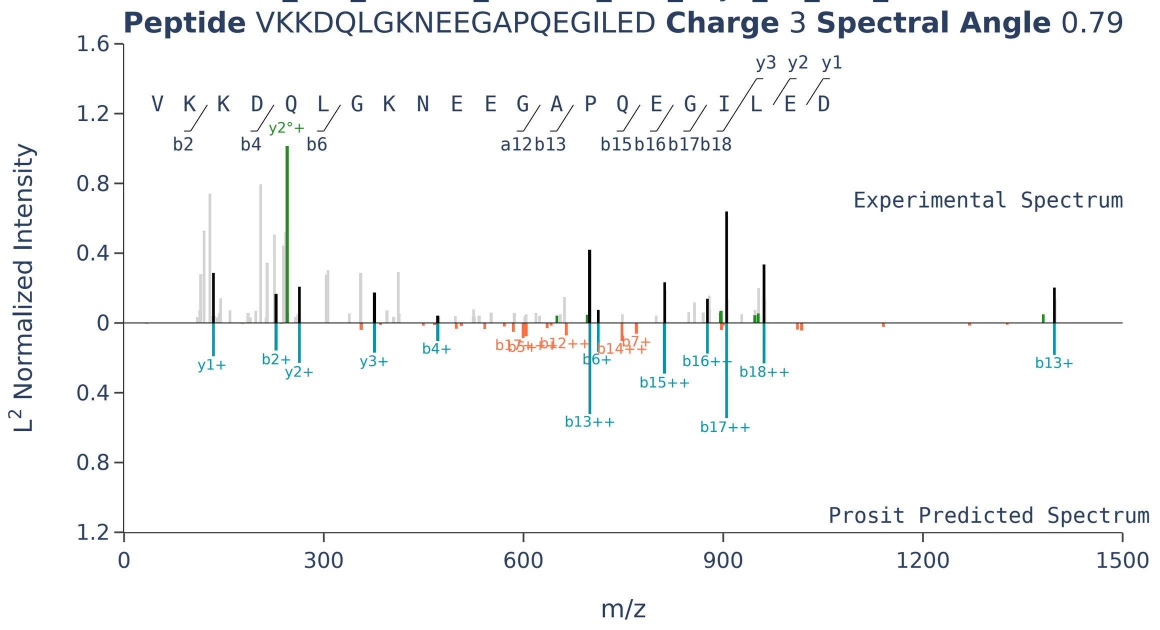


**Source** W_Soh_160821_170921_Goe_aSyn_A2_30'_R1 **Scan** 20043
**Peptide** GVATVAEKTKEQVTNVGGAVVTGVTAVA **Charge** 3 **Spectral Angle** 0.68

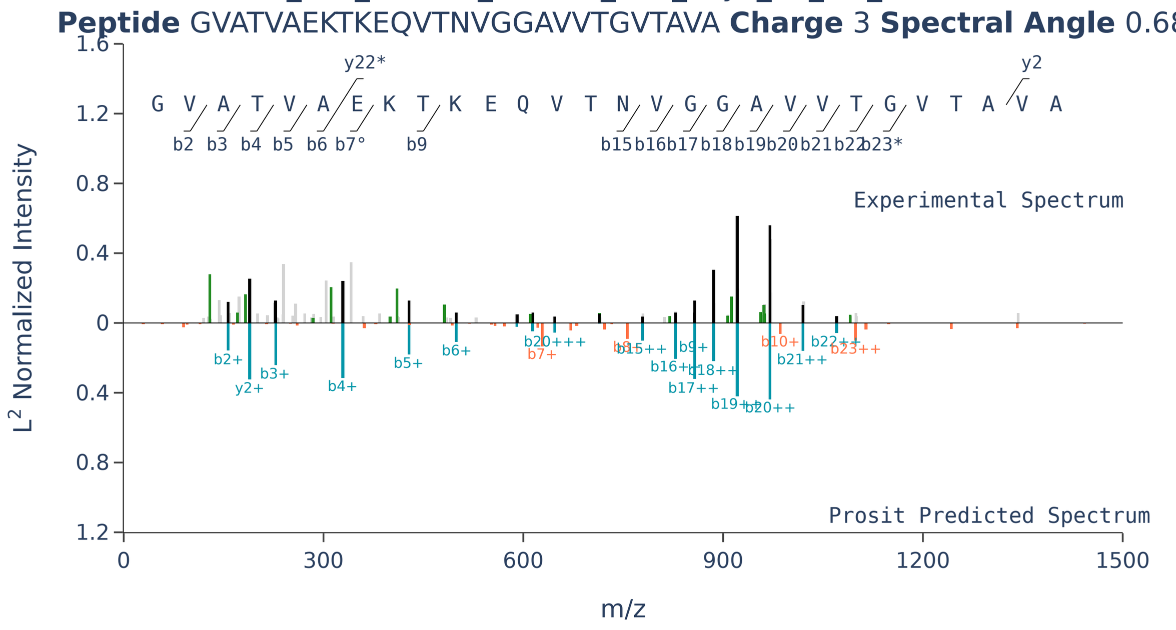


**Source** W_Soh_160821_170921_Goe_aSyn_A2_24h_R2 **Scan** 8119
**Peptide** ATGFVKKDQLGKNEEGAPQEG **Charge** 3 **Spectral Angle** 0.77

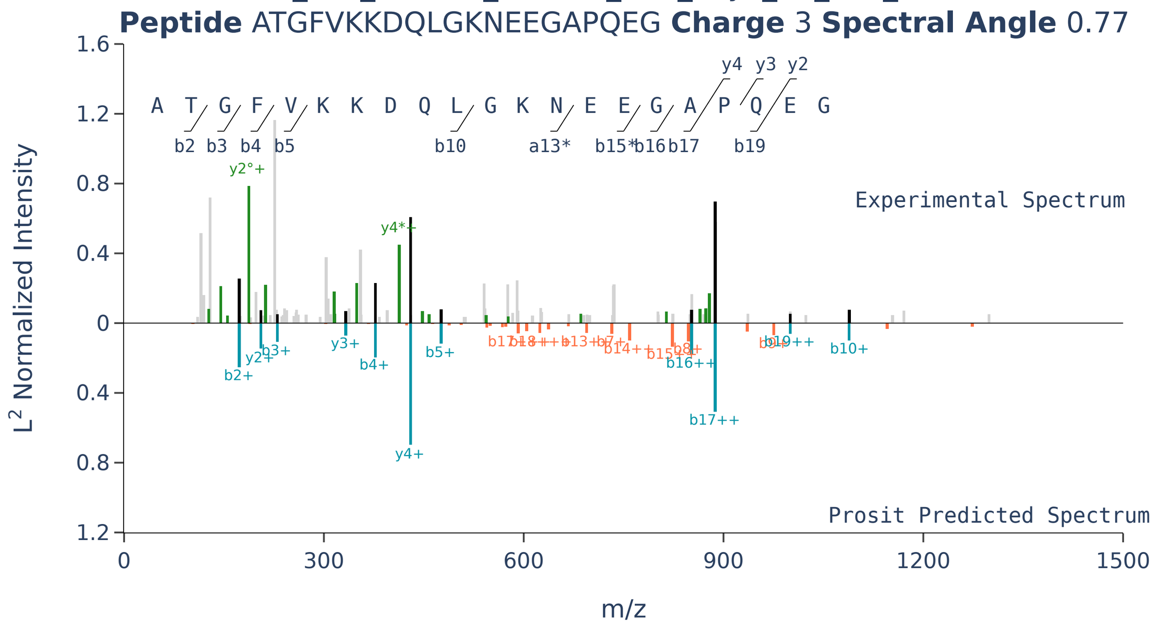


**Source** W_Soh_160821_170921_Goe_aSyn_A1_24h_R1 **Scan** 6050
**Peptide** VAAAEKTKQGVAEAAGKTKEG **Charge** 3 **Spectral Angle** 0.75

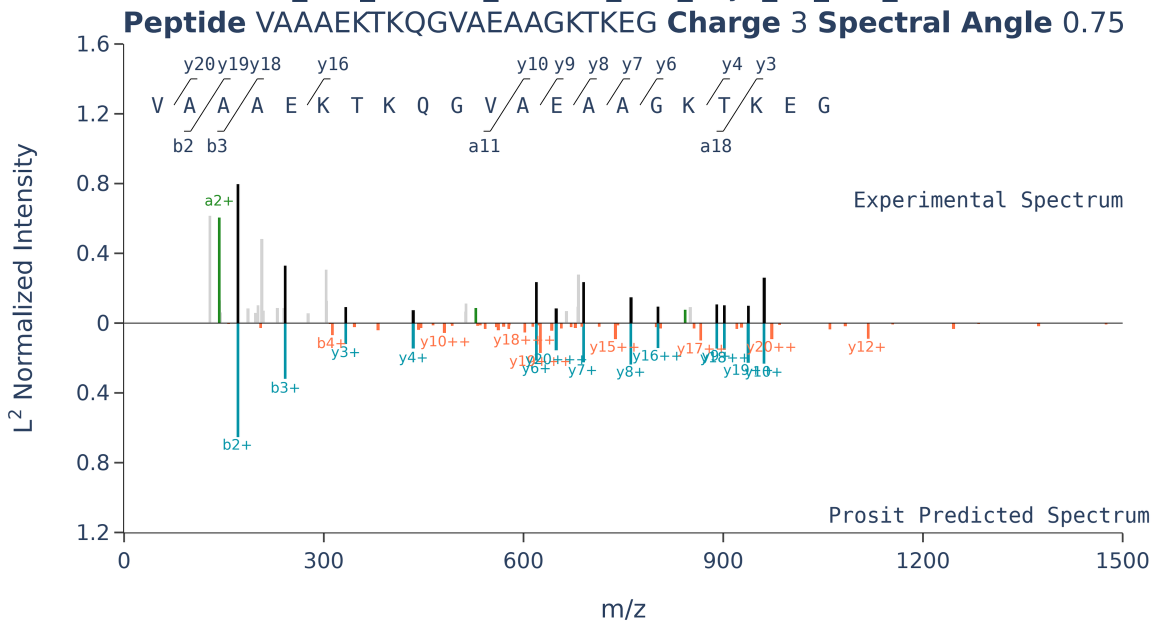


**Source** W_Soh_160821_170921_Goe_aSyn_A1_24h_R2 **Scan** 7075
**Peptide** SKAKEGVVAAAEKTKQGVA **Charge** 4 **Spectral Angle** 0.68

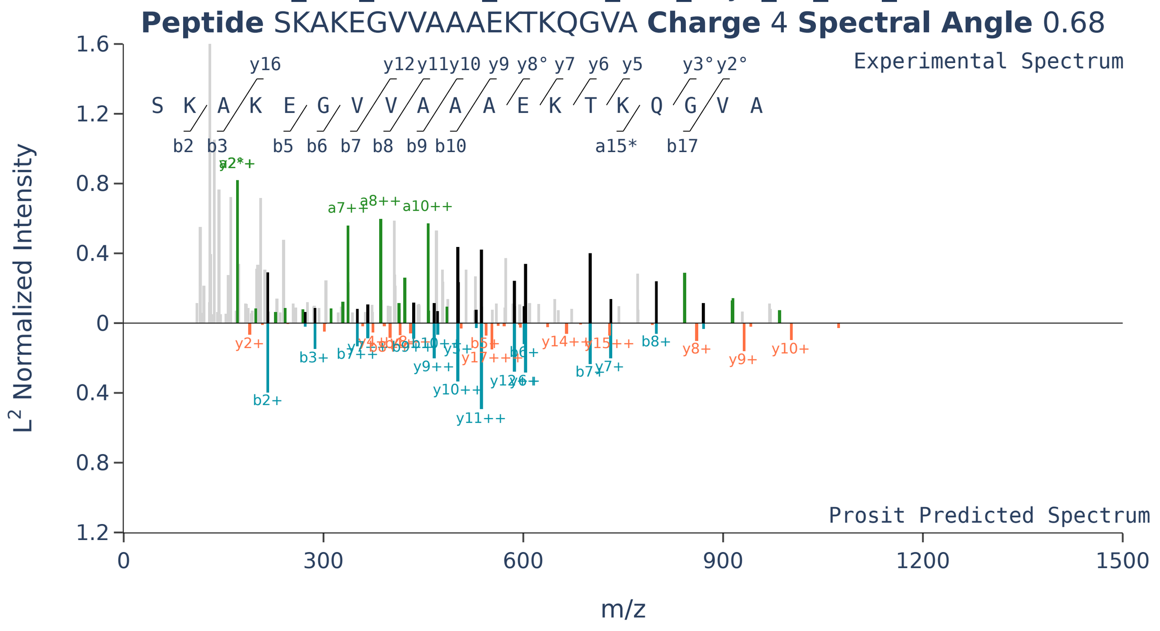


**Source** W_Soh_160821_170921_Goe_aSyn_A1_24h_R2 **Scan** 8052
**Peptide** VVHGVATVAEKT **Charge** 2 **Spectral Angle** 0.73

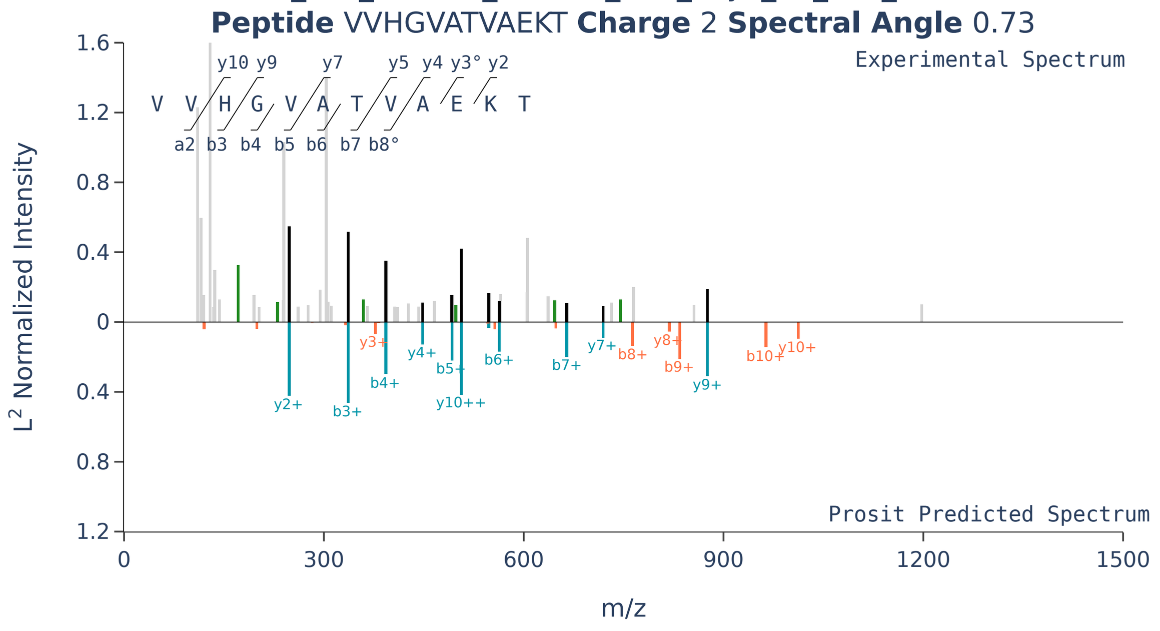


**Source** W_Soh_160821_170921_Goe_aSyn_A1_24h_R1 **Scan** 10436
**Peptide** QKTVEGAGSIAAATG **Charge** 2 **Spectral Angle** 0.83

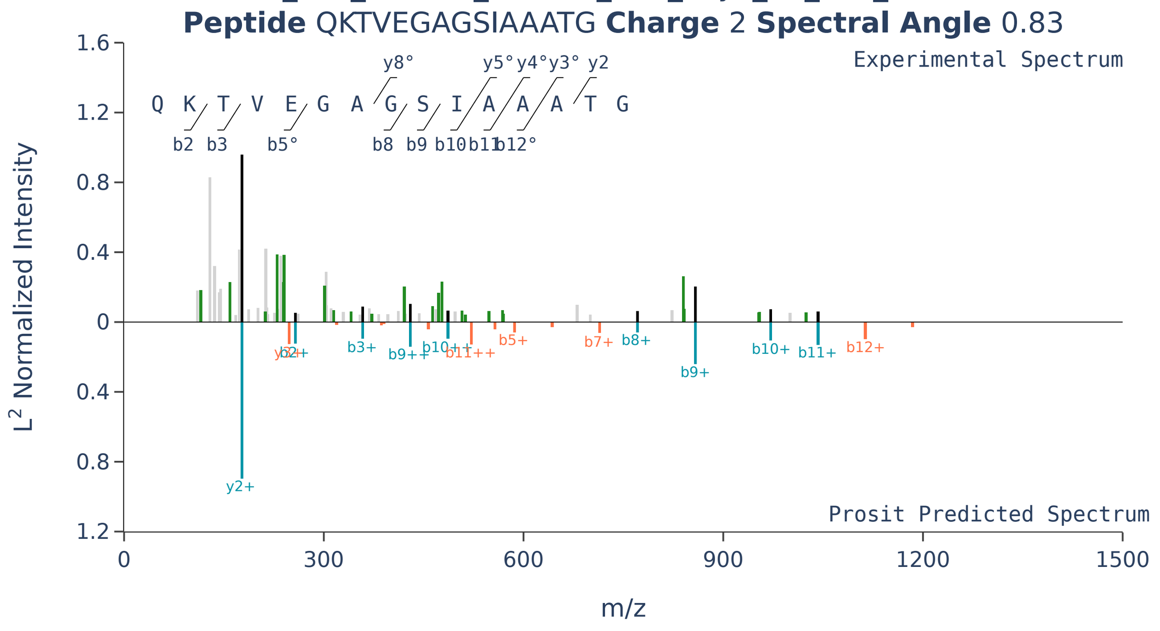


**Source** W_Soh_160821_170921_Goe_aSyn_A1_24h_R2 **Scan** 23695
**Peptide** QVTNVGGAVVTGVTAVAQ **Charge** 2 **Spectral Angle** 0.79

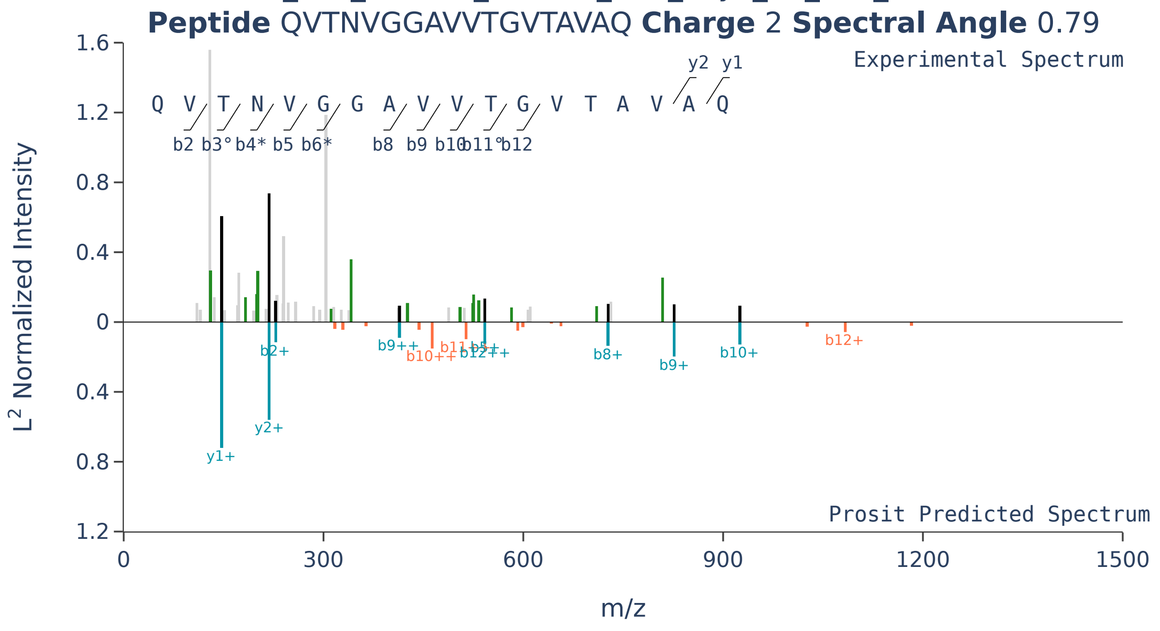


**Source** W_Soh_160821_170921_Goe_aSyn_A1_24h_R2 **Scan** 12250
**Peptide** GLSKAKEGVVAAAEKTKQGVAEAAGKTK **Charge** 4 **Spectral Angle** 0.67

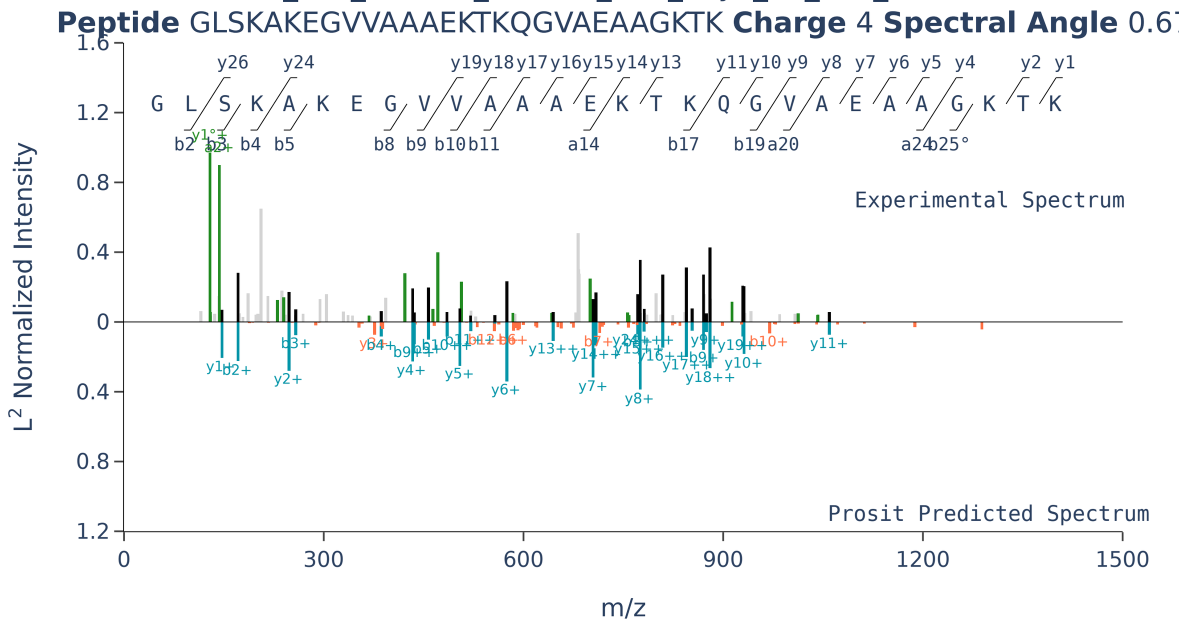


**Source** W_Soh_160821_170921_Goe_aSyn_A2_24h_R1 **Scan** 9510
**Peptide** KQGVAEAAGKTKEGVLY **Charge** 4 **Spectral Angle** 0.87

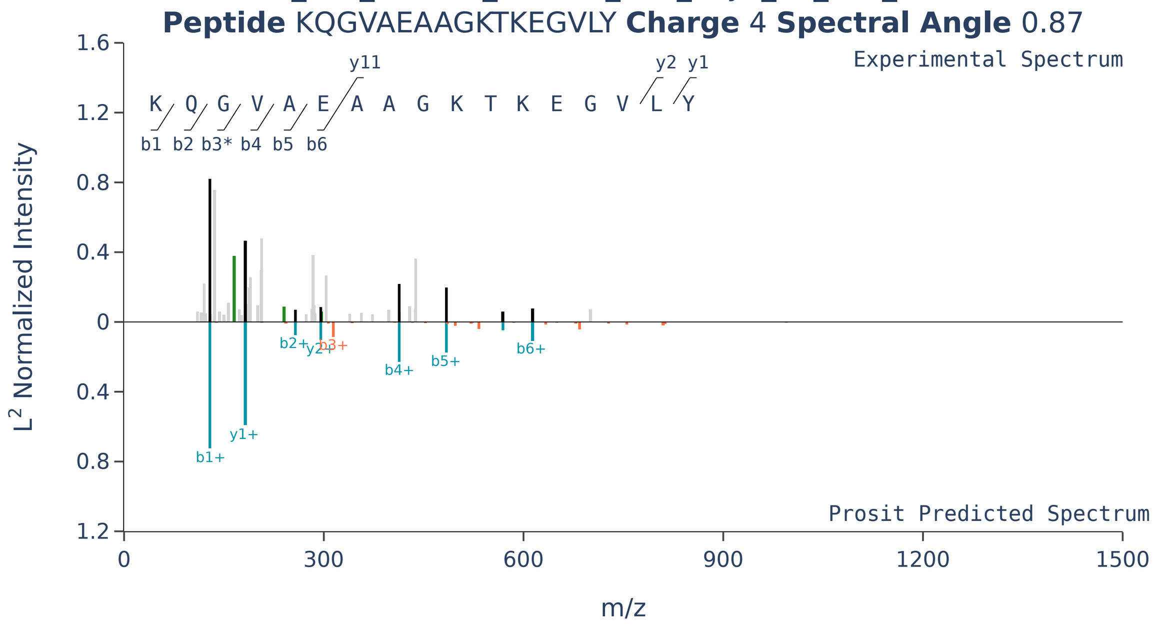


**Source** W_Soh_160821_170921_Goe_aSyn_A1_24h_R1 **Scan** 5975
**Peptide** KGLSKAKEGVV **Charge** 3 **Spectral Angle** 0.85

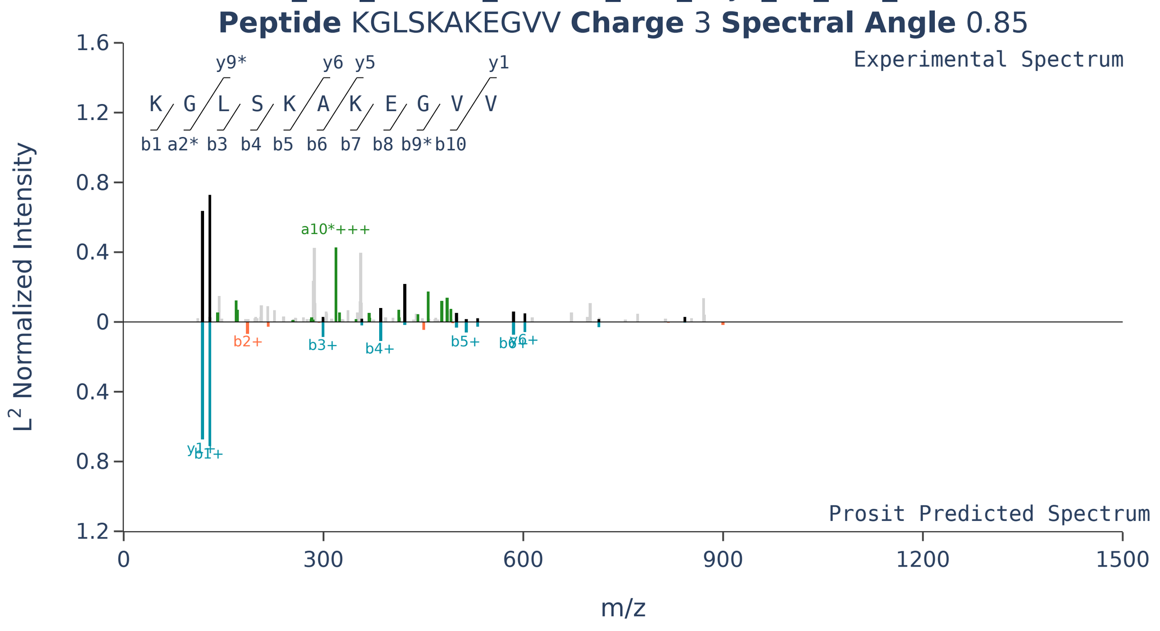

**Source** W_Soh_160821_170921_Goe_aSyn_A2_24h_R1 **Scan** 12045
**Peptide** GVTAVAQKTVEGA **Charge** 2 **Spectral Angle** 0.7

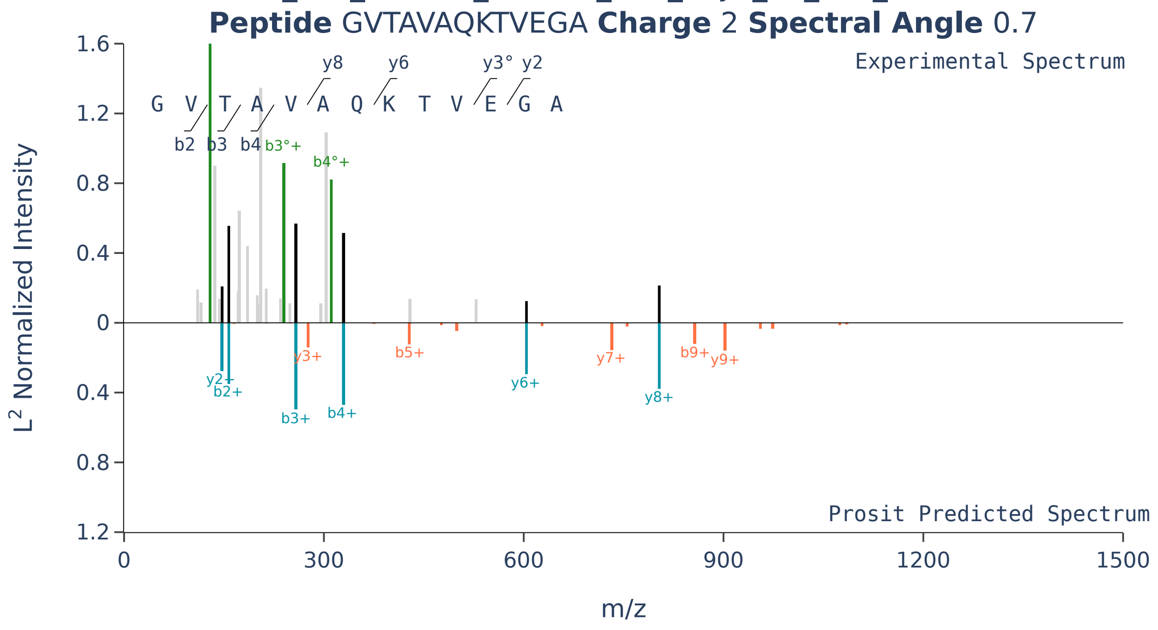


**Source** W_Soh_160821_170921_Goe_aSyn_A1_24h_R1 **Scan** 8453
**Peptide** VAEAAGKTKEGVLYVGSKTKEG **Charge** 3 **Spectral Angle** 0.81

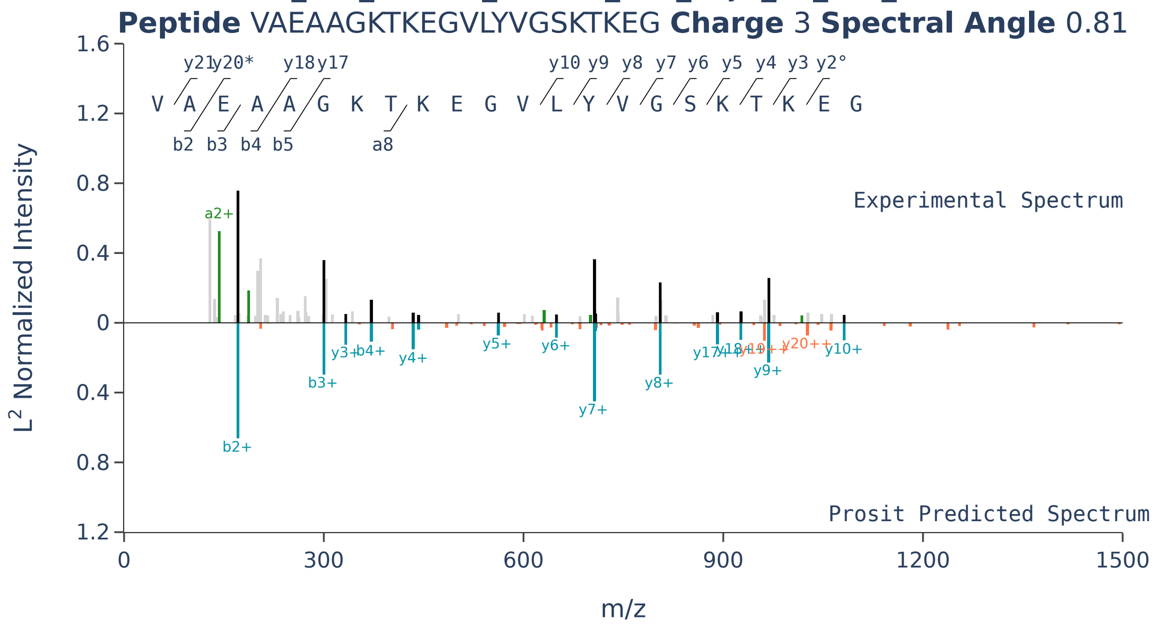


**Source** W_Soh_160821_170921_Goe_aSyn_A1_24h_R1 **Scan** 17018
**Peptide** KTKEGVVHGVATVAEKTKEQVTNVGGAVV **Charge** 4 **Spectral Angle** 0.72

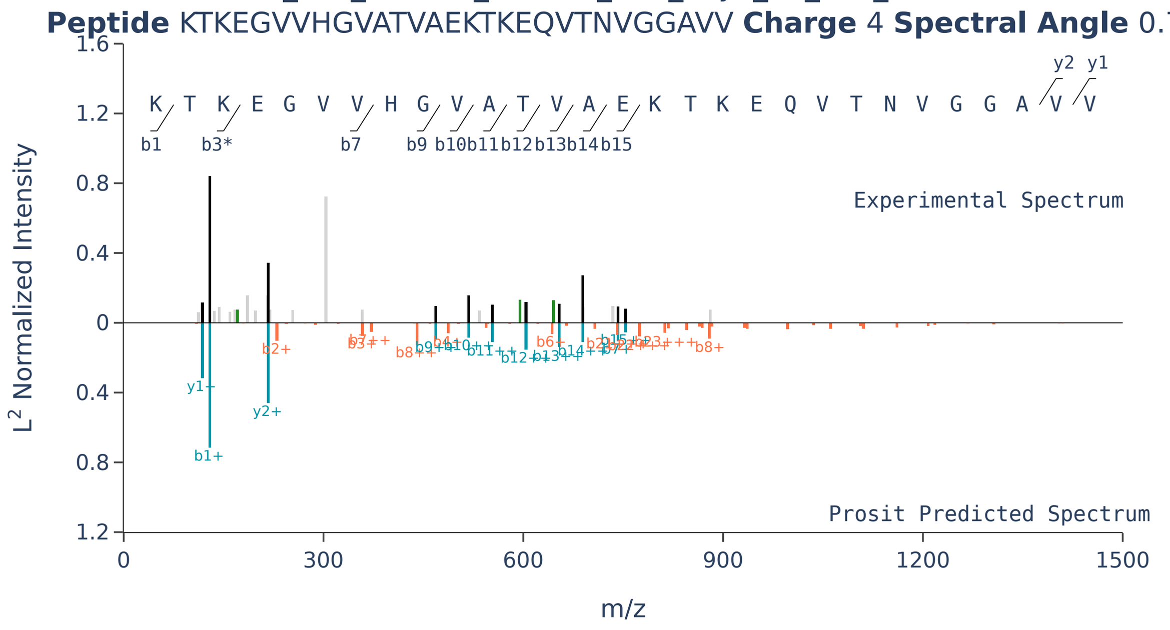


**Source** W_Soh_160821_170921_Goe_aSyn_A1_24h_R2 **Scan** 8185
**Peptide** VGSKTKEGVVHGVATVAEKTKEQ **Charge** 5 **Spectral Angle** 0.67

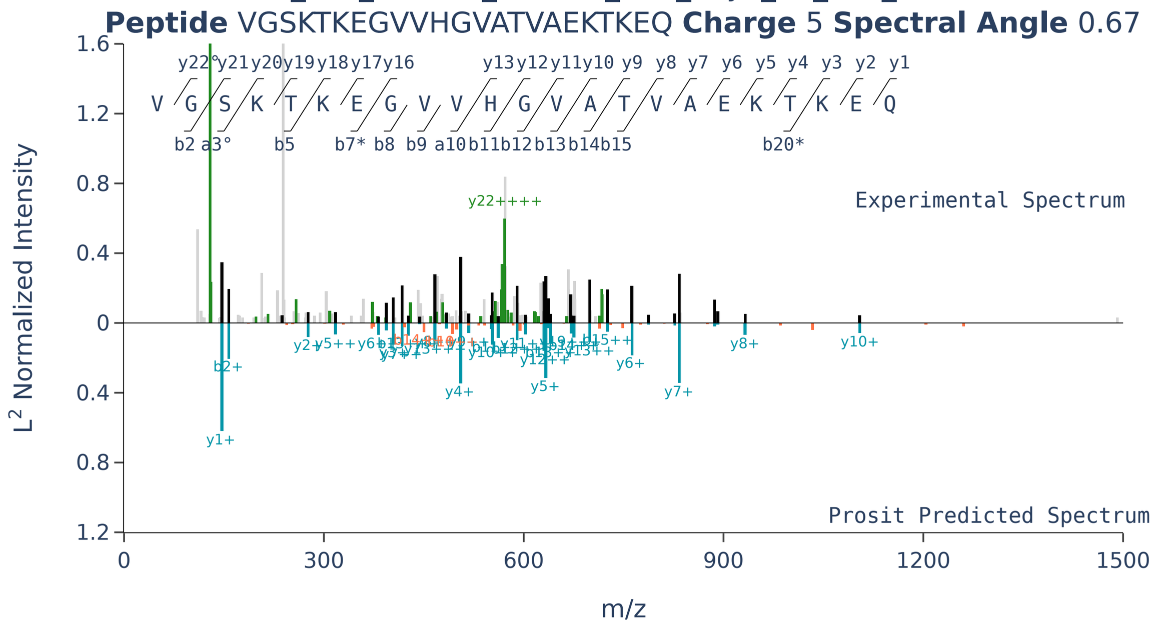


**Source** W_Soh_160821_170921_Goe_aSyn_A2_6h_R1 **Scan** 21762
**Peptide** KTKEQVTNVGGAVVTGVTAVAQKTVEG **Charge** 4 **Spectral Angle** 0.74

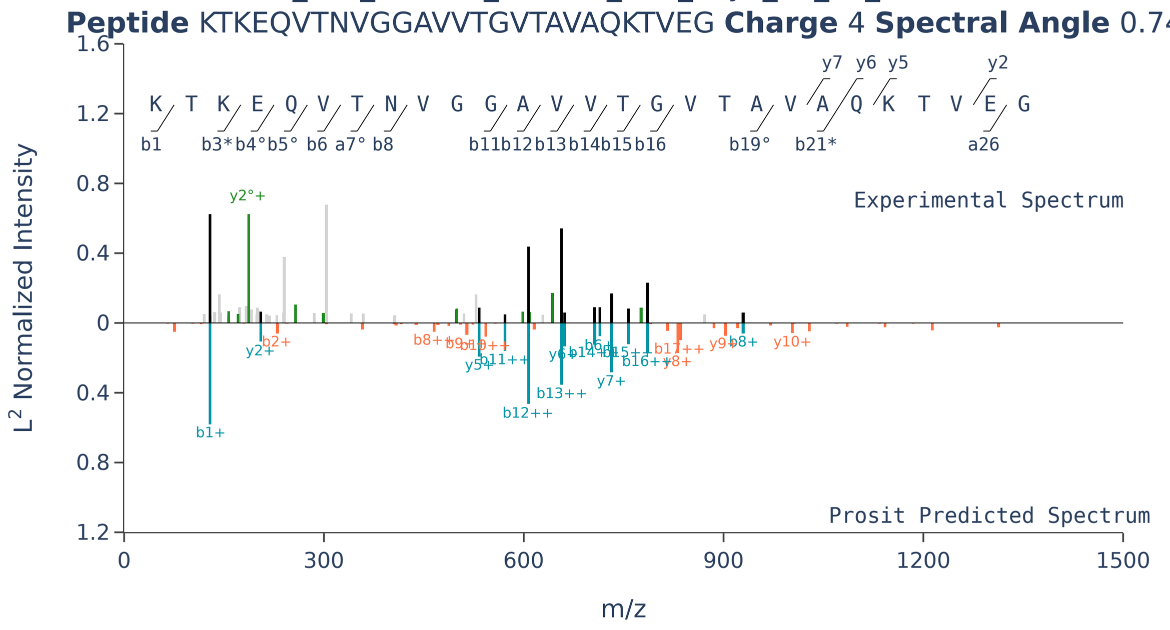


**Source** W_Soh_160821_170921_Goe_aSyn_A1_24h_R1 **Scan** 10993
**Peptide** KQGVAEAAGKTKEGVLYVGSKTKEGVVHG **Charge** 4 **Spectral Angle** 0.86

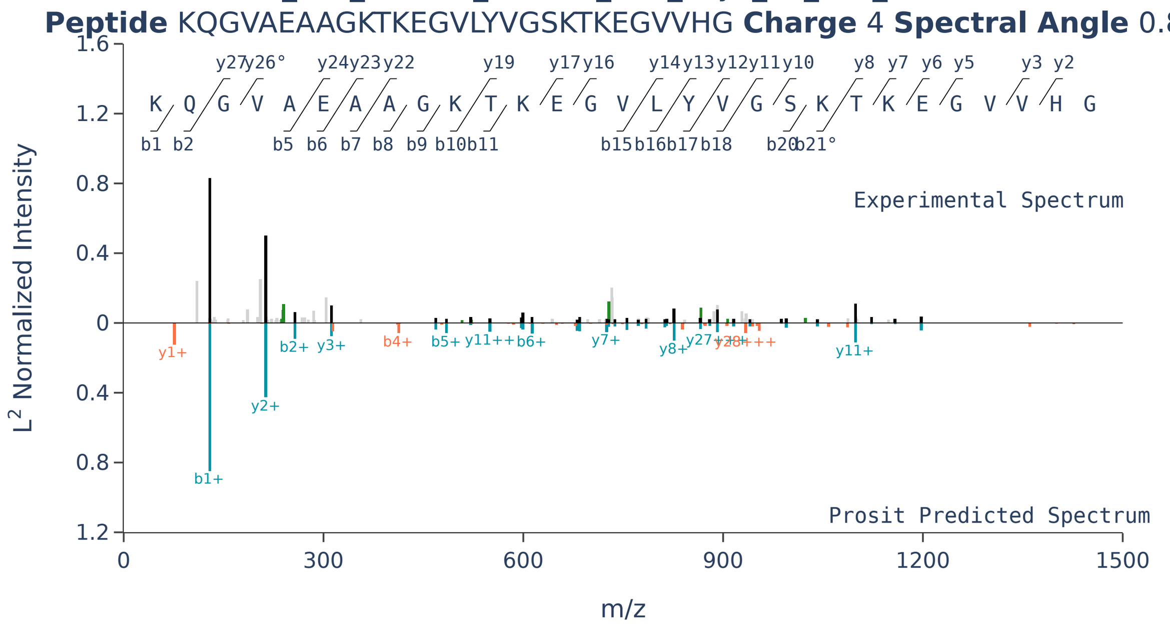


**Source** W_Soh_160821_170921_Goe_aSyn_A1_24h_R2 **Scan** 8430
**Peptide** AAGKTKEGVLYVGSKT **Charge** 2 **Spectral Angle** 0.69

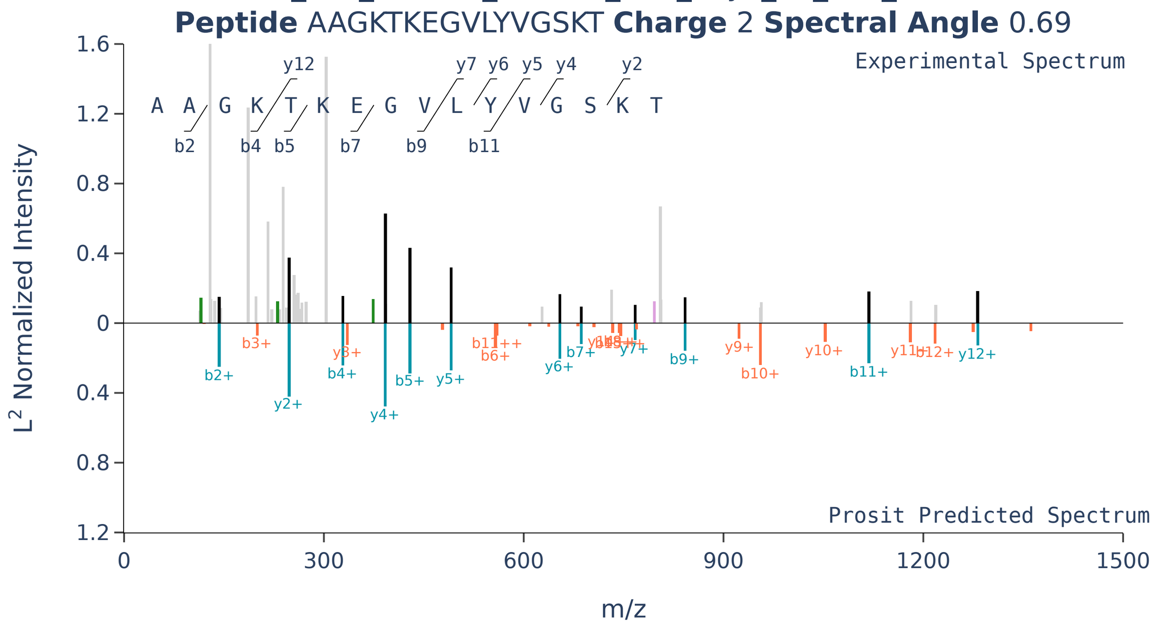


**Source** W_Soh_160821_170921_Goe_aSyn_A2_24h_R1 **Scan** 27380
**Peptide** QVTNVGGAVVTGVTAVAQKTVEG **Charge** 2 **Spectral Angle** 0.77

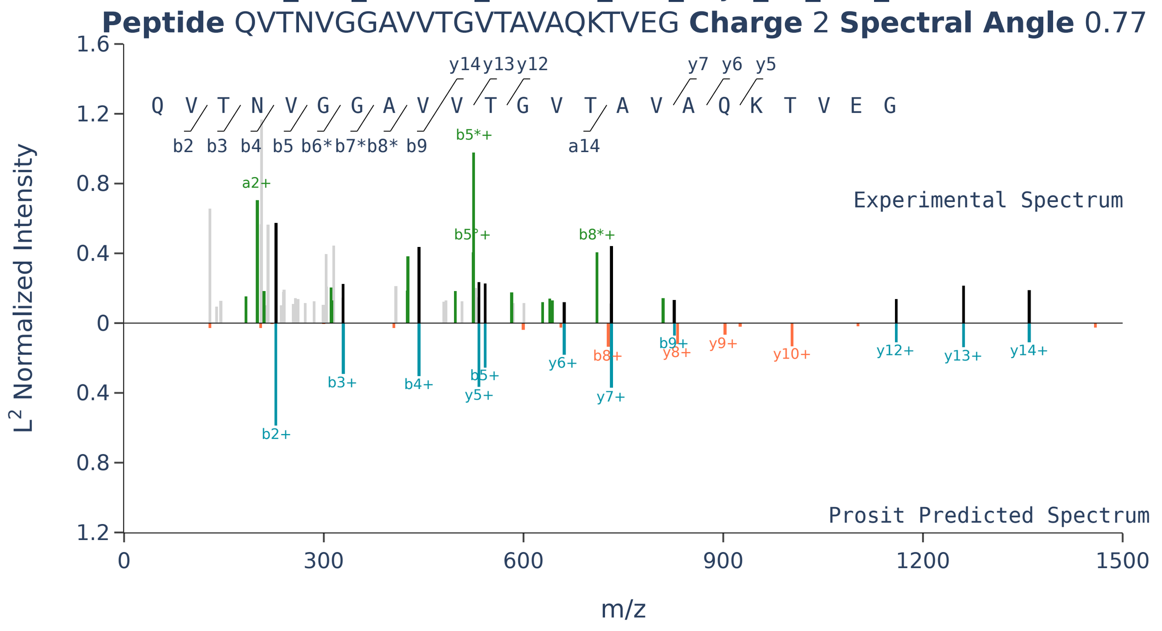


**Source** W_Soh_160821_170921_Goe_aSyn_A1_24h_R1 **Scan** 19436
**Peptide** VVHGVATVAEKTKEQVTNVGGAVVTGV **Charge** 3 **Spectral Angle** 0.7

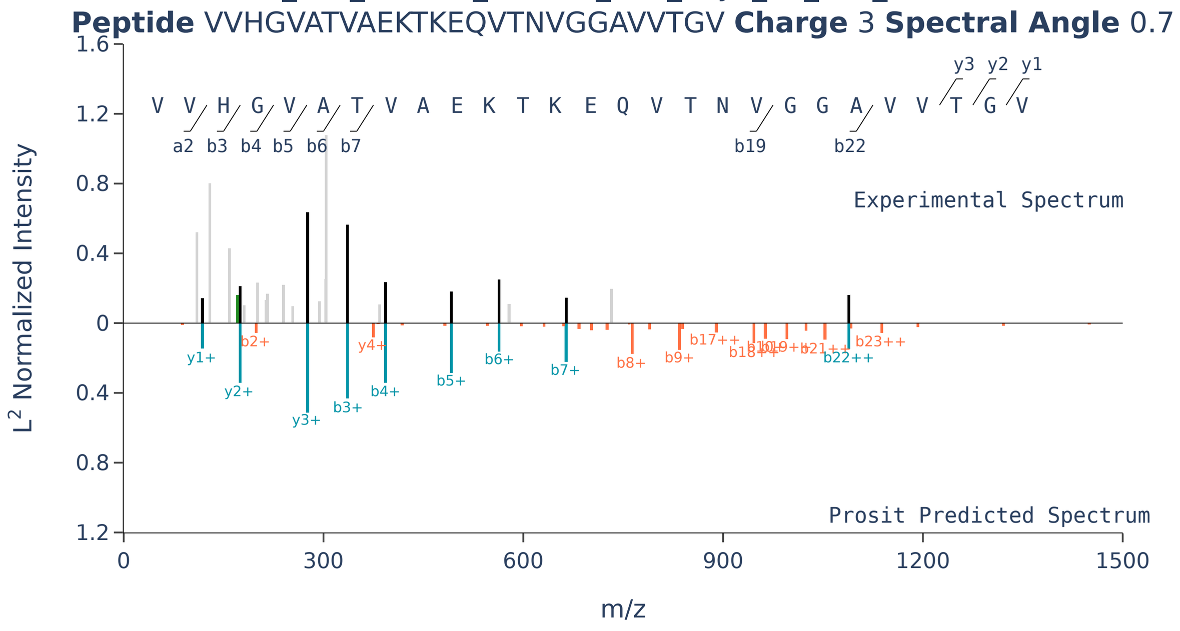


**Source** W_Soh_160821_170921_Goe_aSyn_A2_24h_R1 **Scan** 19282
**Peptide** TGVTAVAQKTVEGAGSIAAA **Charge** 2 **Spectral Angle** 0.68

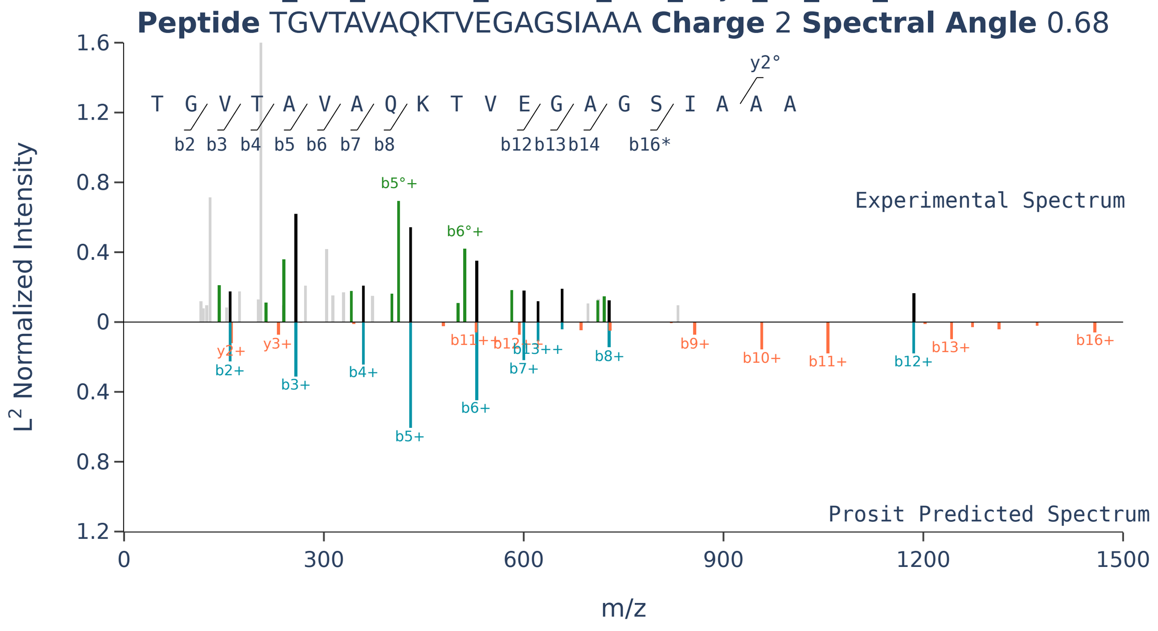


**Source** W_Soh_160821_170921_Goe_aSyn_A1_24h_R2 **Scan** 12802
**Peptide** TKQGVAEAAGKTKEGVLYV **Charge** 2 **Spectral Angle** 0.89

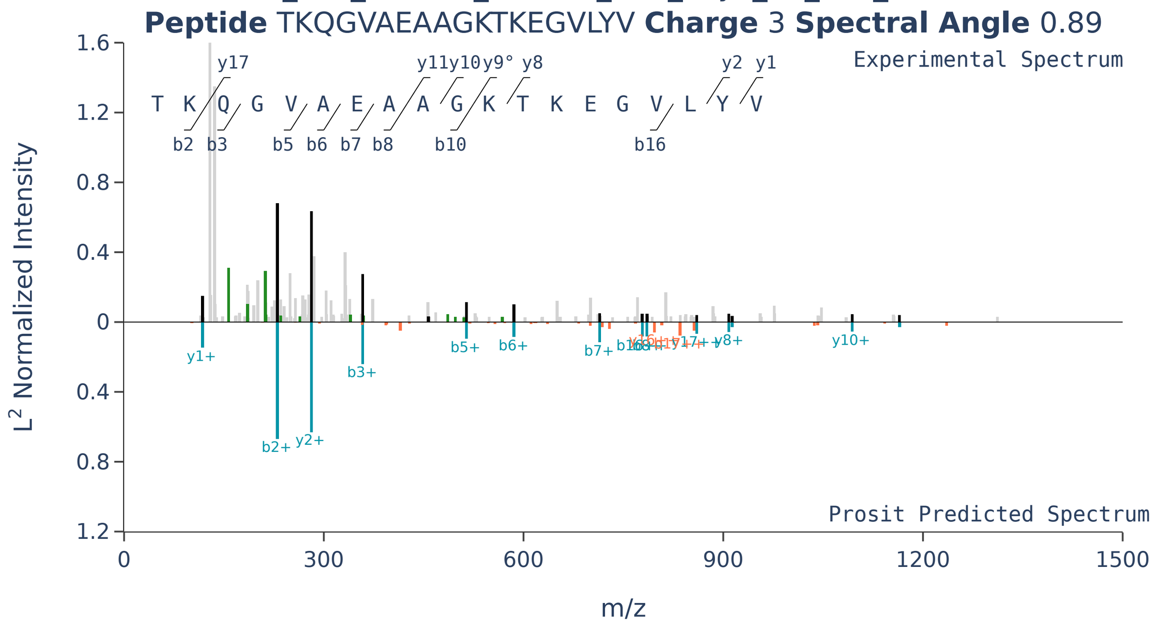


**Source** W_Soh_160821_170921_Goe_aSyn_A1_6h_R2 **Scan** 21433
**Peptide** ATVAEKTKEQVTNVGGAVVTGVTAVA **Charge** 3 **Spectral Angle** 0.76

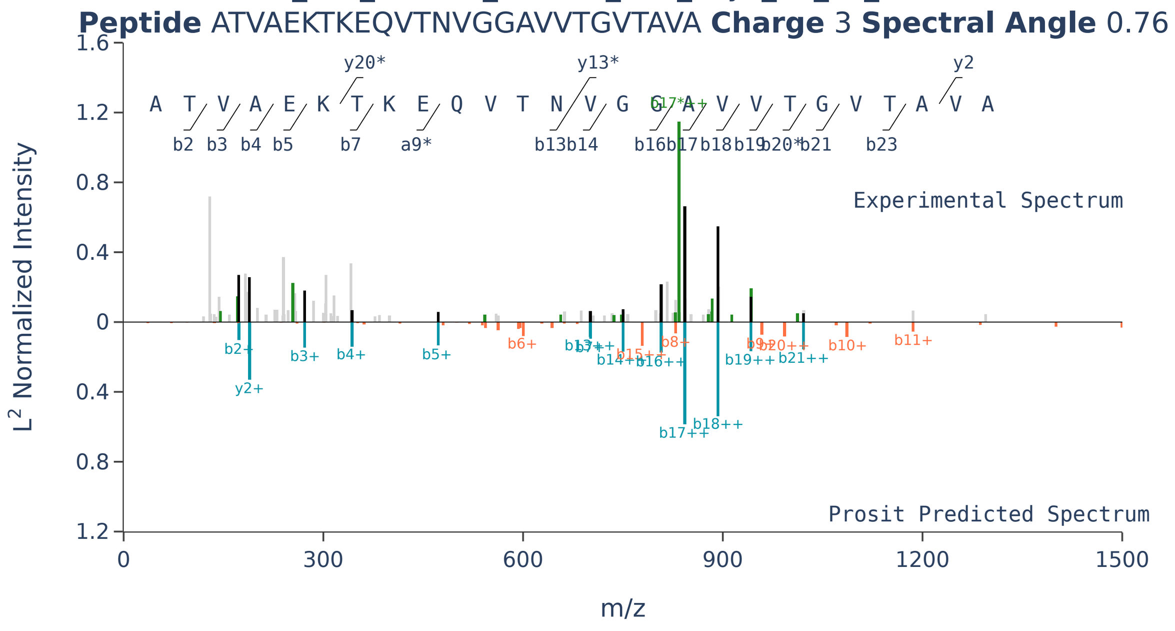


**Source** W_Soh_160821_170921_Goe_aSyn_A2_24h_R1 **Scan** 16707
**Peptide** EGAPQEGILED **Charge** 2 **Spectral Angle** 0.58

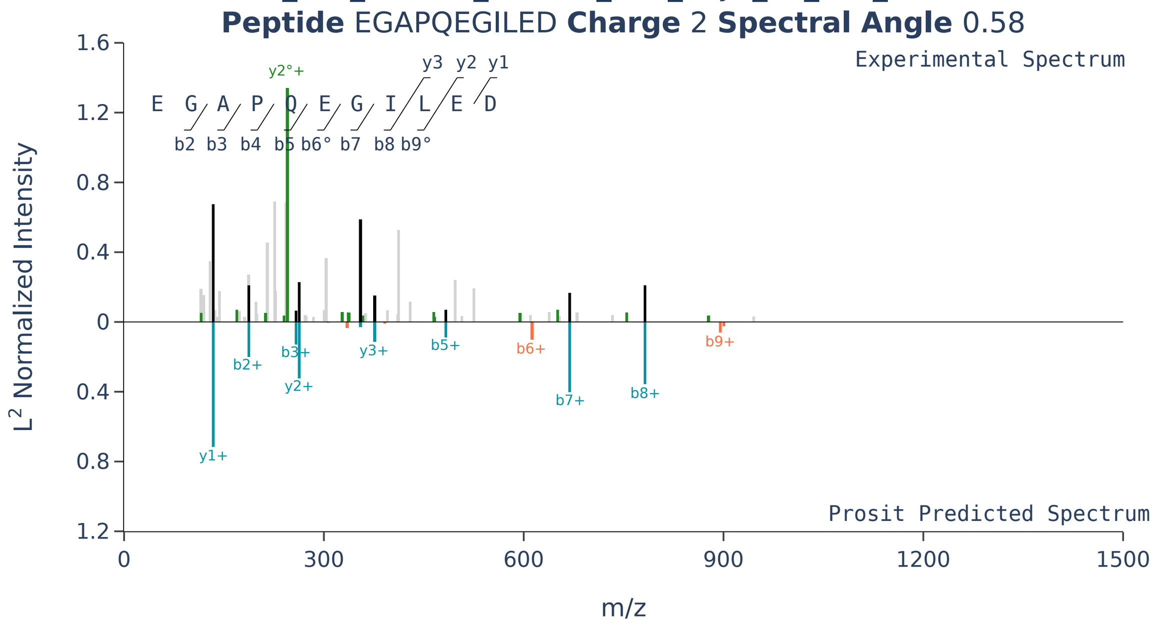


**Source** W_Soh_160821_170921_Goe_aSyn_A2_24h_R1 **Scan** 9697
**Peptide** FVKKDQLGKNEEGAPQEGI **Charge** 4 **Spectral Angle** 0.85

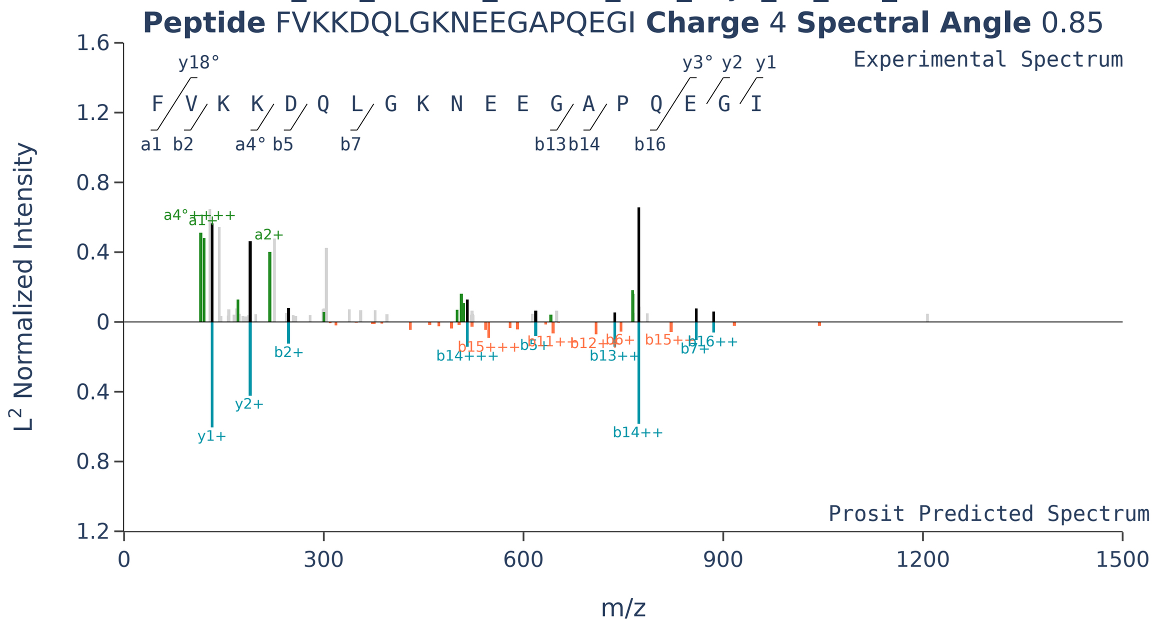


**Source** W_Soh_160821_170921_Goe_aSyn_A1_24h_R2 **Scan** 10833
**Peptide** SKTKEGVVHGVATVAEKTKEQVT **Charge** 4 **Spectral Angle** 0.75

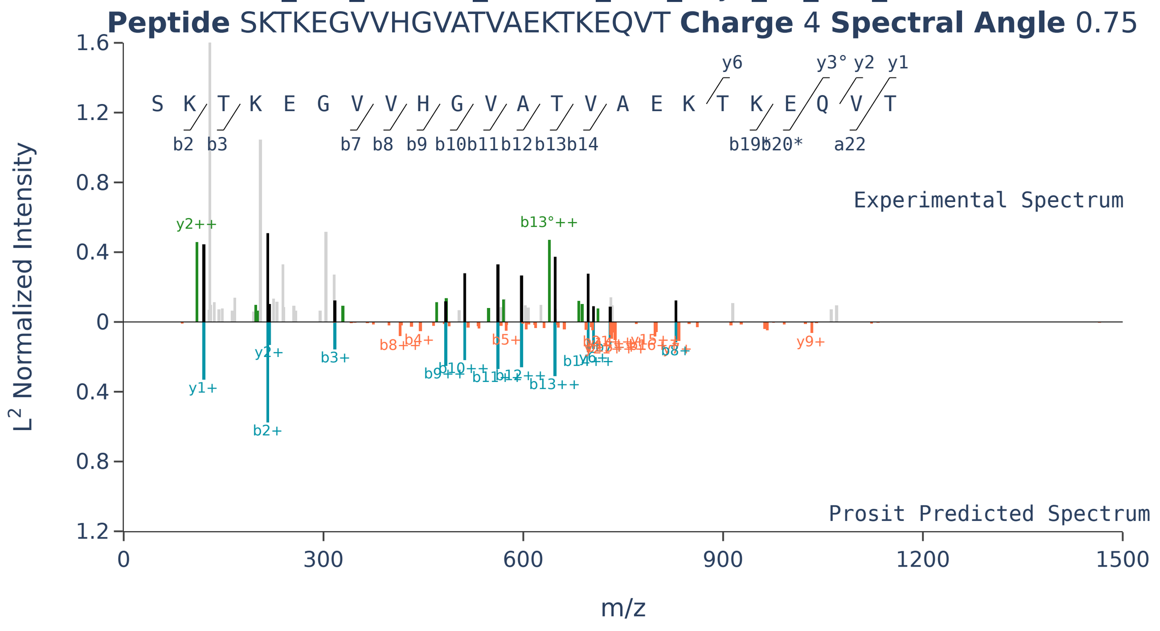

**Source** W_Soh_160821_170921_Goe_aSyn_A1_24h_R1 **Scan** 30064
**Peptide** TNVGGAVVTGVTAVAQKTVEGAGSIAA **Charge** 3 **Spectral Angle** 0.87

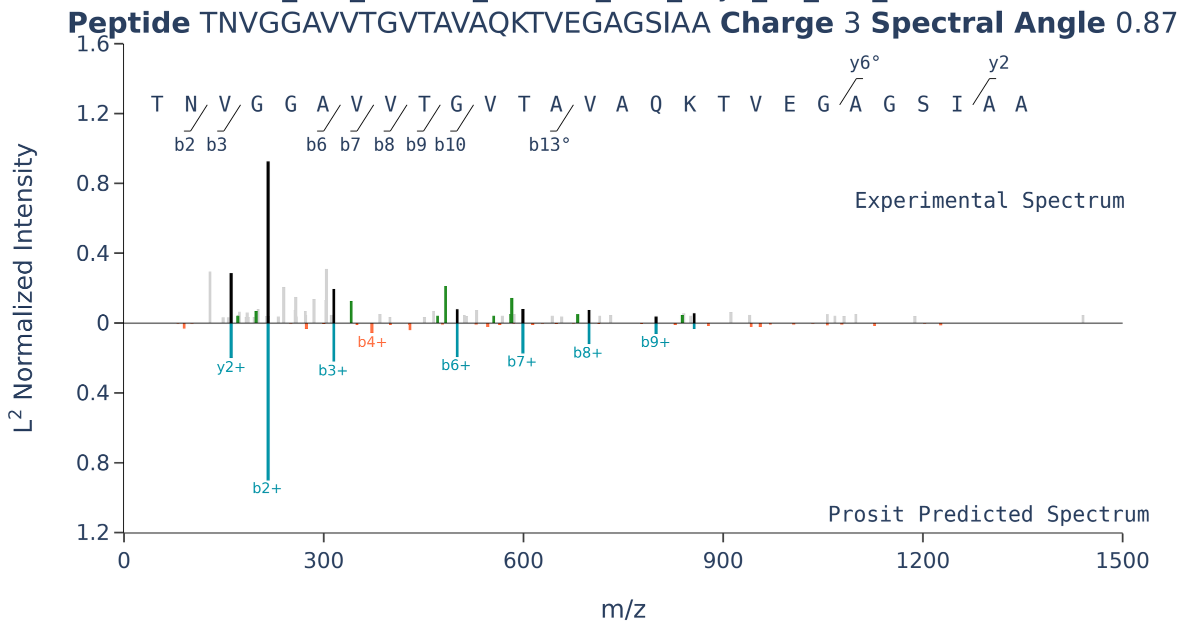


**Source** W_Soh_160821_170921_Goe_aSyn_A1_24h_R1 **Scan** 11183
**Peptide** AAAEKTKQGVAEAAGKTKEGVLYVGS **Charge** 4 **Spectral Angle** 0.62

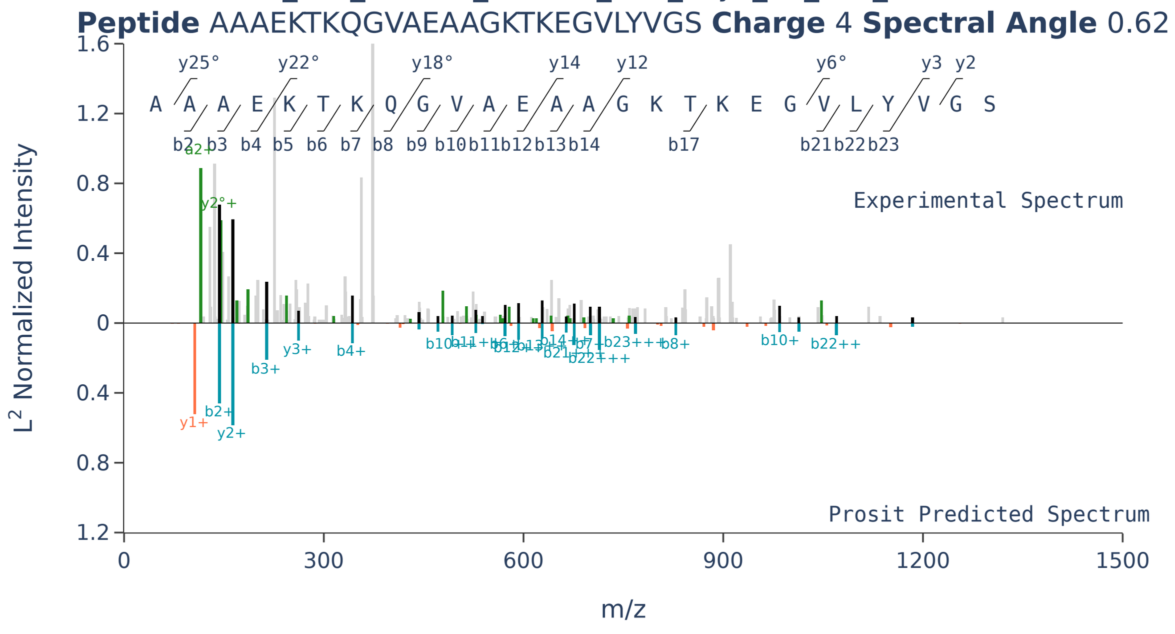


**Source** W_Soh_160821_170921_Goe_aSyn_A1_24h_R2 **Scan** 14398
**Peptide** AGSIAAATGFVKKDQLGKNEEGAPQEG **Charge** 3 **Spectral Angle** 0.85

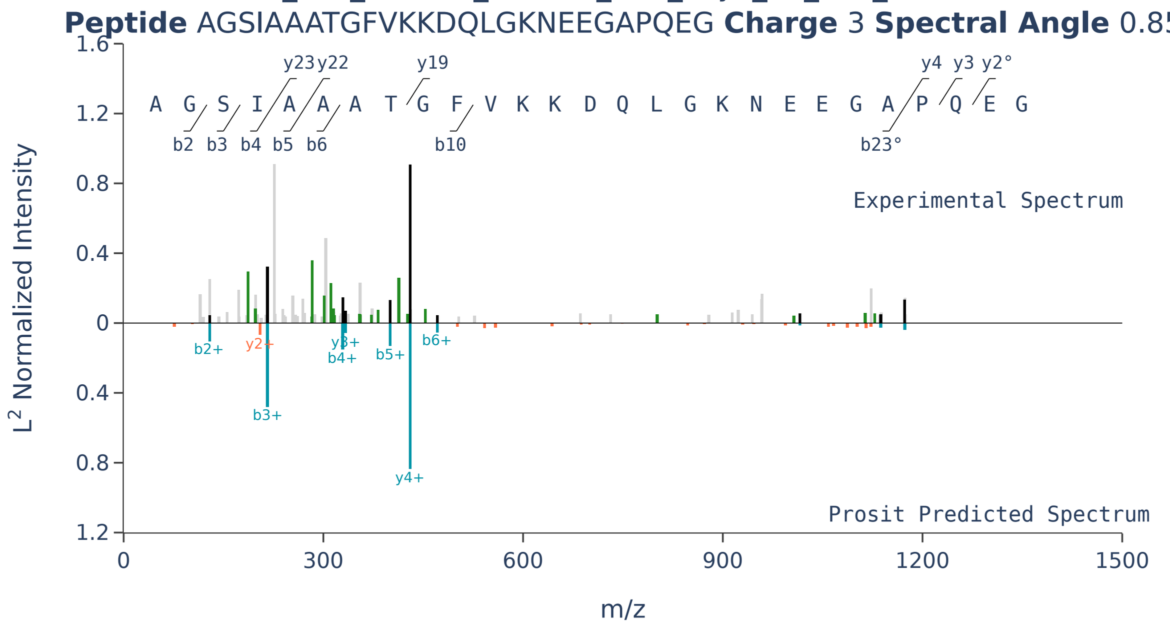


**Source** W_Soh_160821_170921_Goe_aSyn_A2_24h_R1 **Scan** 18821
**Peptide** EKTKEQVTNVGGAVVTGVTAVAQ **Charge** 3 **Spectral Angle** 0.72

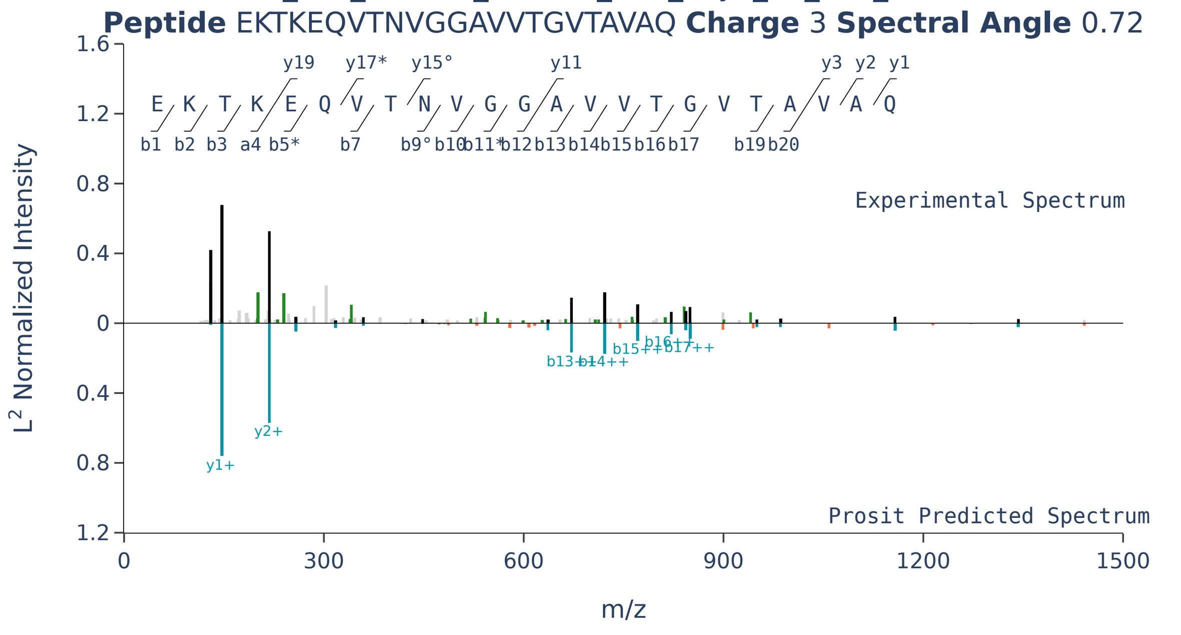


**Source** W_Soh_160821_170921_Goe_aSyn_A1_24h_R2 **Scan** 18962
**Peptide** VTAVAQKTVEGAGSIAAATGFVKK **Charge** 4 **Spectral Angle** 0.79

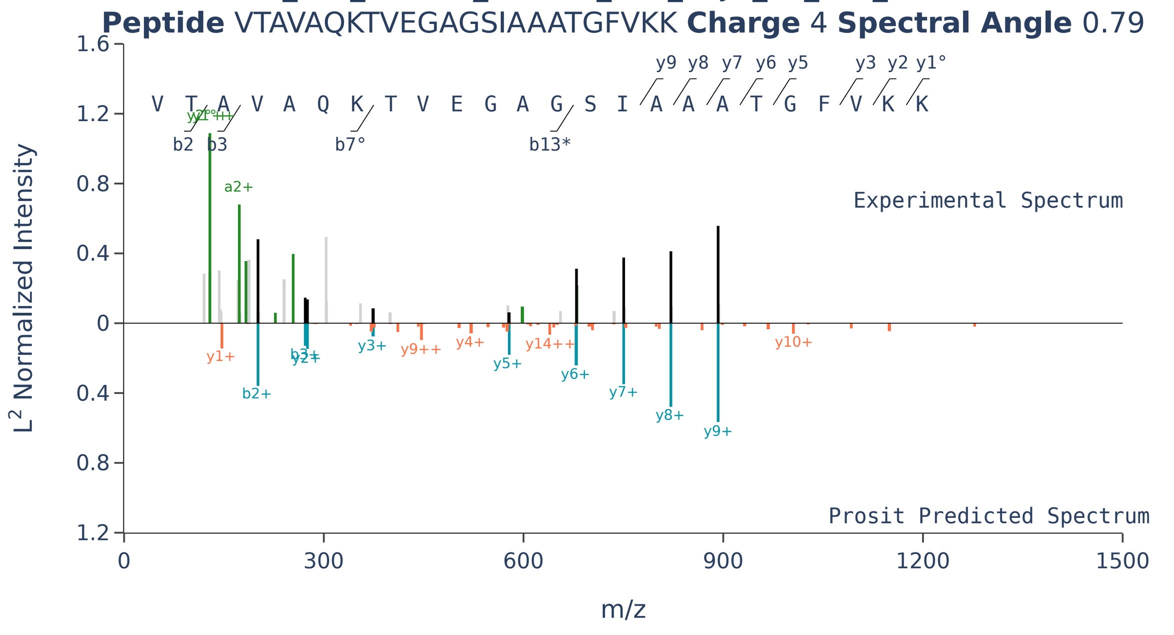


**Source** W_Soh_160821_170921_Goe_aSyn_A1_24h_R2 **Scan** 13382
**Peptide** KDQLGKNEEGAPQEGILED **Charge** 3 **Spectral Angle** 0.72

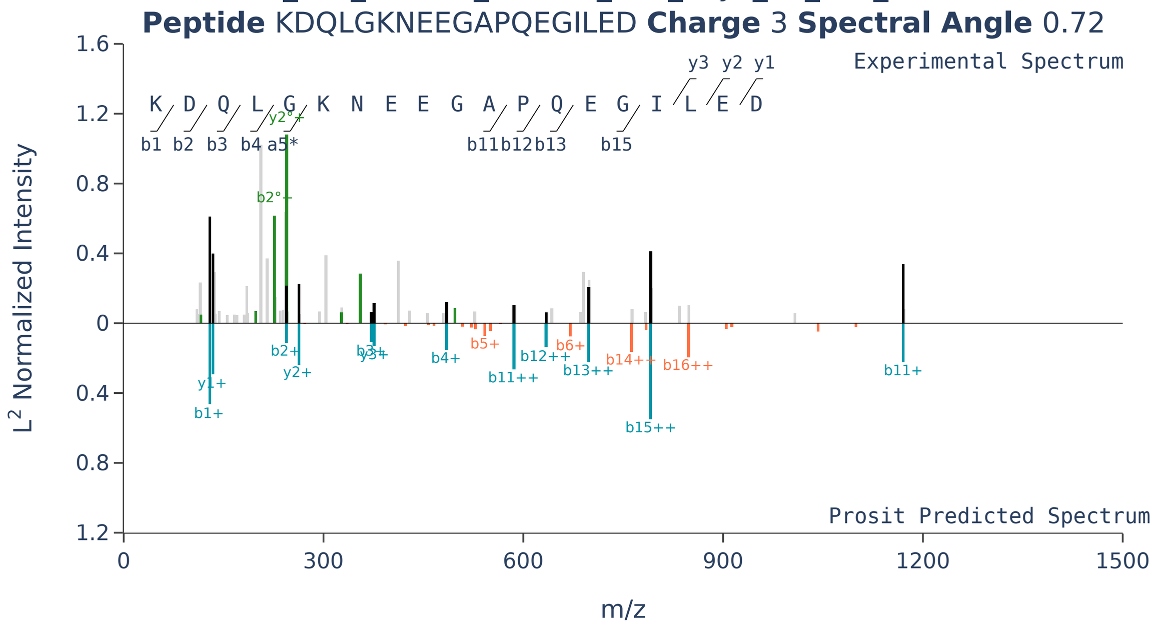


**Source** W_Soh_160821_170921_Goe_aSyn_A2_24h_R2 **Scan** 9727
**Peptide** VVHGVATV **Charge** 2 **Spectral Angle** 0.73

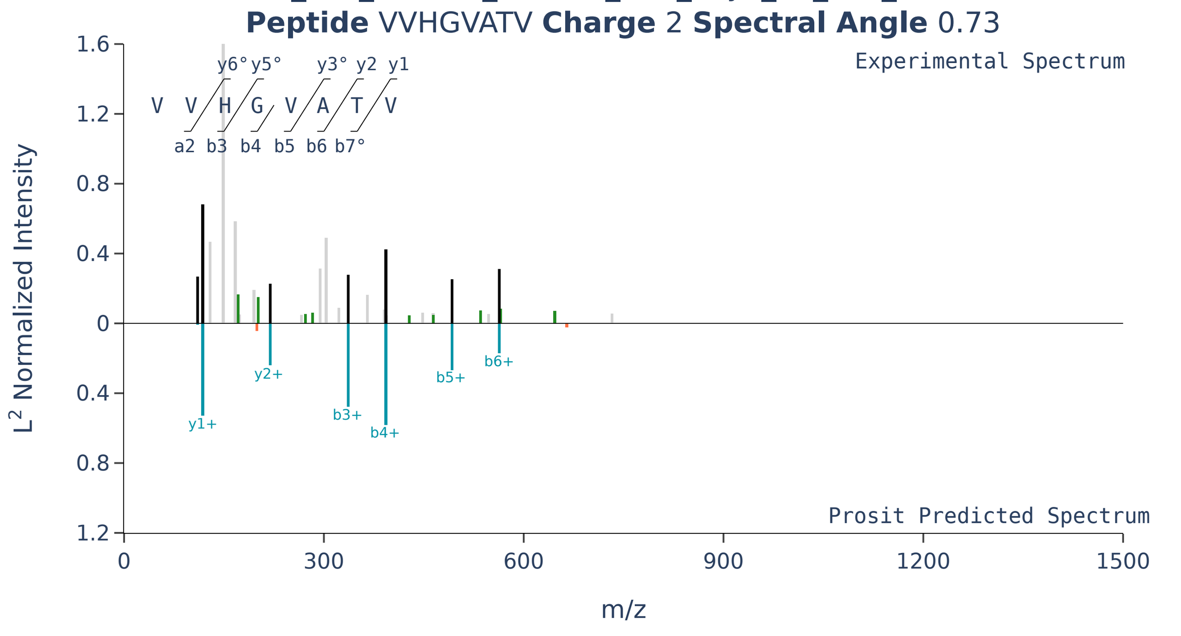


**Source** W_Soh_160821_170921_Goe_aSyn_A1_24h_R1 **Scan** 13414
**Peptide** KEGVLYVGSKTKEGVVHGVATV **Charge** 4 **Spectral Angle** 0.84

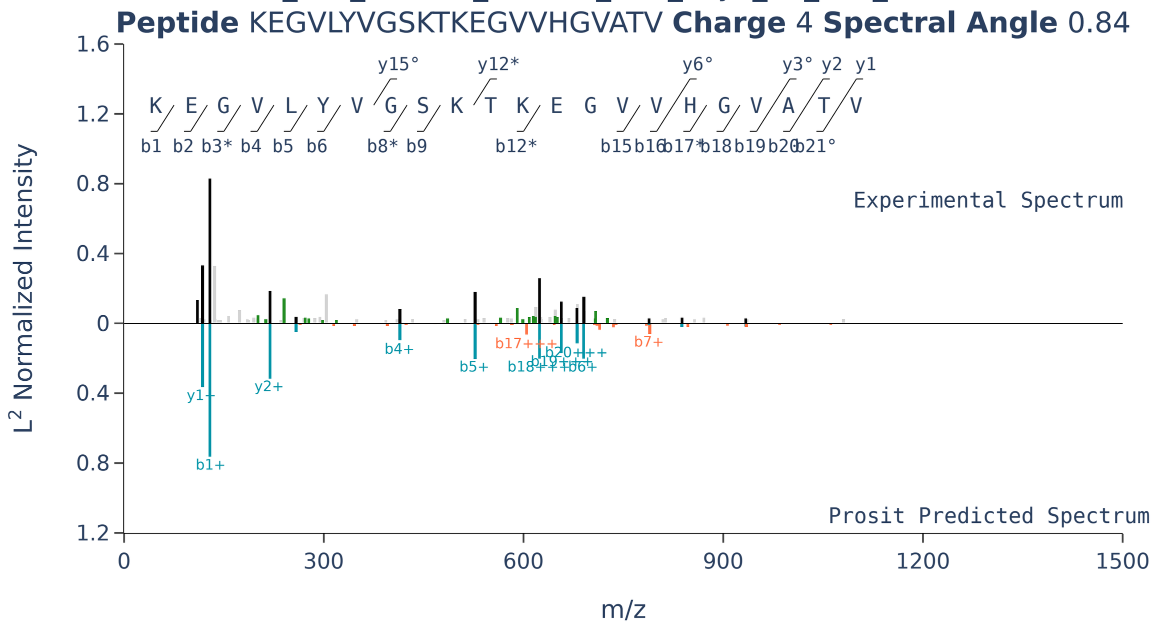


**Source** W_Soh_160821_170921_Goe_aSyn_A2_24h_R1 **Scan** 10397
**Peptide** EKTKEQVTNVGGAVVTG **Charge** 3 **Spectral Angle** 0.85

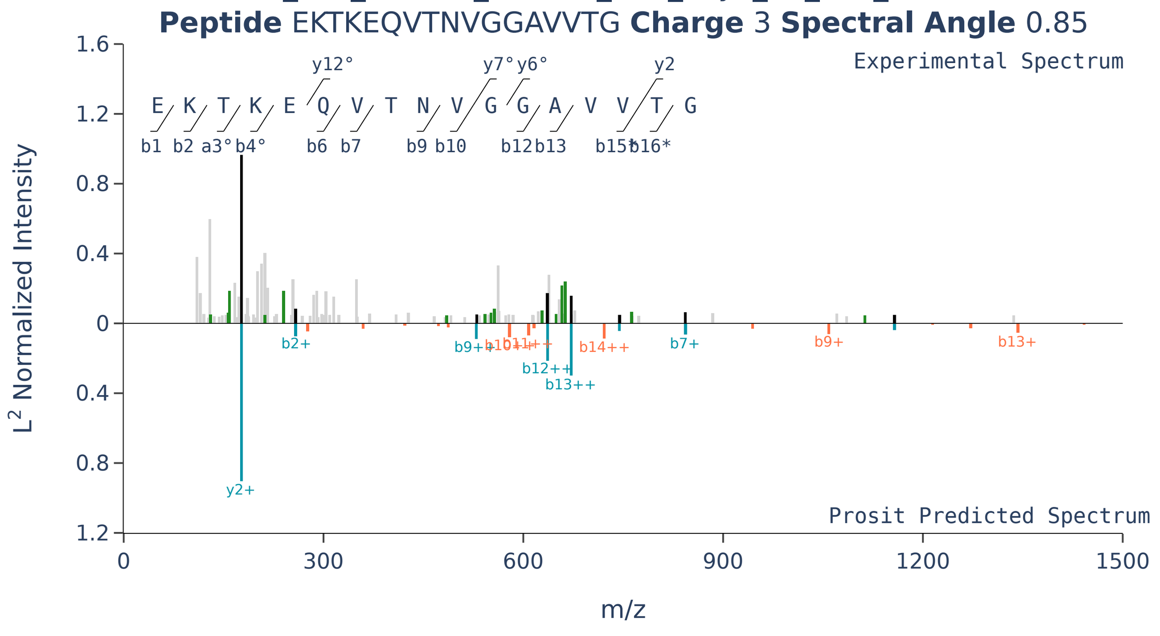


**Source** W_Soh_160821_170921_Goe_aSyn_A1_24h_R2 **Scan** 22500
**Peptide** EKTKEQVTNVGGAVVTGVTAVAQKTV **Charge** 4 **Spectral Angle** 0.73

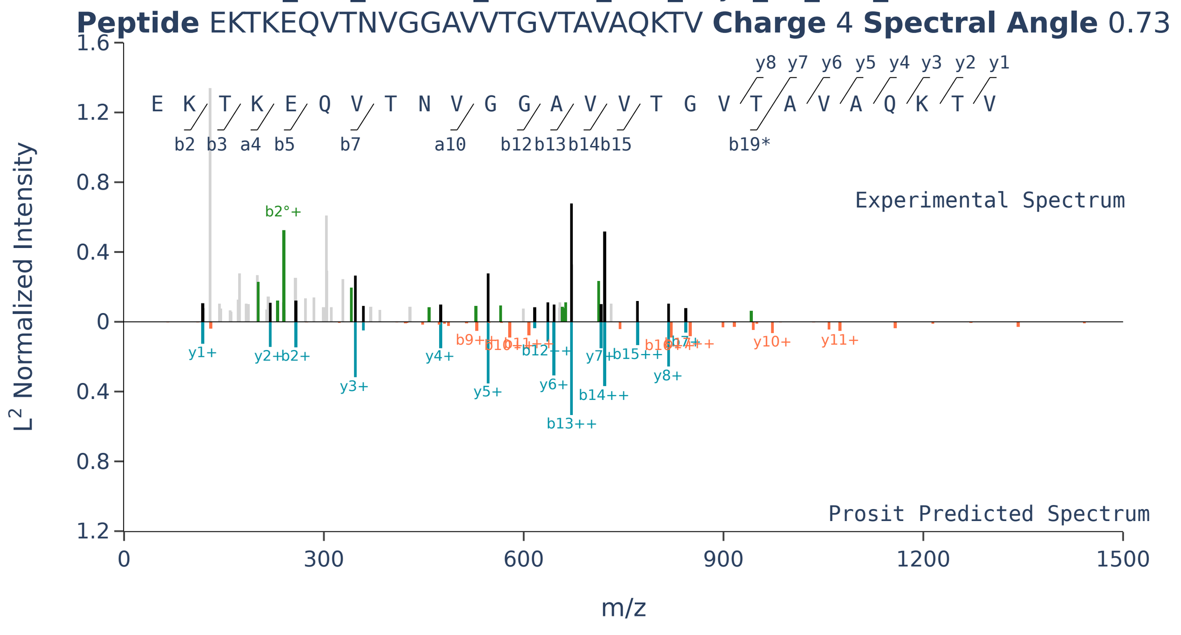


**Source** W_Soh_160821_170921_Goe_aSyn_A1_24h_R2 **Scan** 11168
**Peptide** KGLSKAKEGVVAAAEKTKQGVAEAAGKTKE **Charge** 4 **Spectral Angle** 0.73

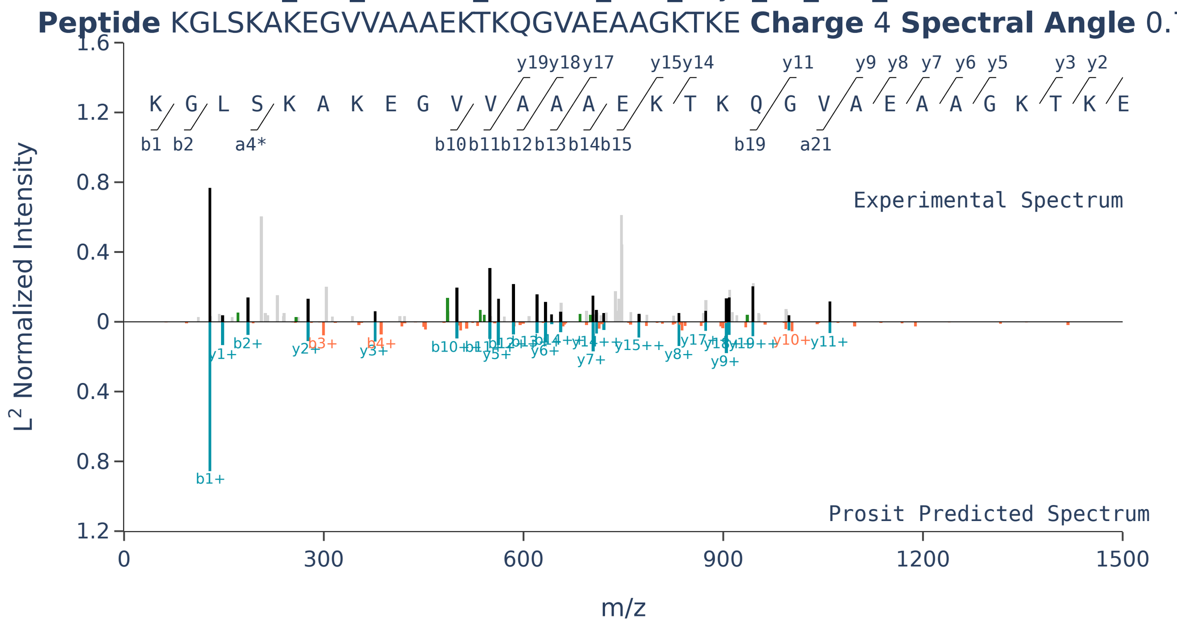


**Source** W_Soh_160821_170921_Goe_aSyn_A1_24h_R2 **Scan** 9906
**Peptide** GKTKEGVLYVGSKTKEGVVHGVA **Charge** 4 **Spectral Angle** 0.79

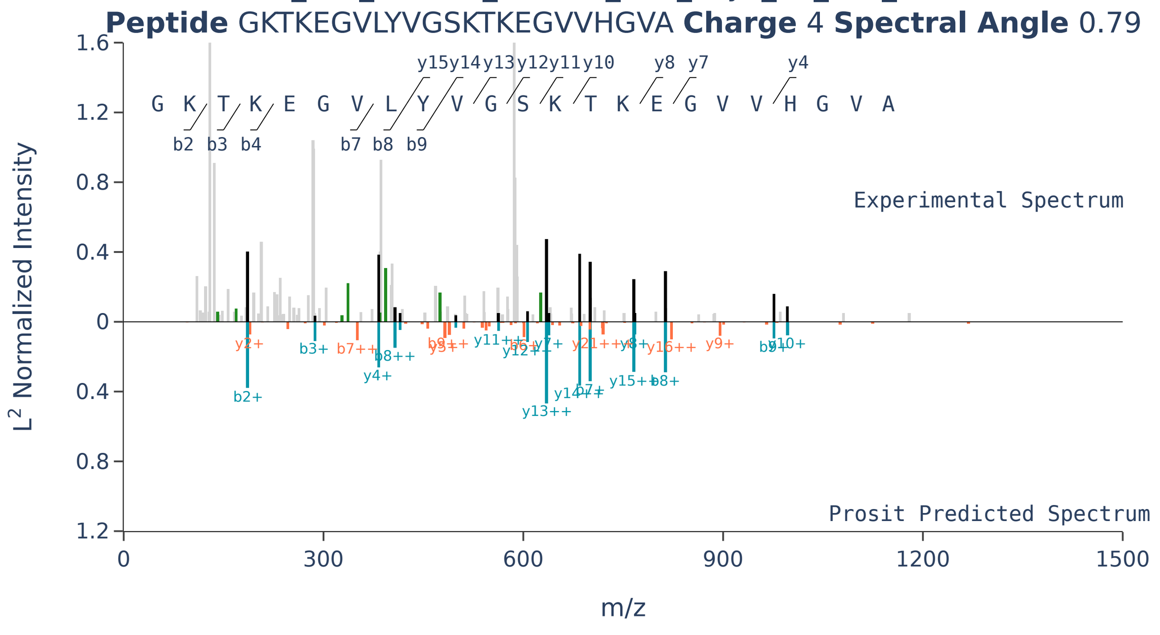


**Source** W_Soh_160821_170921_Goe_aSyn_A1_24h_R1 **Scan** 10068
**Peptide** AKEGVVAAAEKTKQGVAEAAGKTKEG **Charge** 5 **Spectral Angle** 0.73

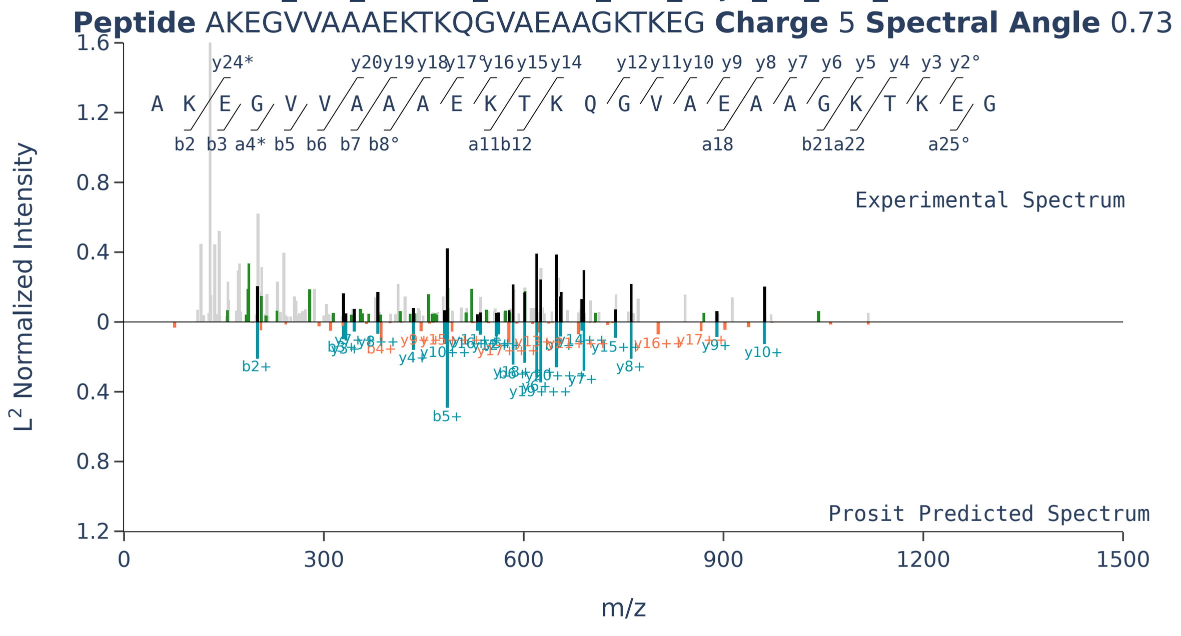


**Source** W_Soh_160821_170921_Goe_aSyn_A1_24h_R1 **Scan** 21020
**Peptide** TVEGAGSIAAATGFVK **Charge** 2 **Spectral Angle** 0.63

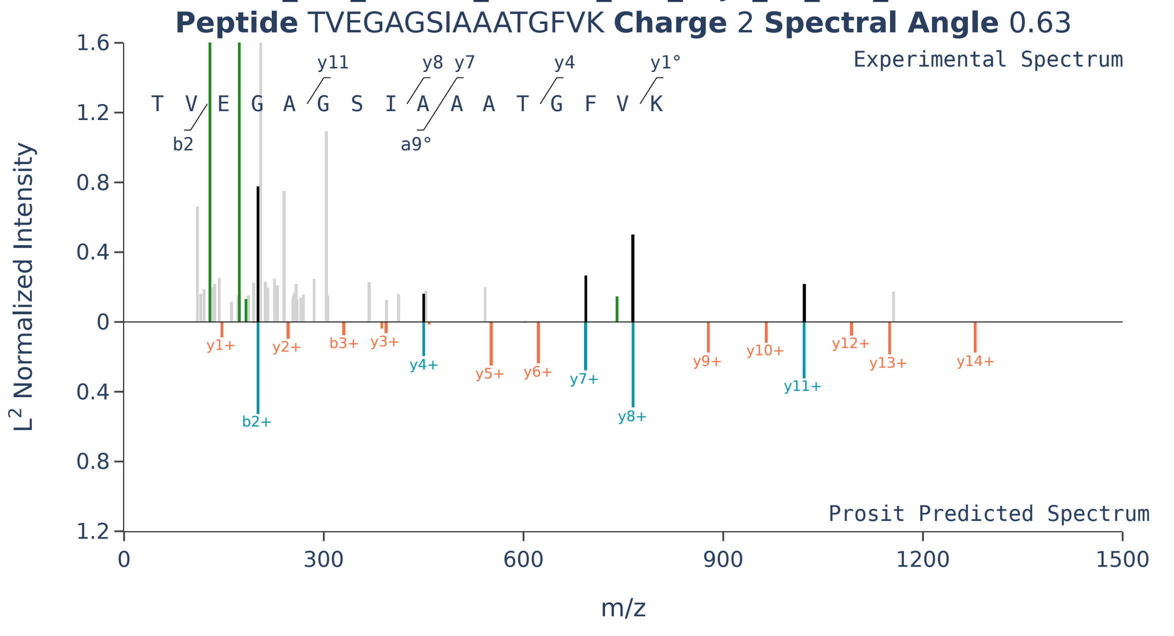


**Source** W_Soh_160821_170921_Goe_aSyn_A1_24h_R2 **Scan** 5685
**Peptide** EKTKEQVTNVGG **Charge** 2 **Spectral Angle** 0.55

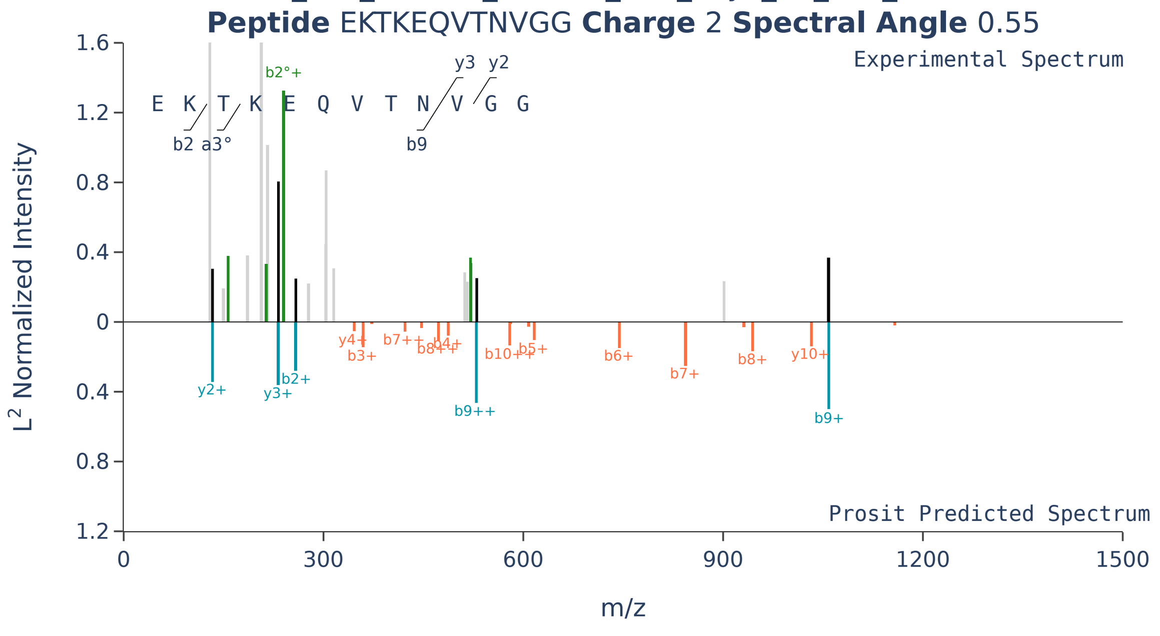

**Source** W_Soh_160821_170921_Goe_aSyn_A2_24h_R2 **Scan** 19480
**Peptide** DVFMKGLSKAKEGVVAAAEKTKQGVAE **Charge** 5 **Spectral Angle** 0.74

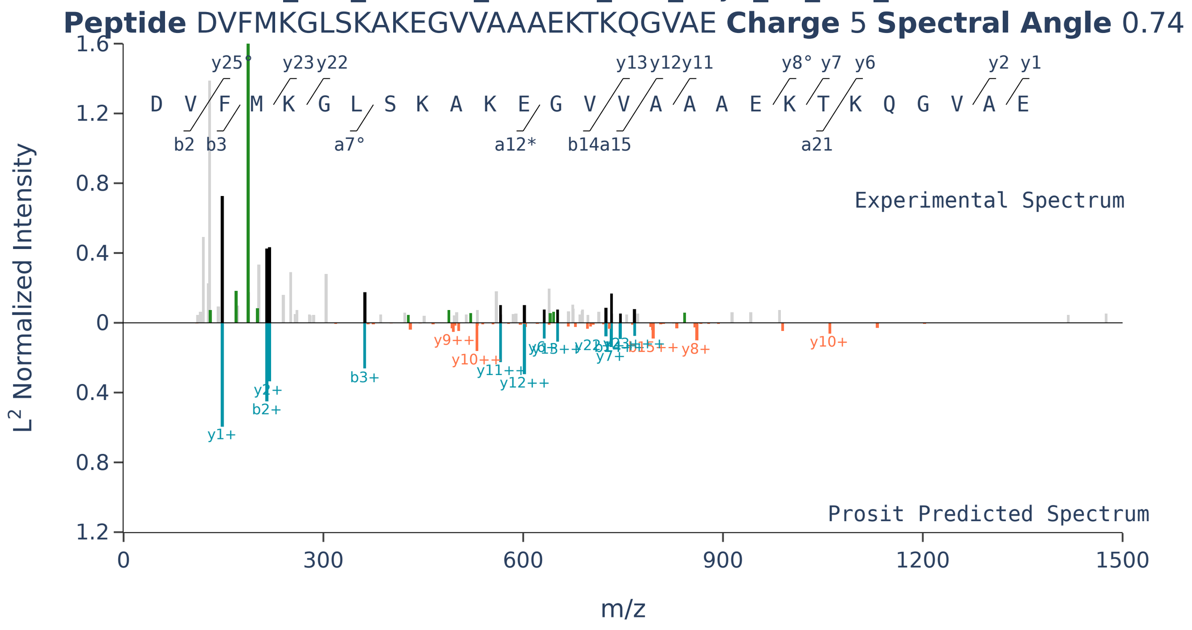


**Source** W_Soh_160821_170921_Goe_aSyn_A2_24h_R1 **Scan** 14000
**Peptide** VAEAAGKTKEGVLYV **Charge** 3 **Spectral Angle** 0.86

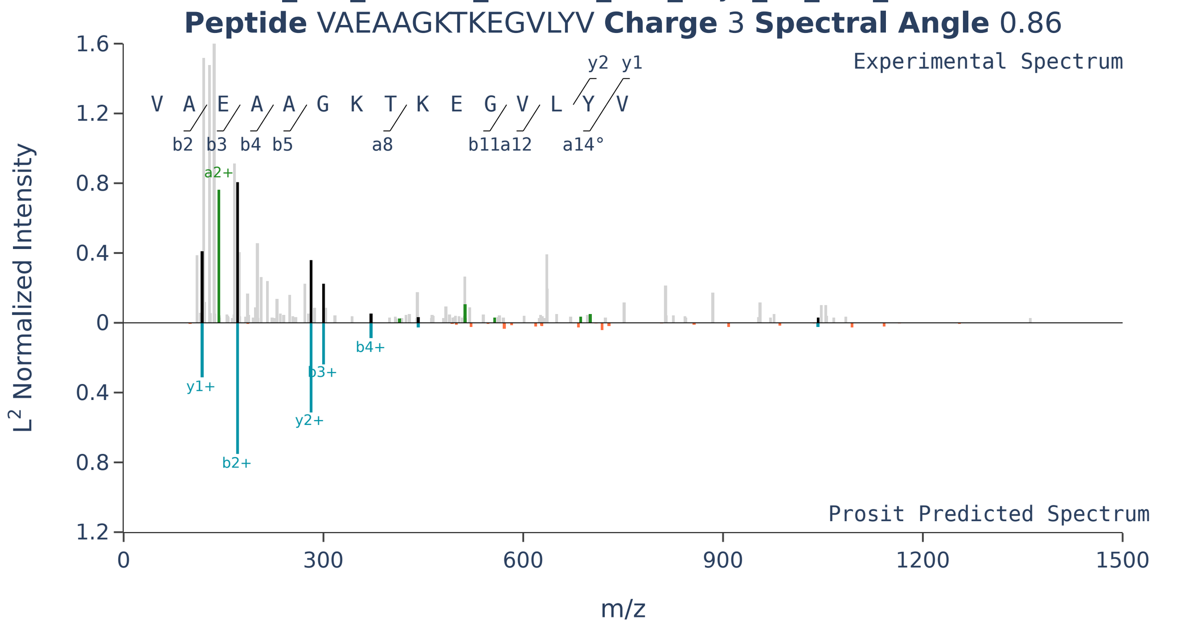


**Source** W_Soh_160821_170921_Goe_aSyn_A2_24h_R1 **Scan** 7540
**Peptide** AEAAGKTKEGVLYVGSKTKE **Charge** 4 **Spectral Angle** 0.83

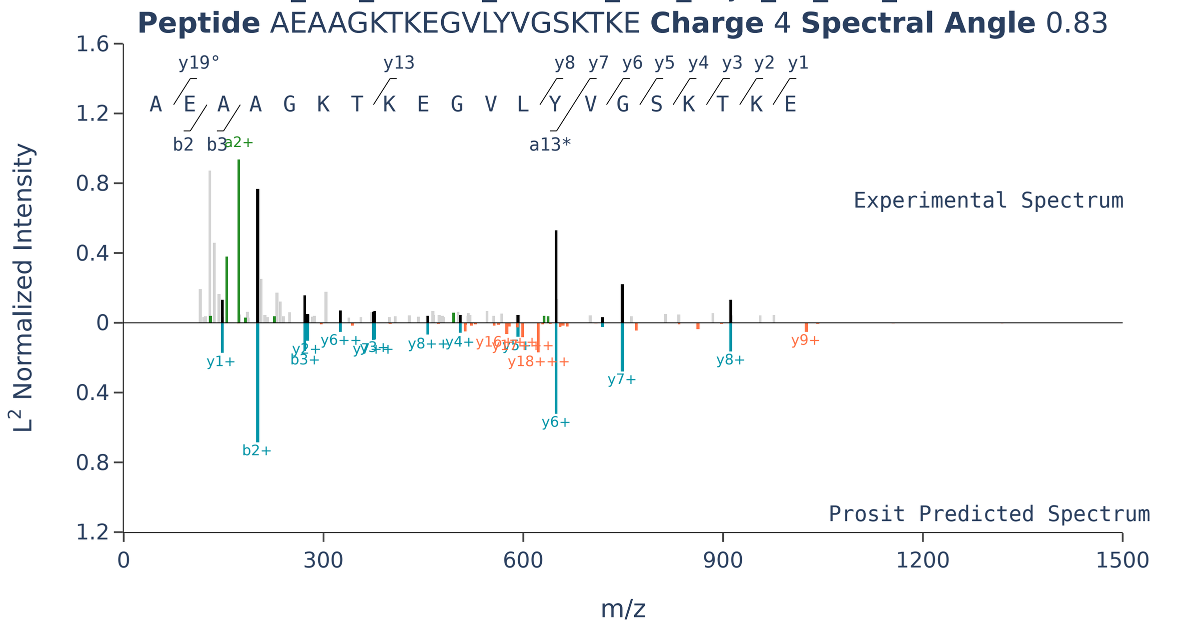


**Source** W_Soh_160821_170921_Goe_aSyn_A1_24h_R1 **Scan** 9773
**Peptide** IAAATGFVKKD **Charge** 3 **Spectral Angle** 0.69

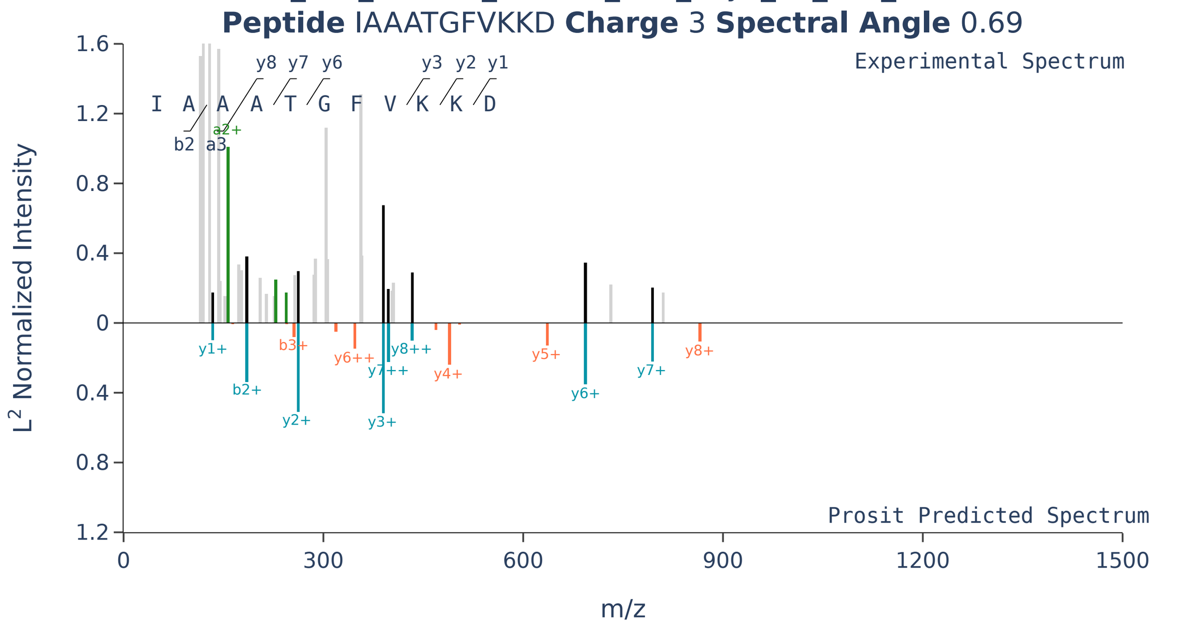


**Source** W_Soh_160821_170921_Goe_aSyn_A2_24h_R2 **Scan** 11559
**Peptide** KKDQLGKNEEGAPQEGIL **Charge** 3 **Spectral Angle** 0.83

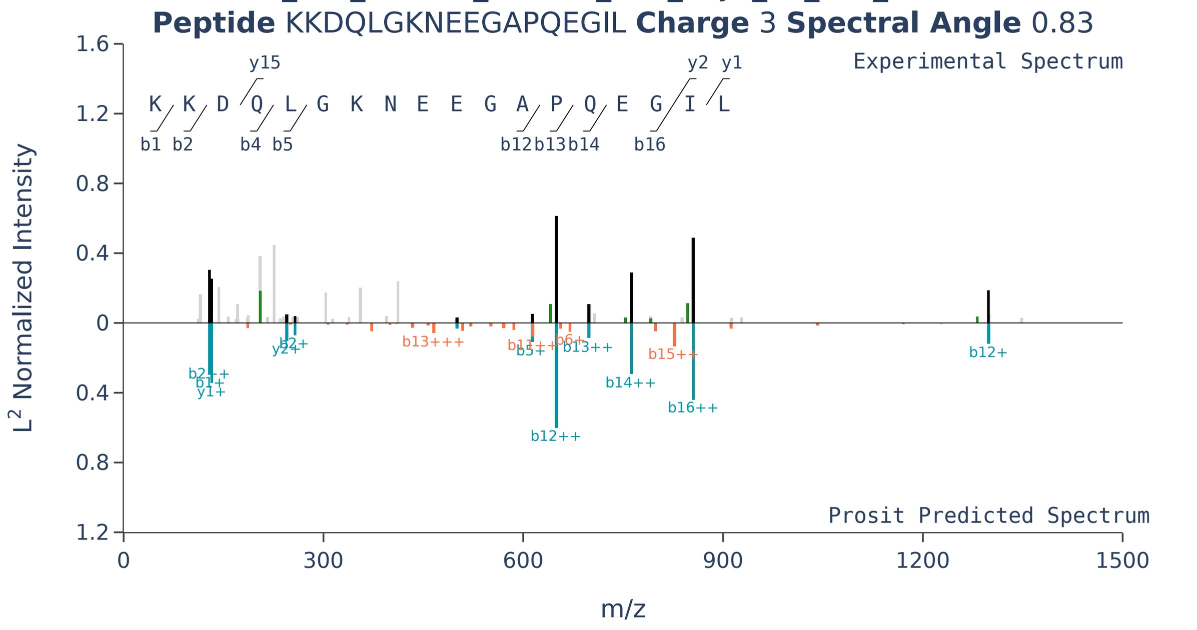


**Source** W_Soh_160821_170921_Goe_aSyn_A2_24h_R1 **Scan** 11981
**Peptide** VAEAAGKTKEGVLYVGSKTKEGVVHGVAT **Charge** 5 **Spectral Angle** 0.8

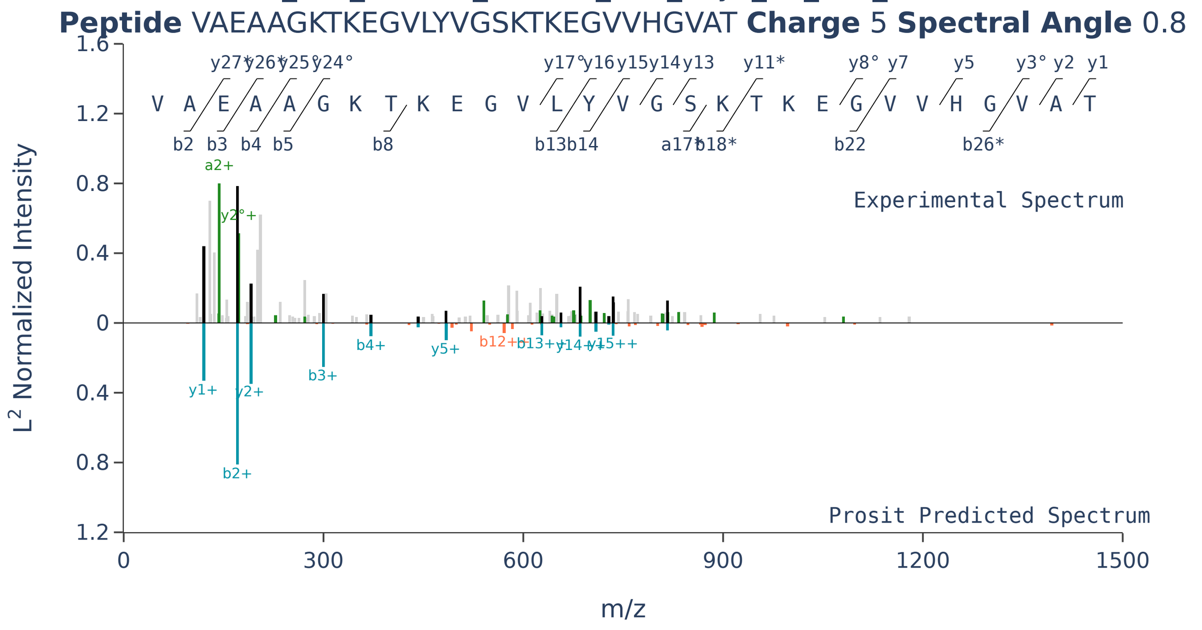


**Source** W_Soh_160821_170921_Goe_aSyn_A1_24h_R1 **Scan** 6647
**Peptide** GKTKEGVLYVGSKTKEG **Charge** 3 **Spectral Angle** 0.75

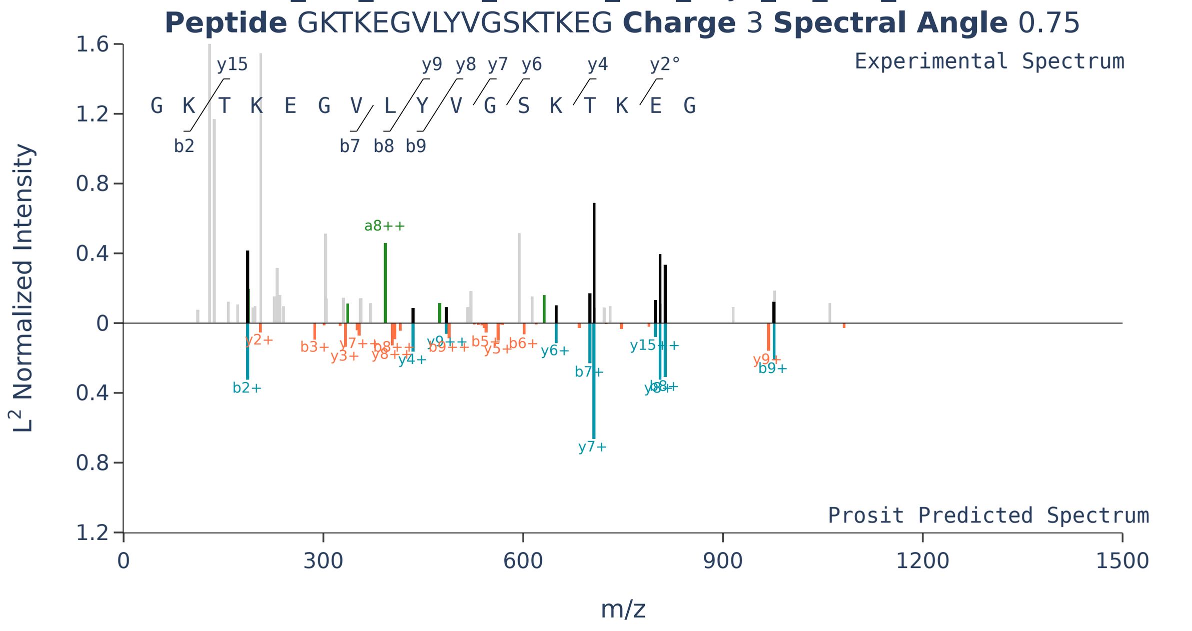


**Source** W_Soh_160821_170921_Goe_aSyn_A1_24h_R2 **Scan** 17388
**Peptide** SKTKEGVVHGVATVAEKTKEQVTNVGGAVV **Charge** 4 **Spectral Angle** 0.69

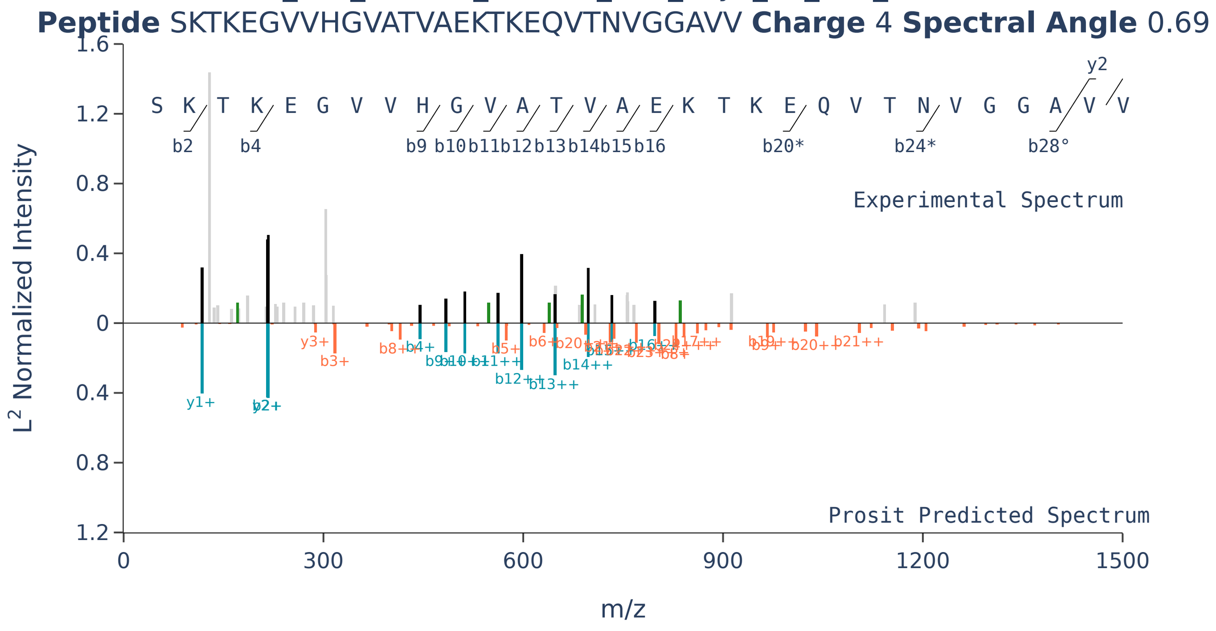


**Source** W_Soh_160821_170921_Goe_aSyn_A2_24h_R1 **Scan** 11337
**Peptide** GVVHGVATVAEKTKEQV **Charge** 3 **Spectral Angle** 0.66

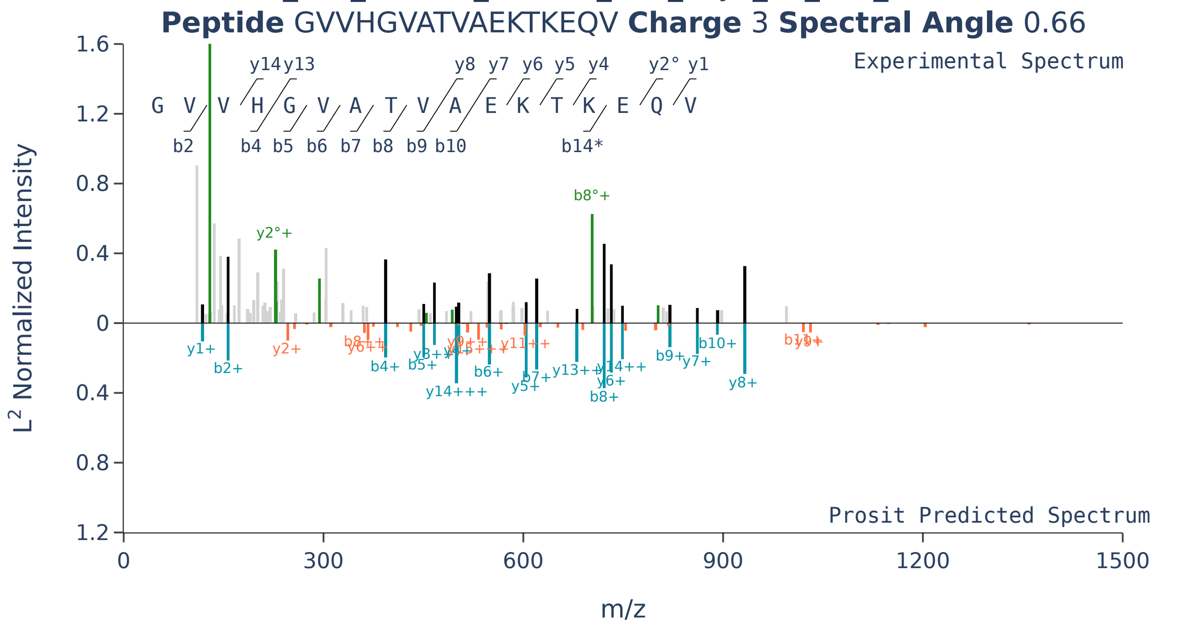


**Source** W_Soh_160821_170921_Goe_aSyn_A1_24h_R2 **Scan** 16700
**Peptide** AGSIAAATGFVKKDQLG **Charge** 3 **Spectral Angle** 0.72

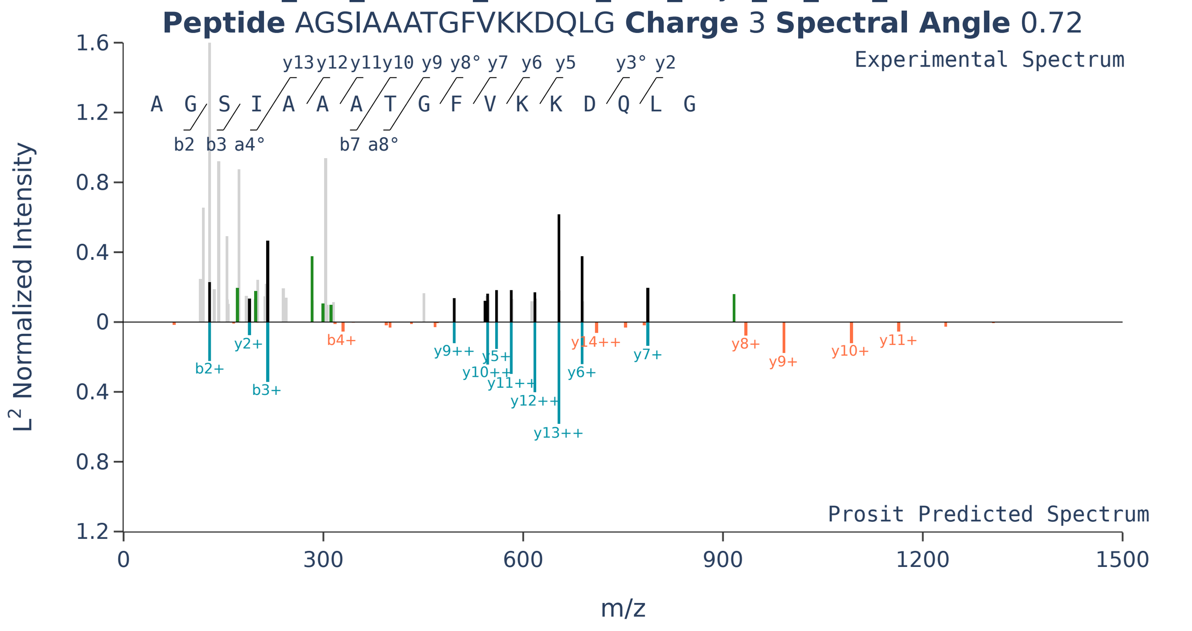


**Source** W_Soh_160821_170921_Goe_aSyn_A1_24h_R2 **Scan** 9548
**Peptide** EGVVHGVATVAEKTKEQ **Charge** 3 **Spectral Angle** 0.71

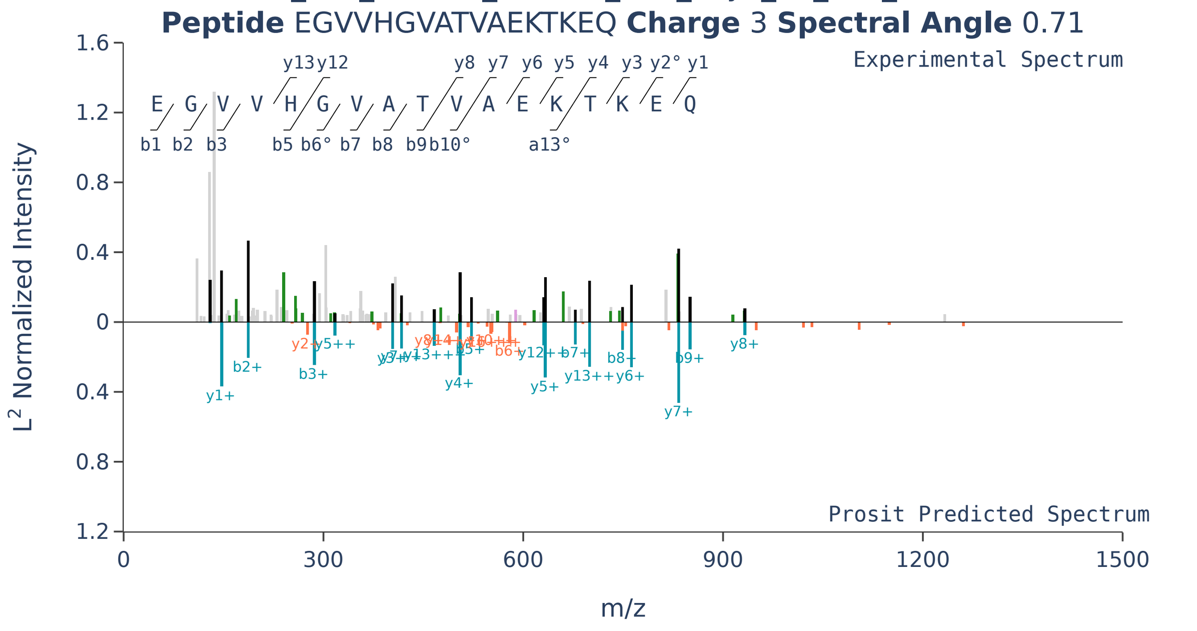


**Source** W_Soh_160821_170921_Goe_aSyn_A1_24h_R1 **Scan** 14675
**Peptide** GAGSIAAATGFVKKDQLGKNEEG **Charge** 3 **Spectral Angle** 0.68

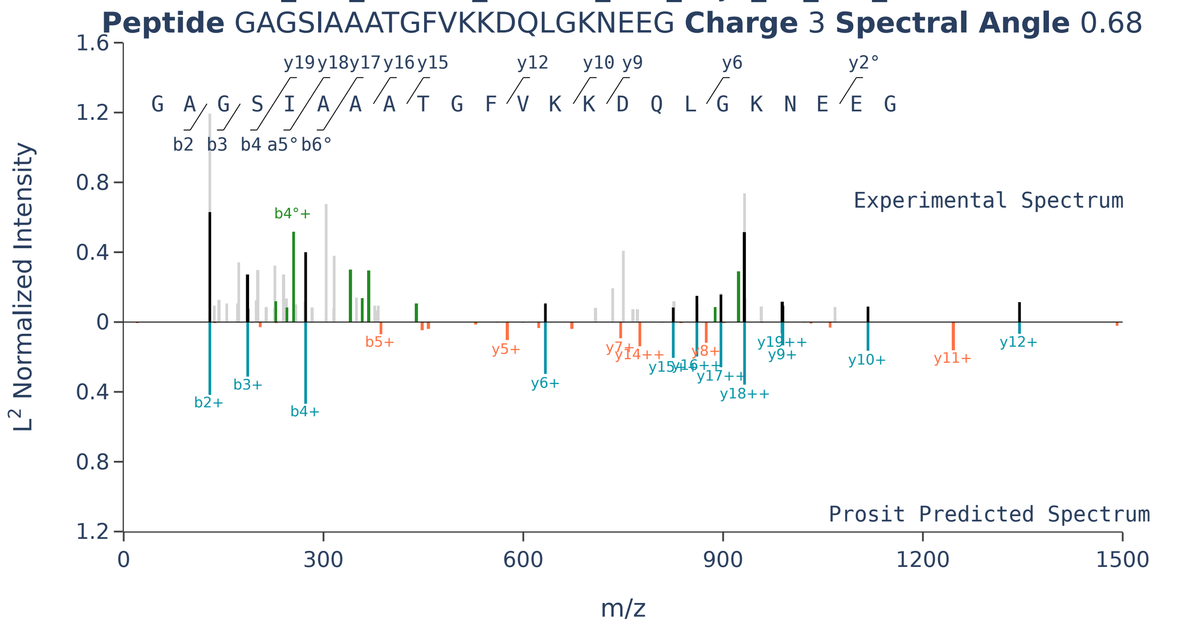


**Source** W_Soh_160821_170921_Goe_aSyn_A1_24h_R1 **Scan** 12309
**Peptide** QGVAEAAGKTKEGVLYVGSKT **Charge** 3 **Spectral Angle** 0.65

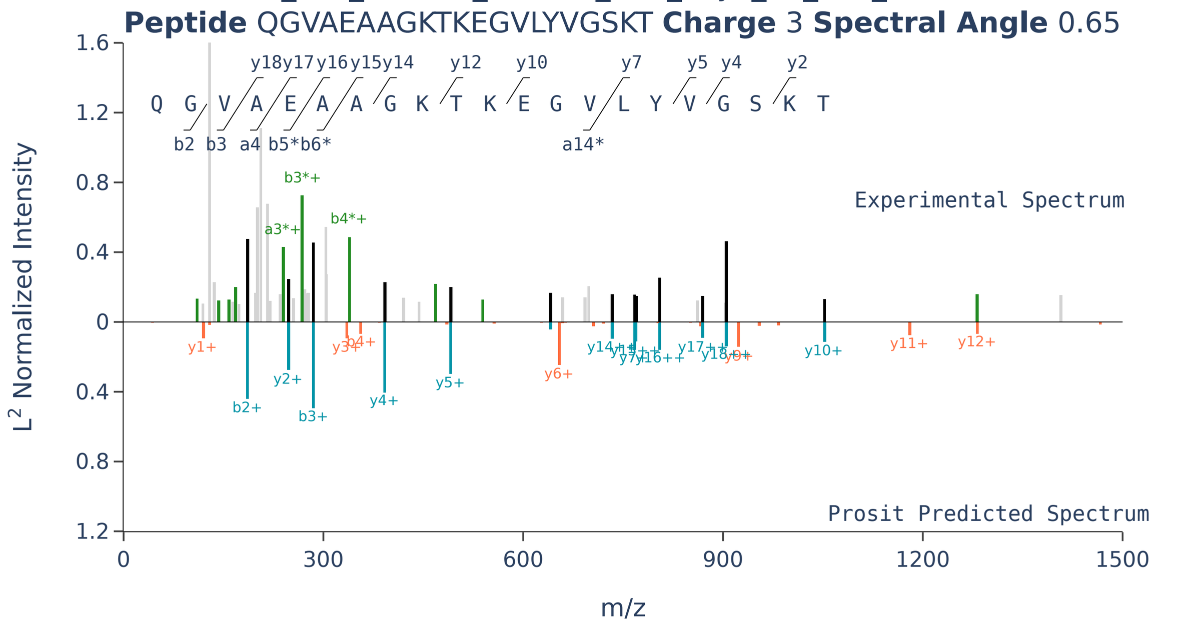


**Source** W_Soh_160821_170921_Goe_aSyn_A2_24h_R2 **Scan** 12943
**Peptide** GKTKEGVLYVGSKTKEGVVHGVATVAEKT **Charge** 5 **Spectral Angle** 0.61

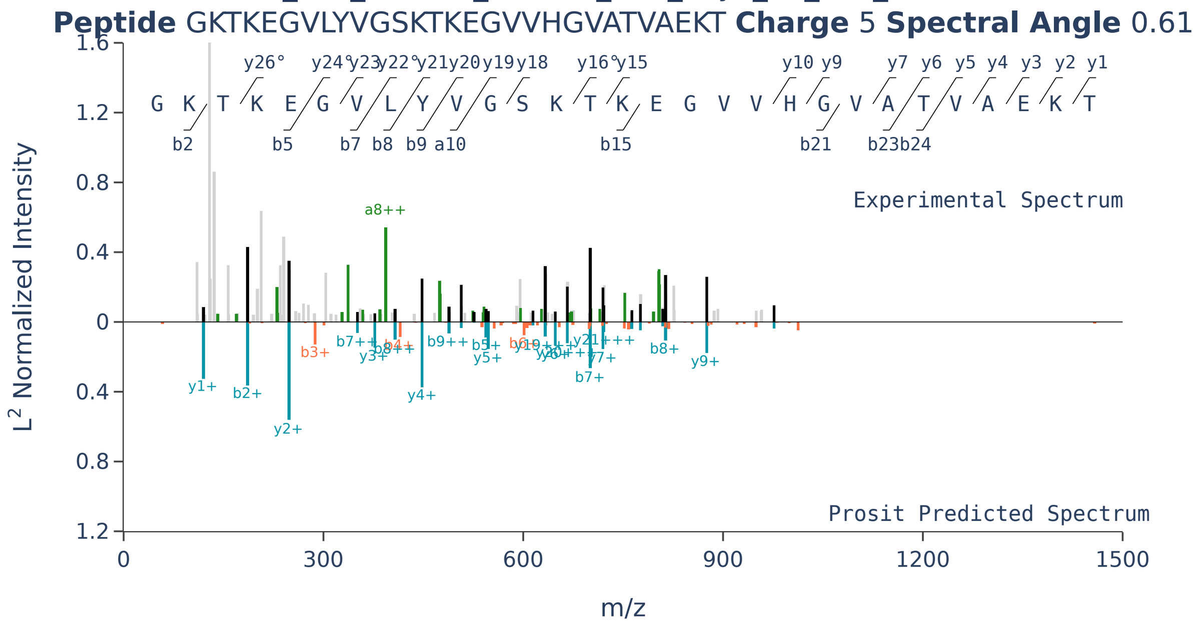


**Source** W_Soh_160821_170921_Goe_aSyn_A2_24h_R2 **Scan** 8669
**Peptide** KQGVAEAAGKTKEGVLYVGSKTK **Charge** 5 **Spectral Angle** 0.85

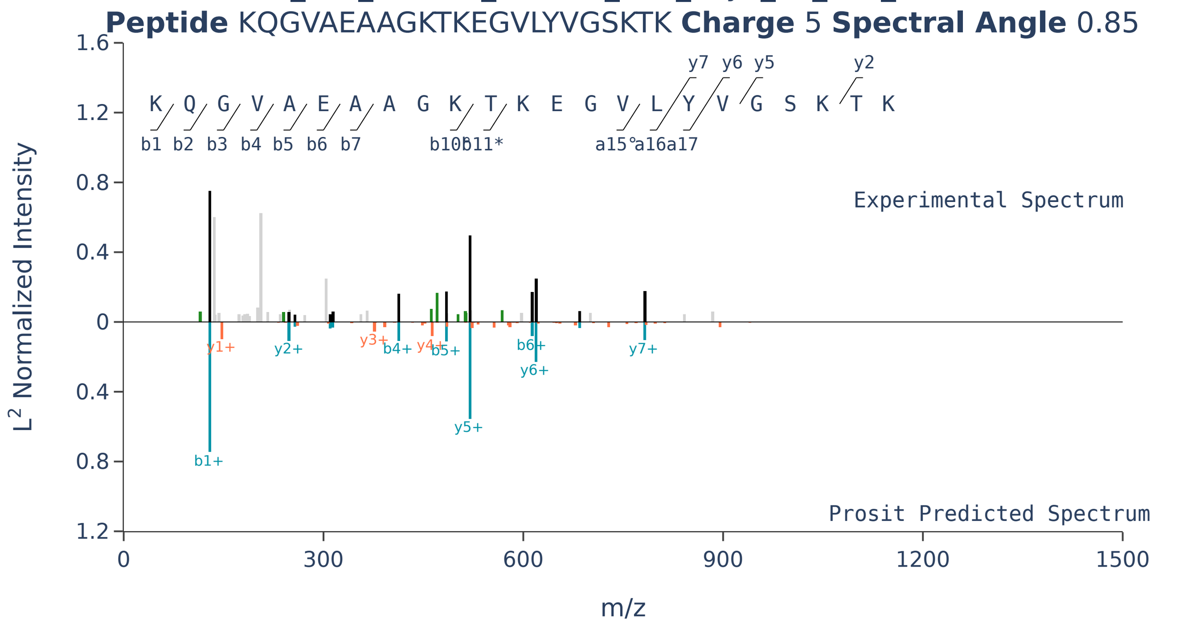

**Source** W_Soh_160821_170921_Goe_aSyn_A1_24h_R2 **Scan** 7563
**Peptide** KEGVLYVGSKTK **Charge** 3 **Spectral Angle** 0.5

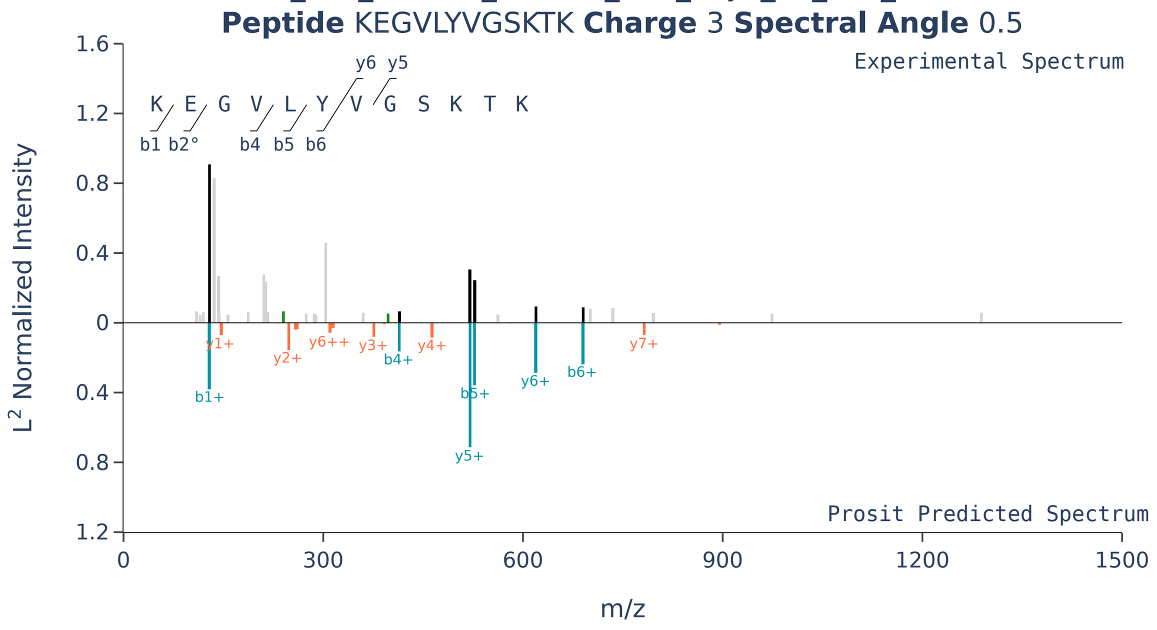


**Source** W_Soh_160821_170921_Goe_aSyn_A2_24h_R2 **Scan** 19600
**Peptide** VAEKTKEQVTNVGGAVVTGVTAVAQKT **Charge** 4 **Spectral Angle** 0.73

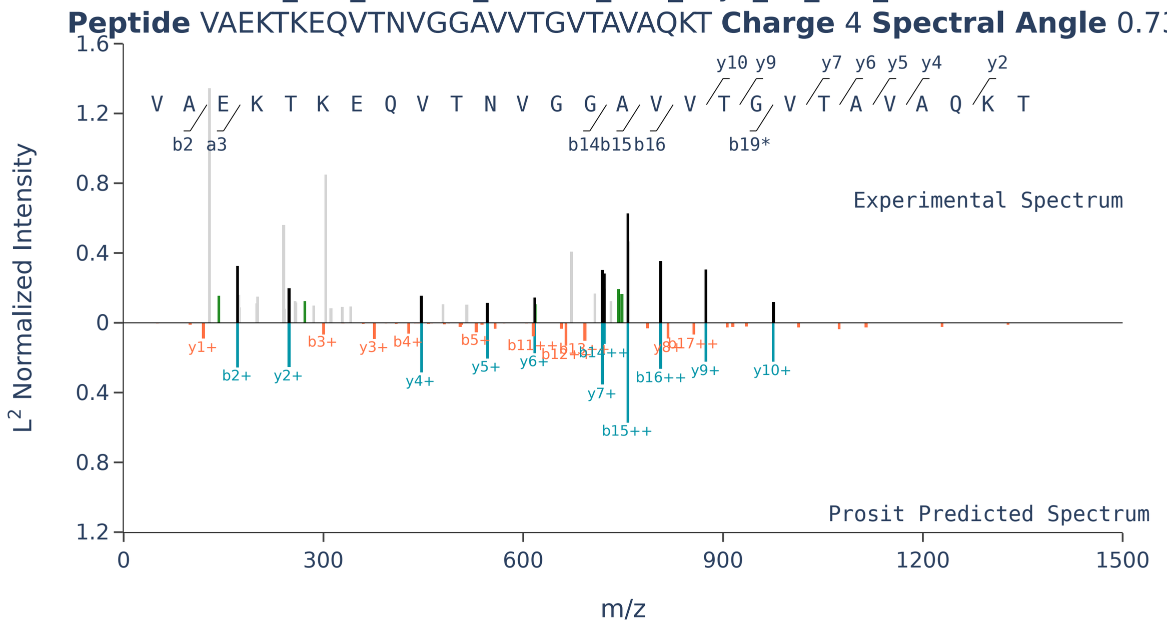


**Source** W_Soh_160821_170921_Goe_aSyn_A1_24h_R2 **Scan** 12155
**Peptide** GKTKEGVLYVGSKTKEGVVHGVATV **Charge** 5 **Spectral Angle** 0.58

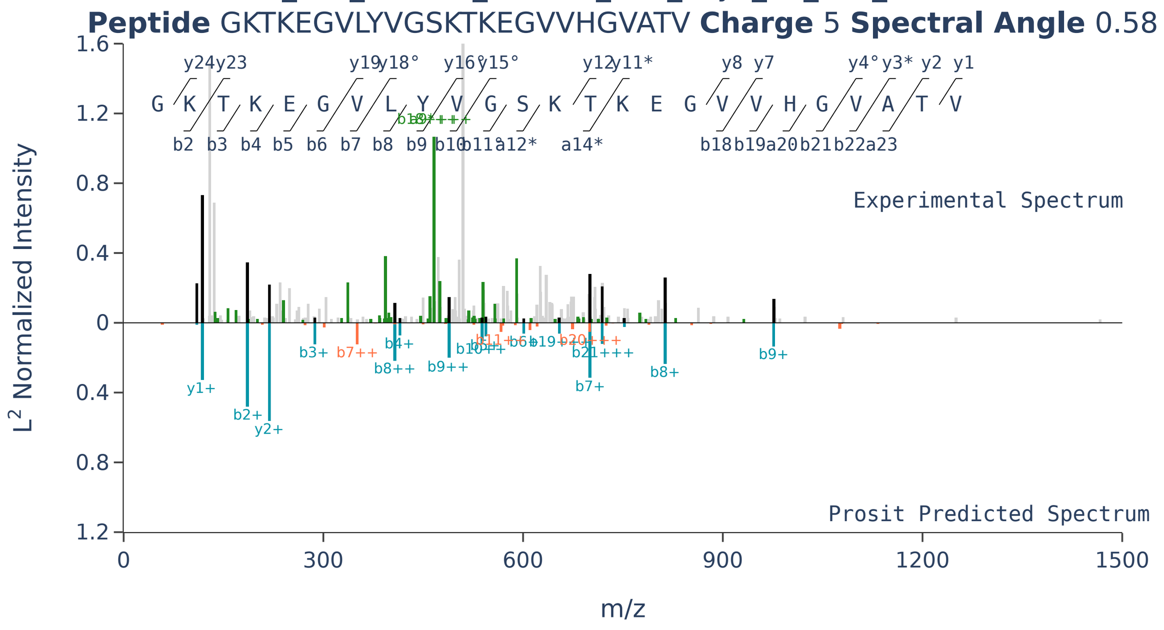


**Source** W_Soh_160821_170921_Goe_aSyn_A1_24h_R2 **Scan** 11945
**Peptide** TAVAQKTVEGAGSI **Charge** 2 **Spectral Angle** 0.83

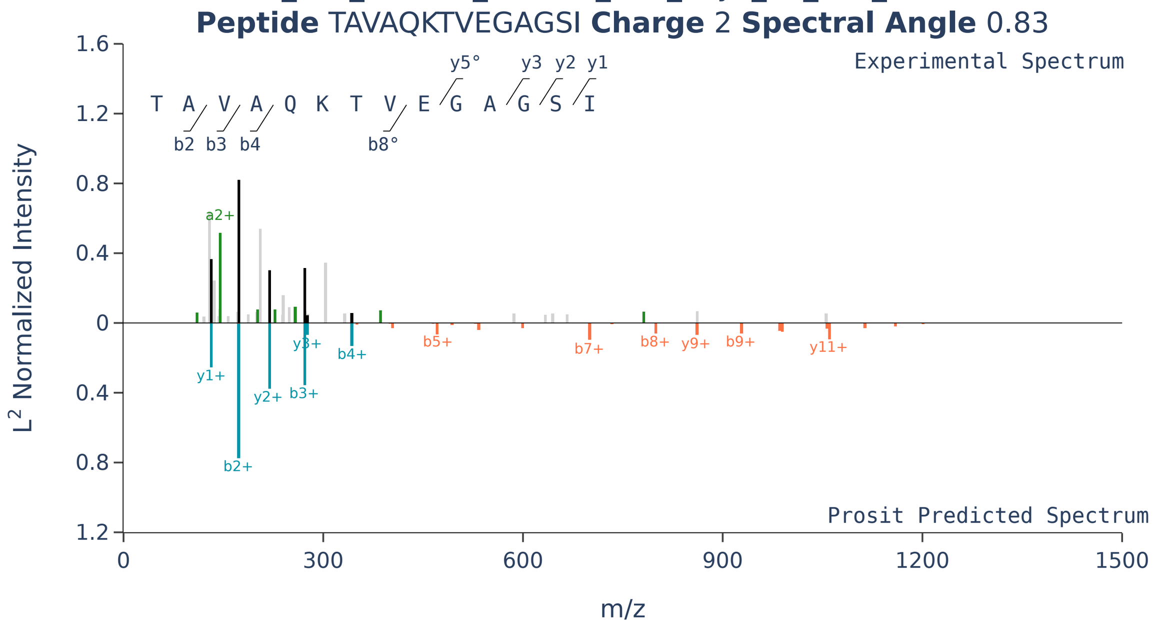


**Source** W_Soh_160821_170921_Goe_aSyn_A1_24h_R2 **Scan** 12661
**Peptide** VAEAAGKTKEGVLYVGS **Charge** 2 **Spectral Angle** 0.83

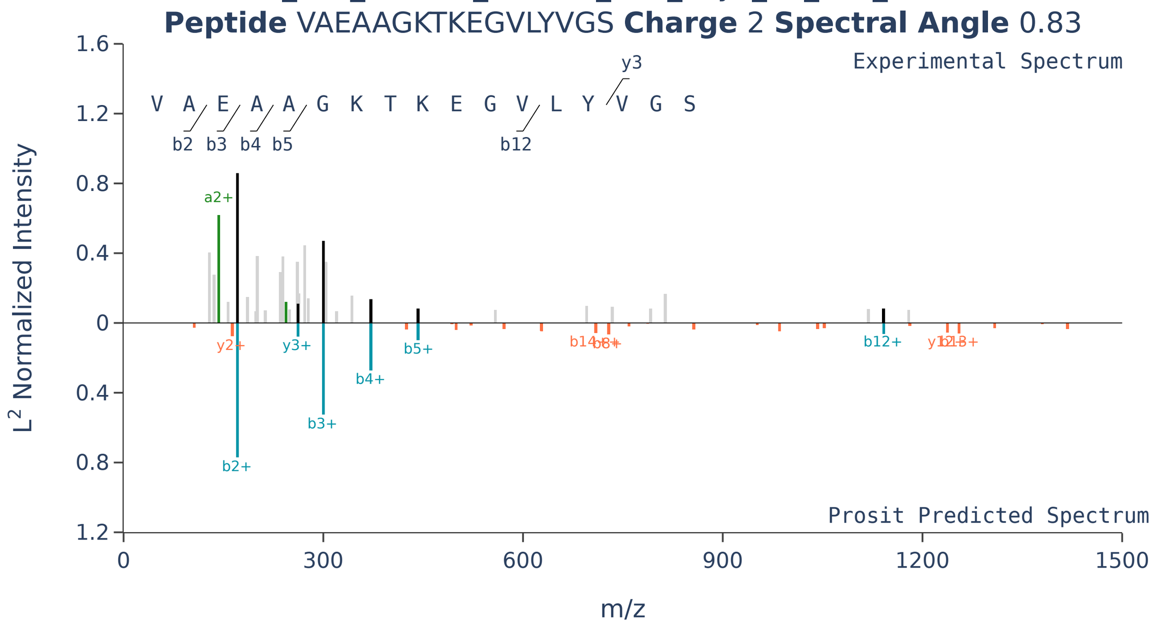


**Source** W_Soh_160821_170921_Goe_aSyn_A1_6h_R2 **Scan** 14823
**Peptide** GAGSIAAATGFVKKDQLGKNEEGAPQEG **Charge** 3 **Spectral Angle** 0.81

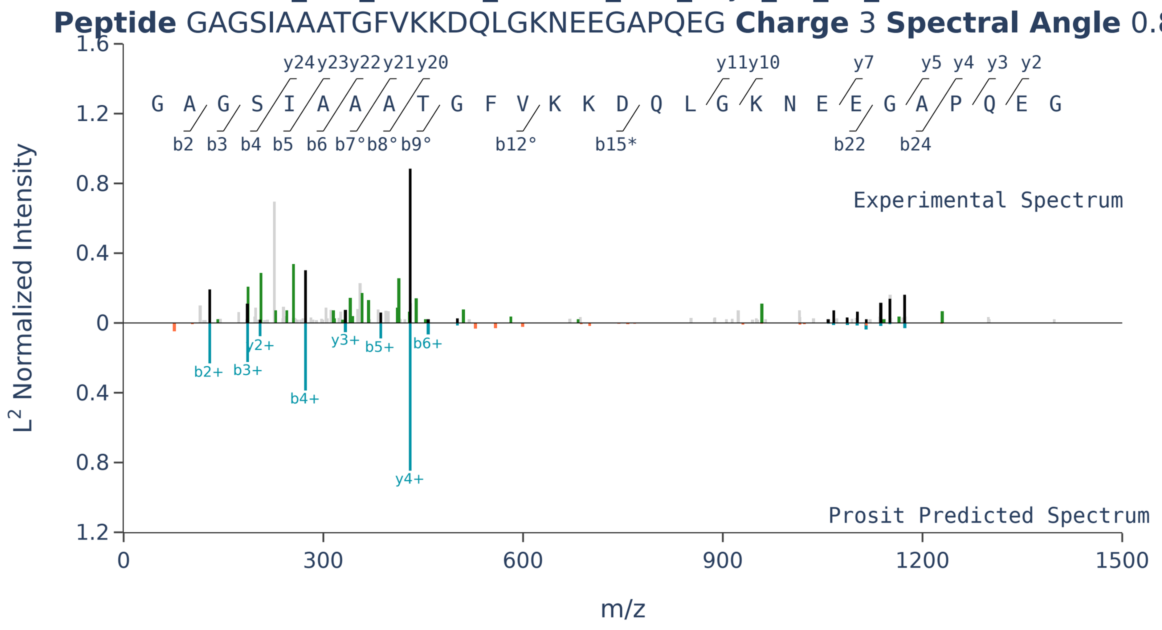


**Source** W_Soh_160821_170921_Goe_aSyn_A1_24h_R1 **Scan** 5811
**Peptide** VAAAEKTKQG **Charge** 2 **Spectral Angle** 0.69

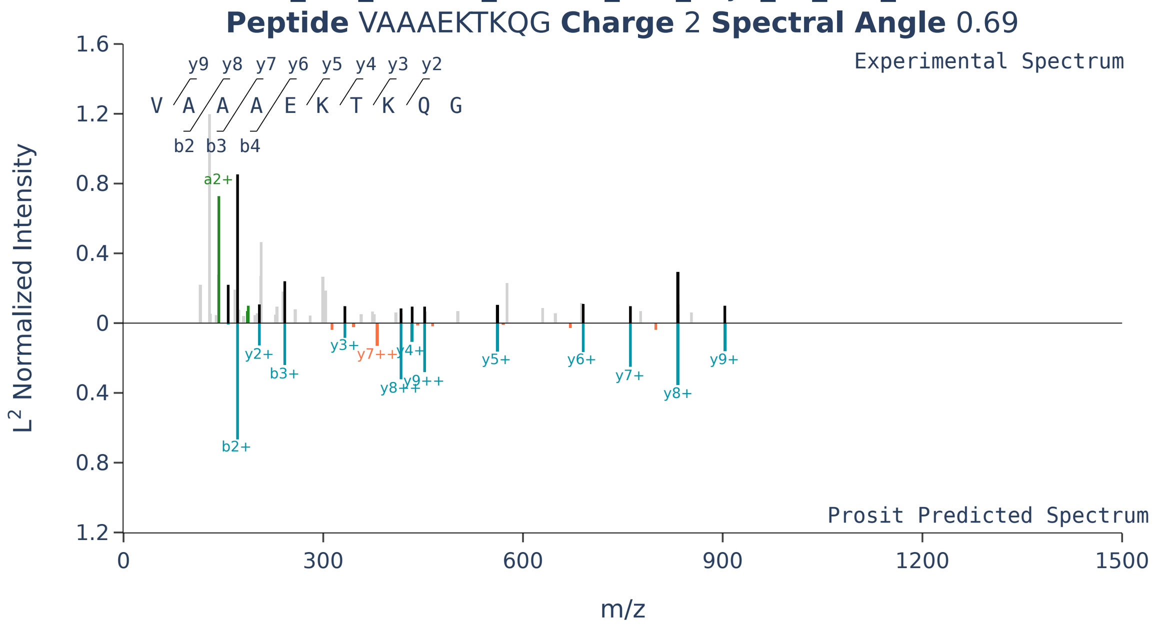


**Source** W_Soh_160821_170921_Goe_aSyn_A1_24h_R1 **Scan** 13692
**Peptide** AGSIAAATGFVKKDQLGKN **Charge** 3 **Spectral Angle** 0.77

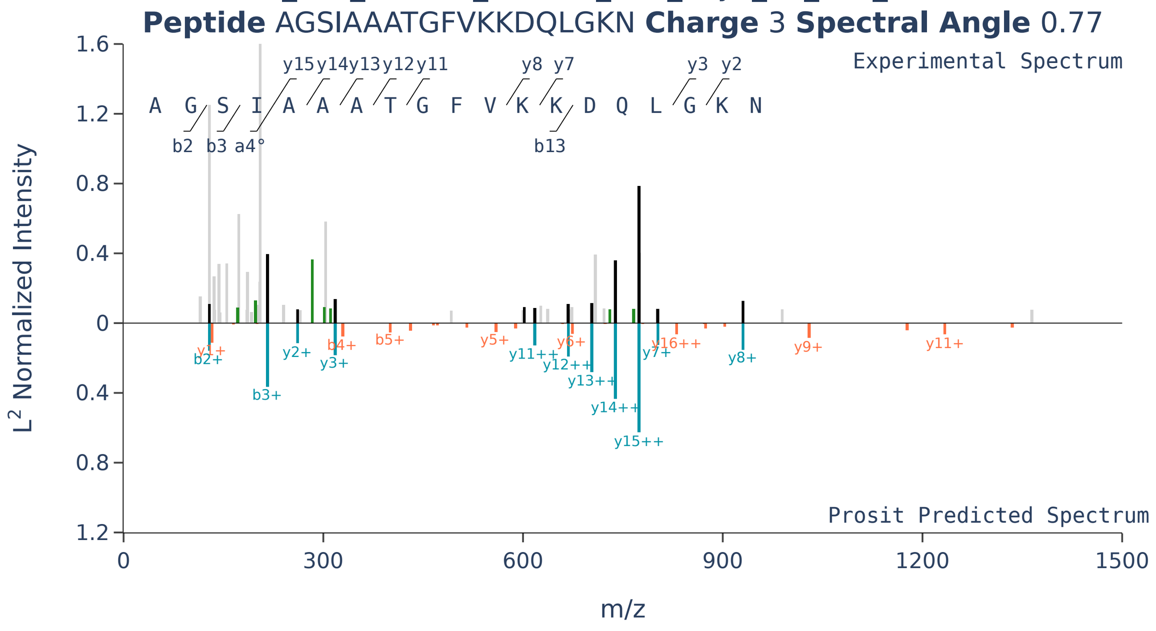


**Source** W_Soh_160821_170921_Goe_aSyn_A1_24h_R1 **Scan** 6341
**Peptide** KGLSKAKEGVVAAAEKTKQ **Charge** 4 **Spectral Angle** 0.78

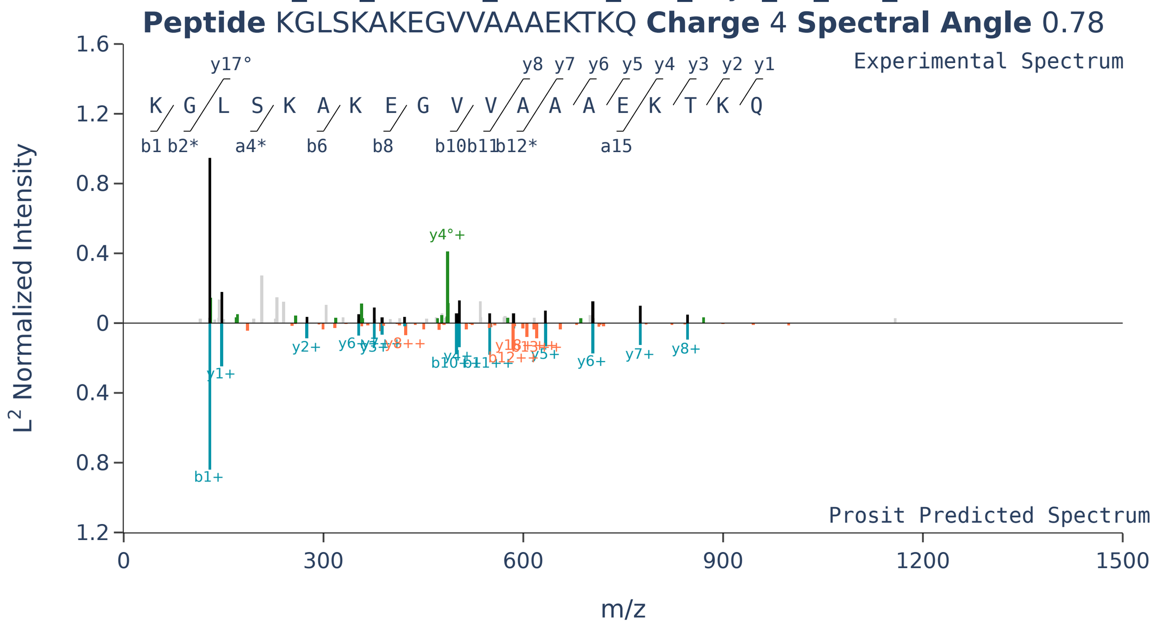


**Source** W_Soh_160821_170921_Goe_aSyn_A1_24h_R1 **Scan** 18899
**Peptide** GSIAAATGFVKKDQLGKNEEGAPQEGILE **Charge** 3 **Spectral Angle** 0.74

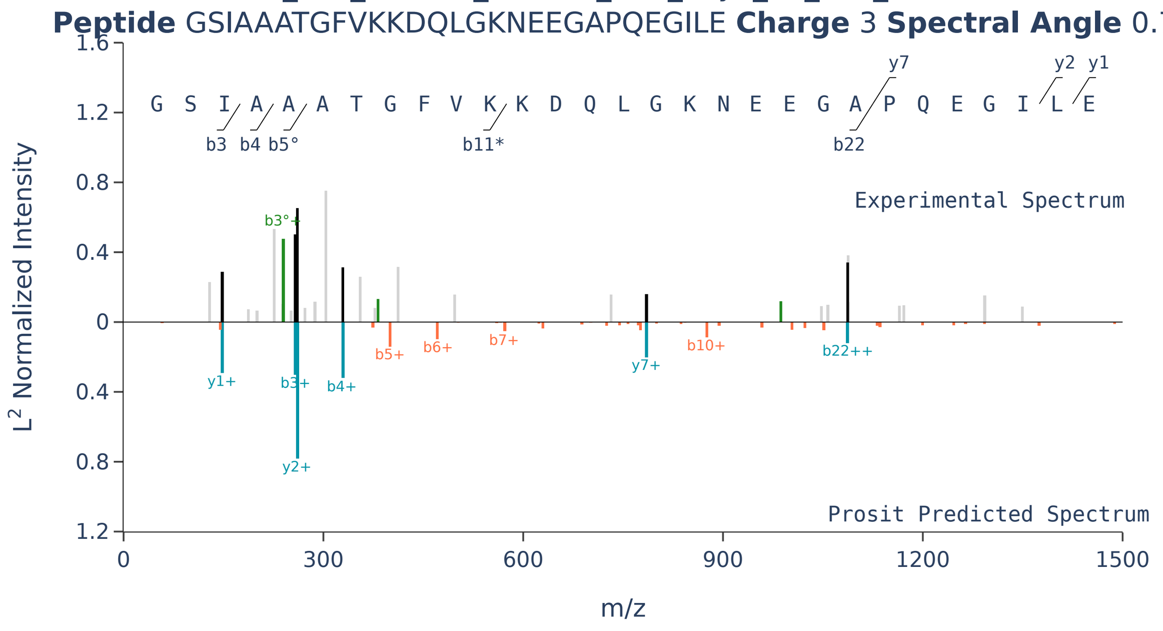


**Source** W_Soh_160821_170921_Goe_aSyn_A1_6h_R2 **Scan** 16653
**Peptide** GVLYVGSKTKEGVVHGVATVAEKTKEQVT **Charge** 4 **Spectral Angle** 0.83

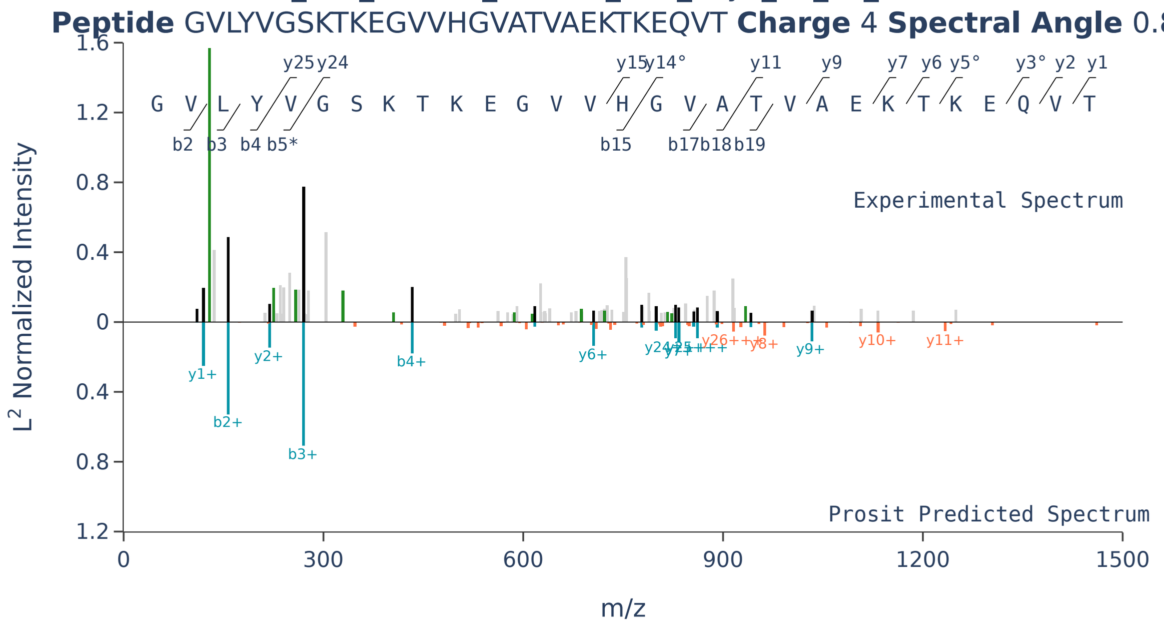


**Source** W_Soh_160821_170921_Goe_aSyn_A1_24h_R1 **Scan** 5325
**Peptide** SKAKEGVVAAAEKTKQ **Charge** 4 **Spectral Angle** 0.61

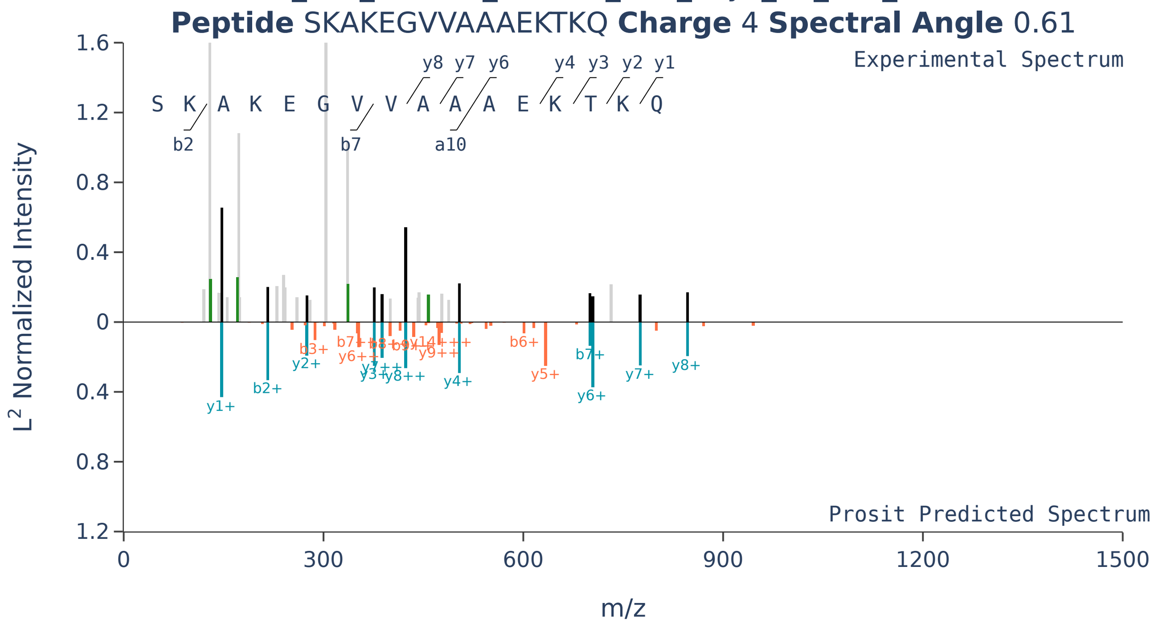


**Source** W_Soh_160821_170921_Goe_aSyn_A1_24h_R2 **Scan** 13591
**Peptide** SKTKEGVVHGVATVAEKTKEQVTNVGG **Charge** 4 **Spectral Angle** 0.63

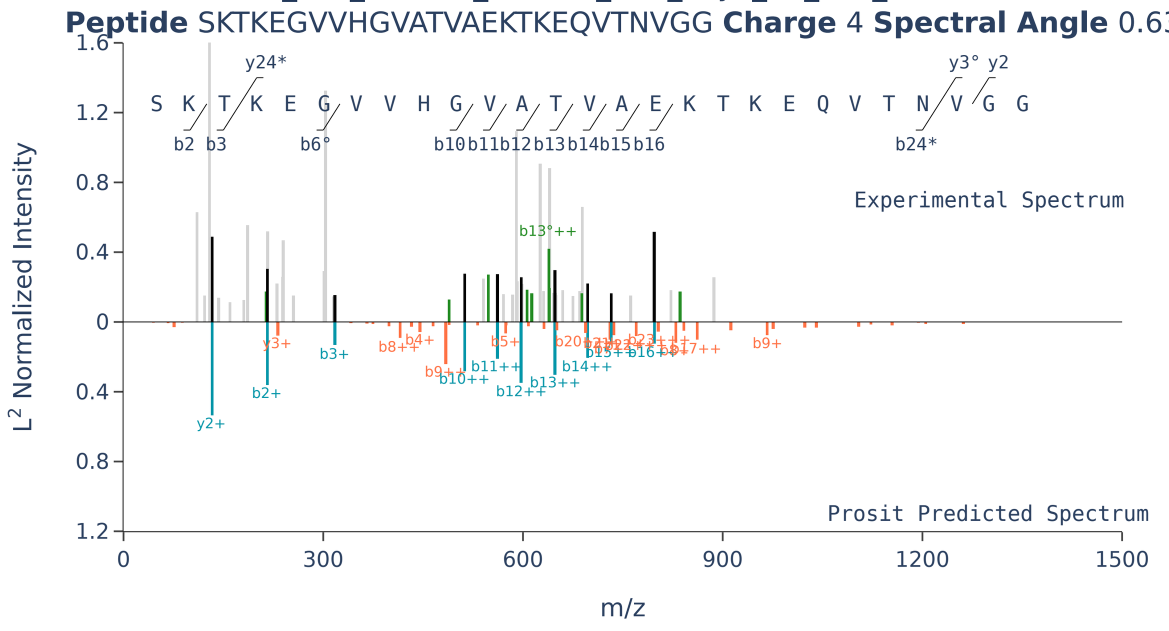


**Source** W_Soh_160821_170921_Goe_aSyn_A1_24h_R2 **Scan** 24753
**Peptide** TGVTAVAQKTVEGAGSIAAATGFVKKDQL **Charge** 3 **Spectral Angle** 0.63

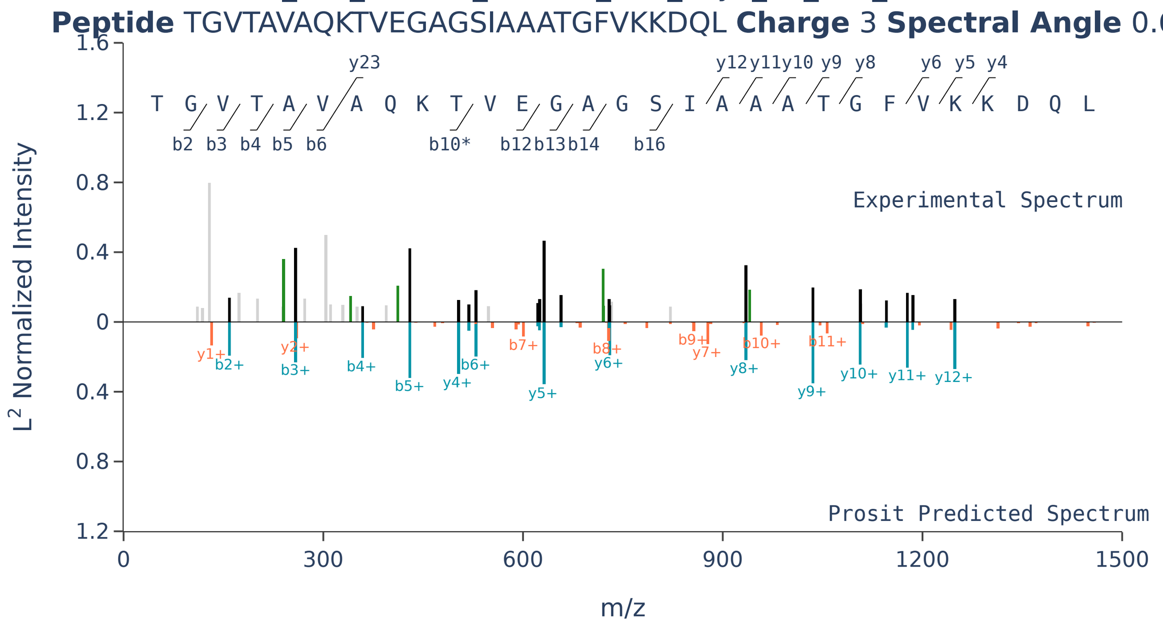


**Source** W_Soh_160821_170921_Goe_aSyn_A1_24h_R2 **Scan** 15278
**Peptide** GVVAAAEKTKQGVAEAAGKTKEGVLY **Charge** 4 **Spectral Angle** 0.76

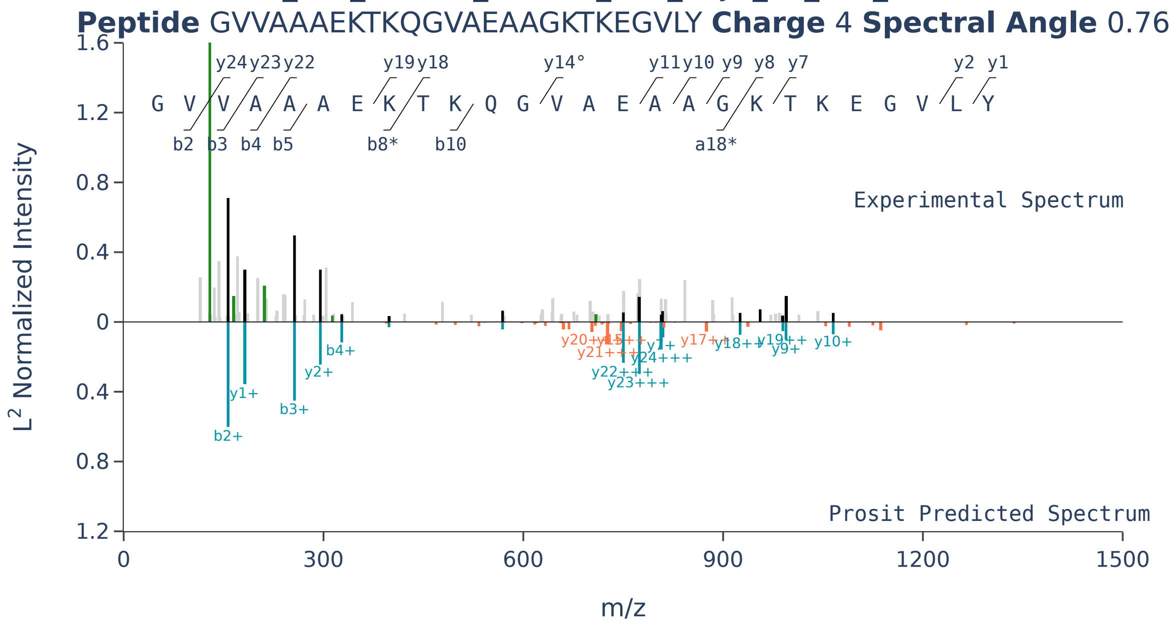

**Source** W_Soh_160821_170921_Goe_aSyn_A1_24h_R1 **Scan** 8986
**Peptide** KQGVAEAAGKTKEGVLYVGSKTKE **Charge** 5 **Spectral Angle** 0.83

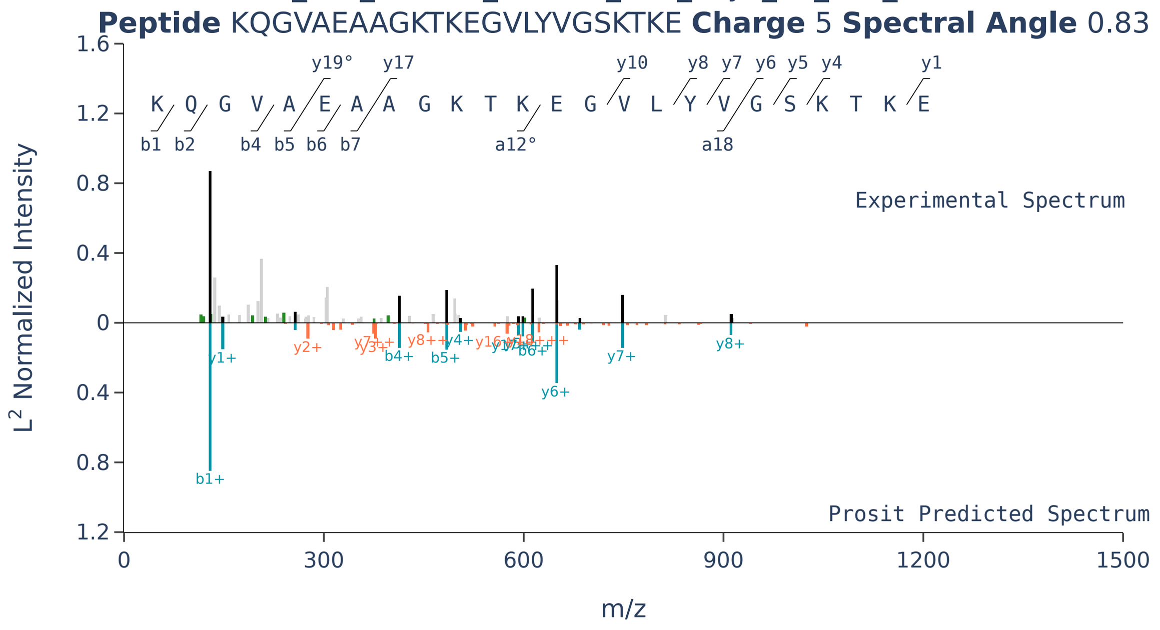


**Source** W_Soh_160821_170921_Goe_aSyn_A1_1h_R1 **Scan** 27239
**Peptide** GILEDMPVDPDNEAYEMPSEEGYQDYEPEA **Charge** 3 **Spectral Angle** 0.8

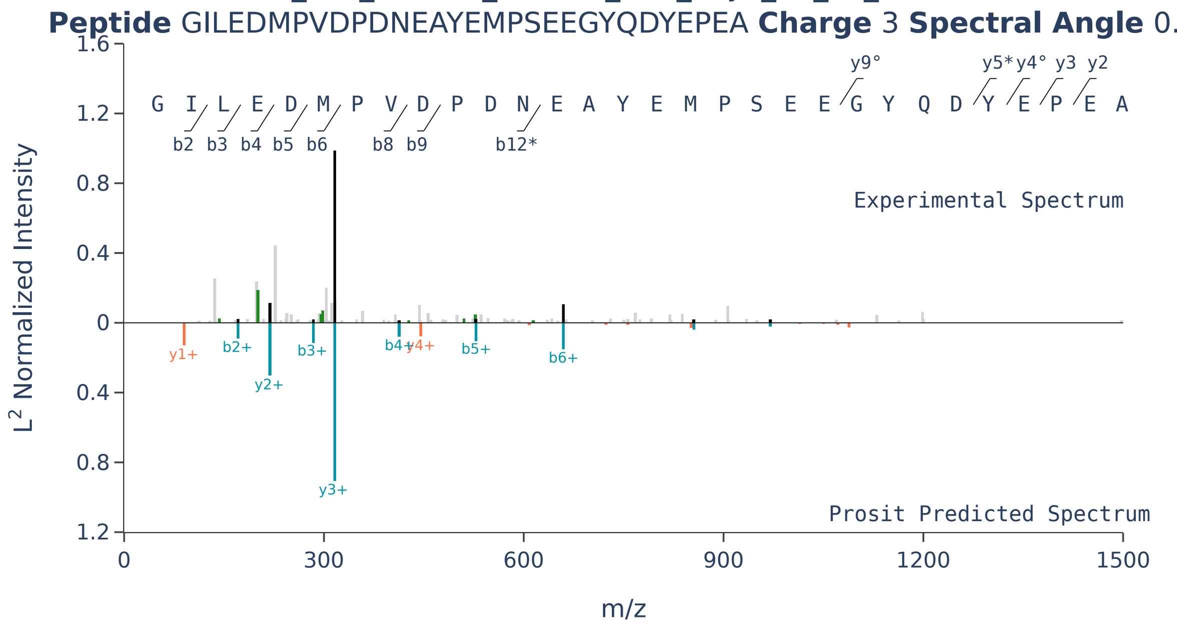


**Source** W_Soh_160821_170921_Goe_aSyn_A1_6h_R1 **Scan** 25578
**Peptide** GGAVVTGVTAVAQKTVEGAGSIAAA **Charge** 2 **Spectral Angle** 0.75

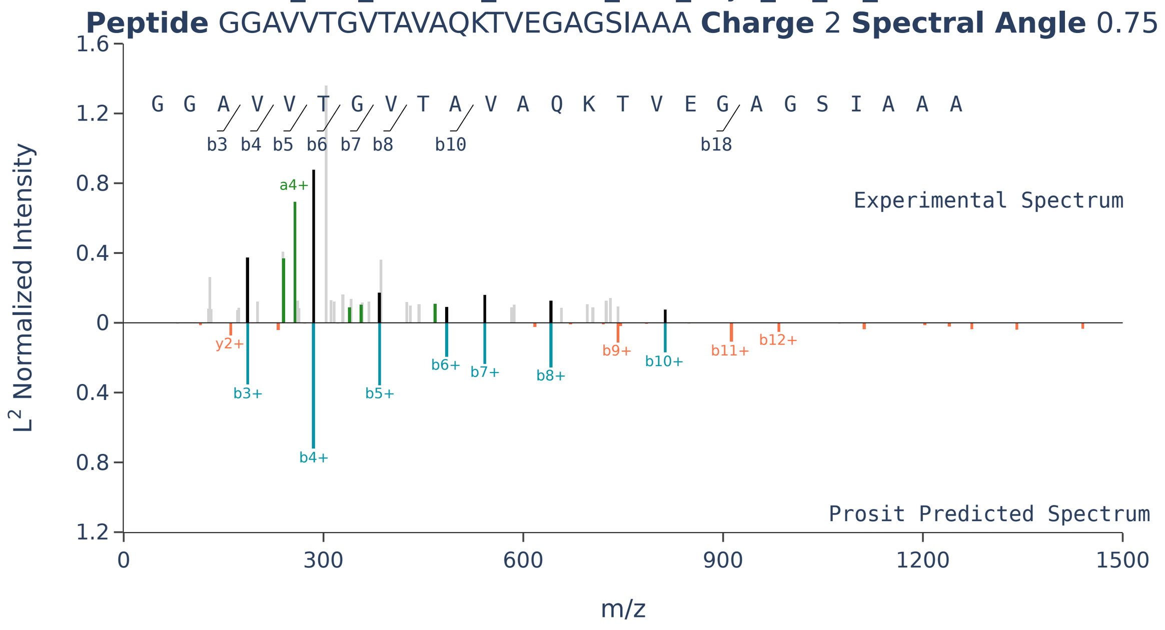


**Source** W_Soh_160821_170921_Goe_aSyn_A1_6h_R2 **Scan** 15120
**Peptide** YVGSKTKEGVVHGVATVAEKTKEQVTNV **Charge** 4 **Spectral Angle** 0.64

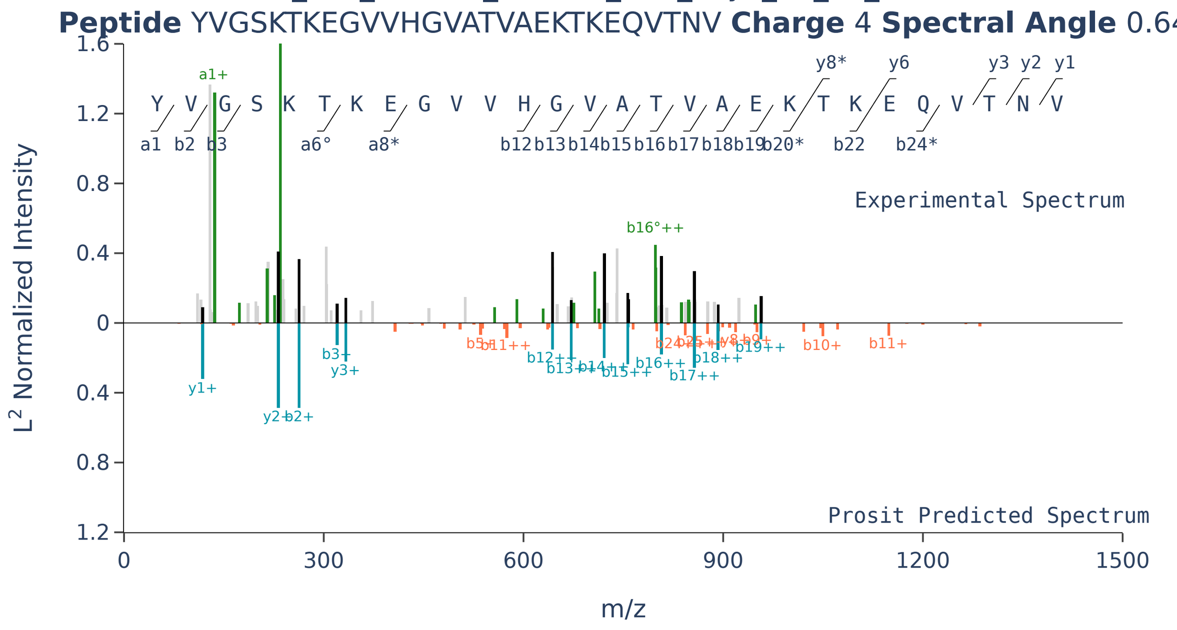


**Source** W_Soh_160821_170921_Goe_aSyn_A1_6h_R1 **Scan** 19721
**Peptide** VVHGVATVAEKTKEQVTNVGGAVVTGVTA **Charge** 3 **Spectral Angle** 0.73

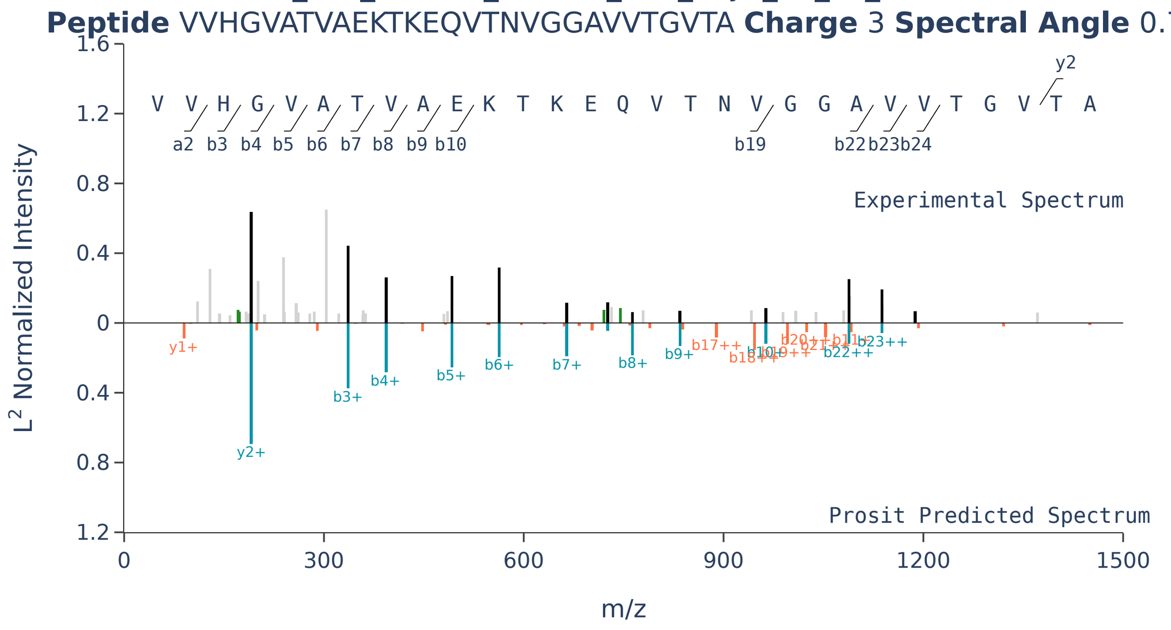


**Source** W_Soh_160821_170921_Goe_aSyn_A1_6h_R2 **Scan** 30700
**Peptide** GLLEDMPVDPDNEAYEMPSEEGYQDYEPEA **Charge** 3 **Spectral Angle** 0.81

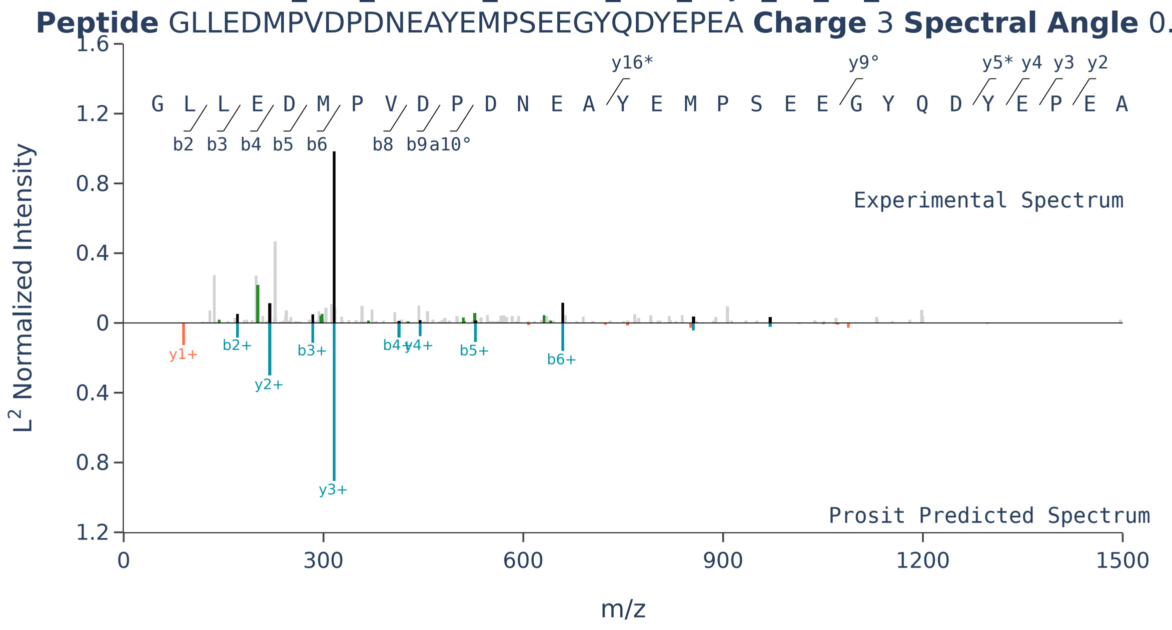


**Source** W_Soh_160821_170921_Goe_aSyn_A2_24h_R1 **Scan** 10765
**Peptide** EGVVAAAEKTKQGVAEA **Charge** 3 **Spectral Angle** 0.73

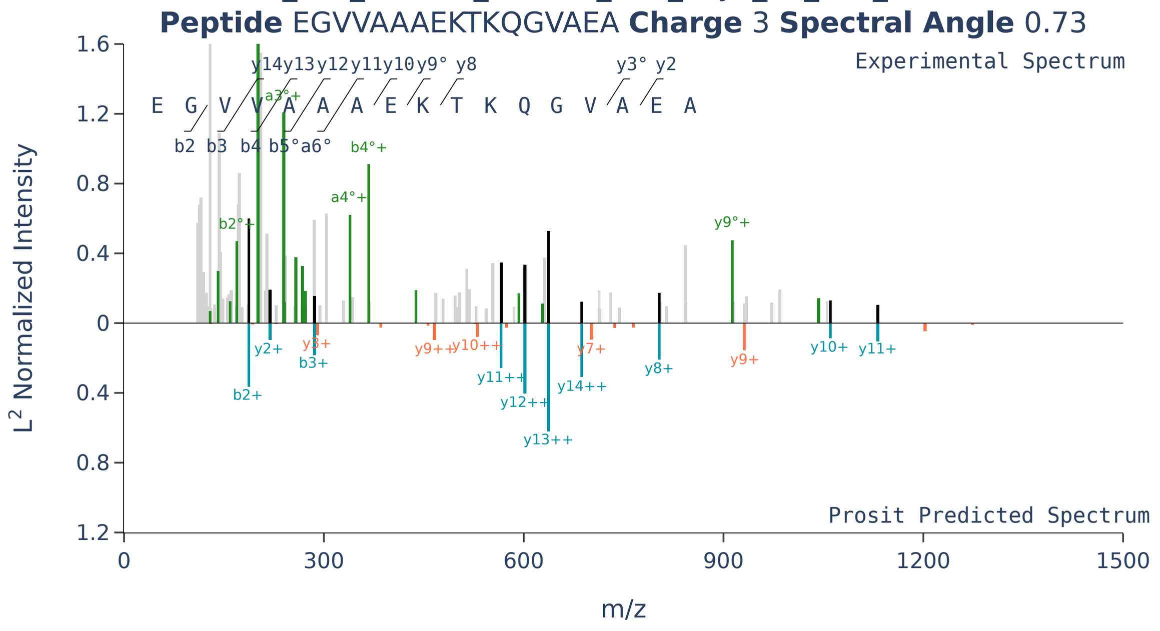


**Source** W_Soh_160821_170921_Goe_aSyn_A1_3h_R1 **Scan** 29970
**Peptide** VTNVGGAVVTGVTAVAQKTVEGAGSIAA **Charge** 3 **Spectral Angle** 0.76

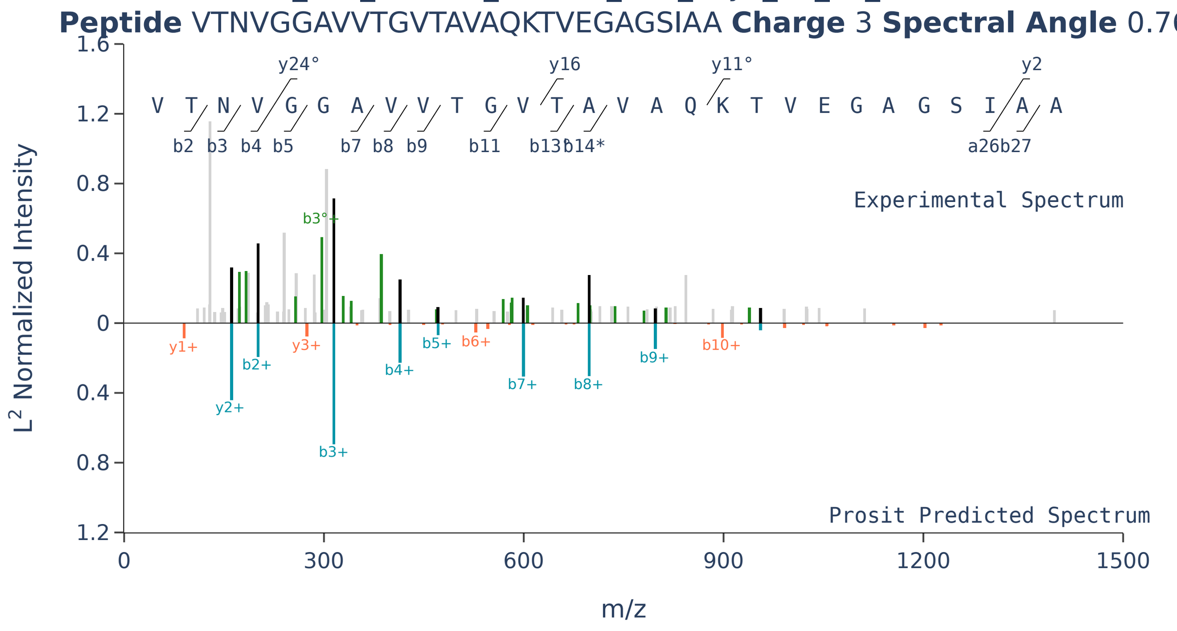


**Source** W_Soh_160821_170921_Goe_aSyn_A1_24h_R2 **Scan** 9562
**Peptide** VATVAEKTKEQVTNVG **Charge** 3 **Spectral Angle** 0.87

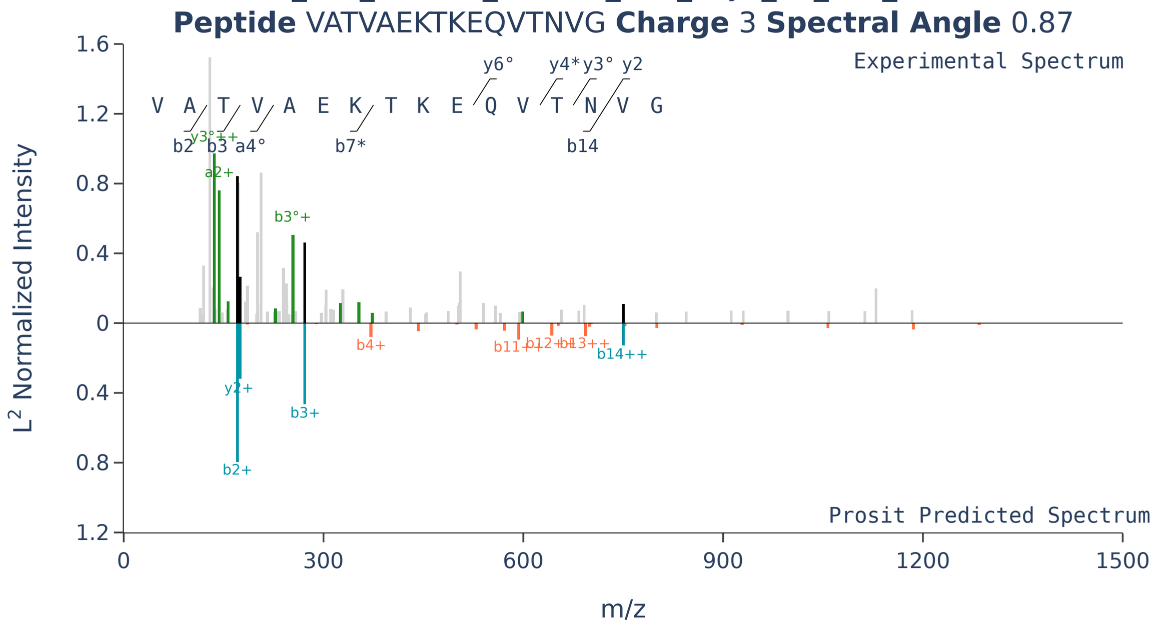


**Source** W_Soh_160821_170921_Goe_aSyn_A1_6h_R2 **Scan** 26240
**Peptide** DMPVDPDNEAYEMPSEEGYQDYEPEA **Charge** 3 **Spectral Angle** 0.93

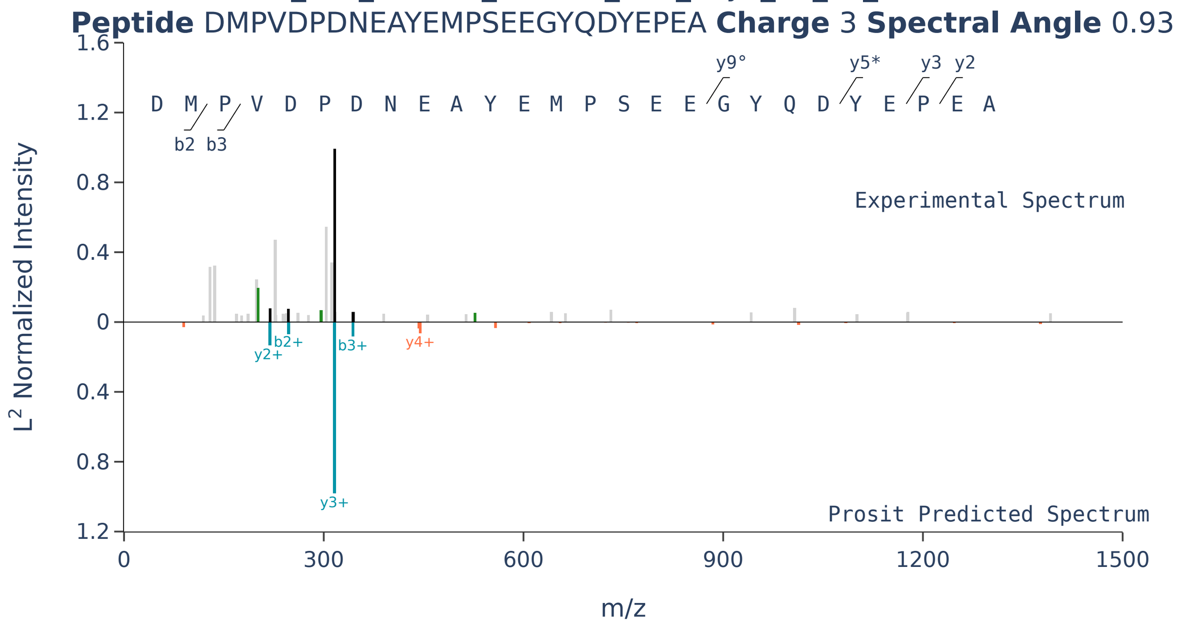


**Source** W_Soh_160821_170921_Goe_aSyn_A2_24h_R2 **Scan** 7274
**Peptide** HGVATVAEKTKEQVT **Charge** 3 **Spectral Angle** 0.69

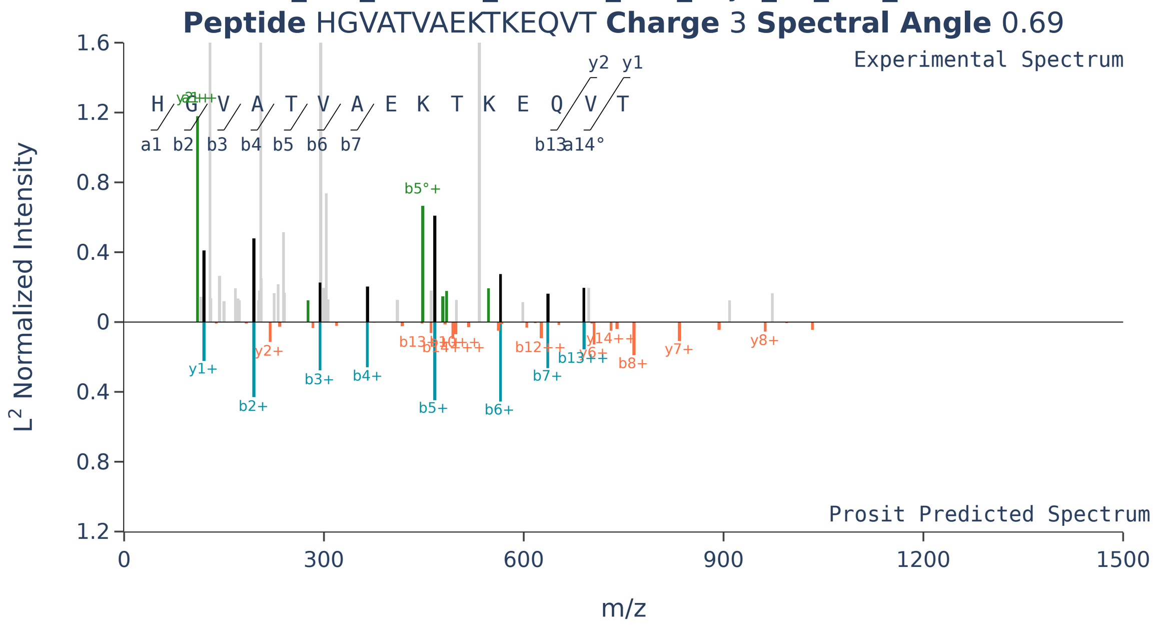


**Source** W_Soh_160821_170921_Goe_aSyn_A1_24h_R2 **Scan** 7526
**Peptide** AEAAGKTKEGVL **Charge** 2 **Spectral Angle** 0.77

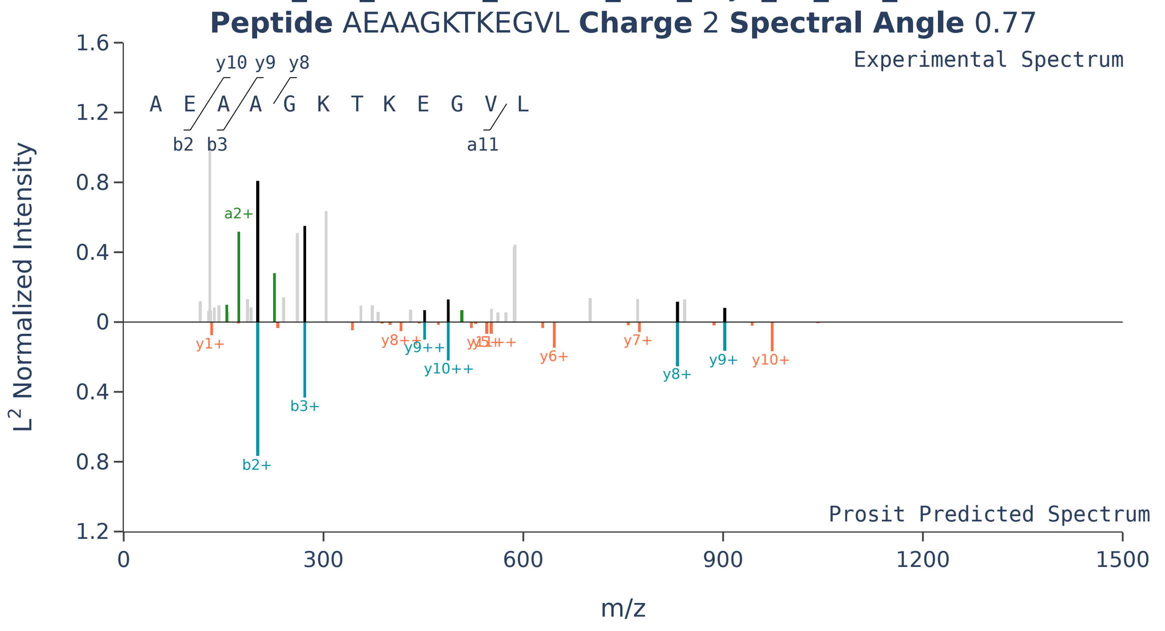


**Source** W_Soh_160821_170921_Goe_aSyn_A1_24h_R1 **Scan** 11668
**Peptide** TKEGVLYVGSKTKEGVVHGVAT **Charge** 4 **Spectral Angle** 0.76

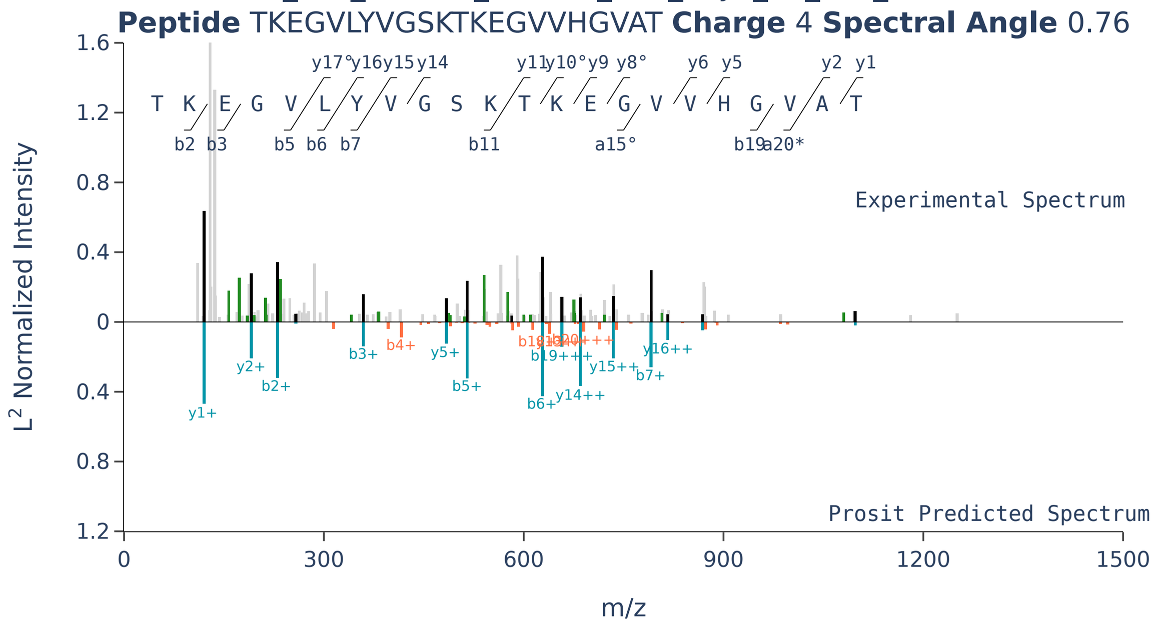


**Source** W_Soh_160821_170921_Goe_aSyn_A1_24h_R1 **Scan** 16358
**Peptide** EGVLYVGSKTKEGVVHGVATVA **Charge** 3 **Spectral Angle** 0.85

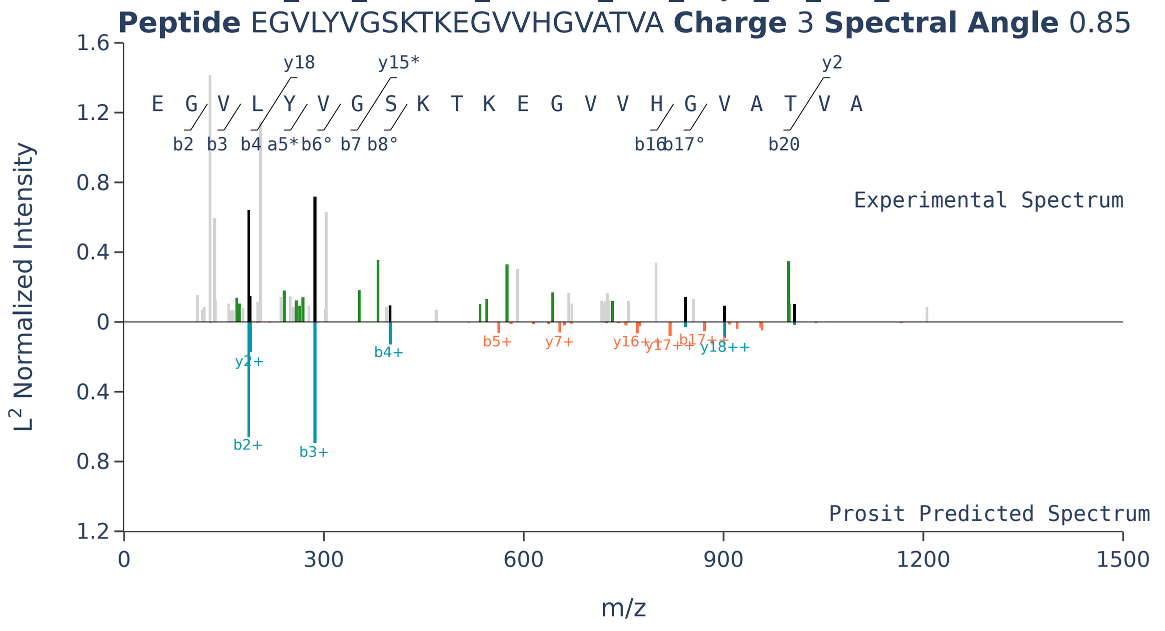


**Source** W_Soh_160821_170921_Goe_aSyn_A2_24h_R1 **Scan** 9266
**Peptide** EAAGKTKEGVLY **Charge** 2 **Spectral Angle** 0.8

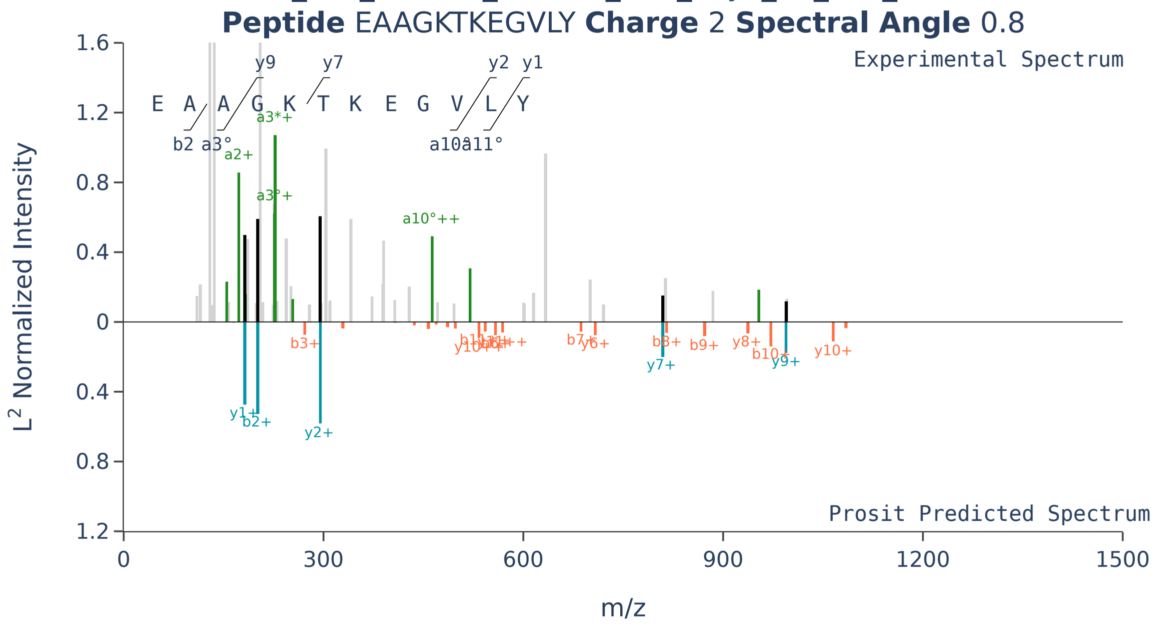

**Source** W_Soh_160821_170921_Goe_aSyn_A1_24h_R2 **Scan** 13088
**Peptide** AGKTKEGVLYVGSKTKEGVVHGVATVAEKT **Charge** 5 **Spectral Angle** 0.5

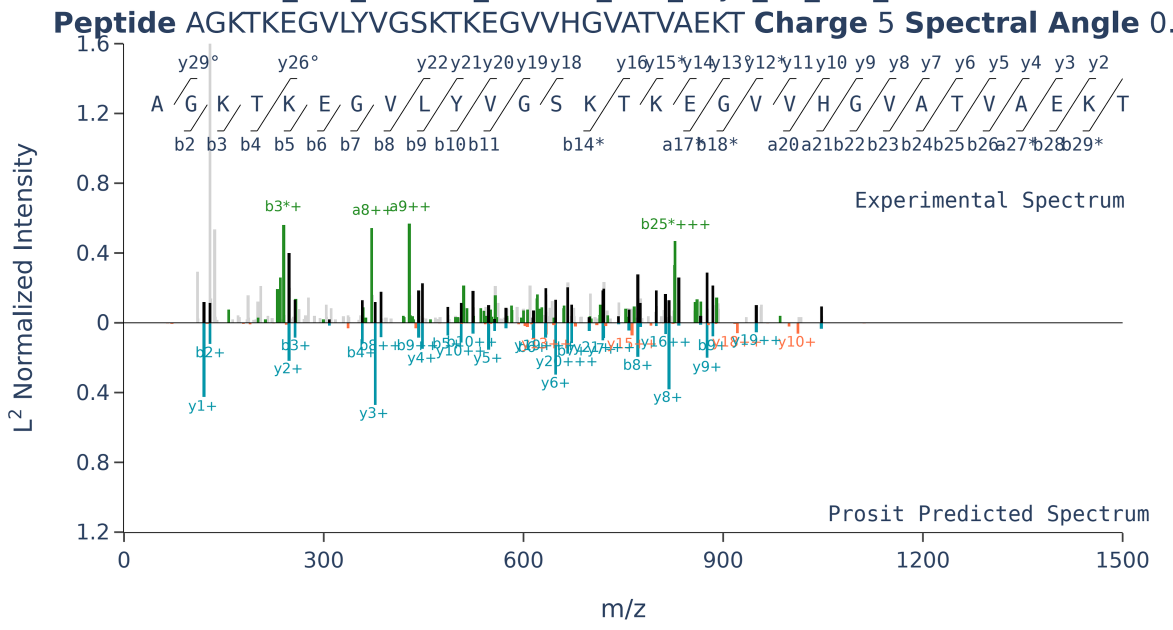


**Source** W_Soh_160821_170921_Goe_aSyn_A1_6h_R2 **Scan** 9420
**Peptide** GVVHGVATVAEKT **Charge** 3 **Spectral Angle** 0.72

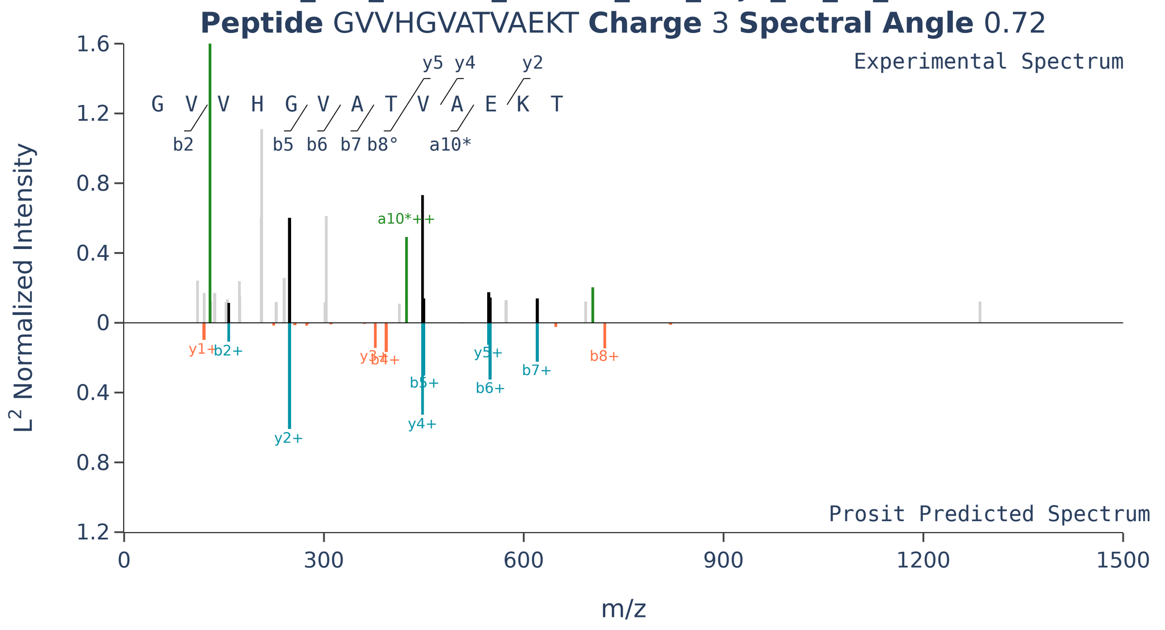


**Source** W_Soh_160821_170921_Goe_aSyn_A2_24h_R1 **Scan** 16201
**Peptide** SIAAATGFVKKDQLGKNEEGAPQEGI **Charge** 3 **Spectral Angle** 0.83

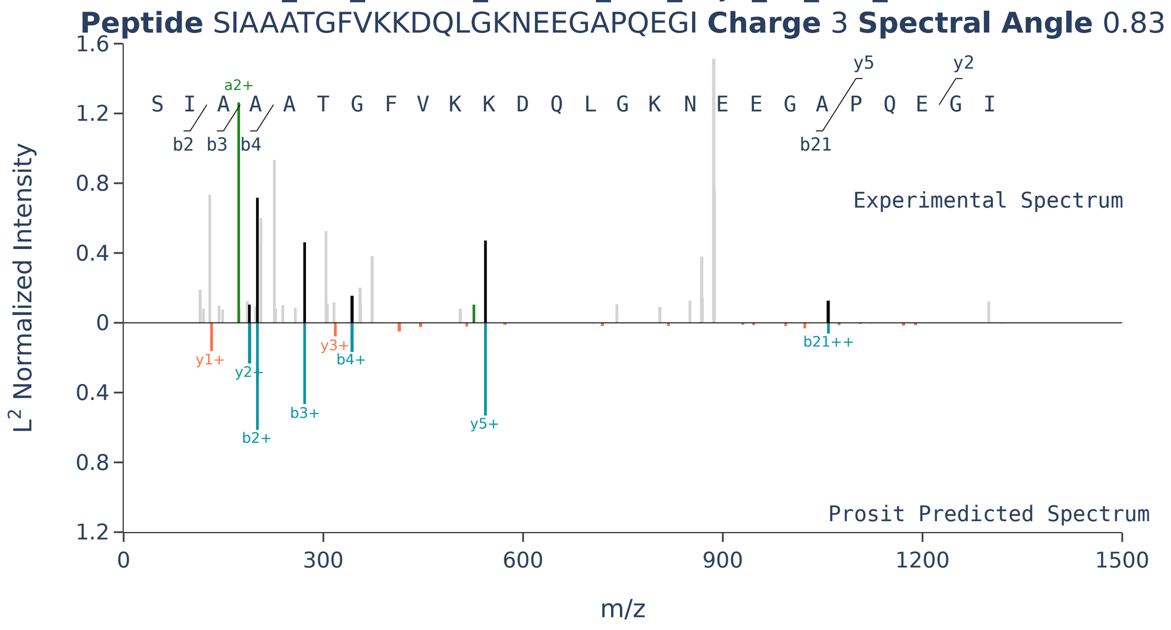


**Source** W_Soh_160821_170921_Goe_aSyn_A2_24h_R2 **Scan** 16706
**Peptide** VVHGVATVAEKTKEQVTNVGGAVVTG **Charge** 4 **Spectral Angle** 0.73

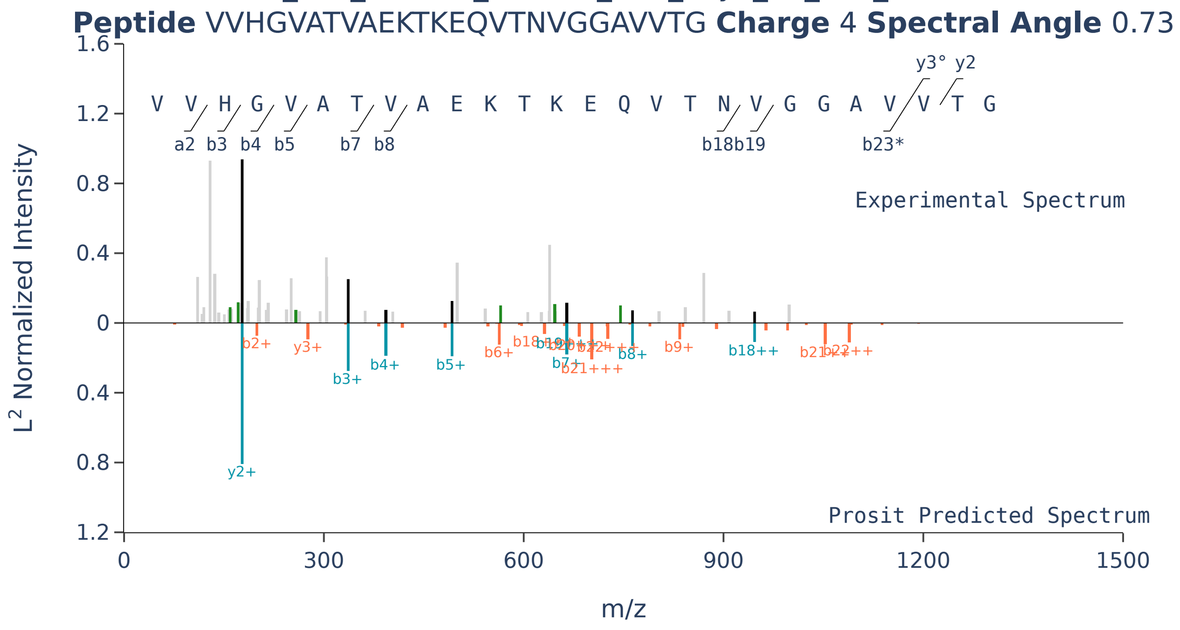


**Source** W_Soh_160821_170921_Goe_aSyn_A1_24h_R1 **Scan** 7450
**Peptide** VGSKTKEGVVHGVATVAEK **Charge** 3 **Spectral Angle** 0.64

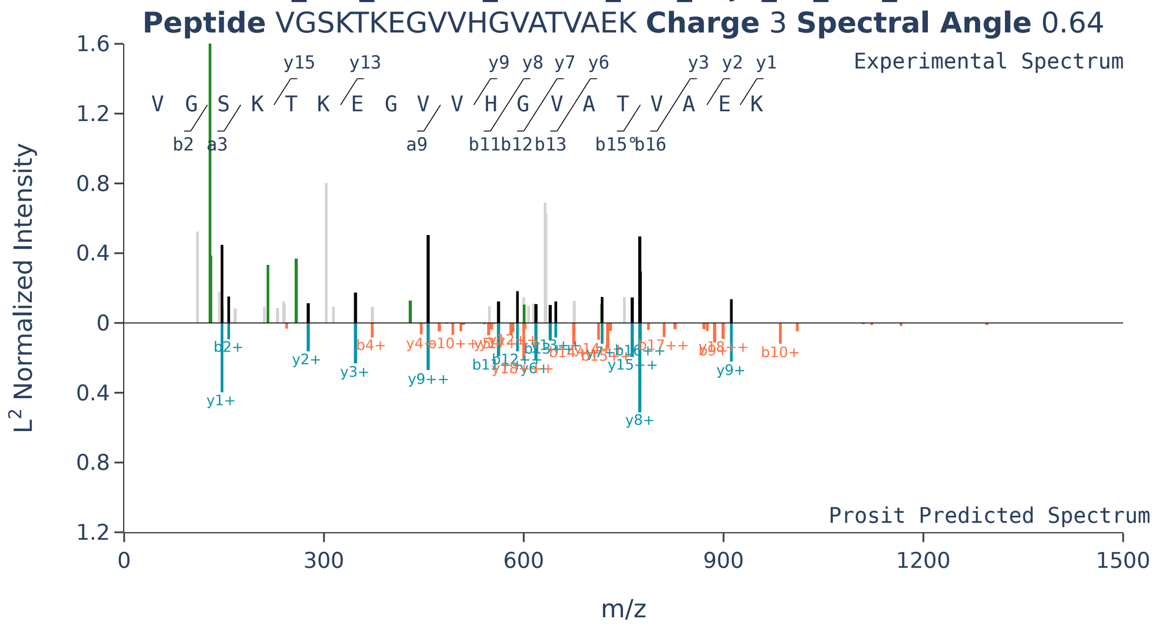


**Source** W_Soh_160821_170921_Goe_aSyn_A2_24h_R1 **Scan** 11527
**Peptide** FMKGLSKAKEGVVAAAEKT **Charge** 3 **Spectral Angle** 0.74

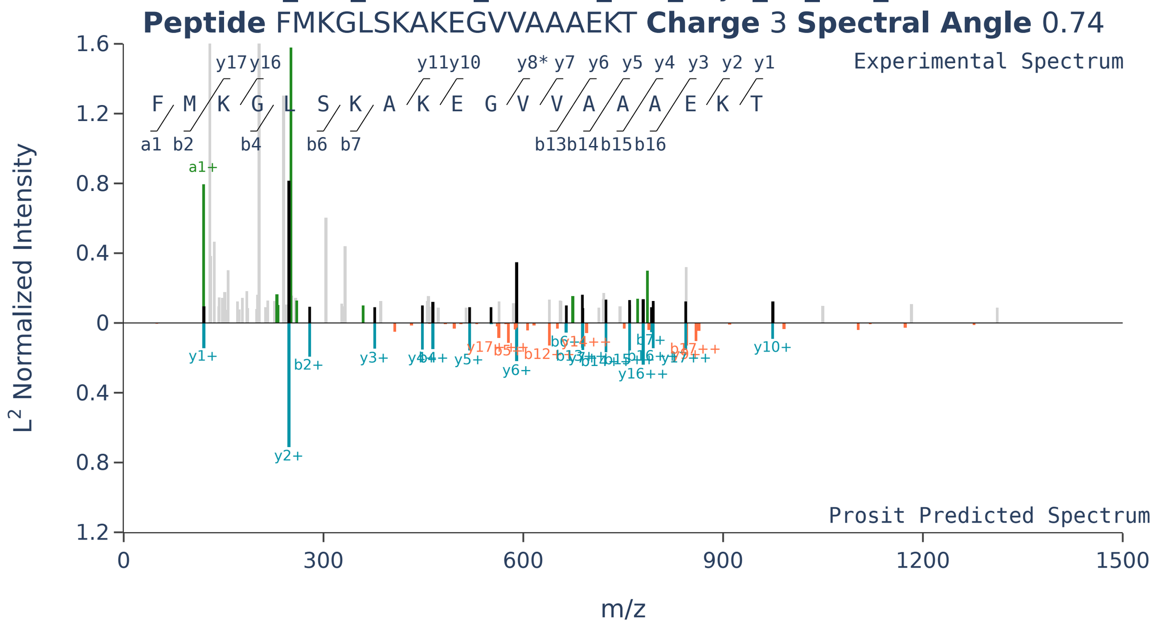


**Source** W_Soh_160821_170921_Goe_aSyn_A1_6h_R1 **Scan** 23174
**Peptide** EKTKEQVTNVGGAVVTGVTAVAQKTVEG **Charge** 3 **Spectral Angle** 0.64

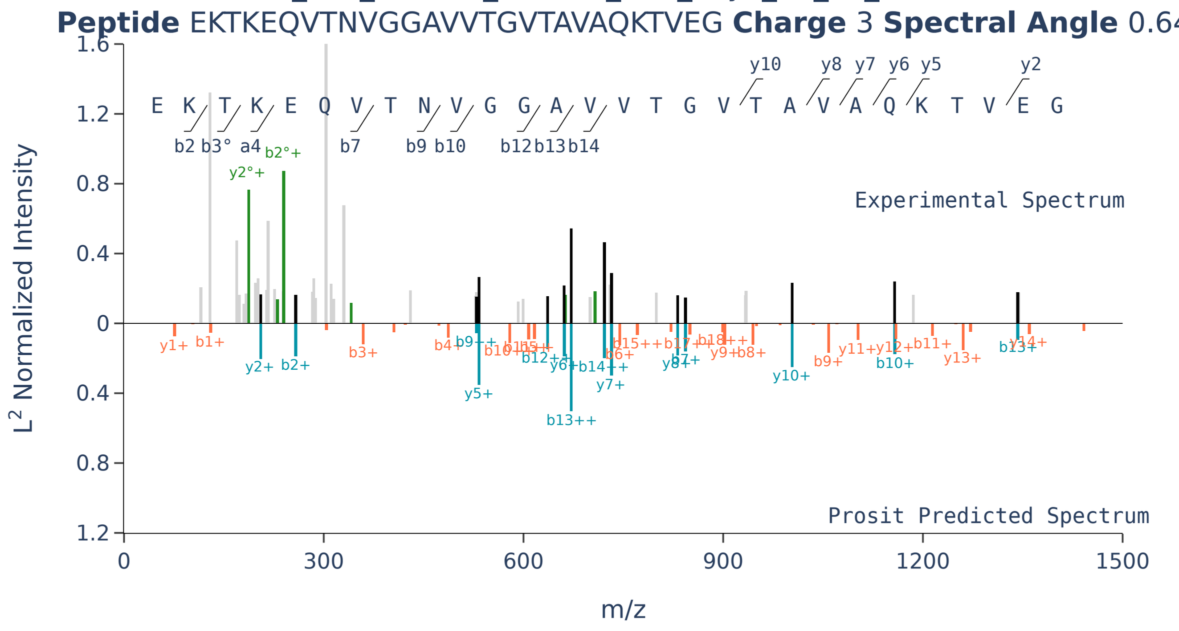


**Source** W_Soh_160821_170921_Goe_aSyn_A1_24h_R2 **Scan** 10326
**Peptide** GVAEAAGKTKEGVLYVGSKTK **Charge** 3 **Spectral Angle** 0.7

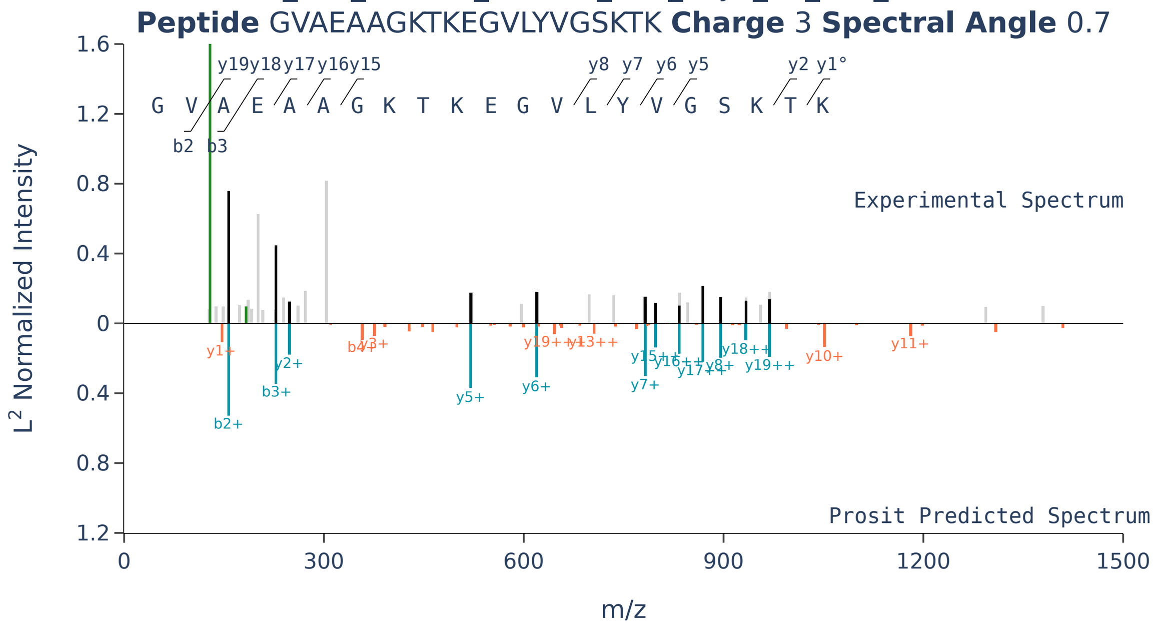


**Source** W_Soh_160821_170921_Goe_aSyn_A1_24h_R2 **Scan** 14394
**Peptide** FMKGLSKAKEGVVAAAEKTKQGVAEA **Charge** 5 **Spectral Angle** 0.58

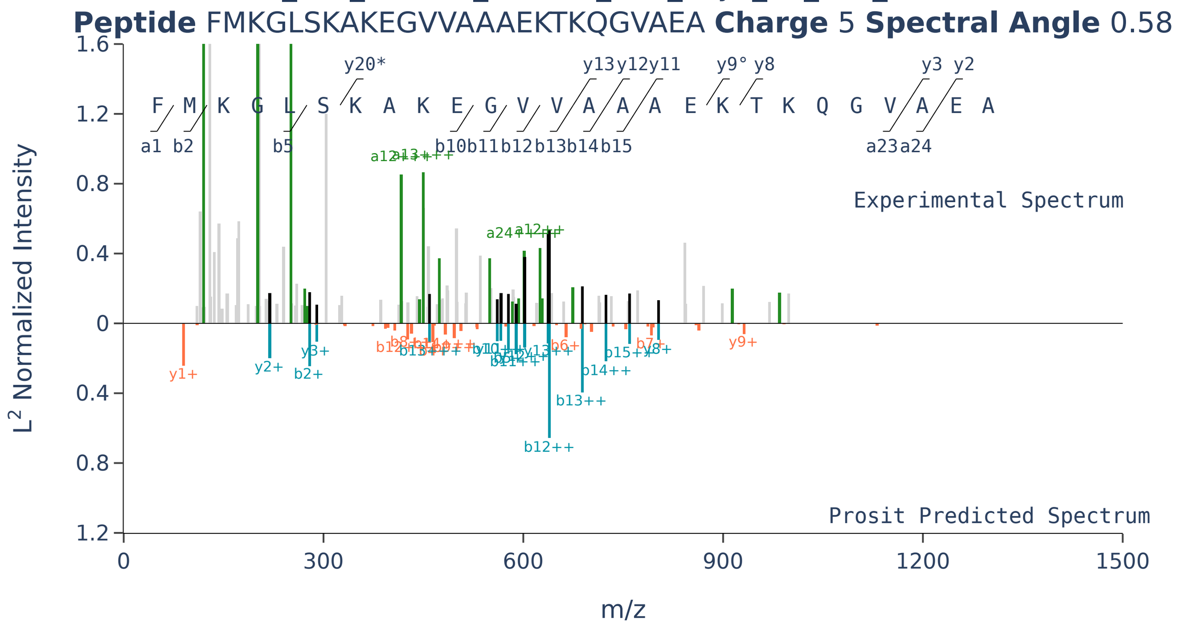


**Source** W_Soh_160821_170921_Goe_aSyn_A1_24h_R2 **Scan** 8202
**Peptide** LSKAKEGVVAAAEKTKQGVAE **Charge** 3 **Spectral Angle** 0.69

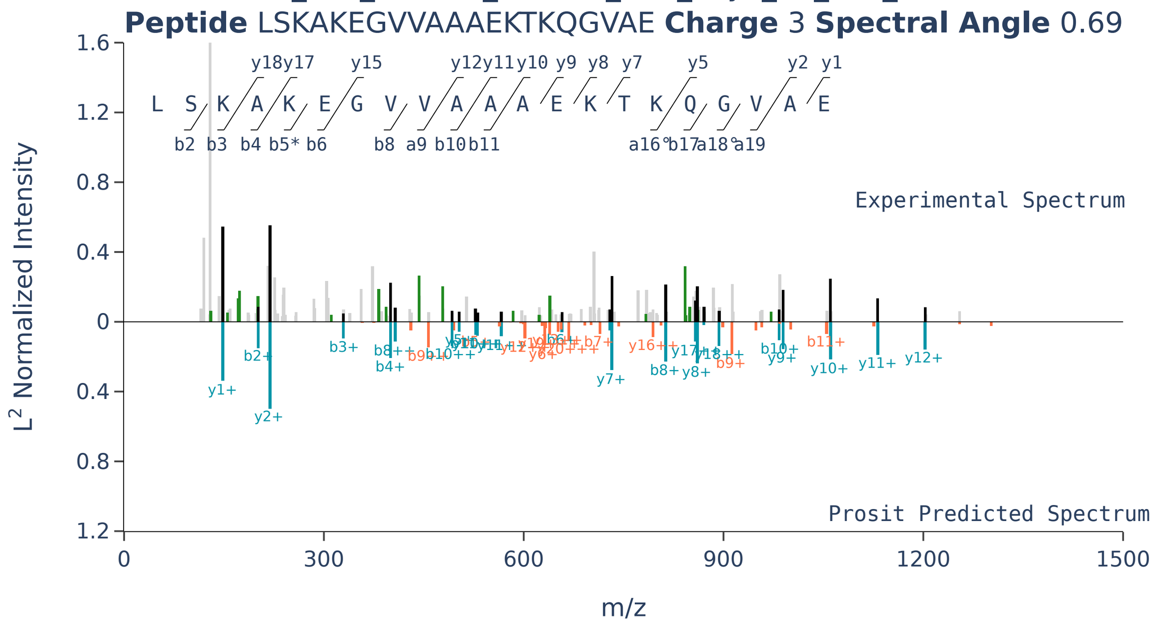


**Source** W_Soh_160821_170921_Goe_aSyn_A2_24h_R1 **Scan** 13744
**Peptide** VTAVAQKTVEGAGSI **Charge** 2 **Spectral Angle** 0.67

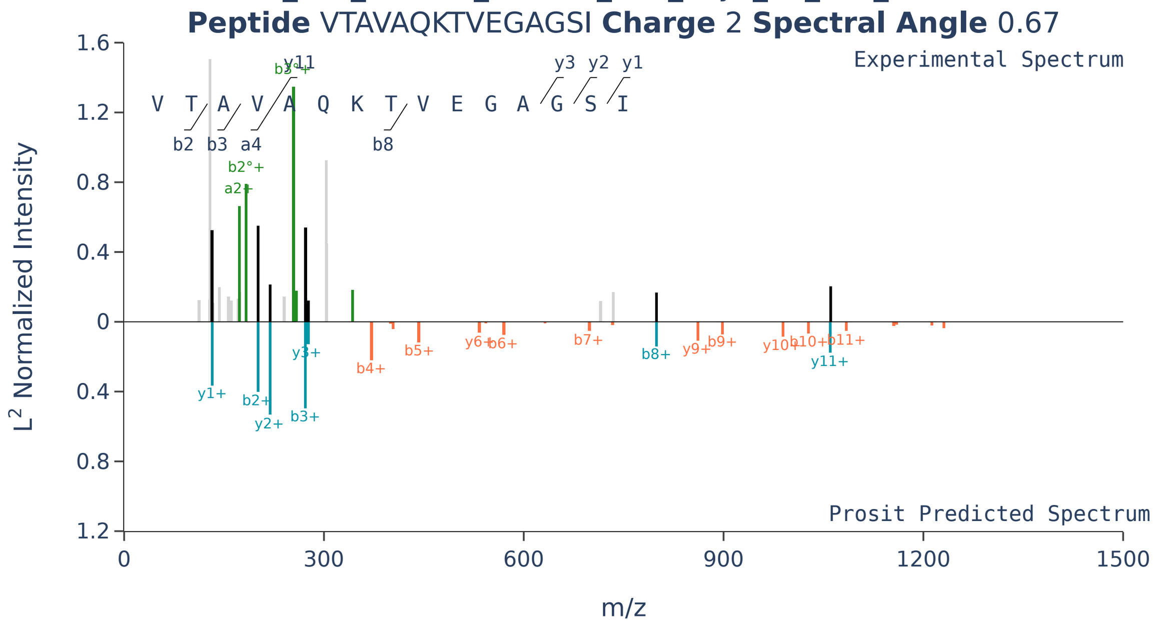


**Source** W_Soh_160821_170921_Goe_aSyn_A1_24h_R2 **Scan** 12103
**Peptide** EMPSEEGY **Charge** 1 **Spectral Angle** 0.54

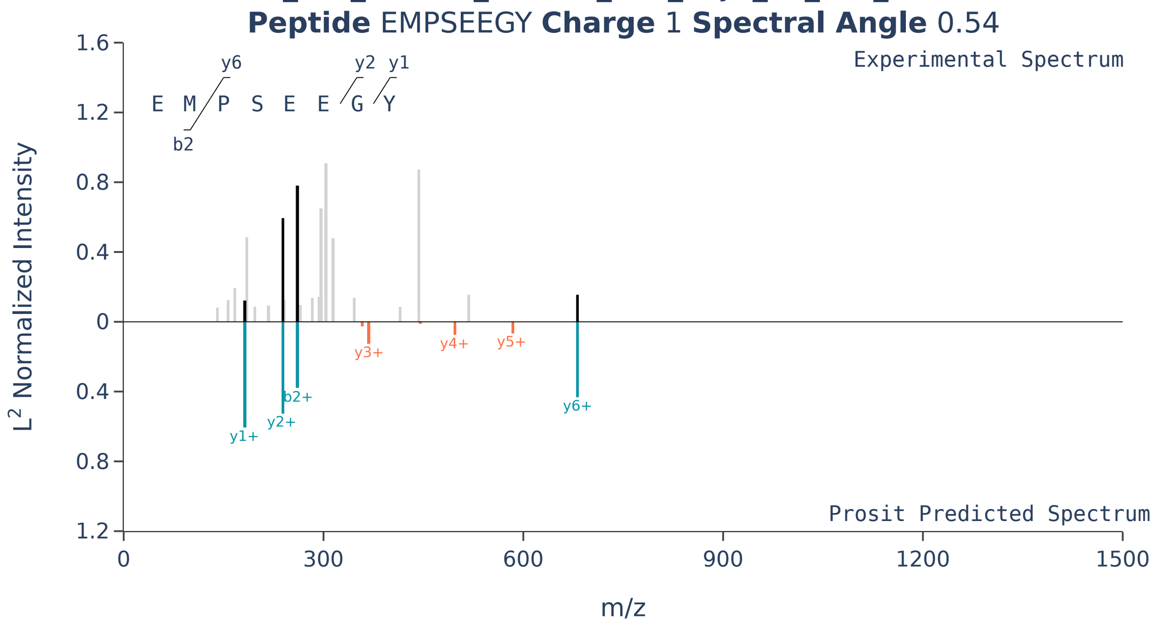


**Source** W_Soh_160821_170921_Goe_aSyn_A1_6h_R1 **Scan** 14362
**Peptide** QGVAEAAGKTKEGVLYVG **Charge** 3 **Spectral Angle** 0.88

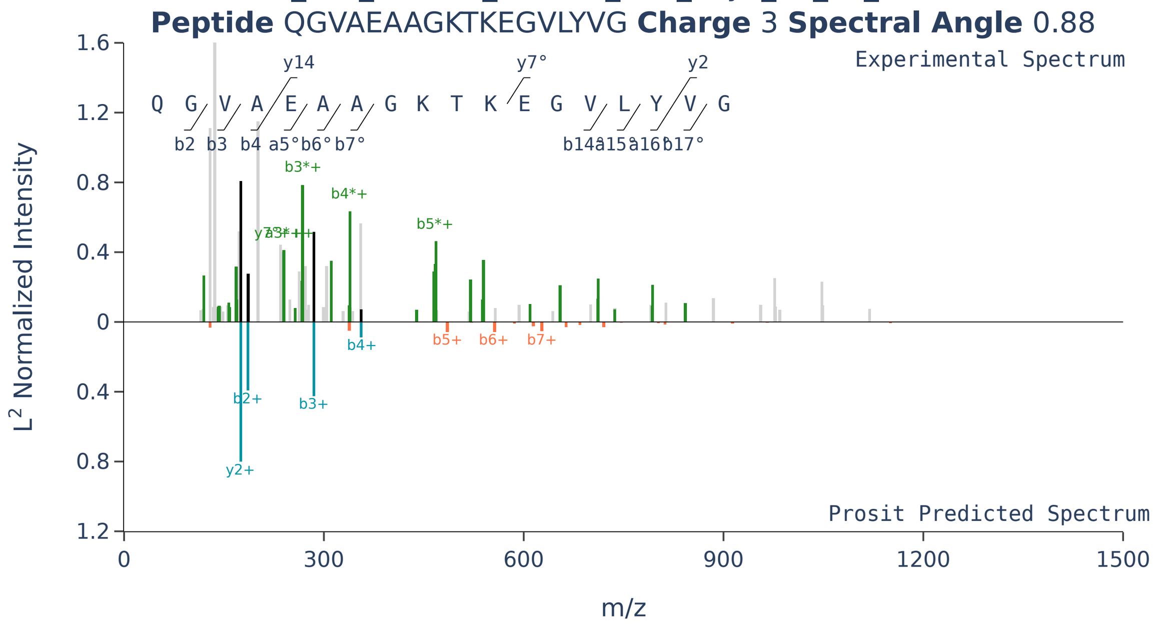


**Source** W_Soh_160821_170921_Goe_aSyn_A1_24h_R2 **Scan** 10490
**Peptide** AEKTKQGVAEAAGKTKEGVLYVGS **Charge** 3 **Spectral Angle** 0.56

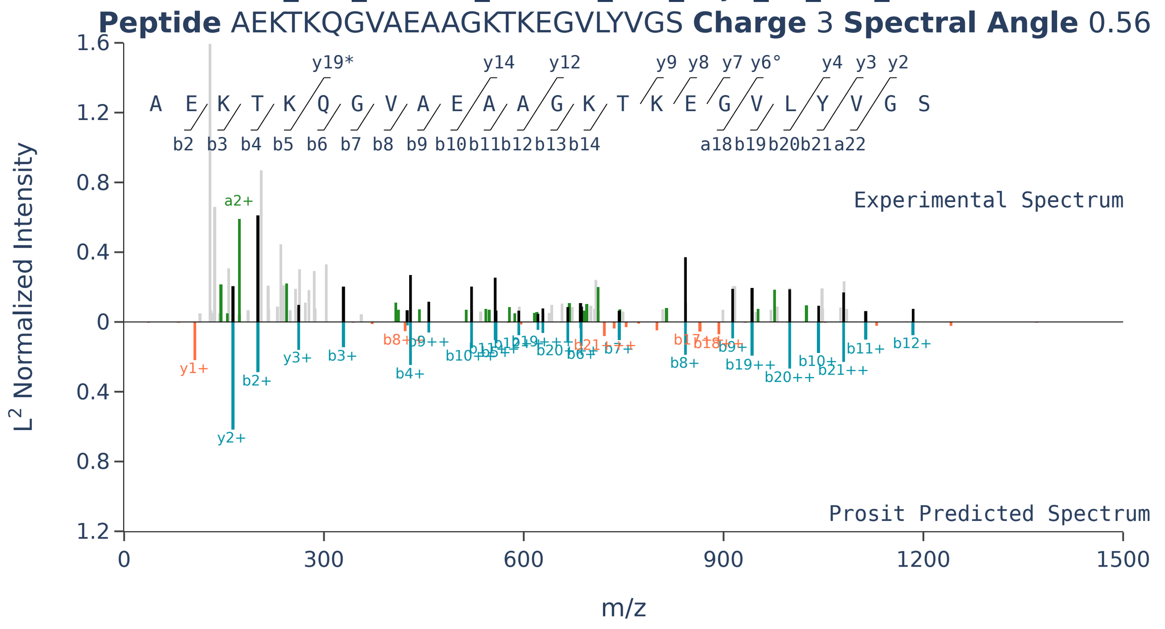


**Source** W_Soh_160821_170921_Goe_aSyn_A2_24h_R2 **Scan** 15215
**Peptide** QKTVEGAGSIAAATGFVKKDQLGKNEE **Charge** 4 **Spectral Angle** 0.64

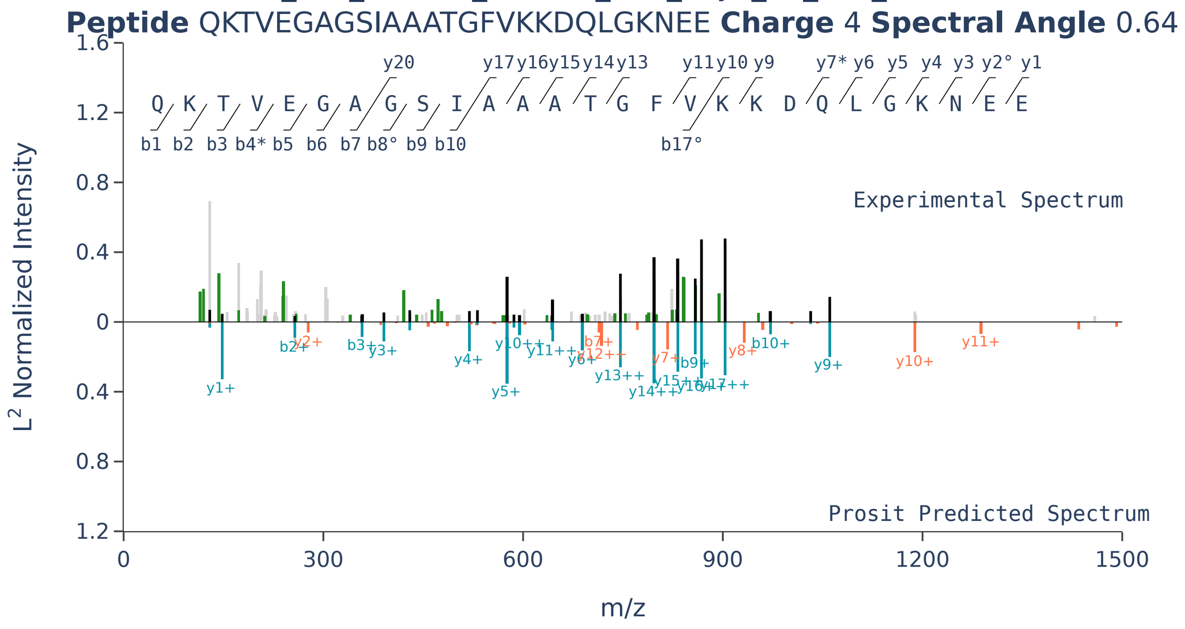

**Source** W_Soh_160821_170921_Goe_aSyn_A2_24h_R2 **Scan** 5006
**Peptide** AAAEKTKQGVAEAAGKTK **Charge** 3 **Spectral Angle** 0.66

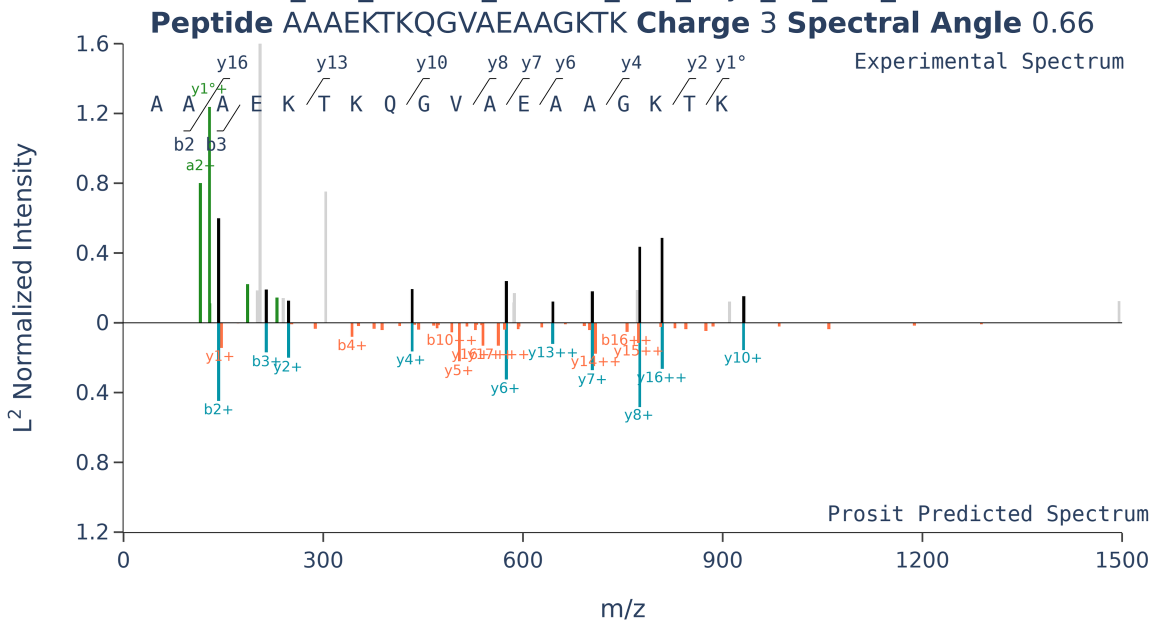


**Source** W_Soh_160821_170921_Goe_aSyn_A1_24h_R2 **Scan** 11787
**Peptide** GVVHGVATVAEKTKEQVTN **Charge** 3 **Spectral Angle** 0.69

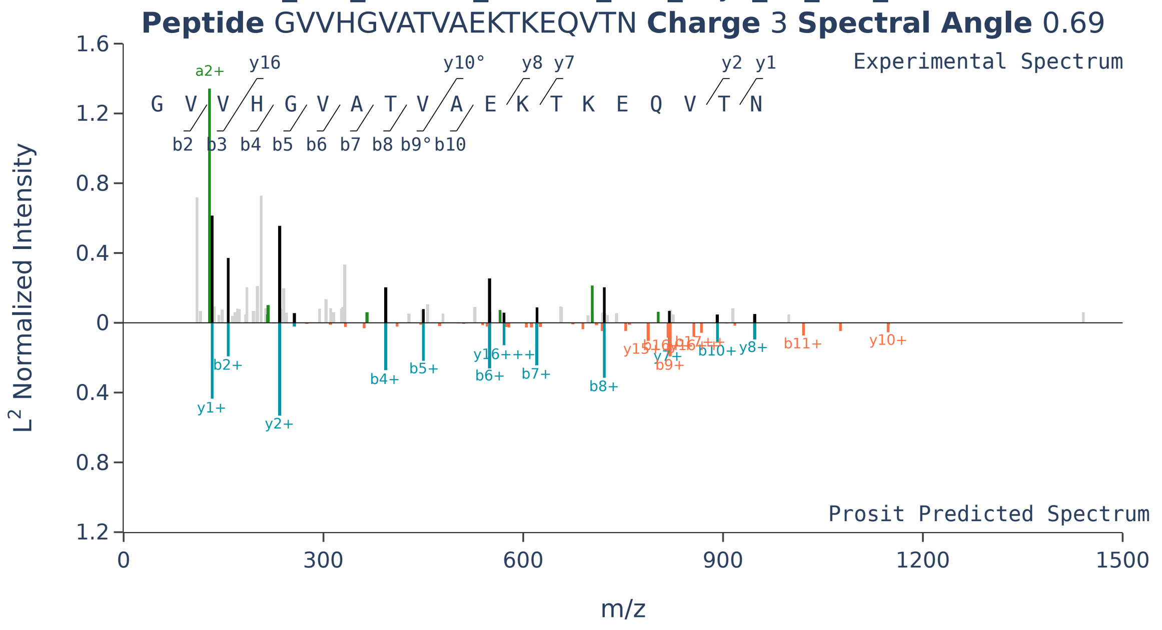


**Source** W_Soh_160821_170921_Goe_aSyn_A2_6h_R1 **Scan** 10610
**Peptide** AAGKTKEGVLYVGS **Charge** 2 **Spectral Angle** 0.62

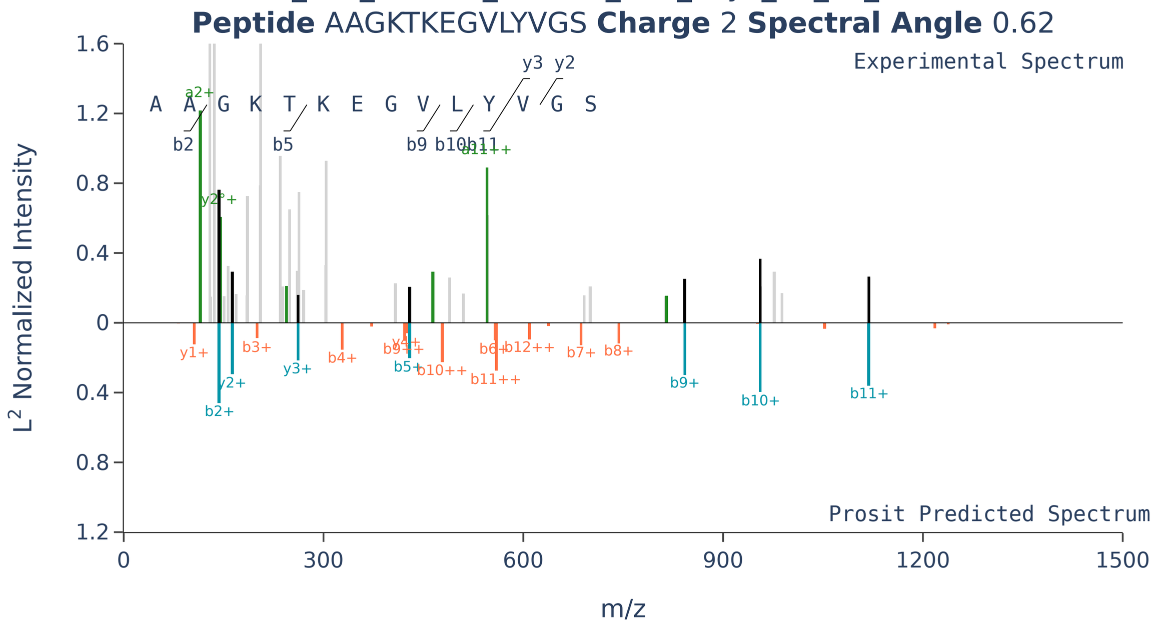


**Source** W_Soh_160821_170921_Goe_aSyn_A1_24h_R2 **Scan** 7216
**Peptide** HGVATVAEKTKEQVTN **Charge** 3 **Spectral Angle** 0.72

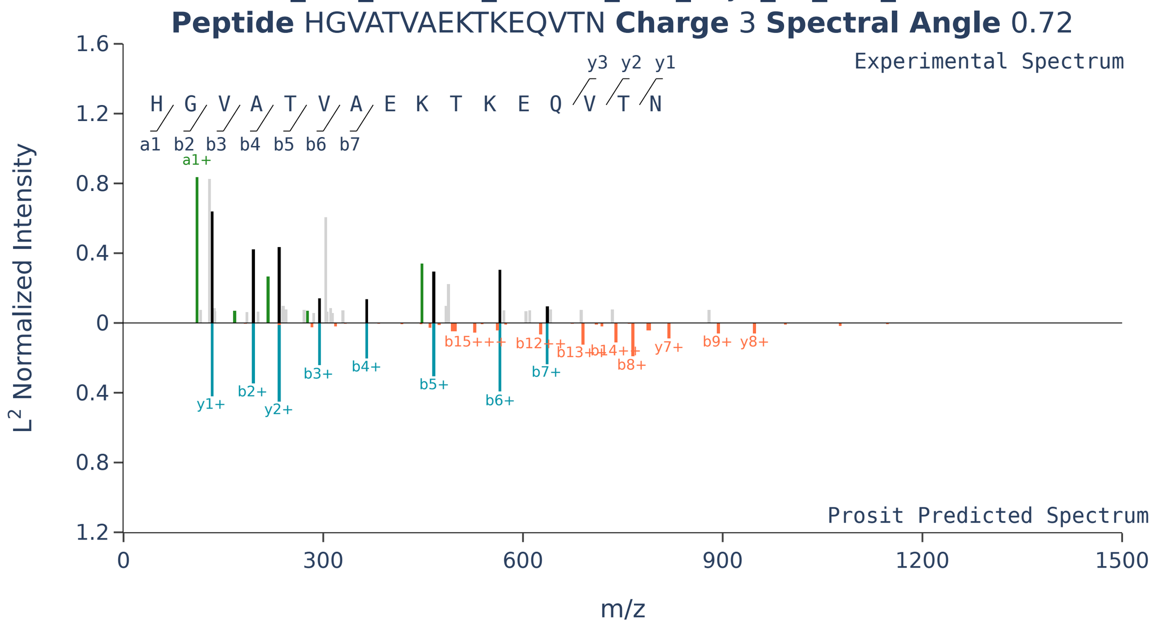


**Source** W_Soh_160821_170921_Goe_aSyn_A2_24h_R2 **Scan** 11952
**Peptide** KTKEGVLYVGSKTKEGVVHGVATV **Charge** 4 **Spectral Angle** 0.82

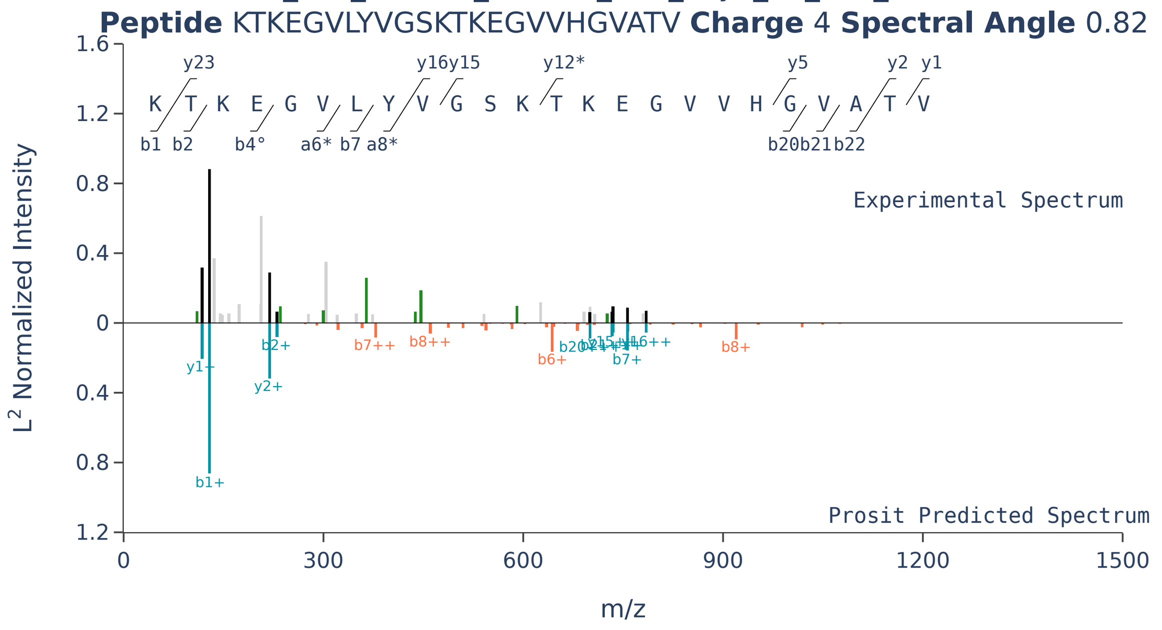


**Source** W_Soh_160821_170921_Goe_aSyn_A2_24h_R2 **Scan** 8199
**Peptide** ATVAEKTKEQVTNVG **Charge** 2 **Spectral Angle** 0.65

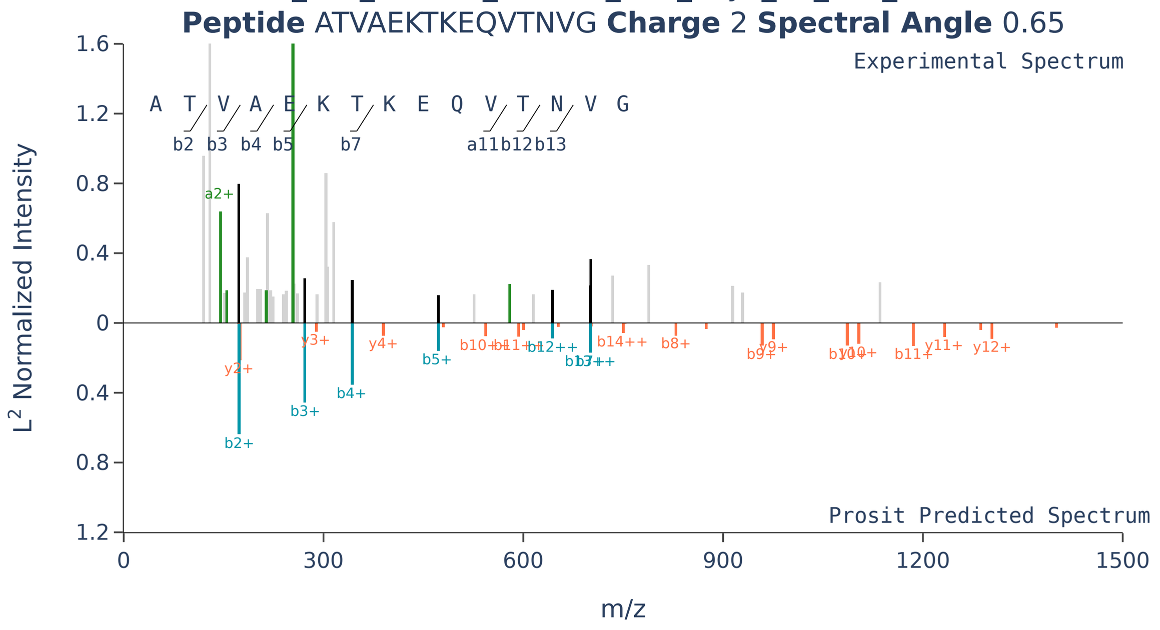


**Source** W_Soh_160821_170921_Goe_aSyn_A2_24h_R1 **Scan** 16797
**Peptide** GVLYVGSKTKEGVVHGVATVAEKTKEQVTN **Charge** 4 **Spectral Angle** 0.79

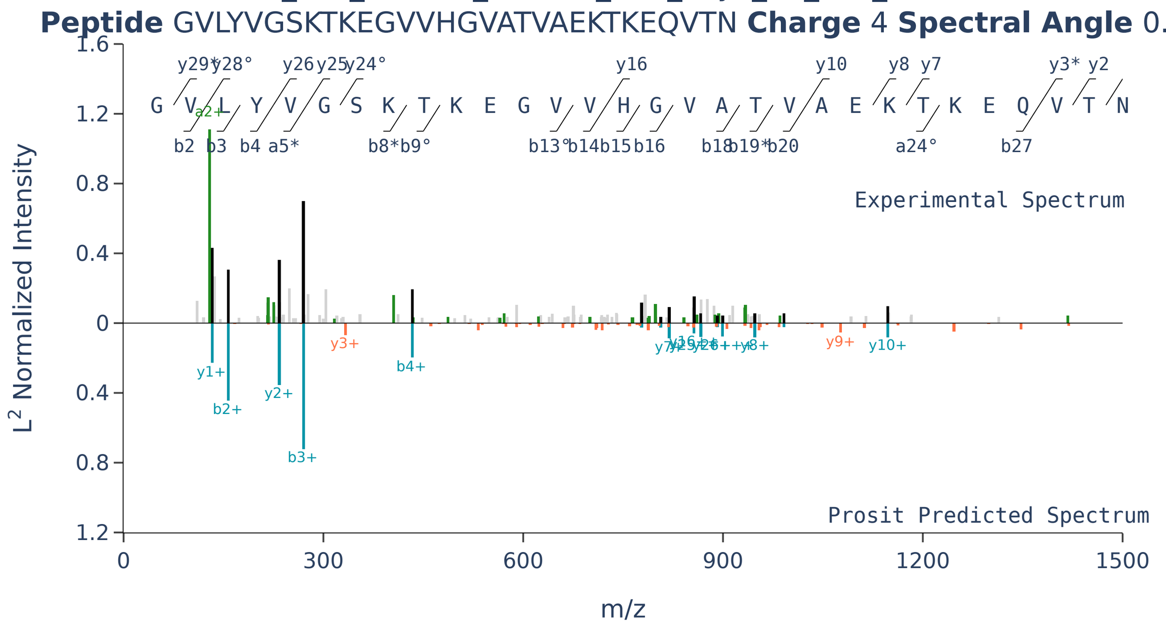


**Source** W_Soh_160821_170921_Goe_aSyn_A1_6h_R2 **Scan** 18234
**Peptide** EGAGSIAAATGFVKKDQLGKNEEGAPQEGI **Charge** 4 **Spectral Angle** 0.69

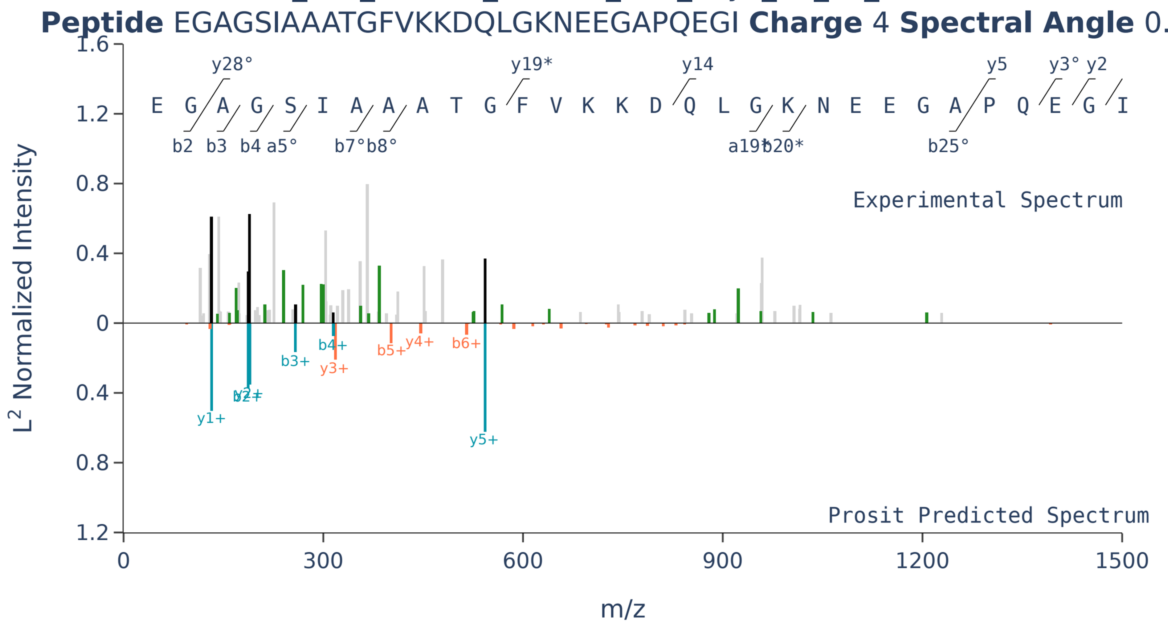


**Source** W_Soh_160821_170921_Goe_aSyn_A1_24h_R2 **Scan** 10008
**Peptide** GKTKEGVLYVGSKTKEGVVHGVAT **Charge** 4 **Spectral Angle** 0.75

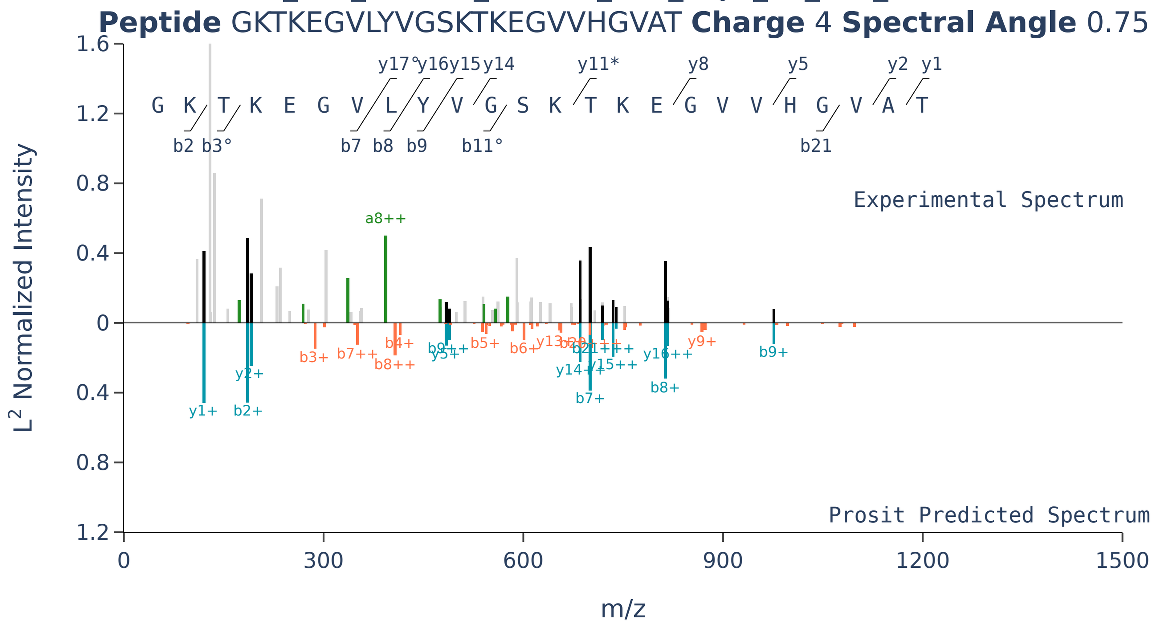


**Source** W_Soh_160821_170921_Goe_aSyn_A1_24h_R1 **Scan** 5997
**Peptide** VAAAEKTKQGVAEAAGKTKE **Charge** 4 **Spectral Angle** 0.6

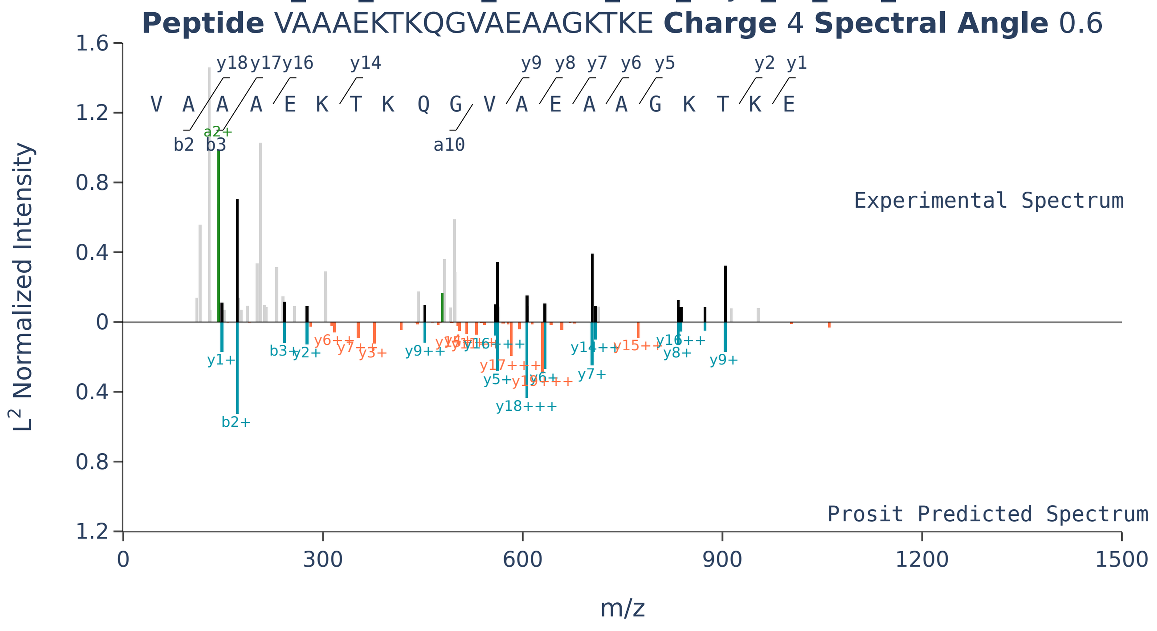


**Source** W_Soh_160821_170921_Goe_aSyn_A1_1h_R1 **Scan** 23397
**Peptide** GAPQEGILEDMPVD **Charge** 2 **Spectral Angle** 0.73

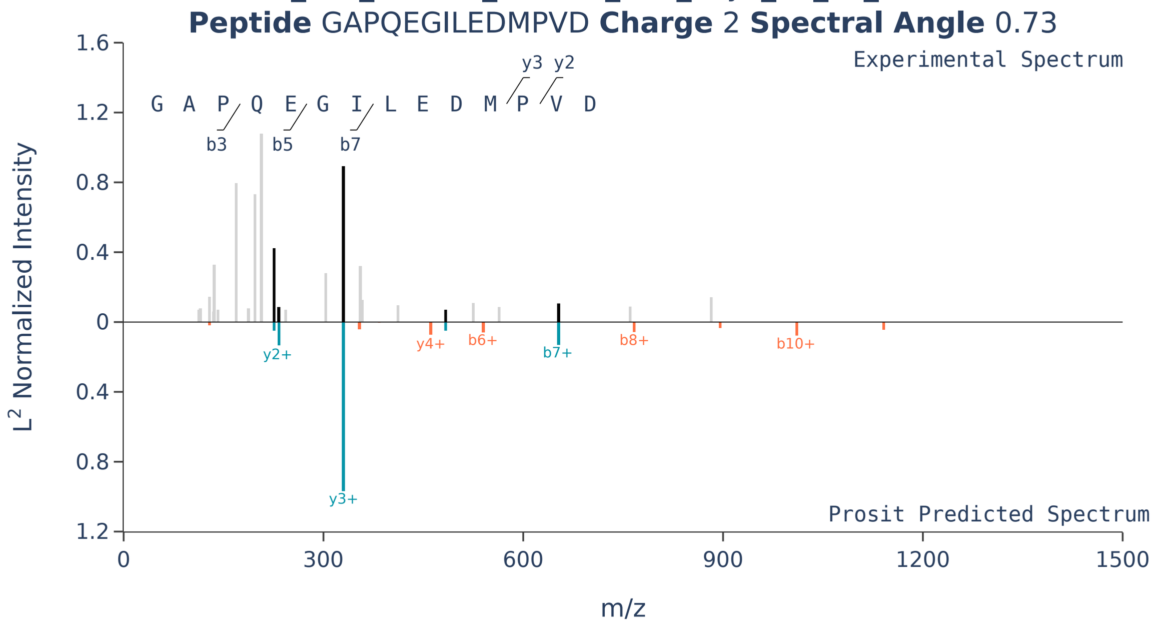


**Source** W_Soh_160821_170921_Goe_aSyn_A2_24h_R2 **Scan** 13367
**Peptide** KNEEGAPQEGILE **Charge** 2 **Spectral Angle** 0.53

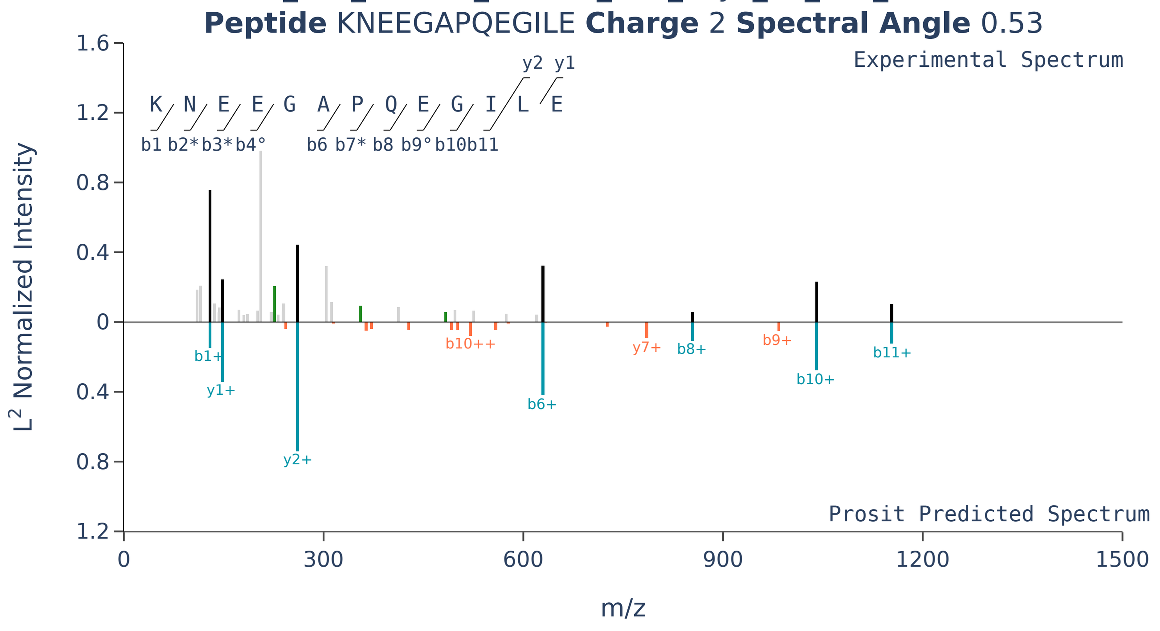


**Source** W_Soh_160821_170921_Goe_aSyn_A1_24h_R1 **Scan** 10321
**Peptide** FMKGLSKAKEGVV **Charge** 3 **Spectral Angle** 0.74

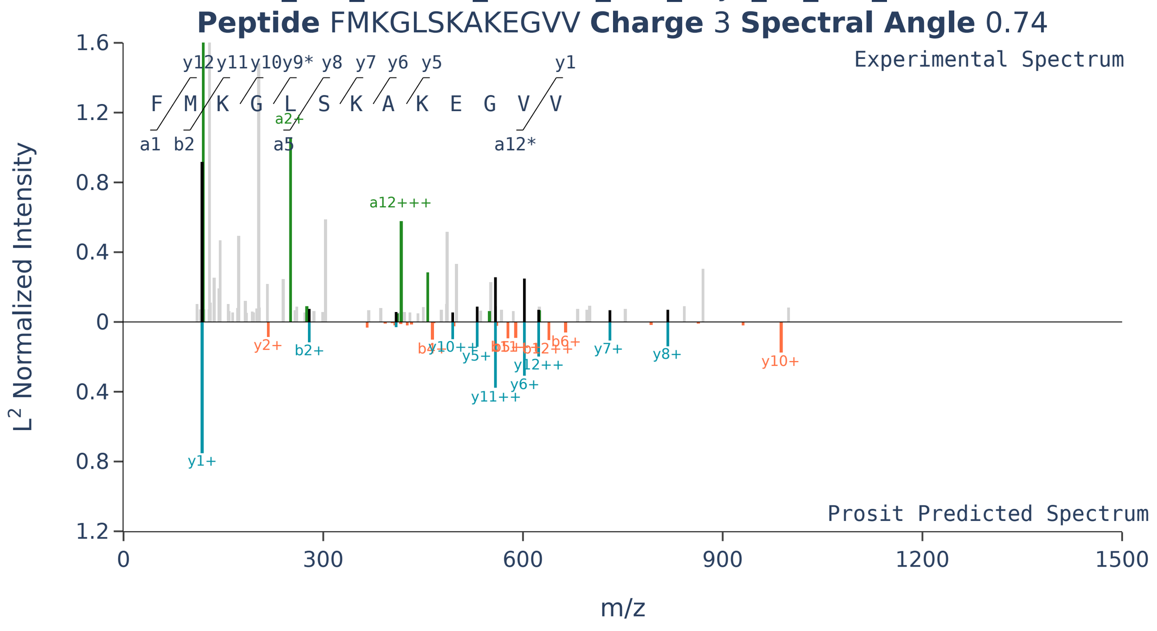


**Source** W_Soh_160821_170921_Goe_aSyn_A2_24h_R2 **Scan** 13283
**Peptide** TGVTAVAQKTVEGA **Charge** 2 **Spectral Angle** 0.61

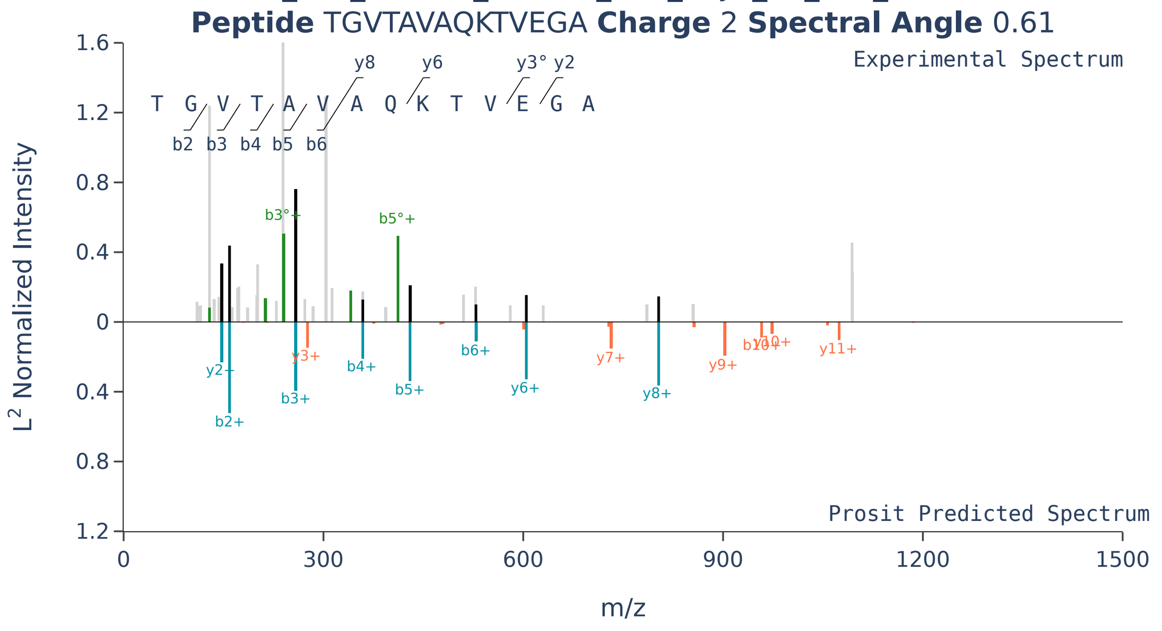


**Source** W_Soh_160821_170921_Goe_aSyn_A2_24h_R1 **Scan** 10450
**Peptide** LSKAKEGVVAAAEKTKQGVAEAAGKTKE **Charge** 5 **Spectral Angle** 0.67

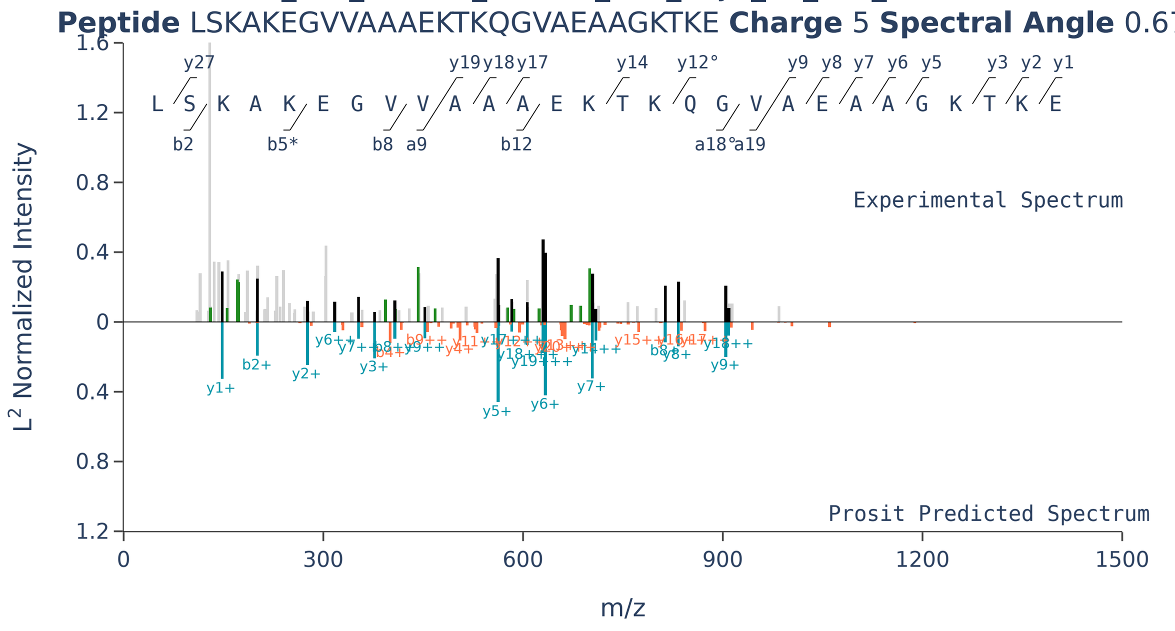

**Source** W_Soh_160821_170921_Goe_aSyn_A2_24h_R1 **Scan** 21126
**Peptide** DVFMKGLSKAKEGVVAAAEKTKQGVAEAA **Charge** 4 **Spectral Angle** 0.76

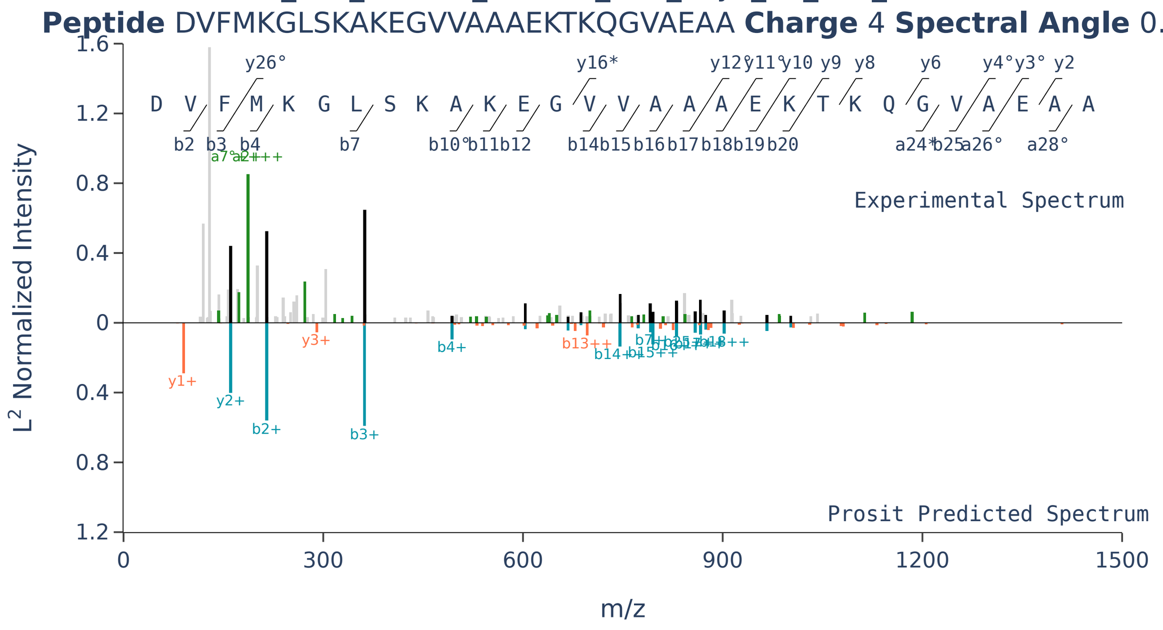


**Source** W_Soh_160821_170921_Goe_aSyn_A1_6h_R1 **Scan** 11292
**Peptide** KTKEGVLYVG **Charge** 2 **Spectral Angle** 0.61

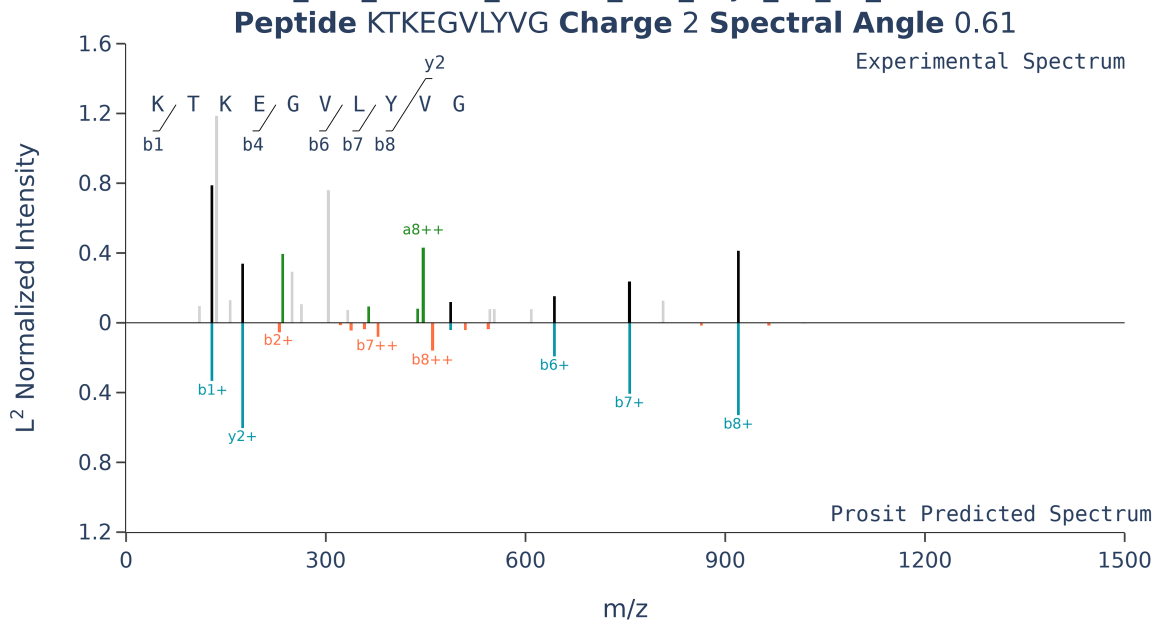


**Source** W_Soh_160821_170921_Goe_aSyn_A1_24h_R2 **Scan** 14380
**Peptide** GVAEAAGKTKEGVLYVGSKTKEGVVHGVA **Charge** 4 **Spectral Angle** 0.75

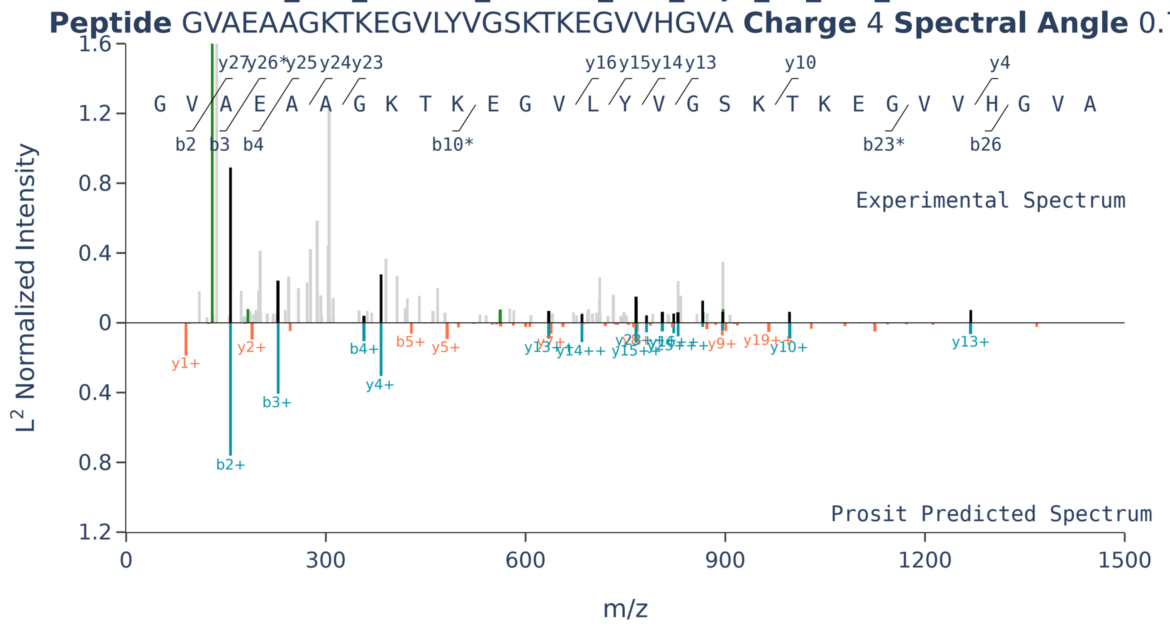


**Source** W_Soh_160821_170921_Goe_aSyn_A1_24h_R1 **Scan** 8934
**Peptide** SKAKEGVVAAAEKTKQGVAEAAGKTK **Charge** 4 **Spectral Angle** 0.67

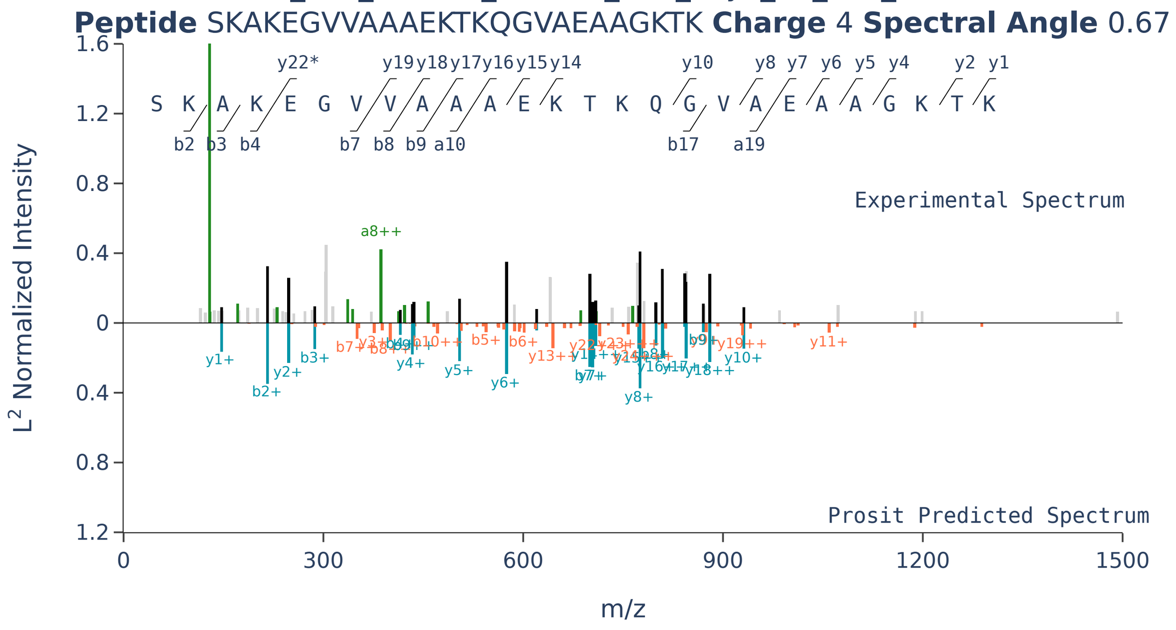


**Source** W_Soh_160821_170921_Goe_aSyn_A2_1h_R1 **Scan** 14608
**Peptide** GVTAVAQKTVEGAGSL **Charge** 2 **Spectral Angle** 0.74

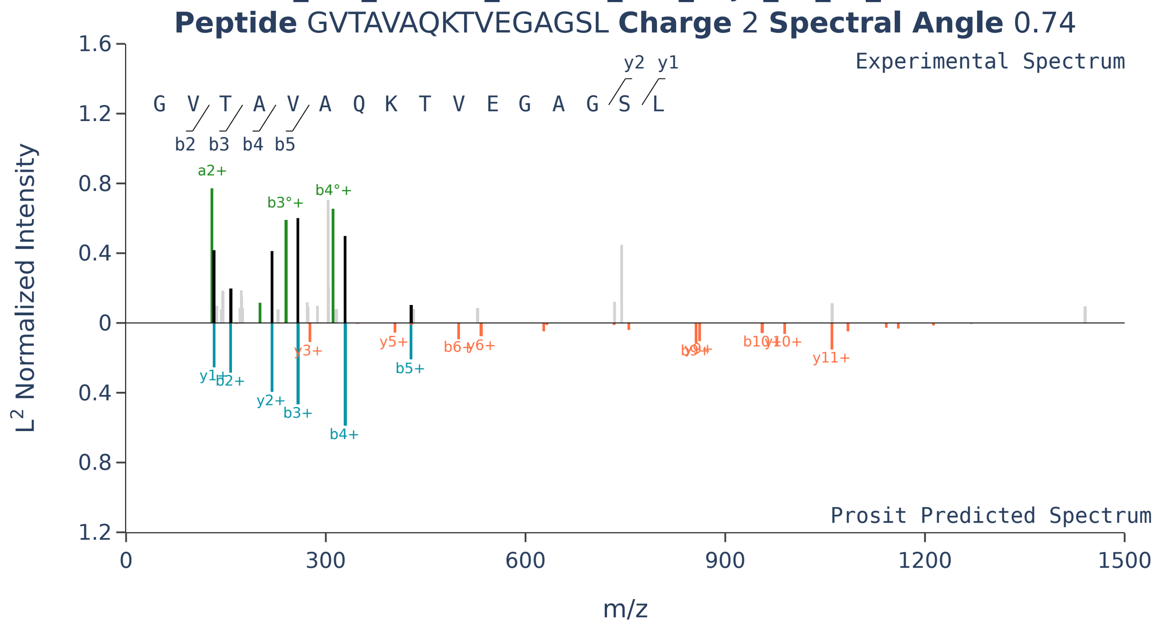


**Source** W_Soh_160821_170921_Goe_aSyn_A1_6h_R1 **Scan** 8889
**Peptide** AEAAGKTKEGVLYVGSKT **Charge** 3 **Spectral Angle** 0.86

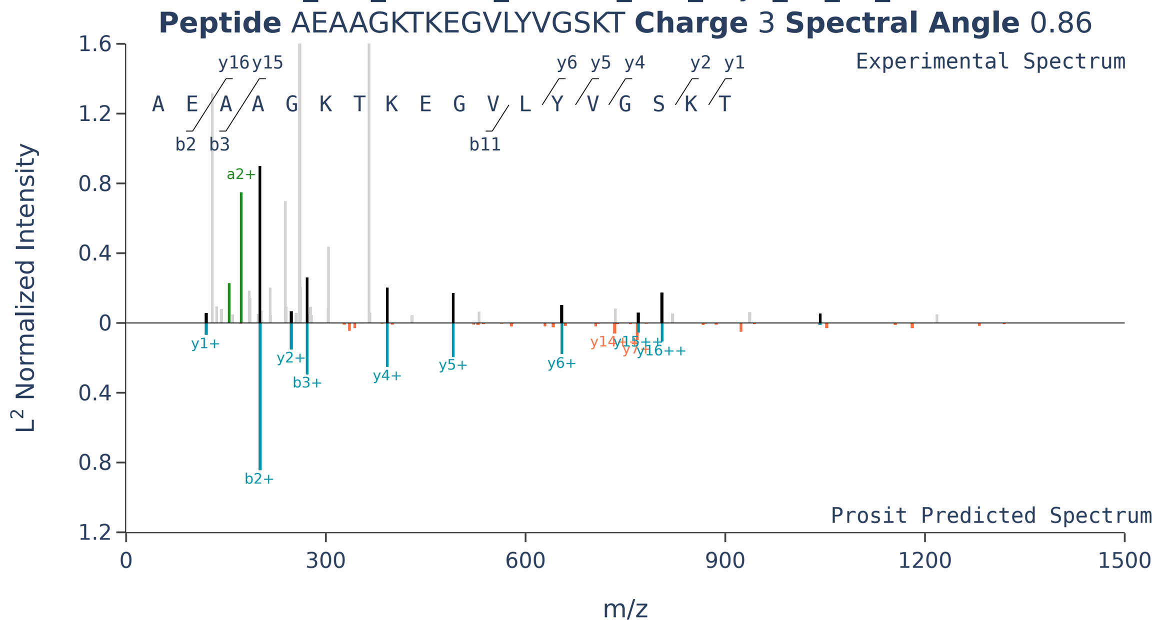


**Source** W_Soh_160821_170921_Goe_aSyn_A2_6h_R1 **Scan** 19700
**Peptide** GAGSIAAATGFVKKDQLGKNEEGAPQEGIL **Charge** 4 **Spectral Angle** 0.75

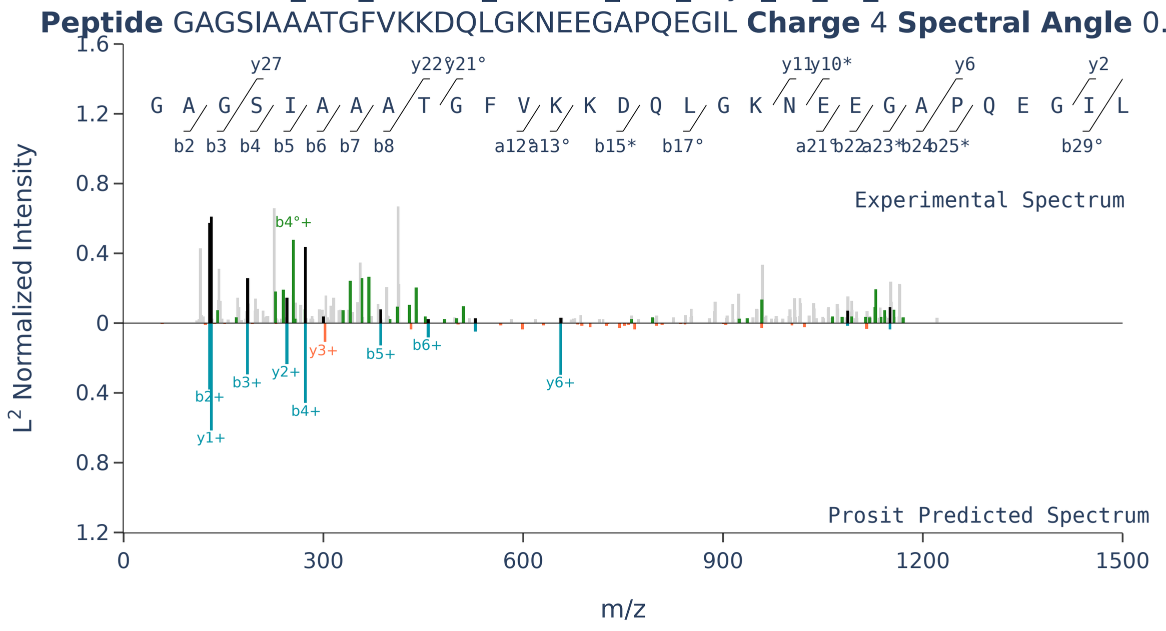


**Source** W_Soh_160821_170921_Goe_aSyn_A2_24h_R2 **Scan** 16358
**Peptide** EGVLYVGSKTKEGVVHGVATVAE **Charge** 3 **Spectral Angle** 0.81

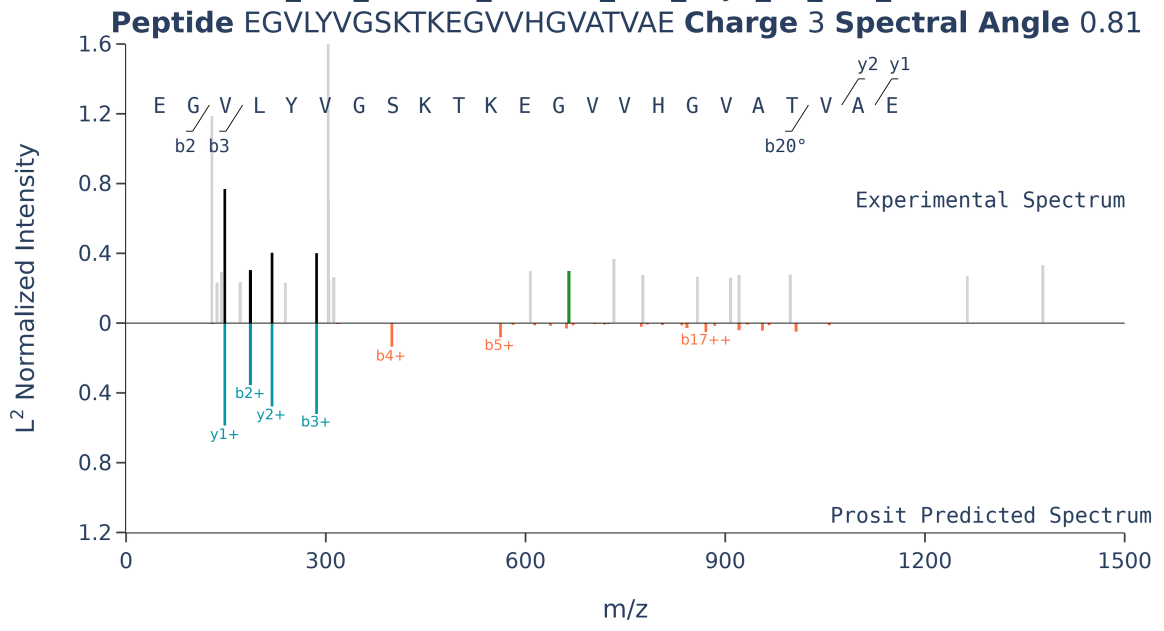


**Source** W_Soh_160821_170921_Goe_aSyn_A1_24h_R1 **Scan** 9744
**Peptide** GLSKAKEGVVAAAEKTKQGV **Charge** 4 **Spectral Angle** 0.64

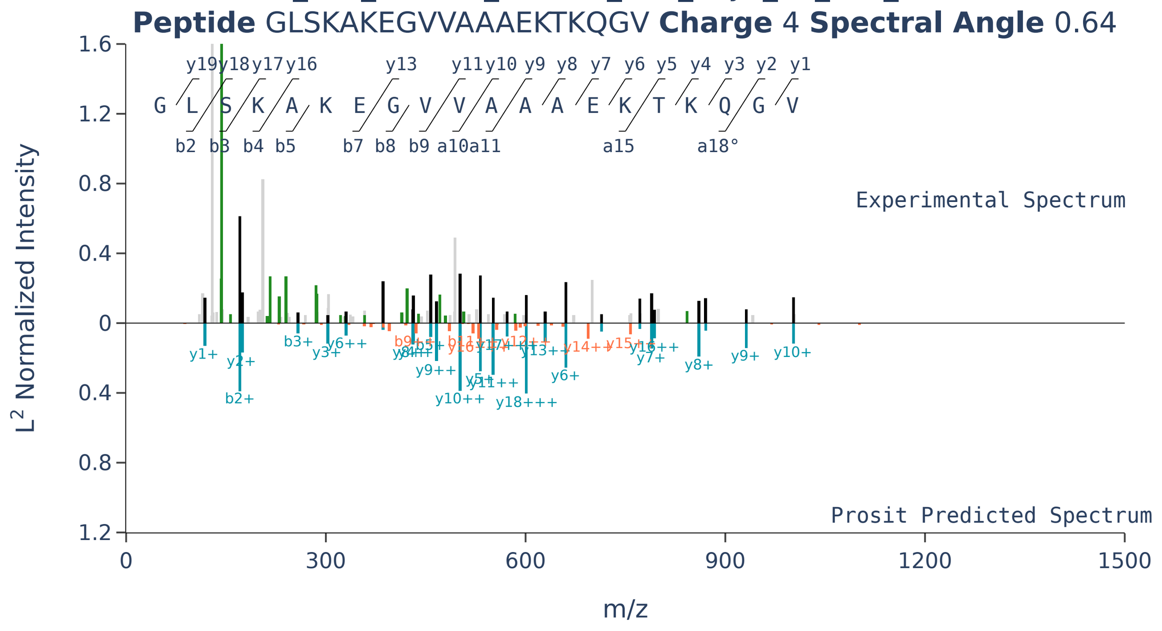


**Source** W_Soh_160821_170921_Goe_aSyn_A1_6h_R2 **Scan** 21042
**Peptide** VTAVAQKTVEGAGSIAAATGFVKKDQL **Charge** 3 **Spectral Angle** 0.65

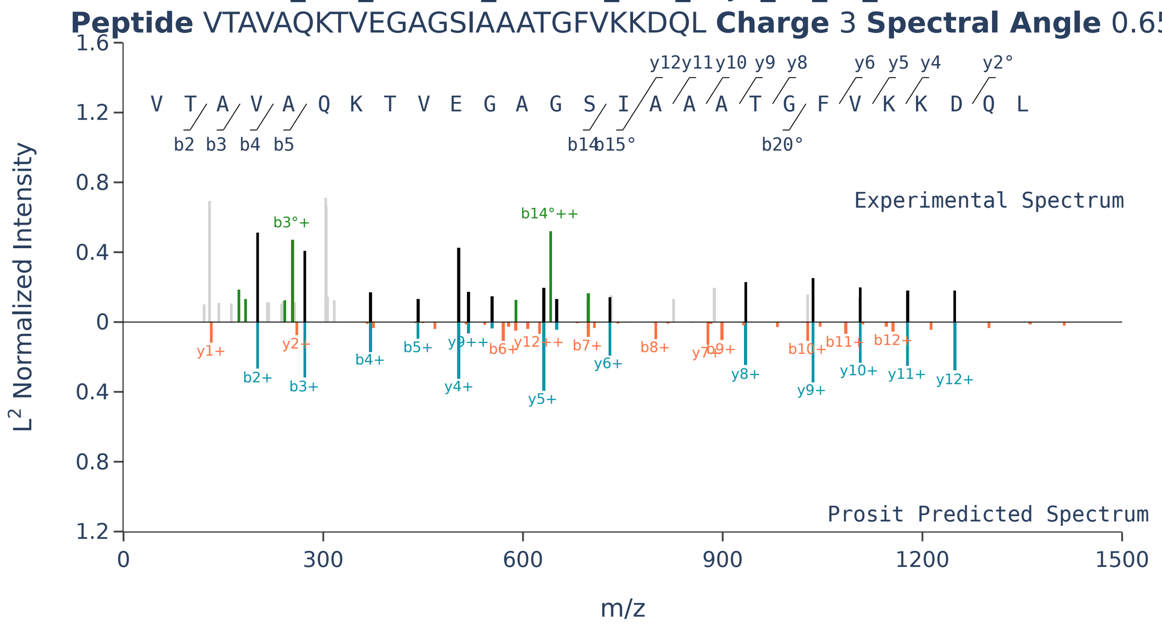


**Source** W_Soh_160821_170921_Goe_aSyn_A2_24h_R2 **Scan** 19490
**Peptide** GSIAAATGFVKKDQLGKNEEGAPQEGIL **Charge** 3 **Spectral Angle** 0.71

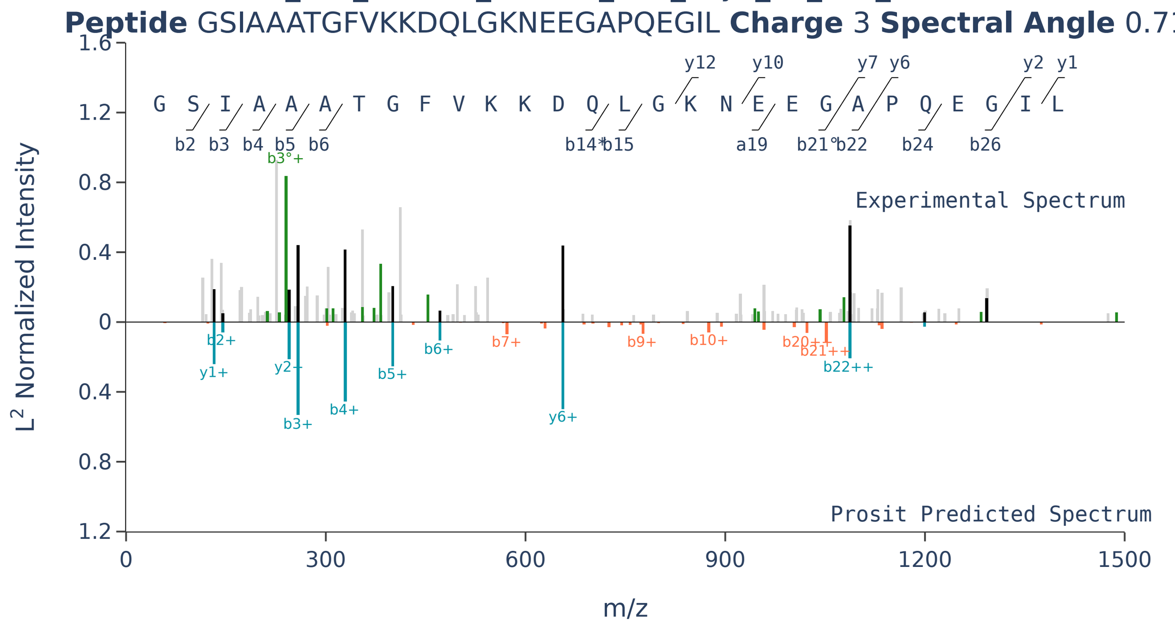


**Source** W_Soh_160821_170921_Goe_aSyn_A1_6h_R1 **Scan** 5096
**Peptide** TVAEKTKEQVTN **Charge** 2 **Spectral Angle** 0.72

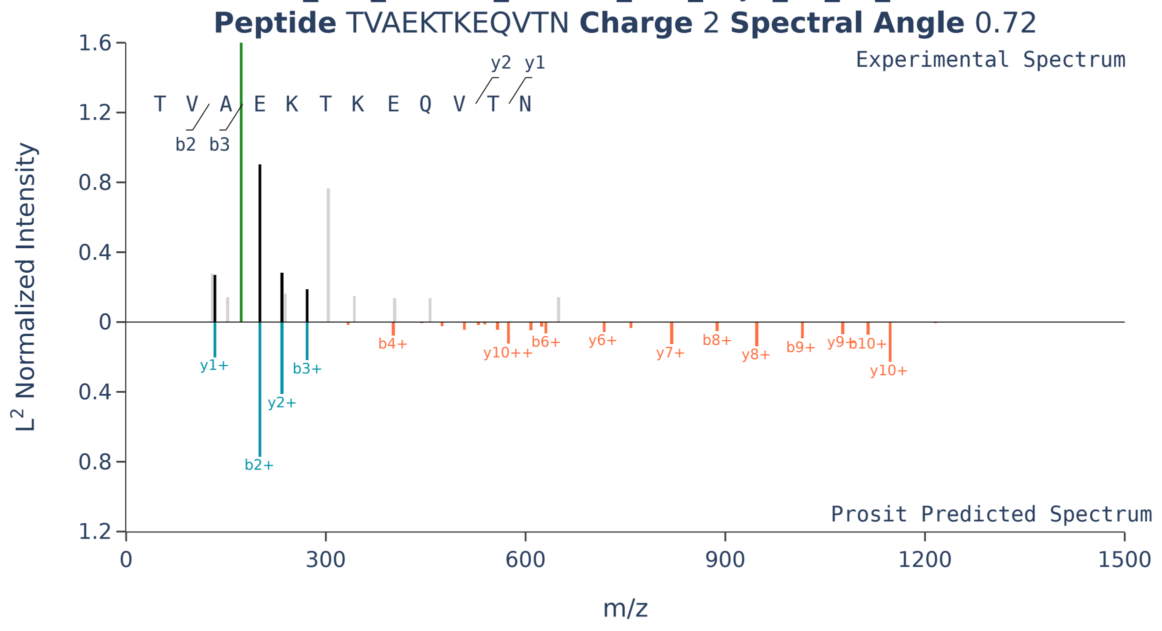


**Source** W_Soh_160821_170921_Goe_aSyn_A2_24h_R2 **Scan** 11204
**Peptide** GLSKAKEGVVAAAEKTKQGVAEA **Charge** 2 **Spectral Angle** 0.67

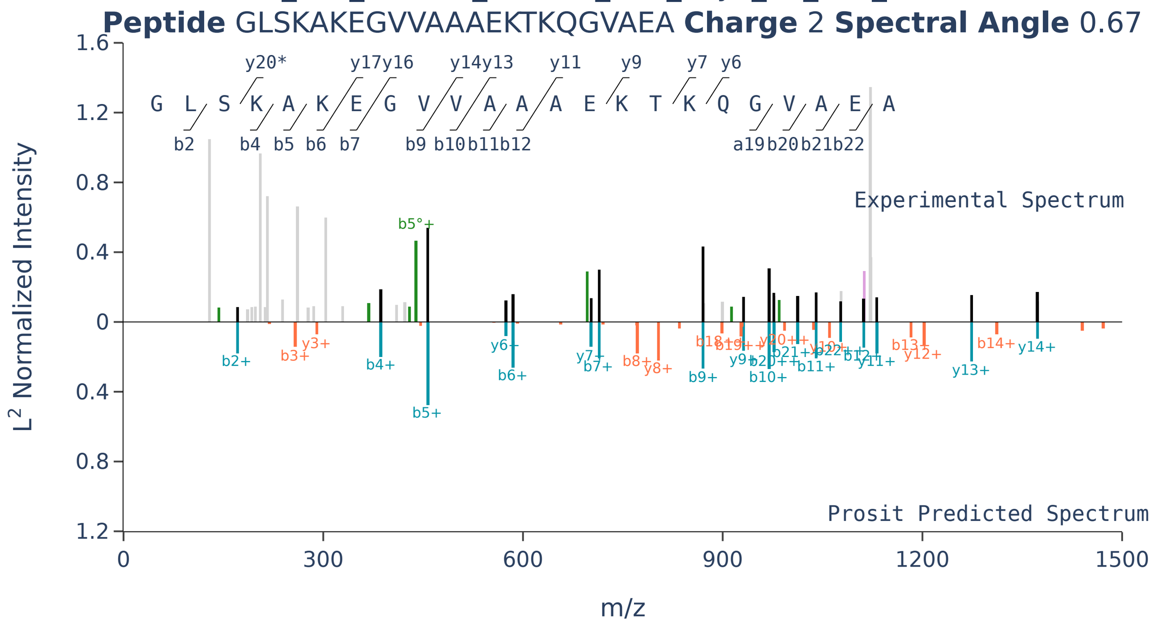


**Source** W_Soh_160821_170921_Goe_aSyn_A2_24h_R2 **Scan** 10945
**Peptide** FMKGLSKAKEGVVAAAEKTKQG **Charge** 4 **Spectral Angle** 0.71

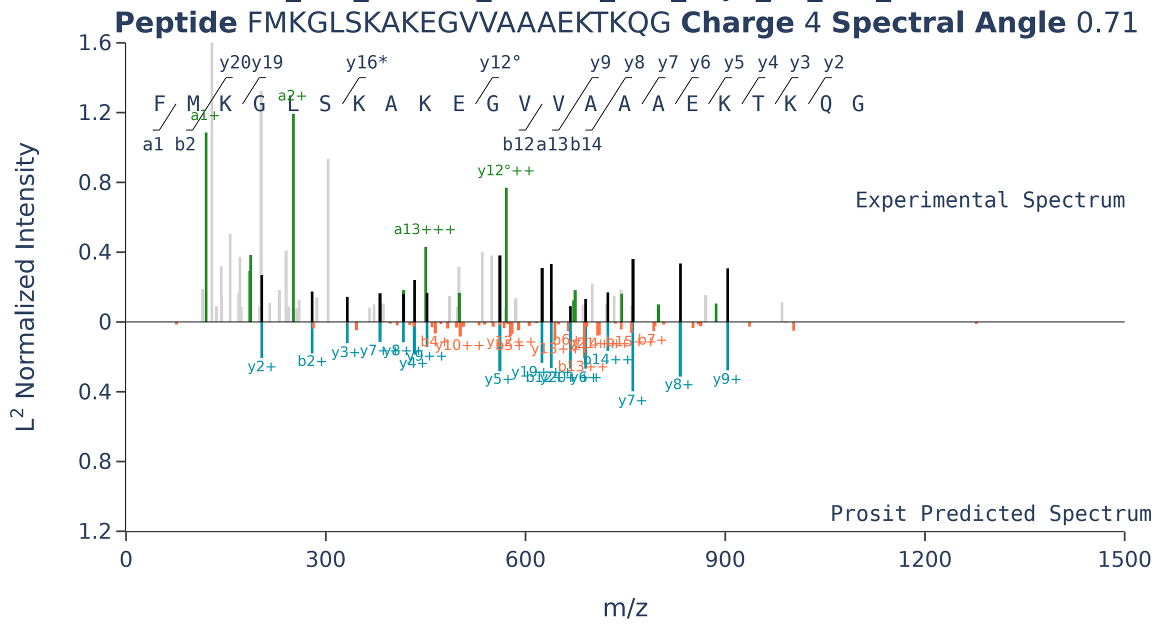


**Source** W_Soh_160821_170921_Goe_aSyn_A1_24h_R2 **Scan** 12996
**Peptide** FVKKDQLGKNEEGAPQEGILE **Charge** 4 **Spectral Angle** 0.79

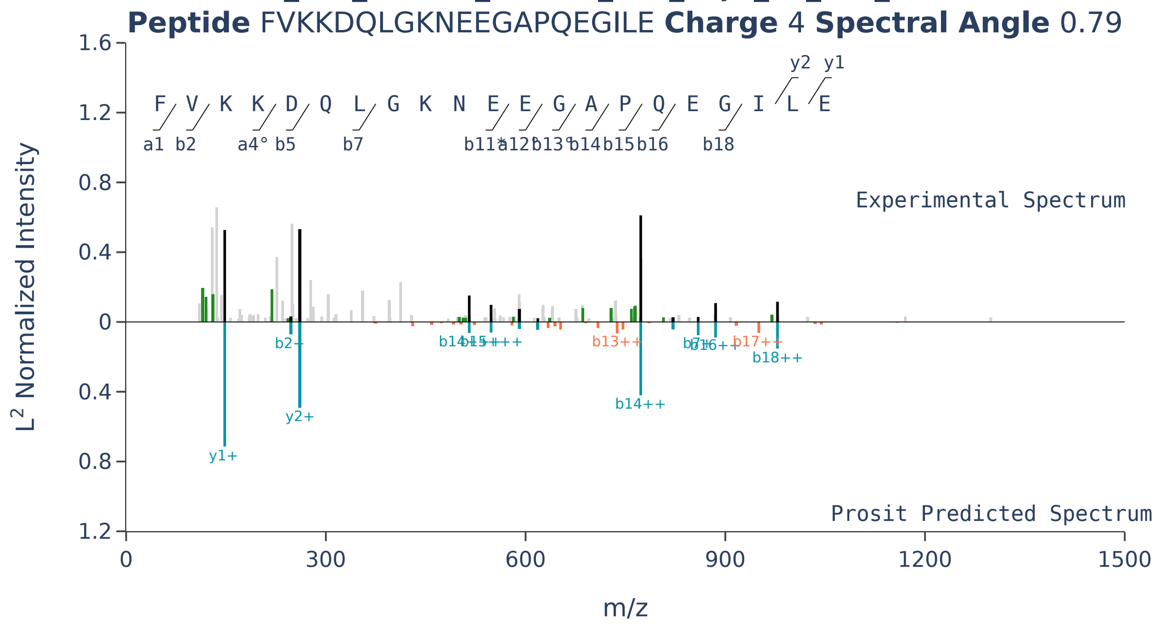

**Source** W_Soh_160821_170921_Goe_aSyn_A1_24h_R1 **Scan** 8972
**Peptide** EAAGKTKEGVLYVGSKT **Charge** 3 **Spectral Angle** 0.8

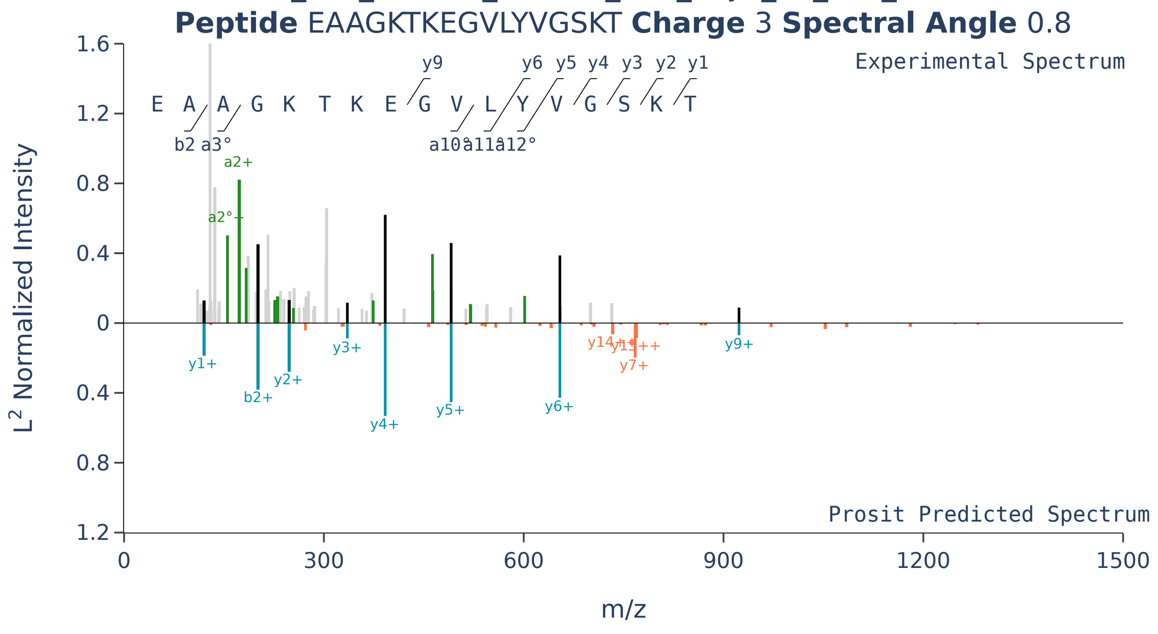


**Source** W_Soh_160821_170921_Goe_aSyn_A1_24h_R1 **Scan** 8041
**Peptide** VKKDQLGKNEEGAPQEGI **Charge** 3 **Spectral Angle** 0.77

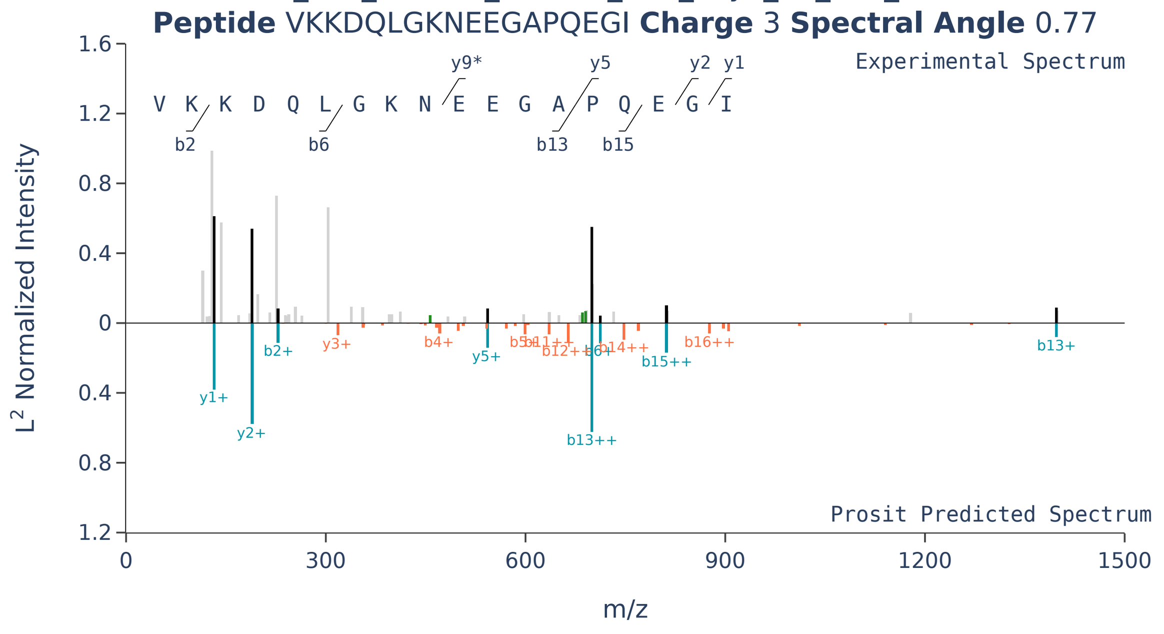


**Source** W_Soh_160821_170921_Goe_aSyn_A1_24h_R2 **Scan** 12600
**Peptide** GLSKAKEGVVAAAEKTKQGVAEAAGKTKEG **Charge** 5 **Spectral Angle** 0.59

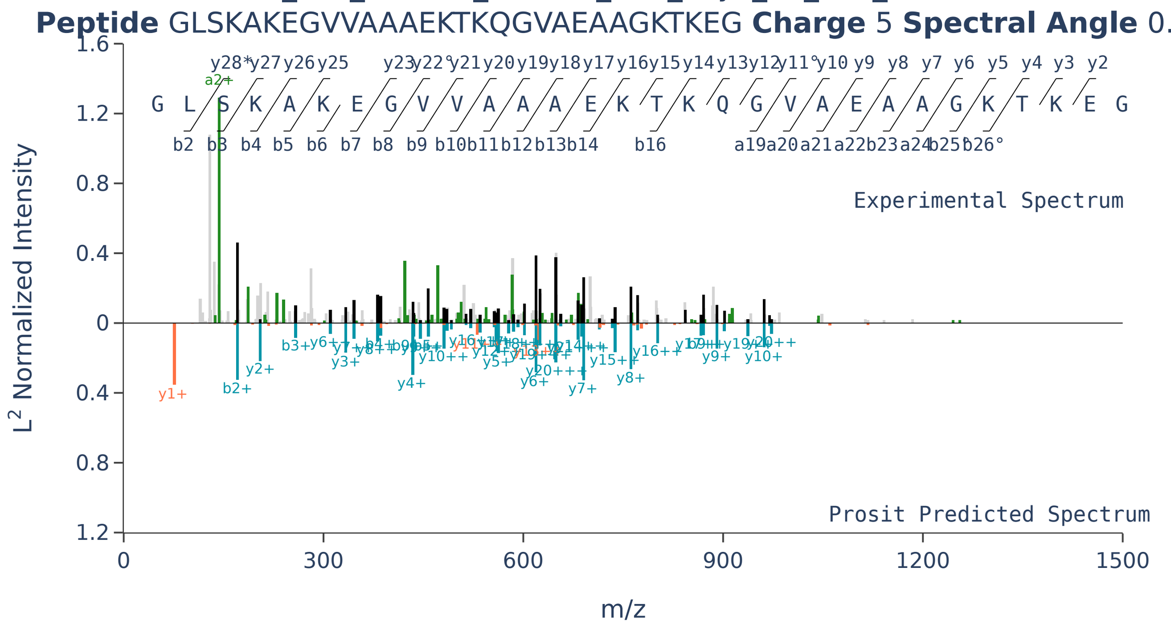


**Source** W_Soh_160821_170921_Goe_aSyn_A1_24h_R2 **Scan** 13157
**Peptide** KTKEGVVHGVATVAEKTKEQVTNVGG **Charge** 4 **Spectral Angle** 0.66

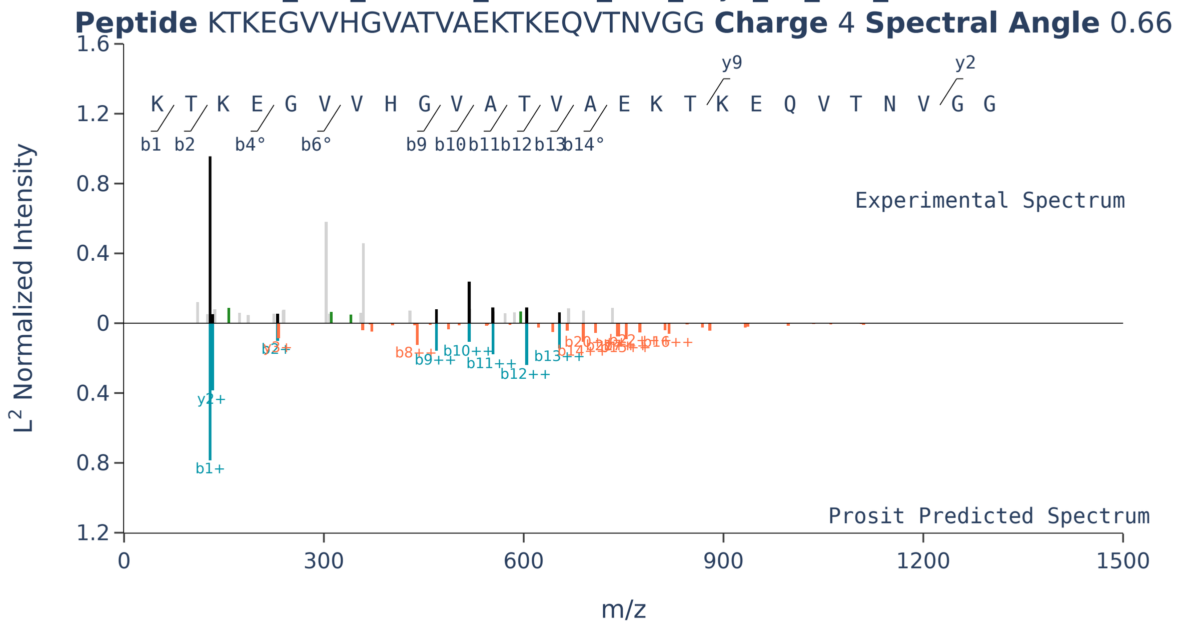


**Source** W_Soh_160821_170921_Goe_aSyn_A1_24h_R1 **Scan** 11514
**Peptide** TKQGVAEAAGKTKEGVLYVGSKTKEGVVHG **Charge** 5 **Spectral Angle** 0.64

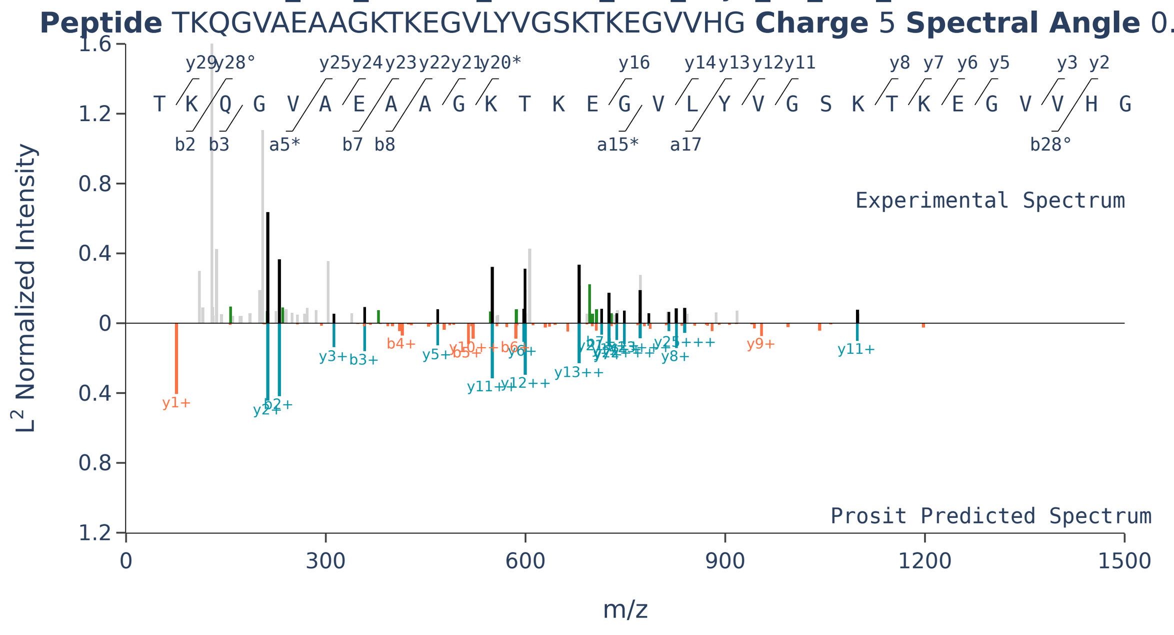


**Source** W_Soh_160821_170921_Goe_aSyn_A1_24h_R2 **Scan** 8652
**Peptide** YVGSKTKEGVVHGVATVAEK **Charge** 4 **Spectral Angle** 0.69

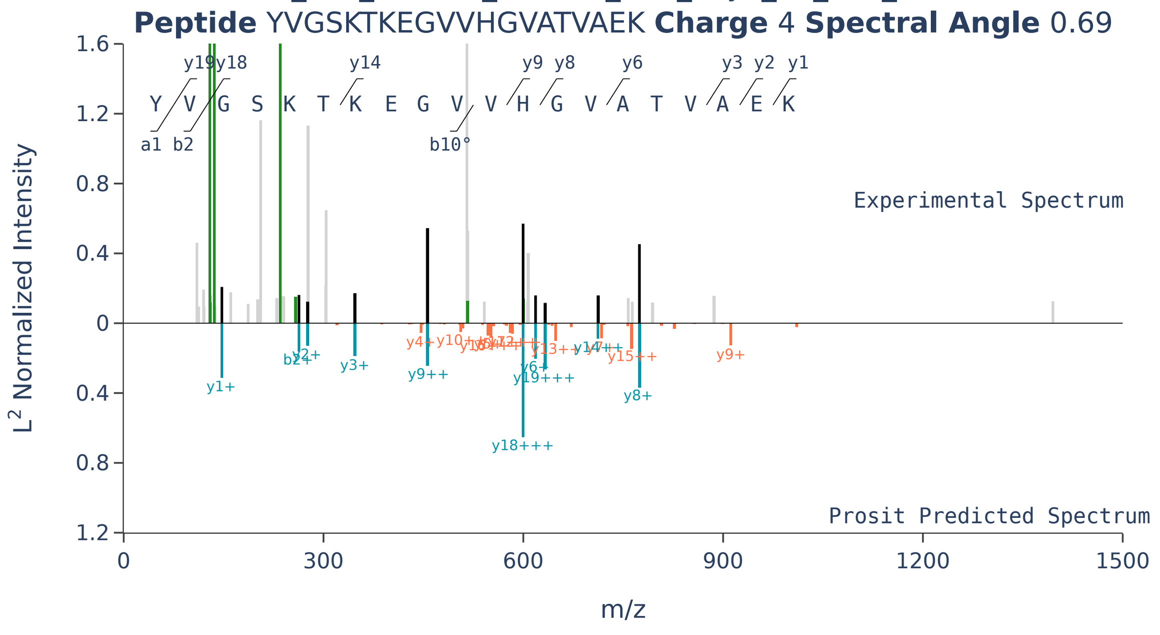


**Source** W_Soh_160821_170921_Goe_aSyn_A2_24h_R2 **Scan** 14723
**Peptide** VVAAAEKTKQGVAEAAGKTKEGVLYV **Charge** 3 **Spectral Angle** 0.86

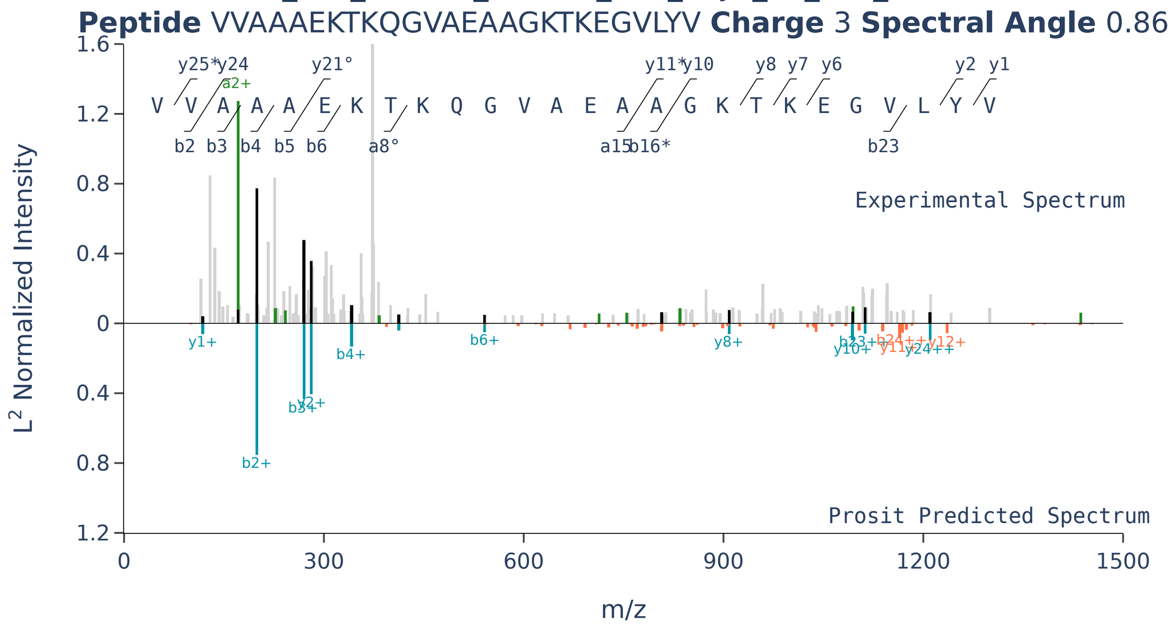


**Source** W_Soh_160821_170921_Goe_aSyn_A1_24h_R2 **Scan** 4907
**Peptide** AEKTKQGVAEAAGKTKEG **Charge** 2 **Spectral Angle** 0.62

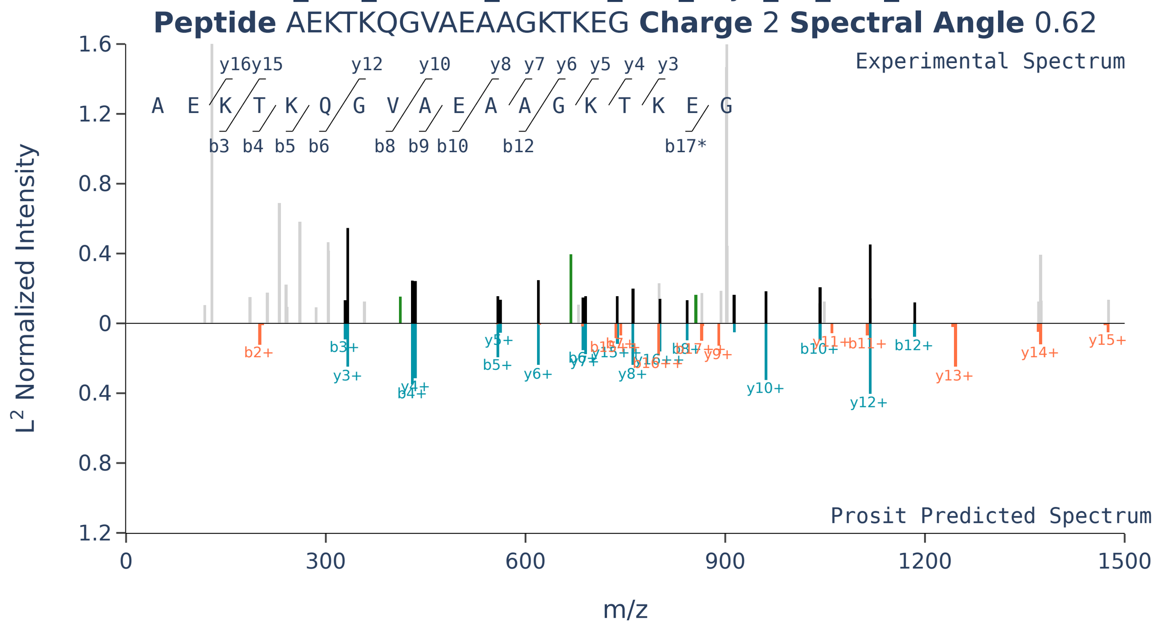


**Source** W_Soh_160821_170921_Goe_aSyn_A1_24h_R1 **Scan** 7559
**Peptide** AEKTKQGVAEAAGKTKEGVL **Charge** 3 **Spectral Angle** 0.59

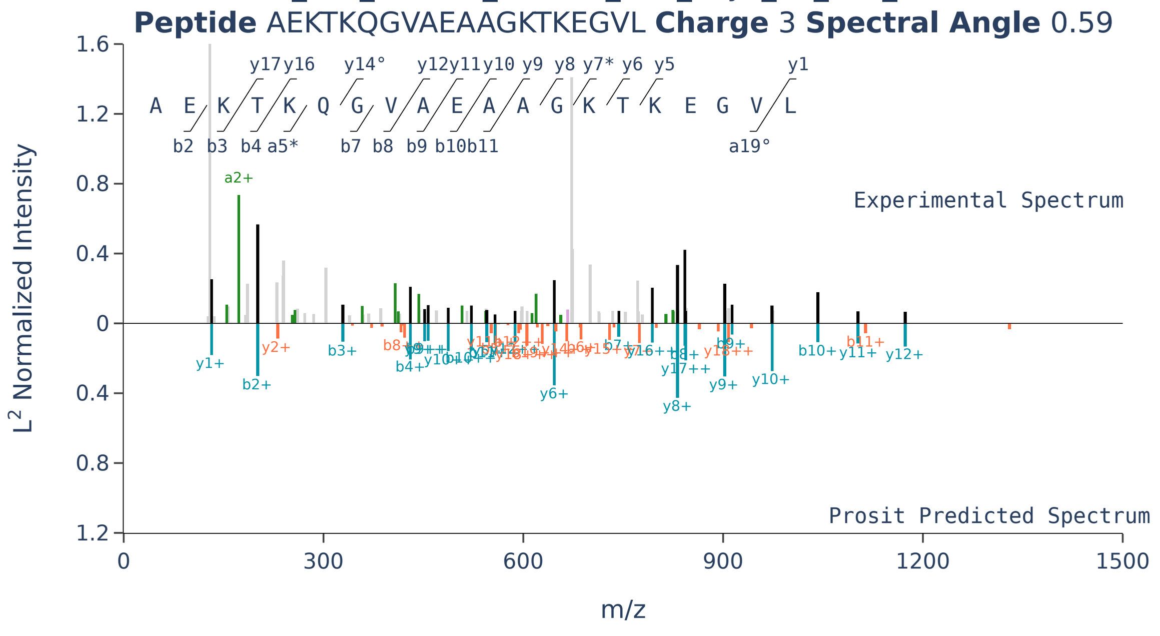


**Source** W_Soh_160821_170921_Goe_aSyn_A1_6h_R1 **Scan** 21094
**Peptide** VHGVATVAEKTKEQVTNVGGAVVTGVTAV **Charge** 3 **Spectral Angle** 0.67

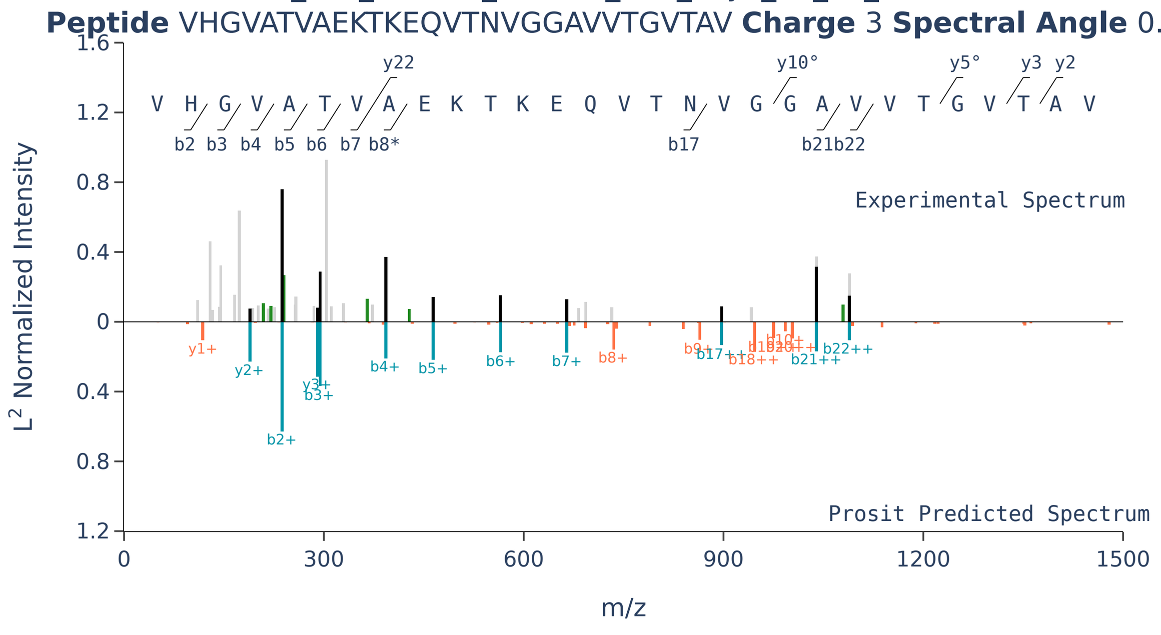


**Source** W_Soh_160821_170921_Goe_aSyn_A1_24h_R1 **Scan** 17673
**Peptide** GAGSIAAATGFVKKDQLGKNEEGAPQEGL **Charge** 3 **Spectral Angle** 0.8

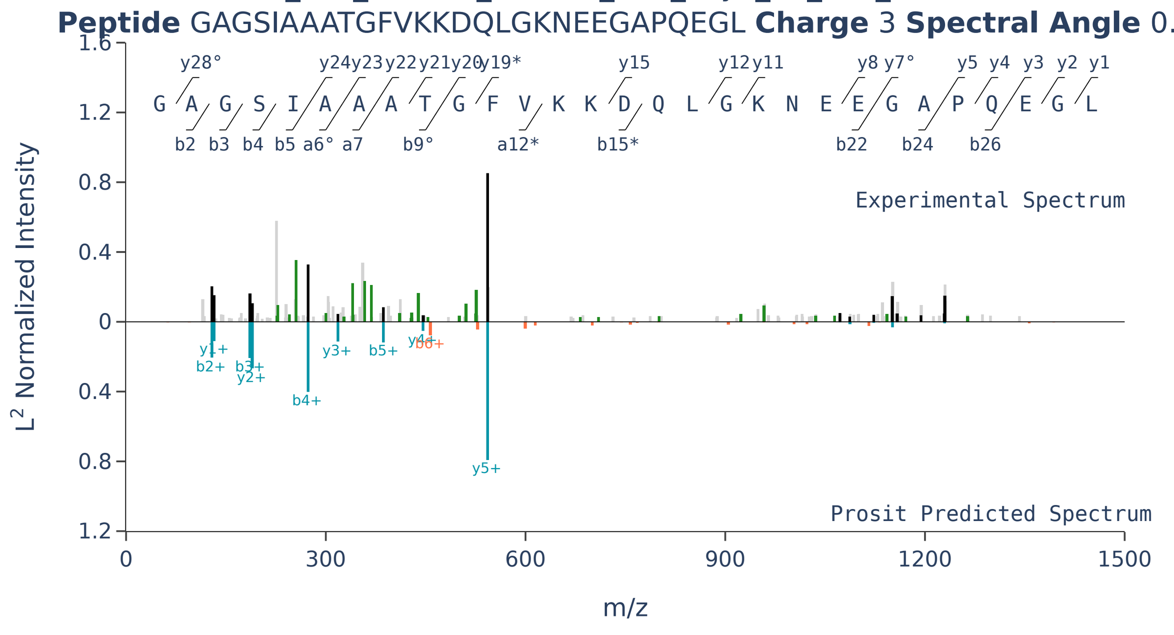


**Source** W_Soh_160821_170921_Goe_aSyn_A1_24h_R1 **Scan** 5817
**Peptide** KAKEGVVAAAEKT **Charge** 2 **Spectral Angle** 0.82

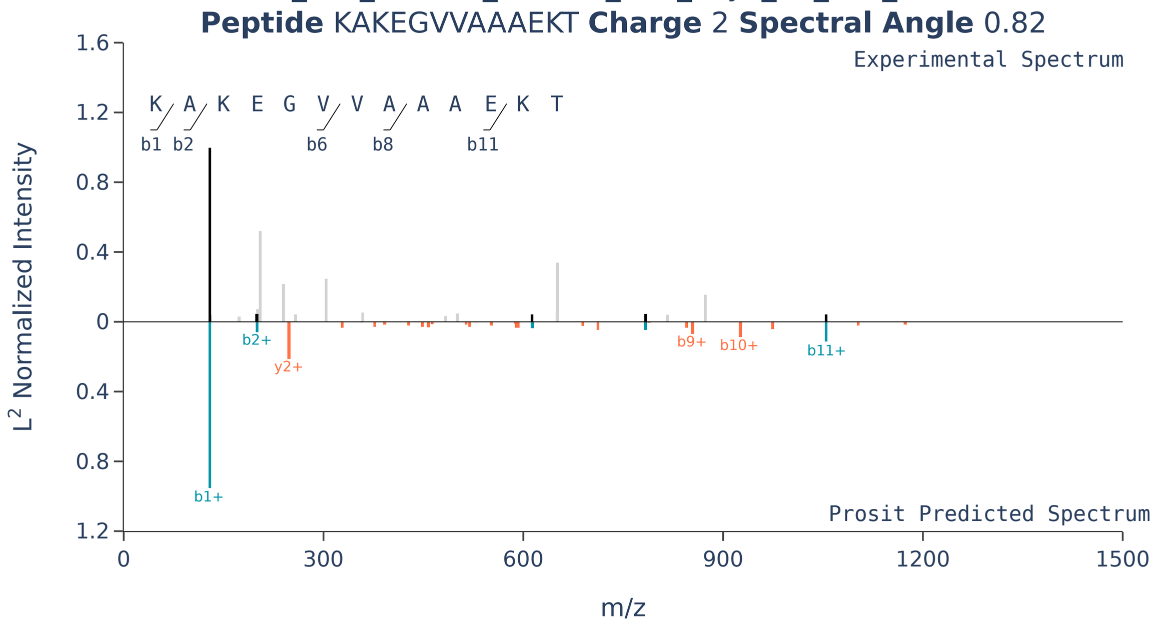


**Source** W_Soh_160821_170921_Goe_aSyn_A2_24h_R1 **Scan** 19176
**Peptide** SIAAATGFVKKDQLGKNEEGAPQEGIL **Charge** 4 **Spectral Angle** 0.78

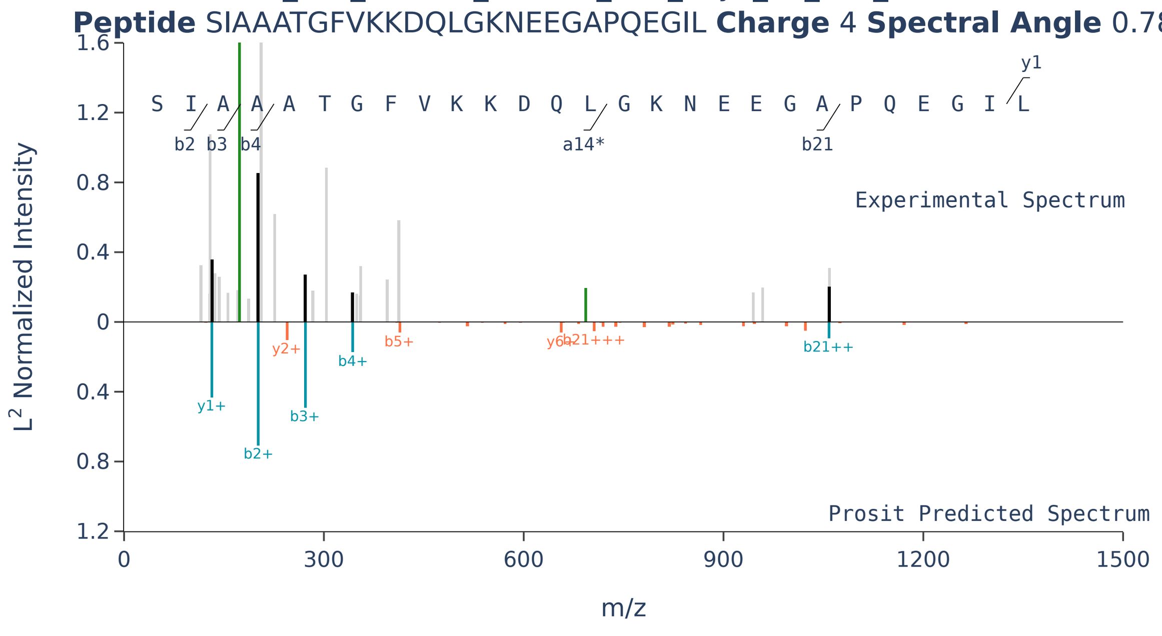


**Source** W_Soh_160821_170921_Goe_aSyn_A1_24h_R2 **Scan** 11972
**Peptide** VKKDQLGKNEEGAPQEGIL **Charge** 3 **Spectral Angle** 0.8

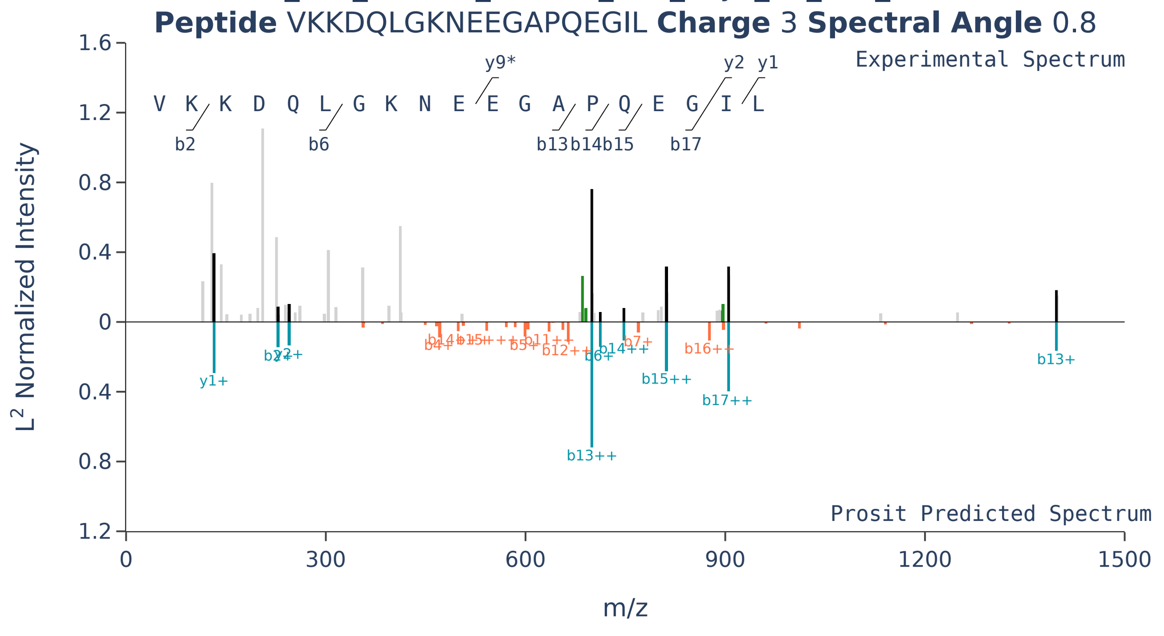


**Source** W_Soh_160821_170921_Goe_aSyn_A2_1h_R2 **Scan** 10580
**Peptide** AEAAGKTKEGVLYVG **Charge** 3 **Spectral Angle** 0.9

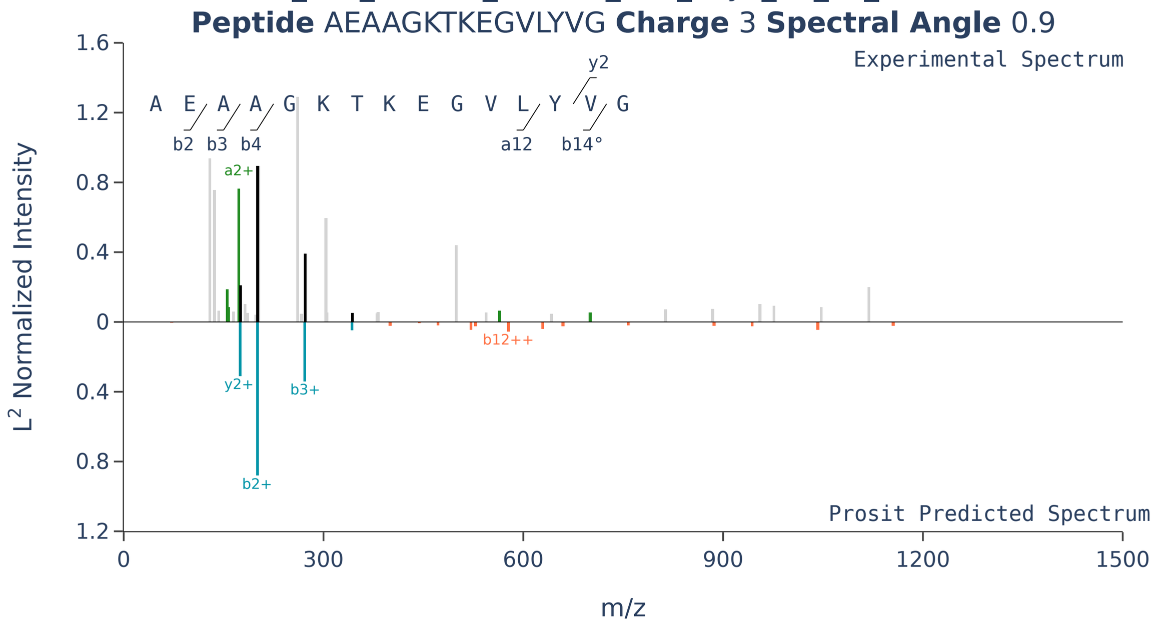

**Source** W_Soh_160821_170921_Goe_aSyn_A1_24h_R1 **Scan** 17158
**Peptide** DVFMKGLSKAKEGVVAAAEK **Charge** 3 **Spectral Angle** 0.74

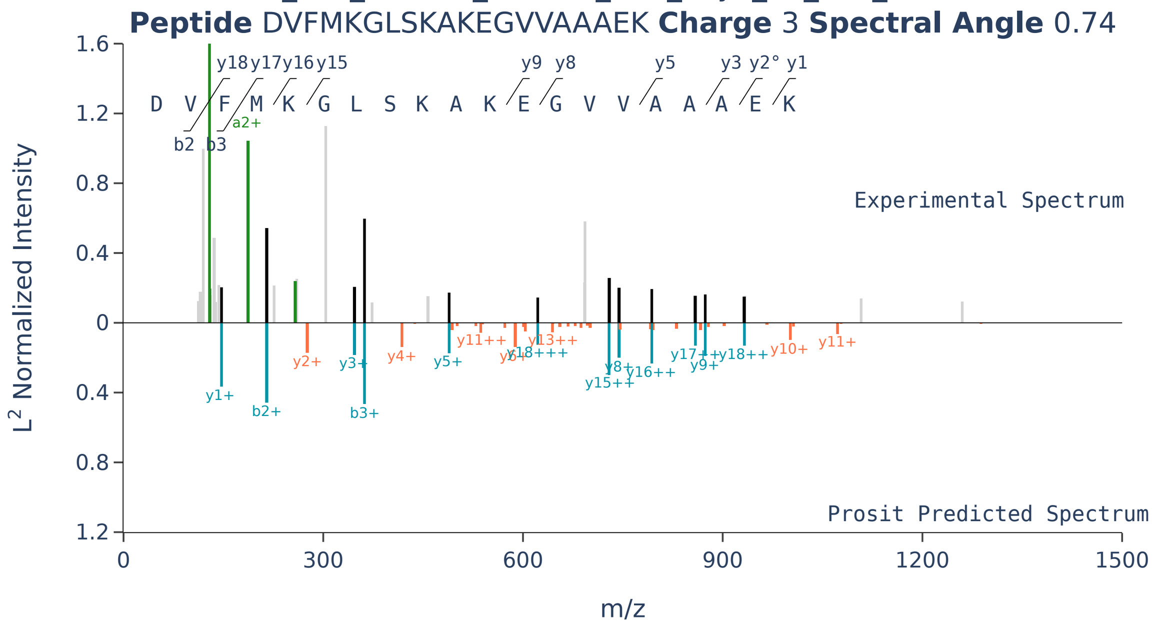


**Source** W_Soh_160821_170921_Goe_aSyn_A1_24h_R2 **Scan** 15452
**Peptide** AQKTVEGAGSIAAATGFVKKDQLGKN **Charge** 5 **Spectral Angle** 0.69

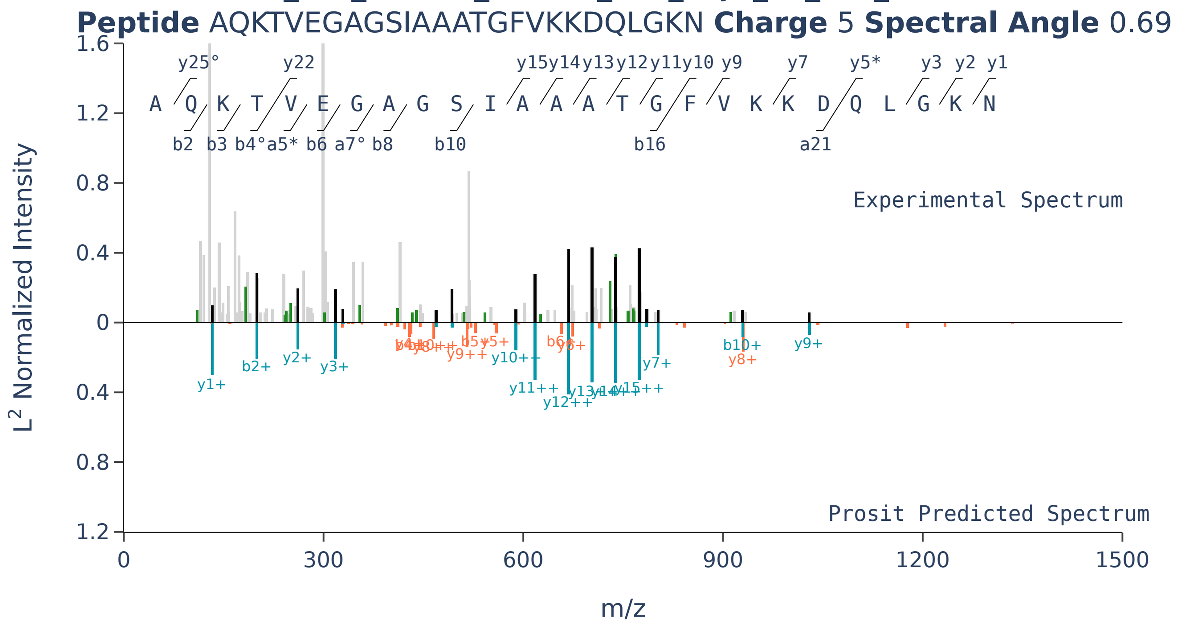


**Source** W_Soh_160821_170921_Goe_aSyn_A1_24h_R1 **Scan** 22950
**Peptide** TGVTAVAQKTVEGAGSIAAATGFVKKDQ **Charge** 3 **Spectral Angle** 0.65

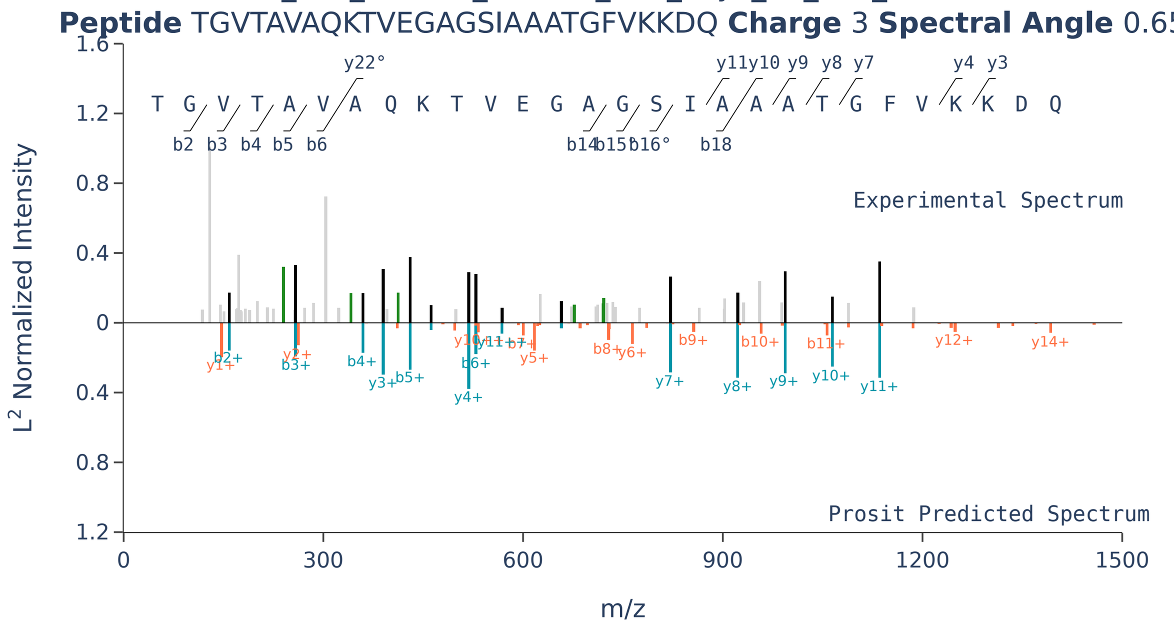


**Source** W_Soh_160821_170921_Goe_aSyn_A2_24h_R2 **Scan** 18355
**Peptide** DVFMKGLSKAKEGVVAAAE **Charge** 3 **Spectral Angle** 0.84

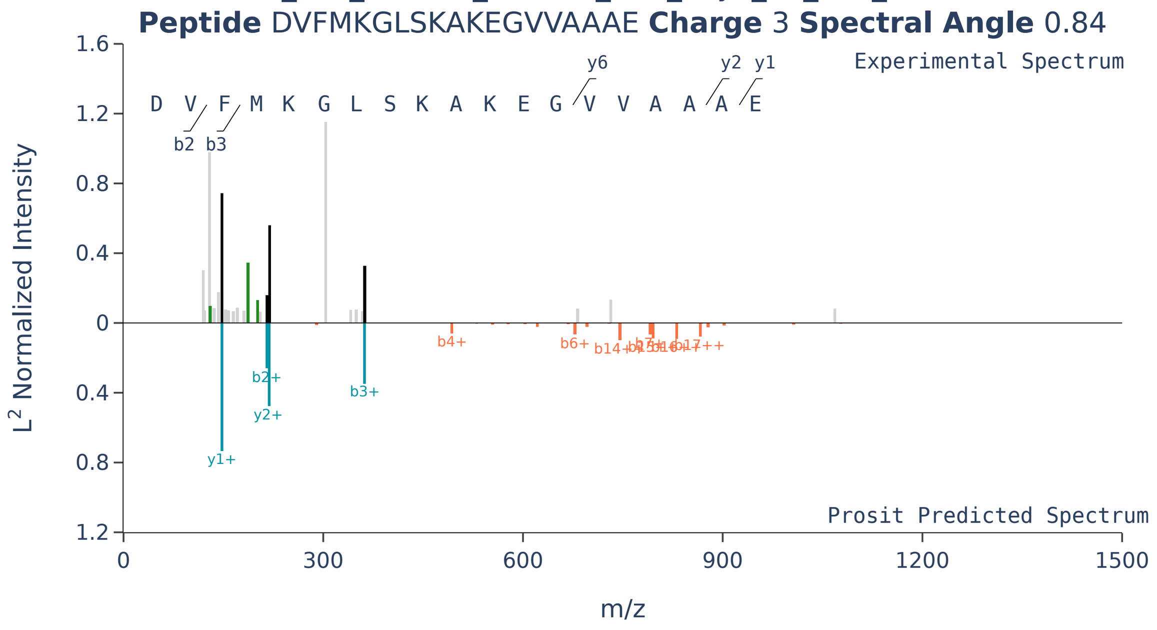


**Source** W_Soh_160821_170921_Goe_aSyn_A1_24h_R2 **Scan** 7924
**Peptide** AAEKTKQGVAEAAGKTKEGVL **Charge** 3 **Spectral Angle** 0.7

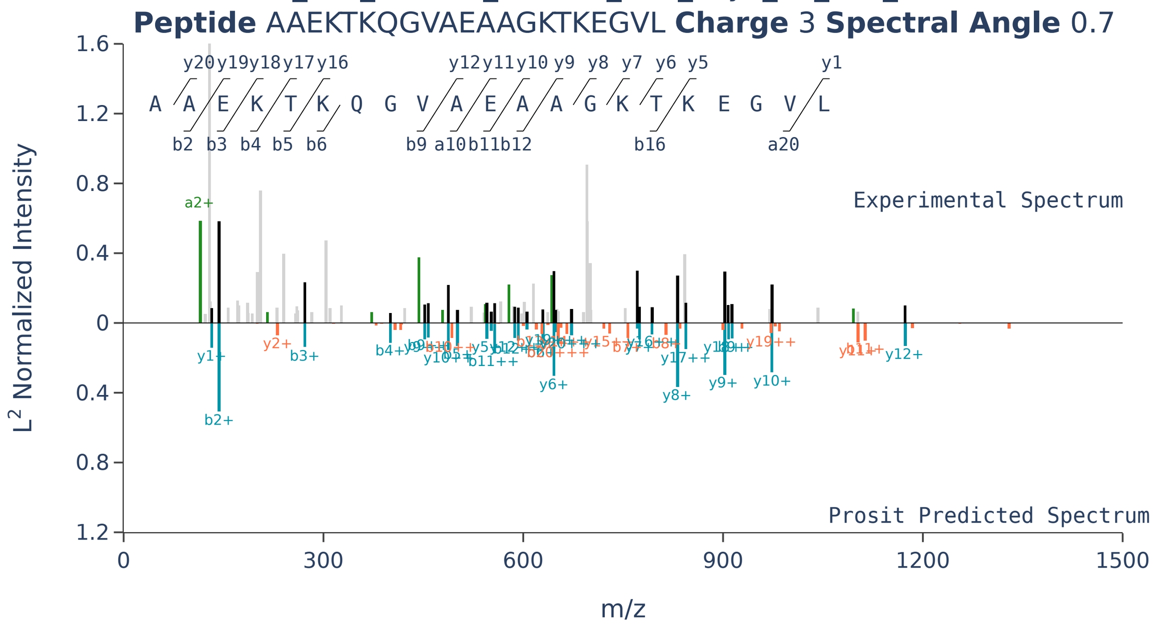


**Source** W_Soh_160821_170921_Goe_aSyn_A1_24h_R1 **Scan** 5819
**Peptide** QLGKNEEGAPQ **Charge** 2 **Spectral Angle** 0.83

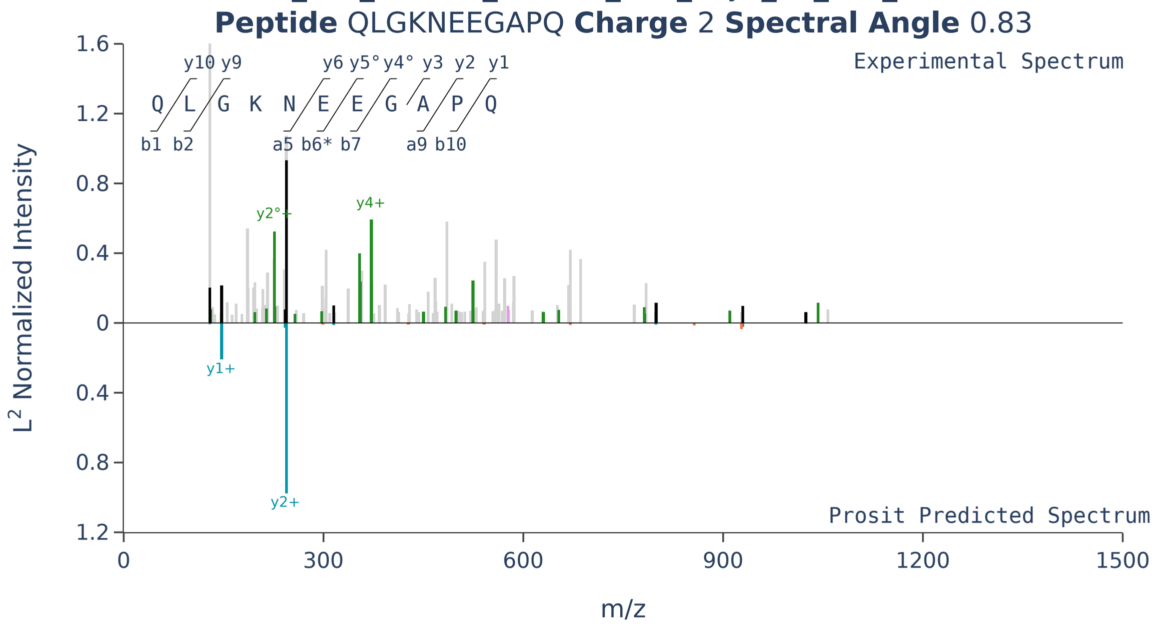


**Source** W_Soh_160821_170921_Goe_aSyn_A1_3h_R1 **Scan** 5231
**Peptide** AAAEKTKQGVAEAA **Charge** 2 **Spectral Angle** 0.69

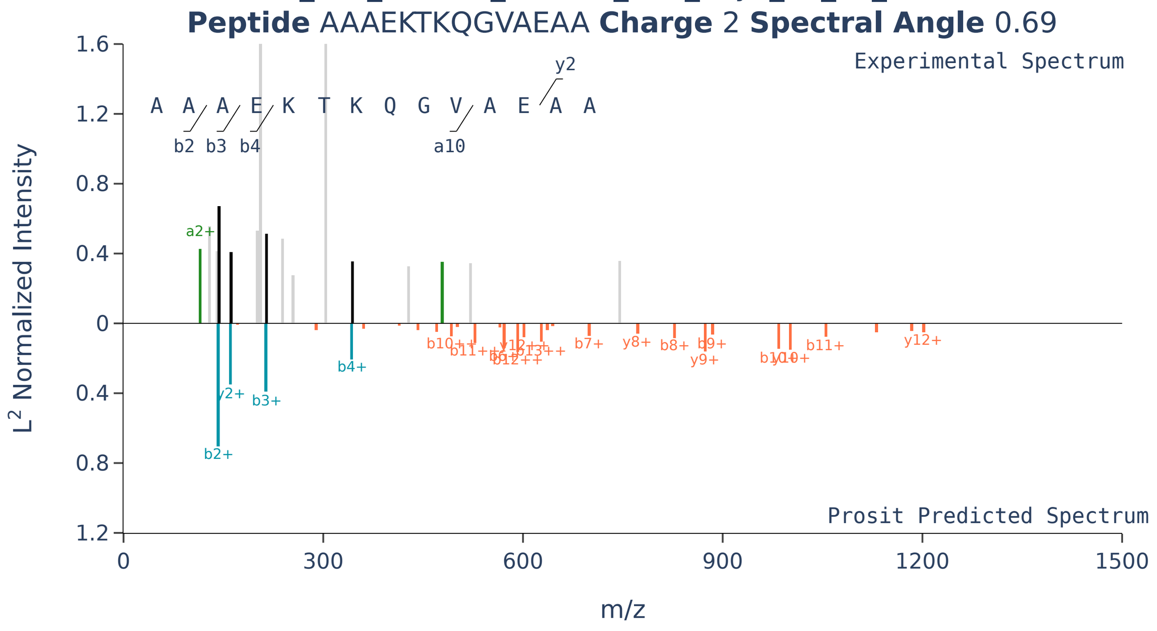


**Source** W_Soh_160821_170921_Goe_aSyn_A2_24h_R1 **Scan** 18508
**Peptide** VFMKGLSKAKEGVVA **Charge** 3 **Spectral Angle** 0.77

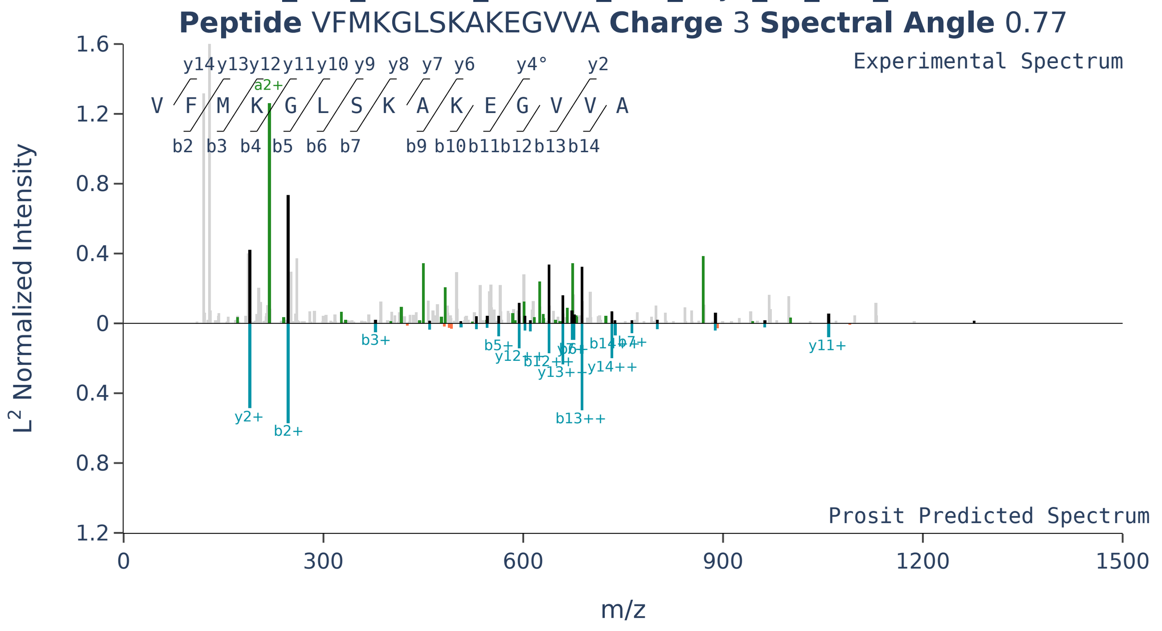


**Source** W_Soh_160821_170921_Goe_aSyn_A1_24h_R2 **Scan** 10148
**Peptide** AAGKTKEGVLYVGSKTKEGVVHGVA **Charge** 4 **Spectral Angle** 0.71

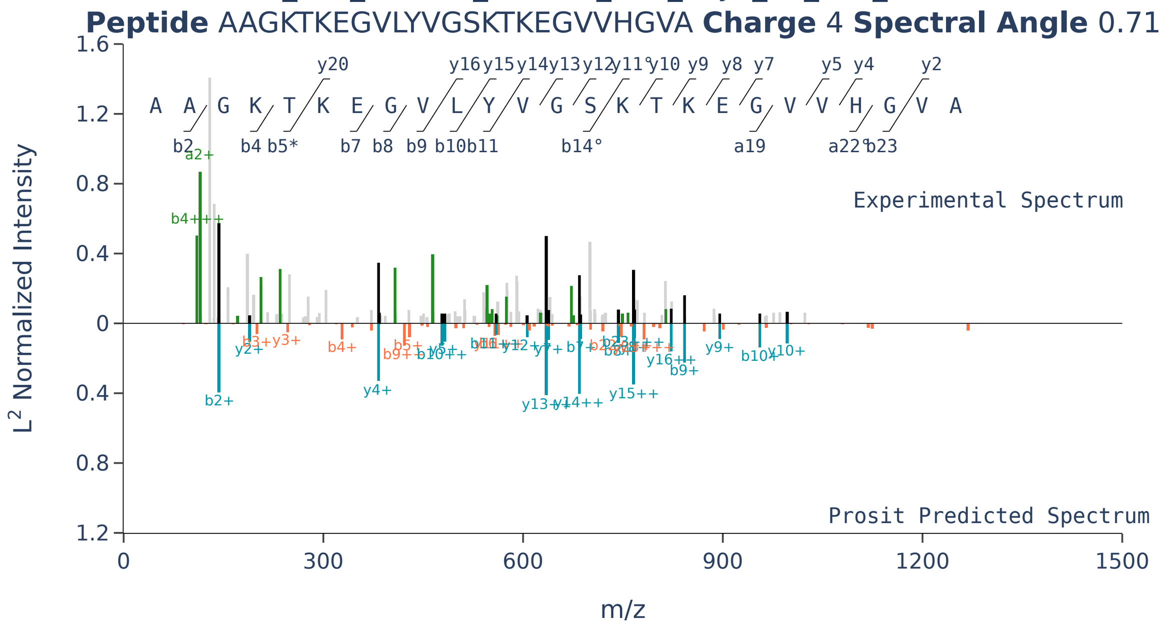


**Source** W_Soh_160821_170921_Goe_aSyn_A2_24h_R1 **Scan** 16660
**Peptide** GSIAAATGFVKKDQLGKNEEGAPQEGI **Charge** 3 **Spectral Angle** 0.82

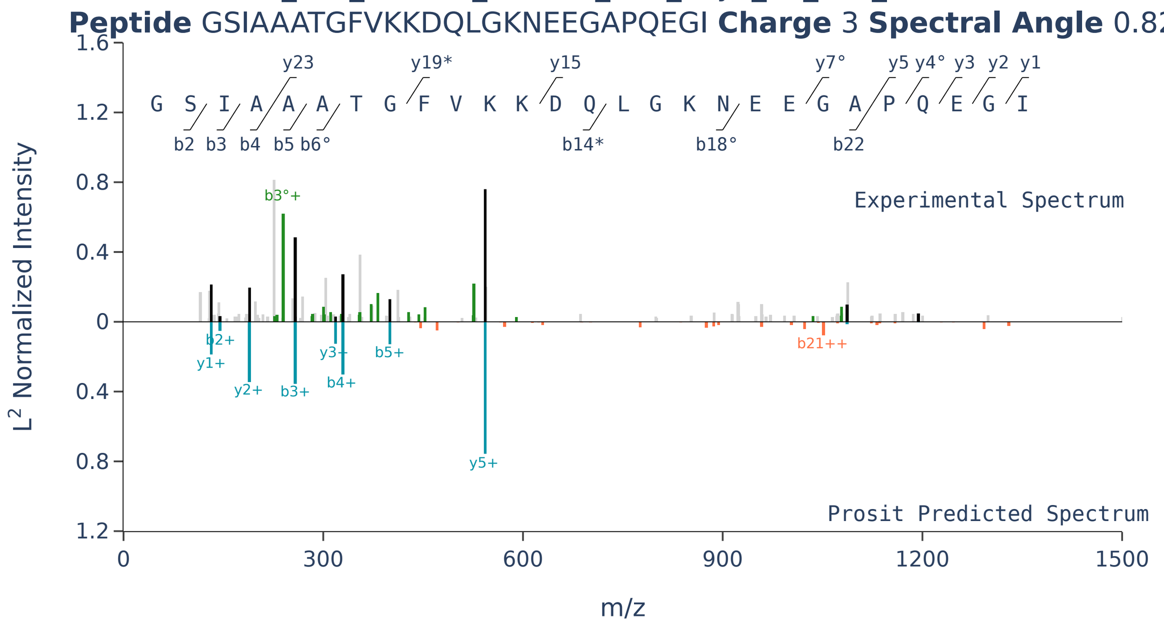


**Source** W_Soh_160821_170921_Goe_aSyn_A1_24h_R1 **Scan** 11100
**Peptide** VGSKTKEGVVHGVATVAEKTKEQV **Charge** 4 **Spectral Angle** 0.62

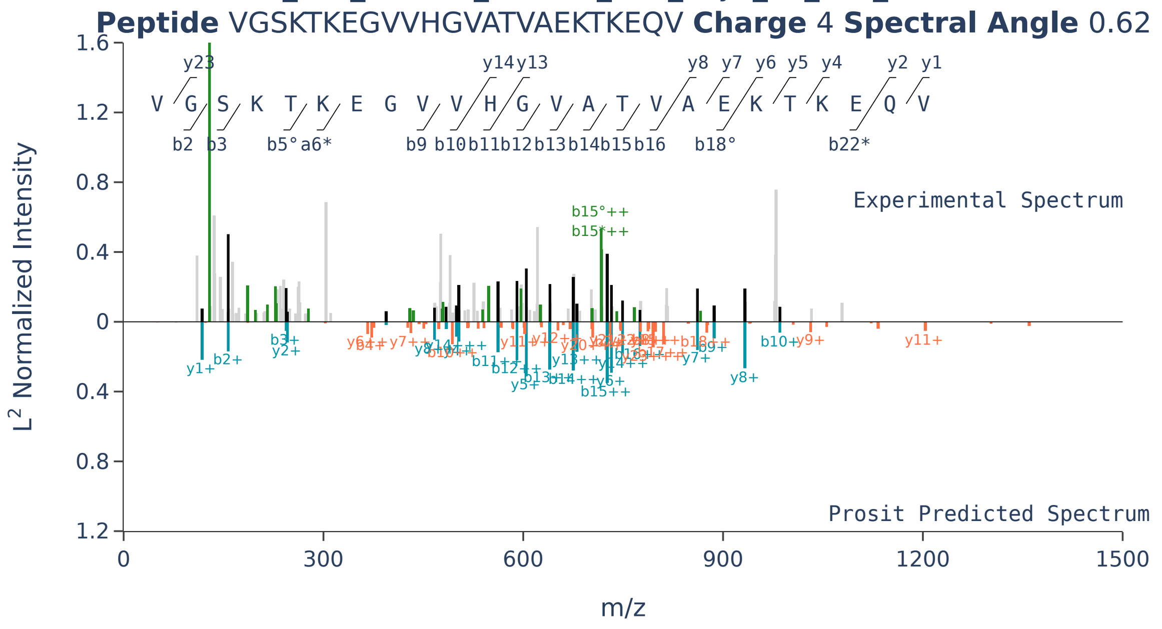


**Source** W_Soh_160821_170921_Goe_aSyn_A2_24h_R2 **Scan** 16929
**Peptide** AGSIAAATGFVKKDQLGKNEEGAPQEGI **Charge** 3 **Spectral Angle** 0.75

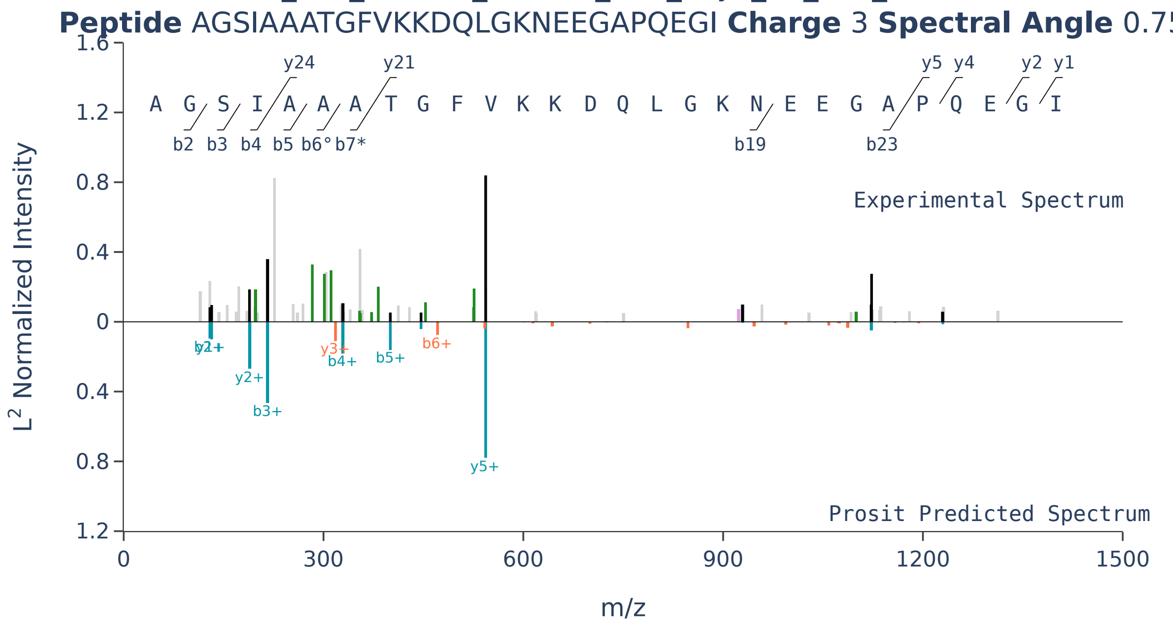


**Source** W_Soh_160821_170921_Goe_aSyn_A2_24h_R1 **Scan** 16856
**Peptide** GVTAVAQKTVEGAGSI **Charge** 2 **Spectral Angle** 0.7

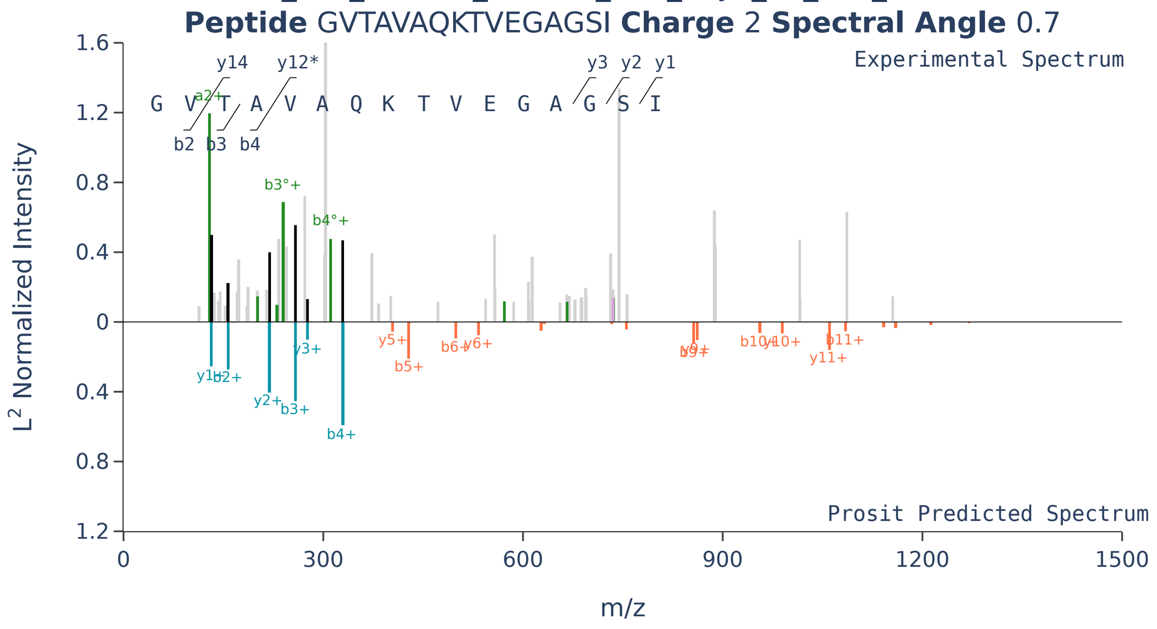


**Source** W_Soh_160821_170921_Goe_aSyn_A1_6h_R1 **Scan** 22144
**Peptide** PVDPDNEAYEMPSEEGYQDYEPEA **Charge** 3 **Spectral Angle** 0.85

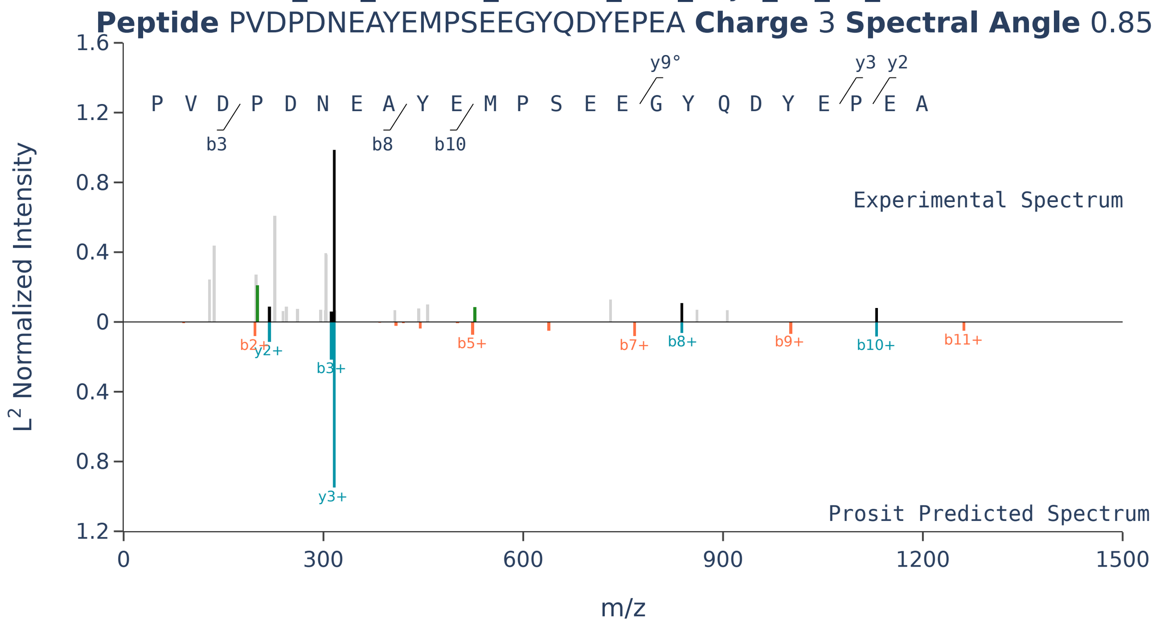


**Source** W_Soh_160821_170921_Goe_aSyn_A1_24h_R1 **Scan** 15767
**Peptide** VLYVGSKTKEGVVHGVATVAEKTKEQV **Charge** 5 **Spectral Angle** 0.66

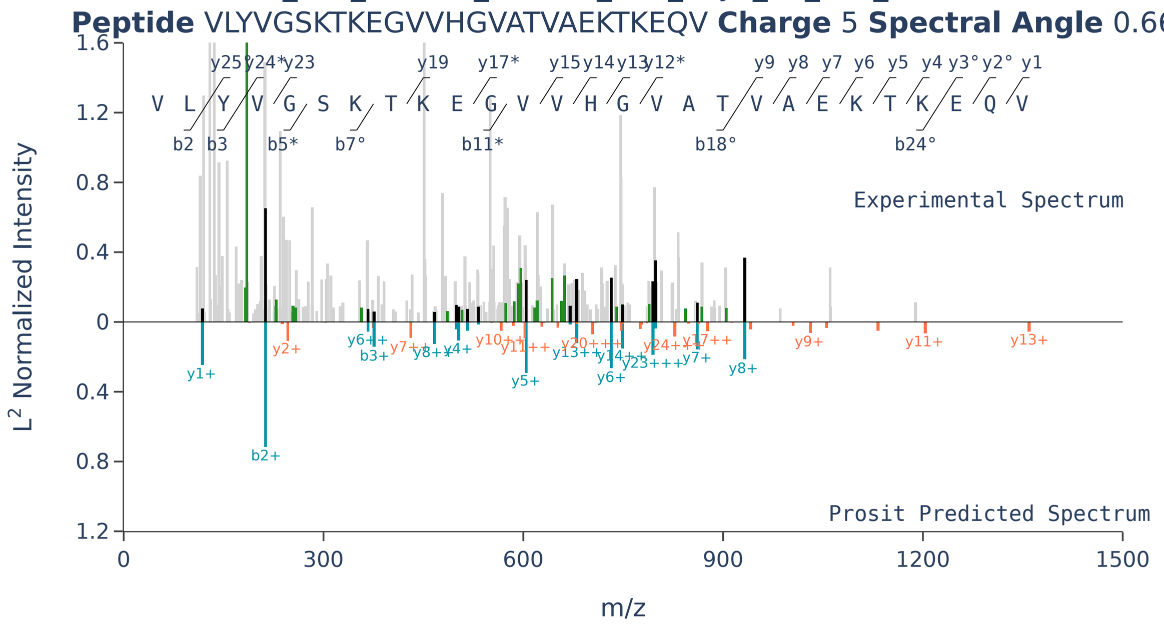

**Source** W_Soh_160821_170921_Goe_aSyn_A1_6h_R1 **Scan** 19637
**Peptide** EGVVHGVATVAEKTKEQVTNVGGAVVT **Charge** 4 **Spectral Angle** 0.72

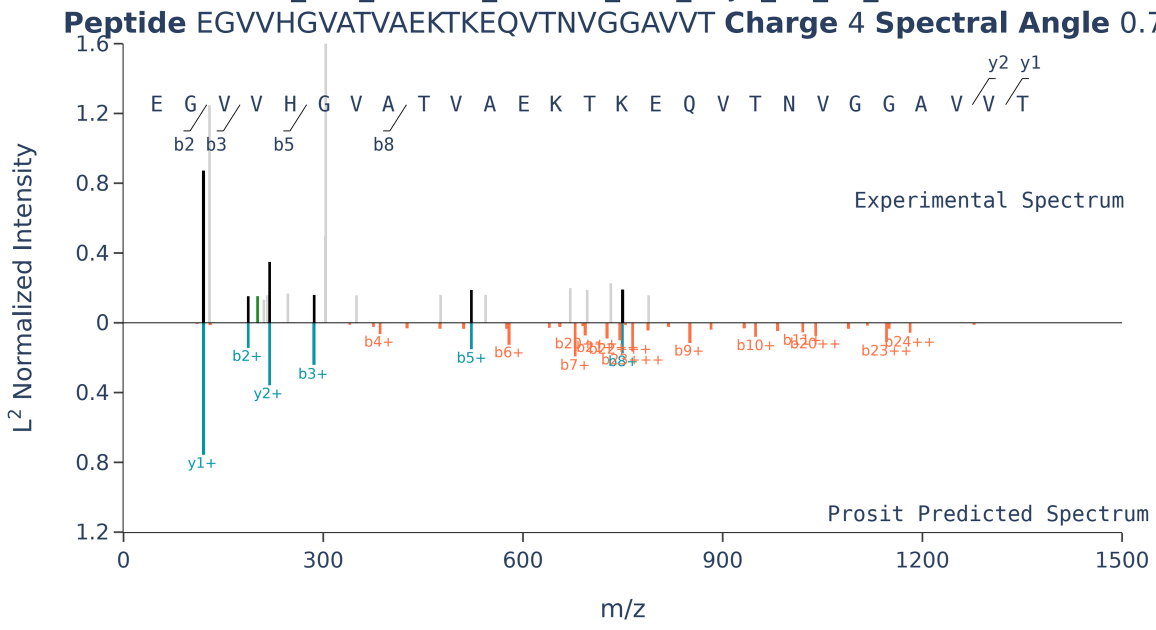


**Source** W_Soh_160821_170921_Goe_aSyn_A2_24h_R1 **Scan** 8108
**Peptide** GLSKAKEGVVAAA **Charge** 2 **Spectral Angle** 0.62

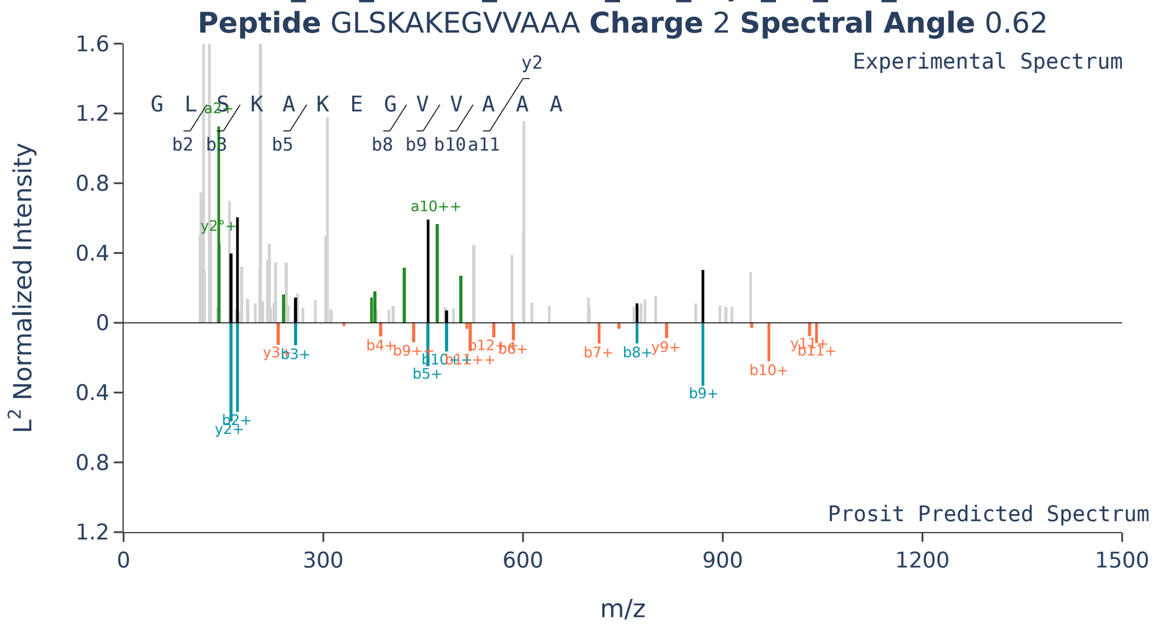


**Source** W_Soh_160821_170921_Goe_aSyn_A1_24h_R1 **Scan** 19361
**Peptide** KTVEGAGSIAAATGF **Charge** 2 **Spectral Angle** 0.36

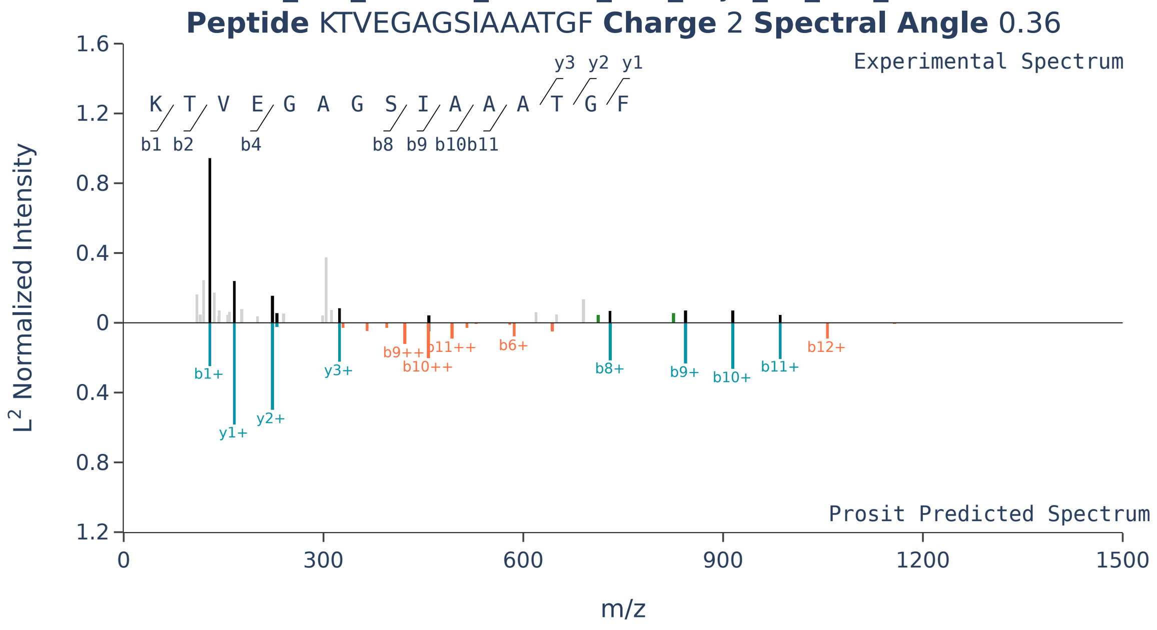


**Source** W_Soh_160821_170921_Goe_aSyn_A1_24h_R1 **Scan** 10655
**Peptide** SKTKEGVVHGVATVAEKTKEQVTN **Charge** 4 **Spectral Angle** 0.7

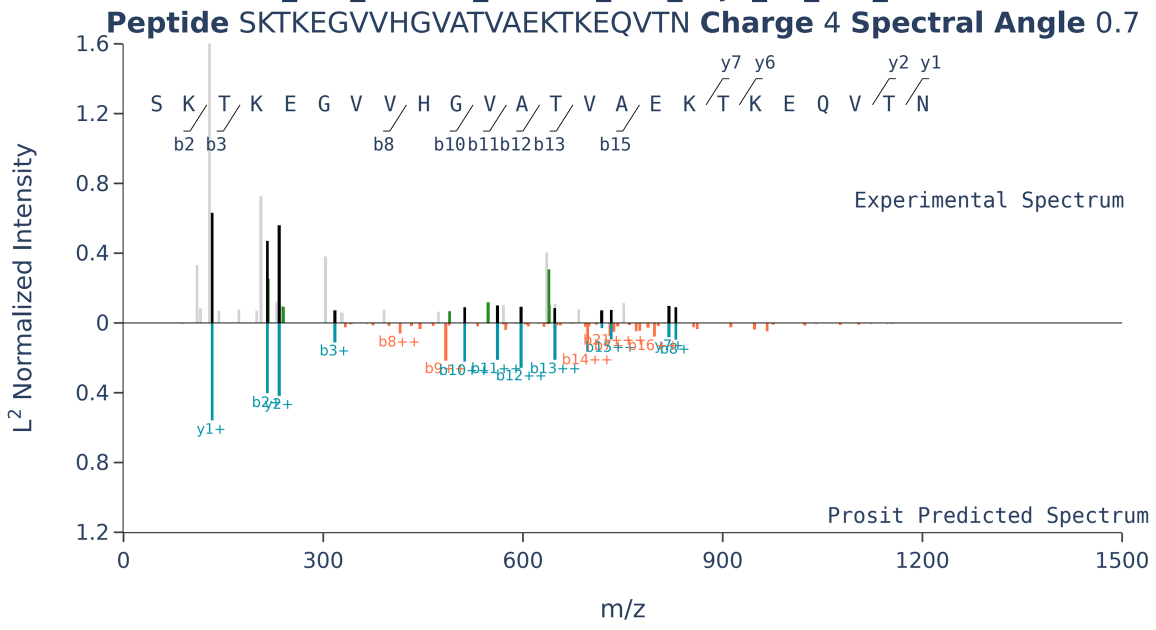


**Source** W_Soh_160821_170921_Goe_aSyn_A1_6h_R2 **Scan** 16455
**Peptide** AVAQKTVEGAGSIAAATGFVKKDQLGKNEE **Charge** 4 **Spectral Angle** 0.58

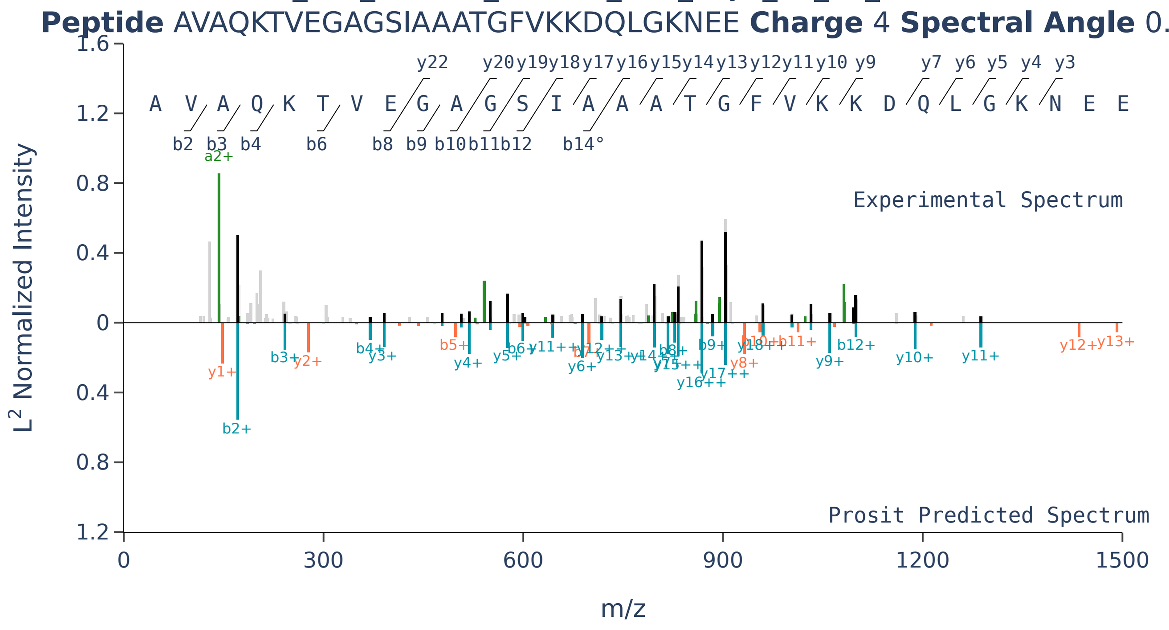


**Source** W_Soh_160821_170921_Goe_aSyn_A2_24h_R2 **Scan** 24716
**Peptide** KEQVTNVGGAVVTGVTAVAQKTVEG **Charge** 3 **Spectral Angle** 0.72

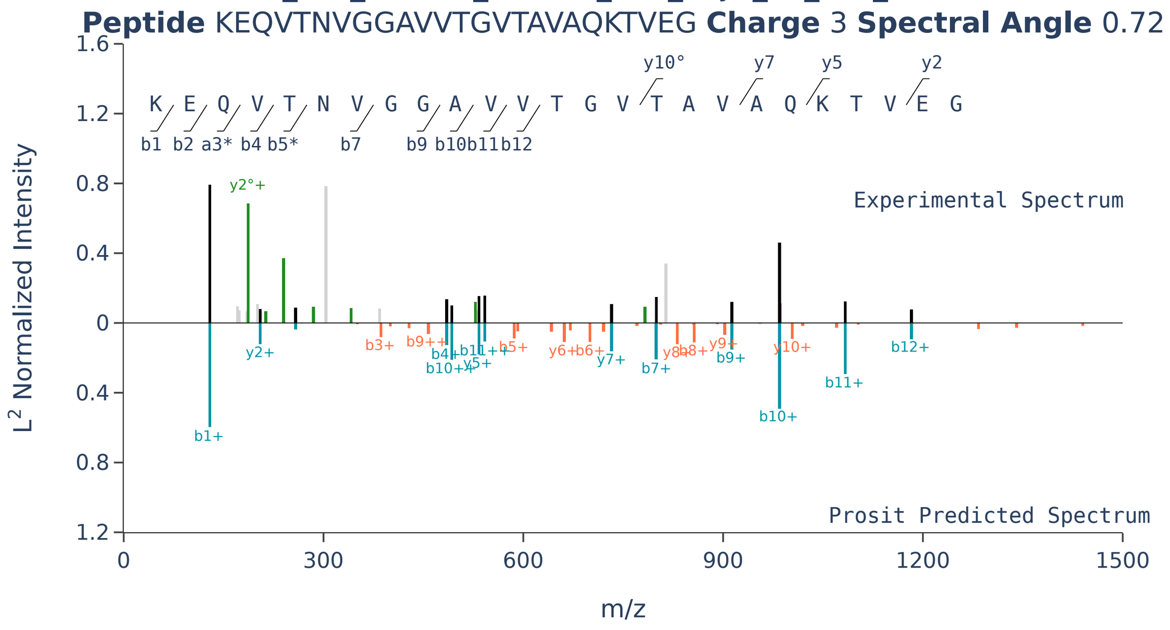


**Source** W_Soh_160821_170921_Goe_aSyn_A1_24h_R2 **Scan** 13219
**Peptide** EGVVHGVATVAEKTKEQV **Charge** 3 **Spectral Angle** 0.61

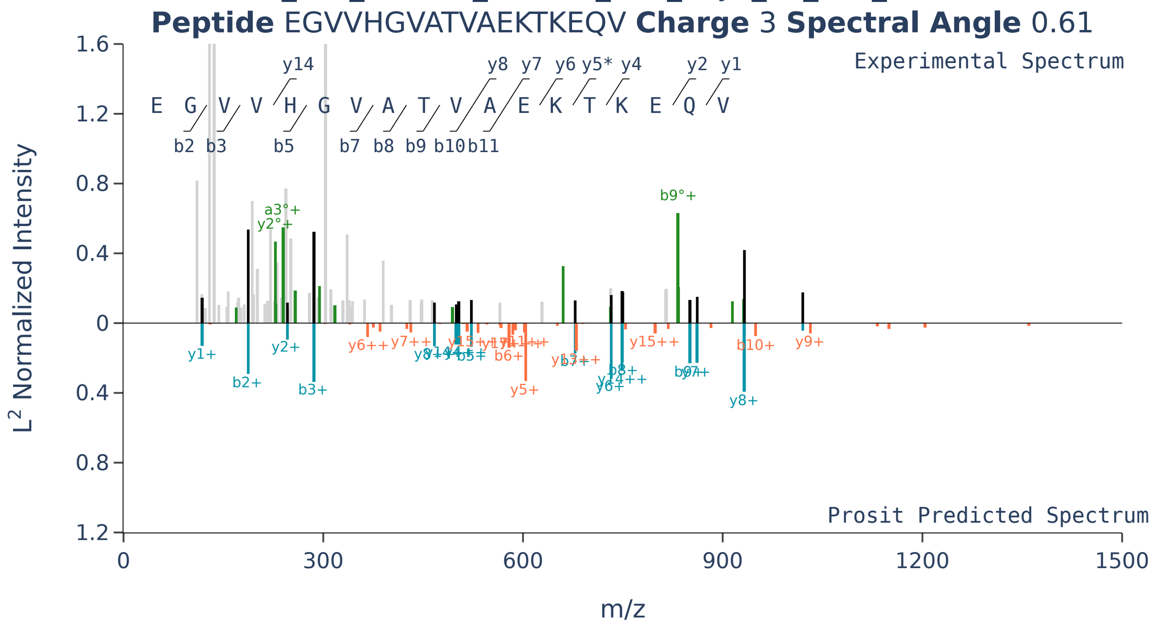


**Source** W_Soh_160821_170921_Goe_aSyn_A2_24h_R2 **Scan** 20036
**Peptide** KEGVVHGVATVAEKTKEQVTNVGGAVVTGV **Charge** 4 **Spectral Angle** 0.68

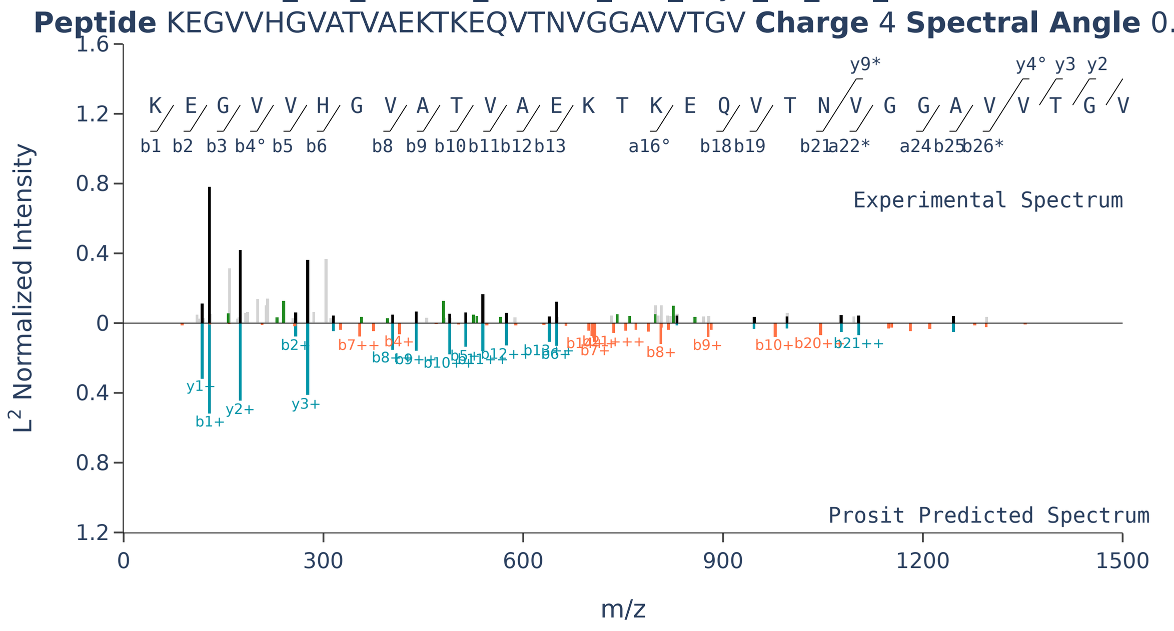


**Source** W_Soh_160821_170921_Goe_aSyn_A1_3h_R2 **Scan** 9781
**Peptide** AAGKTKEGVLYVGSKTKEGVV **Charge** 4 **Spectral Angle** 0.73

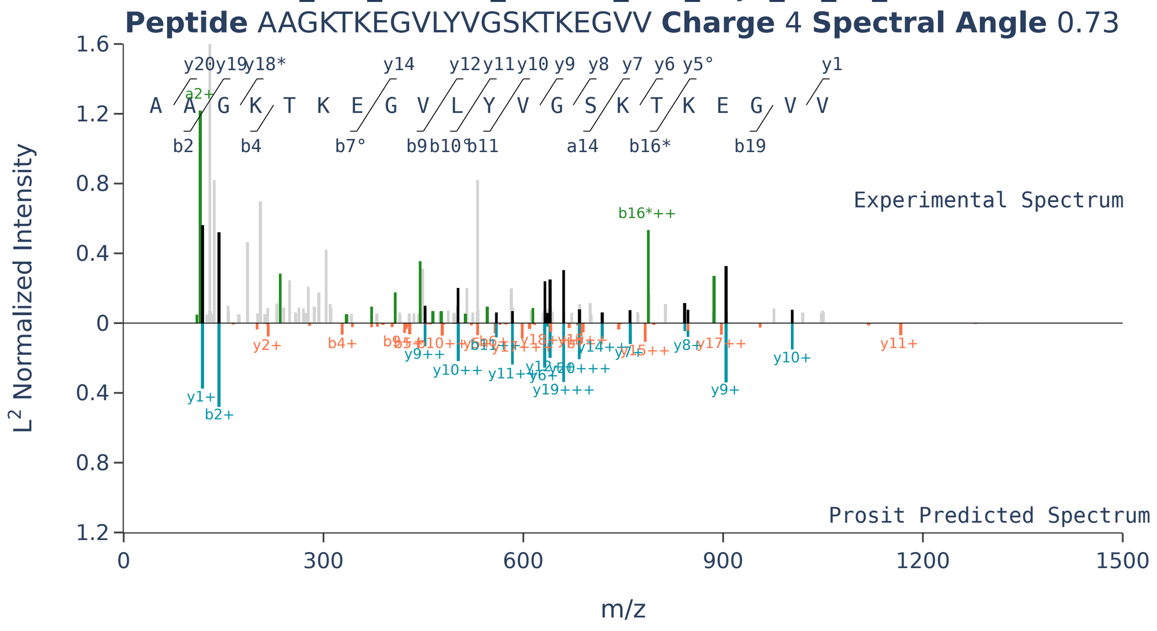


**Source** W_Soh_160821_170921_Goe_aSyn_A1_24h_R1 **Scan** 20531
**Peptide** EQVTNVGGAVVTGV **Charge** 2 **Spectral Angle** 0.64

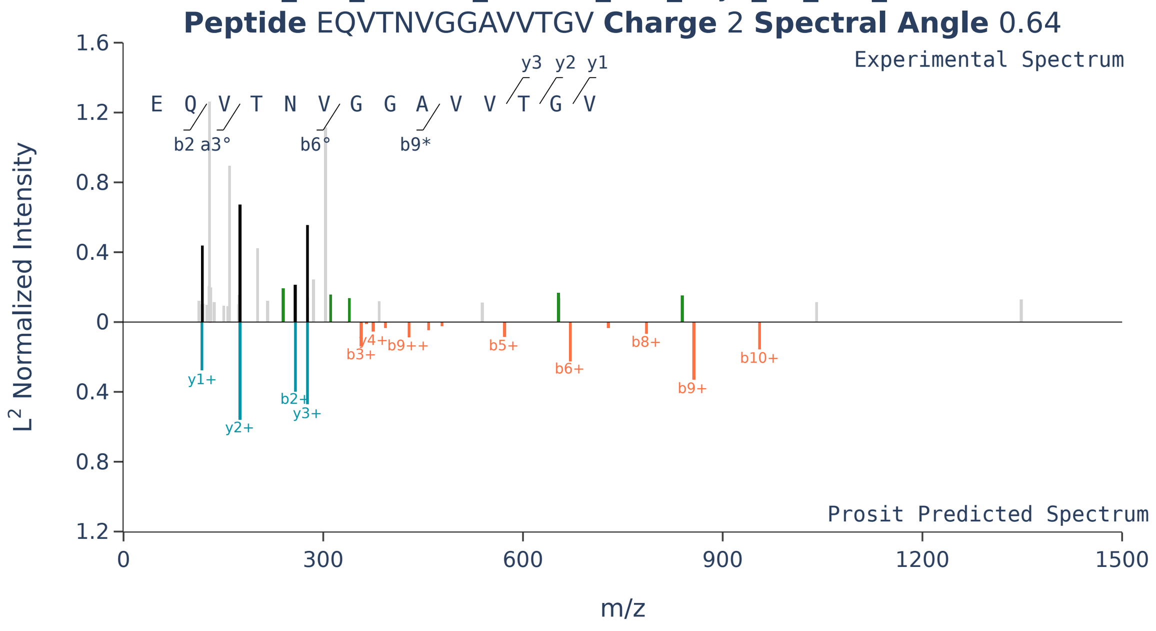


**Source** W_Soh_160821_170921_Goe_aSyn_A1_24h_R1 **Scan** 12663
**Peptide** MKGLSKAKEGVVAAAEKTKQGVAEAAGK **Charge** 5 **Spectral Angle** 0.64

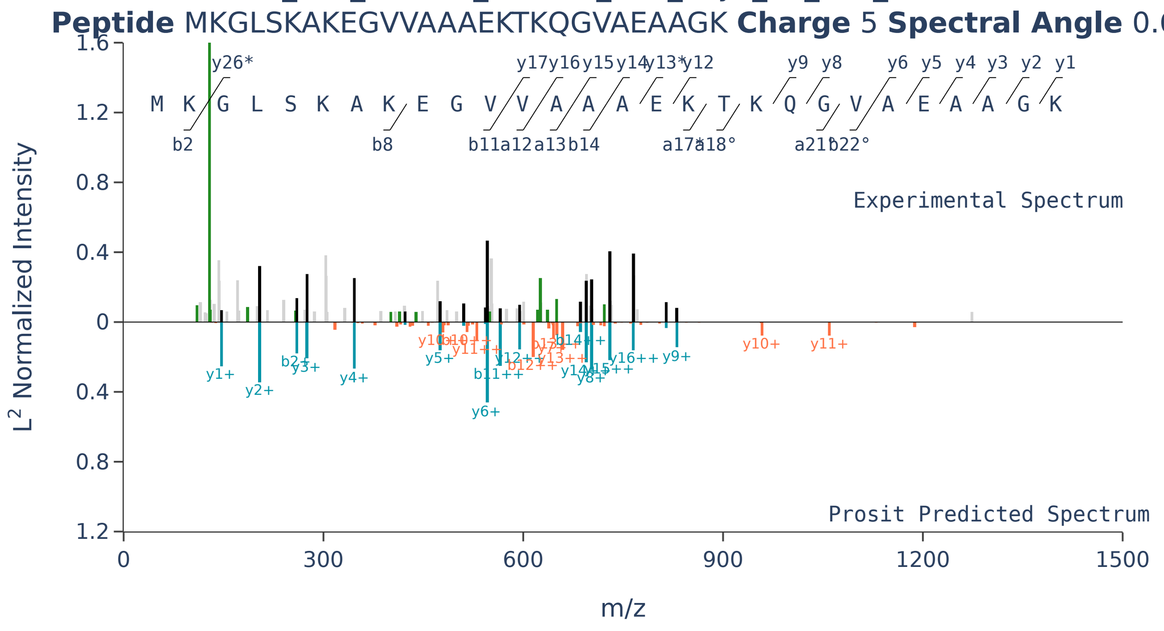


**Source** W_Soh_160821_170921_Goe_aSyn_A2_24h_R2 **Scan** 15862
**Peptide** EGVLYVGSKTKEGVVHGVATVAEKT **Charge** 3 **Spectral Angle** 0.8

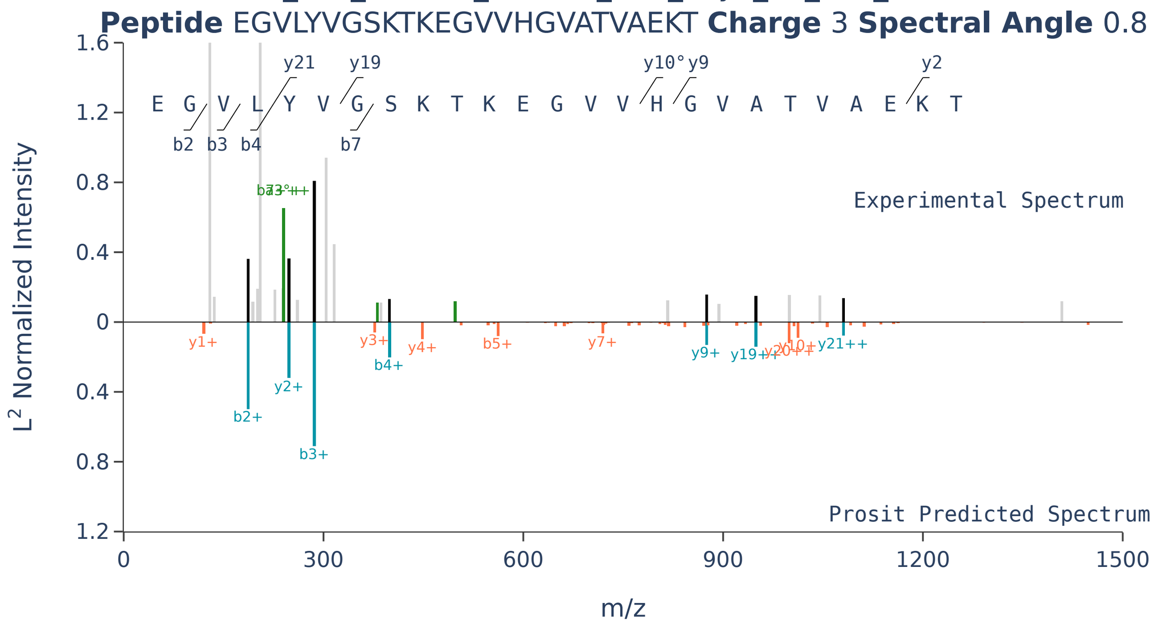


**Source** W_Soh_160821_170921_Goe_aSyn_A2_3h_R1 **Scan** 22645
**Peptide** TVAEKTKEQVTNVGGAVVTGVTAVAQKTVE **Charge** 3 **Spectral Angle** 0.69

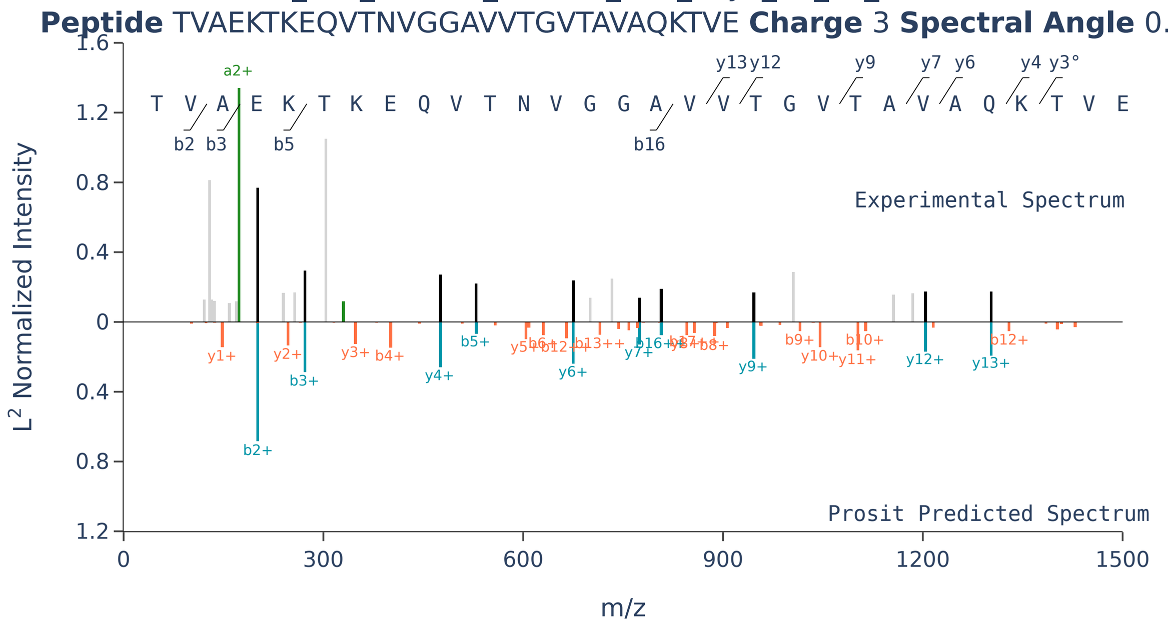


**Source** W_Soh_160821_170921_Goe_aSyn_A1_24h_R1 **Scan** 7434
**Peptide** GSKTKEGVVHGVATVAEKTKEQ **Charge** 5 **Spectral Angle** 0.59

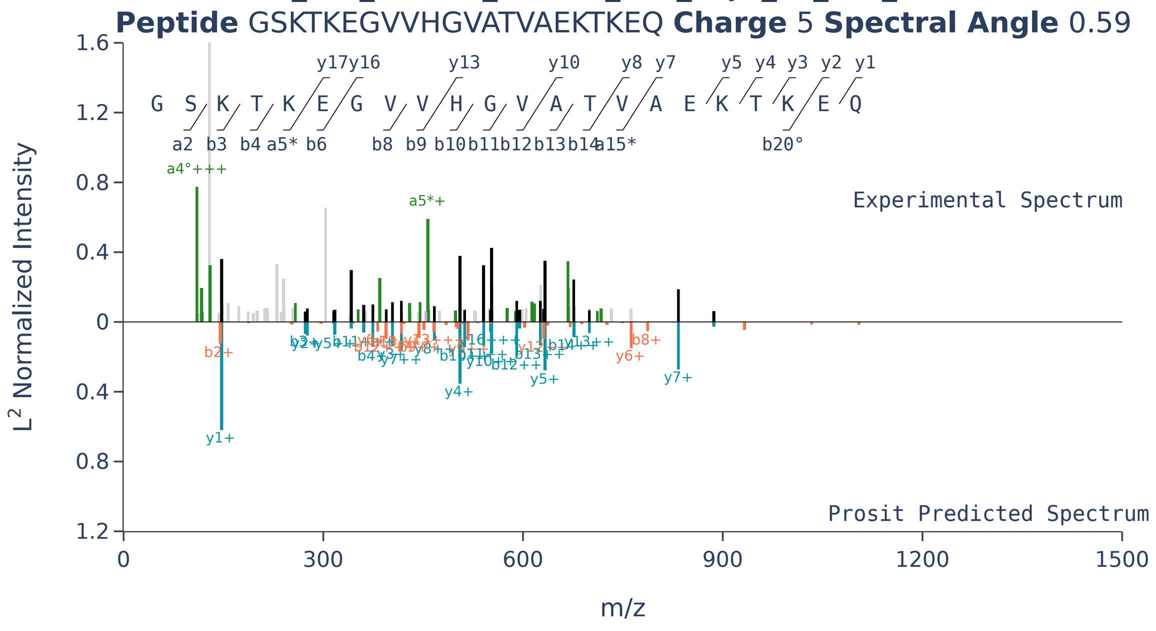


**Source** W_Soh_160821_170921_Goe_aSyn_A1_24h_R1 **Scan** 14064
**Peptide** KEGVLYVGS **Charge** 2 **Spectral Angle** 0.51

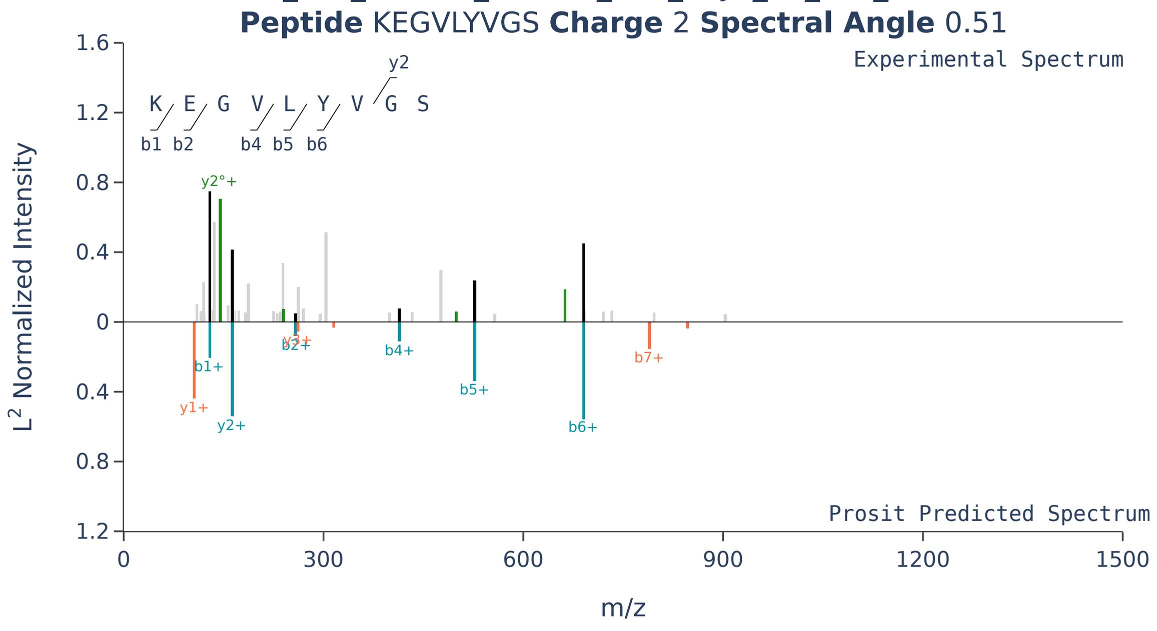

**Source** W_Soh_160821_170921_Goe_aSyn_A1_24h_R2 **Scan** 9702
**Peptide** EGVVAAAEKTKQGVAE **Charge** 2 **Spectral Angle** 0.79

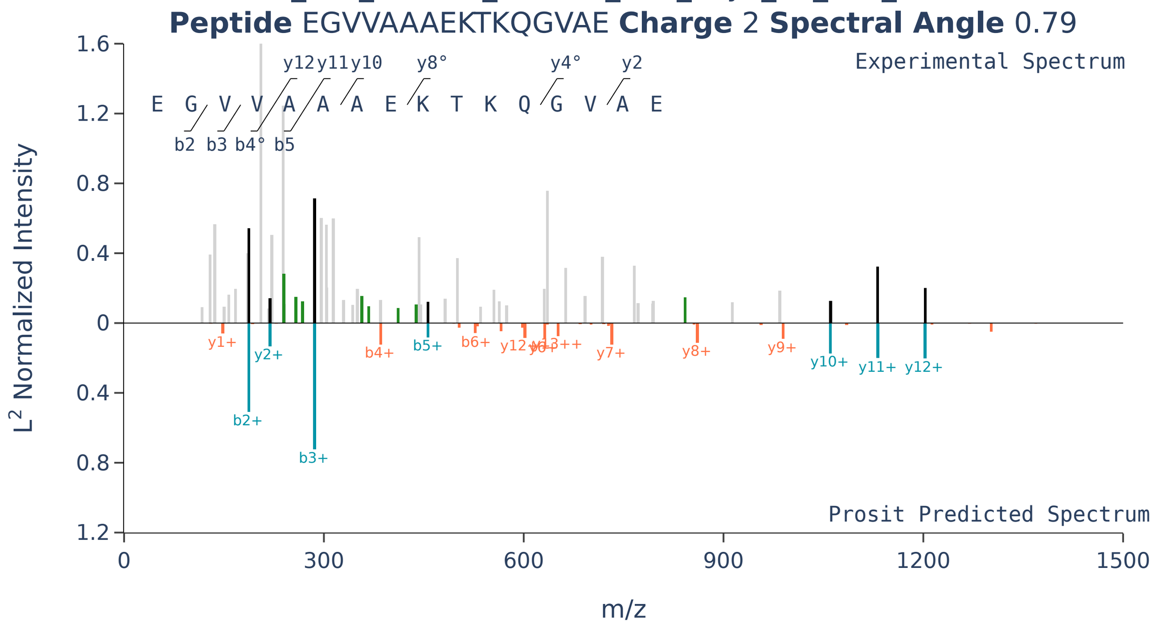


**Source** W_Soh_160821_170921_Goe_aSyn_A2_6h_R2 **Scan** 21535
**Peptide** VATVAEKTKEQVTNVGGAVVTGVTAVAQ **Charge** 3 **Spectral Angle** 0.76

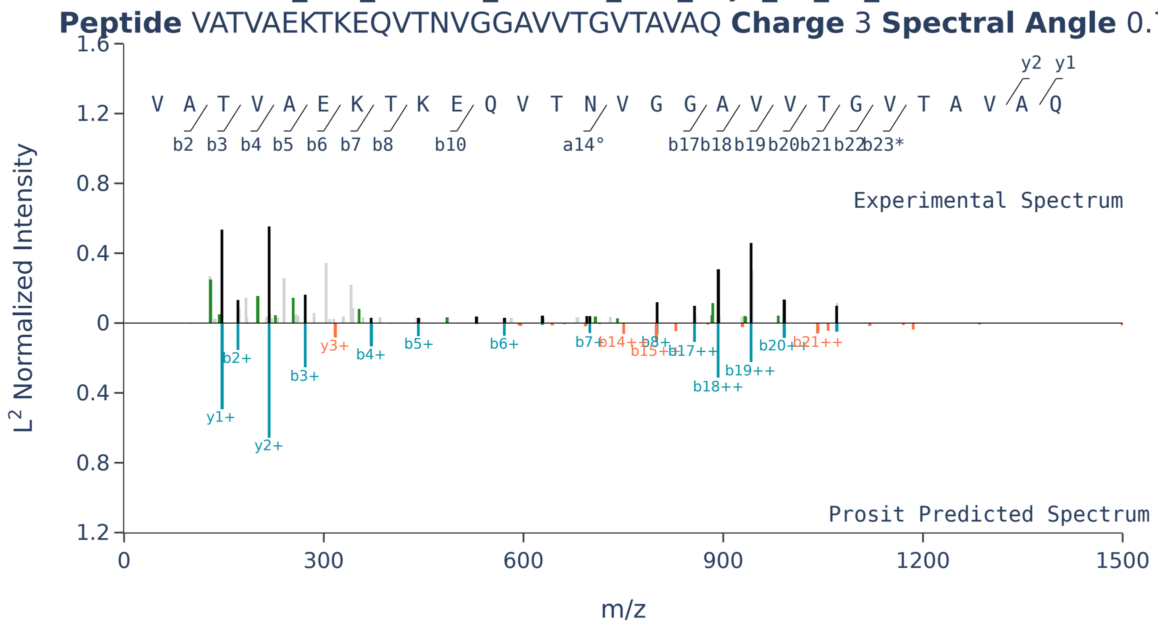


**Source** W_Soh_160821_170921_Goe_aSyn_A1_24h_R1 **Scan** 9333
**Peptide** YVGSKTKEGVVHGVATVAEKTKEQ **Charge** 5 **Spectral Angle** 0.69

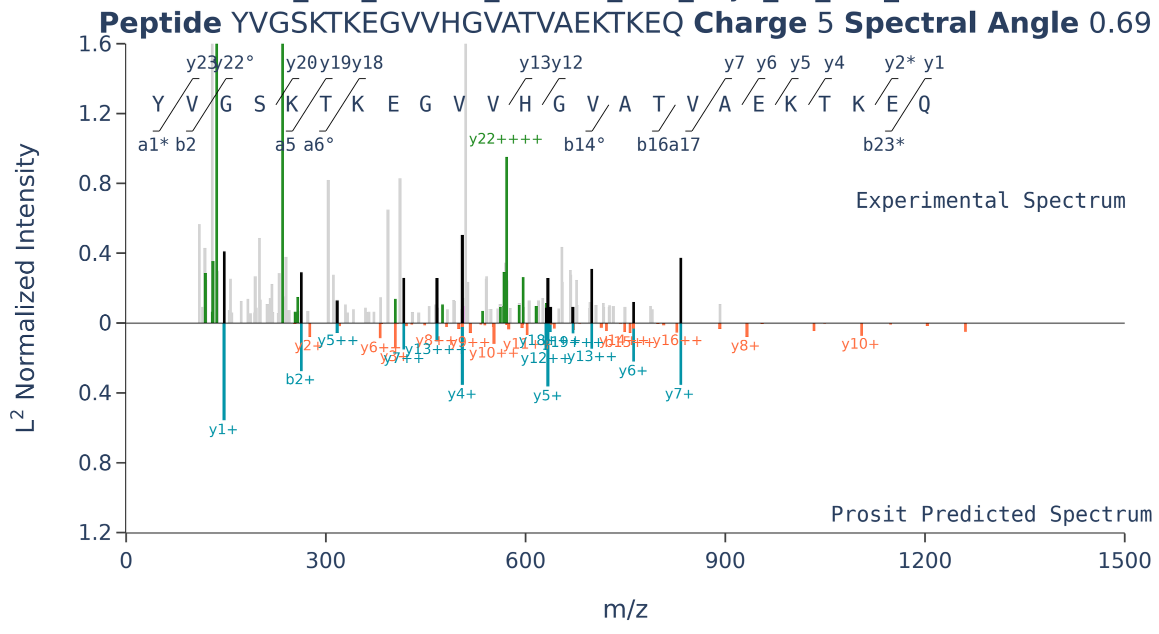


**Source** W_Soh_160821_170921_Goe_aSyn_A1_3h_R2 **Scan** 25821
**Peptide** LEDMPVDPDNEAYEMPSEEGYQDYEPEA **Charge** 3 **Spectral Angle** 0.91

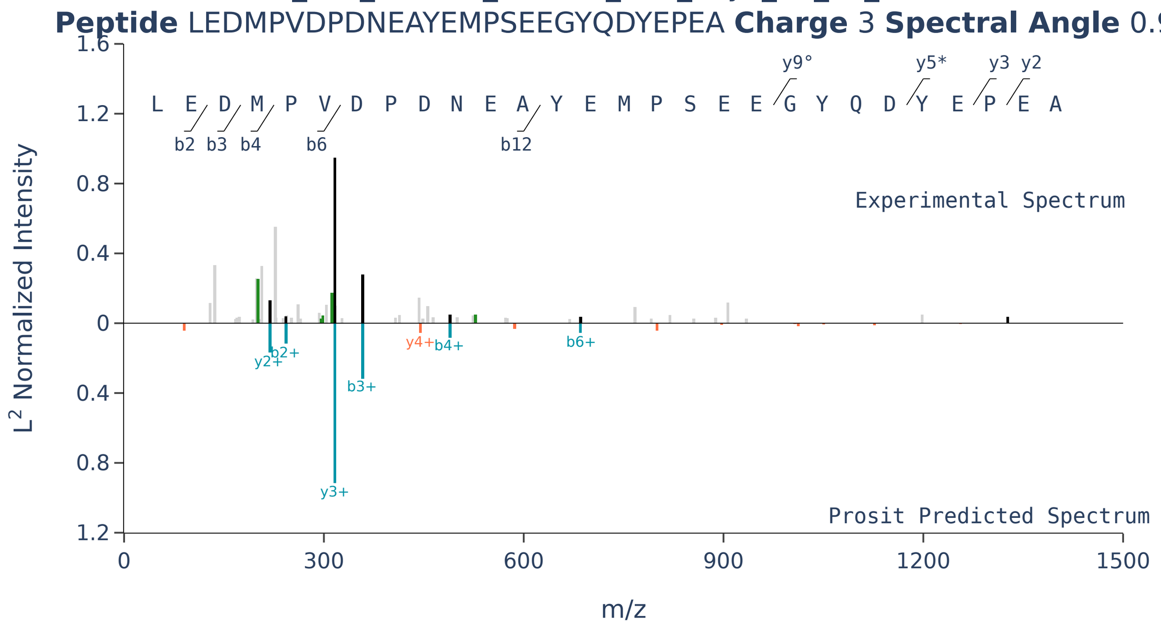


**Source** W_Soh_160821_170921_Goe_aSyn_A2_24h_R1 **Scan** 11267
**Peptide** VKKDQLGKNEEGAPQEGILE **Charge** 3 **Spectral Angle** 0.81

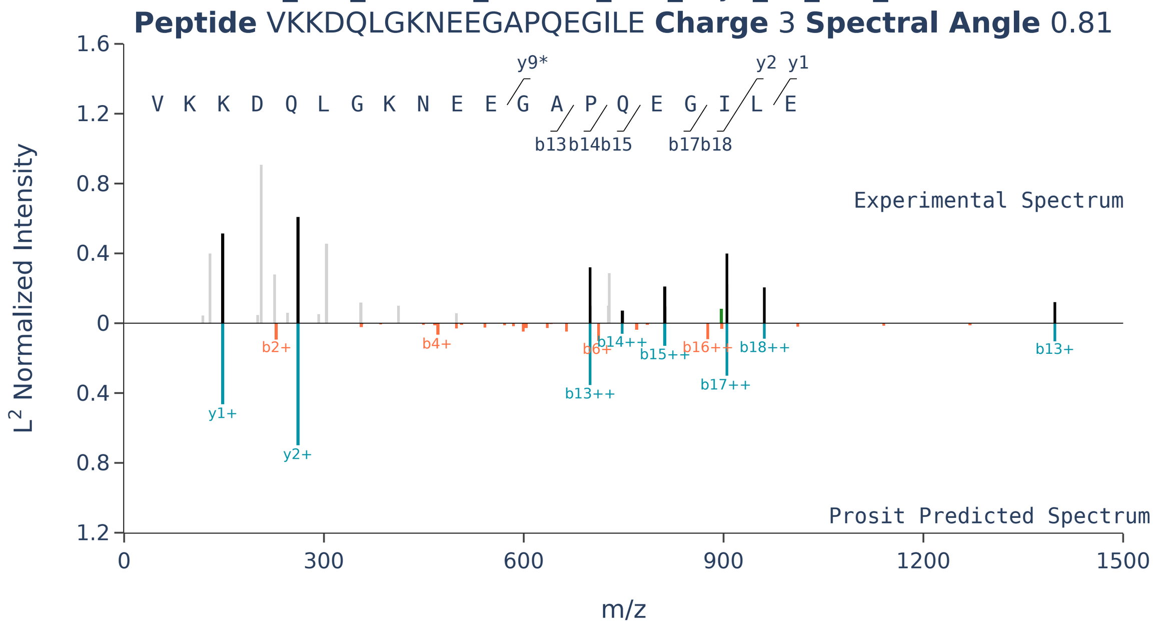


**Source** W_Soh_160821_170921_Goe_aSyn_A1_24h_R2 **Scan** 16505
**Peptide** KTKEGVVHGVATVAEKTKEQVTNVGGAVVT **Charge** 4 **Spectral Angle** 0.72

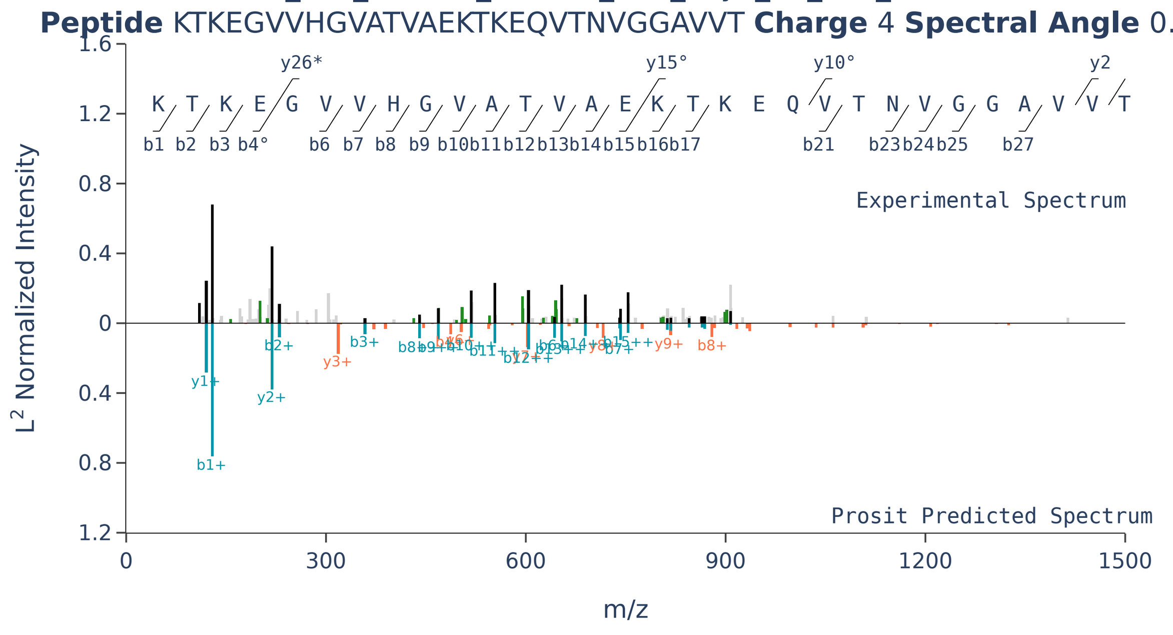


**Source** W_Soh_160821_170921_Goe_aSyn_A2_24h_R2 **Scan** 6101
**Peptide** VVAAAEKTKQGVA **Charge** 2 **Spectral Angle** 0.67

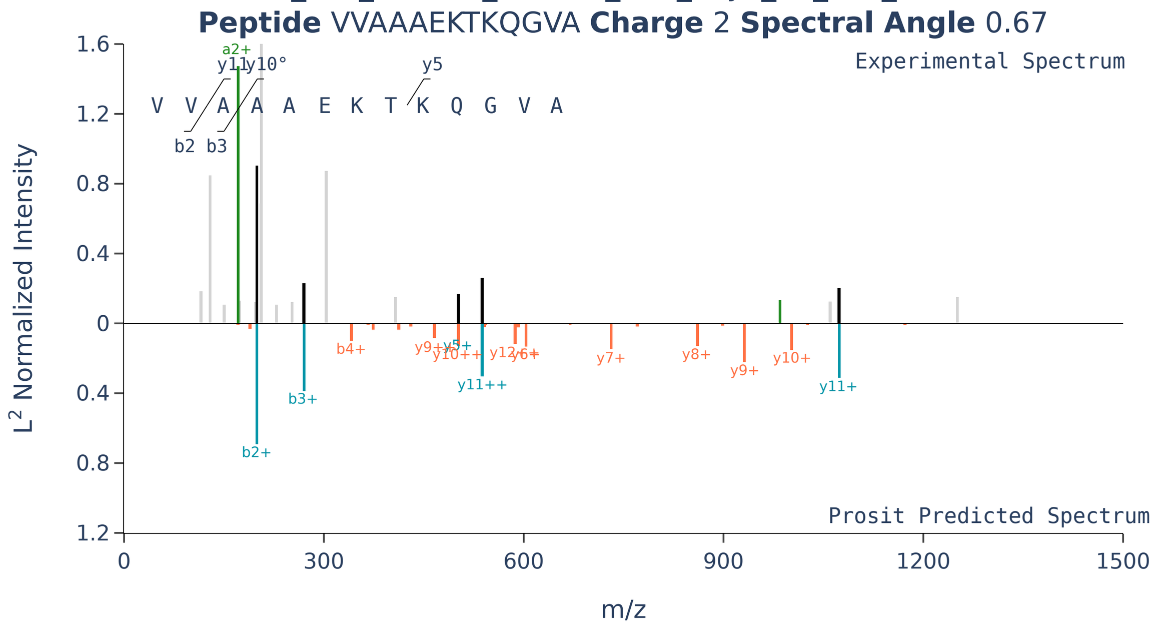


**Source** W_Soh_160821_170921_Goe_aSyn_A1_6h_R2 **Scan** 22955
**Peptide** GKNEEGAPQEGILEDMPVDPDNE **Charge** 3 **Spectral Angle** 0.7

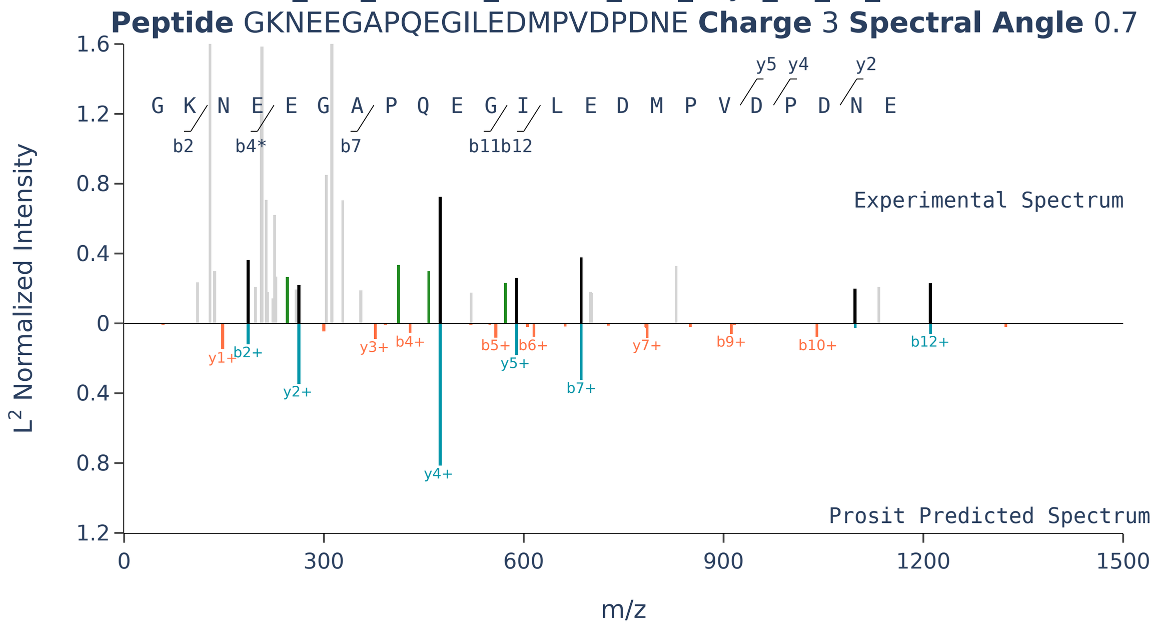


**Source** W_Soh_160821_170921_Goe_aSyn_A2_24h_R1 **Scan** 20895
**Peptide** GVVHGVATVAEKTKEQVTNVGGAVVTGVT **Charge** 3 **Spectral Angle** 0.56

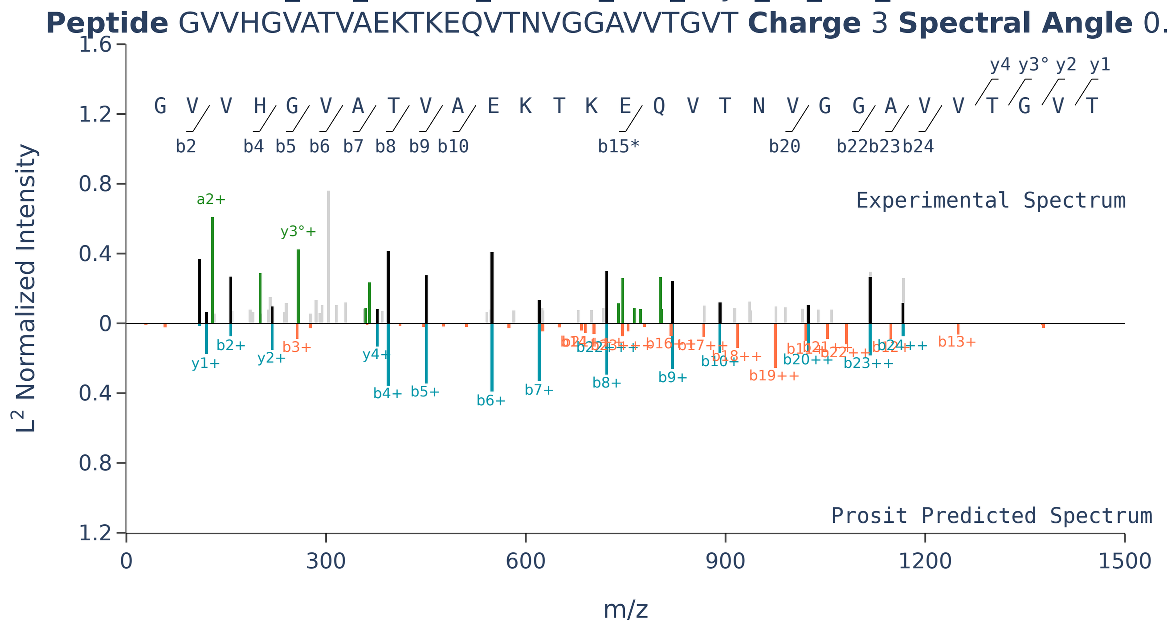


**Source** W_Soh_160821_170921_Goe_aSyn_A1_24h_R1 **Scan** 16483
**Peptide** VVHGVATVAEKTKEQVTNVGGAVVT **Charge** 4 **Spectral Angle** 0.68

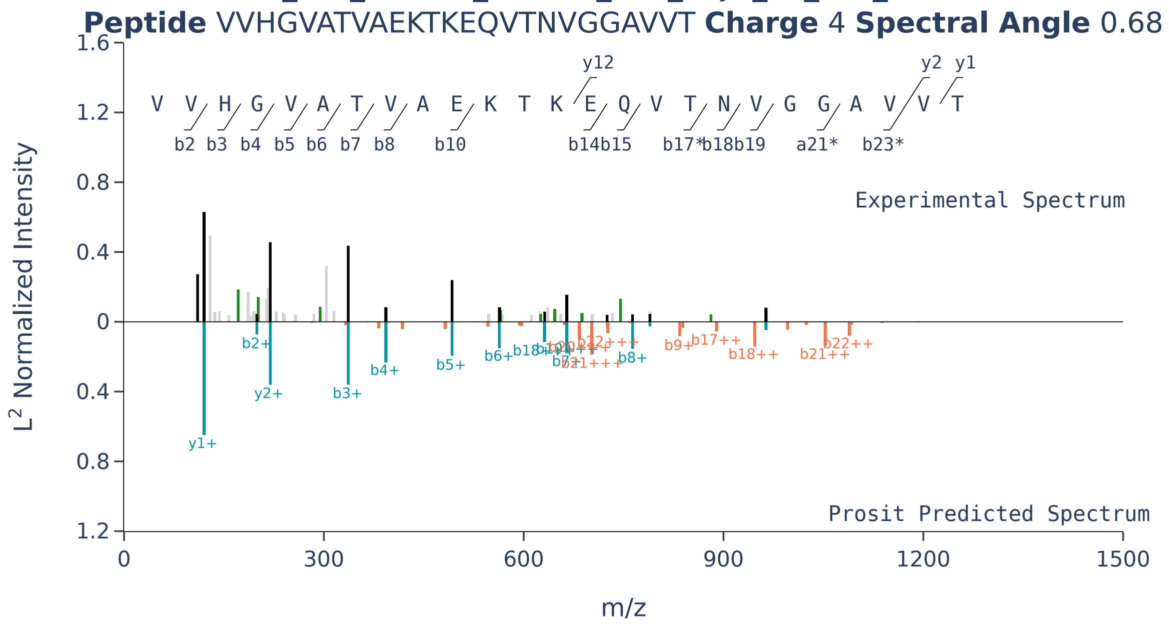


**Source** W_Soh_160821_170921_Goe_aSyn_A1_24h_R1 **Scan** 17033
**Peptide** AGSIAAATGFVKKDQLGKNEEGAPQEGL **Charge** 3 **Spectral Angle** 0.75

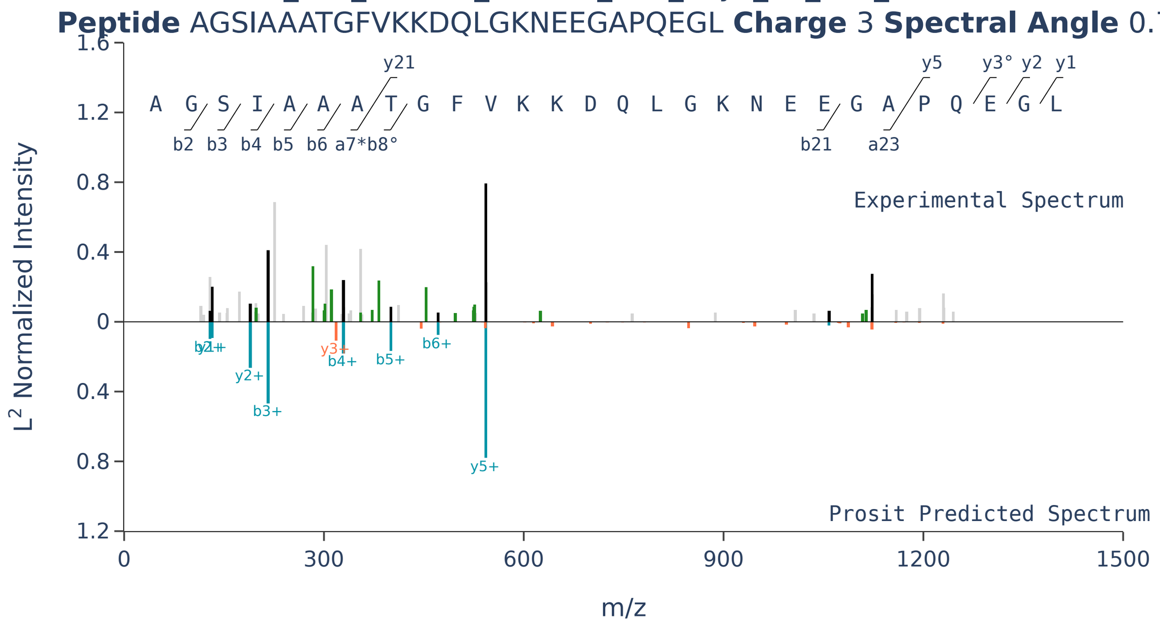


**Source** W_Soh_160821_170921_Goe_aSyn_A1_6h_R1 **Scan** 5028
**Peptide** AAEKTKQGVAEA **Charge** 2 **Spectral Angle** 0.66

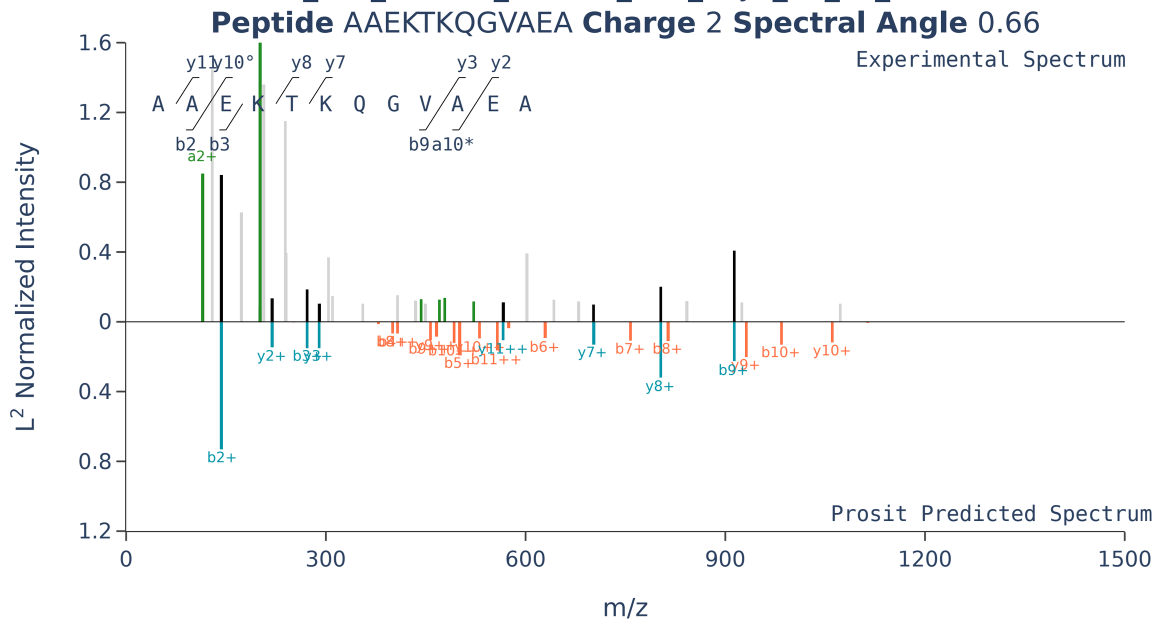


**Source** W_Soh_160821_170921_Goe_aSyn_A2_6h_R2 **Scan** 17877
**Peptide** SIAAATGFVKKDQLGKNEEGAPQEGILE **Charge** 4 **Spectral Angle** 0.73

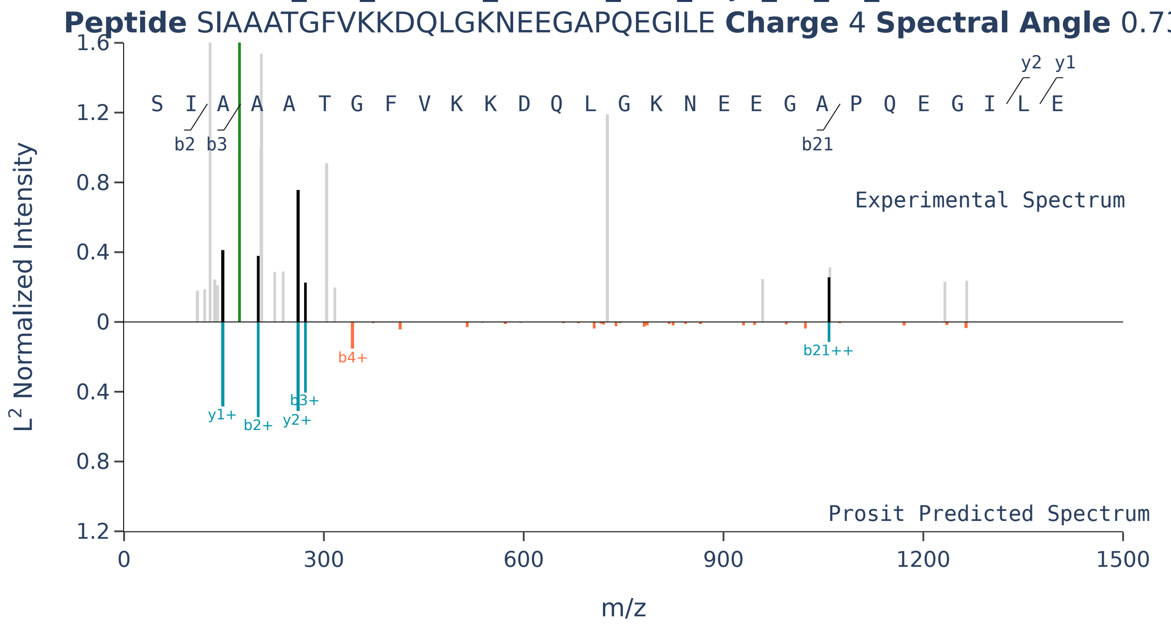


**Source** W_Soh_160821_170921_Goe_aSyn_A2_24h_R2 **Scan** 10664
**Peptide** GVAEAAGKTKEGVLYVGSKTKE **Charge** 4 **Spectral Angle** 0.68

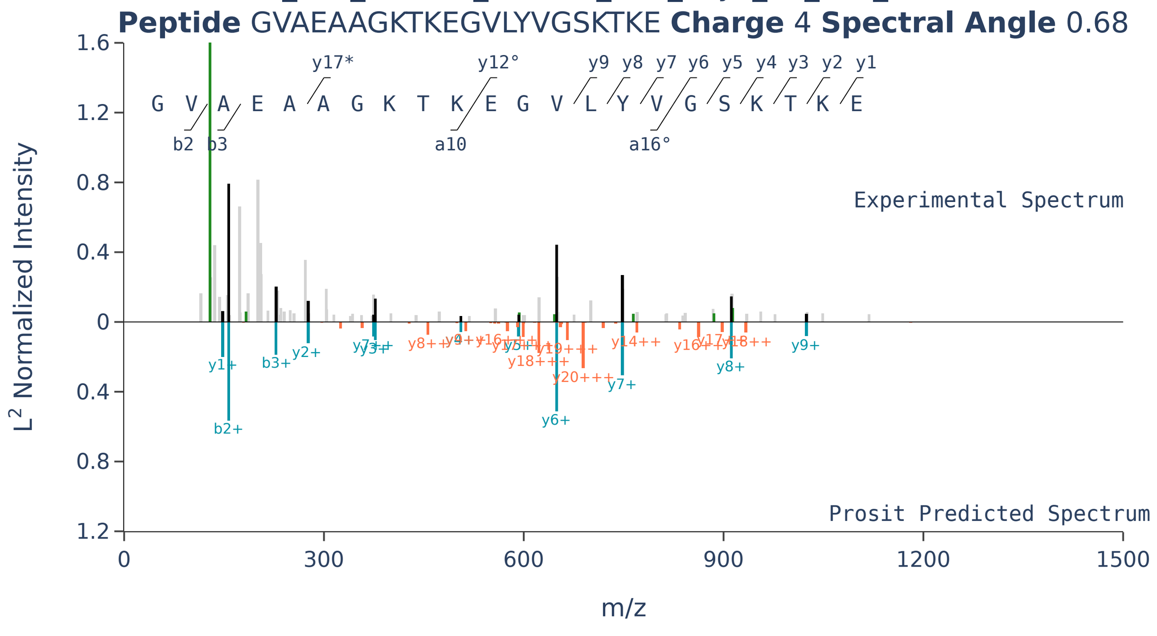


**Source** W_Soh_160821_170921_Goe_aSyn_A1_24h_R1 **Scan** 16138
**Peptide** LGKNEEGAPQEGIL **Charge** 2 **Spectral Angle** 0.72

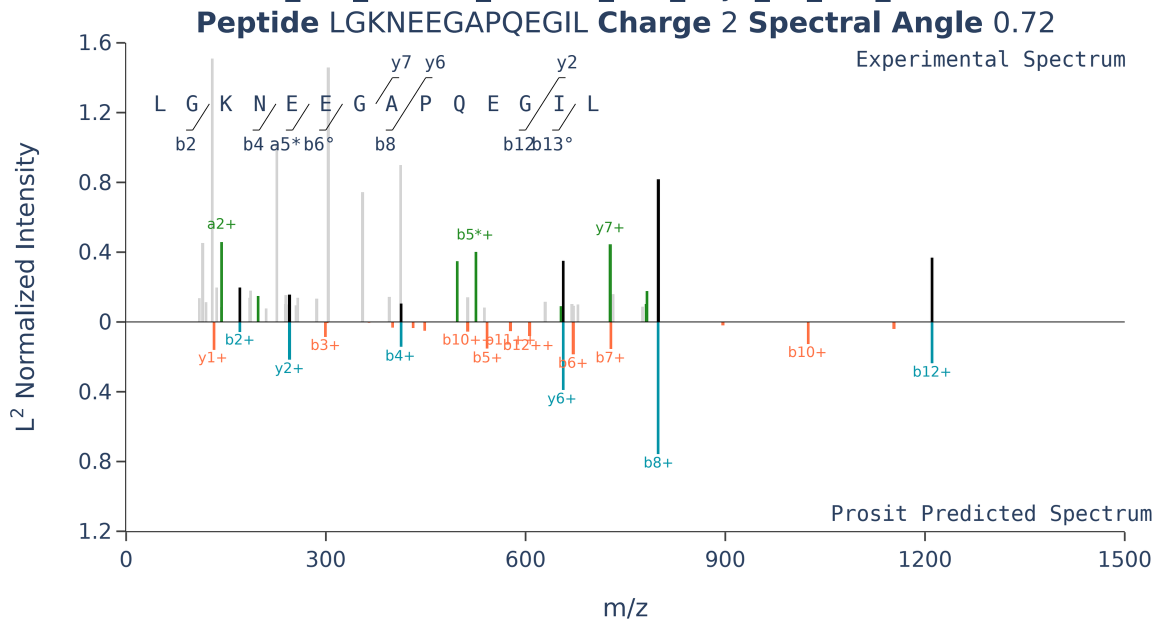

**Source** W_Soh_160821_170921_Goe_aSyn_A1_6h_R2 **Scan** 16255
**Peptide** GVVAAAEKTKQGVAEAAGKTKEGVLYVG **Charge** 3 **Spectral Angle** 0.76

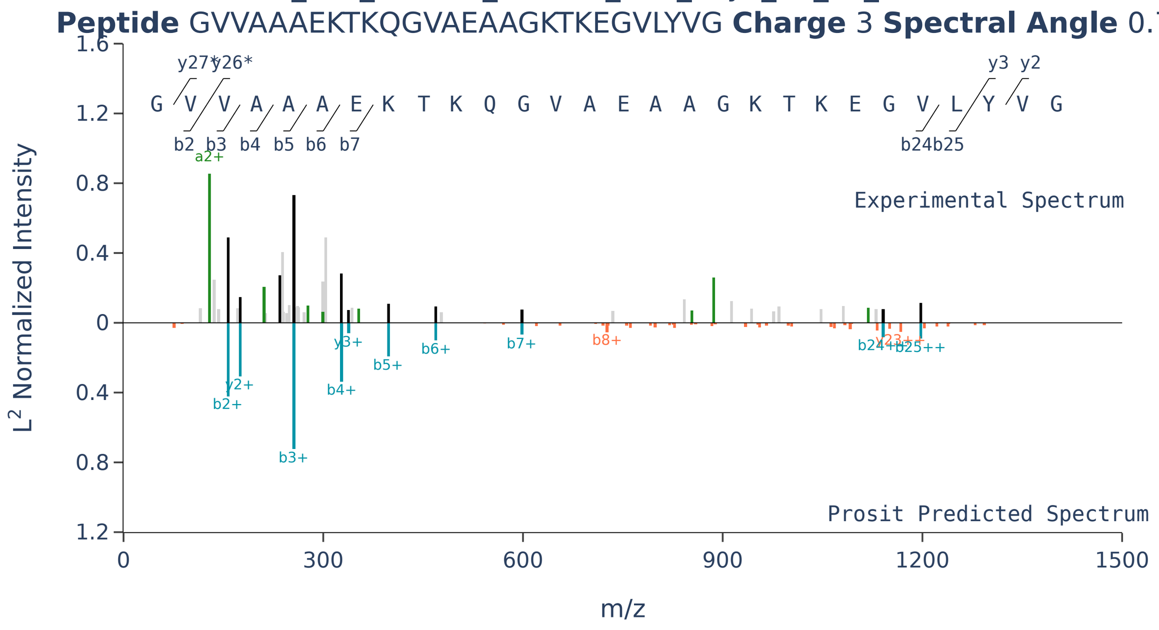


**Source** W_Soh_160821_170921_Goe_aSyn_A1_24h_R1 **Scan** 25376
**Peptide** TKEQVTNVGGAVVTGVTAVAQKTVEG **Charge** 3 **Spectral Angle** 0.63

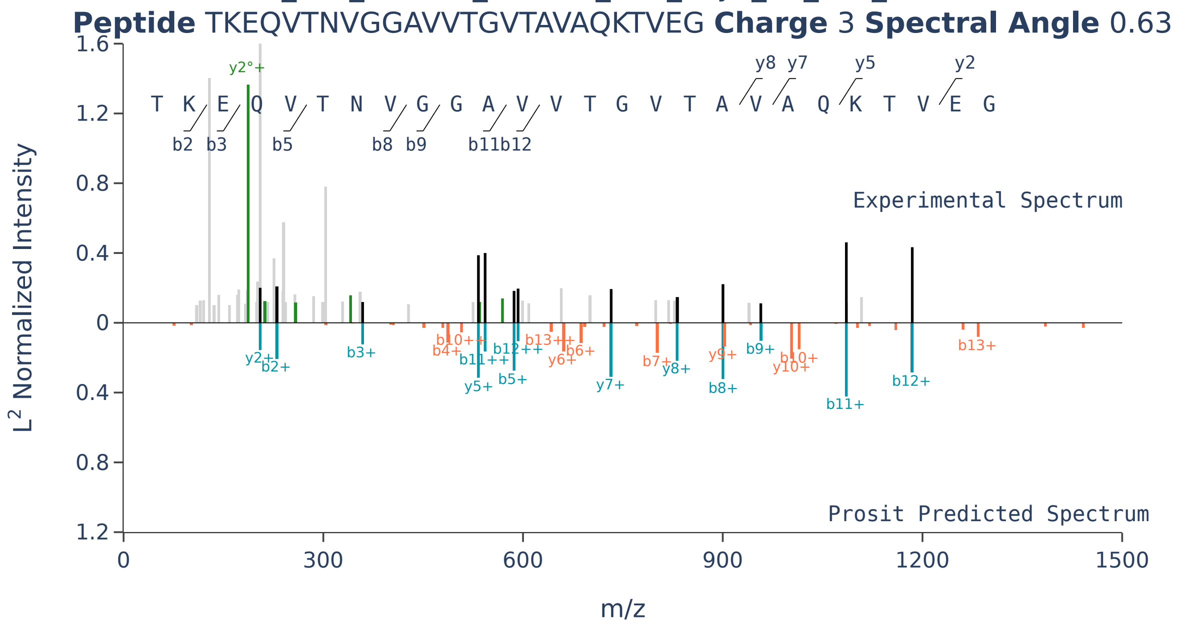


**Source** W_Soh_160821_170921_Goe_aSyn_A1_24h_R2 **Scan** 14143
**Peptide** TKEGVLYVGSKTKEGVVHGVATVAEKTKEQ **Charge** 5 **Spectral Angle** 0.59

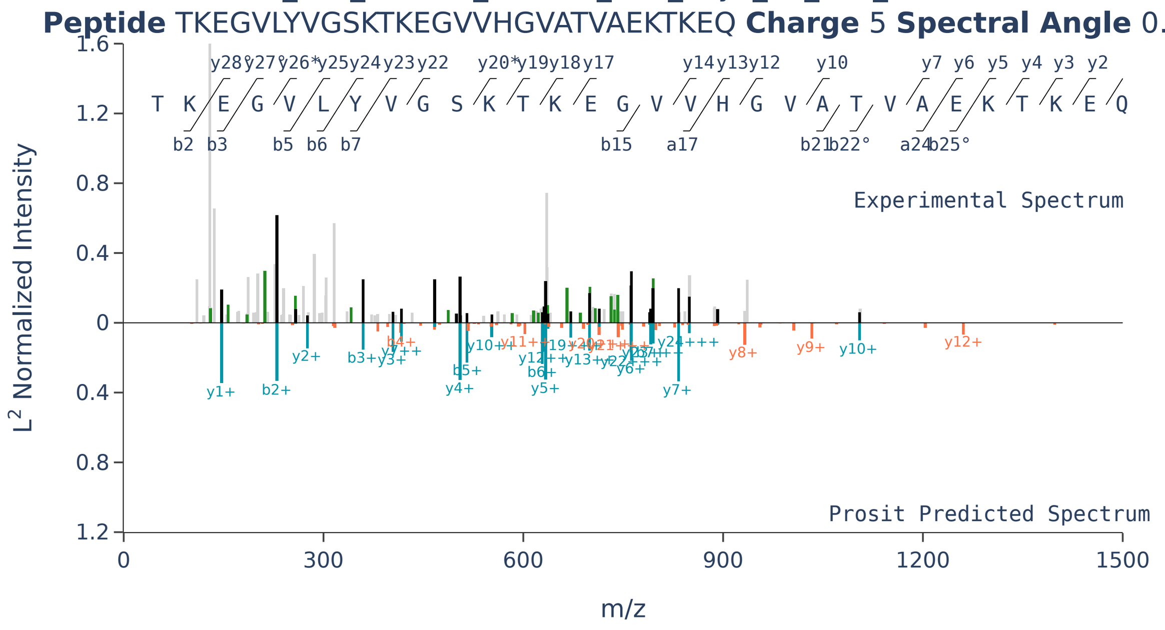


**Source** W_Soh_160821_170921_Goe_aSyn_A1_24h_R1 **Scan** 10722
**Peptide** AVAQKTVEGAGSI **Charge** 2 **Spectral Angle** 0.74

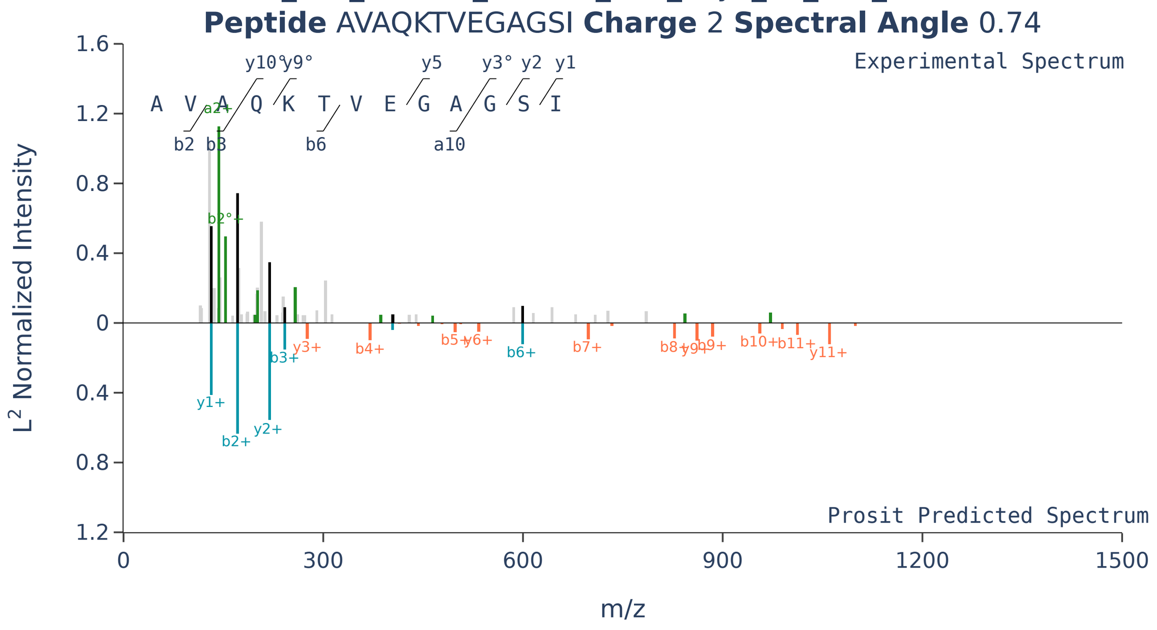


**Source** W_Soh_160821_170921_Goe_aSyn_A1_24h_R2 **Scan** 11527
**Peptide** VHGVATVAEKTKEQVTNV **Charge** 3 **Spectral Angle** 0.85

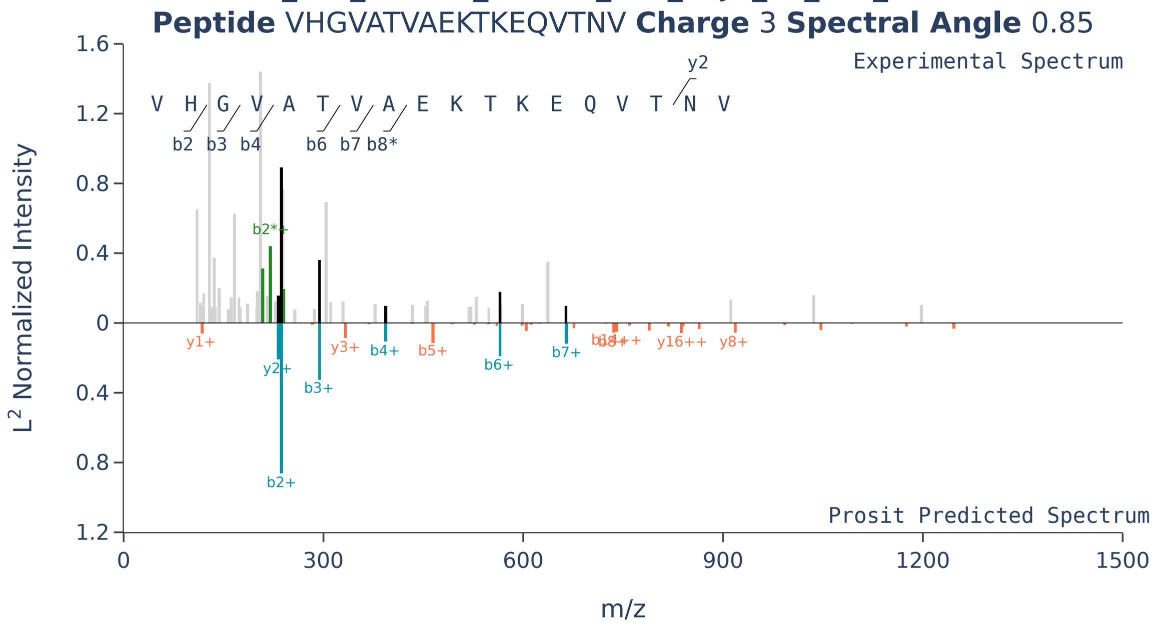


**Source** W_Soh_160821_170921_Goe_aSyn_A1_24h_R2 **Scan** 6980
**Peptide** SKAKEGVVAAAEKTKQGV **Charge** 3 **Spectral Angle** 0.54

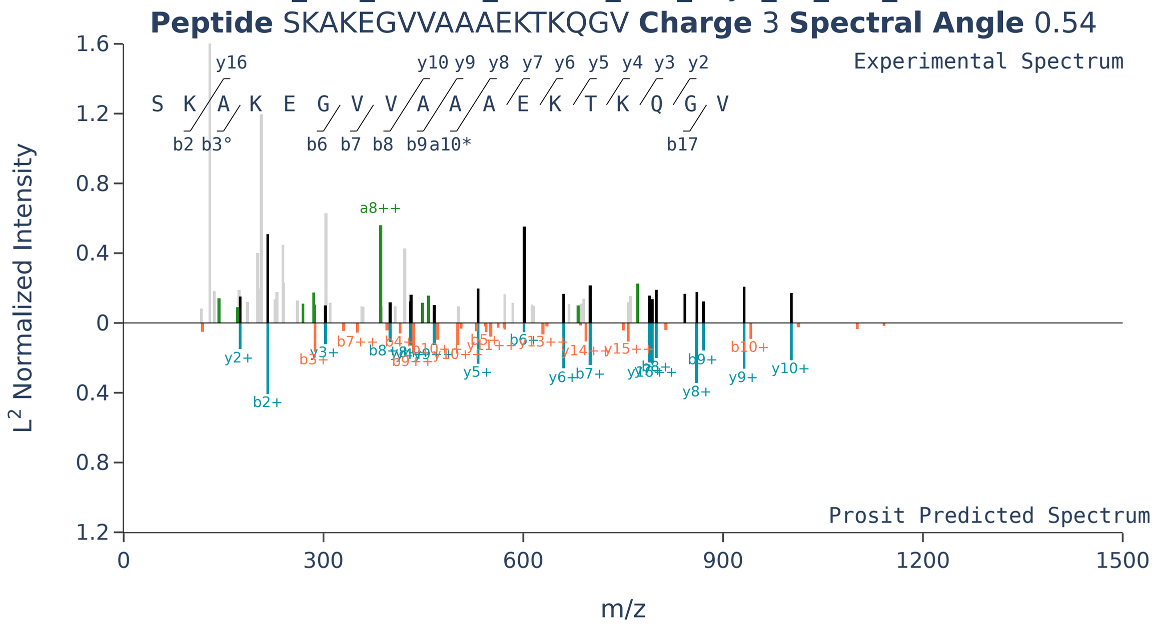


**Source** W_Soh_160821_170921_Goe_aSyn_A2_6h_R2 **Scan** 9356
**Peptide** VAQKTVEGAGSL **Charge** 2 **Spectral Angle** 0.76

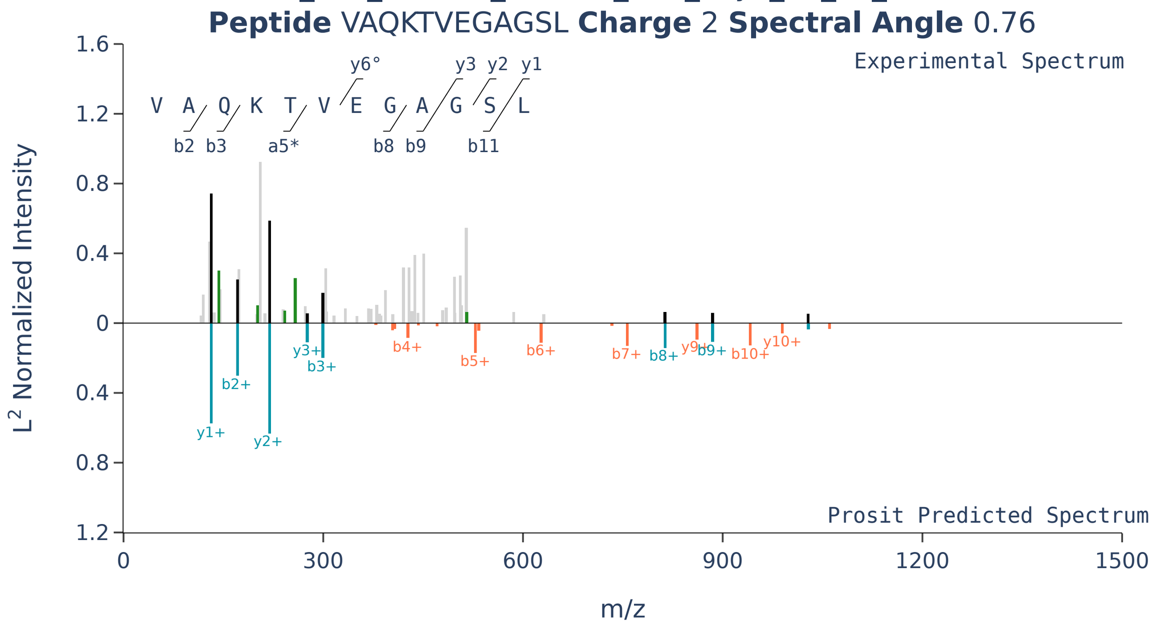


**Source** W_Soh_160821_170921_Goe_aSyn_A1_24h_R2 **Scan** 13344
**Peptide** FMKGLSKAKEGVVAAAEKTKQGVAE **Charge** 5 **Spectral Angle** 0.75

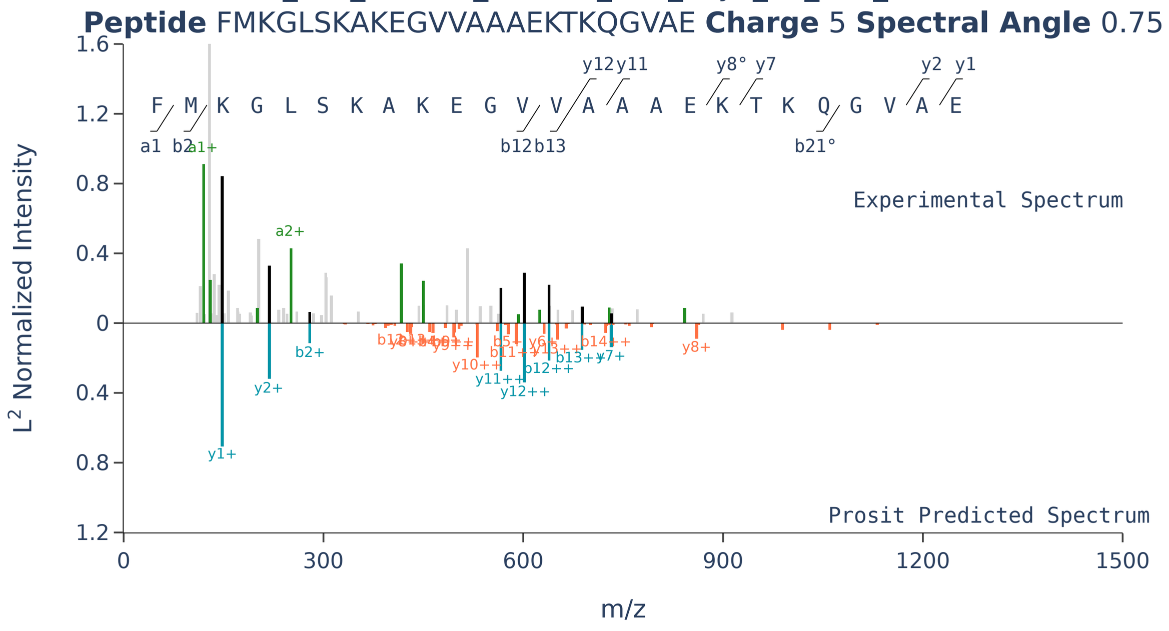


**Source** W_Soh_160821_170921_Goe_aSyn_A1_24h_R2 **Scan** 10697
**Peptide** LSKAKEGVVAAAEKTKQGVAEAAGKTKEG **Charge** 5 **Spectral Angle** 0.6

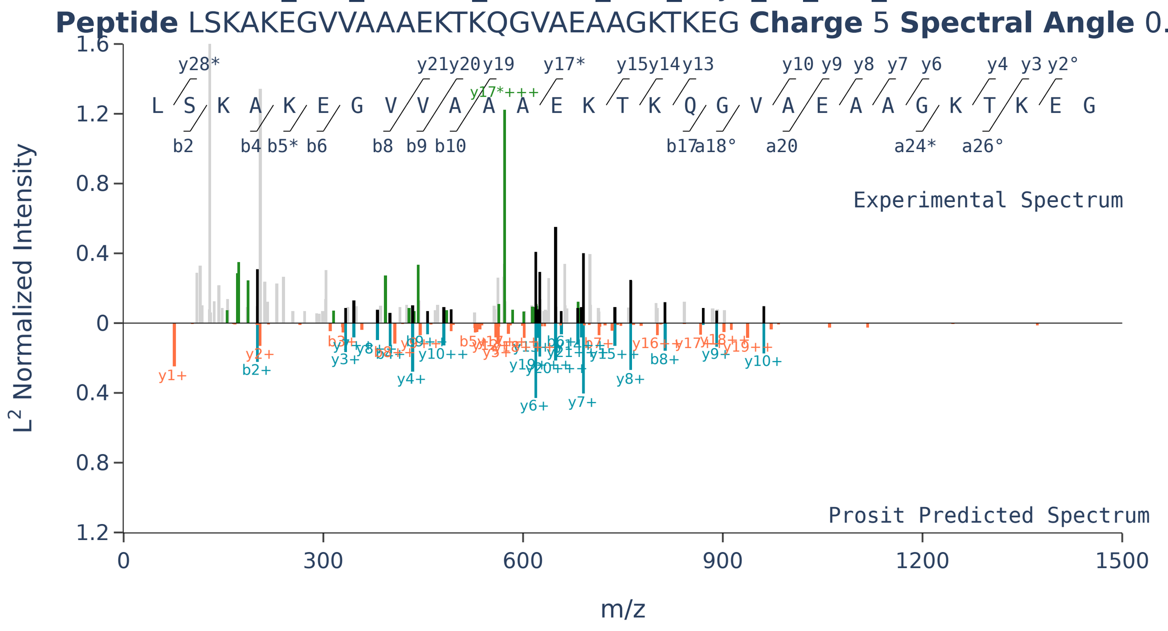


**Source** W_Soh_160821_170921_Goe_aSyn_A2_24h_R1 **Scan** 13141
**Peptide** GLSKAKEGVVAAAEKTKQGVAEAAGKT **Charge** 4 **Spectral Angle** 0.63

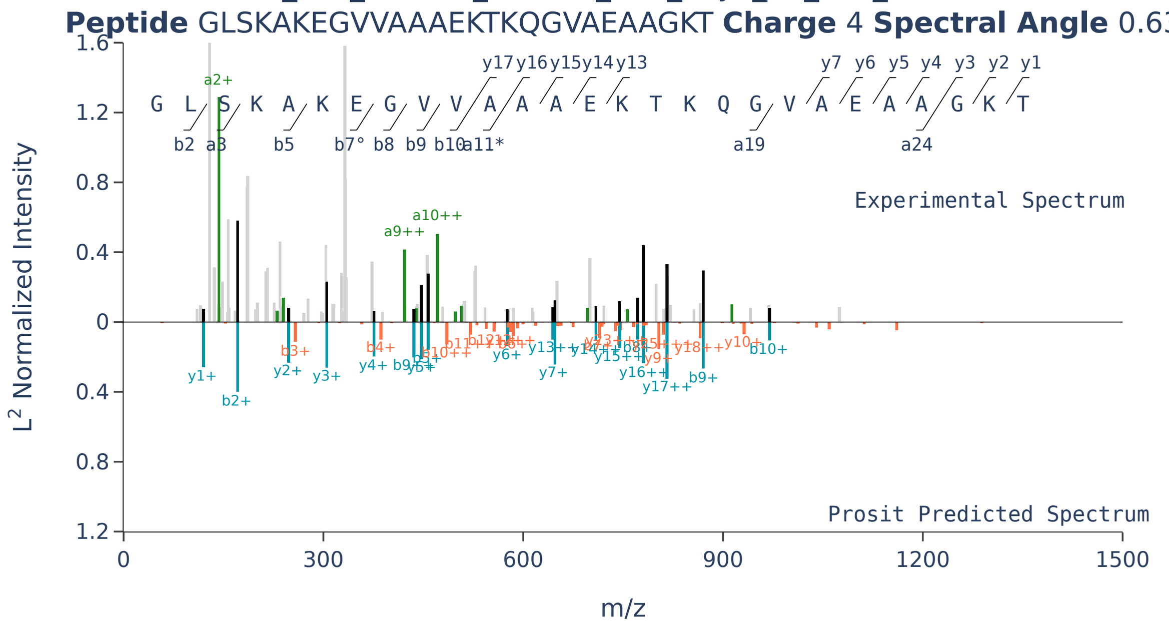


**Source** W_Soh_160821_170921_Goe_aSyn_A2_24h_R1 **Scan** 11382
**Peptide** EKTKQGVAEAAGKTKEGVLYV **Charge** 4 **Spectral Angle** 0.81

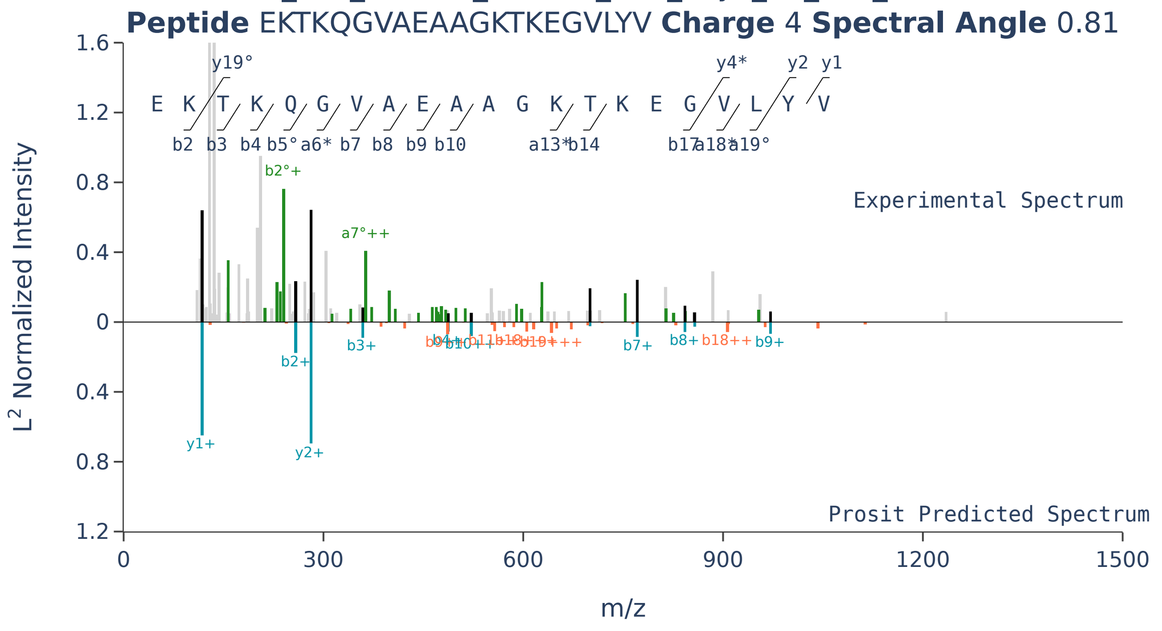


**Source** W_Soh_160821_170921_Goe_aSyn_A2_24h_R1 **Scan** 7415
**Peptide** EAAGKTKEGVLYVGSKTKE **Charge** 4 **Spectral Angle** 0.73

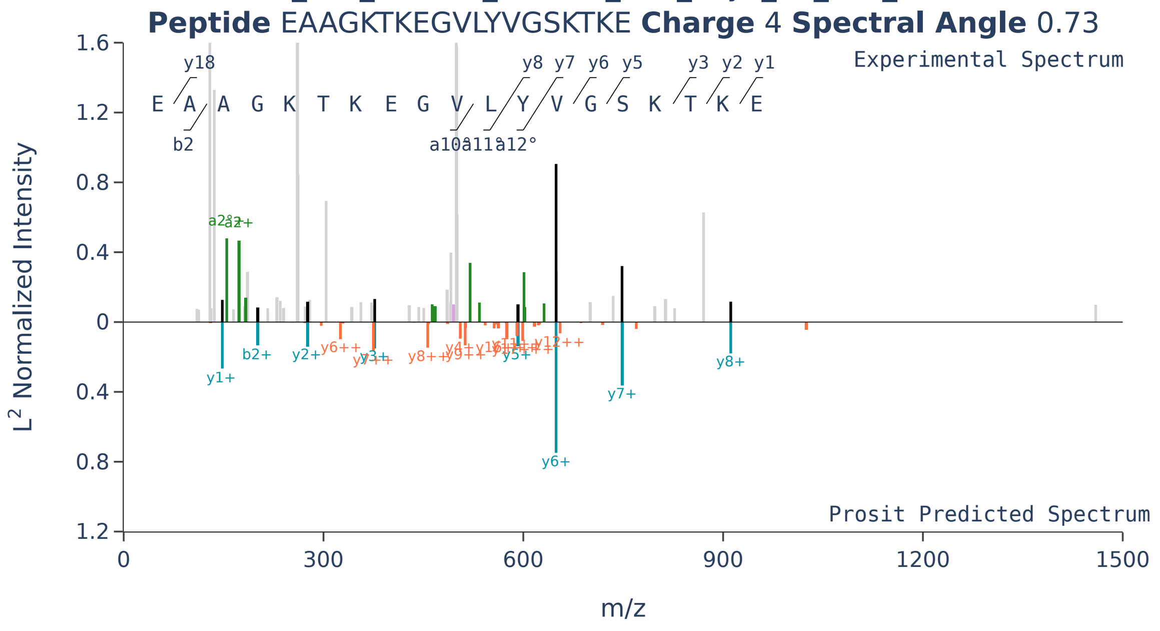


**Source** W_Soh_160821_170921_Goe_aSyn_A2_24h_R1 **Scan** 21706
**Peptide** KTVEGAGSIAAATGFV **Charge** 2 **Spectral Angle** 0.45

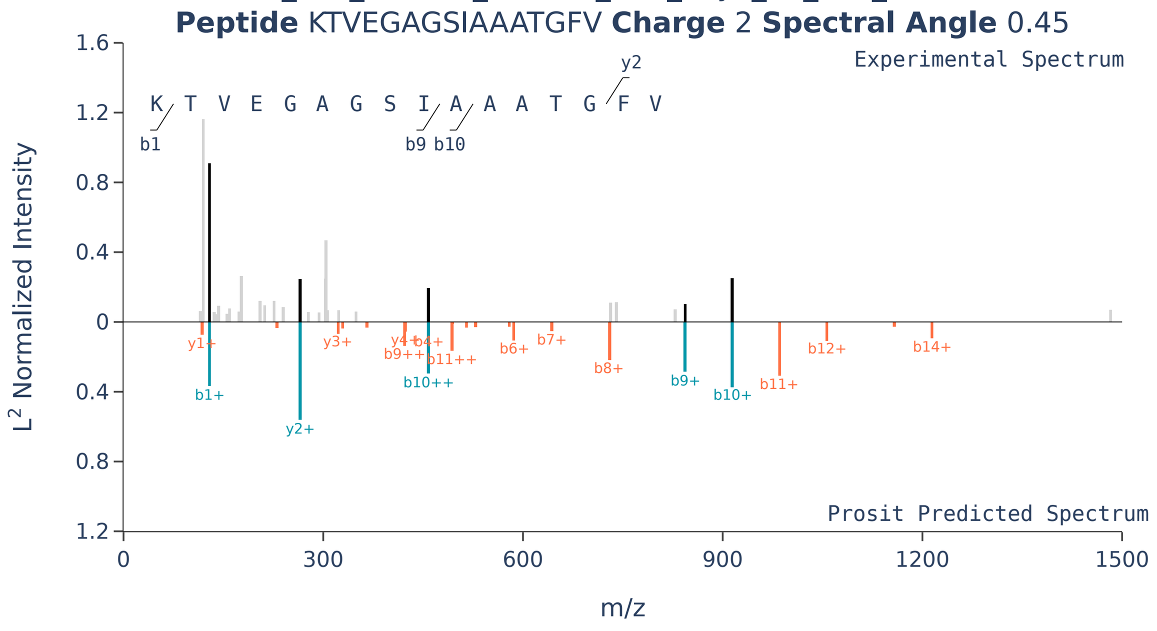


**Source** W_Soh_160821_170921_Goe_aSyn_A1_24h_R2 **Scan** 11508
**Peptide** EGVVAAAEKTKQGVAEAAGKTKEG **Charge** 4 **Spectral Angle** 0.54

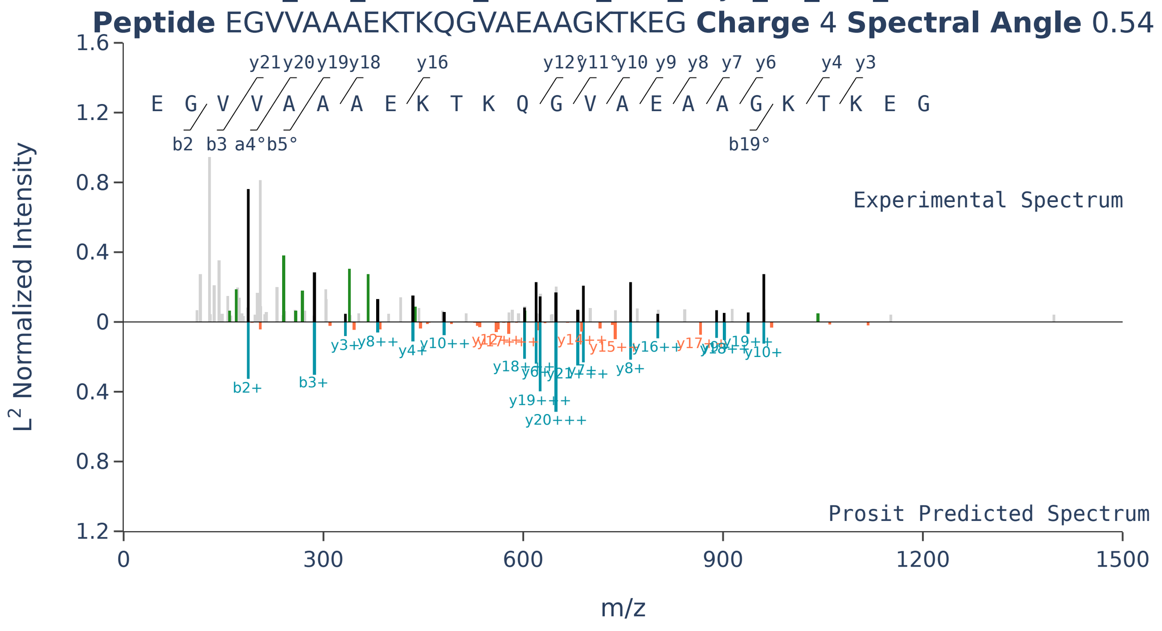


**Source** W_Soh_160821_170921_Goe_aSyn_A2_24h_R1 **Scan** 11310
**Peptide** KTKEGVLYVGS **Charge** 2 **Spectral Angle** 0.58

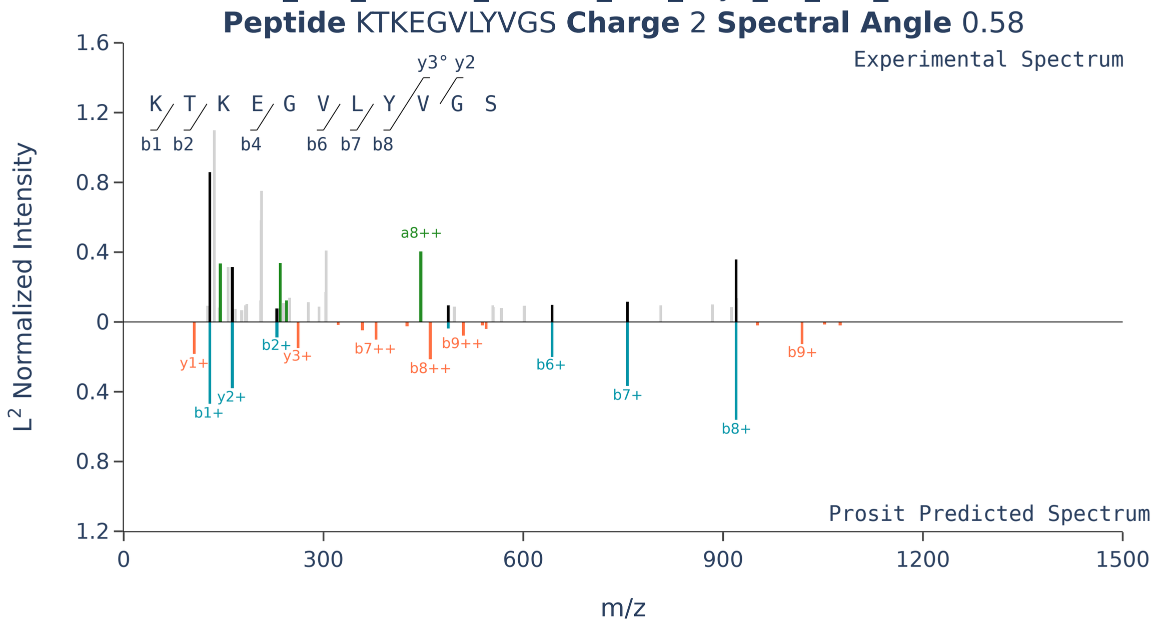

**Source** W_Soh_160821_170921_Goe_aSyn_A1_6h_R2 **Scan** 11888
**Peptide** AAAEKTKQGVAEAAGKTKEGVLYV **Charge** 3 **Spectral Angle** 0.81

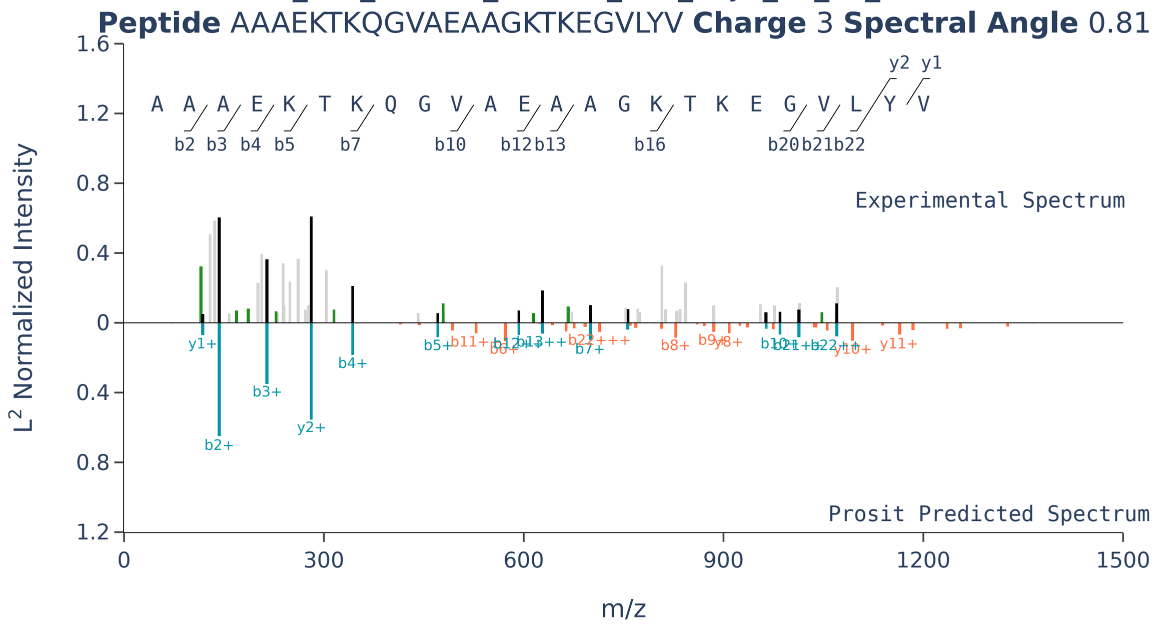


**Source** W_Soh_160821_170921_Goe_aSyn_A2_24h_R2 **Scan** 10557
**Peptide** KTKQGVAEAAGKTKEGVLYV **Charge** 4 **Spectral Angle** 0.74

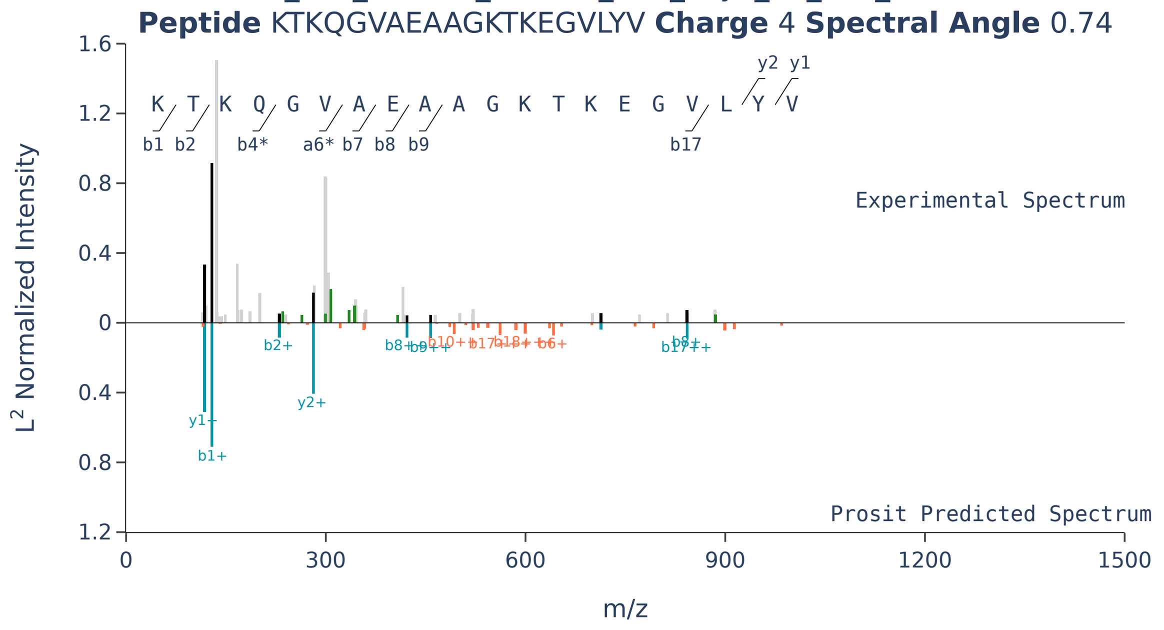


**Source** W_Soh_160821_170921_Goe_aSyn_A2_24h_R2 **Scan** 9984
**Peptide** GKTKEGVLYVGSKTKEGVV **Charge** 3 **Spectral Angle** 0.72

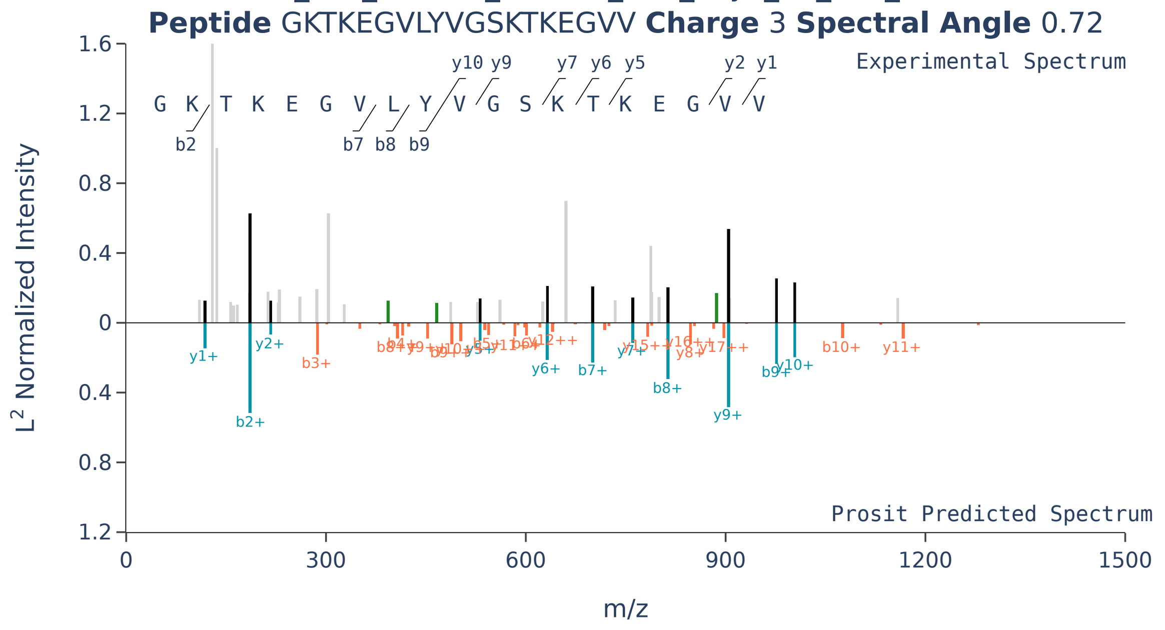


**Source** W_Soh_160821_170921_Goe_aSyn_A1_24h_R1 **Scan** 10988
**Peptide** EAAGKTKEGVLYVGSKTKEGVVHGV **Charge** 4 **Spectral Angle** 0.72

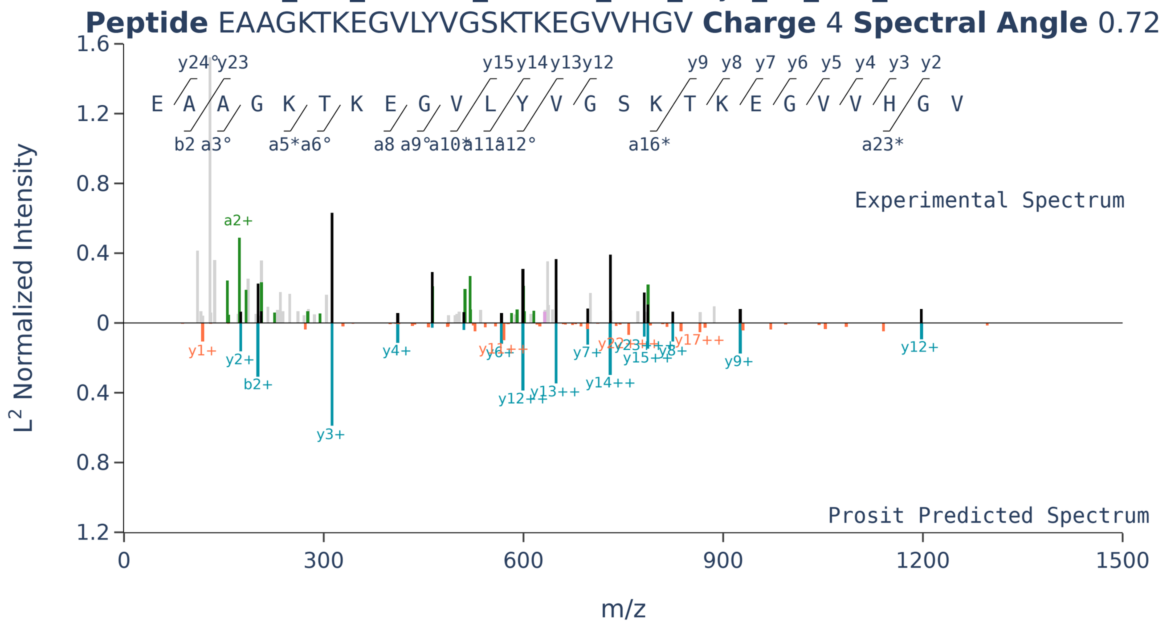


**Source** W_Soh_160821_170921_Goe_aSyn_A2_24h_R2 **Scan** 7737
**Peptide** MKGLSKAKEGVVAAAEKTK **Charge** 4 **Spectral Angle** 0.66

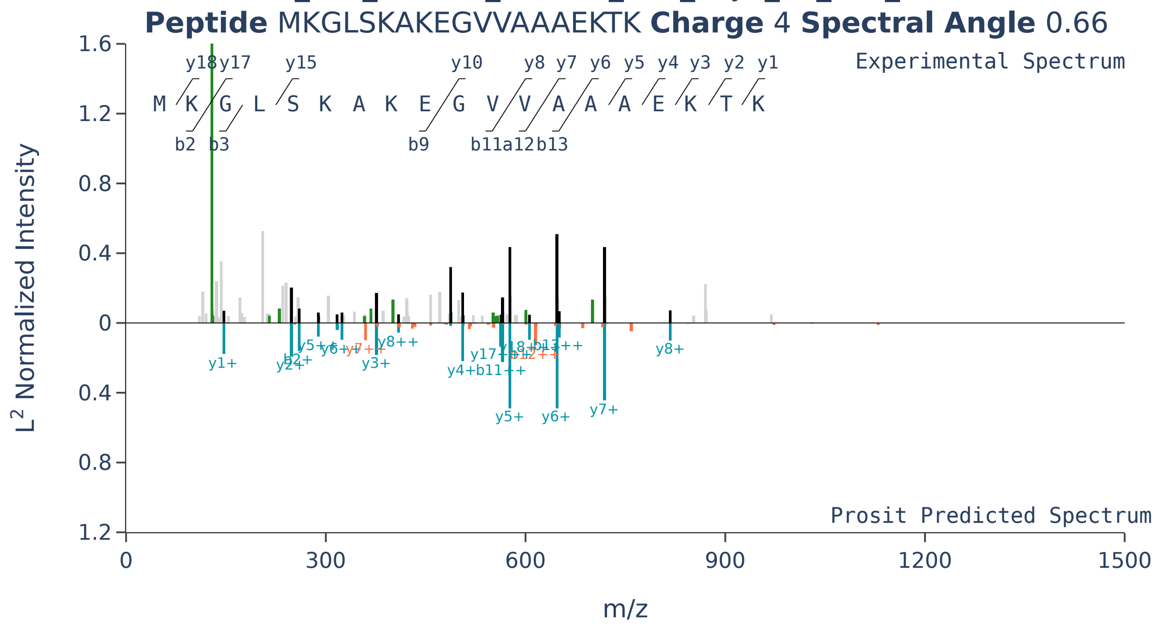


**Source** W_Soh_160821_170921_Goe_aSyn_A2_24h_R2 **Scan** 14321
**Peptide** GVAEAAGKTKEGVLYVGSKTKEGVVHGVAT **Charge** 4 **Spectral Angle** 0.67

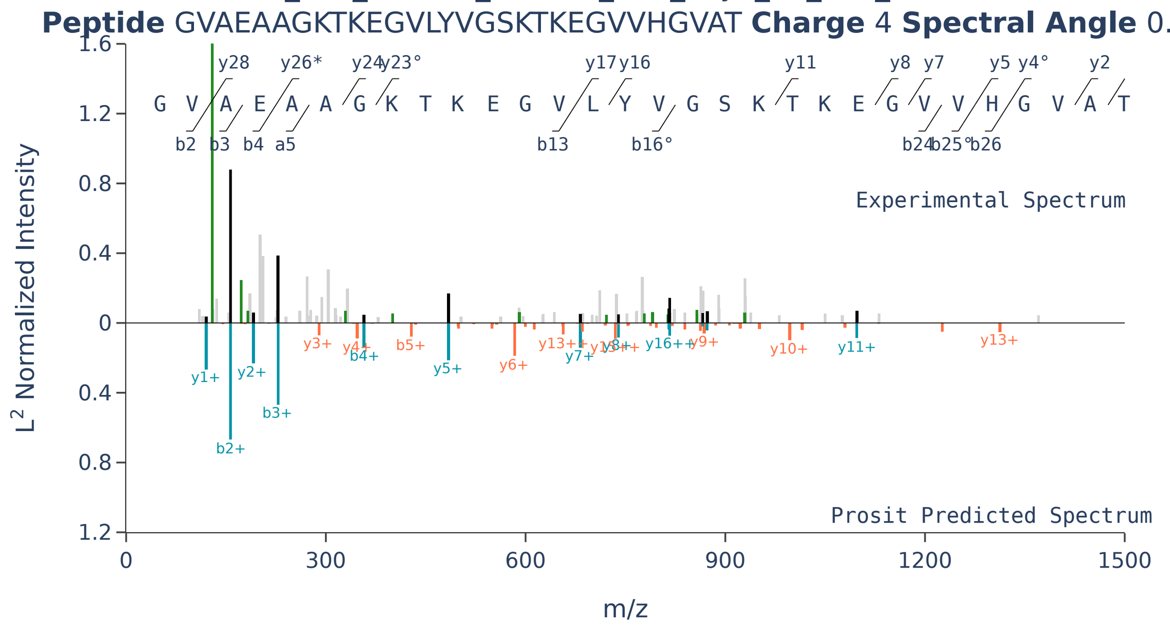


**Source** W_Soh_160821_170921_Goe_aSyn_A1_24h_R1 **Scan** 13797
**Peptide** TAVAQKTVEGAGSIAAAT **Charge** 2 **Spectral Angle** 0.73

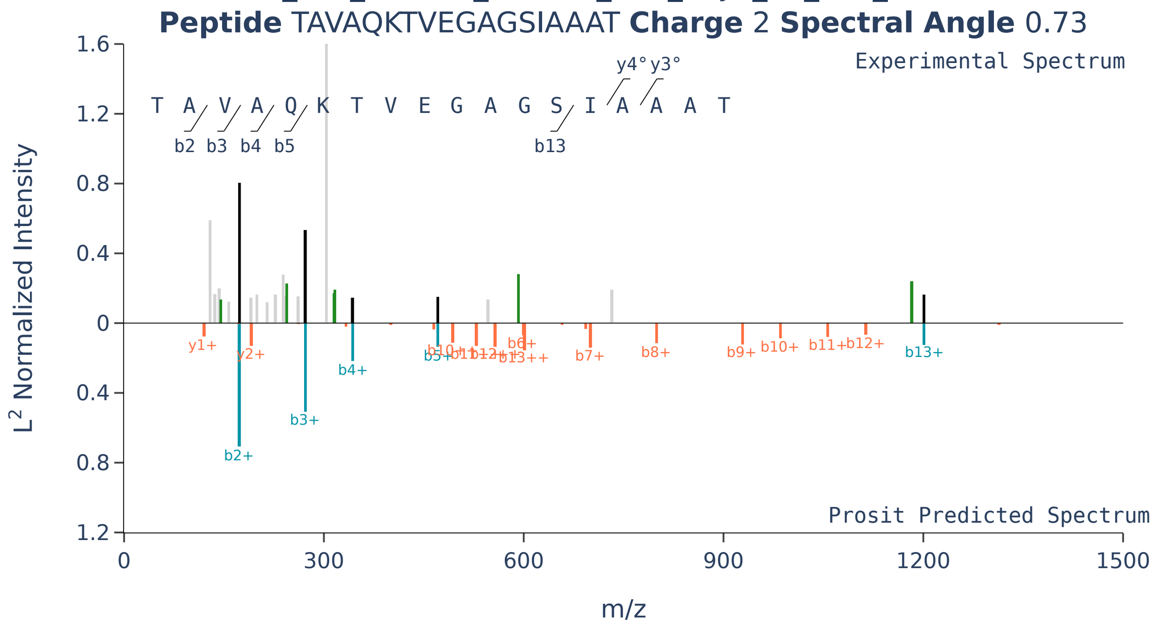


**Source** W_Soh_160821_170921_Goe_aSyn_A2_24h_R2 **Scan** 13943
**Peptide** VAEAAGKTKEGVLYVGSKTKEGVVHGVATV **Charge** 4 **Spectral Angle** 0.75

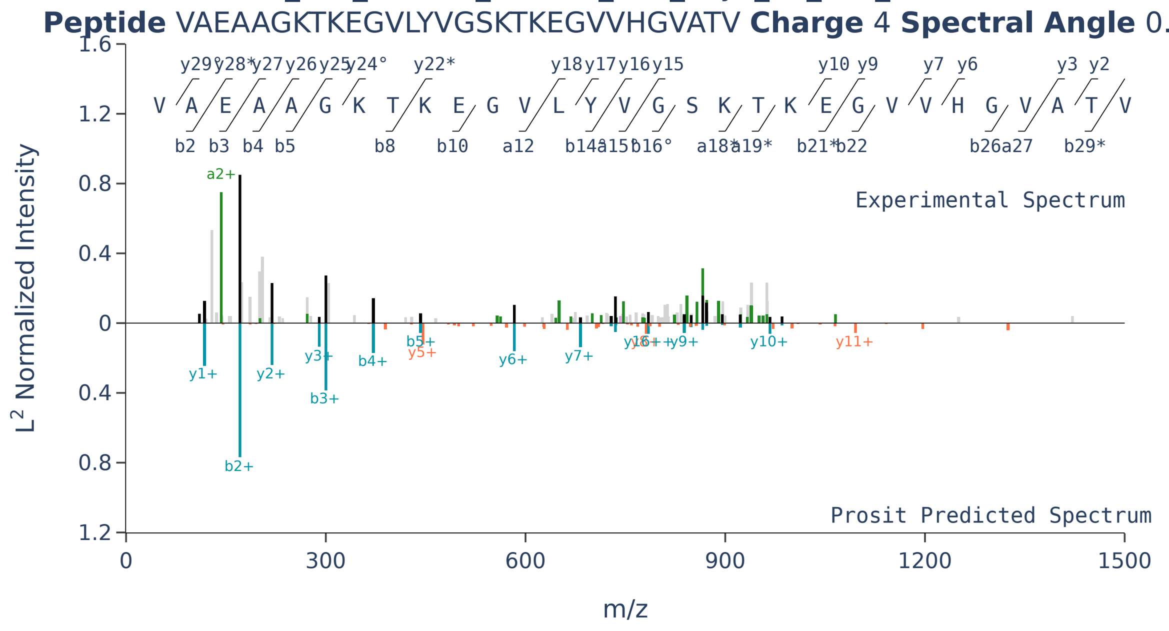


**Source** W_Soh_160821_170921_Goe_aSyn_A1_24h_R2 **Scan** 12626
**Peptide** MKGLSKAKEGVVAAAEKTKQGVAEAA **Charge** 5 **Spectral Angle** 0.66

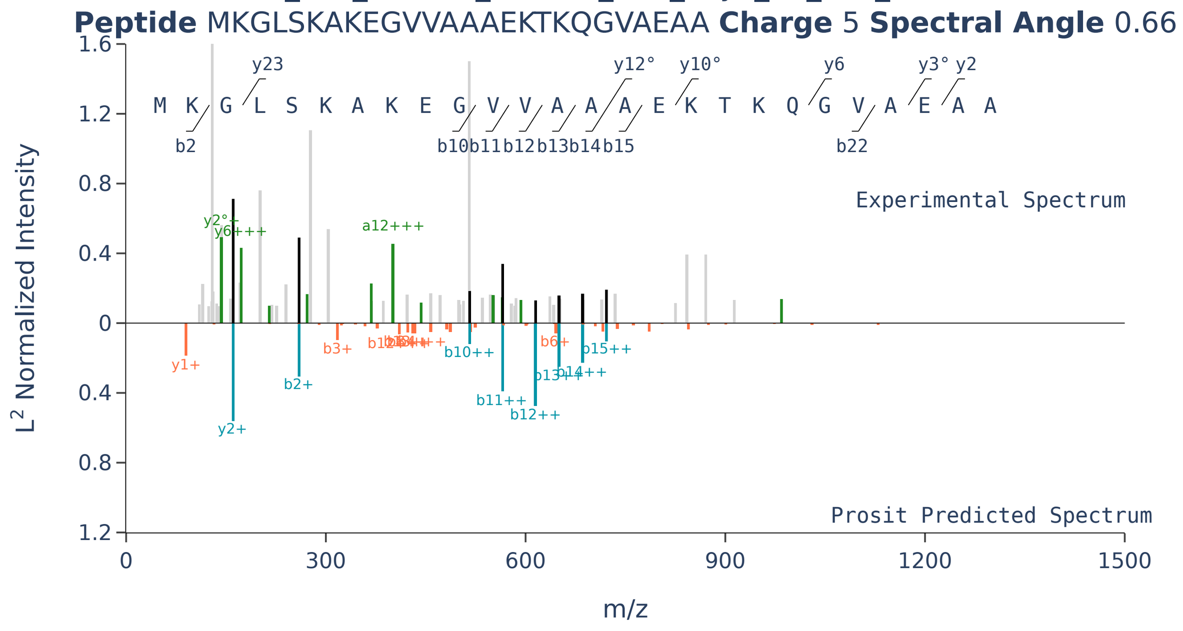


**Source** W_Soh_160821_170921_Goe_aSyn_A1_24h_R2 **Scan** 20108
**Peptide** TAVAQKTVEGAGSIAAATGFVK **Charge** 3 **Spectral Angle** 0.7

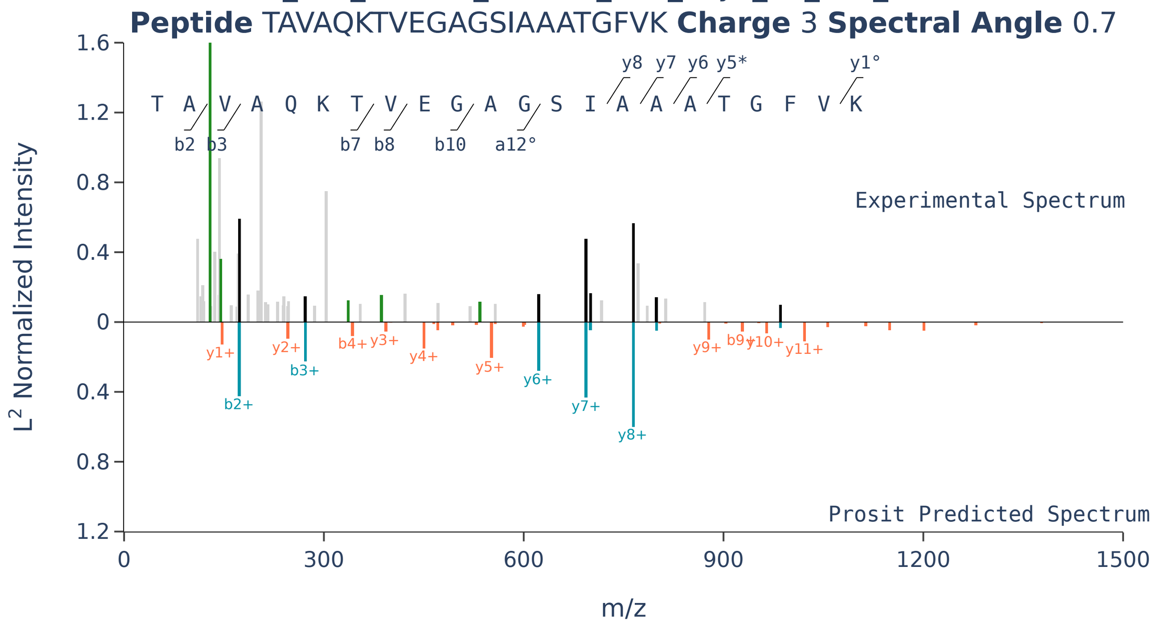


**Source** W_Soh_160821_170921_Goe_aSyn_A1_24h_R1 **Scan** 10191
**Peptide** SKTKEGVVH **Charge** 2 **Spectral Angle** 0.83

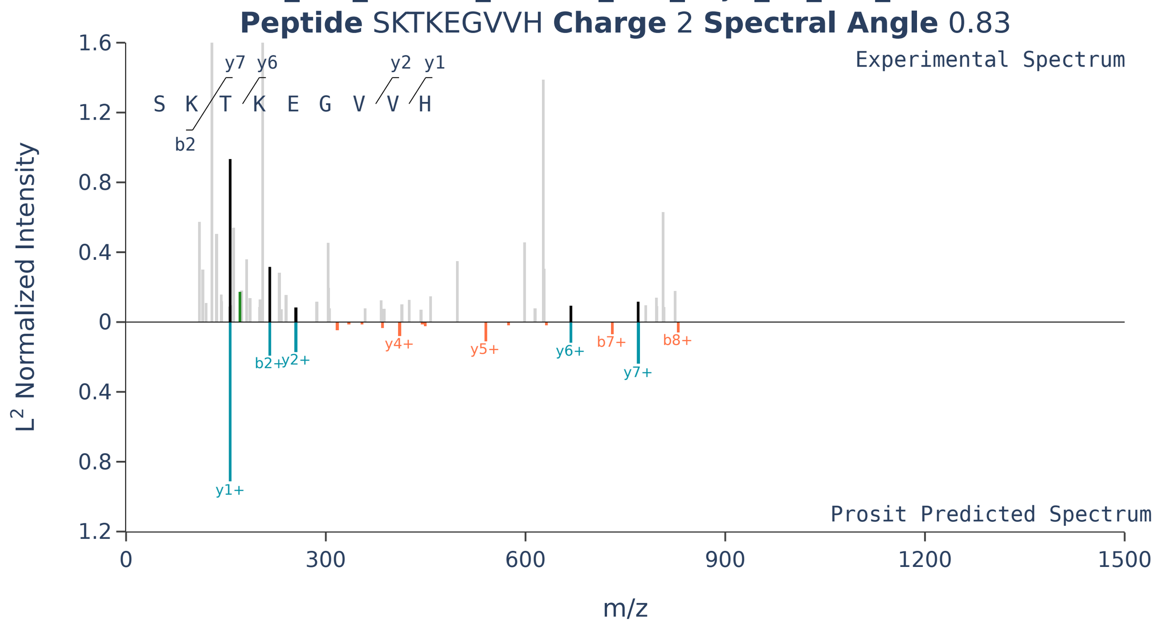


**Source** W_Soh_160821_170921_Goe_aSyn_A1_6h_R2 **Scan** 20326
**Peptide** AATGFVKKDQLGKNEEGAPQEGILEDMPVD **Charge** 4 **Spectral Angle** 0.63

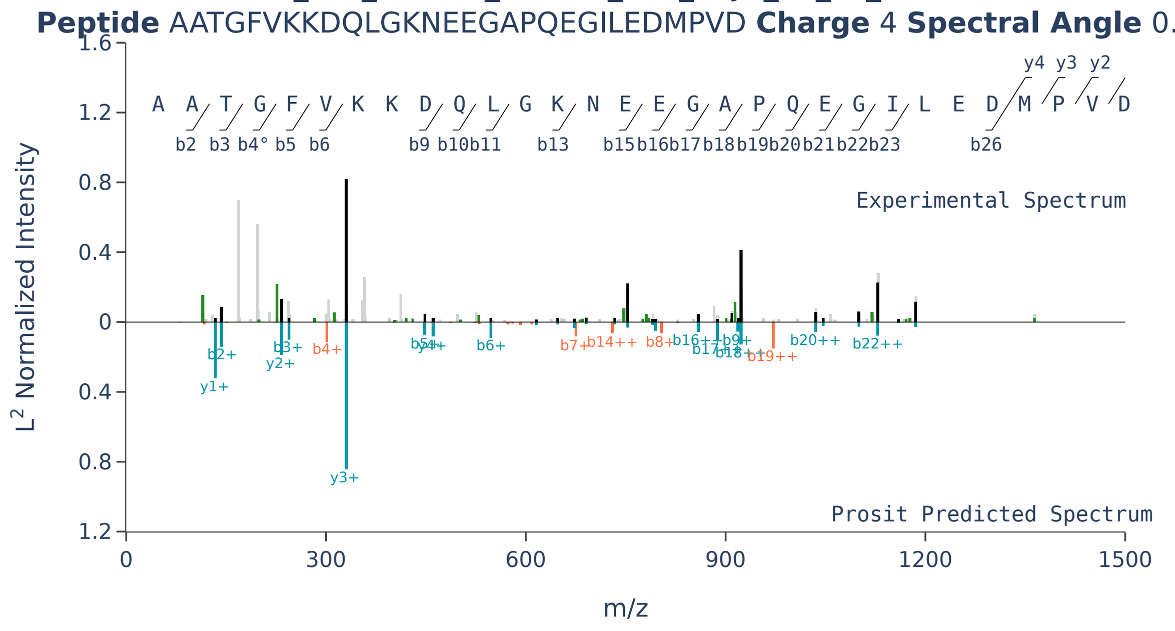


**Source** W_Soh_160821_170921_Goe_aSyn_A1_6h_R2 **Scan** 28562
**Peptide** KEQVTNVGGAVVTGVTAVAQKTVEGAGSL **Charge** 3 **Spectral Angle** 0.59

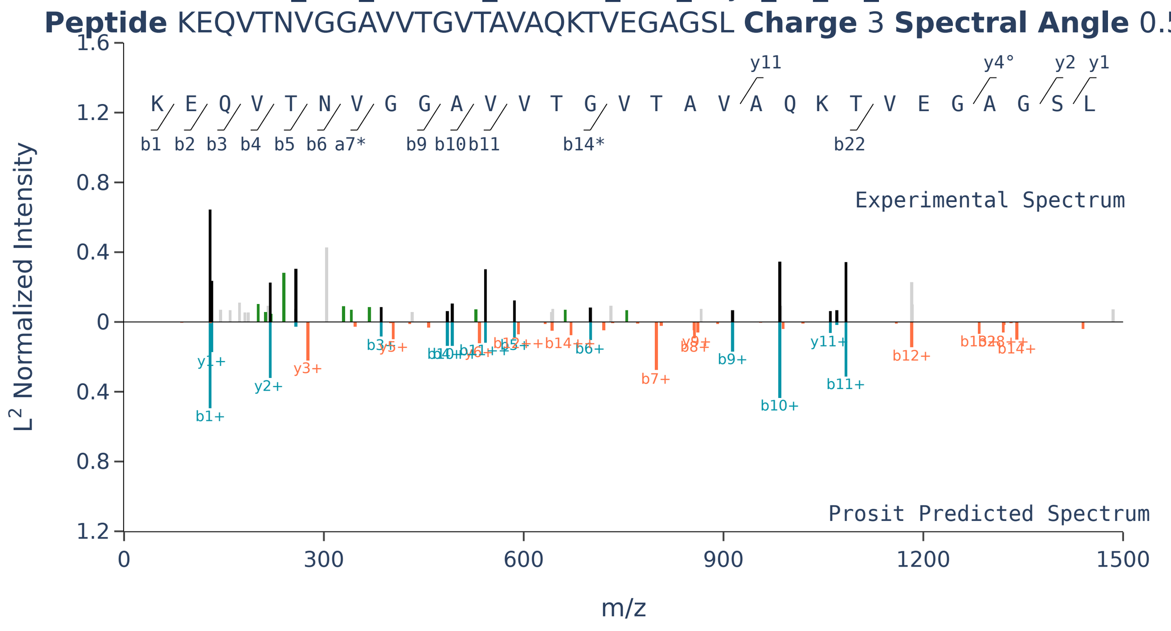


**Source** W_Soh_160821_170921_Goe_aSyn_A1_24h_R1 **Scan** 18544
**Peptide** EGAGSIAAATGFVKKDQLGKNEEGAPQEGL **Charge** 4 **Spectral Angle** 0.65

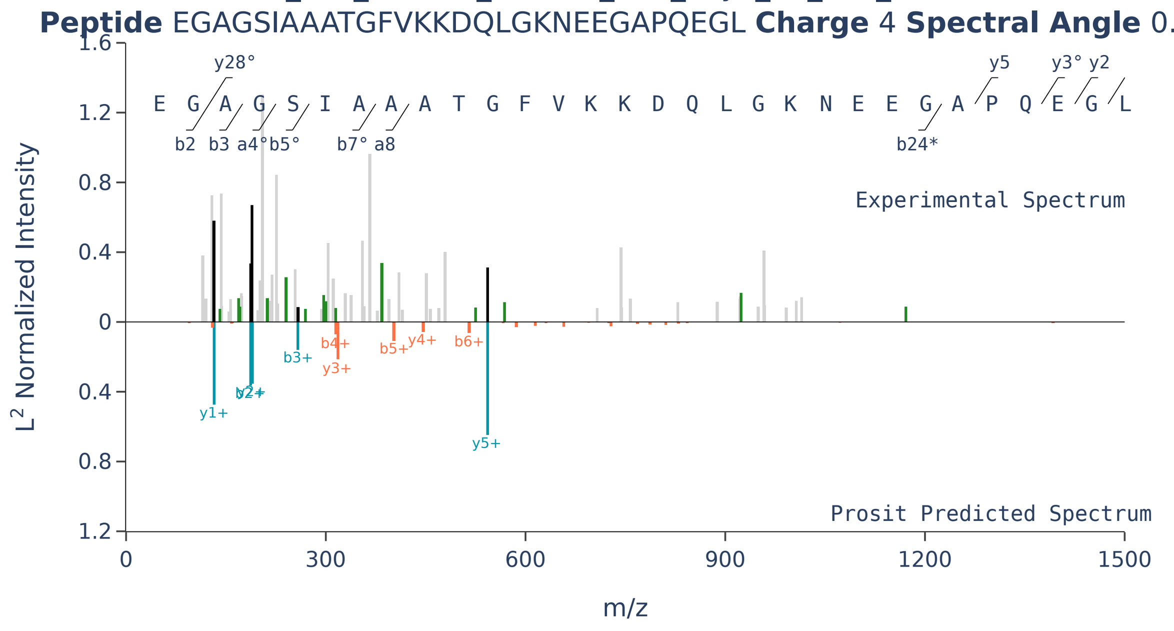


**Source** W_Soh_160821_170921_Goe_aSyn_A1_24h_R1 **Scan** 22845
**Peptide** VAEKTKEQVTNVGGAVVTGVTAVAQKTV **Charge** 3 **Spectral Angle** 0.64

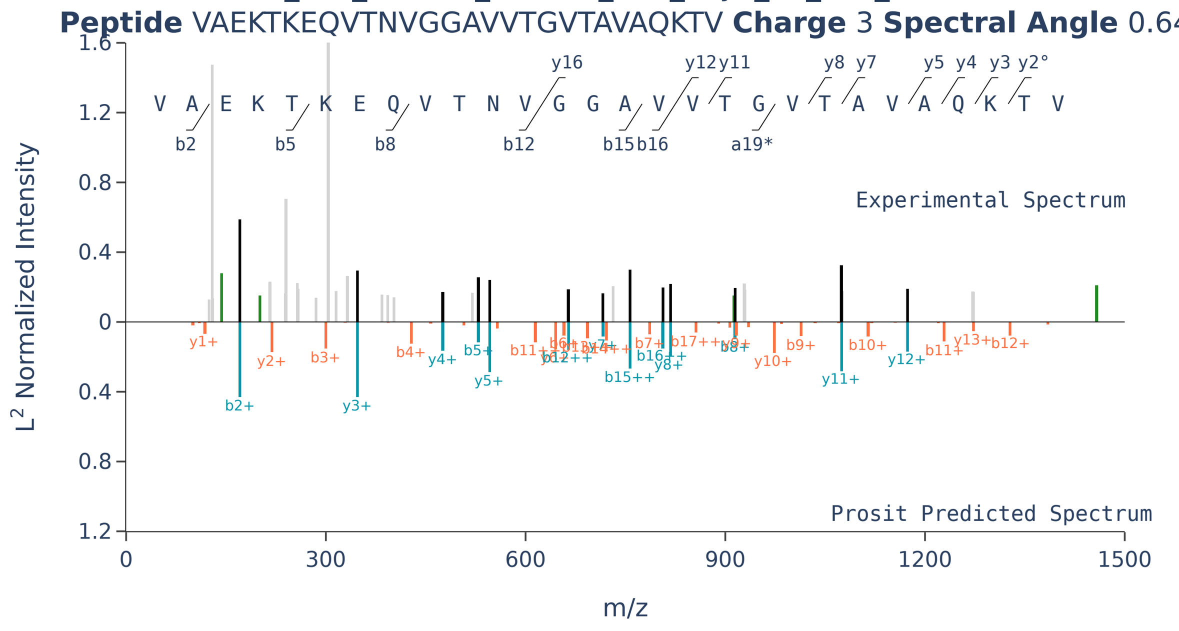

**Source** W_Soh_160821_170921_Goe_aSyn_A1_24h_R1 **Scan** 18901
**Peptide** GSIAAATGFVKKDQLGKNEEGAPQEGILED **Charge** 3 **Spectral Angle** 0.43

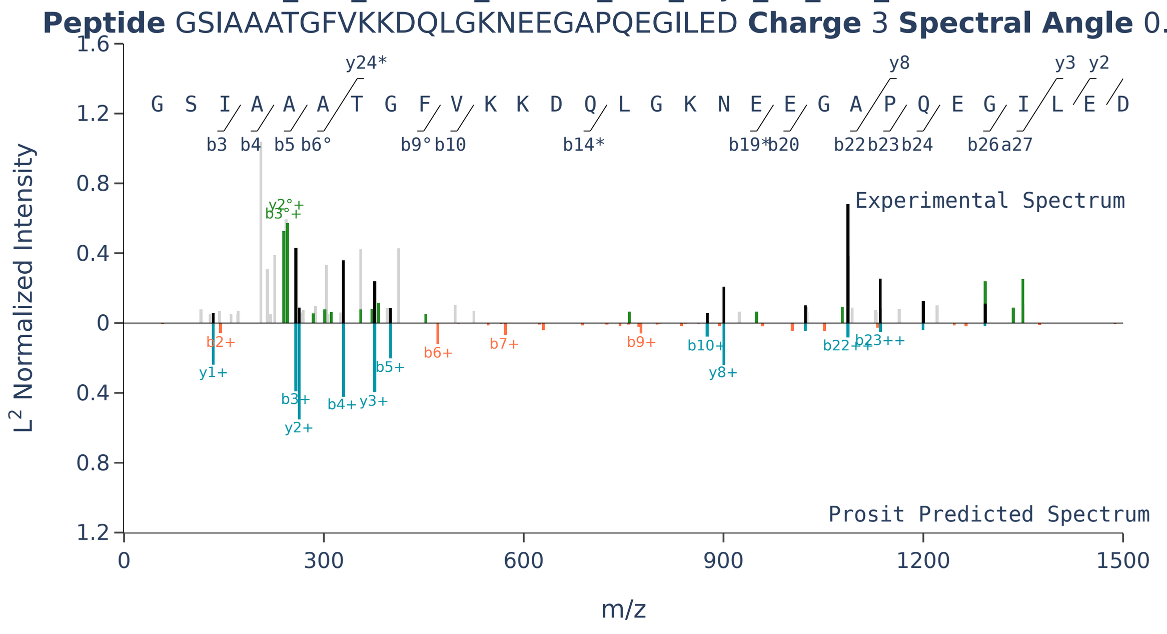


**Source** W_Soh_160821_170921_Goe_aSyn_A1_24h_R2 **Scan** 20553
**Peptide** VTGVTAVAQKTVEGAGSIAA **Charge** 2 **Spectral Angle** 0.59

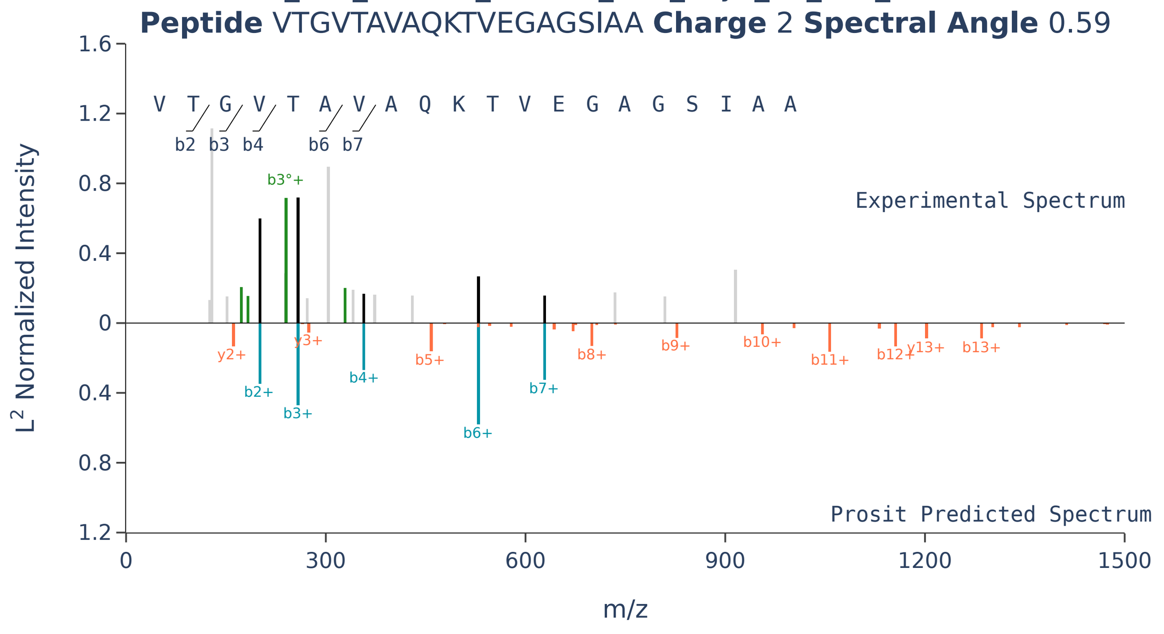


**Source** W_Soh_160821_170921_Goe_aSyn_A2_24h_R1 **Scan** 8238
**Peptide** MKGLSKAKEGVVAAA **Charge** 3 **Spectral Angle** 0.62

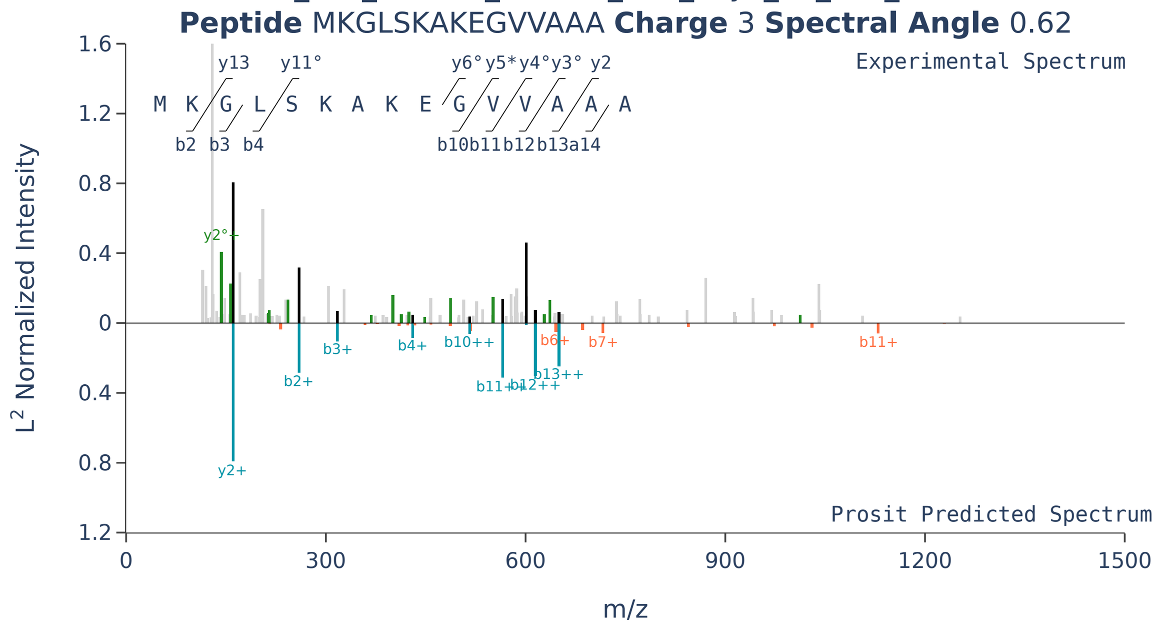


**Source** W_Soh_160821_170921_Goe_aSyn_A2_24h_R2 **Scan** 20808
**Peptide** ATVAEKTKEQVTNVGGAVVTGVTAVAQK **Charge** 4 **Spectral Angle** 0.77

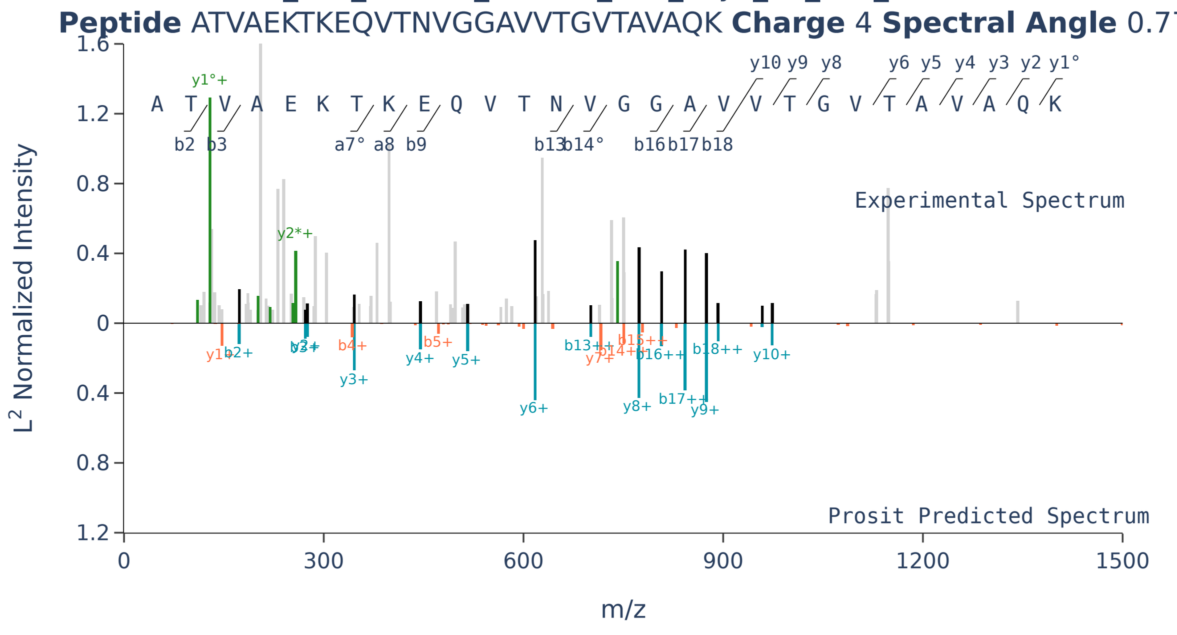


**Source** W_Soh_160821_170921_Goe_aSyn_A1_24h_R2 **Scan** 19292
**Peptide** EKTKEQVTNVGGAVVTGVTAVAQKT **Charge** 4 **Spectral Angle** 0.66

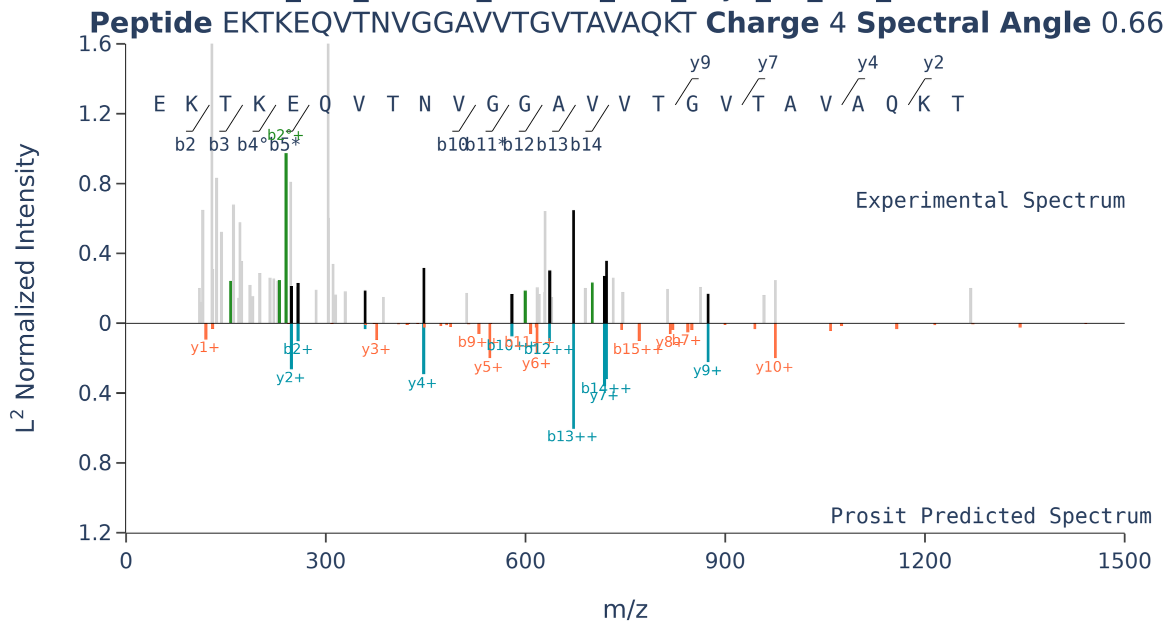


**Source** W_Soh_160821_170921_Goe_aSyn_A1_24h_R1 **Scan** 5680
**Peptide** VAAAEKTKQGVAEA **Charge** 2 **Spectral Angle** 0.76

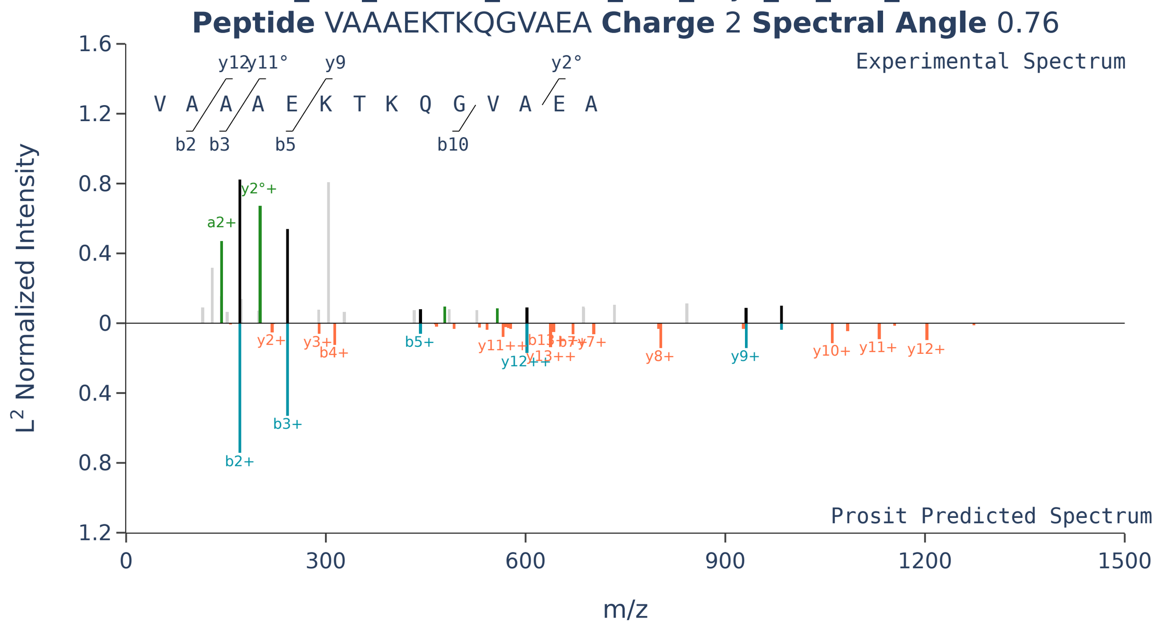


**Source** W_Soh_160821_170921_Goe_aSyn_A2_6h_R1 **Scan** 12166
**Peptide** LYVGSKTKEGVVHGVAT **Charge** 3 **Spectral Angle** 0.74

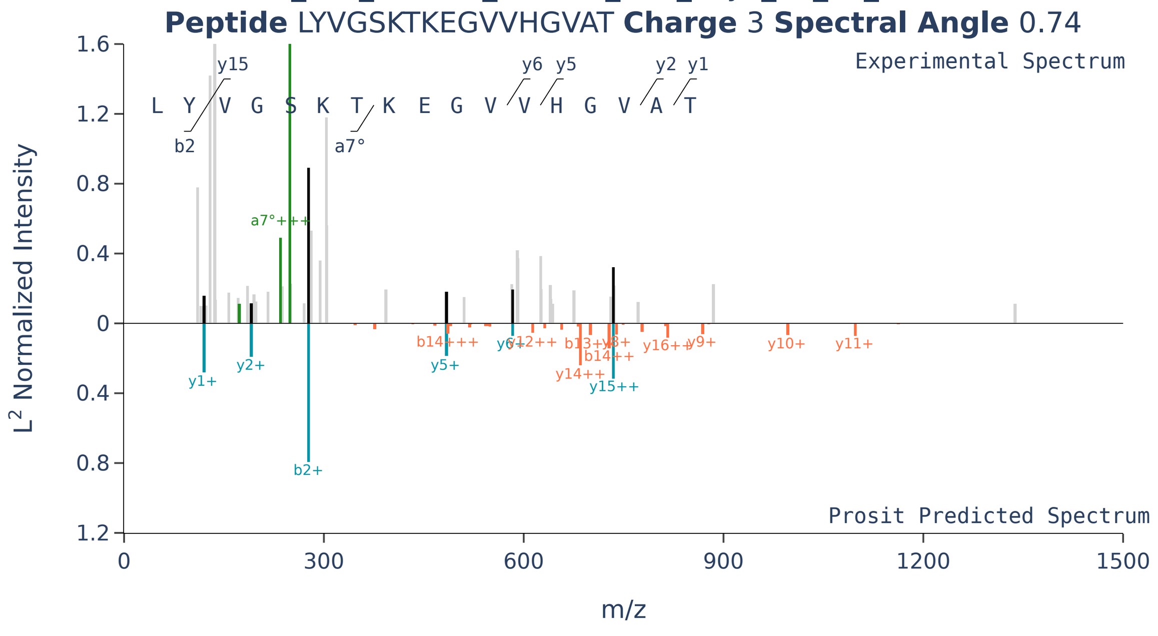


**Source** W_Soh_160821_170921_Goe_aSyn_A1_24h_R1 **Scan** 22999
**Peptide** GKNEEGAPQEGILEDMPVDPDN **Charge** 3 **Spectral Angle** 0.66

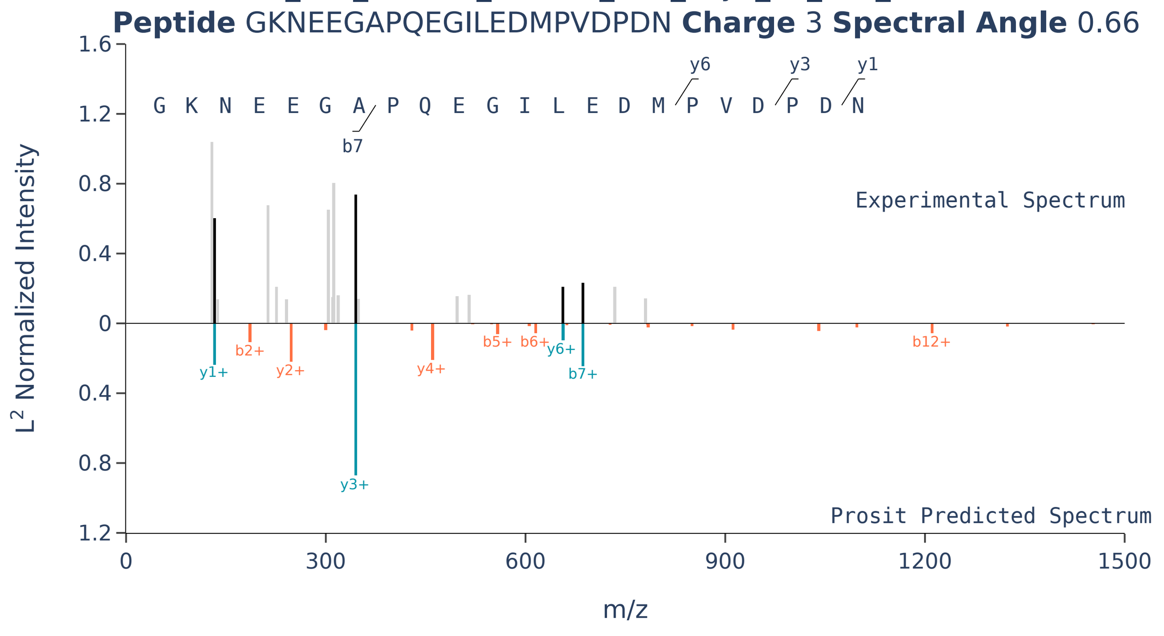


**Source** W_Soh_160821_170921_Goe_aSyn_A1_24h_R1 **Scan** 29723
**Peptide** NVGGAVVTGVTAVAQKTVEGAGSIAAA **Charge** 3 **Spectral Angle** 0.68

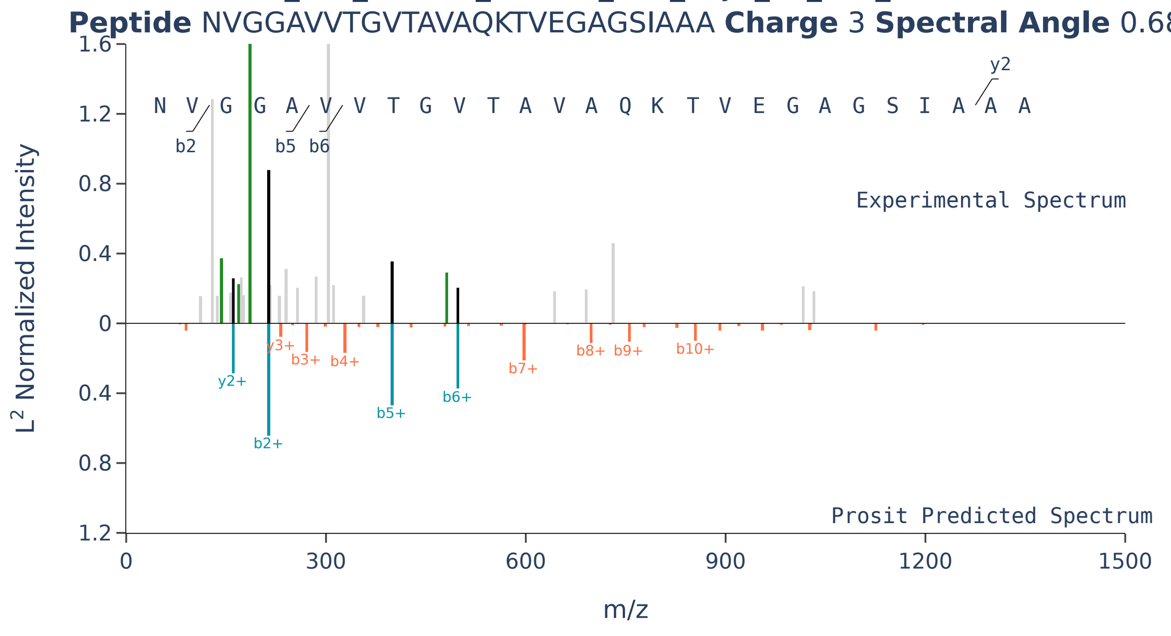


**Source** W_Soh_160821_170921_Goe_aSyn_A1_3h_R1 **Scan** 10300
**Peptide** KKDQLGKNEEGAPQEGILE **Charge** 3 **Spectral Angle** 0.83

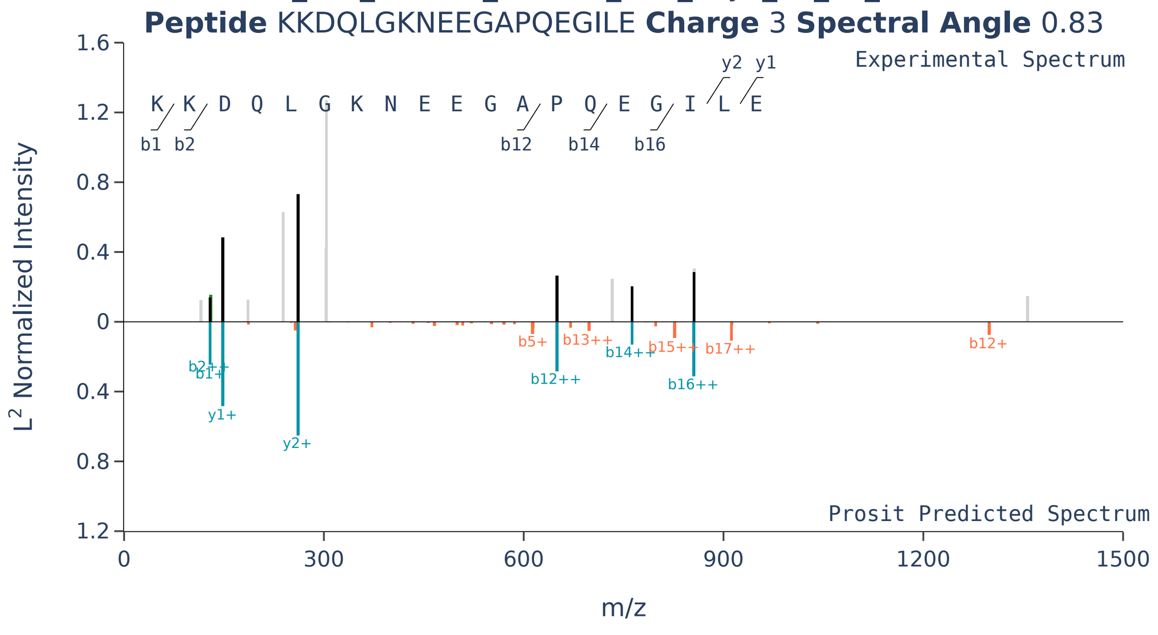


**Source** W_Soh_160821_170921_Goe_aSyn_A2_24h_R2 **Scan** 9927
**Peptide** SKAKEGVVAAAEKTKQGVAEAAGKT **Charge** 4 **Spectral Angle** 0.63

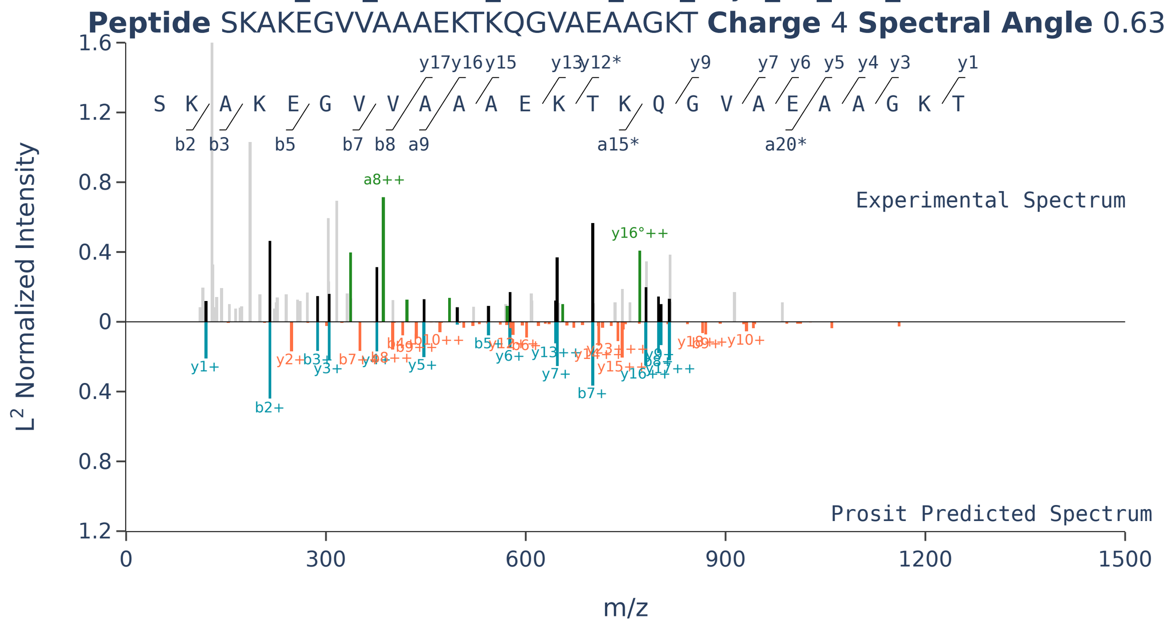


**Source** W_Soh_160821_170921_Goe_aSyn_A1_24h_R2 **Scan** 11326
**Peptide** KEGVLYVGSKTKEGVVHGVAT **Charge** 4 **Spectral Angle** 0.71

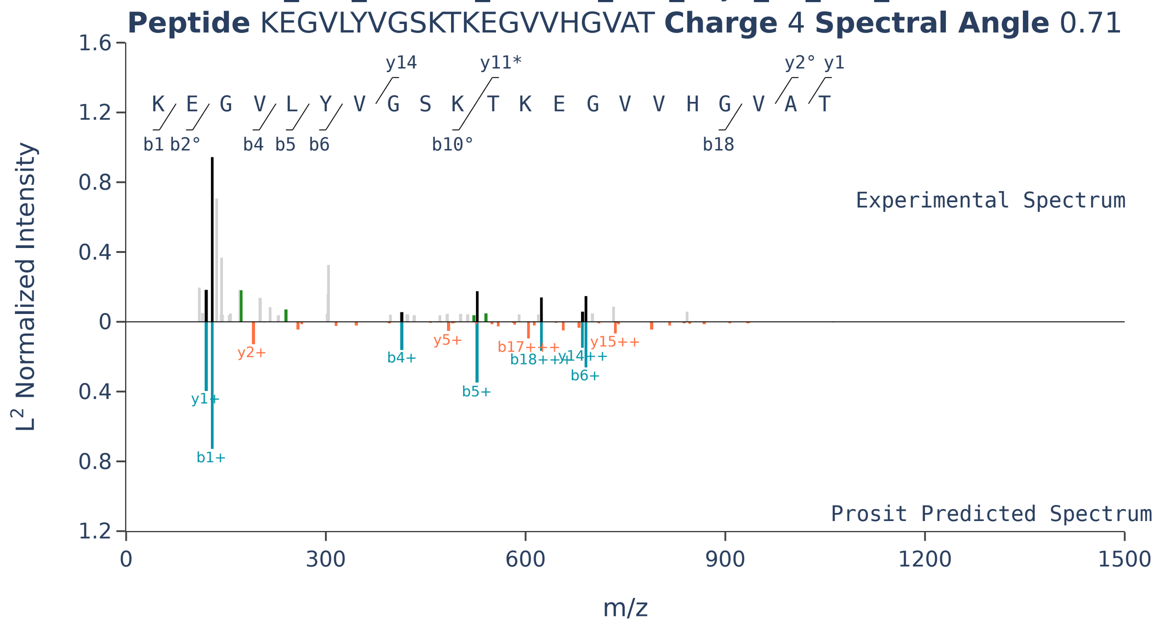


**Source** W_Soh_160821_170921_Goe_aSyn_A1_6h_R1 **Scan** 22896
**Peptide** VATVAEKTKEQVTNVGGAVVTGVTAVAQKT **Charge** 3 **Spectral Angle** 0.66

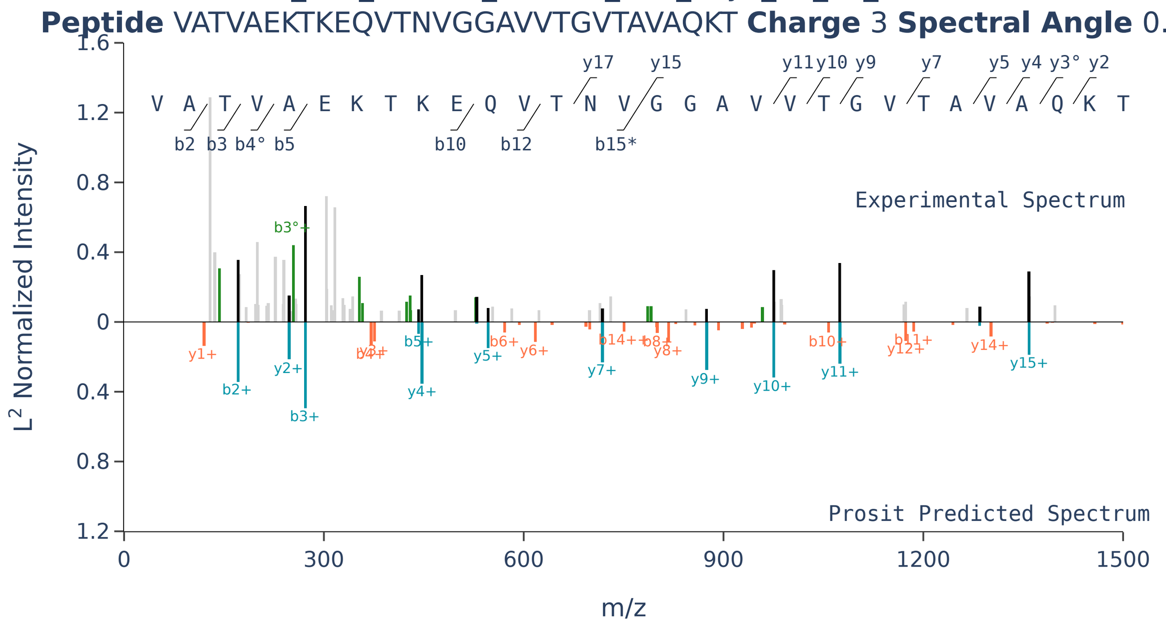


**Source** W_Soh_160821_170921_Goe_aSyn_A1_24h_R1 **Scan** 11877
**Peptide** AVAQKTVEGAGSIAAA **Charge** 2 **Spectral Angle** 0.66

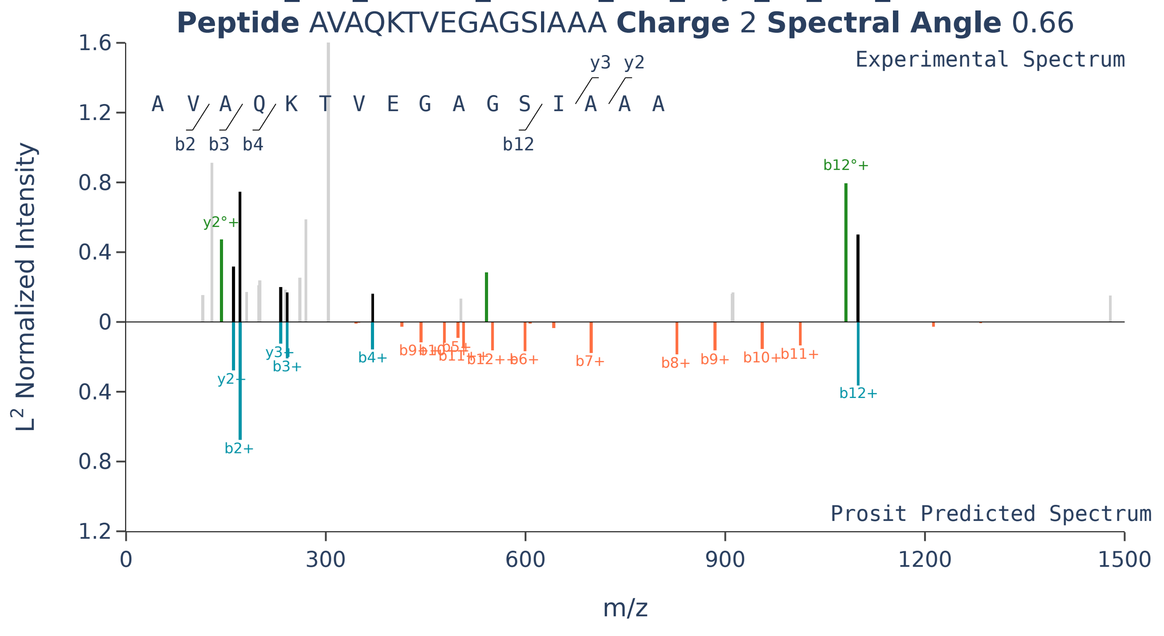


**Source** W_Soh_160821_170921_Goe_aSyn_A2_24h_R2 **Scan** 14738
**Peptide** VFMKGLSKAKEGVVAAAEKTKQGVAE **Charge** 5 **Spectral Angle** 0.73

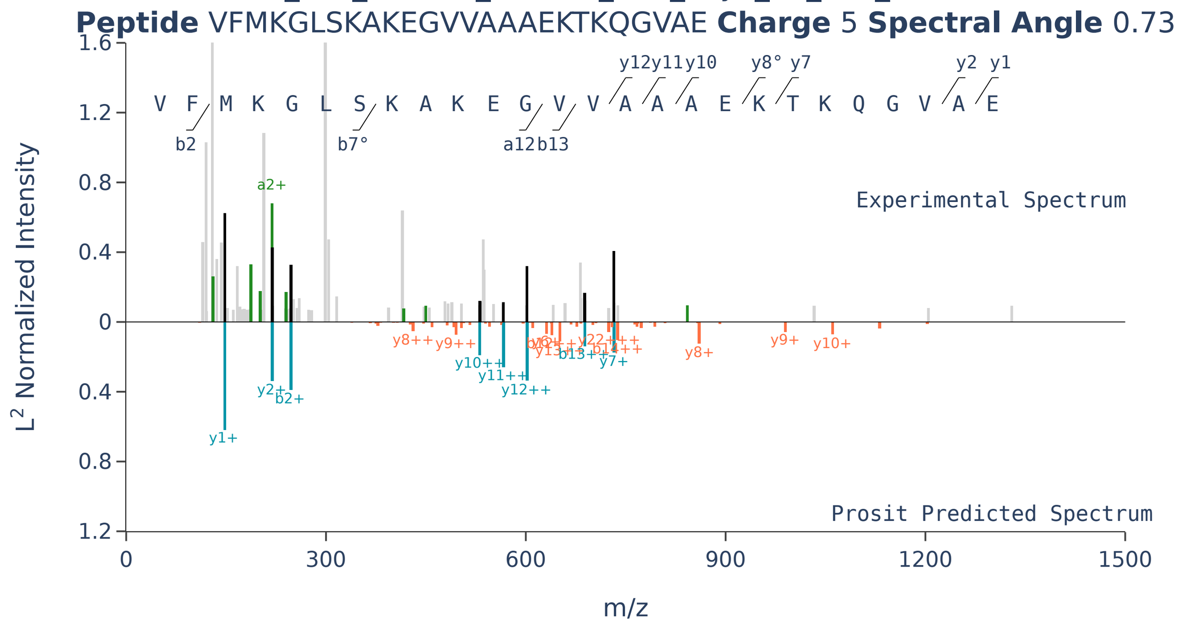

**Source** W_Soh_160821_170921_Goe_aSyn_A1_24h_R1 **Scan** 5843
**Peptide** GVVAAAEKTK **Charge** 2 **Spectral Angle** 0.57

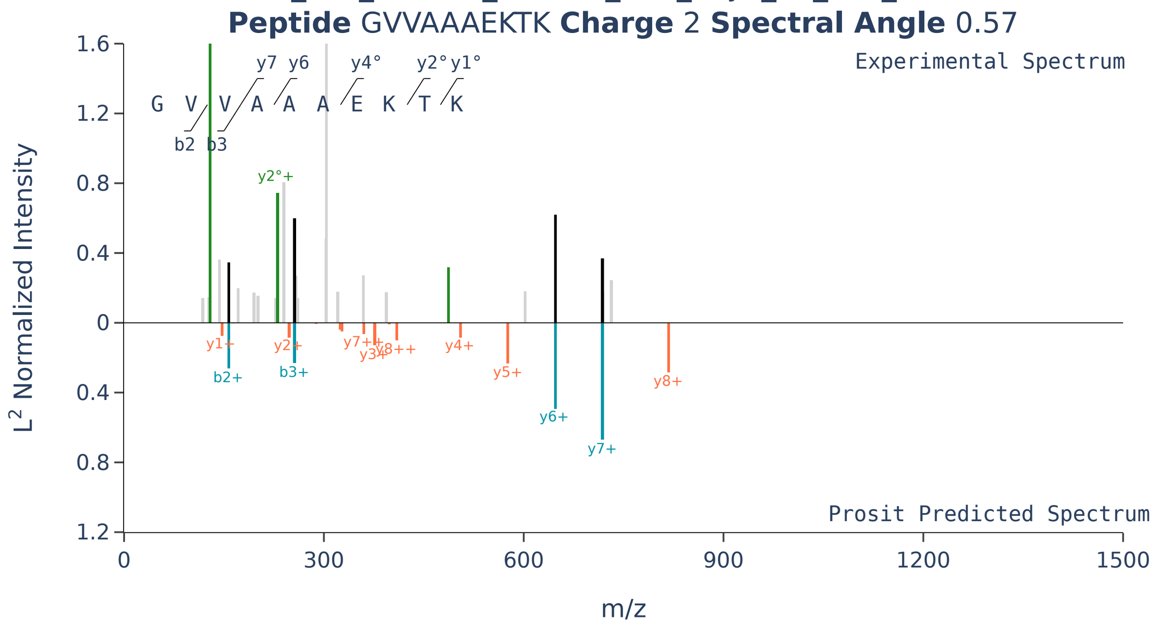


**Source** W_Soh_160821_170921_Goe_aSyn_A2_24h_R1 **Scan** 24708
**Peptide** EKTKEQVTNVGGAVVTGVTAVAQKTVEGA **Charge** 4 **Spectral Angle** 0.66

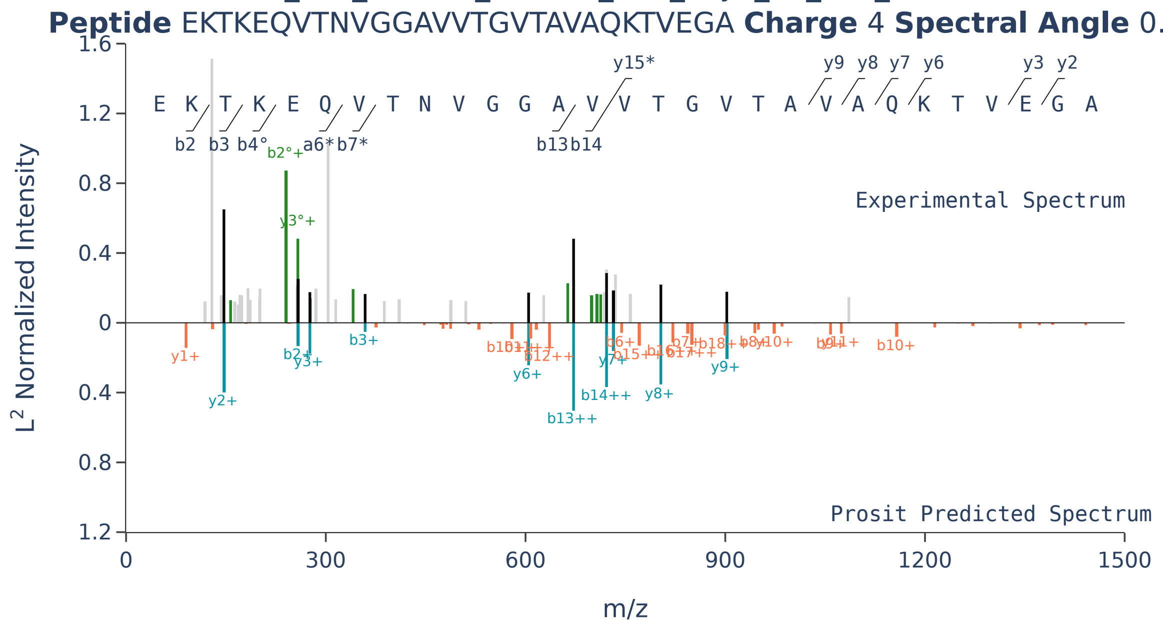


**Source** W_Soh_160821_170921_Goe_aSyn_A1_24h_R1 **Scan** 10921
**Peptide** GSKTKEGVVHGVATVAEKTKEQVT **Charge** 5 **Spectral Angle** 0.58

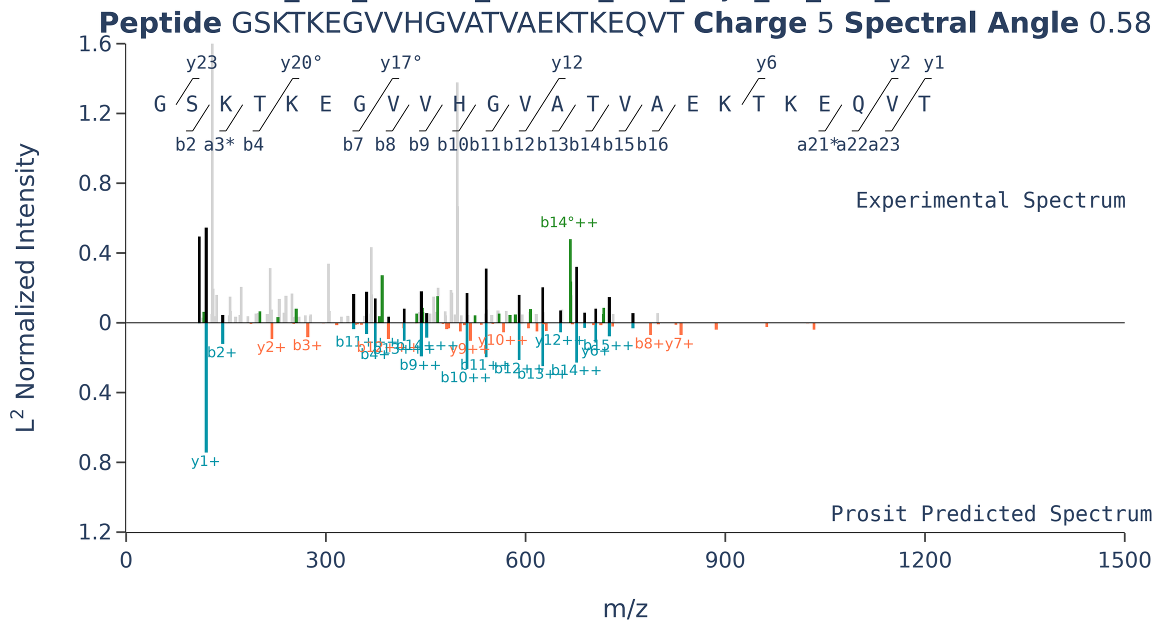


**Source** W_Soh_160821_170921_Goe_aSyn_A1_24h_R2 **Scan** 10294
**Peptide** FMKGLSKAKEGVVA **Charge** 4 **Spectral Angle** 0.65

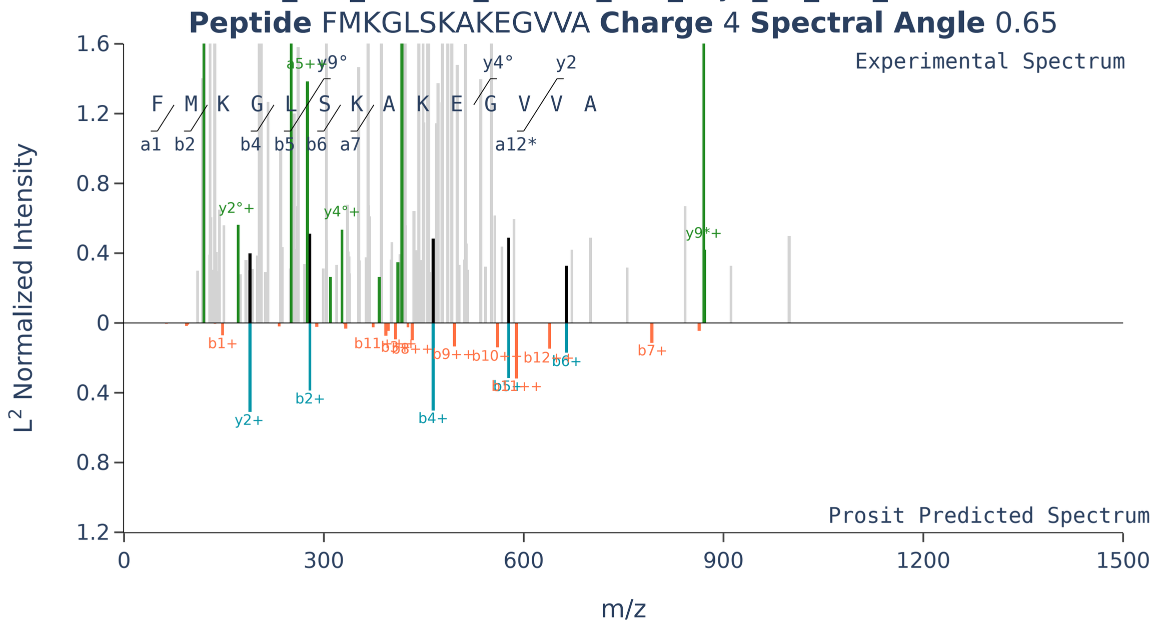


**Source** W_Soh_160821_170921_Goe_aSyn_A1_24h_R1 **Scan** 13390
**Peptide** KEGVLYVGSKTKEGVVHGVATVAEKTKE **Charge** 5 **Spectral Angle** 0.8

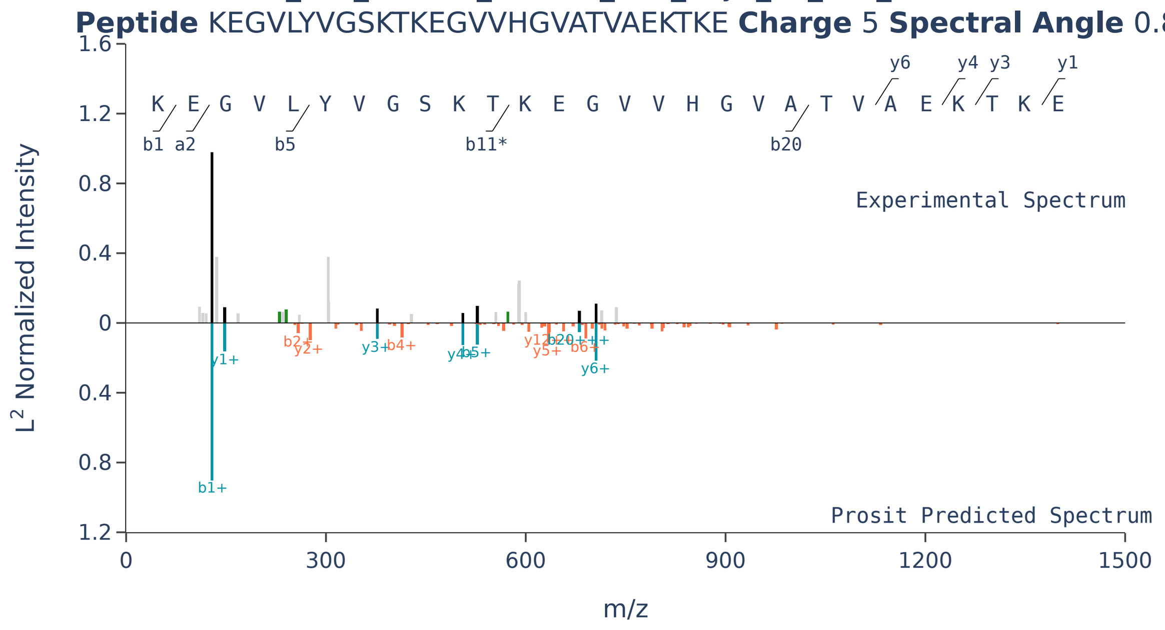


**Source** W_Soh_160821_170921_Goe_aSyn_A2_24h_R2 **Scan** 12678
**Peptide** YVGSKTKEGVVHGVATVAEKTKEQVTN **Charge** 4 **Spectral Angle** 0.7

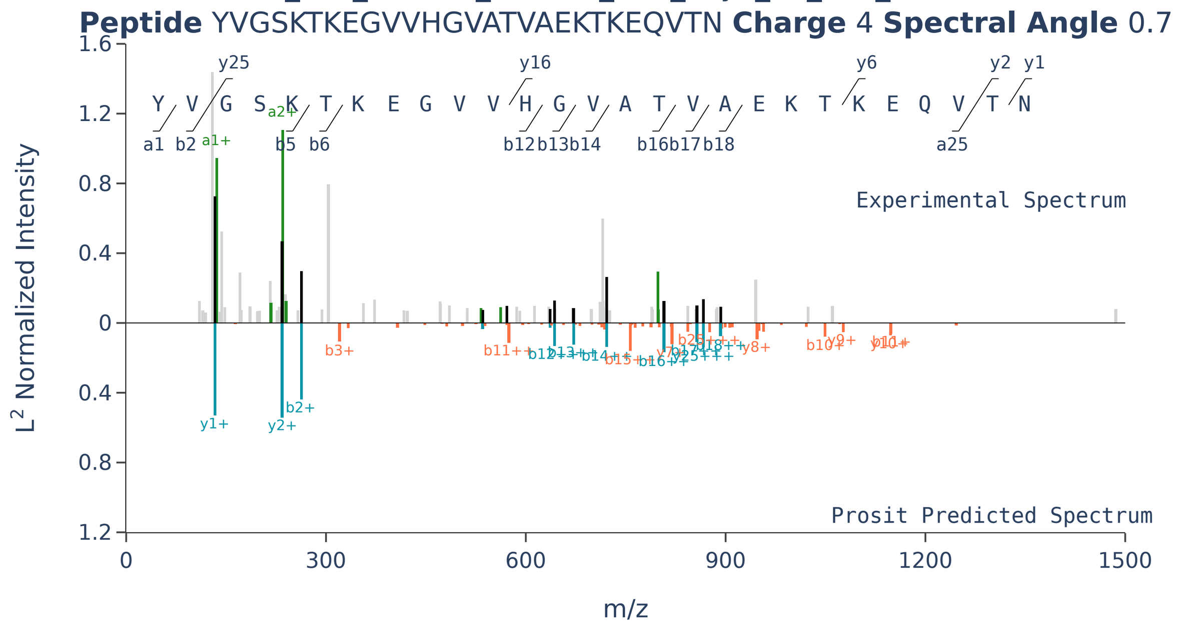


**Source** W_Soh_160821_170921_Goe_aSyn_A2_24h_R2 **Scan** 7377
**Peptide** VVAAAEKTKQGVAEAA **Charge** 2 **Spectral Angle** 0.8

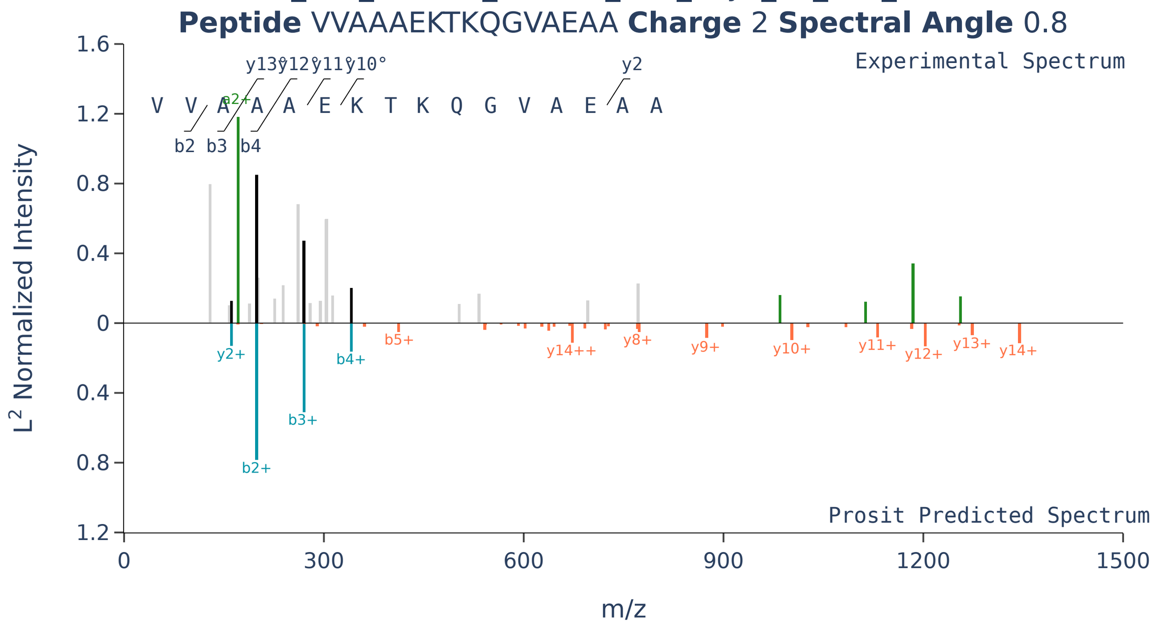


**Source** W_Soh_160821_170921_Goe_aSyn_A2_24h_R1 **Scan** 12773
**Peptide** GKTKEGVLYVGSKTKEGVVHGVATVAE **Charge** 4 **Spectral Angle** 0.78

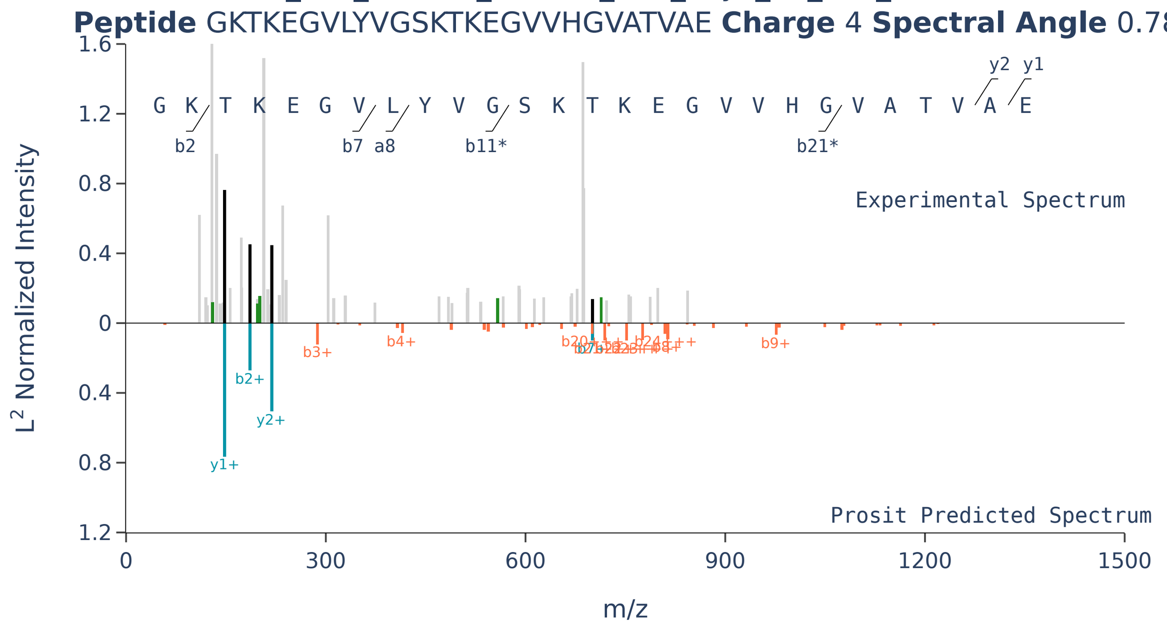


**Source** W_Soh_160821_170921_Goe_aSyn_A1_24h_R1 **Scan** 10628
**Peptide** GVAEAAGKTKEGVLYVGSKTKEG **Charge** 4 **Spectral Angle** 0.69

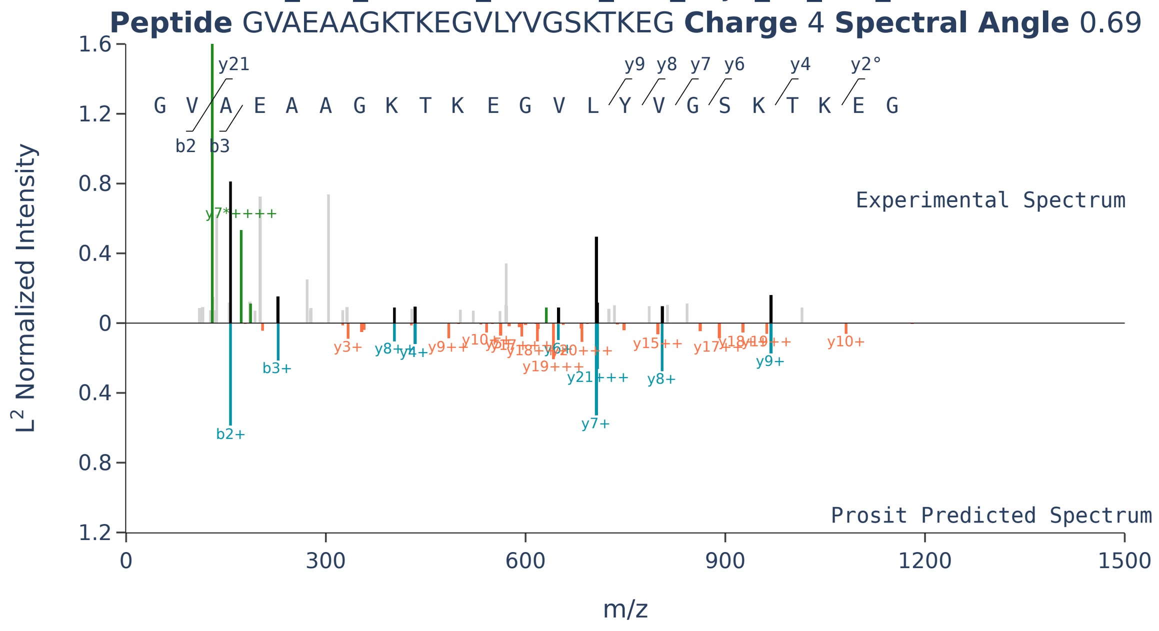


**Source** W_Soh_160821_170921_Goe_aSyn_A1_24h_R2 **Scan** 7724
**Peptide** KKDQLGKNEEGAPQEGI **Charge** 3 **Spectral Angle** 0.53

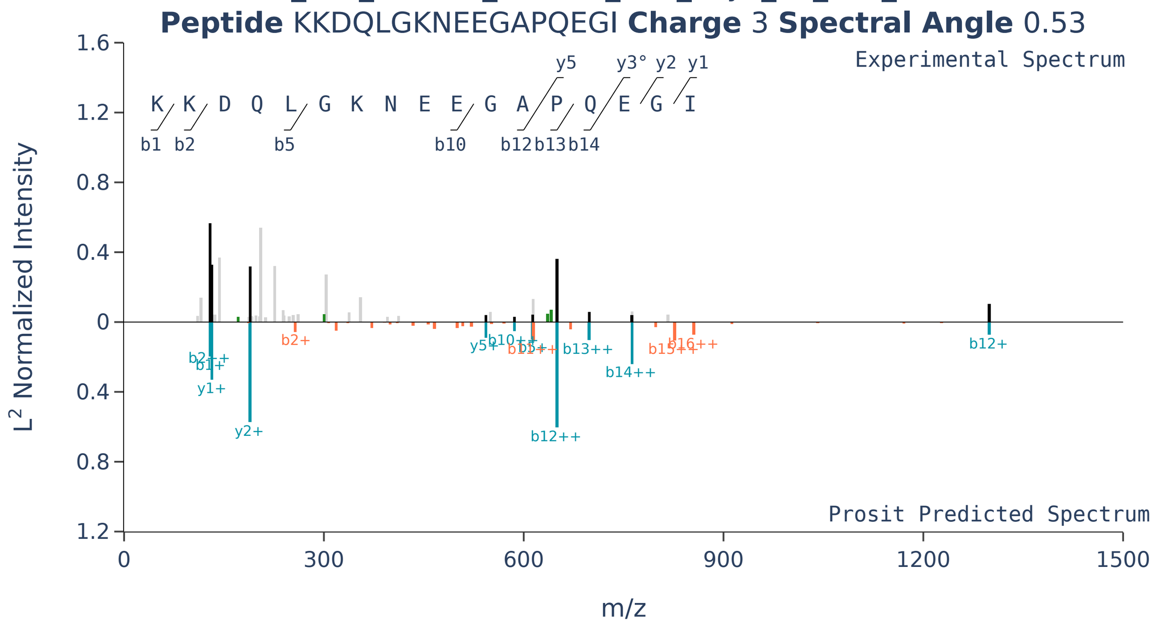


**Source** W_Soh_160821_170921_Goe_aSyn_A2_24h_R2 **Scan** 11485
**Peptide** GVTAVAQKTVEGAGS **Charge** 2 **Spectral Angle** 0.69

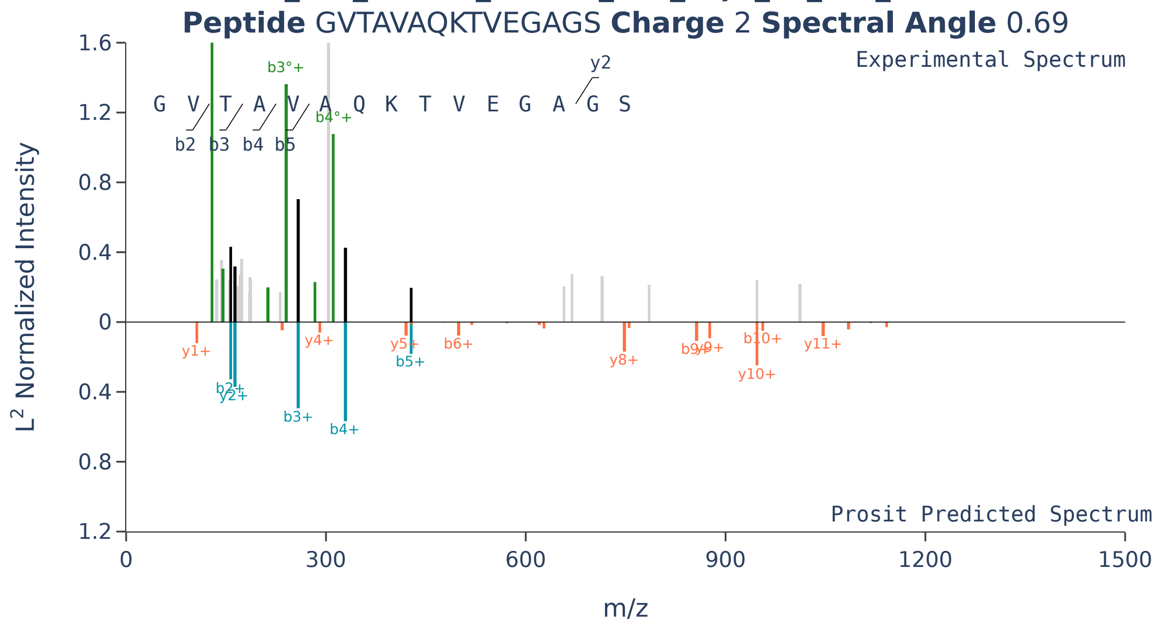


**Source** W_Soh_160821_170921_Goe_aSyn_A1_24h_R2 **Scan** 13324
**Peptide** EAAGKTKEGVLYV **Charge** 2 **Spectral Angle** 0.81

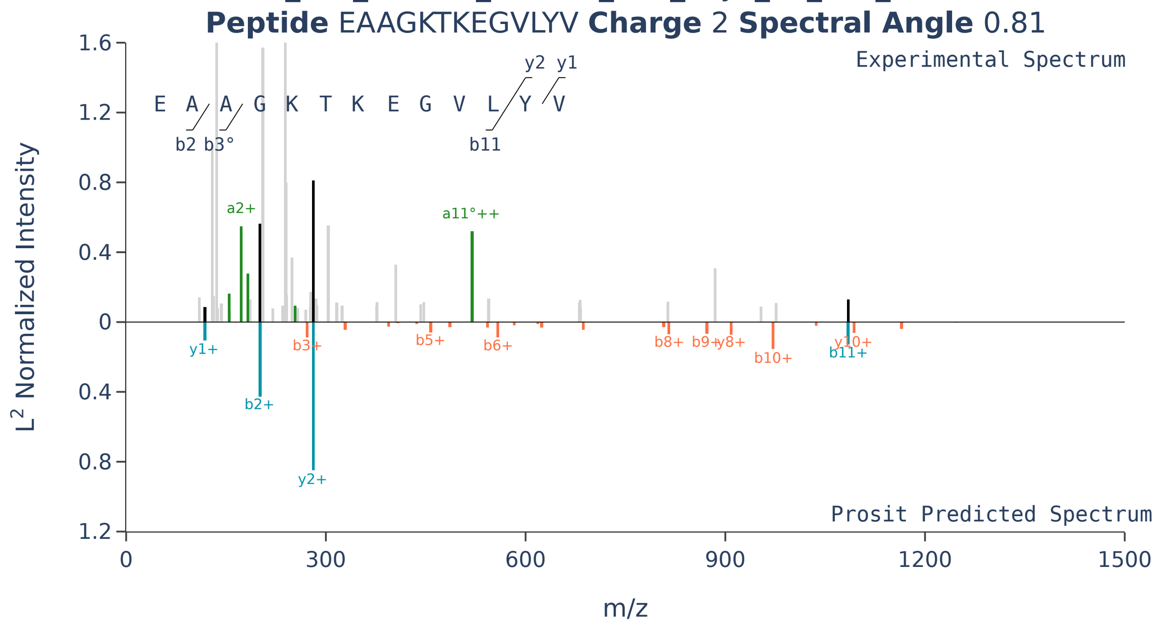


**Source** W_Soh_160821_170921_Goe_aSyn_A1_24h_R2 **Scan** 8461
**Peptide** AAAEKTKQGVAEA **Charge** 2 **Spectral Angle** 0.74

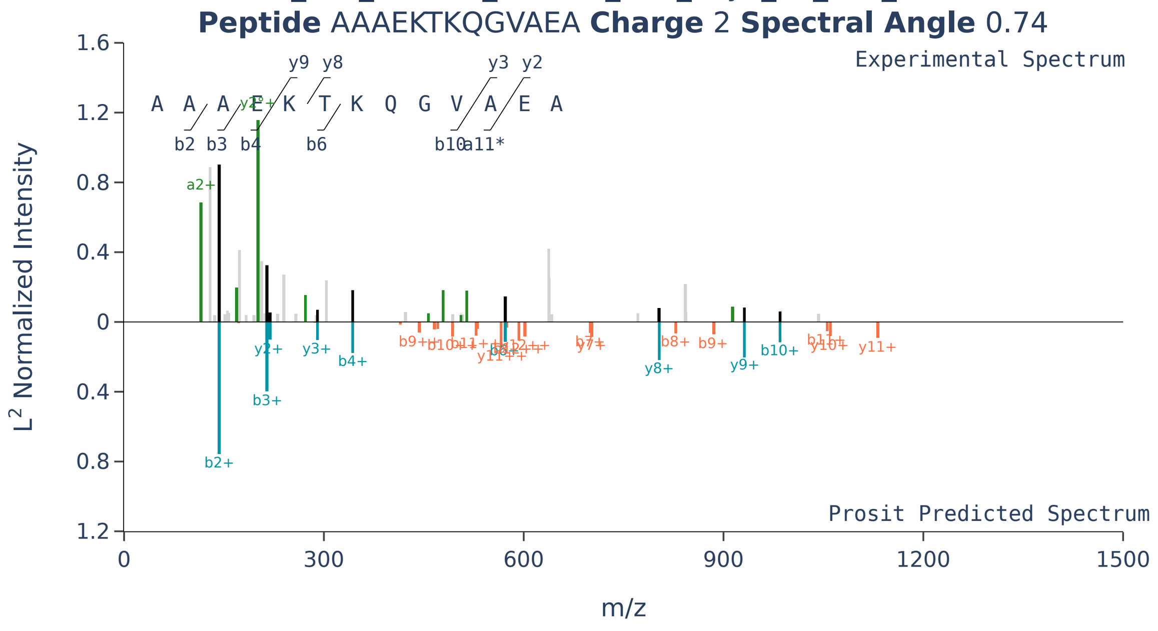


**Source** W_Soh_160821_170921_Goe_aSyn_A1_24h_R2 **Scan** 14160
**Peptide** VVAAAEKTKQGVAEAAGKTKEGVLYVGS **Charge** 4 **Spectral Angle** 0.84

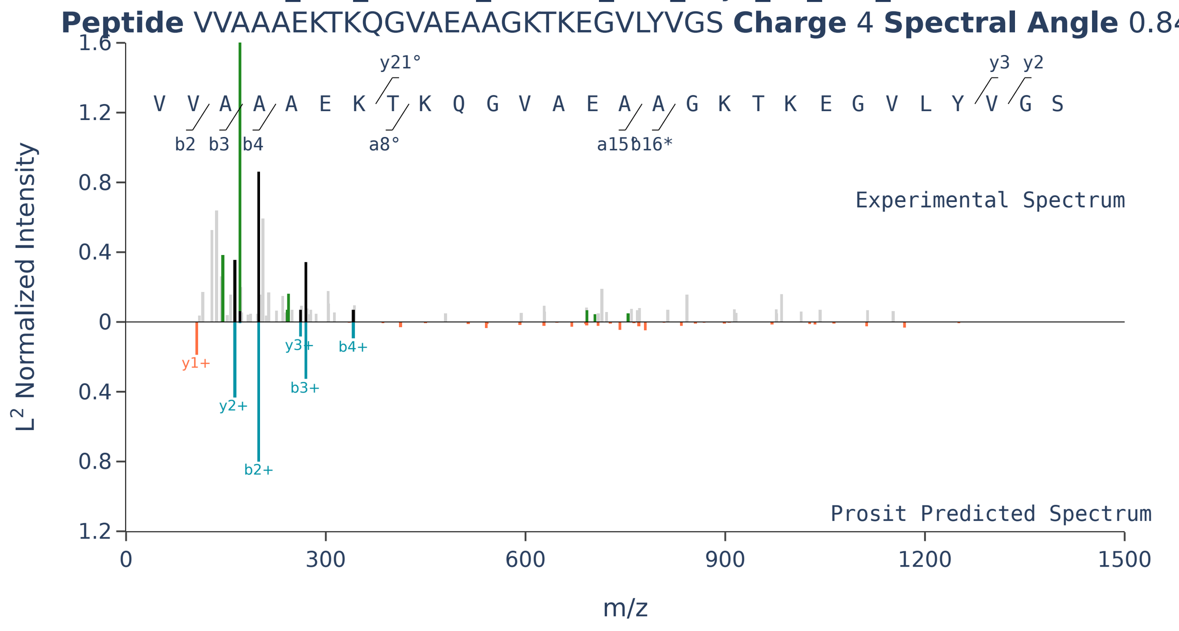


**Source** W_Soh_160821_170921_Goe_aSyn_A1_24h_R1 **Scan** 14246
**Peptide** TKEGVLYVGSKTKEGVVHGVATVA **Charge** 3 **Spectral Angle** 0.76

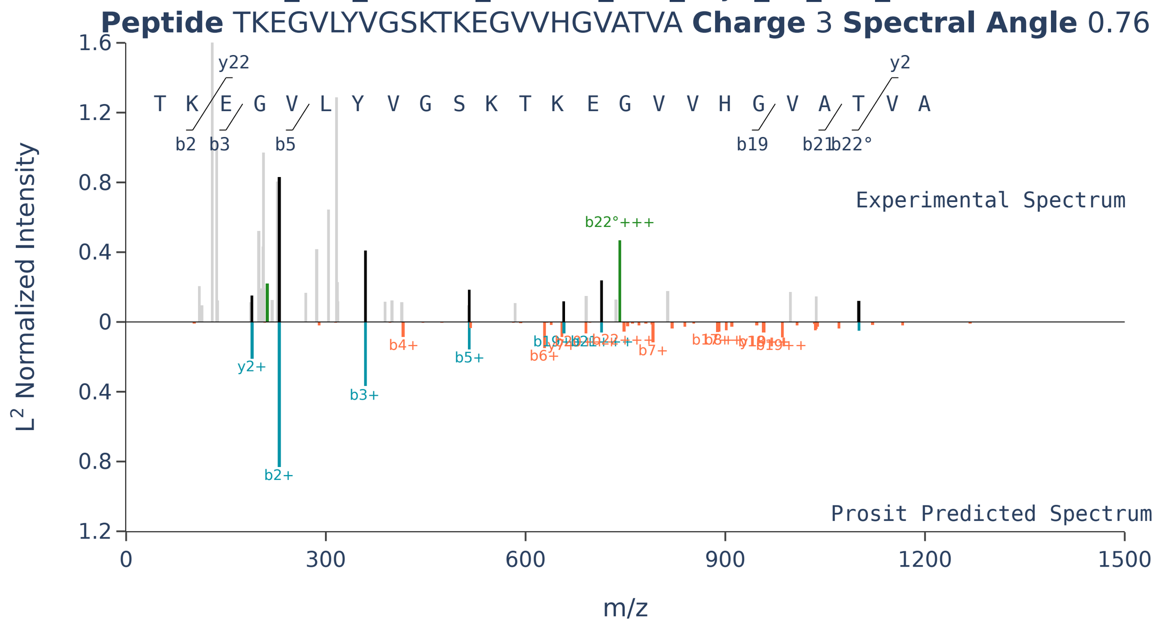

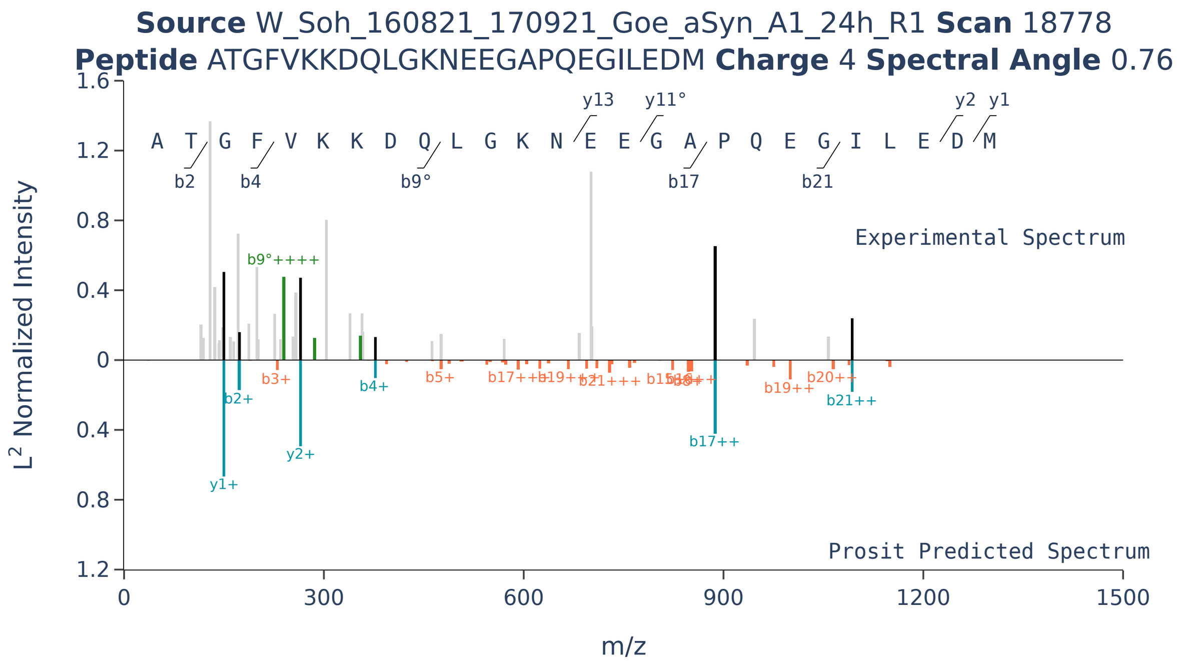
Source W_Soh_160821_170921_Goe_aSyn_A1_24h_R1 Scan 18778
Peptide ATGFVKKDQLGKNEEGAPQEGILEDM Charge 4 Spectral Angle 0.76
Experimental Spectrum
Prosit Predicted Spectrum
L² Normalized Intensity
m/z

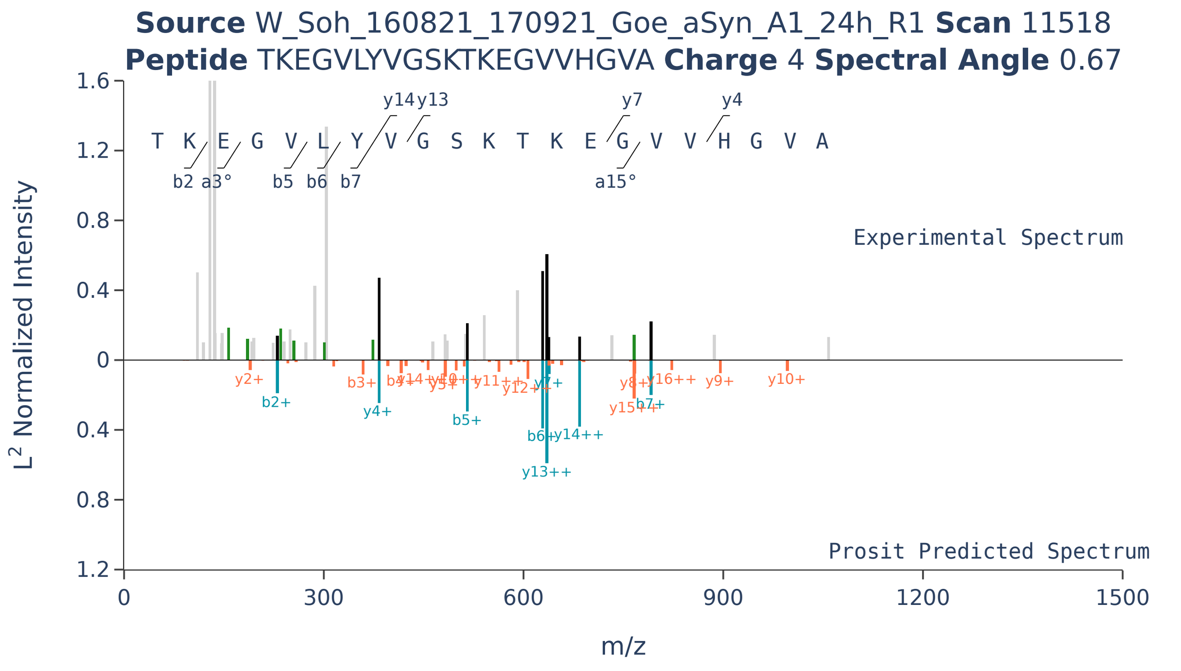
Source W_Soh_160821_170921_Goe_aSyn_A1_24h_R1 Scan 11518
Peptide TKEGVLYVGSKTKEGVVHGVA Charge 4 Spectral Angle 0.67
Experimental Spectrum
Prosit Predicted Spectrum
L² Normalized Intensity
m/z

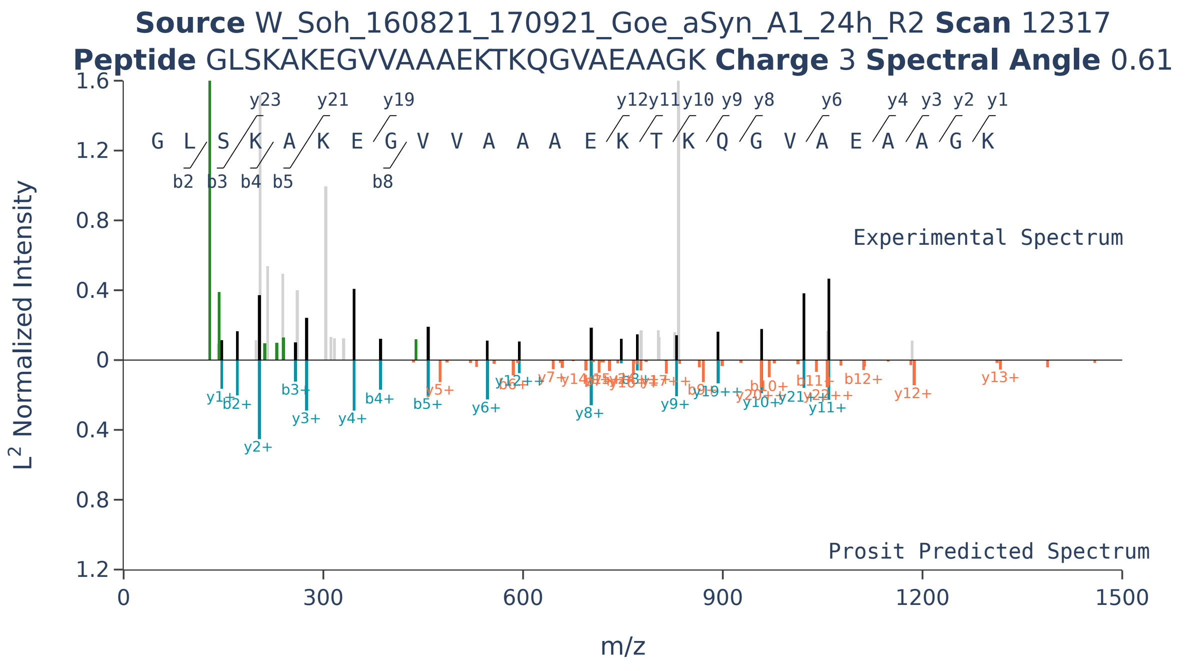
Source W_Soh_160821_170921_Goe_aSyn_A1_24h_R2 Scan 12317
Peptide GLSKAKEGVVAAAEKTKQGVAEAAGK Charge 3 Spectral Angle 0.61
Experimental Spectrum
Prosit Predicted Spectrum
L² Normalized Intensity
m/z

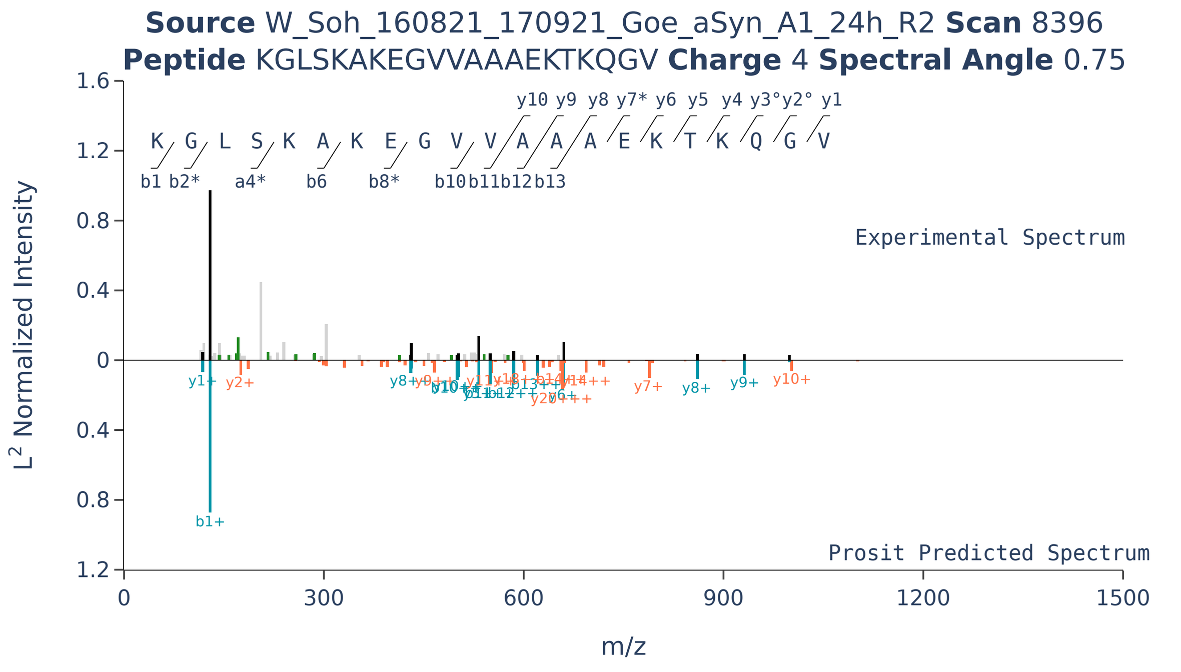
Source W_Soh_160821_170921_Goe_aSyn_A1_24h_R2 Scan 8396
Peptide KGLSKAKEGVVAAAEKTKQGV Charge 4 Spectral Angle 0.75
Experimental Spectrum
Prosit Predicted Spectrum
L² Normalized Intensity
m/z

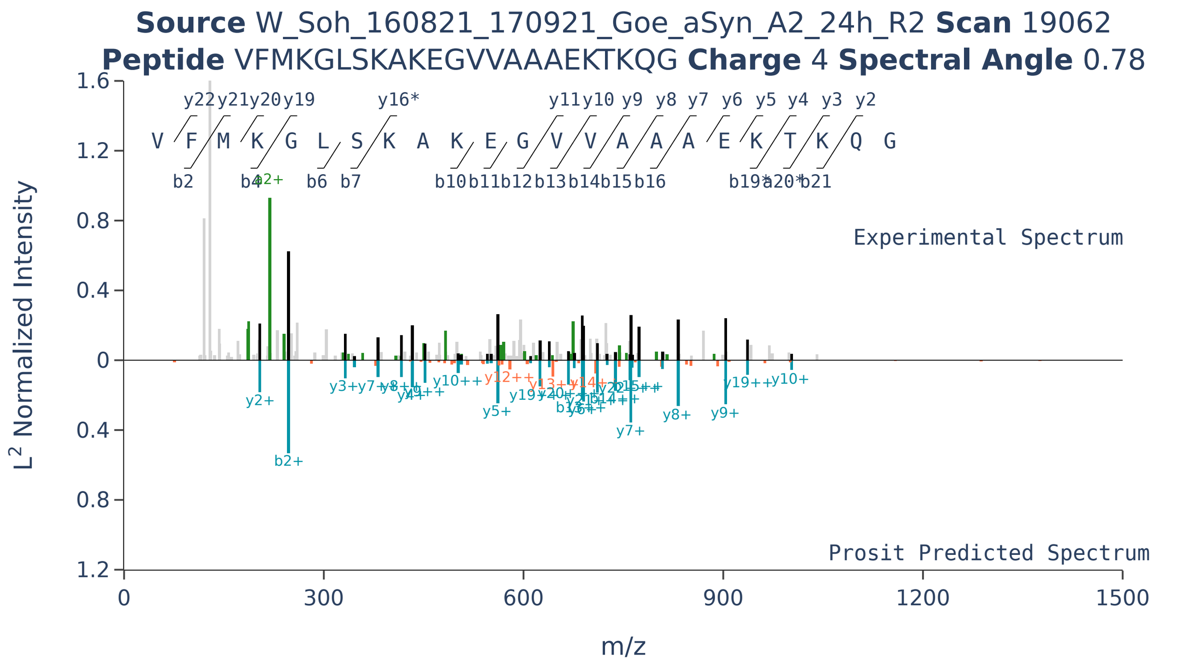
Source W_Soh_160821_170921_Goe_aSyn_A2_24h_R2 Scan 19062
Peptide VFMKGLSKAKEGVVAAAEKTKQG Charge 4 Spectral Angle 0.78
Experimental Spectrum
Prosit Predicted Spectrum
L² Normalized Intensity
m/z

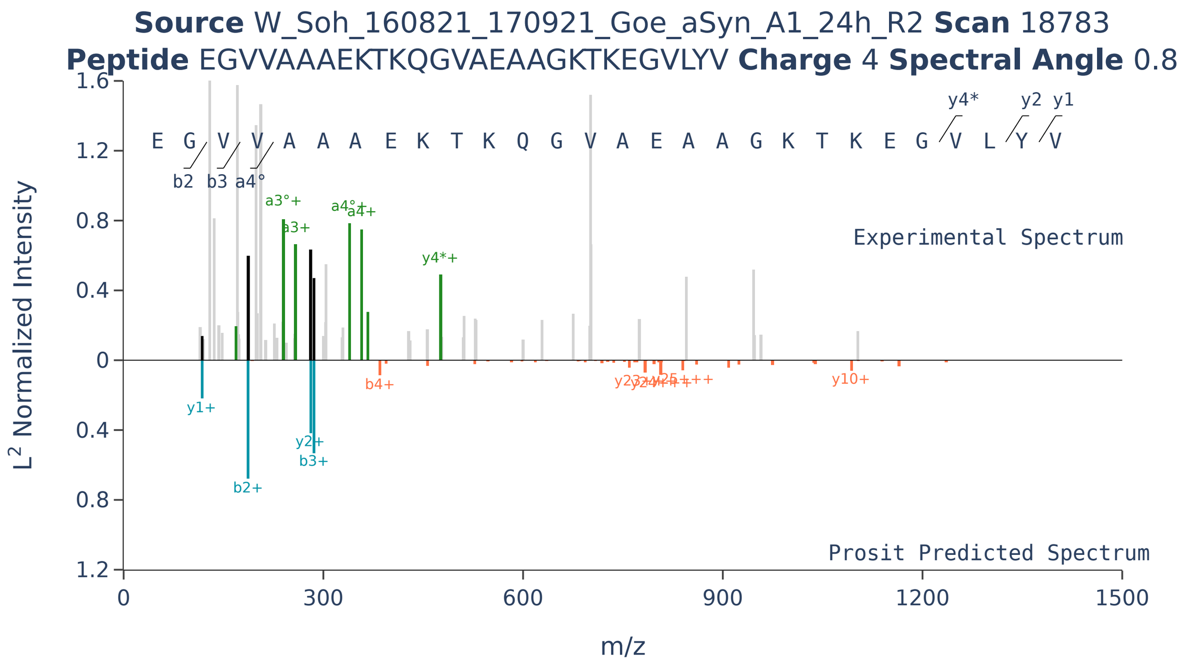
Source W_Soh_160821_170921_Goe_aSyn_A1_24h_R2 Scan 18783
Peptide EGVVAAAEKTKQGVAEAAGKTKEGVLYV Charge 4 Spectral Angle 0.8
Experimental Spectrum
Prosit Predicted Spectrum
L² Normalized Intensity
m/z

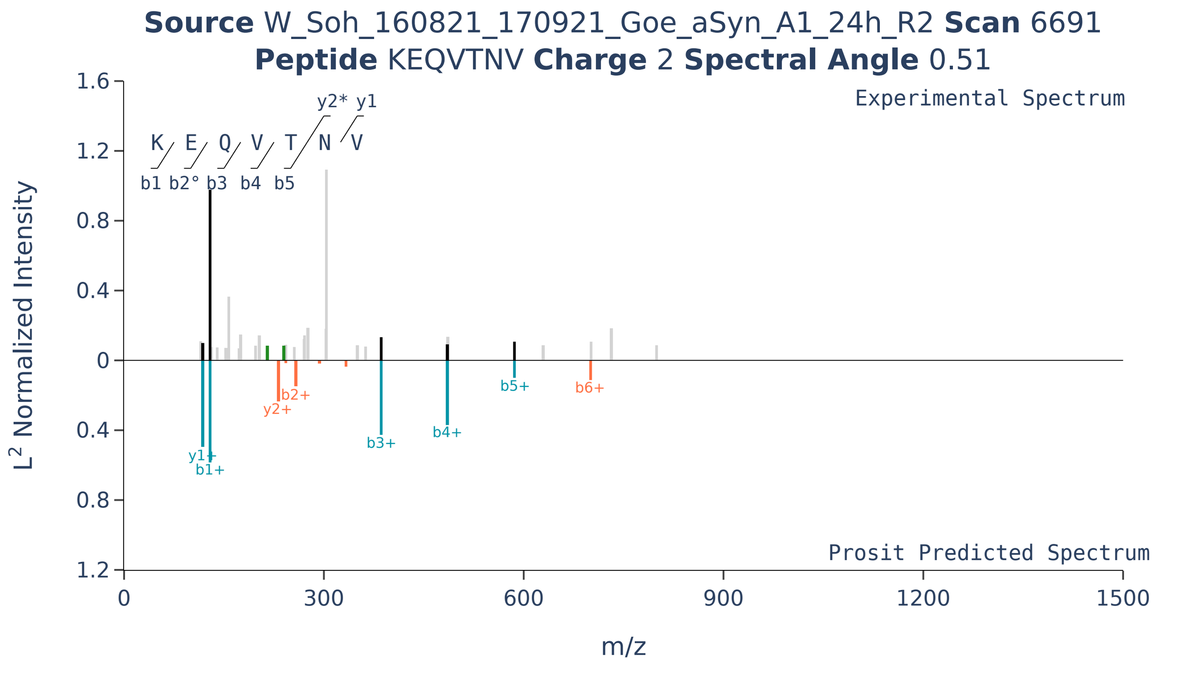
Source W_Soh_160821_170921_Goe_aSyn_A1_24h_R2 Scan 6691
Peptide KEQVTNV Charge 2 Spectral Angle 0.51
Experimental Spectrum
Prosit Predicted Spectrum
L² Normalized Intensity
m/z

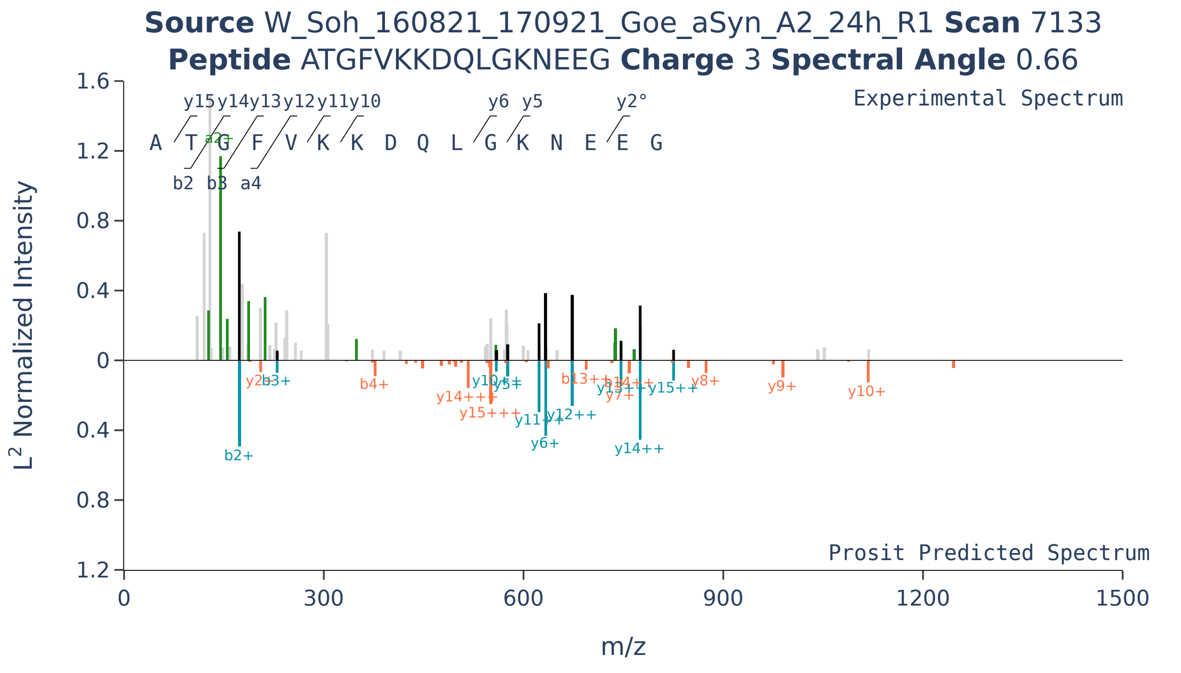
Source W_Soh_160821_170921_Goe_aSyn_A2_24h_R1 Scan 7133
Peptide ATGFVKKDQLGKNEEG Charge 3 Spectral Angle 0.66
Experimental Spectrum
Prosit Predicted Spectrum
L² Normalized Intensity
m/z

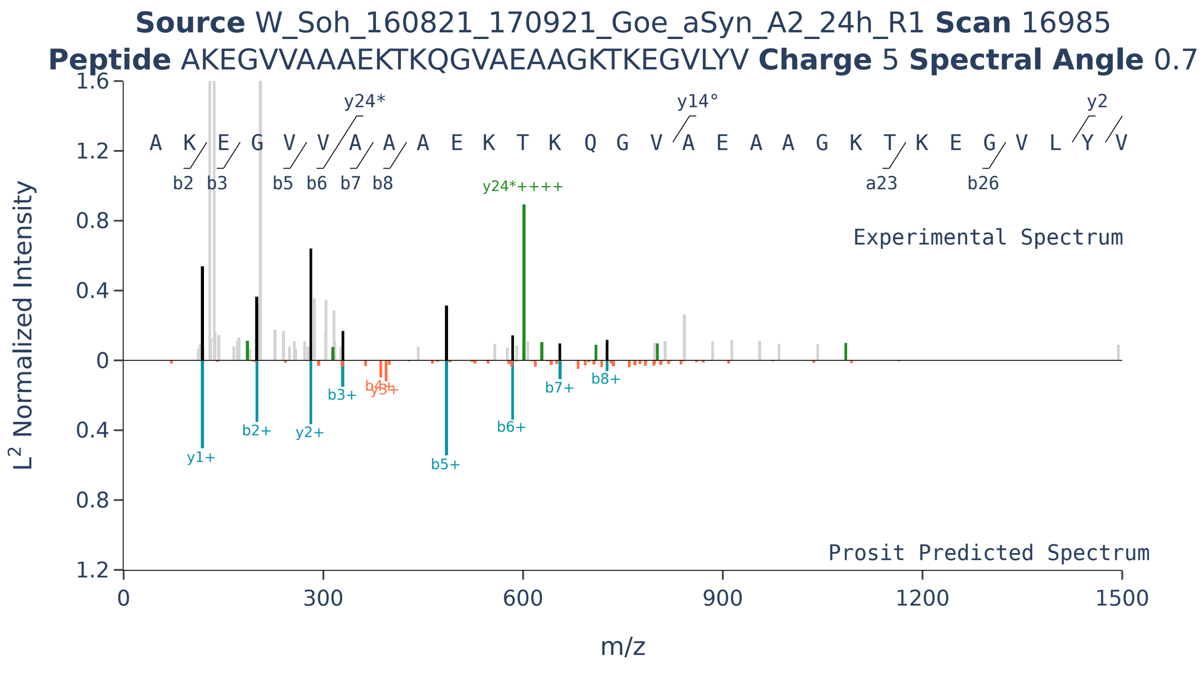
Source W_Soh_160821_170921_Goe_aSyn_A2_24h_R1 Scan 16985
Peptide AKEGVVAAAEKTKQGVAEAAGKTKEGVLYV Charge 5 Spectral Angle 0.7
Experimental Spectrum
Prosit Predicted Spectrum
L² Normalized Intensity
m/z

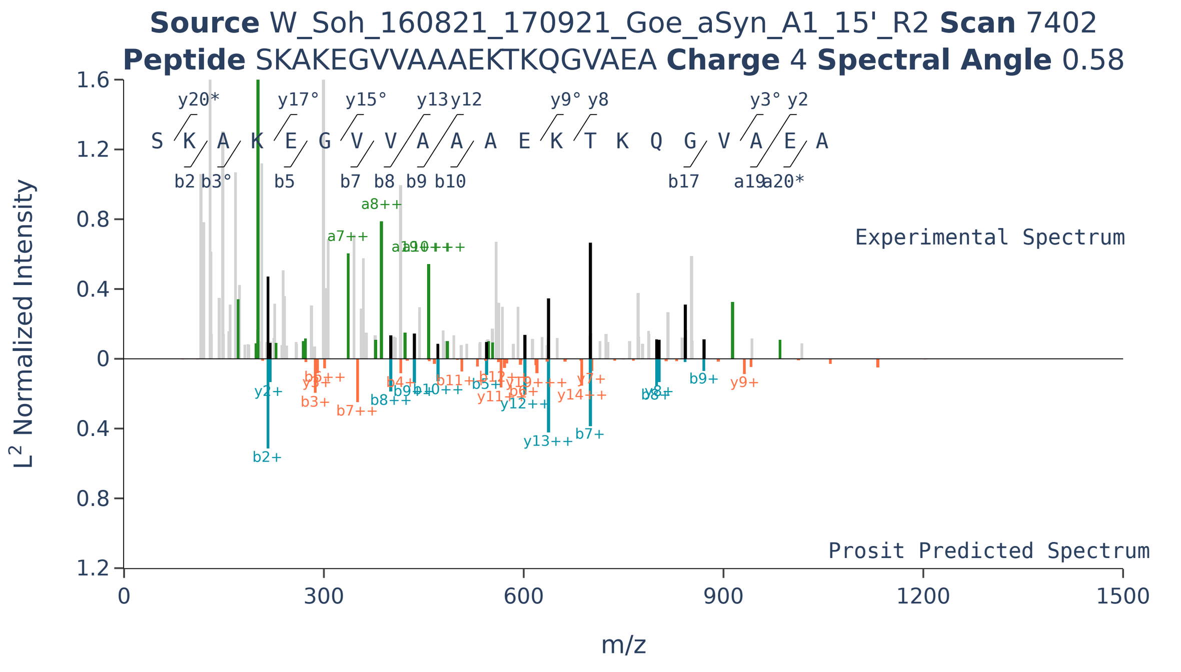
Source W_Soh_160821_170921_Goe_aSyn_A1_15'_R2 Scan 7402
Peptide SKAKEGVVAAAEKTKQGVAEA Charge 4 Spectral Angle 0.58
Experimental Spectrum
Prosit Predicted Spectrum
L² Normalized Intensity
m/z

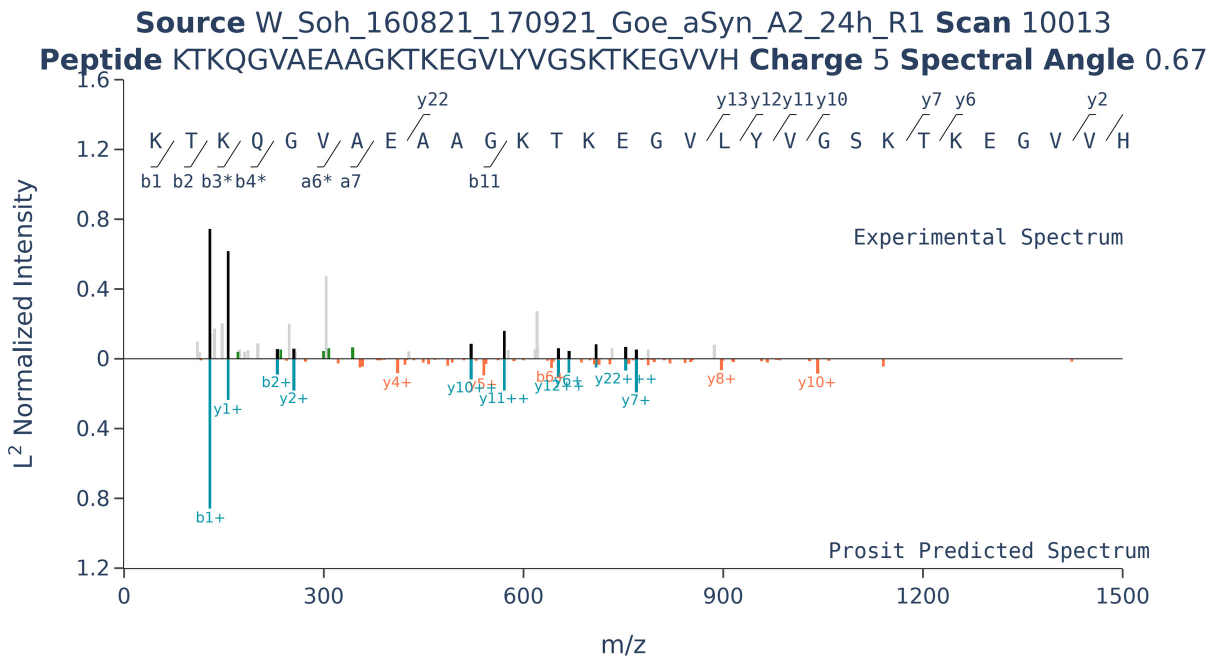
Source W_Soh_160821_170921_Goe_aSyn_A2_24h_R1 Scan 10013
Peptide KTKQGVAEAAGKTKEGVLYVGSKTKEGVVH Charge 5 Spectral Angle 0.67
Experimental Spectrum
Prosit Predicted Spectrum
L² Normalized Intensity
m/z

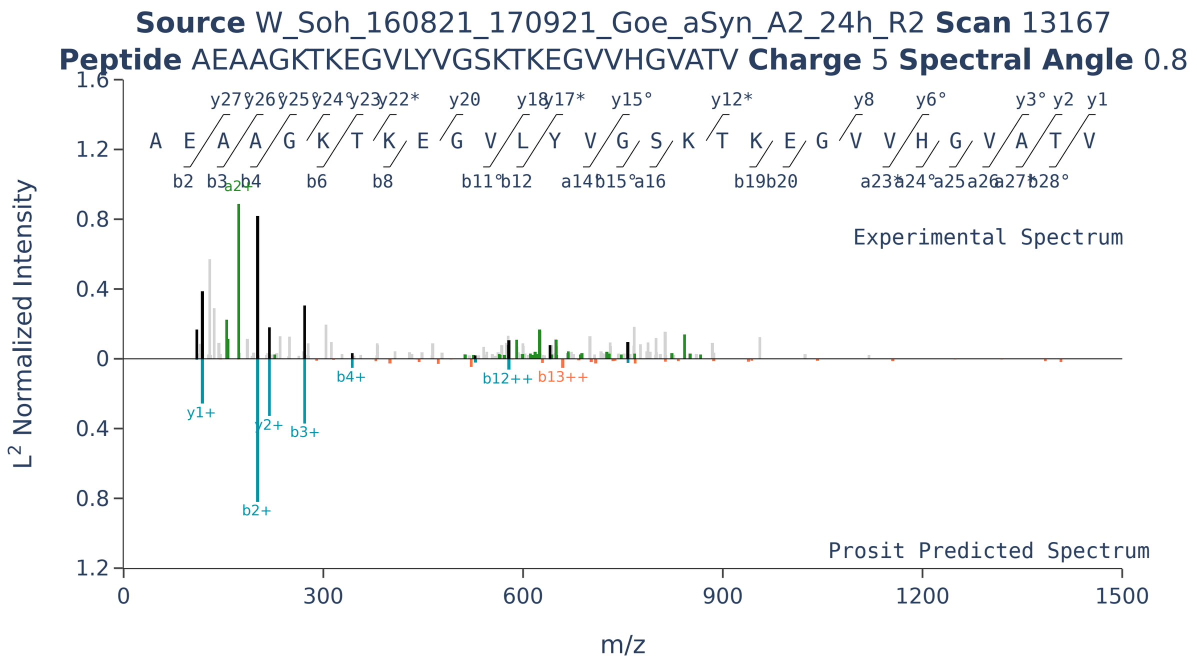
Source W_Soh_160821_170921_Goe_aSyn_A2_24h_R2 Scan 13167
Peptide AEAAGKTKEGVLYVGSKTKEGVVHGVATV Charge 5 Spectral Angle 0.8
Experimental Spectrum
Prosit Predicted Spectrum
L² Normalized Intensity
m/z

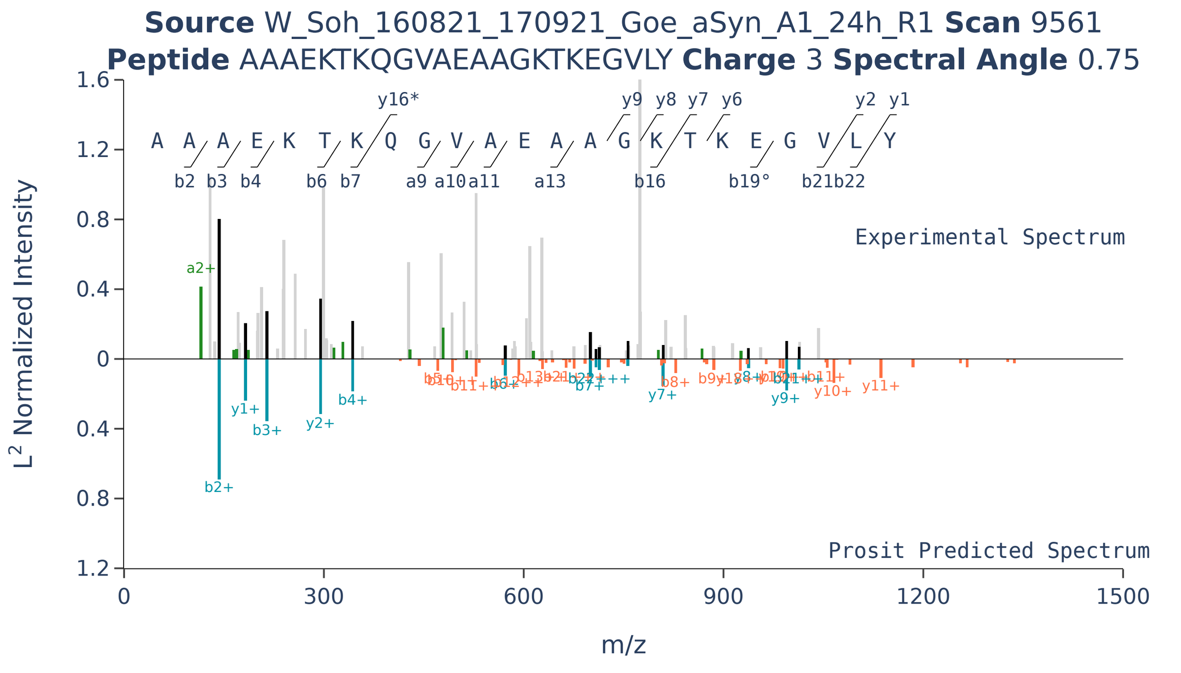
Source W_Soh_160821_170921_Goe_aSyn_A1_24h_R1 Scan 9561
Peptide AAAEKTKQGVAEAAGKTKEGVLY Charge 3 Spectral Angle 0.75
Experimental Spectrum
Prosit Predicted Spectrum
L² Normalized Intensity
m/z

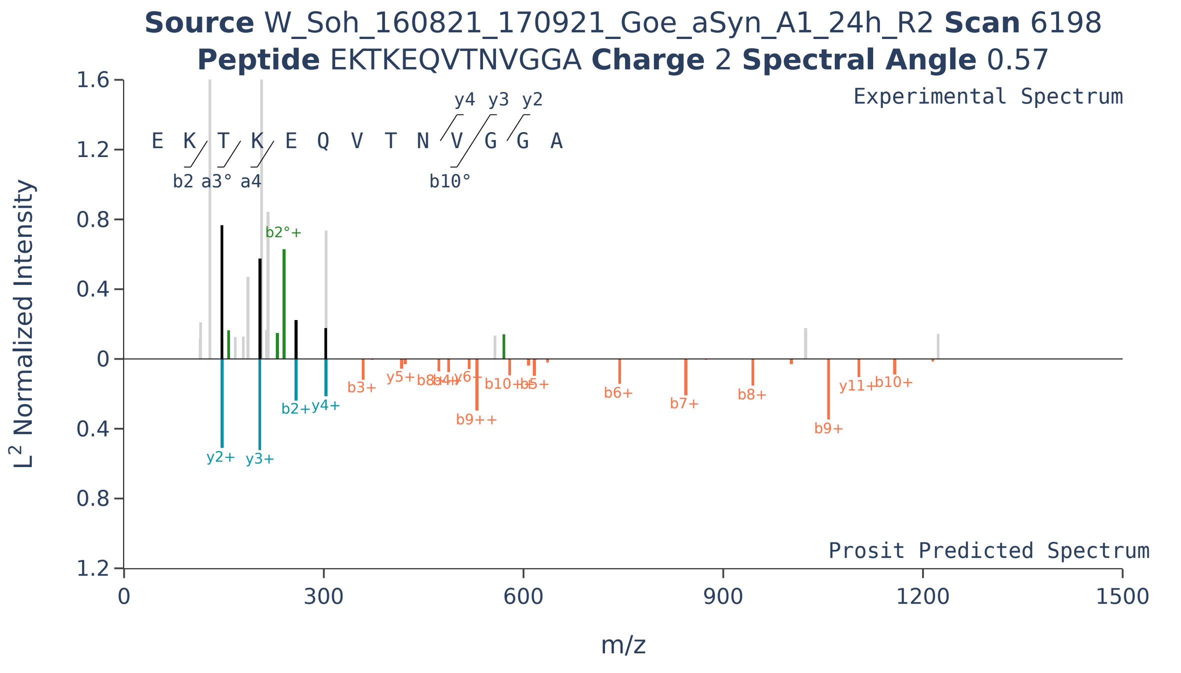
Source W_Soh_160821_170921_Goe_aSyn_A1_24h_R2 Scan 6198
Peptide EKTKEQVTNVGGA Charge 2 Spectral Angle 0.57
Experimental Spectrum
Prosit Predicted Spectrum
L² Normalized Intensity
m/z

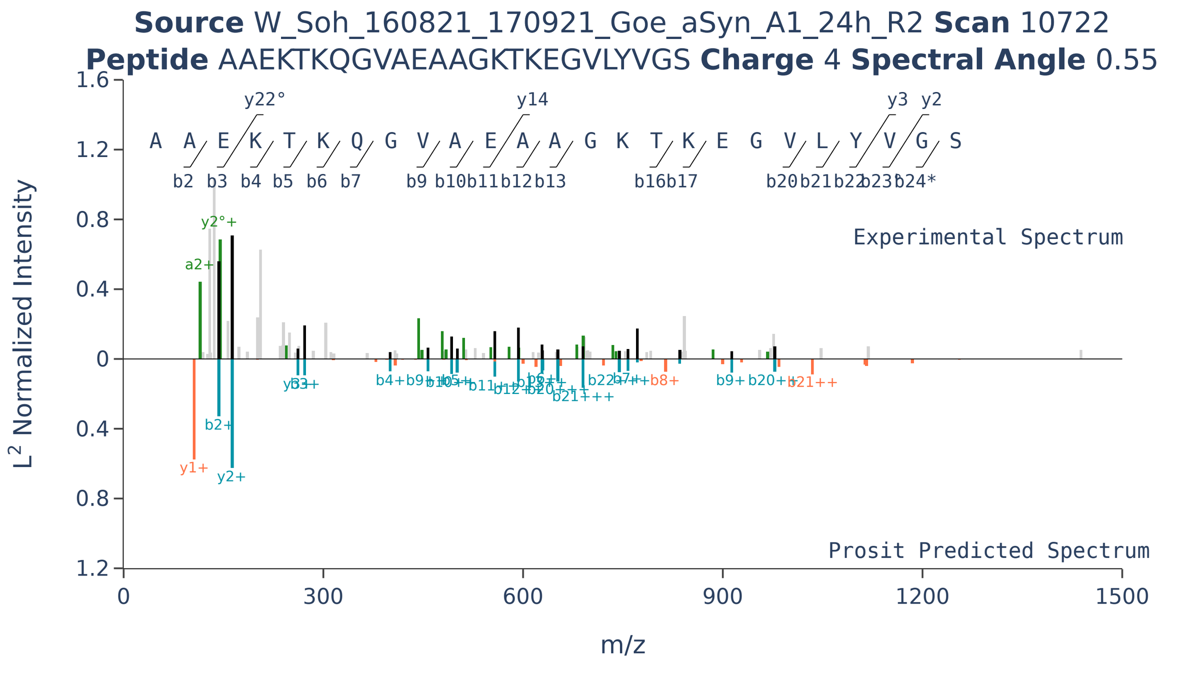
Source W_Soh_160821_170921_Goe_aSyn_A1_24h_R2 Scan 10722
Peptide AAEKTKQGVAEAAGKTKEGVLYVGS Charge 4 Spectral Angle 0.55
Experimental Spectrum
Prosit Predicted Spectrum
L² Normalized Intensity
m/z

**Source** W_Soh_160821_170921_Goe_aSyn_A2_24h_R1 **Scan** 13715
**Peptide** VTGVTAVAQKTVEG **Charge** 2 **Spectral Angle** 0.67

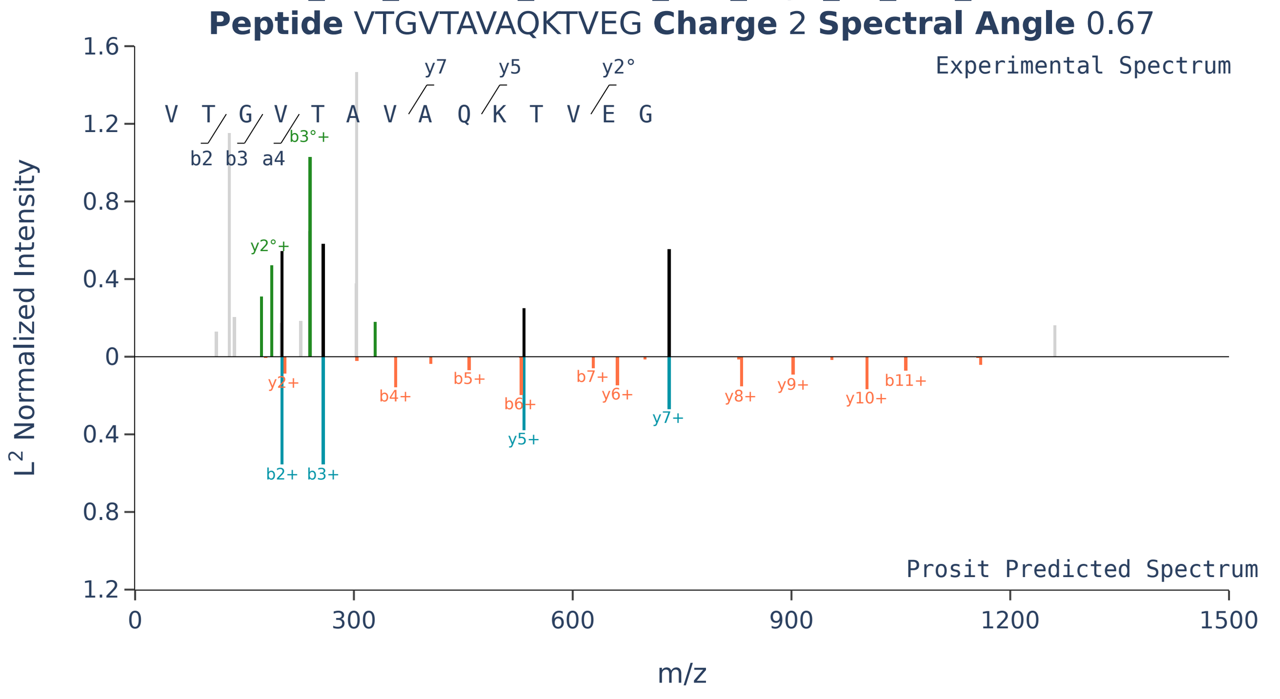


**Source** W_Soh_160821_170921_Goe_aSyn_A1_24h_R2 **Scan** 10525
**Peptide** AAAEKTKQGVAEAAGKTKEGVLYVGSKTKE **Charge** 5 **Spectral Angle** 0.69

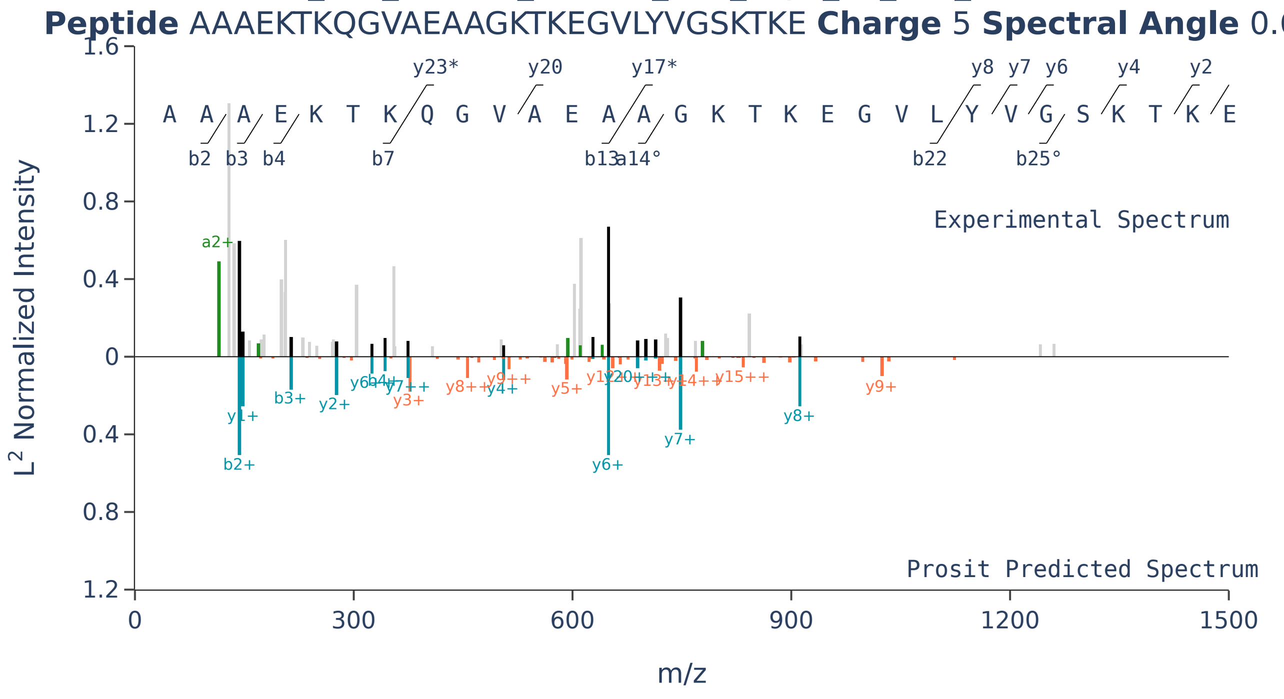


**Source** W_Soh_160821_170921_Goe_aSyn_A2_6h_R1 **Scan** 11272
**Peptide** GVLYVGSKTKEGV **Charge** 2 **Spectral Angle** 0.66

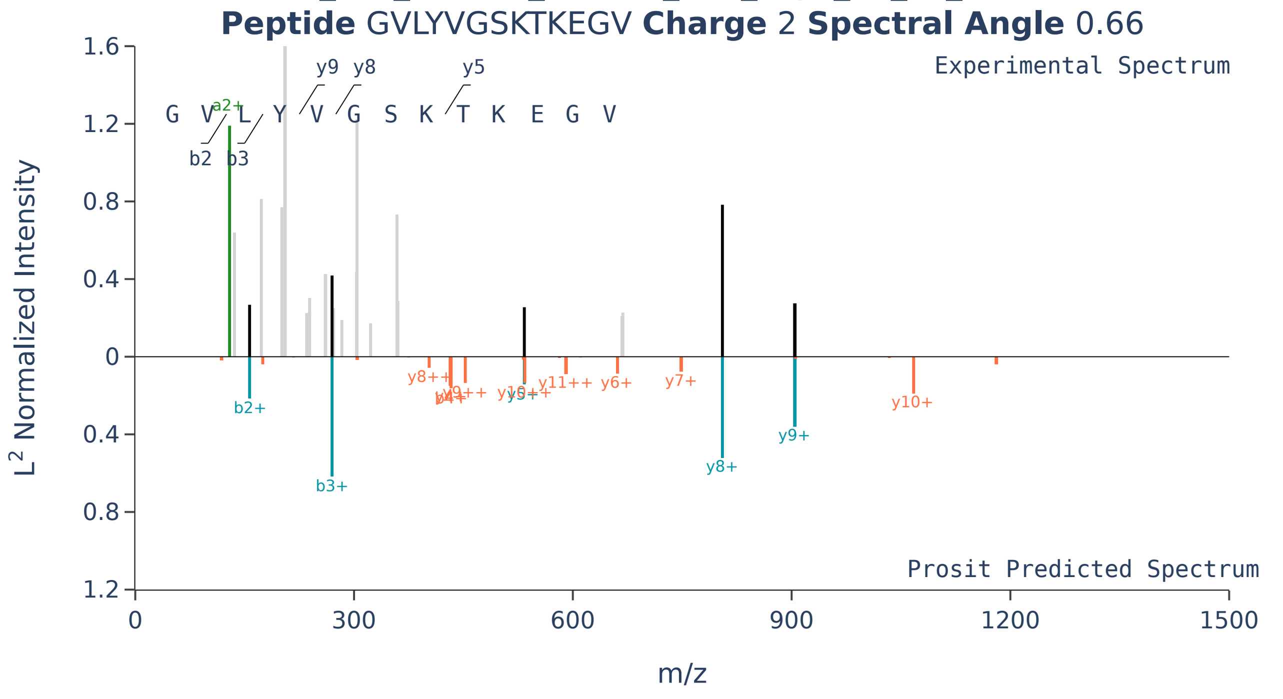


**Source** W_Soh_160821_170921_Goe_aSyn_A2_24h_R1 **Scan** 14157
**Peptide** VVAAAEKTKQGVAEAAGKTKEGVLYVG **Charge** 3 **Spectral Angle** 0.78

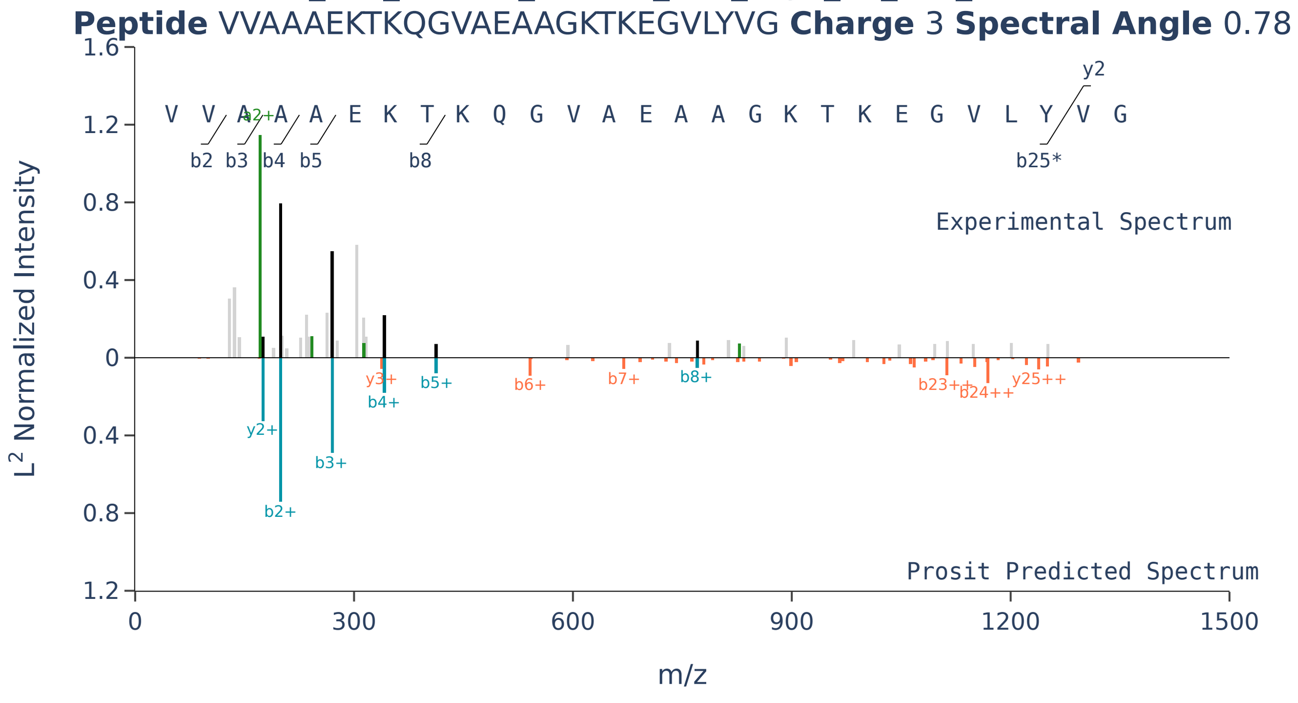


**Source** W_Soh_160821_170921_Goe_aSyn_A2_24h_R1 **Scan** 5700
**Peptide** MKGLSKA **Charge** 2 **Spectral Angle** 0.86

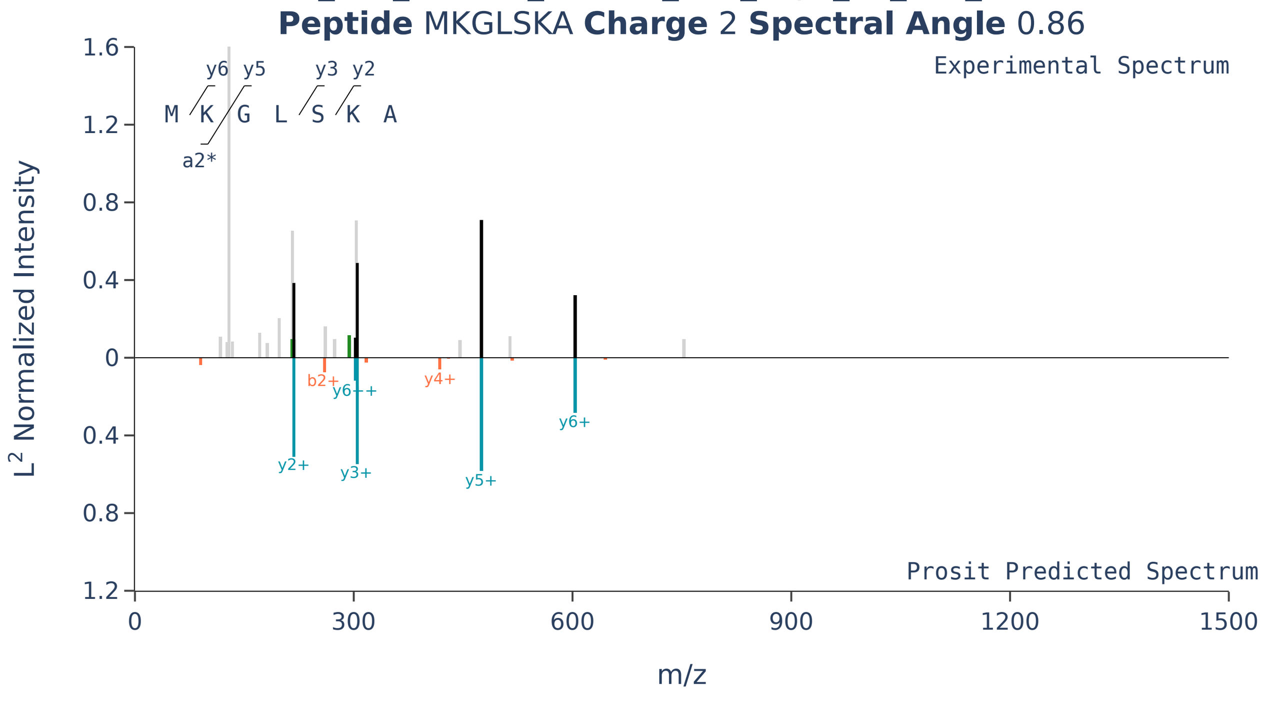


**Source** W_Soh_160821_170921_Goe_aSyn_A1_24h_R1 **Scan** 8709
**Peptide** AEKTKQGVAEAAGKTKEGVLY **Charge** 5 **Spectral Angle** 0.77

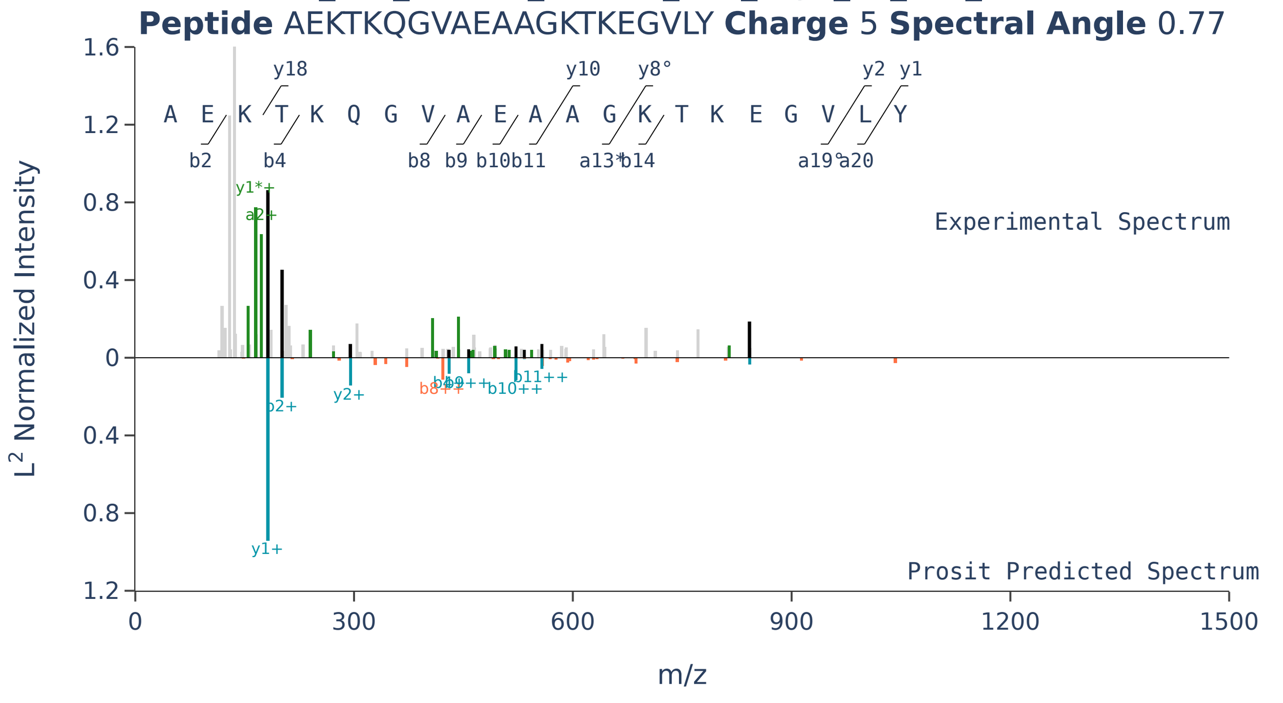


**Source** W_Soh_160821_170921_Goe_aSyn_A1_24h_R2 **Scan** 12337
**Peptide** VFMKGLSKAKEGVVAAAEKTKQ **Charge** 5 **Spectral Angle** 0.68

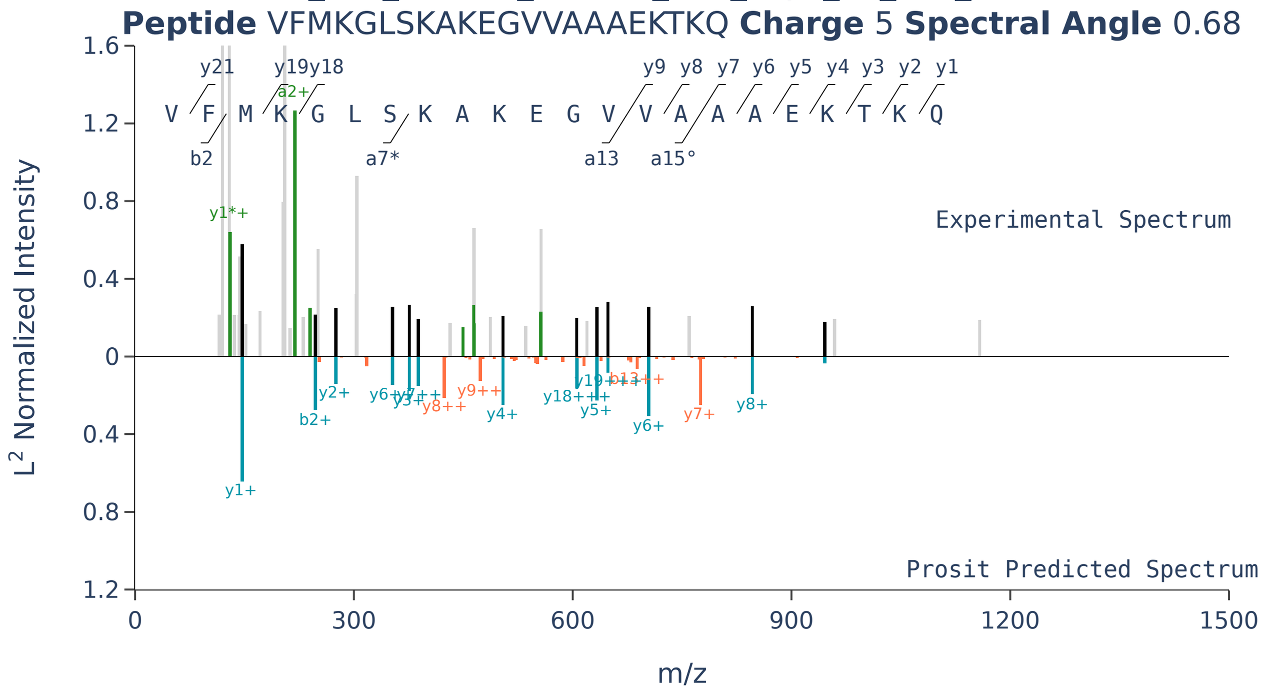


**Source** W_Soh_160821_170921_Goe_aSyn_A1_24h_R2 **Scan** 6179
**Peptide** KGLSKAKEGVVAAAEK **Charge** 3 **Spectral Angle** 0.65

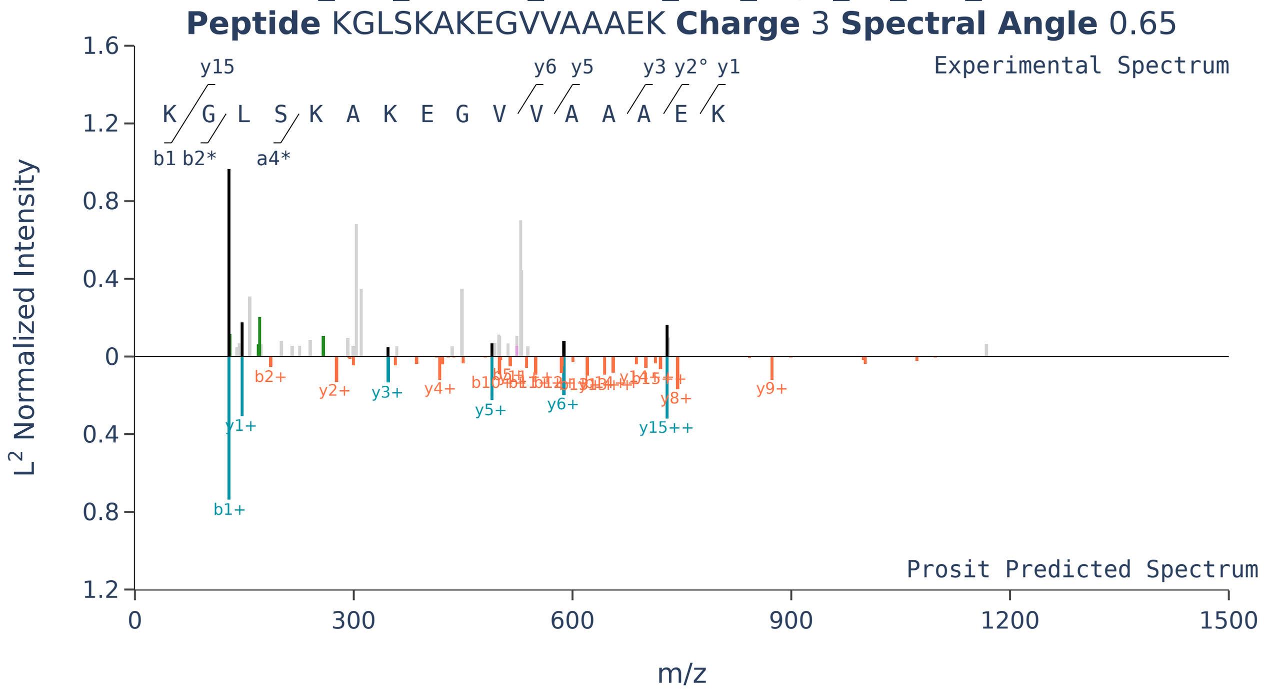


**Source** W_Soh_160821_170921_Goe_aSyn_A2_24h_R1 **Scan** 10913
**Peptide** QGVAEAAGKTKEGVLYVGSKTKE **Charge** 4 **Spectral Angle** 0.71

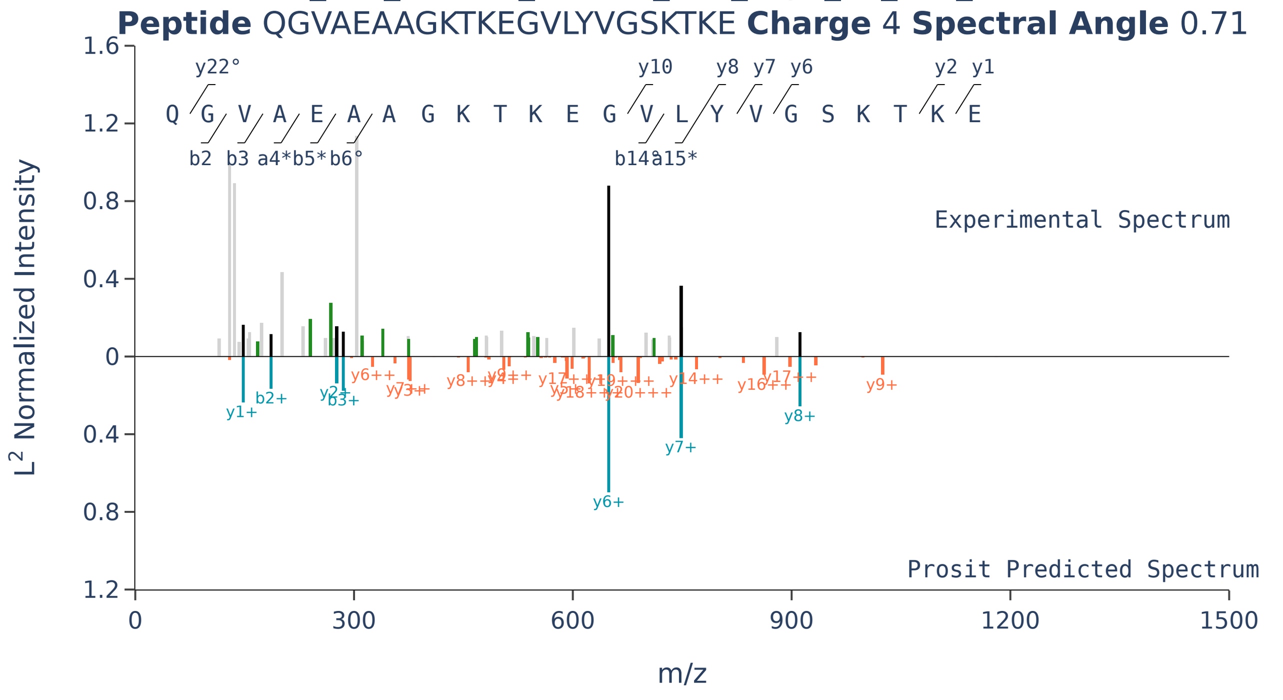


**Source** W_Soh_160821_170921_Goe_aSyn_A1_6h_R1 **Scan** 19119
**Peptide** AVAQKTVEGAGSIAAATGFVKKDQL **Charge** 4 **Spectral Angle** 0.65

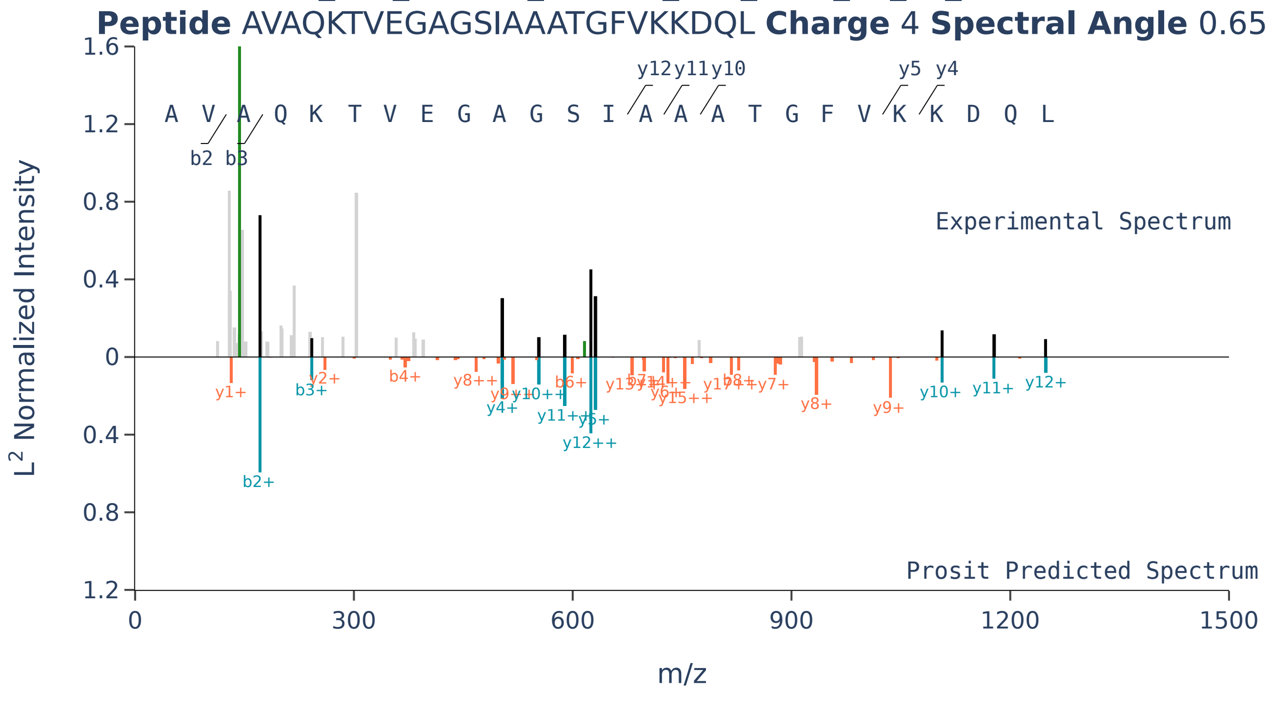


**Source** W_Soh_160821_170921_Goe_aSyn_A1_6h_R2 **Scan** 25497
**Peptide** EDMPVDPDNEAYEMPSEEGYQDYEPEA **Charge** 3 **Spectral Angle** 0.87

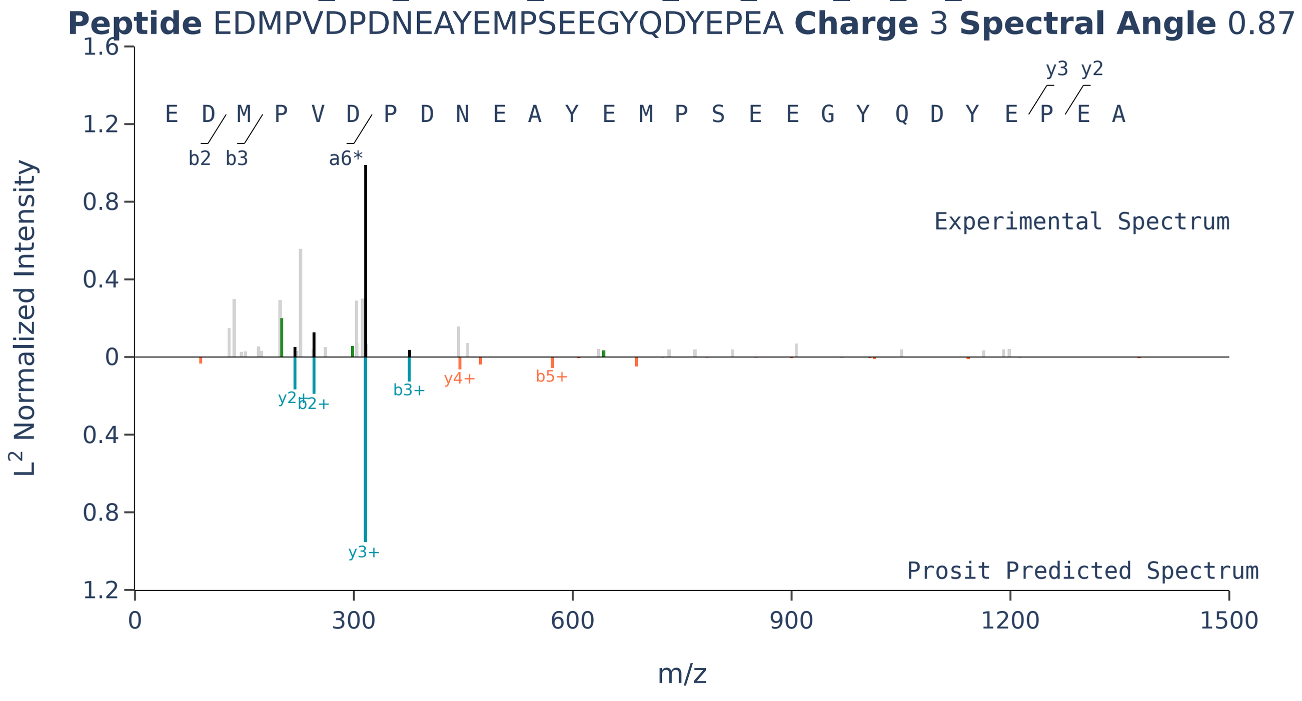


**Source** W_Soh_160821_170921_Goe_aSyn_A1_24h_R1 **Scan** 6884
**Peptide** KGLSKAKEGVVAAAE **Charge** 2 **Spectral Angle** 0.59

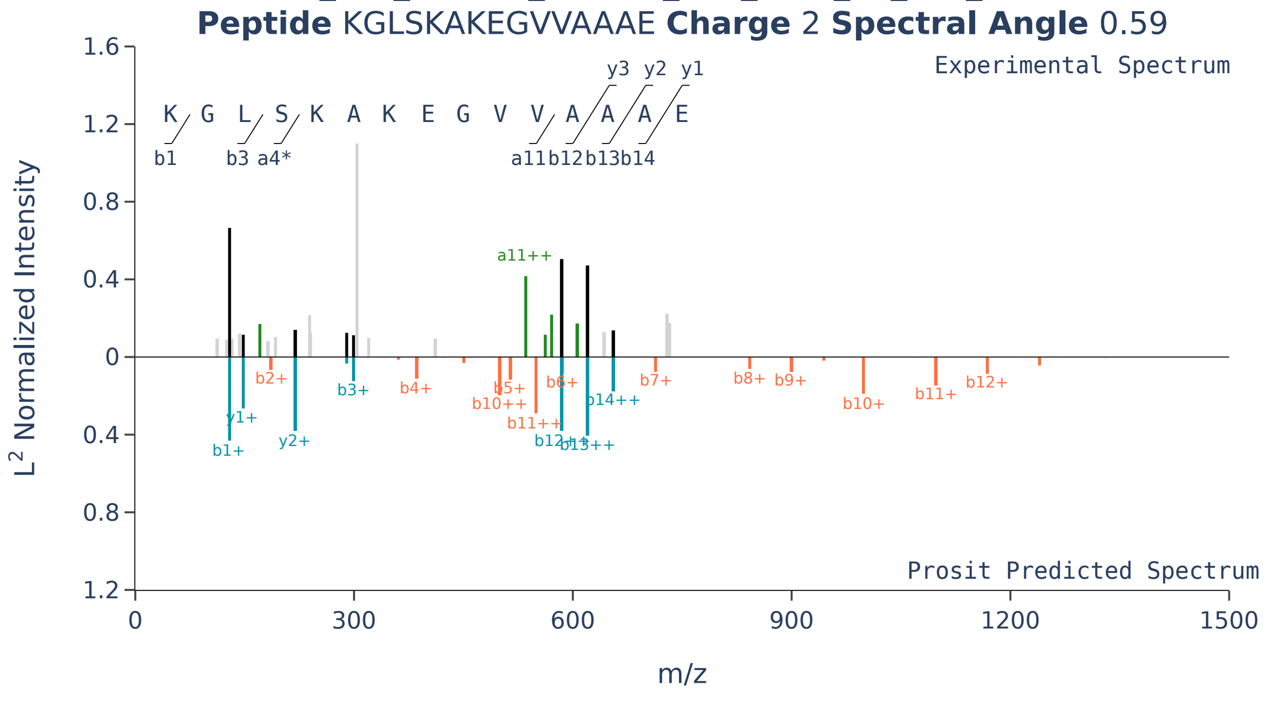


**Source** W_Soh_160821_170921_Goe_aSyn_A2_6h_R1 **Scan** 19760
**Peptide** KEQVTNVGGAVVTGVTAVAQKT **Charge** 3 **Spectral Angle** 0.67

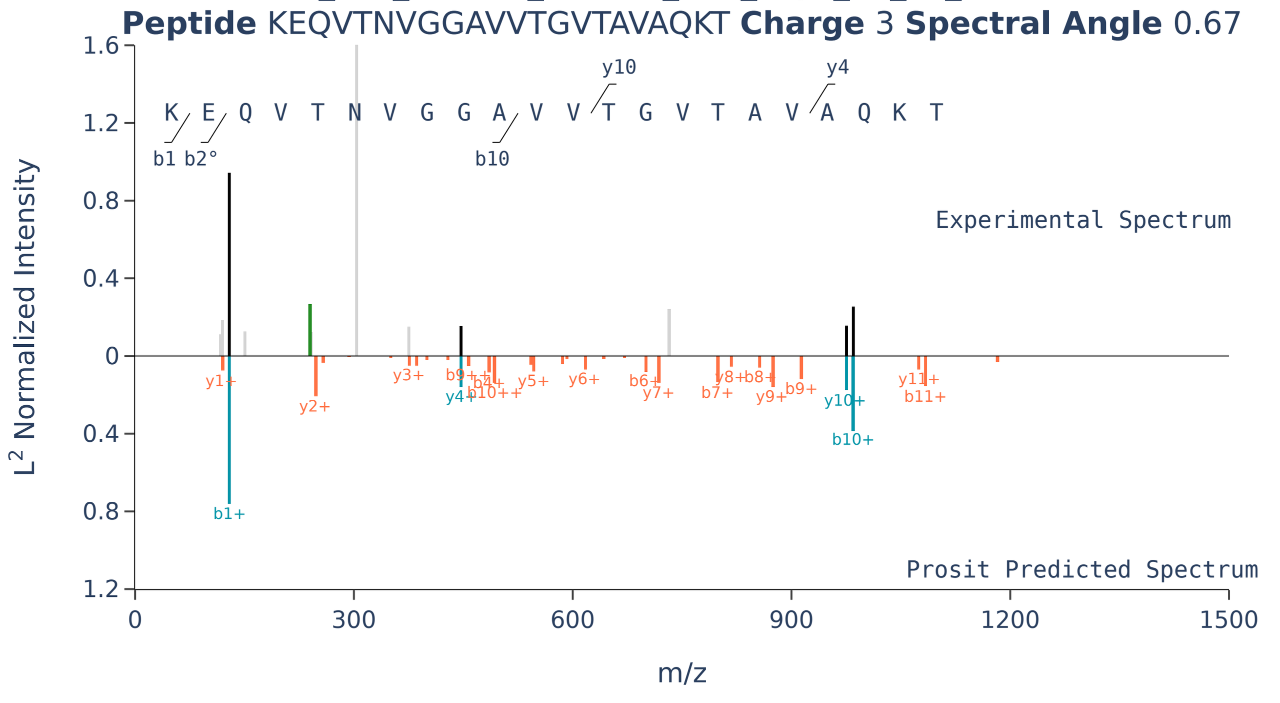

**Source** HRoetschke_291222_040123_Fu_CaM_b1R1_24h **Scan** 2654
**Peptide** RSLGQNPTE **Charge** 2 **Spectral Angle** 0.95

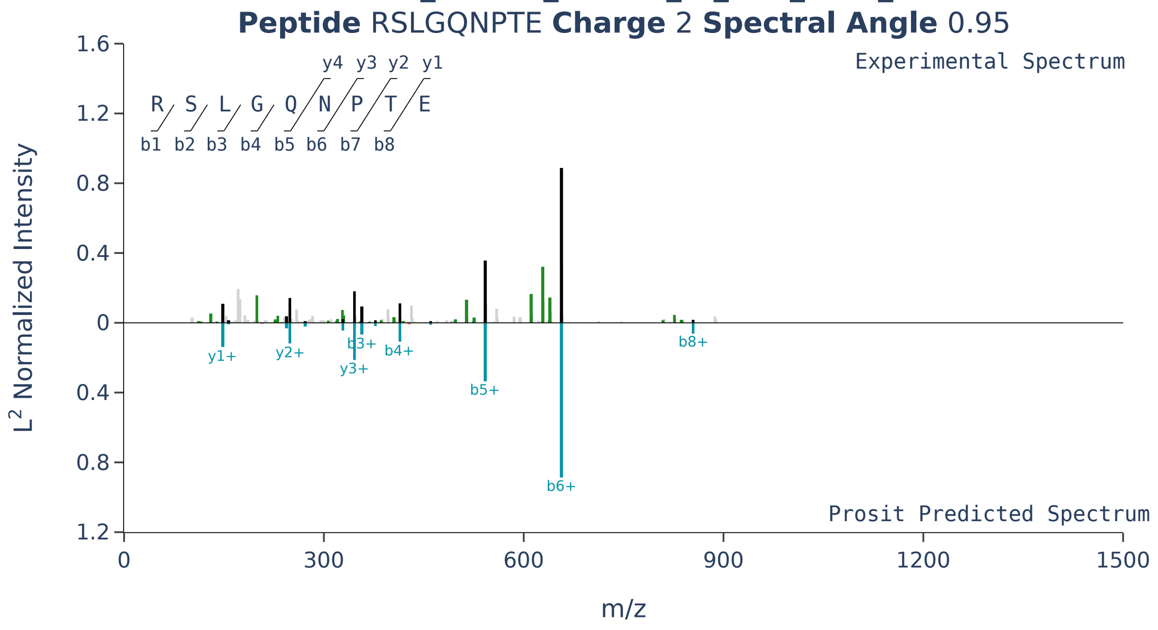


**Source** HRoetschke_291222_040123_Fu_CaM_b1R2_4h **Scan** 3269
**Peptide** VMRSLGQNPTE **Charge** 2 **Spectral Angle** 0.94

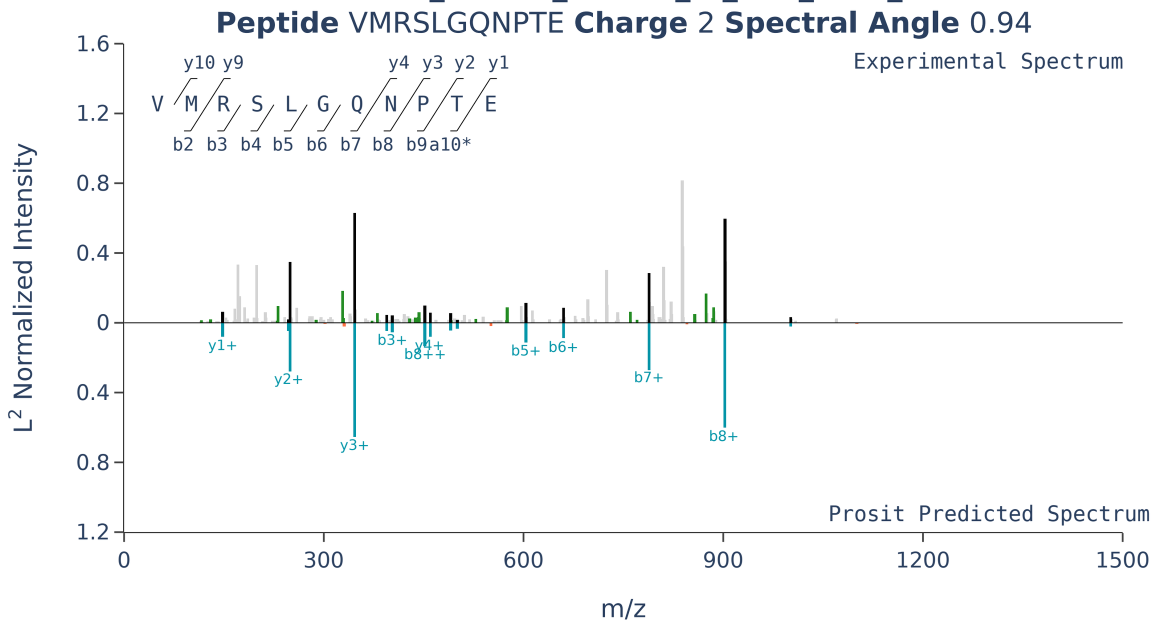


**Source** HRoetschke_291222_040123_Fu_CaM_b1R1_1h **Scan** 5321
**Peptide** MTNLGEKLTD **Charge** 2 **Spectral Angle** 0.89

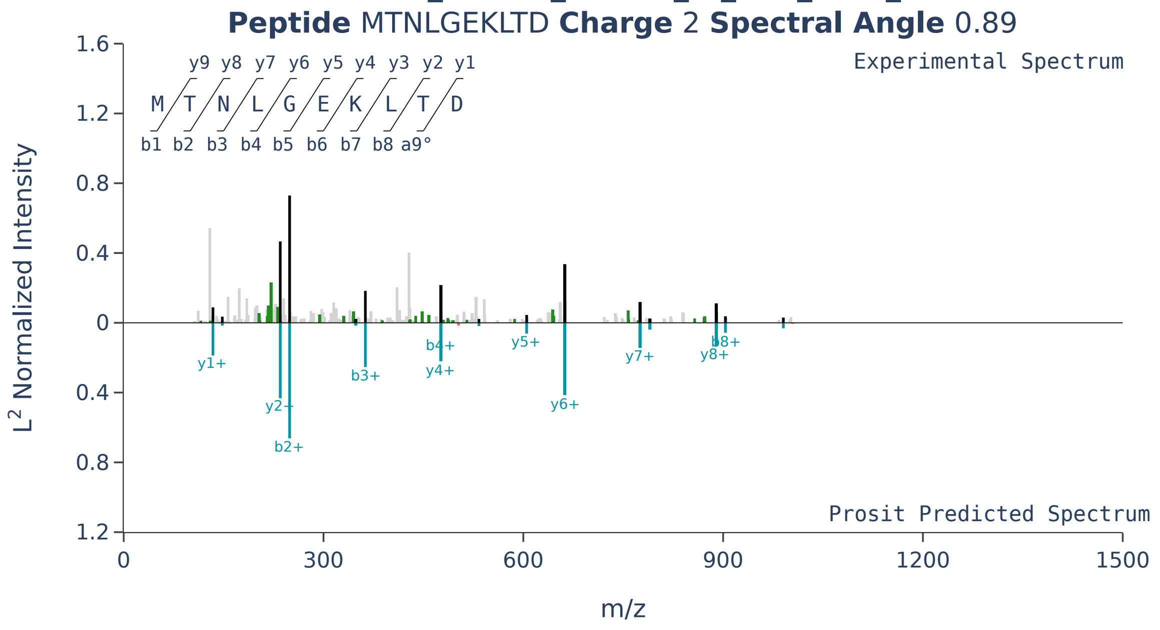


**Source** HRoetschke_291222_040123_Fu_CaM_b1R1_1h **Scan** 8302
**Peptide** SLFDKDGDGTITT **Charge** 2 **Spectral Angle** 0.9

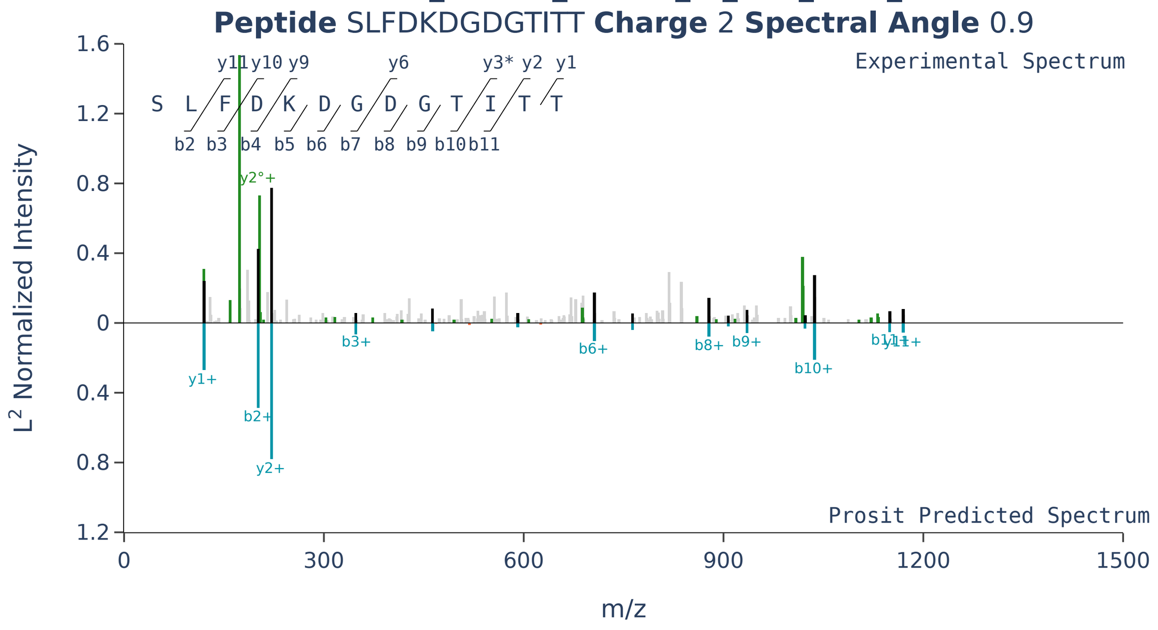


**Source** HRoetschke_291222_040123_Fu_CaM_b1R1_1h **Scan** 4021
**Peptide** DKDGNGYISA **Charge** 2 **Spectral Angle** 0.96

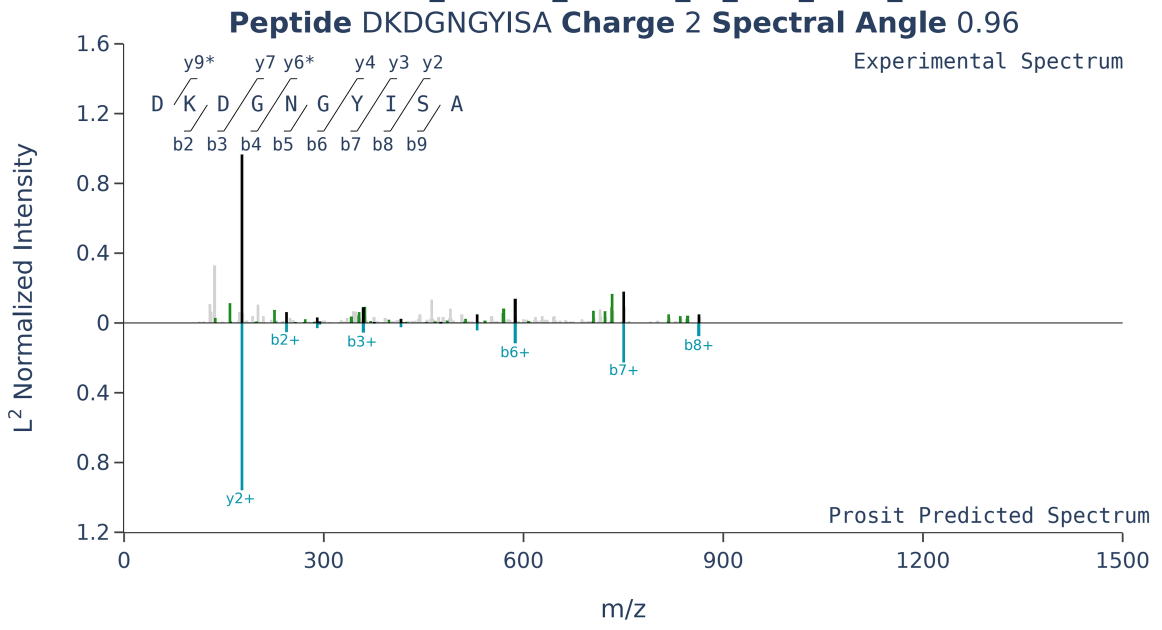


**Source** HRoetschke_291222_040123_Fu_CaM_b1R1_1h **Scan** 9782
**Peptide** EQIAEFKEAF **Charge** 2 **Spectral Angle** 0.94

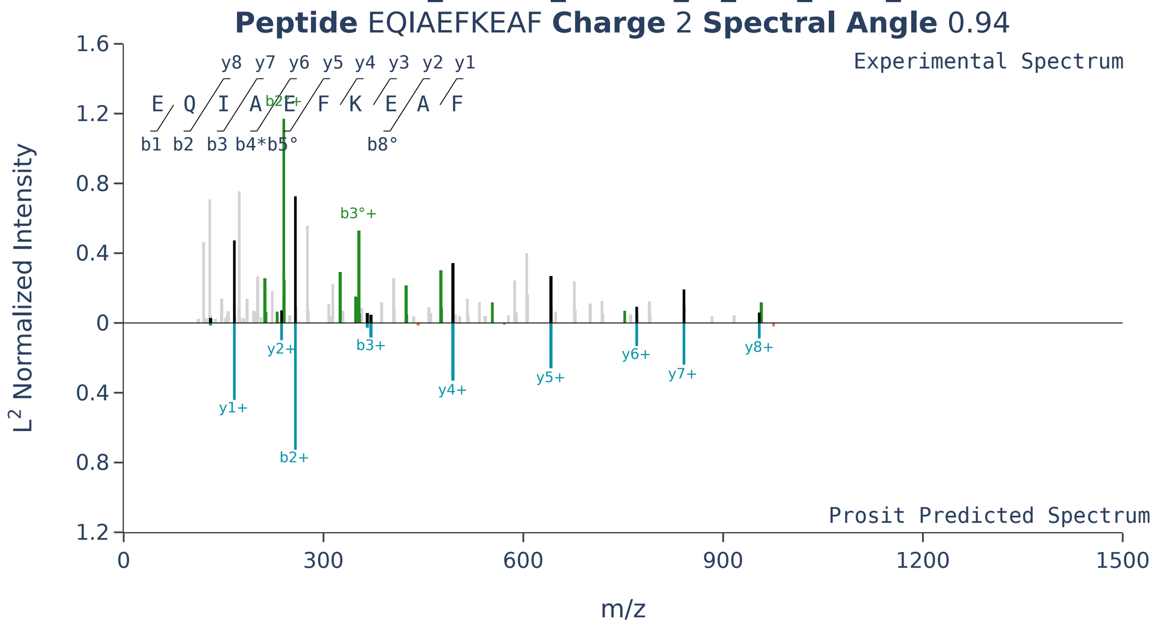


**Source** HRoetschke_291222_040123_Fu_CaM_b2R2_1h **Scan** 5557
**Peptide** LGEKLTDEEVD **Charge** 2 **Spectral Angle** 0.86

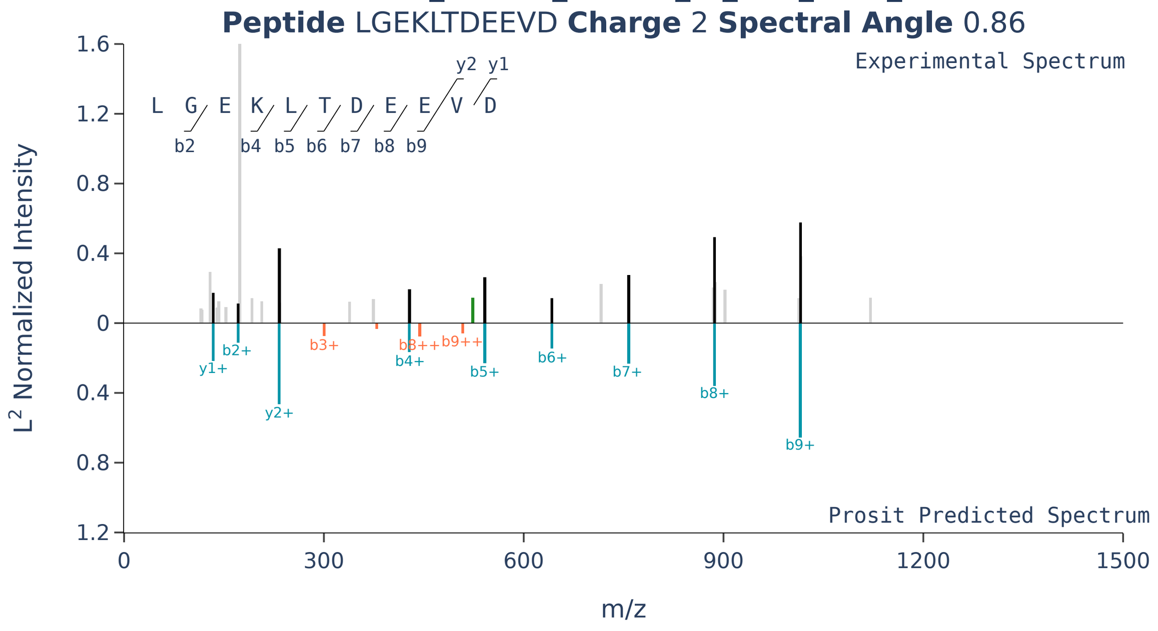


**Source** HRoetschke_291222_040123_Fu_CaM_b2R1_4h **Scan** 3575
**Peptide** GTVMRSLGQNPTE **Charge** 2 **Spectral Angle** 0.92

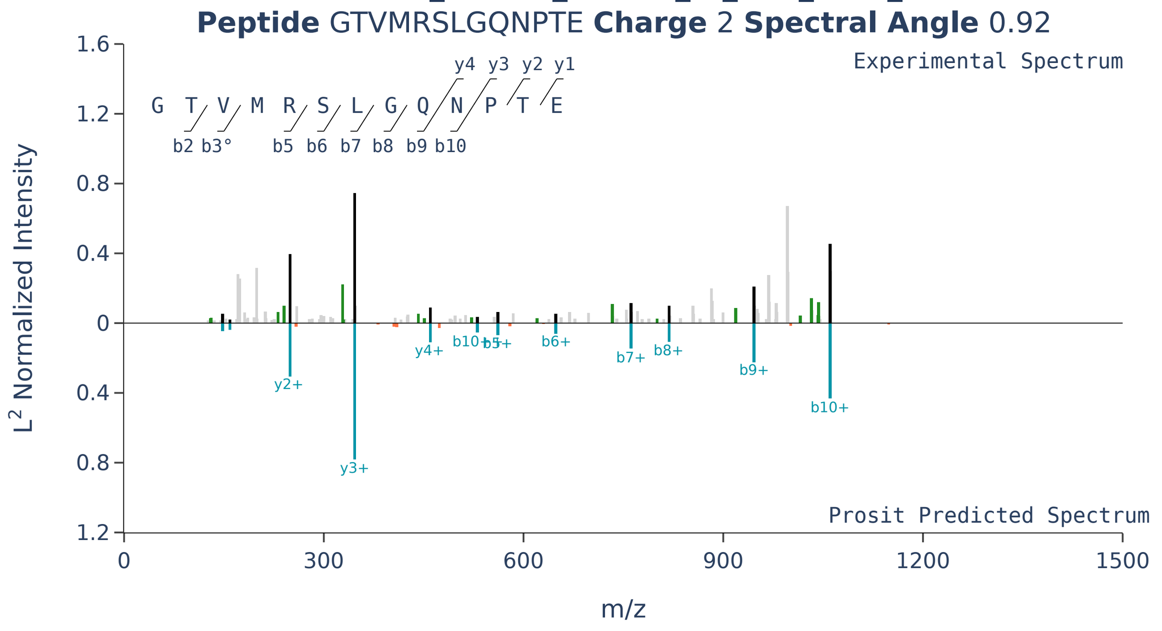


**Source** HRoetschke_291222_040123_Fu_CaM_b2R1_2h **Scan** 7900
**Peptide** MTNLGEKLTDEEVD **Charge** 2 **Spectral Angle** 0.9

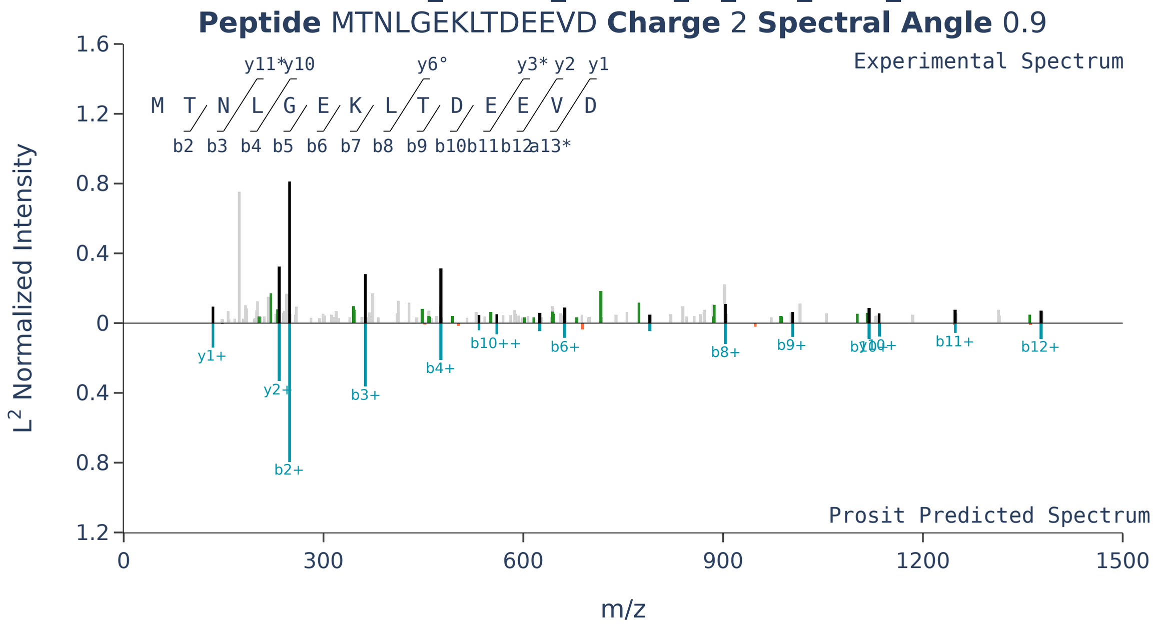


**Source** HRoetschke_291222_040123_Fu_CaM_b1R1_24h **Scan** 8177
**Peptide** MINEVDADGNGTID **Charge** 2 **Spectral Angle** 0.93

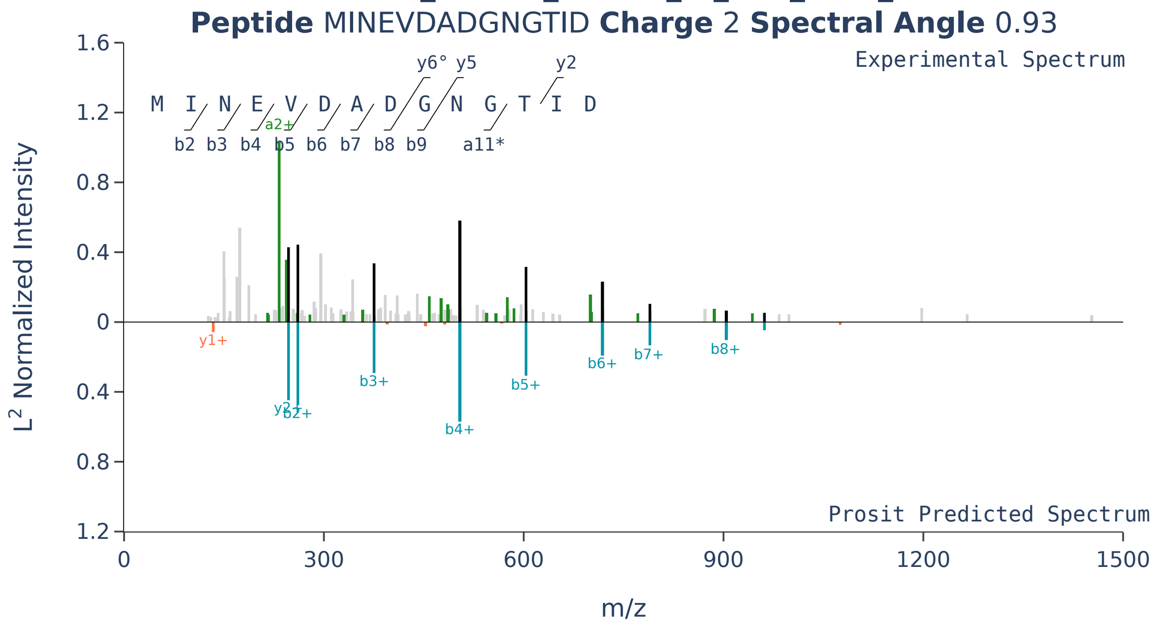


**Source** HRoetschke_291222_040123_Fu_CaM_b1R2_2h **Scan** 5731
**Peptide** FDKDGNGYISA **Charge** 2 **Spectral Angle** 0.92

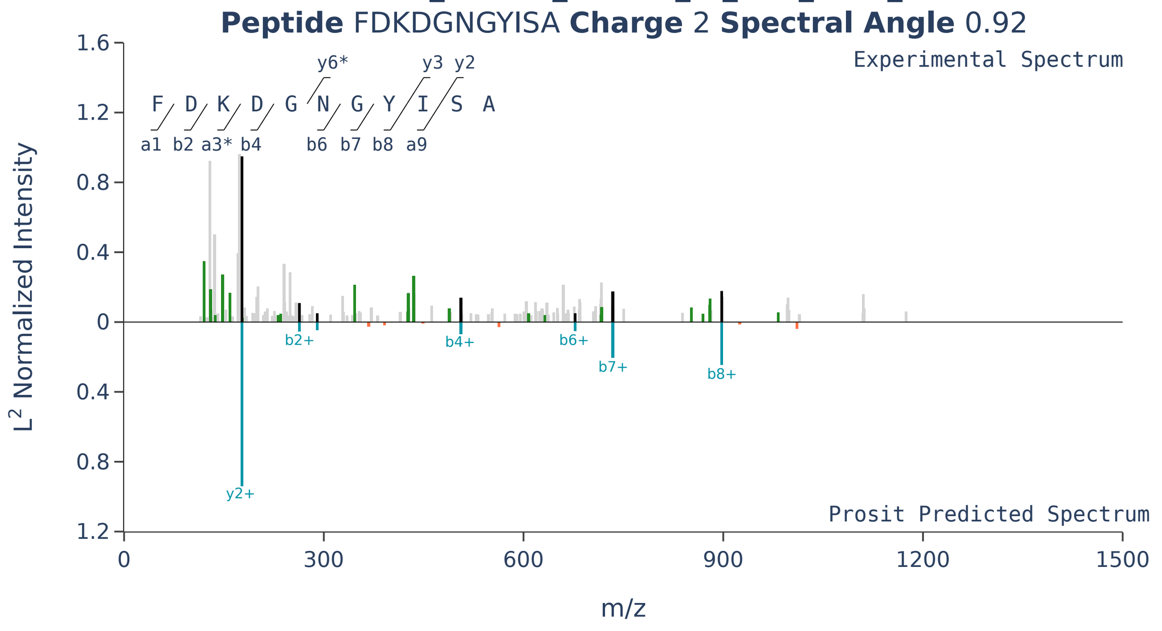


**Source** HRoetschke_291222_040123_Fu_CaM_b1R1_24h **Scan** 5377
**Peptide** KDGDGTITTKEL **Charge** 2 **Spectral Angle** 0.94

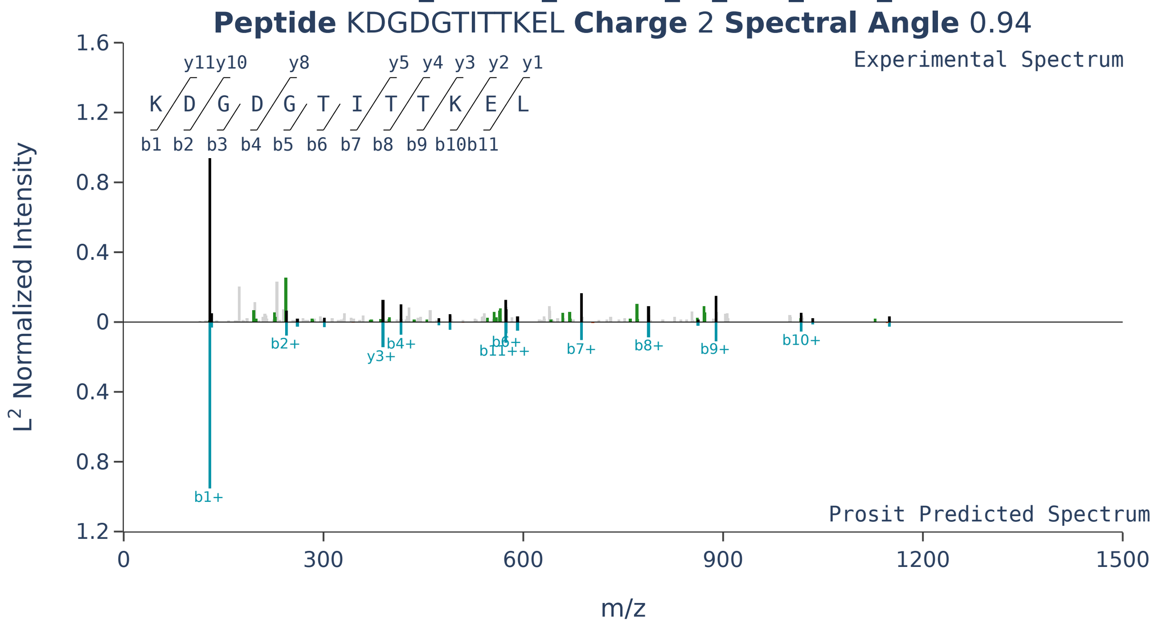


**Source** HRoetschke_291222_040123_Fu_CaM_b2R1_1h **Scan** 6379
**Peptide** NLGEKLTDEEVD **Charge** 2 **Spectral Angle** 0.89

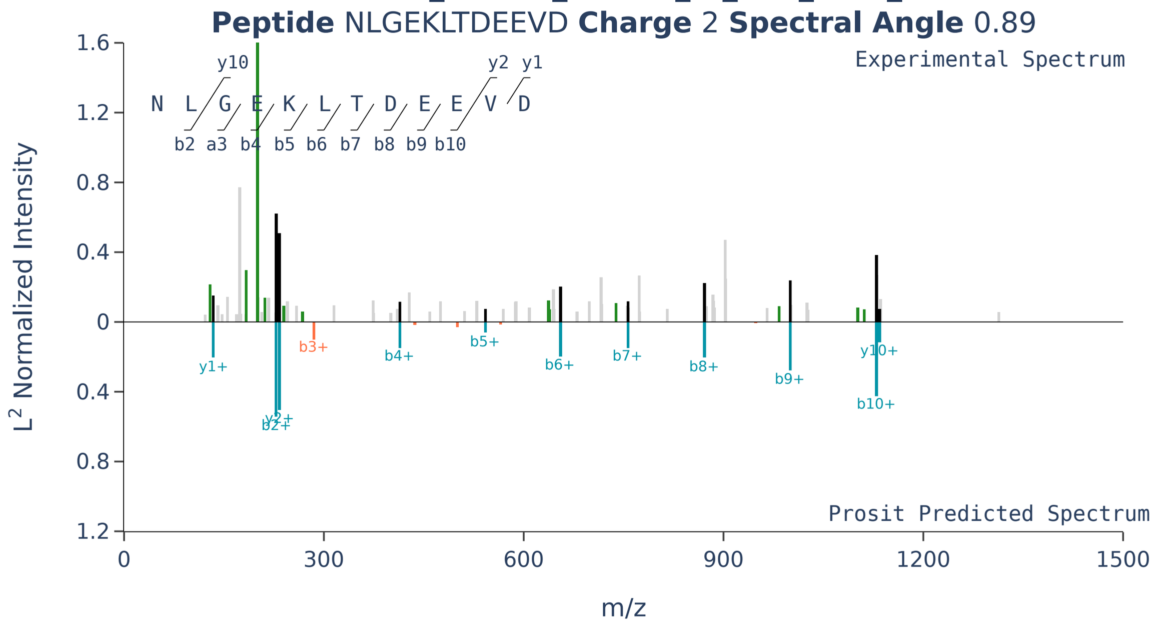


**Source** HRoetschke_291222_040123_Fu_CaM_b2R1_1h **Scan** 6672
**Peptide** SEEEIREAF **Charge** 2 **Spectral Angle** 0.93

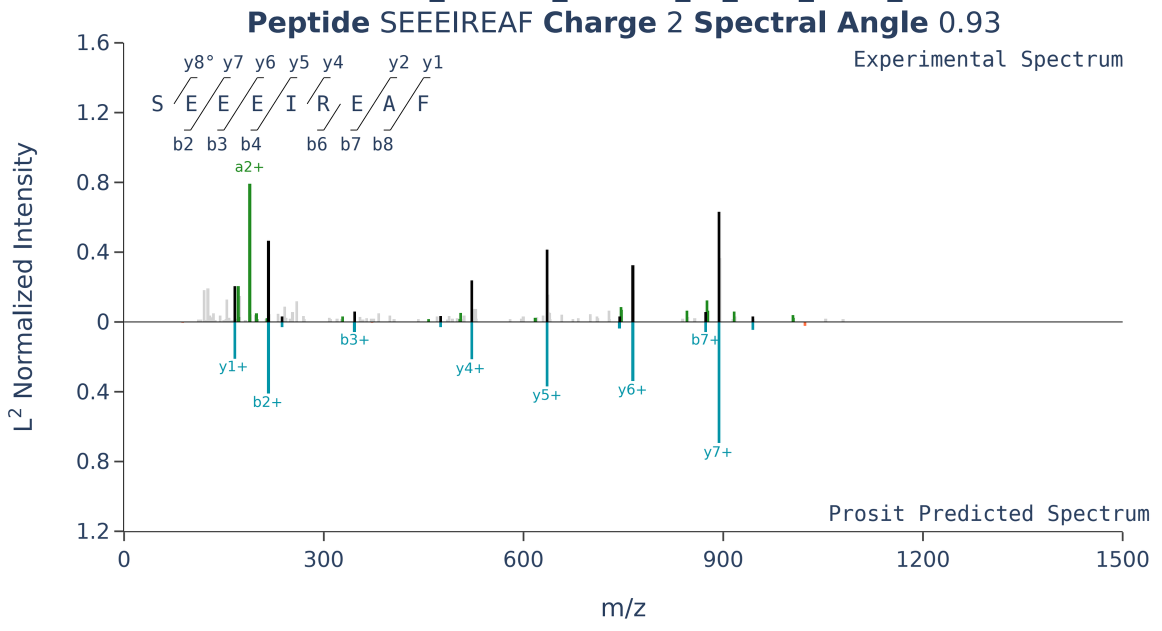


**Source** HRoetschke_291222_040123_Fu_CaM_b2R2_1h **Scan** 7504
**Peptide** KDGNGYISAAEL **Charge** 2 **Spectral Angle** 0.86

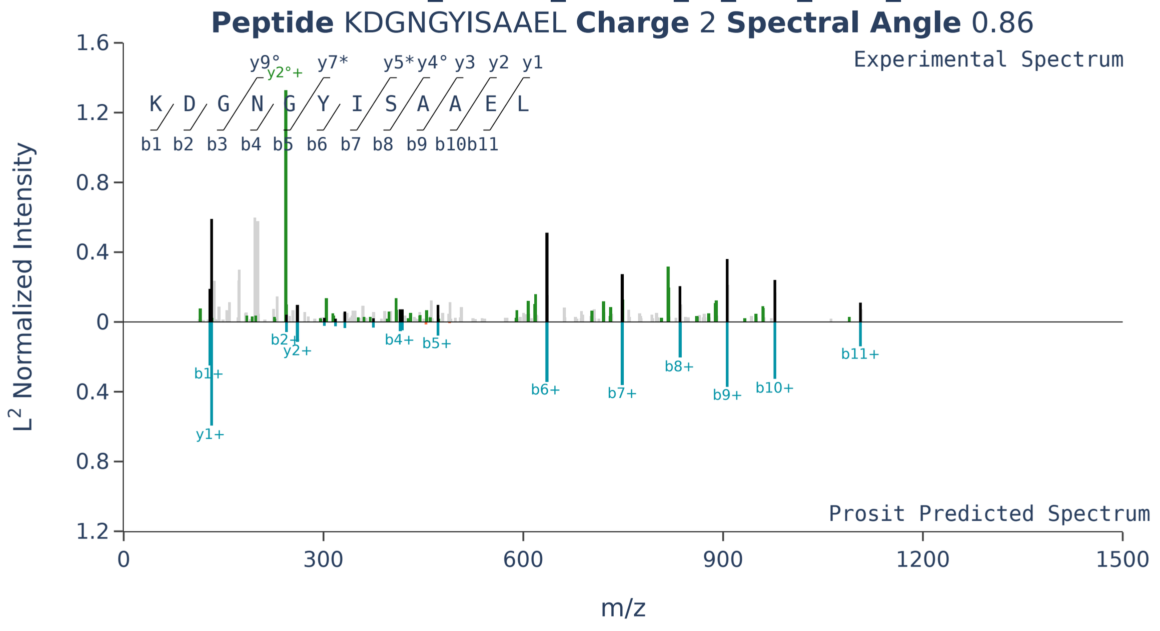

**Source** HRoetschke_291222_040123_Fu_CaM_b1R1_1h **Scan** 6184
**Peptide** KDGNGYISAAELR **Charge** 3 **Spectral Angle** 0.9

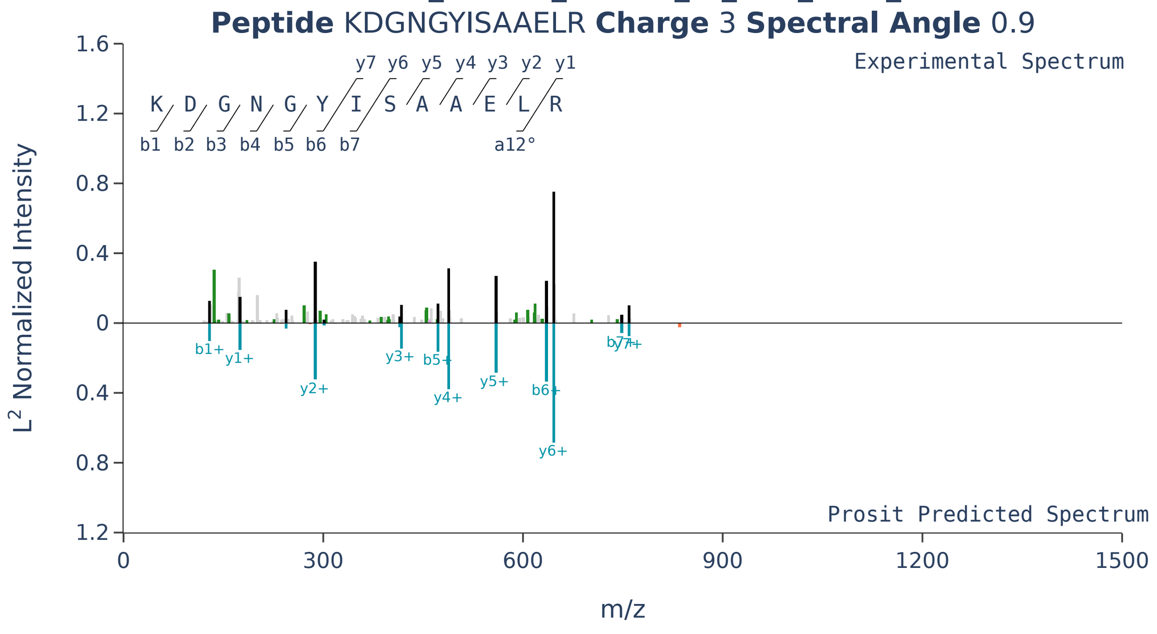


**Source** HRoetschke_291222_040123_Fu_CaM_b2R1_2h **Scan** 3249
**Peptide** KELGTVMR **Charge** 2 **Spectral Angle** 0.91

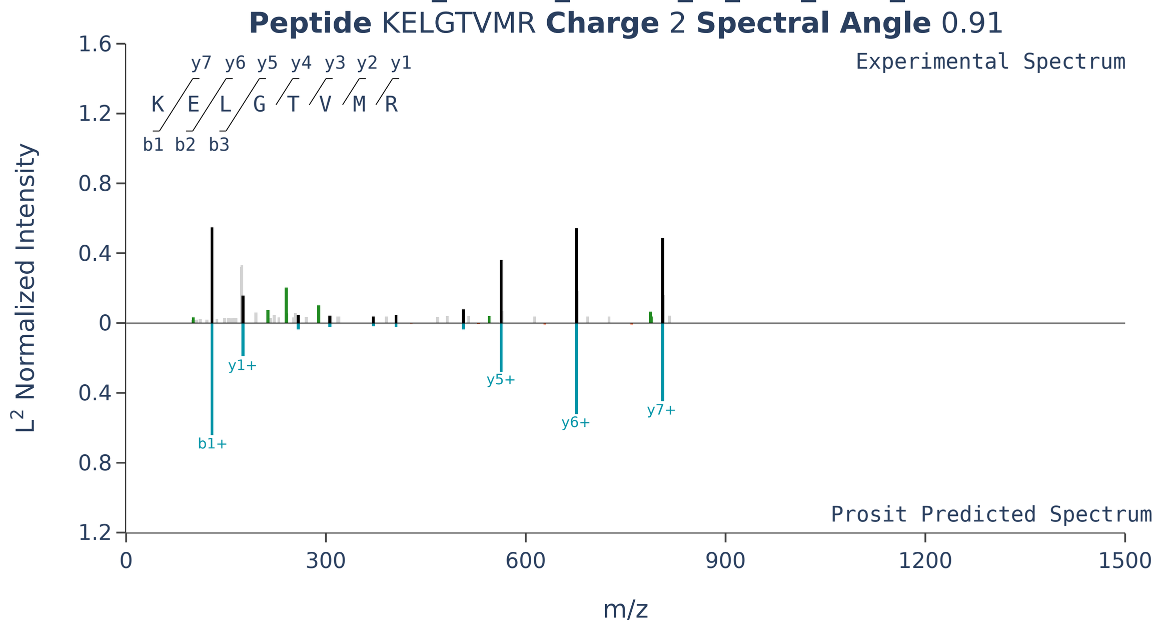


**Source** HRoetschke_291222_040123_Fu_CaM_b1R2_1h **Scan** 3969
**Peptide** KDGNGYISA **Charge** 2 **Spectral Angle** 0.91

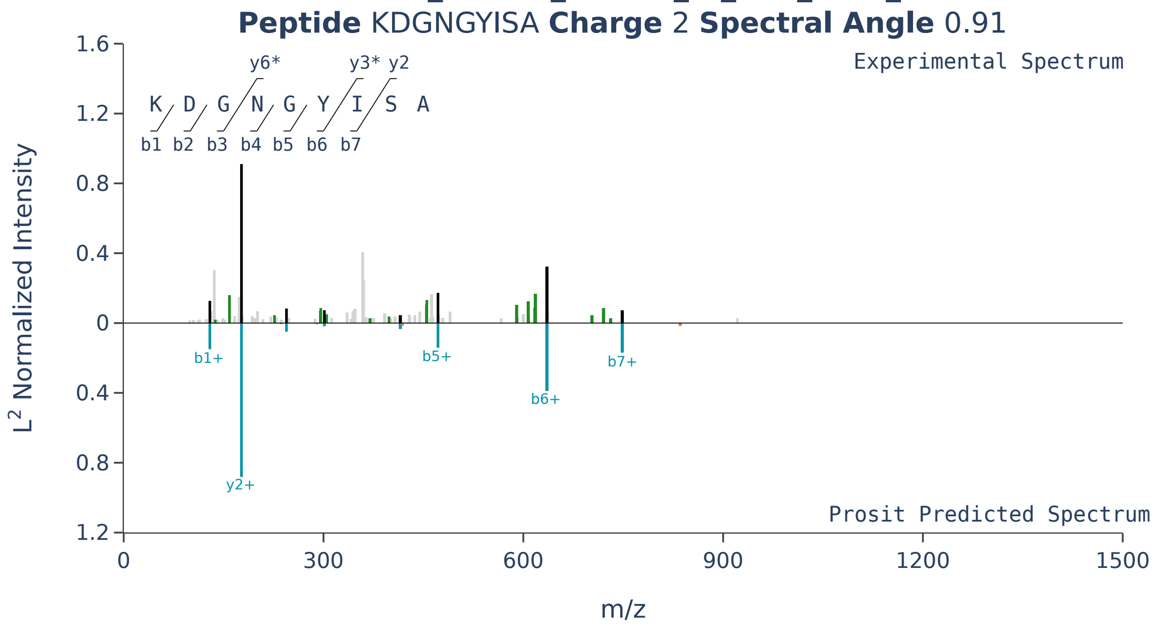


**Source** HRoetschke_291222_040123_Fu_CaM_b2R1_2h **Scan** 4481
**Peptide** EEVDEMIRE **Charge** 2 **Spectral Angle** 0.93

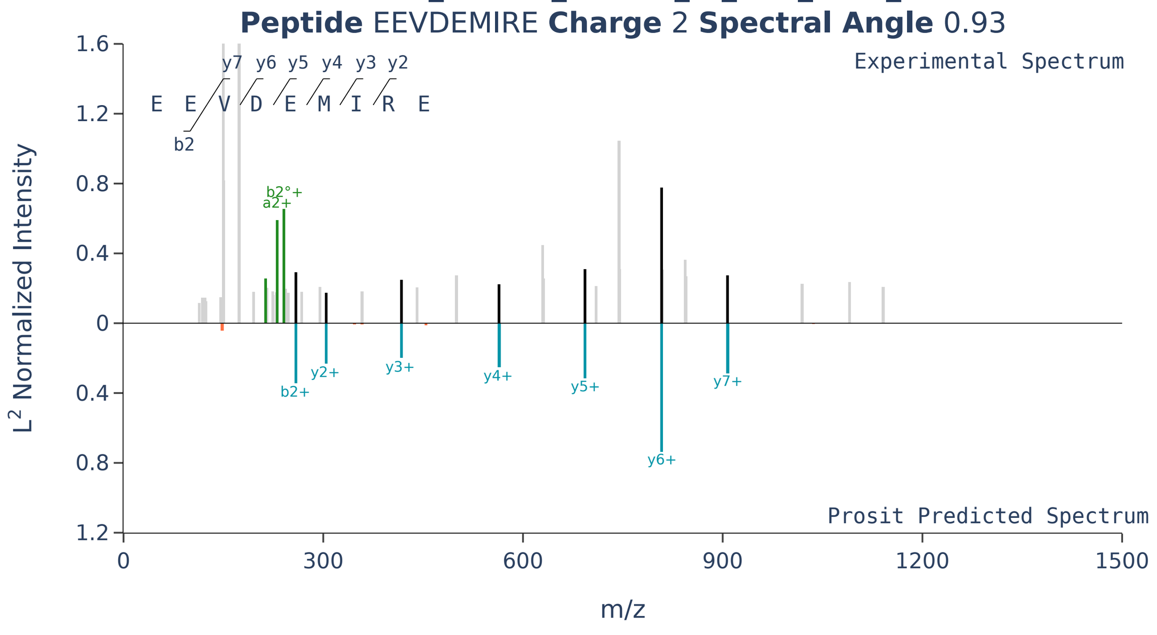


**Source** HRoetschke_291222_040123_Fu_CaM_b1R2_1h **Scan** 5612
**Peptide** EQIAEFKE **Charge** 2 **Spectral Angle** 0.88

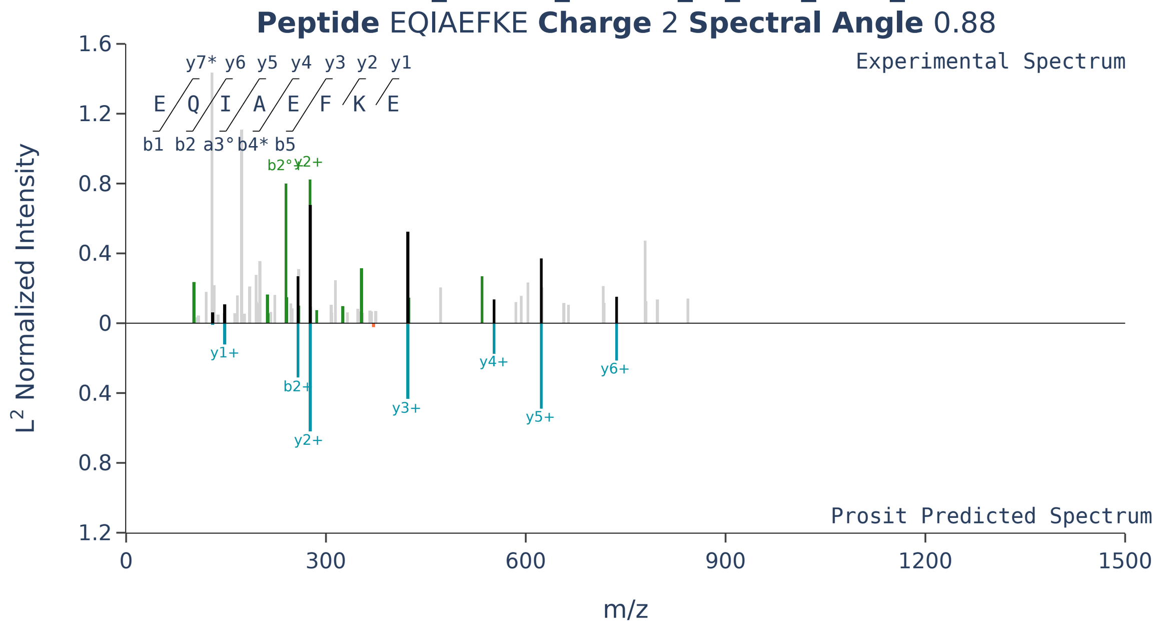


**Source** HRoetschke_291222_040123_Fu_CaM_b1R2_1h **Scan** 3952
**Peptide** KDGNGYISAA **Charge** 2 **Spectral Angle** 0.87

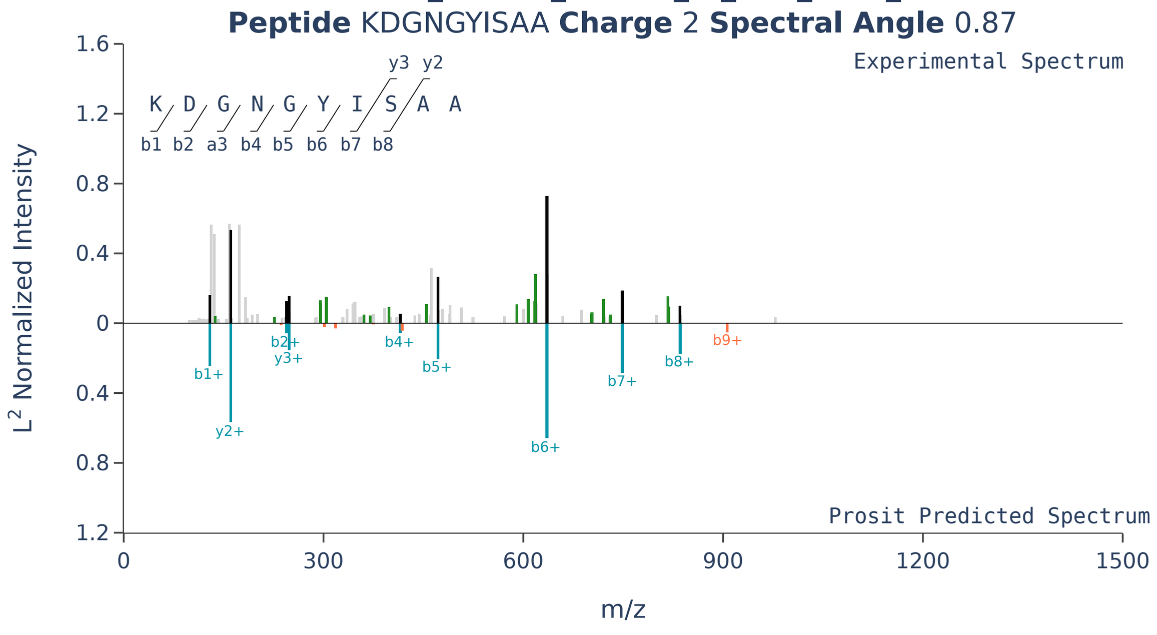


**Source** HRoetschke_291222_040123_Fu_CaM_b1R2_24h **Scan** 7723
**Peptide** VFDKDGNGYISA **Charge** 2 **Spectral Angle** 0.91

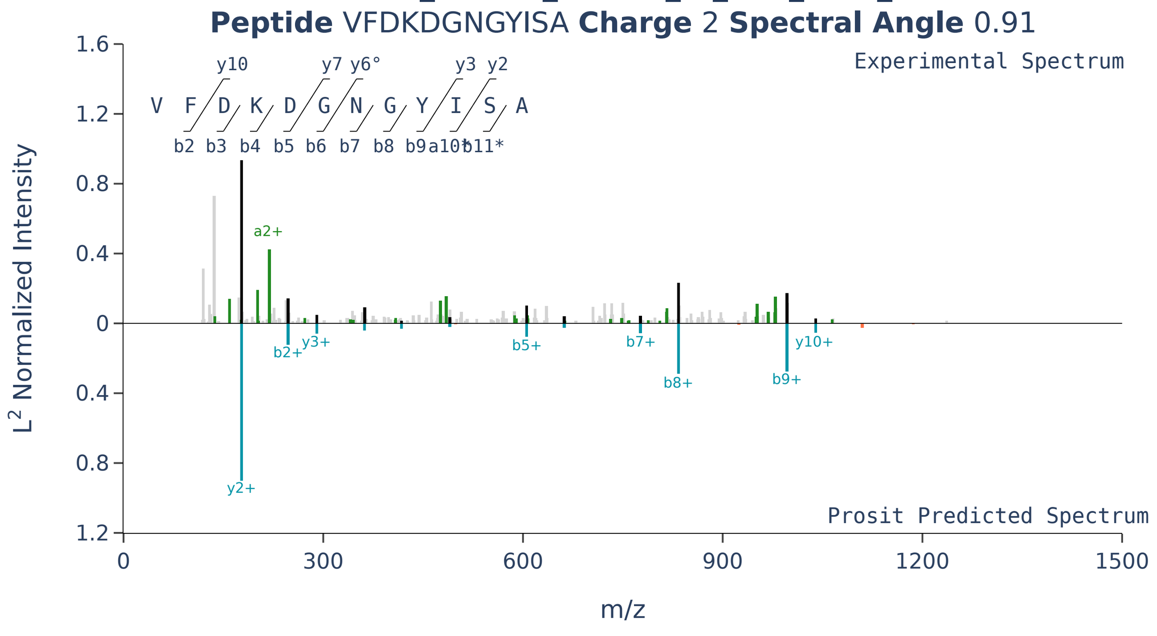


**Source** HRoetschke_291222_040123_Fu_CaM_b2R1_4h **Scan** 4768
**Peptide** AELRHVMTNL **Charge** 2 **Spectral Angle** 0.87

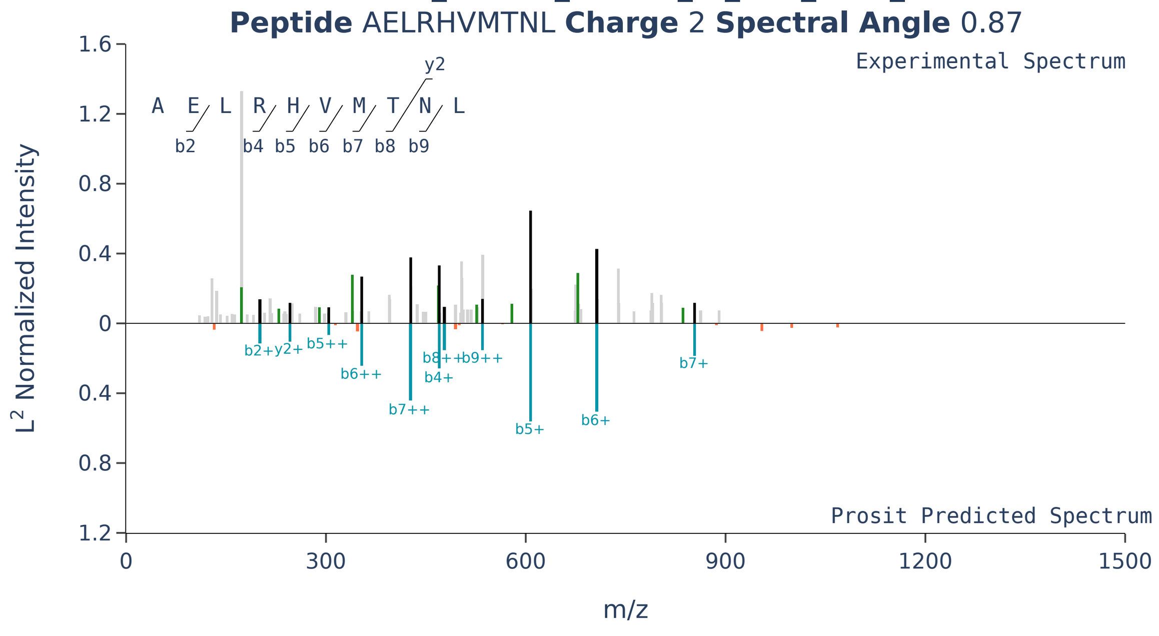


**Source** HRoetschke_291222_040123_Fu_CaM_b2R1_2h **Scan** 8195
**Peptide** REAFRVFD **Charge** 3 **Spectral Angle** 0.87

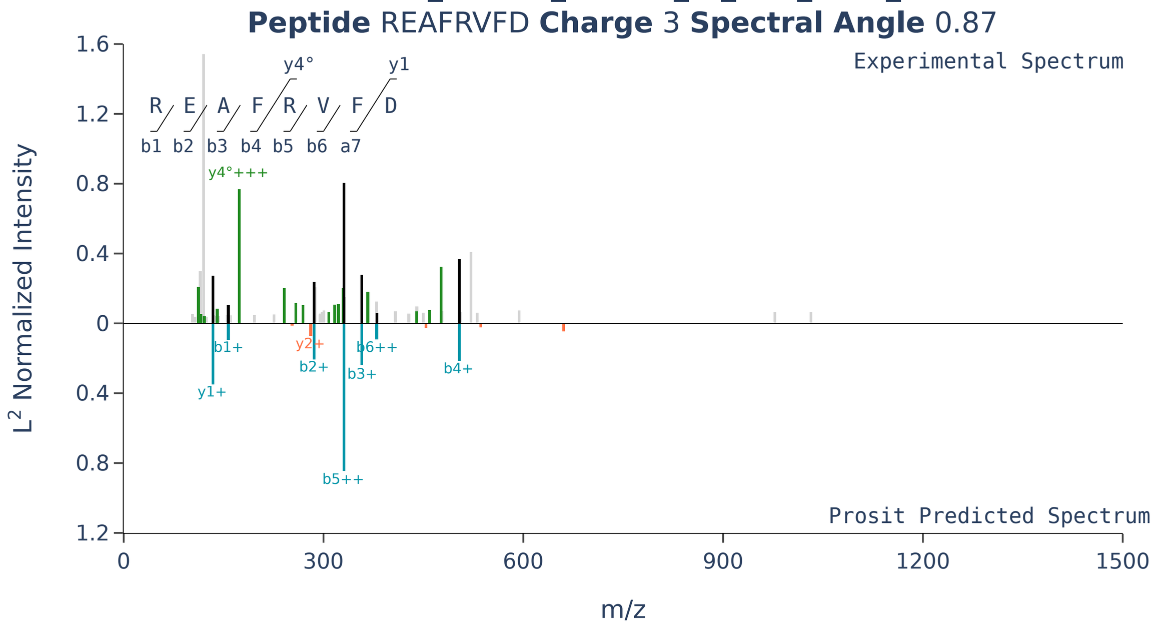


**Source** HRoetschke_291222_040123_Fu_CaM_b2R1_1h **Scan** 6053
**Peptide** QDMINEVD **Charge** 2 **Spectral Angle** 0.84

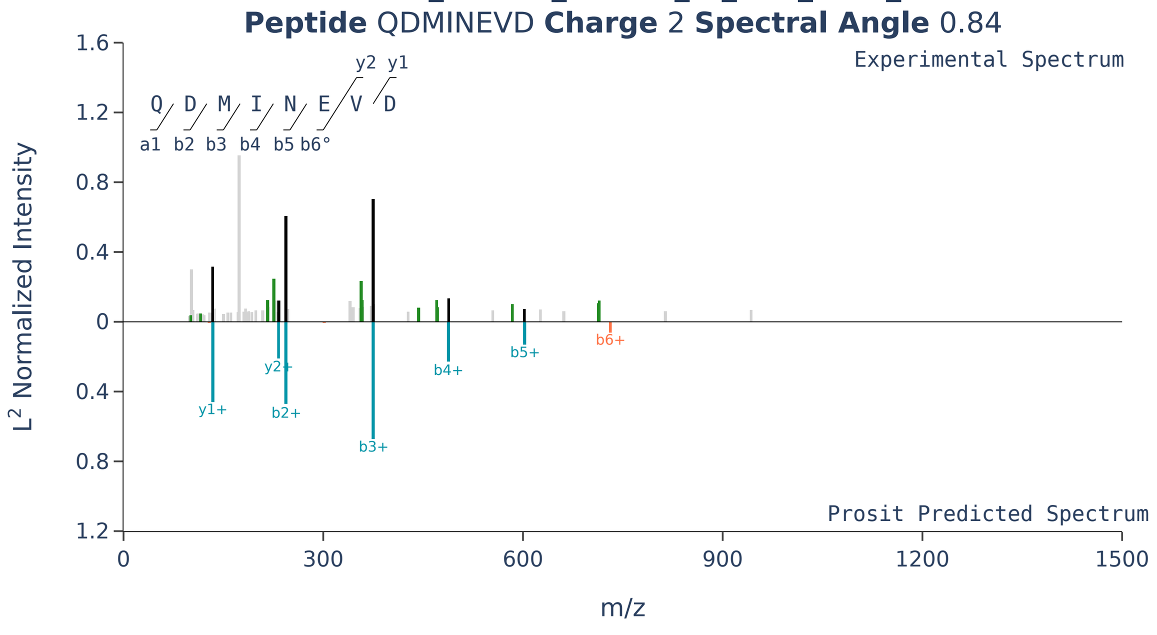


**Source** HRoetschke_291222_040123_Fu_CaM_b1R2_2h **Scan** 8428
**Peptide** DGNGYISAAELR **Charge** 2 **Spectral Angle** 0.91

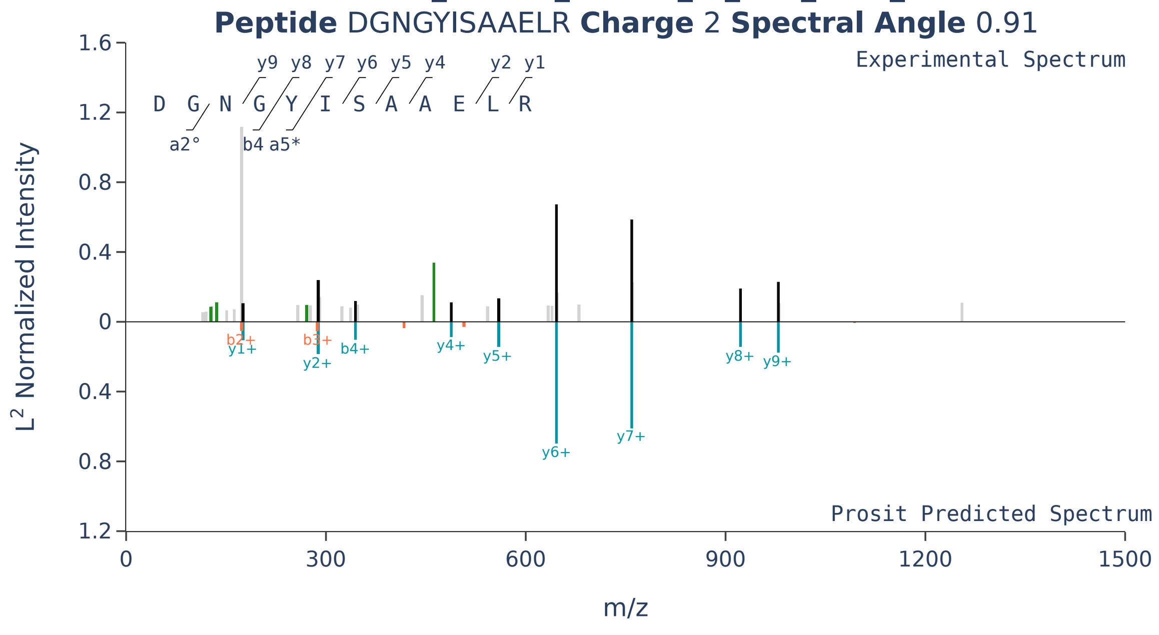


**Source** HRoetschke_291222_040123_Fu_CaM_b1R2_24h **Scan** 6023
**Peptide** MTNLGEKL **Charge** 2 **Spectral Angle** 0.91

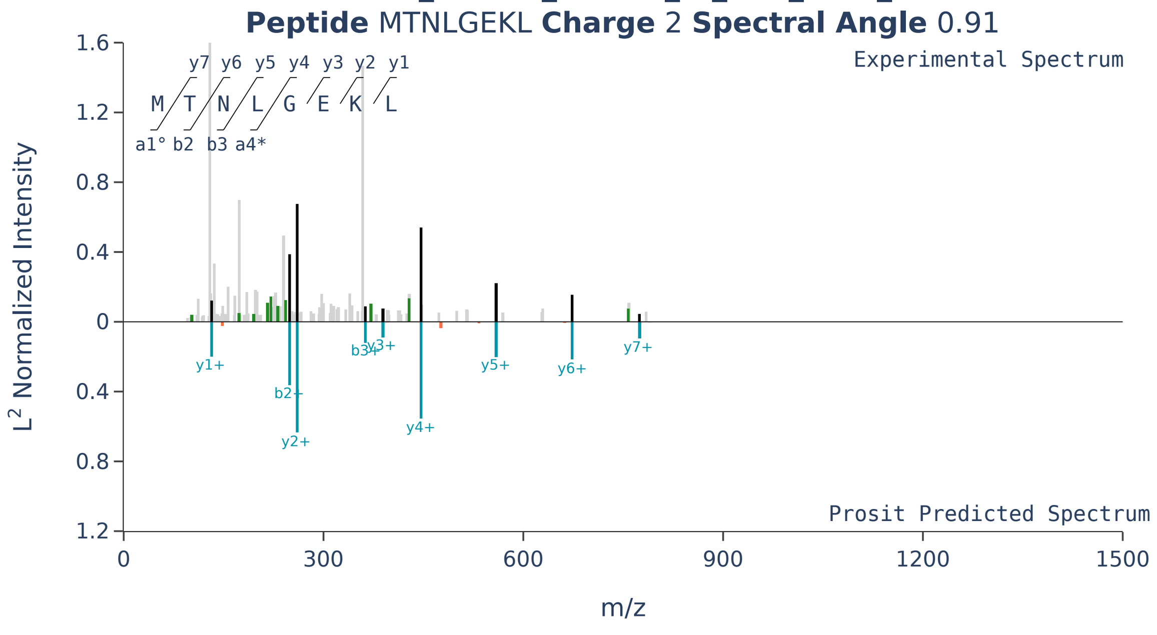


**Source** HRoetschke_291222_040123_Fu_CaM_b1R1_4h **Scan** 5636
**Peptide** RSLGQNPTEAELQD **Charge** 2 **Spectral Angle** 0.9

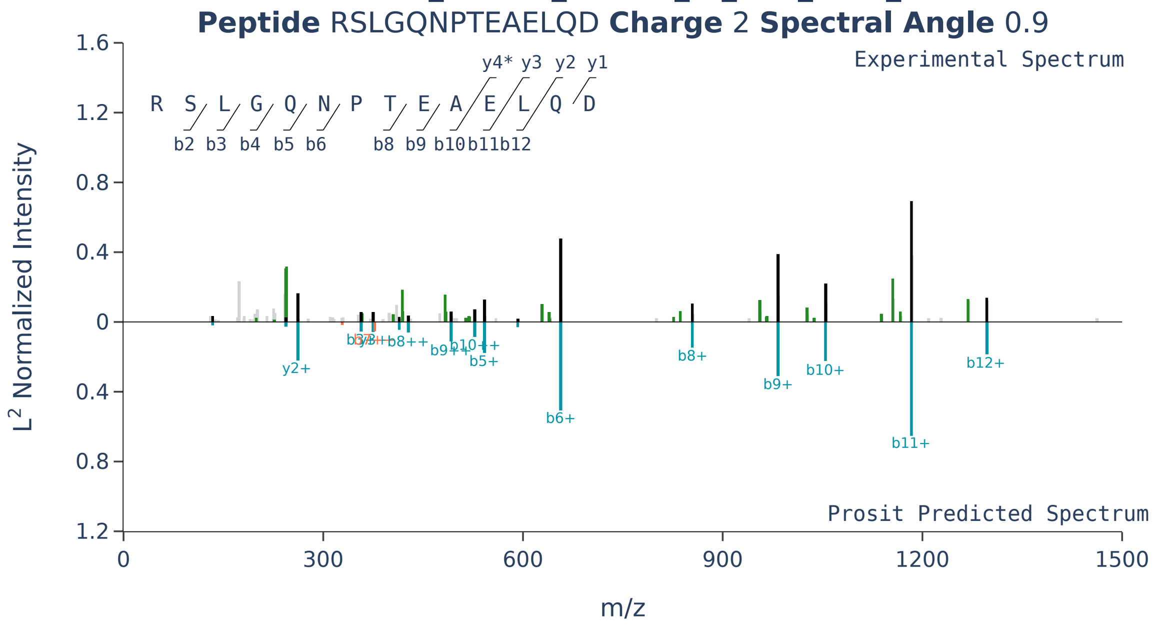


**Source** HRoetschke_291222_040123_Fu_CaM_b1R2_2h **Scan** 4494
**Peptide** GEKLTDEEVD **Charge** 2 **Spectral Angle** 0.87

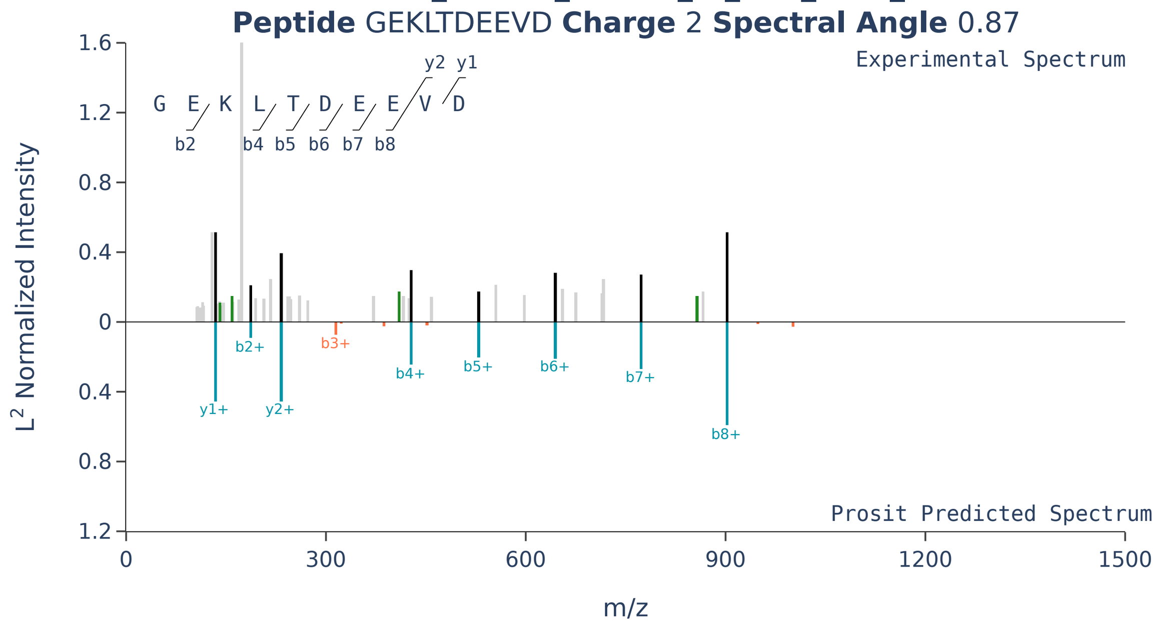


**Source** HRoetschke_291222_040123_Fu_CaM_b2R1_1h **Scan** 2965
**Peptide** SLGQNPTE **Charge** 2 **Spectral Angle** 0.87

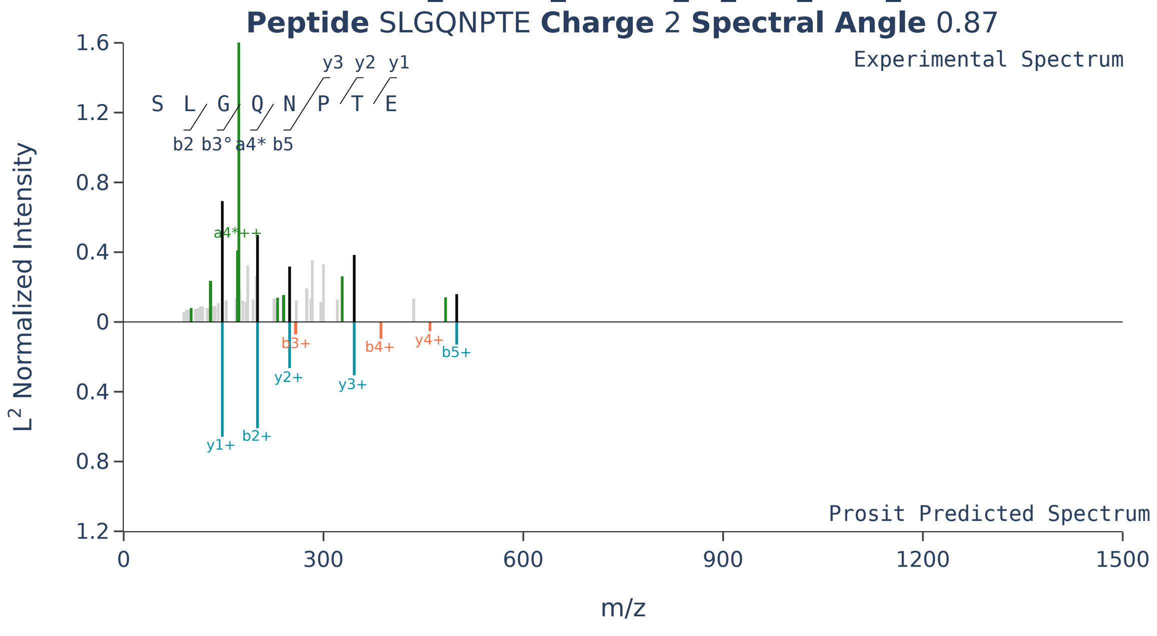

**Source** HRoetschke_291222_040123_Fu_CaM_b2R2_24h **Scan** 3381
**Peptide** AELRHVMT **Charge** 2 **Spectral Angle** 0.85

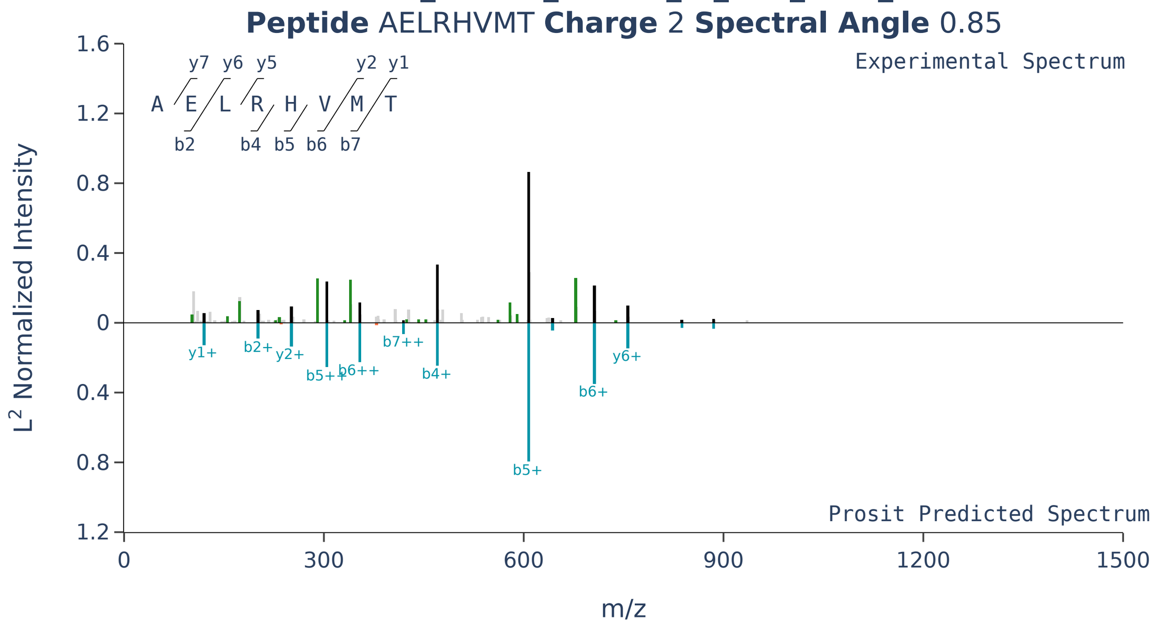


**Source** HRoetschke_291222_040123_Fu_CaM_b2R2_24h **Scan** 2327
**Peptide** KELGTVMRS **Charge** 2 **Spectral Angle** 0.93

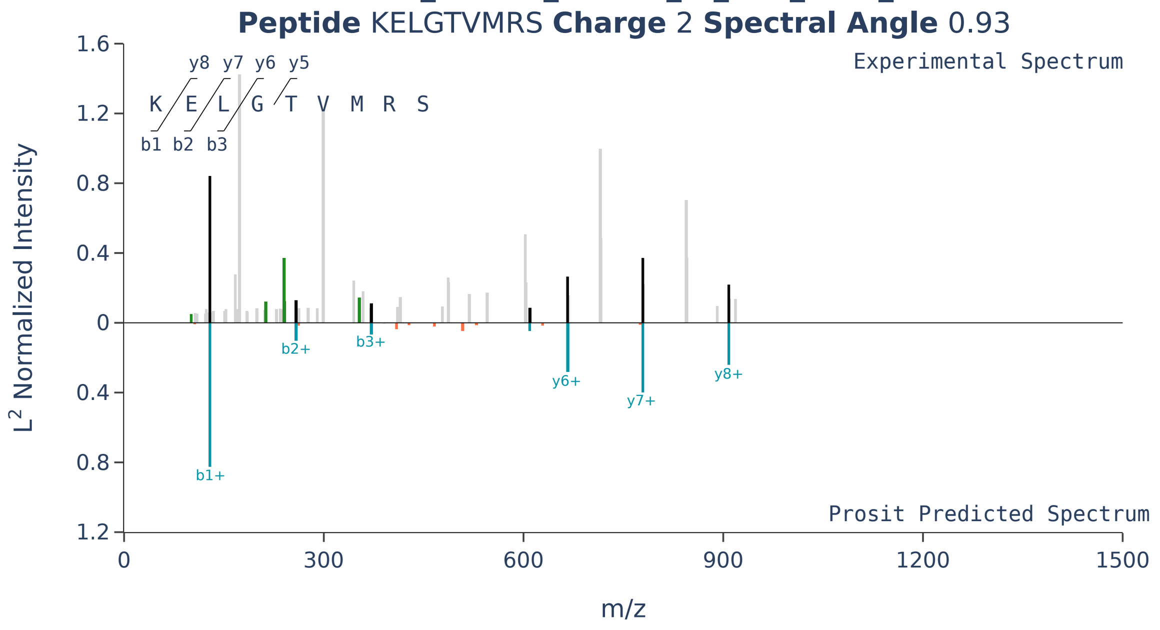


**Source** HRoetschke_291222_040123_Fu_CaM_b1R2_24h **Scan** 5819
**Peptide** KDGNGYISAAELRH **Charge** 3 **Spectral Angle** 0.83

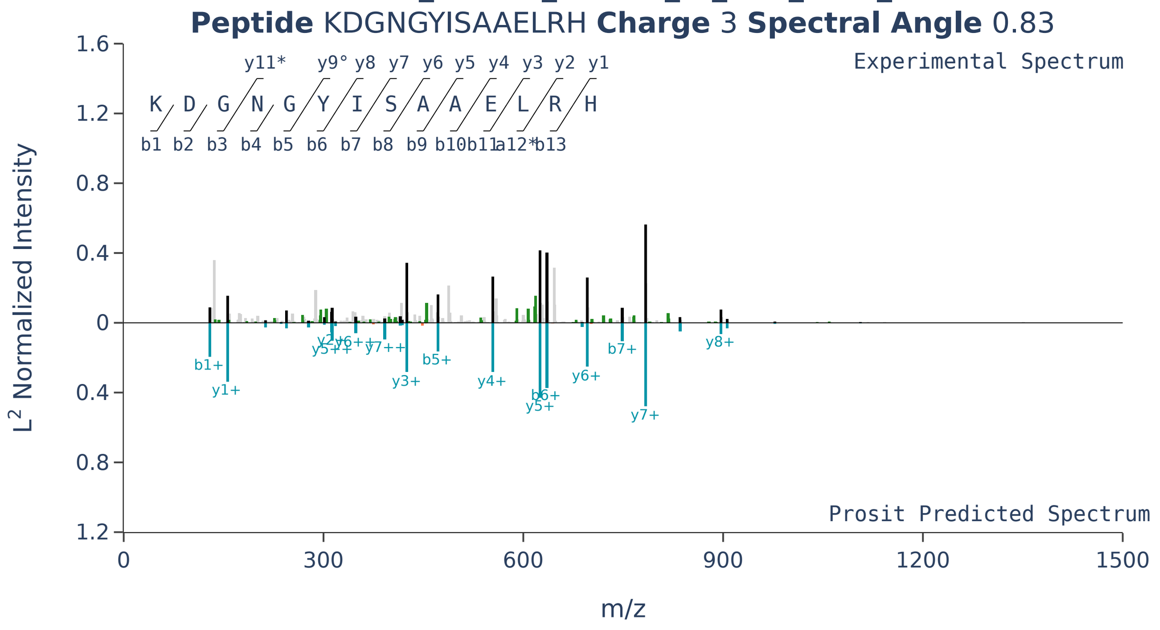


**Source** HRoetschke_291222_040123_Fu_CaM_b2R2_24h **Scan** 10598
**Peptide** SLFDKDGDGTITTKELGTVM **Charge** 3 **Spectral Angle** 0.9

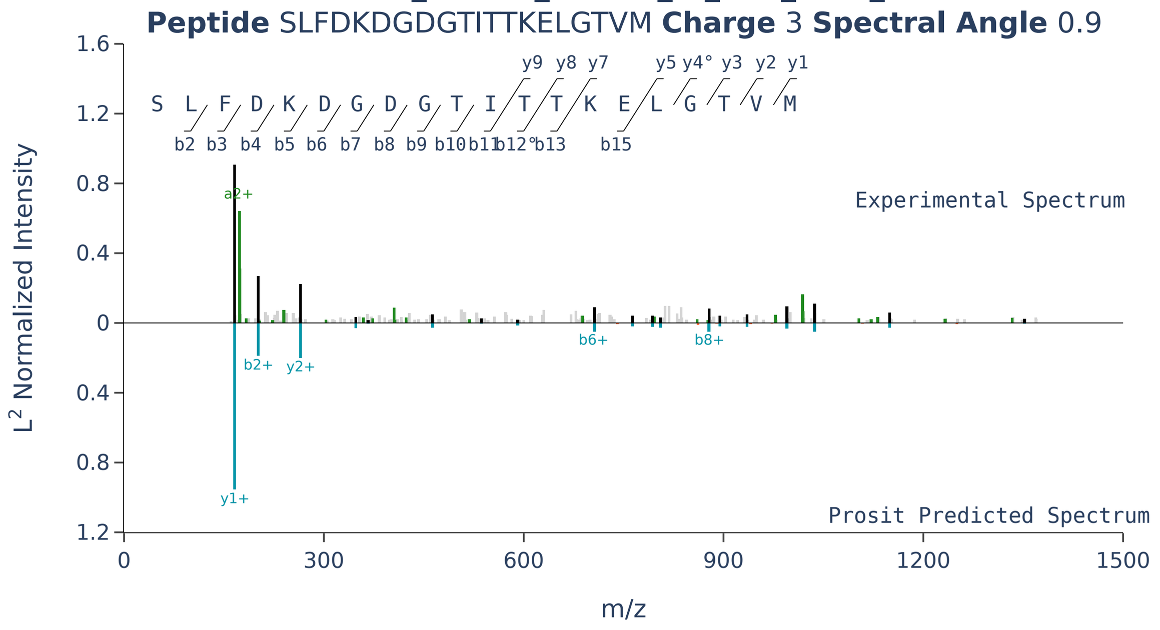


**Source** HRoetschke_291222_040123_Fu_CaM_b2R2_1h **Scan** 6159
**Peptide** SLGQNPTEAELQD **Charge** 2 **Spectral Angle** 0.89

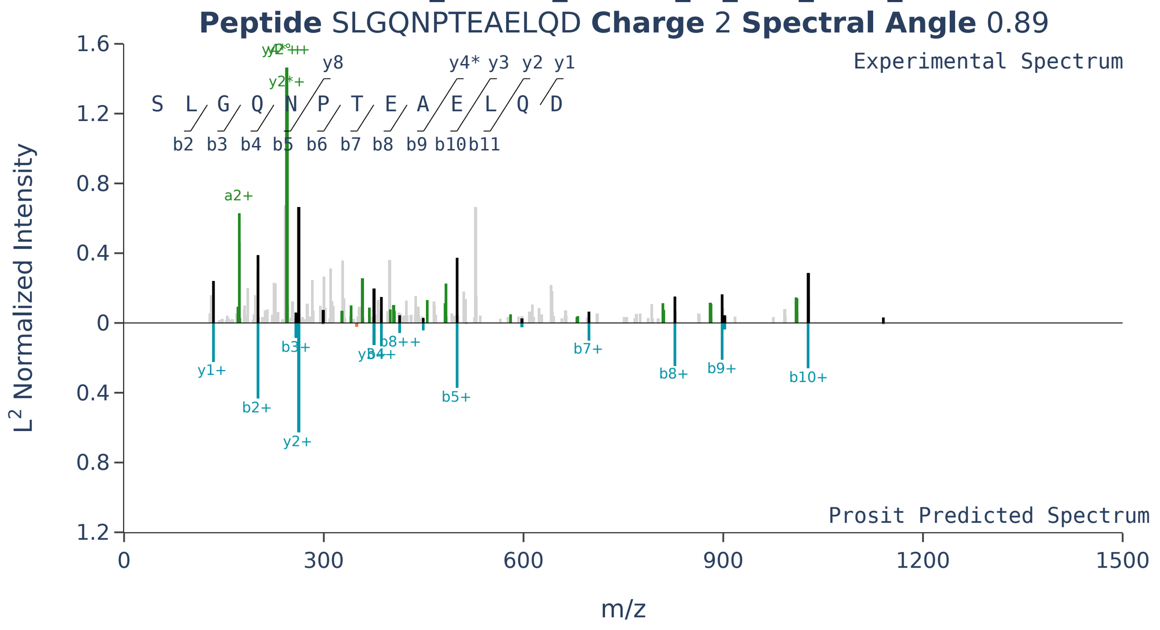


**Source** HRoetschke_291222_040123_Fu_CaM_b1R1_2h **Scan** 7066
**Peptide** DKDGNGYISAAELR **Charge** 3 **Spectral Angle** 0.9

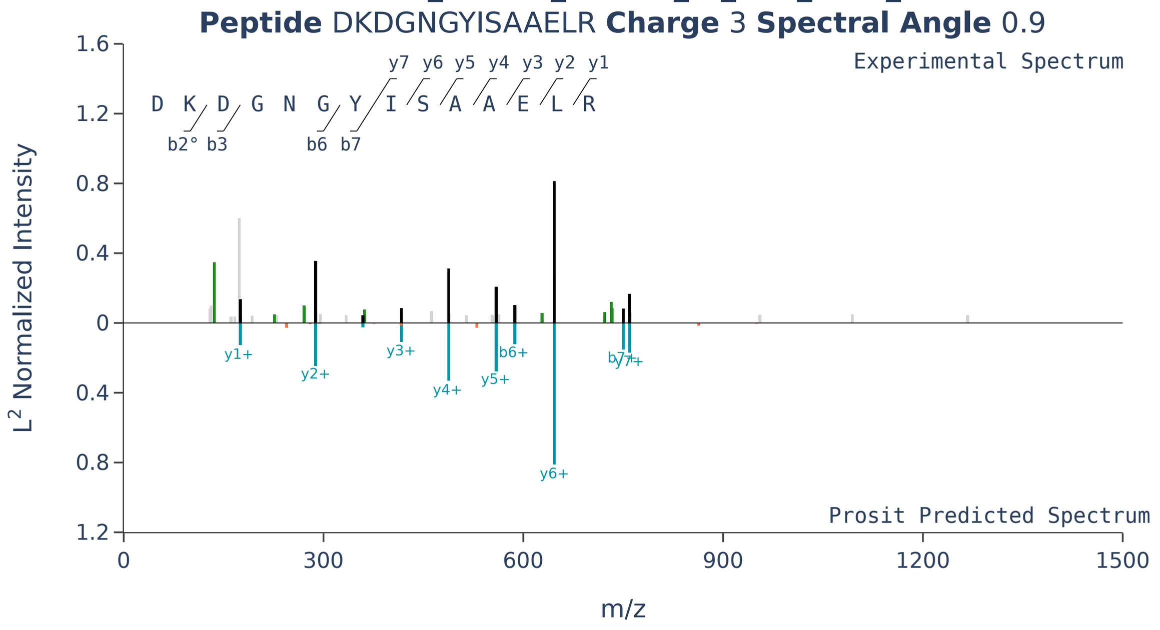


**Source** HRoetschke_291222_040123_Fu_CaM_b1R2_1h **Scan** 6232
**Peptide** RSLGQNPTEAEL **Charge** 2 **Spectral Angle** 0.89

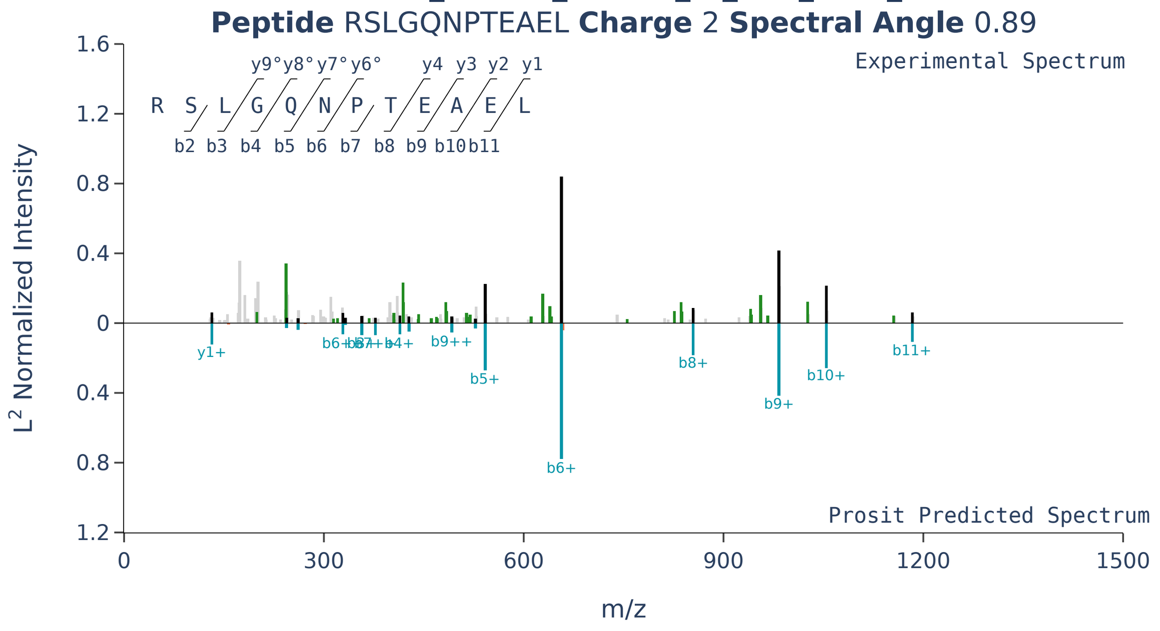


**Source** HRoetschke_291222_040123_Fu_CaM_b2R2_24h **Scan** 5631
**Peptide** RKMKDTDSEEEIREAF **Charge** 3 **Spectral Angle** 0.85

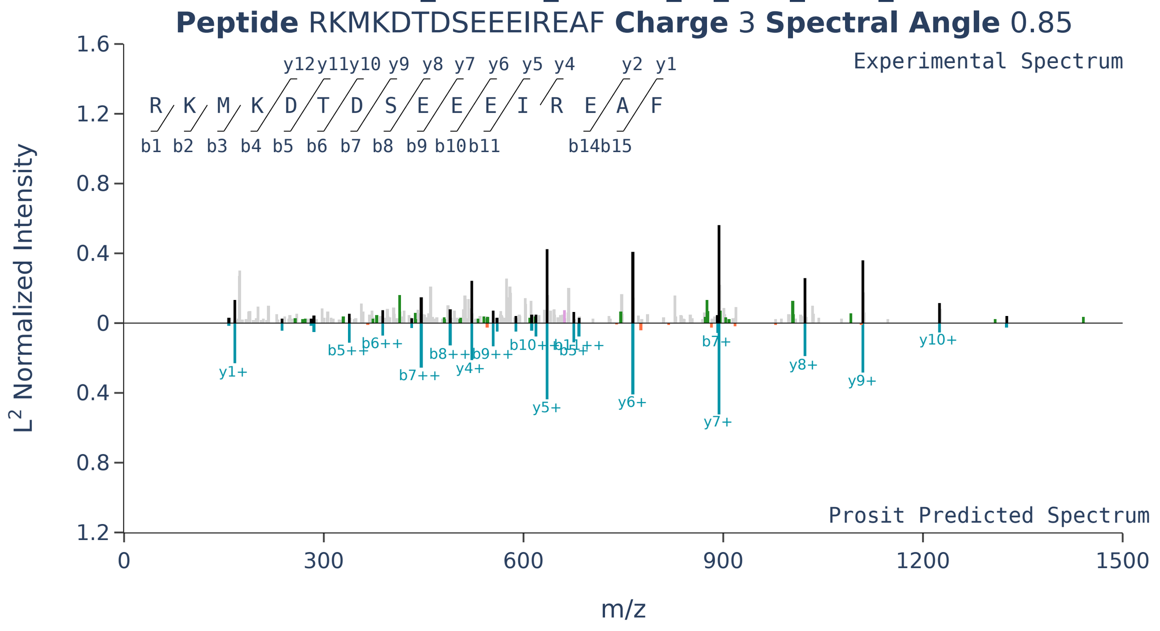


**Source** HRoetschke_291222_040123_Fu_CaM_b2R2_4h **Scan** 2629
**Peptide** ELRHVMTN **Charge** 2 **Spectral Angle** 0.84

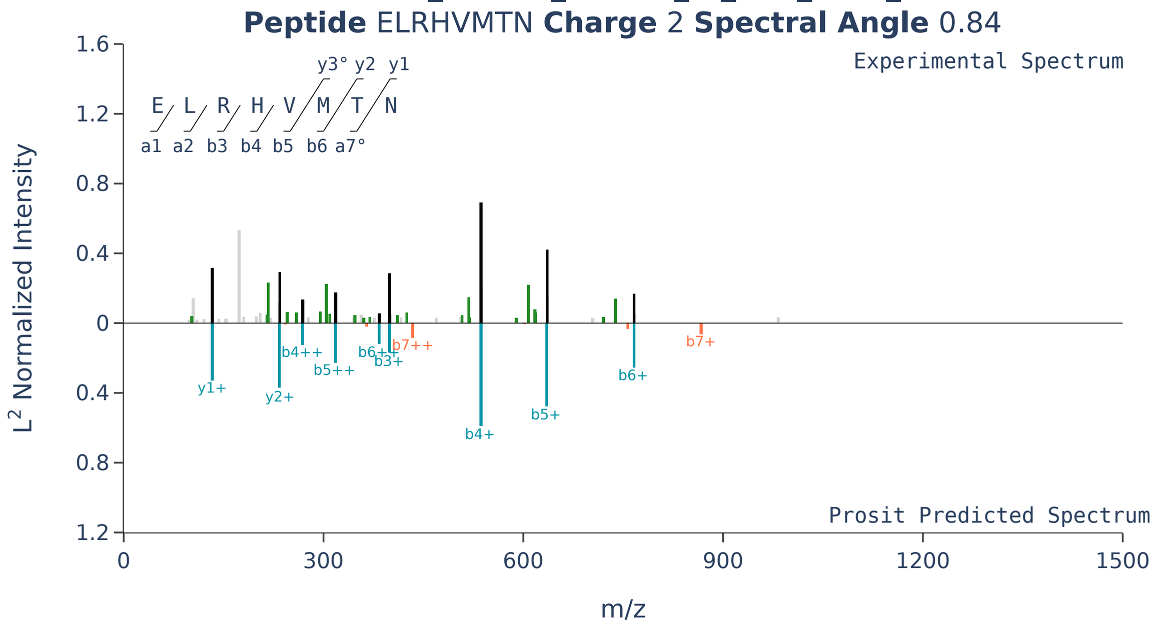


**Source** HRoetschke_291222_040123_Fu_CaM_b1R1_24h **Scan** 9875
**Peptide** REAFRVF **Charge** 2 **Spectral Angle** 0.83

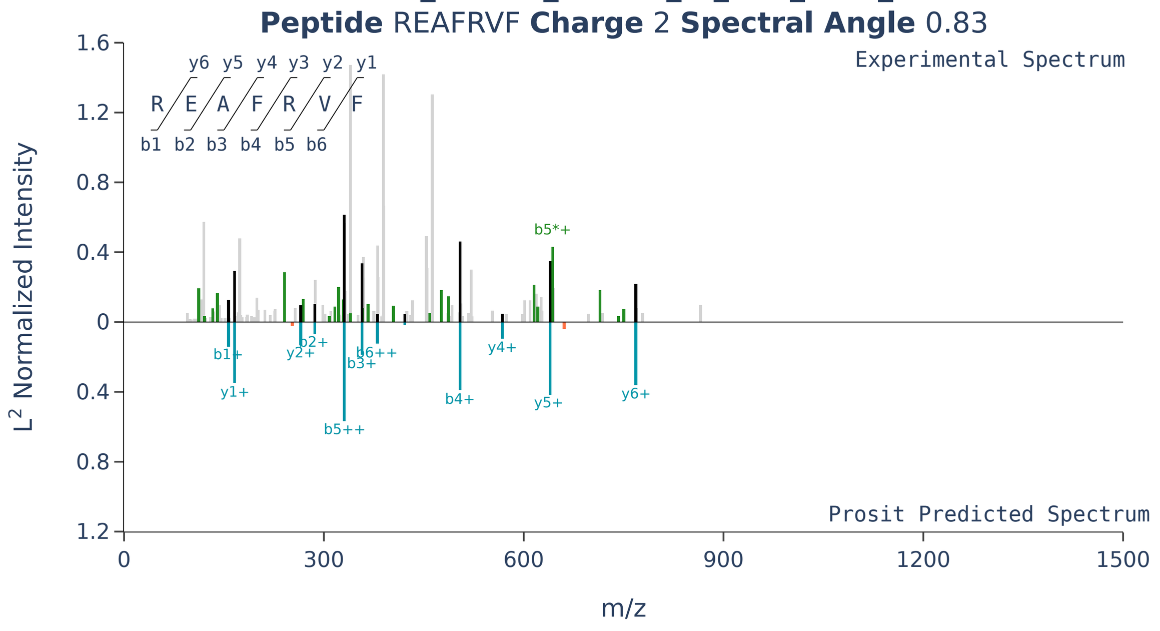


**Source** HRoetschke_291222_040123_Fu_CaM_b1R1_24h **Scan** 2332
**Peptide** ELRHVMT **Charge** 2 **Spectral Angle** 0.8

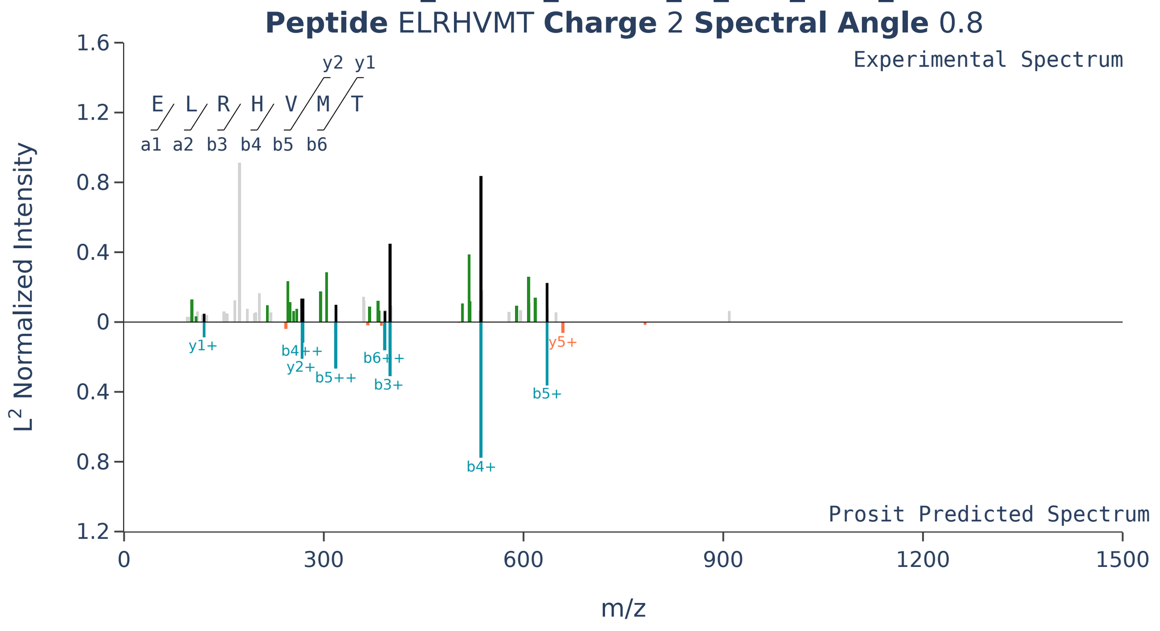


**Source** HRoetschke_291222_040123_Fu_CaM_b1R1_4h **Scan** 6533
**Peptide** LGQNPTEAELQD **Charge** 2 **Spectral Angle** 0.93

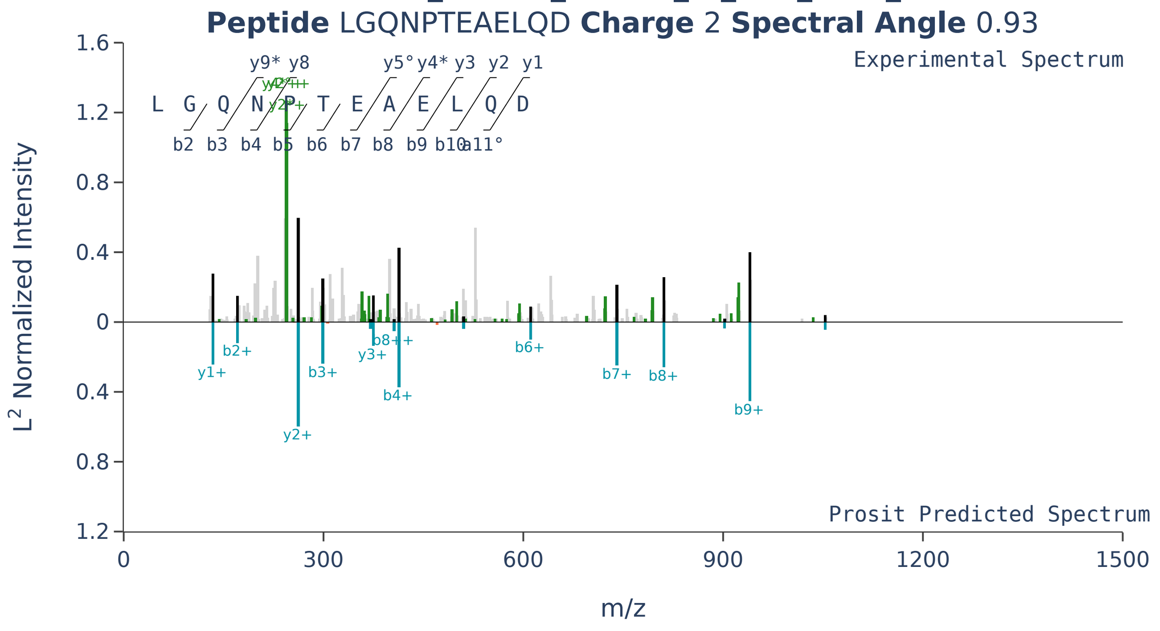


**Source** HRoetschke_291222_040123_Fu_CaM_b1R1_24h **Scan** 3745
**Peptide** RHVMTNLGE **Charge** 3 **Spectral Angle** 0.88

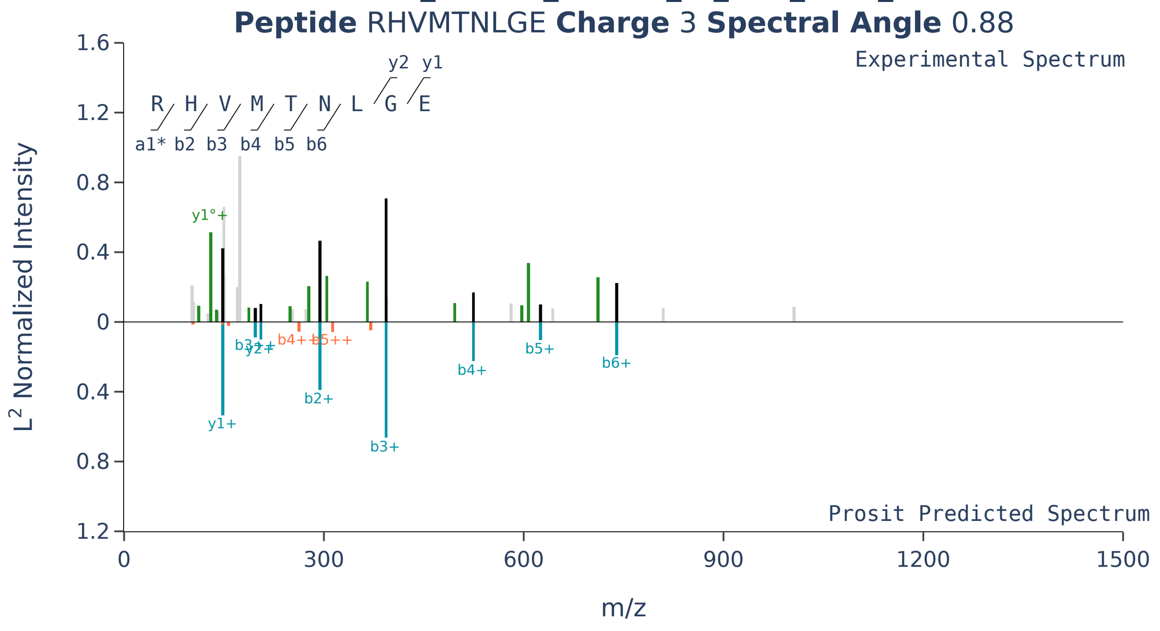


**Source** HRoetschke_291222_040123_Fu_CaM_b2R1_24h **Scan** 10084
**Peptide** READIDGDGQVNYEEFV **Charge** 2 **Spectral Angle** 0.85

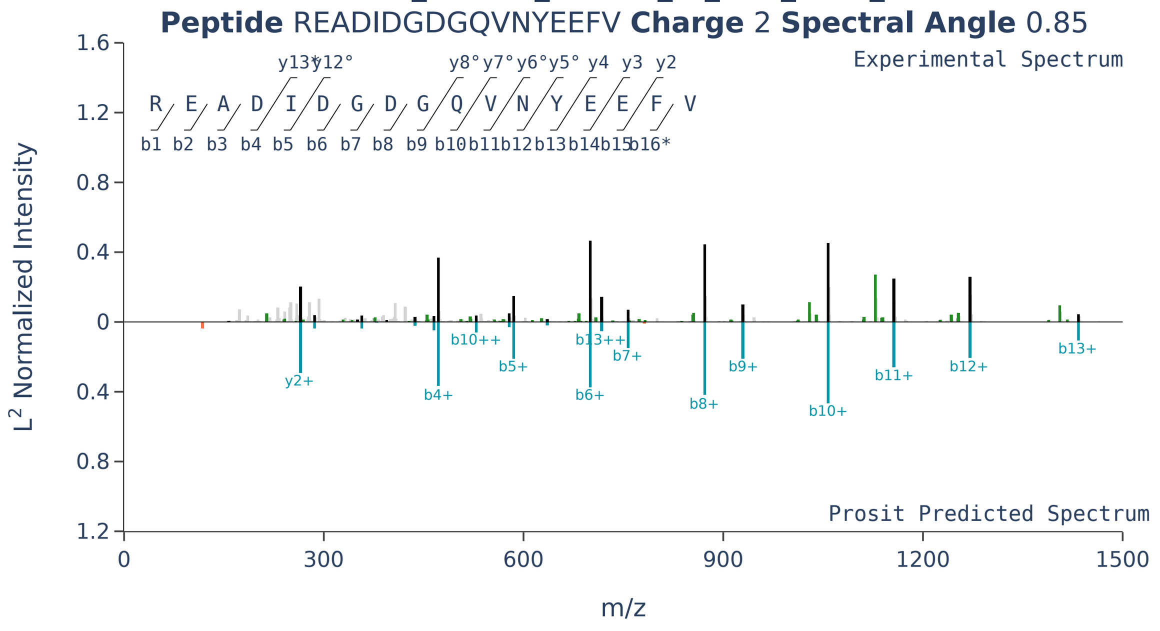


**Source** HRoetschke_291222_040123_Fu_CaM_b1R1_24h **Scan** 10317
**Peptide** SEEEIREAFRV **Charge** 3 **Spectral Angle** 0.88

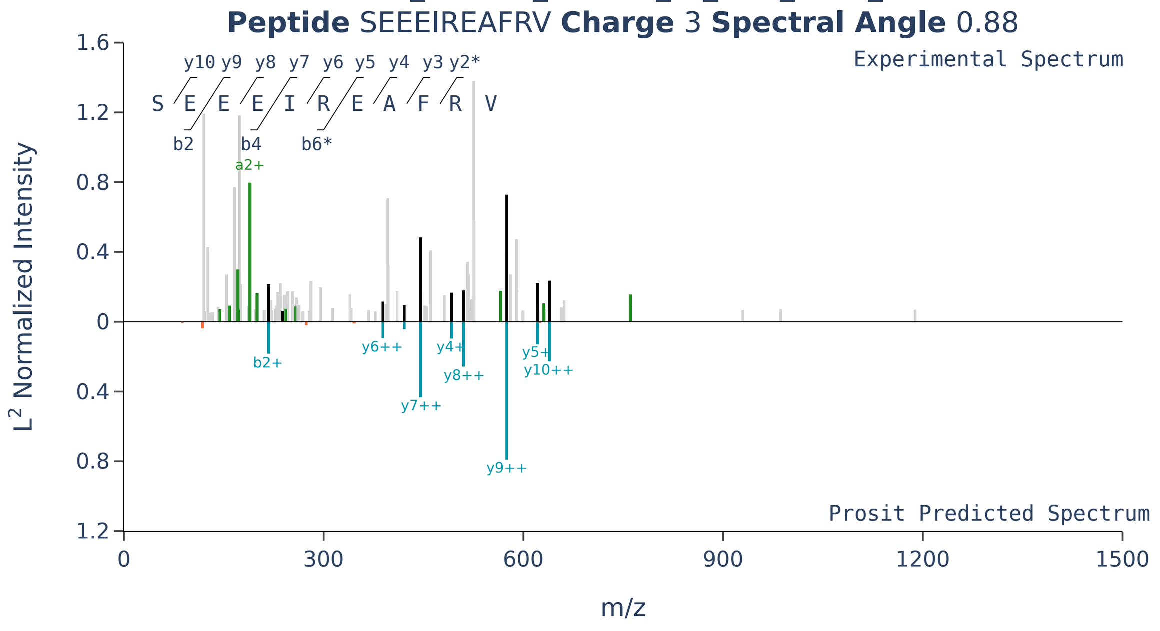

**Source** HRoetschke_291222_040123_Fu_CaM_b2R1_2h **Scan** 4881
**Peptide** READIDGDGQVNYE **Charge** 2 **Spectral Angle** 0.89

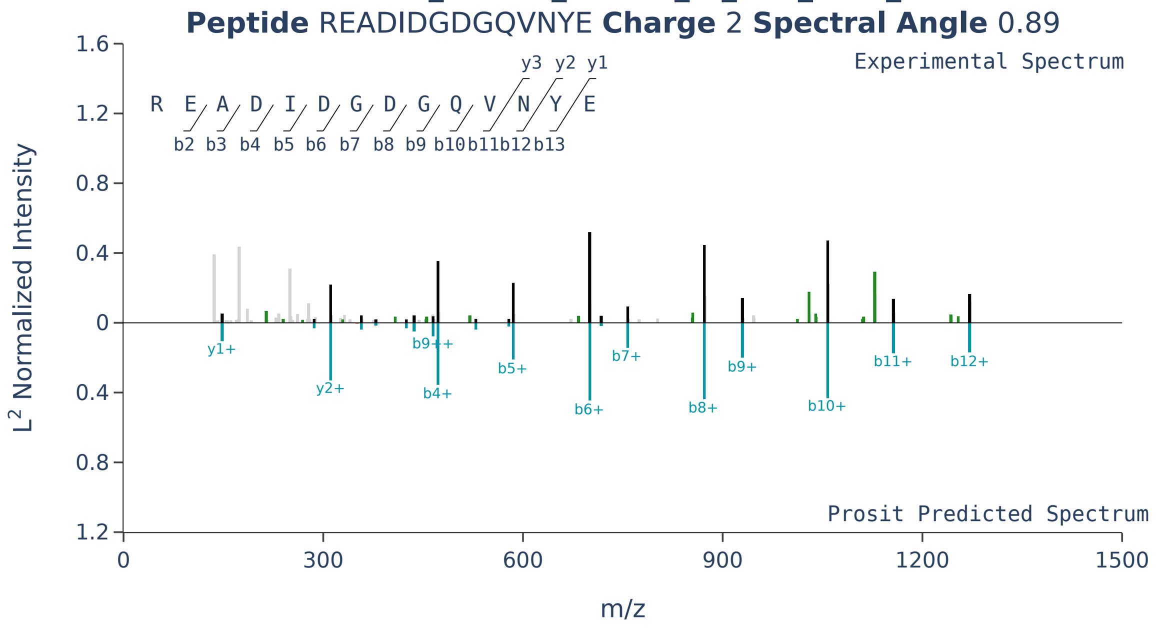


**Source** HRoetschke_291222_040123_Fu_CaM_b1R2_1h **Scan** 5287
**Peptide** EMIREADL **Charge** 2 **Spectral Angle** 0.9

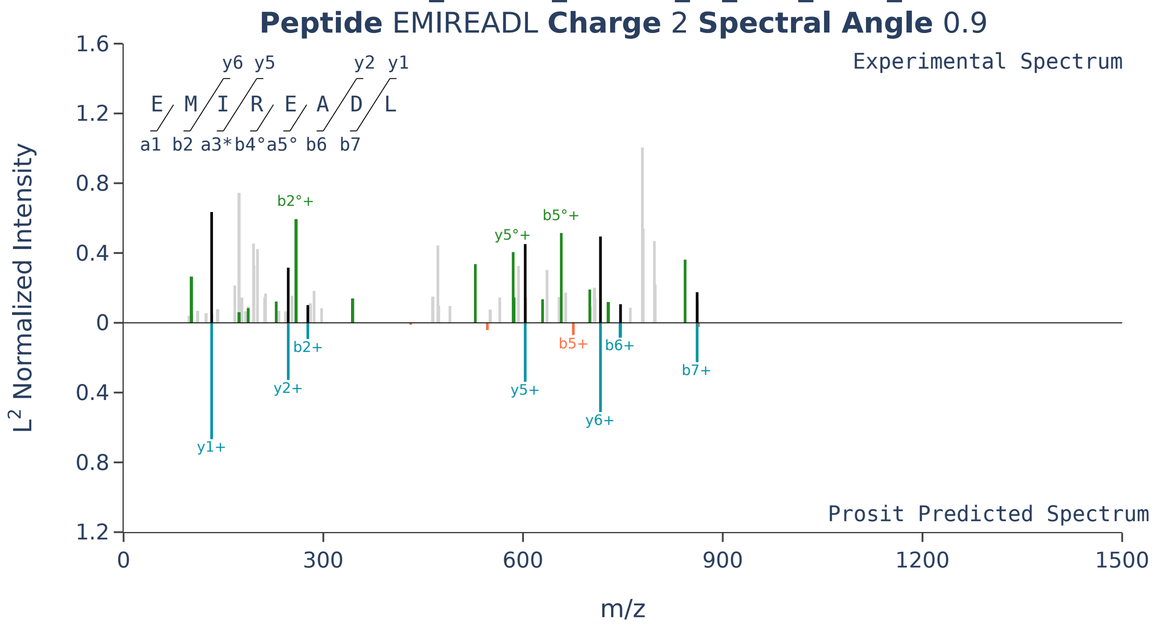


**Source** HRoetschke_291222_040123_Fu_CaM_b2R1_4h **Scan** 6118
**Peptide** VMTNLGEKLTD **Charge** 2 **Spectral Angle** 0.89

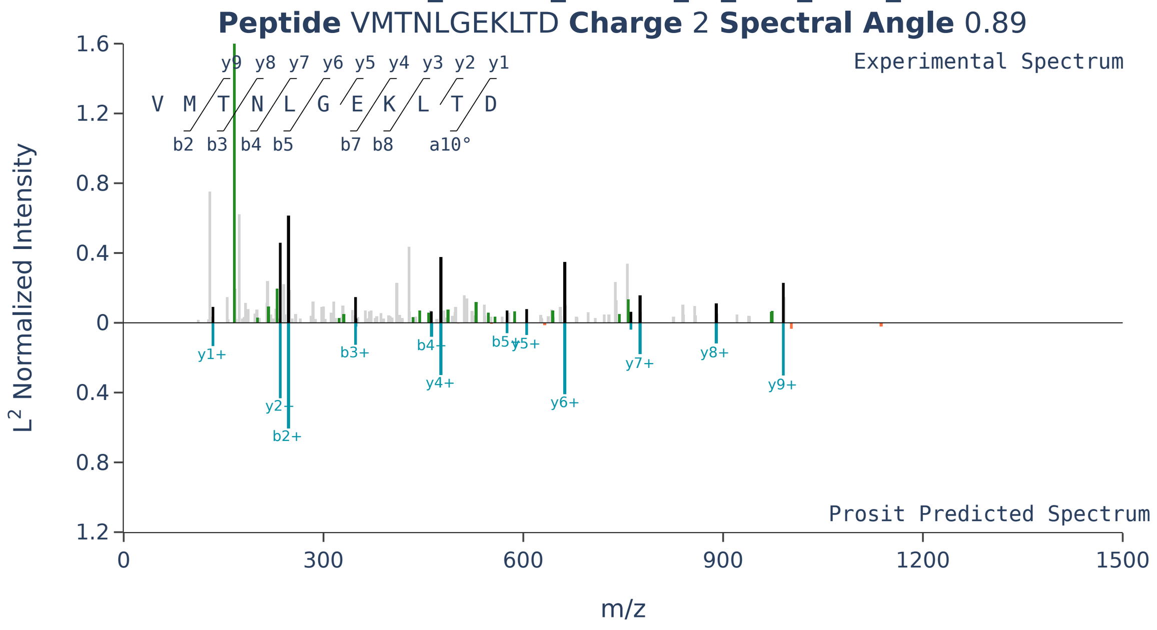


**Source** HRoetschke_291222_040123_Fu_CaM_b2R2_4h **Scan** 9145
**Peptide** FSLFDKDGDGTITTK **Charge** 3 **Spectral Angle** 0.78

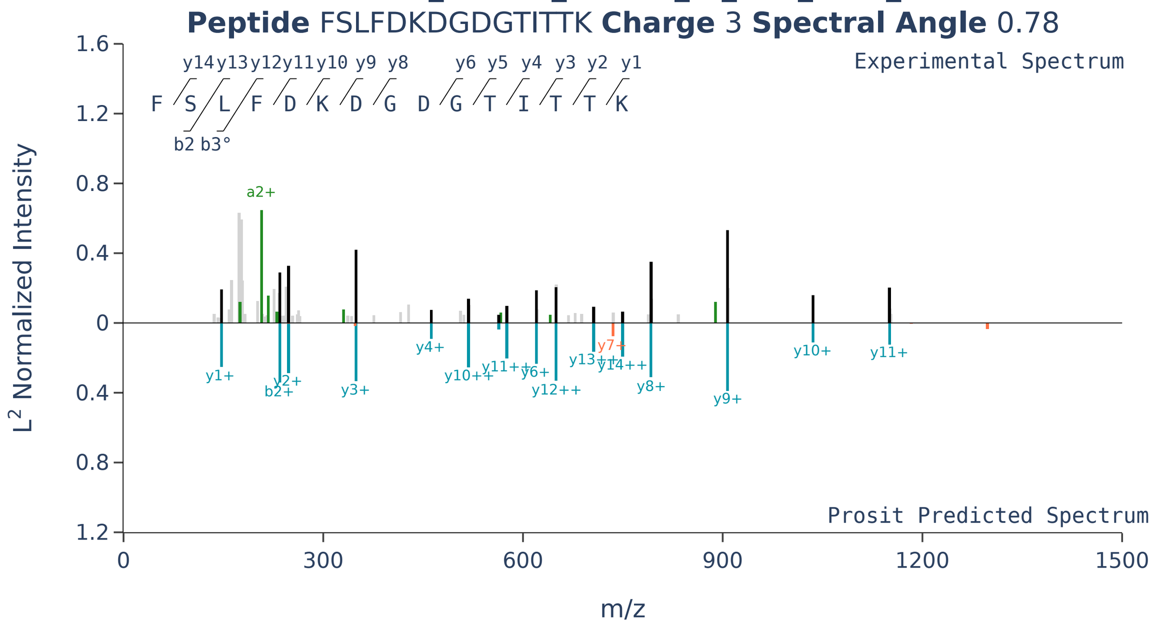


**Source** HRoetschke_291222_040123_Fu_CaM_b1R2_24h **Scan** 3211
**Peptide** KELGTVM **Charge** 2 **Spectral Angle** 0.94

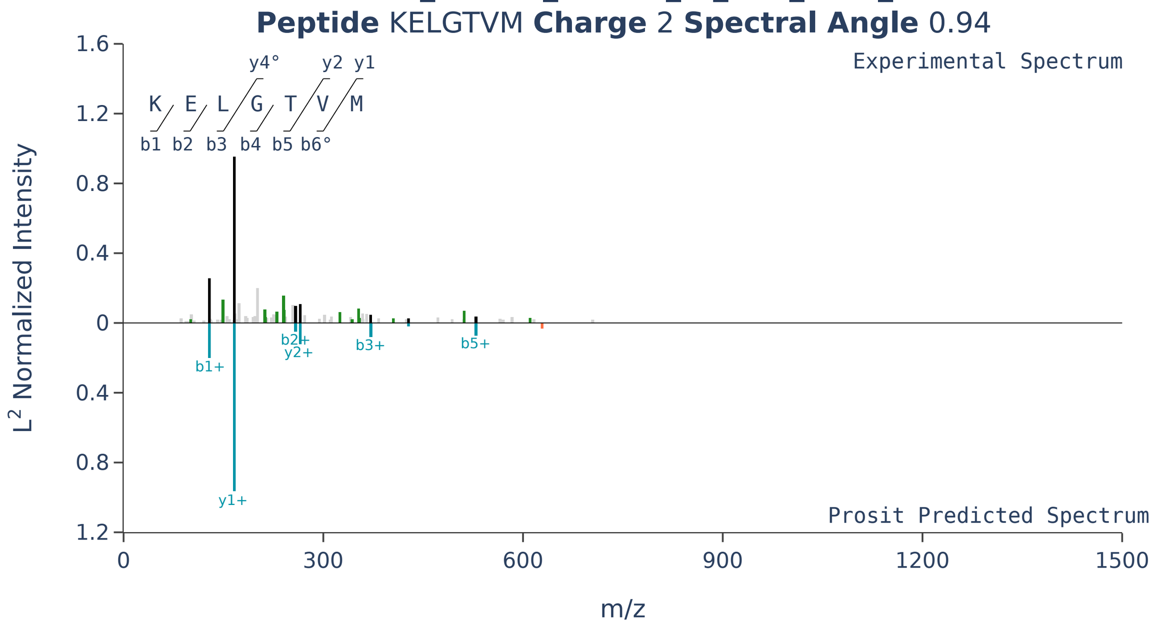


**Source** HRoetschke_291222_040123_Fu_CaM_b1R1_1h **Scan** 2146
**Peptide** AELRHVMTN **Charge** 3 **Spectral Angle** 0.91

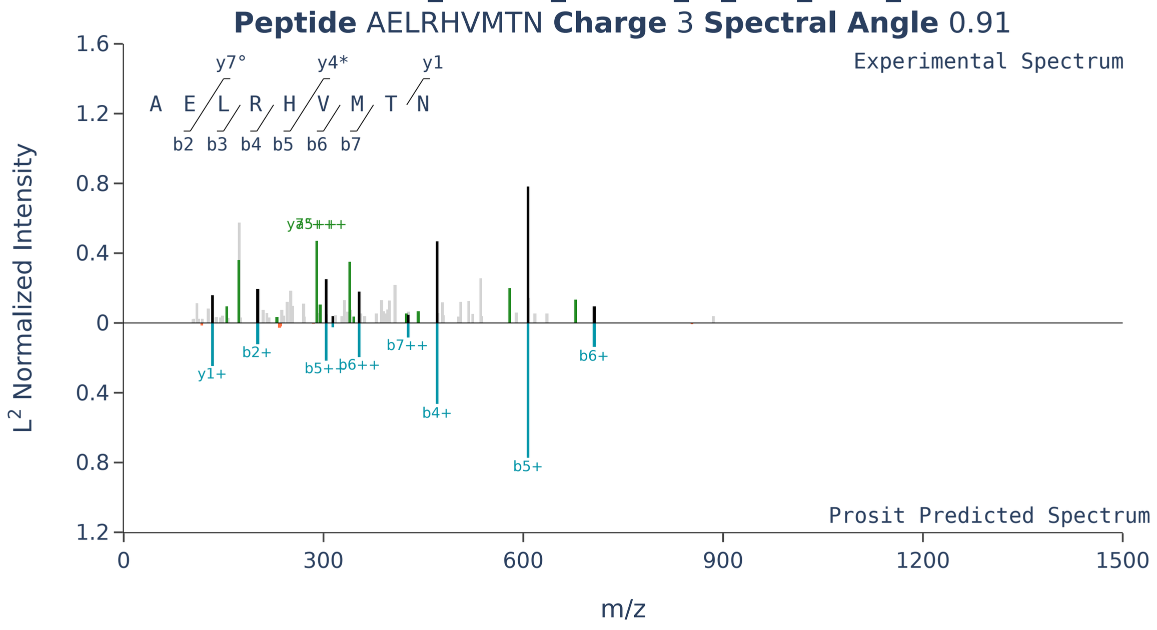


**Source** HRoetschke_291222_040123_Fu_CaM_b1R2_24h **Scan** 2936
**Peptide** KDTDSEEEIREA **Charge** 3 **Spectral Angle** 0.9

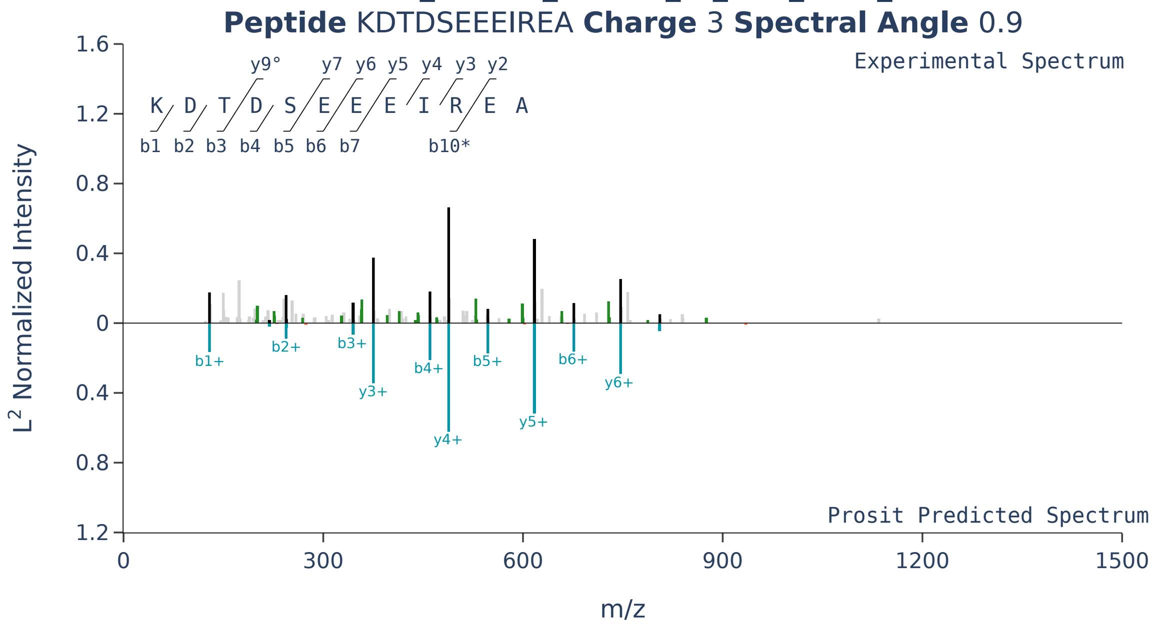


**Source** HRoetschke_291222_040123_Fu_CaM_b2R1_24h **Scan** 9239
**Peptide** ADIDGDGQVNYEEF **Charge** 2 **Spectral Angle** 0.94

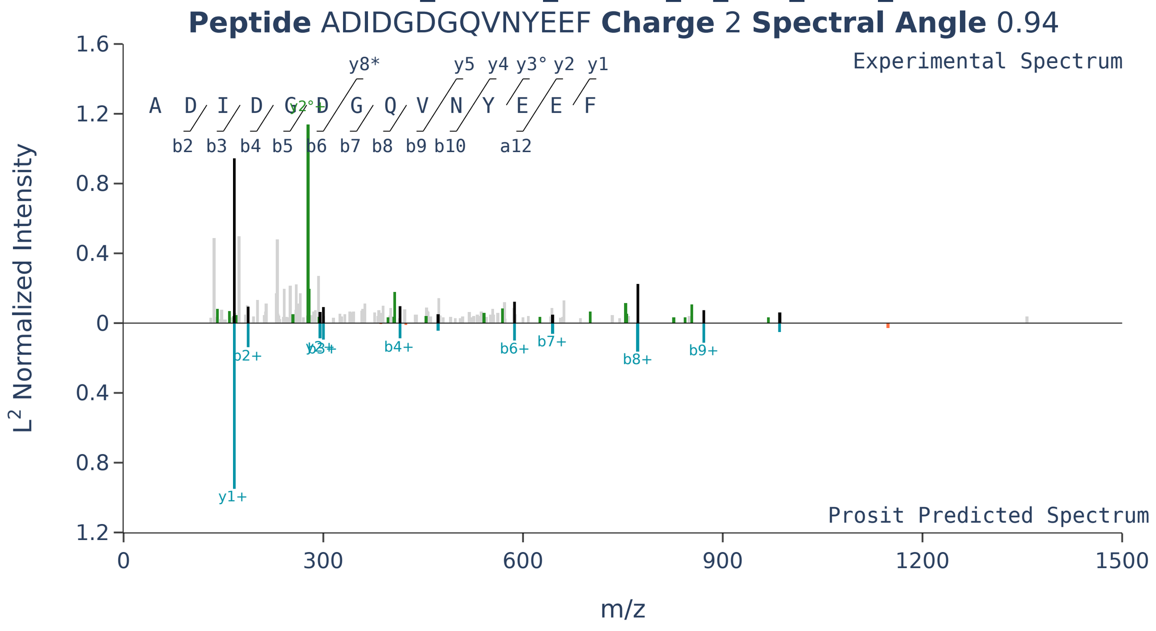


**Source** HRoetschke_291222_040123_Fu_CaM_b1R1_24h **Scan** 7002
**Peptide** RVFDKDGNGYISA **Charge** 2 **Spectral Angle** 0.89

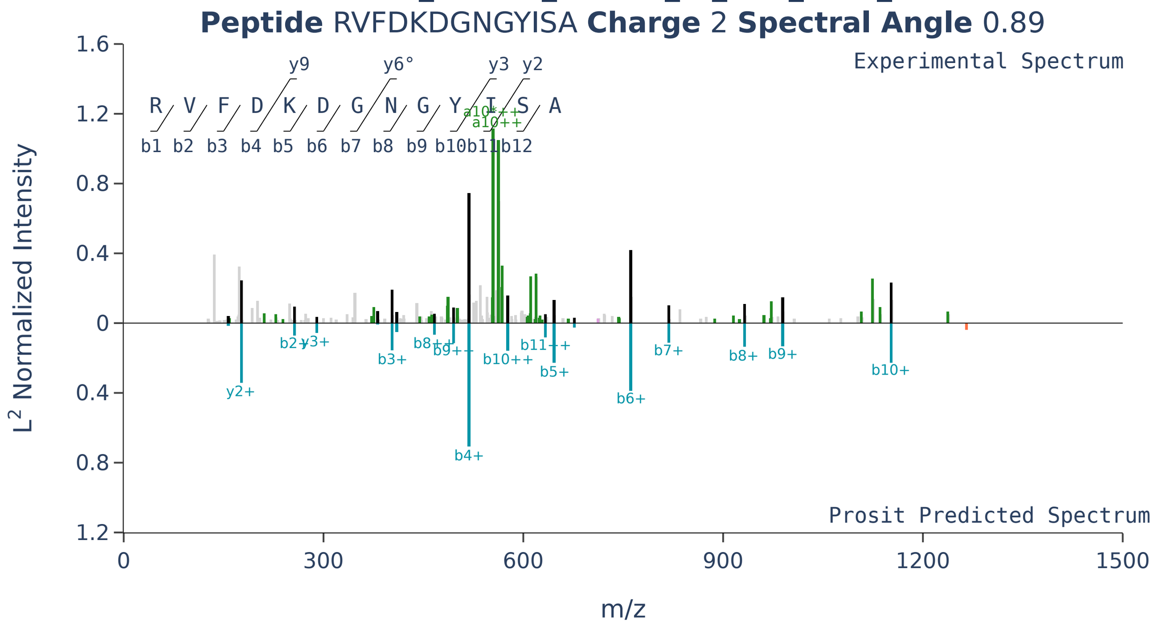


**Source** HRoetschke_291222_040123_Fu_CaM_b1R2_1h **Scan** 5073
**Peptide** RHVMTNLGEKLTD **Charge** 3 **Spectral Angle** 0.81

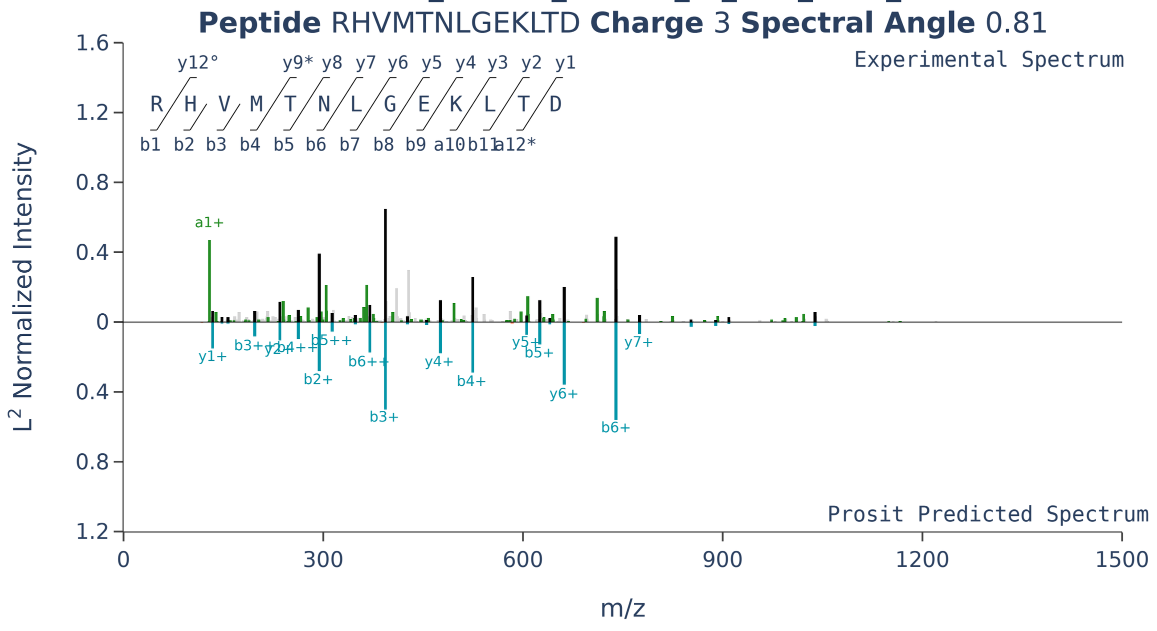


**Source** HRoetschke_291222_040123_Fu_CaM_b1R2_24h **Scan** 8912
**Peptide** DKDGDGTITTKELGTVMR **Charge** 3 **Spectral Angle** 0.79

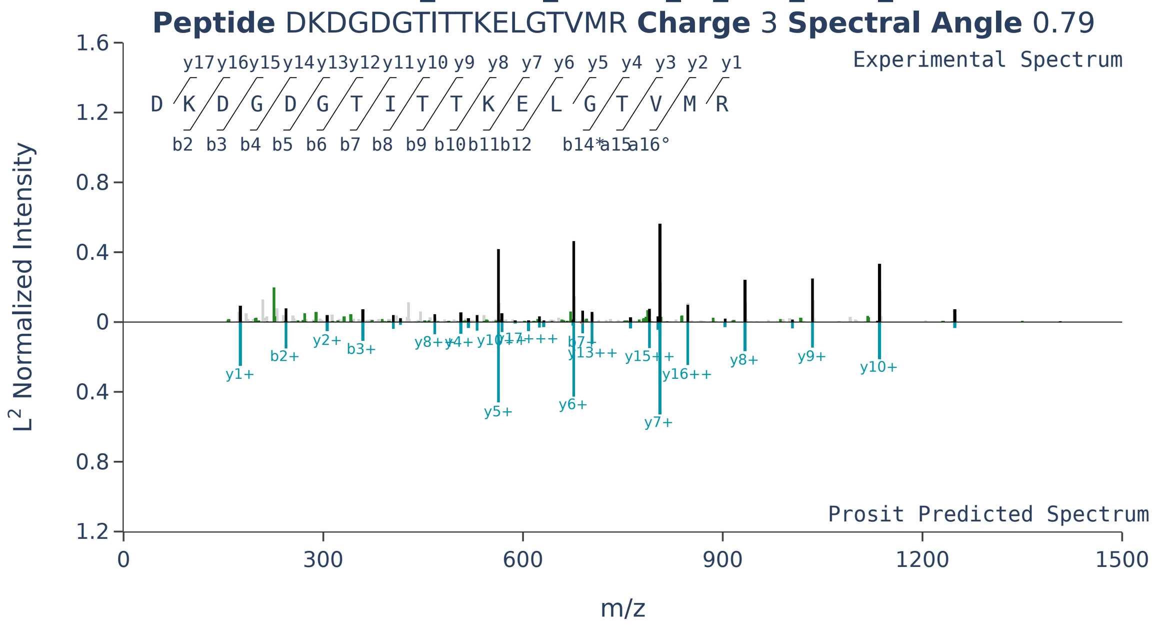


**Source** HRoetschke_291222_040123_Fu_CaM_b2R1_24h **Scan** 12201
**Peptide** ADQLTEEQIAEFKEAF **Charge** 2 **Spectral Angle** 0.87

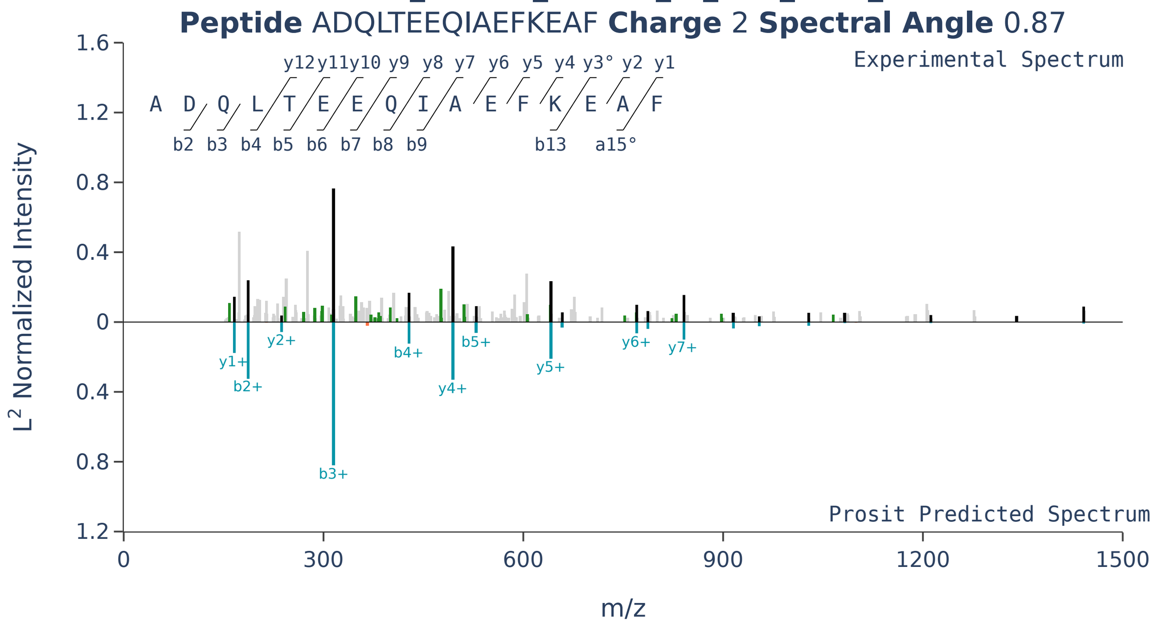


**Source** HRoetschke_291222_040123_Fu_CaM_b1R1_4h **Scan** 9721
**Peptide** FRVFDKDGNGYISA **Charge** 2 **Spectral Angle** 0.85

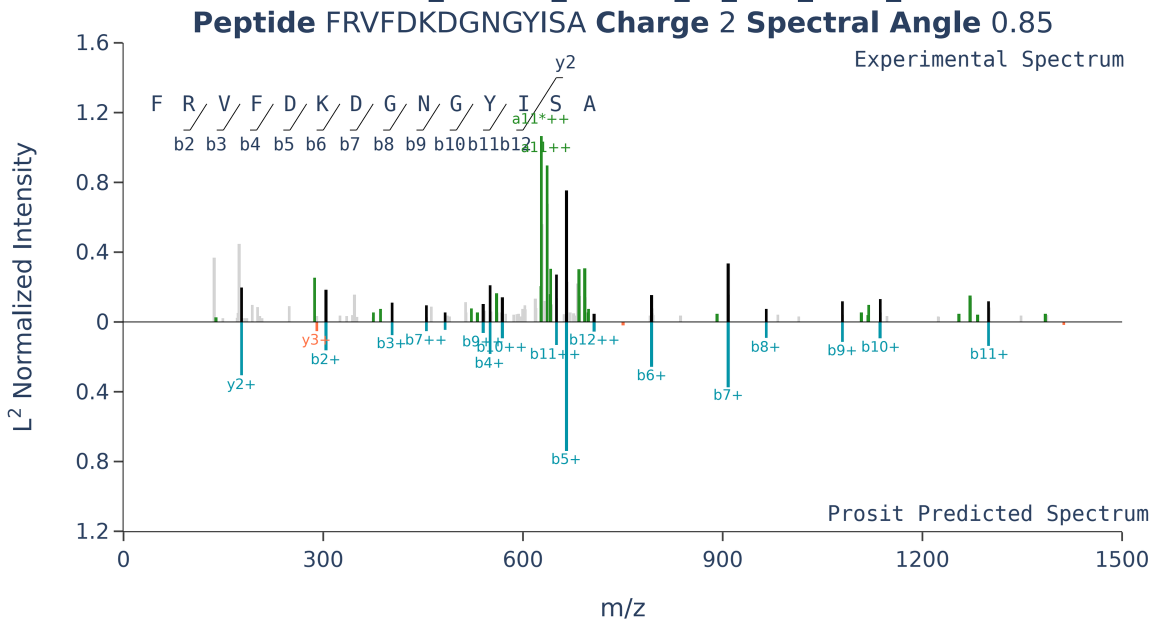


**Source** HRoetschke_291222_040123_Fu_CaM_b1R1_24h **Scan** 7922
**Peptide** ADQLTEEQIAE **Charge** 2 **Spectral Angle** 0.92

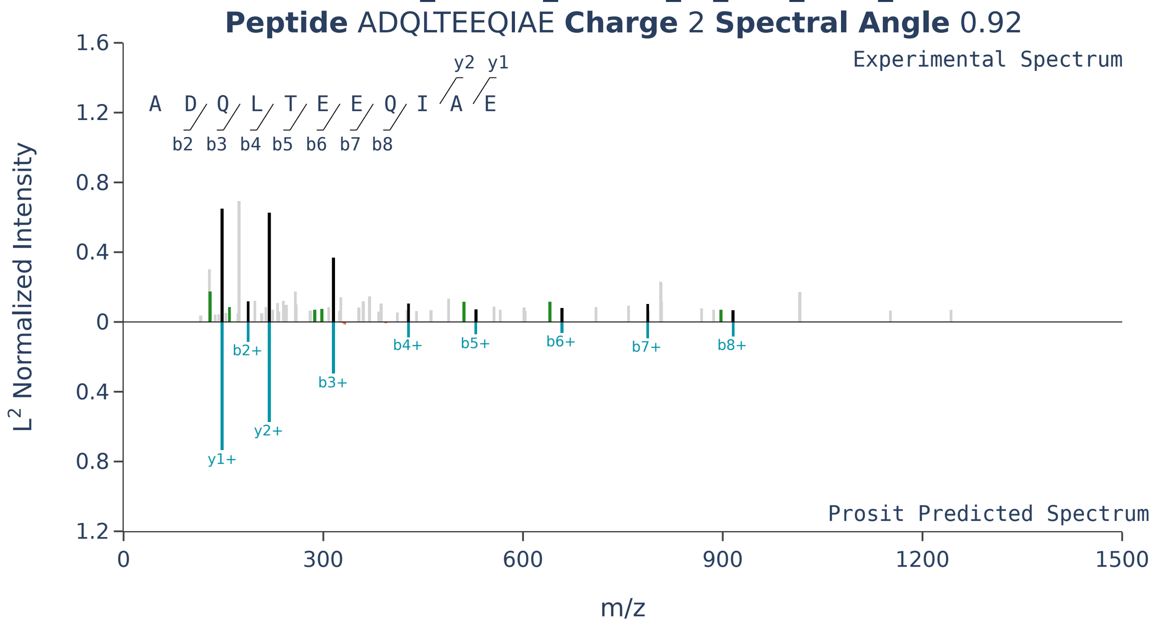


**Source** HRoetschke_291222_040123_Fu_CaM_b1R1_1h **Scan** 3656
**Peptide** RHVMTNL **Charge** 2 **Spectral Angle** 0.91

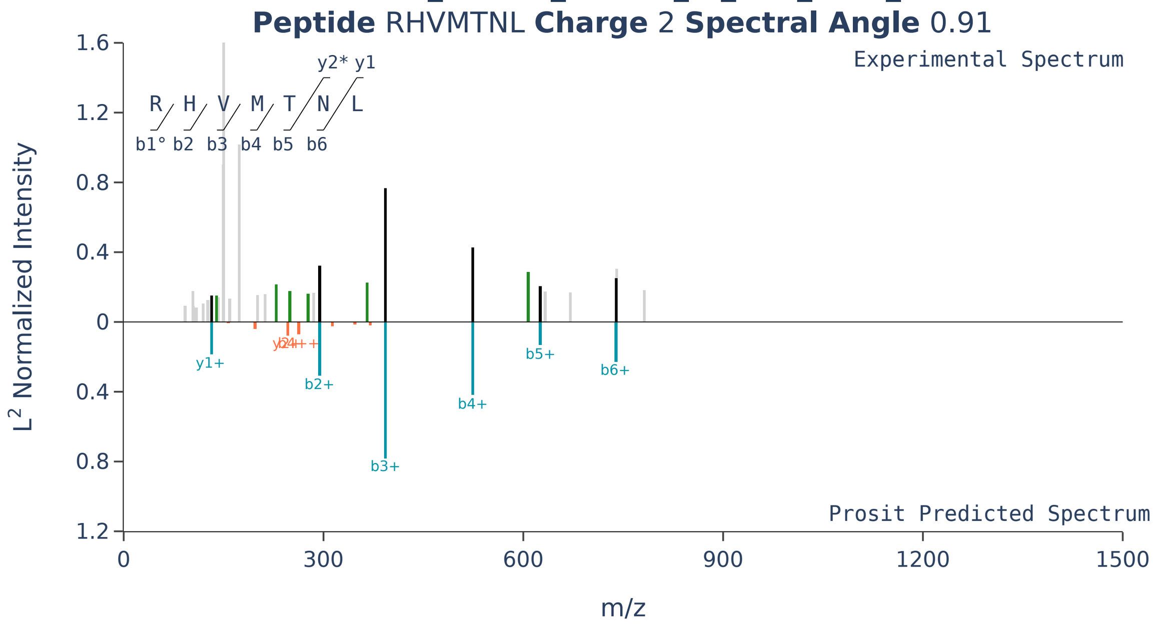

**Source** HRoetschke_291222_040123_Fu_CaM_b1R2_1h **Scan** 8984
**Peptide** DKDGNGYISAAEL **Charge** 2 **Spectral Angle** 0.81

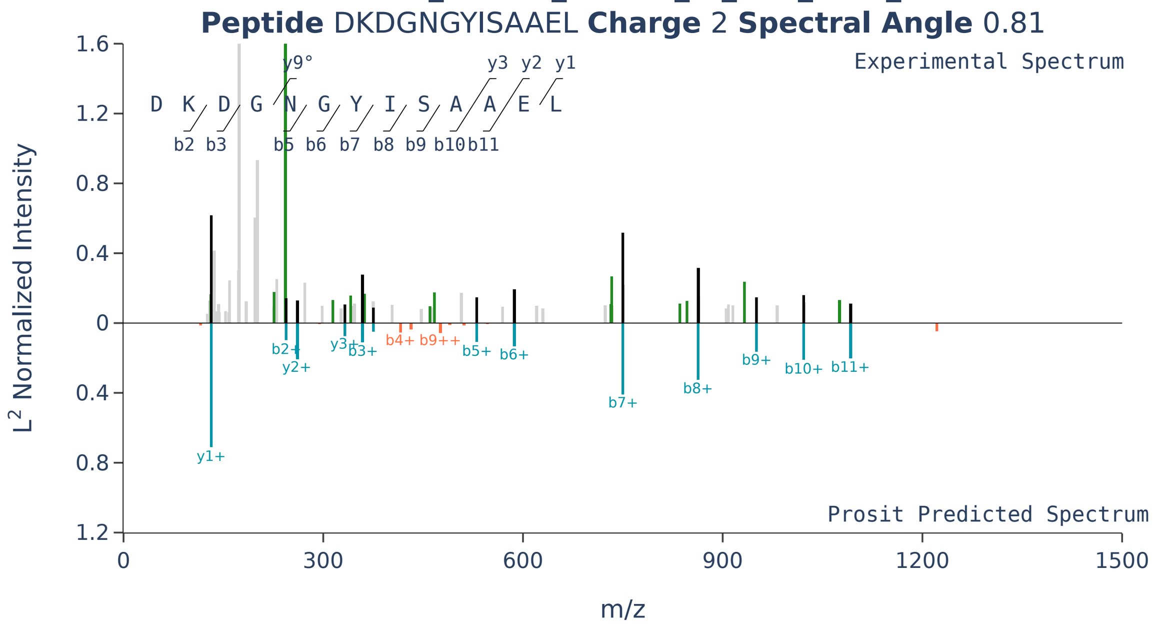


**Source** HRoetschke_291222_040123_Fu_CaM_b2R1_1h **Scan** 6740
**Peptide** ADQLTEEQL **Charge** 2 **Spectral Angle** 0.8

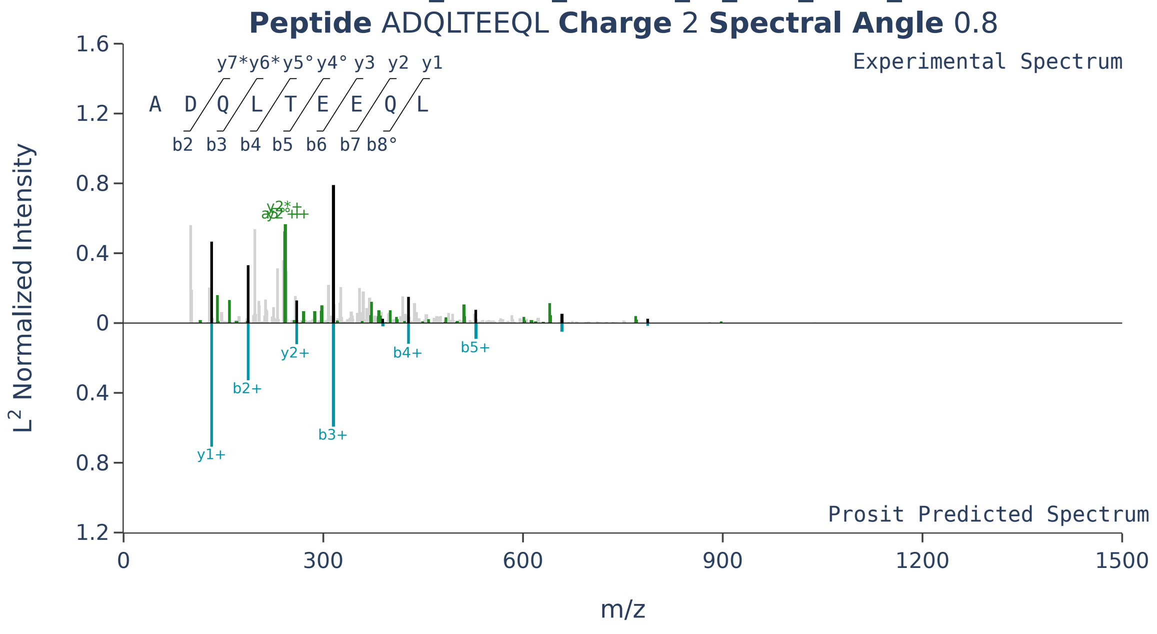


**Source** HRoetschke_291222_040123_Fu_CaM_b1R1_1h **Scan** 7457
**Peptide** LGQNPTEAEL **Charge** 2 **Spectral Angle** 0.9

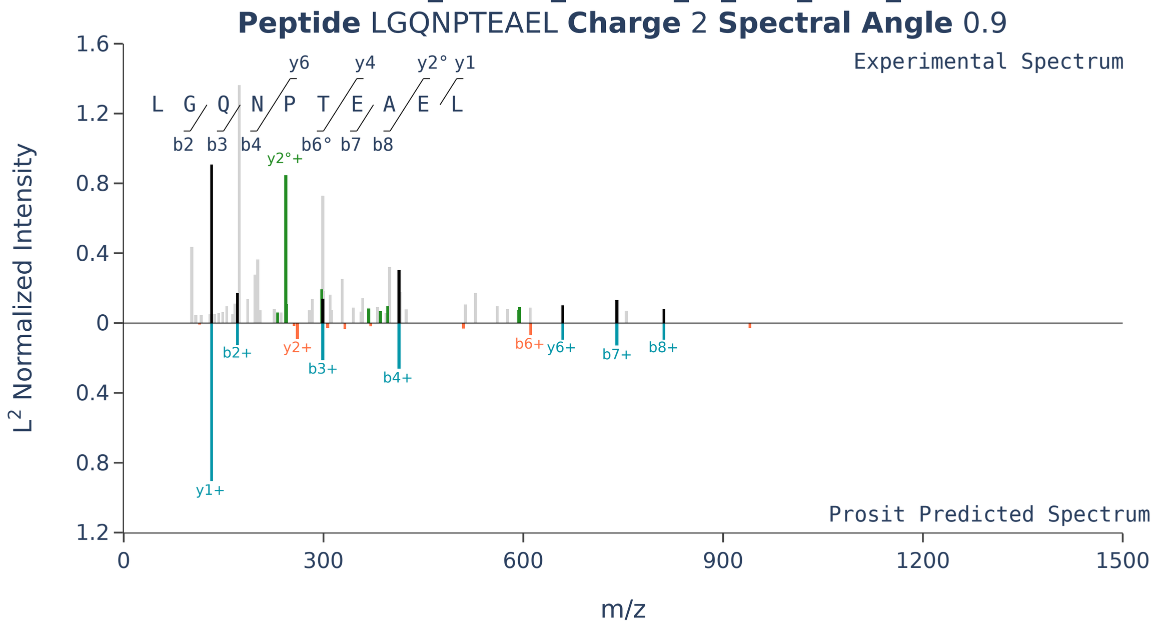


**Source** HRoetschke_291222_040123_Fu_CaM_b1R2_24h **Scan** 8597
**Peptide** KELGTVMRSL **Charge** 2 **Spectral Angle** 0.87

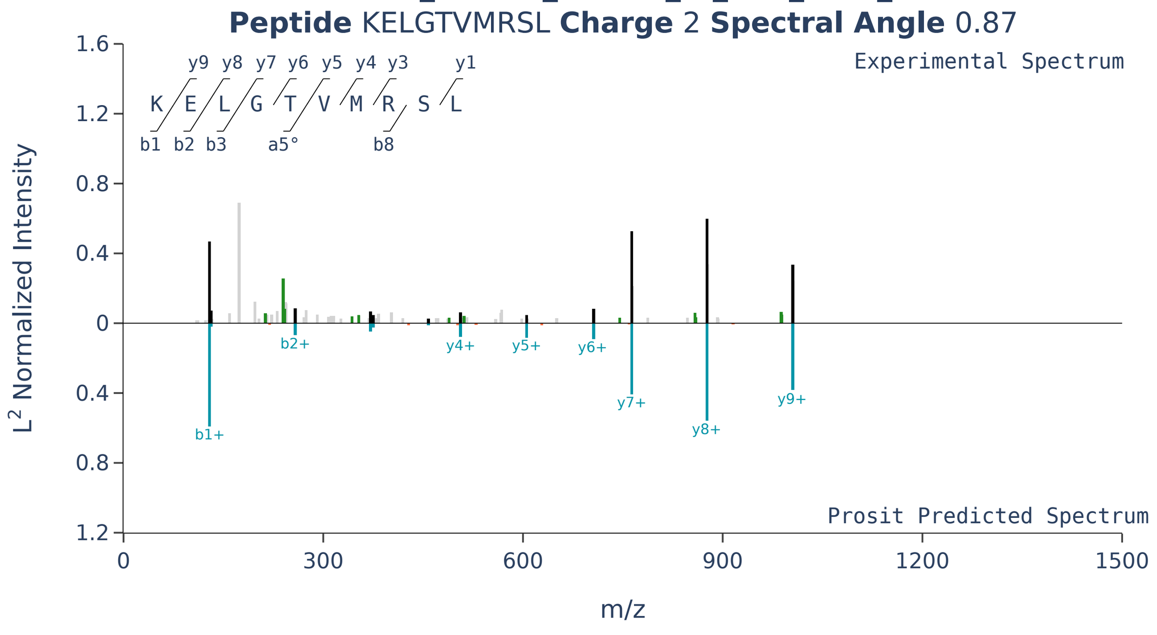


**Source** HRoetschke_291222_040123_Fu_CaM_b1R2_24h **Scan** 8798
**Peptide** DKDGNGYISAAELRHV **Charge** 3 **Spectral Angle** 0.87

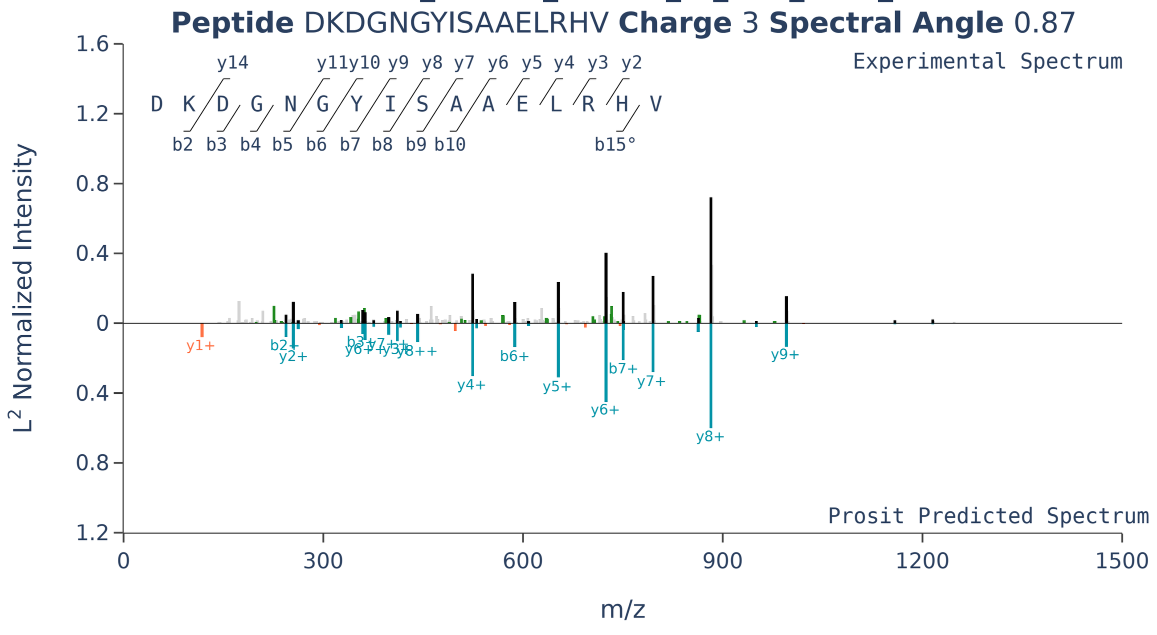


**Source** HRoetschke_291222_040123_Fu_CaM_b1R1_24h **Scan** 3551
**Peptide** VDADGNGTID **Charge** 2 **Spectral Angle** 0.92

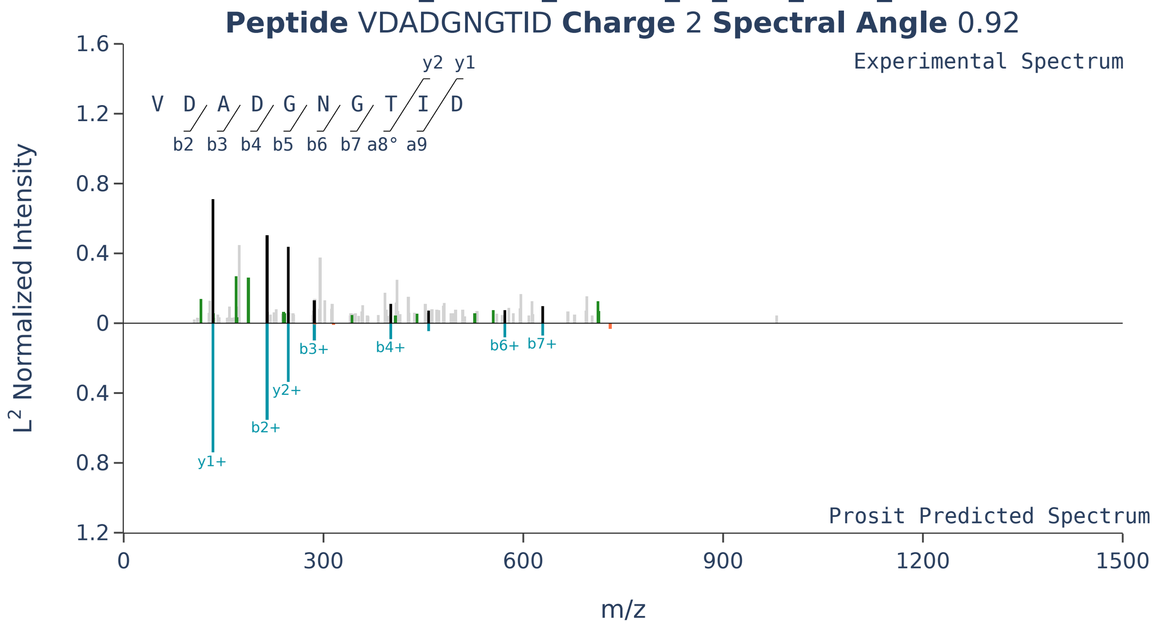


**Source** HRoetschke_291222_040123_Fu_CaM_b1R2_4h **Scan** 11630
**Peptide** KEAFSLFD **Charge** 2 **Spectral Angle** 0.81

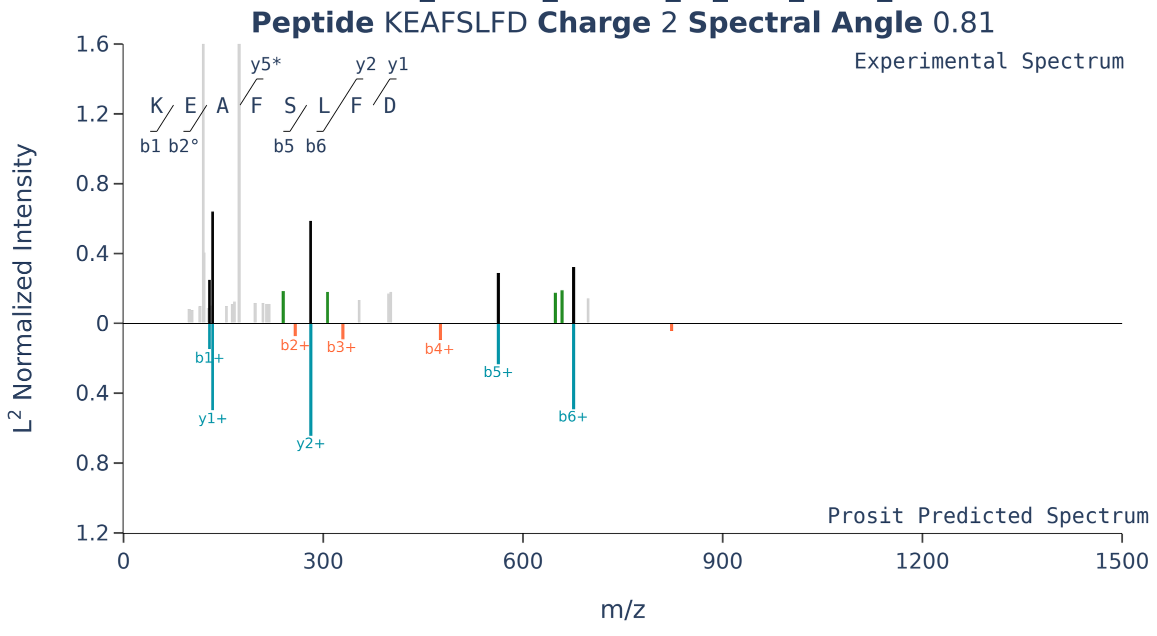


**Source** HRoetschke_291222_040123_Fu_CaM_b1R1_4h **Scan** 10111
**Peptide** AFRVFDKDGNGYISA **Charge** 3 **Spectral Angle** 0.77

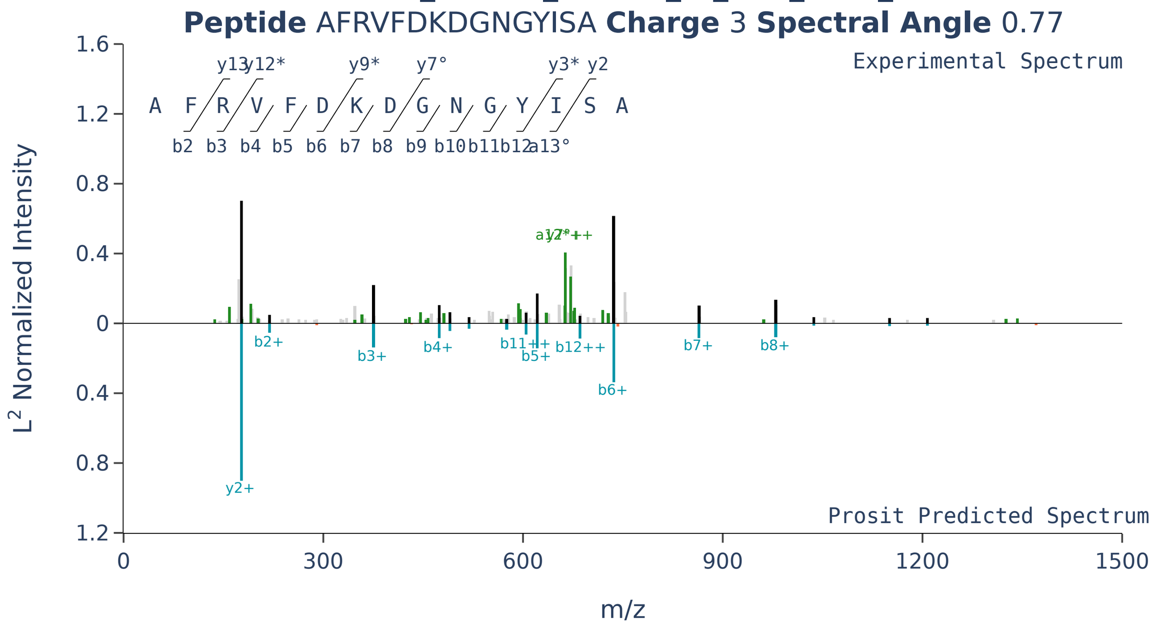


**Source** HRoetschke_291222_040123_Fu_CaM_b1R1_4h **Scan** 7531
**Peptide** DKDGDGTITTKELGTVM **Charge** 3 **Spectral Angle** 0.92

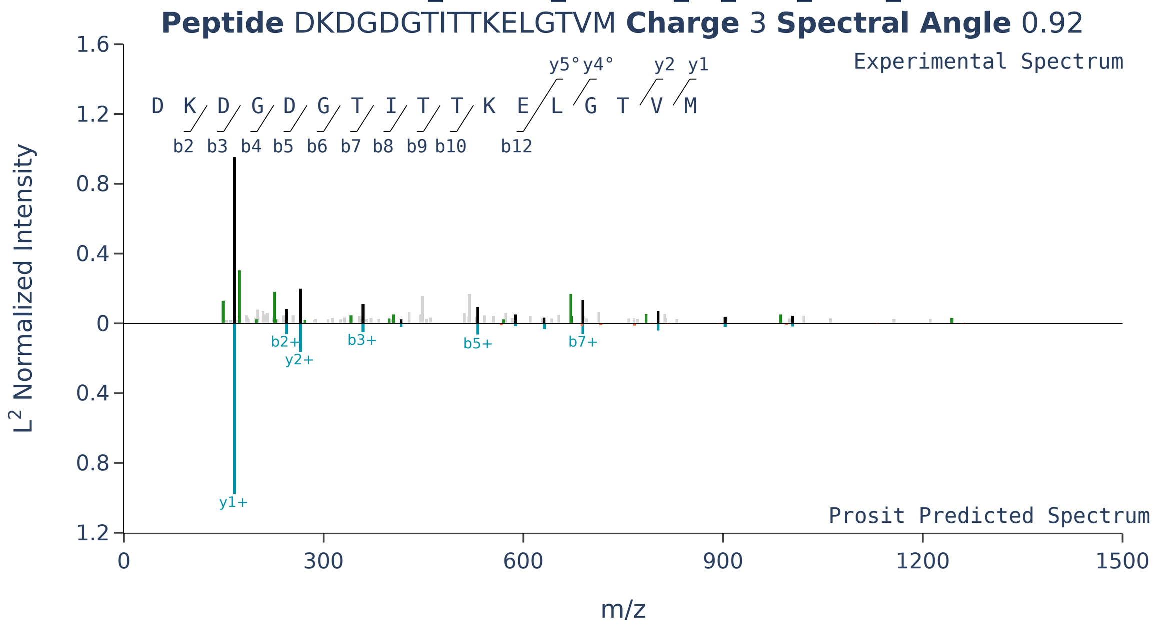


**Source** HRoetschke_291222_040123_Fu_CaM_b2R2_24h **Scan** 13735
**Peptide** AEFKEAFSLFD **Charge** 2 **Spectral Angle** 0.9

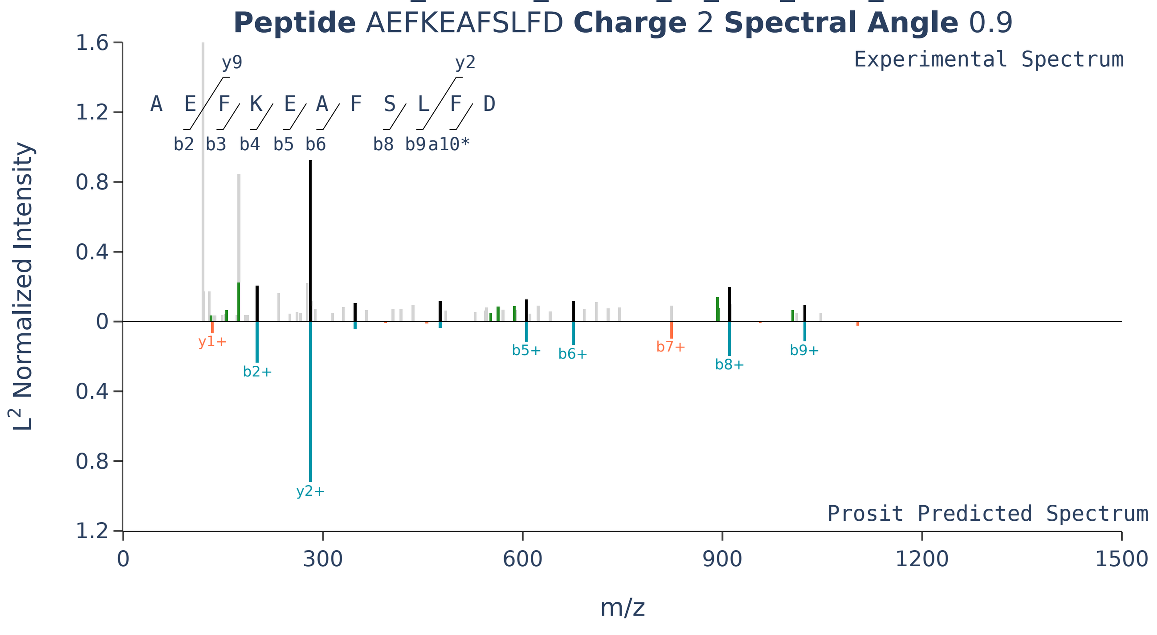


**Source** HRoetschke_291222_040123_Fu_CaM_b2R2_24h **Scan** 2218
**Peptide** RKMKDTDSEEEL **Charge** 3 **Spectral Angle** 0.87

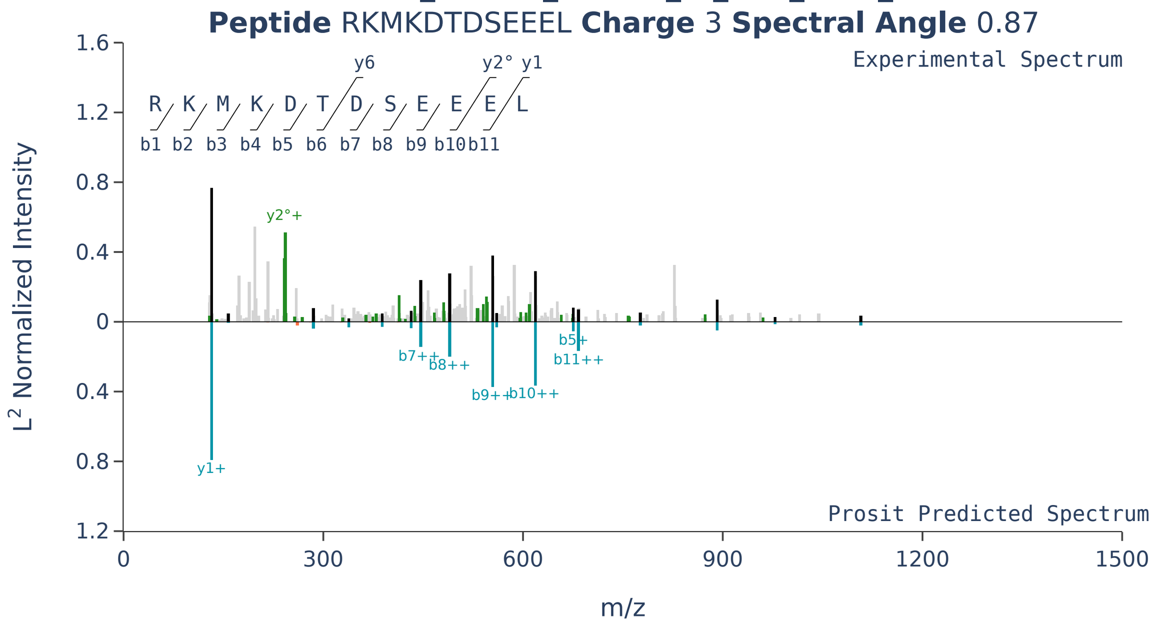


**Source** HRoetschke_291222_040123_Fu_CaM_b1R1_1h **Scan** 6768
**Peptide** TNLGEKLTDEEVD **Charge** 2 **Spectral Angle** 0.92

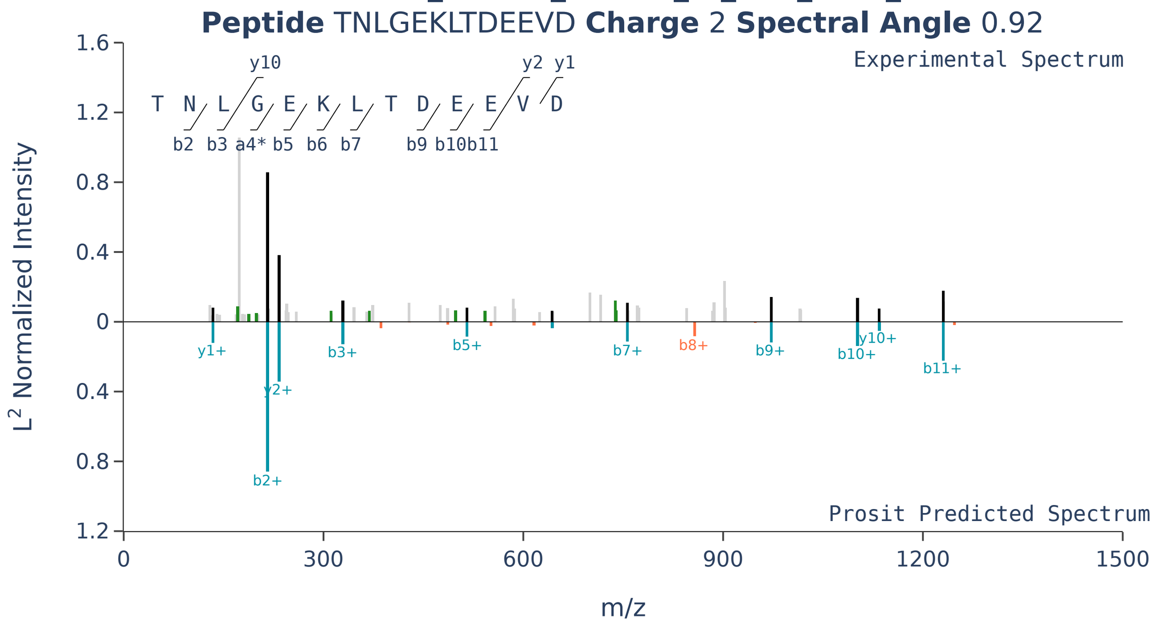


**Source** HRoetschke_291222_040123_Fu_CaM_b1R2_24h **Scan** 5901
**Peptide** READIDGDGQVNY **Charge** 2 **Spectral Angle** 0.88

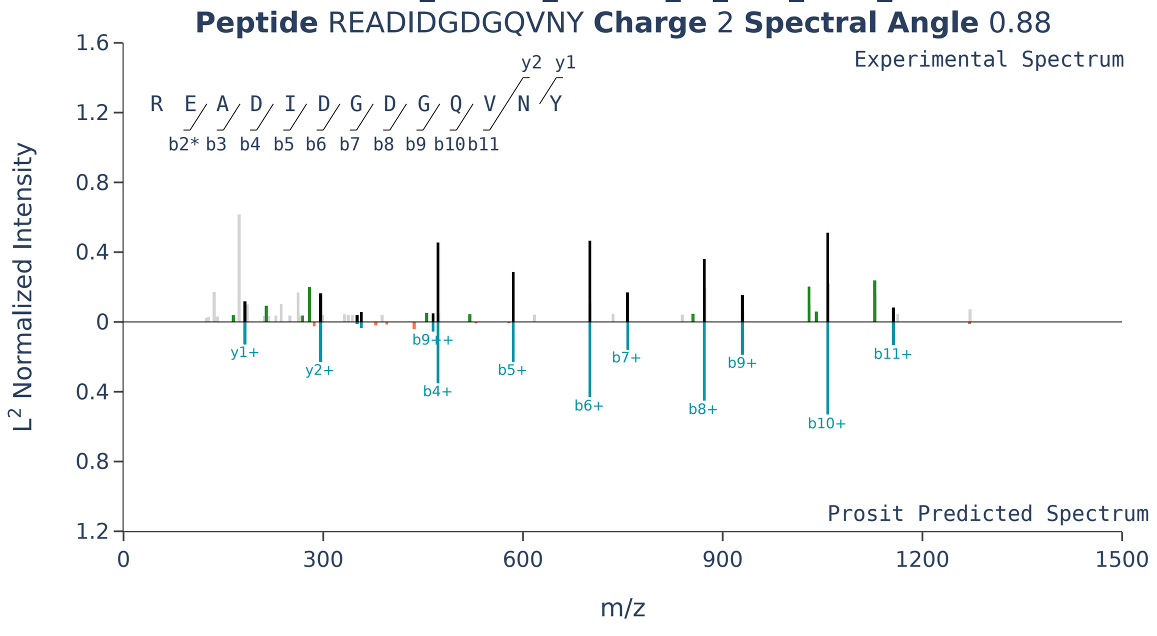


**Source** HRoetschke_291222_040123_Fu_CaM_b2R1_4h **Scan** 2156
**Peptide** RKMKDTDSEEEI **Charge** 3 **Spectral Angle** 0.88

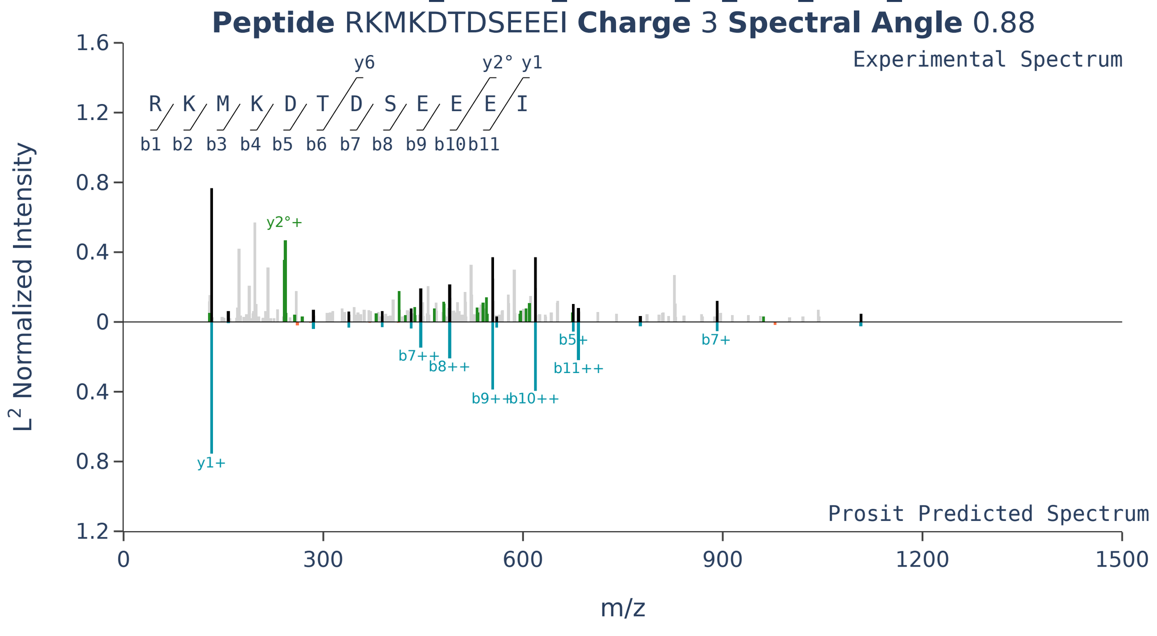


**Source** HRoetschke_291222_040123_Fu_CaM_b1R1_24h **Scan** 4549
**Peptide** FDKDGDGTITT **Charge** 2 **Spectral Angle** 0.89

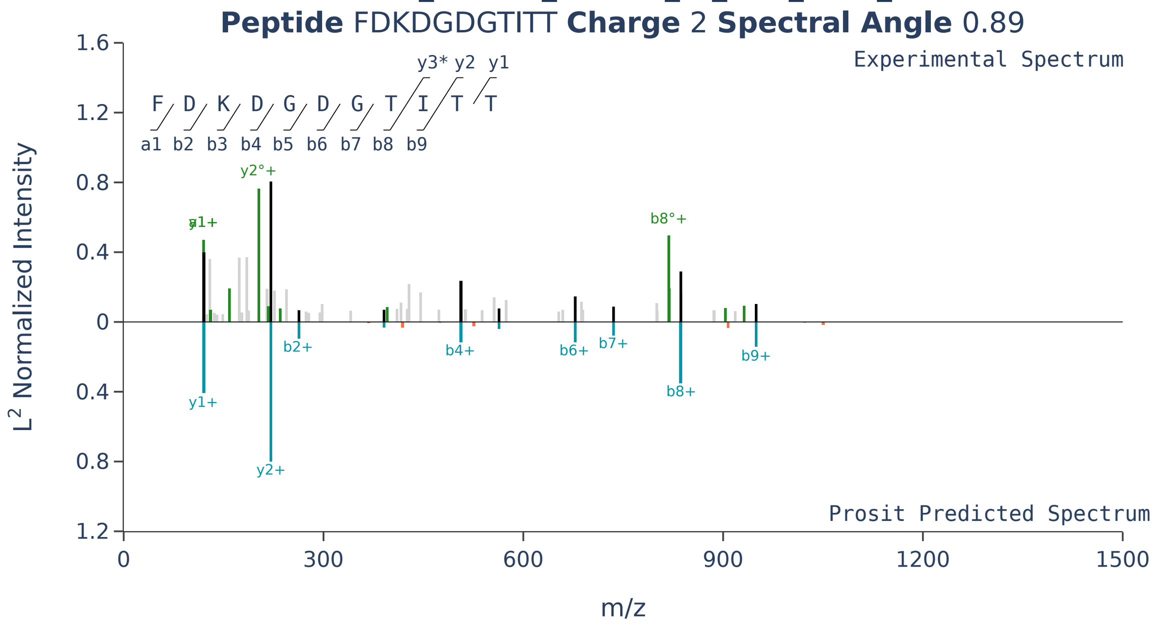

**Source** HRoetschke_291222_040123_Fu_CaM_b2R1_24h **Scan** 8387
**Peptide** SLFDKDGDGTITTKELGTVMR **Charge** 3 **Spectral Angle** 0.81

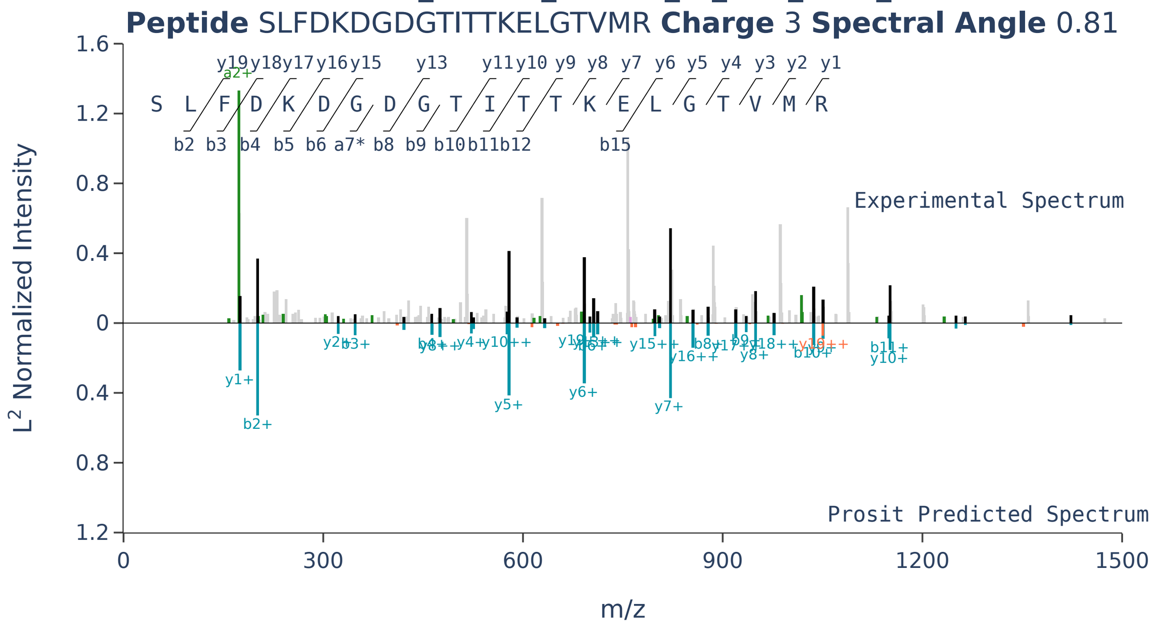


**Source** HRoetschke_291222_040123_Fu_CaM_b2R1_2h **Scan** 5567
**Peptide** ADIDGDGQVNYE **Charge** 2 **Spectral Angle** 0.84

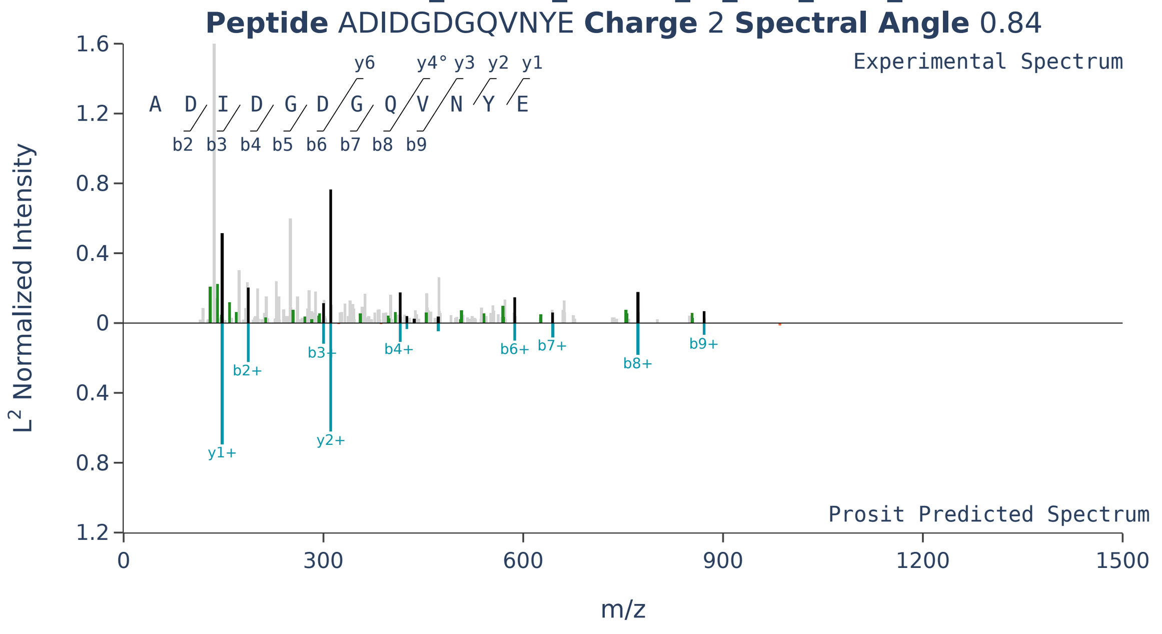


**Source** HRoetschke_291222_040123_Fu_CaM_b1R1_1h **Scan** 2201
**Peptide** ARKMKDTDSEEEL **Charge** 3 **Spectral Angle** 0.9

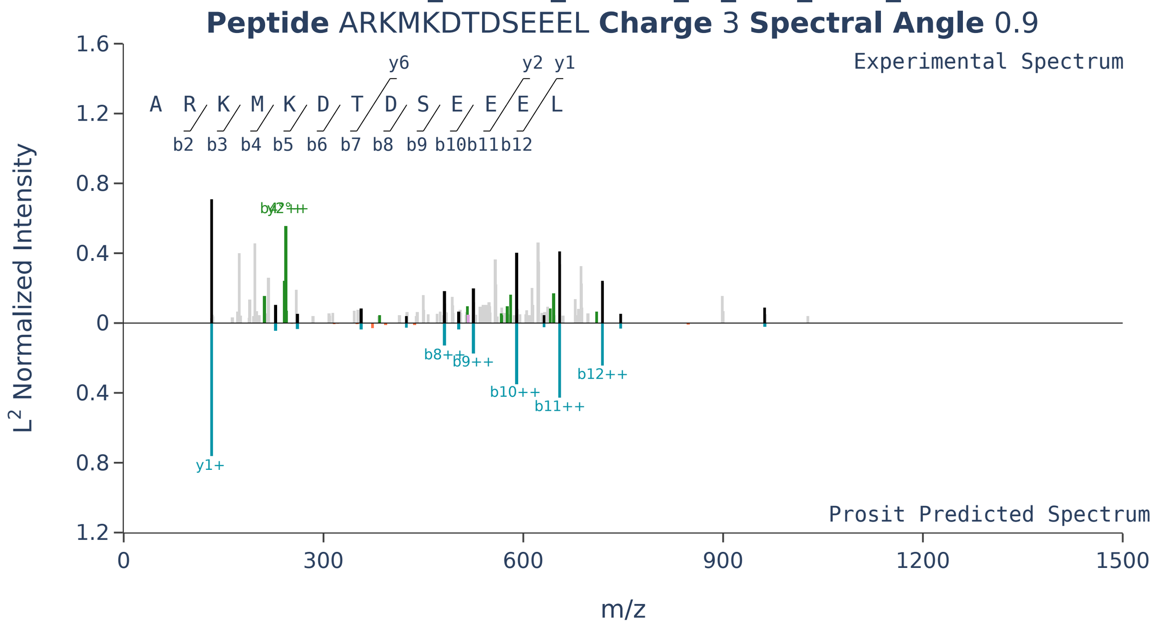


**Source** HRoetschke_291222_040123_Fu_CaM_b1R1_24h **Scan** 9478
**Peptide** FDKDGNGYISAAELR **Charge** 3 **Spectral Angle** 0.9

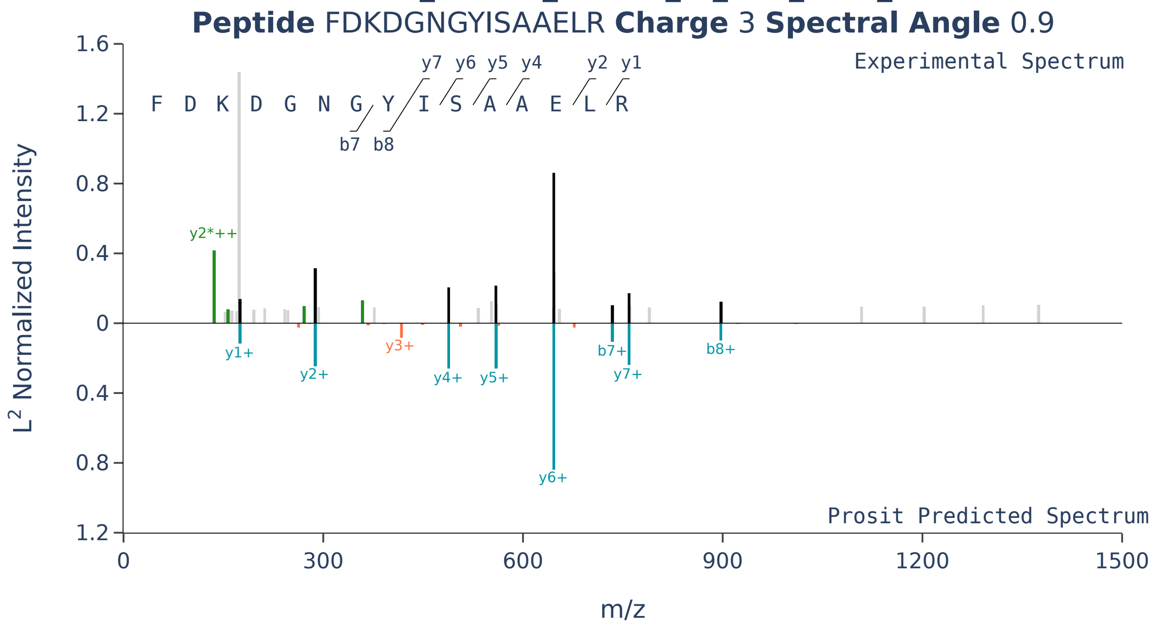


**Source** HRoetschke_291222_040123_Fu_CaM_b1R1_24h **Scan** 5532
**Peptide** ELGTVMR **Charge** 2 **Spectral Angle** 0.87

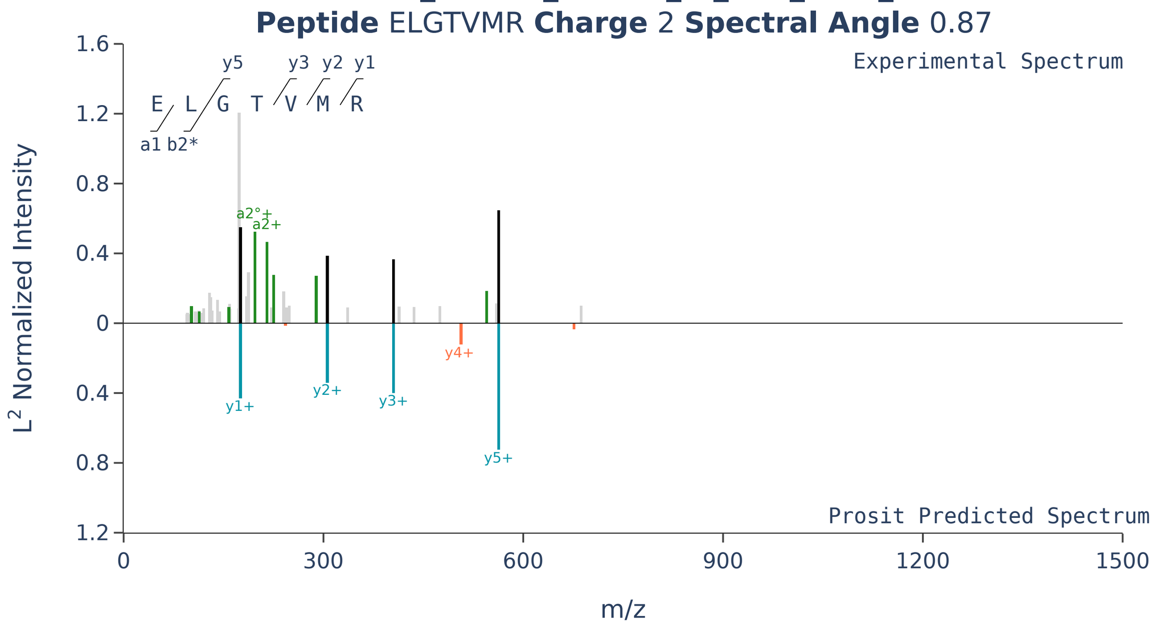


**Source** HRoetschke_291222_040123_Fu_CaM_b2R2_2h **Scan** 2753
**Peptide** SAAELRHV **Charge** 2 **Spectral Angle** 0.86

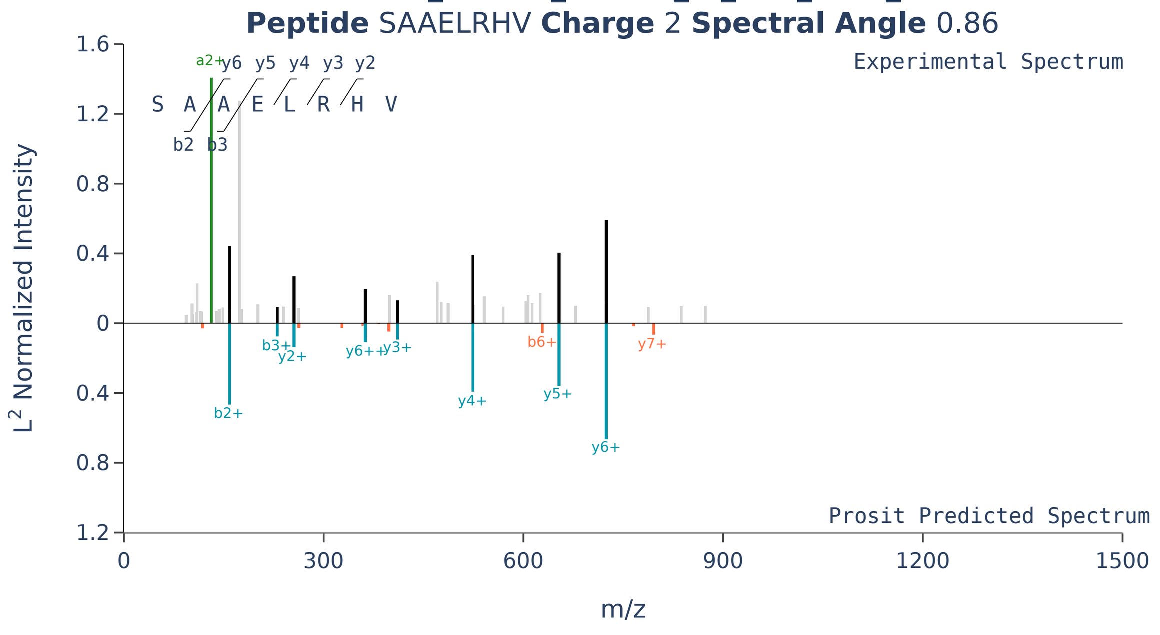


**Source** HRoetschke_291222_040123_Fu_CaM_b1R2_2h **Scan** 2960
**Peptide** EMIREAD **Charge** 2 **Spectral Angle** 0.9

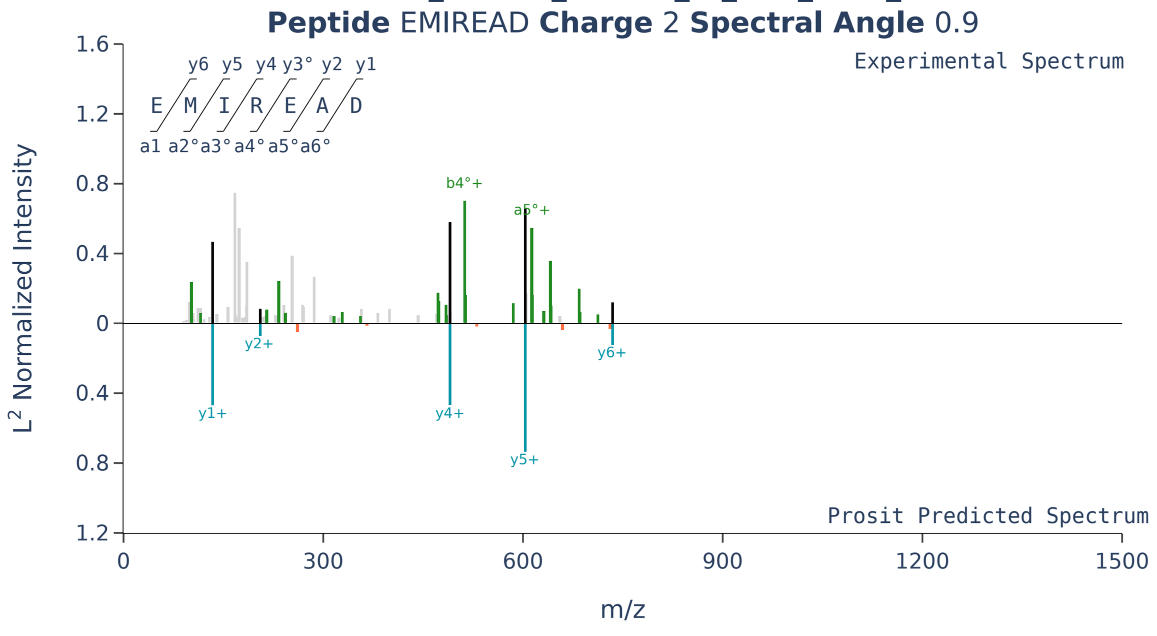


**Source** HRoetschke_291222_040123_Fu_CaM_b1R2_1h **Scan** 5152
**Peptide** EMIREADI **Charge** 2 **Spectral Angle** 0.87

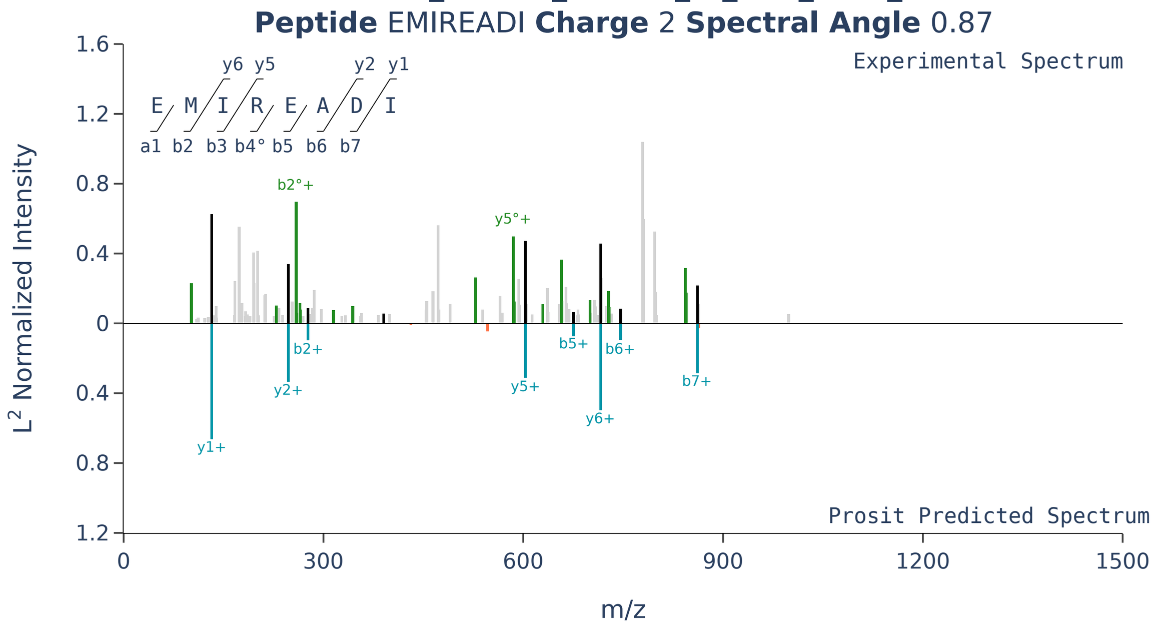


**Source** HRoetschke_291222_040123_Fu_CaM_b2R1_2h **Scan** 11175
**Peptide** SEEEIREAFRVFD **Charge** 3 **Spectral Angle** 0.88

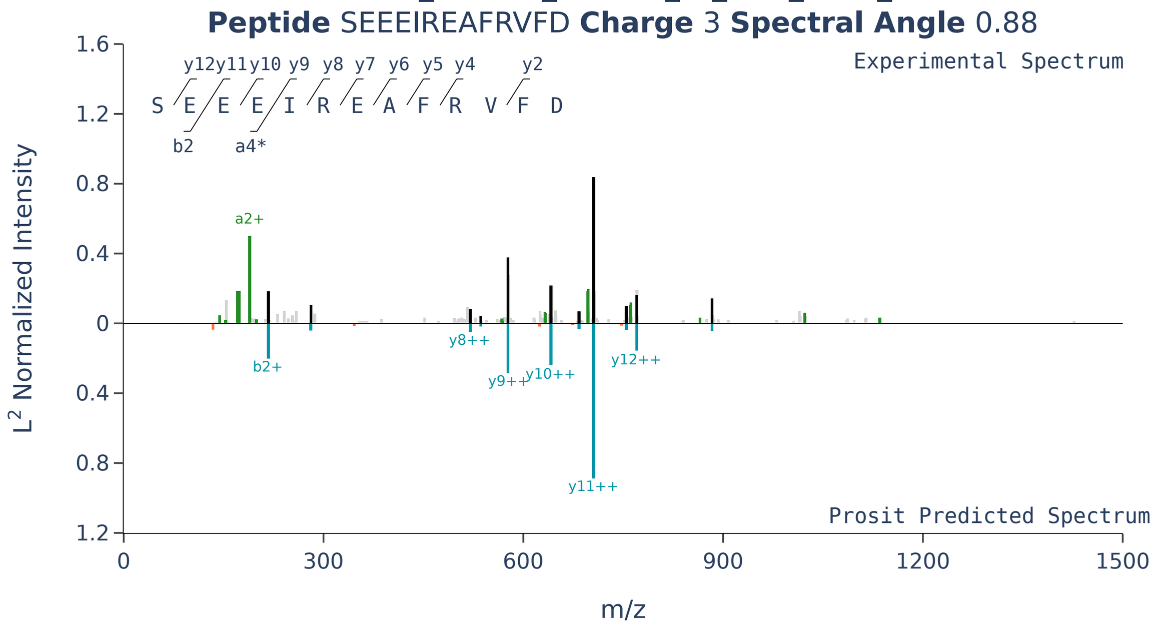


**Source** HRoetschke_291222_040123_Fu_CaM_b2R1_1h **Scan** 2340
**Peptide** KDTDSEEEIRE **Charge** 3 **Spectral Angle** 0.86

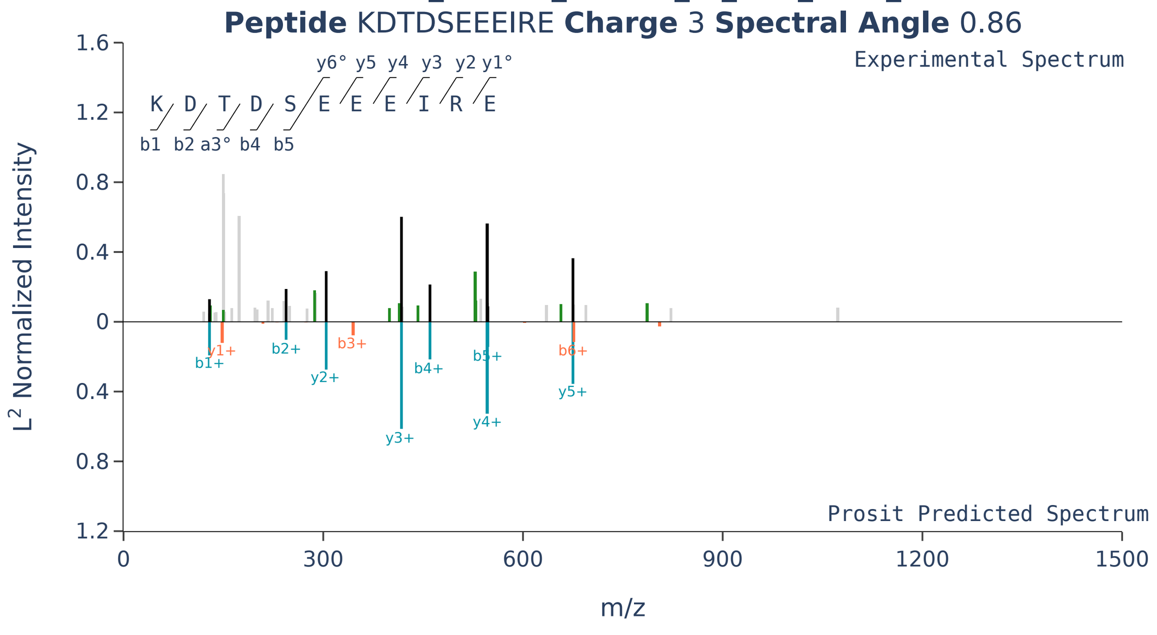


**Source** HRoetschke_291222_040123_Fu_CaM_b1R2_4h **Scan** 4225
**Peptide** RHVMTNLGEKL **Charge** 3 **Spectral Angle** 0.78

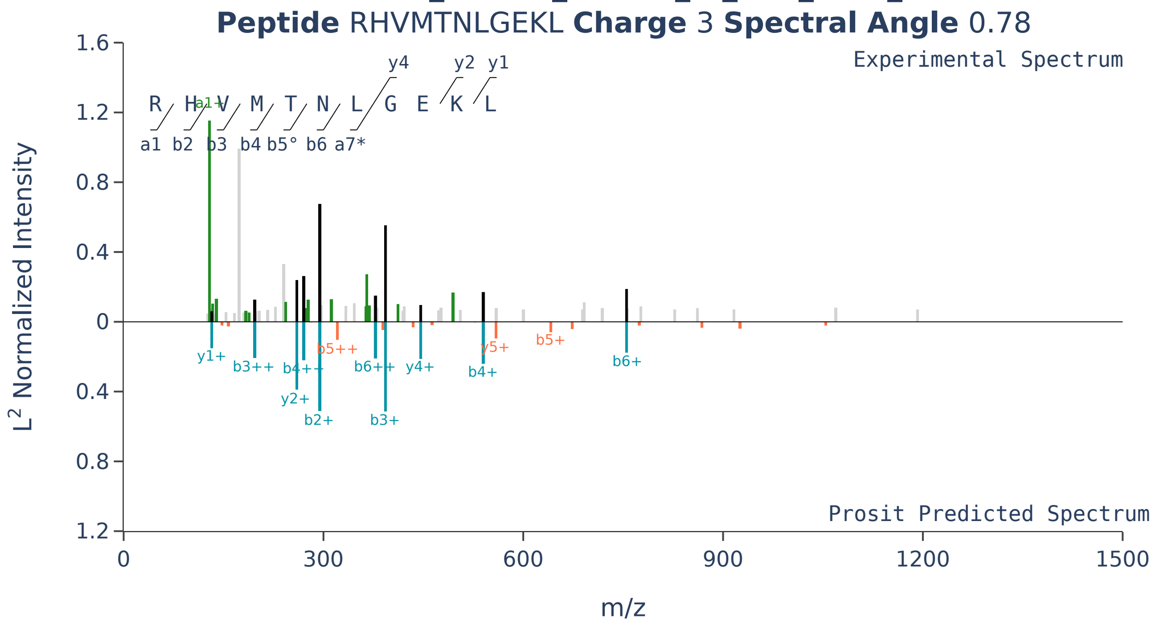


**Source** HRoetschke_291222_040123_Fu_CaM_b1R2_1h **Scan** 4390
**Peptide** AELRHVMTNLGE **Charge** 3 **Spectral Angle** 0.67

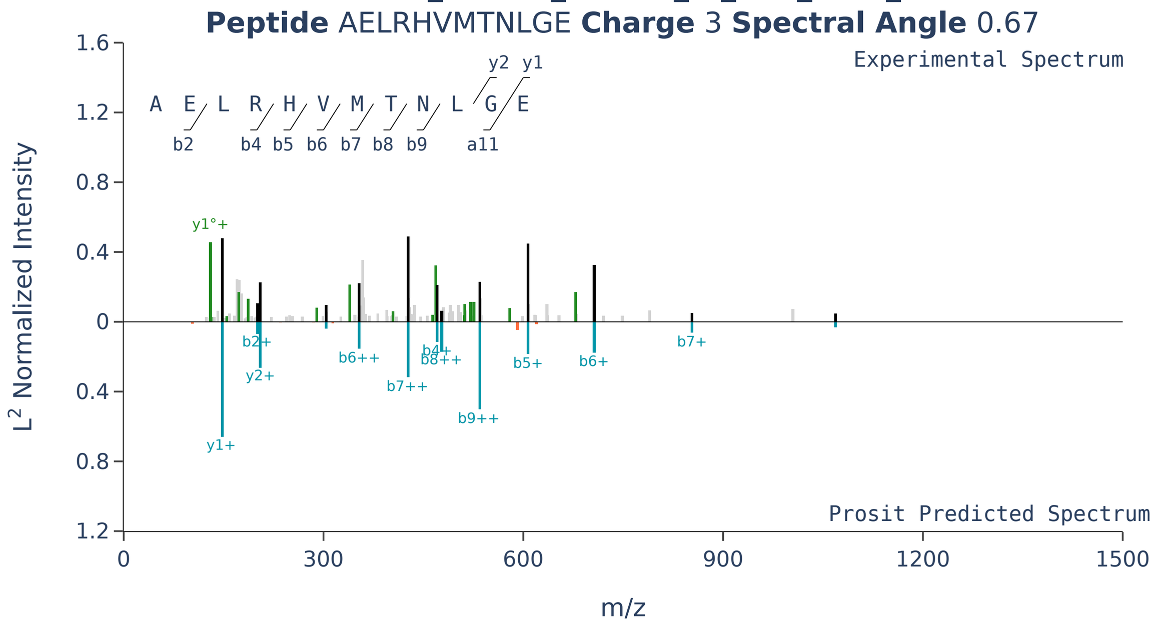


**Source** HRoetschke_291222_040123_Fu_CaM_b1R1_24h **Scan** 6035
**Peptide** DKDGDGTITTKEL **Charge** 3 **Spectral Angle** 0.78

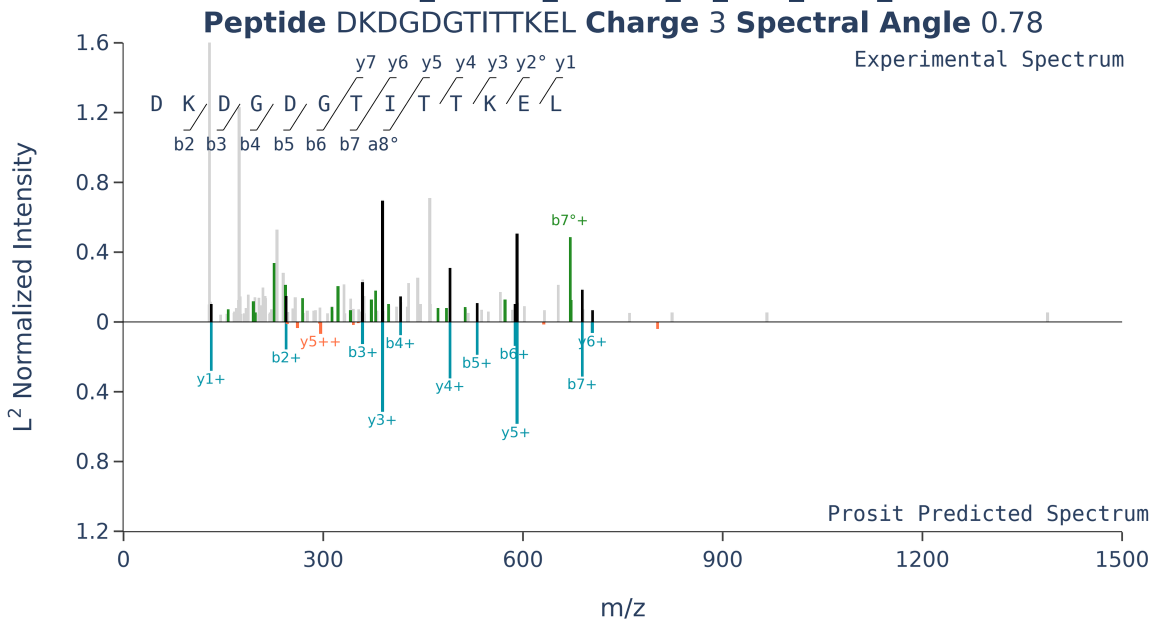


**Source** HRoetschke_291222_040123_Fu_CaM_b2R1_1h **Scan** 4155
**Peptide** NLGEKLTD **Charge** 2 **Spectral Angle** 0.77

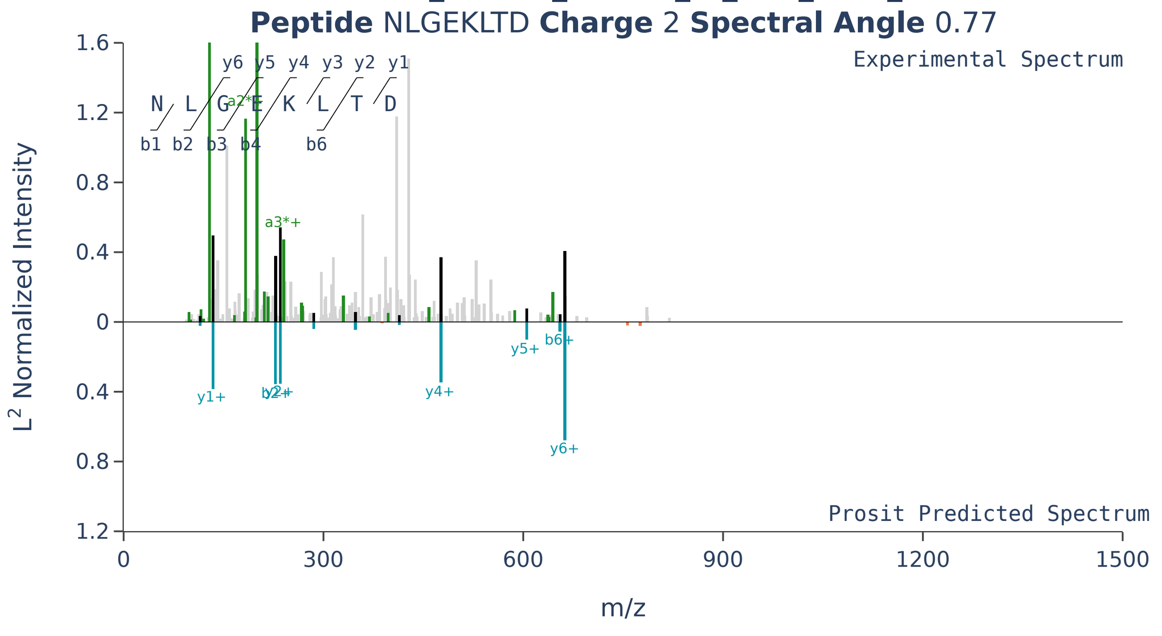


**Source** HRoetschke_291222_040123_Fu_CaM_b1R1_4h **Scan** 8641
**Peptide** EEVDEMIREA **Charge** 2 **Spectral Angle** 0.76

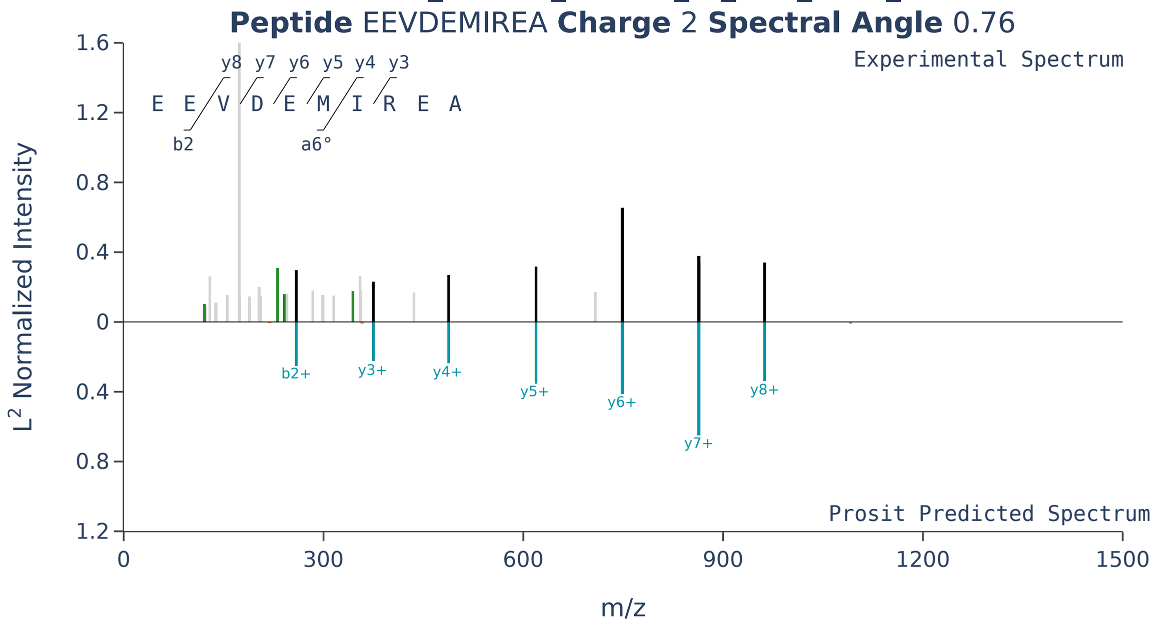

**Source** HRoetschke_291222_040123_Fu_CaM_b1R2_4h **Scan** 2739
**Peptide** SEEEIREA **Charge** 2 **Spectral Angle** 0.9

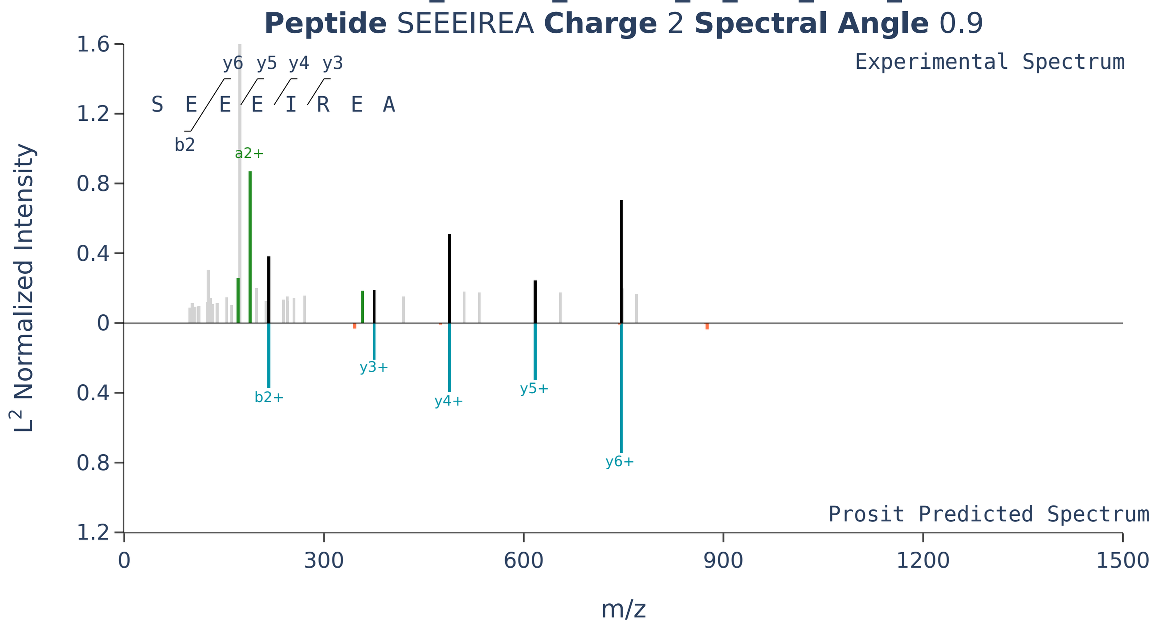


**Source** HRoetschke_291222_040123_Fu_CaM_b2R1_24h **Scan** 8166
**Peptide** READIDGDGQVNYEEF **Charge** 2 **Spectral Angle** 0.87

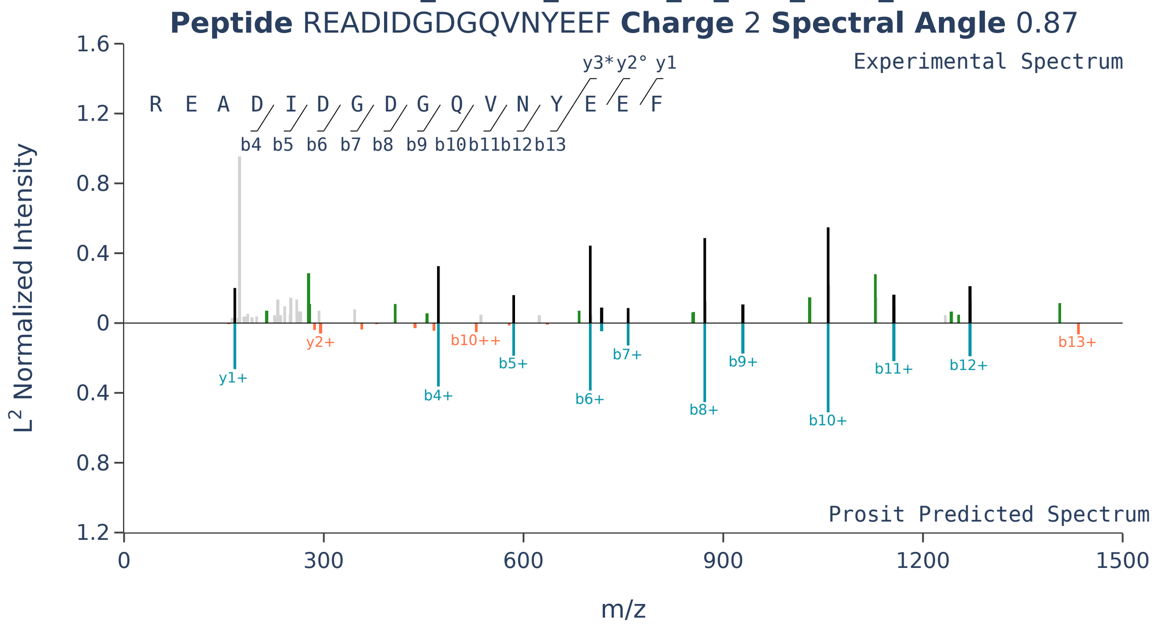


**Source** HRoetschke_291222_040123_Fu_CaM_b1R1_24h **Scan** 4949
**Peptide** DKDGNGYISAA **Charge** 2 **Spectral Angle** 0.83

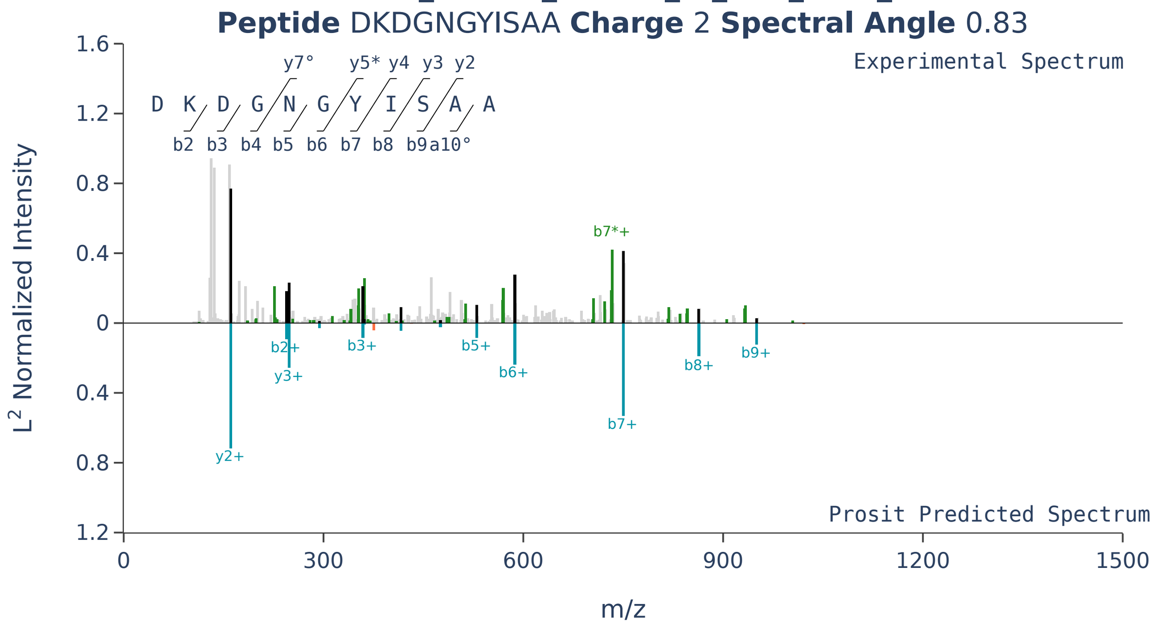


**Source** HRoetschke_291222_040123_Fu_CaM_b1R2_4h **Scan** 7908
**Peptide** GNGYISAAELR **Charge** 2 **Spectral Angle** 0.83

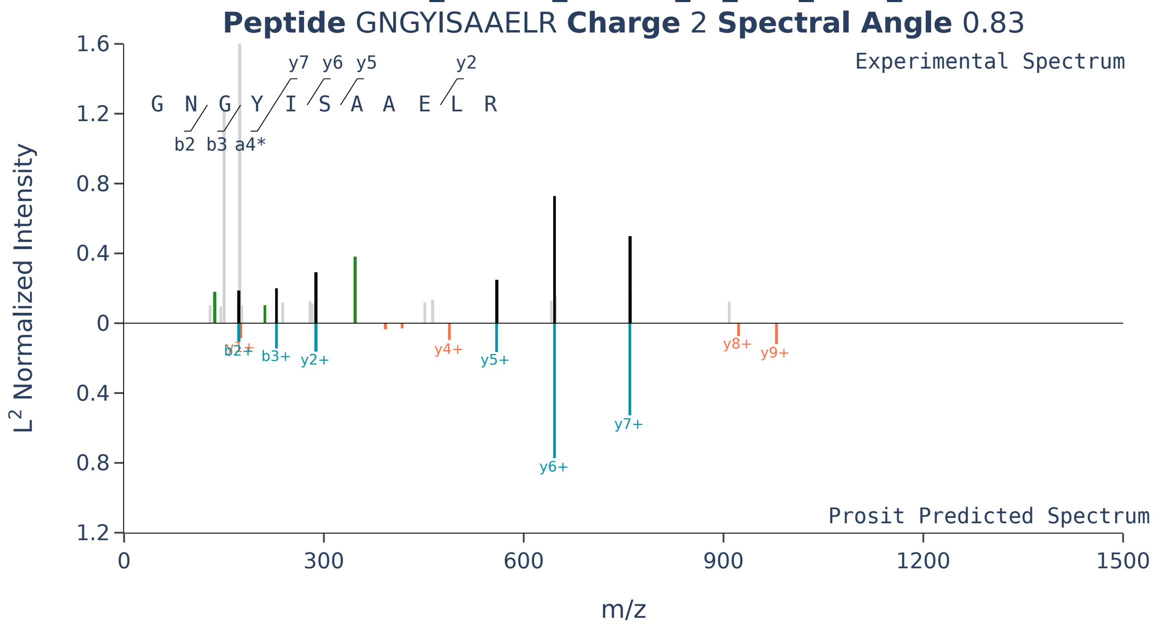


**Source** HRoetschke_291222_040123_Fu_CaM_b2R1_4h **Scan** 6219
**Peptide** EQIAEFKEA **Charge** 2 **Spectral Angle** 0.87

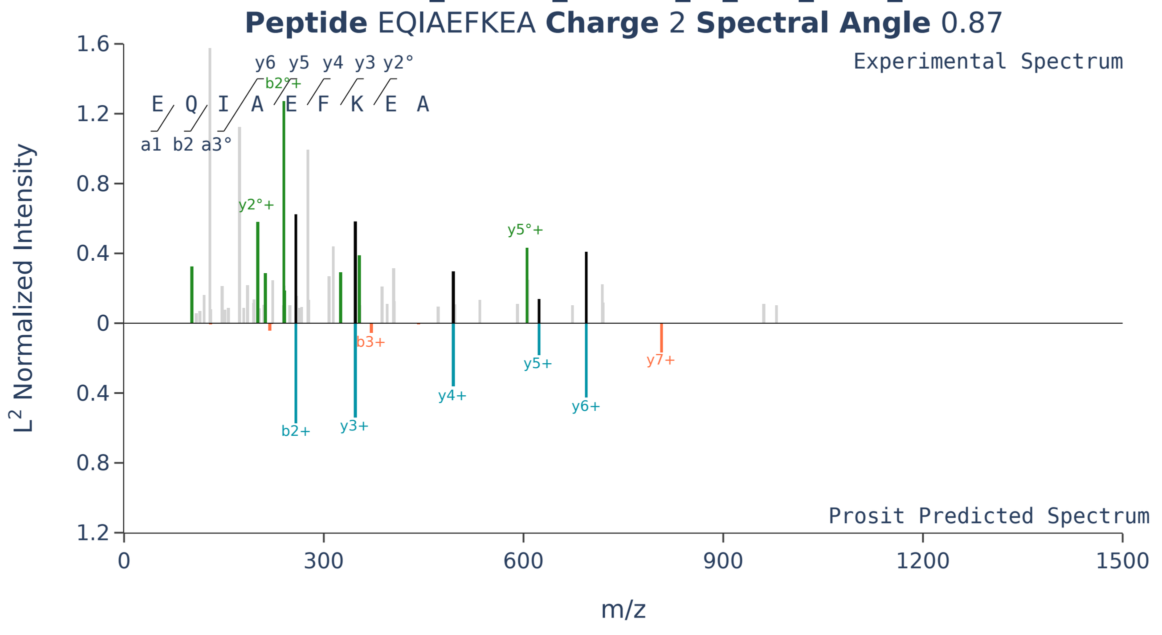


**Source** HRoetschke_291222_040123_Fu_CaM_b1R2_2h **Scan** 6129
**Peptide** GNGYISAAELRH **Charge** 3 **Spectral Angle** 0.8

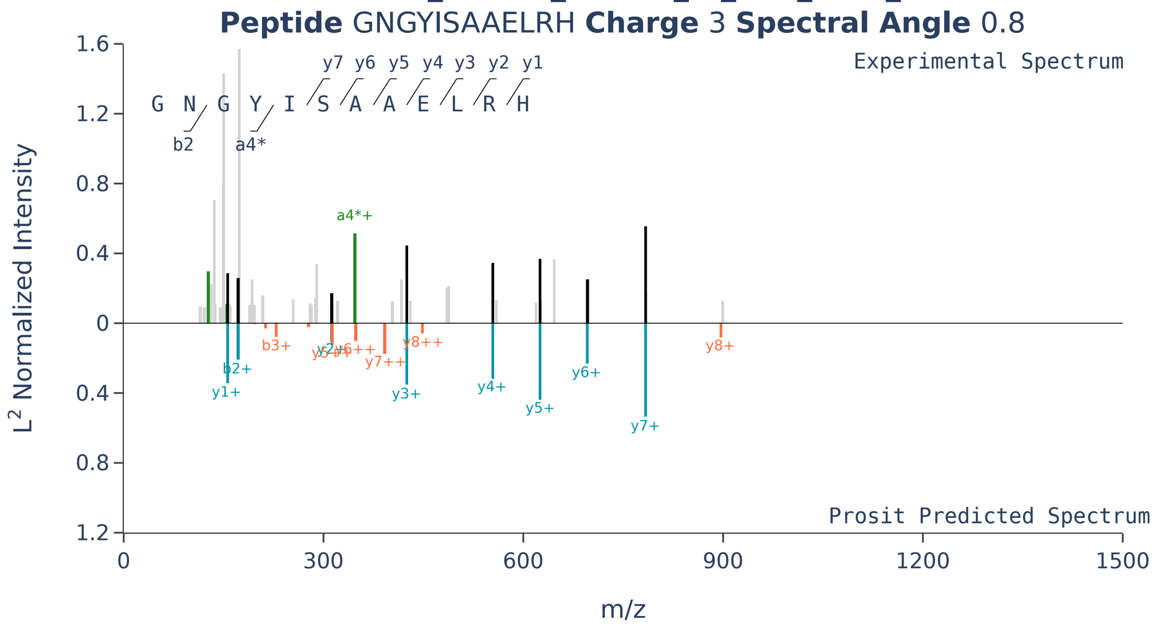


**Source** HRoetschke_291222_040123_Fu_CaM_b1R2_24h **Scan** 4451
**Peptide** KDGDGTITTKELG **Charge** 3 **Spectral Angle** 0.87

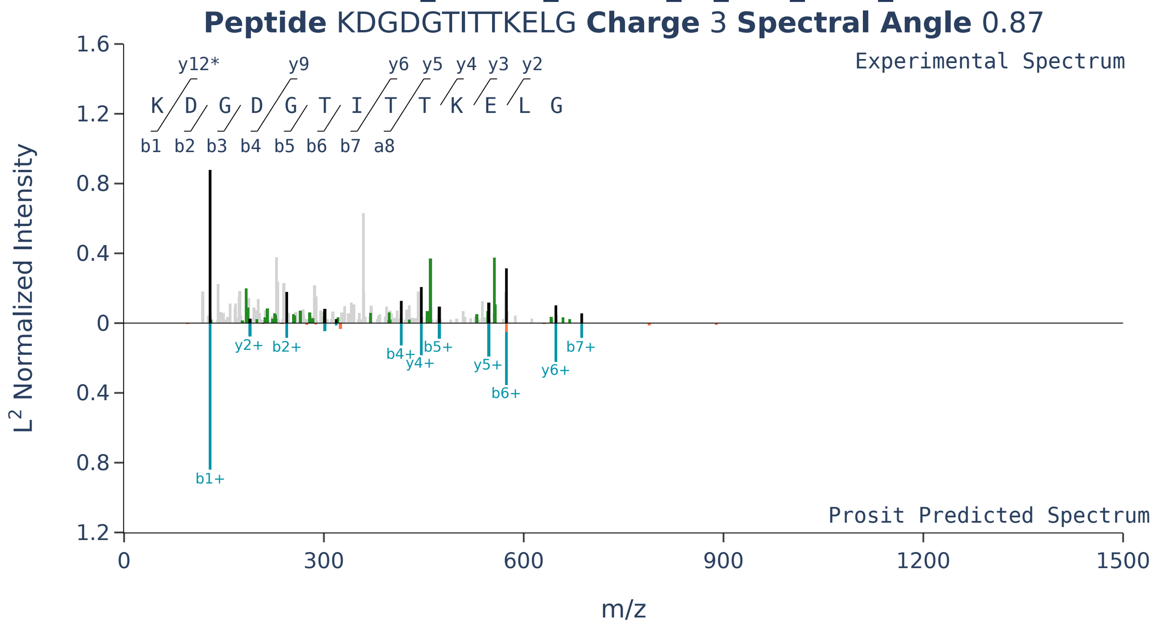


**Source** HRoetschke_291222_040123_Fu_CaM_b1R2_2h **Scan** 3296
**Peptide** KLTDEEVD **Charge** 2 **Spectral Angle** 0.86

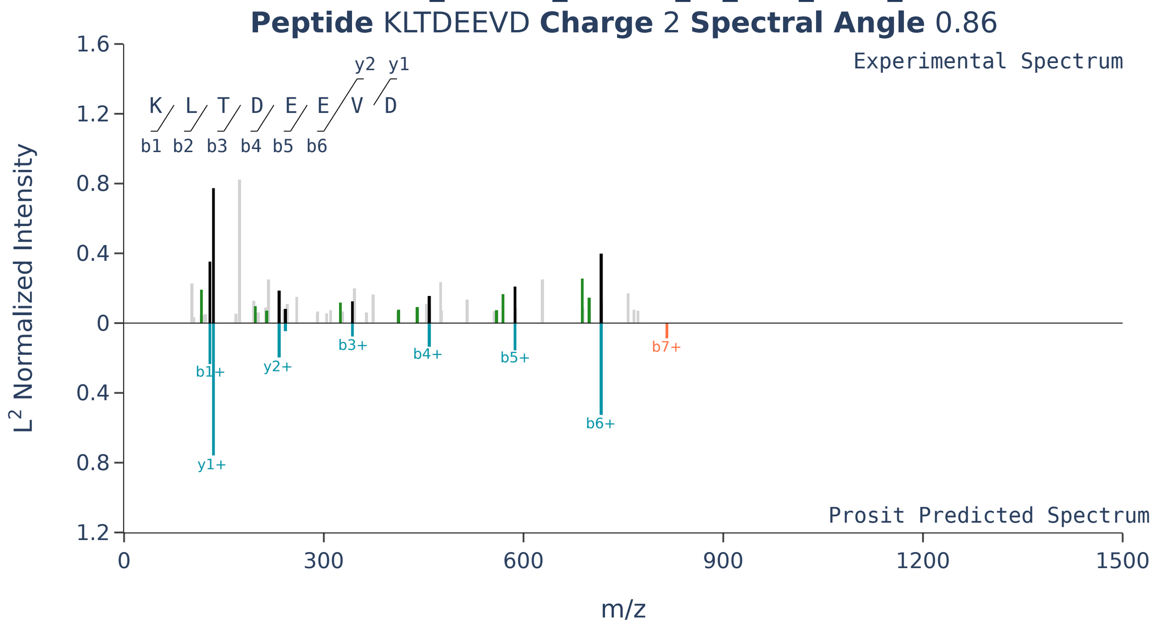


**Source** HRoetschke_291222_040123_Fu_CaM_b2R2_24h **Scan** 12442
**Peptide** DKDGDGTITTKELGTVMRSL **Charge** 3 **Spectral Angle** 0.83

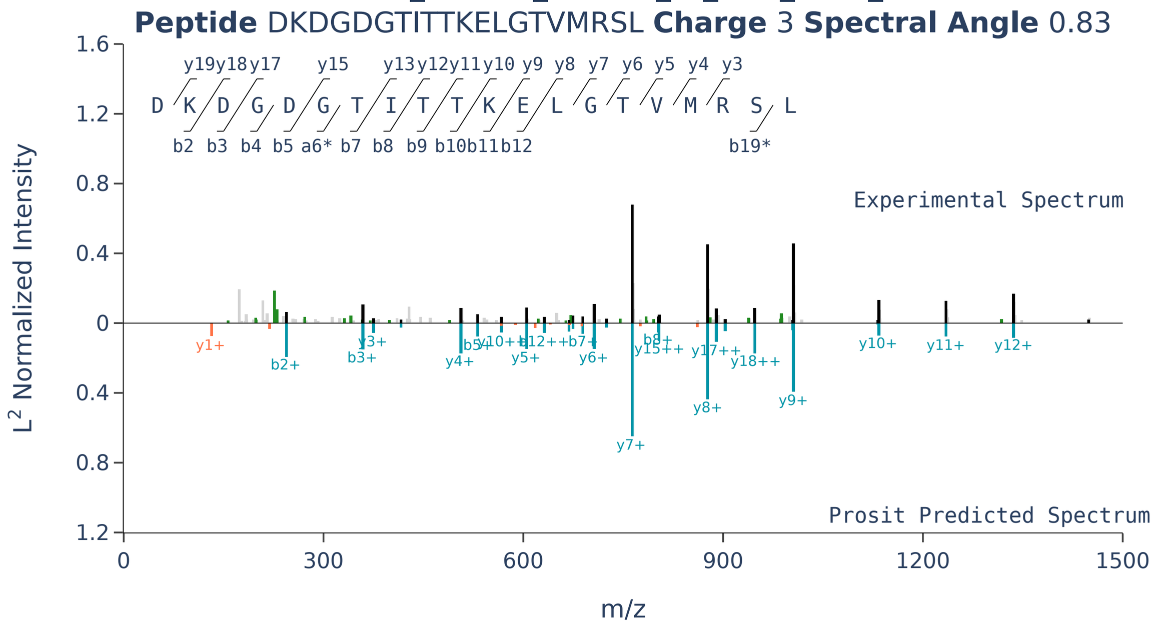


**Source** HRoetschke_291222_040123_Fu_CaM_b2R2_24h **Scan** 6965
**Peptide** SLFDKDGDGTITTK **Charge** 3 **Spectral Angle** 0.76

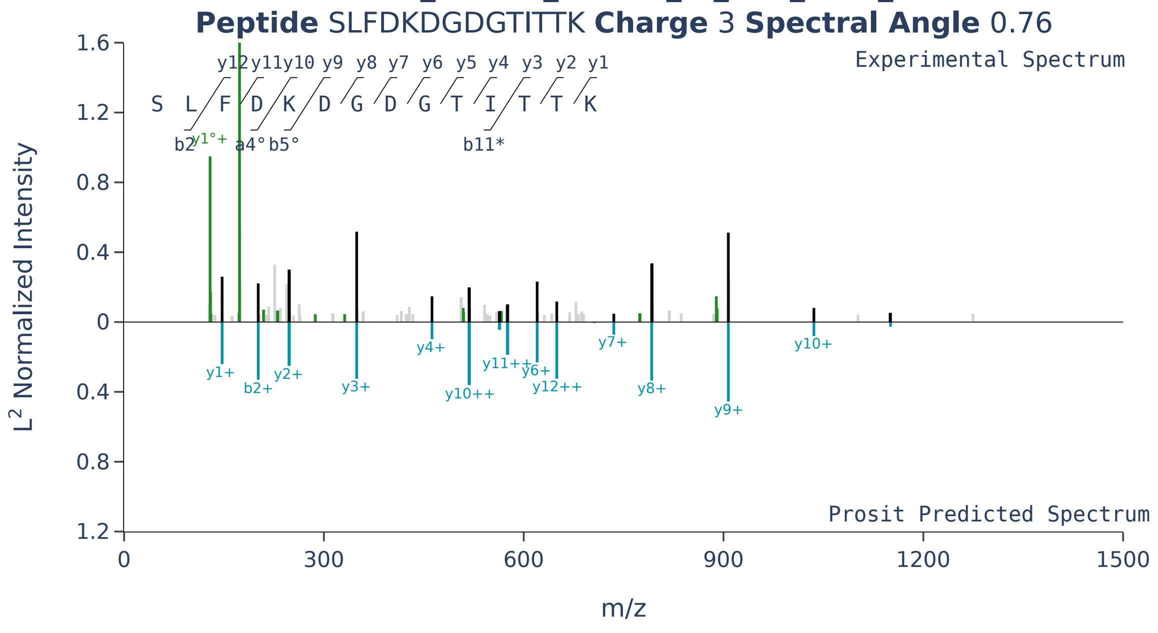


**Source** HRoetschke_291222_040123_Fu_CaM_b1R1_2h **Scan** 4185
**Peptide** KDGNGYISAAE **Charge** 2 **Spectral Angle** 0.89

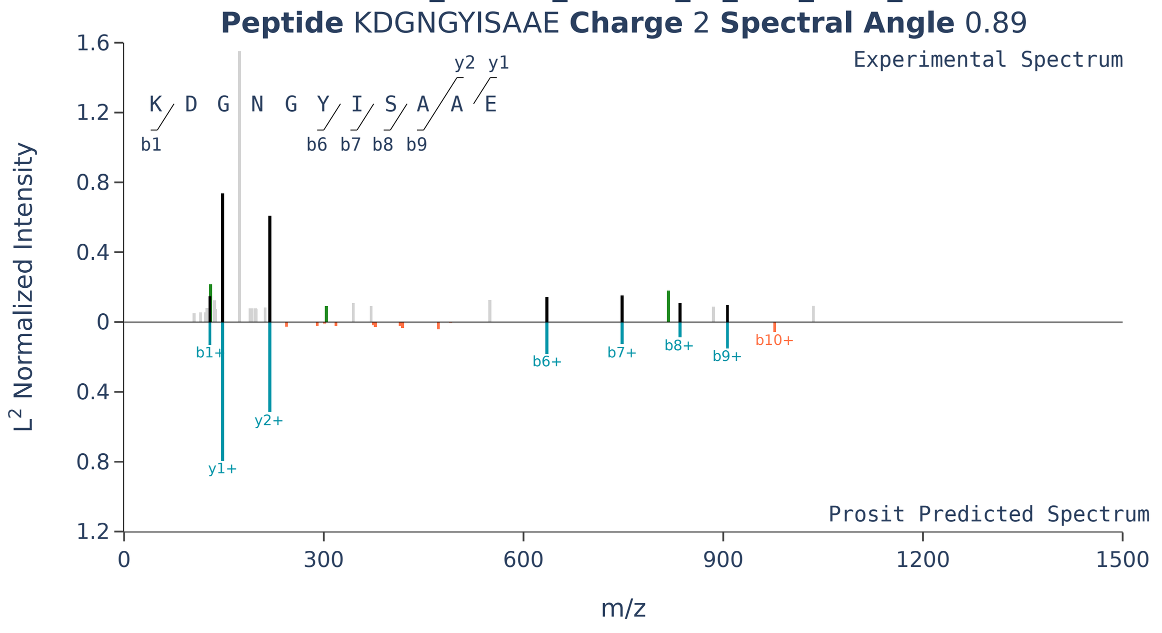


**Source** HRoetschke_291222_040123_Fu_CaM_b1R1_1h **Scan** 7508
**Peptide** GTITTKELGTVMR **Charge** 3 **Spectral Angle** 0.83

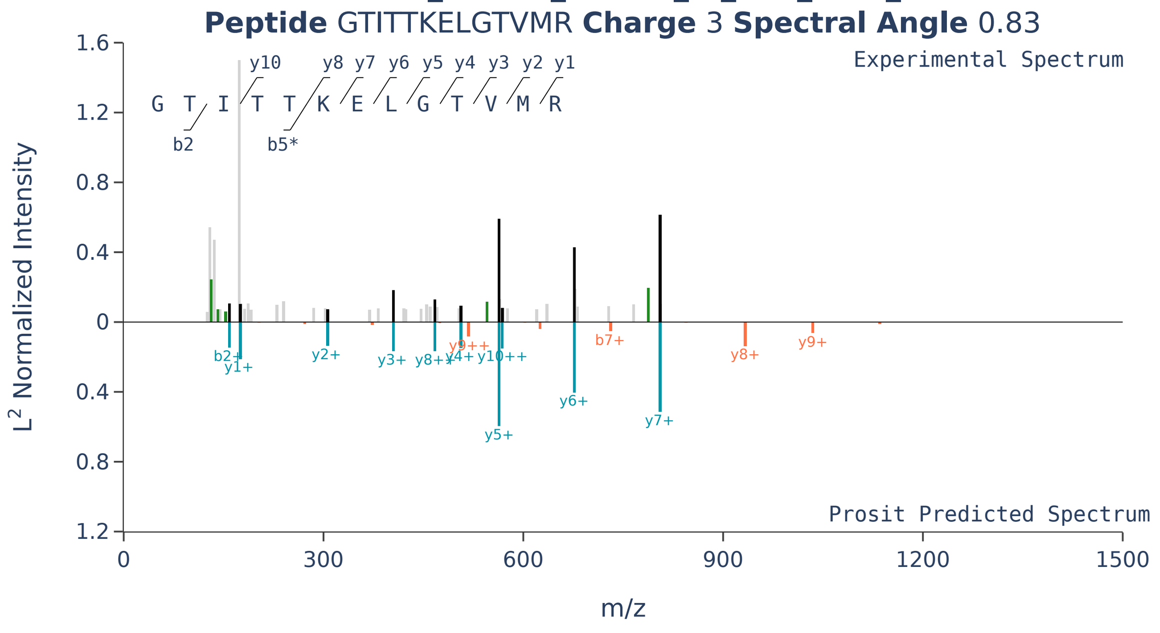


**Source** HRoetschke_291222_040123_Fu_CaM_b1R2_24h **Scan** 6863
**Peptide** ADIDGDGQVNY **Charge** 2 **Spectral Angle** 0.81

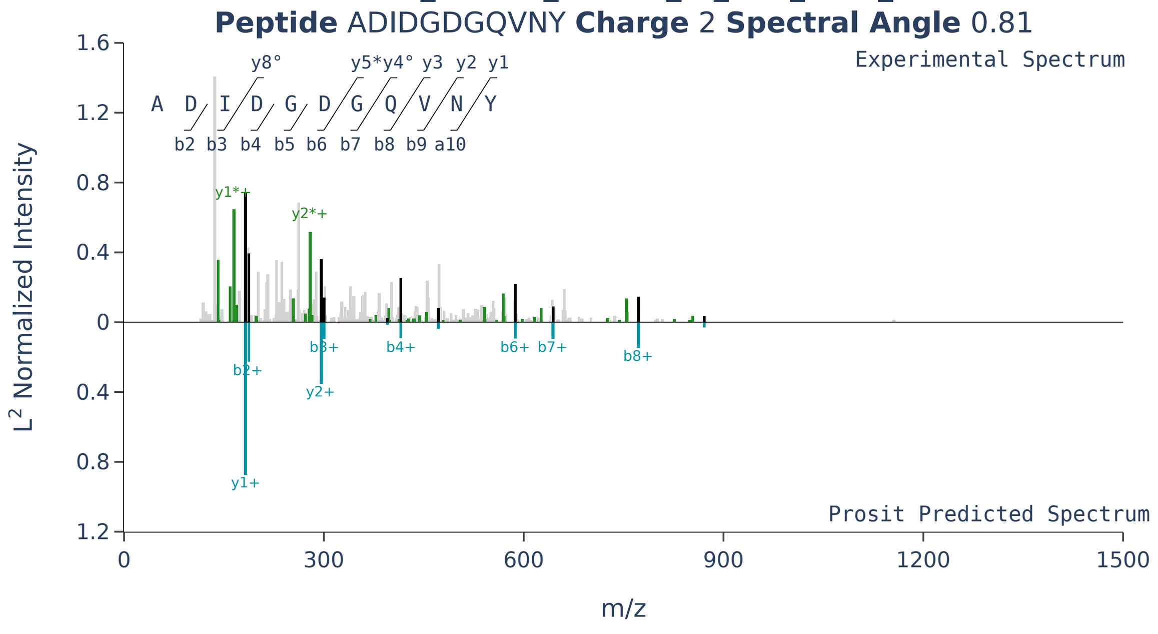


**Source** HRoetschke_291222_040123_Fu_CaM_b1R1_2h **Scan** 5569
**Peptide** ARKMKDTDSEEEIREAF **Charge** 4 **Spectral Angle** 0.7

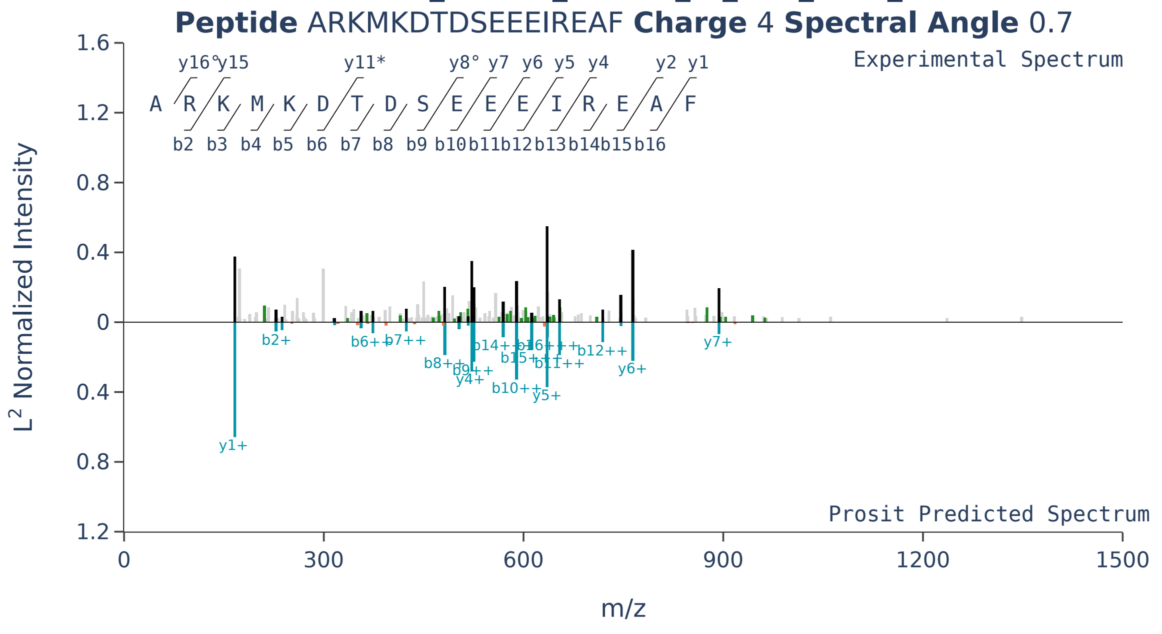


**Source** HRoetschke_291222_040123_Fu_CaM_b1R2_24h **Scan** 3827
**Peptide** MINEVDADGNG **Charge** 2 **Spectral Angle** 0.83

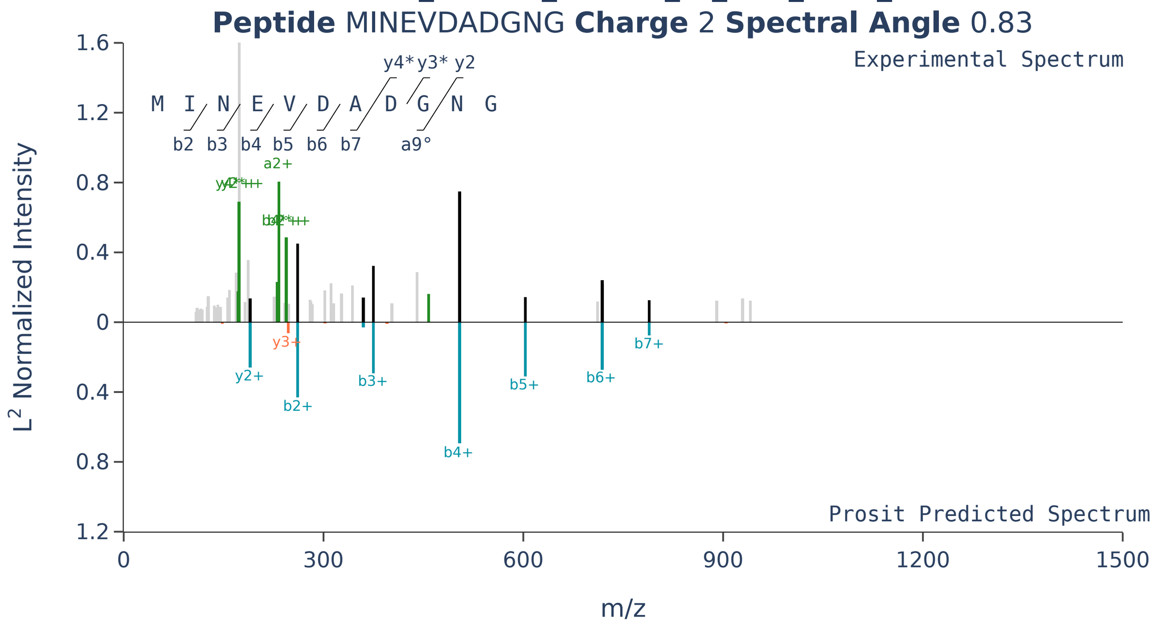

**Source** HRoetschke_291222_040123_Fu_CaM_b2R2_2h **Scan** 8548
**Peptide** GEKLTDEEVDEMIRE **Charge** 3 **Spectral Angle** 0.88

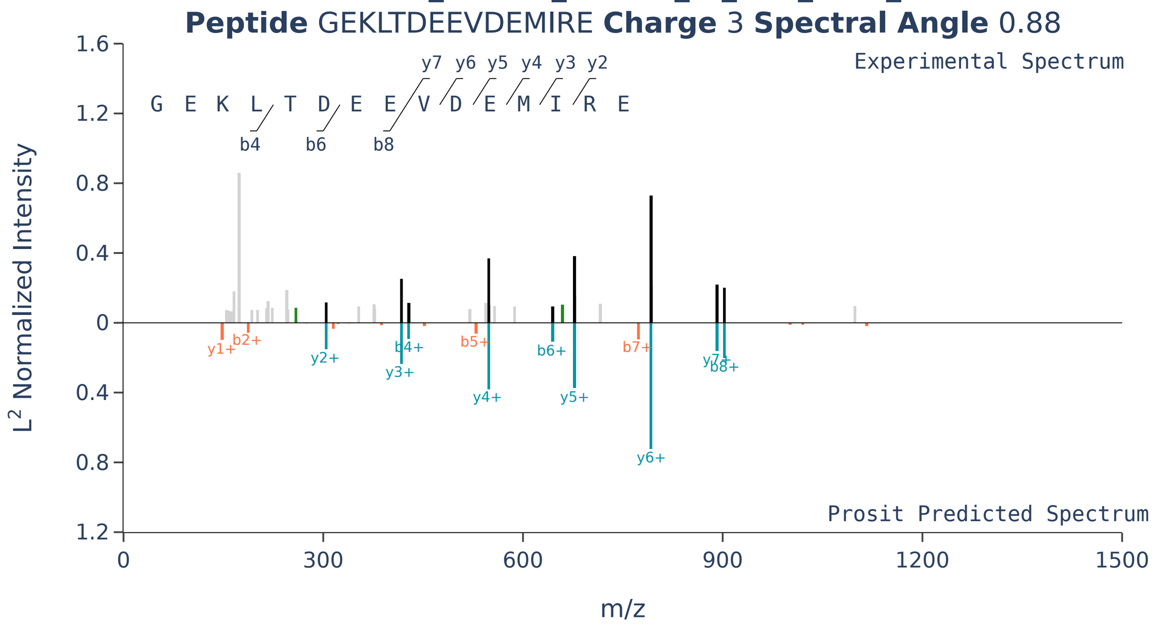


**Source** HRoetschke_291222_040123_Fu_CaM_b1R2_2h **Scan** 4865
**Peptide** TNLGEKLTD **Charge** 2 **Spectral Angle** 0.89

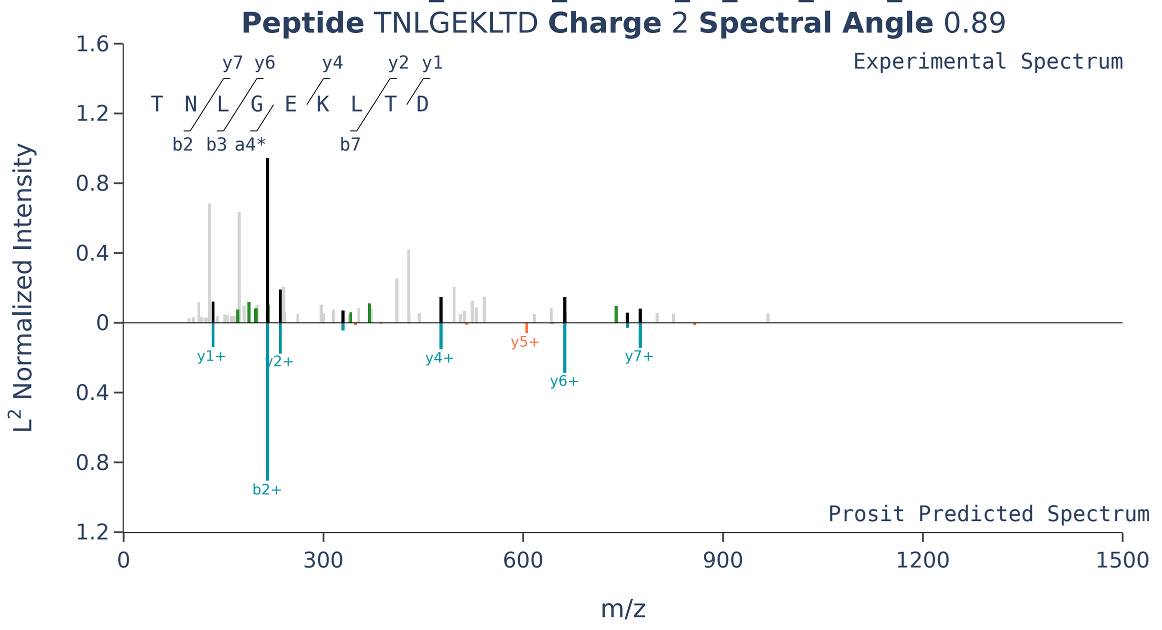


**Source** HRoetschke_291222_040123_Fu_CaM_b1R1_24h **Scan** 10617
**Peptide** DGDGQVNYEEF **Charge** 2 **Spectral Angle** 0.88

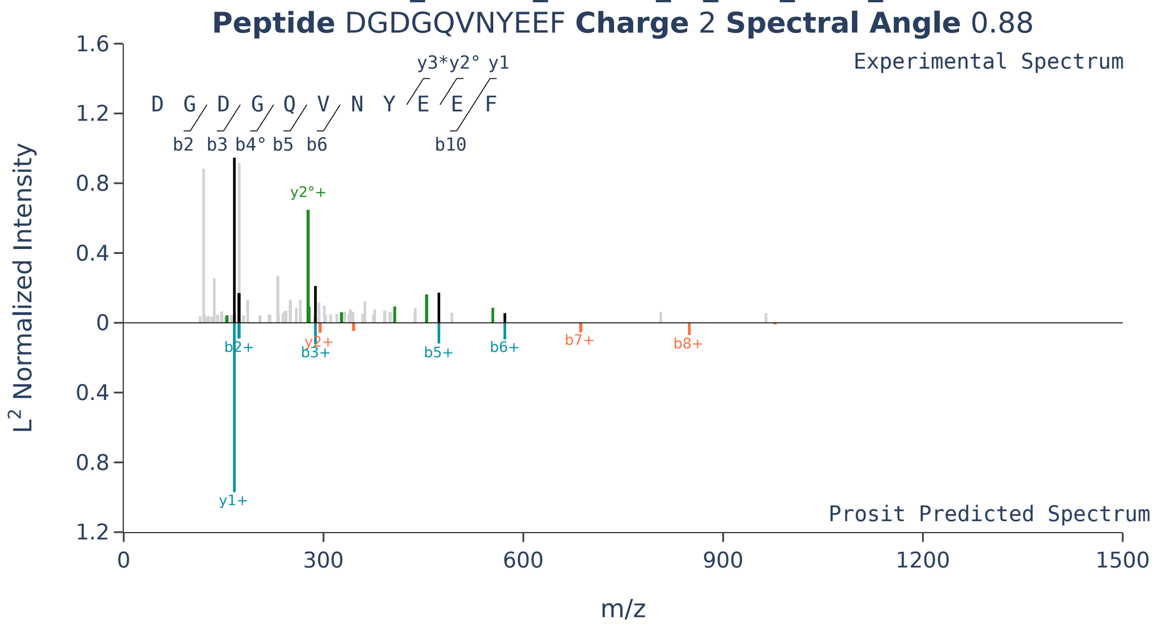


**Source** HRoetschke_291222_040123_Fu_CaM_b2R2_24h **Scan** 11509
**Peptide** ADQLTEEQIAEF **Charge** 2 **Spectral Angle** 0.88

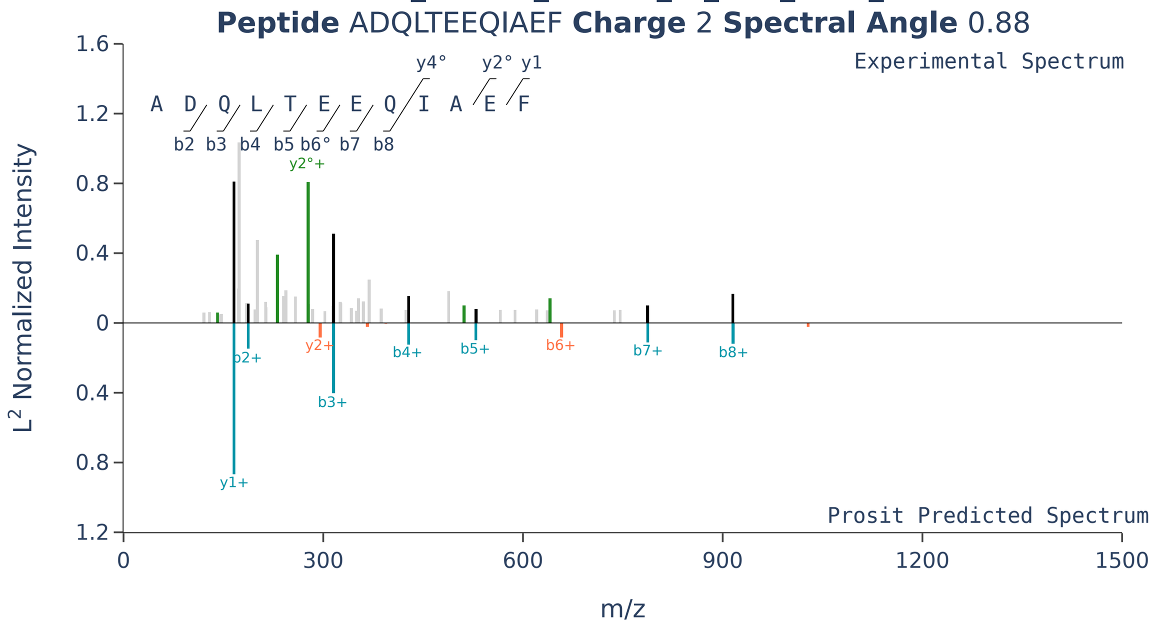


**Source** HRoetschke_291222_040123_Fu_CaM_b1R1_24h **Scan** 4971
**Peptide** KDGDGTITTKELGT **Charge** 2 **Spectral Angle** 0.83

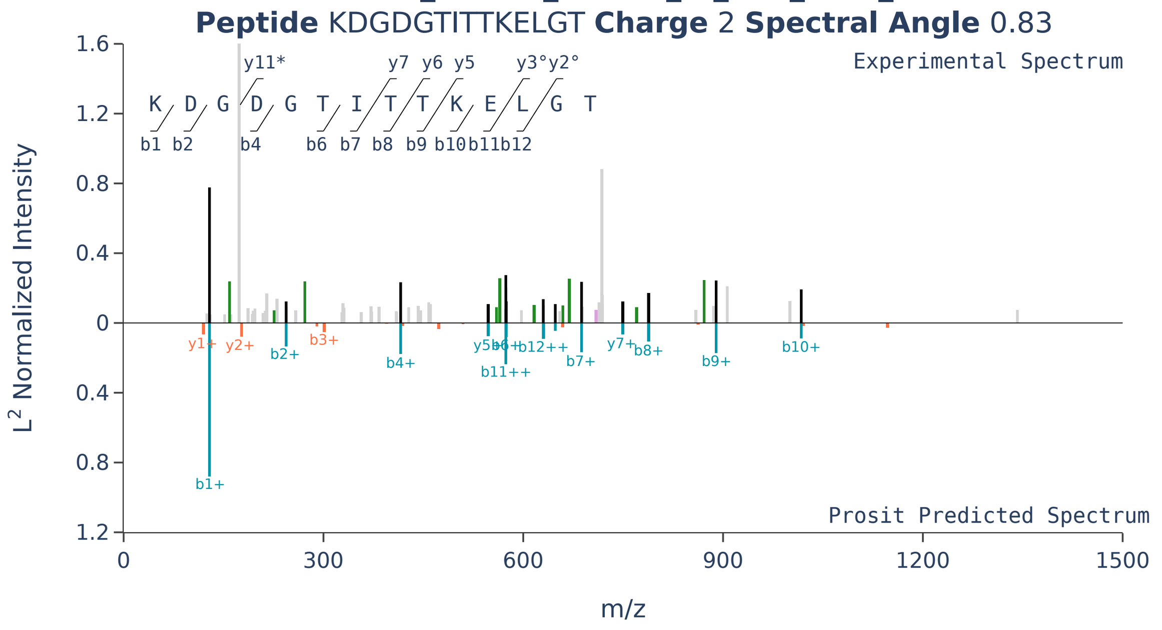


**Source** HRoetschke_291222_040123_Fu_CaM_b1R1_24h **Scan** 4996
**Peptide** LDGDGQVNYE **Charge** 2 **Spectral Angle** 0.82

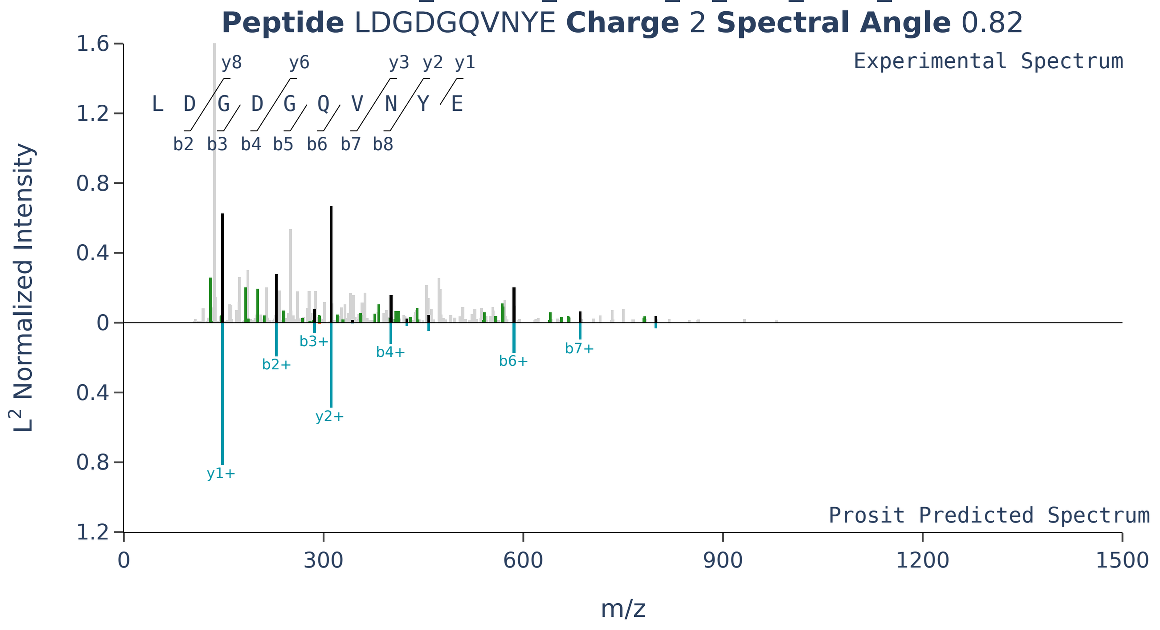


**Source** HRoetschke_291222_040123_Fu_CaM_b1R2_4h **Scan** 7524
**Peptide** KDGNGYISAAELRHV **Charge** 3 **Spectral Angle** 0.81

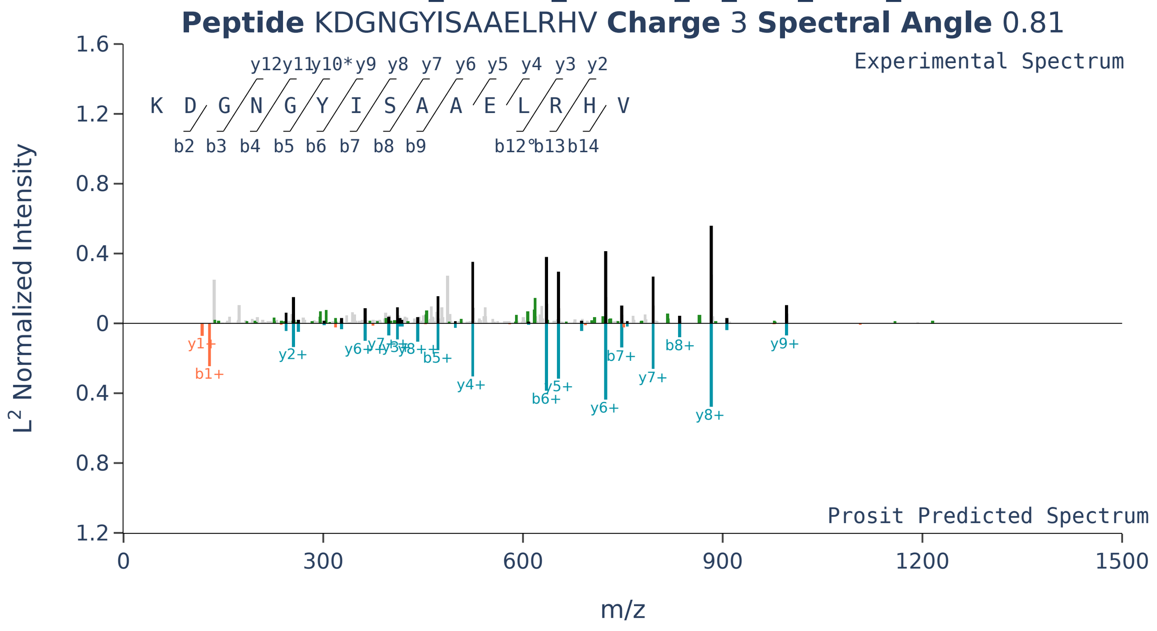


**Source** HRoetschke_291222_040123_Fu_CaM_b2R1_4h **Scan** 11825
**Peptide** FPEFLTM **Charge** 2 **Spectral Angle** 0.69

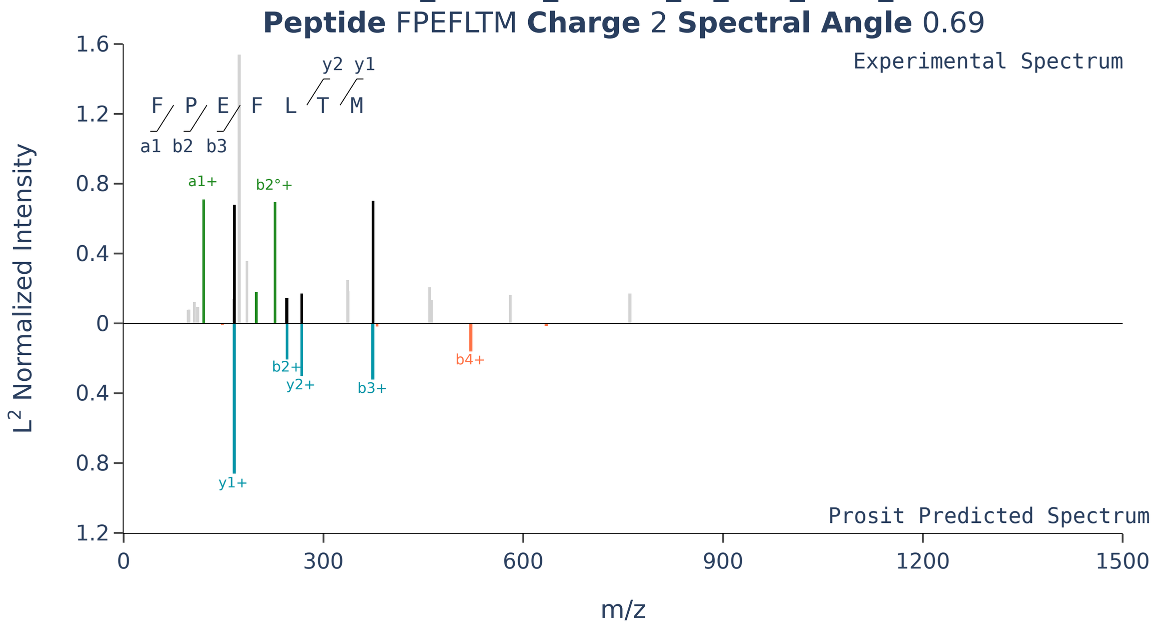


**Source** HRoetschke_291222_040123_Fu_CaM_b1R2_24h **Scan** 3349
**Peptide** FDKDGDGTITTKE **Charge** 3 **Spectral Angle** 0.84

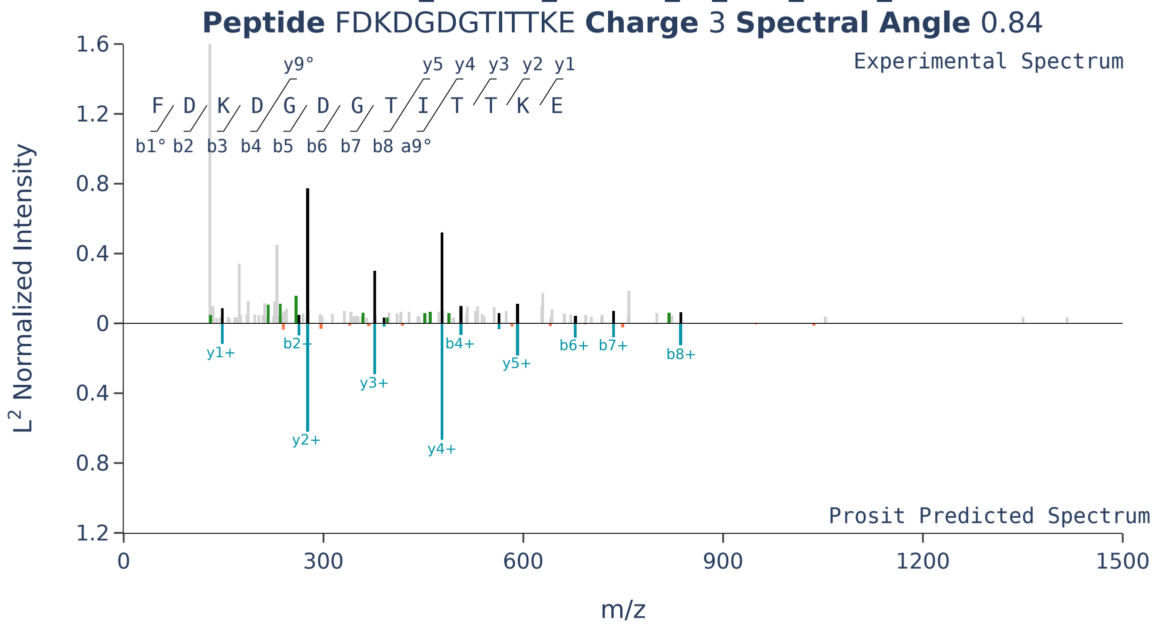


**Source** HRoetschke_291222_040123_Fu_CaM_b1R1_24h **Scan** 11329
**Peptide** SLFDKDGDGTITTKEL **Charge** 3 **Spectral Angle** 0.82

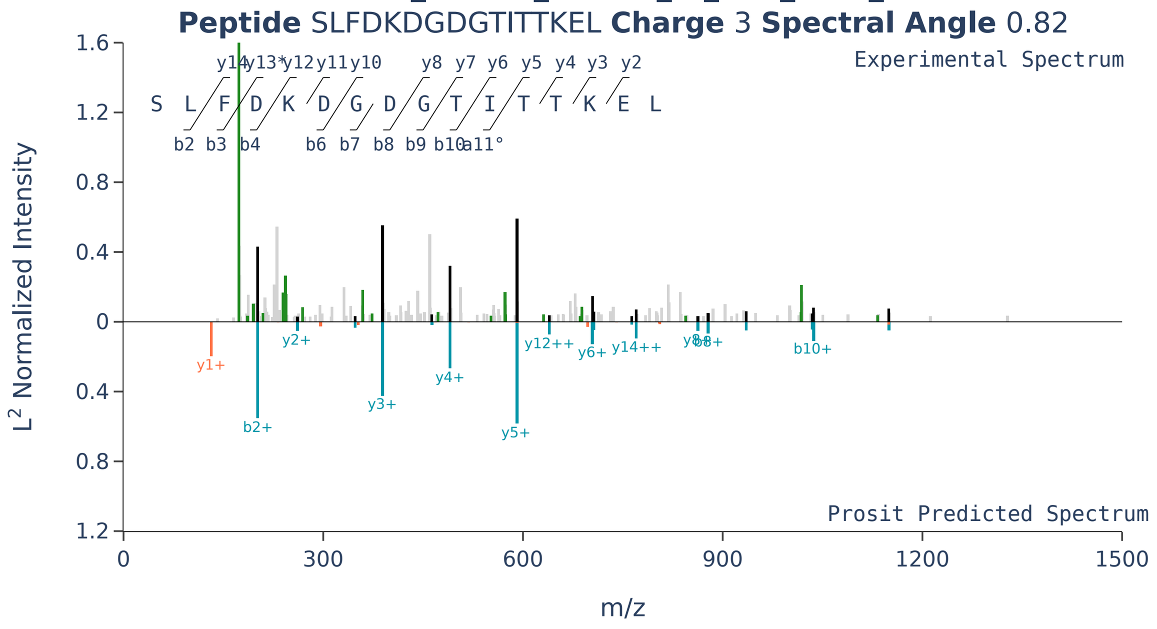


**Source** HRoetschke_291222_040123_Fu_CaM_b1R2_4h **Scan** 3513
**Peptide** RVFDKDGNGY **Charge** 3 **Spectral Angle** 0.76

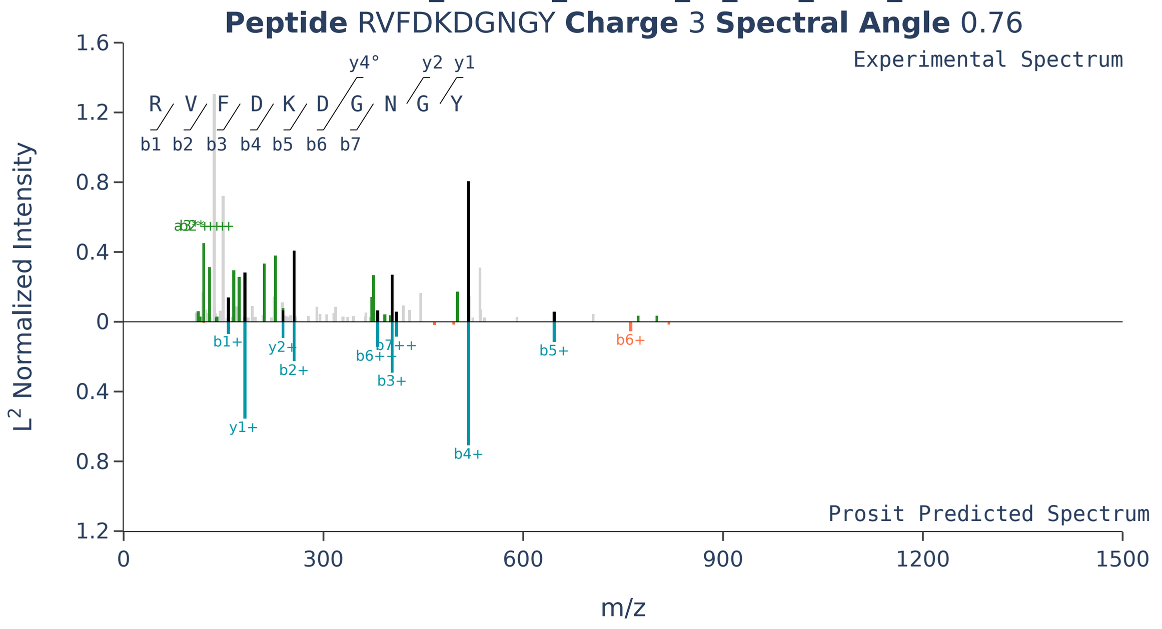


**Source** HRoetschke_291222_040123_Fu_CaM_b2R1_24h **Scan** 10815
**Peptide** SEEEIREAFRVF **Charge** 3 **Spectral Angle** 0.88

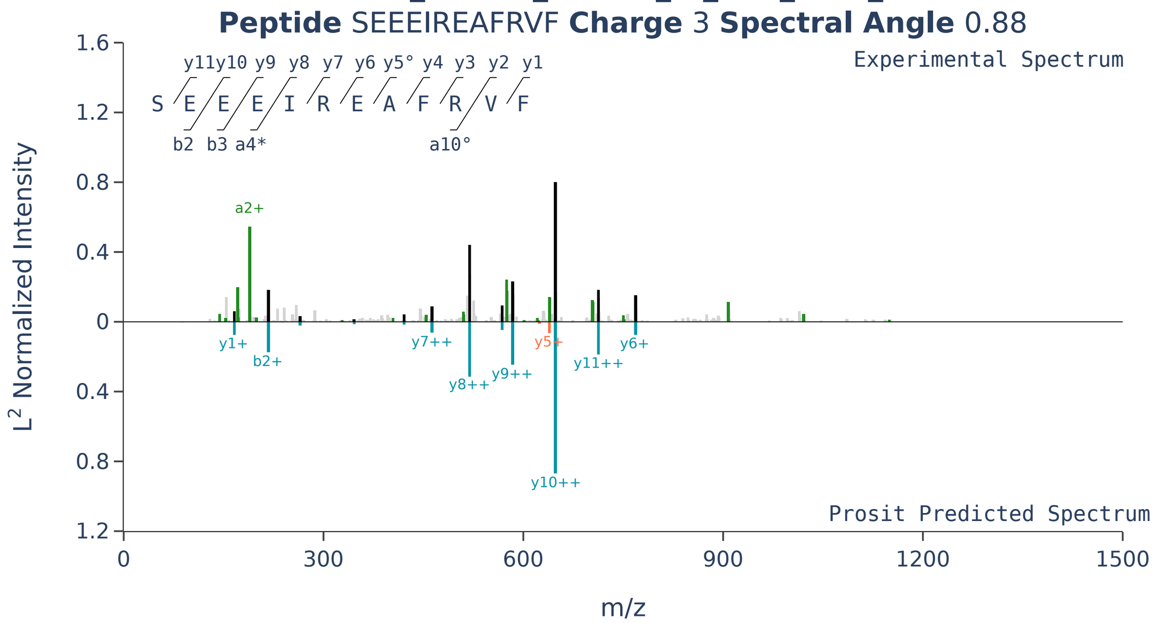


**Source** HRoetschke_291222_040123_Fu_CaM_b1R1_4h **Scan** 13029
**Peptide** TDSEEEIREAFRVFD **Charge** 3 **Spectral Angle** 0.82

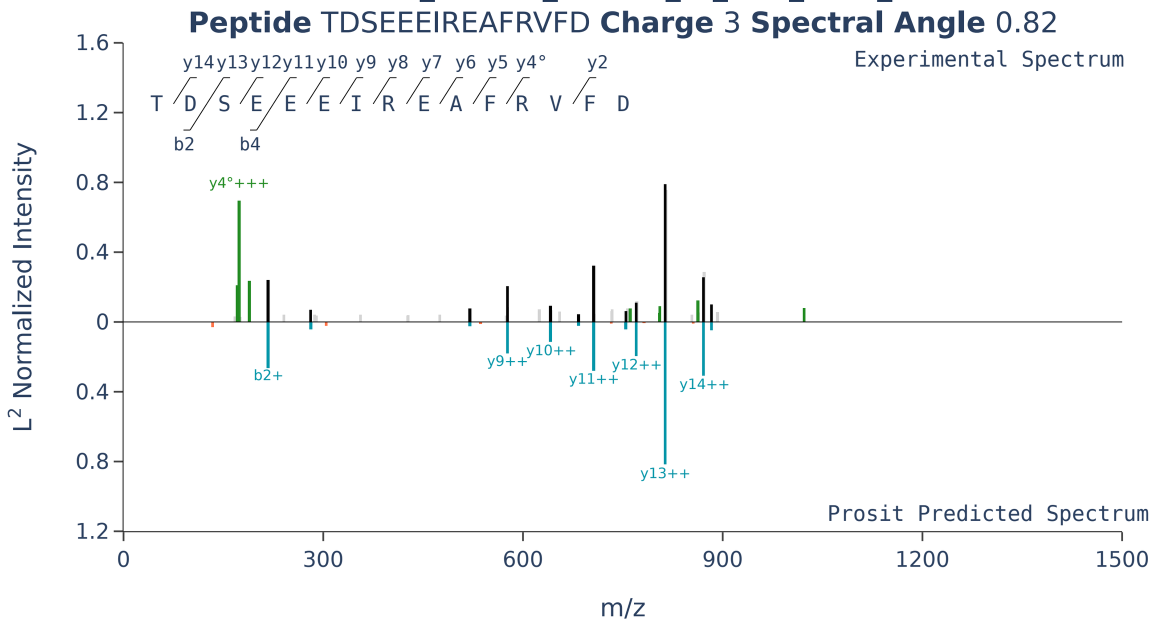


**Source** HRoetschke_291222_040123_Fu_CaM_b1R1_1h **Scan** 6714
**Peptide** LFDKDGDGTITT **Charge** 2 **Spectral Angle** 0.87

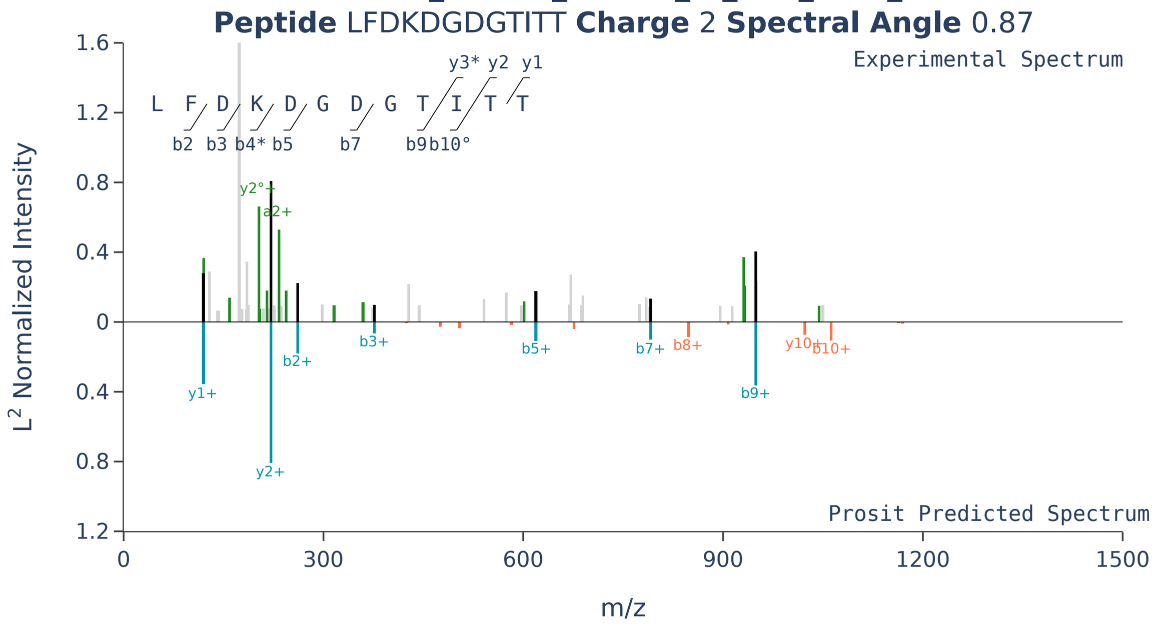


**Source** HRoetschke_291222_040123_Fu_CaM_b1R1_4h **Scan** 2972
**Peptide** RSLGQNPTEAE **Charge** 2 **Spectral Angle** 0.85

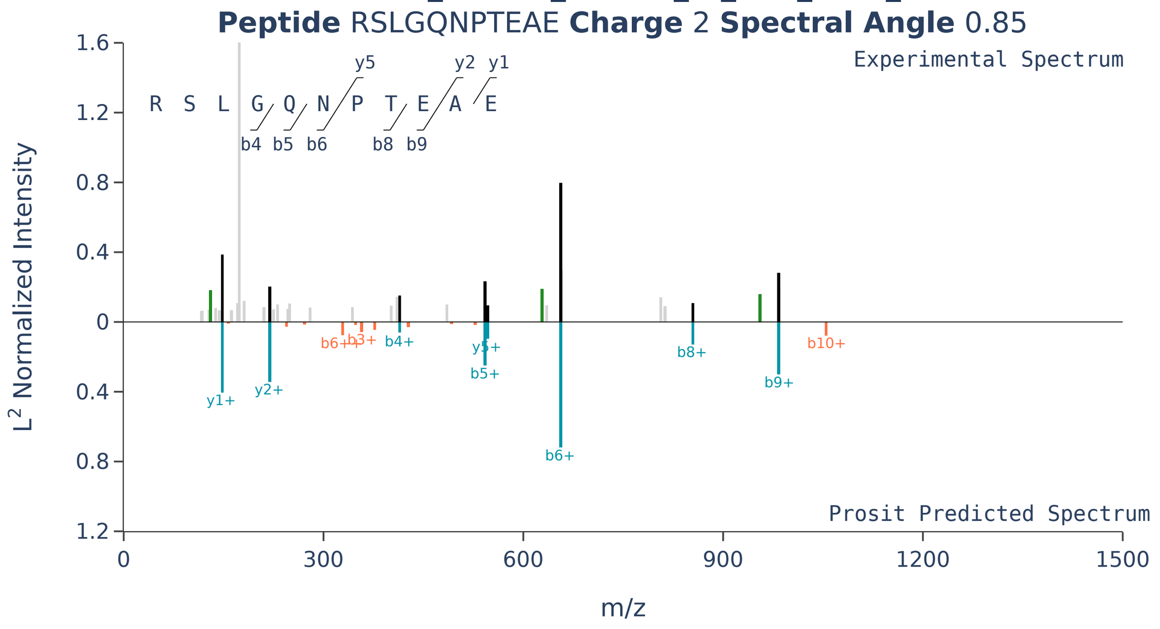

**Source** HRoetschke_291222_040123_Fu_CaM_b1R1_24h **Scan** 12606
**Peptide** FSLFDKDGDGTITT **Charge** 2 **Spectral Angle** 0.8

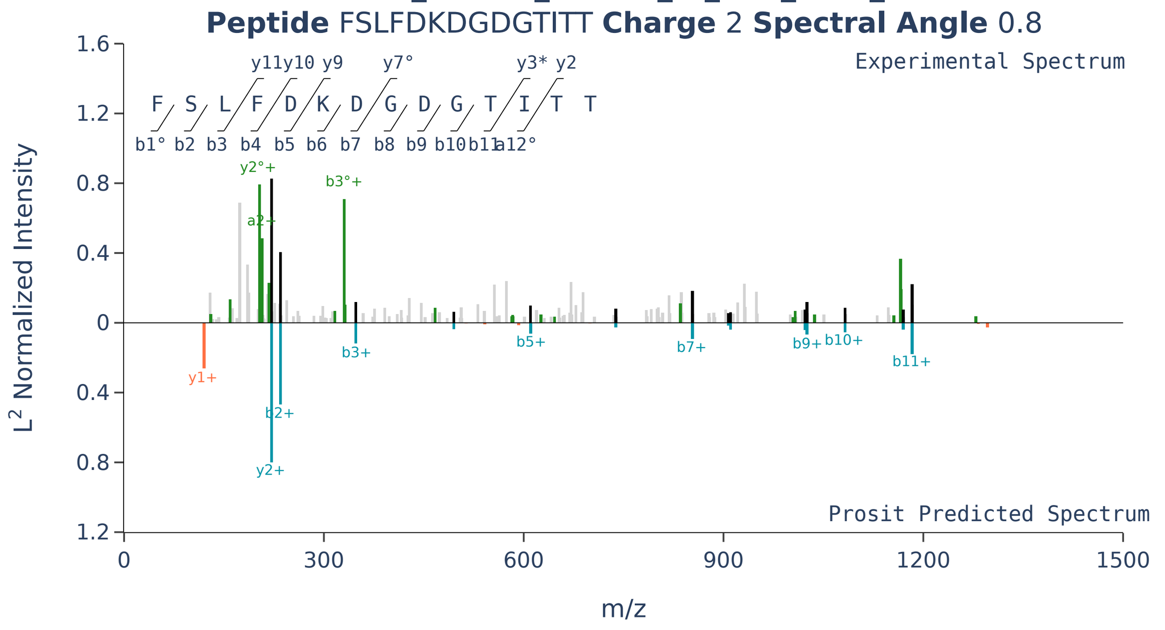


**Source** HRoetschke_291222_040123_Fu_CaM_b1R1_24h **Scan** 6724
**Peptide** DKDGNGYISAAELRH **Charge** 3 **Spectral Angle** 0.8

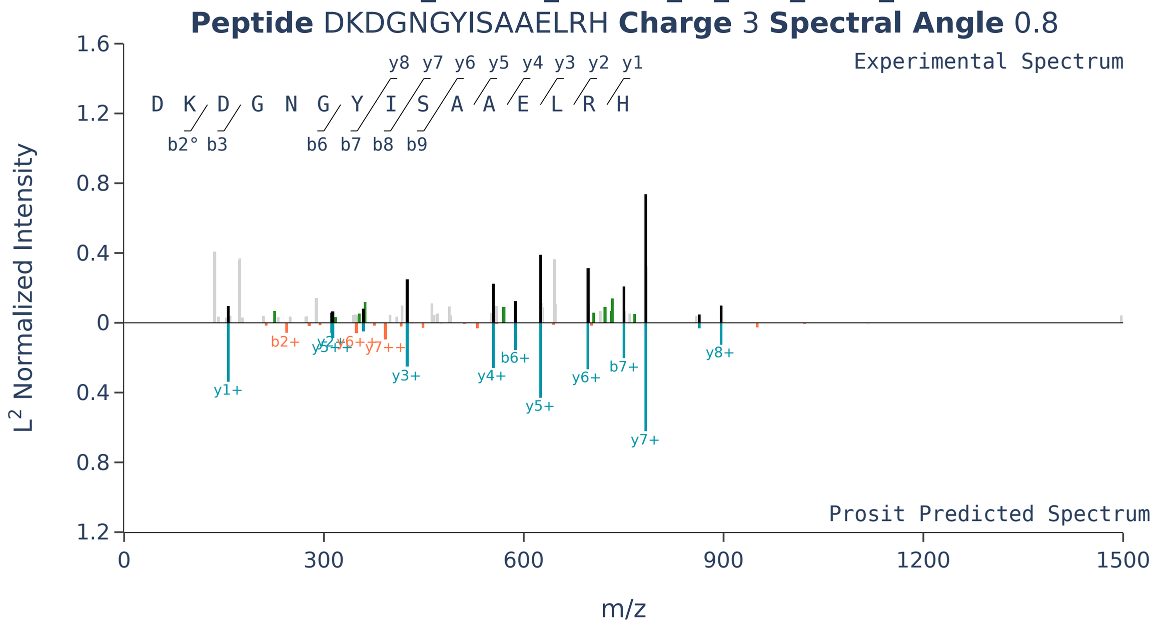


**Source** HRoetschke_291222_040123_Fu_CaM_b1R1_1h **Scan** 7857
**Peptide** GNGYISAAELRHV **Charge** 3 **Spectral Angle** 0.74

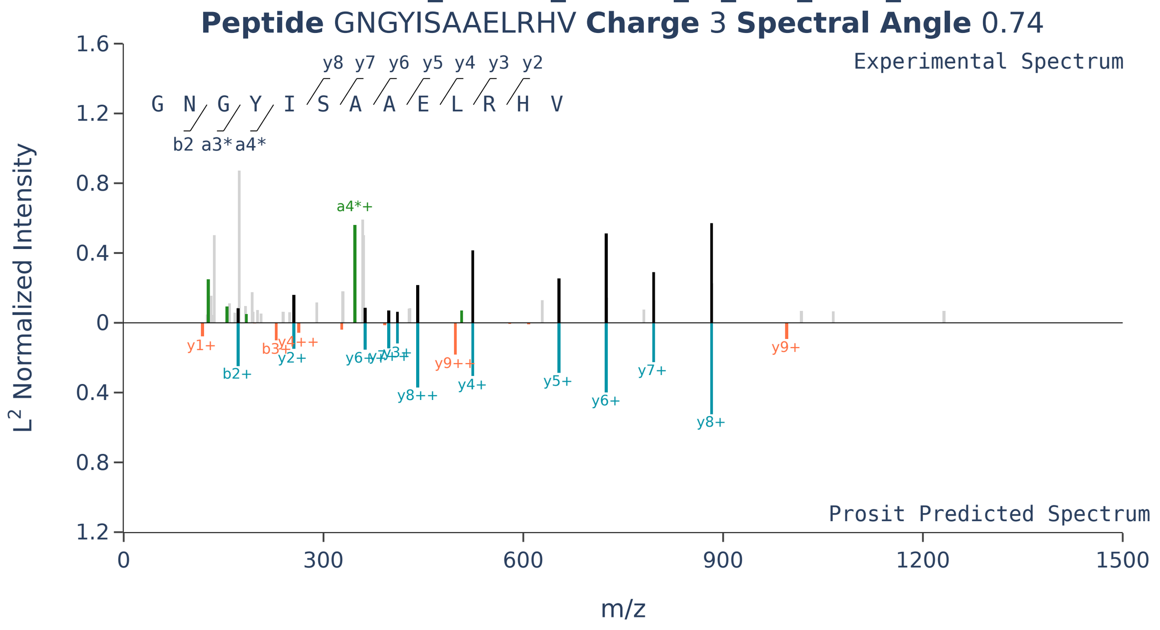


**Source** HRoetschke_291222_040123_Fu_CaM_b1R2_24h **Scan** 3638
**Peptide** AELRHVM **Charge** 2 **Spectral Angle** 0.68

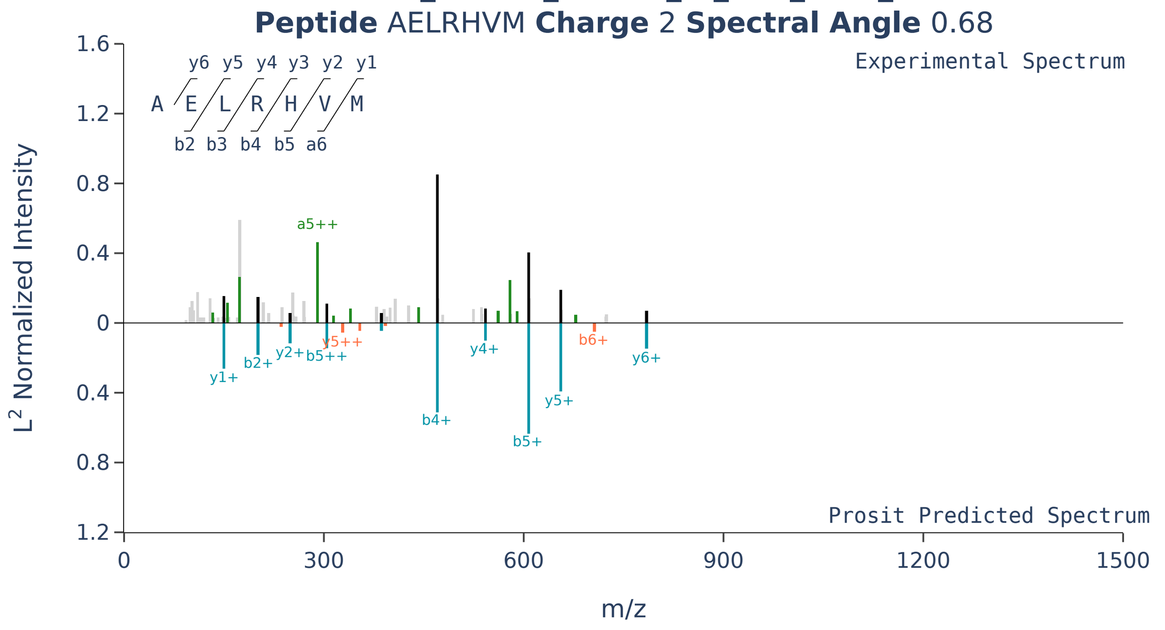


**Source** HRoetschke_291222_040123_Fu_CaM_b2R2_4h **Scan** 6330
**Peptide** TEEQIAEFKE **Charge** 2 **Spectral Angle** 0.83

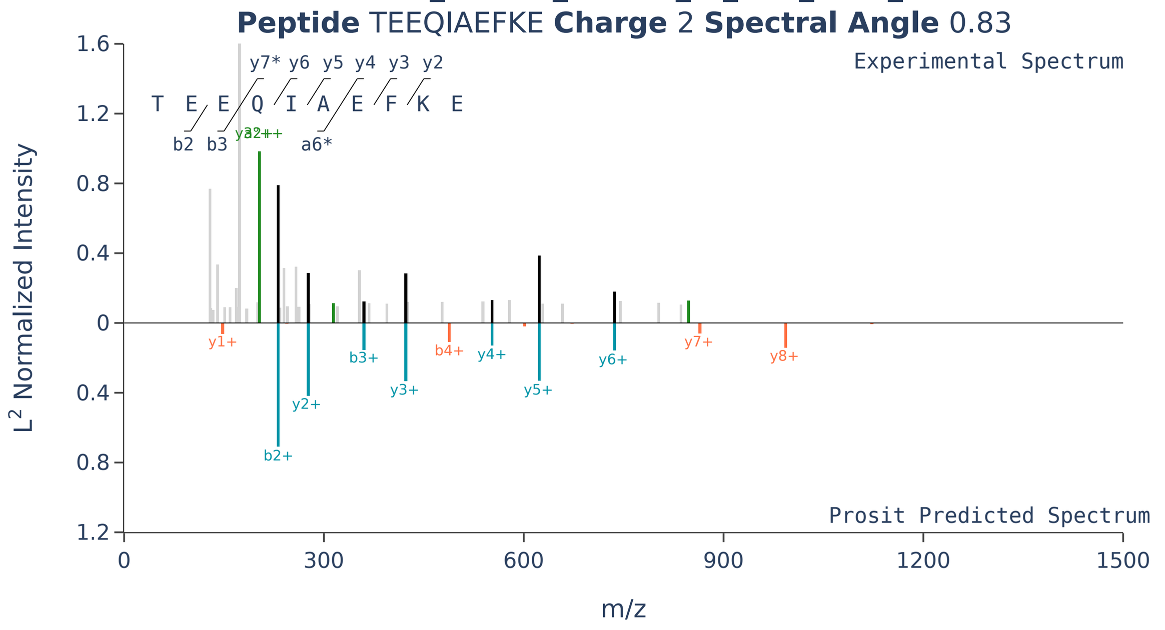


**Source** HRoetschke_291222_040123_Fu_CaM_b1R1_4h **Scan** 3614
**Peptide** MRSLGQNPTE **Charge** 2 **Spectral Angle** 0.83

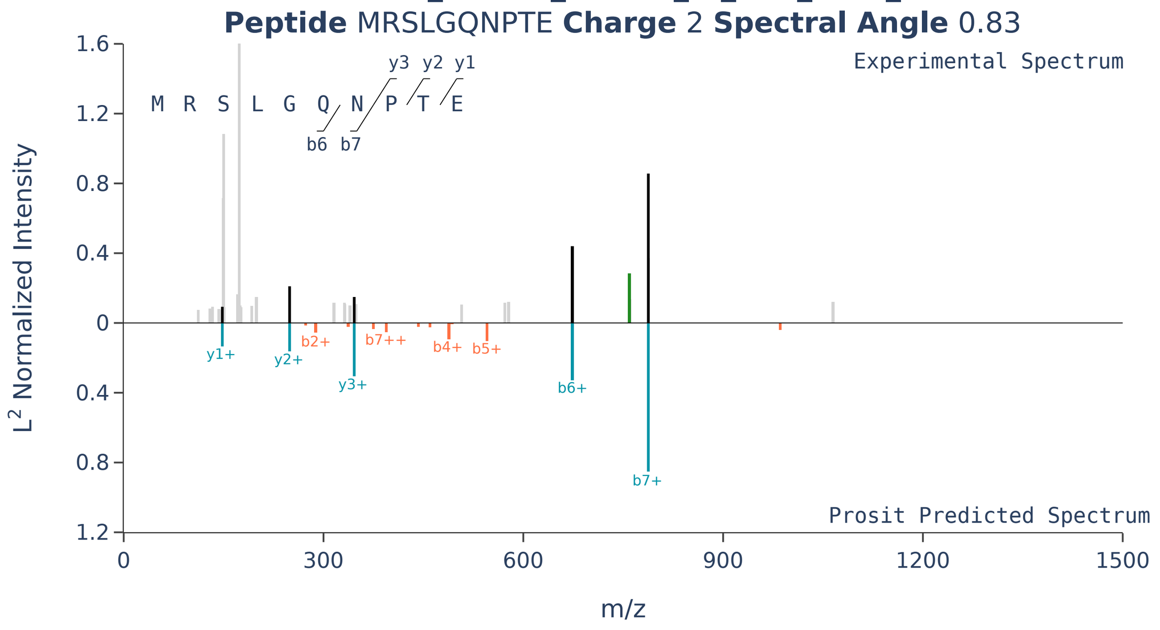


**Source** HRoetschke_291222_040123_Fu_CaM_b2R2_2h **Scan** 7457
**Peptide** EEVDEMIREAD **Charge** 2 **Spectral Angle** 0.9

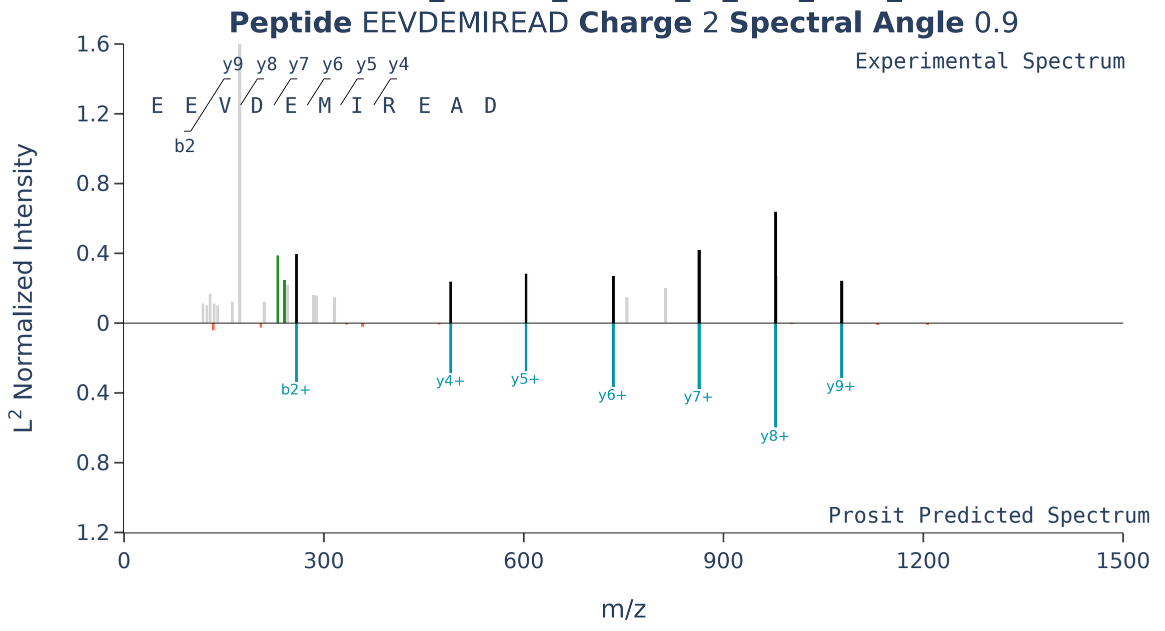


**Source** HRoetschke_291222_040123_Fu_CaM_b2R1_24h **Scan** 8739
**Peptide** LDGDGQVNYEEF **Charge** 2 **Spectral Angle** 0.93

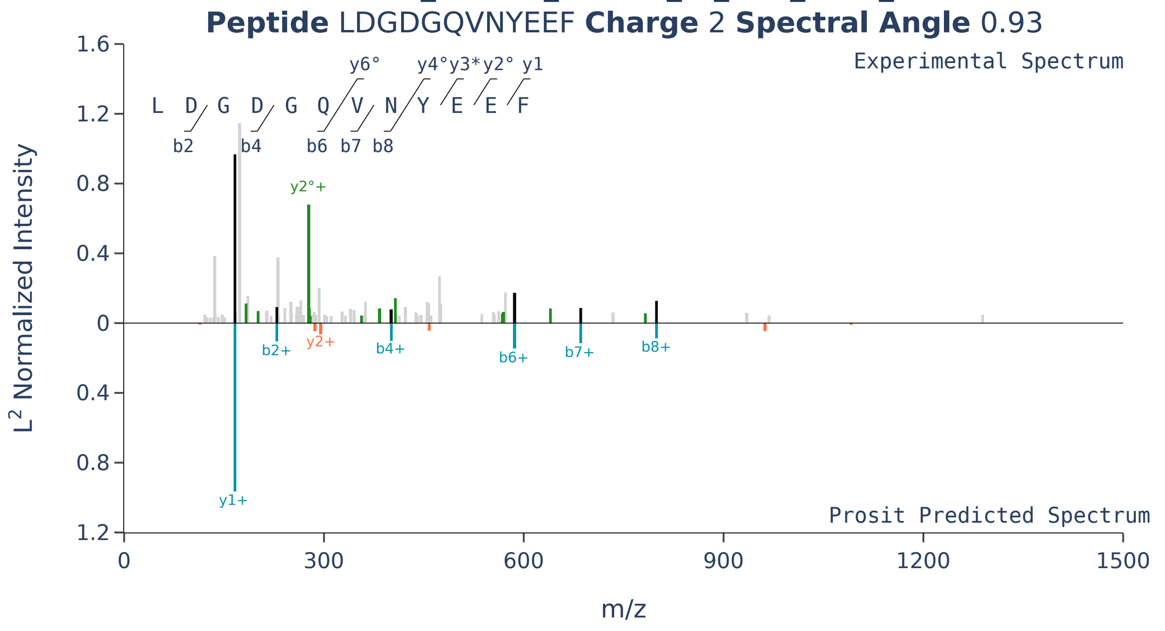


**Source** HRoetschke_291222_040123_Fu_CaM_b2R2_24h **Scan** 11131
**Peptide** TEEQIAEFKEAF **Charge** 2 **Spectral Angle** 0.86

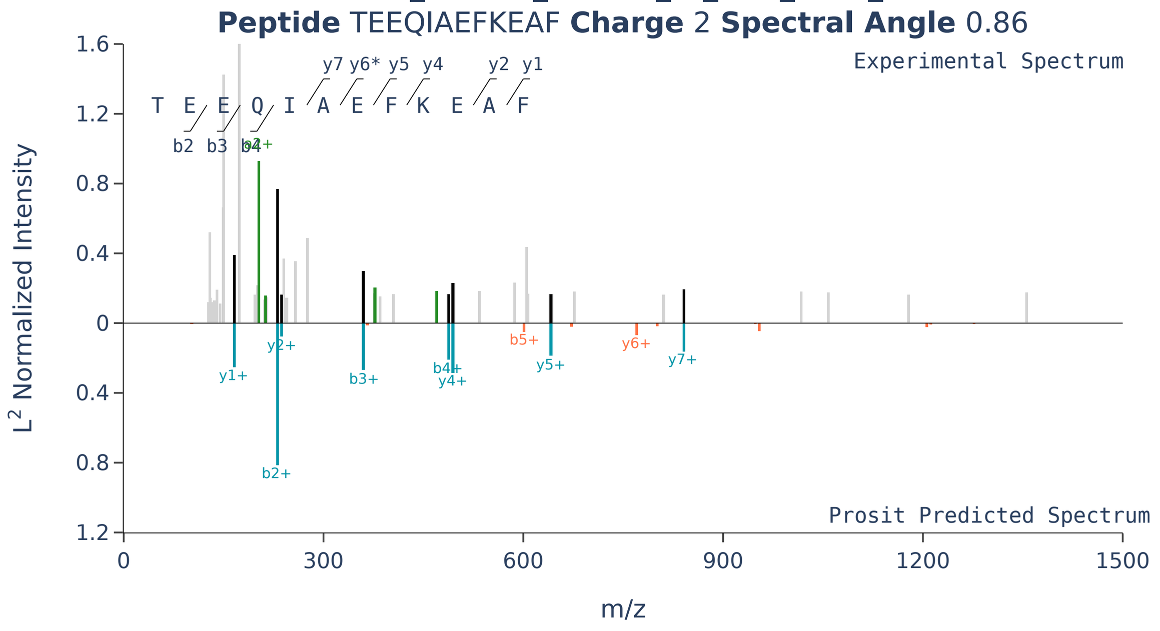


**Source** HRoetschke_291222_040123_Fu_CaM_b1R1_2h **Scan** 9919
**Peptide** READIDGDGQVNYEEFVQMMTAK **Charge** 3 **Spectral Angle** 0.81

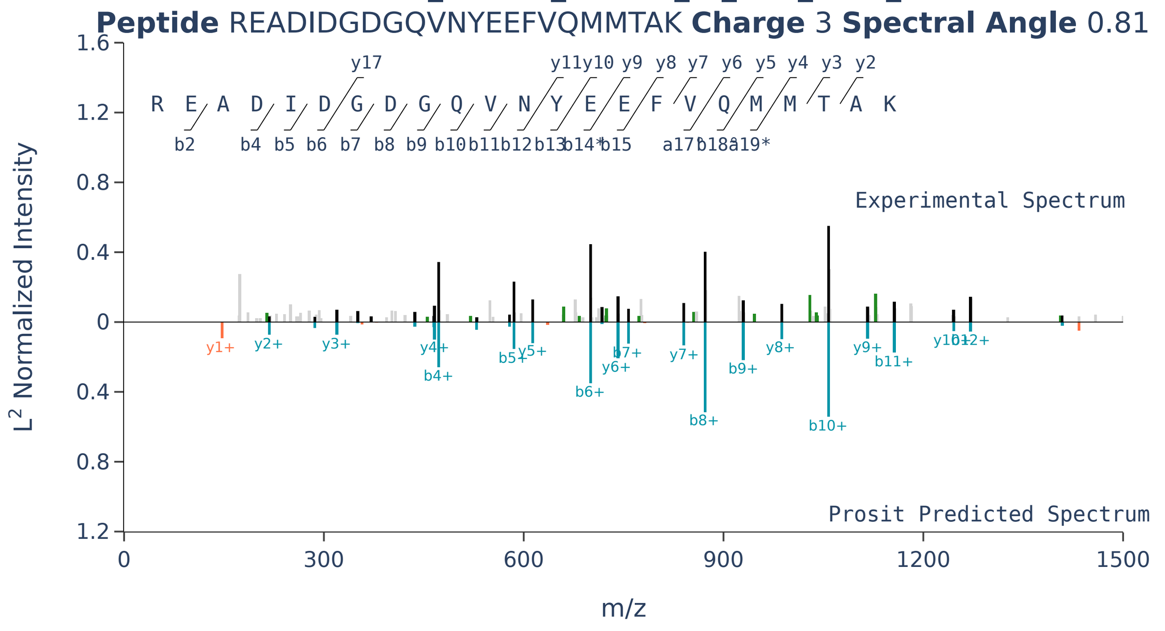


**Source** HRoetschke_291222_040123_Fu_CaM_b2R1_1h **Scan** 3927
**Peptide** NPTEAELQD **Charge** 2 **Spectral Angle** 0.75

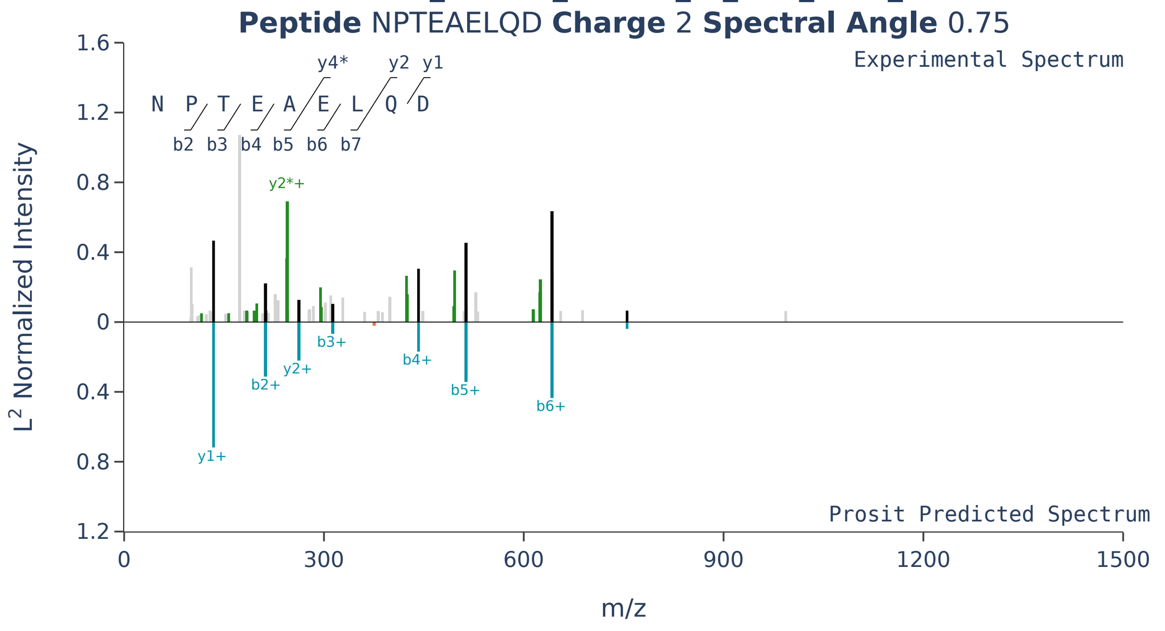


**Source** HRoetschke_291222_040123_Fu_CaM_b1R1_1h **Scan** 5282
**Peptide** SEEEIREAFR **Charge** 3 **Spectral Angle** 0.81

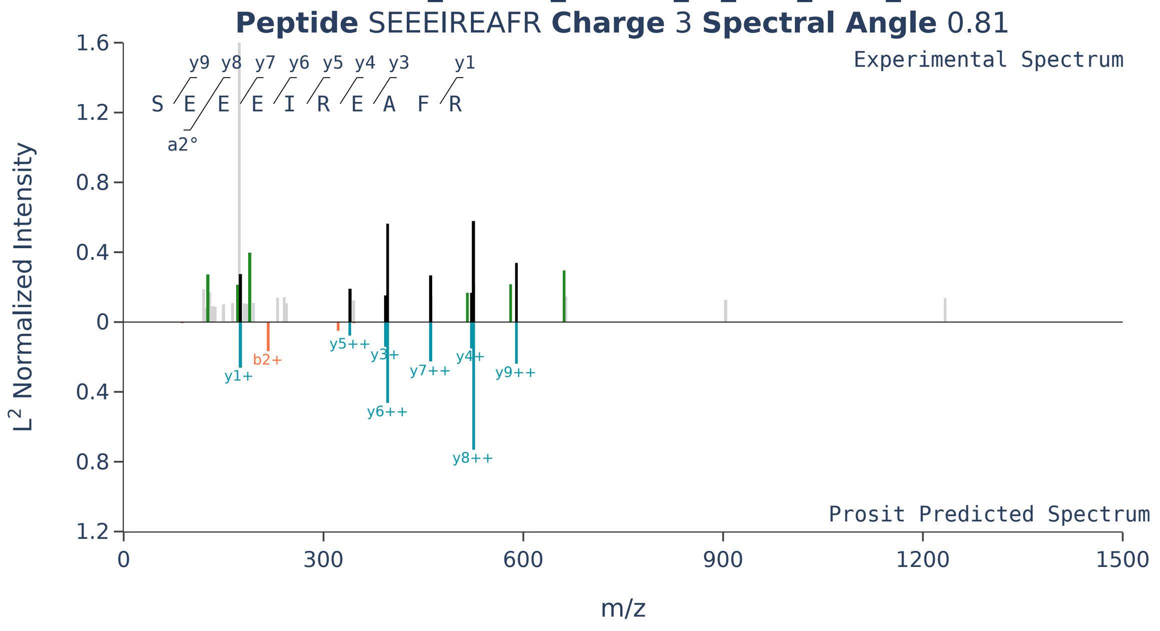


**Source** HRoetschke_291222_040123_Fu_CaM_b2R1_2h **Scan** 7364
**Peptide** TDSEEEIREAFR **Charge** 3 **Spectral Angle** 0.8

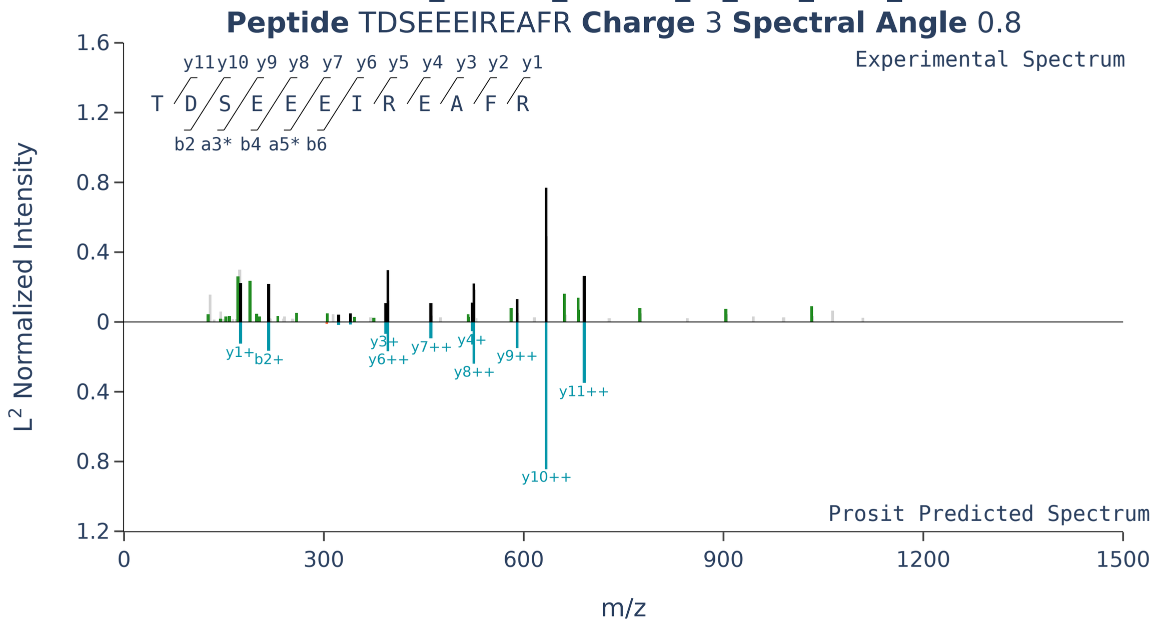


**Source** HRoetschke_291222_040123_Fu_CaM_b2R1_24h **Scan** 12929
**Peptide** ADIDGDGQVNYEEFVQMM **Charge** 2 **Spectral Angle** 0.88

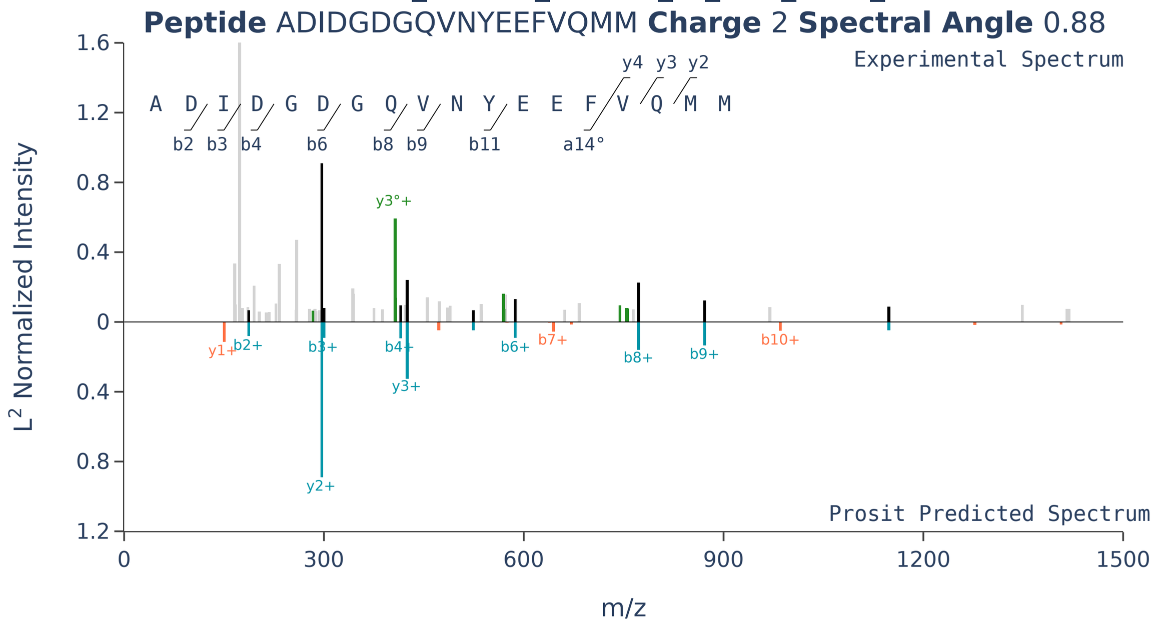


**Source** HRoetschke_291222_040123_Fu_CaM_b1R2_4h **Scan** 4603
**Peptide** QNPTEAELQD **Charge** 2 **Spectral Angle** 0.87

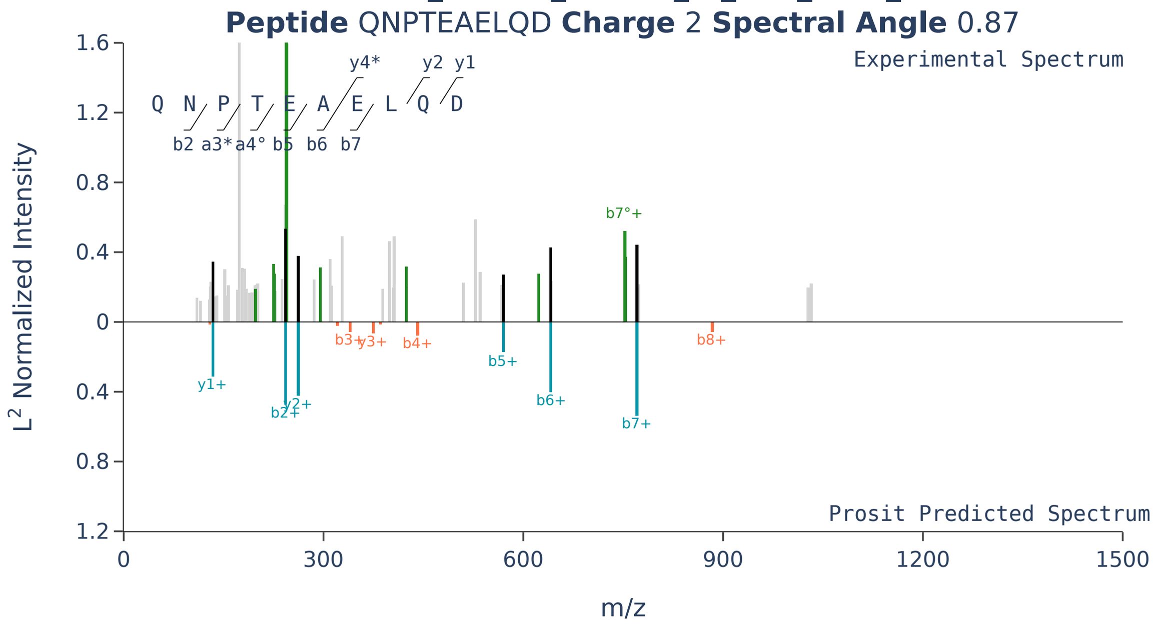

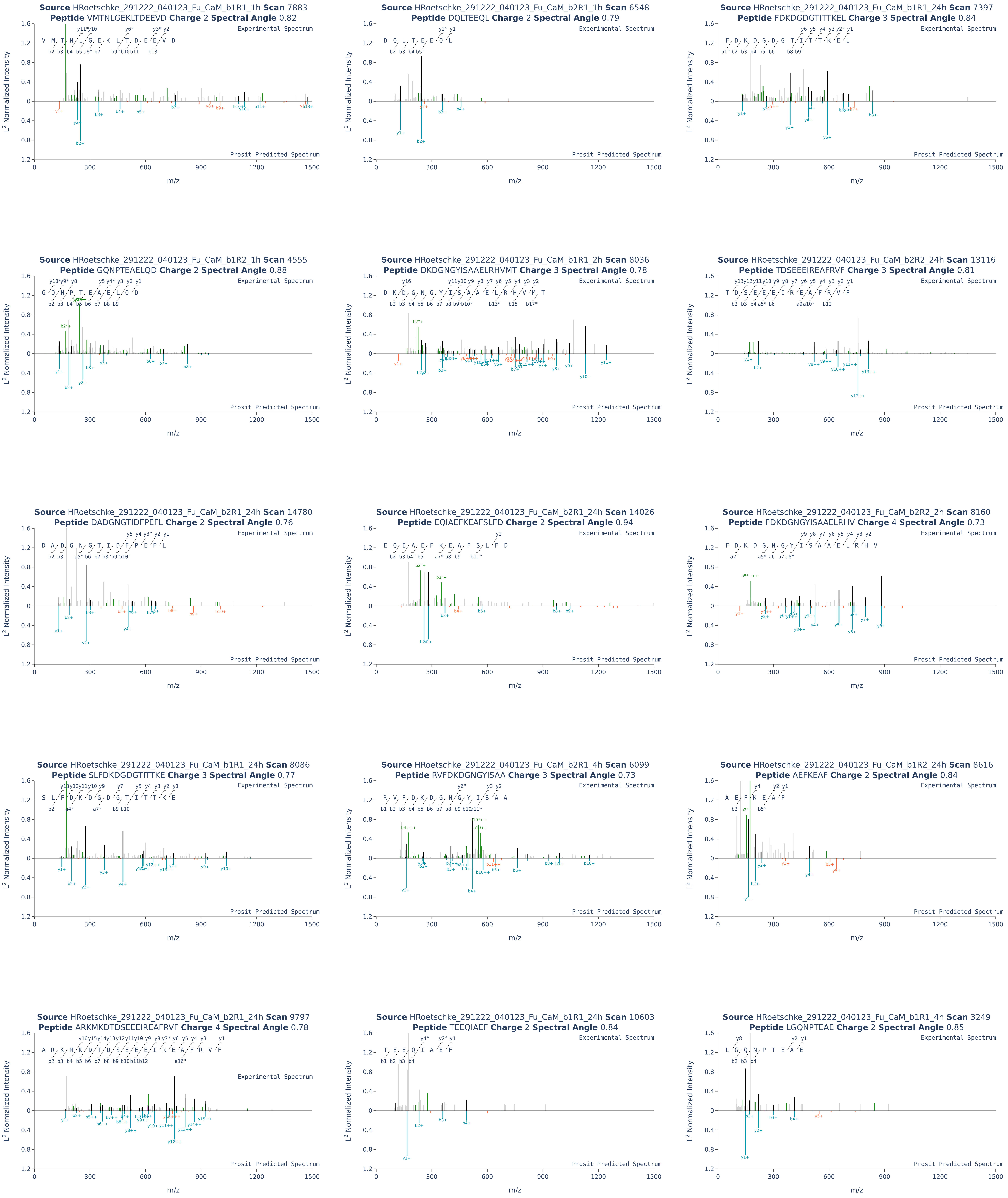
Source HRoetschke_291222_040123_Fu_CaM_b1R1_1h Scan 7883
Peptide VMTNLGEKLTDEEVD Charge 2 Spectral Angle 0.82
Source HRoetschke_291222_040123_Fu_CaM_b2R1_1h Scan 6548
Peptide DQLTEEQL Charge 2 Spectral Angle 0.79
Source HRoetschke_291222_040123_Fu_CaM_b1R1_24h Scan 7397
Peptide FDKDGDGTITTKEL Charge 3 Spectral Angle 0.84
Source HRoetschke_291222_040123_Fu_CaM_b1R2_1h Scan 4555
Peptide GQNPTEAELQD Charge 2 Spectral Angle 0.88
Source HRoetschke_291222_040123_Fu_CaM_b1R1_2h Scan 8036
Peptide DKDGNGYISAAELRHVMT Charge 3 Spectral Angle 0.78
Source HRoetschke_291222_040123_Fu_CaM_b2R2_24h Scan 13116
Peptide TDSEEEIREAFRVF Charge 3 Spectral Angle 0.81
Source HRoetschke_291222_040123_Fu_CaM_b2R1_24h Scan 14780
Peptide DADGNGTIDFPEFL Charge 2 Spectral Angle 0.76
Source HRoetschke_291222_040123_Fu_CaM_b2R1_24h Scan 14026
Peptide EQIAEFKEAFSLFD Charge 2 Spectral Angle 0.94
Source HRoetschke_291222_040123_Fu_CaM_b2R2_2h Scan 8160
Peptide FDKDGNGYISAAELRHV Charge 4 Spectral Angle 0.73
Source HRoetschke_291222_040123_Fu_CaM_b1R1_24h Scan 8086
Peptide SLFDKDGDGTITTKE Charge 3 Spectral Angle 0.77
Source HRoetschke_291222_040123_Fu_CaM_b2R1_4h Scan 6099
Peptide RVFDKDGNGYISAA Charge 3 Spectral Angle 0.73
Source HRoetschke_291222_040123_Fu_CaM_b1R2_24h Scan 8616
Peptide AEFKEAF Charge 2 Spectral Angle 0.84
Source HRoetschke_291222_040123_Fu_CaM_b2R1_24h Scan 9797
Peptide ARKMKDTDSEEEIREAFRVF Charge 4 Spectral Angle 0.78
Source HRoetschke_291222_040123_Fu_CaM_b1R1_24h Scan 10603
Peptide TEEQIAEF Charge 2 Spectral Angle 0.84
Source HRoetschke_291222_040123_Fu_CaM_b1R1_4h Scan 3249
Peptide LGQNPTEAE Charge 2 Spectral Angle 0.85
Experimental Spectrum
Prosit Predicted Spectrum
L² Normalized Intensity
m/z

**Source** HRoetschke_291222_040123_Fu_CaM_b1R1_24h **Scan** 3340
**Peptide** DTDSEEEIRE **Charge** 2 **Spectral Angle** 0.77

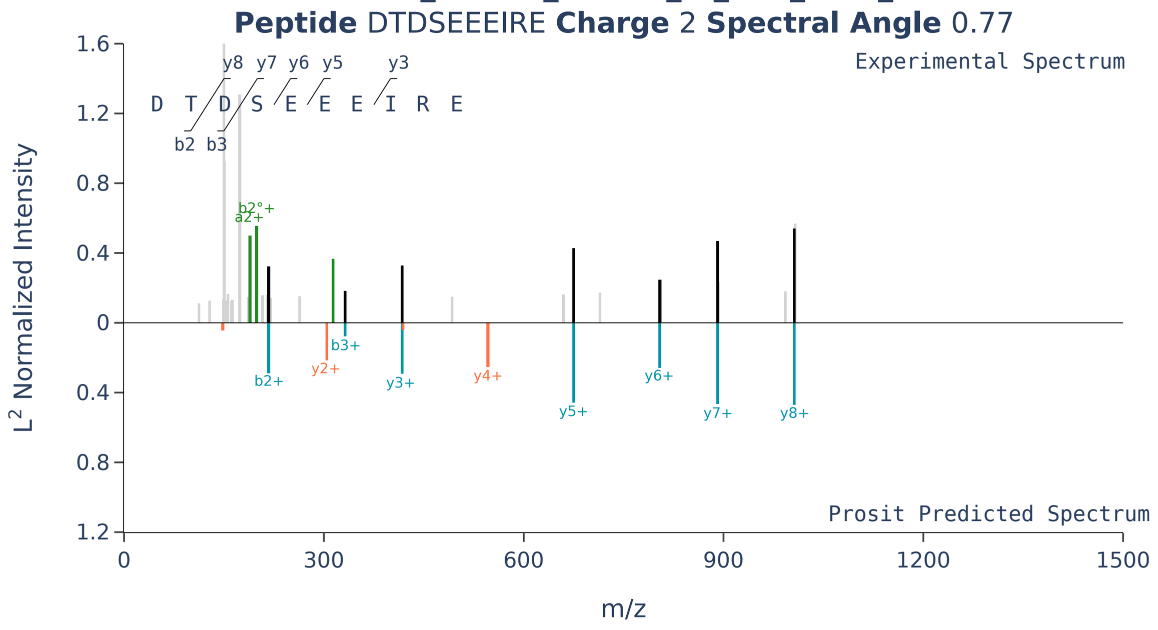


**Source** HRoetschke_291222_040123_Fu_CaM_b1R2_2h **Scan** 7140
**Peptide** REAFRVFDKDGNGY **Charge** 3 **Spectral Angle** 0.85

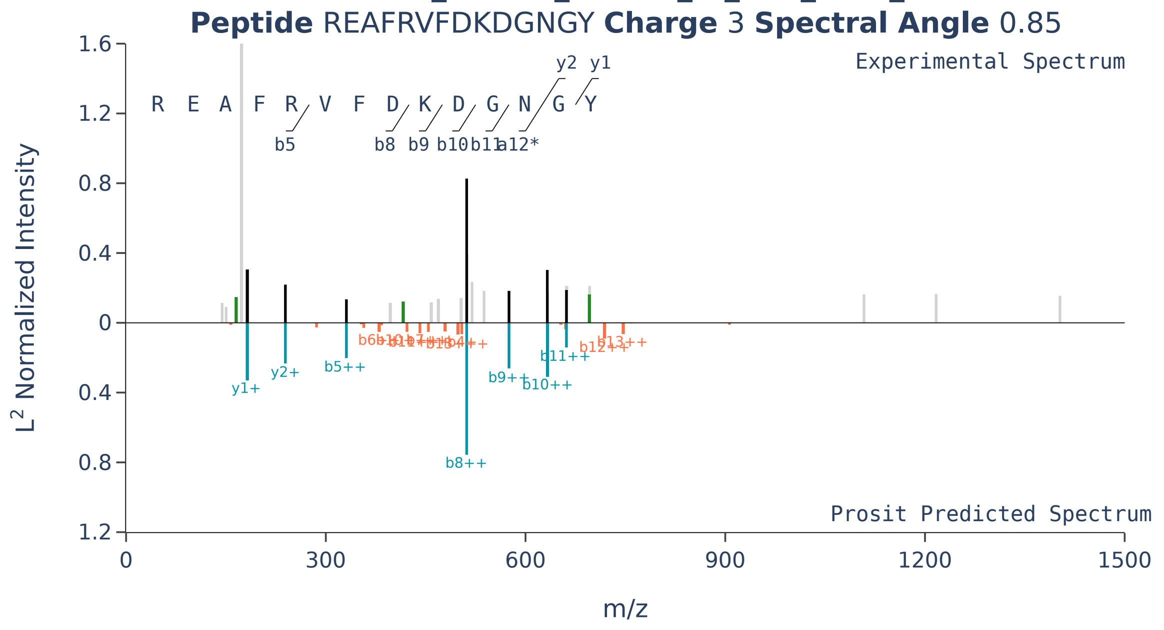


**Source** HRoetschke_291222_040123_Fu_CaM_b2R2_24h **Scan** 8943
**Peptide** AELRHVMTNLGEKLTD **Charge** 3 **Spectral Angle** 0.75

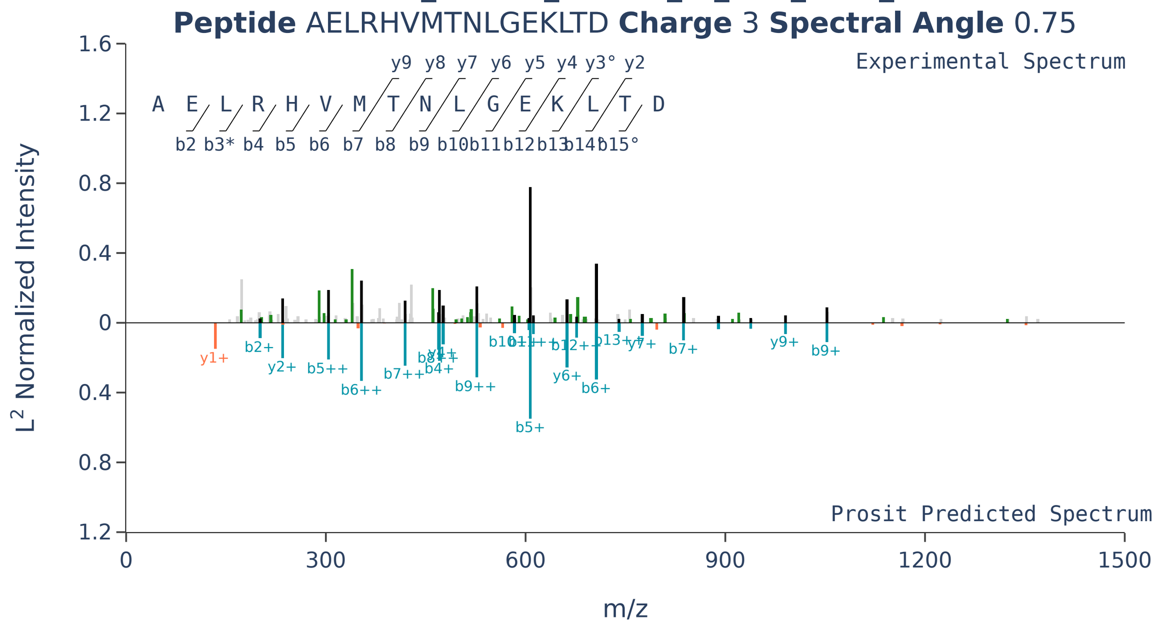


**Source** HRoetschke_291222_040123_Fu_CaM_b1R1_4h **Scan** 9143
**Peptide** AELRHVMTNLGEKL **Charge** 3 **Spectral Angle** 0.73

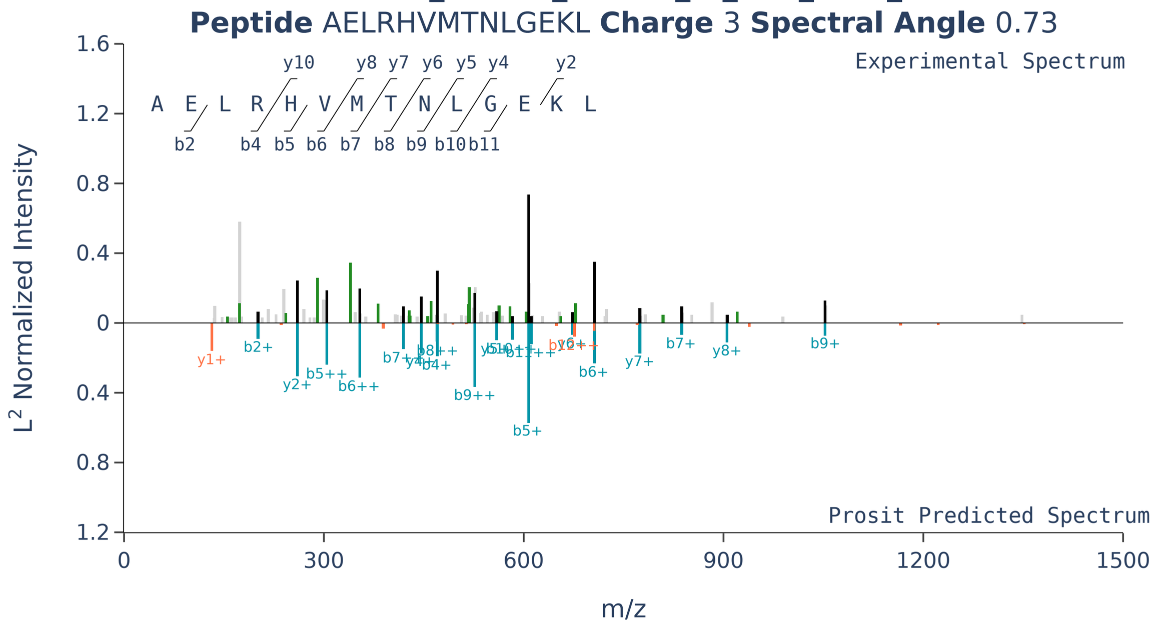


**Source** HRoetschke_291222_040123_Fu_CaM_b1R1_2h **Scan** 11889
**Peptide** ADIDGDGQVNYEEFV **Charge** 2 **Spectral Angle** 0.81

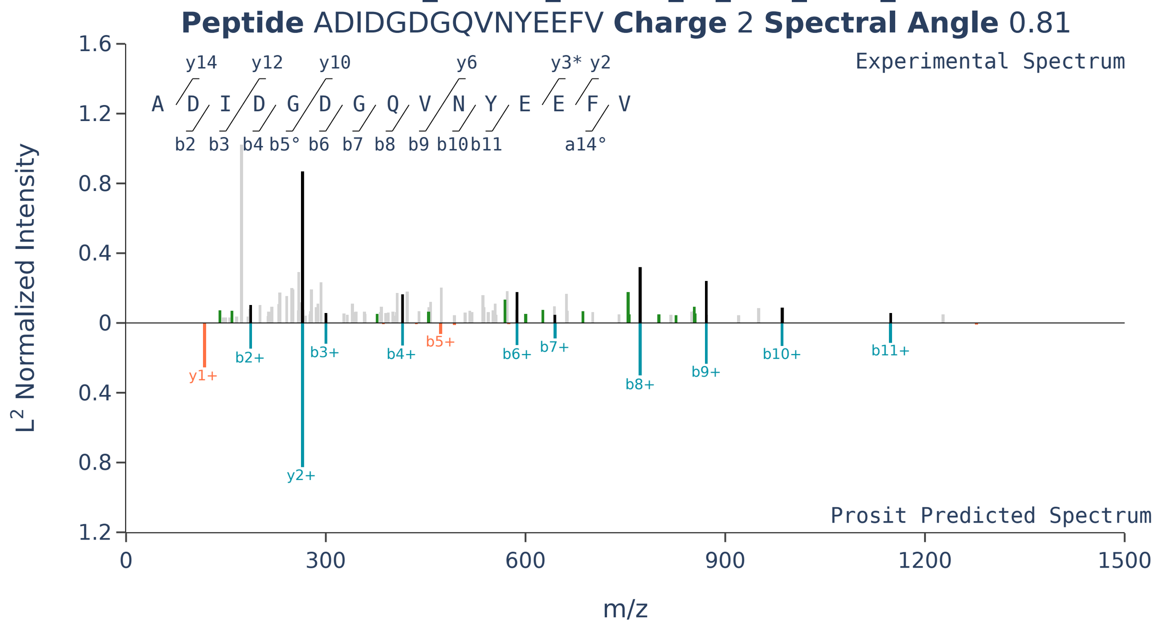


**Source** HRoetschke_291222_040123_Fu_CaM_b2R2_24h **Scan** 12386
**Peptide** SEEEIREAFRVFDKDGNGYISA **Charge** 4 **Spectral Angle** 0.64

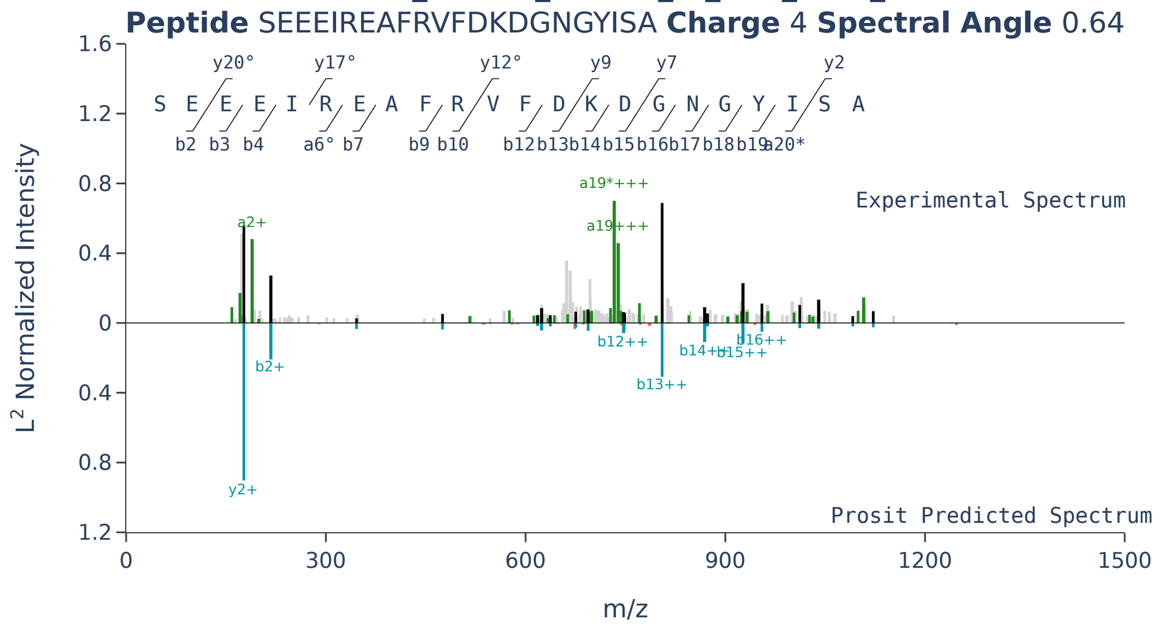


**Source** HRoetschke_291222_040123_Fu_CaM_b1R1_24h **Scan** 10911
**Peptide** SLFDKDGDGTITTKELGT **Charge** 3 **Spectral Angle** 0.7

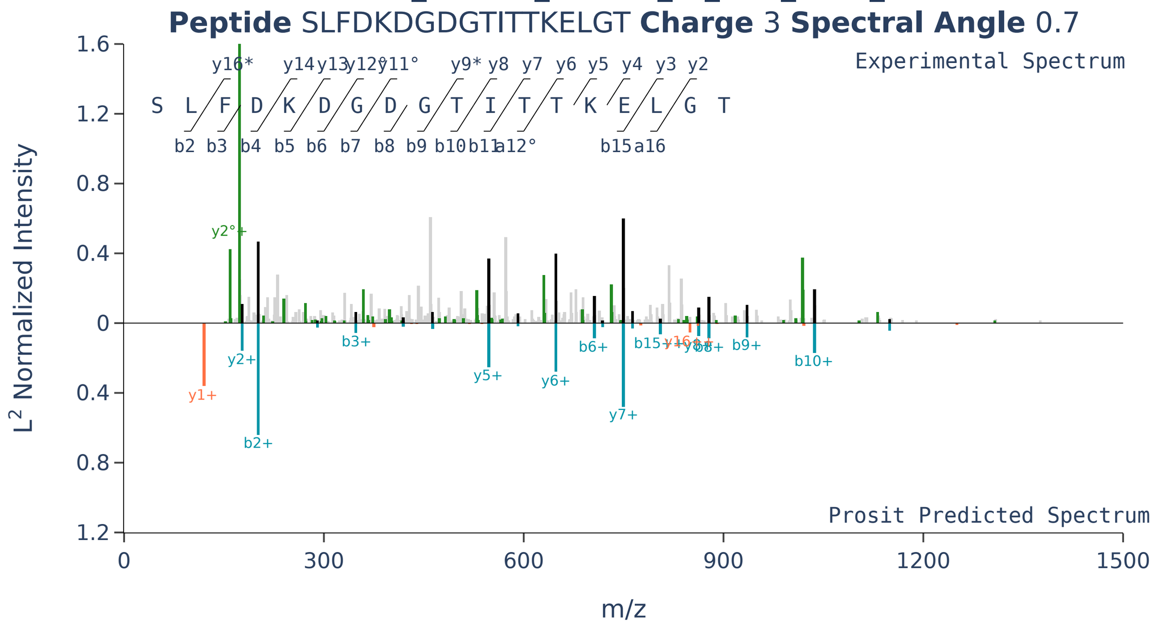


**Source** HRoetschke_291222_040123_Fu_CaM_b1R1_4h **Scan** 4930
**Peptide** NLGEKLTDE **Charge** 2 **Spectral Angle** 0.81

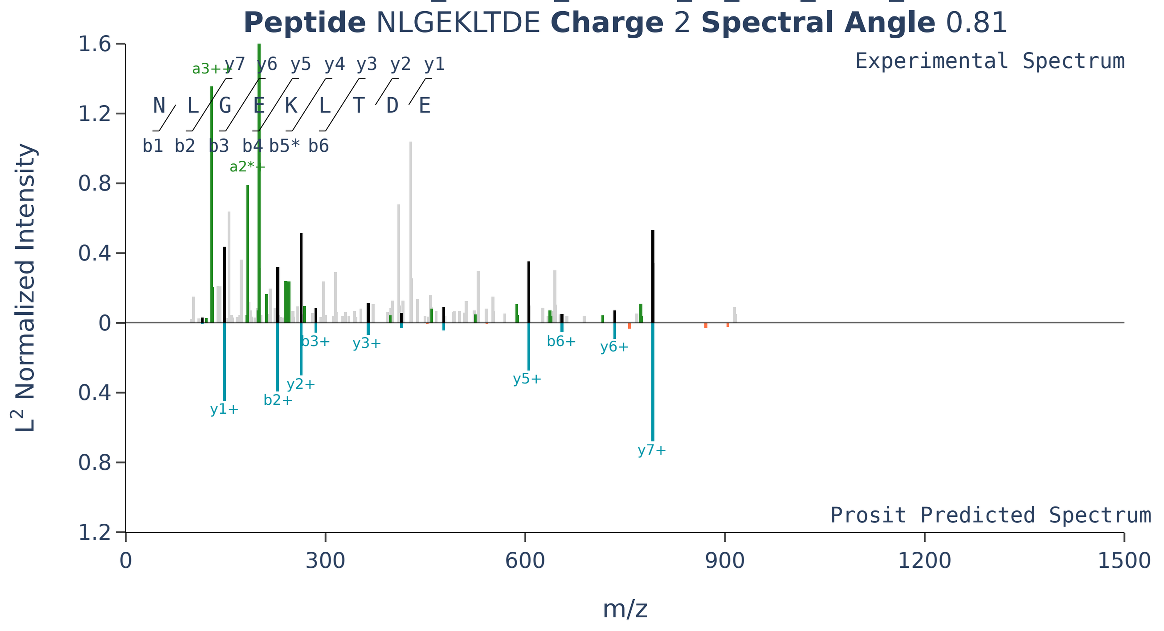


**Source** HRoetschke_291222_040123_Fu_CaM_b1R2_1h **Scan** 3089
**Peptide** KDTDSEEEL **Charge** 2 **Spectral Angle** 0.85

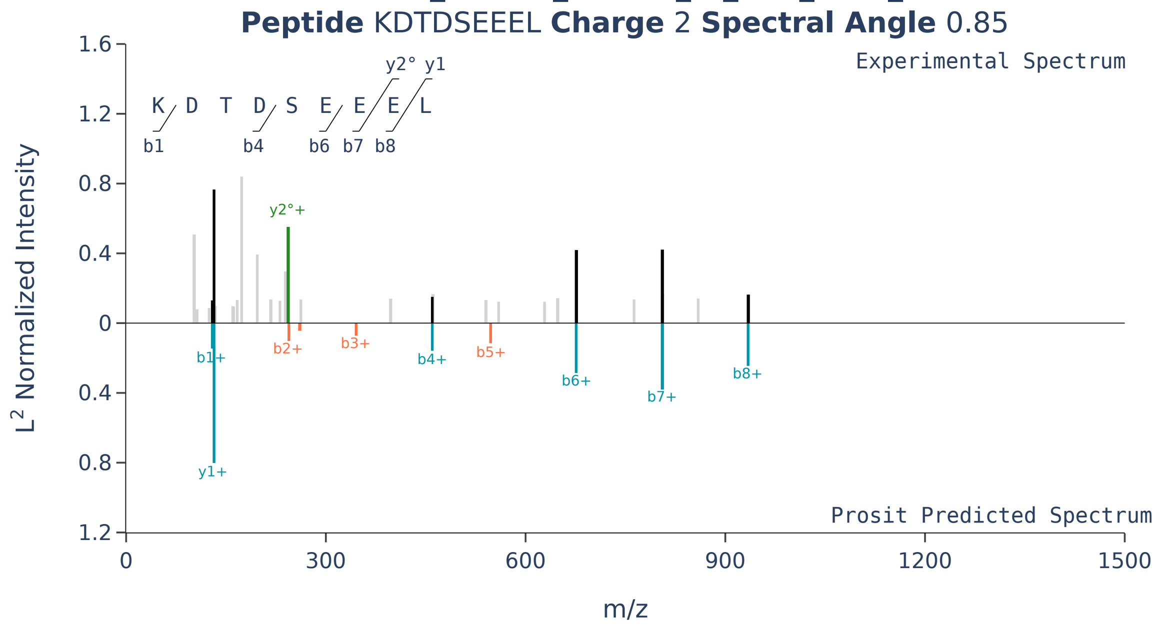


**Source** HRoetschke_291222_040123_Fu_CaM_b1R1_2h **Scan** 6697
**Peptide** EEQIAEFKEA **Charge** 2 **Spectral Angle** 0.82

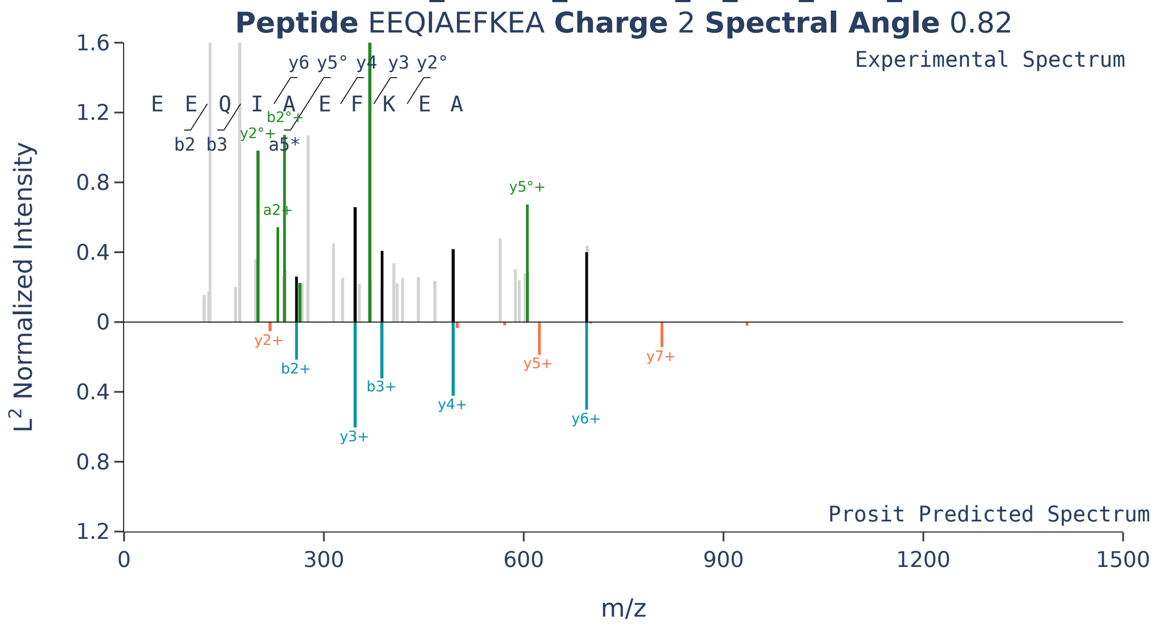


**Source** HRoetschke_291222_040123_Fu_CaM_b2R1_24h **Scan** 10190
**Peptide** ADIDGDGQVNYEEFVQM **Charge** 2 **Spectral Angle** 0.76

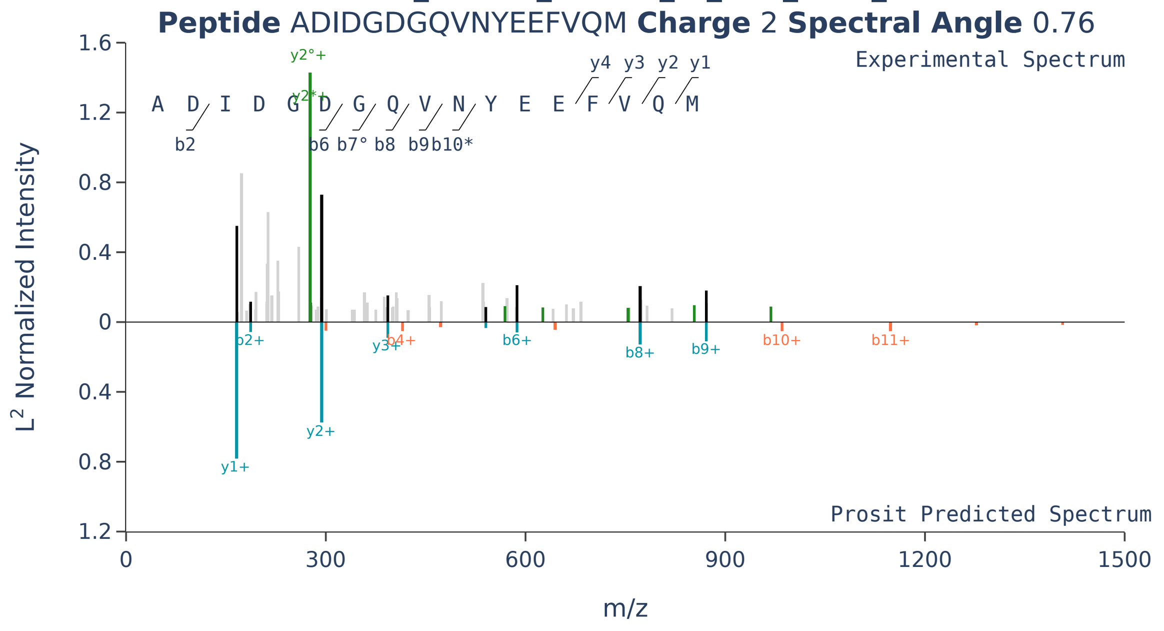


**Source** HRoetschke_291222_040123_Fu_CaM_b2R2_24h **Scan** 10415
**Peptide** READIDGDGQVNYEEFVQM **Charge** 2 **Spectral Angle** 0.72

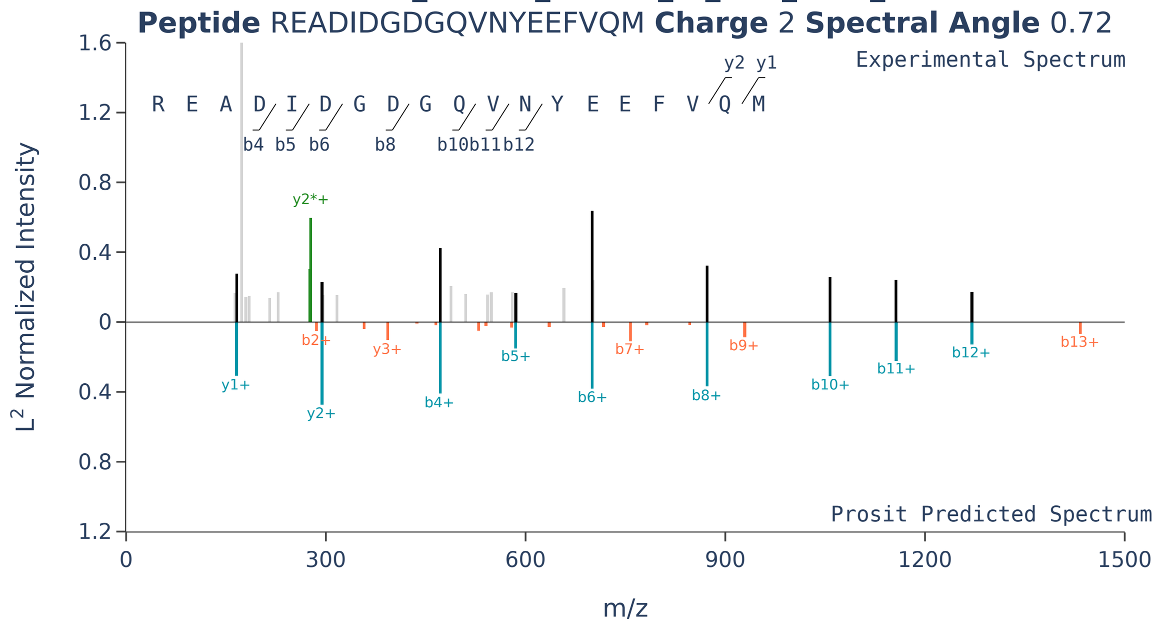


**Source** HRoetschke_291222_040123_Fu_CaM_b1R1_24h **Scan** 7321
**Peptide** KDGDGTITTKELGTVM **Charge** 3 **Spectral Angle** 0.77

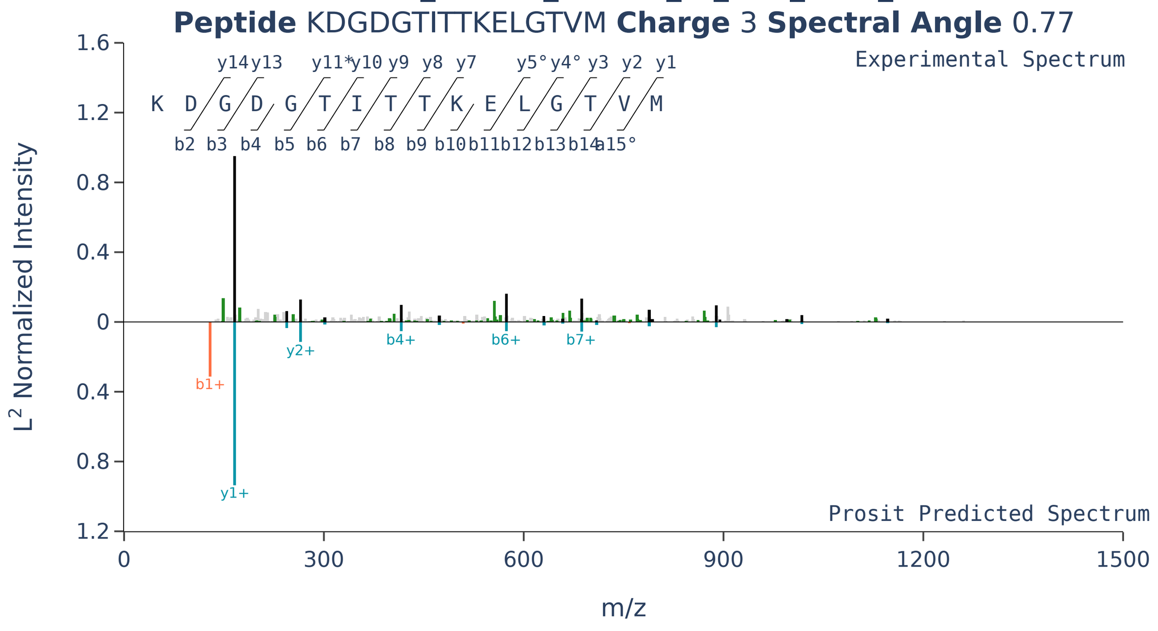


**Source** HRoetschke_291222_040123_Fu_CaM_b2R1_24h **Scan** 9684
**Peptide** RKMKDTDSEEEIREAFRVF **Charge** 4 **Spectral Angle** 0.78

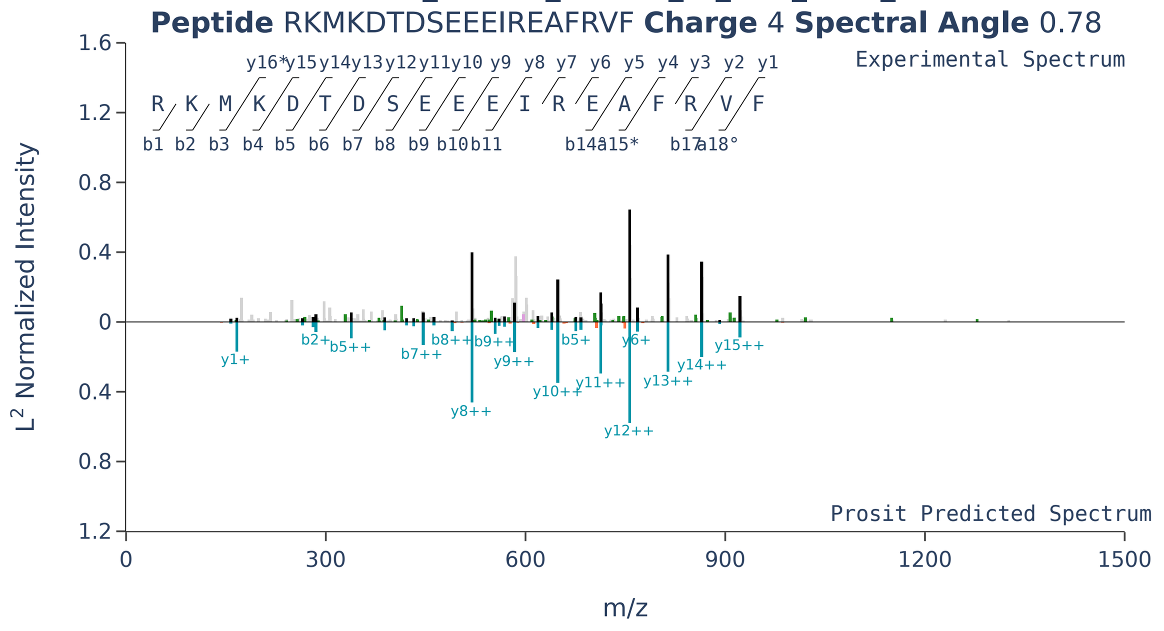


**Source** HRoetschke_291222_040123_Fu_CaM_b1R1_24h **Scan** 12317
**Peptide** AEFKEAFSL **Charge** 2 **Spectral Angle** 0.82

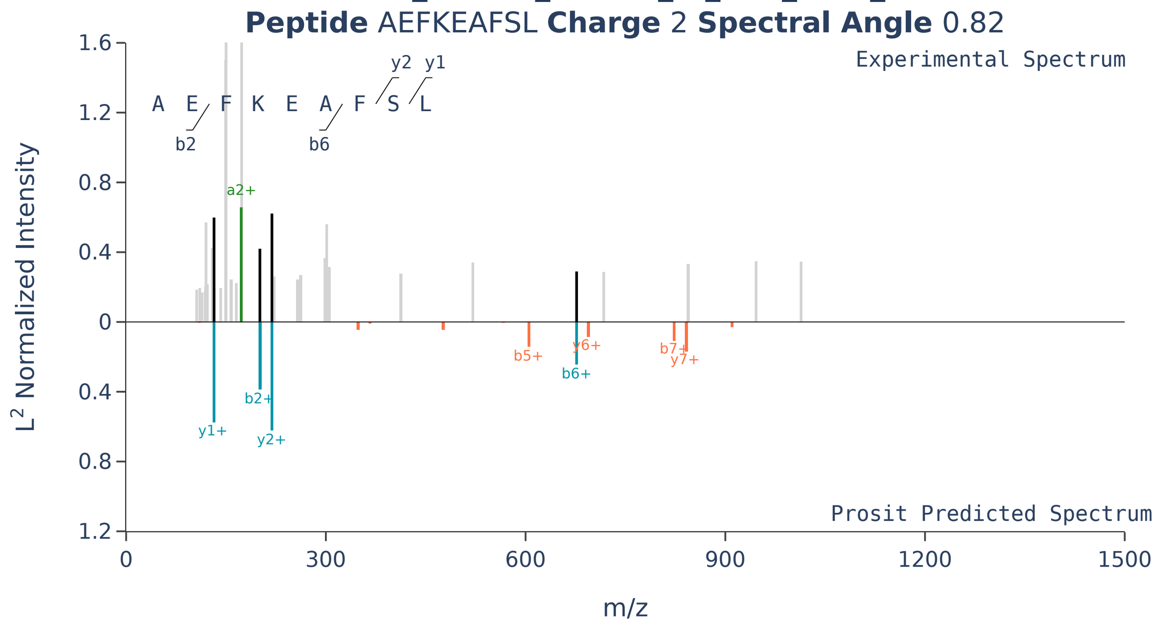

**Source** HRoetschke_291222_040123_Fu_CaM_b2R1_24h **Scan** 9257
**Peptide** READIDGDGQVNYEEFVQMM **Charge** 3 **Spectral Angle** 0.65

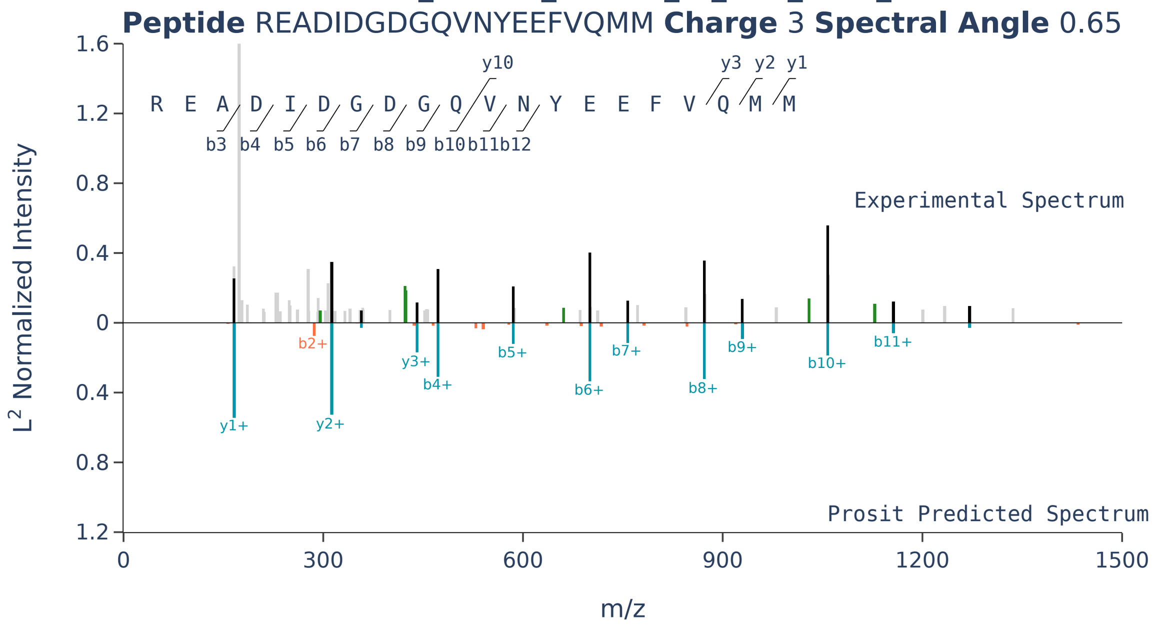


**Source** HRoetschke_291222_040123_Fu_CaM_b1R1_2h **Scan** 11891
**Peptide** KDTDSEEEIREAFRVF **Charge** 4 **Spectral Angle** 0.77

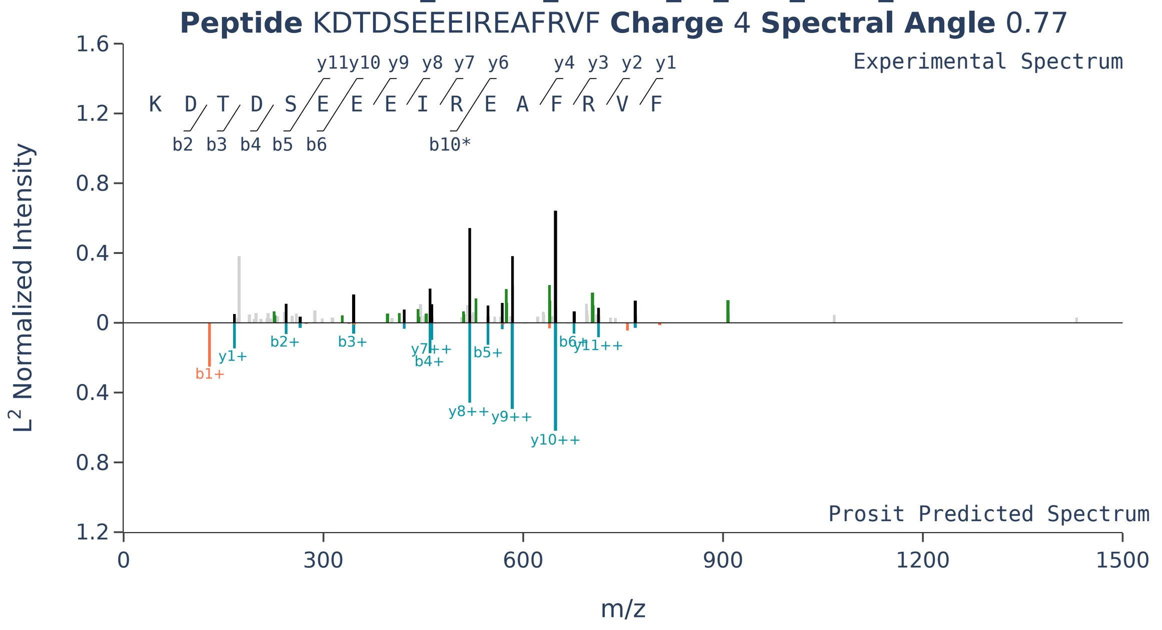


**Source** HRoetschke_291222_040123_Fu_CaM_b2R1_4h **Scan** 6399
**Peptide** FDKDGDGTITTKELGTVMR **Charge** 4 **Spectral Angle** 0.71

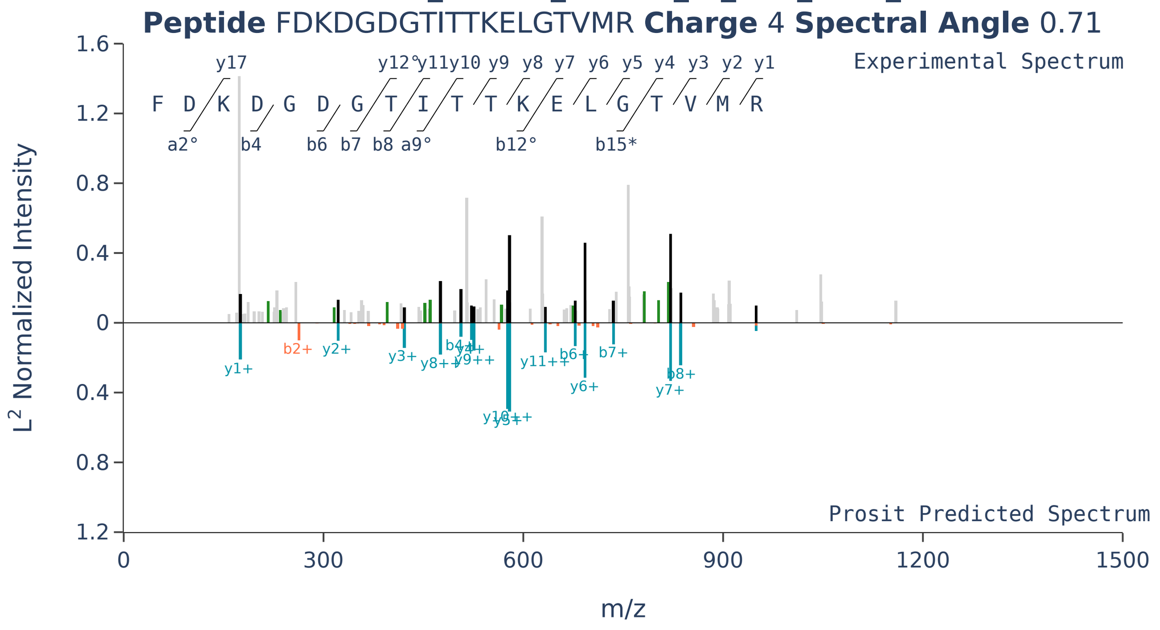


**Source** HRoetschke_291222_040123_Fu_CaM_b1R1_4h **Scan** 9037
**Peptide** RVFDKDGNGYISAAELRHV **Charge** 4 **Spectral Angle** 0.82

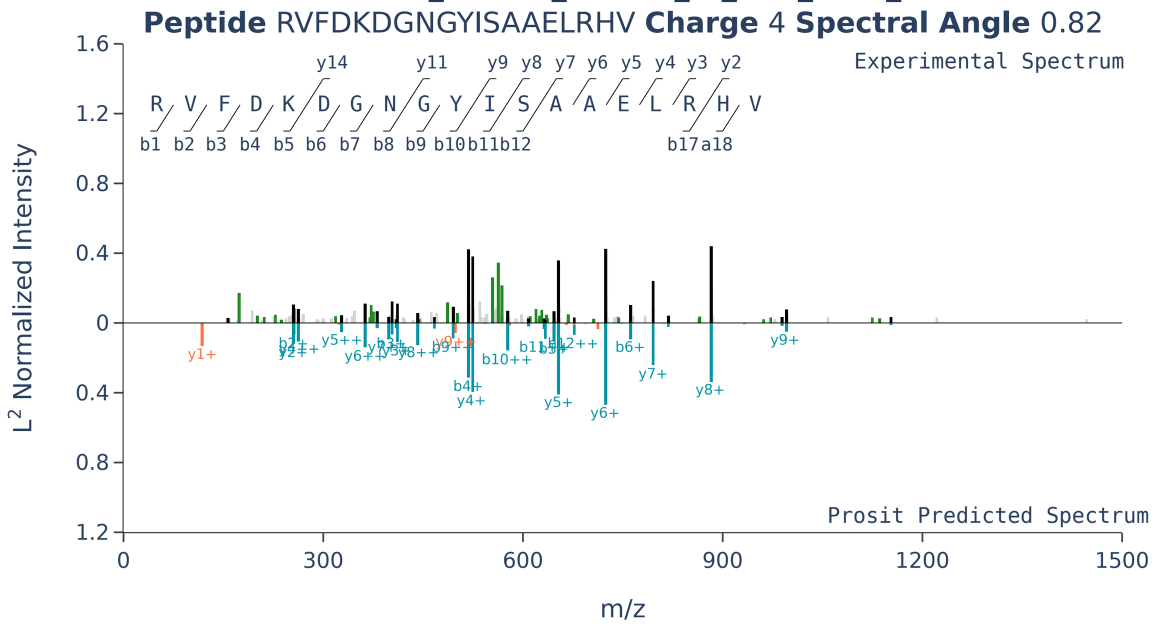


**Source** HRoetschke_291222_040123_Fu_CaM_b2R2_2h **Scan** 5148
**Peptide** SLFDKDGD **Charge** 2 **Spectral Angle** 0.87

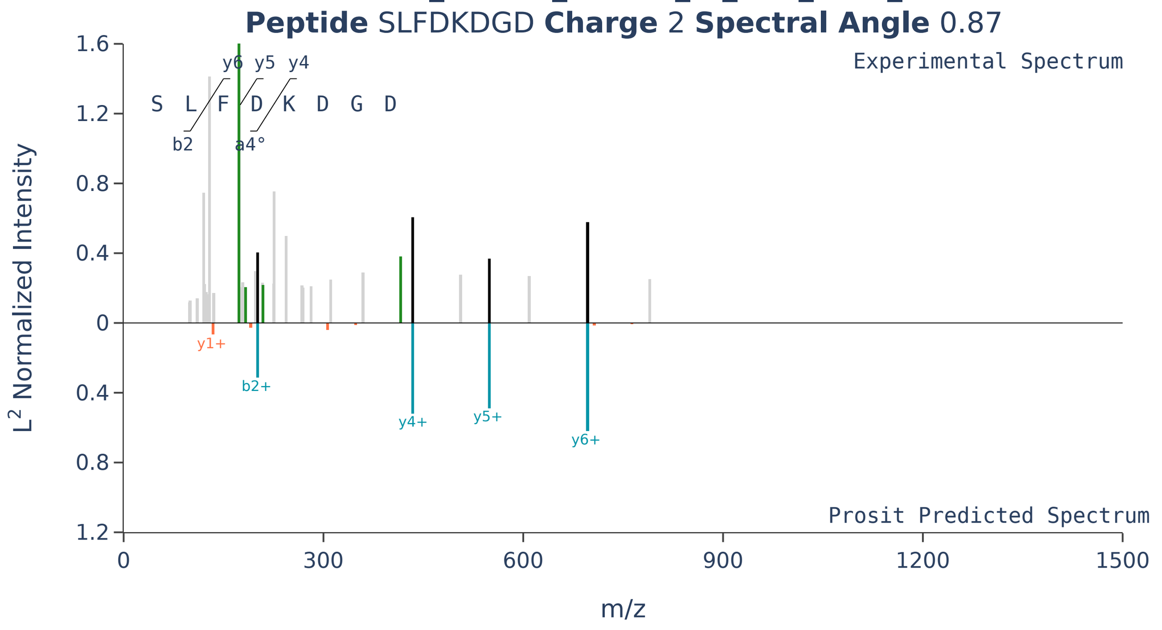


**Source** HRoetschke_291222_040123_Fu_CaM_b1R1_24h **Scan** 5665
**Peptide** RKMKDTDSEEEIREAFR **Charge** 4 **Spectral Angle** 0.8

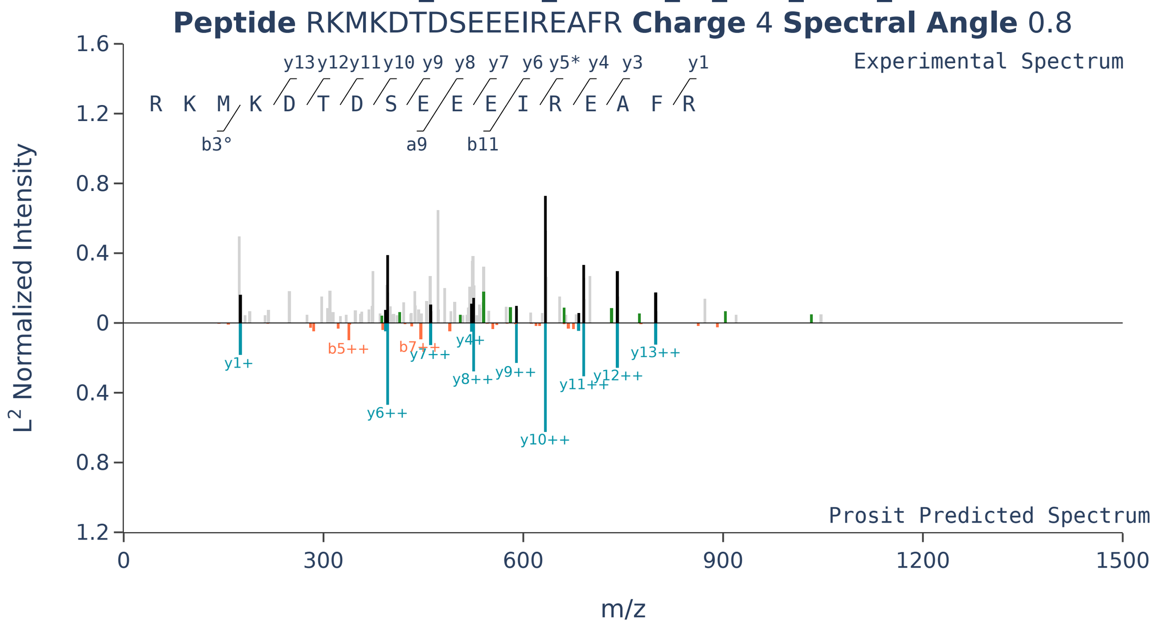


**Source** HRoetschke_291222_040123_Fu_CaM_b1R1_24h **Scan** 1933
**Peptide** RKMKDTDSEEEIRE **Charge** 4 **Spectral Angle** 0.8

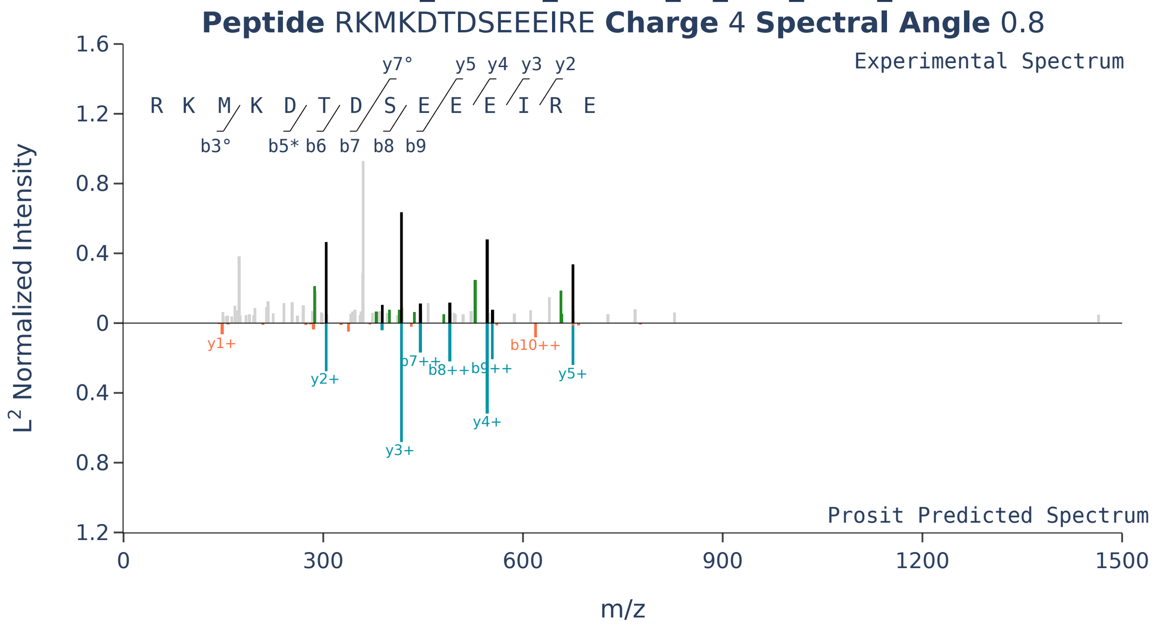


**Source** HRoetschke_291222_040123_Fu_CaM_b1R1_2h **Scan** 9424
**Peptide** AELQDMINE **Charge** 2 **Spectral Angle** 0.75

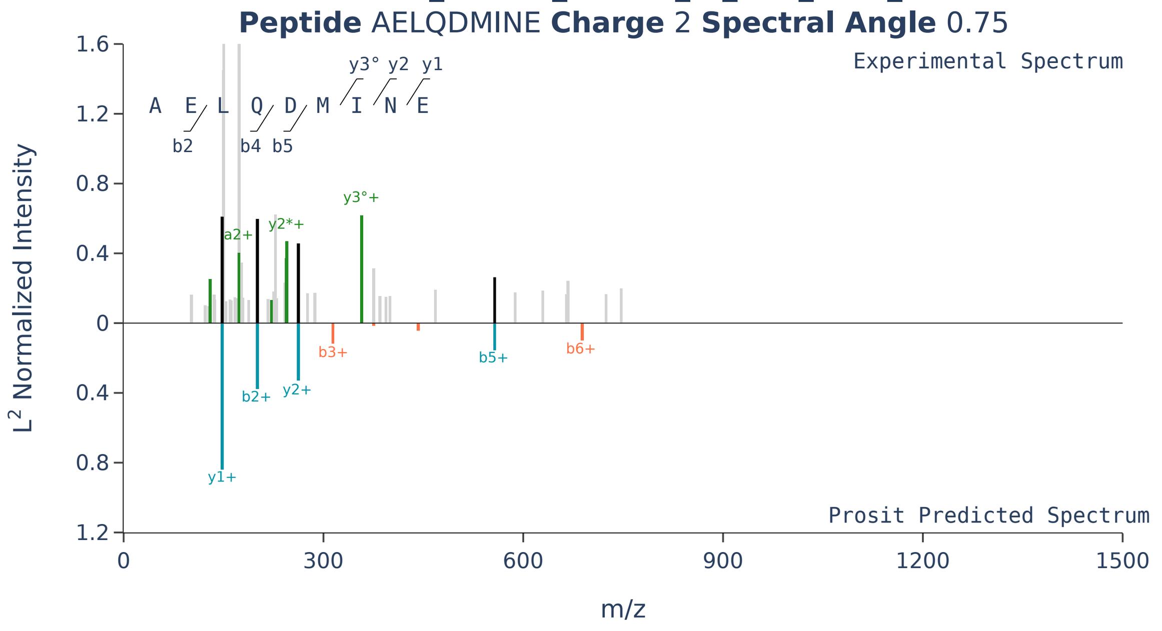


**Source** HRoetschke_291222_040123_Fu_CaM_b2R1_2h **Scan** 9647
**Peptide** ADQLTEEQIAEFKEA **Charge** 3 **Spectral Angle** 0.75

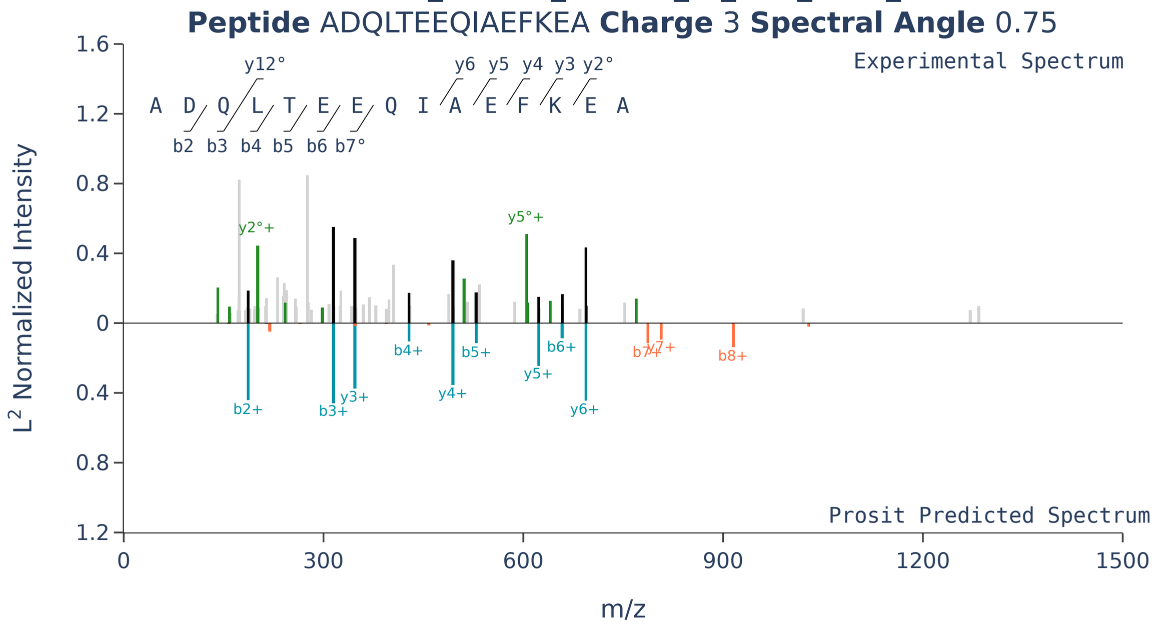


**Source** HRoetschke_291222_040123_Fu_CaM_b1R1_4h **Scan** 5280
**Peptide** QIAEFKE **Charge** 2 **Spectral Angle** 0.76

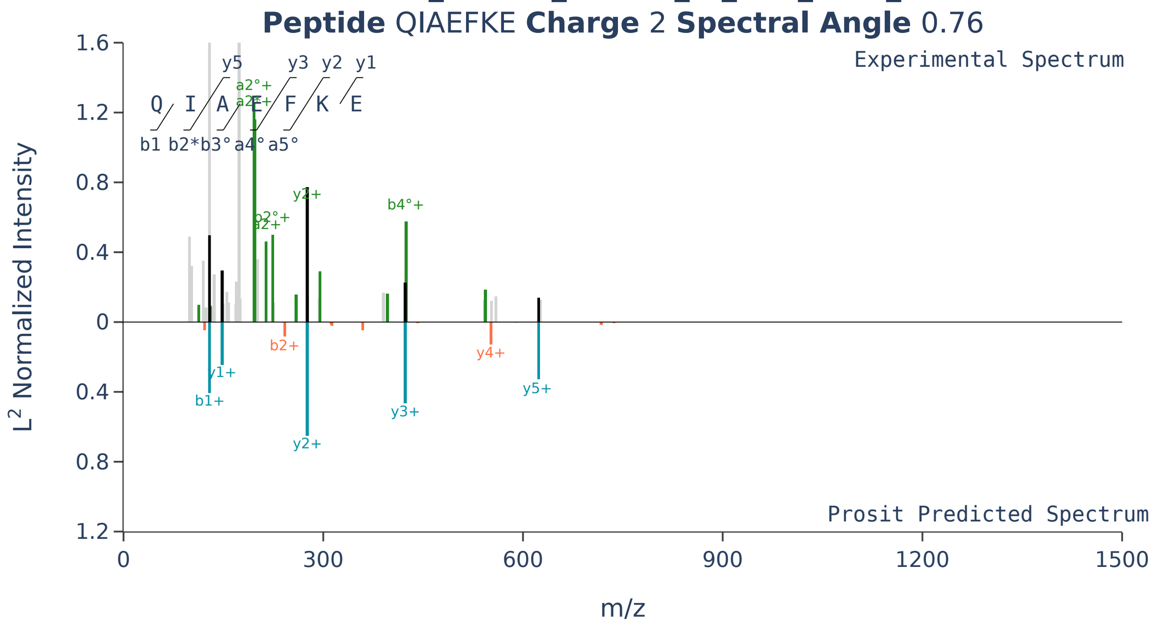


**Source** HRoetschke_291222_040123_Fu_CaM_b2R1_24h **Scan** 14476
**Peptide** ADGNGTIDFPEFL **Charge** 2 **Spectral Angle** 0.74

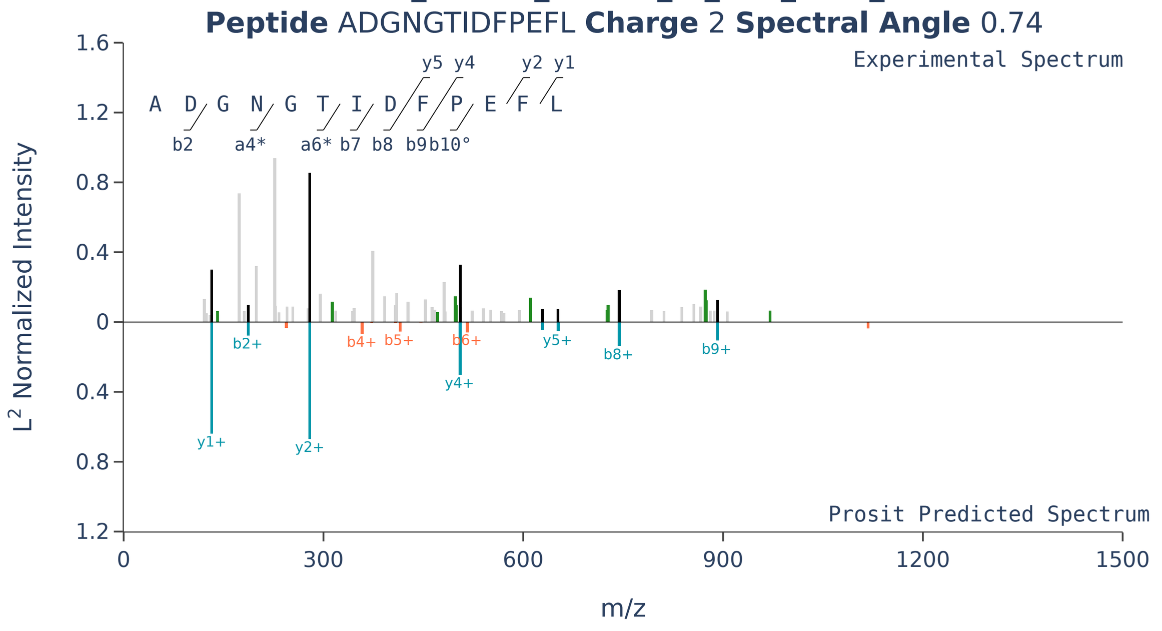


**Source** HRoetschke_291222_040123_Fu_CaM_b1R2_4h **Scan** 10552
**Peptide** TDSEEEIREAFRV **Charge** 3 **Spectral Angle** 0.79

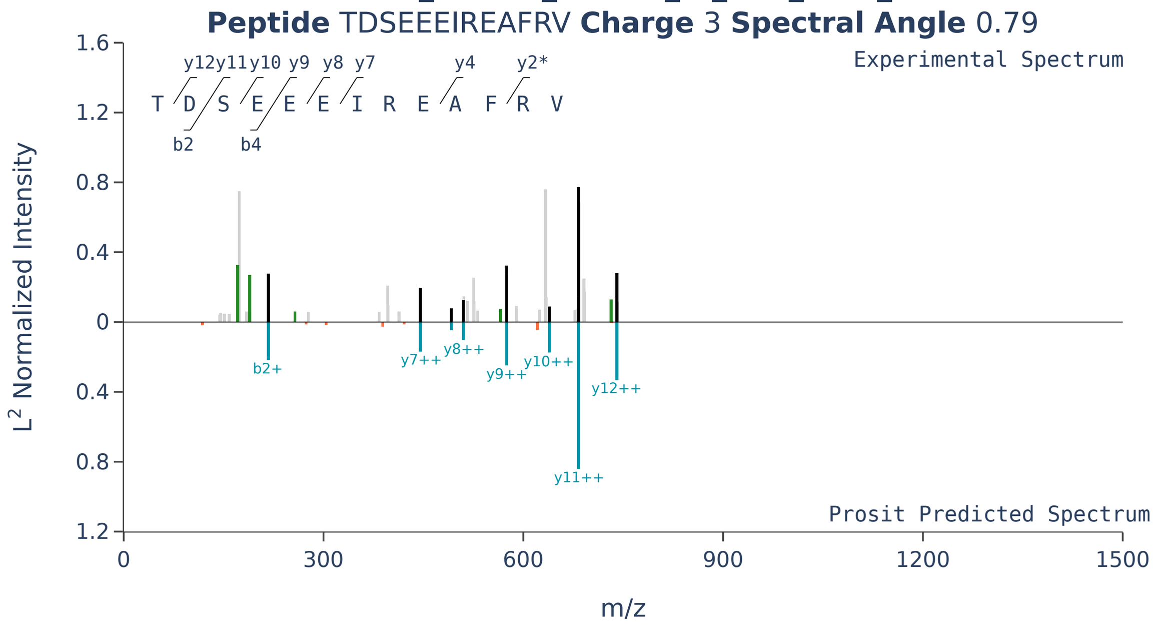


**Source** HRoetschke_291222_040123_Fu_CaM_b1R2_4h **Scan** 5336
**Peptide** TVMRSLGQNPTE **Charge** 2 **Spectral Angle** 0.83

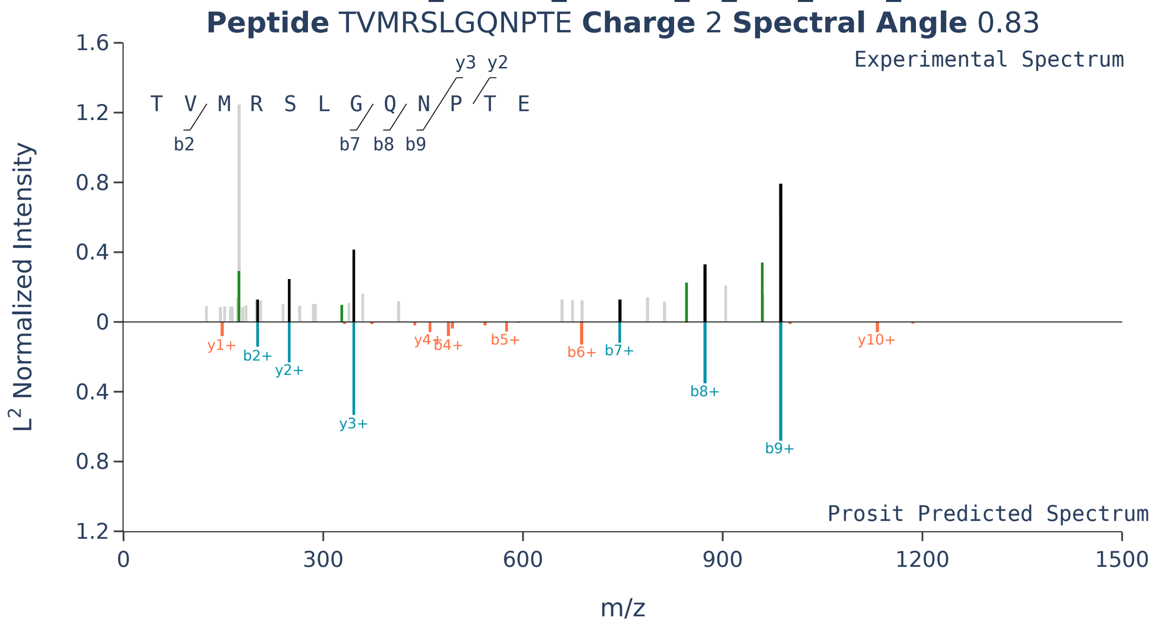


**Source** HRoetschke_291222_040123_Fu_CaM_b1R1_1h **Scan** 3494
**Peptide** READIDGDGQV **Charge** 2 **Spectral Angle** 0.82

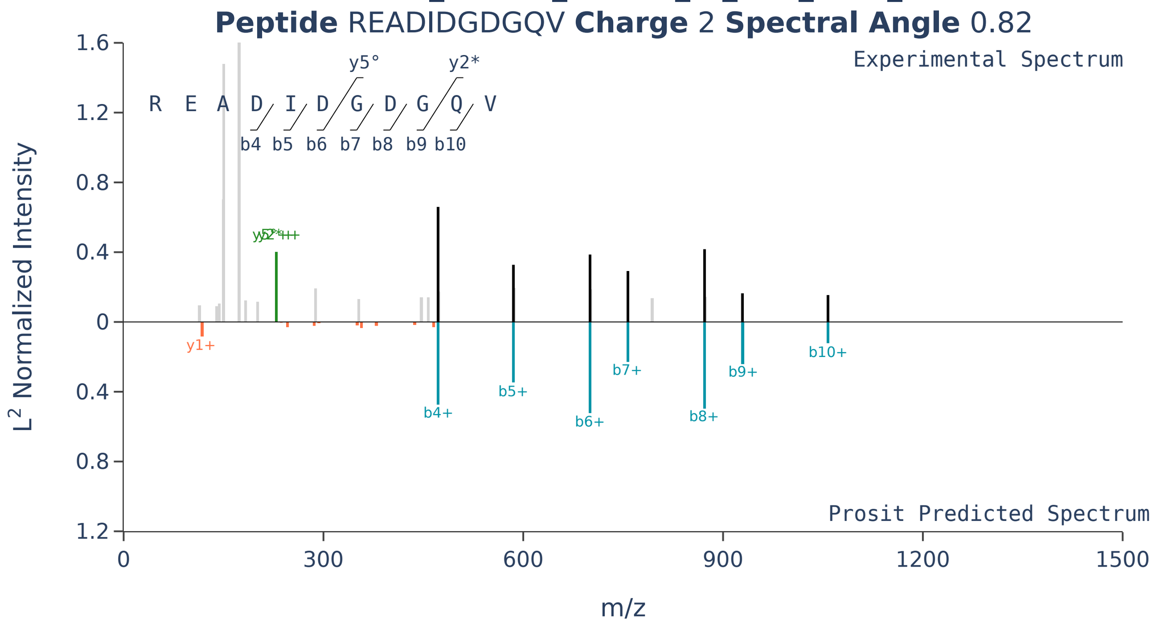


**Source** HRoetschke_291222_040123_Fu_CaM_b2R1_4h **Scan** 3925
**Peptide** DKDGNGYL **Charge** 2 **Spectral Angle** 0.9

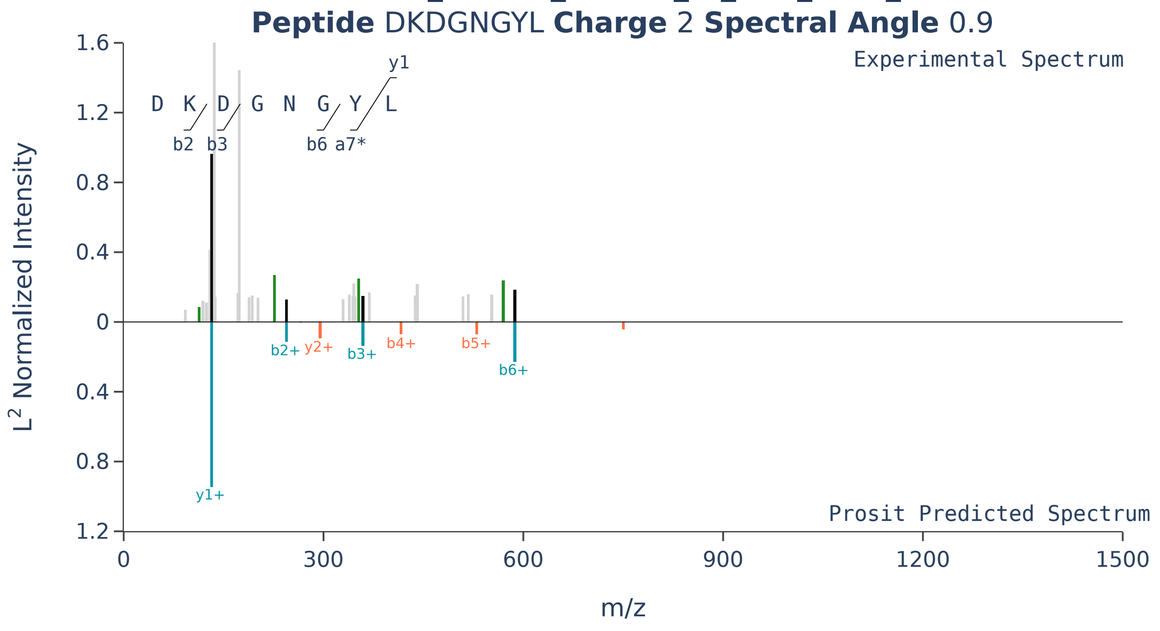

**Source** HRoetschke_291222_040123_Fu_CaM_b2R2_2h **Scan** 6515
**Peptide** KDGNGYISAAELRHVM **Charge** 4 **Spectral Angle** 0.71

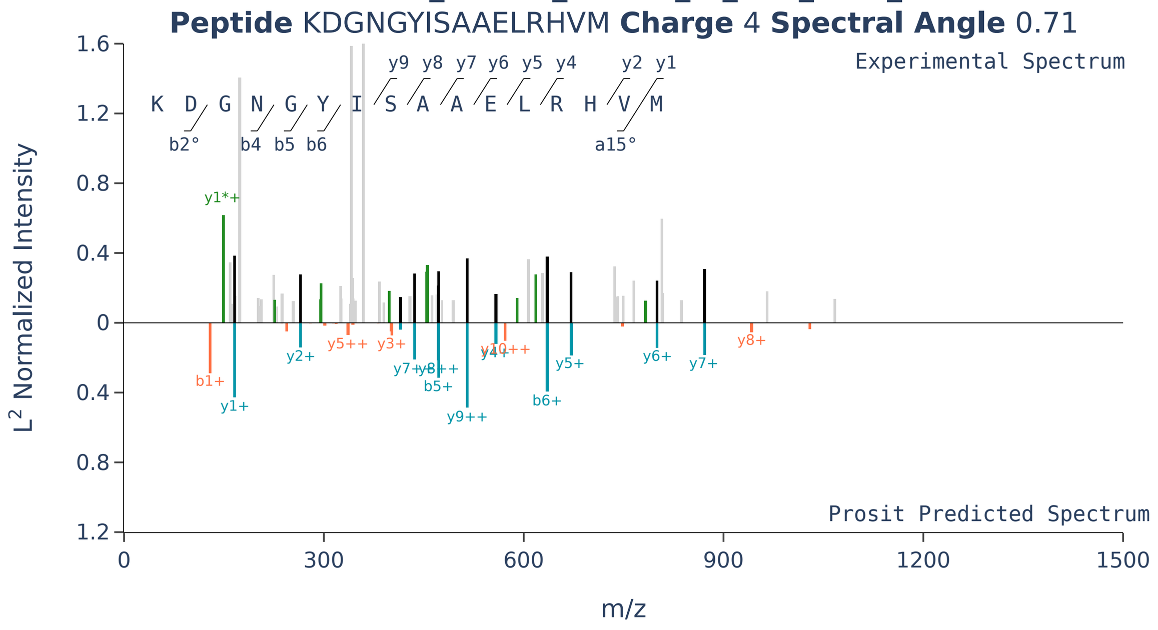


**Source** HRoetschke_291222_040123_Fu_CaM_b1R1_24h **Scan** 3964
**Peptide** LGEKLTD **Charge** 1 **Spectral Angle** 0.71

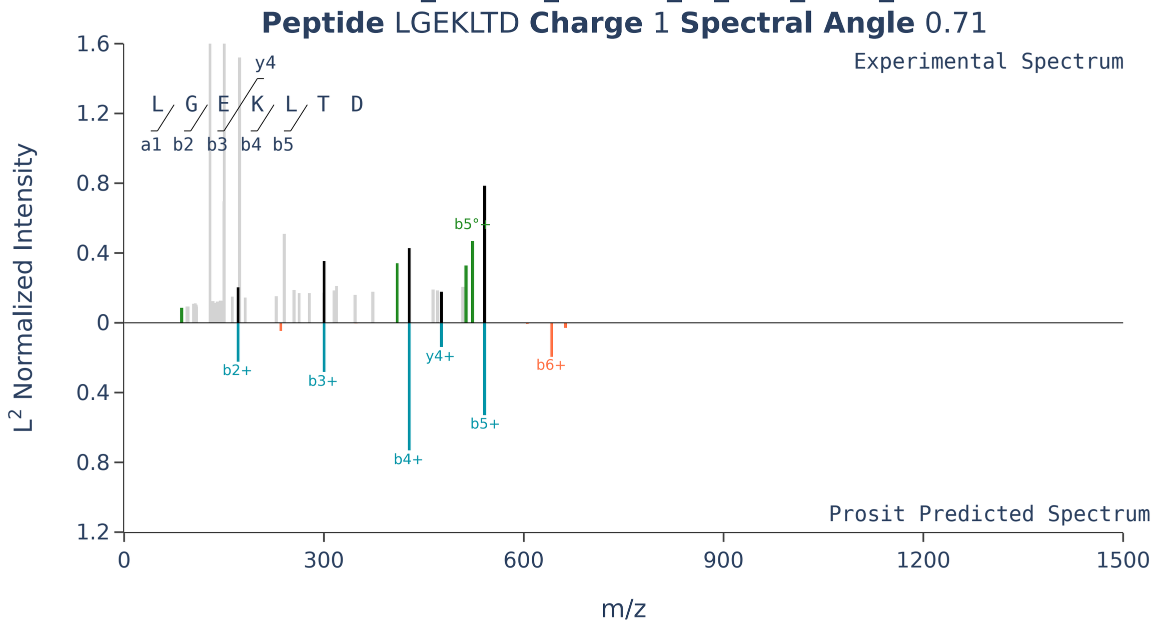


**Source** HRoetschke_291222_040123_Fu_CaM_b2R2_4h **Scan** 5147
**Peptide** RSLGQNPTEAELQ **Charge** 2 **Spectral Angle** 0.79

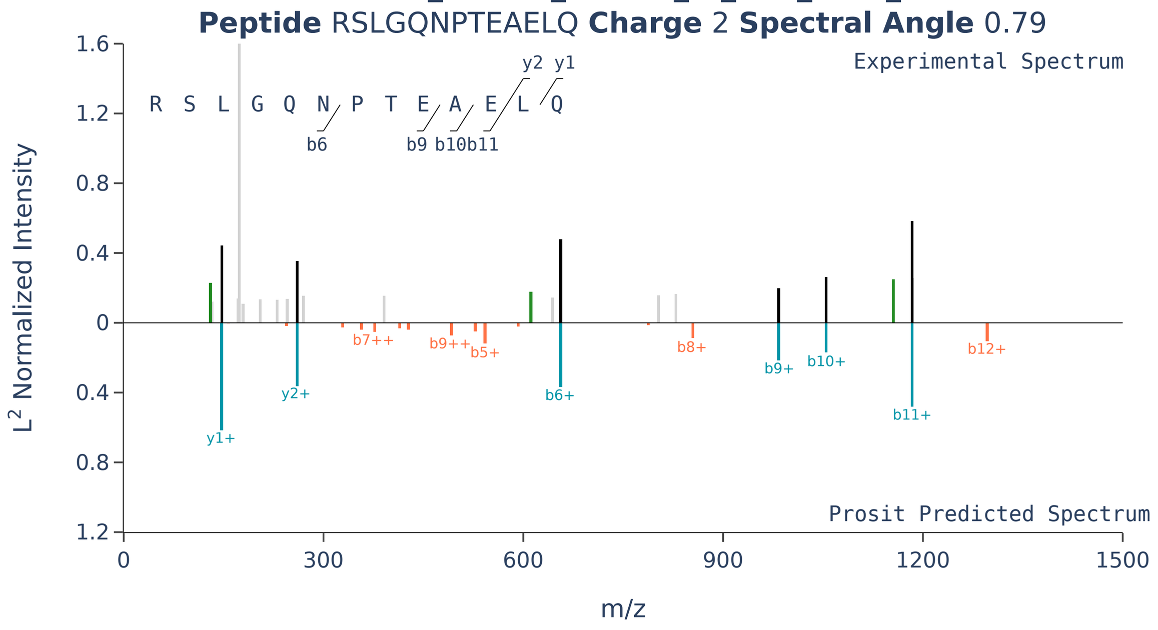


**Source** HRoetschke_291222_040123_Fu_CaM_b2R1_24h **Scan** 4759
**Peptide** DKDGDGTITTKELGTVMRS **Charge** 4 **Spectral Angle** 0.72

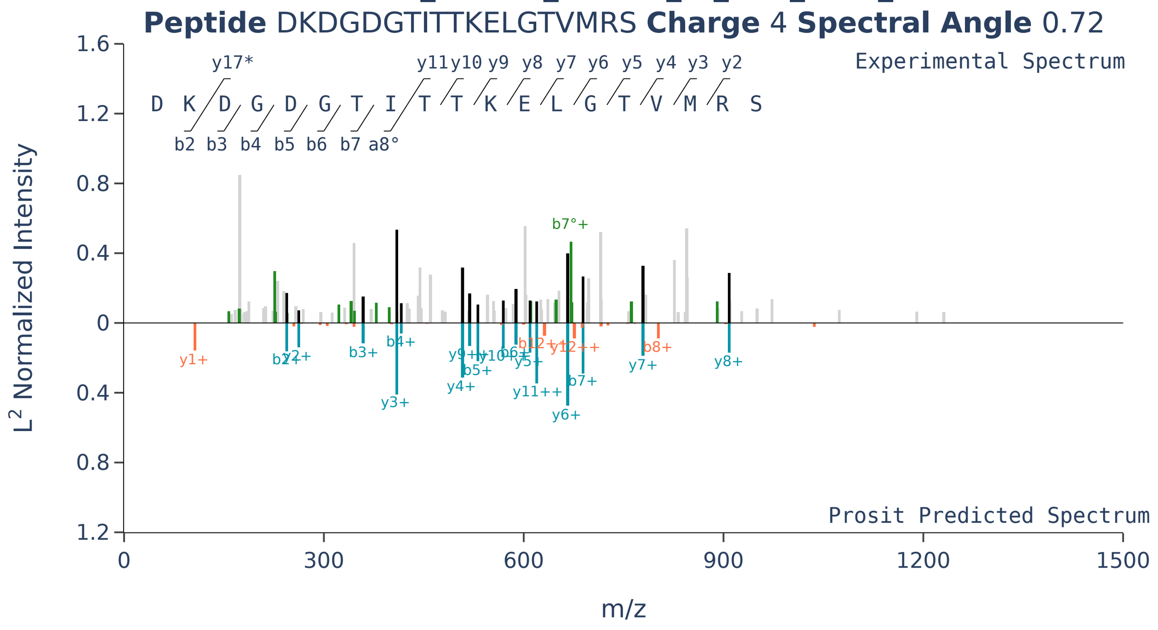


**Source** HRoetschke_291222_040123_Fu_CaM_b2R1_4h **Scan** 4983
**Peptide** GTITTKEL **Charge** 2 **Spectral Angle** 0.81

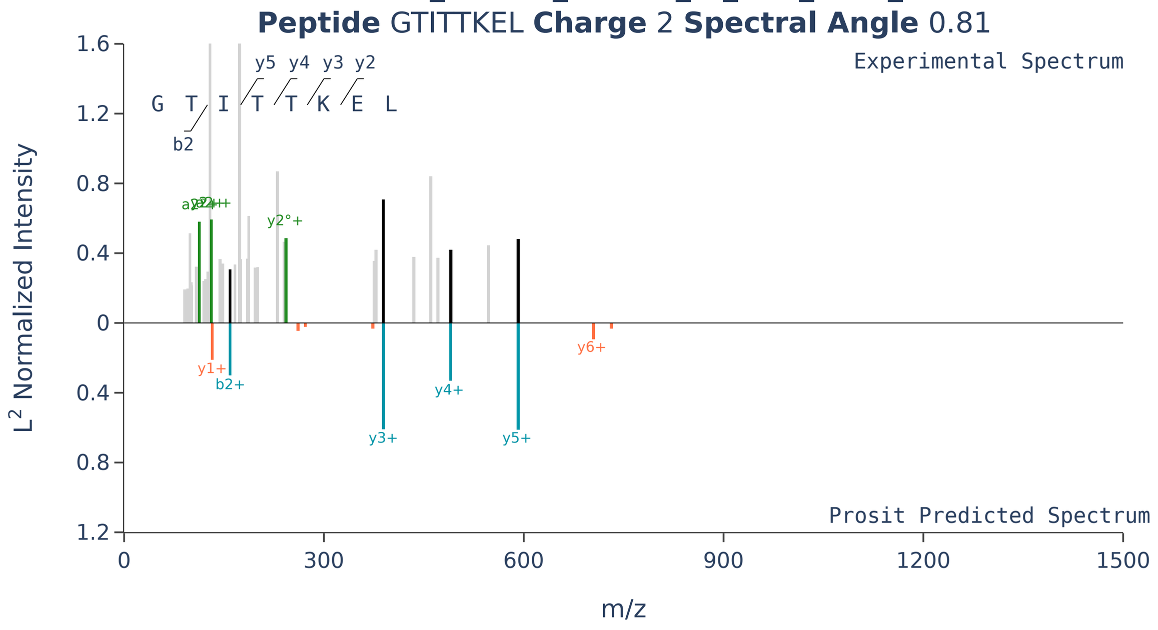


**Source** HRoetschke_291222_040123_Fu_CaM_b1R1_2h **Scan** 6748
**Peptide** DGNGYISAAELRH **Charge** 3 **Spectral Angle** 0.74

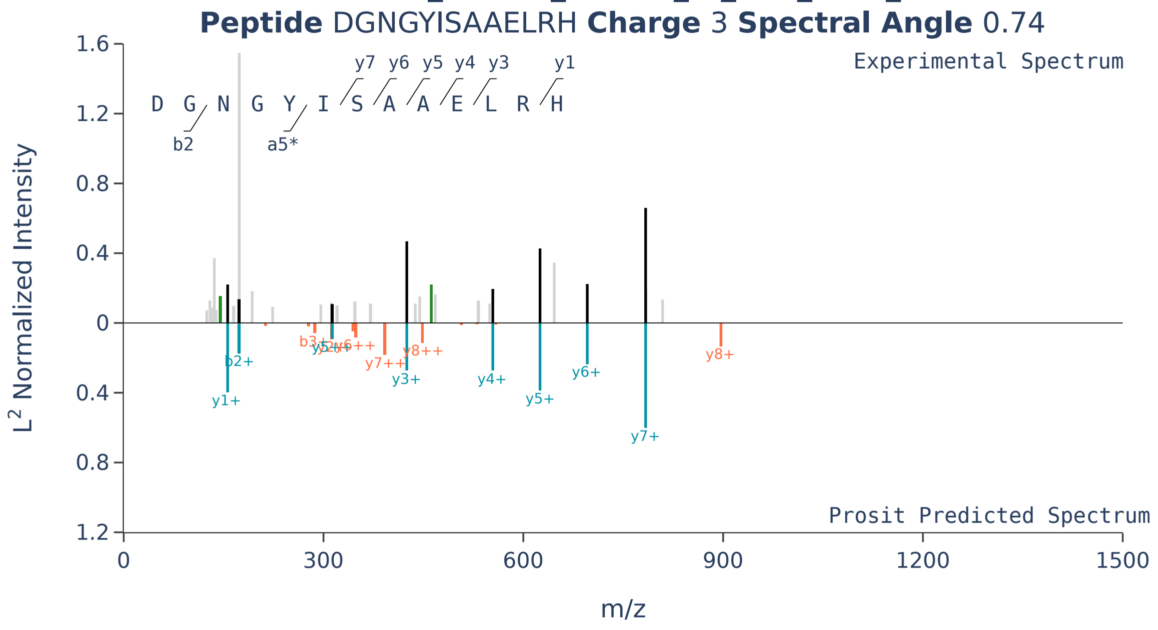


**Source** HRoetschke_291222_040123_Fu_CaM_b2R1_1h **Scan** 6814
**Peptide** KDTDSEEEIREAF **Charge** 3 **Spectral Angle** 0.69

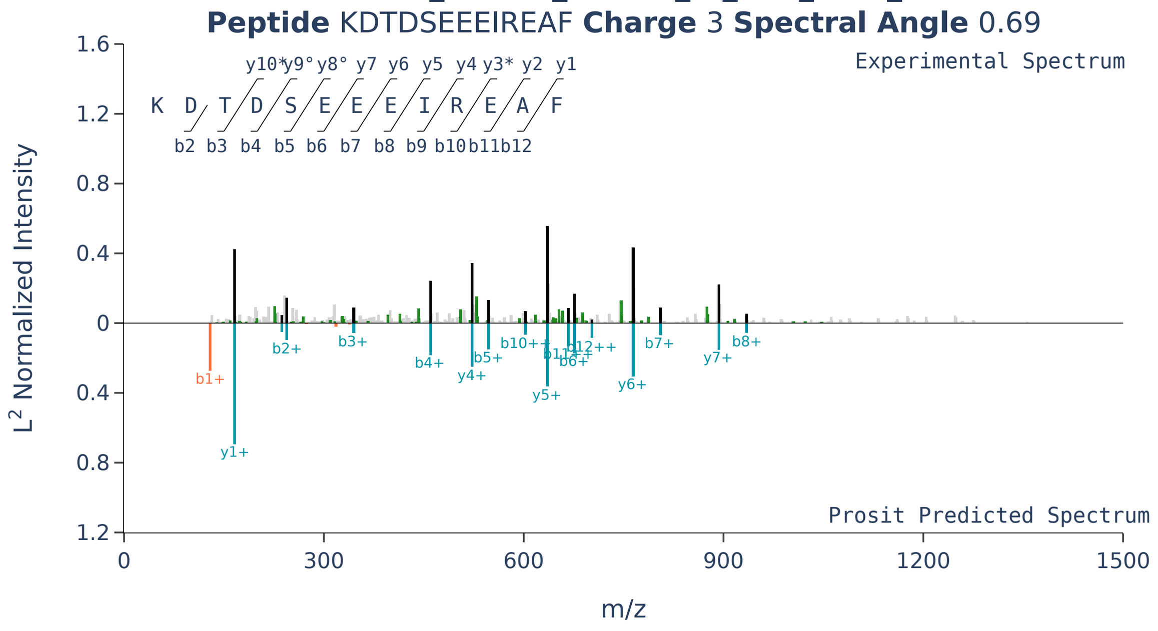


**Source** HRoetschke_291222_040123_Fu_CaM_b1R1_1h **Scan** 5946
**Peptide** VMRSLGQNPTEAELQD **Charge** 2 **Spectral Angle** 0.75

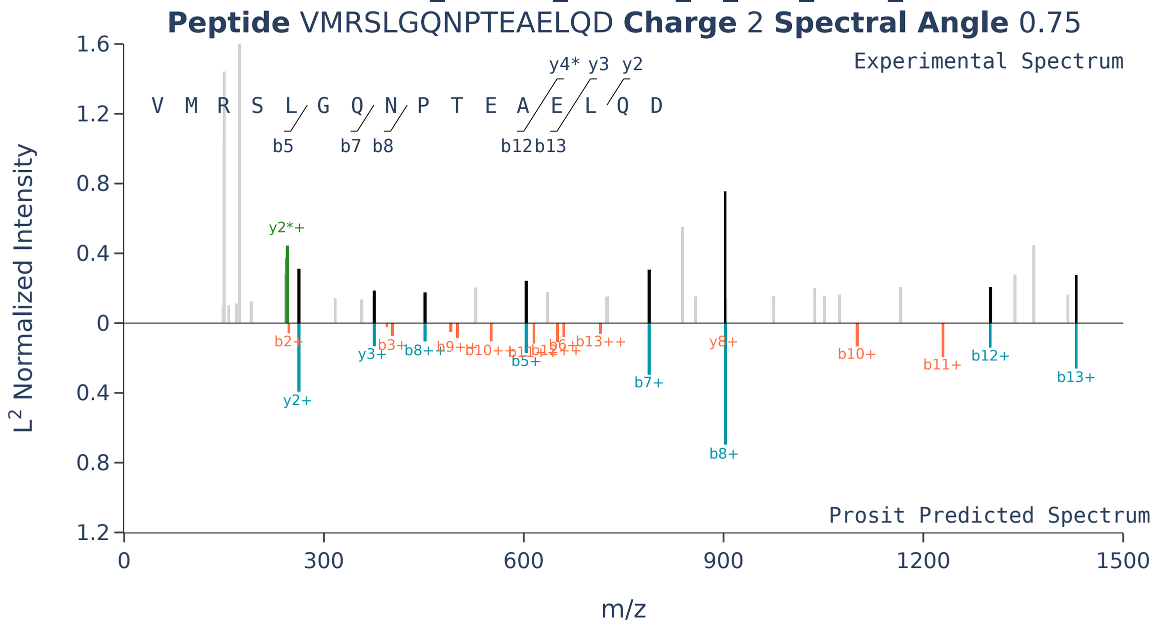


**Source** HRoetschke_291222_040123_Fu_CaM_b2R1_24h **Scan** 13859
**Peptide** ADGNGTIDFPEFLTM **Charge** 2 **Spectral Angle** 0.93

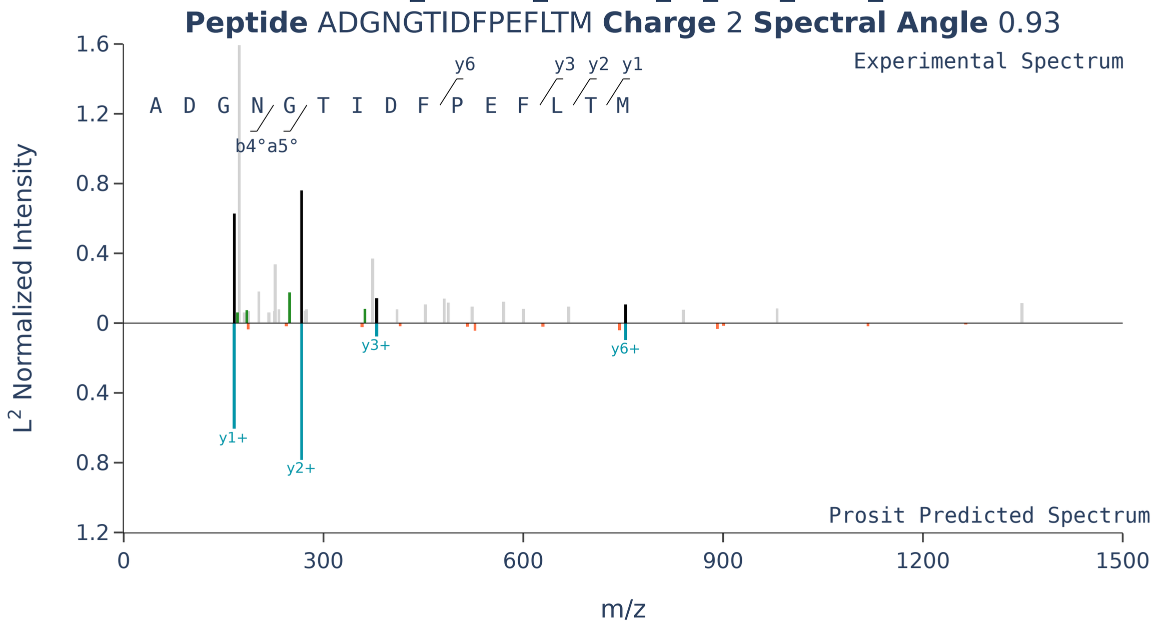


**Source** HRoetschke_291222_040123_Fu_CaM_b1R1_2h **Scan** 9120
**Peptide** REAFRVFDKDGNGYISA **Charge** 3 **Spectral Angle** 0.7

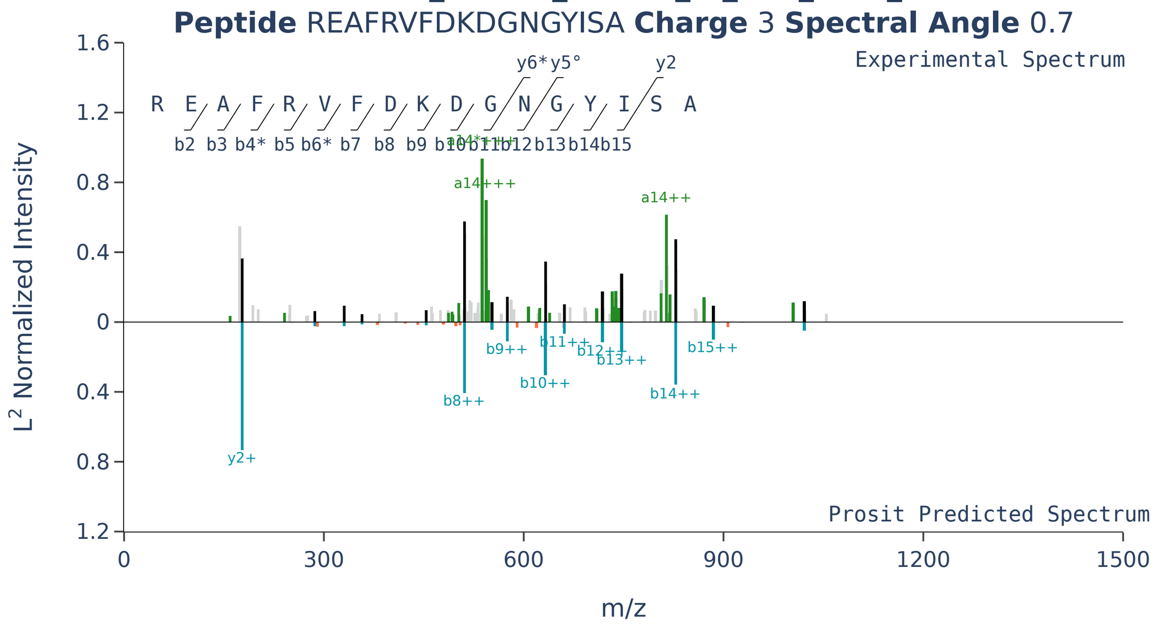


**Source** HRoetschke_291222_040123_Fu_CaM_b1R2_4h **Scan** 11441
**Peptide** AELQDMINEVD **Charge** 2 **Spectral Angle** 0.77

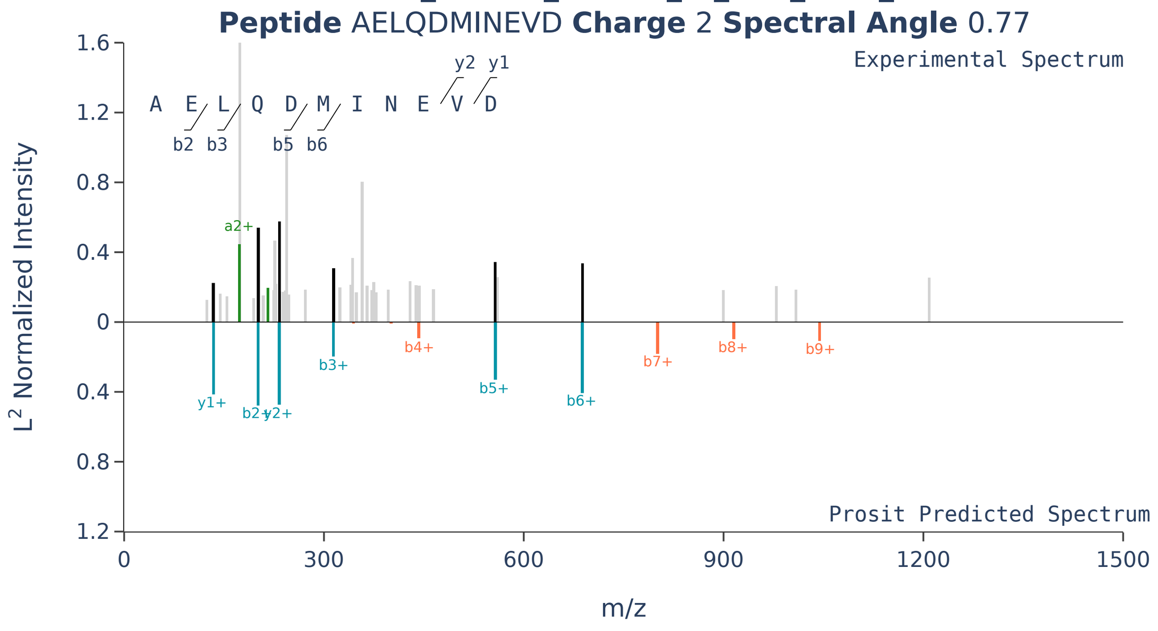


**Source** HRoetschke_291222_040123_Fu_CaM_b1R1_24h **Scan** 5155
**Peptide** MINEVDADGN **Charge** 2 **Spectral Angle** 0.85

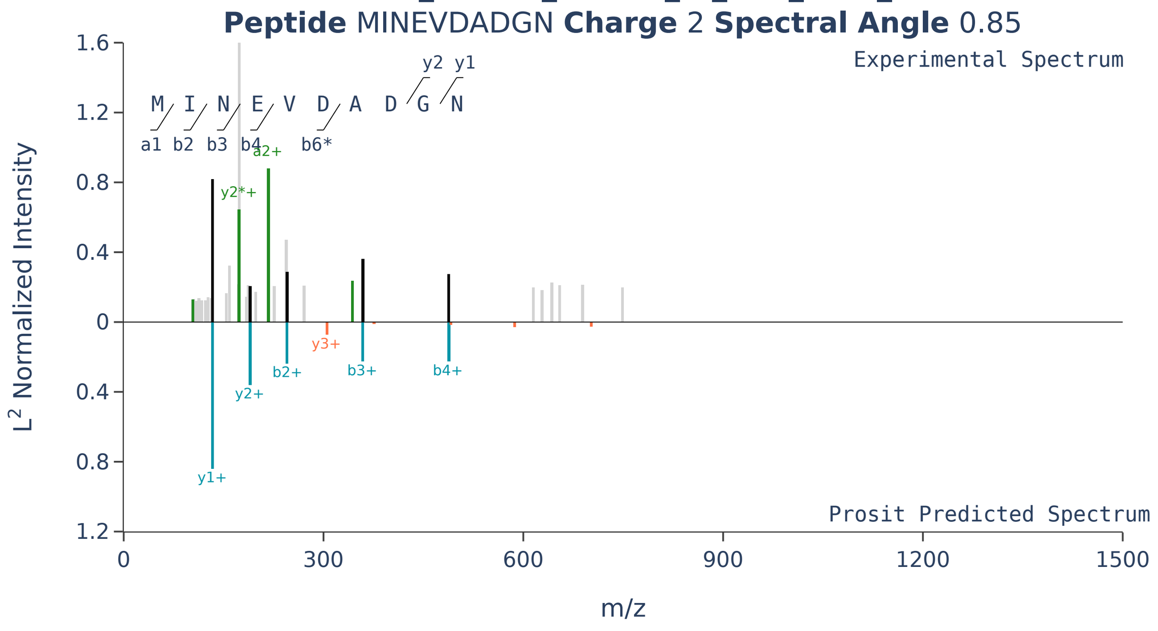


**Source** HRoetschke_291222_040123_Fu_CaM_b1R1_24h **Scan** 11474
**Peptide** FSLFDKDGDGTITTKE **Charge** 3 **Spectral Angle** 0.79

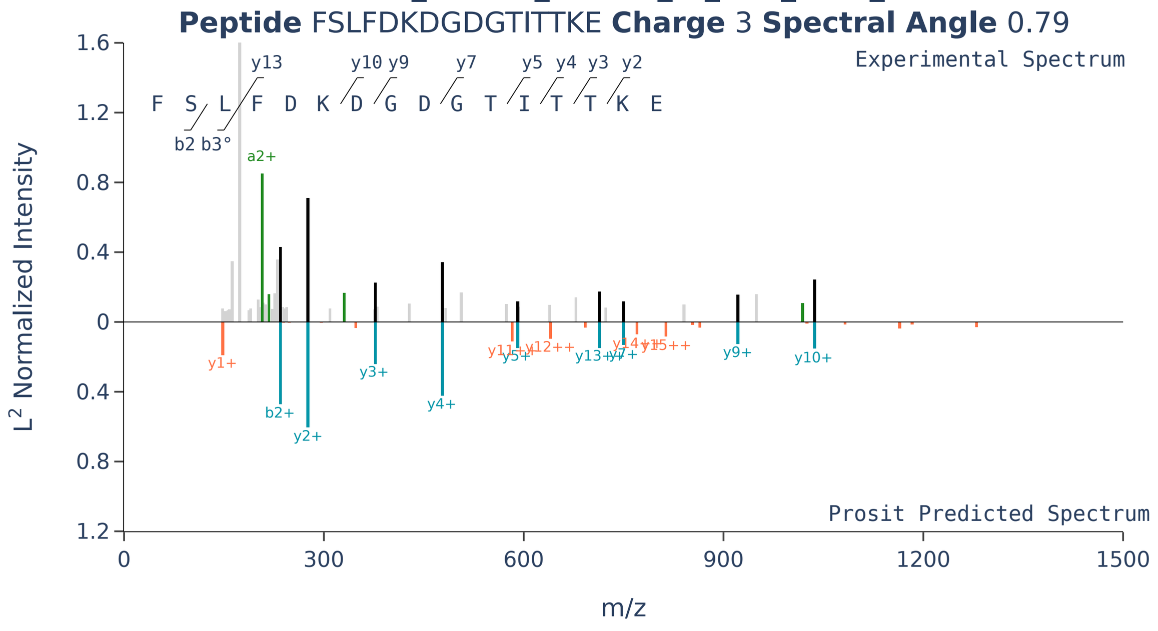


**Source** HRoetschke_291222_040123_Fu_CaM_b1R1_24h **Scan** 3749
**Peptide** VQMMTAK **Charge** 2 **Spectral Angle** 0.72

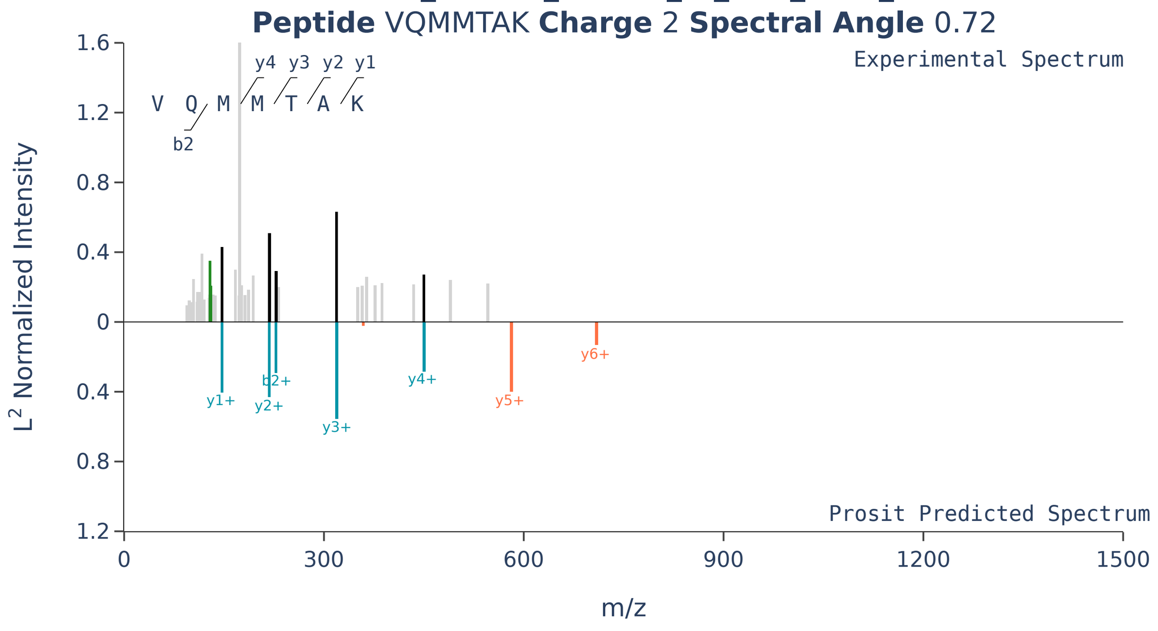


**Source** HRoetschke_291222_040123_Fu_CaM_b1R1_2h **Scan** 2124
**Peptide** ARKMKDTDSEEEIRE **Charge** 3 **Spectral Angle** 0.74

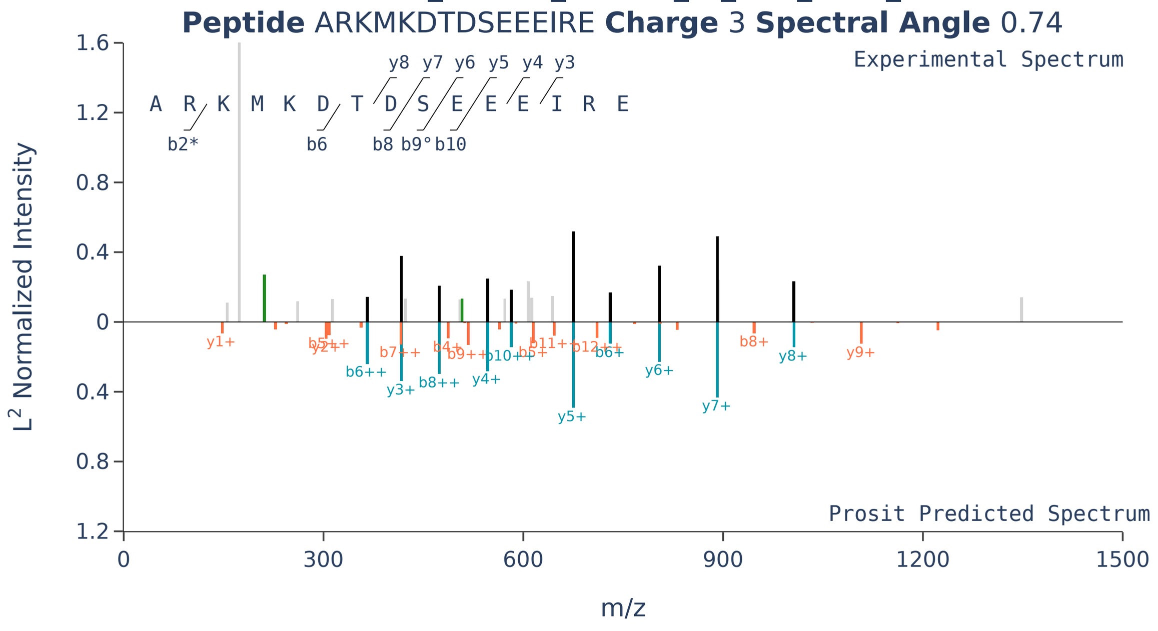

**Source** HRoetschke_291222_040123_Fu_CaM_b1R2_2h **Scan** 6486
**Peptide** HVMTNLGEKLTD **Charge** 3 **Spectral Angle** 0.83

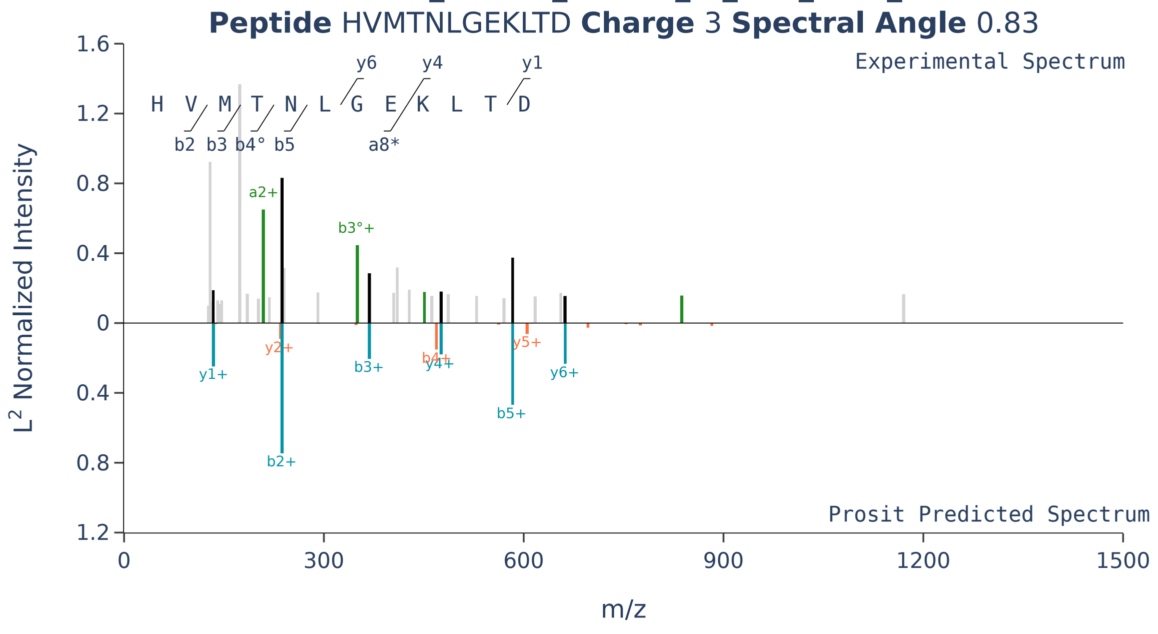


**Source** HRoetschke_291222_040123_Fu_CaM_b1R1_2h **Scan** 9058
**Peptide** REAFRVFDKDGNGYISAA **Charge** 3 **Spectral Angle** 0.75

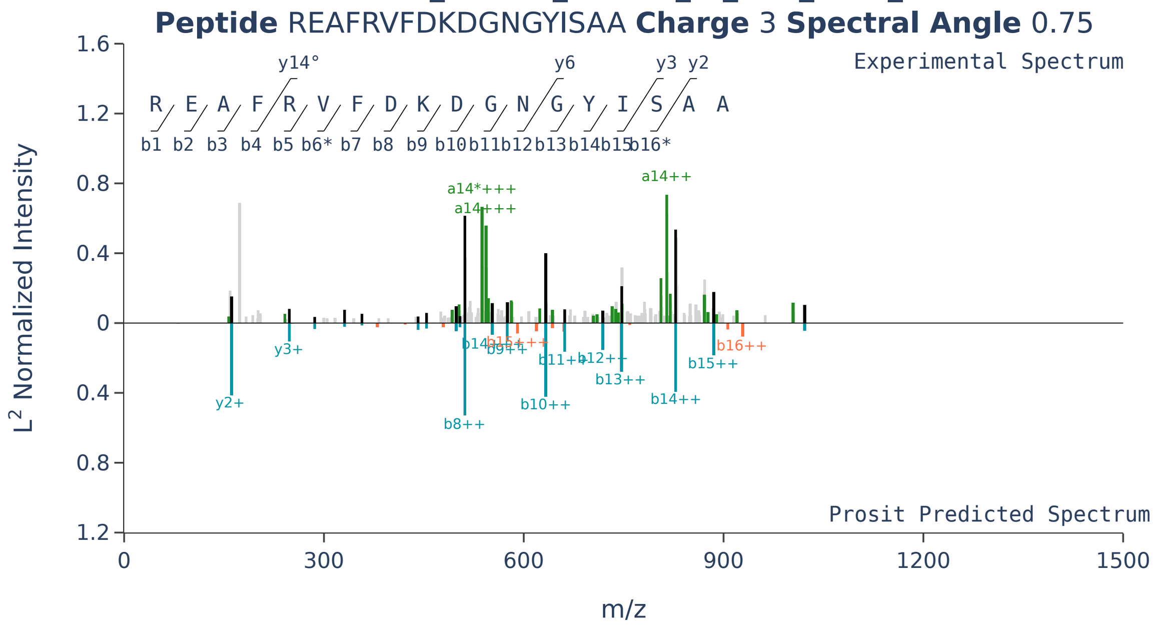


**Source** HRoetschke_291222_040123_Fu_CaM_b1R2_4h **Scan** 7882
**Peptide** EMIREADIDGDGQV **Charge** 2 **Spectral Angle** 0.85

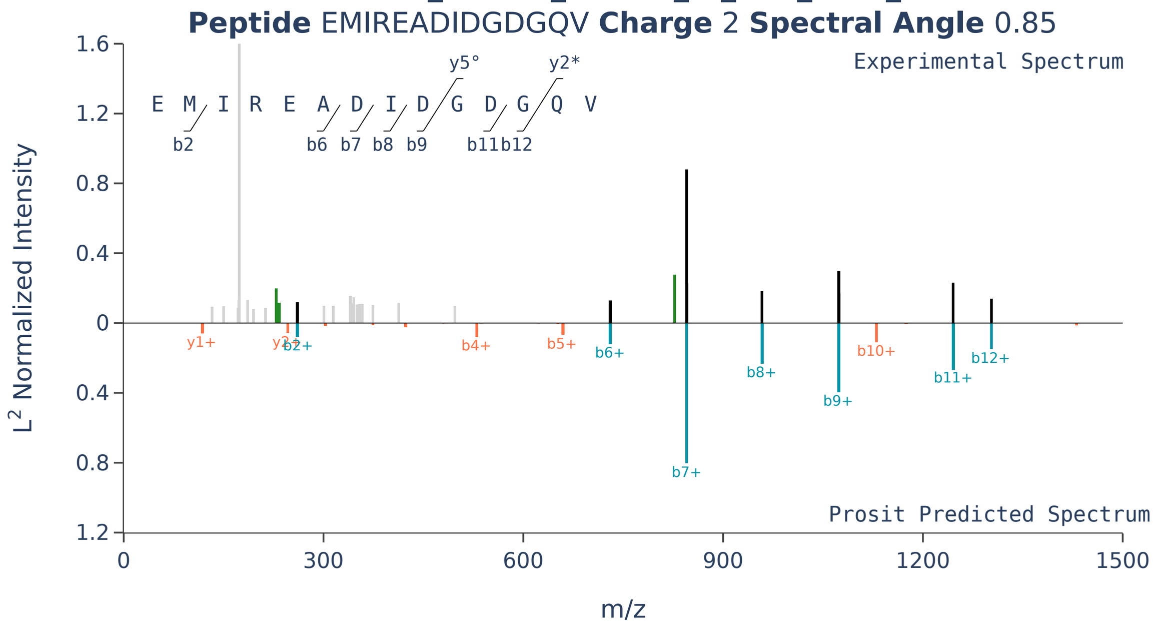


**Source** HRoetschke_291222_040123_Fu_CaM_b2R2_24h **Scan** 6533
**Peptide** RVFDKDGNGYL **Charge** 3 **Spectral Angle** 0.7

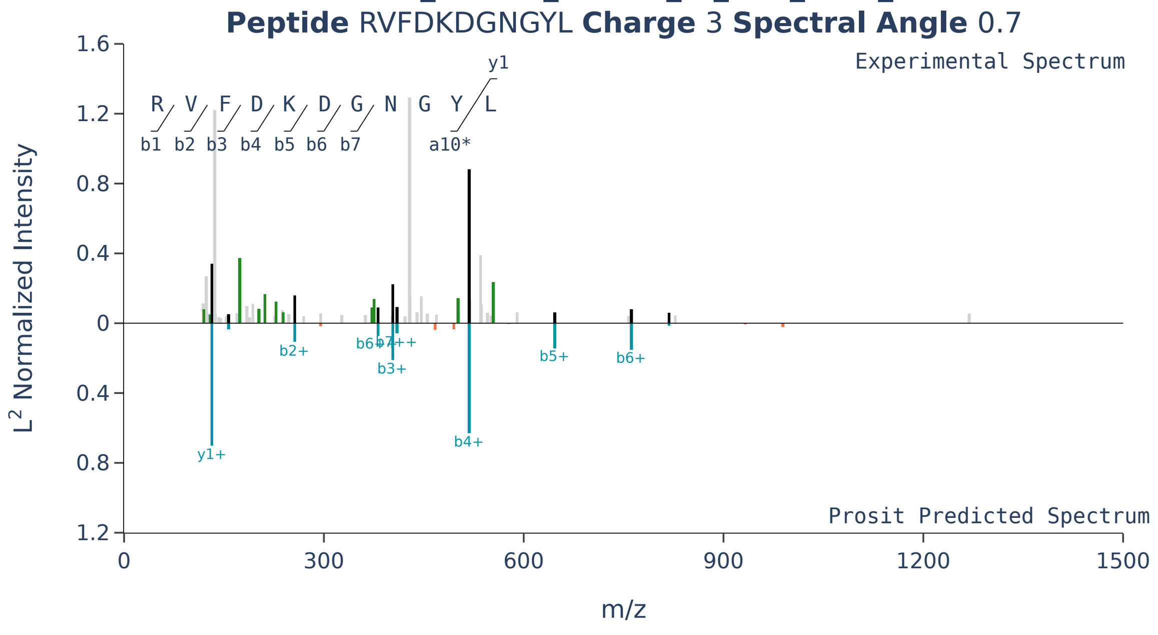


**Source** HRoetschke_291222_040123_Fu_CaM_b1R2_4h **Scan** 4632
**Peptide** SAAELRHVMT **Charge** 2 **Spectral Angle** 0.73

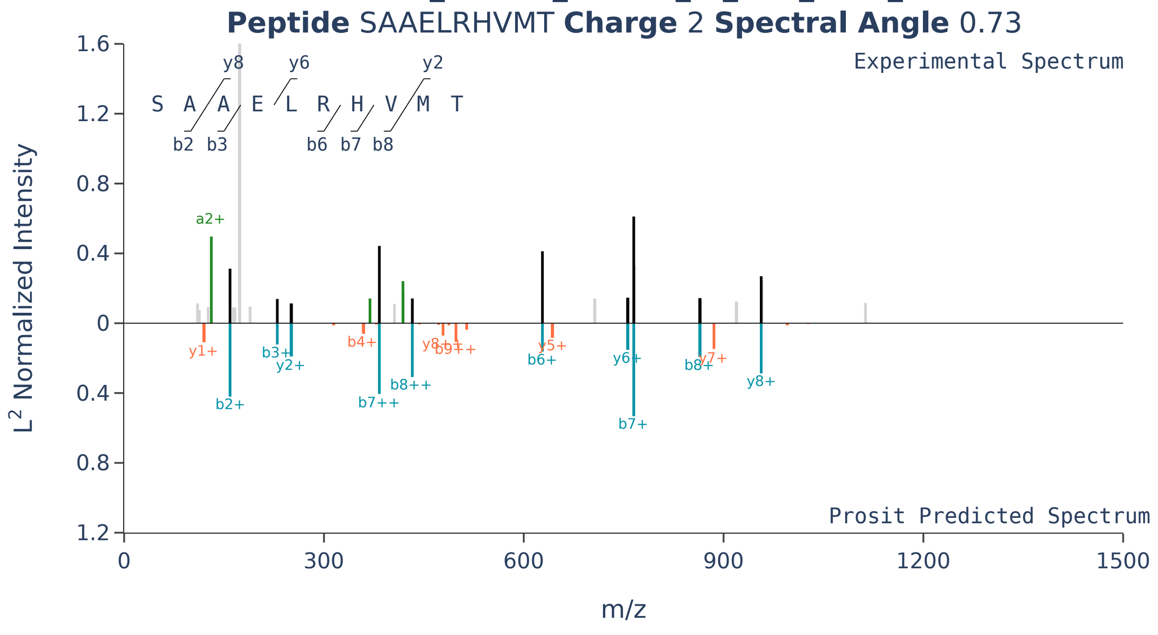


**Source** HRoetschke_291222_040123_Fu_CaM_b2R2_24h **Scan** 12159
**Peptide** AELQDMINEV **Charge** 2 **Spectral Angle** 0.81

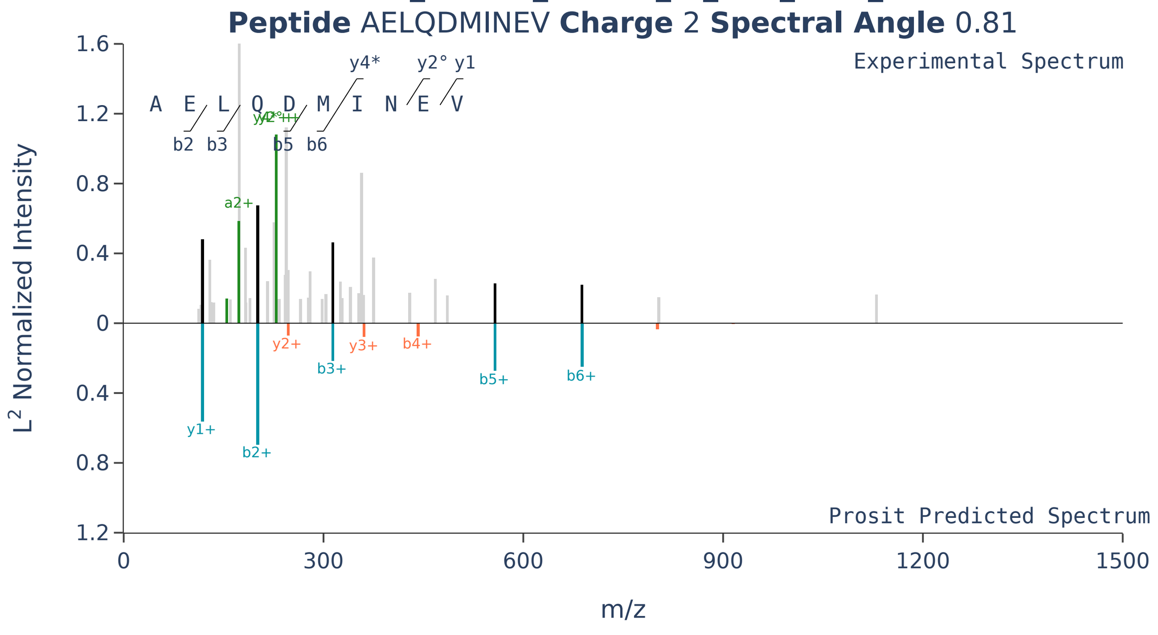


**Source** HRoetschke_291222_040123_Fu_CaM_b1R1_4h **Scan** 6053
**Peptide** REAFRVFDKD **Charge** 3 **Spectral Angle** 0.72

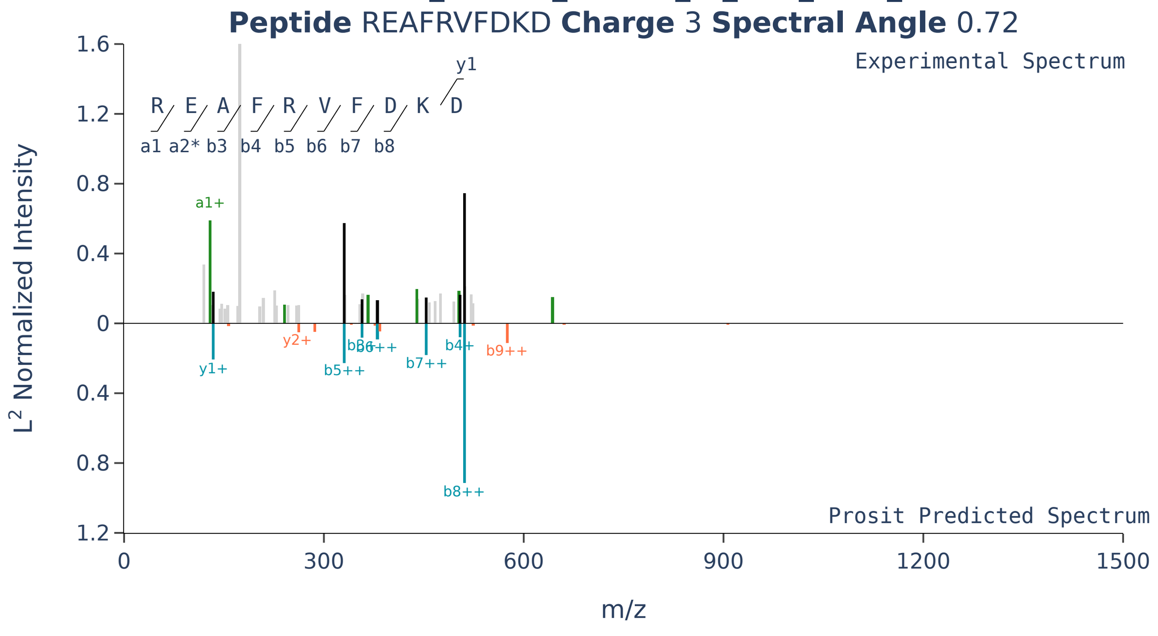


**Source** HRoetschke_291222_040123_Fu_CaM_b1R1_24h **Scan** 9090
**Peptide** AFRVFDKDGNGY **Charge** 3 **Spectral Angle** 0.79

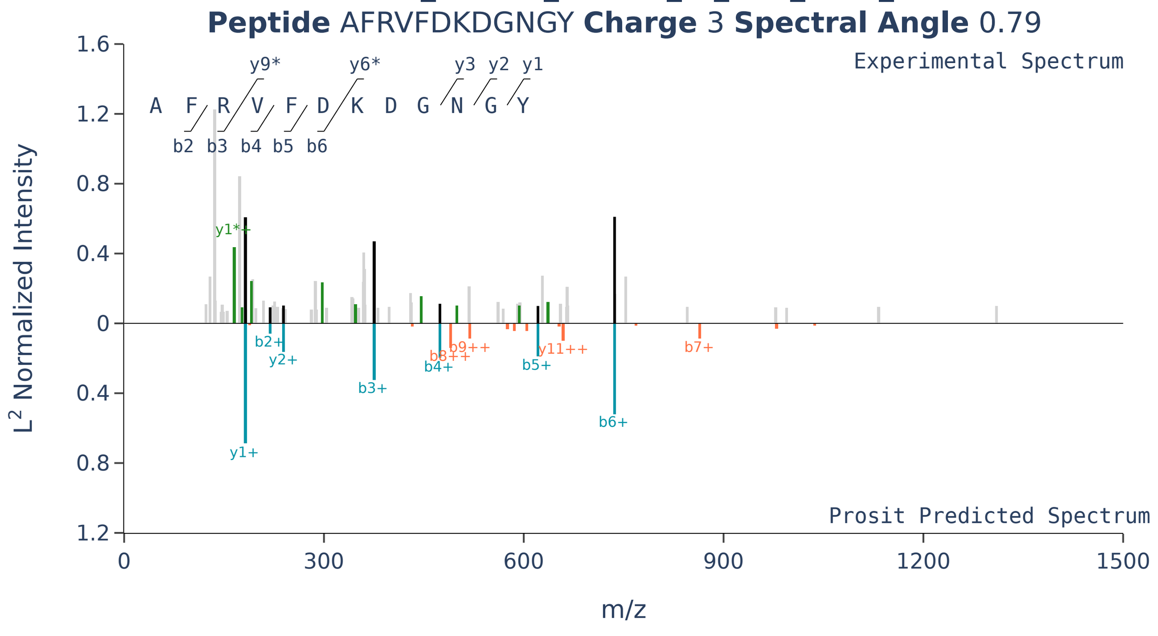


**Source** HRoetschke_291222_040123_Fu_CaM_b1R1_1h **Scan** 5387
**Peptide** LSAAELRHV **Charge** 3 **Spectral Angle** 0.8

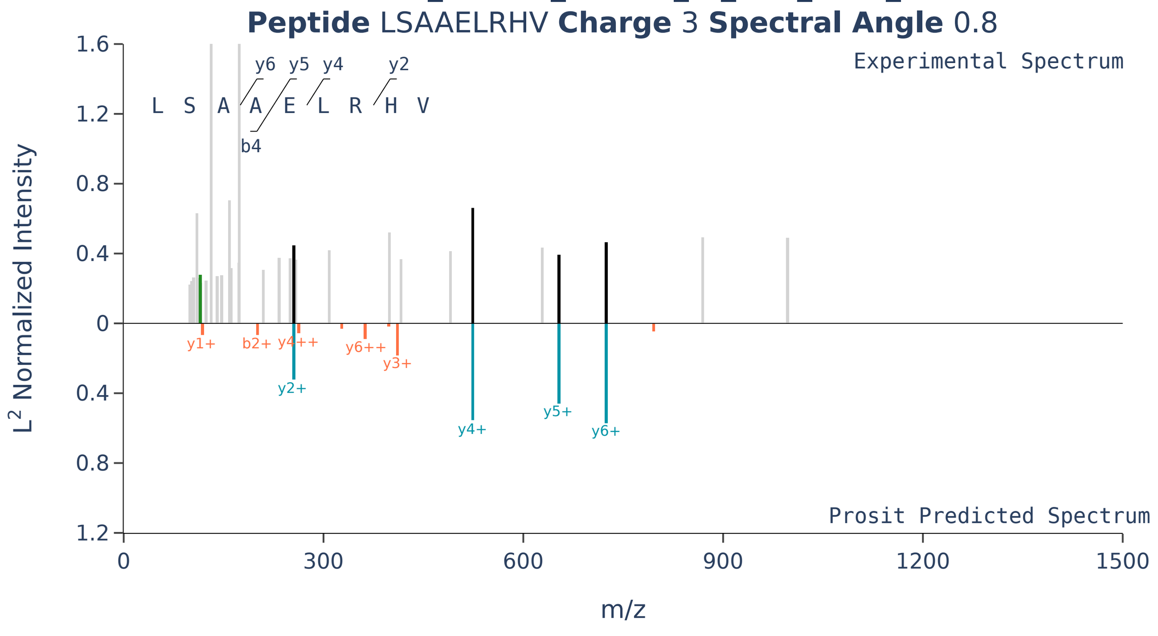


**Source** HRoetschke_291222_040123_Fu_CaM_b1R1_2h **Scan** 2632
**Peptide** KMKDTDSEEEL **Charge** 3 **Spectral Angle** 0.86

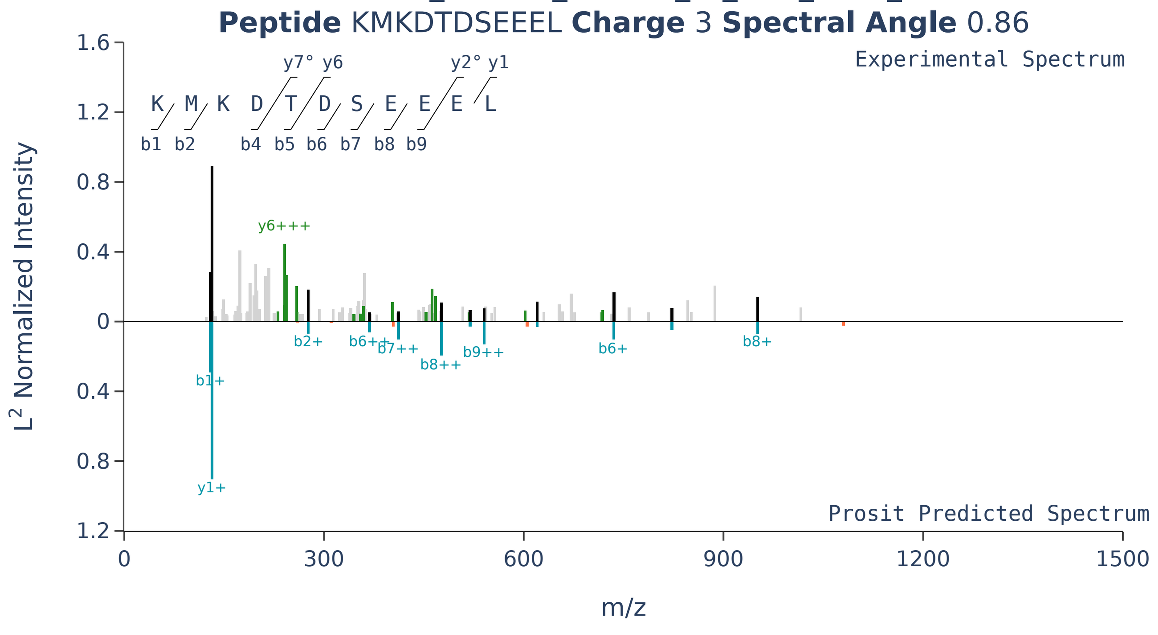


**Source** HRoetschke_291222_040123_Fu_CaM_b1R1_24h **Scan** 2542
**Peptide** ARKMKDTDSEEEIREA **Charge** 4 **Spectral Angle** 0.8

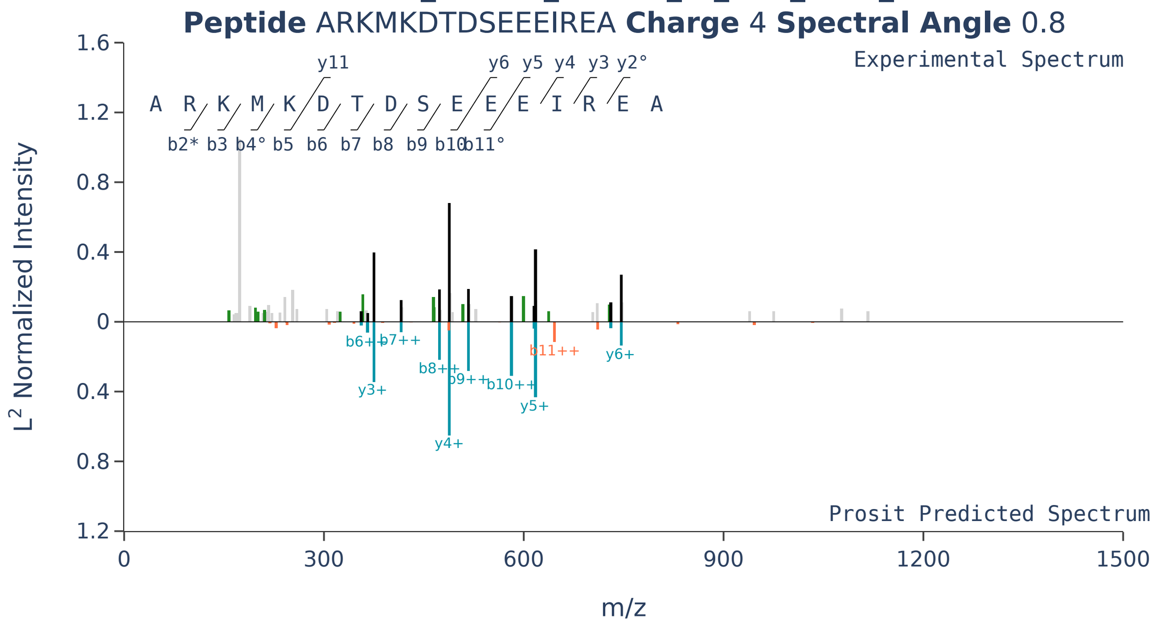


**Source** HRoetschke_291222_040123_Fu_CaM_b2R2_1h **Scan** 4767
**Peptide** QLAEFKE **Charge** 2 **Spectral Angle** 0.72

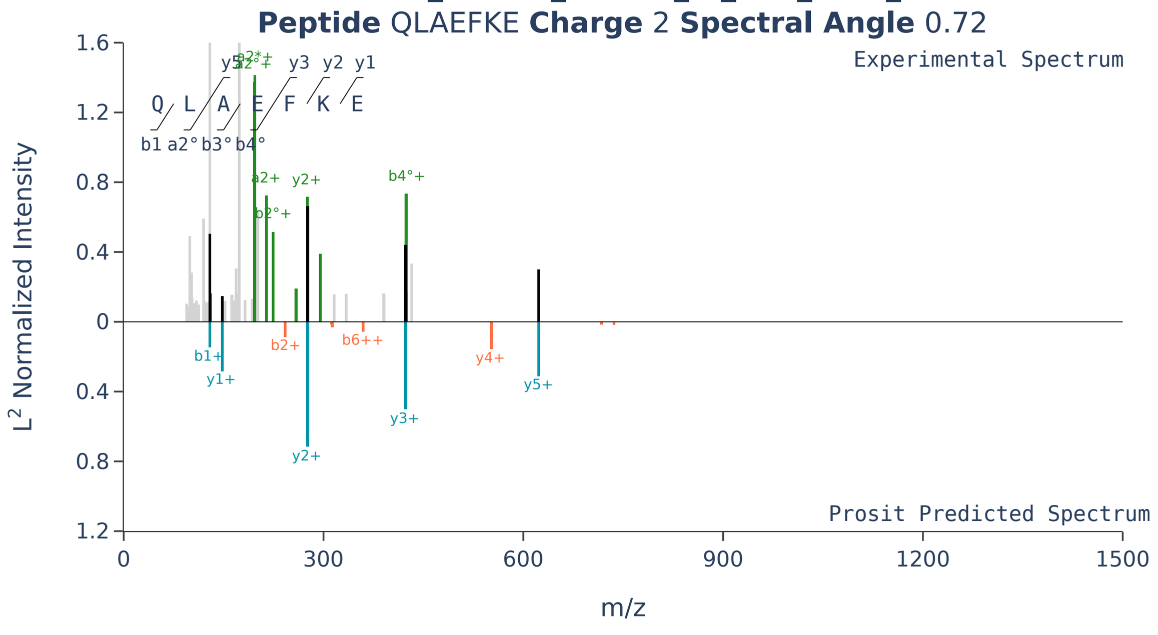


**Source** HRoetschke_291222_040123_Fu_CaM_b1R1_2h **Scan** 9445
**Peptide** REAFRVFDKDGNGYL **Charge** 3 **Spectral Angle** 0.7

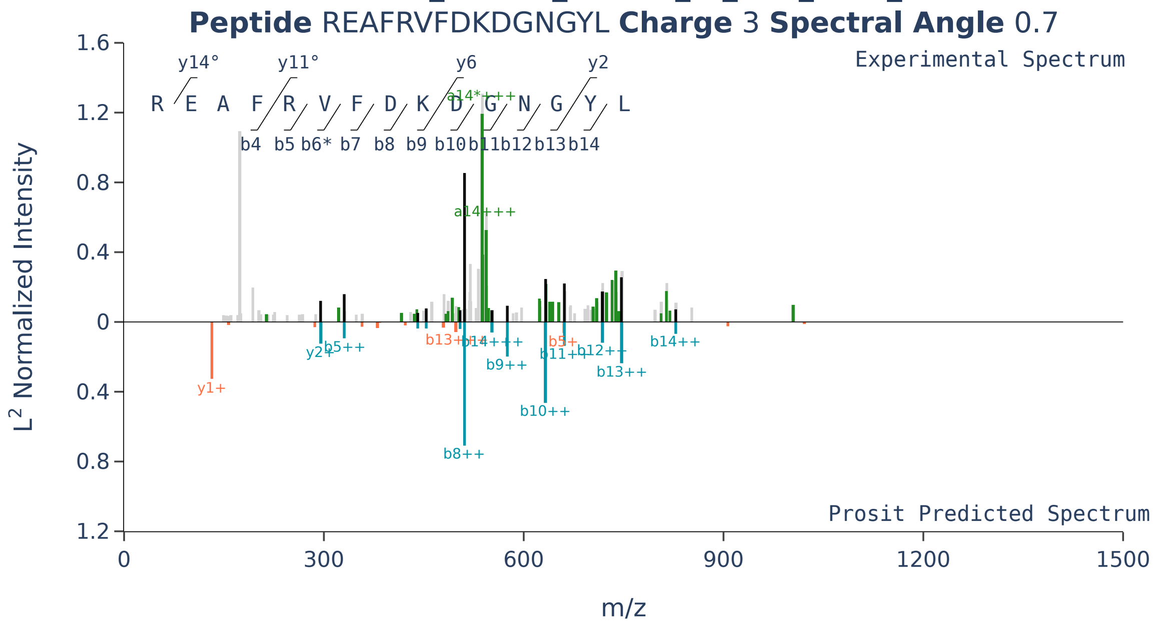


**Source** HRoetschke_291222_040123_Fu_CaM_b1R1_24h **Scan** 5952
**Peptide** LFDKDGDGTITTK **Charge** 3 **Spectral Angle** 0.68

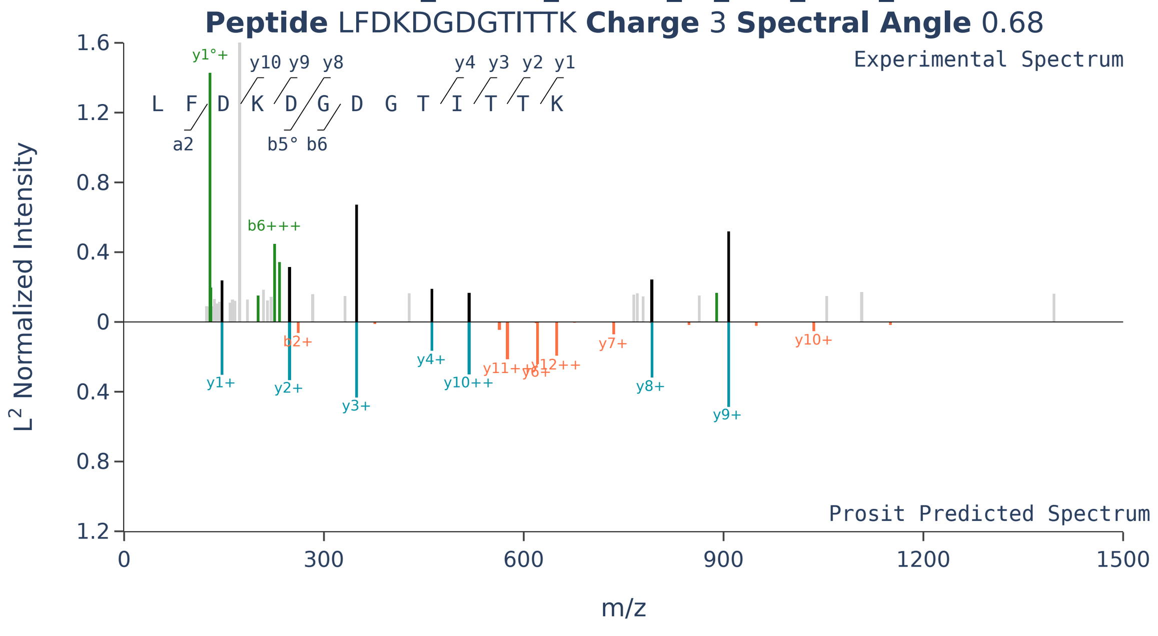


**Source** HRoetschke_291222_040123_Fu_CaM_b1R1_24h **Scan** 4194
**Peptide** AAELRHVMT **Charge** 2 **Spectral Angle** 0.78

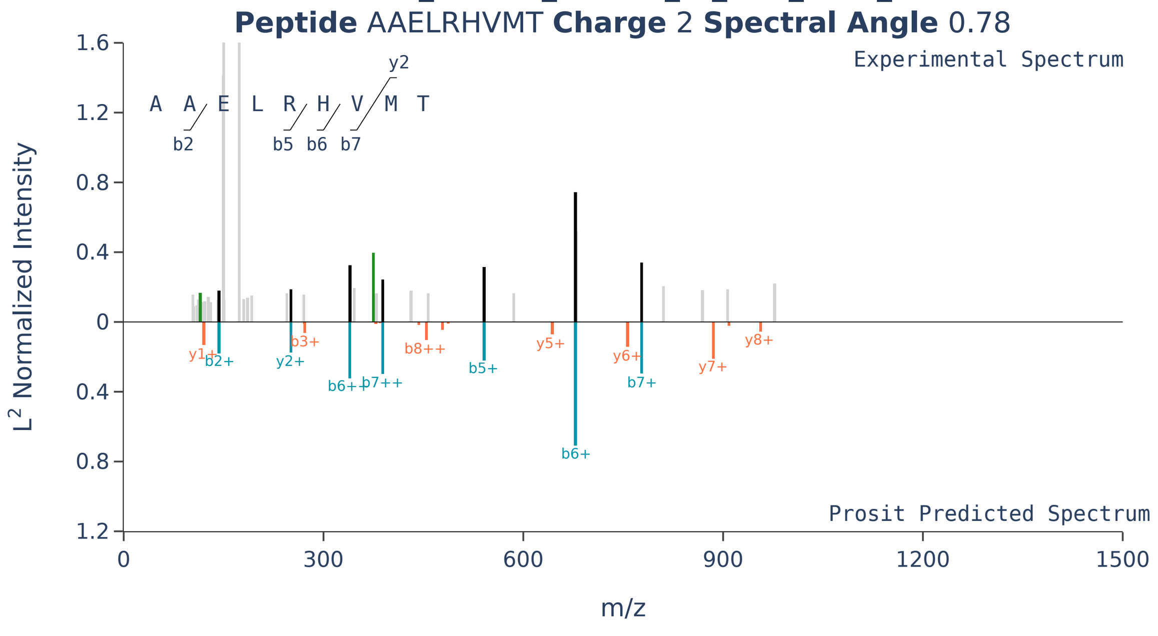

**Source** HRoetschke_291222_040123_Fu_CaM_b2R2_24h **Scan** 9817
**Peptide** AFRVFDKDGNGYISAA **Charge** 3 **Spectral Angle** 0.75

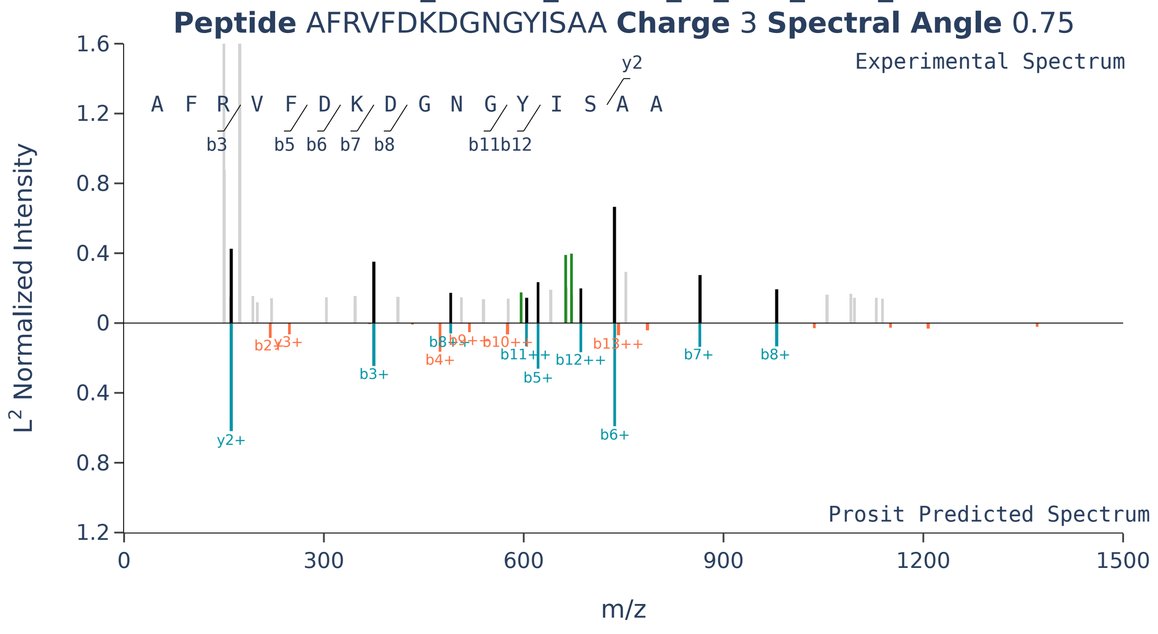


**Source** HRoetschke_291222_040123_Fu_CaM_b1R1_1h **Scan** 7783
**Peptide** ELRHVMTNLGEKLTD **Charge** 3 **Spectral Angle** 0.71

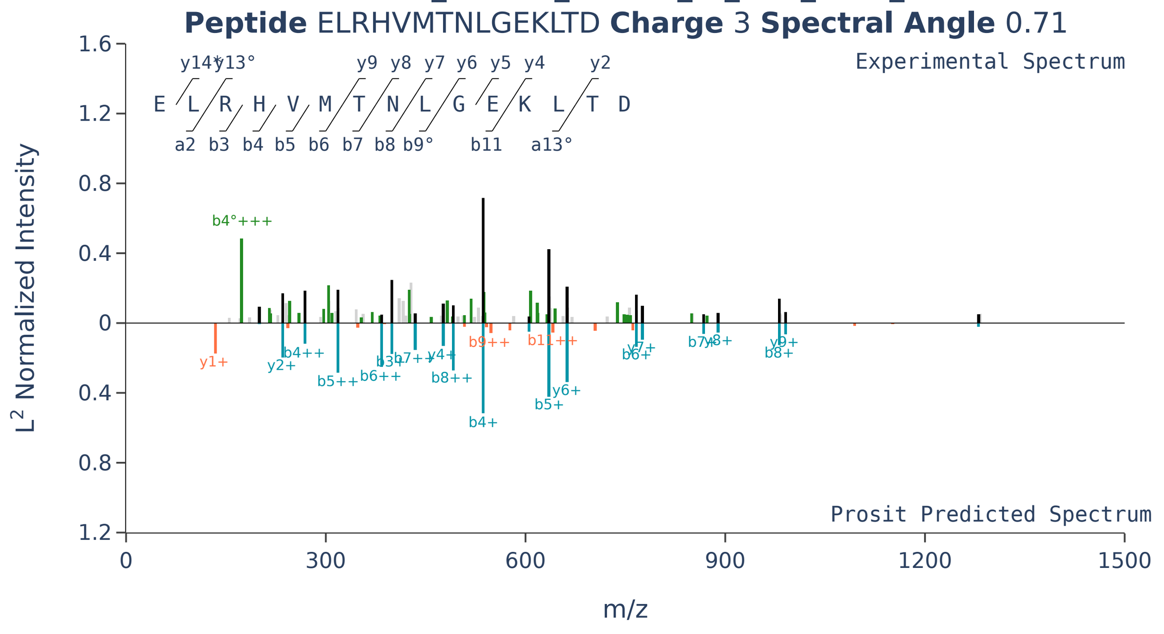


**Source** HRoetschke_291222_040123_Fu_CaM_b2R1_24h **Scan** 9319
**Peptide** AFSLFDKDGDGTITTKE **Charge** 3 **Spectral Angle** 0.74

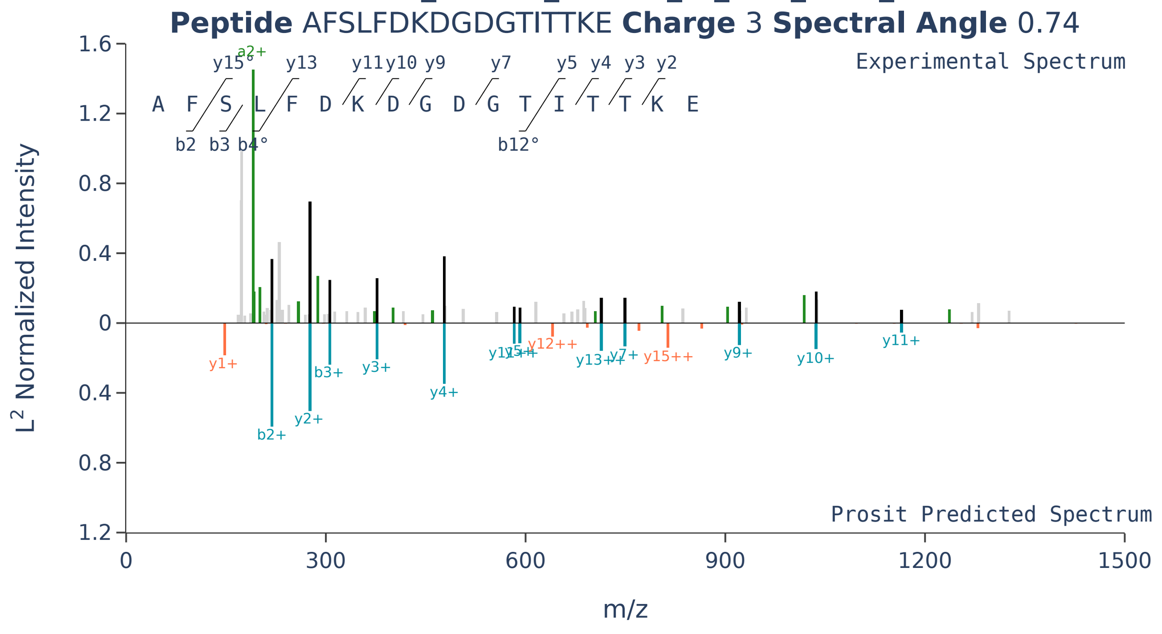


**Source** HRoetschke_291222_040123_Fu_CaM_b2R2_4h **Scan** 7750
**Peptide** GDGTITTKELGTVMR **Charge** 3 **Spectral Angle** 0.76

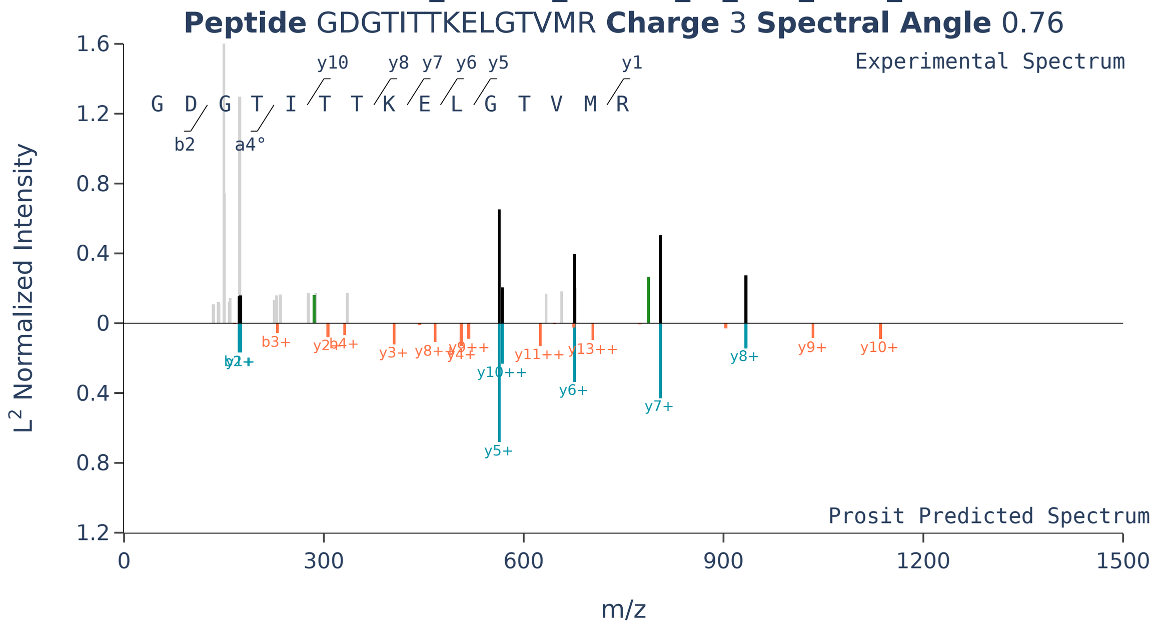


**Source** HRoetschke_291222_040123_Fu_CaM_b1R2_2h **Scan** 11721
**Peptide** KDTDSEEEIREAFRVFD **Charge** 4 **Spectral Angle** 0.73

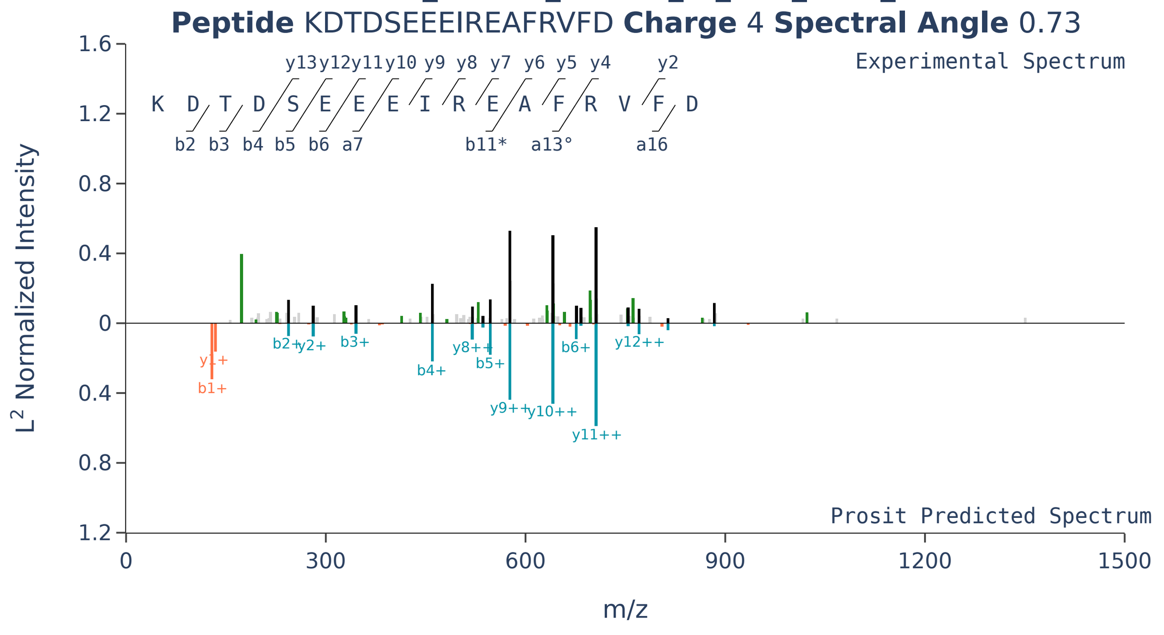


**Source** HRoetschke_291222_040123_Fu_CaM_b1R2_2h **Scan** 2843
**Peptide** TDSEEEIRE **Charge** 2 **Spectral Angle** 0.78

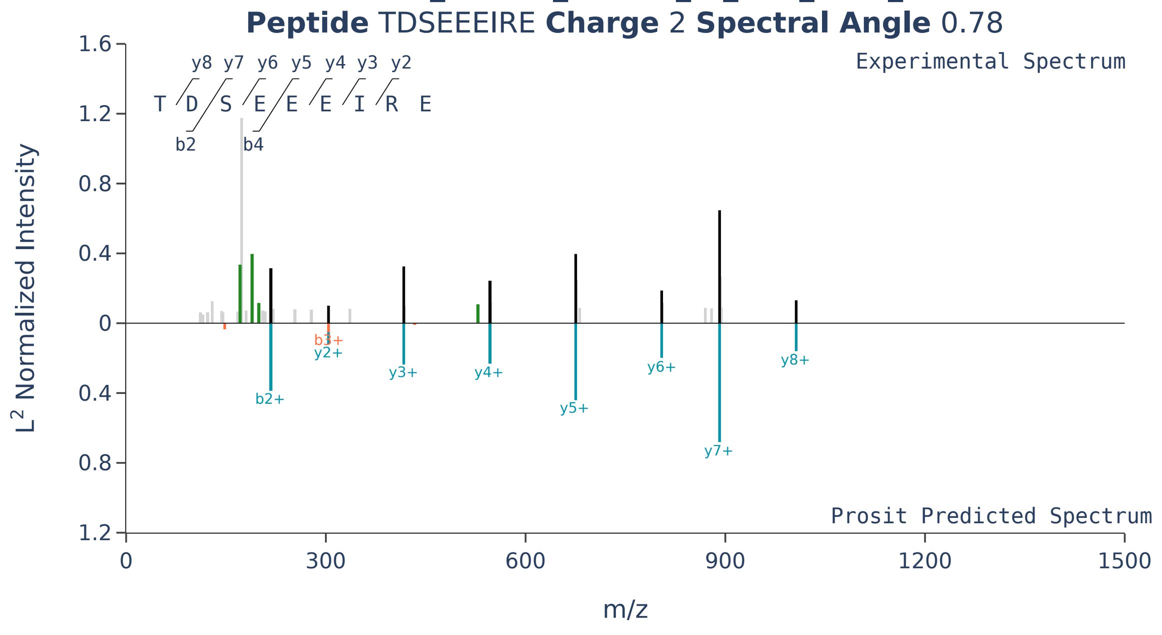


**Source** HRoetschke_291222_040123_Fu_CaM_b1R2_4h **Scan** 2896
**Peptide** RSLGQNPTEA **Charge** 2 **Spectral Angle** 0.88

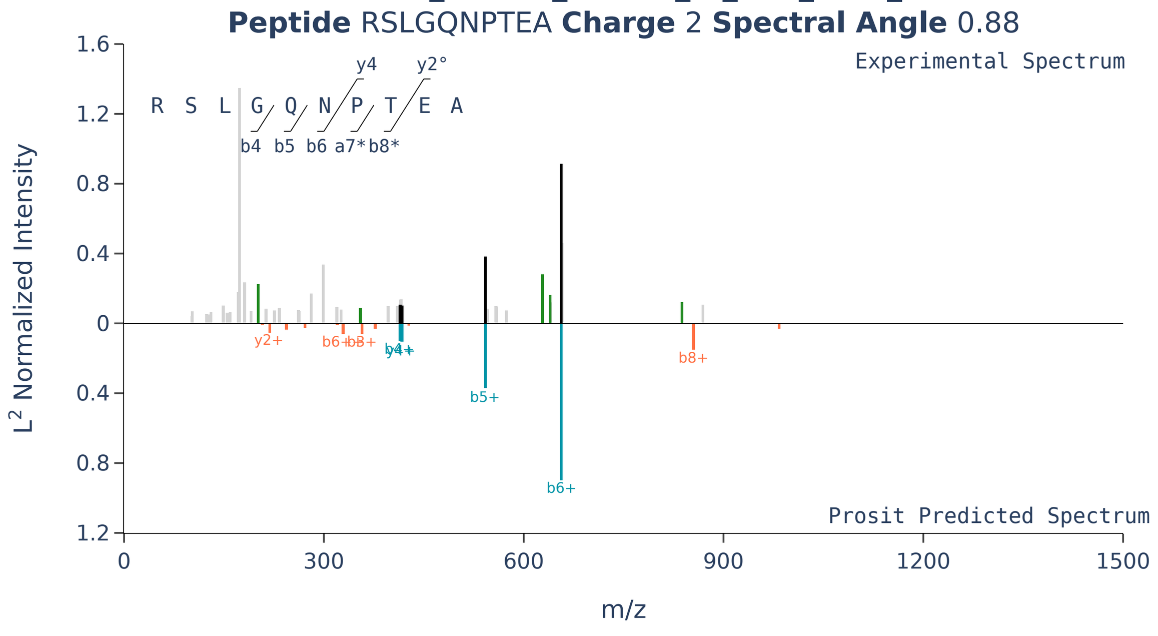


**Source** HRoetschke_291222_040123_Fu_CaM_b1R1_24h **Scan** 6688
**Peptide** FDKDGDGTITTKELG **Charge** 3 **Spectral Angle** 0.77

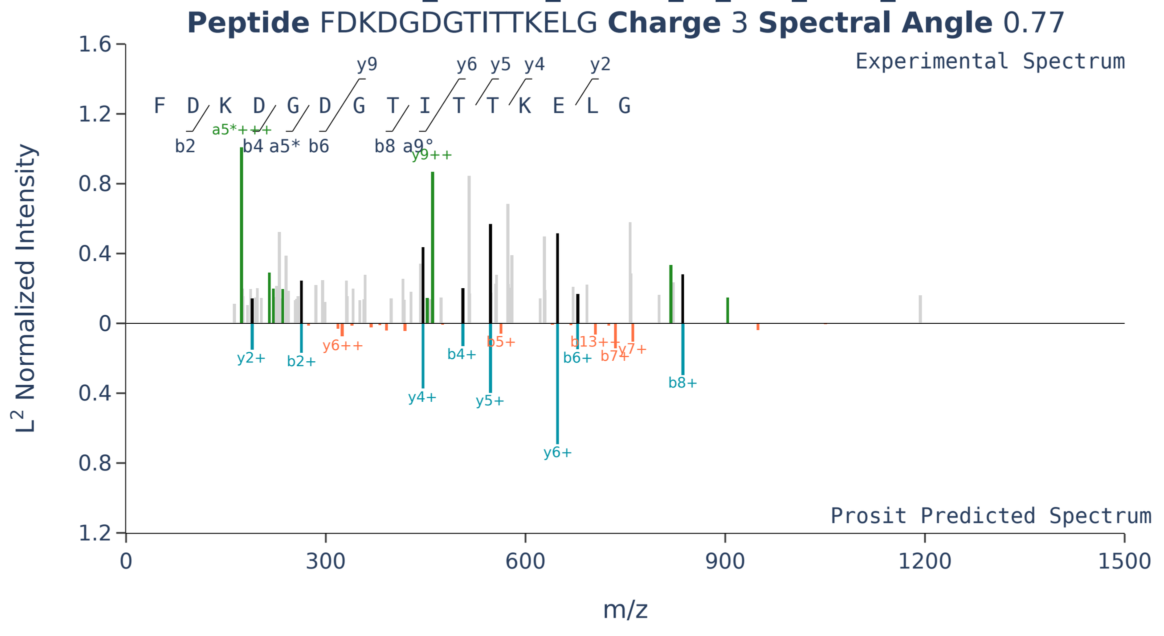


**Source** HRoetschke_291222_040123_Fu_CaM_b2R2_24h **Scan** 13547
**Peptide** DTDSEEEIREAFRVF **Charge** 3 **Spectral Angle** 0.78

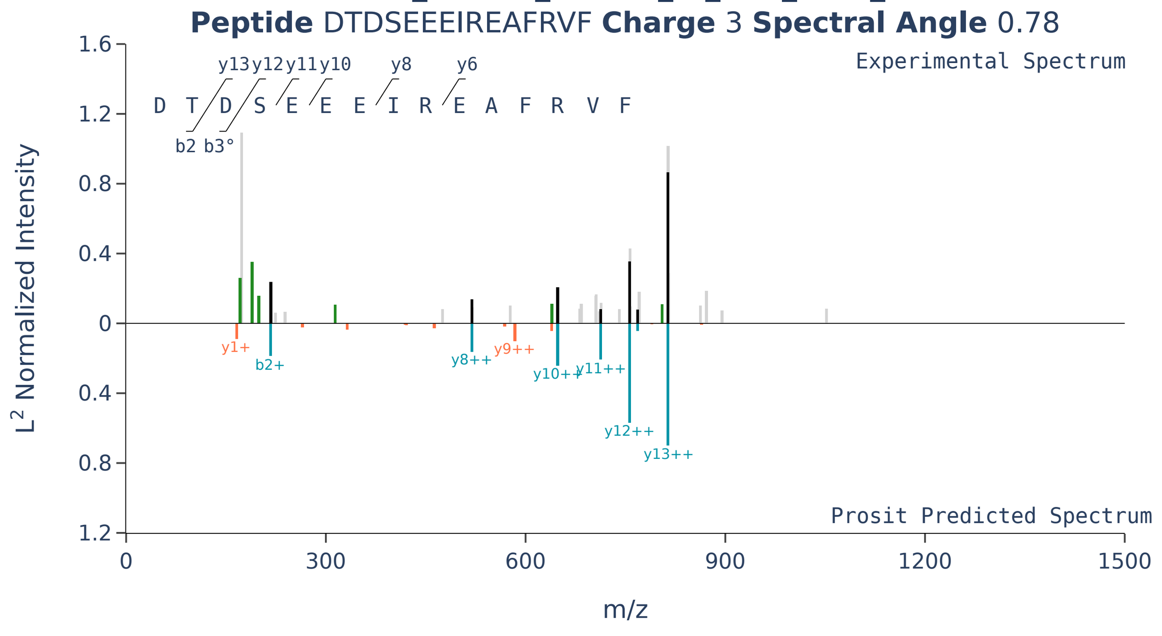


**Source** HRoetschke_291222_040123_Fu_CaM_b1R1_24h **Scan** 5169
**Peptide** FDKDGNGYIS **Charge** 2 **Spectral Angle** 0.81

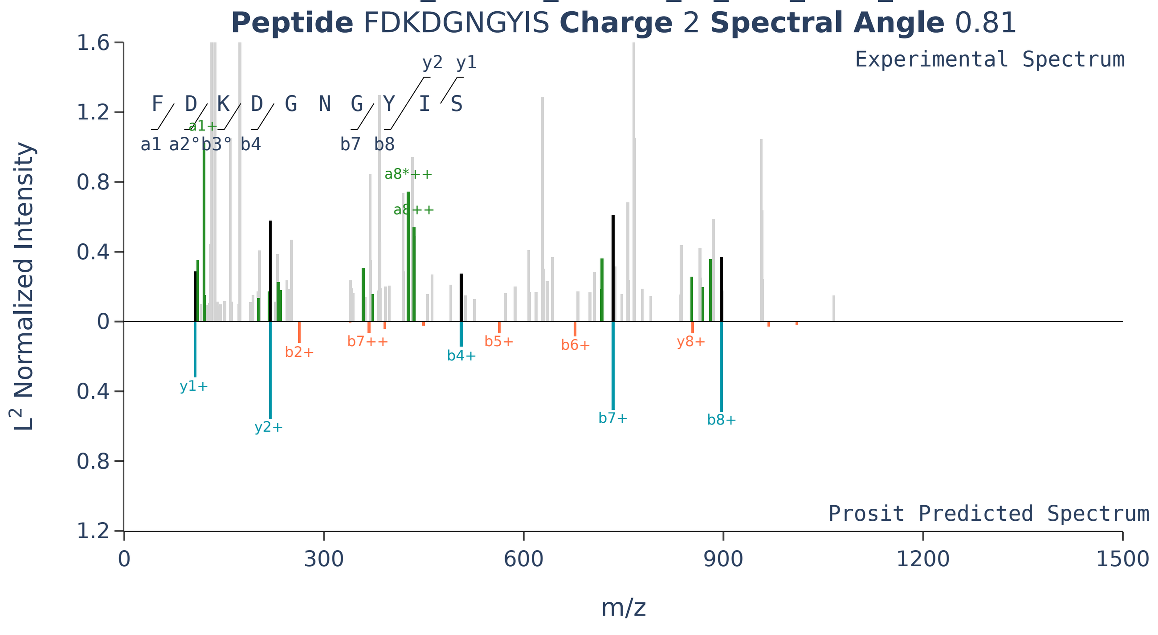


**Source** HRoetschke_291222_040123_Fu_CaM_b1R2_24h **Scan** 7214
**Peptide** EEIREAF **Charge** 2 **Spectral Angle** 0.86

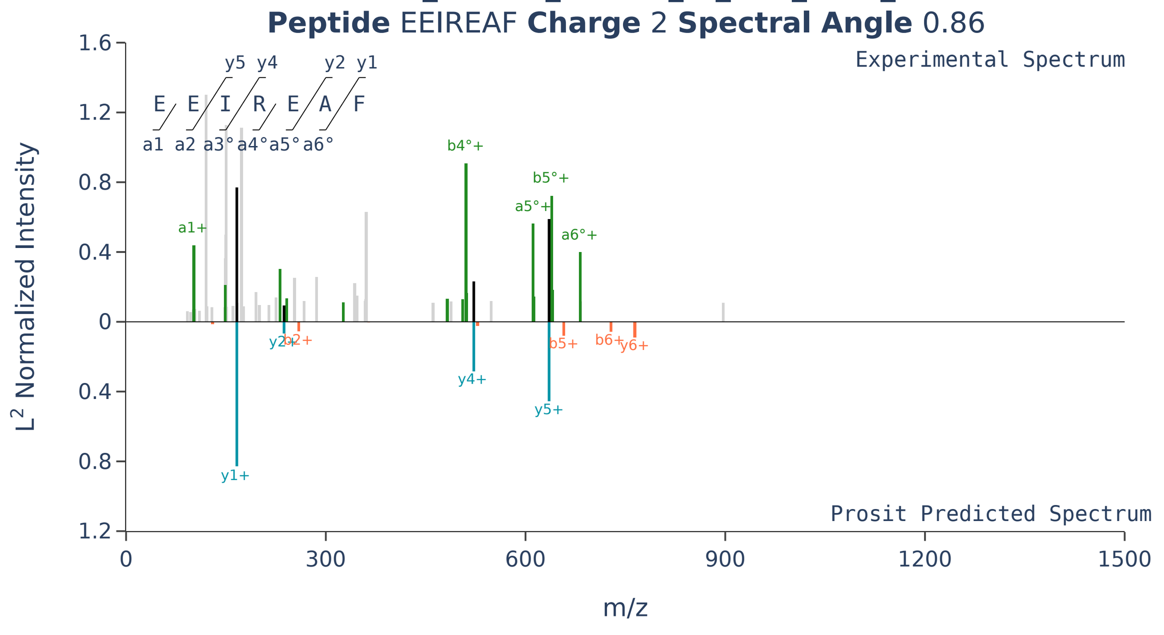


**Source** HRoetschke_291222_040123_Fu_CaM_b2R1_24h **Scan** 9702
**Peptide** RKMKDTDSEEEIREAFRVFD **Charge** 4 **Spectral Angle** 0.75

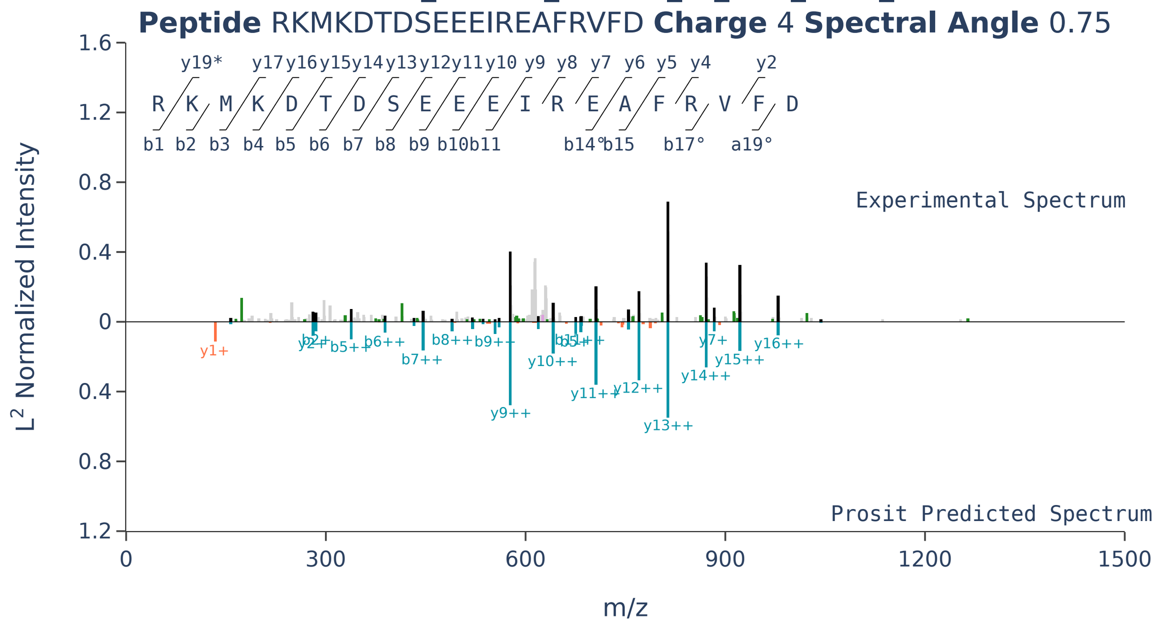


**Source** HRoetschke_291222_040123_Fu_CaM_b1R2_1h **Scan** 6749
**Peptide** MTNLGEKLTDE **Charge** 2 **Spectral Angle** 0.76

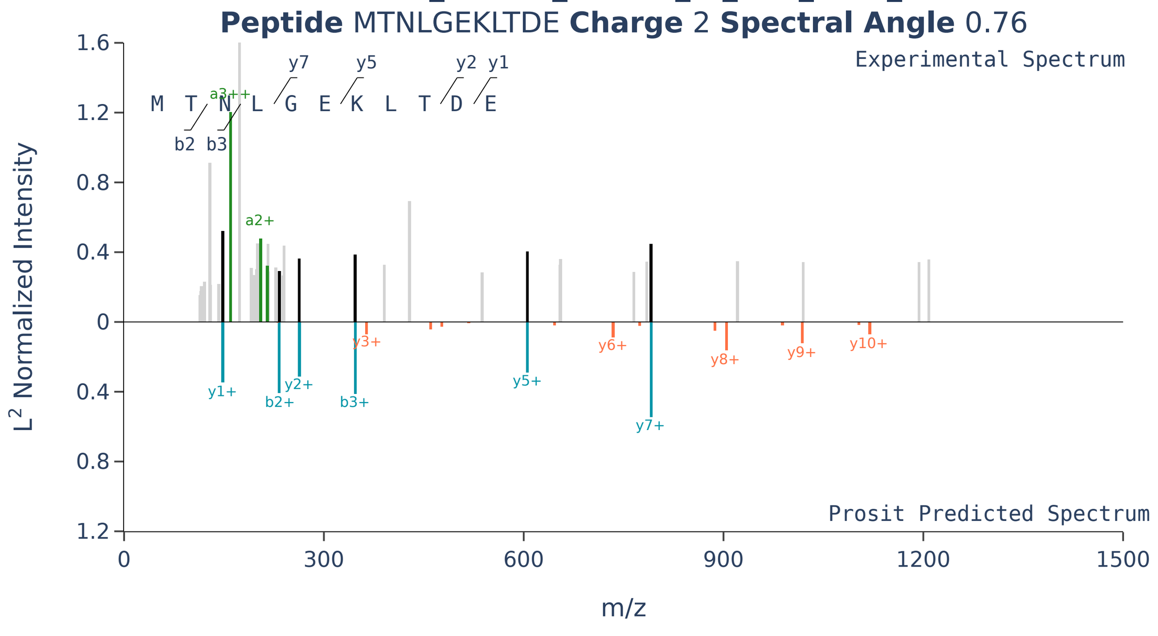


**Source** HRoetschke_291222_040123_Fu_CaM_b1R1_2h **Scan** 6940
**Peptide** TEEQIAEFKEA **Charge** 2 **Spectral Angle** 0.78

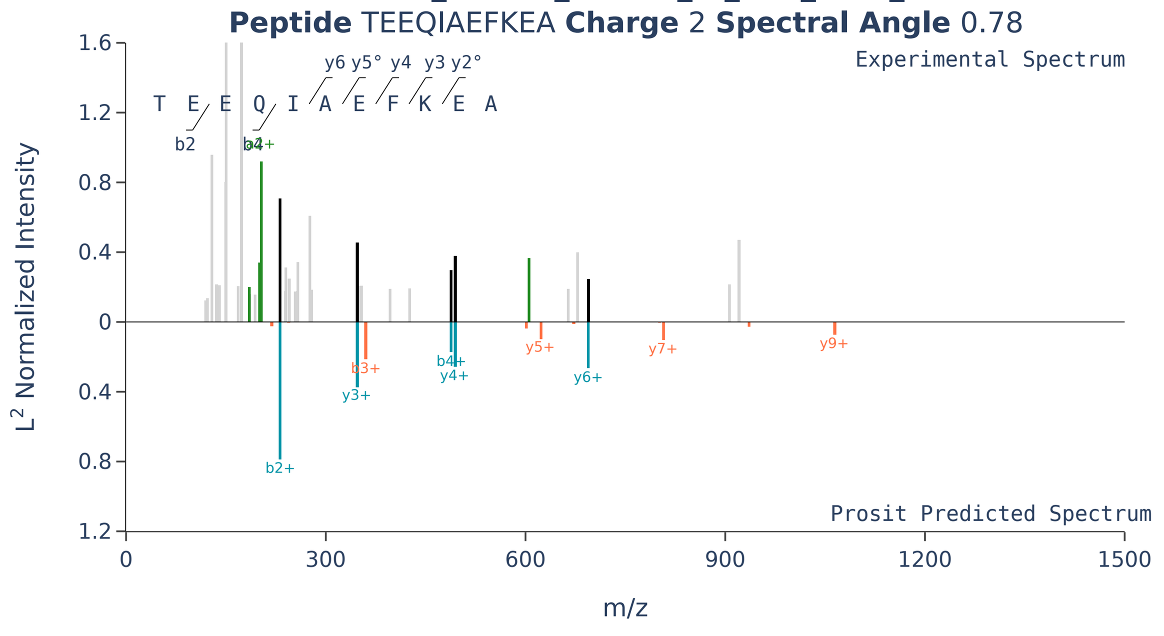


**Source** HRoetschke_291222_040123_Fu_CaM_b2R2_24h **Scan** 7344
**Peptide** FRVFDKDGNGY **Charge** 3 **Spectral Angle** 0.68

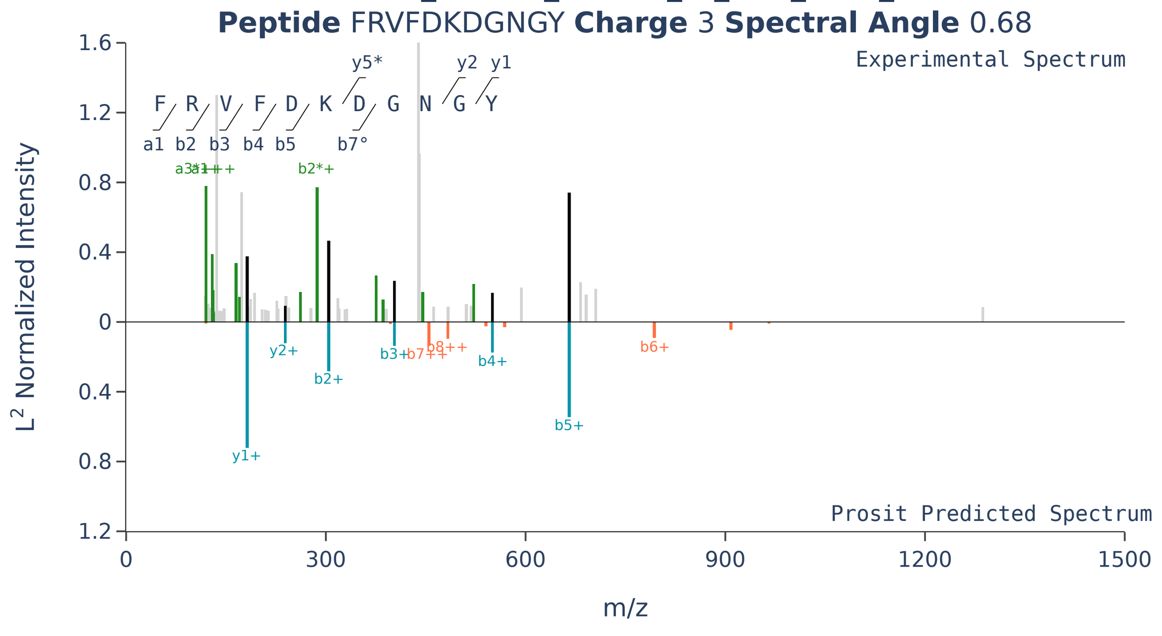

**Source** HRoetschke_291222_040123_Fu_CaM_b1R1_4h **Scan** 8635
**Peptide** ELRHVMTNLGEKL **Charge** 3 **Spectral Angle** 0.71

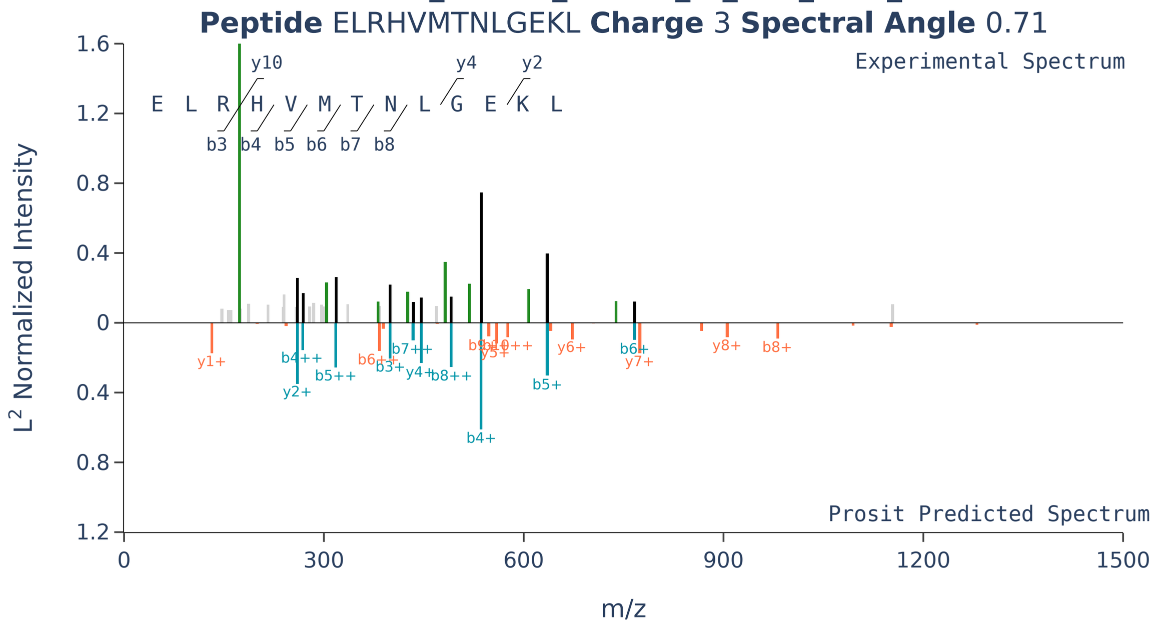


**Source** HRoetschke_291222_040123_Fu_CaM_b1R2_24h **Scan** 10169
**Peptide** ADQLTEEQIAEFKE **Charge** 2 **Spectral Angle** 0.78

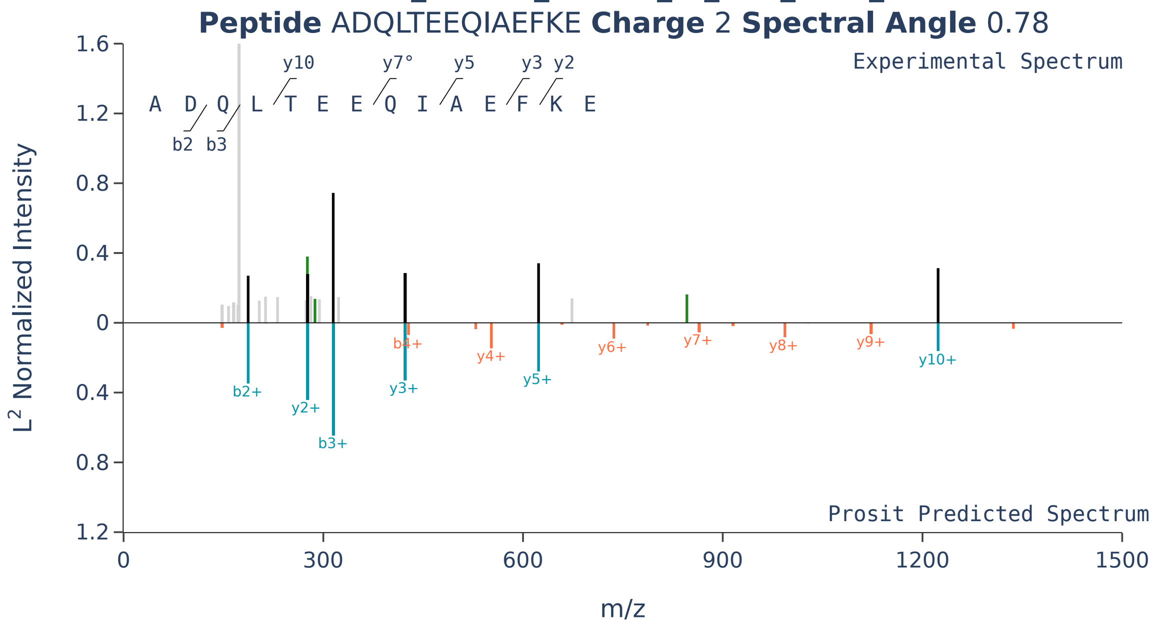


**Source** HRoetschke_291222_040123_Fu_CaM_b2R1_24h **Scan** 11238
**Peptide** DKDGDGTITTKELGTVMRSLGQNPTE **Charge** 3 **Spectral Angle** 0.69

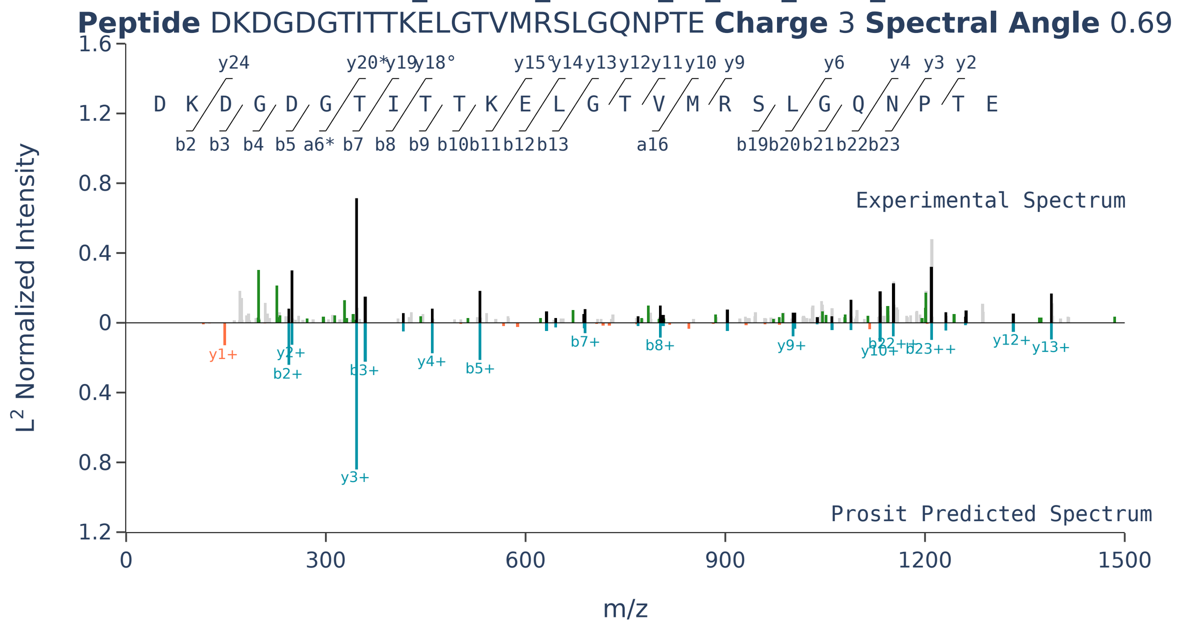


**Source** HRoetschke_291222_040123_Fu_CaM_b2R1_24h **Scan** 9779
**Peptide** SLFDKDGDGTITTKELGTV **Charge** 3 **Spectral Angle** 0.66

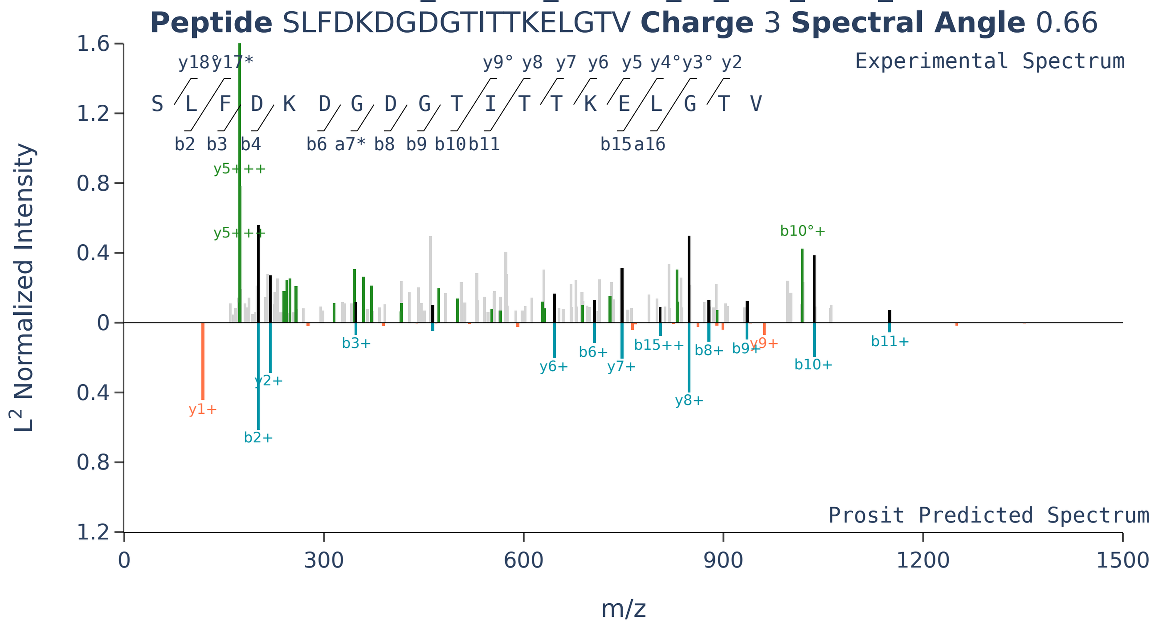


**Source** HRoetschke_291222_040123_Fu_CaM_b1R1_24h **Scan** 9417
**Peptide** RVFDKDGNGYISAAELR **Charge** 3 **Spectral Angle** 0.61

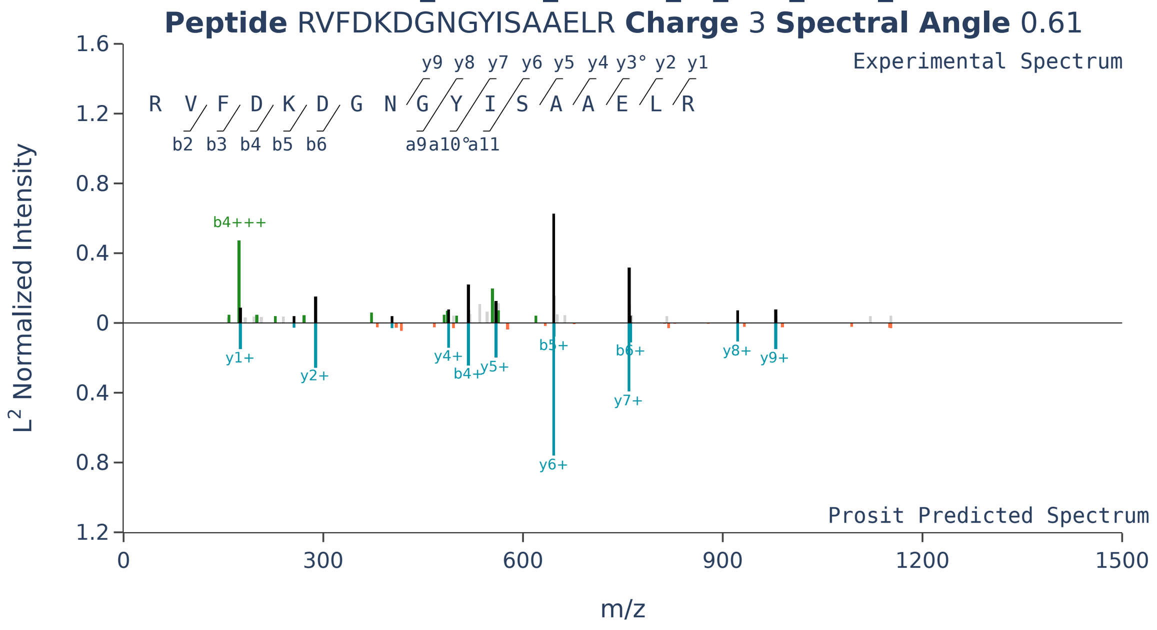


**Source** HRoetschke_291222_040123_Fu_CaM_b1R1_24h **Scan** 3009
**Peptide** READIDGDGQVN **Charge** 2 **Spectral Angle** 0.76

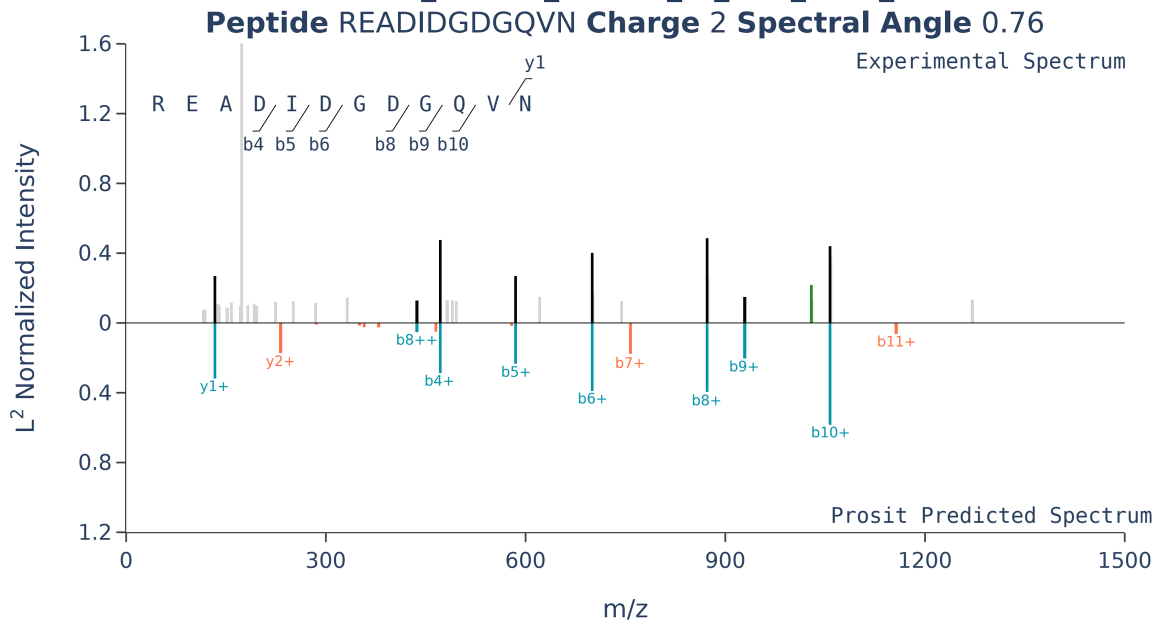


**Source** HRoetschke_291222_040123_Fu_CaM_b2R2_24h **Scan** 5006
**Peptide** TNLGEKL **Charge** 2 **Spectral Angle** 0.81

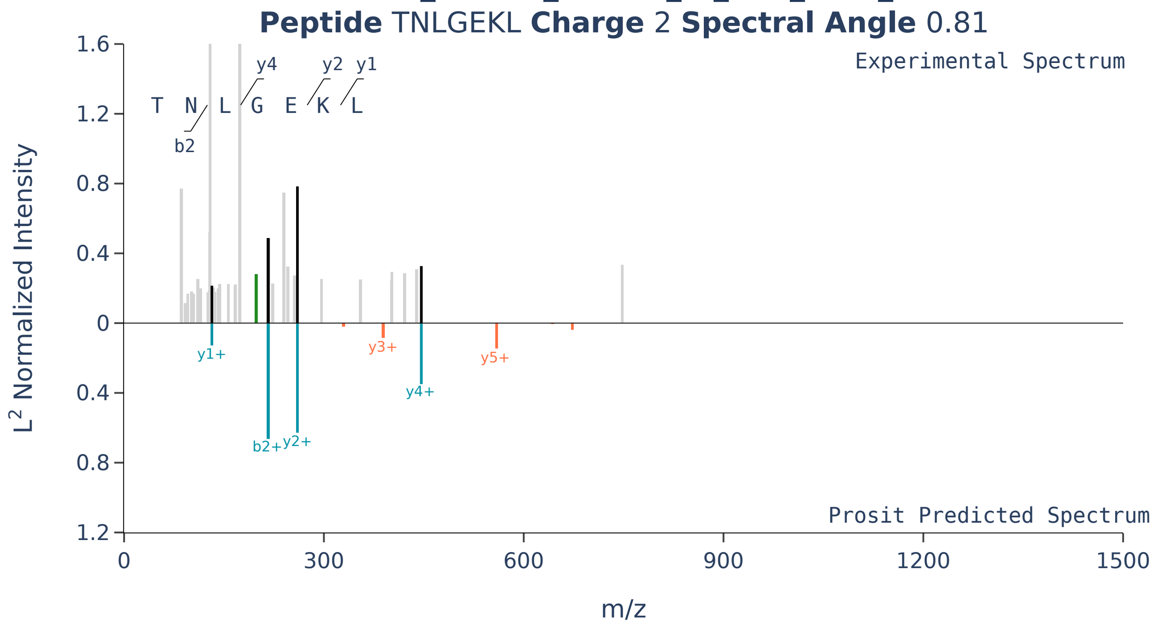


**Source** HRoetschke_291222_040123_Fu_CaM_b2R1_24h **Scan** 8589
**Peptide** KDTDSEEEIREAFRV **Charge** 3 **Spectral Angle** 0.68

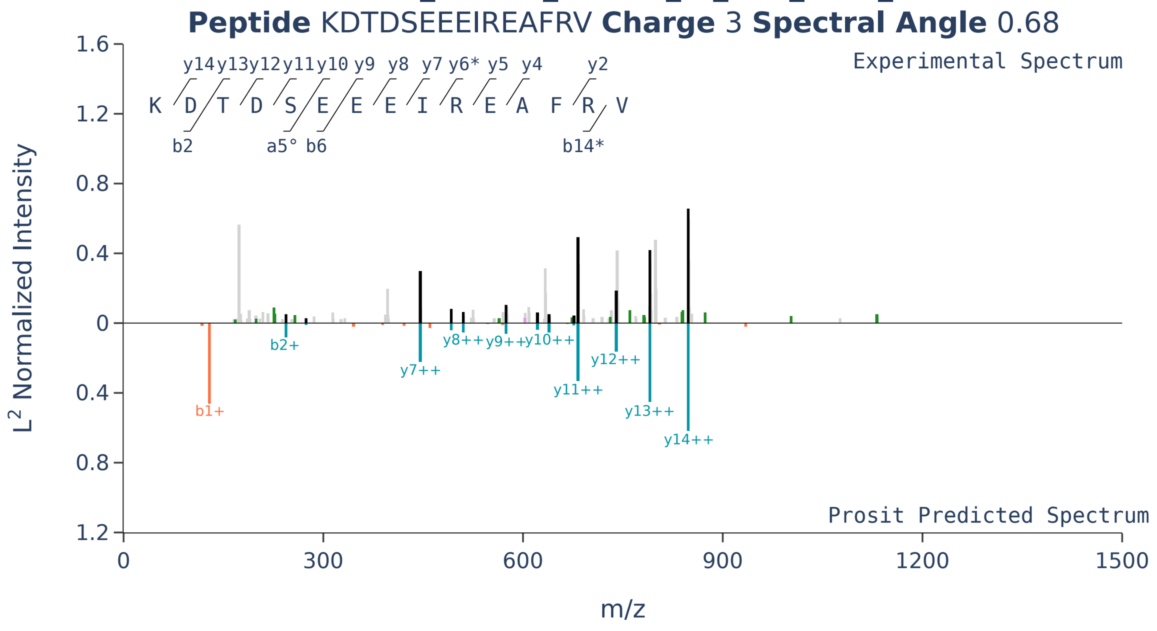


**Source** HRoetschke_291222_040123_Fu_CaM_b1R2_1h **Scan** 2650
**Peptide** TMMARKM **Charge** 2 **Spectral Angle** 0.87

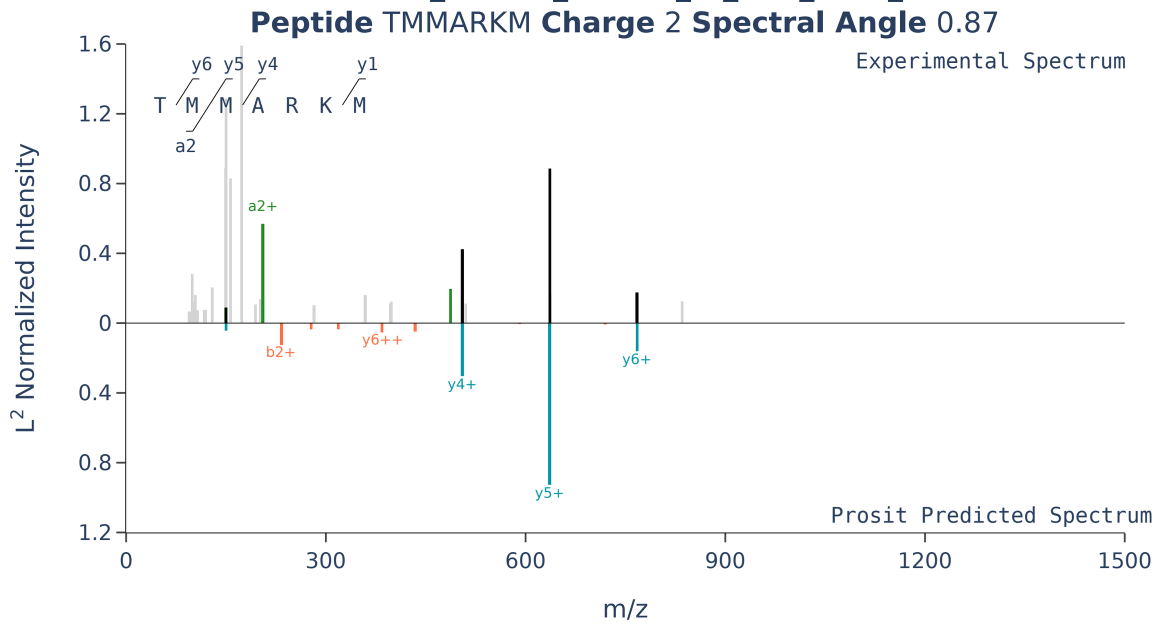


**Source** HRoetschke_291222_040123_Fu_CaM_b1R2_1h **Scan** 7917
**Peptide** EMIREADIDGDGQVNYE **Charge** 2 **Spectral Angle** 0.8

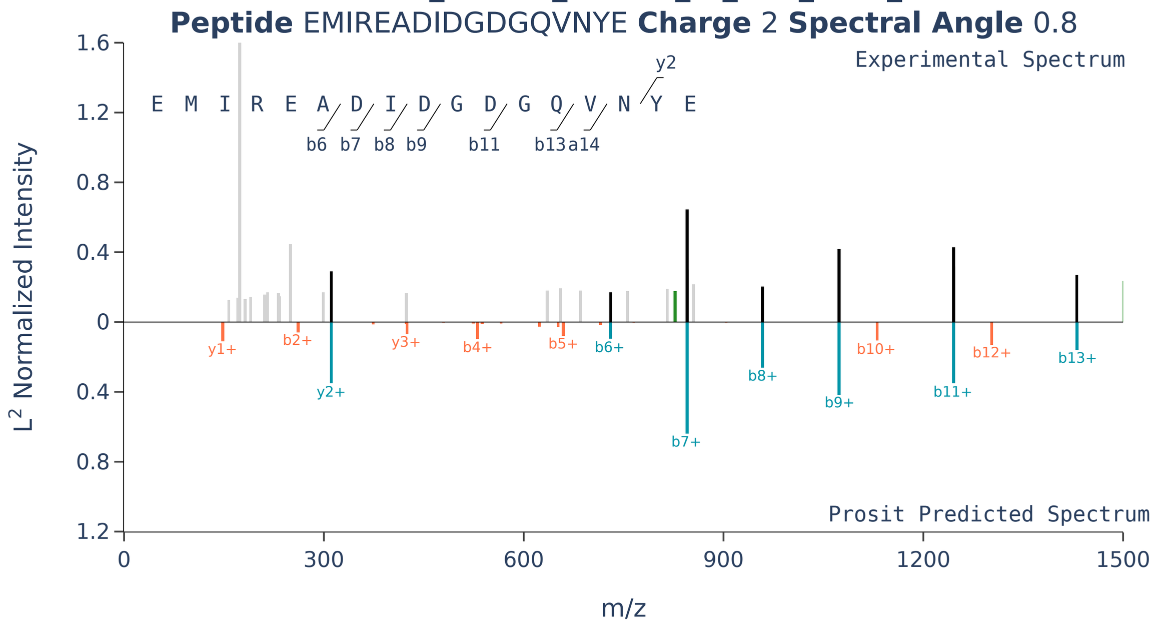


**Source** HRoetschke_291222_040123_Fu_CaM_b1R1_24h **Scan** 9052
**Peptide** TDEEVDEMIRE **Charge** 2 **Spectral Angle** 0.73

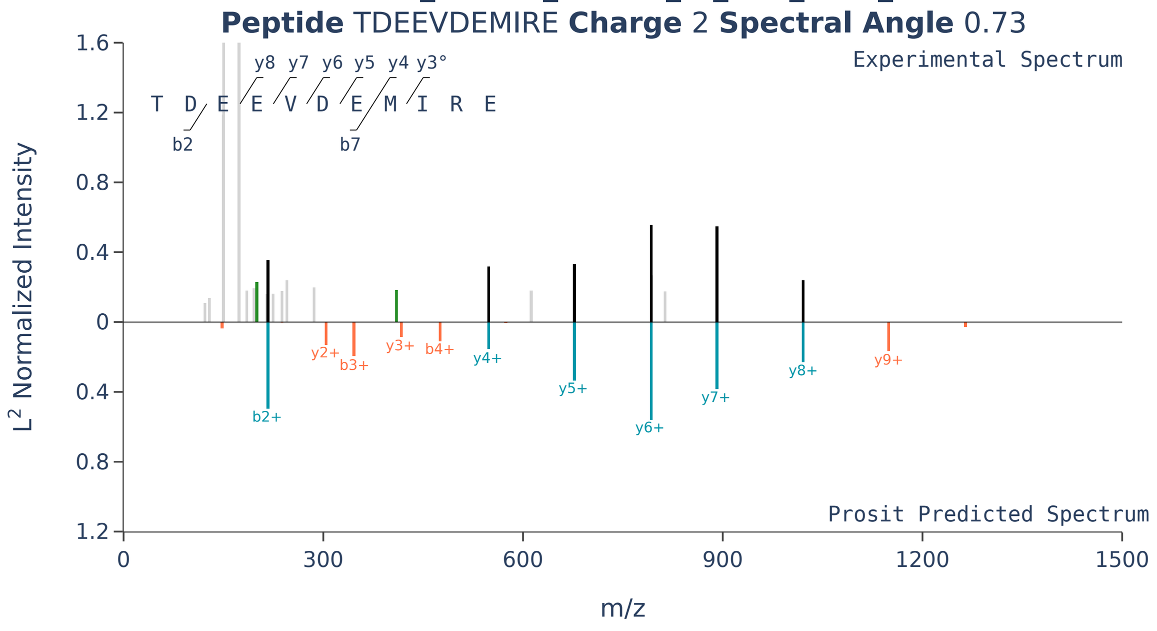


**Source** HRoetschke_291222_040123_Fu_CaM_b2R1_2h **Scan** 10120
**Peptide** AFRVFDKDGNGYISAAELR **Charge** 4 **Spectral Angle** 0.71

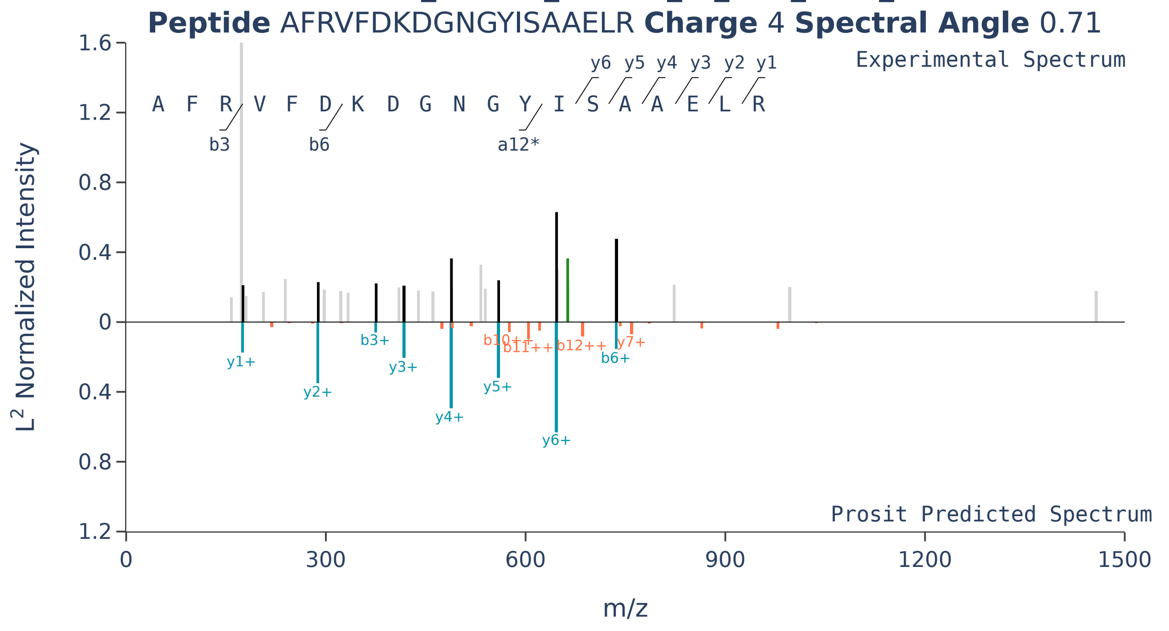


**Source** HRoetschke_291222_040123_Fu_CaM_b1R2_1h **Scan** 5722
**Peptide** FDKDGNGYL **Charge** 2 **Spectral Angle** 0.84

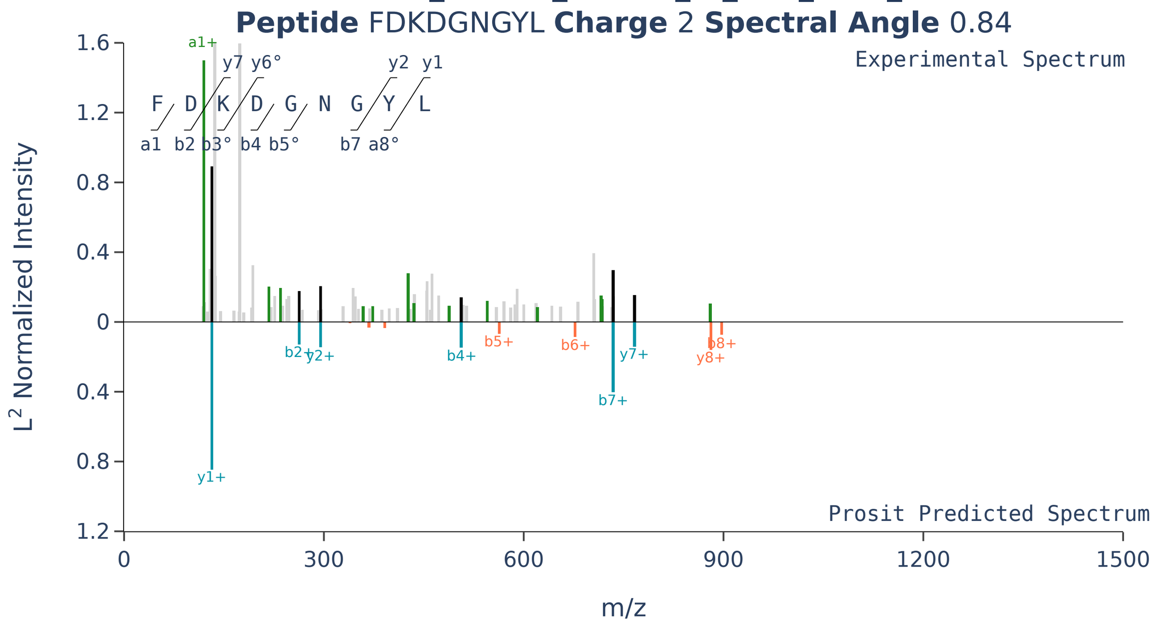


**Source** HRoetschke_291222_040123_Fu_CaM_b1R1_24h **Scan** 10181
**Peptide** GDGQVNYEEF **Charge** 2 **Spectral Angle** 0.9

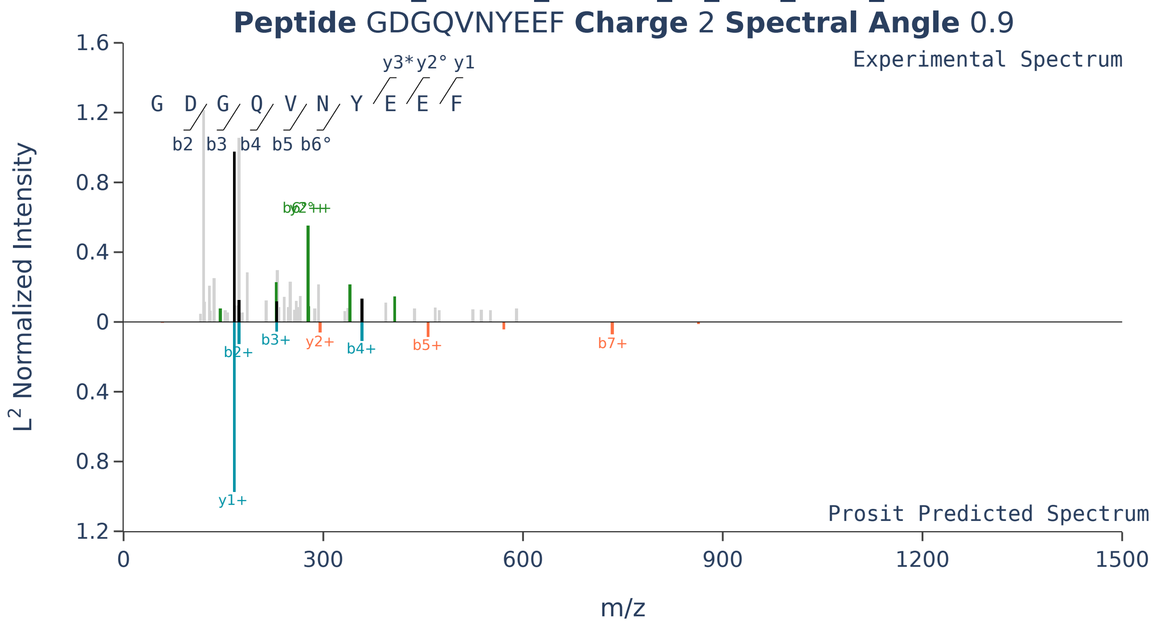


**Source** HRoetschke_291222_040123_Fu_CaM_b2R2_24h **Scan** 13478
**Peptide** READIDGDGQVNYEEFVQMMTA **Charge** 3 **Spectral Angle** 0.76

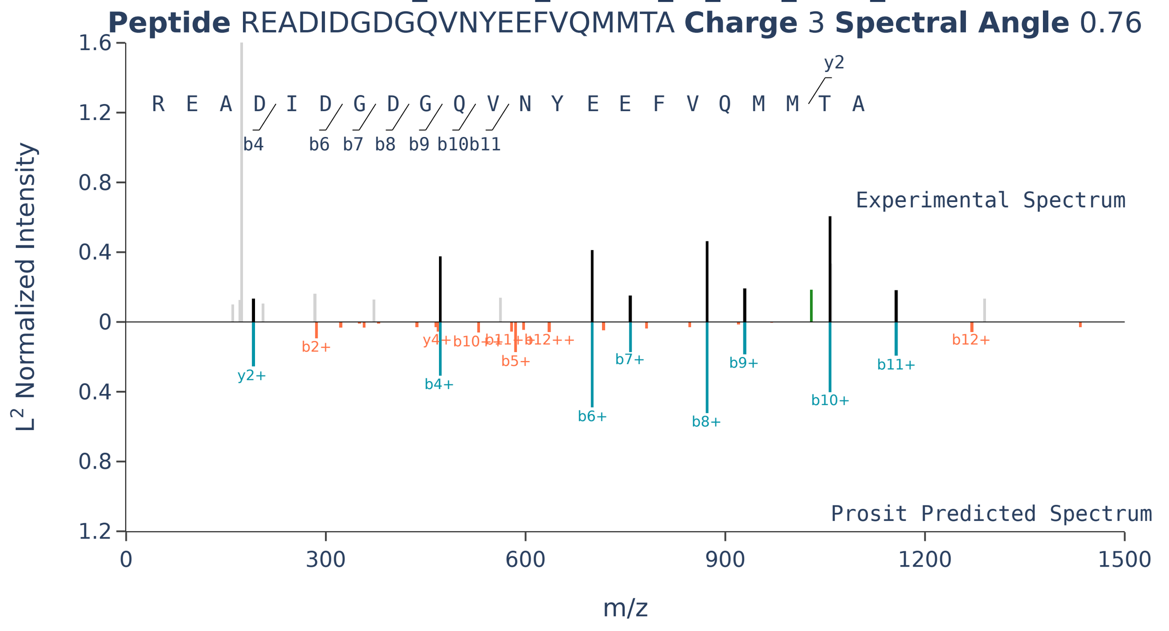

**Source** HRoetschke_291222_040123_Fu_CaM_b2R1_24h **Scan** 10915
**Peptide** LDGDGQVNYEEFV **Charge** 2 **Spectral Angle** 0.7

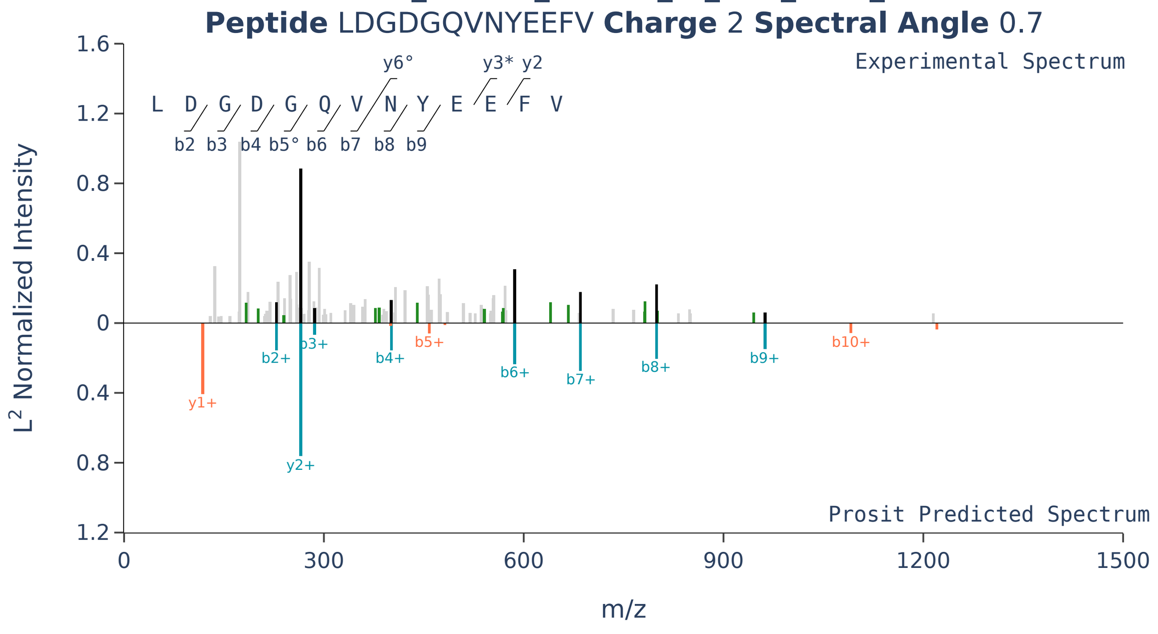


**Source** HRoetschke_291222_040123_Fu_CaM_b1R1_4h **Scan** 5463
**Peptide** ARKMKDTDSEEEIREAFR **Charge** 4 **Spectral Angle** 0.79

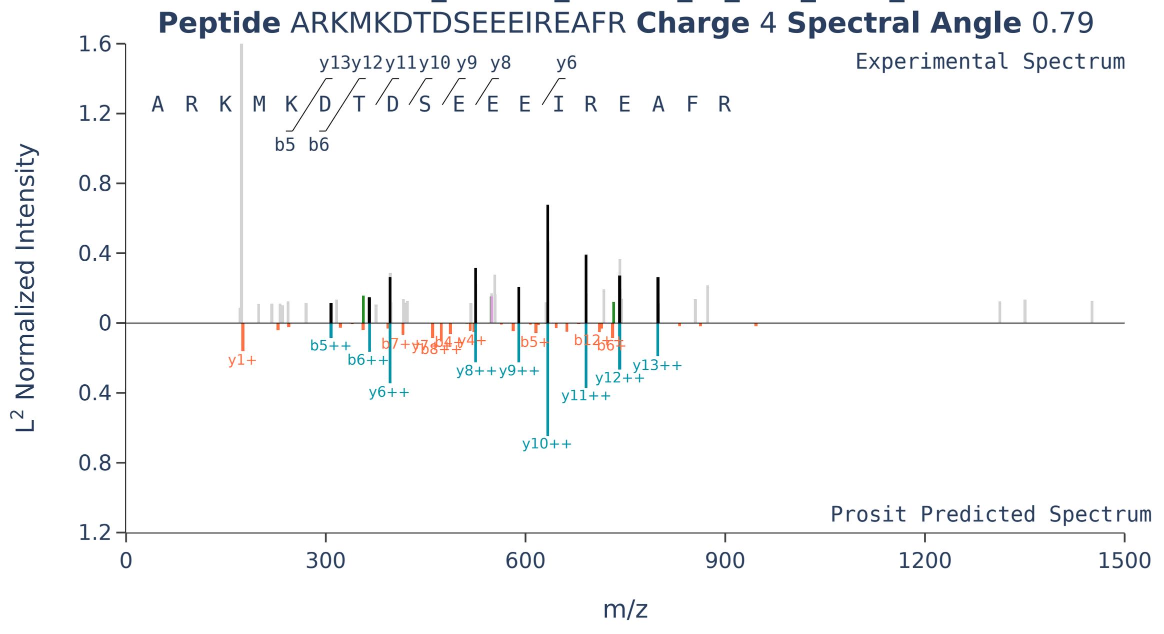


**Source** HRoetschke_291222_040123_Fu_CaM_b1R2_24h **Scan** 5895
**Peptide** KELGTVMRSLGQNPTE **Charge** 3 **Spectral Angle** 0.62

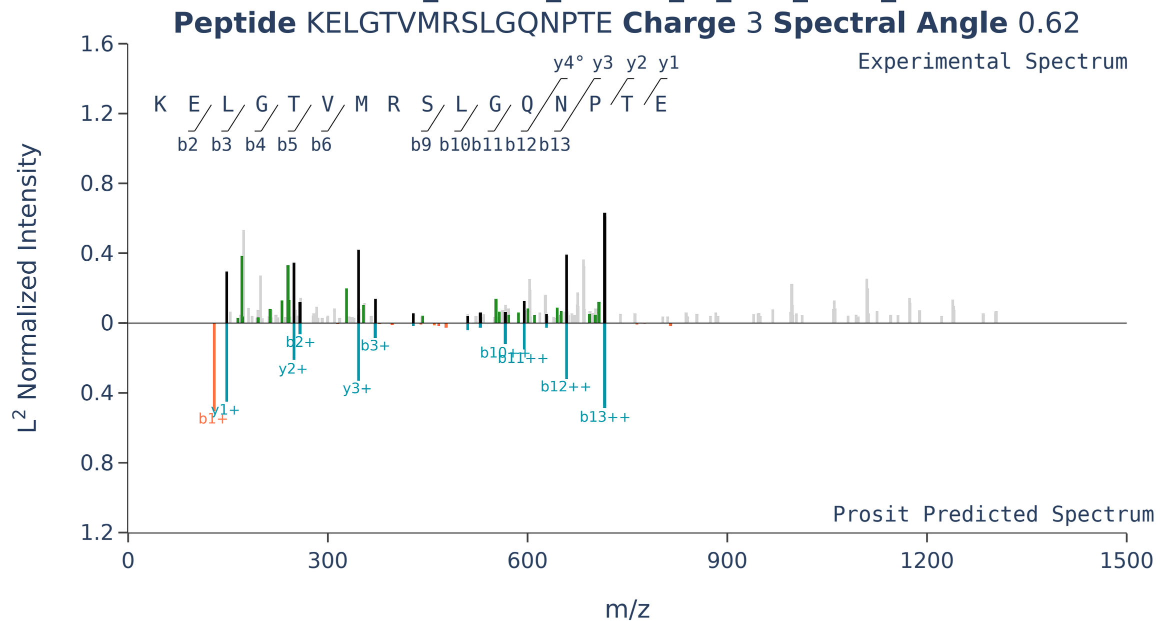


**Source** HRoetschke_291222_040123_Fu_CaM_b2R1_1h **Scan** 6006
**Peptide** KDTDSEEEIREAFR **Charge** 3 **Spectral Angle** 0.73

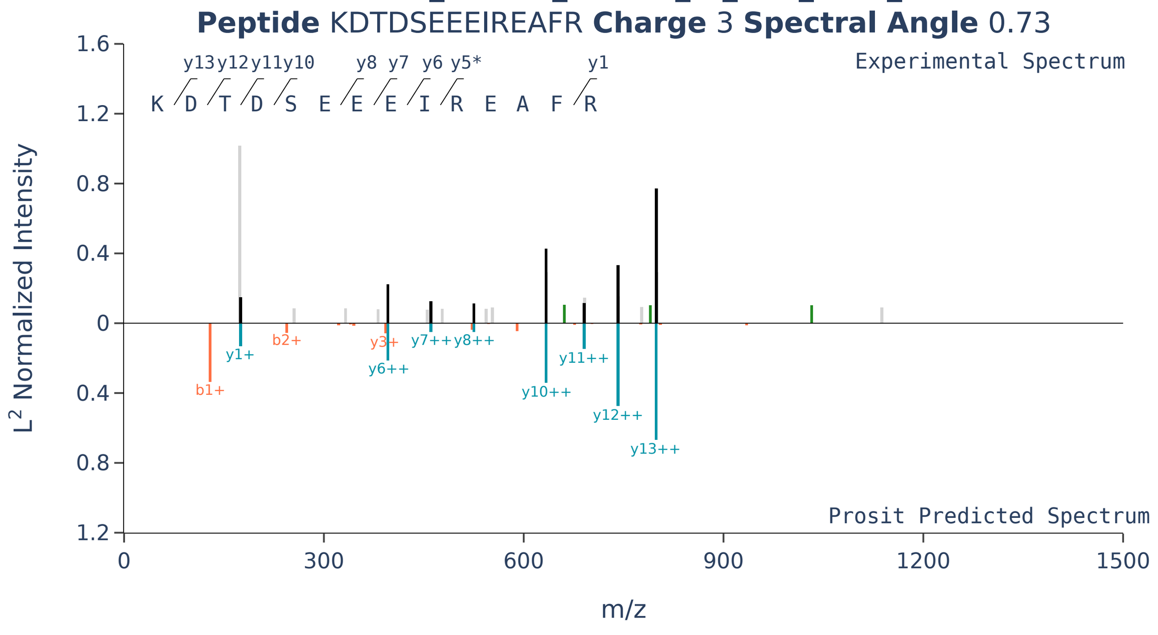


**Source** HRoetschke_291222_040123_Fu_CaM_b1R1_4h **Scan** 9030
**Peptide** TDSEEEIREAF **Charge** 3 **Spectral Angle** 0.83

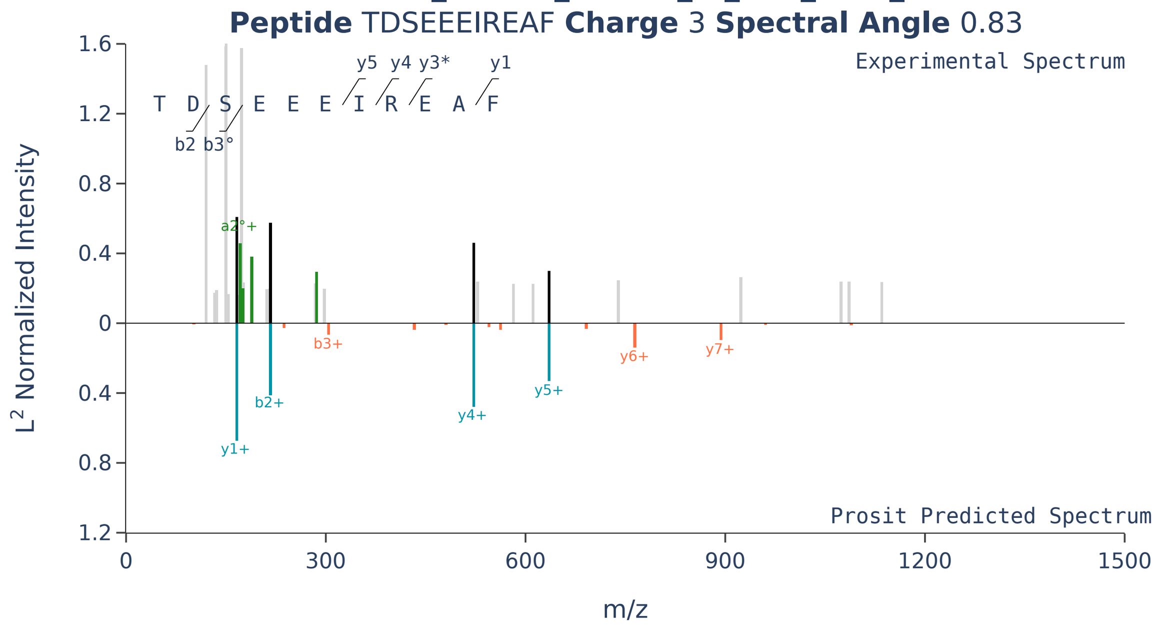


**Source** HRoetschke_291222_040123_Fu_CaM_b1R1_24h **Scan** 6014
**Peptide** GDGTITTKELGT **Charge** 2 **Spectral Angle** 0.71

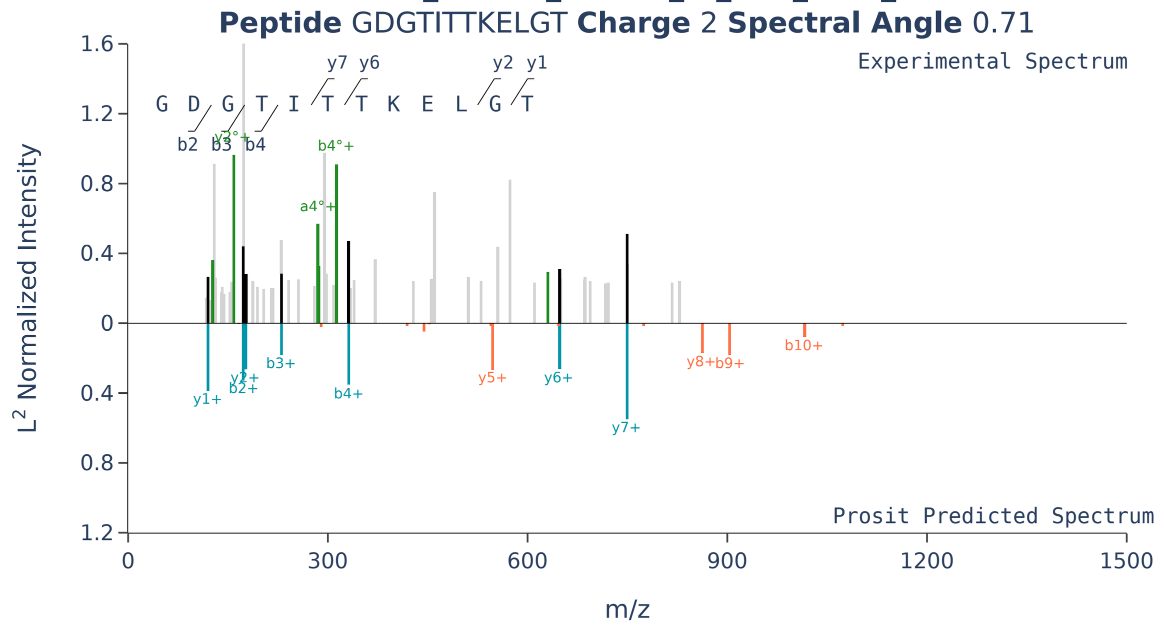


**Source** HRoetschke_291222_040123_Fu_CaM_b1R2_24h **Scan** 3073
**Peptide** MKDTDSEEEIRE **Charge** 3 **Spectral Angle** 0.86

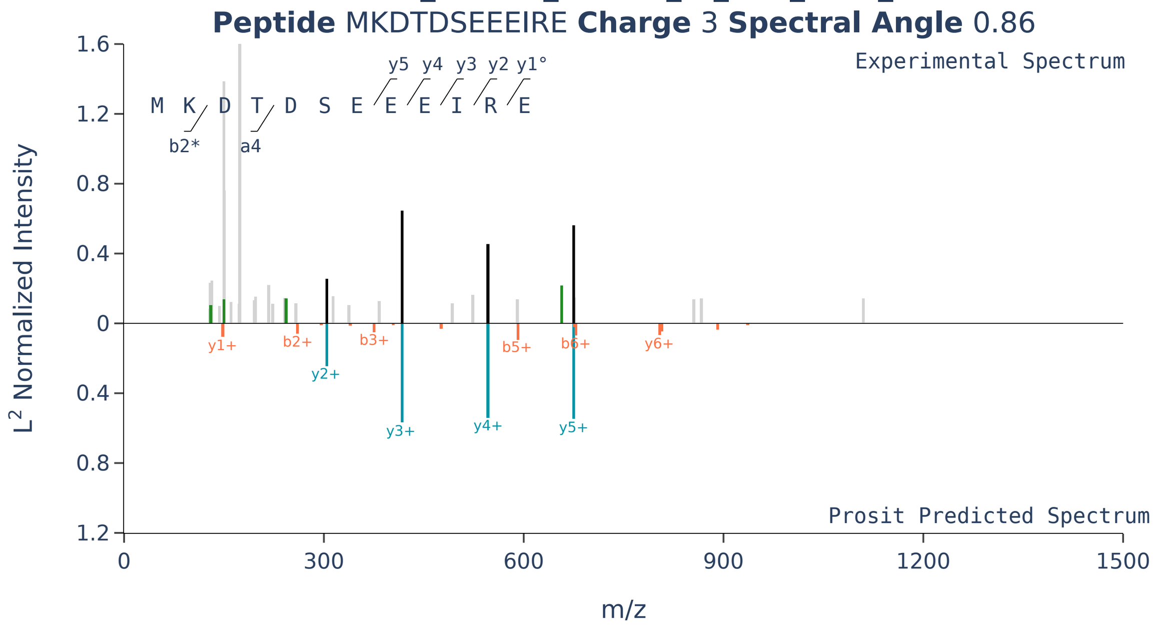


**Source** HRoetschke_291222_040123_Fu_CaM_b1R1_4h **Scan** 8726
**Peptide** GTVMRSLGQNPTEAELQD **Charge** 2 **Spectral Angle** 0.77

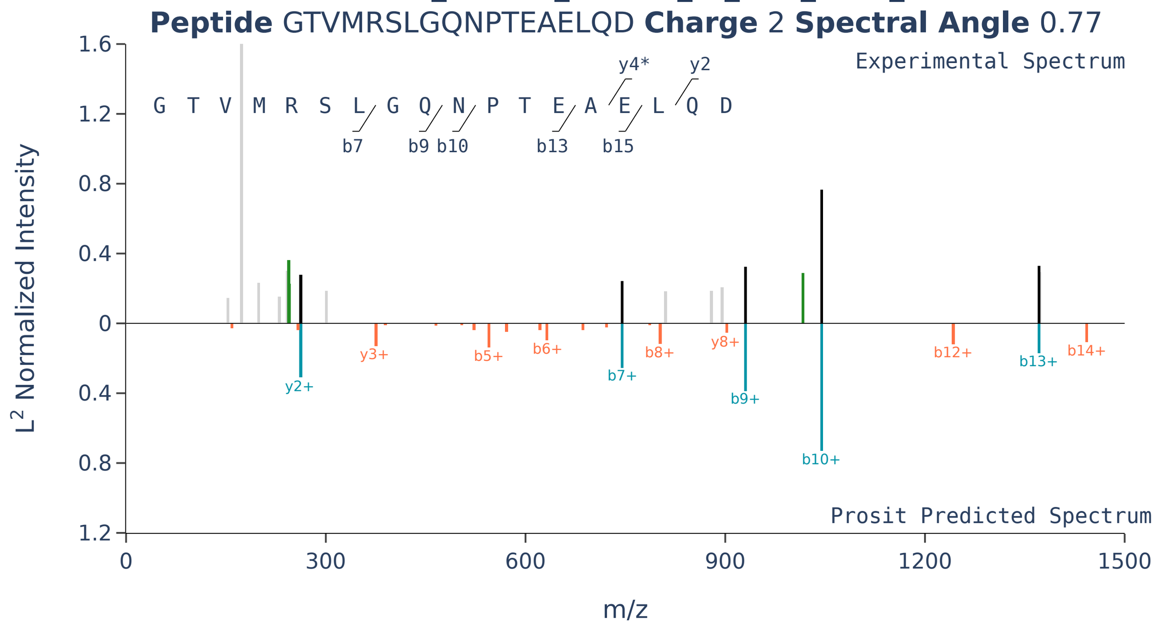


**Source** HRoetschke_291222_040123_Fu_CaM_b1R2_2h **Scan** 8546
**Peptide** REAFRVFDKDGNGYIS **Charge** 3 **Spectral Angle** 0.75

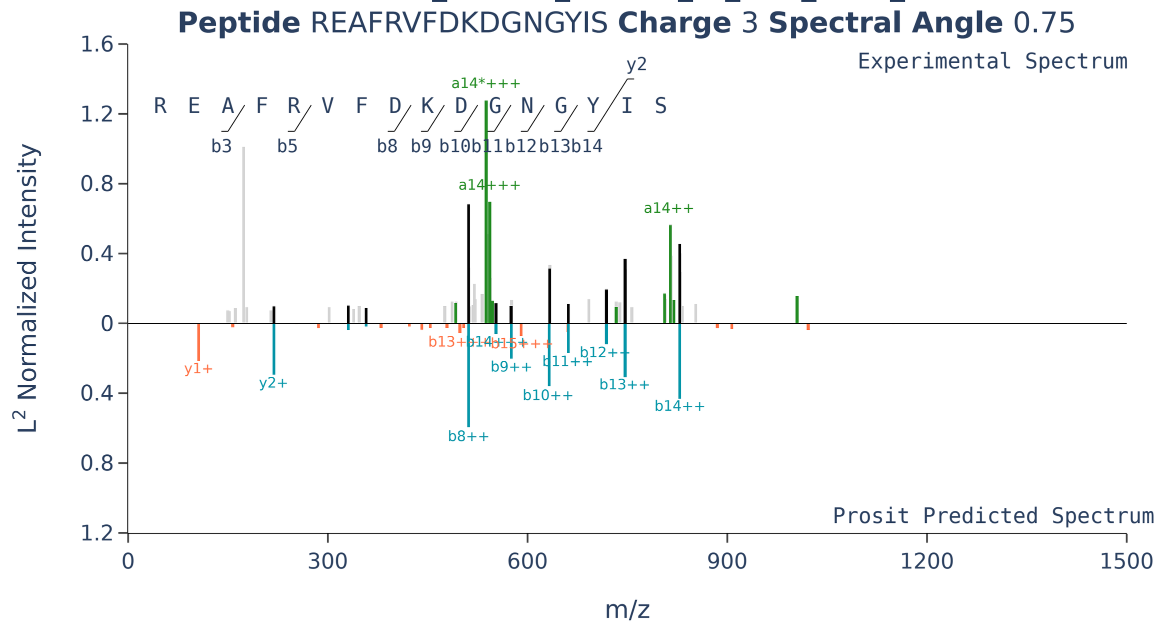


**Source** HRoetschke_291222_040123_Fu_CaM_b1R2_24h **Scan** 7886
**Peptide** KELGTVMRSLGQ **Charge** 3 **Spectral Angle** 0.67

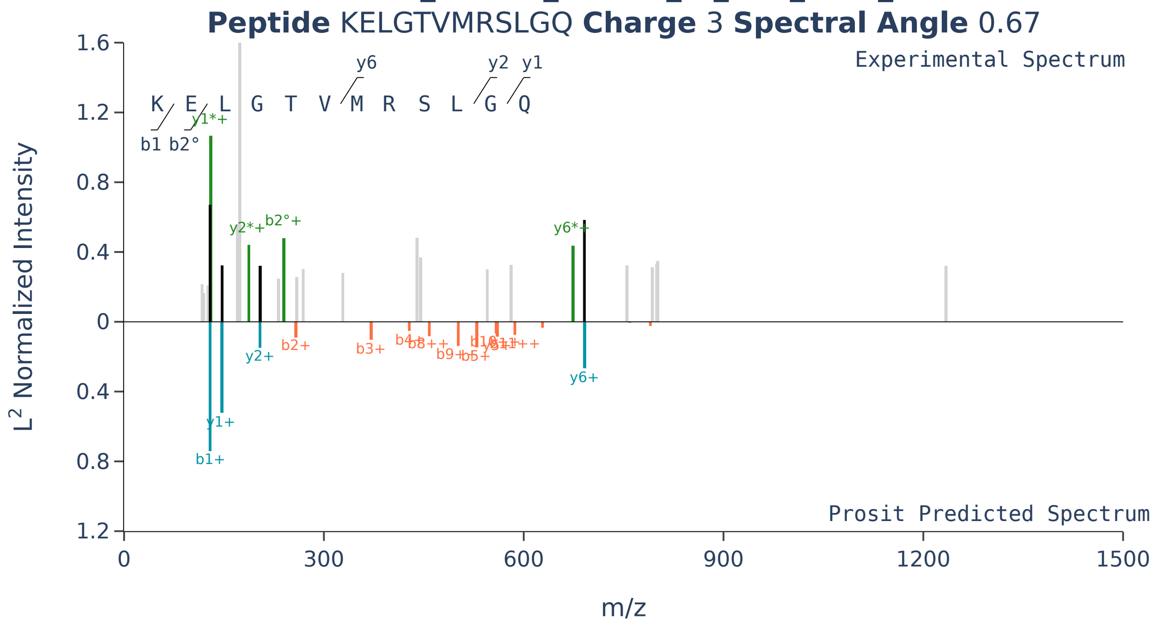


**Source** HRoetschke_291222_040123_Fu_CaM_b2R1_4h **Scan** 4877
**Peptide** READIDGDGQVNYEE **Charge** 2 **Spectral Angle** 0.76

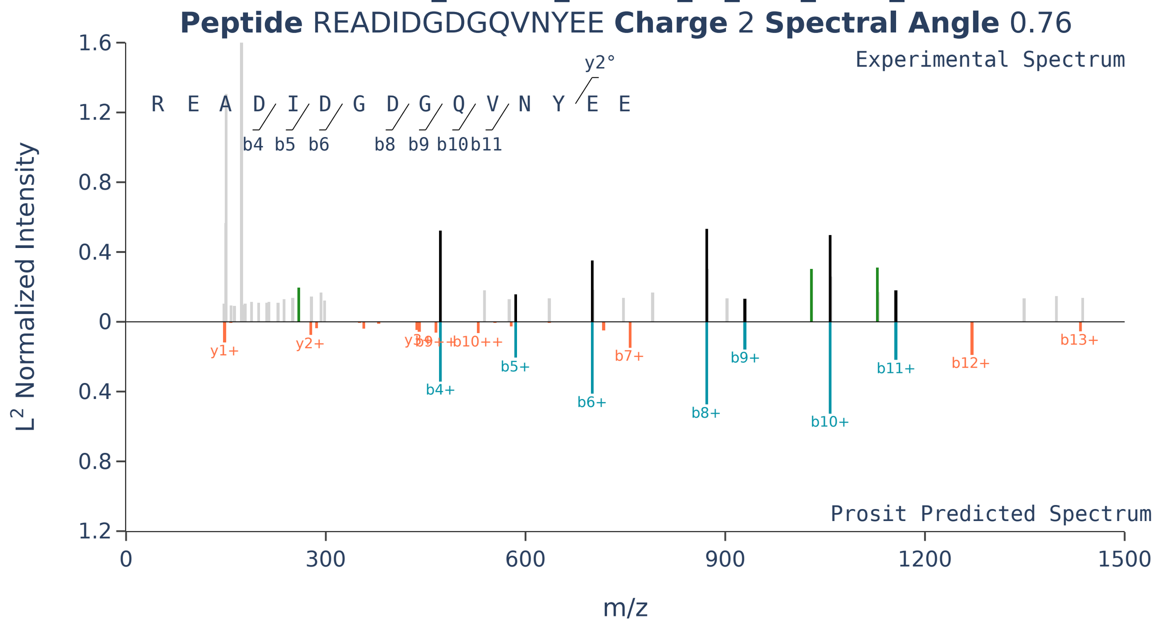


**Source** HRoetschke_291222_040123_Fu_CaM_b2R1_24h **Scan** 9039
**Peptide** QIAEFKEAF **Charge** 2 **Spectral Angle** 0.75

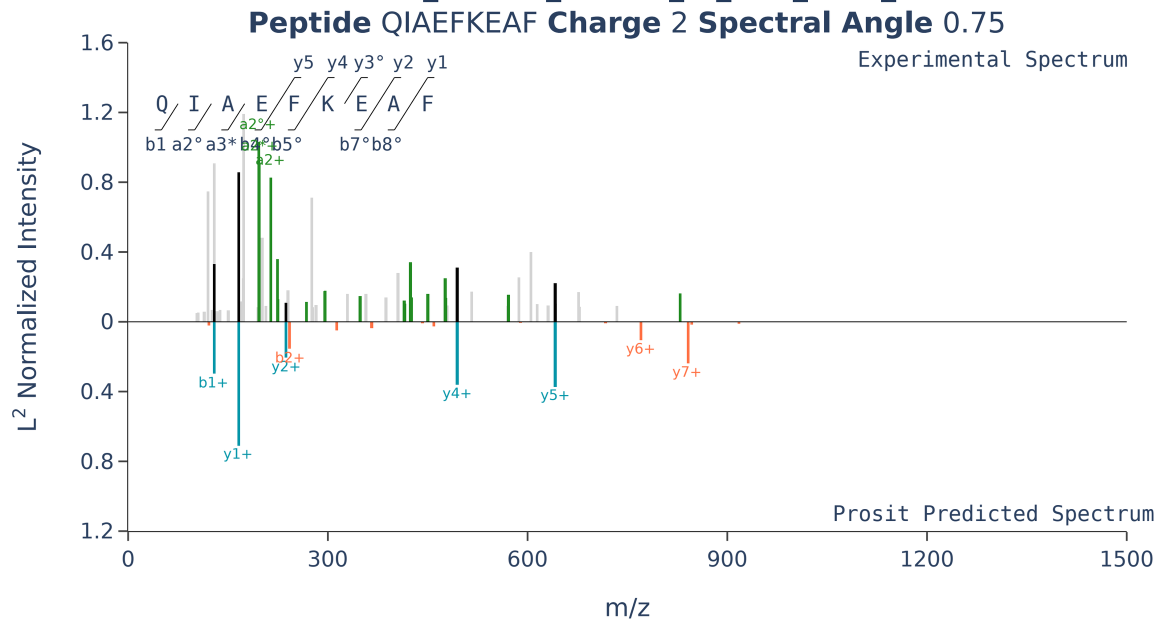


**Source** HRoetschke_291222_040123_Fu_CaM_b1R1_2h **Scan** 6859
**Peptide** RVFDKDGNGYISAAELRH **Charge** 4 **Spectral Angle** 0.66

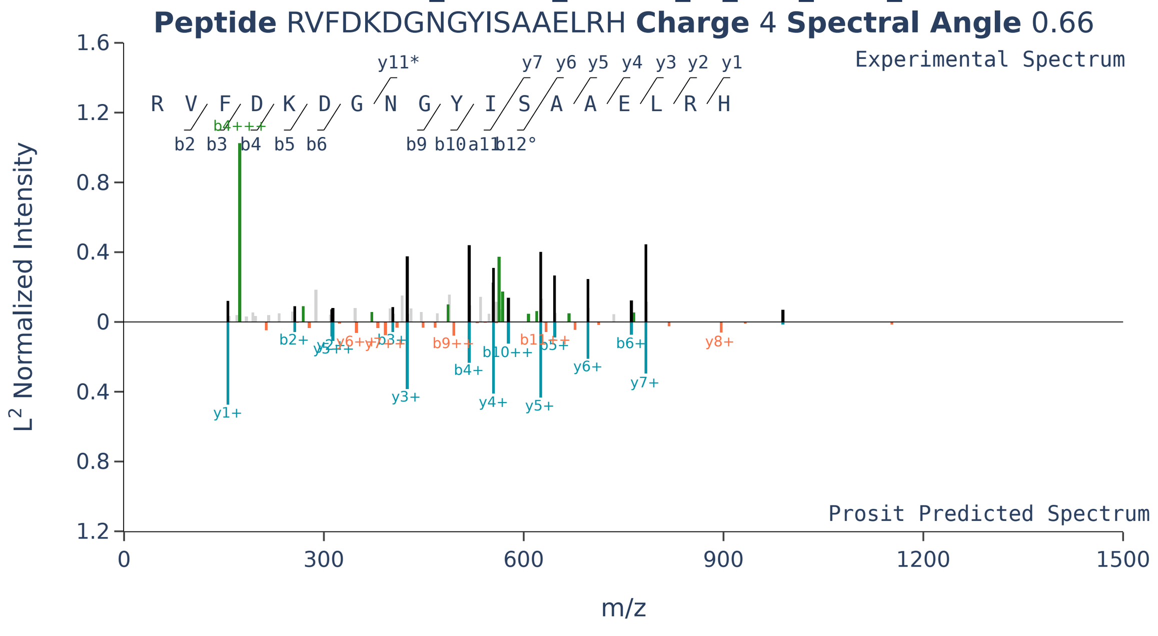


**Source** HRoetschke_291222_040123_Fu_CaM_b2R2_24h **Scan** 5828
**Peptide** FDKDGNGYISAA **Charge** 2 **Spectral Angle** 0.73

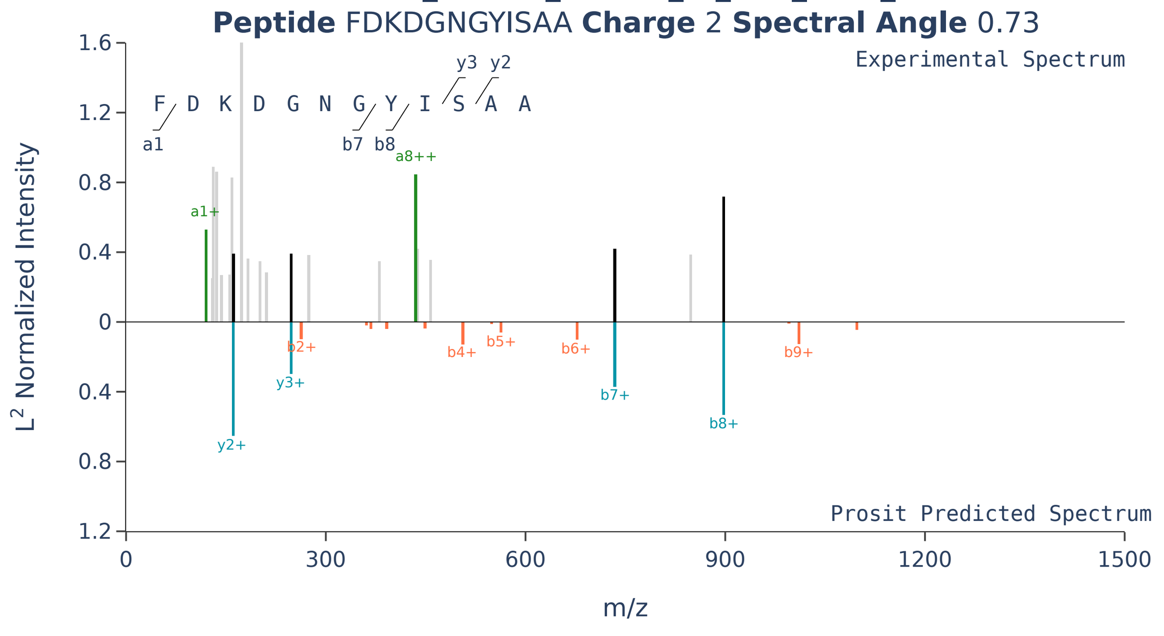


**Source** HRoetschke_291222_040123_Fu_CaM_b1R1_4h **Scan** 10678
**Peptide** FSLFDKDGD **Charge** 2 **Spectral Angle** 0.73

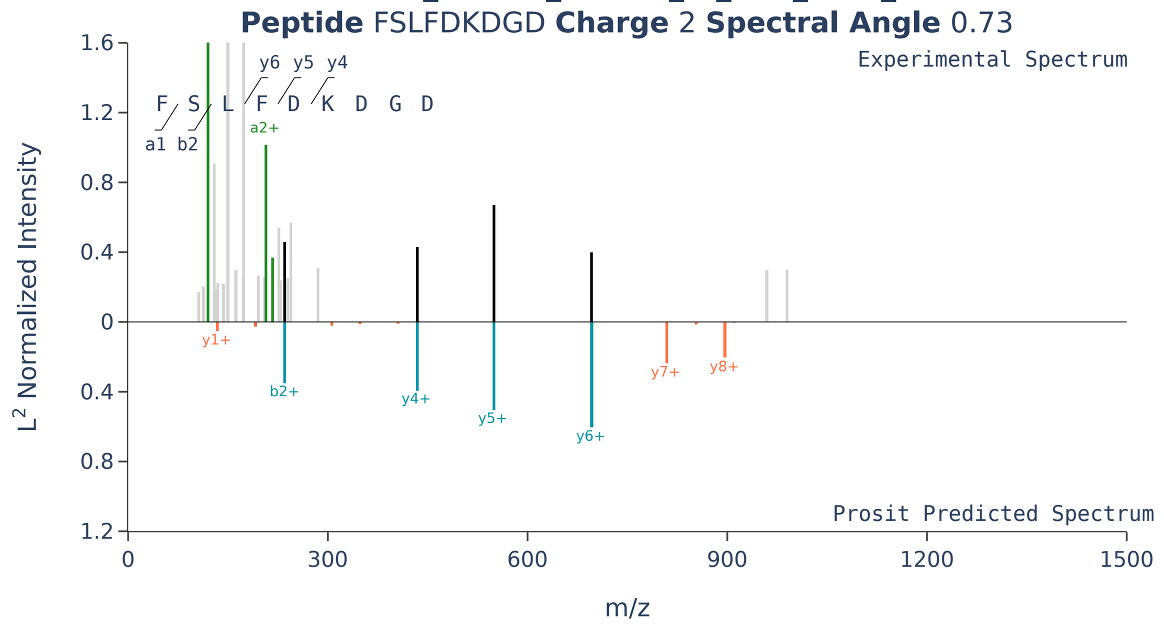

**Source** HRoetschke_291222_040123_Fu_CaM_b1R2_24h **Scan** 7144
**Peptide** QLTEEQL **Charge** 2 **Spectral Angle** 0.82

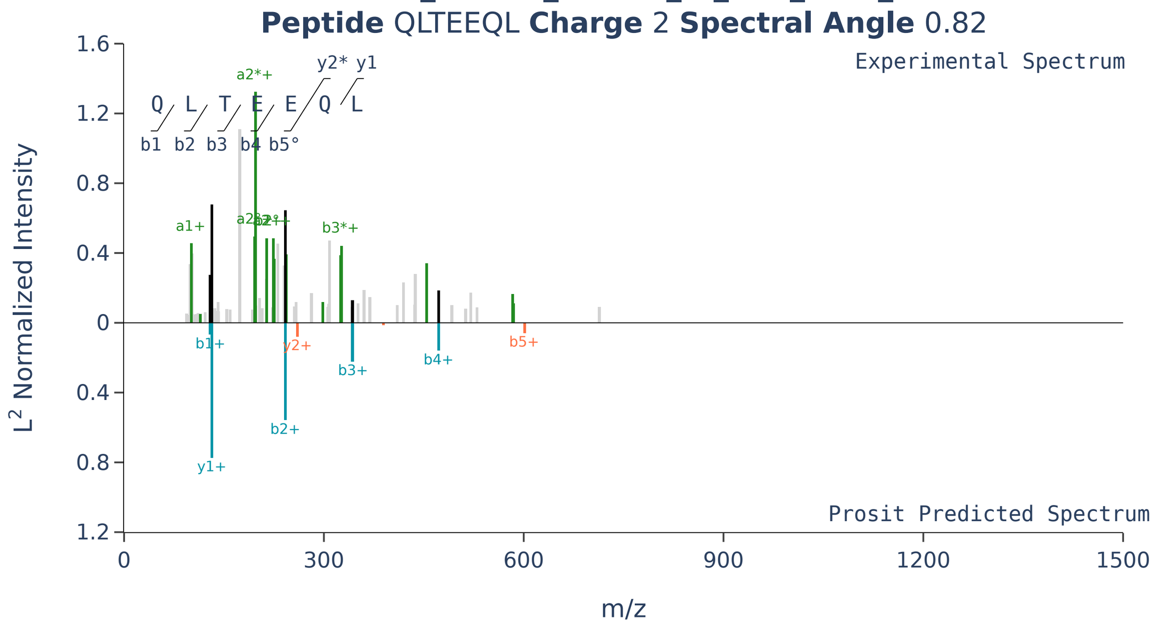


**Source** HRoetschke_291222_040123_Fu_CaM_b2R1_24h **Scan** 11764
**Peptide** AEFKEAFSLFDKDGDGTITT **Charge** 3 **Spectral Angle** 0.66

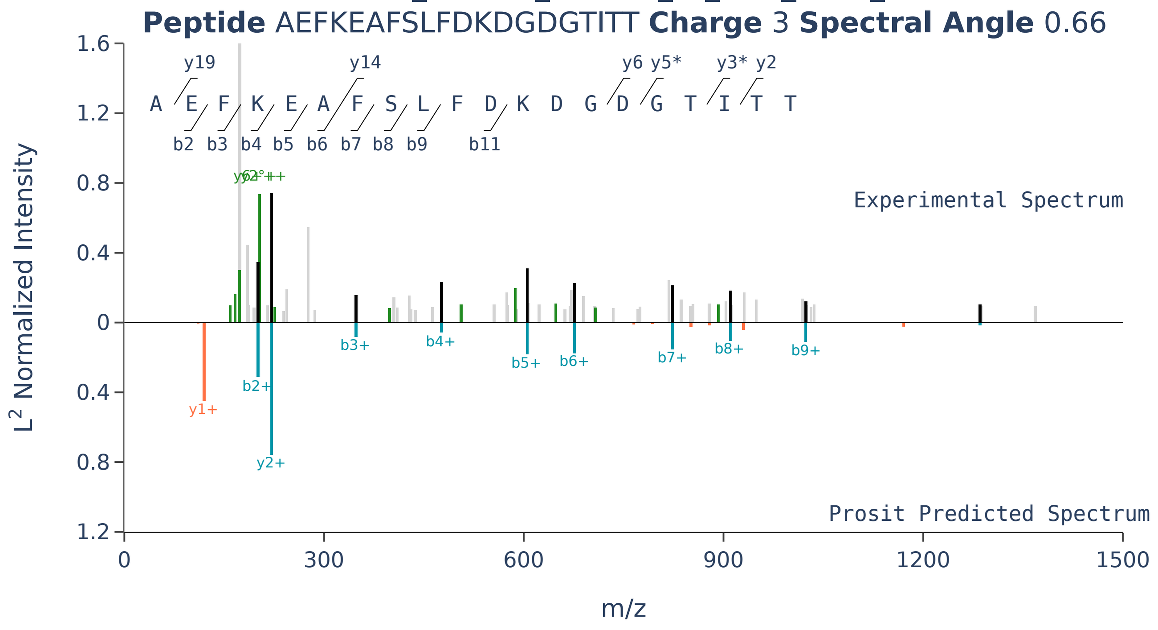


**Source** HRoetschke_291222_040123_Fu_CaM_b1R2_24h **Scan** 11778
**Peptide** DGDGQVNYEEFV **Charge** 2 **Spectral Angle** 0.64

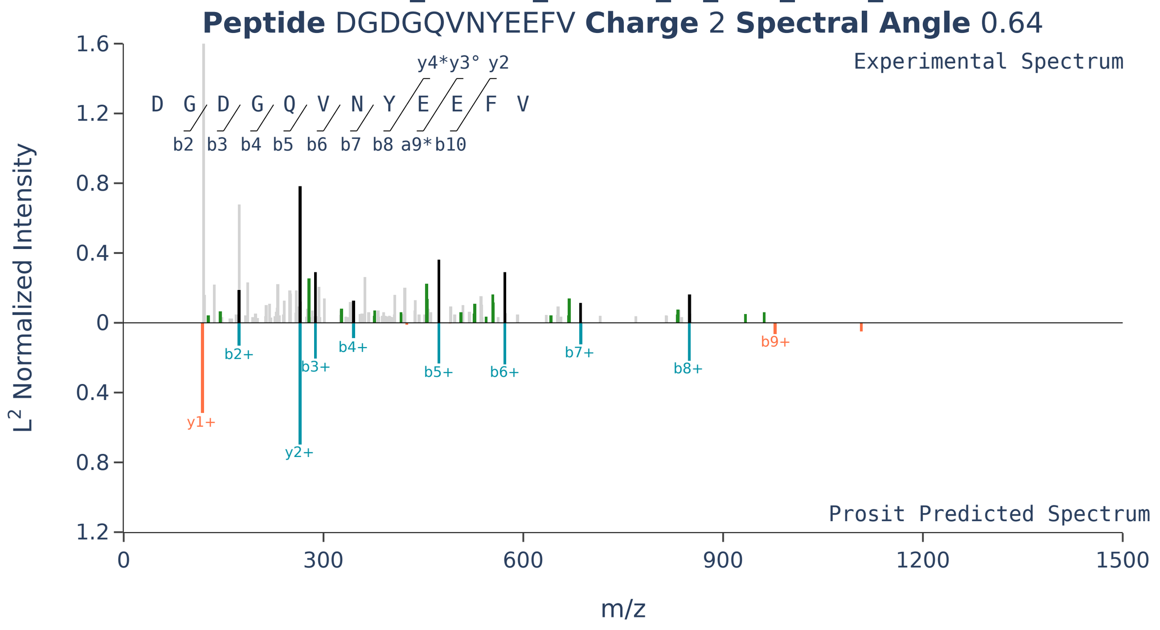


**Source** HRoetschke_291222_040123_Fu_CaM_b1R1_24h **Scan** 3876
**Peptide** AAELRHVMTN **Charge** 2 **Spectral Angle** 0.67

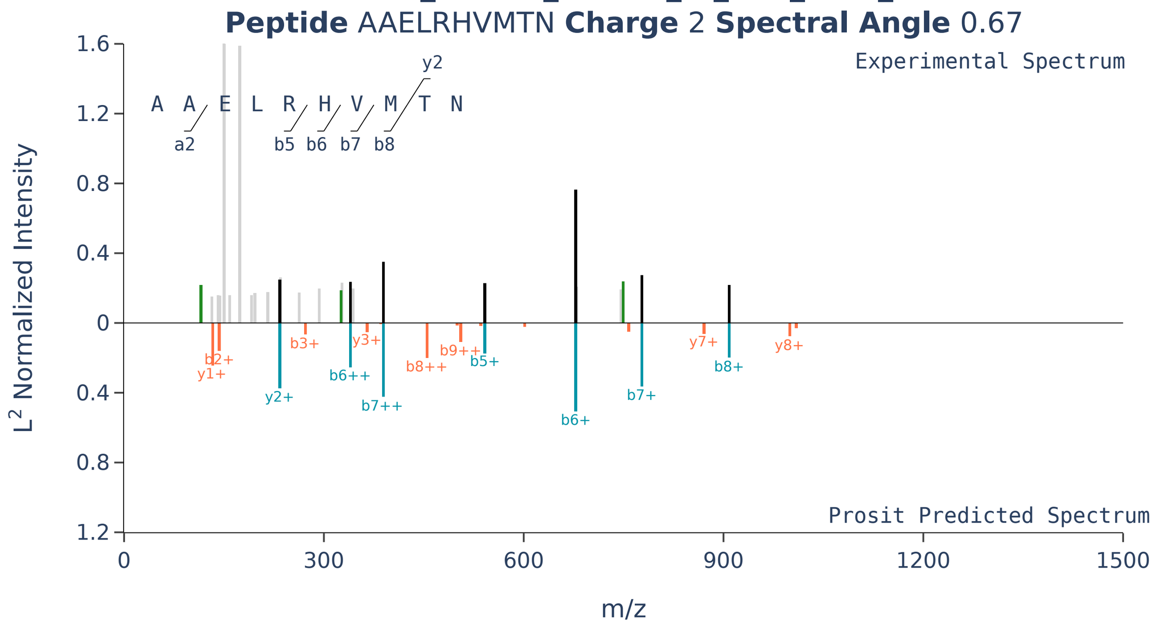


**Source** HRoetschke_291222_040123_Fu_CaM_b1R1_24h **Scan** 9299
**Peptide** LGTVMRSLGQNPTE **Charge** 2 **Spectral Angle** 0.82

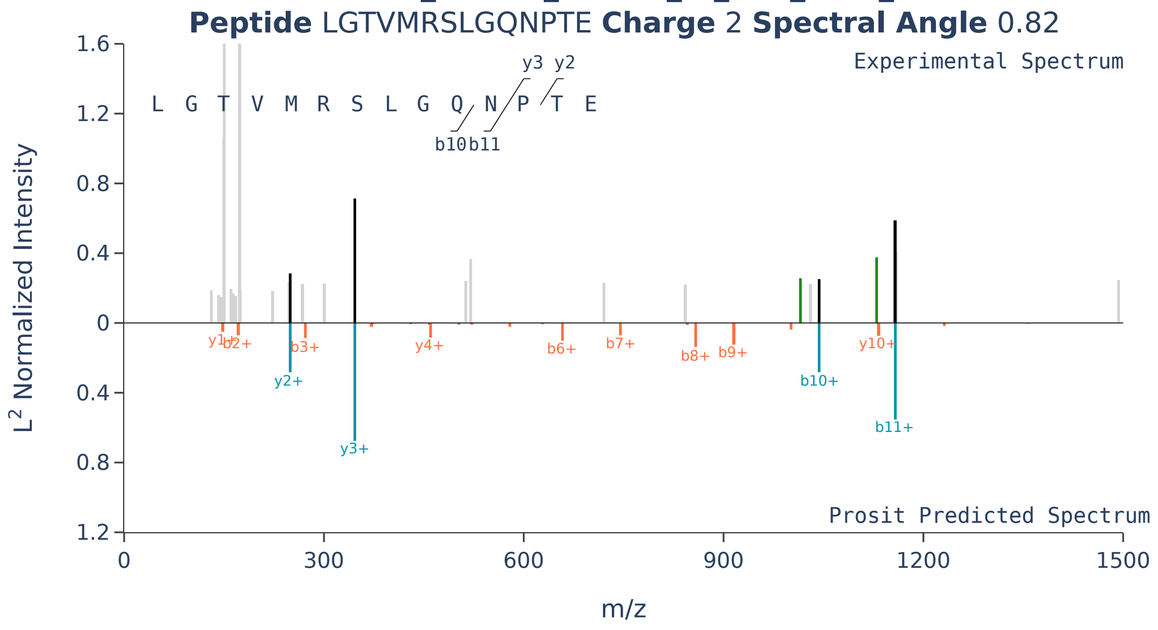


**Source** HRoetschke_291222_040123_Fu_CaM_b1R2_2h **Scan** 10572
**Peptide** ARKMKDTDSEEEIREAFRVFD **Charge** 4 **Spectral Angle** 0.79

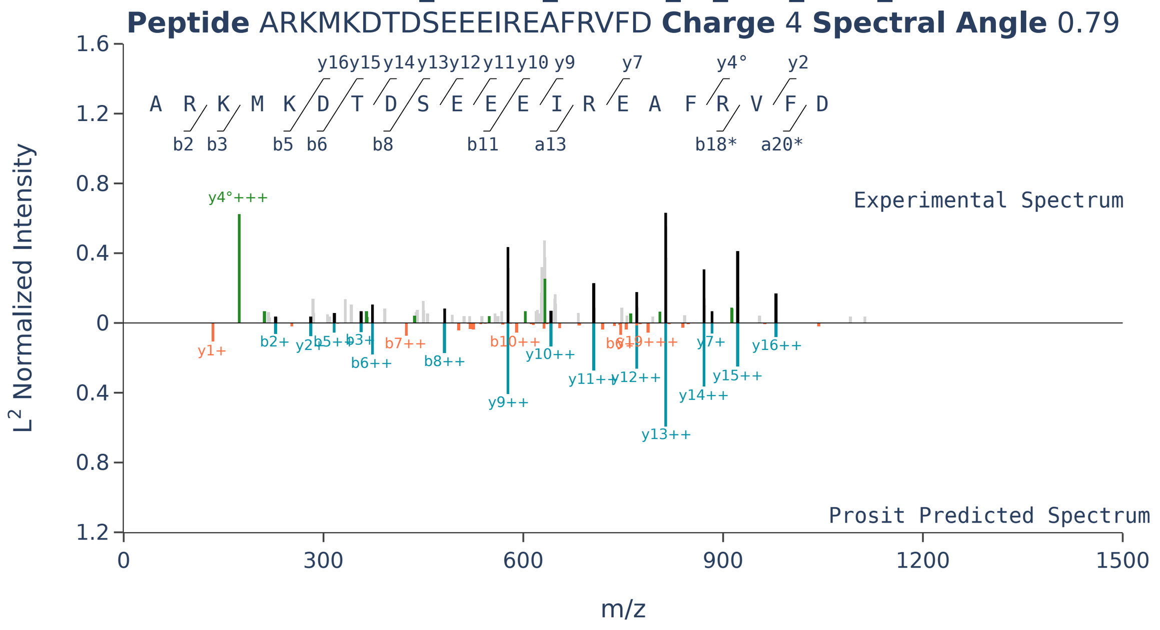


**Source** HRoetschke_291222_040123_Fu_CaM_b2R2_24h **Scan** 5047
**Peptide** LDGDGQVNY **Charge** 2 **Spectral Angle** 0.83

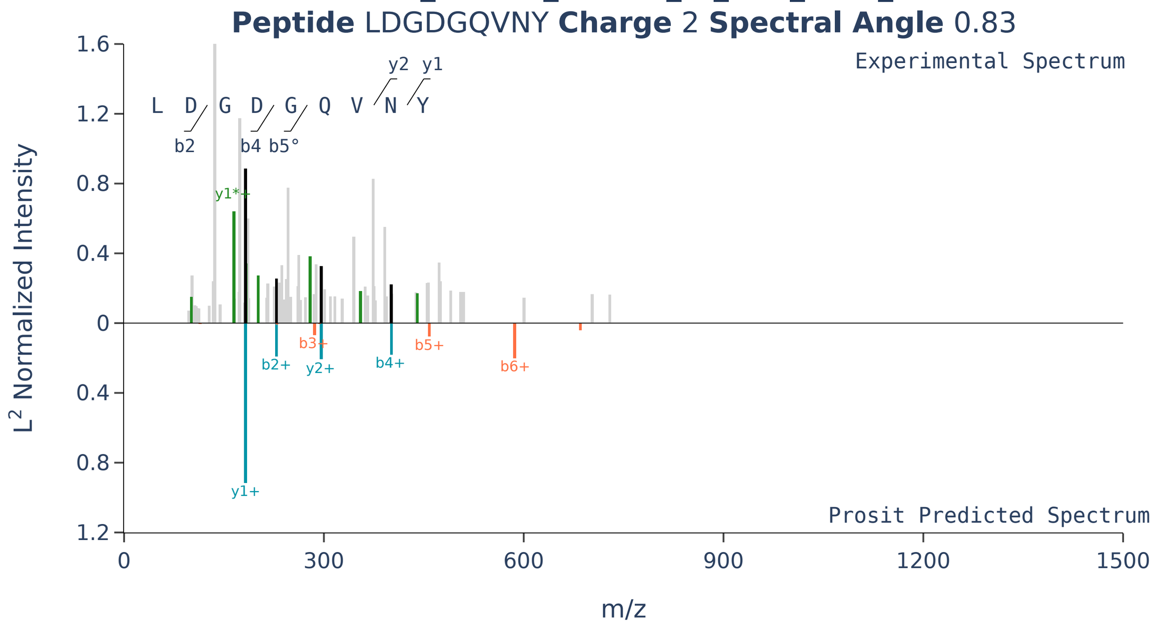


**Source** HRoetschke_291222_040123_Fu_CaM_b2R1_24h **Scan** 12609
**Peptide** DTDSEEEIREAFRVFD **Charge** 3 **Spectral Angle** 0.74

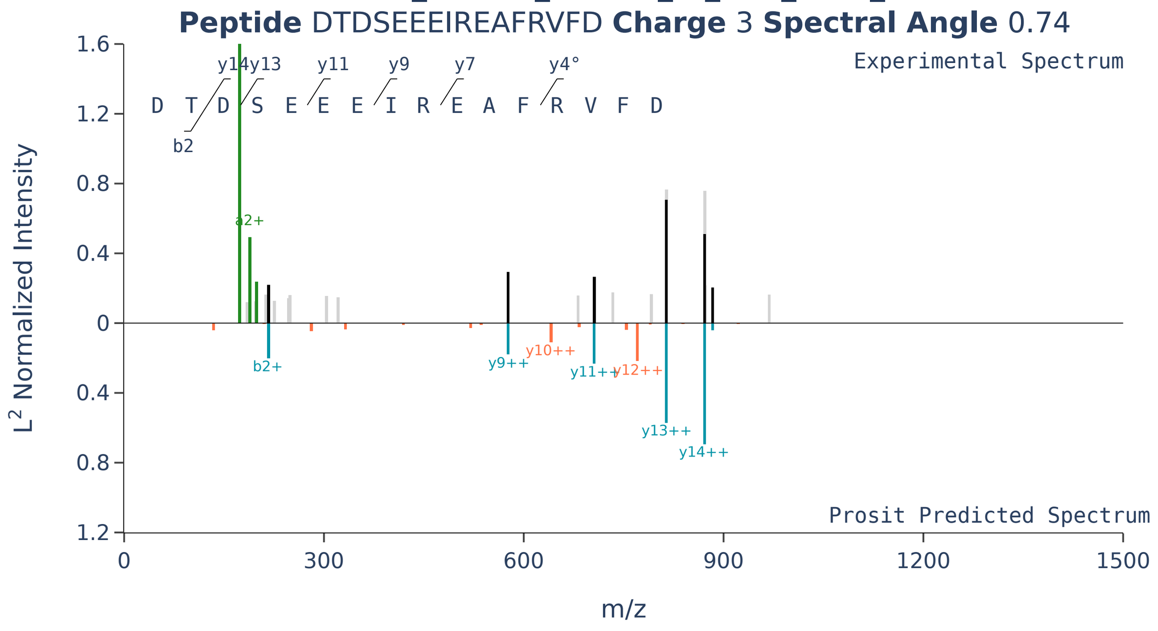


**Source** HRoetschke_291222_040123_Fu_CaM_b1R1_24h **Scan** 3023
**Peptide** FDKDGDGTITTK **Charge** 3 **Spectral Angle** 0.82

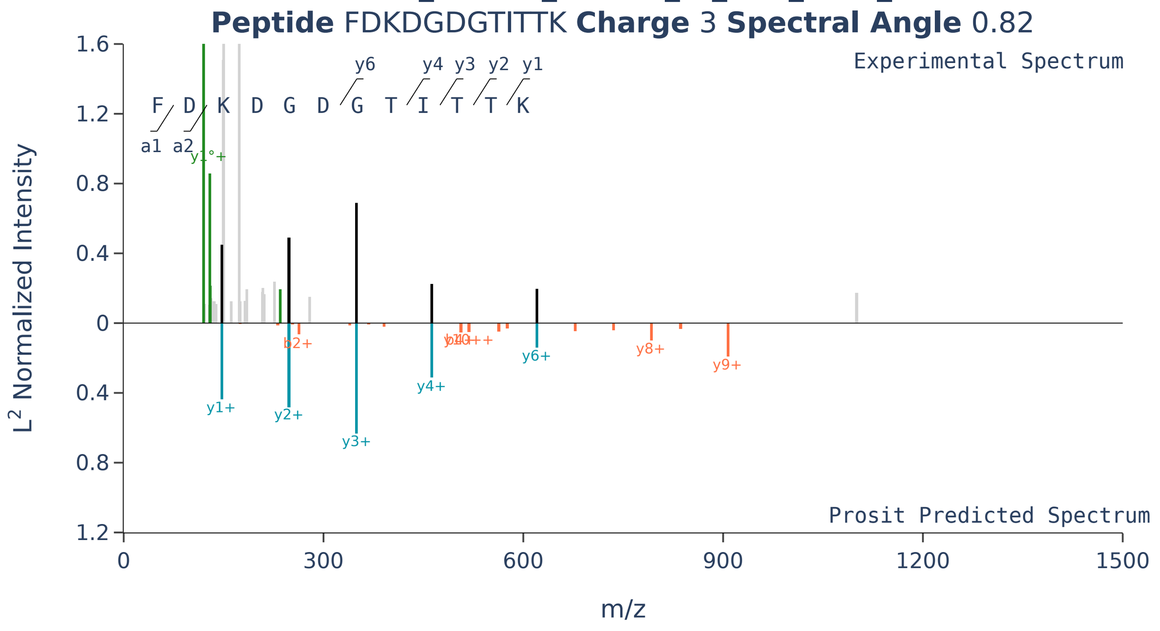


**Source** HRoetschke_291222_040123_Fu_CaM_b1R2_4h **Scan** 2419
**Peptide** RKMKDTDSEEEIREA **Charge** 3 **Spectral Angle** 0.7

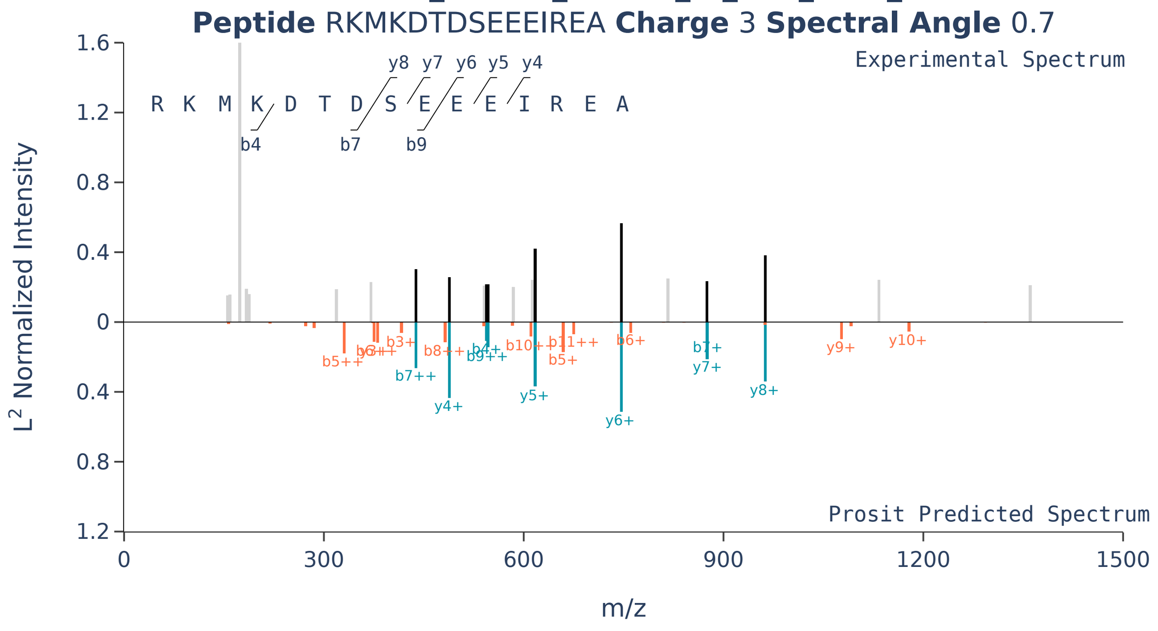


**Source** HRoetschke_291222_040123_Fu_CaM_b1R1_4h **Scan** 6353
**Peptide** DIDGDGQVNY **Charge** 2 **Spectral Angle** 0.76

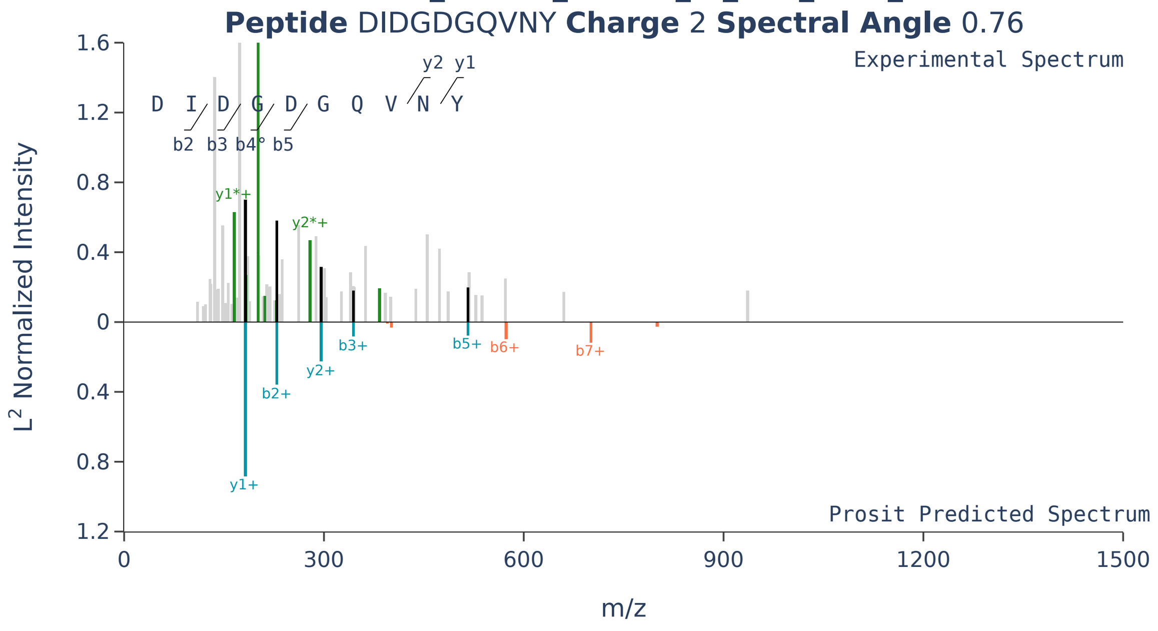


**Source** HRoetschke_291222_040123_Fu_CaM_b1R1_2h **Scan** 7266
**Peptide** KDGNGYISAAELRHVMT **Charge** 4 **Spectral Angle** 0.63

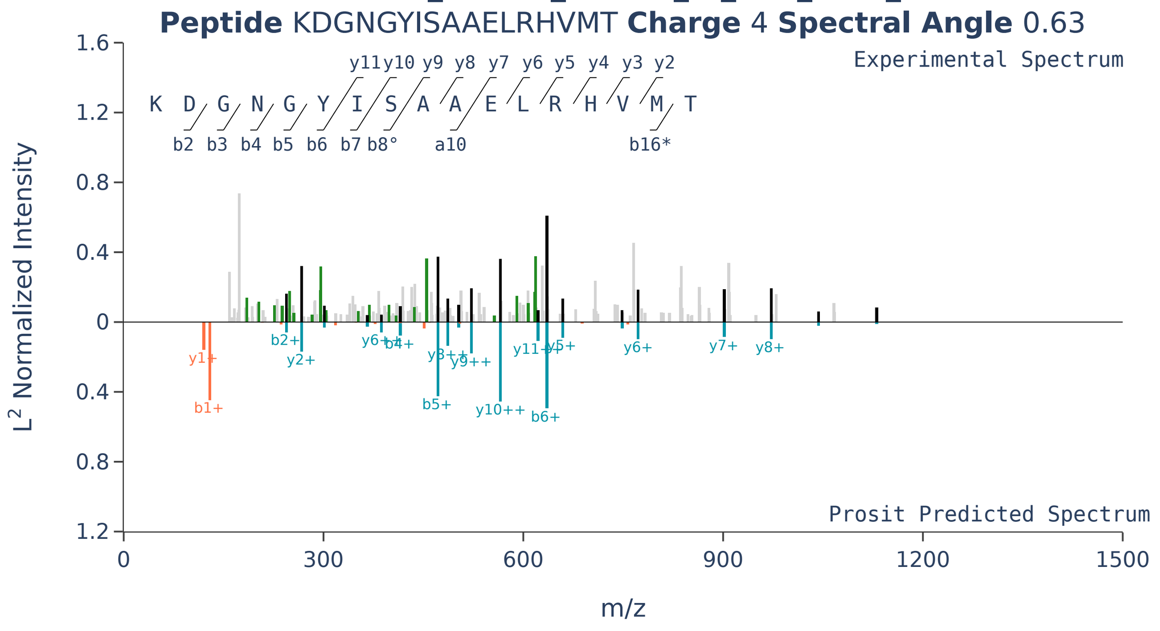


**Source** HRoetschke_291222_040123_Fu_CaM_b2R1_24h **Scan** 8990
**Peptide** AELRHVMTNLGEKLTDEEVD **Charge** 3 **Spectral Angle** 0.63

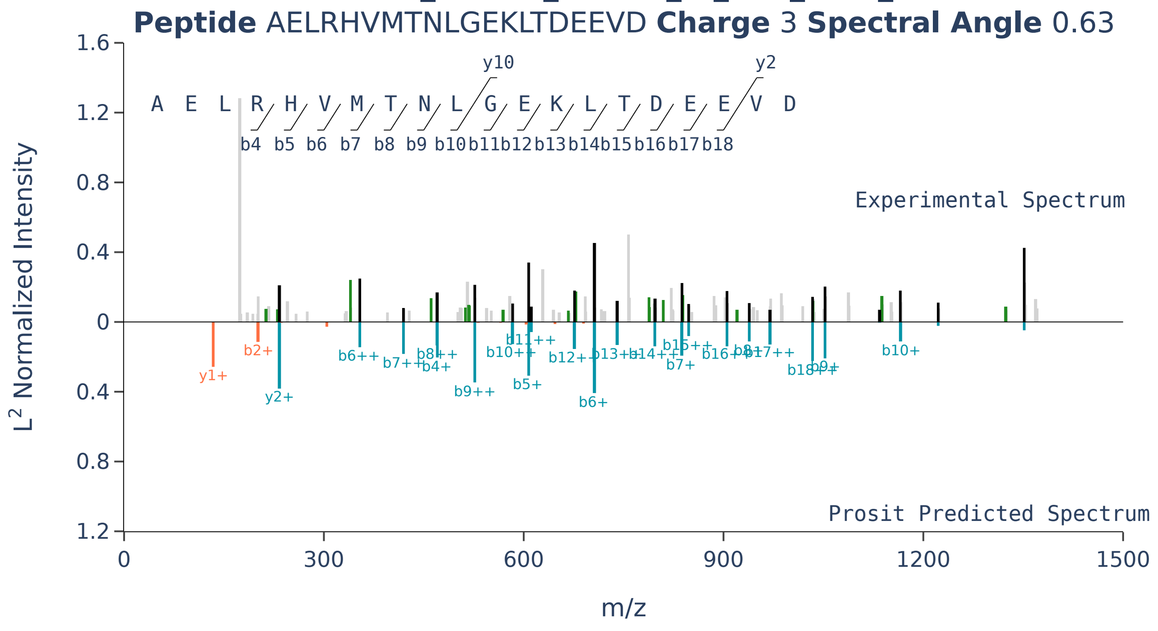


**Source** HRoetschke_291222_040123_Fu_CaM_b2R1_24h **Scan** 10057
**Peptide** SLFDKDGDGTITTKELGTVMRS **Charge** 4 **Spectral Angle** 0.75

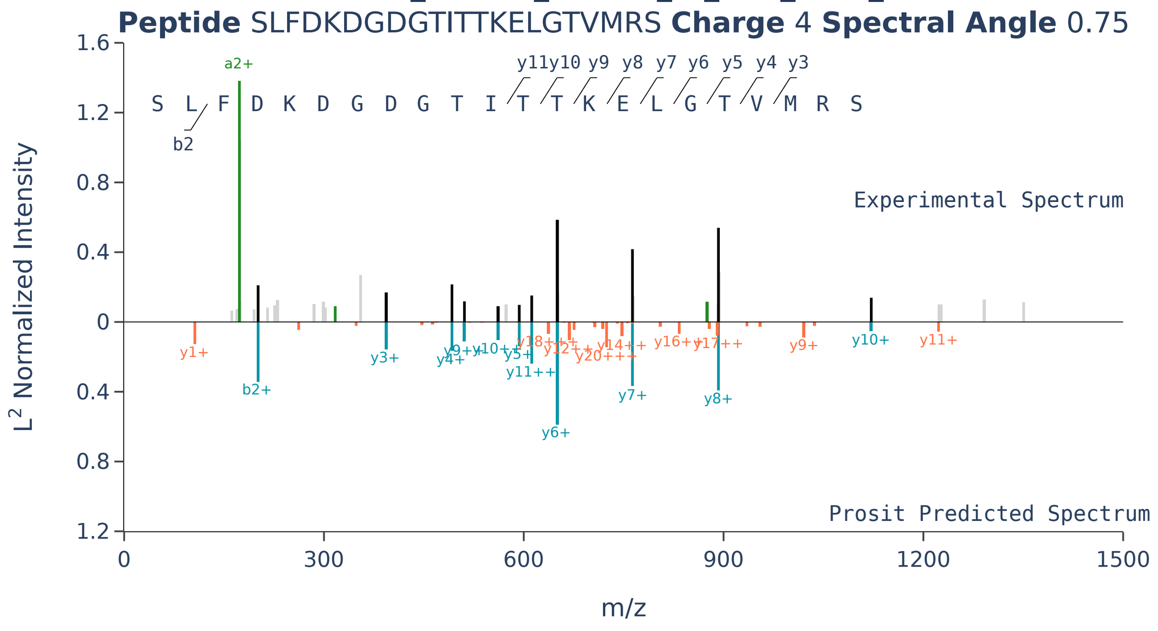


**Source** HRoetschke_291222_040123_Fu_CaM_b2R2_24h **Scan** 12157
**Peptide** AFSLFDKDGDGTITTKELGTVM **Charge** 3 **Spectral Angle** 0.92

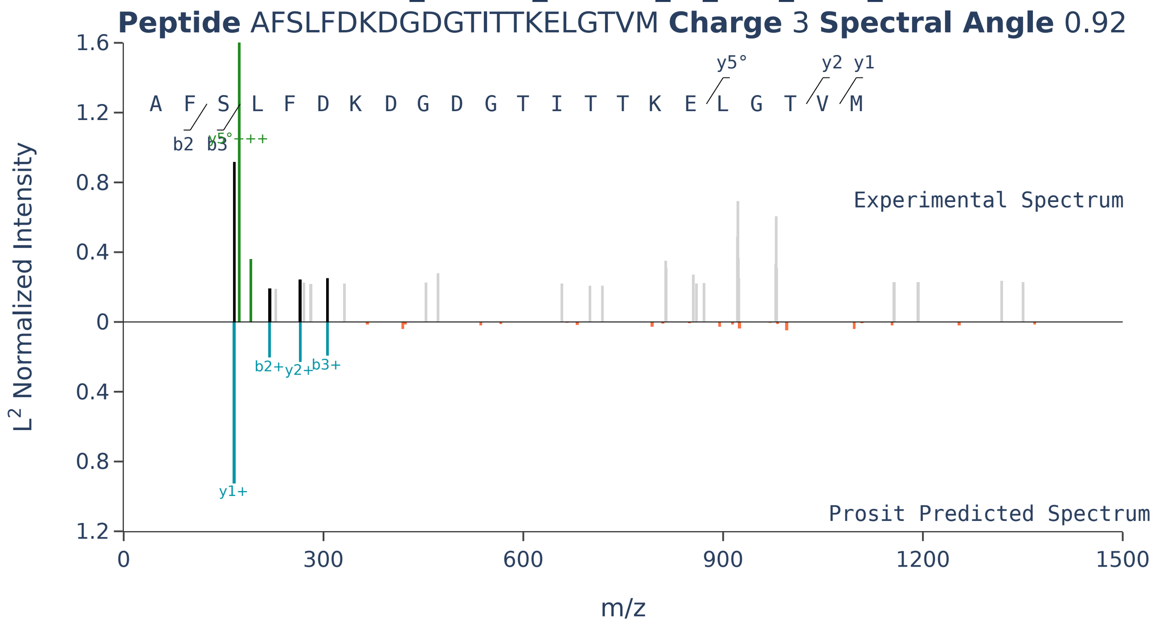

**Source** HRoetschke_291222_040123_Fu_CaM_b2R2_24h **Scan** 11594
**Peptide** AEFKEAFSLFDKDGDGTITTK **Charge** 4 **Spectral Angle** 0.67

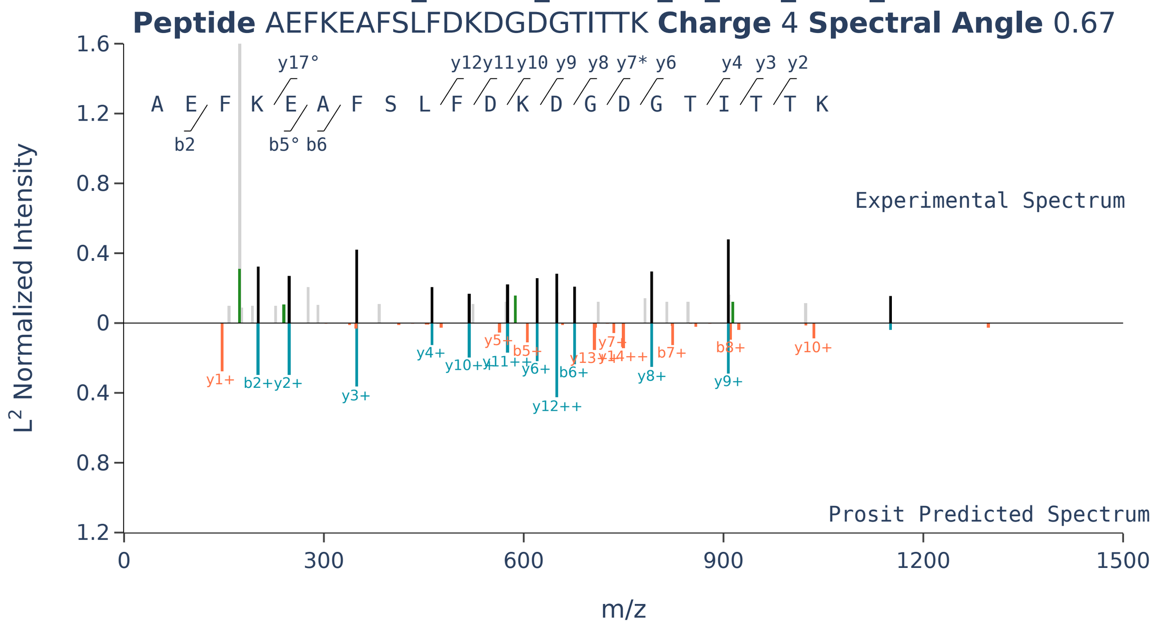

**Source** HRoetschke_291222_040123_Fu_CaM_b1R2_2h **Scan** 11621
**Peptide** ADGNGTIDFPEFLTMMARKMKD **Charge** 4 **Spectral Angle** 0.71

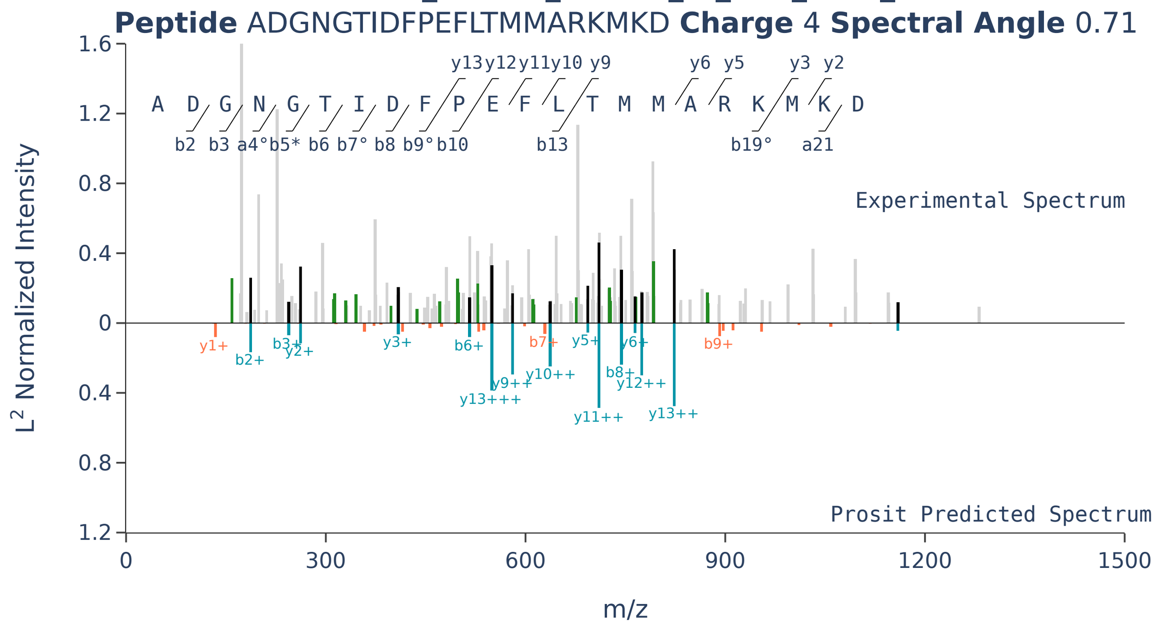

**Source** HRoetschke_291222_040123_Fu_CaM_b1R1_24h **Scan** 1942
**Peptide** RKMKDTDSEEELR **Charge** 3 **Spectral Angle** 0.67

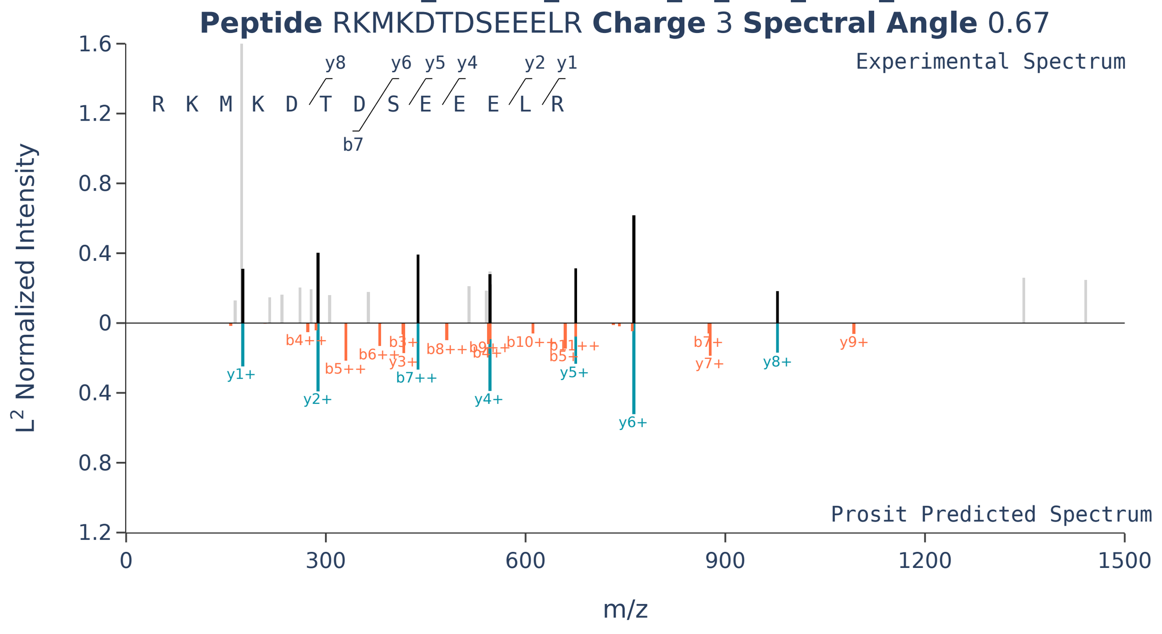

**Source** HRoetschke_291222_040123_Fu_CaM_b1R1_4h **Scan** 7023
**Peptide** FDKDGNGYISAAELRH **Charge** 4 **Spectral Angle** 0.69

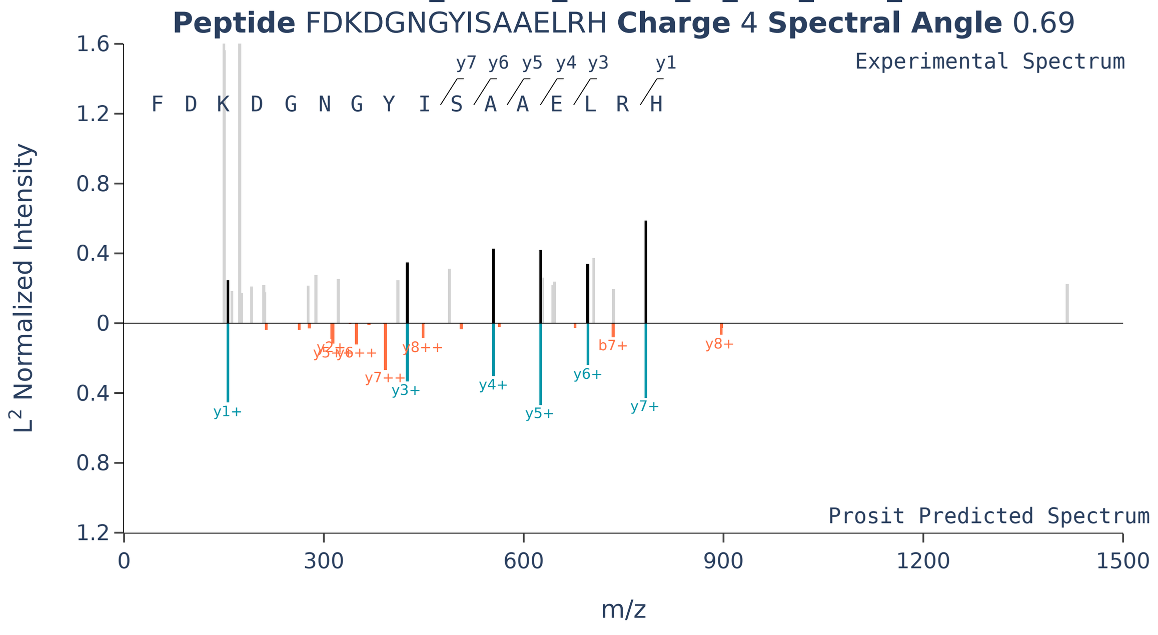

**Source** HRoetschke_291222_040123_Fu_CaM_b2R1_24h **Scan** 8797
**Peptide** FRVFDKDGNGYL **Charge** 3 **Spectral Angle** 0.72

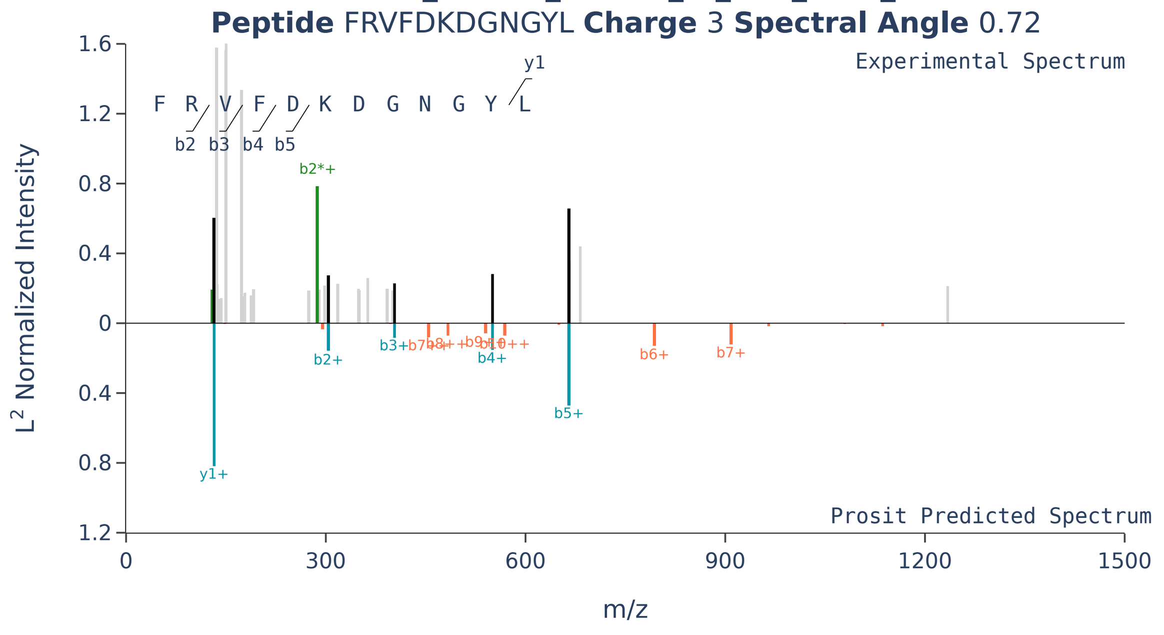

**Source** HRoetschke_291222_040123_Fu_CaM_b2R1_2h **Scan** 5023
**Peptide** RVFDKDGNGYIS **Charge** 2 **Spectral Angle** 0.71

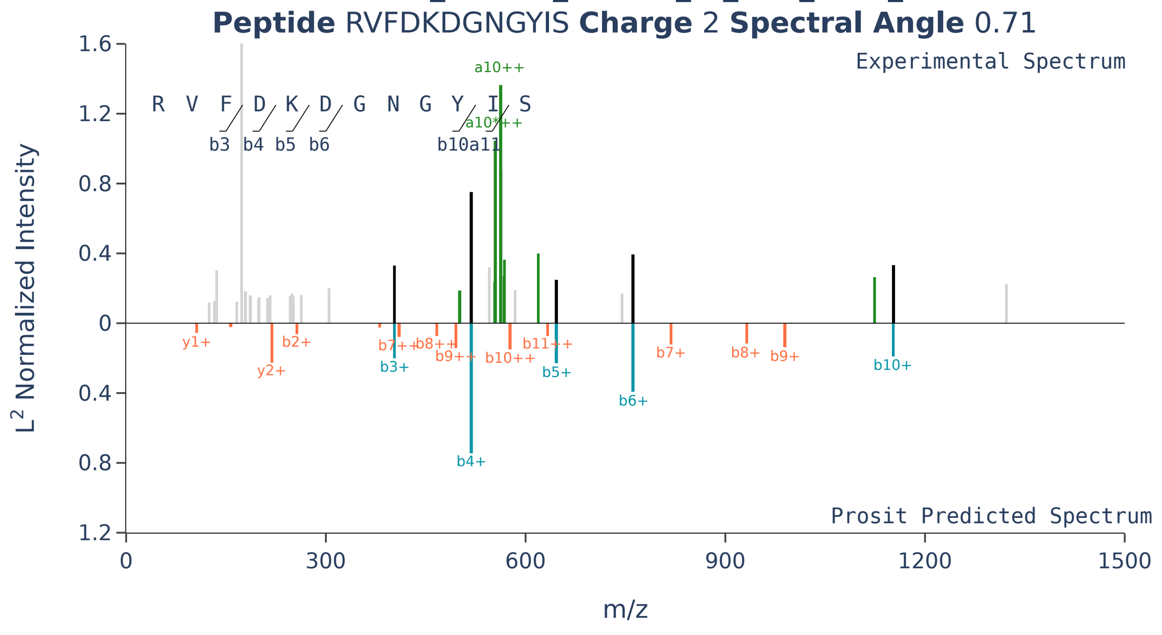

**Source** HRoetschke_291222_040123_Fu_CaM_b1R1_24h **Scan** 5743
**Peptide** DKDGDGTITTKELGT **Charge** 3 **Spectral Angle** 0.55

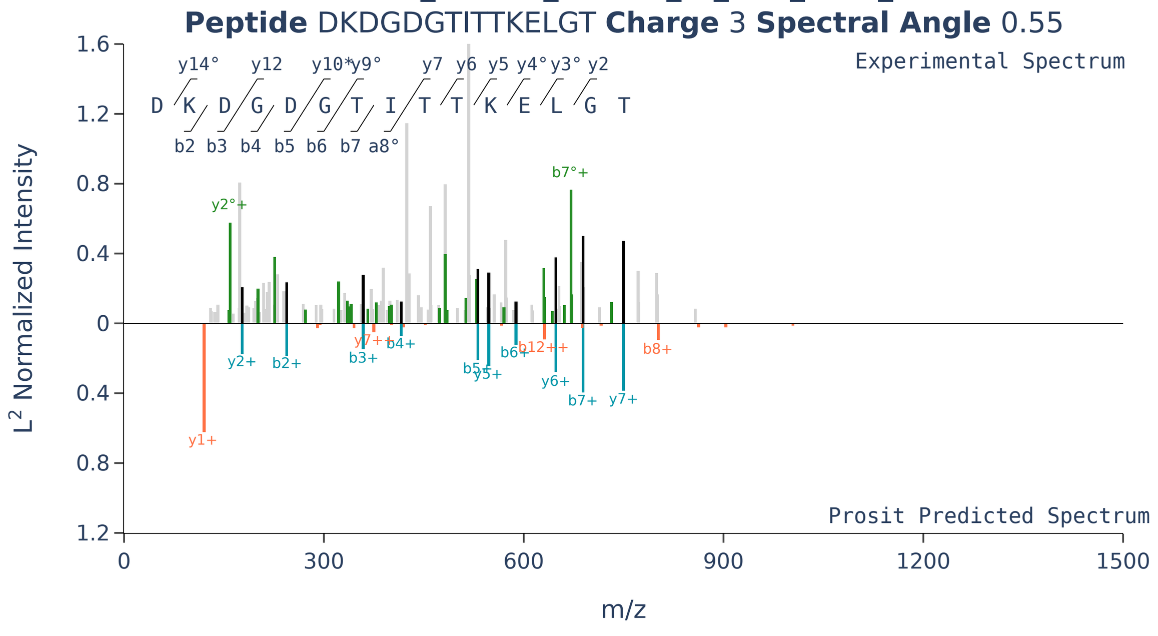

**Source** HRoetschke_291222_040123_Fu_CaM_b1R2_4h **Scan** 3363
**Peptide** TDSEEEIREA **Charge** 2 **Spectral Angle** 0.71

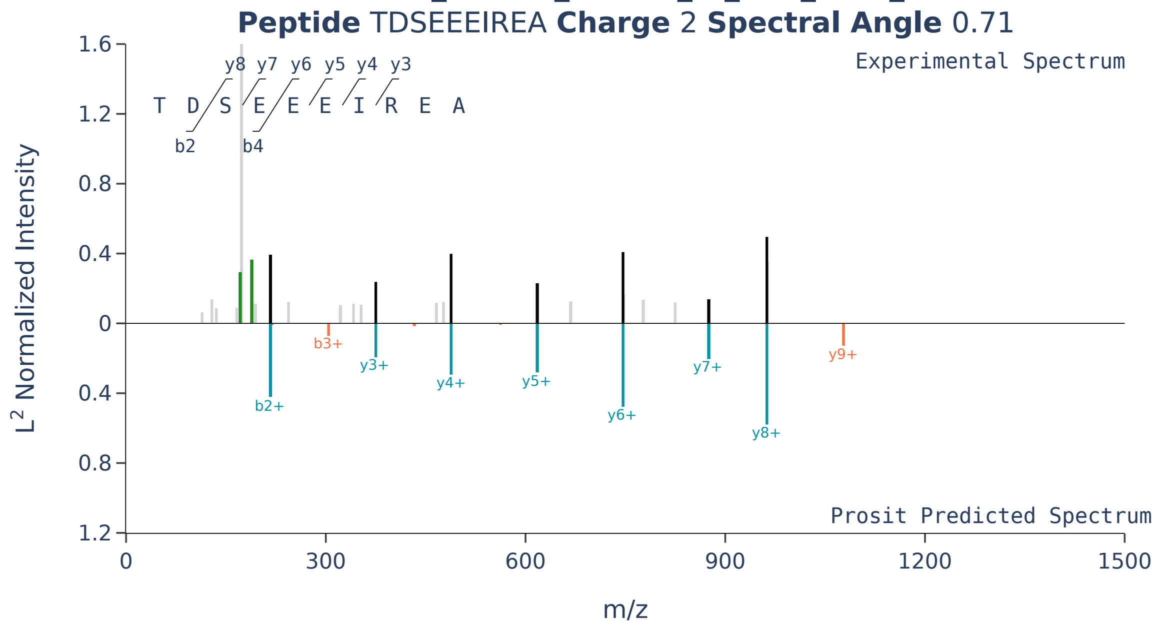

**Source** HRoetschke_291222_040123_Fu_CaM_b1R2_4h **Scan** 4635
**Peptide** DKDGNGYISAAE **Charge** 2 **Spectral Angle** 0.83

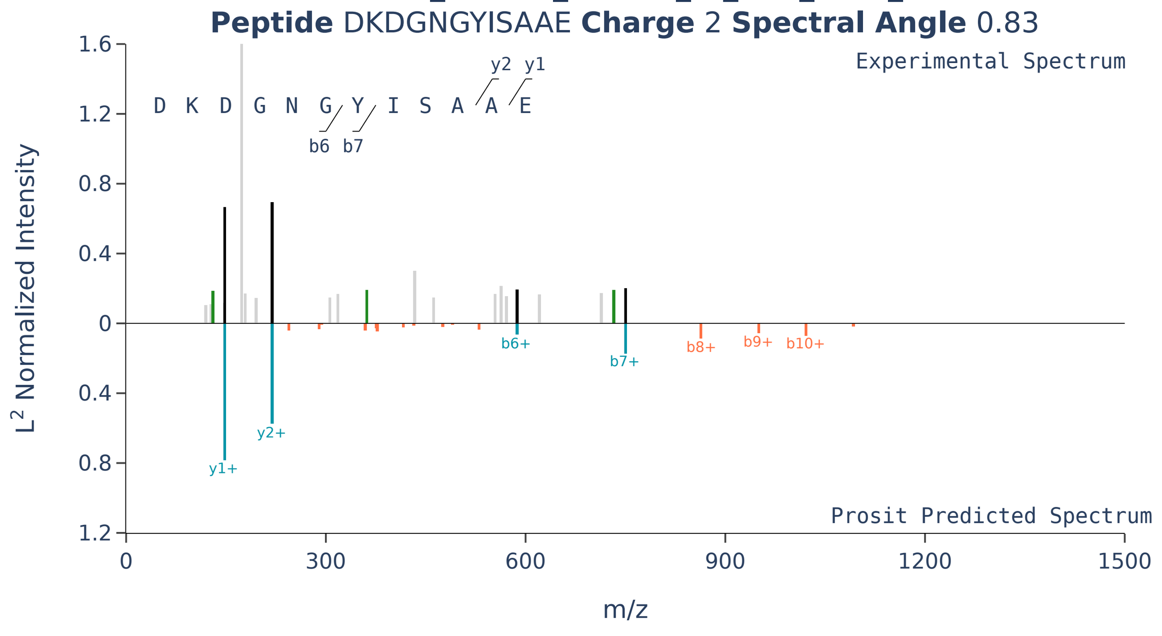

**Source** HRoetschke_291222_040123_Fu_CaM_b2R1_24h **Scan** 11370
**Peptide** EMIREADIDGDGQVNYEEFV **Charge** 2 **Spectral Angle** 0.71

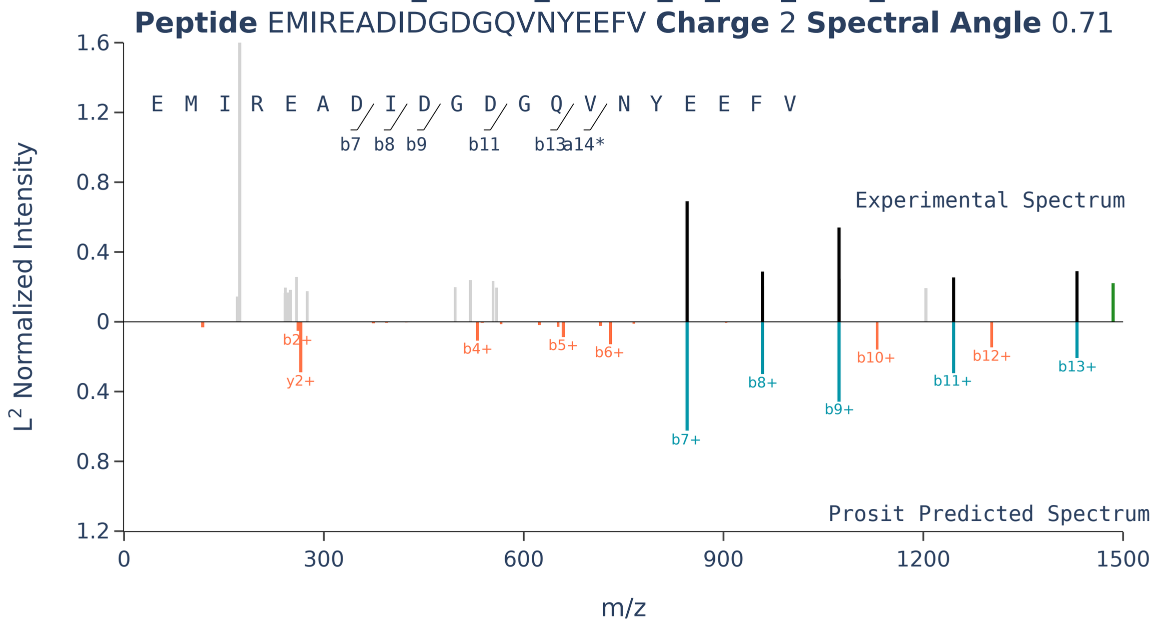

**Source** HRoetschke_291222_040123_Fu_CaM_b2R2_24h **Scan** 5278
**Peptide** KDGDGTITTKELGTVMR **Charge** 3 **Spectral Angle** 0.31

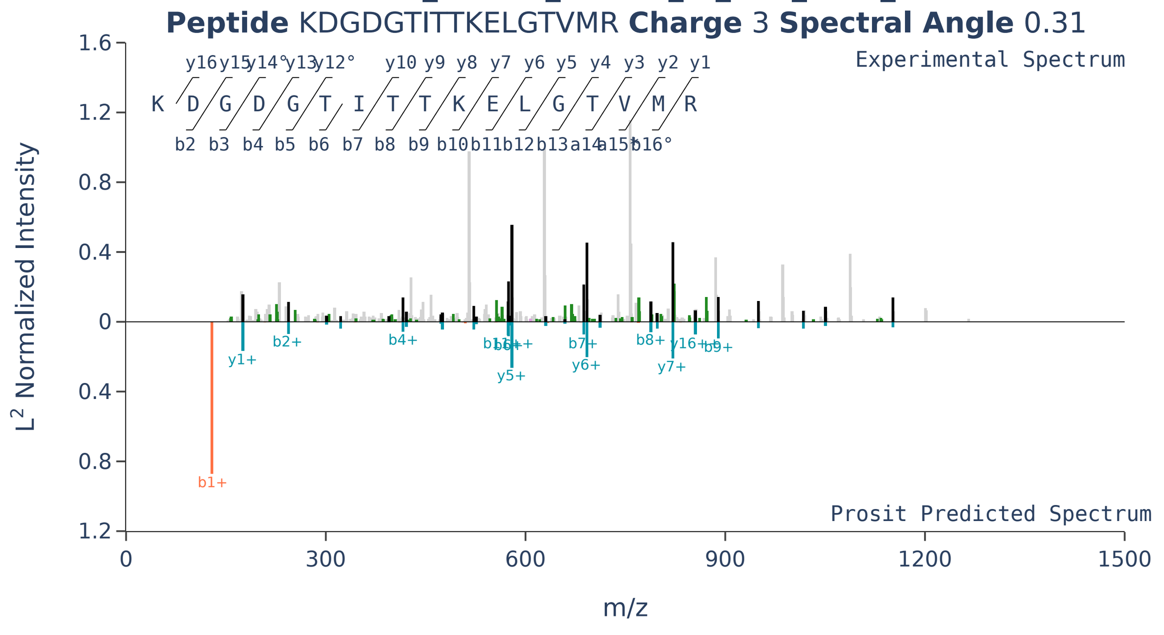

**Source** HRoetschke_291222_040123_Fu_CaM_b2R2_24h **Scan** 10195
**Peptide** EAFRVFDKDGNGYISA **Charge** 3 **Spectral Angle** 0.8

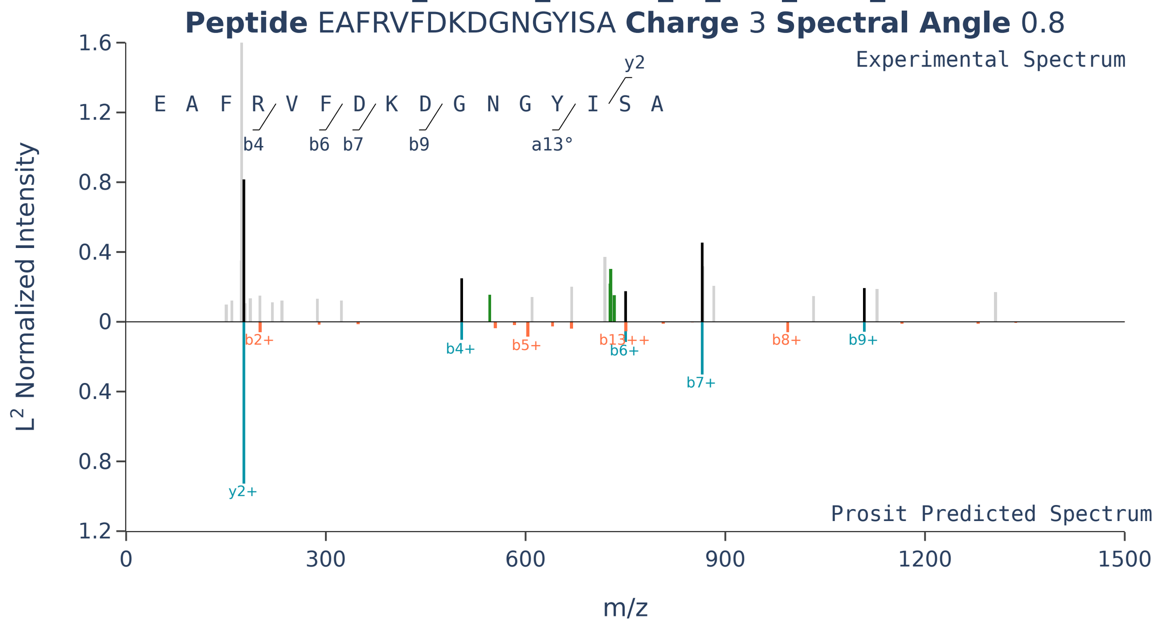

**Source** HRoetschke_291222_040123_Fu_CaM_b1R1_24h **Scan** 2275
**Peptide** MARKMKDTDSEEEIRE **Charge** 4 **Spectral Angle** 0.78

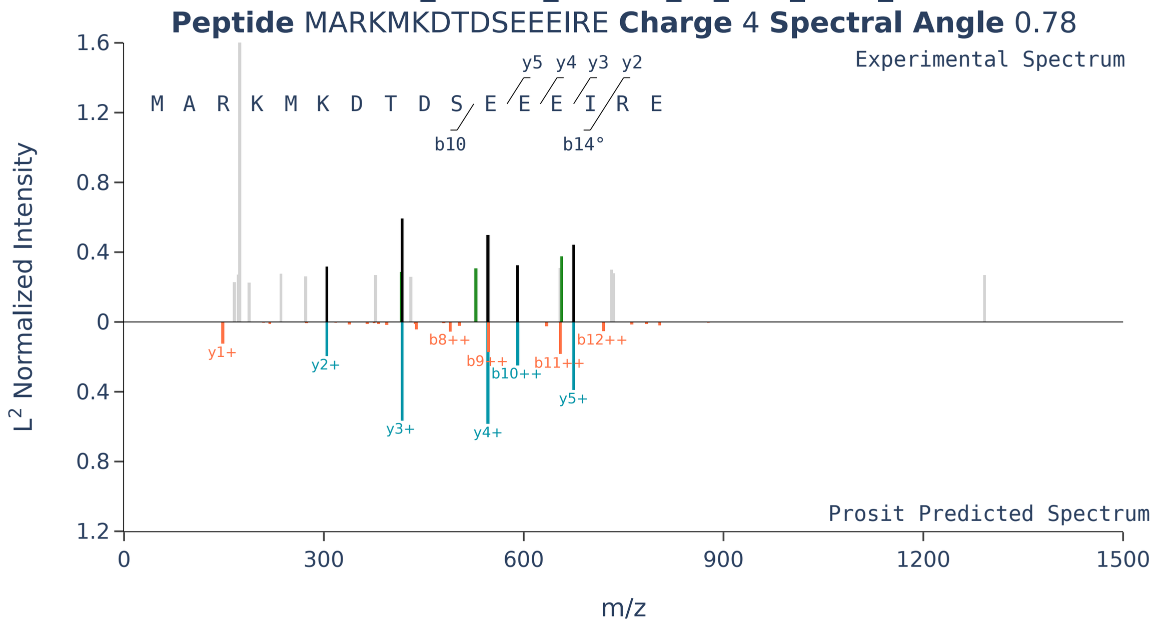

**Source** HRoetschke_291222_040123_Fu_CaM_b2R2_4h **Scan** 5630
**Peptide** FDKDGNGYISAAE **Charge** 2 **Spectral Angle** 0.84

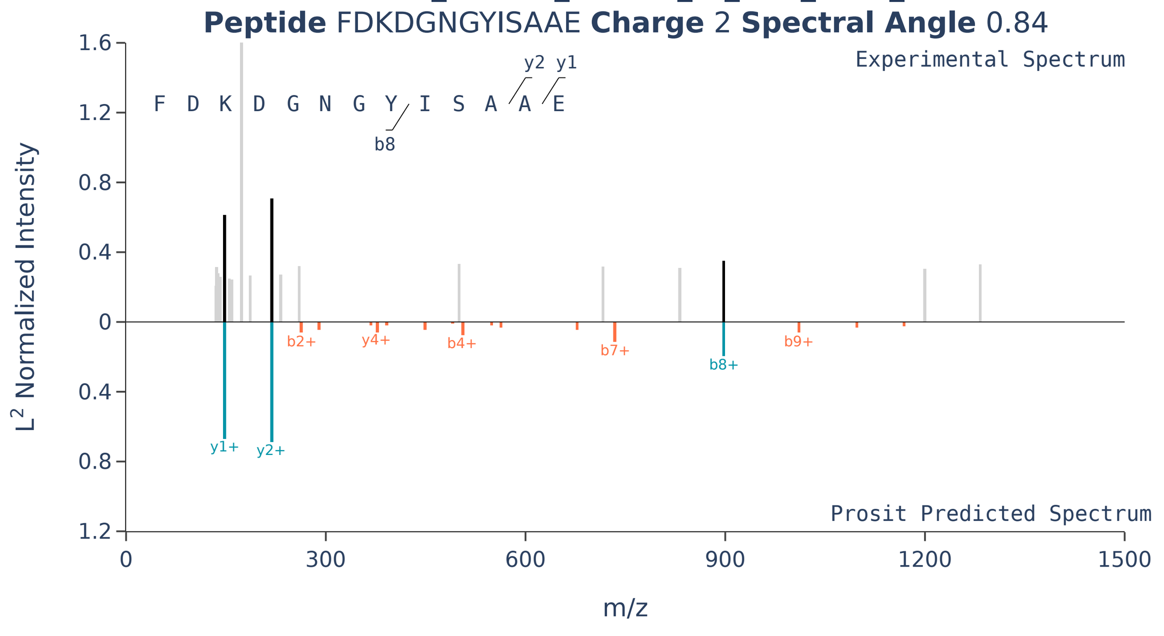

**Source** HRoetschke_291222_040123_Fu_CaM_b1R2_4h **Scan** 7923
**Peptide** KDGDGTITTKELGTVMRS **Charge** 2 **Spectral Angle** 0.63

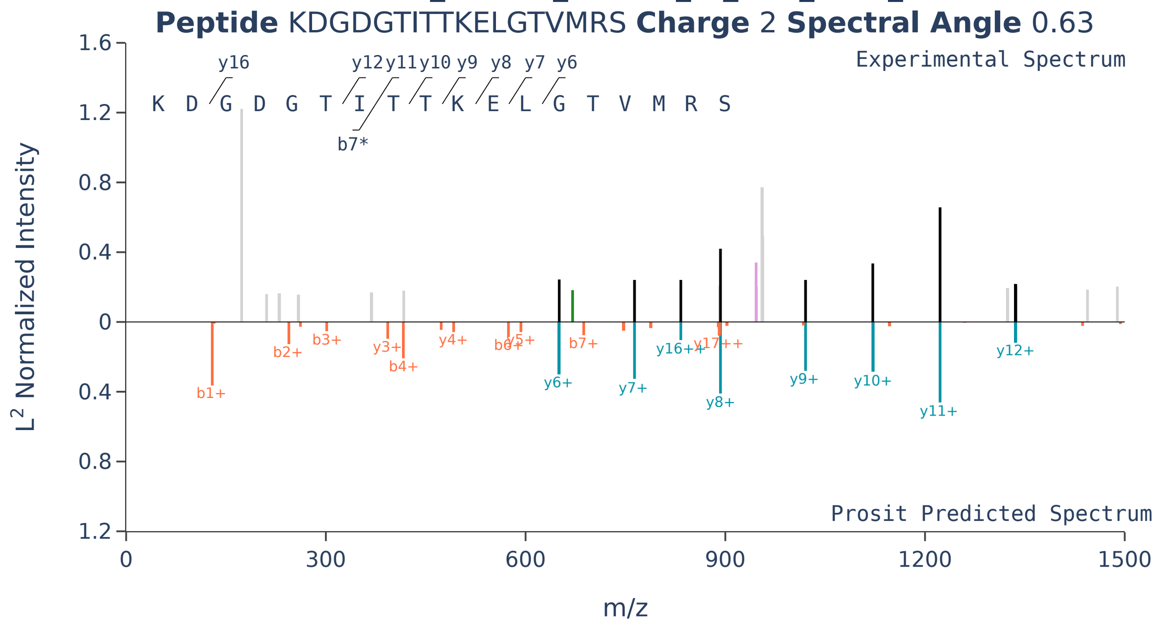

**Source** HRoetschke_291222_040123_Fu_CaM_b2R1_4h **Scan** 11164
**Peptide** AFSLFDKDGDGTITT **Charge** 2 **Spectral Angle** 0.76

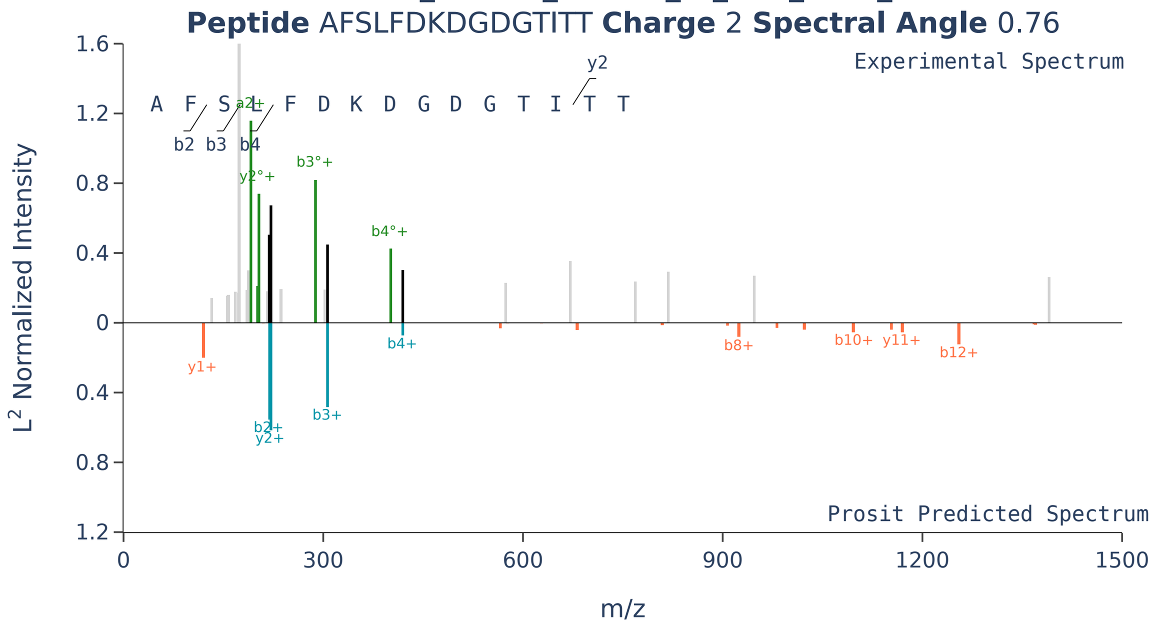


**Source** HRoetschke_291222_040123_Fu_CaM_b2R1_24h **Scan** 6496
**Peptide** FDKDGDGTITTKELGTVM **Charge** 3 **Spectral Angle** 0.88

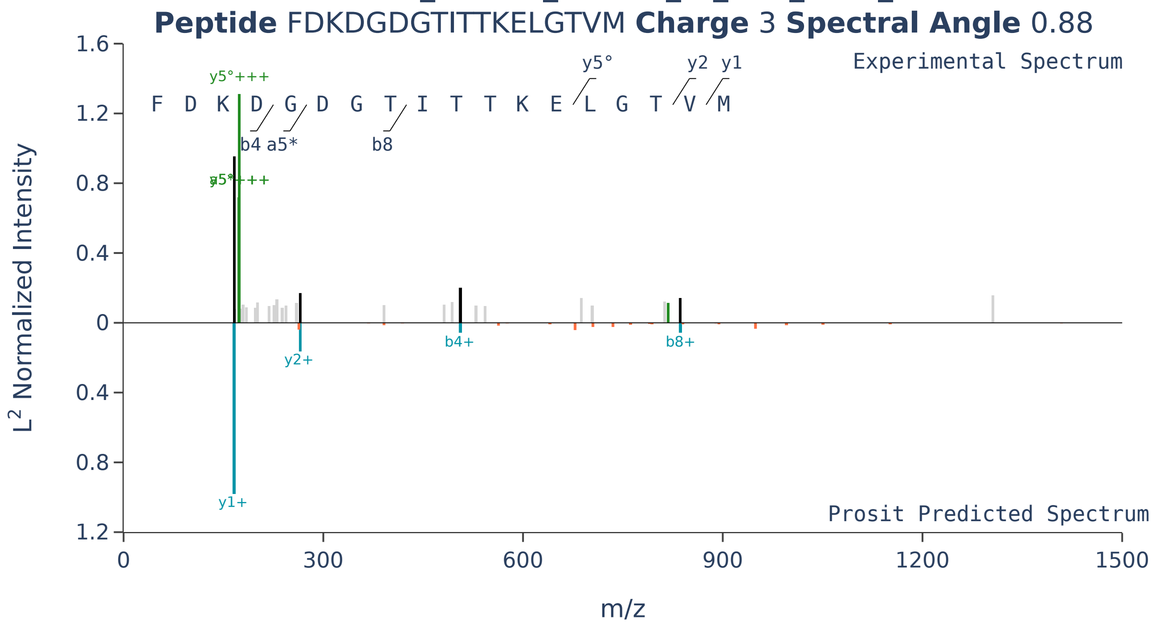


**Source** HRoetschke_291222_040123_Fu_CaM_b1R1_2h **Scan** 5655
**Peptide** MARKMKDTDSEEEIREAF **Charge** 4 **Spectral Angle** 0.59

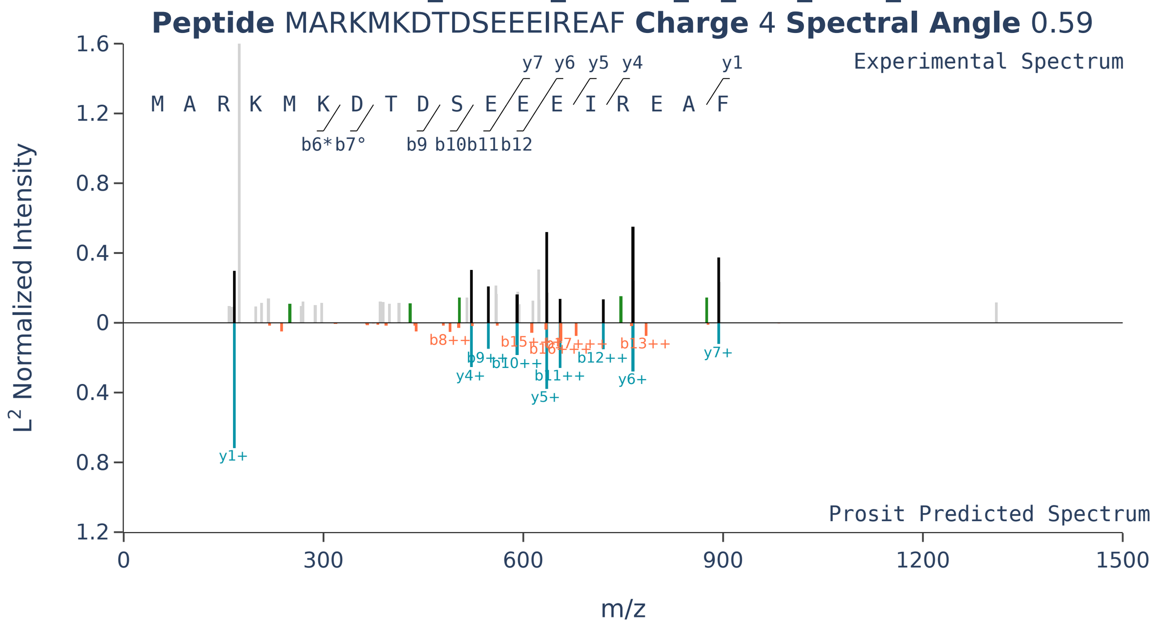


**Source** HRoetschke_291222_040123_Fu_CaM_b2R2_2h **Scan** 5448
**Peptide** GQNPTEAEL **Charge** 2 **Spectral Angle** 0.89

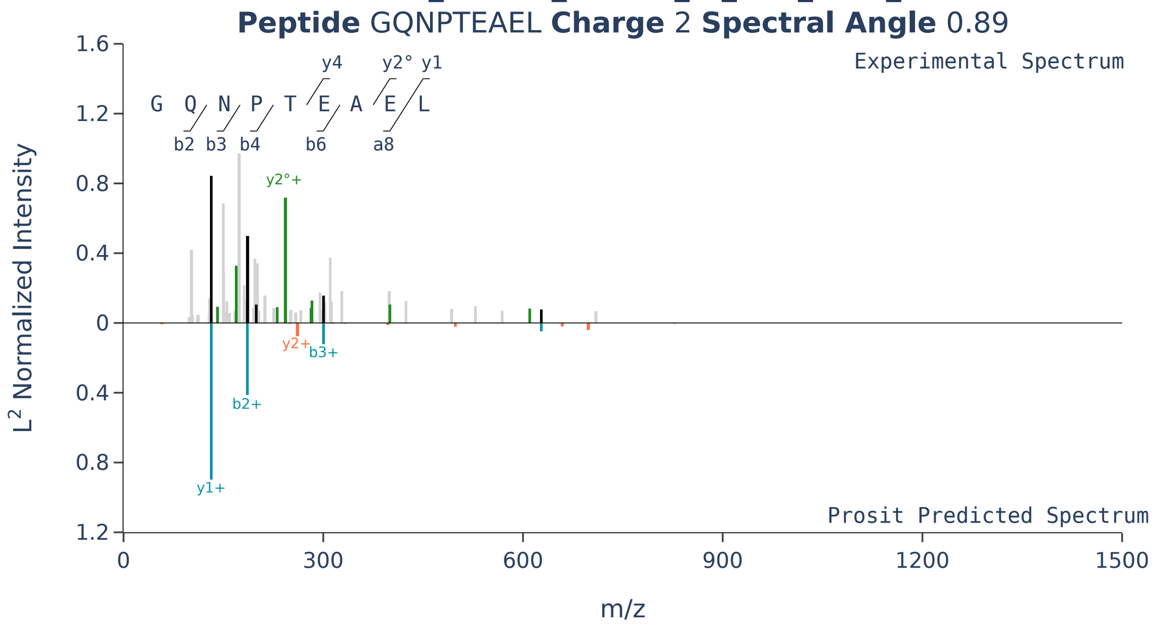


**Source** HRoetschke_291222_040123_Fu_CaM_b2R1_24h **Scan** 10281
**Peptide** SLFDKDGDGTITTKELGTVMRSLGQNPTE **Charge** 4 **Spectral Angle** 0.66

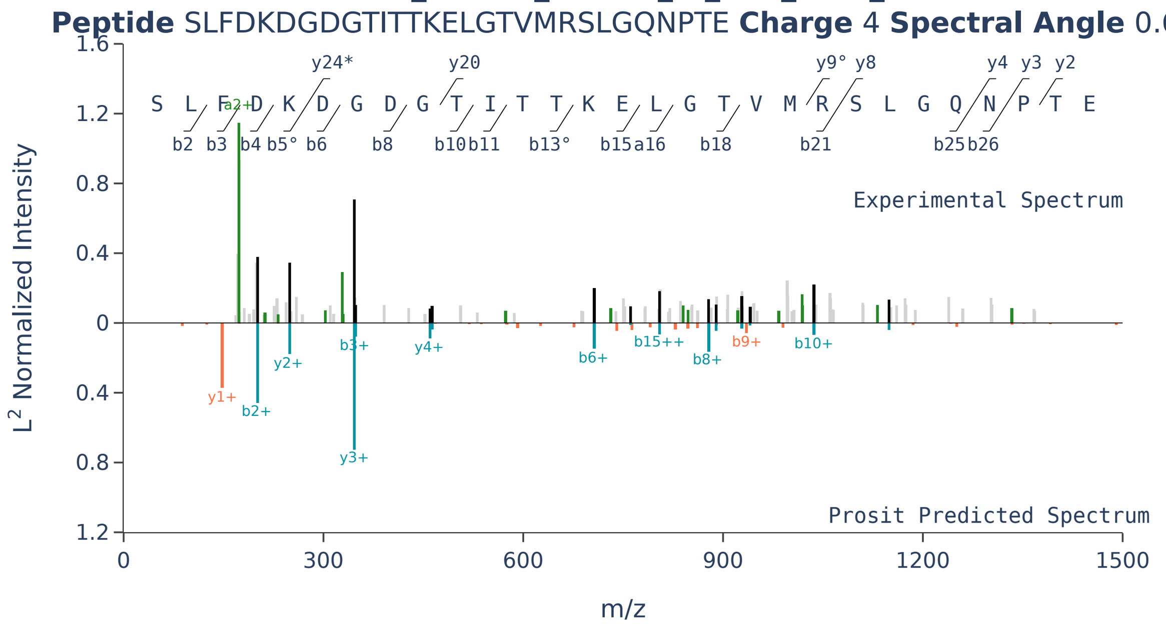


**Source** HRoetschke_291222_040123_Fu_CaM_b2R1_2h **Scan** 6900
**Peptide** RHVMTNLGEKLTDEEVD **Charge** 3 **Spectral Angle** 0.56

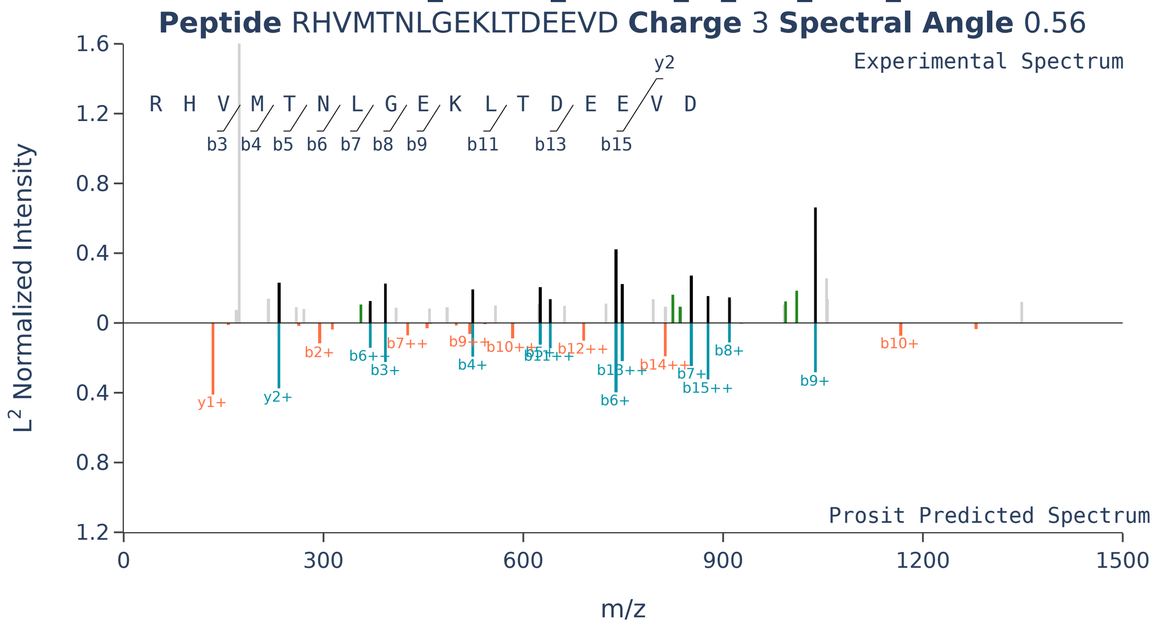


**Source** HRoetschke_291222_040123_Fu_CaM_b1R1_24h **Scan** 7020
**Peptide** EADIDGDGQVNYE **Charge** 2 **Spectral Angle** 0.74

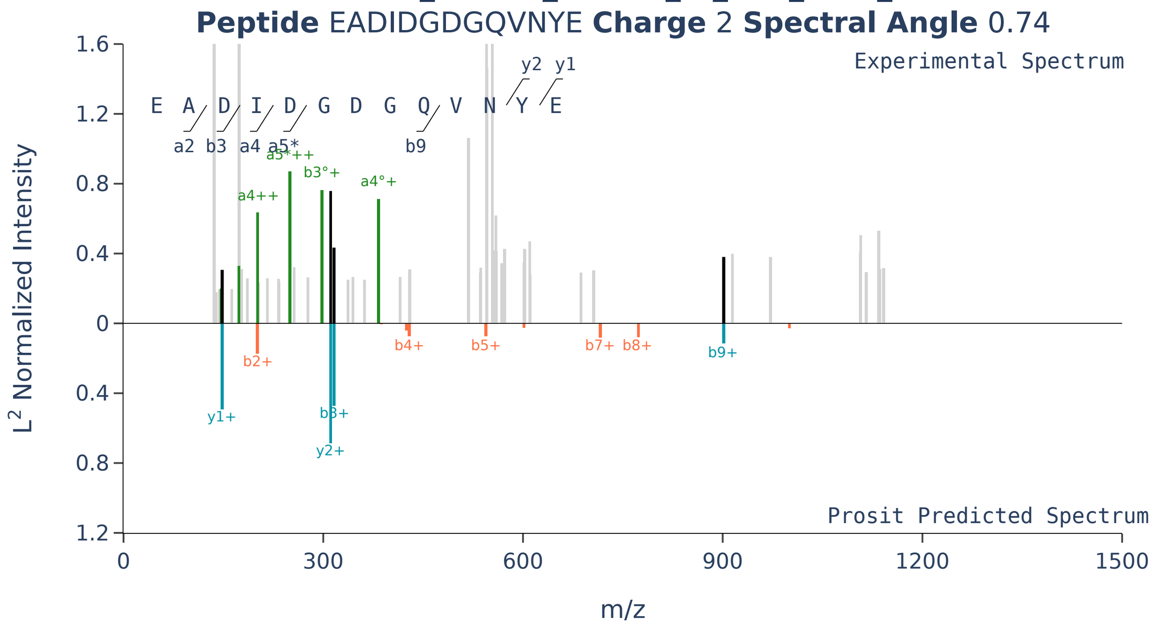


**Source** HRoetschke_291222_040123_Fu_CaM_b2R2_24h **Scan** 8115
**Peptide** ARKMKDTDSEEEIREAFRV **Charge** 4 **Spectral Angle** 0.8

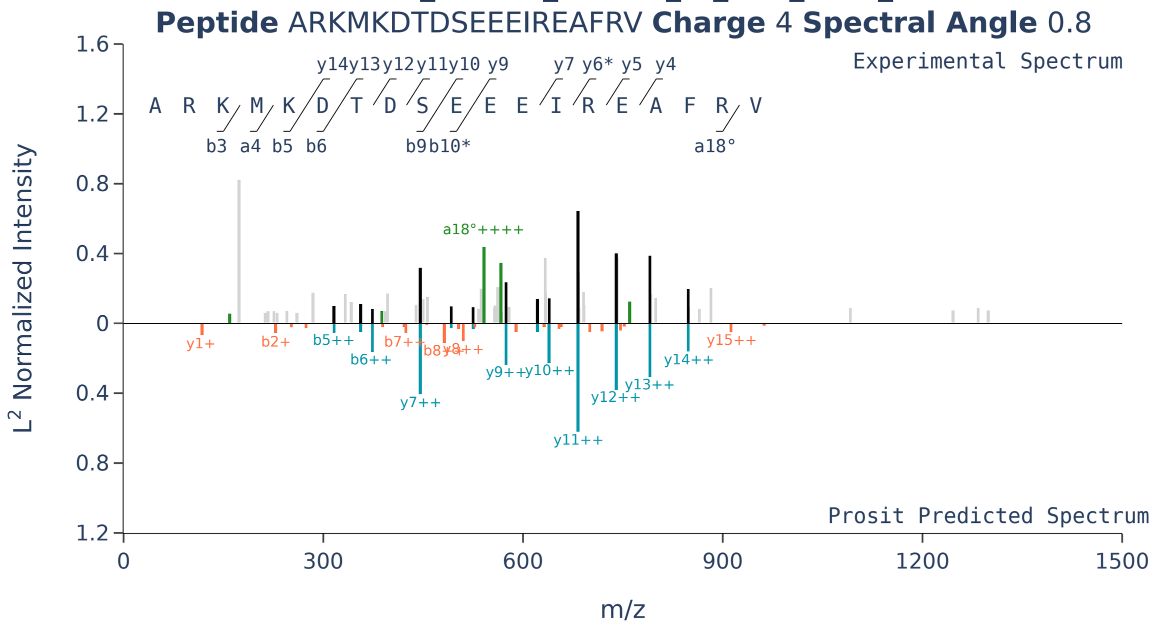


**Source** HRoetschke_291222_040123_Fu_CaM_b1R2_4h **Scan** 9513
**Peptide** VFDKDGNGYISAAELRHV **Charge** 4 **Spectral Angle** 0.72

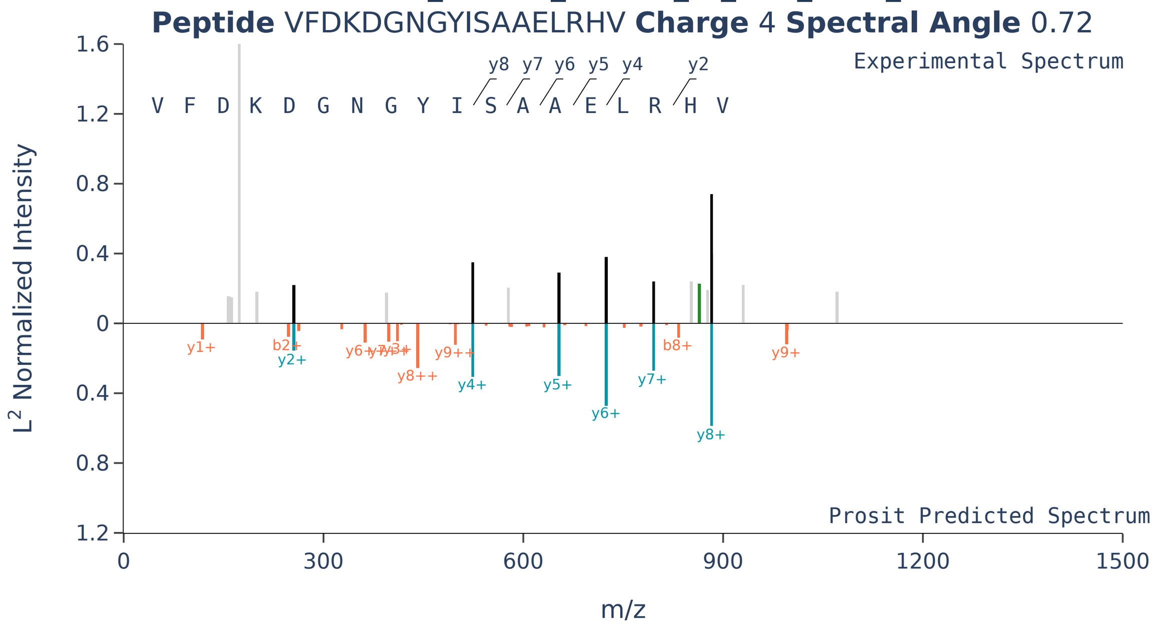


**Source** HRoetschke_291222_040123_Fu_CaM_b1R2_24h **Scan** 1947
**Peptide** ARKMKDTDSEEELR **Charge** 4 **Spectral Angle** 0.74

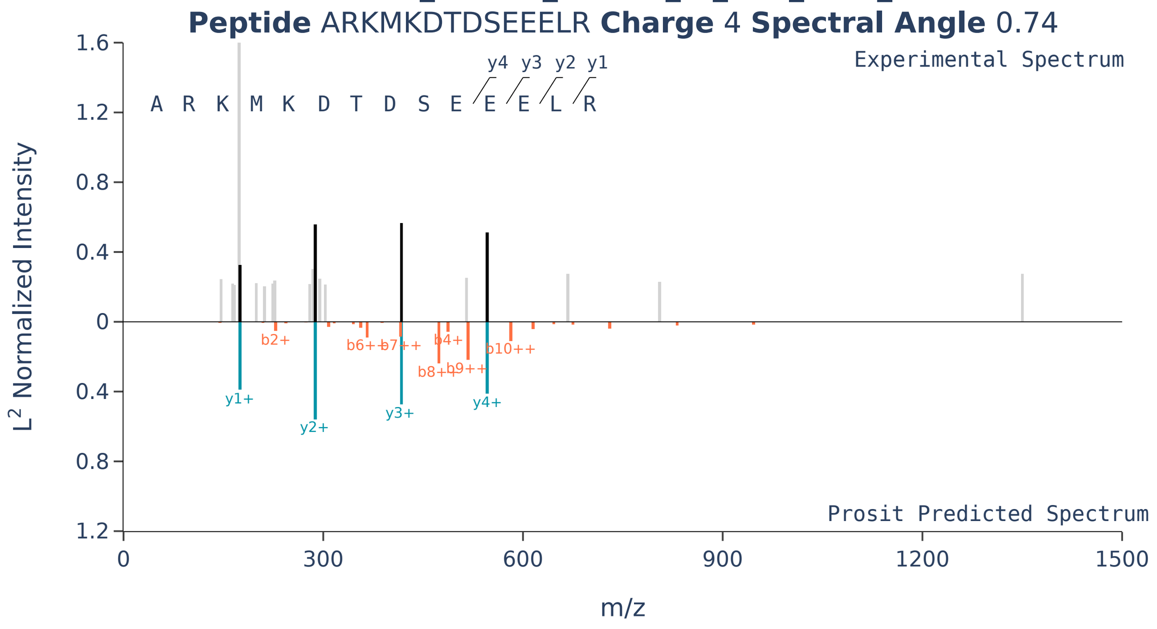


**Source** HRoetschke_291222_040123_Fu_CaM_b1R1_4h **Scan** 2452
**Peptide** KMKDTDSEEEIRE **Charge** 3 **Spectral Angle** 0.72

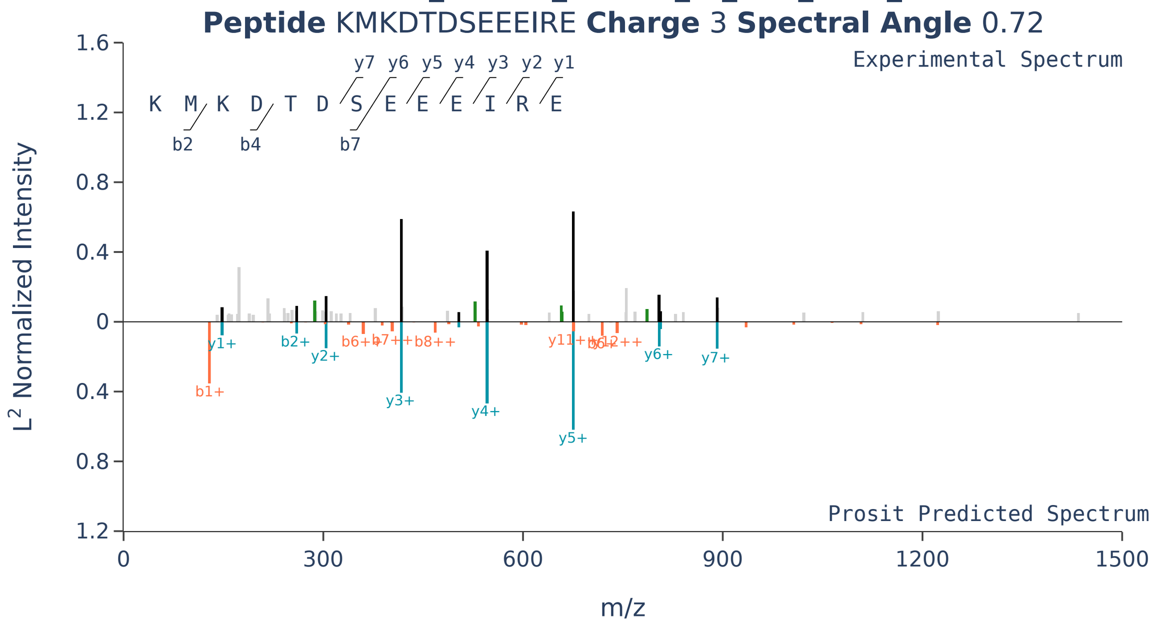


**Source** HRoetschke_291222_040123_Fu_CaM_b1R1_24h **Scan** 10997
**Peptide** ELGTVMRSLGQNPTE **Charge** 2 **Spectral Angle** 0.72

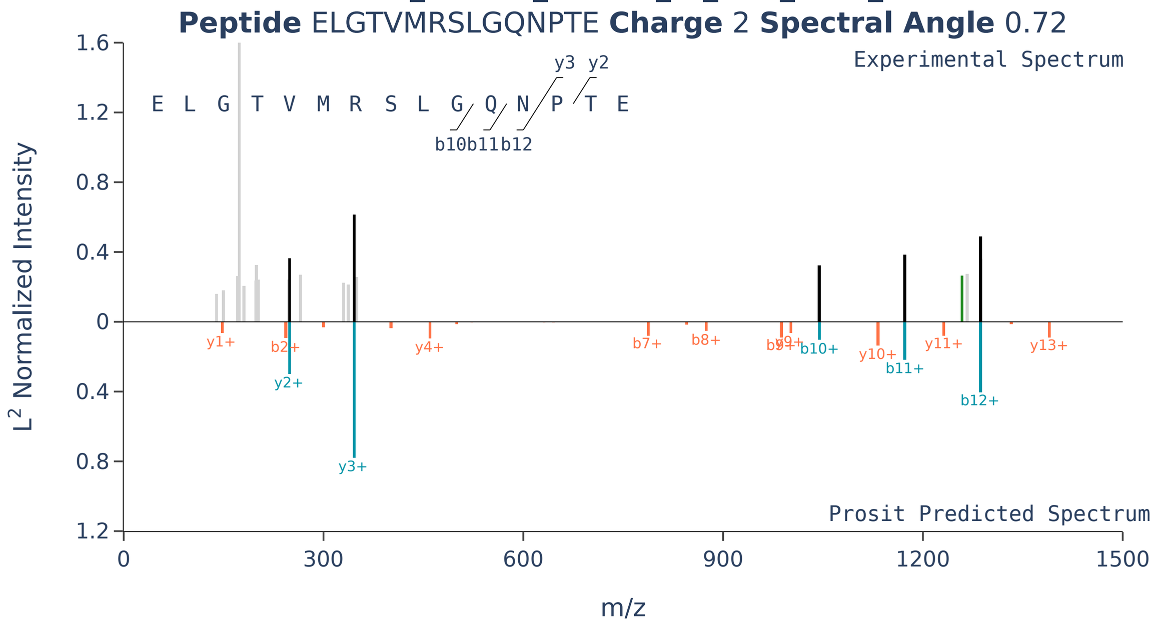


**Source** HRoetschke_291222_040123_Fu_CaM_b2R2_1h **Scan** 8245
**Peptide** QLAEFKEAF **Charge** 2 **Spectral Angle** 0.64

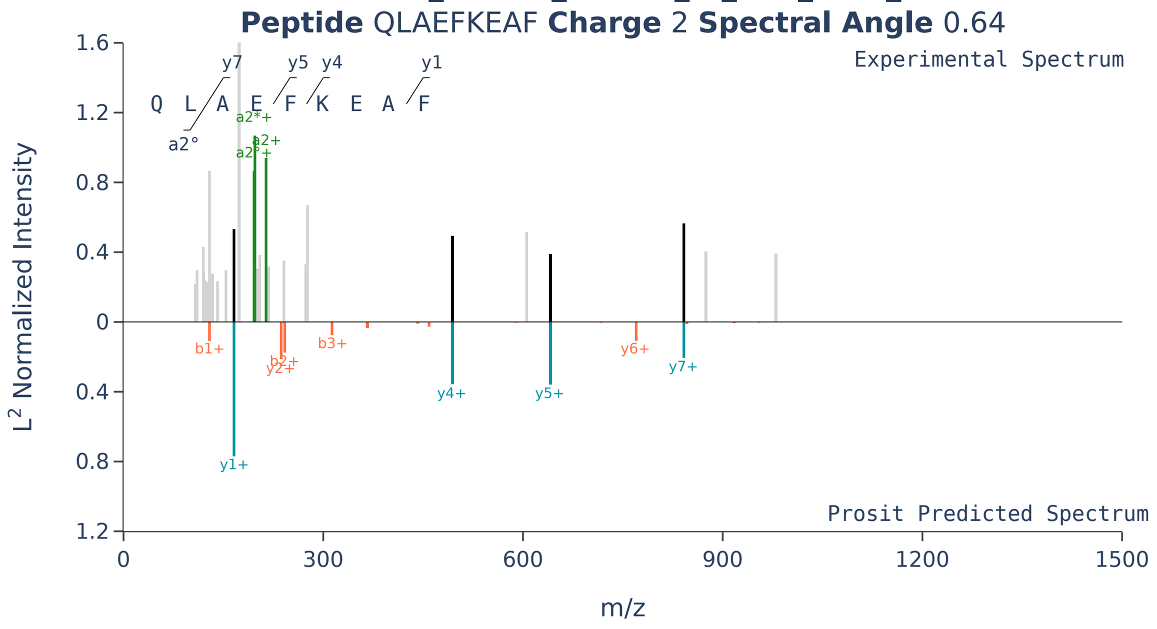


**Source** HRoetschke_291222_040123_Fu_CaM_b2R2_24h **Scan** 12475
**Peptide** FSLFDKDGDGTITTKELGTVMR **Charge** 4 **Spectral Angle** 0.67

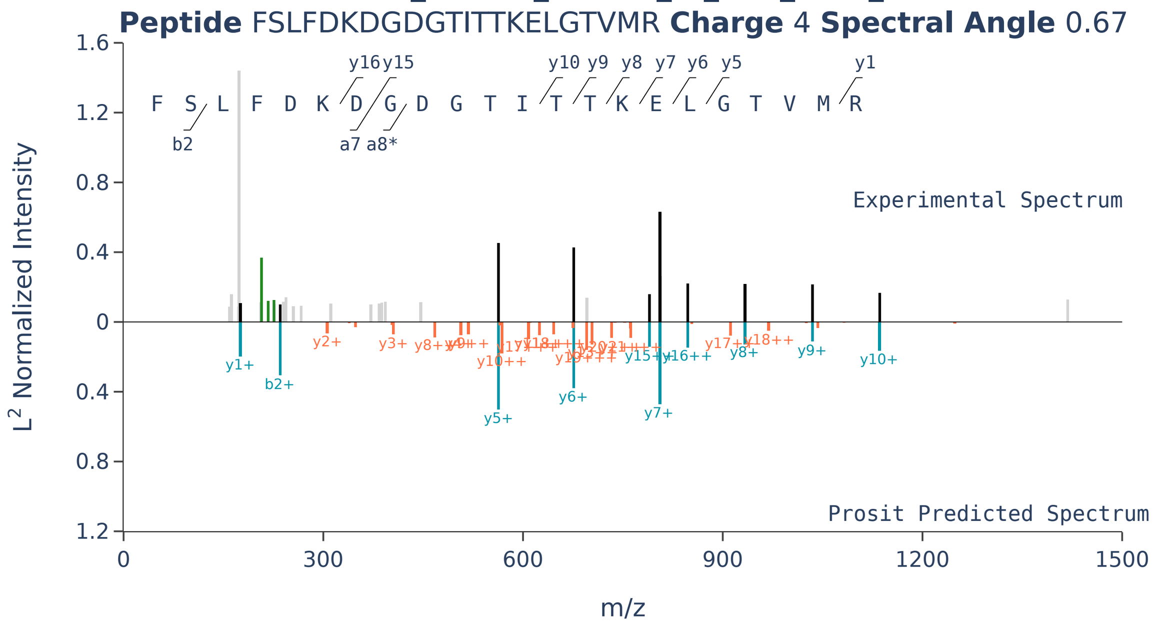


**Source** HRoetschke_291222_040123_Fu_CaM_b2R2_4h **Scan** 6105
**Peptide** AEFKEAFS **Charge** 2 **Spectral Angle** 0.71

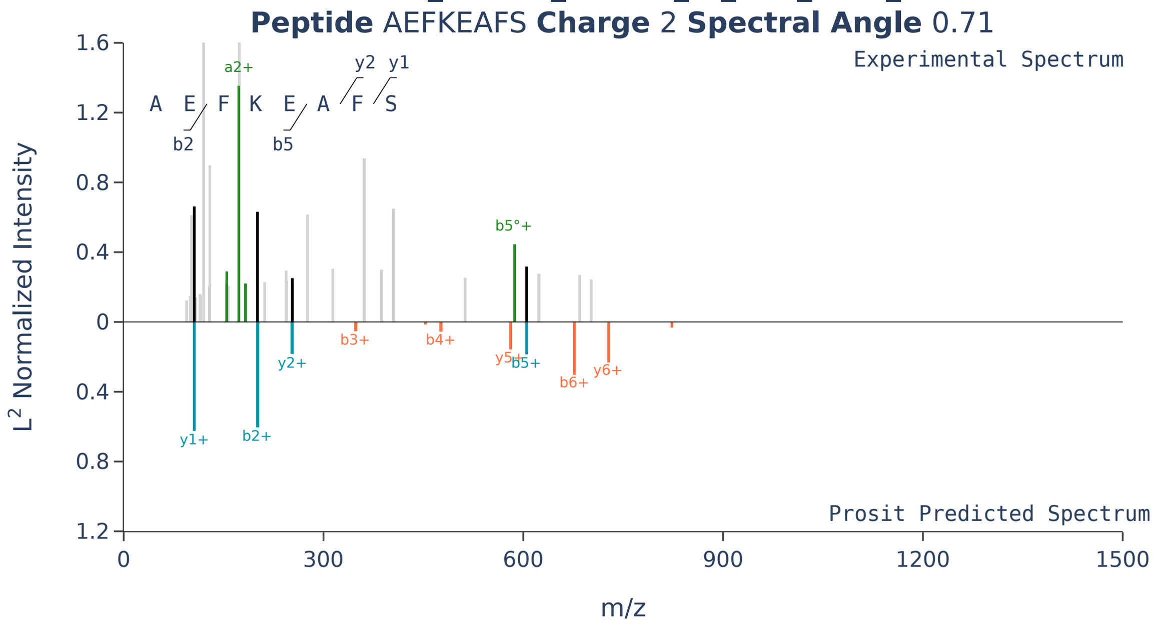

**Source** HRoetschke_291222_040123_Fu_CaM_b1R1_4h **Scan** 9320
**Peptide** KDGDGTITTKELGTVMRSLGQNPTE **Charge** 3 **Spectral Angle** 0.55

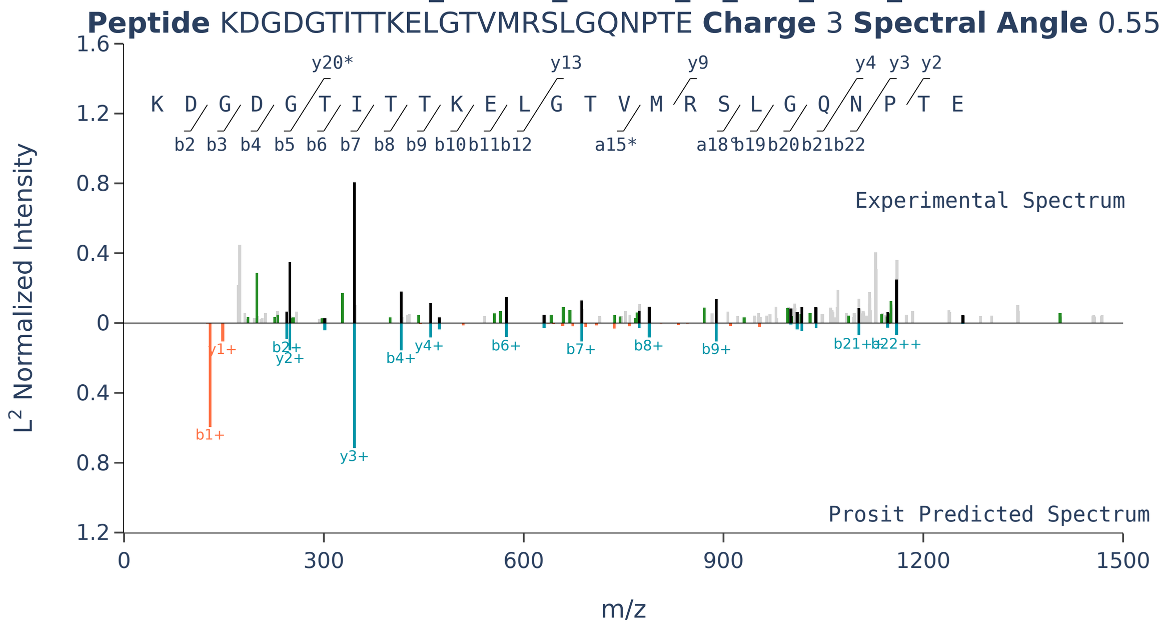


**Source** HRoetschke_291222_040123_Fu_CaM_b1R2_4h **Scan** 12652
**Peptide** DGDGQVNYEEFVQMMTAK **Charge** 2 **Spectral Angle** 0.55

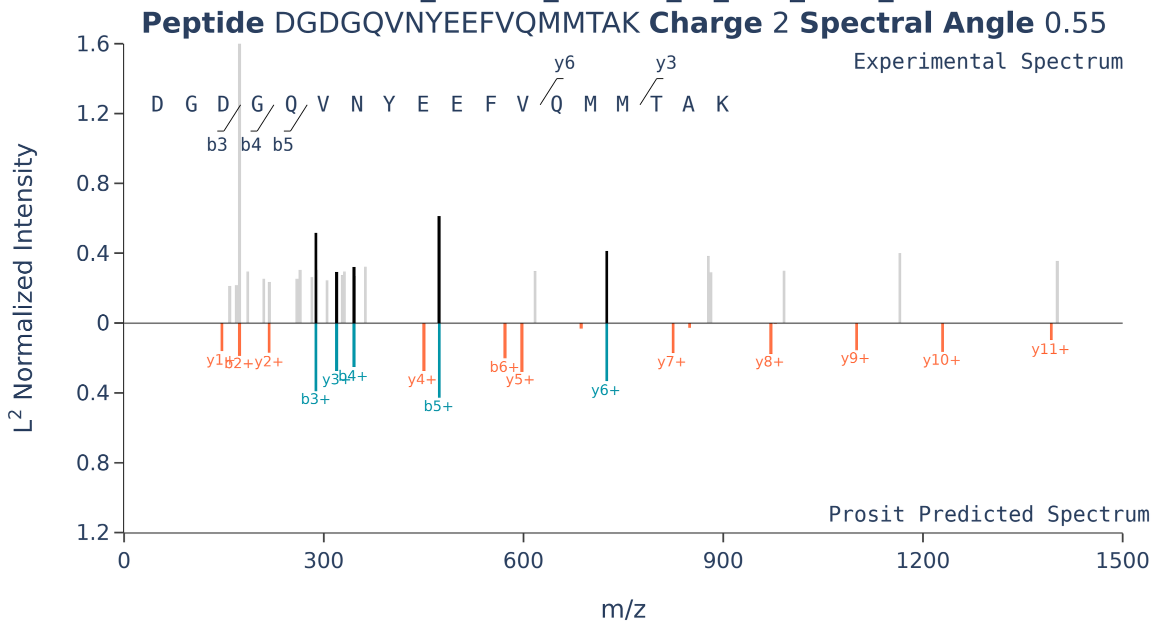


**Source** HRoetschke_291222_040123_Fu_CaM_b1R2_2h **Scan** 9866
**Peptide** DKDGNGYISAAELRHVM **Charge** 3 **Spectral Angle** 0.66

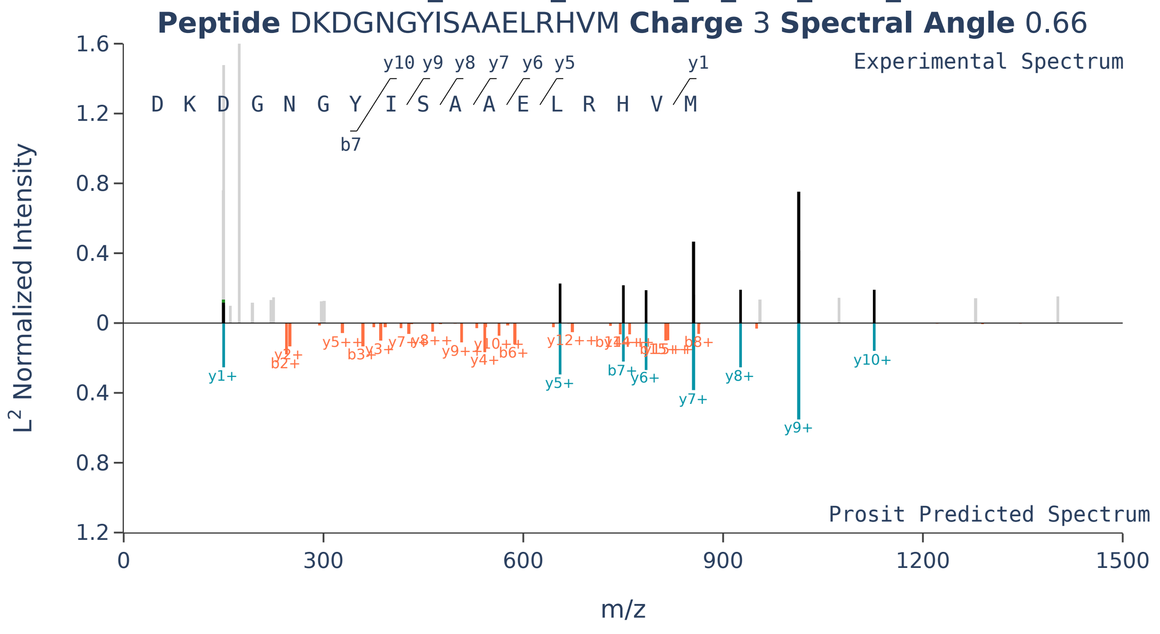


**Source** HRoetschke_291222_040123_Fu_CaM_b2R1_4h **Scan** 8445
**Peptide** EMIREADIDGDGQVNY **Charge** 2 **Spectral Angle** 0.69

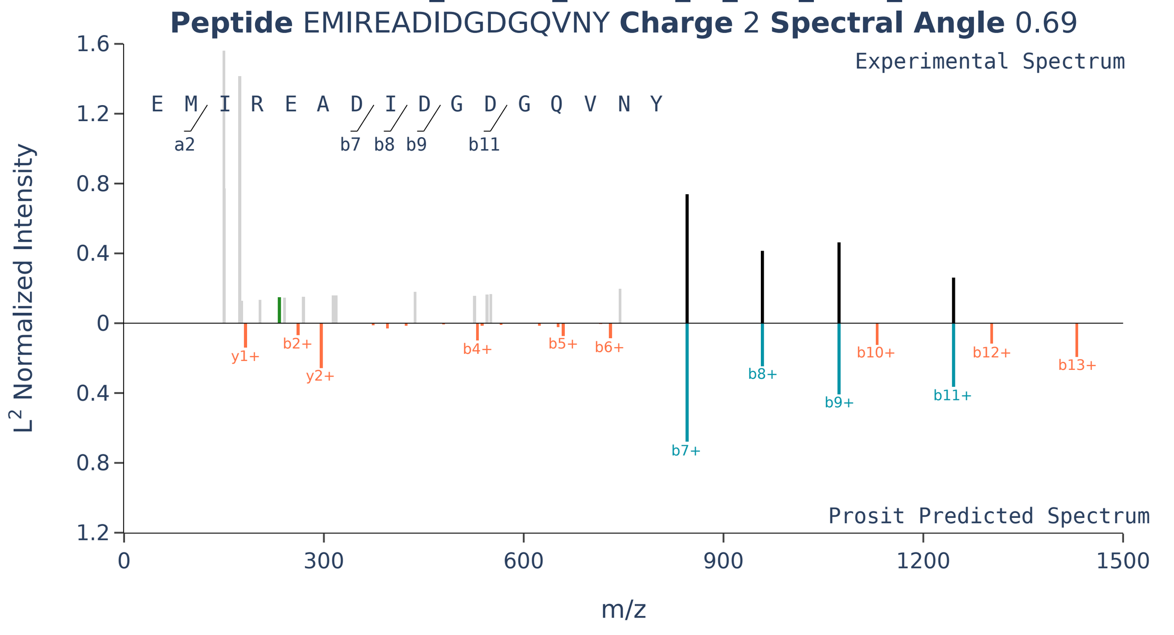


**Source** HRoetschke_291222_040123_Fu_CaM_b1R1_4h **Scan** 6946
**Peptide** KDGDGTITTKELGTV **Charge** 3 **Spectral Angle** 0.55

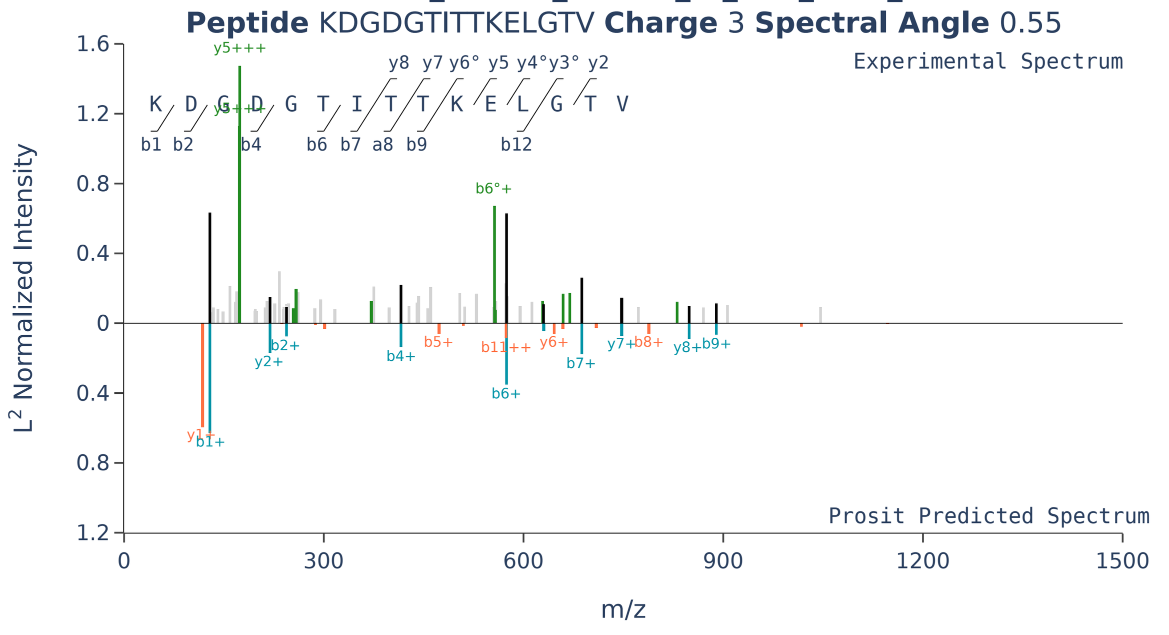


**Source** HRoetschke_291222_040123_Fu_CaM_b2R1_24h **Scan** 13220
**Peptide** AEFKEAFSLF **Charge** 2 **Spectral Angle** 0.77

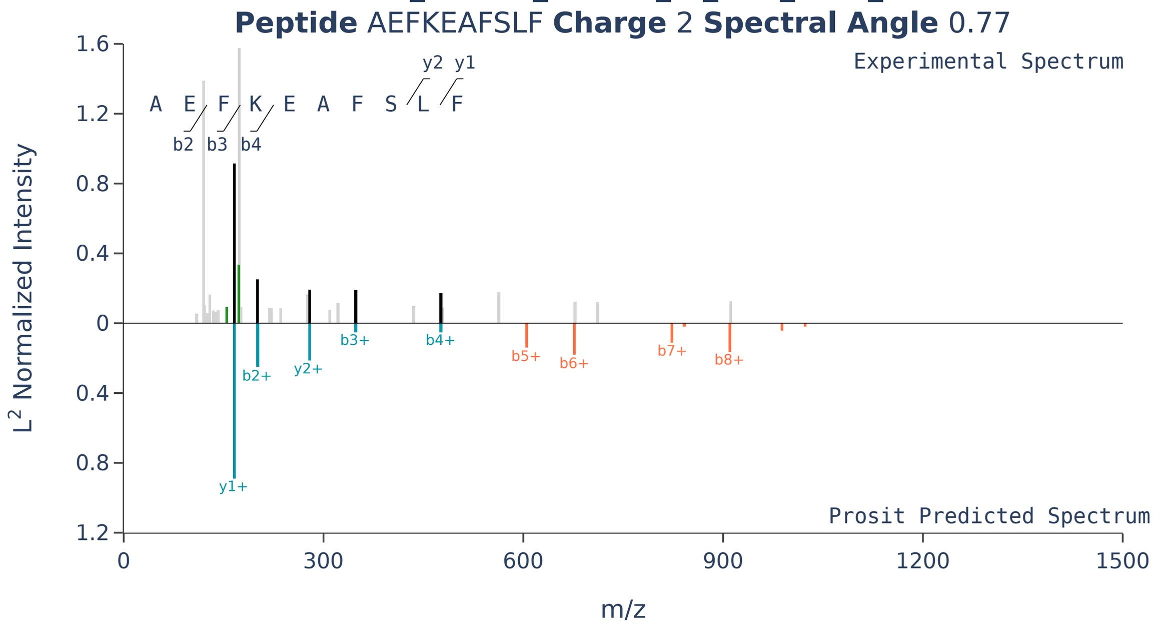


**Source** HRoetschke_291222_040123_Fu_CaM_b1R1_24h **Scan** 10109
**Peptide** LFDKDGDGTITTKEL **Charge** 3 **Spectral Angle** 0.71

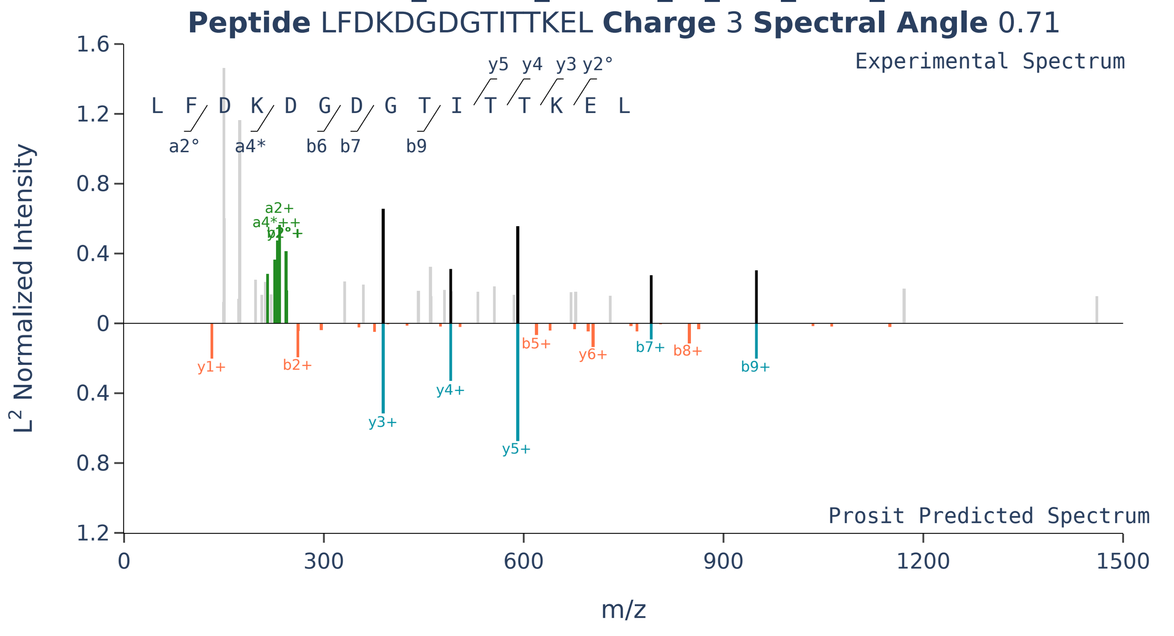


**Source** HRoetschke_291222_040123_Fu_CaM_b1R1_24h **Scan** 6386
**Peptide** SLFDKDGDGT **Charge** 2 **Spectral Angle** 0.57

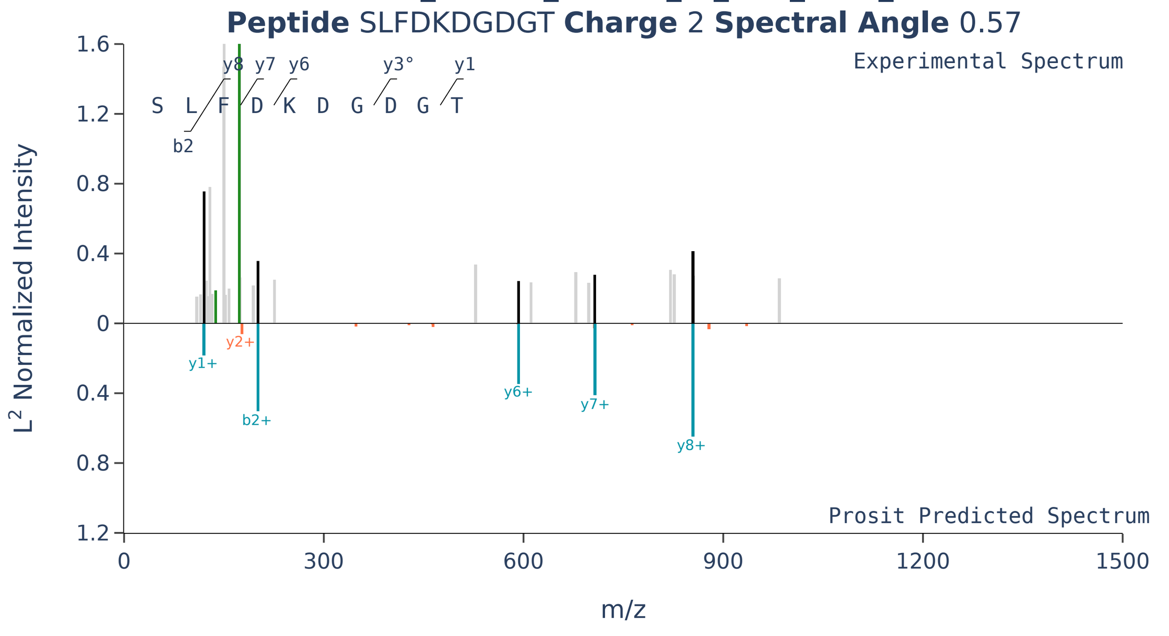


**Source** HRoetschke_291222_040123_Fu_CaM_b2R1_24h **Scan** 11612
**Peptide** DIDGDGQVNYEEFV **Charge** 2 **Spectral Angle** 0.71

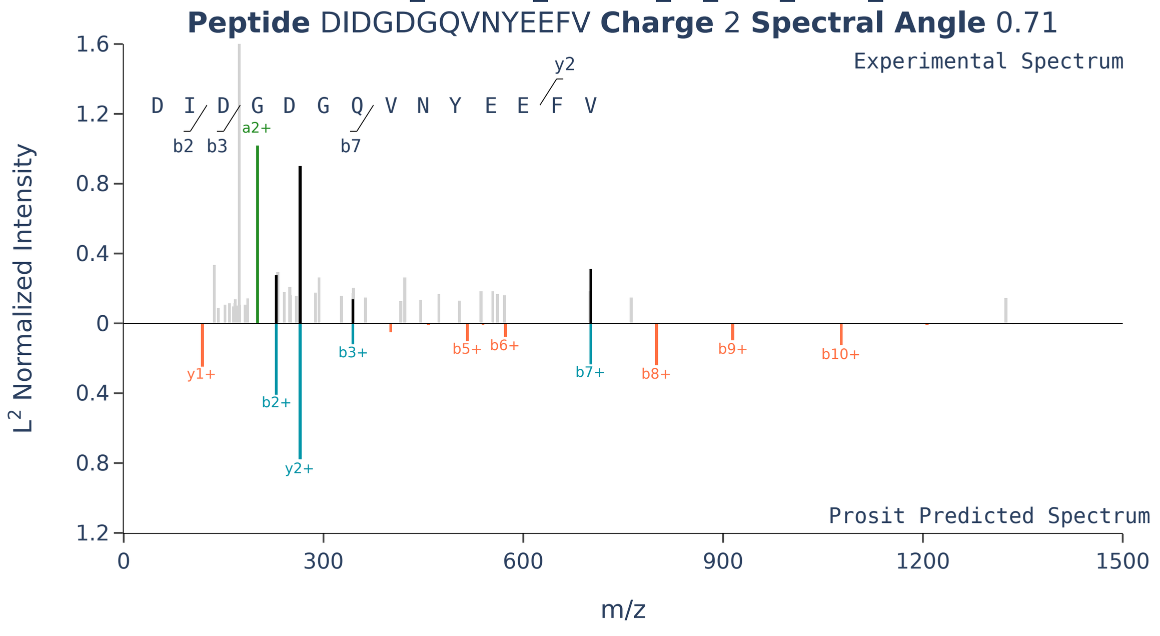


**Source** HRoetschke_291222_040123_Fu_CaM_b2R1_24h **Scan** 12589
**Peptide** RSLGQNPTEAELQDMINEV **Charge** 2 **Spectral Angle** 0.64

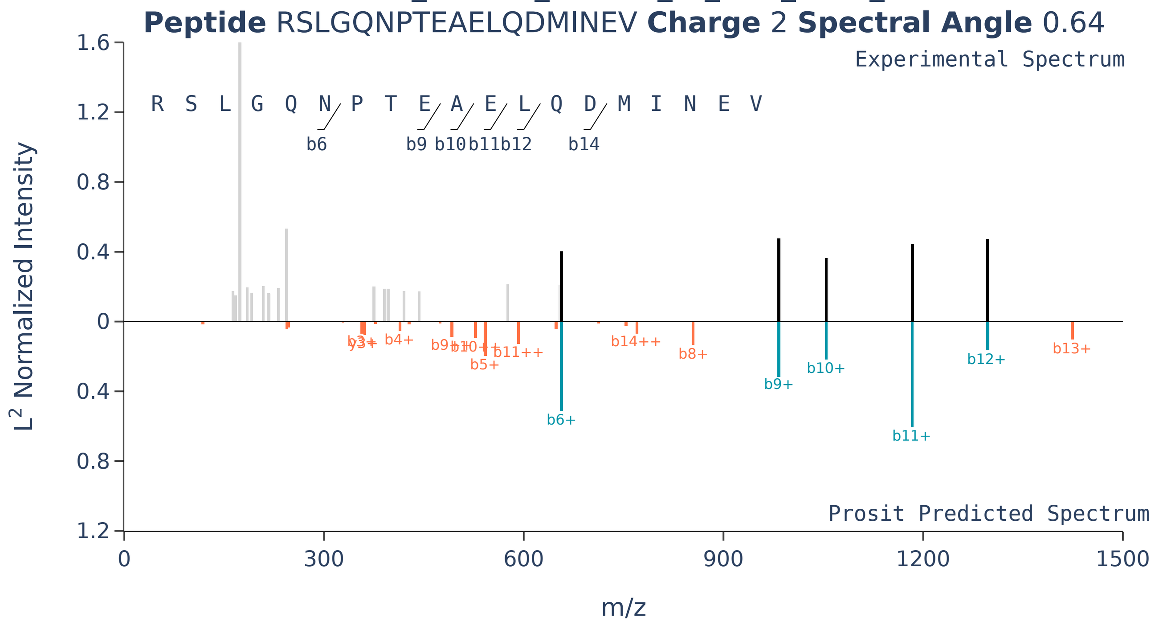


**Source** HRoetschke_291222_040123_Fu_CaM_b1R1_24h **Scan** 9534
**Peptide** MKDTDSEEEIREAF **Charge** 3 **Spectral Angle** 0.64

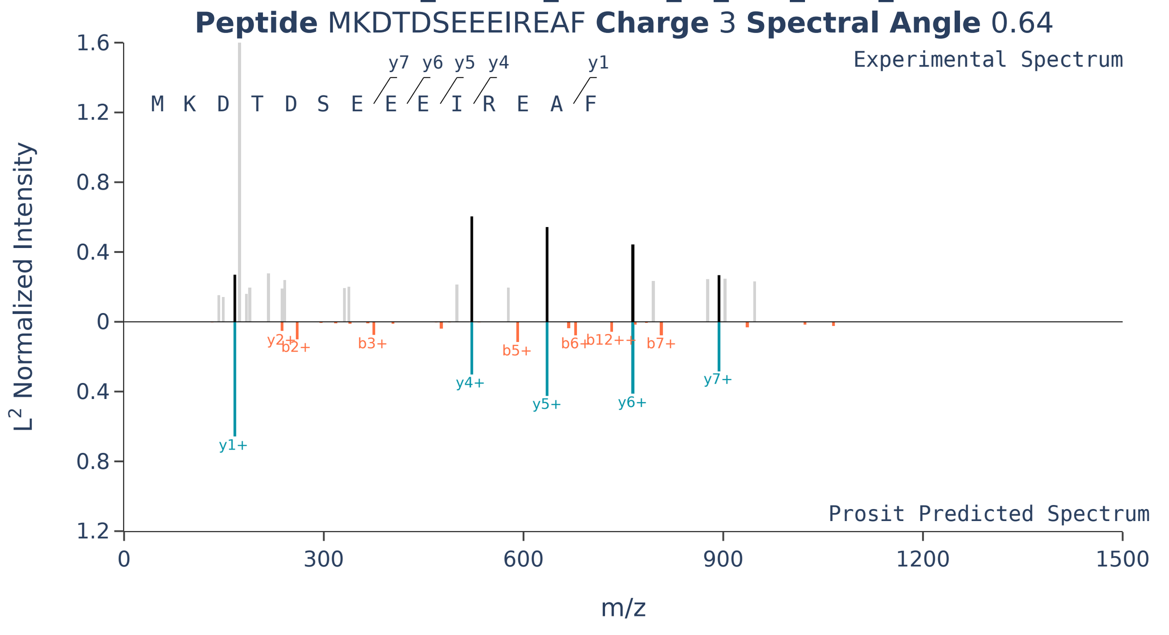


**Source** HRoetschke_291222_040123_Fu_CaM_b2R2_4h **Scan** 5279
**Peptide** GDGTITTKEL **Charge** 2 **Spectral Angle** 0.63

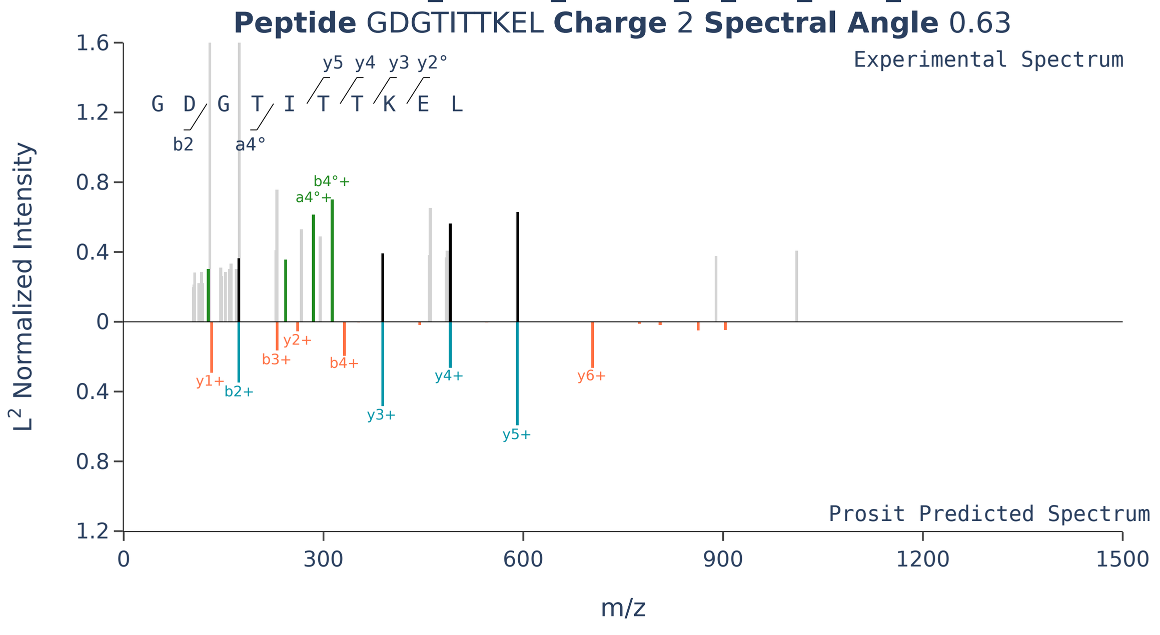


**Source** HRoetschke_291222_040123_Fu_CaM_b2R2_24h **Scan** 9101
**Peptide** FDKDGDGTITTKELGTVMRS **Charge** 4 **Spectral Angle** 0.73

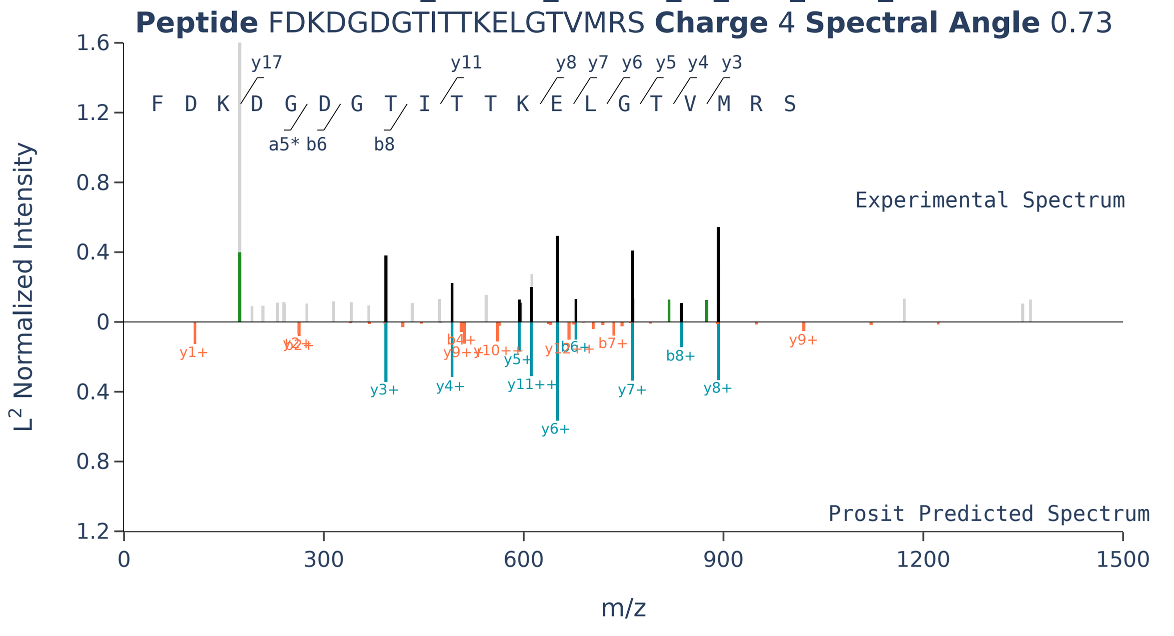


**Source** HRoetschke_291222_040123_Fu_CaM_b1R1_24h **Scan** 6360
**Peptide** QLAEFKEA **Charge** 2 **Spectral Angle** 0.6

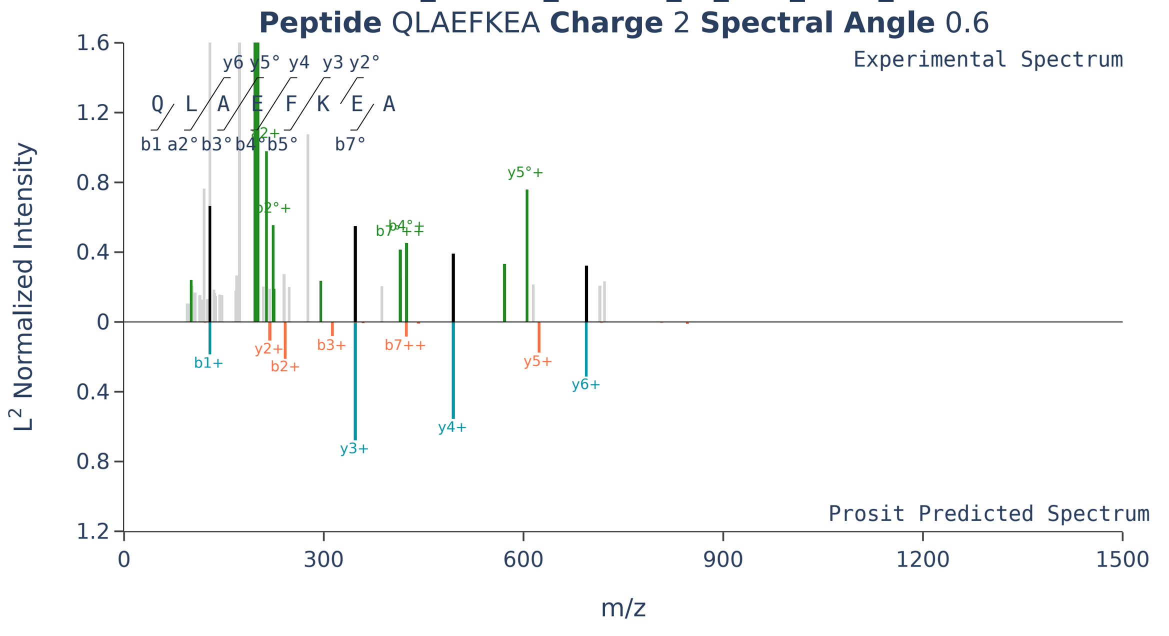


**Source** HRoetschke_291222_040123_Fu_CaM_b1R1_24h **Scan** 7121
**Peptide** RVFDKDGNGYISAAE **Charge** 3 **Spectral Angle** 0.81

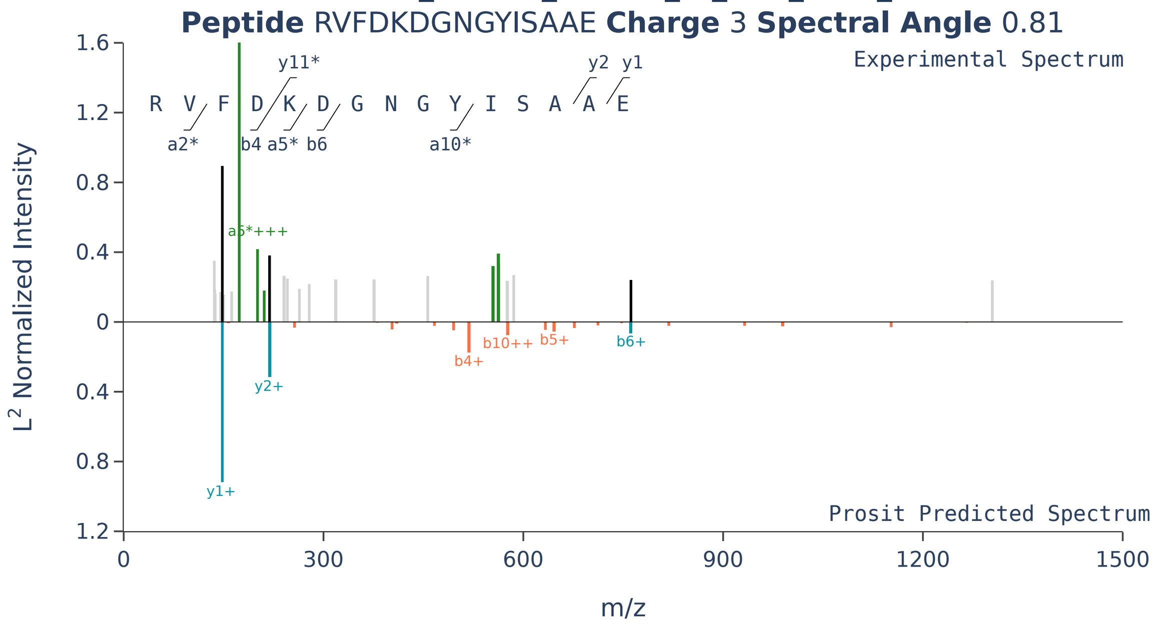

**Source** HRoetschke_291222_040123_Fu_CaM_b2R2_4h **Scan** 8312
**Peptide** DGNGYISAAELRHV **Charge** 3 **Spectral Angle** 0.59

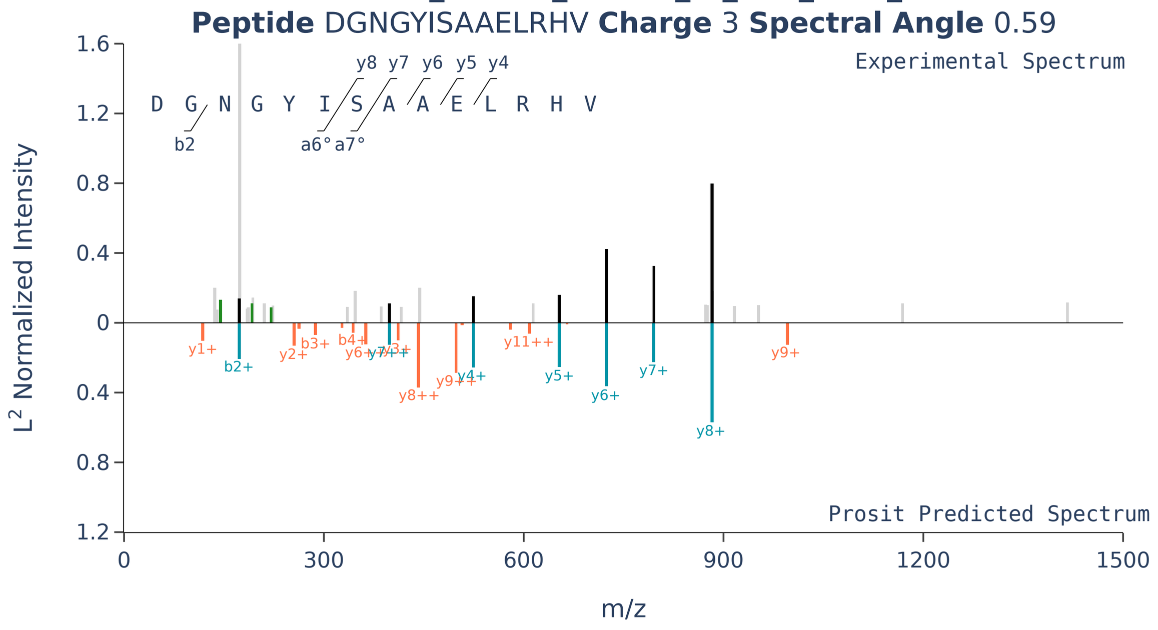


**Source** HRoetschke_291222_040123_Fu_CaM_b1R2_4h **Scan** 6233
**Peptide** ELRHVMTNLGE **Charge** 3 **Spectral Angle** 0.59

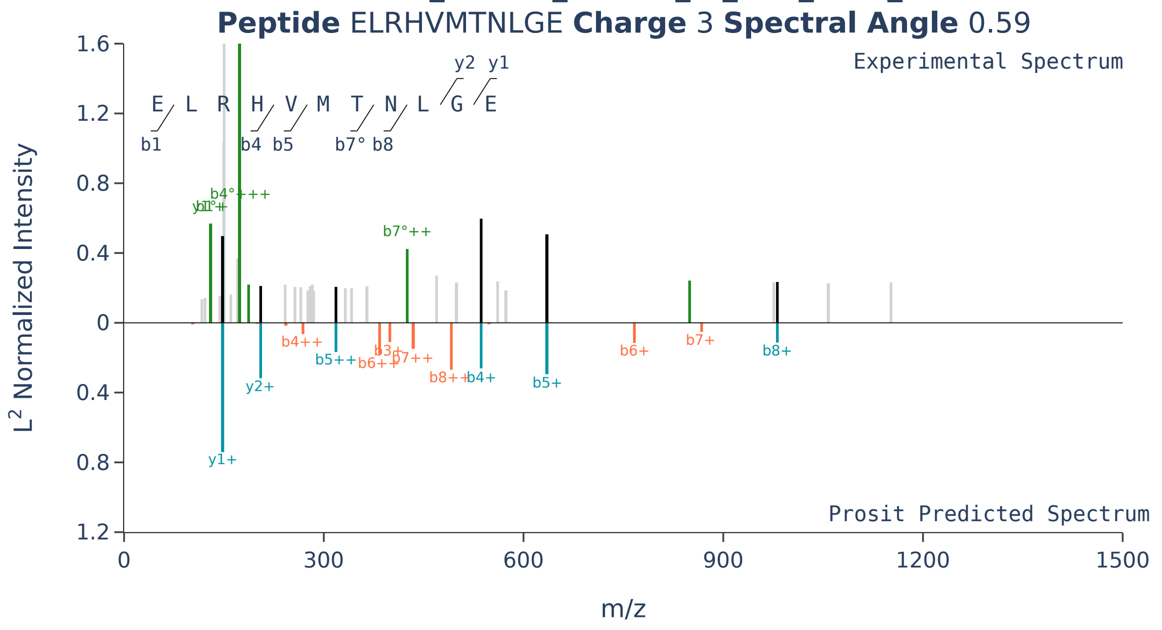


**Source** HRoetschke_291222_040123_Fu_CaM_b2R2_24h **Scan** 13153
**Peptide** READIDGDGQVNYEEFVQMMT **Charge** 3 **Spectral Angle** 0.69

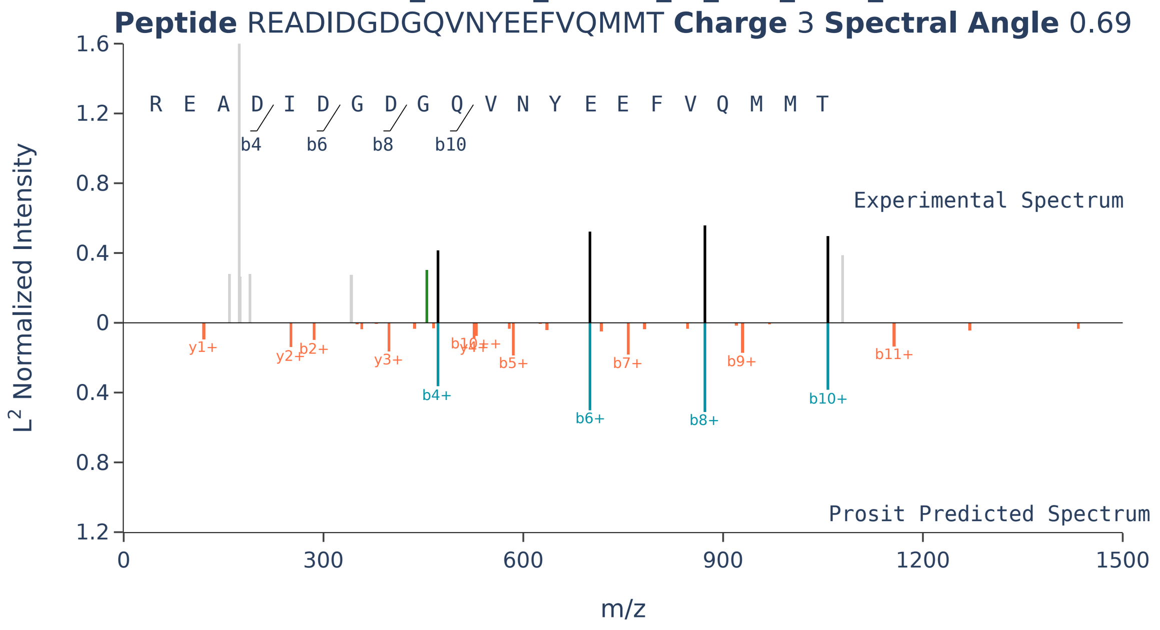


**Source** HRoetschke_291222_040123_Fu_CaM_b1R1_4h **Scan** 7007
**Peptide** KDGNGYISAAELRHVMTN **Charge** 4 **Spectral Angle** 0.6

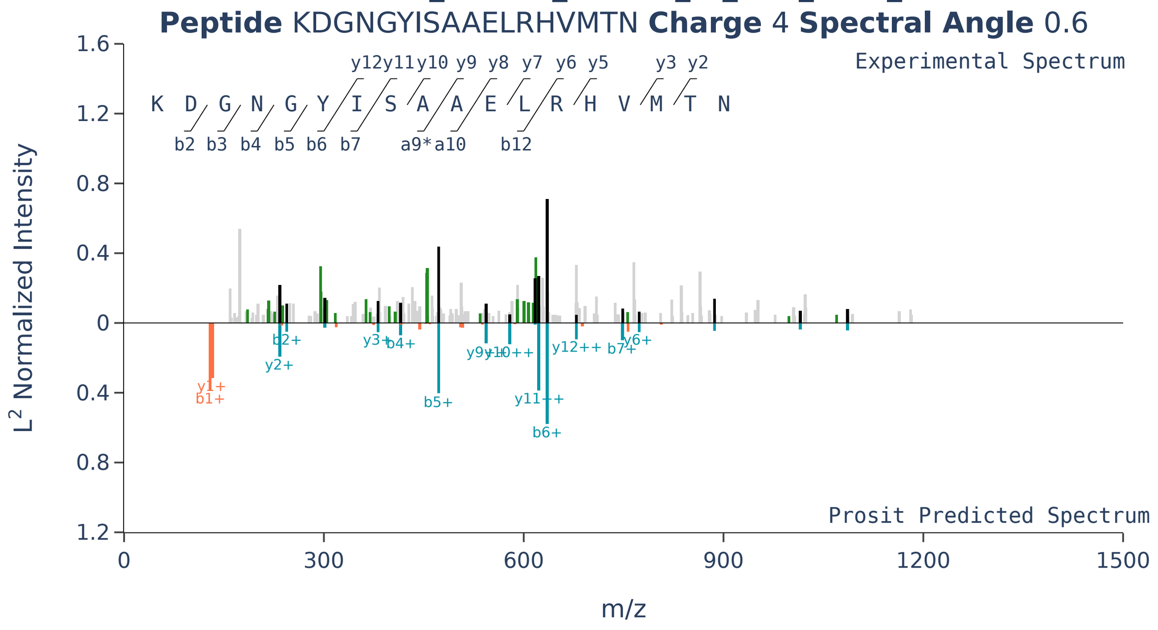


**Source** HRoetschke_291222_040123_Fu_CaM_b1R2_4h **Scan** 10756
**Peptide** MARKMKDTDSEEEIREAFRVFD **Charge** 4 **Spectral Angle** 0.76

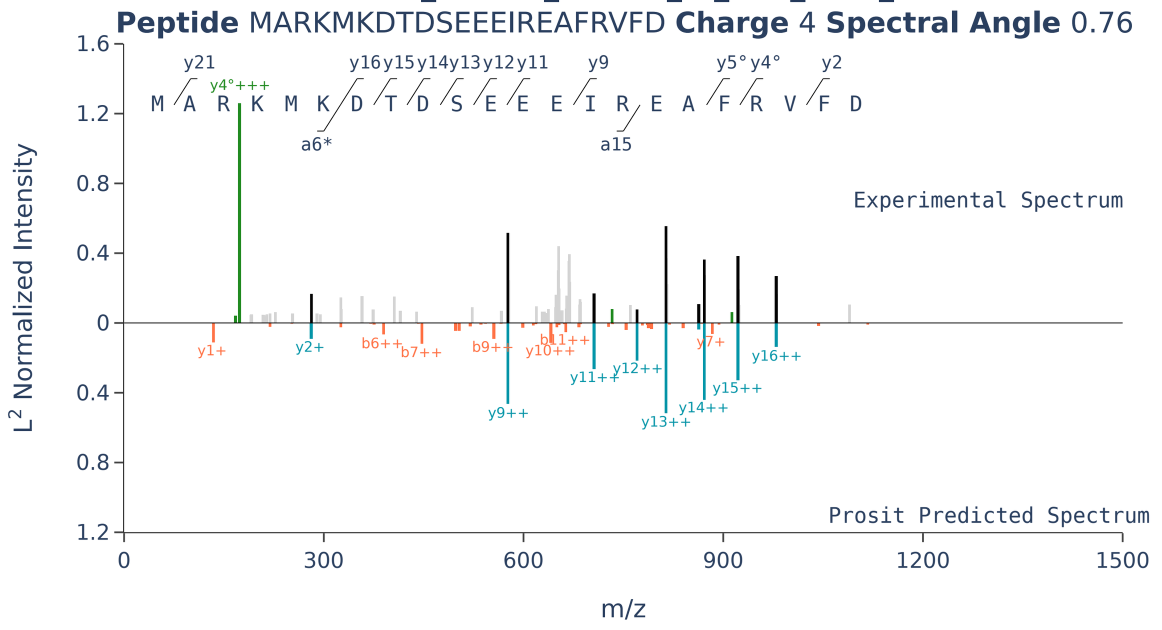


**Source** HRoetschke_291222_040123_Fu_CaM_b2R1_24h **Scan** 10991
**Peptide** FSLFDKDGDGTITTKEL **Charge** 3 **Spectral Angle** 0.74

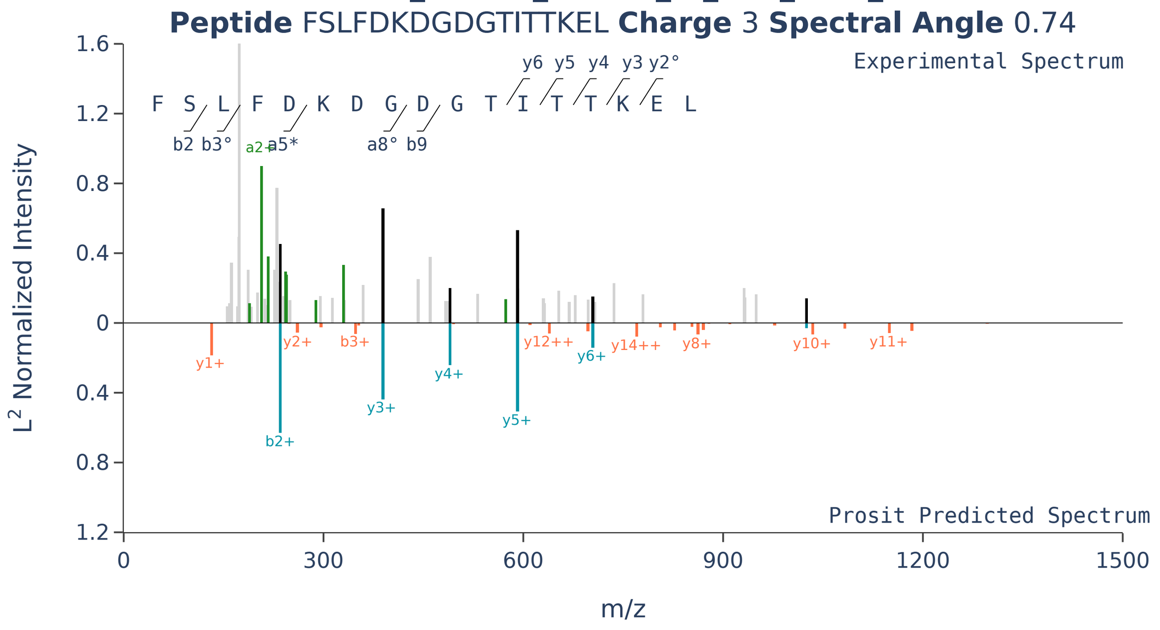


**Source** HRoetschke_291222_040123_Fu_CaM_b1R1_2h **Scan** 10544
**Peptide** ADIDGDGQVNYEEFVQMMTAK **Charge** 3 **Spectral Angle** 0.59

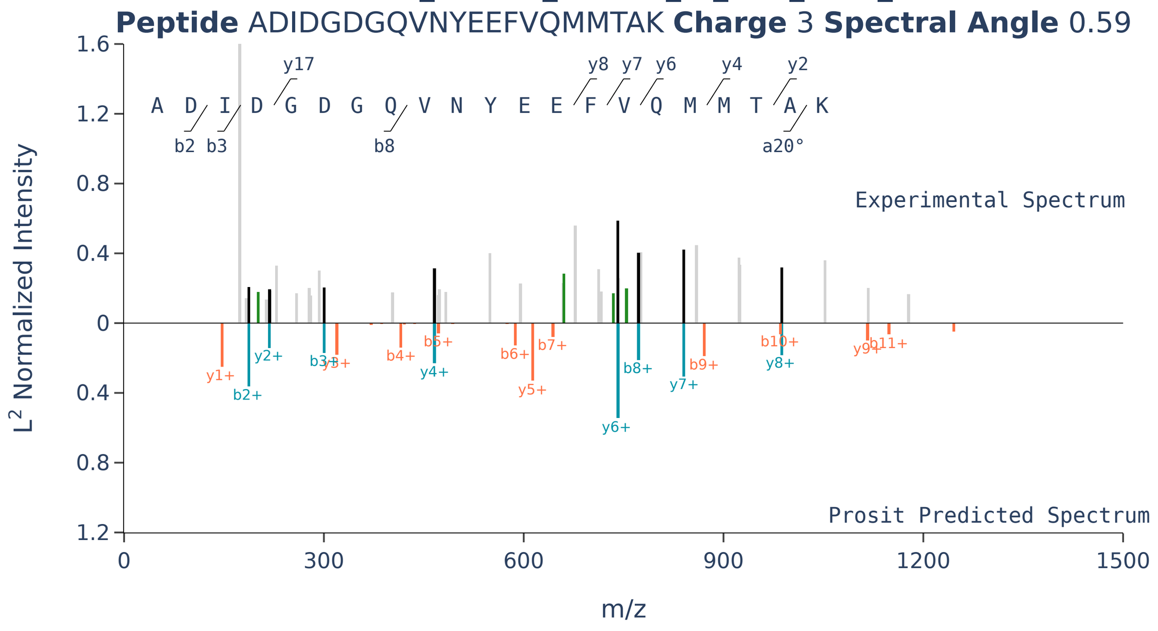


**Source** HRoetschke_291222_040123_Fu_CaM_b1R2_2h **Scan** 9463
**Peptide** SLFDKDGDGTITTKELG **Charge** 3 **Spectral Angle** 0.8

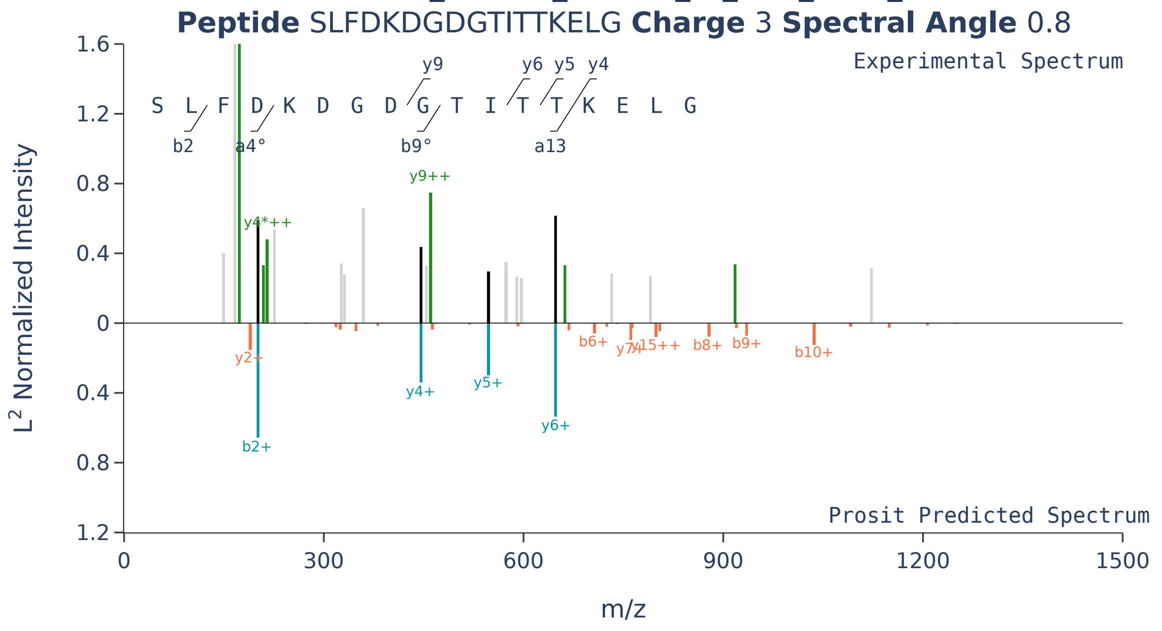


**Source** HRoetschke_291222_040123_Fu_CaM_b1R2_24h **Scan** 4983
**Peptide** DKDGDGTITTKELG **Charge** 3 **Spectral Angle** 0.65

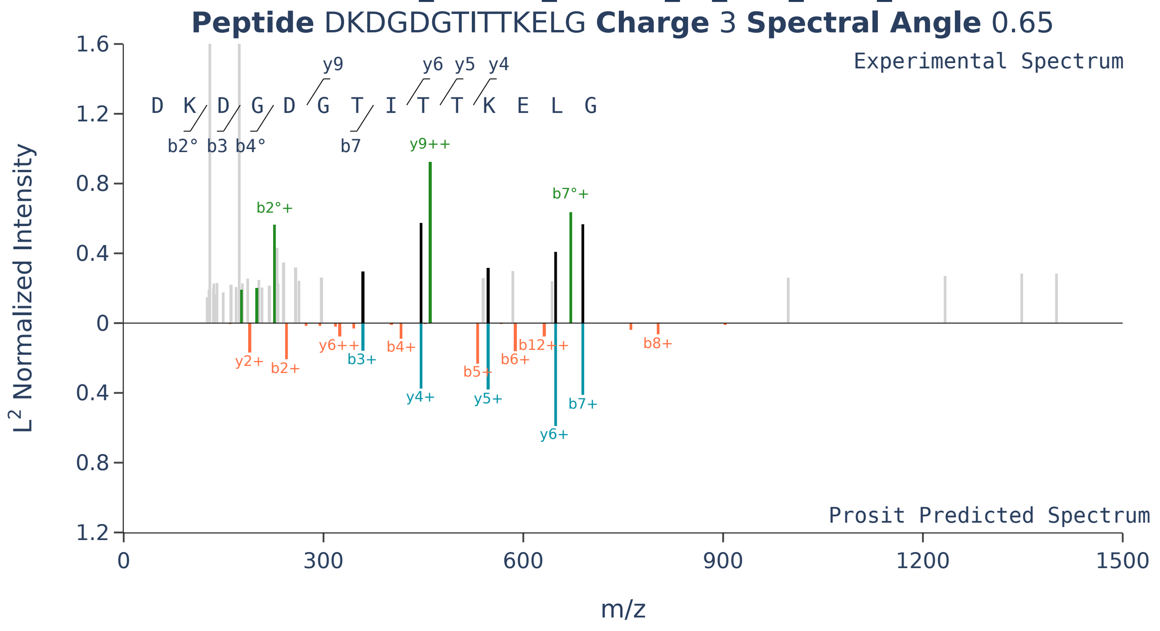


**Source** HRoetschke_291222_040123_Fu_CaM_b2R1_24h **Scan** 9247
**Peptide** READIDGDGQVNYEEFVQ **Charge** 2 **Spectral Angle** 0.55

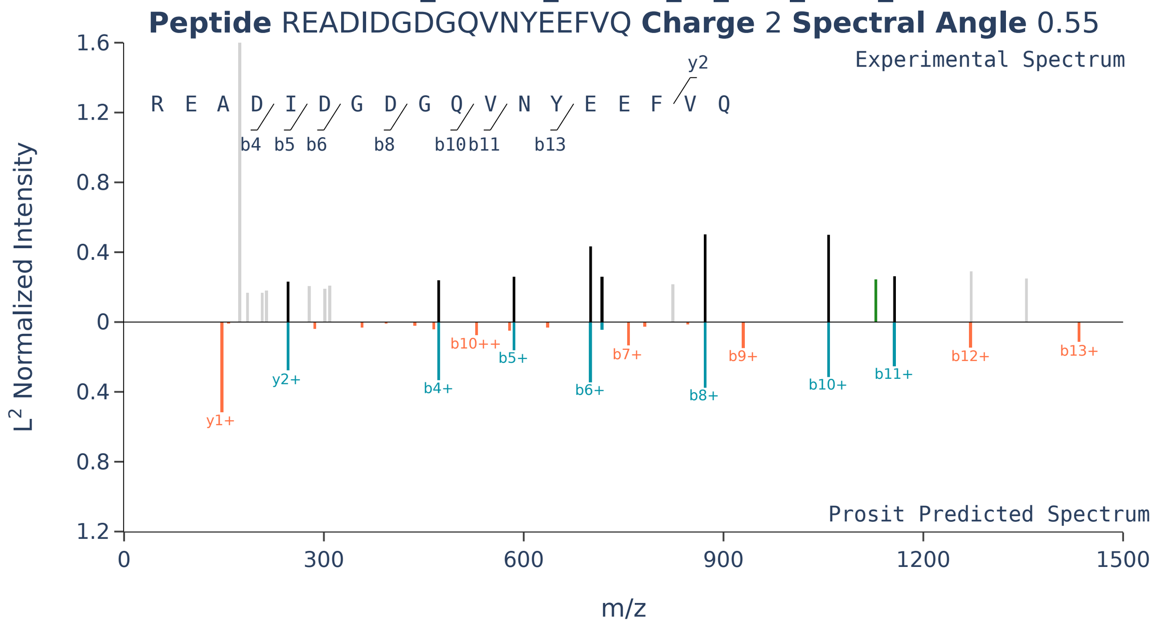


**Source** HRoetschke_291222_040123_Fu_CaM_b1R1_2h **Scan** 9924
**Peptide** REAFRVFDKDGNGYISAAELR **Charge** 5 **Spectral Angle** 0.73

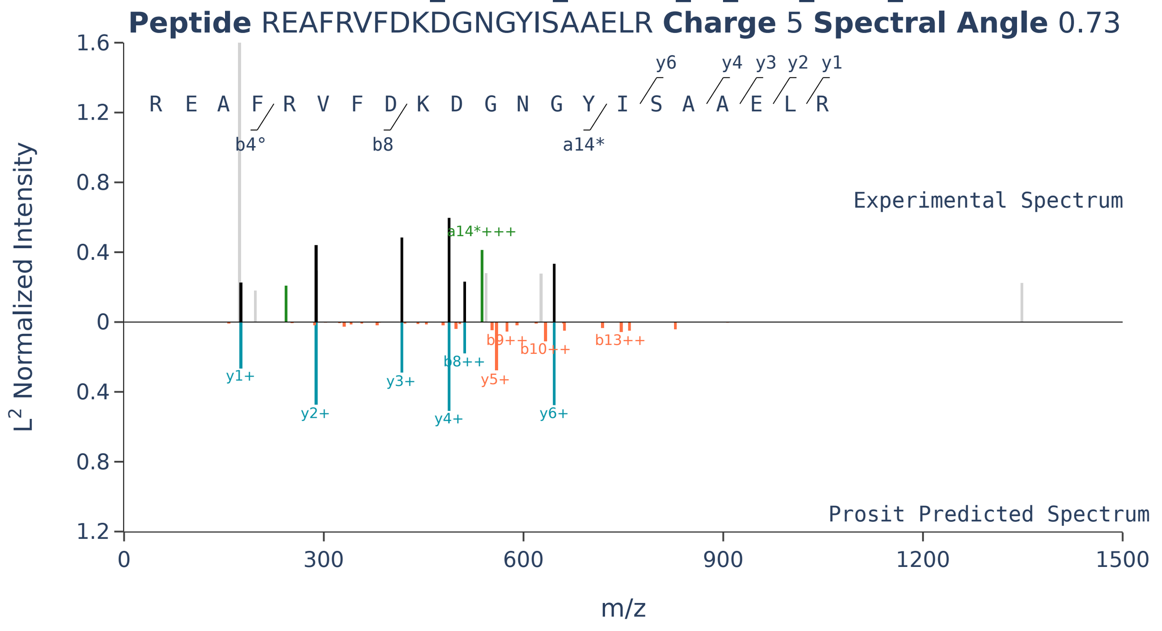


**Source** HRoetschke_291222_040123_Fu_CaM_b2R2_24h **Scan** 6601
**Peptide** KMKDTDSEEEIREAF **Charge** 3 **Spectral Angle** 0.68

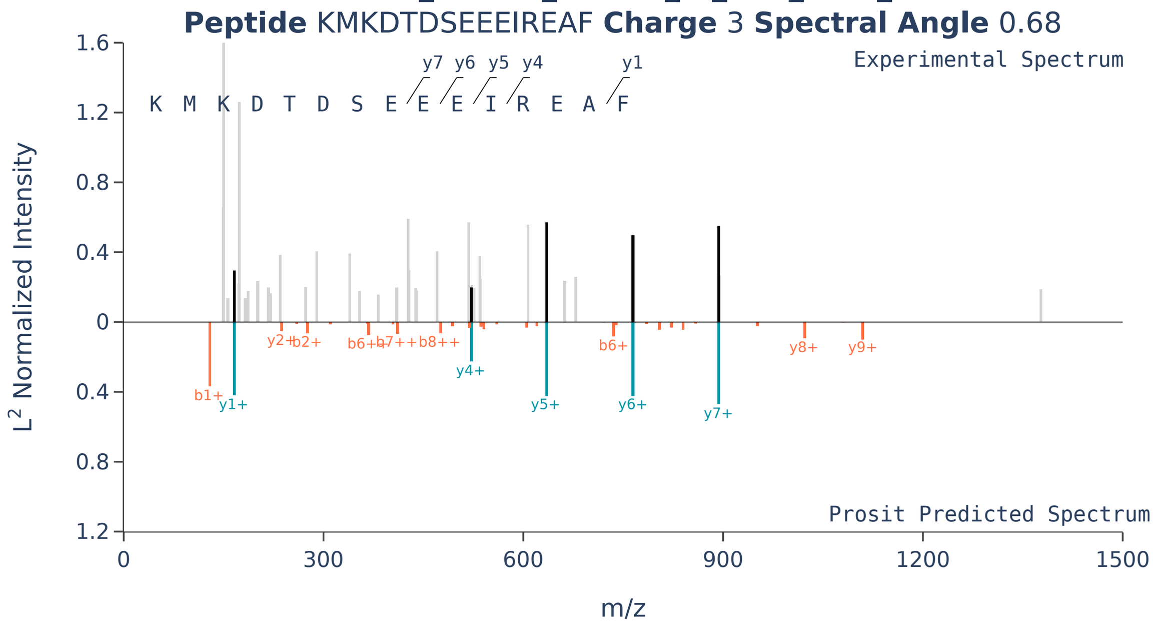


**Source** HRoetschke_291222_040123_Fu_CaM_b1R1_24h **Scan** 4273
**Peptide** VFDKDGNGY **Charge** 2 **Spectral Angle** 0.59

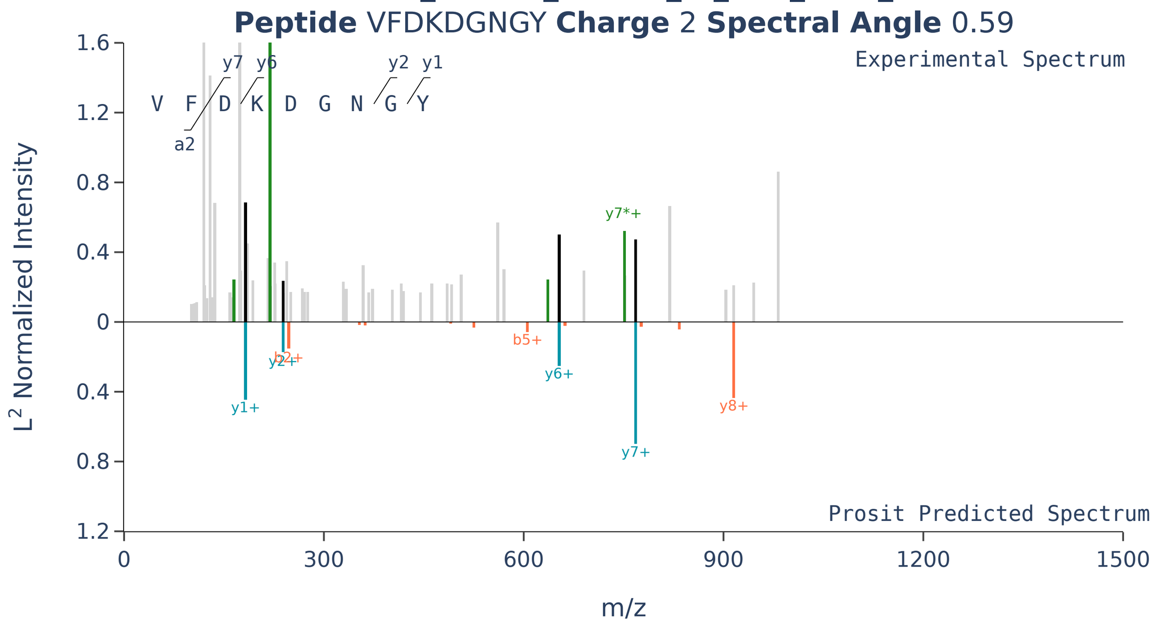


**Source** HRoetschke_291222_040123_Fu_CaM_b2R1_24h **Scan** 11584
**Peptide** GDGTITTKELGTVMRSL **Charge** 3 **Spectral Angle** 0.66

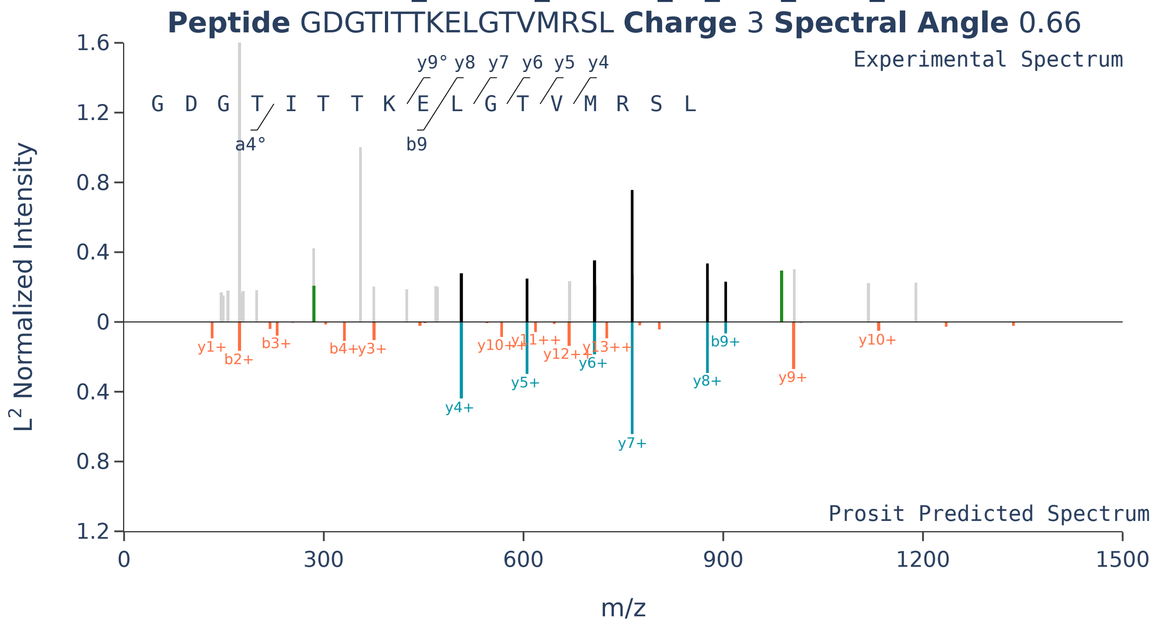


**Source** HRoetschke_291222_040123_Fu_CaM_b1R1_4h **Scan** 11524
**Peptide** EEVDEMIREADL **Charge** 2 **Spectral Angle** 0.57

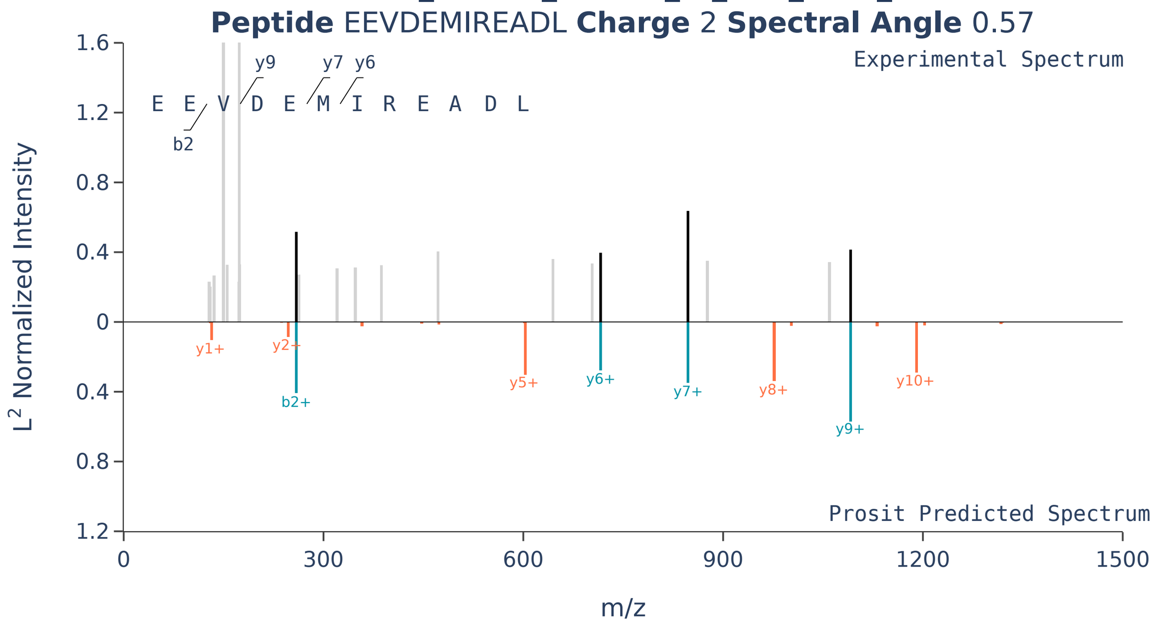

**Source** HRoetschke_291222_040123_Fu_CaM_b2R1_24h **Scan** 10415
**Peptide** SLFDKDGDGTITTKELGTVMRSL **Charge** 4 **Spectral Angle** 0.67

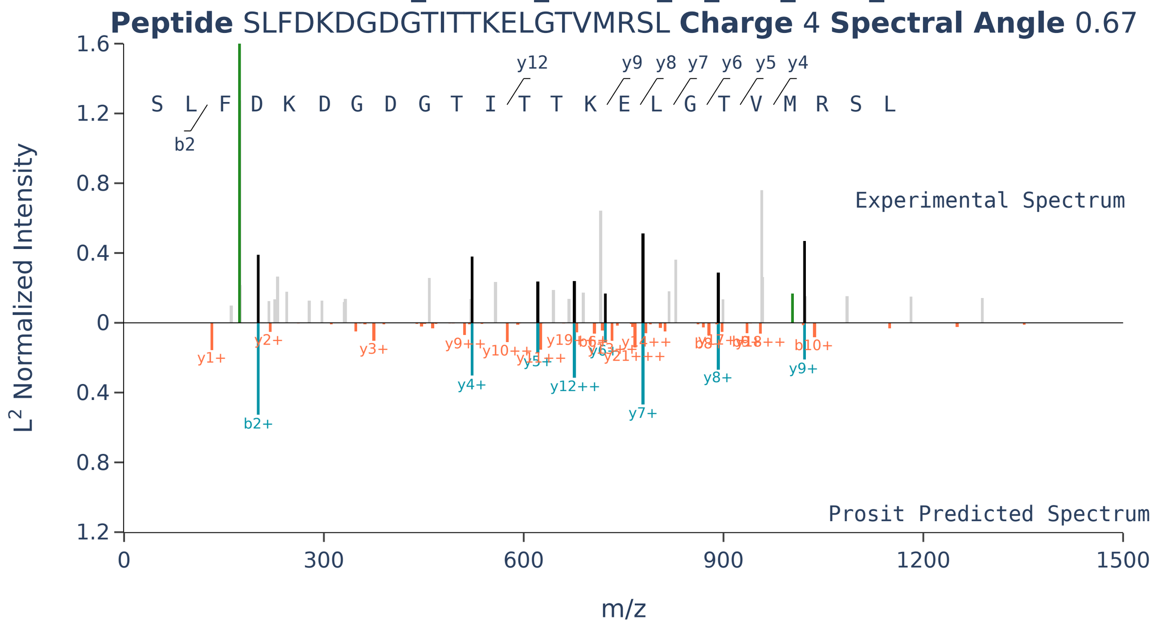


**Source** HRoetschke_291222_040123_Fu_CaM_b1R1_24h **Scan** 6475
**Peptide** REAFRVFDK **Charge** 3 **Spectral Angle** 0.65

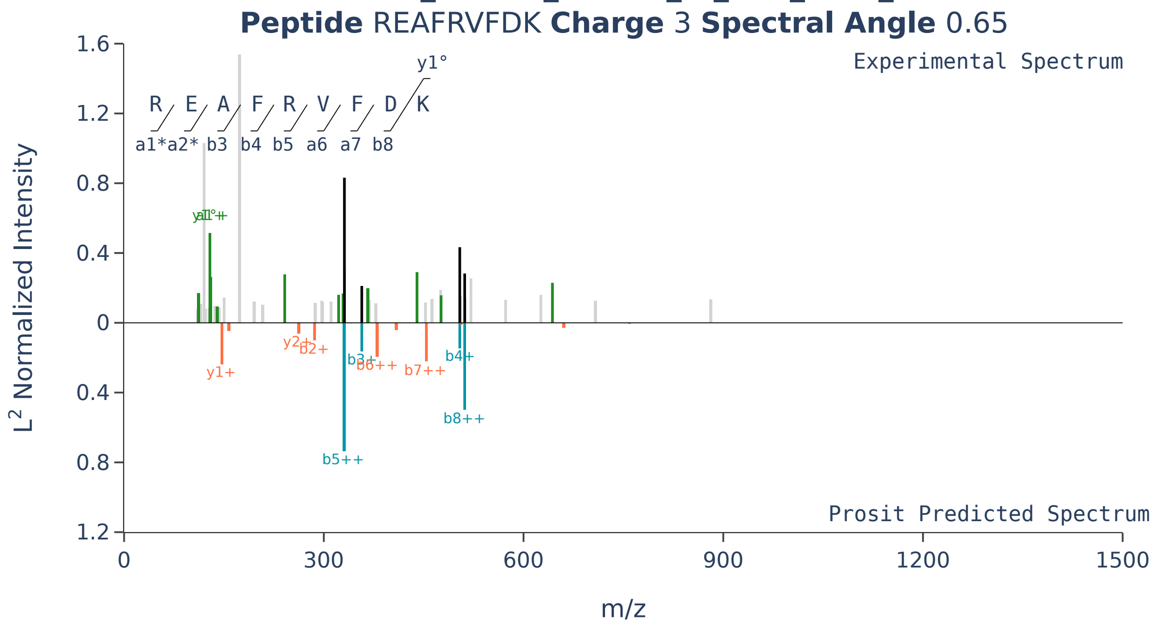


**Source** HRoetschke_291222_040123_Fu_CaM_b2R2_2h **Scan** 4219
**Peptide** SAAELRHVMTN **Charge** 3 **Spectral Angle** 0.65

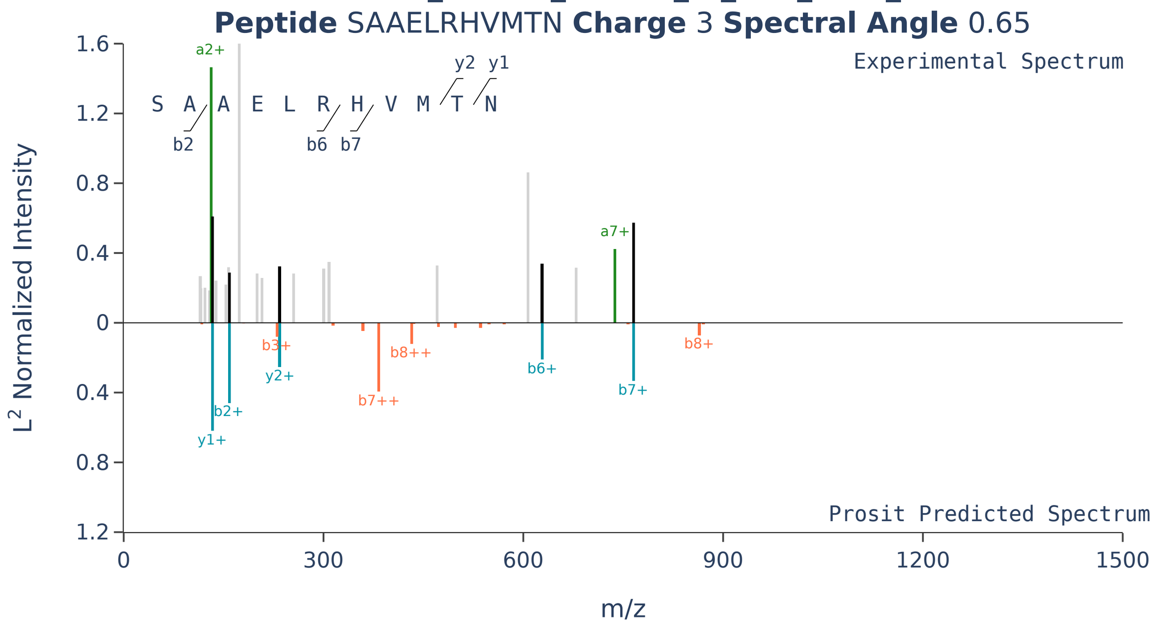


**Source** HRoetschke_291222_040123_Fu_CaM_b1R1_24h **Scan** 7824
**Peptide** EMIREADIDGDGQVN **Charge** 2 **Spectral Angle** 0.63

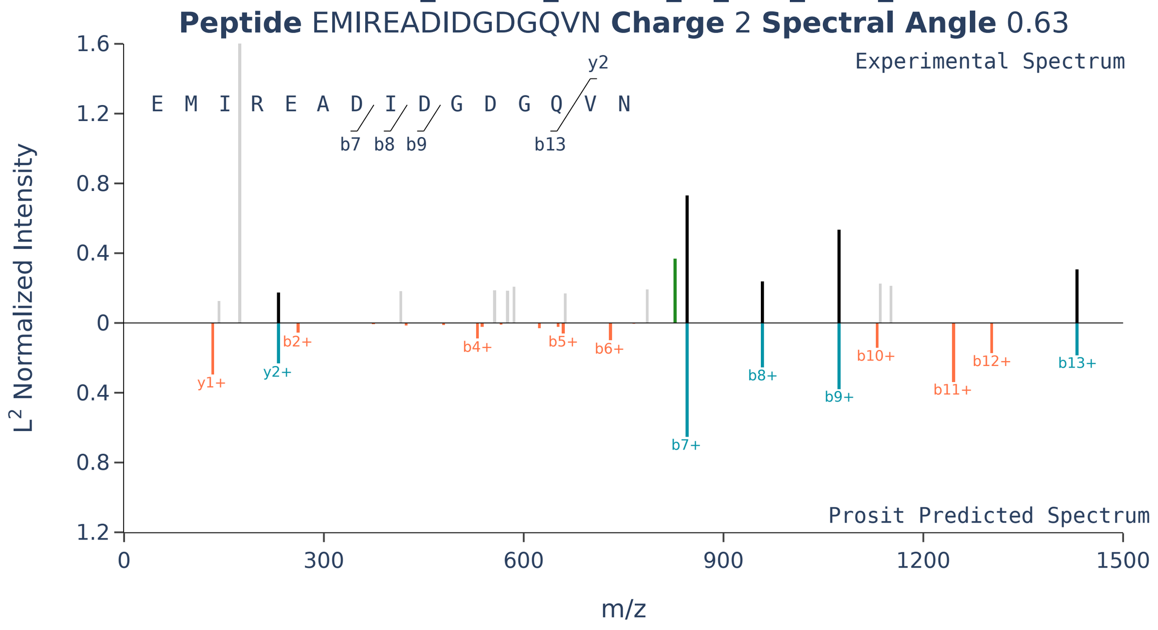


**Source** HRoetschke_291222_040123_Fu_CaM_b2R2_24h **Scan** 7622
**Peptide** DKDGNGYISAAELRHVMTN **Charge** 4 **Spectral Angle** 0.58

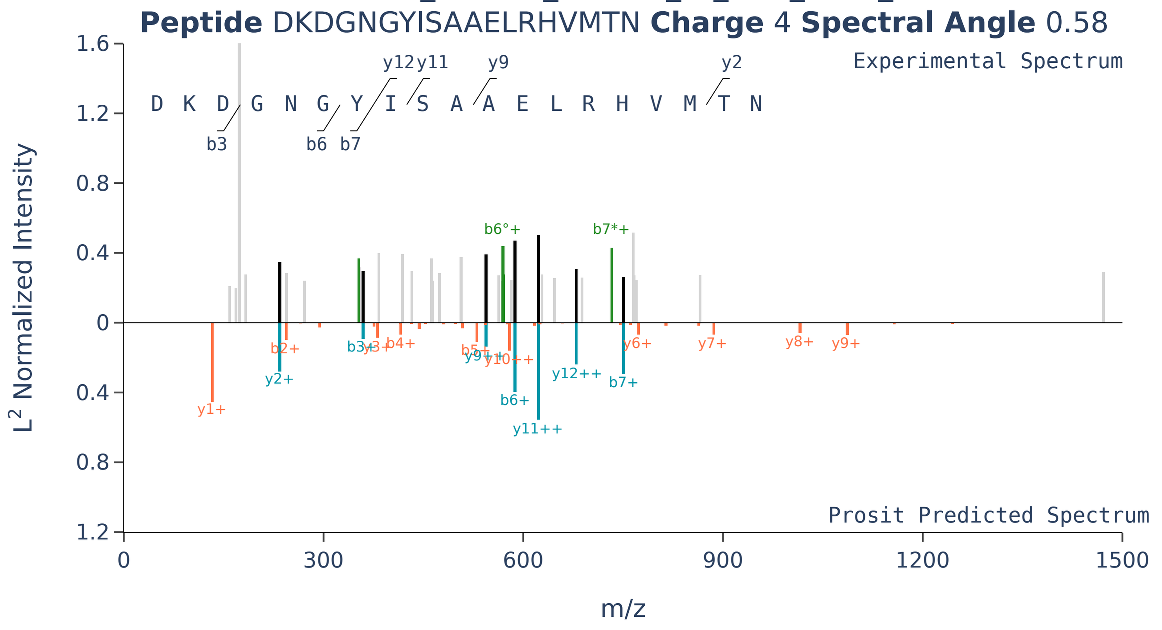


**Source** HRoetschke_291222_040123_Fu_CaM_b2R1_24h **Scan** 12536
**Peptide** EMIREADIDGDGQVNYEEFVQMMTAK **Charge** 3 **Spectral Angle** 0.73

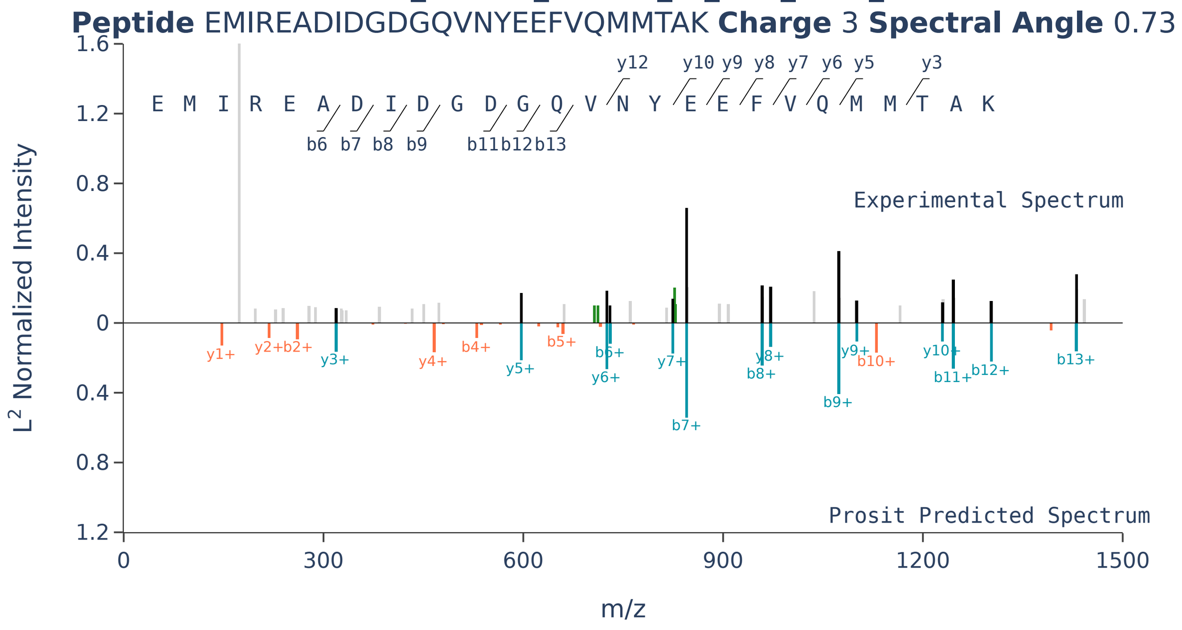


**Source** HRoetschke_291222_040123_Fu_CaM_b2R1_24h **Scan** 12563
**Peptide** TDSEEEIREAFRVFDKDGNGYISA **Charge** 3 **Spectral Angle** 0.53

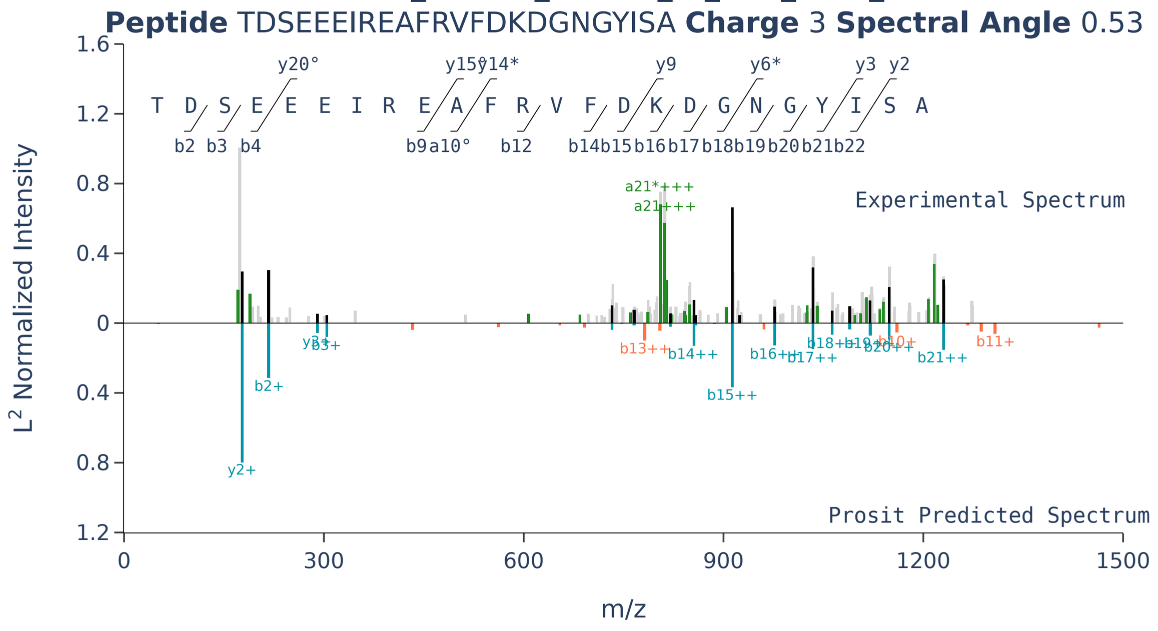


**Source** HRoetschke_291222_040123_Fu_CaM_b1R1_4h **Scan** 11620
**Peptide** KMKDTDSEEEIREAFRVFD **Charge** 4 **Spectral Angle** 0.65

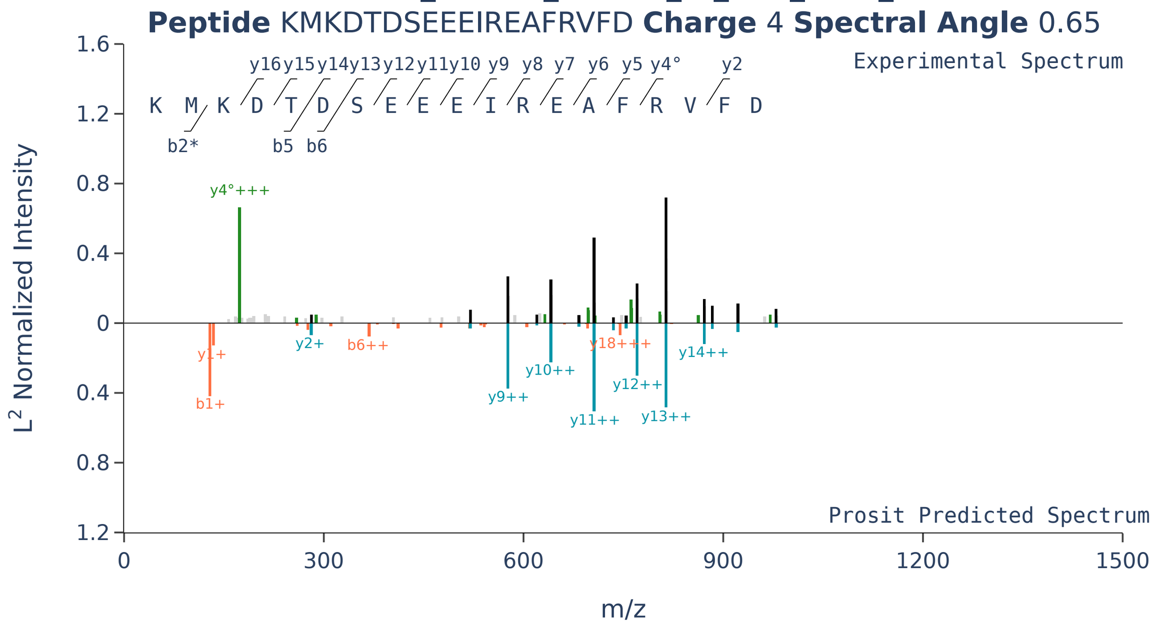


**Source** HRoetschke_291222_040123_Fu_CaM_b2R1_24h **Scan** 10630
**Peptide** FSLFDKDGDGTITTKELGT **Charge** 3 **Spectral Angle** 0.65

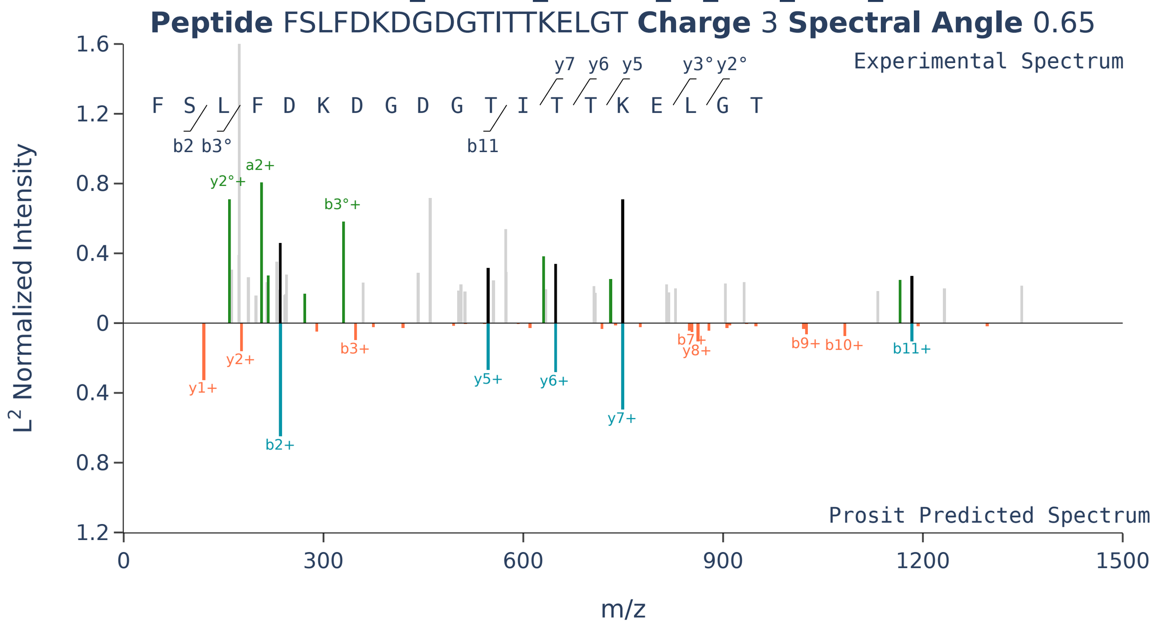


**Source** HRoetschke_291222_040123_Fu_CaM_b1R1_24h **Scan** 7033
**Peptide** FDKDGDGTITTKELGT **Charge** 3 **Spectral Angle** 0.56

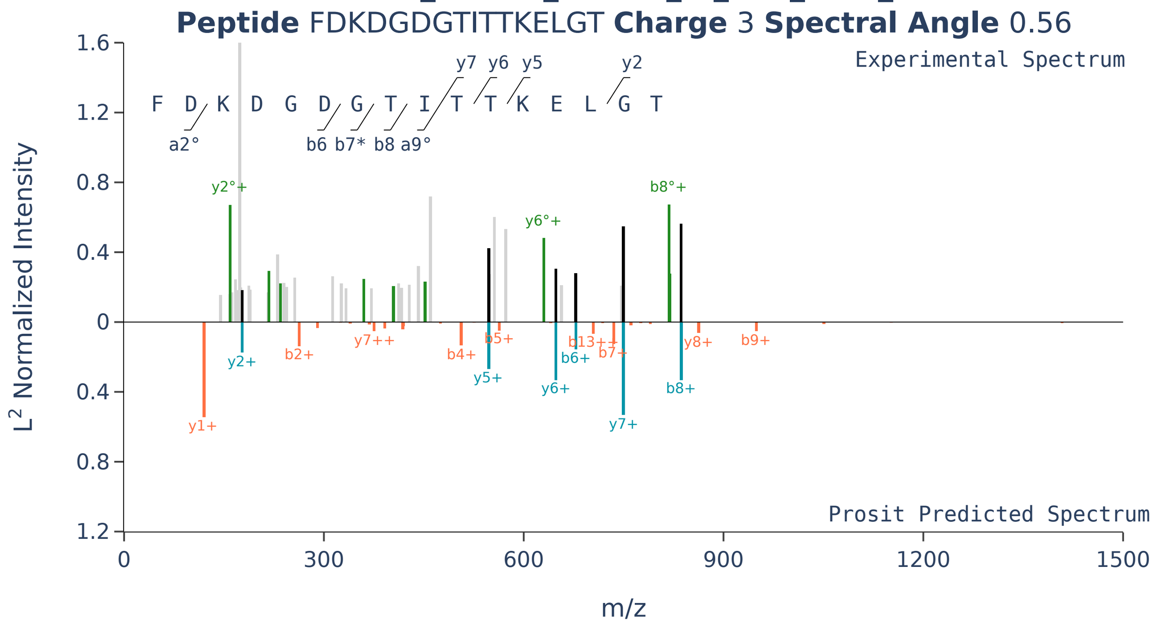


**Source** HRoetschke_291222_040123_Fu_CaM_b1R1_24h **Scan** 10682
**Peptide** FRVFDKDGNGYISAA **Charge** 3 **Spectral Angle** 0.6

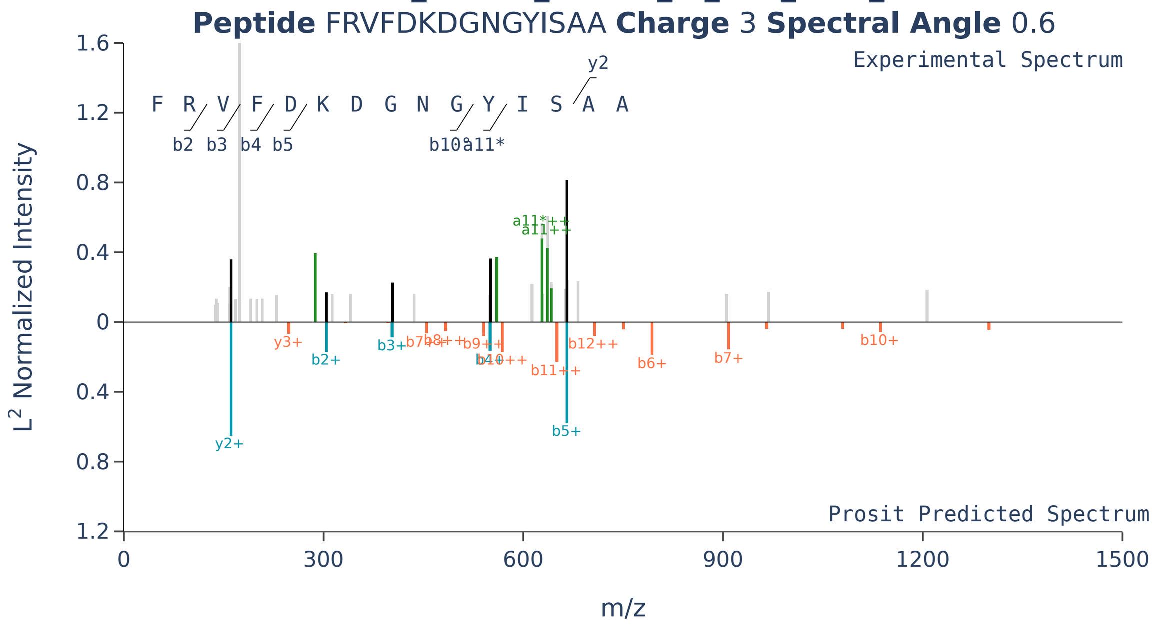


**Source** HRoetschke_291222_040123_Fu_CaM_b1R1_4h **Scan** 8803
**Peptide** RVFDKDGNGYISAAELRHVMT **Charge** 4 **Spectral Angle** 0.6

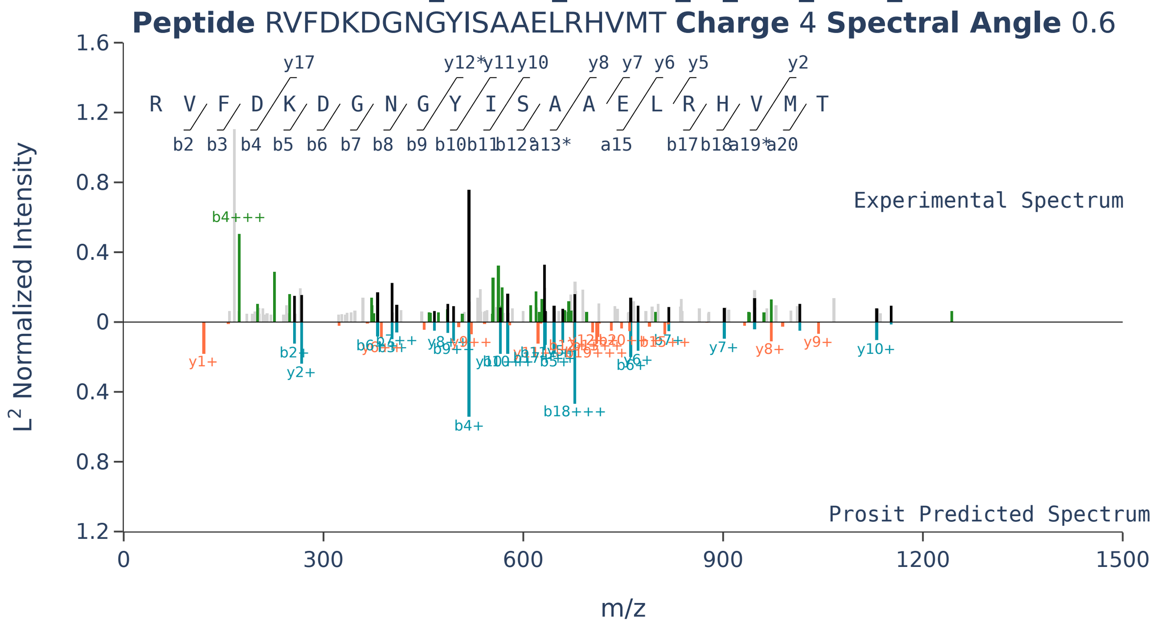


**Source** HRoetschke_291222_040123_Fu_CaM_b2R1_2h **Scan** 9389
**Peptide** AFRVFDKDGNGYISAAE **Charge** 3 **Spectral Angle** 0.6

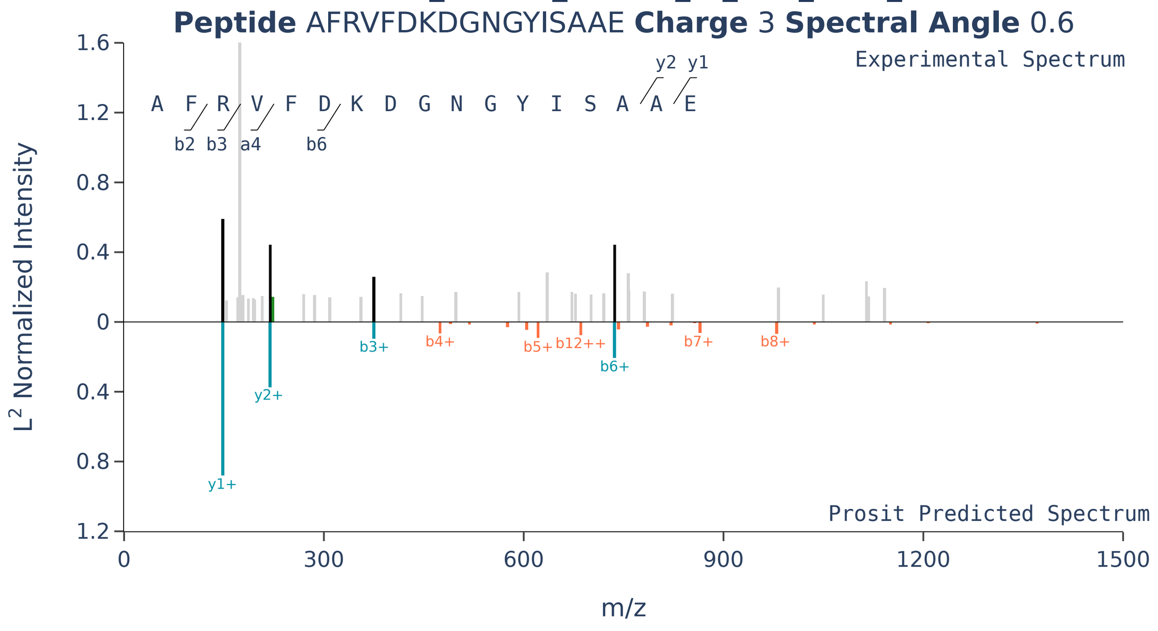


**Source** HRoetschke_291222_040123_Fu_CaM_b1R2_4h **Scan** 2363
**Peptide** KDGDGTITT **Charge** 2 **Spectral Angle** 0.72

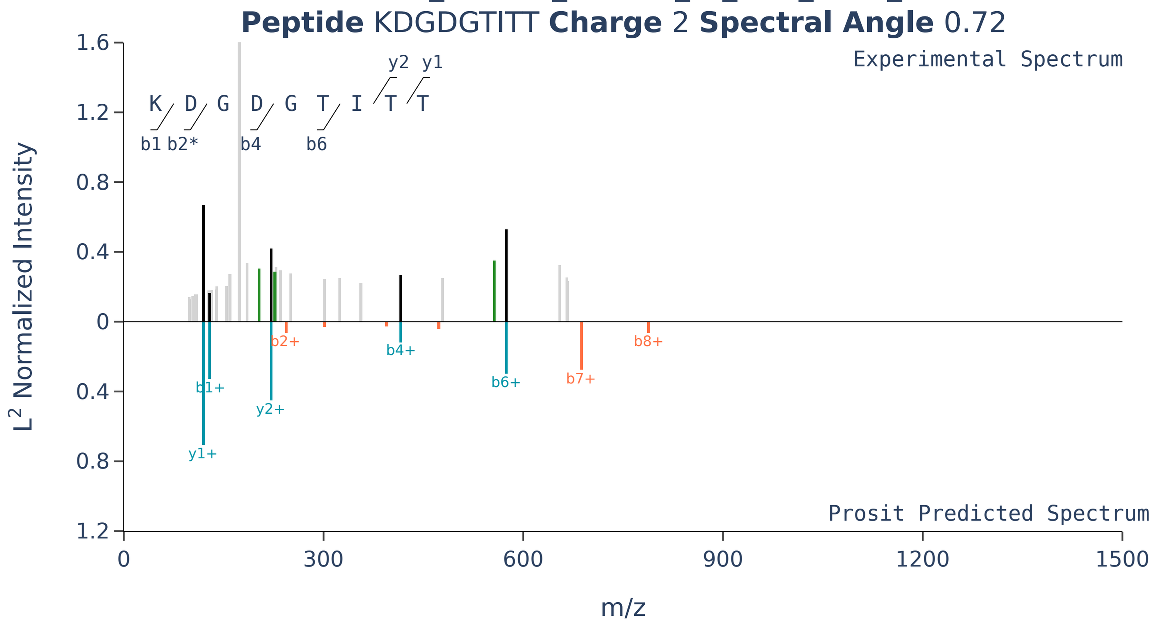


**Source** HRoetschke_291222_040123_Fu_CaM_b2R1_2h **Scan** 11155
**Peptide** AFSLFDKDGDGTITTKELG **Charge** 3 **Spectral Angle** 0.7

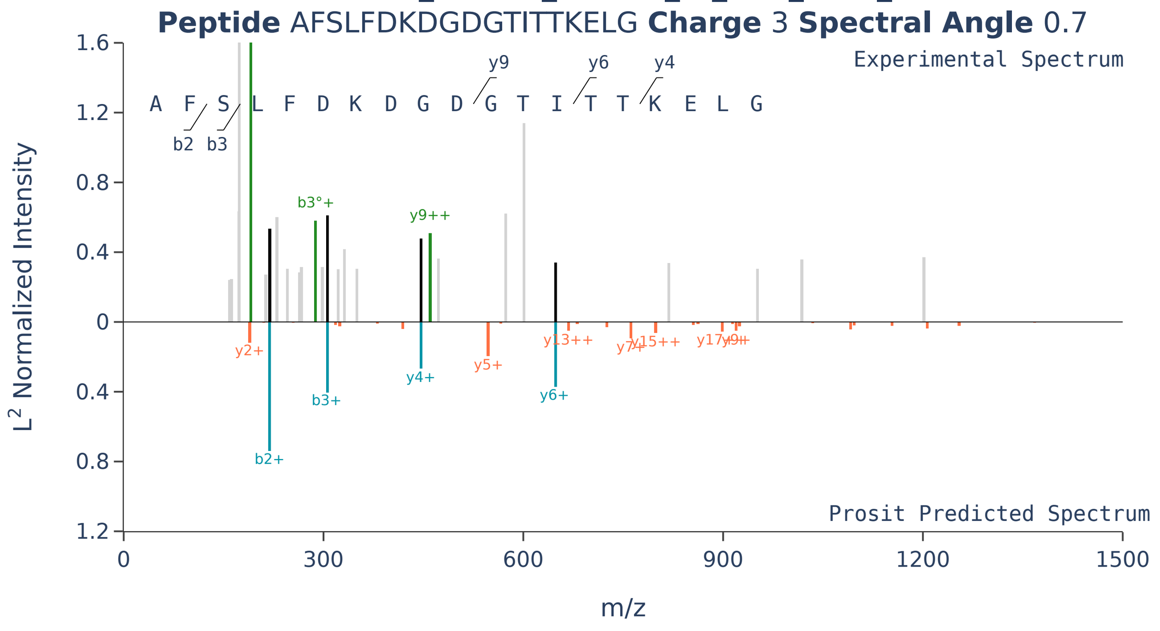

**Source** HRoetschke_291222_040123_Fu_CaM_b2R1_24h **Scan** 7011
**Peptide** RKMKDTDSEEEIREAFRV **Charge** 4 **Spectral Angle** 0.74

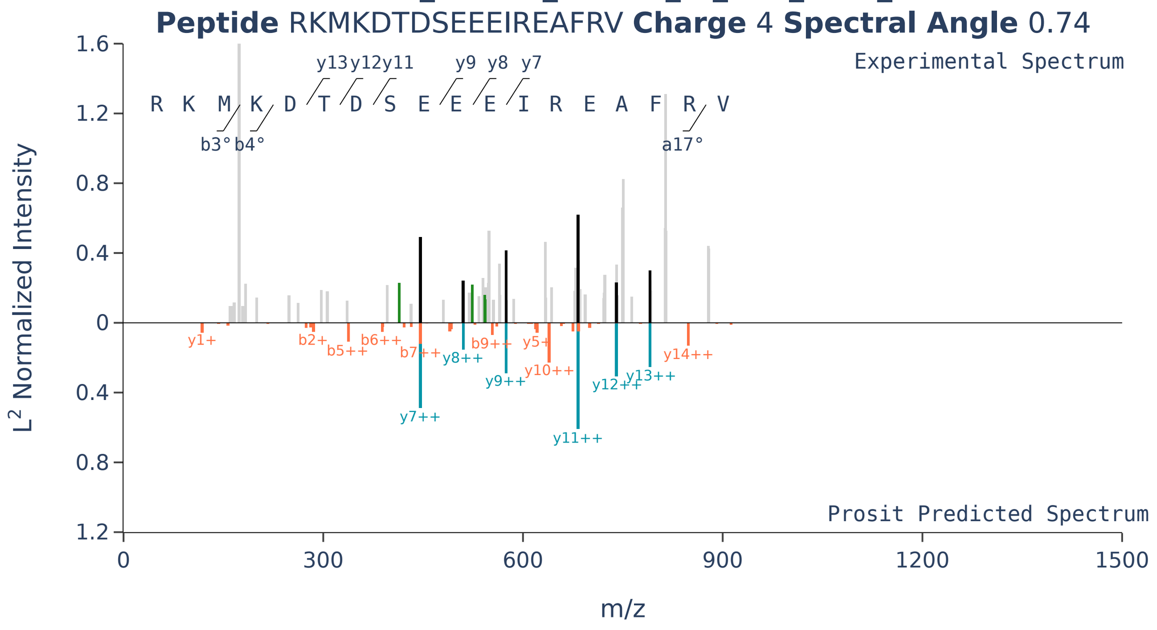


**Source** HRoetschke_291222_040123_Fu_CaM_b1R1_4h **Scan** 10936
**Peptide** MARKMKDTDSEEEIREAFRVF **Charge** 4 **Spectral Angle** 0.75

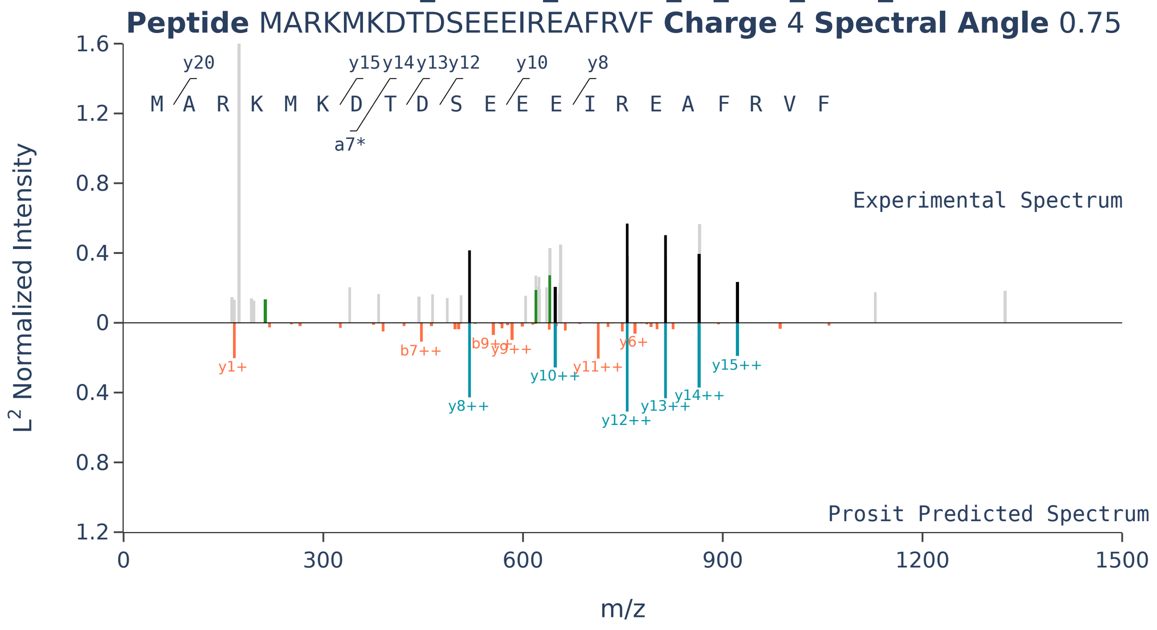


**Source** HRoetschke_291222_040123_Fu_CaM_b2R1_2h **Scan** 10395
**Peptide** KEAFSLFDKDGDGTITT **Charge** 3 **Spectral Angle** 0.45

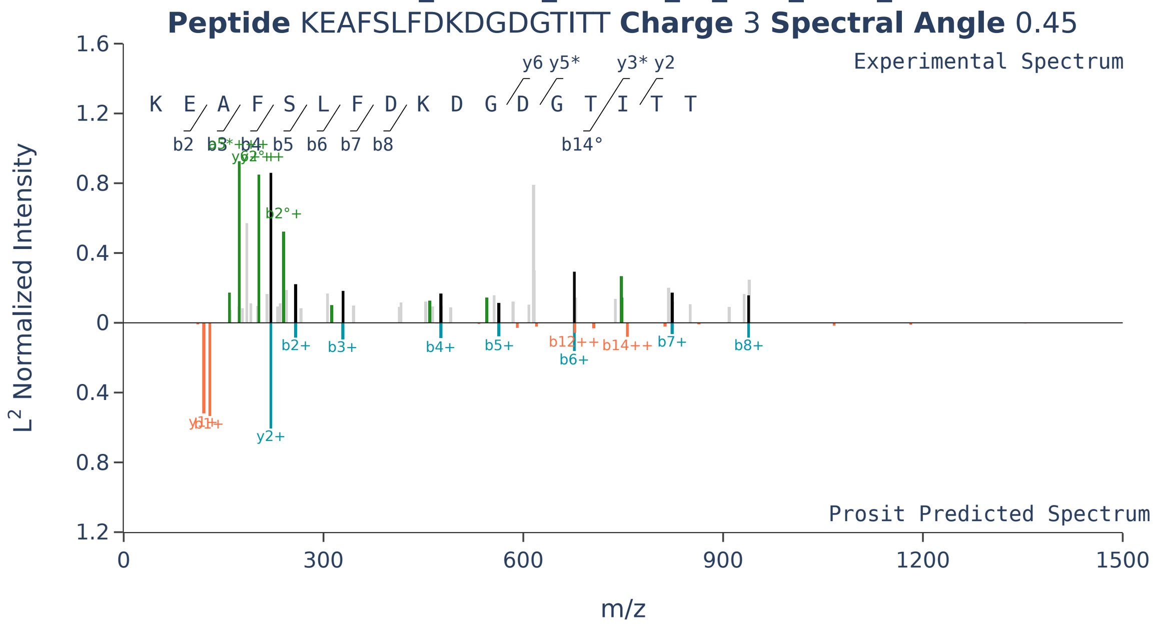


**Source** HRoetschke_291222_040123_Fu_CaM_b1R2_4h **Scan** 9594
**Peptide** KEAFSLFDKDGDGTITTK **Charge** 4 **Spectral Angle** 0.38

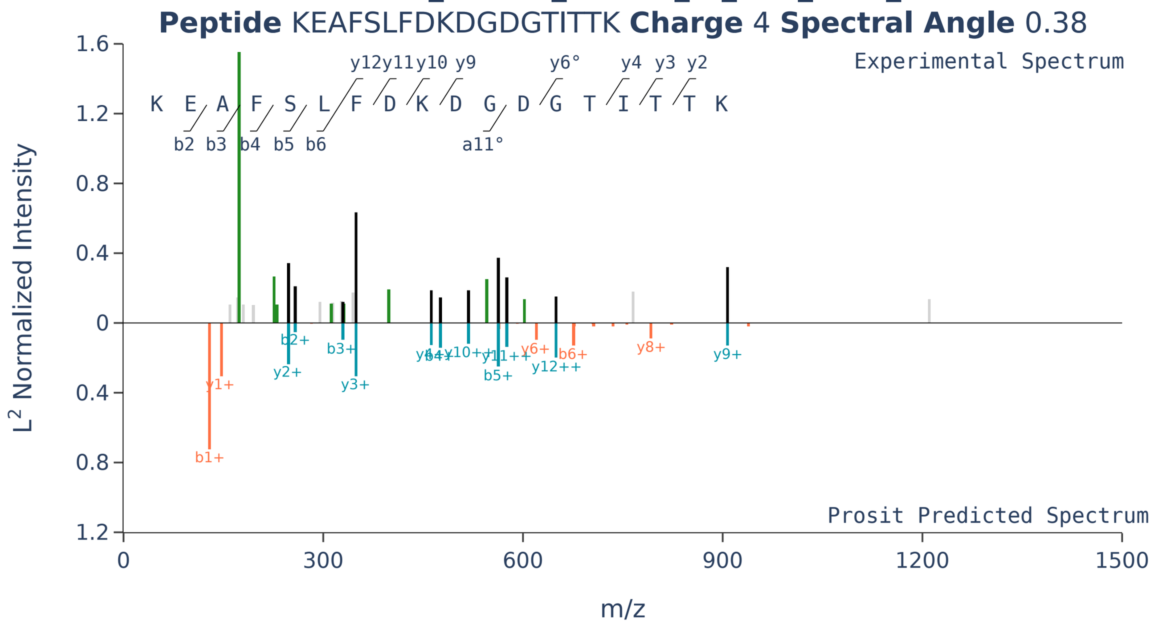


**Source** HRoetschke_291222_040123_Fu_CaM_b2R1_24h **Scan** 11032
**Peptide** DKDGDGTITTKELGTVMRSLGQ **Charge** 3 **Spectral Angle** 0.66

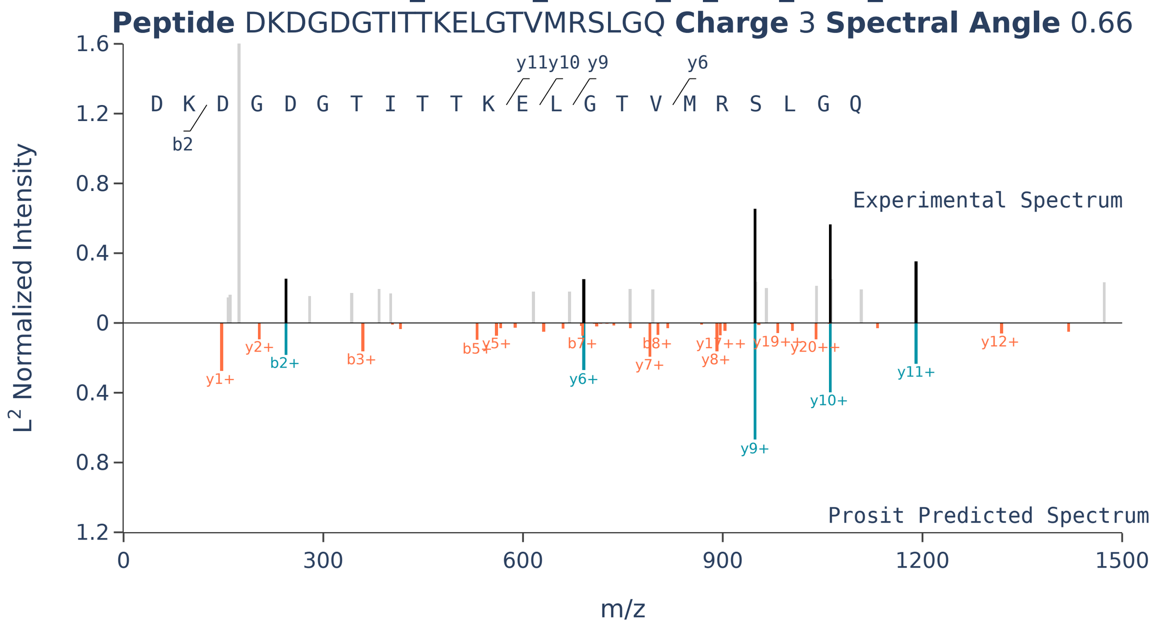


**Source** HRoetschke_291222_040123_Fu_CaM_b1R2_2h **Scan** 8396
**Peptide** HVMTNLGEKLTDEEVD **Charge** 3 **Spectral Angle** 0.58

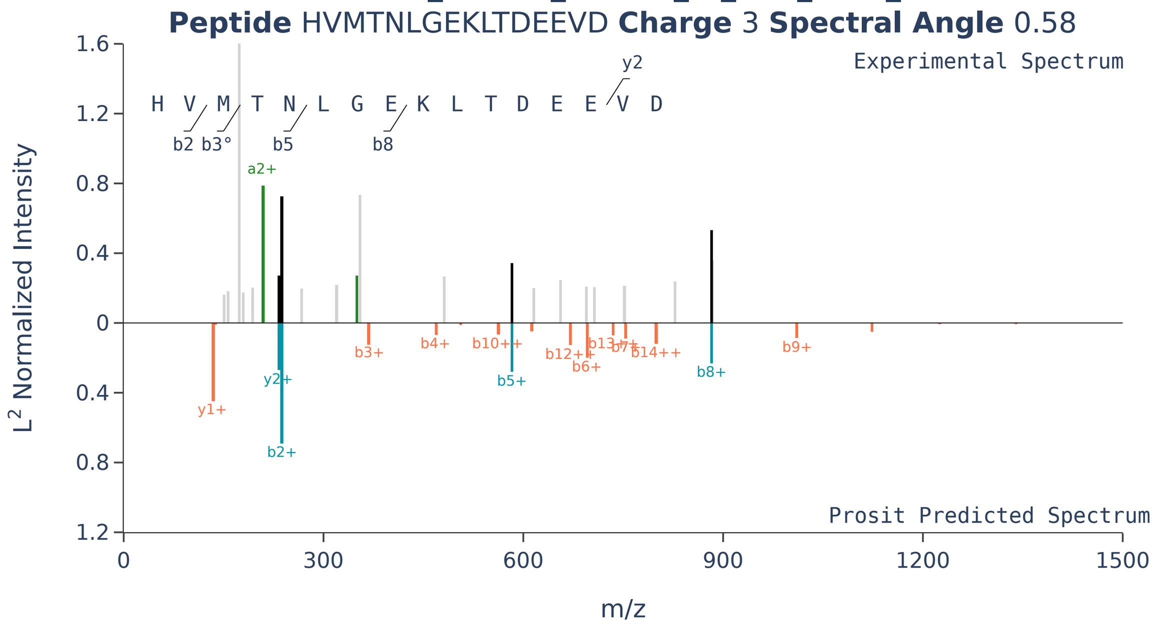


**Source** HRoetschke_291222_040123_Fu_CaM_b2R2_24h **Scan** 12219
**Peptide** AEFKEAFSLFDKD **Charge** 3 **Spectral Angle** 0.6

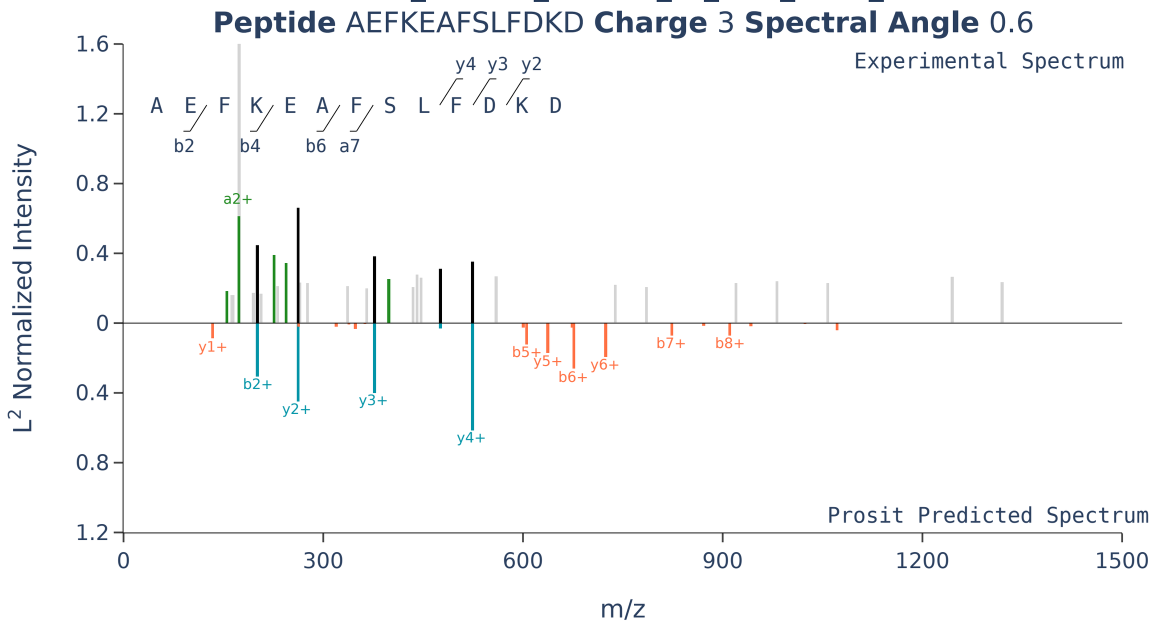


**Source** HRoetschke_291222_040123_Fu_CaM_b1R1_4h **Scan** 5804
**Peptide** RHVMTNLGEKLTDE **Charge** 3 **Spectral Angle** 0.69

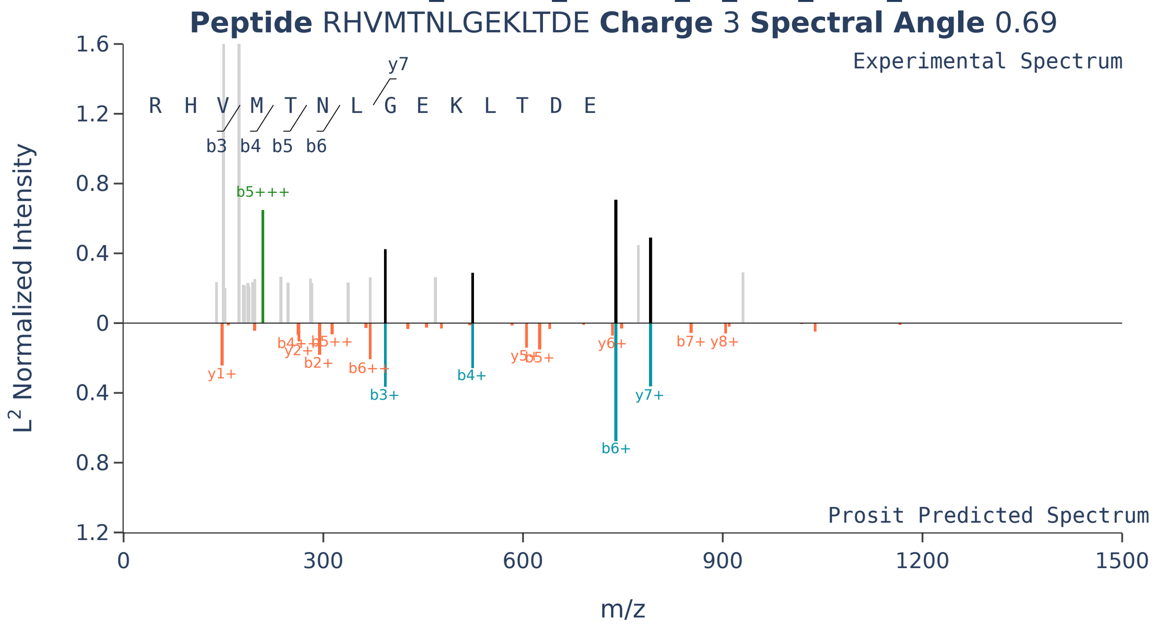


**Source** HRoetschke_291222_040123_Fu_CaM_b2R2_24h **Scan** 11759
**Peptide** AFSLFDKDGDGTITTKELGT **Charge** 3 **Spectral Angle** 0.65

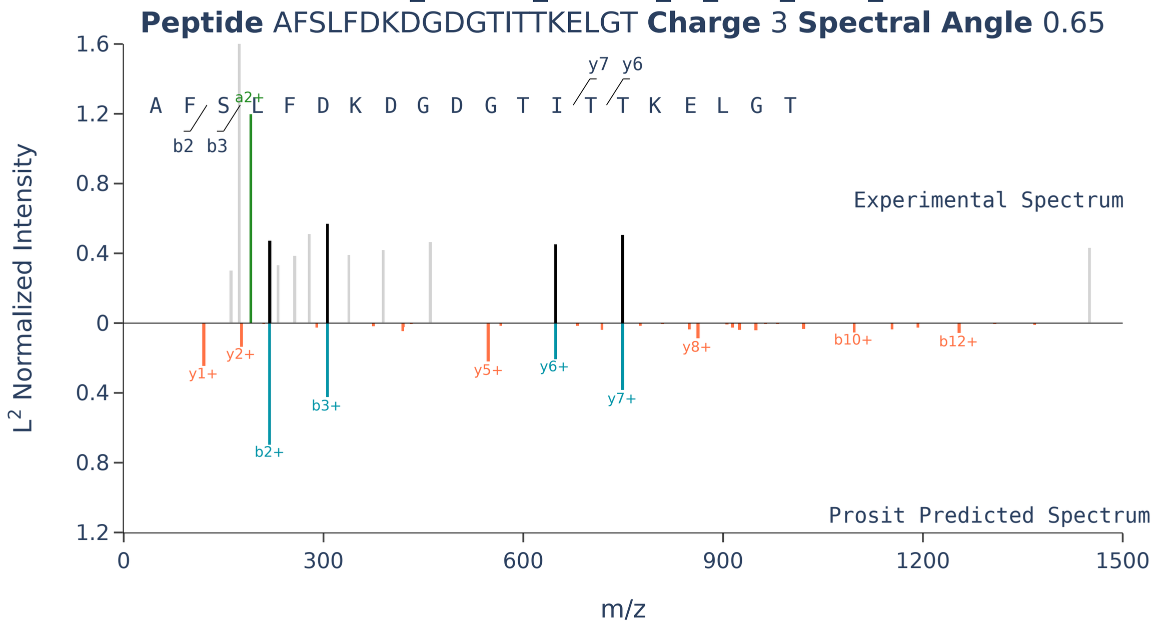


**Source** HRoetschke_291222_040123_Fu_CaM_b2R1_24h **Scan** 9126
**Peptide** AFRVFDKDGNGYL **Charge** 3 **Spectral Angle** 0.53

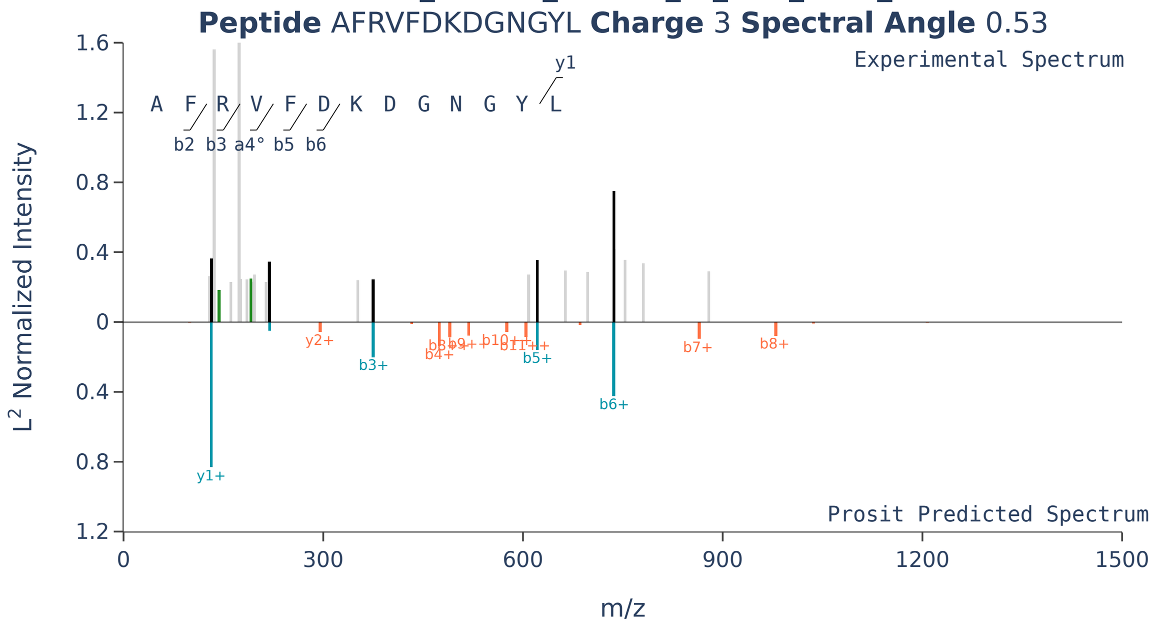


**Source** HRoetschke_291222_040123_Fu_CaM_b2R2_24h **Scan** 9619
**Peptide** KEAFSLFDKDGDGTITTKE **Charge** 4 **Spectral Angle** 0.32

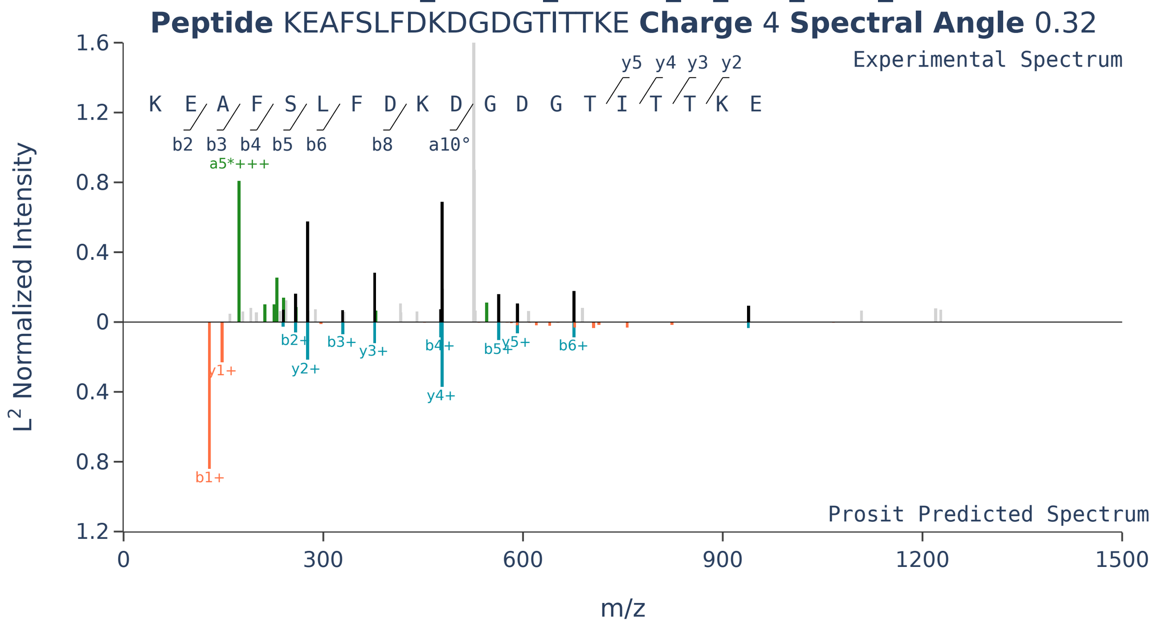


**Source** HRoetschke_291222_040123_Fu_CaM_b2R2_24h **Scan** 10976
**Peptide** KEAFSLFDKDGDGTITTKELGT **Charge** 4 **Spectral Angle** 0.32

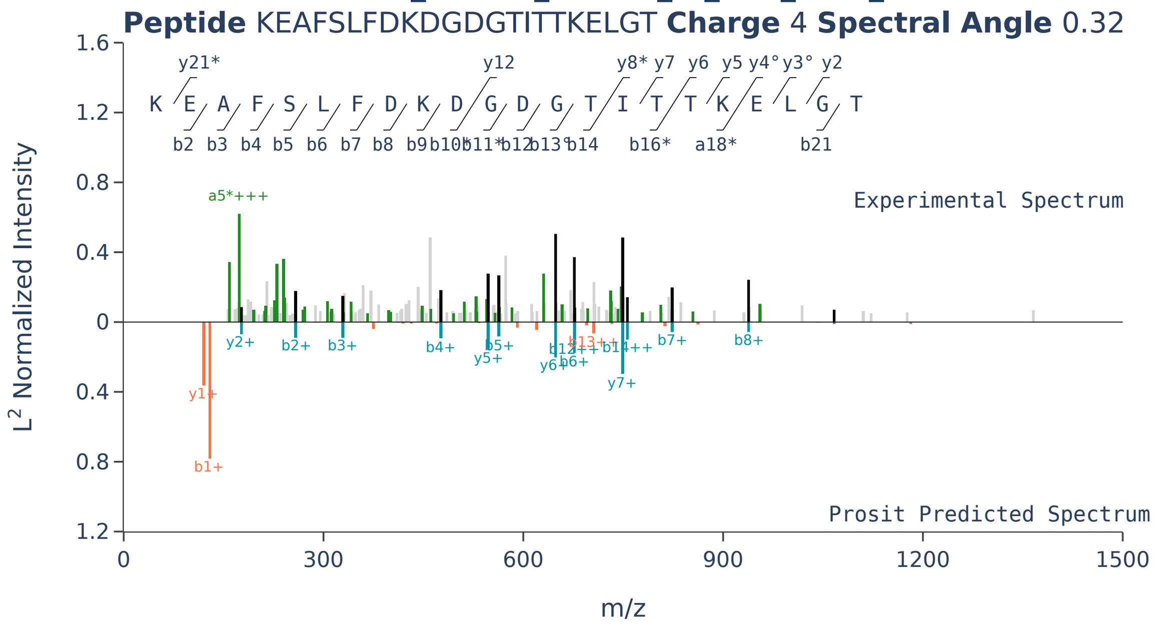


**Source** HRoetschke_291222_040123_Fu_CaM_b1R2_2h **Scan** 8926
**Peptide** KDGDGTITTKELGTVMRSL **Charge** 3 **Spectral Angle** 0.24

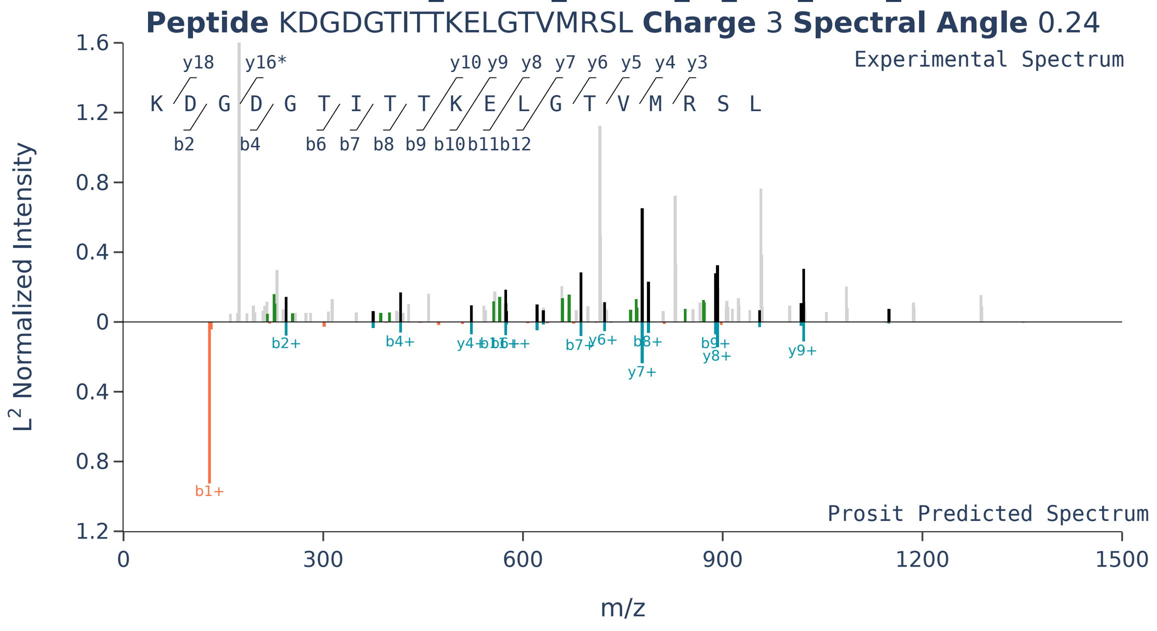


**Source** HRoetschke_291222_040123_Fu_CaM_b1R1_2h **Scan** 9215
**Peptide** REAFRVFDKDGNGYISAAELRH **Charge** 5 **Spectral Angle** 0.61

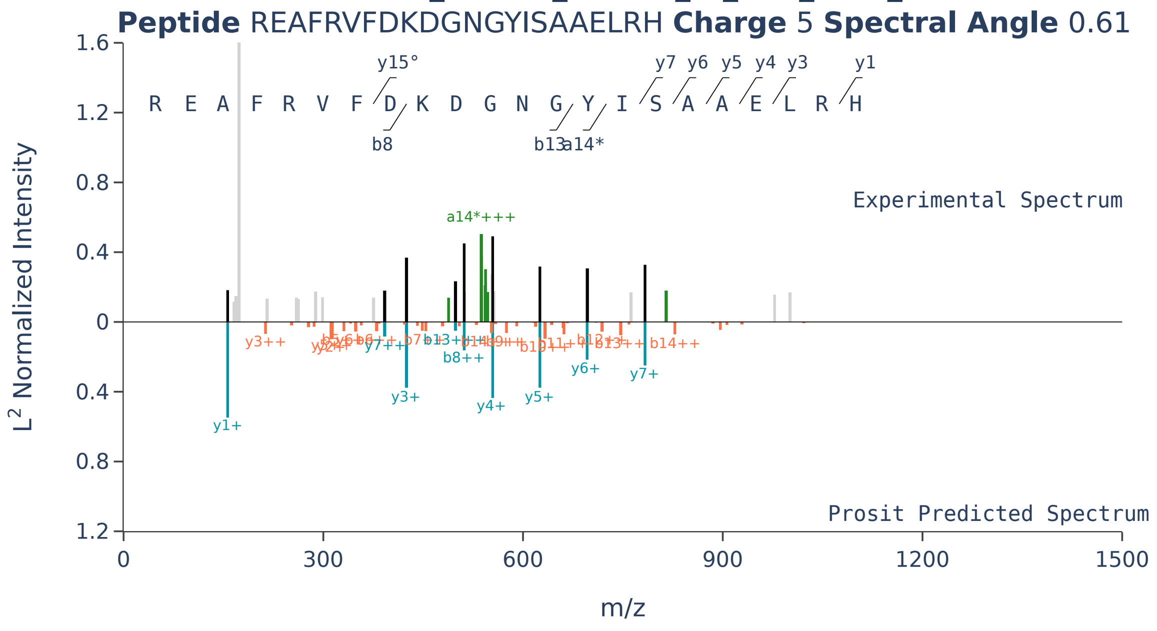


**Source** HRoetschke_291222_040123_Fu_CaM_b2R1_4h **Scan** 9910
**Peptide** AFSLFDKDGDGTITTK **Charge** 3 **Spectral Angle** 0.49

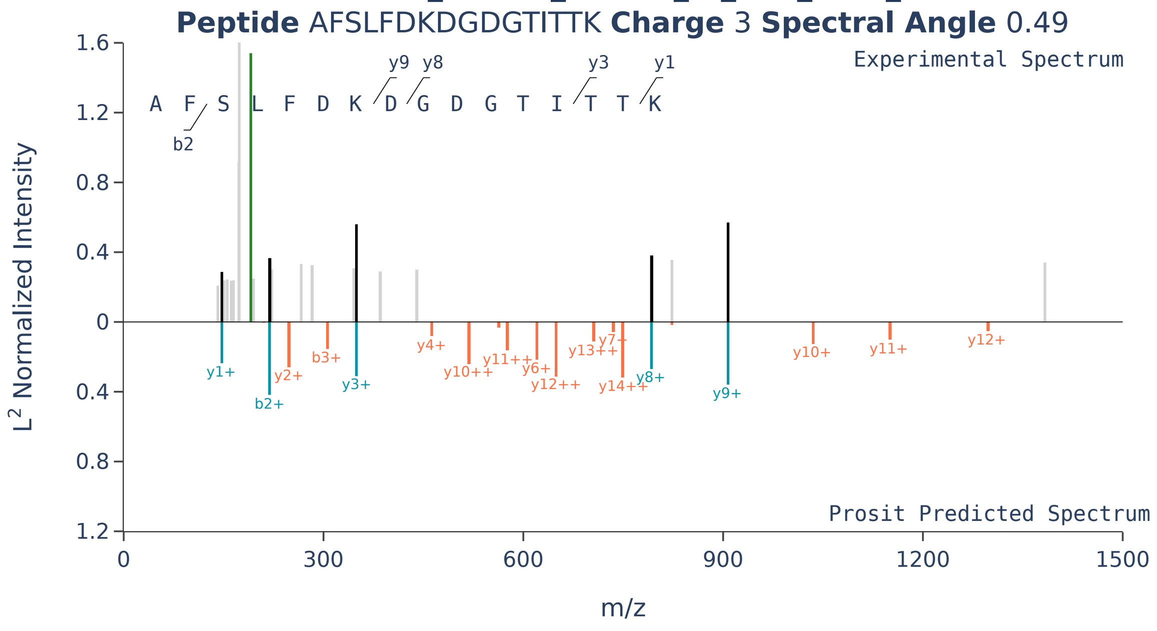

**Source** Ncheng_210623_230623_HFGoe_FFH_20S_25_1_A1_1h_R1 **Scan** 16643
**Peptide** MFDNLTD **Charge** 1 **Spectral Angle** 0.98

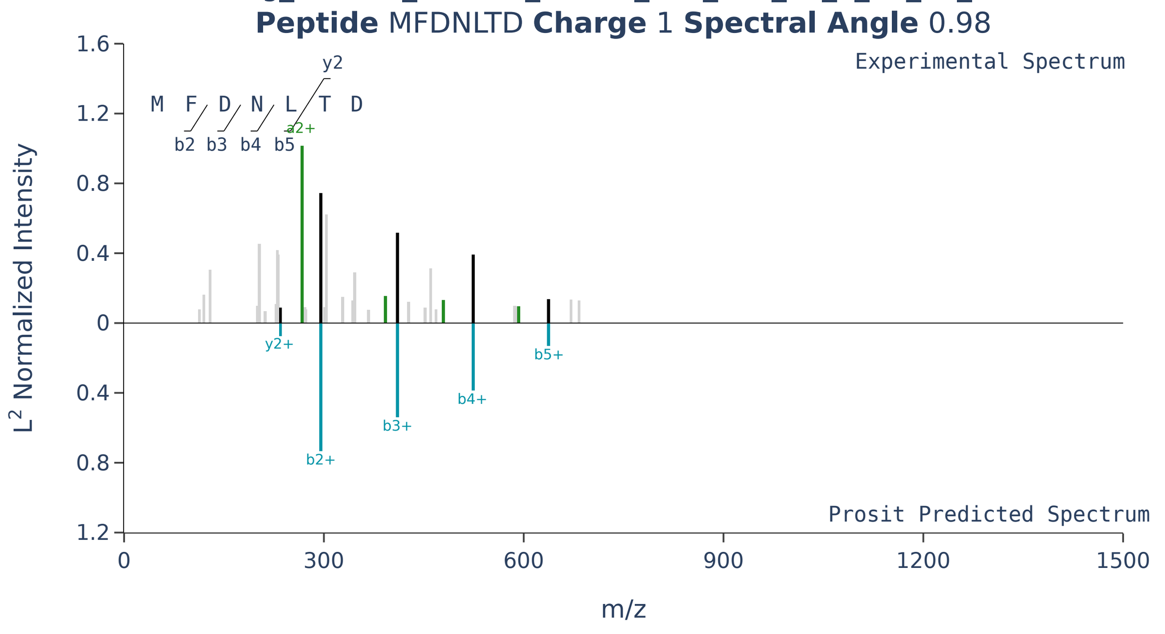

**Source** Ncheng_210623_230623_HFGoe_FFH_20S_25_1_A1_2h_R1 **Scan** 33053
**Peptide** TLAEQVGVDFFPSD **Charge** 2 **Spectral Angle** 0.95

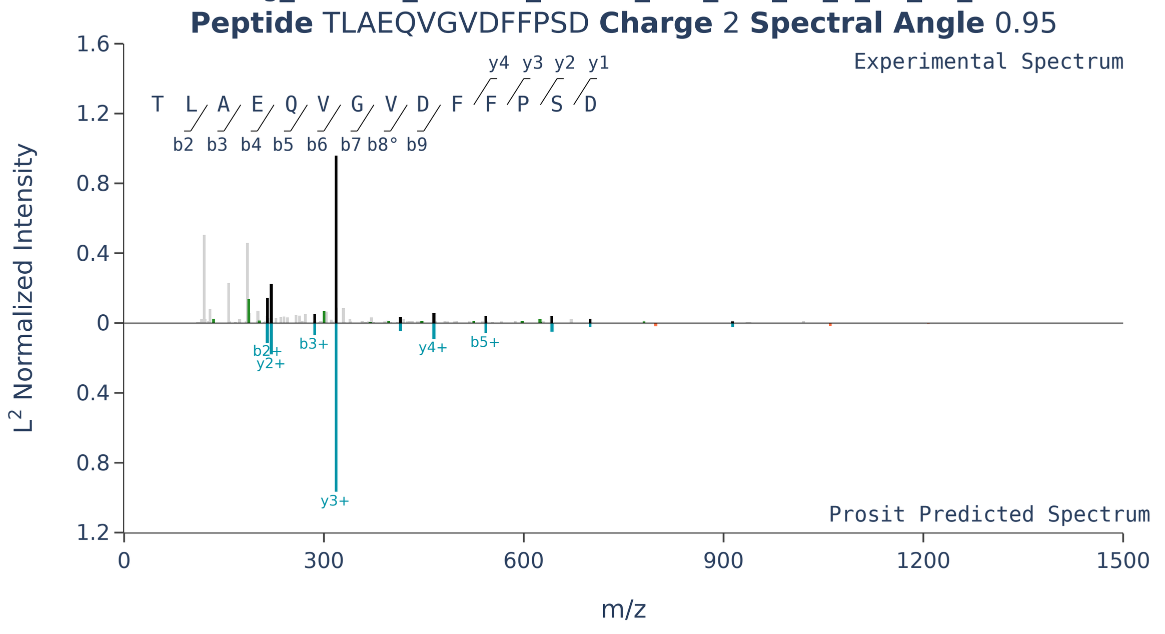

**Source** Ncheng_210623_230623_HFGoe_FFH_20S_25_1_A2_4h_R2 **Scan** 27293
**Peptide** VDFFPSD **Charge** 1 **Spectral Angle** 0.96

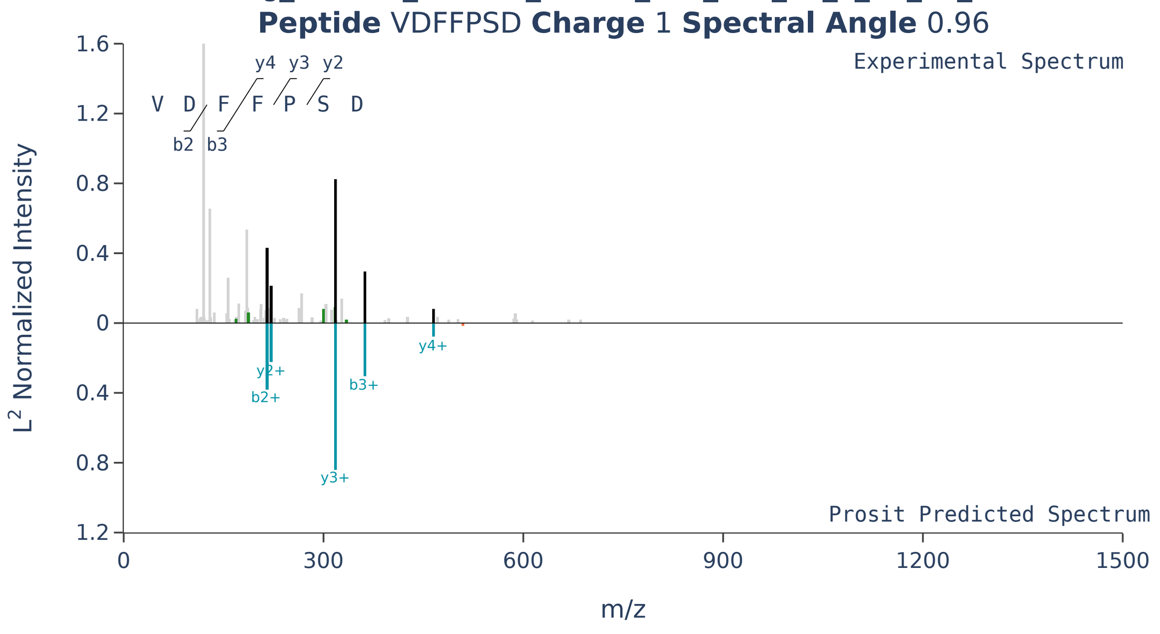

**Source** Ncheng_210623_230623_HFGoe_FFH_20S_25_1_A2_24h_R1 **Scan** 16574
**Peptide** MEAIINS **Charge** 1 **Spectral Angle** 0.93

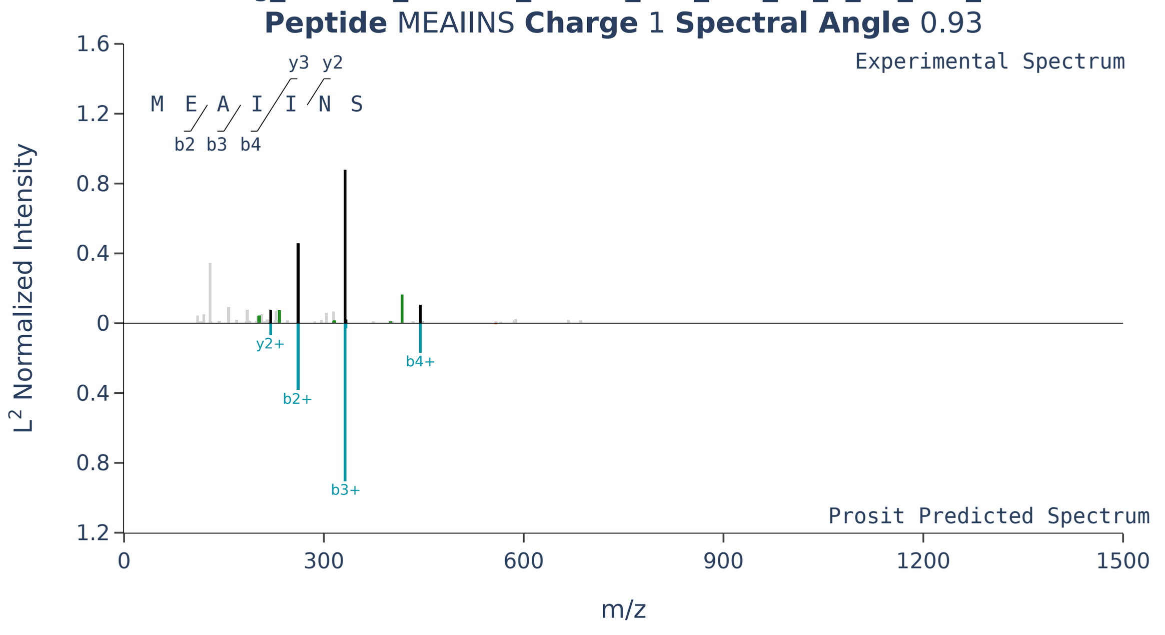

**Source** Ncheng_210623_230623_HFGoe_FFH_20S_25_1_A1_24h_R1 **Scan** 13871
**Peptide** GQIPDNV **Charge** 1 **Spectral Angle** 0.93

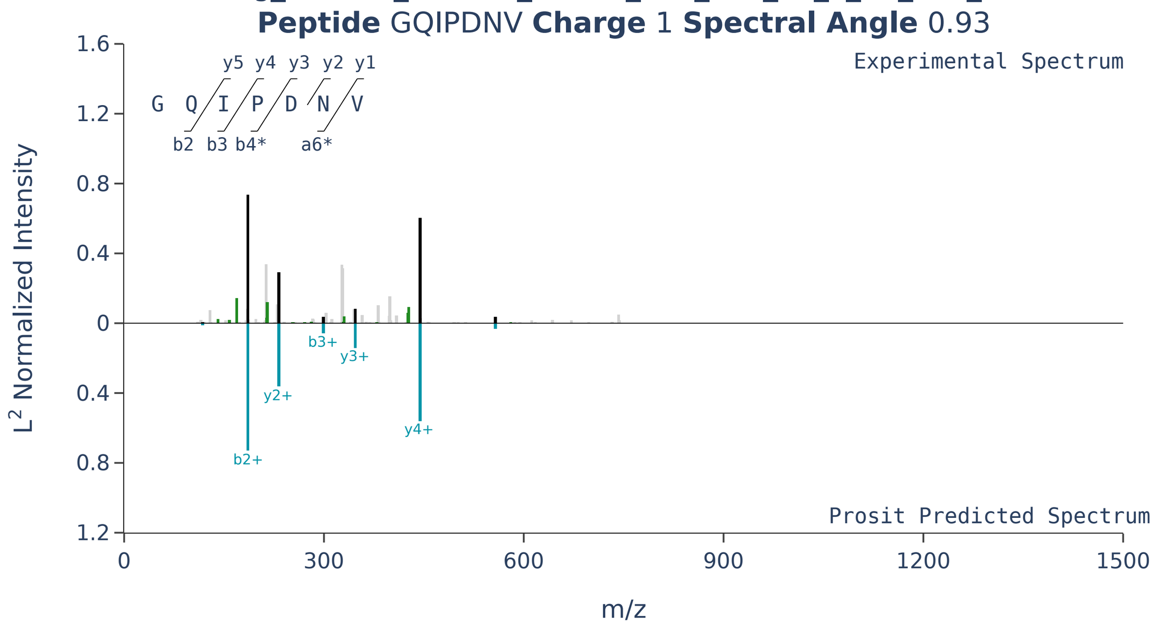

**Source** Ncheng_210623_230623_HFGoe_FFH_20S_25_1_A2_24h_R1 **Scan** 19130
**Peptide** GEENQTLNL **Charge** 1 **Spectral Angle** 0.94

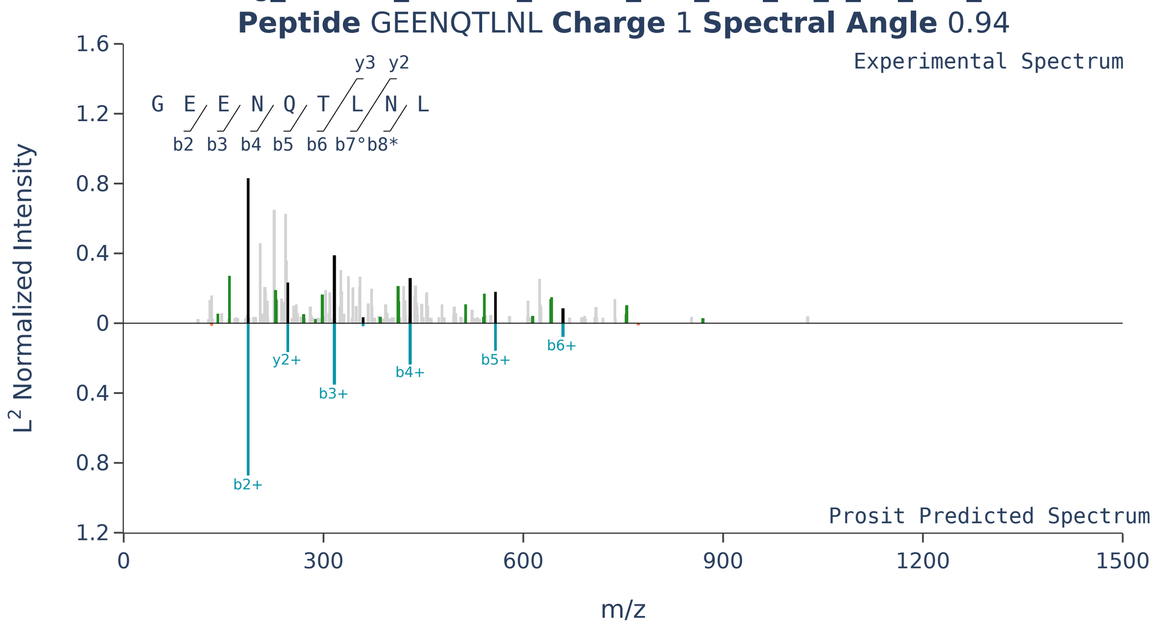

**Source** Ncheng_210623_230623_HFGoe_FFH_20S_25_1_A2_24h_R1 **Scan** 18224
**Peptide** AIINSMTMKE **Charge** 1 **Spectral Angle** 0.91

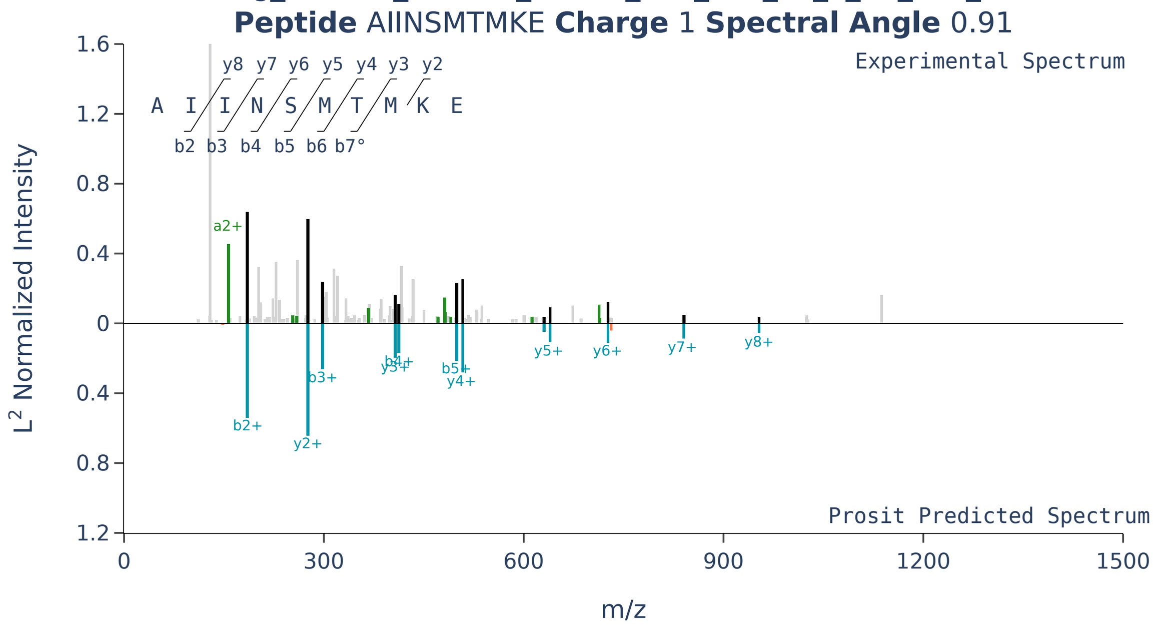

**Source** Ncheng_210623_230623_HFGoe_FFH_20S_25_1_A2_24h_R2 **Scan** 7625
**Peptide** ANTAKAFNE **Charge** 1 **Spectral Angle** 0.9

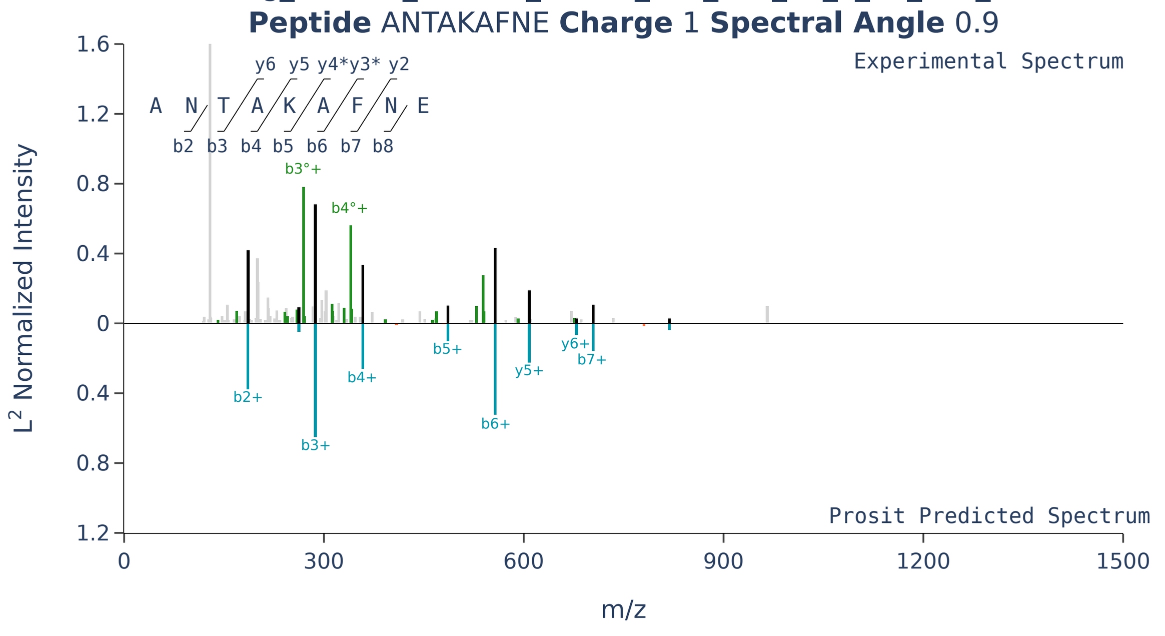

**Source** Ncheng_210623_230623_HFGoe_FFH_20S_25_1_A1_4h_R1 **Scan** 29784
**Peptide** QVGVDFFPSD **Charge** 1 **Spectral Angle** 0.95

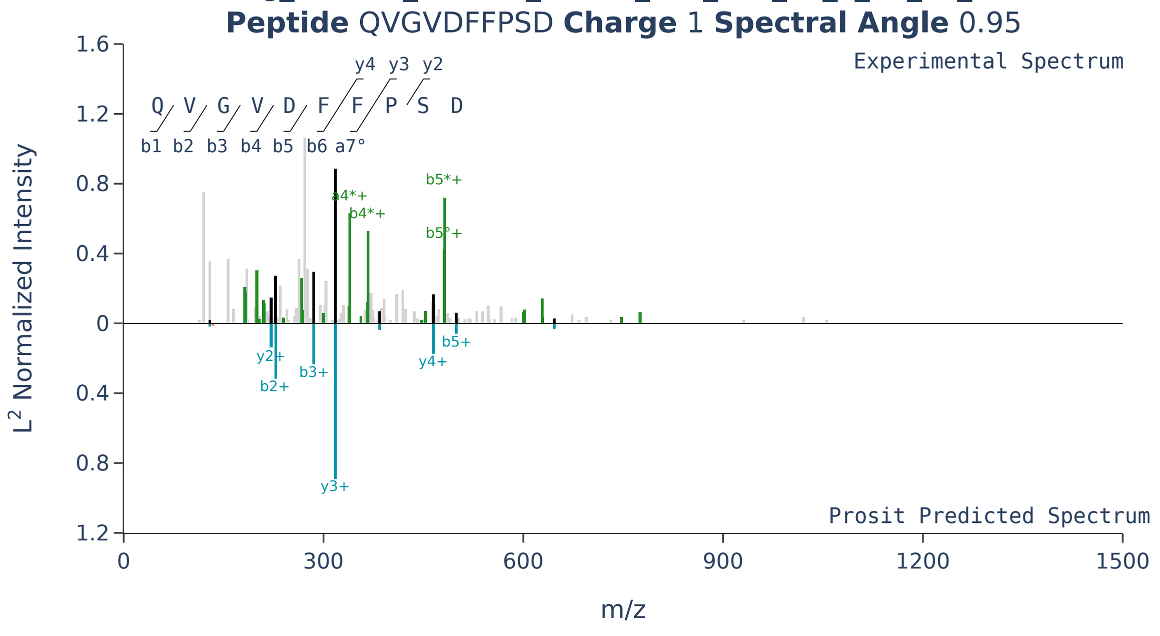

**Source** Ncheng_210623_230623_HFGoe_FFH_20S_25_1_A2_4h_R2 **Scan** 10732
**Peptide** IVNAALKE **Charge** 1 **Spectral Angle** 0.95

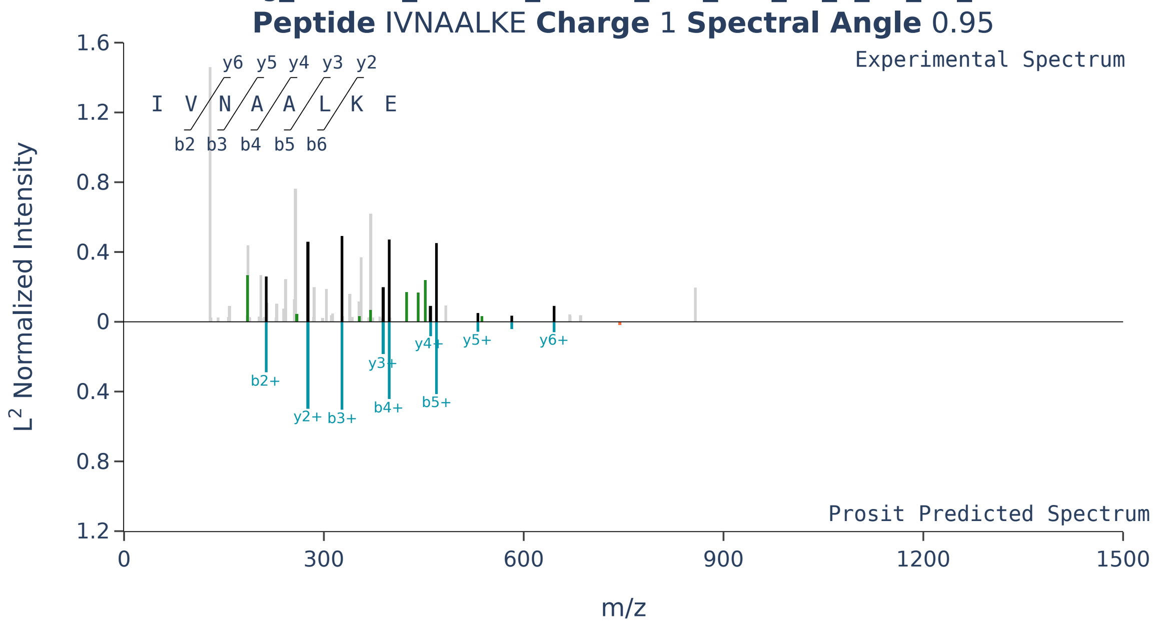

**Source** Ncheng_210623_230623_HFGoe_FFH_20S_25_1_A1_4h_R1 **Scan** 7543
**Peptide** VNAALKE **Charge** 2 **Spectral Angle** 0.87

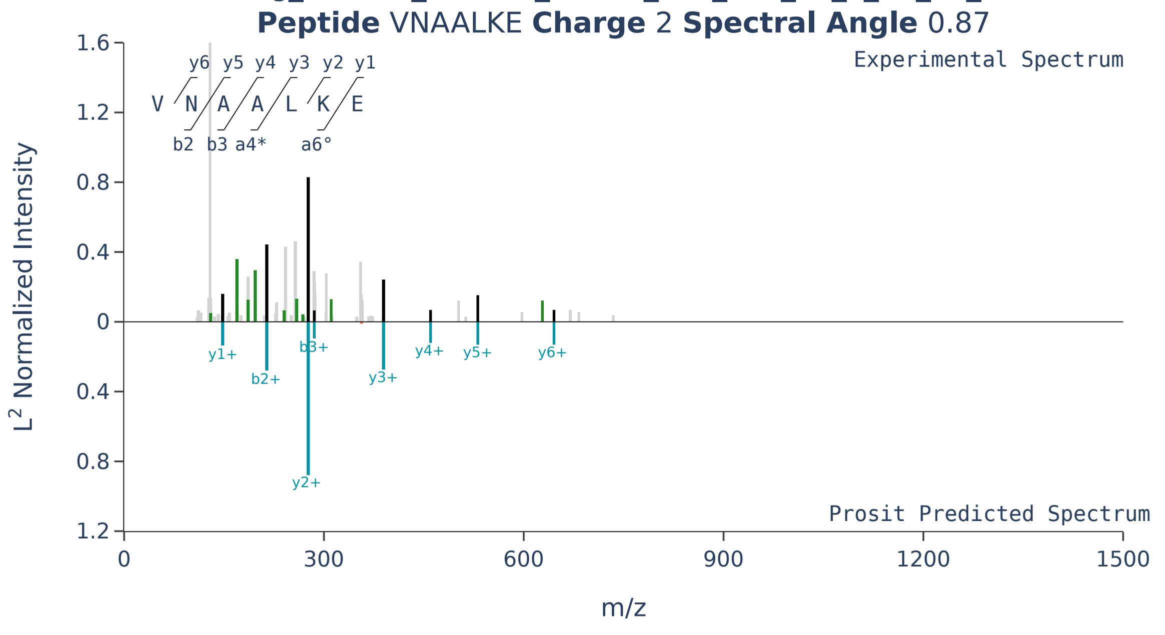

**Source** Ncheng_210623_230623_HFGoe_FFH_20S_25_1_A1_24h_R2 **Scan** 28695
**Peptide** ADVALPVV **Charge** 1 **Spectral Angle** 0.9

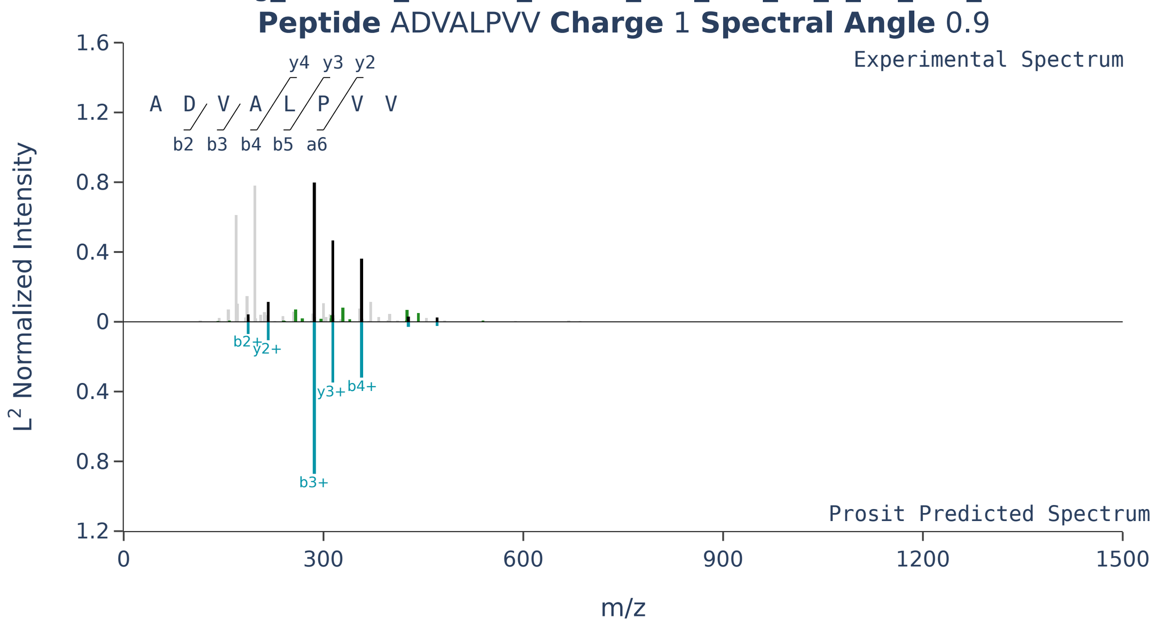

**Source** Ncheng_210623_230623_HFGoe_FFH_20S_25_1_A1_4h_R1 **Scan** 32270
**Peptide** GDVLSLIE **Charge** 1 **Spectral Angle** 0.91

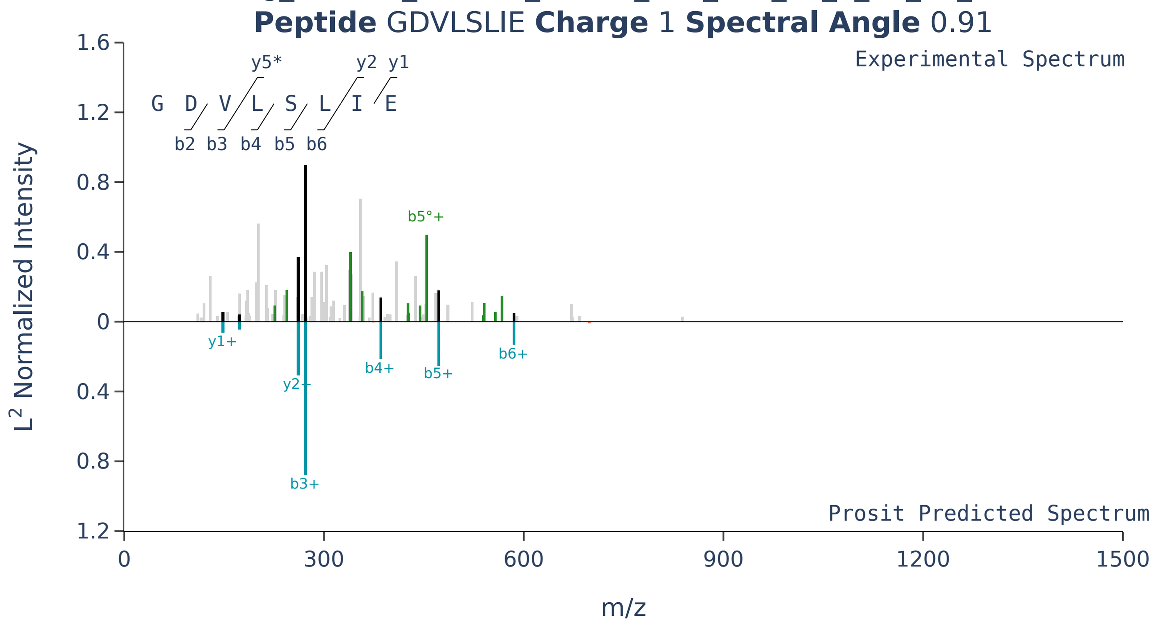

**Source** Ncheng_210623_230623_HFGoe_FFH_20S_25_1_A2_24h_R1 **Scan** 17388
**Peptide** GEENQTLNLA **Charge** 1 **Spectral Angle** 0.94

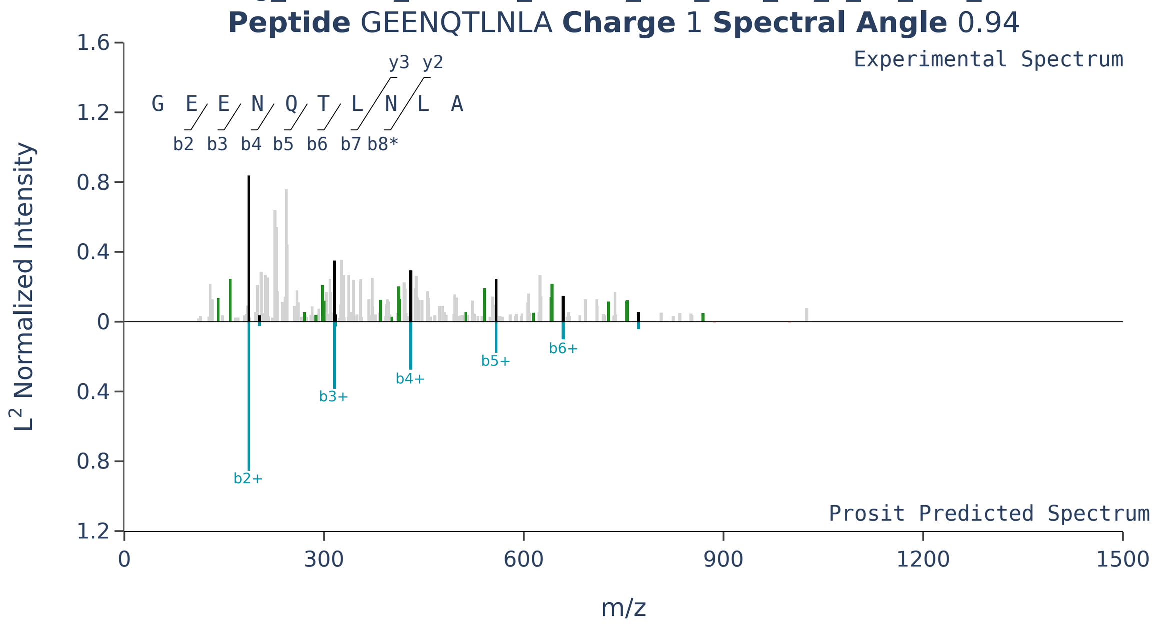

**Source** Ncheng_210623_230623_HFGoe_FFH_20S_25_1_A2_24h_R1 **Scan** 8800
**Peptide** VNAALKEA **Charge** 1 **Spectral Angle** 0.92

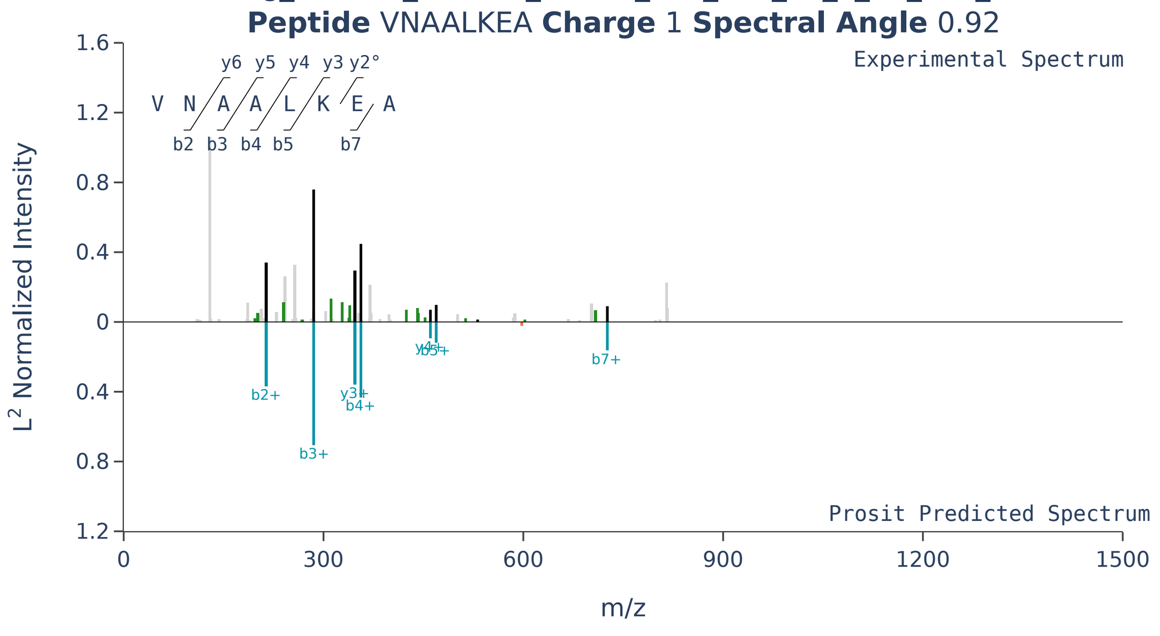

**Source** Ncheng_210623_230623_HFGoe_FFH_20S_25_1_A2_24h_R1 **Scan** 18609
**Peptide** ENQTLNL **Charge** 1 **Spectral Angle** 0.92

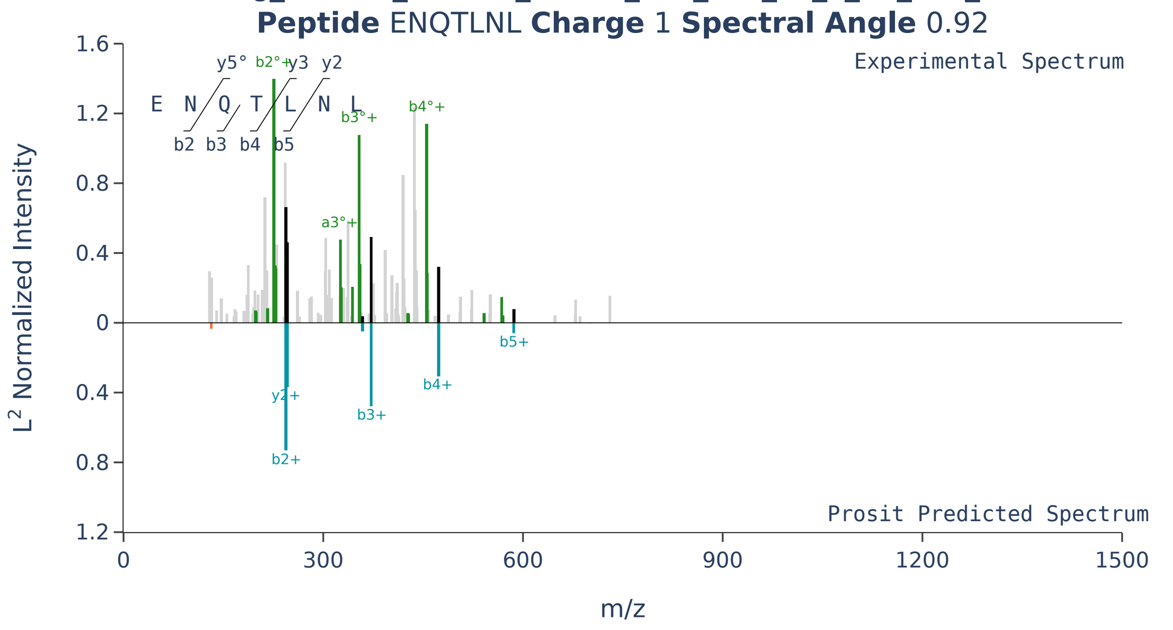


**Source** Ncheng_210623_230623_HFGoe_FFH_20S_25_1_A1_24h_R1 **Scan** 12550
**Peptide** VHASINPVE **Charge** 1 **Spectral Angle** 0.94

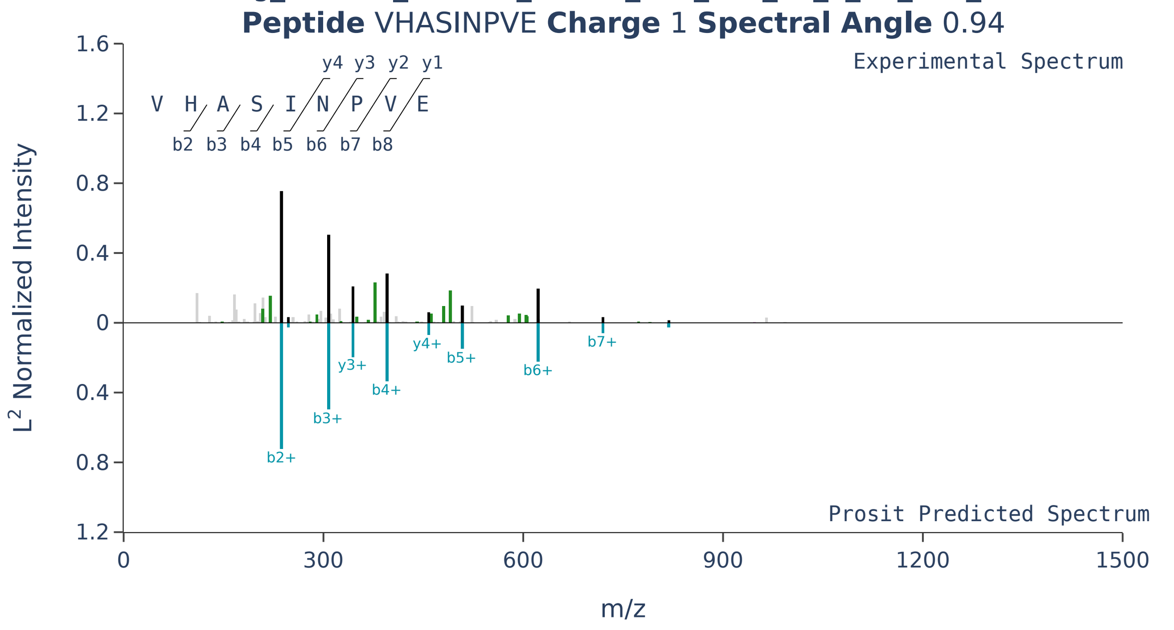


**Source** Ncheng_210623_230623_HFGoe_FFH_20S_25_1_A2_24h_R1 **Scan** 24930
**Peptide** PLTGVVLT **Charge** 1 **Spectral Angle** 0.95

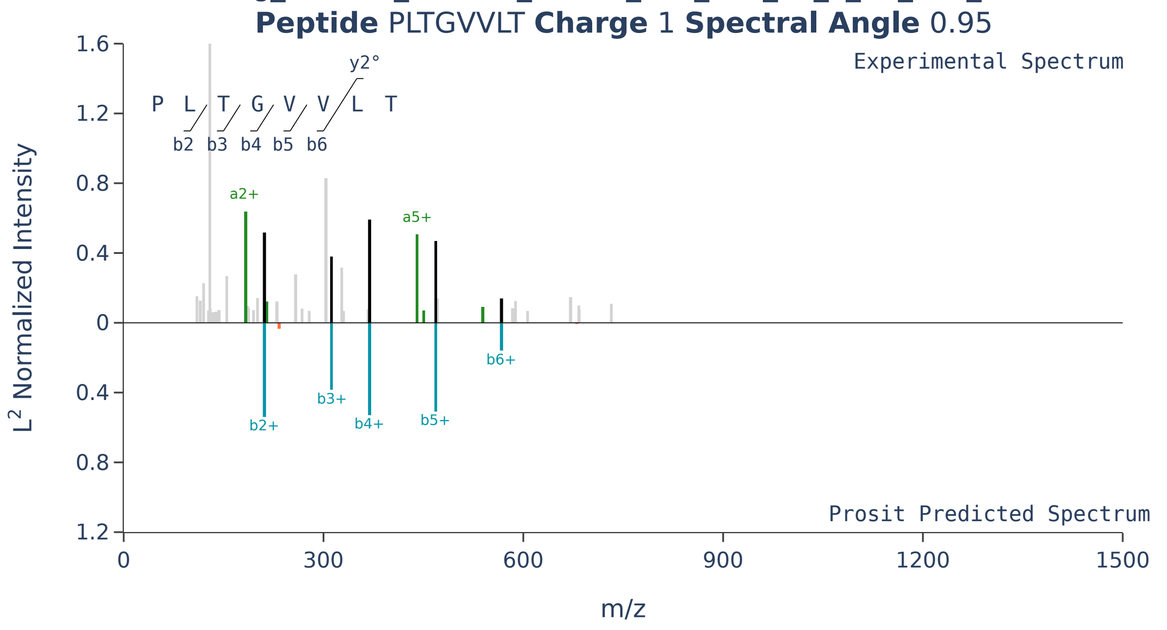


**Source** Ncheng_210623_230623_HFGoe_FFH_20S_25_1_A2_24h_R2 **Scan** 17971
**Peptide** NLAAQPPAV **Charge** 1 **Spectral Angle** 0.93

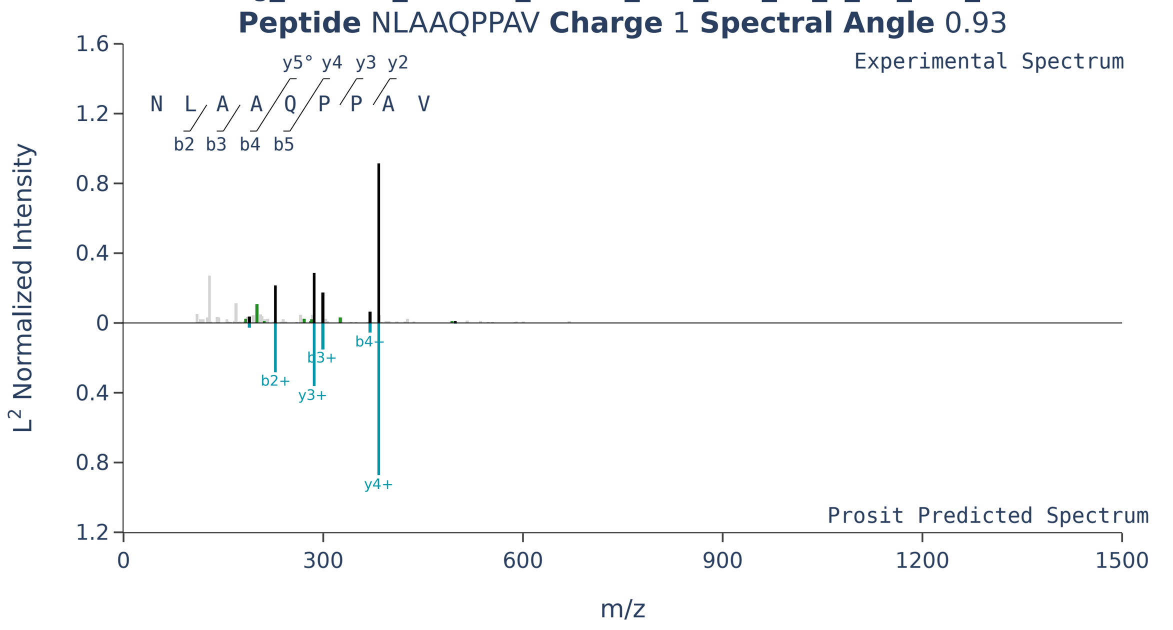


**Source** Ncheng_210623_230623_HFGoe_FFH_20S_25_1_A1_24h_R2 **Scan** 8035
**Peptide** AANTAKAFNE **Charge** 1 **Spectral Angle** 0.91

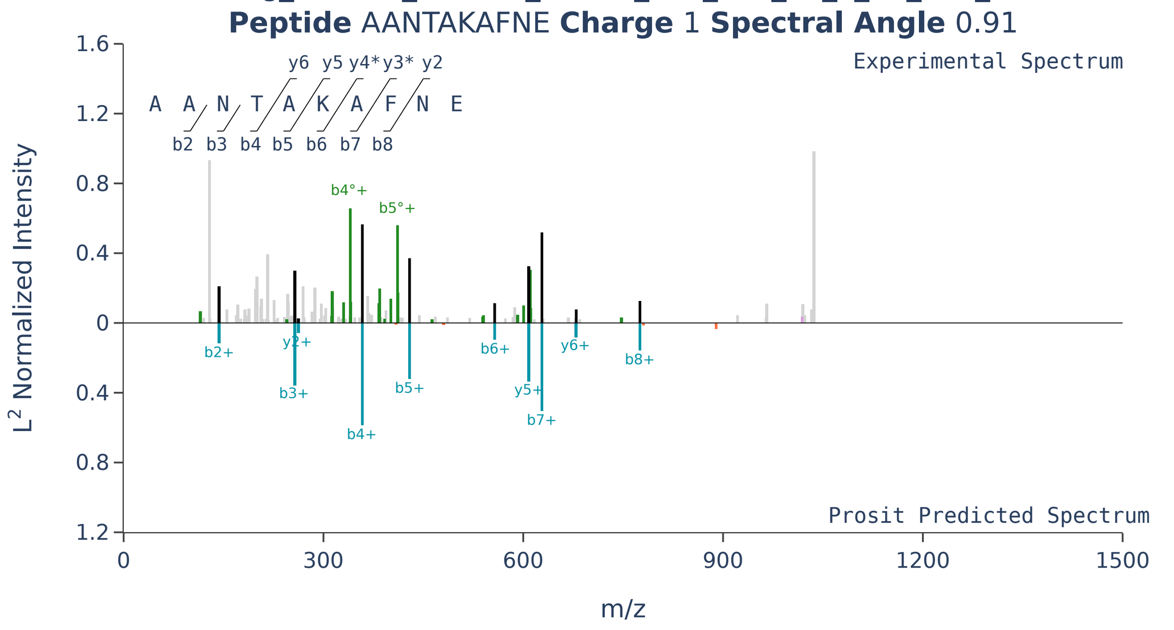


**Source** Ncheng_210623_230623_HFGoe_FFH_20S_25_1_A2_24h_R2 **Scan** 10665
**Peptide** HASINPVE **Charge** 2 **Spectral Angle** 0.93

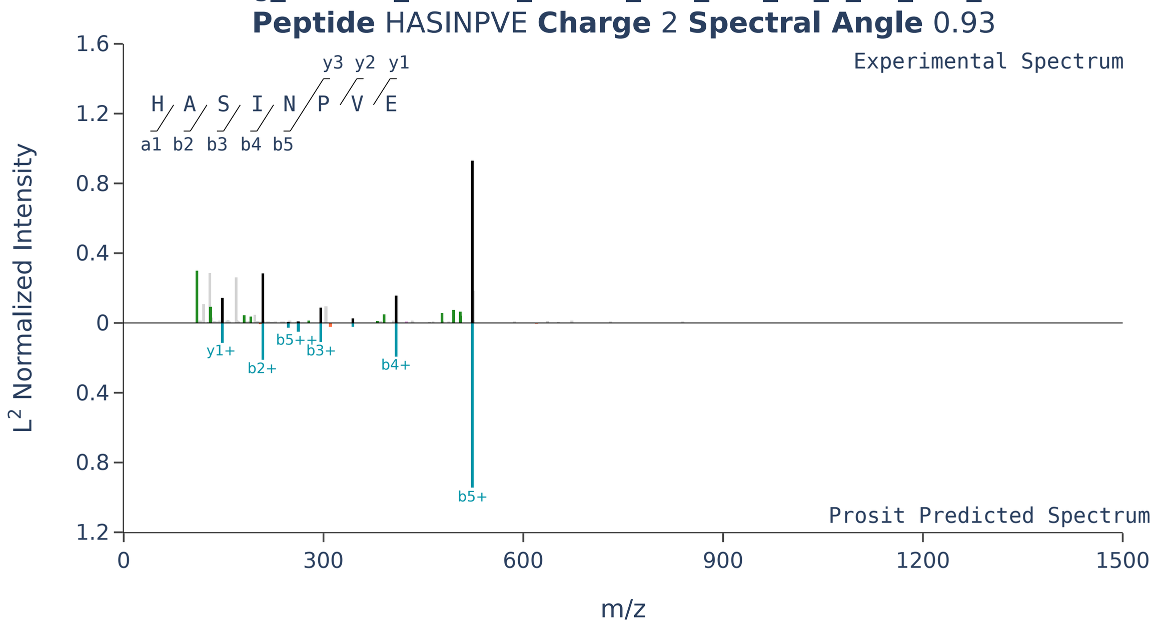


**Source** Ncheng_210623_230623_HFGoe_FFH_20S_25_1_A1_4h_R2 **Scan** 8753
**Peptide** ALKEAKL **Charge** 1 **Spectral Angle** 0.95

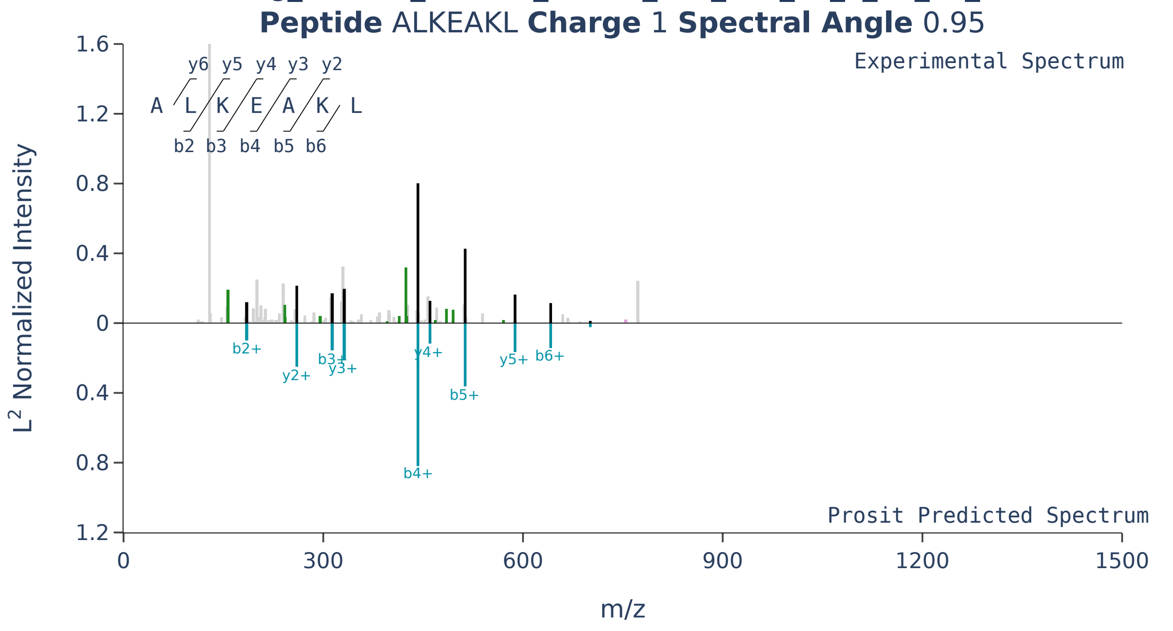


**Source** Ncheng_210623_230623_HFGoe_FFH_20S_25_1_A2_24h_R1 **Scan** 17363
**Peptide** GMQVQDVNRL **Charge** 1 **Spectral Angle** 0.96

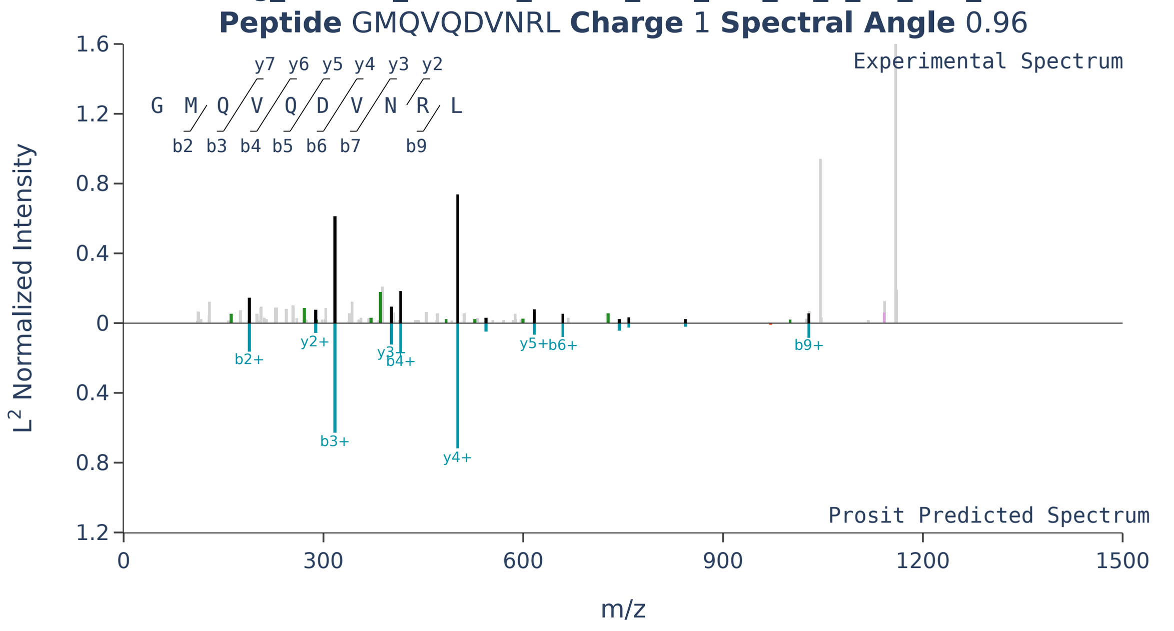


**Source** Ncheng_210623_230623_HFGoe_FFH_20S_25_1_A1_24h_R2 **Scan** 16116
**Peptide** VDAMTGQDAANTAKAFN **Charge** 2 **Spectral Angle** 0.93

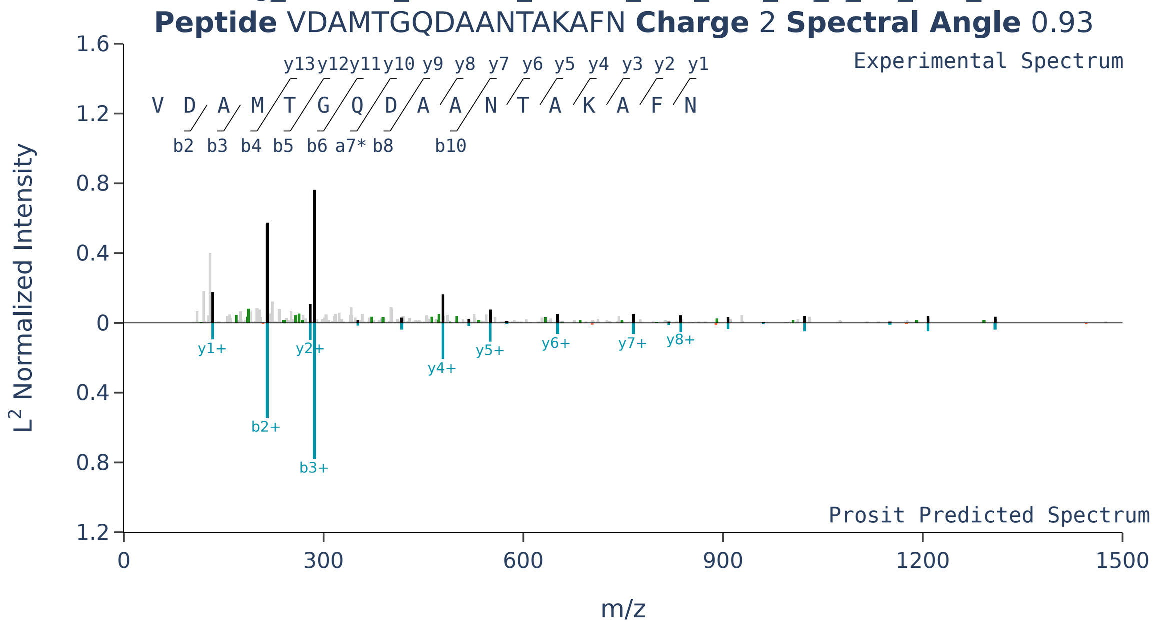


**Source** Ncheng_210623_230623_HFGoe_FFH_20S_25_1_A1_4h_R1 **Scan** 29840
**Peptide** AEQVGVDFFPSD **Charge** 2 **Spectral Angle** 0.94

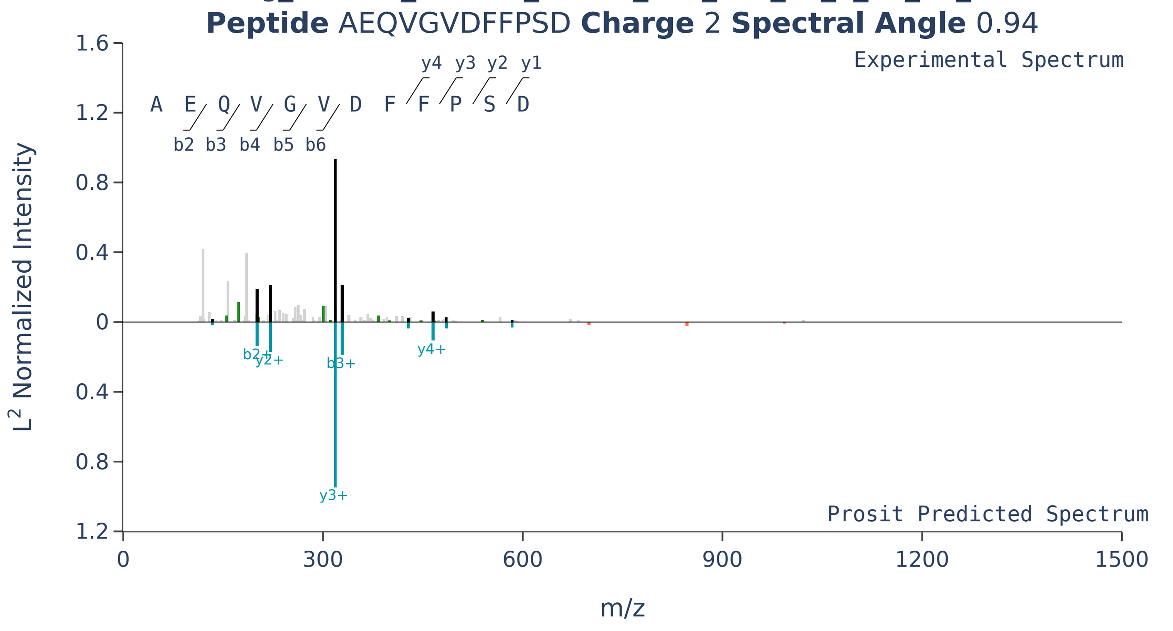


**Source** Ncheng_210623_230623_HFGoe_FFH_20S_25_1_A2_4h_R2 **Scan** 9320
**Peptide** EDIESKV **Charge** 2 **Spectral Angle** 0.95

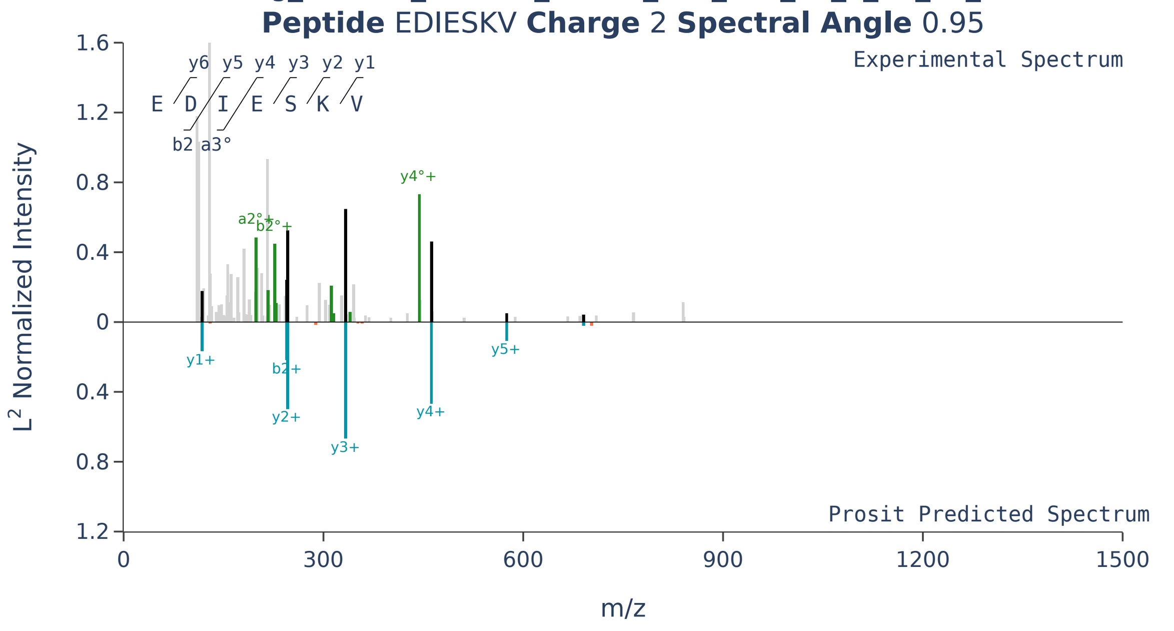


**Source** Ncheng_210623_230623_HFGoe_FFH_20S_25_1_A2_4h_R1 **Scan** 12188
**Peptide** PAAIKQLE **Charge** 1 **Spectral Angle** 0.92

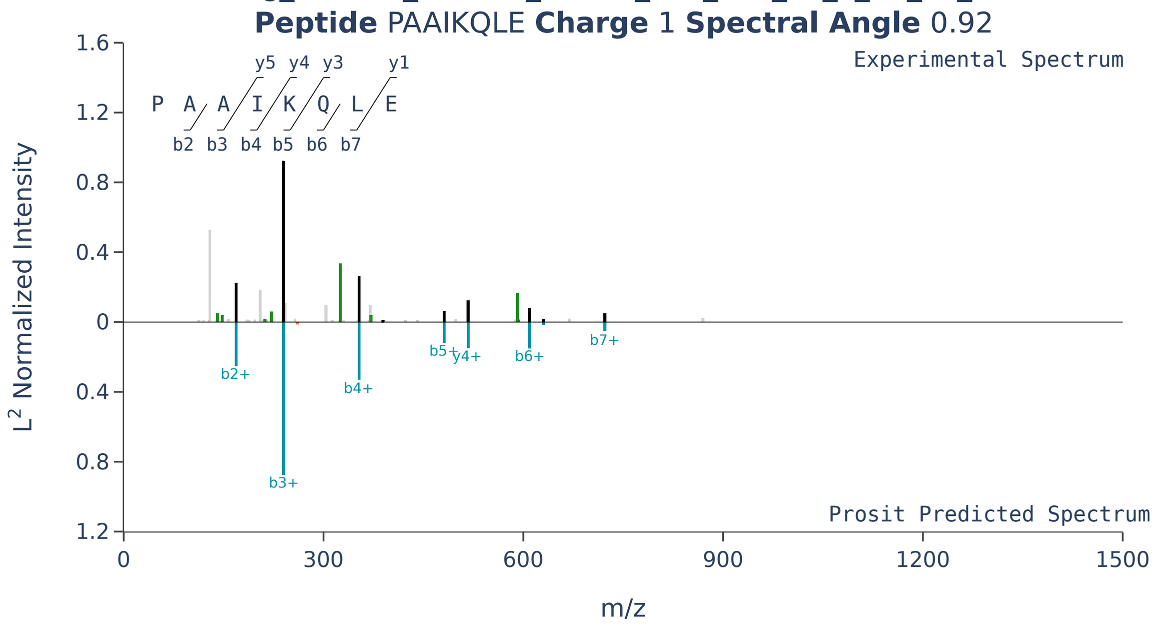


**Source** Ncheng_210623_230623_HFGoe_FFH_20S_25_1_A1_1h_R2 **Scan** 7149
**Peptide** GVGEKTE **Charge** 1 **Spectral Angle** 0.93

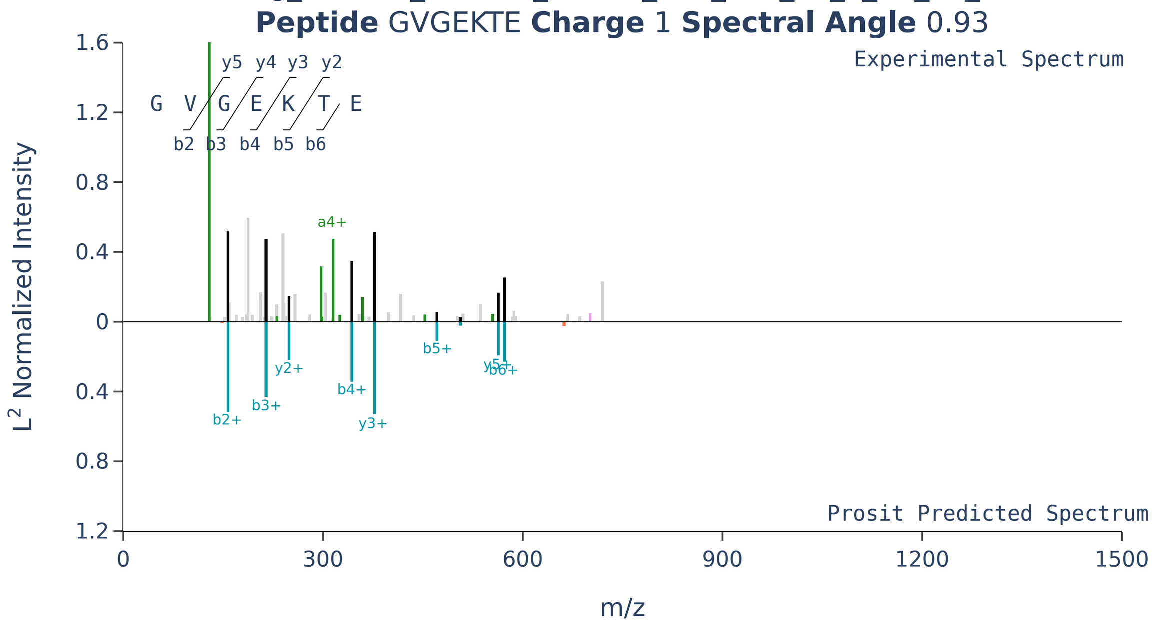


**Source** Ncheng_210623_230623_HFGoe_FFH_20S_25_1_A1_4h_R1 **Scan** 16426
**Peptide** FLEQLRQ **Charge** 2 **Spectral Angle** 0.91

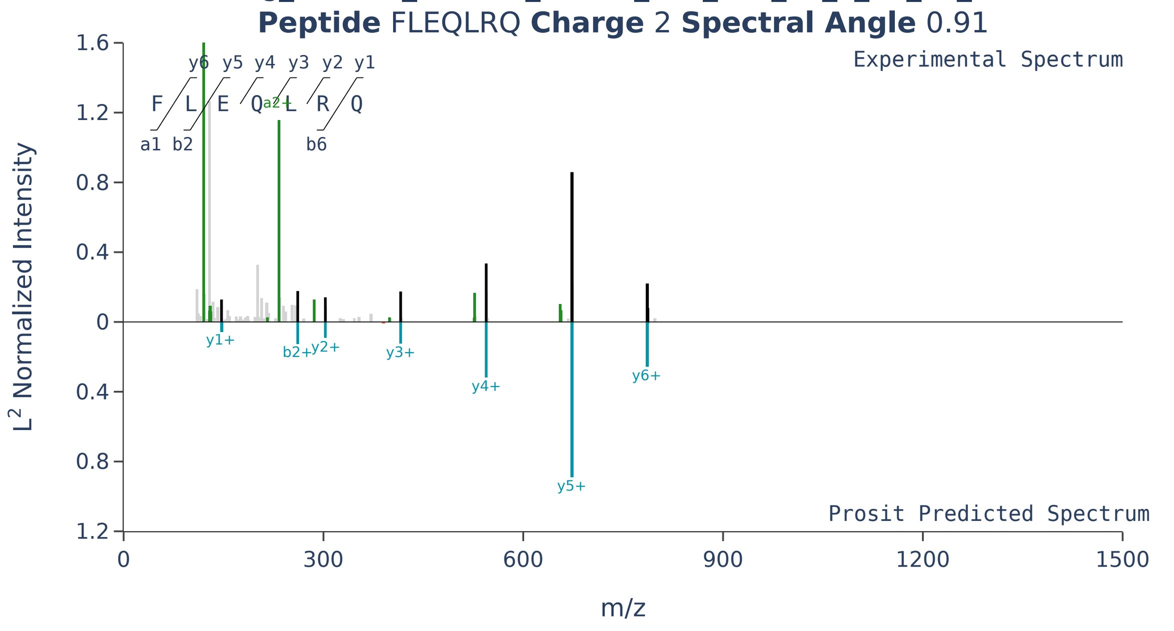


**Source** Ncheng_210623_230623_HFGoe_FFH_20S_25_1_A2_2h_R2 **Scan** 10663
**Peptide** TGKPIKF **Charge** 1 **Spectral Angle** 0.92

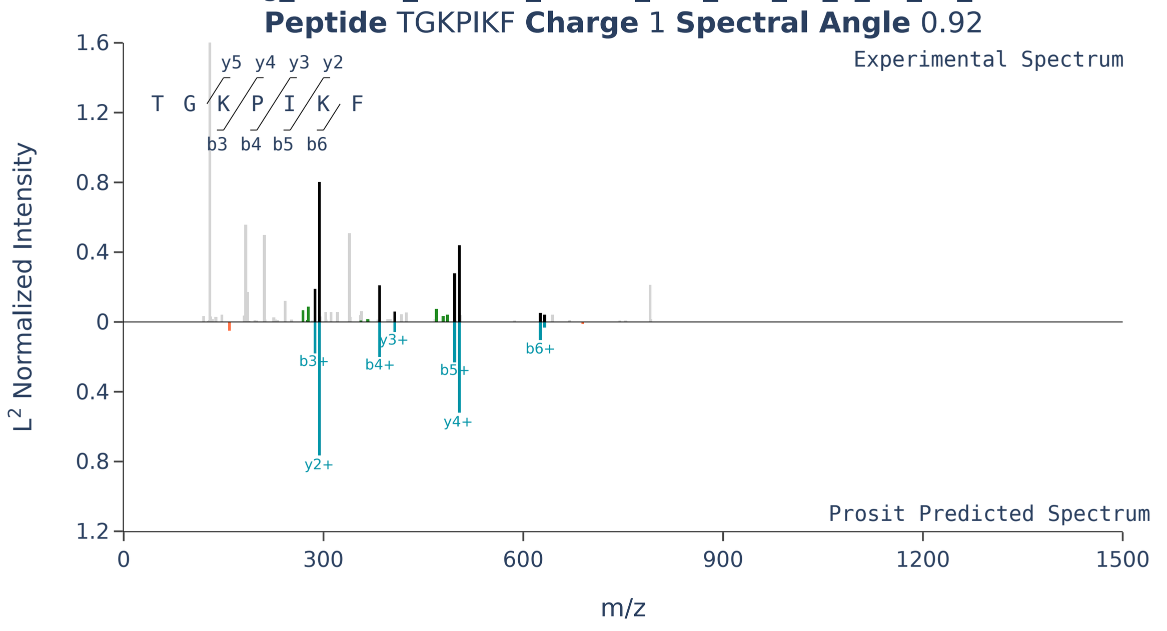

**Source** Ncheng_210623_230623_HFGoe_FFH_20S_25_1_A1_2h_R2 **Scan** 29413
**Peptide** LFVVDAMTGQDAANTAKAFN **Charge** 3 **Spectral Angle** 0.94

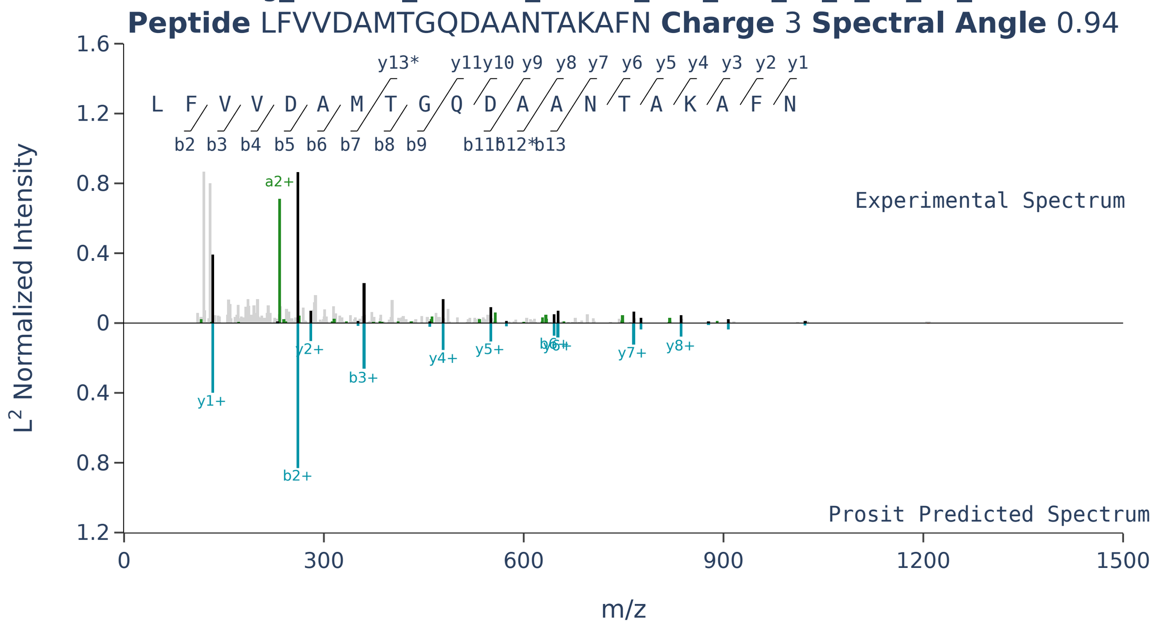


**Source** Ncheng_210623_230623_HFGoe_FFH_20S_25_1_A1_4h_R1 **Scan** 7329
**Peptide** LGVGEKTE **Charge** 1 **Spectral Angle** 0.9

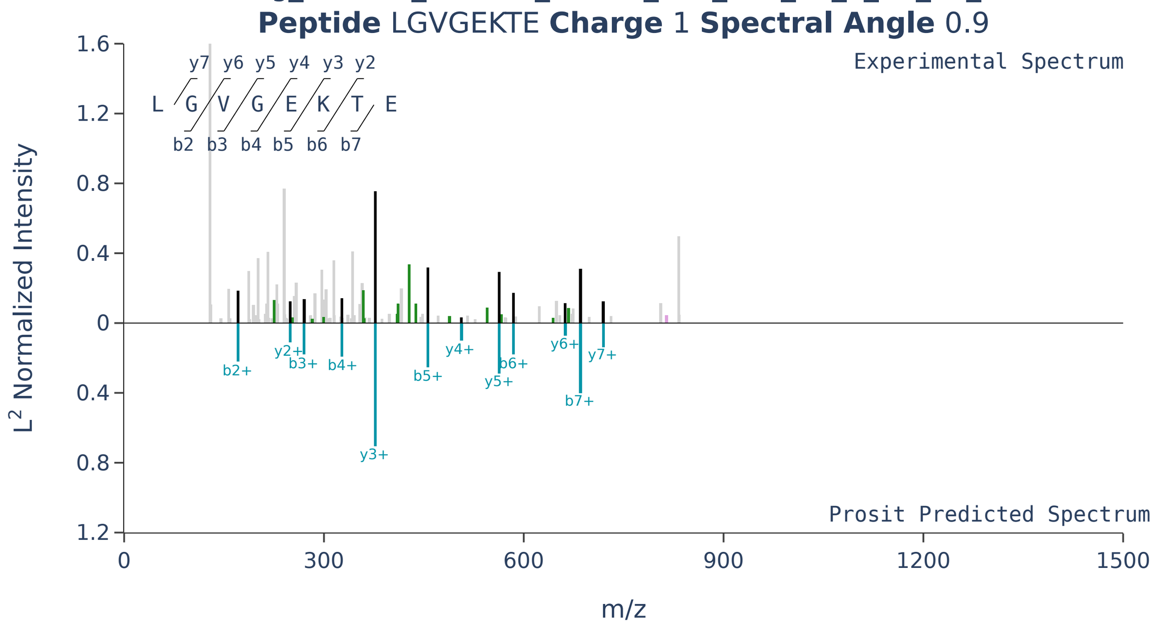


**Source** Ncheng_210623_230623_HFGoe_FFH_20S_25_1_A1_24h_R1 **Scan** 8851
**Peptide** SADVYRPA **Charge** 1 **Spectral Angle** 0.89

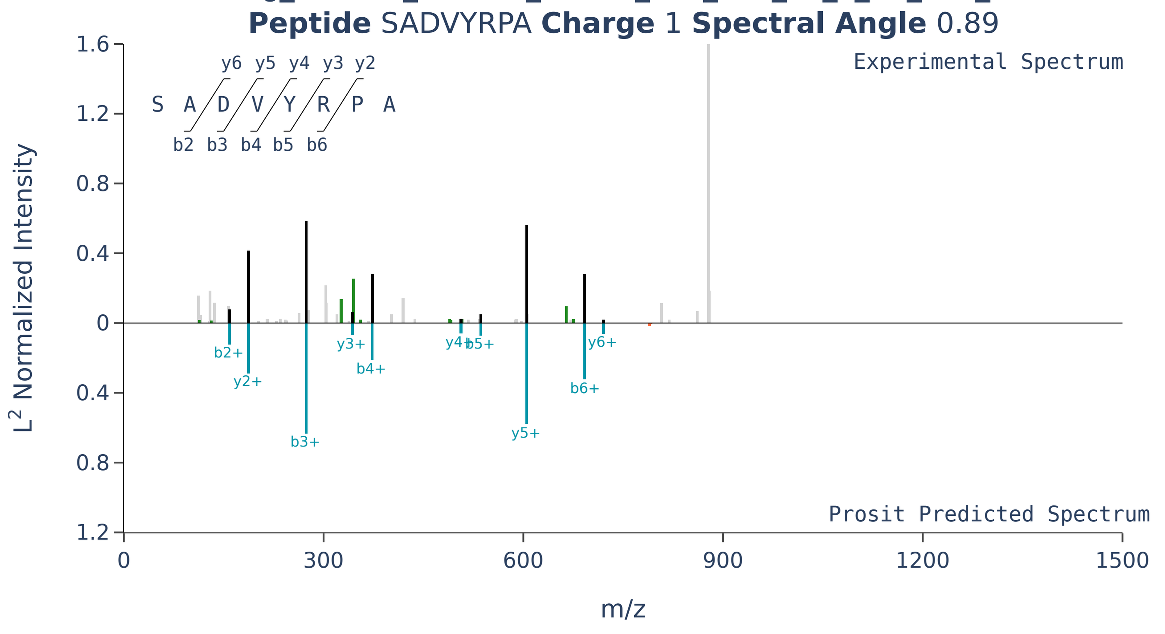


**Source** Ncheng_210623_230623_HFGoe_FFH_20S_25_1_A1_4h_R2 **Scan** 9008
**Peptide** AANTAKAFNEA **Charge** 1 **Spectral Angle** 0.9

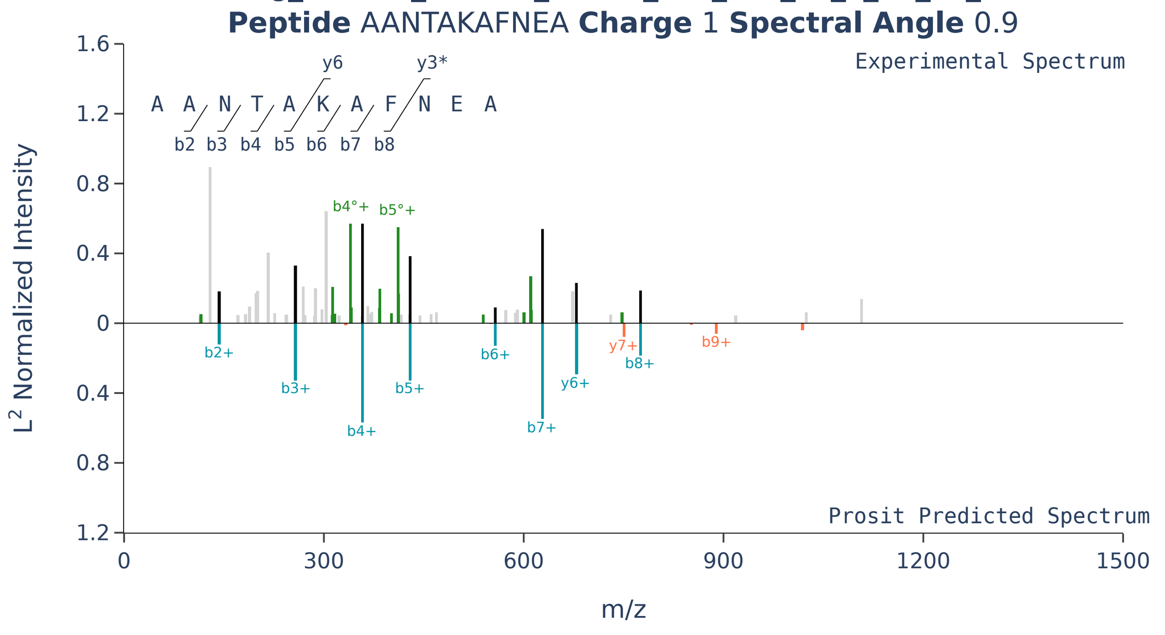


**Source** Ncheng_210623_230623_HFGoe_FFH_20S_25_1_A2_24h_R2 **Scan** 17680
**Peptide** GHEVNKSLTPGQEFV **Charge** 2 **Spectral Angle** 0.92

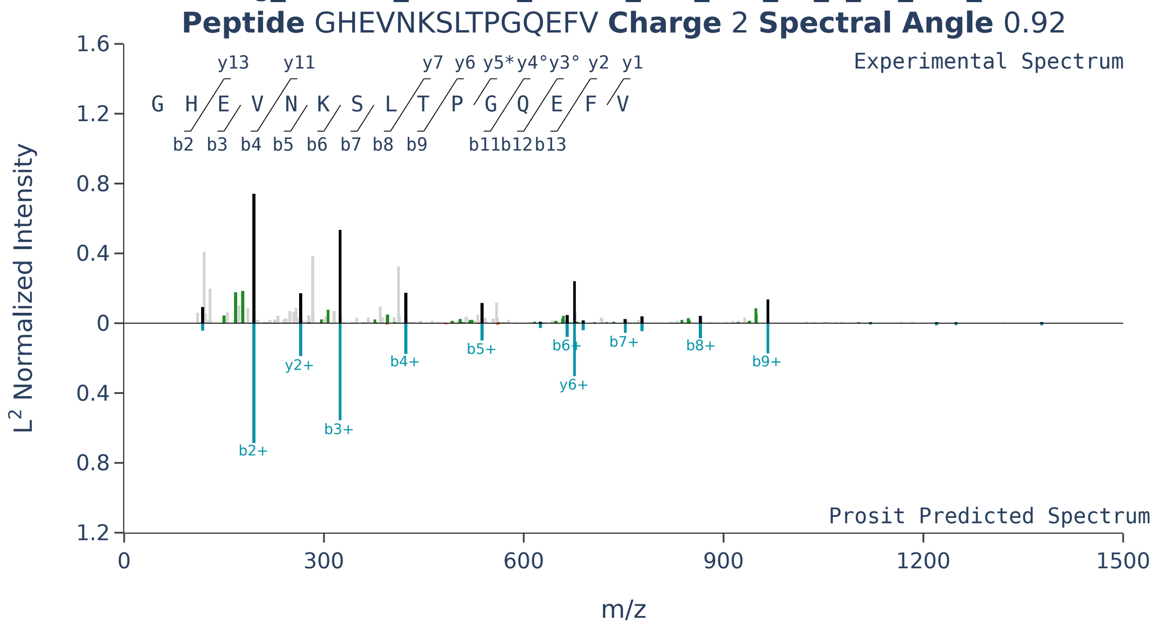


**Source** Ncheng_210623_230623_HFGoe_FFH_20S_25_1_A2_24h_R2 **Scan** 8209
**Peptide** SLTPGQE **Charge** 1 **Spectral Angle** 0.94

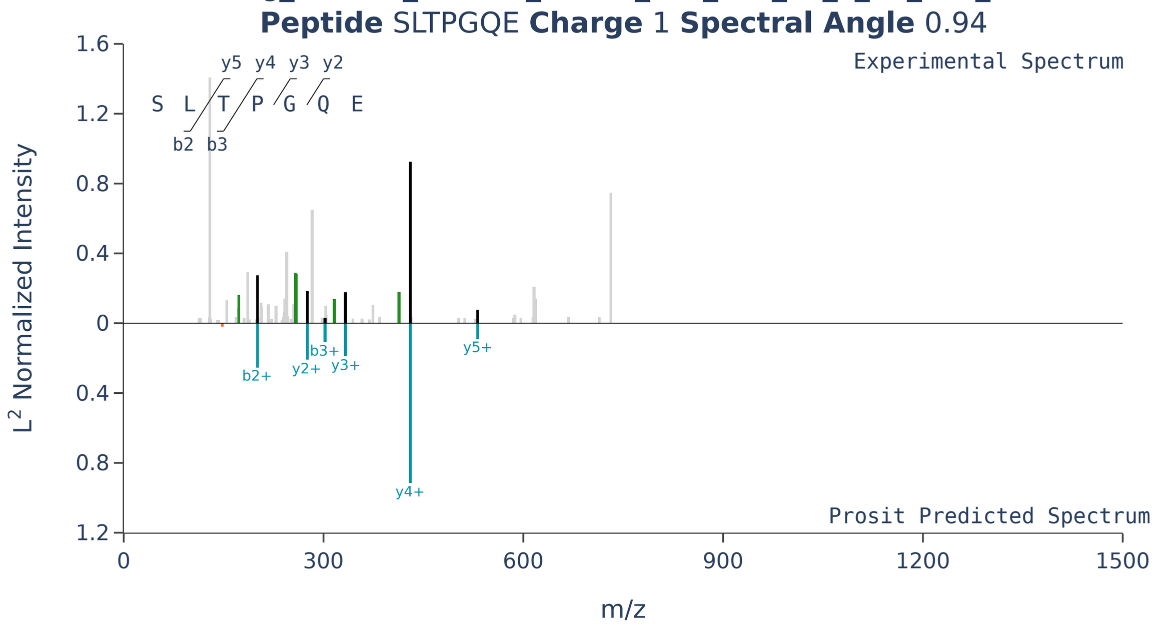


**Source** Ncheng_210623_230623_HFGoe_FFH_20S_25_1_A1_4h_R2 **Scan** 10765
**Peptide** HASINPVET **Charge** 1 **Spectral Angle** 0.85

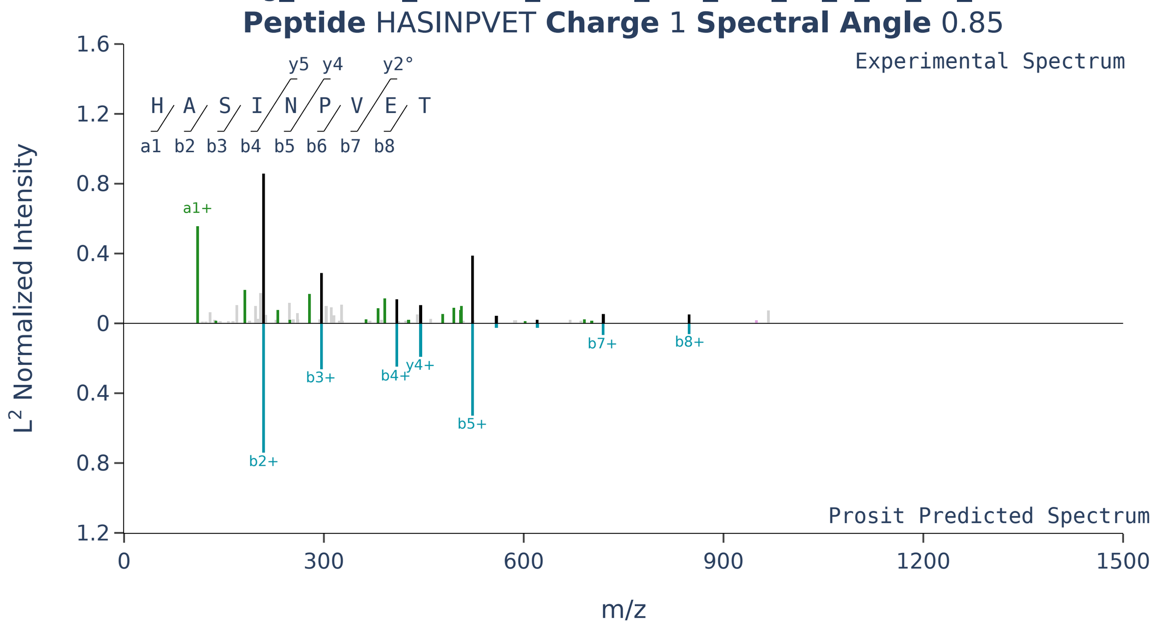


**Source** Ncheng_210623_230623_HFGoe_FFH_20S_25_1_A1_24h_R1 **Scan** 8895
**Peptide** EVNKSLTPGQE **Charge** 1 **Spectral Angle** 0.86

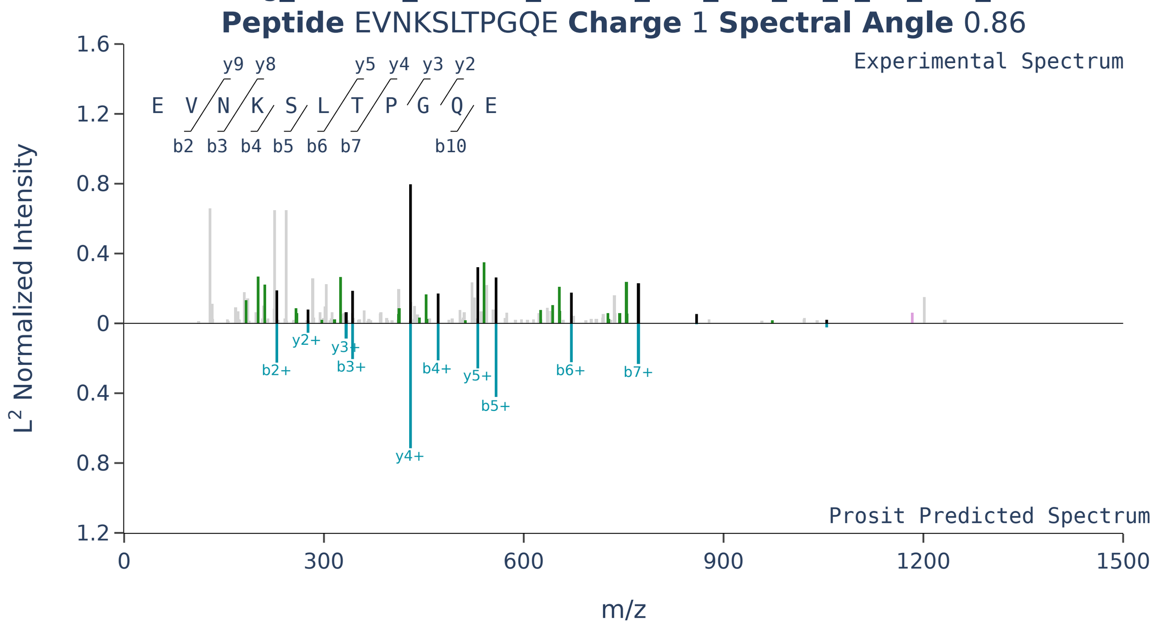


**Source** Ncheng_210623_230623_HFGoe_FFH_20S_25_1_A2_24h_R1 **Scan** 7801
**Peptide** GMAKMMR **Charge** 2 **Spectral Angle** 0.86

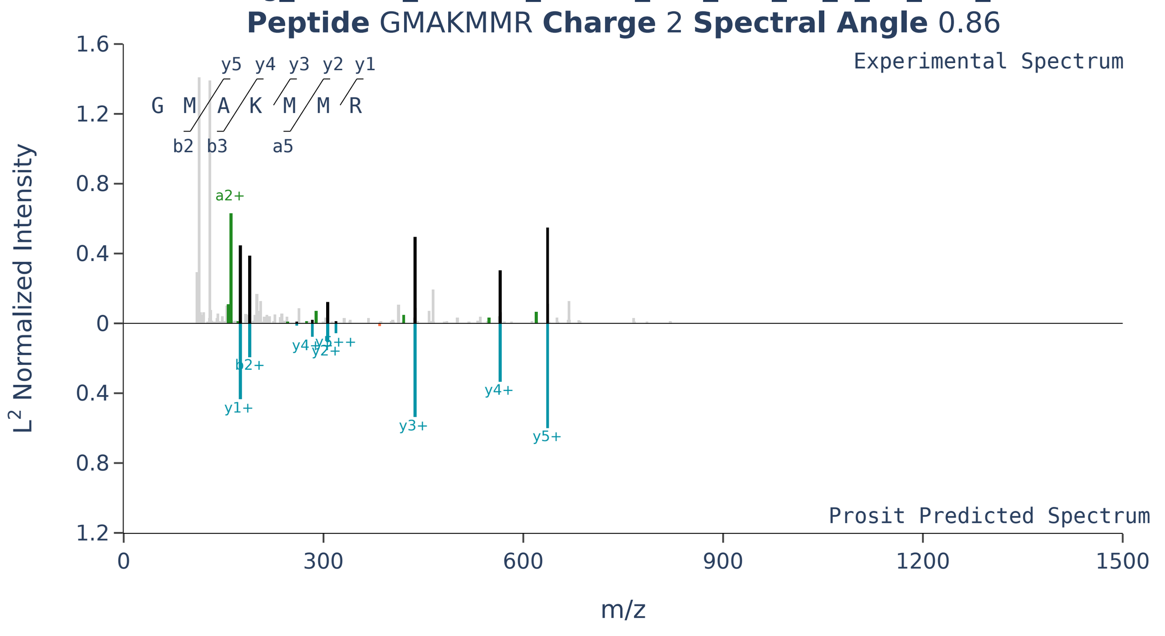


**Source** Ncheng_210623_230623_HFGoe_FFH_20S_25_1_A2_4h_R1 **Scan** 8275
**Peptide** CGMQVQD **Charge** 1 **Spectral Angle** 0.9

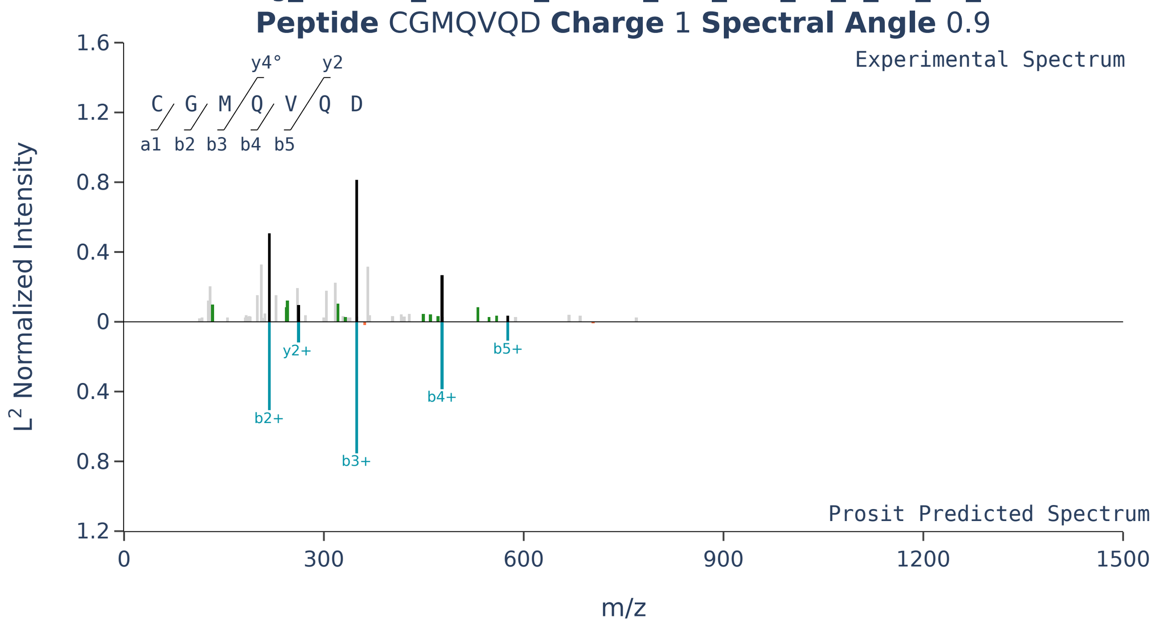


**Source** Ncheng_210623_230623_HFGoe_FFH_20S_25_1_A2_24h_R2 **Scan** 17441
**Peptide** AANTAKAFNEAL **Charge** 1 **Spectral Angle** 0.92

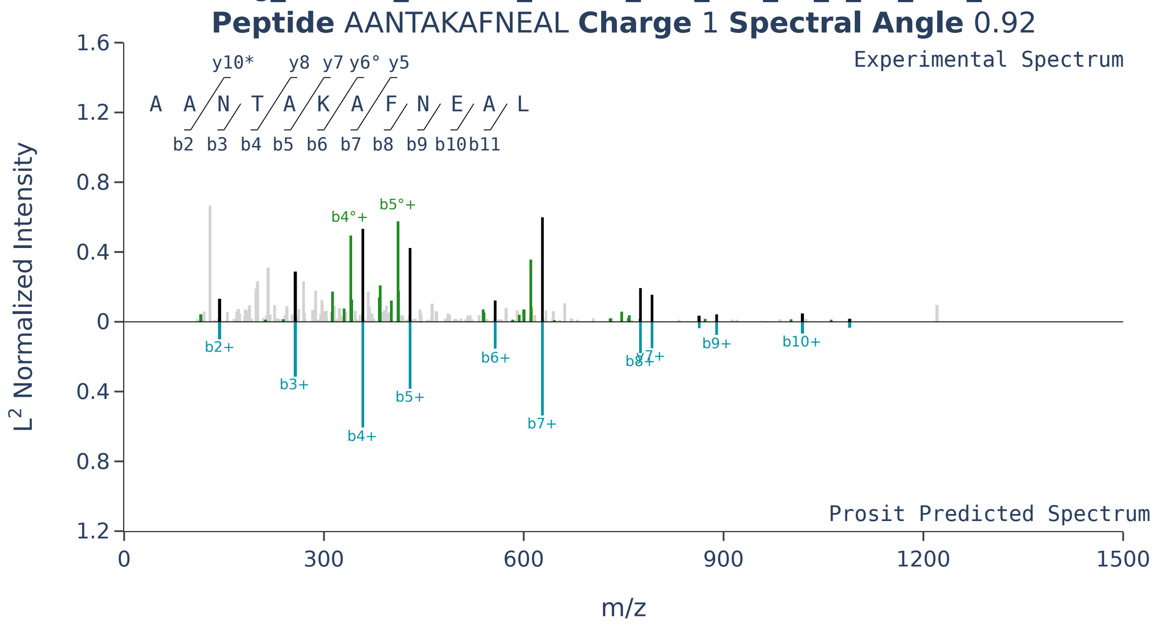


**Source** Ncheng_210623_230623_HFGoe_FFH_20S_25_1_A2_2h_R2 **Scan** 18279
**Peptide** EAIINSM **Charge** 1 **Spectral Angle** 0.94

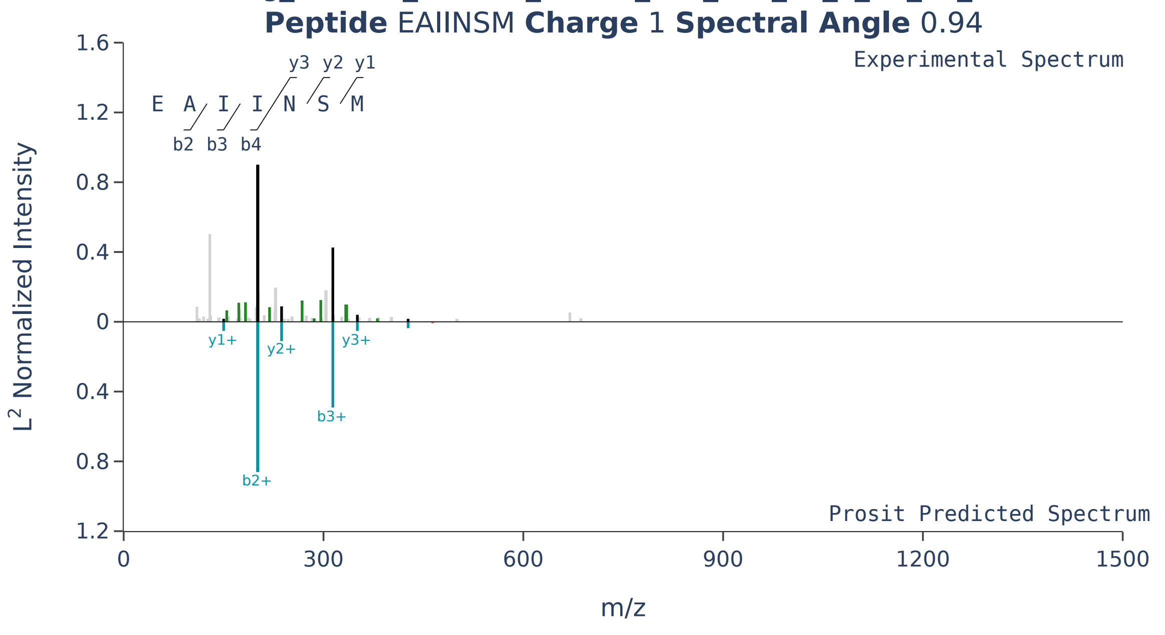


**Source** Ncheng_210623_230623_HFGoe_FFH_20S_25_1_A1_24h_R1 **Scan** 15148
**Peptide** AAQPPAVV **Charge** 1 **Spectral Angle** 0.95

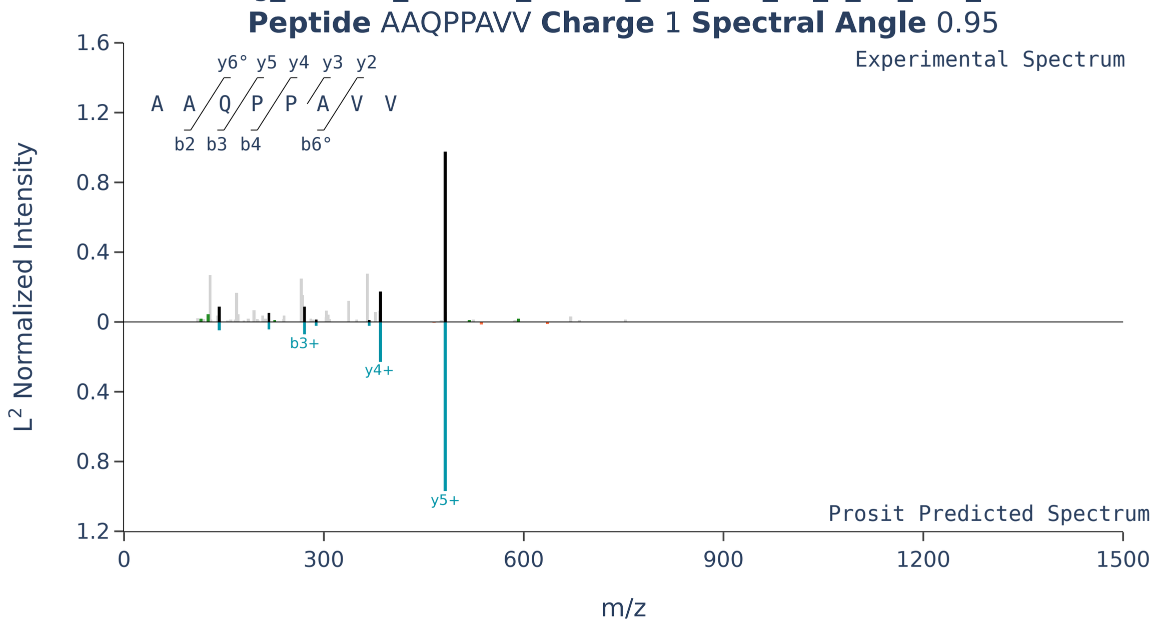


**Source** Ncheng_210623_230623_HFGoe_FFH_20S_25_1_A2_4h_R1 **Scan** 13303
**Peptide** DKVLVRME **Charge** 2 **Spectral Angle** 0.93

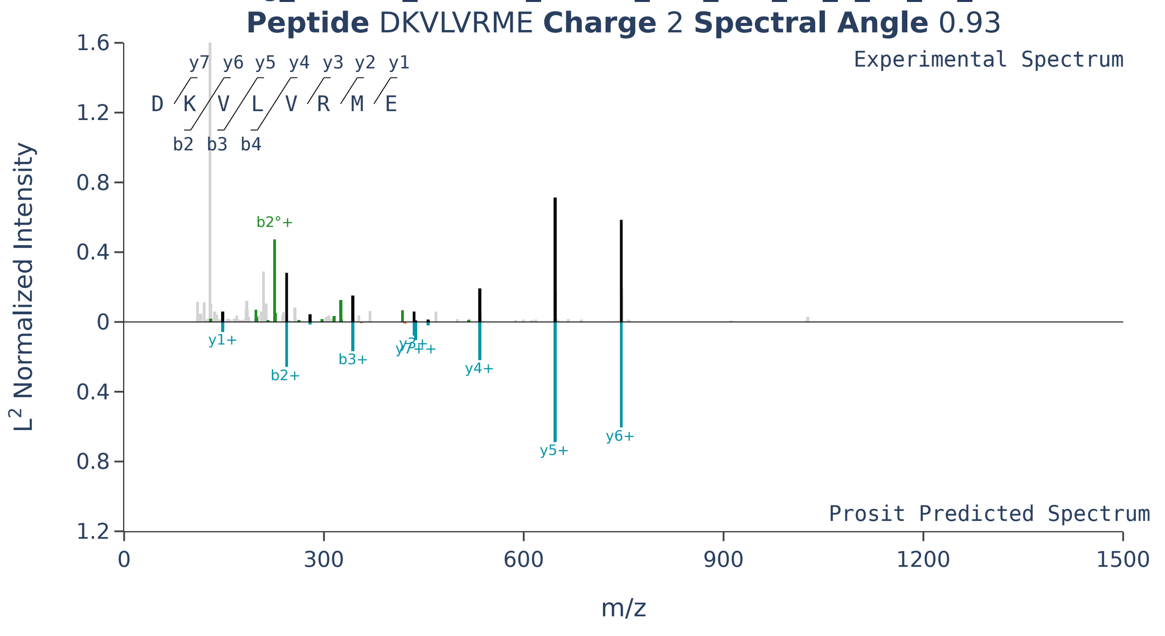


**Source** Ncheng_210623_230623_HFGoe_FFH_20S_25_1_A1_24h_R2 **Scan** 14785
**Peptide** PGMGQIPDNVKS **Charge** 1 **Spectral Angle** 0.9

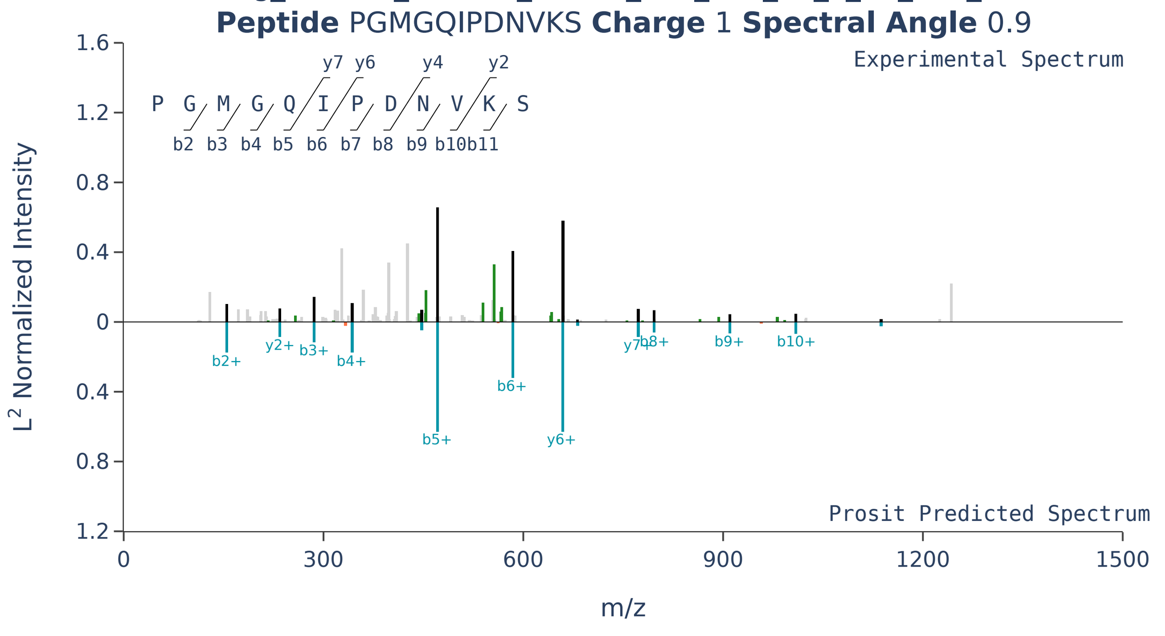

**Source** Ncheng_210623_230623_HFGoe_FFH_20S_25_1_A1_24h_R1 **Scan** 16911
**Peptide** PVDIVNA **Charge** 1 **Spectral Angle** 0.98

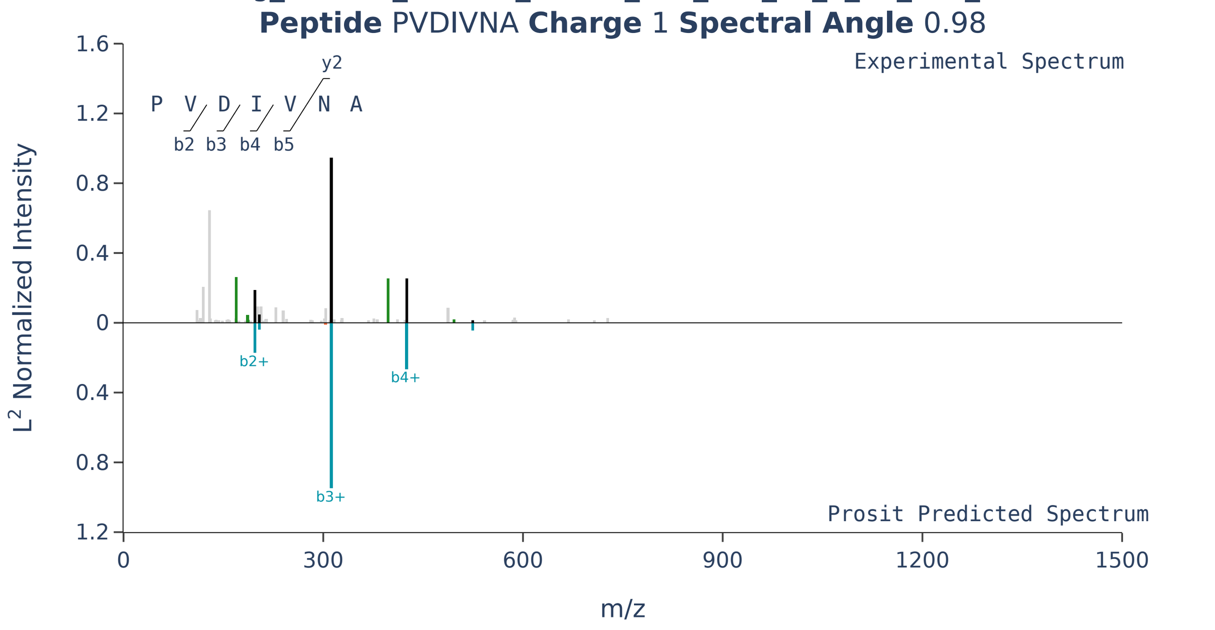

**Source** Ncheng_210623_230623_HFGoe_FFH_20S_25_1_A1_24h_R1 **Scan** 10496
**Peptide** VQDVNRL **Charge** 2 **Spectral Angle** 0.91

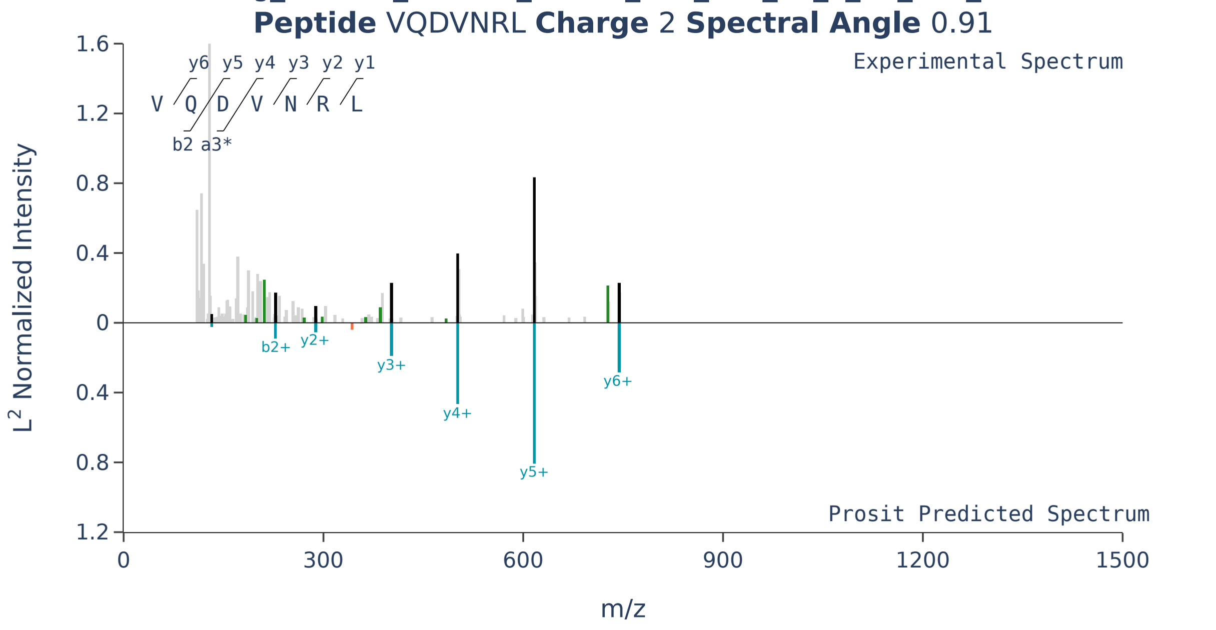

**Source** Ncheng_210623_230623_HFGoe_FFH_20S_25_1_A2_24h_R1 **Scan** 14752
**Peptide** DKVLVRMEA **Charge** 1 **Spectral Angle** 0.89

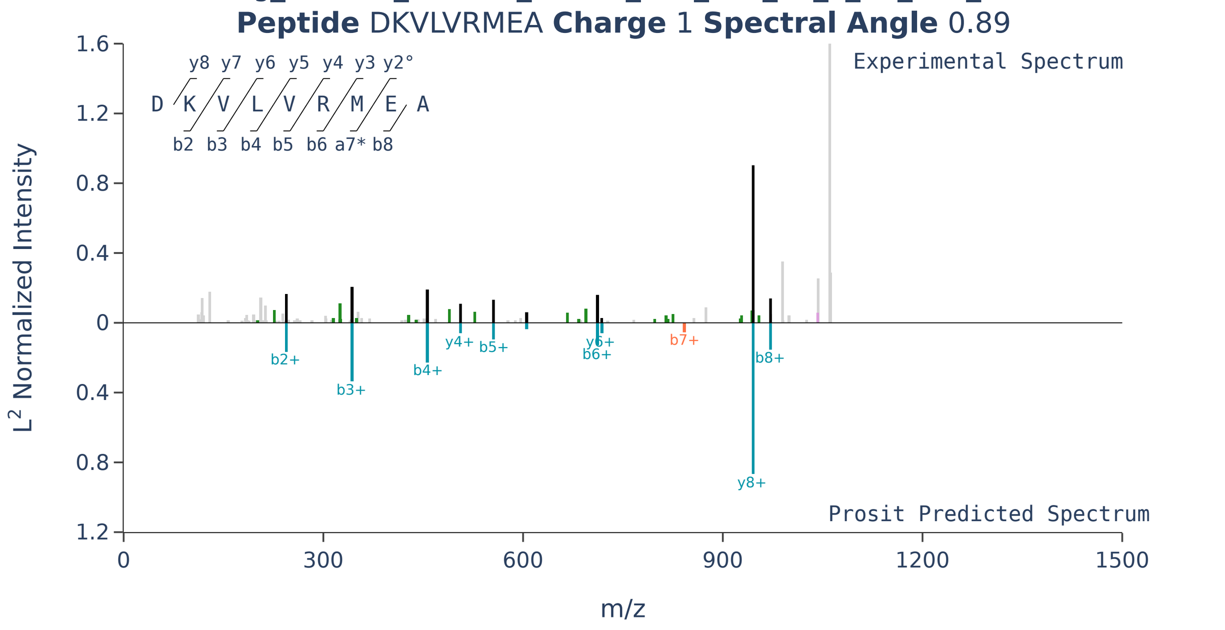

**Source** Ncheng_210623_230623_HFGoe_FFH_20S_25_1_A2_4h_R1 **Scan** 10779
**Peptide** TGKPLKF **Charge** 1 **Spectral Angle** 0.86

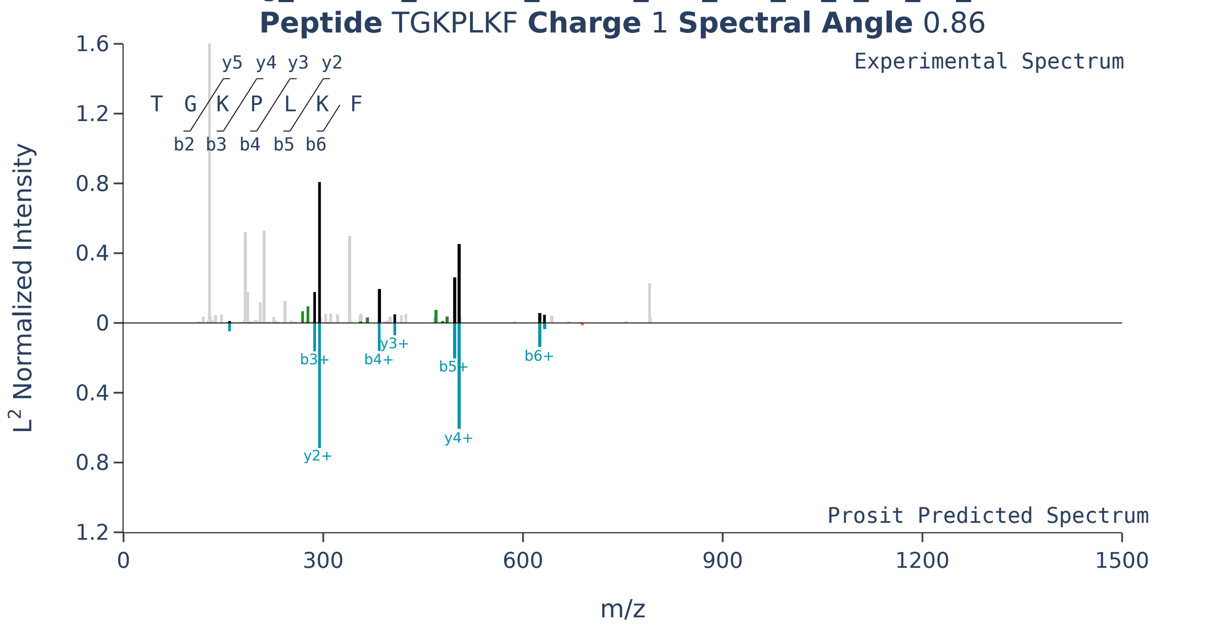

**Source** Ncheng_210623_230623_HFGoe_FFH_20S_25_1_A2_4h_R2 **Scan** 26174
**Peptide** EALPLTGV **Charge** 1 **Spectral Angle** 0.88

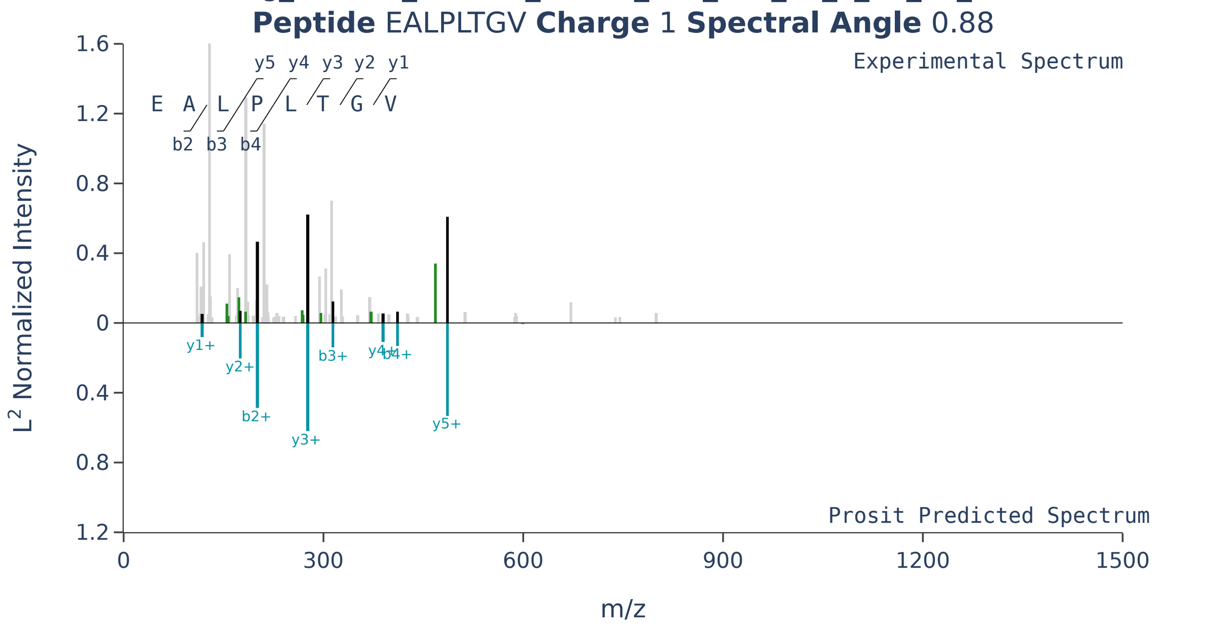

**Source** Ncheng_210623_230623_HFGoe_FFH_20S_25_1_A2_24h_R2 **Scan** 9454
**Peptide** MAGLQGAGKTT **Charge** 1 **Spectral Angle** 0.87

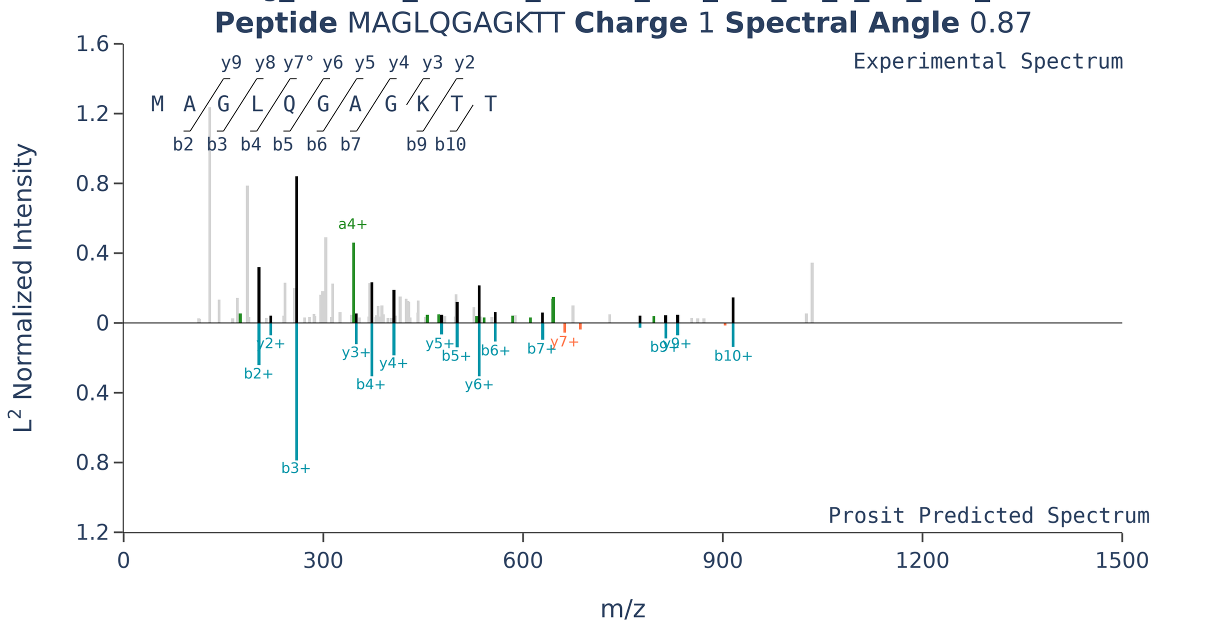

**Source** Ncheng_210623_230623_HFGoe_FFH_20S_25_1_A2_2h_R2 **Scan** 8493
**Peptide** KGGMAKMM **Charge** 2 **Spectral Angle** 0.92

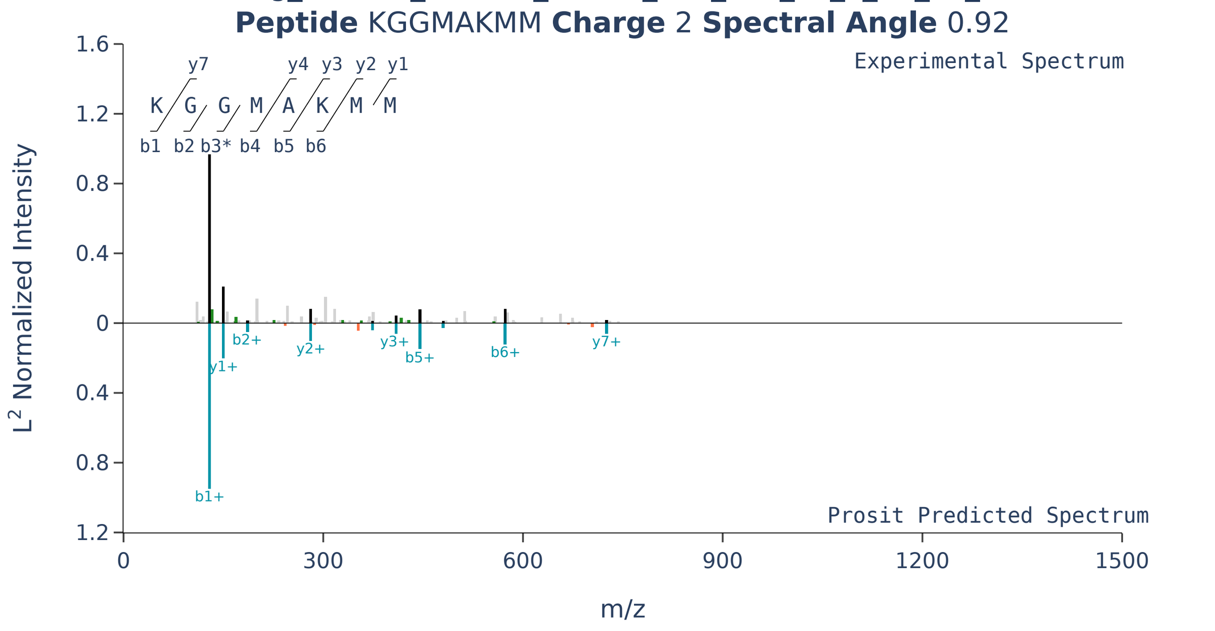

**Source** Ncheng_210623_230623_HFGoe_FFH_20S_25_1_A1_1h_R2 **Scan** 10049
**Peptide** AAQPPAV **Charge** 1 **Spectral Angle** 0.93

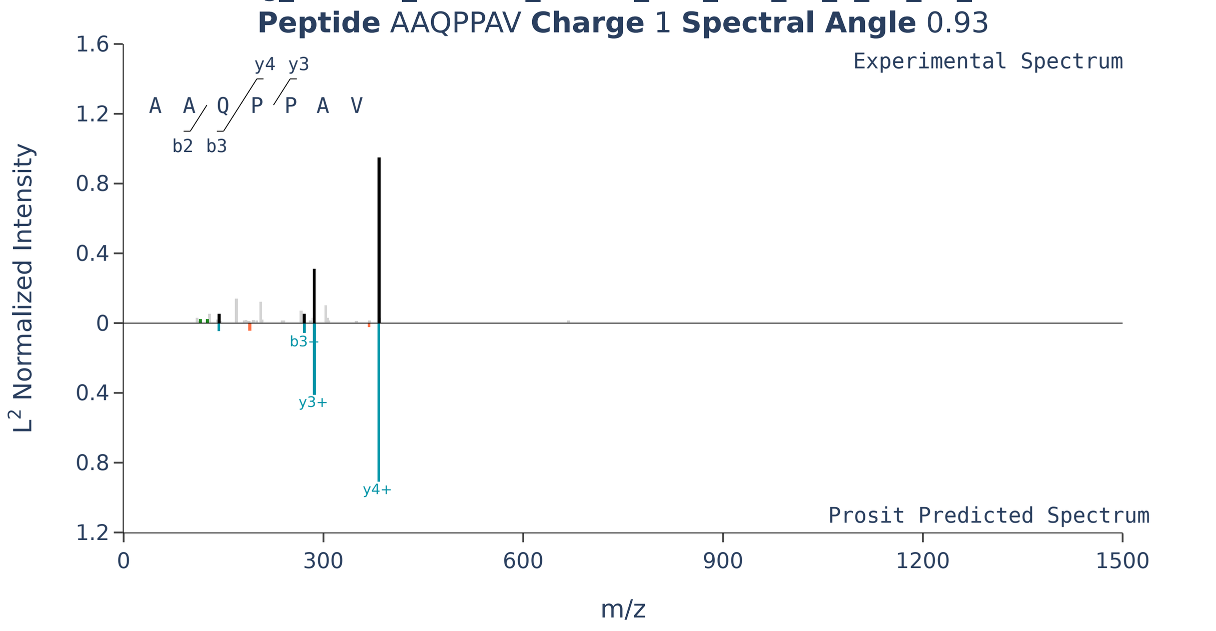

**Source** Ncheng_210623_230623_HFGoe_FFH_20S_25_1_A1_2h_R2 **Scan** 18804
**Peptide** GEKTEALEPFHPDRL **Charge** 2 **Spectral Angle** 0.87

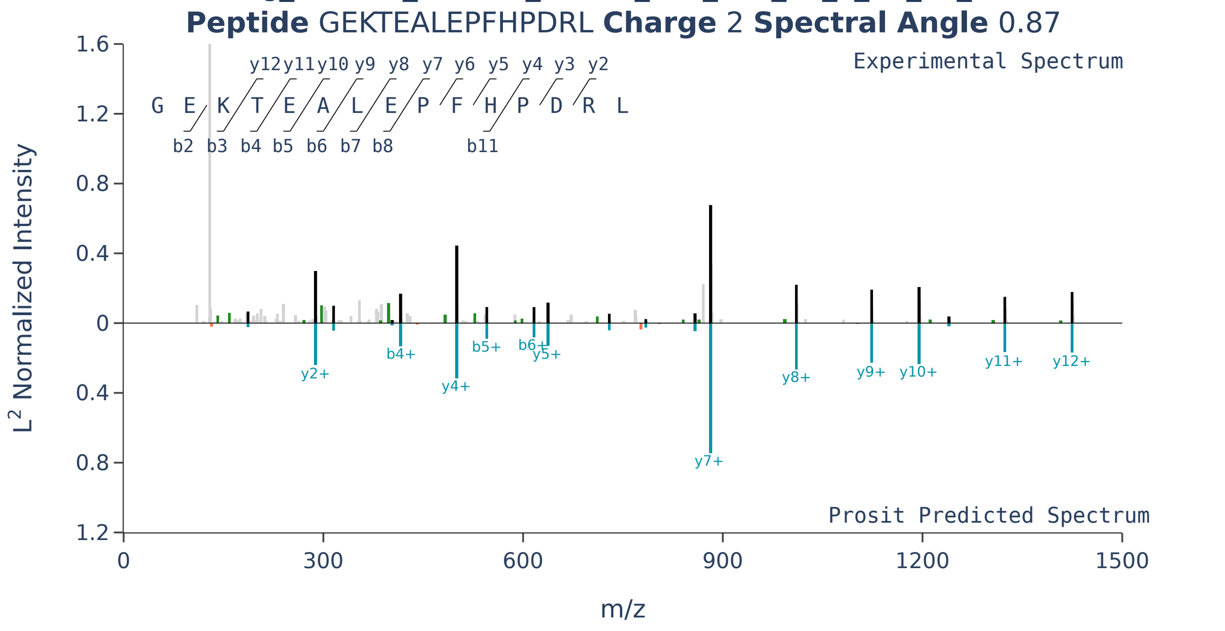

**Source** Ncheng_210623_230623_HFGoe_FFH_20S_25_1_A1_24h_R1 **Scan** 16207
**Peptide** PDRIASR **Charge** 2 **Spectral Angle** 0.87

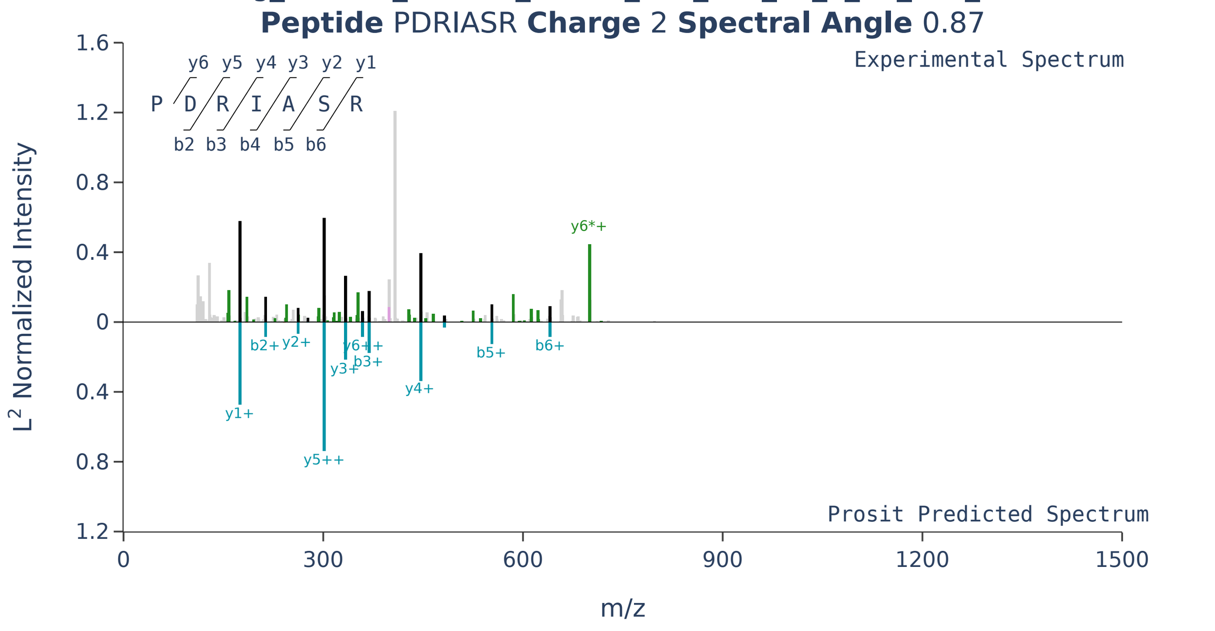

**Source** Ncheng_210623_230623_HFGoe_FFH_20S_25_1_A1_24h_R2 **Scan** 18628
**Peptide** NKSLTPGQEFV **Charge** 1 **Spectral Angle** 0.9

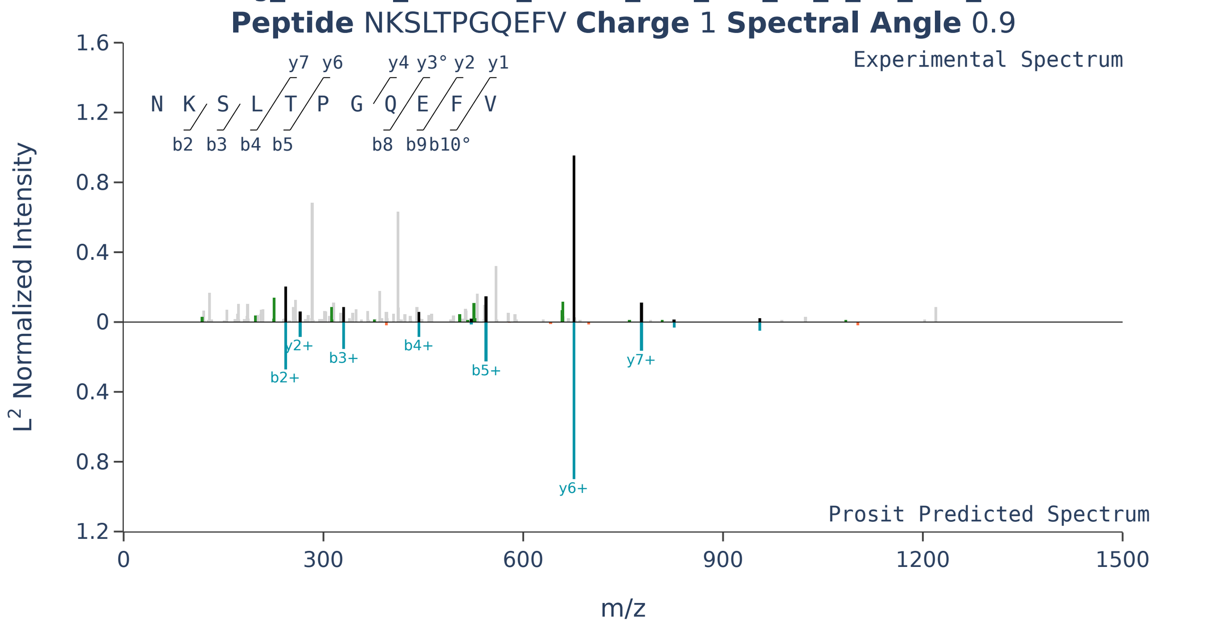

**Source** Ncheng_210623_230623_HFGoe_FFH_20S_25_1_A1_24h_R2 **Scan** 8574
**Peptide** ANTAKAFNEA **Charge** 1 **Spectral Angle** 0.89

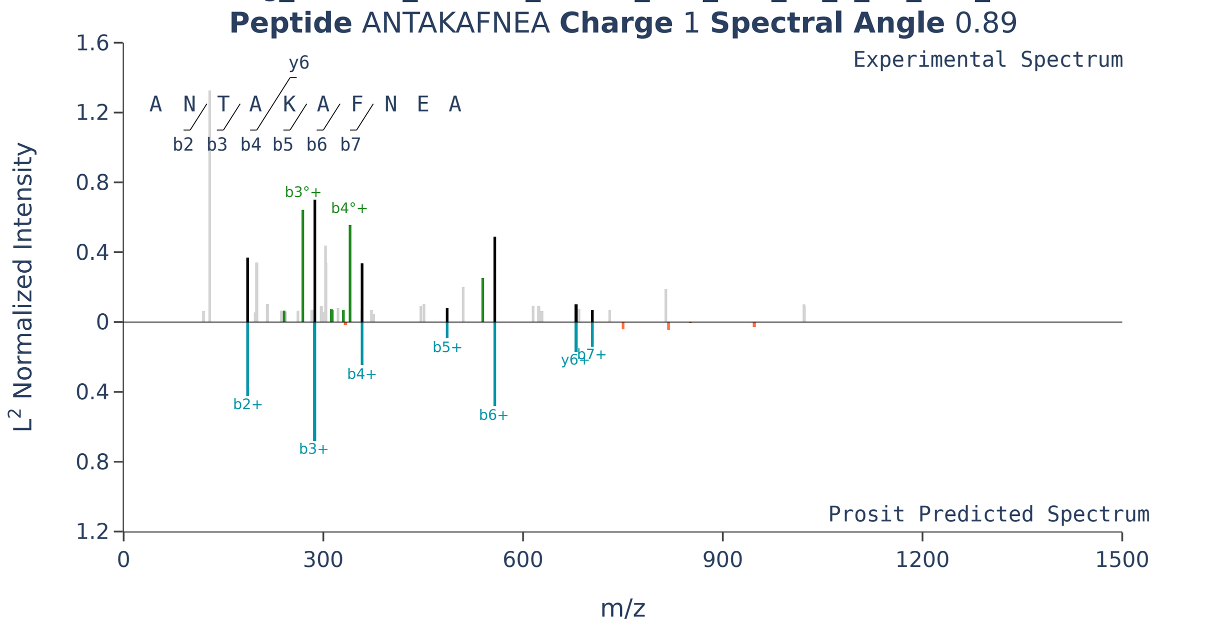

**Source** Ncheng_210623_230623_HFGoe_FFH_20S_25_1_A2_4h_R1 **Scan** 8466
**Peptide** GCGMQVQD **Charge** 1 **Spectral Angle** 0.92

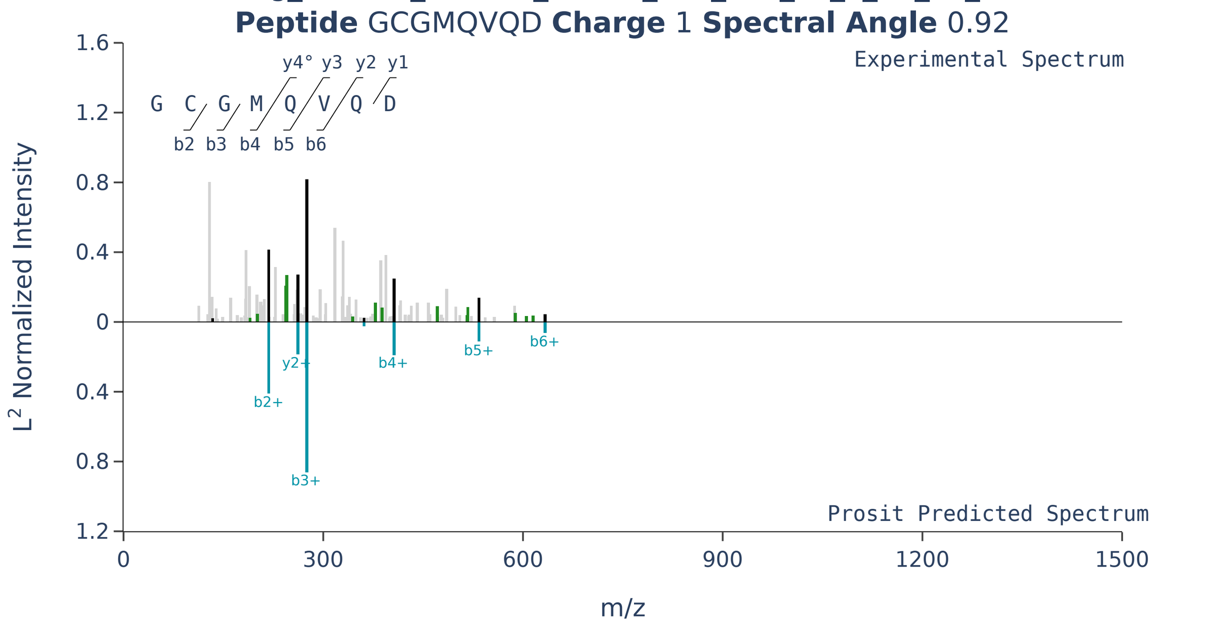

**Source** Ncheng_210623_230623_HFGoe_FFH_20S_25_1_A2_4h_R1 **Scan** 14729
**Peptide** MDDKVLVRME **Charge** 3 **Spectral Angle** 0.92

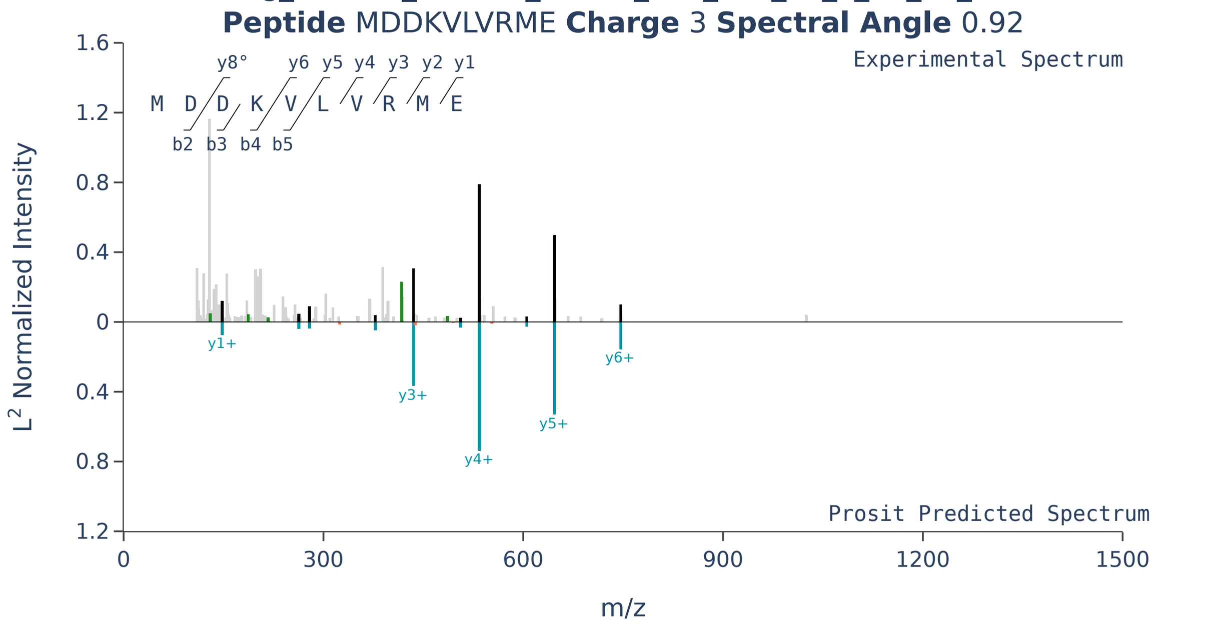

**Source** Ncheng_210623_230623_HFGoe_FFH_20S_25_1_A1_2h_R2 **Scan** 24989
**Peptide** GEENQTLNLAAQPPAV **Charge** 2 **Spectral Angle** 0.96

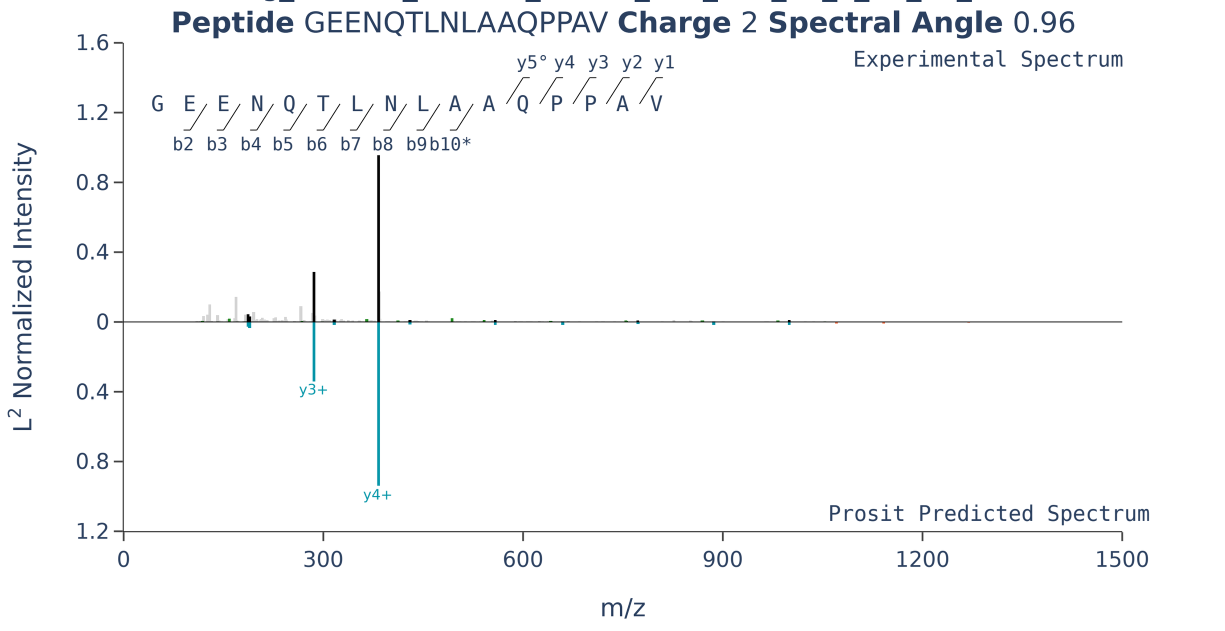

**Source** Ncheng_210623_230623_HFGoe_FFH_20S_25_1_A2_24h_R2 **Scan** 20990
**Peptide** ADVALPVVR **Charge** 2 **Spectral Angle** 0.84

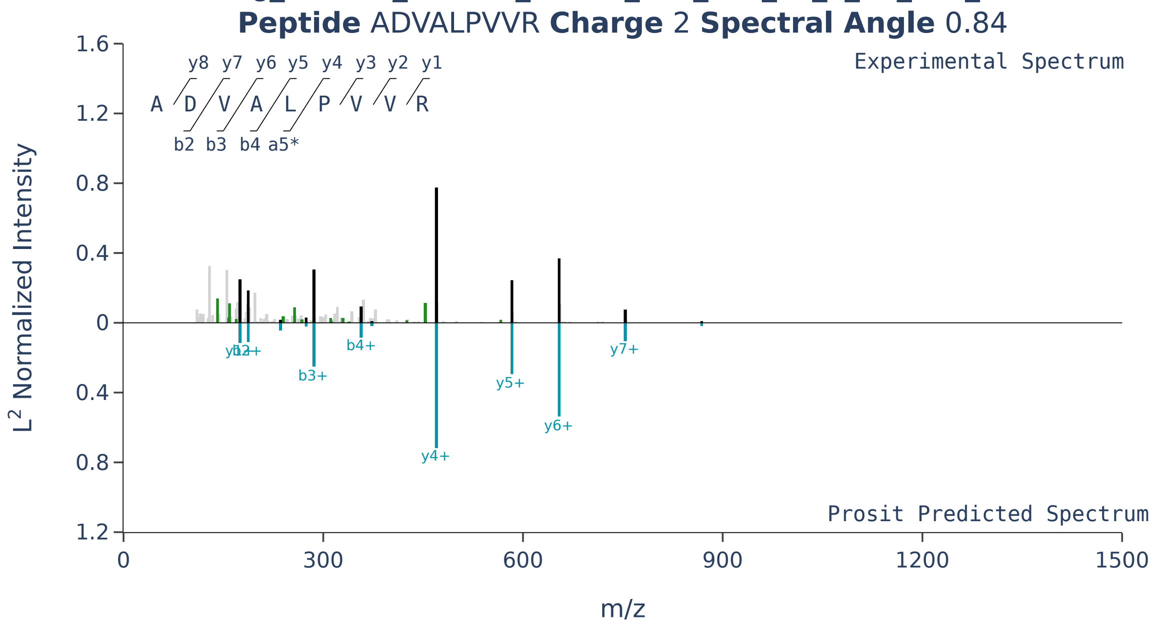


**Source** Ncheng_210623_230623_HFGoe_FFH_20S_25_1_A2_24h_R1 **Scan** 6859
**Peptide** GRLHVDE **Charge** 2 **Spectral Angle** 0.91

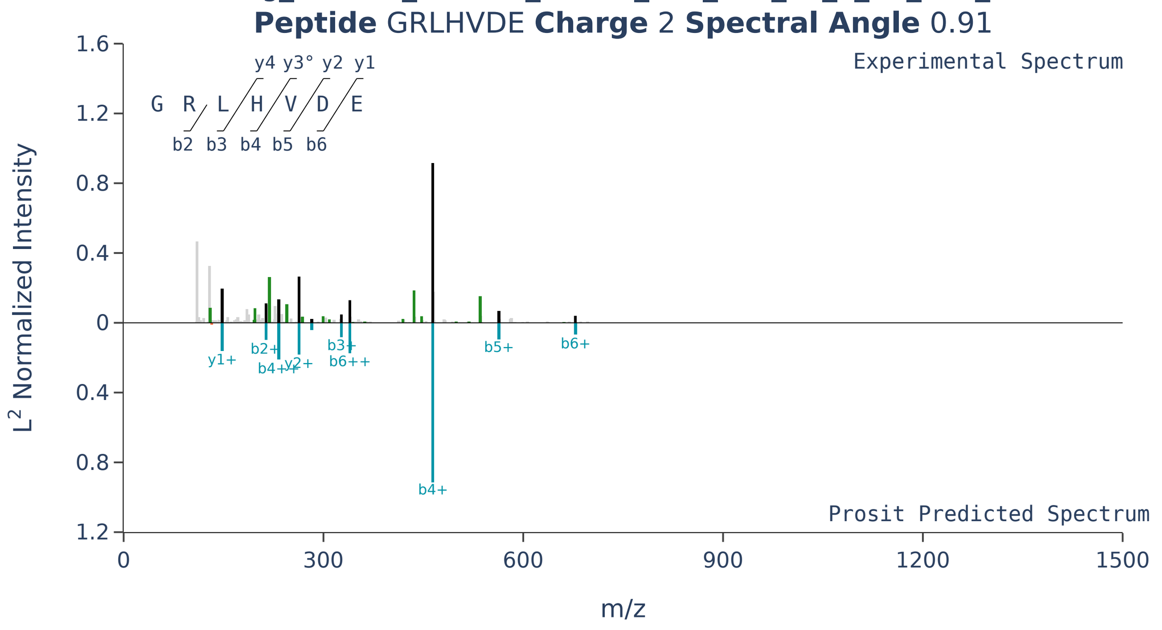


**Source** Ncheng_210623_230623_HFGoe_FFH_20S_25_1_A2_1h_R1 **Scan** 13243
**Peptide** KTEALEPFH **Charge** 3 **Spectral Angle** 0.93

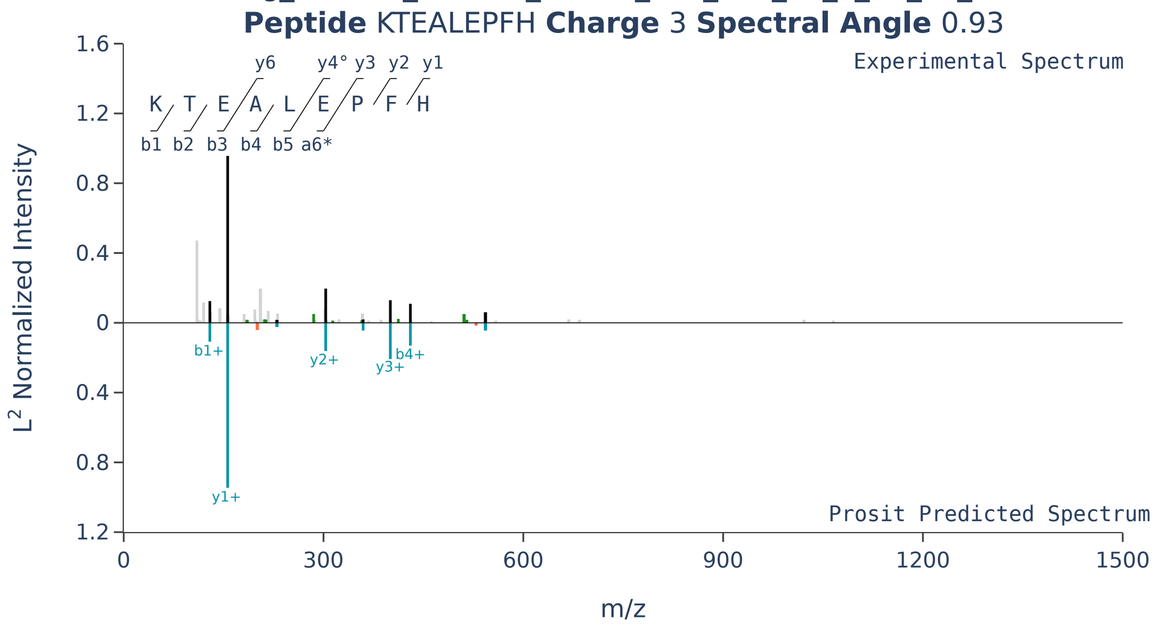


**Source** Ncheng_210623_230623_HFGoe_FFH_20S_25_1_A2_4h_R2 **Scan** 37798
**Peptide** GMGDVLSLIE **Charge** 1 **Spectral Angle** 0.87

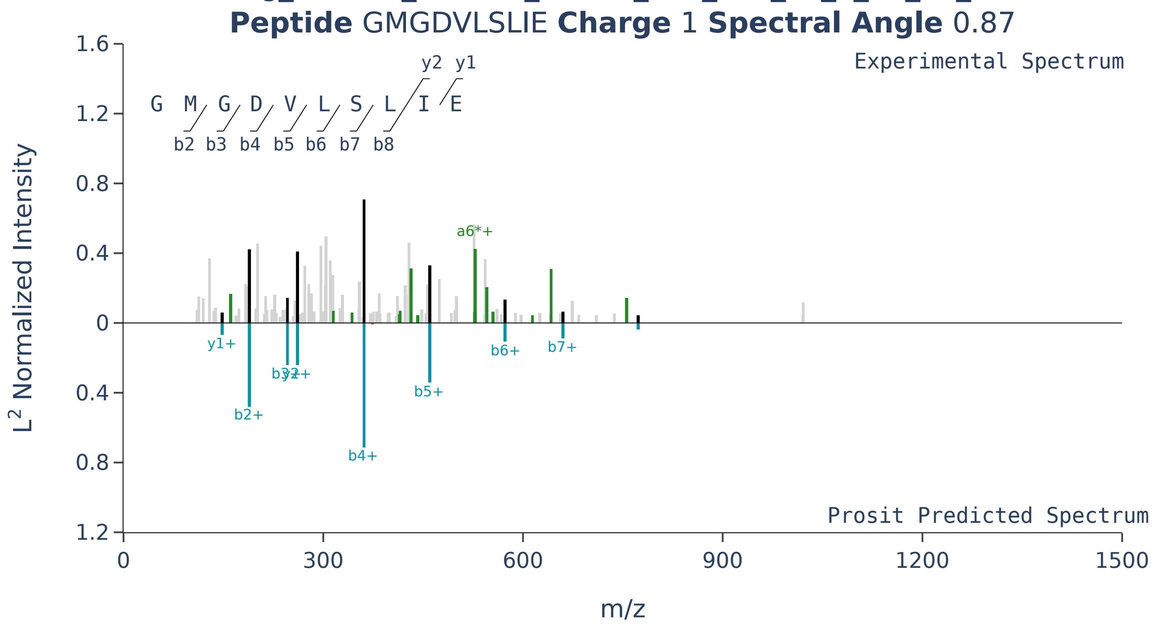


**Source** Ncheng_210623_230623_HFGoe_FFH_20S_25_1_A1_2h_R2 **Scan** 23516
**Peptide** AAQPPAVVL **Charge** 1 **Spectral Angle** 0.95

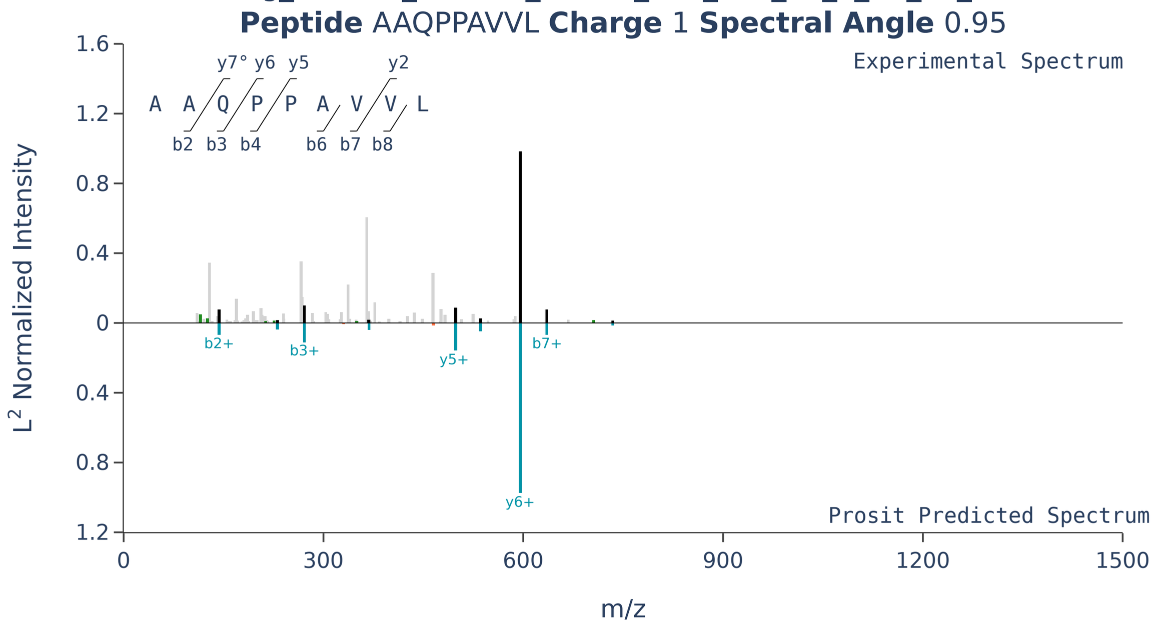


**Source** Ncheng_210623_230623_HFGoe_FFH_20S_25_1_A1_4h_R2 **Scan** 6687
**Peptide** DRAQAEKL **Charge** 2 **Spectral Angle** 0.91

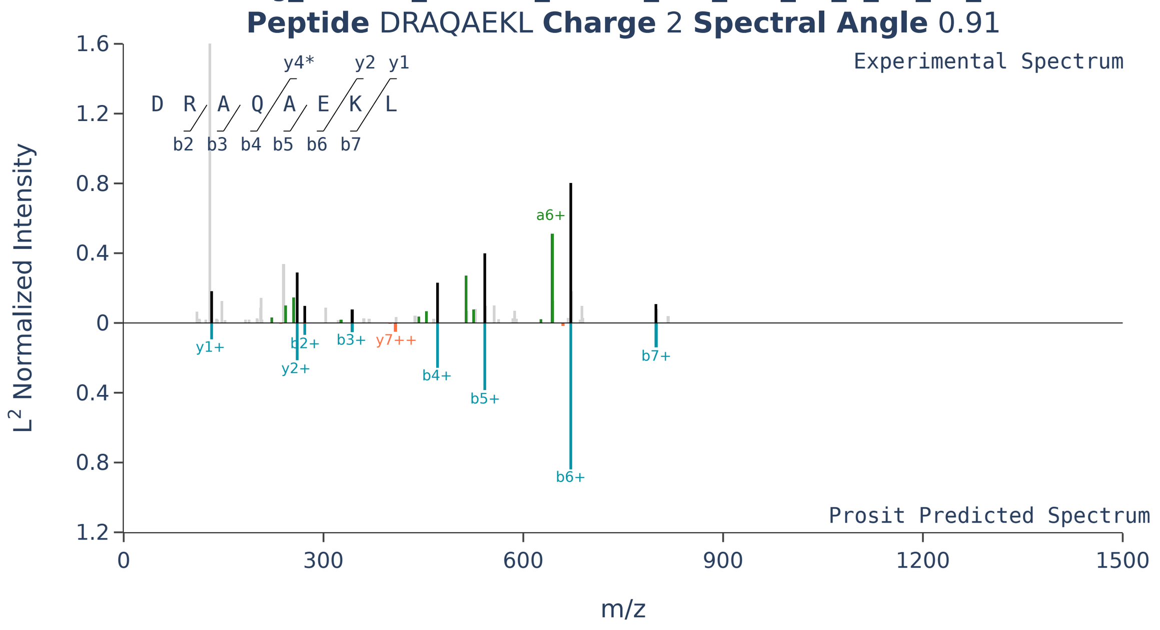


**Source** Ncheng_210623_230623_HFGoe_FFH_20S_25_1_A1_24h_R1 **Scan** 13378
**Peptide** AMTGQDAANTAKAFN **Charge** 1 **Spectral Angle** 0.89

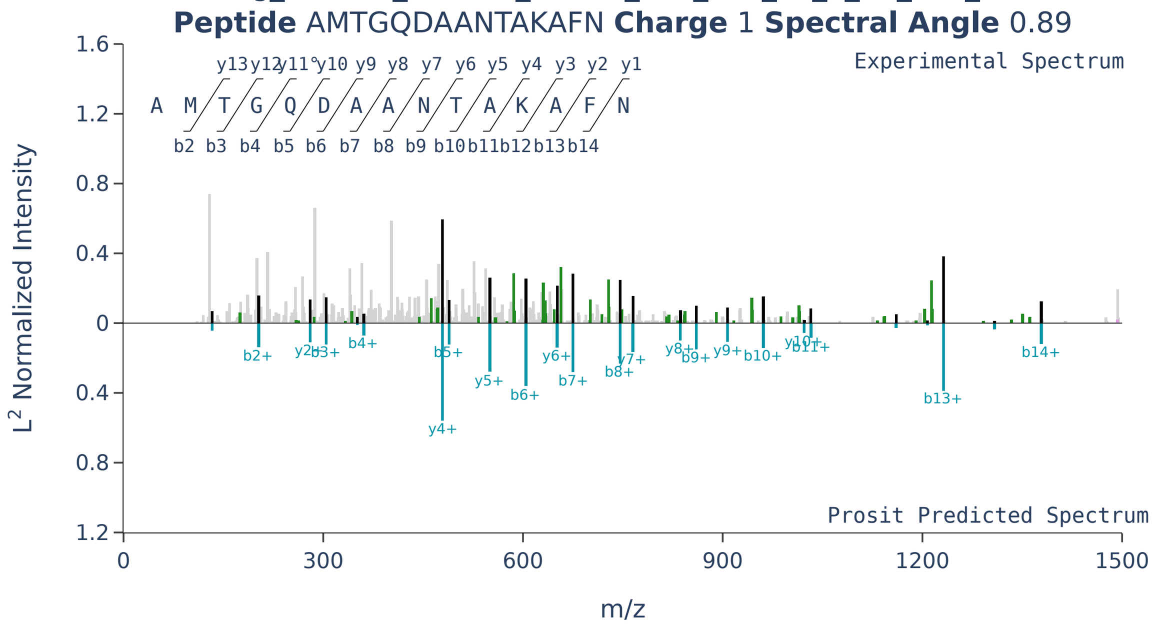


**Source** Ncheng_210623_230623_HFGoe_FFH_20S_25_1_A1_2h_R1 **Scan** 18595
**Peptide** GEKTEALEPFHPDRI **Charge** 2 **Spectral Angle** 0.88

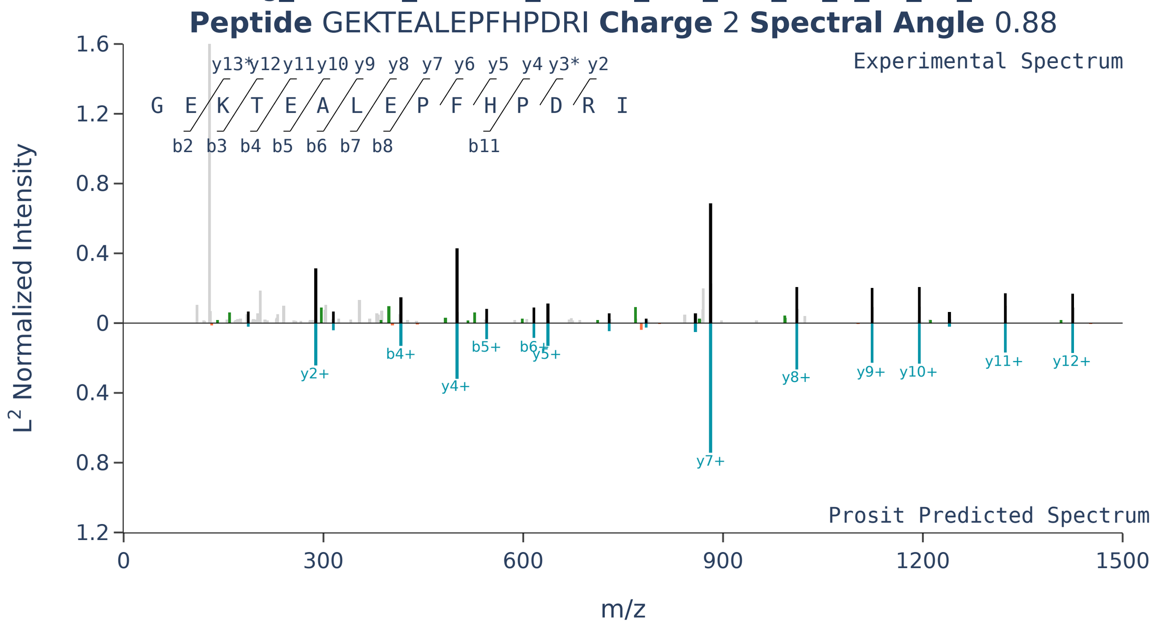


**Source** Ncheng_210623_230623_HFGoe_FFH_20S_25_1_A1_4h_R1 **Scan** 31924
**Peptide** LAEQVGVDFFPSD **Charge** 2 **Spectral Angle** 0.93

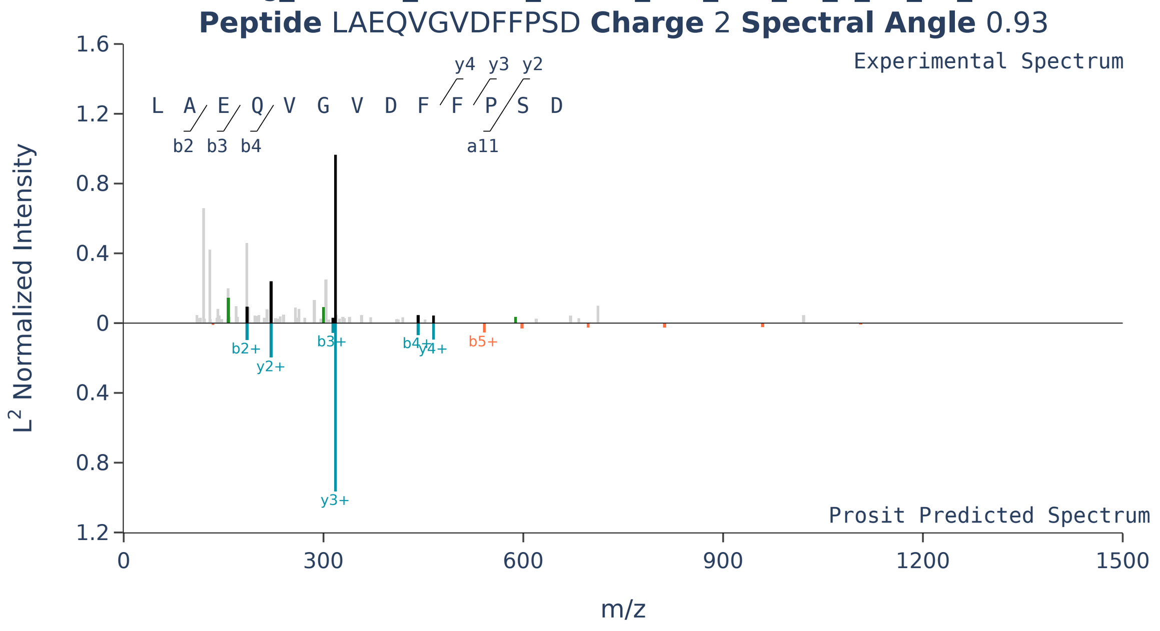


**Source** Ncheng_210623_230623_HFGoe_FFH_20S_25_1_A1_24h_R2 **Scan** 15016
**Peptide** DDKVLVRME **Charge** 3 **Spectral Angle** 0.91

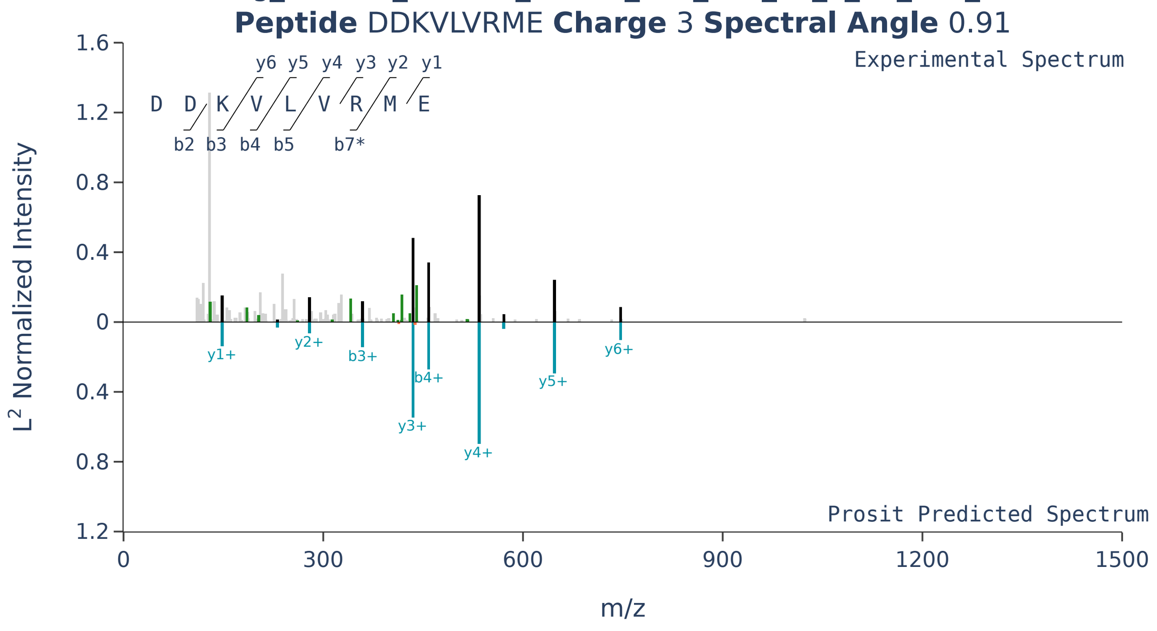


**Source** Ncheng_210623_230623_HFGoe_FFH_20S_25_1_A2_24h_R1 **Scan** 18435
**Peptide** EENQTLNL **Charge** 2 **Spectral Angle** 0.88

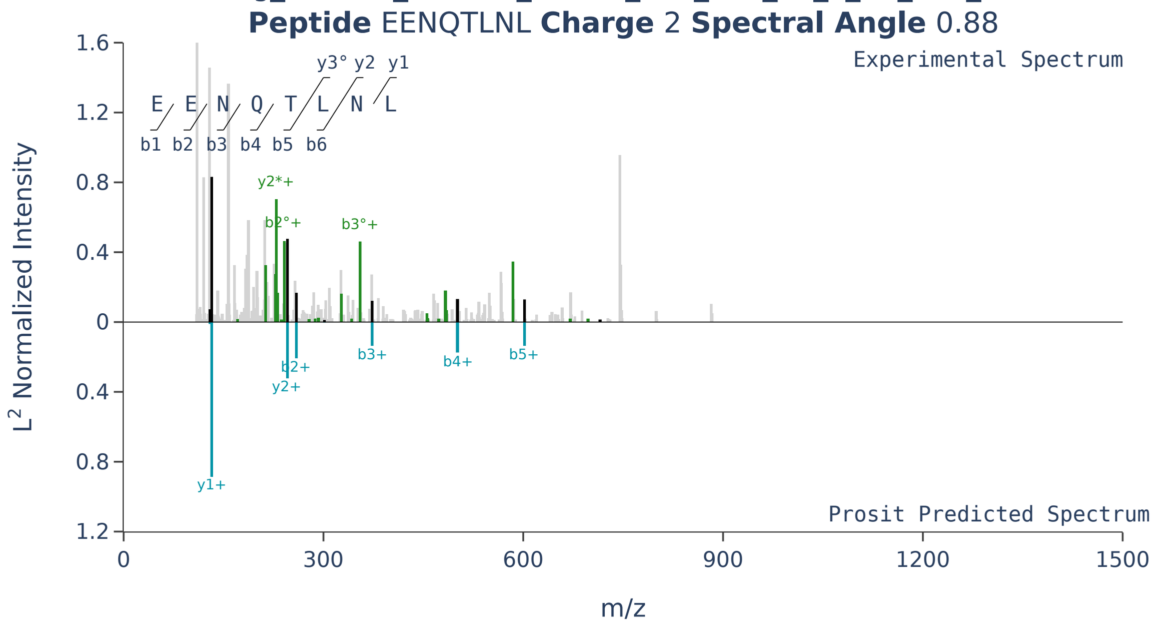


**Source** Ncheng_210623_230623_HFGoe_FFH_20S_25_1_A1_2h_R1 **Scan** 29283
**Peptide** ADVALPVVREF **Charge** 2 **Spectral Angle** 0.87

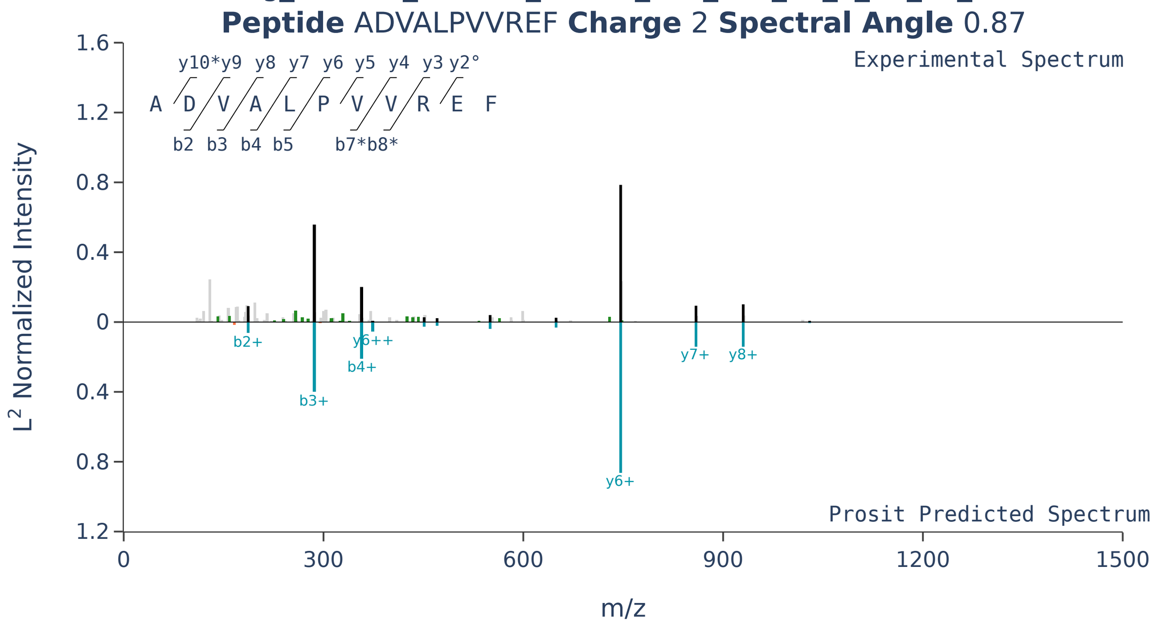


**Source** Ncheng_210623_230623_HFGoe_FFH_20S_25_1_A1_1h_R2 **Scan** 30878
**Peptide** TLFVVDAMTGQD **Charge** 2 **Spectral Angle** 0.92

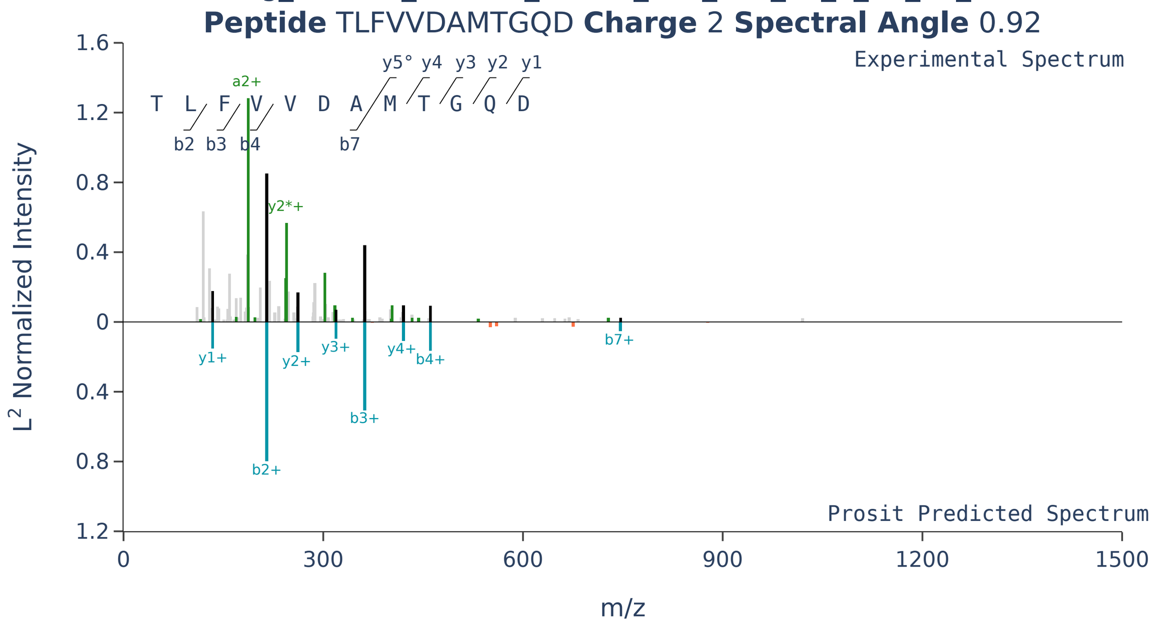


**Source** Ncheng_210623_230623_HFGoe_FFH_20S_25_1_A2_2h_R1 **Scan** 25069
**Peptide** MGKLPGMGQIPDNV **Charge** 2 **Spectral Angle** 0.9

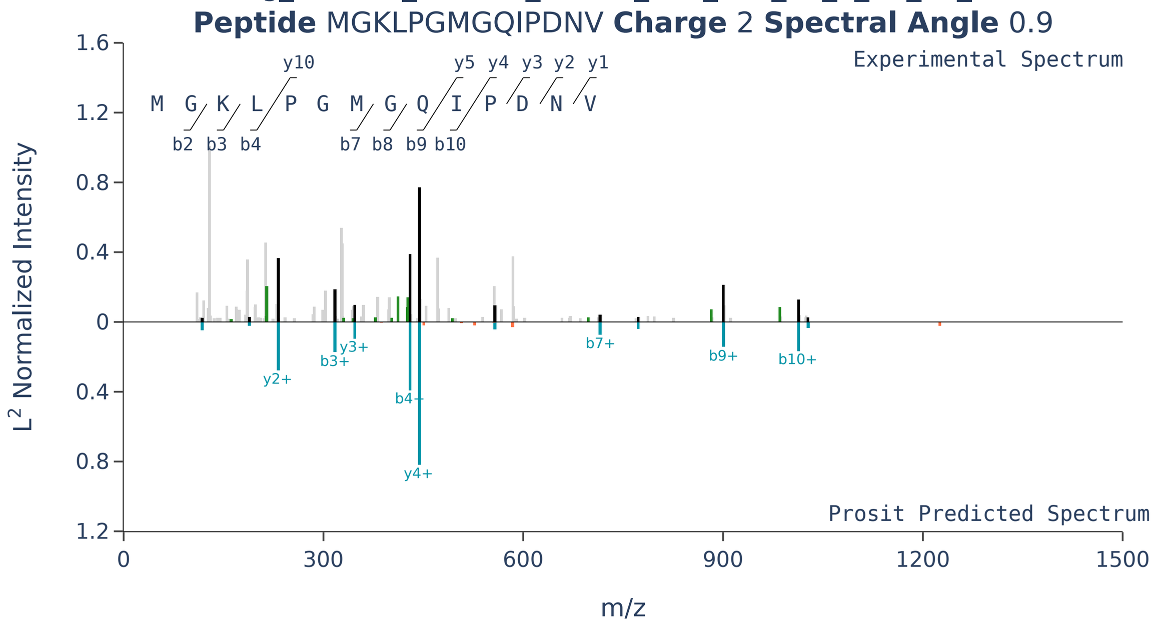


**Source** Ncheng_210623_230623_HFGoe_FFH_20S_25_1_A2_24h_R1 **Scan** 7053
**Peptide** AANTAKAFN **Charge** 1 **Spectral Angle** 0.9

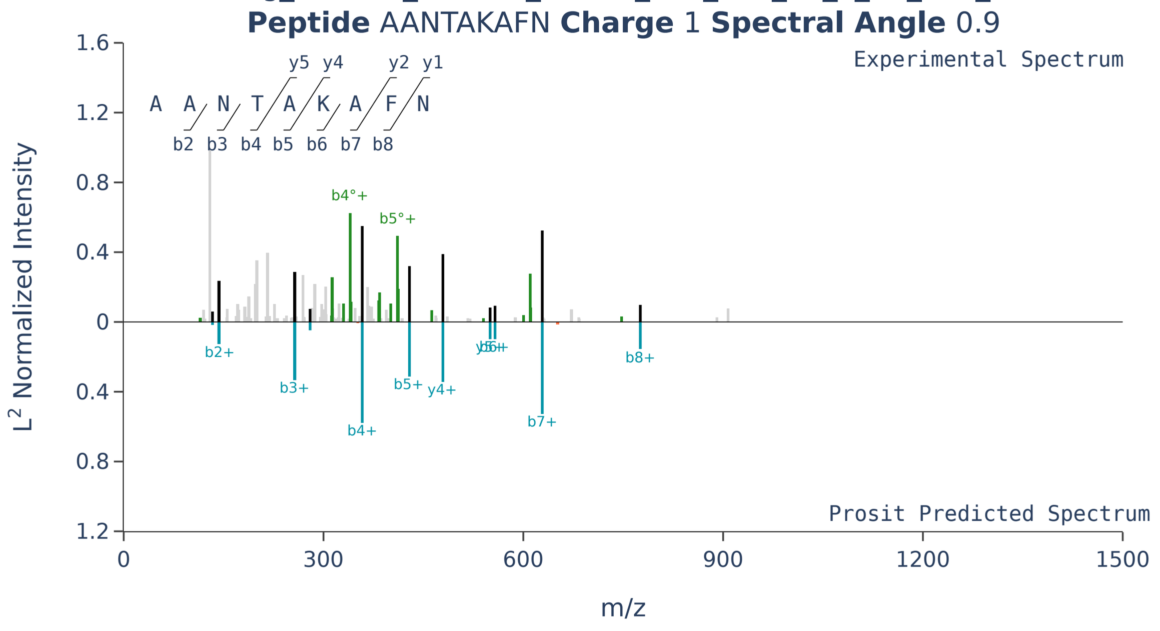

**Source** Ncheng_210623_230623_HFGoe_FFH_20S_25_1_A1_24h_R2 **Scan** 19147
**Peptide** LTEDNVKDTLREV **Charge** 2 **Spectral Angle** 0.85

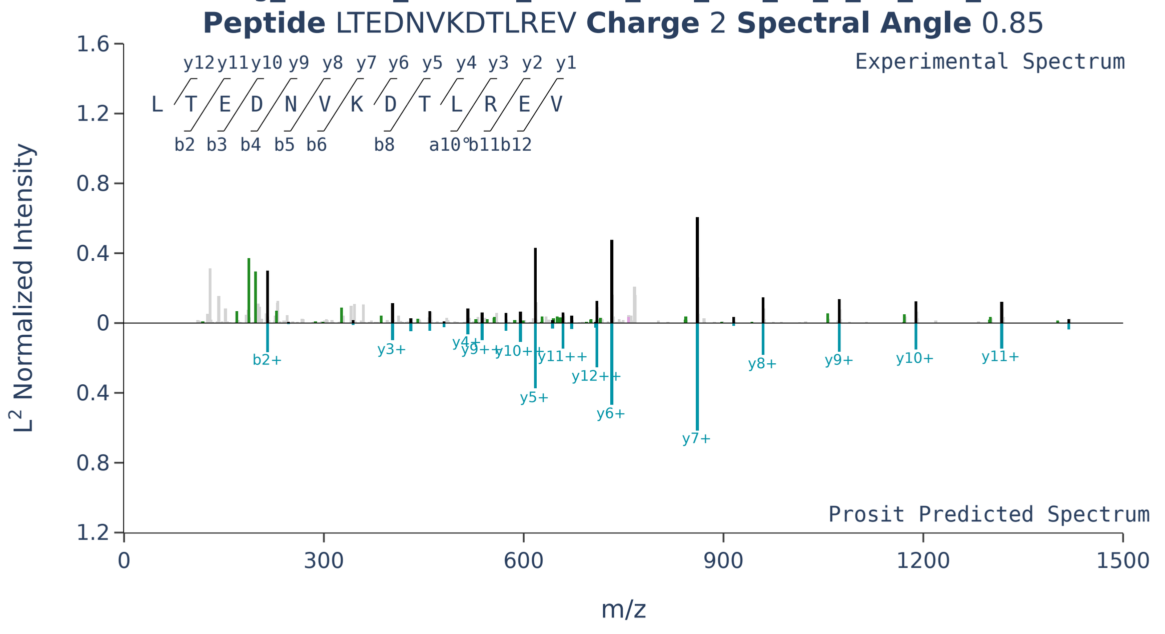


**Source** Ncheng_210623_230623_HFGoe_FFH_20S_25_1_A2_1h_R1 **Scan** 10146
**Peptide** KVLVRME **Charge** 2 **Spectral Angle** 0.83

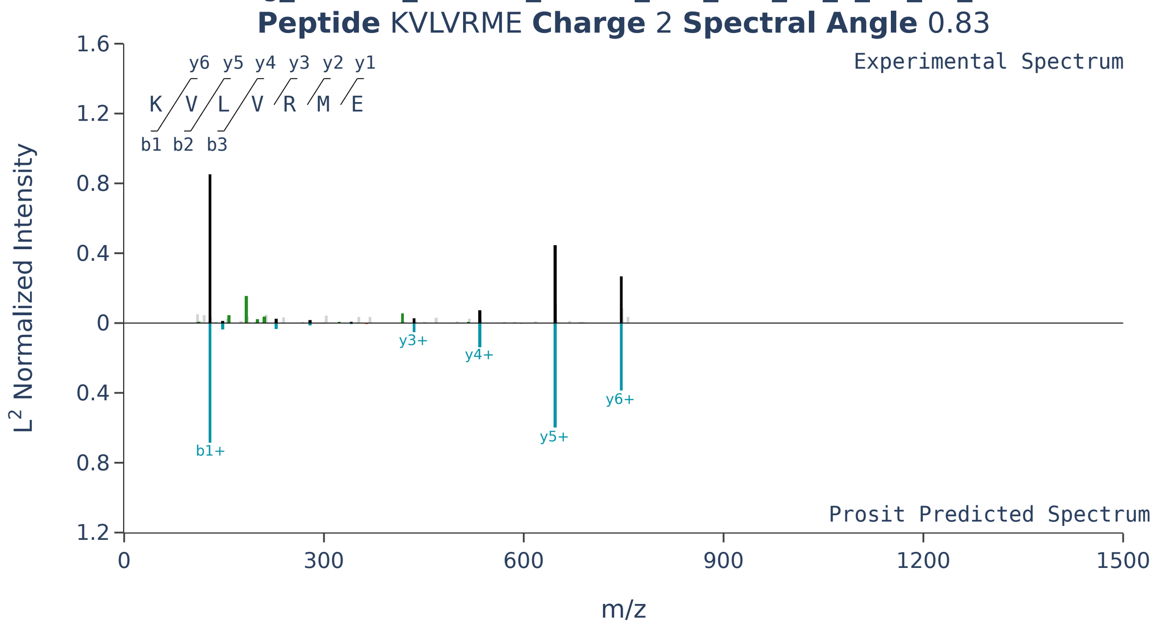


**Source** Ncheng_210623_230623_HFGoe_FFH_20S_25_1_A2_24h_R2 **Scan** 31559
**Peptide** AEQVGVDFF **Charge** 2 **Spectral Angle** 0.95

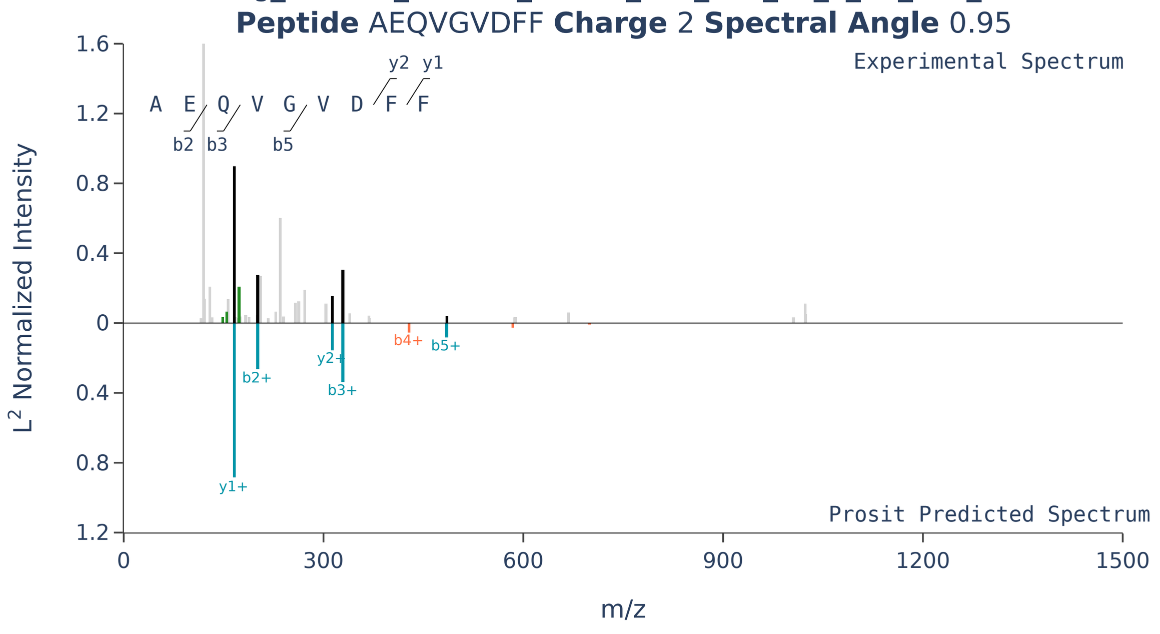


**Source** Ncheng_210623_230623_HFGoe_FFH_20S_25_1_A1_24h_R1 **Scan** 10104
**Peptide** MDEIKQVHA **Charge** 1 **Spectral Angle** 0.9

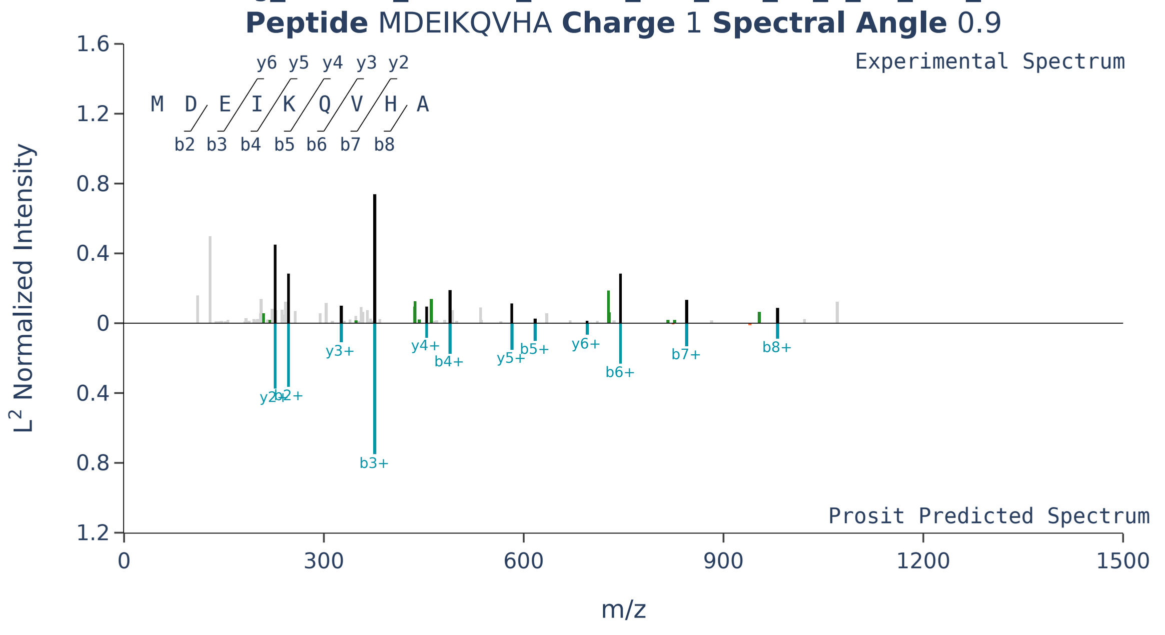


**Source** Ncheng_210623_230623_HFGoe_FFH_20S_25_1_A2_24h_R1 **Scan** 20703
**Peptide** RMEAIINSMT **Charge** 2 **Spectral Angle** 0.89

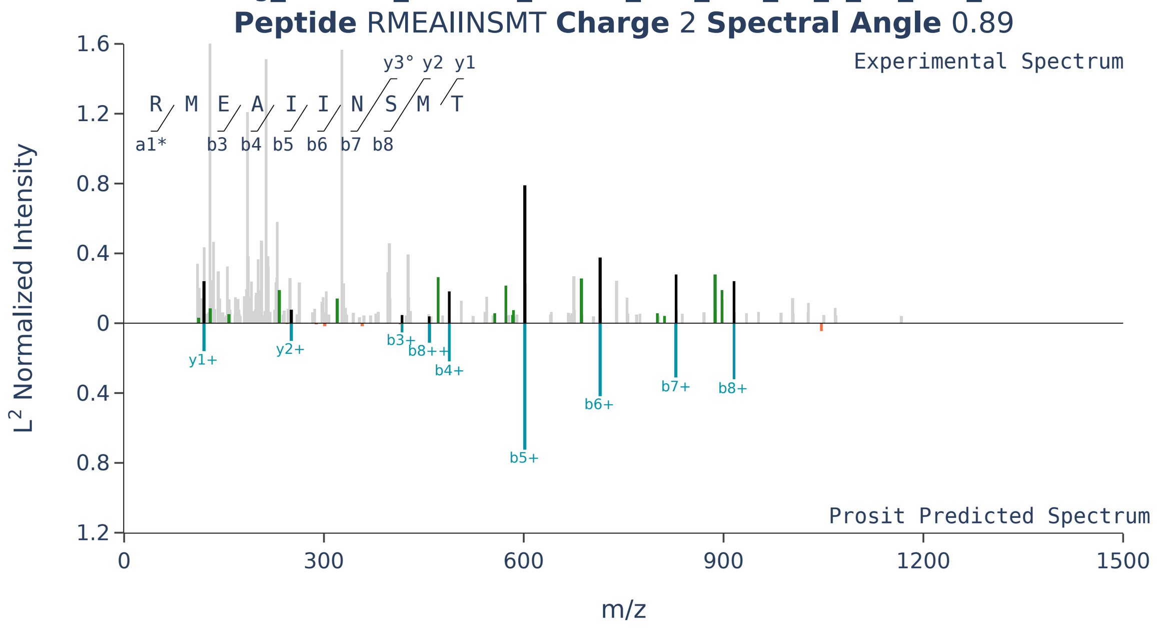


**Source** Ncheng_210623_230623_HFGoe_FFH_20S_25_1_A1_4h_R1 **Scan** 28155
**Peptide** YDVLLVD **Charge** 1 **Spectral Angle** 0.91

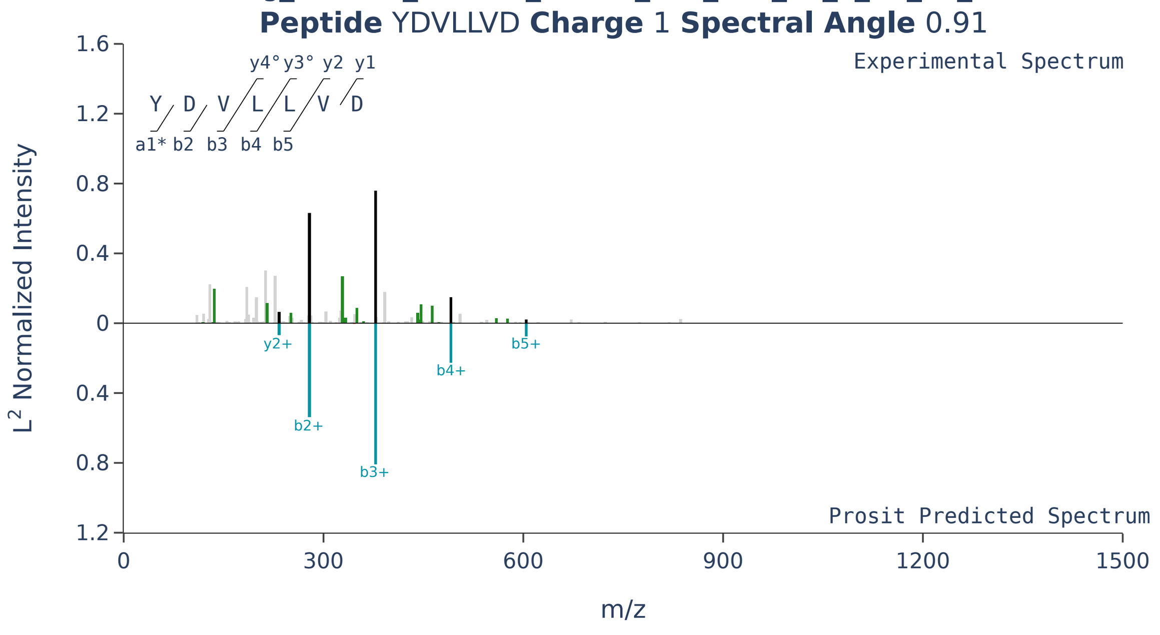


**Source** Ncheng_210623_230623_HFGoe_FFH_20S_25_1_A1_4h_R1 **Scan** 14641
**Peptide** AMTGQDAANTAKAF **Charge** 1 **Spectral Angle** 0.85

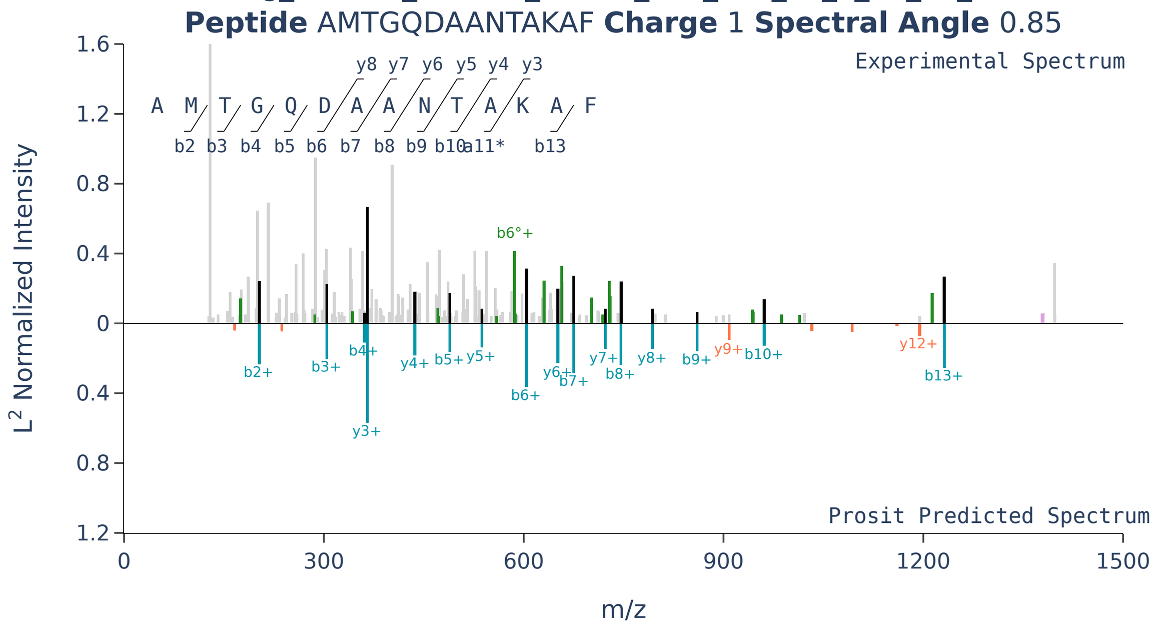


**Source** Ncheng_210623_230623_HFGoe_FFH_20S_25_1_A2_24h_R1 **Scan** 20936
**Peptide** PGMGQIPDNV **Charge** 1 **Spectral Angle** 0.9

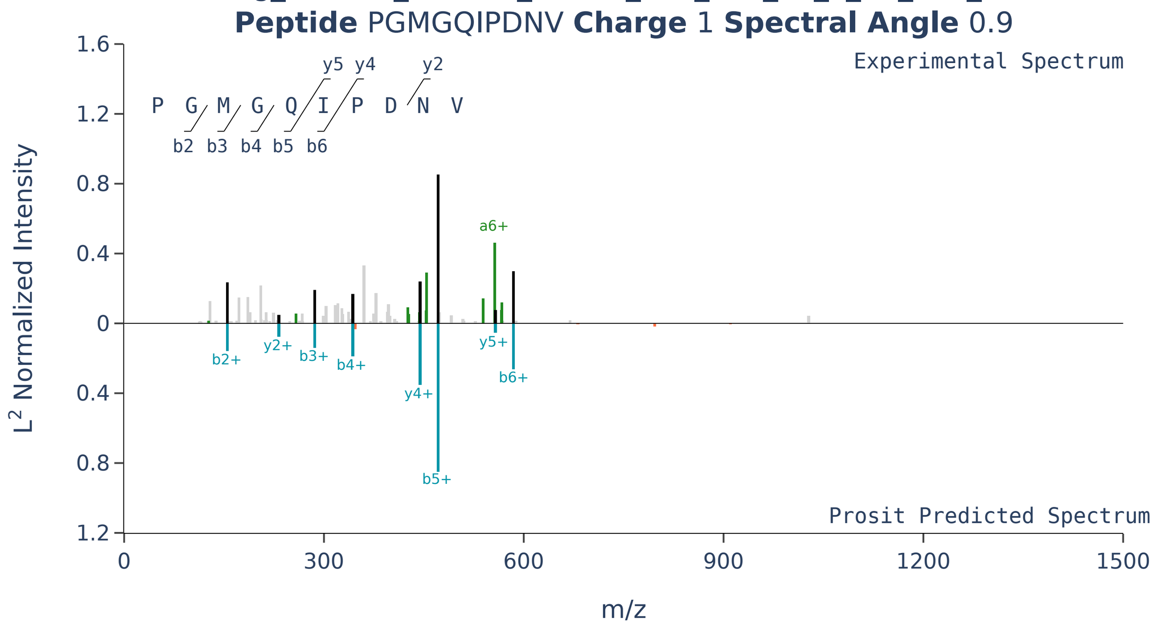


**Source** Ncheng_210623_230623_HFGoe_FFH_20S_25_1_A2_4h_R2 **Scan** 13275
**Peptide** ASINPVET **Charge** 1 **Spectral Angle** 0.88

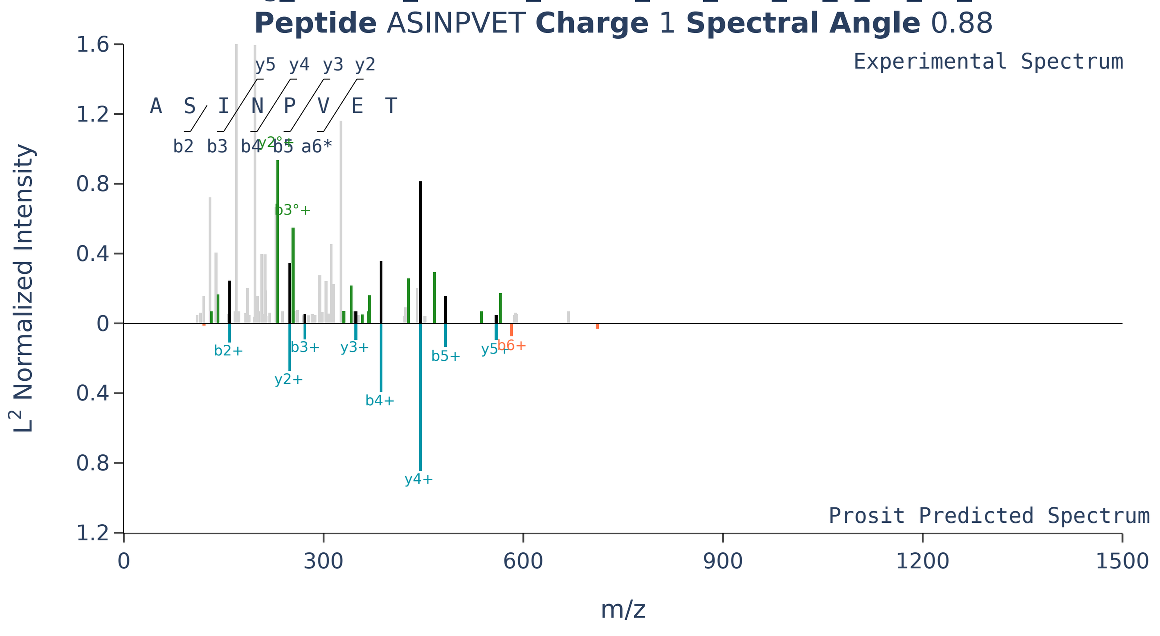


**Source** Ncheng_210623_230623_HFGoe_FFH_20S_25_1_A2_24h_R2 **Scan** 17156
**Peptide** ELVAAMGEENQ **Charge** 2 **Spectral Angle** 0.92

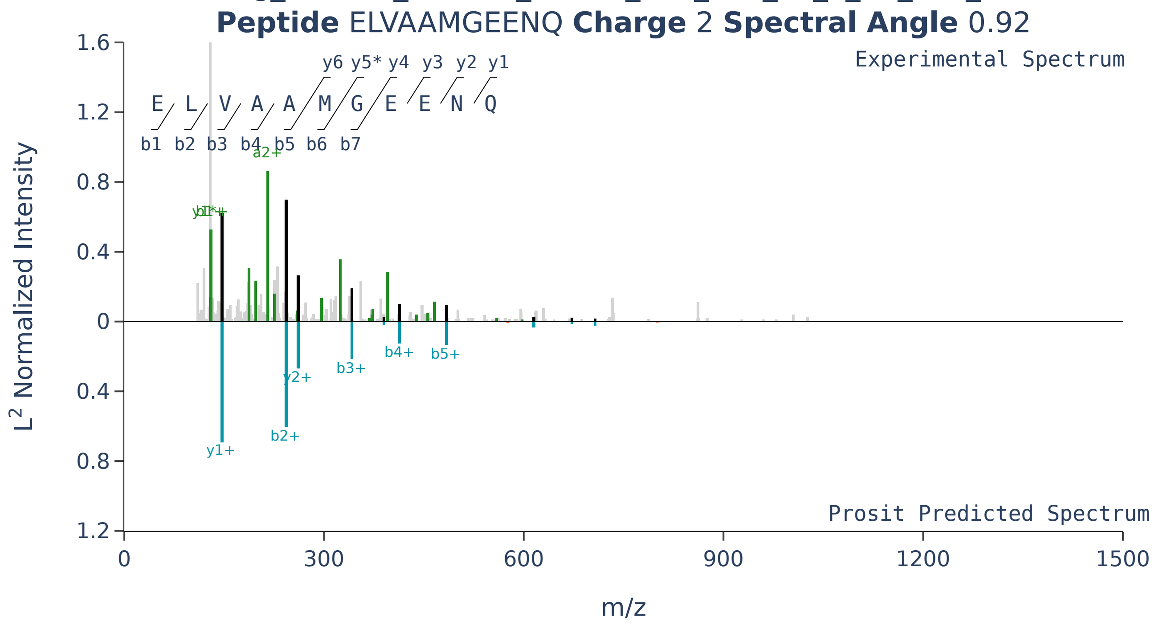


**Source** Ncheng_210623_230623_HFGoe_FFH_20S_25_1_A1_2h_R1 **Scan** 10499
**Peptide** TLAEQVG **Charge** 1 **Spectral Angle** 0.9

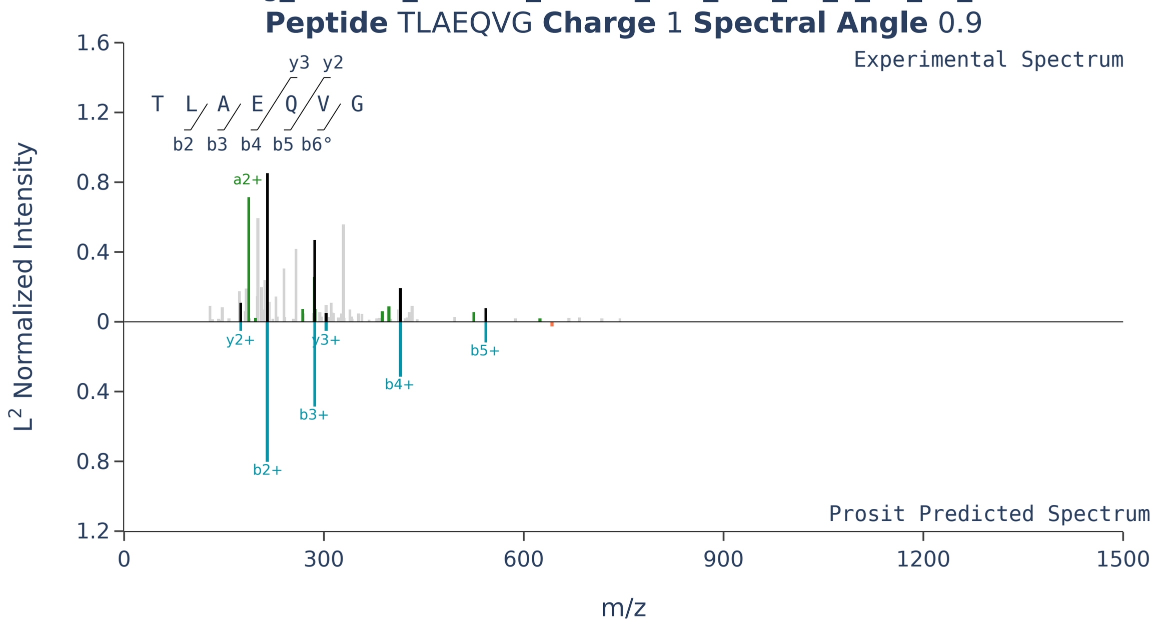


**Source** Ncheng_210623_230623_HFGoe_FFH_20S_25_1_A1_2h_R1 **Scan** 7875
**Peptide** ESKVDRA **Charge** 2 **Spectral Angle** 0.93

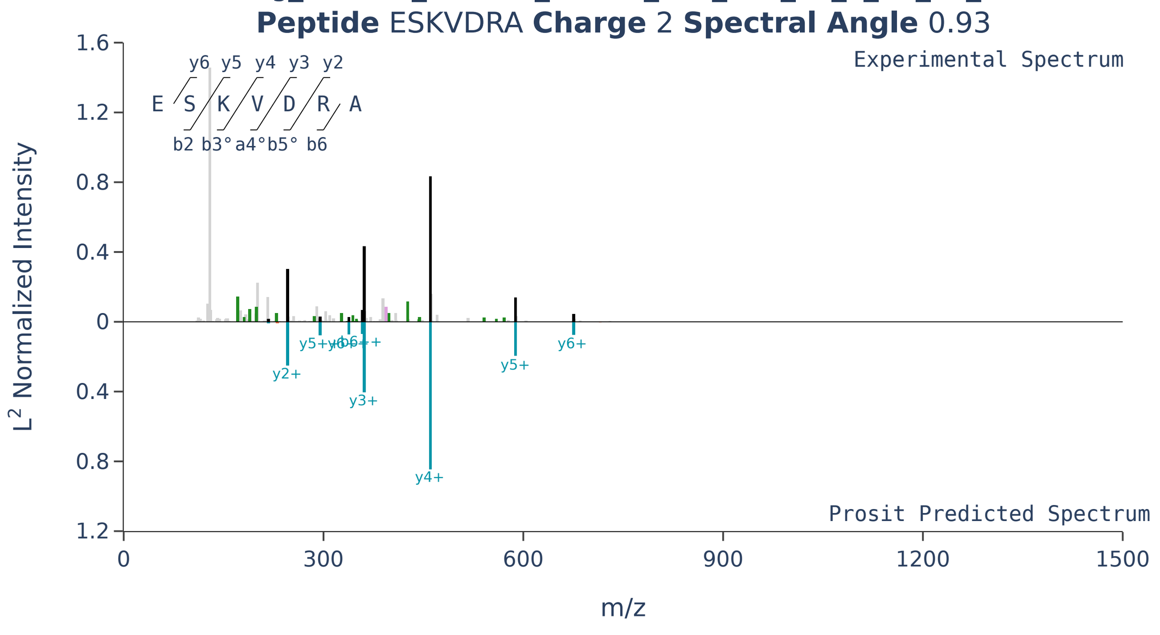


**Source** Ncheng_210623_230623_HFGoe_FFH_20S_25_1_A2_24h_R2 **Scan** 9252
**Peptide** AVGHEVNKSLTPGQE **Charge** 2 **Spectral Angle** 0.92

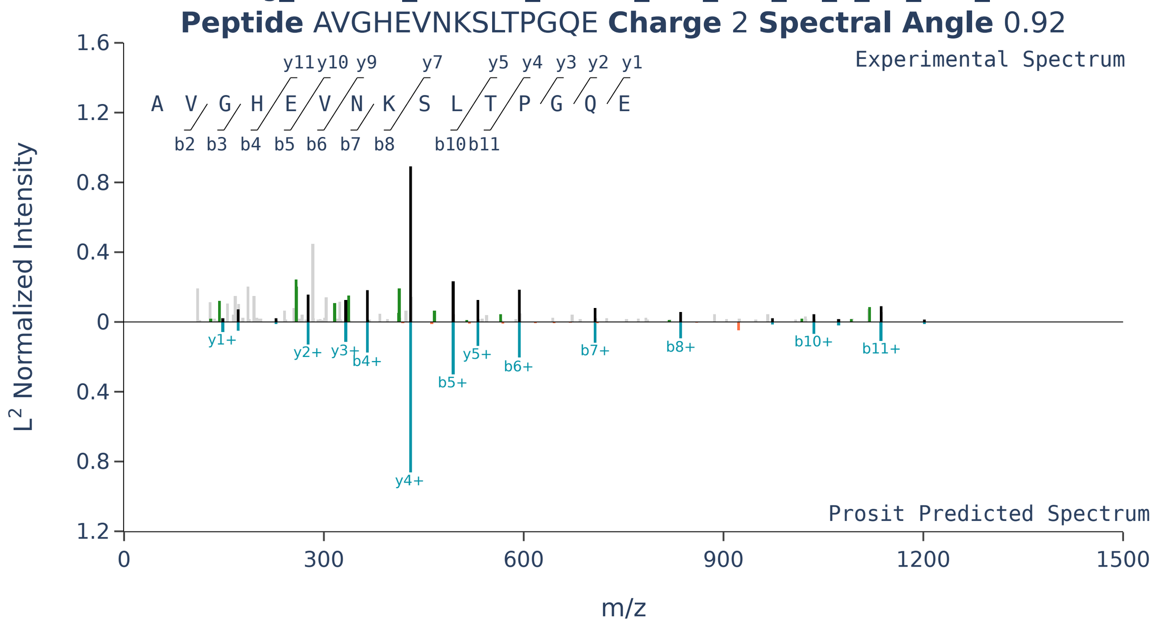


**Source** Ncheng_210623_230623_HFGoe_FFH_20S_25_1_A2_24h_R2 **Scan** 21450
**Peptide** ADVALPVVRE **Charge** 1 **Spectral Angle** 0.92

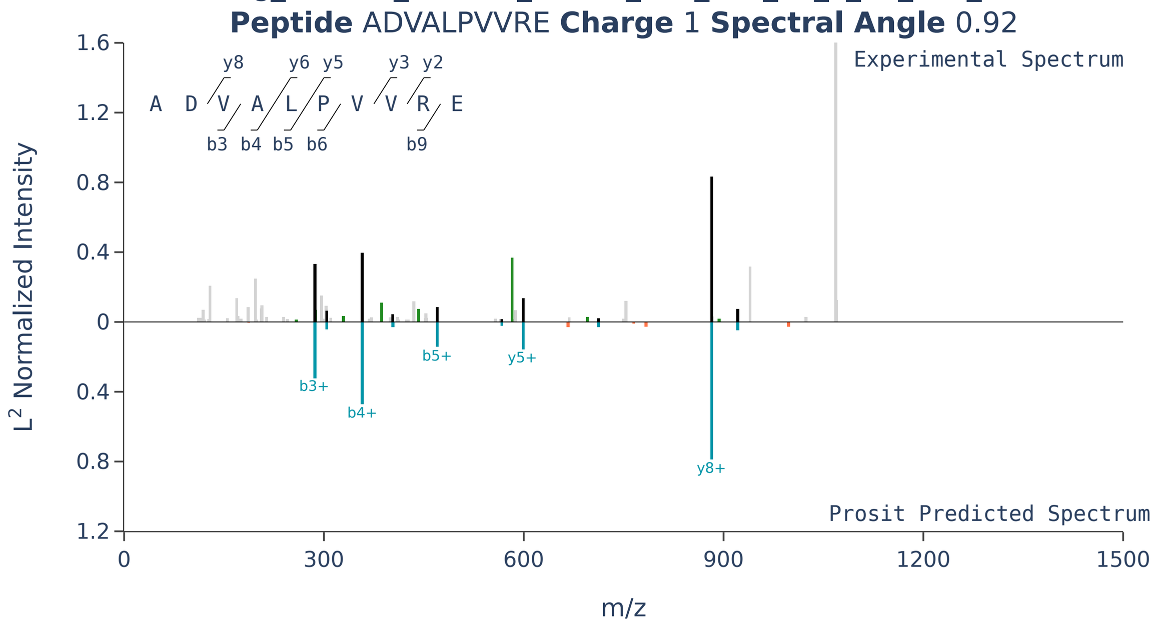


**Source** Ncheng_210623_230623_HFGoe_FFH_20S_25_1_A2_4h_R2 **Scan** 13940
**Peptide** ARGGAALSIRHL **Charge** 3 **Spectral Angle** 0.9

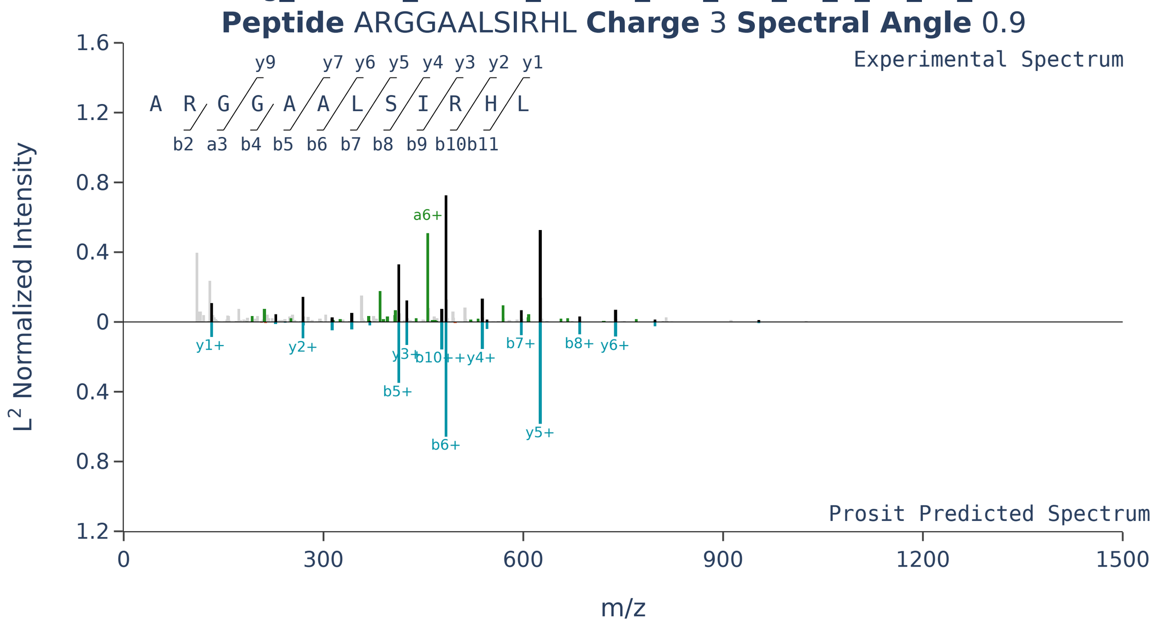

**Source** Ncheng_210623_230623_HFGoe_FFH_20S_25_1_A2_1h_R2 **Scan** 21432
**Peptide** FLEQLRQM **Charge** 2 **Spectral Angle** 0.94

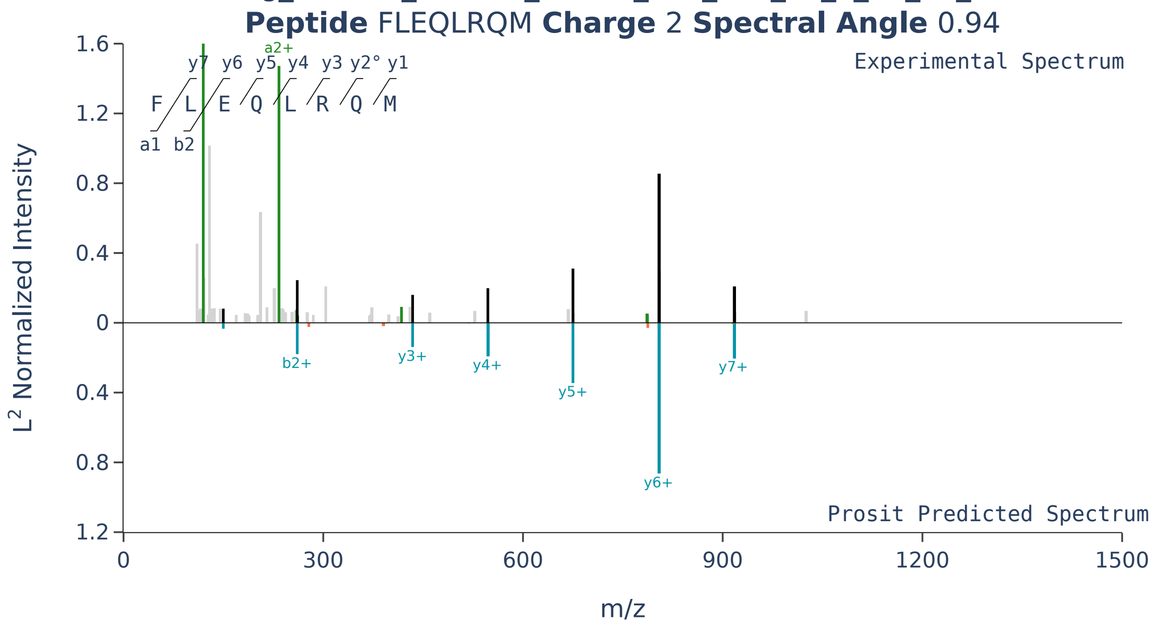


**Source** Ncheng_210623_230623_HFGoe_FFH_20S_25_1_A1_4h_R2 **Scan** 25543
**Peptide** FNEALPLTG **Charge** 1 **Spectral Angle** 0.88

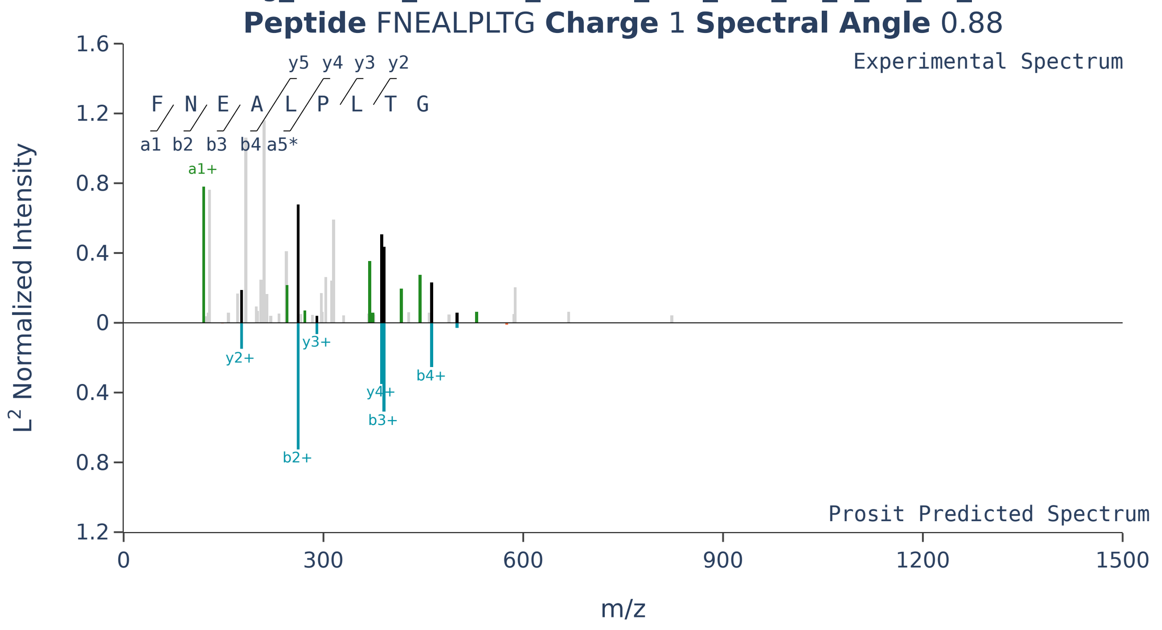


**Source** Ncheng_210623_230623_HFGoe_FFH_20S_25_1_A1_24h_R1 **Scan** 19809
**Peptide** EALPLTG **Charge** 1 **Spectral Angle** 0.85

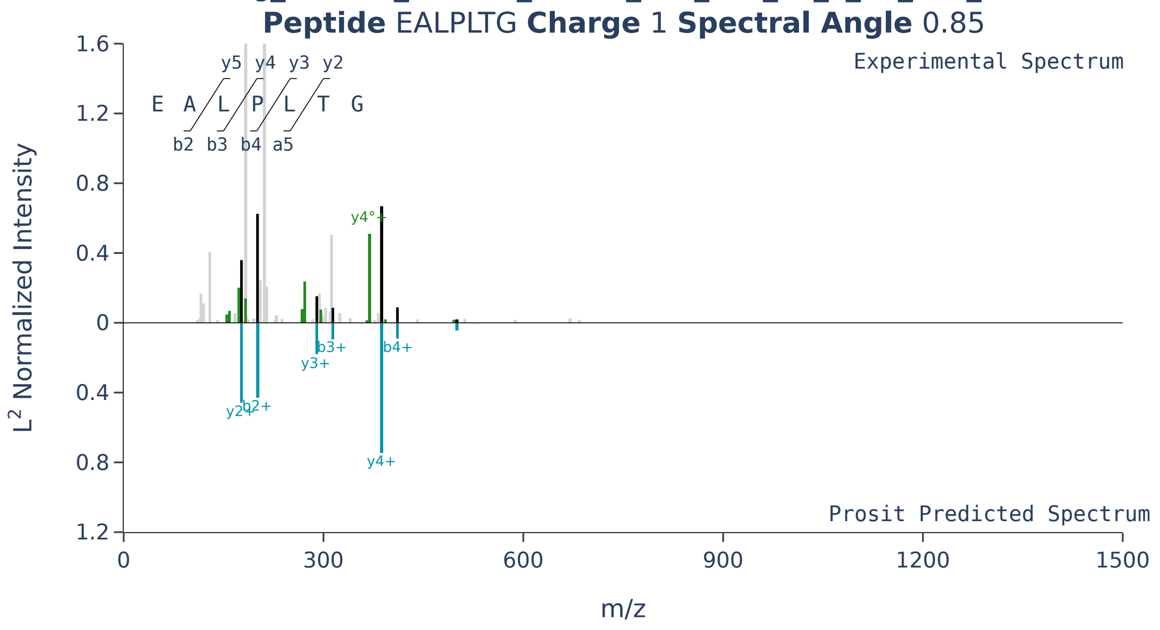


**Source** Ncheng_210623_230623_HFGoe_FFH_20S_25_1_A1_24h_R1 **Scan** 13768
**Peptide** DKVLVRM **Charge** 2 **Spectral Angle** 0.94

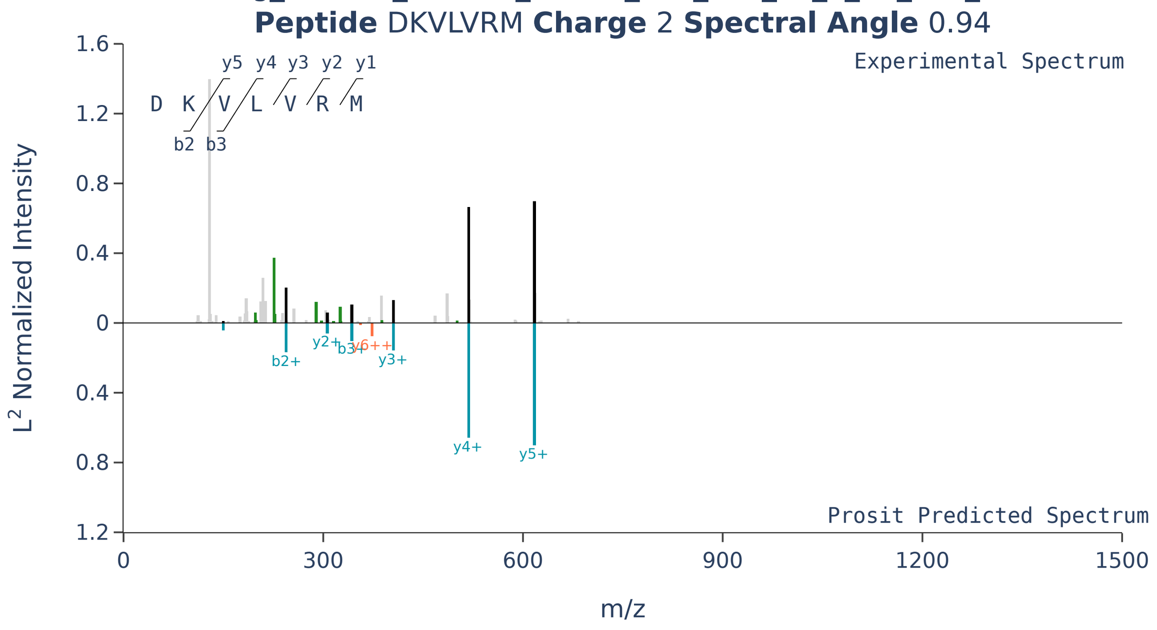


**Source** Ncheng_210623_230623_HFGoe_FFH_20S_25_1_A1_24h_R2 **Scan** 18584
**Peptide** LEPFHPDRL **Charge** 3 **Spectral Angle** 0.87

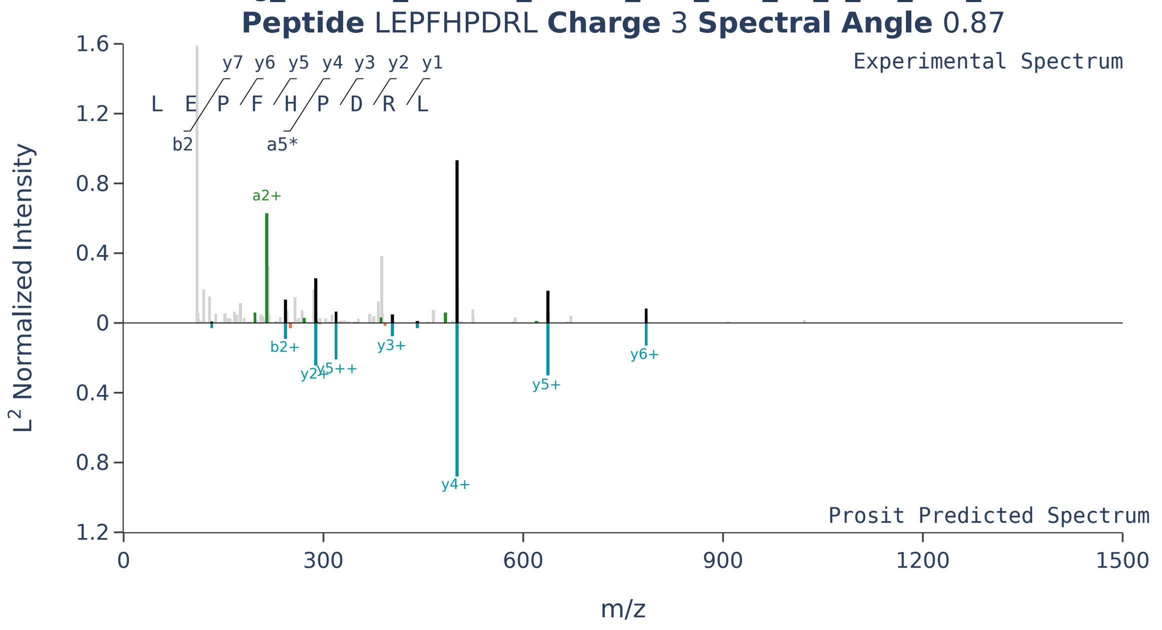


**Source** Ncheng_210623_230623_HFGoe_FFH_20S_25_1_A2_4h_R1 **Scan** 10917
**Peptide** QVQDVNRL **Charge** 1 **Spectral Angle** 0.89

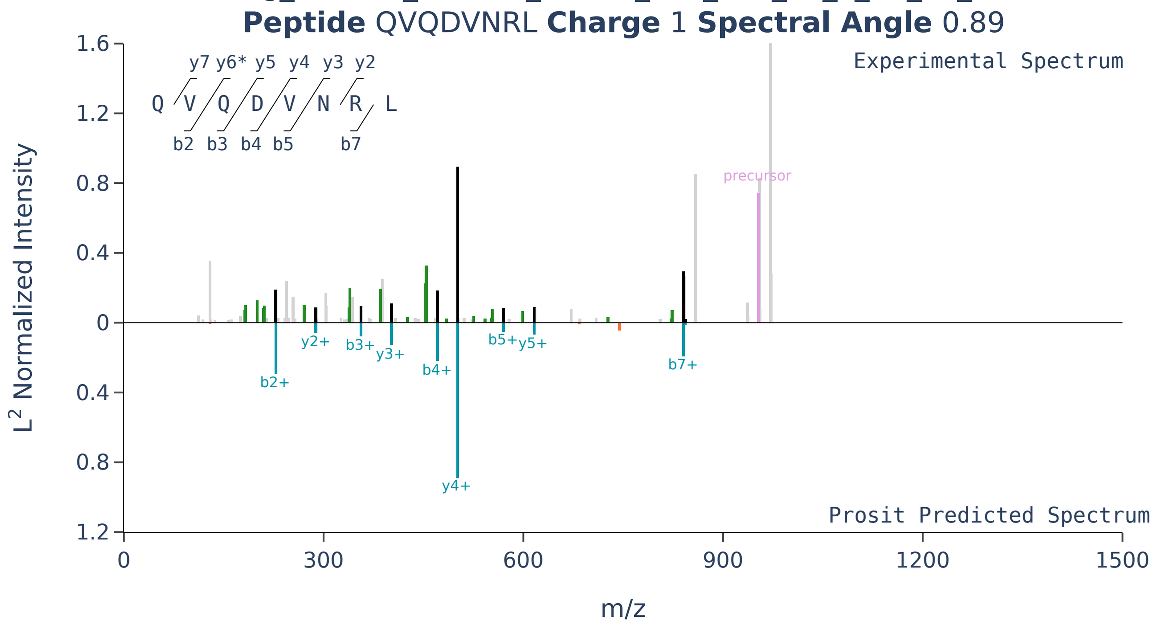


**Source** Ncheng_210623_230623_HFGoe_FFH_20S_25_1_A2_24h_R1 **Scan** 6373
**Peptide** GHEVNKSL **Charge** 1 **Spectral Angle** 0.9

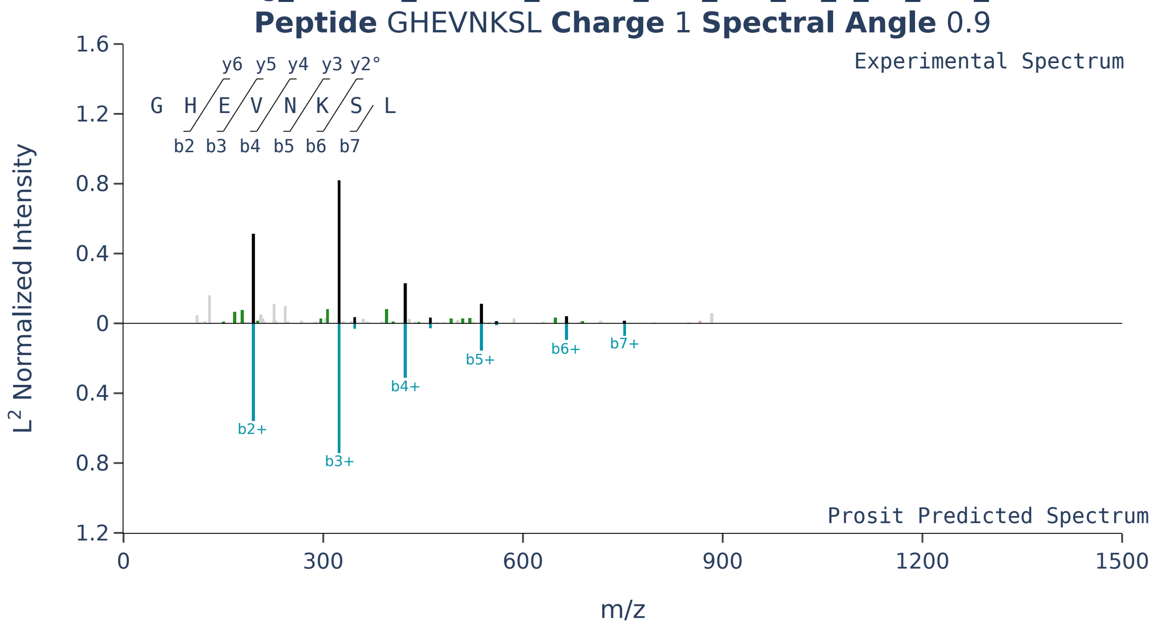


**Source** Ncheng_210623_230623_HFGoe_FFH_20S_25_1_A1_24h_R1 **Scan** 20046
**Peptide** ALEPFHPDRL **Charge** 3 **Spectral Angle** 0.86

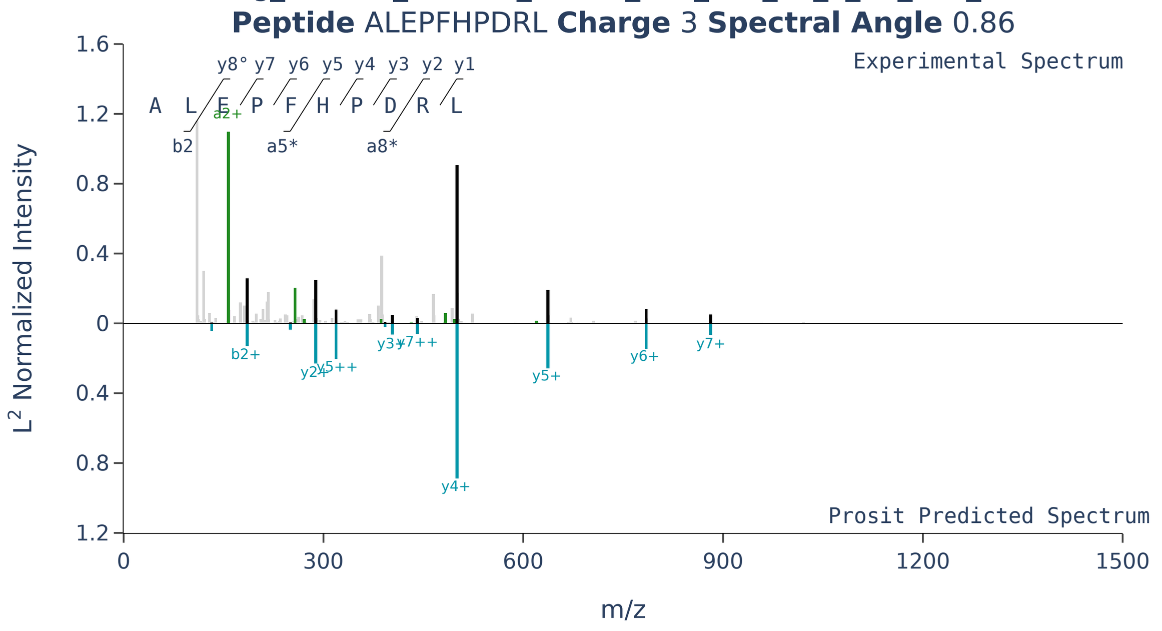


**Source** Ncheng_210623_230623_HFGoe_FFH_20S_25_1_A2_24h_R2 **Scan** 9737
**Peptide** AMGEENQTL **Charge** 1 **Spectral Angle** 0.89

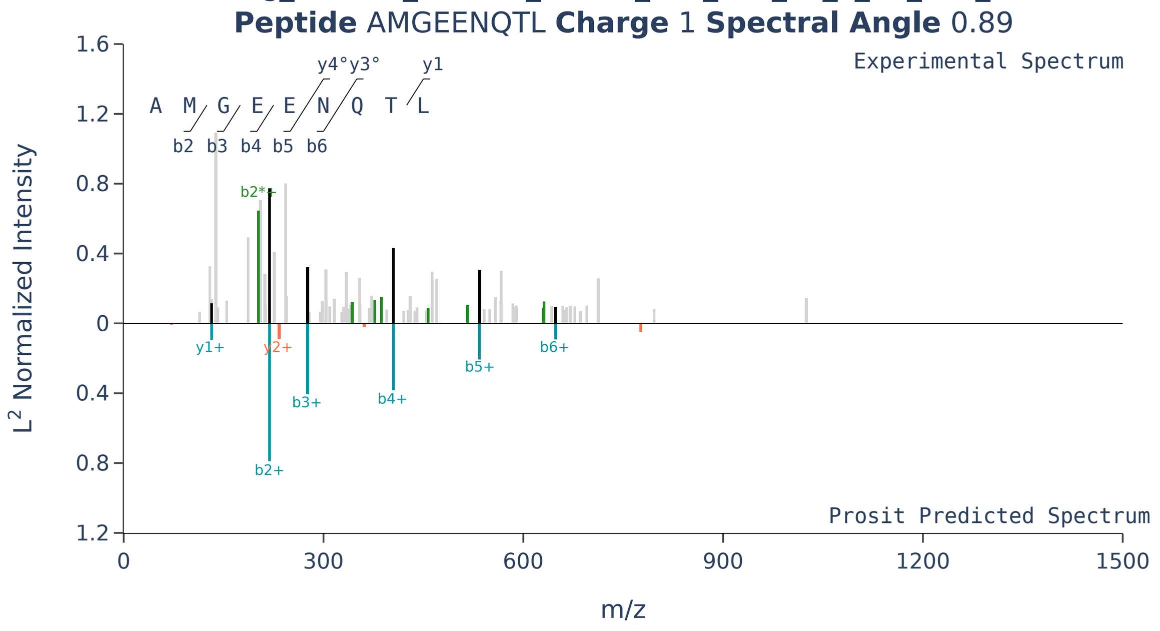


**Source** Ncheng_210623_230623_HFGoe_FFH_20S_25_1_A1_24h_R1 **Scan** 6833
**Peptide** KSLTPGQE **Charge** 1 **Spectral Angle** 0.92

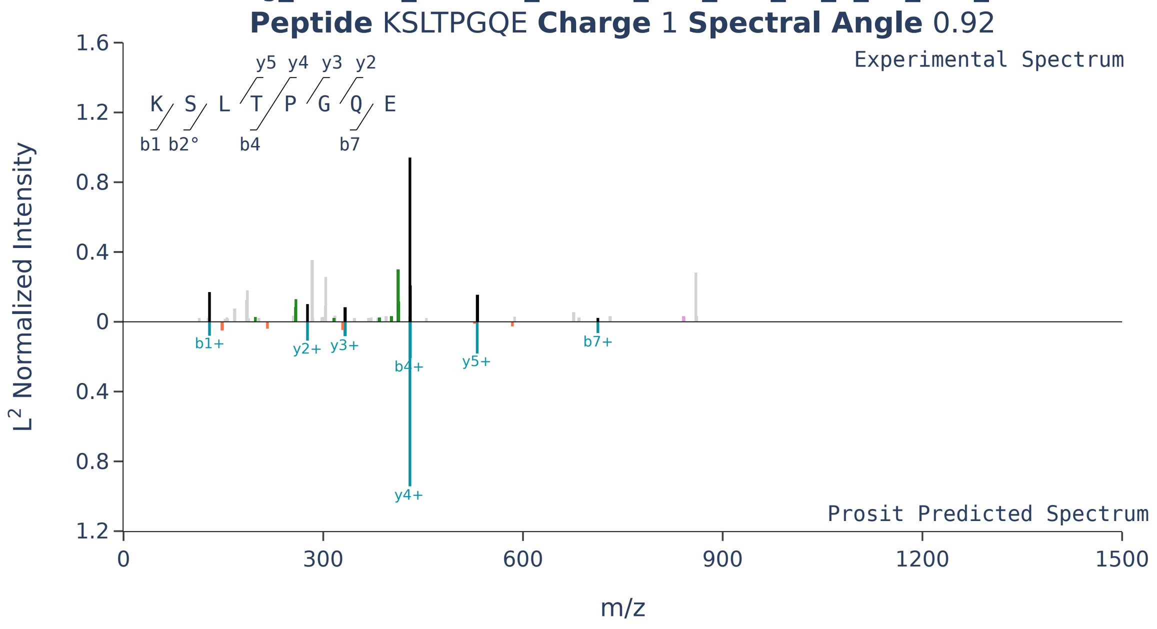


**Source** Ncheng_210623_230623_HFGoe_FFH_20S_25_1_A1_24h_R1 **Scan** 12721
**Peptide** MTGQDAANTAKAFN **Charge** 2 **Spectral Angle** 0.79

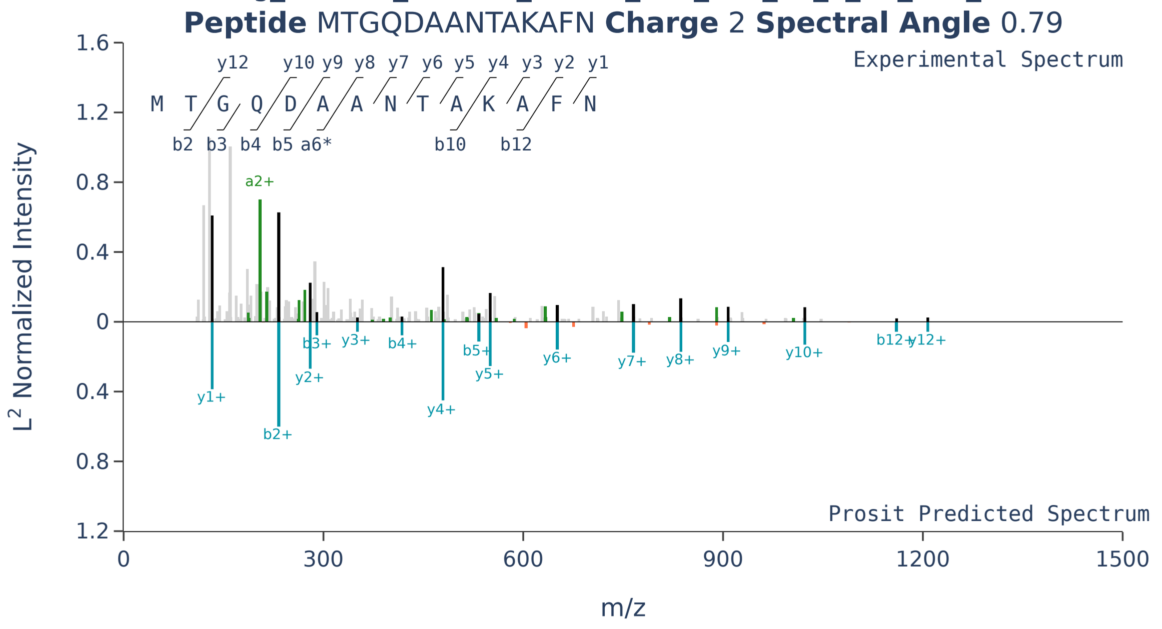


**Source** Ncheng_210623_230623_HFGoe_FFH_20S_25_1_A2_24h_R2 **Scan** 27312
**Peptide** GVDFFPSD **Charge** 1 **Spectral Angle** 0.91

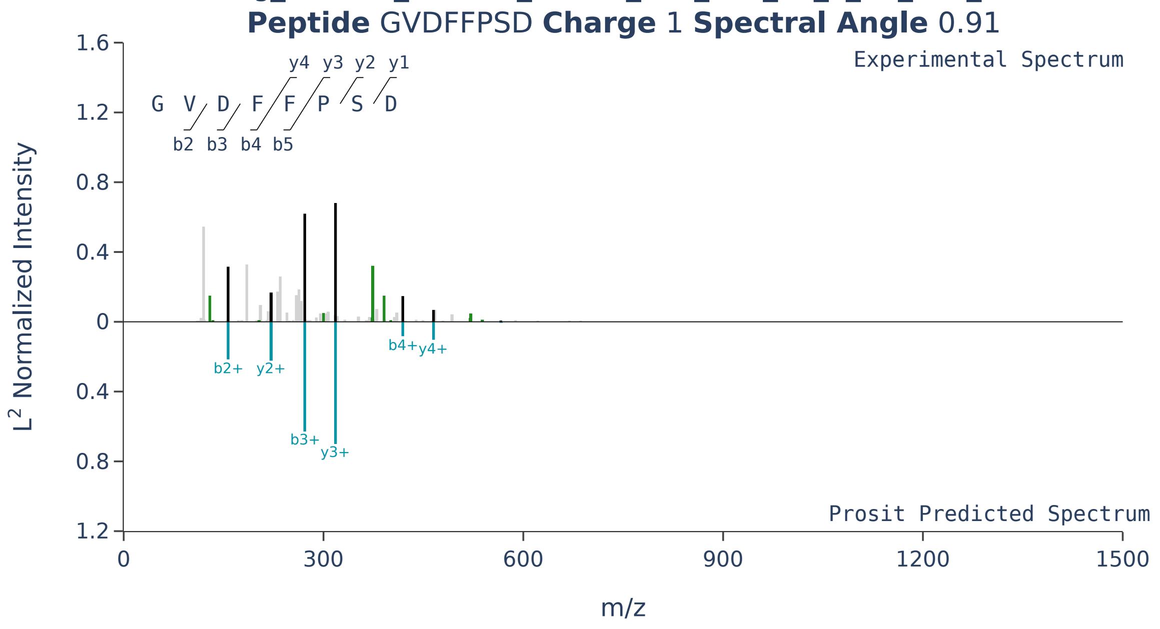


**Source** Ncheng_210623_230623_HFGoe_FFH_20S_25_1_A2_2h_R2 **Scan** 17842
**Peptide** PGMGQIPDNVKSQMDD **Charge** 2 **Spectral Angle** 0.85

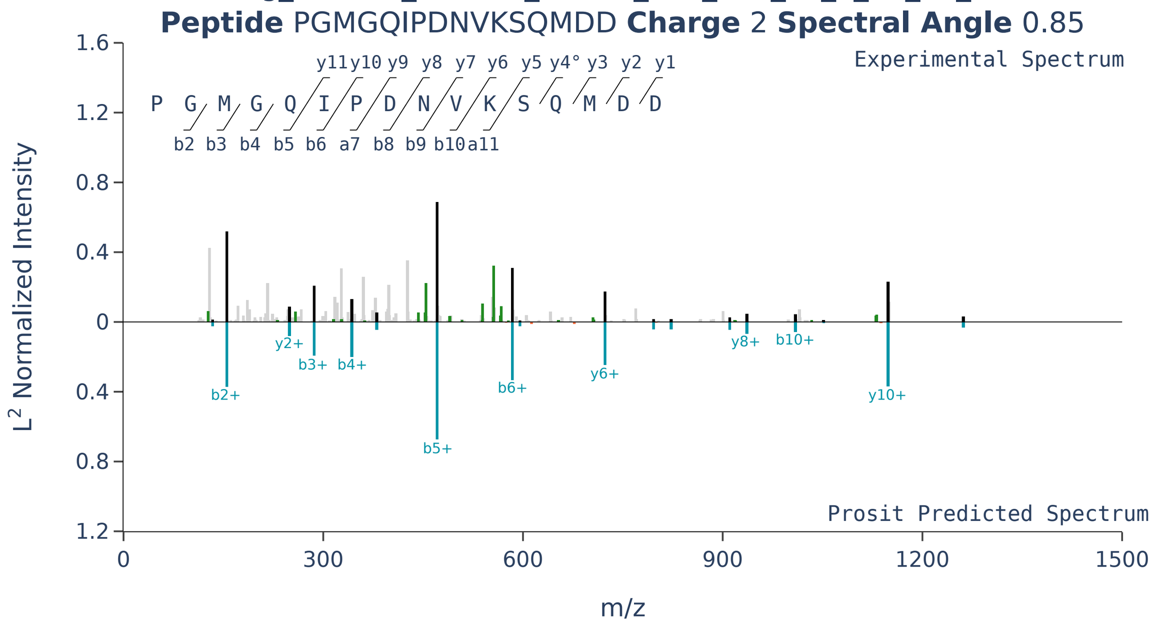


**Source** Ncheng_210623_230623_HFGoe_FFH_20S_25_1_A1_4h_R2 **Scan** 14869
**Peptide** GEKTEALEPFH **Charge** 3 **Spectral Angle** 0.91

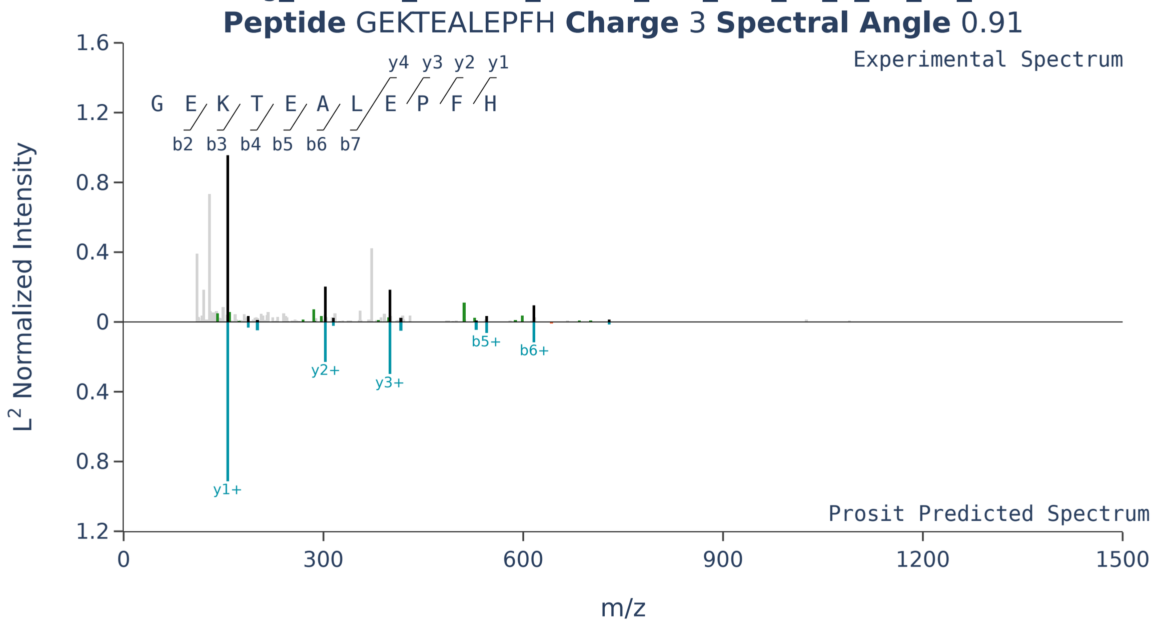


**Source** Ncheng_210623_230623_HFGoe_FFH_20S_25_1_A2_24h_R1 **Scan** 6314
**Peptide** LTEDNVKD **Charge** 1 **Spectral Angle** 0.91

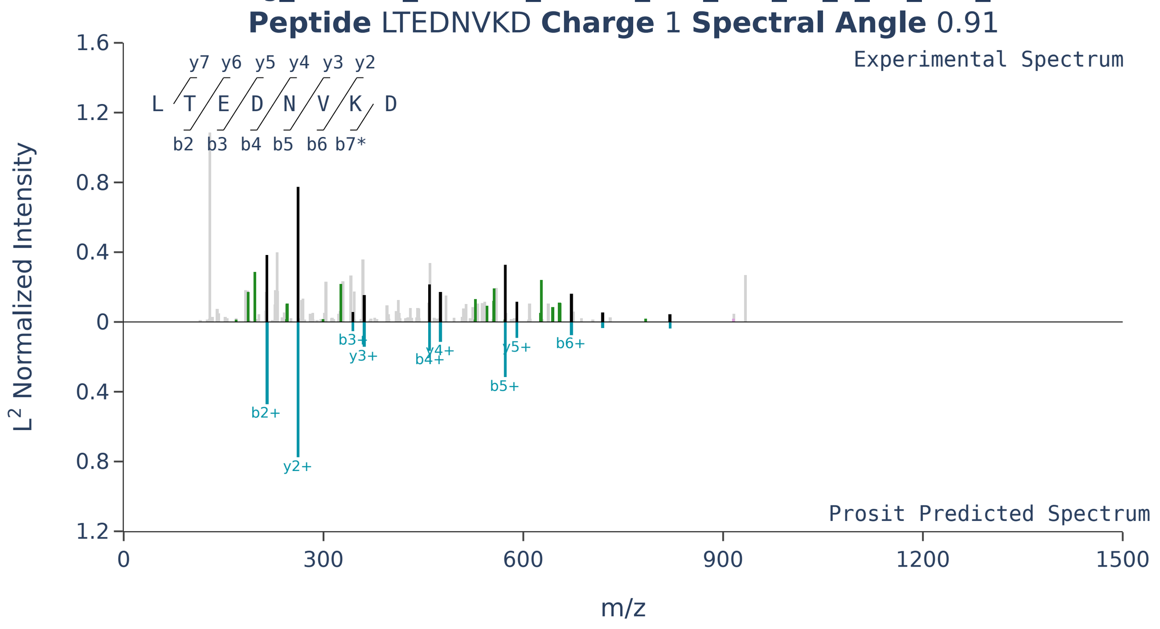

**Source** Ncheng_210623_230623_HFGoe_FFH_20S_25_1_A1_2h_R1 **Scan** 31069
**Peptide** TLFVVDAMTGQDAANTAKAFN **Charge** 3 **Spectral Angle** 0.94

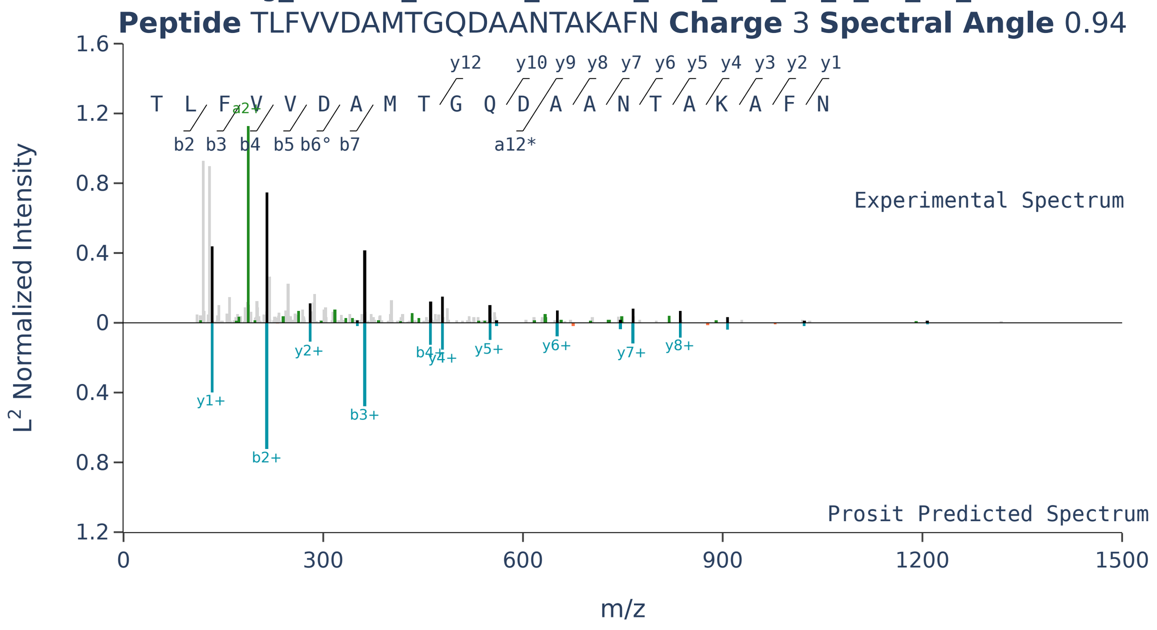


**Source** Ncheng_210623_230623_HFGoe_FFH_20S_25_1_A1_2h_R2 **Scan** 7490
**Peptide** GDARGGAAL **Charge** 2 **Spectral Angle** 0.75

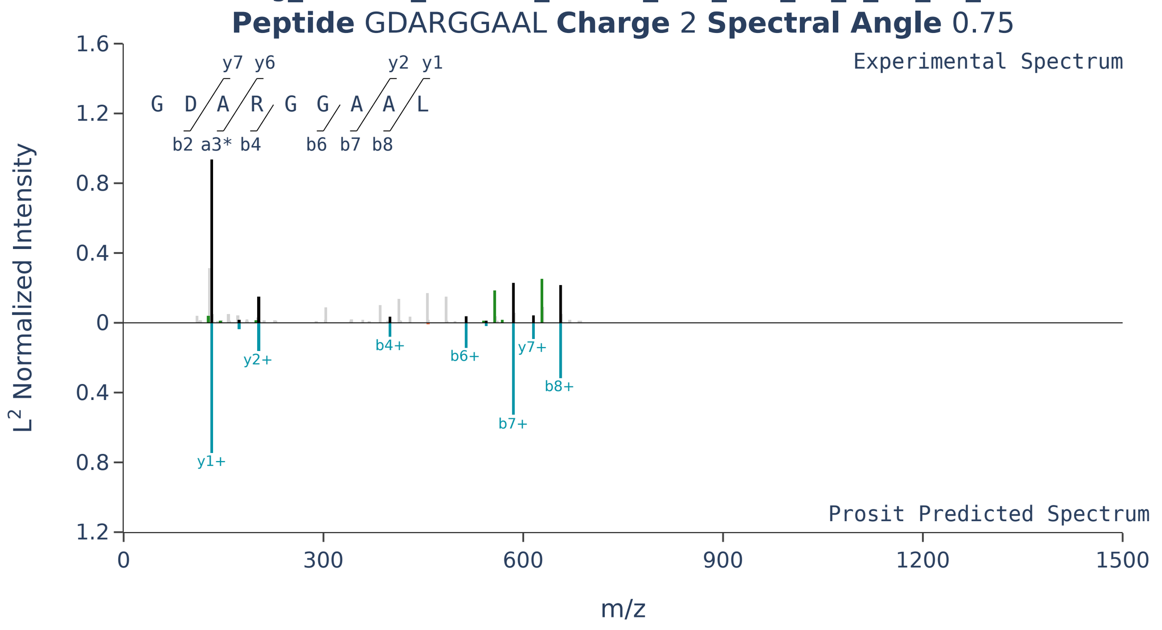


**Source** Ncheng_210623_230623_HFGoe_FFH_20S_25_1_A1_2h_R1 **Scan** 21055
**Peptide** TAGRLHVDEAMMDEIKQVH **Charge** 5 **Spectral Angle** 0.89

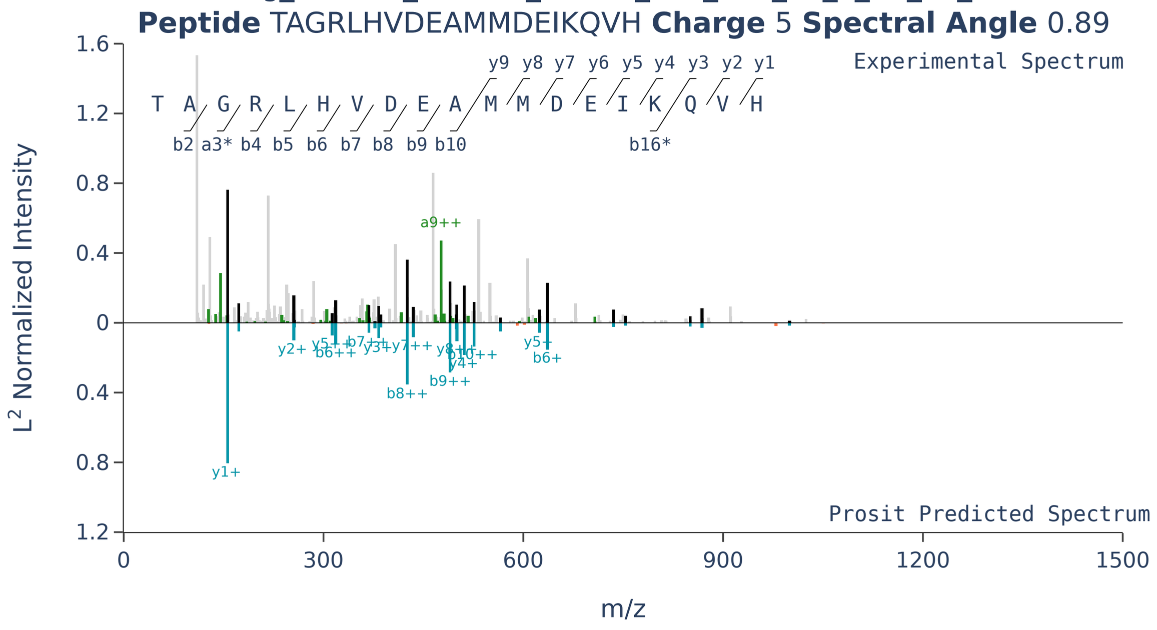


**Source** Ncheng_210623_230623_HFGoe_FFH_20S_25_1_A1_4h_R1 **Scan** 16030
**Peptide** TGKPIKFLGVGEKTE **Charge** 2 **Spectral Angle** 0.86

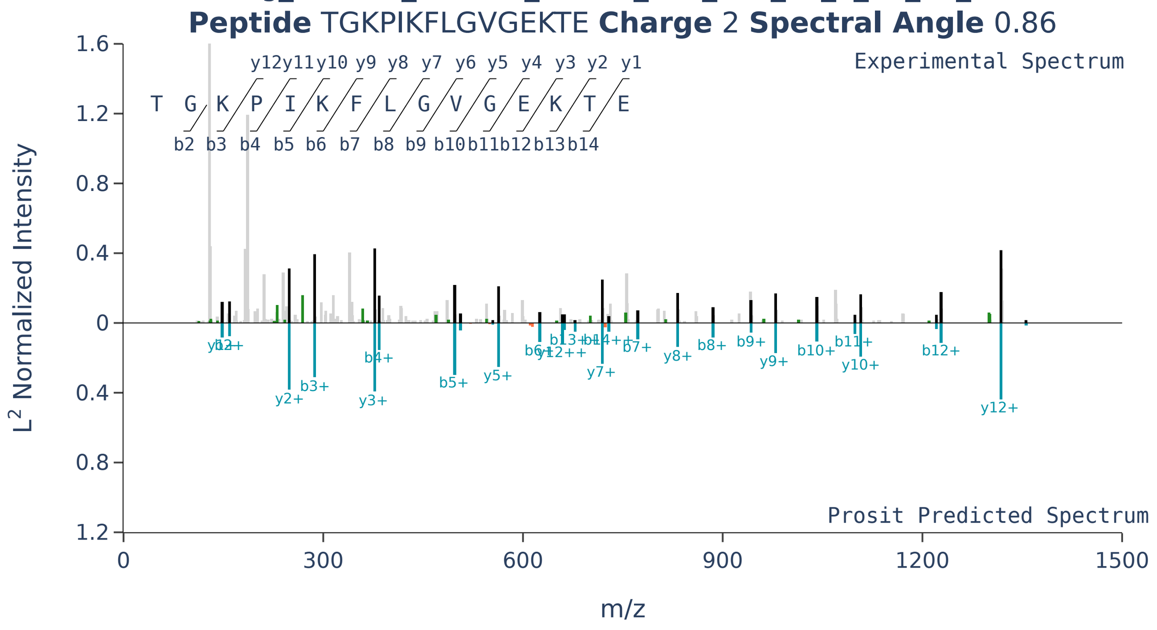


**Source** Ncheng_210623_230623_HFGoe_FFH_20S_25_1_A2_4h_R2 **Scan** 8996
**Peptide** MKNMGGMA **Charge** 2 **Spectral Angle** 0.91

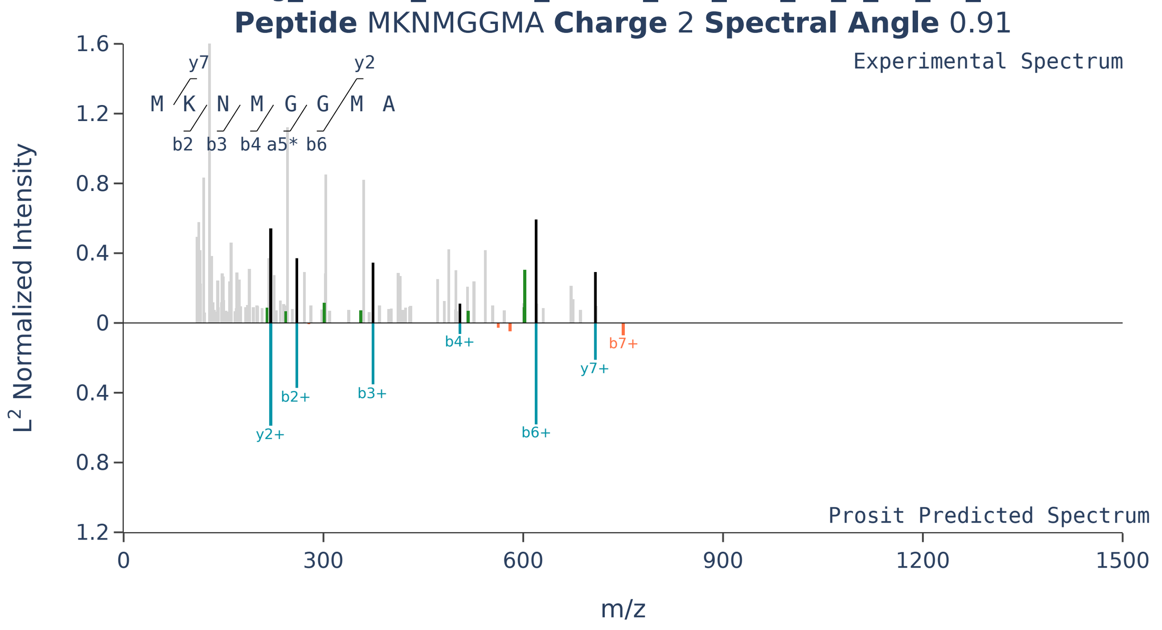


**Source** Ncheng_210623_230623_HFGoe_FFH_20S_25_1_A1_2h_R2 **Scan** 27810
**Peptide** VLVVSADVYRPAAI **Charge** 2 **Spectral Angle** 0.83

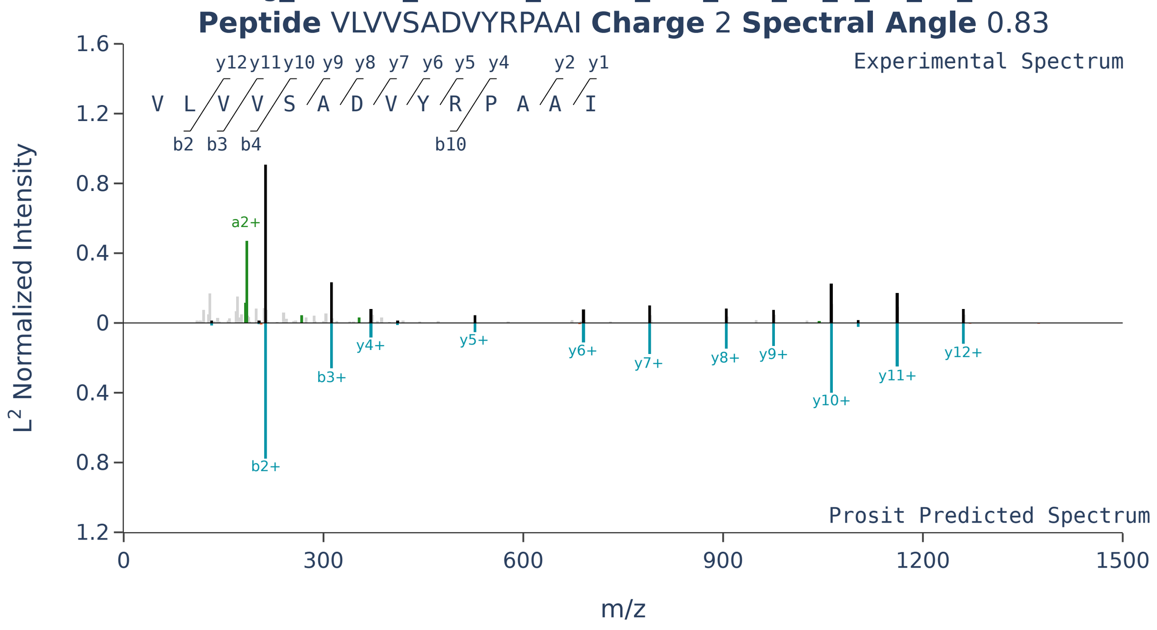


**Source** Ncheng_210623_230623_HFGoe_FFH_20S_25_1_A2_24h_R2 **Scan** 18063
**Peptide** AGCGMQVQDVNRL **Charge** 2 **Spectral Angle** 0.87

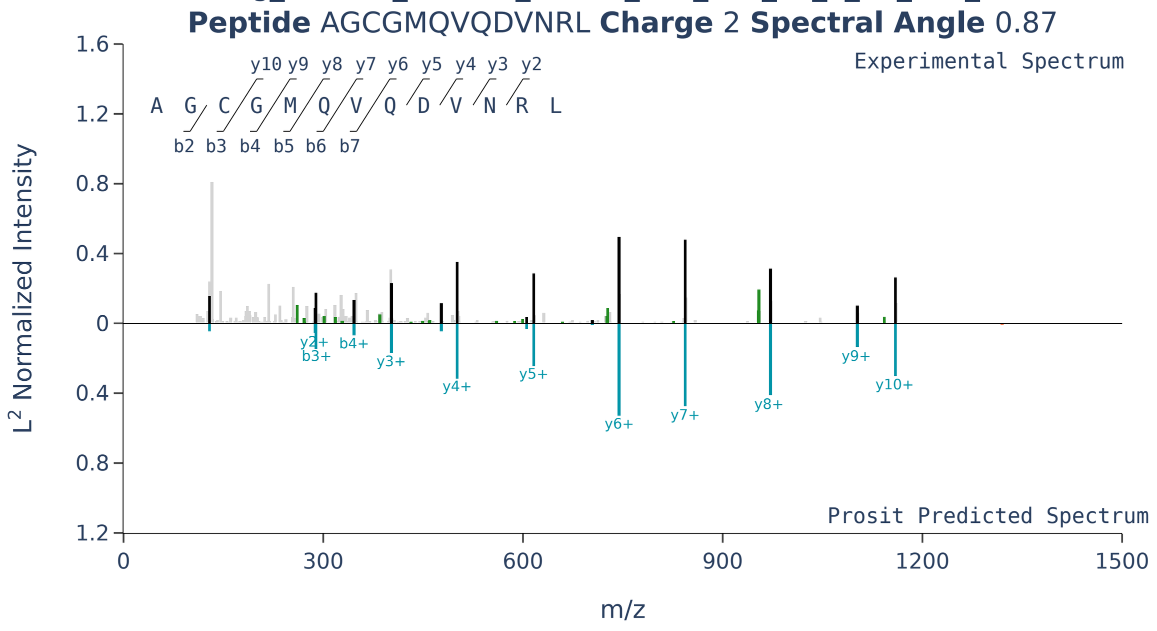


**Source** Ncheng_210623_230623_HFGoe_FFH_20S_25_1_A2_2h_R2 **Scan** 11174
**Peptide** KQFDDMQRM **Charge** 3 **Spectral Angle** 0.9

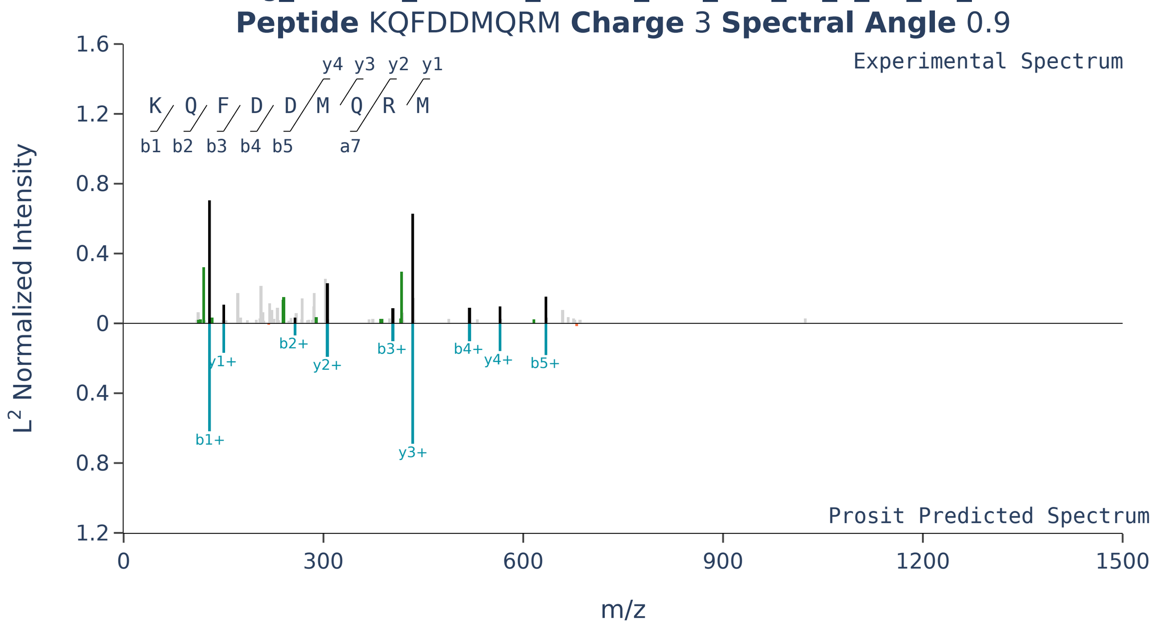


**Source** Ncheng_210623_230623_HFGoe_FFH_20S_25_1_A2_4h_R1 **Scan** 8632
**Peptide** EDIESKVDRA **Charge** 3 **Spectral Angle** 0.88

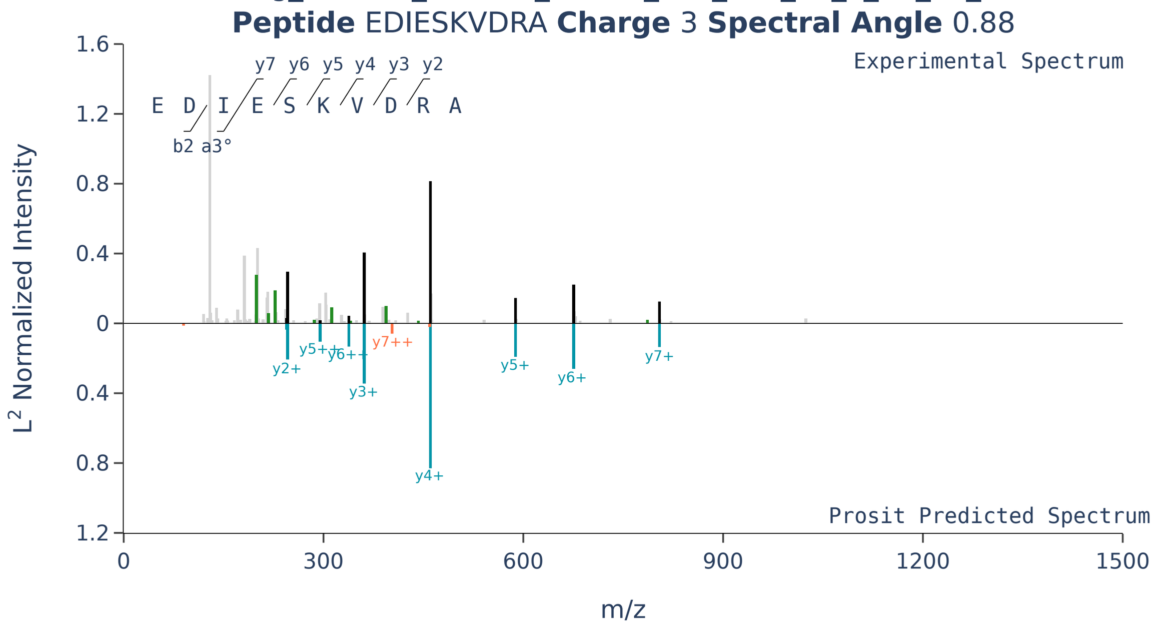


**Source** Ncheng_210623_230623_HFGoe_FFH_20S_25_1_A1_1h_R1 **Scan** 34504
**Peptide** ETLAEQVGVDFFPSD **Charge** 2 **Spectral Angle** 0.95

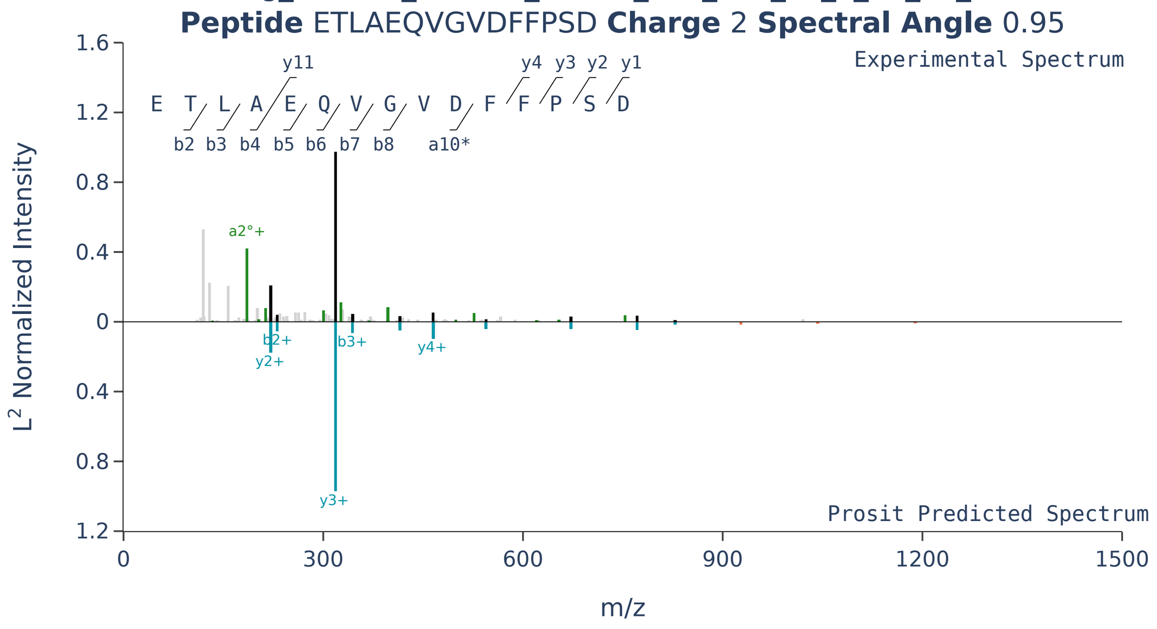


**Source** Ncheng_210623_230623_HFGoe_FFH_20S_25_1_A2_24h_R2 **Scan** 16855
**Peptide** RNELVAAMGEENQ **Charge** 2 **Spectral Angle** 0.88

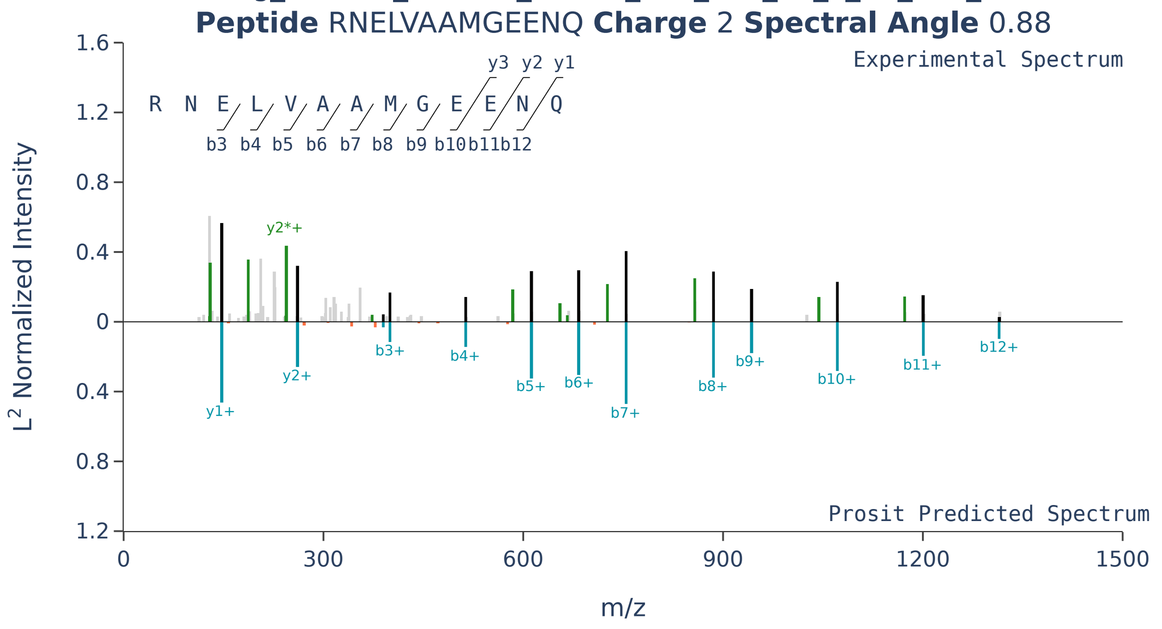


**Source** Ncheng_210623_230623_HFGoe_FFH_20S_25_1_A1_24h_R1 **Scan** 12133
**Peptide** IEDIESKVD **Charge** 1 **Spectral Angle** 0.95

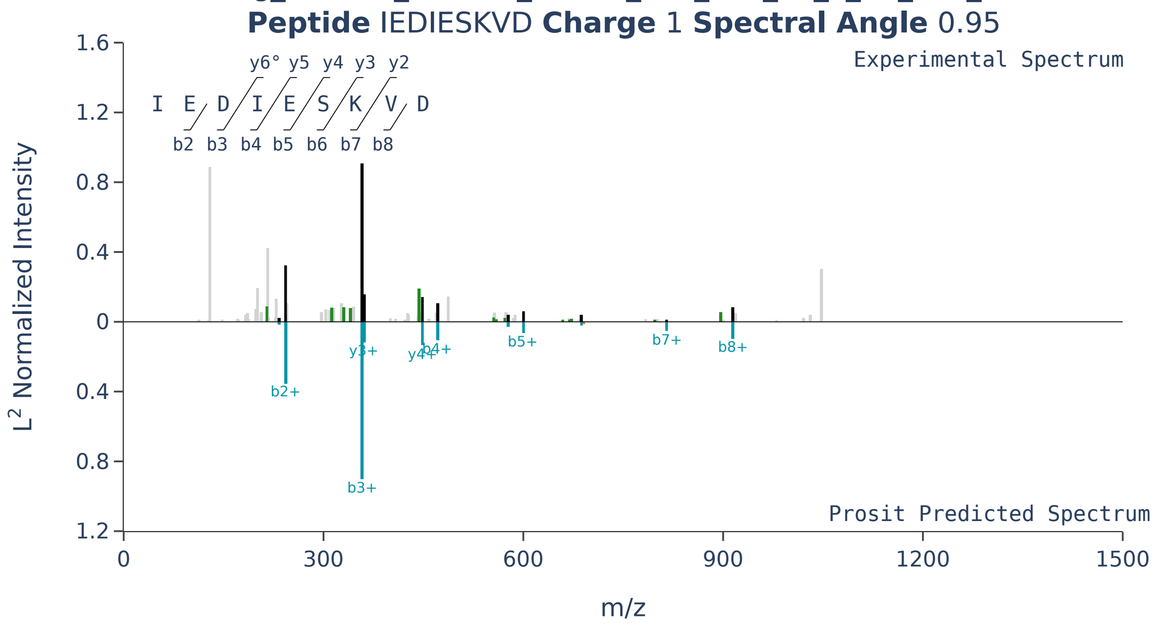


**Source** Ncheng_210623_230623_HFGoe_FFH_20S_25_1_A2_24h_R2 **Scan** 18024
**Peptide** VVDAMTGQDAANTAKAFN **Charge** 2 **Spectral Angle** 0.91

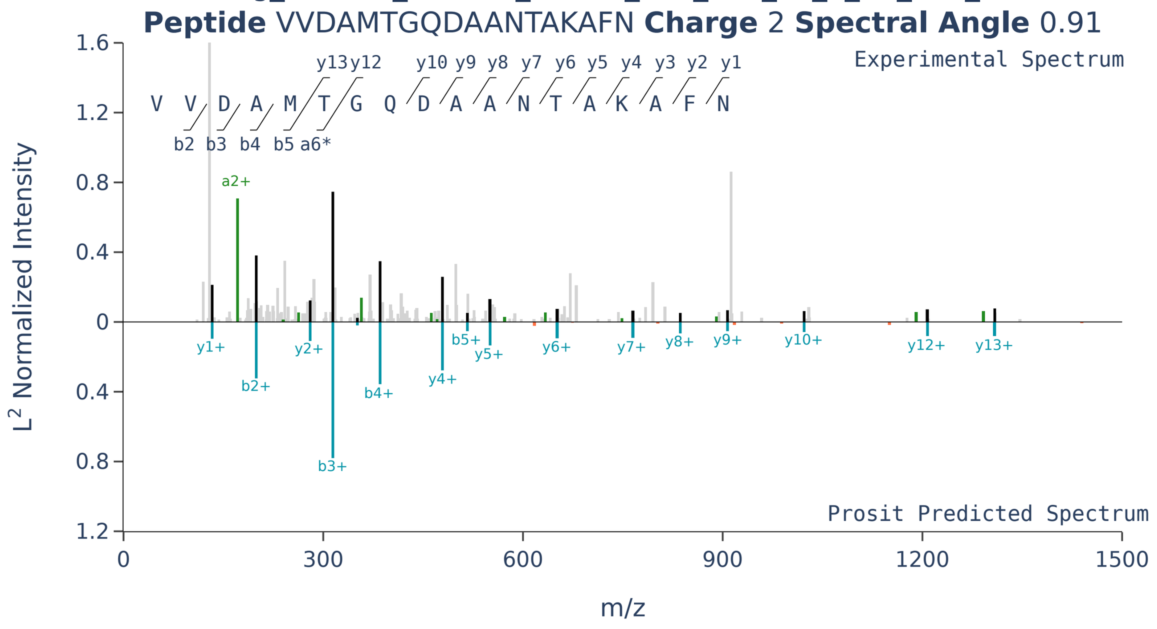


**Source** Ncheng_210623_230623_HFGoe_FFH_20S_25_1_A2_1h_R1 **Scan** 25018
**Peptide** AMTGQDAANTAKAFNEALPLTG **Charge** 2 **Spectral Angle** 0.89

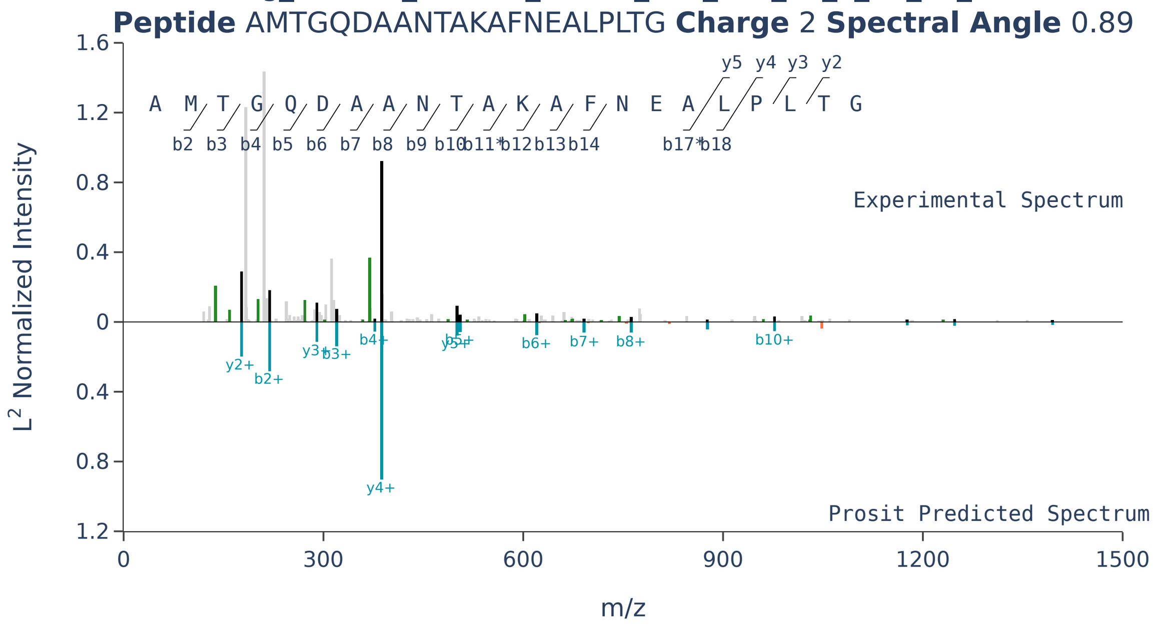


**Source** Ncheng_210623_230623_HFGoe_FFH_20S_25_1_A2_4h_R1 **Scan** 15247
**Peptide** DARGGAALSIRHI **Charge** 3 **Spectral Angle** 0.92

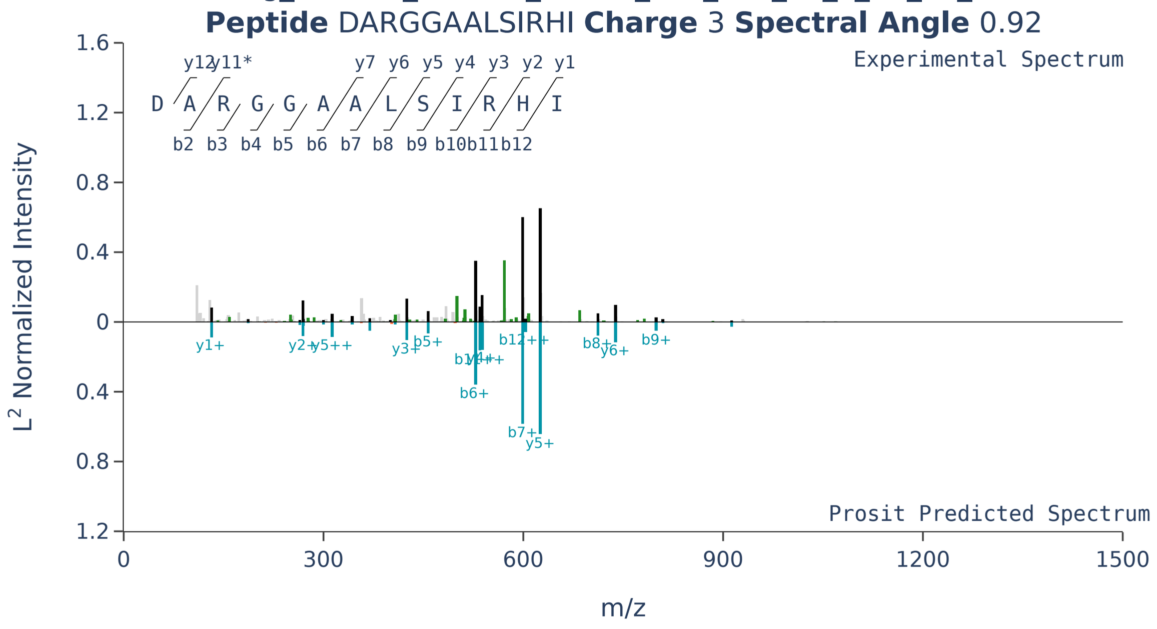

**Source** Ncheng_210623_230623_HFGoe_FFH_20S_25_1_A1_1h_R1 **Scan** 36521
**Peptide** LETLAEQVGVDFFPSD **Charge** 2 **Spectral Angle** 0.96

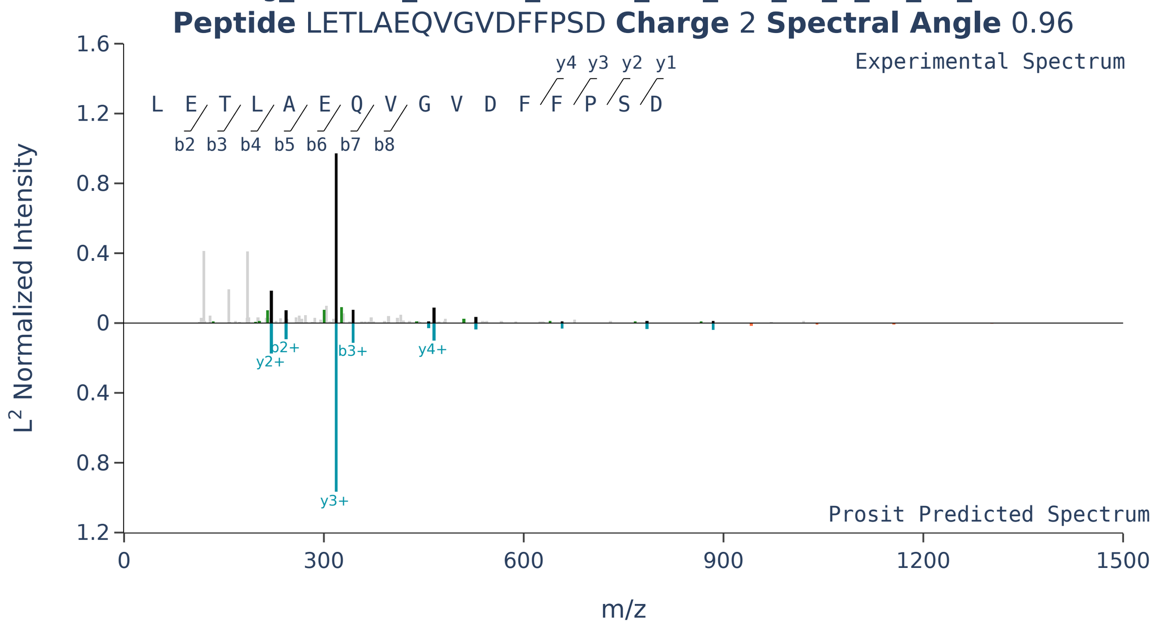


**Source** Ncheng_210623_230623_HFGoe_FFH_20S_25_1_A2_24h_R2 **Scan** 8182
**Peptide** AKPEIIKG **Charge** 1 **Spectral Angle** 0.82

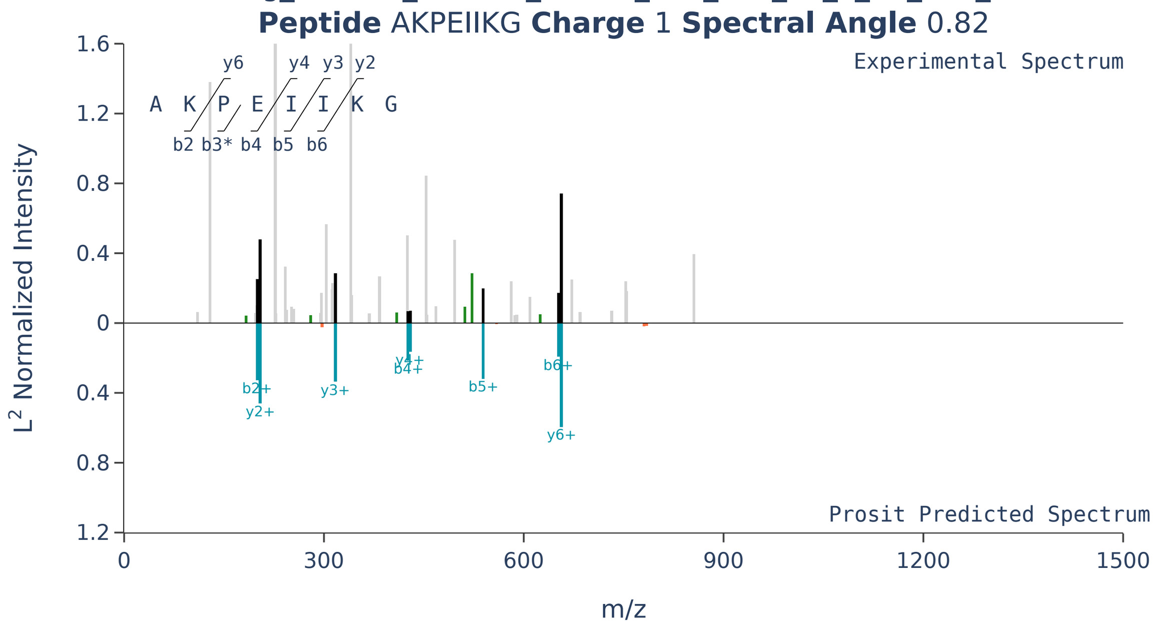


**Source** Ncheng_210623_230623_HFGoe_FFH_20S_25_1_A1_4h_R1 **Scan** 9766
**Peptide** RILGMGD **Charge** 2 **Spectral Angle** 0.81

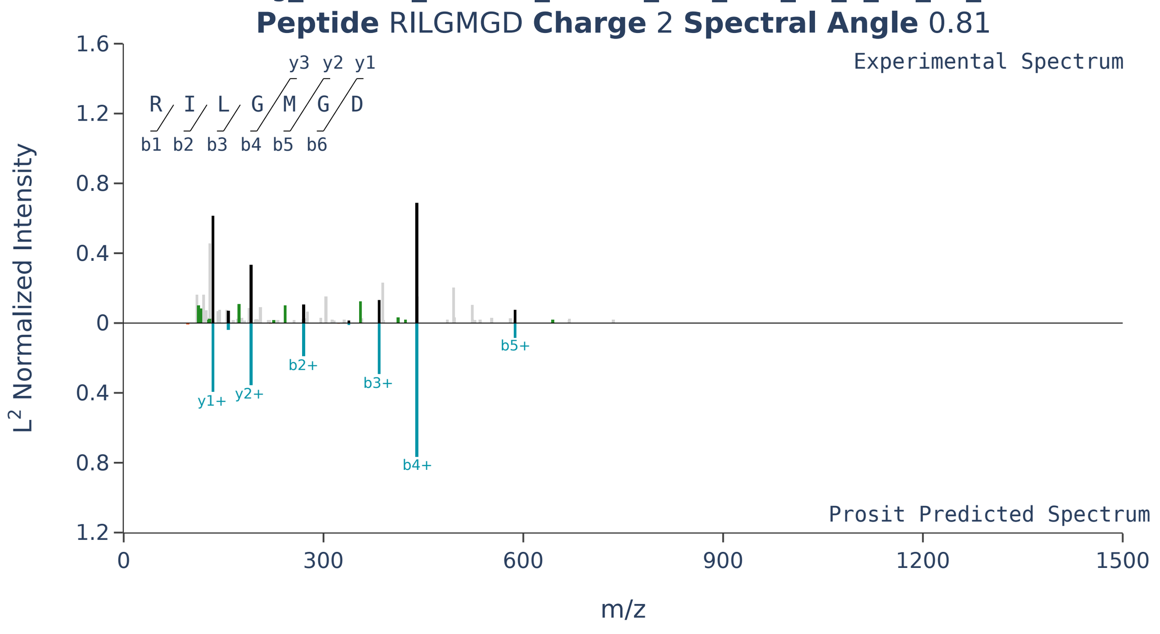


**Source** Ncheng_210623_230623_HFGoe_FFH_20S_25_1_A2_4h_R2 **Scan** 9613
**Peptide** EFINRVK **Charge** 2 **Spectral Angle** 0.92

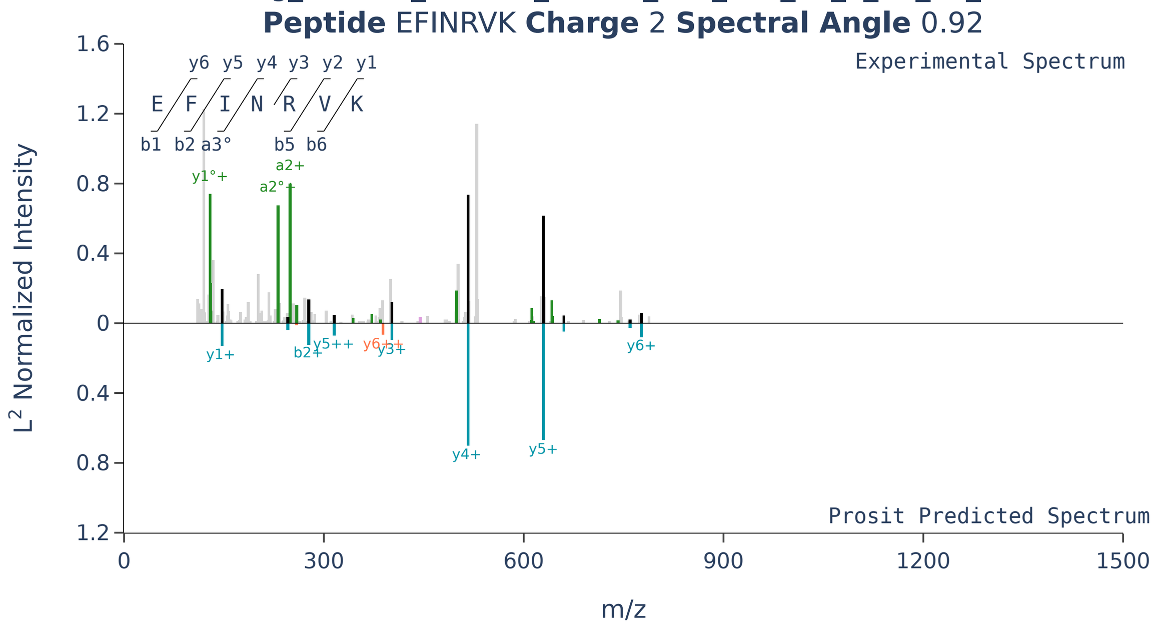


**Source** Ncheng_210623_230623_HFGoe_FFH_20S_25_1_A2_24h_R2 **Scan** 17102
**Peptide** RNELVAAMGEENQT **Charge** 2 **Spectral Angle** 0.9

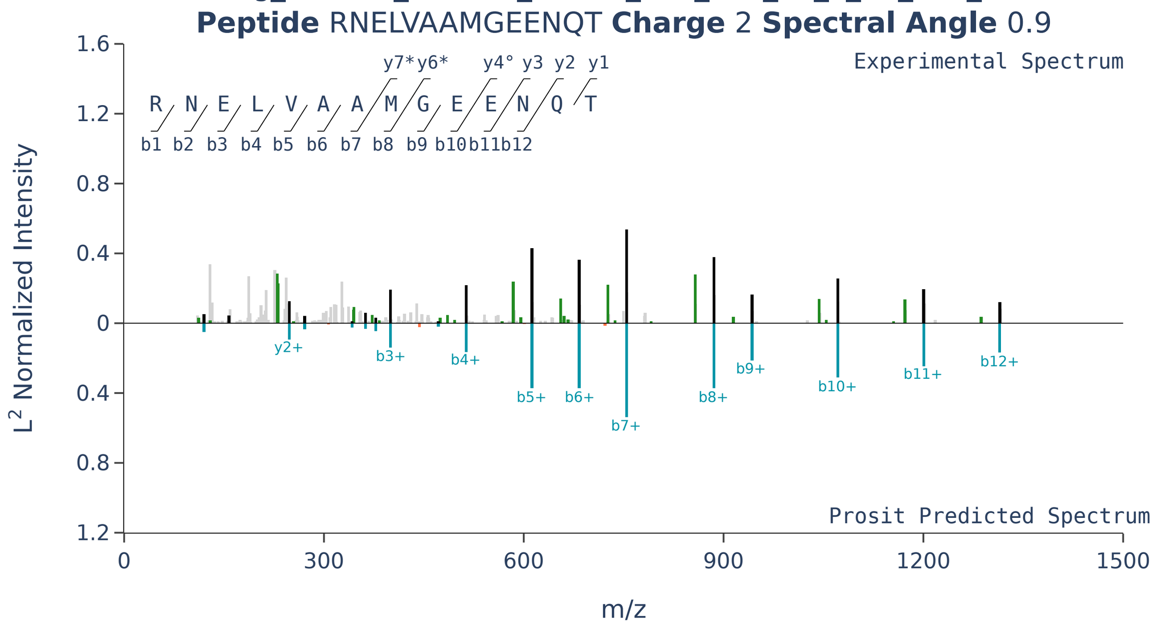


**Source** Ncheng_210623_230623_HFGoe_FFH_20S_25_1_A1_1h_R2 **Scan** 30911
**Peptide** TLAEQVGVDFFPSDVGQKPVD **Charge** 2 **Spectral Angle** 0.92

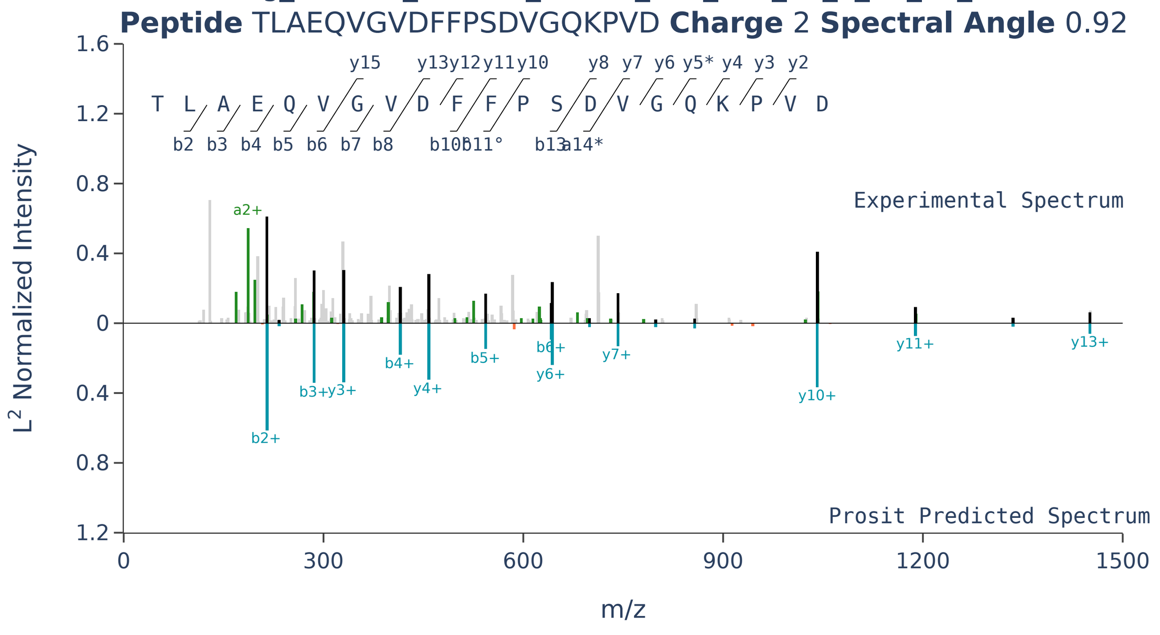


**Source** Ncheng_210623_230623_HFGoe_FFH_20S_25_1_A2_24h_R1 **Scan** 14824
**Peptide** SVGKLGKFLR **Charge** 3 **Spectral Angle** 0.89

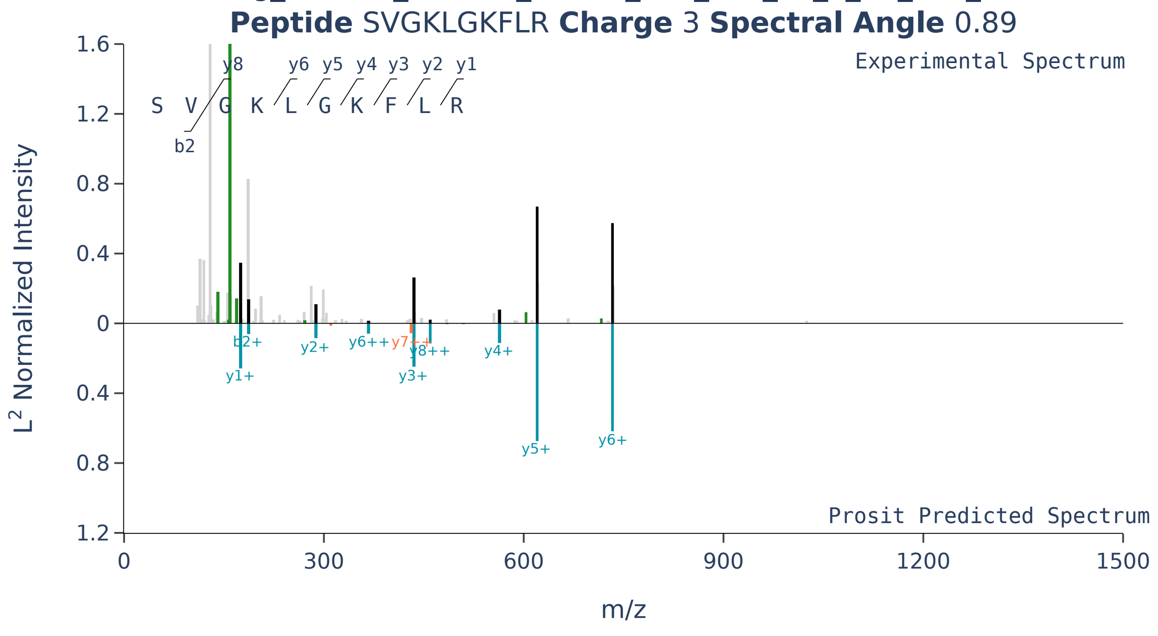


**Source** Ncheng_210623_230623_HFGoe_FFH_20S_25_1_A2_24h_R2 **Scan** 8666
**Peptide** GQDAANTAKAFN **Charge** 1 **Spectral Angle** 0.89

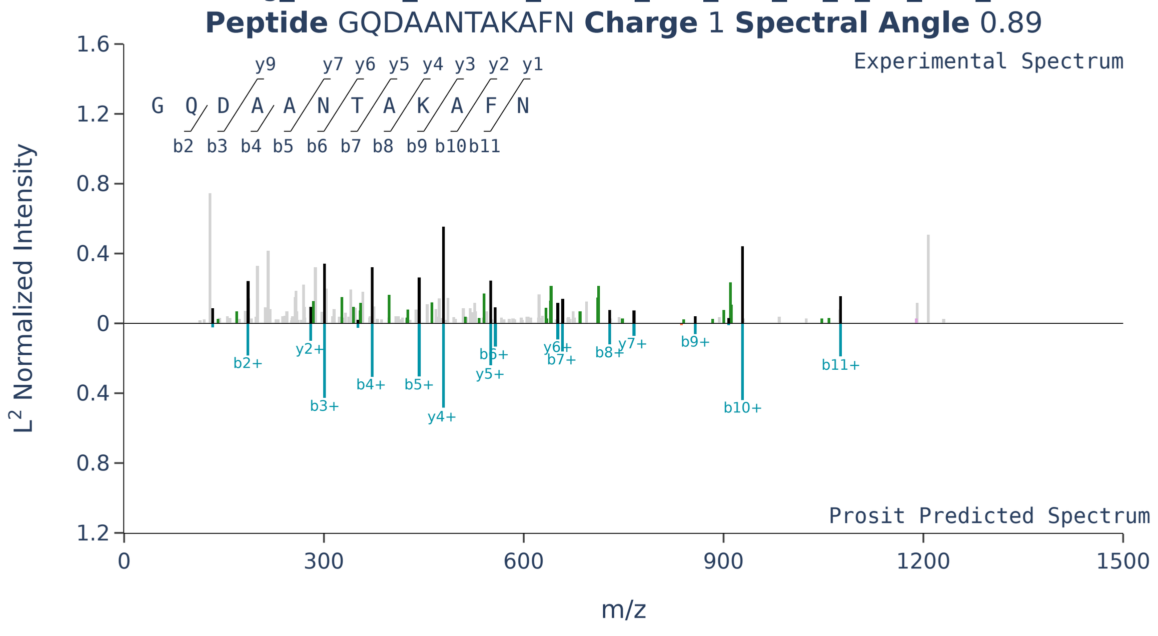


**Source** Ncheng_210623_230623_HFGoe_FFH_20S_25_1_A1_24h_R1 **Scan** 14191
**Peptide** EFVKIVR **Charge** 2 **Spectral Angle** 0.82

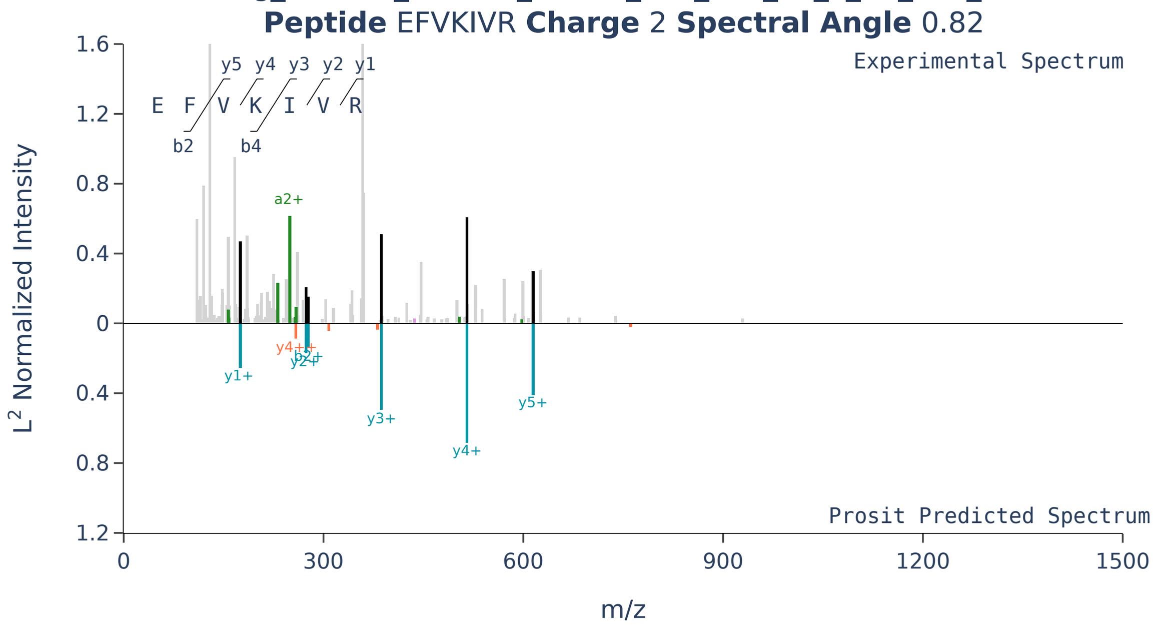


**Source** Ncheng_210623_230623_HFGoe_FFH_20S_25_1_A1_1h_R2 **Scan** 11802
**Peptide** SINPVET **Charge** 1 **Spectral Angle** 0.82

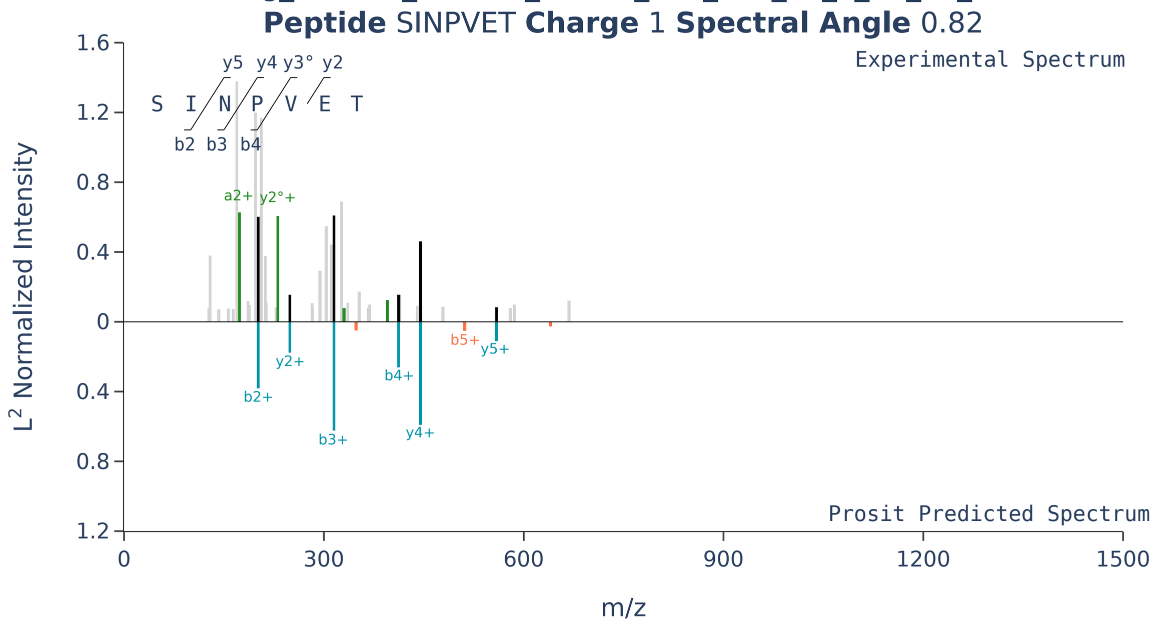


**Source** Ncheng_210623_230623_HFGoe_FFH_20S_25_1_A2_4h_R1 **Scan** 18124
**Peptide** KTEALEPFHPDRL **Charge** 2 **Spectral Angle** 0.84

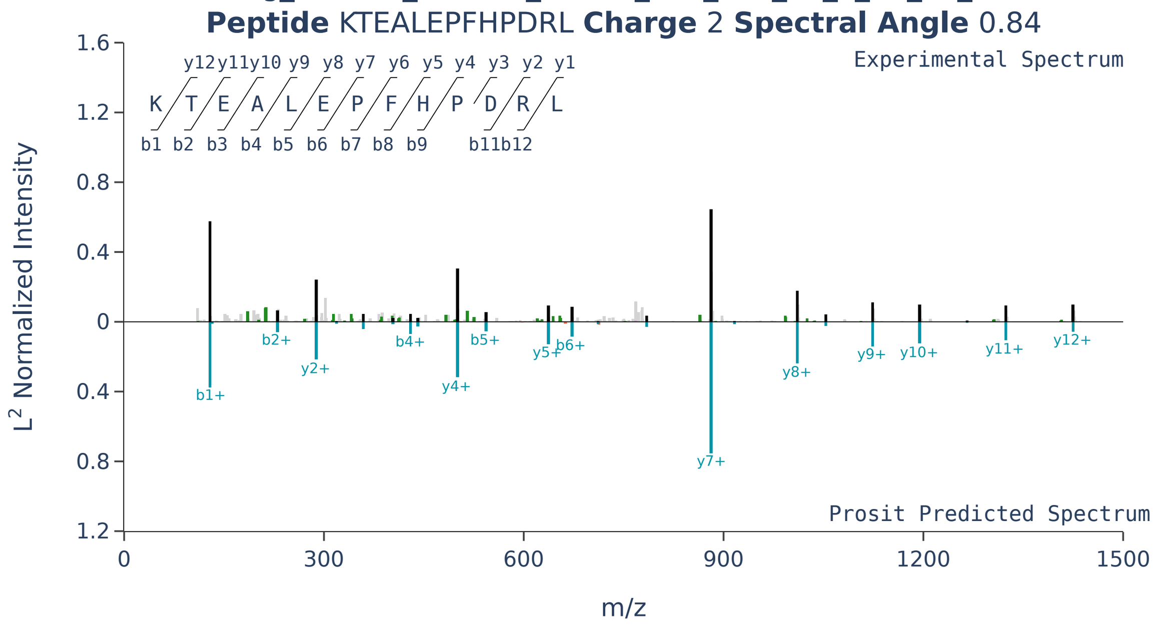


**Source** Ncheng_210623_230623_HFGoe_FFH_20S_25_1_A2_24h_R1 **Scan** 14253
**Peptide** MTGQDAANTAKAF **Charge** 2 **Spectral Angle** 0.79

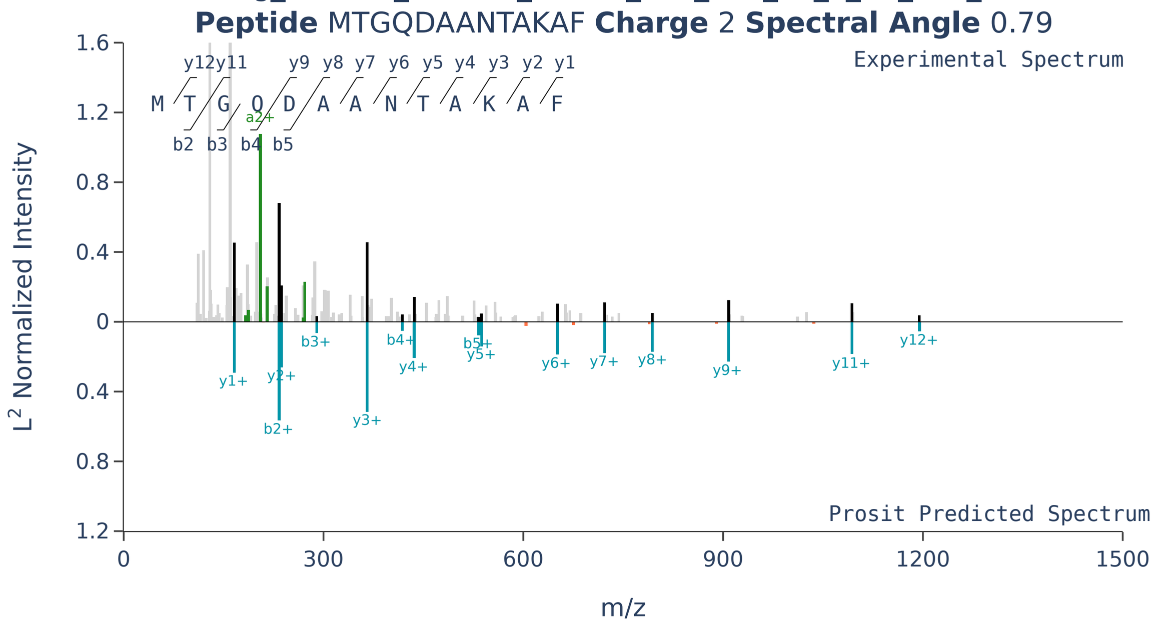


**Source** Ncheng_210623_230623_HFGoe_FFH_20S_25_1_A1_2h_R1 **Scan** 8215
**Peptide** KPEIIKG **Charge** 2 **Spectral Angle** 0.87

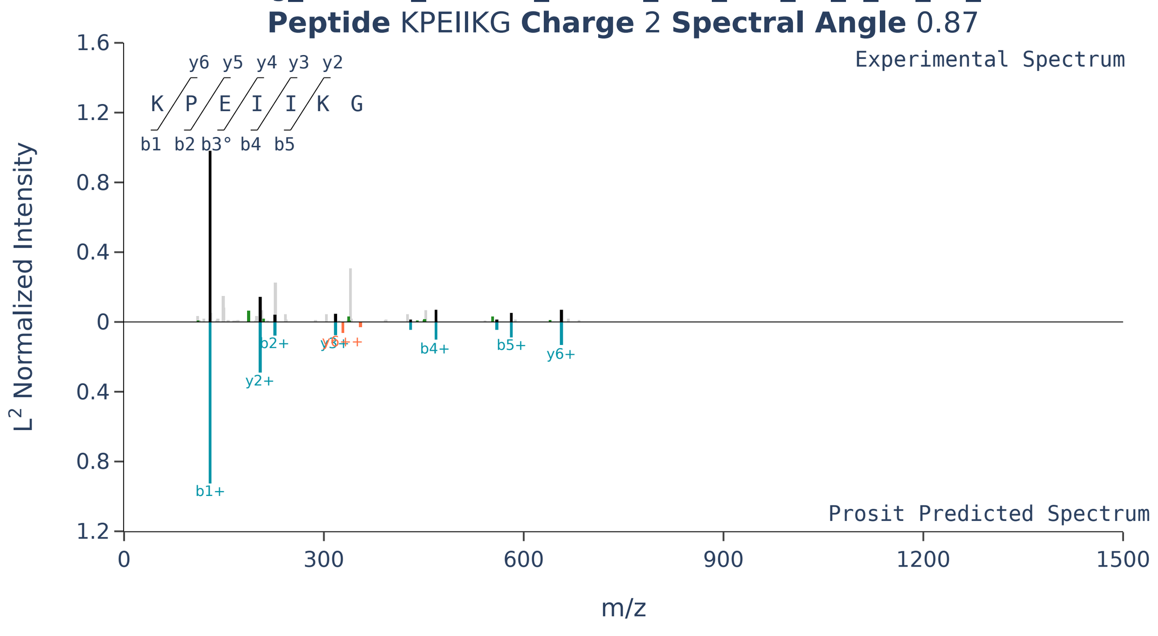


**Source** Ncheng_210623_230623_HFGoe_FFH_20S_25_1_A2_24h_R2 **Scan** 10772
**Peptide** VRNELVA **Charge** 1 **Spectral Angle** 0.91

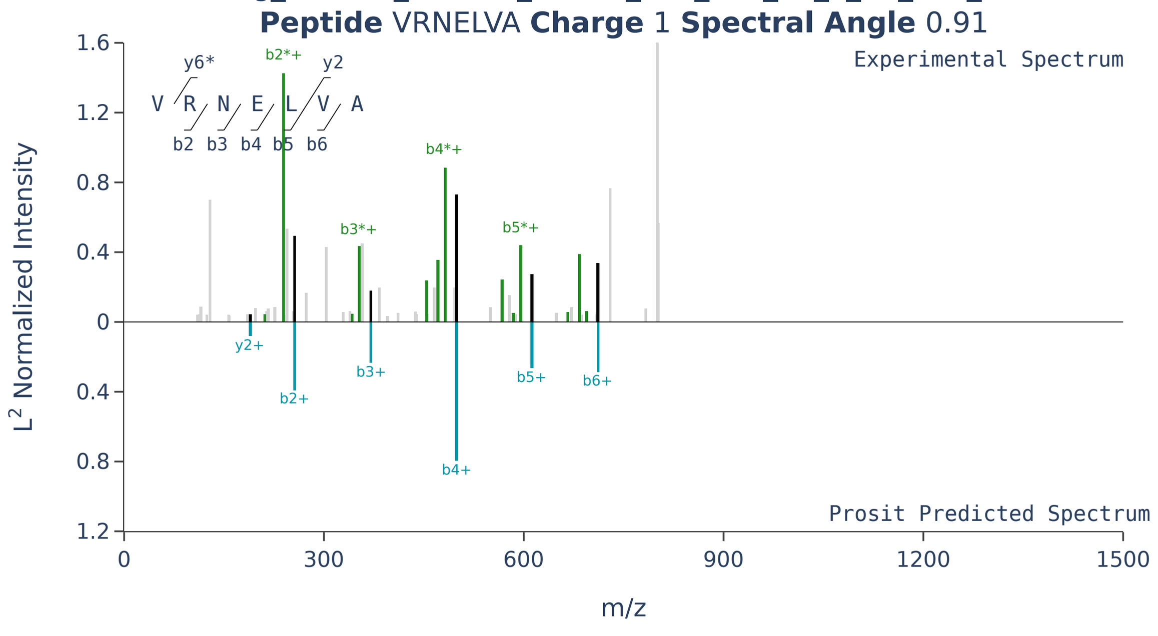


**Source** Ncheng_210623_230623_HFGoe_FFH_20S_25_1_A2_24h_R1 **Scan** 13659
**Peptide** IEDIESKV **Charge** 2 **Spectral Angle** 0.85

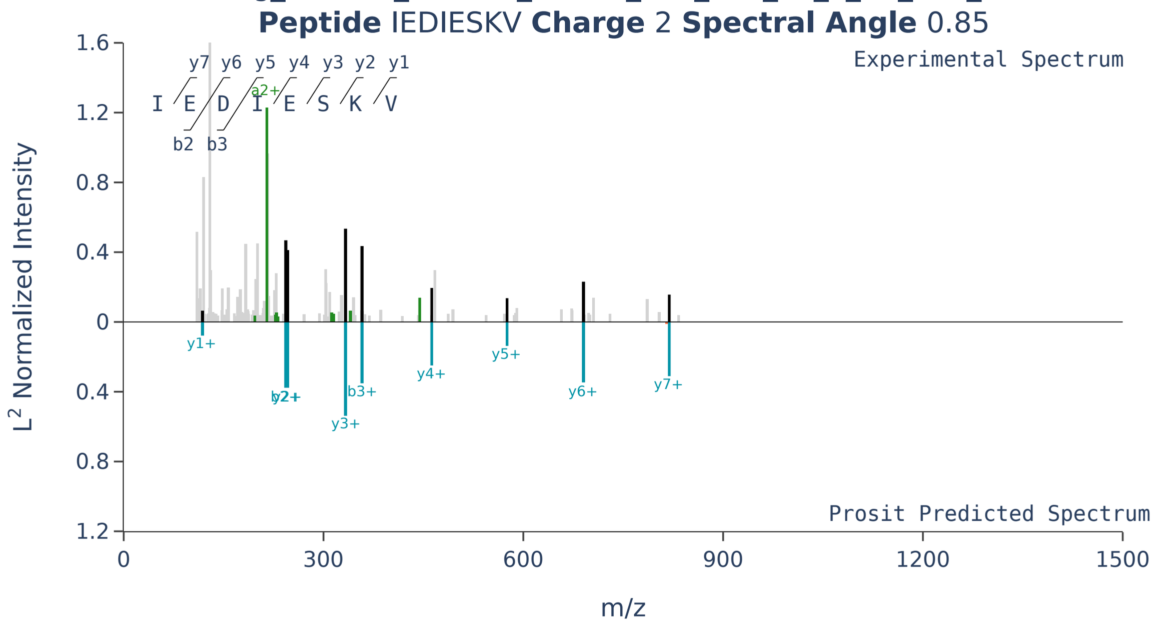

**Source** Ncheng_210623_230623_HFGoe_FFH_20S_25_1_A2_2h_R2 **Scan** 17877
**Peptide** KLPGMGQIPDNVKS **Charge** 2 **Spectral Angle** 0.87

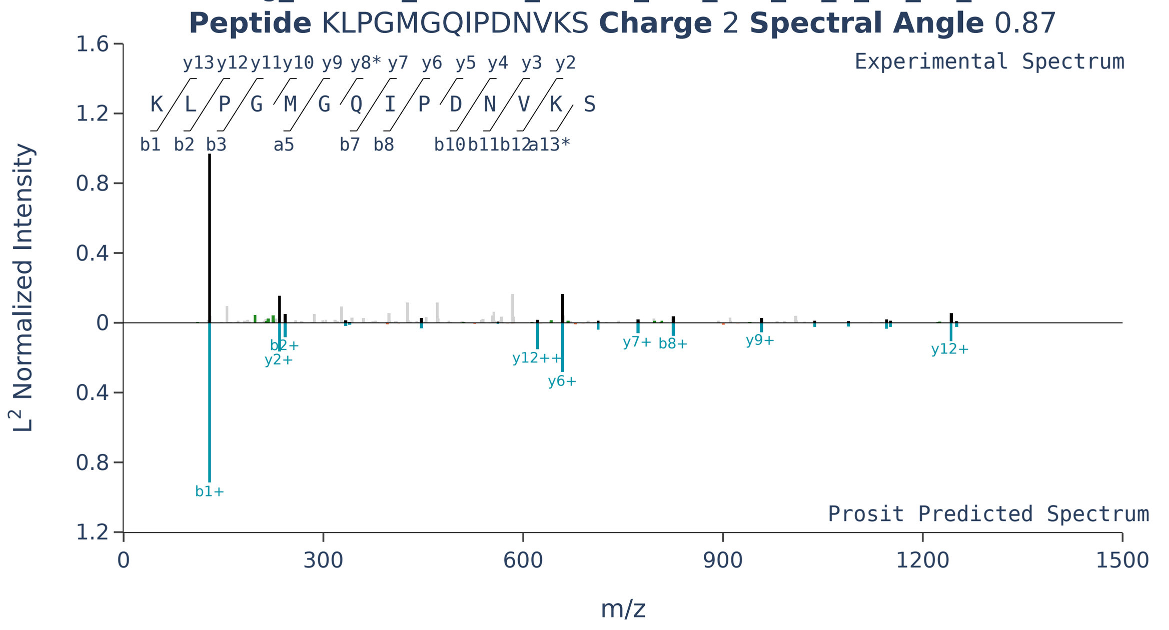


**Source** Ncheng_210623_230623_HFGoe_FFH_20S_25_1_A1_24h_R1 **Scan** 8681
**Peptide** TAGRLHV **Charge** 2 **Spectral Angle** 0.83

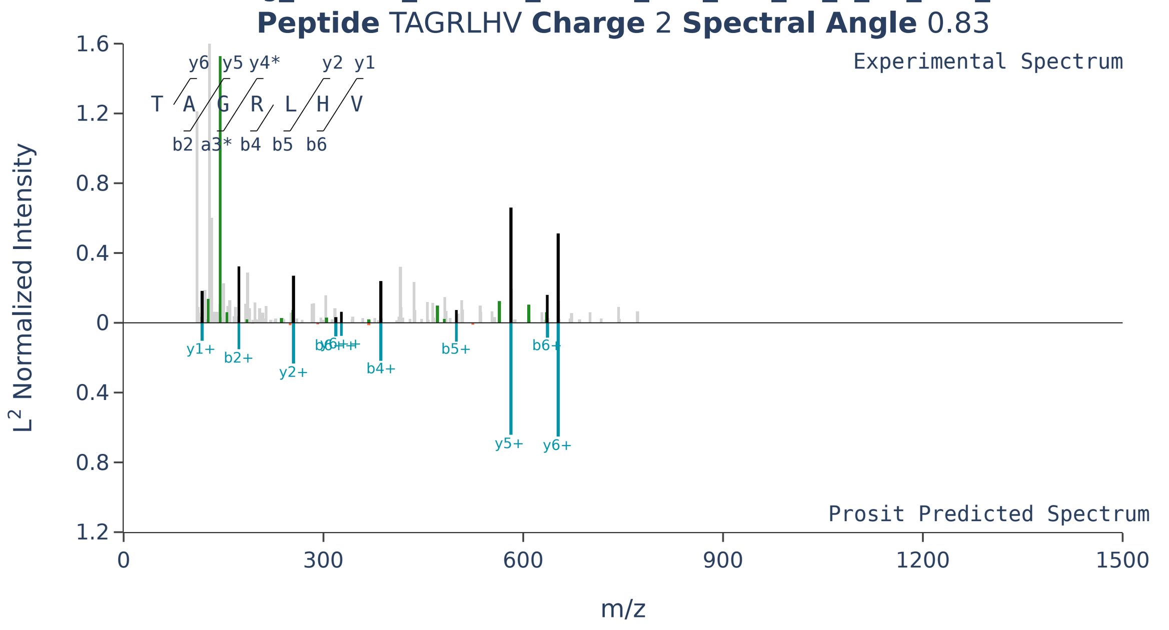


**Source** Ncheng_210623_230623_HFGoe_FFH_20S_25_1_A1_4h_R2 **Scan** 10128
**Peptide** RNELVAA **Charge** 2 **Spectral Angle** 0.85

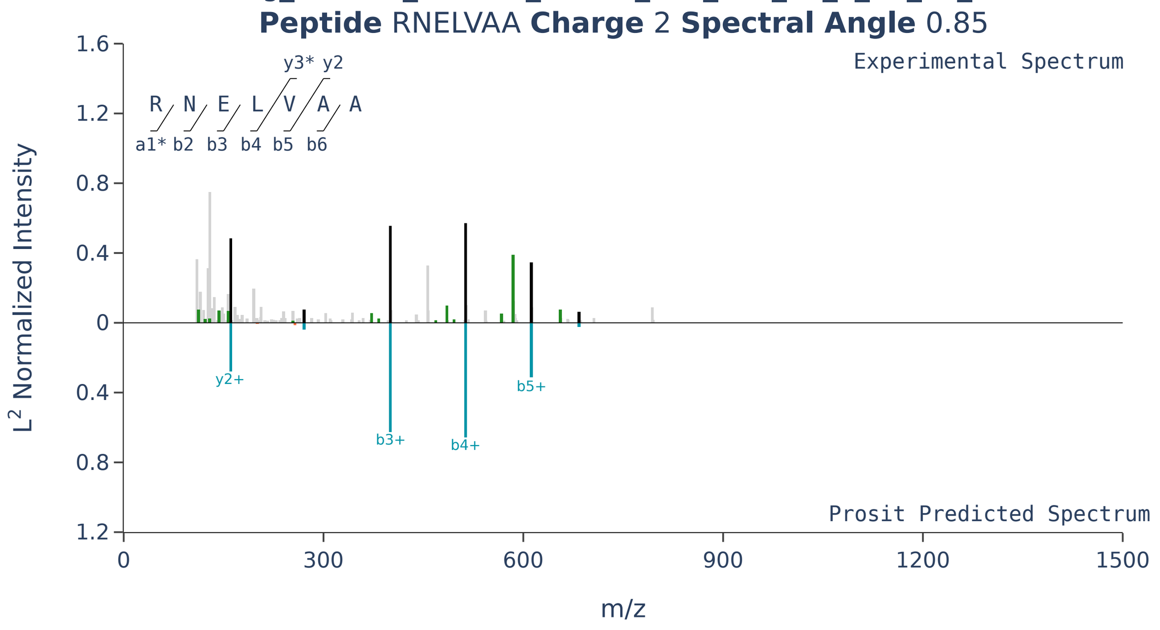


**Source** Ncheng_210623_230623_HFGoe_FFH_20S_25_1_A1_4h_R2 **Scan** 21639
**Peptide** DVALPVVRE **Charge** 2 **Spectral Angle** 0.81

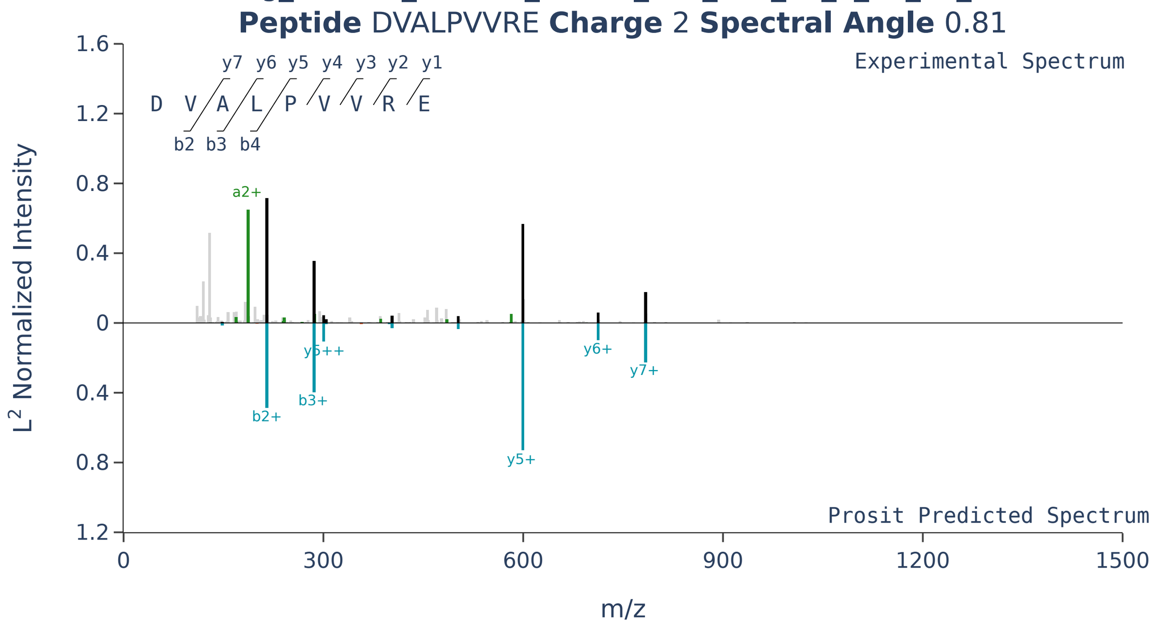


**Source** Ncheng_210623_230623_HFGoe_FFH_20S_25_1_A2_24h_R2 **Scan** 21313
**Peptide** MGEENQTLNL **Charge** 1 **Spectral Angle** 0.82

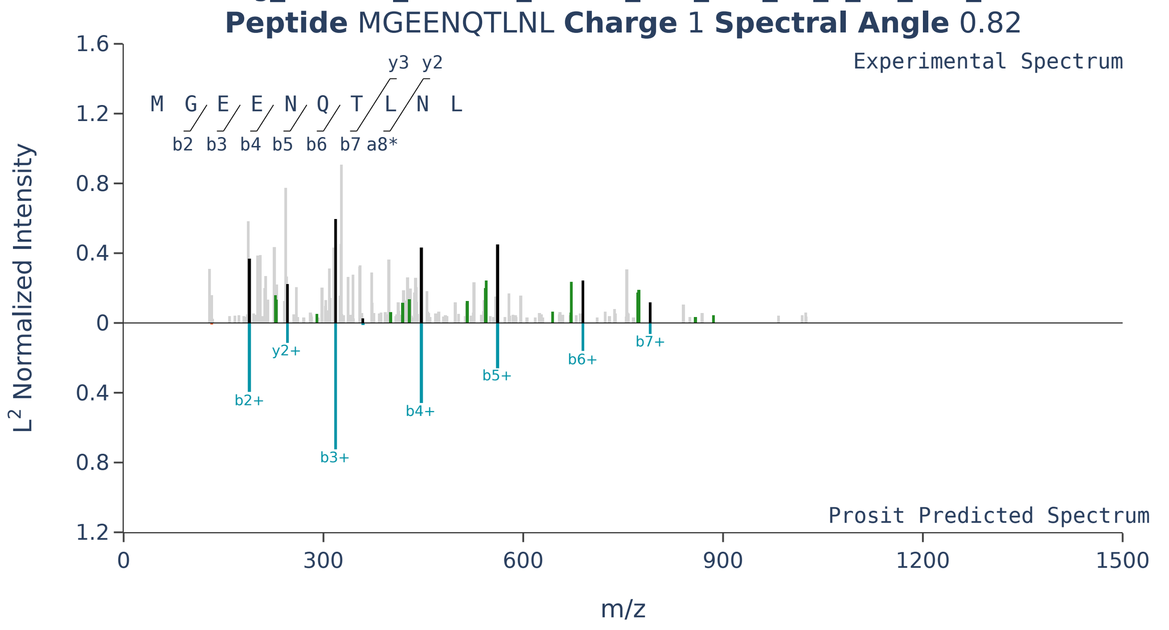


**Source** Ncheng_210623_230623_HFGoe_FFH_20S_25_1_A2_1h_R1 **Scan** 11393
**Peptide** ASINPVE **Charge** 1 **Spectral Angle** 0.89

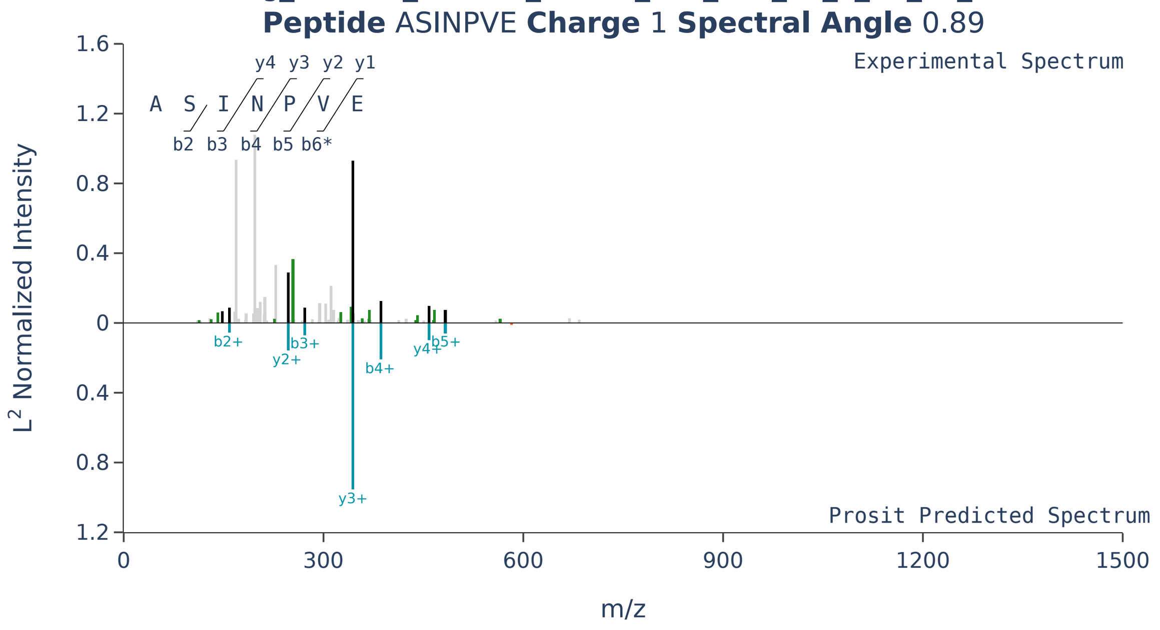


**Source** Ncheng_210623_230623_HFGoe_FFH_20S_25_1_A1_4h_R2 **Scan** 20385
**Peptide** VVSADVYRPAAI **Charge** 2 **Spectral Angle** 0.83

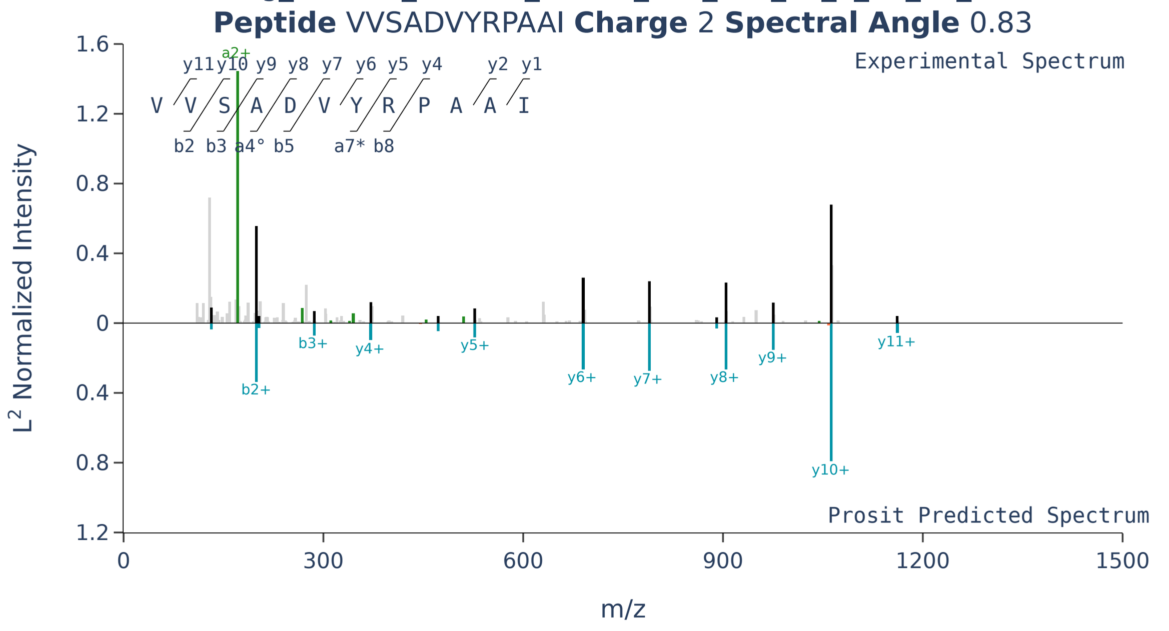


**Source** Ncheng_210623_230623_HFGoe_FFH_20S_25_1_A1_2h_R2 **Scan** 25272
**Peptide** VDFFPSDVGQKPVD **Charge** 2 **Spectral Angle** 0.83

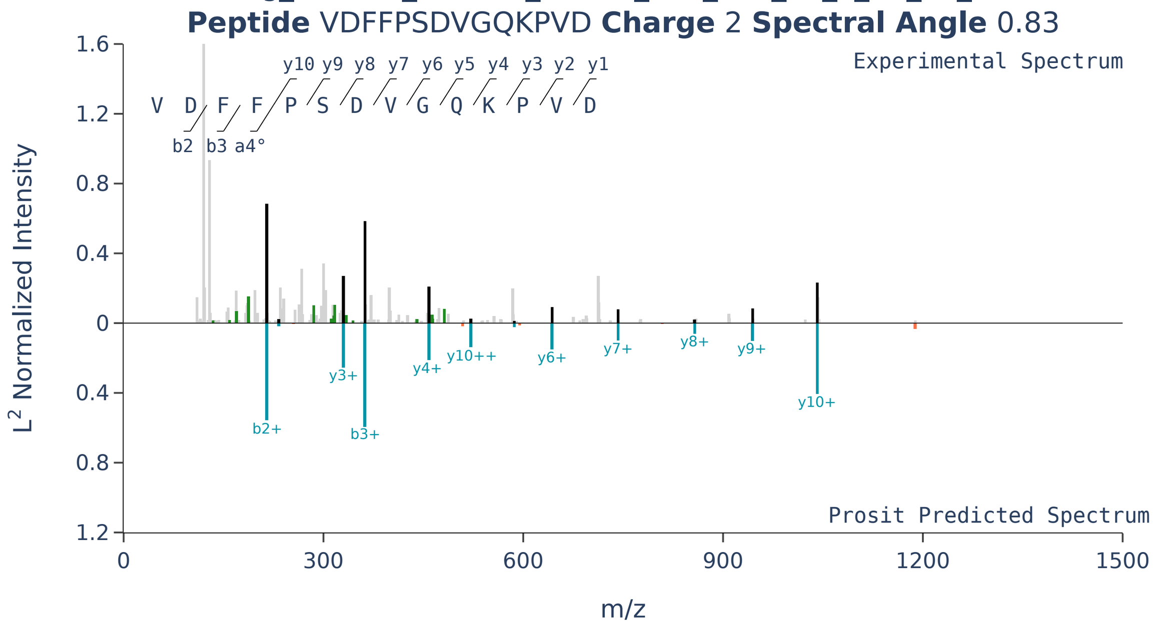


**Source** Ncheng_210623_230623_HFGoe_FFH_20S_25_1_A1_2h_R2 **Scan** 13869
**Peptide** PGMGQIPDNVKSQMD **Charge** 2 **Spectral Angle** 0.9

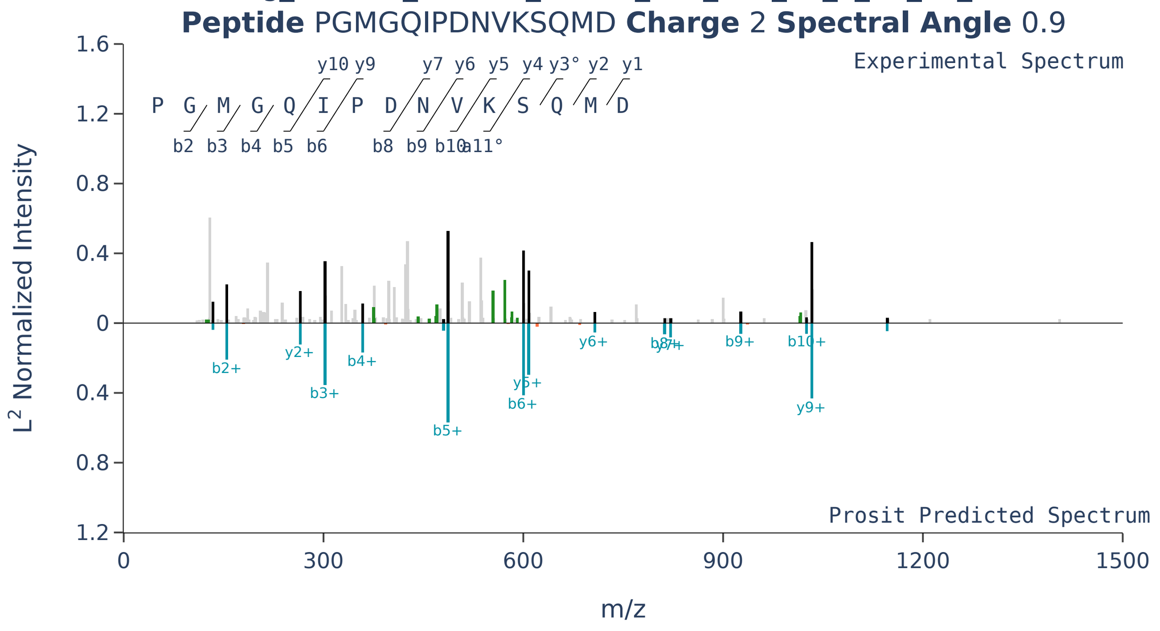


**Source** Ncheng_210623_230623_HFGoe_FFH_20S_25_1_A1_1h_R2 **Scan** 23827
**Peptide** EENQTLNLAAQPPAV **Charge** 2 **Spectral Angle** 0.97

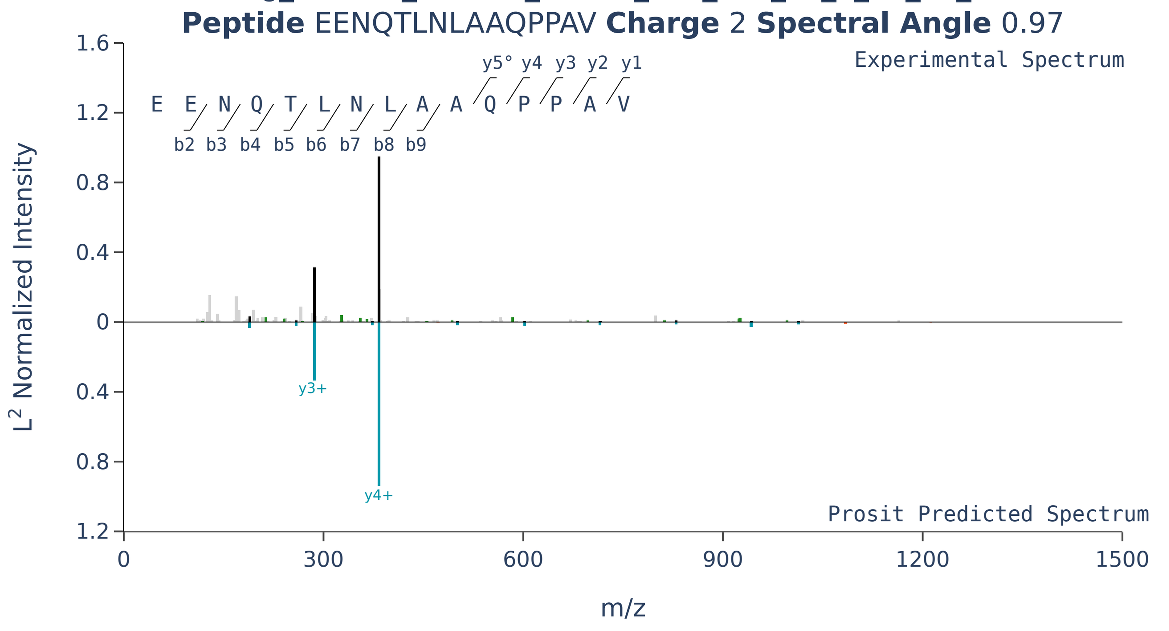


**Source** Ncheng_210623_230623_HFGoe_FFH_20S_25_1_A2_24h_R2 **Scan** 21851
**Peptide** AMGEENQTLNL **Charge** 1 **Spectral Angle** 0.85

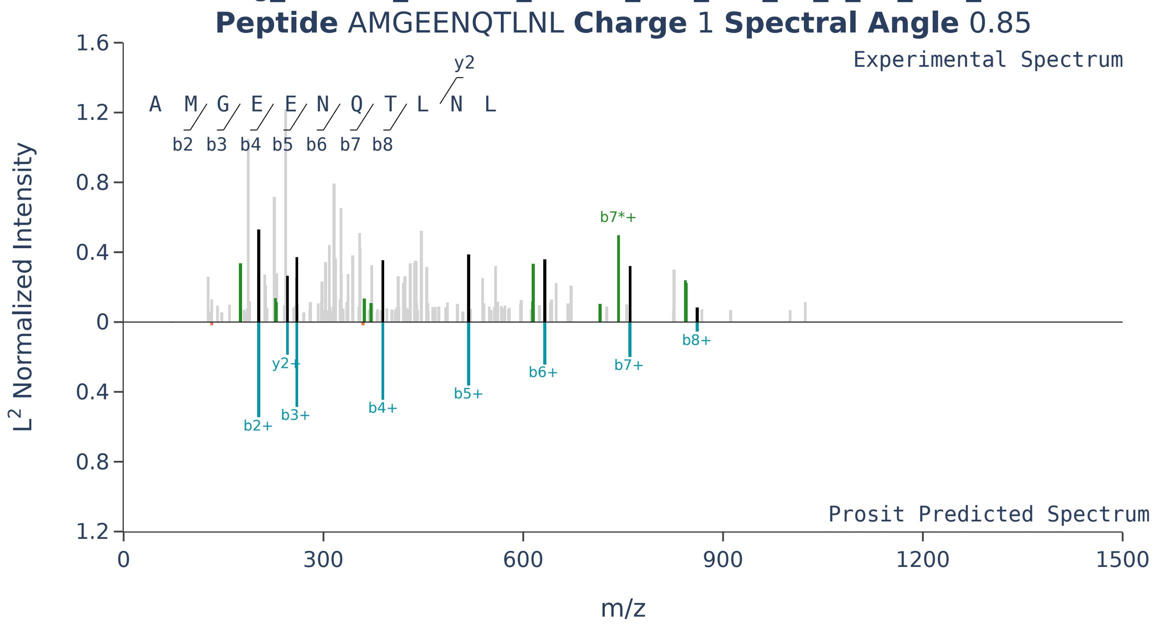


**Source** Ncheng_210623_230623_HFGoe_FFH_20S_25_1_A2_24h_R2 **Scan** 16918
**Peptide** MGKLPGM **Charge** 2 **Spectral Angle** 0.93

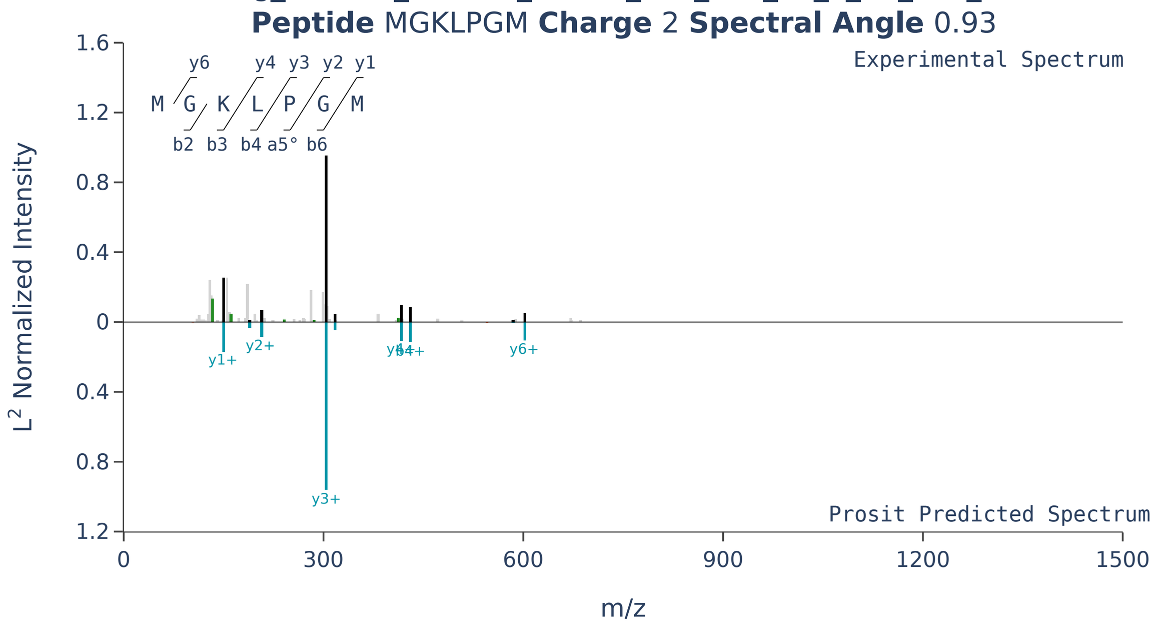


**Source** Ncheng_210623_230623_HFGoe_FFH_20S_25_1_A1_4h_R1 **Scan** 29943
**Peptide** EQVGVDFFPSD **Charge** 1 **Spectral Angle** 0.86

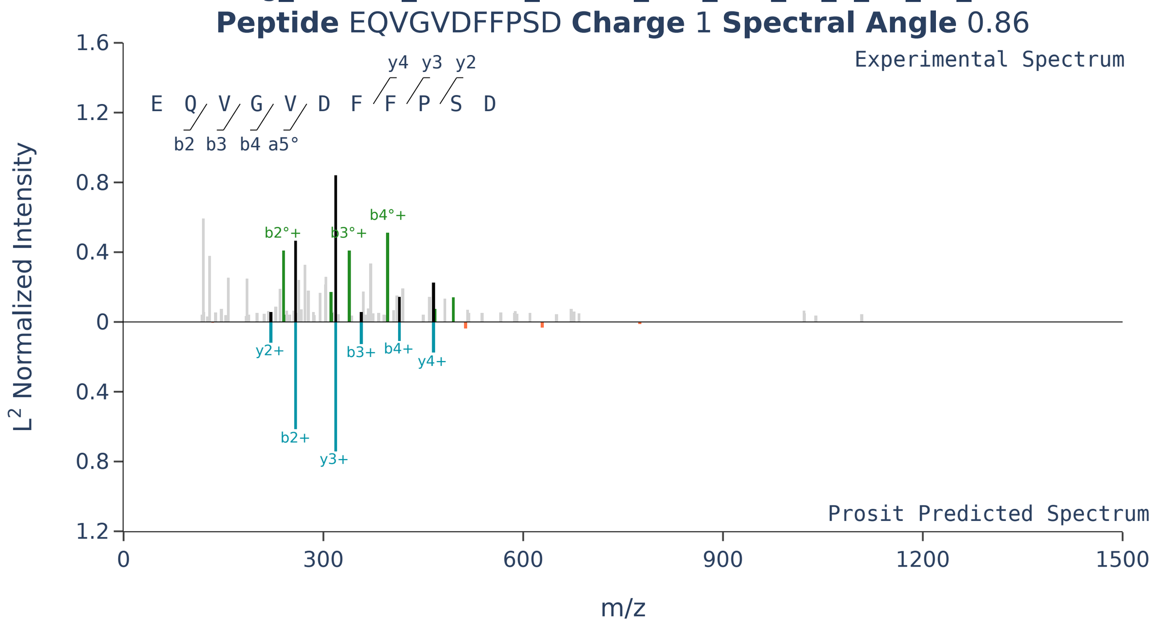


**Source** Ncheng_210623_230623_HFGoe_FFH_20S_25_1_A1_2h_R2 **Scan** 30007
**Peptide** LAEQVGVDFFPSDVGQKPVD **Charge** 2 **Spectral Angle** 0.9

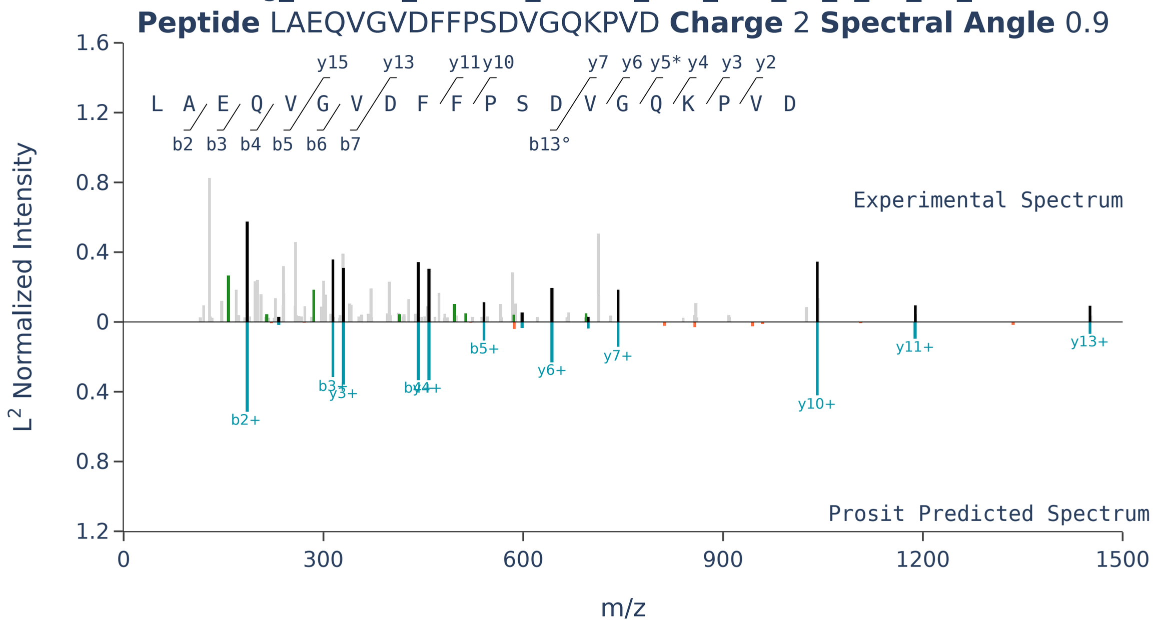


**Source** Ncheng_210623_230623_HFGoe_FFH_20S_25_1_A1_1h_R2 **Scan** 16322
**Peptide** VDAMTGQDAANTAKAFNE **Charge** 2 **Spectral Angle** 0.93

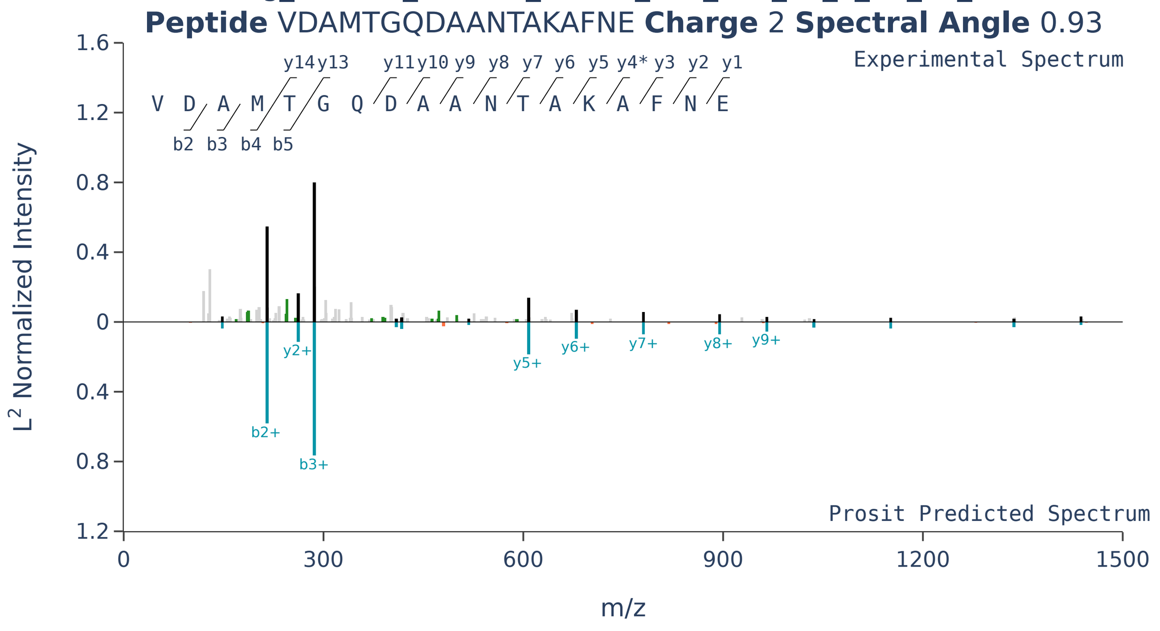

**Source** Ncheng_210623_230623_HFGoe_FFH_20S_25_1_A1_4h_R2 **Scan** 25849
**Peptide** AGCGMQVQDVNRLL **Charge** 2 **Spectral Angle** 0.85

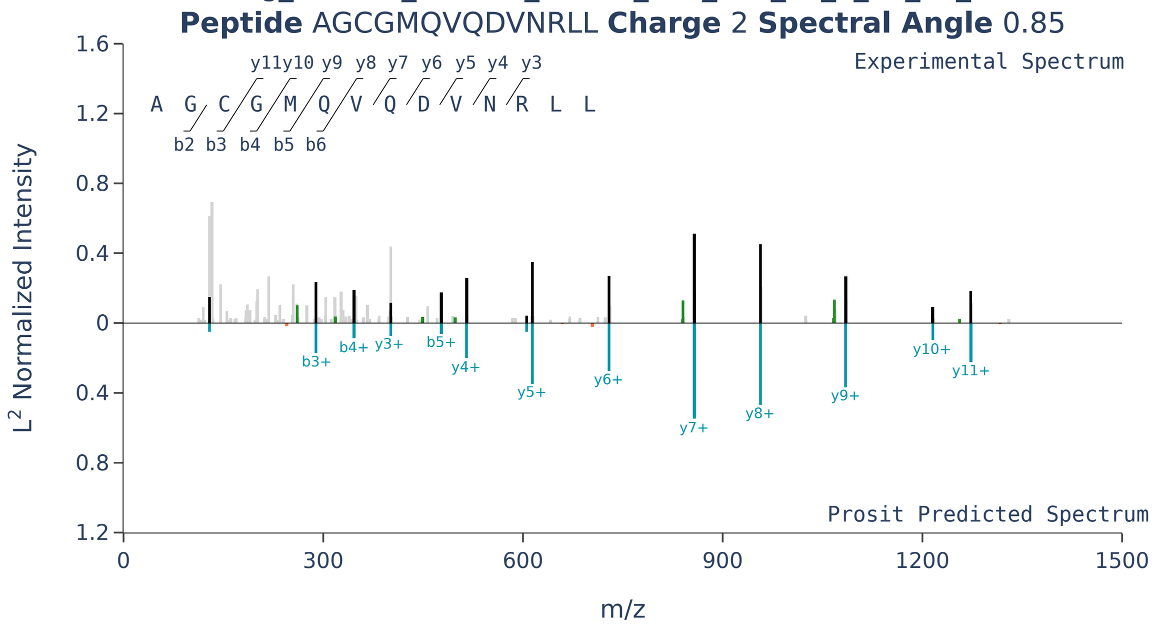


**Source** Ncheng_210623_230623_HFGoe_FFH_20S_25_1_A2_4h_R2 **Scan** 27957
**Peptide** ENQTLNLAAQPPAVV **Charge** 2 **Spectral Angle** 0.94

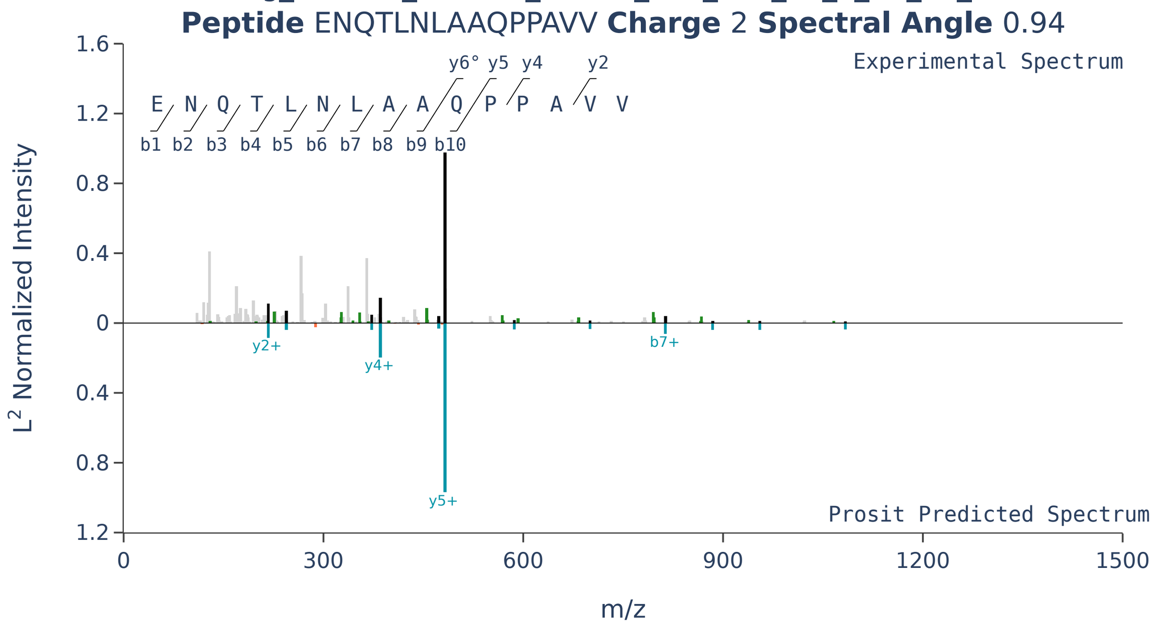


**Source** Ncheng_210623_230623_HFGoe_FFH_20S_25_1_A1_4h_R2 **Scan** 22396
**Peptide** VLVVSADVYRPAALKQ **Charge** 2 **Spectral Angle** 0.86

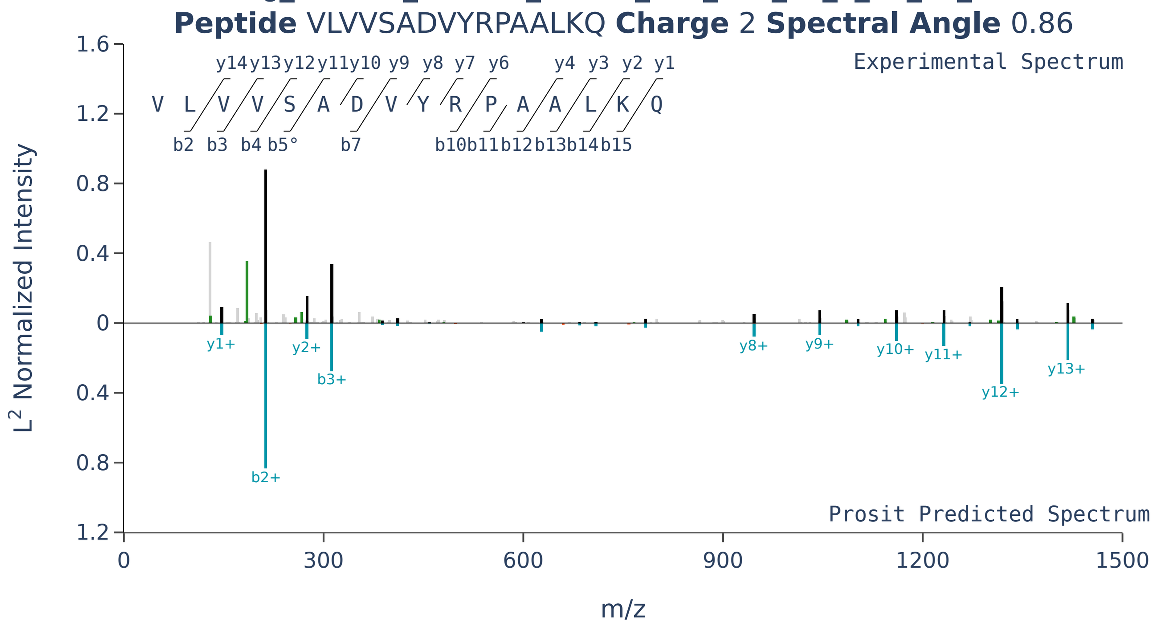


**Source** Ncheng_210623_230623_HFGoe_FFH_20S_25_1_A1_24h_R2 **Scan** 13731
**Peptide** NRLLKQF **Charge** 2 **Spectral Angle** 0.89

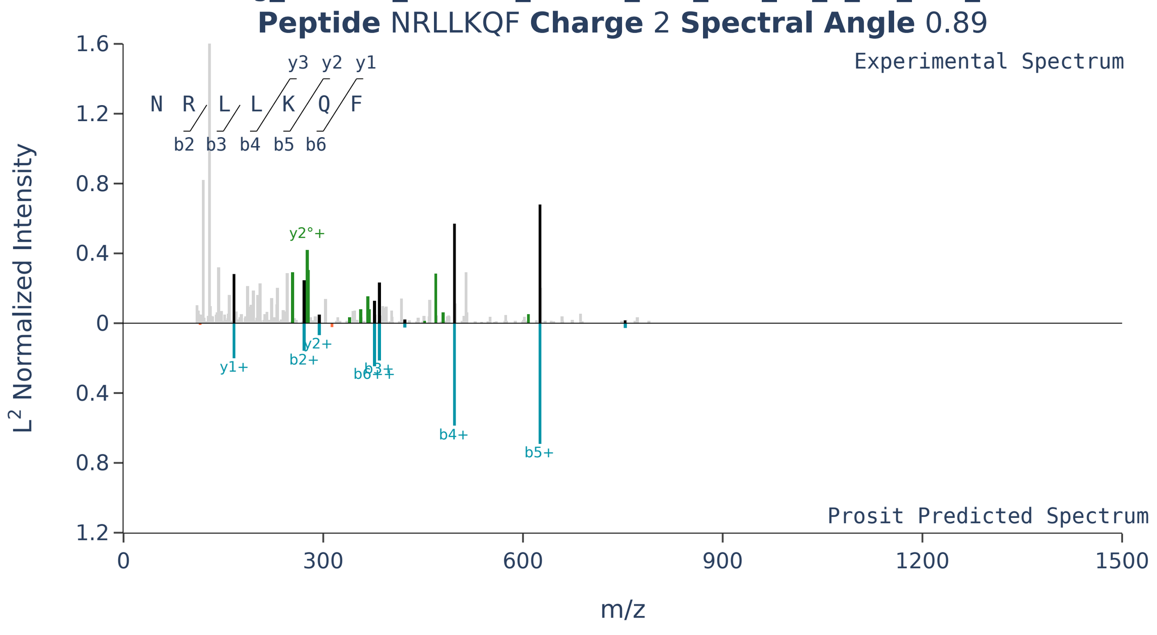


**Source** Ncheng_210623_230623_HFGoe_FFH_20S_25_1_A2_2h_R1 **Scan** 28683
**Peptide** ELVAAMGEENQTLNL **Charge** 2 **Spectral Angle** 0.91

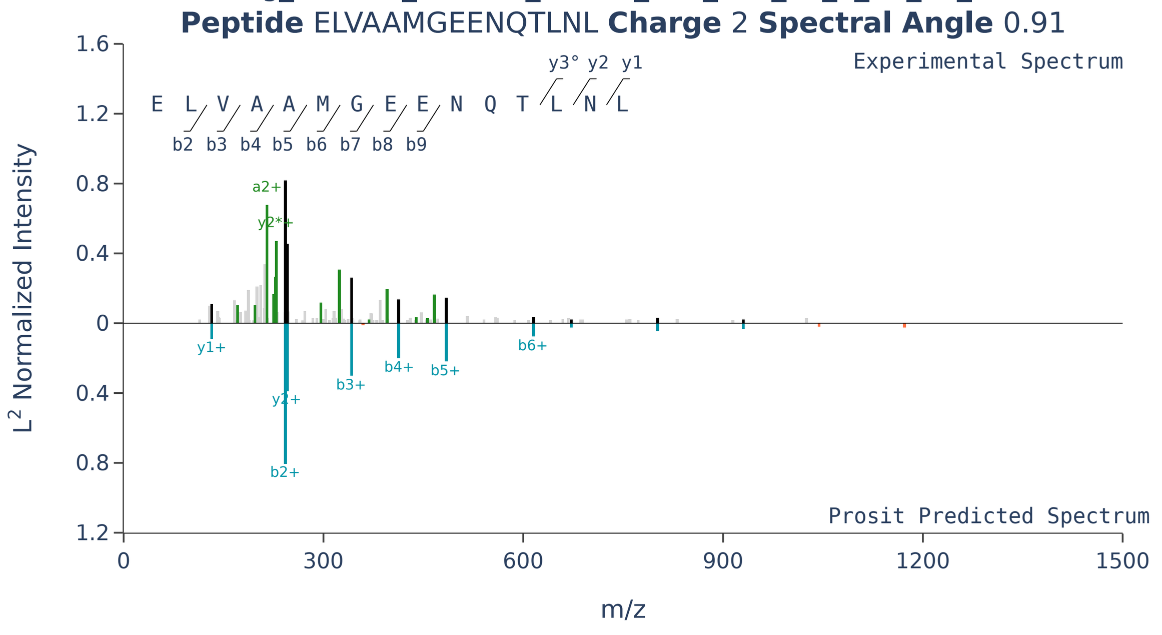


**Source** Ncheng_210623_230623_HFGoe_FFH_20S_25_1_A2_4h_R1 **Scan** 19606
**Peptide** LETLAEQV **Charge** 1 **Spectral Angle** 0.86

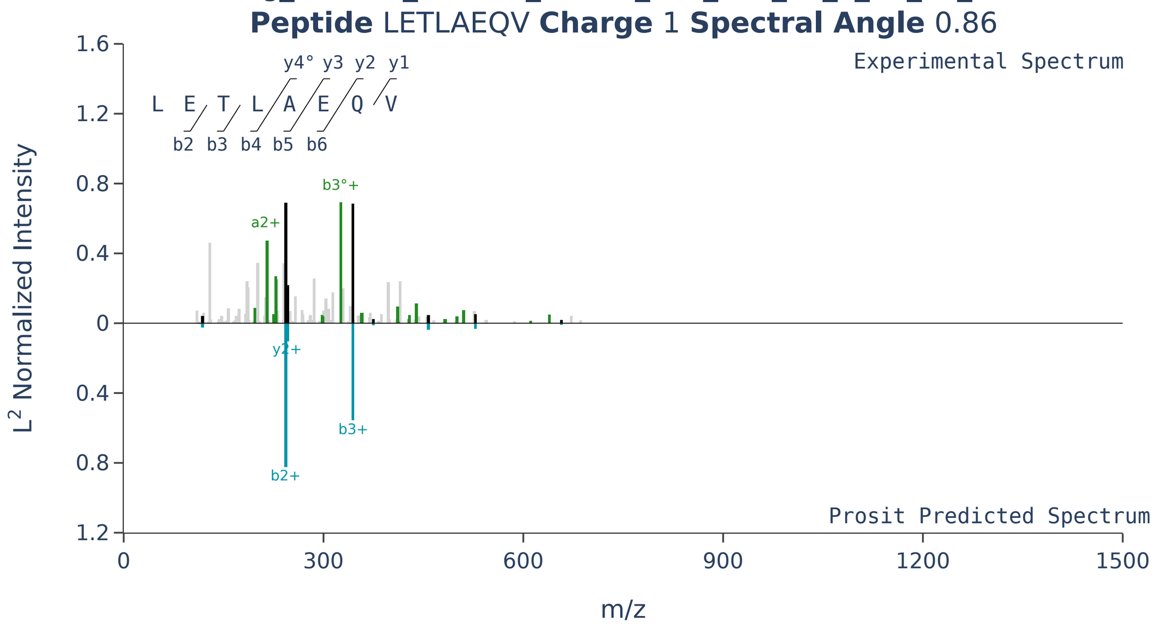


**Source** Ncheng_210623_230623_HFGoe_FFH_20S_25_1_A2_24h_R2 **Scan** 12197
**Peptide** VVLTKVD **Charge** 1 **Spectral Angle** 0.86

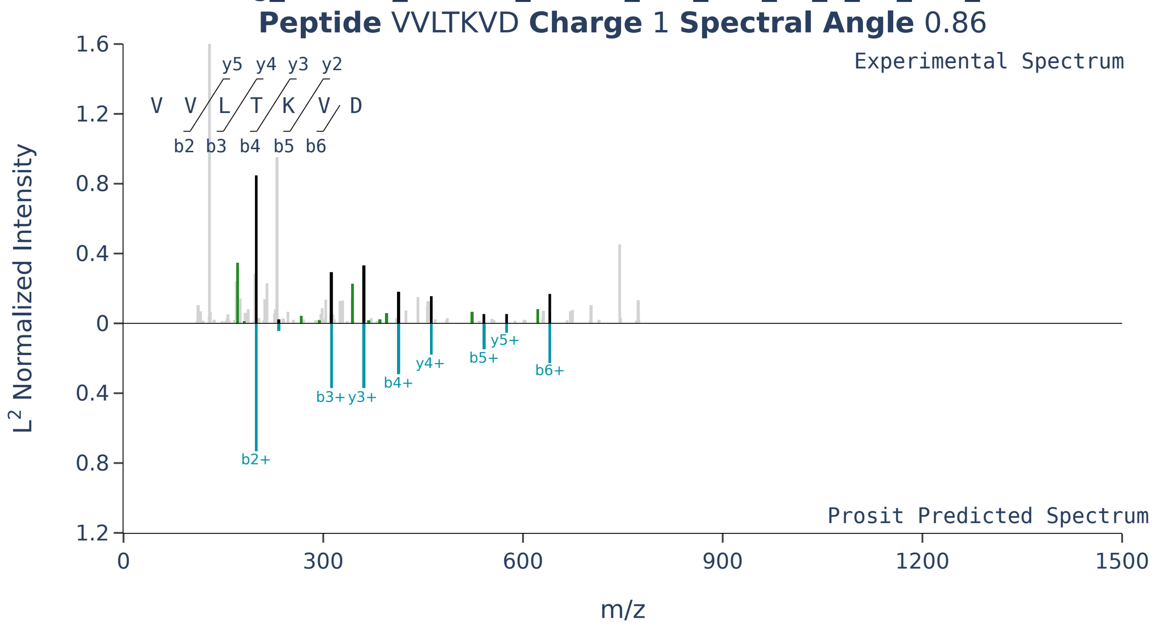


**Source** Ncheng_210623_230623_HFGoe_FFH_20S_25_1_A1_24h_R1 **Scan** 6414
**Peptide** GRLTEDNVKD **Charge** 3 **Spectral Angle** 0.88

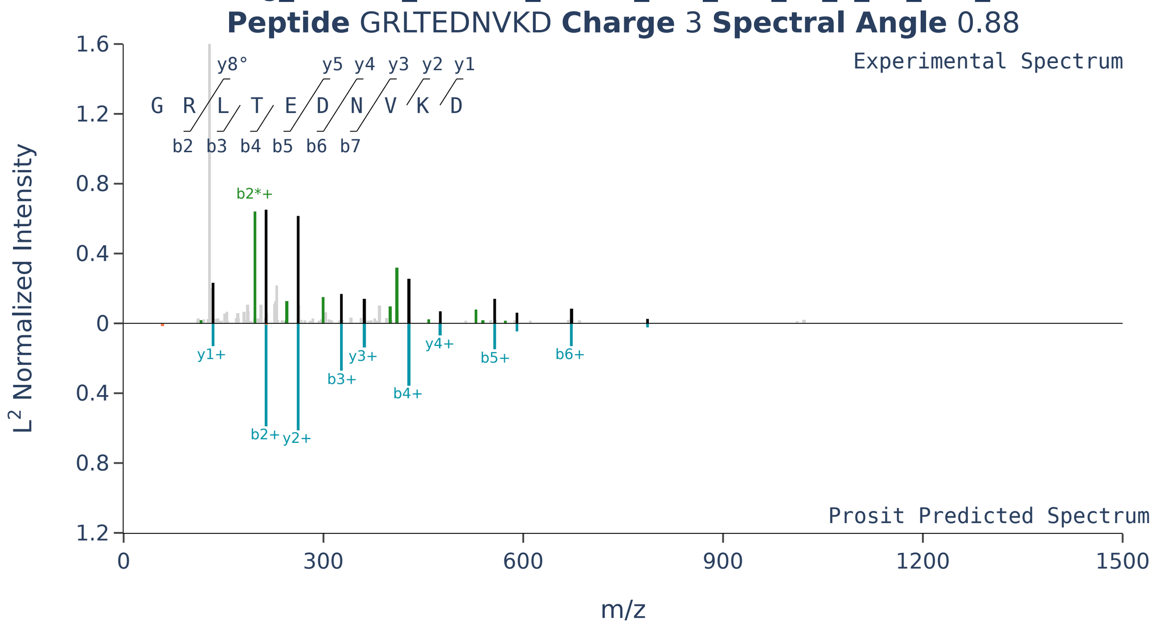


**Source** Ncheng_210623_230623_HFGoe_FFH_20S_25_1_A2_4h_R2 **Scan** 13572
**Peptide** EPFHPDRI **Charge** 2 **Spectral Angle** 0.91

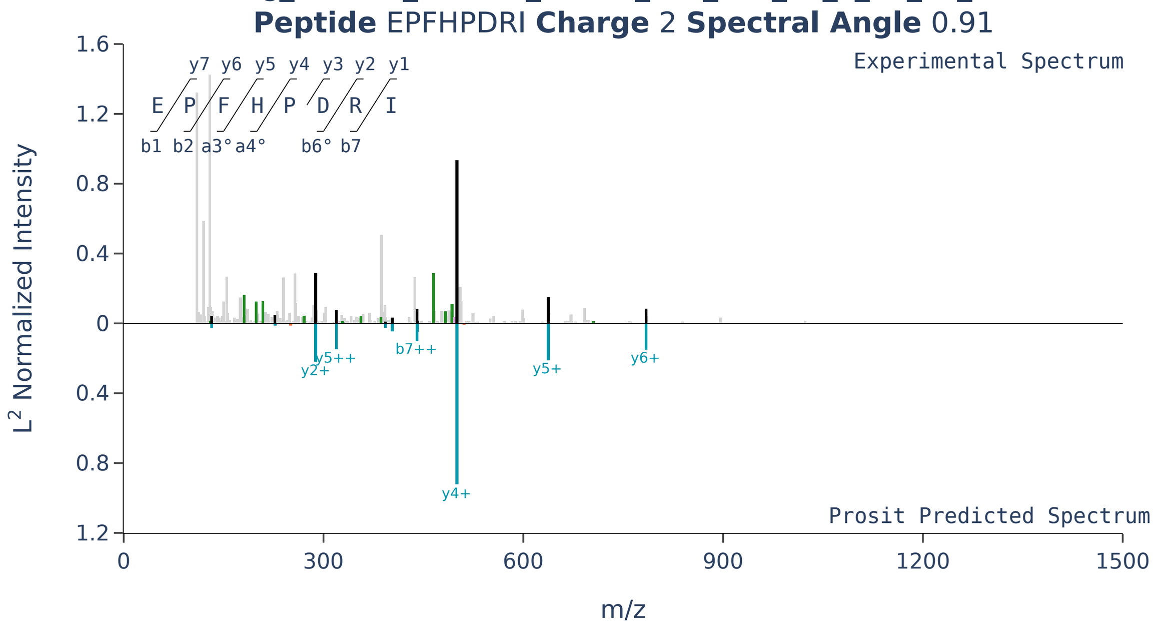


**Source** Ncheng_210623_230623_HFGoe_FFH_20S_25_1_A2_4h_R1 **Scan** 8339
**Peptide** DAANTAKAFN **Charge** 1 **Spectral Angle** 0.86

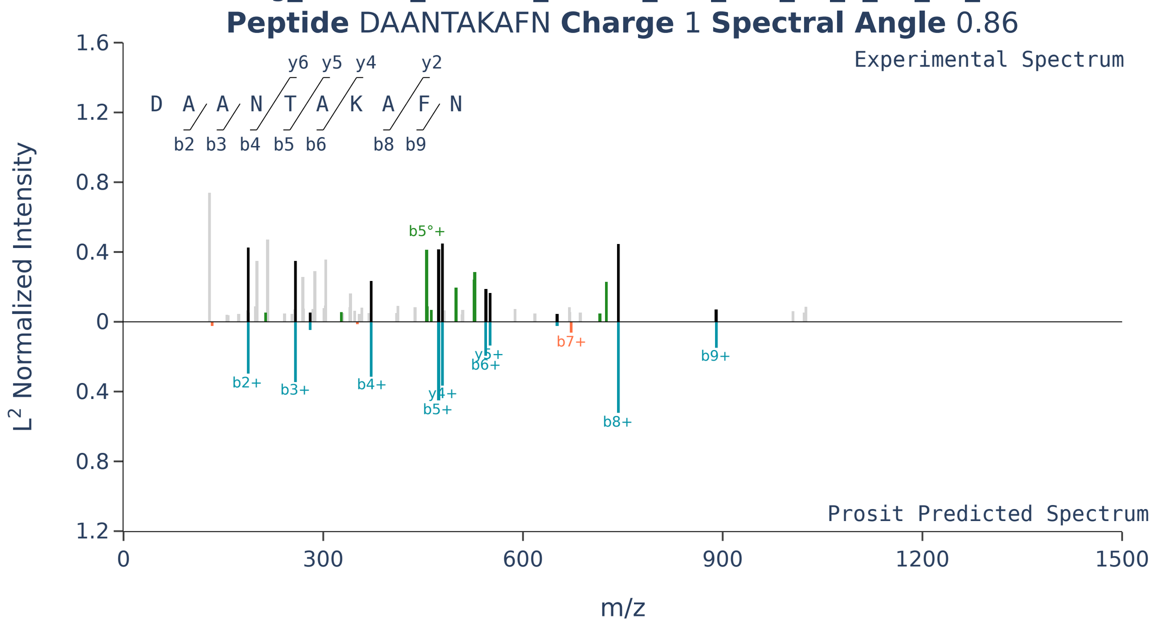


**Source** Ncheng_210623_230623_HFGoe_FFH_20S_25_1_A1_24h_R1 **Scan** 6259
**Peptide** ELKQVHA **Charge** 1 **Spectral Angle** 0.94

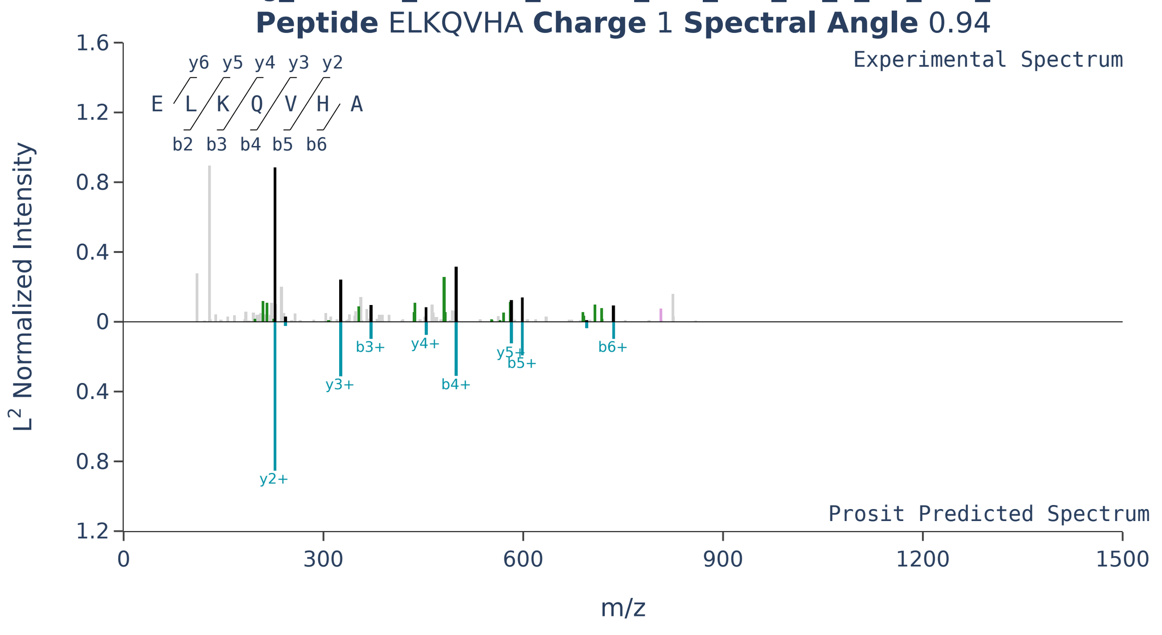


**Source** Ncheng_210623_230623_HFGoe_FFH_20S_25_1_A1_2h_R2 **Scan** 26383
**Peptide** VLVVSADVYRPAAIKQLE **Charge** 2 **Spectral Angle** 0.91

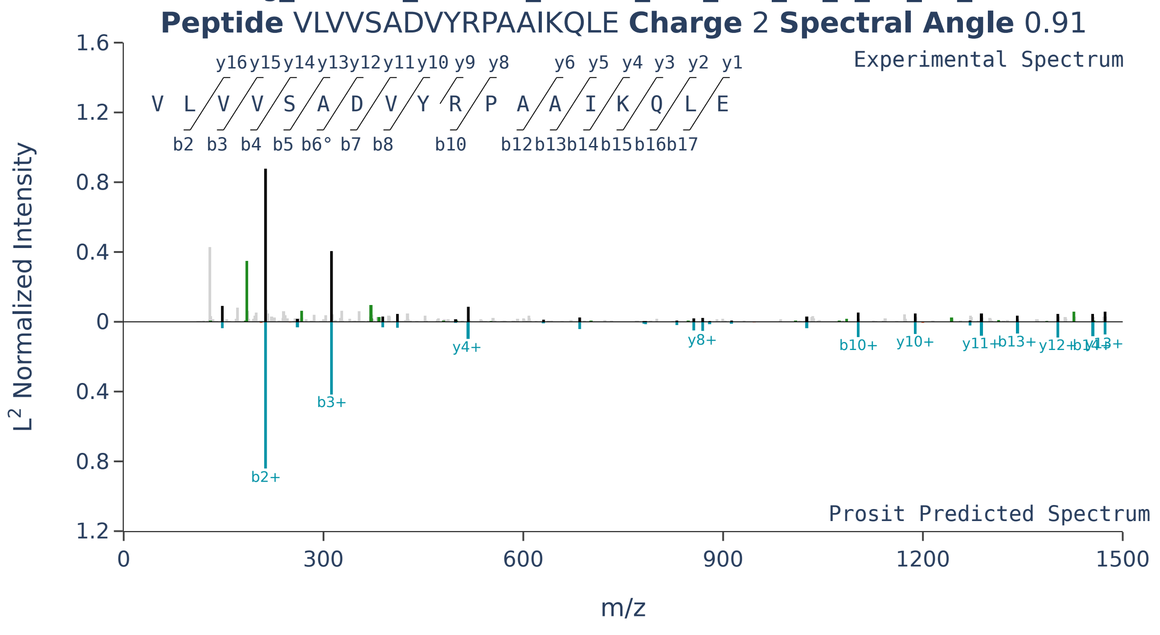


**Source** Ncheng_210623_230623_HFGoe_FFH_20S_25_1_A2_24h_R2 **Scan** 23758
**Peptide** MEAIINSMTMKE **Charge** 2 **Spectral Angle** 0.85

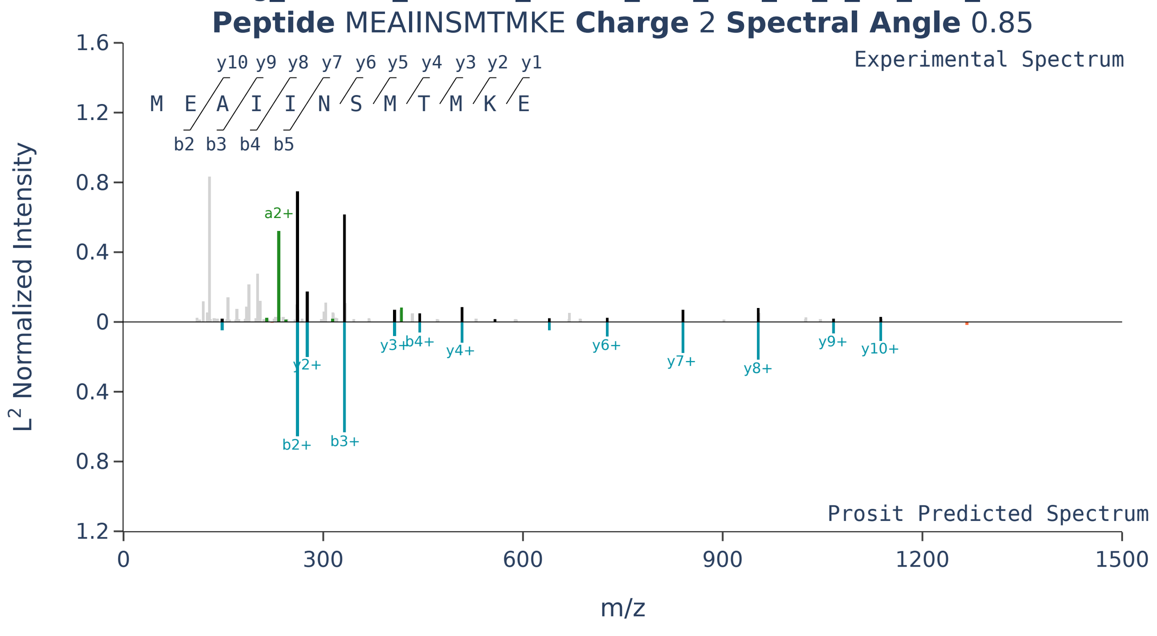


**Source** Ncheng_210623_230623_HFGoe_FFH_20S_25_1_A2_4h_R1 **Scan** 24987
**Peptide** PIKFLGVGE **Charge** 2 **Spectral Angle** 0.86

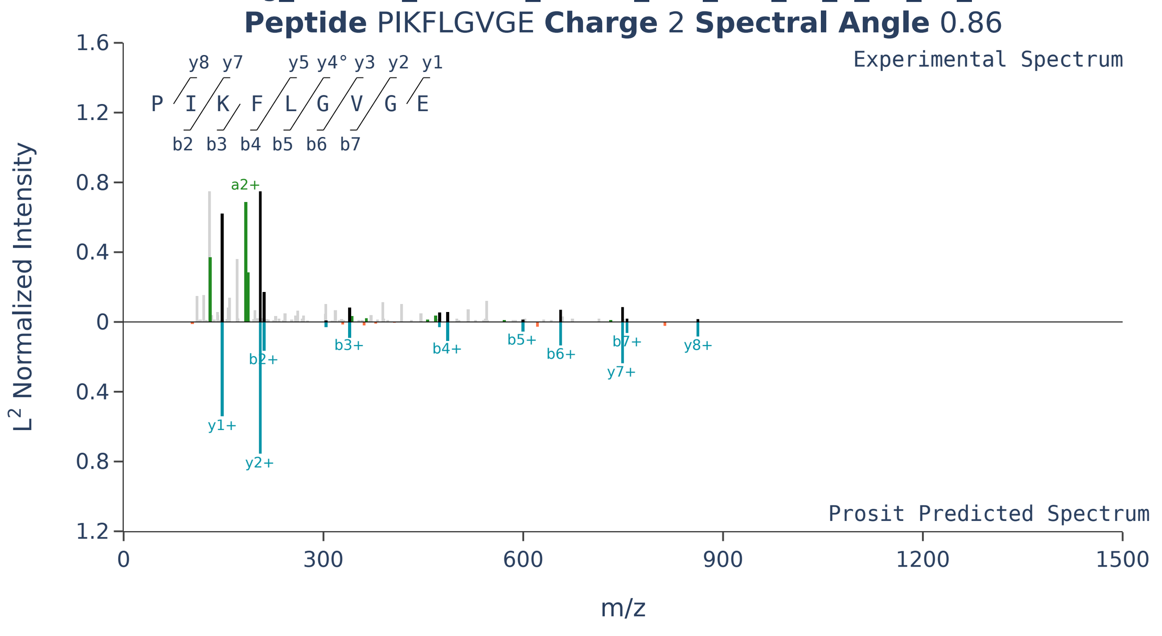


**Source** Ncheng_210623_230623_HFGoe_FFH_20S_25_1_A1_4h_R2 **Scan** 26200
**Peptide** MFDNLTDRL **Charge** 2 **Spectral Angle** 0.78

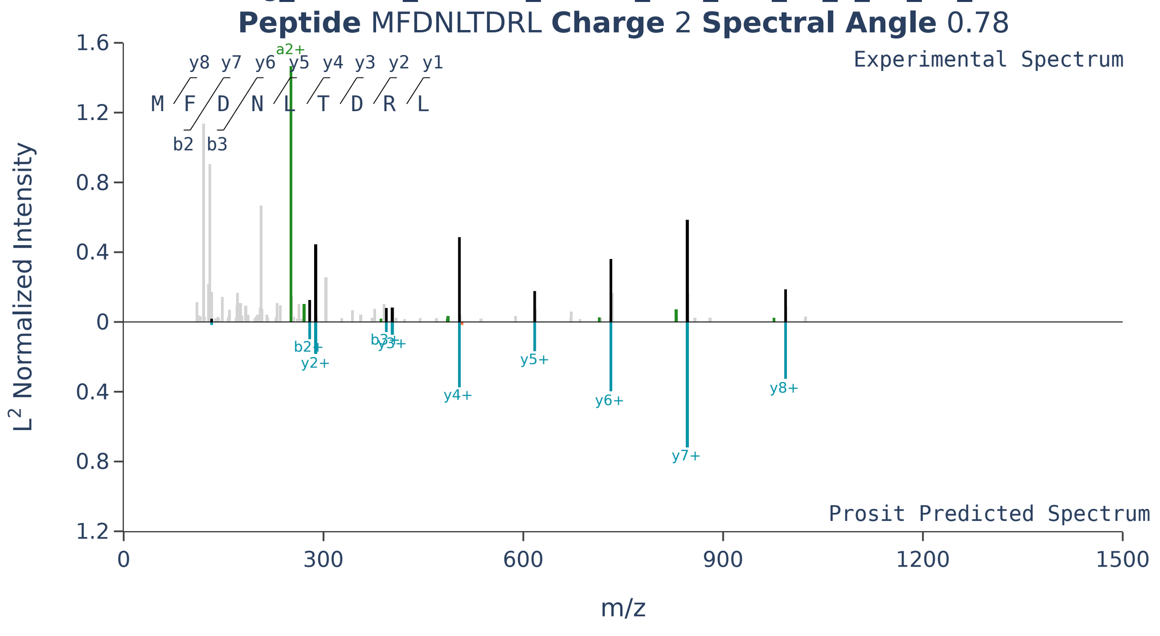

**Source** Ncheng_210623_230623_HFGoe_FFH_20S_25_1_A2_4h_R2 **Scan** 8566
**Peptide** GQIPDNVKS **Charge** 1 **Spectral Angle** 0.83

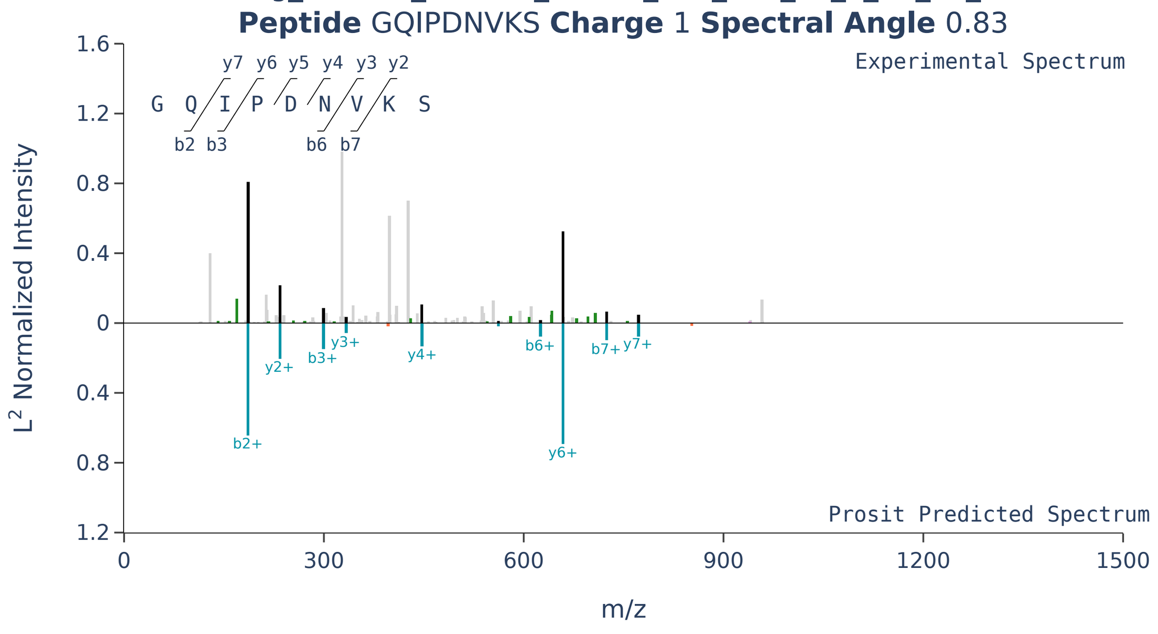


**Source** Ncheng_210623_230623_HFGoe_FFH_20S_25_1_A1_2h_R1 **Scan** 5622
**Peptide** DEIKQVH **Charge** 2 **Spectral Angle** 0.9

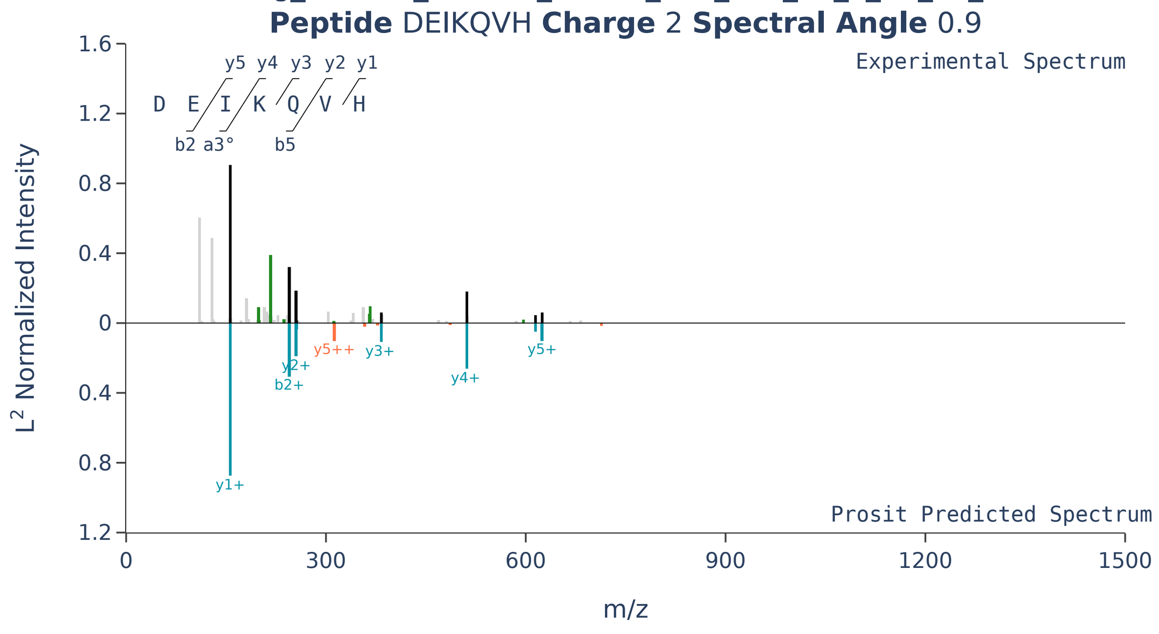


**Source** Ncheng_210623_230623_HFGoe_FFH_20S_25_1_A2_4h_R2 **Scan** 25275
**Peptide** ENQTLNLAAQPPAV **Charge** 2 **Spectral Angle** 0.98

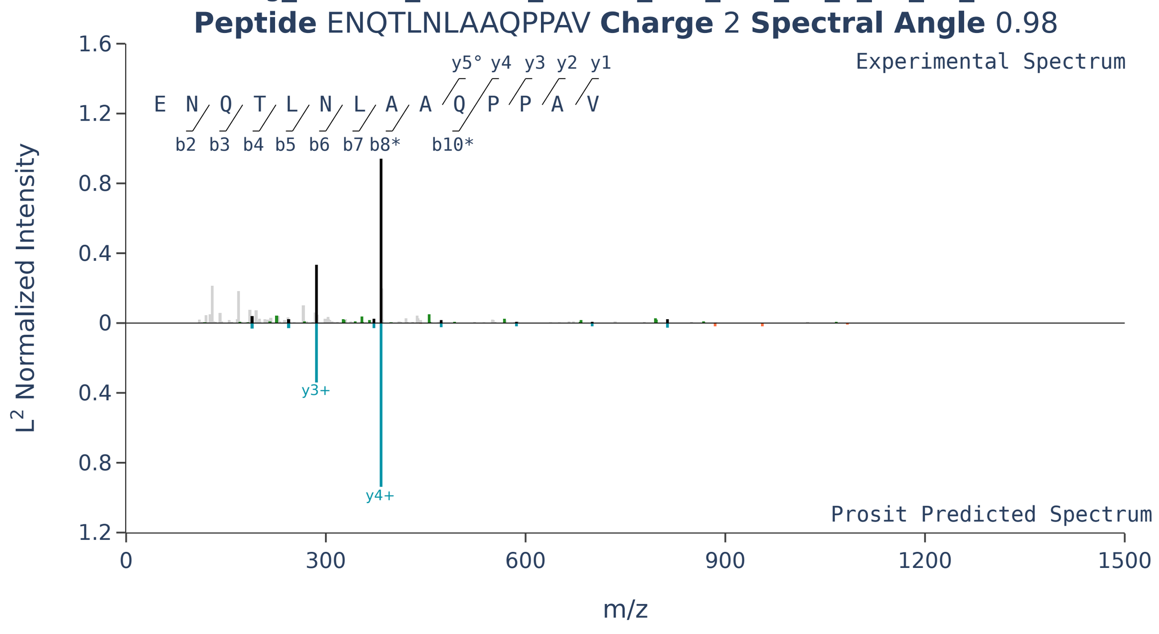


**Source** Ncheng_210623_230623_HFGoe_FFH_20S_25_1_A2_4h_R1 **Scan** 30482
**Peptide** NELVAAMGEENQTLNL **Charge** 2 **Spectral Angle** 0.95

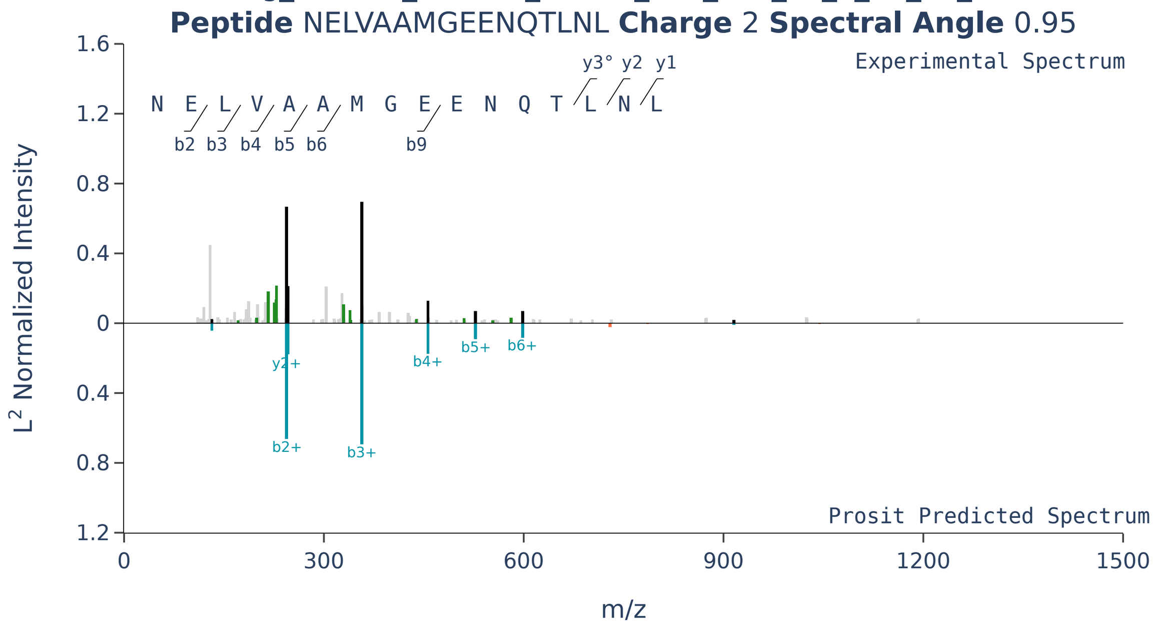


**Source** Ncheng_210623_230623_HFGoe_FFH_20S_25_1_A2_4h_R2 **Scan** 21663
**Peptide** EVNKSLTPGQEFVKIVRN **Charge** 2 **Spectral Angle** 0.91

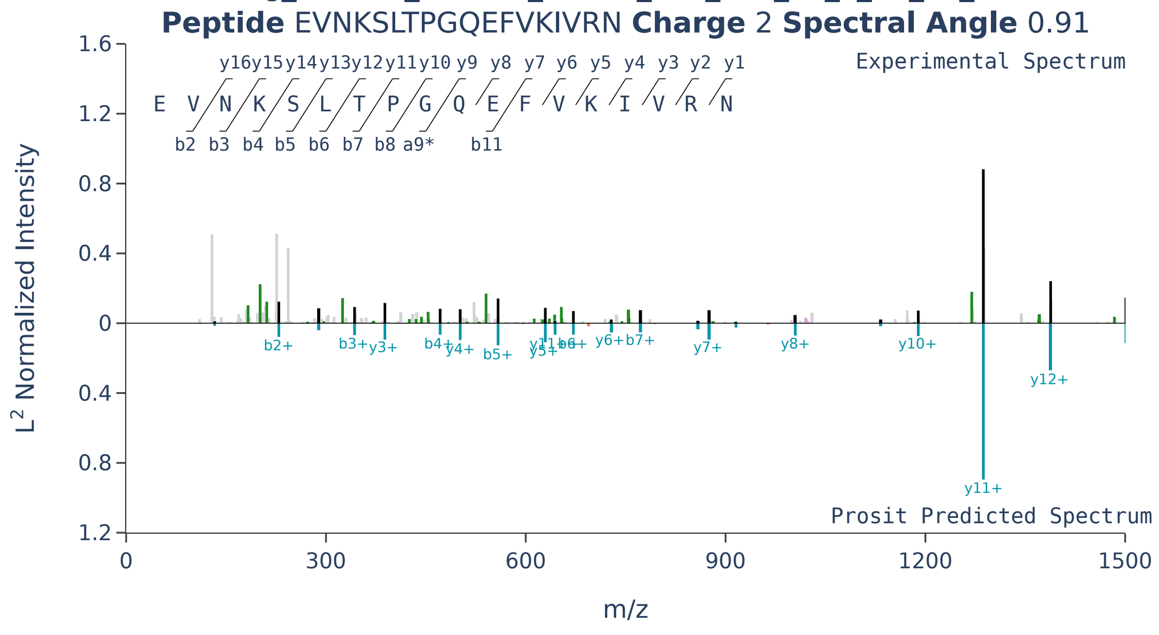


**Source** Ncheng_210623_230623_HFGoe_FFH_20S_25_1_A1_4h_R1 **Scan** 21316
**Peptide** RMEAIINSMTMKE **Charge** 3 **Spectral Angle** 0.87

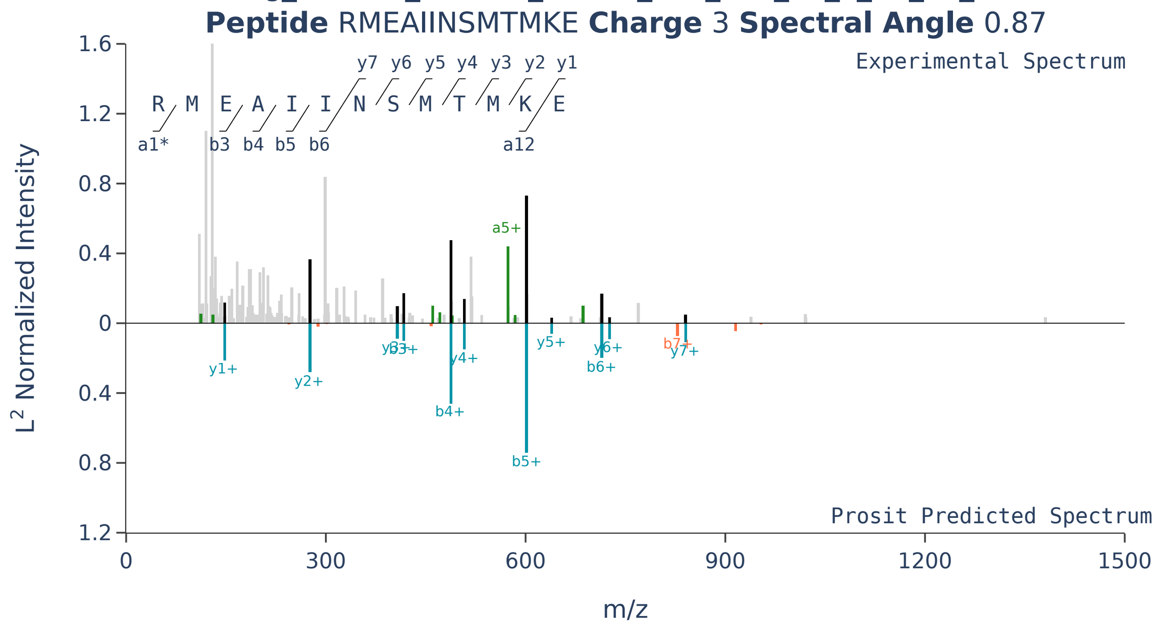


**Source** Ncheng_210623_230623_HFGoe_FFH_20S_25_1_A1_24h_R1 **Scan** 14398
**Peptide** VVSADVY **Charge** 1 **Spectral Angle** 0.82

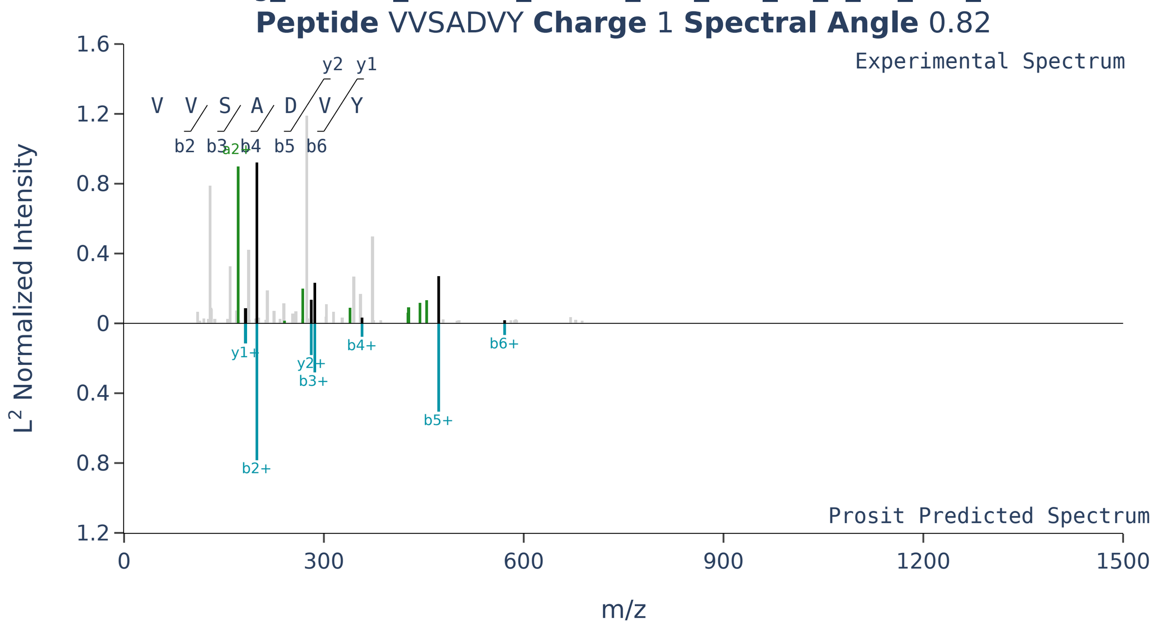


**Source** Ncheng_210623_230623_HFGoe_FFH_20S_25_1_A1_4h_R2 **Scan** 16959
**Peptide** PGQEFVKIVRNE **Charge** 2 **Spectral Angle** 0.85

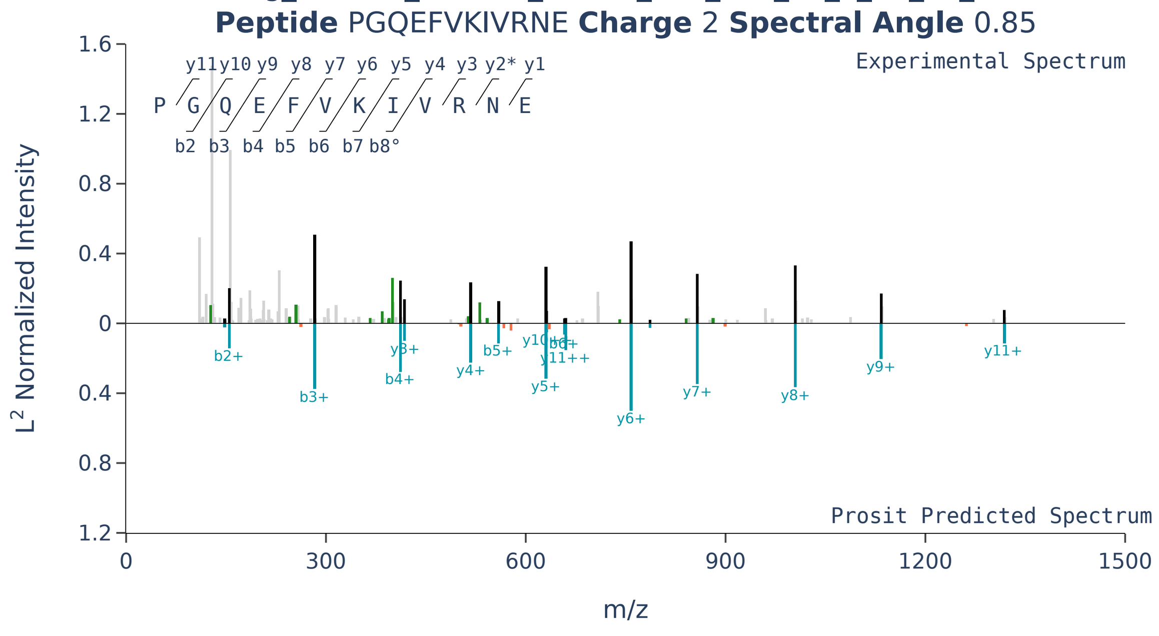


**Source** Ncheng_210623_230623_HFGoe_FFH_20S_25_1_A2_4h_R2 **Scan** 10708
**Peptide** LVNAALKE **Charge** 2 **Spectral Angle** 0.91

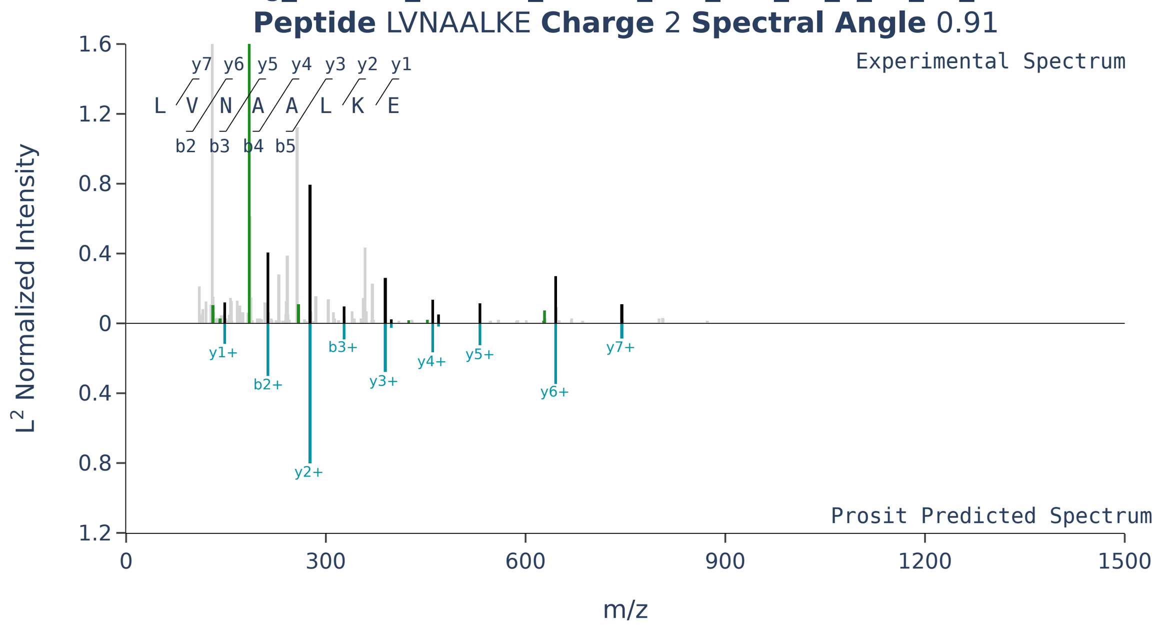


**Source** Ncheng_210623_230623_HFGoe_FFH_20S_25_1_A2_24h_R1 **Scan** 10325
**Peptide** AALKEAKL **Charge** 1 **Spectral Angle** 0.93

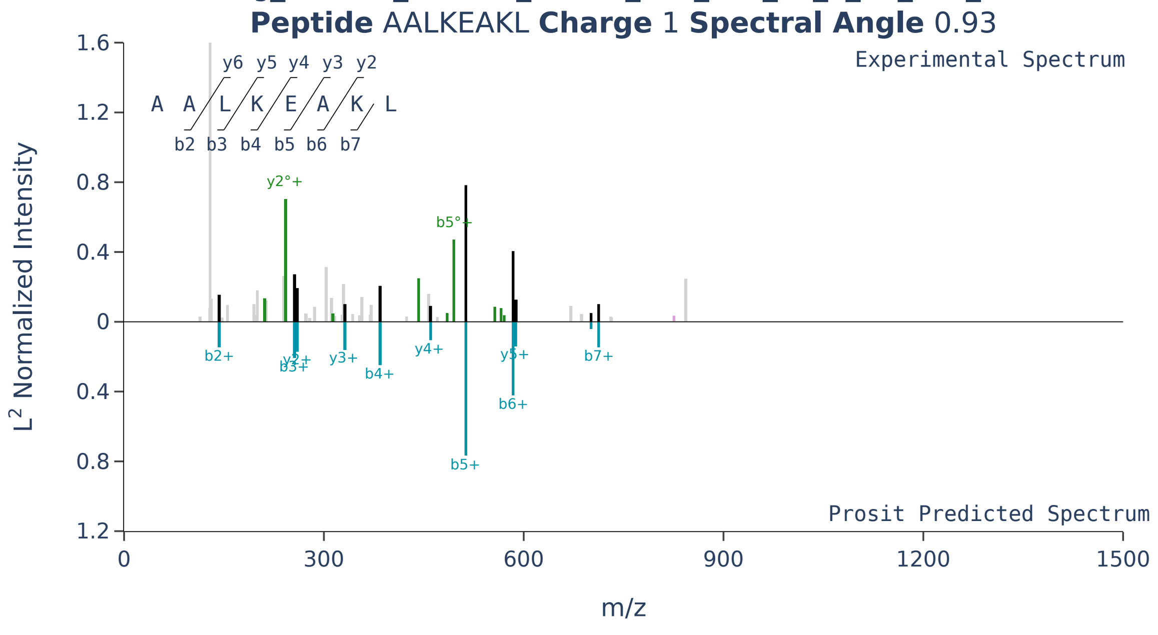


**Source** Ncheng_210623_230623_HFGoe_FFH_20S_25_1_A2_24h_R1 **Scan** 14868
**Peptide** GCGMQVQDV **Charge** 1 **Spectral Angle** 0.91

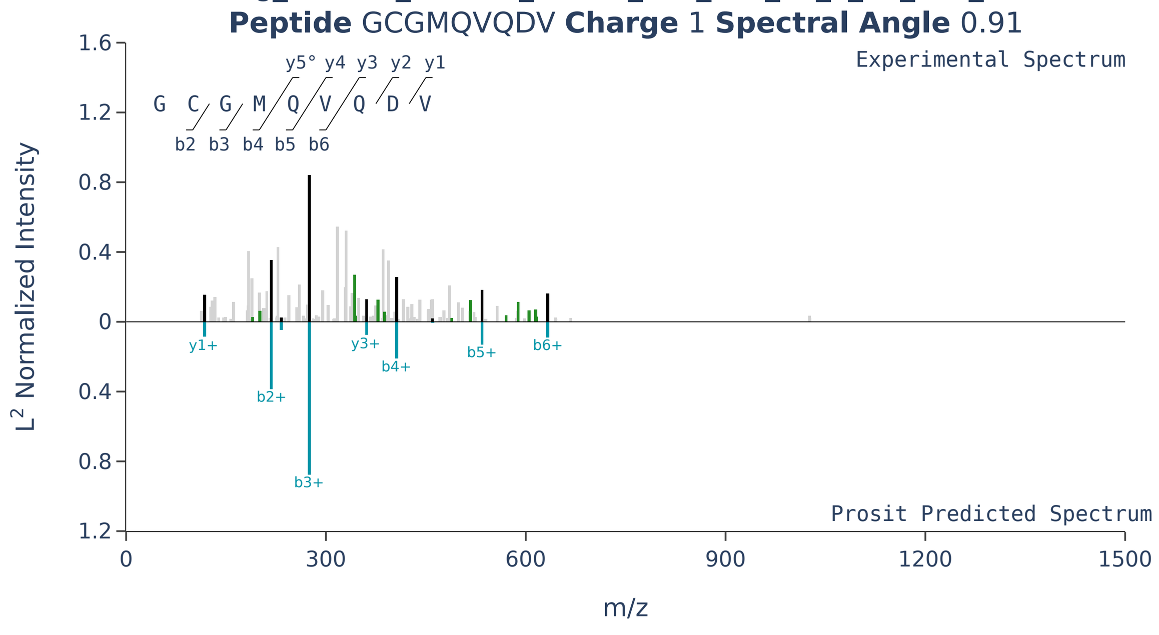


**Source** Ncheng_210623_230623_HFGoe_FFH_20S_25_1_A1_24h_R2 **Scan** 11925
**Peptide** DARGGAALSIR **Charge** 3 **Spectral Angle** 0.88

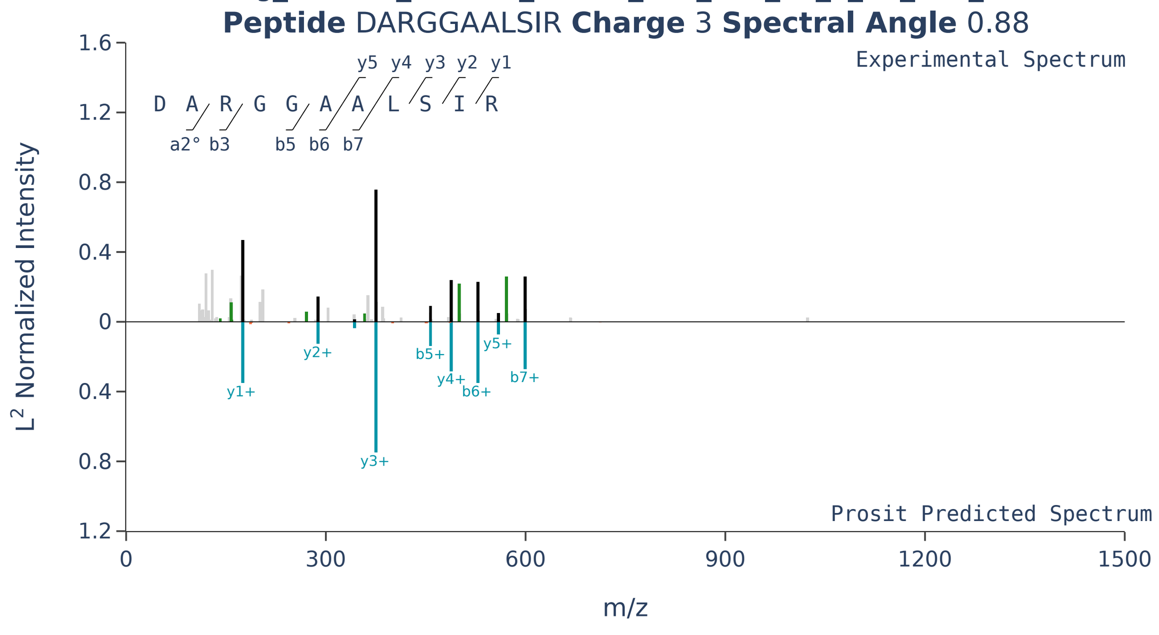


**Source** Ncheng_210623_230623_HFGoe_FFH_20S_25_1_A2_24h_R1 **Scan** 6302
**Peptide** INRVKEK **Charge** 2 **Spectral Angle** 0.88

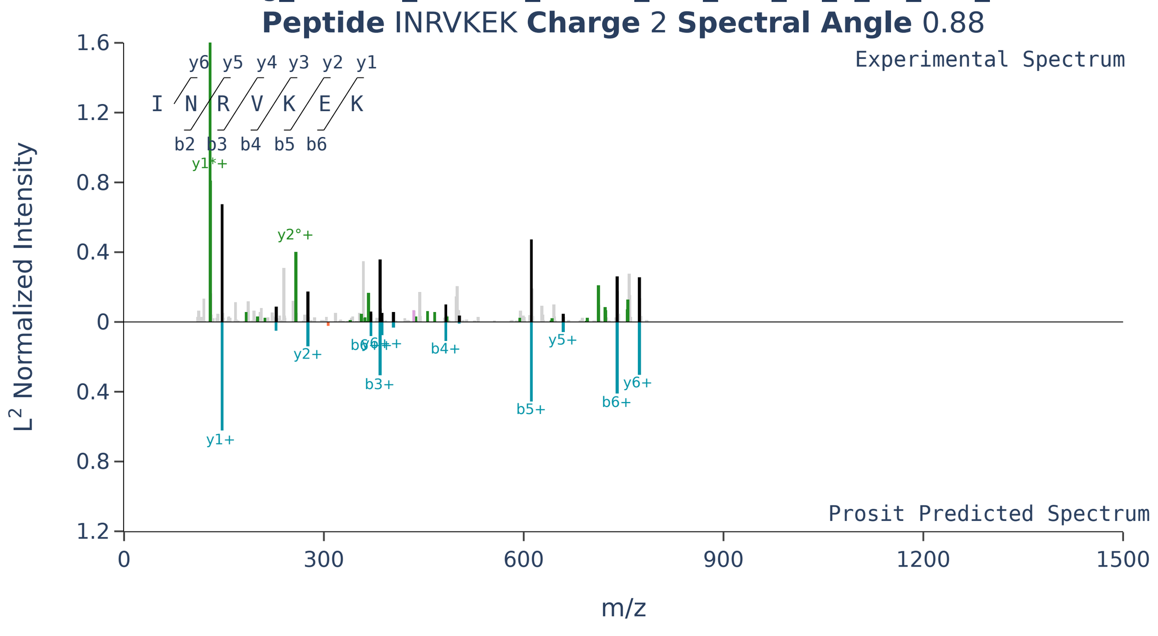


**Source** Ncheng_210623_230623_HFGoe_FFH_20S_25_1_A1_4h_R1 **Scan** 30202
**Peptide** VGVDFFPSD **Charge** 1 **Spectral Angle** 0.91

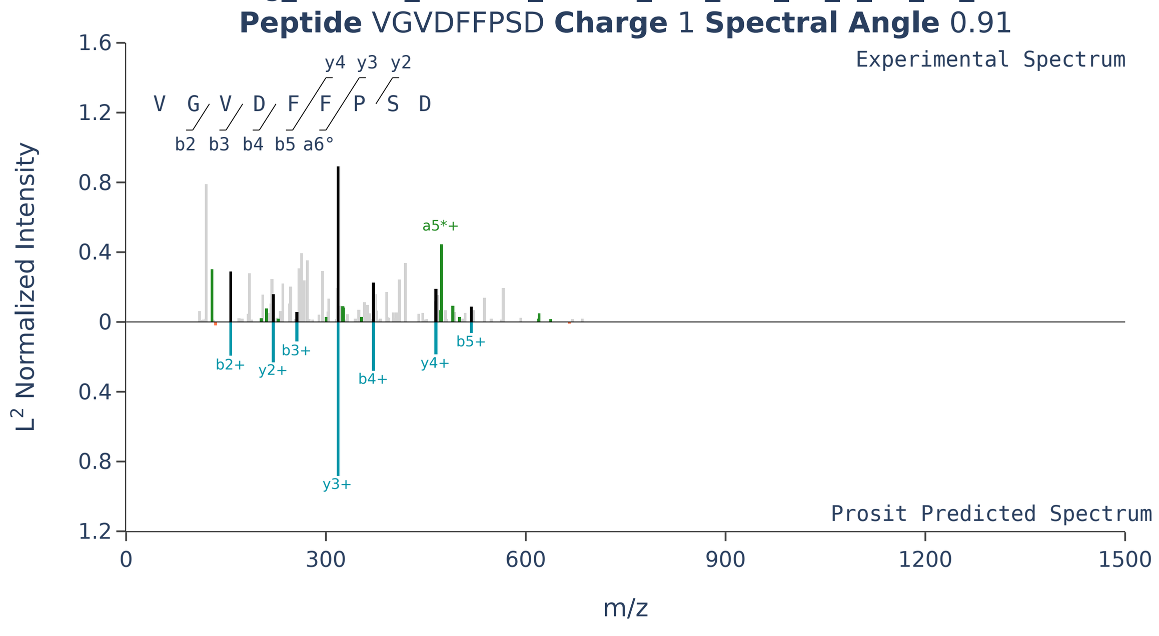


**Source** Ncheng_210623_230623_HFGoe_FFH_20S_25_1_A2_24h_R2 **Scan** 21639
**Peptide** MGKLPGMGQIPDN **Charge** 2 **Spectral Angle** 0.9

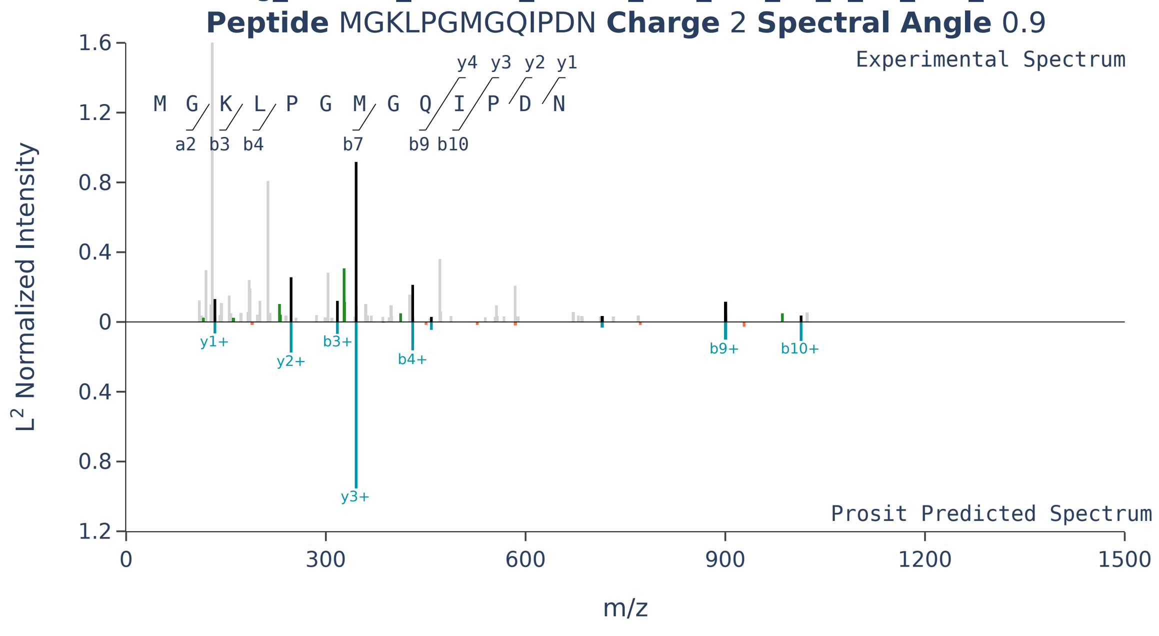

**Source** Ncheng_210623_230623_HFGoe_FFH_20S_25_1_A2_4h_R2 **Scan** 12125
**Peptide** IVNAALKEA **Charge** 1 **Spectral Angle** 0.82

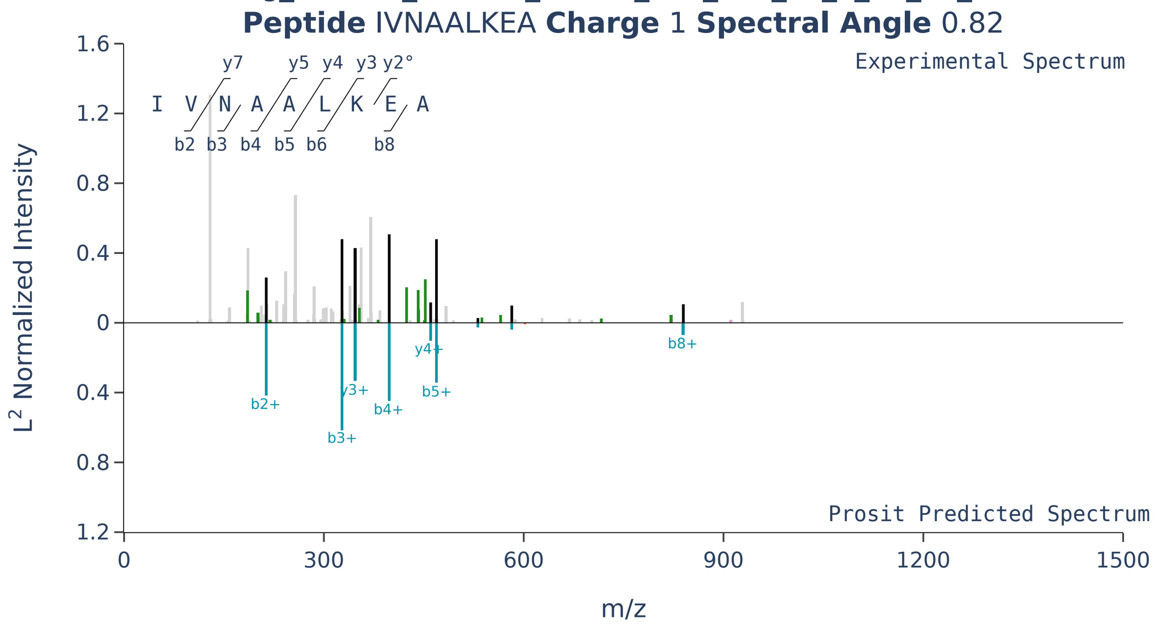


**Source** Ncheng_210623_230623_HFGoe_FFH_20S_25_1_A2_4h_R1 **Scan** 12240
**Peptide** SLRHITGKPIKF **Charge** 4 **Spectral Angle** 0.9

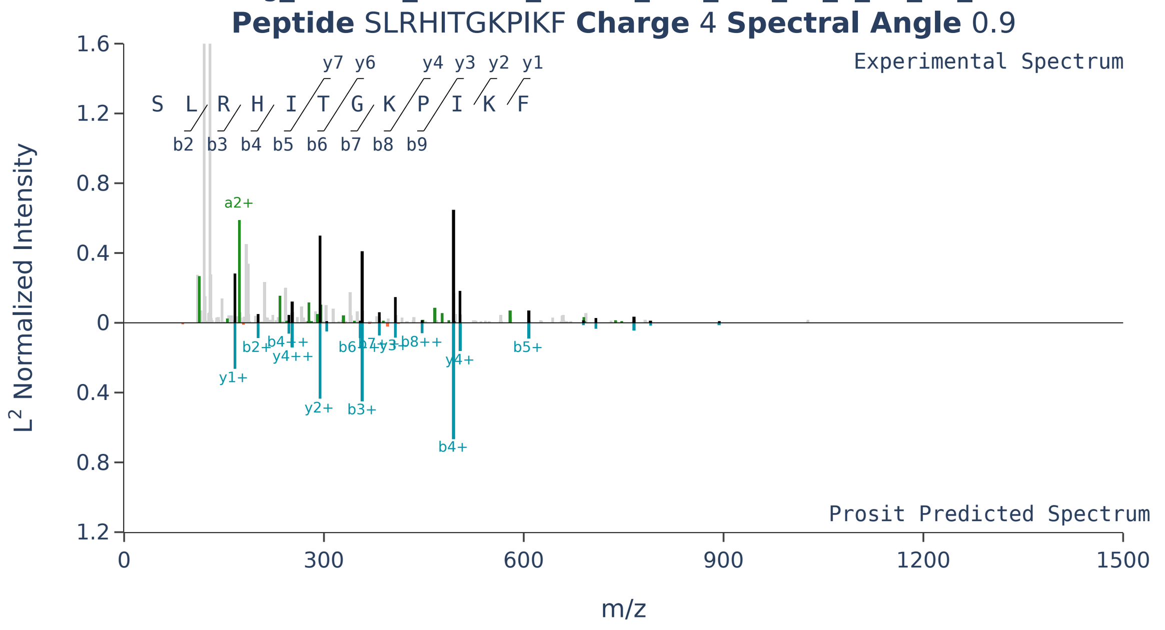


**Source** Ncheng_210623_230623_HFGoe_FFH_20S_25_1_A2_1h_R2 **Scan** 14879
**Peptide** PGQEFVKIVRN **Charge** 3 **Spectral Angle** 0.84

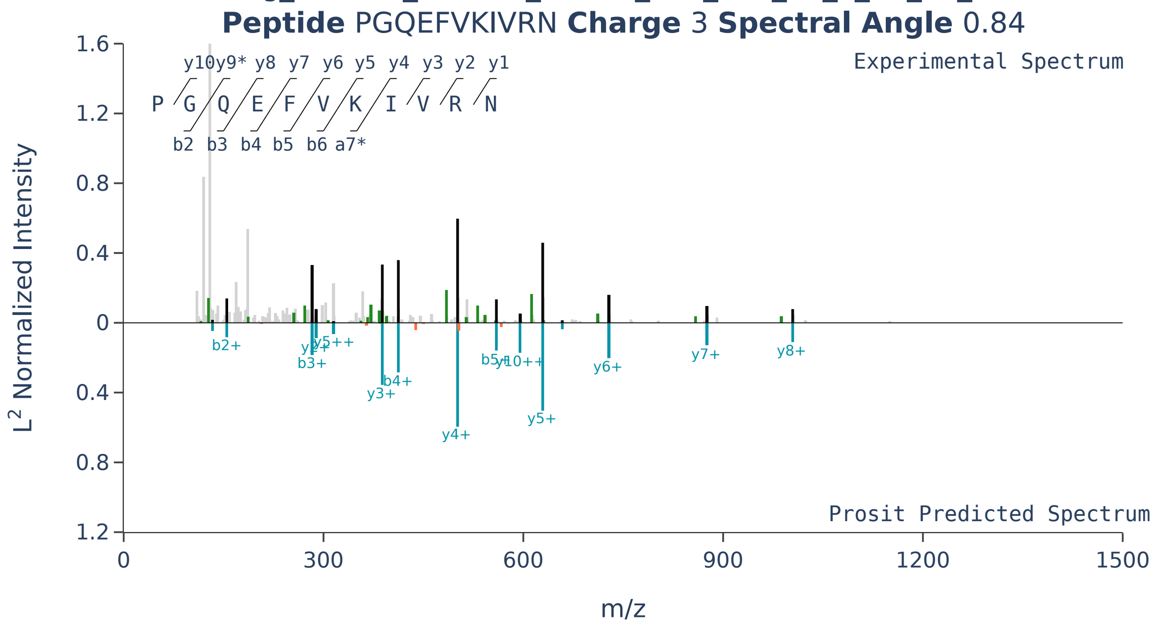


**Source** Ncheng_210623_230623_HFGoe_FFH_20S_25_1_A1_2h_R2 **Scan** 10421
**Peptide** VVLTKVDGDARG **Charge** 3 **Spectral Angle** 0.85

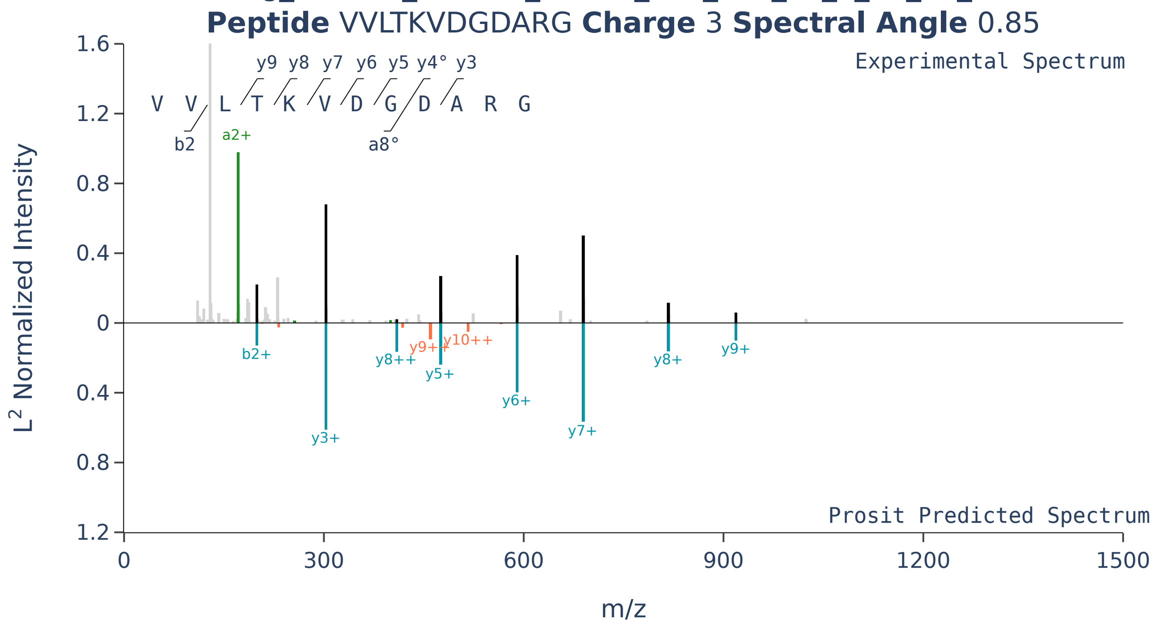


**Source** Ncheng_210623_230623_HFGoe_FFH_20S_25_1_A1_1h_R2 **Scan** 31733
**Peptide** GEENQTLNLAAQPPAVVL **Charge** 2 **Spectral Angle** 0.9

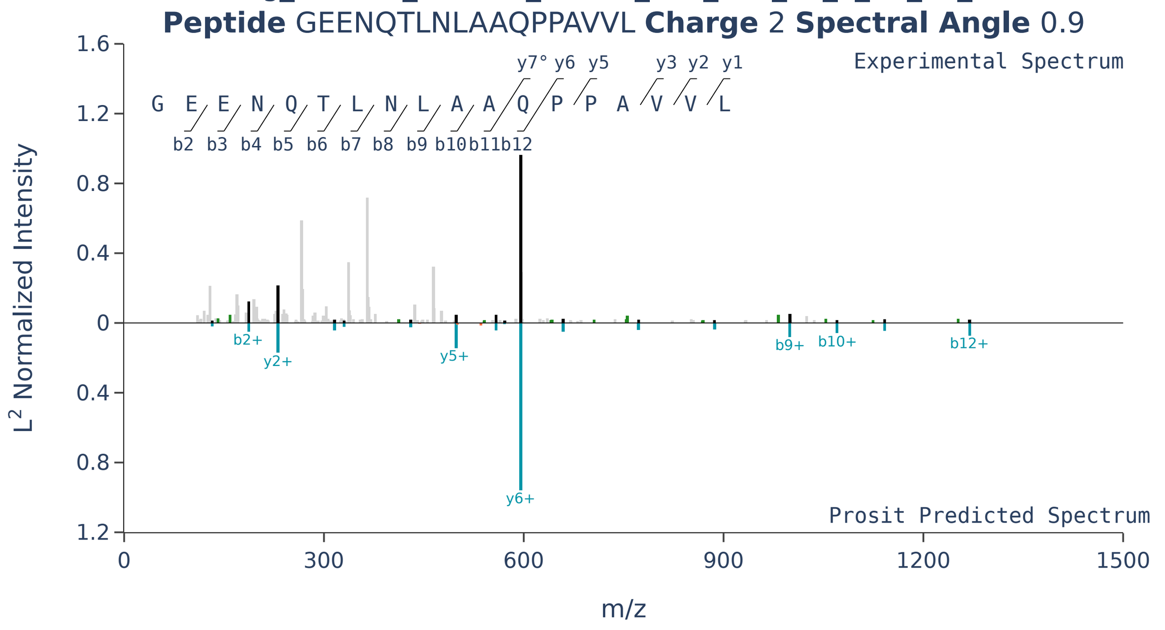


**Source** Ncheng_210623_230623_HFGoe_FFH_20S_25_1_A2_4h_R2 **Scan** 30645
**Peptide** EADVALPVVREF **Charge** 2 **Spectral Angle** 0.88

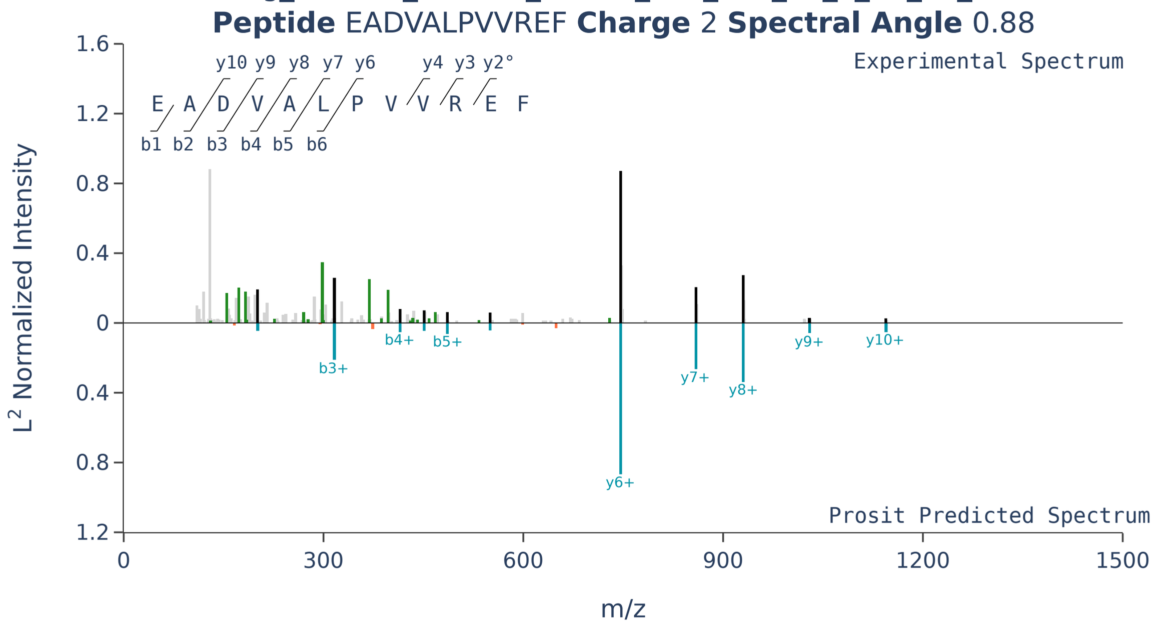


**Source** Ncheng_210623_230623_HFGoe_FFH_20S_25_1_A2_4h_R2 **Scan** 26067
**Peptide** AAGCGMQVQDVNRLL **Charge** 2 **Spectral Angle** 0.86

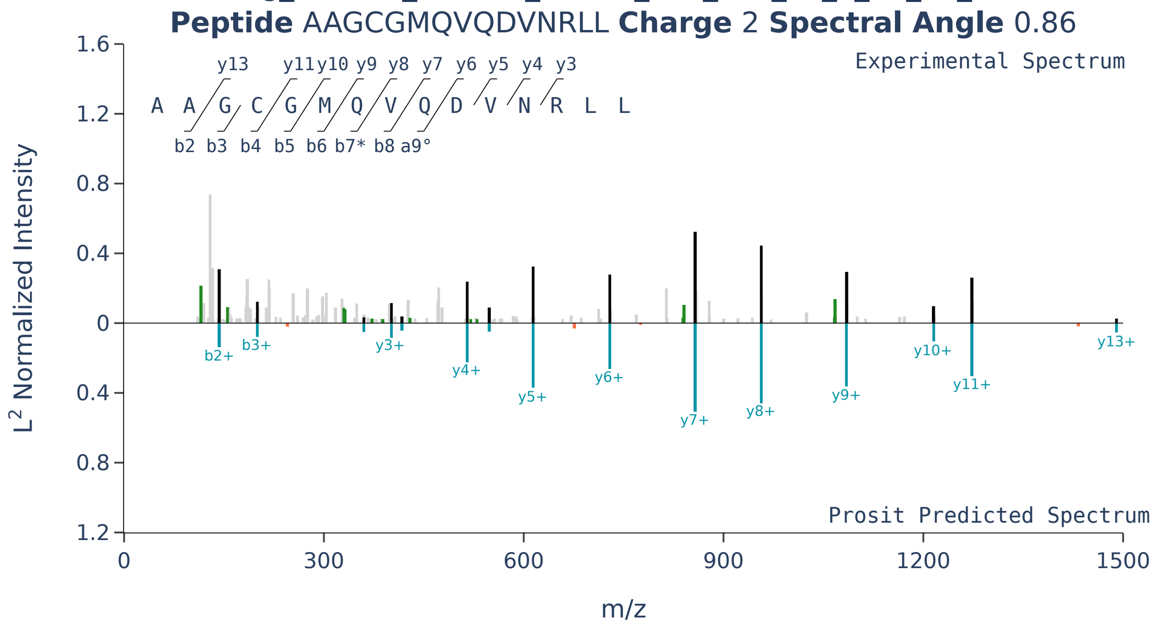


**Source** Ncheng_210623_230623_HFGoe_FFH_20S_25_1_A2_24h_R2 **Scan** 13371
**Peptide** MAGLQGAGKTTSV **Charge** 1 **Spectral Angle** 0.85

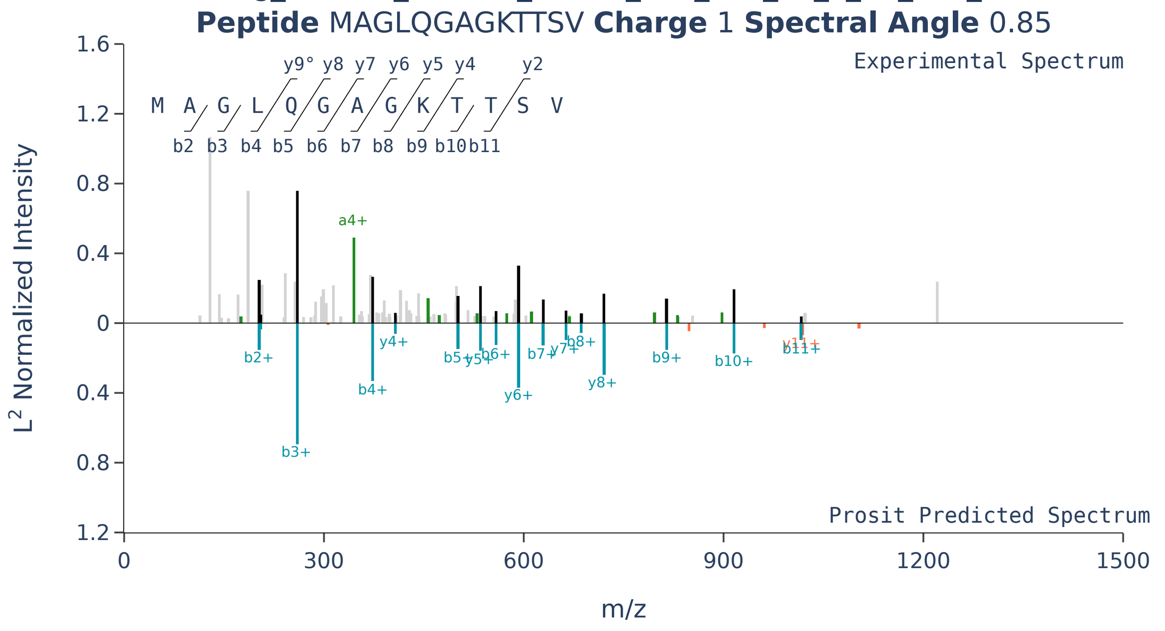


**Source** Ncheng_210623_230623_HFGoe_FFH_20S_25_1_A2_2h_R1 **Scan** 13143
**Peptide** EFVKIVRNE **Charge** 3 **Spectral Angle** 0.9

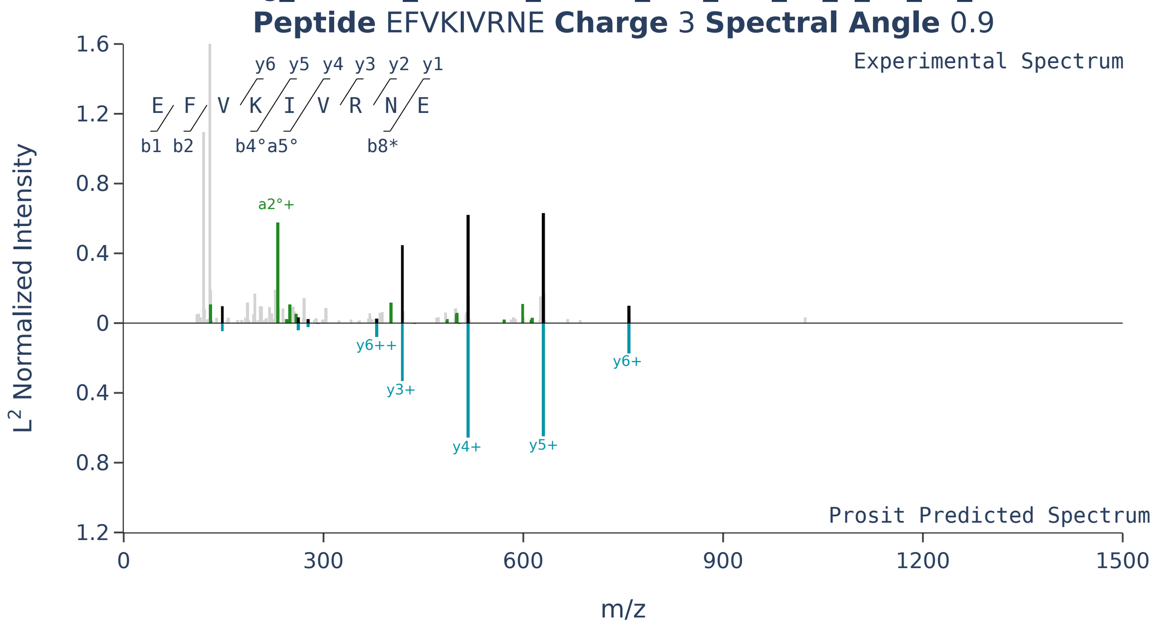


**Source** Ncheng_210623_230623_HFGoe_FFH_20S_25_1_A1_4h_R1 **Scan** 14365
**Peptide** AIINSMTMKER **Charge** 3 **Spectral Angle** 0.81

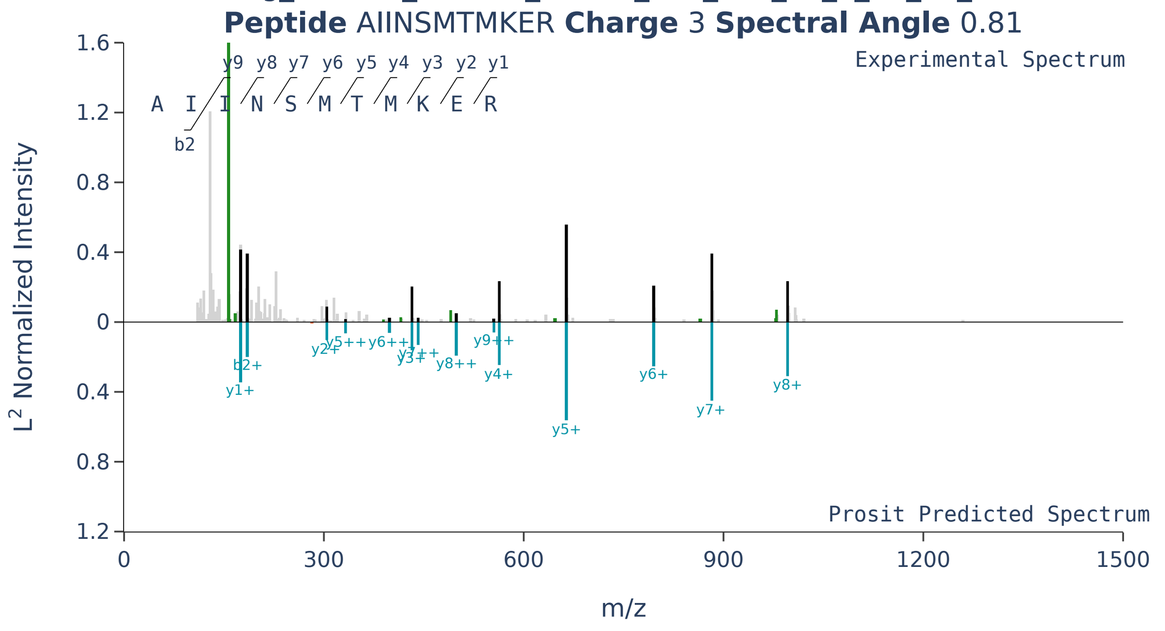


**Source** Ncheng_210623_230623_HFGoe_FFH_20S_25_1_A2_24h_R2 **Scan** 21529
**Peptide** DVALPVVR **Charge** 2 **Spectral Angle** 0.85

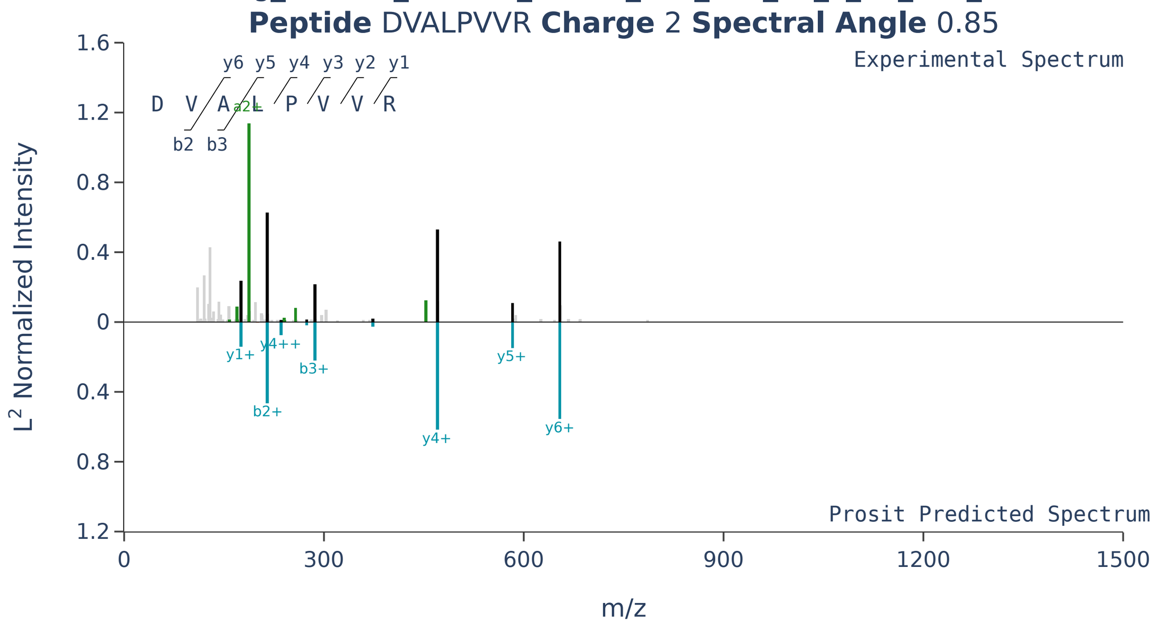


**Source** Ncheng_210623_230623_HFGoe_FFH_20S_25_1_A2_4h_R1 **Scan** 7987
**Peptide** GEENQTL **Charge** 2 **Spectral Angle** 0.93

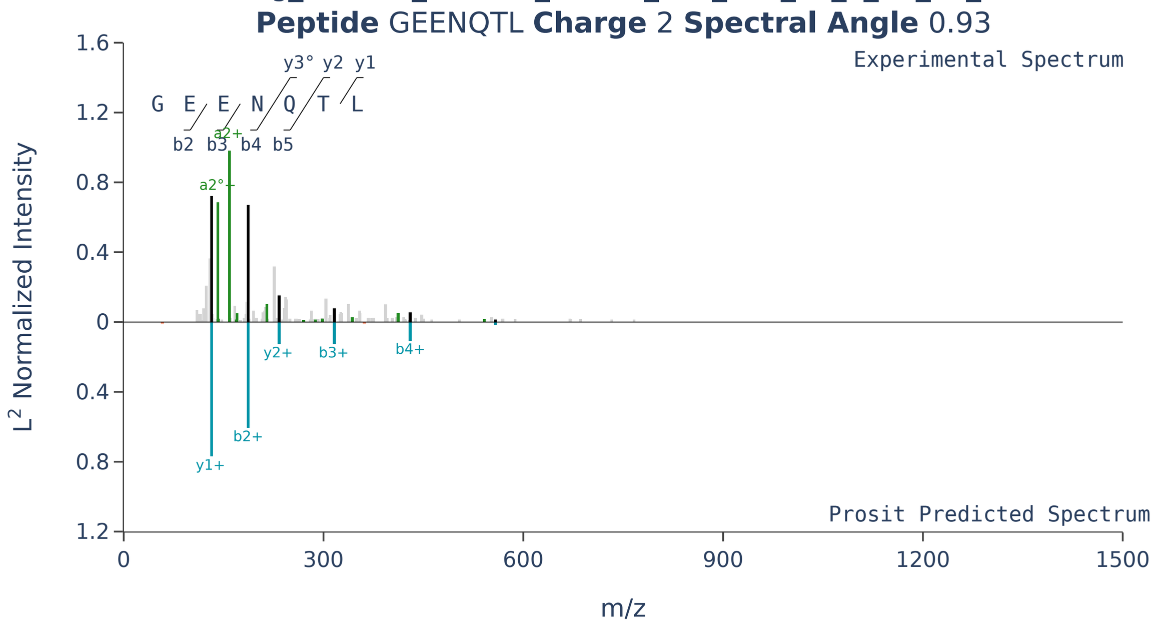


**Source** Ncheng_210623_230623_HFGoe_FFH_20S_25_1_A1_1h_R1 **Scan** 7363
**Peptide** KDTLREV **Charge** 2 **Spectral Angle** 0.83

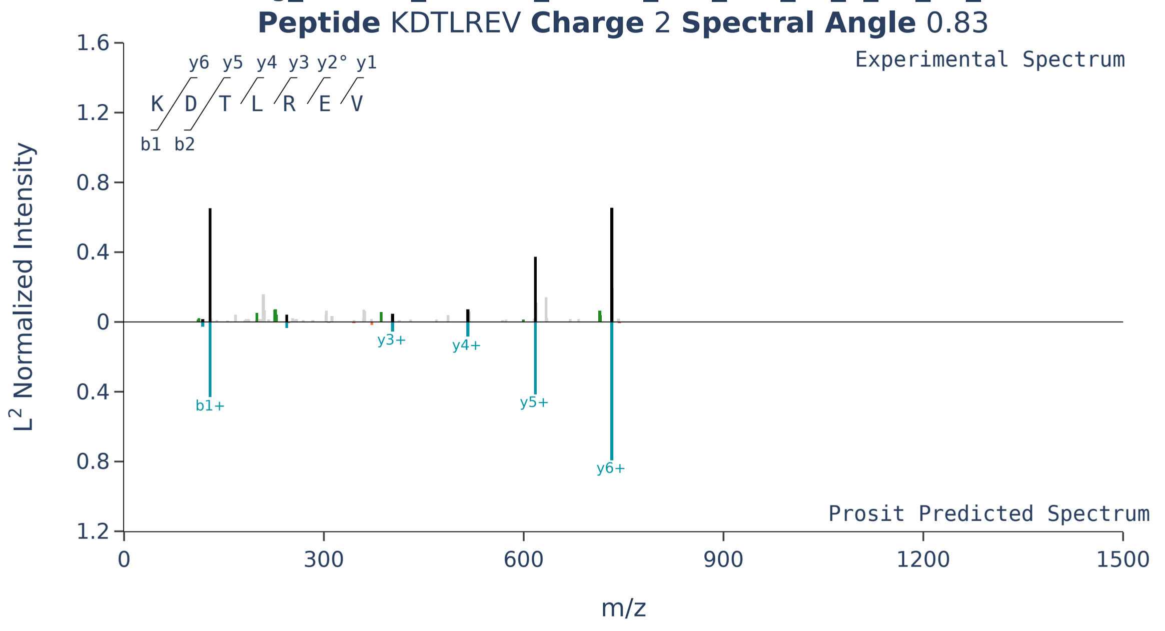


**Source** Ncheng_210623_230623_HFGoe_FFH_20S_25_1_A1_1h_R1 **Scan** 13767
**Peptide** RMEAIINSM **Charge** 2 **Spectral Angle** 0.81

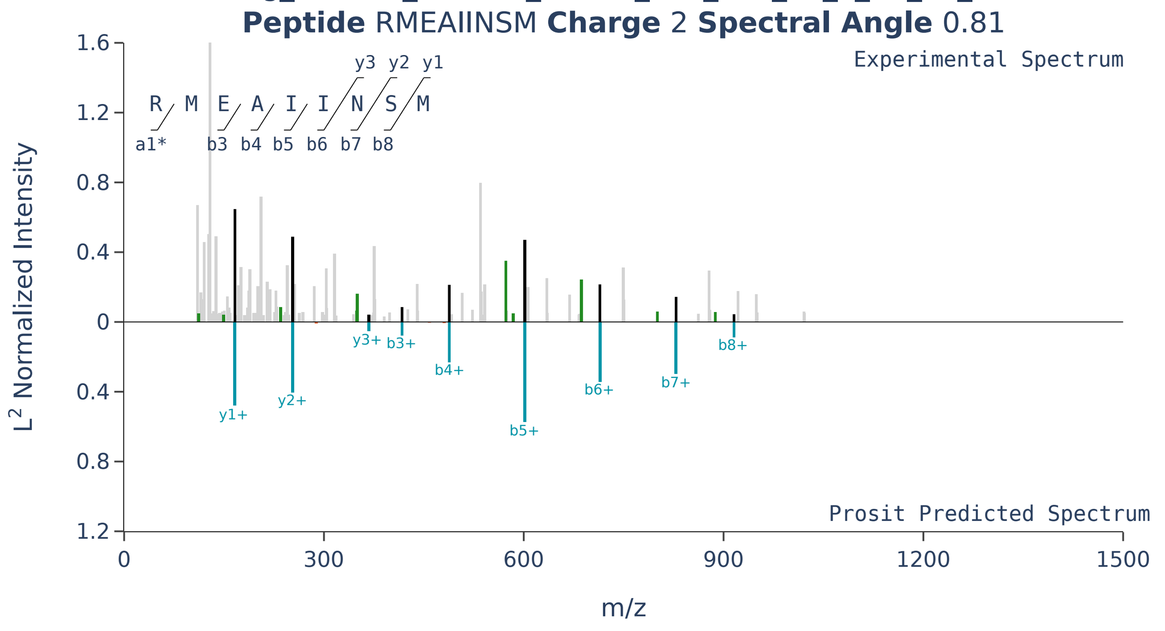


**Source** Ncheng_210623_230623_HFGoe_FFH_20S_25_1_A1_24h_R1 **Scan** 16367
**Peptide** EAIINSMTMKER **Charge** 3 **Spectral Angle** 0.83

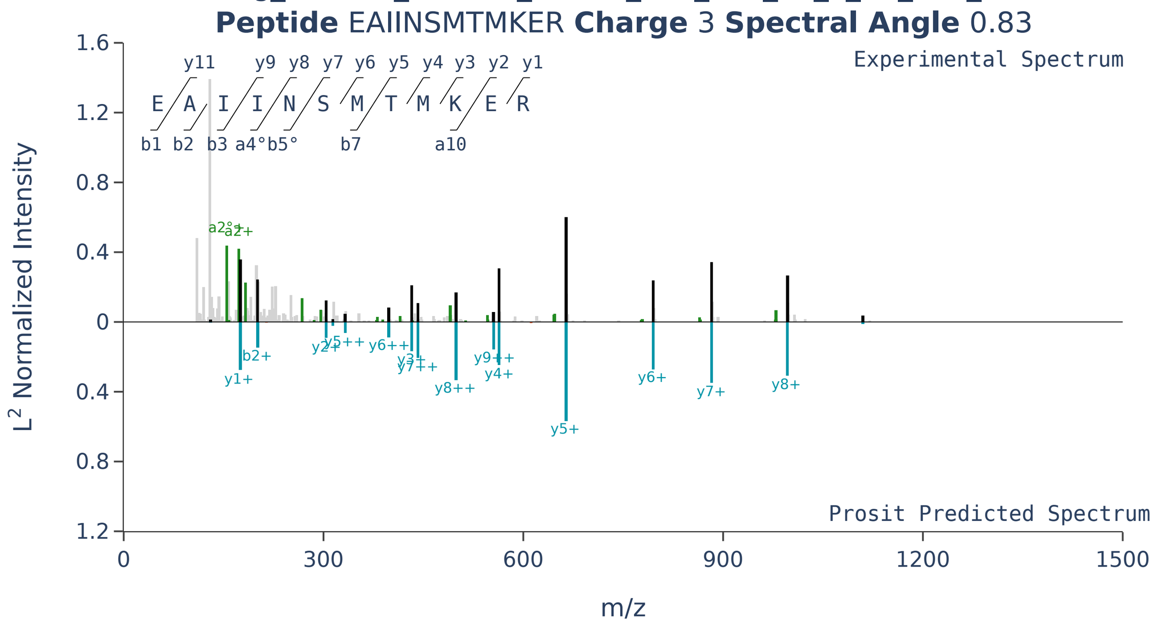

**Source** Ncheng_210623_230623_HFGoe_FFH_20S_25_1_A1_24h_R1 **Scan** 8173
**Peptide** GCGMQVQ **Charge** 1 **Spectral Angle** 0.89

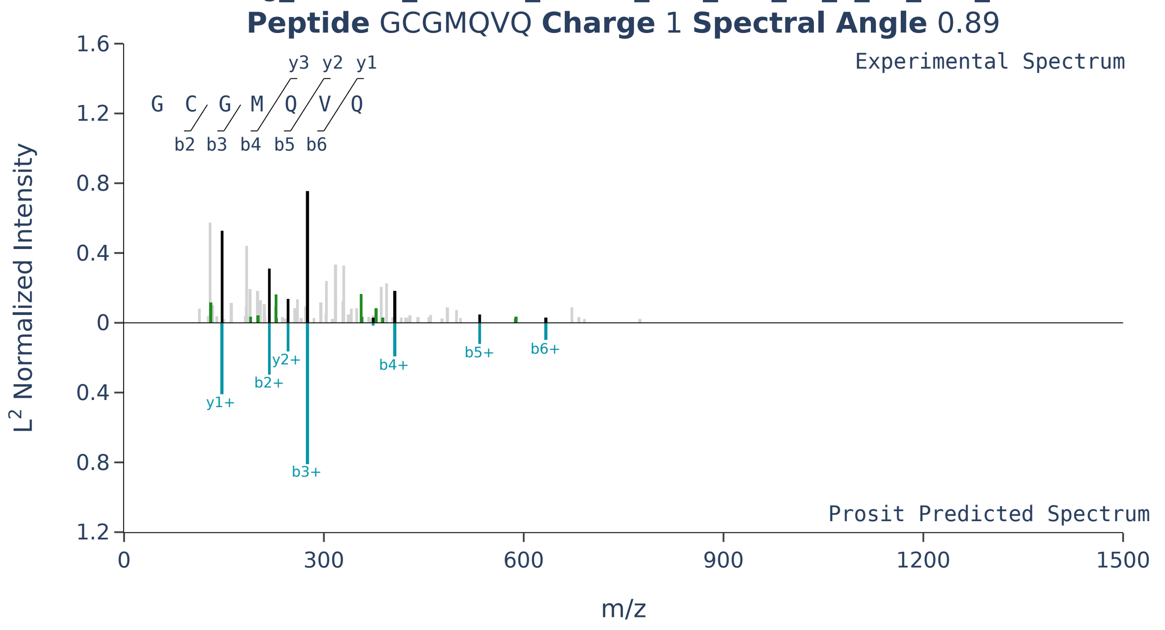

**Source** Ncheng_210623_230623_HFGoe_FFH_20S_25_1_A1_24h_R1 **Scan** 23836
**Peptide** AQPPAVVL **Charge** 1 **Spectral Angle** 0.94

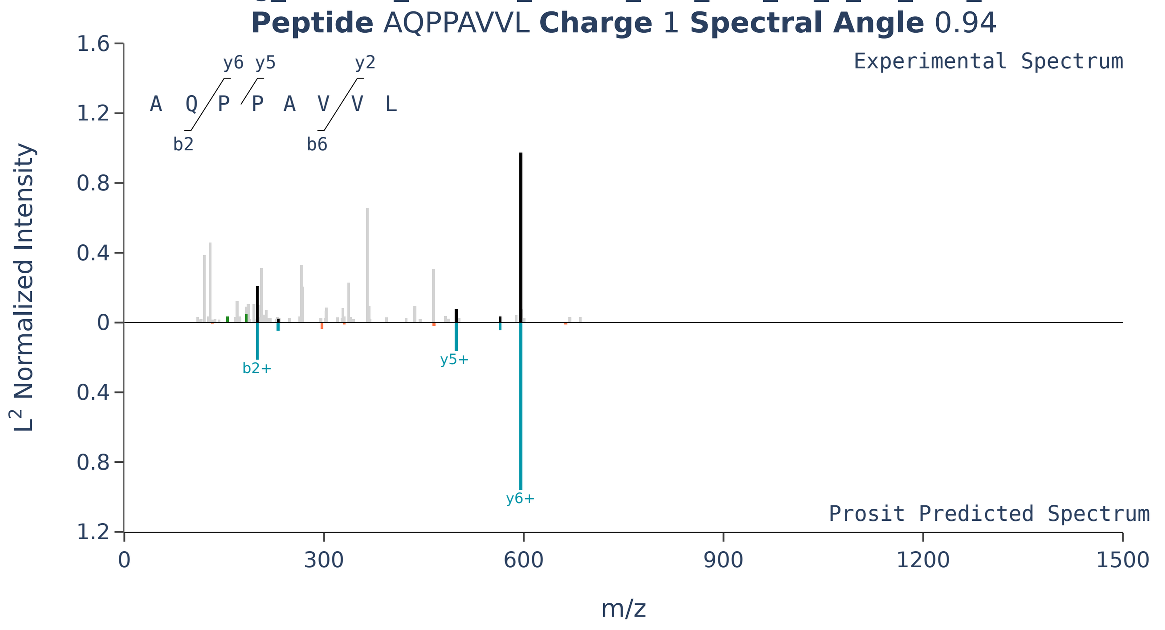

**Source** Ncheng_210623_230623_HFGoe_FFH_20S_25_1_A2_24h_R1 **Scan** 12934
**Peptide** AMTGQDAANTAKAFNE **Charge** 2 **Spectral Angle** 0.88

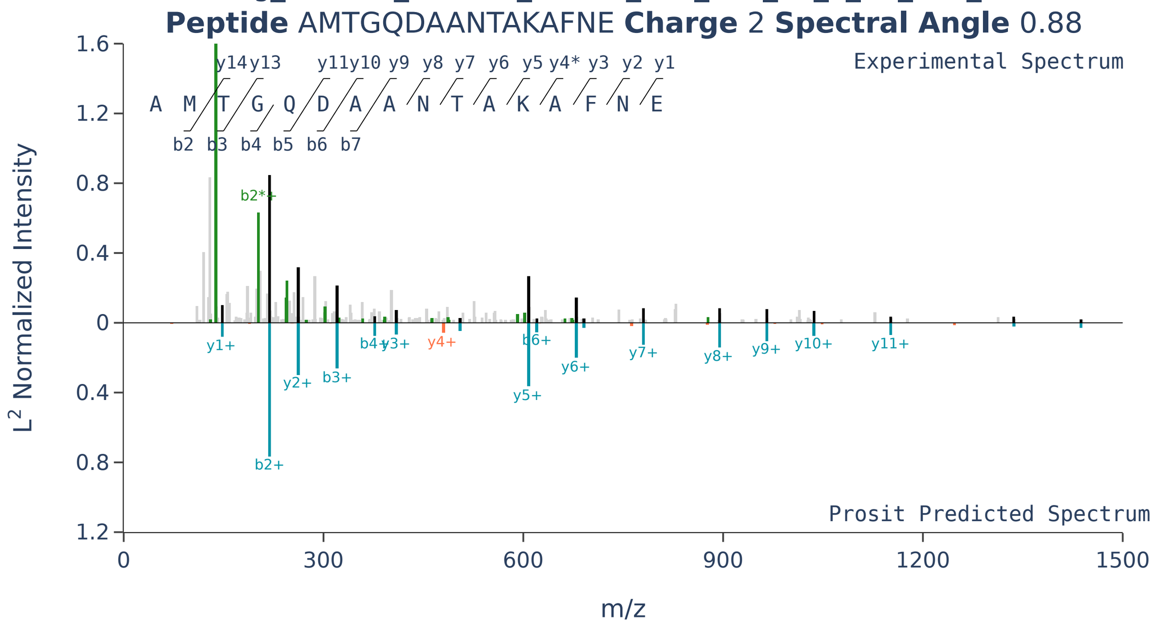

**Source** Ncheng_210623_230623_HFGoe_FFH_20S_25_1_A2_4h_R1 **Scan** 18070
**Peptide** RIAAGCGMQVQDVNRL **Charge** 3 **Spectral Angle** 0.9

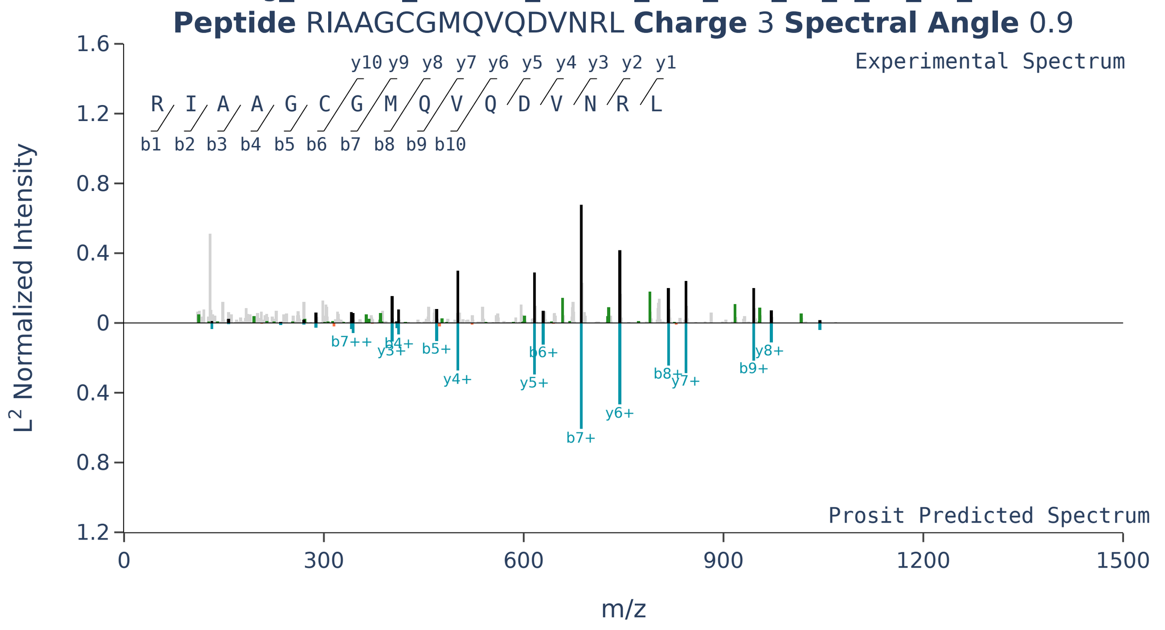

**Source** Ncheng_210623_230623_HFGoe_FFH_20S_25_1_A1_4h_R1 **Scan** 23062
**Peptide** EVNKSLTPGQEFVKL **Charge** 2 **Spectral Angle** 0.87

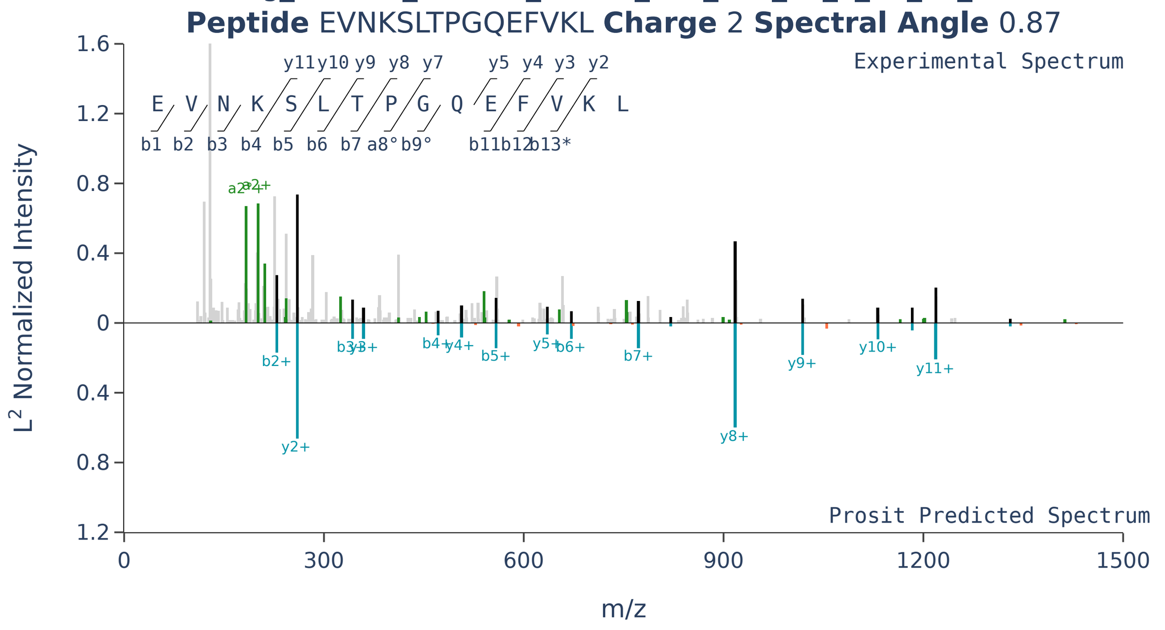

**Source** Ncheng_210623_230623_HFGoe_FFH_20S_25_1_A2_24h_R1 **Scan** 9586
**Peptide** VKDTLREV **Charge** 2 **Spectral Angle** 0.88

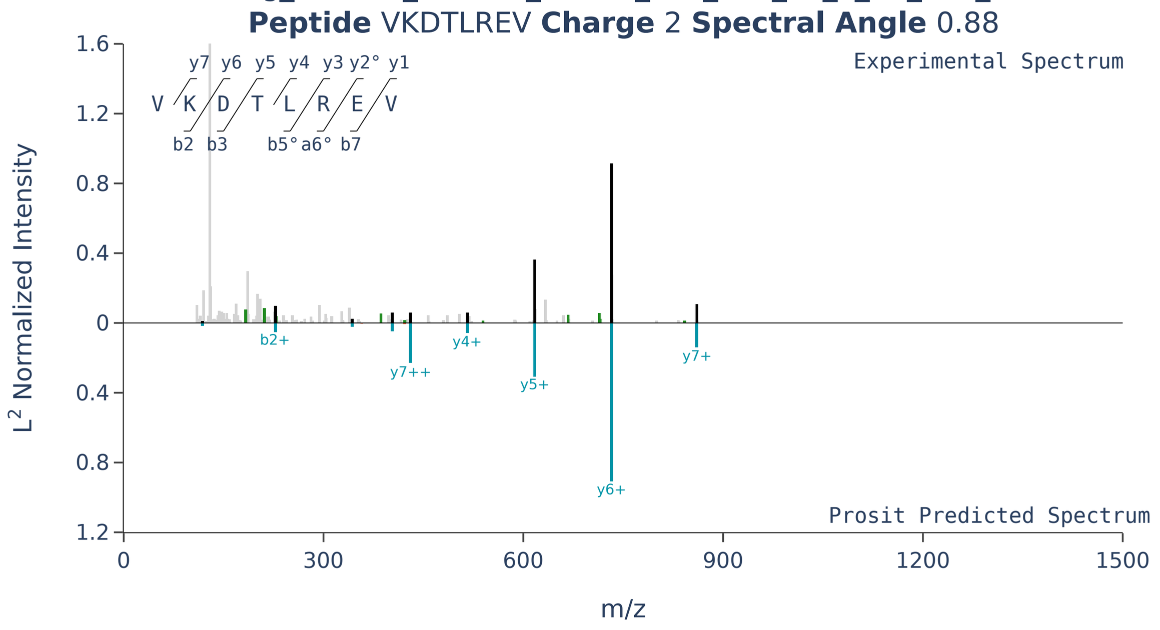

**Source** Ncheng_210623_230623_HFGoe_FFH_20S_25_1_A1_24h_R2 **Scan** 17692
**Peptide** TGKPIKFL **Charge** 1 **Spectral Angle** 0.85

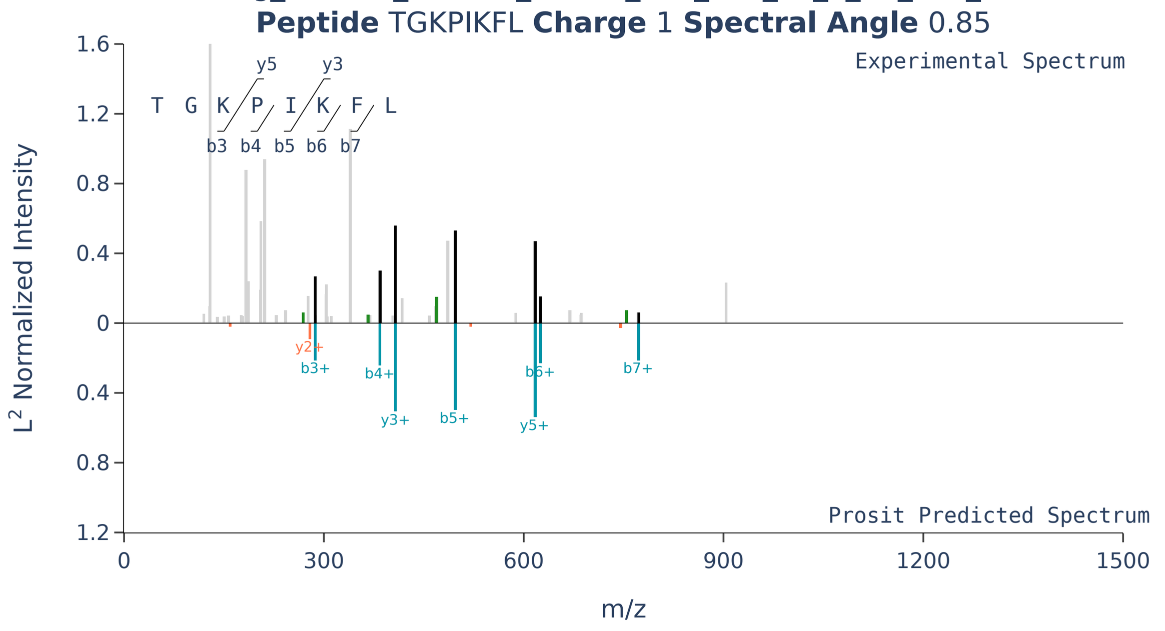

**Source** Ncheng_210623_230623_HFGoe_FFH_20S_25_1_A2_4h_R2 **Scan** 20920
**Peptide** DGFDLND **Charge** 1 **Spectral Angle** 0.8

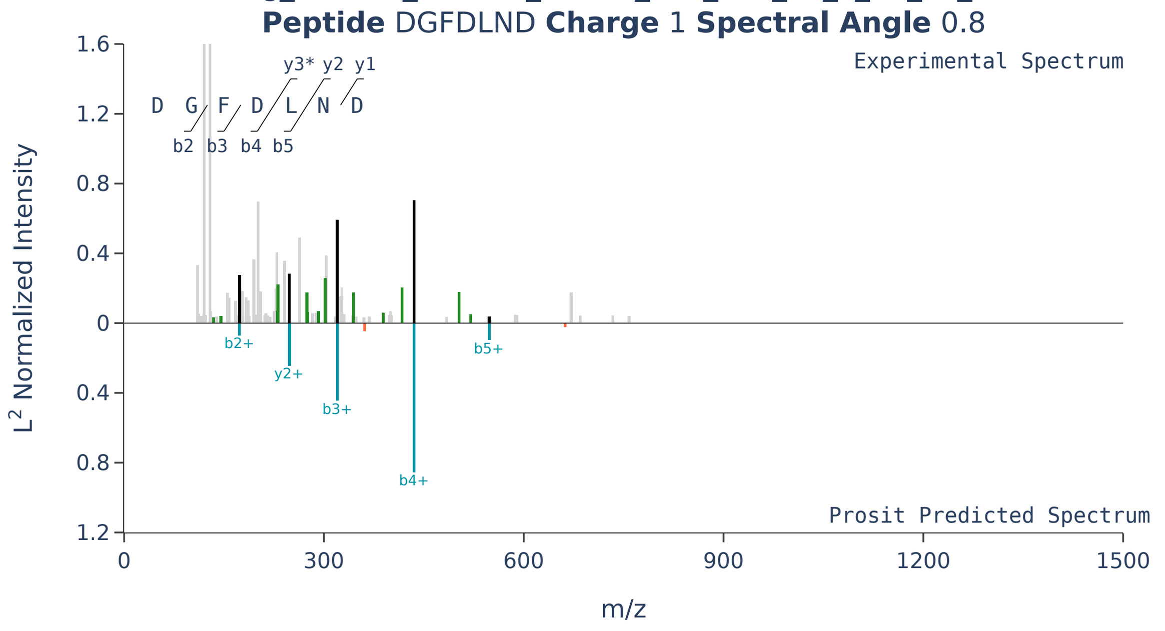

**Source** Ncheng_210623_230623_HFGoe_FFH_20S_25_1_A1_2h_R2 **Scan** 30351
**Peptide** DVALPVVREF **Charge** 2 **Spectral Angle** 0.8

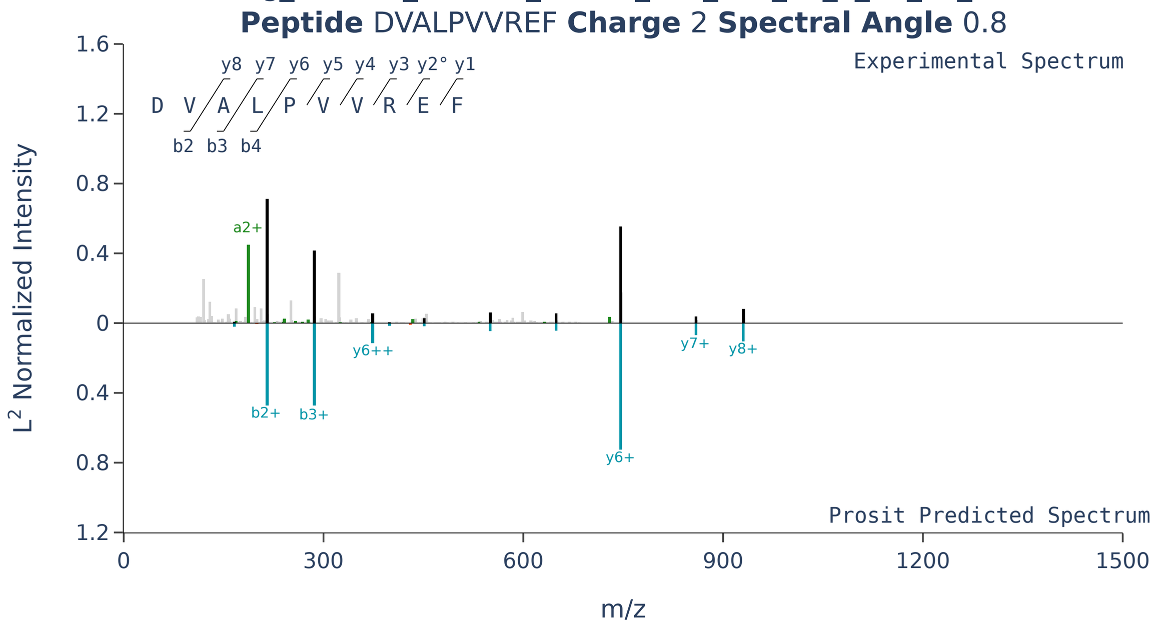

**Source** Ncheng_210623_230623_HFGoe_FFH_20S_25_1_A2_4h_R2 **Scan** 17025
**Peptide** ELVAAMGEENQT **Charge** 1 **Spectral Angle** 0.89

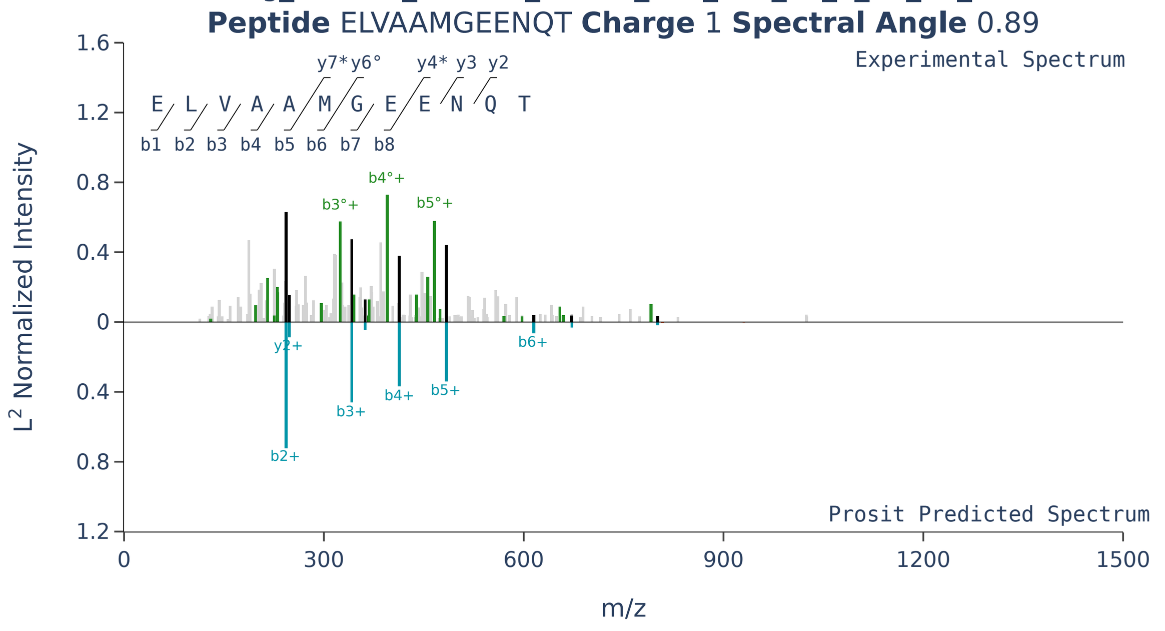

**Source** Ncheng_210623_230623_HFGoe_FFH_20S_25_1_A1_24h_R1 **Scan** 16011
**Peptide** DNLTDRL **Charge** 2 **Spectral Angle** 0.89

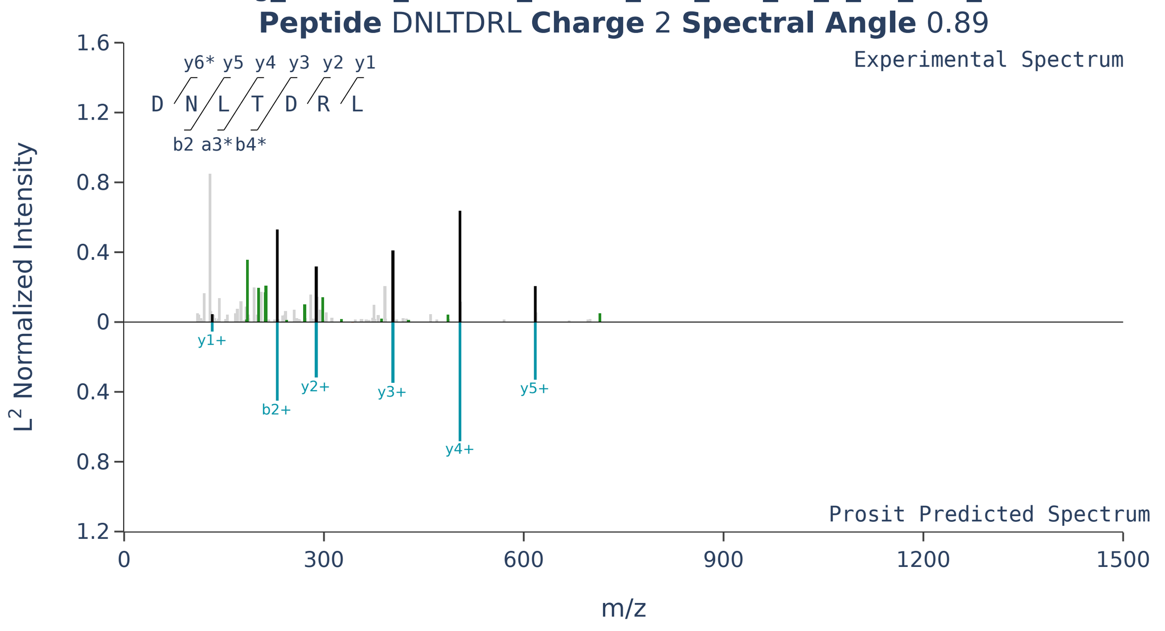

**Source** Ncheng_210623_230623_HFGoe_FFH_20S_25_1_A2_4h_R1 **Scan** 15214
**Peptide** SVGKLGKFLRE **Charge** 3 **Spectral Angle** 0.85

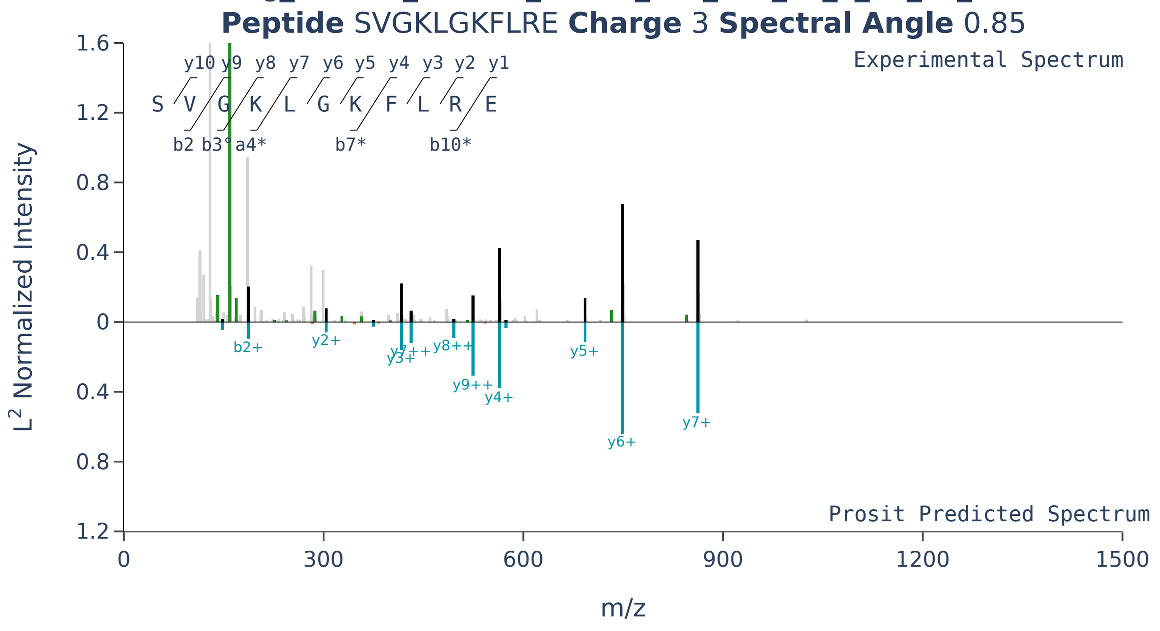

**Source** Ncheng_210623_230623_HFGoe_FFH_20S_25_1_A2_24h_R2 **Scan** 16217
**Peptide** DAMTGQDAANTAKAFNE **Charge** 2 **Spectral Angle** 0.88

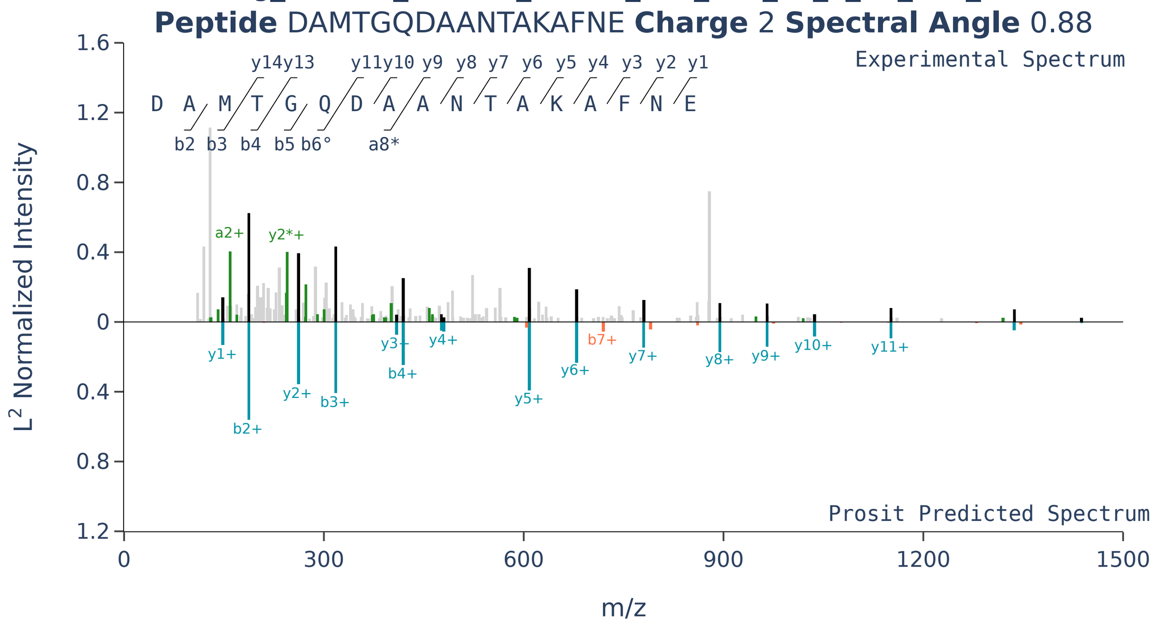

**Source** Ncheng_210623_230623_HFGoe_FFH_20S_25_1_A1_24h_R1 **Scan** 16474
**Peptide** QDVNRLL **Charge** 1 **Spectral Angle** 0.9

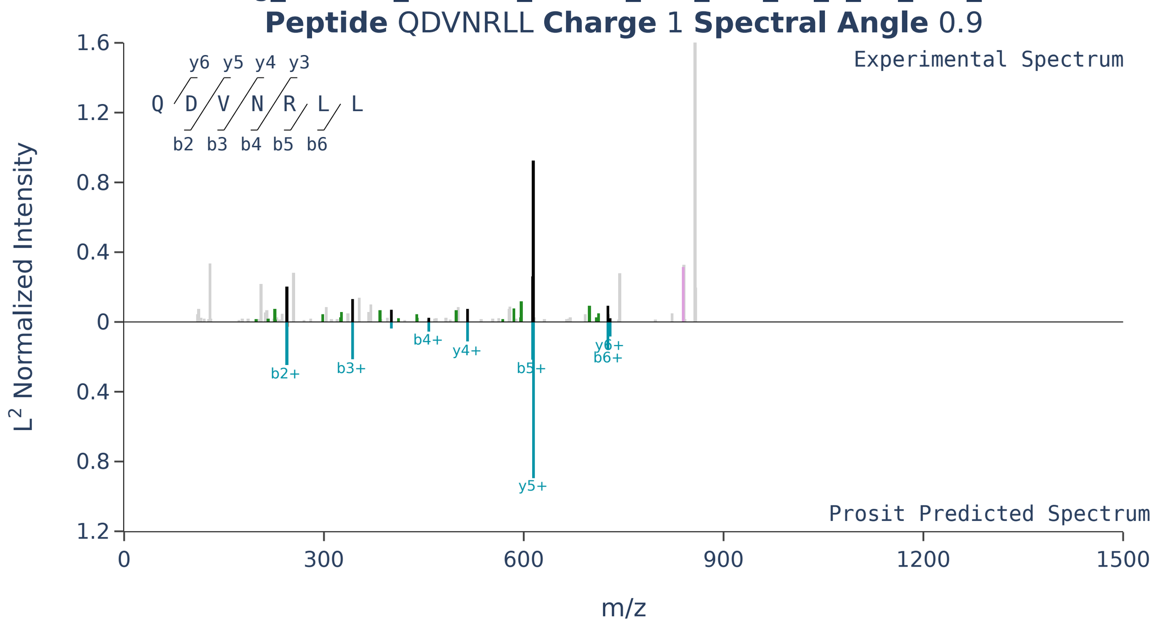

**Source** Ncheng_210623_230623_HFGoe_FFH_20S_25_1_A1_24h_R2 **Scan** 7994
**Peptide** EDIESKVD **Charge** 1 **Spectral Angle** 0.88

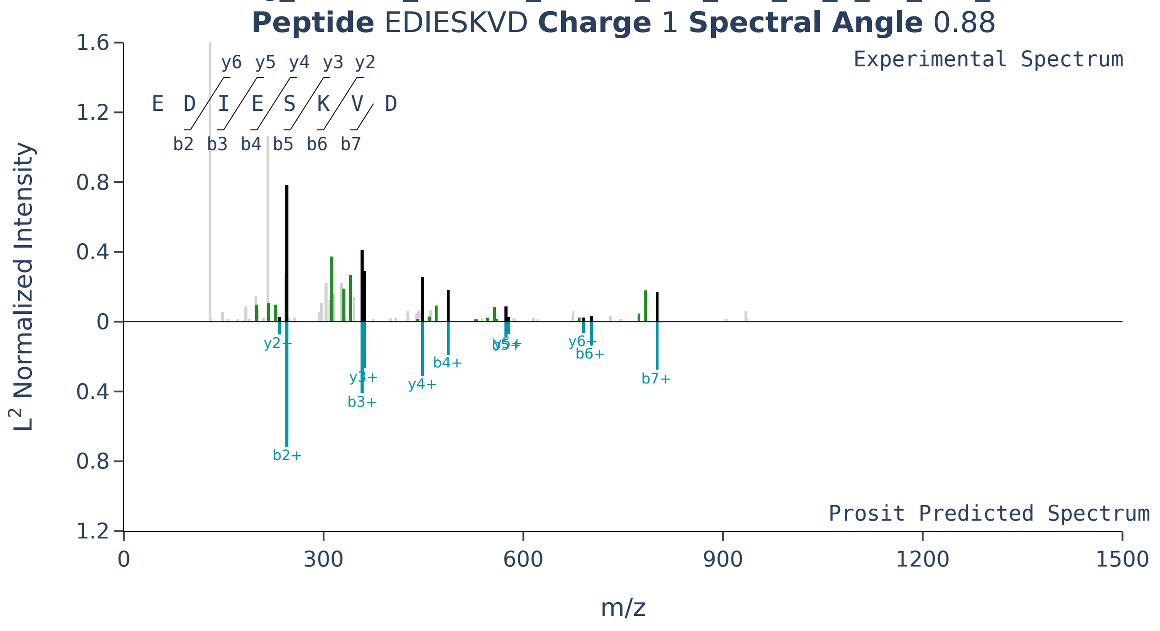

**Source** Ncheng_210623_230623_HFGoe_FFH_20S_25_1_A2_24h_R2 **Scan** 15377
**Peptide** VGQKPVDIVNA **Charge** 1 **Spectral Angle** 0.85

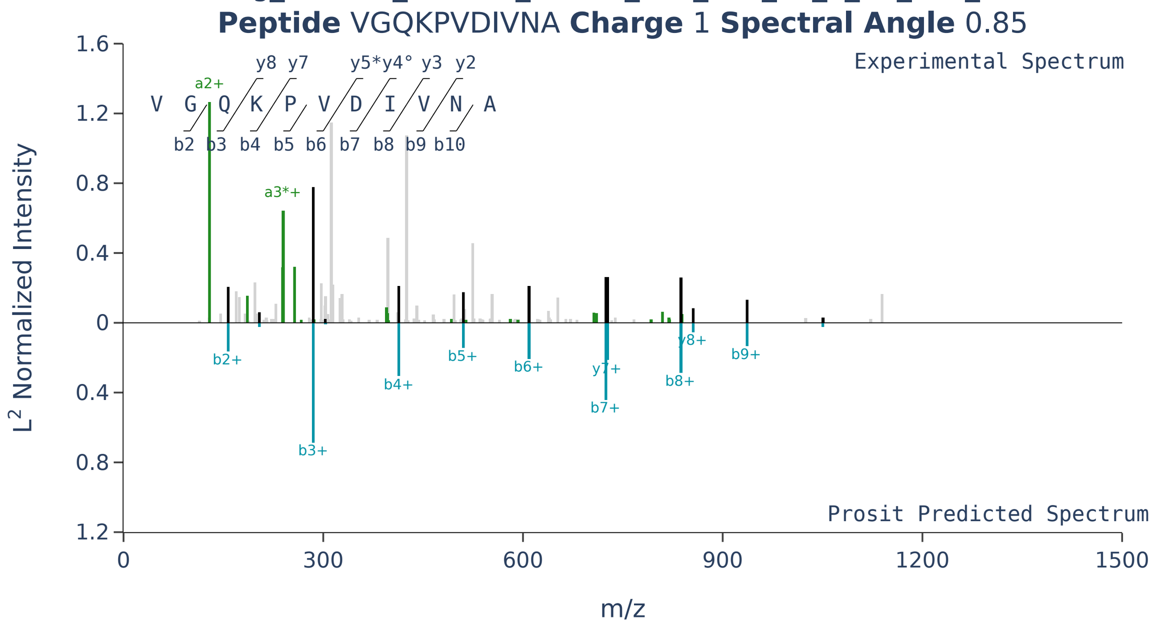


**Source** Ncheng_210623_230623_HFGoe_FFH_20S_25_1_A1_2h_R1 **Scan** 17949
**Peptide** KTEALEPFHPDRI **Charge** 2 **Spectral Angle** 0.81

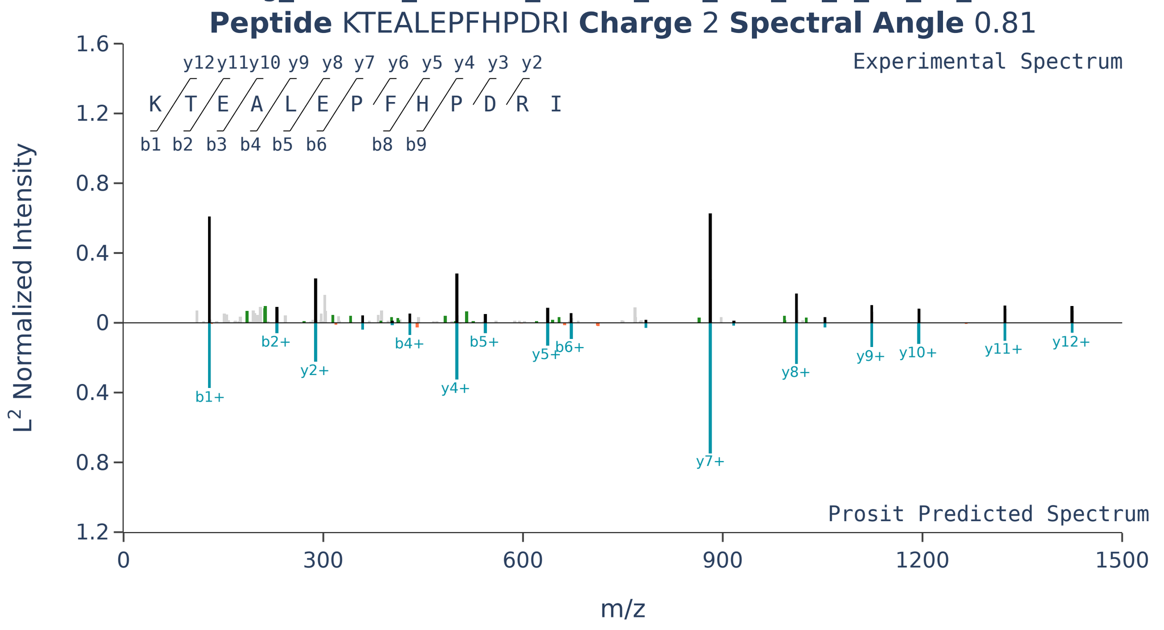


**Source** Ncheng_210623_230623_HFGoe_FFH_20S_25_1_A1_24h_R2 **Scan** 18112
**Peptide** MDDKVLVRM **Charge** 3 **Spectral Angle** 0.92

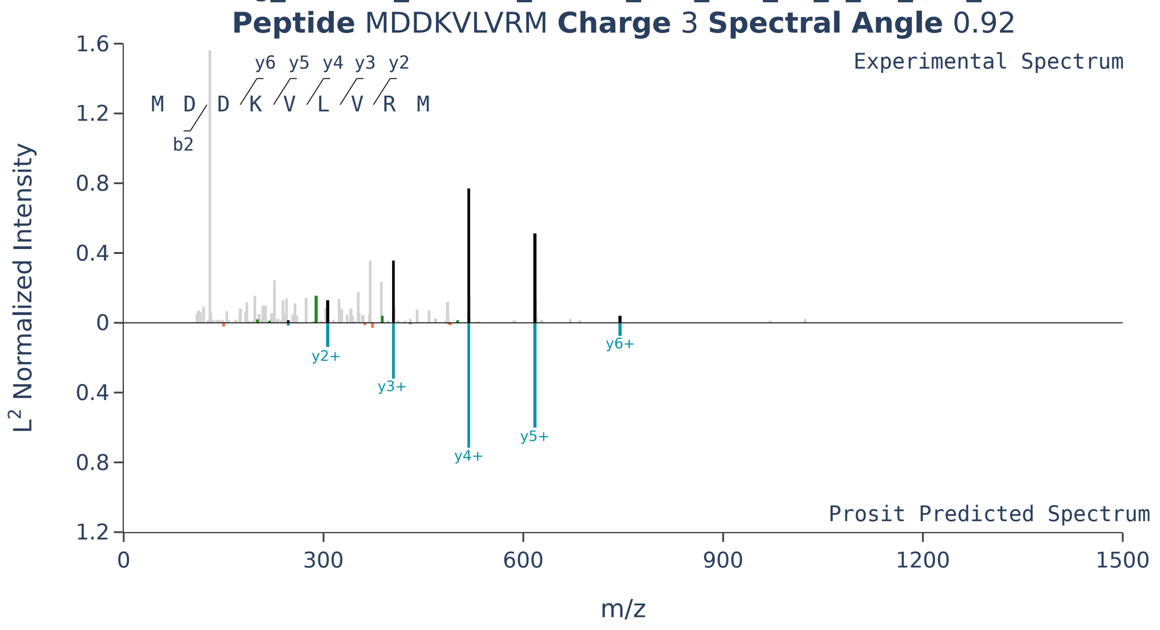


**Source** Ncheng_210623_230623_HFGoe_FFH_20S_25_1_A2_4h_R1 **Scan** 9763
**Peptide** DNVKDTL **Charge** 2 **Spectral Angle** 0.89

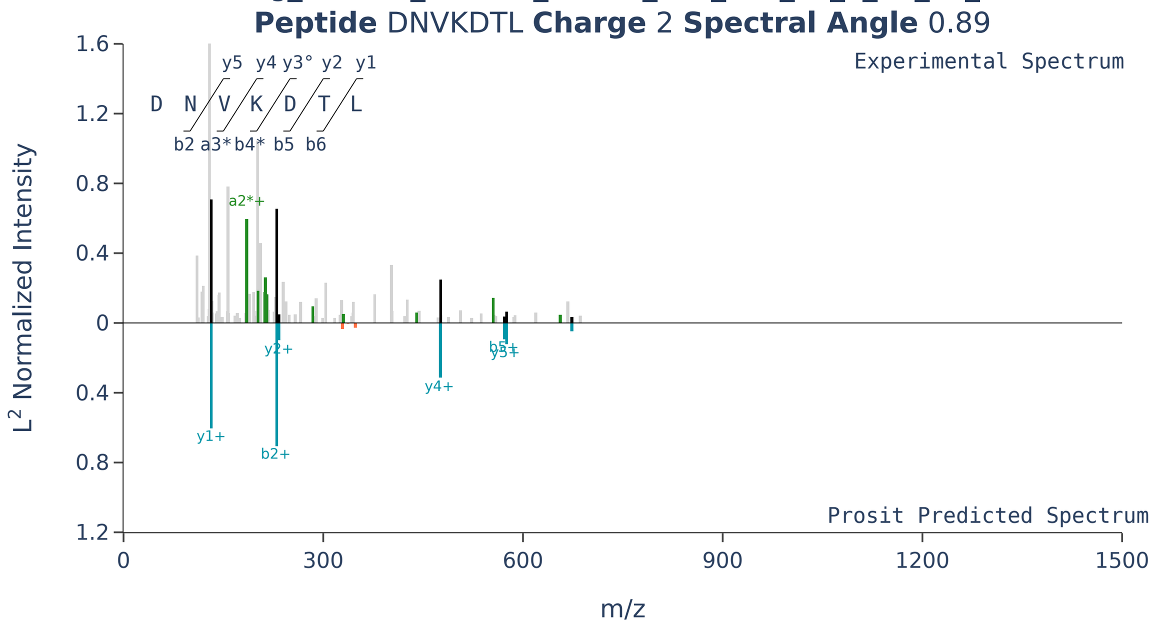


**Source** Ncheng_210623_230623_HFGoe_FFH_20S_25_1_A2_24h_R1 **Scan** 19730
**Peptide** VALPVVRE **Charge** 2 **Spectral Angle** 0.76

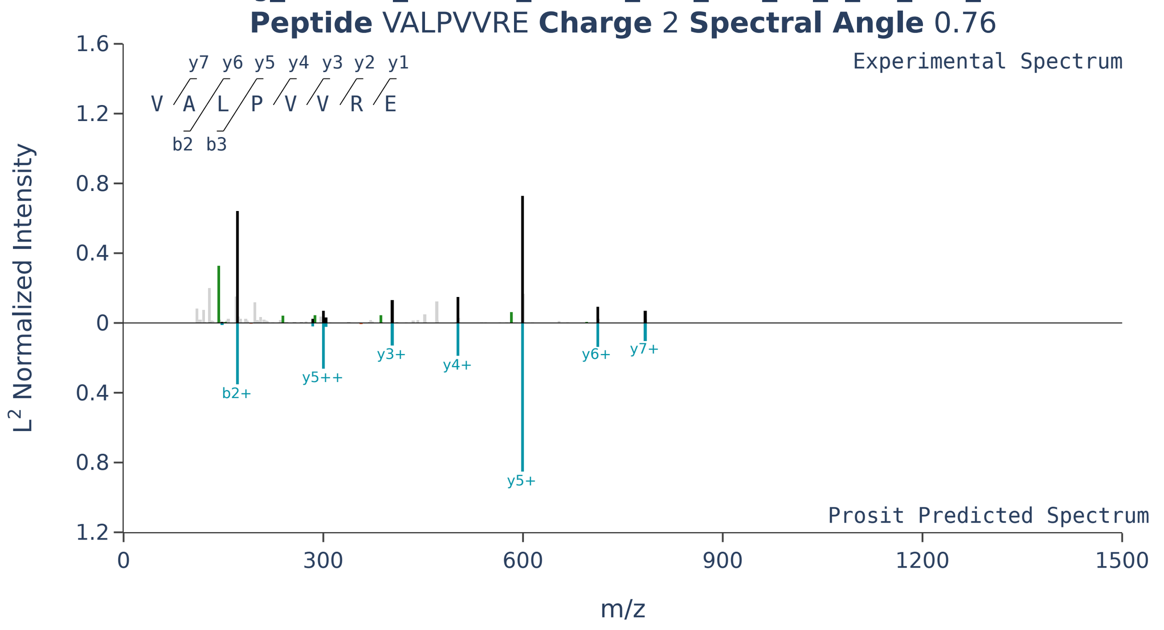


**Source** Ncheng_210623_230623_HFGoe_FFH_20S_25_1_A2_24h_R1 **Scan** 13899
**Peptide** LTGKPIKF **Charge** 2 **Spectral Angle** 0.86

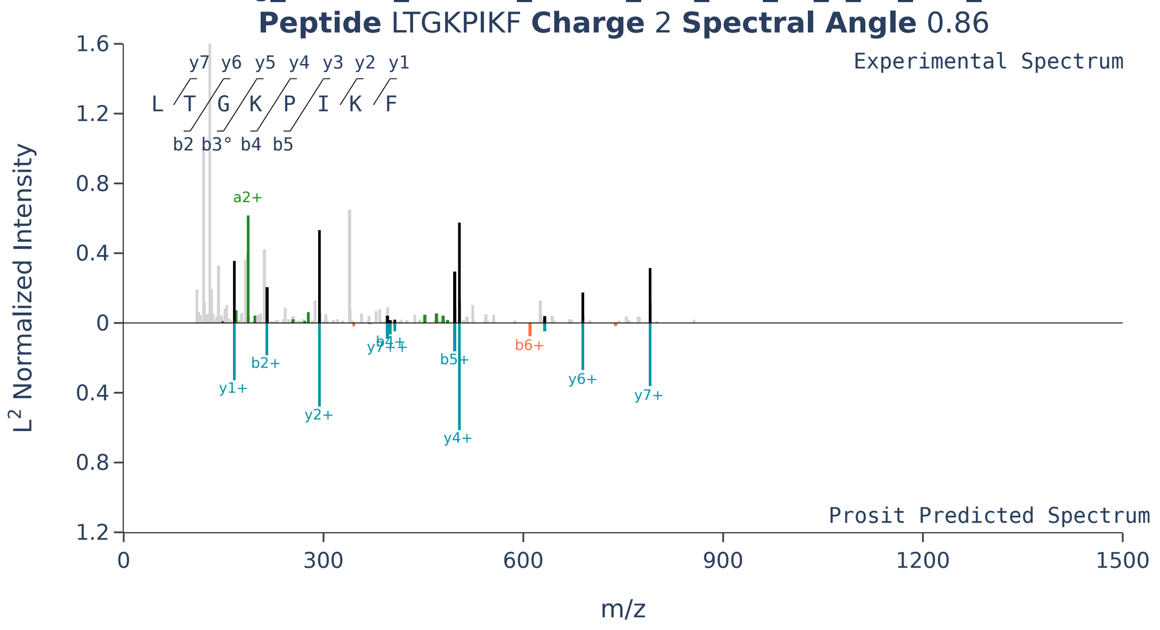


**Source** Ncheng_210623_230623_HFGoe_FFH_20S_25_1_A2_4h_R1 **Scan** 9995
**Peptide** AGCGMQVQDVNR **Charge** 2 **Spectral Angle** 0.82

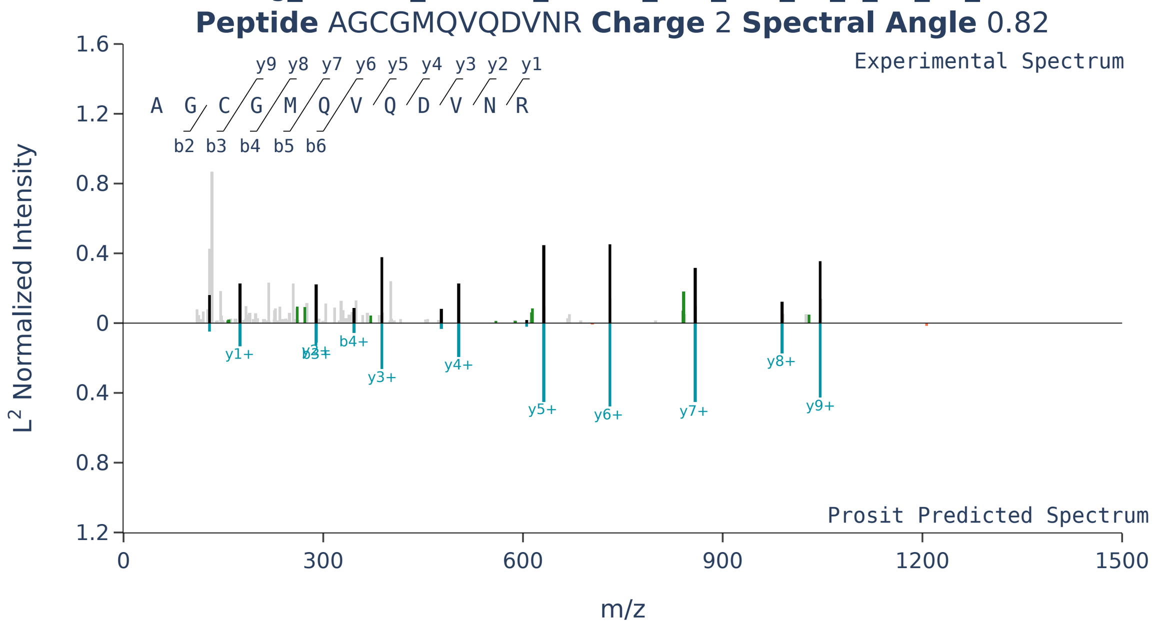


**Source** Ncheng_210623_230623_HFGoe_FFH_20S_25_1_A1_4h_R1 **Scan** 30663
**Peptide** NDFLEQLRQM **Charge** 2 **Spectral Angle** 0.8

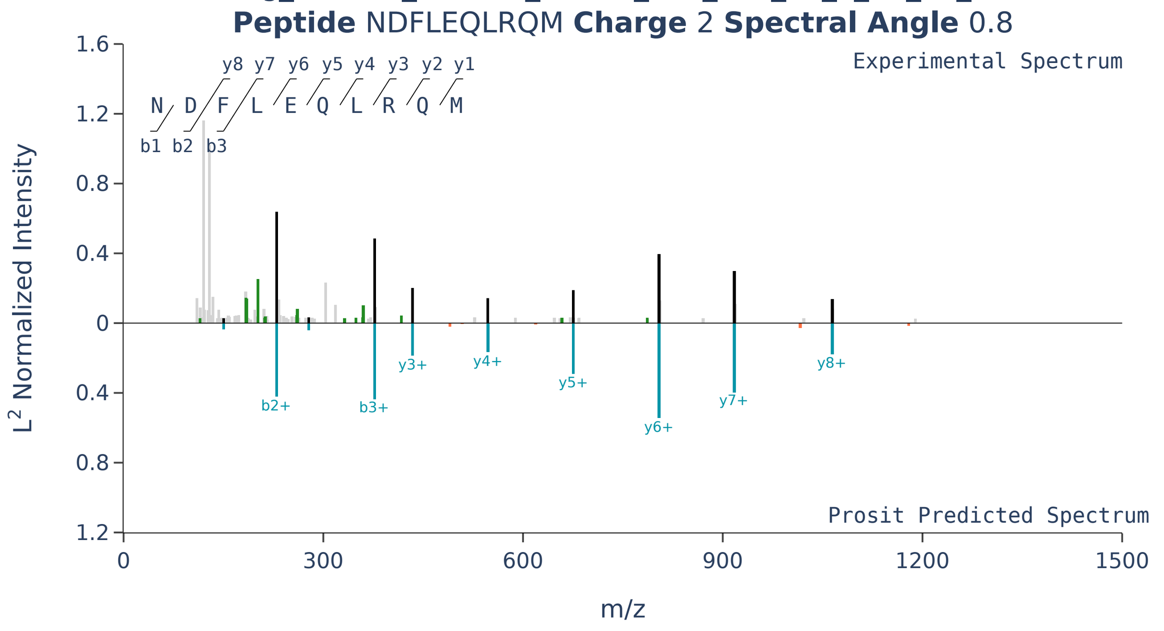


**Source** Ncheng_210623_230623_HFGoe_FFH_20S_25_1_A2_24h_R1 **Scan** 12211
**Peptide** LEDIESKVD **Charge** 1 **Spectral Angle** 0.95

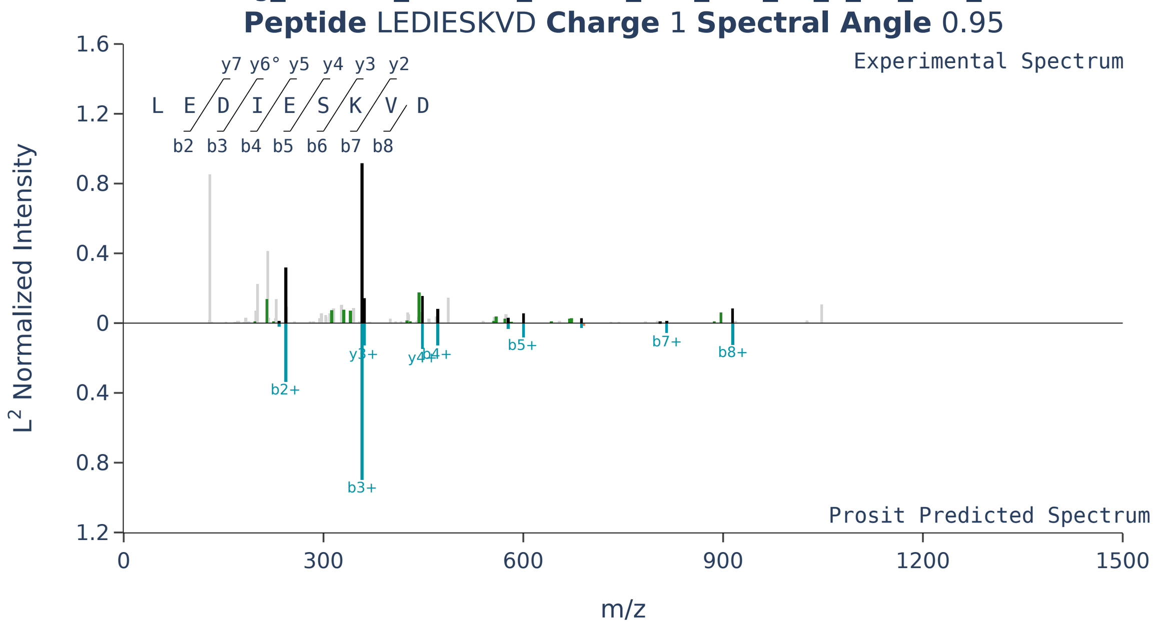


**Source** Ncheng_210623_230623_HFGoe_FFH_20S_25_1_A1_24h_R2 **Scan** 11819
**Peptide** PFHPDRI **Charge** 2 **Spectral Angle** 0.87

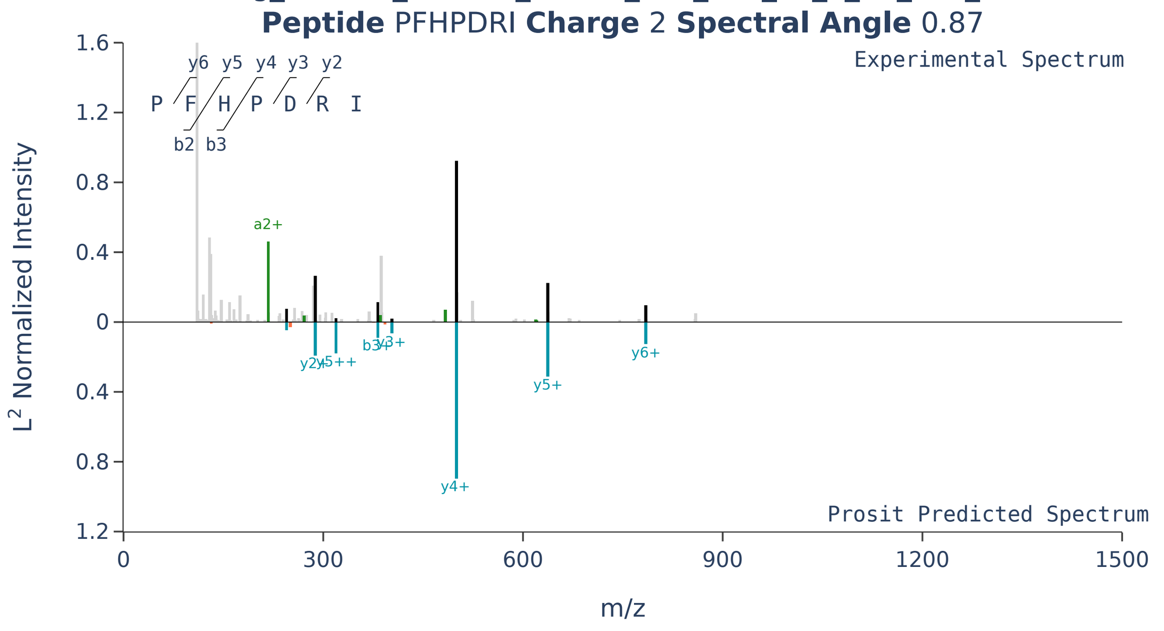


**Source** Ncheng_210623_230623_HFGoe_FFH_20S_25_1_A1_24h_R1 **Scan** 6518
**Peptide** PSDVGQKPVD **Charge** 1 **Spectral Angle** 0.88

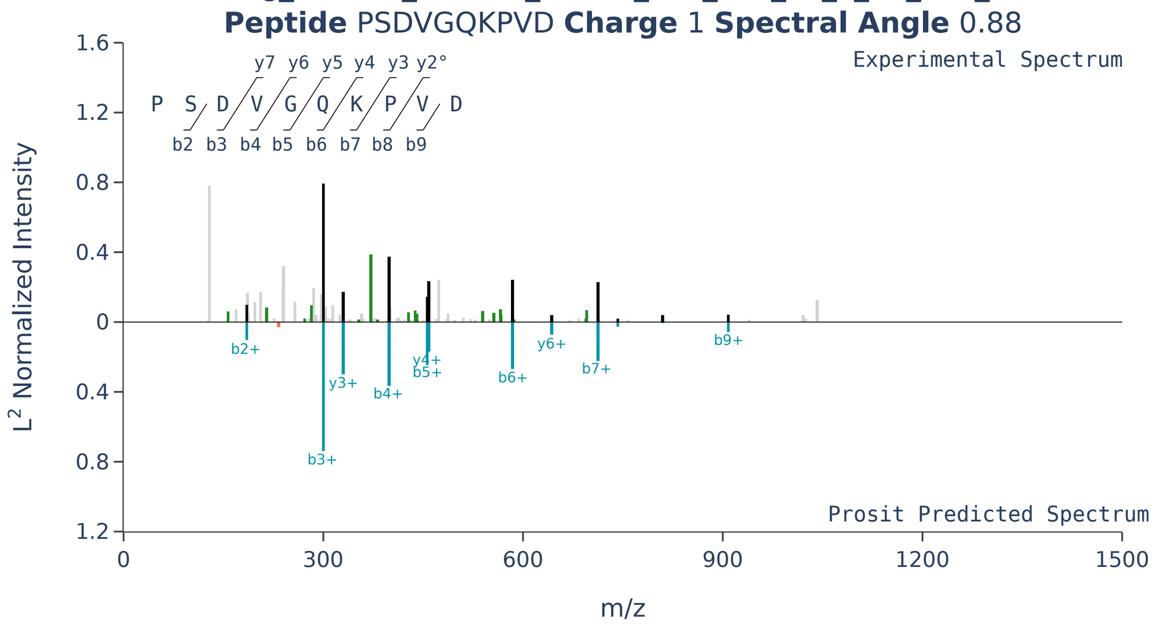


**Source** Ncheng_210623_230623_HFGoe_FFH_20S_25_1_A2_24h_R1 **Scan** 18319
**Peptide** AAGCGMQVQDVNRL **Charge** 2 **Spectral Angle** 0.84

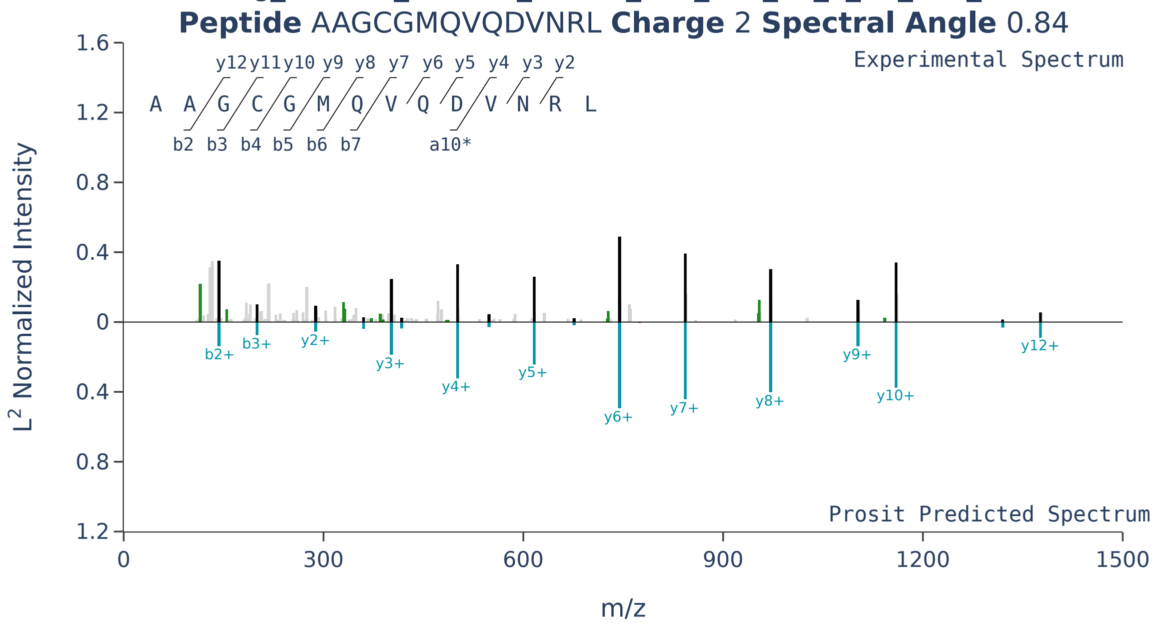


**Source** Ncheng_210623_230623_HFGoe_FFH_20S_25_1_A1_24h_R1 **Scan** 12266
**Peptide** VKIVRNEL **Charge** 2 **Spectral Angle** 0.84

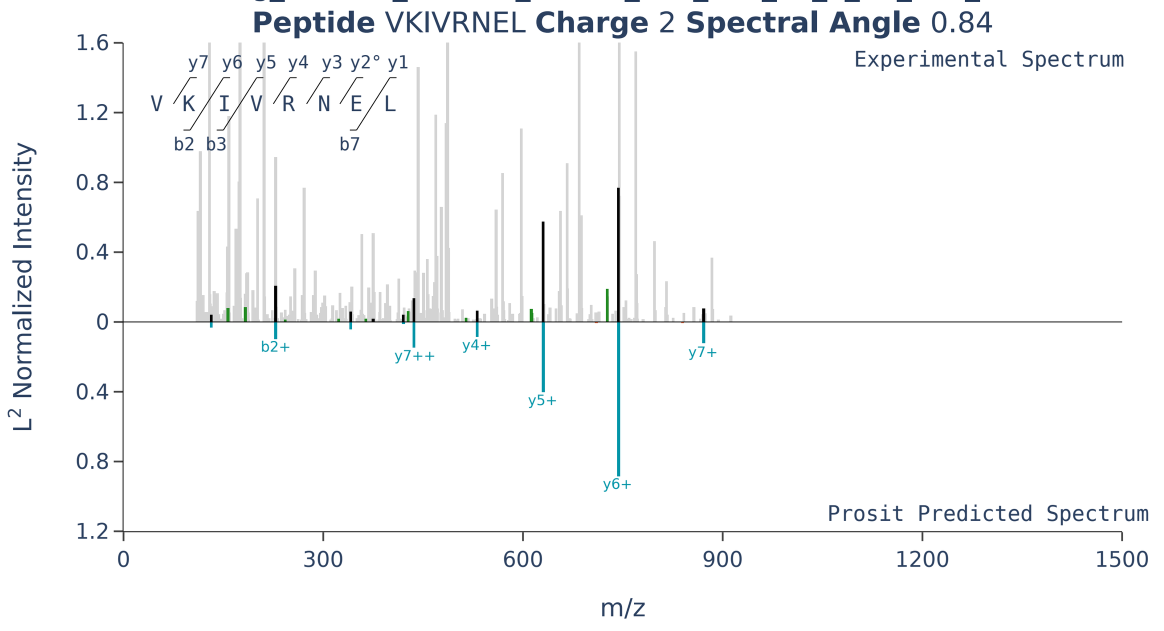


**Source** Ncheng_210623_230623_HFGoe_FFH_20S_25_1_A1_2h_R2 **Scan** 7264
**Peptide** SGRGRLTE **Charge** 2 **Spectral Angle** 0.85

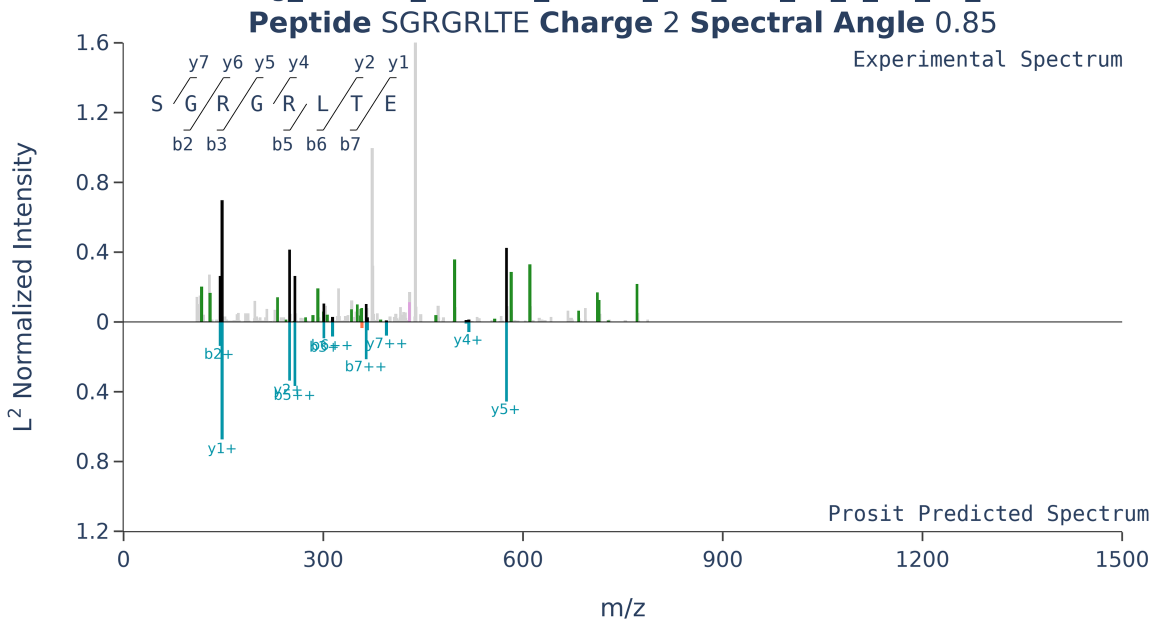


**Source** Ncheng_210623_230623_HFGoe_FFH_20S_25_1_A1_1h_R1 **Scan** 26837
**Peptide** VLVVSADVYRPAAL **Charge** 2 **Spectral Angle** 0.86

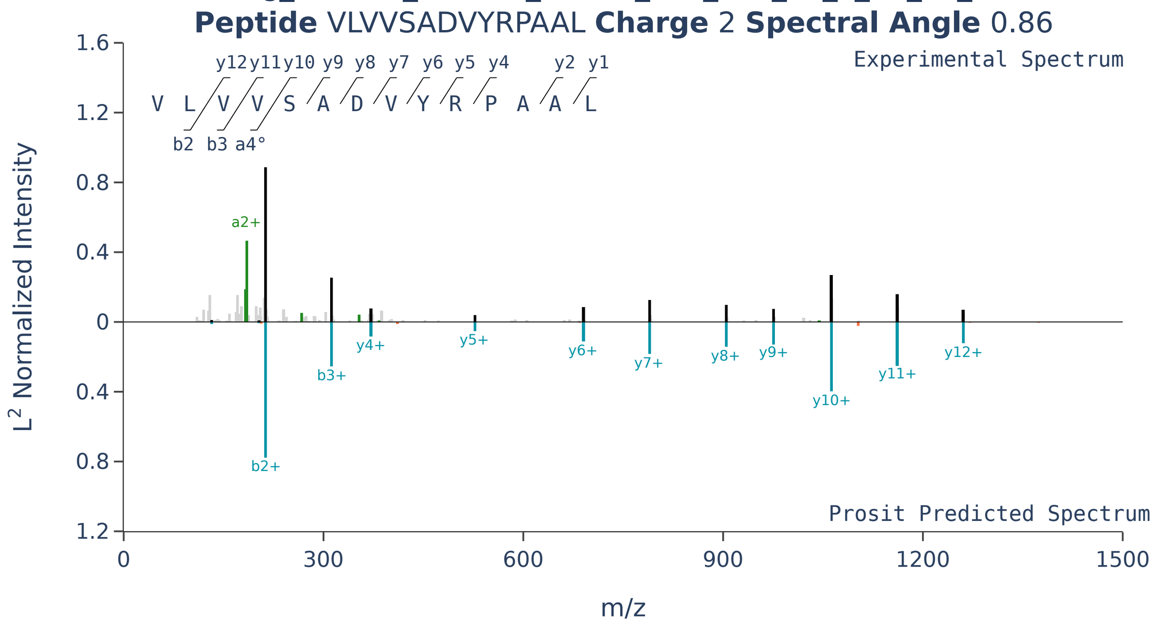

**Source** Ncheng_210623_230623_HFGoe_FFH_20S_25_1_A1_24h_R1 **Scan** 12889
**Peptide** FDDMQRM **Charge** 2 **Spectral Angle** 0.9

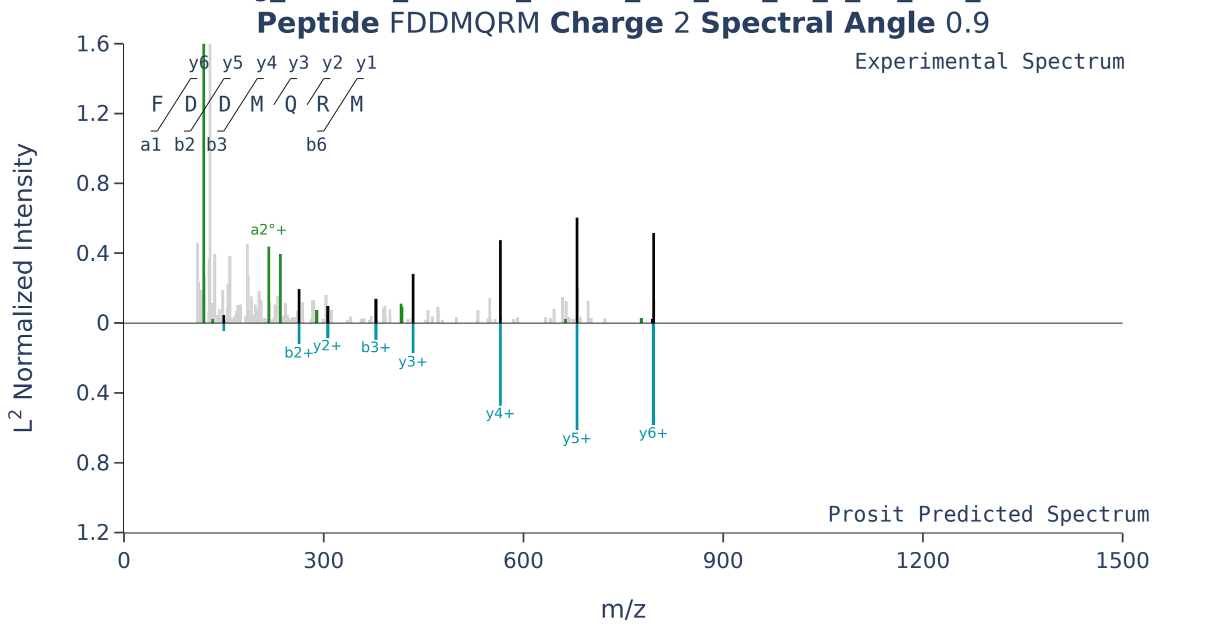


**Source** Ncheng_210623_230623_HFGoe_FFH_20S_25_1_A2_4h_R1 **Scan** 17719
**Peptide** ADVYRPAAIKQL **Charge** 2 **Spectral Angle** 0.84

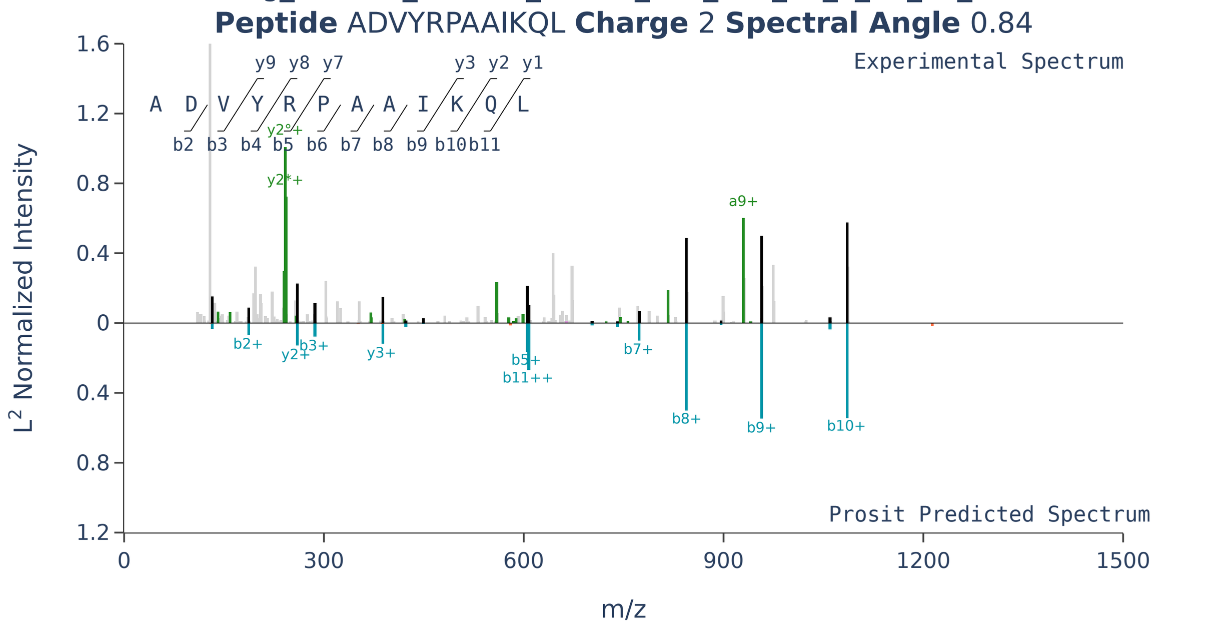


**Source** Ncheng_210623_230623_HFGoe_FFH_20S_25_1_A1_2h_R2 **Scan** 11561
**Peptide** QMDDKVLVR **Charge** 3 **Spectral Angle** 0.95

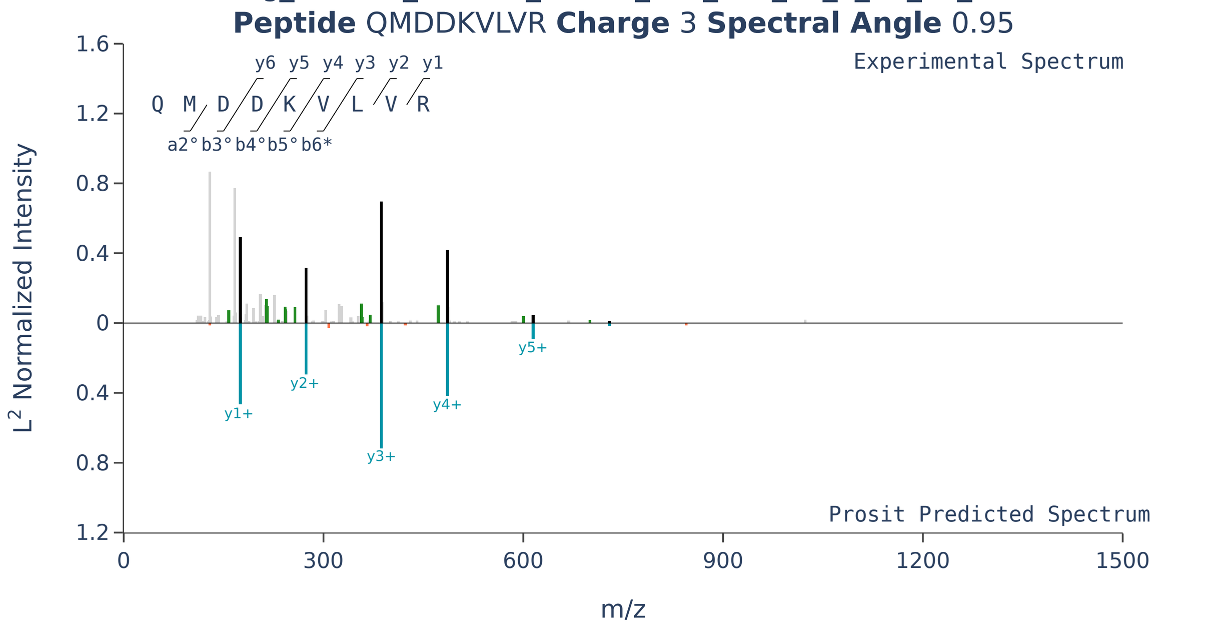


**Source** Ncheng_210623_230623_HFGoe_FFH_20S_25_1_A2_24h_R2 **Scan** 11278
**Peptide** MGEENQTL **Charge** 1 **Spectral Angle** 0.84

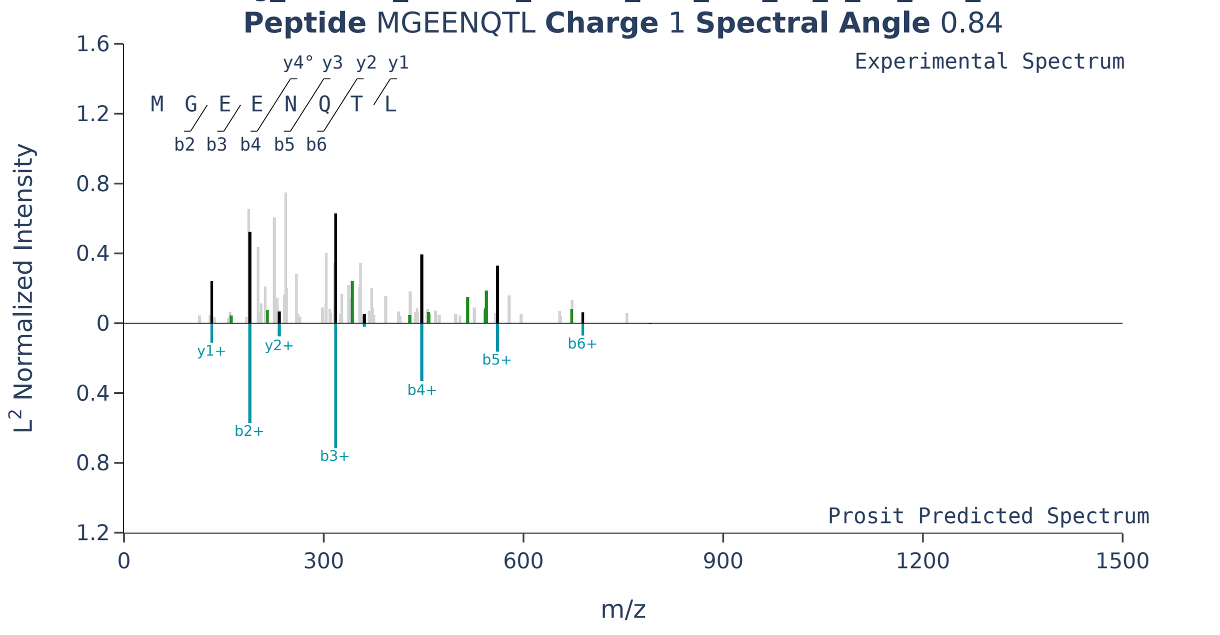


**Source** Ncheng_210623_230623_HFGoe_FFH_20S_25_1_A2_4h_R2 **Scan** 9823
**Peptide** GEKTEAL **Charge** 1 **Spectral Angle** 0.9

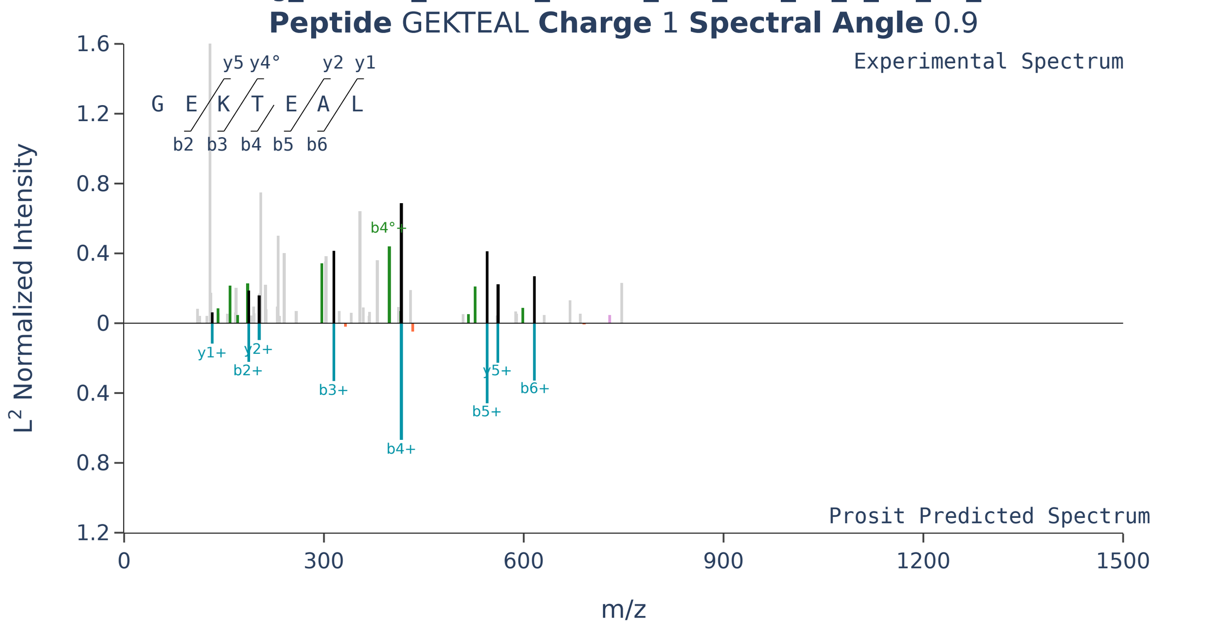


**Source** Ncheng_210623_230623_HFGoe_FFH_20S_25_1_A2_4h_R1 **Scan** 11840
**Peptide** PFHPDRL **Charge** 2 **Spectral Angle** 0.88

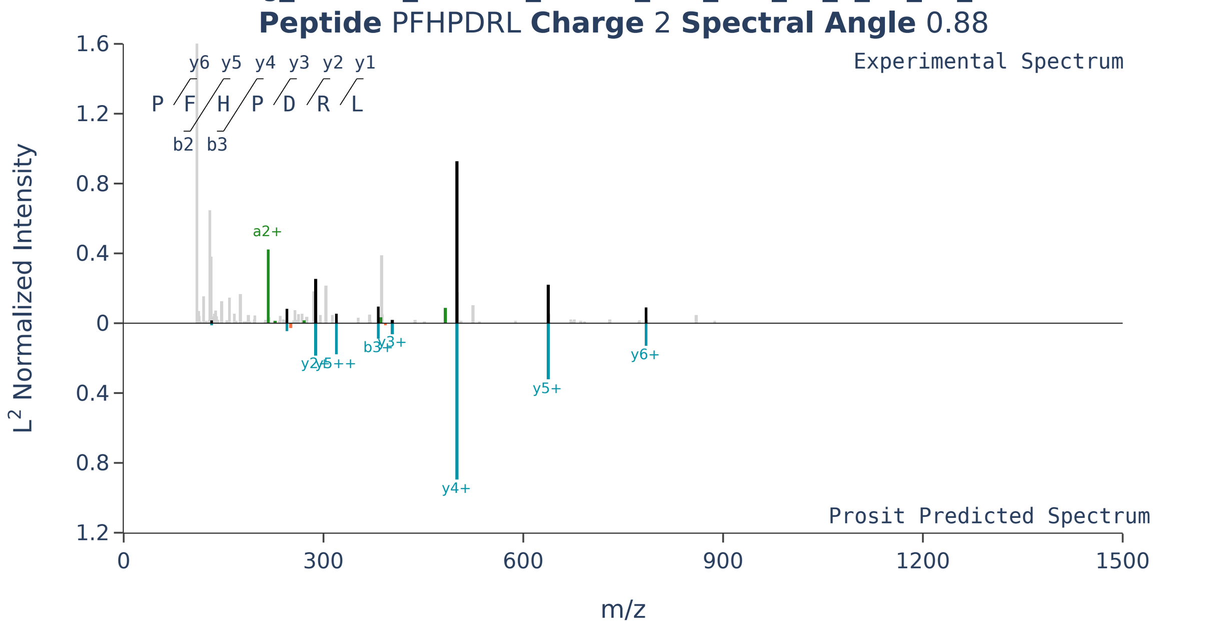


**Source** Ncheng_210623_230623_HFGoe_FFH_20S_25_1_A2_2h_R1 **Scan** 24875
**Peptide** SLMGKLPGMGQIPDNVKS **Charge** 2 **Spectral Angle** 0.83

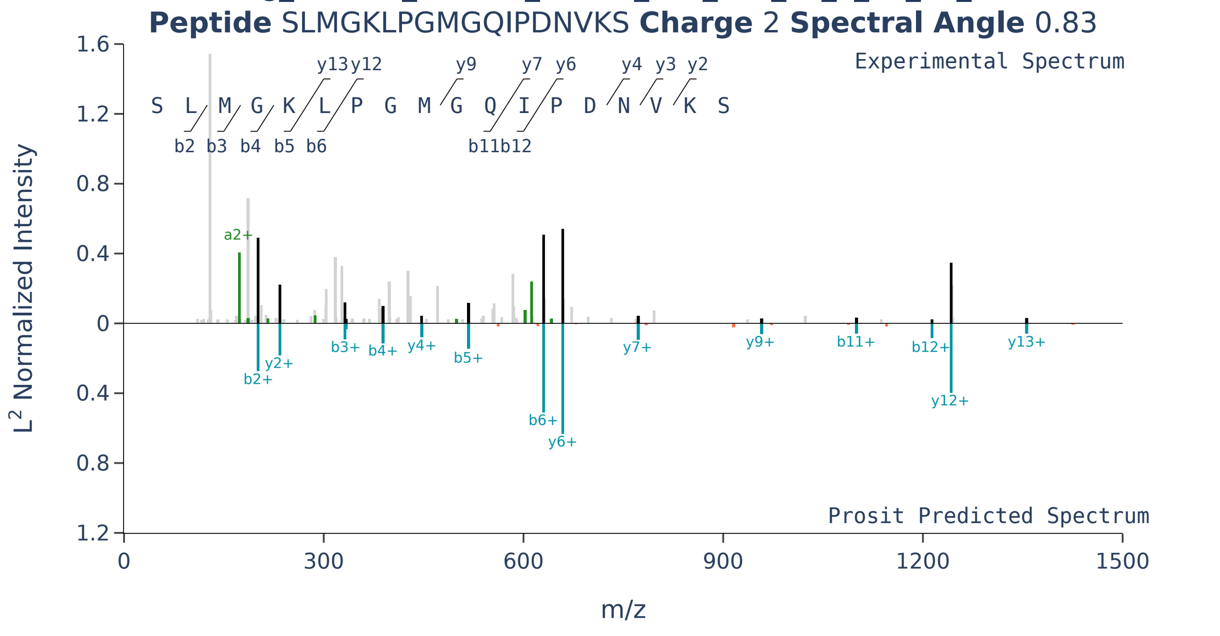


**Source** Ncheng_210623_230623_HFGoe_FFH_20S_25_1_A1_2h_R2 **Scan** 25969
**Peptide** GVDFFPSDVGQKPVD **Charge** 2 **Spectral Angle** 0.87

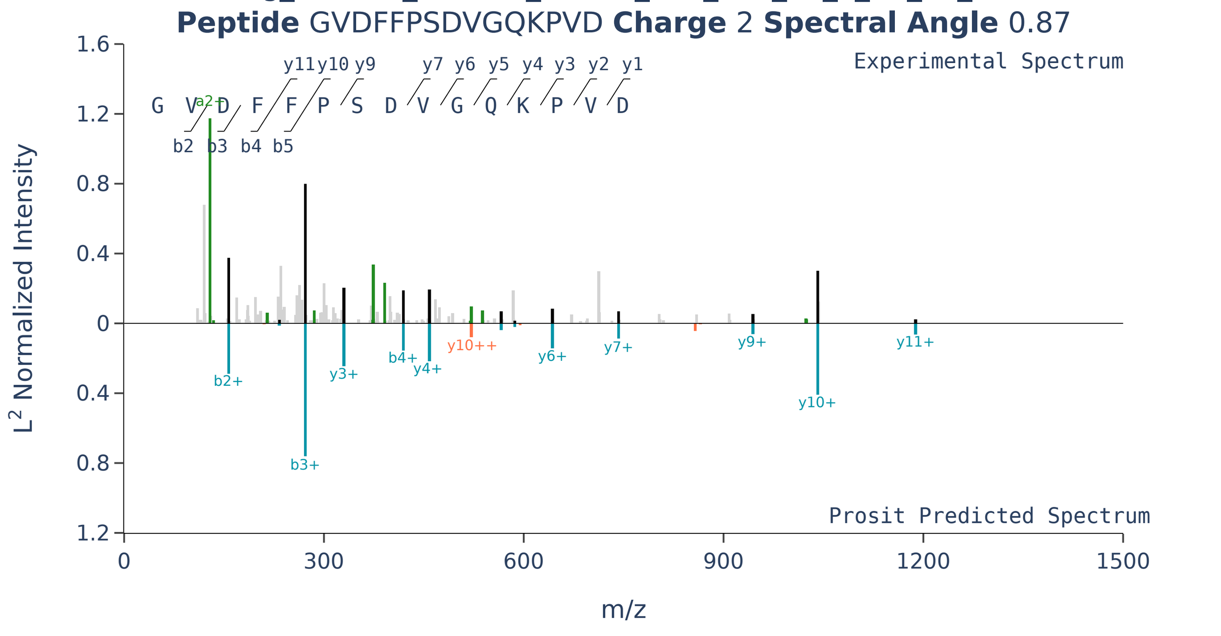


**Source** Ncheng_210623_230623_HFGoe_FFH_20S_25_1_A1_1h_R2 **Scan** 27480
**Peptide** AEQVGVDFFPSDVGQKPVD **Charge** 2 **Spectral Angle** 0.87

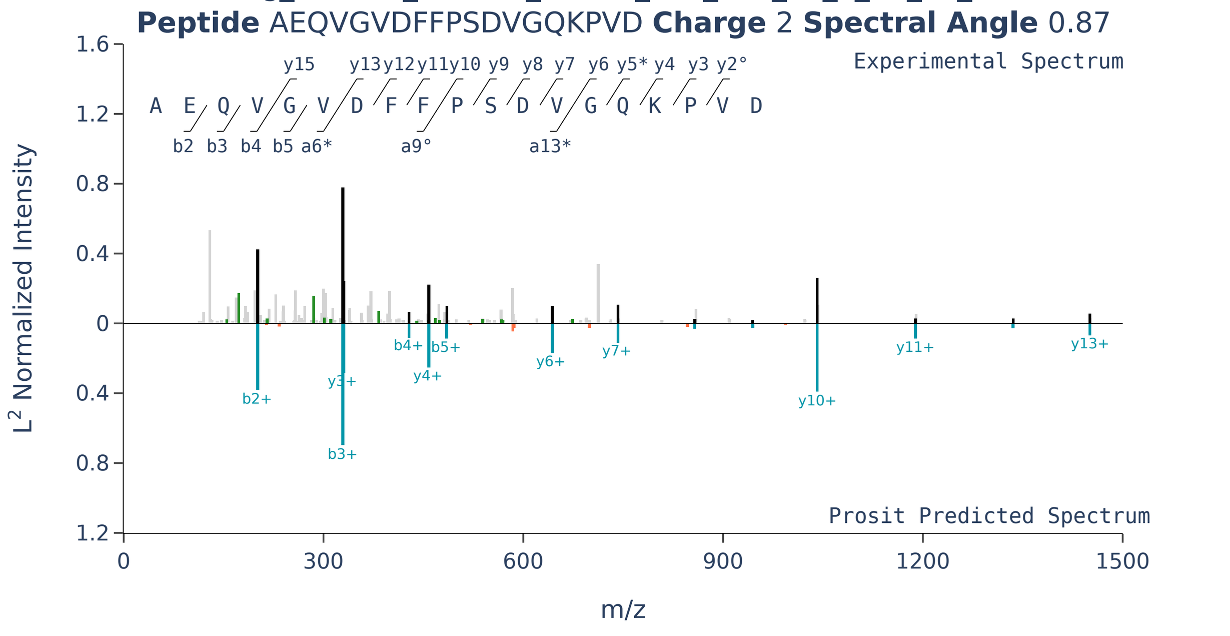


**Source** Ncheng_210623_230623_HFGoe_FFH_20S_25_1_A1_24h_R2 **Scan** 8773
**Peptide** GQIPDNVKSQMD **Charge** 2 **Spectral Angle** 0.83

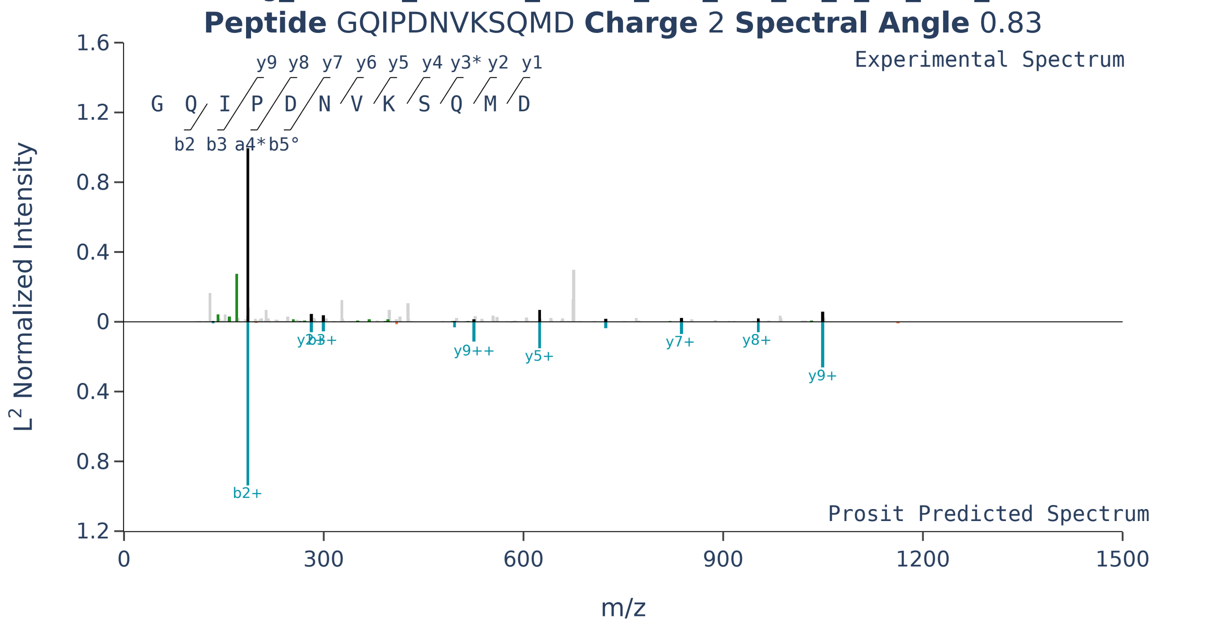


**Source** Ncheng_210623_230623_HFGoe_FFH_20S_25_1_A2_24h_R2 **Scan** 15100
**Peptide** AKAFNEAL **Charge** 2 **Spectral Angle** 0.87

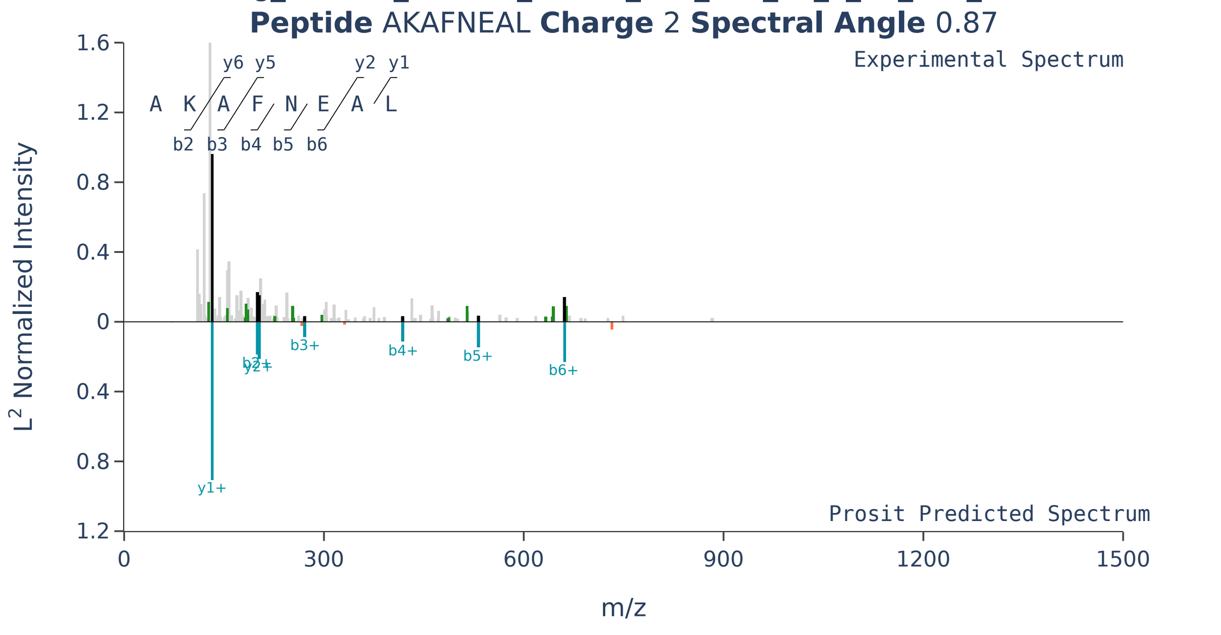


**Source** Ncheng_210623_230623_HFGoe_FFH_20S_25_1_A2_24h_R1 **Scan** 11612
**Peptide** AQAEKLASKL **Charge** 3 **Spectral Angle** 0.87

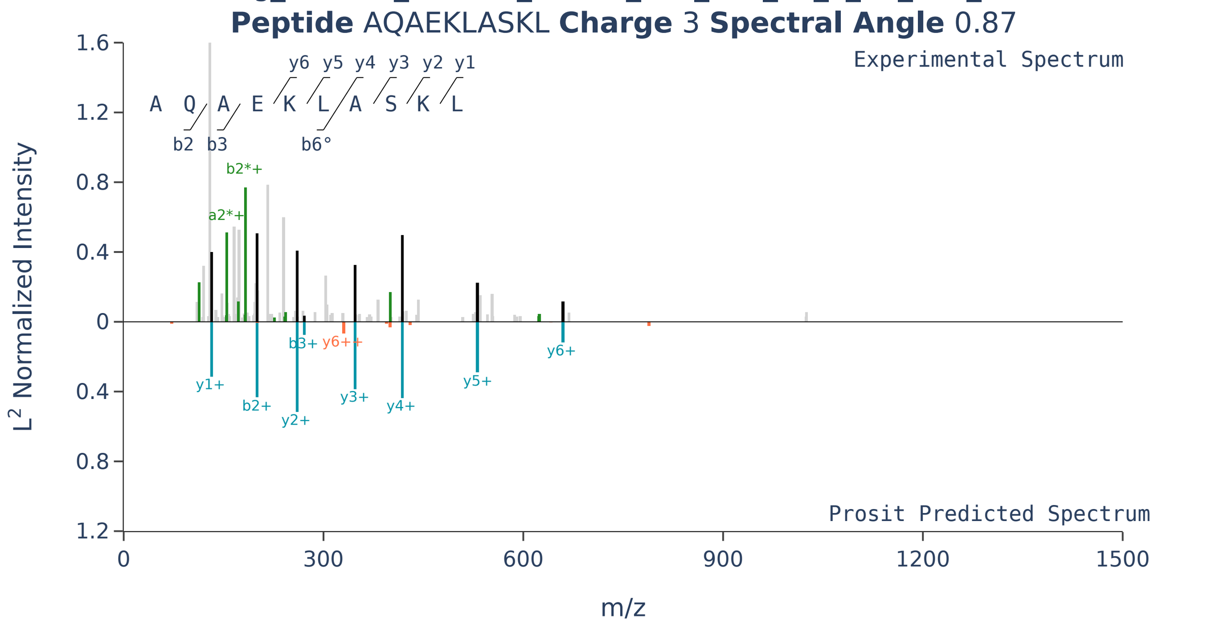


**Source** Ncheng_210623_230623_HFGoe_FFH_20S_25_1_A2_24h_R1 **Scan** 13721
**Peptide** KSQMDDKVLV **Charge** 1 **Spectral Angle** 0.81

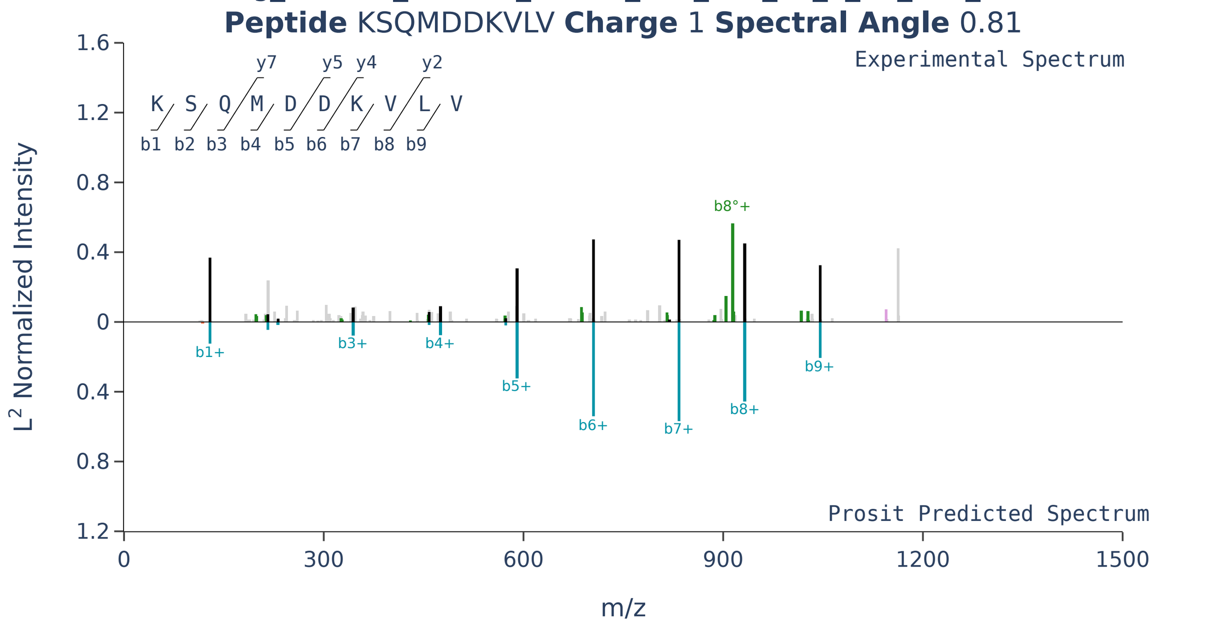


**Source** Ncheng_210623_230623_HFGoe_FFH_20S_25_1_A1_4h_R2 **Scan** 23950
**Peptide** ALPVVREF **Charge** 2 **Spectral Angle** 0.86

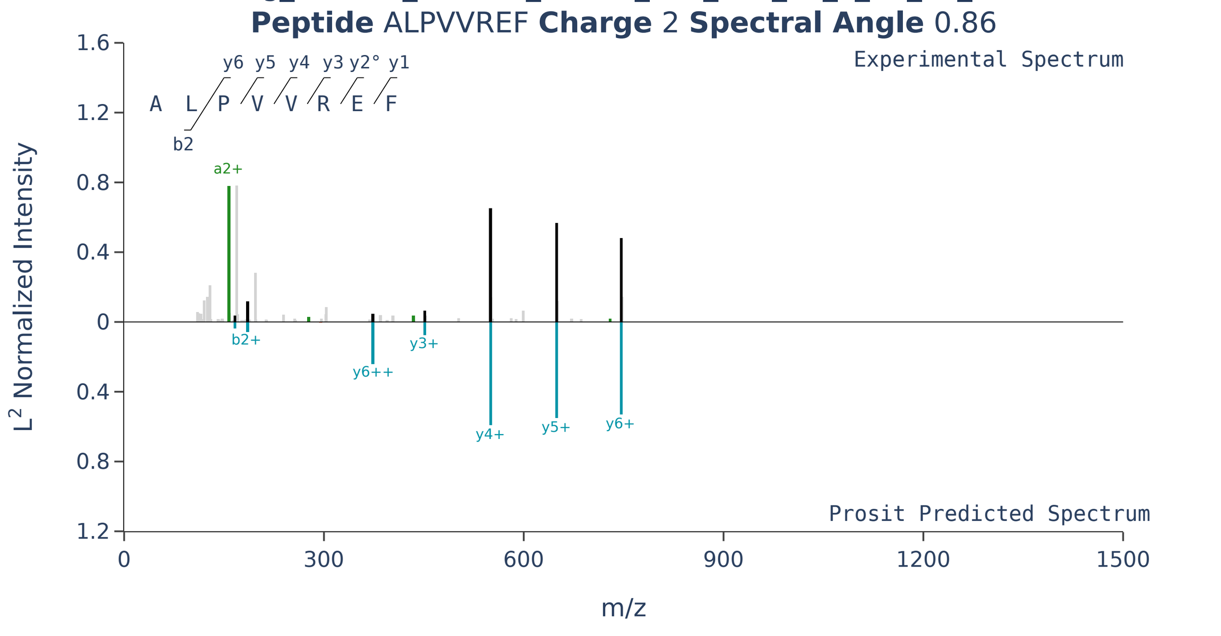


**Source** Ncheng_210623_230623_HFGoe_FFH_20S_25_1_A2_24h_R1 **Scan** 13653
**Peptide** LVDTAGRL **Charge** 2 **Spectral Angle** 0.92

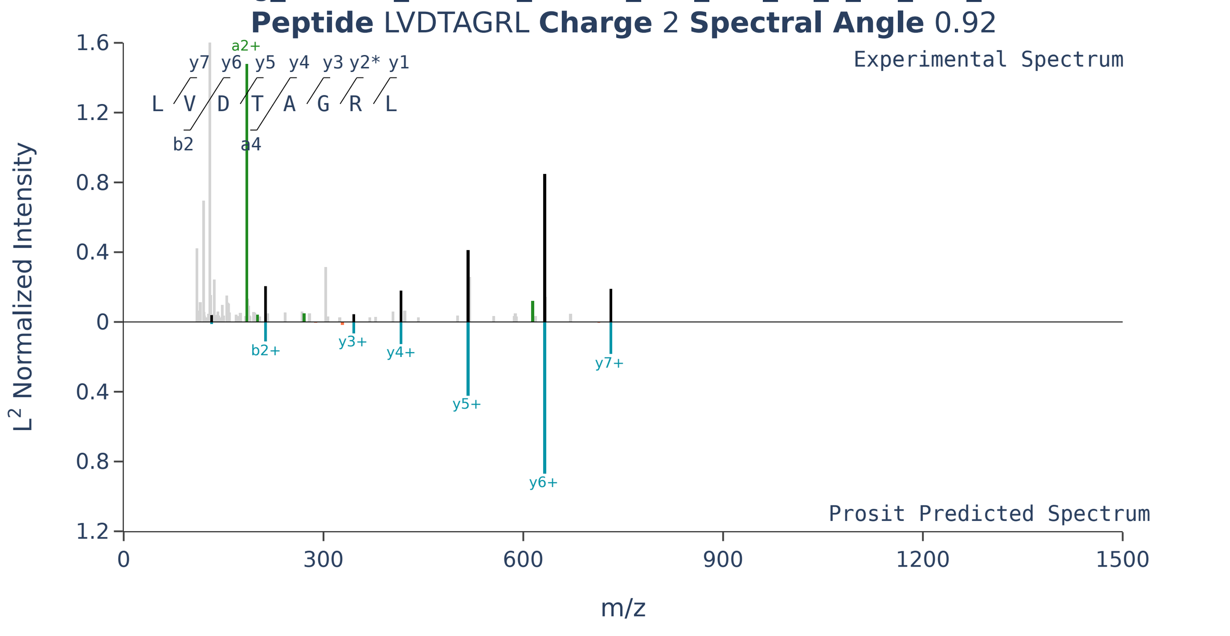

**Source** Ncheng_210623_230623_HFGoe_FFH_20S_25_1_A1_24h_R2 **Scan** 8757
**Peptide** AALSIRH **Charge** 2 **Spectral Angle** 0.83

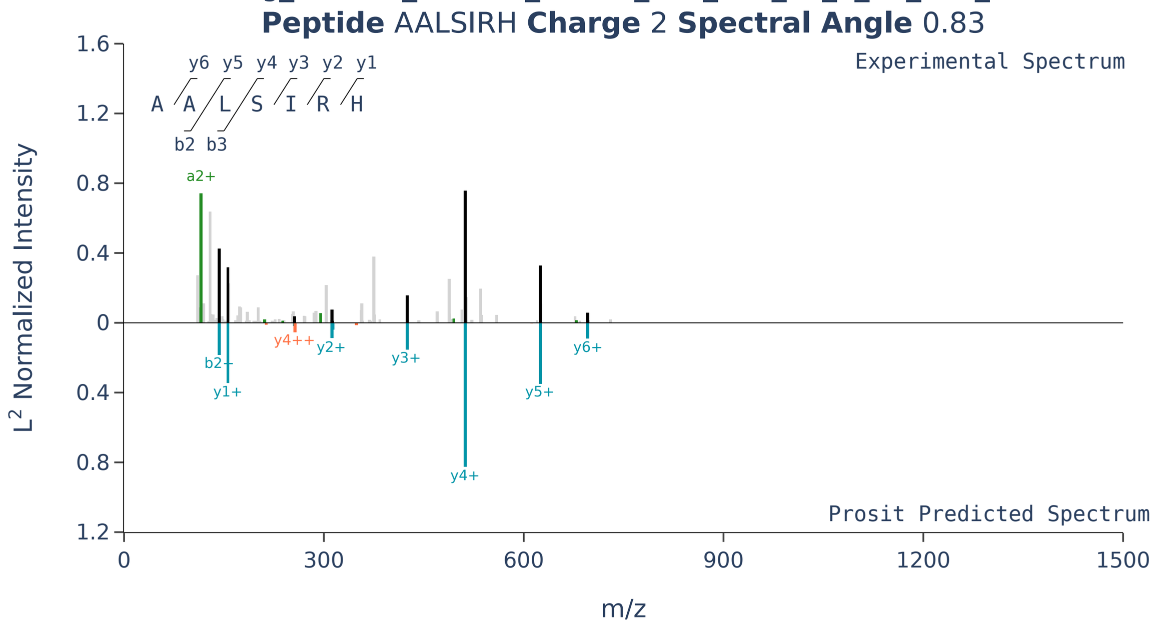


**Source** Ncheng_210623_230623_HFGoe_FFH_20S_25_1_A2_24h_R2 **Scan** 6208
**Peptide** AGLQGAGKTT **Charge** 2 **Spectral Angle** 0.85

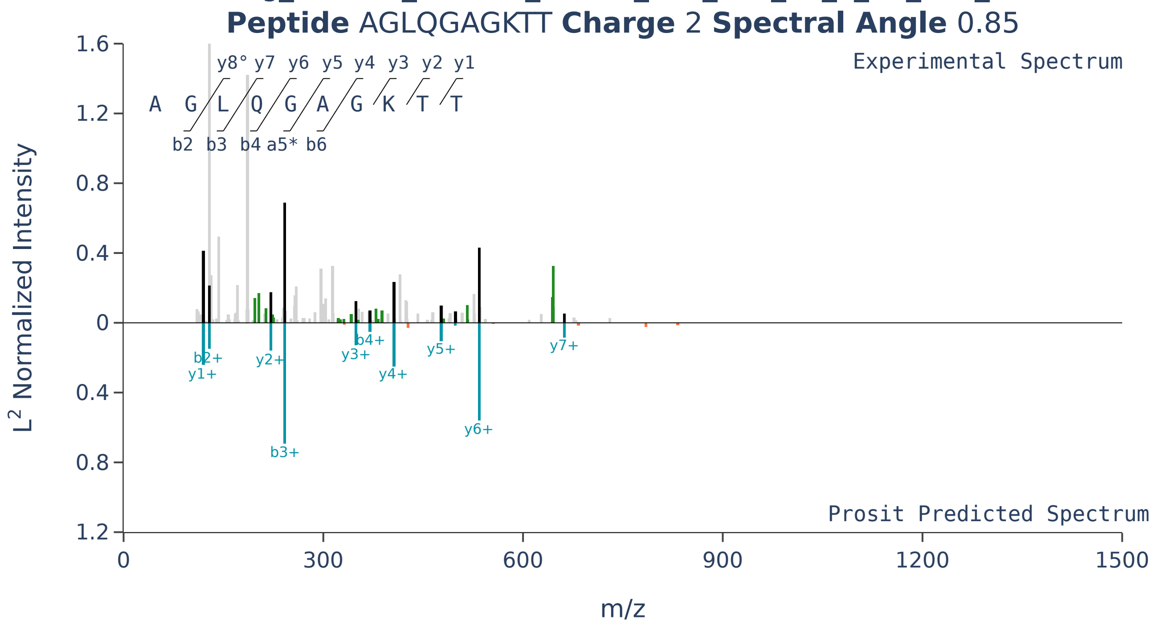


**Source** Ncheng_210623_230623_HFGoe_FFH_20S_25_1_A2_24h_R2 **Scan** 13853
**Peptide** MTGQDAANTAKAFNE **Charge** 2 **Spectral Angle** 0.83

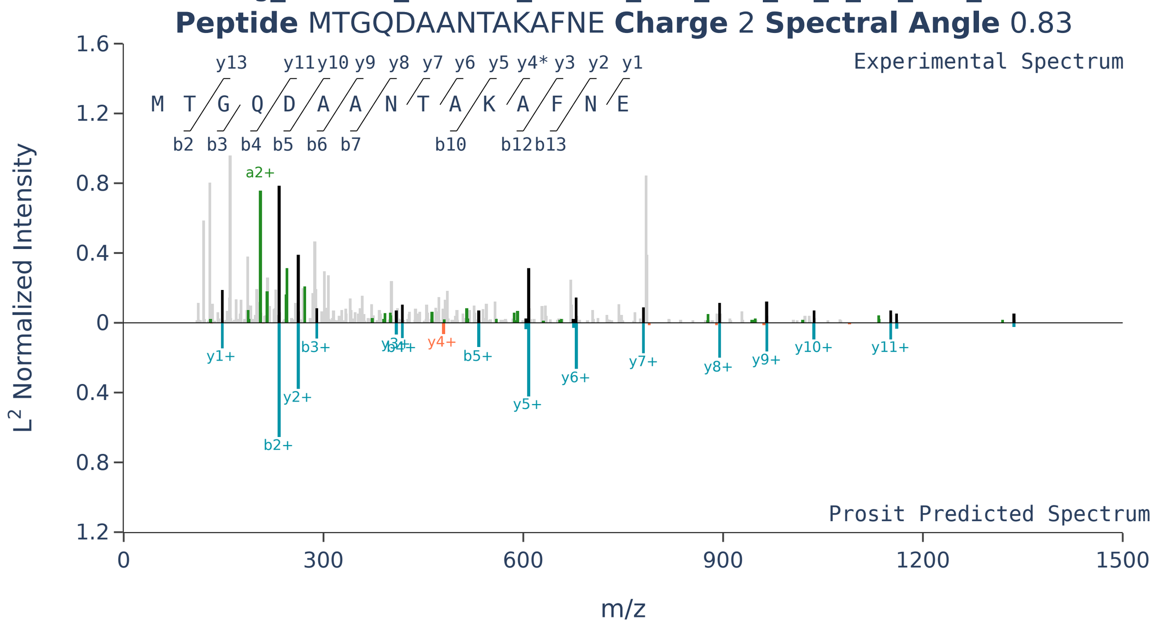


**Source** Ncheng_210623_230623_HFGoe_FFH_20S_25_1_A1_2h_R2 **Scan** 16875
**Peptide** FVKIVRNEL **Charge** 2 **Spectral Angle** 0.9

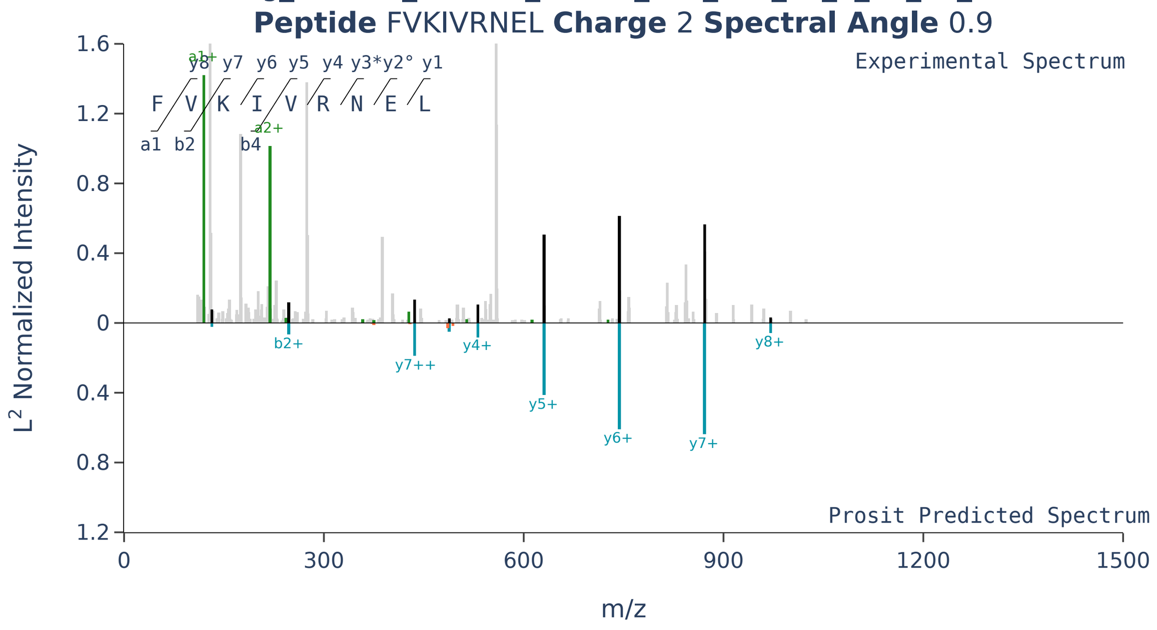


**Source** Ncheng_210623_230623_HFGoe_FFH_20S_25_1_A1_24h_R1 **Scan** 19302
**Peptide** VALPVVR **Charge** 2 **Spectral Angle** 0.84

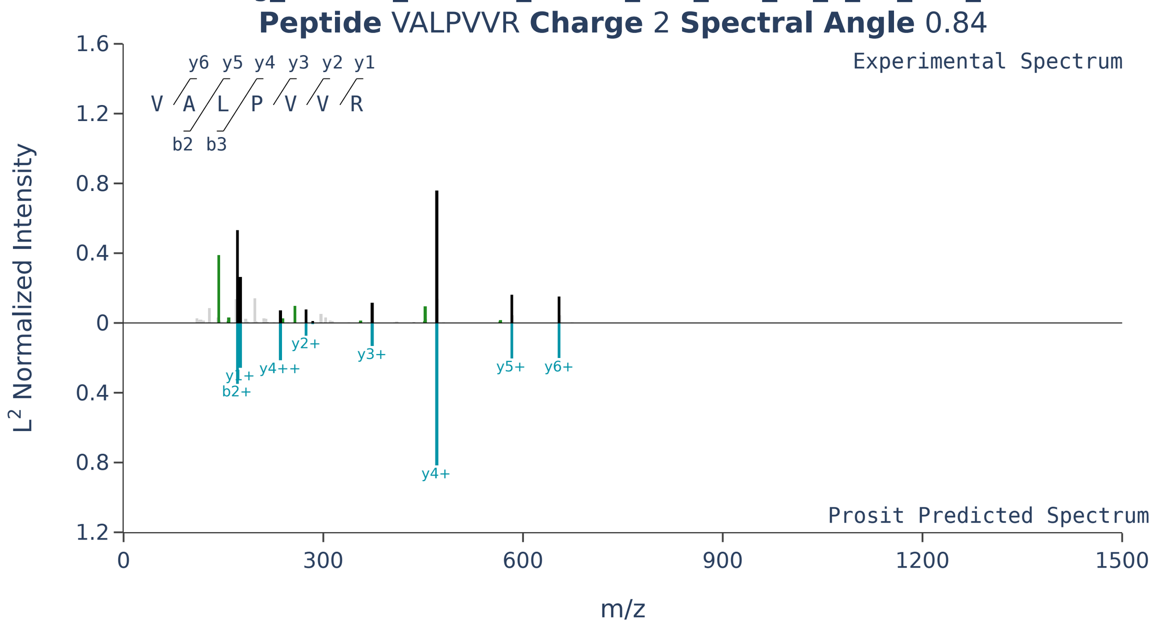


**Source** Ncheng_210623_230623_HFGoe_FFH_20S_25_1_A1_24h_R2 **Scan** 8100
**Peptide** EVNKSLTPGQ **Charge** 1 **Spectral Angle** 0.87

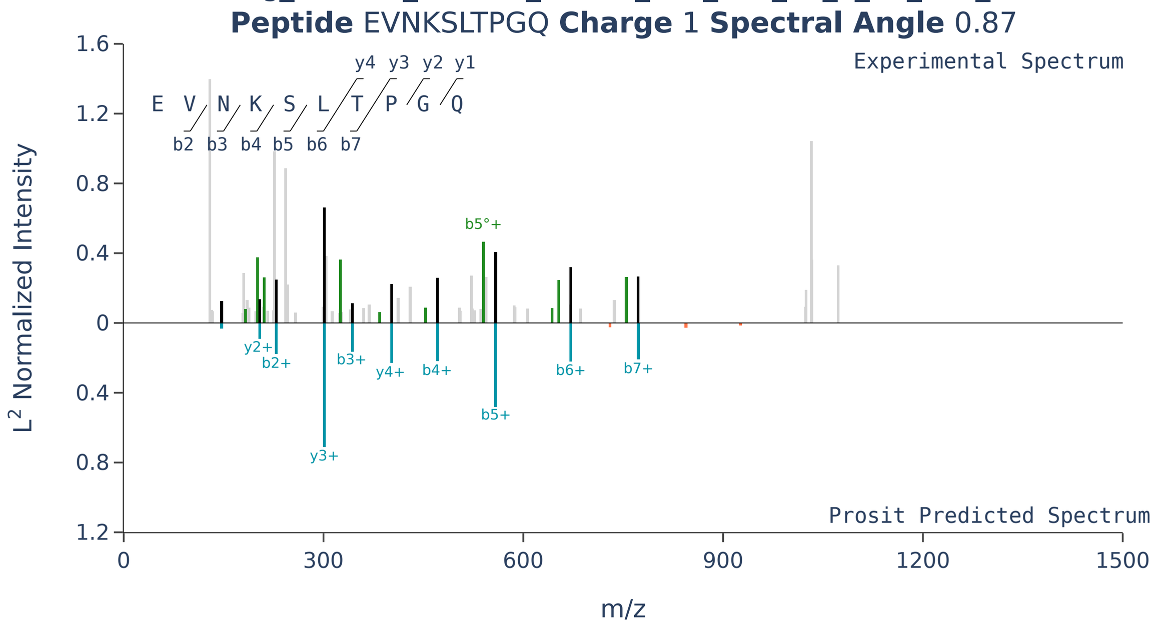


**Source** Ncheng_210623_230623_HFGoe_FFH_20S_25_1_A1_24h_R1 **Scan** 21831
**Peptide** KSLTPGQEFVKL **Charge** 2 **Spectral Angle** 0.86

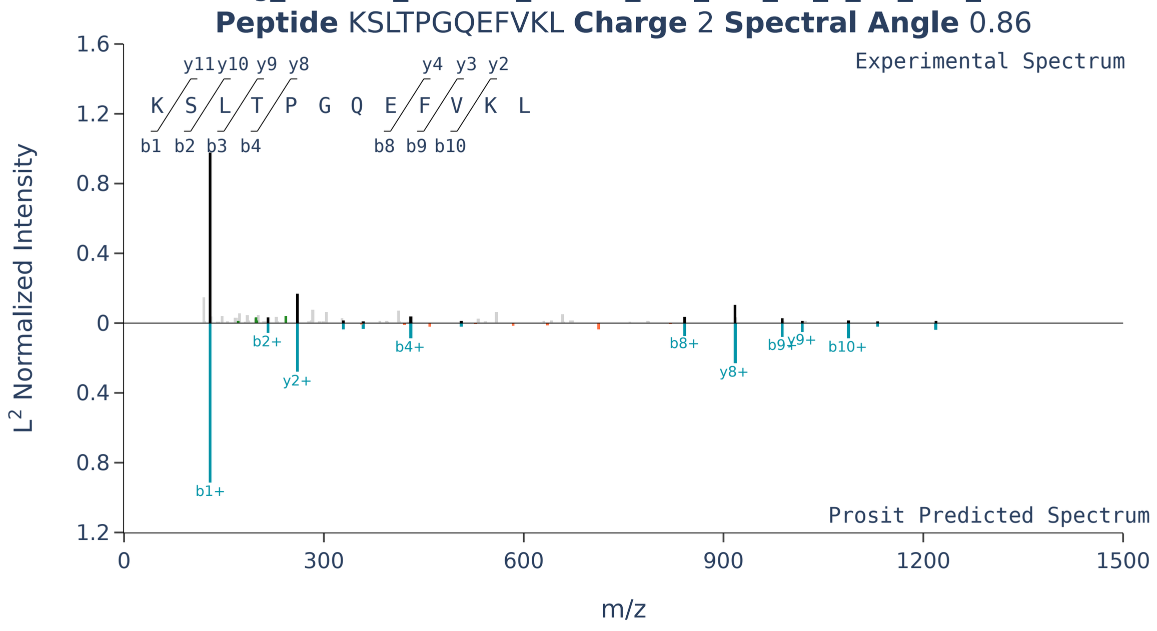


**Source** Ncheng_210623_230623_HFGoe_FFH_20S_25_1_A2_4h_R2 **Scan** 19065
**Peptide** GHEVNKSLTPGQEFVKIVRN **Charge** 2 **Spectral Angle** 0.79

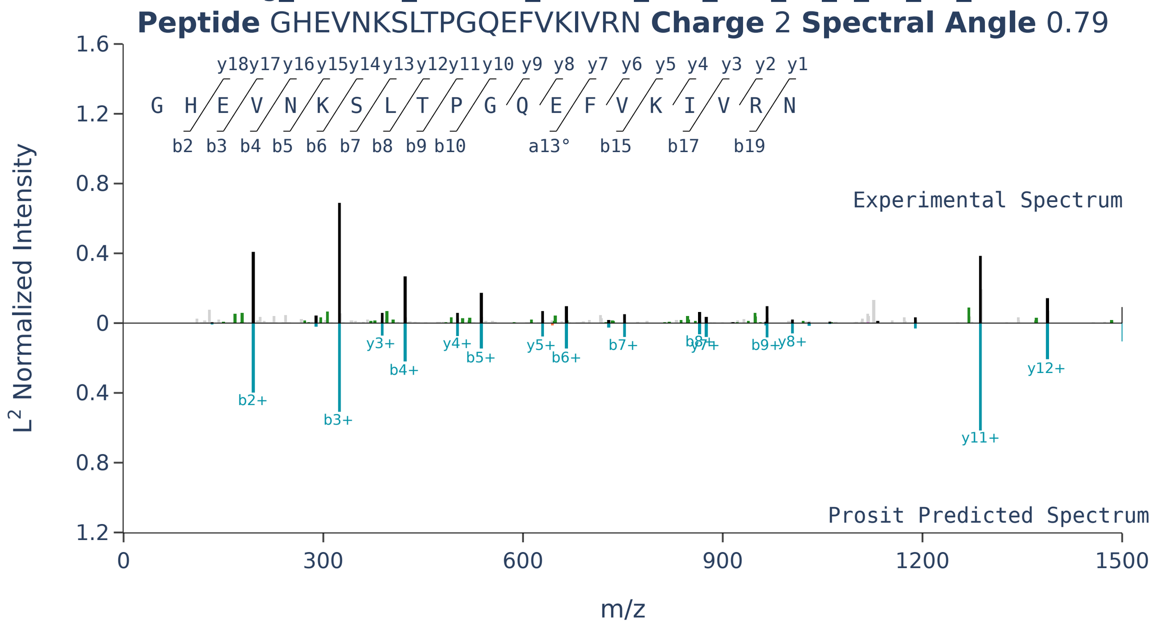


**Source** Ncheng_210623_230623_HFGoe_FFH_20S_25_1_A2_24h_R2 **Scan** 19329
**Peptide** AMMDEIKQV **Charge** 2 **Spectral Angle** 0.81

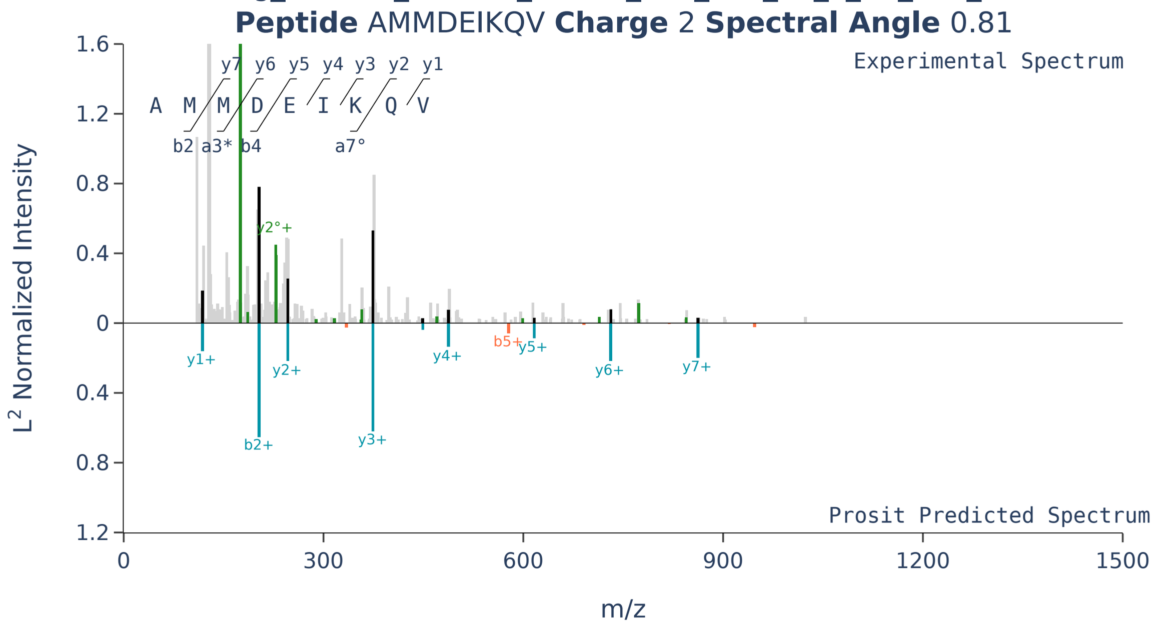


**Source** Ncheng_210623_230623_HFGoe_FFH_20S_25_1_A1_24h_R1 **Scan** 14767
**Peptide** DAMTGQDAANTAKAFN **Charge** 2 **Spectral Angle** 0.84

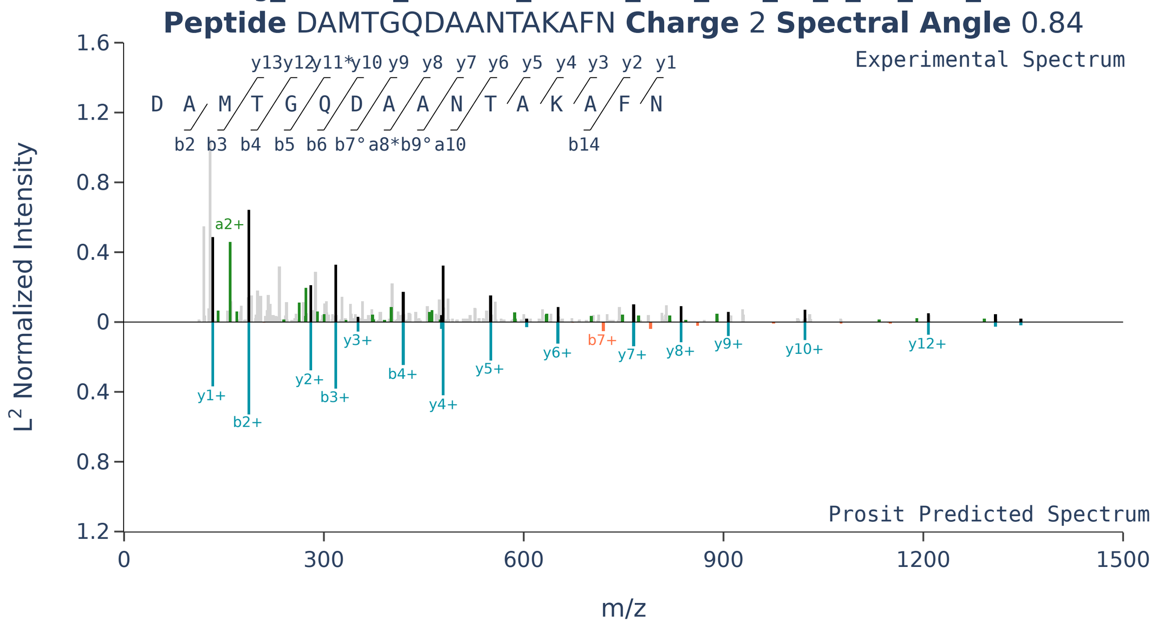


**Source** Ncheng_210623_230623_HFGoe_FFH_20S_25_1_A2_24h_R1 **Scan** 9198
**Peptide** DGDARGGAAL **Charge** 2 **Spectral Angle** 0.76

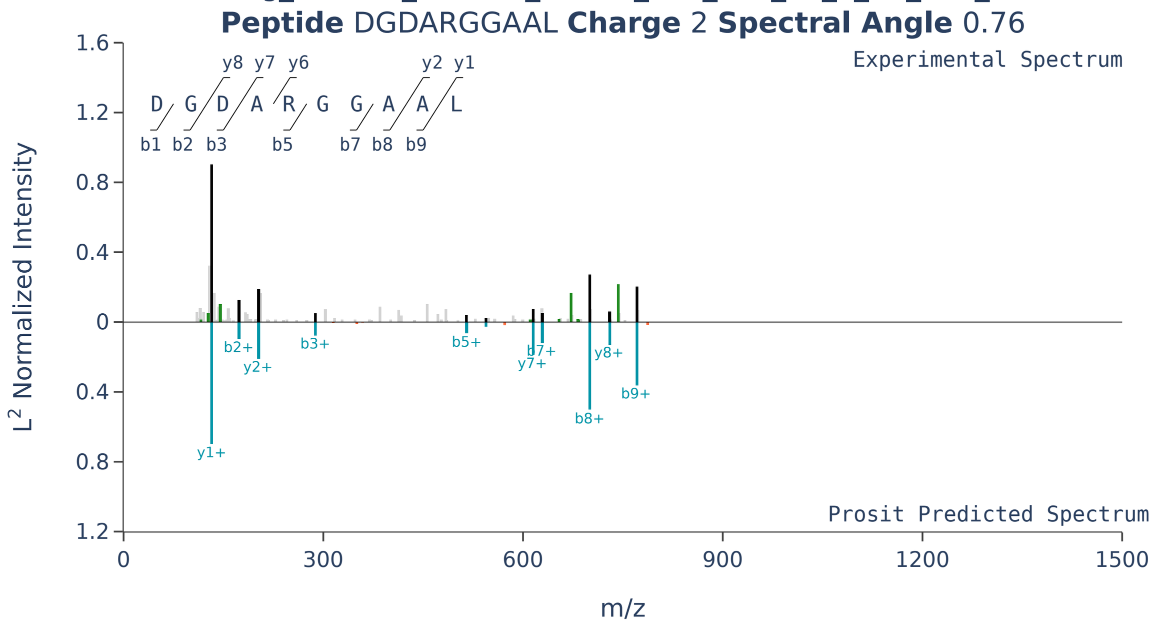


**Source** Ncheng_210623_230623_HFGoe_FFH_20S_25_1_A2_24h_R2 **Scan** 18270
**Peptide** VGHEVNKSLTPGQEFV **Charge** 2 **Spectral Angle** 0.87

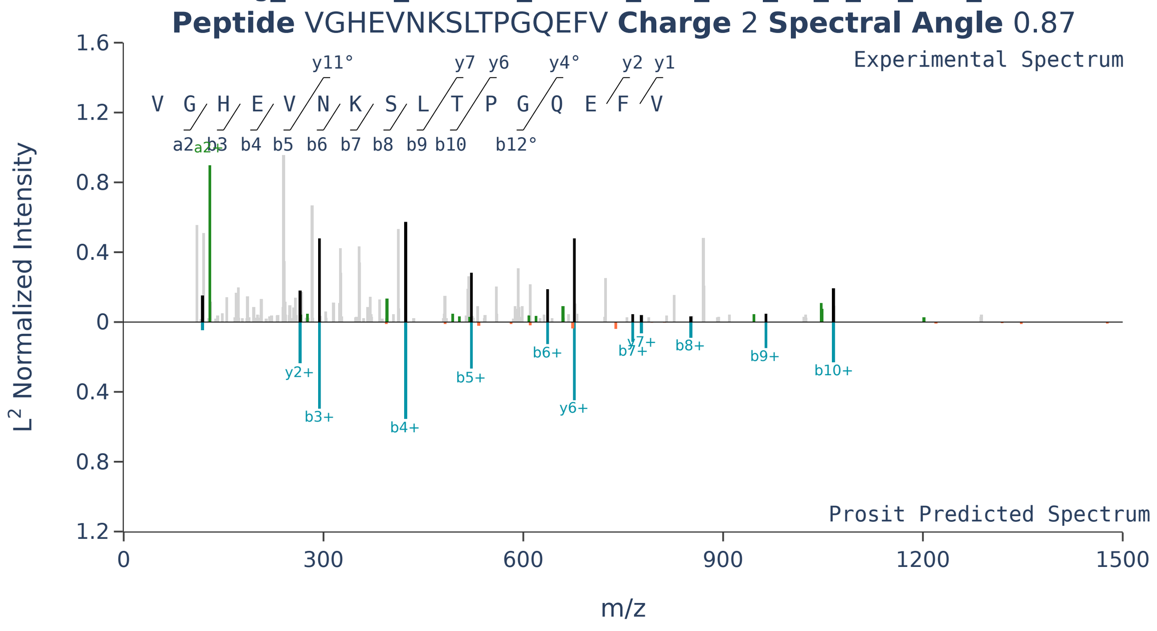


**Source** Ncheng_210623_230623_HFGoe_FFH_20S_25_1_A1_2h_R2 **Scan** 11204
**Peptide** KQLETLAE **Charge** 2 **Spectral Angle** 0.86

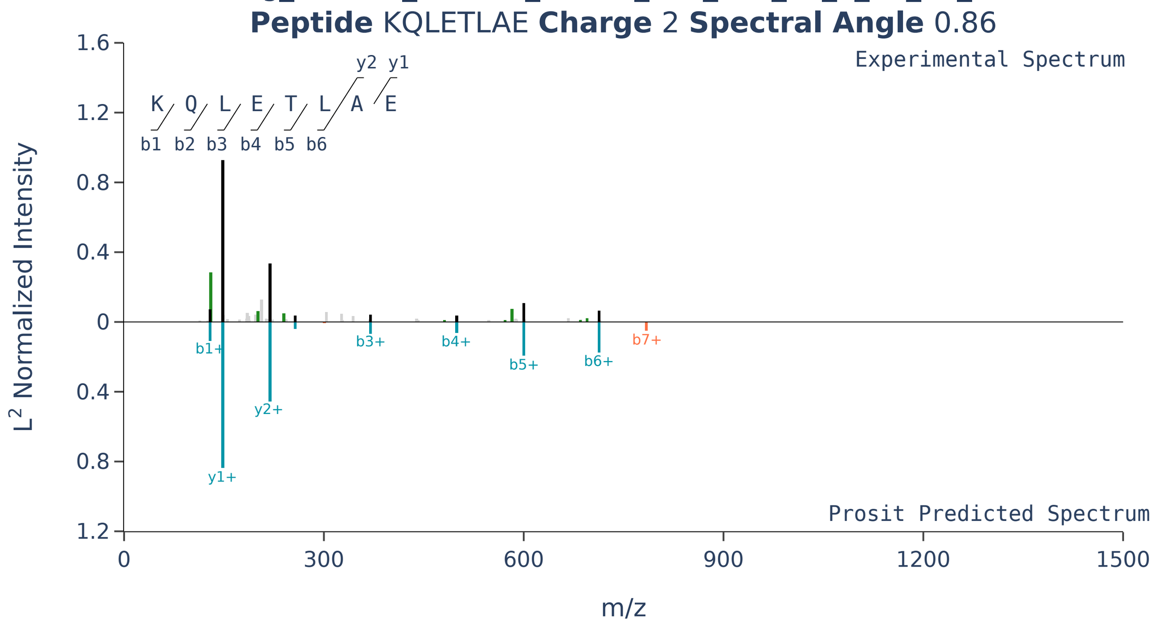


**Source** Ncheng_210623_230623_HFGoe_FFH_20S_25_1_A2_24h_R2 **Scan** 21761
**Peptide** EADVALPVVR **Charge** 2 **Spectral Angle** 0.81

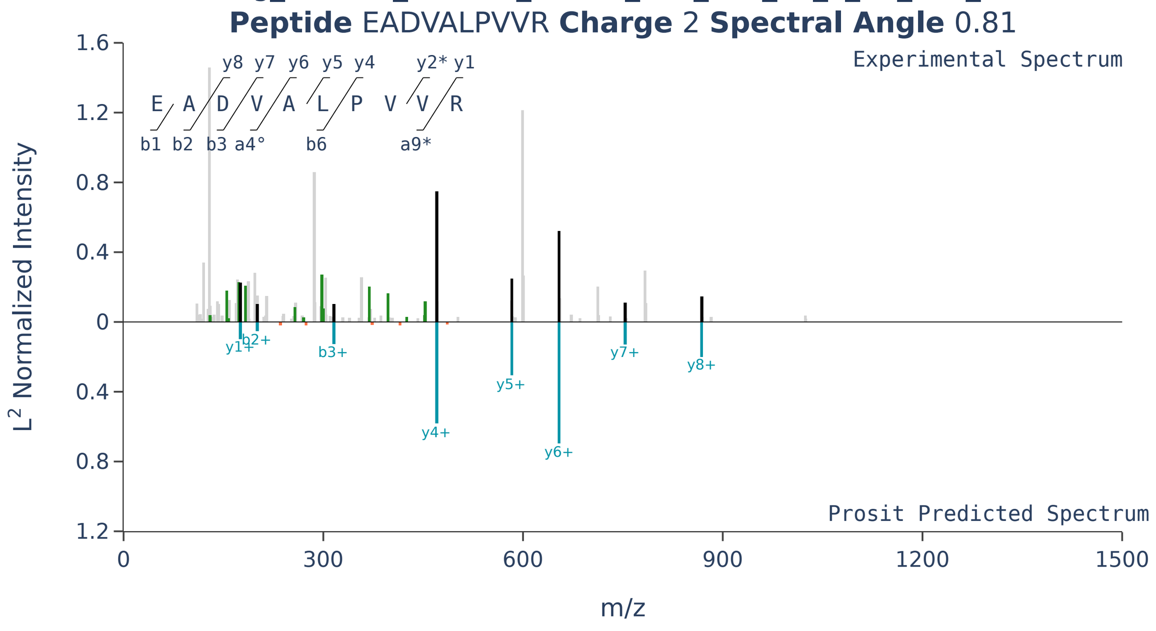


**Source** Ncheng_210623_230623_HFGoe_FFH_20S_25_1_A1_2h_R2 **Scan** 31084
**Peptide** DDKVLVRMEAII **Charge** 2 **Spectral Angle** 0.88

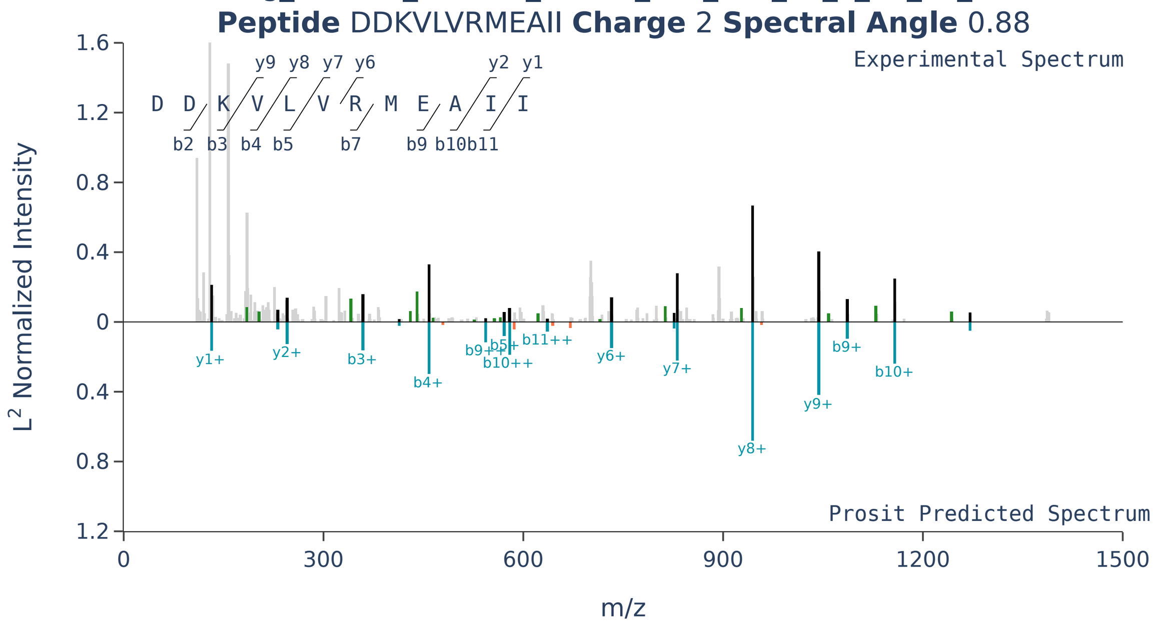

**Source** Ncheng_210623_230623_HFGoe_FFH_20S_25_1_A2_24h_R2 **Scan** 10074
**Peptide** RPAAIKQL **Charge** 2 **Spectral Angle** 0.83

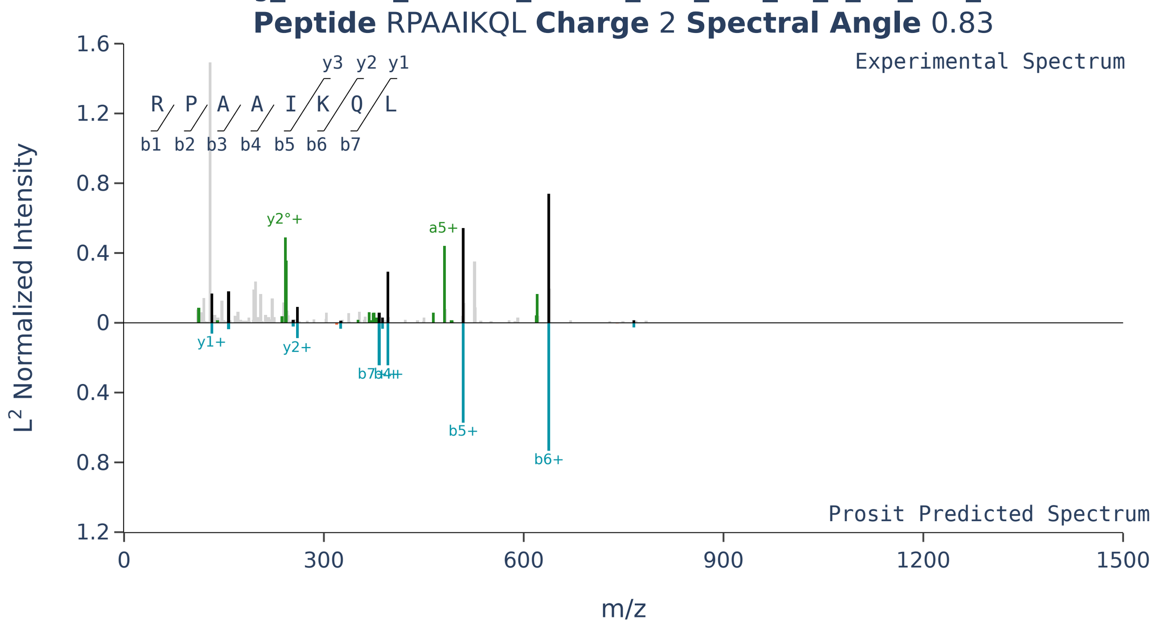


**Source** Ncheng_210623_230623_HFGoe_FFH_20S_25_1_A2_1h_R1 **Scan** 17826
**Peptide** KLKFYDV **Charge** 2 **Spectral Angle** 0.83

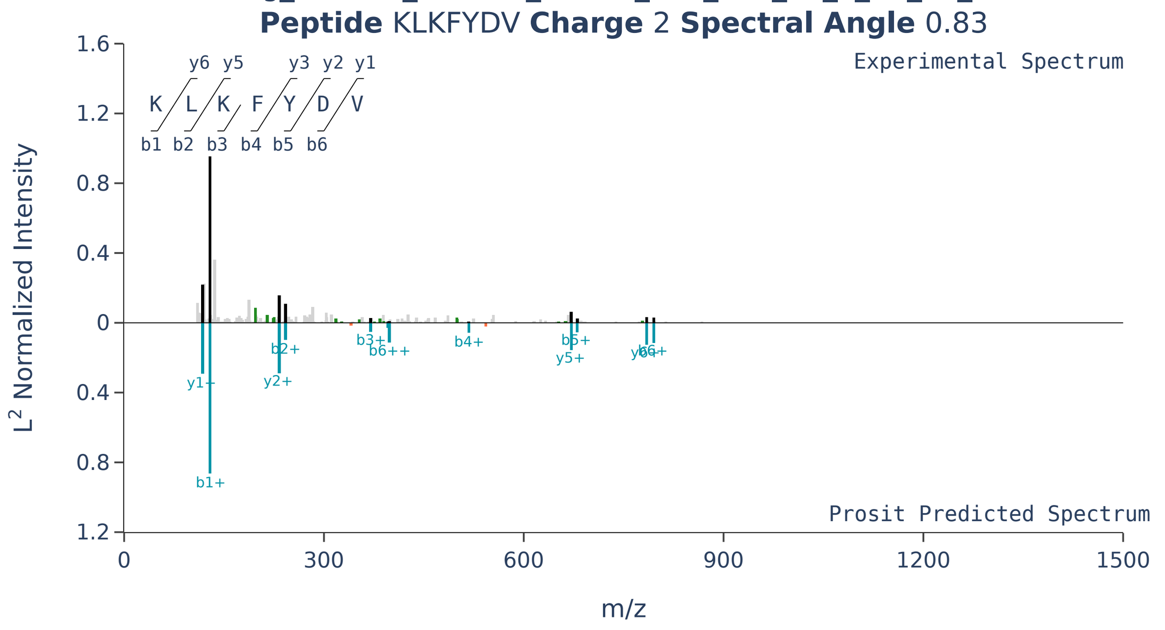


**Source** Ncheng_210623_230623_HFGoe_FFH_20S_25_1_A1_4h_R2 **Scan** 19919
**Peptide** MEAIINSMTMKER **Charge** 2 **Spectral Angle** 0.81

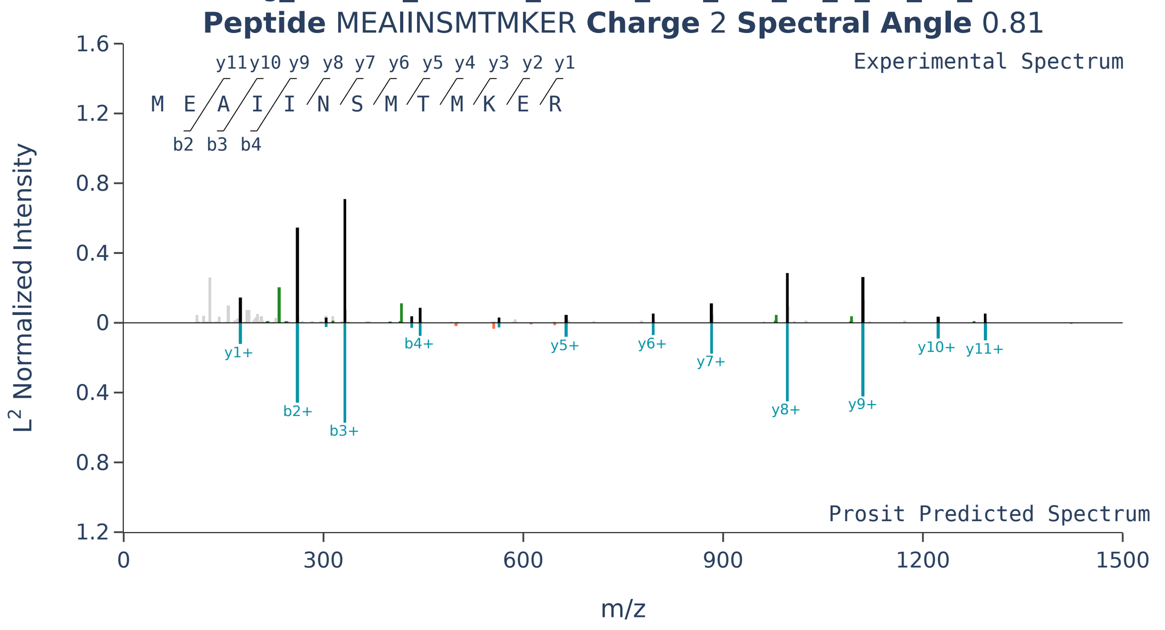


**Source** Ncheng_210623_230623_HFGoe_FFH_20S_25_1_A1_24h_R2 **Scan** 6646
**Peptide** AMTGQDAANTAKA **Charge** 1 **Spectral Angle** 0.86

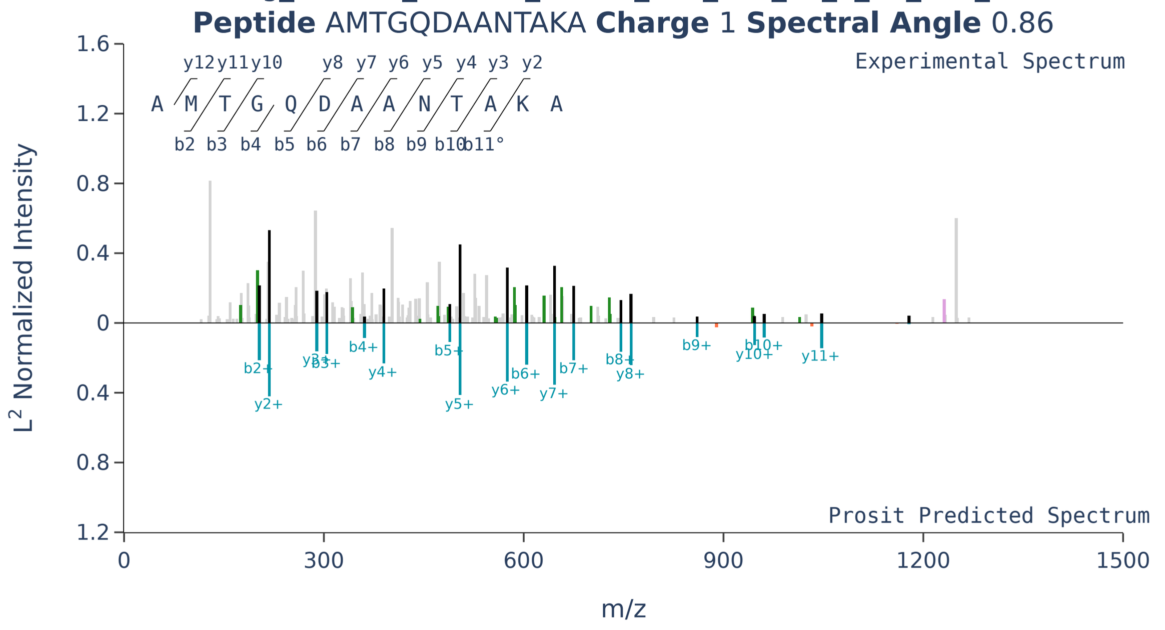


**Source** Ncheng_210623_230623_HFGoe_FFH_20S_25_1_A2_24h_R1 **Scan** 26863
**Peptide** NAALKEA **Charge** 1 **Spectral Angle** 0.86

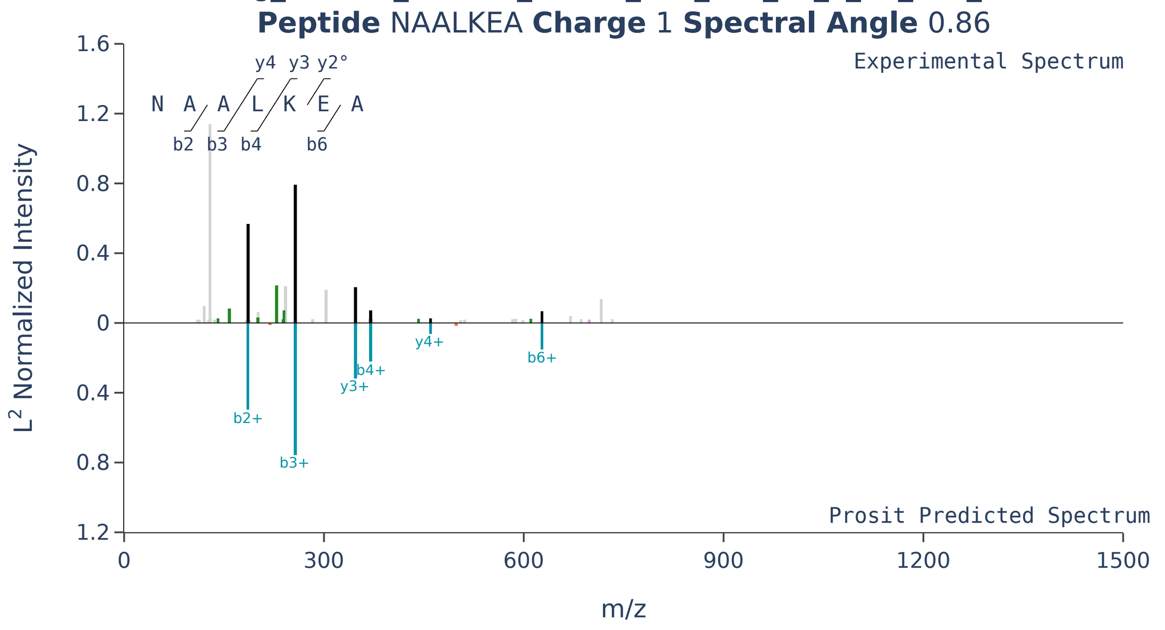


**Source** Ncheng_210623_230623_HFGoe_FFH_20S_25_1_A1_1h_R1 **Scan** 17408
**Peptide** RMEAIINSMTMKER **Charge** 3 **Spectral Angle** 0.86

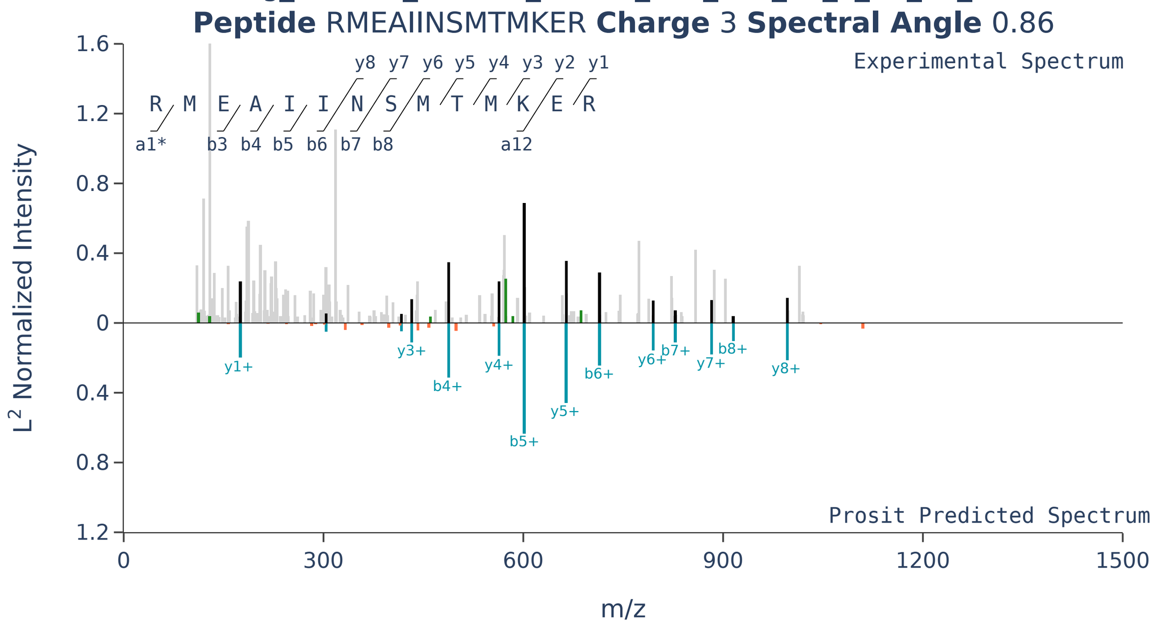


**Source** Ncheng_210623_230623_HFGoe_FFH_20S_25_1_A1_2h_R2 **Scan** 32520
**Peptide** AEQVGVDFFPSDVGQKPVDIVNA **Charge** 2 **Spectral Angle** 0.9

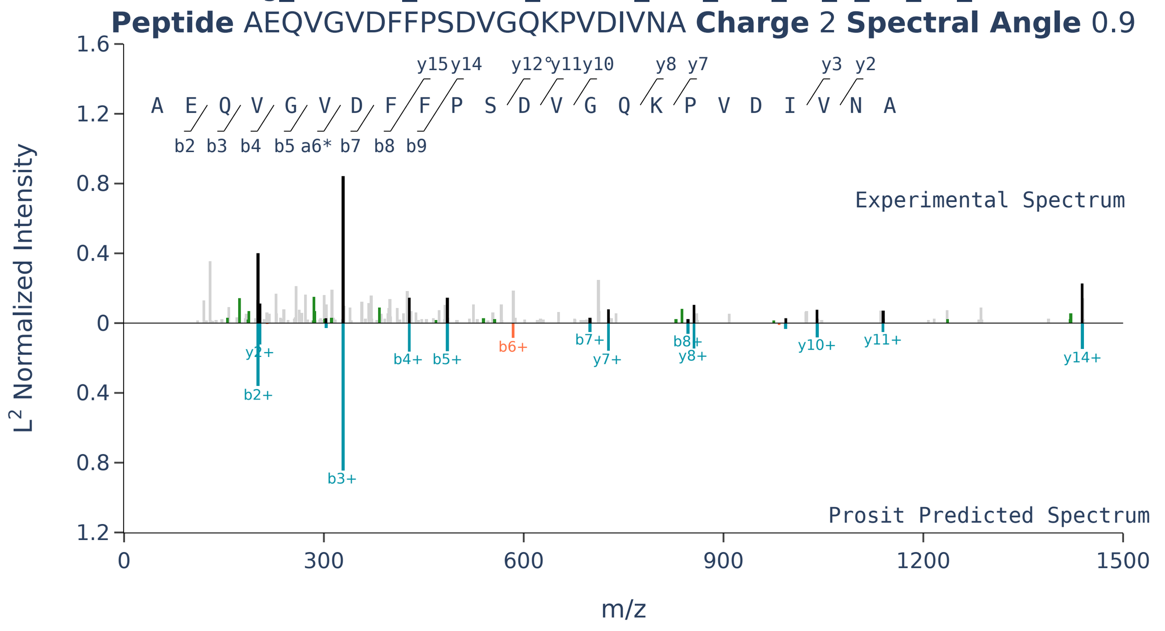


**Source** Ncheng_210623_230623_HFGoe_FFH_20S_25_1_A2_24h_R2 **Scan** 13686
**Peptide** EPFHPDRL **Charge** 2 **Spectral Angle** 0.89

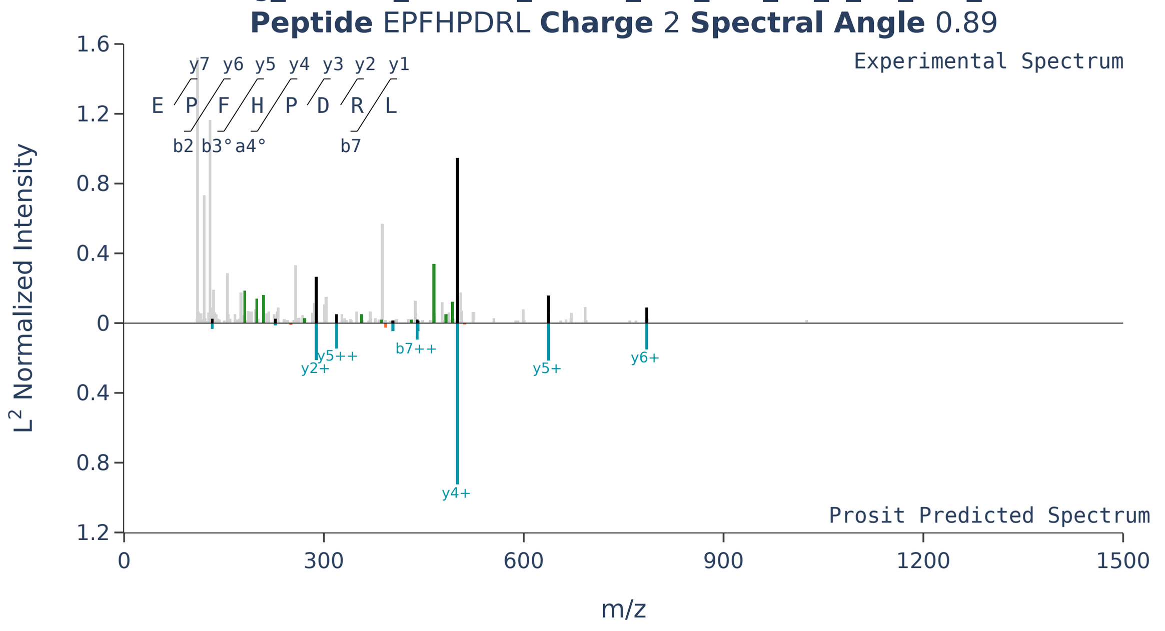


**Source** Ncheng_210623_230623_HFGoe_FFH_20S_25_1_A2_24h_R1 **Scan** 16917
**Peptide** TPGQEFV **Charge** 1 **Spectral Angle** 0.9

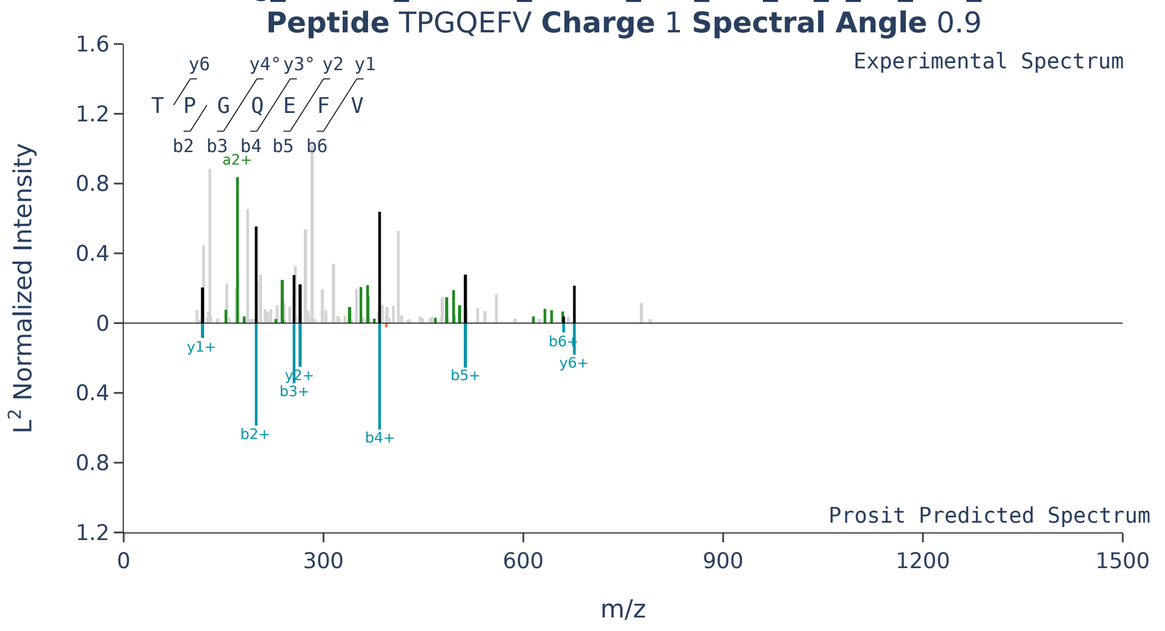


**Source** Ncheng_210623_230623_HFGoe_FFH_20S_25_1_A1_4h_R1 **Scan** 17749
**Peptide** CGMQVQDVNRL **Charge** 2 **Spectral Angle** 0.75

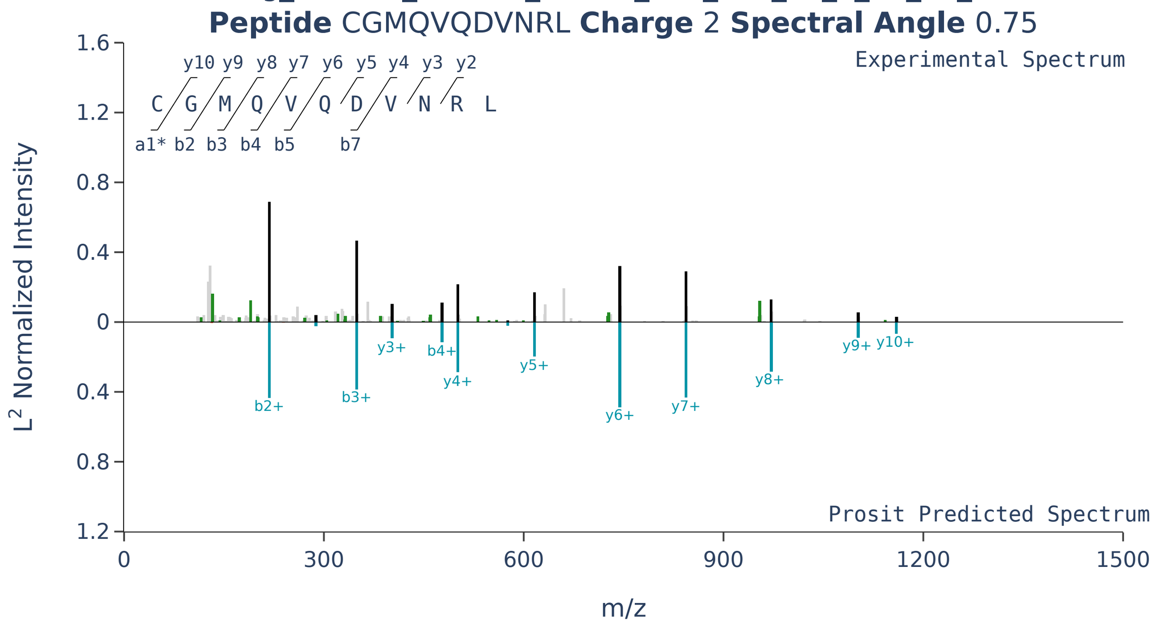


**Source** Ncheng_210623_230623_HFGoe_FFH_20S_25_1_A2_24h_R1 **Scan** 14260
**Peptide** ALPVVRE **Charge** 2 **Spectral Angle** 0.89

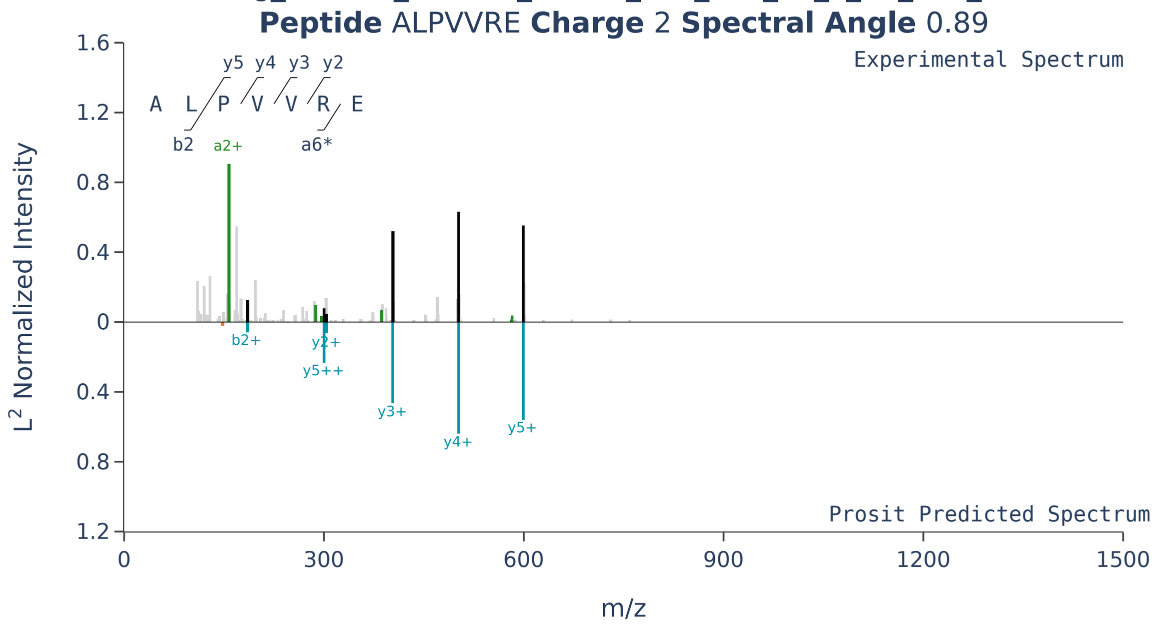


**Source** Ncheng_210623_230623_HFGoe_FFH_20S_25_1_A2_4h_R1 **Scan** 22396
**Peptide** RNELVAAMGEENQTL **Charge** 2 **Spectral Angle** 0.89

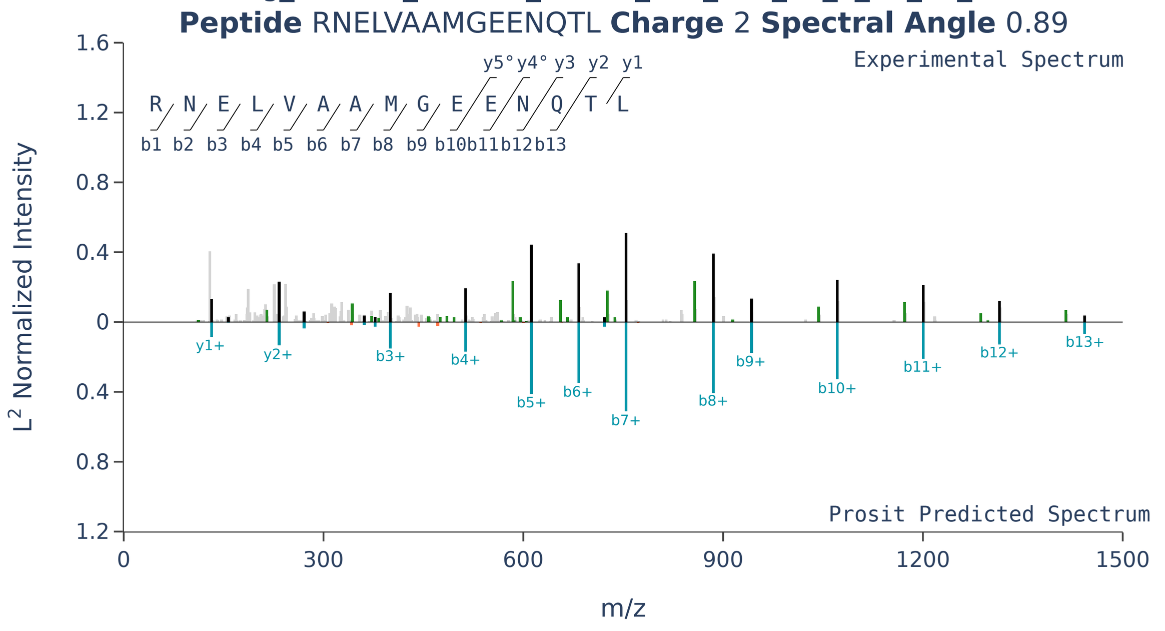


**Source** Ncheng_210623_230623_HFGoe_FFH_20S_25_1_A1_24h_R2 **Scan** 24052
**Peptide** MEAIINSM **Charge** 2 **Spectral Angle** 0.91

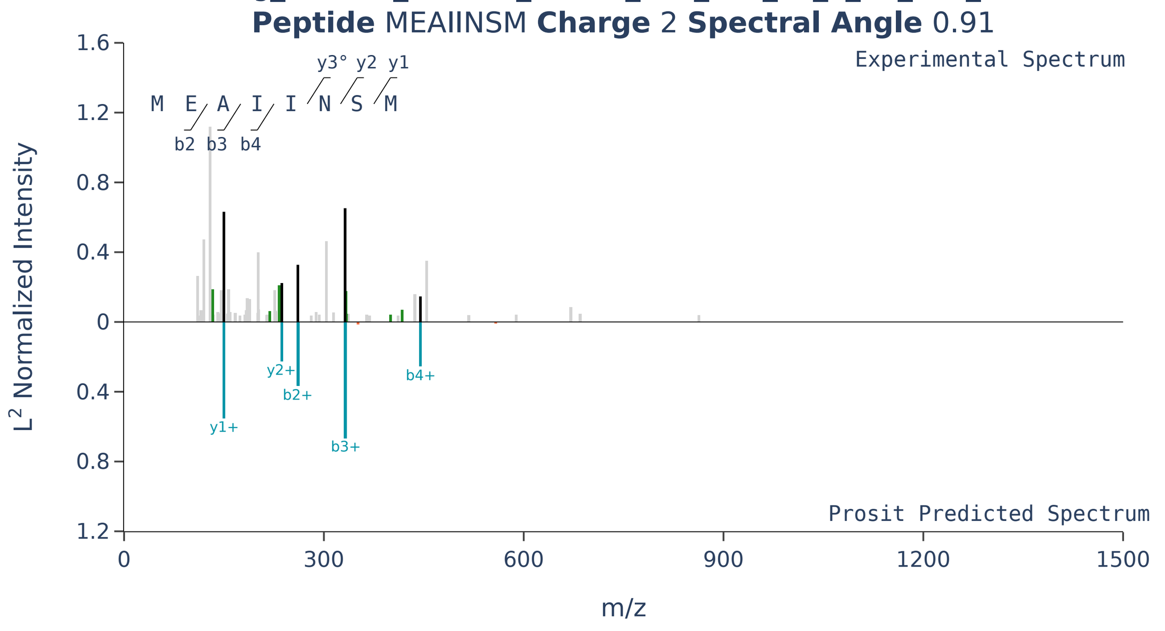


**Source** Ncheng_210623_230623_HFGoe_FFH_20S_25_1_A1_2h_R2 **Scan** 7143
**Peptide** DIESKVD **Charge** 1 **Spectral Angle** 0.86

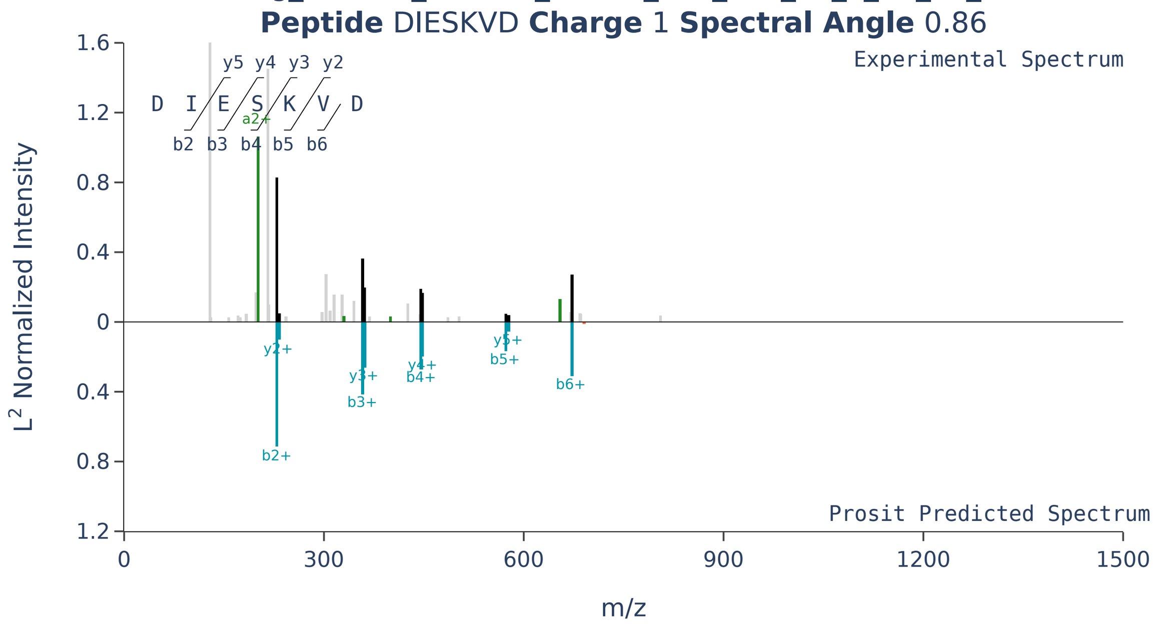


**Source** Ncheng_210623_230623_HFGoe_FFH_20S_25_1_A2_2h_R1 **Scan** 34658
**Peptide** TLAEQVGVDFF **Charge** 2 **Spectral Angle** 0.87

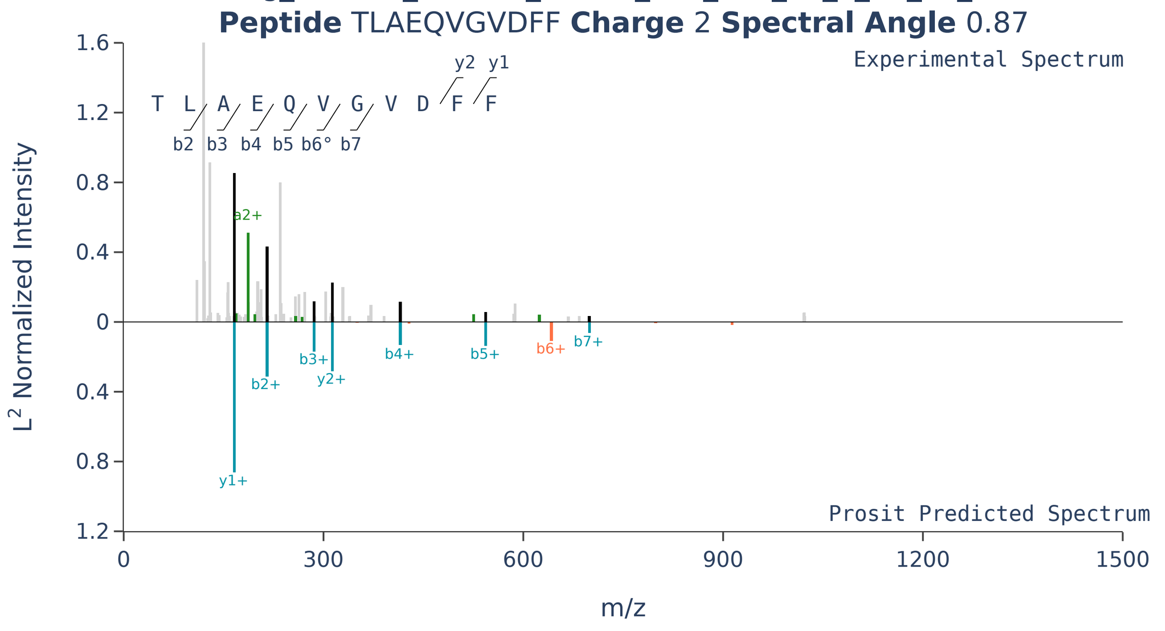

**Source** Ncheng_210623_230623_HFGoe_FFH_20S_25_1_A1_24h_R1 **Scan** 13304
**Peptide** AKLKFYD **Charge** 1 **Spectral Angle** 0.79

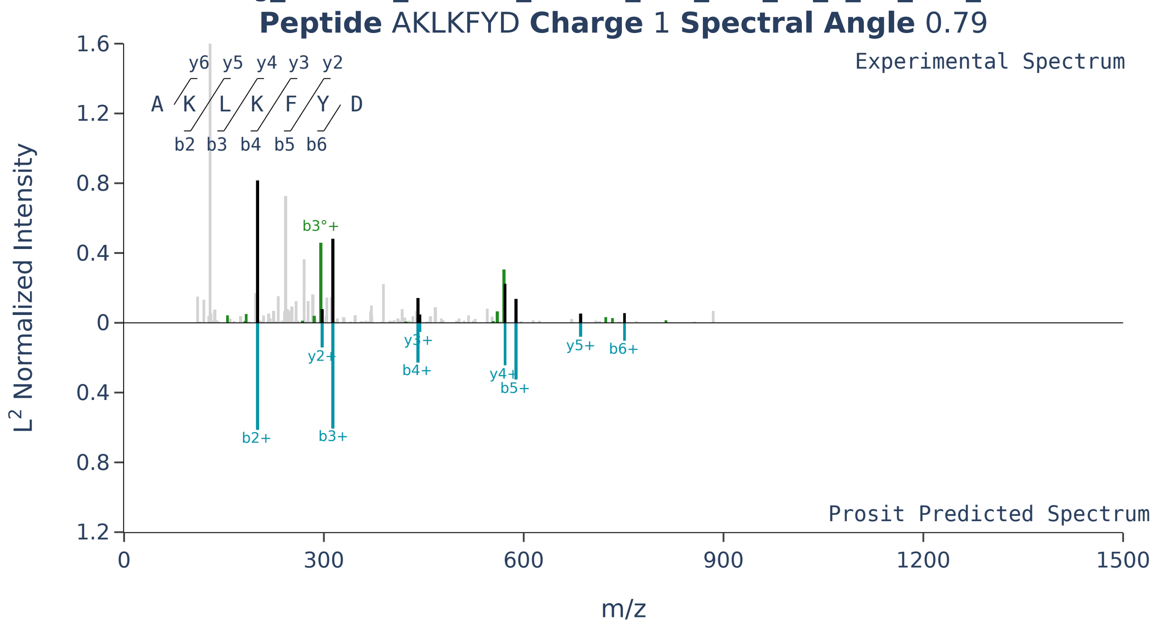


**Source** Ncheng_210623_230623_HFGoe_FFH_20S_25_1_A2_24h_R1 **Scan** 18743
**Peptide** LEPFHPDRI **Charge** 3 **Spectral Angle** 0.88

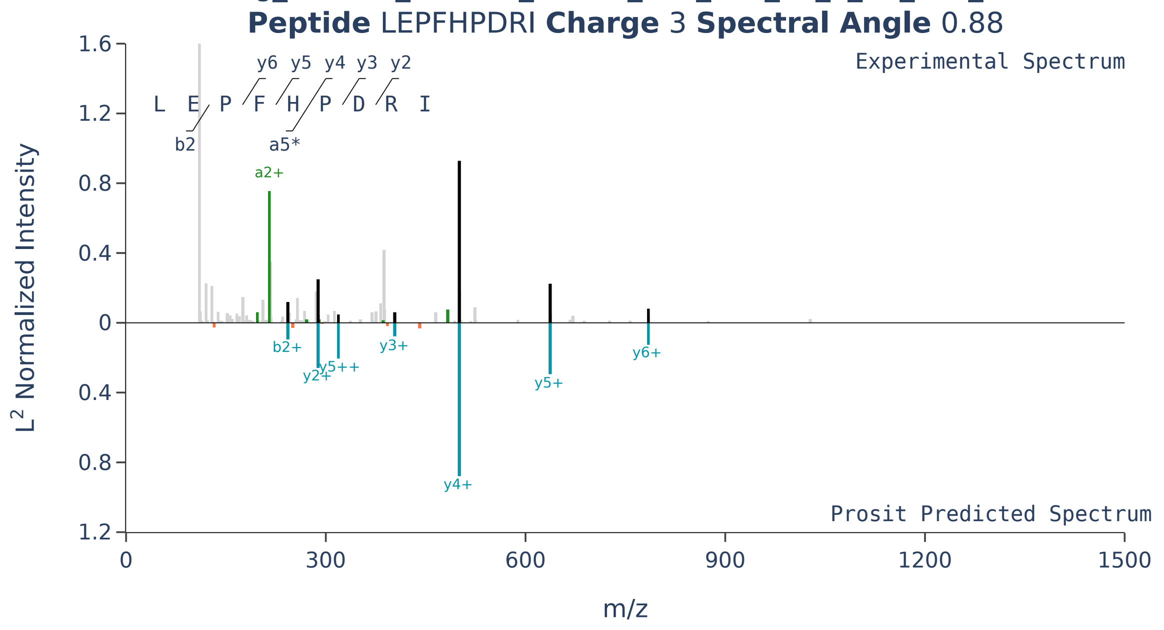


**Source** Ncheng_210623_230623_HFGoe_FFH_20S_25_1_A2_24h_R1 **Scan** 5471
**Peptide** KFLREKH **Charge** 2 **Spectral Angle** 0.88

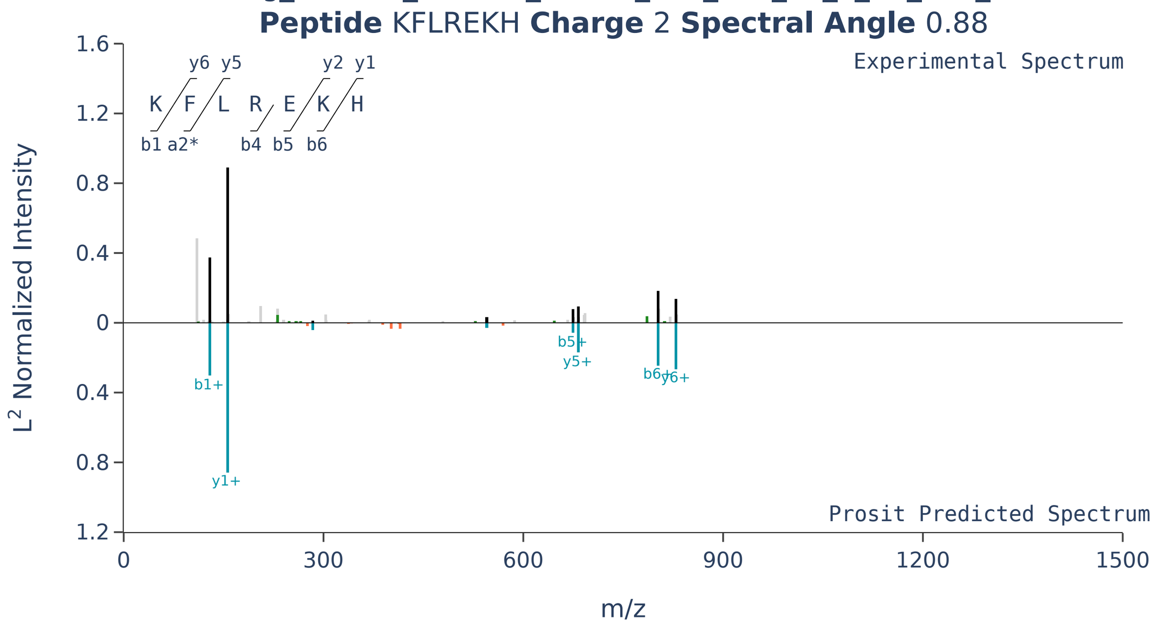


**Source** Ncheng_210623_230623_HFGoe_FFH_20S_25_1_A1_2h_R1 **Scan** 18767
**Peptide** HEVNKSLTPGQEFVKIVRN **Charge** 2 **Spectral Angle** 0.8

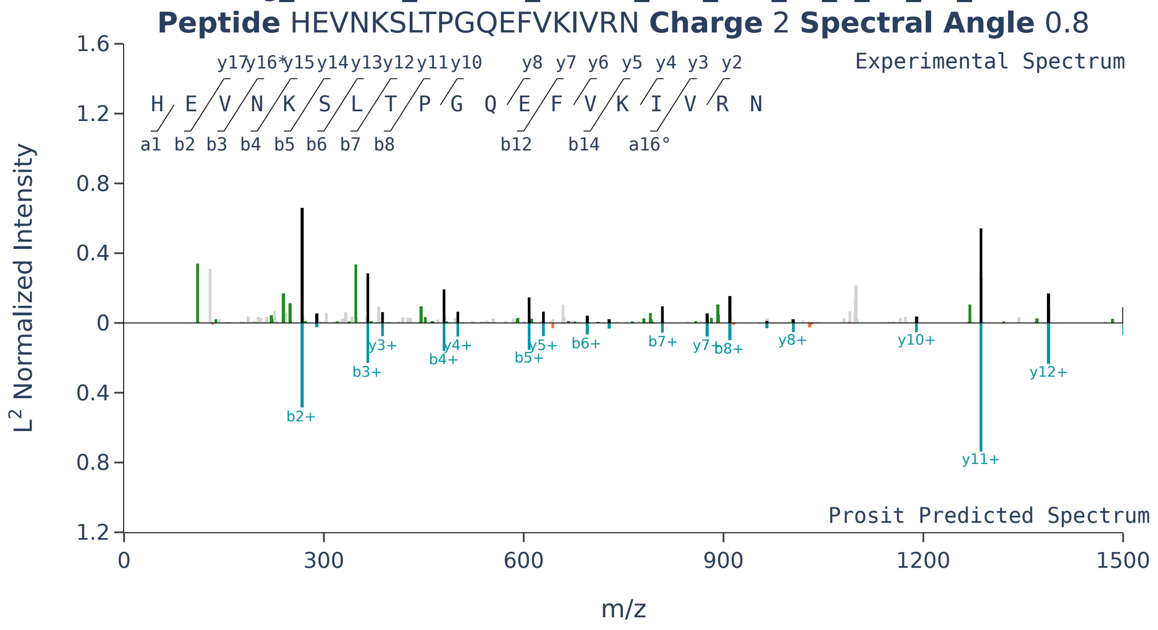


**Source** Ncheng_210623_230623_HFGoe_FFH_20S_25_1_A2_24h_R2 **Scan** 18181
**Peptide** LTGKPIKFLGVGEKTE **Charge** 2 **Spectral Angle** 0.86

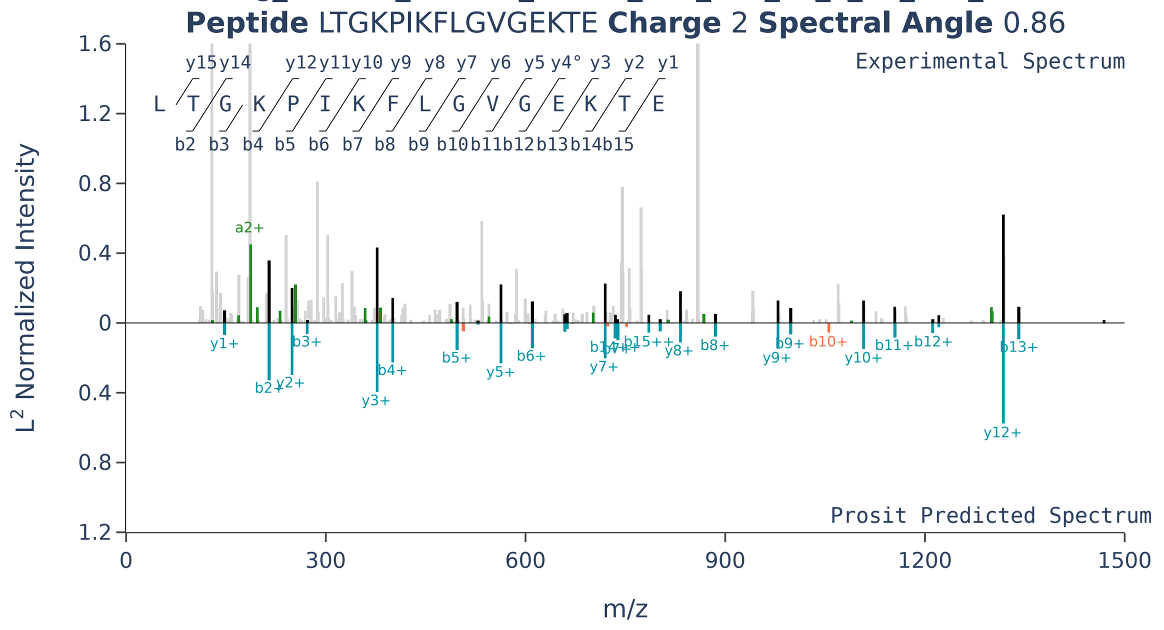


**Source** Ncheng_210623_230623_HFGoe_FFH_20S_25_1_A2_24h_R2 **Scan** 6432
**Peptide** AMTGQDAANTA **Charge** 1 **Spectral Angle** 0.83

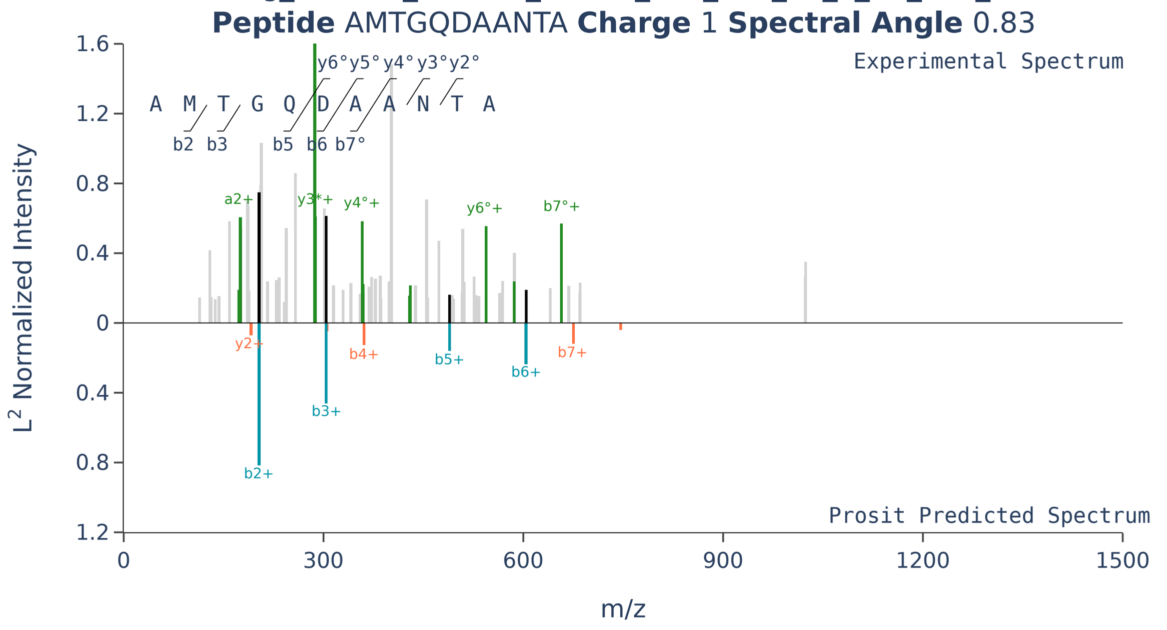


**Source** Ncheng_210623_230623_HFGoe_FFH_20S_25_1_A2_24h_R1 **Scan** 8868
**Peptide** DIESKVDRA **Charge** 2 **Spectral Angle** 0.82

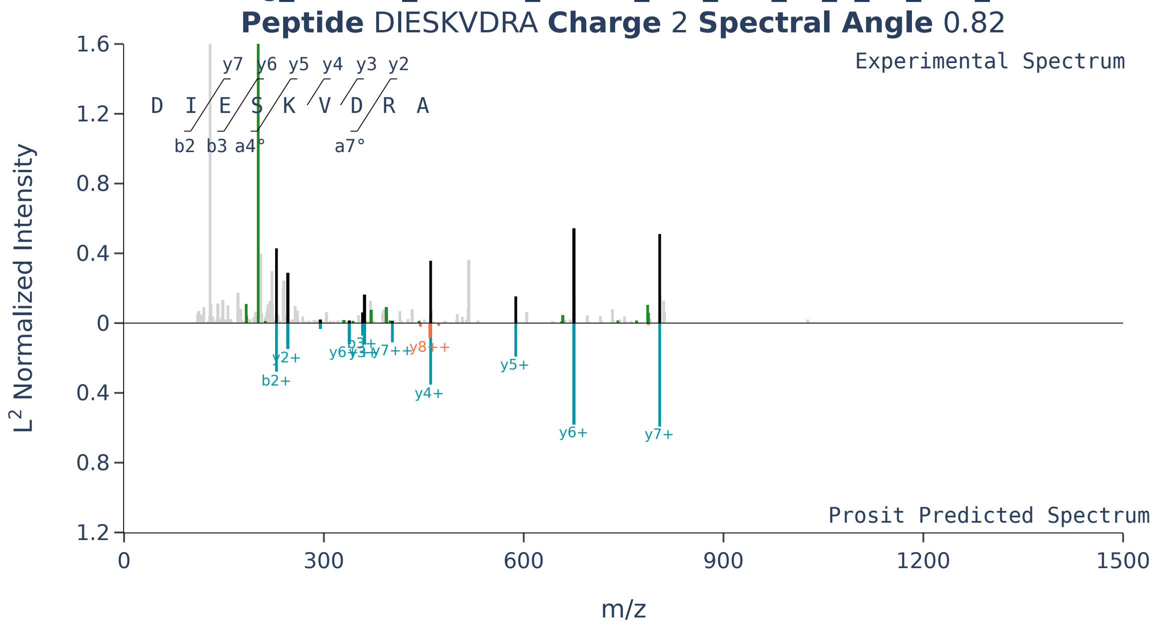


**Source** Ncheng_210623_230623_HFGoe_FFH_20S_25_1_A1_4h_R1 **Scan** 24706
**Peptide** PLTGVVLTKV **Charge** 2 **Spectral Angle** 0.73

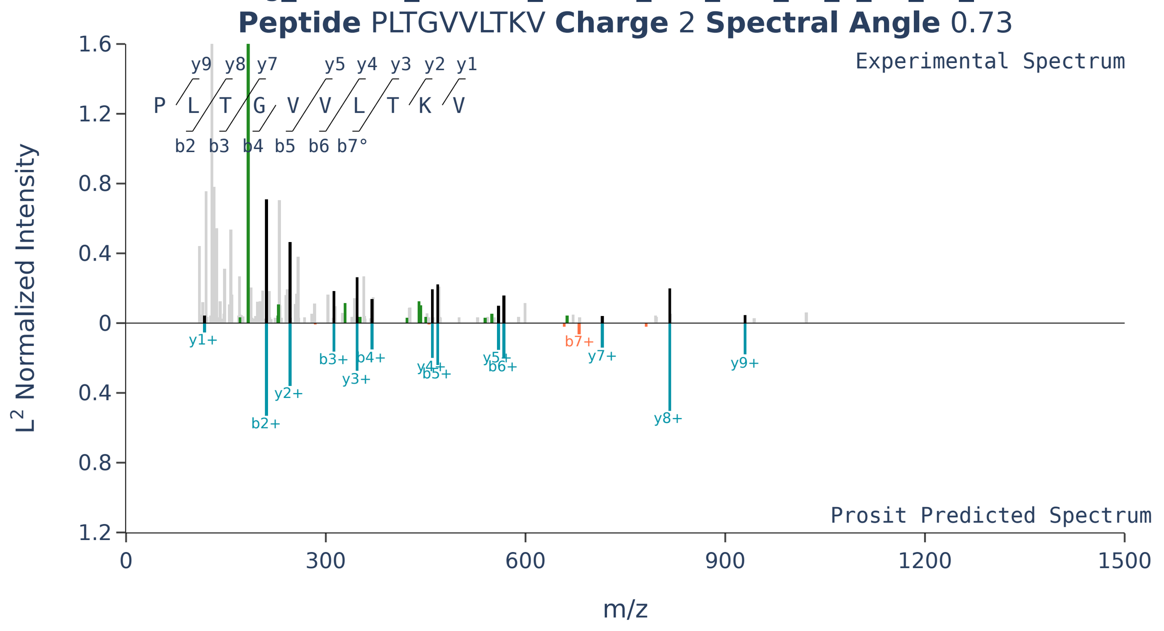


**Source** Ncheng_210623_230623_HFGoe_FFH_20S_25_1_A2_24h_R2 **Scan** 13547
**Peptide** ARGGAALSIRHI **Charge** 3 **Spectral Angle** 0.88

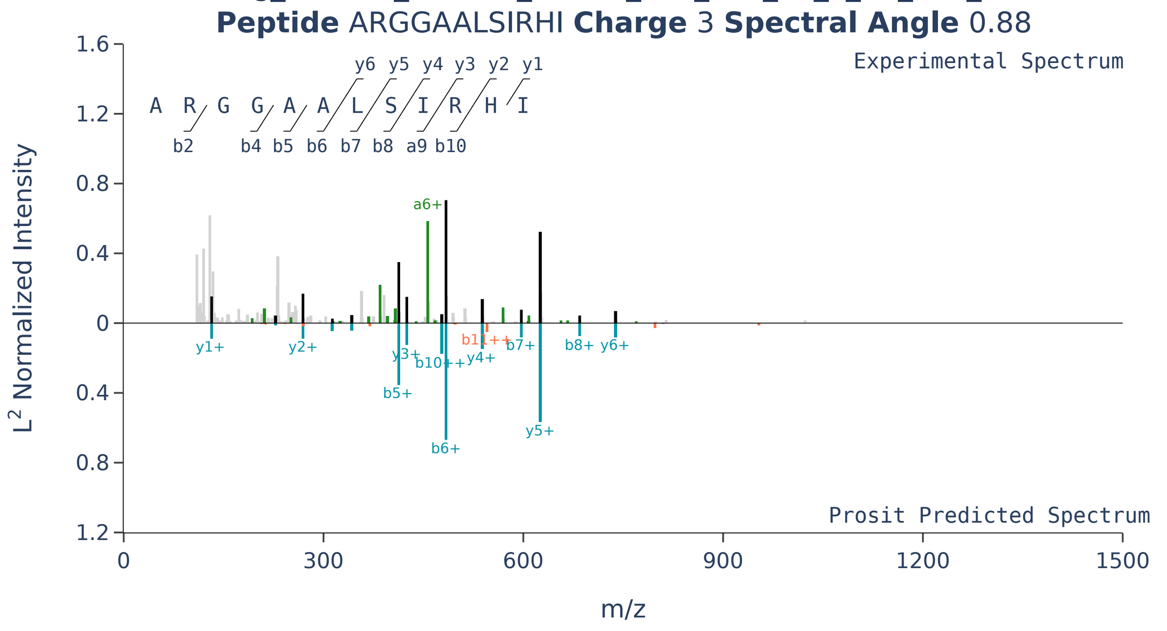


**Source** Ncheng_210623_230623_HFGoe_FFH_20S_25_1_A1_2h_R1 **Scan** 28602
**Peptide** AMGEENQTLNLAAQPPAVV **Charge** 2 **Spectral Angle** 0.93

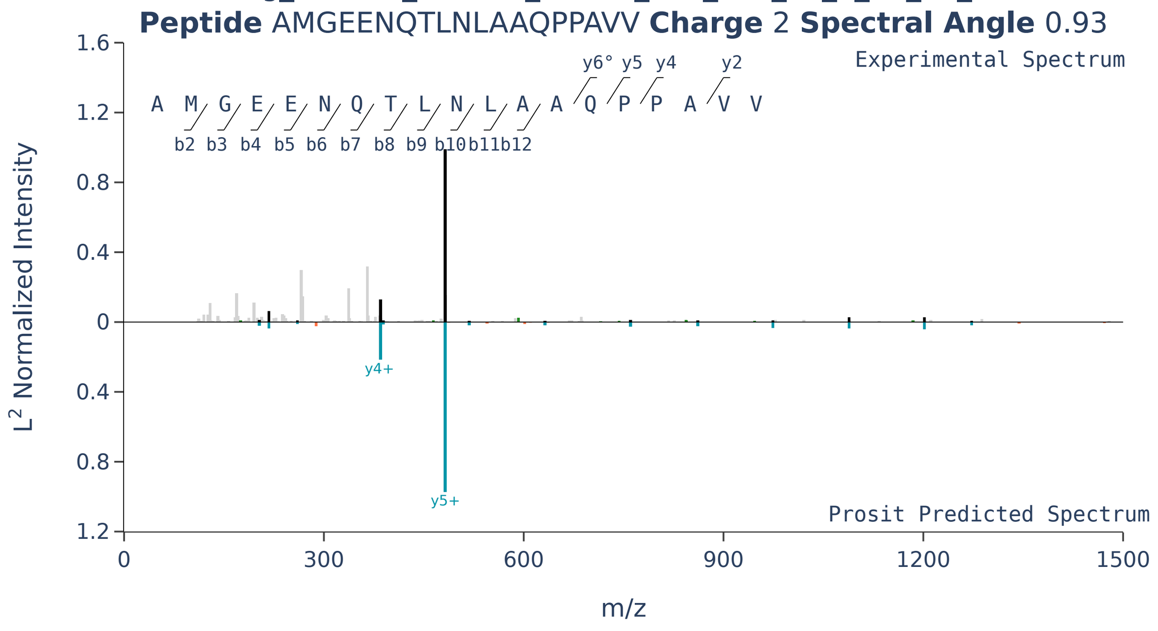


**Source** Ncheng_210623_230623_HFGoe_FFH_20S_25_1_A1_2h_R2 **Scan** 27423
**Peptide** EENQTLNLAAQPPAVV **Charge** 2 **Spectral Angle** 0.94

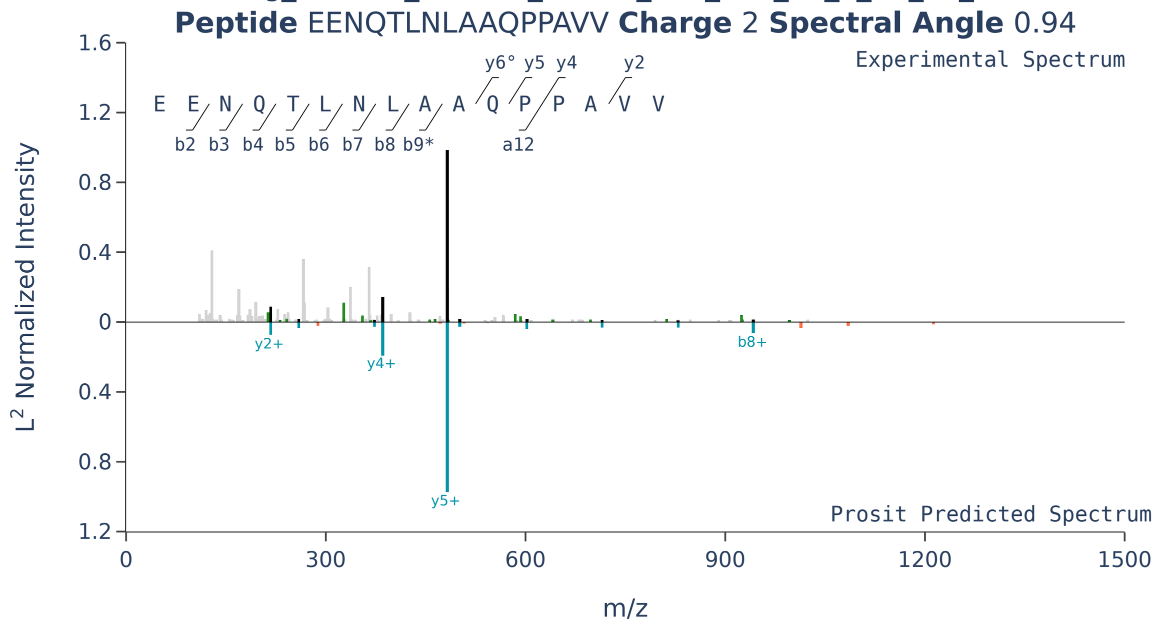


**Source** Ncheng_210623_230623_HFGoe_FFH_20S_25_1_A2_24h_R2 **Scan** 20462
**Peptide** TGQDAANTAKAFNEAL **Charge** 2 **Spectral Angle** 0.82

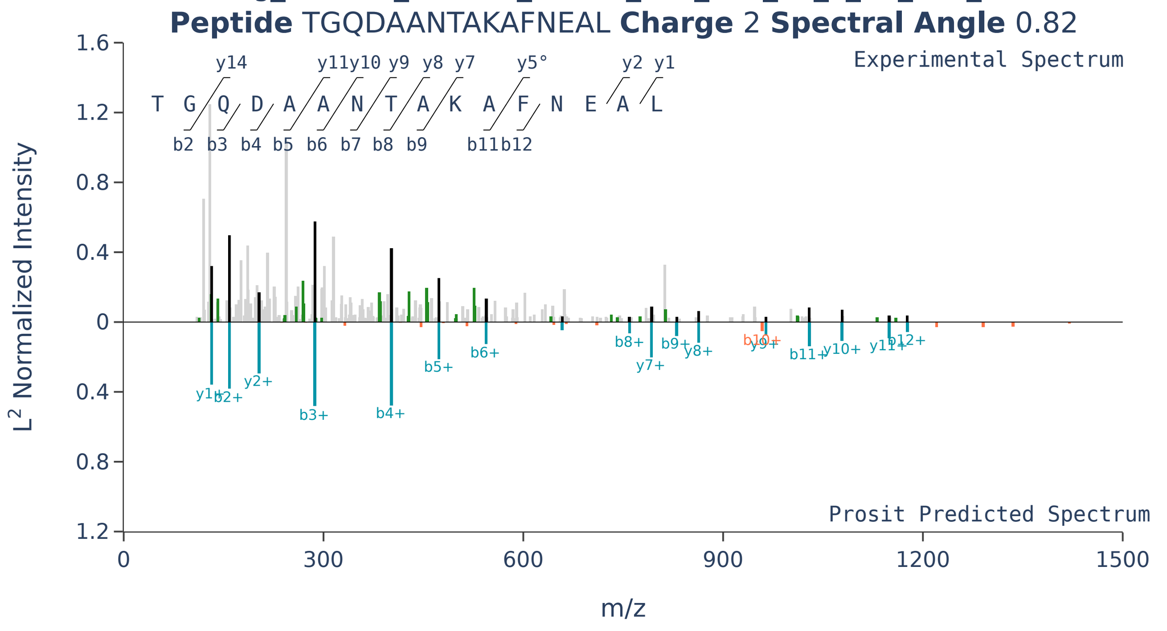


**Source** Ncheng_210623_230623_HFGoe_FFH_20S_25_1_A1_24h_R2 **Scan** 14683
**Peptide** GQEFVKIVRN **Charge** 3 **Spectral Angle** 0.85

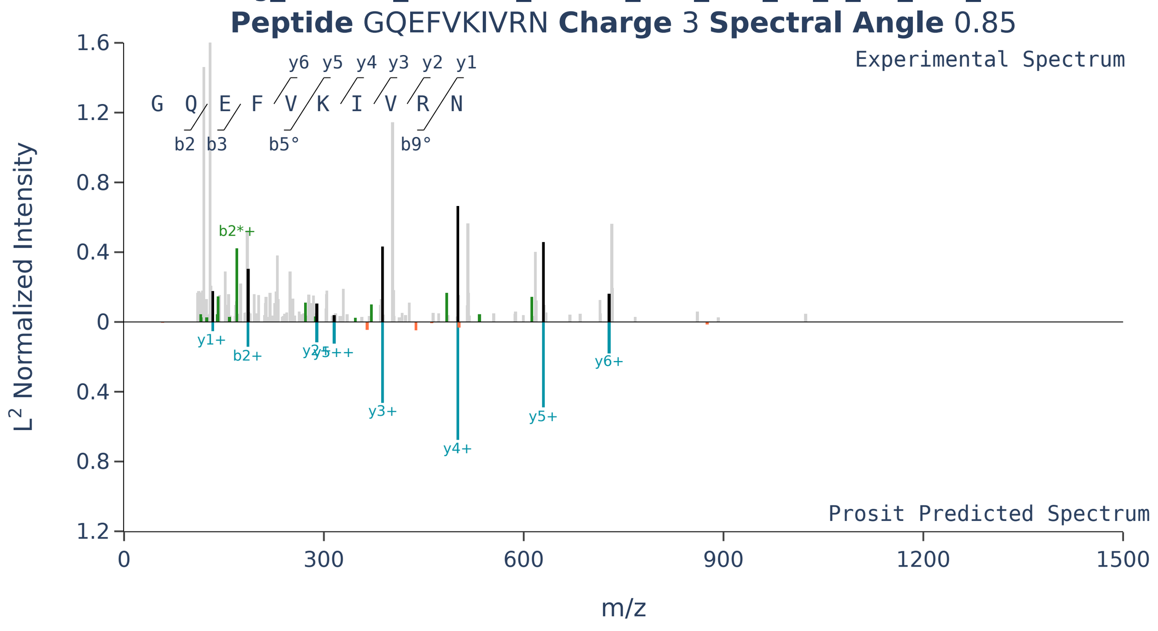


**Source** Ncheng_210623_230623_HFGoe_FFH_20S_25_1_A1_24h_R2 **Scan** 19891
**Peptide** ALEPFHPDRI **Charge** 3 **Spectral Angle** 0.85

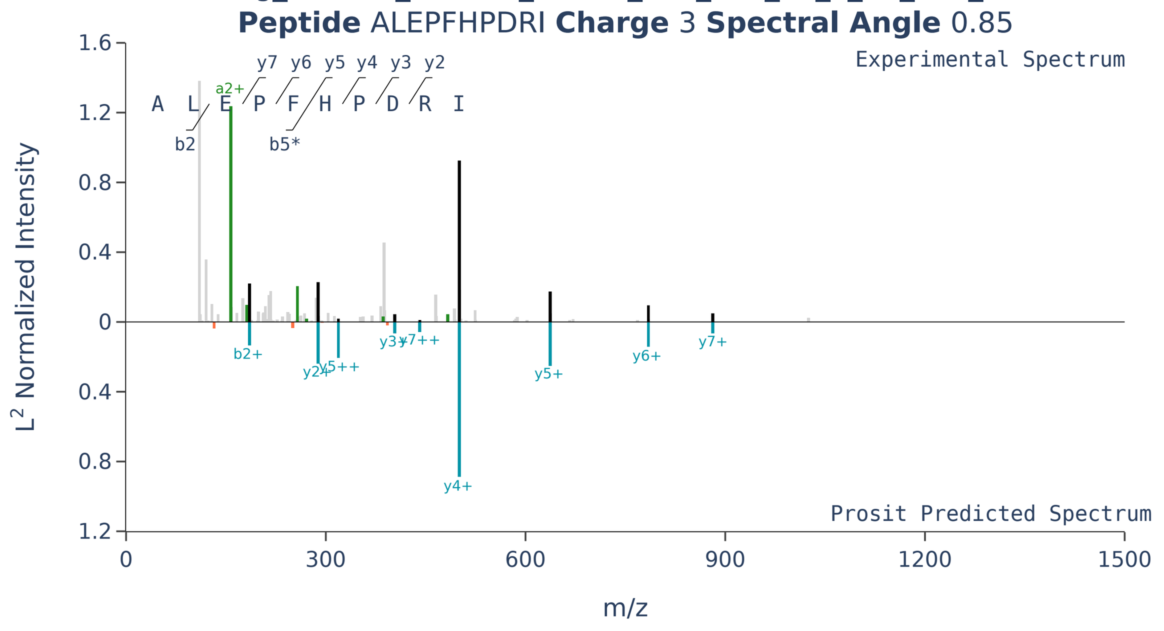


**Source** Ncheng_210623_230623_HFGoe_FFH_20S_25_1_A1_4h_R2 **Scan** 33474
**Peptide** DVLSLIE **Charge** 1 **Spectral Angle** 0.78

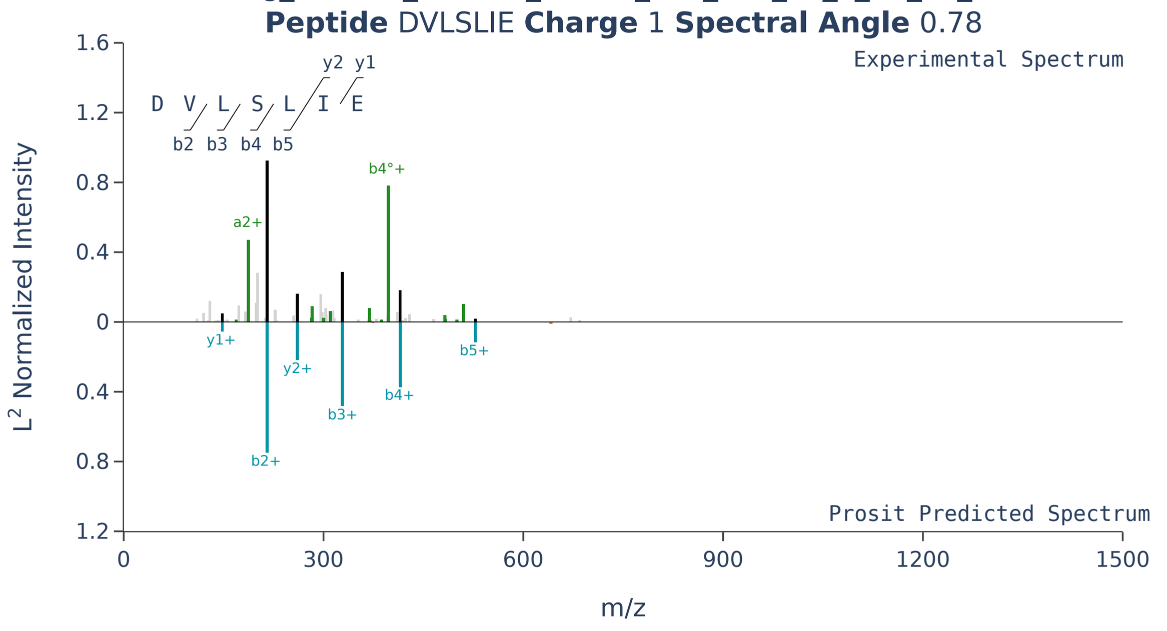

**Source** Ncheng_210623_230623_HFGoe_FFH_20S_25_1_A2_24h_R2 **Scan** 10075
**Peptide** PGMGQIPDNVKSQ **Charge** 2 **Spectral Angle** 0.84

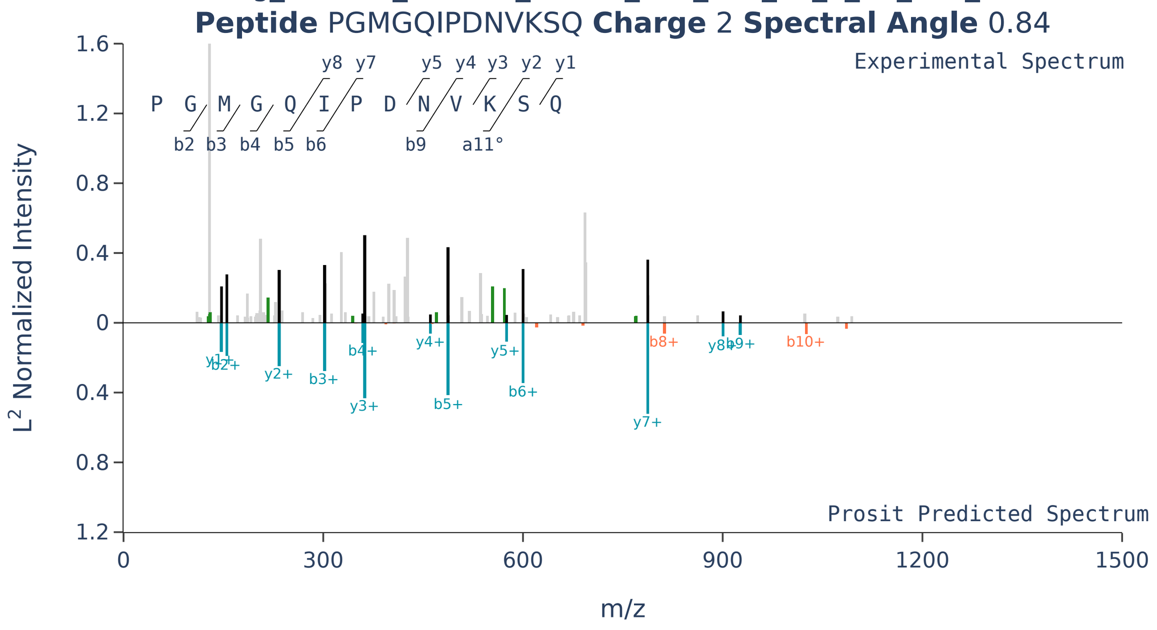


**Source** Ncheng_210623_230623_HFGoe_FFH_20S_25_1_A1_2h_R2 **Scan** 22117
**Peptide** VGQKPVDIVNAALKE **Charge** 3 **Spectral Angle** 0.78

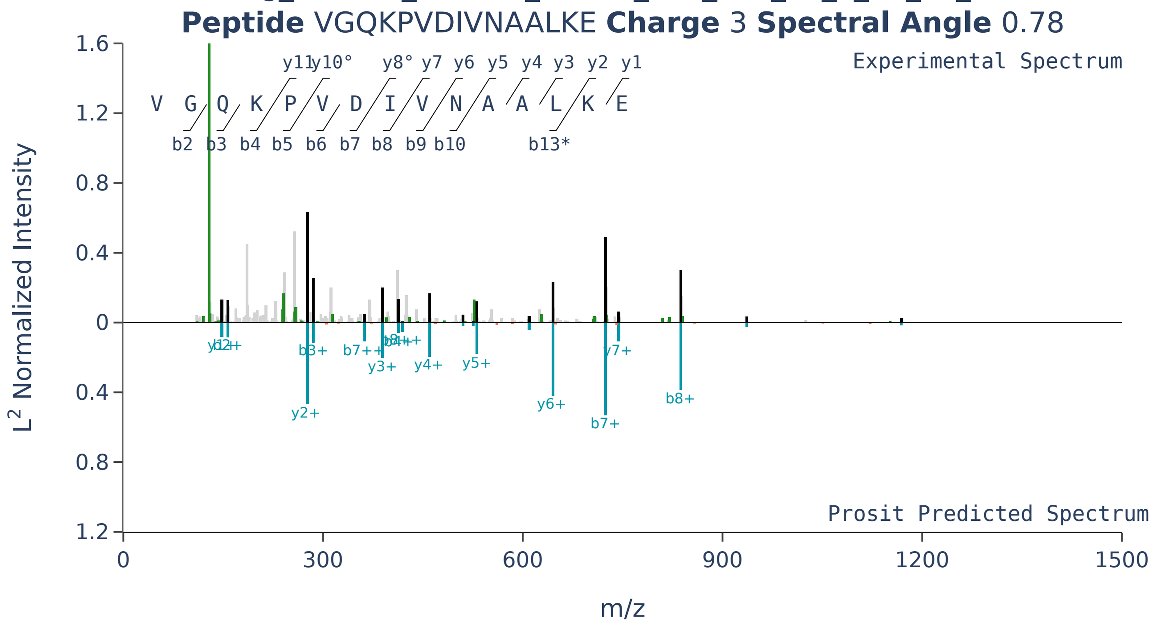


**Source** Ncheng_210623_230623_HFGoe_FFH_20S_25_1_A1_2h_R1 **Scan** 17472
**Peptide** GEKTEALEPFHPDRIAS **Charge** 2 **Spectral Angle** 0.87

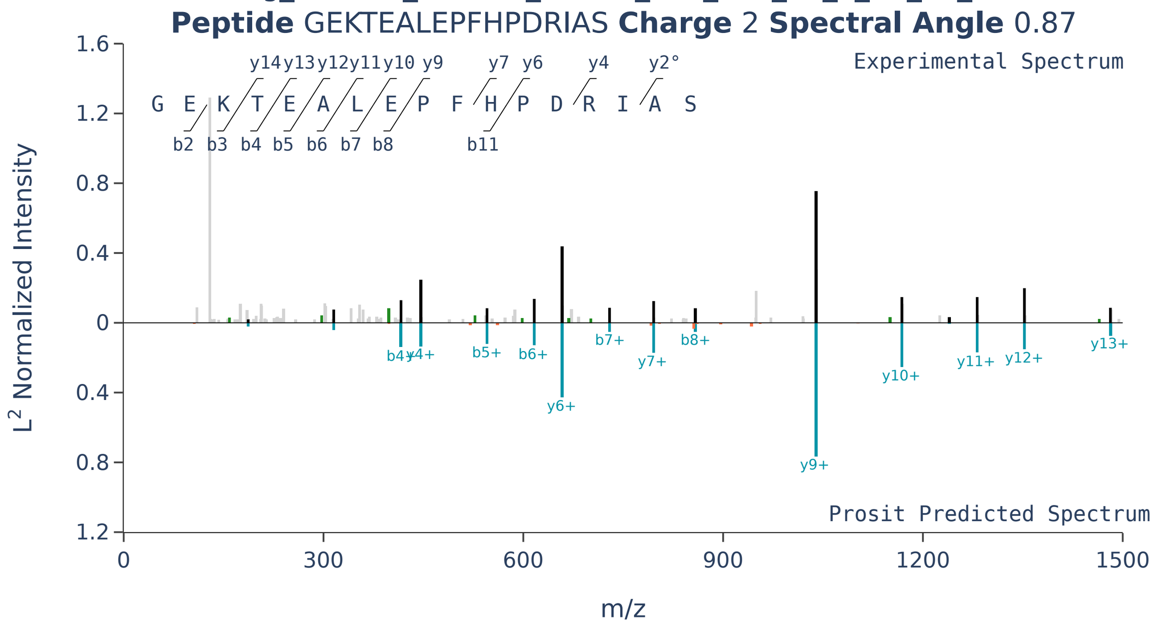


**Source** Ncheng_210623_230623_HFGoe_FFH_20S_25_1_A2_1h_R1 **Scan** 8631
**Peptide** GQIPDNVKSQMDD **Charge** 2 **Spectral Angle** 0.88

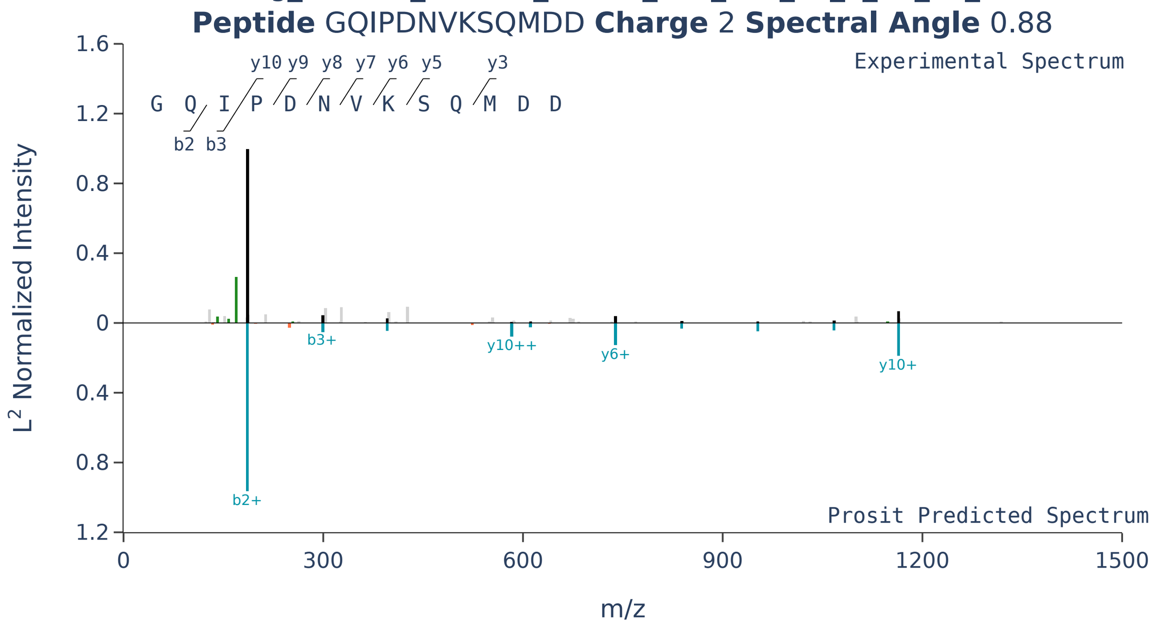


**Source** Ncheng_210623_230623_HFGoe_FFH_20S_25_1_A1_4h_R2 **Scan** 7736
**Peptide** AVGHEVNKSL **Charge** 1 **Spectral Angle** 0.86

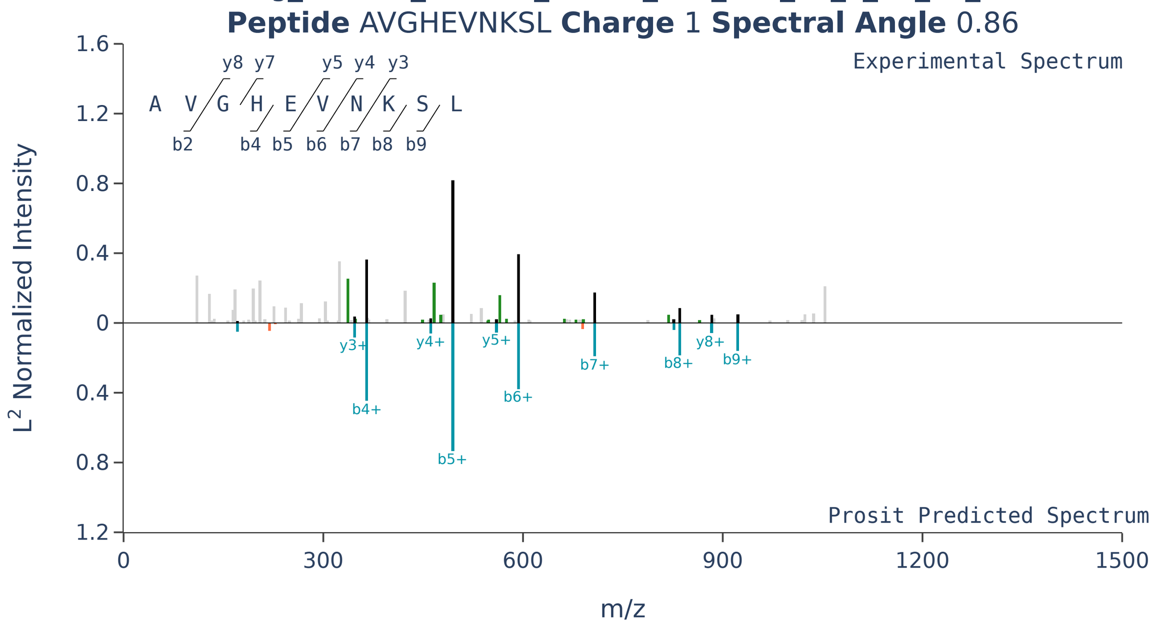


**Source** Ncheng_210623_230623_HFGoe_FFH_20S_25_1_A1_4h_R2 **Scan** 16000
**Peptide** RNELVAAMG **Charge** 2 **Spectral Angle** 0.88

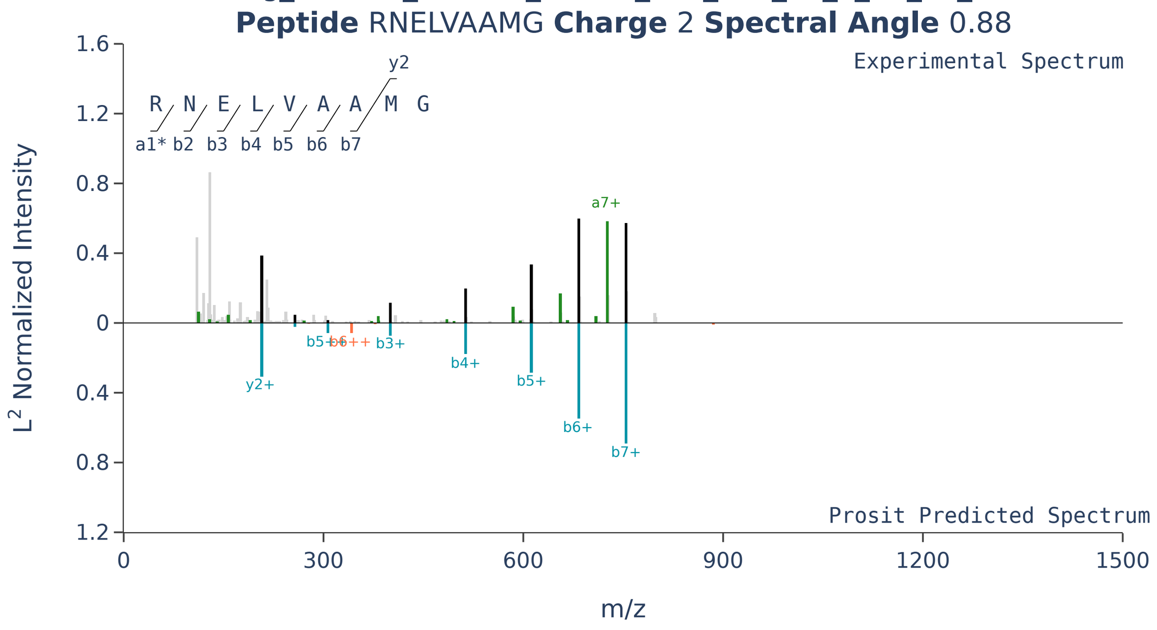


**Source** Ncheng_210623_230623_HFGoe_FFH_20S_25_1_A2_2h_R1 **Scan** 6845
**Peptide** AGRLHVDE **Charge** 2 **Spectral Angle** 0.8

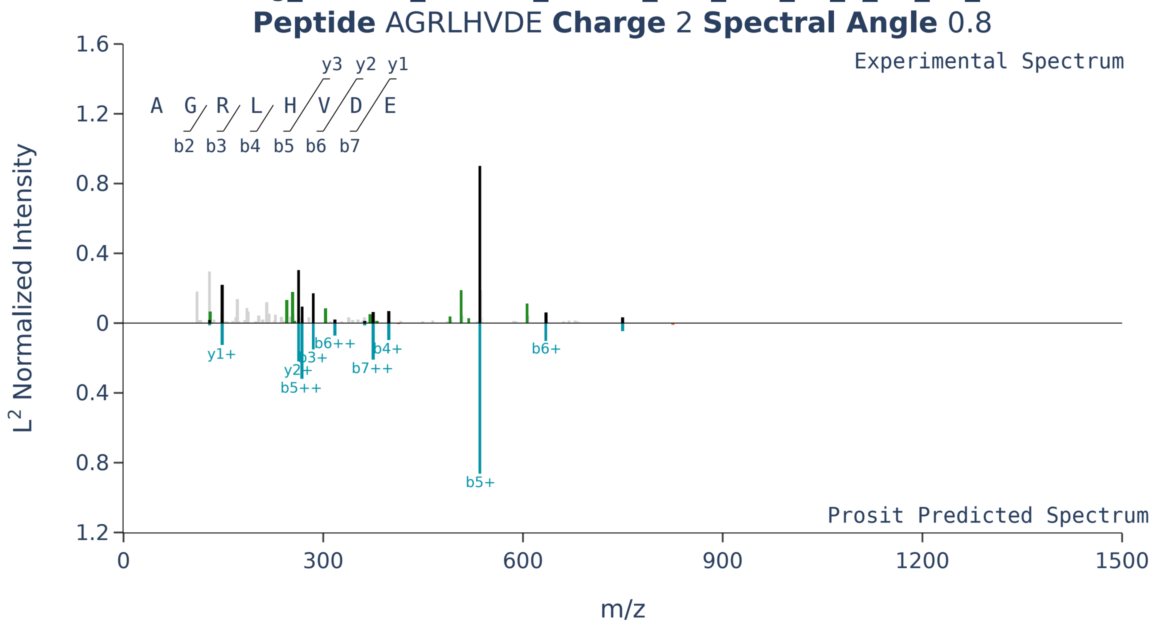


**Source** Ncheng_210623_230623_HFGoe_FFH_20S_25_1_A2_4h_R1 **Scan** 22931
**Peptide** LPGMGQIPDNVKSQMDD **Charge** 2 **Spectral Angle** 0.8

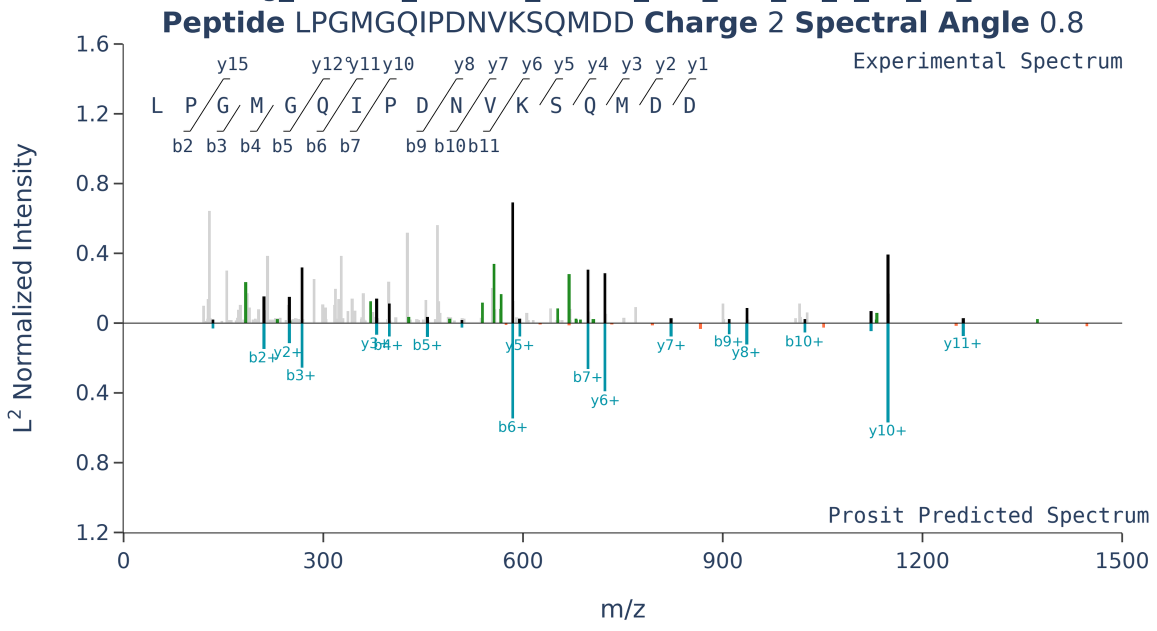


**Source** Ncheng_210623_230623_HFGoe_FFH_20S_25_1_A1_24h_R1 **Scan** 7623
**Peptide** AVGHEVNKSLT **Charge** 1 **Spectral Angle** 0.85

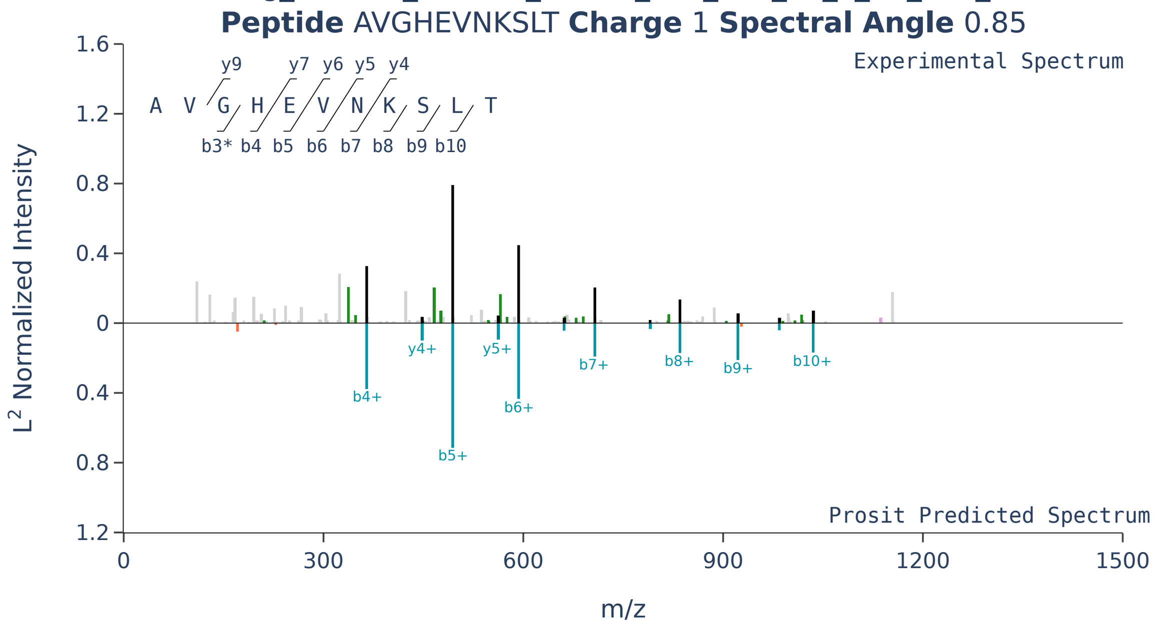


**Source** Ncheng_210623_230623_HFGoe_FFH_20S_25_1_A1_24h_R1 **Scan** 9153
**Peptide** VDTAGRL **Charge** 2 **Spectral Angle** 0.85

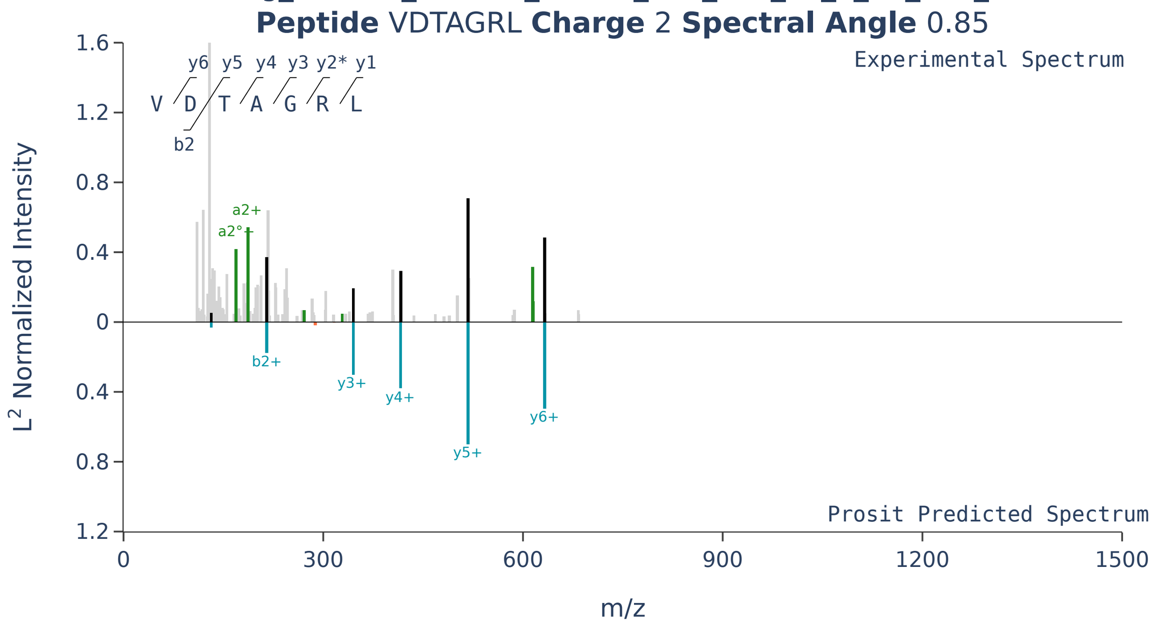


**Source** Ncheng_210623_230623_HFGoe_FFH_20S_25_1_A2_2h_R1 **Scan** 26972
**Peptide** GEENQTLNLAAQPPAVV **Charge** 2 **Spectral Angle** 0.93

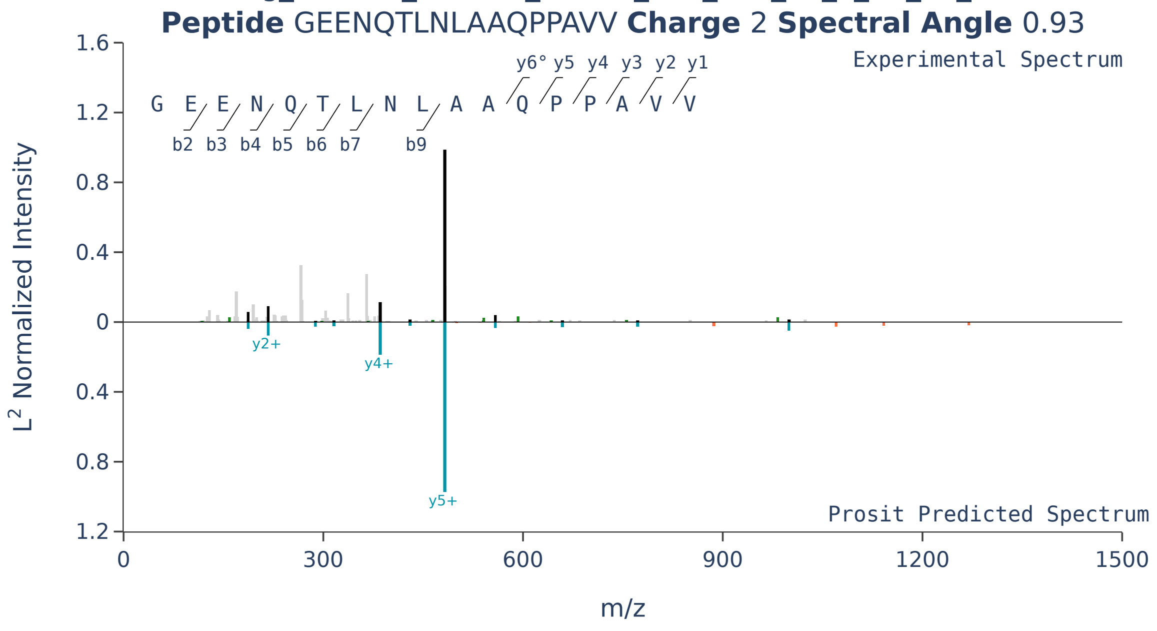


**Source** Ncheng_210623_230623_HFGoe_FFH_20S_25_1_A2_4h_R2 **Scan** 23278
**Peptide** LMGKLPGMGQIPDNVKS **Charge** 2 **Spectral Angle** 0.87

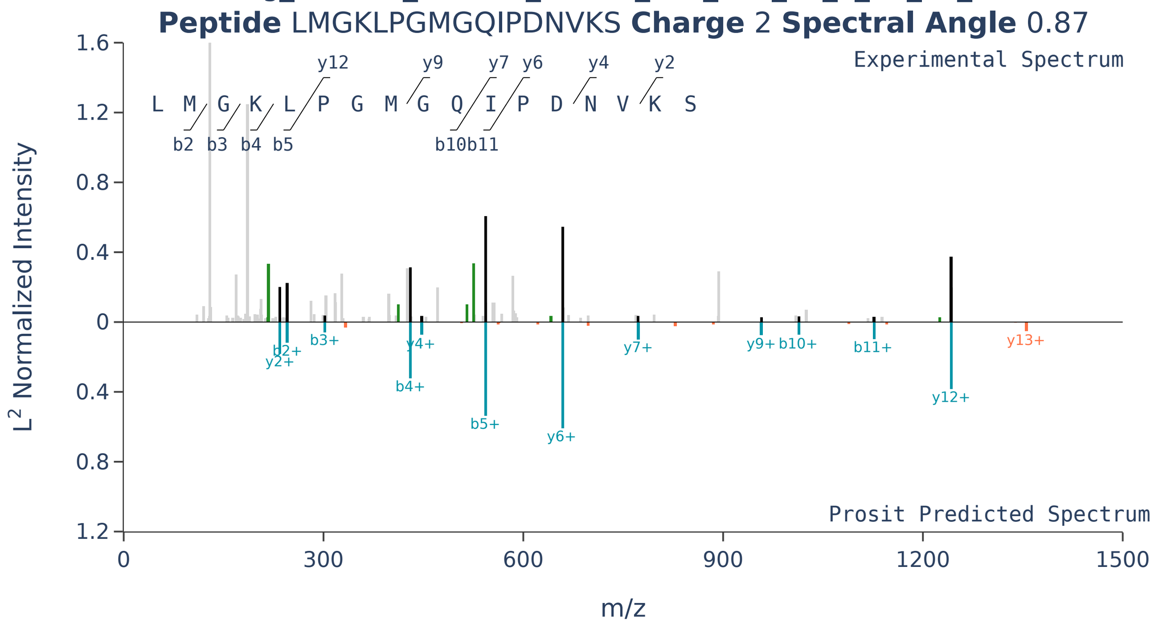


**Source** Ncheng_210623_230623_HFGoe_FFH_20S_25_1_A2_24h_R1 **Scan** 8094
**Peptide** VAAMGEENQT **Charge** 2 **Spectral Angle** 0.94

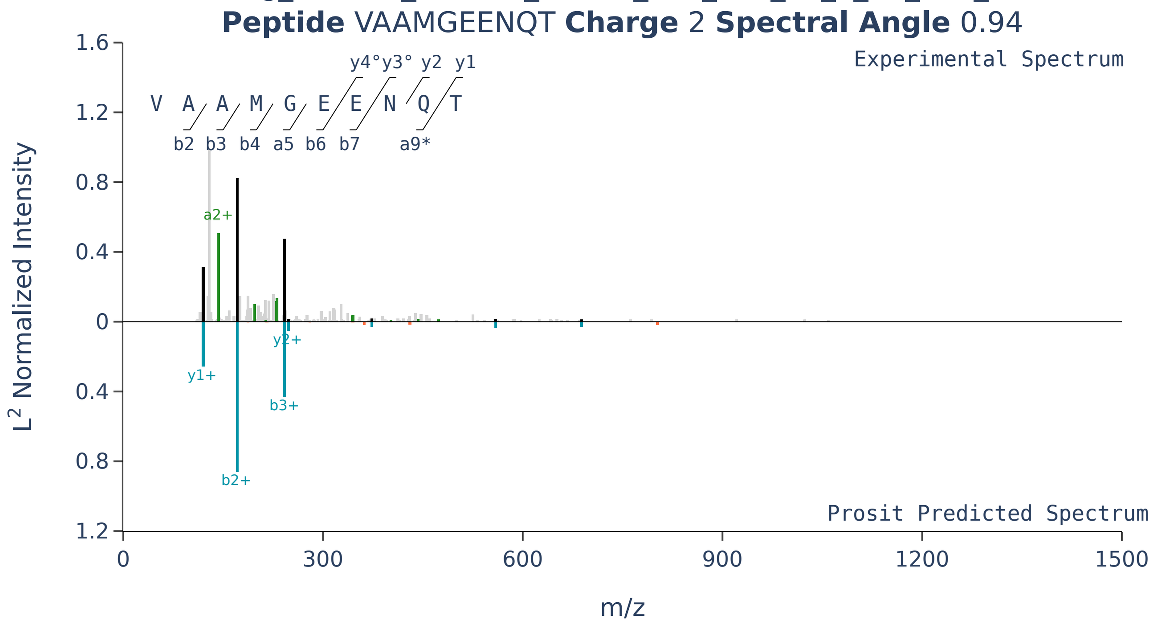


**Source** Ncheng_210623_230623_HFGoe_FFH_20S_25_1_A2_4h_R1 **Scan** 23988
**Peptide** ELVAAMGEENQTL **Charge** 2 **Spectral Angle** 0.9

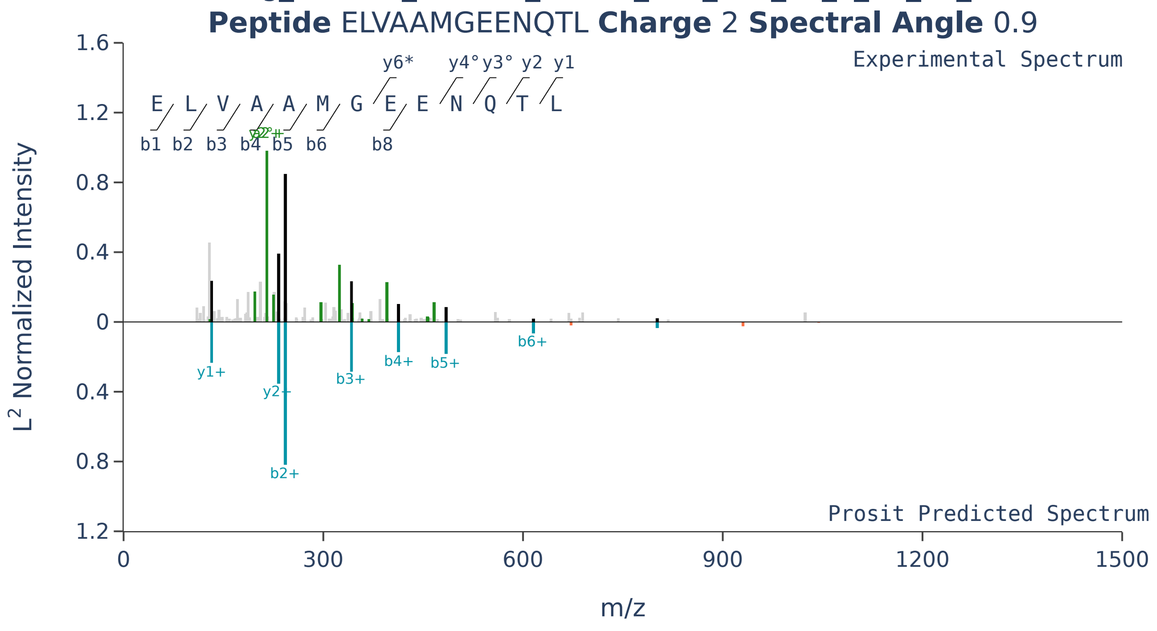


**Source** Ncheng_210623_230623_HFGoe_FFH_20S_25_1_A2_24h_R1 **Scan** 13323
**Peptide** GQIPDNVKSQM **Charge** 2 **Spectral Angle** 0.84

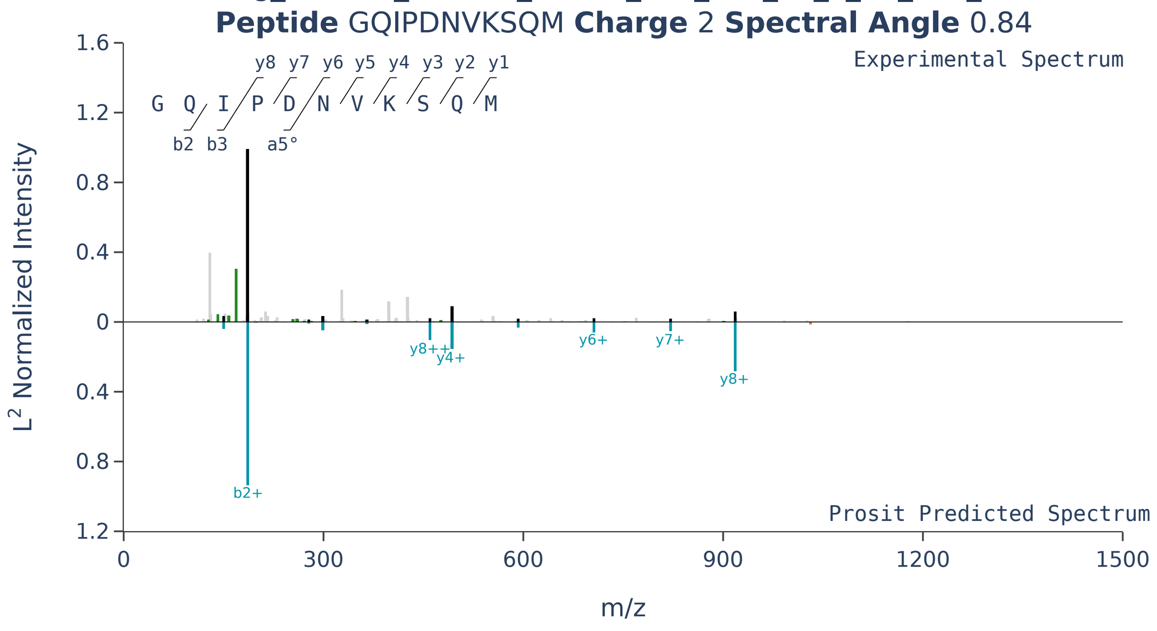

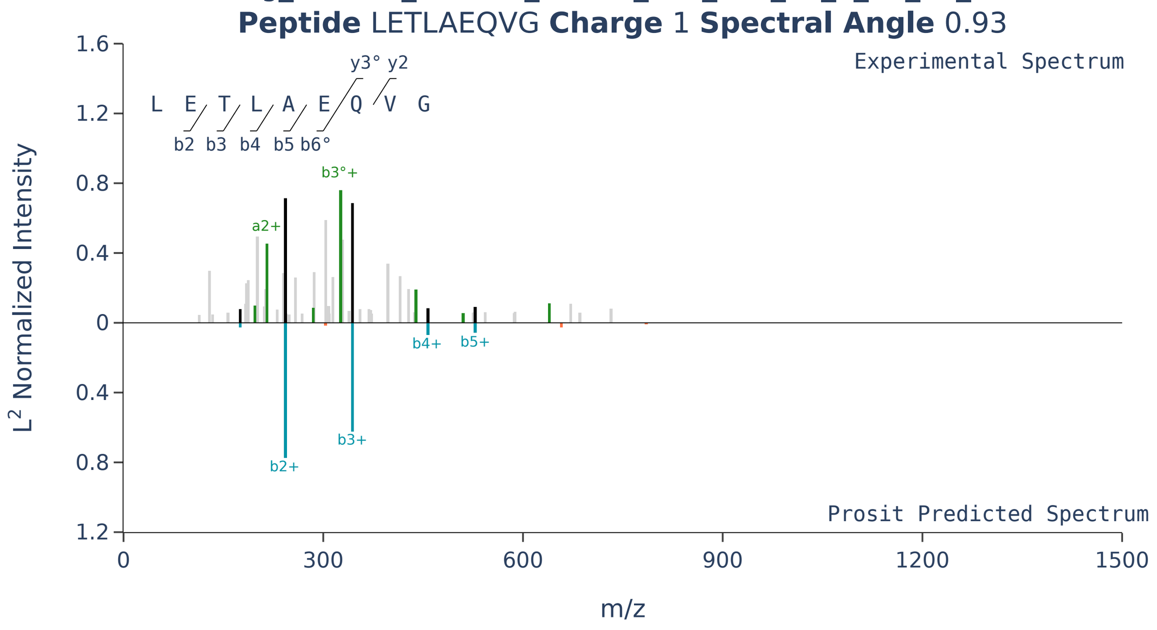


**Source** Ncheng_210623_230623_HFGoe_FFH_20S_25_1_A1_1h_R2 **Scan** 17364
**Peptide** LETLAEQVG **Charge** 1 **Spectral Angle** 0.93

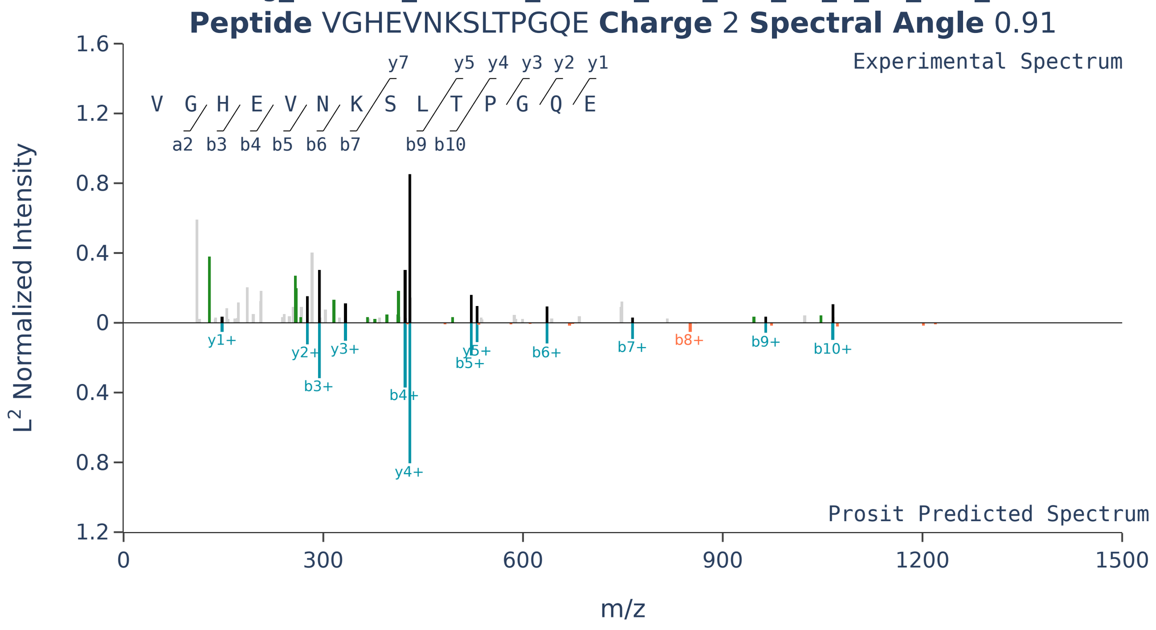


**Source** Ncheng_210623_230623_HFGoe_FFH_20S_25_1_A1_24h_R2 **Scan** 8684
**Peptide** VGHEVNKSLTPGQE **Charge** 2 **Spectral Angle** 0.91

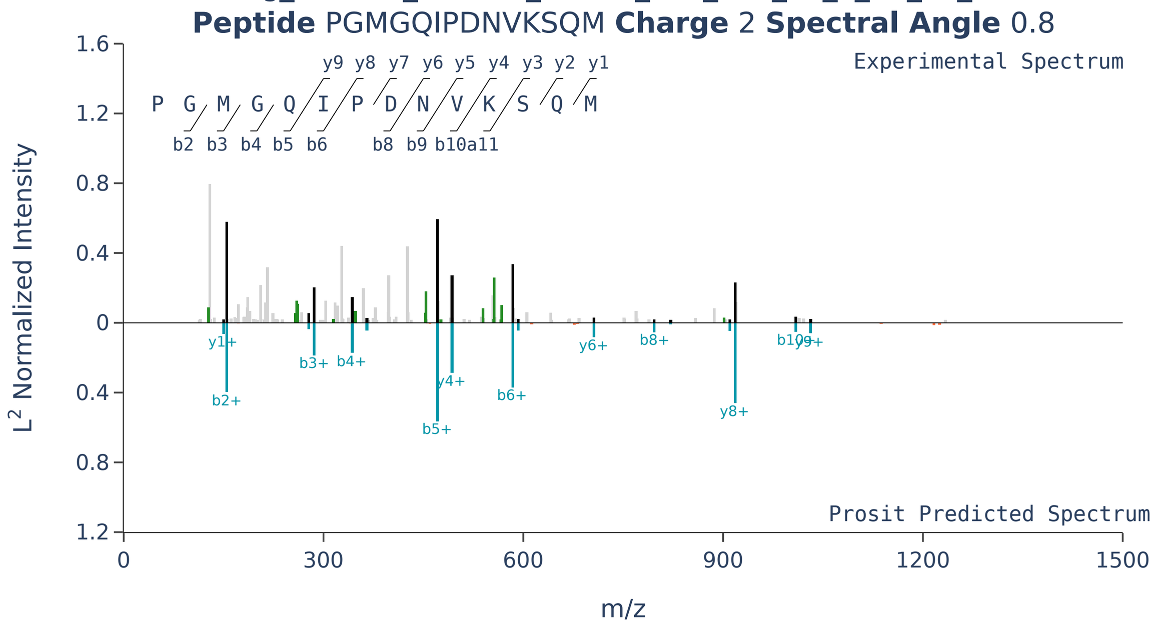


**Source** Ncheng_210623_230623_HFGoe_FFH_20S_25_1_A1_1h_R1 **Scan** 18037
**Peptide** PGMGQIPDNVKSQM **Charge** 2 **Spectral Angle** 0.8

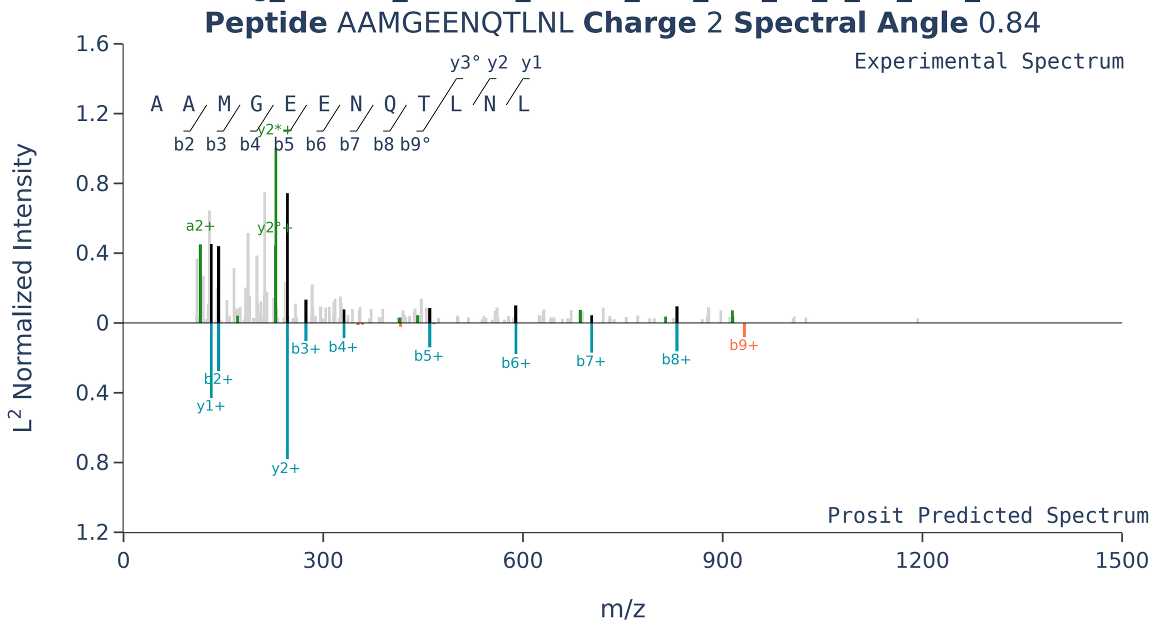


**Source** Ncheng_210623_230623_HFGoe_FFH_20S_25_1_A2_24h_R1 **Scan** 22044
**Peptide** AAMGEENQTLNL **Charge** 2 **Spectral Angle** 0.84

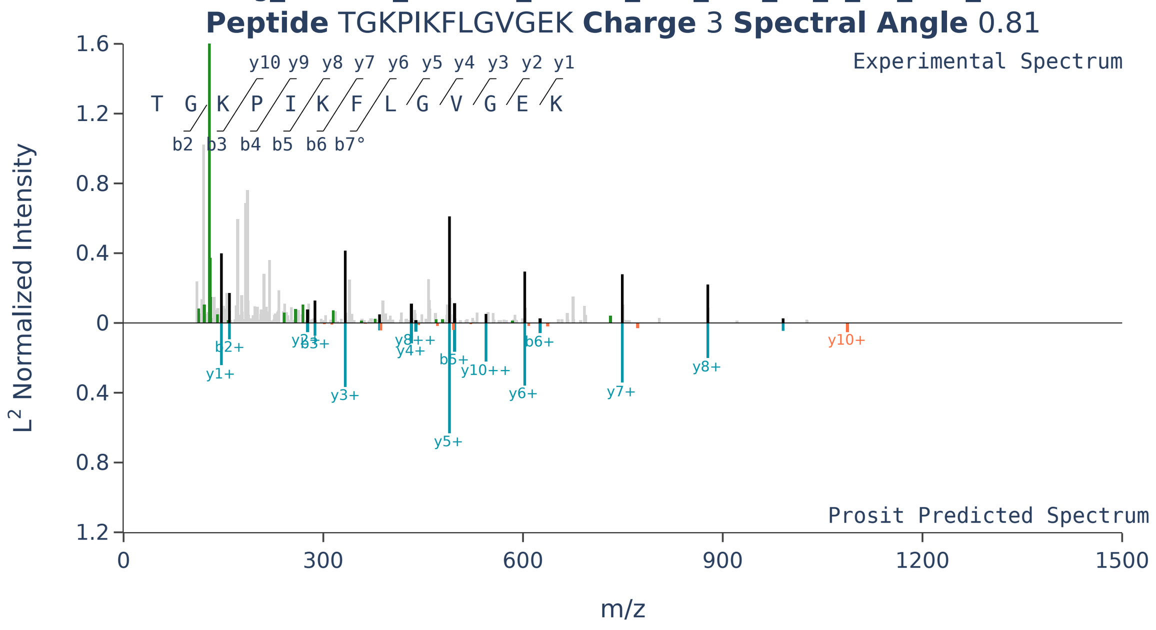


**Source** Ncheng_210623_230623_HFGoe_FFH_20S_25_1_A2_24h_R2 **Scan** 15686
**Peptide** TGKPIKFLGVGEK **Charge** 3 **Spectral Angle** 0.81

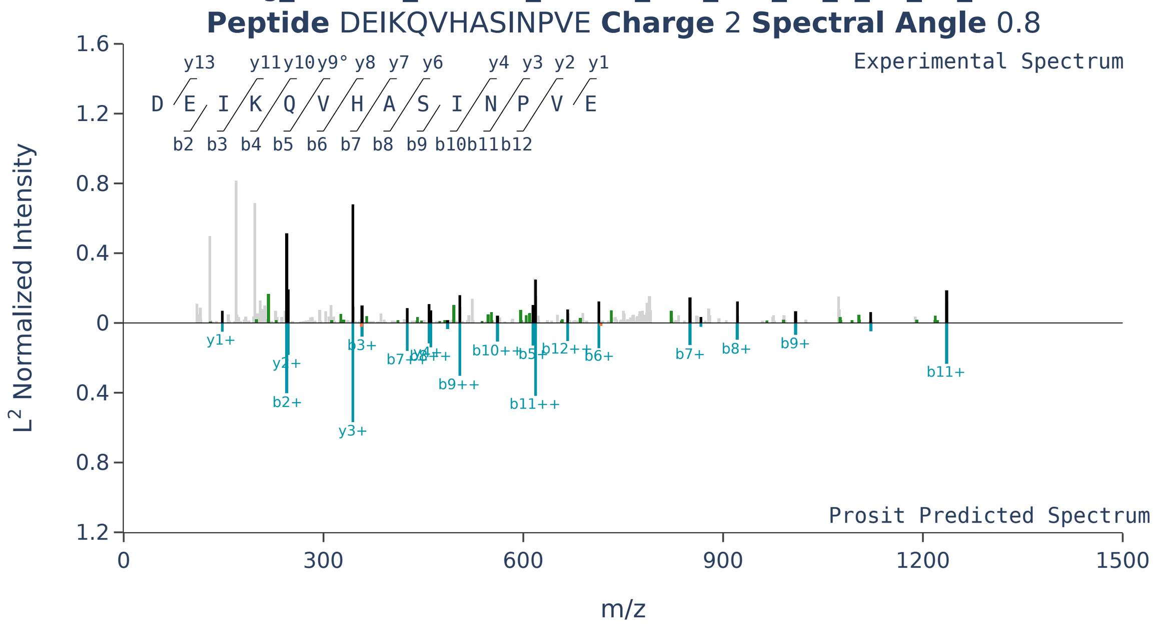


**Source** Ncheng_210623_230623_HFGoe_FFH_20S_25_1_A2_4h_R2 **Scan** 17050
**Peptide** DEIKQVHASINPVE **Charge** 2 **Spectral Angle** 0.8

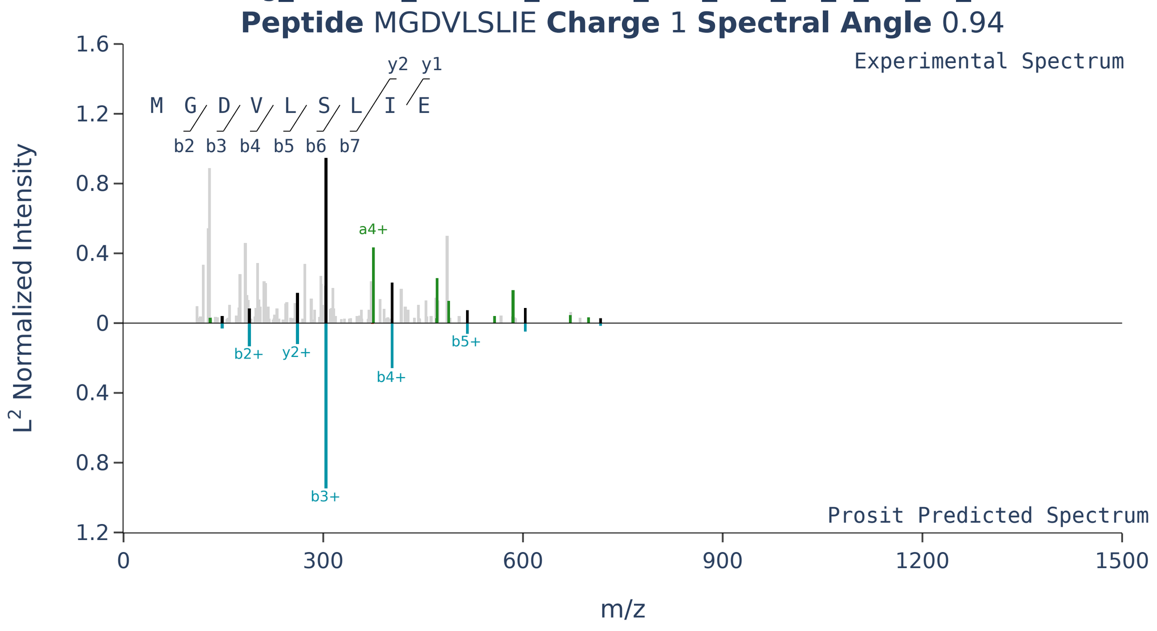


**Source** Ncheng_210623_230623_HFGoe_FFH_20S_25_1_A1_1h_R2 **Scan** 33873
**Peptide** MGDVLSLIE **Charge** 1 **Spectral Angle** 0.94

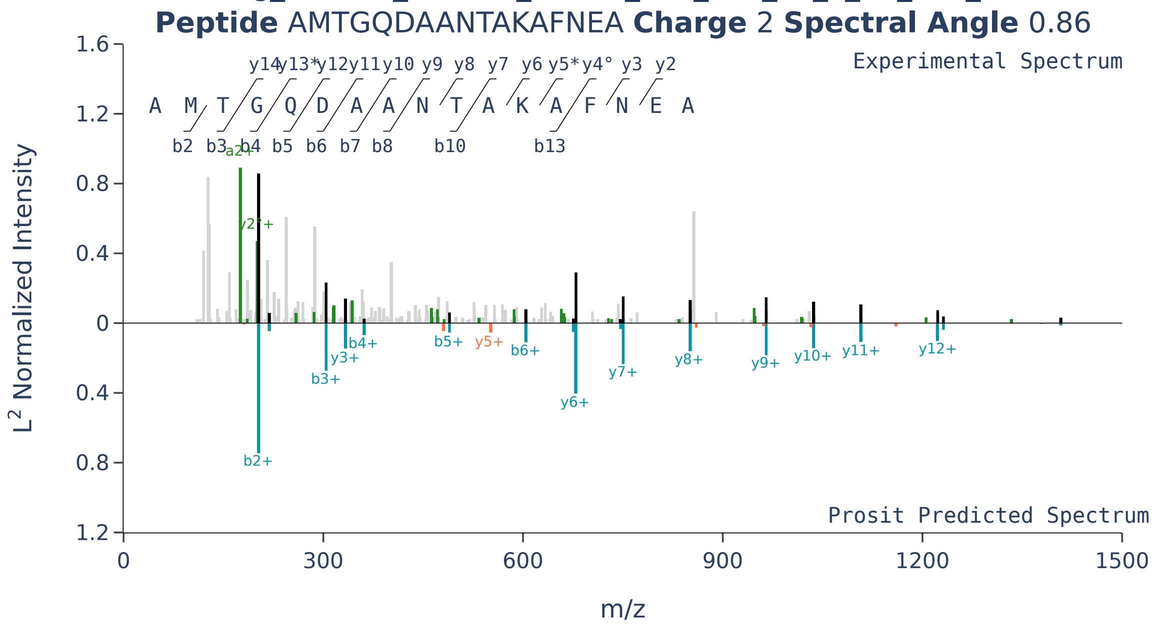


**Source** Ncheng_210623_230623_HFGoe_FFH_20S_25_1_A1_24h_R1 **Scan** 15728
**Peptide** AMTGQDAANTAKAFNEA **Charge** 2 **Spectral Angle** 0.86

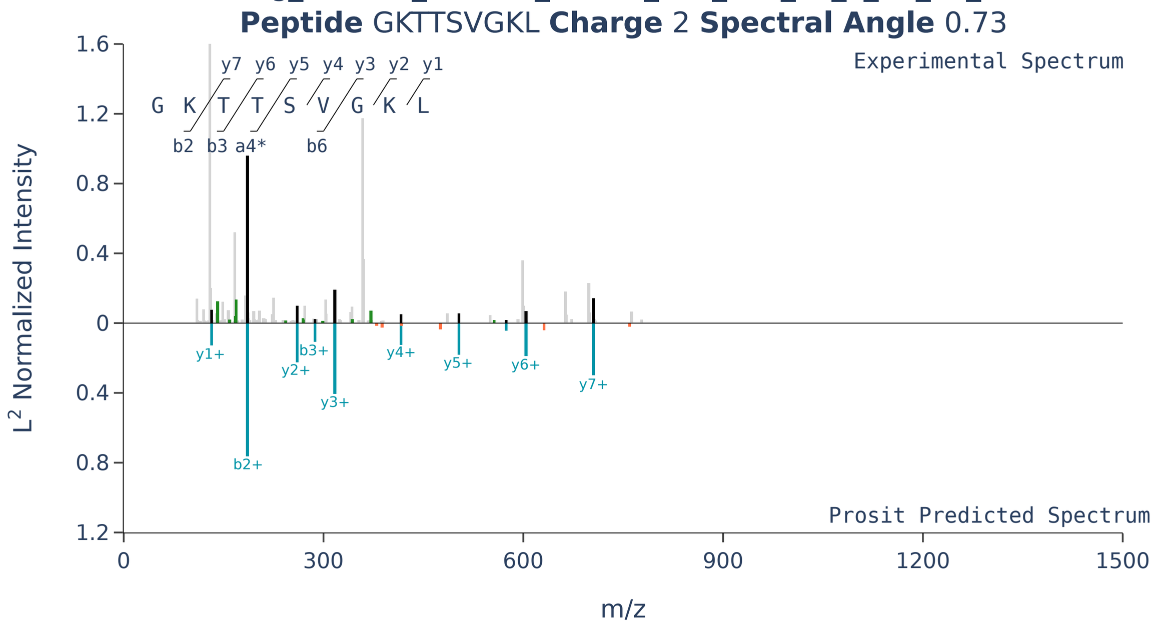


**Source** Ncheng_210623_230623_HFGoe_FFH_20S_25_1_A1_4h_R1 **Scan** 7852
**Peptide** GKTTSVGKL **Charge** 2 **Spectral Angle** 0.73

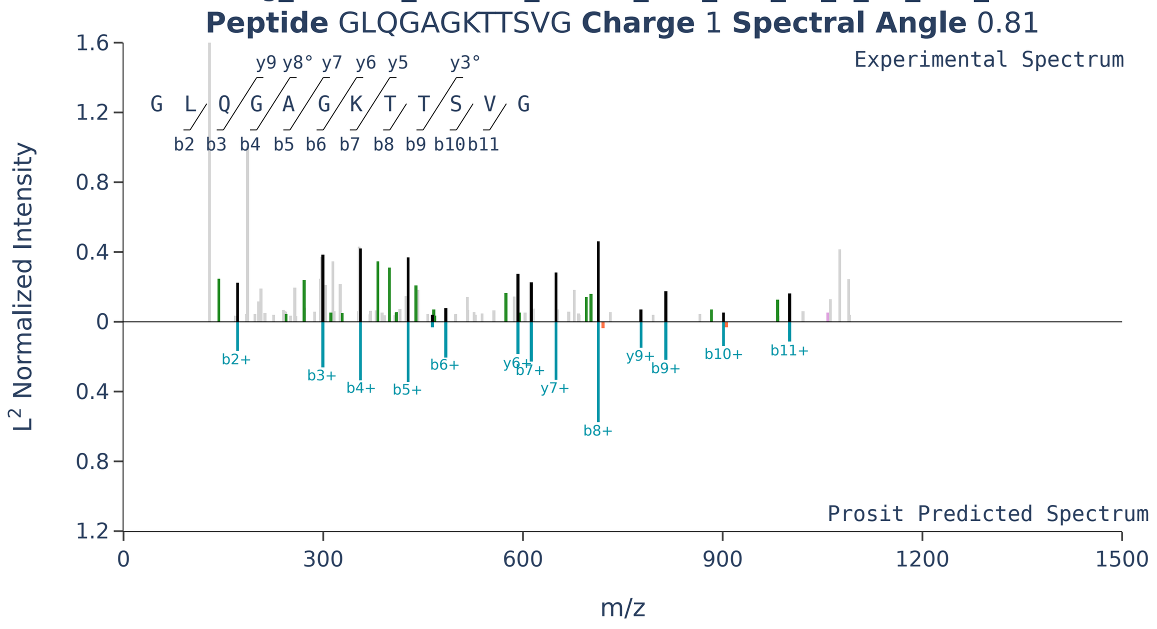


**Source** Ncheng_210623_230623_HFGoe_FFH_20S_25_1_A1_24h_R1 **Scan** 8024
**Peptide** GLQGAGKTTSVG **Charge** 1 **Spectral Angle** 0.81

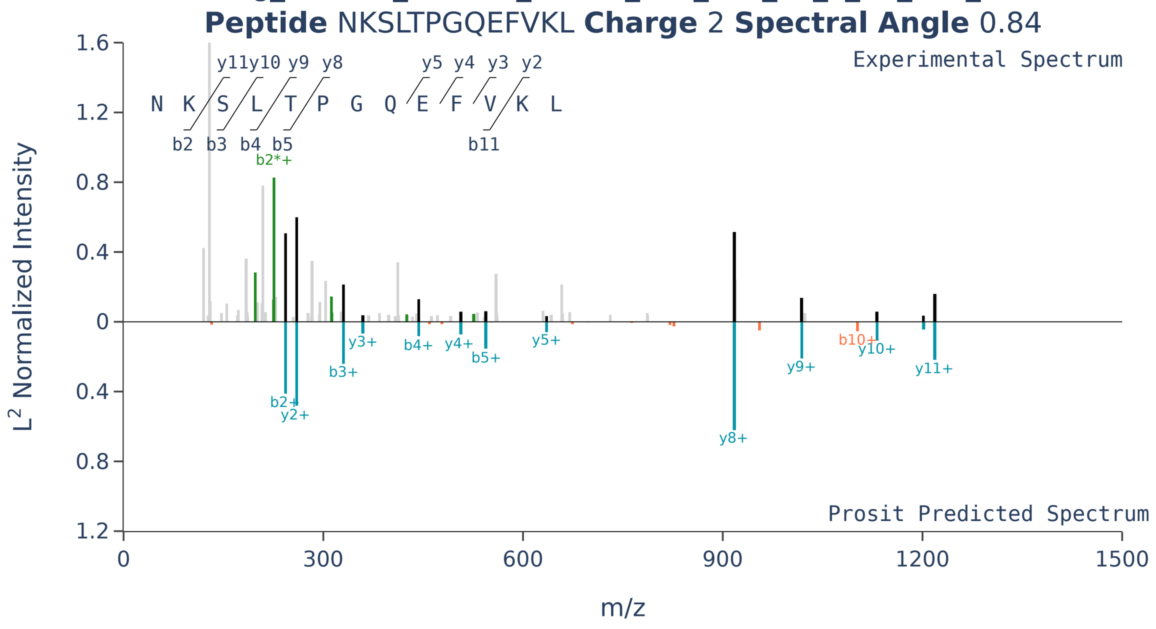


**Source** Ncheng_210623_230623_HFGoe_FFH_20S_25_1_A2_24h_R2 **Scan** 21875
**Peptide** NKSLTPGQEFVKL **Charge** 2 **Spectral Angle** 0.84

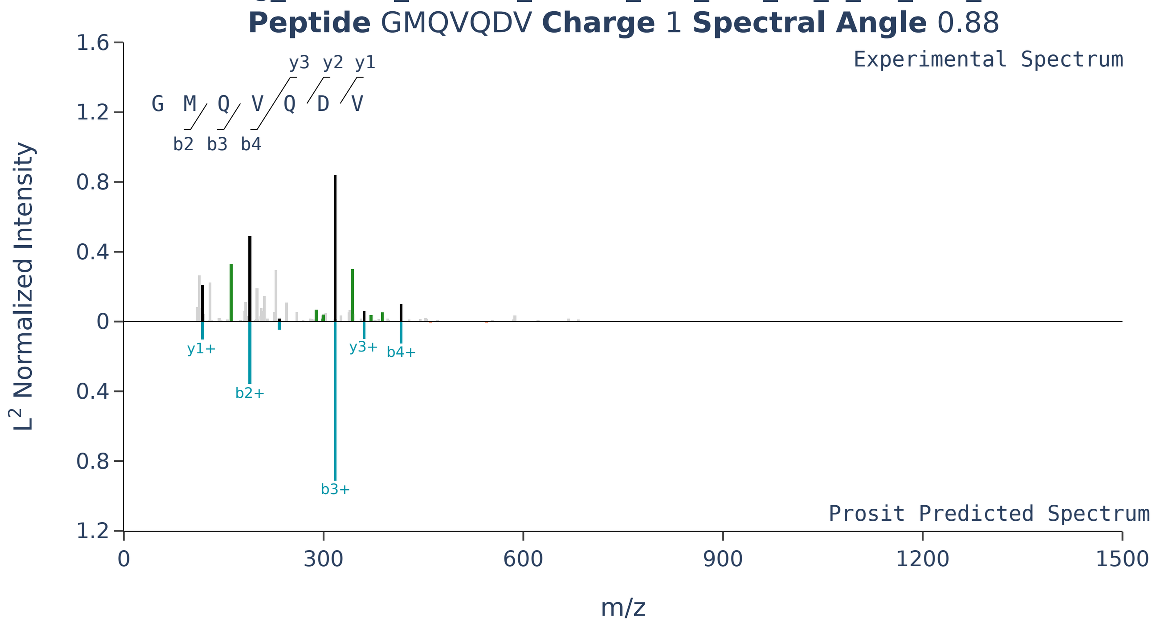


**Source** Ncheng_210623_230623_HFGoe_FFH_20S_25_1_A1_24h_R1 **Scan** 13672
**Peptide** GMQVQDV **Charge** 1 **Spectral Angle** 0.88

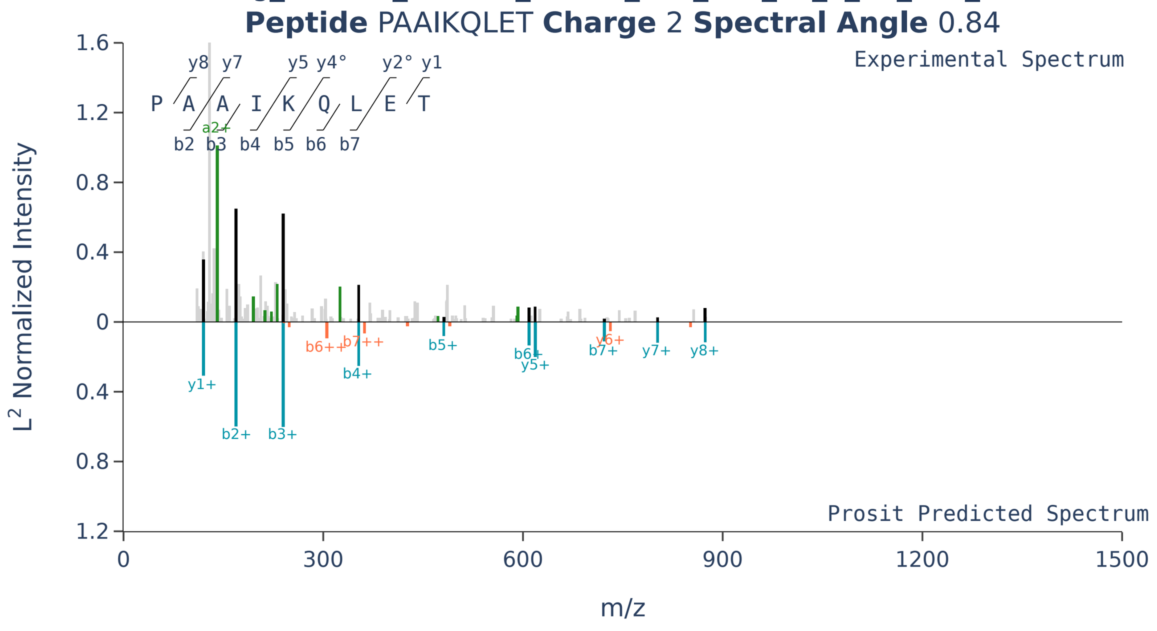


**Source** Ncheng_210623_230623_HFGoe_FFH_20S_25_1_A1_24h_R2 **Scan** 13104
**Peptide** PAAIKQLET **Charge** 2 **Spectral Angle** 0.84

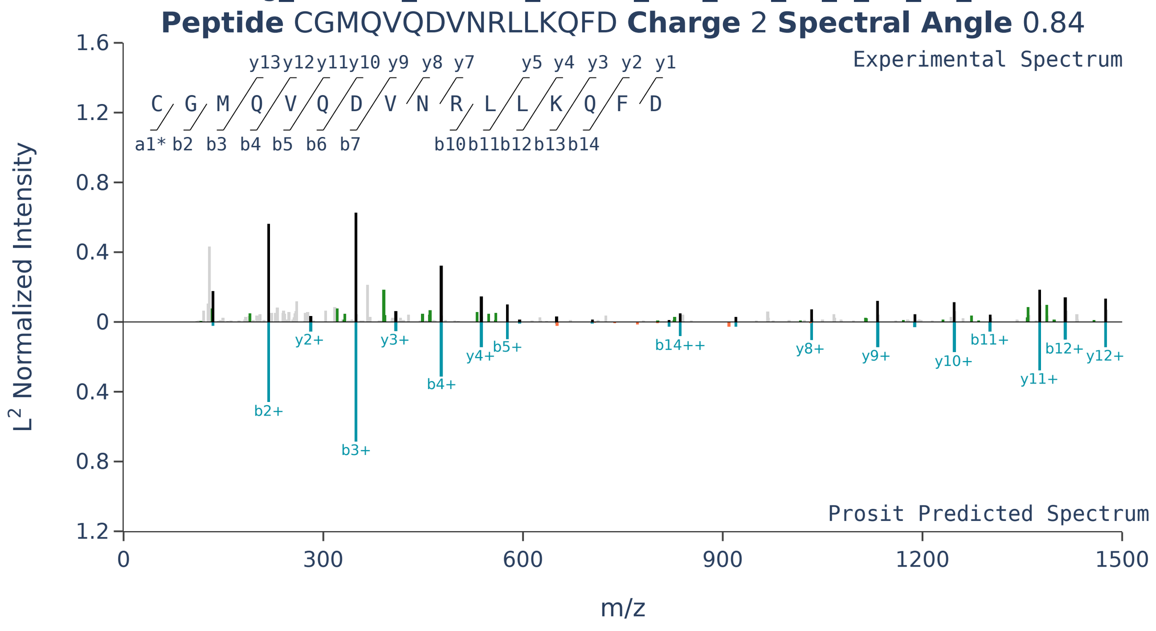


**Source** Ncheng_210623_230623_HFGoe_FFH_20S_25_1_A2_1h_R1 **Scan** 28903
**Peptide** CGMQVQDVNRLLKQFD **Charge** 2 **Spectral Angle** 0.84

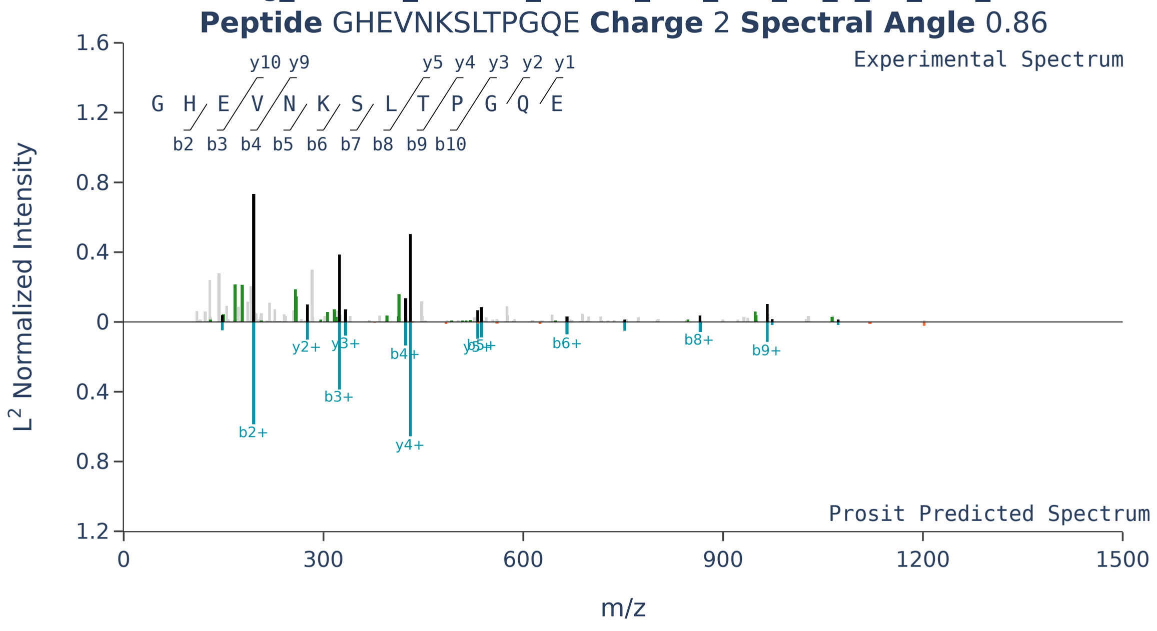


**Source** Ncheng_210623_230623_HFGoe_FFH_20S_25_1_A1_24h_R2 **Scan** 7648
**Peptide** GHEVNKSLTPGQE **Charge** 2 **Spectral Angle** 0.86

**Source** Ncheng_210623_230623_HFGoe_FFH_20S_25_1_A1_2h_R2 **Scan** 27040
**Peptide** PGQEFVKIVRNELVA **Charge** 2 **Spectral Angle** 0.79

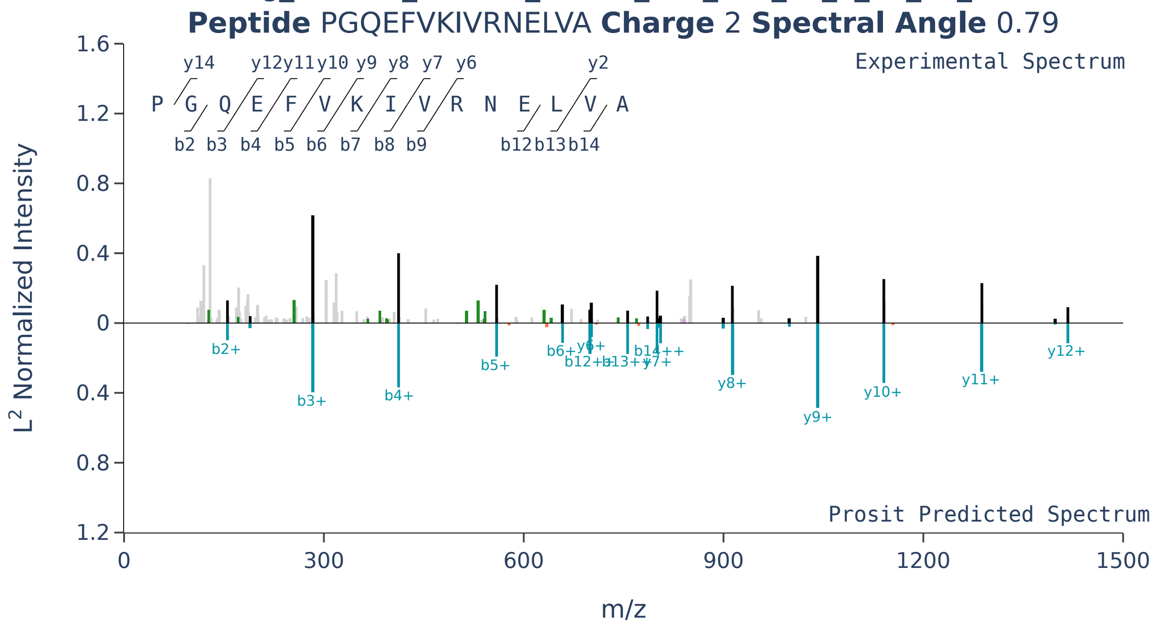


**Source** Ncheng_210623_230623_HFGoe_FFH_20S_25_1_A1_2h_R1 **Scan** 25029
**Peptide** GEKTEALEPFHPDRIASRLL **Charge** 3 **Spectral Angle** 0.8

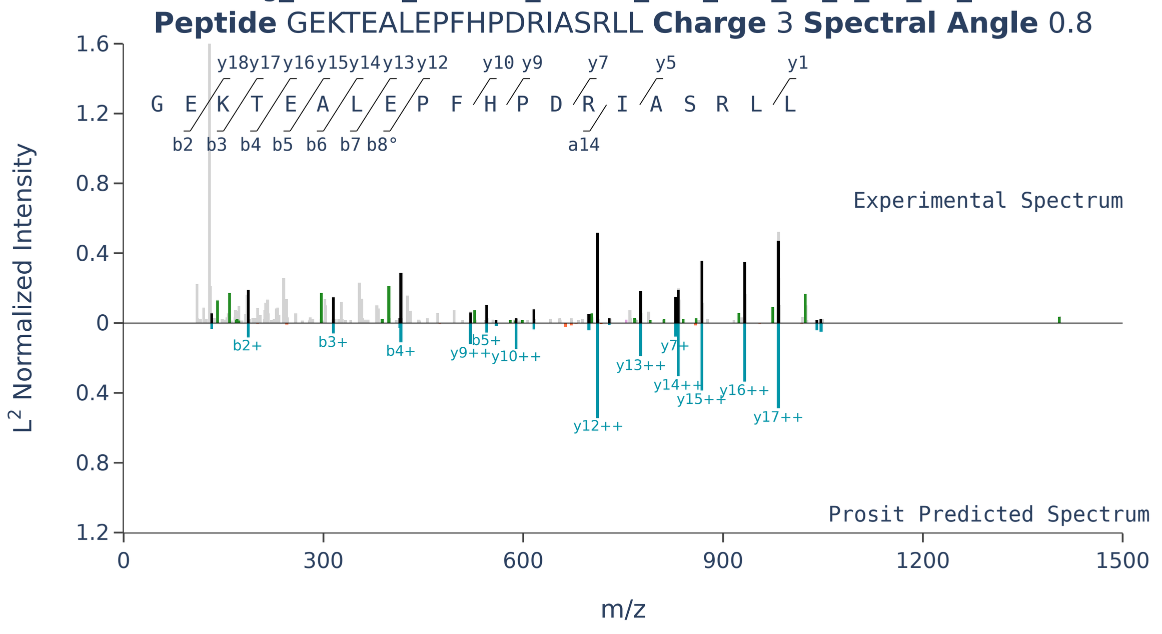


**Source** Ncheng_210623_230623_HFGoe_FFH_20S_25_1_A2_4h_R1 **Scan** 20988
**Peptide** AMTGQDAANTAKAFNEAL **Charge** 2 **Spectral Angle** 0.92

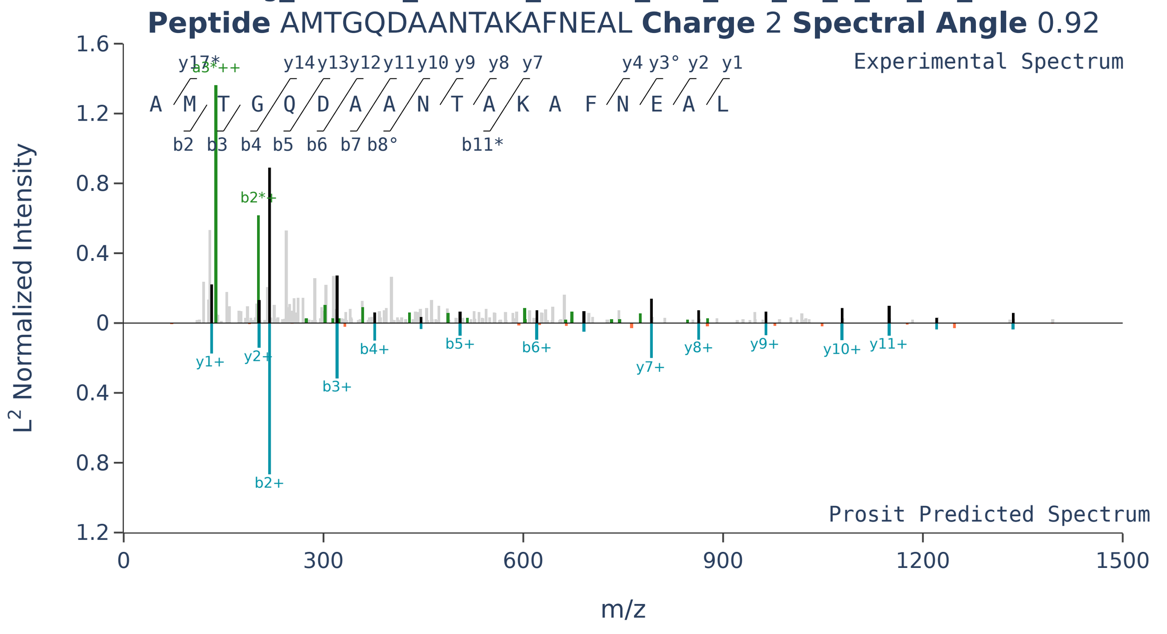


**Source** Ncheng_210623_230623_HFGoe_FFH_20S_25_1_A2_24h_R1 **Scan** 16850
**Peptide** SADVYRPAAI **Charge** 1 **Spectral Angle** 0.88

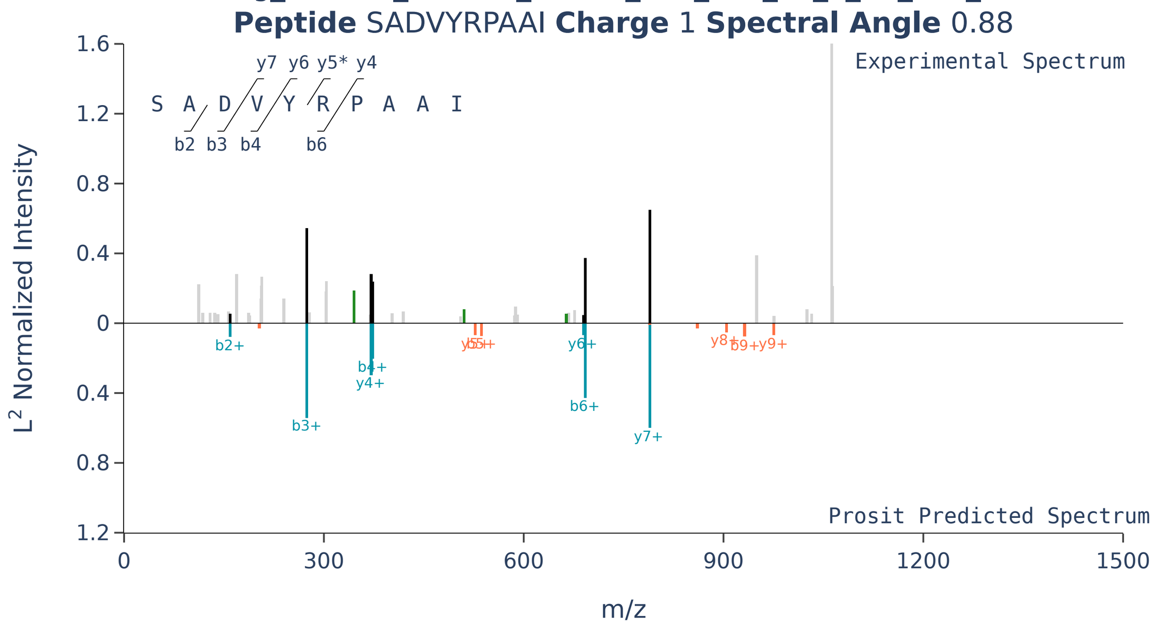


**Source** Ncheng_210623_230623_HFGoe_FFH_20S_25_1_A2_4h_R1 **Scan** 18499
**Peptide** AVGHEVNKSLTPGQEFV **Charge** 2 **Spectral Angle** 0.89

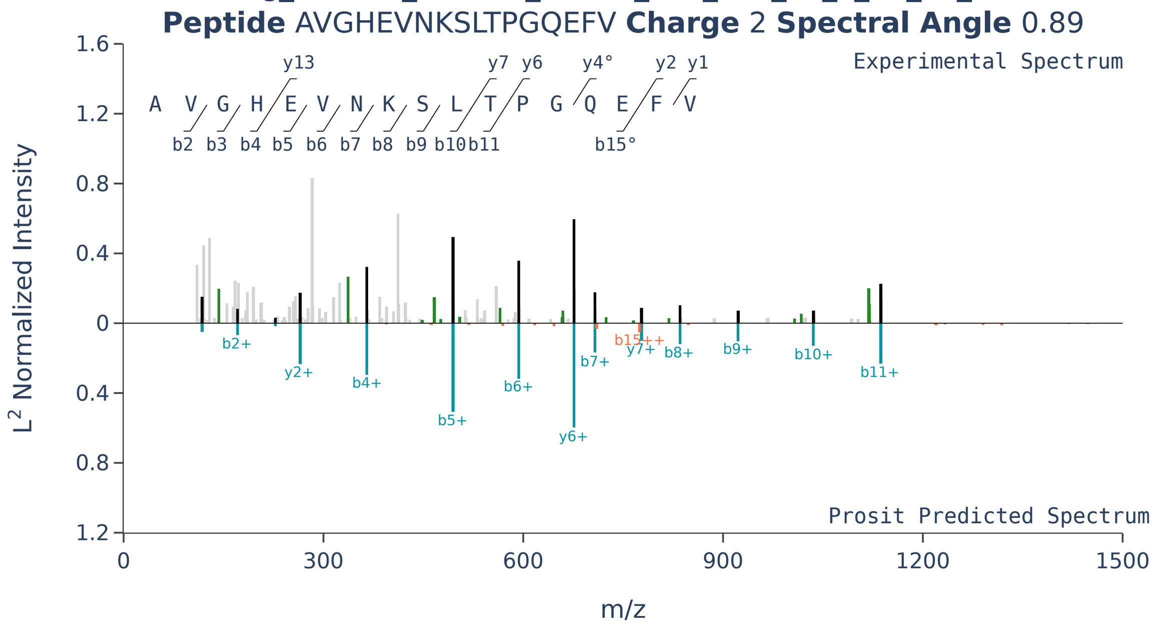


**Source** Ncheng_210623_230623_HFGoe_FFH_20S_25_1_A1_4h_R1 **Scan** 12405
**Peptide** EPFHPDRIAS **Charge** 1 **Spectral Angle** 0.82

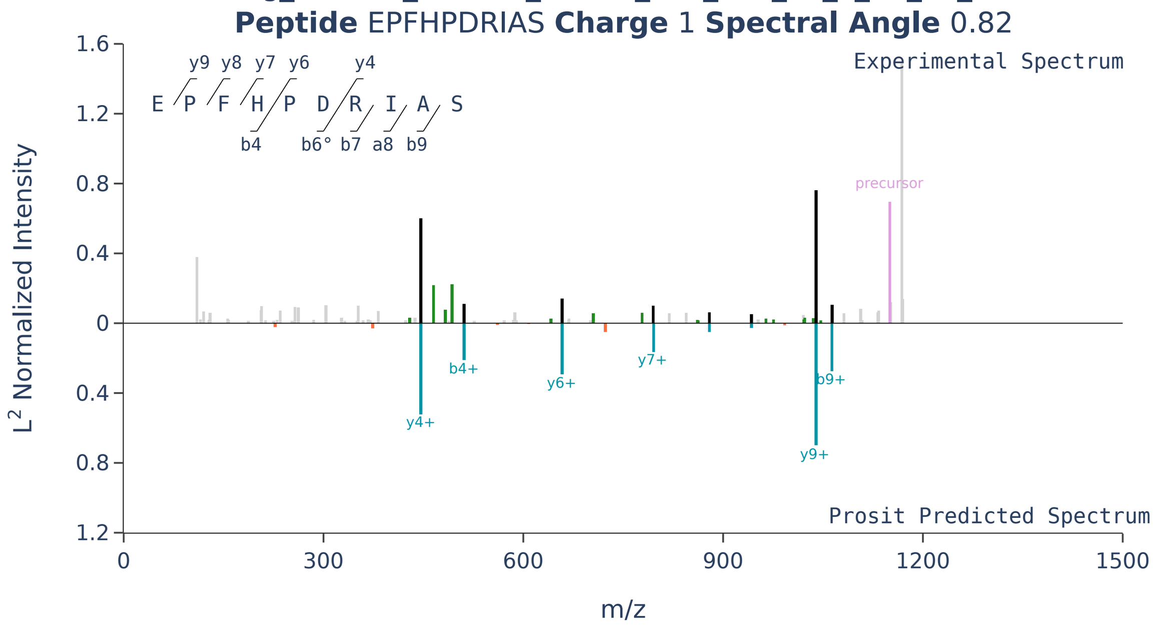


**Source** Ncheng_210623_230623_HFGoe_FFH_20S_25_1_A1_24h_R2 **Scan** 18418
**Peptide** ALEPFHPDRLAS **Charge** 3 **Spectral Angle** 0.89

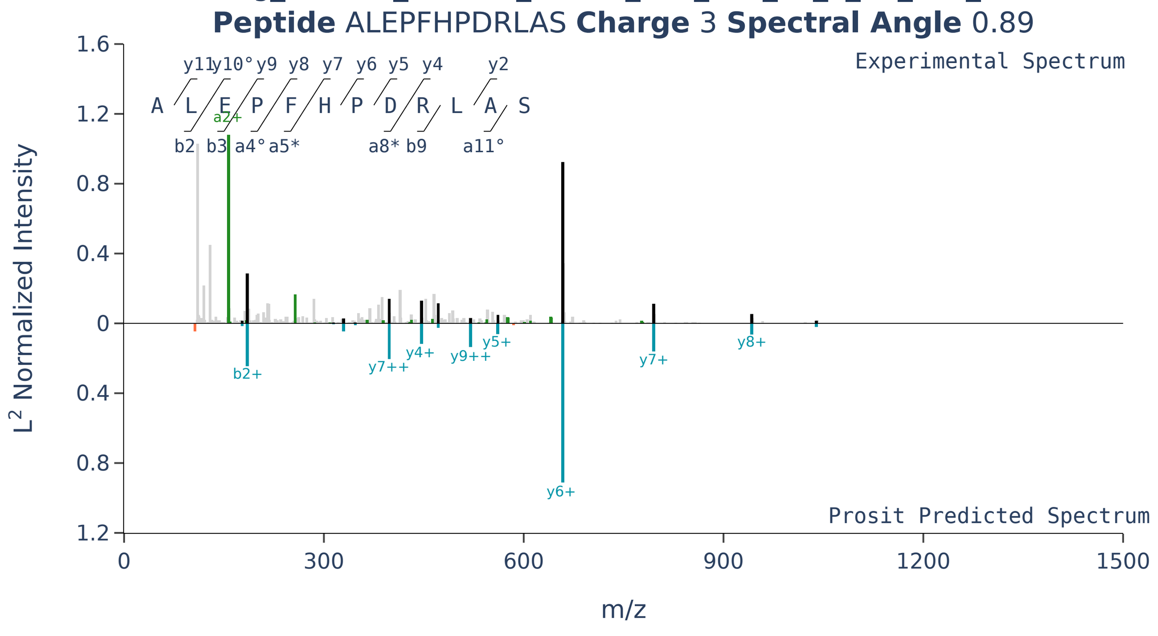


**Source** Ncheng_210623_230623_HFGoe_FFH_20S_25_1_A2_4h_R2 **Scan** 21620
**Peptide** AVGHEVNKSLTPGQEFVKIVRNE **Charge** 2 **Spectral Angle** 0.76

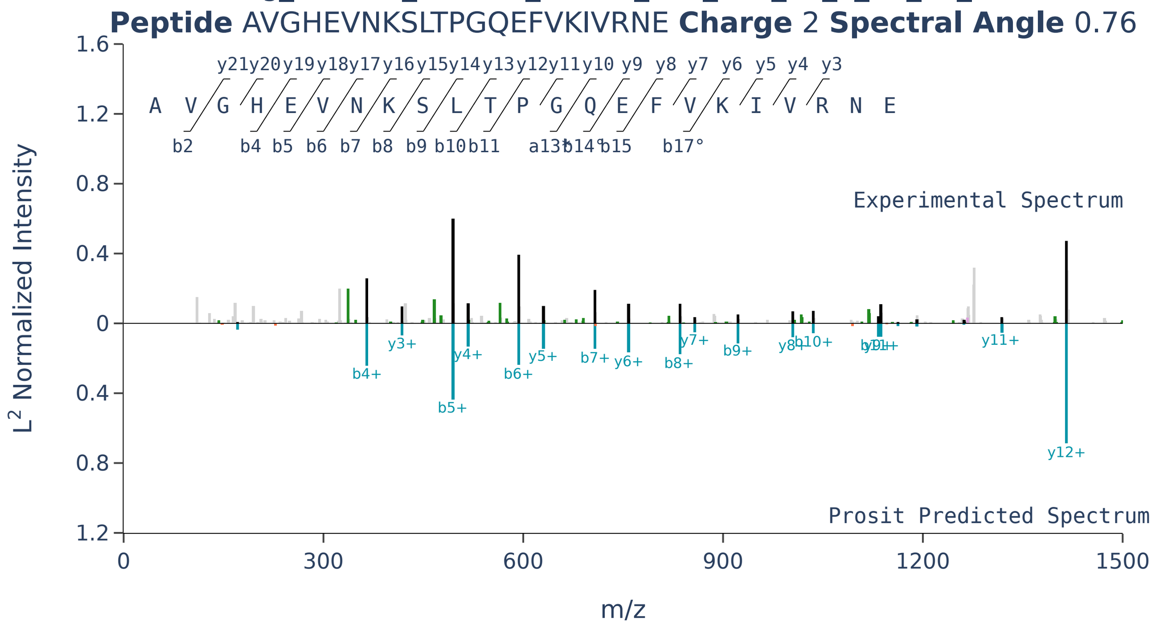


**Source** Ncheng_210623_230623_HFGoe_FFH_20S_25_1_A1_2h_R1 **Scan** 19433
**Peptide** KPIKFLGVGE **Charge** 3 **Spectral Angle** 0.82

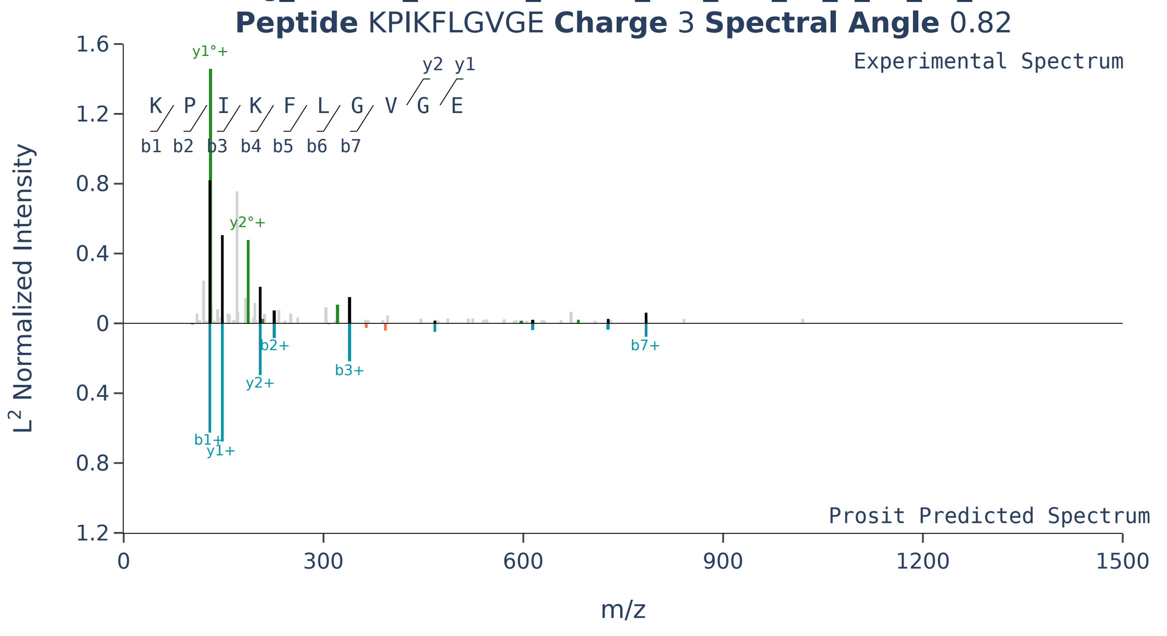


**Source** Ncheng_210623_230623_HFGoe_FFH_20S_25_1_A2_4h_R2 **Scan** 29636
**Peptide** NDFLEQL **Charge** 2 **Spectral Angle** 0.89

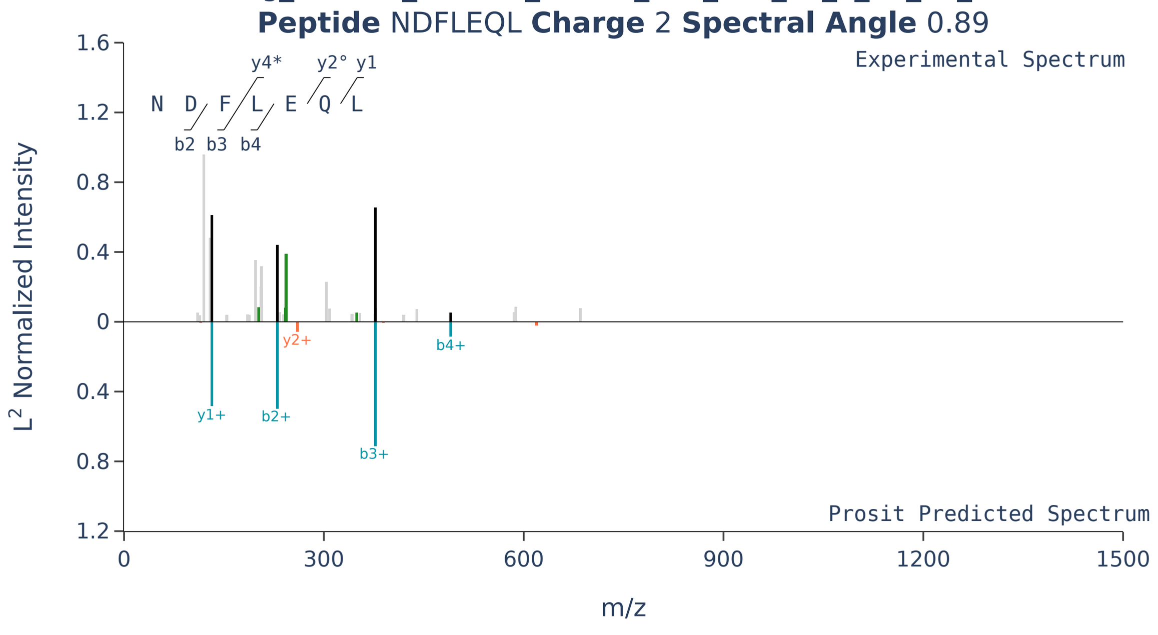


**Source** Ncheng_210623_230623_HFGoe_FFH_20S_25_1_A2_4h_R1 **Scan** 20251
**Peptide** GHEVNKSLTPGQEFVKI **Charge** 2 **Spectral Angle** 0.88

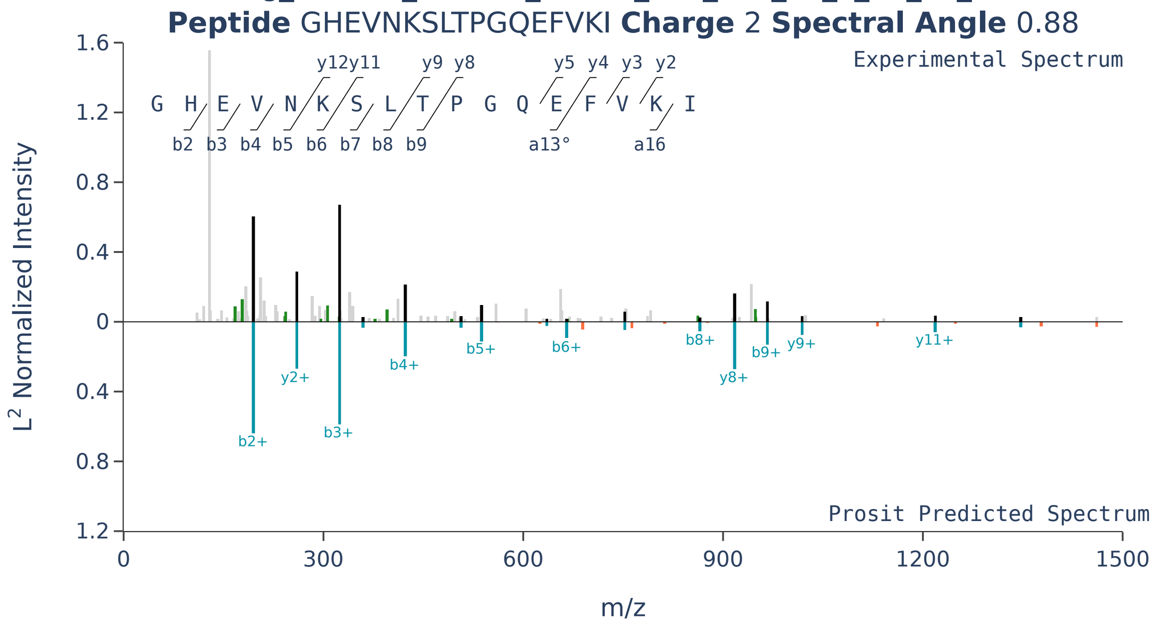


**Source** Ncheng_210623_230623_HFGoe_FFH_20S_25_1_A1_2h_R2 **Scan** 28074
**Peptide** TLFVVDAMTGQDAANTAKA **Charge** 2 **Spectral Angle** 0.87

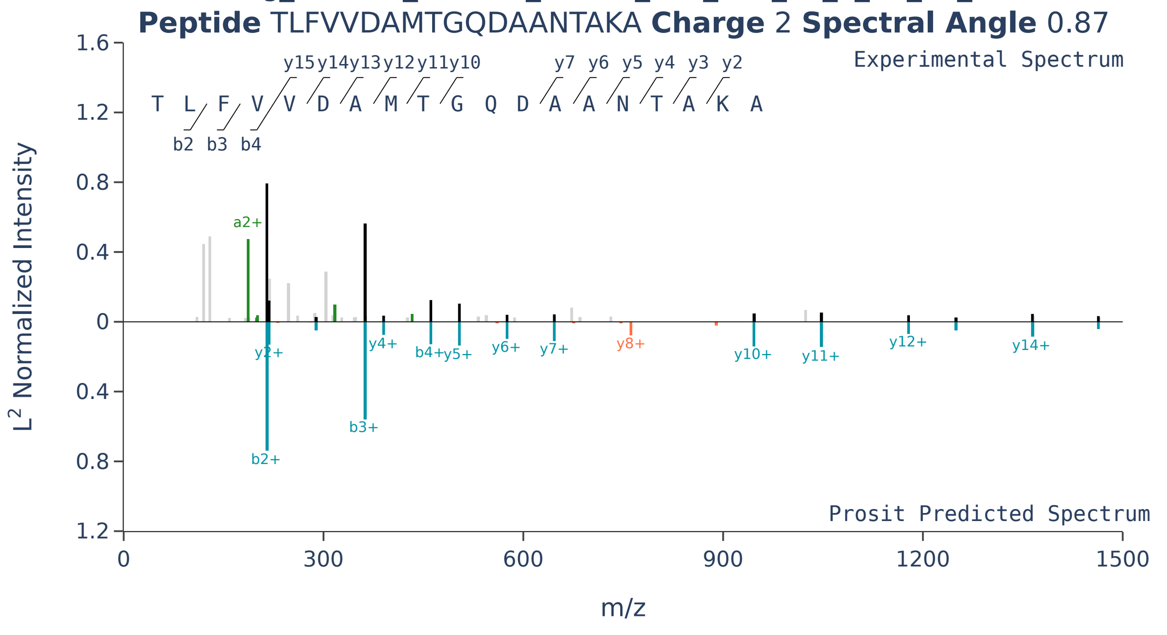


**Source** Ncheng_210623_230623_HFGoe_FFH_20S_25_1_A1_24h_R1 **Scan** 11726
**Peptide** DVYRPAALKQ **Charge** 2 **Spectral Angle** 0.74

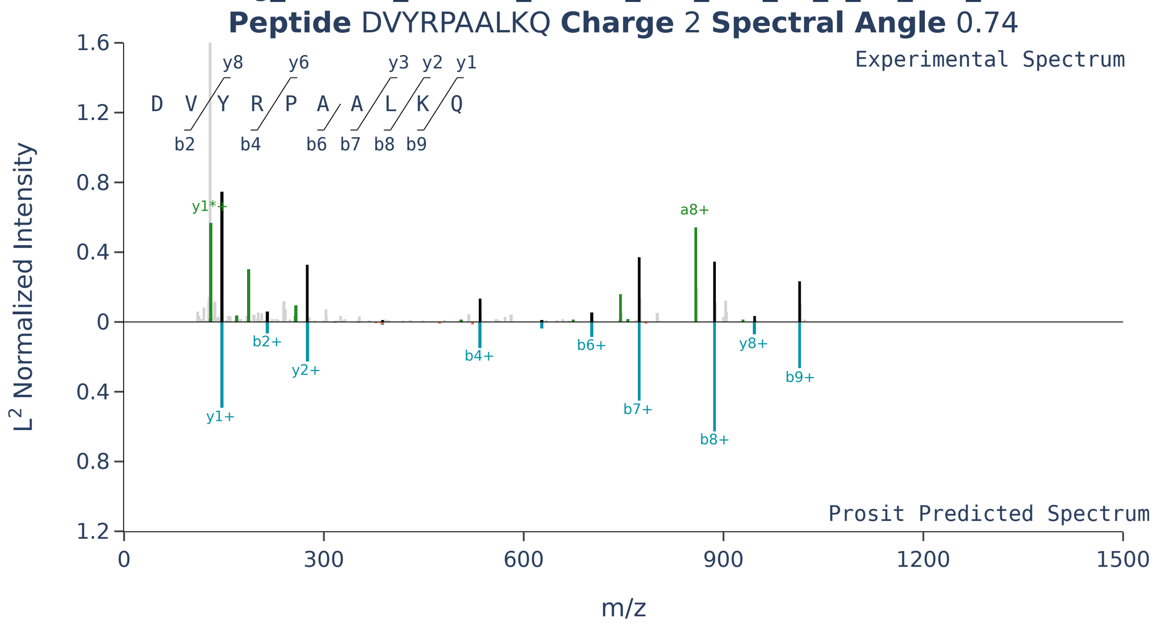


**Source** Ncheng_210623_230623_HFGoe_FFH_20S_25_1_A2_24h_R2 **Scan** 17967
**Peptide** DVYRPAAIKQLE **Charge** 2 **Spectral Angle** 0.8

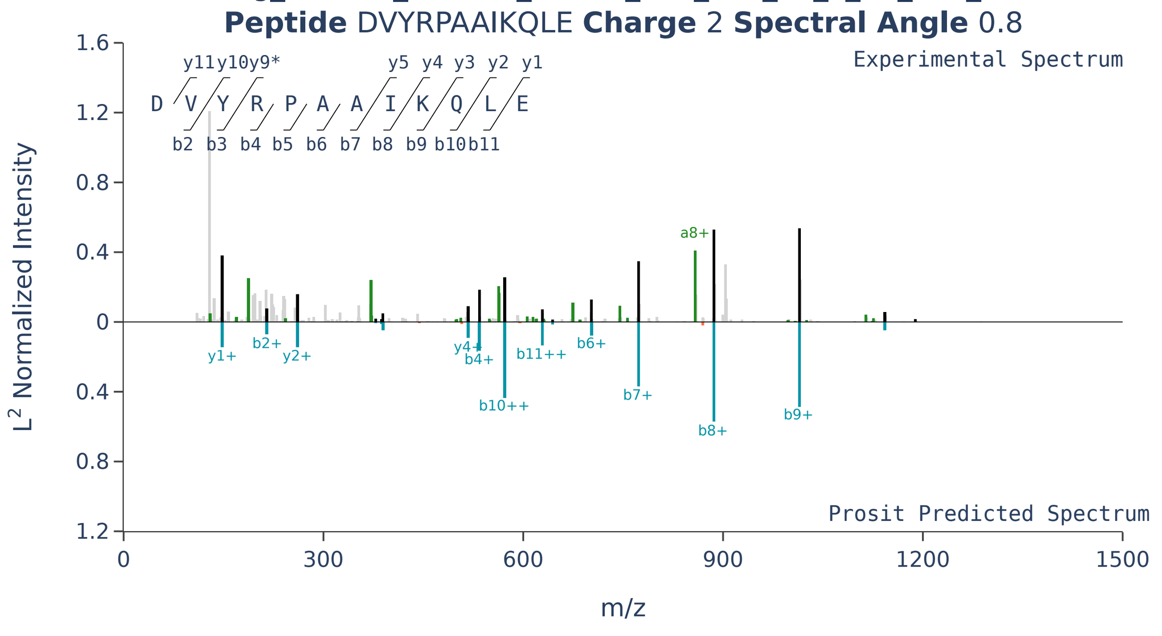


**Source** Ncheng_210623_230623_HFGoe_FFH_20S_25_1_A1_4h_R2 **Scan** 27496
**Peptide** LVAAMGEENQTLNL **Charge** 2 **Spectral Angle** 0.9

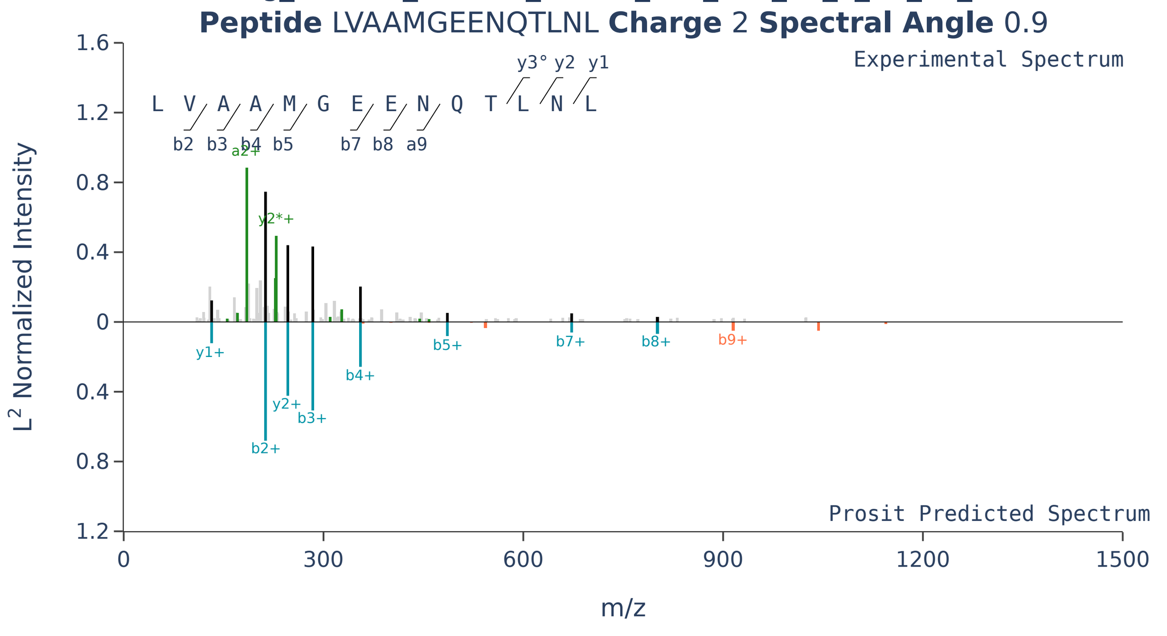

**Source** Ncheng_210623_230623_HFGoe_FFH_20S_25_1_A1_4h_R1 **Scan** 24901
**Peptide** VLSLIED **Charge** 1 **Spectral Angle** 0.83

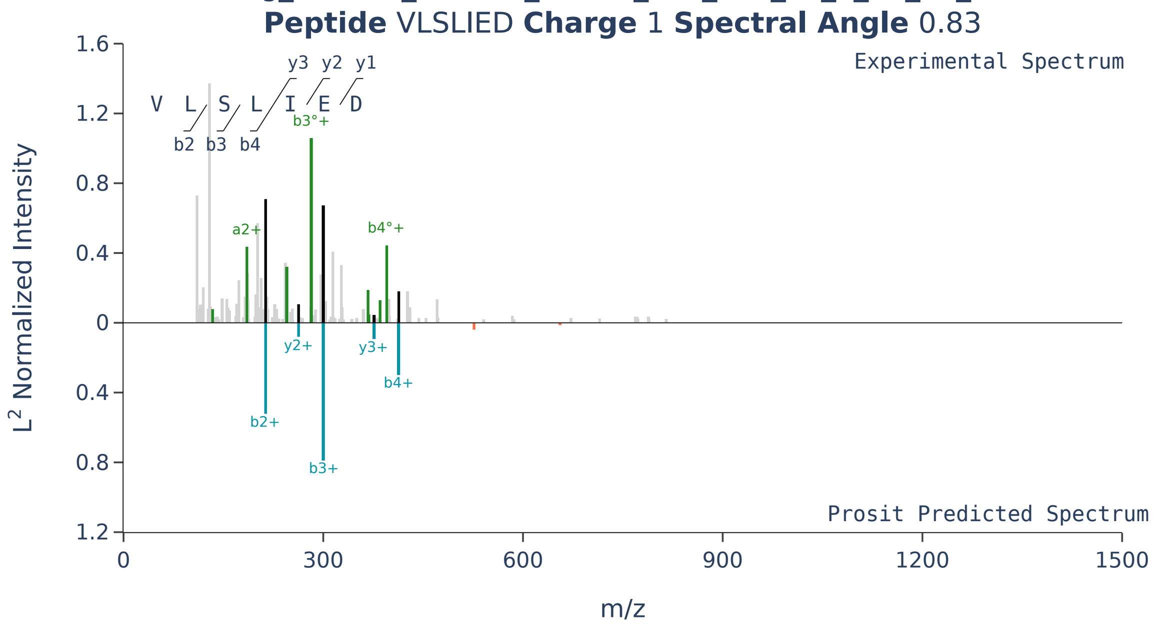

**Source** Ncheng_210623_230623_HFGoe_FFH_20S_25_1_A1_2h_R1 **Scan** 31554
**Peptide** TLFVVDAMTGQDAANTAKAFNE **Charge** 3 **Spectral Angle** 0.89

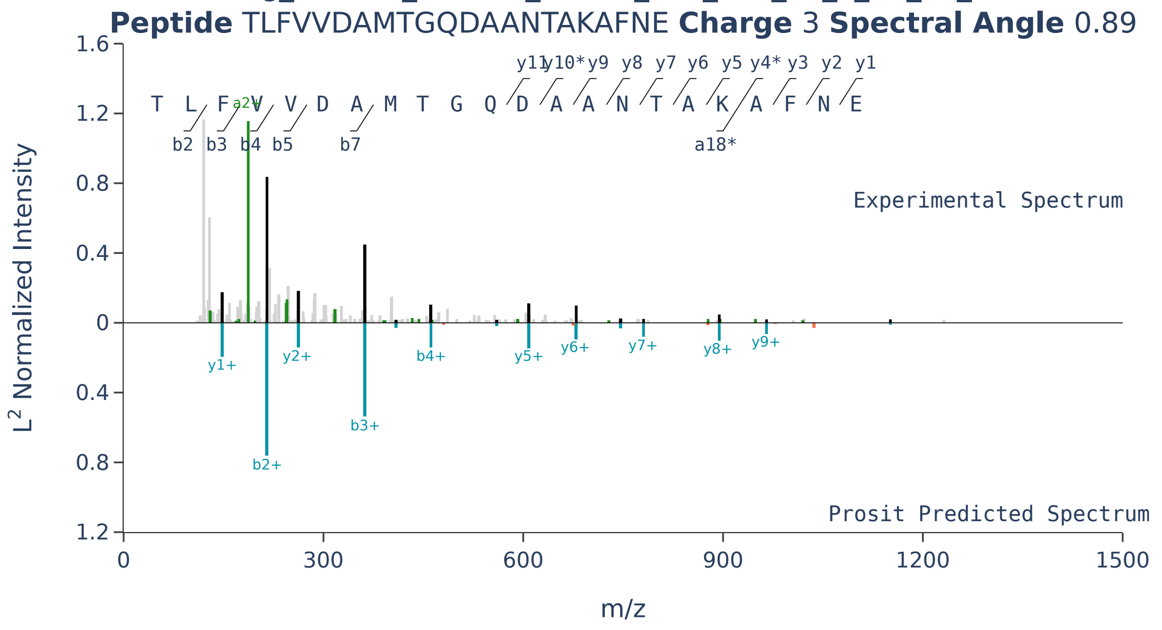

**Source** Ncheng_210623_230623_HFGoe_FFH_20S_25_1_A2_24h_R2 **Scan** 7757
**Peptide** REFINRVK **Charge** 3 **Spectral Angle** 0.92

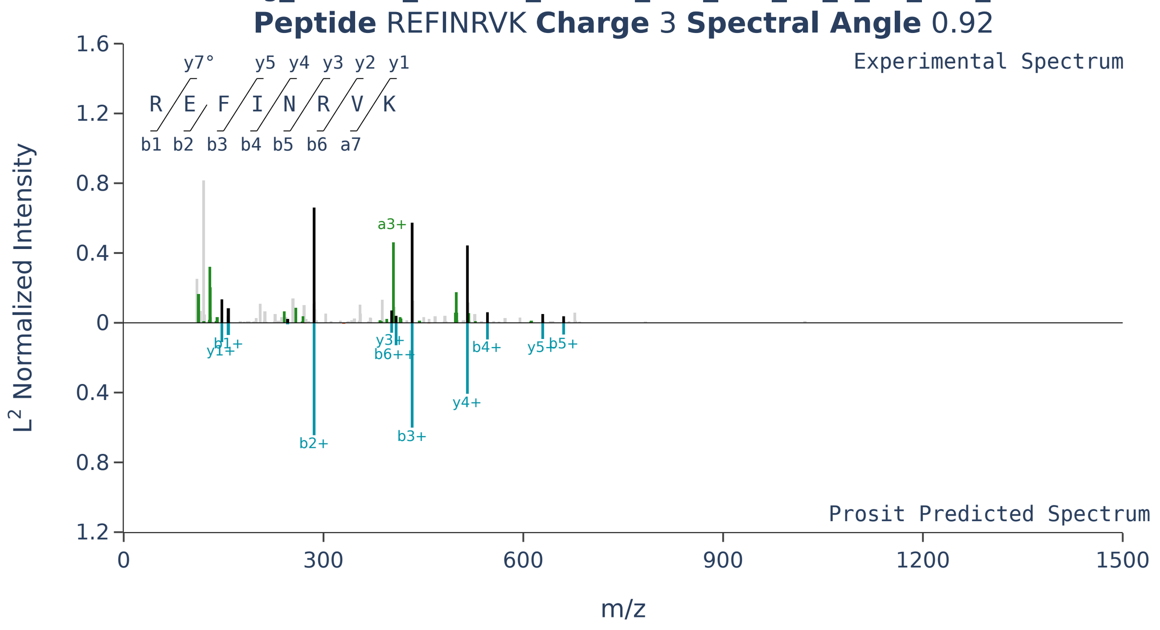

**Source** Ncheng_210623_230623_HFGoe_FFH_20S_25_1_A2_24h_R2 **Scan** 26122
**Peptide** GCGMQVQDVNRLL **Charge** 2 **Spectral Angle** 0.79

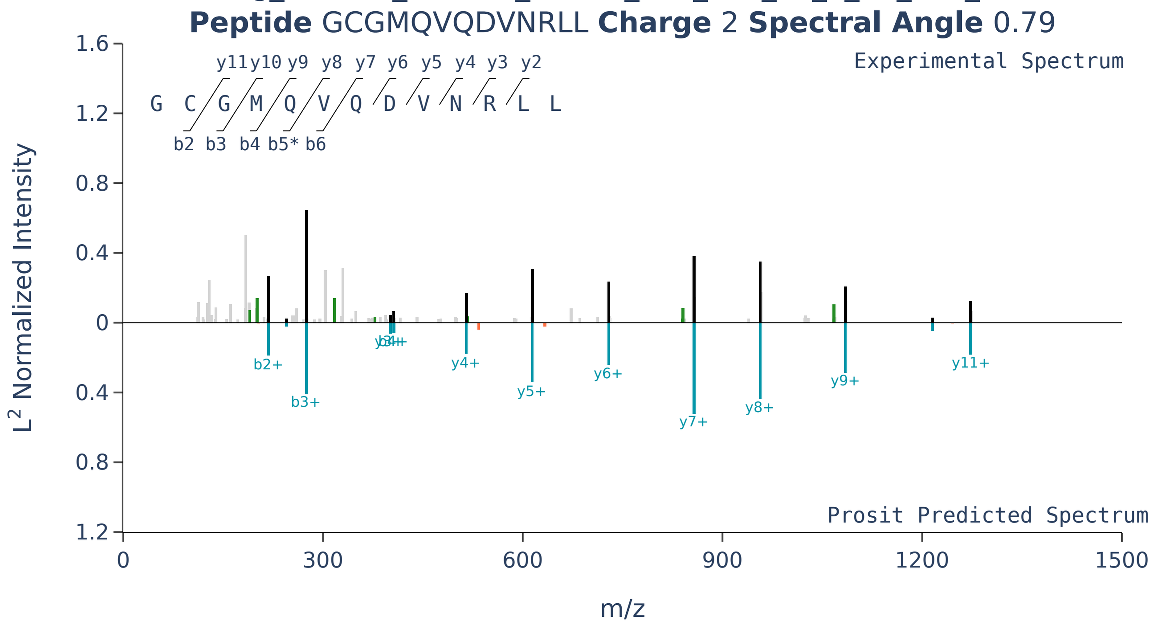

**Source** Ncheng_210623_230623_HFGoe_FFH_20S_25_1_A1_24h_R2 **Scan** 8350
**Peptide** GQIPDNVKSQ **Charge** 1 **Spectral Angle** 0.87

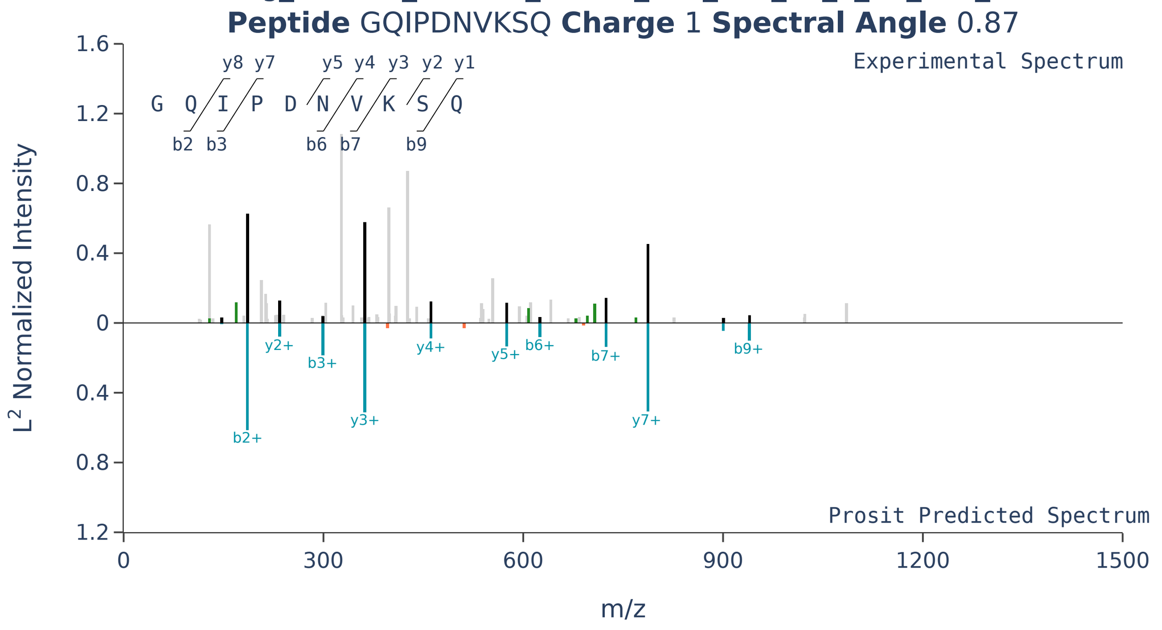

**Source** Ncheng_210623_230623_HFGoe_FFH_20S_25_1_A2_24h_R1 **Scan** 10308
**Peptide** DAANTAKAFNE **Charge** 1 **Spectral Angle** 0.83

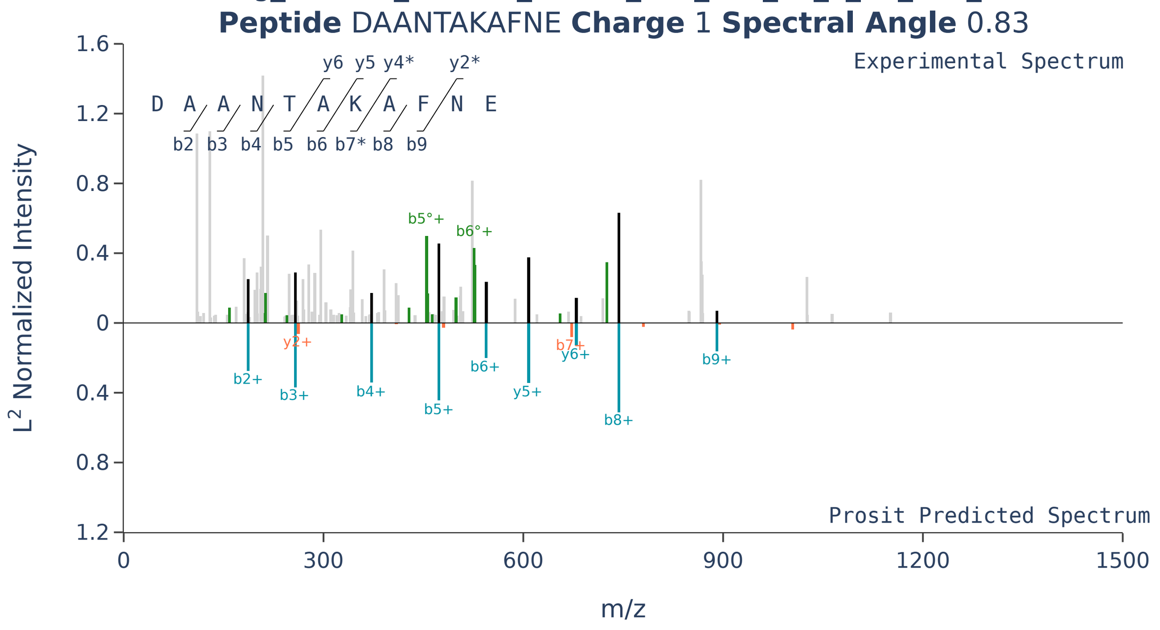

**Source** Ncheng_210623_230623_HFGoe_FFH_20S_25_1_A2_4h_R1 **Scan** 7918
**Peptide** LNRVKEK **Charge** 2 **Spectral Angle** 0.84

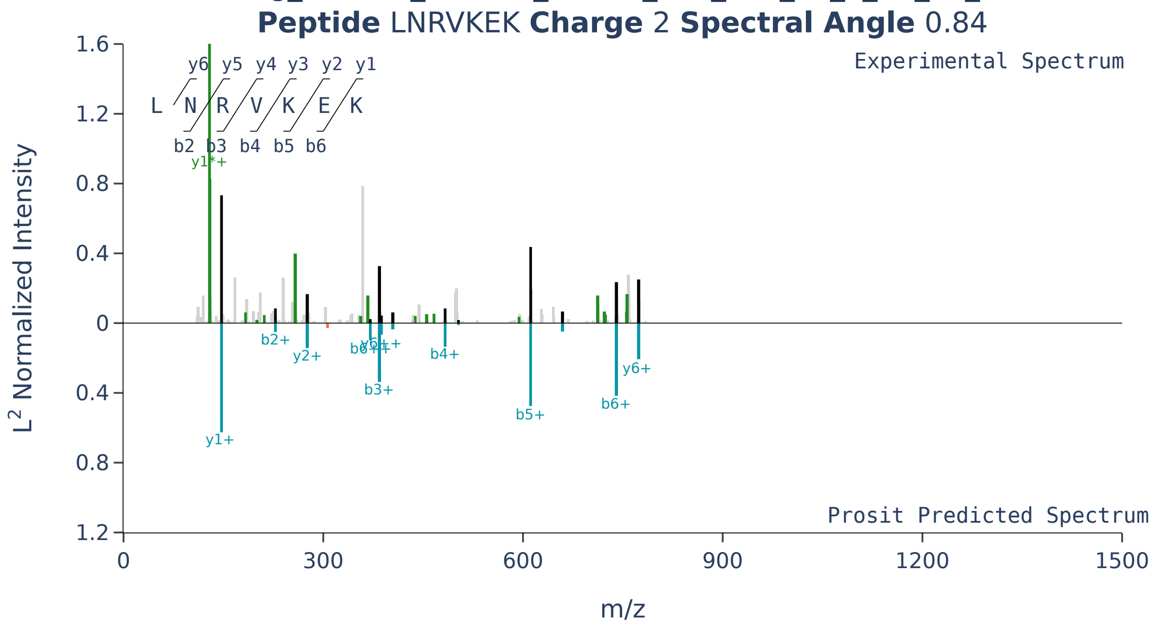

**Source** Ncheng_210623_230623_HFGoe_FFH_20S_25_1_A1_4h_R2 **Scan** 7768
**Peptide** DNVKDTLRE **Charge** 1 **Spectral Angle** 0.8

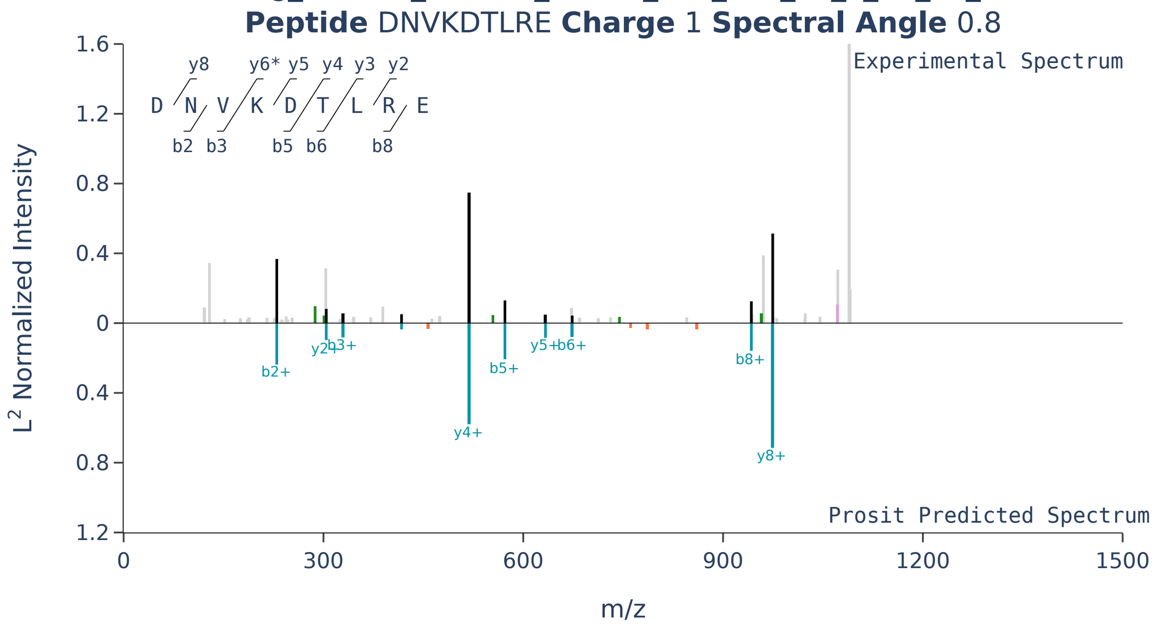

**Source** Ncheng_210623_230623_HFGoe_FFH_20S_25_1_A2_2h_R1 **Scan** 32651
**Peptide** ADVALPVVREFINRVKE **Charge** 2 **Spectral Angle** 0.88

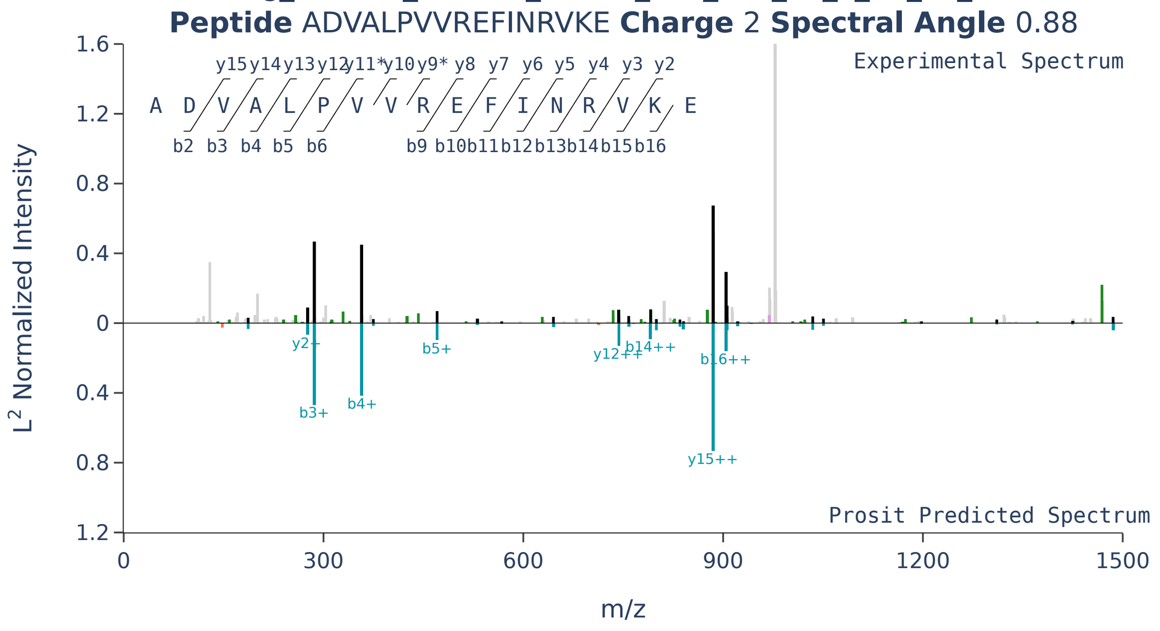

**Source** Ncheng_210623_230623_HFGoe_FFH_20S_25_1_A1_4h_R1 **Scan** 17607
**Peptide** SADVYRPAAIKQLE **Charge** 2 **Spectral Angle** 0.72

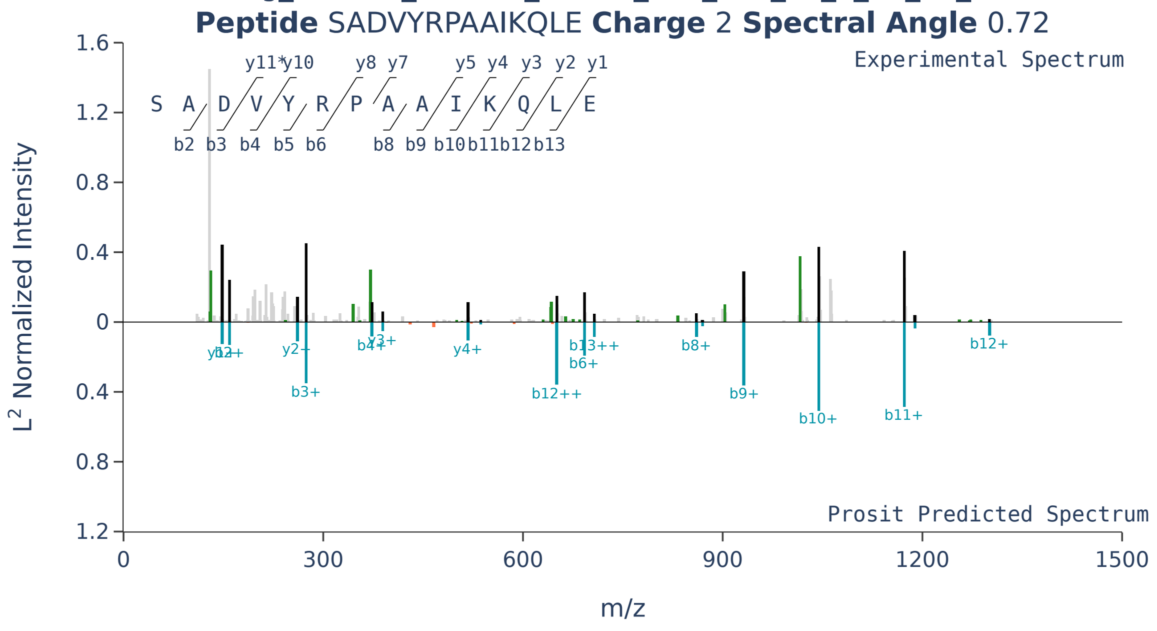

**Source** Ncheng_210623_230623_HFGoe_FFH_20S_25_1_A2_24h_R1 **Scan** 15051
**Peptide** ALSIRHI **Charge** 2 **Spectral Angle** 0.89

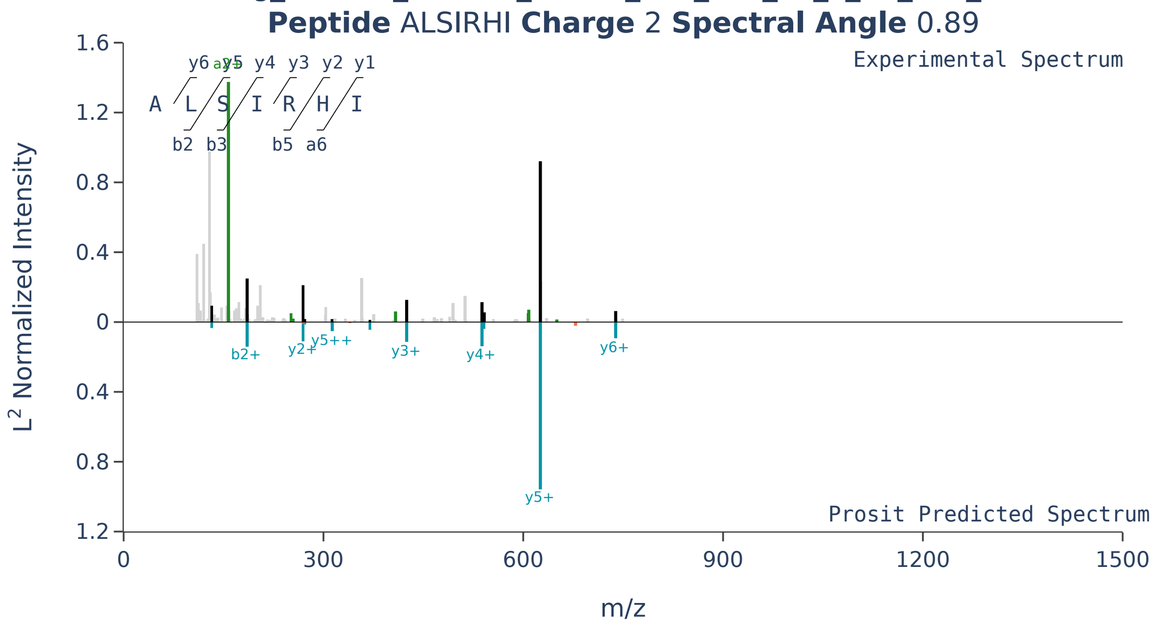

**Source** Ncheng_210623_230623_HFGoe_FFH_20S_25_1_A2_4h_R1 **Scan** 25120
**Peptide** SLMGKLPGMGQIPDNVKSQ **Charge** 2 **Spectral Angle** 0.84

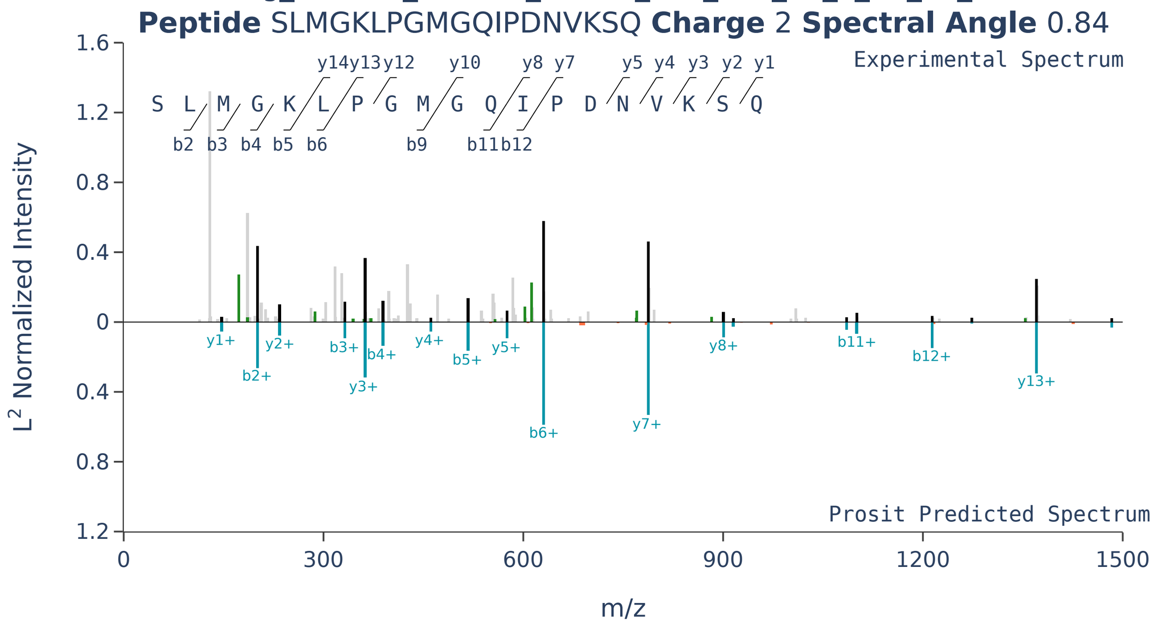

**Source** Ncheng_210623_230623_HFGoe_FFH_20S_25_1_A1_24h_R1 **Scan** 15201
**Peptide** KPIKFLGVGEKTE **Charge** 4 **Spectral Angle** 0.91

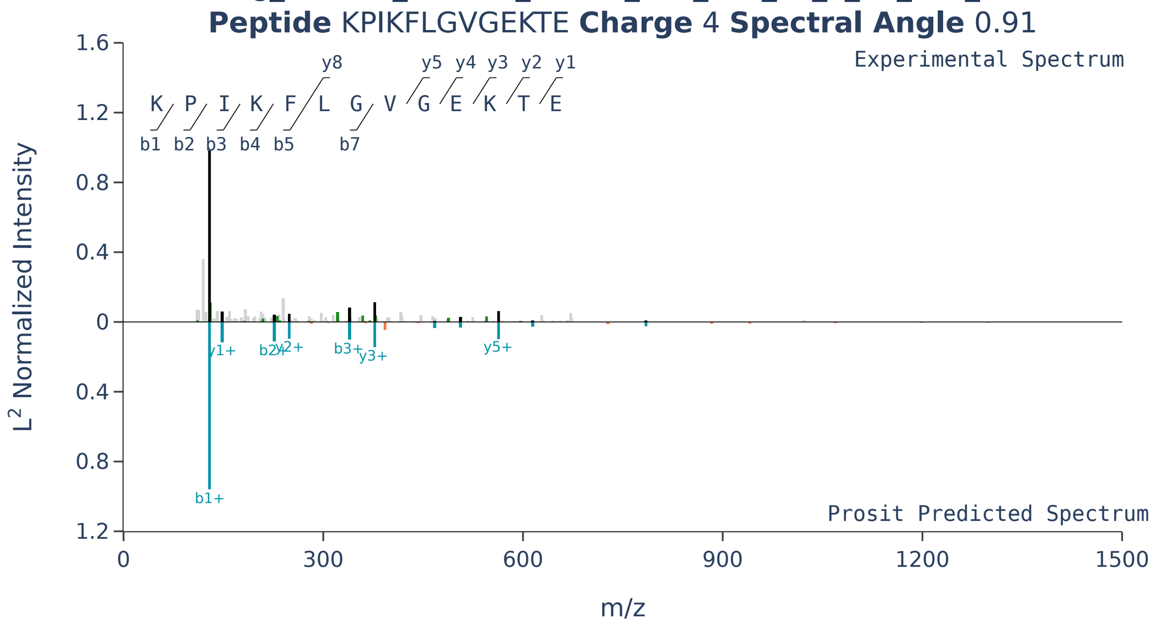

**Source** Ncheng_210623_230623_HFGoe_FFH_20S_25_1_A1_2h_R1 **Scan** 18717
**Peptide** RMEAIINSMTMKERA **Charge** 3 **Spectral Angle** 0.89

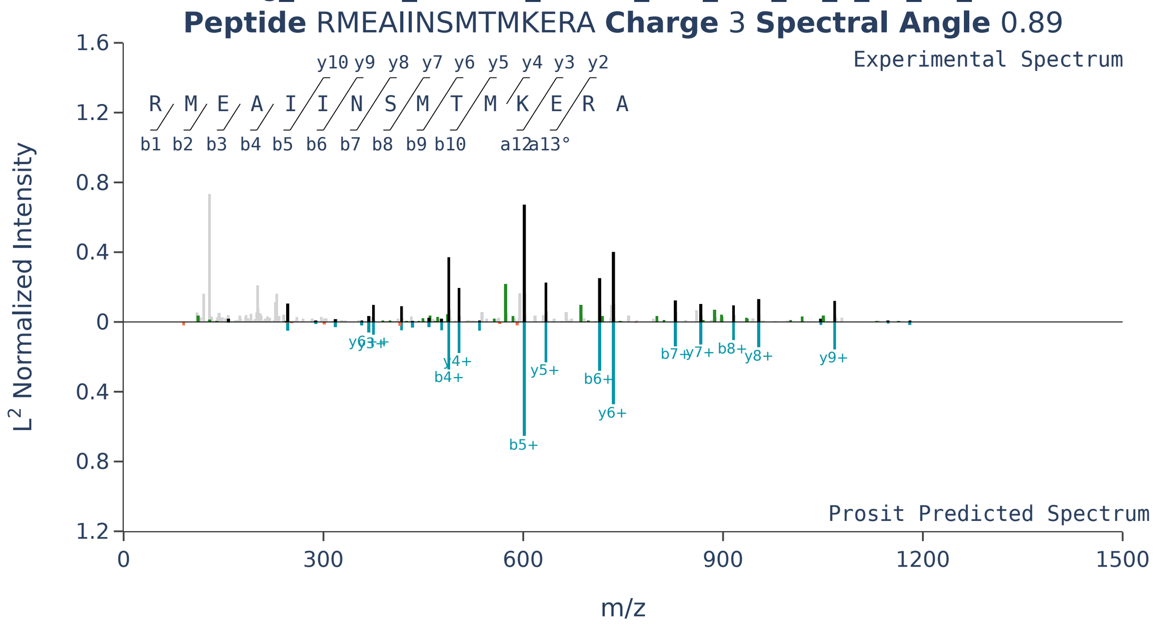

**Source** Ncheng_210623_230623_HFGoe_FFH_20S_25_1_A1_4h_R1 **Scan** 6191
**Peptide** ASKLKKGDGFD **Charge** 1 **Spectral Angle** 0.8

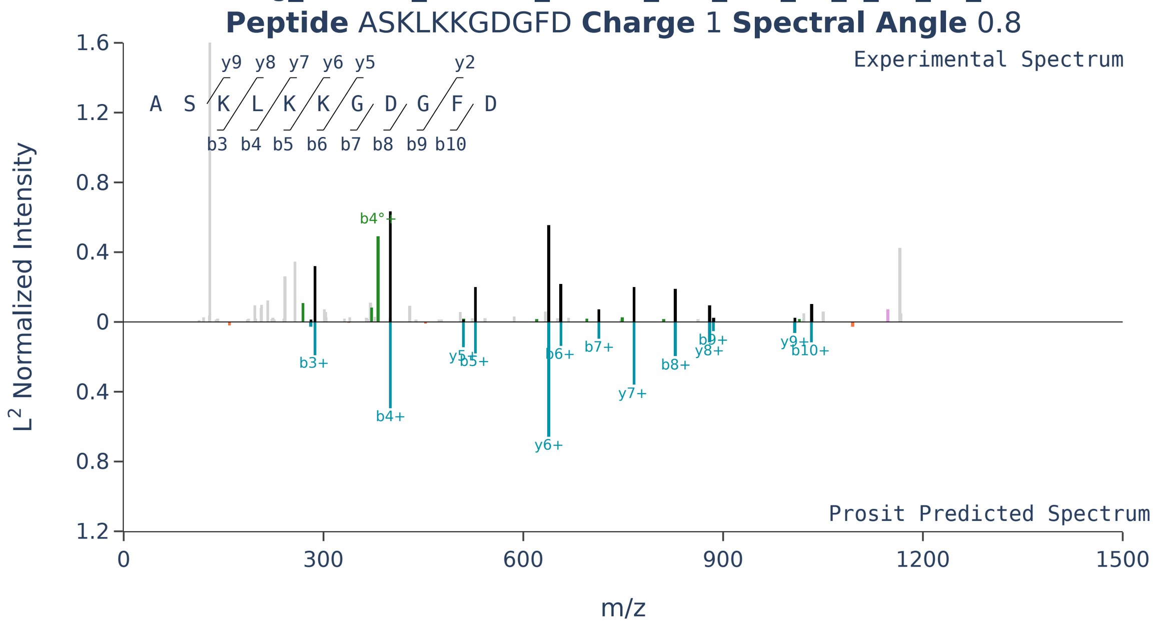

**Source** Ncheng_210623_230623_HFGoe_FFH_20S_25_1_A2_4h_R1 **Scan** 8271
**Peptide** DDKVLVR **Charge** 2 **Spectral Angle** 0.91

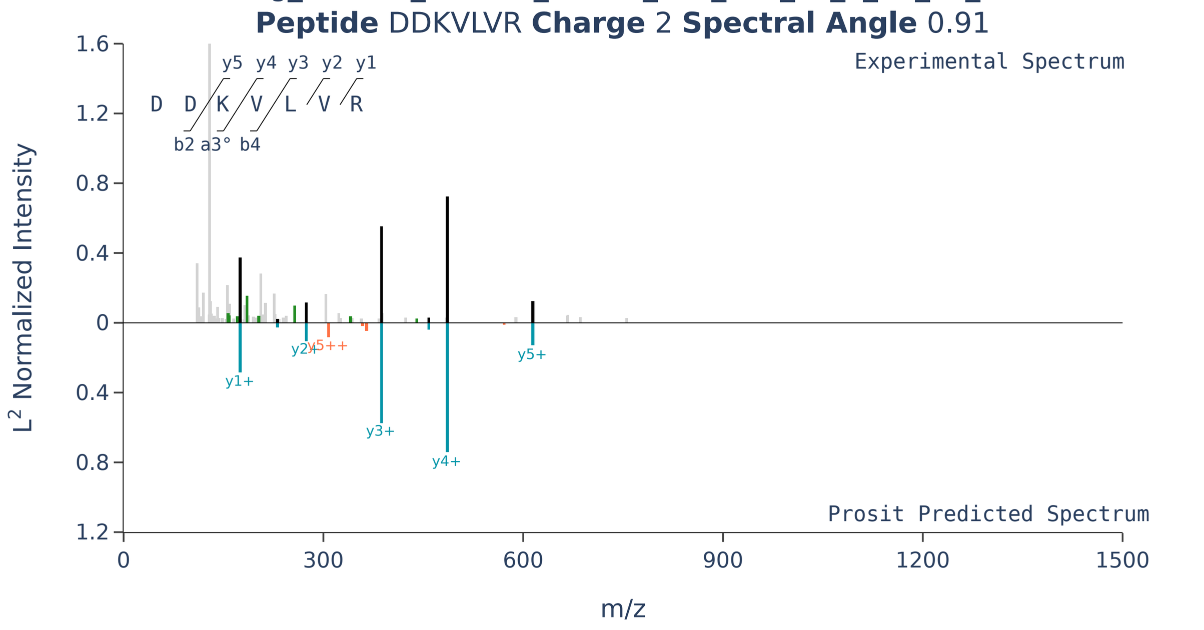


**Source** Ncheng_210623_230623_HFGoe_FFH_20S_25_1_A2_24h_R2 **Scan** 20014
**Peptide** KSLTPGQEFVKLVR **Charge** 2 **Spectral Angle** 0.84

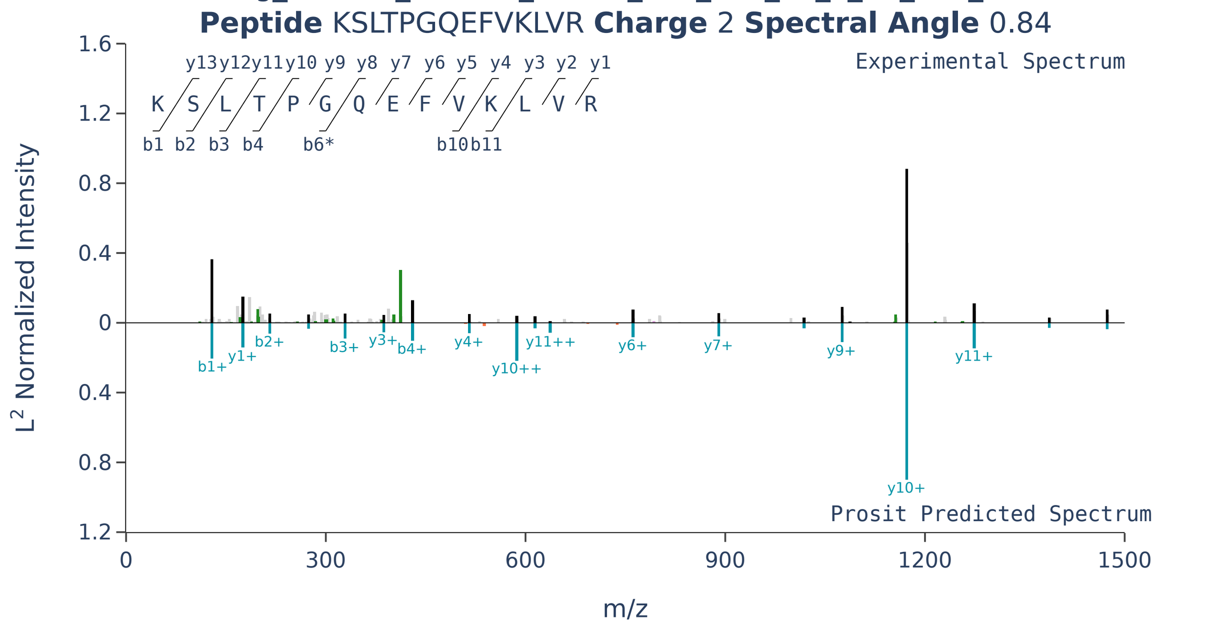


**Source** Ncheng_210623_230623_HFGoe_FFH_20S_25_1_A1_24h_R2 **Scan** 8745
**Peptide** AAGCGMQVQ **Charge** 1 **Spectral Angle** 0.91

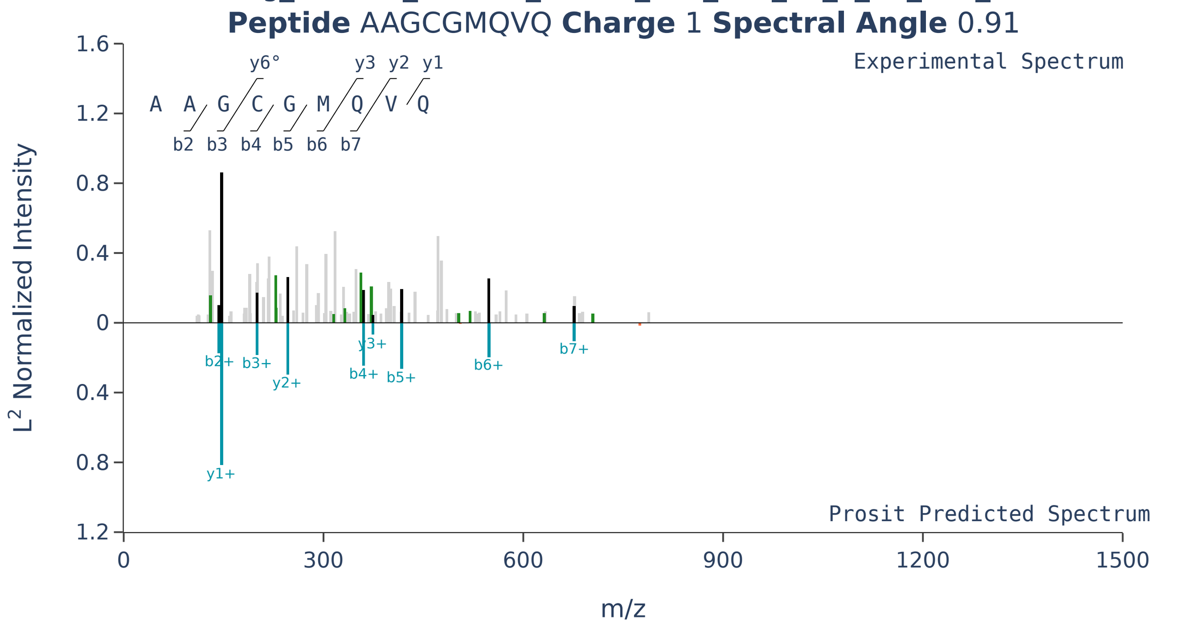


**Source** Ncheng_210623_230623_HFGoe_FFH_20S_25_1_A1_24h_R2 **Scan** 25932
**Peptide** FNEALPLT **Charge** 1 **Spectral Angle** 0.82

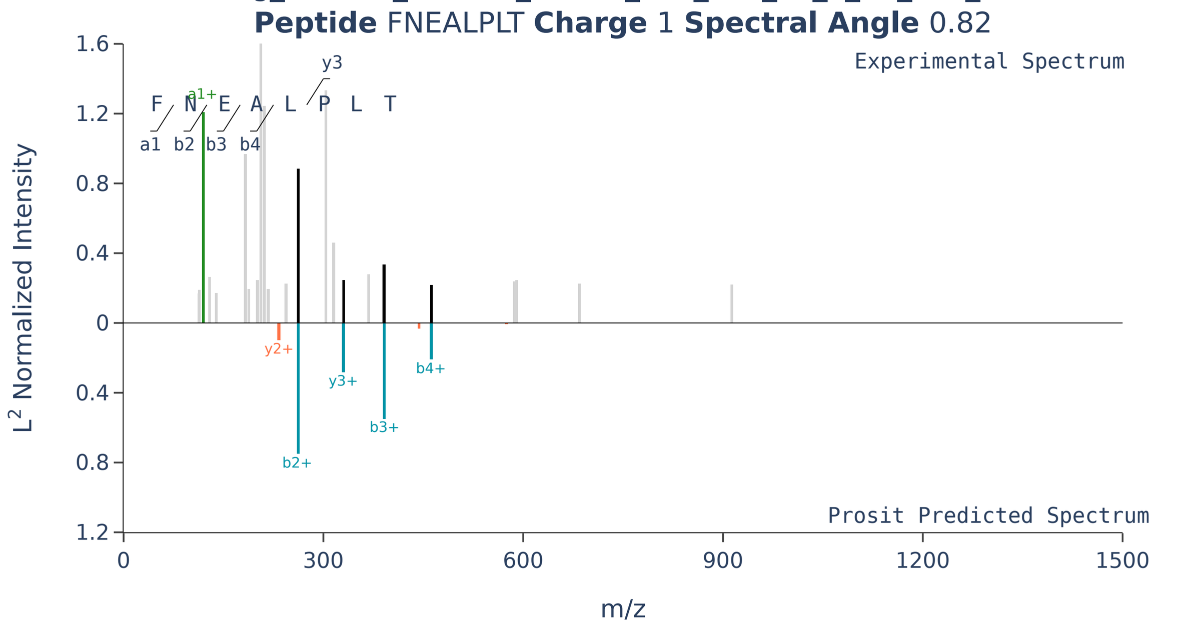


**Source** Ncheng_210623_230623_HFGoe_FFH_20S_25_1_A2_24h_R2 **Scan** 19521
**Peptide** SLTPGQEF **Charge** 1 **Spectral Angle** 0.91

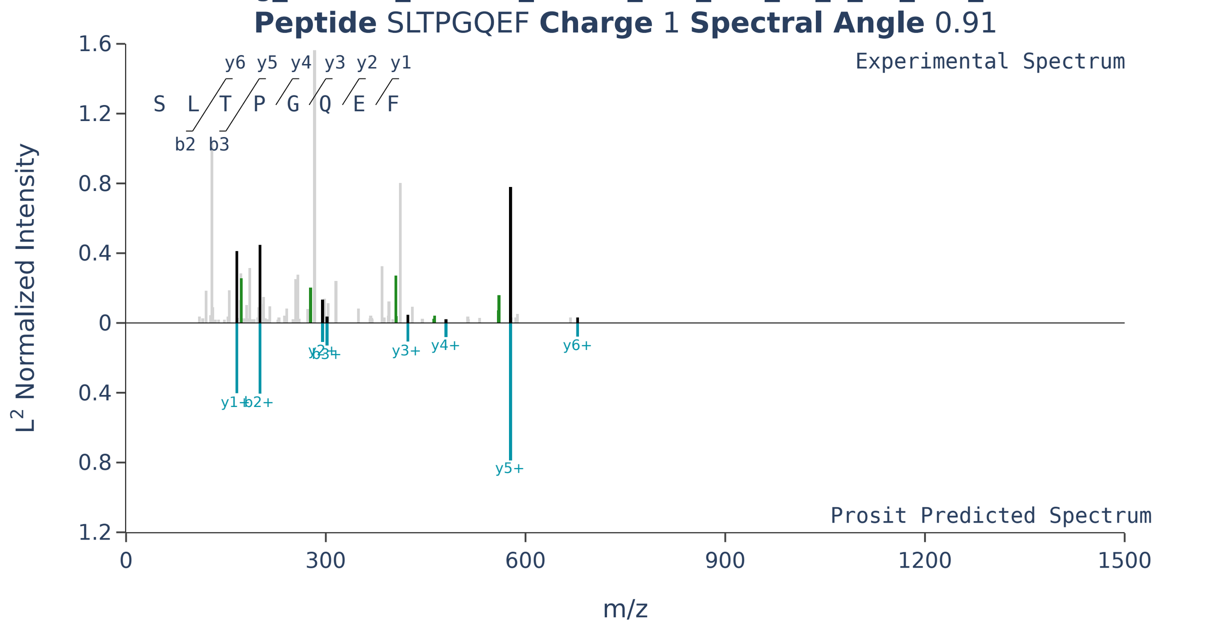


**Source** Ncheng_210623_230623_HFGoe_FFH_20S_25_1_A2_4h_R2 **Scan** 17748
**Peptide** GKLPGMGQIPDNVKSQ **Charge** 2 **Spectral Angle** 0.83

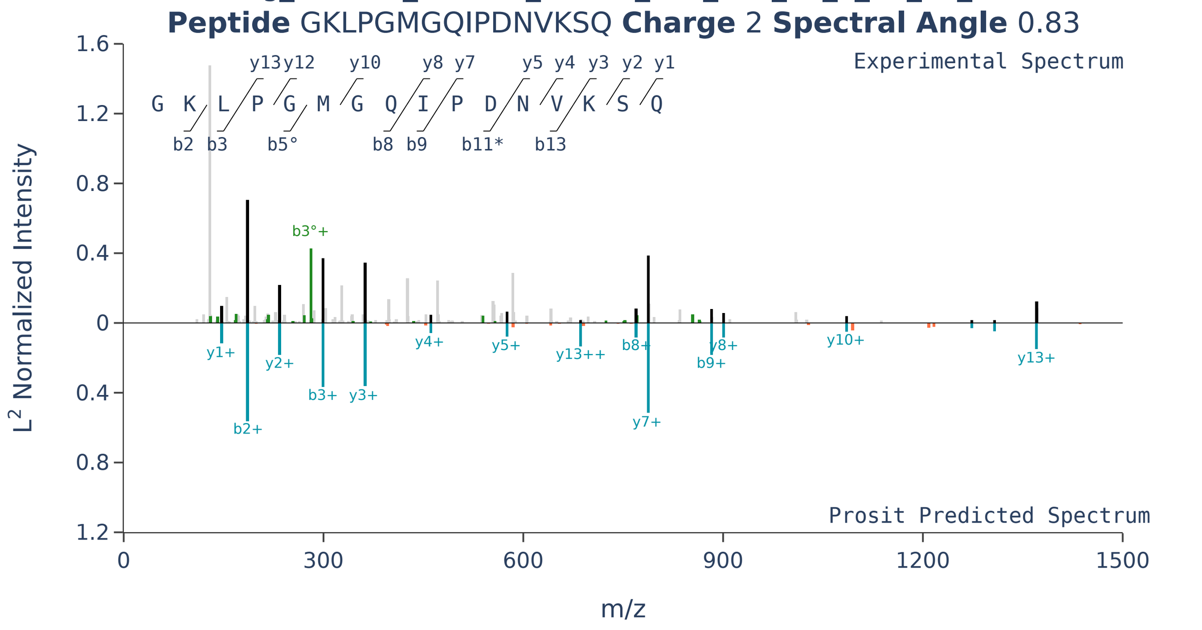


**Source** Ncheng_210623_230623_HFGoe_FFH_20S_25_1_A2_24h_R2 **Scan** 19898
**Peptide** QVQDVNRLL **Charge** 2 **Spectral Angle** 0.84

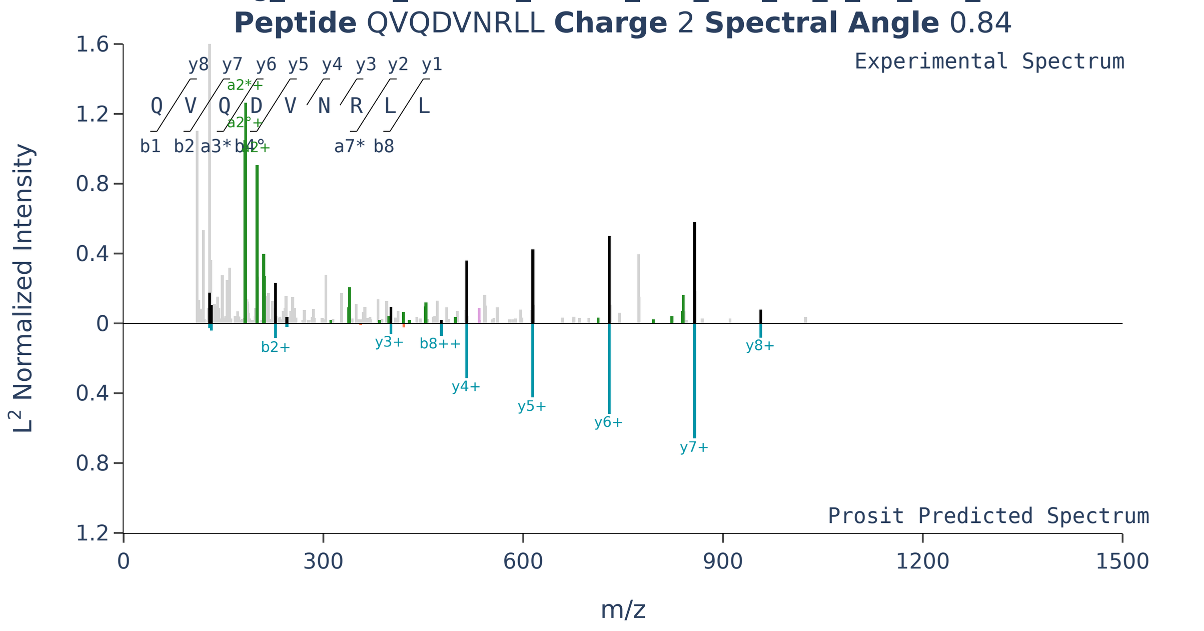


**Source** Ncheng_210623_230623_HFGoe_FFH_20S_25_1_A1_2h_R1 **Scan** 19626
**Peptide** AVGHEVNKSLTPGQEFVKLVR **Charge** 3 **Spectral Angle** 0.86

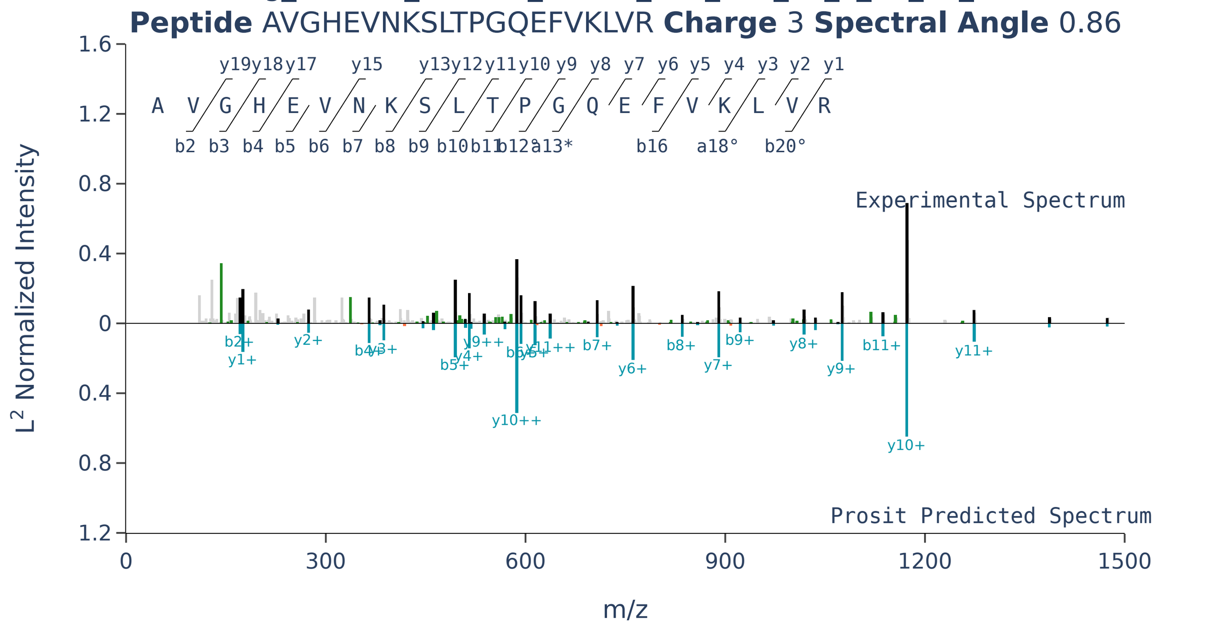


**Source** Ncheng_210623_230623_HFGoe_FFH_20S_25_1_A1_1h_R2 **Scan** 23426
**Peptide** NKSLTPGQEFVKIV **Charge** 2 **Spectral Angle** 0.84

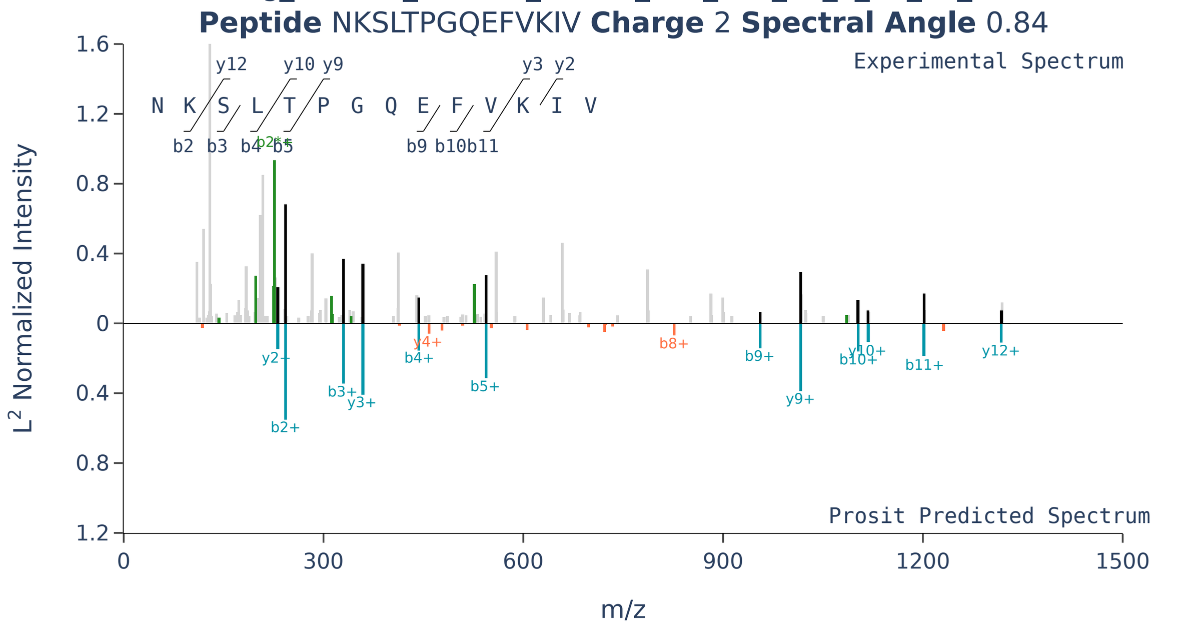


**Source** Ncheng_210623_230623_HFGoe_FFH_20S_25_1_A1_4h_R2 **Scan** 20323
**Peptide** GVGEKTEALEPFHPDRL **Charge** 3 **Spectral Angle** 0.86

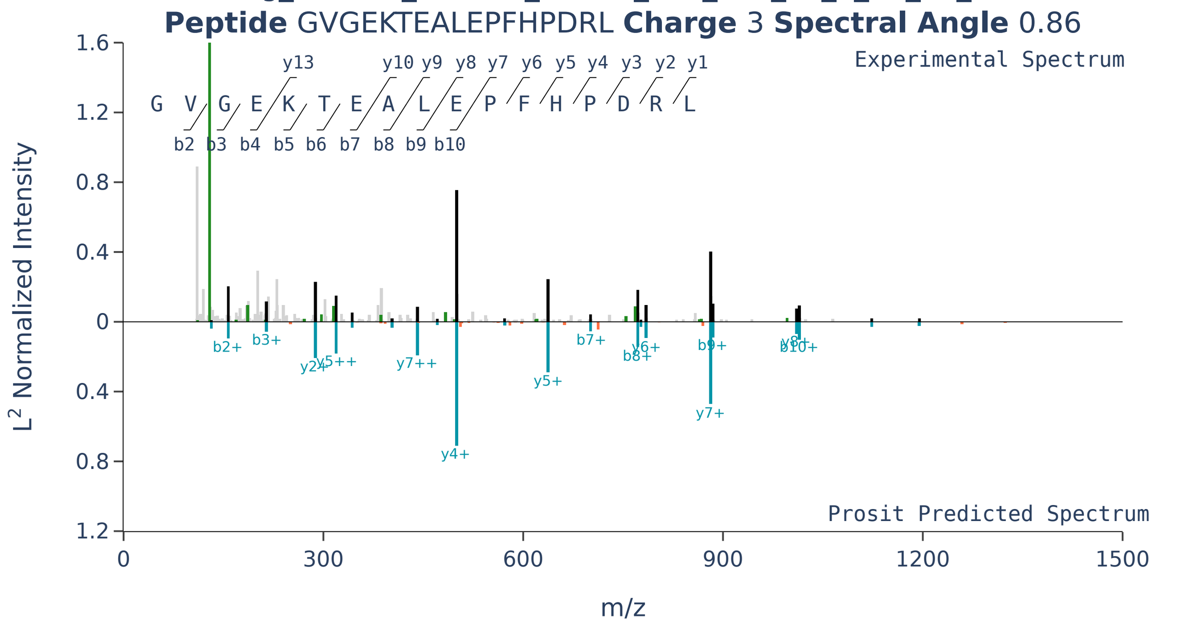


**Source** Ncheng_210623_230623_HFGoe_FFH_20S_25_1_A1_24h_R2 **Scan** 15285
**Peptide** GDARGGAALSIRHL **Charge** 3 **Spectral Angle** 0.88

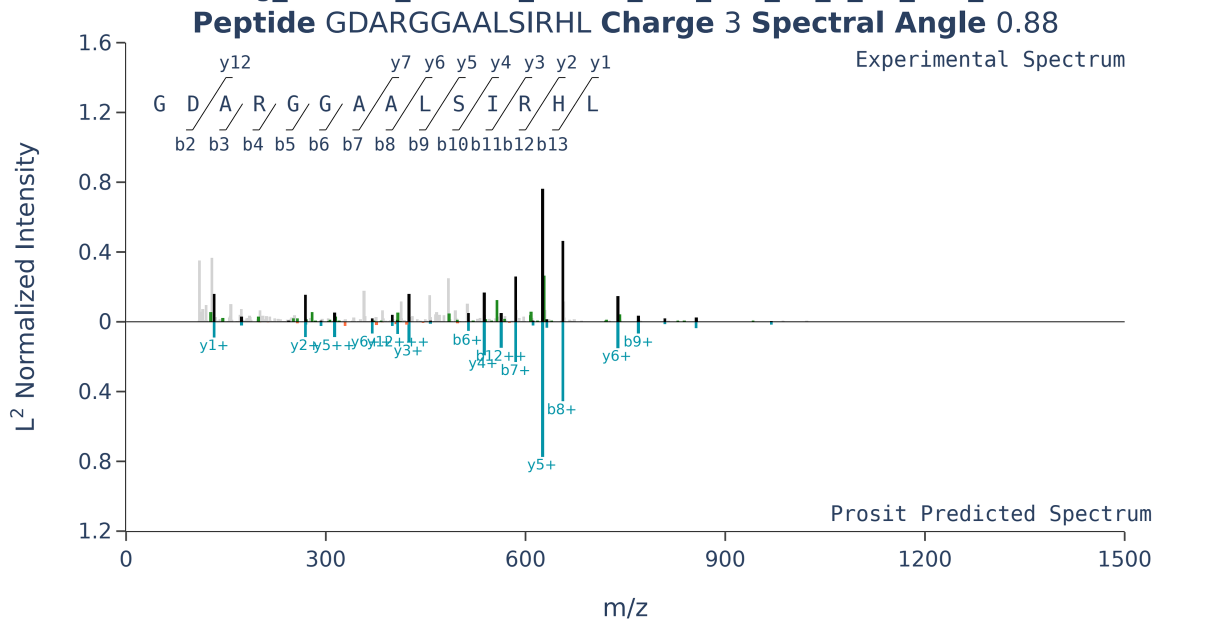


**Source** Ncheng_210623_230623_HFGoe_FFH_20S_25_1_A2_24h_R2 **Scan** 9886
**Peptide** GVGEKTEAL **Charge** 1 **Spectral Angle** 0.86

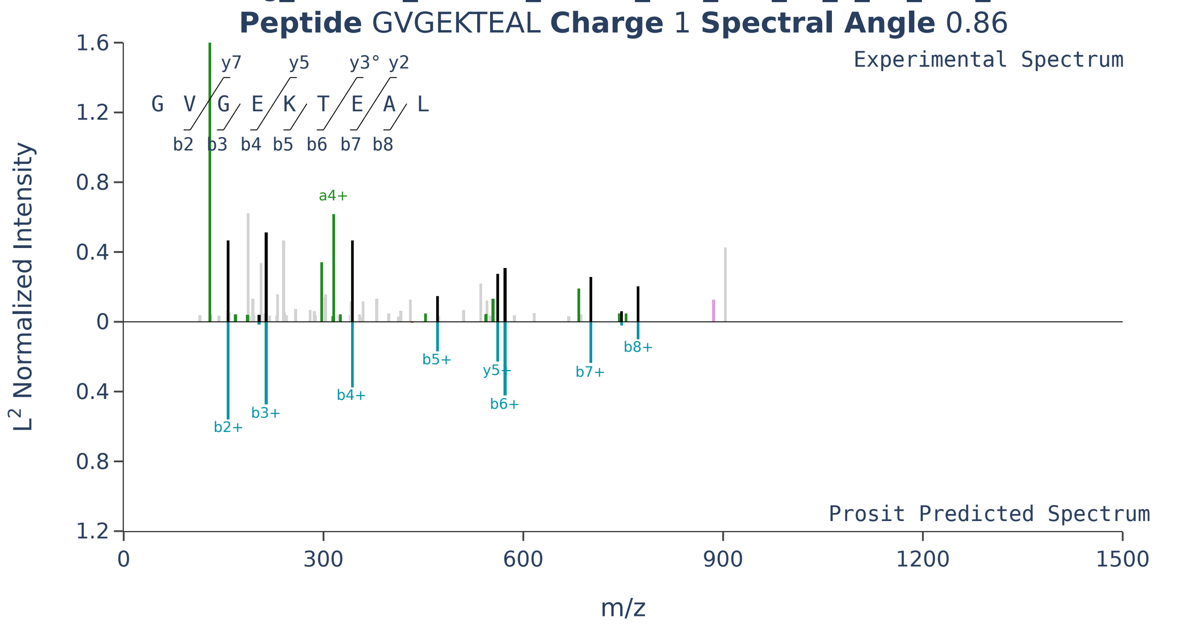


**Source** Ncheng_210623_230623_HFGoe_FFH_20S_25_1_A1_4h_R2 **Scan** 6932
**Peptide** RLHVDEA **Charge** 2 **Spectral Angle** 0.86

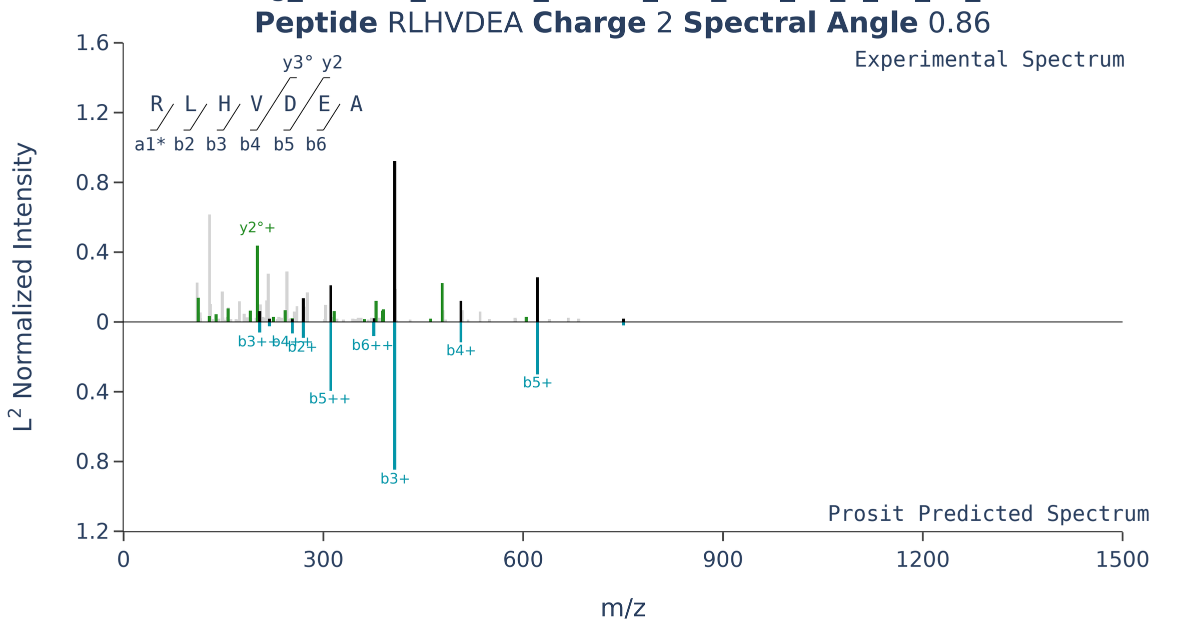


**Source** Ncheng_210623_230623_HFGoe_FFH_20S_25_1_A2_2h_R2 **Scan** 35375
**Peptide** EENQTLNLAAQPPAVVLM **Charge** 2 **Spectral Angle** 0.85

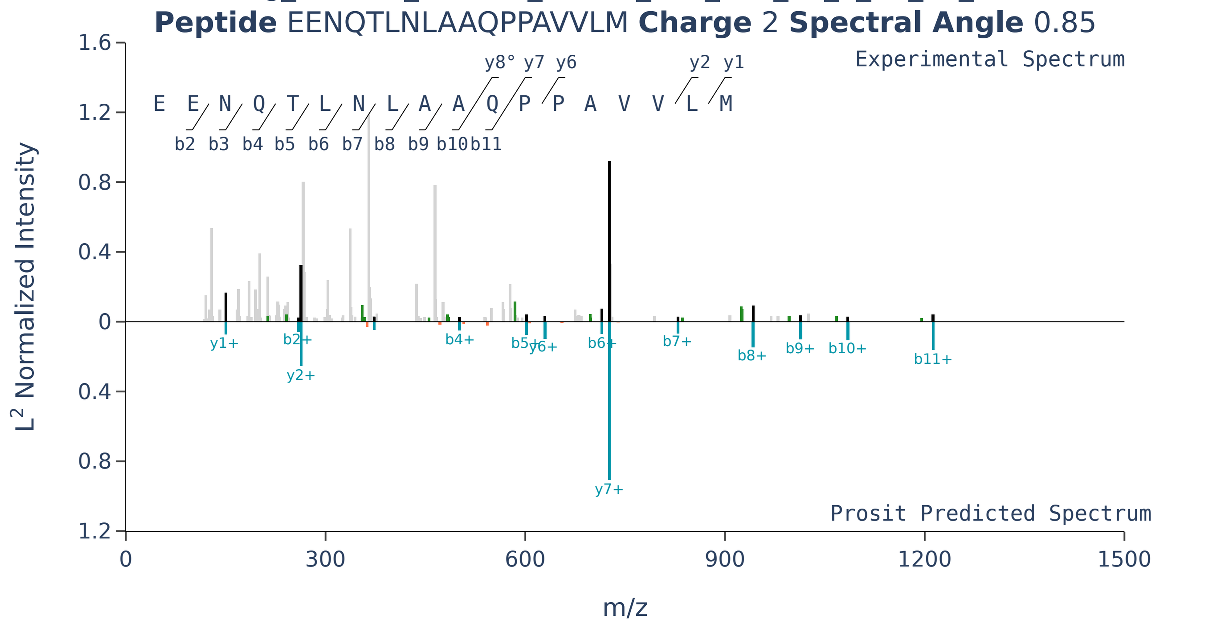


**Source** Ncheng_210623_230623_HFGoe_FFH_20S_25_1_A1_24h_R1 **Scan** 13675
**Peptide** LEDIESKV **Charge** 2 **Spectral Angle** 0.83

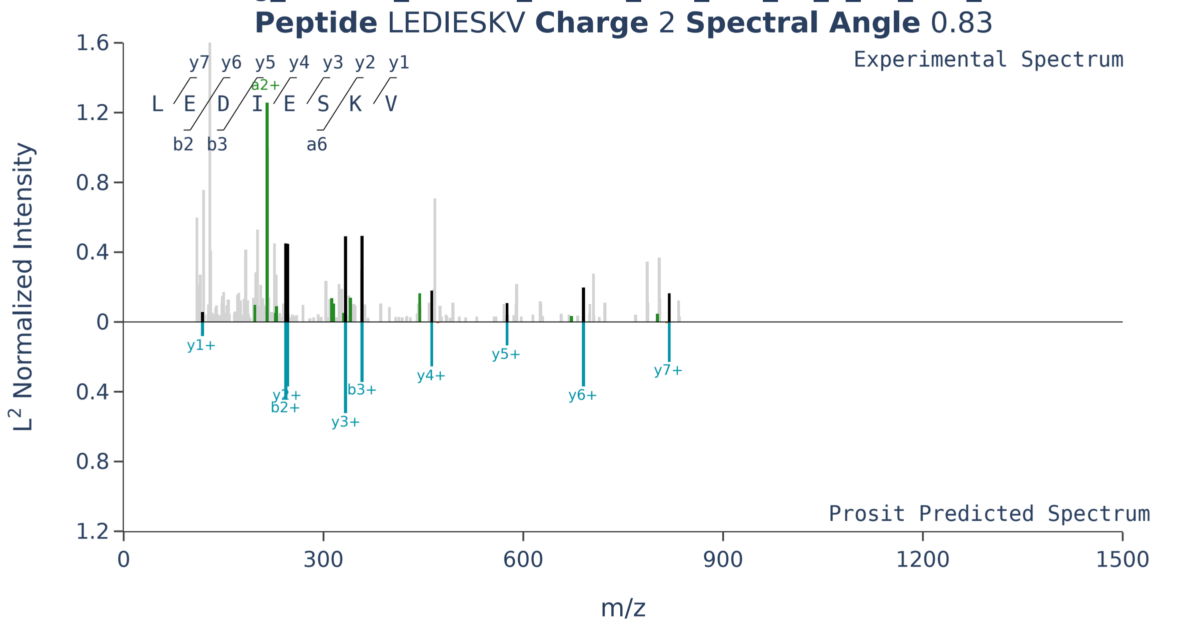

**Source** Ncheng_210623_230623_HFGoe_FFH_20S_25_1_A2_24h_R1 **Scan** 15858
**Peptide** MGQIPDNVKSQMD **Charge** 2 **Spectral Angle** 0.87

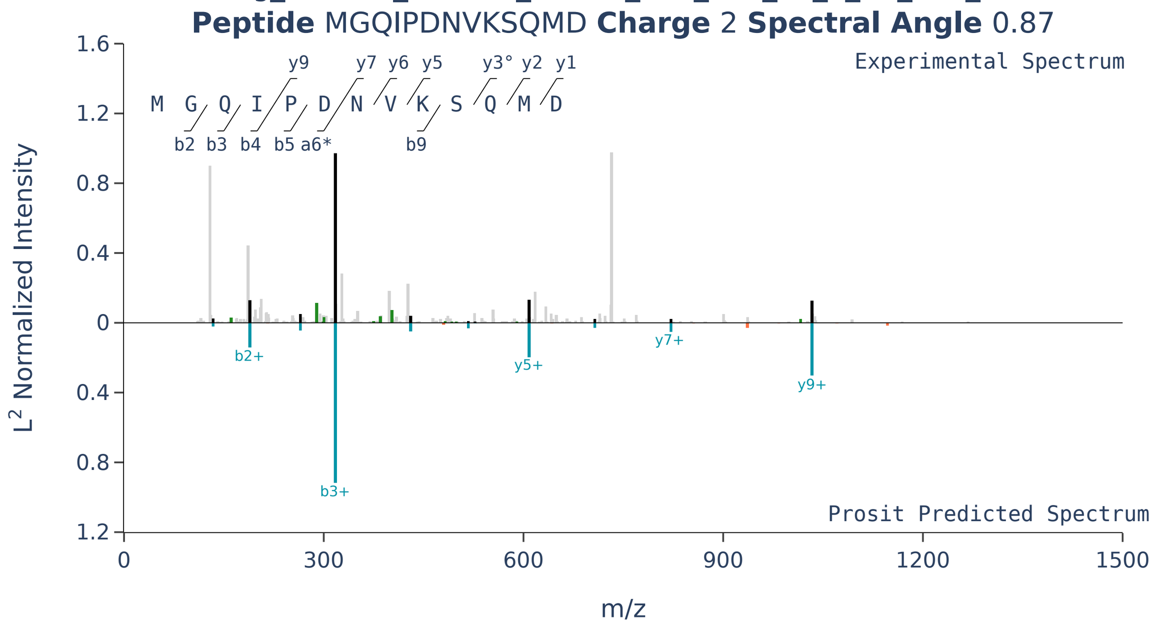


**Source** Ncheng_210623_230623_HFGoe_FFH_20S_25_1_A1_24h_R2 **Scan** 10996
**Peptide** GDARGGAALSLR **Charge** 3 **Spectral Angle** 0.9

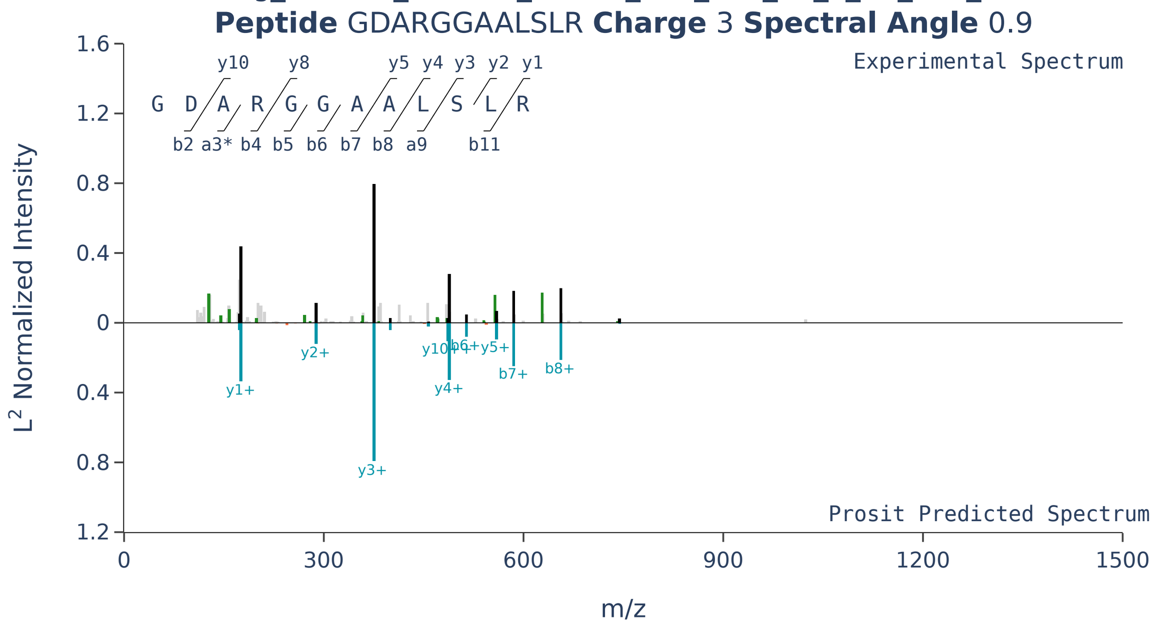


**Source** Ncheng_210623_230623_HFGoe_FFH_20S_25_1_A2_24h_R1 **Scan** 9348
**Peptide** TGQDAANTAKAFN **Charge** 1 **Spectral Angle** 0.82

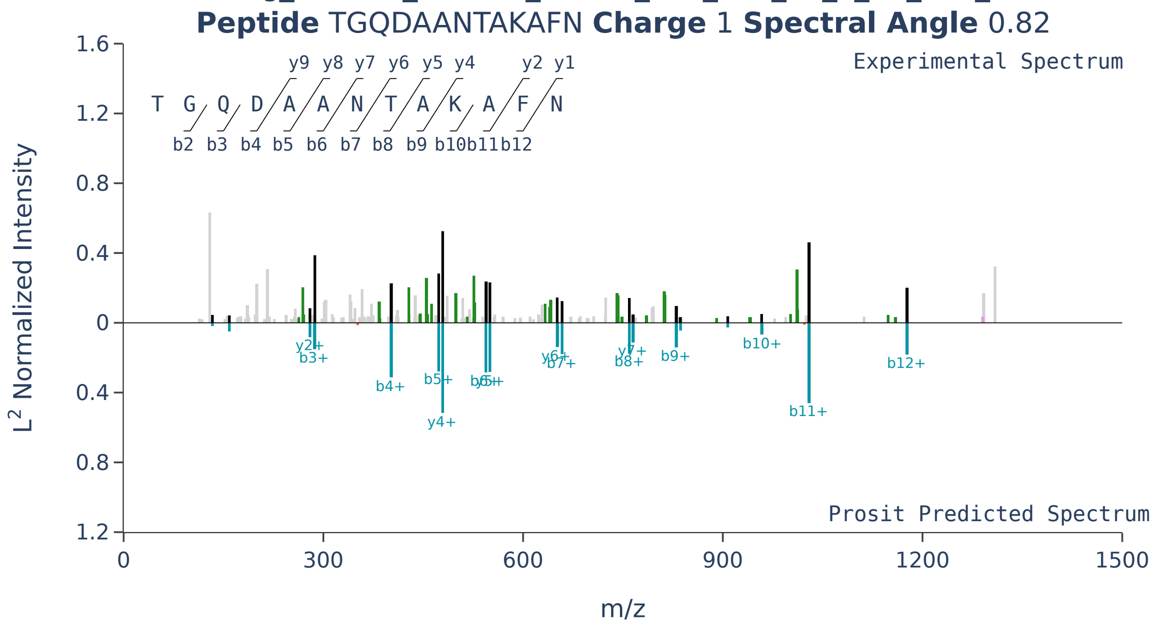


**Source** Ncheng_210623_230623_HFGoe_FFH_20S_25_1_A2_4h_R2 **Scan** 18299
**Peptide** KGMMPPGFPGR **Charge** 3 **Spectral Angle** 0.87

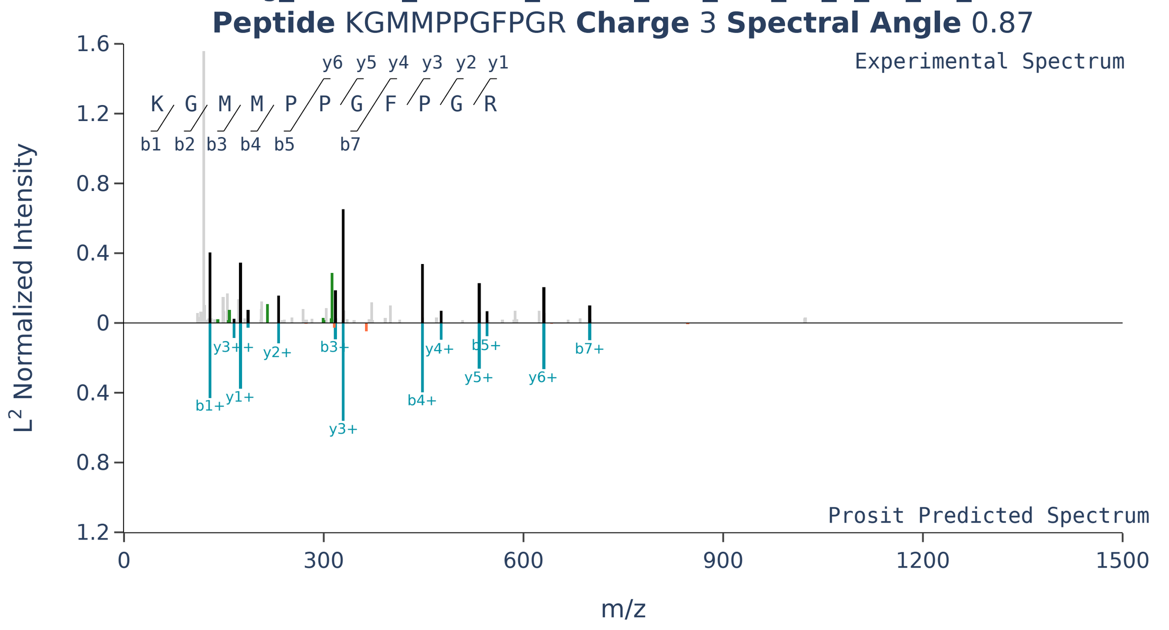


**Source** Ncheng_210623_230623_HFGoe_FFH_20S_25_1_A1_24h_R2 **Scan** 19658
**Peptide** PLKFLGVGEKTE **Charge** 3 **Spectral Angle** 0.78

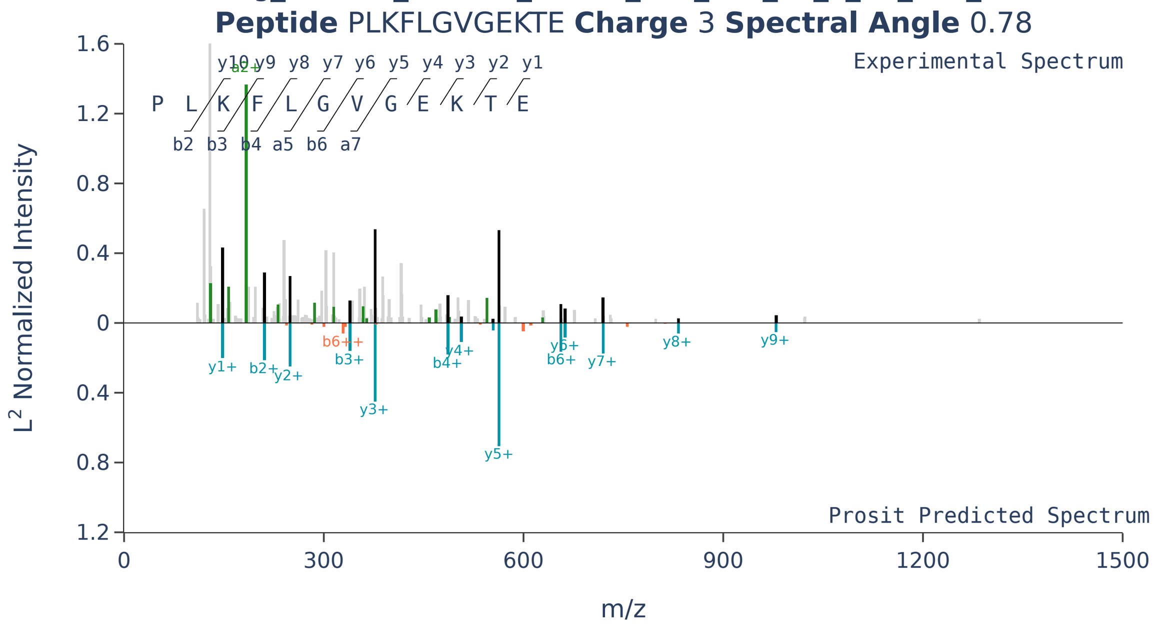


**Source** Ncheng_210623_230623_HFGoe_FFH_20S_25_1_A1_24h_R1 **Scan** 17919
**Peptide** GCGMQVQDVNRL **Charge** 2 **Spectral Angle** 0.78

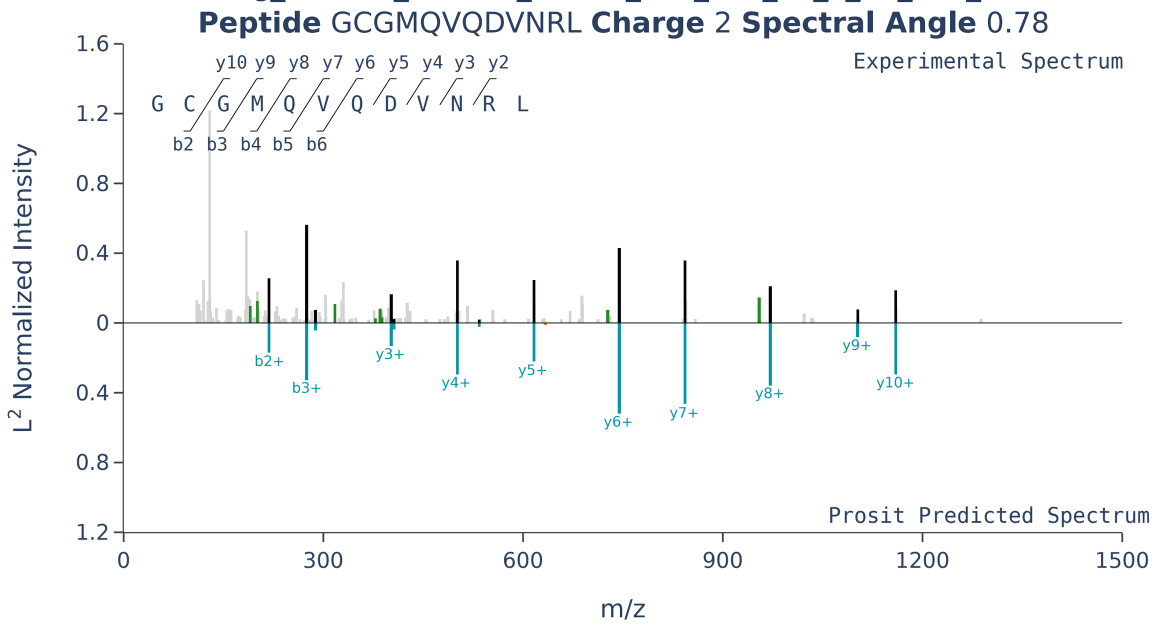


**Source** Ncheng_210623_230623_HFGoe_FFH_20S_25_1_A1_2h_R1 **Scan** 11898
**Peptide** LVNAALKEA **Charge** 2 **Spectral Angle** 0.82

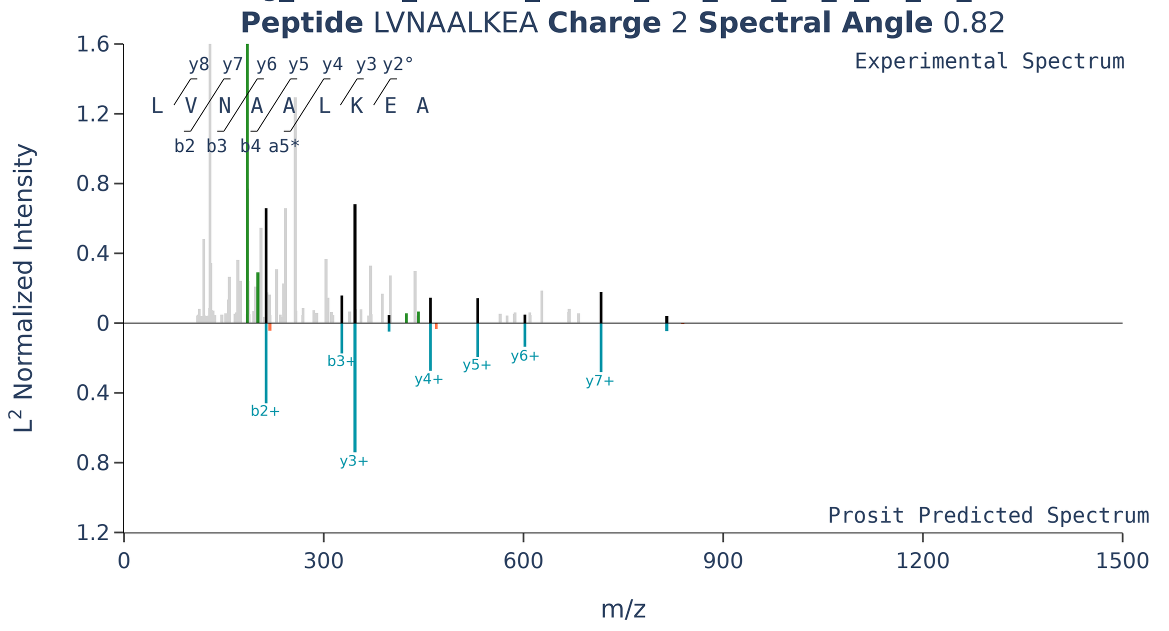


**Source** Ncheng_210623_230623_HFGoe_FFH_20S_25_1_A1_24h_R1 **Scan** 15598
**Peptide** DNVKDTLREV **Charge** 3 **Spectral Angle** 0.86

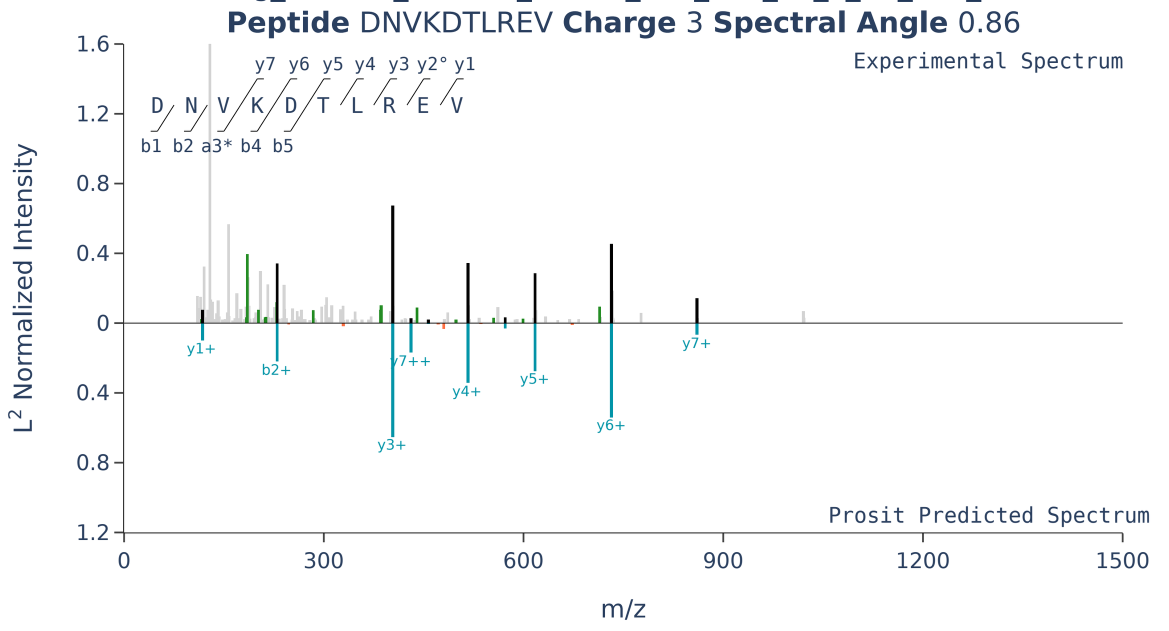


**Source** Ncheng_210623_230623_HFGoe_FFH_20S_25_1_A2_2h_R1 **Scan** 20617
**Peptide** DEAMMDEIKQVHA **Charge** 2 **Spectral Angle** 0.92

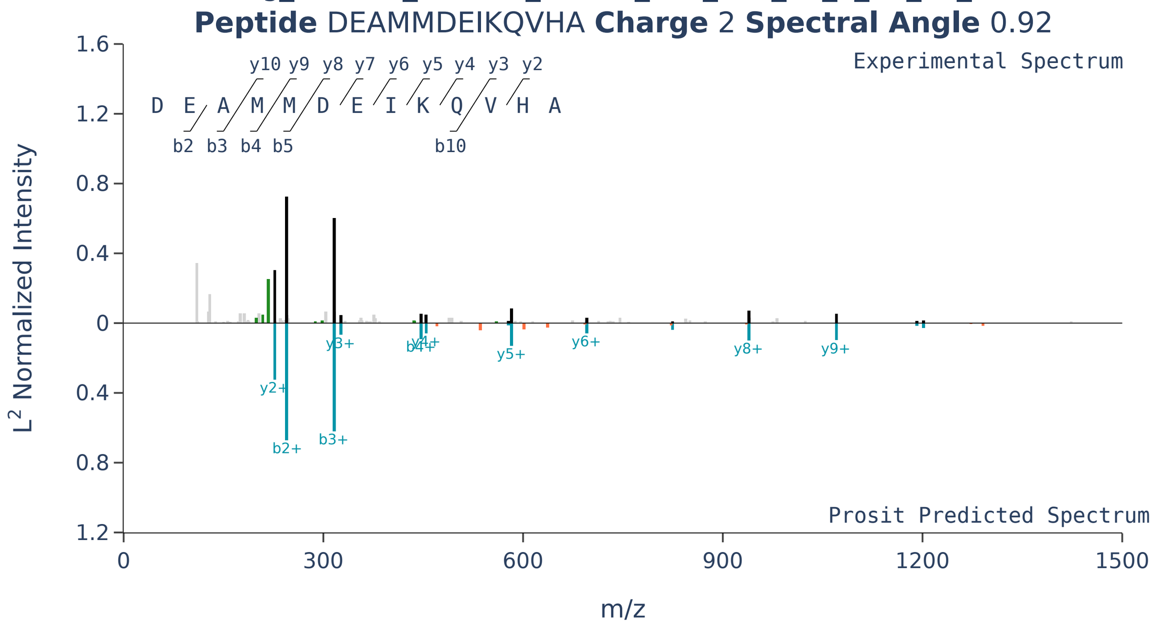


**Source** Ncheng_210623_230623_HFGoe_FFH_20S_25_1_A1_2h_R2 **Scan** 28065
**Peptide** DKVLVRMEAII **Charge** 2 **Spectral Angle** 0.82

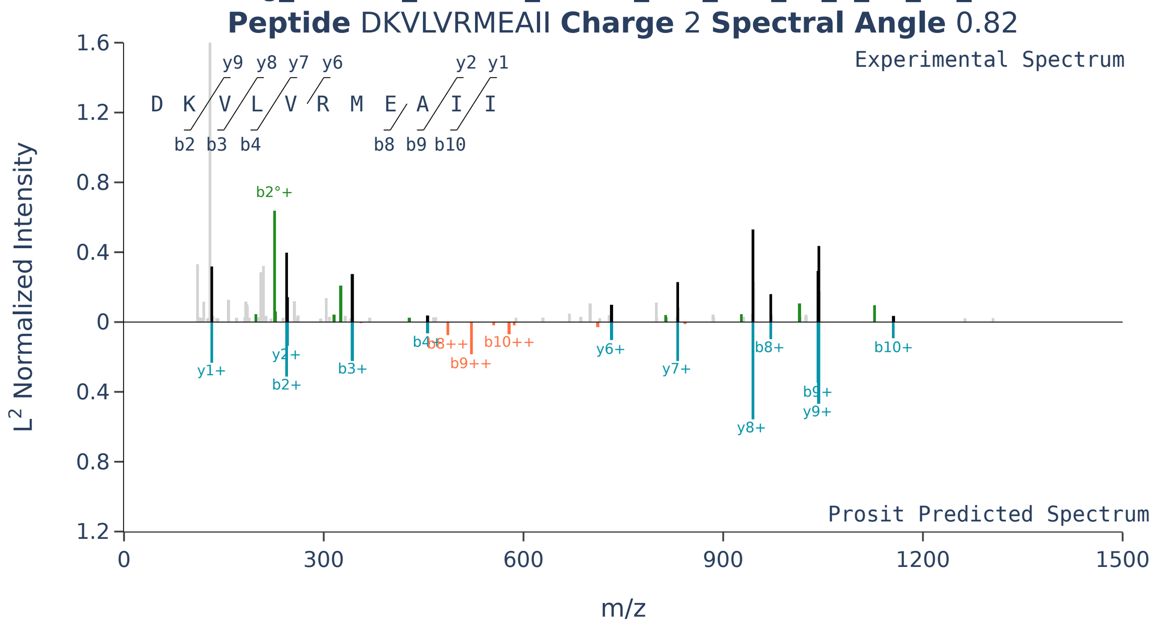


**Source** Ncheng_210623_230623_HFGoe_FFH_20S_25_1_A1_1h_R1 **Scan** 19813
**Peptide** LVAAMGEENQTL **Charge** 2 **Spectral Angle** 0.91

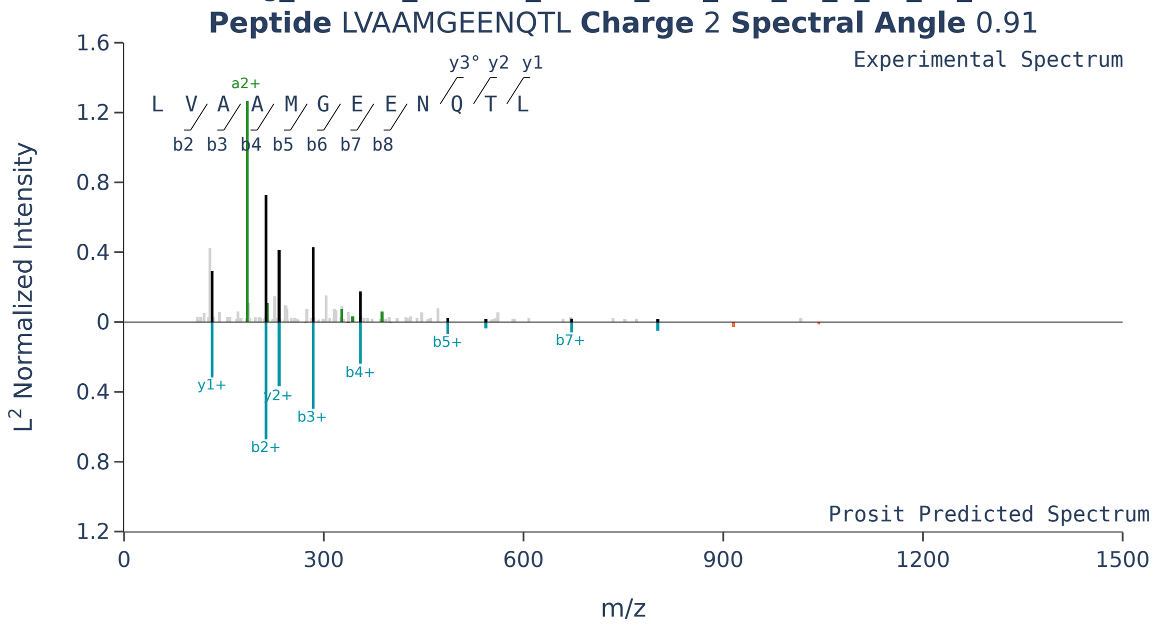


**Source** Ncheng_210623_230623_HFGoe_FFH_20S_25_1_A2_24h_R1 **Scan** 6372
**Peptide** PDNVKSQMDD **Charge** 1 **Spectral Angle** 0.91

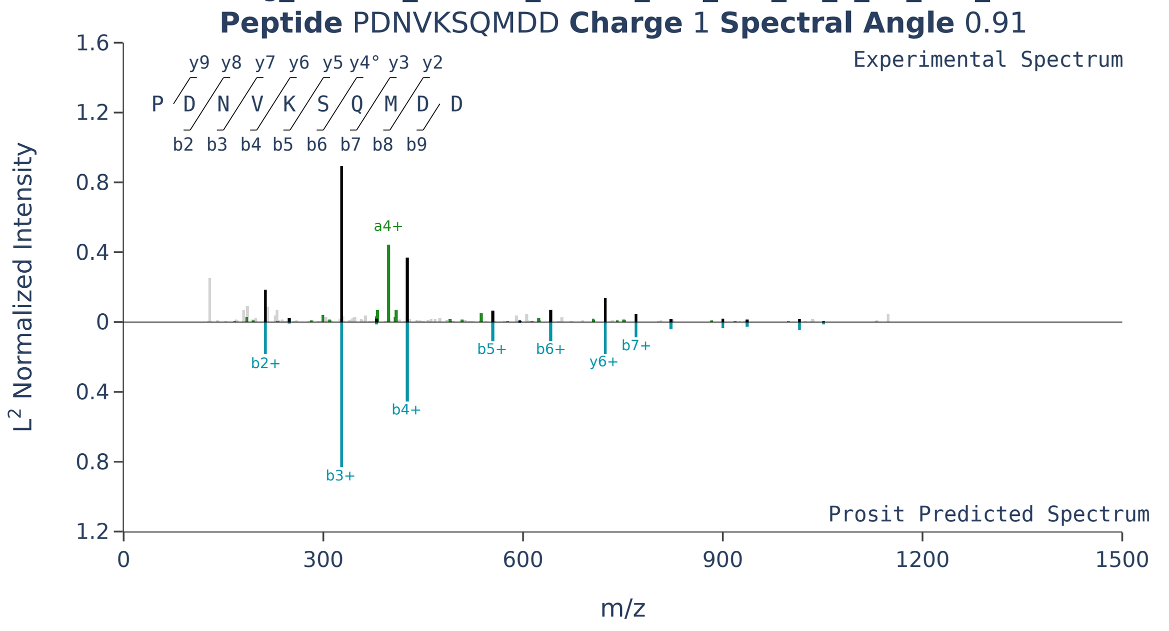


**Source** Ncheng_210623_230623_HFGoe_FFH_20S_25_1_A1_2h_R2 **Scan** 22765
**Peptide** LPGMGQIPDNVKSQMD **Charge** 2 **Spectral Angle** 0.79

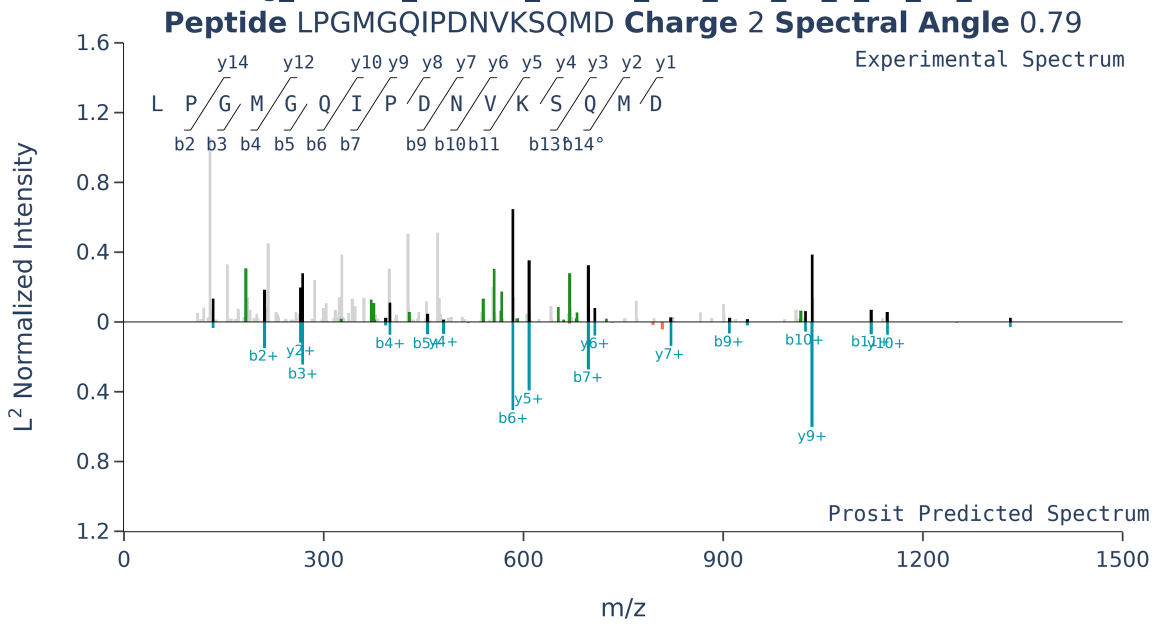


**Source** Ncheng_210623_230623_HFGoe_FFH_20S_25_1_A1_1h_R1 **Scan** 16691
**Peptide** ELVAAMGE **Charge** 1 **Spectral Angle** 0.86

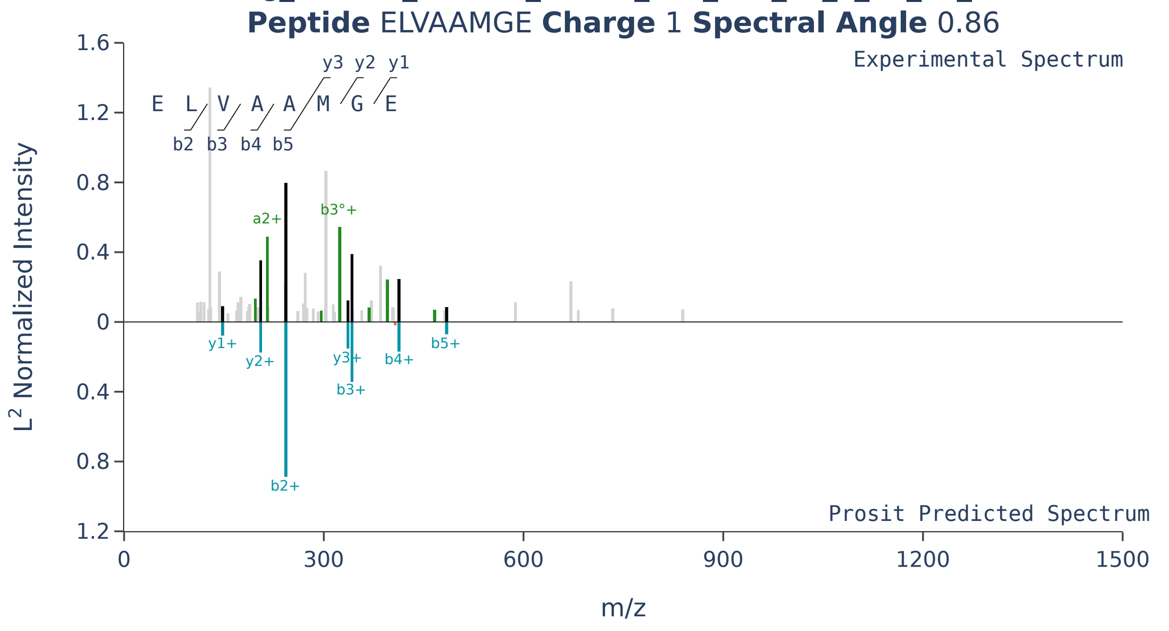


**Source** Ncheng_210623_230623_HFGoe_FFH_20S_25_1_A1_4h_R1 **Scan** 8652
**Peptide** SKVDRAQAE **Charge** 2 **Spectral Angle** 0.82

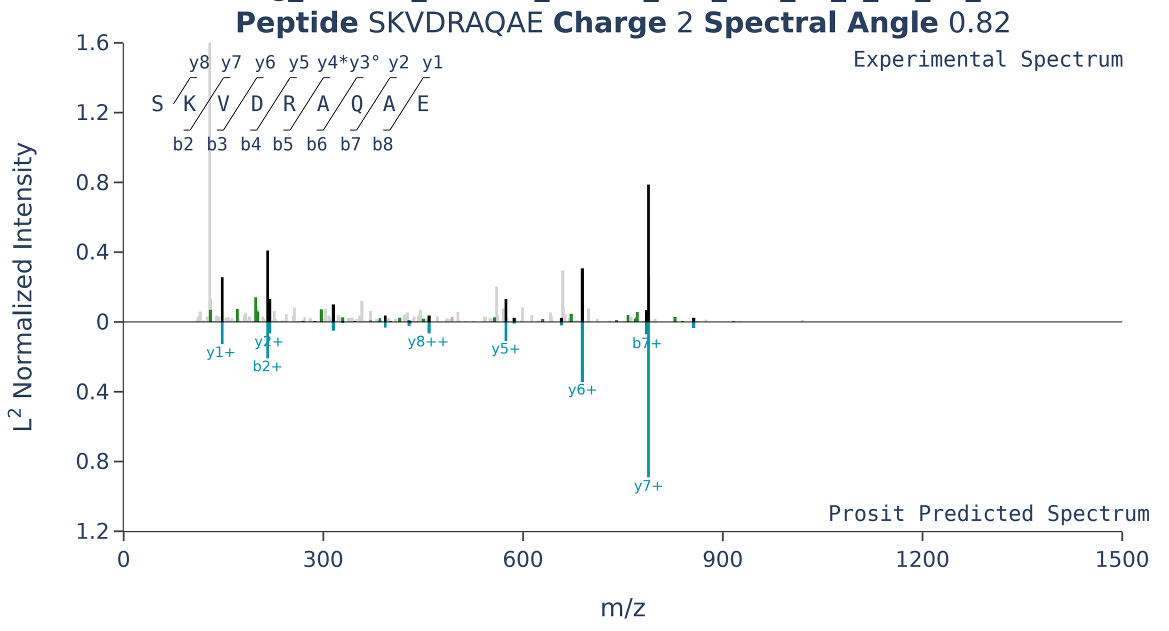

**Source** Ncheng_210623_230623_HFGoe_FFH_20S_25_1_A1_4h_R1 **Scan** 25225
**Peptide** PGQEFVKIVRNEL **Charge** 2 **Spectral Angle** 0.79

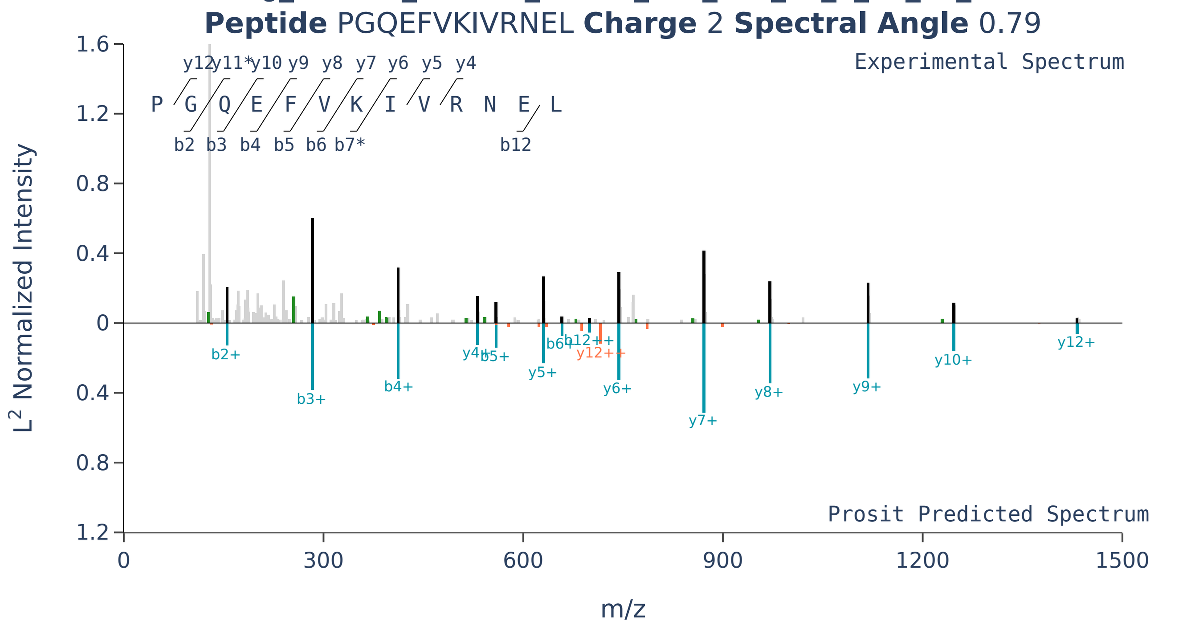


**Source** Ncheng_210623_230623_HFGoe_FFH_20S_25_1_A1_4h_R2 **Scan** 24628
**Peptide** NKSLTPGQEFVKLV **Charge** 2 **Spectral Angle** 0.85

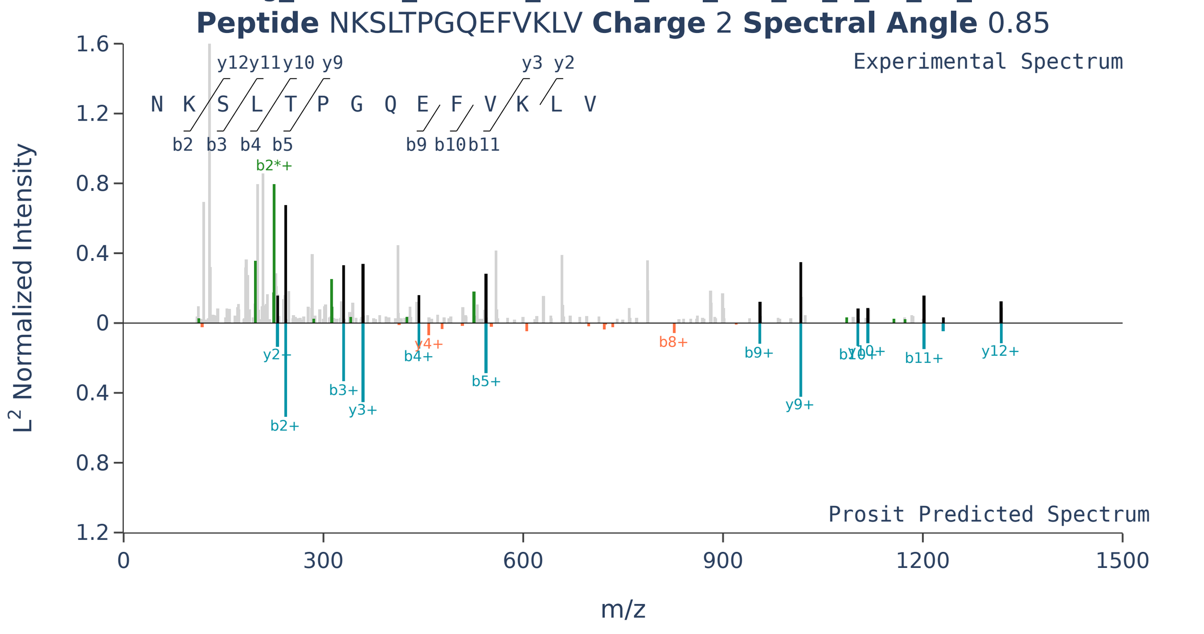


**Source** Ncheng_210623_230623_HFGoe_FFH_20S_25_1_A2_4h_R2 **Scan** 21653
**Peptide** GQIPDNVKSQMDDKVLVRME **Charge** 3 **Spectral Angle** 0.82

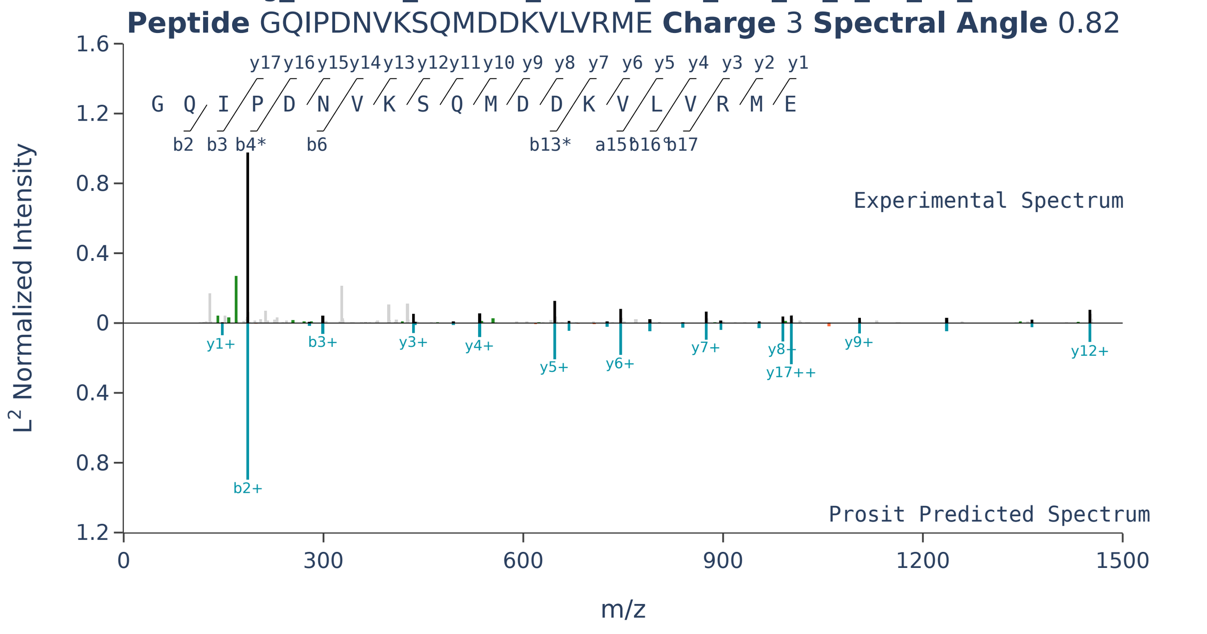


**Source** Ncheng_210623_230623_HFGoe_FFH_20S_25_1_A2_24h_R1 **Scan** 18708
**Peptide** KSLTPGQEFV **Charge** 1 **Spectral Angle** 0.89

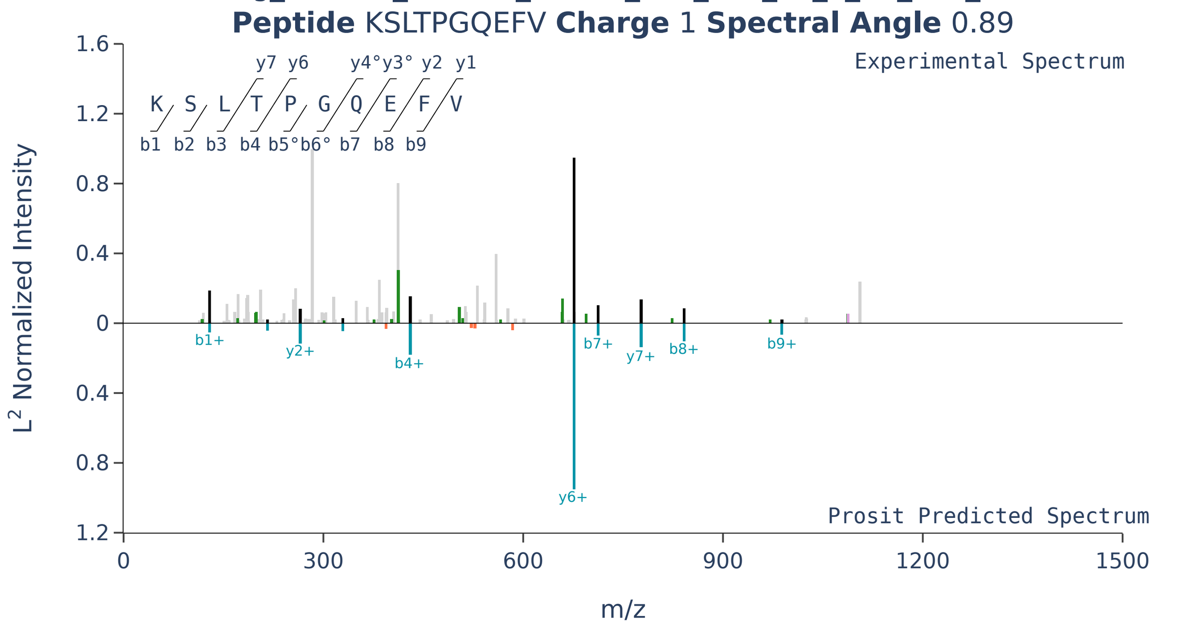


**Source** Ncheng_210623_230623_HFGoe_FFH_20S_25_1_A2_4h_R2 **Scan** 21063
**Peptide** GHEVNKSLTPGQEFVKIVRNE **Charge** 2 **Spectral Angle** 0.82

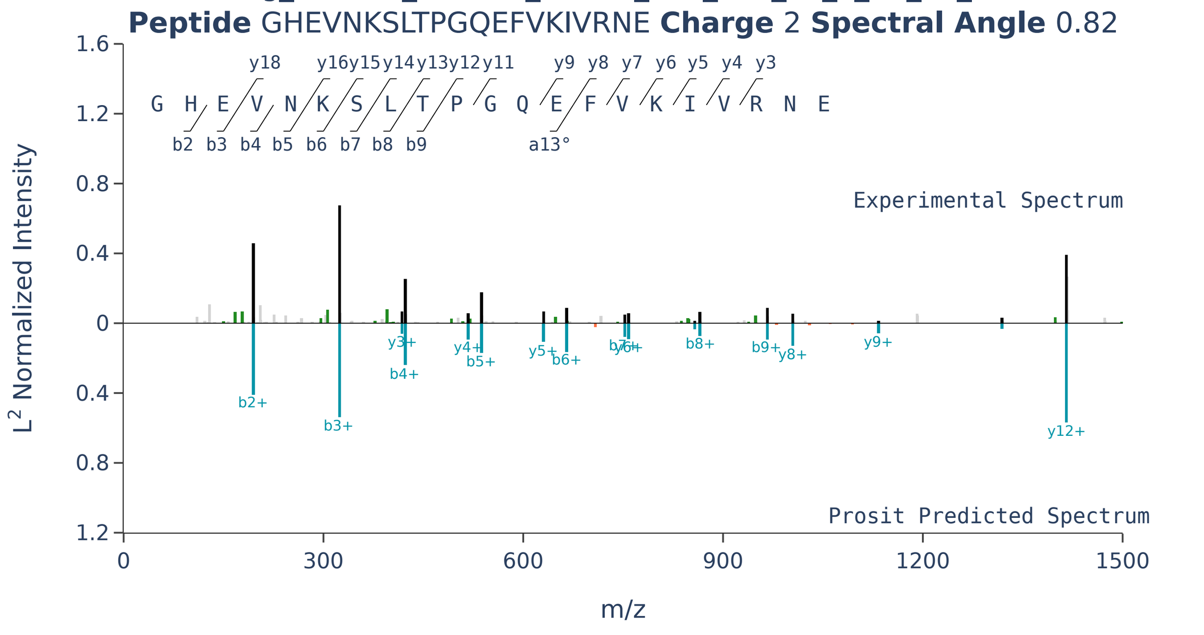


**Source** Ncheng_210623_230623_HFGoe_FFH_20S_25_1_A2_4h_R2 **Scan** 28460
**Peptide** VALPVVREF **Charge** 2 **Spectral Angle** 0.7

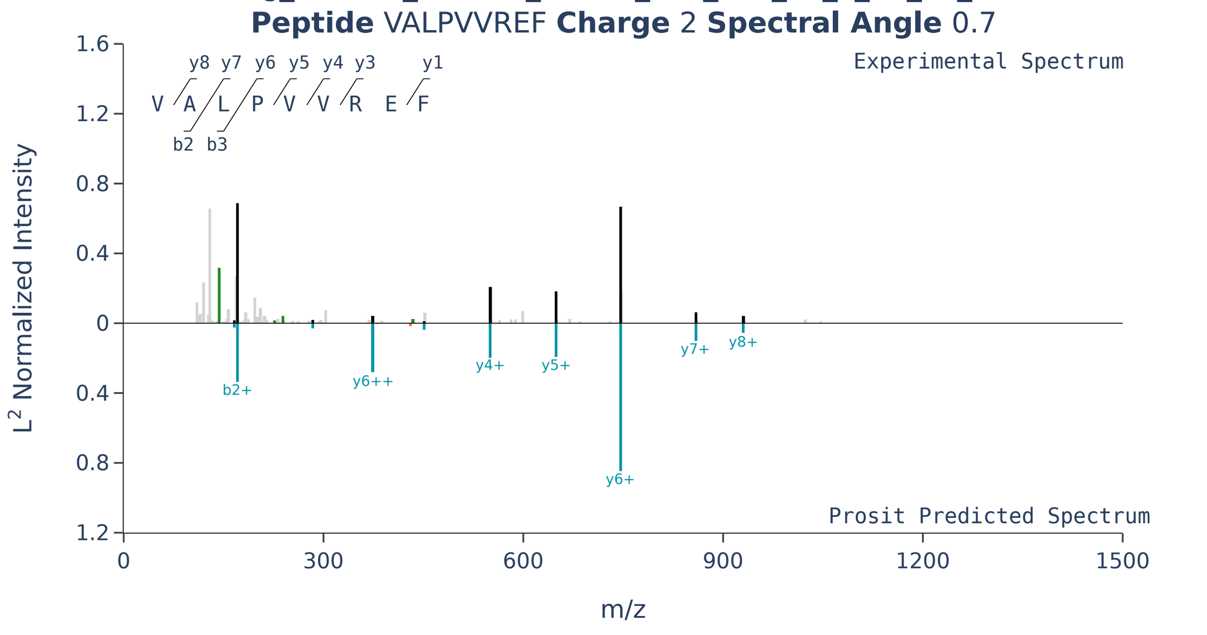


**Source** Ncheng_210623_230623_HFGoe_FFH_20S_25_1_A1_4h_R2 **Scan** 22551
**Peptide** MTGQDAANTAKAFNEAL **Charge** 2 **Spectral Angle** 0.89

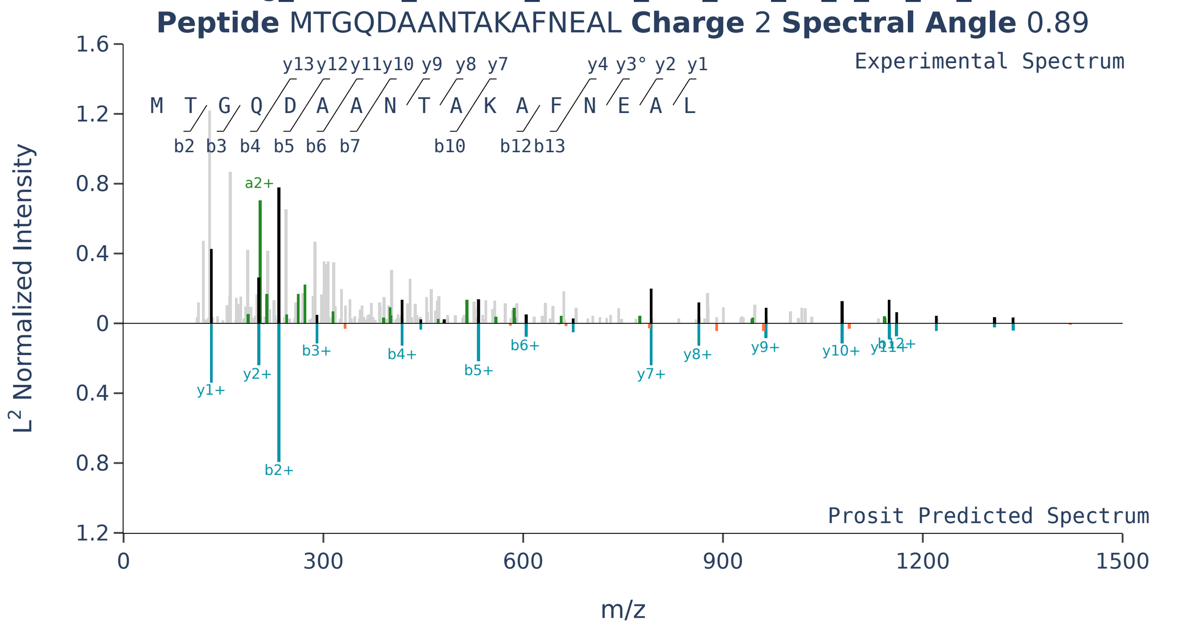


**Source** Ncheng_210623_230623_HFGoe_FFH_20S_25_1_A2_1h_R2 **Scan** 12952
**Peptide** RMEAIINS **Charge** 2 **Spectral Angle** 0.87

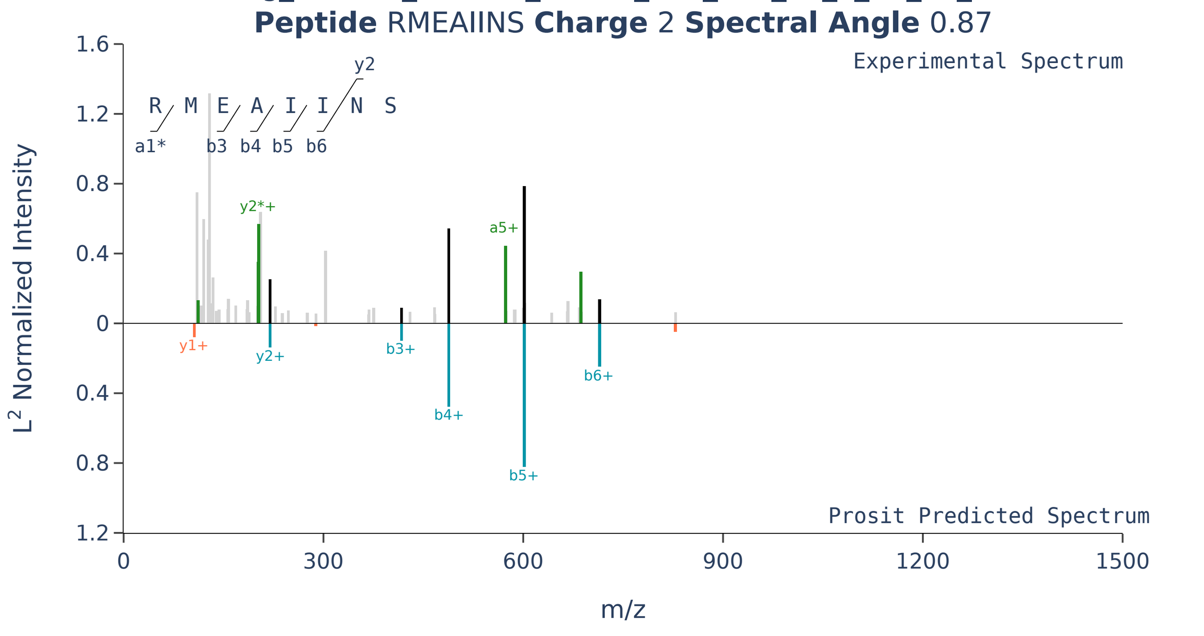


**Source** Ncheng_210623_230623_HFGoe_FFH_20S_25_1_A1_24h_R1 **Scan** 16203
**Peptide** SLRHITGKPIKFLGVGEKTE **Charge** 3 **Spectral Angle** 0.79

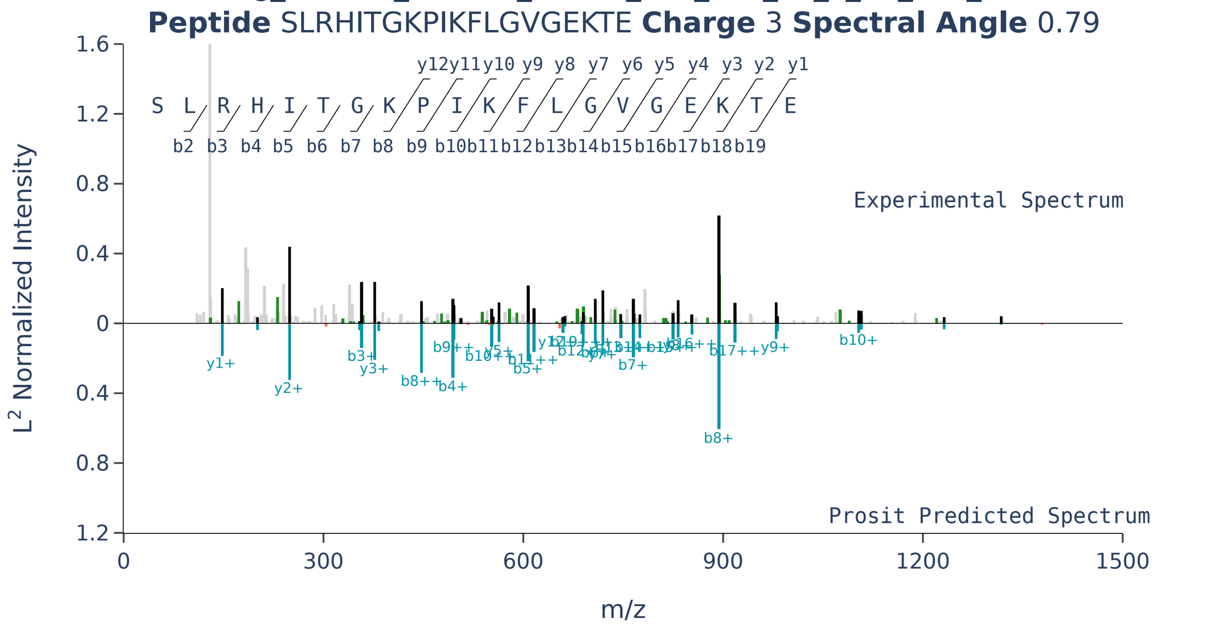


**Source** Ncheng_210623_230623_HFGoe_FFH_20S_25_1_A2_4h_R2 **Scan** 26279
**Peptide** LFVVDAMTGQDAANTAKA **Charge** 2 **Spectral Angle** 0.91

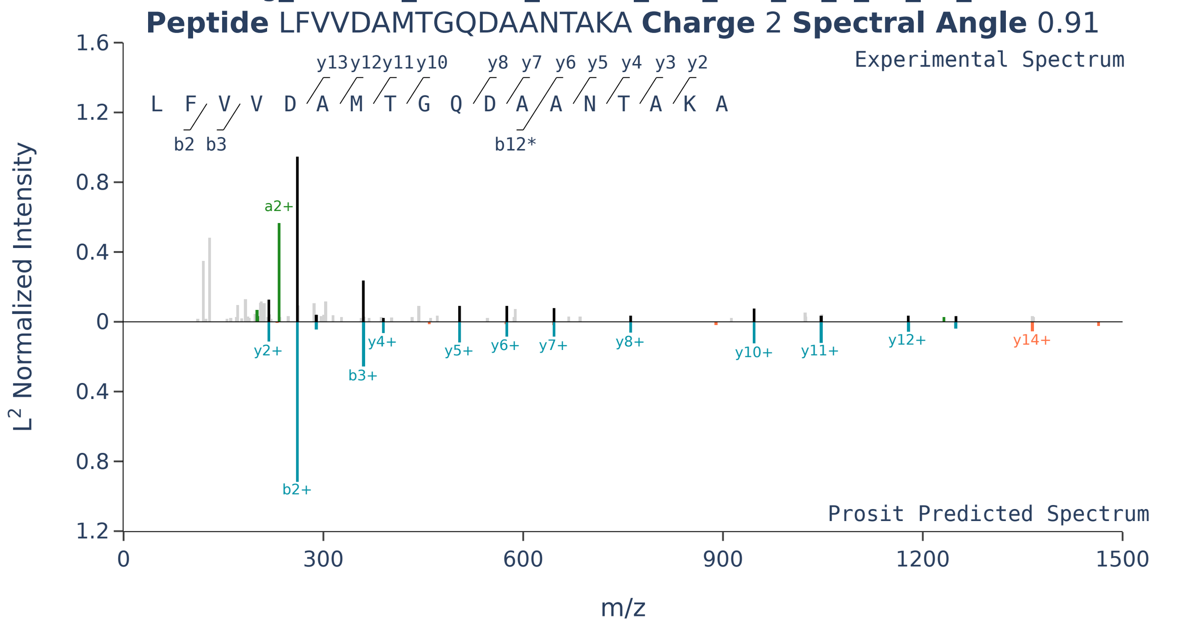


**Source** Ncheng_210623_230623_HFGoe_FFH_20S_25_1_A1_24h_R2 **Scan** 19008
**Peptide** GDGFDLND **Charge** 1 **Spectral Angle** 0.88

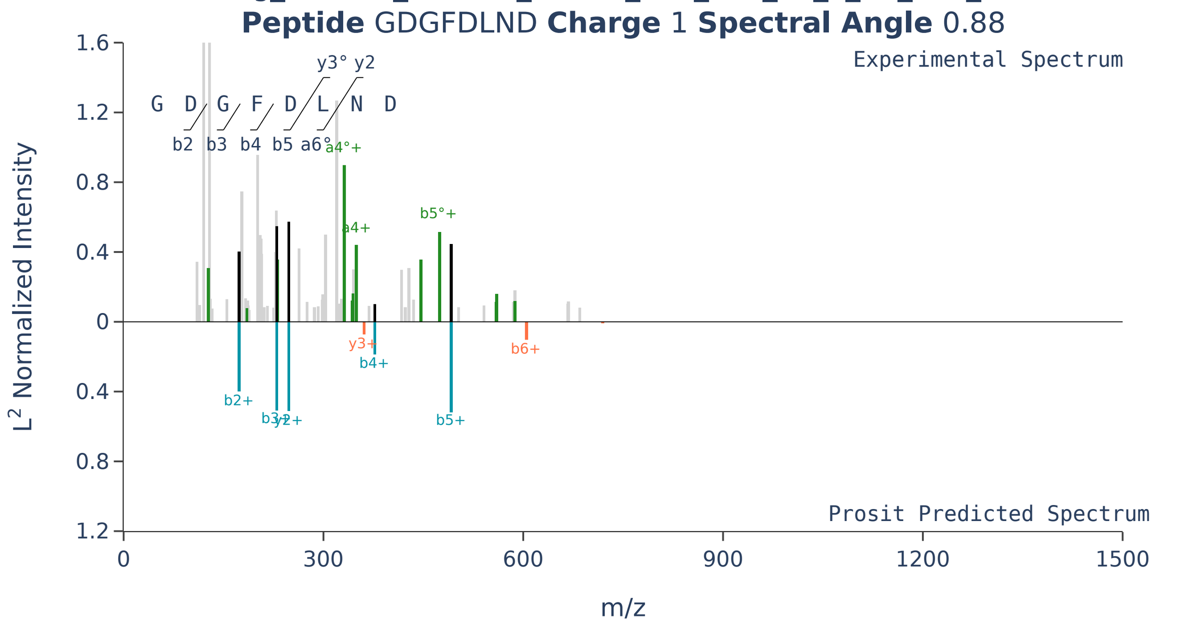


**Source** Ncheng_210623_230623_HFGoe_FFH_20S_25_1_A2_24h_R1 **Scan** 7260
**Peptide** FLNRVKEKAVGH **Charge** 4 **Spectral Angle** 0.89

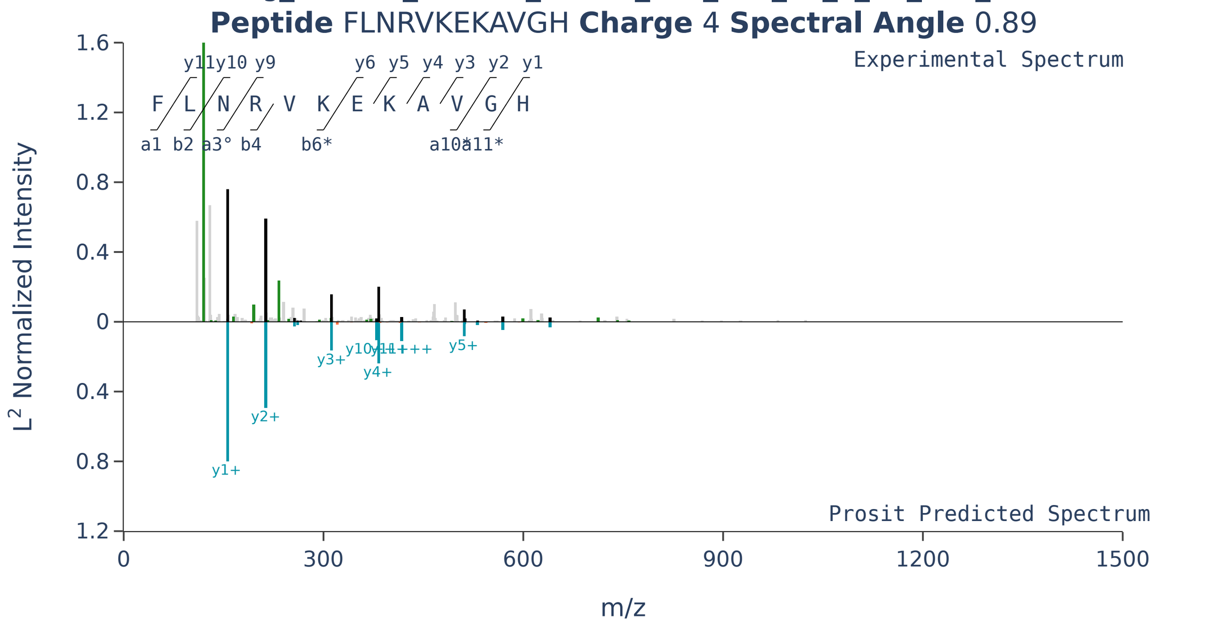


**Source** Ncheng_210623_230623_HFGoe_FFH_20S_25_1_A1_24h_R2 **Scan** 10980
**Peptide** VVLTKVDGDARGGA **Charge** 3 **Spectral Angle** 0.79

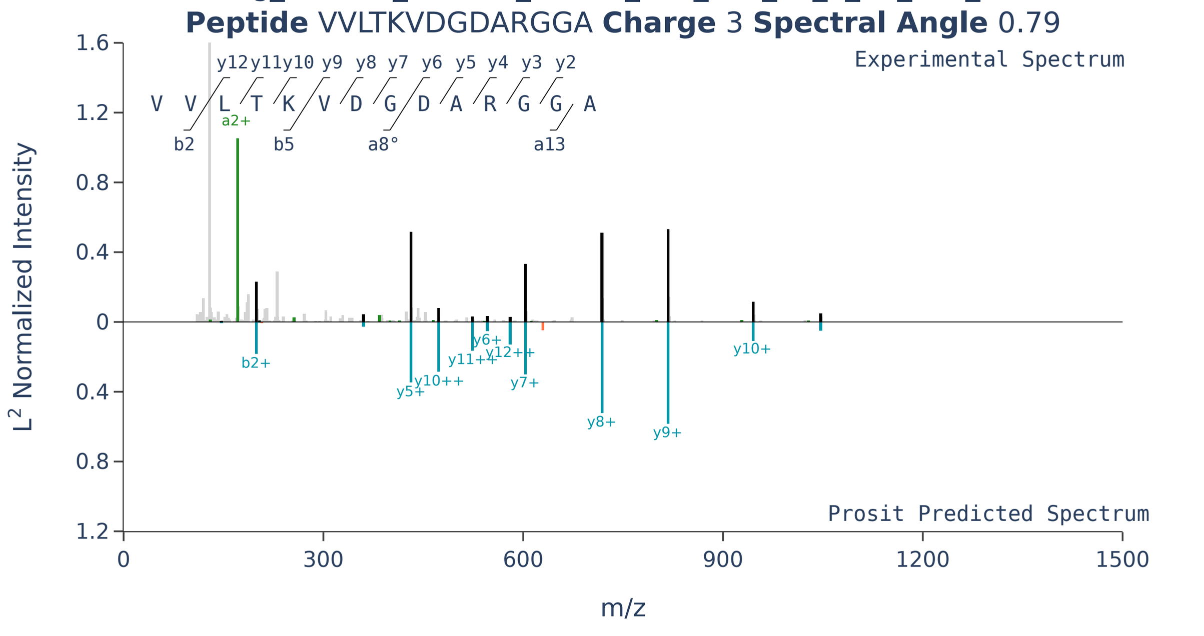


**Source** Ncheng_210623_230623_HFGoe_FFH_20S_25_1_A1_24h_R2 **Scan** 13186
**Peptide** VNAALKEAKL **Charge** 3 **Spectral Angle** 0.74

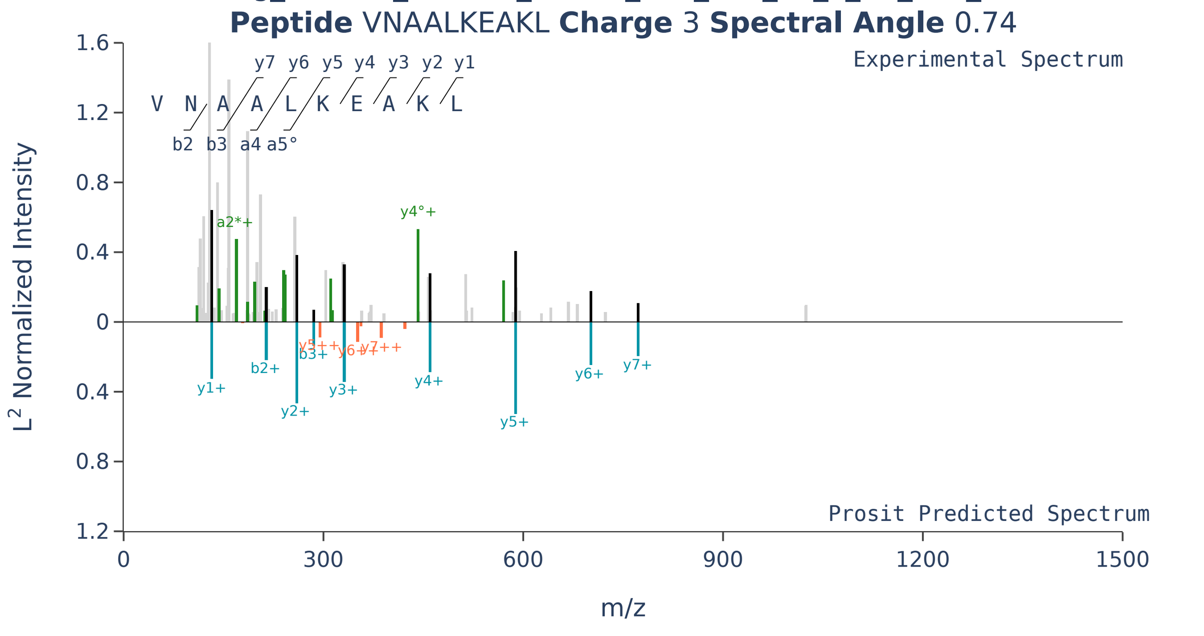


**Source** Ncheng_210623_230623_HFGoe_FFH_20S_25_1_A1_24h_R2 **Scan** 11393
**Peptide** KEAKLKFYD **Charge** 3 **Spectral Angle** 0.93

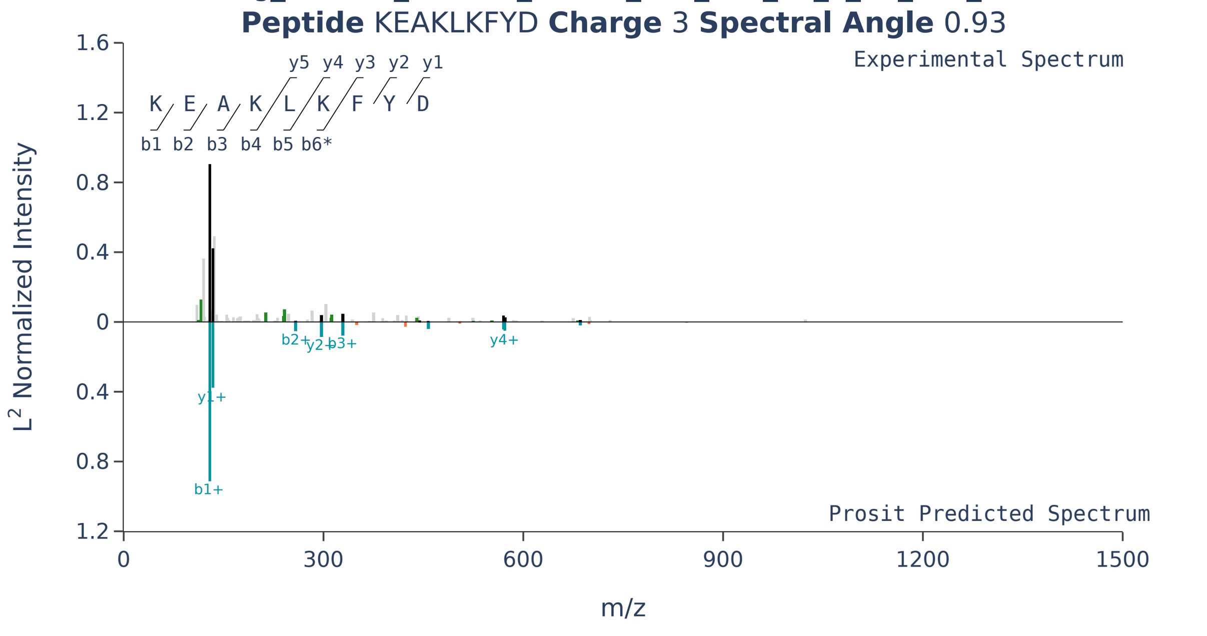

**Source** Ncheng_210623_230623_HFGoe_FFH_20S_25_1_A2_1h_R1 **Scan** 17822
**Peptide** NKSLTPGQEFVKIVRN **Charge** 3 **Spectral Angle** 0.83

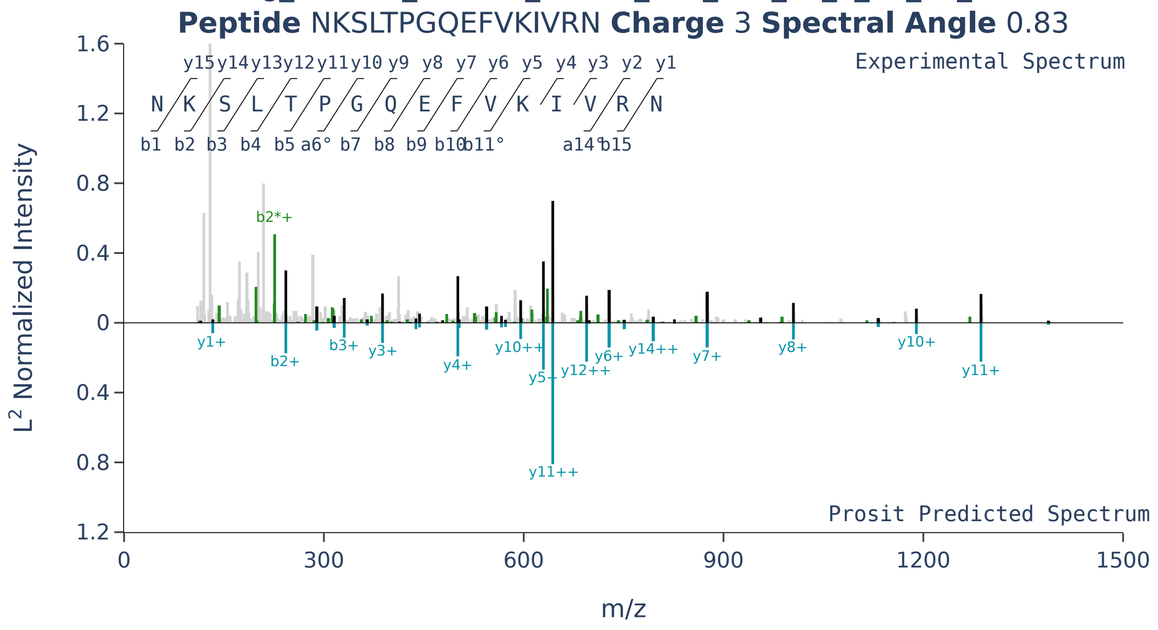


**Source** Ncheng_210623_230623_HFGoe_FFH_20S_25_1_A2_24h_R2 **Scan** 8663
**Peptide** GLQGAGKTTSV **Charge** 1 **Spectral Angle** 0.83

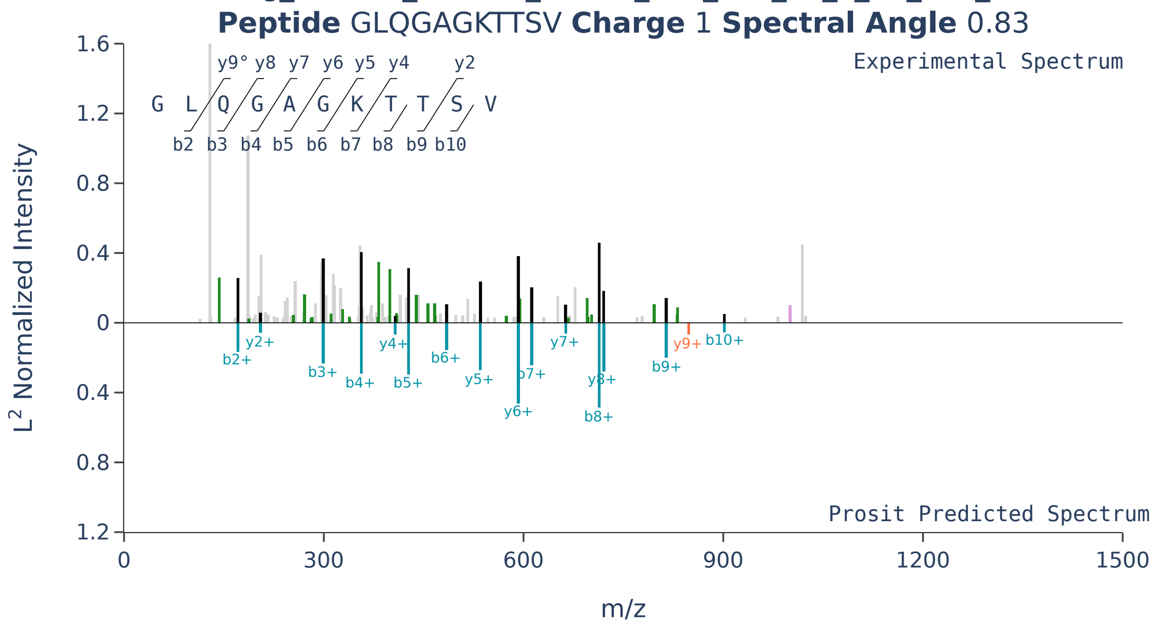


**Source** Ncheng_210623_230623_HFGoe_FFH_20S_25_1_A1_2h_R1 **Scan** 28124
**Peptide** EQVGVDFFPSDVGQKPVD **Charge** 2 **Spectral Angle** 0.88

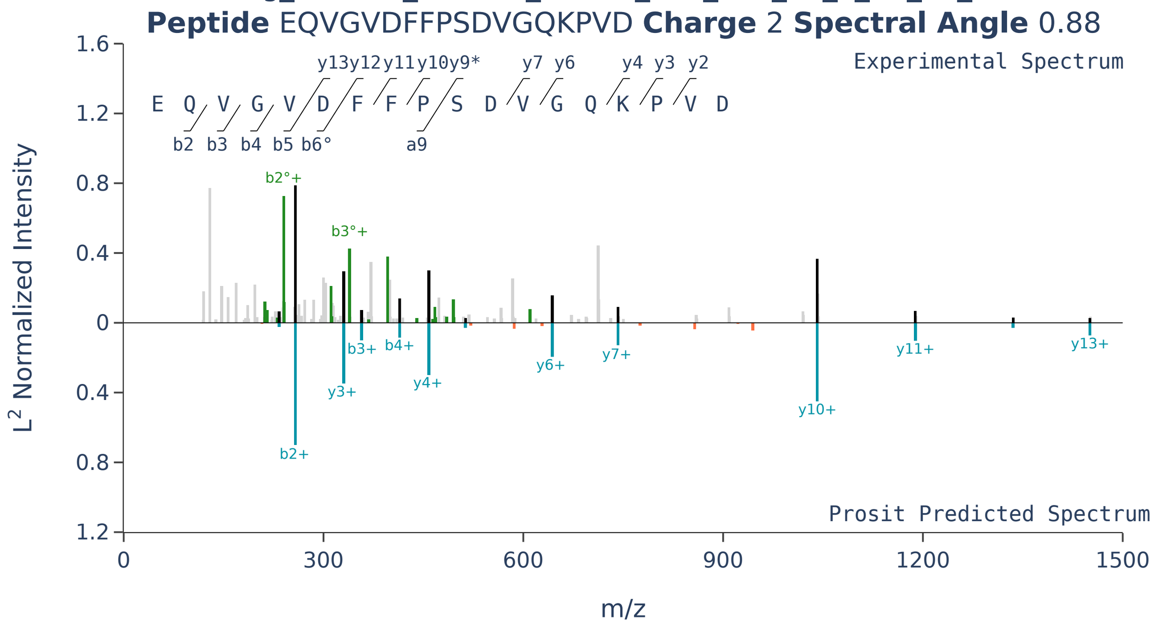


**Source** Ncheng_210623_230623_HFGoe_FFH_20S_25_1_A2_2h_R2 **Scan** 15031
**Peptide** DARGGAALSIRHL **Charge** 3 **Spectral Angle** 0.9

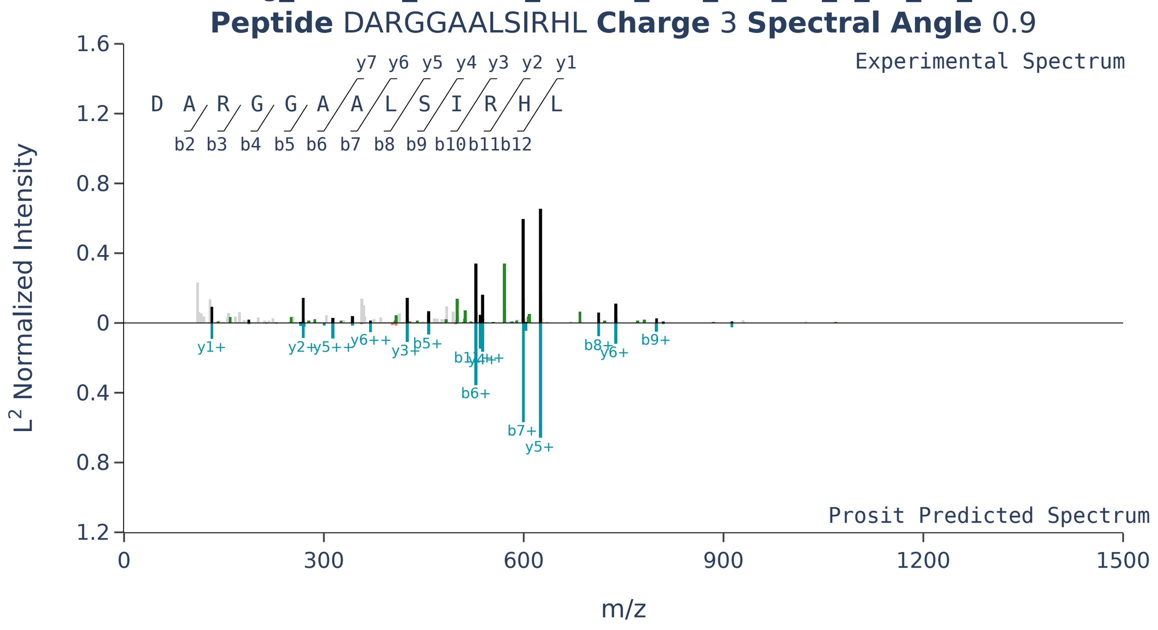


**Source** Ncheng_210623_230623_HFGoe_FFH_20S_25_1_A1_24h_R1 **Scan** 13160
**Peptide** ETLAEQVG **Charge** 1 **Spectral Angle** 0.74

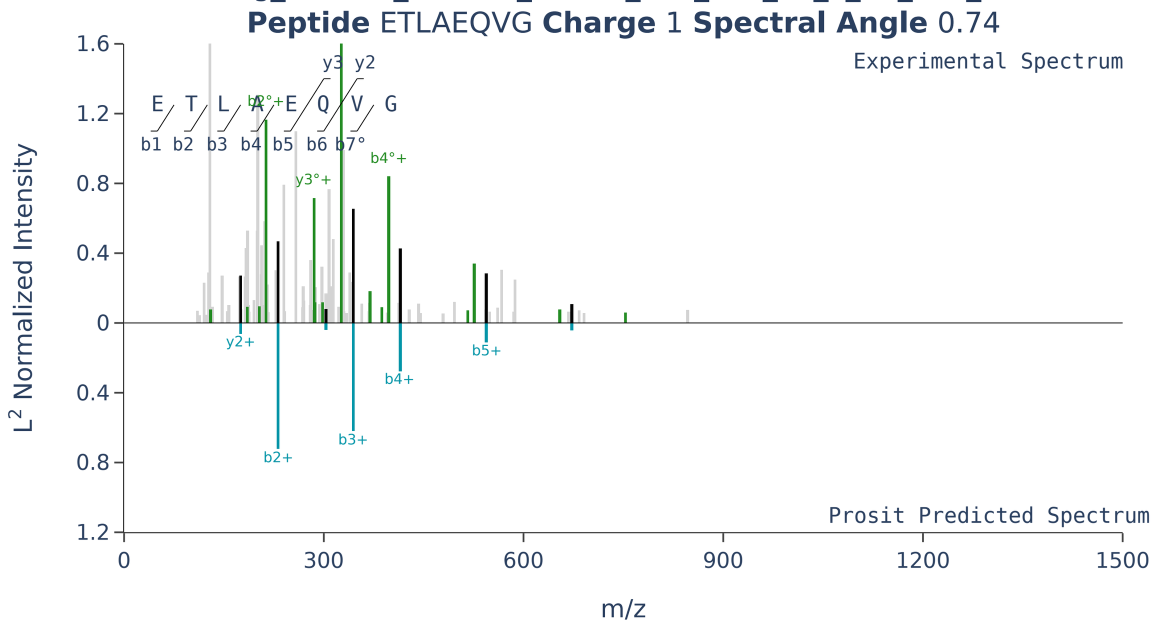


**Source** Ncheng_210623_230623_HFGoe_FFH_20S_25_1_A2_24h_R1 **Scan** 10158
**Peptide** INSMTMKE **Charge** 2 **Spectral Angle** 0.88

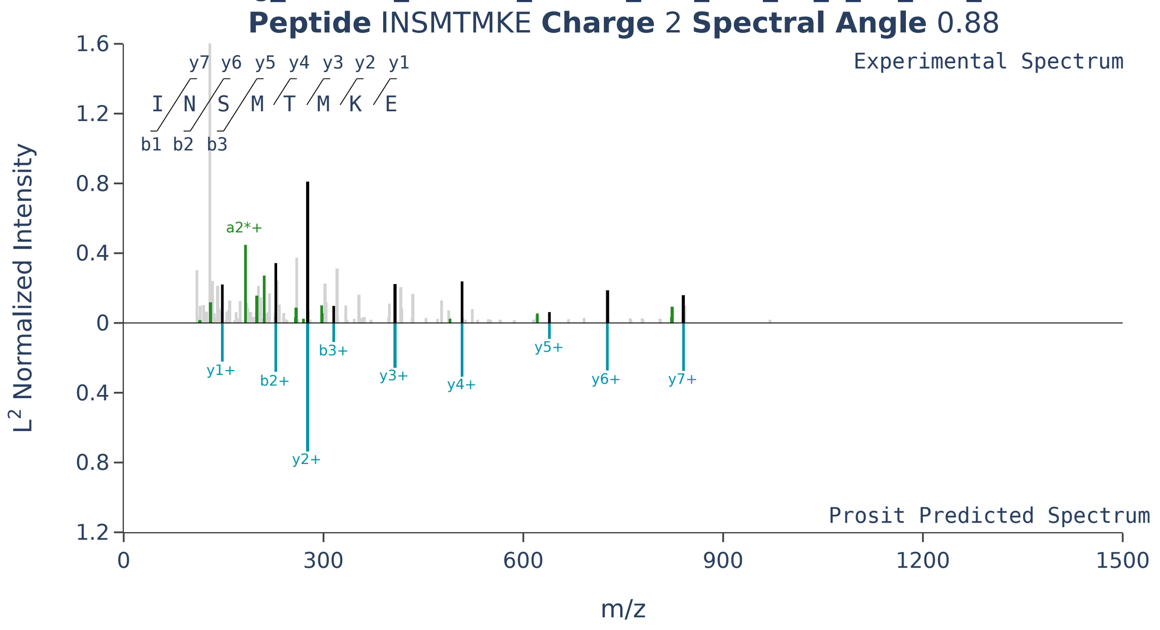


**Source** Ncheng_210623_230623_HFGoe_FFH_20S_25_1_A2_24h_R2 **Scan** 10681
**Peptide** GDARGGAALSIR **Charge** 3 **Spectral Angle** 0.89

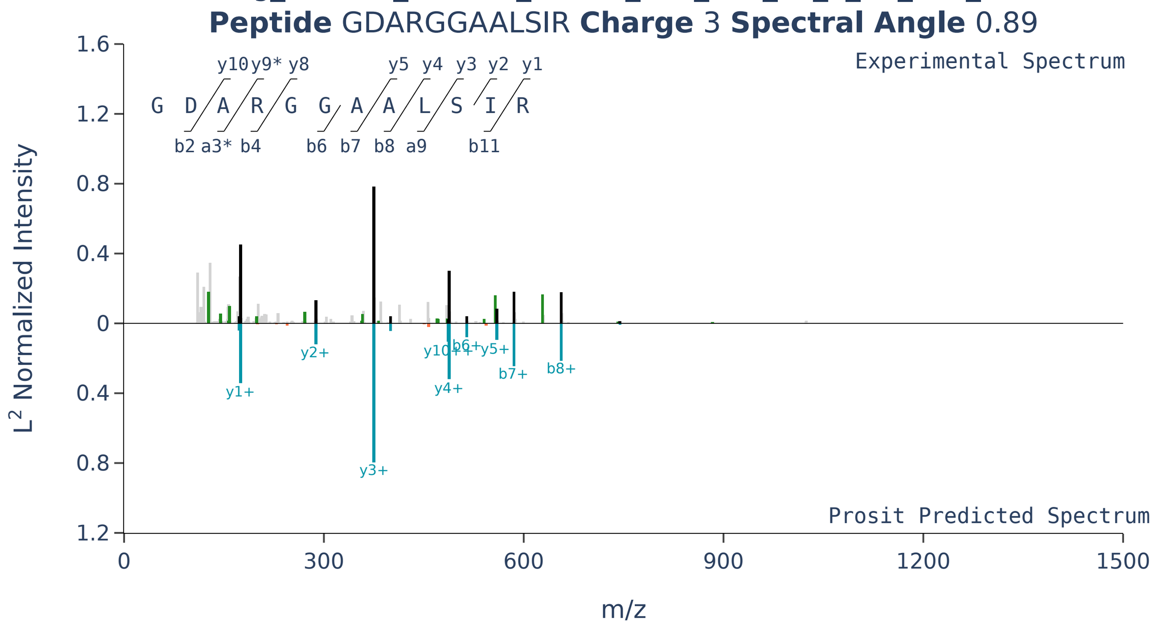


**Source** Ncheng_210623_230623_HFGoe_FFH_20S_25_1_A1_4h_R1 **Scan** 21311
**Peptide** MGEENQTLNLA **Charge** 1 **Spectral Angle** 0.78

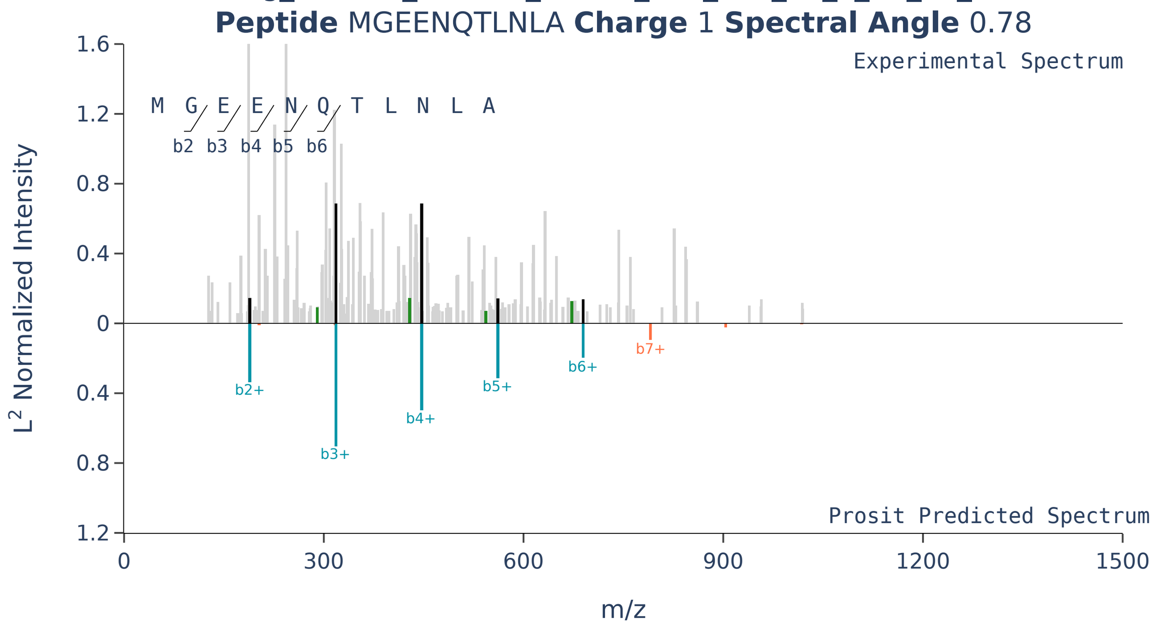


**Source** Ncheng_210623_230623_HFGoe_FFH_20S_25_1_A2_24h_R2 **Scan** 14908
**Peptide** AMMDEIKQVH **Charge** 3 **Spectral Angle** 0.92

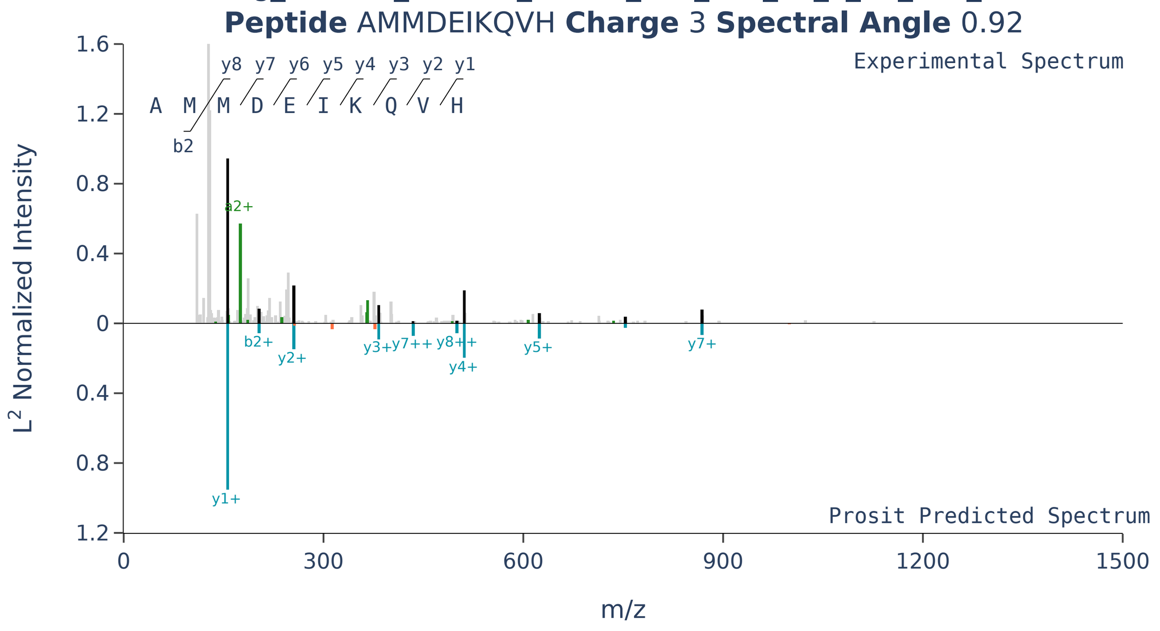


**Source** Ncheng_210623_230623_HFGoe_FFH_20S_25_1_A1_4h_R2 **Scan** 14931
**Peptide** AGKTTSVGKLGKFLRE **Charge** 2 **Spectral Angle** 0.85

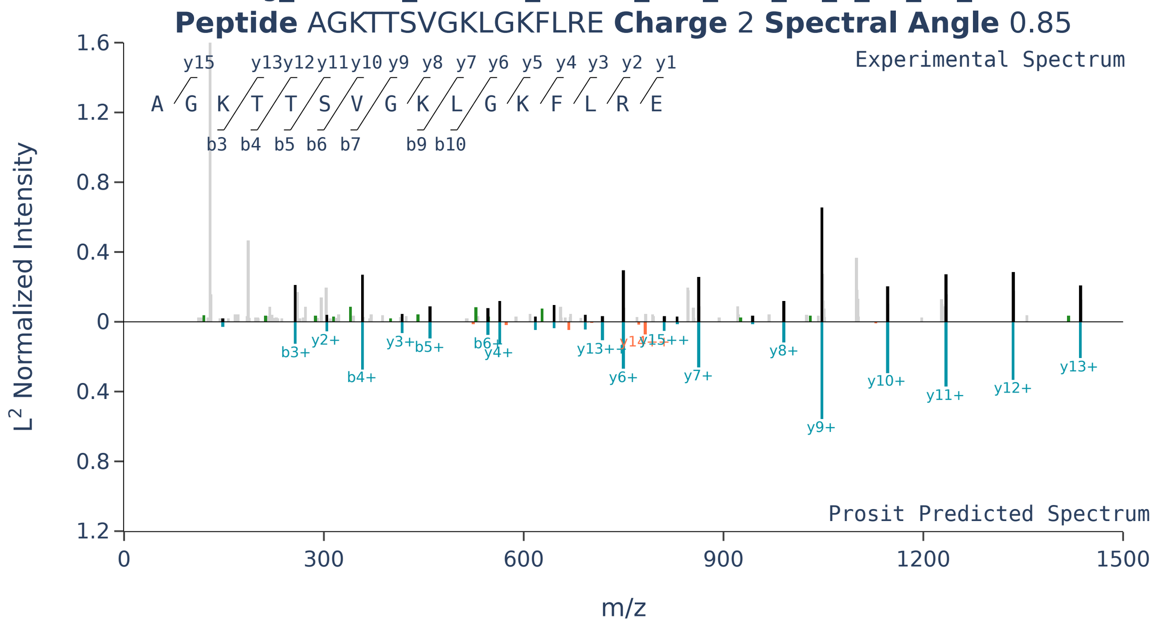


**Source** Ncheng_210623_230623_HFGoe_FFH_20S_25_1_A2_24h_R1 **Scan** 9058
**Peptide** AVGHEVNKSLTPGQ **Charge** 2 **Spectral Angle** 0.81

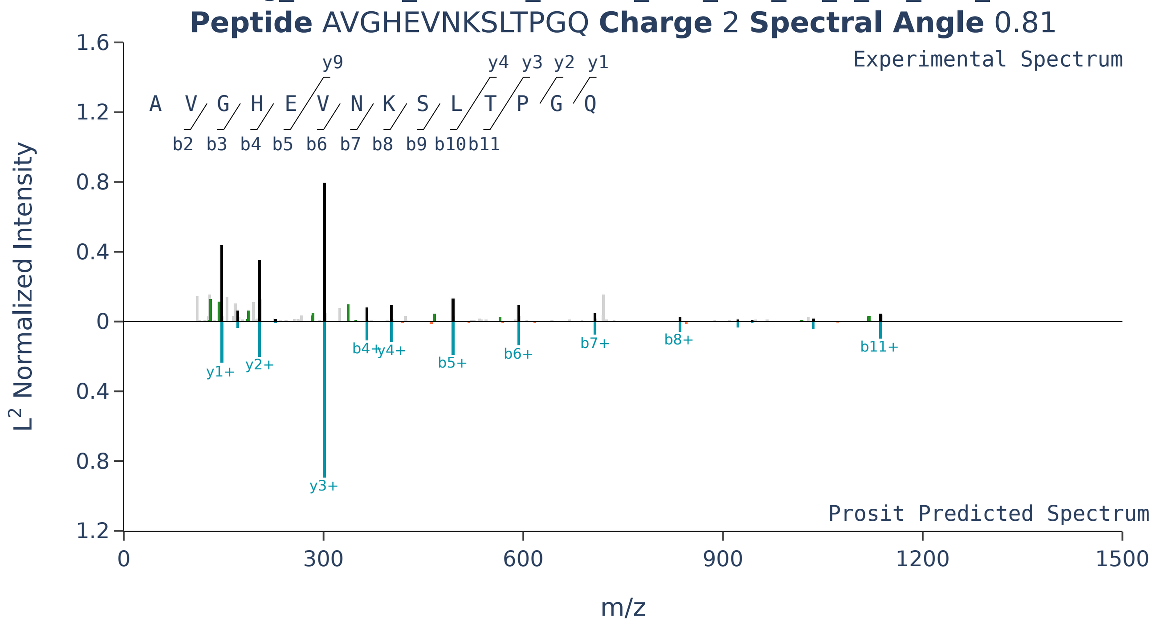


**Source** Ncheng_210623_230623_HFGoe_FFH_20S_25_1_A1_24h_R1 **Scan** 7748
**Peptide** DLESKVD **Charge** 1 **Spectral Angle** 0.9

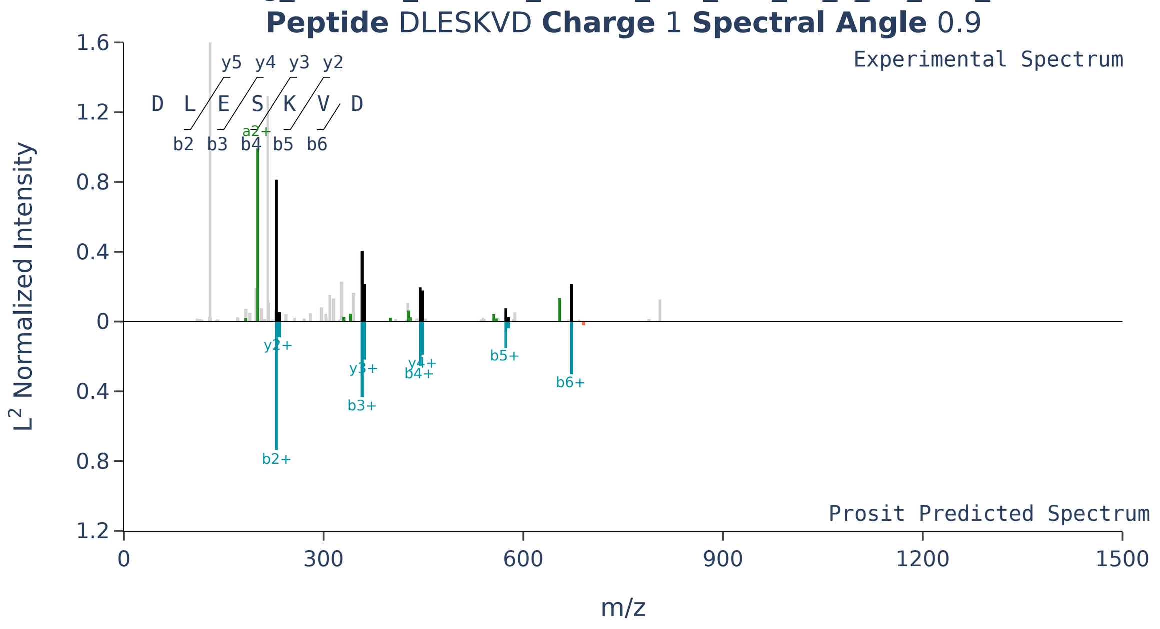


**Source** Ncheng_210623_230623_HFGoe_FFH_20S_25_1_A2_4h_R1 **Scan** 17642
**Peptide** KAVGHEVNKSLTPGQEFVKIVR **Charge** 3 **Spectral Angle** 0.77

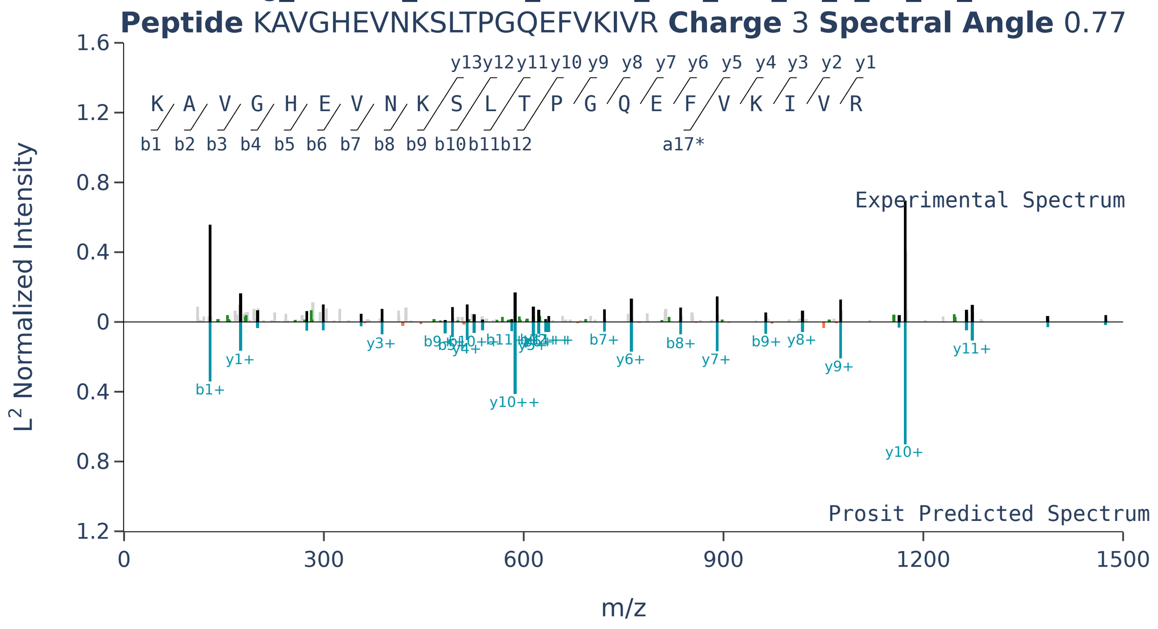


**Source** Ncheng_210623_230623_HFGoe_FFH_20S_25_1_A2_24h_R1 **Scan** 17644
**Peptide** GRLTEDNVKDTLREV **Charge** 2 **Spectral Angle** 0.84

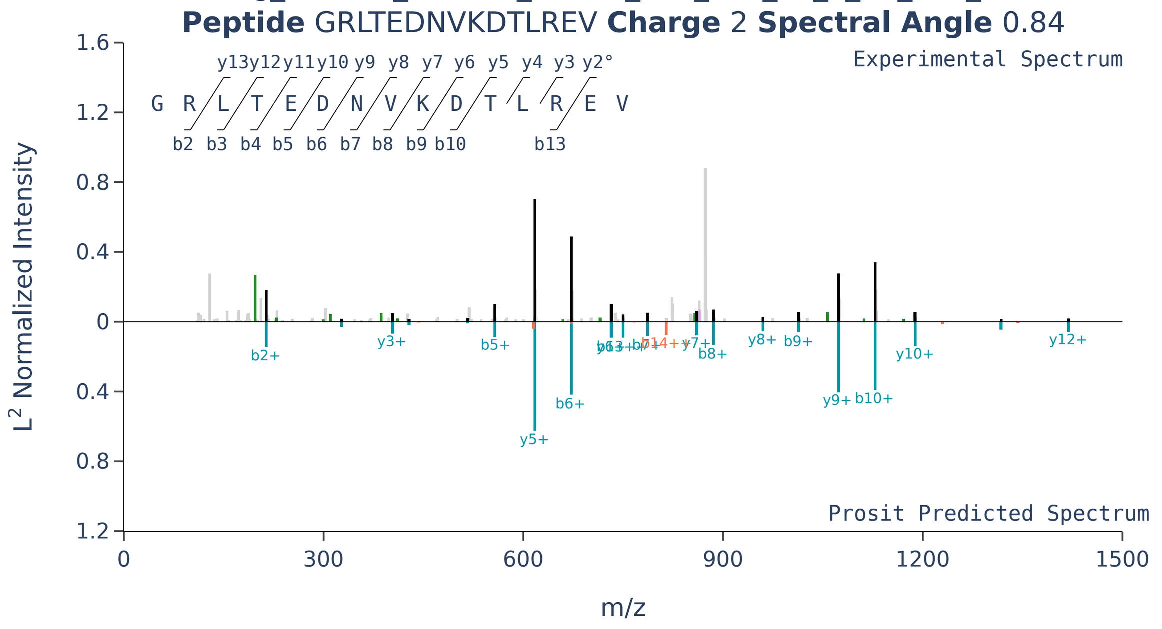


**Source** Ncheng_210623_230623_HFGoe_FFH_20S_25_1_A2_4h_R2 **Scan** 20472
**Peptide** MGKLPGMGQIPDNVKS **Charge** 2 **Spectral Angle** 0.84

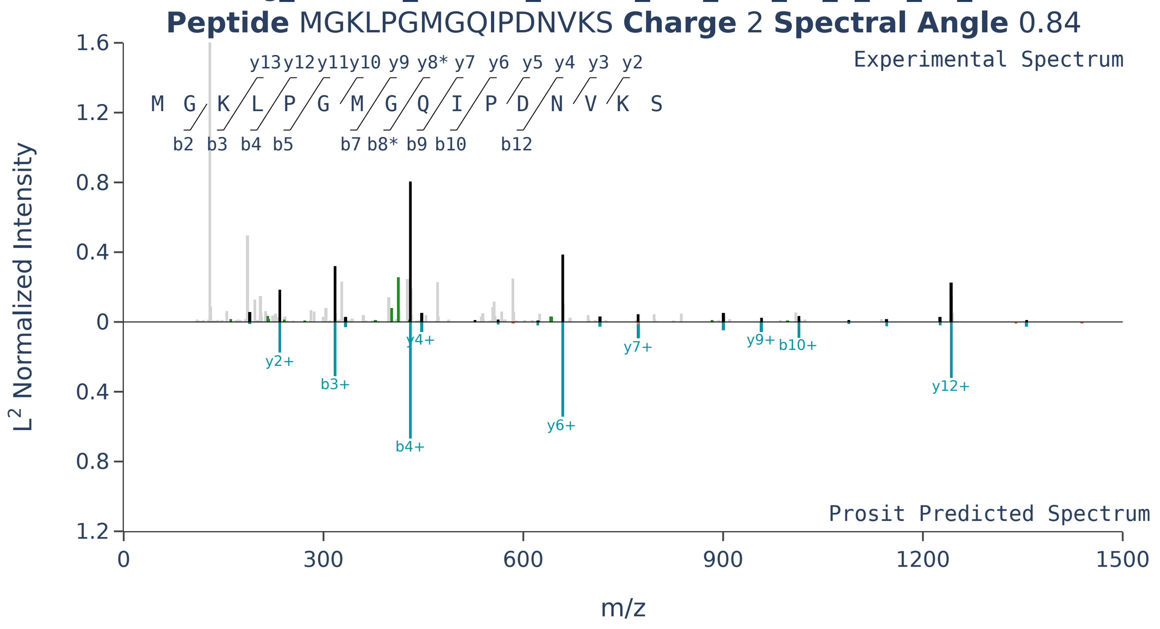

**Source** Ncheng_210623_230623_HFGoe_FFH_20S_25_1_A1_24h_R1 **Scan** 16211
**Peptide** TGKPIKFLGVGEKTEA **Charge** 2 **Spectral Angle** 0.82

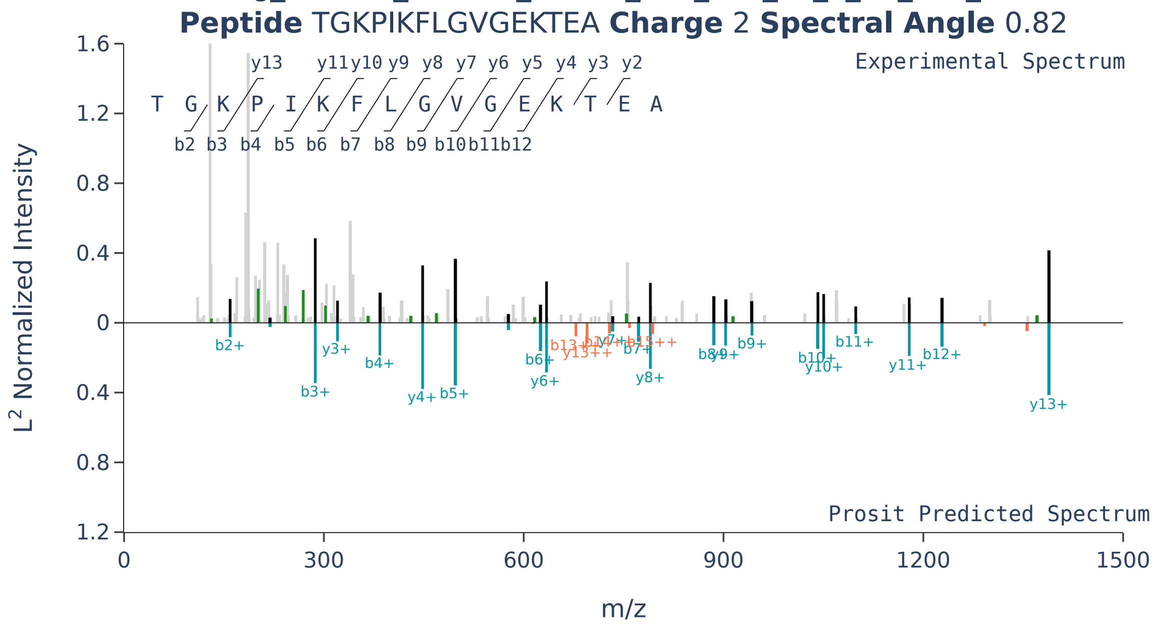


**Source** Ncheng_210623_230623_HFGoe_FFH_20S_25_1_A1_24h_R1 **Scan** 9063
**Peptide** GMQVQDVNR **Charge** 2 **Spectral Angle** 0.82

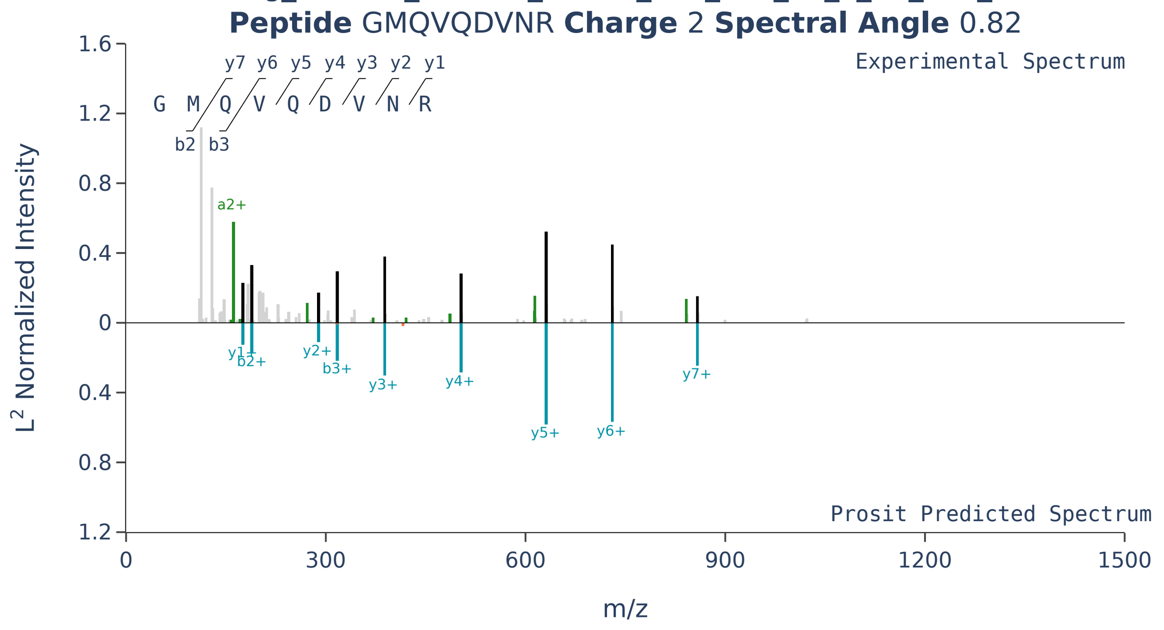


**Source** Ncheng_210623_230623_HFGoe_FFH_20S_25_1_A2_2h_R1 **Scan** 11688
**Peptide** KVLVRMEA **Charge** 2 **Spectral Angle** 0.66

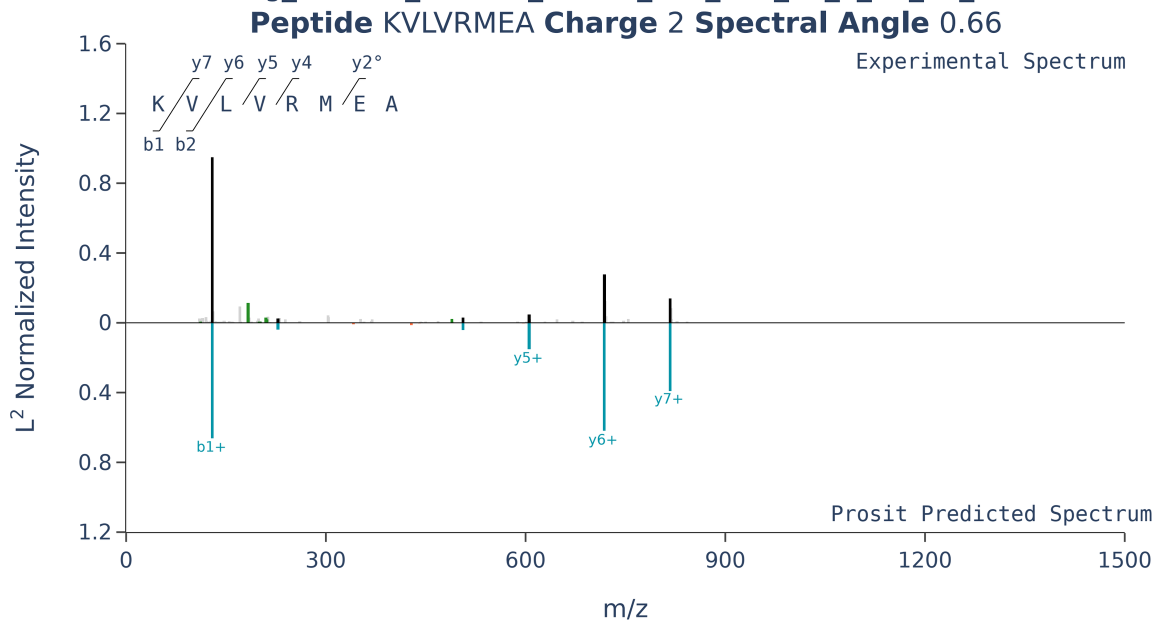


**Source** Ncheng_210623_230623_HFGoe_FFH_20S_25_1_A2_4h_R2 **Scan** 22659
**Peptide** DEAMMDEIKQV **Charge** 2 **Spectral Angle** 0.86

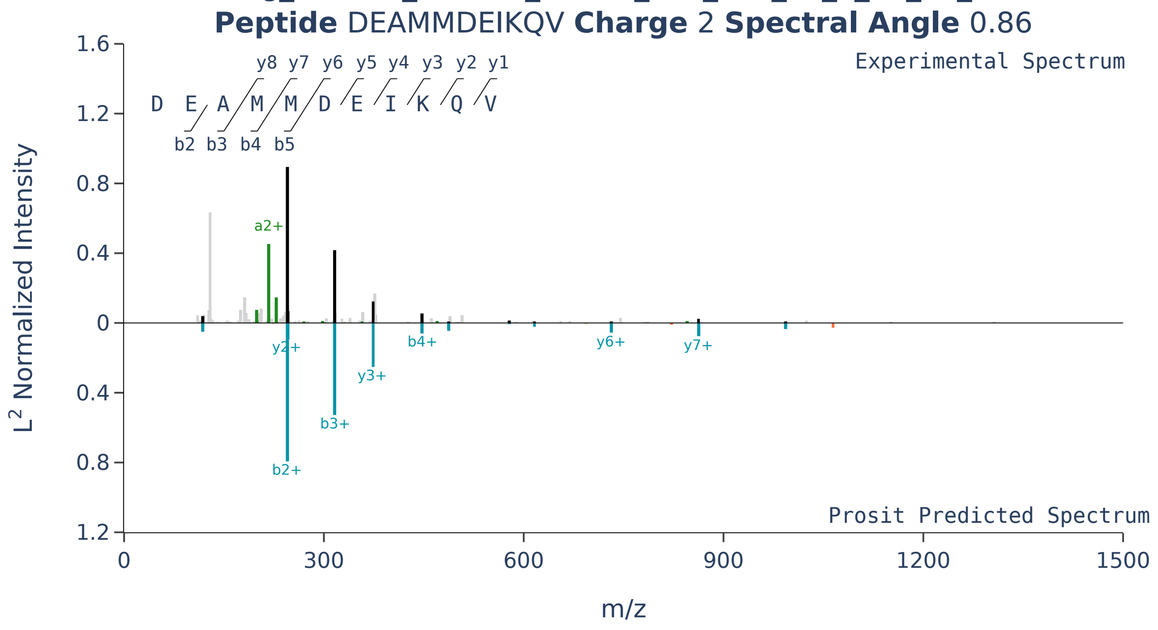


**Source** Ncheng_210623_230623_HFGoe_FFH_20S_25_1_A1_2h_R2 **Scan** 10671
**Peptide** GQKPVDI **Charge** 2 **Spectral Angle** 0.85

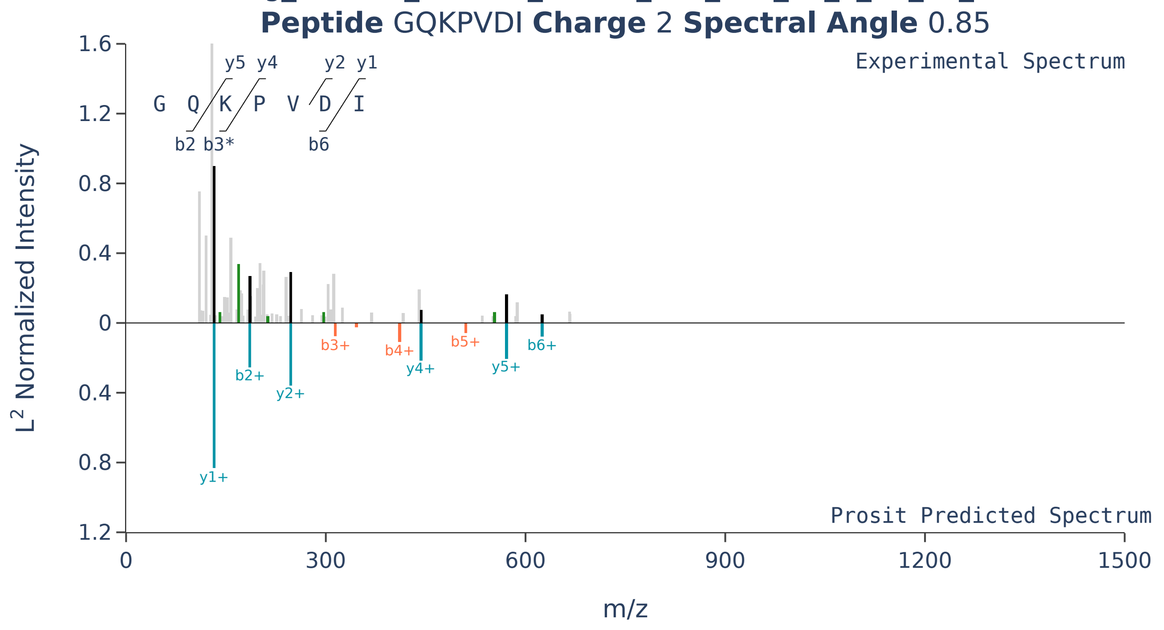


**Source** Ncheng_210623_230623_HFGoe_FFH_20S_25_1_A1_4h_R2 **Scan** 19600
**Peptide** AVGHEVNKSLTPGQEFVKIVRN **Charge** 2 **Spectral Angle** 0.76

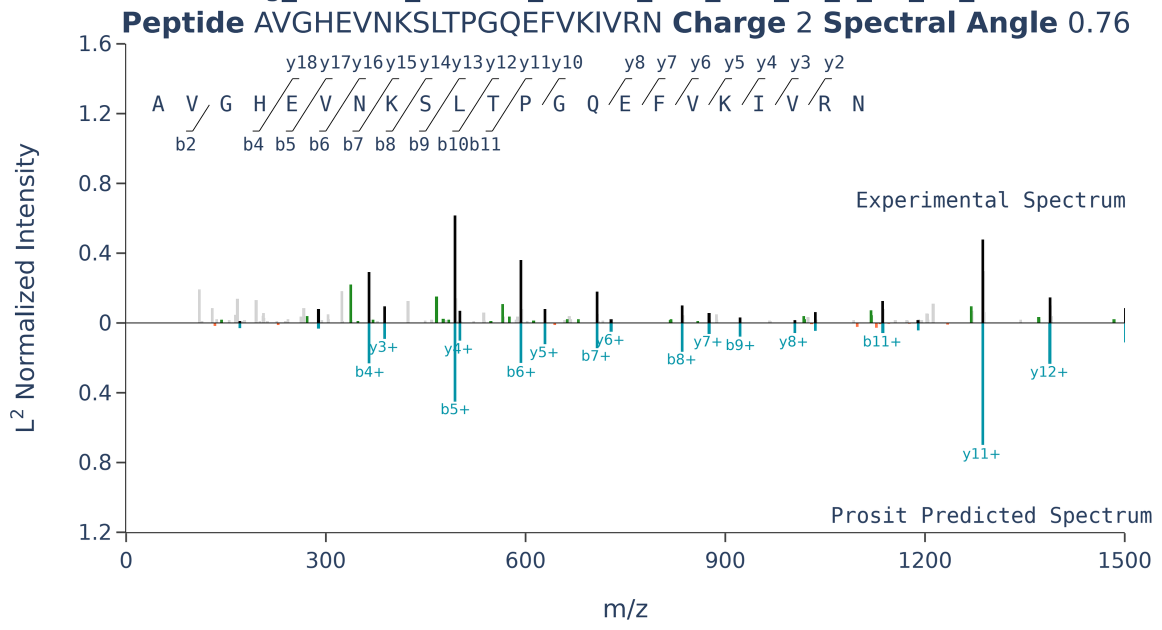


**Source** Ncheng_210623_230623_HFGoe_FFH_20S_25_1_A1_4h_R1 **Scan** 17675
**Peptide** AGKTTSVGKLGKFL **Charge** 4 **Spectral Angle** 0.81

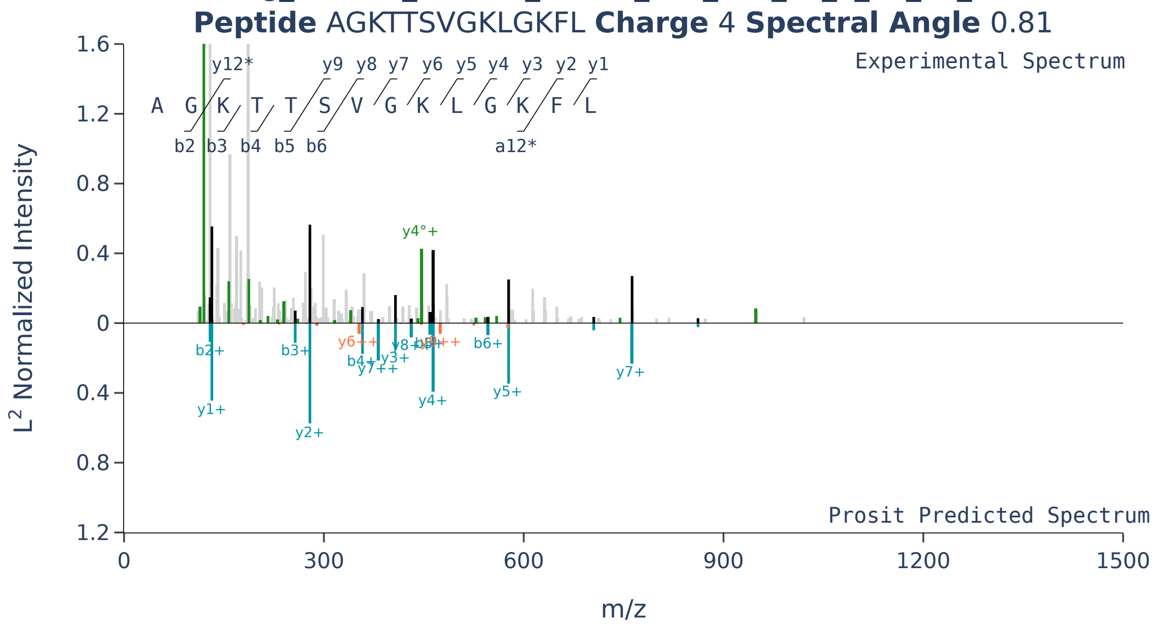


**Source** Ncheng_210623_230623_HFGoe_FFH_20S_25_1_A2_4h_R2 **Scan** 19867
**Peptide** AVGHEVNKSLTPGQEFVKIVR **Charge** 2 **Spectral Angle** 0.76

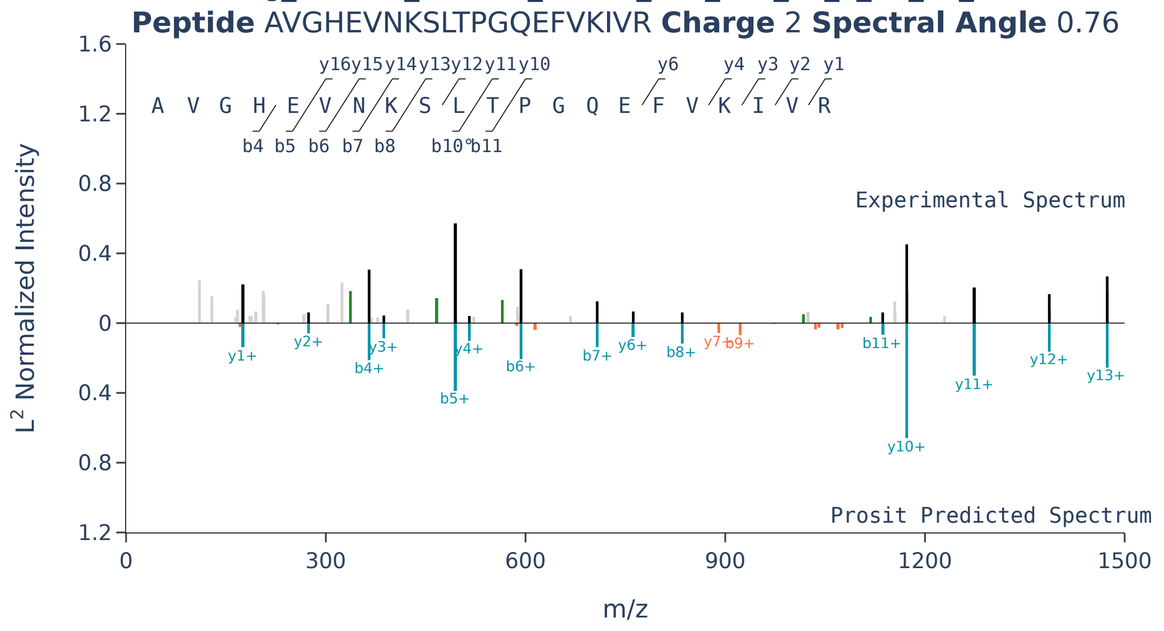


**Source** Ncheng_210623_230623_HFGoe_FFH_20S_25_1_A1_24h_R1 **Scan** 15419
**Peptide** NKSLTPGQEF **Charge** 2 **Spectral Angle** 0.78

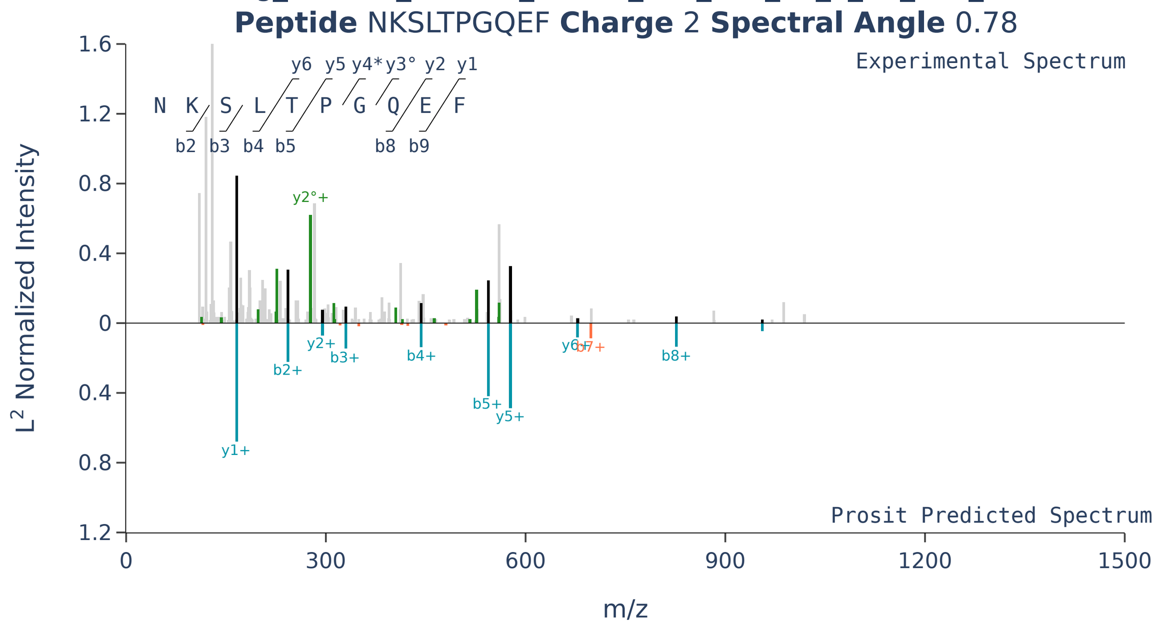


**Source** Ncheng_210623_230623_HFGoe_FFH_20S_25_1_A2_4h_R2 **Scan** 17932
**Peptide** AKLKFYDV **Charge** 1 **Spectral Angle** 0.83

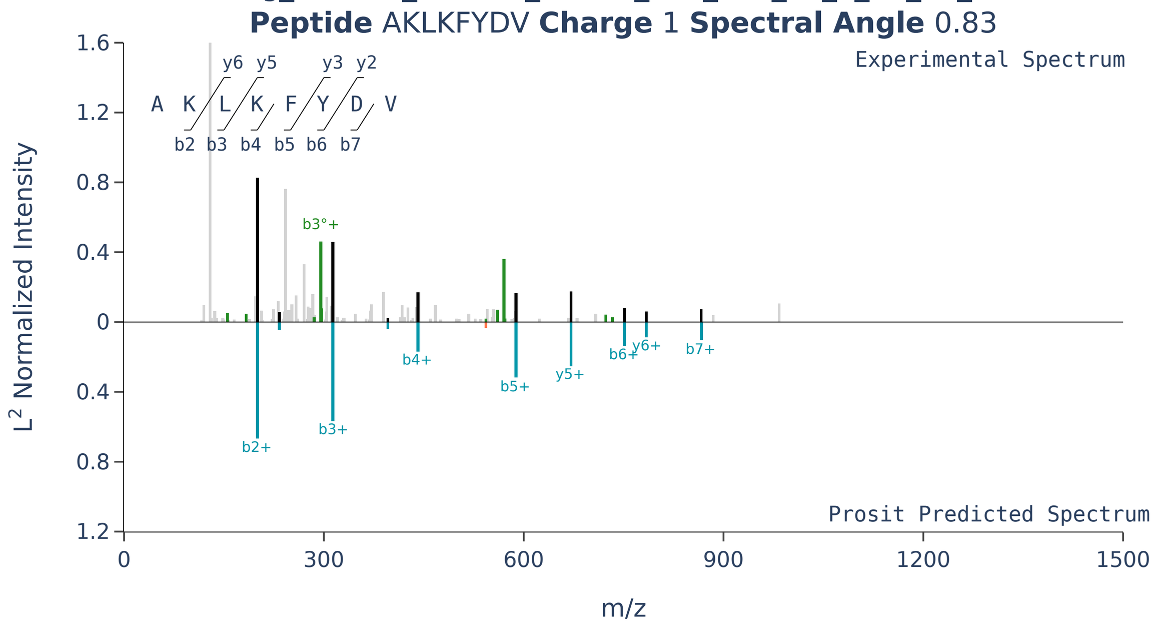


**Source** Ncheng_210623_230623_HFGoe_FFH_20S_25_1_A1_1h_R2 **Scan** 17297
**Peptide** RLAAGCGMQVQDVNRL **Charge** 3 **Spectral Angle** 0.89

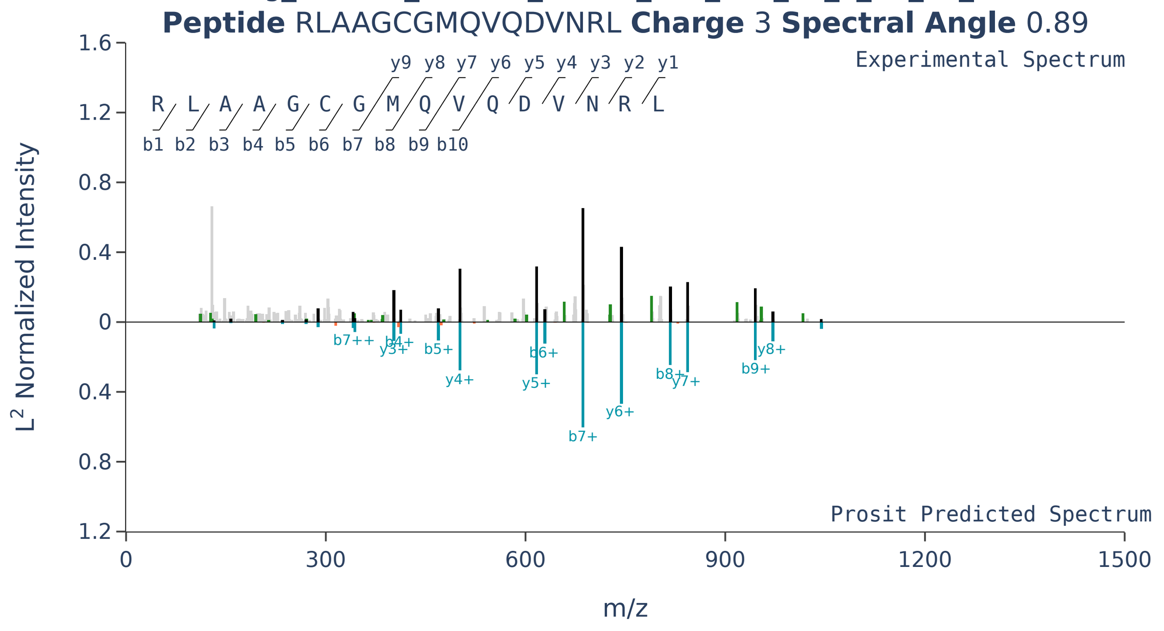


**Source** Ncheng_210623_230623_HFGoe_FFH_20S_25_1_A1_24h_R1 **Scan** 10661
**Peptide** NLTDRLSRT **Charge** 3 **Spectral Angle** 0.8

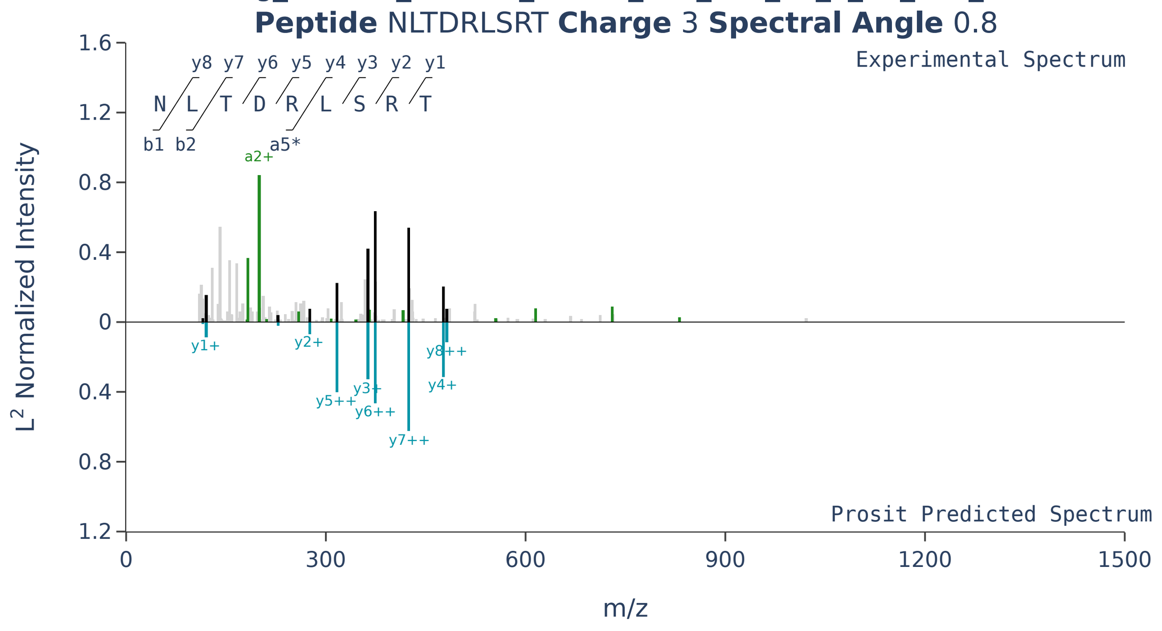


**Source** Ncheng_210623_230623_HFGoe_FFH_20S_25_1_A2_2h_R2 **Scan** 31191
**Peptide** TLFVVDAMTGQDAANTA **Charge** 2 **Spectral Angle** 0.89

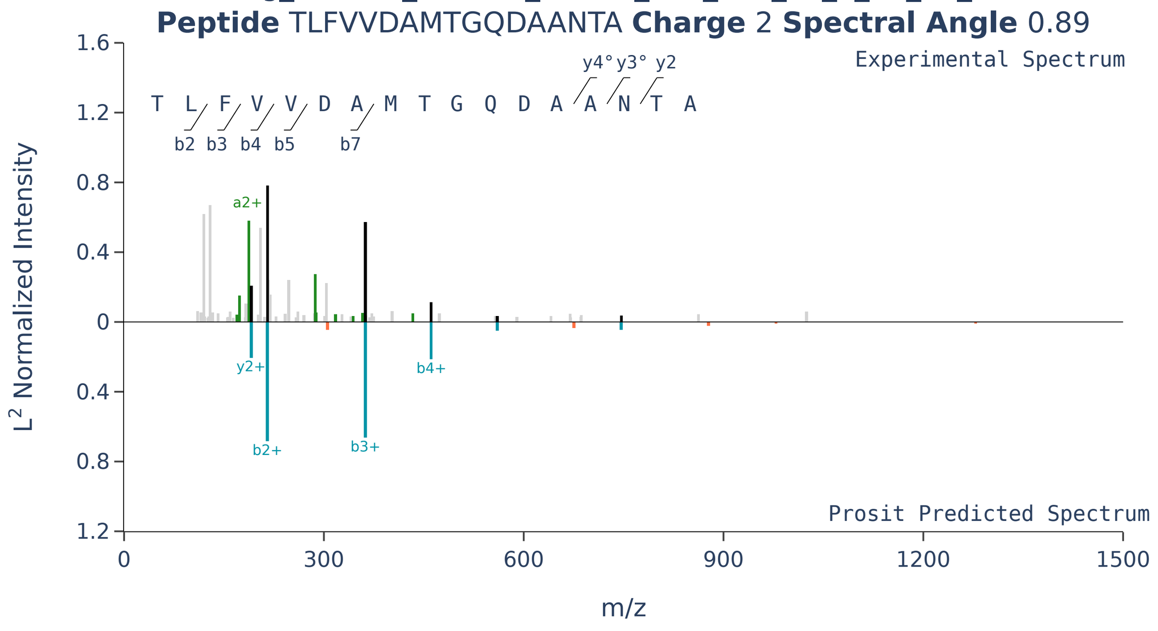


**Source** Ncheng_210623_230623_HFGoe_FFH_20S_25_1_A2_24h_R1 **Scan** 8881
**Peptide** KIVRNEL **Charge** 1 **Spectral Angle** 0.73

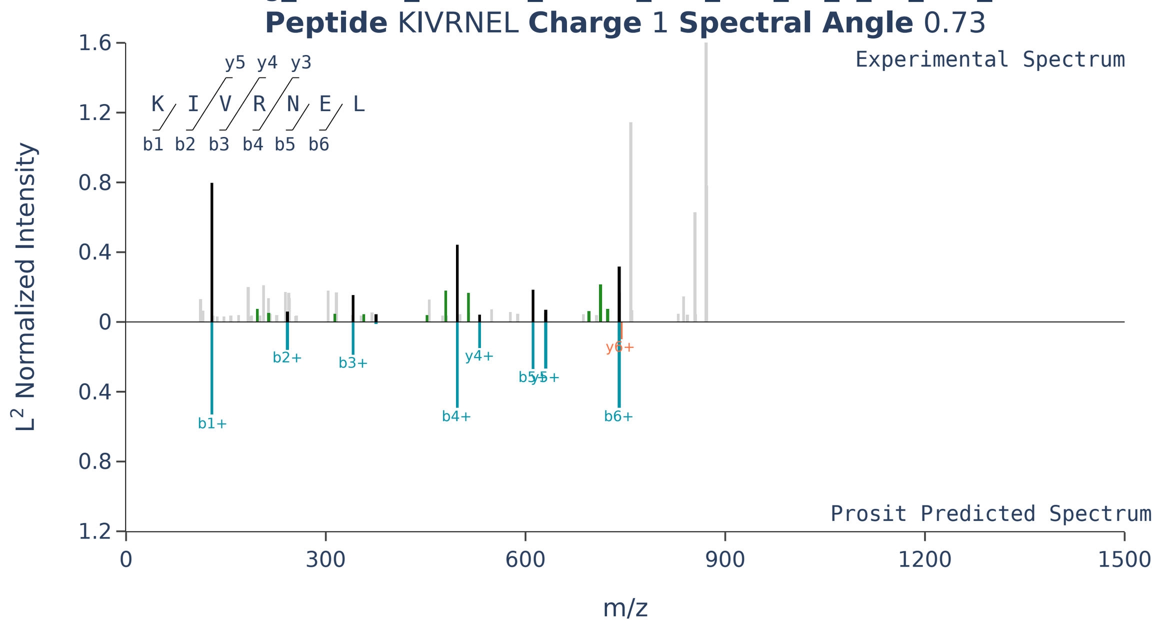


**Source** Ncheng_210623_230623_HFGoe_FFH_20S_25_1_A2_24h_R2 **Scan** 6504
**Peptide** KNMGGMA **Charge** 2 **Spectral Angle** 0.85

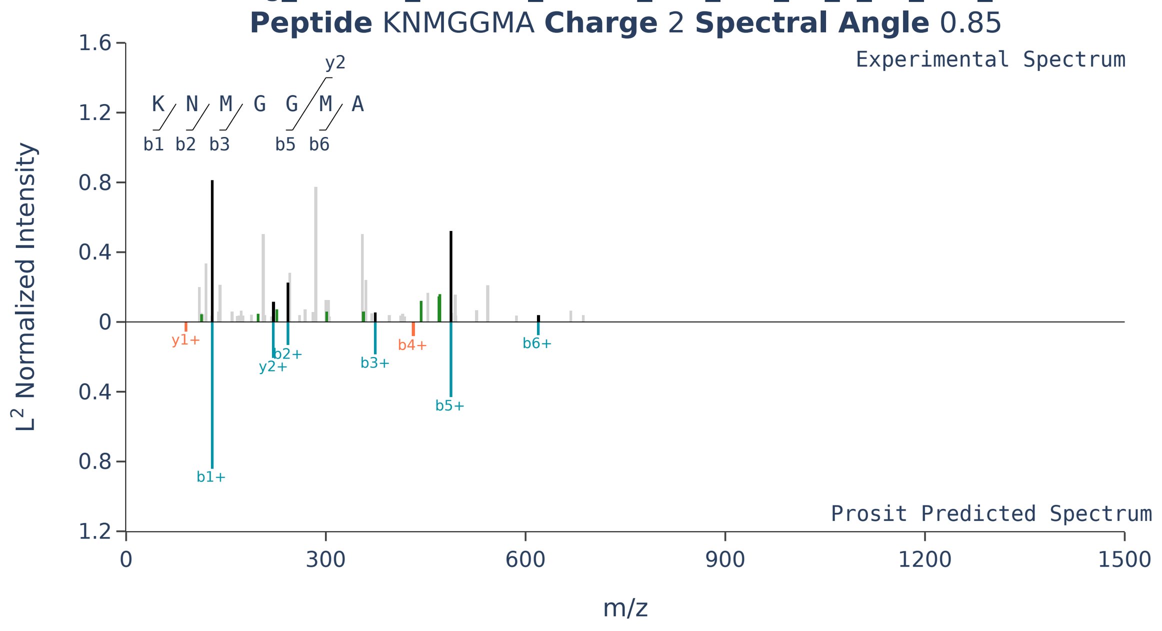

**Source** Ncheng_210623_230623_HFGoe_FFH_20S_25_1_A1_2h_R1 **Scan** 14608
**Peptide** QMDDKVLVRME **Charge** 2 **Spectral Angle** 0.88

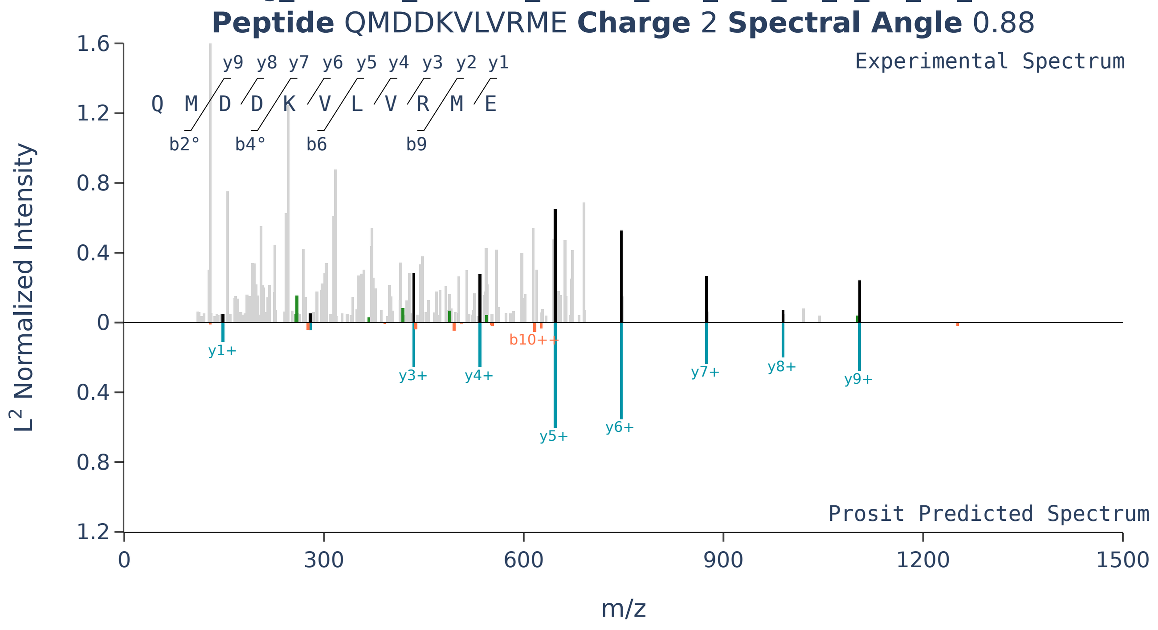


**Source** Ncheng_210623_230623_HFGoe_FFH_20S_25_1_A2_1h_R2 **Scan** 21883
**Peptide** DFFPSDVGQKPVD **Charge** 2 **Spectral Angle** 0.79

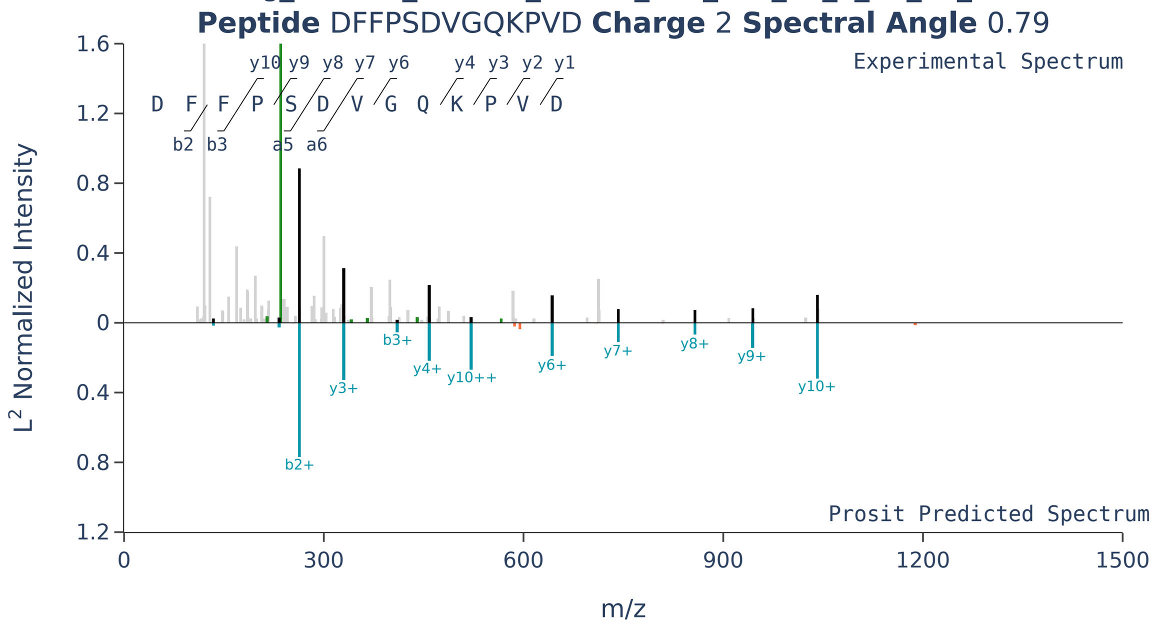


**Source** Ncheng_210623_230623_HFGoe_FFH_20S_25_1_A1_24h_R2 **Scan** 10644
**Peptide** EFINRVKE **Charge** 2 **Spectral Angle** 0.81

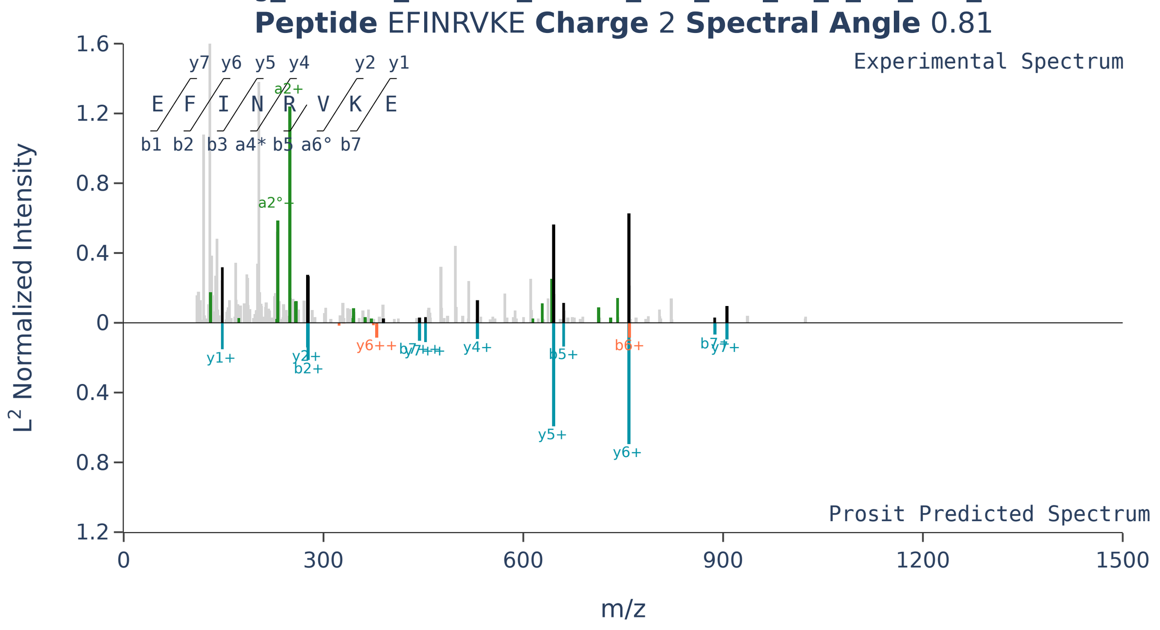


**Source** Ncheng_210623_230623_HFGoe_FFH_20S_25_1_A1_1h_R2 **Scan** 20628
**Peptide** EADVALPVVRE **Charge** 2 **Spectral Angle** 0.84

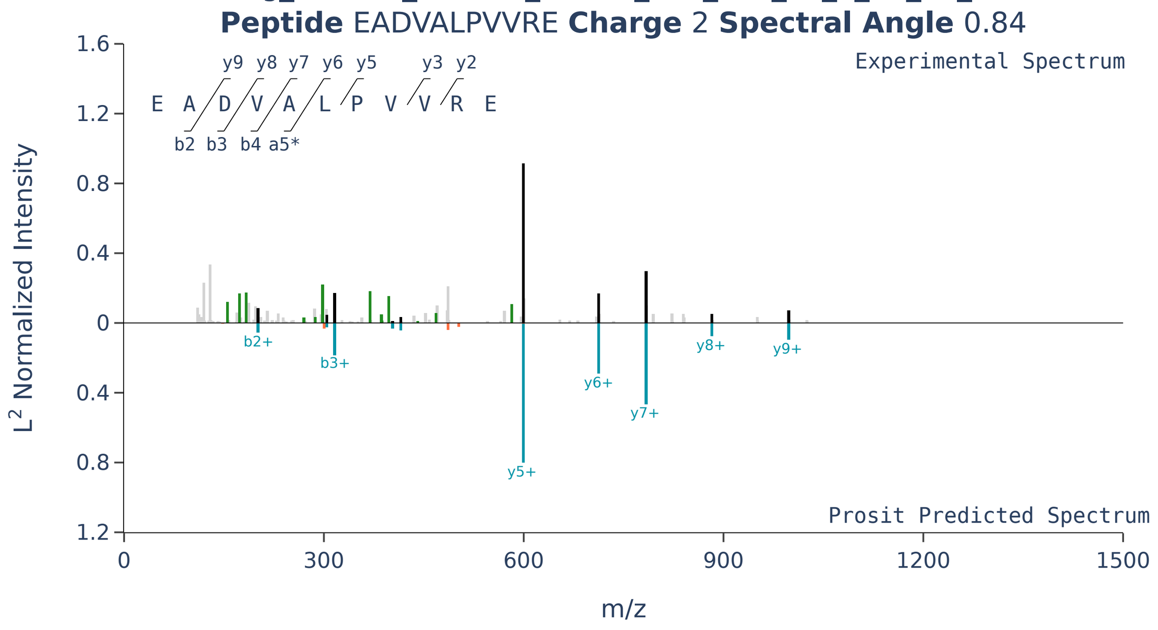


**Source** Ncheng_210623_230623_HFGoe_FFH_20S_25_1_A1_2h_R2 **Scan** 29027
**Peptide** EALPLTGVVLTKVDGDARG **Charge** 2 **Spectral Angle** 0.8

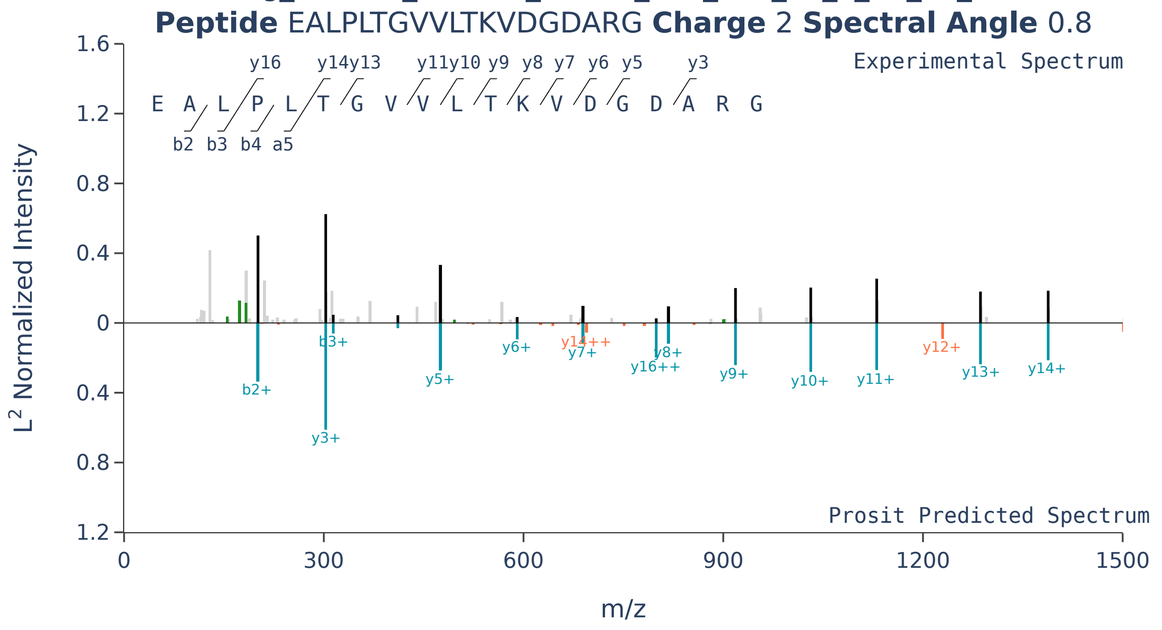


**Source** Ncheng_210623_230623_HFGoe_FFH_20S_25_1_A1_4h_R2 **Scan** 23302
**Peptide** AKAFNEALPLTG **Charge** 2 **Spectral Angle** 0.84

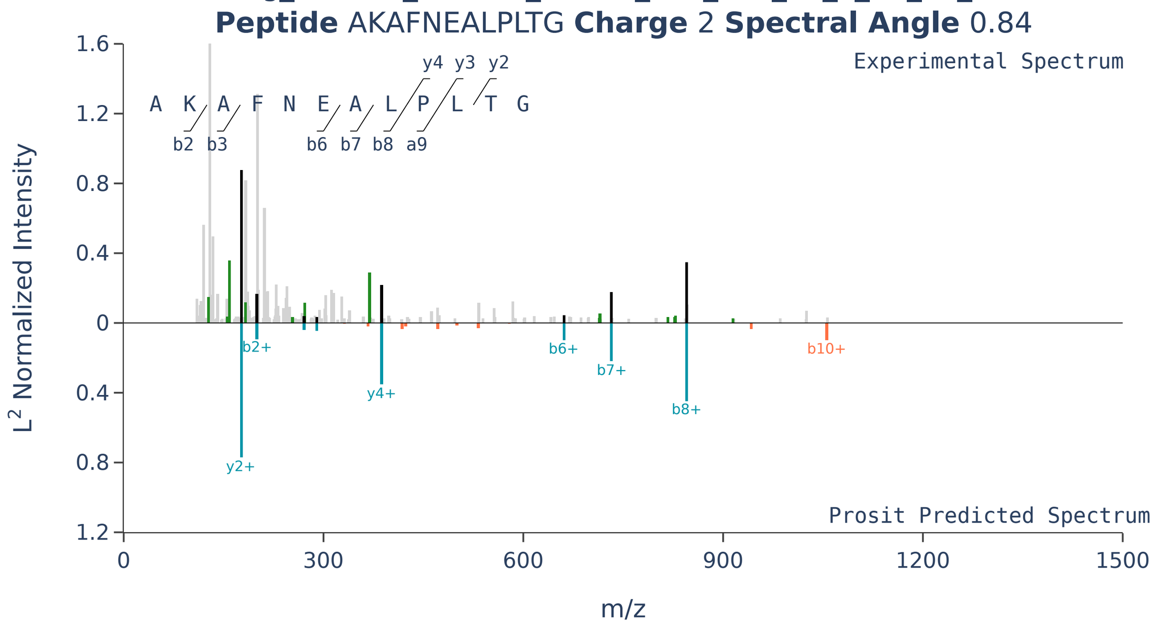


**Source** Ncheng_210623_230623_HFGoe_FFH_20S_25_1_A1_2h_R1 **Scan** 35437
**Peptide** GEENQTLNLAAQPPAVVLM **Charge** 2 **Spectral Angle** 0.88

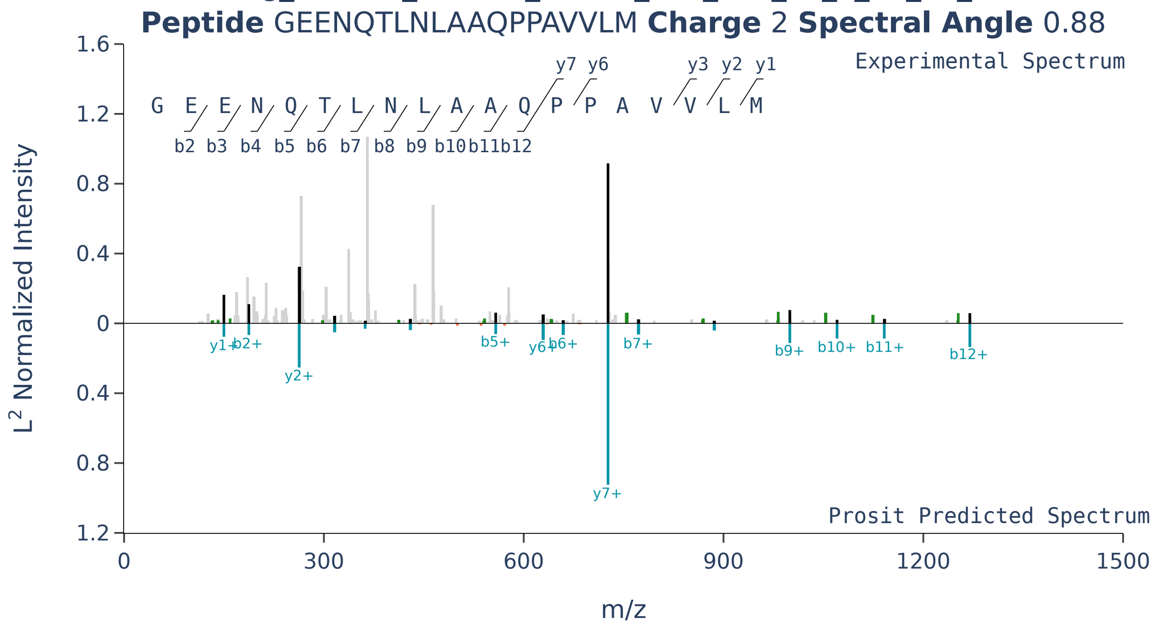


**Source** Ncheng_210623_230623_HFGoe_FFH_20S_25_1_A2_24h_R1 **Scan** 16010
**Peptide** KAVGHEVNKSLTPGQEFV **Charge** 2 **Spectral Angle** 0.82

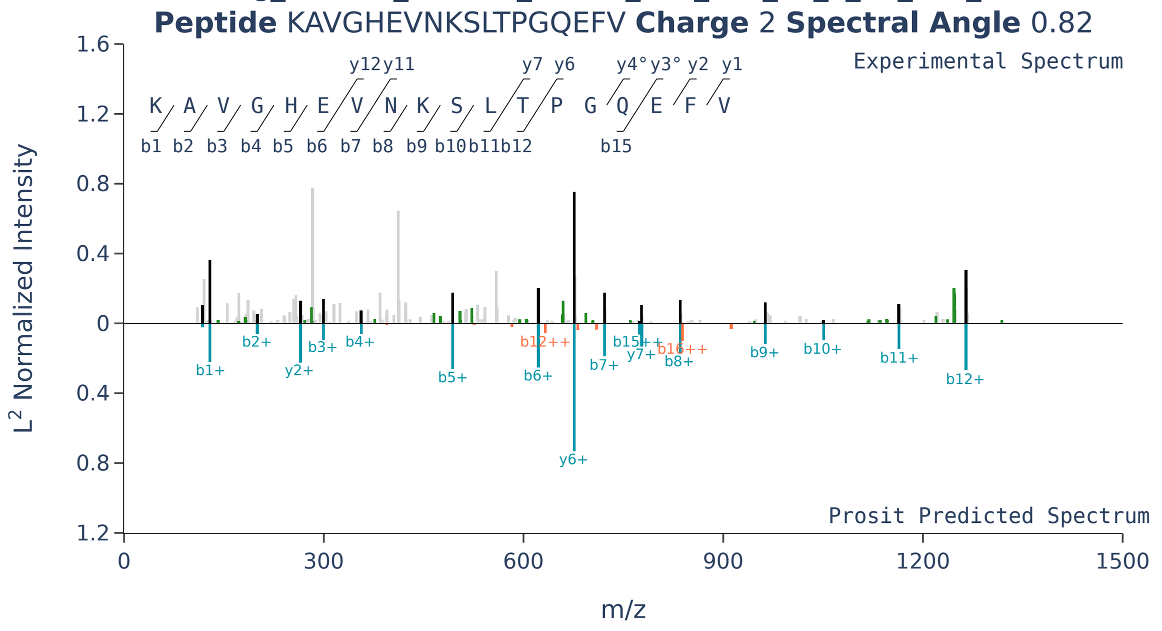


**Source** Ncheng_210623_230623_HFGoe_FFH_20S_25_1_A1_2h_R2 **Scan** 26569
**Peptide** SLMGKLPGMGQIPDNVKSQMD **Charge** 2 **Spectral Angle** 0.84

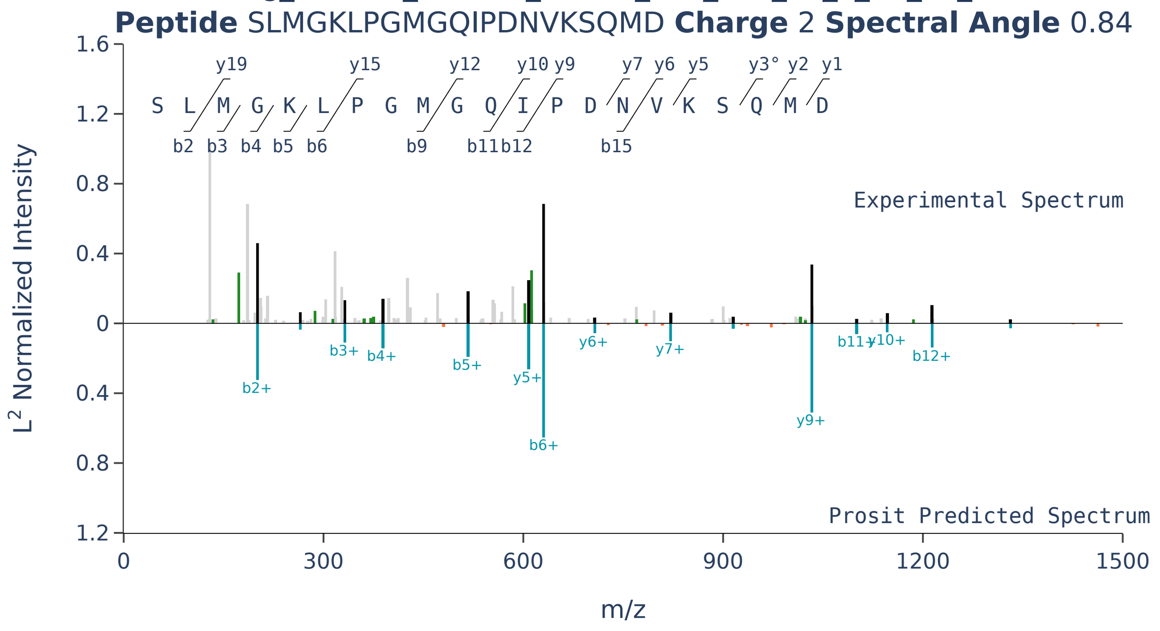


**Source** Ncheng_210623_230623_HFGoe_FFH_20S_25_1_A2_4h_R1 **Scan** 15539
**Peptide** VAAMGEENQTL **Charge** 2 **Spectral Angle** 0.88

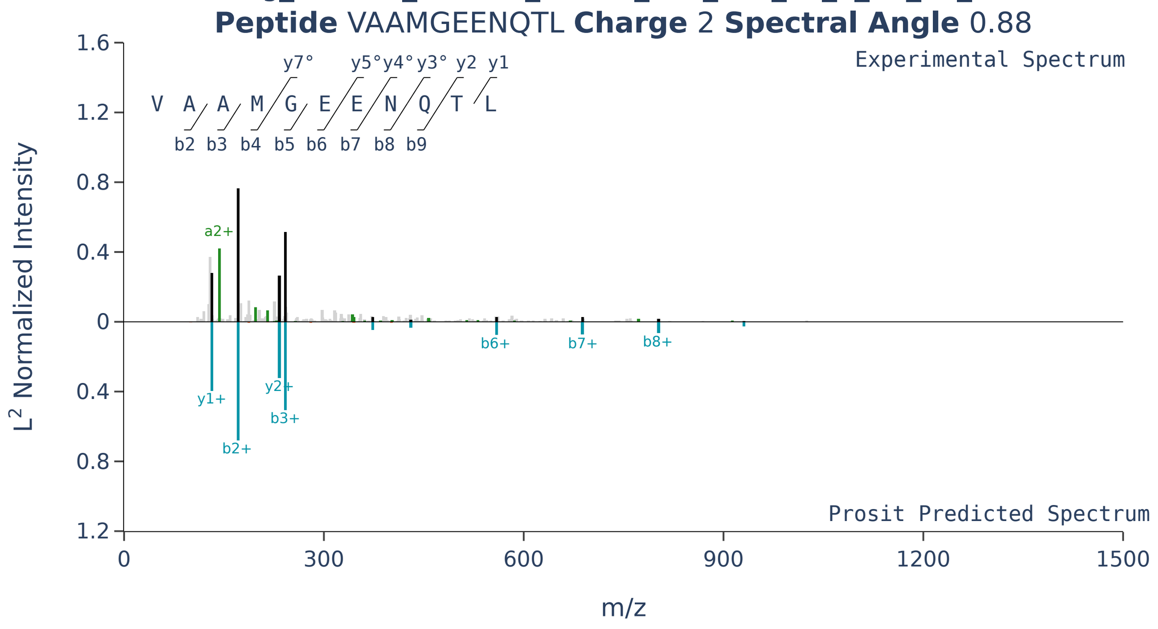


**Source** Ncheng_210623_230623_HFGoe_FFH_20S_25_1_A1_2h_R2 **Scan** 13623
**Peptide** ITGKPIKF **Charge** 2 **Spectral Angle** 0.78

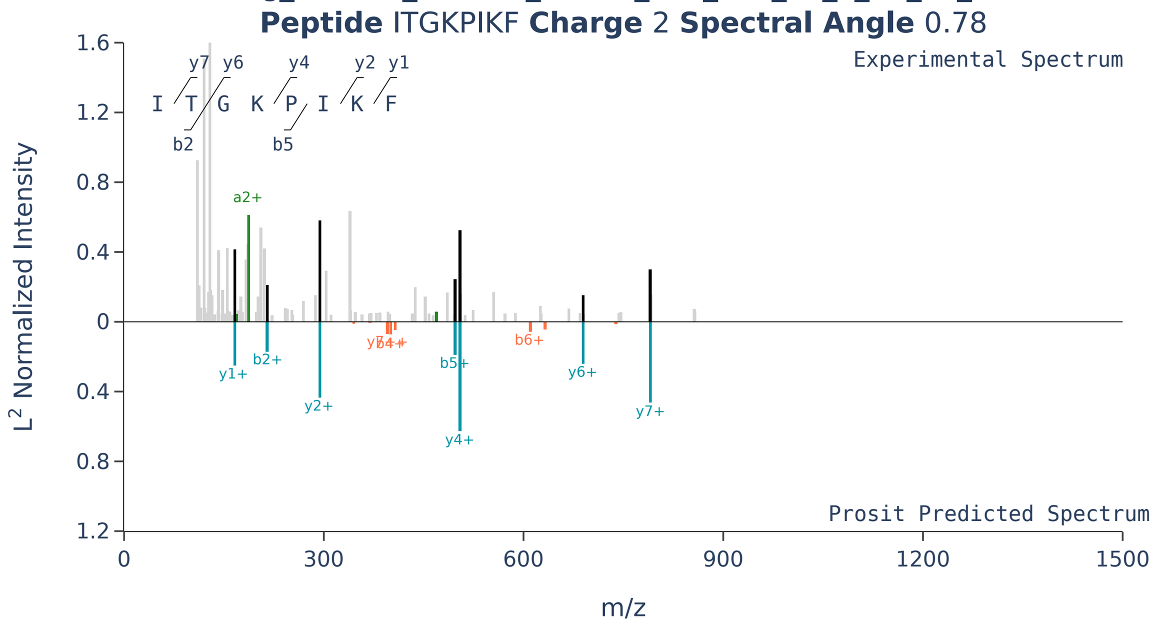


**Source** Ncheng_210623_230623_HFGoe_FFH_20S_25_1_A1_24h_R2 **Scan** 7187
**Peptide** VLTKVDGDARGG **Charge** 3 **Spectral Angle** 0.88

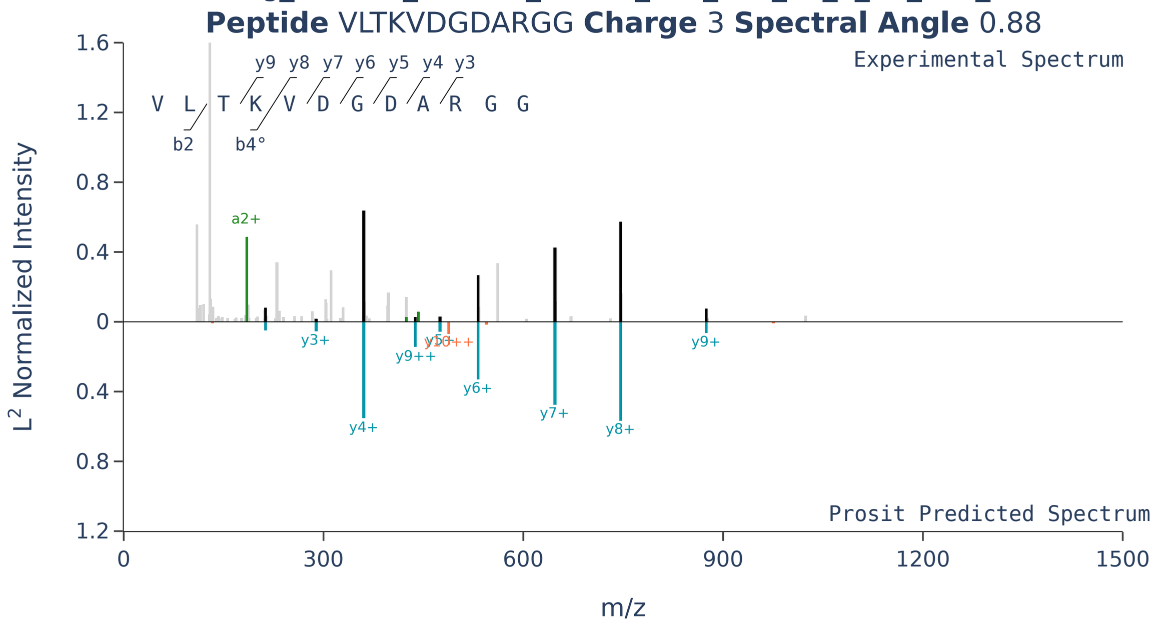


**Source** Ncheng_210623_230623_HFGoe_FFH_20S_25_1_A2_2h_R1 **Scan** 24350
**Peptide** LMGKLPGMGQIPDNVKSQMD **Charge** 3 **Spectral Angle** 0.82

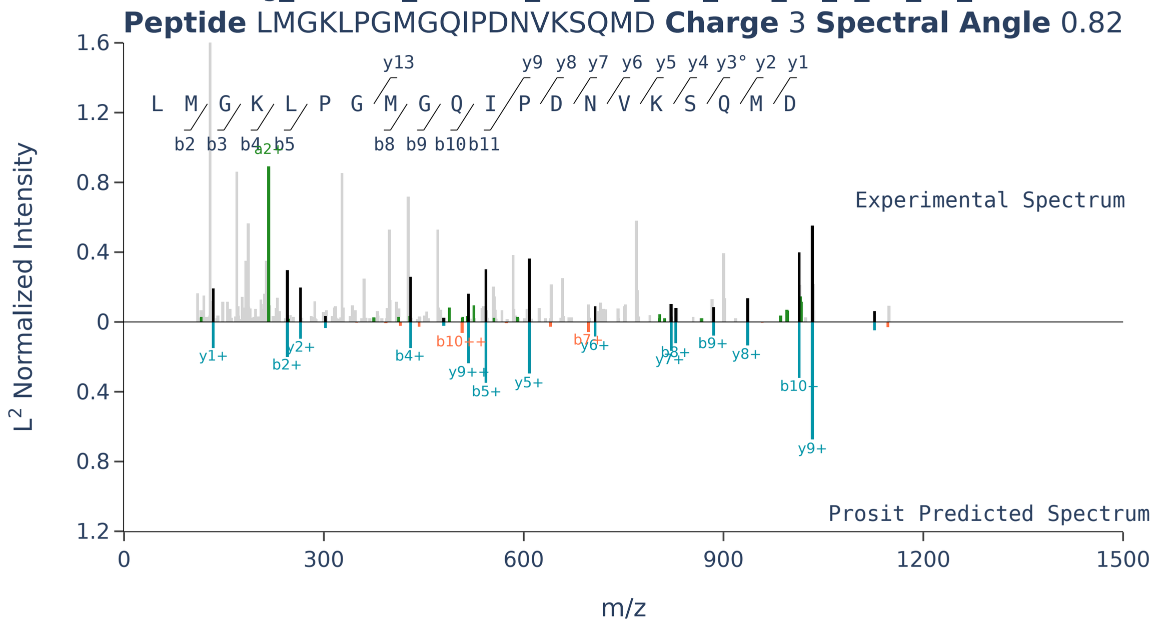


**Source** Ncheng_210623_230623_HFGoe_FFH_20S_25_1_A2_24h_R1 **Scan** 10583
**Peptide** GMQVQDVN **Charge** 2 **Spectral Angle** 0.91

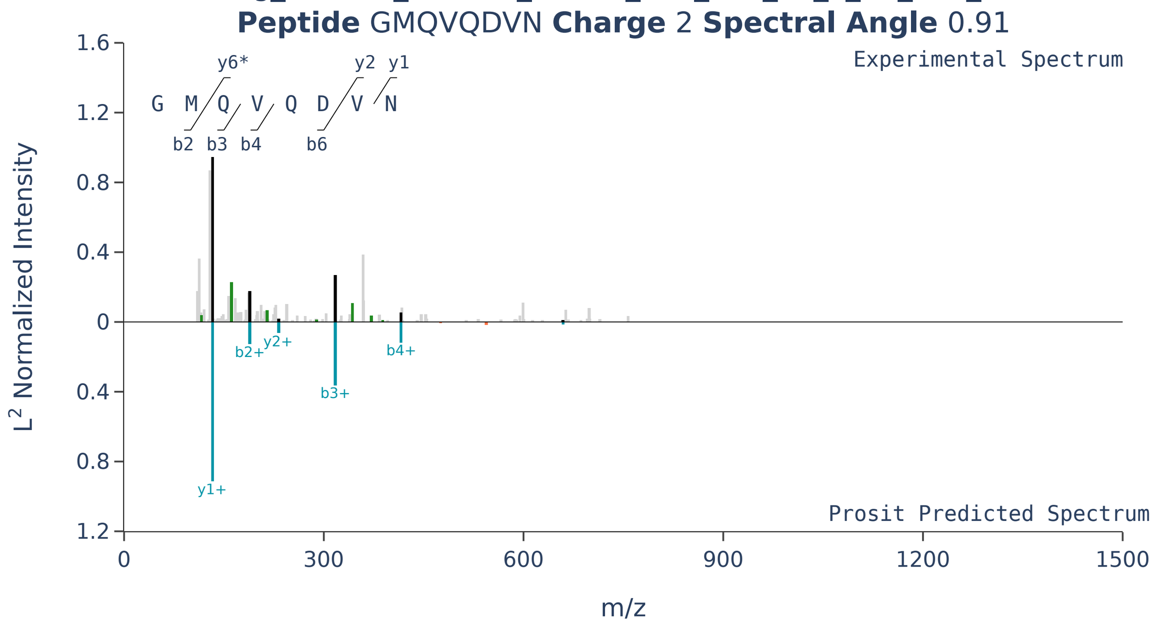


**Source** Ncheng_210623_230623_HFGoe_FFH_20S_25_1_A2_1h_R1 **Scan** 32930
**Peptide** KQLETLAEQVGVDFFPSD **Charge** 2 **Spectral Angle** 0.94

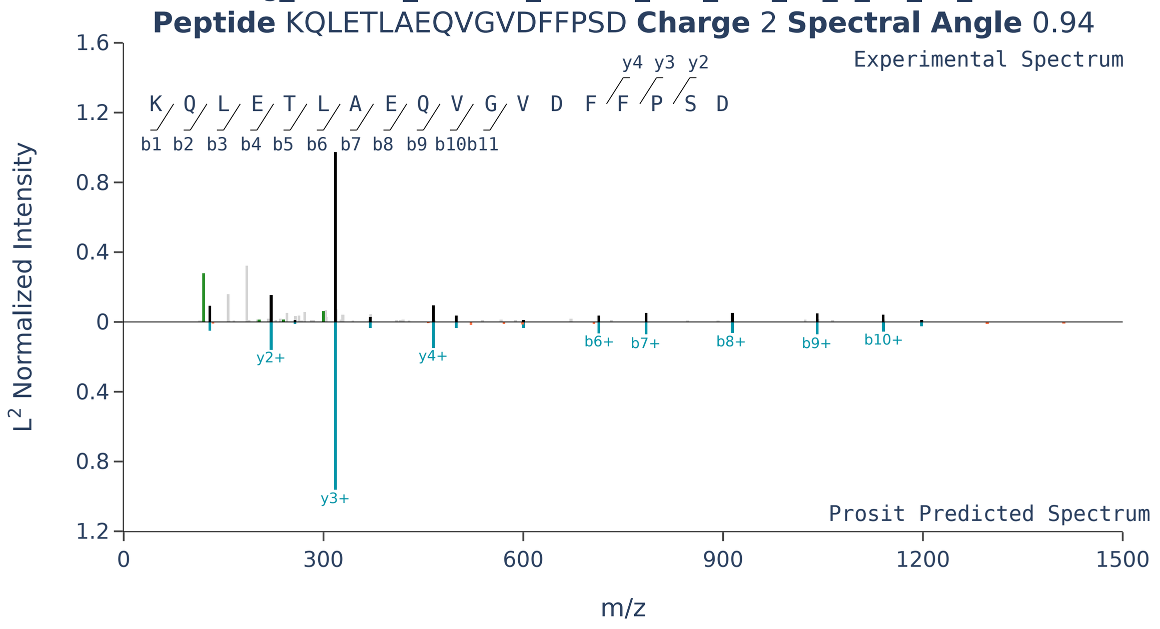

**Source** Ncheng_210623_230623_HFGoe_FFH_20S_25_1_A1_1h_R2 **Scan** 19880
**Peptide** EALEPFHPDRI **Charge** 3 **Spectral Angle** 0.84

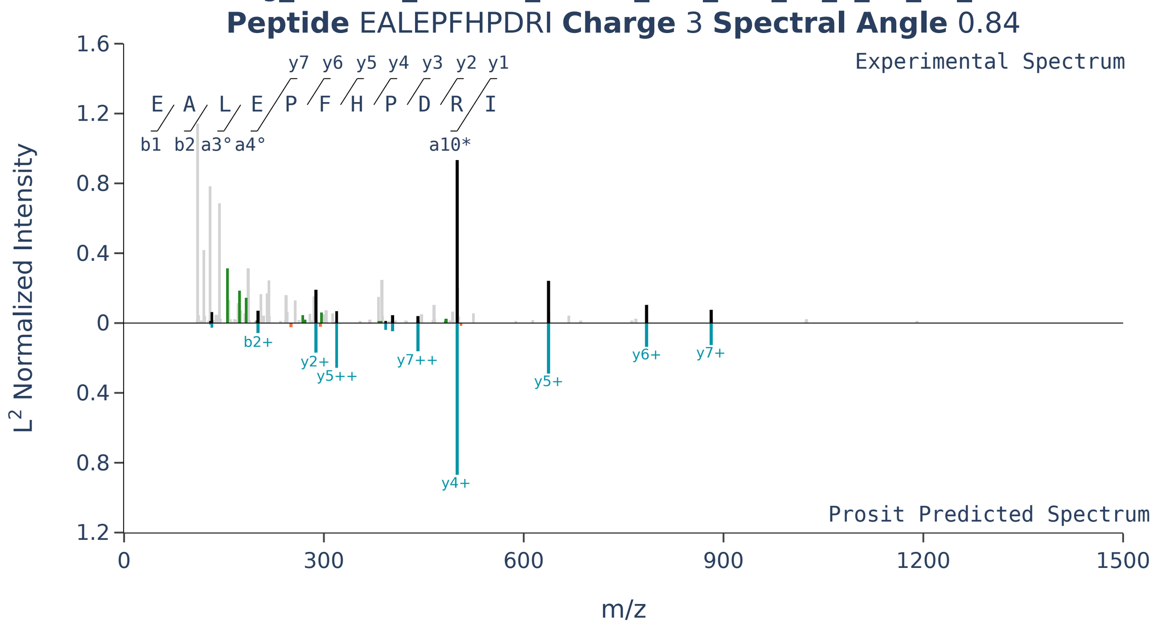


**Source** Ncheng_210623_230623_HFGoe_FFH_20S_25_1_A1_2h_R2 **Scan** 10787
**Peptide** EIKQVHASI **Charge** 1 **Spectral Angle** 0.77

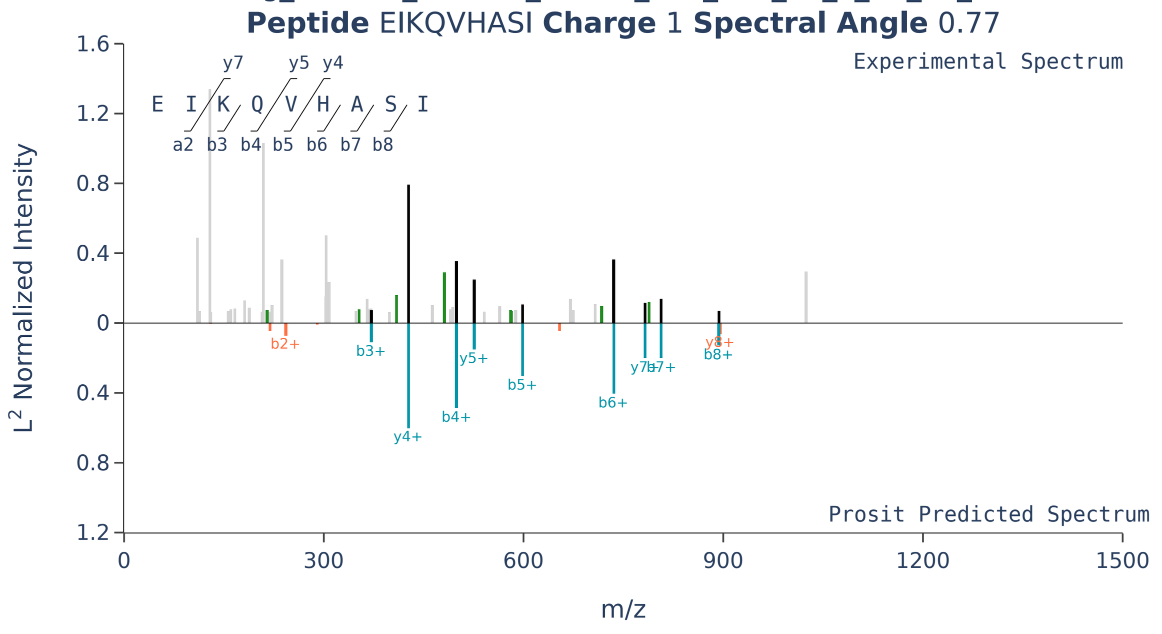


**Source** Ncheng_210623_230623_HFGoe_FFH_20S_25_1_A2_24h_R2 **Scan** 16142
**Peptide** KEAKLKFYDV **Charge** 3 **Spectral Angle** 0.86

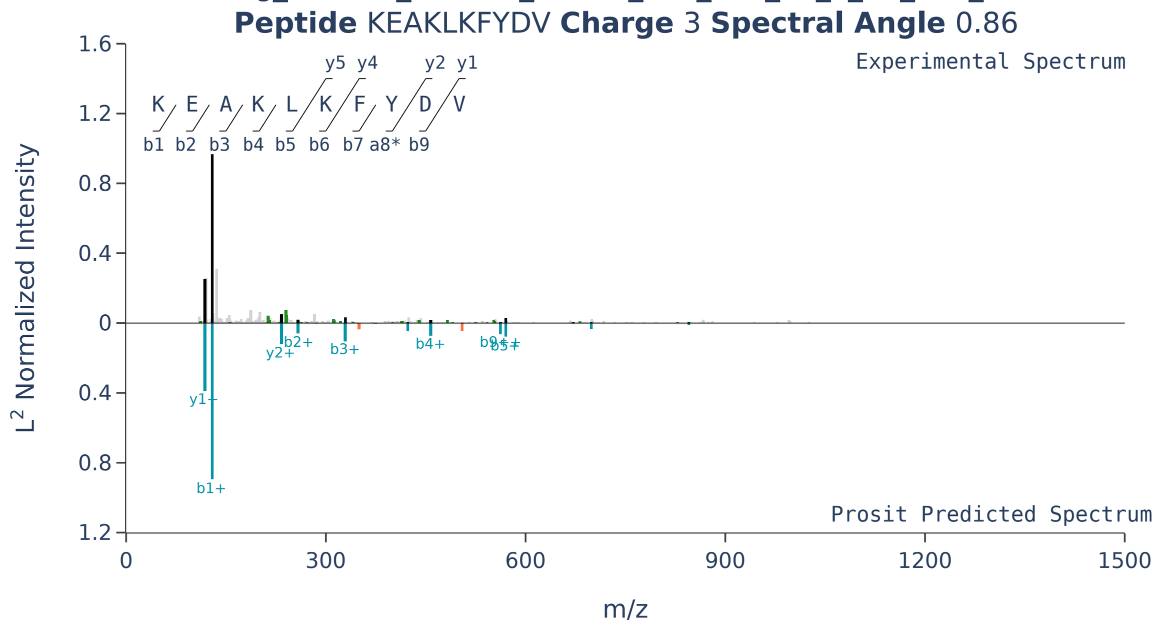


**Source** Ncheng_210623_230623_HFGoe_FFH_20S_25_1_A1_24h_R1 **Scan** 13928
**Peptide** GQKPVDIVNA **Charge** 2 **Spectral Angle** 0.77

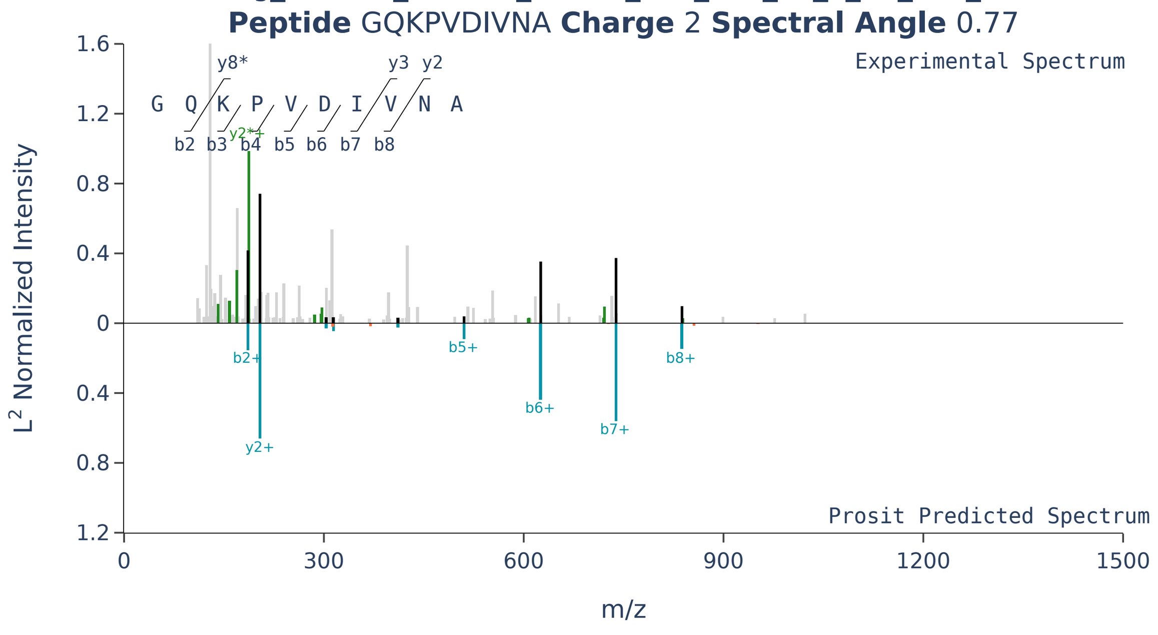


**Source** Ncheng_210623_230623_HFGoe_FFH_20S_25_1_A2_4h_R2 **Scan** 15124
**Peptide** LVDTAGRLHV **Charge** 2 **Spectral Angle** 0.85

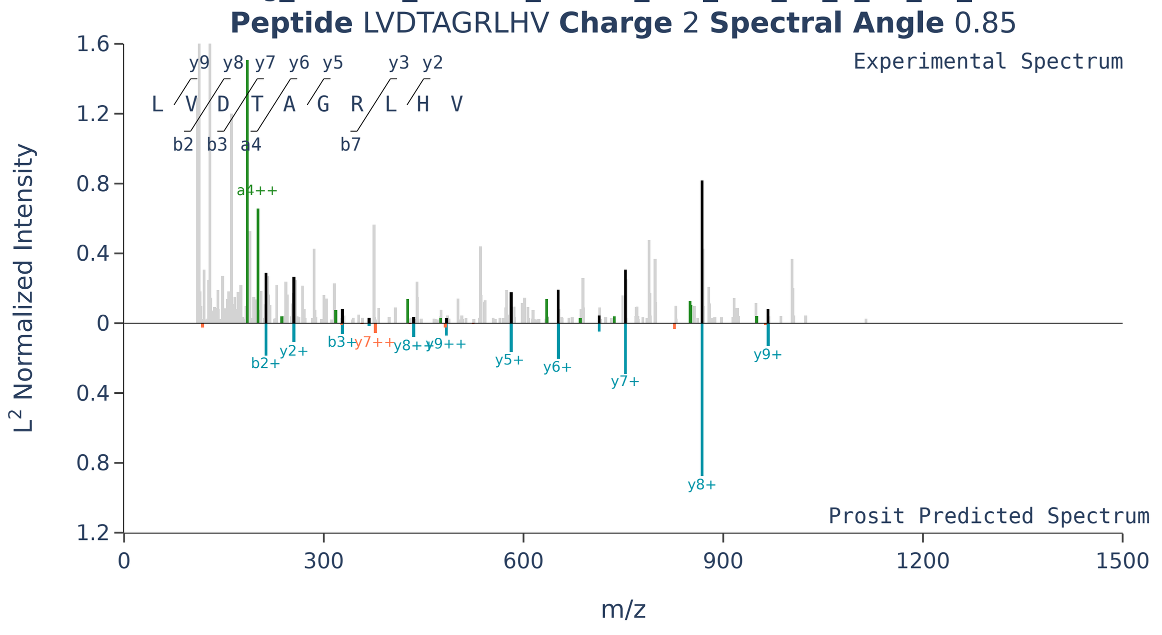


**Source** Ncheng_210623_230623_HFGoe_FFH_20S_25_1_A1_24h_R1 **Scan** 7025
**Peptide** KKGGMAKMM **Charge** 2 **Spectral Angle** 0.89

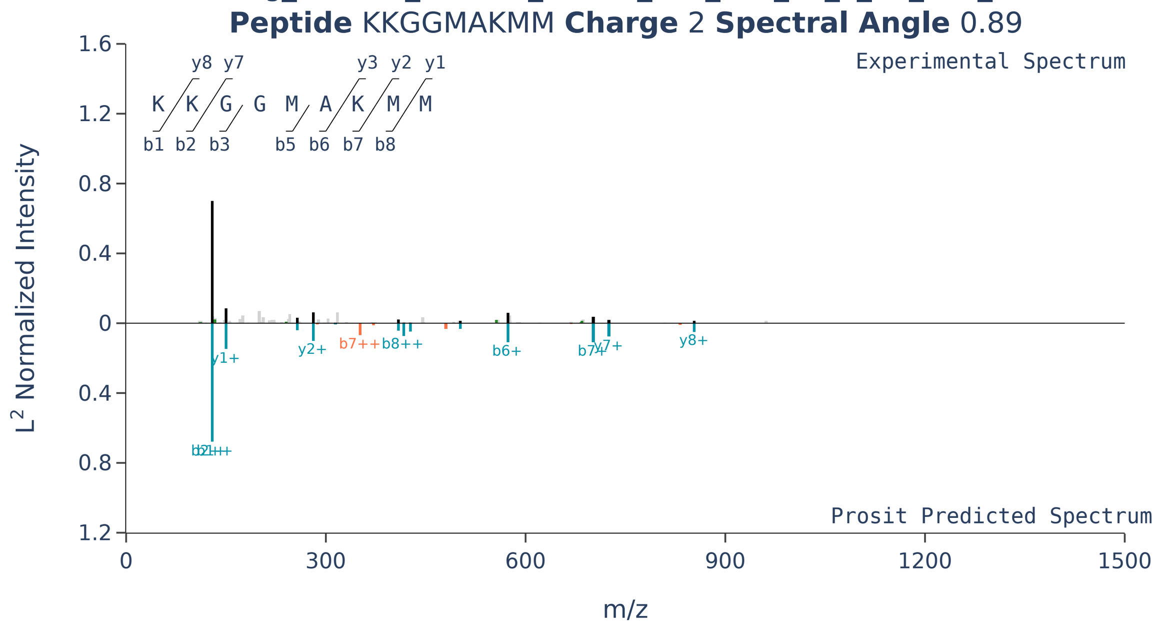


**Source** Ncheng_210623_230623_HFGoe_FFH_20S_25_1_A1_24h_R1 **Scan** 16498
**Peptide** ADVYRPAAI **Charge** 1 **Spectral Angle** 0.92

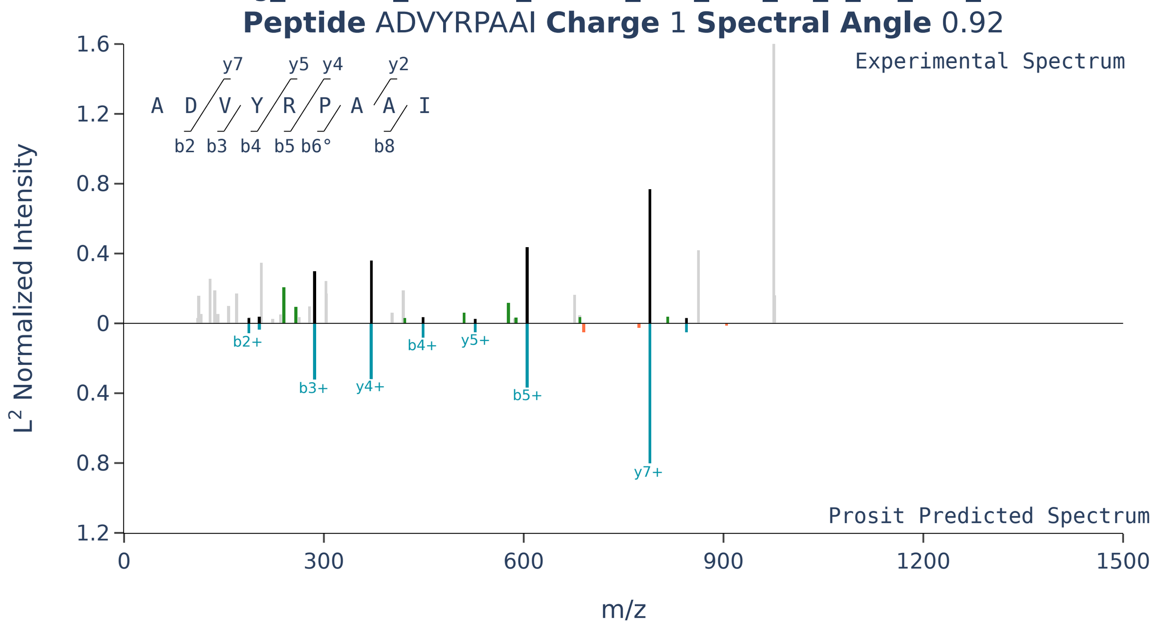


**Source** Ncheng_210623_230623_HFGoe_FFH_20S_25_1_A2_4h_R2 **Scan** 21782
**Peptide** MFDNLTDRLSRT **Charge** 3 **Spectral Angle** 0.78

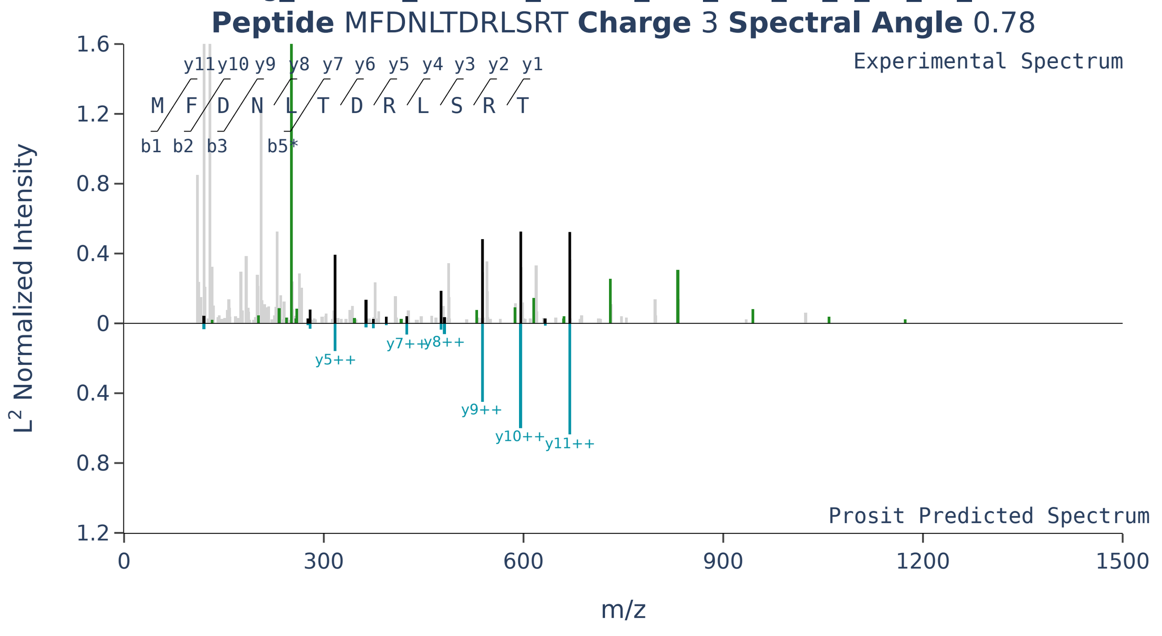


**Source** Ncheng_210623_230623_HFGoe_FFH_20S_25_1_A1_2h_R1 **Scan** 23862
**Peptide** PSDVGQKPVDIVNAALKE **Charge** 3 **Spectral Angle** 0.82

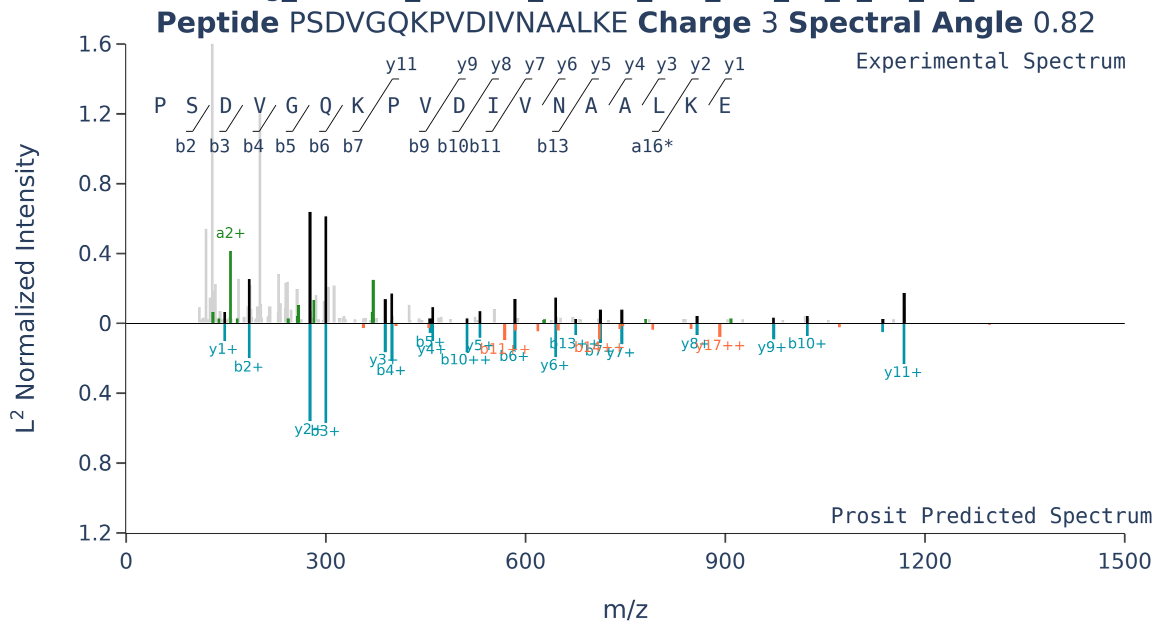


**Source** Ncheng_210623_230623_HFGoe_FFH_20S_25_1_A1_4h_R2 **Scan** 21475
**Peptide** NKSLTPGQEFVKI **Charge** 2 **Spectral Angle** 0.84

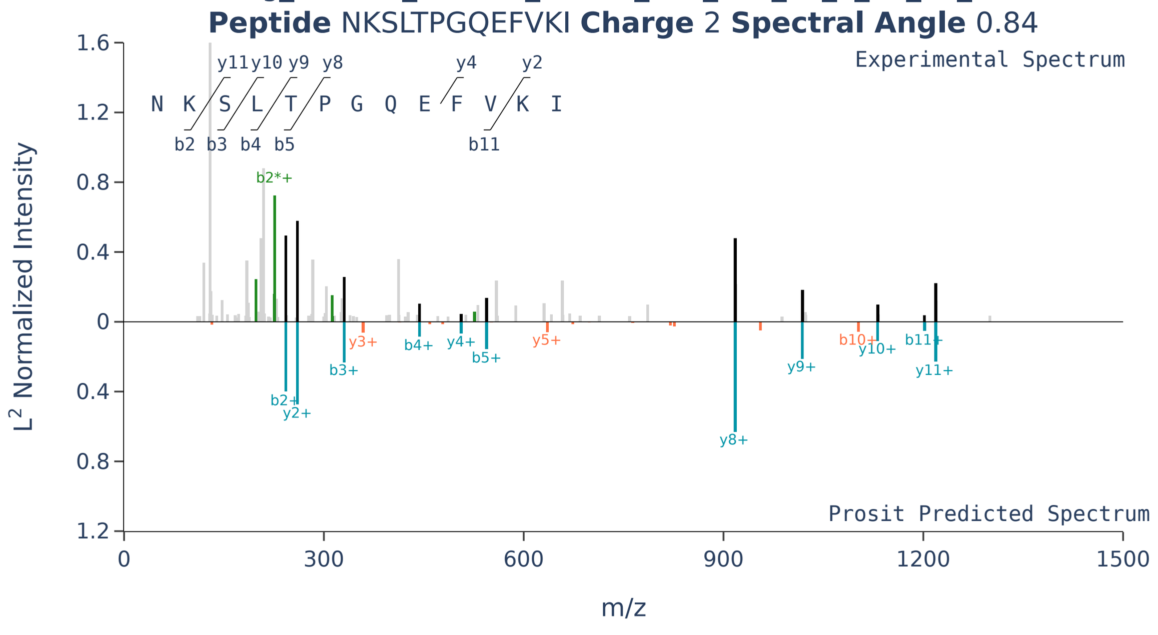


**Source** Ncheng_210623_230623_HFGoe_FFH_20S_25_1_A2_4h_R1 **Scan** 29123
**Peptide** TPGQEFVKIVRNEL **Charge** 2 **Spectral Angle** 0.82

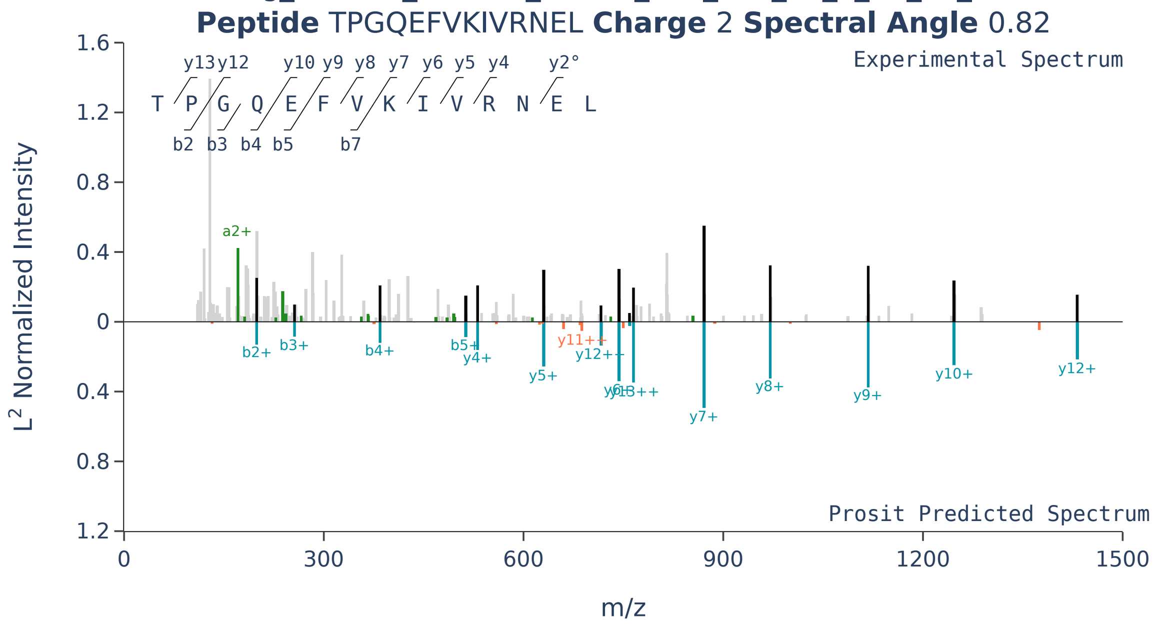


**Source** Ncheng_210623_230623_HFGoe_FFH_20S_25_1_A1_24h_R2 **Scan** 9940
**Peptide** ASRILGMGD **Charge** 2 **Spectral Angle** 0.77

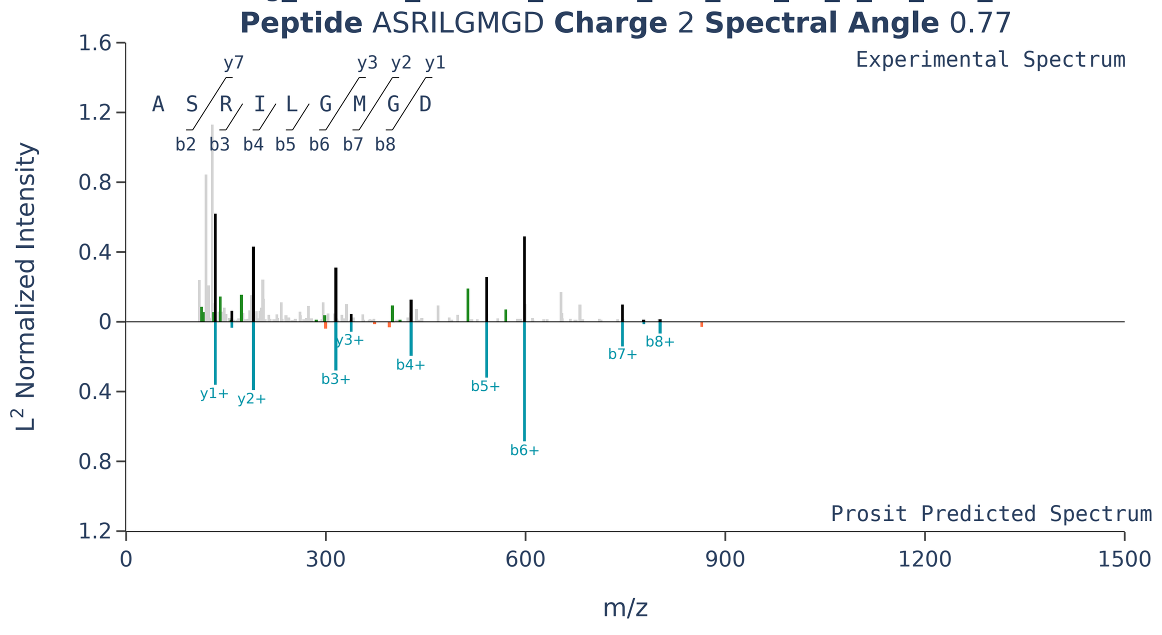


**Source** Ncheng_210623_230623_HFGoe_FFH_20S_25_1_A1_24h_R1 **Scan** 19840
**Peptide** KLPGMGQL **Charge** 2 **Spectral Angle** 0.82

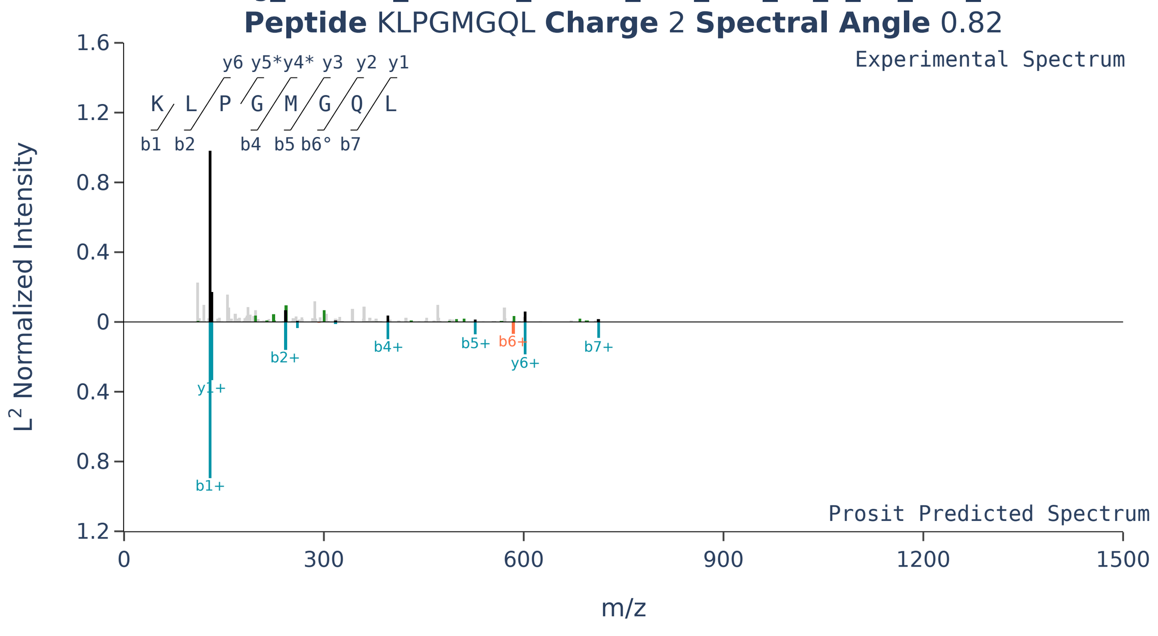


**Source** Ncheng_210623_230623_HFGoe_FFH_20S_25_1_A2_24h_R1 **Scan** 9904
**Peptide** RNELVAAM **Charge** 2 **Spectral Angle** 0.86

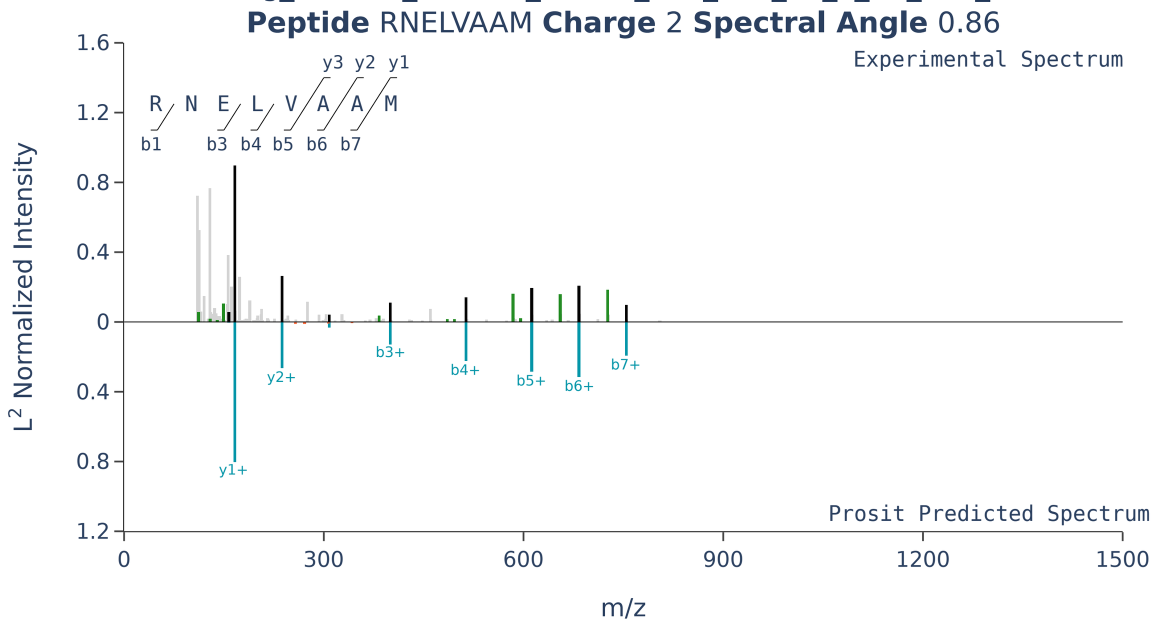


**Source** Ncheng_210623_230623_HFGoe_FFH_20S_25_1_A1_4h_R2 **Scan** 10705
**Peptide** KKVLVVSA **Charge** 2 **Spectral Angle** 0.73

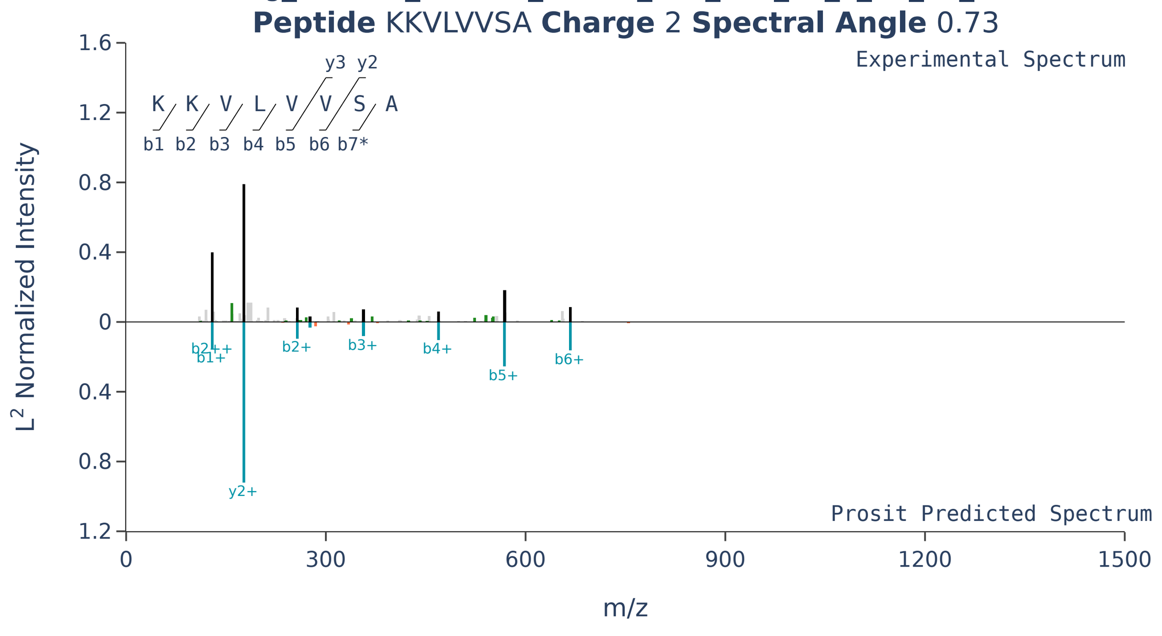

**Source** Ncheng_210623_230623_HFGoe_FFH_20S_25_1_A1_24h_R1 **Scan** 7787
**Peptide** KAVGHEVNKSLTPGQE **Charge** 2 **Spectral Angle** 0.9

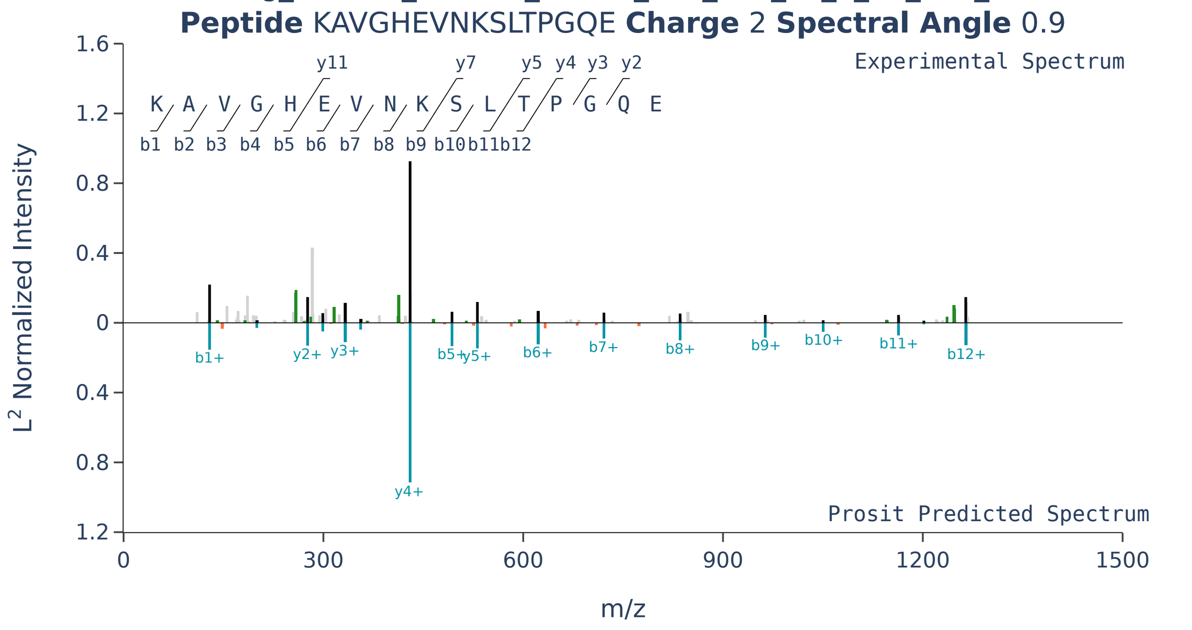


**Source** Ncheng_210623_230623_HFGoe_FFH_20S_25_1_A2_1h_R2 **Scan** 15489
**Peptide** DDKVLVRMEA **Charge** 2 **Spectral Angle** 0.91

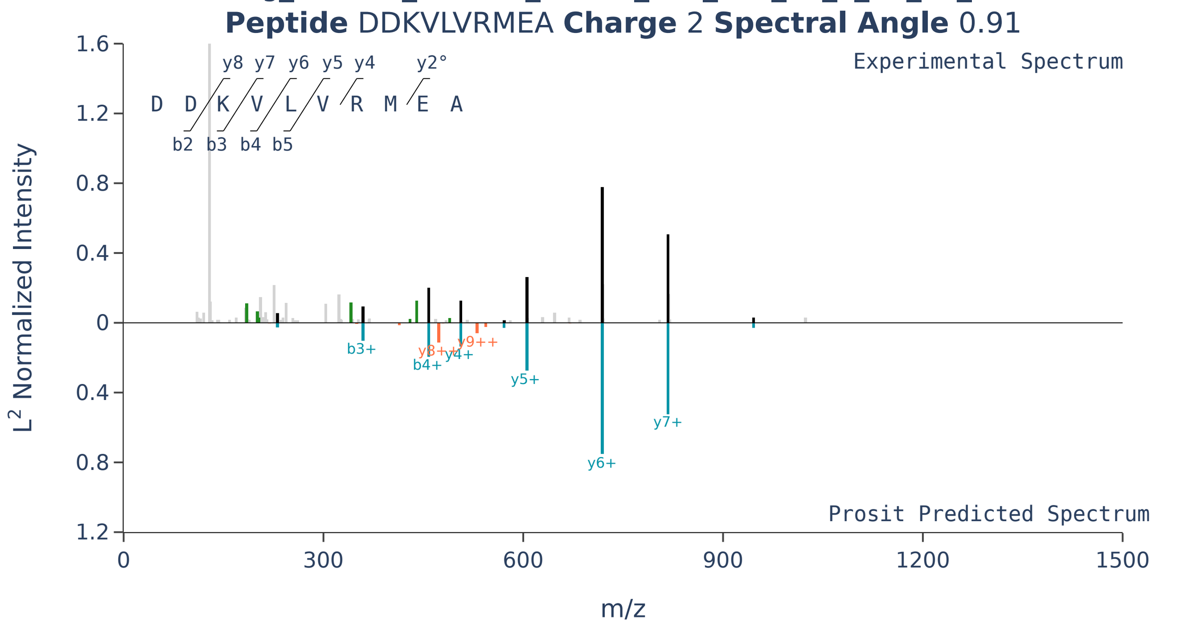


**Source** Ncheng_210623_230623_HFGoe_FFH_20S_25_1_A2_24h_R1 **Scan** 19708
**Peptide** EAIINSMTMKE **Charge** 2 **Spectral Angle** 0.74

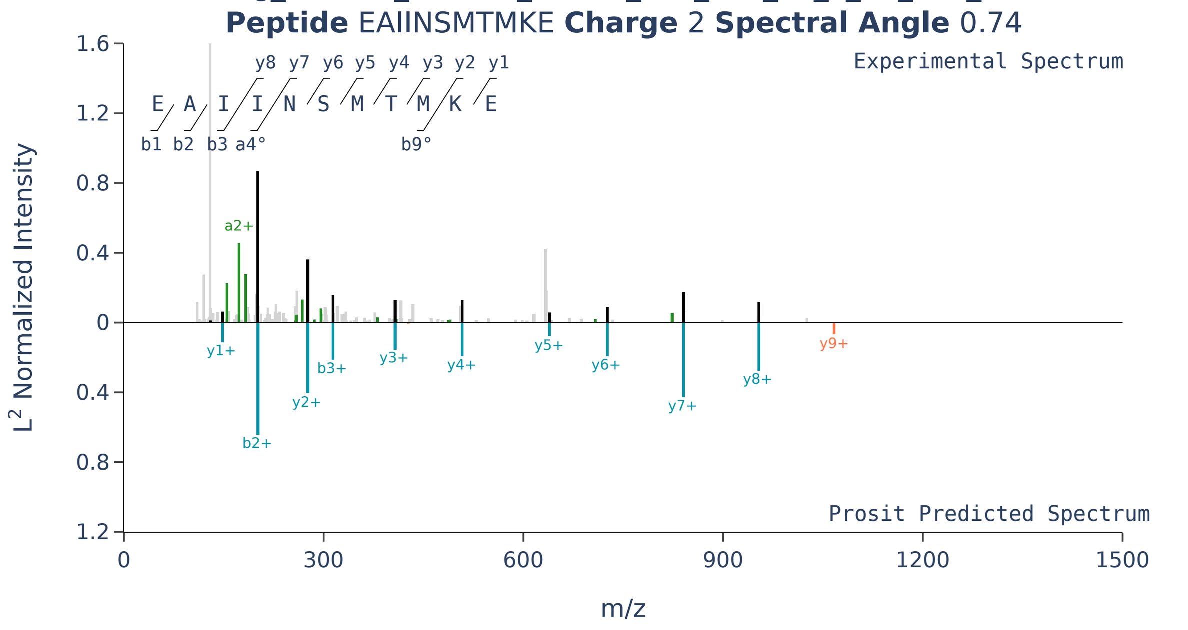


**Source** Ncheng_210623_230623_HFGoe_FFH_20S_25_1_A2_24h_R2 **Scan** 15648
**Peptide** QGAGKTTSVGKLGKFLRE **Charge** 2 **Spectral Angle** 0.83

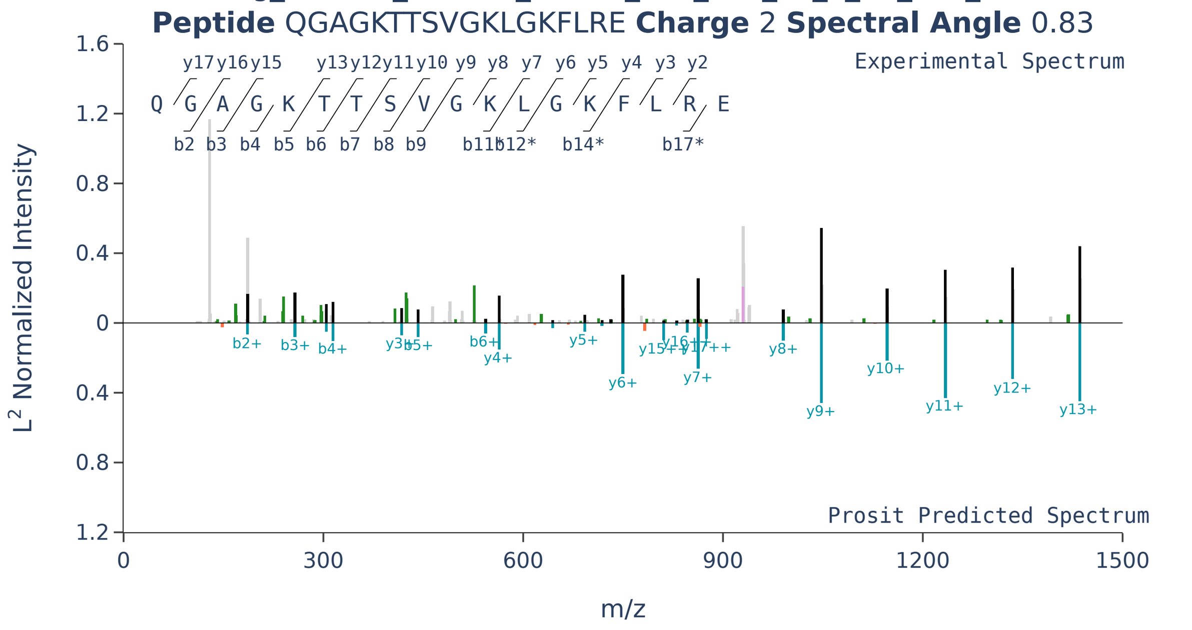


**Source** Ncheng_210623_230623_HFGoe_FFH_20S_25_1_A2_4h_R2 **Scan** 8924
**Peptide** GVGEKTEALE **Charge** 1 **Spectral Angle** 0.85

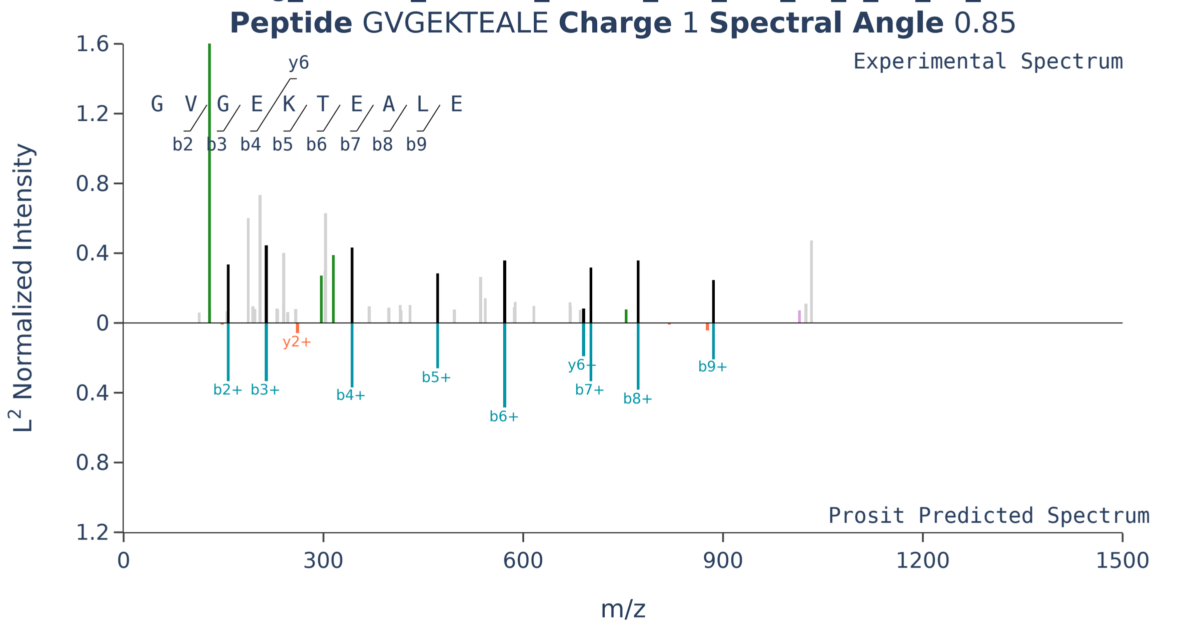


**Source** Ncheng_210623_230623_HFGoe_FFH_20S_25_1_A1_4h_R1 **Scan** 19072
**Peptide** GHEVNKSLTPGQEFVKLVR **Charge** 3 **Spectral Angle** 0.78

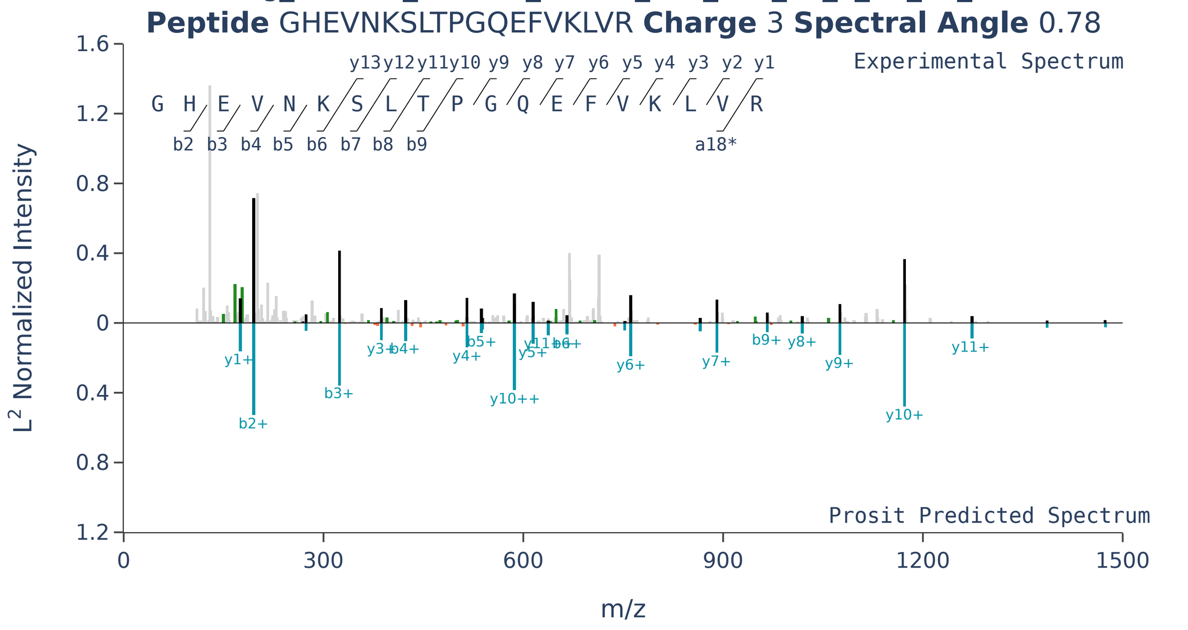


**Source** Ncheng_210623_230623_HFGoe_FFH_20S_25_1_A2_24h_R2 **Scan** 17201
**Peptide** EPFHPDRIASRL **Charge** 4 **Spectral Angle** 0.85

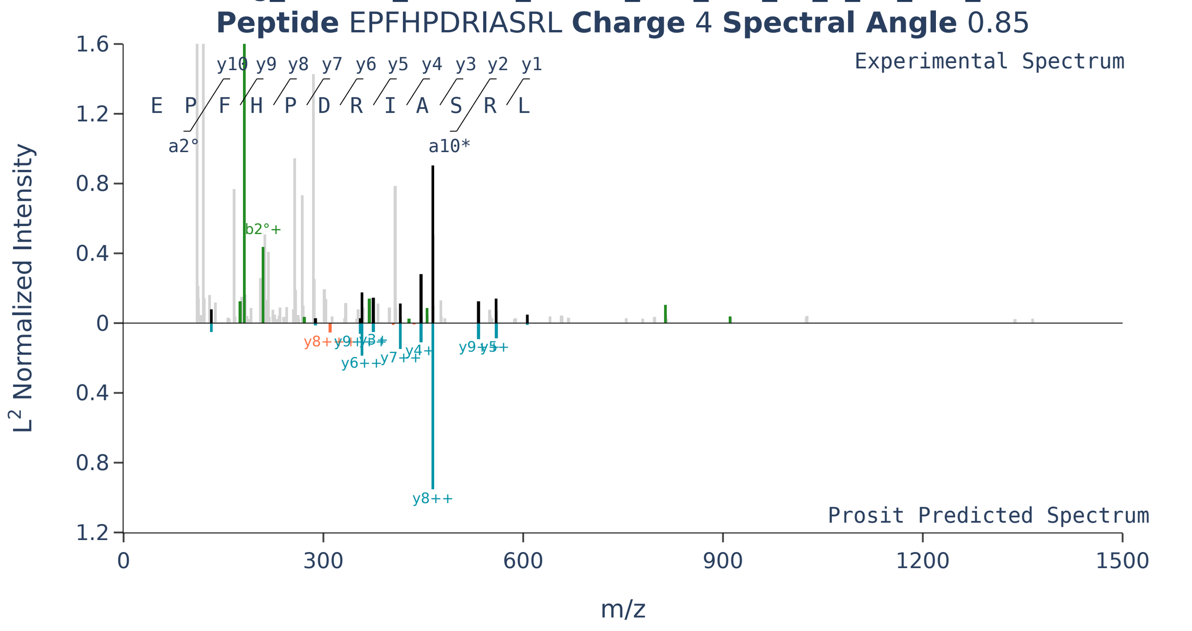


**Source** Ncheng_210623_230623_HFGoe_FFH_20S_25_1_A1_4h_R1 **Scan** 8431
**Peptide** AGRLHVD **Charge** 2 **Spectral Angle** 0.82

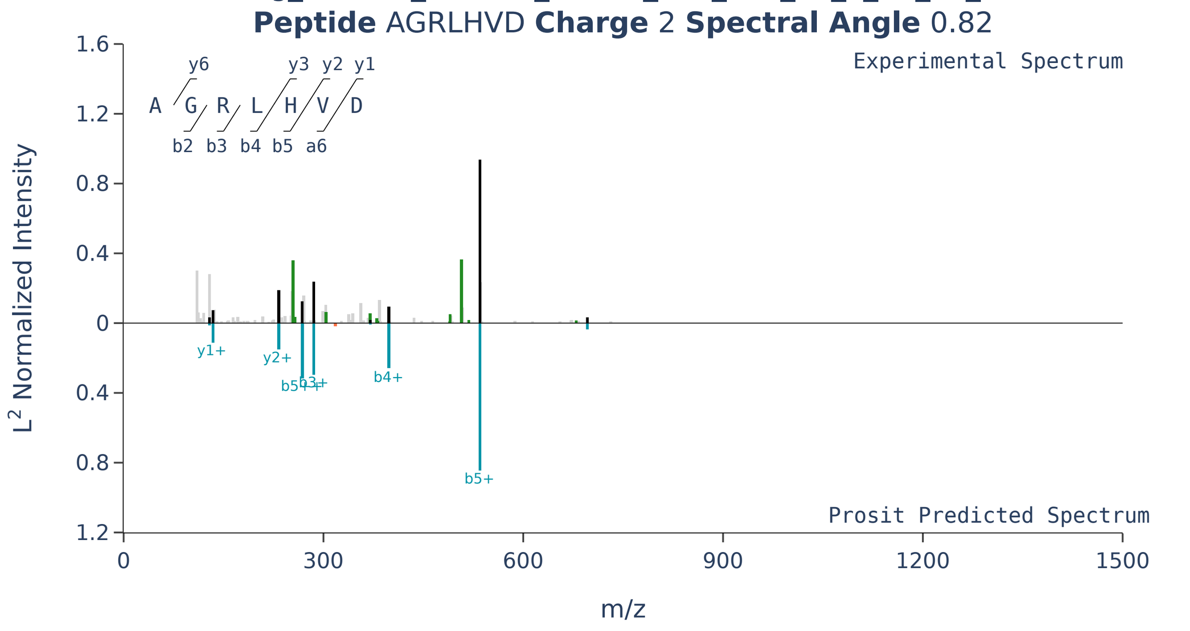


**Source** Ncheng_210623_230623_HFGoe_FFH_20S_25_1_A2_4h_R1 **Scan** 33688
**Peptide** SLTPGQEFVKIVRNEL **Charge** 2 **Spectral Angle** 0.69

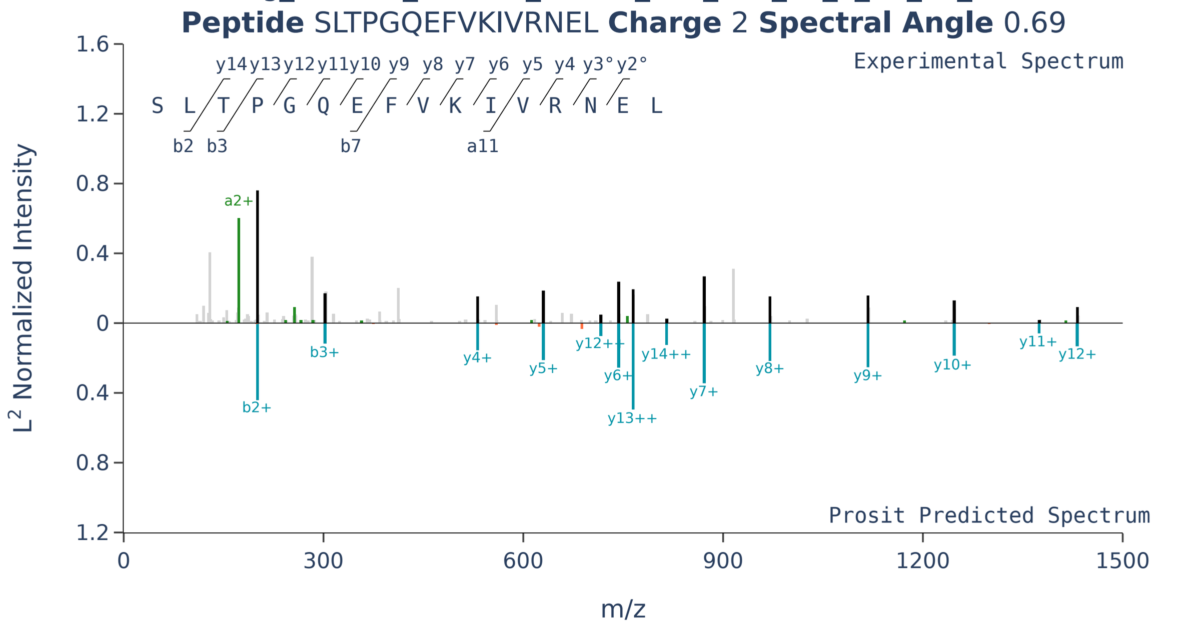


**Source** Ncheng_210623_230623_HFGoe_FFH_20S_25_1_A2_4h_R2 **Scan** 14117
**Peptide** GHEVNKSLTPGQEF **Charge** 2 **Spectral Angle** 0.9

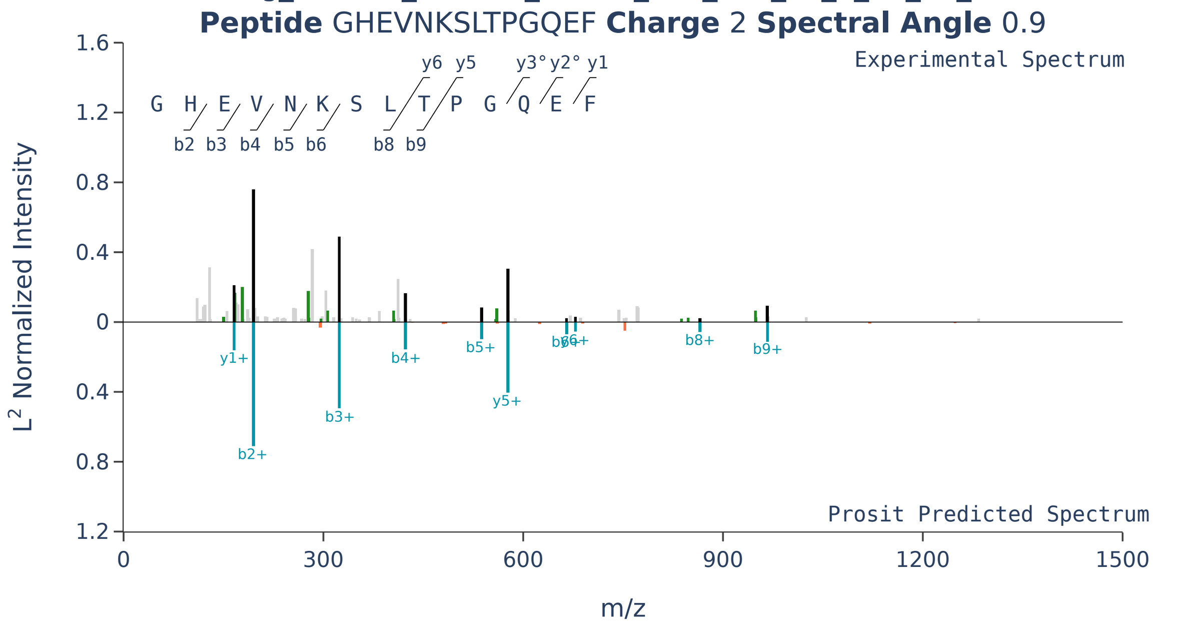


**Source** Ncheng_210623_230623_HFGoe_FFH_20S_25_1_A2_24h_R2 **Scan** 8052
**Peptide** GGMAKMMR **Charge** 1 **Spectral Angle** 0.79

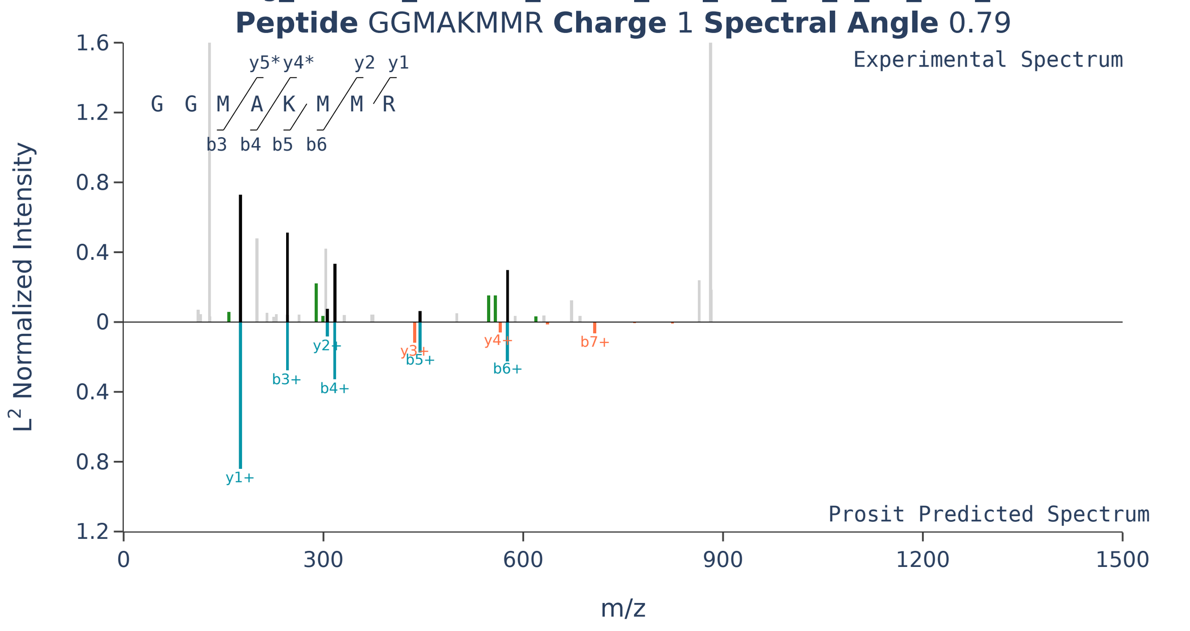


**Source** Ncheng_210623_230623_HFGoe_FFH_20S_25_1_A2_24h_R1 **Scan** 13281
**Peptide** EFVKIVRN **Charge** 1 **Spectral Angle** 0.7

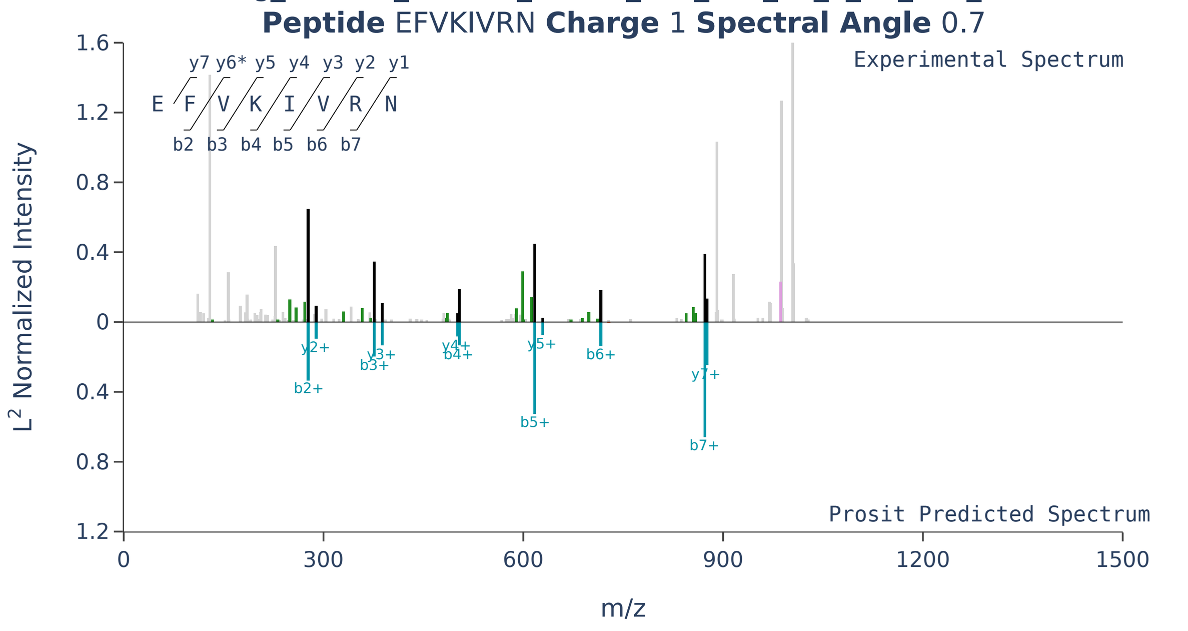


**Source** Ncheng_210623_230623_HFGoe_FFH_20S_25_1_A1_24h_R1 **Scan** 9738
**Peptide** DGDARGGAALSIRH **Charge** 4 **Spectral Angle** 0.94

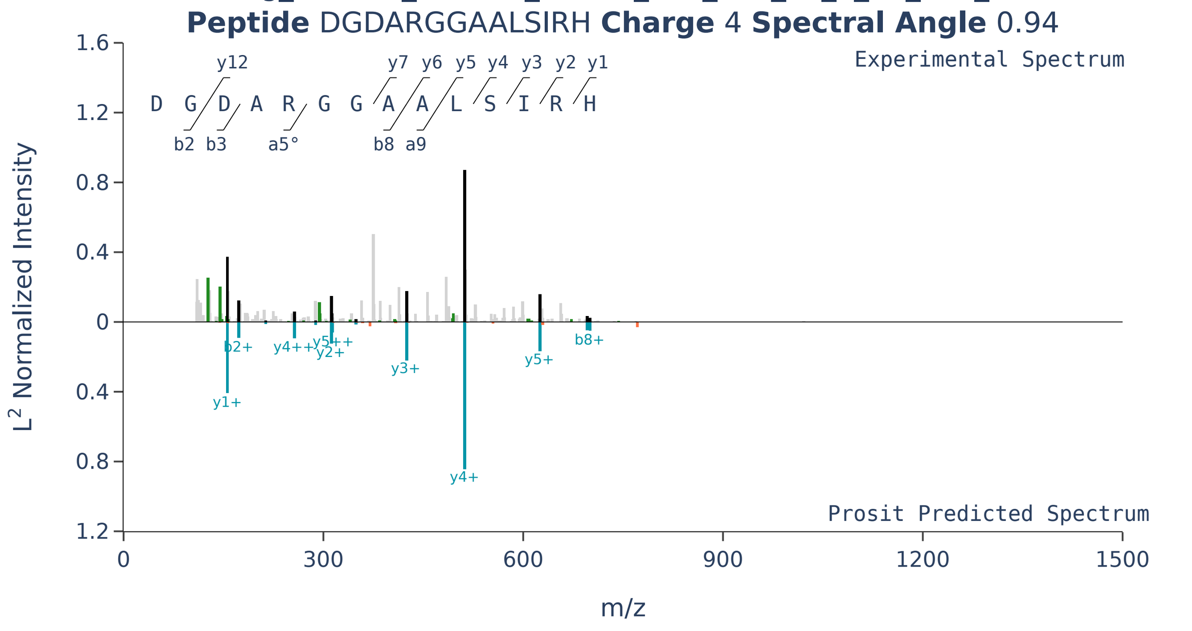


**Source** Ncheng_210623_230623_HFGoe_FFH_20S_25_1_A2_24h_R1 **Scan** 12666
**Peptide** VGQKPVDL **Charge** 1 **Spectral Angle** 0.83

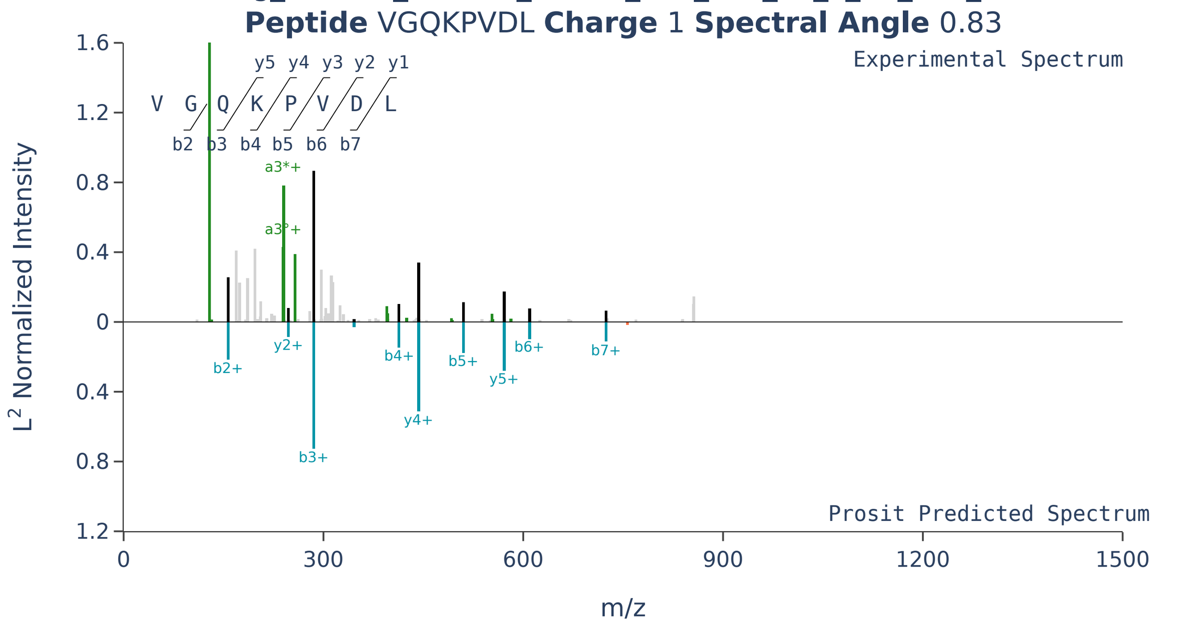


**Source** Ncheng_210623_230623_HFGoe_FFH_20S_25_1_A1_2h_R1 **Scan** 17988
**Peptide** GKLPGMGQIPDNVKS **Charge** 2 **Spectral Angle** 0.82

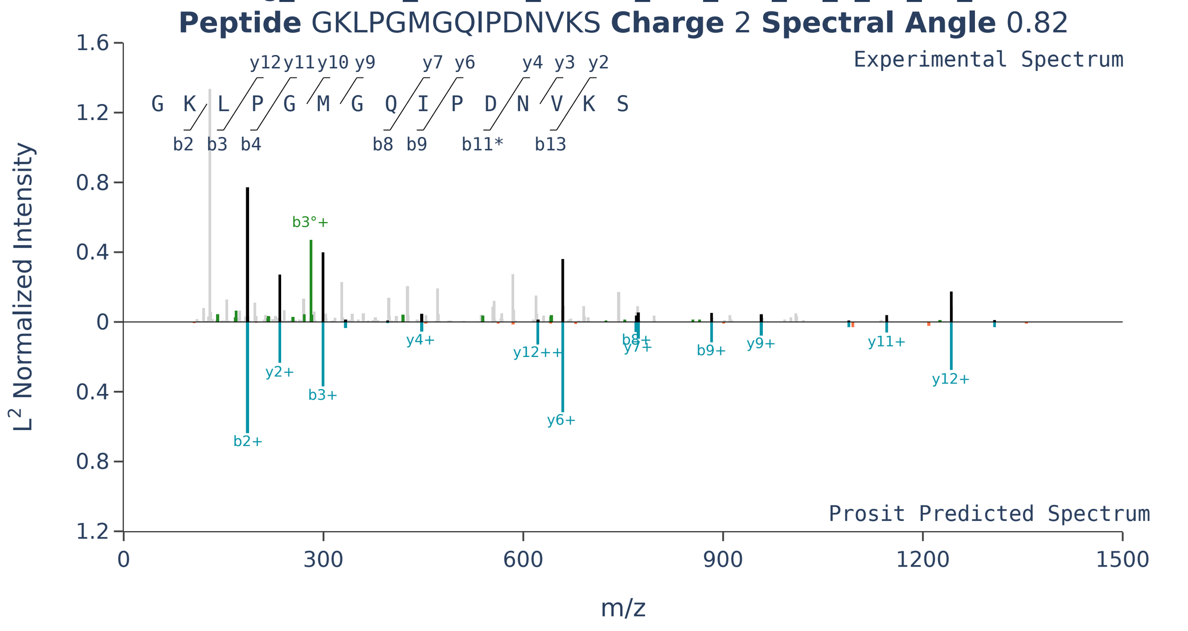

**Source** Ncheng_210623_230623_HFGoe_FFH_20S_25_1_A2_1h_R1 **Scan** 17495
**Peptide** DVYRPAAIKQL **Charge** 2 **Spectral Angle** 0.79

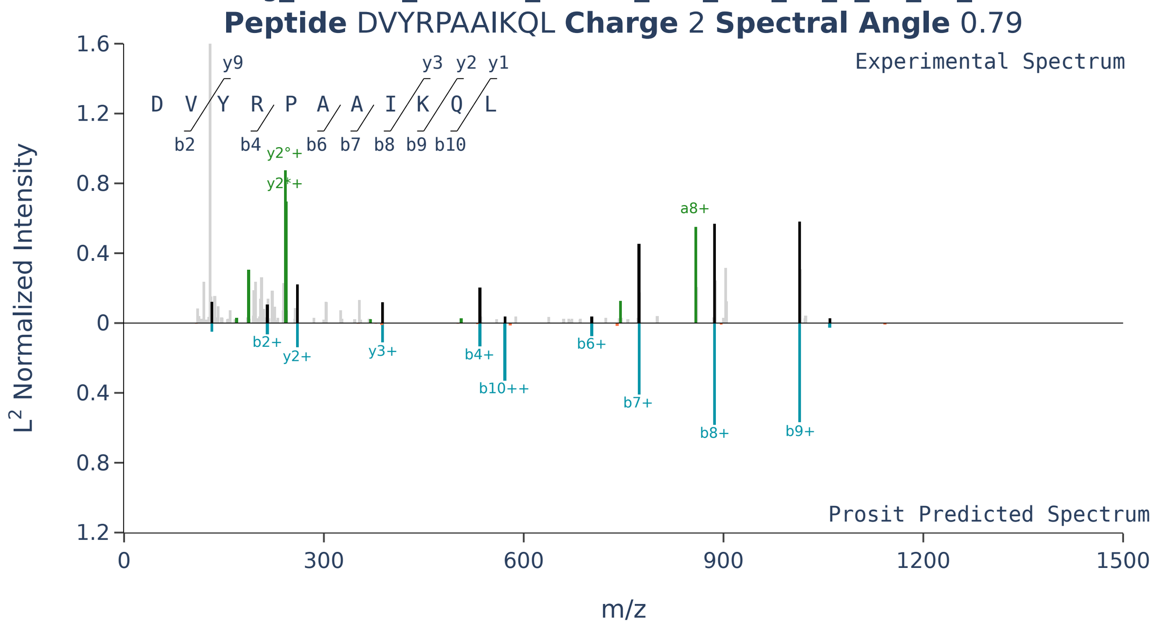

**Source** Ncheng_210623_230623_HFGoe_FFH_20S_25_1_A2_24h_R1 **Scan** 17109
**Peptide** PGQEFVKIVR **Charge** 3 **Spectral Angle** 0.84

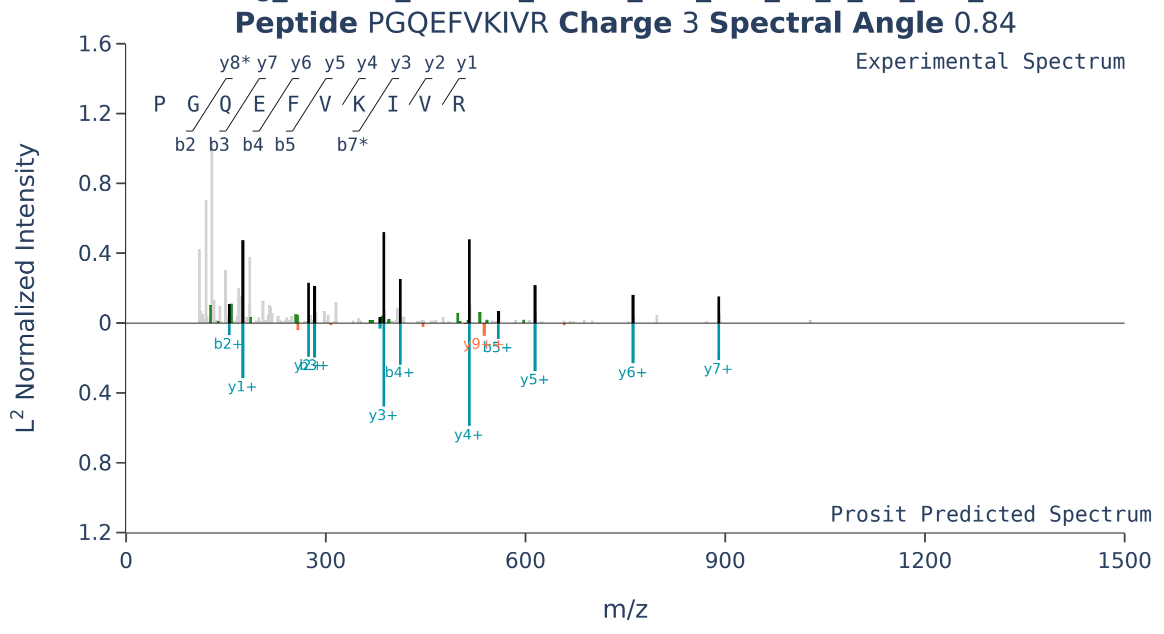

**Source** Ncheng_210623_230623_HFGoe_FFH_20S_25_1_A2_24h_R1 **Scan** 22638
**Peptide** KKGDGFDLNDF **Charge** 3 **Spectral Angle** 0.85

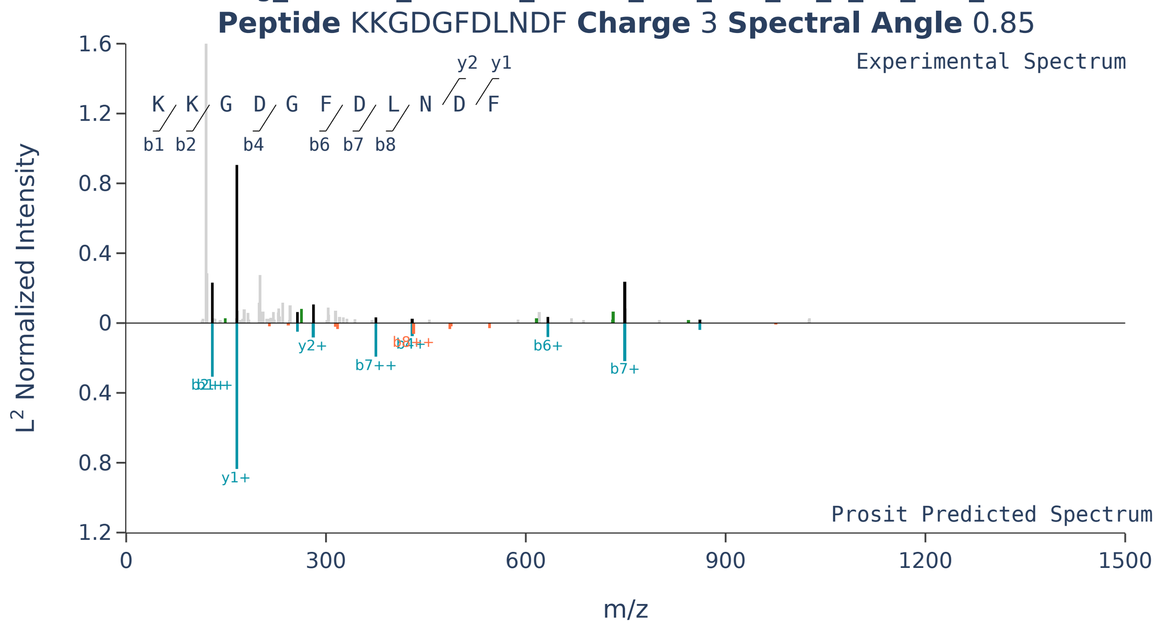

**Source** Ncheng_210623_230623_HFGoe_FFH_20S_25_1_A1_1h_R2 **Scan** 22174
**Peptide** KLPGMGQIPDNV **Charge** 2 **Spectral Angle** 0.73

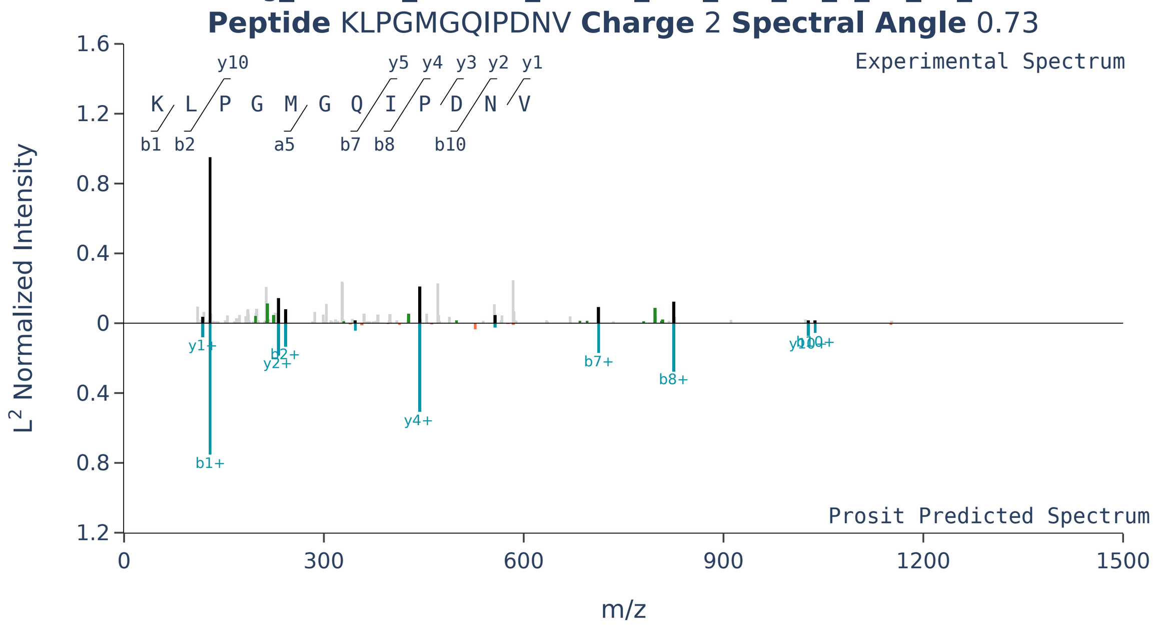

**Source** Ncheng_210623_230623_HFGoe_FFH_20S_25_1_A1_4h_R2 **Scan** 24456
**Peptide** KSLTPGQEFVKLV **Charge** 2 **Spectral Angle** 0.89

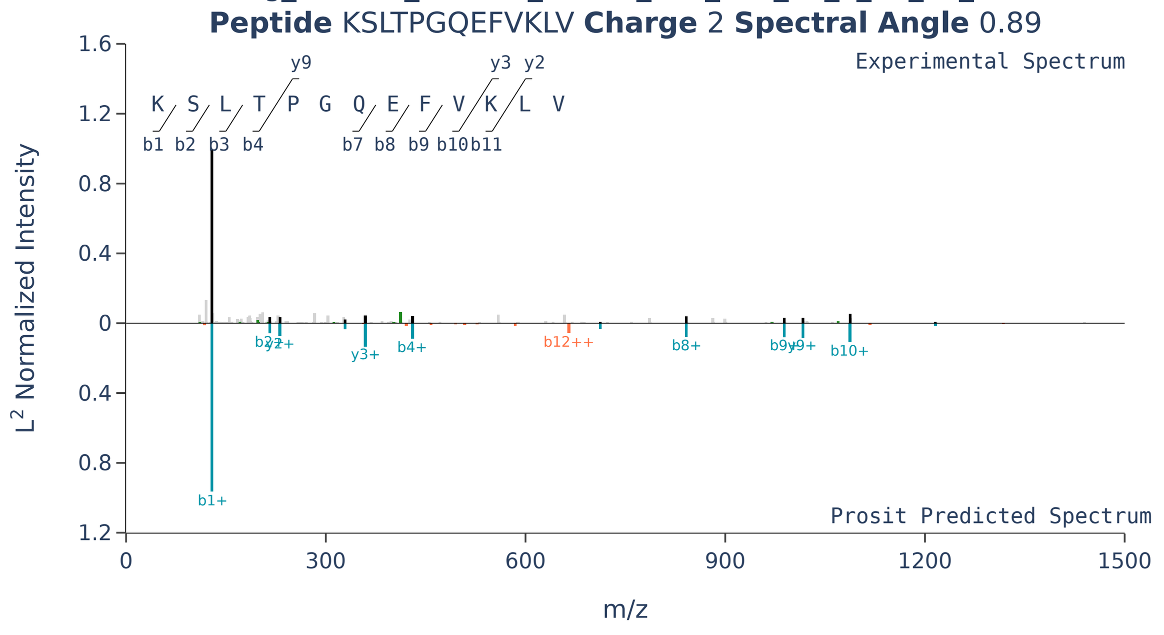

**Source** Ncheng_210623_230623_HFGoe_FFH_20S_25_1_A2_24h_R2 **Scan** 7577
**Peptide** ERAKPEIIKG **Charge** 3 **Spectral Angle** 0.84

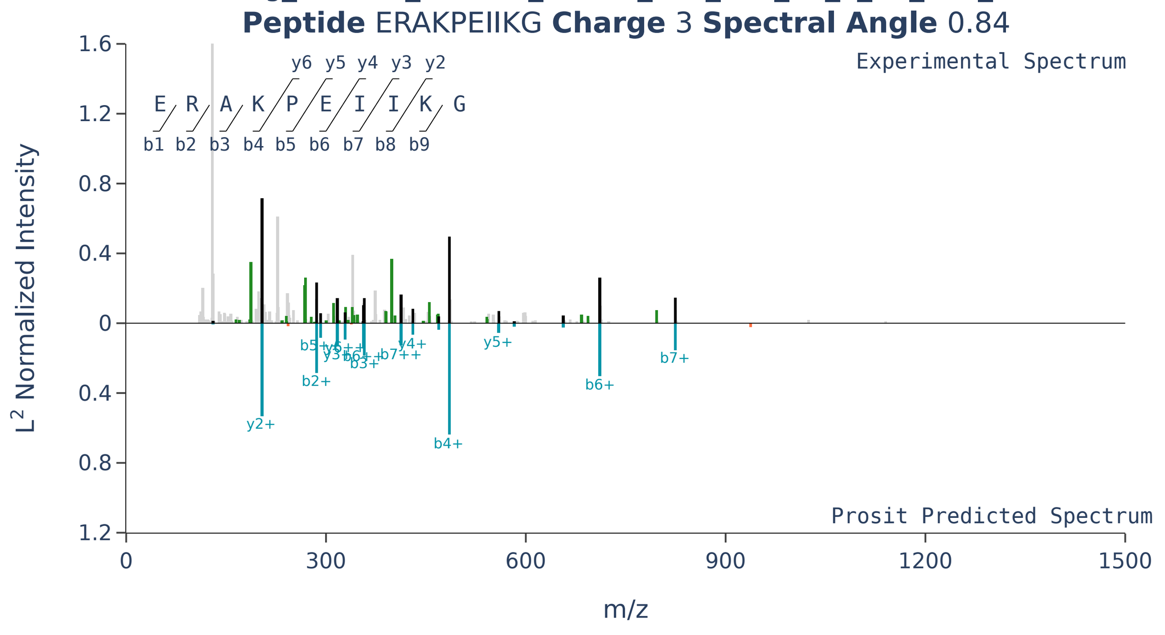

**Source** Ncheng_210623_230623_HFGoe_FFH_20S_25_1_A2_24h_R2 **Scan** 17933
**Peptide** PDNVKSQMDDKVLVRME **Charge** 2 **Spectral Angle** 0.77

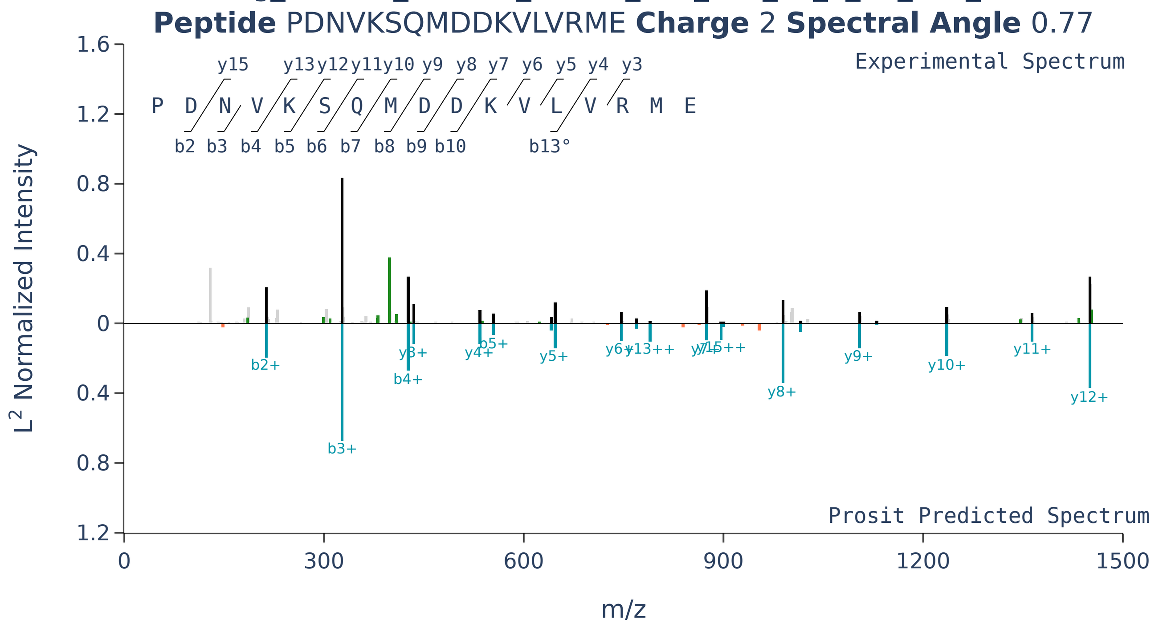

**Source** Ncheng_210623_230623_HFGoe_FFH_20S_25_1_A2_4h_R1 **Scan** 22551
**Peptide** EFVKIVRNELVA **Charge** 2 **Spectral Angle** 0.8

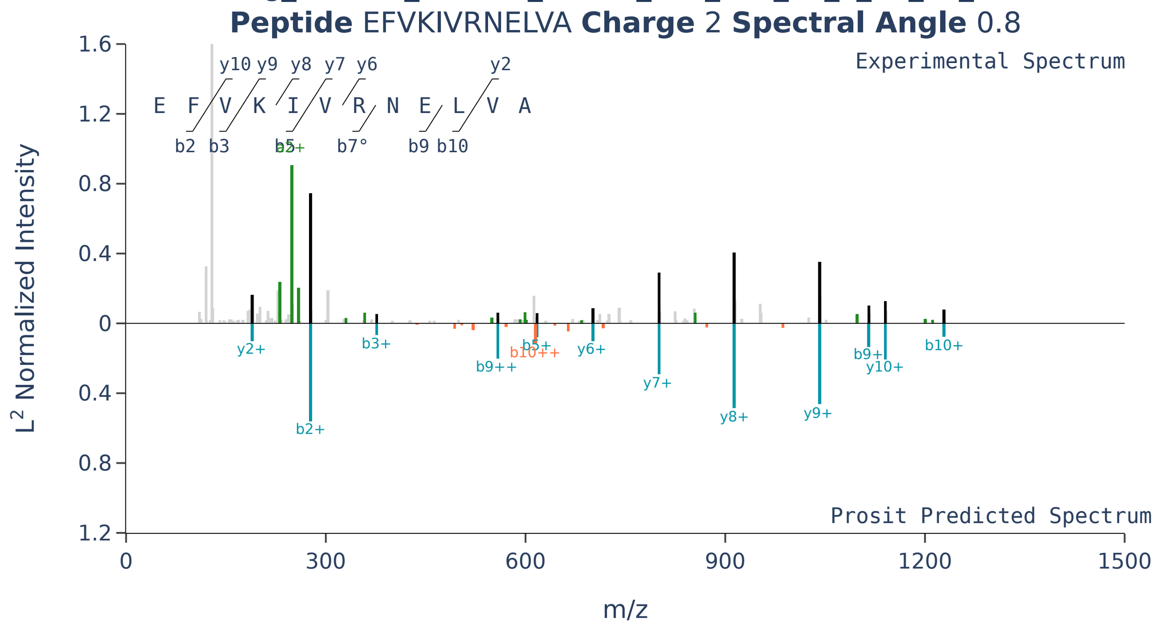

**Source** Ncheng_210623_230623_HFGoe_FFH_20S_25_1_A2_24h_R2 **Scan** 7976
**Peptide** AGKTTSVGKL **Charge** 1 **Spectral Angle** 0.83

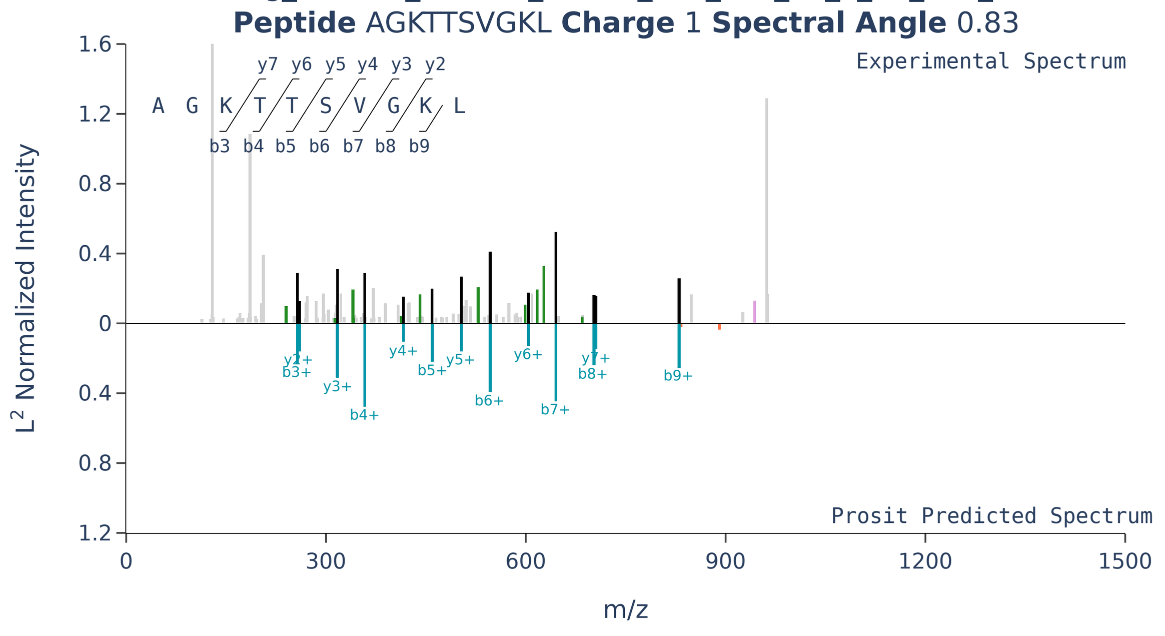

**Source** Ncheng_210623_230623_HFGoe_FFH_20S_25_1_A1_2h_R2 **Scan** 30795
**Peptide** VDFFPSDVGQKPVDIVNA **Charge** 2 **Spectral Angle** 0.92

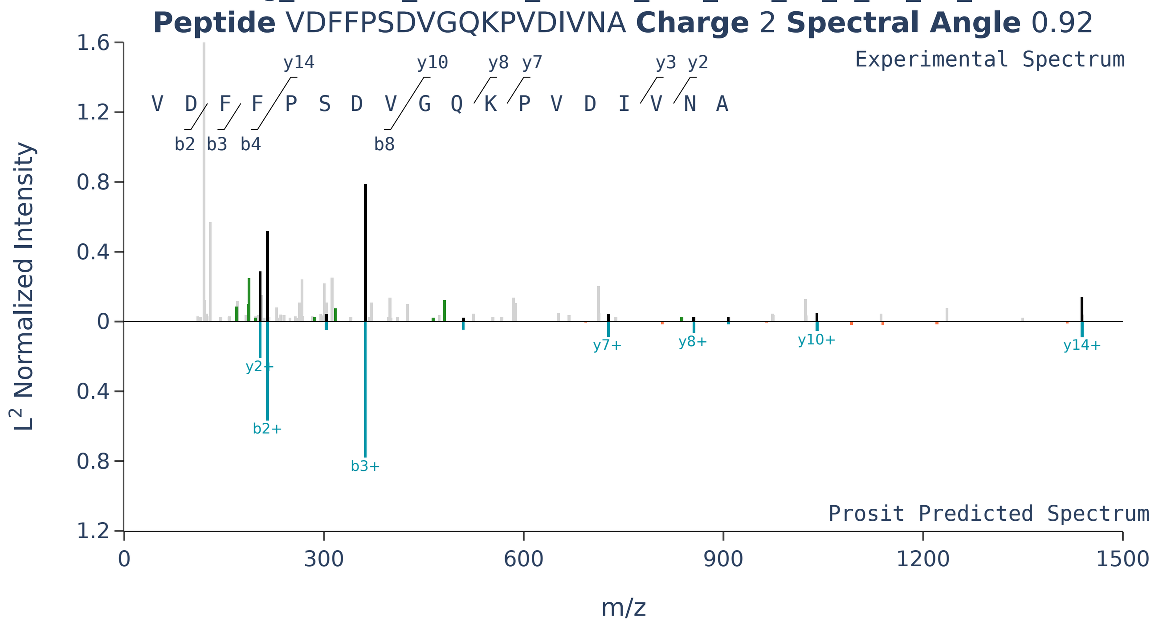

**Source** Ncheng_210623_230623_HFGoe_FFH_20S_25_1_A2_24h_R1 **Scan** 6894
**Peptide** DIESKVDR **Charge** 2 **Spectral Angle** 0.78

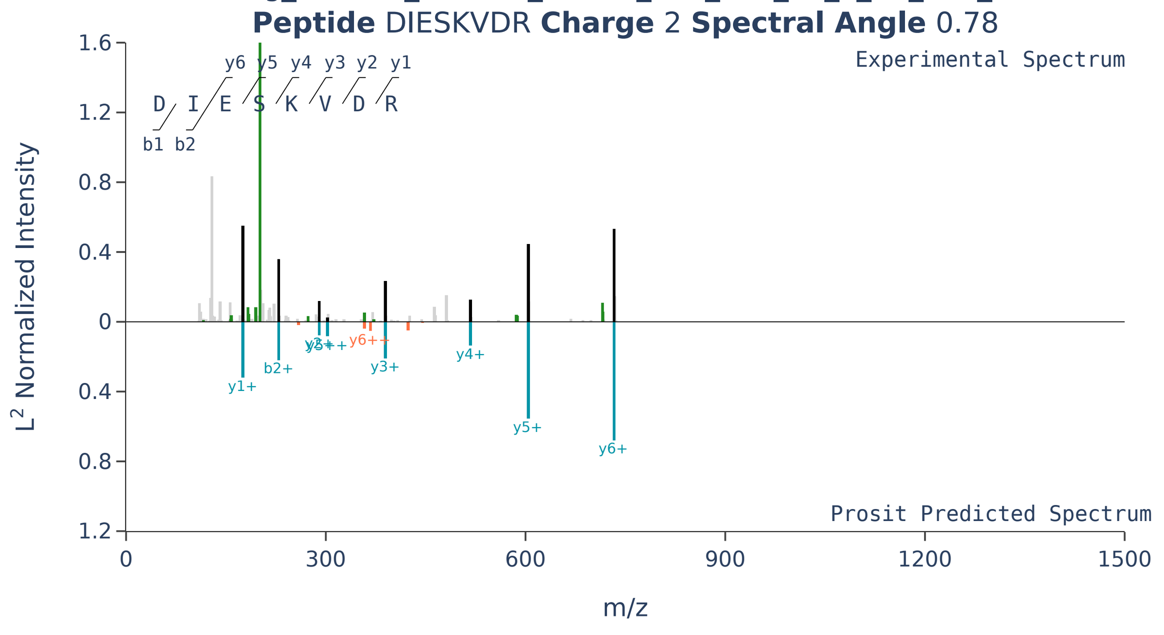

**Source** Ncheng_210623_230623_HFGoe_FFH_20S_25_1_A1_24h_R2 **Scan** 9675
**Peptide** SADVYRPAA **Charge** 2 **Spectral Angle** 0.69

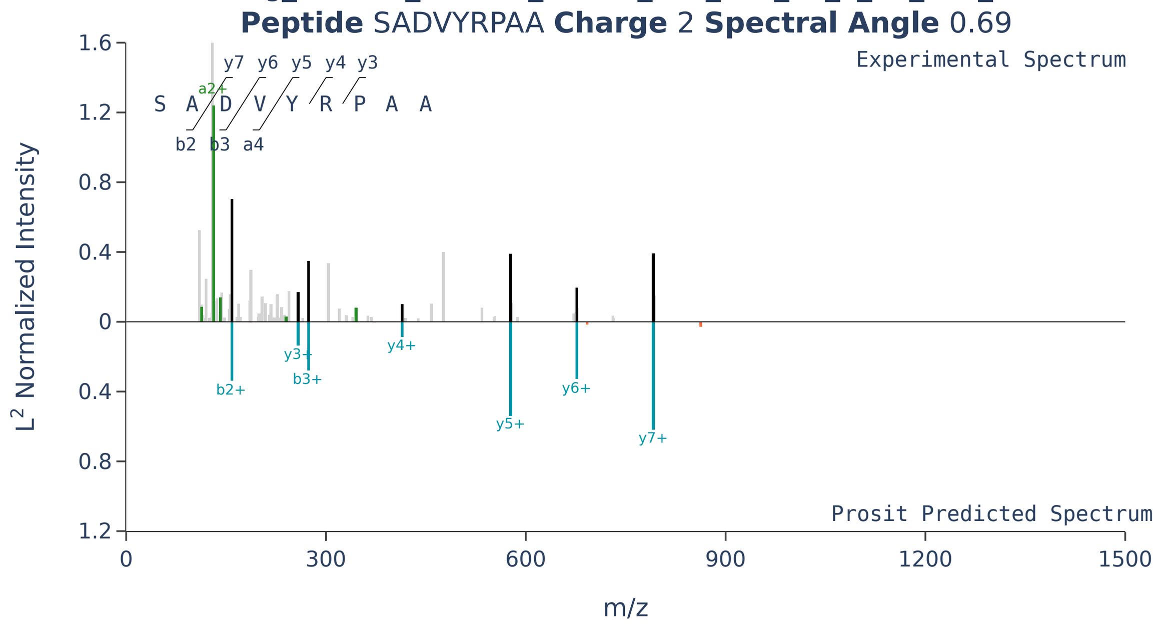

**Source** Ncheng_210623_230623_HFGoe_FFH_20S_25_1_A1_1h_R1 **Scan** 21056
**Peptide** QDVNRLLKQF **Charge** 2 **Spectral Angle** 0.79

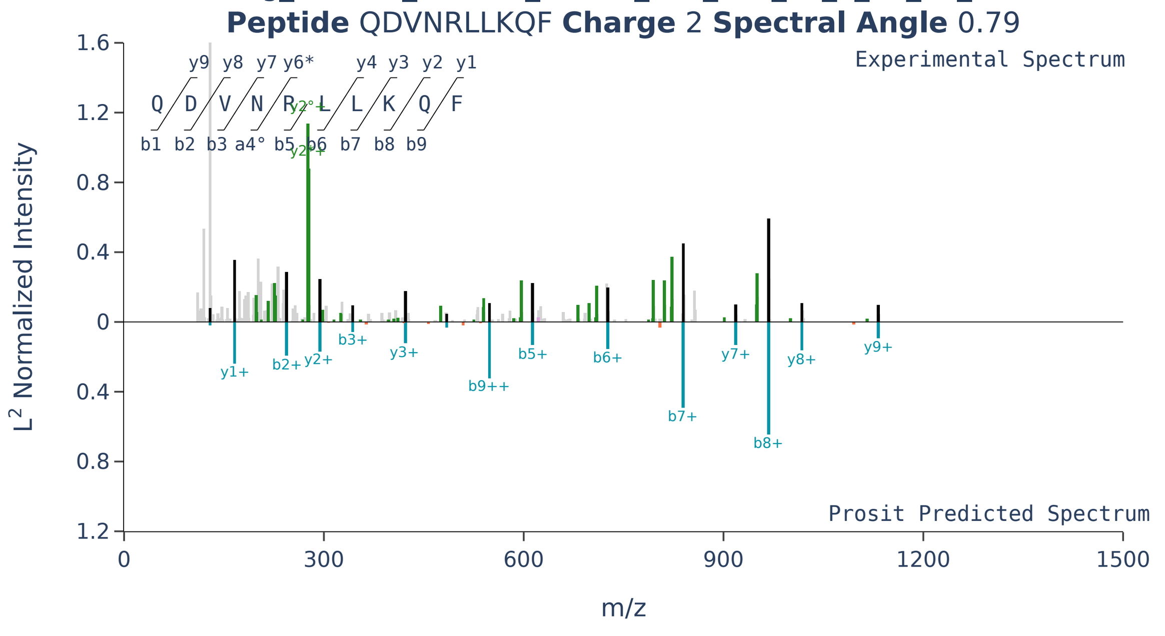

**Source** Ncheng_210623_230623_HFGoe_FFH_20S_25_1_A1_4h_R1 **Scan** 19908
**Peptide** LLVDTAGRLHVDE **Charge** 2 **Spectral Angle** 0.79

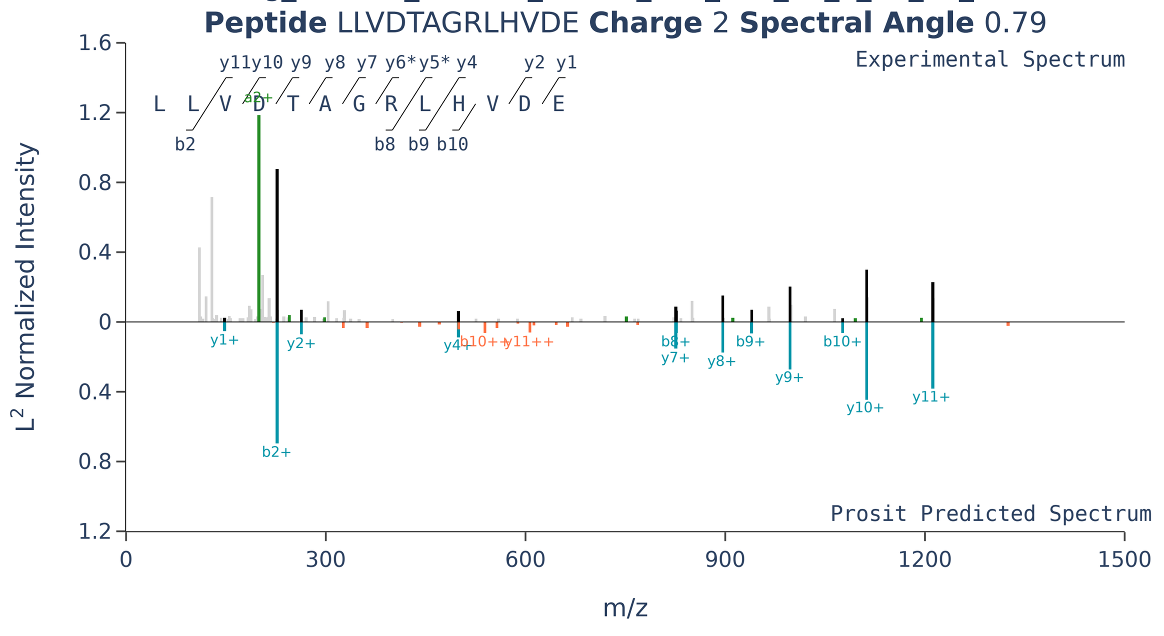

**Source** Ncheng_210623_230623_HFGoe_FFH_20S_25_1_A2_24h_R2 **Scan** 12999
**Peptide** AGLQGAGKTTSVGKL **Charge** 2 **Spectral Angle** 0.83

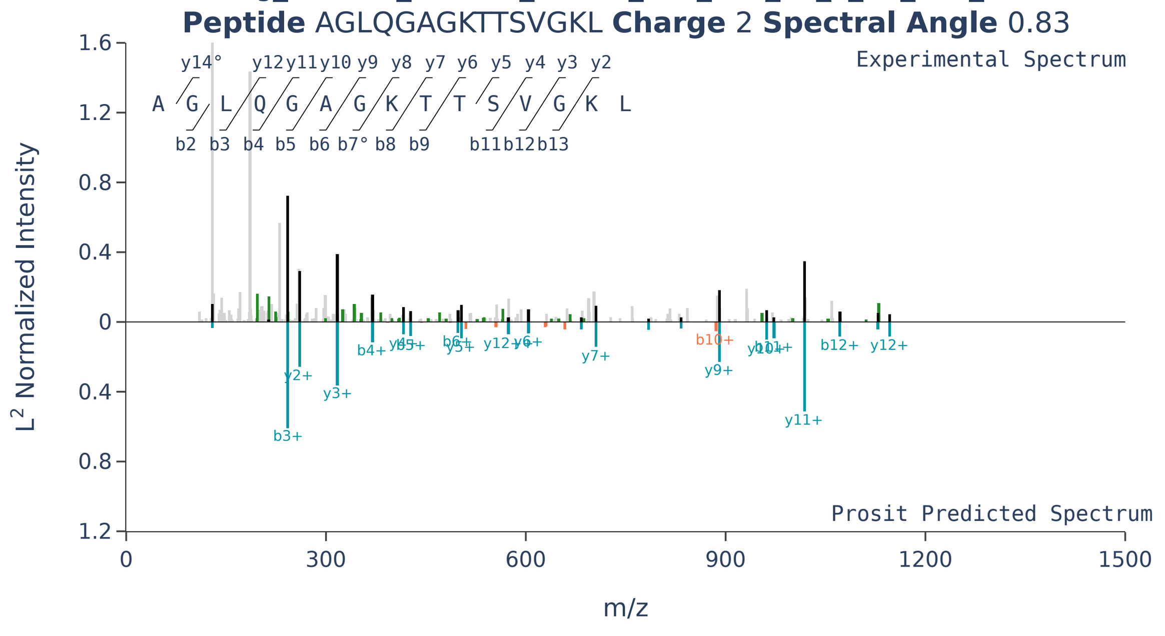

**Source** Ncheng_210623_230623_HFGoe_FFH_20S_25_1_A1_2h_R2 **Scan** 15270
**Peptide** MGKLPGMG **Charge** 2 **Spectral Angle** 0.88

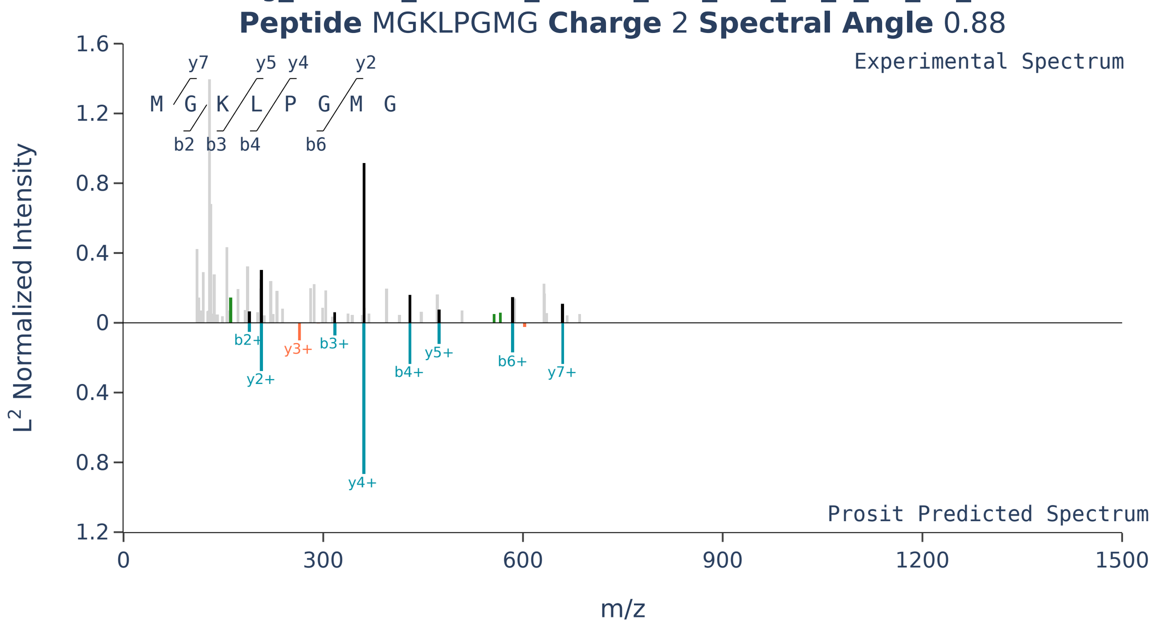


**Source** Ncheng_210623_230623_HFGoe_FFH_20S_25_1_A2_2h_R2 **Scan** 18376
**Peptide** DEAMMDEIKQVH **Charge** 2 **Spectral Angle** 0.9

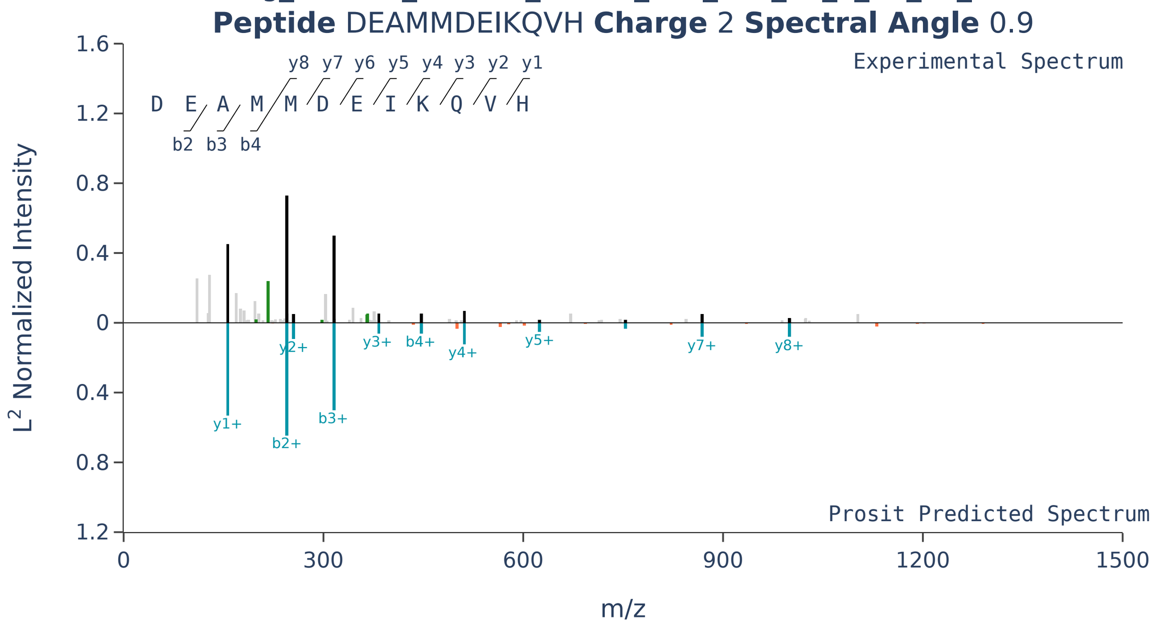


**Source** Ncheng_210623_230623_HFGoe_FFH_20S_25_1_A1_4h_R2 **Scan** 15534
**Peptide** AVGHEVNKSLTPGQEF **Charge** 2 **Spectral Angle** 0.86

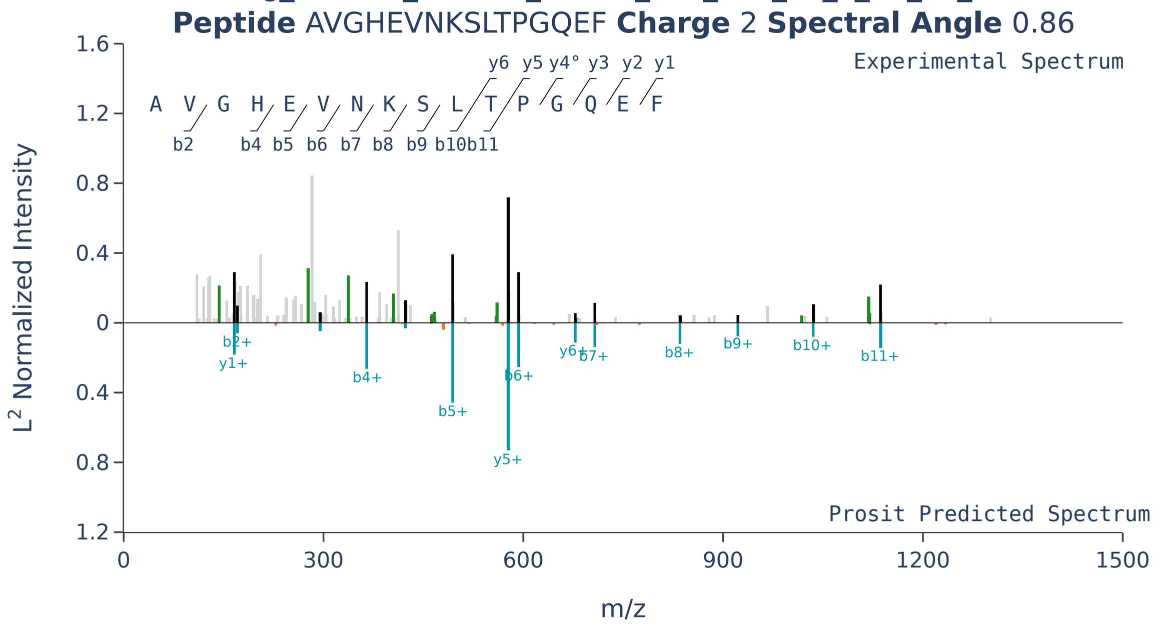


**Source** Ncheng_210623_230623_HFGoe_FFH_20S_25_1_A2_4h_R1 **Scan** 12622
**Peptide** PDNVKSQMDDKVLVR **Charge** 4 **Spectral Angle** 0.88

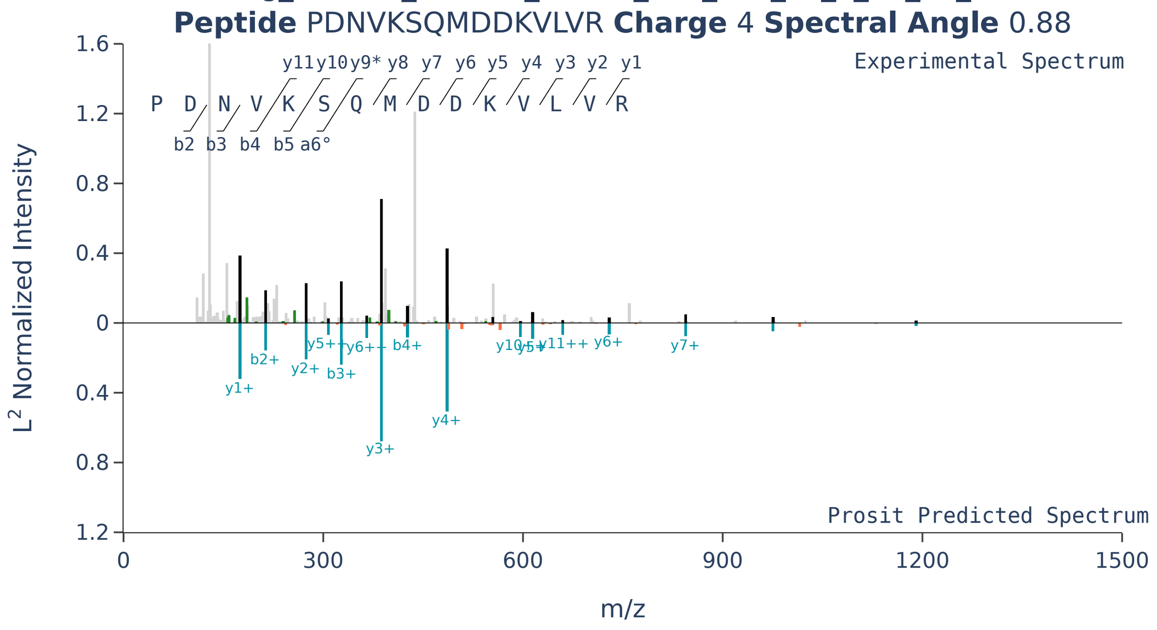


**Source** Ncheng_210623_230623_HFGoe_FFH_20S_25_1_A1_2h_R1 **Scan** 15280
**Peptide** TGKPIKFLGVGEKT **Charge** 2 **Spectral Angle** 0.82

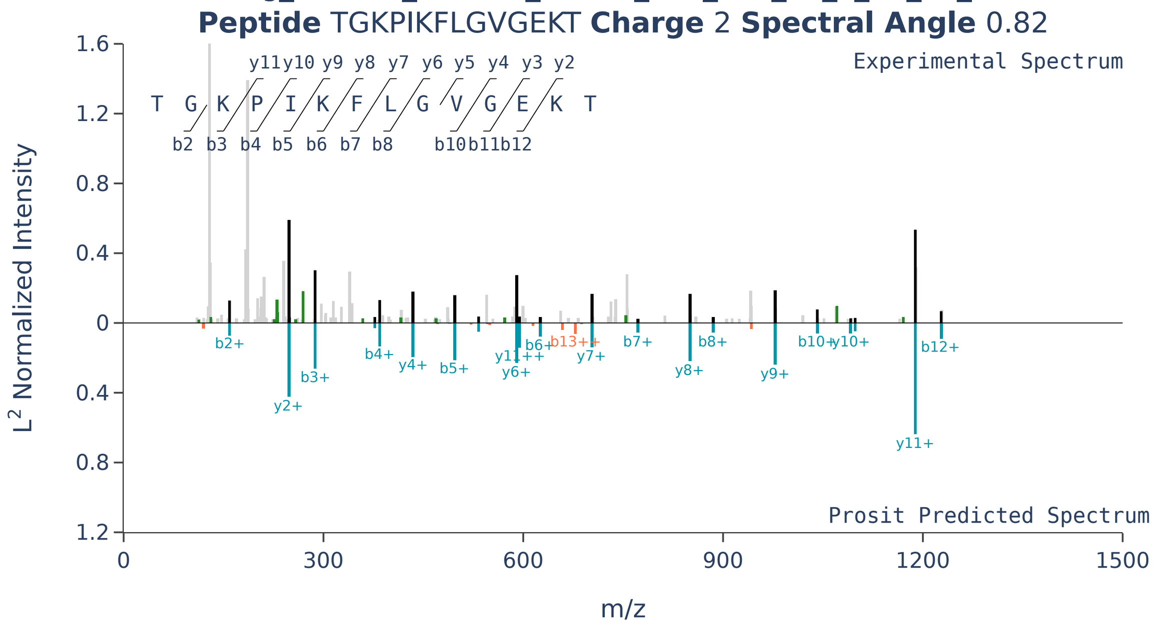


**Source** Ncheng_210623_230623_HFGoe_FFH_20S_25_1_A2_24h_R1 **Scan** 19765
**Peptide** VGHEVNKSLTPGQEFVKIVRN **Charge** 2 **Spectral Angle** 0.81

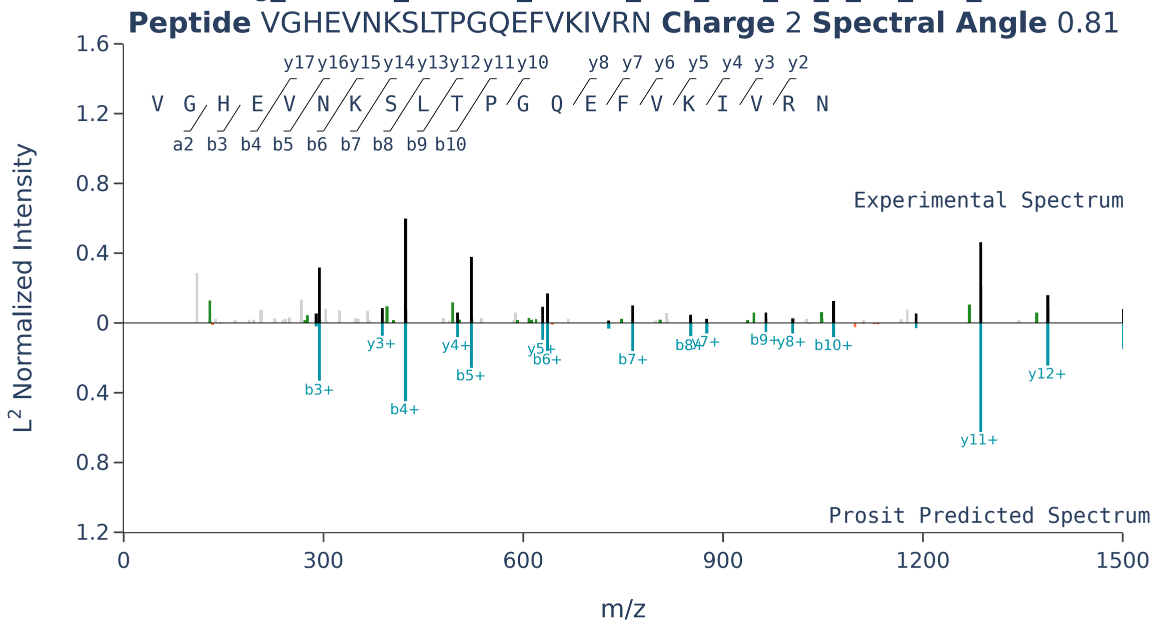


**Source** Ncheng_210623_230623_HFGoe_FFH_20S_25_1_A2_1h_R2 **Scan** 36801
**Peptide** SINPVETLFVVD **Charge** 2 **Spectral Angle** 0.83

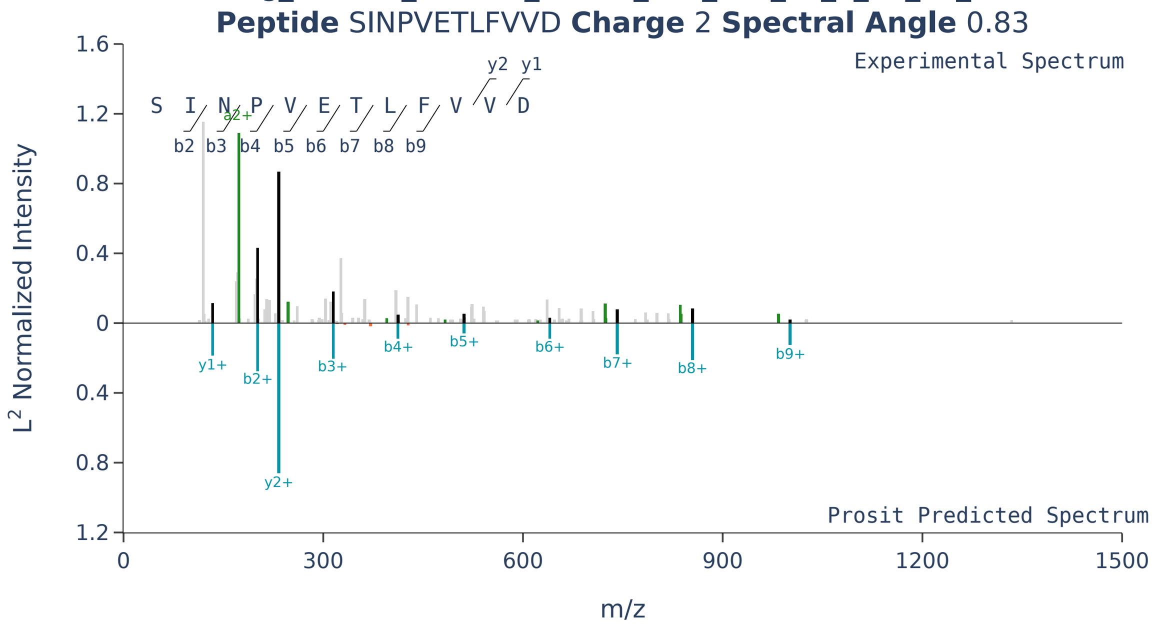


**Source** Ncheng_210623_230623_HFGoe_FFH_20S_25_1_A2_24h_R1 **Scan** 13120
**Peptide** GMGQIPDNVKSQ **Charge** 2 **Spectral Angle** 0.68

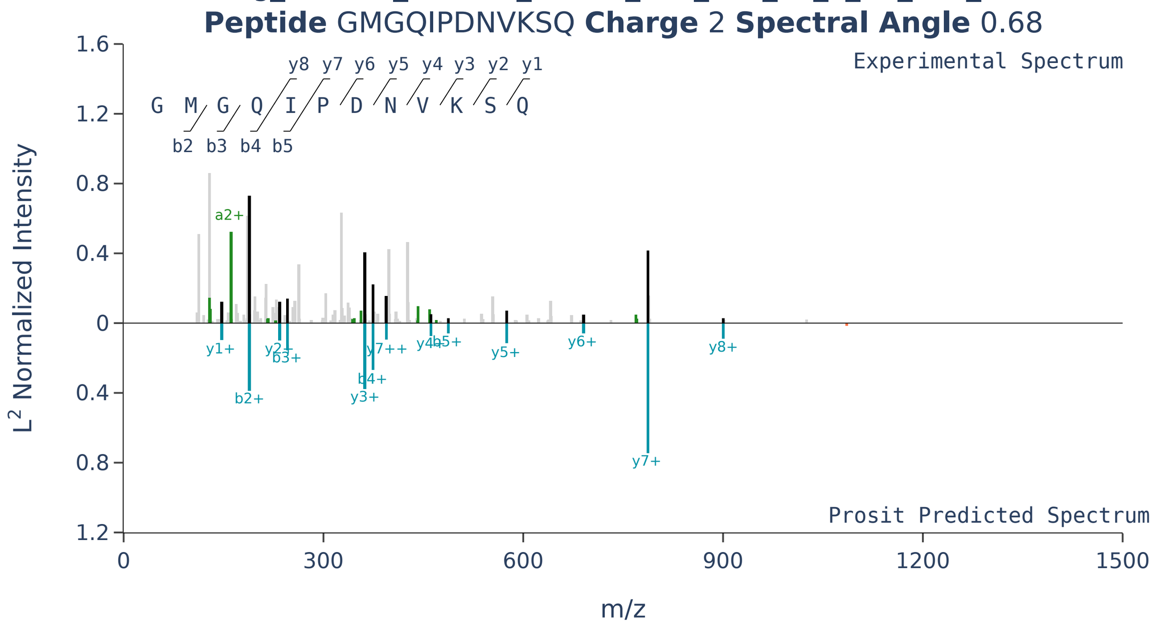


**Source** Ncheng_210623_230623_HFGoe_FFH_20S_25_1_A2_4h_R2 **Scan** 11874
**Peptide** RIAAGCGMQVQDVNR **Charge** 3 **Spectral Angle** 0.81

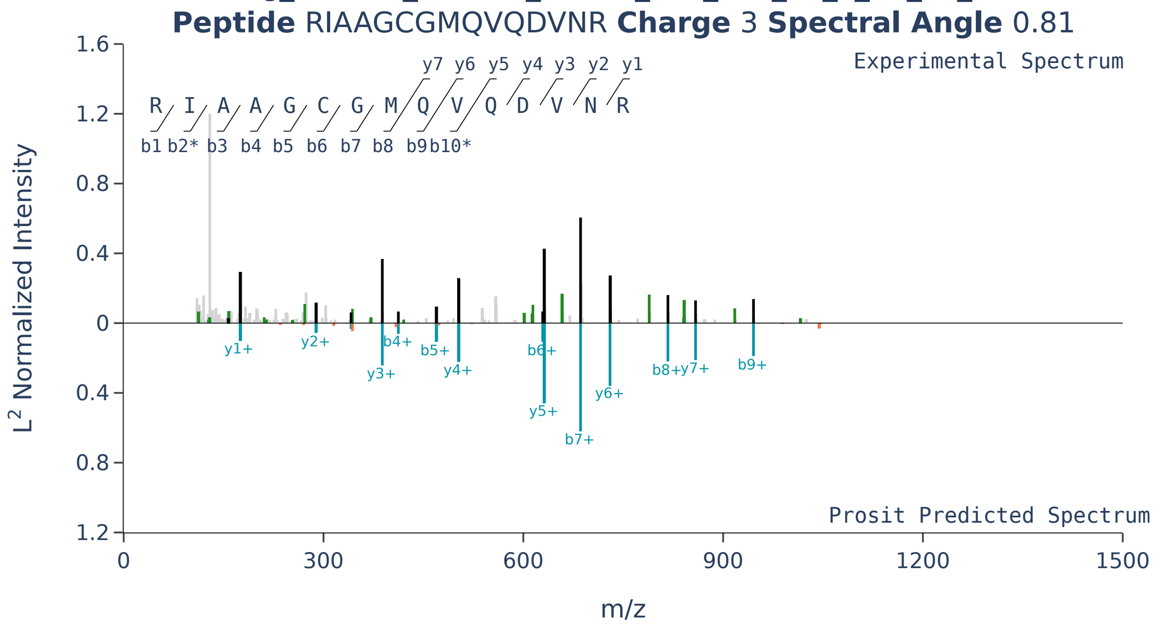


**Source** Ncheng_210623_230623_HFGoe_FFH_20S_25_1_A2_4h_R1 **Scan** 30674
**Peptide** AGCGMQVQDVNRLLKQF **Charge** 2 **Spectral Angle** 0.8

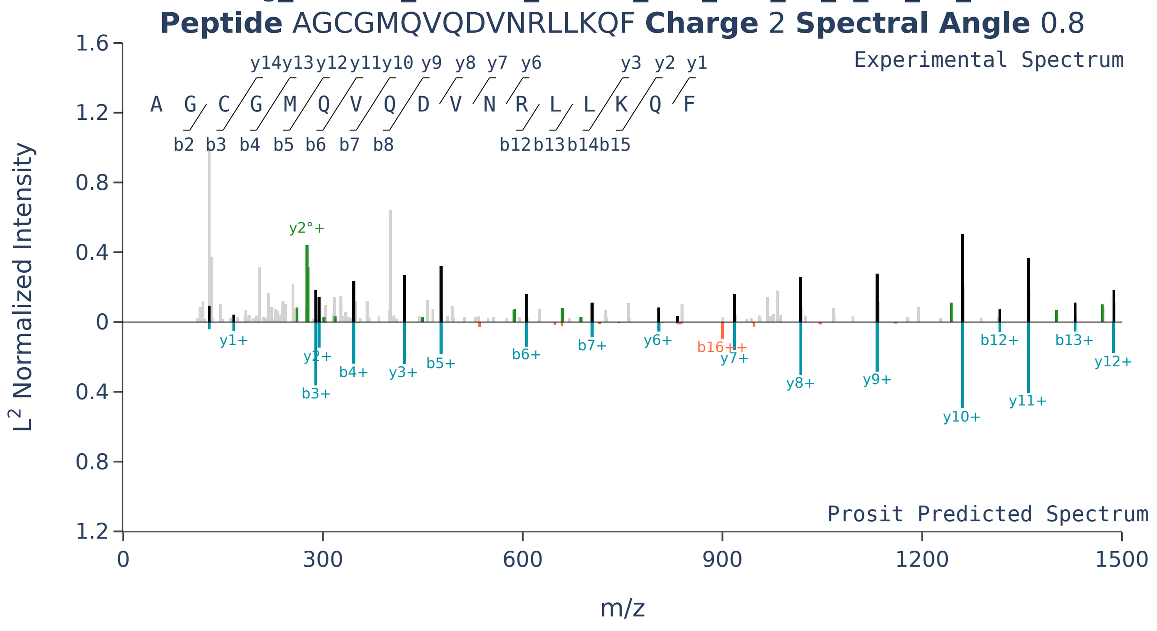


**Source** Ncheng_210623_230623_HFGoe_FFH_20S_25_1_A2_4h_R2 **Scan** 22178
**Peptide** MGKLPGMGQIPDNVKSQMD **Charge** 3 **Spectral Angle** 0.72

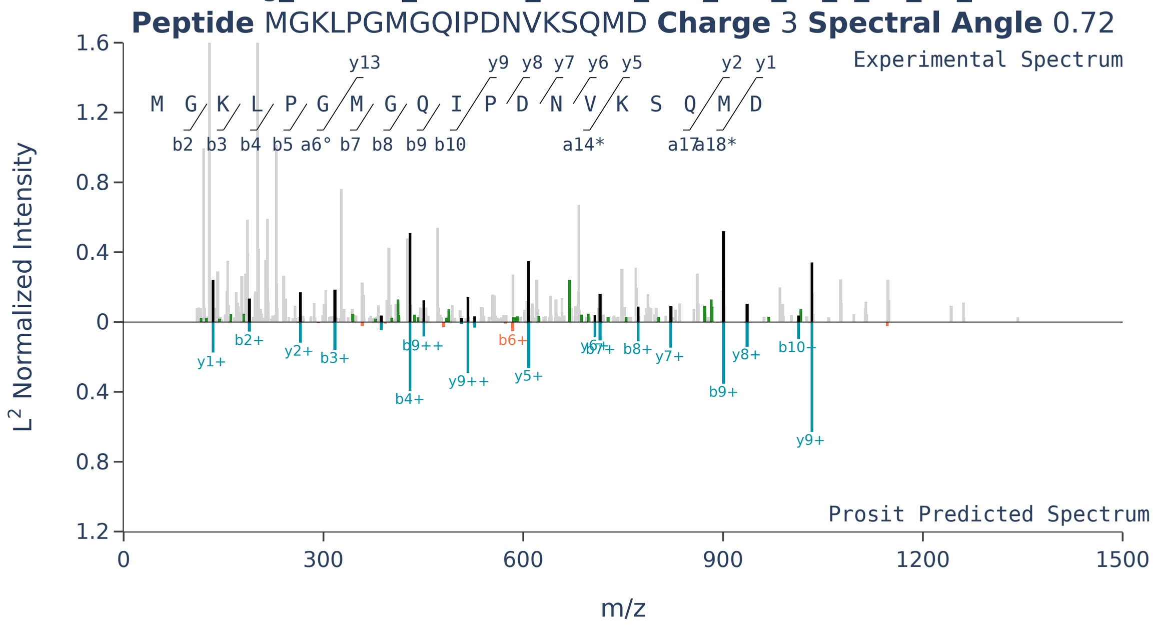


**Source** Ncheng_210623_230623_HFGoe_FFH_20S_25_1_A1_24h_R2 **Scan** 16086
**Peptide** VNRLLKQFD **Charge** 2 **Spectral Angle** 0.79

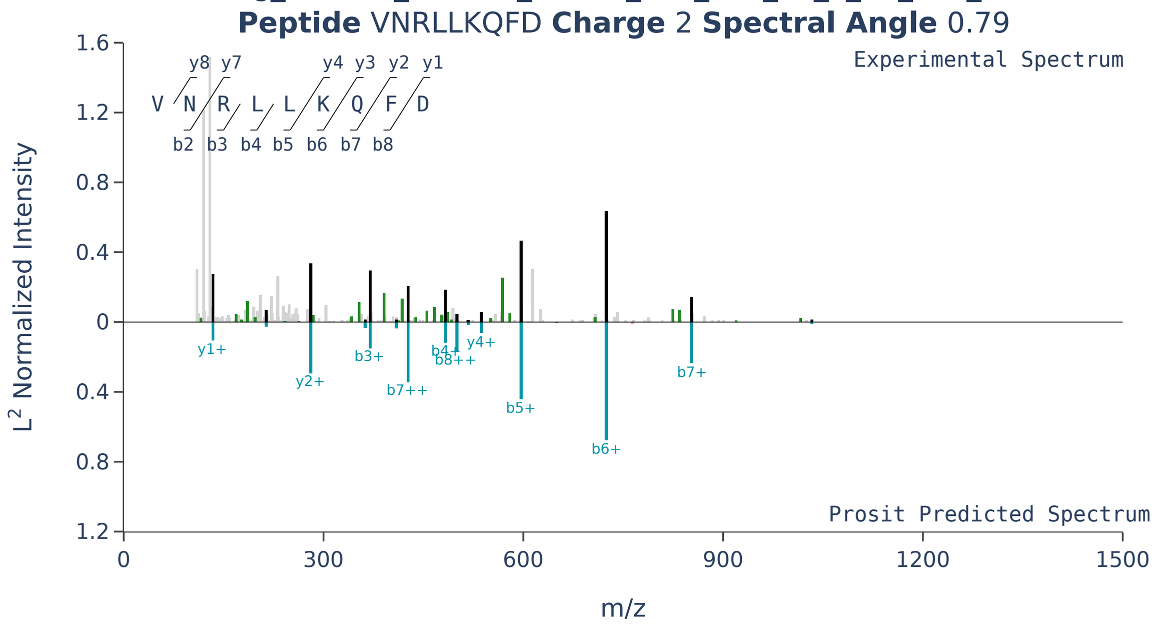


**Source** Ncheng_210623_230623_HFGoe_FFH_20S_25_1_A1_4h_R2 **Scan** 24884
**Peptide** RIAAGCGMQVQDVNRLL **Charge** 3 **Spectral Angle** 0.86

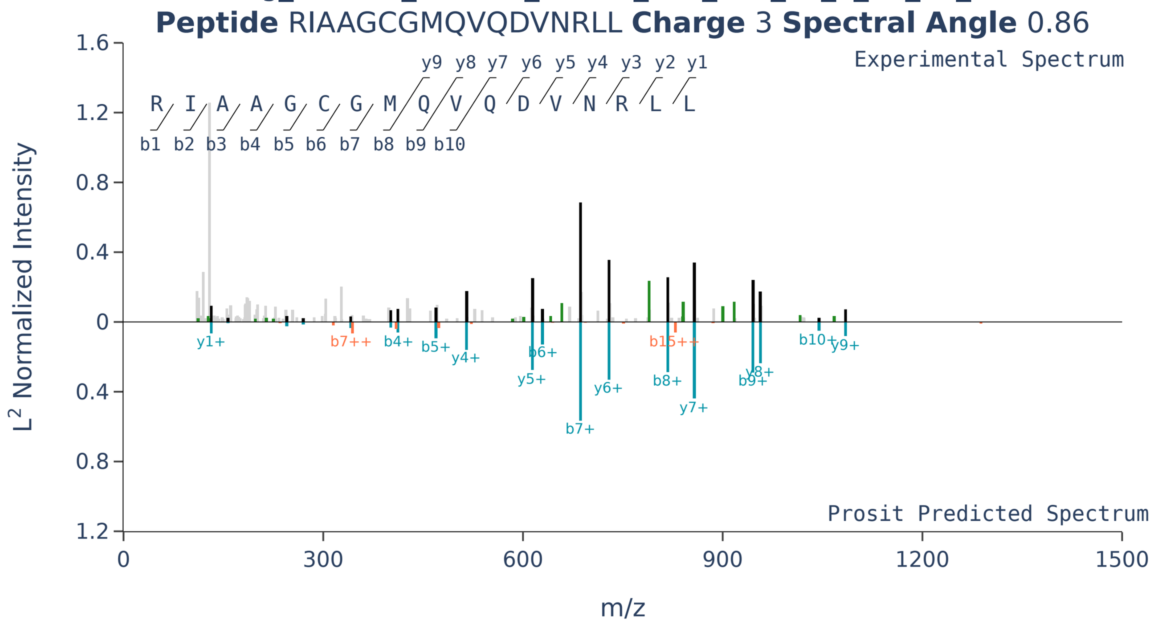


**Source** Ncheng_210623_230623_HFGoe_FFH_20S_25_1_A1_24h_R1 **Scan** 16951
**Peptide** ANTAKAFNEAL **Charge** 2 **Spectral Angle** 0.77

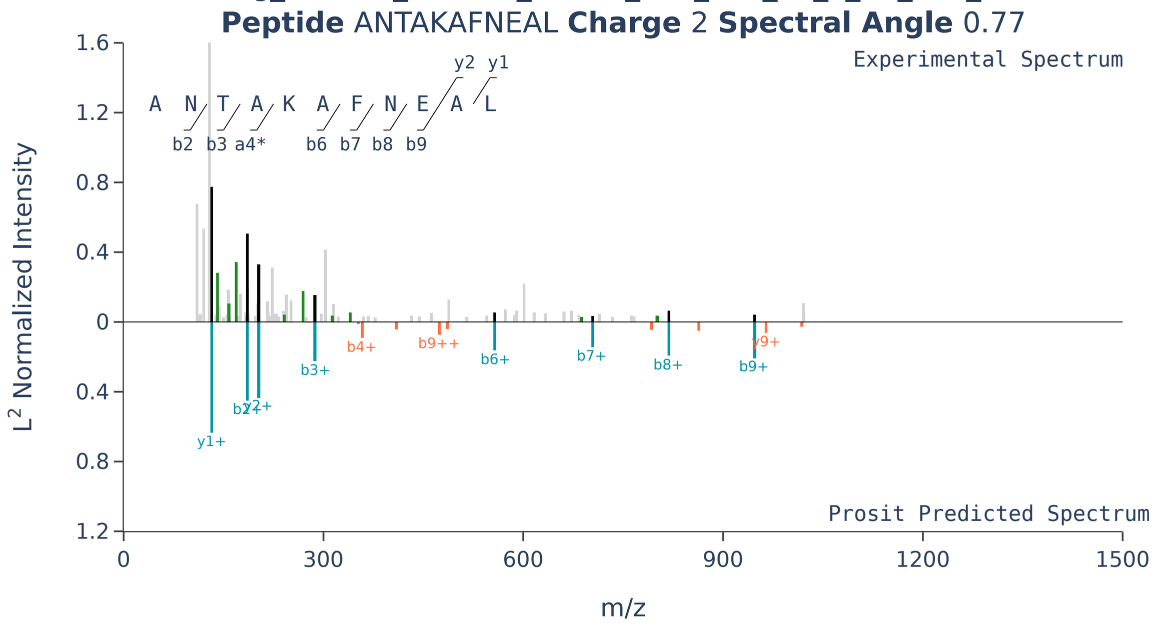


**Source** Ncheng_210623_230623_HFGoe_FFH_20S_25_1_A1_24h_R2 **Scan** 14821
**Peptide** AALSIRHI **Charge** 2 **Spectral Angle** 0.83

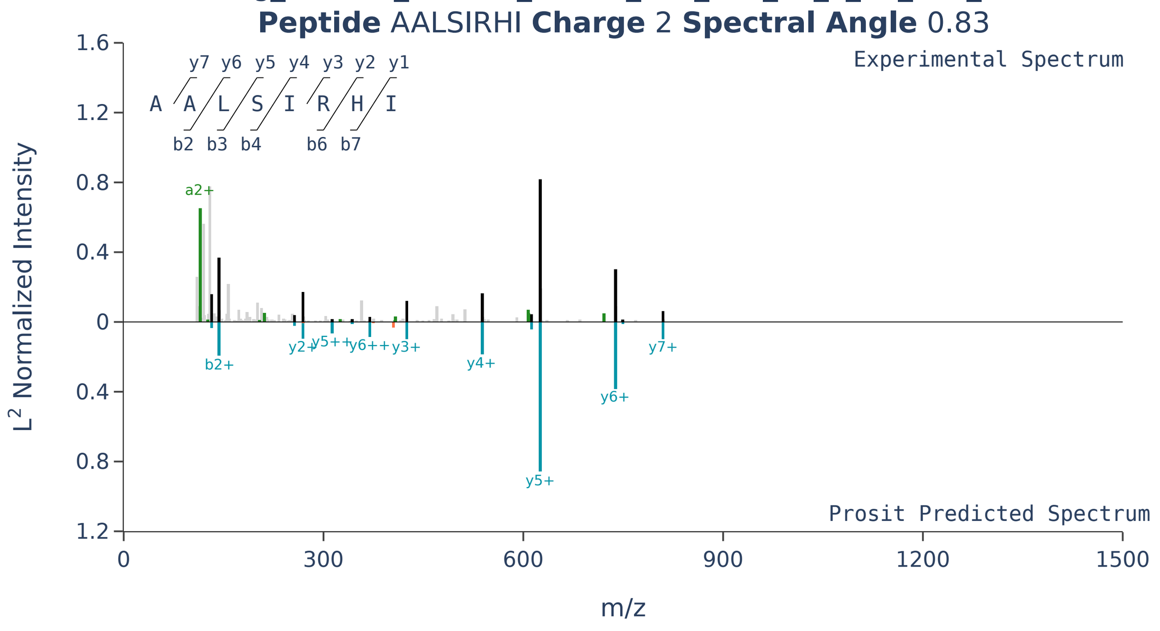

**Source** Ncheng_210623_230623_HFGoe_FFH_20S_25_1_A2_24h_R1 **Scan** 10784
**Peptide** RPAAIKQLET **Charge** 3 **Spectral Angle** 0.75

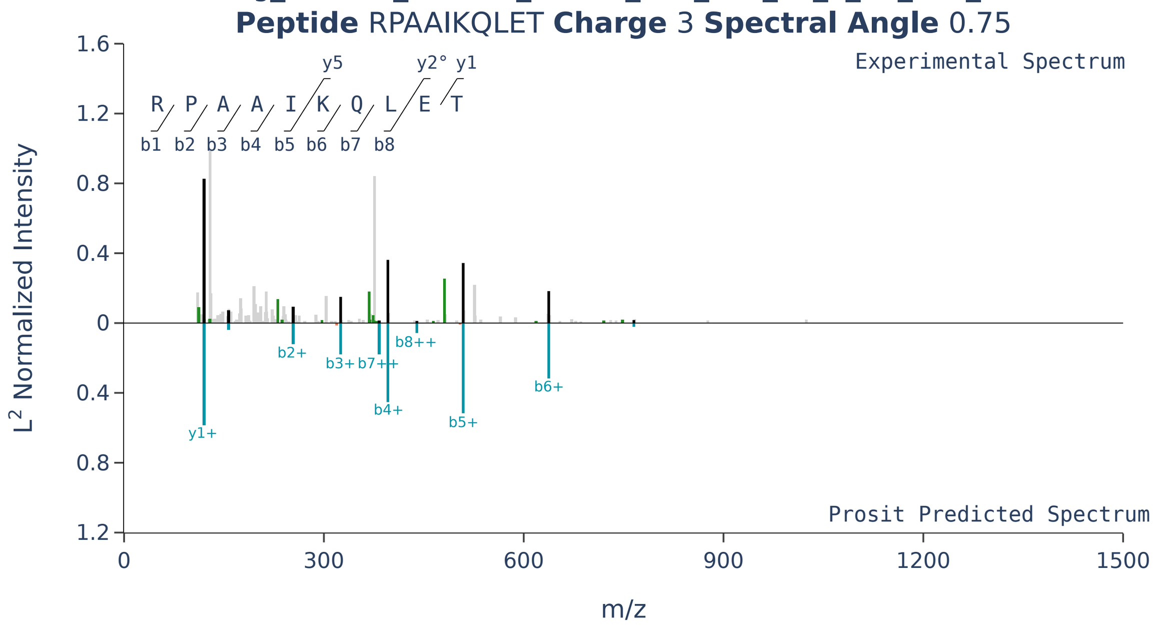


**Source** Ncheng_210623_230623_HFGoe_FFH_20S_25_1_A2_24h_R2 **Scan** 19382
**Peptide** VSADVYRPAAI **Charge** 2 **Spectral Angle** 0.79

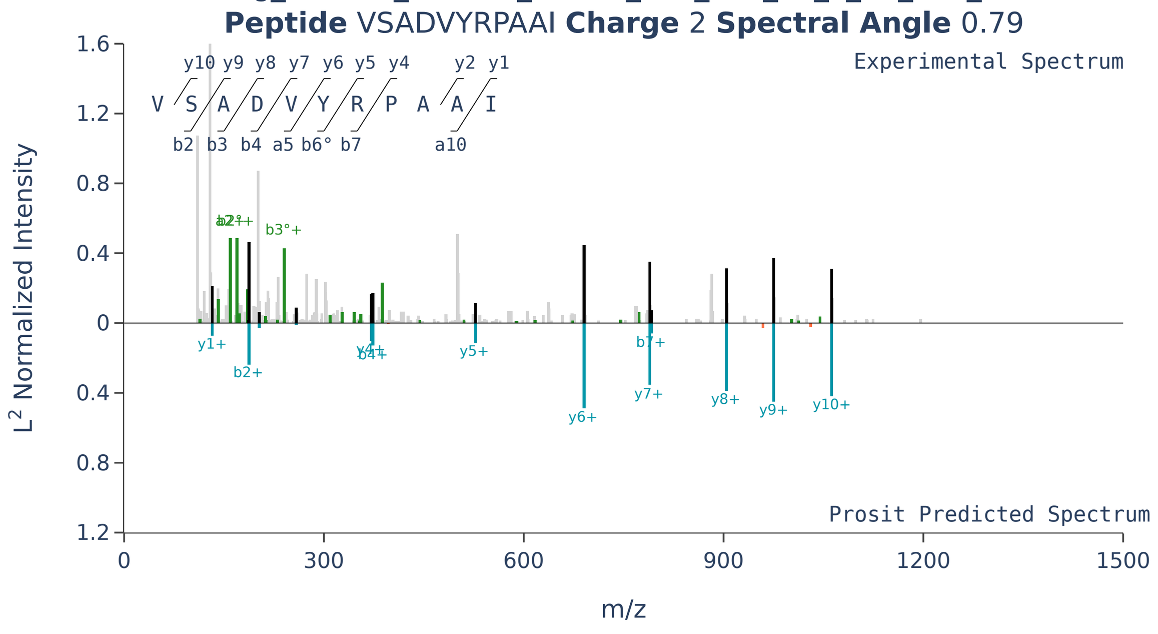


**Source** Ncheng_210623_230623_HFGoe_FFH_20S_25_1_A1_2h_R1 **Scan** 12236
**Peptide** VGQKPVDI **Charge** 1 **Spectral Angle** 0.85

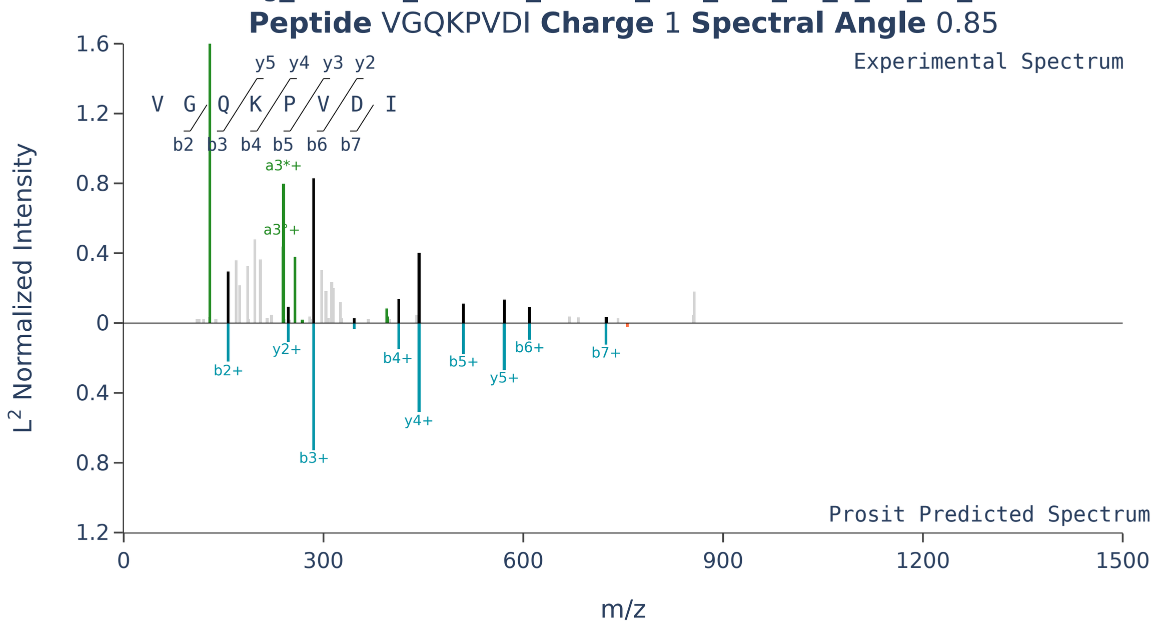


**Source** Ncheng_210623_230623_HFGoe_FFH_20S_25_1_A2_24h_R1 **Scan** 17227
**Peptide** GMGQIPDNVKSQMD **Charge** 2 **Spectral Angle** 0.72

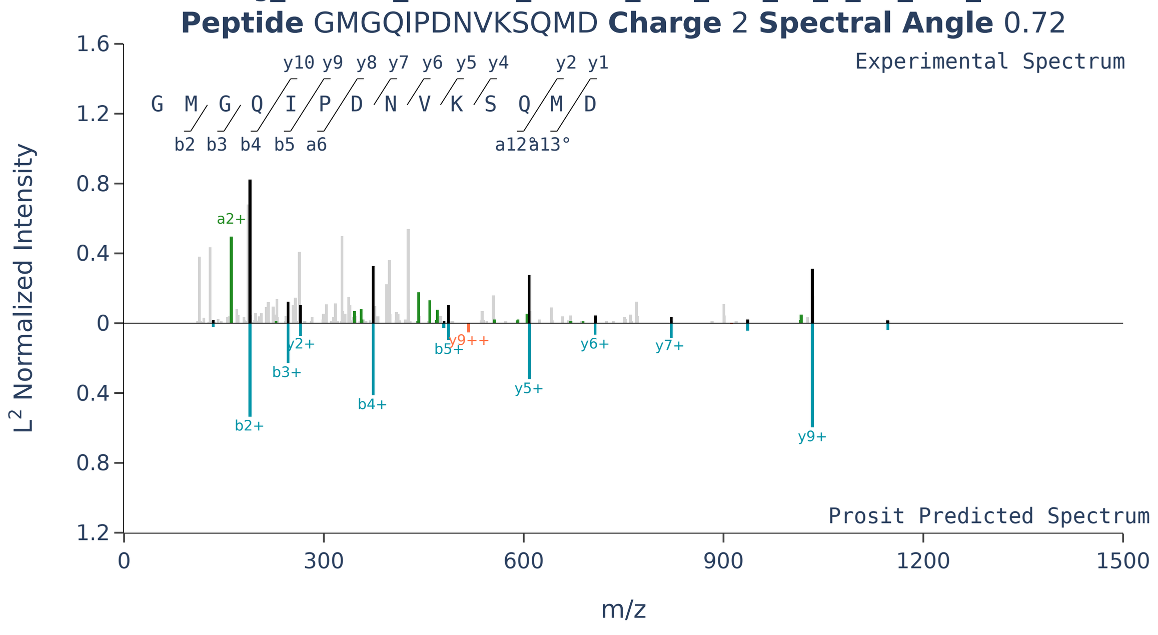


**Source** Ncheng_210623_230623_HFGoe_FFH_20S_25_1_A1_2h_R1 **Scan** 8299
**Peptide** QDAANTAKAFN **Charge** 1 **Spectral Angle** 0.78

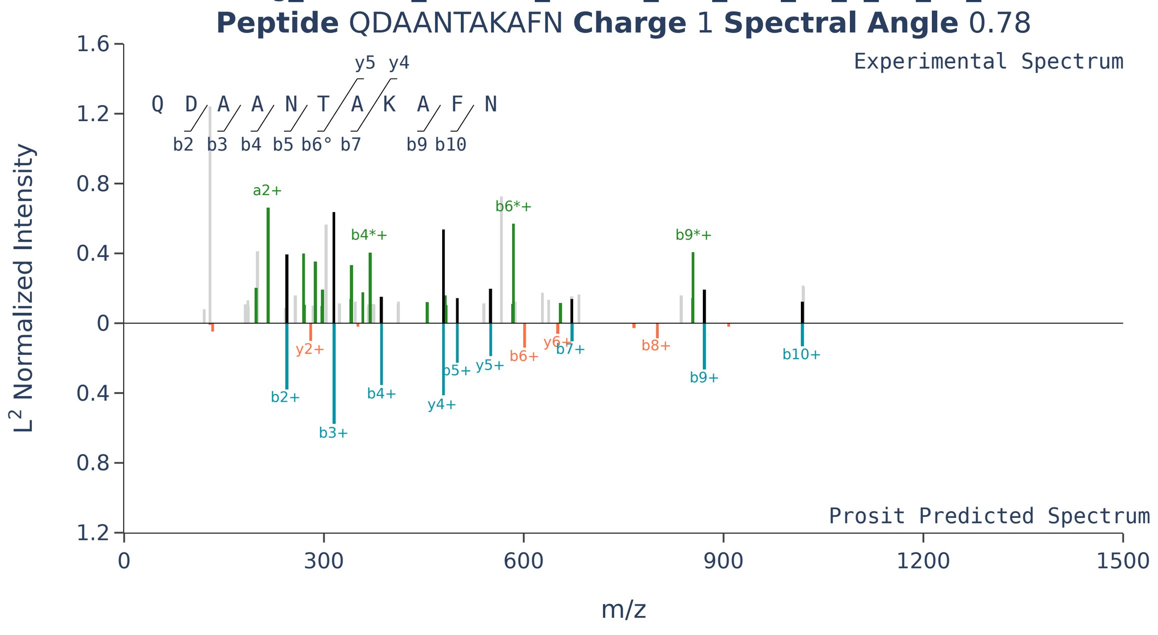


**Source** Ncheng_210623_230623_HFGoe_FFH_20S_25_1_A1_2h_R2 **Scan** 28353
**Peptide** QVGVDFFPSDVGQKPVD **Charge** 2 **Spectral Angle** 0.79

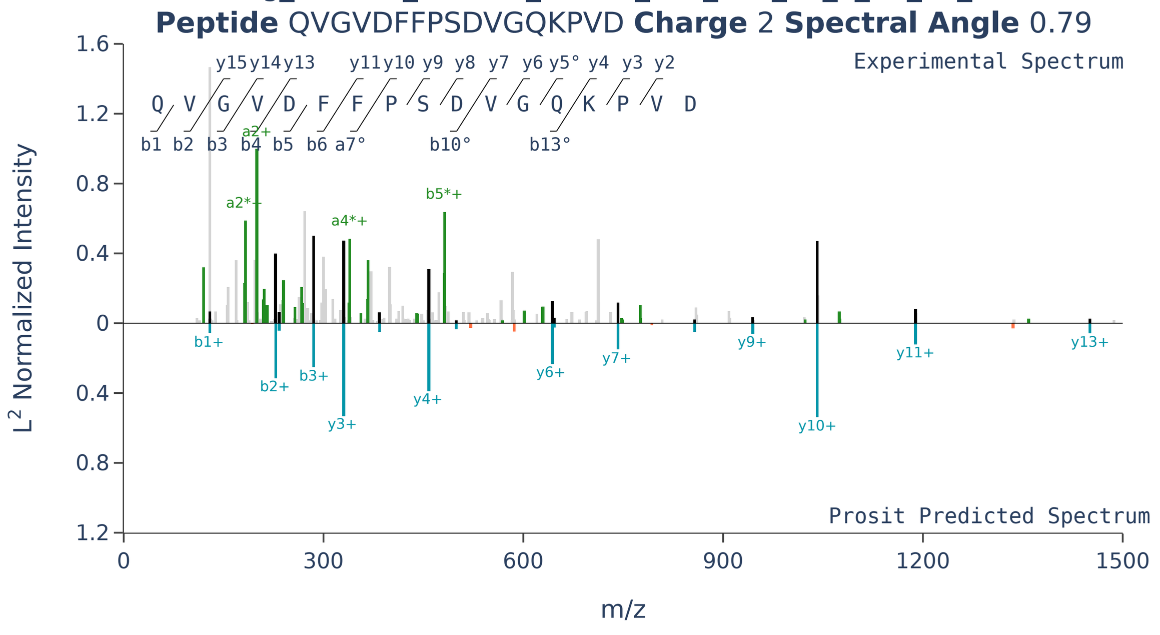


**Source** Ncheng_210623_230623_HFGoe_FFH_20S_25_1_A1_24h_R2 **Scan** 7307
**Peptide** HEVNKSLTPGQE **Charge** 2 **Spectral Angle** 0.9

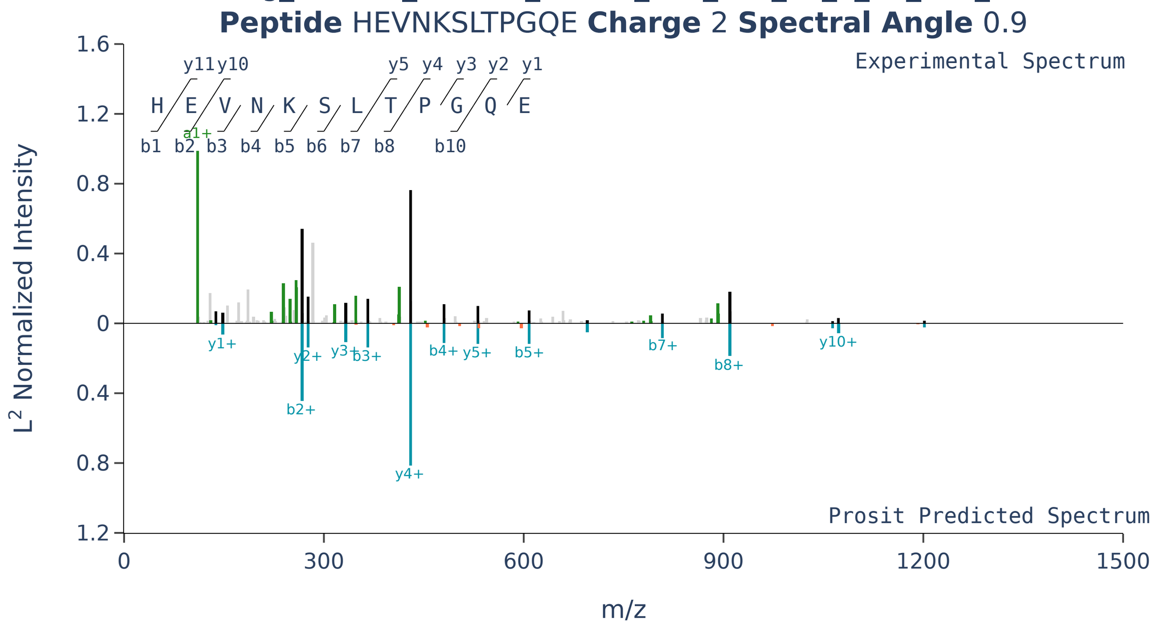


**Source** Ncheng_210623_230623_HFGoe_FFH_20S_25_1_A2_24h_R1 **Scan** 15428
**Peptide** VGHEVNKSLTPGQEF **Charge** 3 **Spectral Angle** 0.91

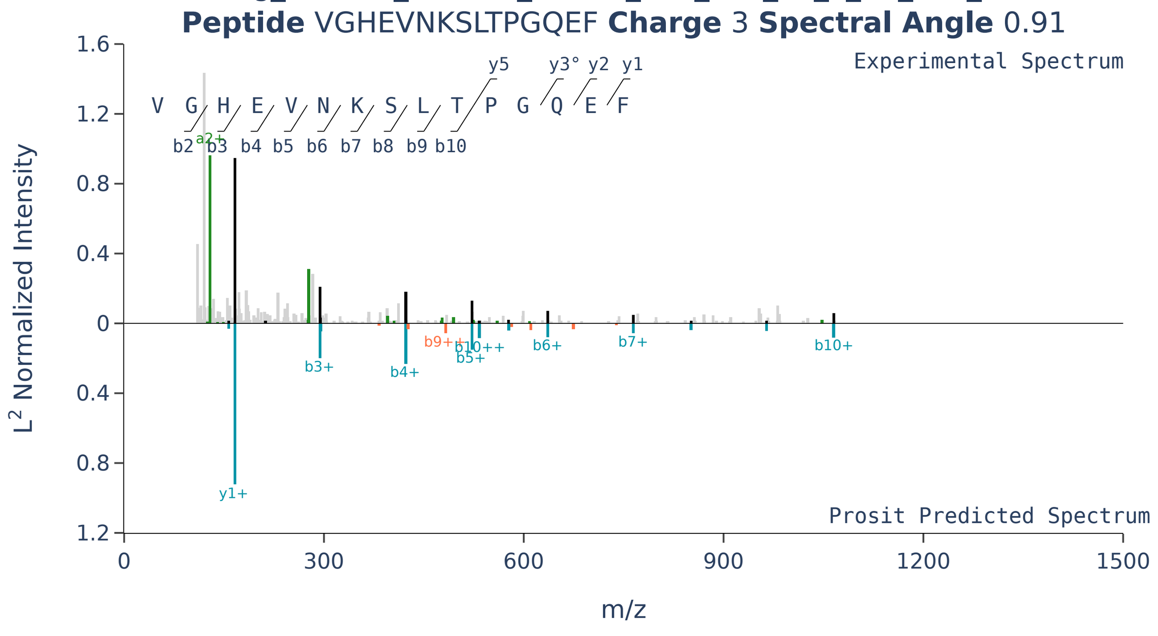


**Source** Ncheng_210623_230623_HFGoe_FFH_20S_25_1_A2_1h_R1 **Scan** 17570
**Peptide** ALEPFHPDRIAS **Charge** 3 **Spectral Angle** 0.84

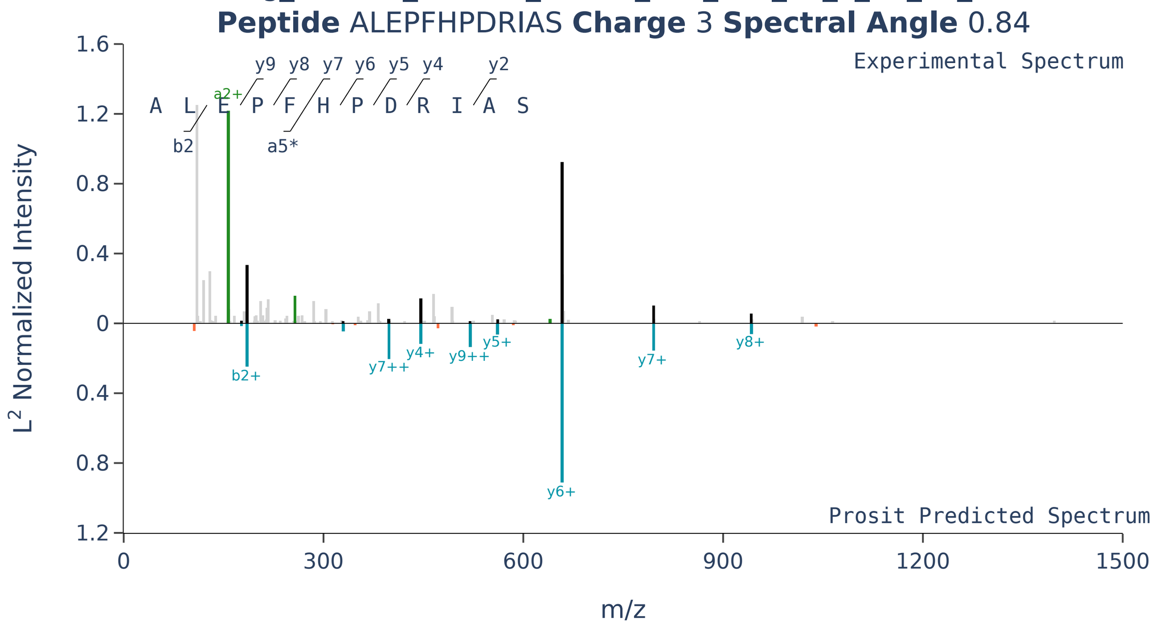


**Source** Ncheng_210623_230623_HFGoe_FFH_20S_25_1_A2_24h_R1 **Scan** 12923
**Peptide** DGDARGGAALSIR **Charge** 3 **Spectral Angle** 0.74

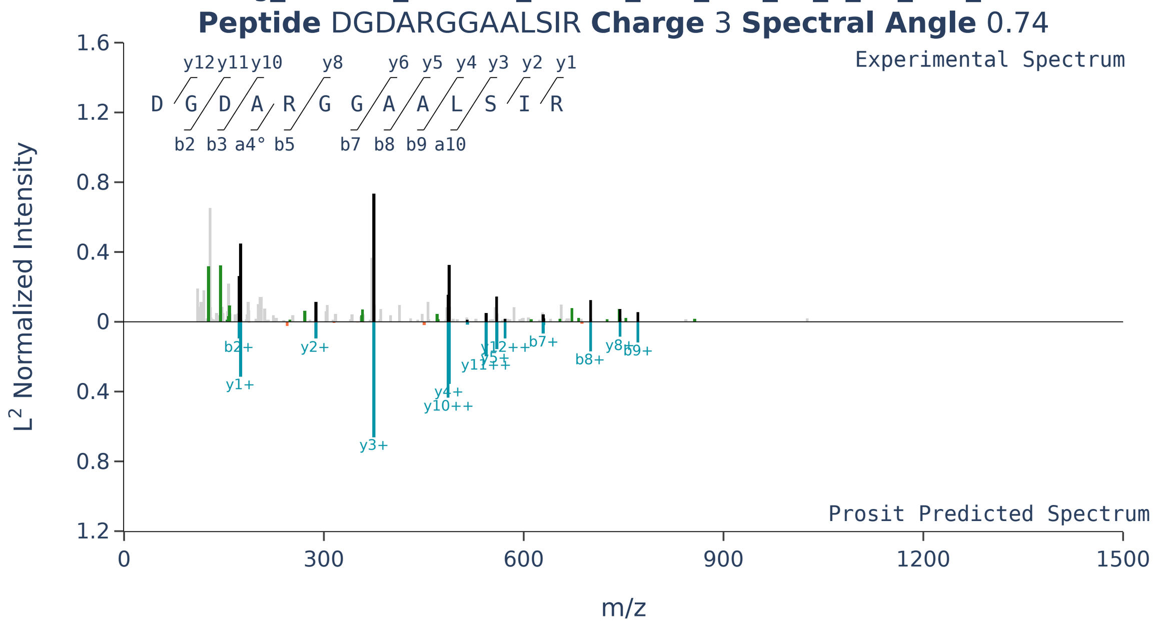


**Source** Ncheng_210623_230623_HFGoe_FFH_20S_25_1_A2_4h_R2 **Scan** 19499
**Peptide** AMGEENQTLNLAAQ **Charge** 2 **Spectral Angle** 0.94

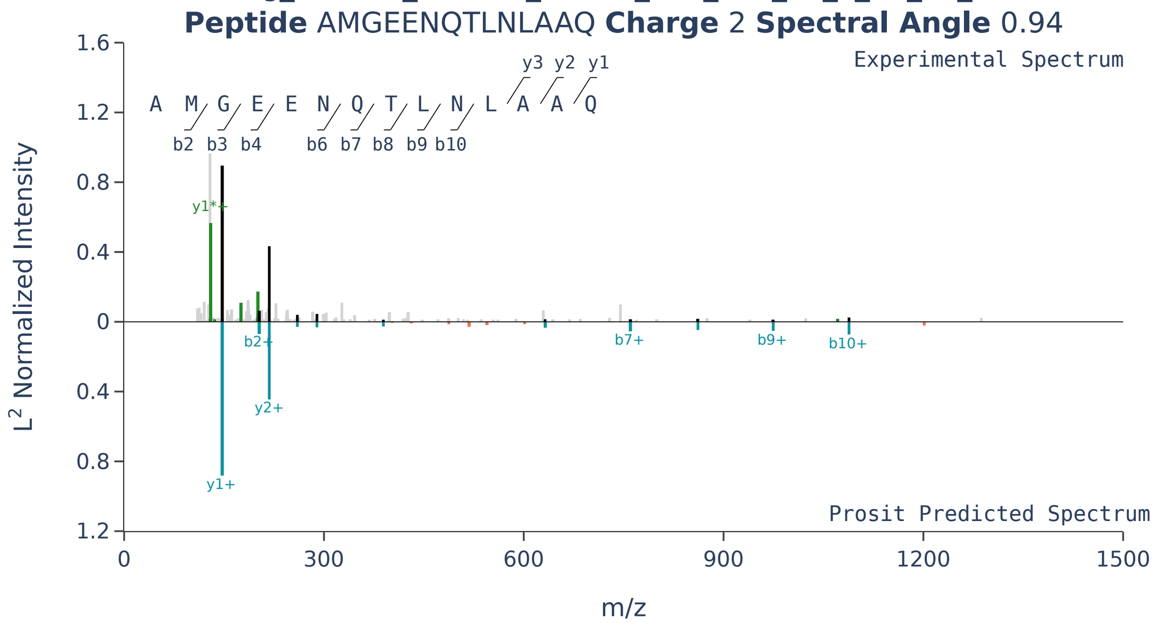


**Source** Ncheng_210623_230623_HFGoe_FFH_20S_25_1_A2_4h_R1 **Scan** 21875
**Peptide** EVNKSLTPGQEFVKIVR **Charge** 2 **Spectral Angle** 0.88

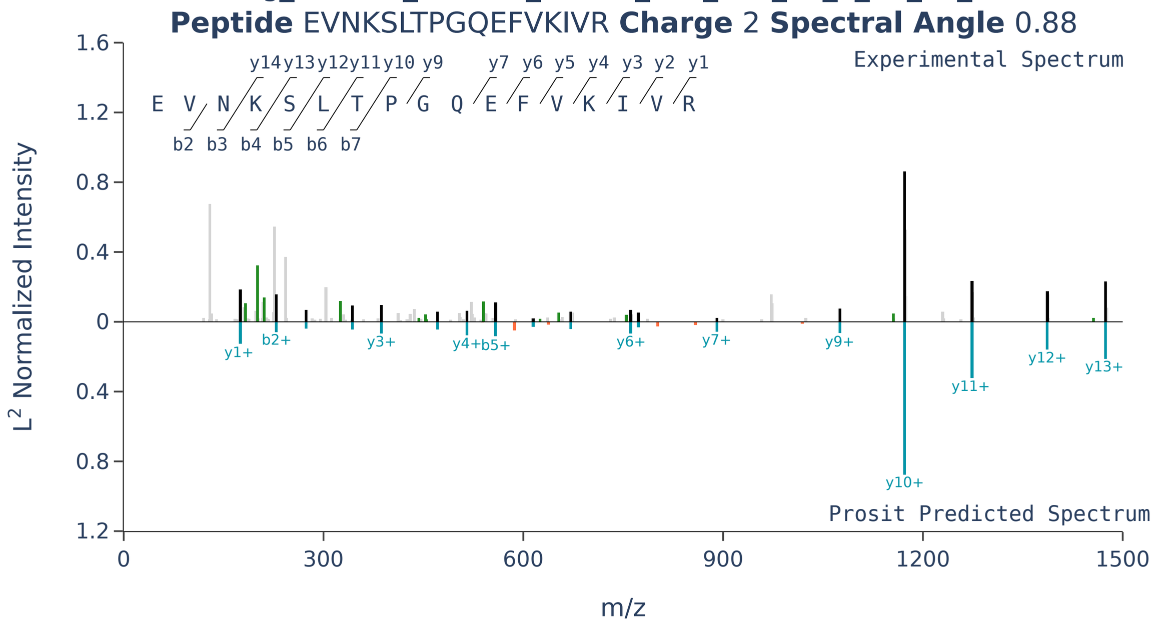


**Source** Ncheng_210623_230623_HFGoe_FFH_20S_25_1_A2_2h_R1 **Scan** 6911
**Peptide** TAGRLHVD **Charge** 1 **Spectral Angle** 0.77

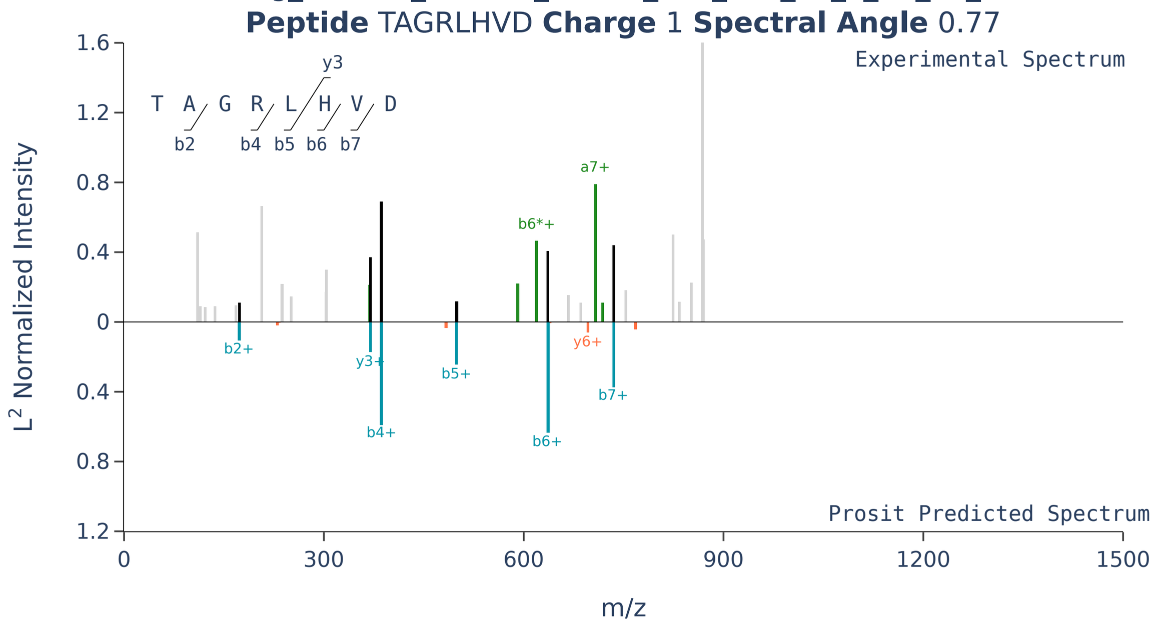


**Source** Ncheng_210623_230623_HFGoe_FFH_20S_25_1_A2_2h_R2 **Scan** 8267
**Peptide** TKVDGDARGGAAL **Charge** 2 **Spectral Angle** 0.83

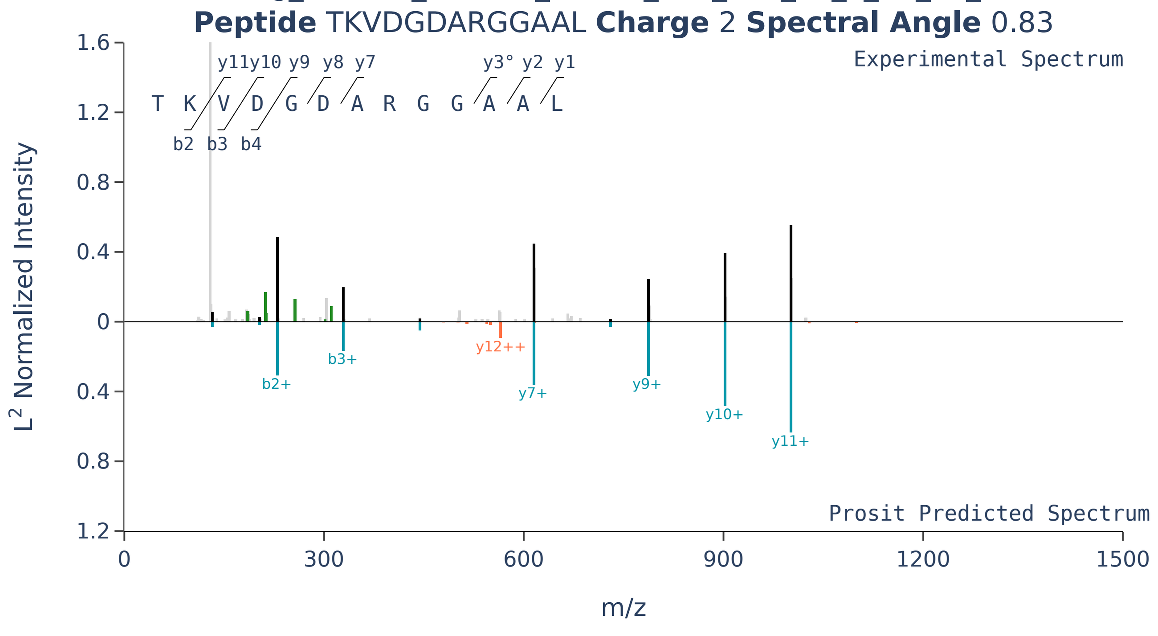


**Source** Ncheng_210623_230623_HFGoe_FFH_20S_25_1_A2_24h_R2 **Scan** 9736
**Peptide** AGLQGAGKTTSV **Charge** 1 **Spectral Angle** 0.76

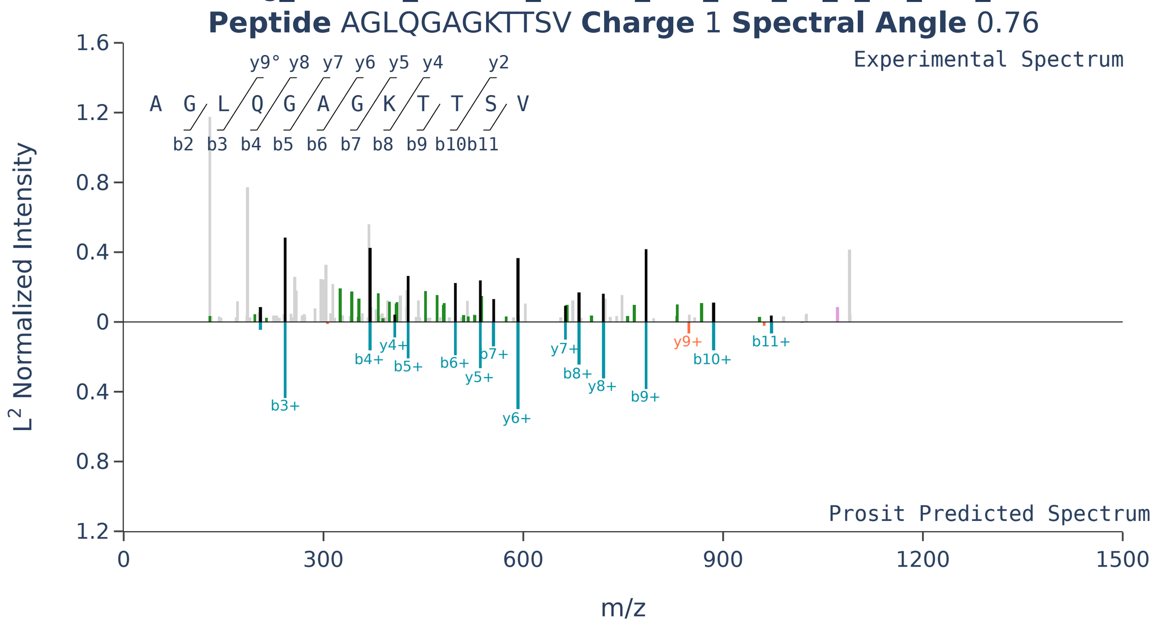

**Source** Ncheng_210623_230623_HFGoe_FFH_20S_25_1_A2_24h_R1 **Scan** 9074
**Peptide** RIAAGCGM **Charge** 2 **Spectral Angle** 0.8

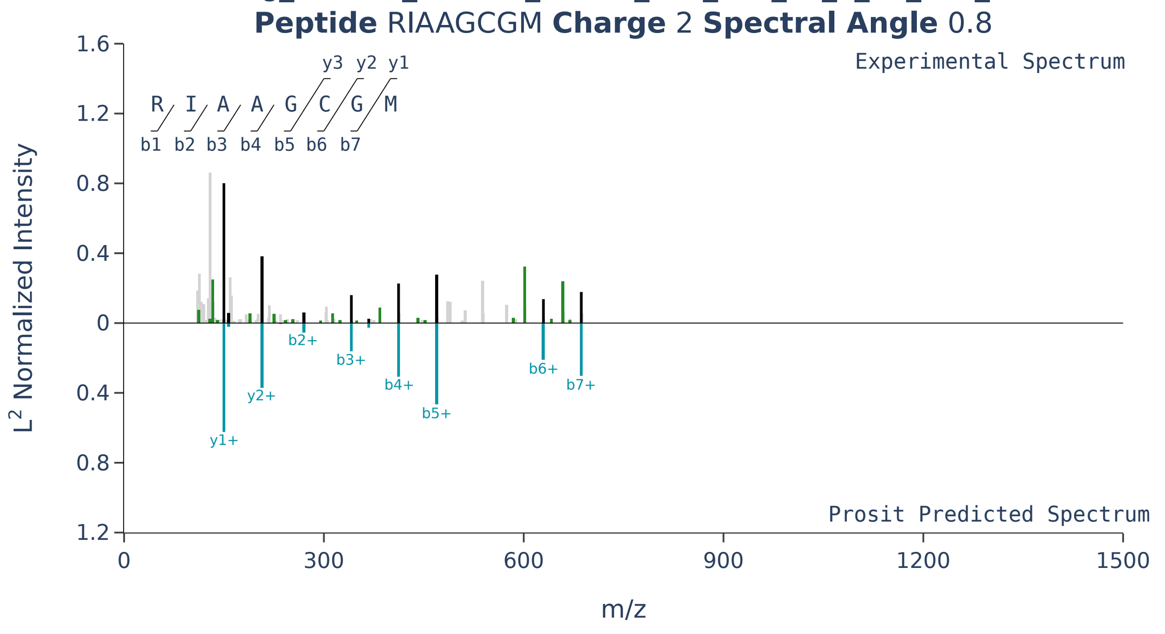


**Source** Ncheng_210623_230623_HFGoe_FFH_20S_25_1_A2_24h_R2 **Scan** 7190
**Peptide** AGKTTSVGKLG **Charge** 1 **Spectral Angle** 0.79

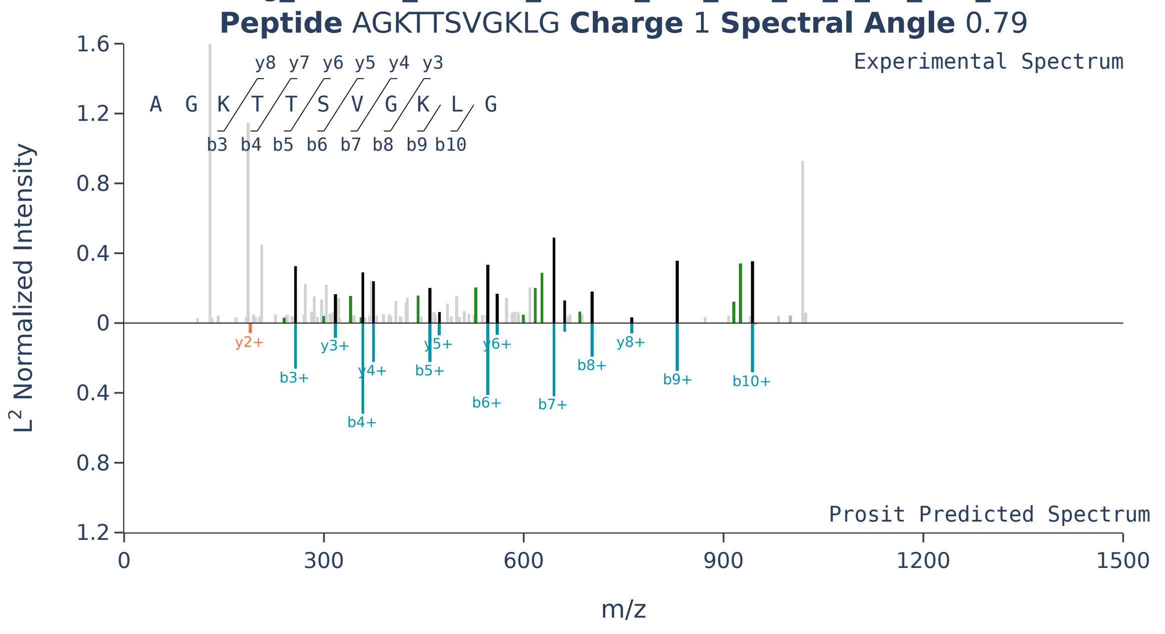


**Source** Ncheng_210623_230623_HFGoe_FFH_20S_25_1_A2_4h_R2 **Scan** 33581
**Peptide** LNDFLEQLRQM **Charge** 2 **Spectral Angle** 0.8

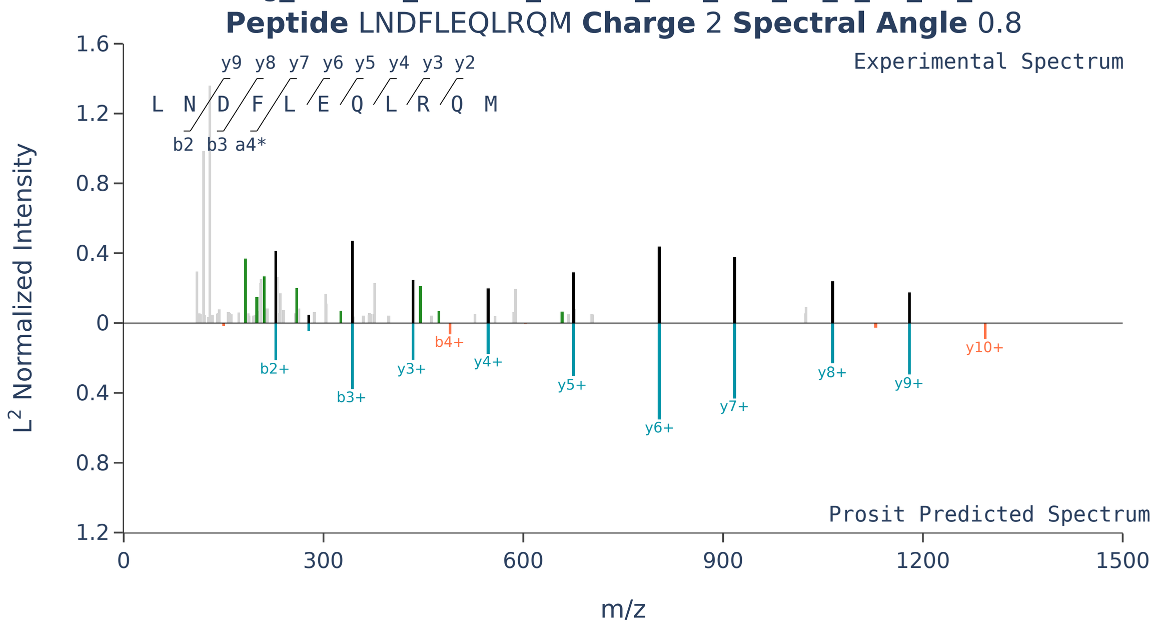


**Source** Ncheng_210623_230623_HFGoe_FFH_20S_25_1_A1_2h_R1 **Scan** 10548
**Peptide** RAKPEII **Charge** 1 **Spectral Angle** 0.83

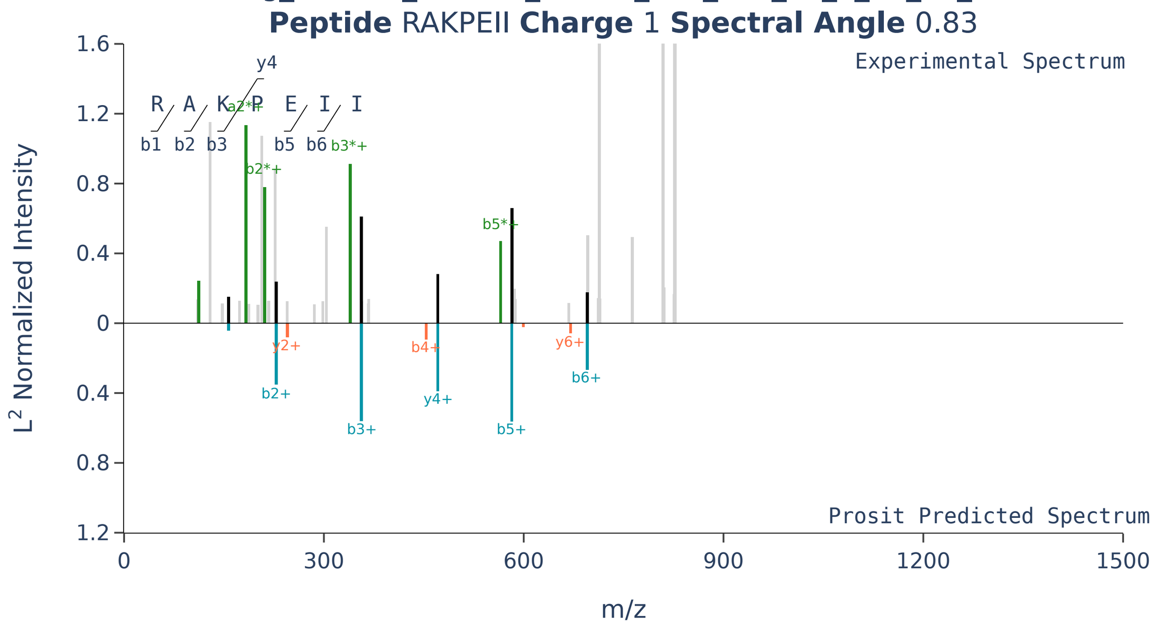


**Source** Ncheng_210623_230623_HFGoe_FFH_20S_25_1_A1_4h_R1 **Scan** 18681
**Peptide** KAVGHEVNKSLTPGQEFVKL **Charge** 3 **Spectral Angle** 0.92

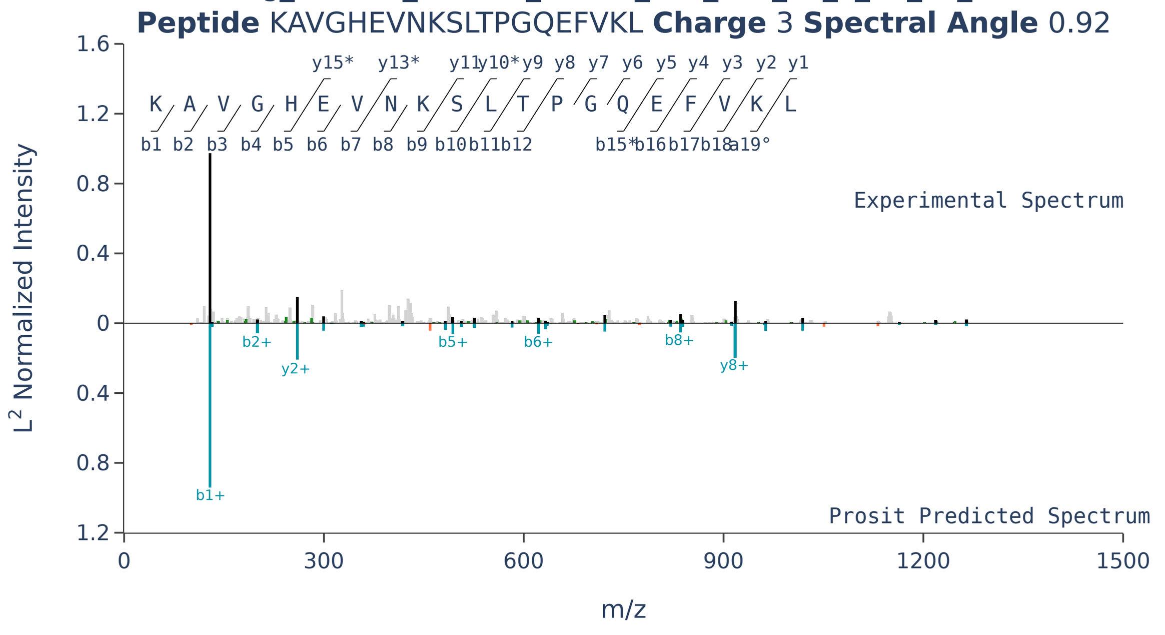


**Source** Ncheng_210623_230623_HFGoe_FFH_20S_25_1_A1_1h_R1 **Scan** 37431
**Peptide** SLNPVETLFVVD **Charge** 2 **Spectral Angle** 0.8

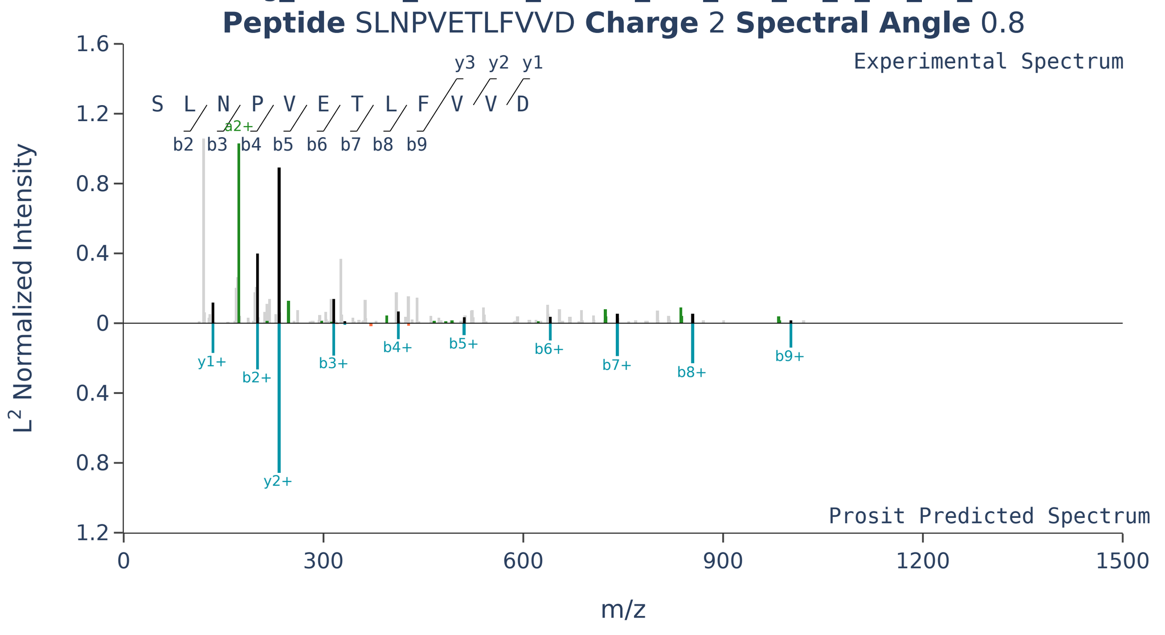


**Source** Ncheng_210623_230623_HFGoe_FFH_20S_25_1_A1_4h_R2 **Scan** 19206
**Peptide** HEVNKSLTPGQEFVKIVR **Charge** 3 **Spectral Angle** 0.81

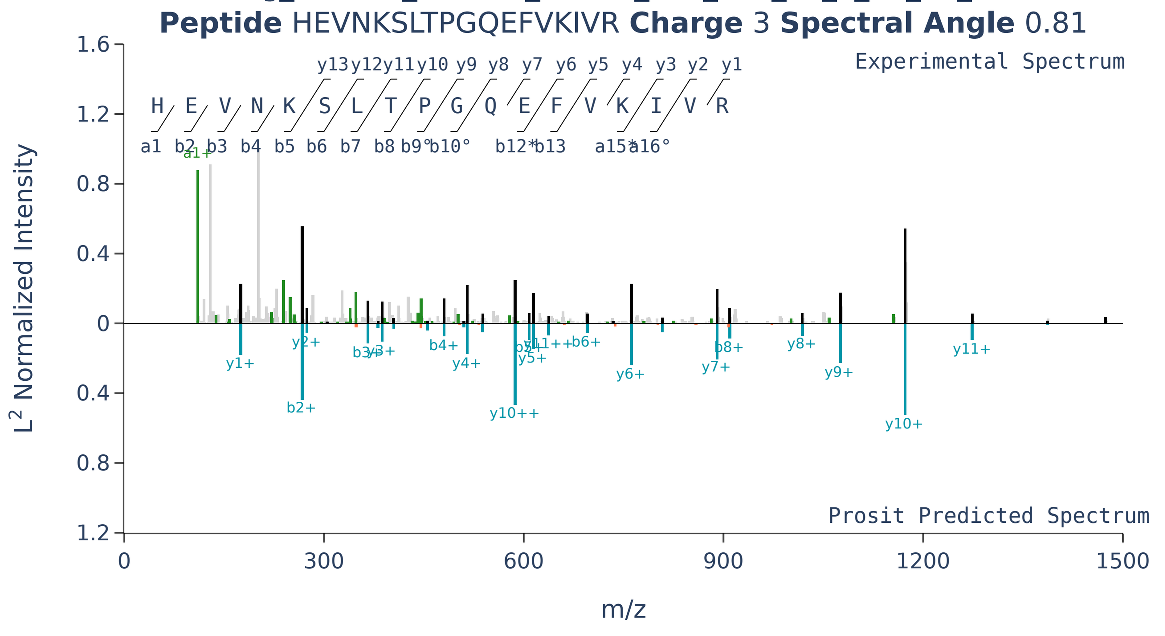


**Source** Ncheng_210623_230623_HFGoe_FFH_20S_25_1_A2_2h_R1 **Scan** 16847
**Peptide** KTEALEPFHPDRIAS **Charge** 2 **Spectral Angle** 0.69

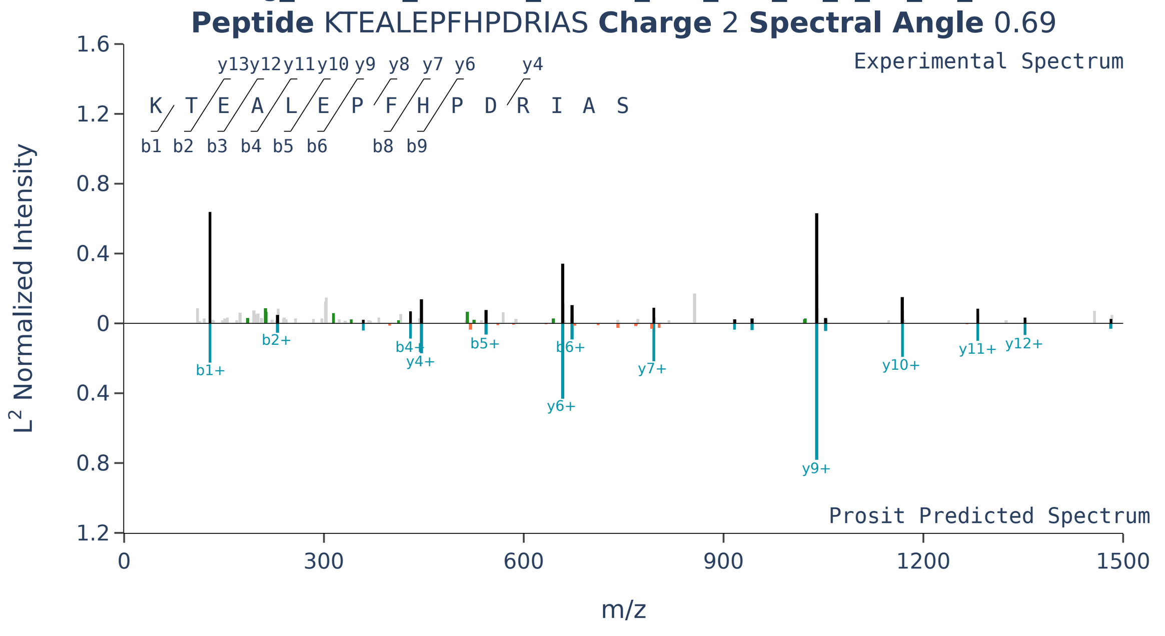


**Source** Ncheng_210623_230623_HFGoe_FFH_20S_25_1_A2_1h_R2 **Scan** 24587
**Peptide** SLTPGQEFVKIVRNE **Charge** 2 **Spectral Angle** 0.72

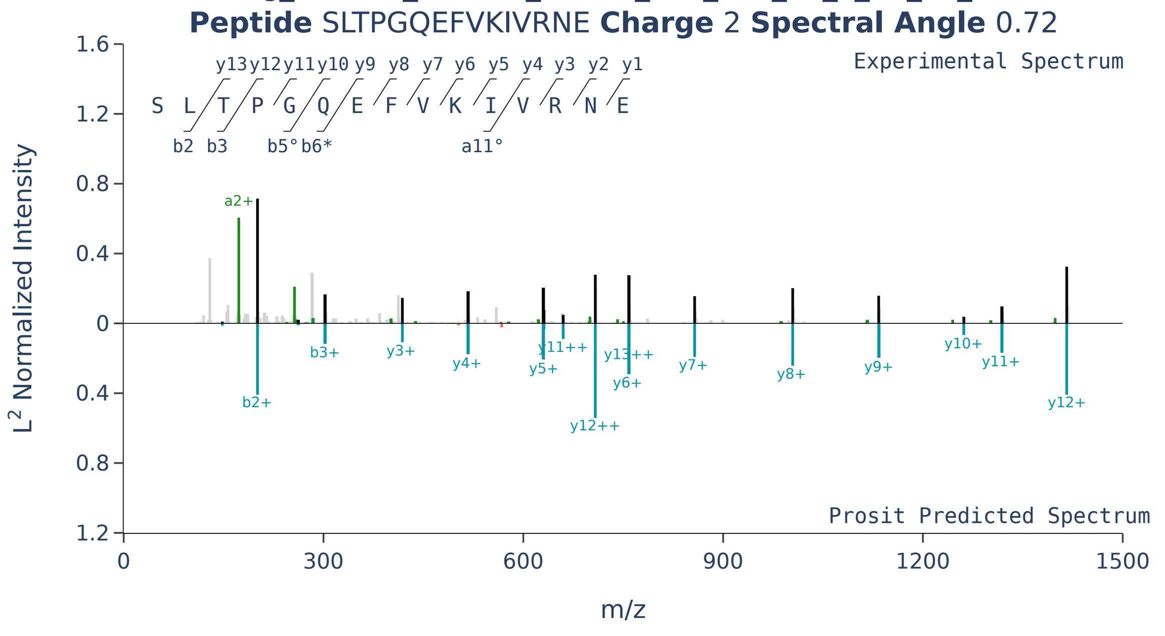


**Source** Ncheng_210623_230623_HFGoe_FFH_20S_25_1_A2_2h_R2 **Scan** 28722
**Peptide** MASLMGKLPGMGQIPDNVKSQMDD **Charge** 3 **Spectral Angle** 0.86

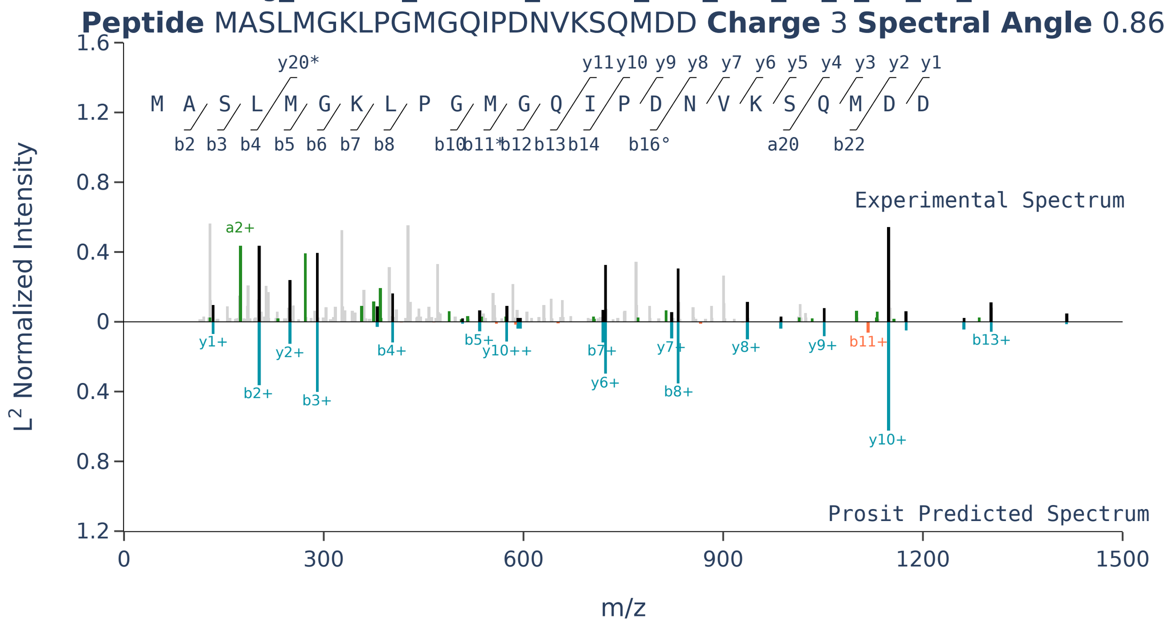


**Source** Ncheng_210623_230623_HFGoe_FFH_20S_25_1_A2_24h_R2 **Scan** 17934
**Peptide** KLPGMGQIPDNVKSQ **Charge** 2 **Spectral Angle** 0.85

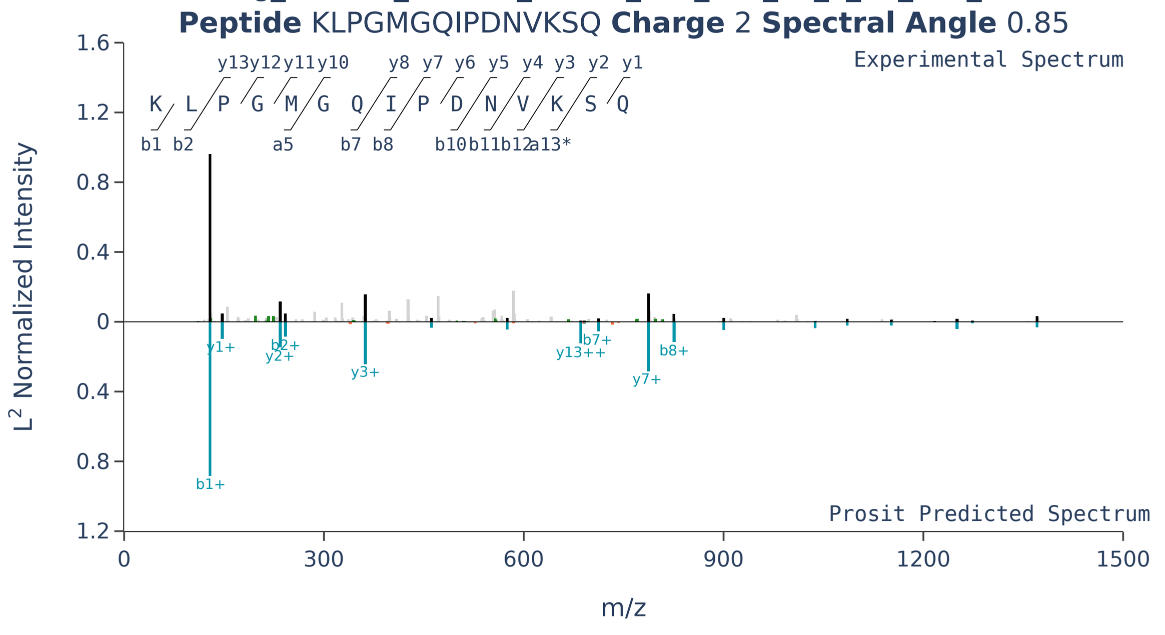


**Source** Ncheng_210623_230623_HFGoe_FFH_20S_25_1_A2_4h_R2 **Scan** 20978
**Peptide** HEVNKSLTPGQEFVKIVRNE **Charge** 2 **Spectral Angle** 0.83

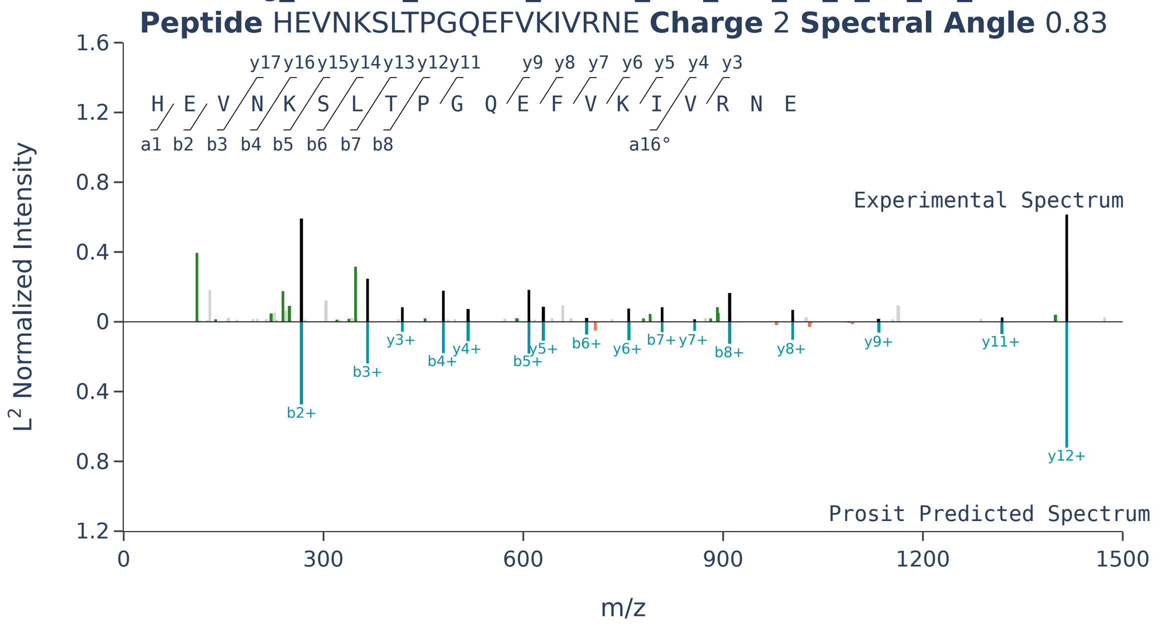


**Source** Ncheng_210623_230623_HFGoe_FFH_20S_25_1_A2_4h_R2 **Scan** 12288
**Peptide** SVGKLGKF **Charge** 2 **Spectral Angle** 0.84

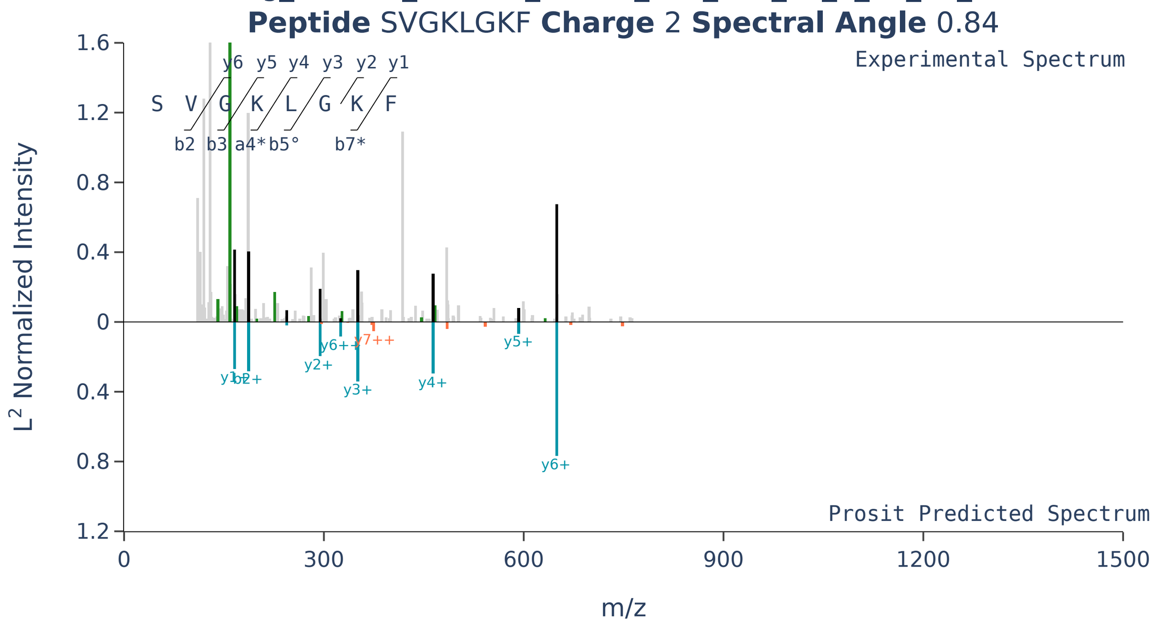


**Source** Ncheng_210623_230623_HFGoe_FFH_20S_25_1_A2_4h_R2 **Scan** 22428
**Peptide** MGKLPGMGQIPDNVKSQMDD **Charge** 3 **Spectral Angle** 0.77

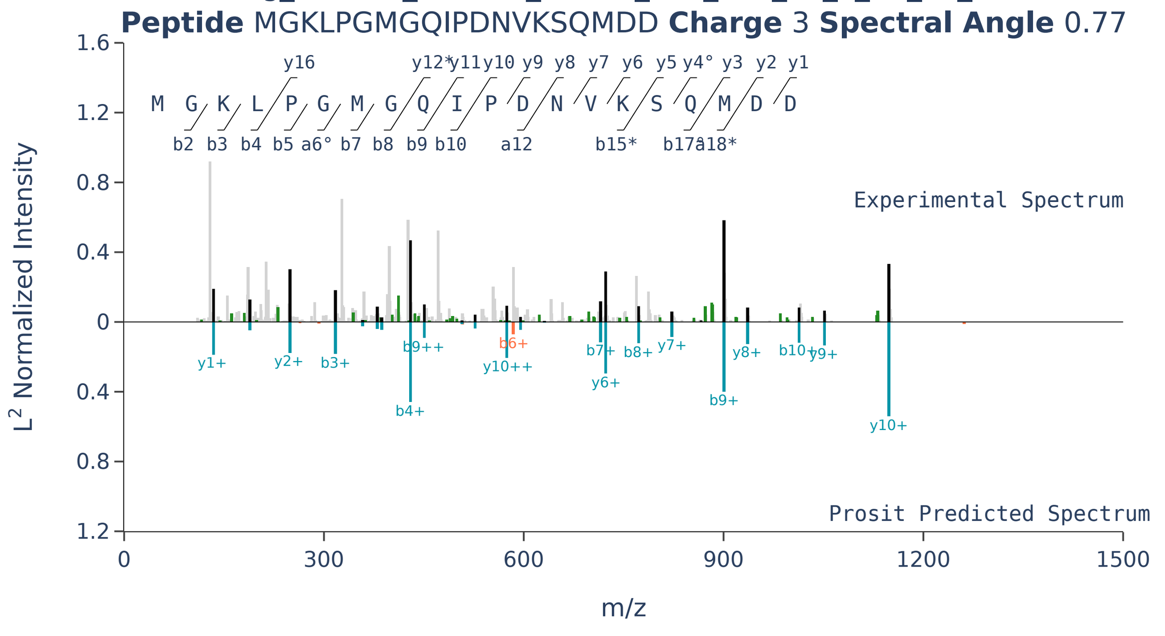


**Source** Ncheng_210623_230623_HFGoe_FFH_20S_25_1_A1_4h_R1 **Scan** 14892
**Peptide** KPIKFLGVGEKT **Charge** 3 **Spectral Angle** 0.9

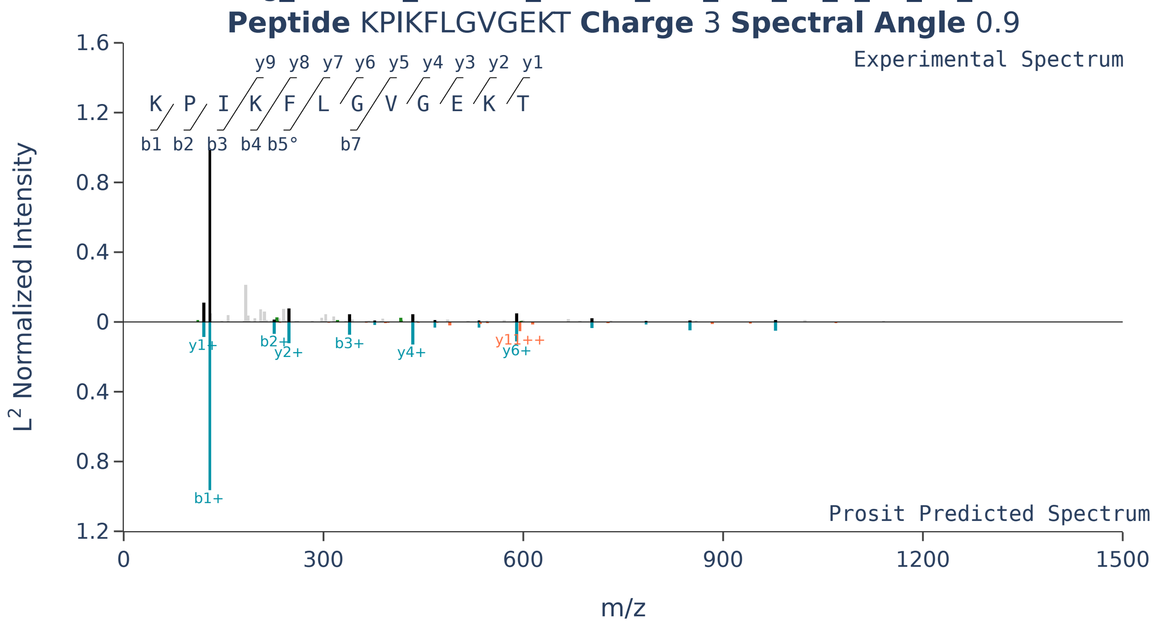

**Source** Ncheng_210623_230623_HFGoe_FFH_20S_25_1_A2_2h_R1 **Scan** 17811
**Peptide** ADVYRPAAIKQLET **Charge** 2 **Spectral Angle** 0.78

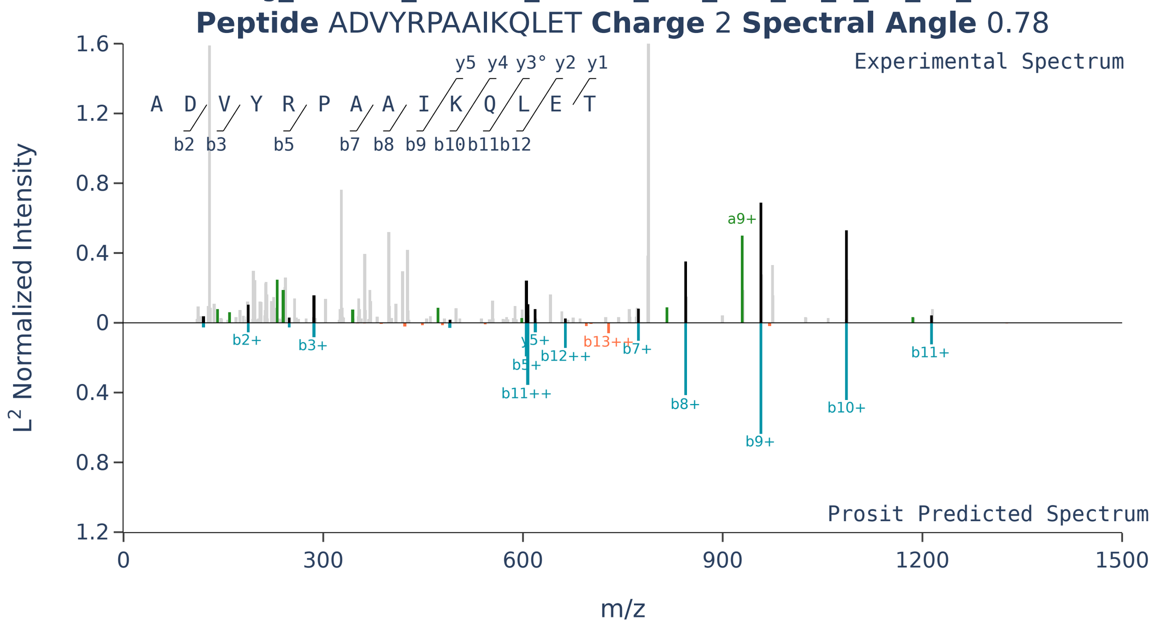


**Source** Ncheng_210623_230623_HFGoe_FFH_20S_25_1_A1_2h_R2 **Scan** 33583
**Peptide** VDFFPSDVGQKPVDIVNAALKE **Charge** 3 **Spectral Angle** 0.84

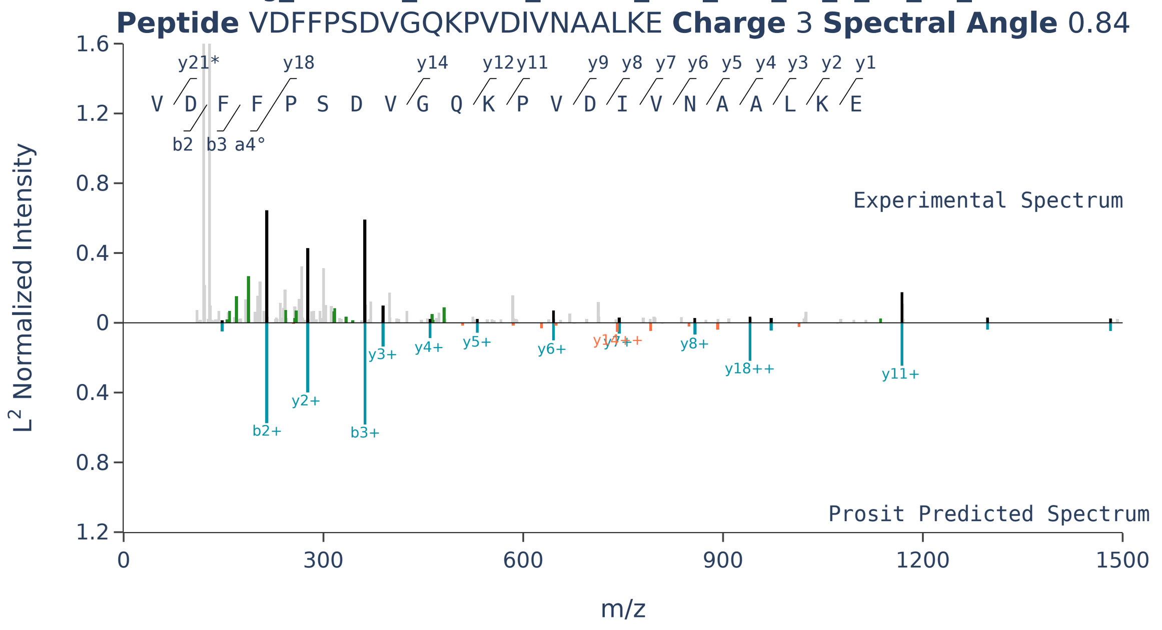


**Source** Ncheng_210623_230623_HFGoe_FFH_20S_25_1_A2_24h_R2 **Scan** 18068
**Peptide** MDEIKQVHASINPVE **Charge** 2 **Spectral Angle** 0.74

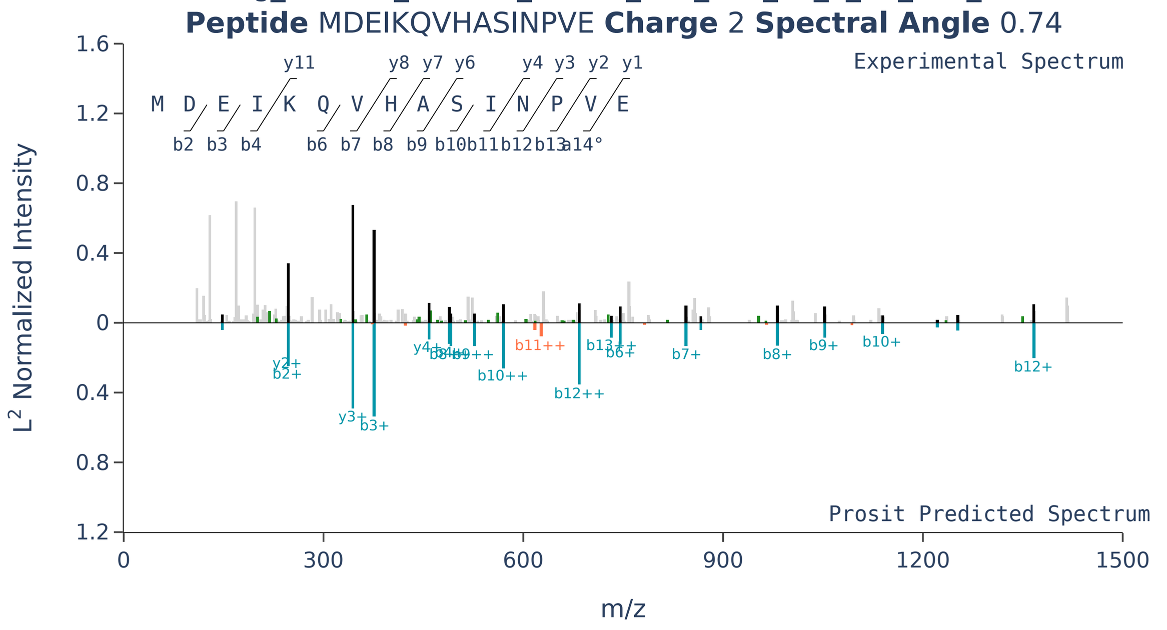


**Source** Ncheng_210623_230623_HFGoe_FFH_20S_25_1_A2_2h_R1 **Scan** 23417
**Peptide** PAAIKQLETLAE **Charge** 2 **Spectral Angle** 0.83

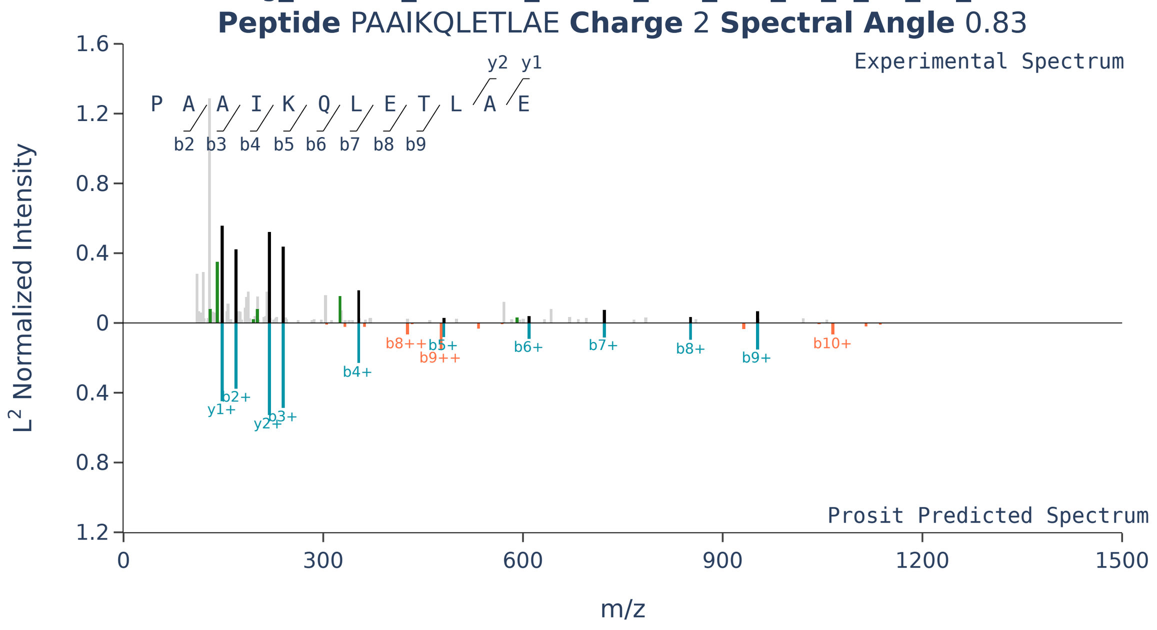


**Source** Ncheng_210623_230623_HFGoe_FFH_20S_25_1_A1_2h_R2 **Scan** 30271
**Peptide** GMASLMGKLPGMGQIPDNVKSQMD **Charge** 3 **Spectral Angle** 0.81

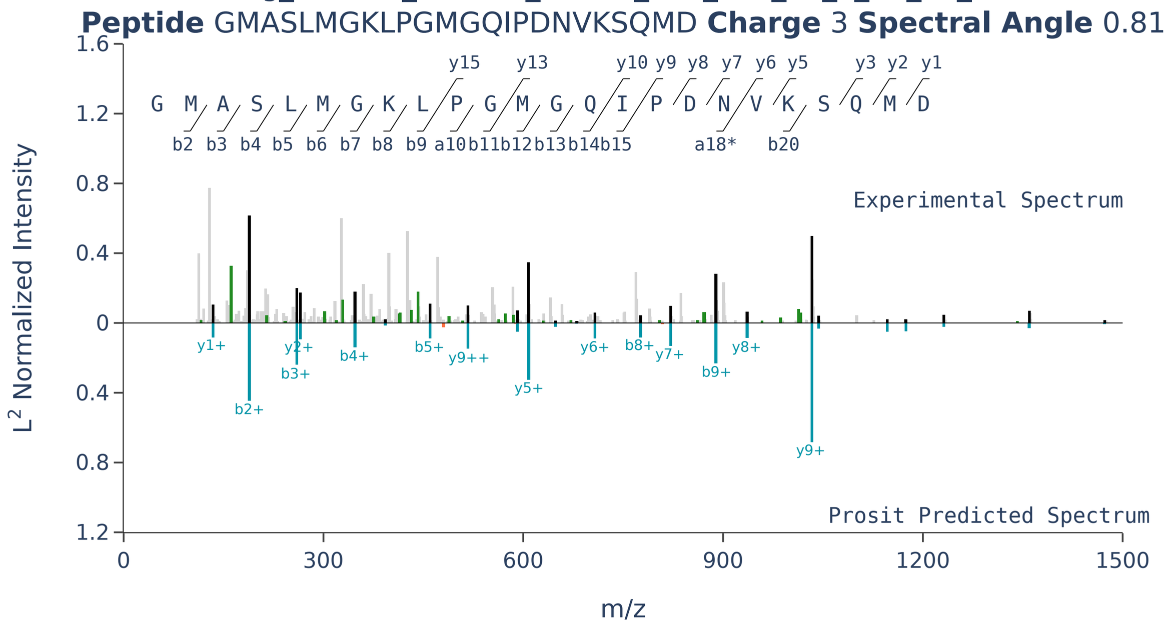


**Source** Ncheng_210623_230623_HFGoe_FFH_20S_25_1_A2_24h_R2 **Scan** 14410
**Peptide** EIKQVHASINPVE **Charge** 1 **Spectral Angle** 0.79

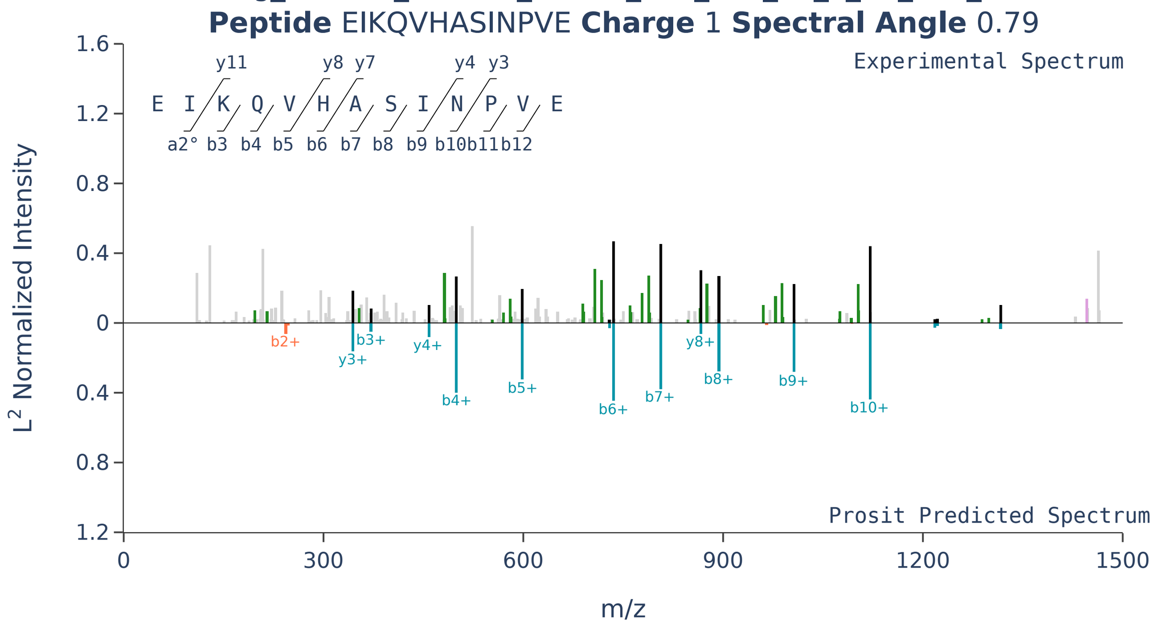


**Source** Ncheng_210623_230623_HFGoe_FFH_20S_25_1_A1_1h_R1 **Scan** 14172
**Peptide** KNMGGMASL **Charge** 2 **Spectral Angle** 0.83

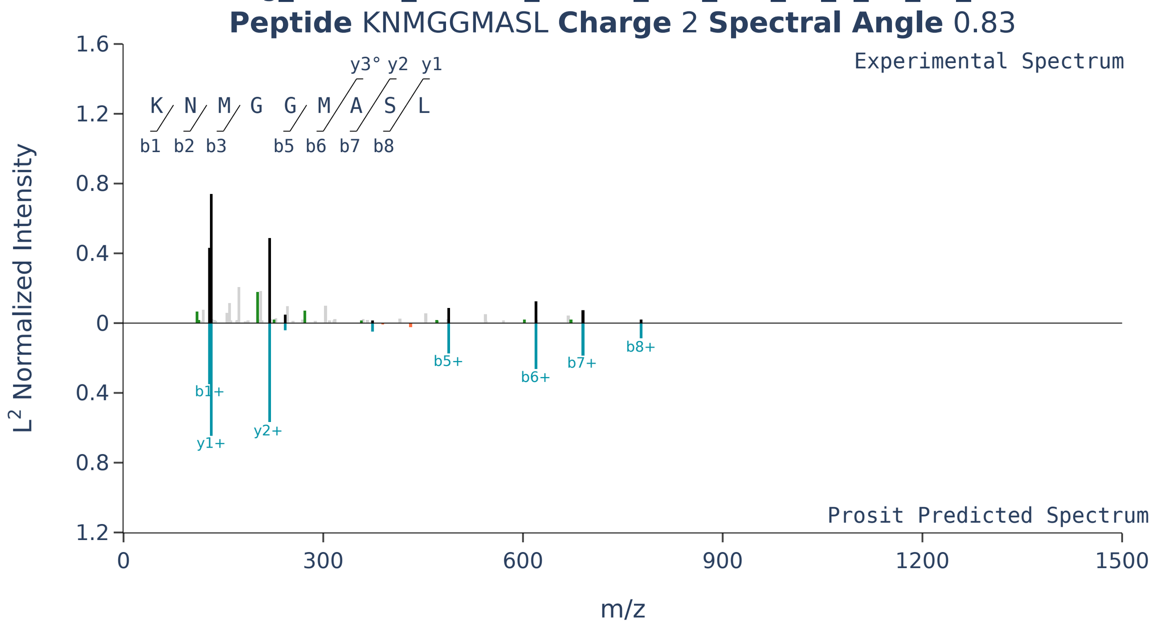


**Source** Ncheng_210623_230623_HFGoe_FFH_20S_25_1_A2_24h_R2 **Scan** 20286
**Peptide** LPGMGQIPDNVKSQ **Charge** 2 **Spectral Angle** 0.78

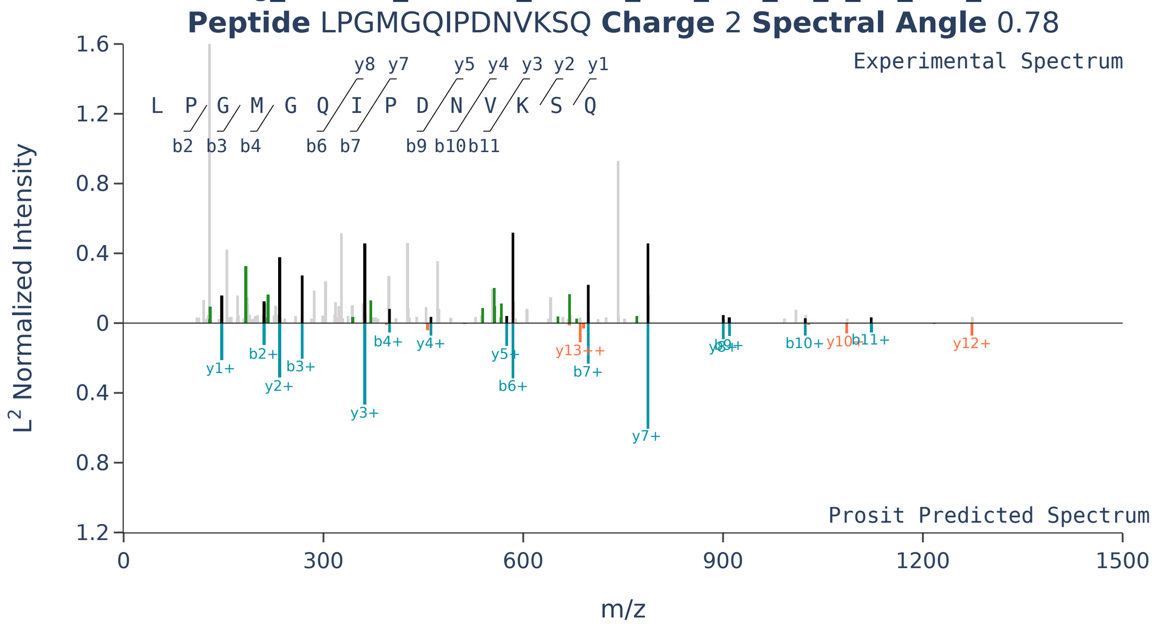


**Source** Ncheng_210623_230623_HFGoe_FFH_20S_25_1_A2_24h_R2 **Scan** 18331
**Peptide** SADVYRPAAIKQL **Charge** 2 **Spectral Angle** 0.78

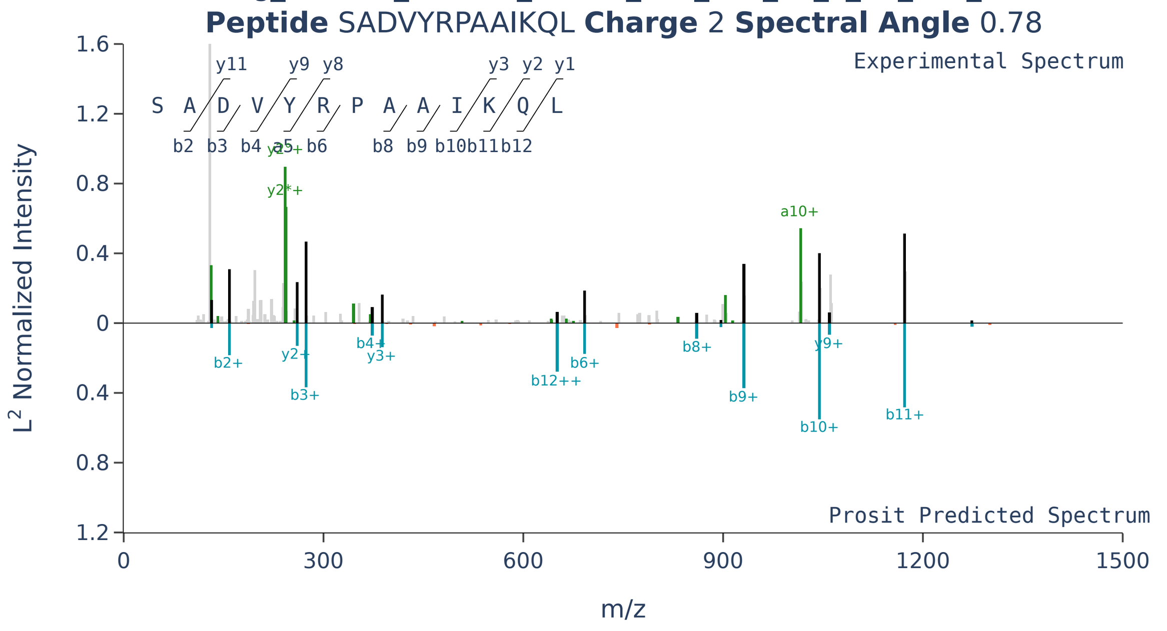


**Source** Ncheng_210623_230623_HFGoe_FFH_20S_25_1_A2_2h_R2 **Scan** 29360
**Peptide** AVGHEVNKSLTPGQEFVKIVRNELV **Charge** 3 **Spectral Angle** 0.87

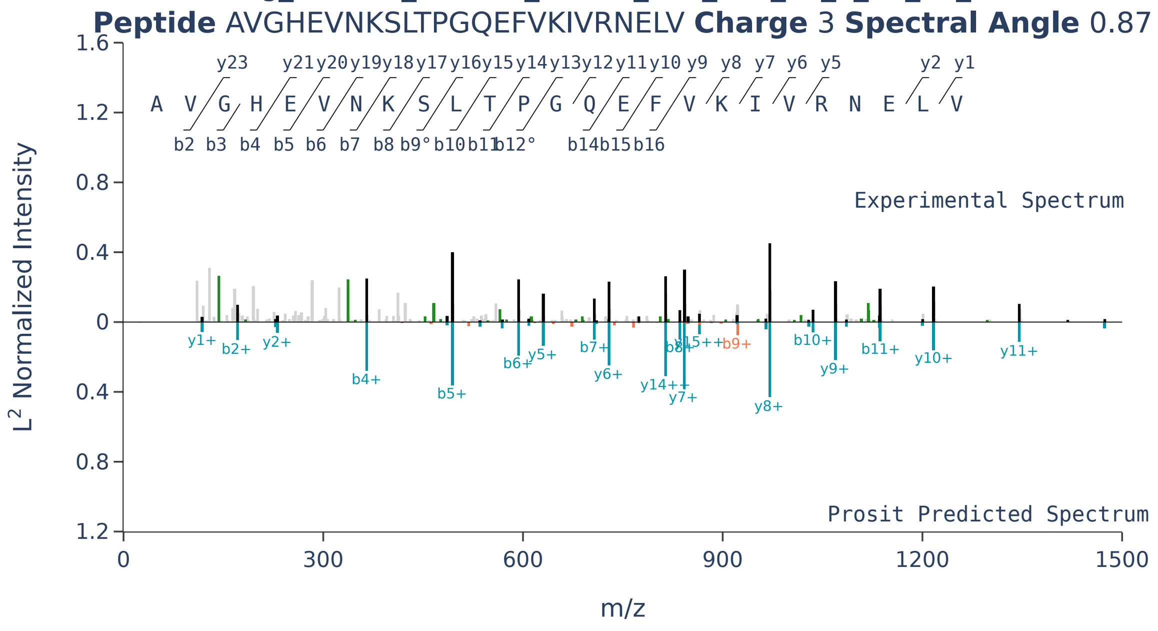


**Source** Ncheng_210623_230623_HFGoe_FFH_20S_25_1_A2_24h_R2 **Scan** 10415
**Peptide** IPDNVKSQMD **Charge** 1 **Spectral Angle** 0.83

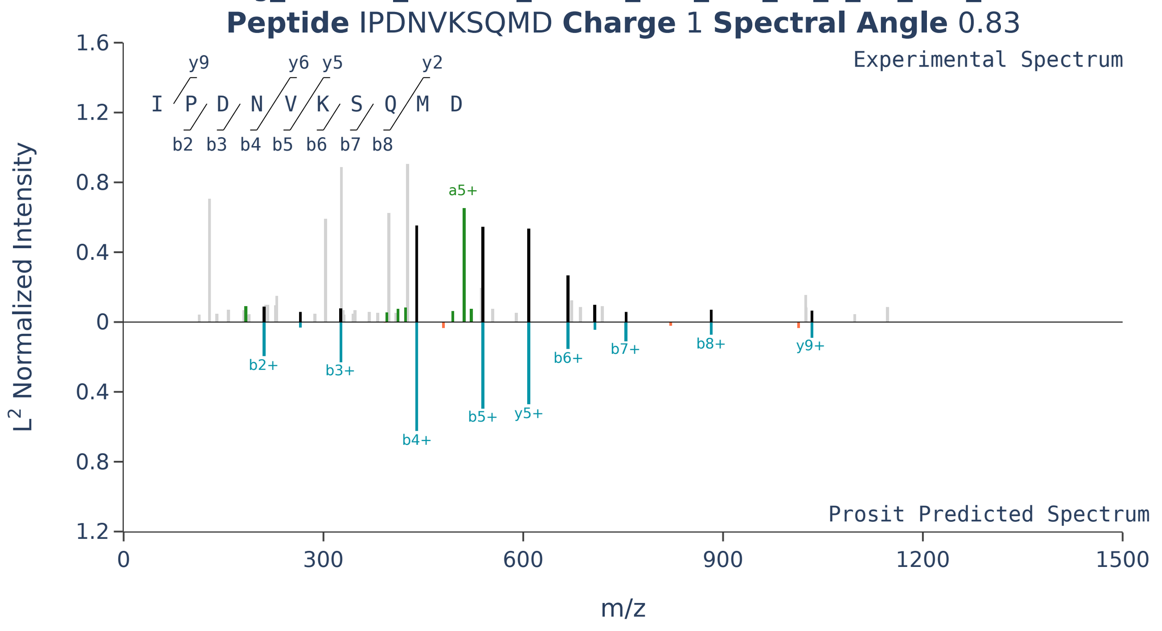


**Source** Ncheng_210623_230623_HFGoe_FFH_20S_25_1_A2_4h_R2 **Scan** 19215
**Peptide** HEVNKSLTPGQEFVKLVR **Charge** 3 **Spectral Angle** 0.83

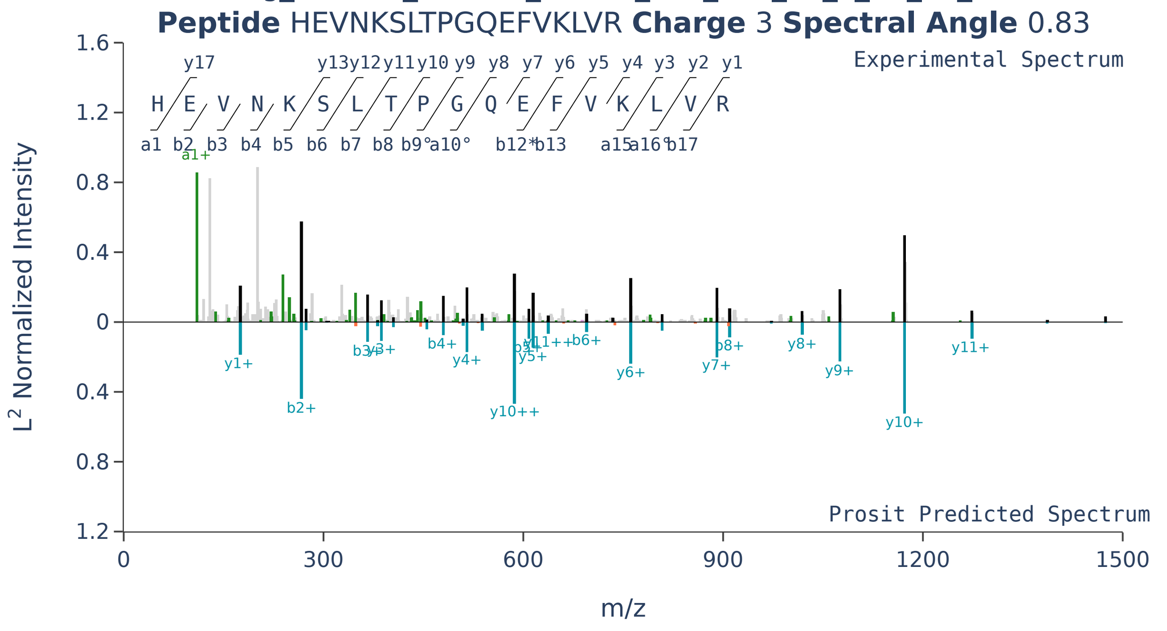


**Source** Ncheng_210623_230623_HFGoe_FFH_20S_25_1_A1_2h_R2 **Scan** 34084
**Peptide** TGQDAANTAKAFNEALPLTGVVLTKVD **Charge** 3 **Spectral Angle** 0.79

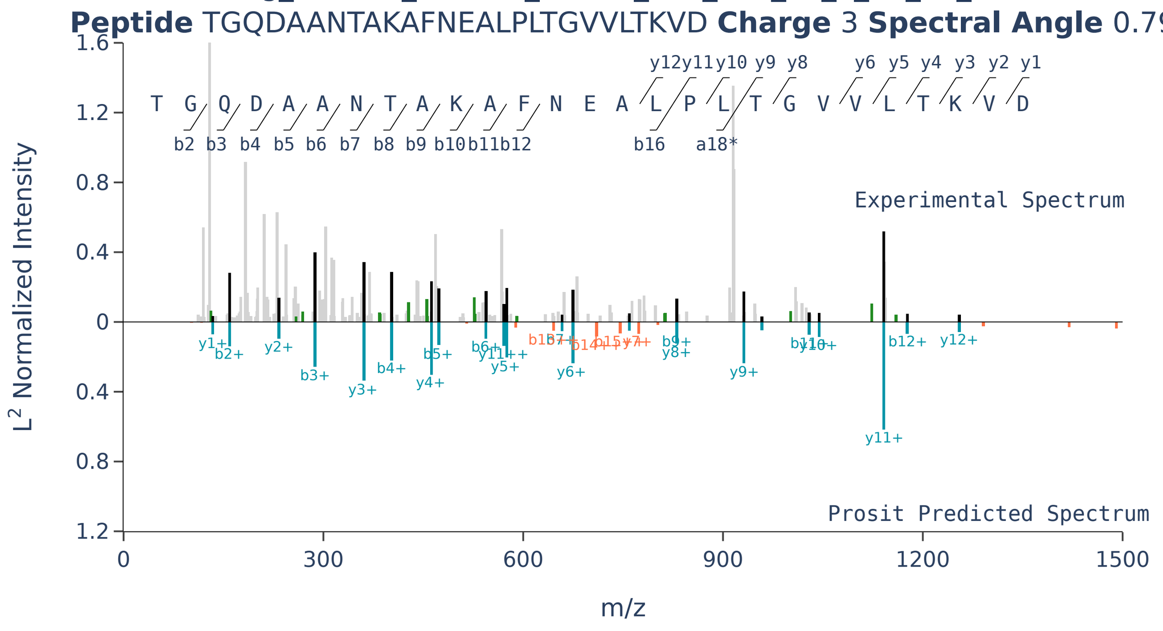


**Source** Ncheng_210623_230623_HFGoe_FFH_20S_25_1_A2_2h_R1 **Scan** 29930
**Peptide** GMASLMGKLPGMGQIPDNVKSQMDD **Charge** 3 **Spectral Angle** 0.87

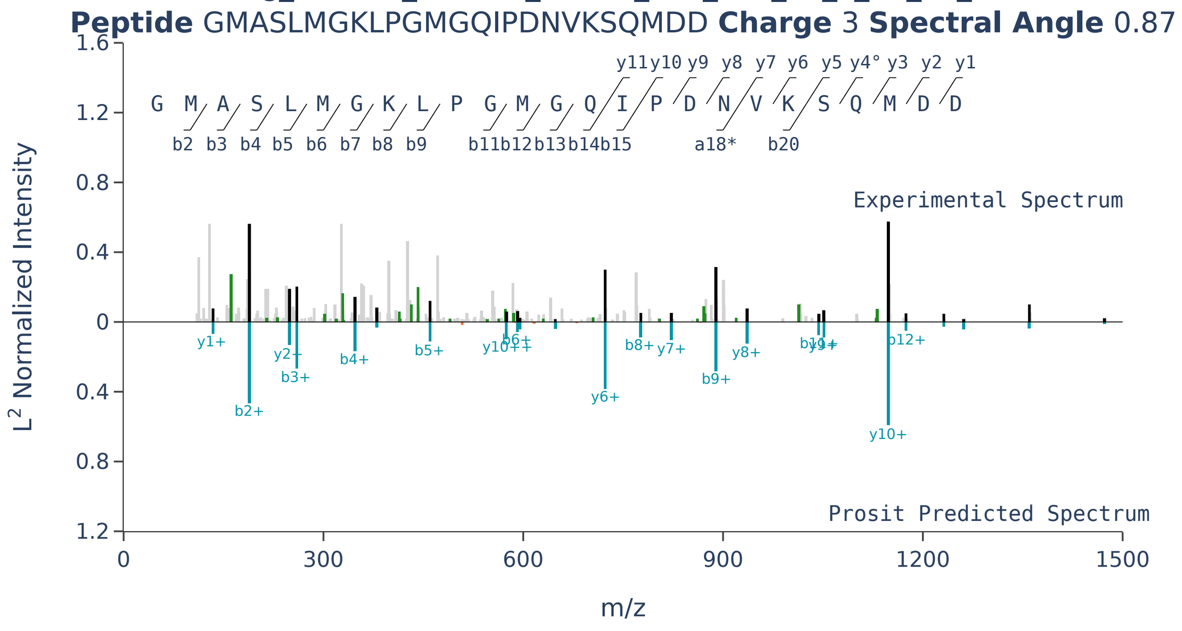


**Source** Ncheng_210623_230623_HFGoe_FFH_20S_25_1_A1_2h_R2 **Scan** 32681
**Peptide** AMTGQDAANTAKAFNEALPLTGVVLTK **Charge** 3 **Spectral Angle** 0.84

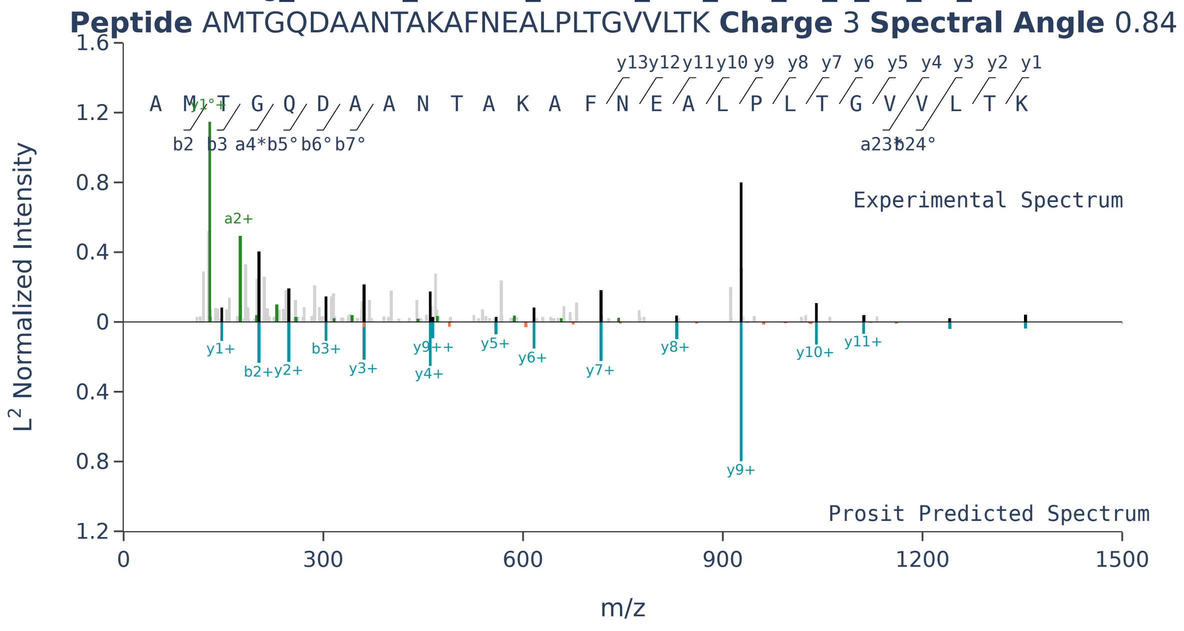

**Source** Ncheng_210623_230623_HFGoe_FFH_20S_25_1_A1_2h_R1 **Scan** 13601
**Peptide** MMDEIKQVHA **Charge** 2 **Spectral Angle** 0.73

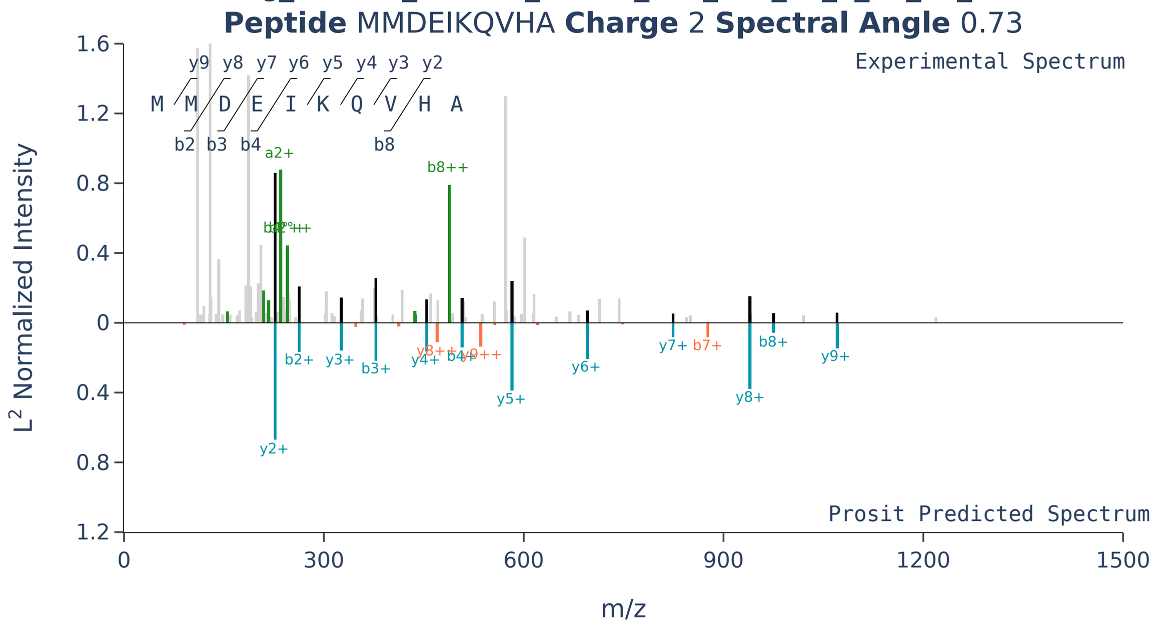


**Source** Ncheng_210623_230623_HFGoe_FFH_20S_25_1_A1_24h_R1 **Scan** 11015
**Peptide** LLNSMTMKERAKPEIIKGSRK **Charge** 6 **Spectral Angle** 0.85

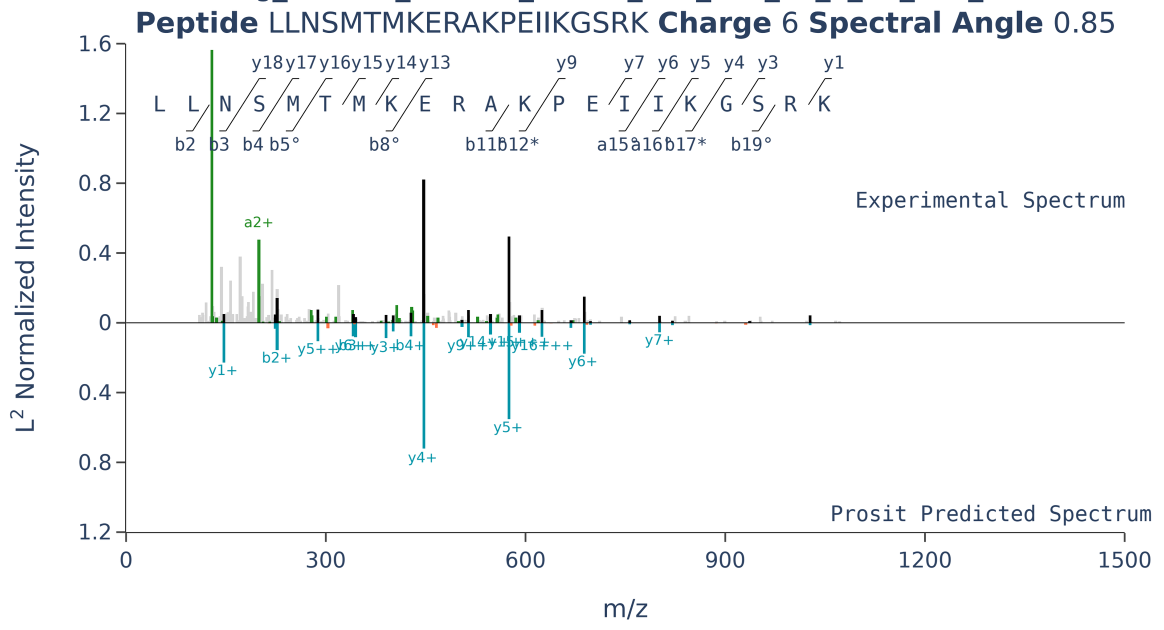


**Source** Ncheng_210623_230623_HFGoe_FFH_20S_25_1_A1_24h_R1 **Scan** 7365
**Peptide** VLTKVDGDARG **Charge** 3 **Spectral Angle** 0.88

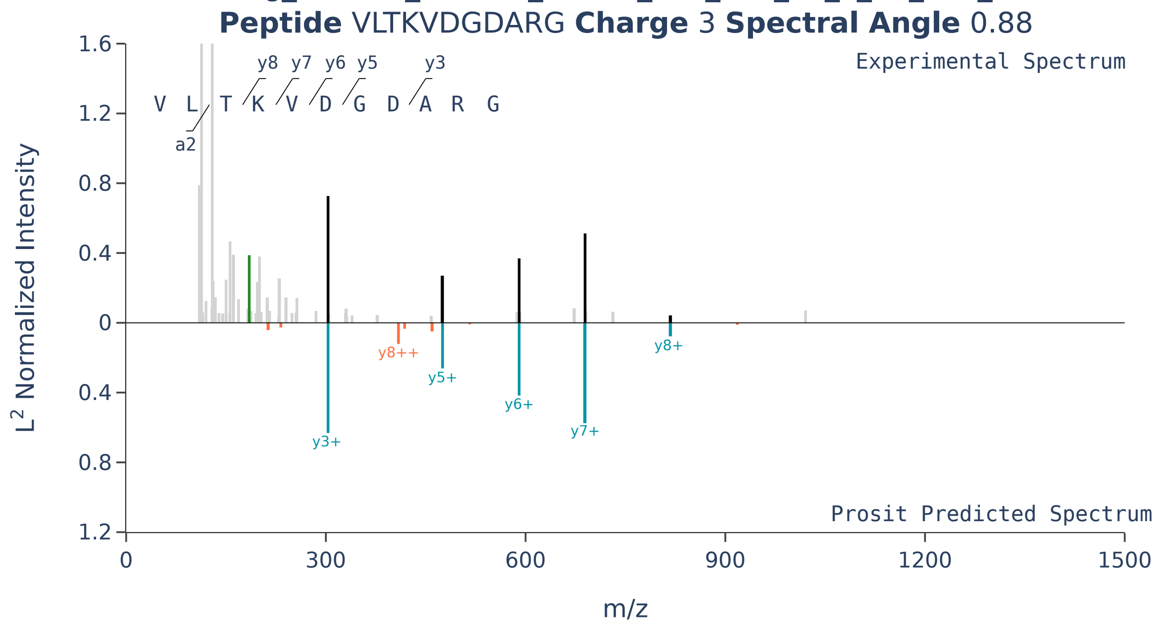


**Source** Ncheng_210623_230623_HFGoe_FFH_20S_25_1_A1_24h_R1 **Scan** 9388
**Peptide** VDGDARGGAAL **Charge** 1 **Spectral Angle** 0.93

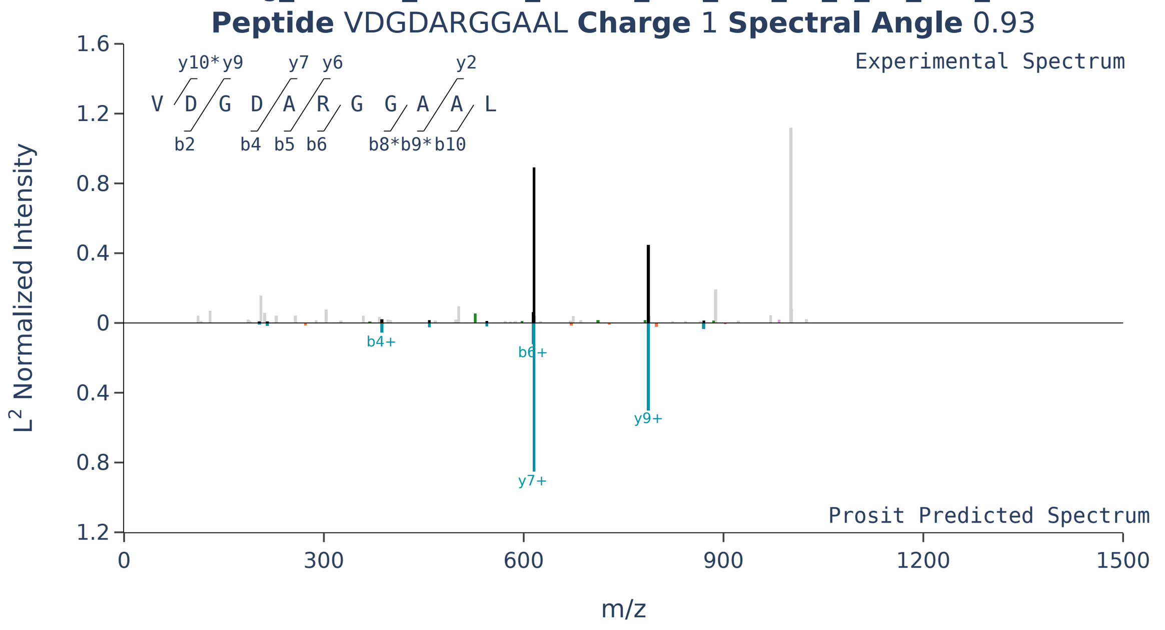


**Source** Ncheng_210623_230623_HFGoe_FFH_20S_25_1_A1_2h_R2 **Scan** 31664
**Peptide** ELVAAMGEENQTLNLAAQPPAV **Charge** 2 **Spectral Angle** 0.96

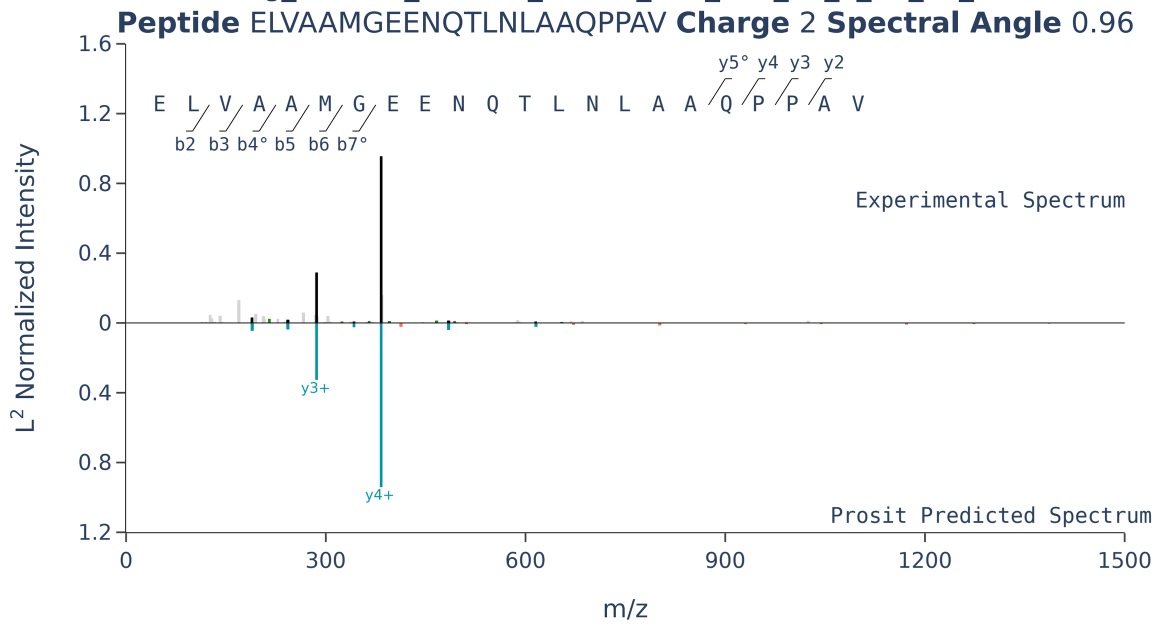


**Source** Ncheng_210623_230623_HFGoe_FFH_20S_25_1_A2_2h_R2 **Scan** 26658
**Peptide** RILGMGDVL **Charge** 2 **Spectral Angle** 0.83

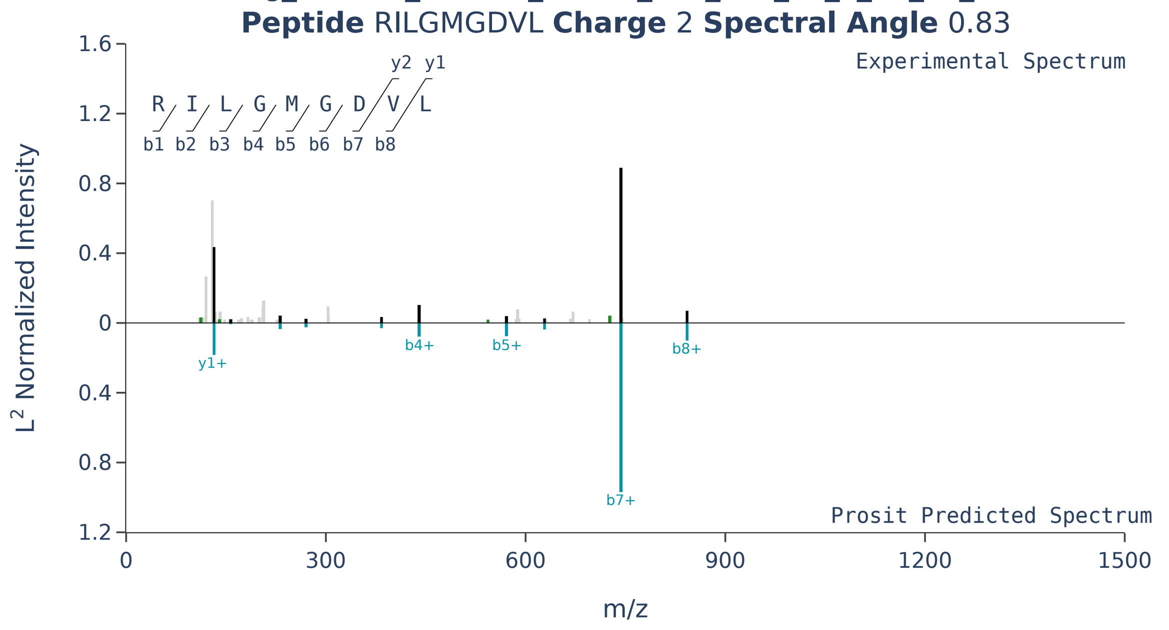


**Source** Ncheng_210623_230623_HFGoe_FFH_20S_25_1_A2_4h_R2 **Scan** 8559
**Peptide** DTAGRLHVD **Charge** 1 **Spectral Angle** 0.75

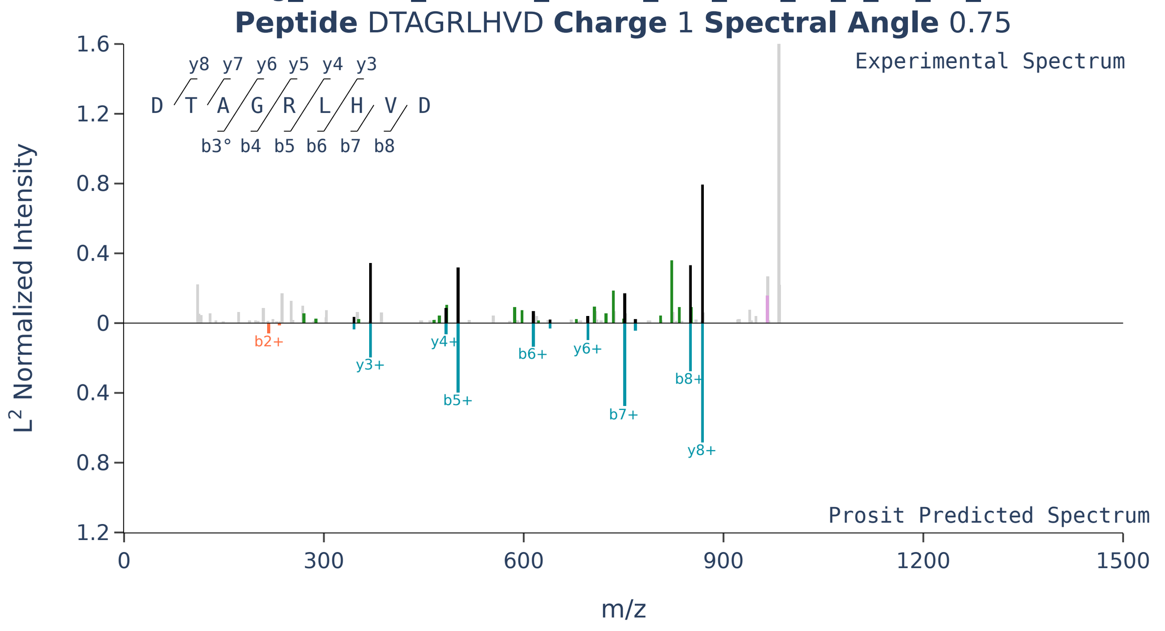


**Source** Ncheng_210623_230623_HFGoe_FFH_20S_25_1_A2_24h_R1 **Scan** 24112
**Peptide** DVNRLLKQF **Charge** 2 **Spectral Angle** 0.74

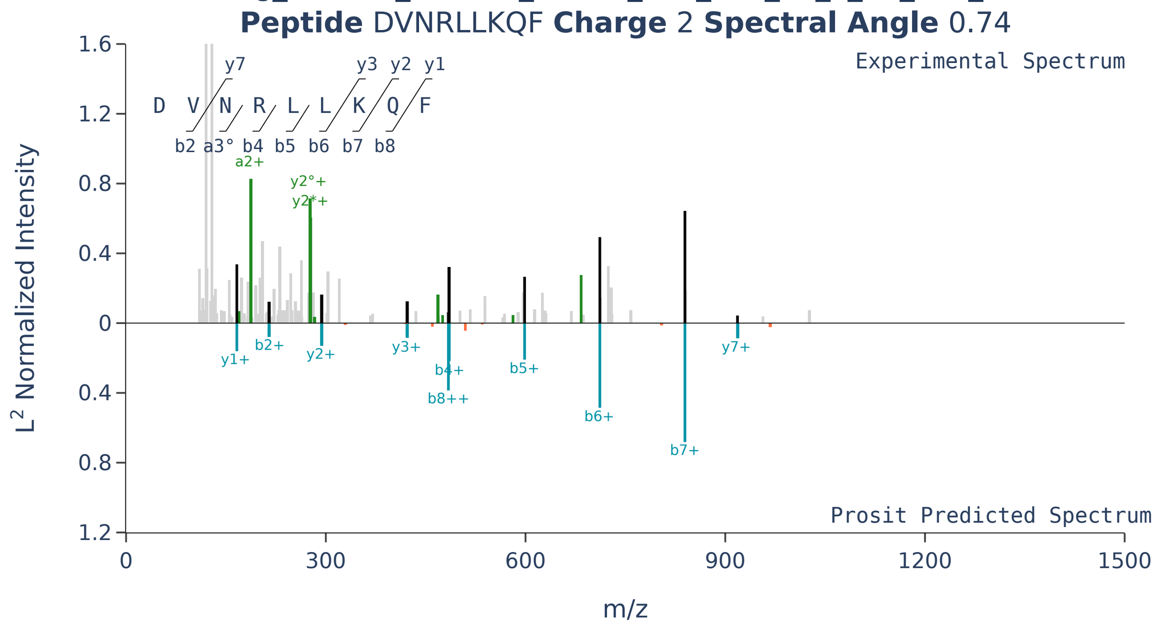


**Source** Ncheng_210623_230623_HFGoe_FFH_20S_25_1_A1_2h_R2 **Scan** 12615
**Peptide** DRAQAEKLASKL **Charge** 3 **Spectral Angle** 0.77

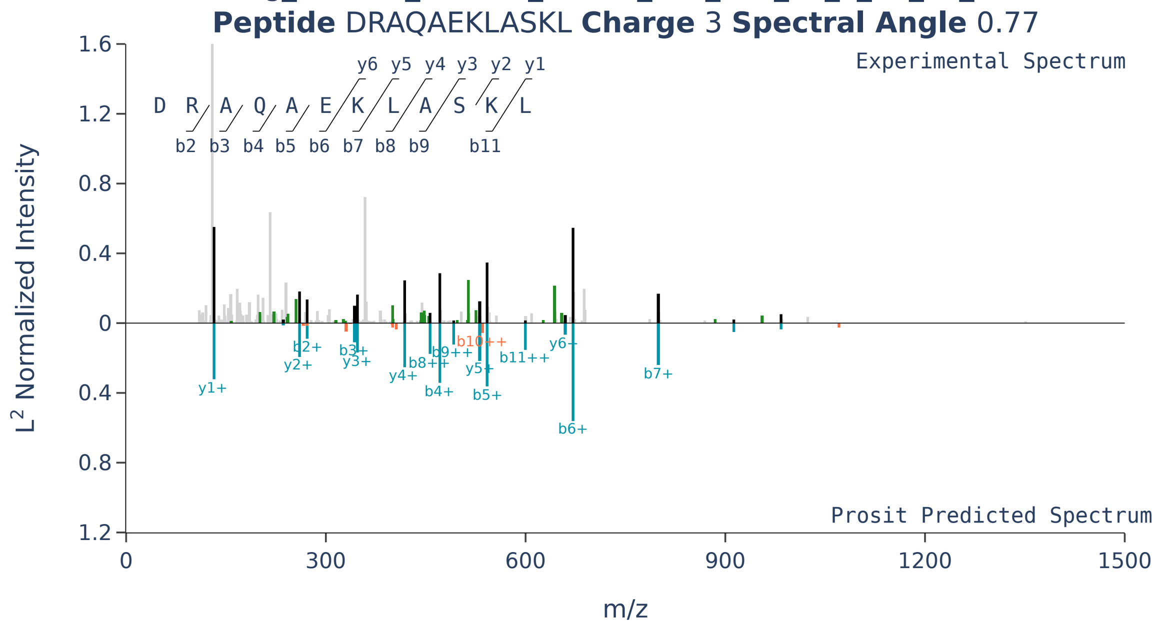


**Source** Ncheng_210623_230623_HFGoe_FFH_20S_25_1_A1_2h_R1 **Scan** 34532
**Peptide** QVGVDFFPSDVGQKPVDIVNAALKE **Charge** 3 **Spectral Angle** 0.79

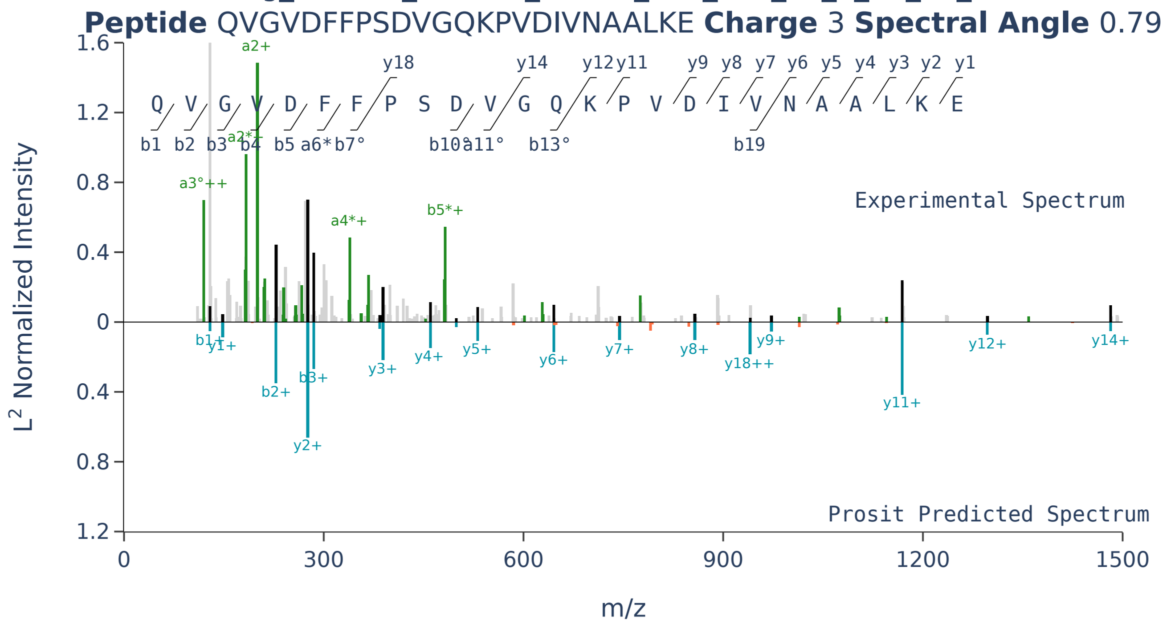


**Source** Ncheng_210623_230623_HFGoe_FFH_20S_25_1_A2_24h_R1 **Scan** 16998
**Peptide** VVLTKVDGDARGGAAL **Charge** 2 **Spectral Angle** 0.73

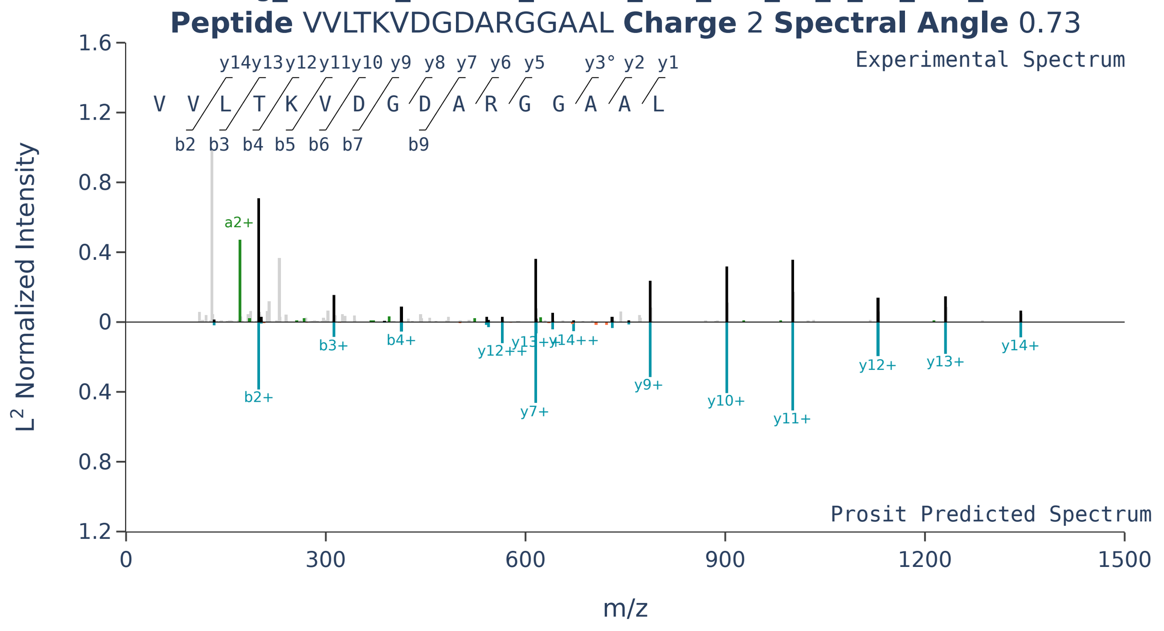


**Source** Ncheng_210623_230623_HFGoe_FFH_20S_25_1_A1_24h_R2 **Scan** 16695
**Peptide** EAMMDEIKQVH **Charge** 2 **Spectral Angle** 0.84

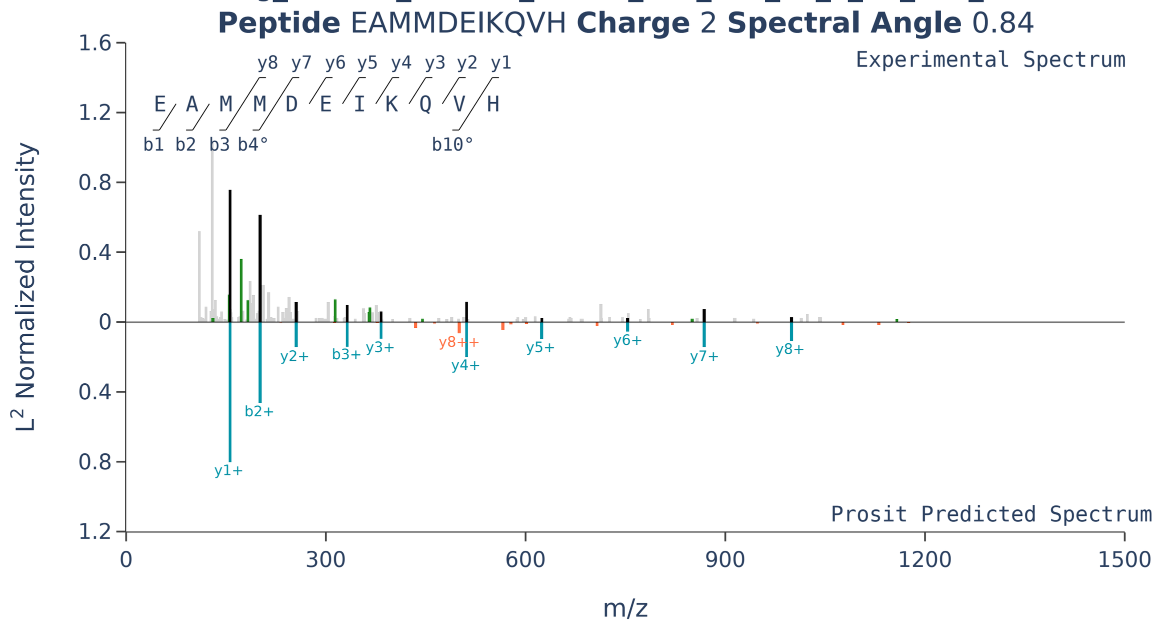


**Source** Ncheng_210623_230623_HFGoe_FFH_20S_25_1_A2_24h_R1 **Scan** 13842
**Peptide** EAIINSMTMKERAK **Charge** 2 **Spectral Angle** 0.74

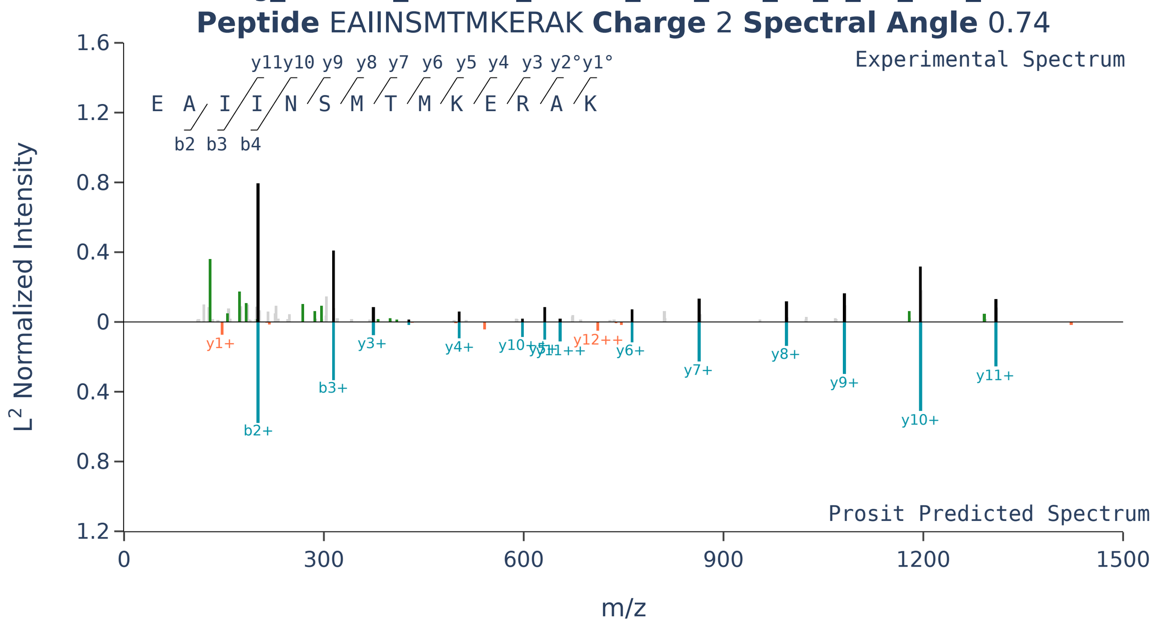


**Source** Ncheng_210623_230623_HFGoe_FFH_20S_25_1_A1_4h_R1 **Scan** 12238
**Peptide** RLLKQFD **Charge** 2 **Spectral Angle** 0.8

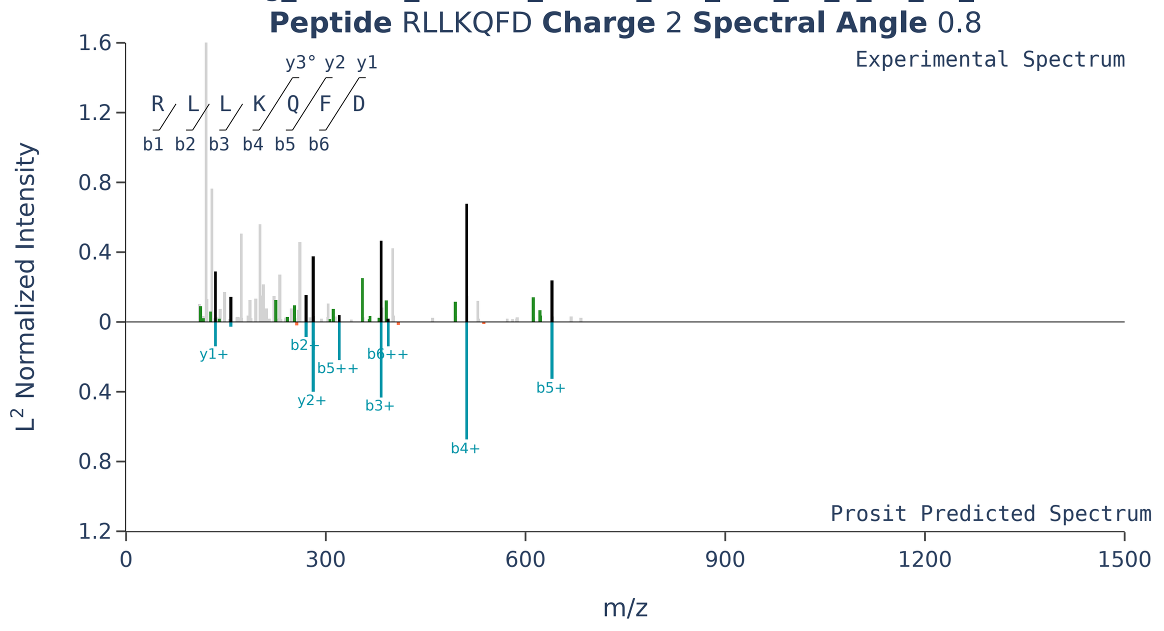


**Source** Ncheng_210623_230623_HFGoe_FFH_20S_25_1_A1_4h_R1 **Scan** 7559
**Peptide** DIESKVDRAQ **Charge** 3 **Spectral Angle** 0.87

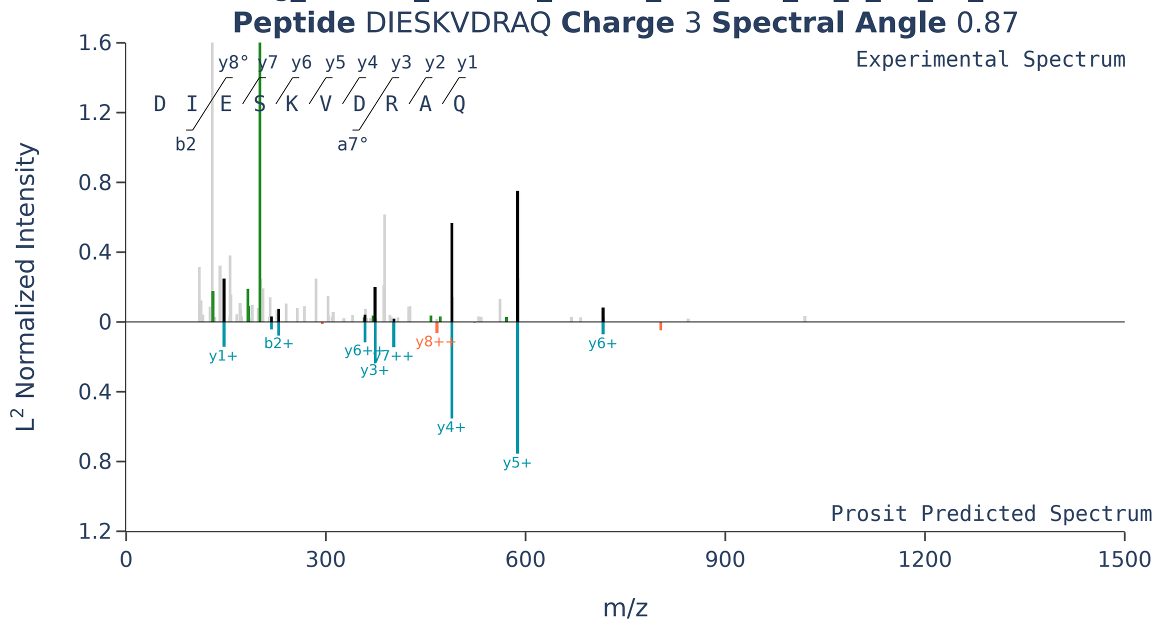

**Source** Ncheng_210623_230623_HFGoe_FFH_20S_25_1_A1_2h_R2 **Scan** 29564
**Peptide** HEVNKSLTPGQEFVKIVRNELVA **Charge** 3 **Spectral Angle** 0.8

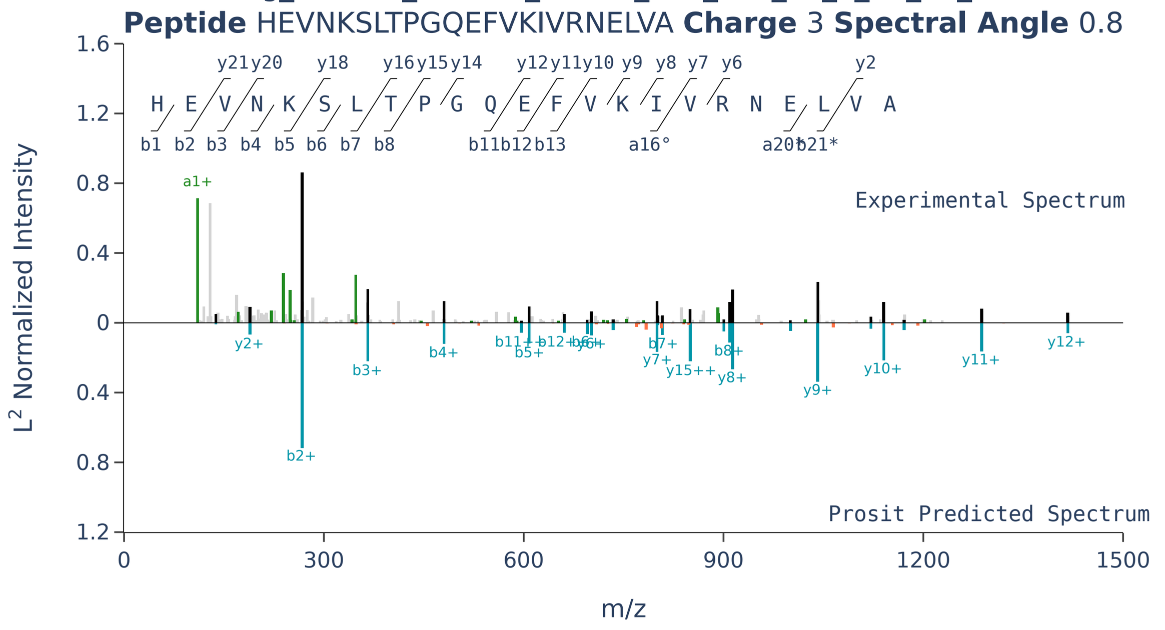


**Source** Ncheng_210623_230623_HFGoe_FFH_20S_25_1_A1_24h_R2 **Scan** 17291
**Peptide** MEAIINSMTMKERAK **Charge** 2 **Spectral Angle** 0.83

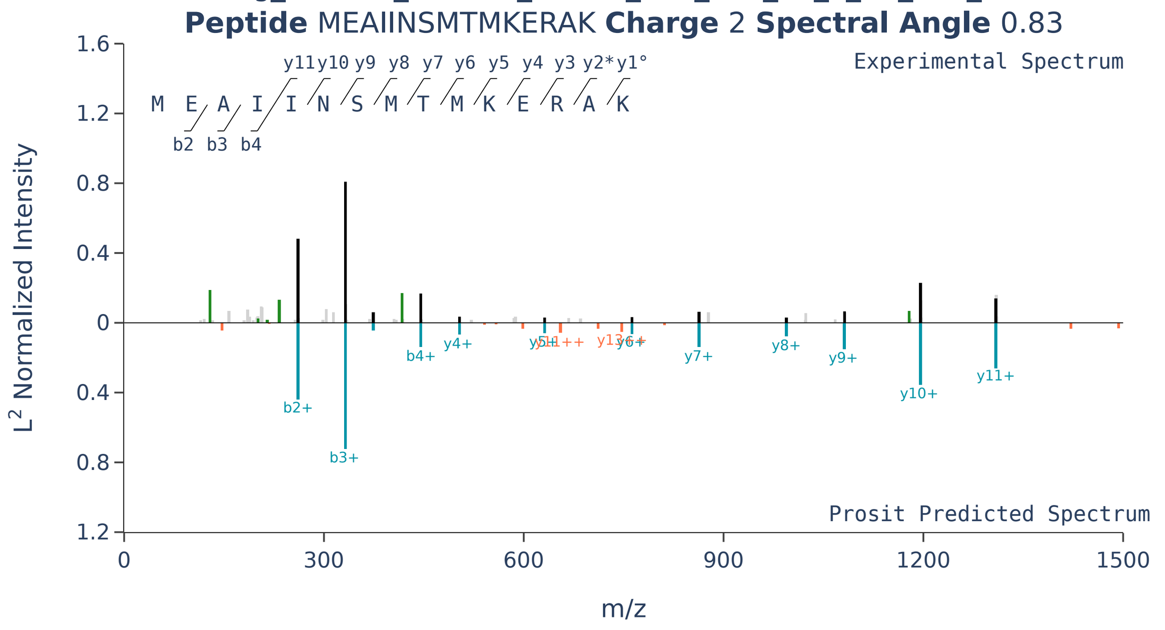


**Source** Ncheng_210623_230623_HFGoe_FFH_20S_25_1_A1_1h_R2 **Scan** 28855
**Peptide** VAAMGEENQTLNLAAQPPAVV **Charge** 2 **Spectral Angle** 0.94

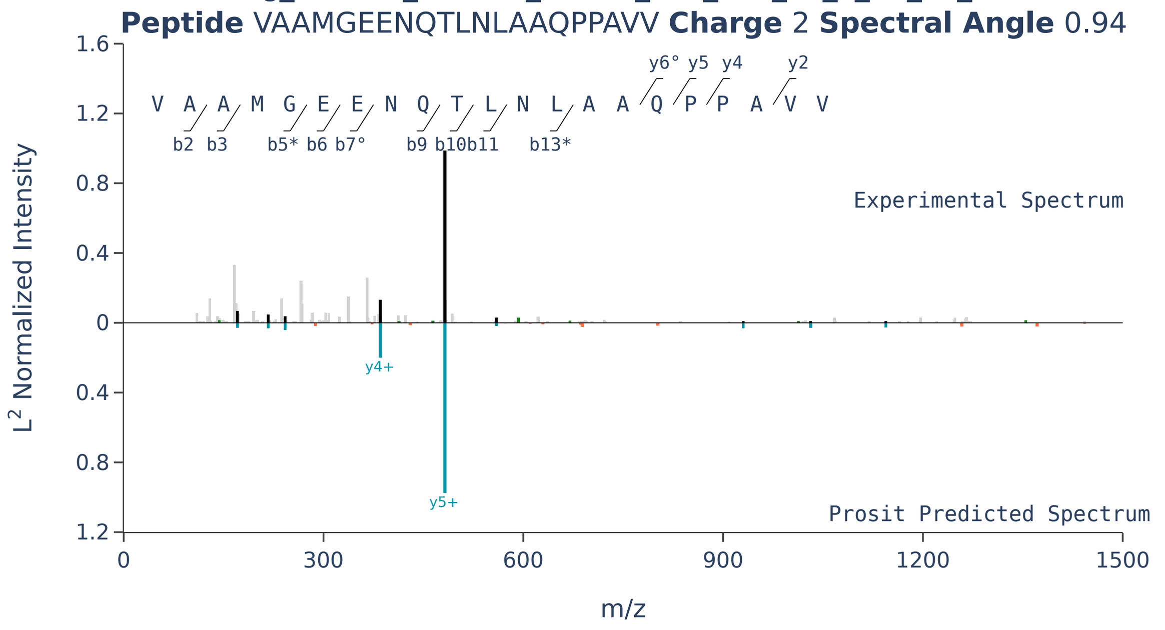


**Source** Ncheng_210623_230623_HFGoe_FFH_20S_25_1_A2_24h_R1 **Scan** 12644
**Peptide** MAGLQGAGKTTSVG **Charge** 2 **Spectral Angle** 0.85

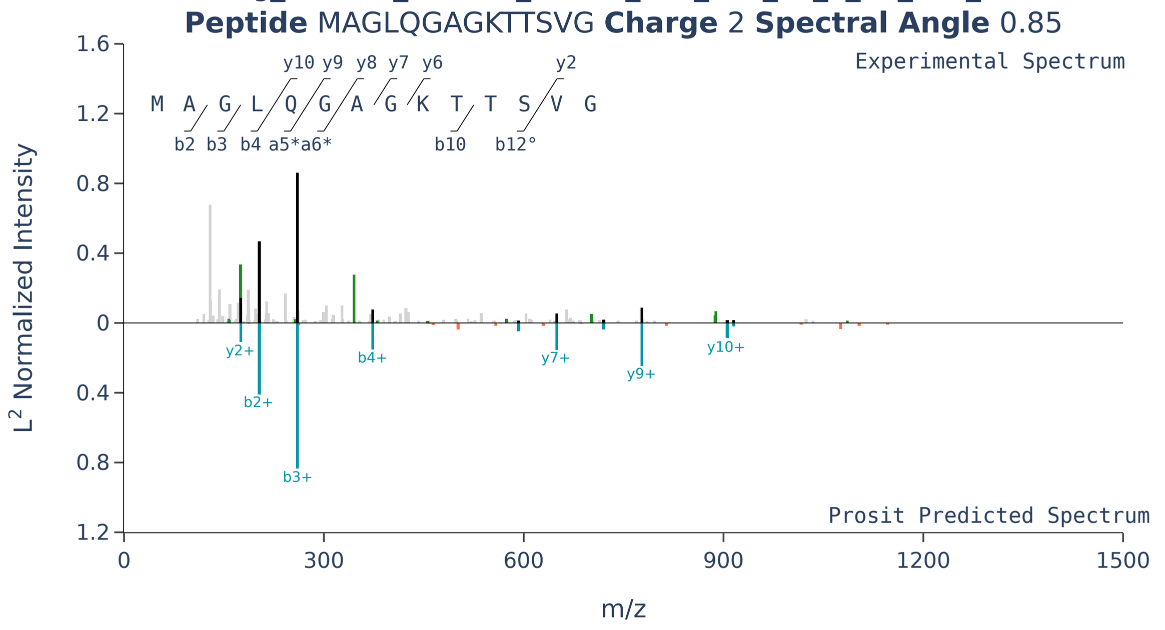


**Source** Ncheng_210623_230623_HFGoe_FFH_20S_25_1_A2_24h_R2 **Scan** 19809
**Peptide** KLPGMGQIPDNVKSQMD **Charge** 3 **Spectral Angle** 0.73

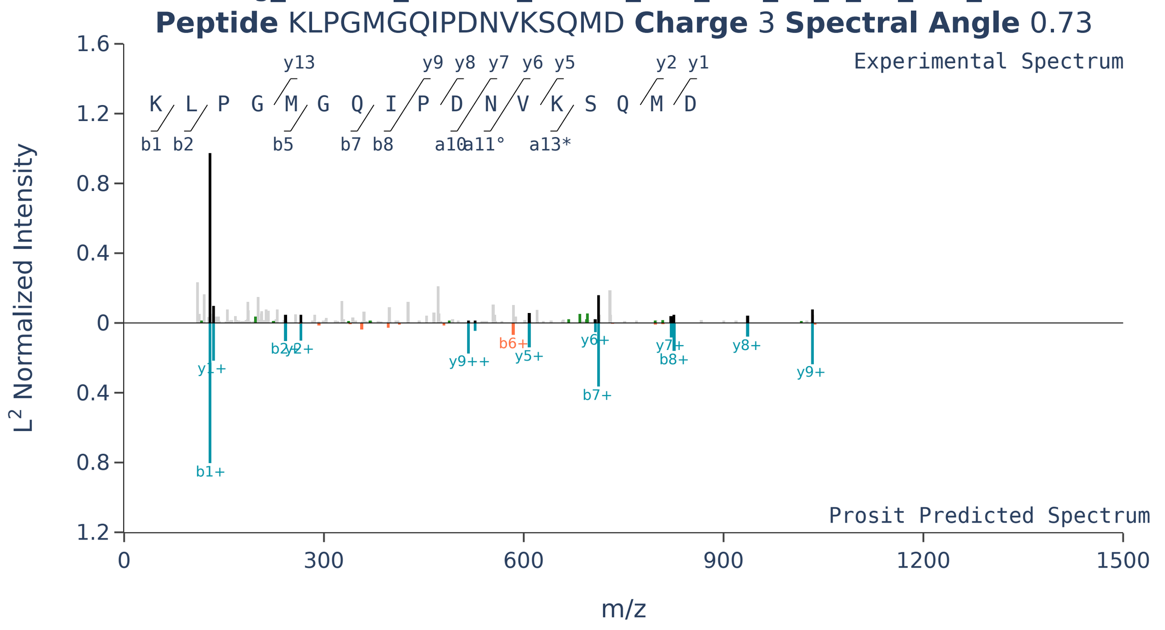


**Source** Ncheng_210623_230623_HFGoe_FFH_20S_25_1_A2_24h_R2 **Scan** 14872
**Peptide** KPIKFLGVGEK **Charge** 3 **Spectral Angle** 0.73

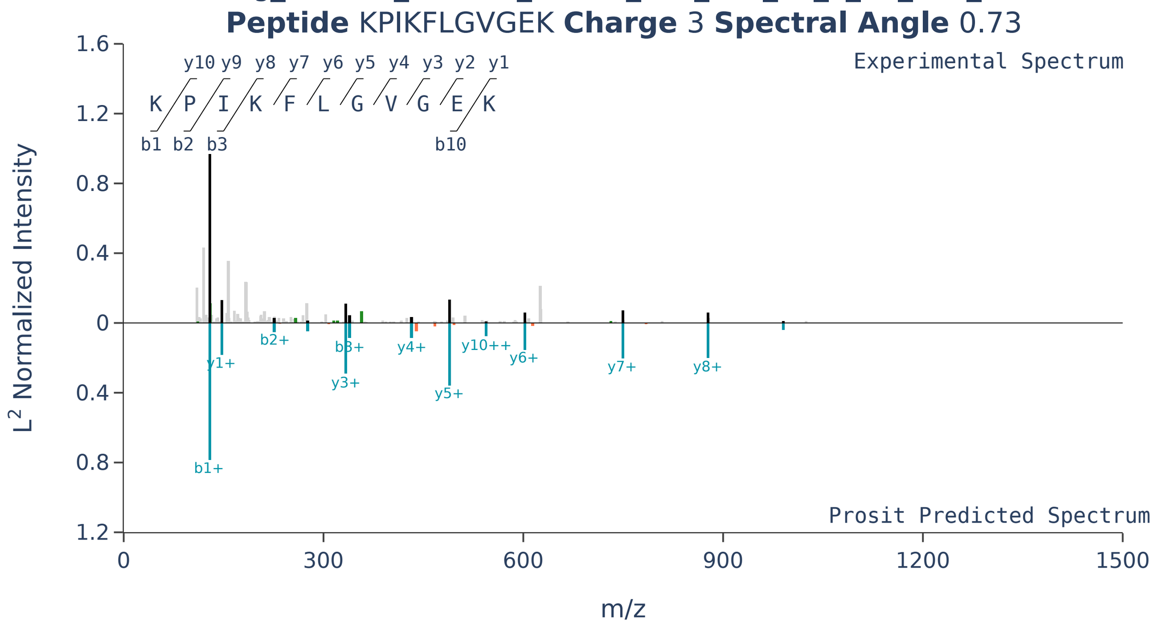


**Source** Ncheng_210623_230623_HFGoe_FFH_20S_25_1_A2_4h_R1 **Scan** 28798
**Peptide** AVGHEVNKSLTPGQEFVKIVRNEL **Charge** 3 **Spectral Angle** 0.81

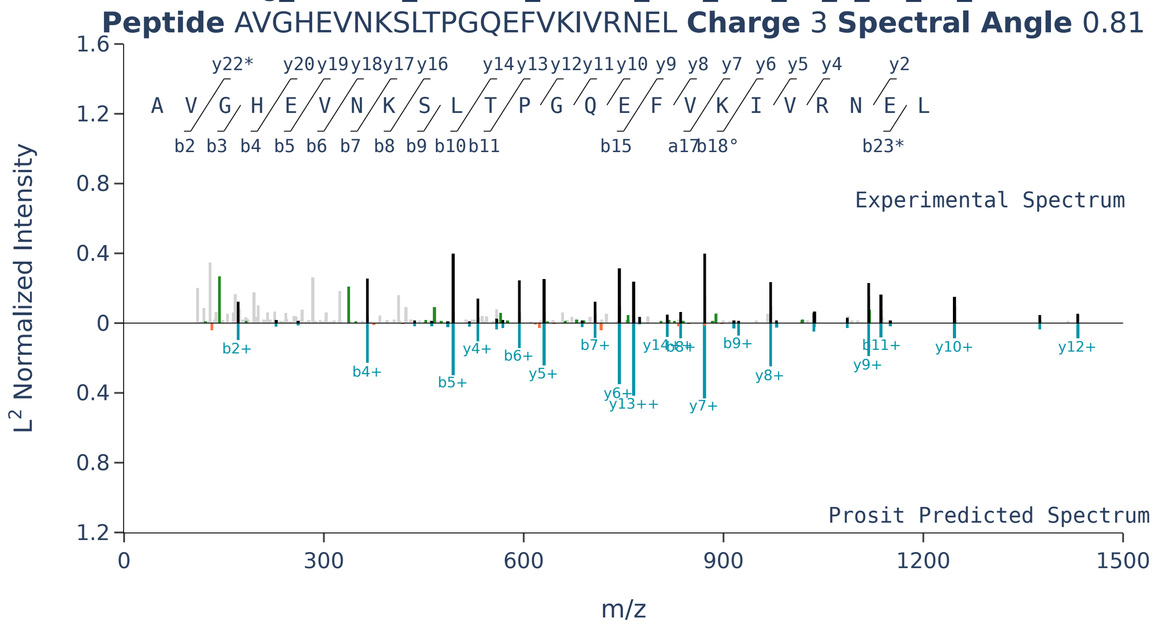


**Source** Ncheng_210623_230623_HFGoe_FFH_20S_25_1_A2_24h_R2 **Scan** 19948
**Peptide** GQDAANTAKAFNEAL **Charge** 2 **Spectral Angle** 0.82

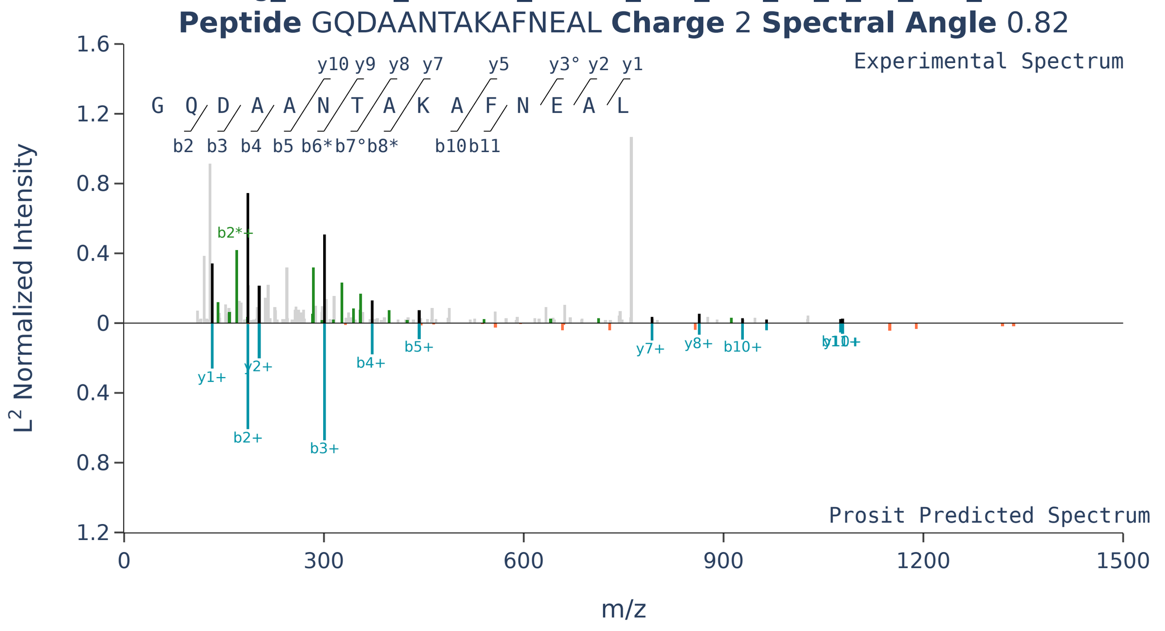


**Source** Ncheng_210623_230623_HFGoe_FFH_20S_25_1_A2_24h_R1 **Scan** 13575
**Peptide** VLTKVDGDARGGAAL **Charge** 2 **Spectral Angle** 0.82

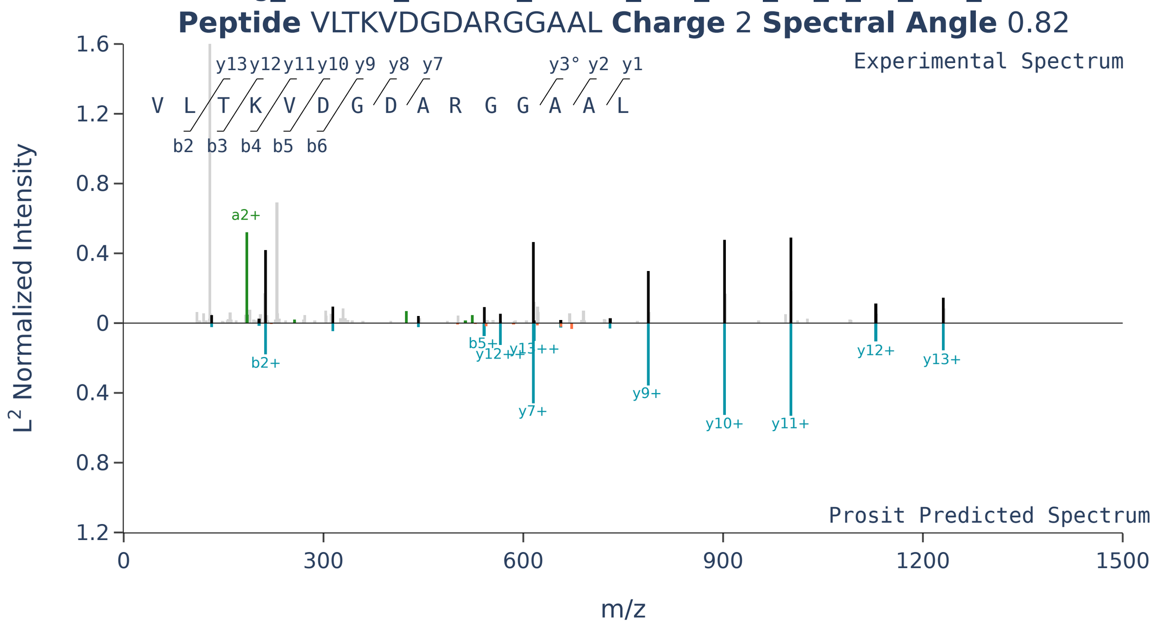


**Source** Ncheng_210623_230623_HFGoe_FFH_20S_25_1_A2_24h_R1 **Scan** 18241
**Peptide** PSDVGQKPVDIVNAA **Charge** 2 **Spectral Angle** 0.73

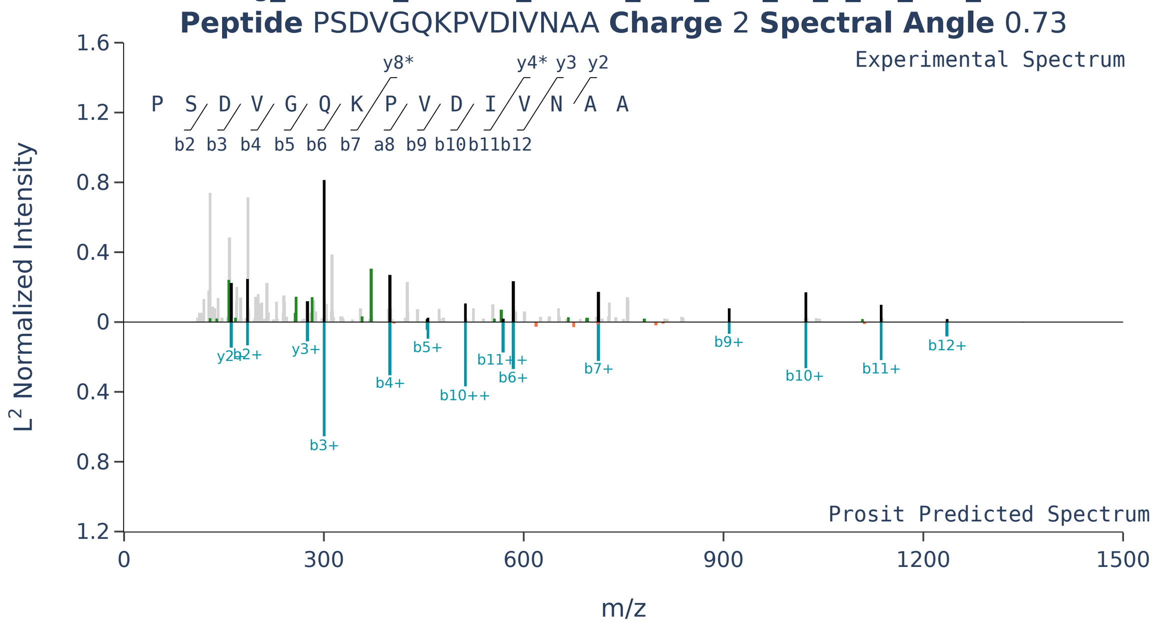


**Source** Ncheng_210623_230623_HFGoe_FFH_20S_25_1_A2_2h_R1 **Scan** 32939
**Peptide** ELVAAMGEENQTLNLAAQPPAVV **Charge** 2 **Spectral Angle** 0.94

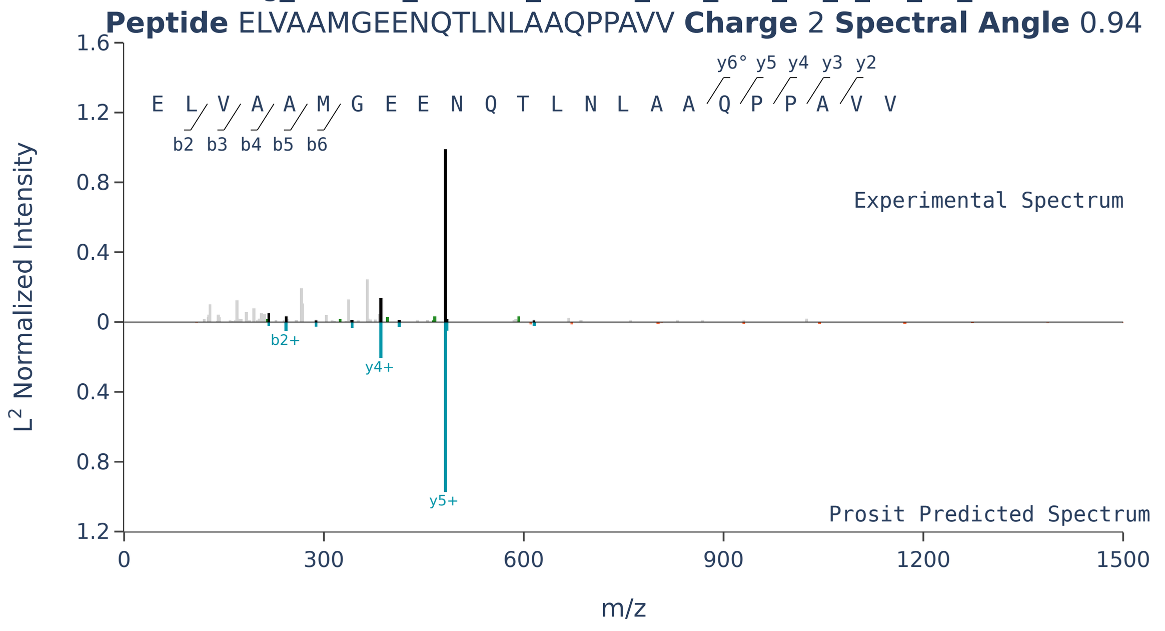


**Source** Ncheng_210623_230623_HFGoe_FFH_20S_25_1_A1_4h_R2 **Scan** 19219
**Peptide** GHEVNKSLTPGQEFVKIVR **Charge** 3 **Spectral Angle** 0.78

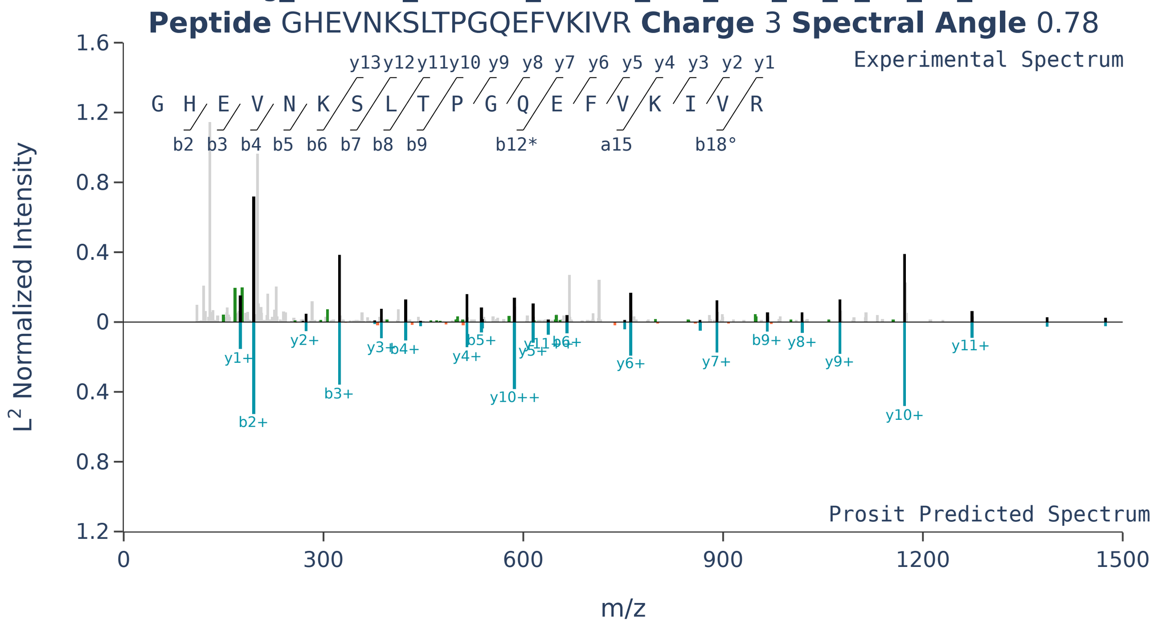


**Source** Ncheng_210623_230623_HFGoe_FFH_20S_25_1_A2_1h_R1 **Scan** 14749
**Peptide** TLAEQVGVD **Charge** 2 **Spectral Angle** 0.84

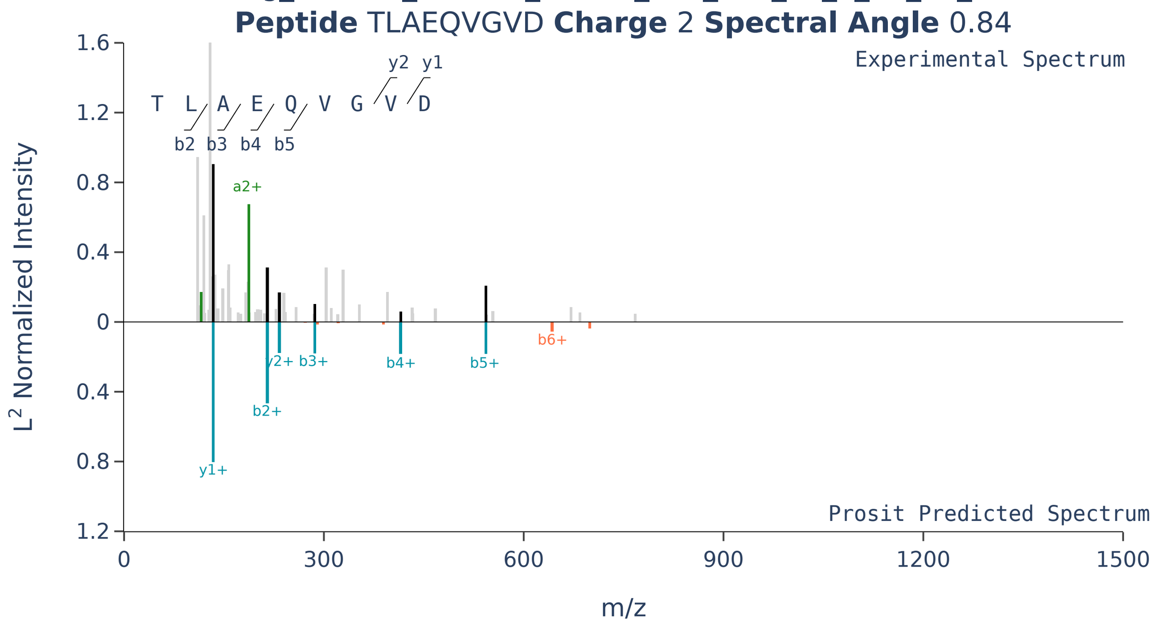


**Source** Ncheng_210623_230623_HFGoe_FFH_20S_25_1_A1_2h_R2 **Scan** 15019
**Peptide** LKQFDDMQRM **Charge** 3 **Spectral Angle** 0.9

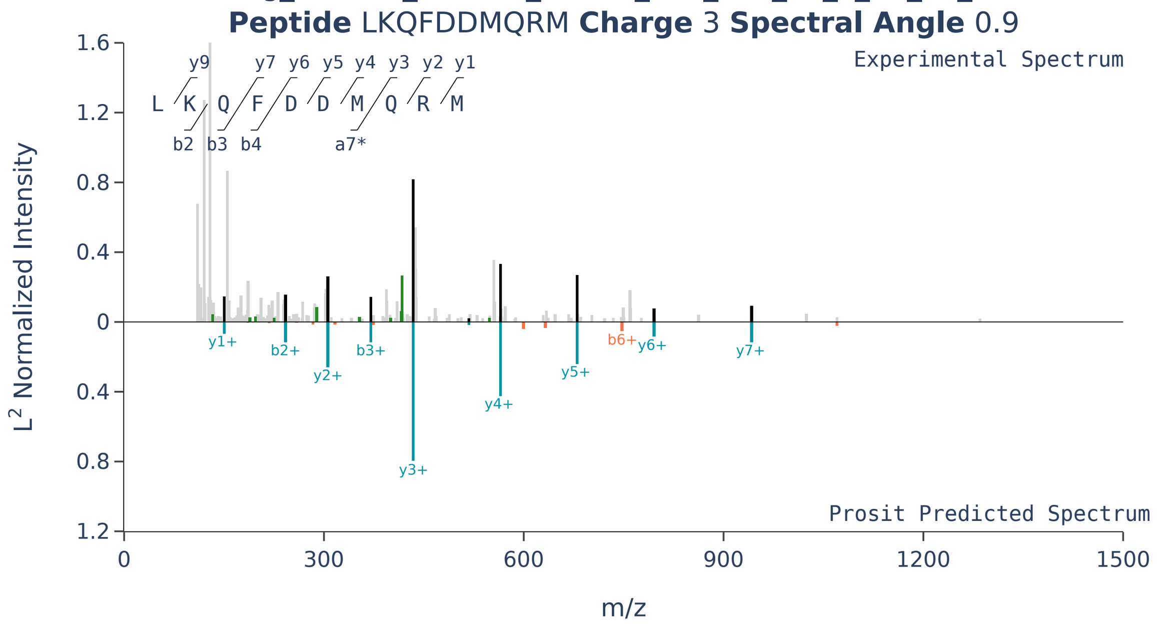


**Source** Ncheng_210623_230623_HFGoe_FFH_20S_25_1_A2_2h_R2 **Scan** 29853
**Peptide** LVAAMGEENQTLNLAAQPPAV **Charge** 2 **Spectral Angle** 0.97

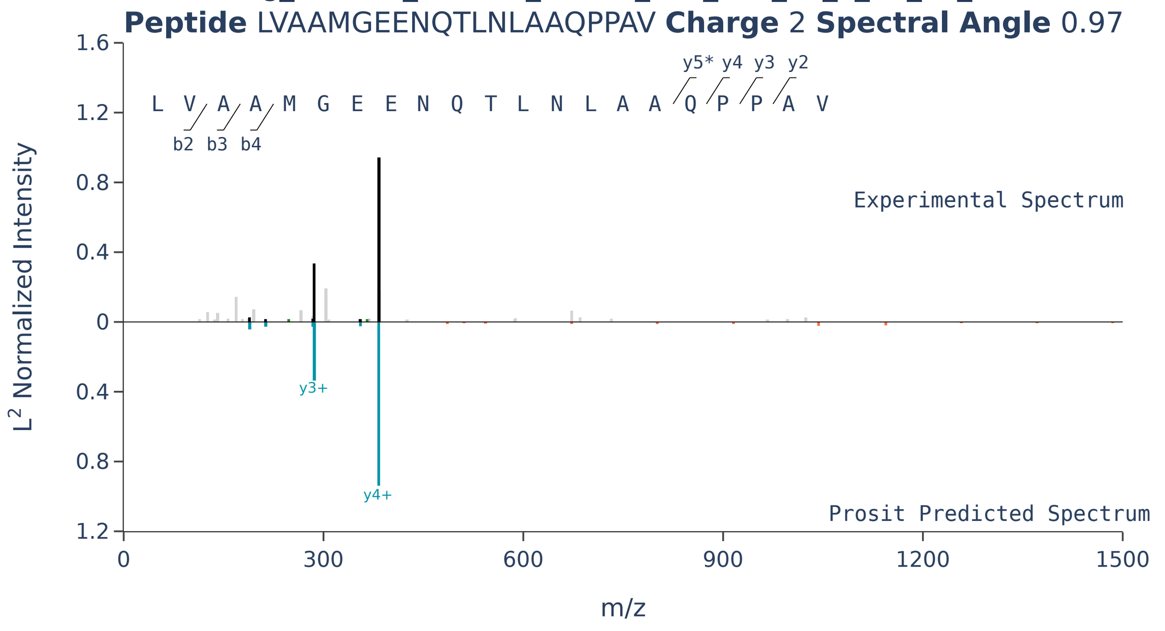

**Source** Ncheng_210623_230623_HFGoe_FFH_20S_25_1_A2_2h_R2 **Scan** 29260
**Peptide** GMQVQDVNRLLKQFD **Charge** 2 **Spectral Angle** 0.72

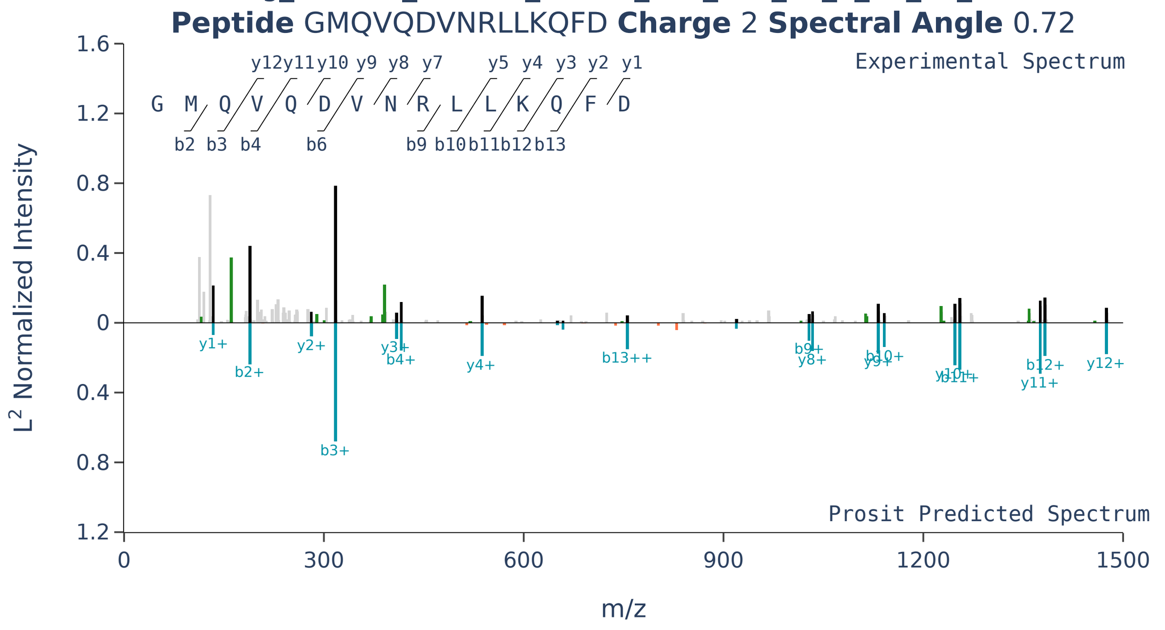


**Source** Ncheng_210623_230623_HFGoe_FFH_20S_25_1_A2_4h_R1 **Scan** 15559
**Peptide** DDKVLVRM **Charge** 2 **Spectral Angle** 0.91

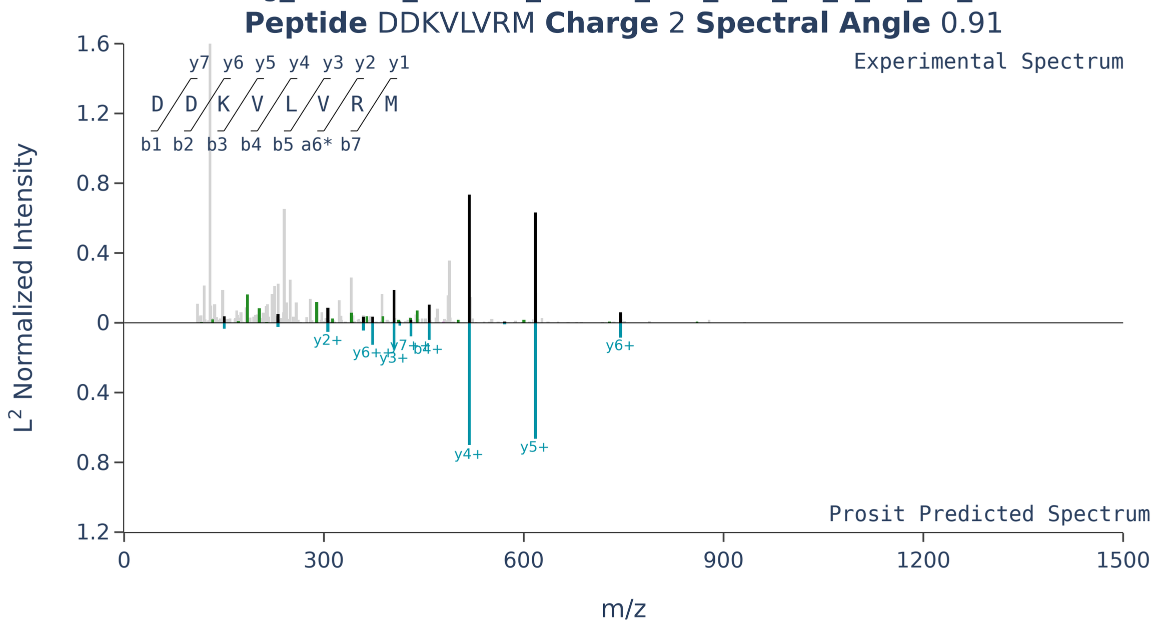


**Source** Ncheng_210623_230623_HFGoe_FFH_20S_25_1_A1_2h_R2 **Scan** 10059
**Peptide** QDAANTAKAF **Charge** 2 **Spectral Angle** 0.83

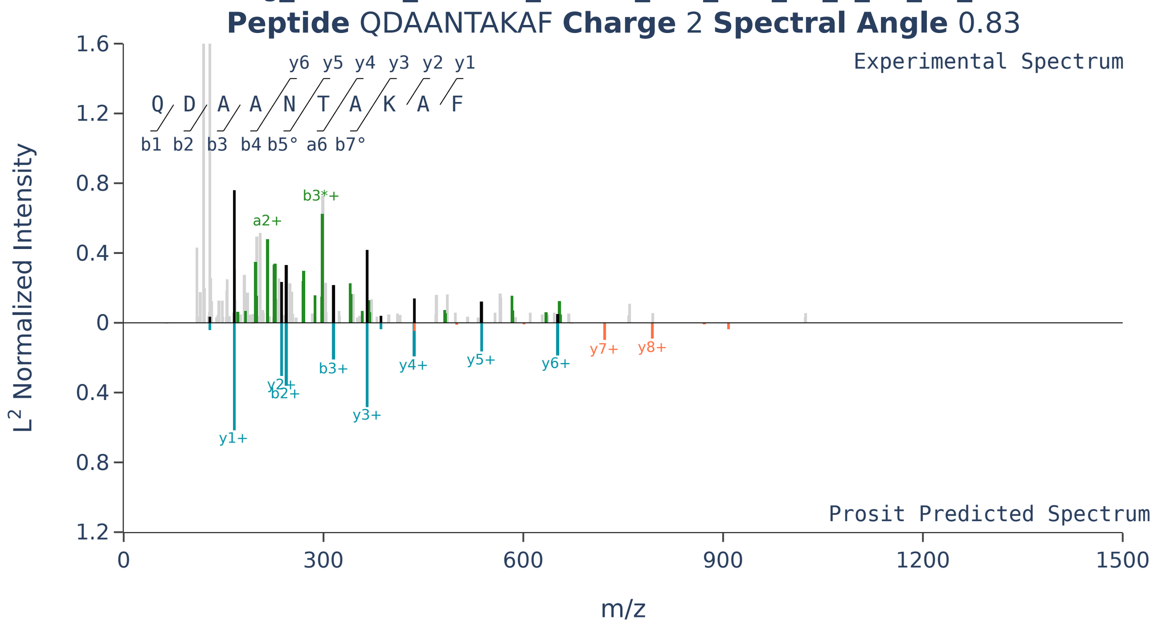


**Source** Ncheng_210623_230623_HFGoe_FFH_20S_25_1_A2_24h_R2 **Scan** 20611
**Peptide** HEVNKSLTPGQEFVKL **Charge** 2 **Spectral Angle** 0.87

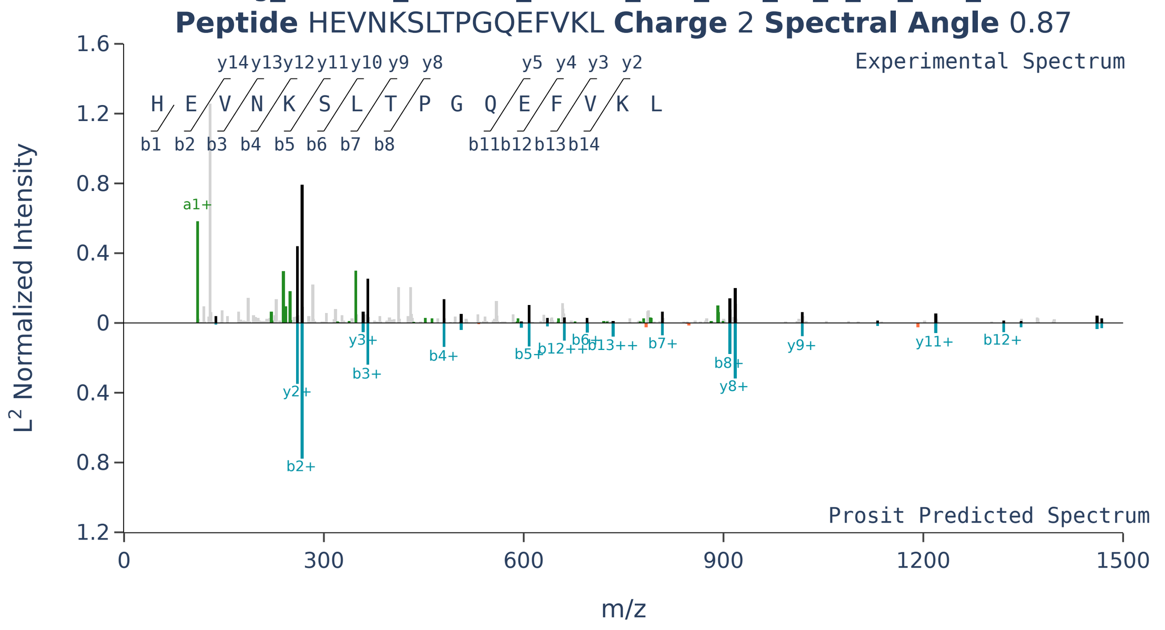


**Source** Ncheng_210623_230623_HFGoe_FFH_20S_25_1_A1_24h_R2 **Scan** 7586
**Peptide** DAMTGQDAANTAKA **Charge** 2 **Spectral Angle** 0.7

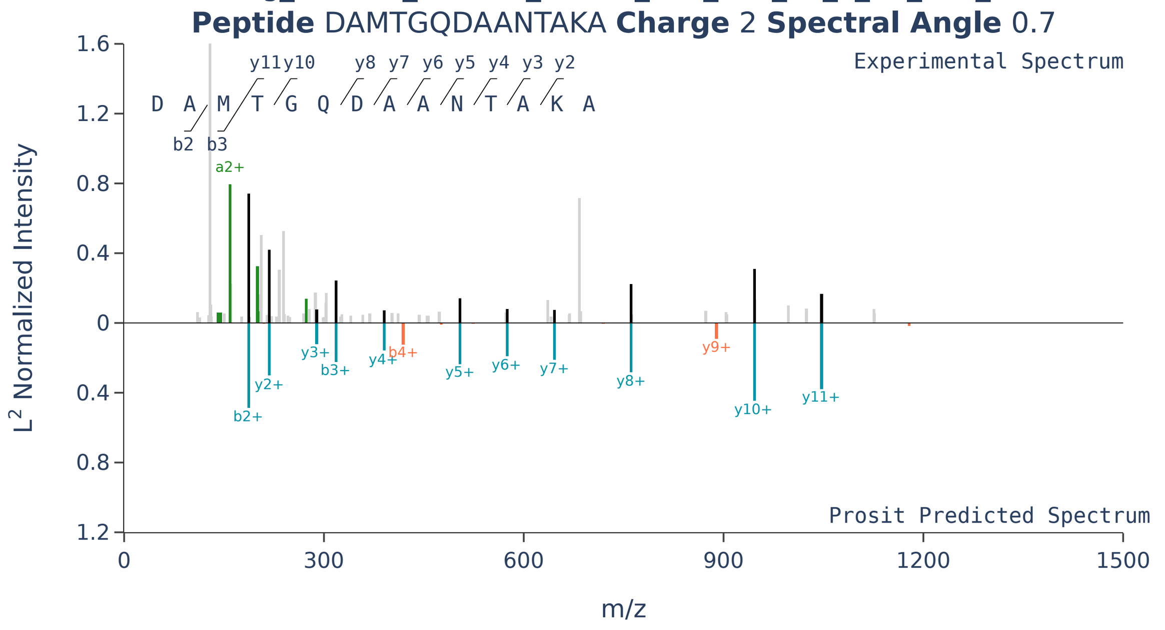


**Source** Ncheng_210623_230623_HFGoe_FFH_20S_25_1_A1_24h_R1 **Scan** 19626
**Peptide** EALINSMTMKE **Charge** 2 **Spectral Angle** 0.73

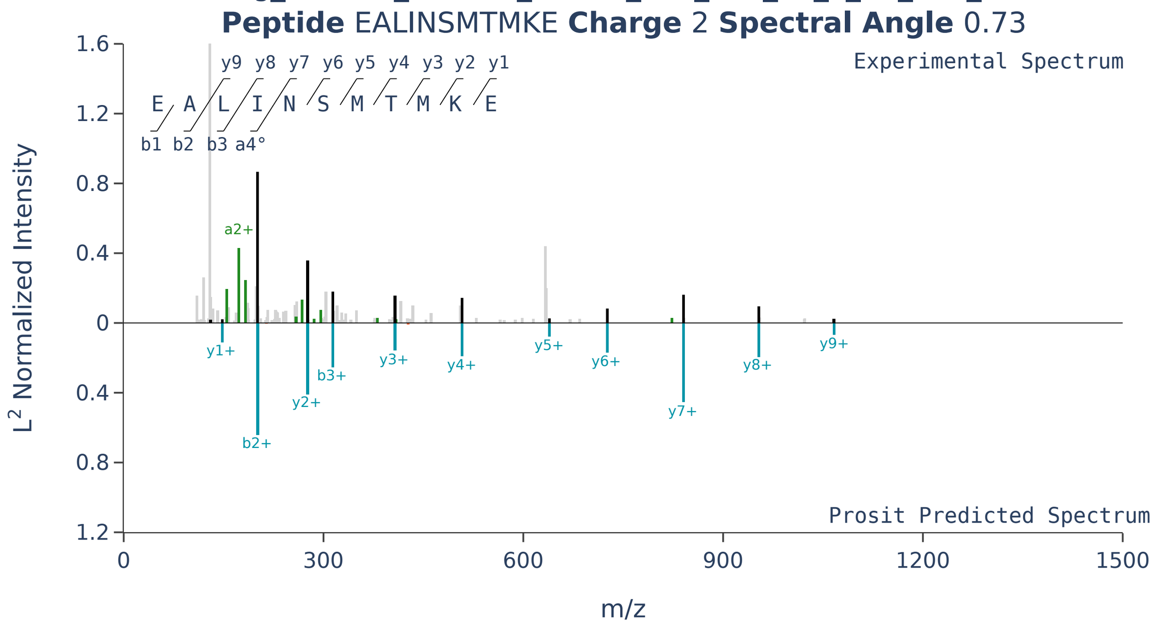


**Source** Ncheng_210623_230623_HFGoe_FFH_20S_25_1_A1_1h_R2 **Scan** 18199
**Peptide** EAMMDEIKQVHA **Charge** 2 **Spectral Angle** 0.76

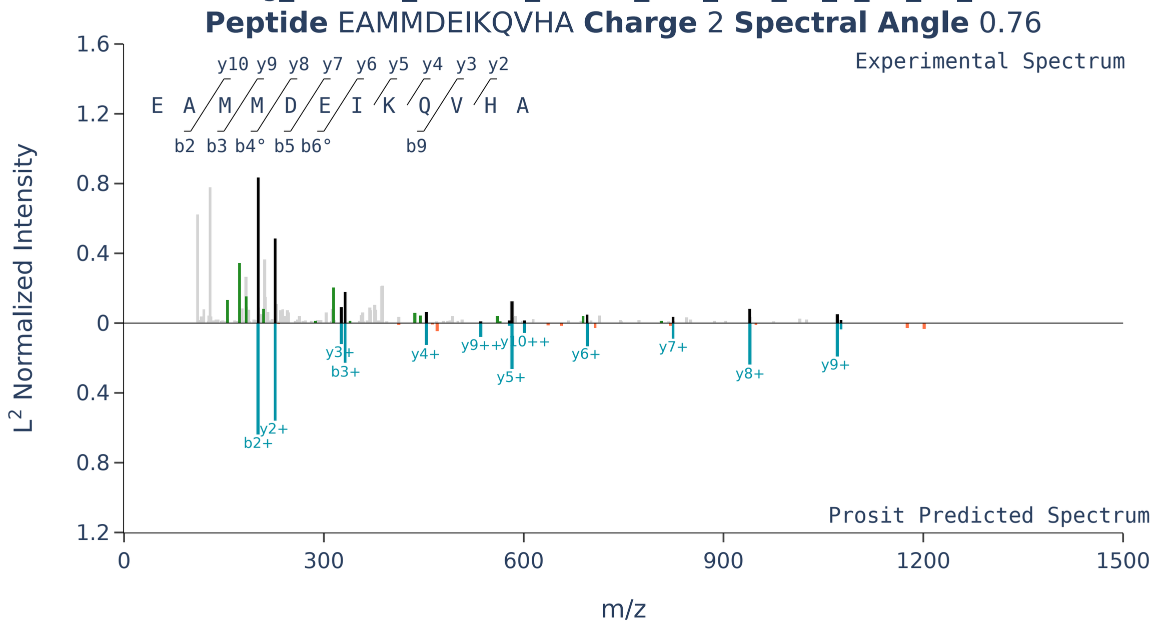


**Source** Ncheng_210623_230623_HFGoe_FFH_20S_25_1_A2_1h_R1 **Scan** 14624
**Peptide** FLGVGEKTE **Charge** 2 **Spectral Angle** 0.77

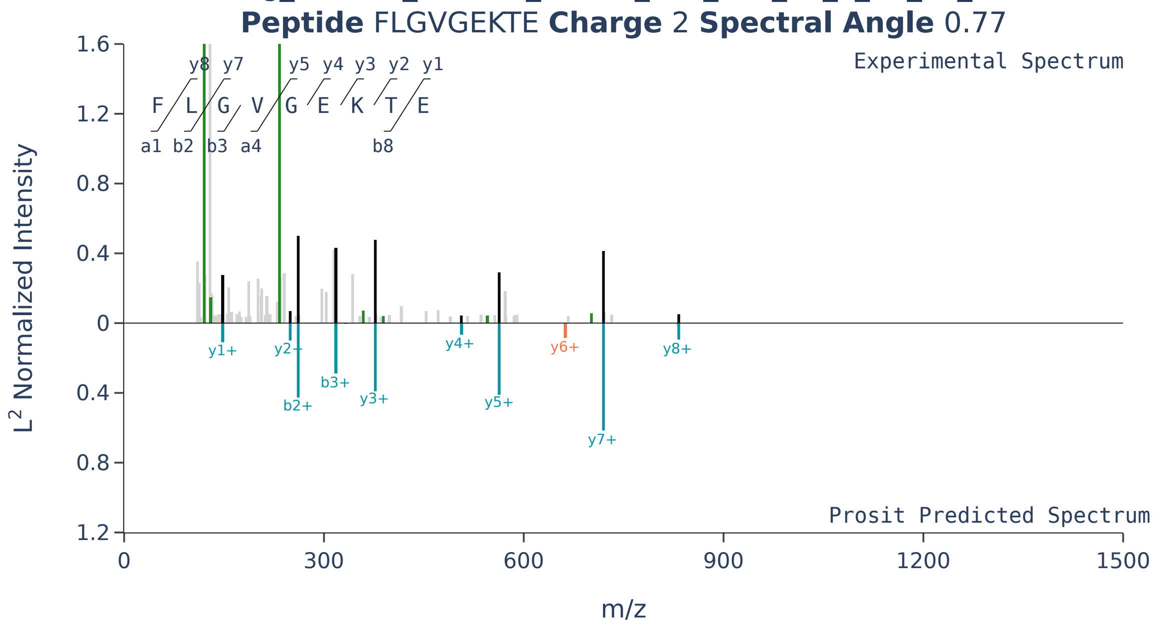


**Source** Ncheng_210623_230623_HFGoe_FFH_20S_25_1_A1_1h_R2 **Scan** 12556
**Peptide** ALKEAKLKF **Charge** 3 **Spectral Angle** 0.78

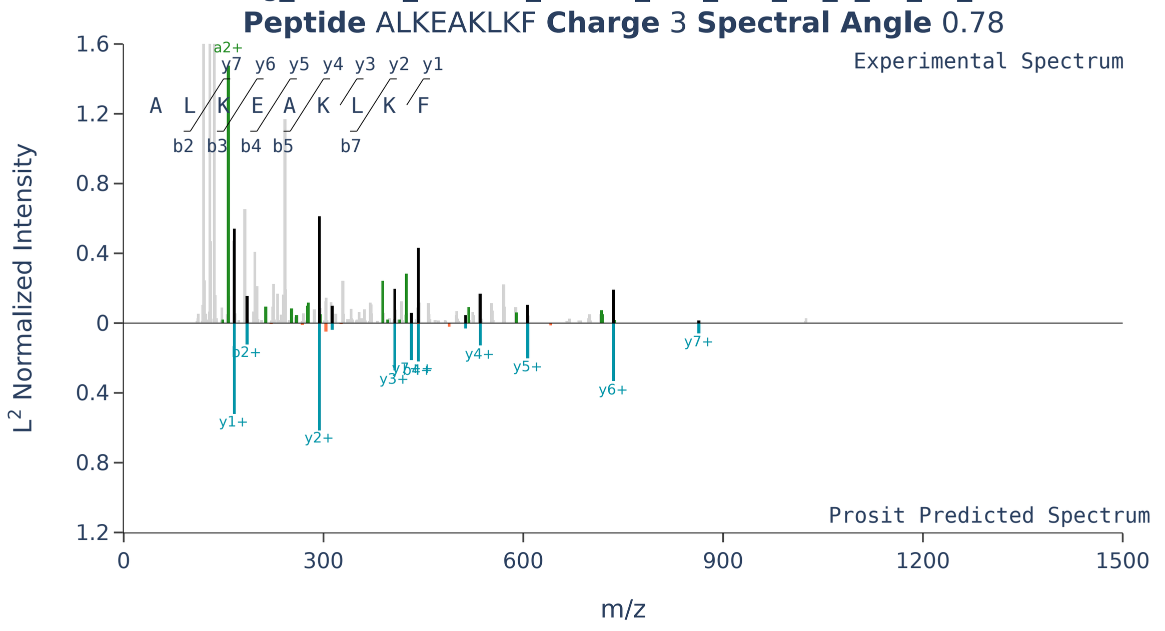


**Source** Ncheng_210623_230623_HFGoe_FFH_20S_25_1_A2_24h_R2 **Scan** 12061
**Peptide** ARGGAALSIR **Charge** 2 **Spectral Angle** 0.84

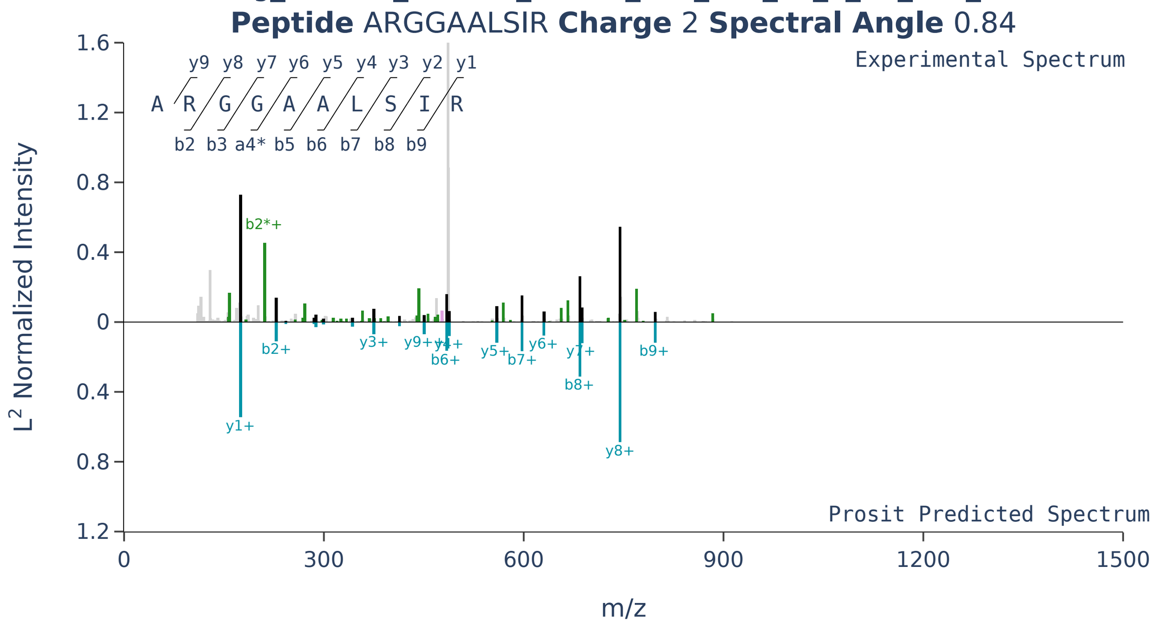


**Source** Ncheng_210623_230623_HFGoe_FFH_20S_25_1_A1_2h_R2 **Scan** 34093
**Peptide** EVNKSLTPGQEFVKIVRNELVAAM **Charge** 3 **Spectral Angle** 0.89

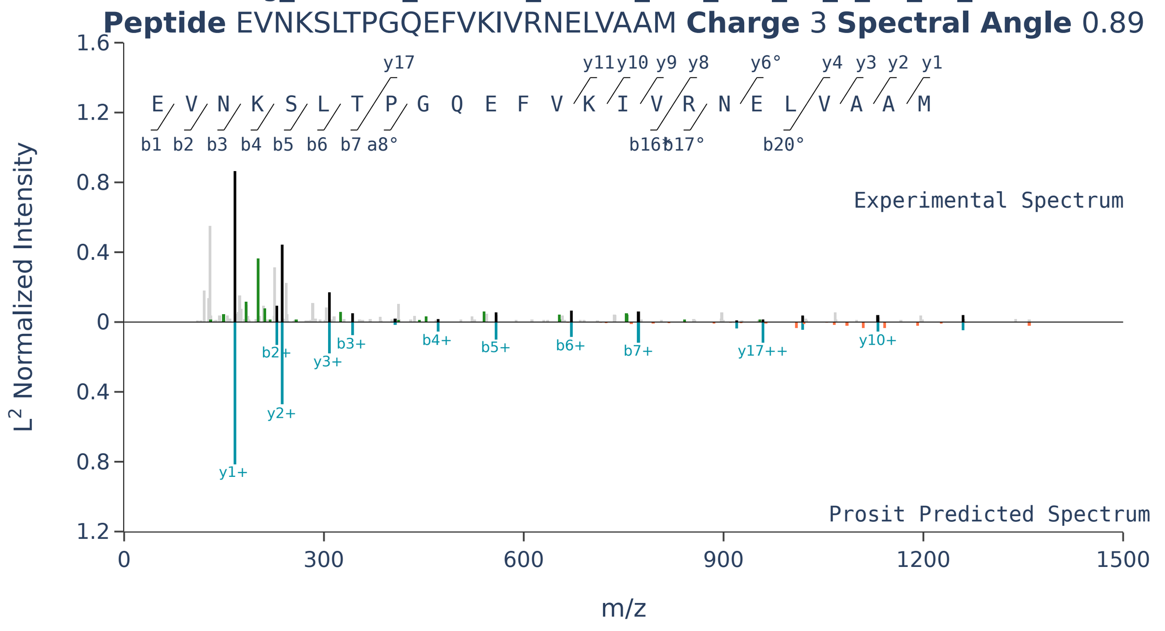


**Source** Ncheng_210623_230623_HFGoe_FFH_20S_25_1_A2_24h_R2 **Scan** 6657
**Peptide** KGGMAKMMR **Charge** 2 **Spectral Angle** 0.84

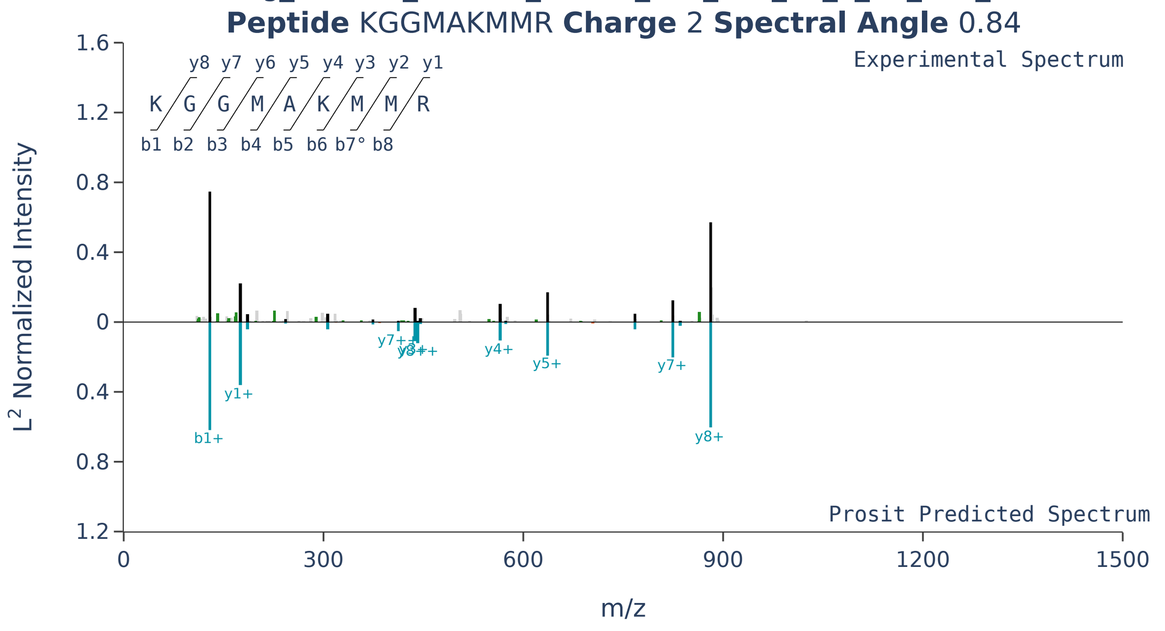


**Source** Ncheng_210623_230623_HFGoe_FFH_20S_25_1_A1_2h_R2 **Scan** 31270
**Peptide** GVDFFPSDVGQKPVDIVNA **Charge** 2 **Spectral Angle** 0.9

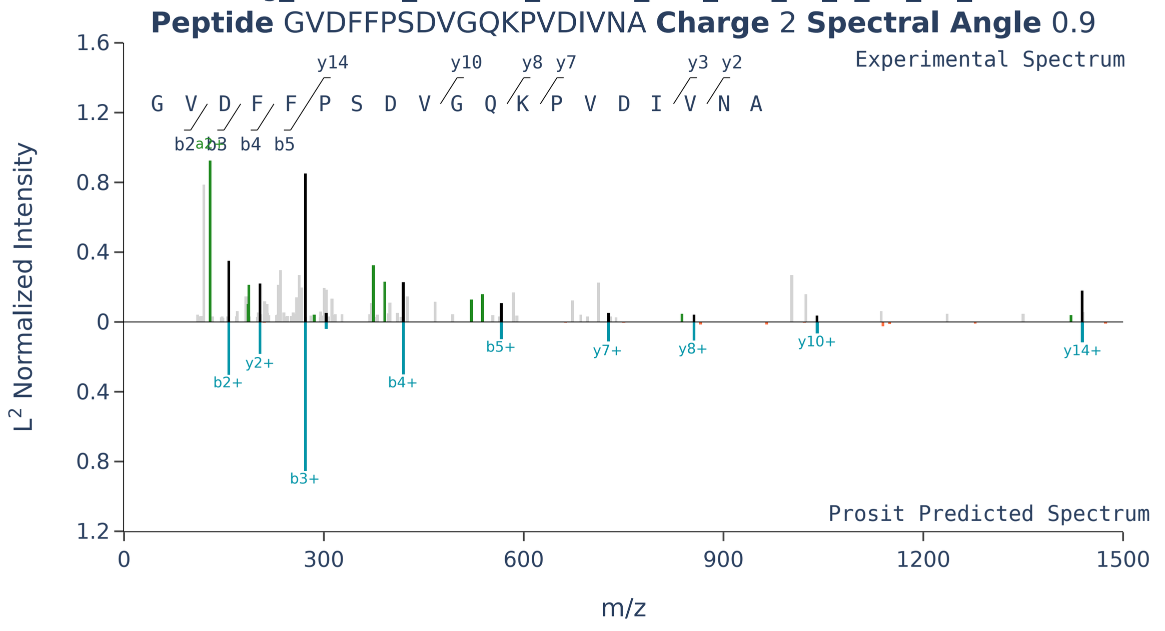


**Source** Ncheng_210623_230623_HFGoe_FFH_20S_25_1_A1_4h_R1 **Scan** 7884
**Peptide** VNKSLTPGQE **Charge** 2 **Spectral Angle** 0.77

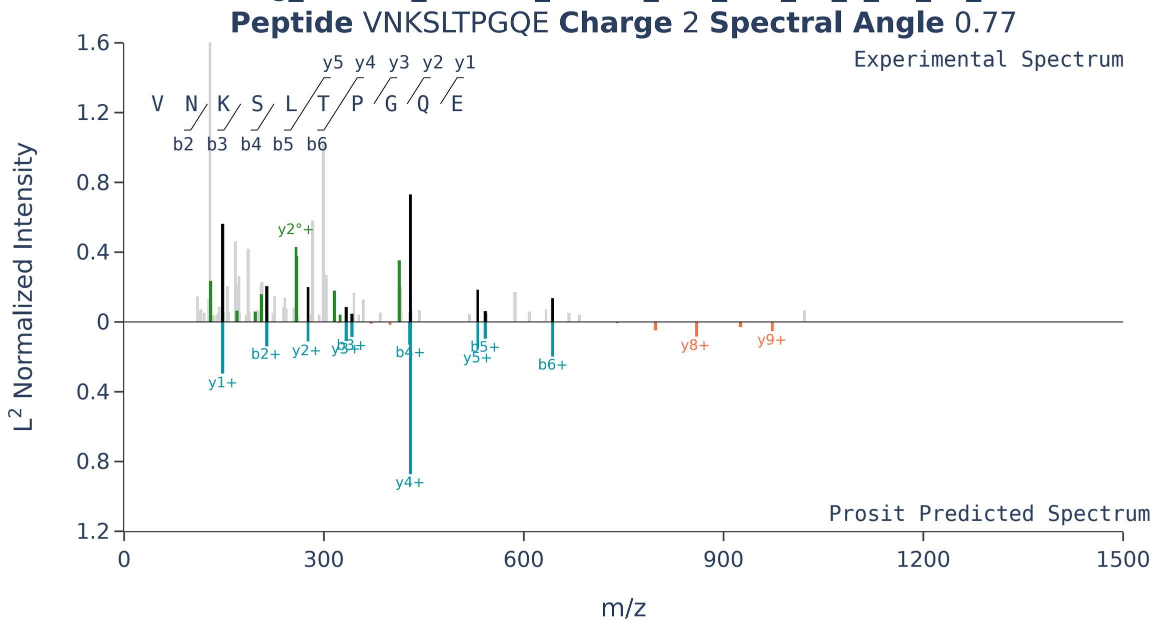


**Source** Ncheng_210623_230623_HFGoe_FFH_20S_25_1_A2_4h_R2 **Scan** 20451
**Peptide** MEAIINSMTMKERA **Charge** 2 **Spectral Angle** 0.82

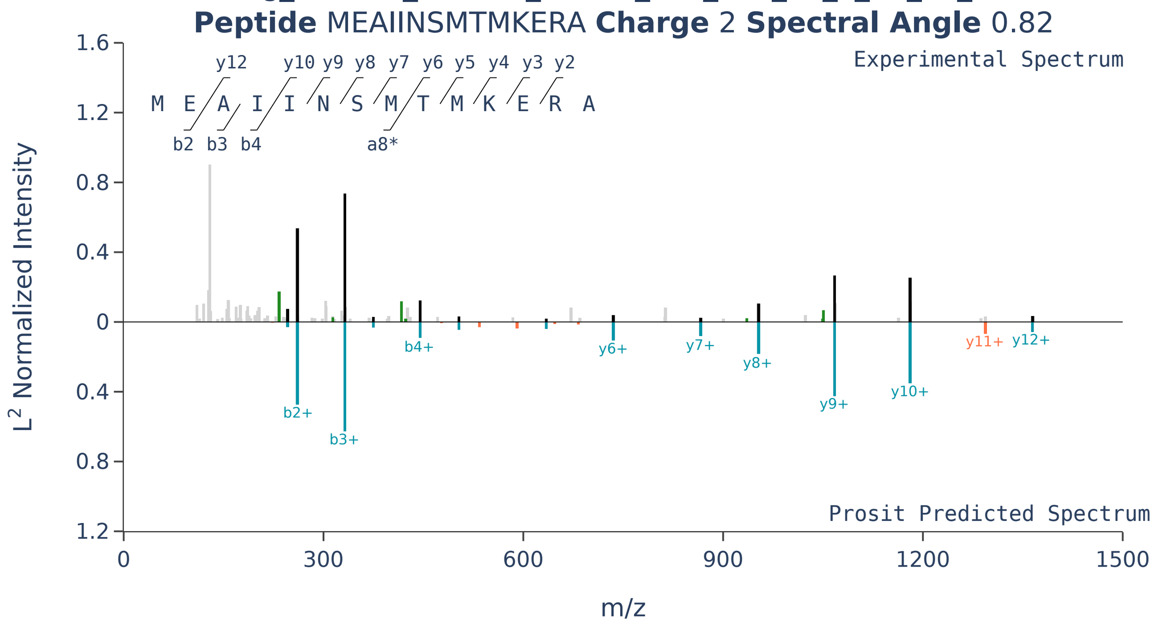

**Source** Ncheng_210623_230623_HFGoe_FFH_20S_25_1_A1_24h_R1 **Scan** 20064
**Peptide** KEKAVGHEVNKSLTPGQEFVKLV **Charge** 3 **Spectral Angle** 0.89

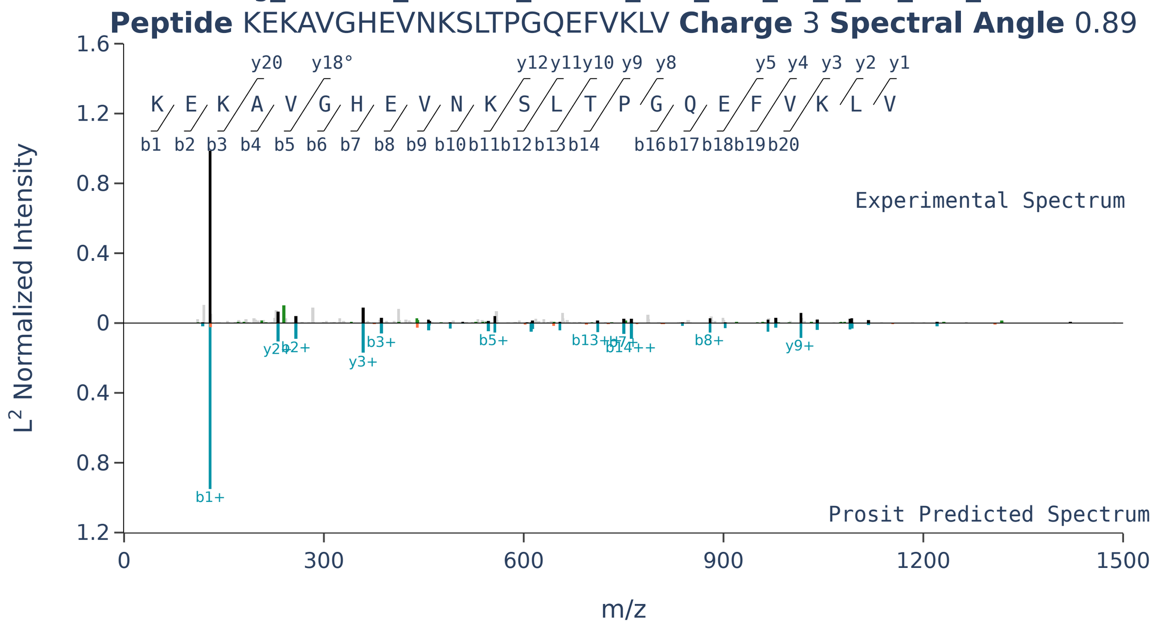


**Source** Ncheng_210623_230623_HFGoe_FFH_20S_25_1_A1_24h_R1 **Scan** 10677
**Peptide** TGQDAANTAKAFNE **Charge** 1 **Spectral Angle** 0.77

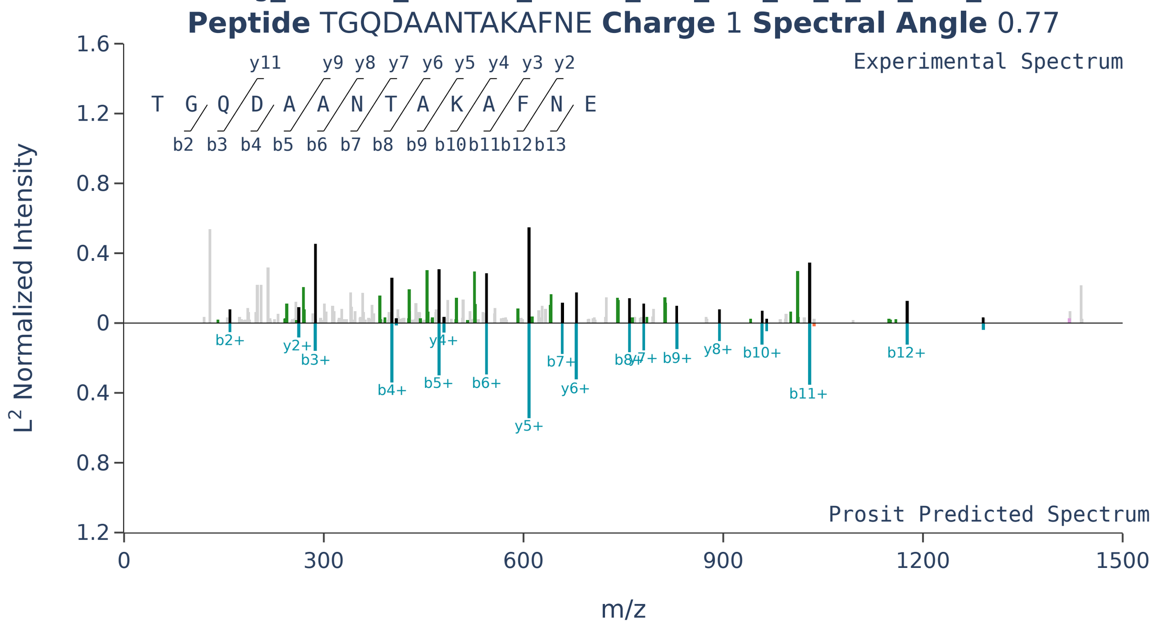


**Source** Ncheng_210623_230623_HFGoe_FFH_20S_25_1_A2_2h_R1 **Scan** 29441
**Peptide** LFVVDAMTGQDAANTAKAFNE **Charge** 2 **Spectral Angle** 0.93

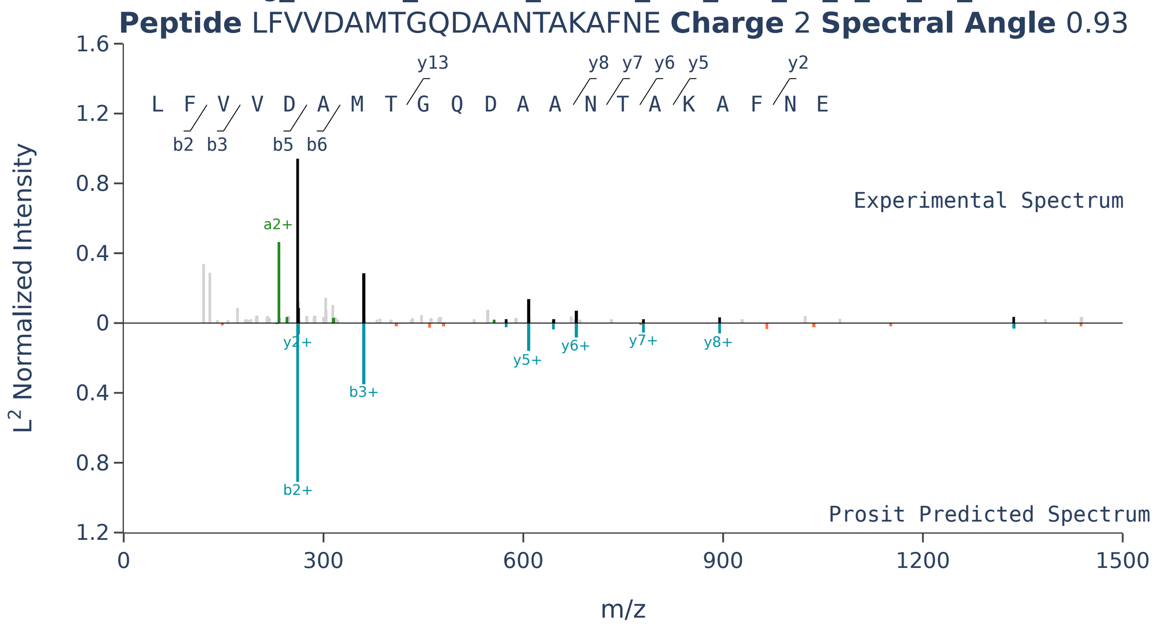


**Source** Ncheng_210623_230623_HFGoe_FFH_20S_25_1_A1_4h_R1 **Scan** 17389
**Peptide** AMMDEIKQVHA **Charge** 3 **Spectral Angle** 0.82

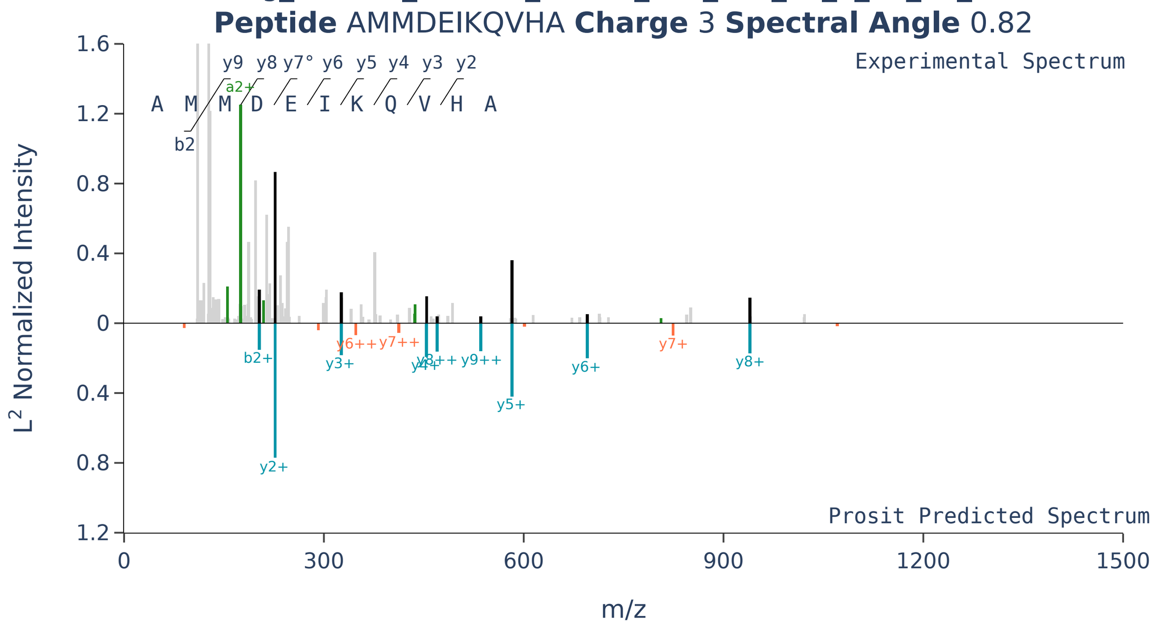


**Source** Ncheng_210623_230623_HFGoe_FFH_20S_25_1_A2_2h_R2 **Scan** 22929
**Peptide** GKLPGMGMGQIPDNV **Charge** 2 **Spectral Angle** 0.8

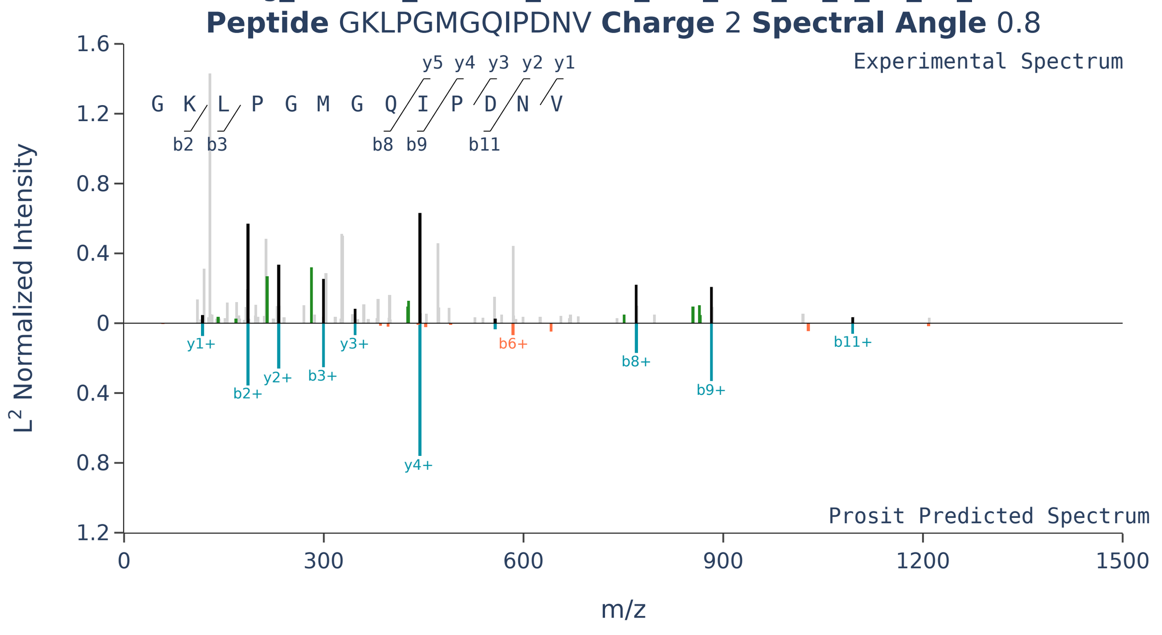


**Source** Ncheng_210623_230623_HFGoe_FFH_20S_25_1_A1_2h_R1 **Scan** 15400
**Peptide** LEPFHPDRIASR **Charge** 3 **Spectral Angle** 0.79

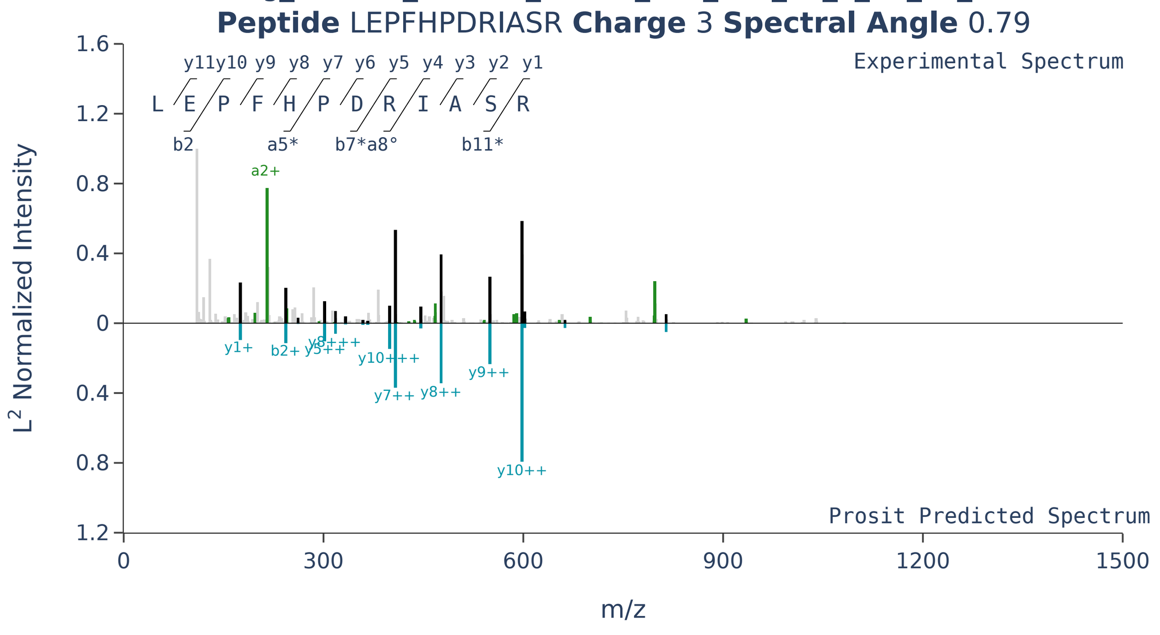


**Source** Ncheng_210623_230623_HFGoe_FFH_20S_25_1_A2_2h_R1 **Scan** 19526
**Peptide** MGKLPGMGQIPDNVKSQ **Charge** 2 **Spectral Angle** 0.87

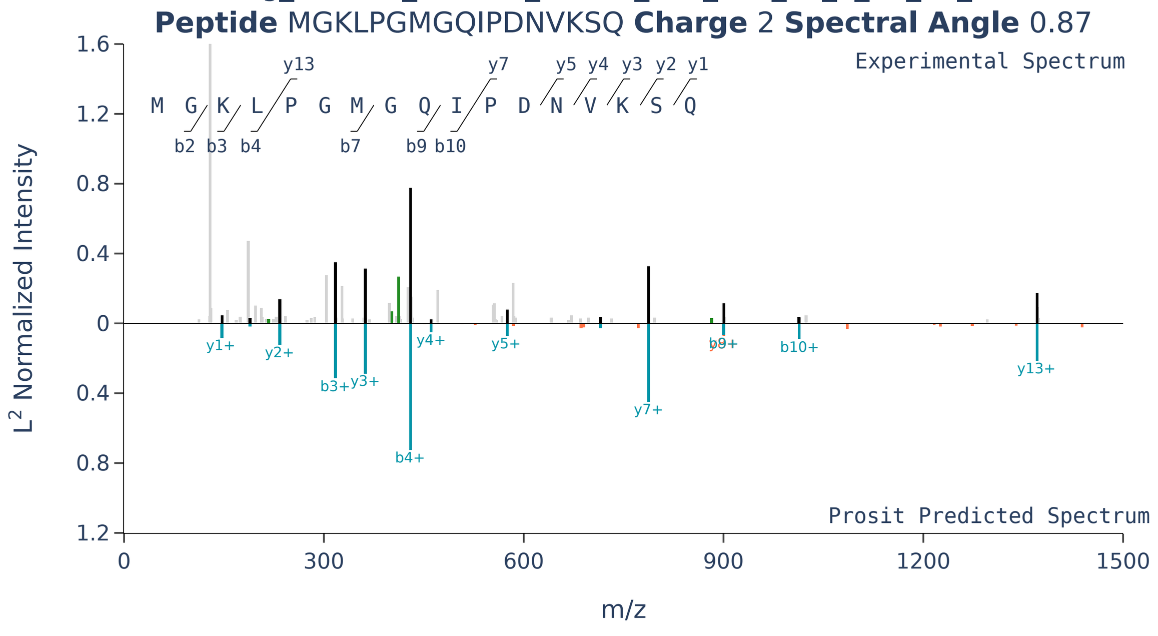


**Source** Ncheng_210623_230623_HFGoe_FFH_20S_25_1_A2_4h_R1 **Scan** 32711
**Peptide** EVNKSLTPGQEFVKIVRNELV **Charge** 3 **Spectral Angle** 0.81

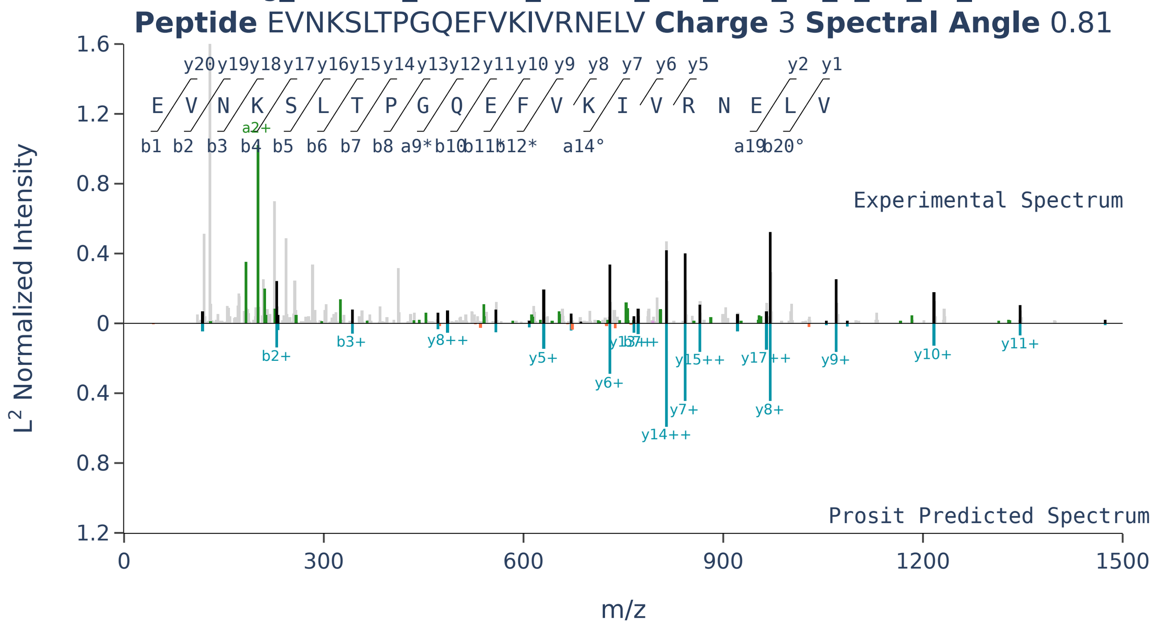


**Source** Ncheng_210623_230623_HFGoe_FFH_20S_25_1_A1_4h_R1 **Scan** 22284
**Peptide** EALEPFHPDRIASRL **Charge** 3 **Spectral Angle** 0.82

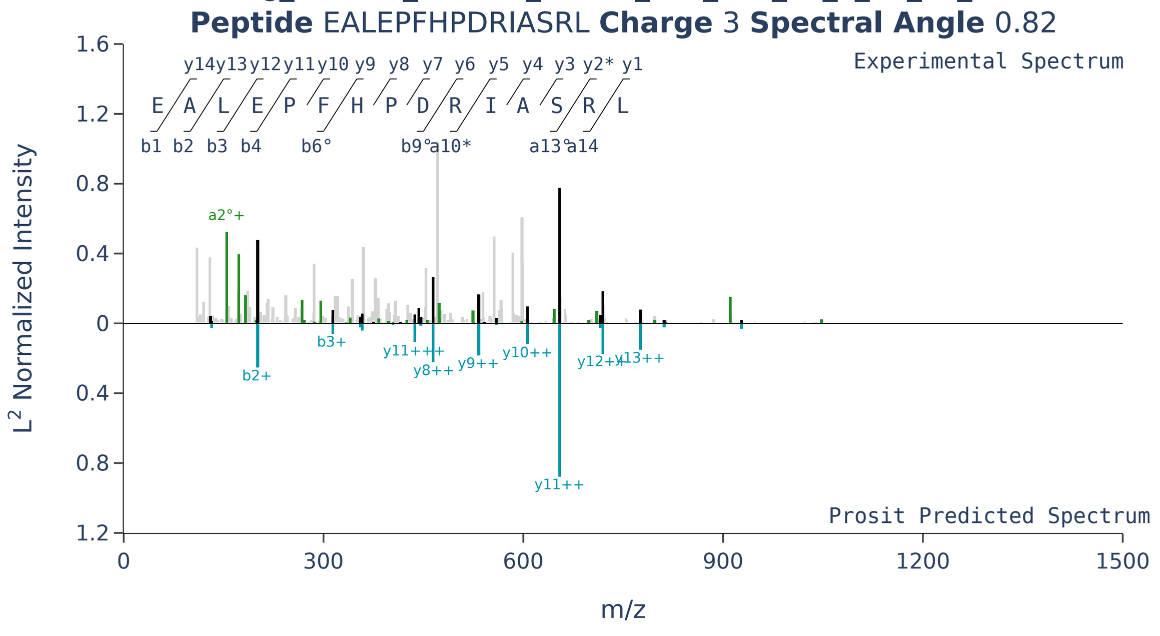


**Source** Ncheng_210623_230623_HFGoe_FFH_20S_25_1_A1_24h_R1 **Scan** 12711
**Peptide** DDMQRMM **Charge** 2 **Spectral Angle** 0.92

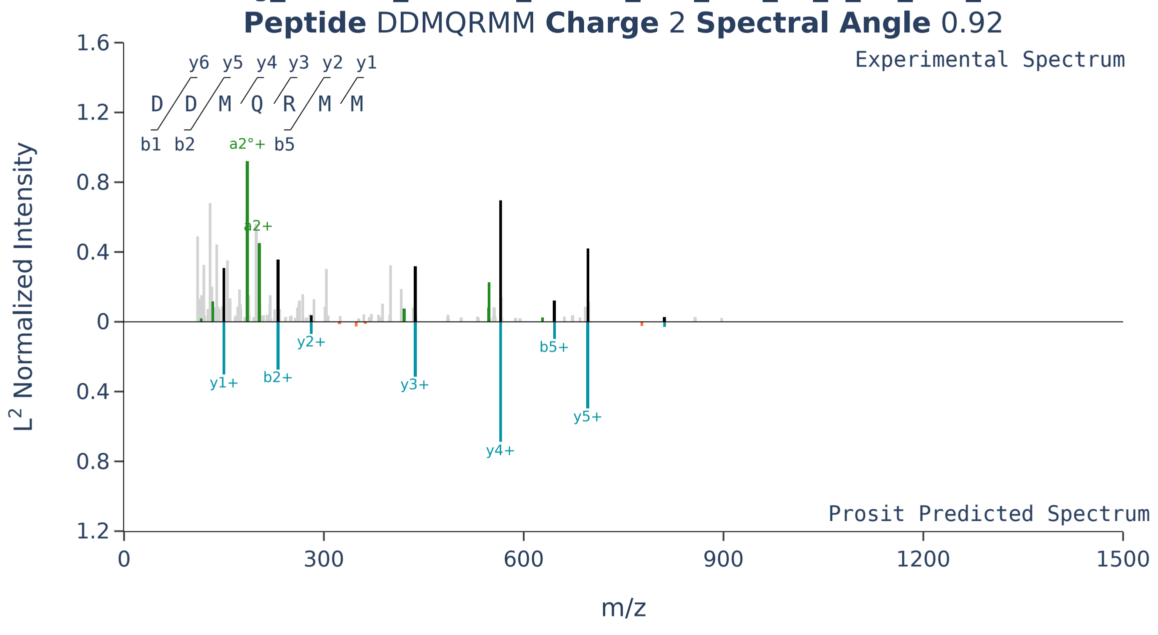


**Source** Ncheng_210623_230623_HFGoe_FFH_20S_25_1_A1_2h_R2 **Scan** 20010
**Peptide** GVGEKTEALEPFHPDRI **Charge** 2 **Spectral Angle** 0.74

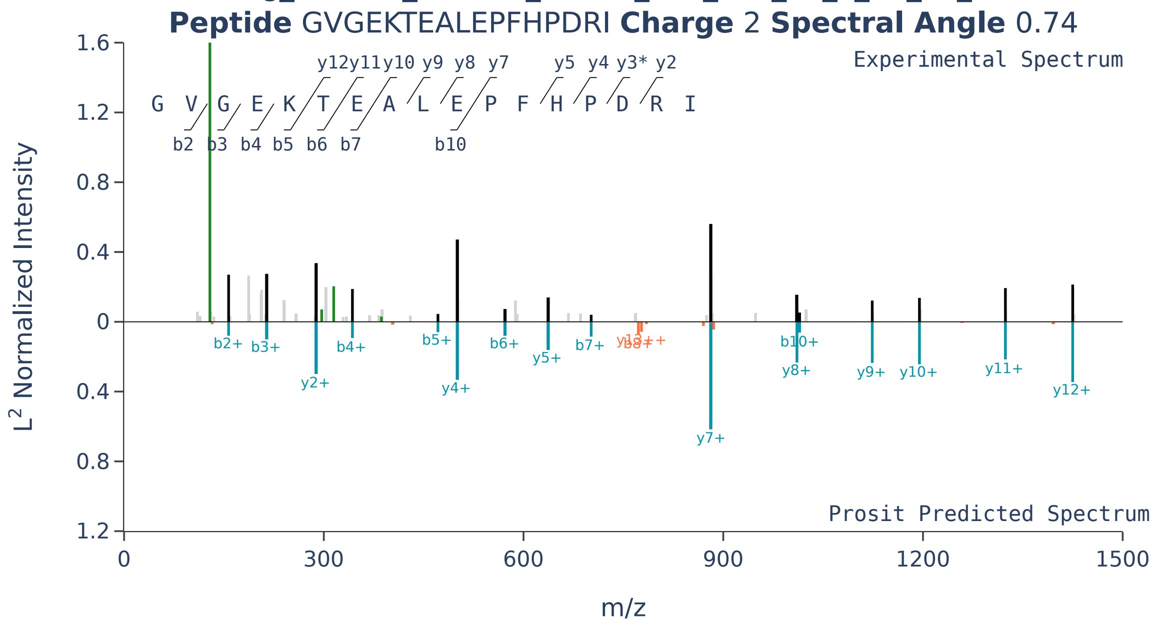


**Source** Ncheng_210623_230623_HFGoe_FFH_20S_25_1_A1_2h_R2 **Scan** 12200
**Peptide** PDNVKSQMD **Charge** 1 **Spectral Angle** 0.9

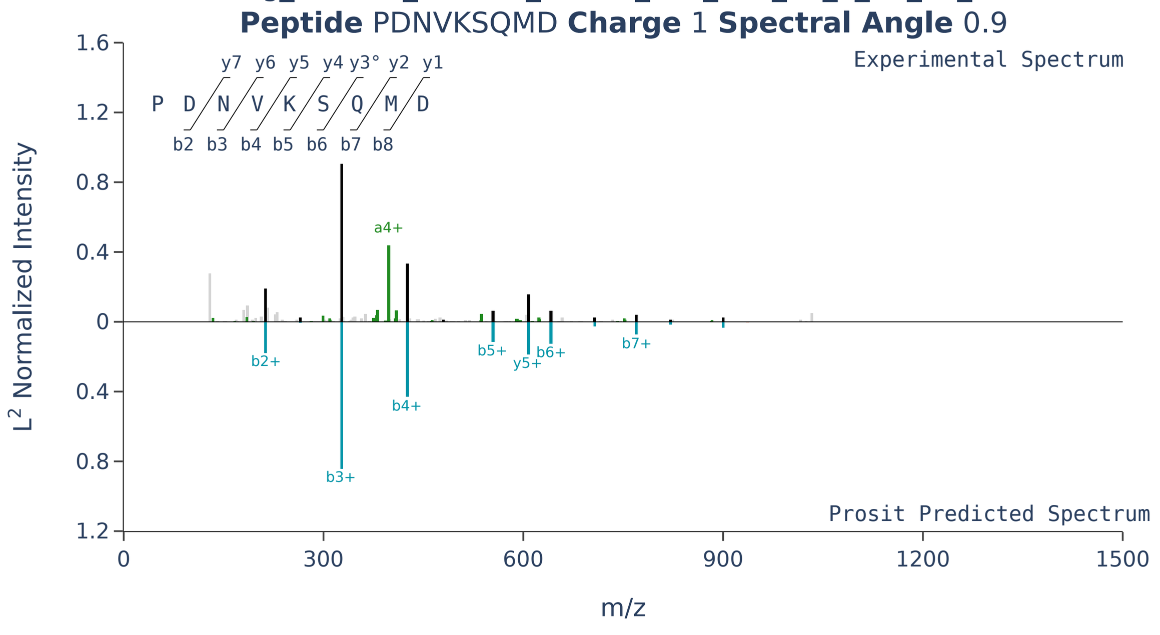


**Source** Ncheng_210623_230623_HFGoe_FFH_20S_25_1_A2_1h_R1 **Scan** 9636
**Peptide** DTAGRLHV **Charge** 2 **Spectral Angle** 0.83

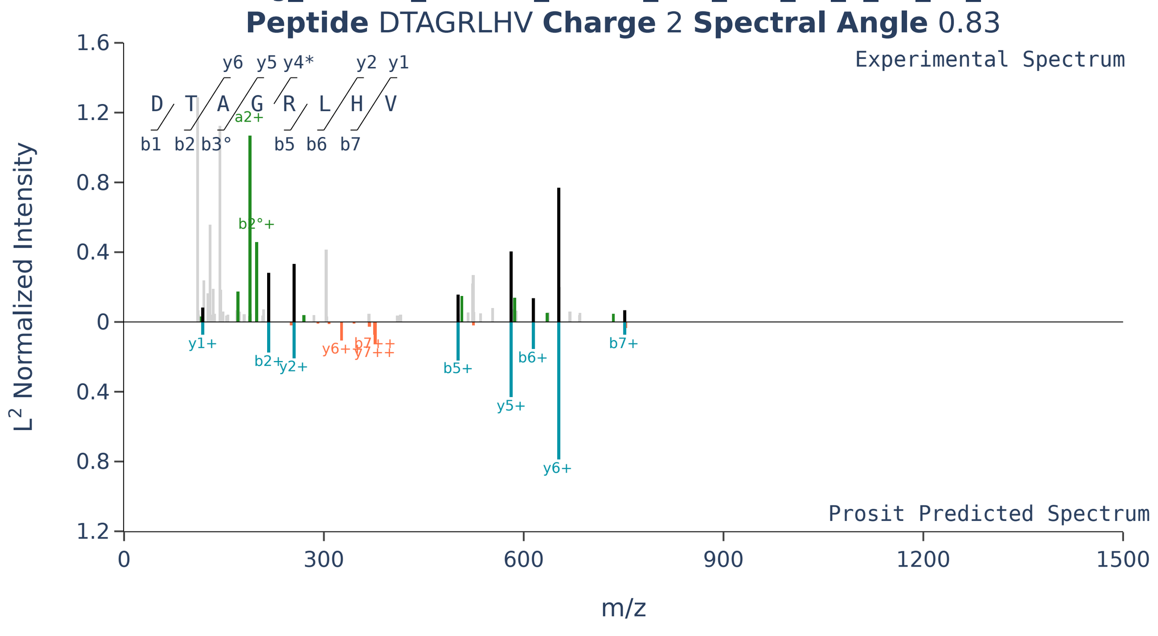


**Source** Ncheng_210623_230623_HFGoe_FFH_20S_25_1_A2_24h_R1 **Scan** 21901
**Peptide** KSLTPGQEFVKI **Charge** 2 **Spectral Angle** 0.84

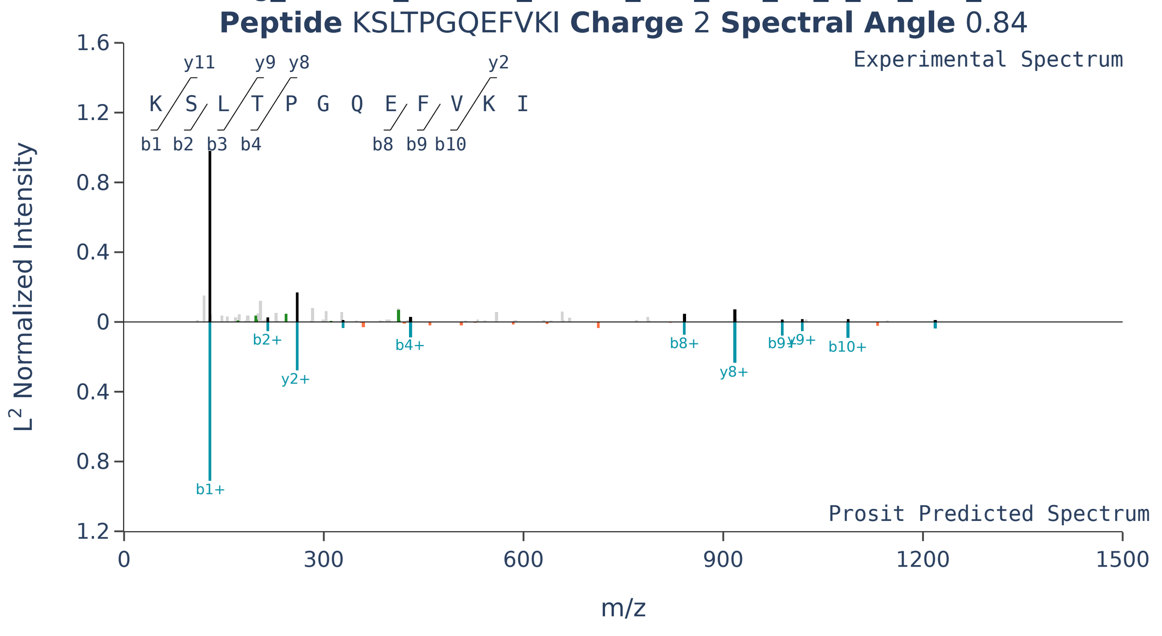


**Source** Ncheng_210623_230623_HFGoe_FFH_20S_25_1_A1_4h_R1 **Scan** 17471
**Peptide** DGDARGGAALSL **Charge** 1 **Spectral Angle** 0.92

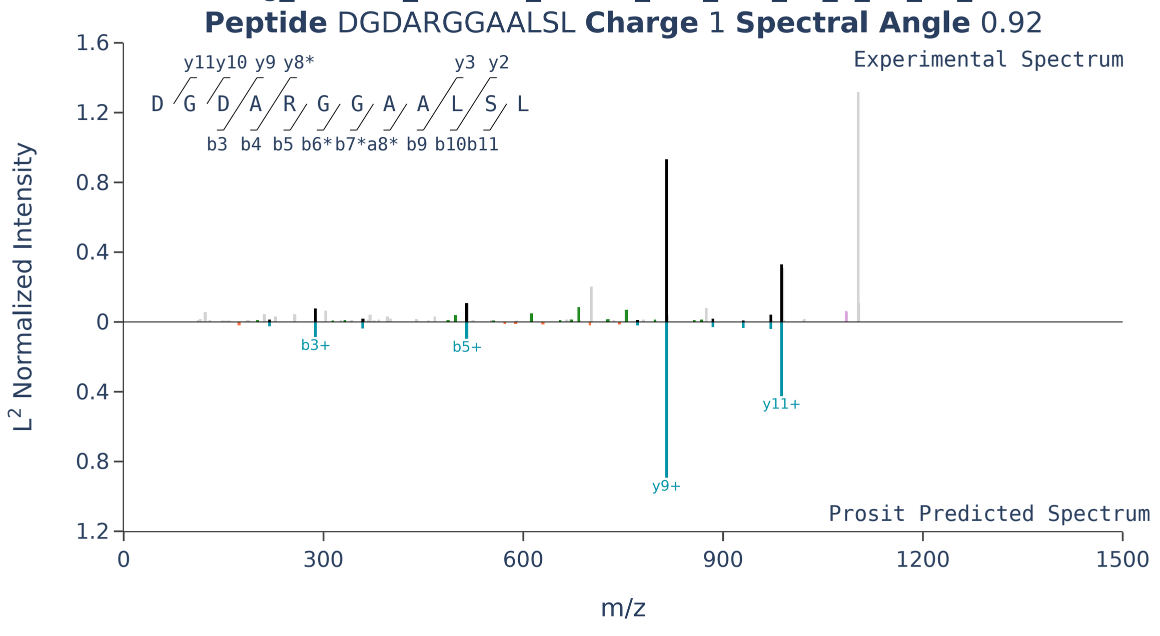

**Source** Ncheng_210623_230623_HFGoe_FFH_20S_25_1_A2_2h_R2 **Scan** 16480
**Peptide** IVNAALKEAKL **Charge** 3 **Spectral Angle** 0.8

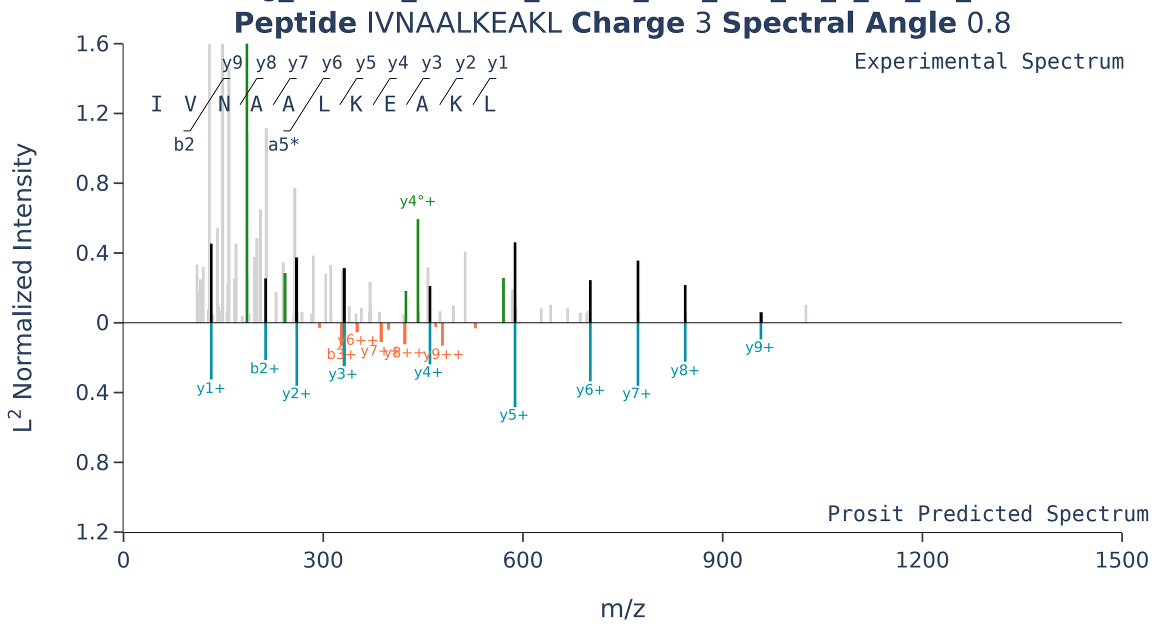


**Source** Ncheng_210623_230623_HFGoe_FFH_20S_25_1_A1_2h_R2 **Scan** 26548
**Peptide** SLMGKLPGMGQIPDNVKSQMDD **Charge** 3 **Spectral Angle** 0.76

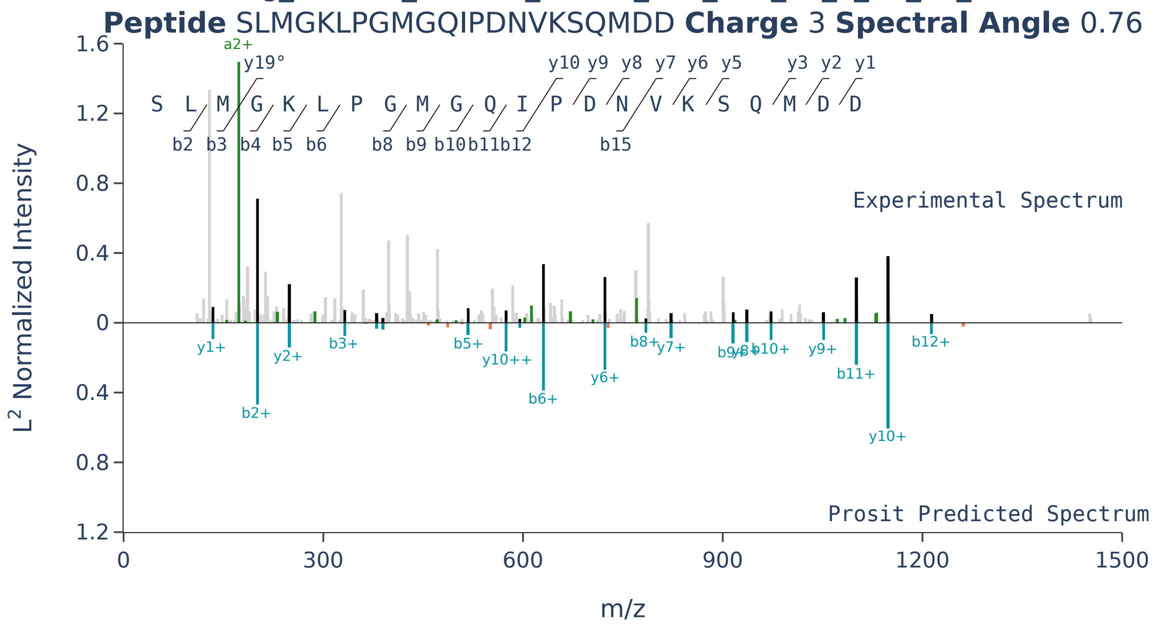


**Source** Ncheng_210623_230623_HFGoe_FFH_20S_25_1_A1_4h_R1 **Scan** 23681
**Peptide** EVNKSLTPGQEFVKIVRNE **Charge** 3 **Spectral Angle** 0.84

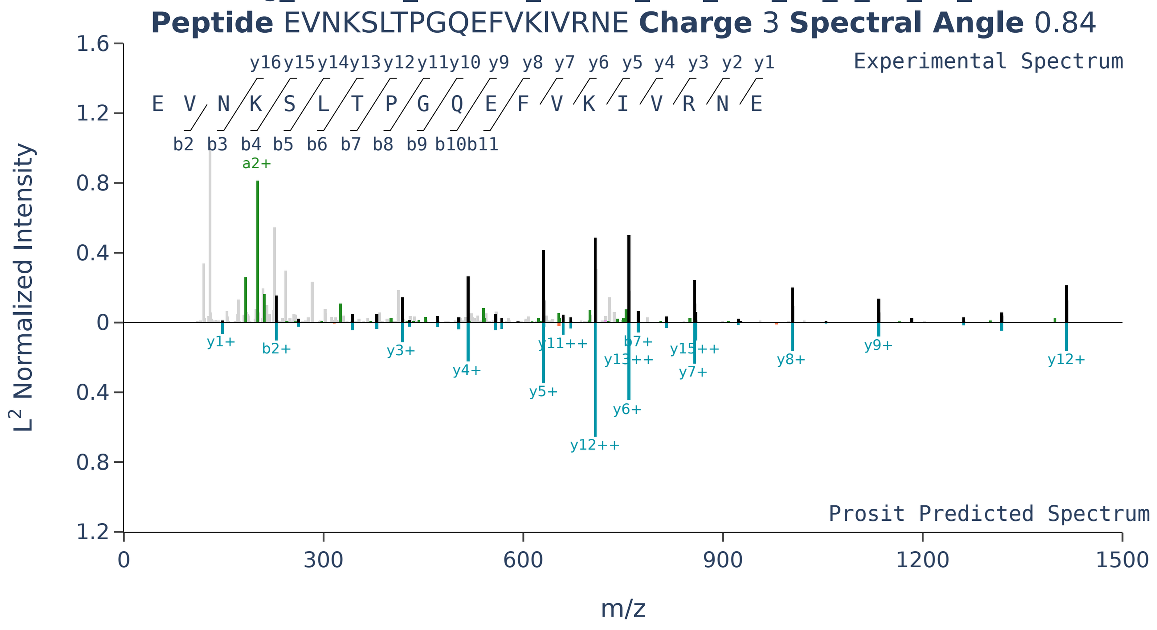


**Source** Ncheng_210623_230623_HFGoe_FFH_20S_25_1_A2_24h_R1 **Scan** 6753
**Peptide** ANTAKAFN **Charge** 1 **Spectral Angle** 0.86

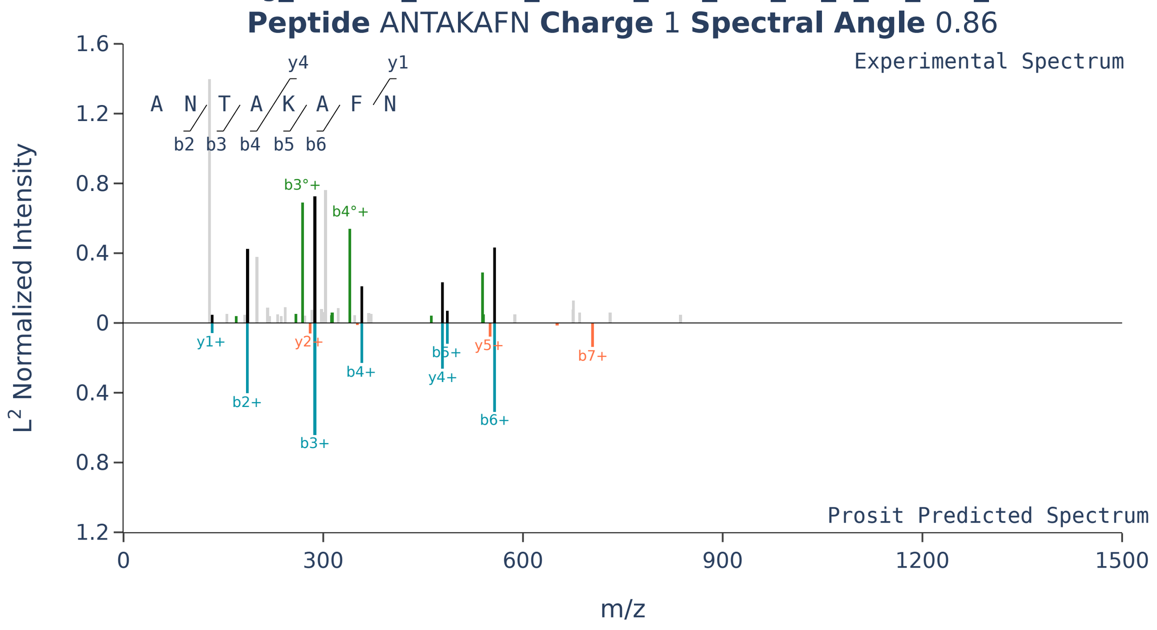


**Source** Ncheng_210623_230623_HFGoe_FFH_20S_25_1_A2_24h_R2 **Scan** 7355
**Peptide** DEIKQVHA **Charge** 1 **Spectral Angle** 0.85

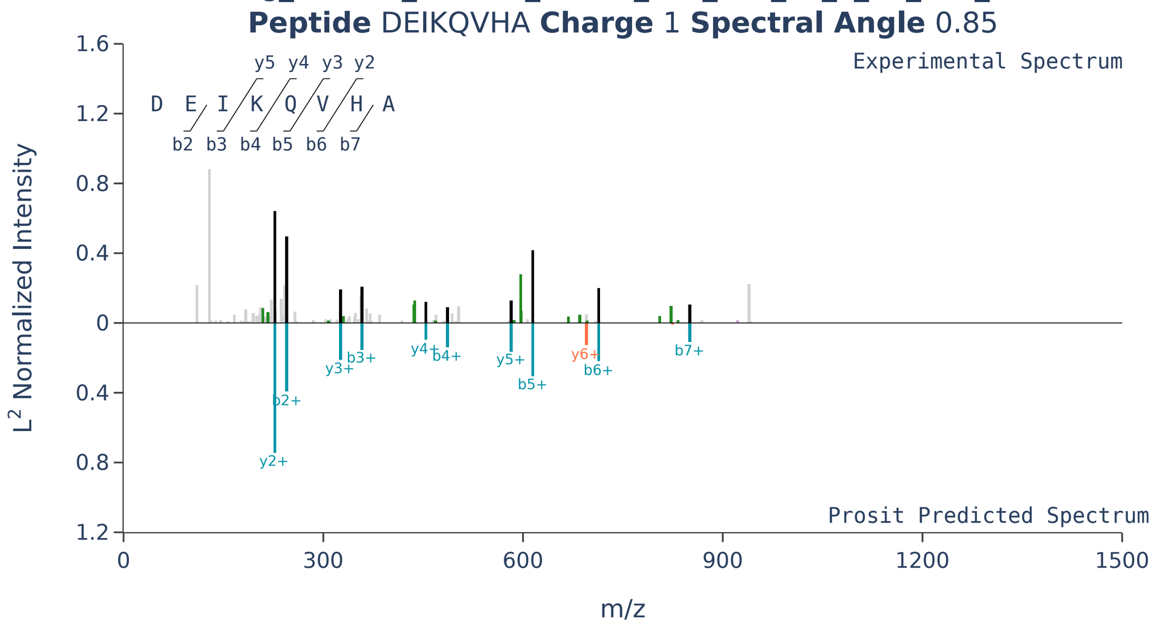


**Source** Ncheng_210623_230623_HFGoe_FFH_20S_25_1_A2_2h_R1 **Scan** 33434
**Peptide** GVDFFPSDVGQKPVDIVNAALKE **Charge** 3 **Spectral Angle** 0.82

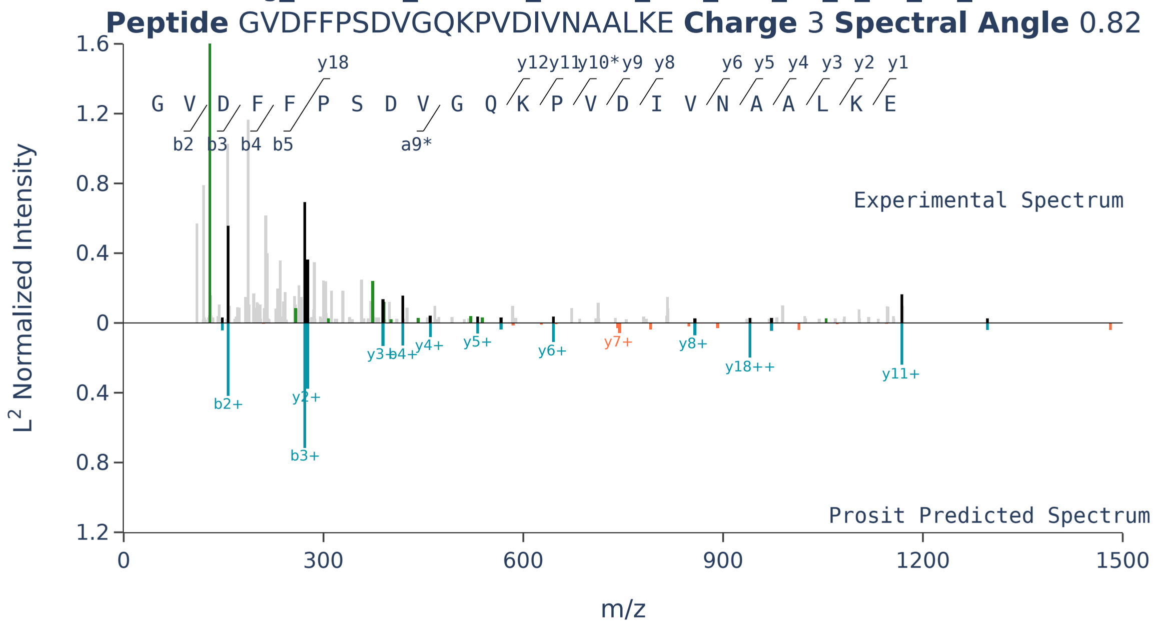


**Source** Ncheng_210623_230623_HFGoe_FFH_20S_25_1_A2_2h_R1 **Scan** 27379
**Peptide** AMTGQDAANTAKAFNEALPLT **Charge** 2 **Spectral Angle** 0.91

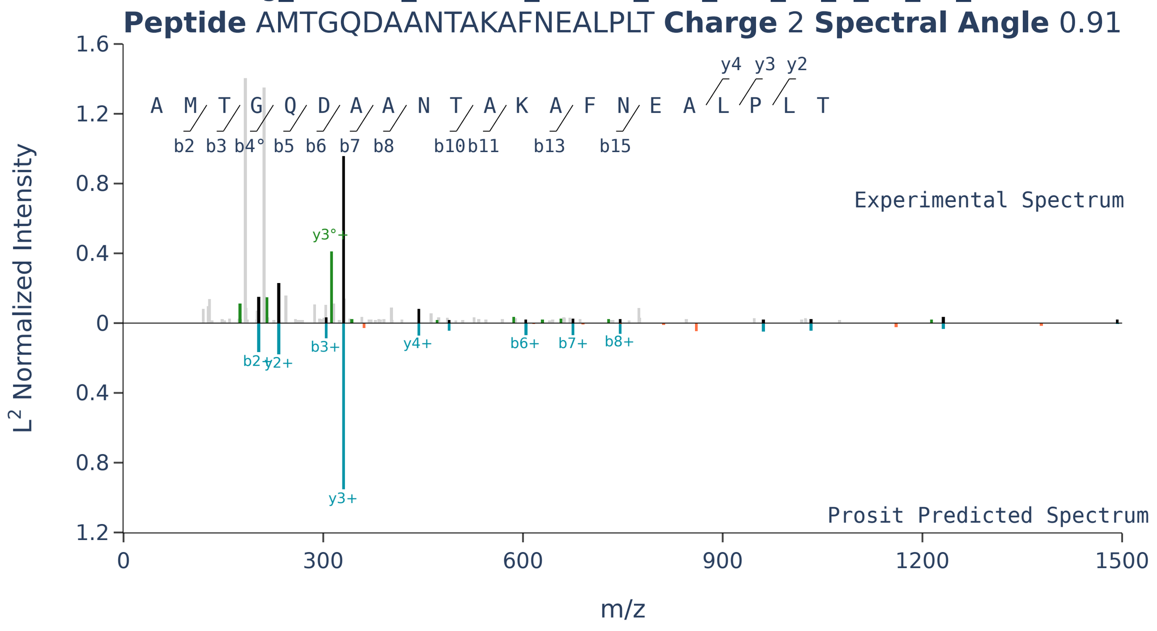


**Source** Ncheng_210623_230623_HFGoe_FFH_20S_25_1_A2_24h_R1 **Scan** 13880
**Peptide** LVAAMGEENQT **Charge** 2 **Spectral Angle** 0.91

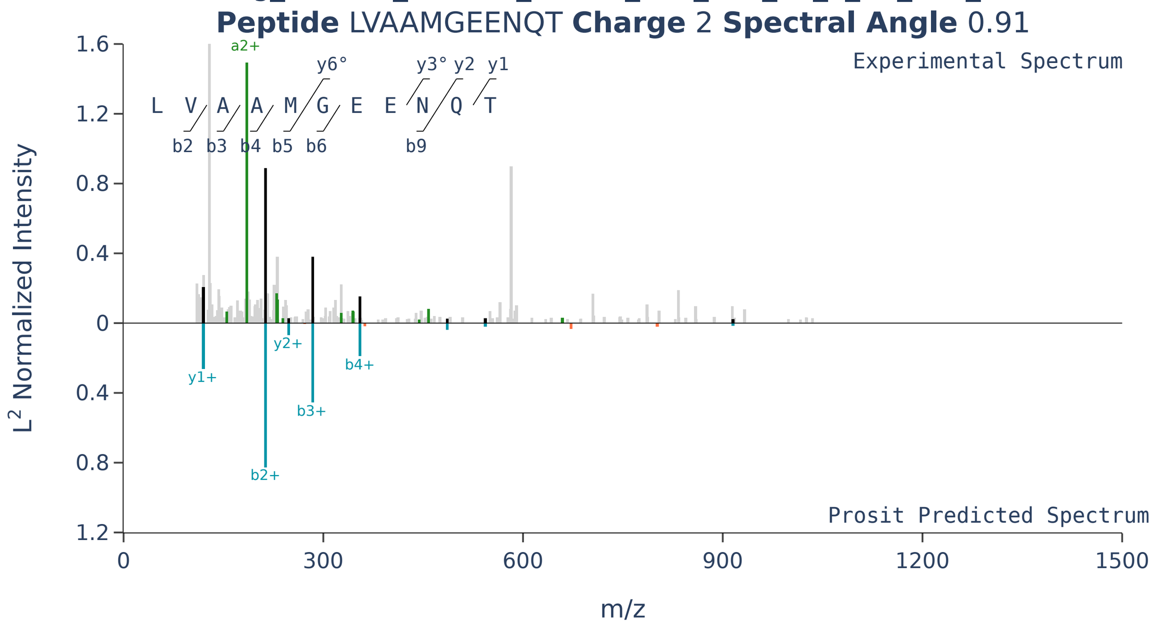


**Source** Ncheng_210623_230623_HFGoe_FFH_20S_25_1_A2_24h_R1 **Scan** 6233
**Peptide** GHEVNKSLT **Charge** 1 **Spectral Angle** 0.83

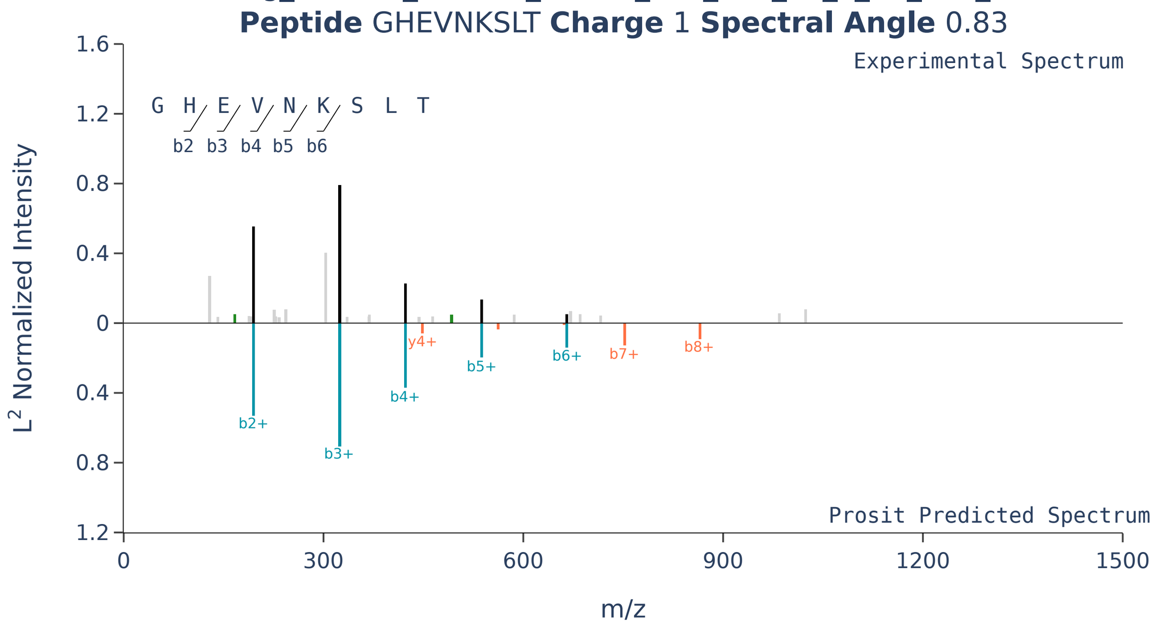


**Source** Ncheng_210623_230623_HFGoe_FFH_20S_25_1_A2_4h_R1 **Scan** 17664
**Peptide** GQIPDNVKSQMDDKVLVR **Charge** 3 **Spectral Angle** 0.76

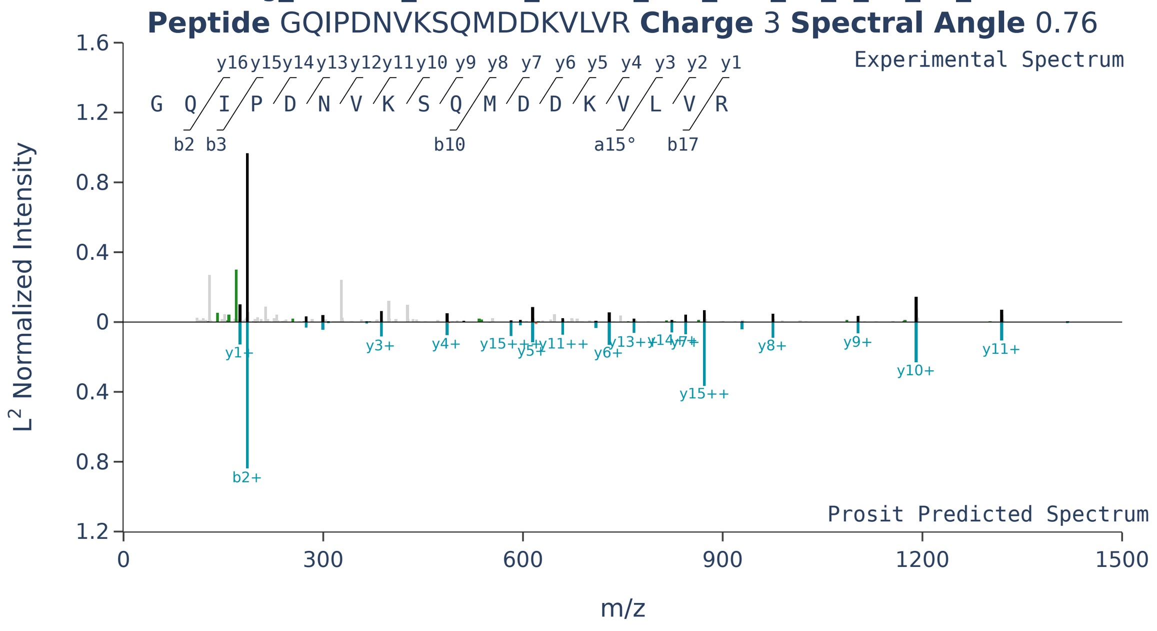


**Source** Ncheng_210623_230623_HFGoe_FFH_20S_25_1_A2_2h_R1 **Scan** 25234
**Peptide** VRMEAIINSMTMKE **Charge** 3 **Spectral Angle** 0.83

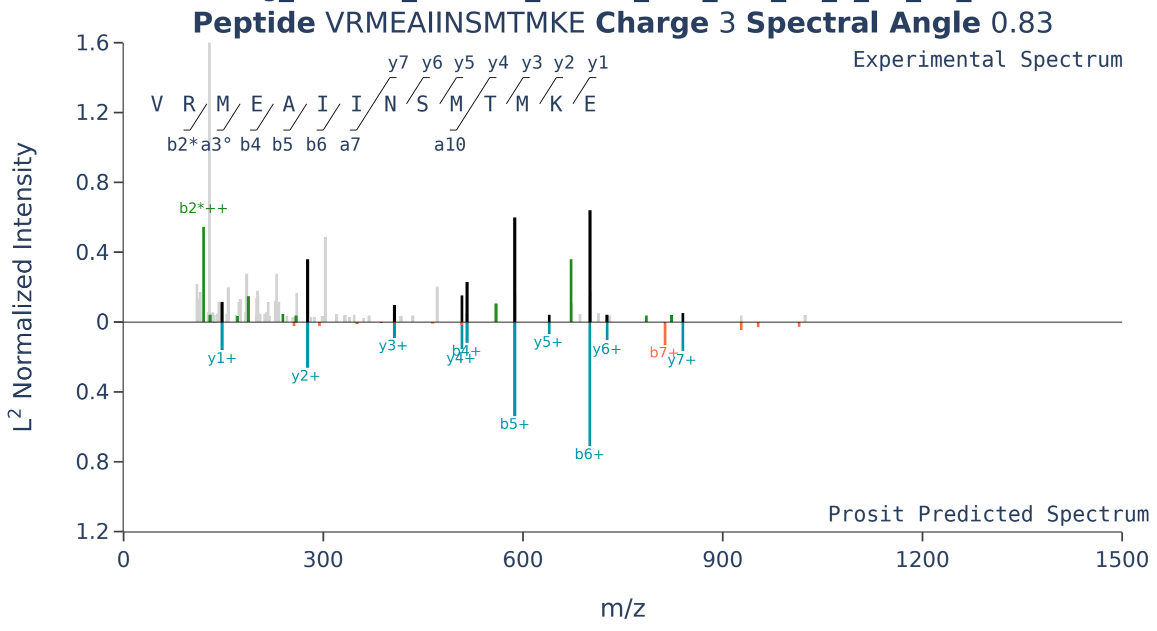


**Source** Ncheng_210623_230623_HFGoe_FFH_20S_25_1_A1_24h_R1 **Scan** 12775
**Peptide** AMMDEIKQ **Charge** 2 **Spectral Angle** 0.8

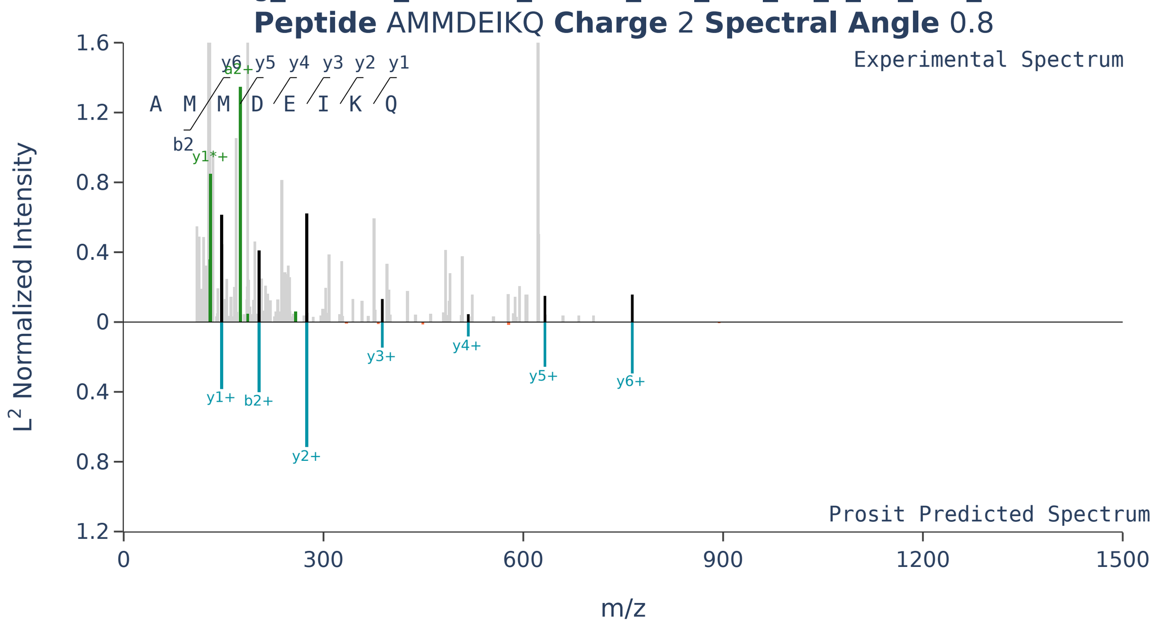


**Source** Ncheng_210623_230623_HFGoe_FFH_20S_25_1_A1_2h_R2 **Scan** 22720
**Peptide** TLREVRMALLE **Charge** 2 **Spectral Angle** 0.76

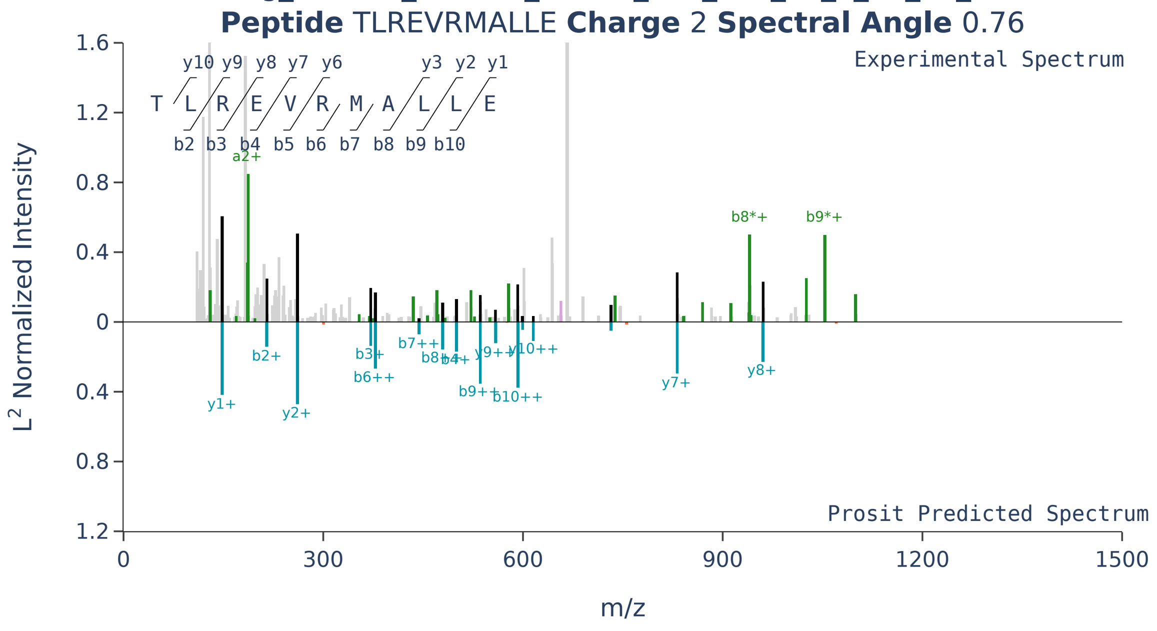


**Source** Ncheng_210623_230623_HFGoe_FFH_20S_25_1_A2_24h_R2 **Scan** 11689
**Peptide** MGQIPDNVKSQ **Charge** 2 **Spectral Angle** 0.79

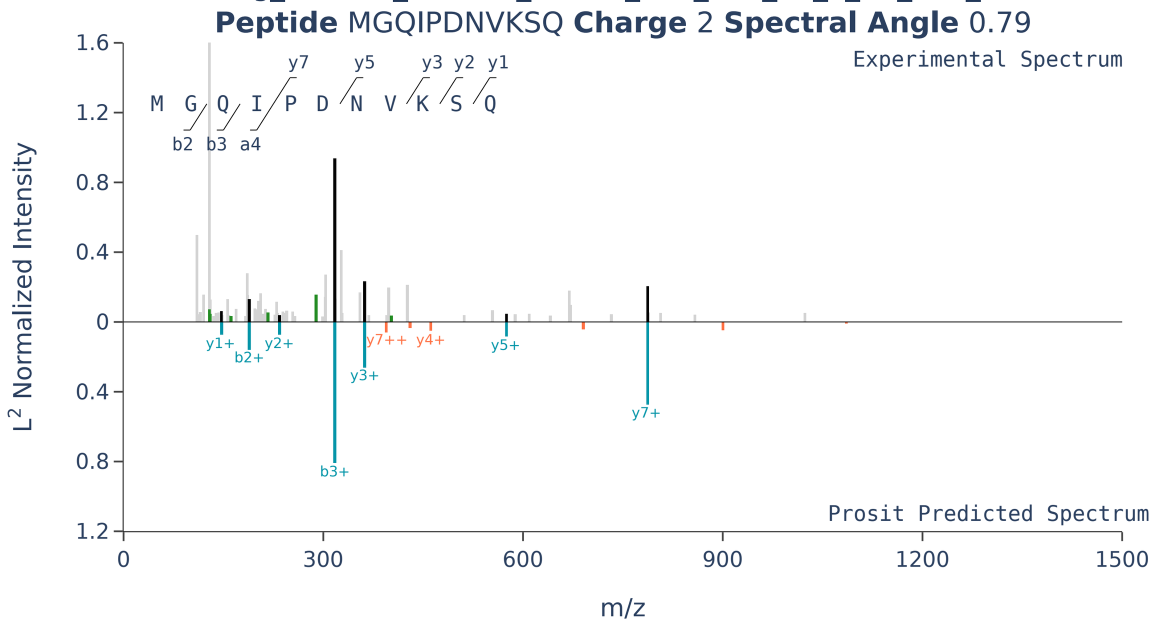


**Source** Ncheng_210623_230623_HFGoe_FFH_20S_25_1_A1_4h_R1 **Scan** 19869
**Peptide** LVVSADVYRPAAIKQ **Charge** 2 **Spectral Angle** 0.8

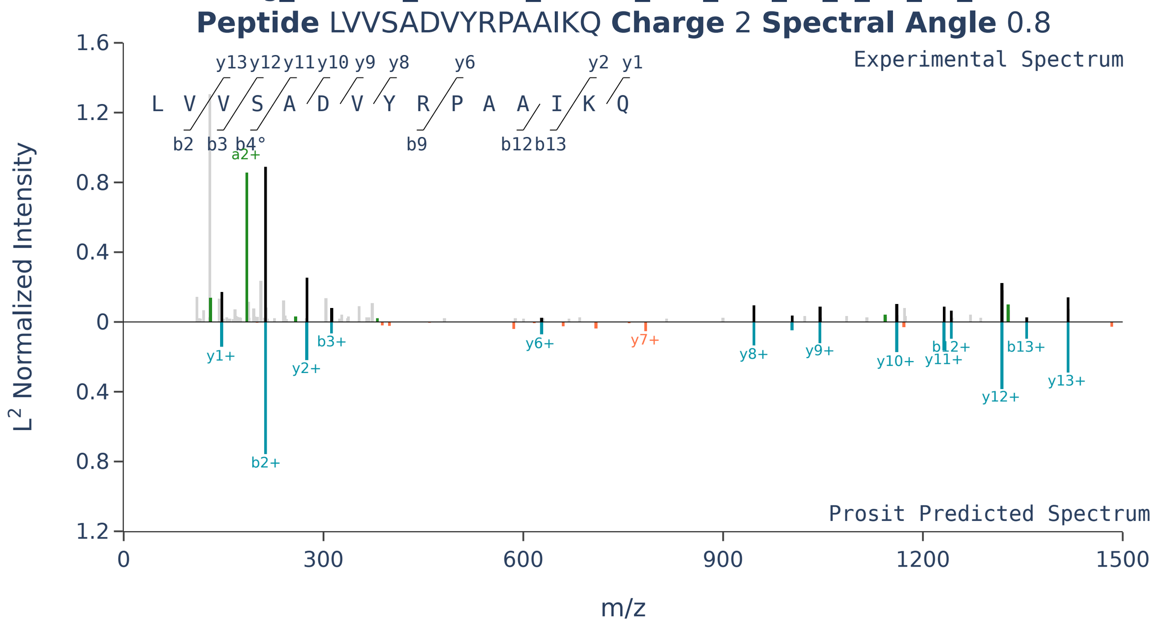

**Source** Ncheng_210623_230623_HFGoe_FFH_20S_25_1_A2_24h_R2 **Scan** 7620
**Peptide** DLESKVDRAQ **Charge** 3 **Spectral Angle** 0.88

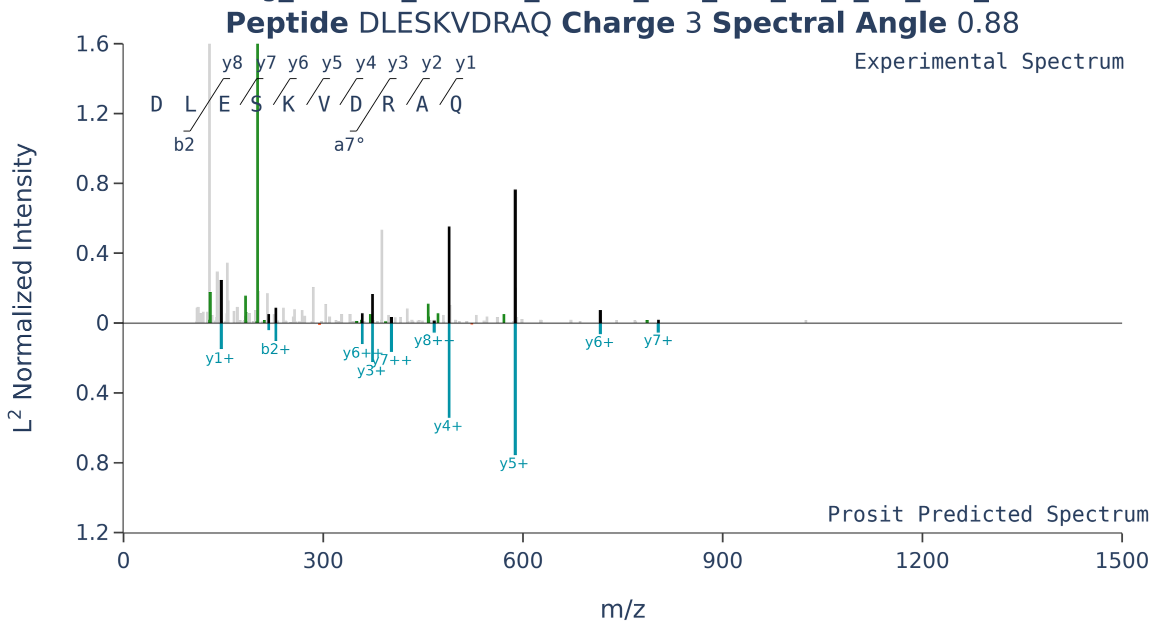


**Source** Ncheng_210623_230623_HFGoe_FFH_20S_25_1_A1_2h_R2 **Scan** 23996
**Peptide** AANTAKAFNEALPLTG **Charge** 2 **Spectral Angle** 0.82

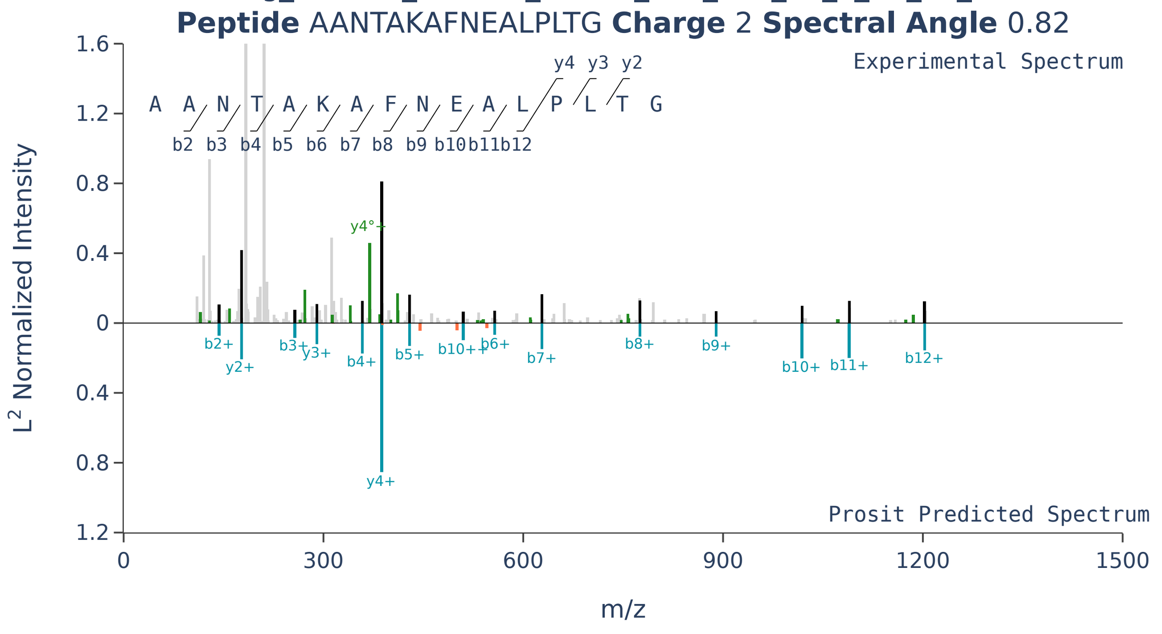


**Source** Ncheng_210623_230623_HFGoe_FFH_20S_25_1_A2_24h_R1 **Scan** 12782
**Peptide** SVGKLGKFLREK **Charge** 2 **Spectral Angle** 0.79

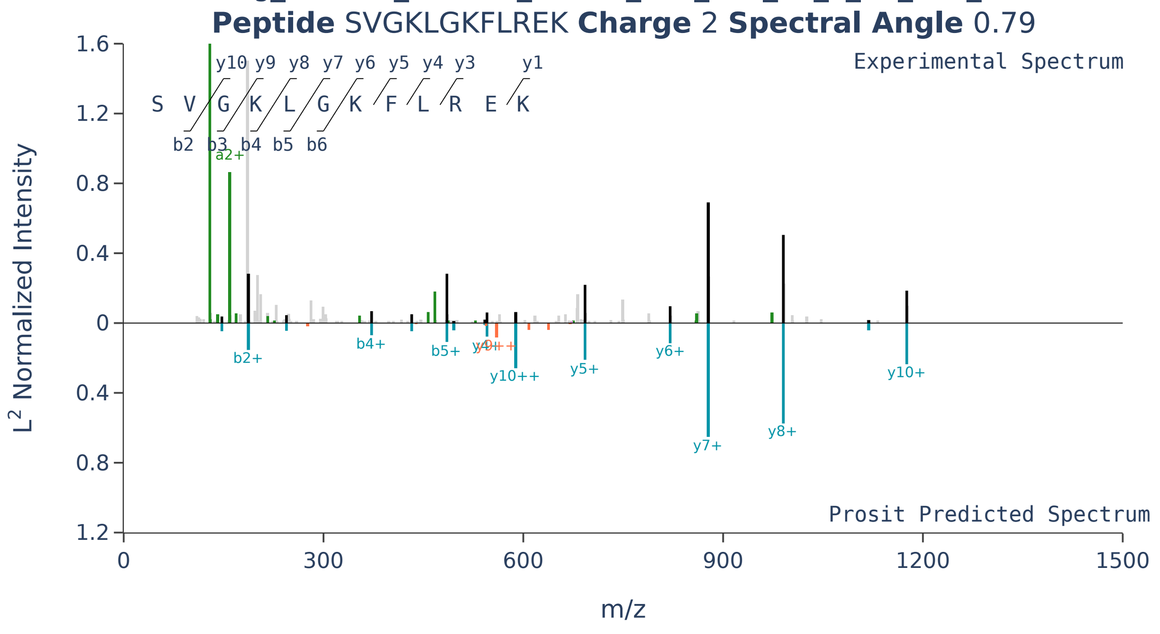


**Source** Ncheng_210623_230623_HFGoe_FFH_20S_25_1_A2_24h_R1 **Scan** 7453
**Peptide** LGVGEKT **Charge** 1 **Spectral Angle** 0.9

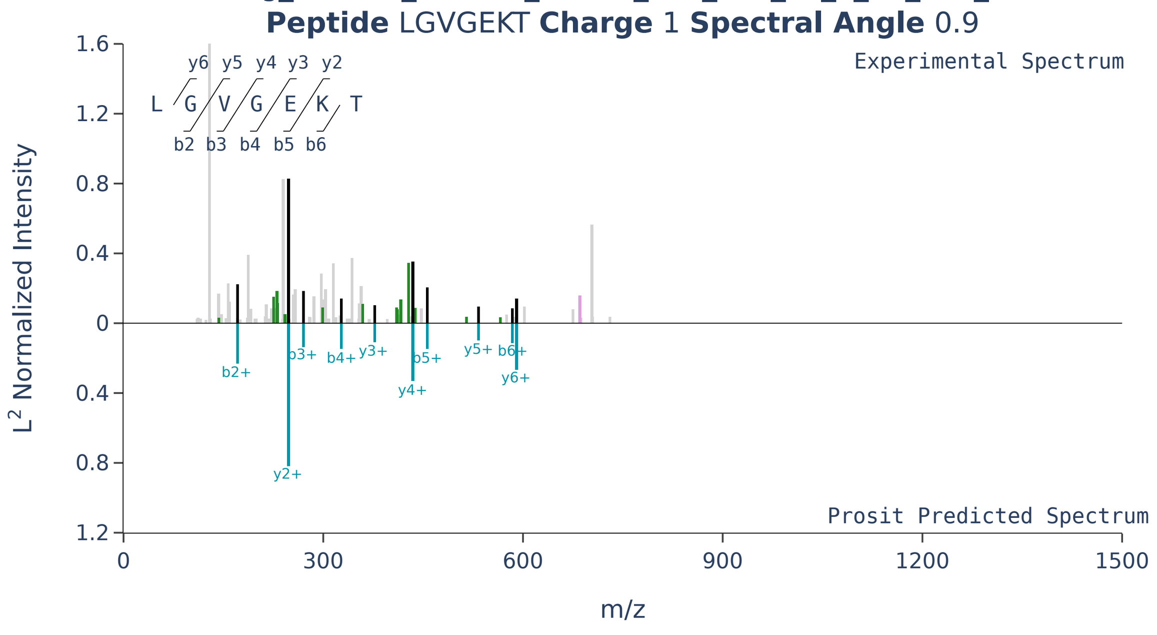


**Source** Ncheng_210623_230623_HFGoe_FFH_20S_25_1_A1_2h_R2 **Scan** 25989
**Peptide** TGQDAANTAKAFNEALPLTG **Charge** 2 **Spectral Angle** 0.85

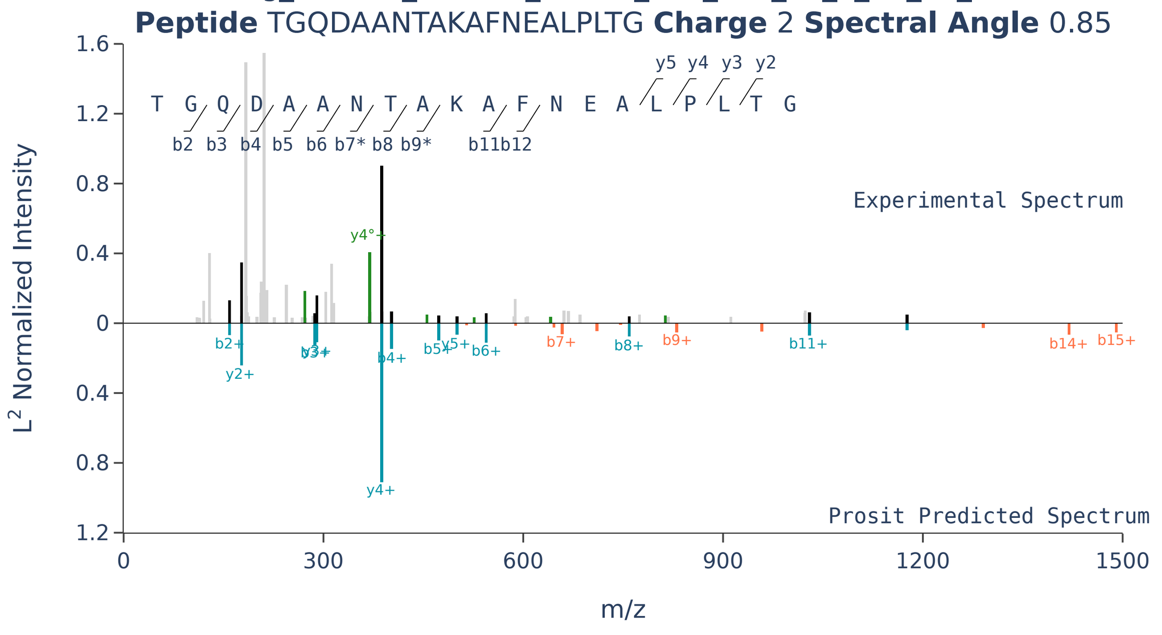


**Source** Ncheng_210623_230623_HFGoe_FFH_20S_25_1_A2_4h_R2 **Scan** 23252
**Peptide** MGKLPGMGQIPDNVKSQM **Charge** 2 **Spectral Angle** 0.86

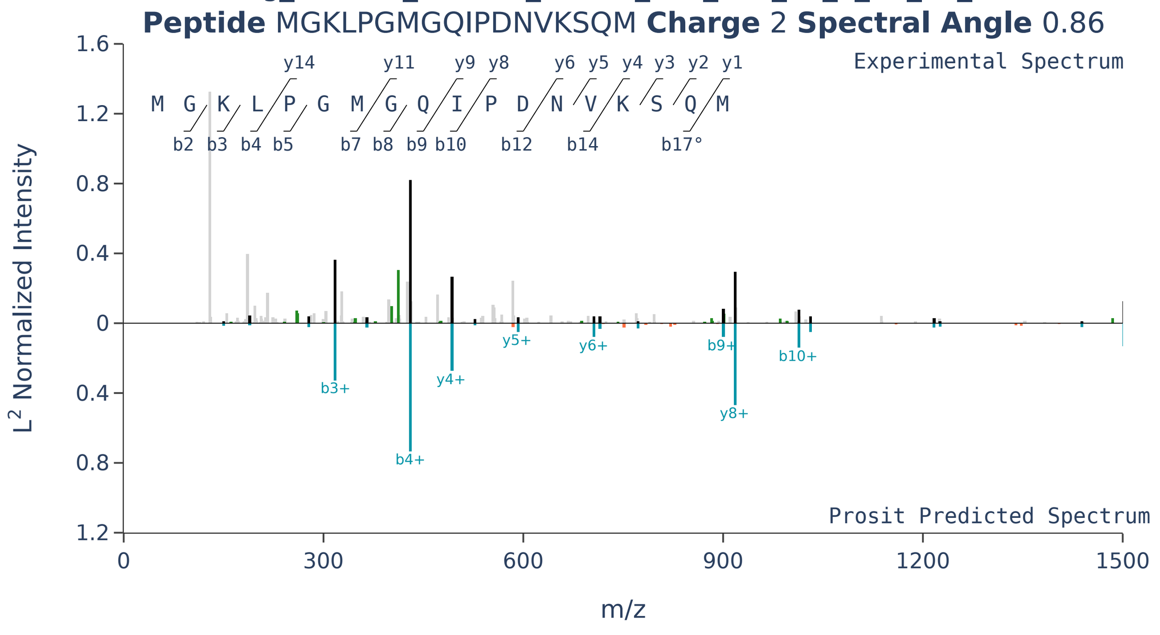


**Source** Ncheng_210623_230623_HFGoe_FFH_20S_25_1_A2_4h_R1 **Scan** 13832
**Peptide** RHITGKPIKFL **Charge** 3 **Spectral Angle** 0.82

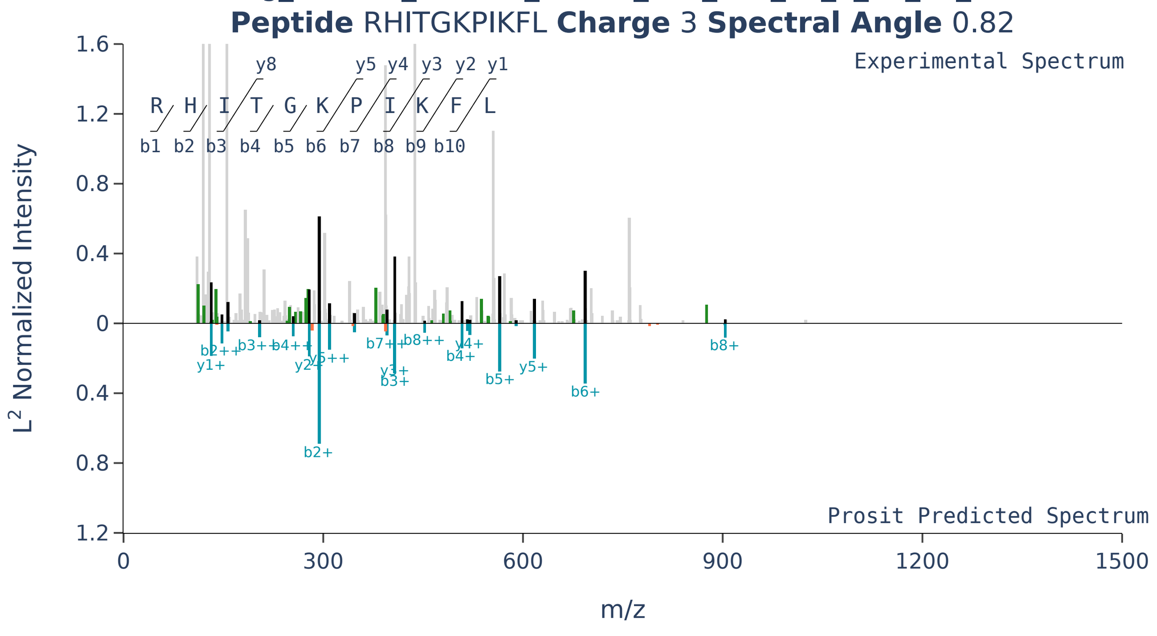


**Source** Ncheng_210623_230623_HFGoe_FFH_20S_25_1_A2_4h_R1 **Scan** 19126
**Peptide** KAVGHEVNKSLTPGQEFVKIVRNE **Charge** 3 **Spectral Angle** 0.74

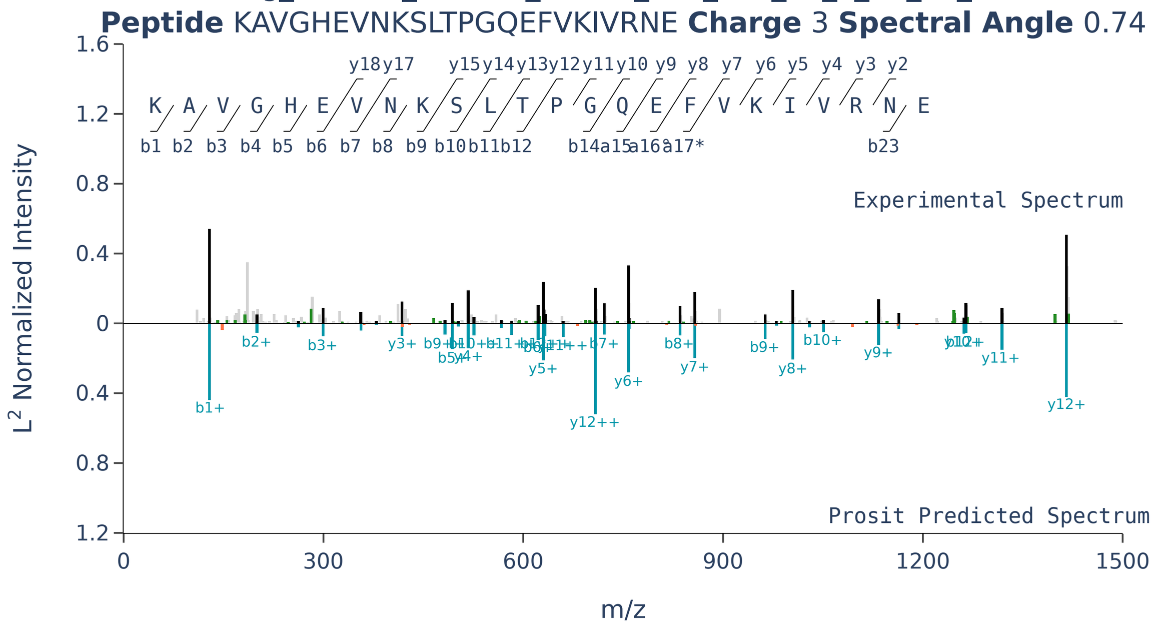


**Source** Ncheng_210623_230623_HFGoe_FFH_20S_25_1_A1_24h_R2 **Scan** 13516
**Peptide** TSVGKLGKF **Charge** 2 **Spectral Angle** 0.78

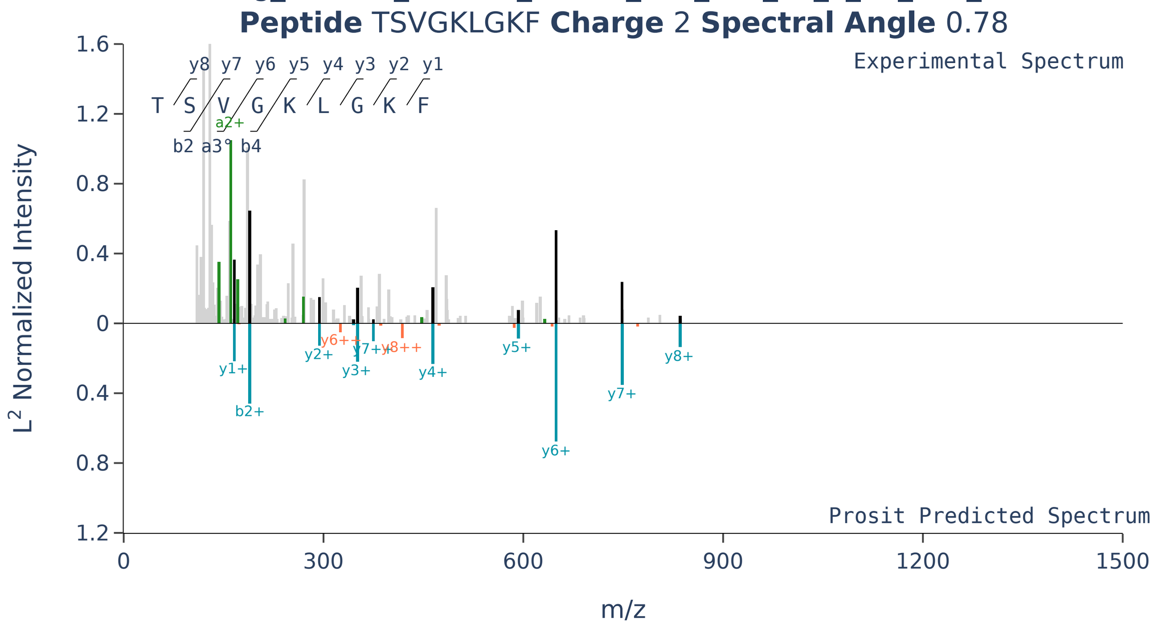


**Source** Ncheng_210623_230623_HFGoe_FFH_20S_25_1_A2_2h_R2 **Scan** 22751
**Peptide** EVNKSLTPGQEFVKI **Charge** 2 **Spectral Angle** 0.81

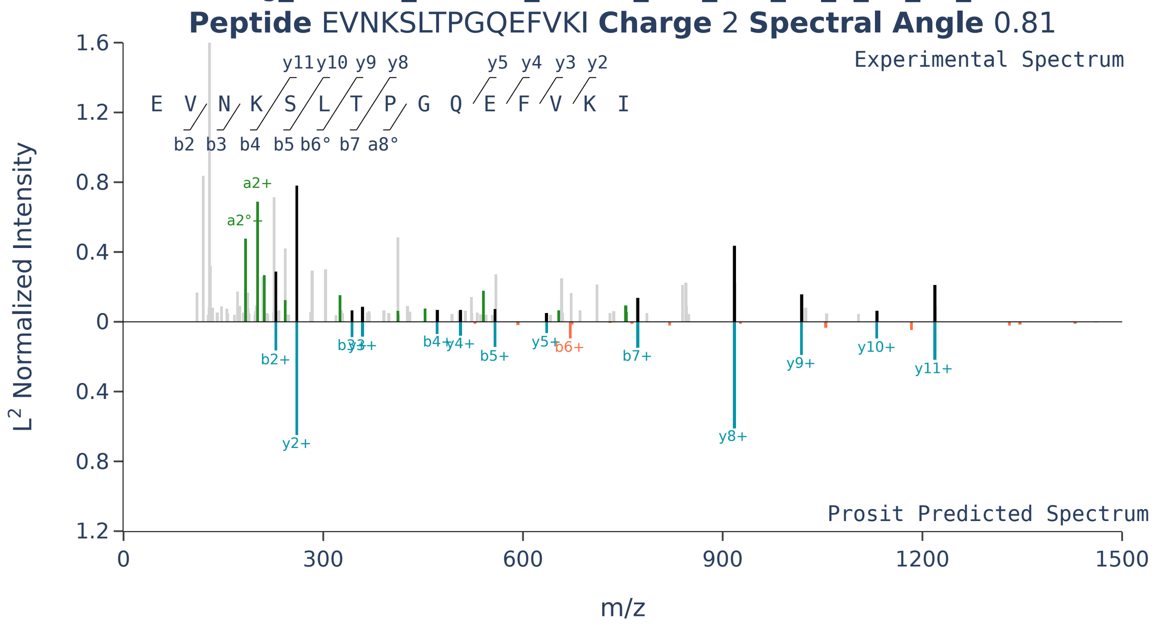


**Source** Ncheng_210623_230623_HFGoe_FFH_20S_25_1_A2_24h_R1 **Scan** 5895
**Peptide** IESKVDRAQA **Charge** 3 **Spectral Angle** 0.93

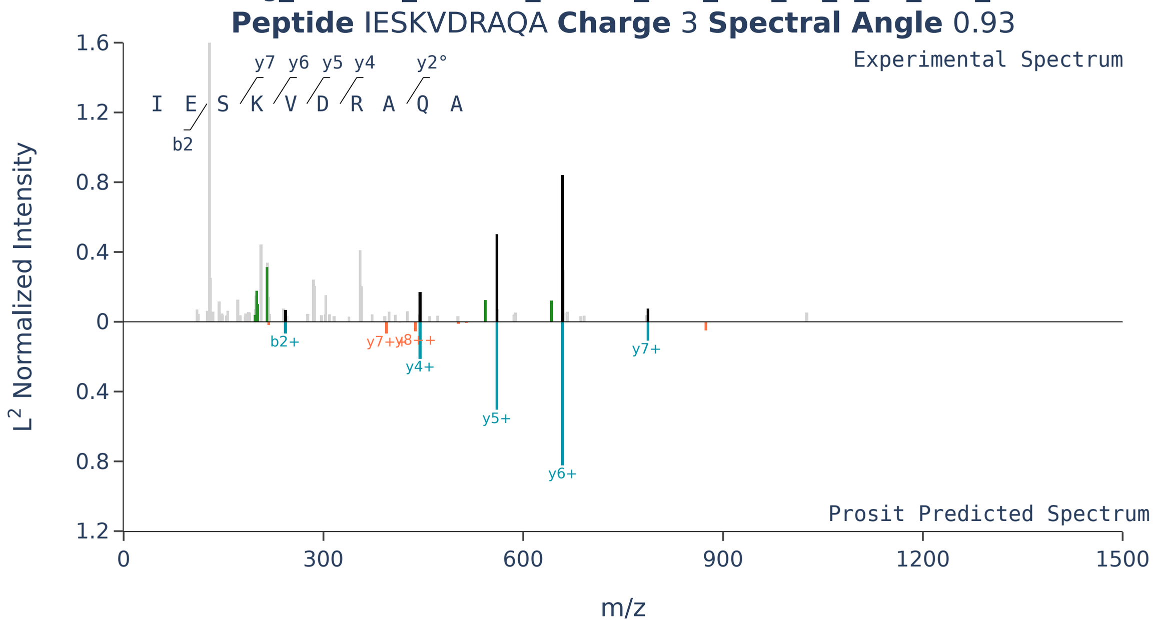


**Source** Ncheng_210623_230623_HFGoe_FFH_20S_25_1_A1_24h_R1 **Scan** 13889
**Peptide** NVKDTLREV **Charge** 2 **Spectral Angle** 0.87

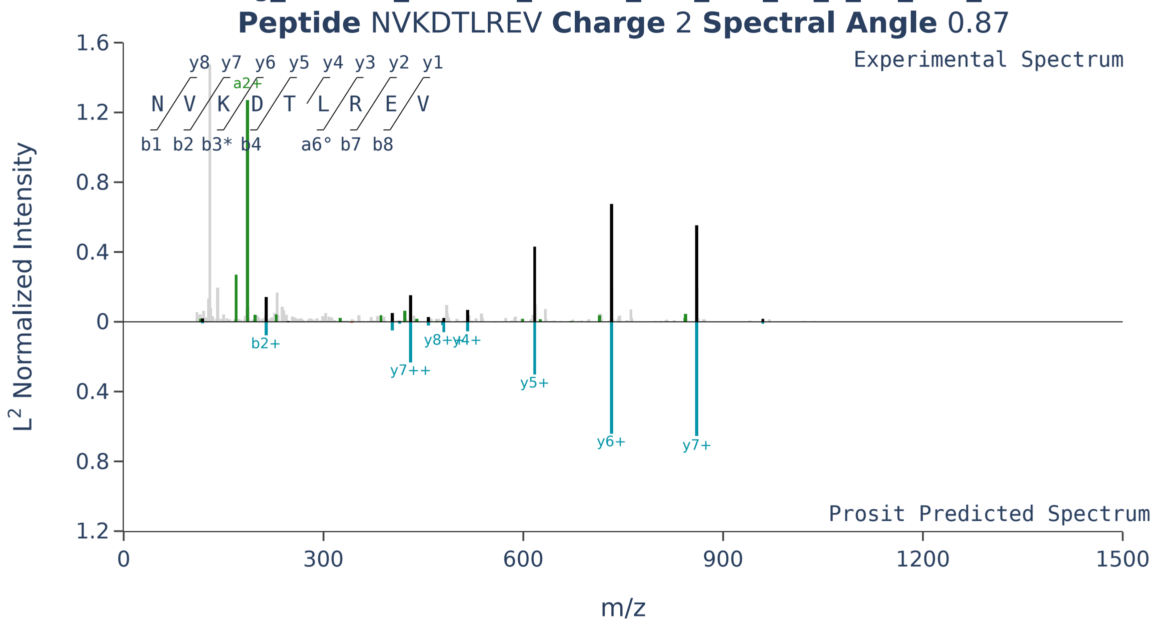


**Source** Ncheng_210623_230623_HFGoe_FFH_20S_25_1_A1_24h_R2 **Scan** 9326
**Peptide** DLESKVDRA **Charge** 2 **Spectral Angle** 0.77

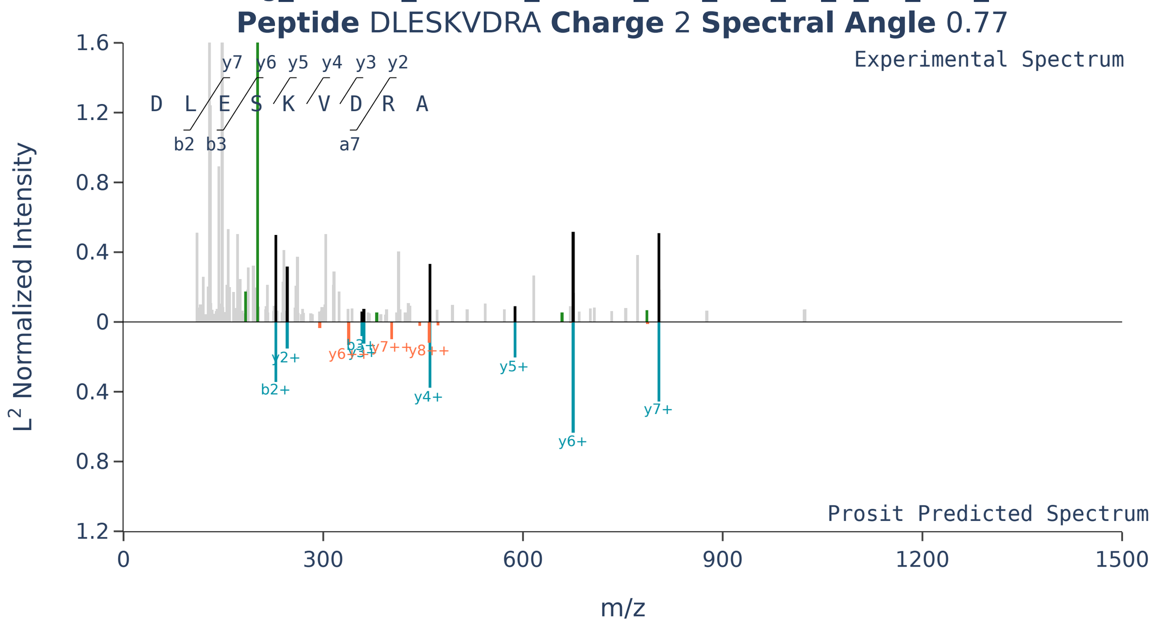


**Source** Ncheng_210623_230623_HFGoe_FFH_20S_25_1_A2_2h_R1 **Scan** 35908
**Peptide** AMTGQDAANTAKAFNEALPLTGVVLTKV **Charge** 3 **Spectral Angle** 0.84

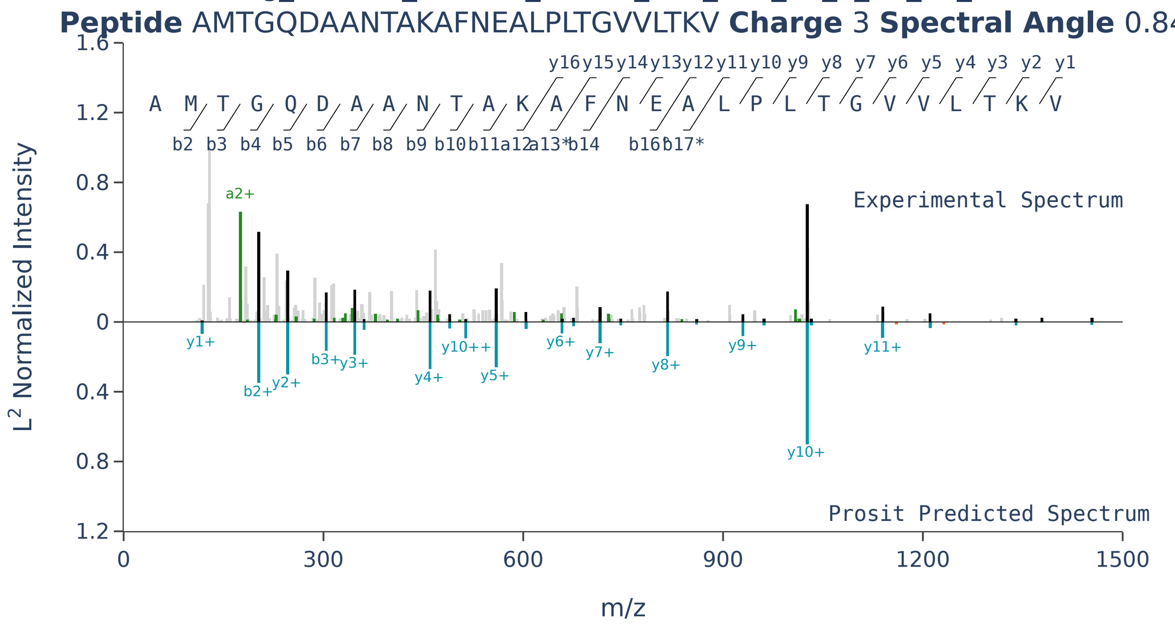


**Source** Ncheng_210623_230623_HFGoe_FFH_20S_25_1_A1_2h_R1 **Scan** 39018
**Peptide** ASRILGMGDVLSLIE **Charge** 2 **Spectral Angle** 0.83

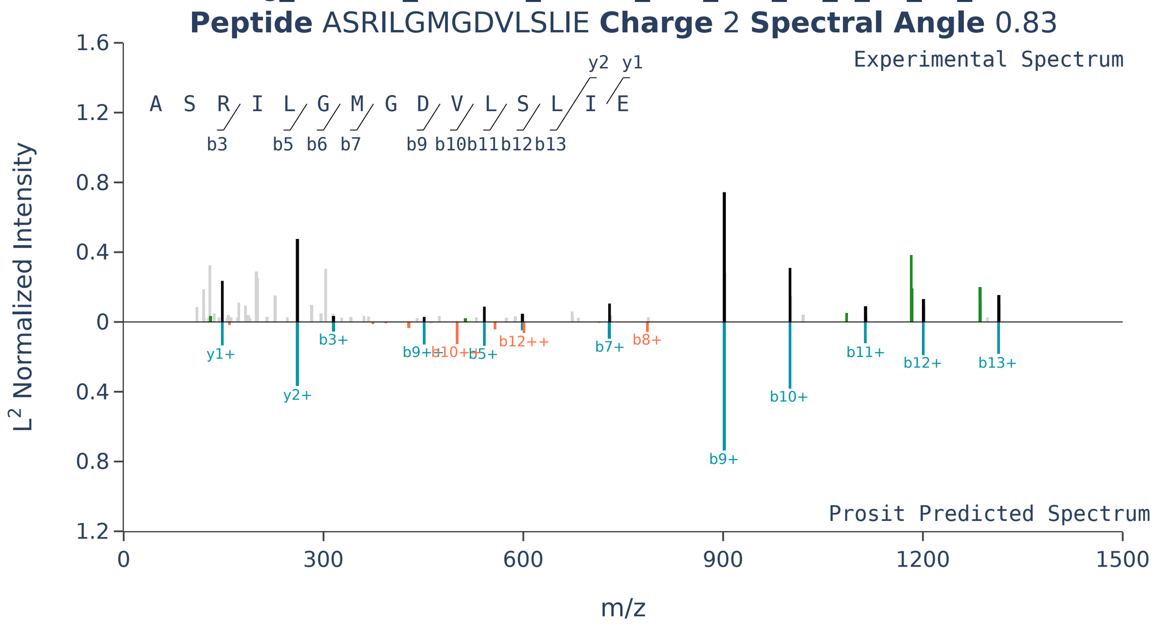

**Source** Ncheng_210623_230623_HFGoe_FFH_20S_25_1_A1_24h_R2 **Scan** 15252
**Peptide** VVSADVYRPAALKQ **Charge** 2 **Spectral Angle** 0.75

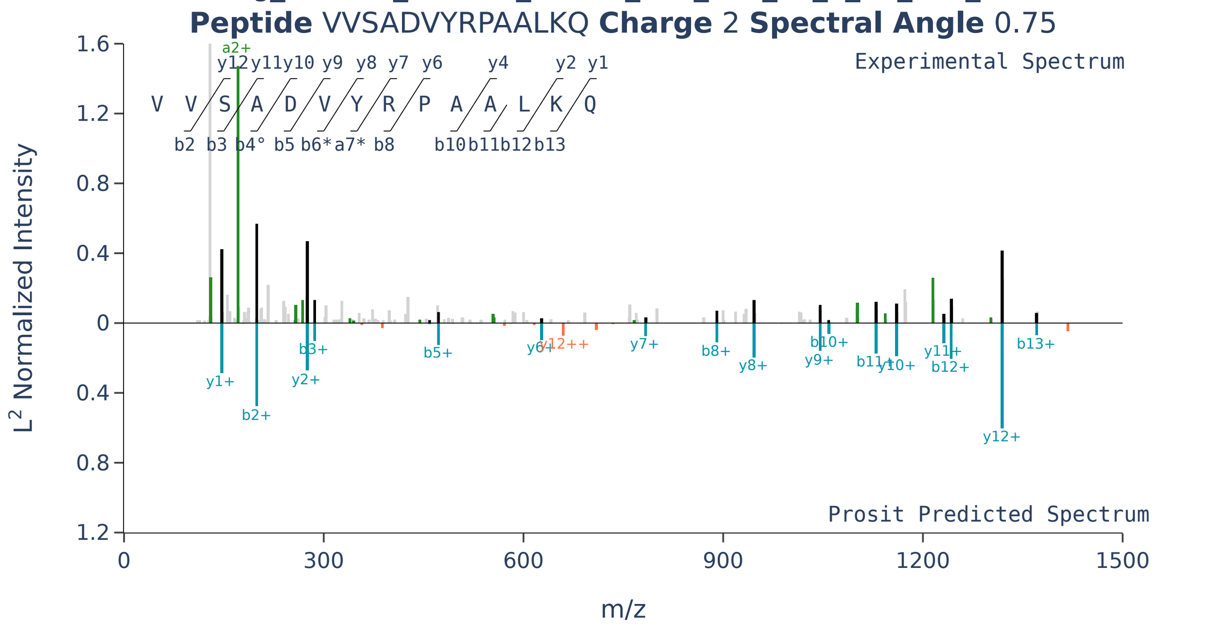


**Source** Ncheng_210623_230623_HFGoe_FFH_20S_25_1_A1_2h_R1 **Scan** 35867
**Peptide** GEENQTLNLAAQPPAVVLMA **Charge** 2 **Spectral Angle** 0.85

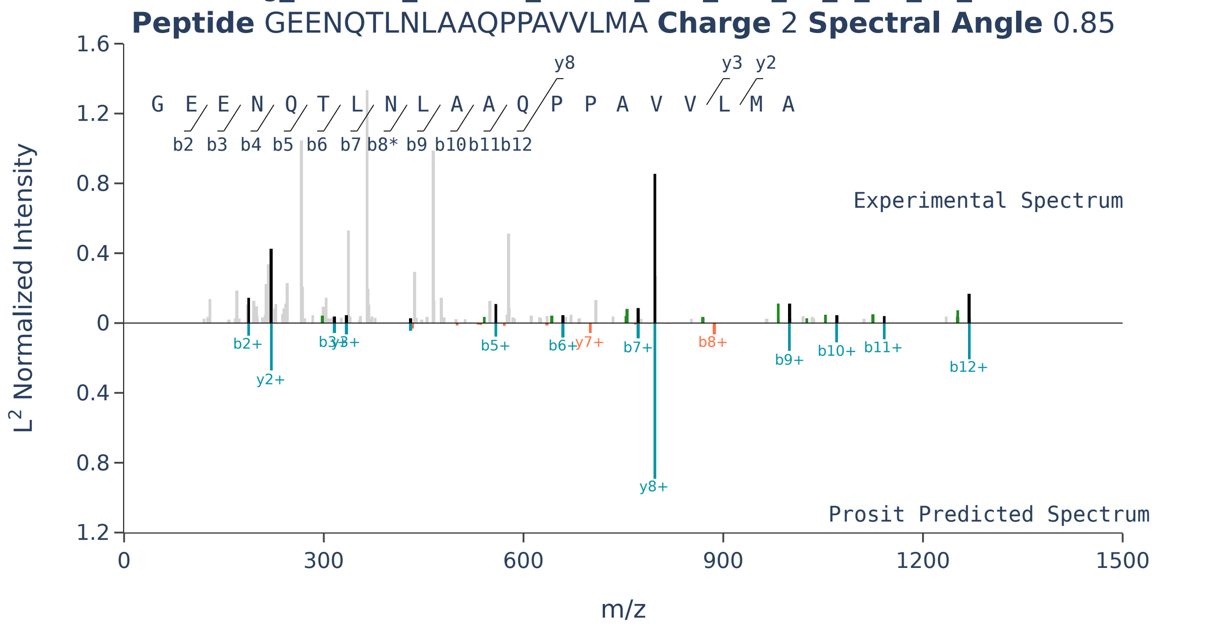


**Source** Ncheng_210623_230623_HFGoe_FFH_20S_25_1_A1_2h_R2 **Scan** 17314
**Peptide** ADVYRPAAIKQLE **Charge** 2 **Spectral Angle** 0.67

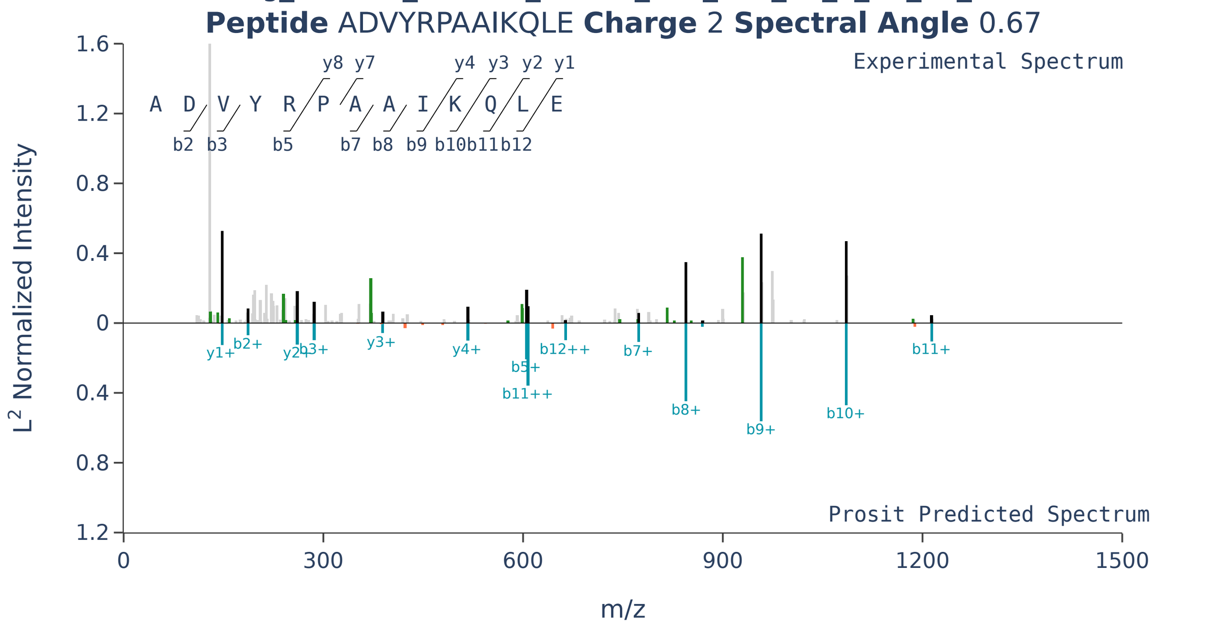


**Source** Ncheng_210623_230623_HFGoe_FFH_20S_25_1_A1_24h_R1 **Scan** 11737
**Peptide** AAGCGMQV **Charge** 1 **Spectral Angle** 0.84

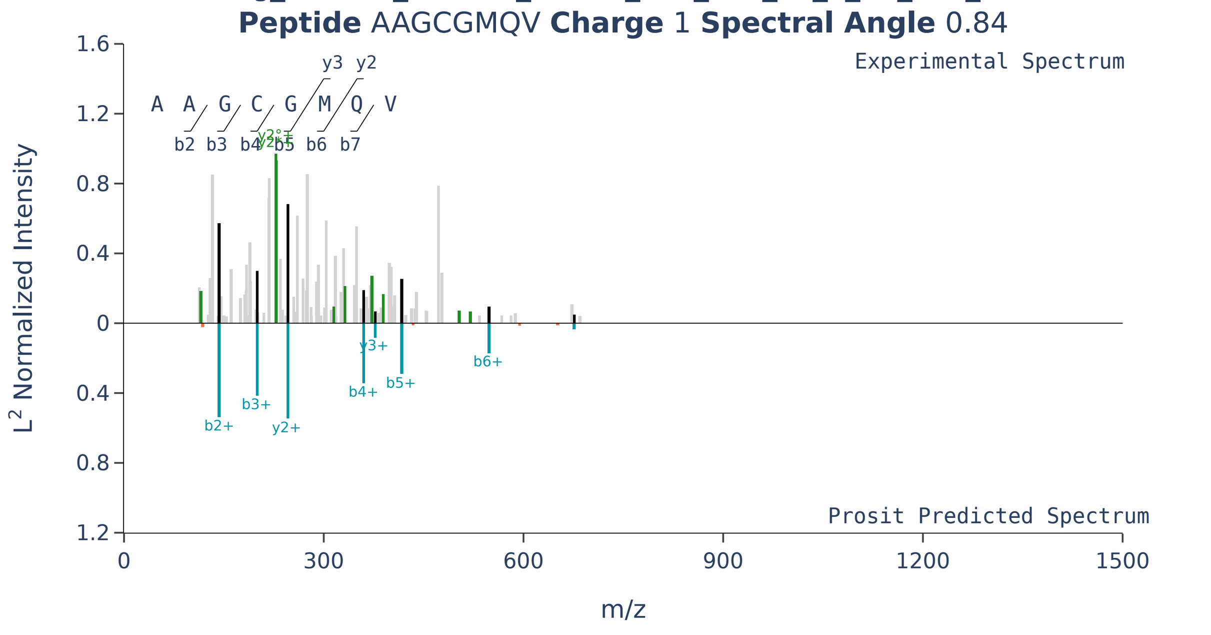


**Source** Ncheng_210623_230623_HFGoe_FFH_20S_25_1_A2_24h_R2 **Scan** 17299
**Peptide** HEVNKSLTPGQEFV **Charge** 2 **Spectral Angle** 0.83

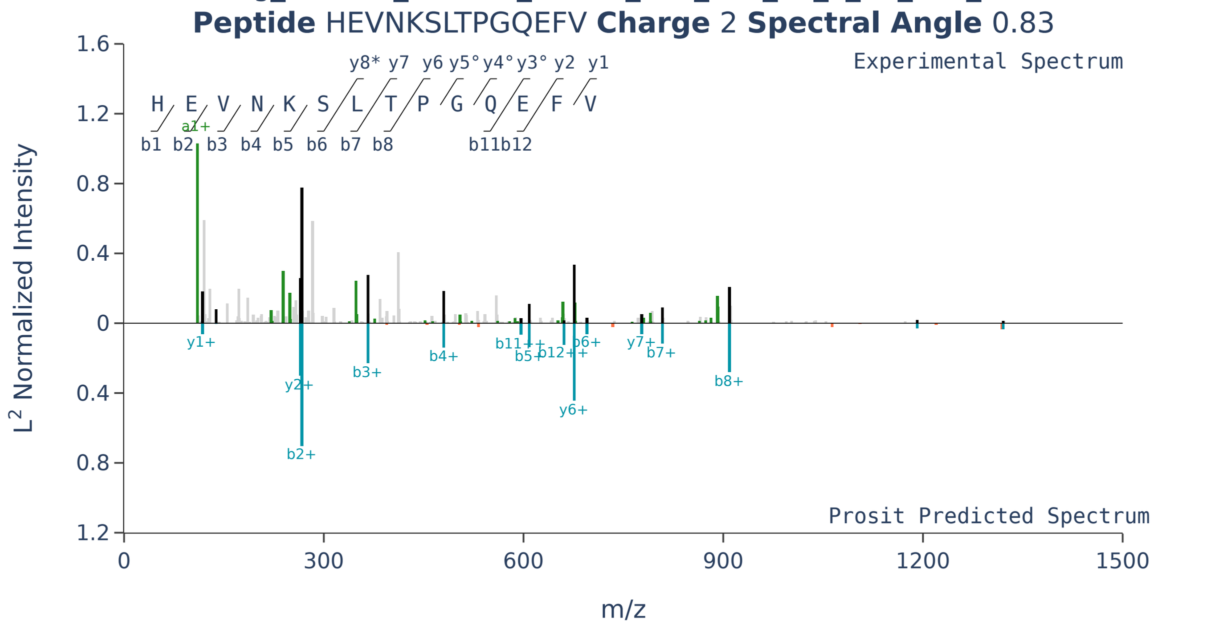


**Source** Ncheng_210623_230623_HFGoe_FFH_20S_25_1_A2_24h_R1 **Scan** 10454
**Peptide** VVLTKVDGDARGG **Charge** 2 **Spectral Angle** 0.74

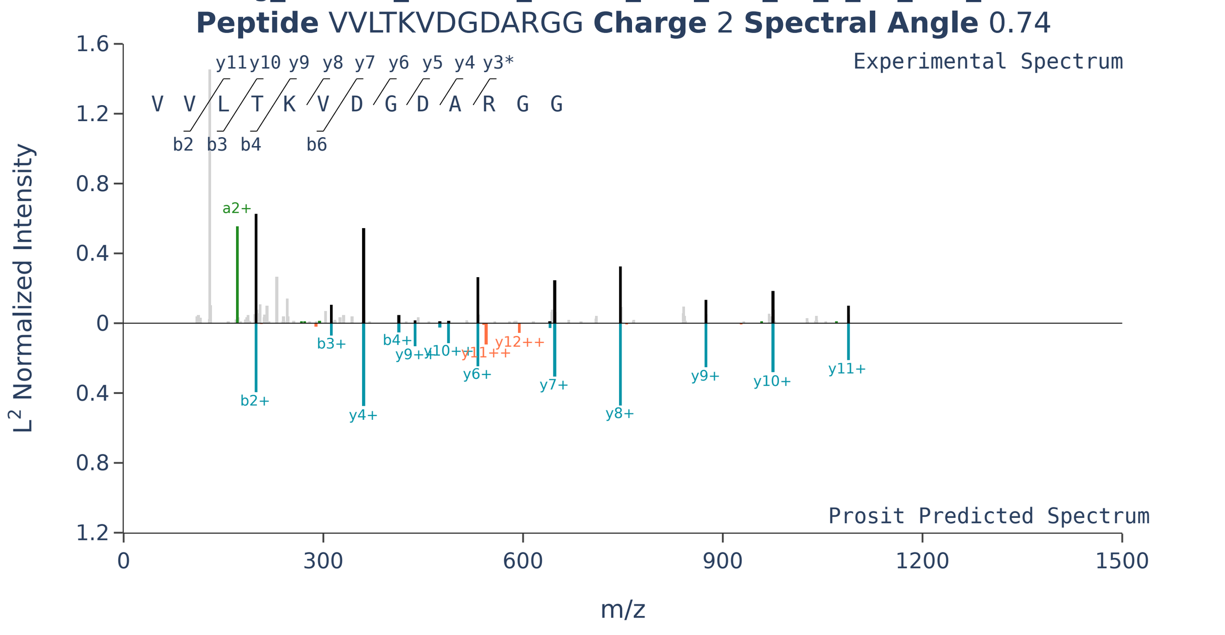


**Source** Ncheng_210623_230623_HFGoe_FFH_20S_25_1_A2_24h_R1 **Scan** 7460
**Peptide** KAVGHEVNKSLTPGQ **Charge** 2 **Spectral Angle** 0.85

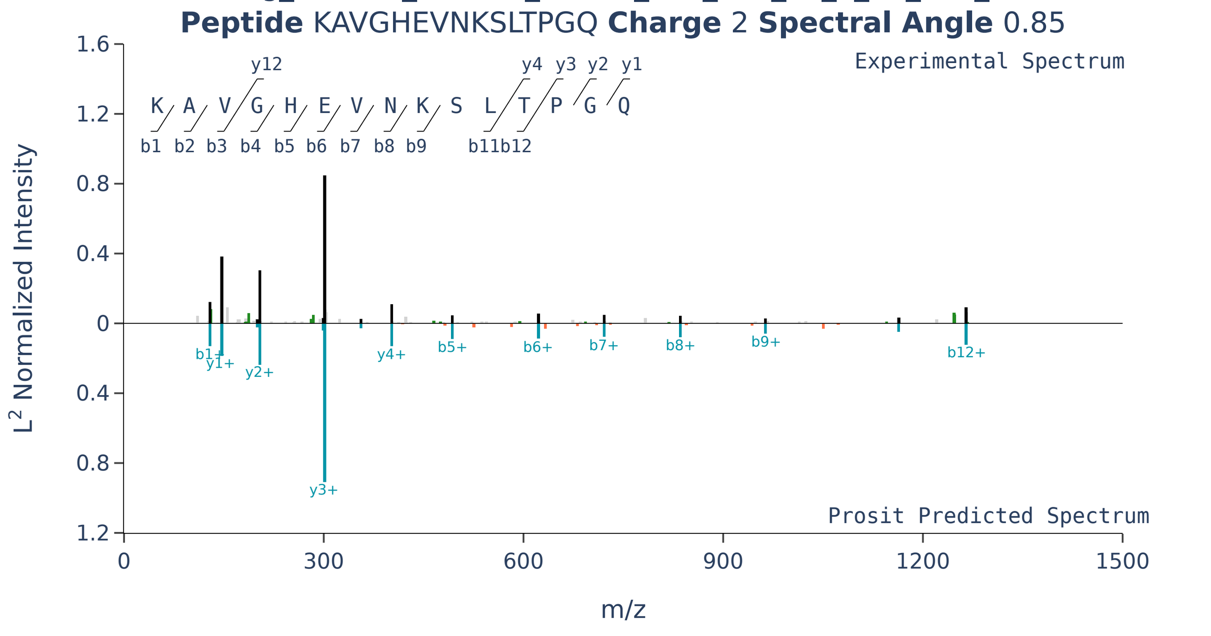


**Source** Ncheng_210623_230623_HFGoe_FFH_20S_25_1_A2_24h_R2 **Scan** 16795
**Peptide** DVYRPAAI **Charge** 1 **Spectral Angle** 0.76

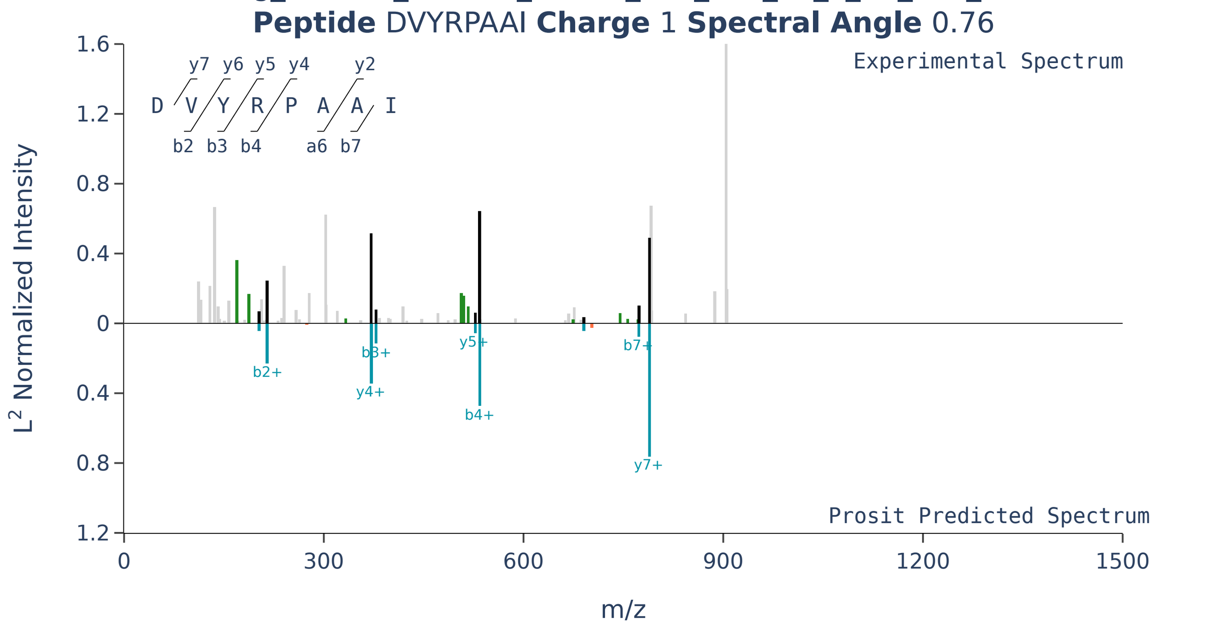


**Source** Ncheng_210623_230623_HFGoe_FFH_20S_25_1_A1_24h_R2 **Scan** 20238
**Peptide** EVNKSLTPGQEFV **Charge** 2 **Spectral Angle** 0.73

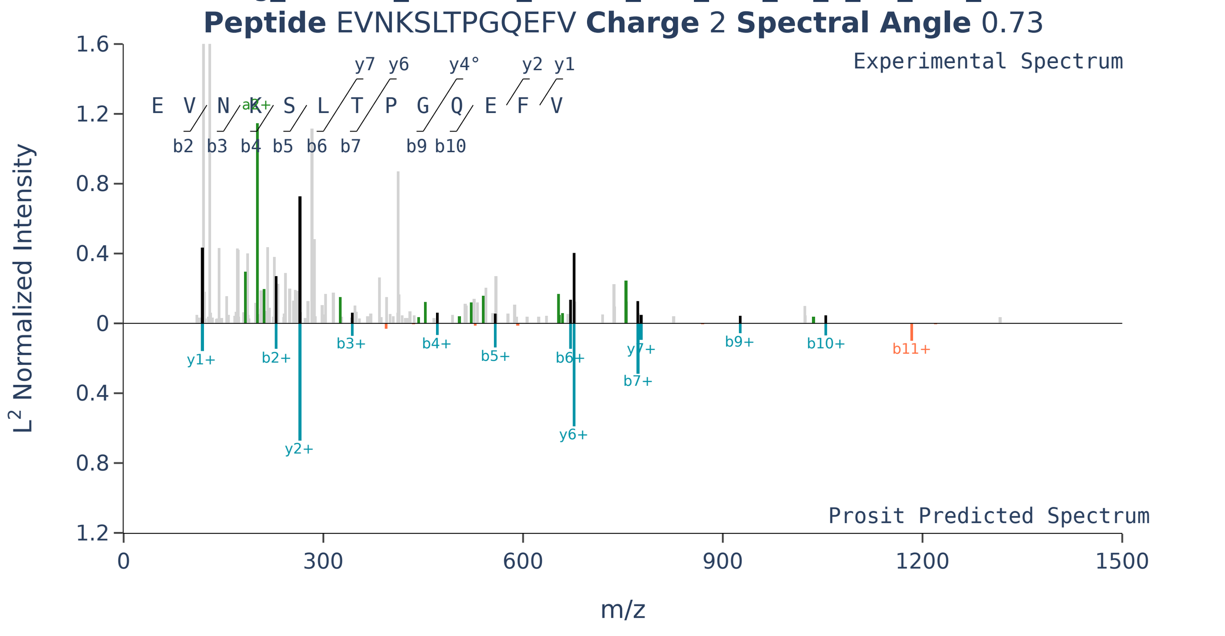


**Source** Ncheng_210623_230623_HFGoe_FFH_20S_25_1_A2_24h_R2 **Scan** 28328
**Peptide** AQPPAVVLM **Charge** 2 **Spectral Angle** 0.78

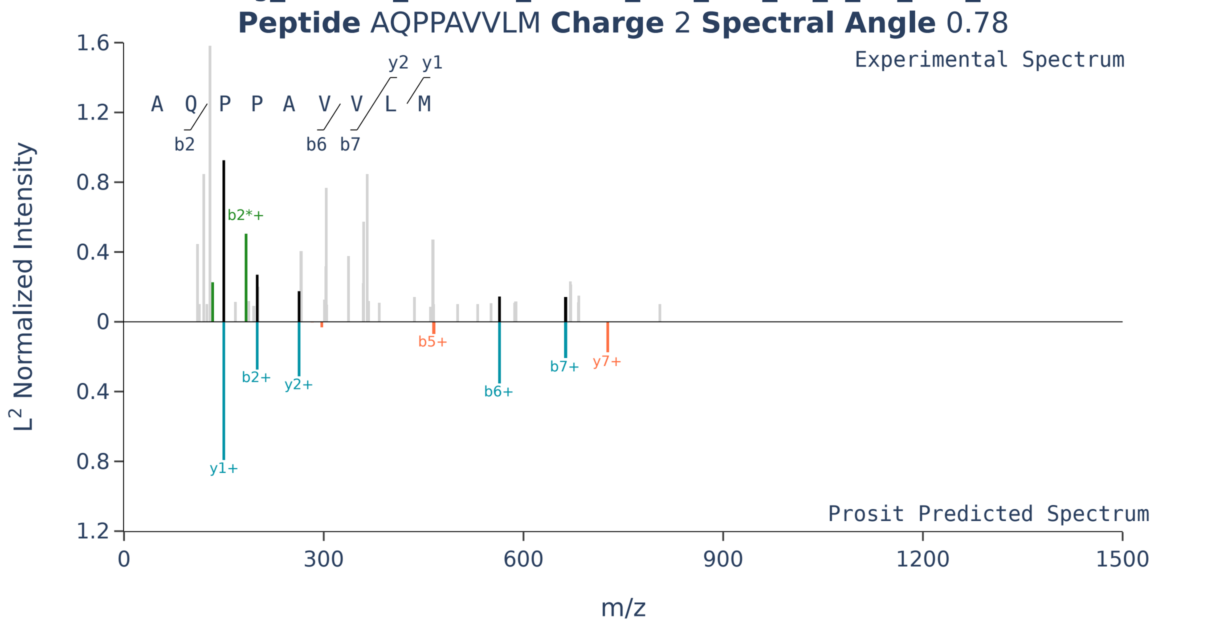


**Source** Ncheng_210623_230623_HFGoe_FFH_20S_25_1_A2_2h_R1 **Scan** 18131
**Peptide** RPAAIKQLETL **Charge** 3 **Spectral Angle** 0.76

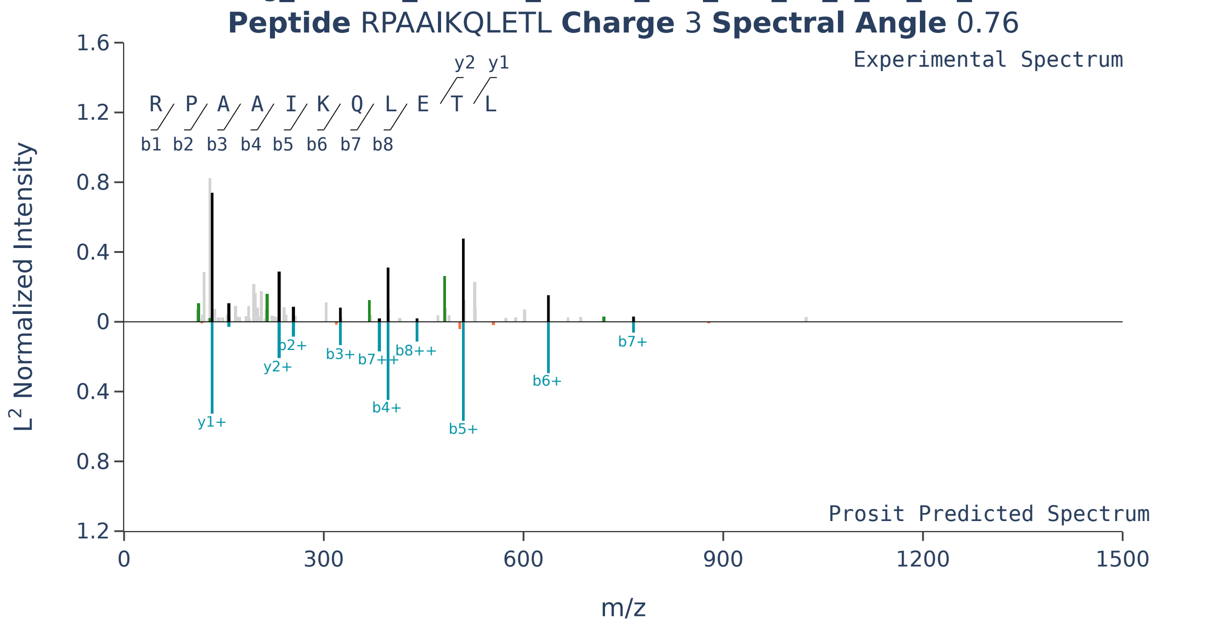


**Source** Ncheng_210623_230623_HFGoe_FFH_20S_25_1_A2_4h_R2 **Scan** 29118
**Peptide** AANTAKAFNEALPLTGV **Charge** 2 **Spectral Angle** 0.84

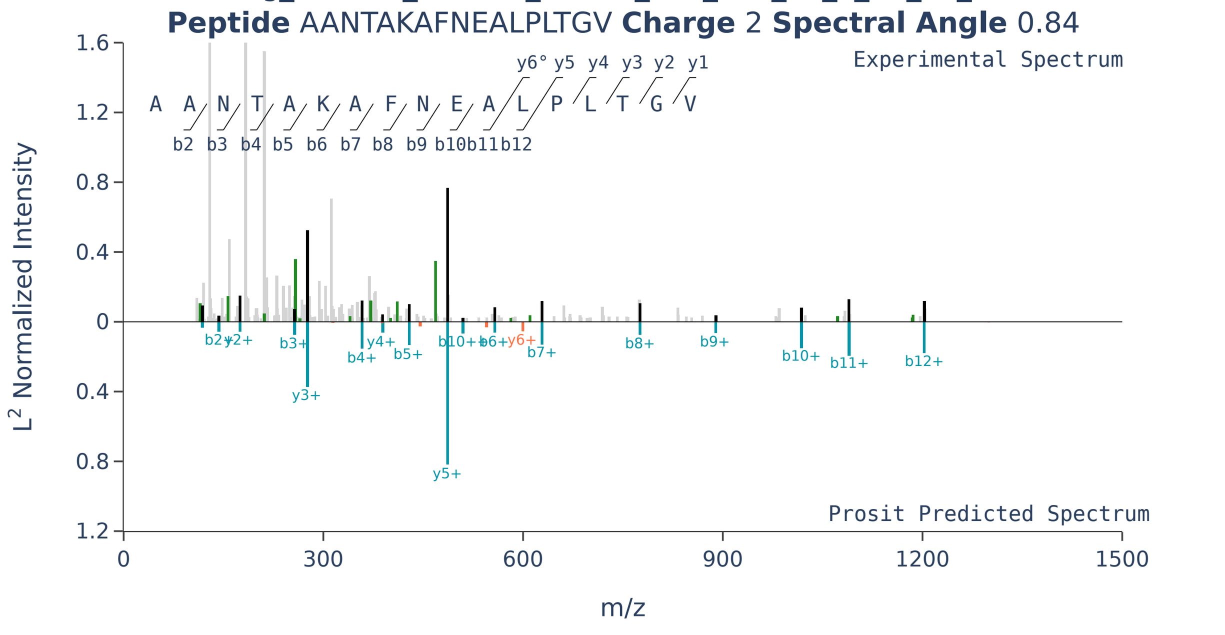


**Source** Ncheng_210623_230623_HFGoe_FFH_20S_25_1_A1_2h_R2 **Scan** 19940
**Peptide** TGKPIKFLGVGE **Charge** 1 **Spectral Angle** 0.75

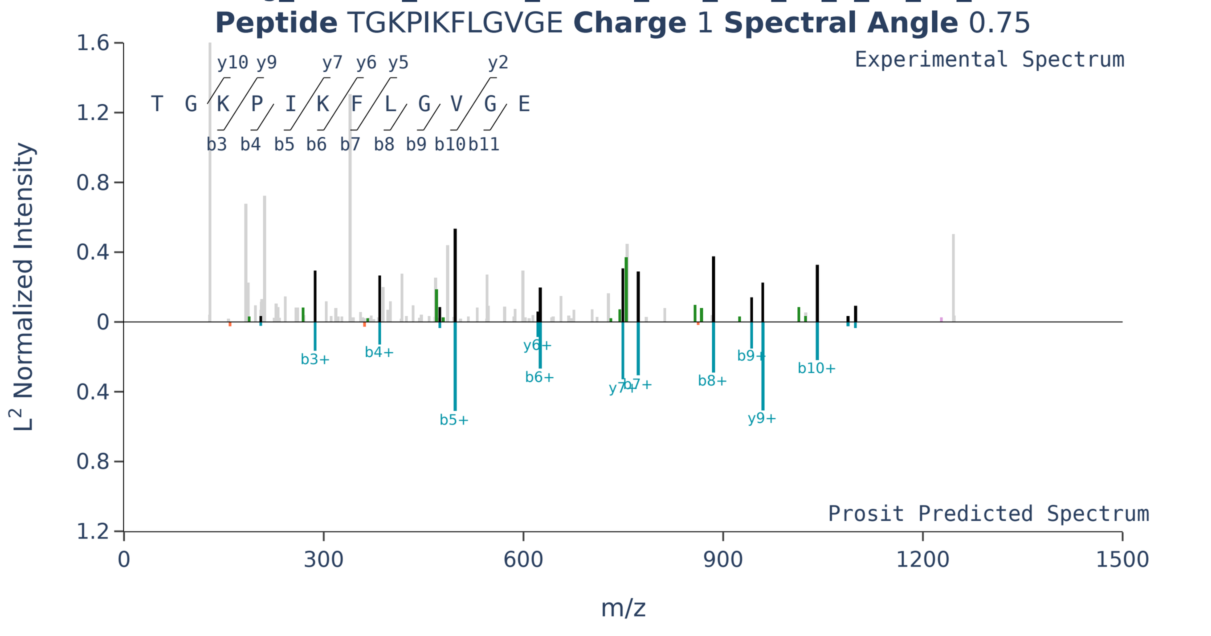


**Source** Ncheng_210623_230623_HFGoe_FFH_20S_25_1_A1_24h_R1 **Scan** 6881
**Peptide** DLESKVDR **Charge** 2 **Spectral Angle** 0.78

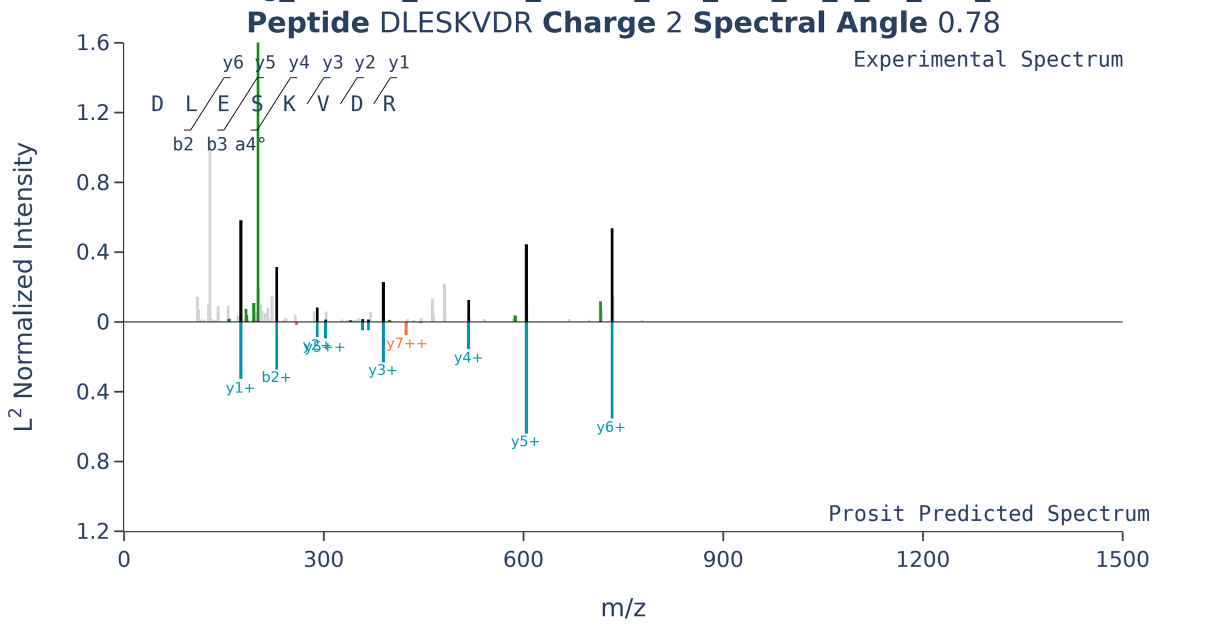


**Source** Ncheng_210623_230623_HFGoe_FFH_20S_25_1_A1_24h_R1 **Scan** 23117
**Peptide** VLVVSADVYRPA **Charge** 2 **Spectral Angle** 0.82

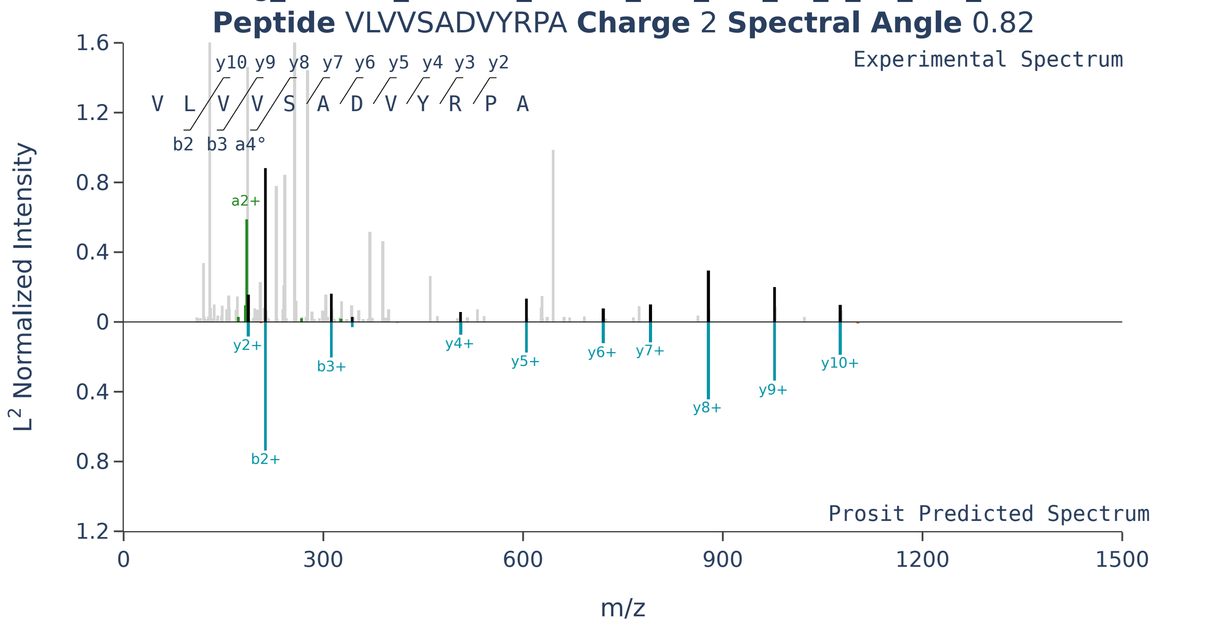

**Source** Ncheng_210623_230623_HFGoe_FFH_20S_25_1_A2_2h_R1 **Scan** 31171
**Peptide** HEVNKSLTPGQEFVKIVRNELVAAM **Charge** 3 **Spectral Angle** 0.89

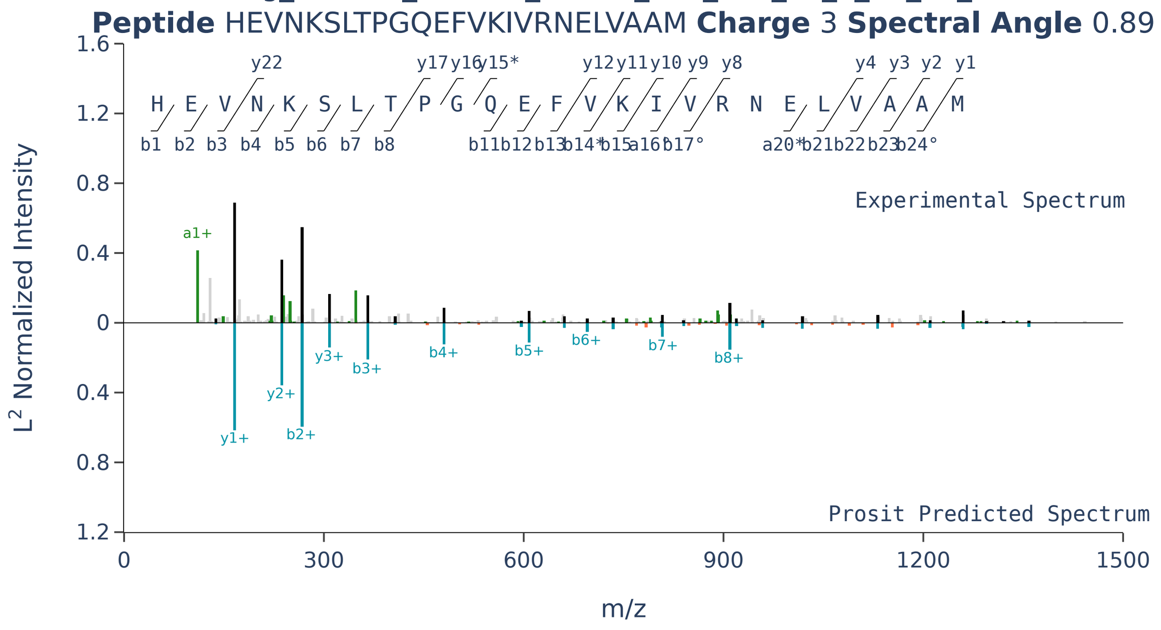


**Source** Ncheng_210623_230623_HFGoe_FFH_20S_25_1_A1_2h_R1 **Scan** 11621
**Peptide** PPGFPGR **Charge** 2 **Spectral Angle** 0.86

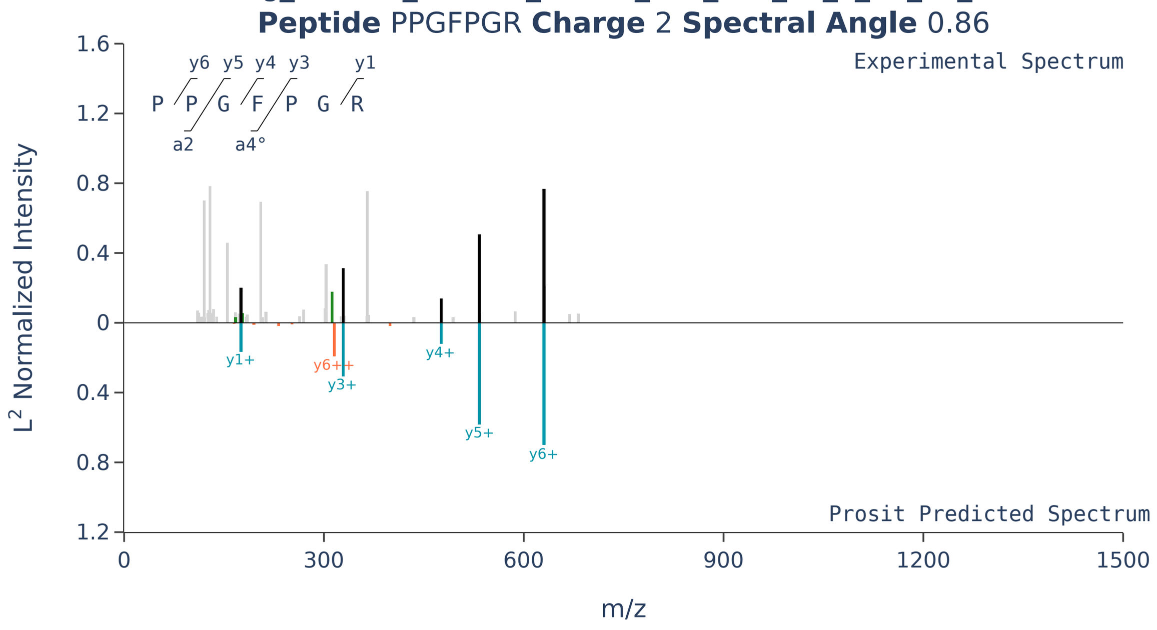


**Source** Ncheng_210623_230623_HFGoe_FFH_20S_25_1_A2_24h_R2 **Scan** 17588
**Peptide** KEKAVGHEVNKSLTPGQEFVKL **Charge** 3 **Spectral Angle** 0.92

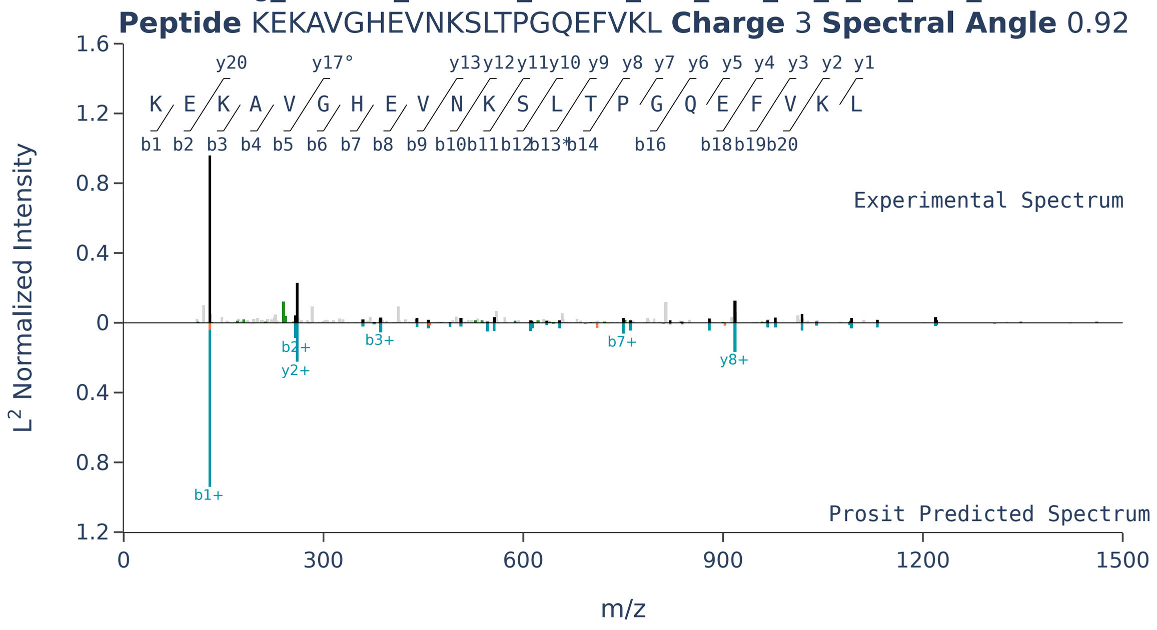


**Source** Ncheng_210623_230623_HFGoe_FFH_20S_25_1_A1_24h_R2 **Scan** 6958
**Peptide** HEVNKSLTPGQ **Charge** 1 **Spectral Angle** 0.83

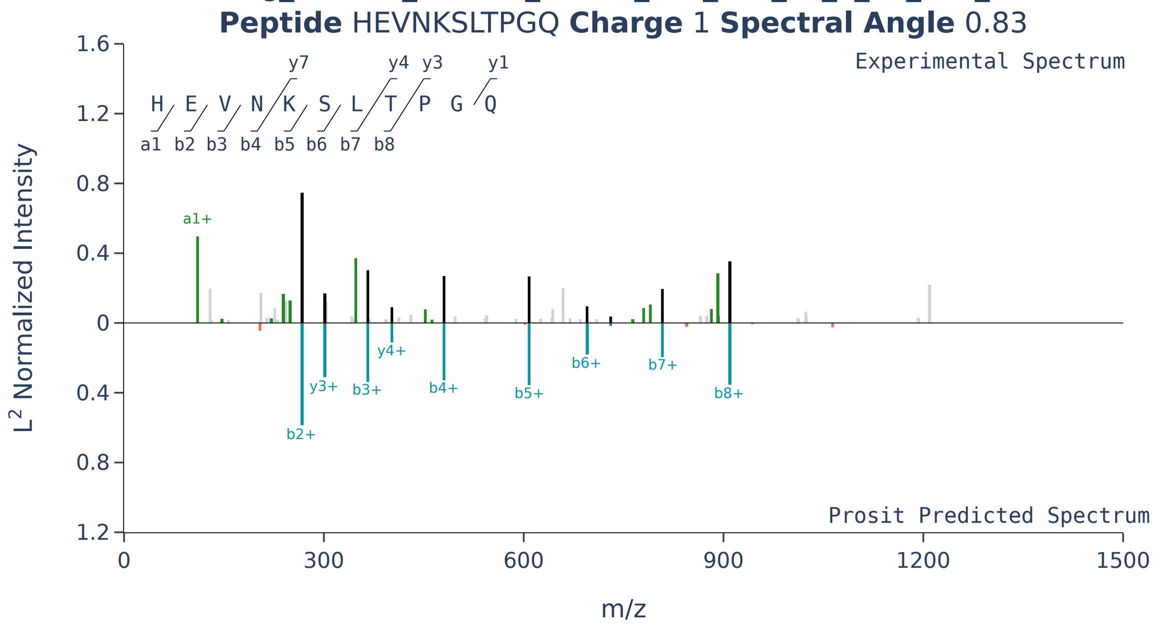


**Source** Ncheng_210623_230623_HFGoe_FFH_20S_25_1_A2_1h_R2 **Scan** 19300
**Peptide** VVSADVYRPAAL **Charge** 2 **Spectral Angle** 0.76

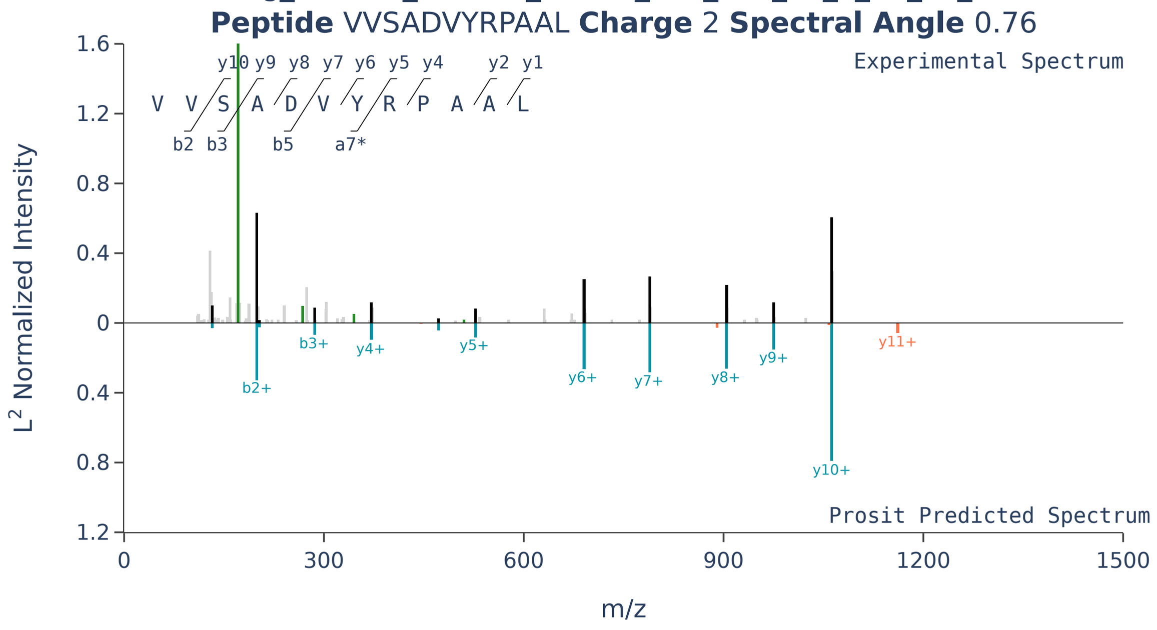


**Source** Ncheng_210623_230623_HFGoe_FFH_20S_25_1_A2_2h_R2 **Scan** 13666
**Peptide** LGVGEKTEALE **Charge** 2 **Spectral Angle** 0.78

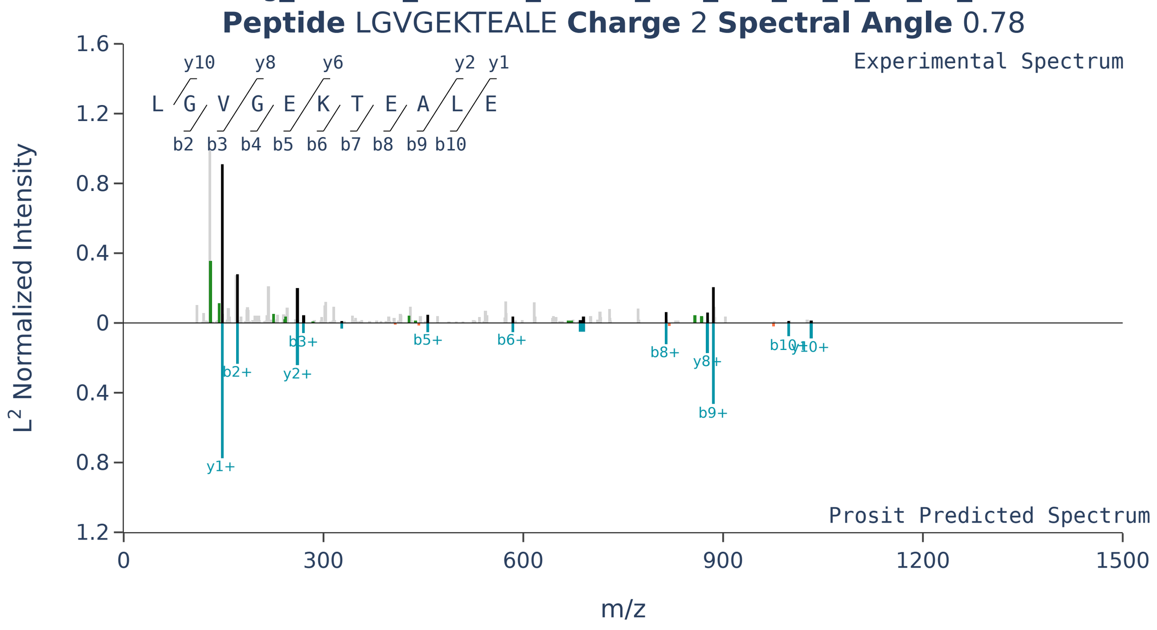


**Source** Ncheng_210623_230623_HFGoe_FFH_20S_25_1_A1_2h_R1 **Scan** 27786
**Peptide** YDVLLVDTAGRLHVDE **Charge** 2 **Spectral Angle** 0.87

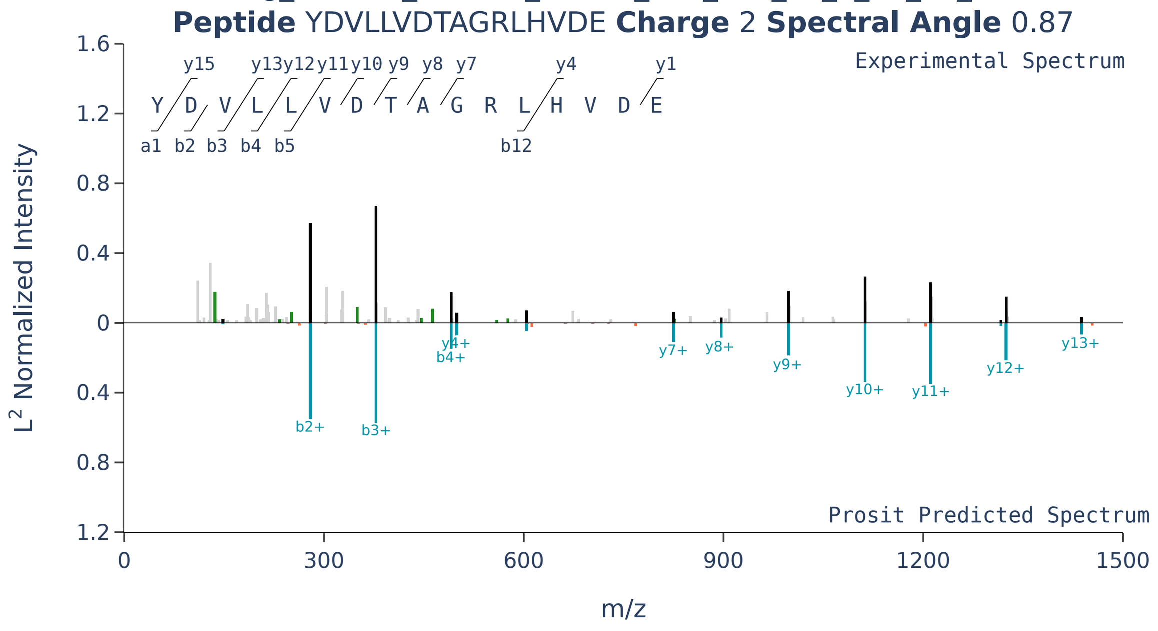


**Source** Ncheng_210623_230623_HFGoe_FFH_20S_25_1_A1_2h_R2 **Scan** 10733
**Peptide** DVYRPAAIKQ **Charge** 2 **Spectral Angle** 0.71

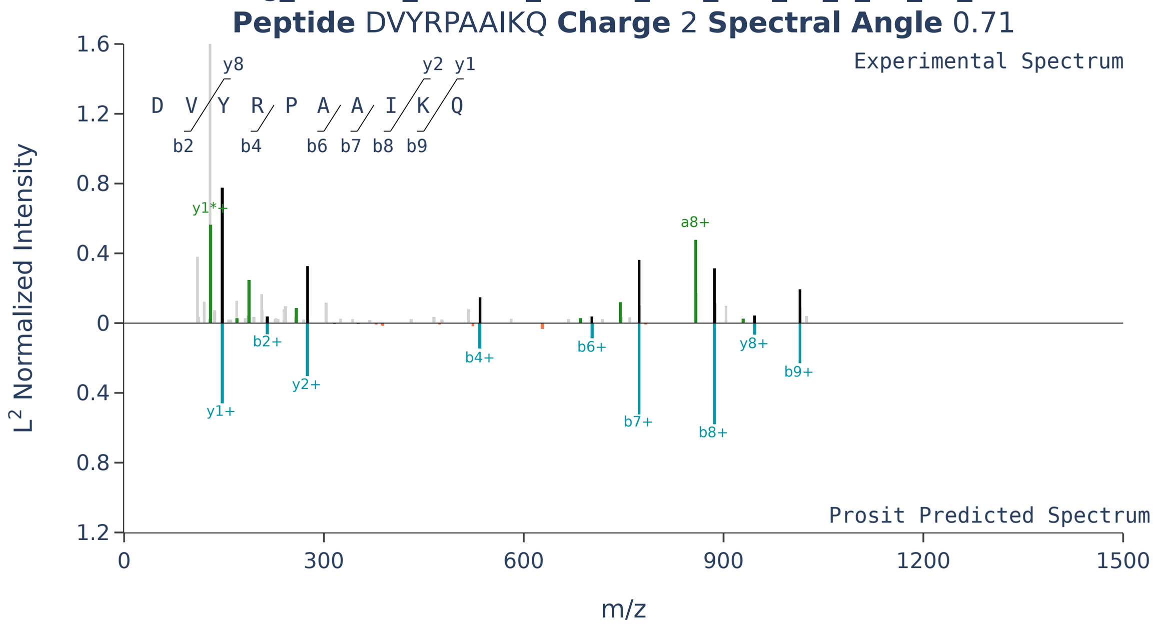


**Source** Ncheng_210623_230623_HFGoe_FFH_20S_25_1_A2_2h_R2 **Scan** 13593
**Peptide** ELKQVHASINPVE **Charge** 1 **Spectral Angle** 0.78

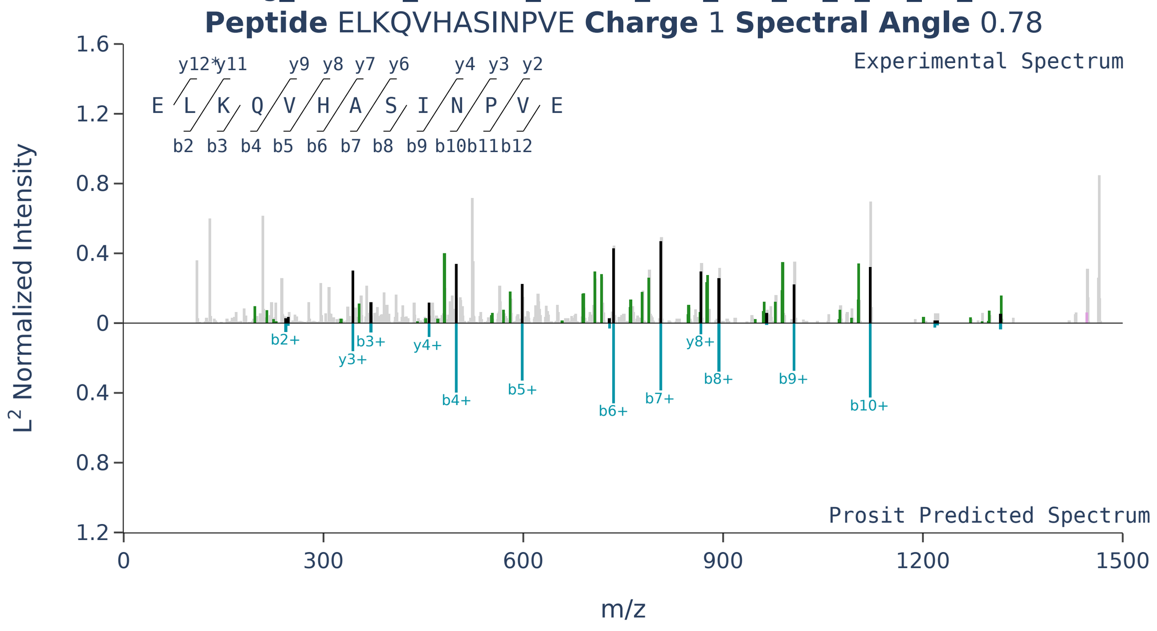


**Source** Ncheng_210623_230623_HFGoe_FFH_20S_25_1_A1_4h_R1 **Scan** 17028
**Peptide** GVGEKTEALEPFHPDRIASR **Charge** 5 **Spectral Angle** 0.85

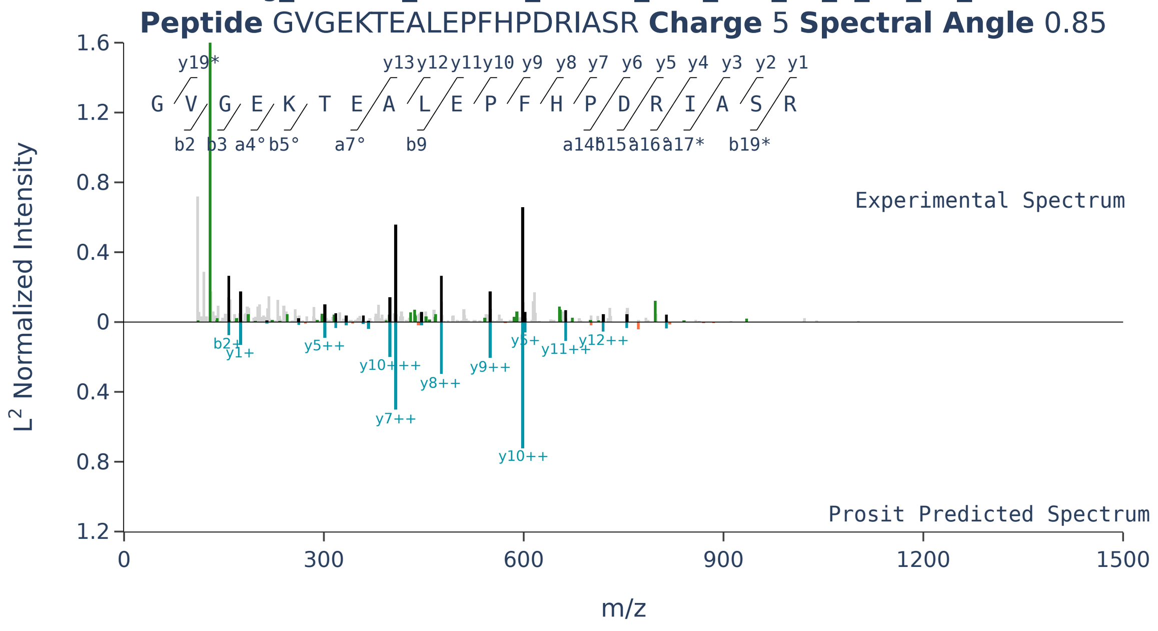


**Source** Ncheng_210623_230623_HFGoe_FFH_20S_25_1_A2_24h_R2 **Scan** 13244
**Peptide** YRPAAIKQLE **Charge** 3 **Spectral Angle** 0.69

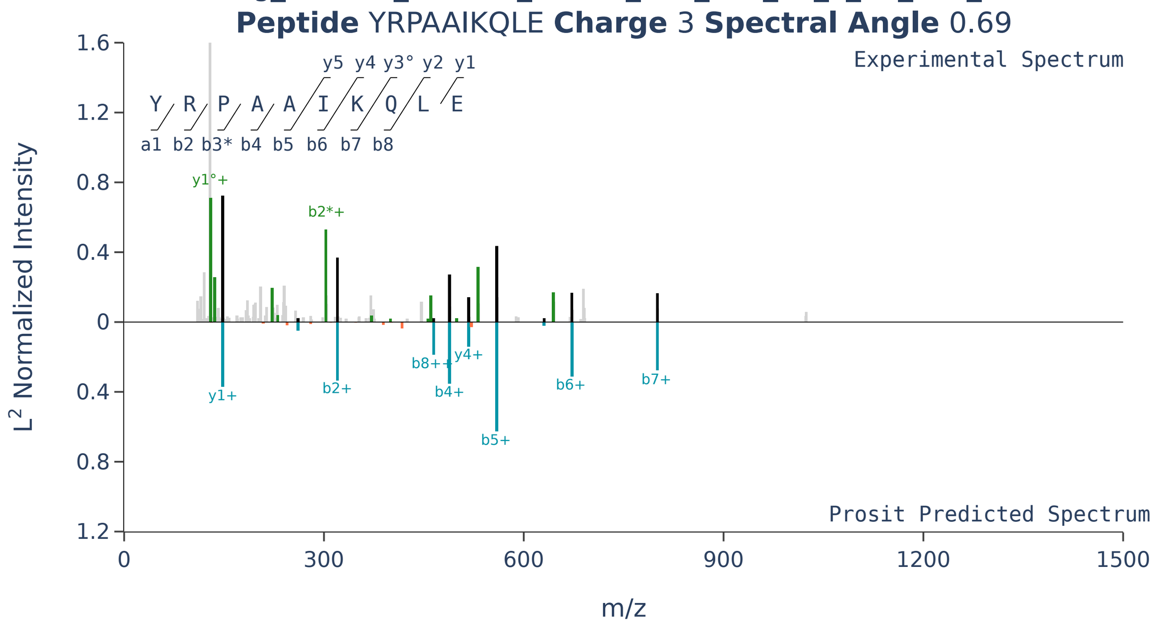


**Source** Ncheng_210623_230623_HFGoe_FFH_20S_25_1_A2_24h_R1 **Scan** 14132
**Peptide** KGDGFDLND **Charge** 2 **Spectral Angle** 0.77

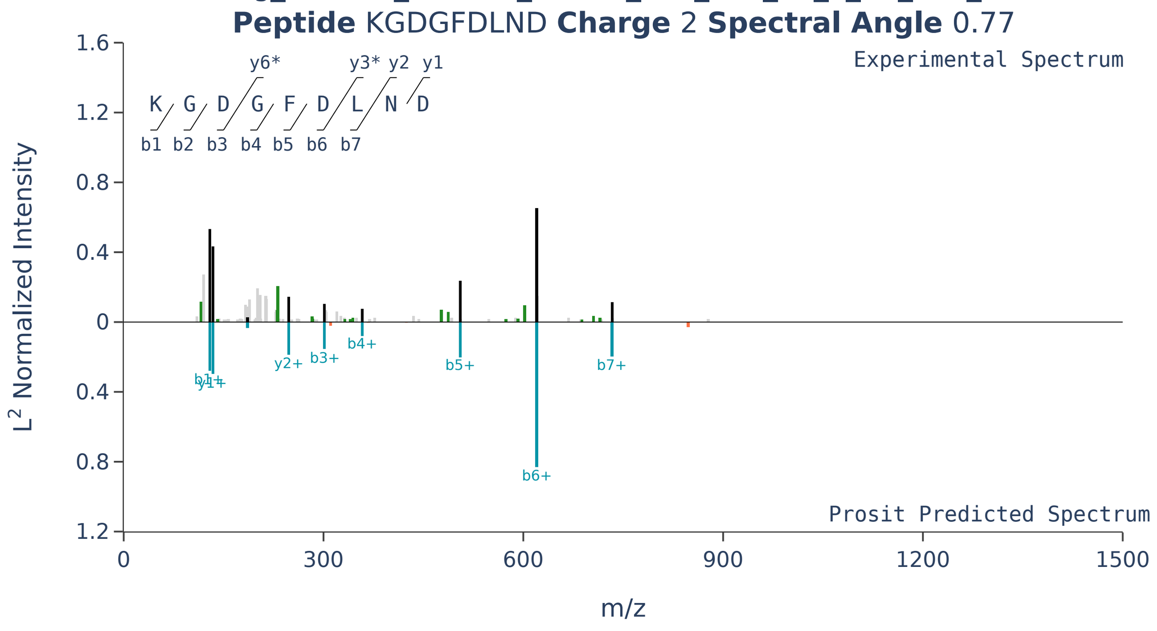


**Source** Ncheng_210623_230623_HFGoe_FFH_20S_25_1_A1_4h_R2 **Scan** 15033
**Peptide** KSQMDDKVLVRME **Charge** 4 **Spectral Angle** 0.83

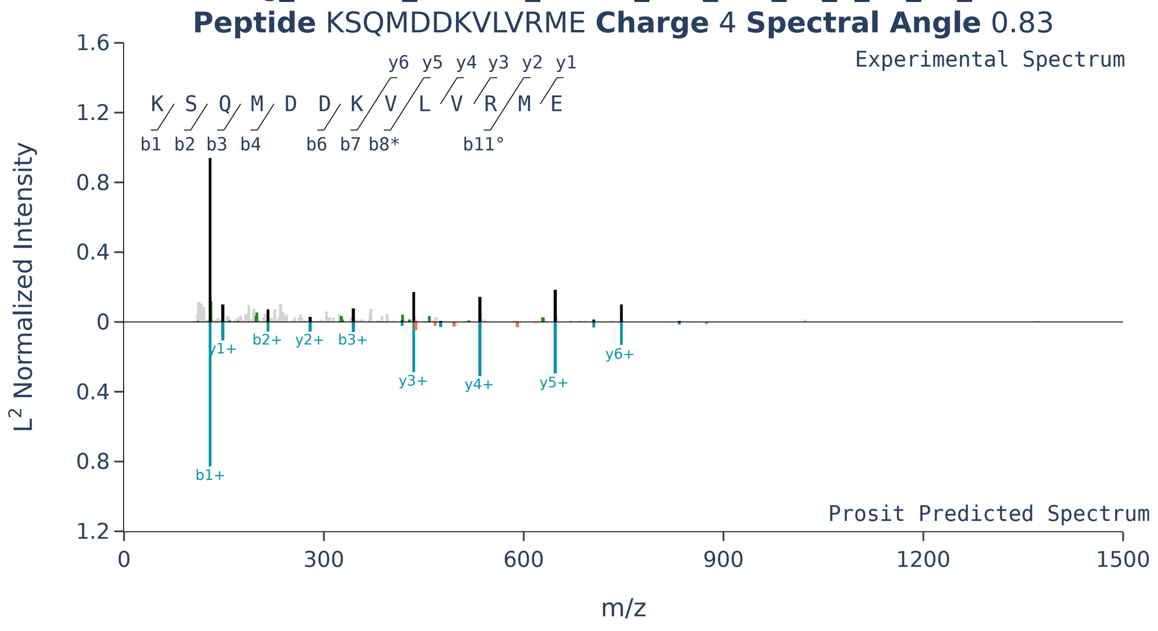


**Source** Ncheng_210623_230623_HFGoe_FFH_20S_25_1_A2_24h_R2 **Scan** 16804
**Peptide** ADVYRPAAL **Charge** 2 **Spectral Angle** 0.74

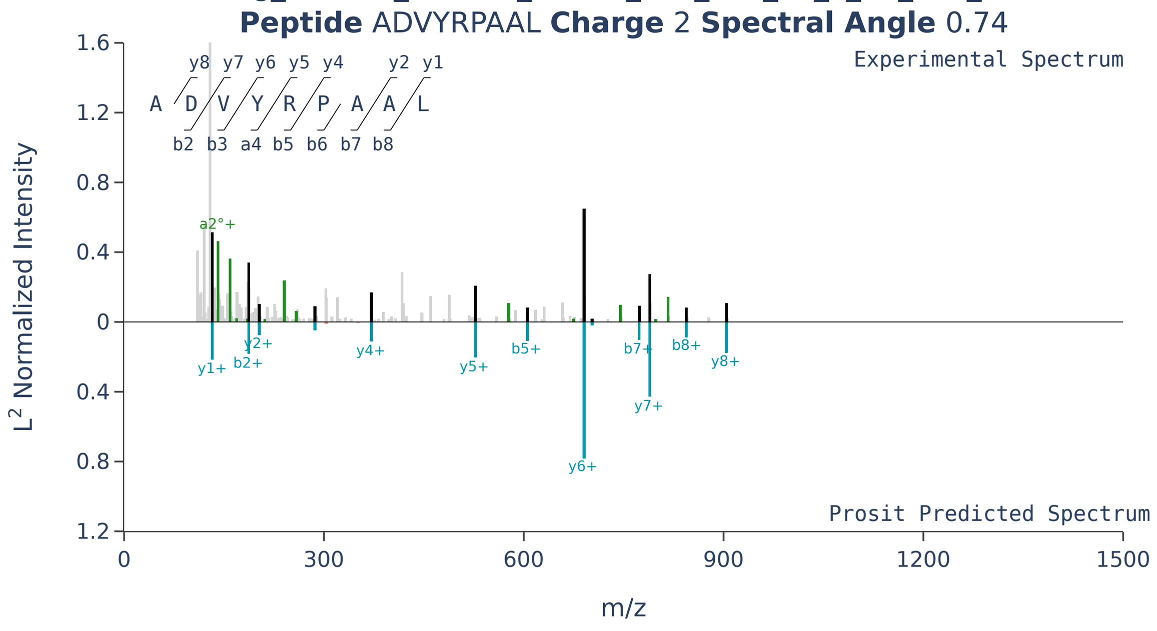


**Source** Ncheng_210623_230623_HFGoe_FFH_20S_25_1_A2_2h_R1 **Scan** 31687
**Peptide** EALPLTGVVLTKVDGDARGGAAL **Charge** 3 **Spectral Angle** 0.72

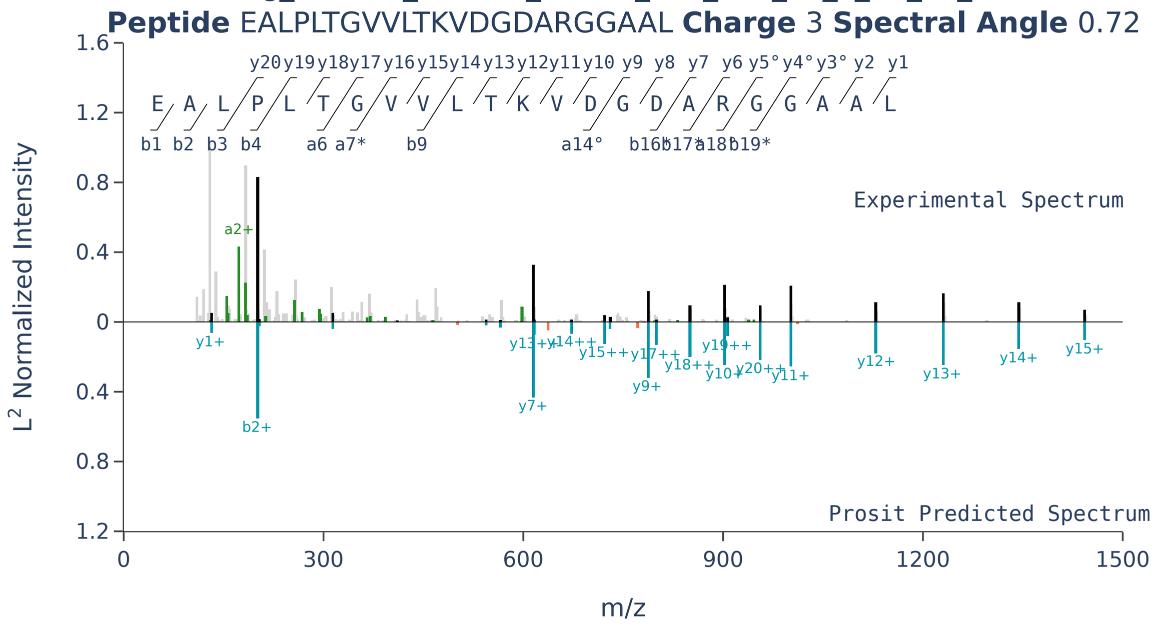

**Source** Ncheng_210623_230623_HFGoe_FFH_20S_25_1_A2_2h_R2 **Scan** 27868
**Peptide** HEVNKSLTPGQEFVKIVRNEL **Charge** 2 **Spectral Angle** 0.78

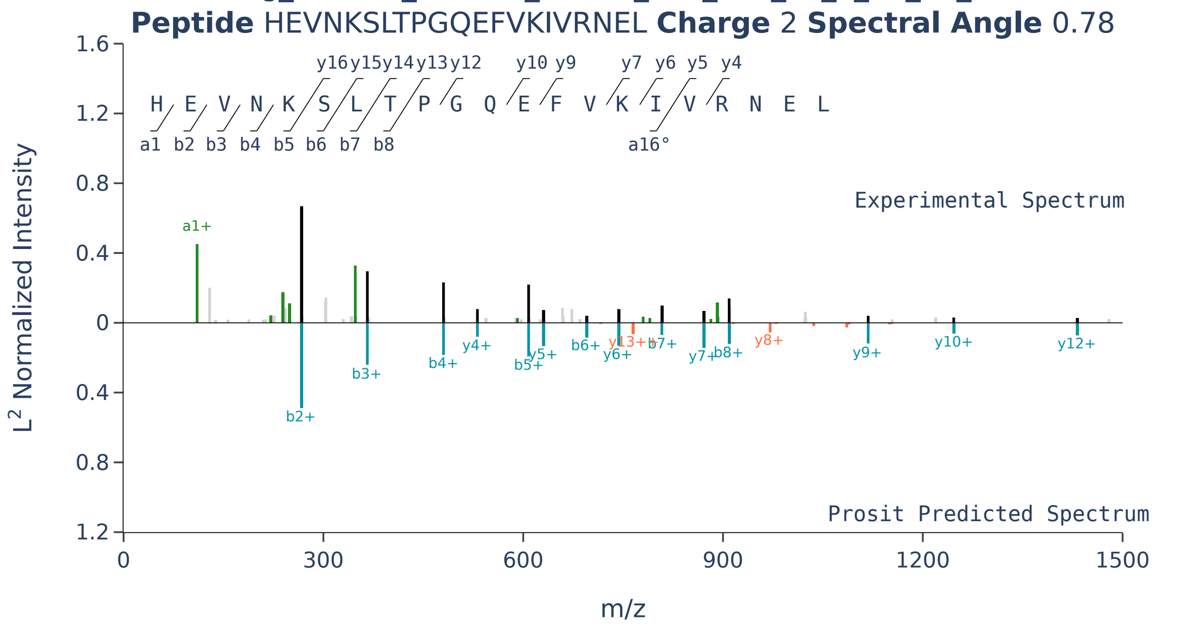


**Source** Ncheng_210623_230623_HFGoe_FFH_20S_25_1_A2_4h_R2 **Scan** 8155
**Peptide** AGCGMQVQ **Charge** 2 **Spectral Angle** 0.91

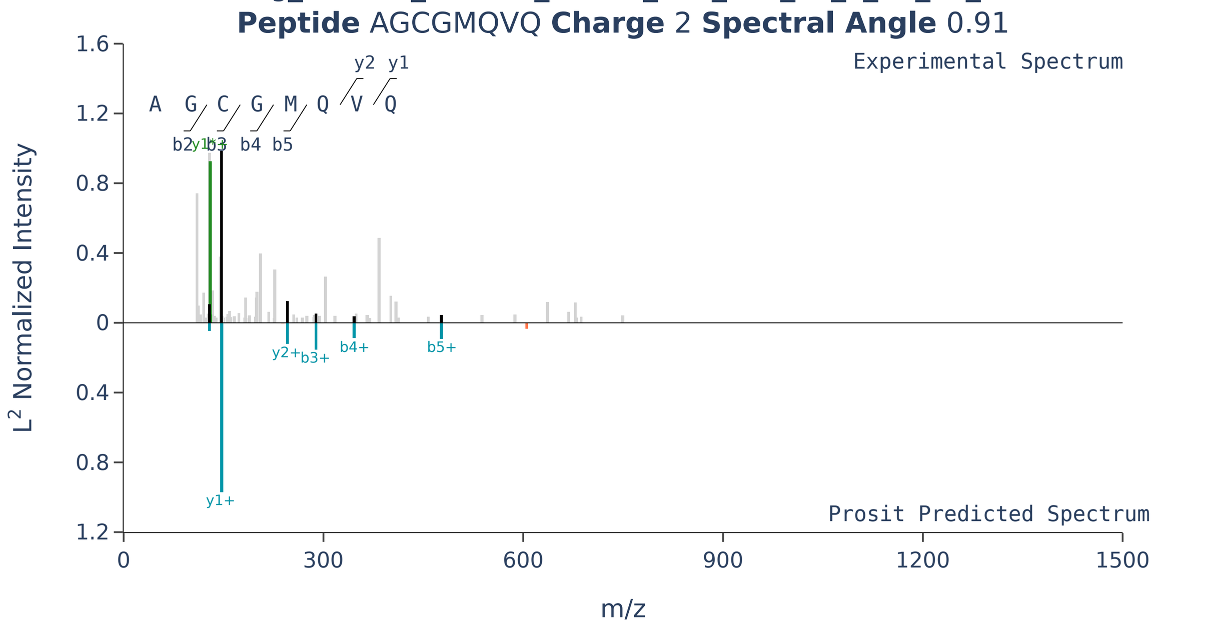


**Source** Ncheng_210623_230623_HFGoe_FFH_20S_25_1_A2_4h_R1 **Scan** 16758
**Peptide** EALEPFHPDRIASR **Charge** 3 **Spectral Angle** 0.83

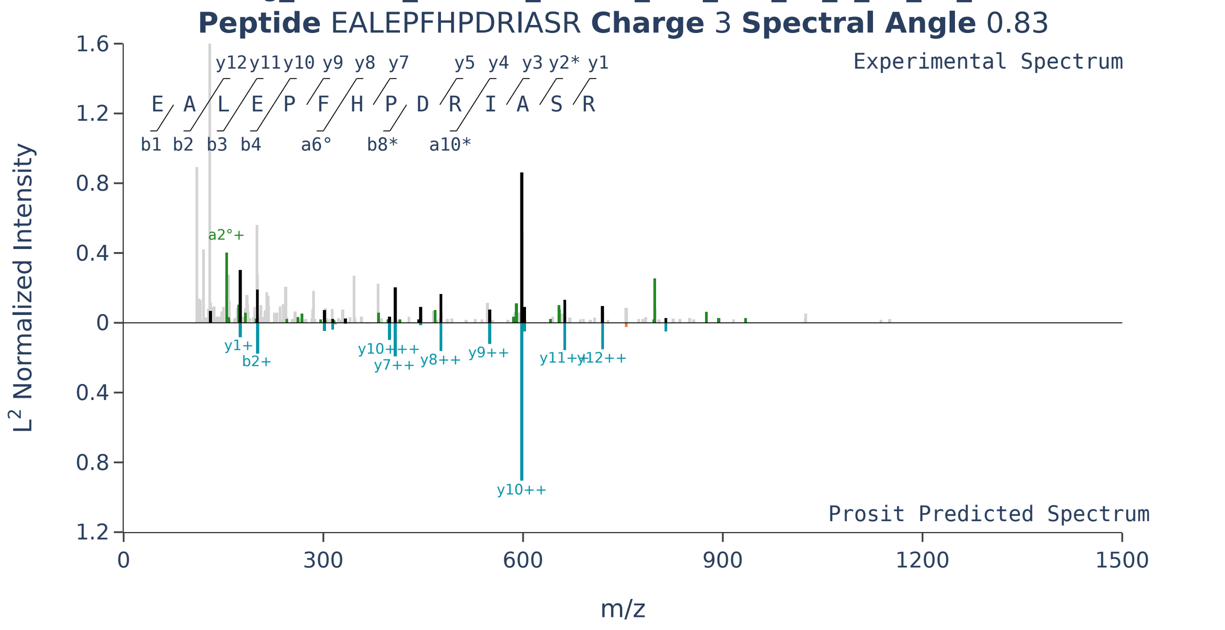


**Source** Ncheng_210623_230623_HFGoe_FFH_20S_25_1_A1_24h_R2 **Scan** 13334
**Peptide** GMGQIPDNVKS **Charge** 2 **Spectral Angle** 0.74

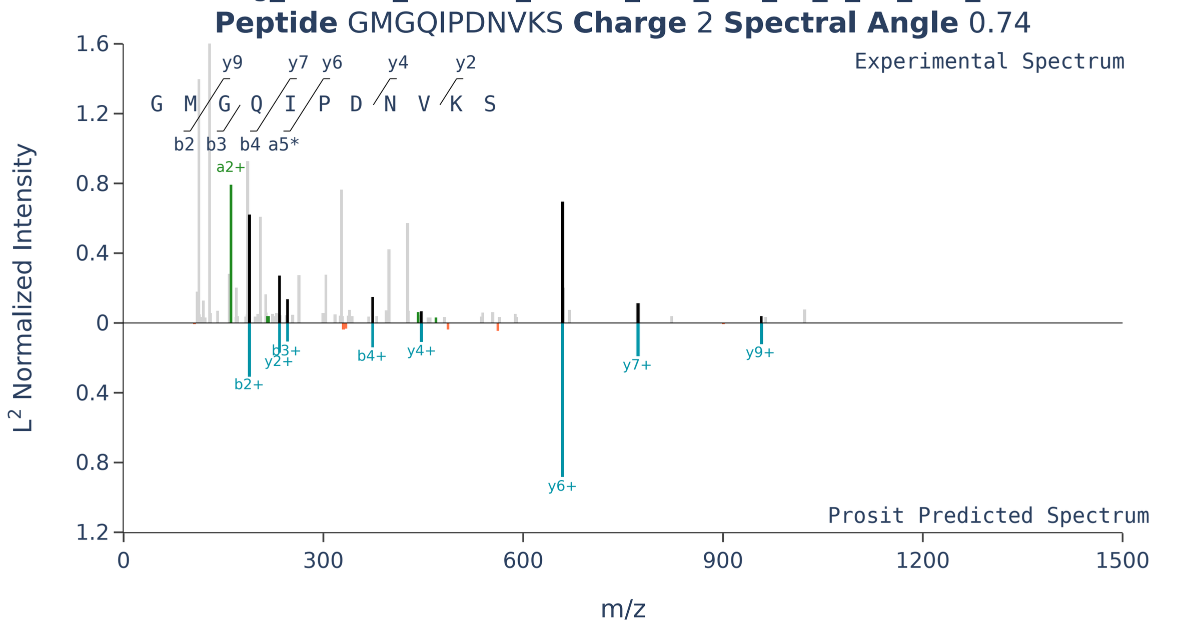


**Source** Ncheng_210623_230623_HFGoe_FFH_20S_25_1_A1_2h_R2 **Scan** 11432
**Peptide** SADVYRPAALKQ **Charge** 2 **Spectral Angle** 0.71

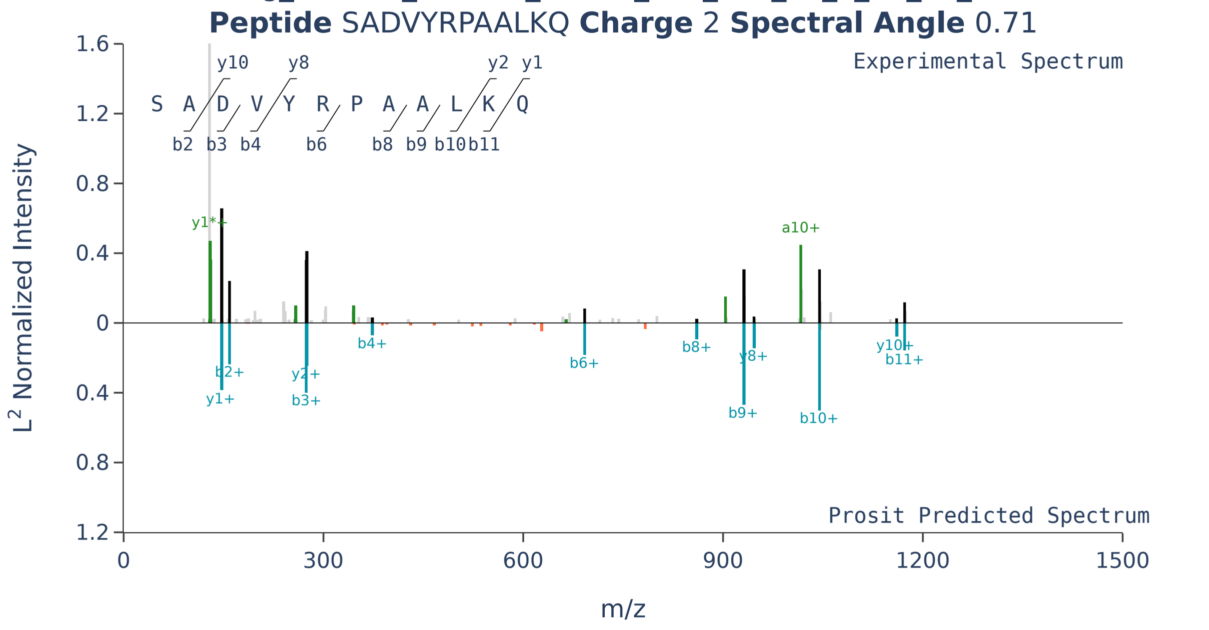


**Source** Ncheng_210623_230623_HFGoe_FFH_20S_25_1_A1_24h_R1 **Scan** 14076
**Peptide** VGKLGKFLRE **Charge** 3 **Spectral Angle** 0.85

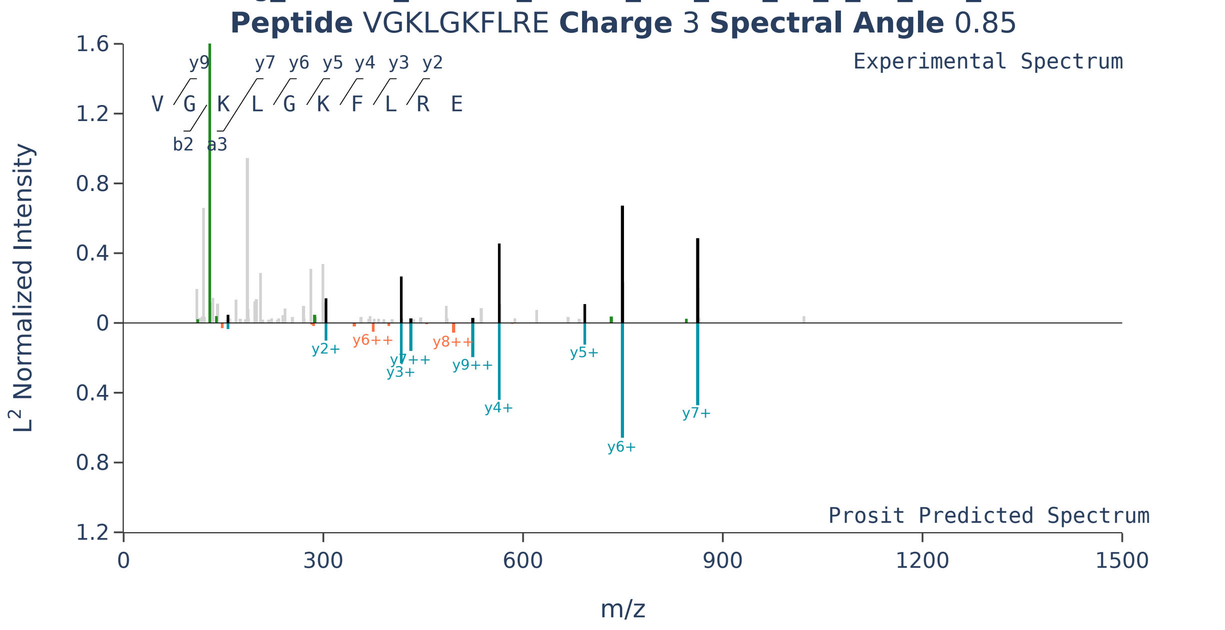


**Source** Ncheng_210623_230623_HFGoe_FFH_20S_25_1_A2_24h_R1 **Scan** 17145
**Peptide** SLRHITGKPIKFL **Charge** 4 **Spectral Angle** 0.81

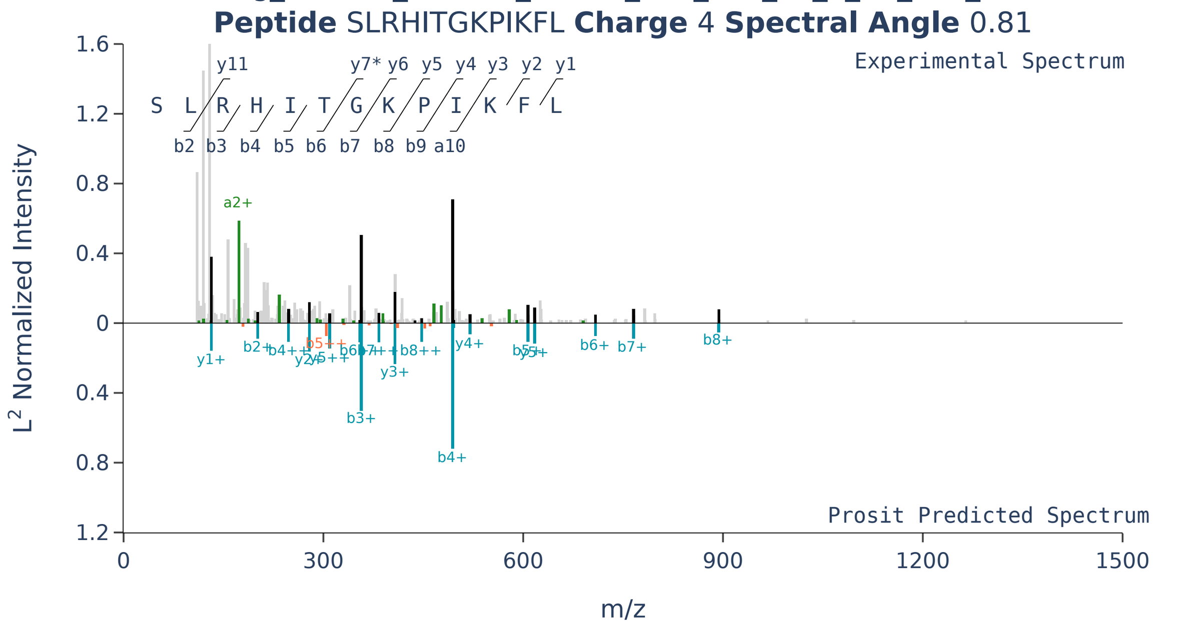


**Source** Ncheng_210623_230623_HFGoe_FFH_20S_25_1_A2_2h_R2 **Scan** 14774
**Peptide** VVSADVYRPAAIKQ **Charge** 2 **Spectral Angle** 0.72

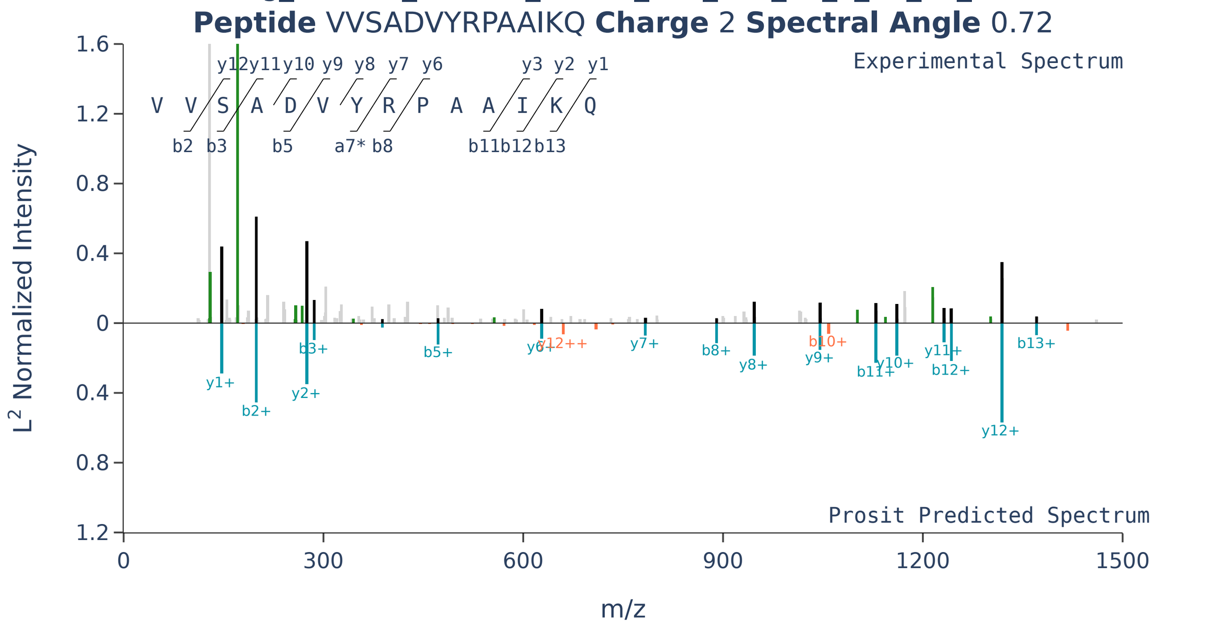


**Source** Ncheng_210623_230623_HFGoe_FFH_20S_25_1_A1_24h_R2 **Scan** 6698
**Peptide** LKKGDGFD **Charge** 2 **Spectral Angle** 0.73

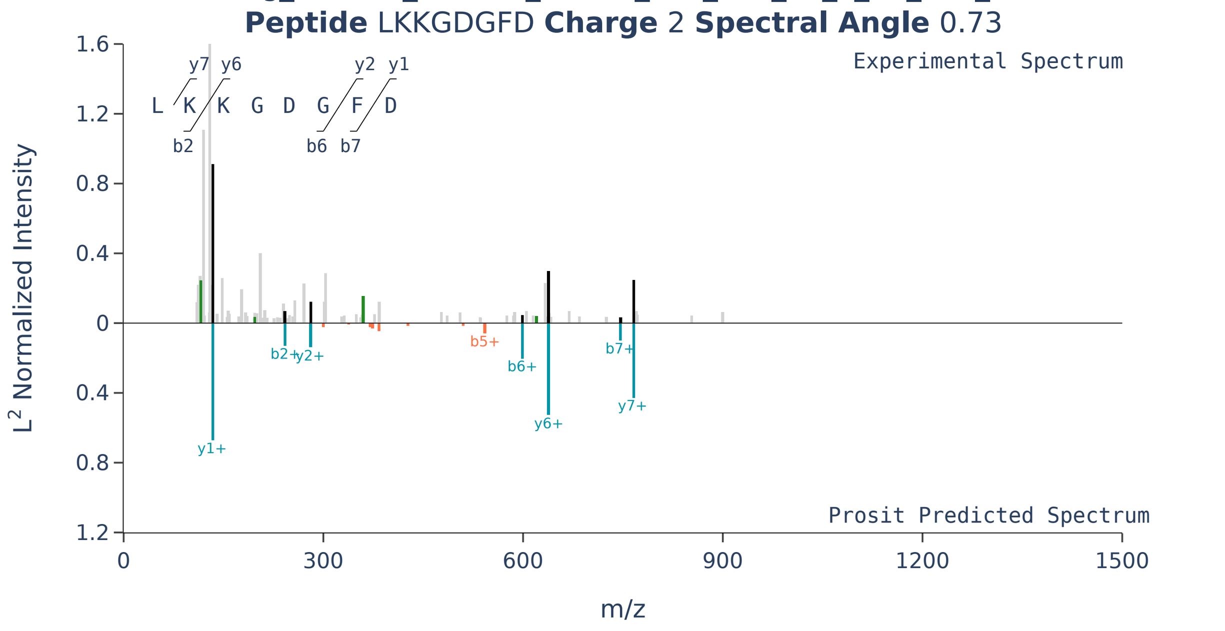


**Source** Ncheng_210623_230623_HFGoe_FFH_20S_25_1_A1_2h_R1 **Scan** 28336
**Peptide** KVLVRMEAIINSMTMKE **Charge** 3 **Spectral Angle** 0.88

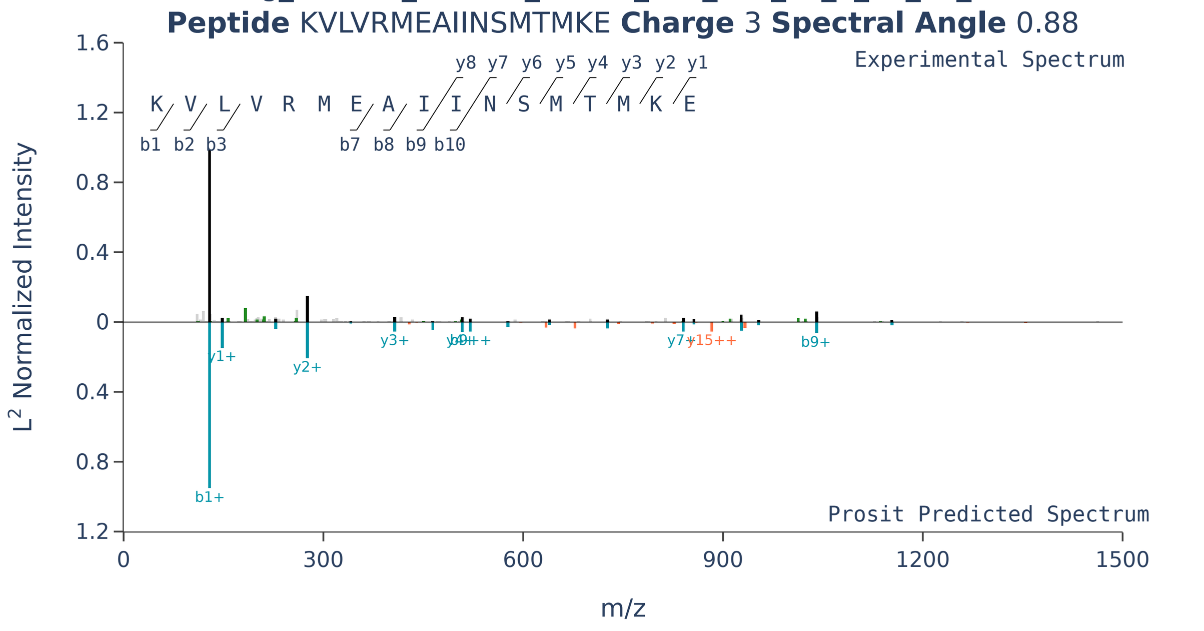


**Source** Ncheng_210623_230623_HFGoe_FFH_20S_25_1_A2_24h_R1 **Scan** 18524
**Peptide** QMDDKVLVRM **Charge** 3 **Spectral Angle** 0.94

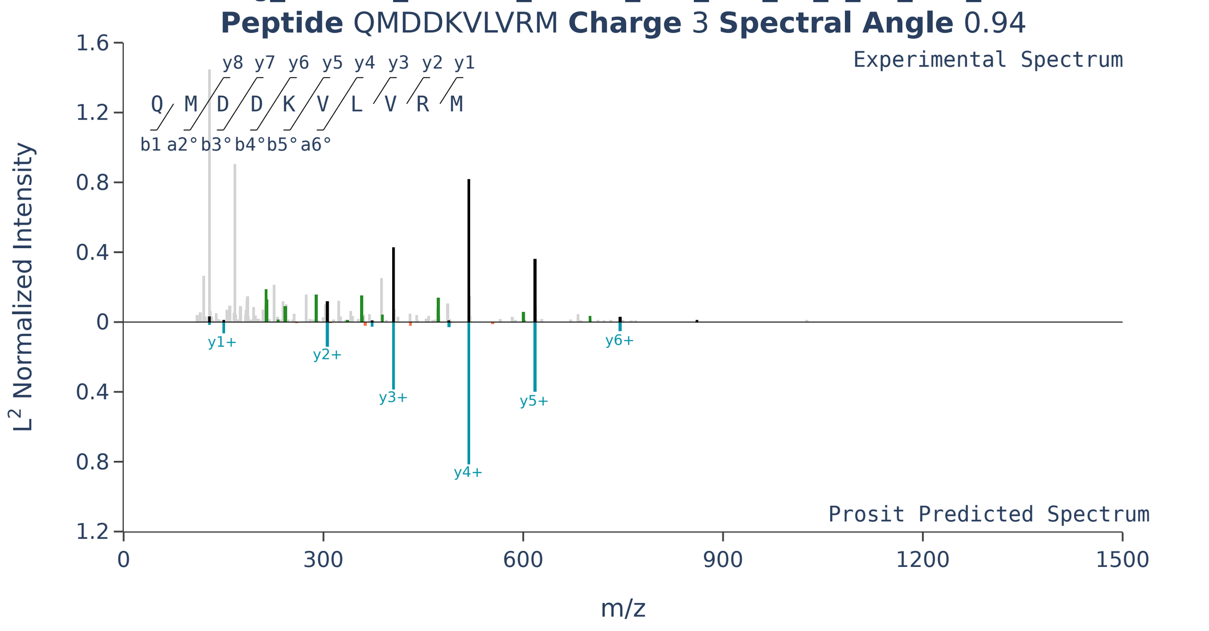


**Source** Ncheng_210623_230623_HFGoe_FFH_20S_25_1_A2_24h_R1 **Scan** 17474
**Peptide** DVGQKPVDIVNA **Charge** 2 **Spectral Angle** 0.82

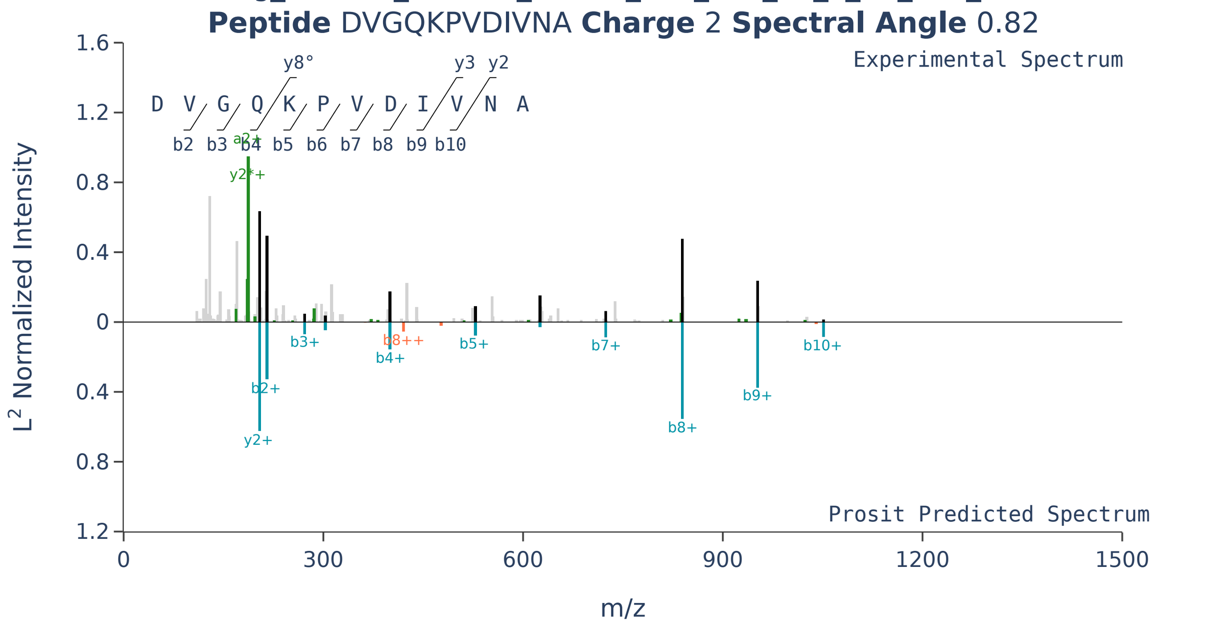


**Source** Ncheng_210623_230623_HFGoe_FFH_20S_25_1_A1_24h_R1 **Scan** 7721
**Peptide** KLASKLKKGDGFD **Charge** 4 **Spectral Angle** 0.96

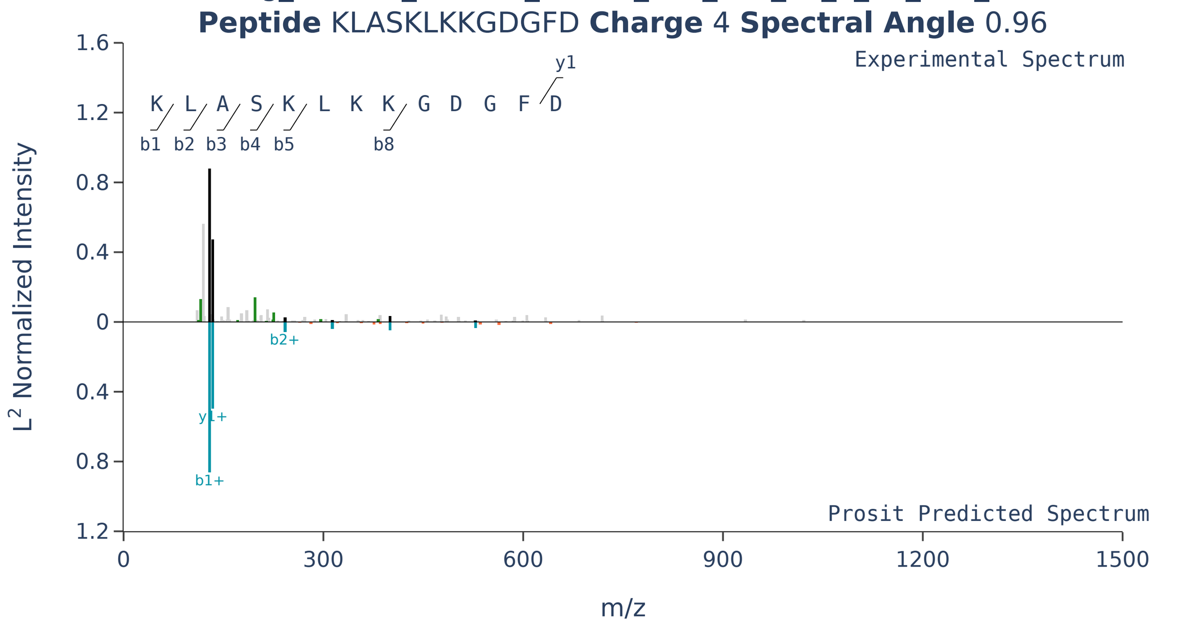


**Source** Ncheng_210623_230623_HFGoe_FFH_20S_25_1_A2_24h_R2 **Scan** 17457
**Peptide** GMGQIPDNVKSQMDD **Charge** 2 **Spectral Angle** 0.73

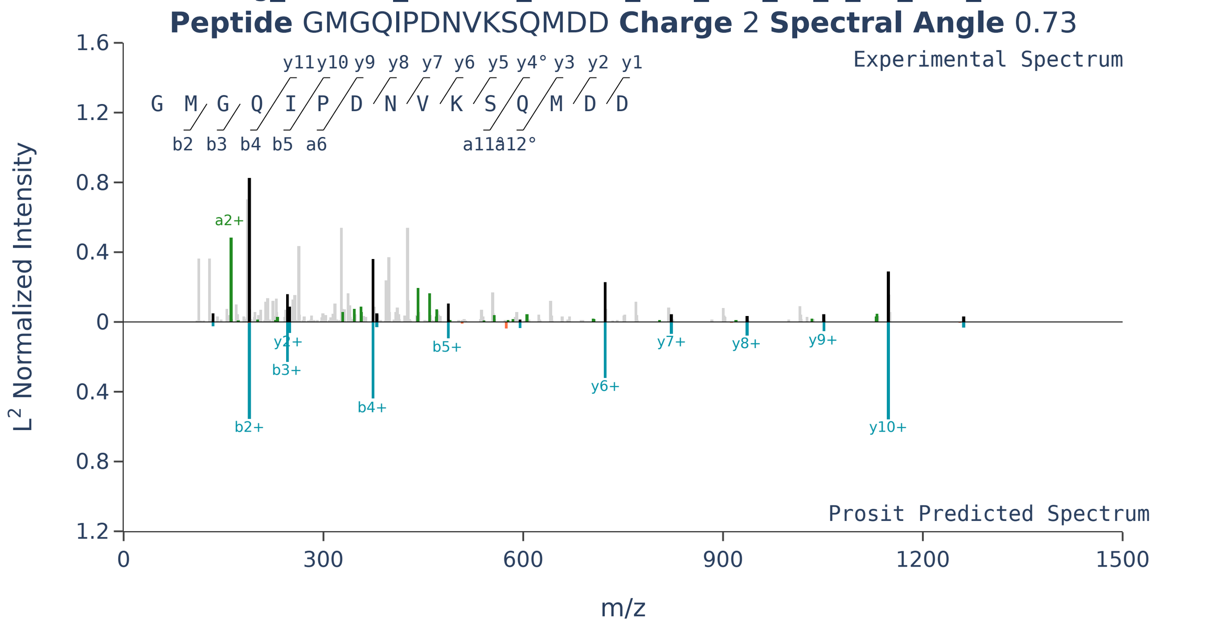


**Source** Ncheng_210623_230623_HFGoe_FFH_20S_25_1_A1_1h_R2 **Scan** 16360
**Peptide** RLTEDNVKDTLREV **Charge** 3 **Spectral Angle** 0.87

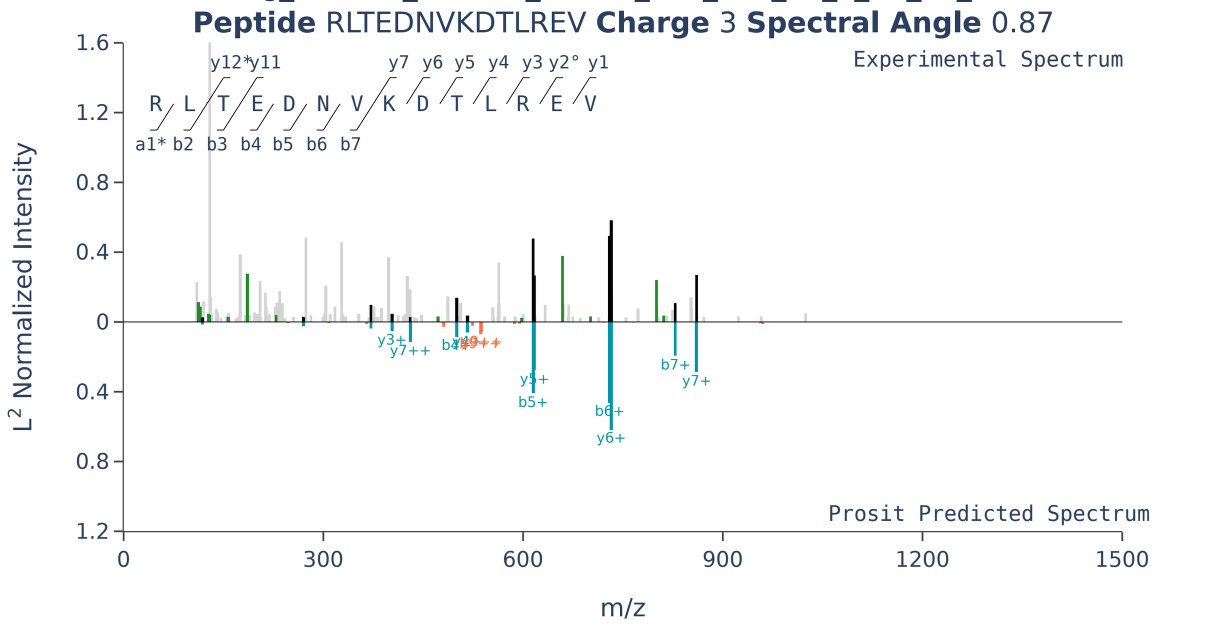

**Source** Ncheng_210623_230623_HFGoe_FFH_20S_25_1_A1_24h_R1 **Scan** 12569
**Peptide** VDGDARGGAALSIR **Charge** 2 **Spectral Angle** 0.78

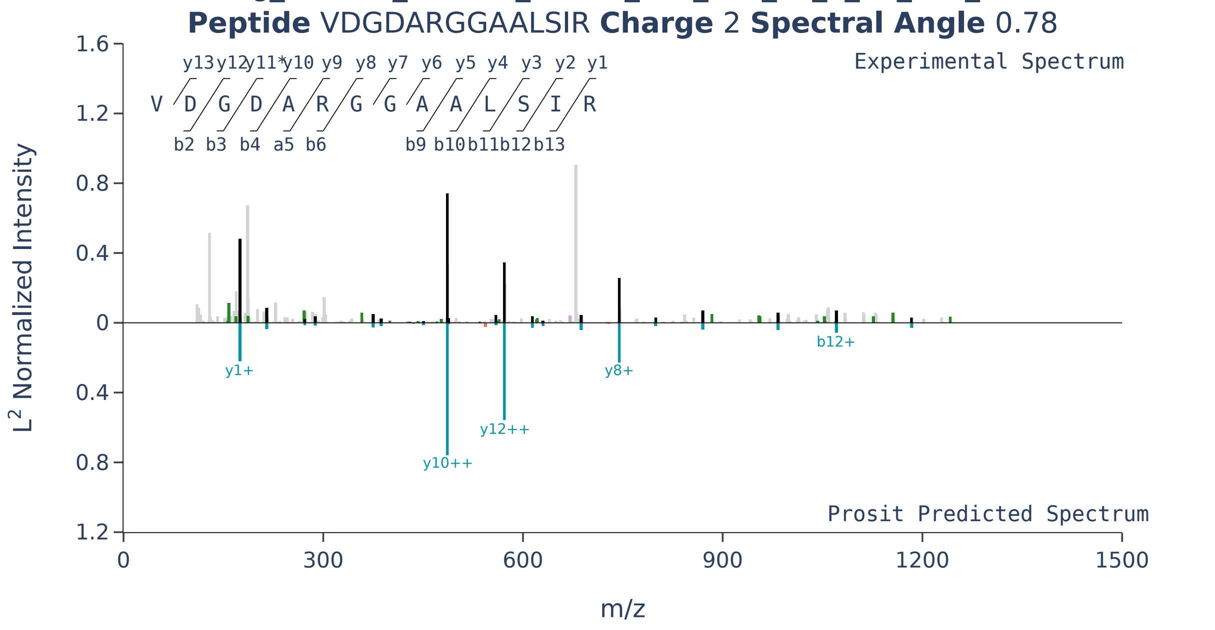


**Source** Ncheng_210623_230623_HFGoe_FFH_20S_25_1_A2_4h_R1 **Scan** 21834
**Peptide** EVNKSLTPGQEFVKLVR **Charge** 3 **Spectral Angle** 0.77

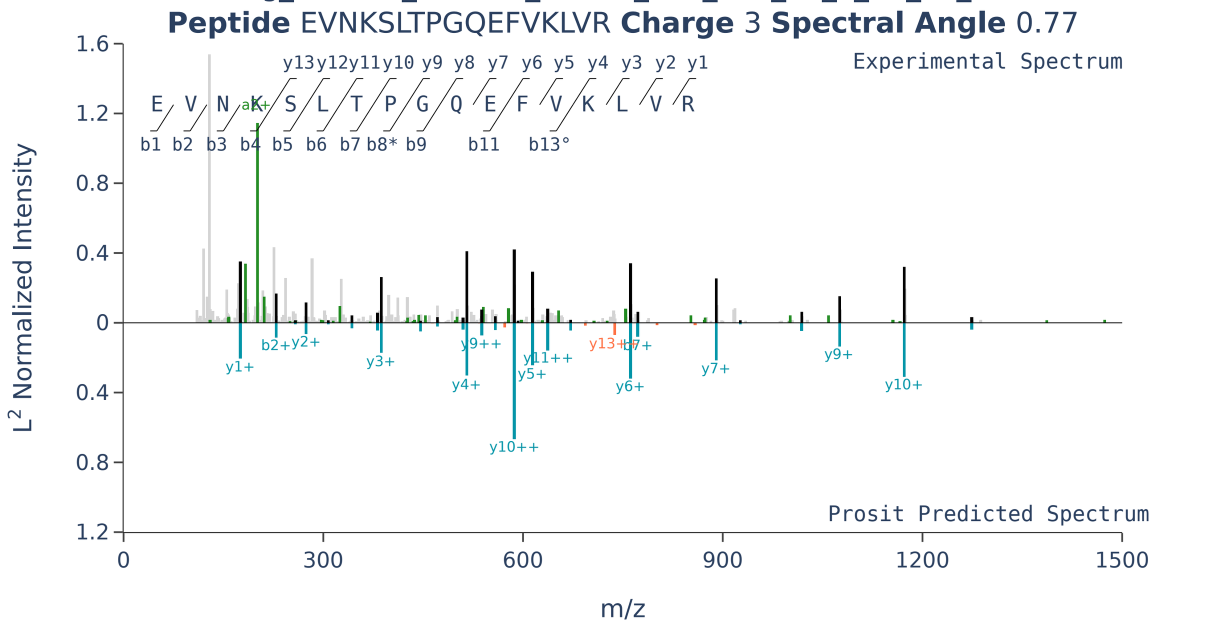


**Source** Ncheng_210623_230623_HFGoe_FFH_20S_25_1_A1_24h_R2 **Scan** 19918
**Peptide** KSLTPGQEFVKIVRN **Charge** 2 **Spectral Angle** 0.79

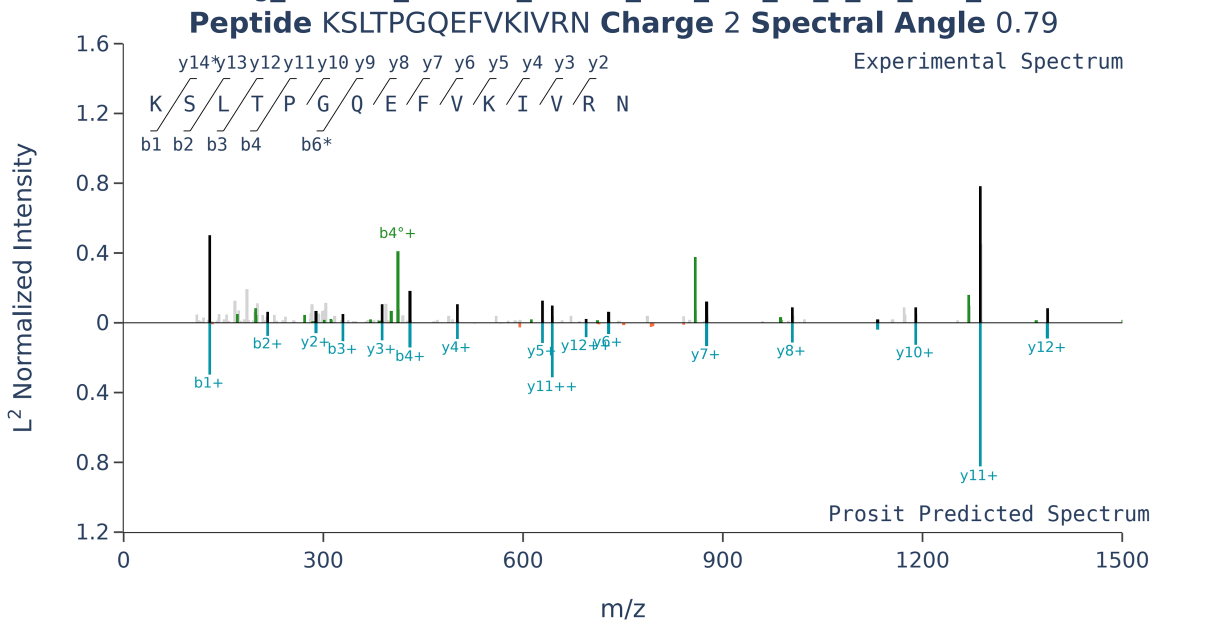


**Source** Ncheng_210623_230623_HFGoe_FFH_20S_25_1_A2_4h_R2 **Scan** 36403
**Peptide** AVGHEVNKSLTPGQEFVKIVRNELVAAM **Charge** 3 **Spectral Angle** 0.75

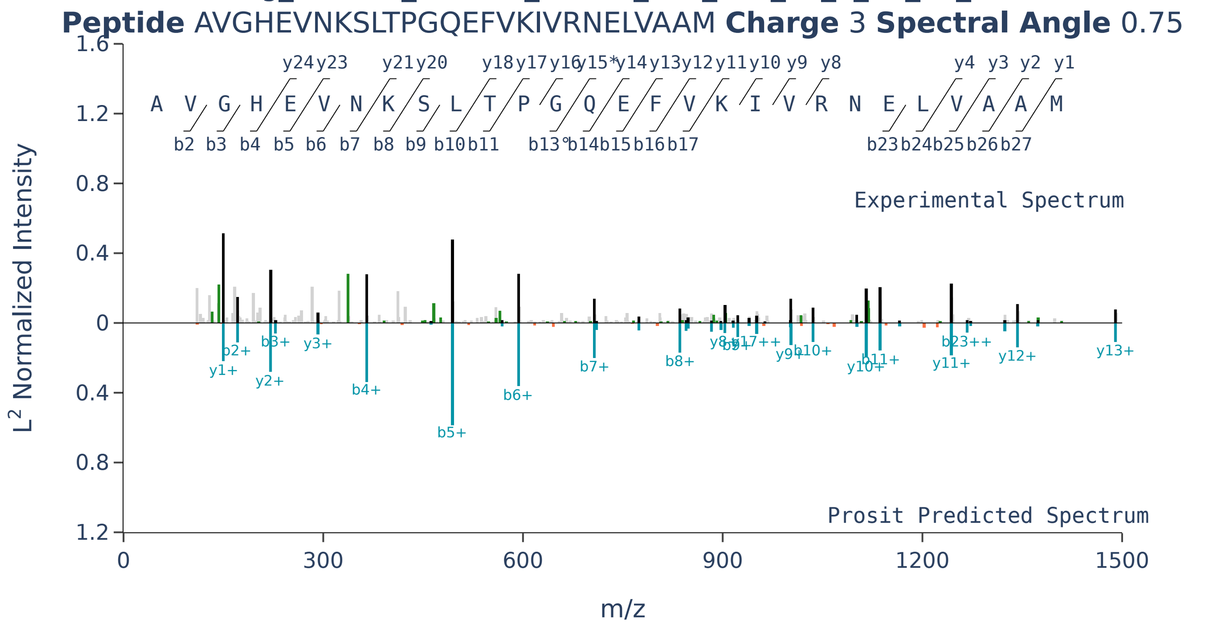


**Source** Ncheng_210623_230623_HFGoe_FFH_20S_25_1_A2_24h_R1 **Scan** 6187
**Peptide** RLTEDNVKD **Charge** 2 **Spectral Angle** 0.84

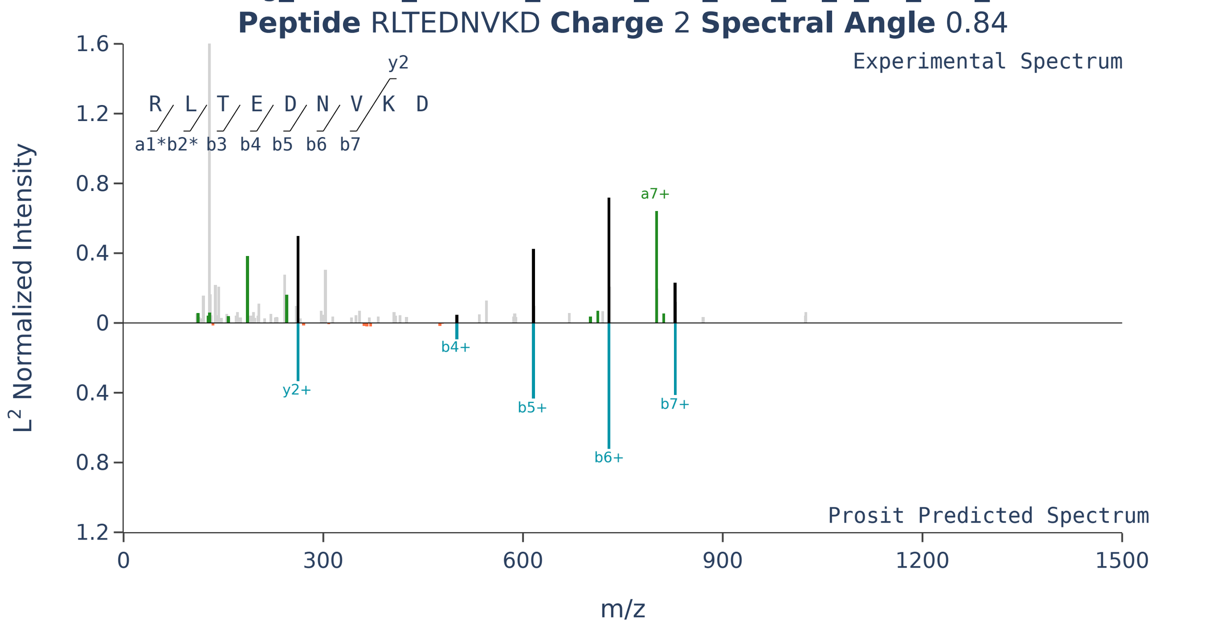


**Source** Ncheng_210623_230623_HFGoe_FFH_20S_25_1_A2_24h_R2 **Scan** 15538
**Peptide** VYRPAAIKQLE **Charge** 2 **Spectral Angle** 0.71

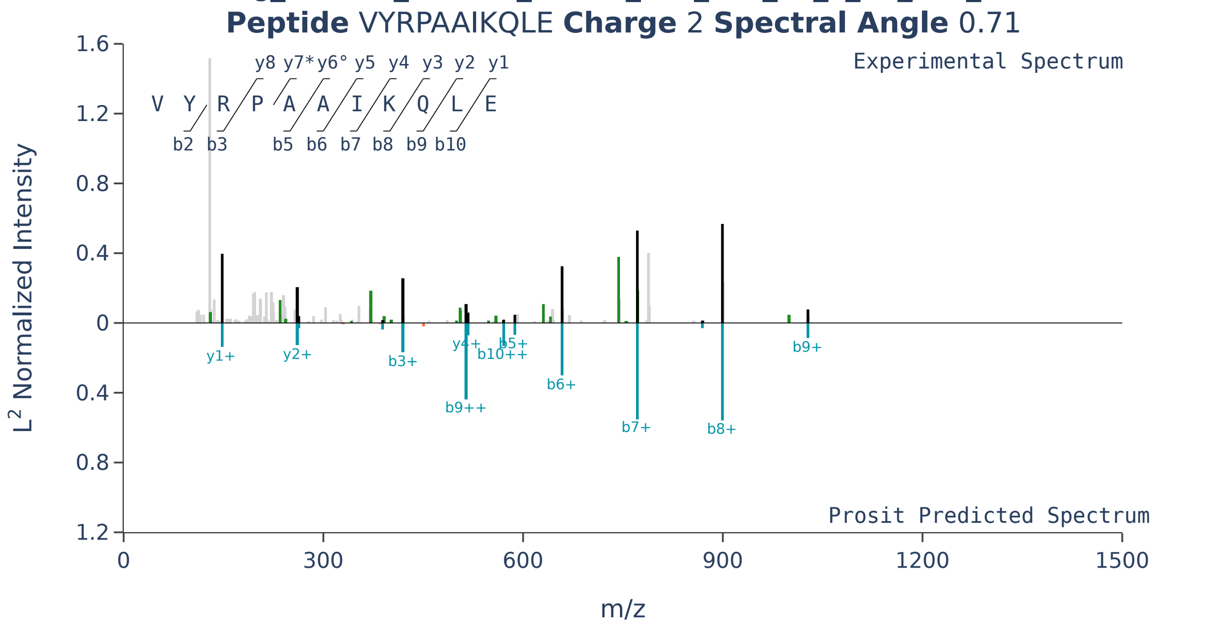


**Source** Ncheng_210623_230623_HFGoe_FFH_20S_25_1_A2_24h_R1 **Scan** 8968
**Peptide** ESKVDRAQA **Charge** 2 **Spectral Angle** 0.9

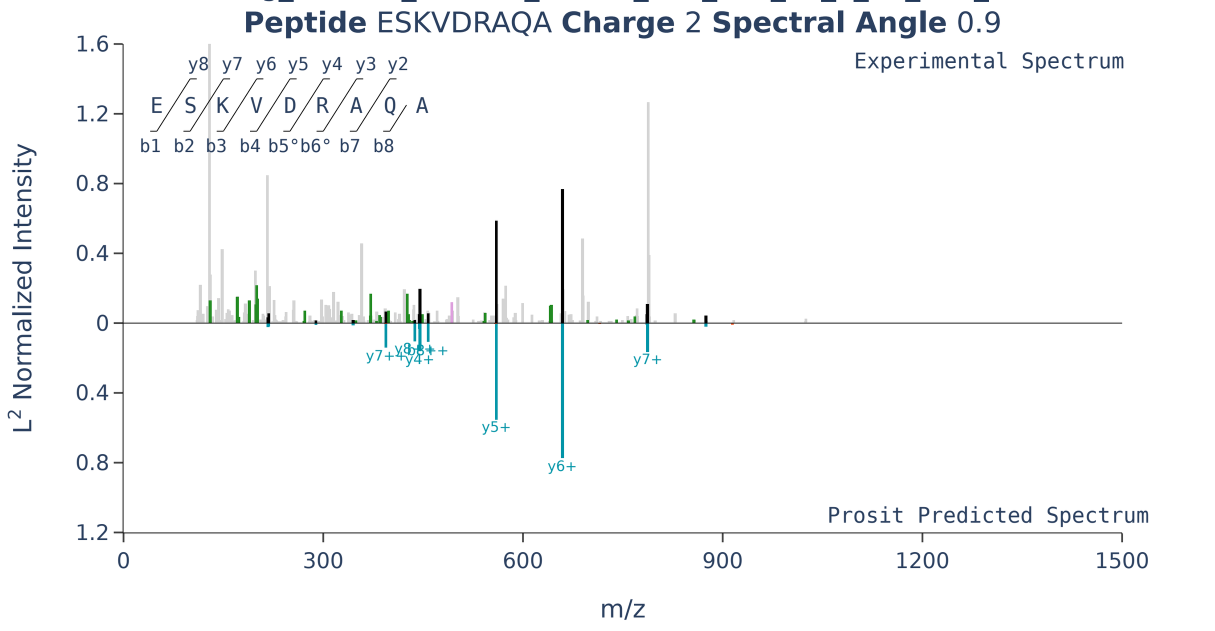


**Source** Ncheng_210623_230623_HFGoe_FFH_20S_25_1_A1_4h_R1 **Scan** 11552
**Peptide** SGRGRLTEDNVKDTL **Charge** 4 **Spectral Angle** 0.75

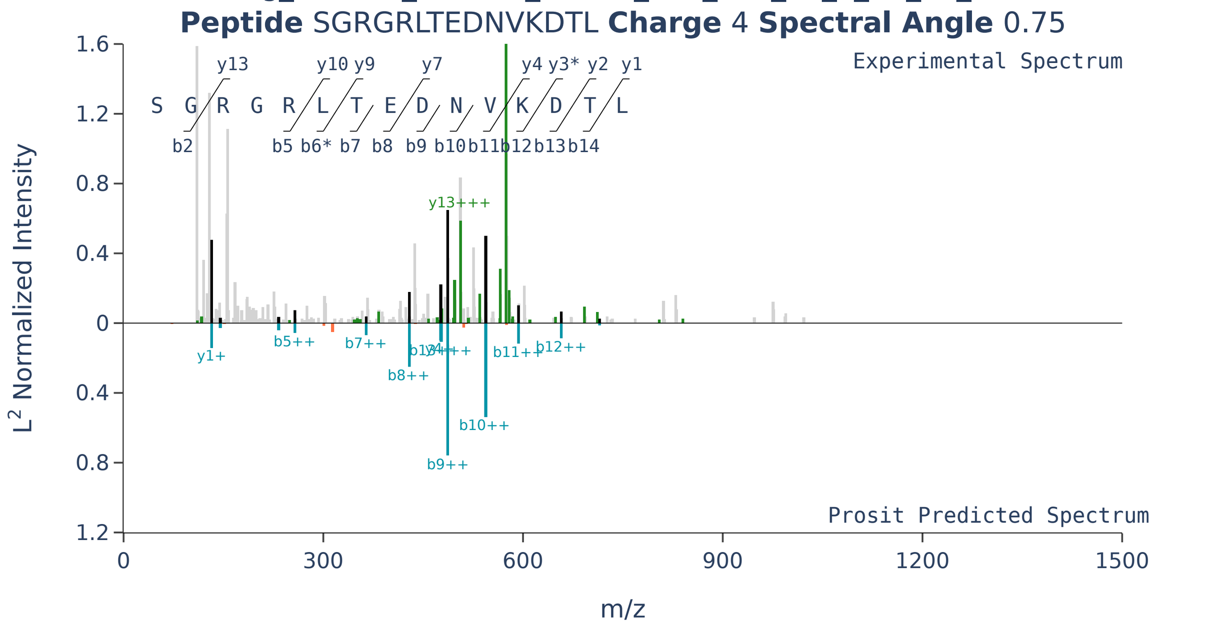


**Source** Ncheng_210623_230623_HFGoe_FFH_20S_25_1_A2_2h_R1 **Scan** 29626
**Peptide** AVGHEVNKSLTPGQEFVKIVRNELVA **Charge** 3 **Spectral Angle** 0.84

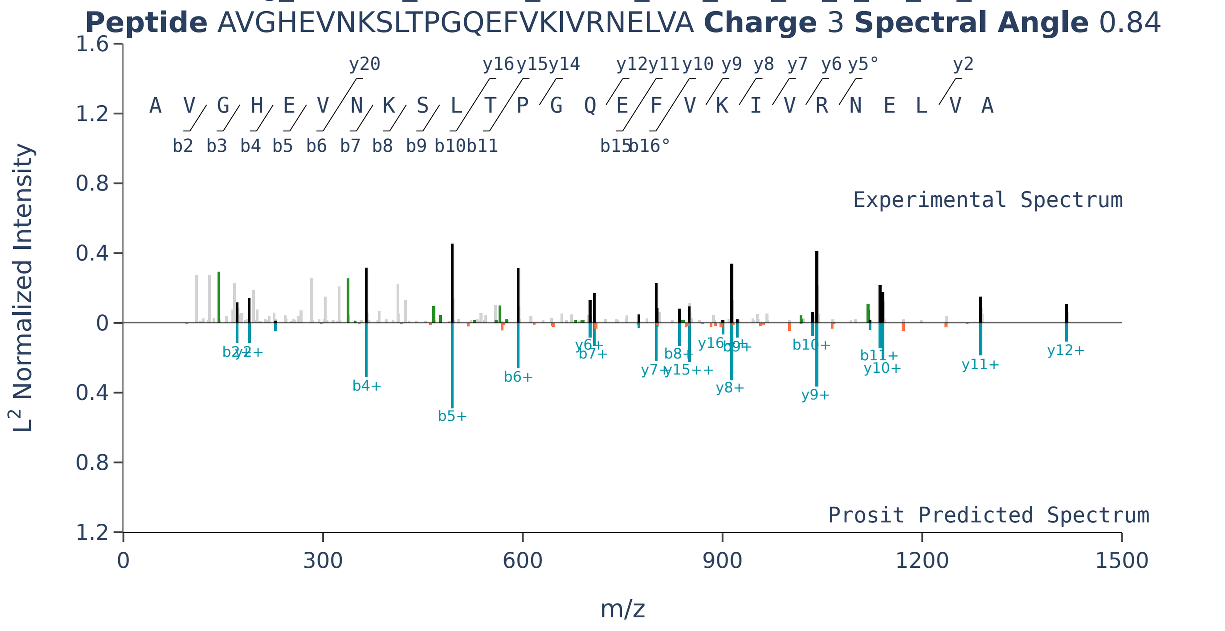


**Source** Ncheng_210623_230623_HFGoe_FFH_20S_25_1_A2_1h_R2 **Scan** 12639
**Peptide** NRLLKQFD **Charge** 2 **Spectral Angle** 0.76

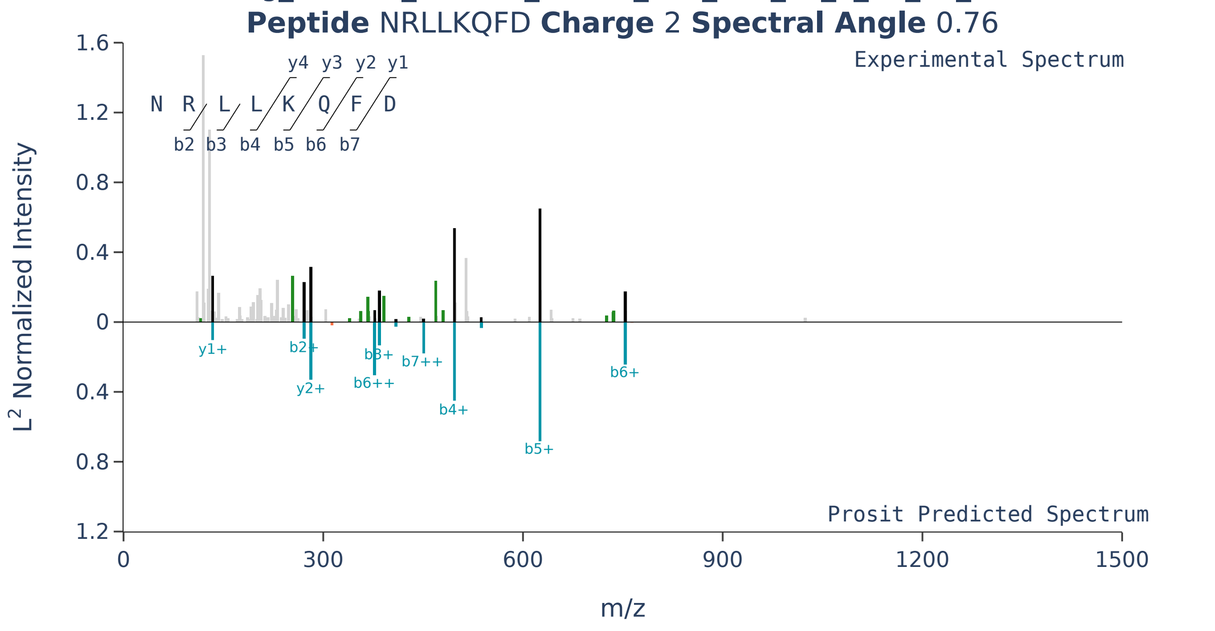


**Source** Ncheng_210623_230623_HFGoe_FFH_20S_25_1_A2_4h_R1 **Scan** 15878
**Peptide** SLRHITGKPIKFLGVGEKT **Charge** 3 **Spectral Angle** 0.8

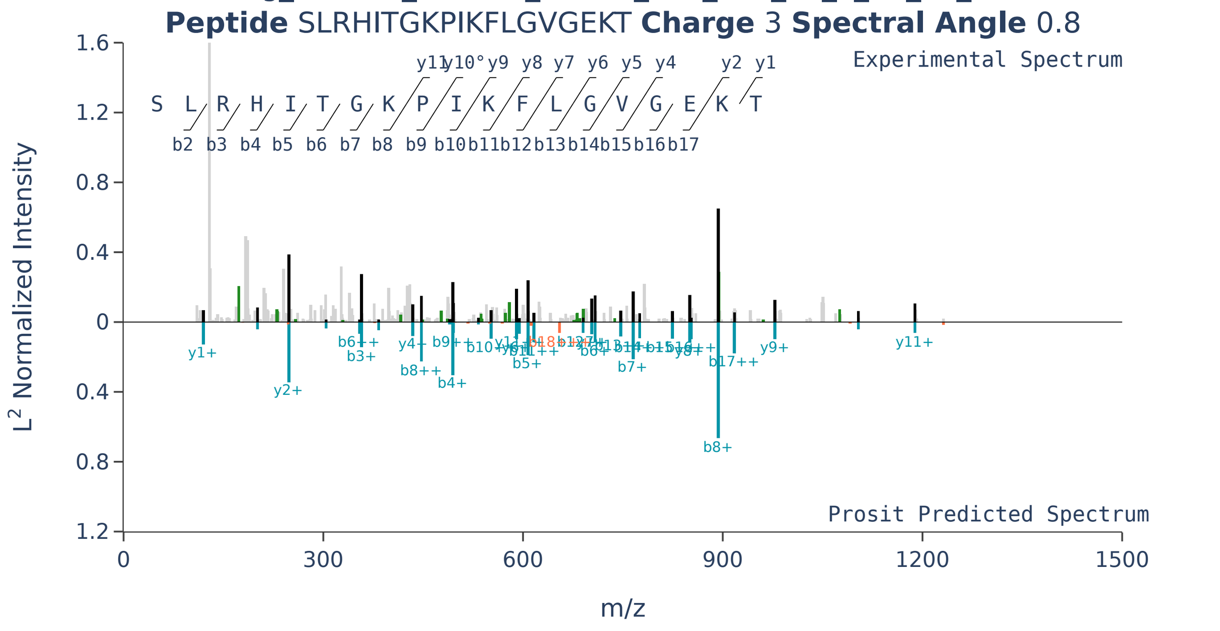


**Source** Ncheng_210623_230623_HFGoe_FFH_20S_25_1_A2_4h_R1 **Scan** 34897
**Peptide** HASINPVETLFVVD **Charge** 2 **Spectral Angle** 0.79

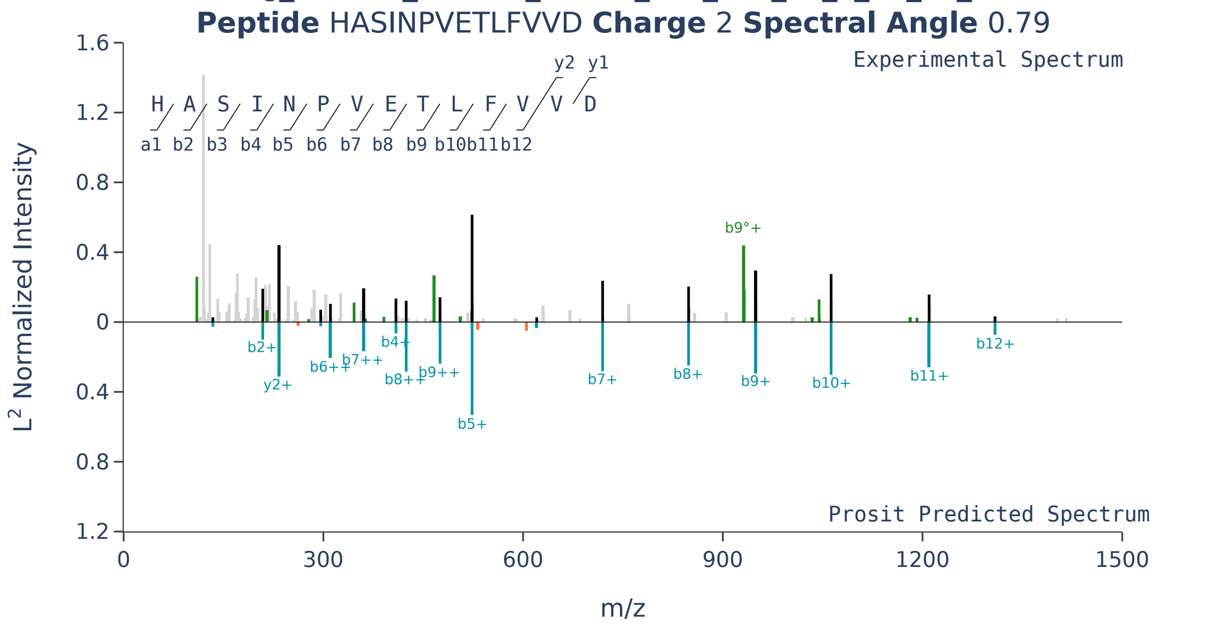


**Source** Ncheng_210623_230623_HFGoe_FFH_20S_25_1_A2_24h_R2 **Scan** 23825
**Peptide** GHEVNKSLTPGQEFVKLV **Charge** 3 **Spectral Angle** 0.82

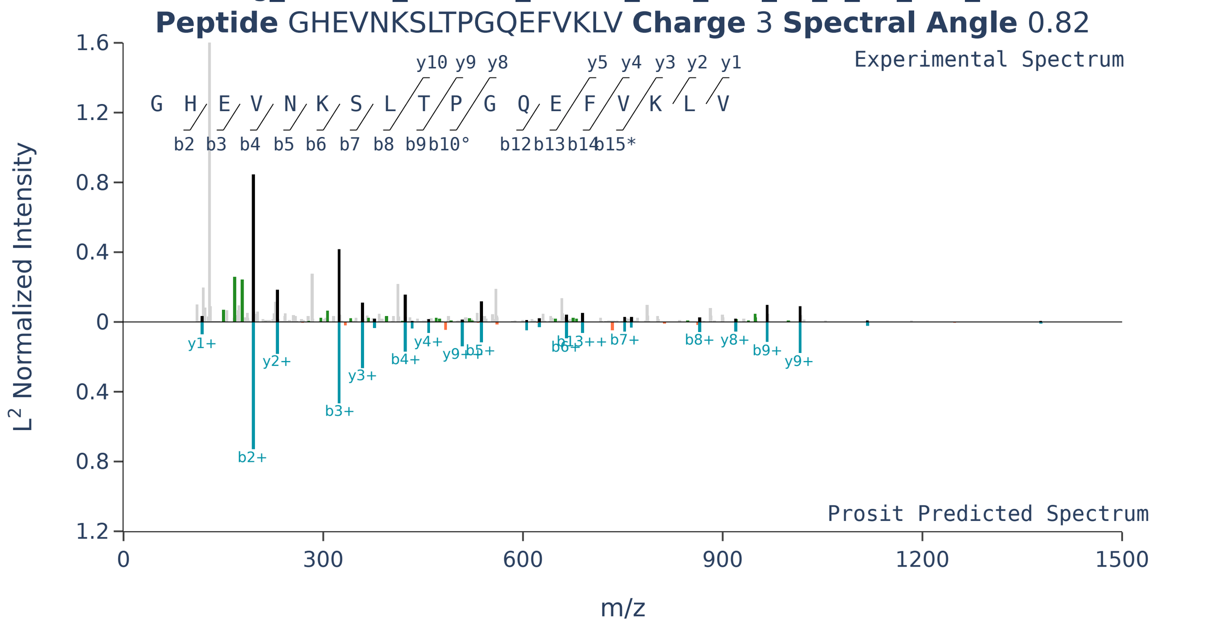


**Source** Ncheng_210623_230623_HFGoe_FFH_20S_25_1_A2_4h_R2 **Scan** 19309
**Peptide** EDIESKVDRAQAEKLASKL **Charge** 3 **Spectral Angle** 0.84

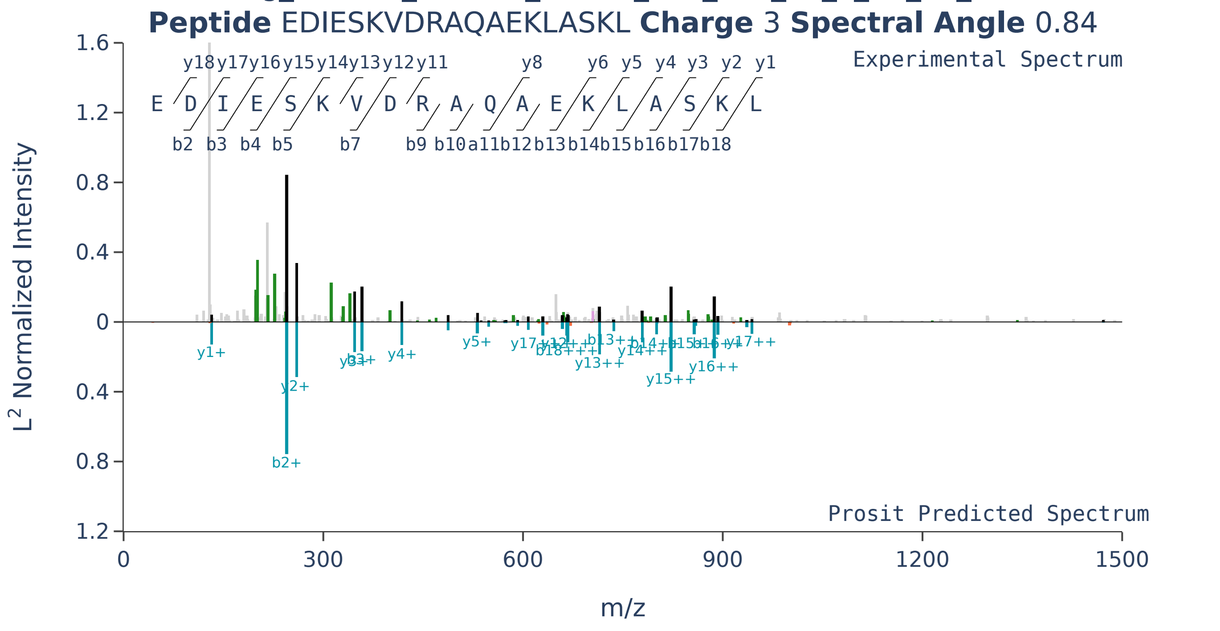


**Source** Ncheng_210623_230623_HFGoe_FFH_20S_25_1_A2_24h_R2 **Scan** 15392
**Peptide** RGRLTEDNVKDTLREV **Charge** 4 **Spectral Angle** 0.83

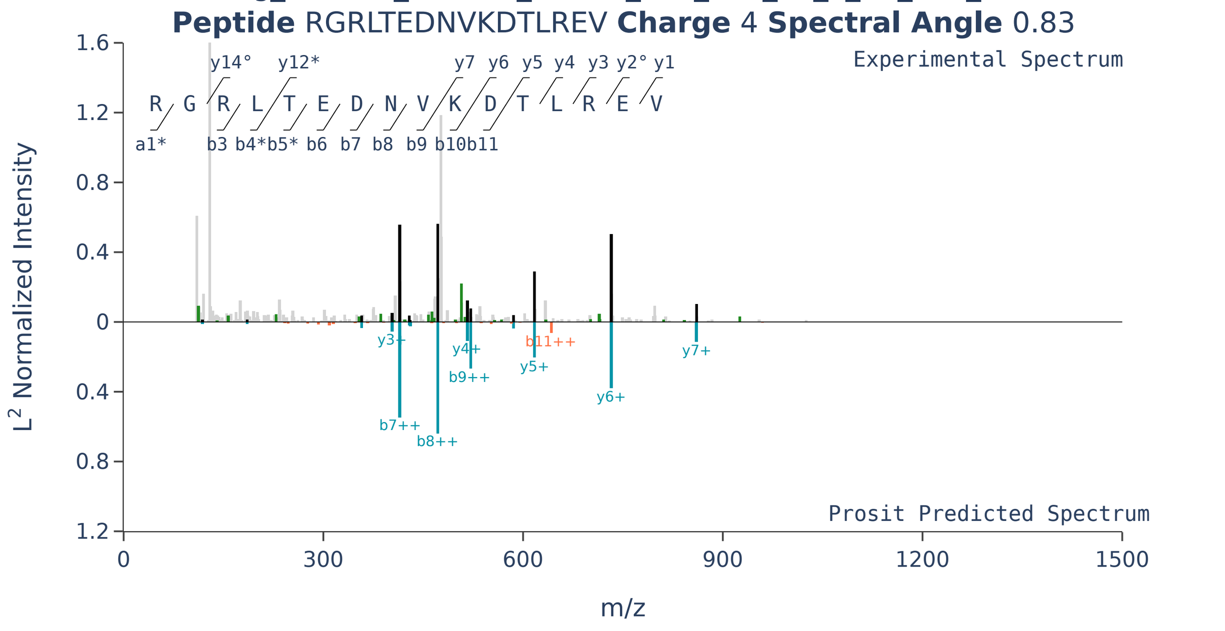

**Source** Ncheng_210623_230623_HFGoe_FFH_20S_25_1_A1_24h_R2 **Scan** 9458
**Peptide** KEAKLKF **Charge** 2 **Spectral Angle** 0.91

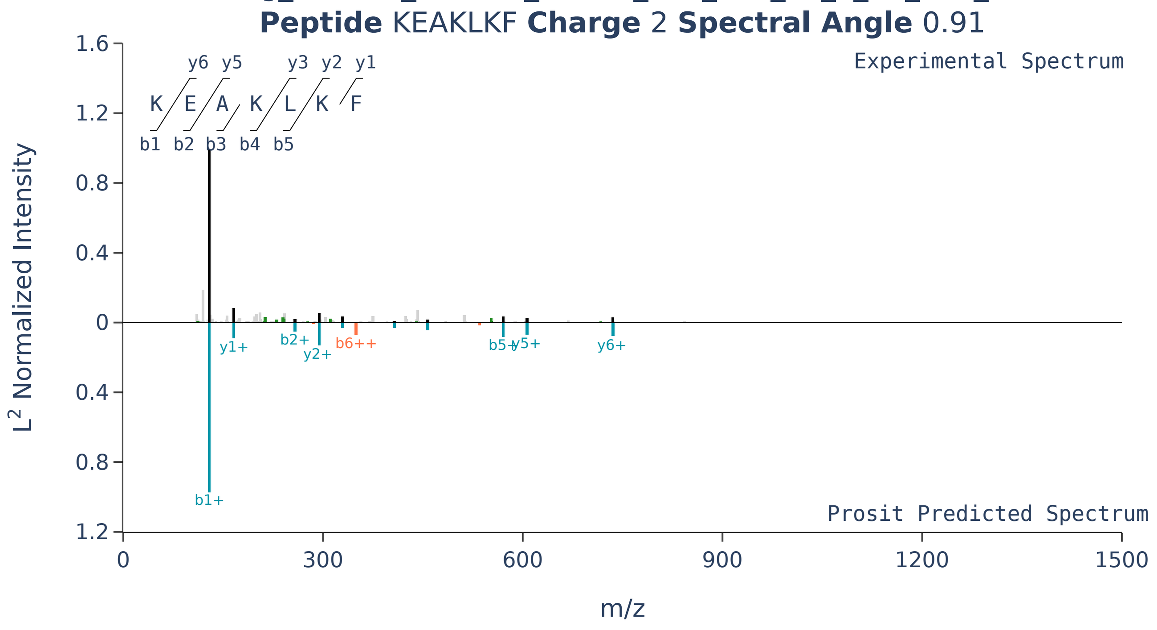


**Source** Ncheng_210623_230623_HFGoe_FFH_20S_25_1_A1_2h_R1 **Scan** 20902
**Peptide** GQIPDNVKSQMDDKVLV **Charge** 2 **Spectral Angle** 0.96

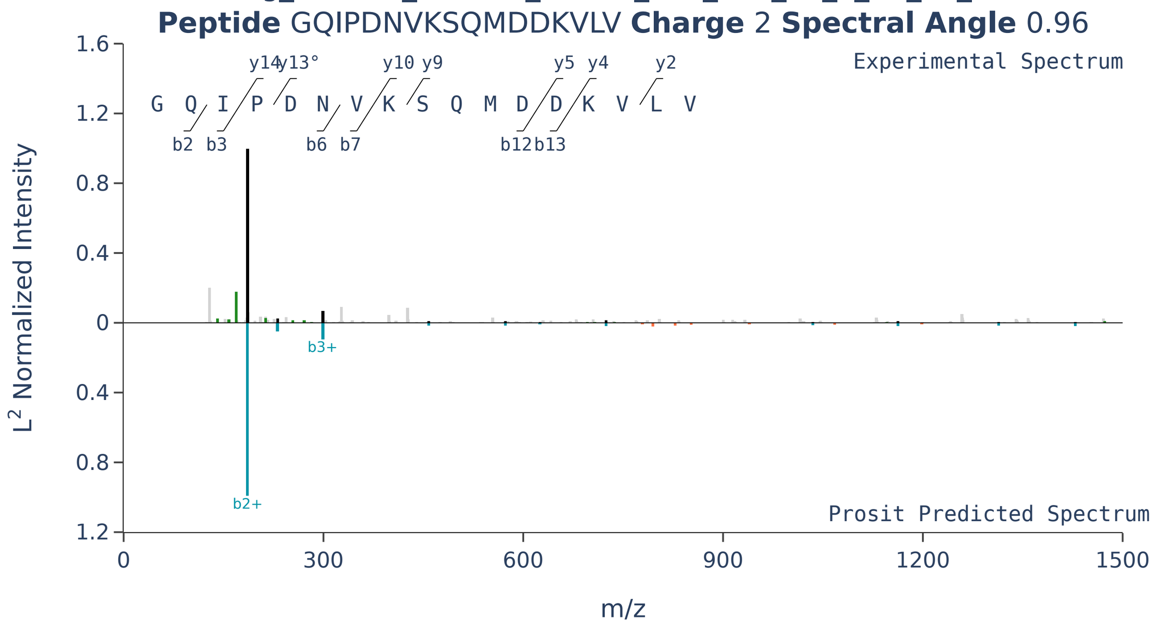


**Source** Ncheng_210623_230623_HFGoe_FFH_20S_25_1_A1_24h_R2 **Scan** 7779
**Peptide** QIPDNVKSQ **Charge** 2 **Spectral Angle** 0.69

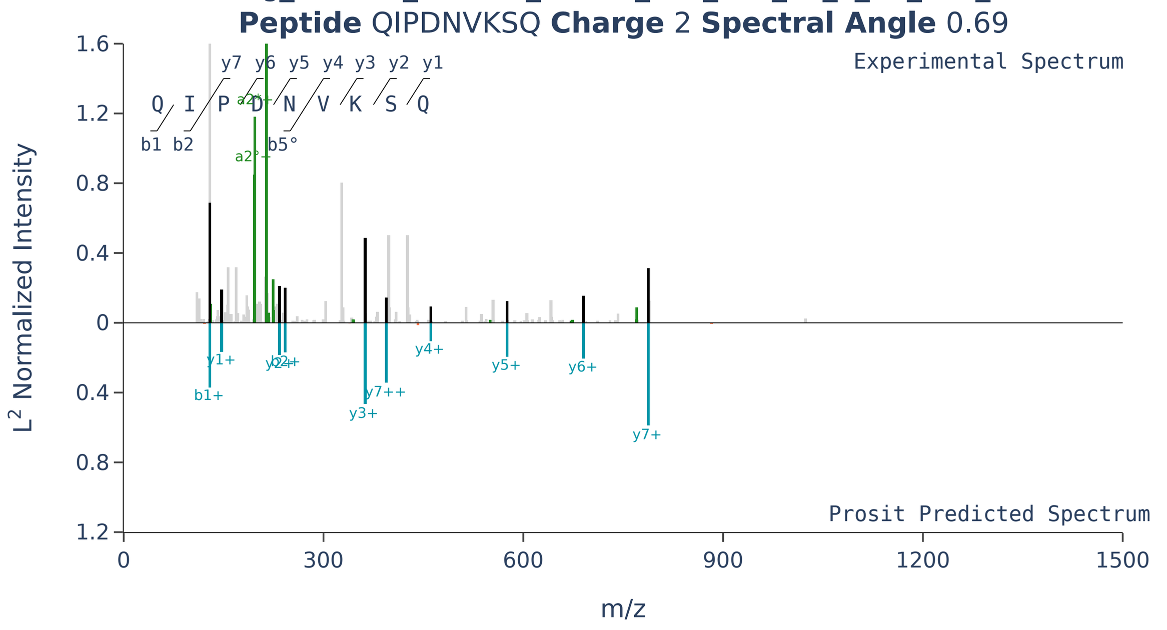


**Source** Ncheng_210623_230623_HFGoe_FFH_20S_25_1_A2_4h_R1 **Scan** 16152
**Peptide** RMEAIINSMTMKERAK **Charge** 4 **Spectral Angle** 0.8

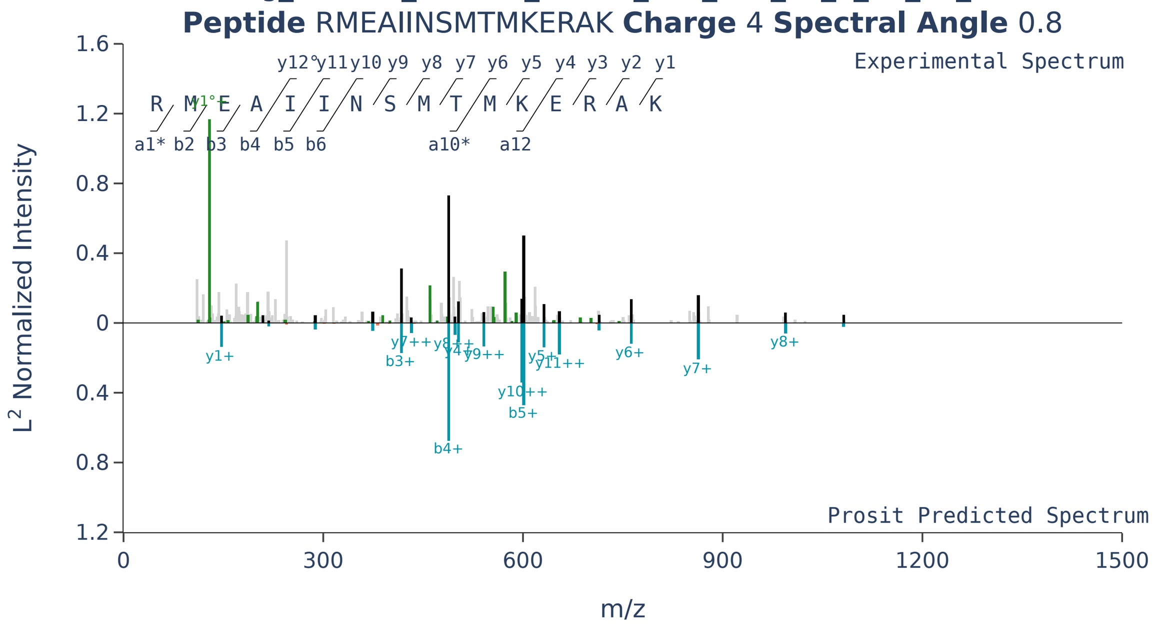


**Source** Ncheng_210623_230623_HFGoe_FFH_20S_25_1_A1_24h_R1 **Scan** 13828
**Peptide** EALINSMTMKERAK **Charge** 2 **Spectral Angle** 0.71

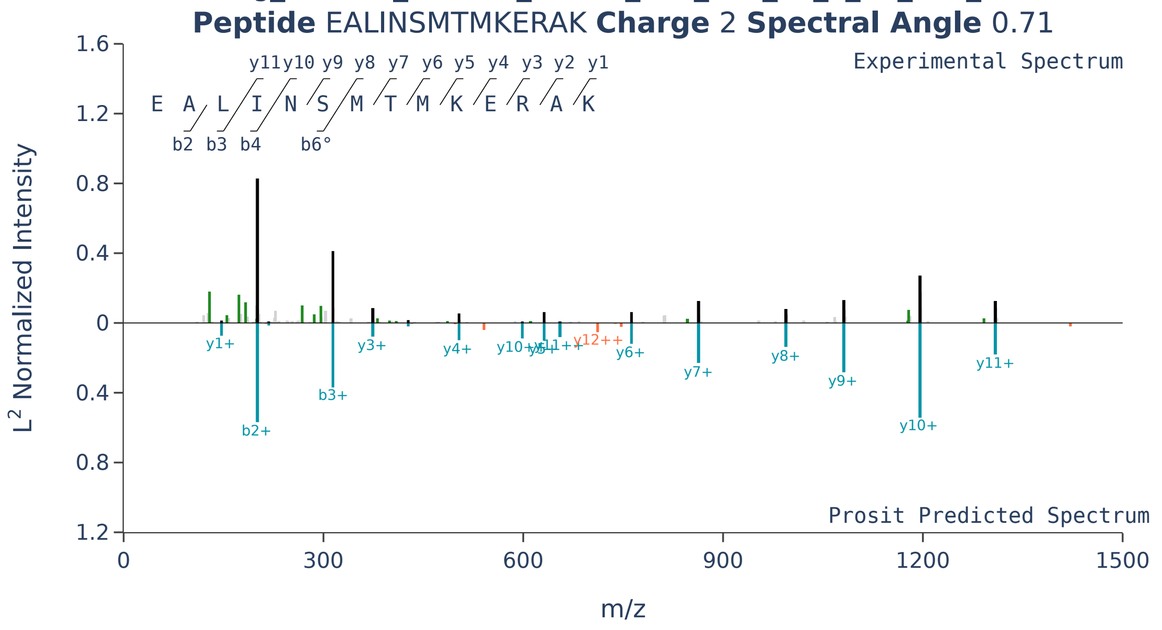


**Source** Ncheng_210623_230623_HFGoe_FFH_20S_25_1_A1_24h_R2 **Scan** 16031
**Peptide** LESKVDRAQAEKLASKL **Charge** 2 **Spectral Angle** 0.79

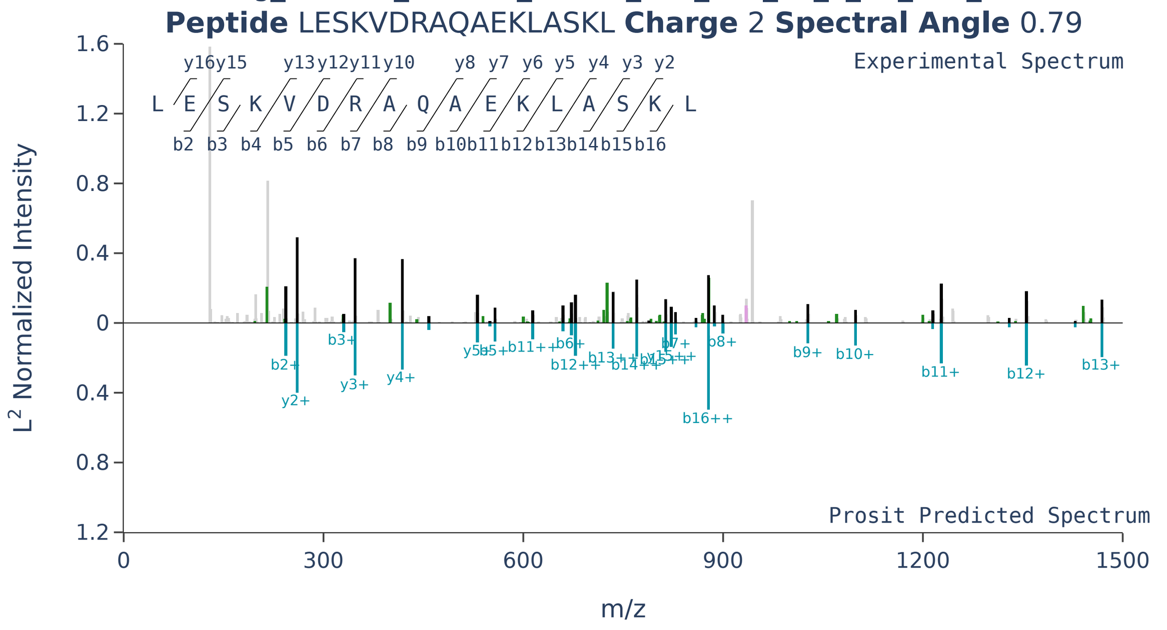


**Source** Ncheng_210623_230623_HFGoe_FFH_20S_25_1_A2_2h_R1 **Scan** 20009
**Peptide** KLPGMGQIPDNVKSQMDD **Charge** 3 **Spectral Angle** 0.76

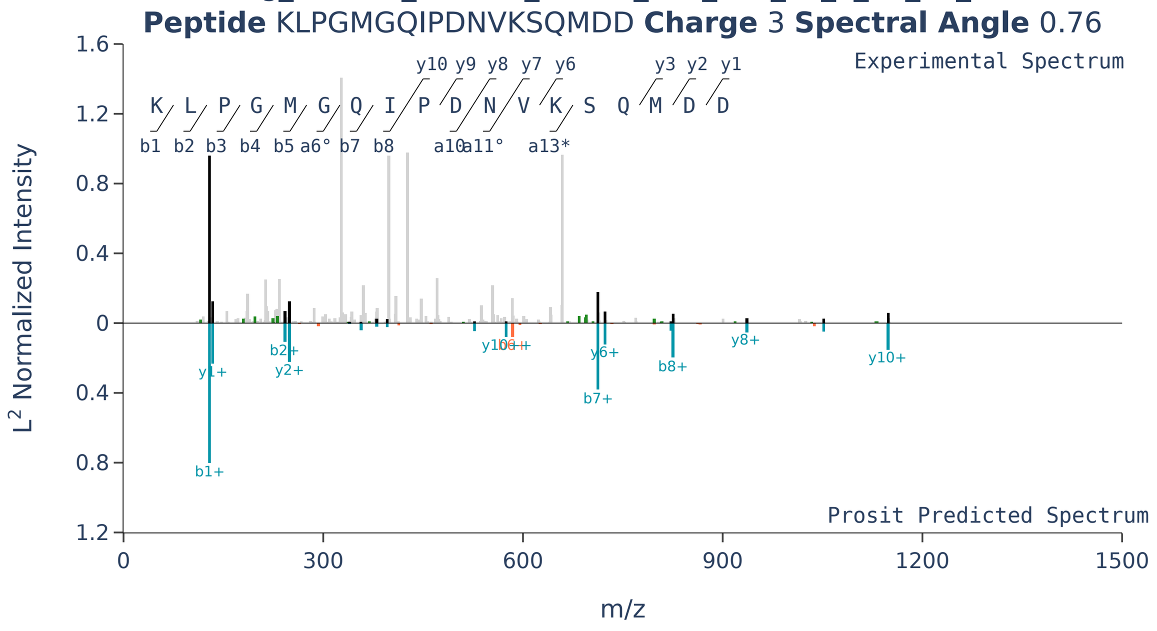


**Source** Ncheng_210623_230623_HFGoe_FFH_20S_25_1_A2_2h_R1 **Scan** 21856
**Peptide** VNKSLTPGQEFVKIVRNE **Charge** 3 **Spectral Angle** 0.74

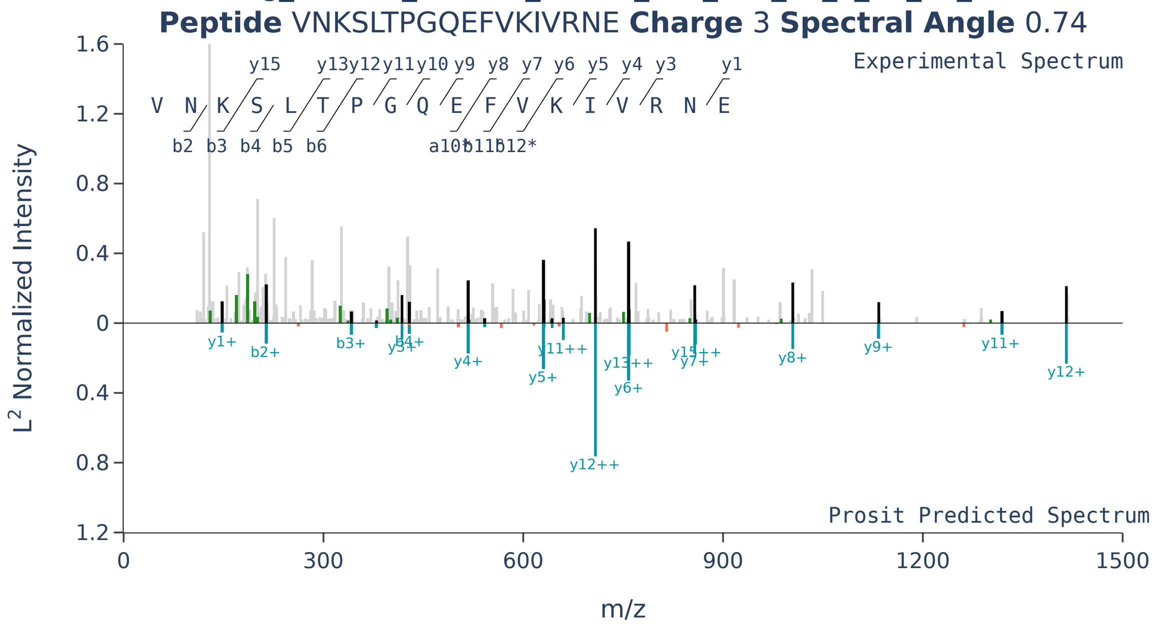


**Source** Ncheng_210623_230623_HFGoe_FFH_20S_25_1_A2_24h_R1 **Scan** 17903
**Peptide** GKPIKFL **Charge** 2 **Spectral Angle** 0.71

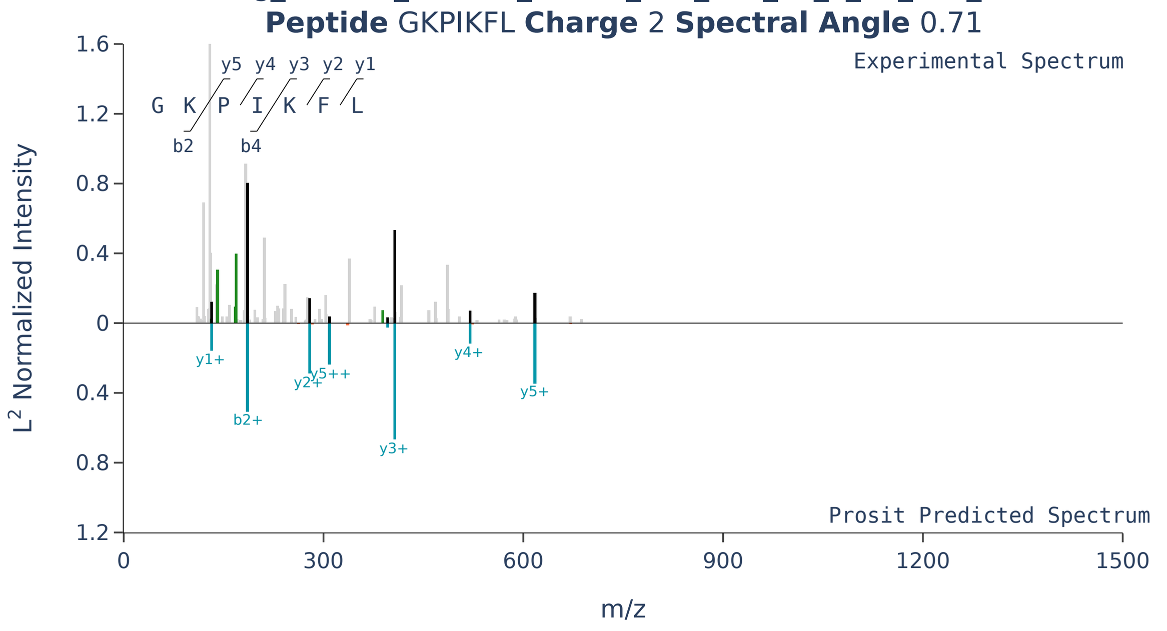


**Source** Ncheng_210623_230623_HFGoe_FFH_20S_25_1_A1_24h_R2 **Scan** 6913
**Peptide** TLRNISGR **Charge** 2 **Spectral Angle** 0.78

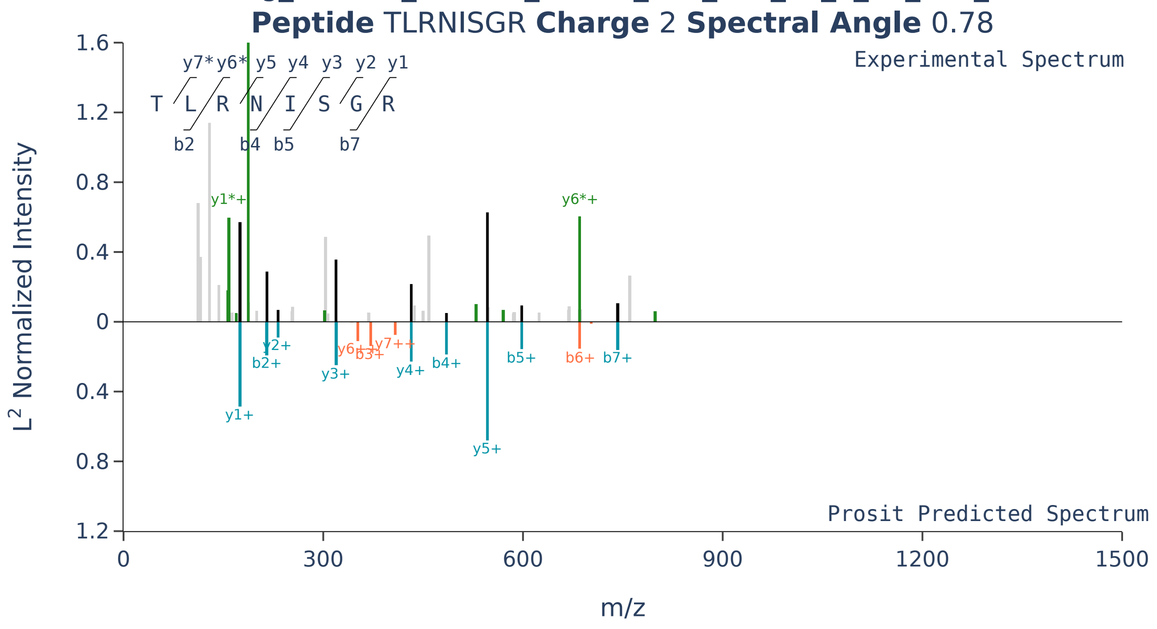


**Source** Ncheng_210623_230623_HFGoe_FFH_20S_25_1_A2_24h_R2 **Scan** 16927
**Peptide** KEKAVGHEVNKSLTPGQEFVKIVRN **Charge** 3 **Spectral Angle** 0.79

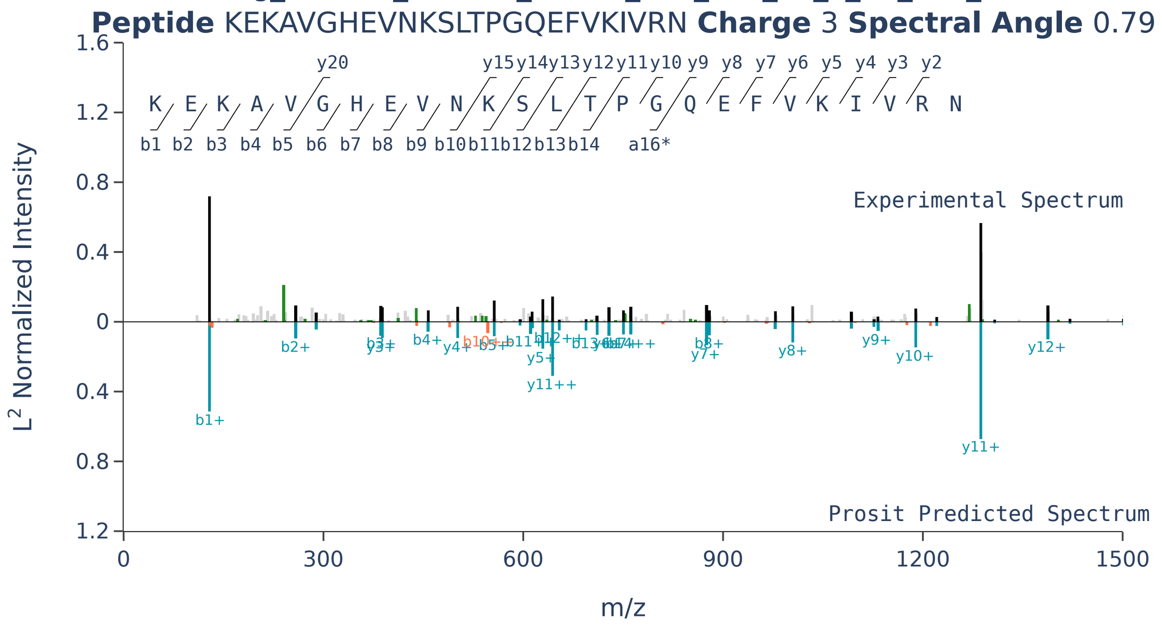


**Source** Ncheng_210623_230623_HFGoe_FFH_20S_25_1_A1_24h_R2 **Scan** 20463
**Peptide** GHEVNKSLTPGQEFVKL **Charge** 4 **Spectral Angle** 0.81

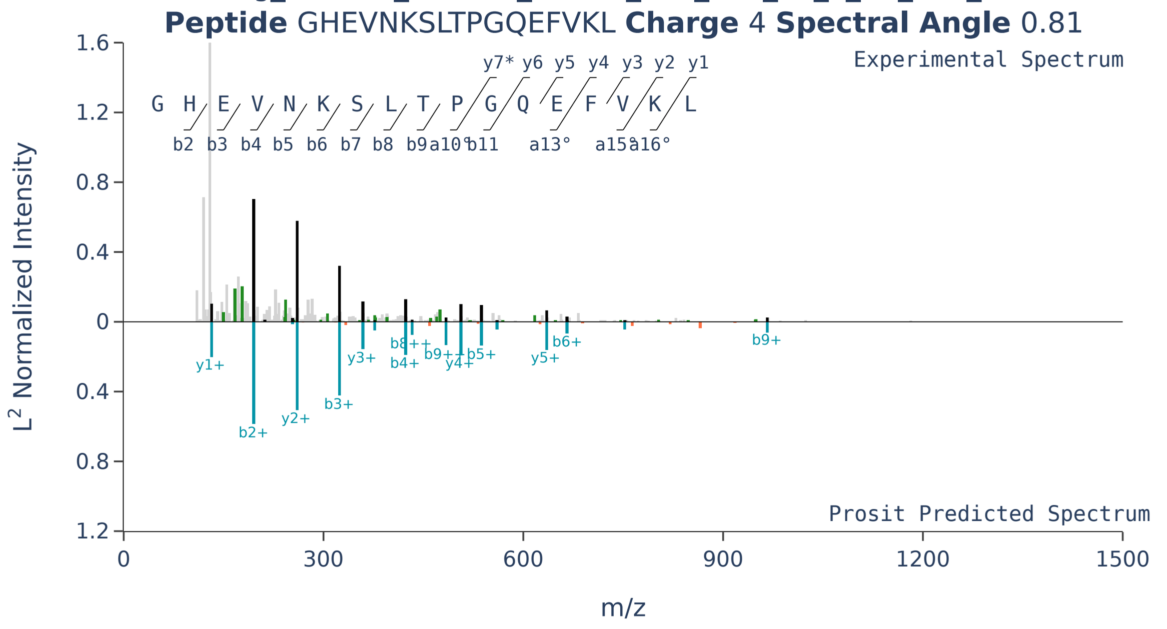


**Source** Ncheng_210623_230623_HFGoe_FFH_20S_25_1_A2_2h_R1 **Scan** 27616
**Peptide** MFDNLTDRLSRTL **Charge** 2 **Spectral Angle** 0.81

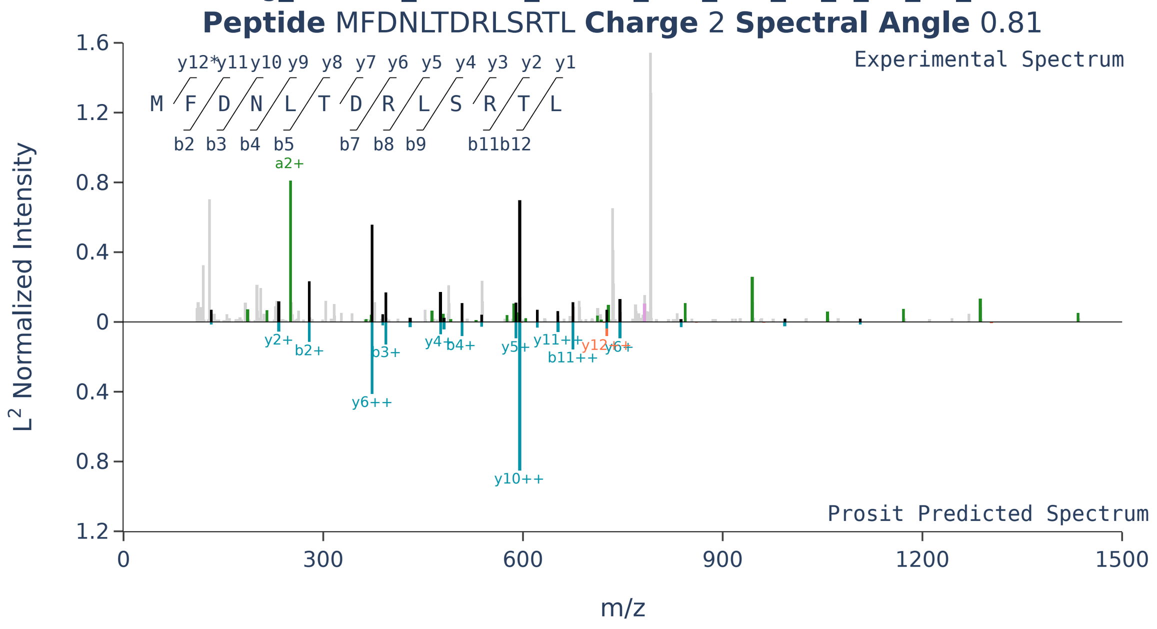


**Source** Ncheng_210623_230623_HFGoe_FFH_20S_25_1_A1_2h_R1 **Scan** 27464
**Peptide** VAAMGEENQTLNLAAQPPAV **Charge** 2 **Spectral Angle** 0.96

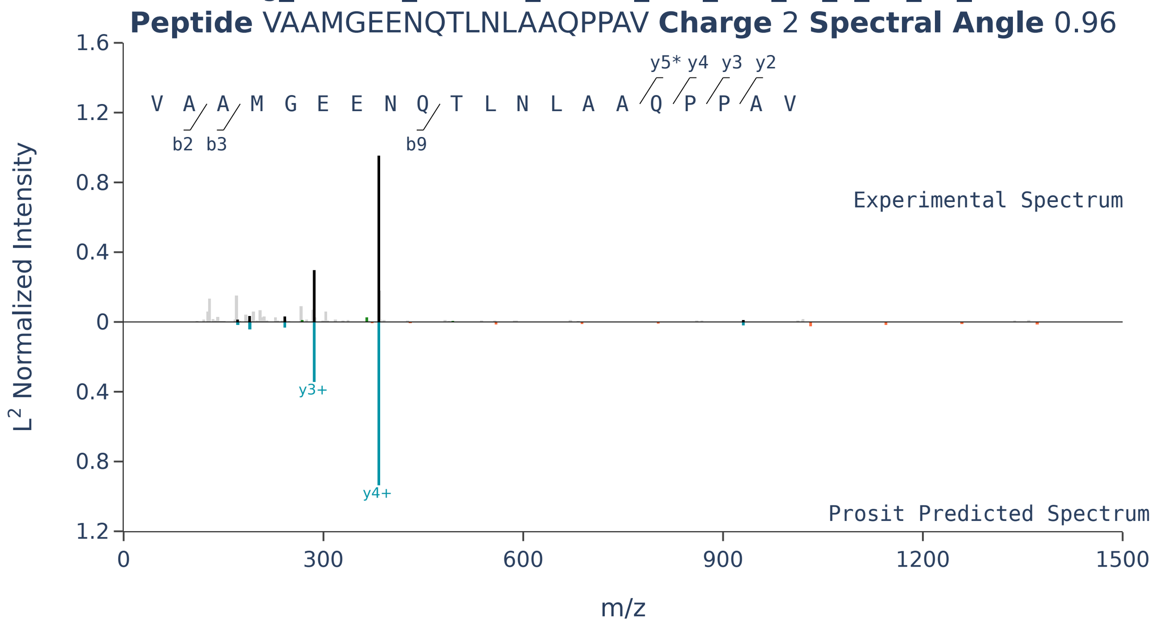


**Source** Ncheng_210623_230623_HFGoe_FFH_20S_25_1_A1_24h_R2 **Scan** 28705
**Peptide** AAQPPAVVLM **Charge** 2 **Spectral Angle** 0.77

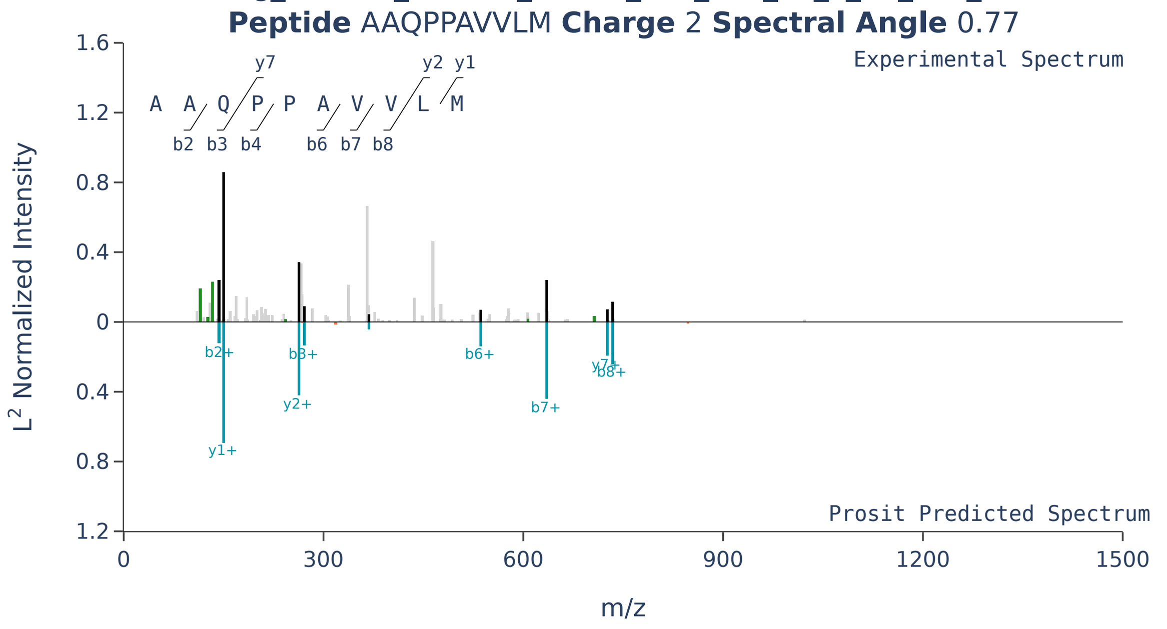

**Source** Ncheng_210623_230623_HFGoe_FFH_20S_25_1_A1_4h_R1 **Scan** 16625
**Peptide** GEENQTLNLAAQ **Charge** 2 **Spectral Angle** 0.94

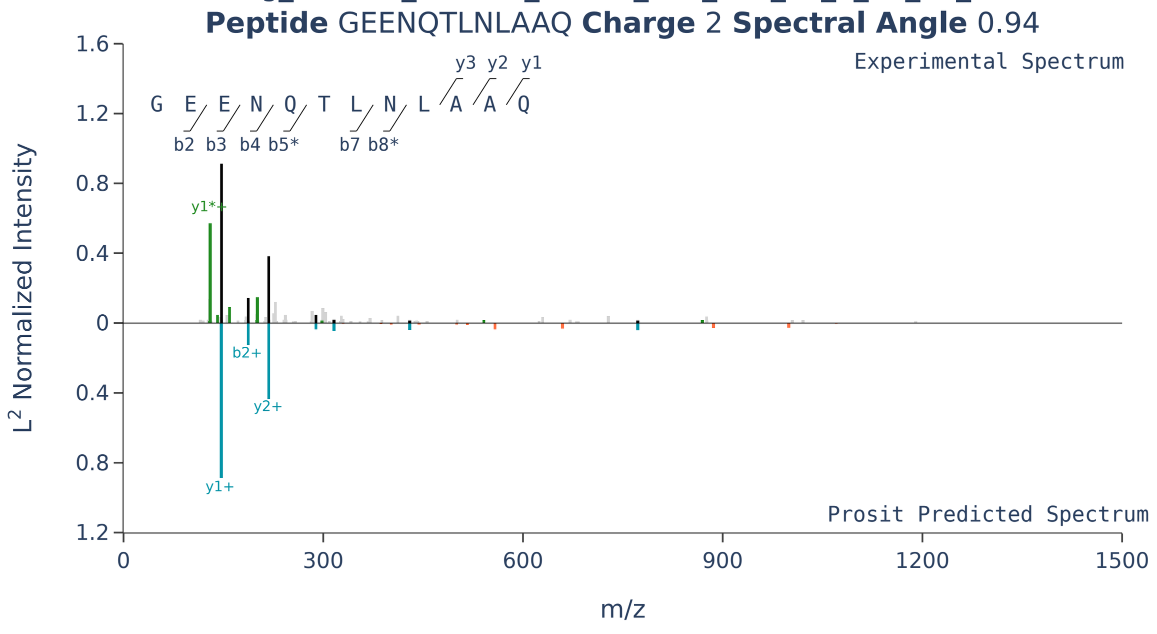


**Source** Ncheng_210623_230623_HFGoe_FFH_20S_25_1_A2_4h_R2 **Scan** 11763
**Peptide** LRHITGKPIKF **Charge** 3 **Spectral Angle** 0.83

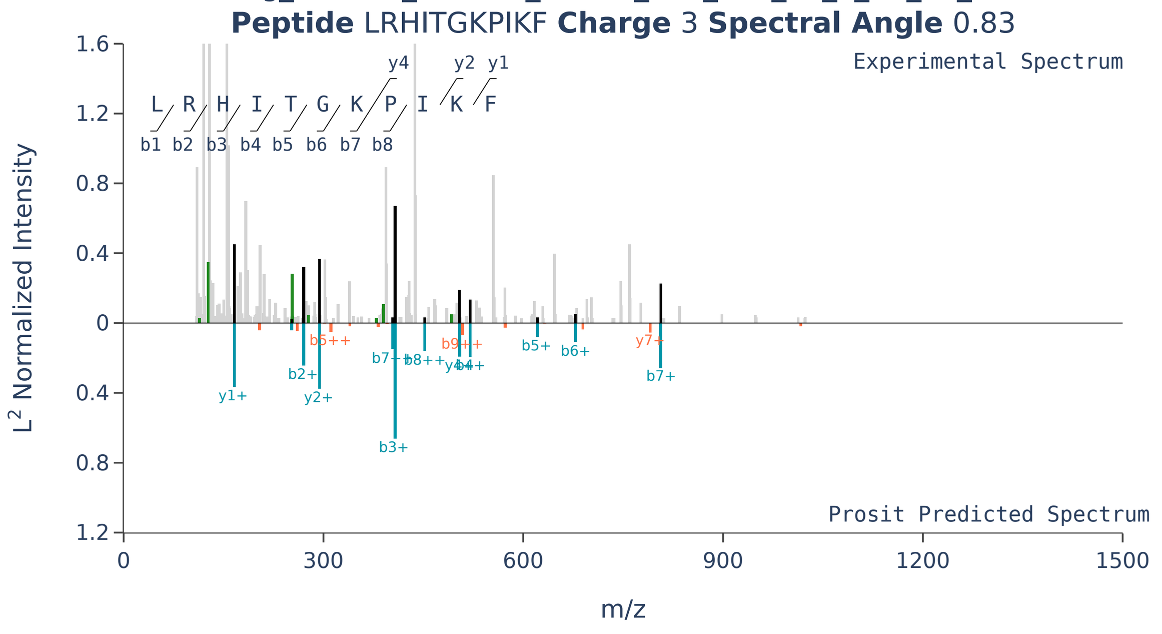


**Source** Ncheng_210623_230623_HFGoe_FFH_20S_25_1_A2_24h_R1 **Scan** 17439
**Peptide** HITGKPIKFL **Charge** 3 **Spectral Angle** 0.84

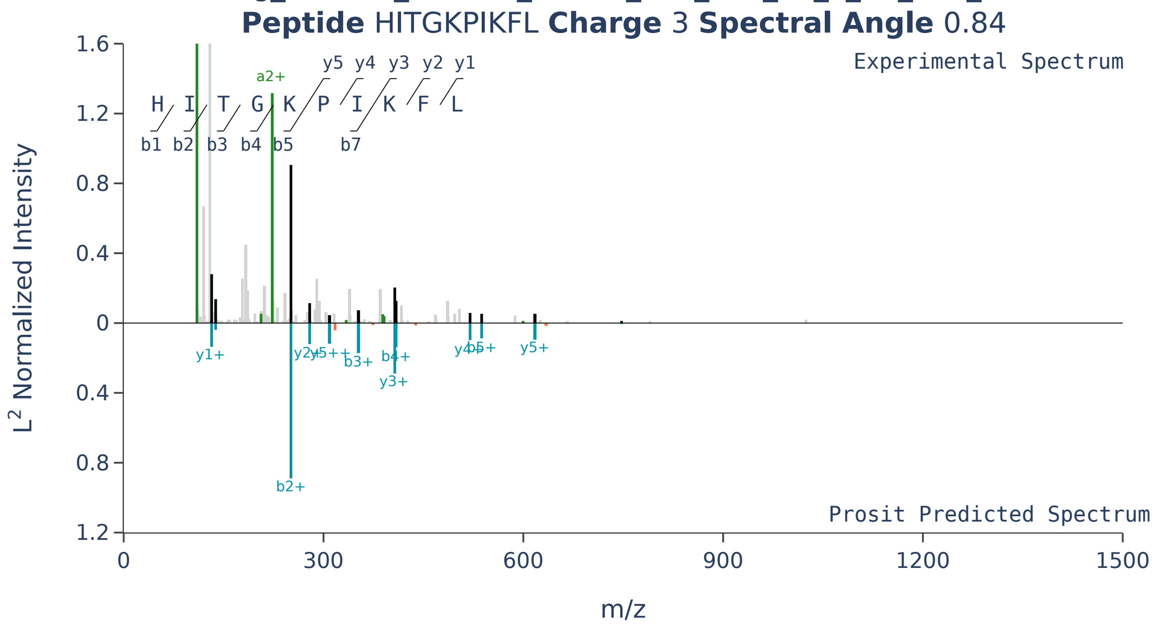


**Source** Ncheng_210623_230623_HFGoe_FFH_20S_25_1_A2_1h_R1 **Scan** 6784
**Peptide** HVDEAMMD **Charge** 2 **Spectral Angle** 0.78

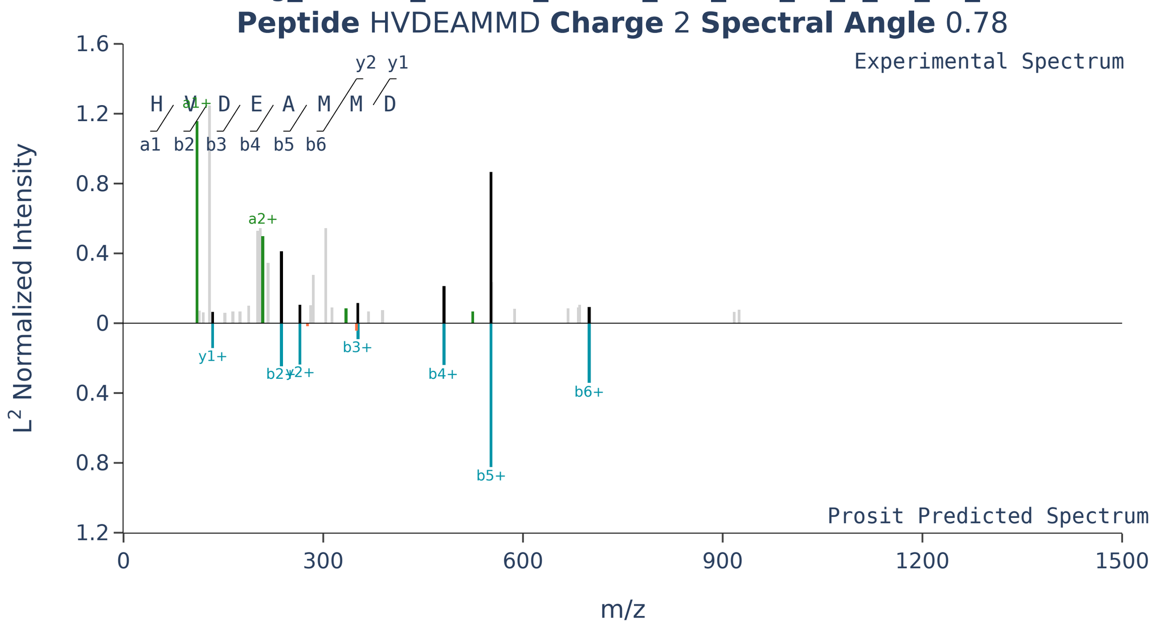


**Source** Ncheng_210623_230623_HFGoe_FFH_20S_25_1_A1_1h_R1 **Scan** 29590
**Peptide** KSLTPGQEFVKIVRNELVA **Charge** 3 **Spectral Angle** 0.84

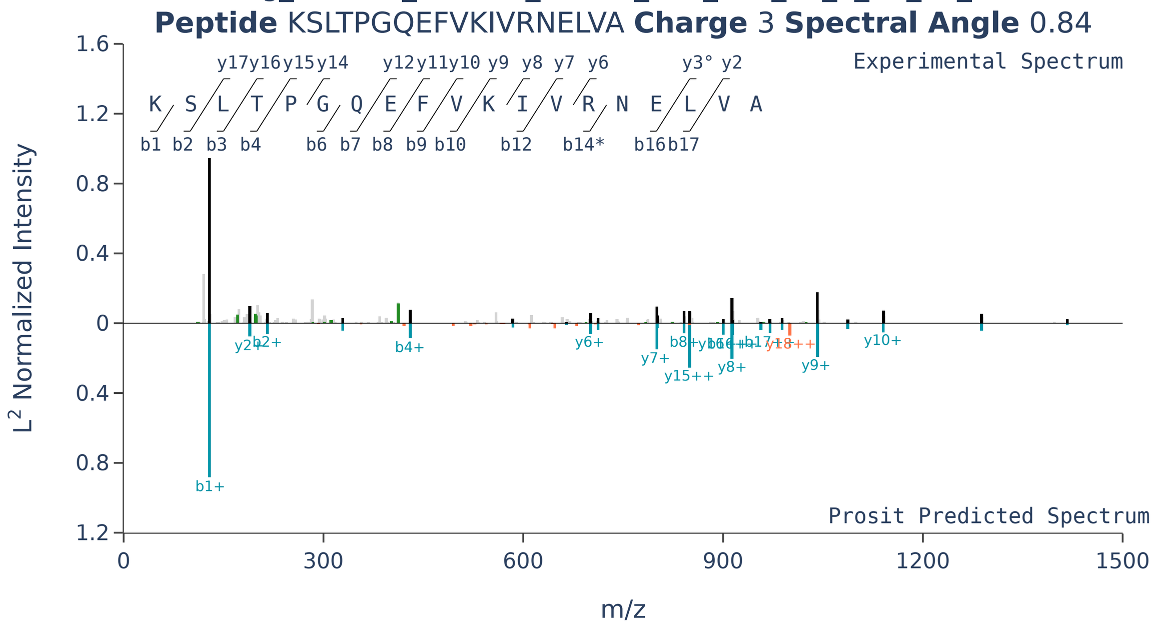


**Source** Ncheng_210623_230623_HFGoe_FFH_20S_25_1_A2_24h_R1 **Scan** 6108
**Peptide** PEIIKGSRK **Charge** 1 **Spectral Angle** 0.91

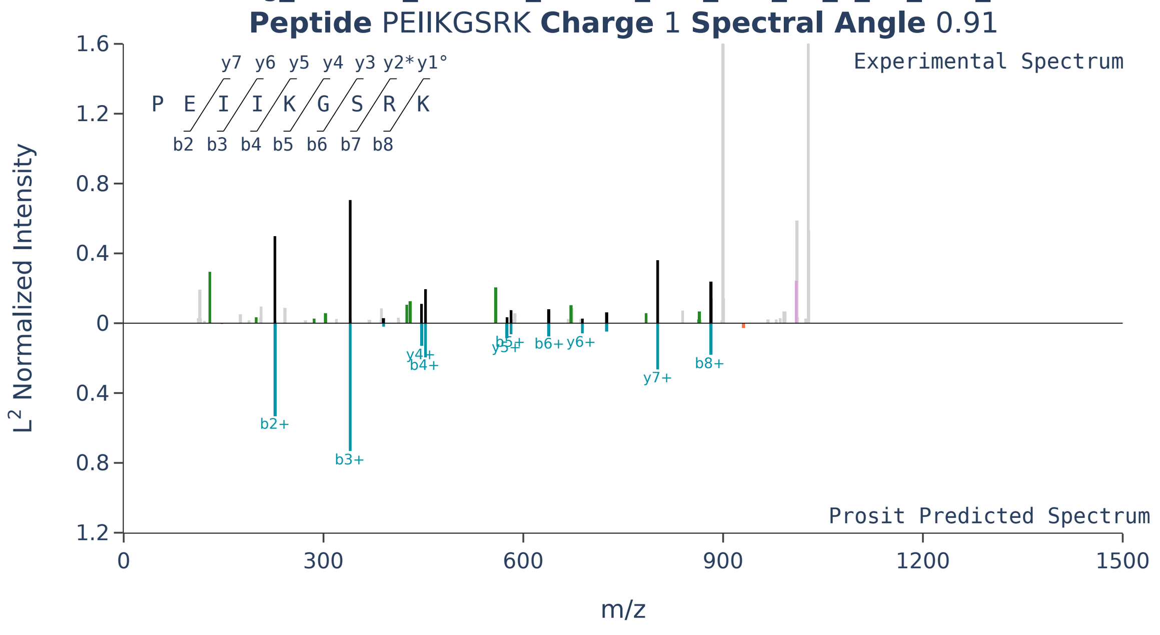


**Source** Ncheng_210623_230623_HFGoe_FFH_20S_25_1_A1_2h_R1 **Scan** 26882
**Peptide** ASLMGKLPGMGQIPDNVKSQMD **Charge** 3 **Spectral Angle** 0.72

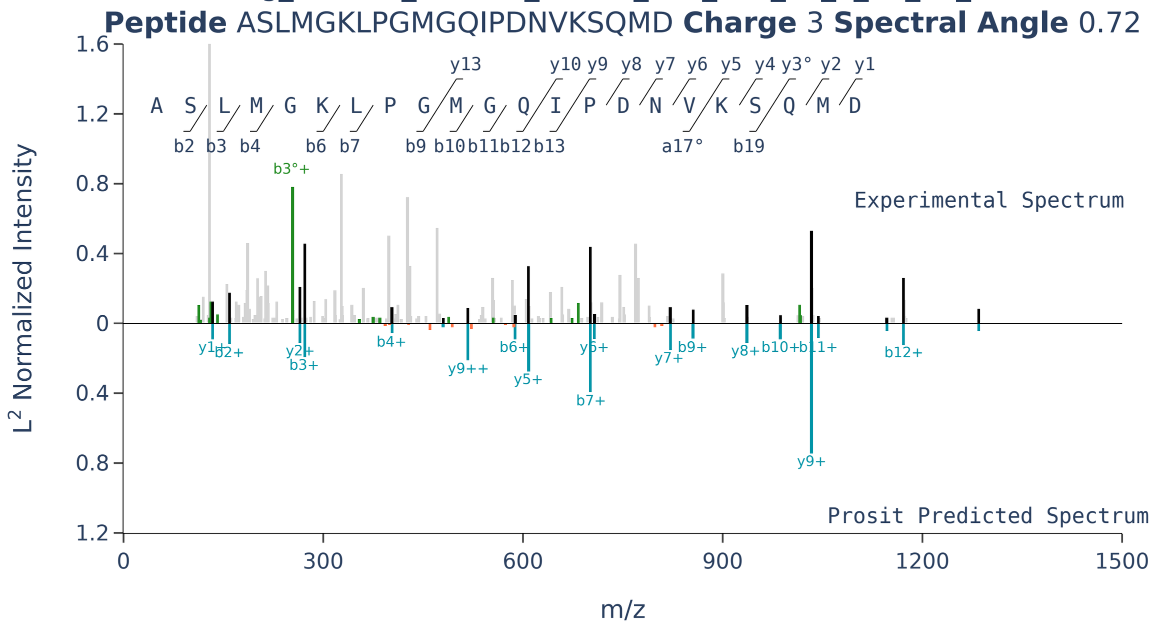


**Source** Ncheng_210623_230623_HFGoe_FFH_20S_25_1_A2_4h_R1 **Scan** 29110
**Peptide** DNVKDTLREVRMALL **Charge** 2 **Spectral Angle** 0.79

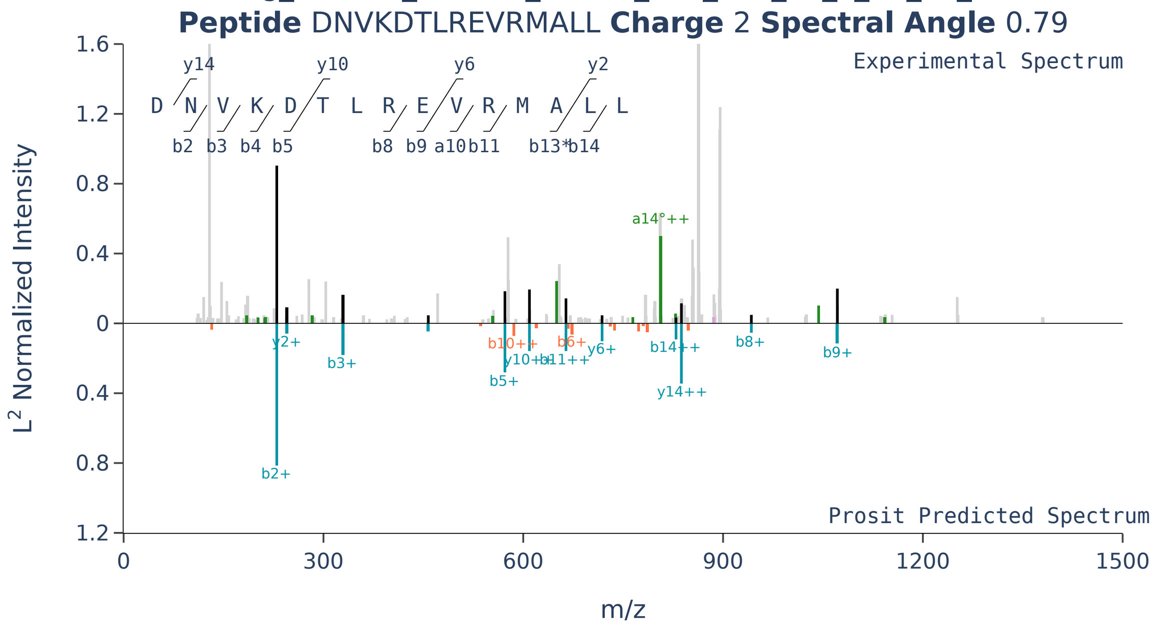


**Source** Ncheng_210623_230623_HFGoe_FFH_20S_25_1_A1_24h_R1 **Scan** 13815
**Peptide** RHITGKPIKFLGVGEKTE **Charge** 3 **Spectral Angle** 0.79

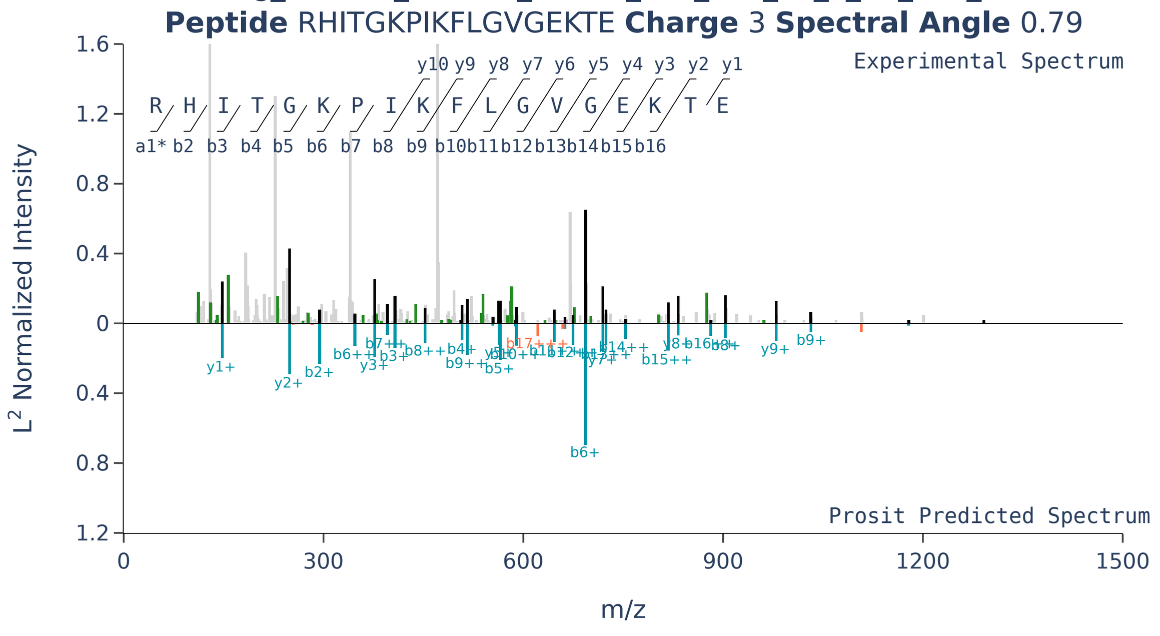


**Source** Ncheng_210623_230623_HFGoe_FFH_20S_25_1_A2_2h_R2 **Scan** 28694
**Peptide** MASLMGKLPGMGQIPDNVKSQMD **Charge** 3 **Spectral Angle** 0.8

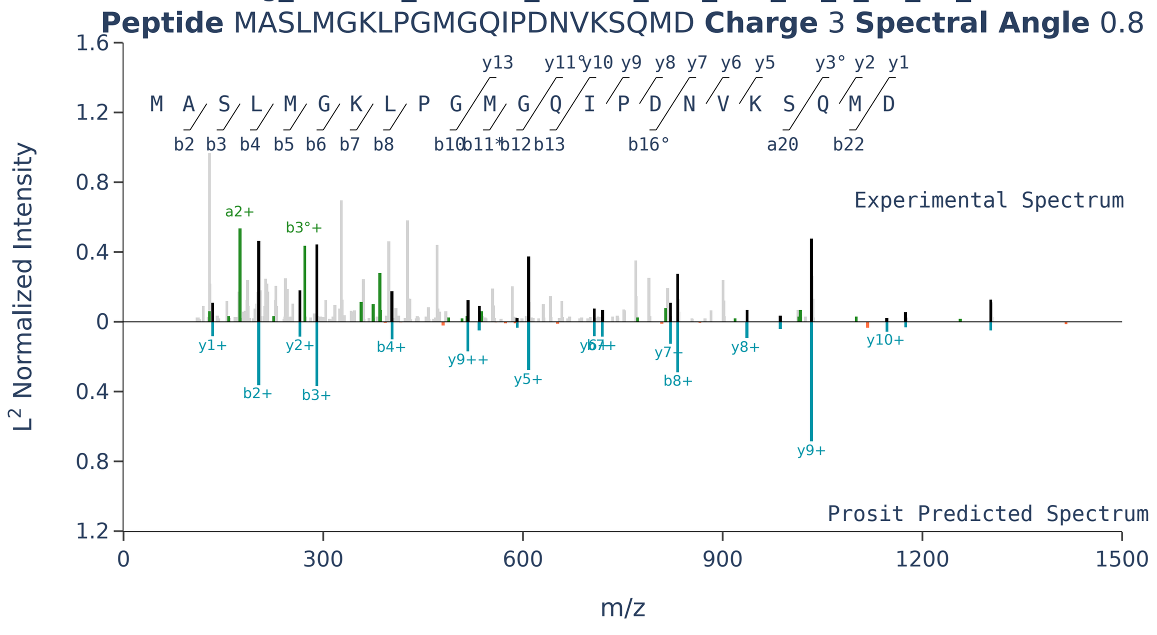


**Source** Ncheng_210623_230623_HFGoe_FFH_20S_25_1_A1_24h_R1 **Scan** 12218
**Peptide** AGLQGAGKTTSVGKLG **Charge** 2 **Spectral Angle** 0.85

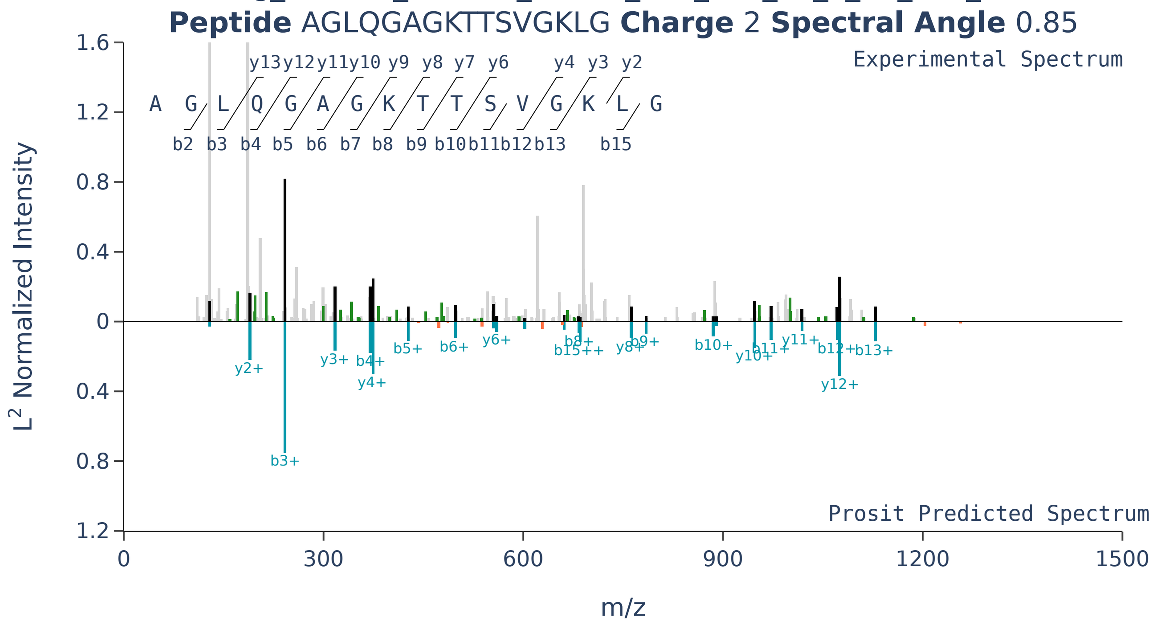


**Source** Ncheng_210623_230623_HFGoe_FFH_20S_25_1_A1_4h_R1 **Scan** 21591
**Peptide** RIAAGCGMQVQDVNRLLKQ **Charge** 3 **Spectral Angle** 0.78

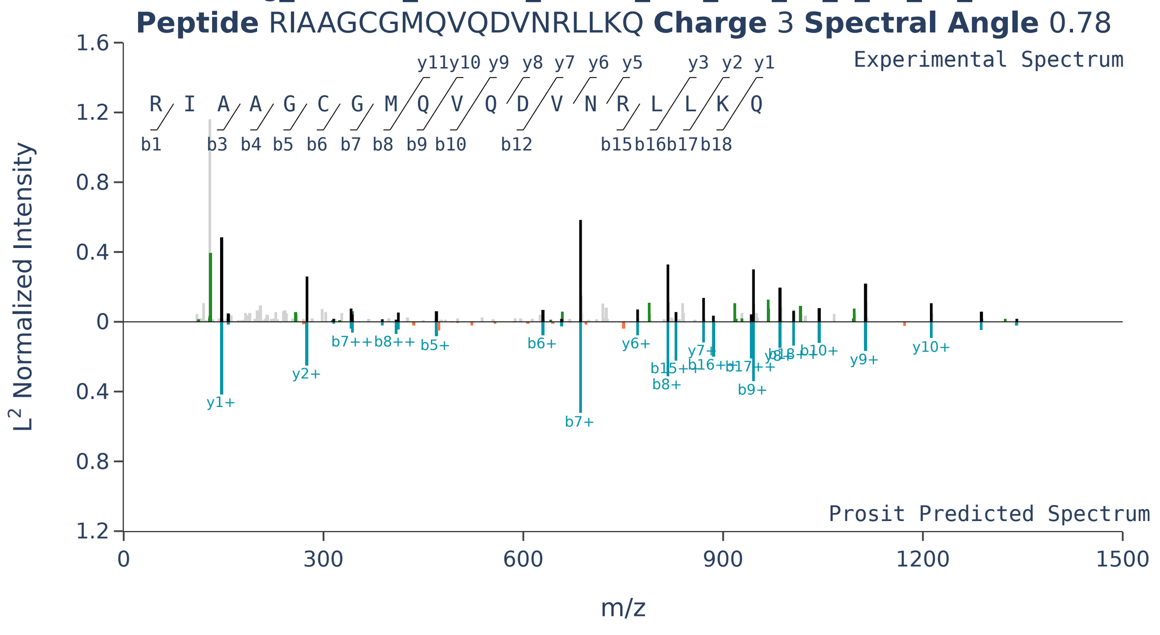


**Source** Ncheng_210623_230623_HFGoe_FFH_20S_25_1_A1_2h_R2 **Scan** 21134
**Peptide** GQKPVDIVNAALKE **Charge** 3 **Spectral Angle** 0.69

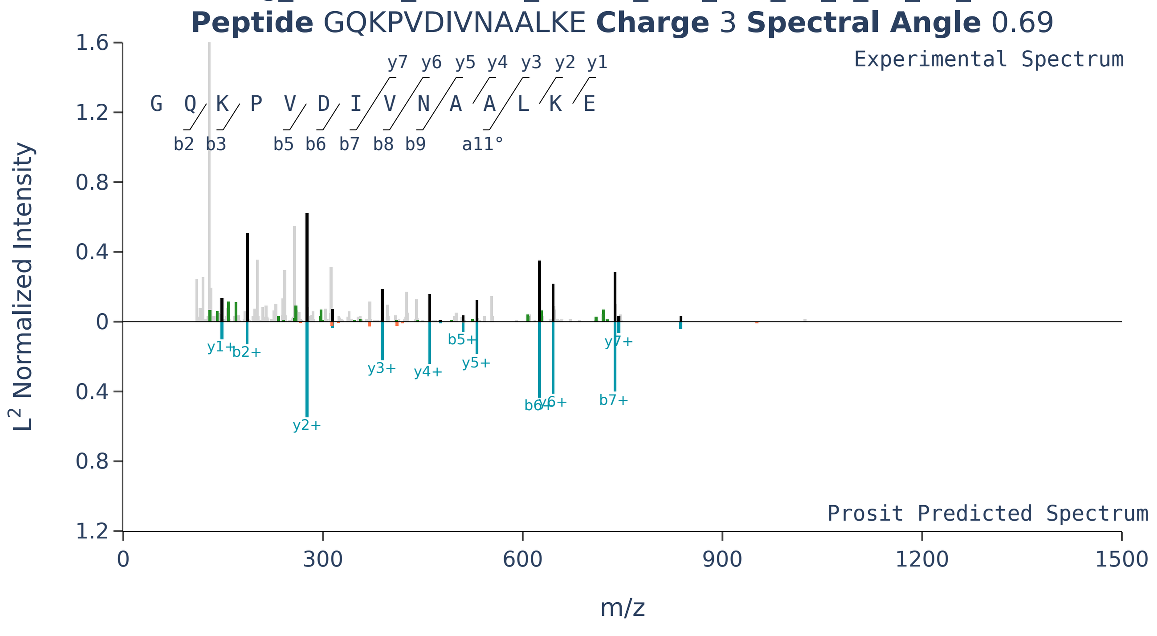


**Source** Ncheng_210623_230623_HFGoe_FFH_20S_25_1_A1_24h_R1 **Scan** 20140
**Peptide** GKPIKFLGVGE **Charge** 3 **Spectral Angle** 0.93

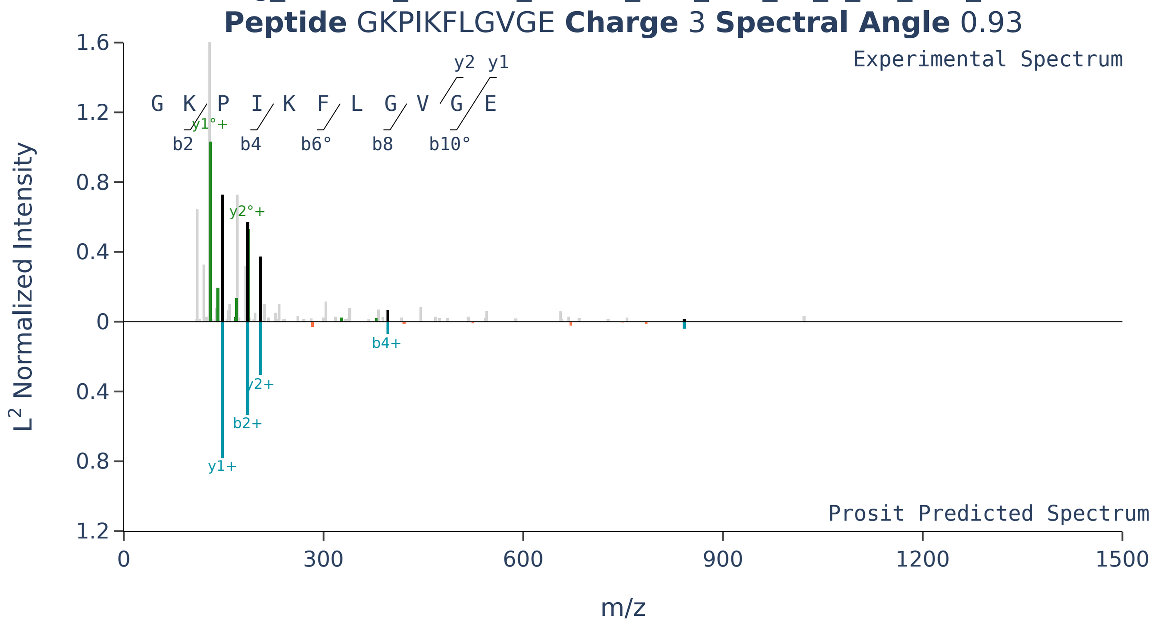


**Source** Ncheng_210623_230623_HFGoe_FFH_20S_25_1_A2_24h_R1 **Scan** 10164
**Peptide** QDAANTAKAFNE **Charge** 1 **Spectral Angle** 0.8

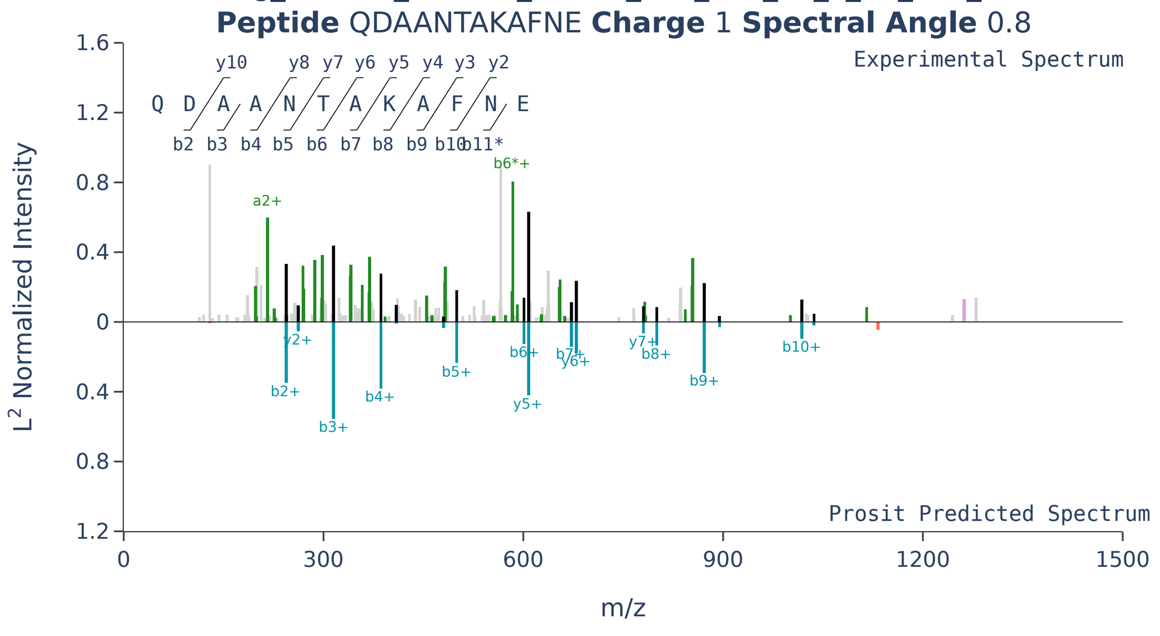

**Source** Ncheng_210623_230623_HFGoe_FFH_20S_25_1_A2_24h_R1 **Scan** 6912
**Peptide** KGGMAKMMRS **Charge** 3 **Spectral Angle** 0.85

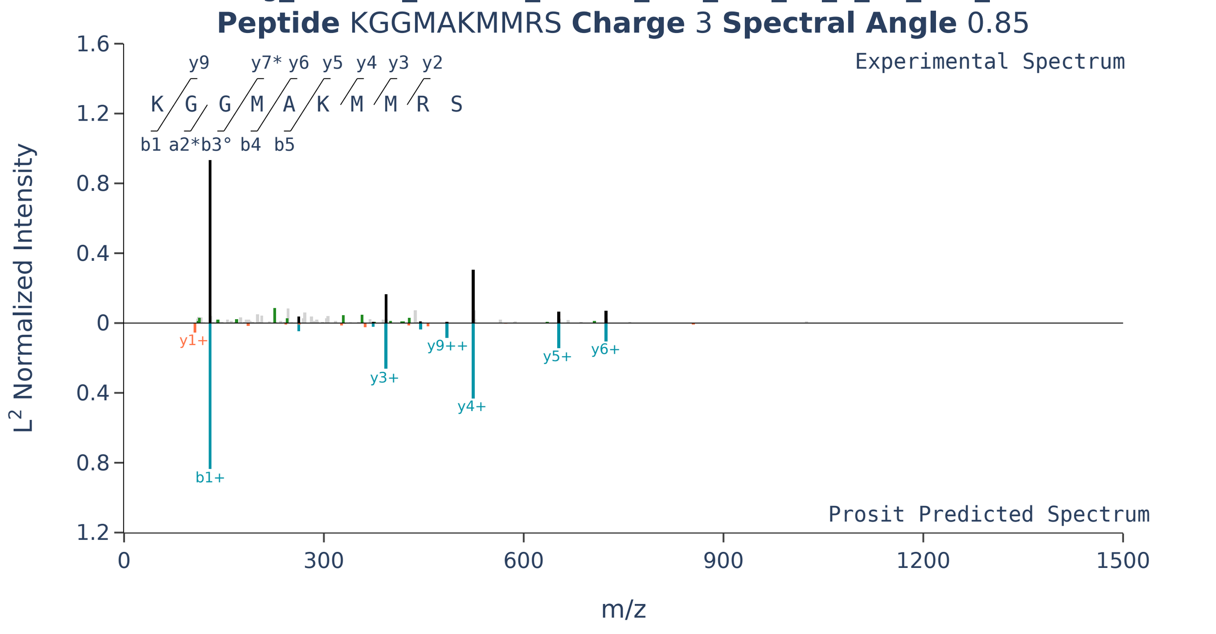


**Source** Ncheng_210623_230623_HFGoe_FFH_20S_25_1_A2_2h_R2 **Scan** 21544
**Peptide** NKSLTPGQEFVKIVRNE **Charge** 2 **Spectral Angle** 0.85

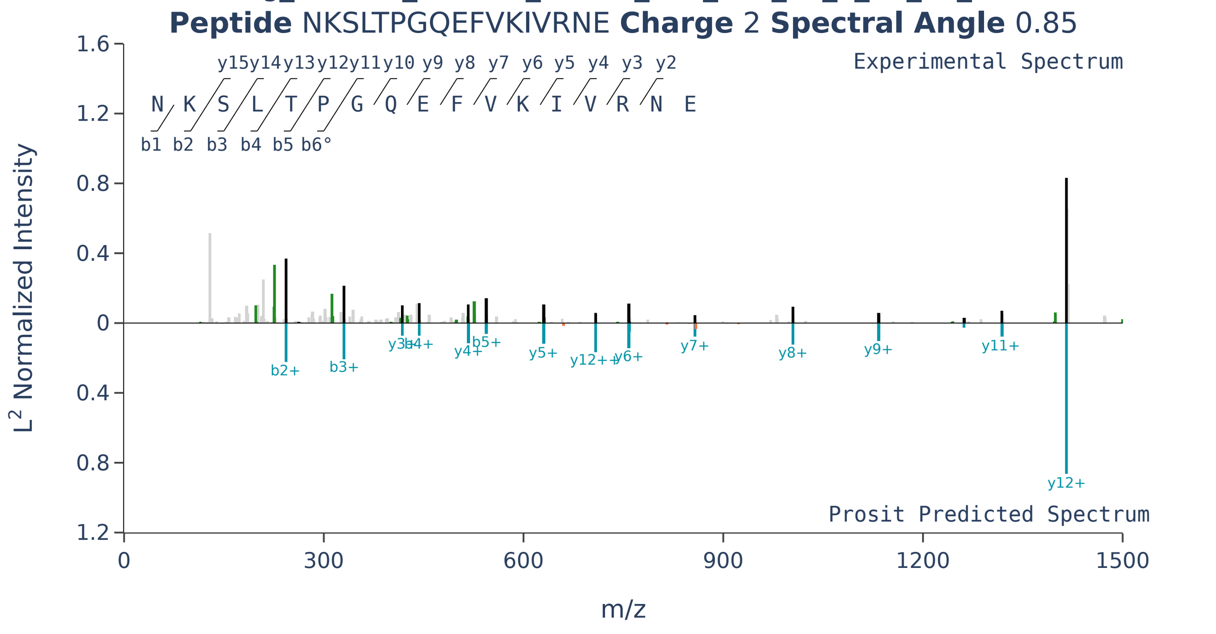


**Source** Ncheng_210623_230623_HFGoe_FFH_20S_25_1_A2_24h_R2 **Scan** 13324
**Peptide** GRLTEDNVKDTL **Charge** 3 **Spectral Angle** 0.77

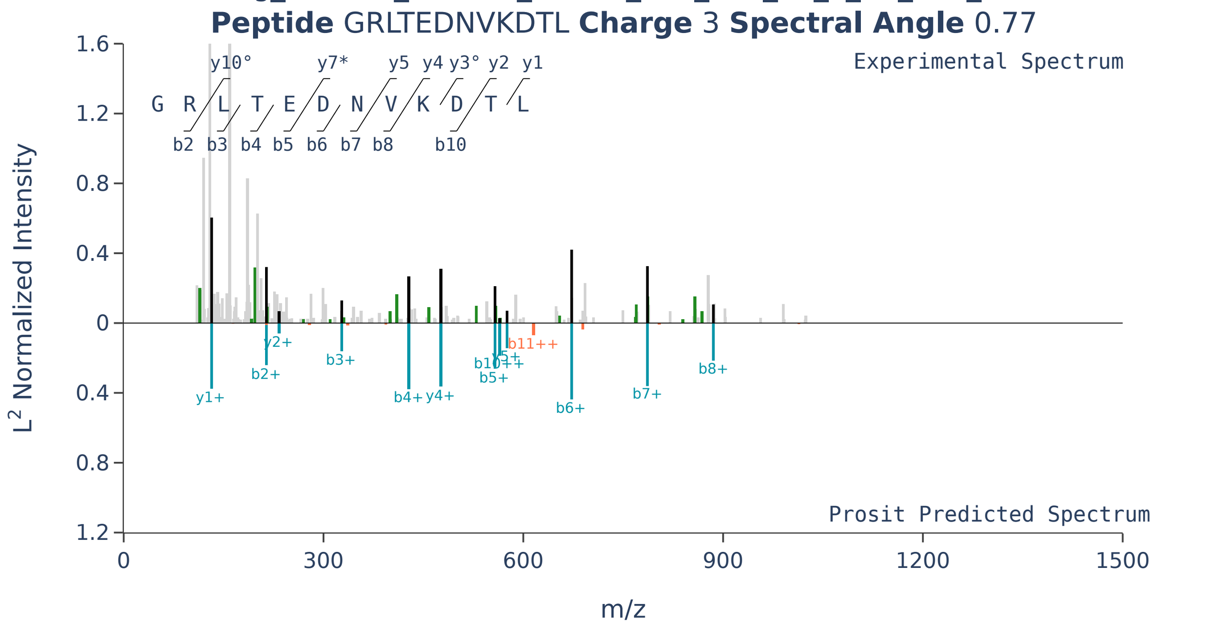


**Source** Ncheng_210623_230623_HFGoe_FFH_20S_25_1_A2_4h_R2 **Scan** 10736
**Peptide** TGQDAANTAKAF **Charge** 1 **Spectral Angle** 0.75

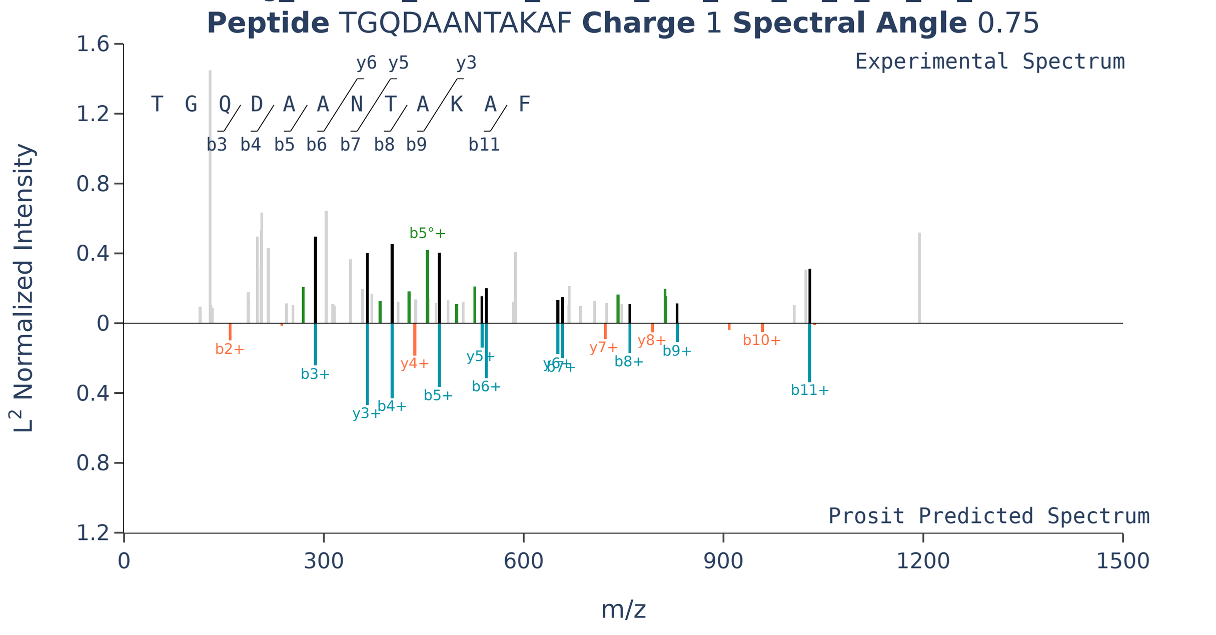


**Source** Ncheng_210623_230623_HFGoe_FFH_20S_25_1_A1_4h_R1 **Scan** 24462
**Peptide** AANTAKAFNEALPLT **Charge** 2 **Spectral Angle** 0.9

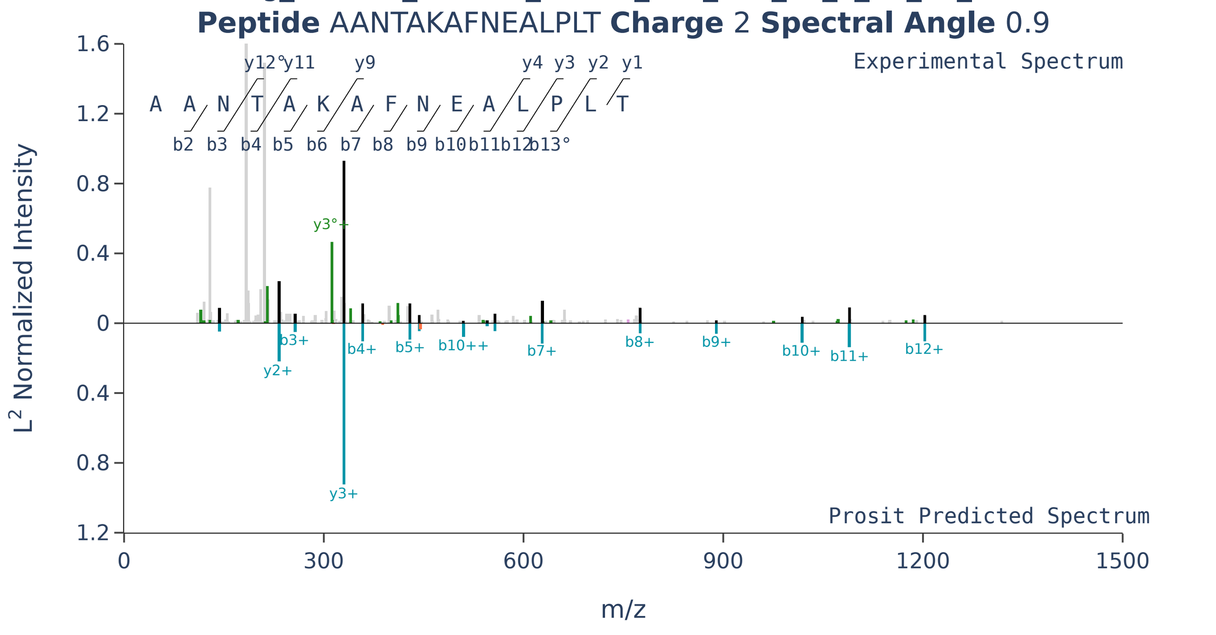


**Source** Ncheng_210623_230623_HFGoe_FFH_20S_25_1_A2_4h_R2 **Scan** 12932
**Peptide** KAVGHEVNKSLTPGQEF **Charge** 2 **Spectral Angle** 0.8

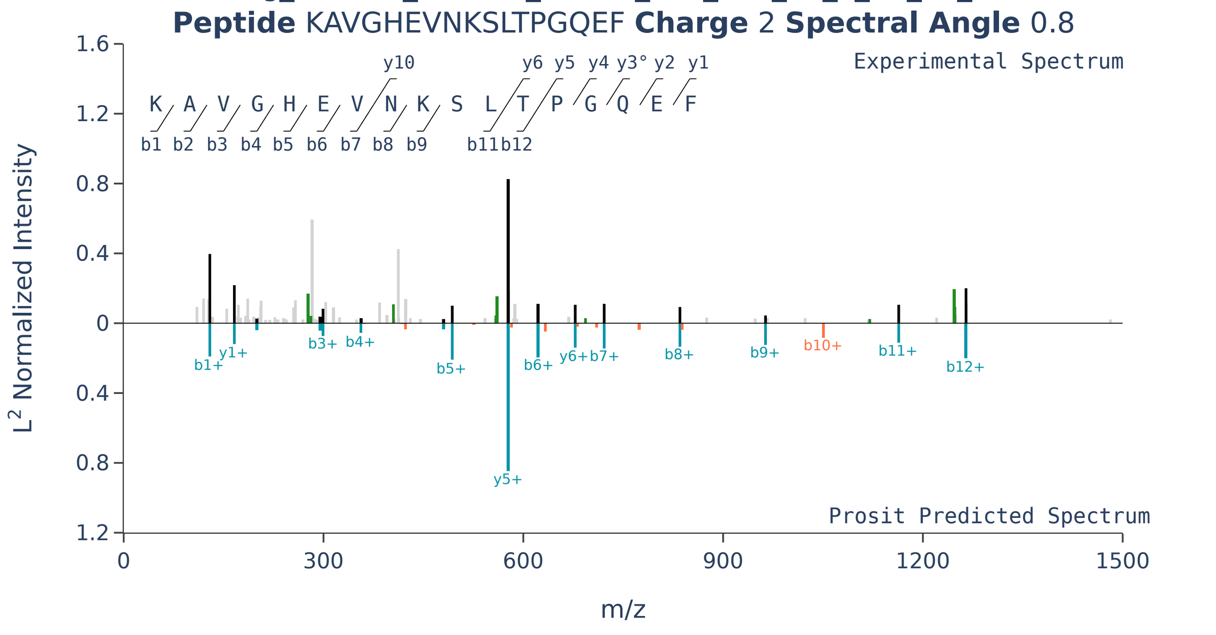


**Source** Ncheng_210623_230623_HFGoe_FFH_20S_25_1_A2_24h_R2 **Scan** 19711
**Peptide** EALEPFHPDRLAS **Charge** 3 **Spectral Angle** 0.8

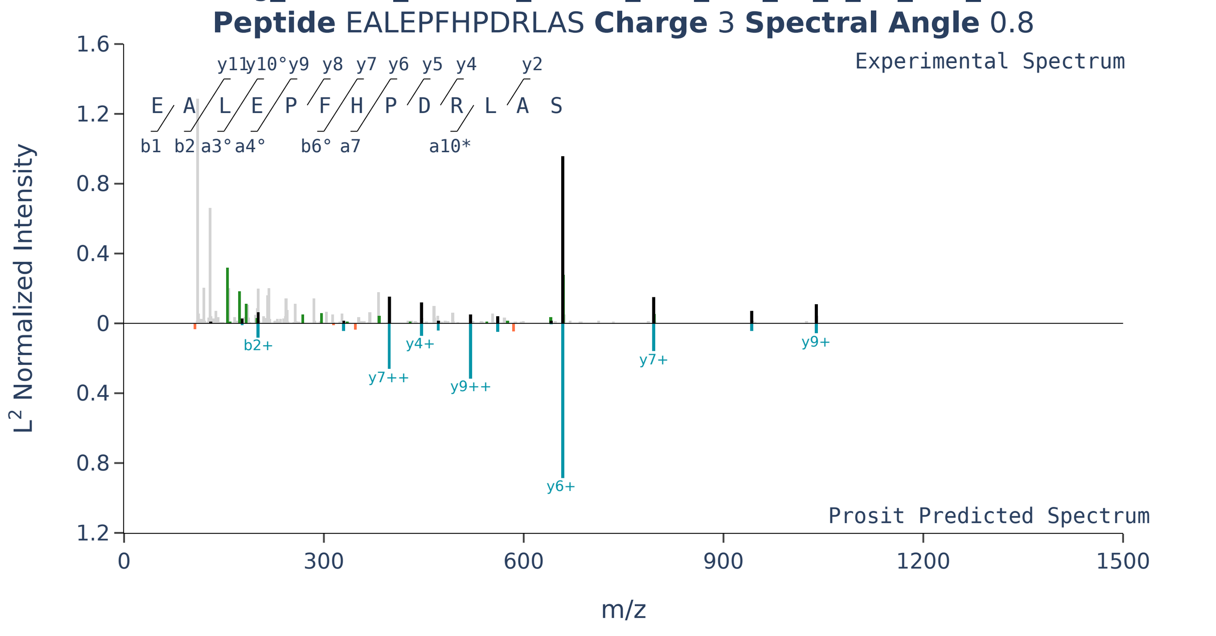


**Source** Ncheng_210623_230623_HFGoe_FFH_20S_25_1_A1_2h_R2 **Scan** 22605
**Peptide** LMGKLPGMGQIPDNVKSQ **Charge** 3 **Spectral Angle** 0.76

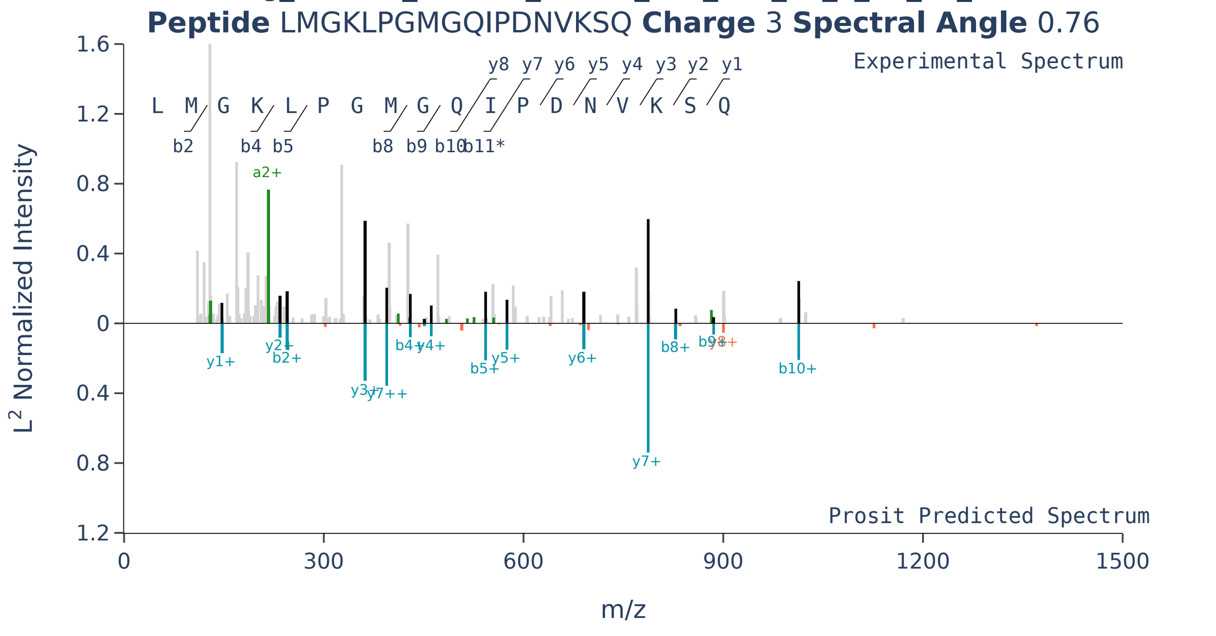


**Source** Ncheng_210623_230623_HFGoe_FFH_20S_25_1_A2_24h_R1 **Scan** 17300
**Peptide** EVNKSLTPGQEF **Charge** 1 **Spectral Angle** 0.8

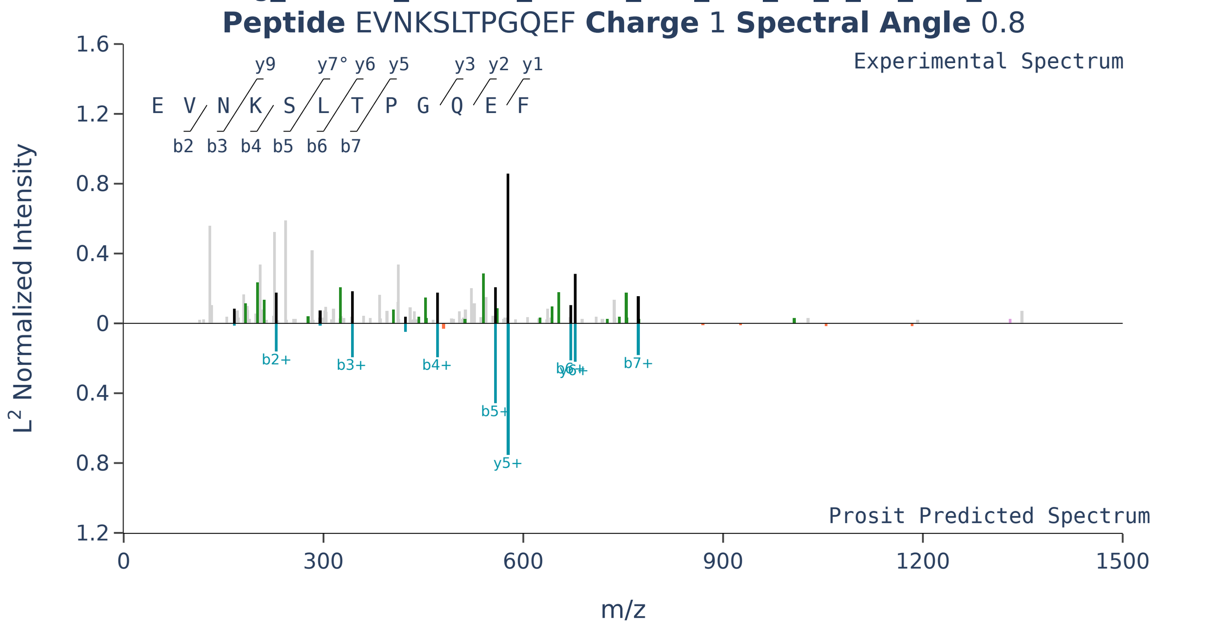


**Source** Ncheng_210623_230623_HFGoe_FFH_20S_25_1_A1_24h_R1 **Scan** 18999
**Peptide** DGDARGGAALSI **Charge** 2 **Spectral Angle** 0.78

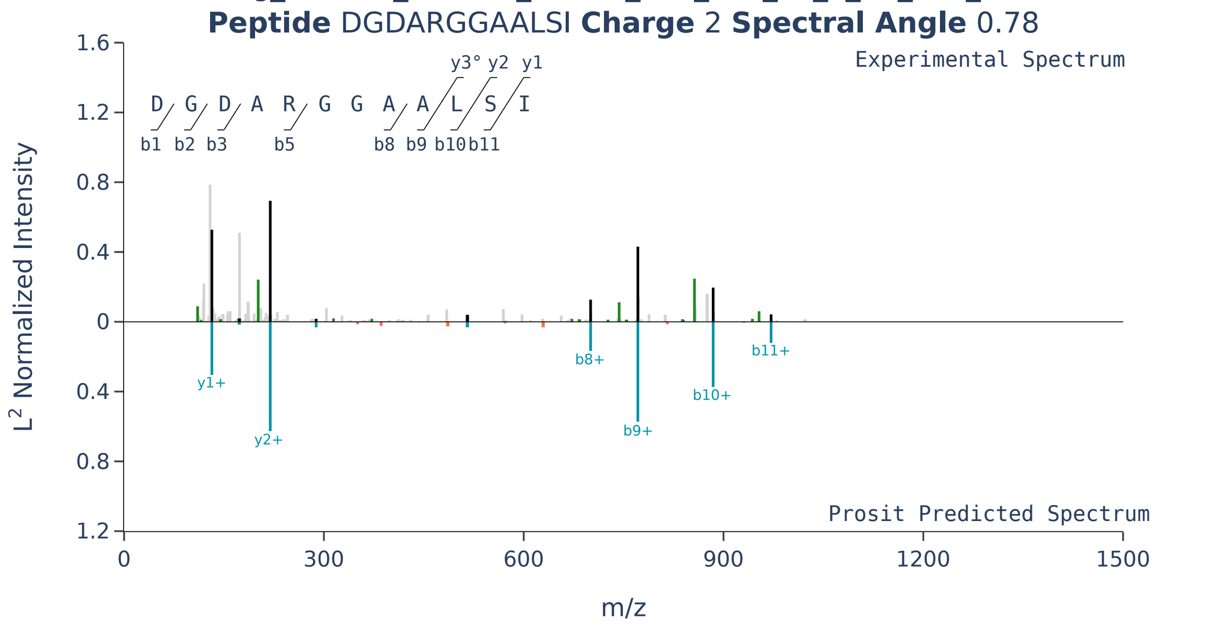


**Source** Ncheng_210623_230623_HFGoe_FFH_20S_25_1_A1_24h_R1 **Scan** 16013
**Peptide** MGQIPDNVKSQMDD **Charge** 2 **Spectral Angle** 0.87

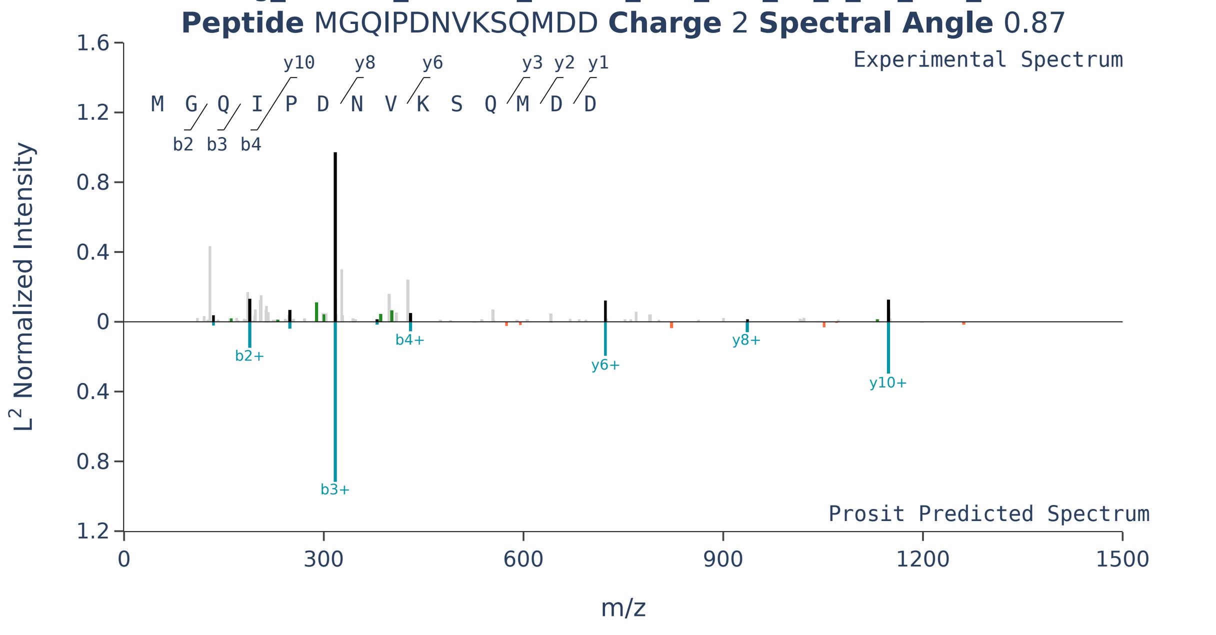


**Source** Ncheng_210623_230623_HFGoe_FFH_20S_25_1_A1_24h_R1 **Scan** 14269
**Peptide** GDARGGAALSIRHI **Charge** 2 **Spectral Angle** 0.74

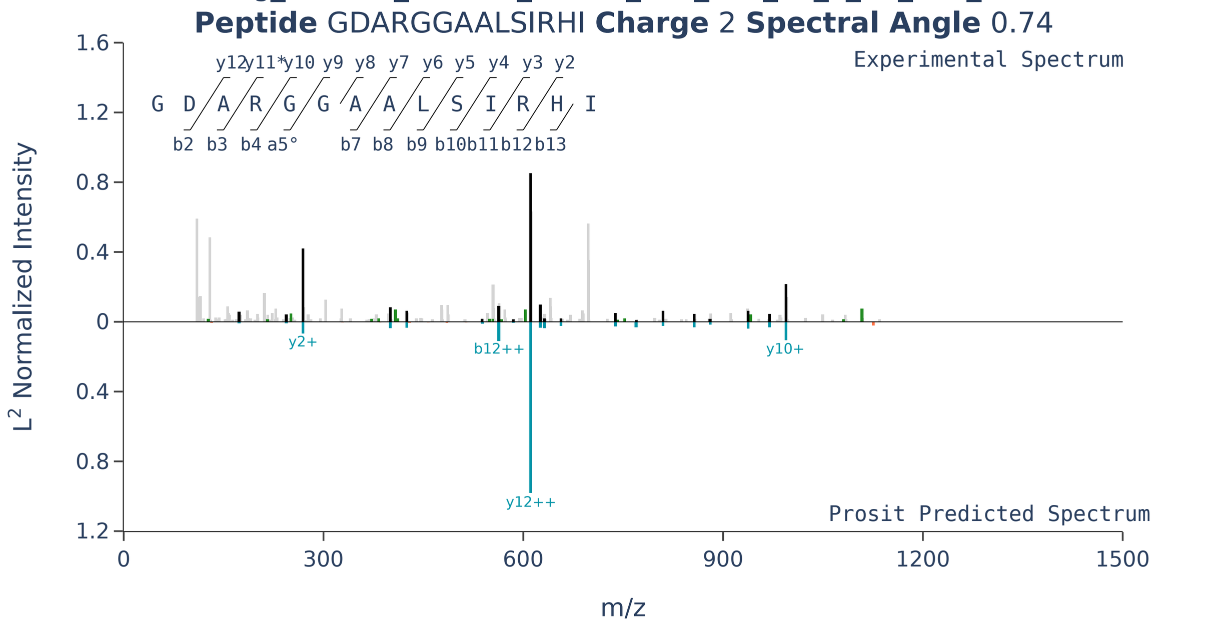


**Source** Ncheng_210623_230623_HFGoe_FFH_20S_25_1_A2_24h_R1 **Scan** 17330
**Peptide** LEPFHPDRLAS **Charge** 2 **Spectral Angle** 0.83

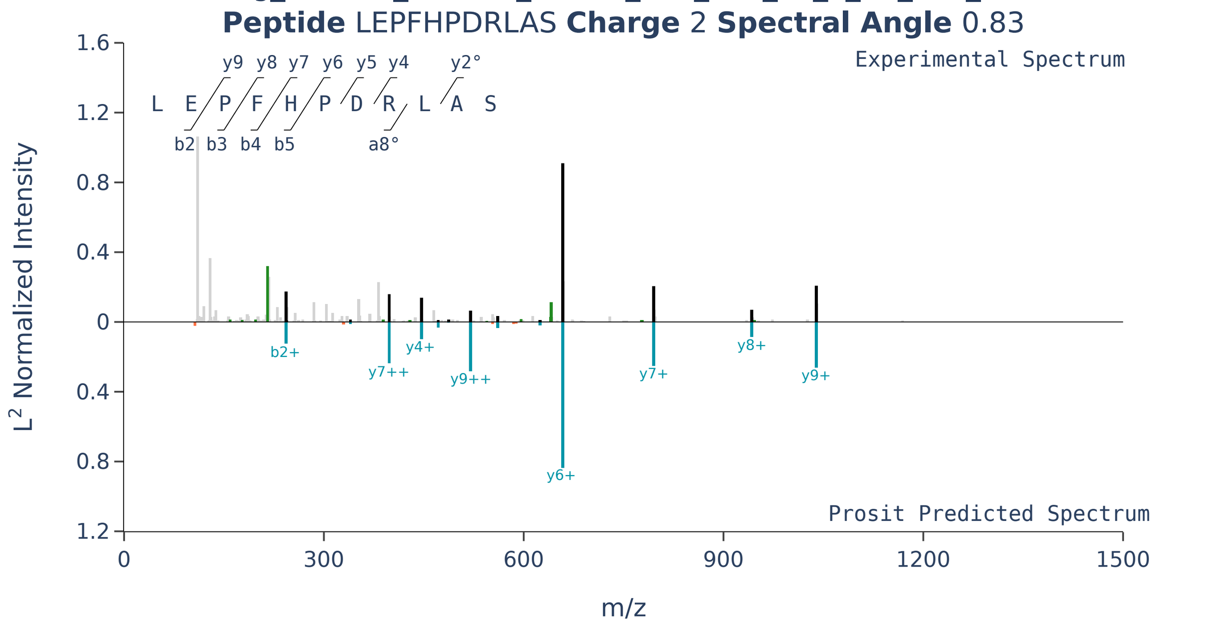


**Source** Ncheng_210623_230623_HFGoe_FFH_20S_25_1_A1_24h_R1 **Scan** 9728
**Peptide** RAQAEKLASKL **Charge** 3 **Spectral Angle** 0.79

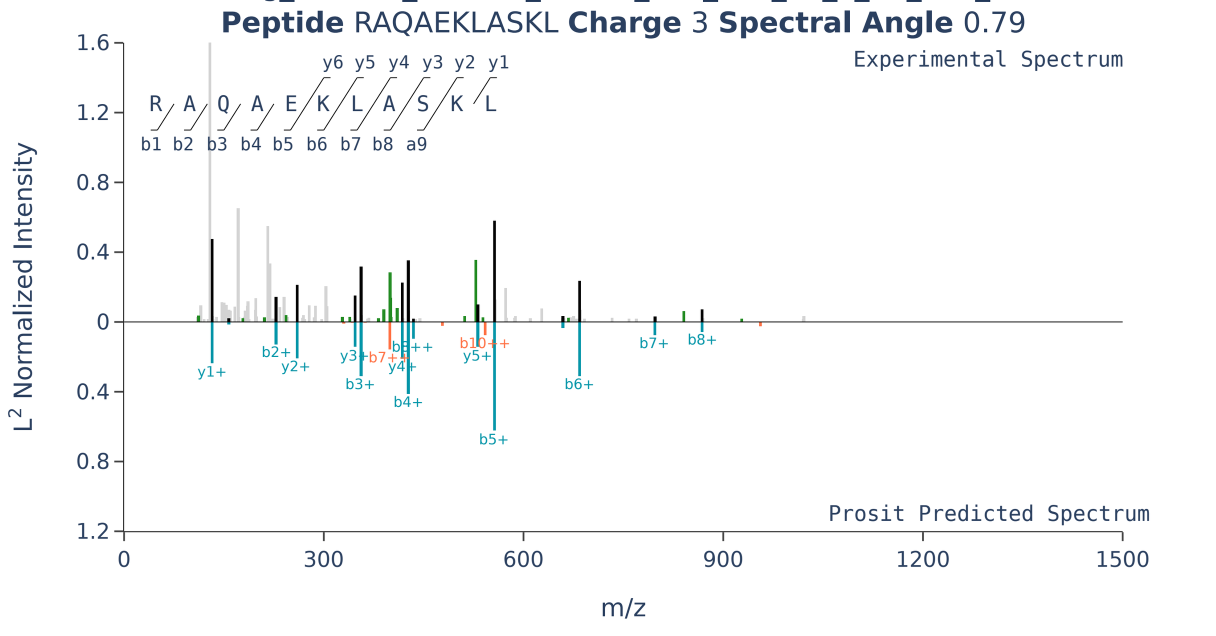


**Source** Ncheng_210623_230623_HFGoe_FFH_20S_25_1_A2_2h_R2 **Scan** 5524
**Peptide** SIRHITGK **Charge** 2 **Spectral Angle** 0.82

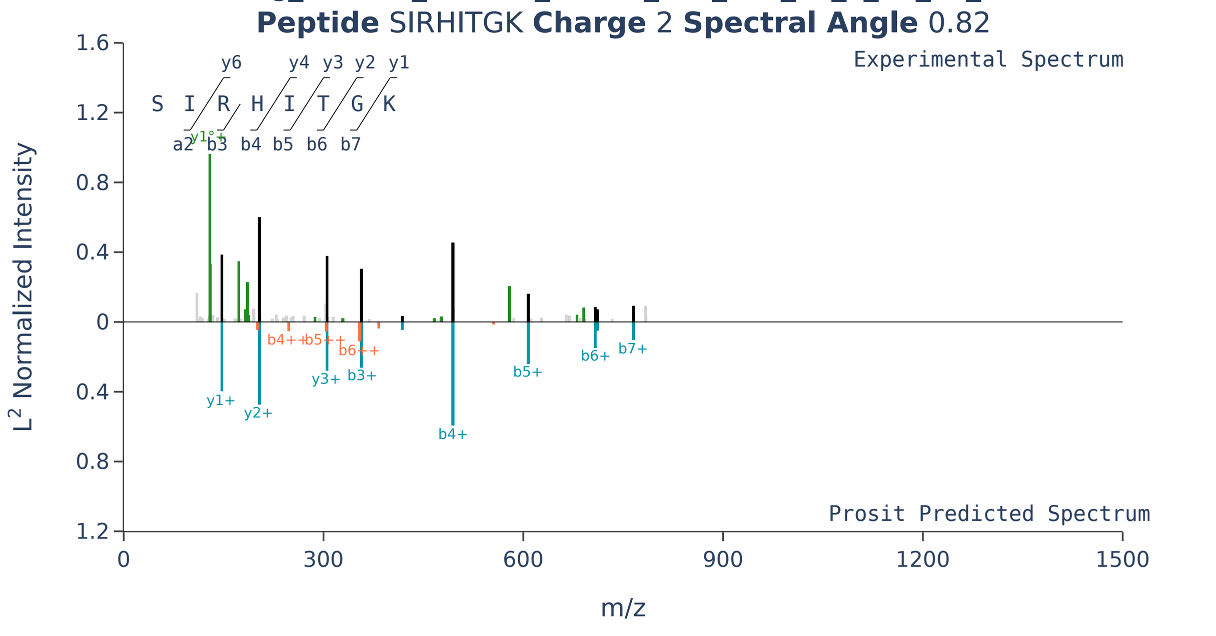

**Source** Ncheng_210623_230623_HFGoe_FFH_20S_25_1_A1_2h_R2 **Scan** 20371
**Peptide** GKLPGMGQIPDNVKSQMDD **Charge** 3 **Spectral Angle** 0.74

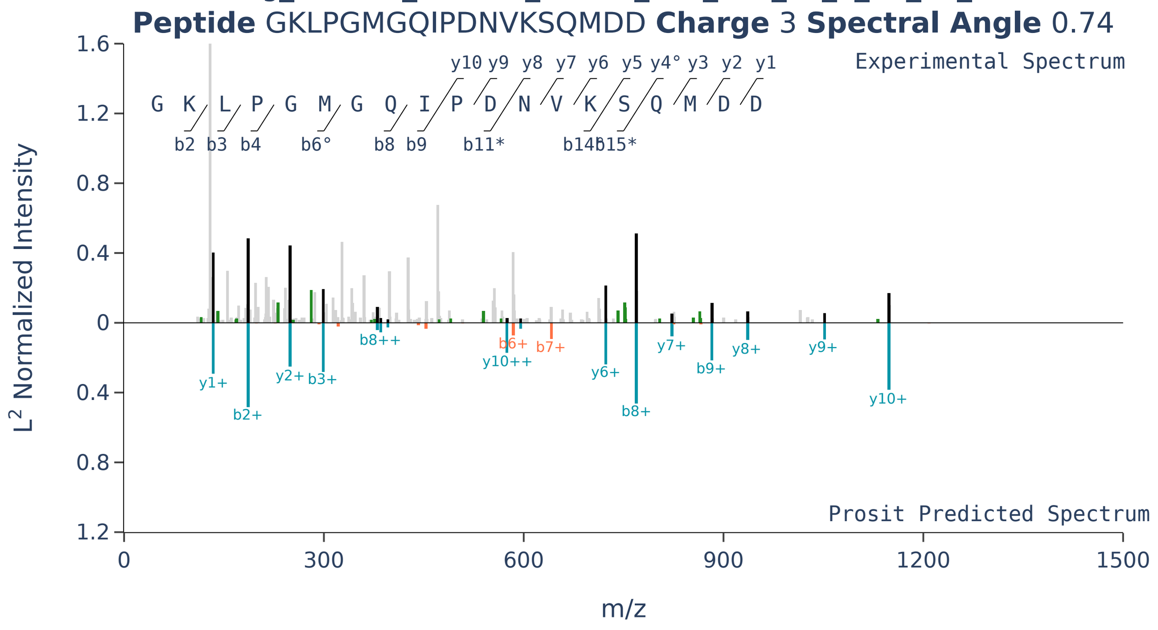


**Source** Ncheng_210623_230623_HFGoe_FFH_20S_25_1_A1_2h_R2 **Scan** 24876
**Peptide** PLTGVVLTKVDGDARGGAAL **Charge** 2 **Spectral Angle** 0.85

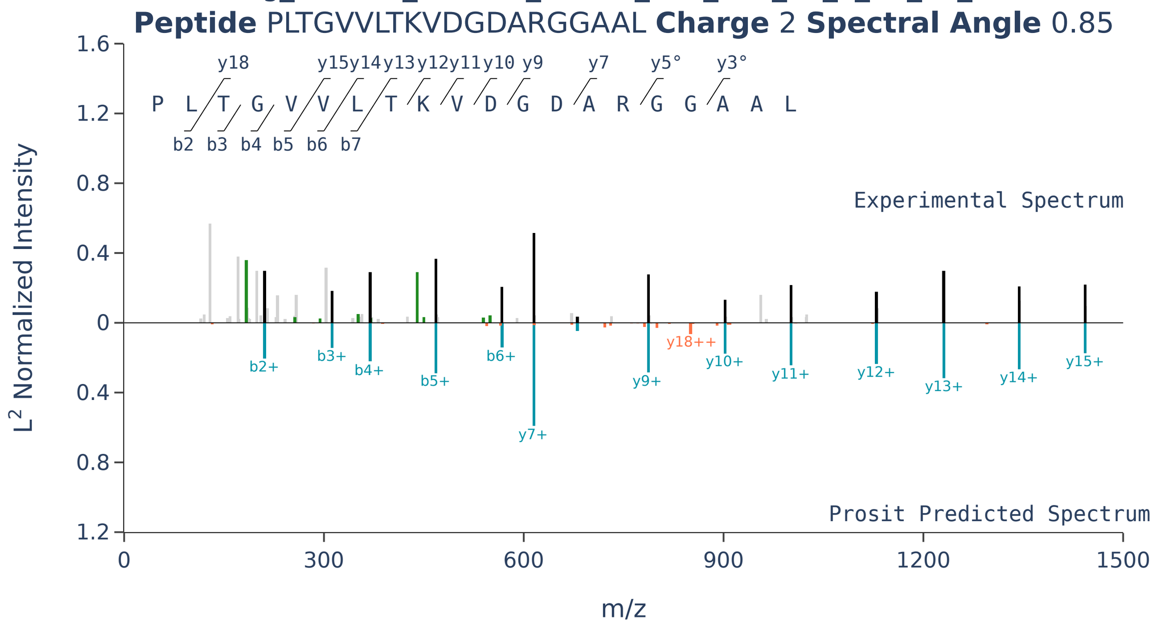


**Source** Ncheng_210623_230623_HFGoe_FFH_20S_25_1_A1_24h_R1 **Scan** 6956
**Peptide** GRLTEDNVKDT **Charge** 3 **Spectral Angle** 0.76

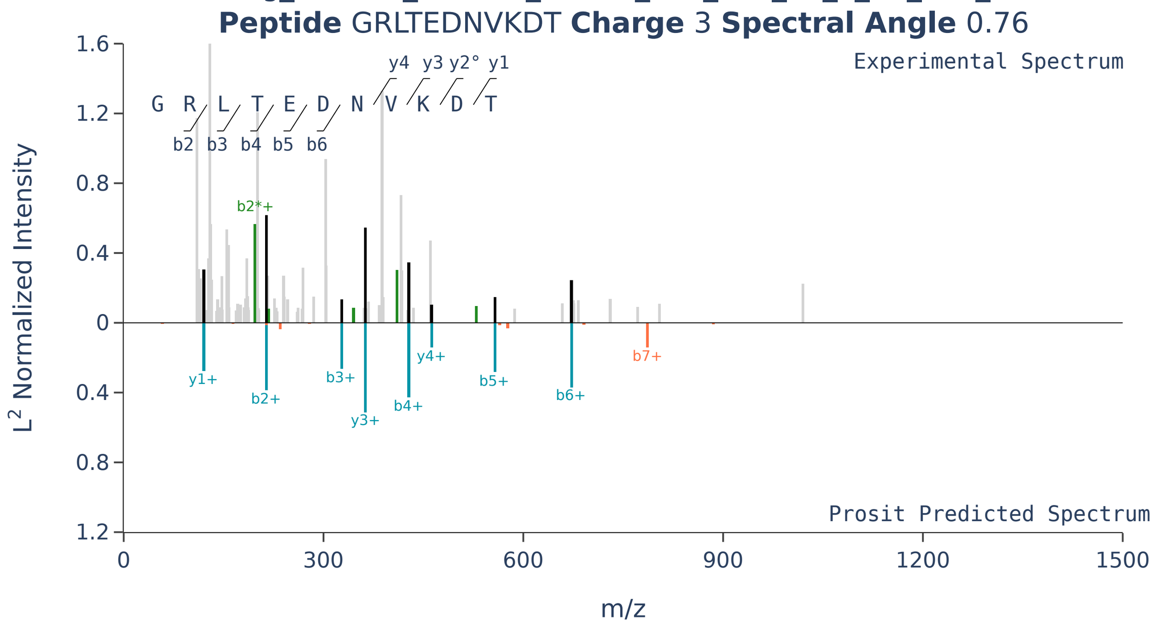


**Source** Ncheng_210623_230623_HFGoe_FFH_20S_25_1_A1_2h_R2 **Scan** 34508
**Peptide** DKVLVRMEAIINSM **Charge** 2 **Spectral Angle** 0.78

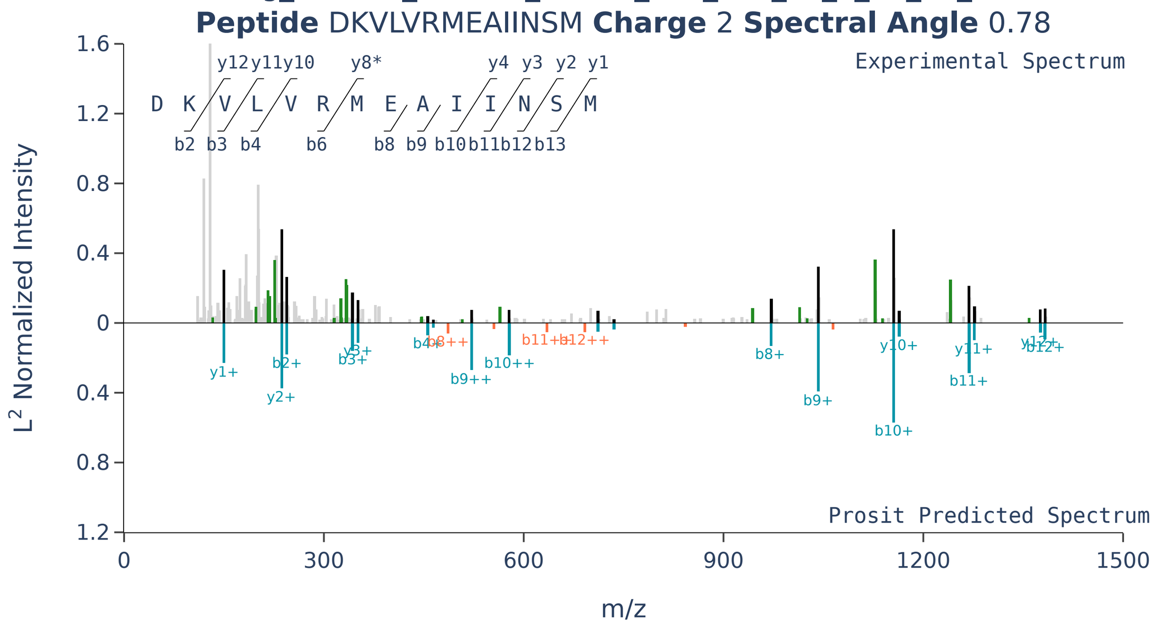


**Source** Ncheng_210623_230623_HFGoe_FFH_20S_25_1_A1_2h_R1 **Scan** 28460
**Peptide** KNMGGMASLMGKLPGMGQIPDNVKS **Charge** 3 **Spectral Angle** 0.81

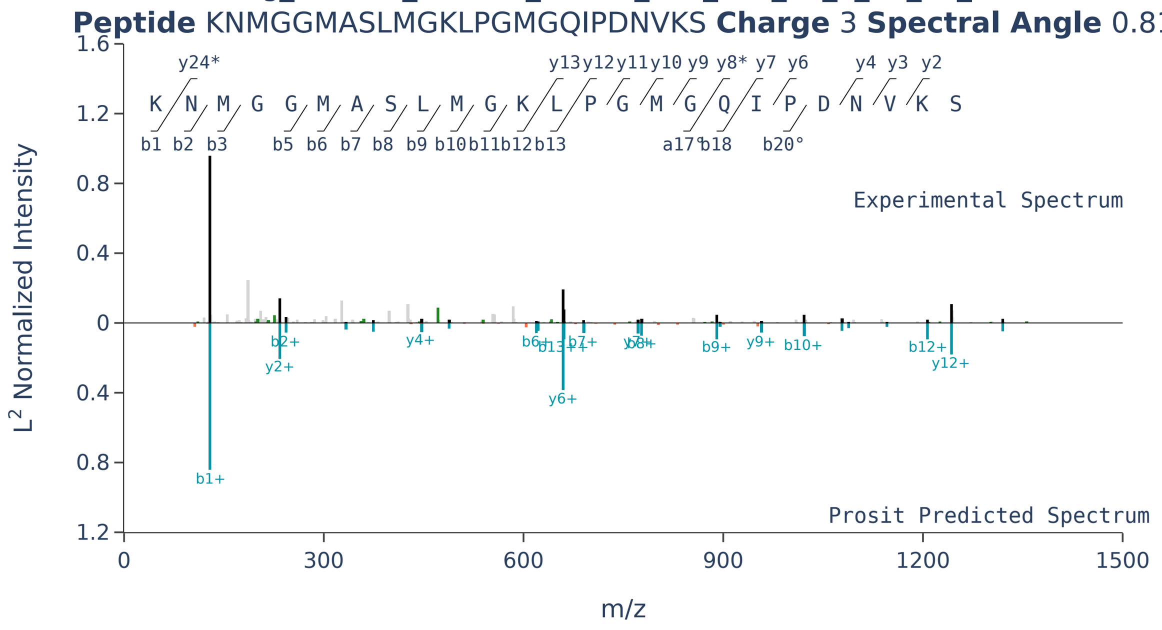


**Source** Ncheng_210623_230623_HFGoe_FFH_20S_25_1_A1_4h_R2 **Scan** 25304
**Peptide** KNMGGMASLMGKL **Charge** 2 **Spectral Angle** 0.89

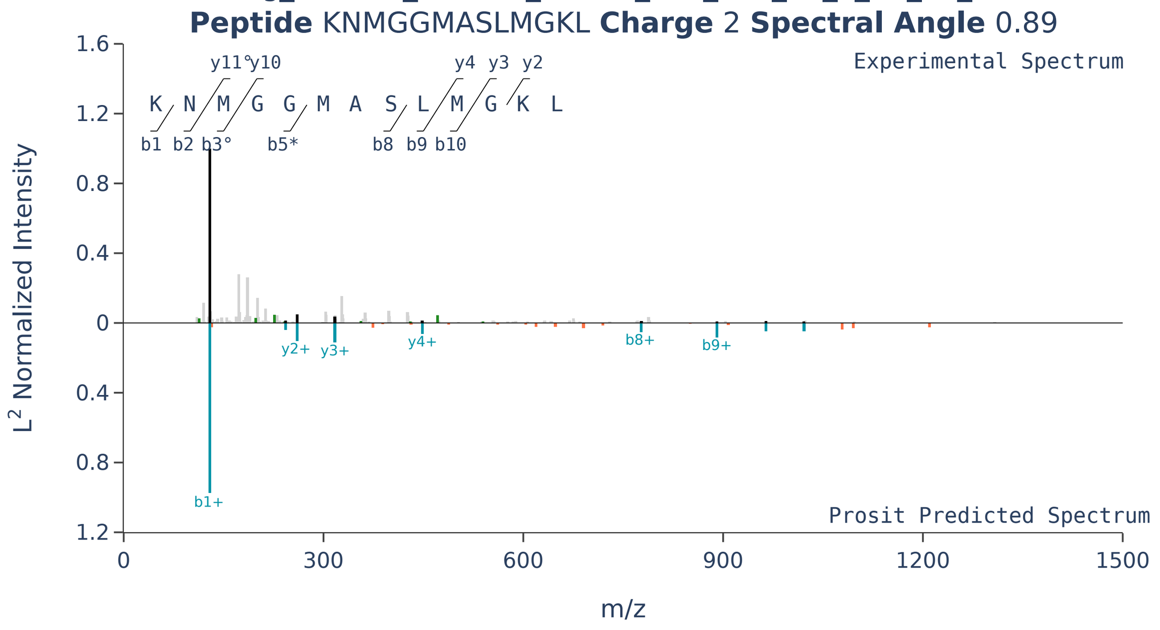


**Source** Ncheng_210623_230623_HFGoe_FFH_20S_25_1_A2_24h_R2 **Scan** 17107
**Peptide** LVNAALKEAKL **Charge** 2 **Spectral Angle** 0.7

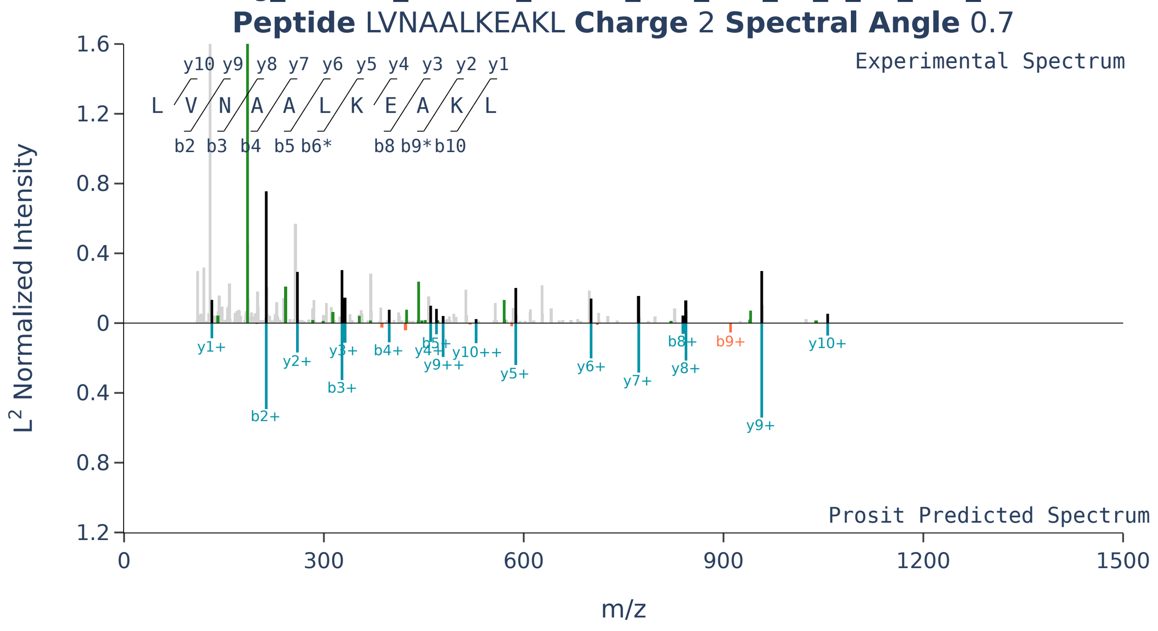


**Source** Ncheng_210623_230623_HFGoe_FFH_20S_25_1_A1_24h_R2 **Scan** 5482
**Peptide** KPEIIKGSRK **Charge** 2 **Spectral Angle** 0.75

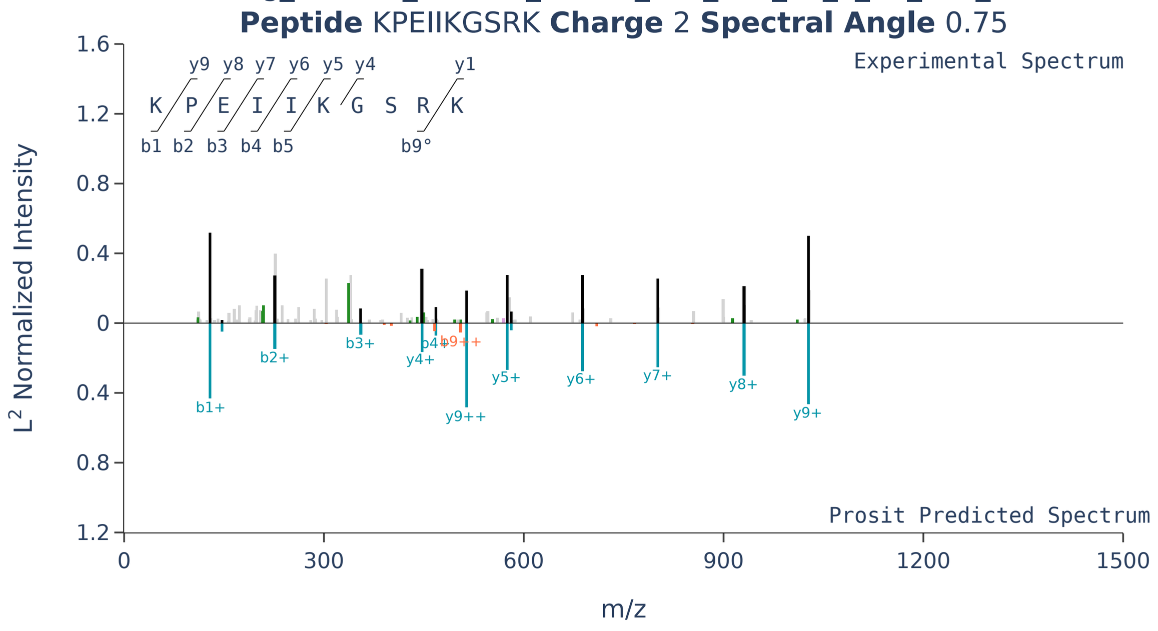


**Source** Ncheng_210623_230623_HFGoe_FFH_20S_25_1_A2_2h_R1 **Scan** 31317
**Peptide** GHEVNKSLTPGQEFVKIVRNELVAAM **Charge** 4 **Spectral Angle** 0.92

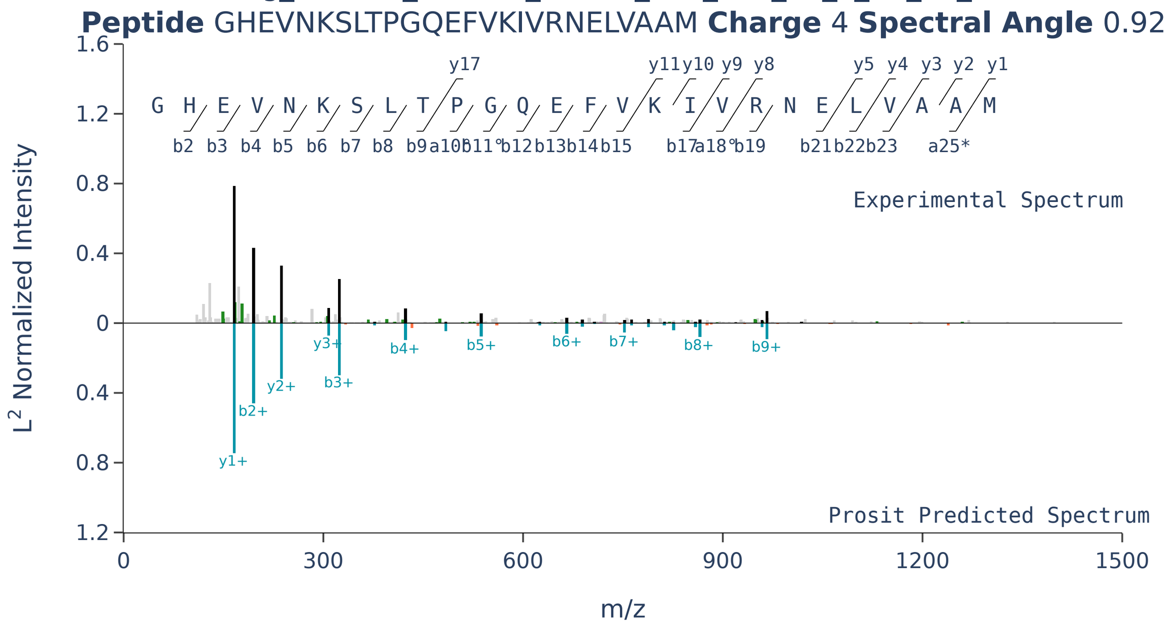


**Source** Ncheng_210623_230623_HFGoe_FFH_20S_25_1_A2_4h_R2 **Scan** 21816
**Peptide** ALEPFHPDRIASRL **Charge** 3 **Spectral Angle** 0.68

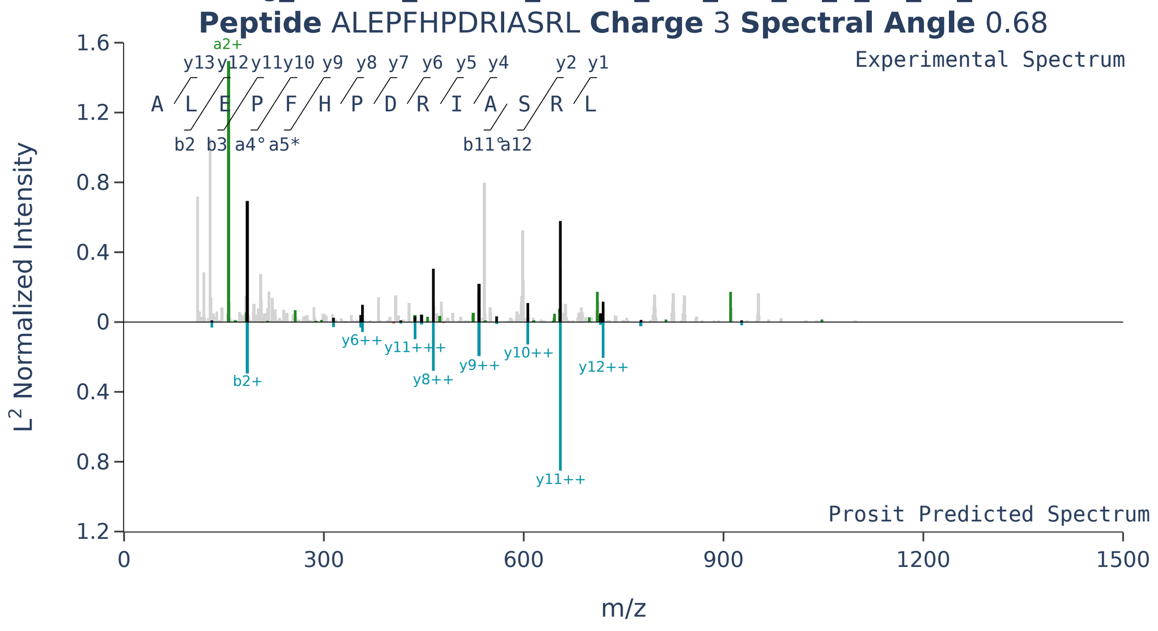


**Source** Ncheng_210623_230623_HFGoe_FFH_20S_25_1_A1_24h_R1 **Scan** 9547
**Peptide** GKLGKFLREK **Charge** 2 **Spectral Angle** 0.72

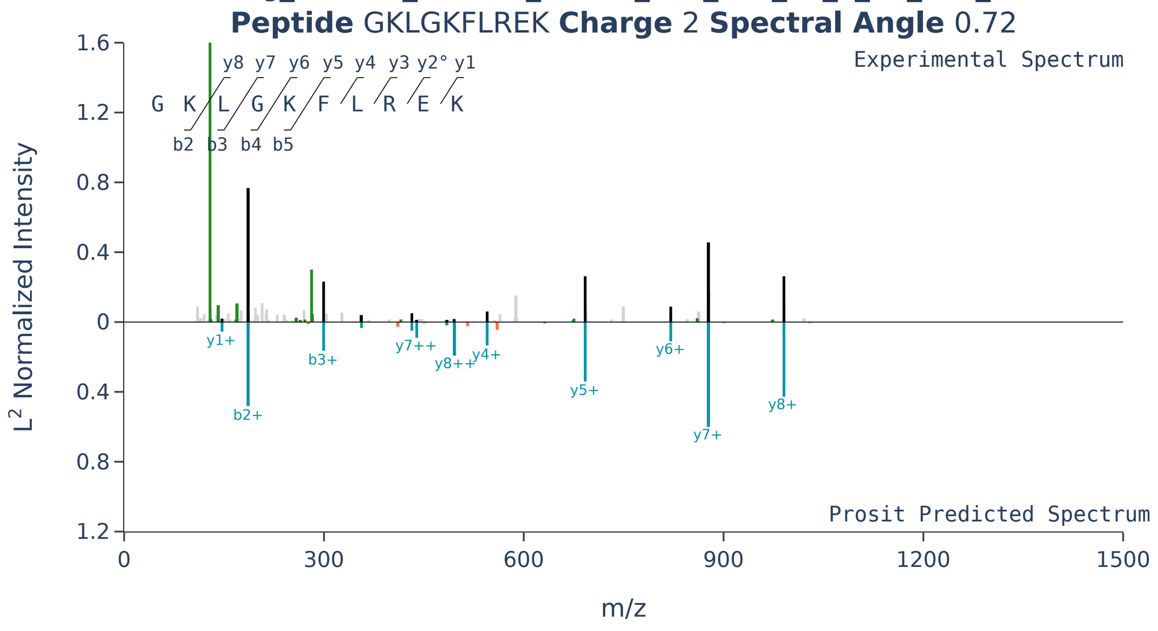


**Source** Ncheng_210623_230623_HFGoe_FFH_20S_25_1_A1_24h_R2 **Scan** 11453
**Peptide** VDTAGRLHV **Charge** 1 **Spectral Angle** 0.75

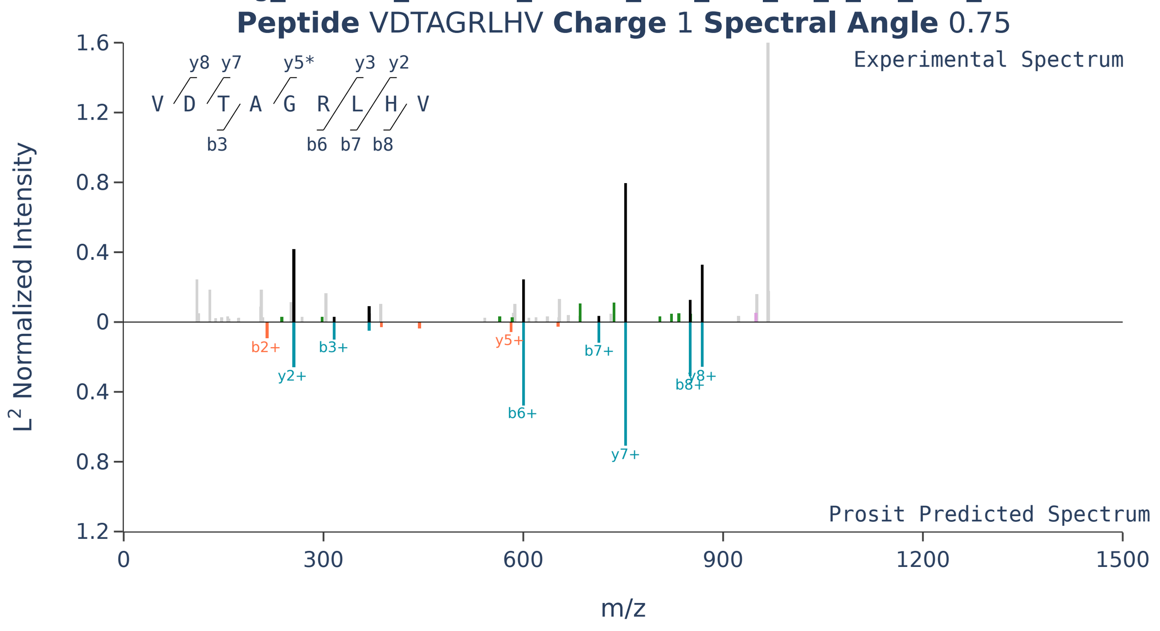


**Source** Ncheng_210623_230623_HFGoe_FFH_20S_25_1_A2_2h_R1 **Scan** 7460
**Peptide** GMAKMMRS **Charge** 2 **Spectral Angle** 0.77

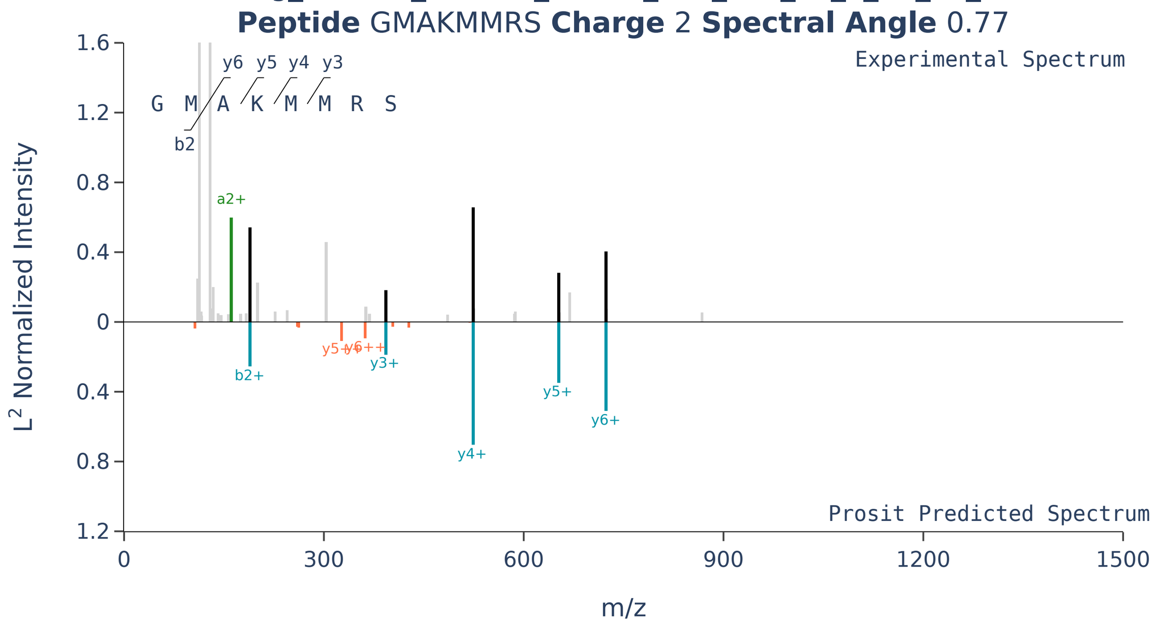


**Source** Ncheng_210623_230623_HFGoe_FFH_20S_25_1_A2_4h_R2 **Scan** 15823
**Peptide** GEKTEALEPFHPDRIASR **Charge** 5 **Spectral Angle** 0.84

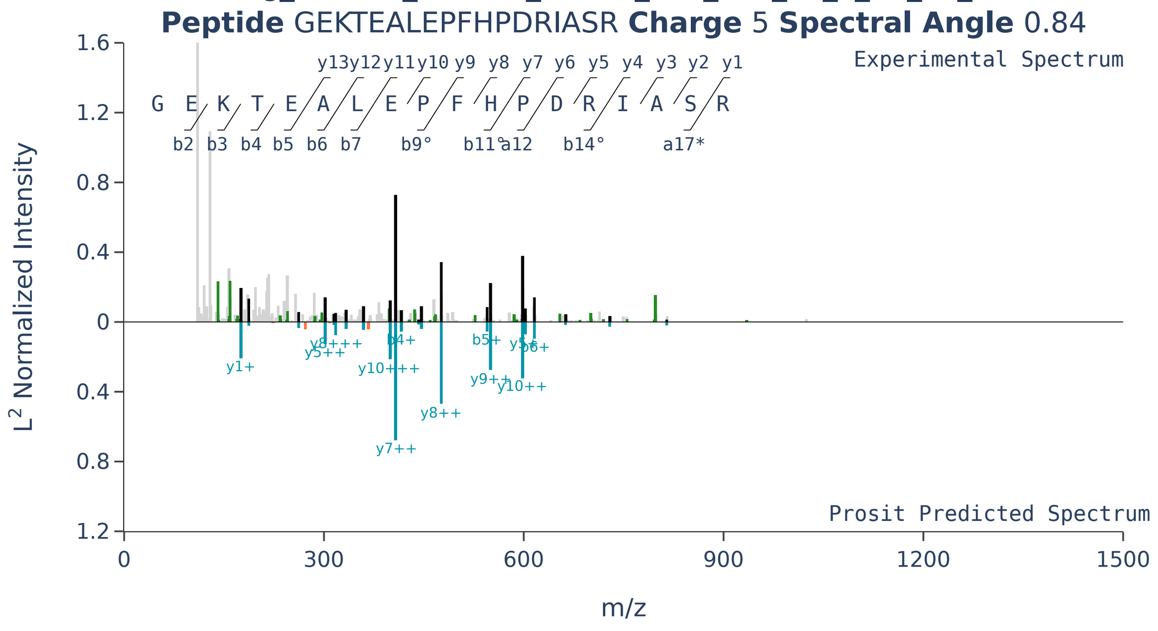


**Source** Ncheng_210623_230623_HFGoe_FFH_20S_25_1_A2_2h_R2 **Scan** 11179
**Peptide** ADVYRPAALKQ **Charge** 2 **Spectral Angle** 0.65

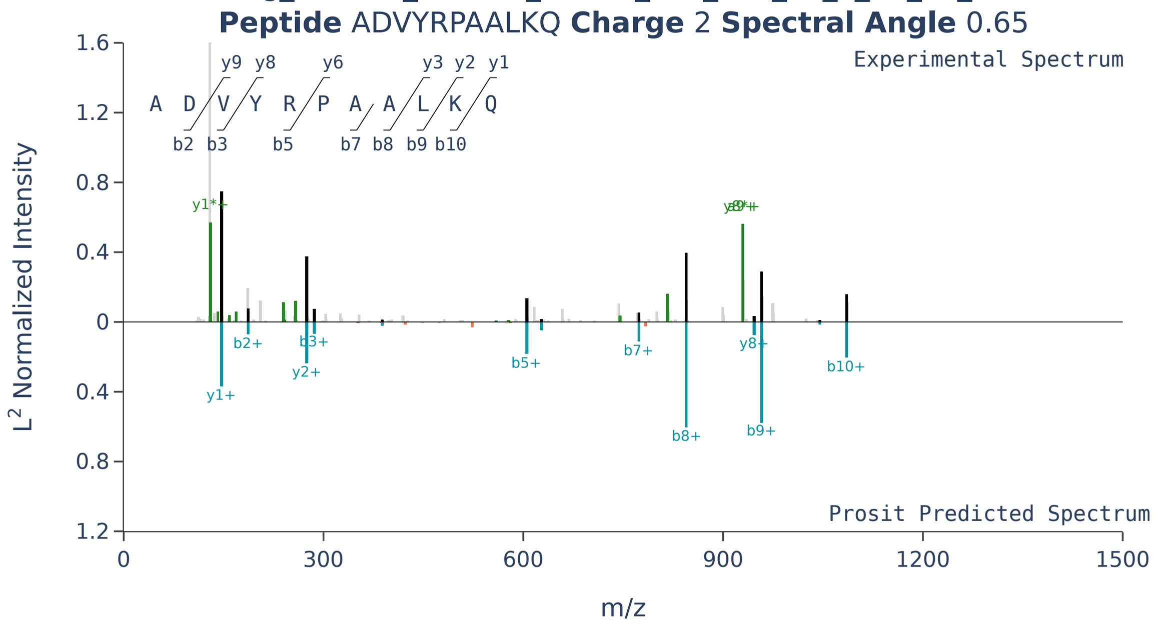

**Source** Ncheng_210623_230623_HFGoe_FFH_20S_25_1_A2_4h_R1 **Scan** 25868
**Peptide** GQDAANTAKAFNEALPLTG **Charge** 2 **Spectral Angle** 0.85

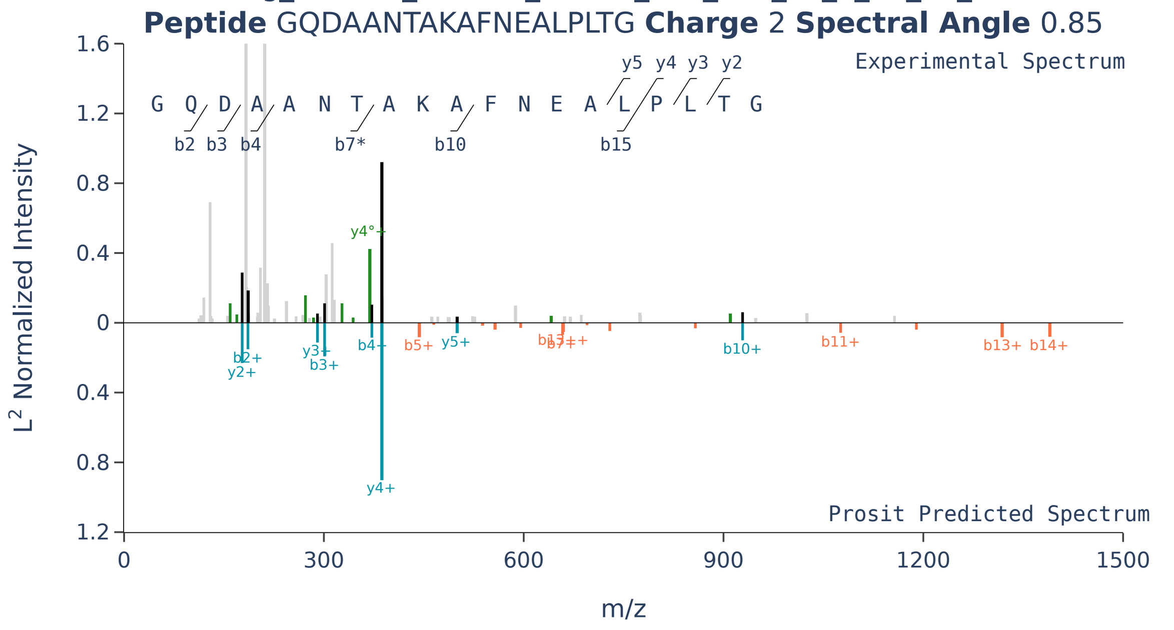


**Source** Ncheng_210623_230623_HFGoe_FFH_20S_25_1_A2_24h_R2 **Scan** 14274
**Peptide** HEVNKSLTPGQEF **Charge** 2 **Spectral Angle** 0.84

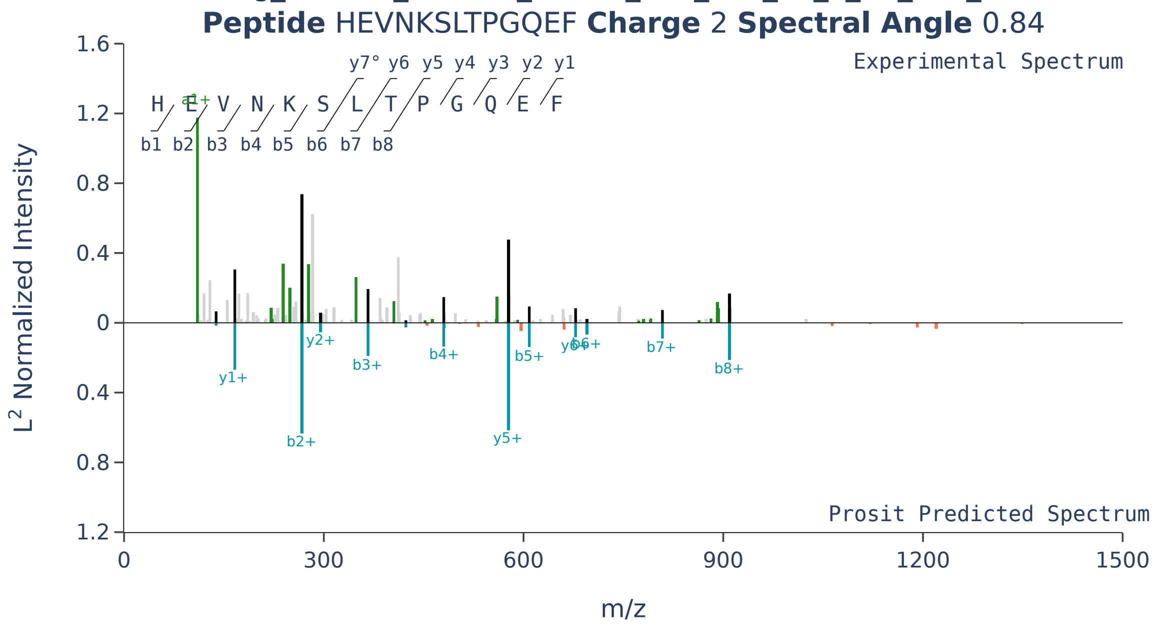


**Source** Ncheng_210623_230623_HFGoe_FFH_20S_25_1_A2_24h_R2 **Scan** 15575
**Peptide** NLTDRLSRTLRN **Charge** 4 **Spectral Angle** 0.73

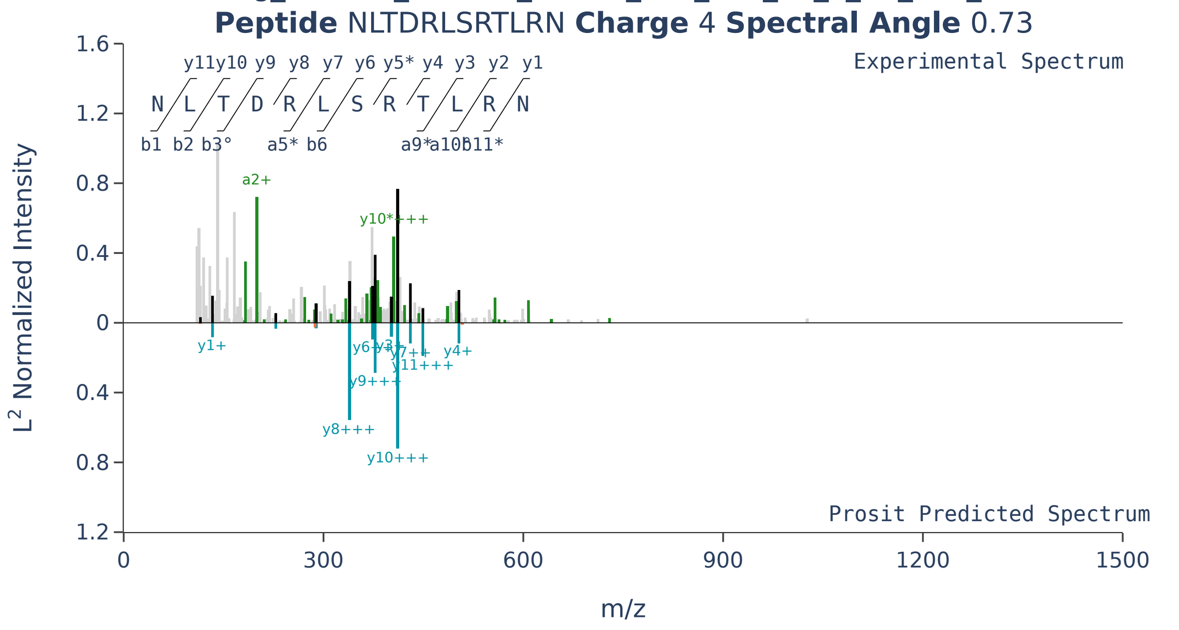


**Source** Ncheng_210623_230623_HFGoe_FFH_20S_25_1_A1_24h_R2 **Scan** 9249
**Peptide** AAGCGMQVQD **Charge** 2 **Spectral Angle** 0.84

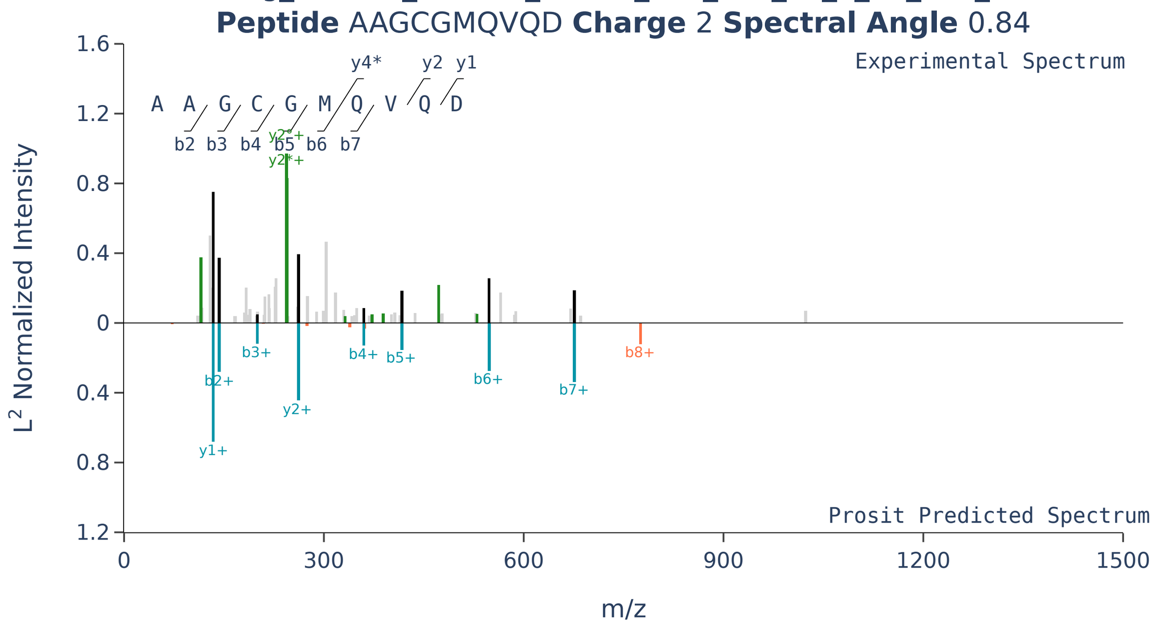


**Source** Ncheng_210623_230623_HFGoe_FFH_20S_25_1_A2_4h_R2 **Scan** 28866
**Peptide** ELVAAMGEENQTLNLA **Charge** 2 **Spectral Angle** 0.84

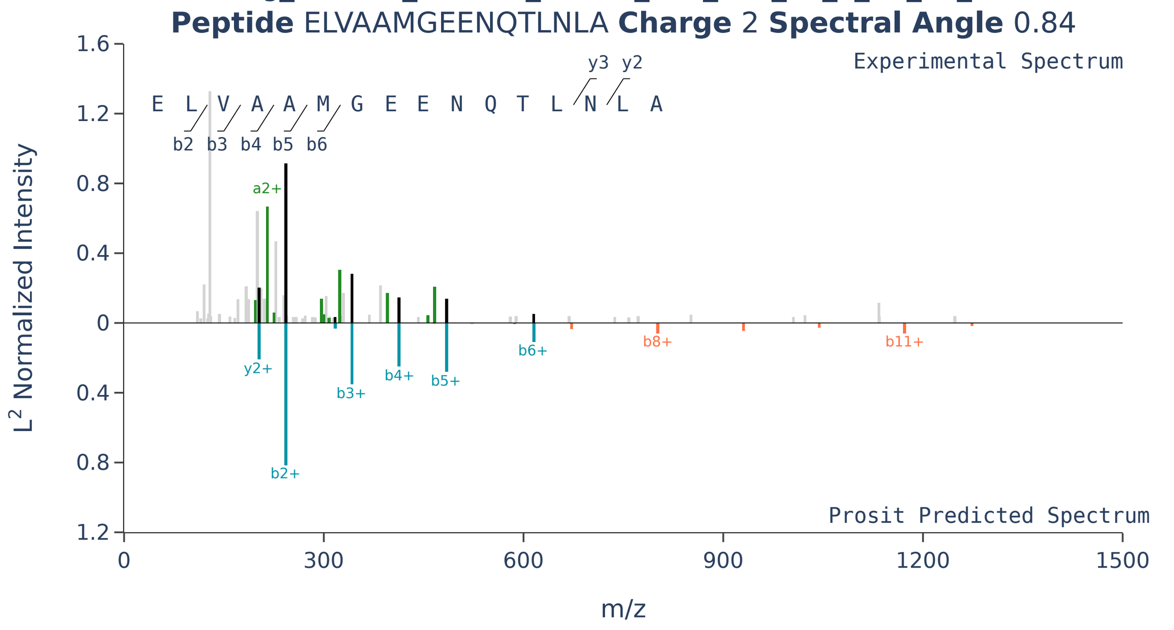


**Source** Ncheng_210623_230623_HFGoe_FFH_20S_25_1_A1_2h_R1 **Scan** 29634
**Peptide** AGCGMQVQDVNRLLKQFD **Charge** 2 **Spectral Angle** 0.72

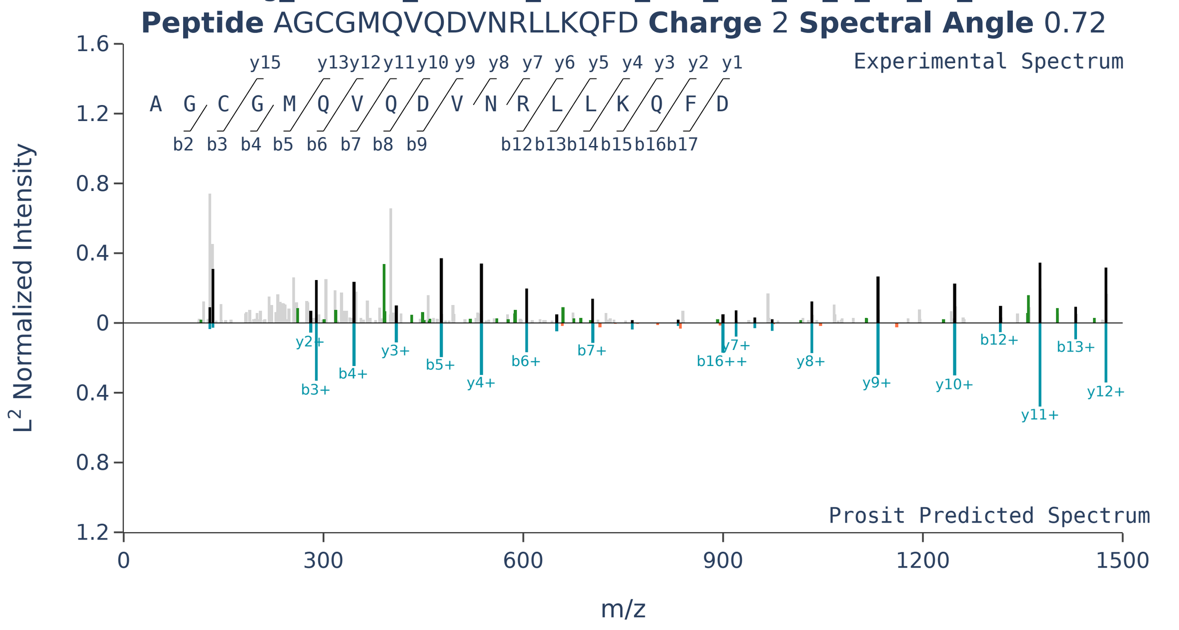


**Source** Ncheng_210623_230623_HFGoe_FFH_20S_25_1_A1_2h_R2 **Scan** 34695
**Peptide** AEQVGVDFFPSDVGQKPVDIVNAALKE **Charge** 3 **Spectral Angle** 0.88

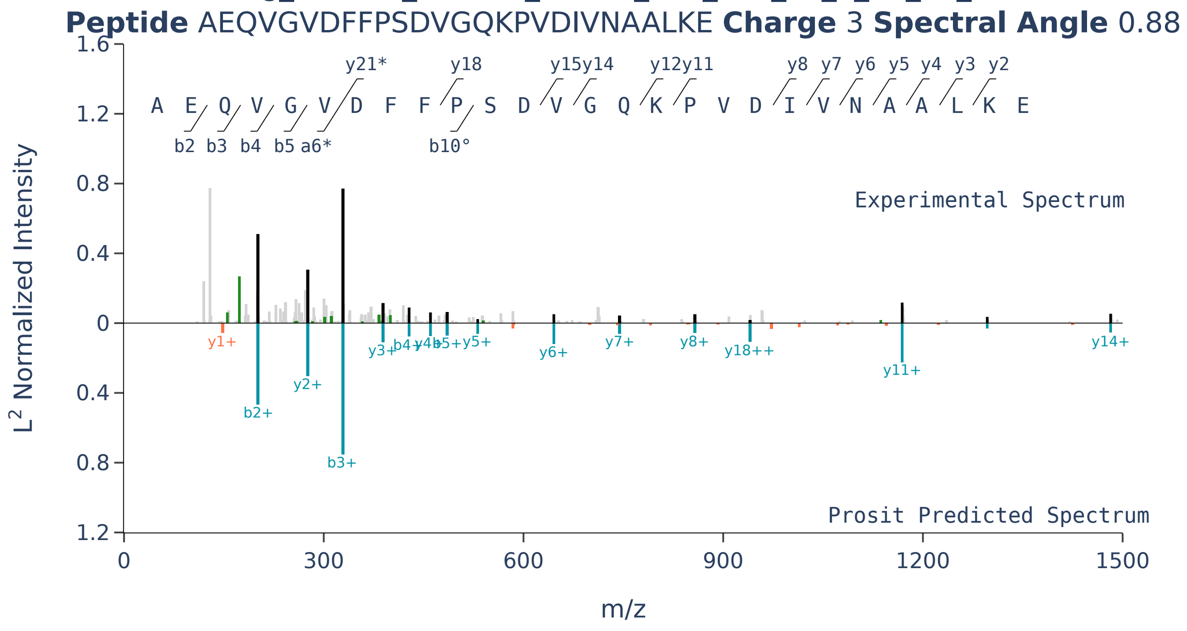


**Source** Ncheng_210623_230623_HFGoe_FFH_20S_25_1_A1_4h_R2 **Scan** 23423
**Peptide** VAAMGEENQTLNL **Charge** 2 **Spectral Angle** 0.86

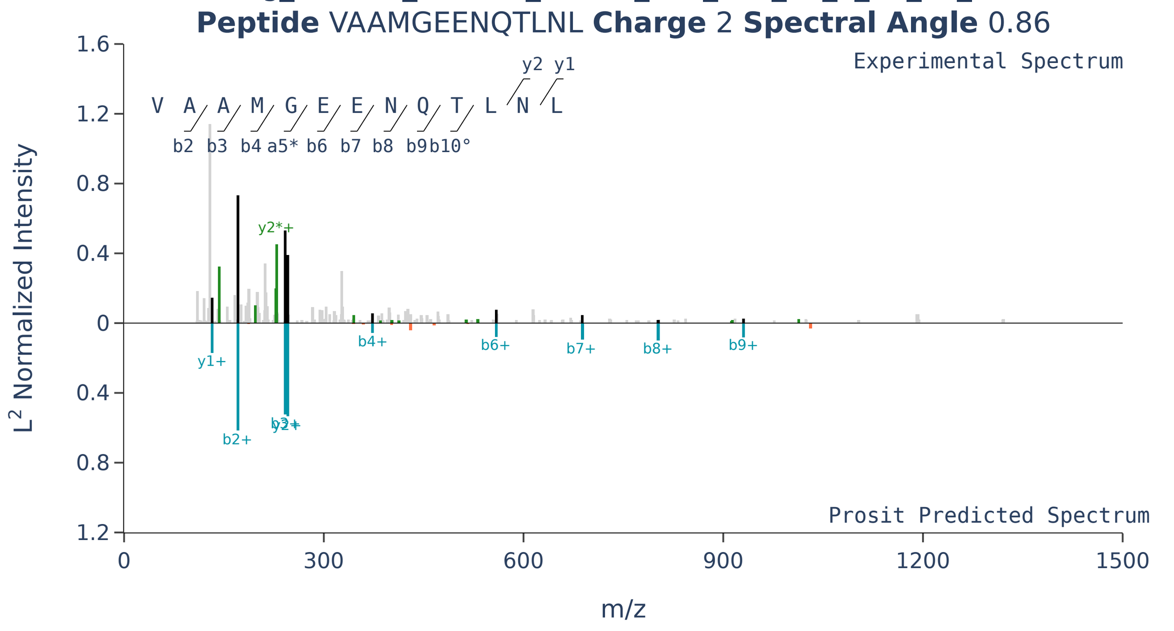


**Source** Ncheng_210623_230623_HFGoe_FFH_20S_25_1_A2_4h_R1 **Scan** 24951
**Peptide** DVGQKPVDIVNAALKE **Charge** 2 **Spectral Angle** 0.73

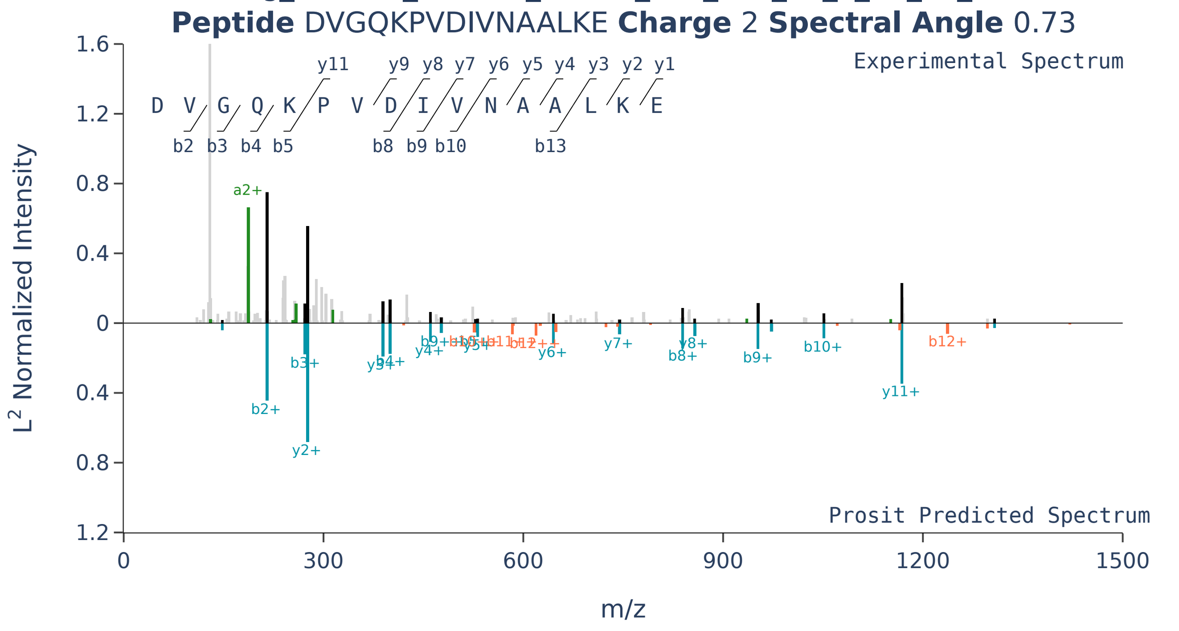


**Source** Ncheng_210623_230623_HFGoe_FFH_20S_25_1_A1_24h_R2 **Scan** 11309
**Peptide** NLTDRLS **Charge** 2 **Spectral Angle** 0.8

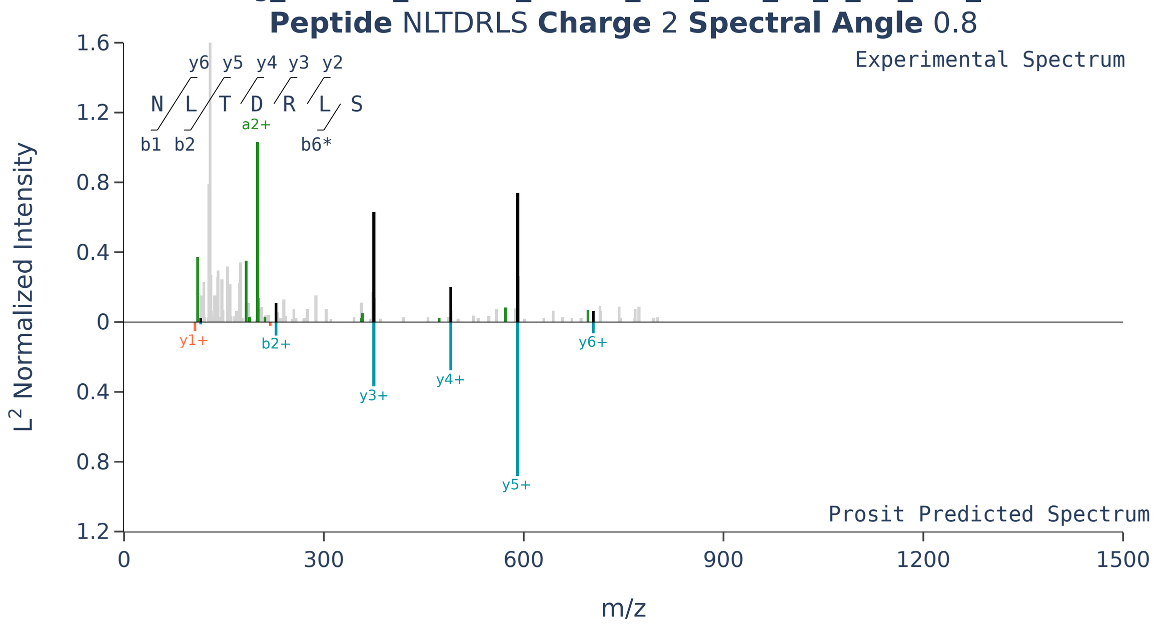


**Source** Ncheng_210623_230623_HFGoe_FFH_20S_25_1_A1_2h_R2 **Scan** 31060
**Peptide** DNVKDTLREVRMALLE **Charge** 2 **Spectral Angle** 0.75

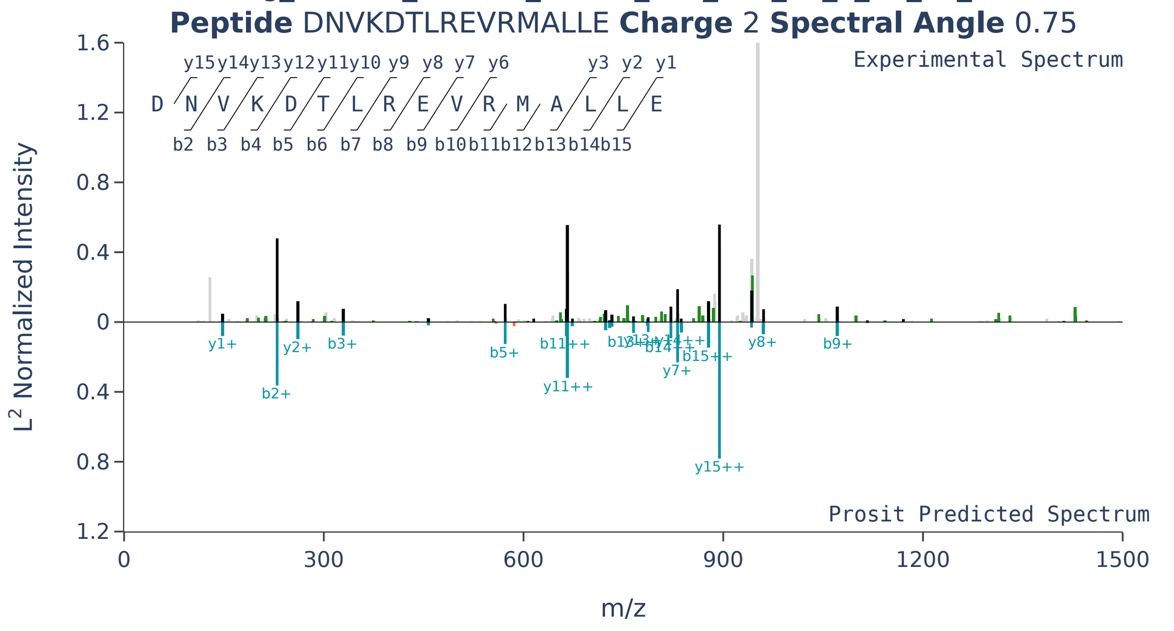


**Source** Ncheng_210623_230623_HFGoe_FFH_20S_25_1_A1_2h_R2 **Scan** 13312
**Peptide** YRPAAIKQL **Charge** 3 **Spectral Angle** 0.89

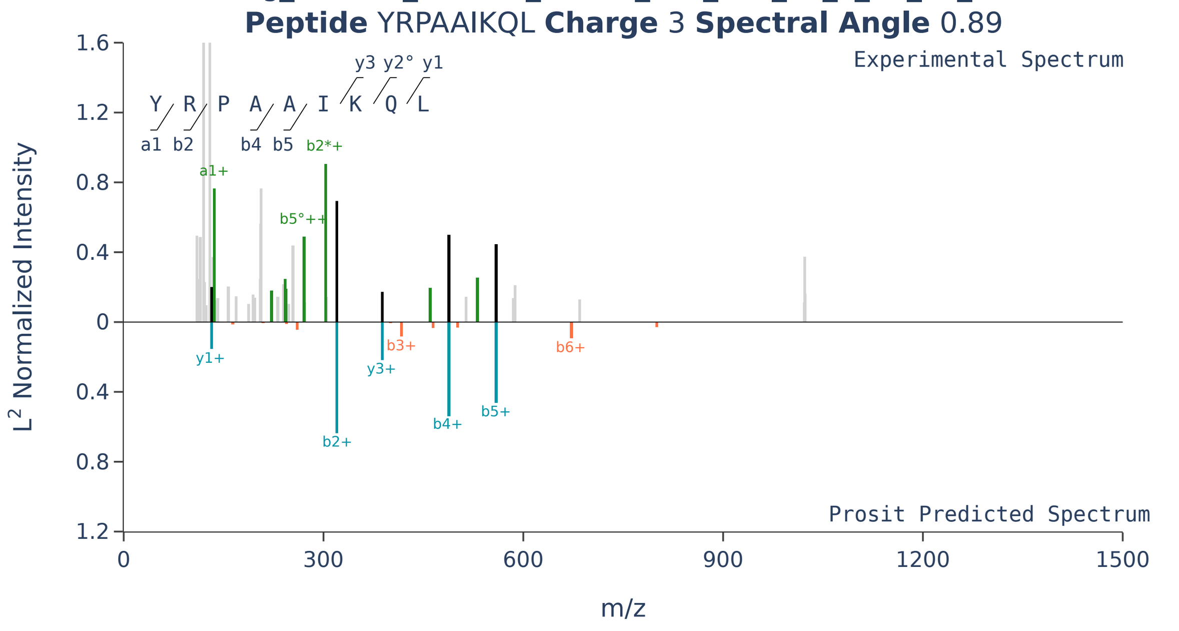


**Source** Ncheng_210623_230623_HFGoe_FFH_20S_25_1_A1_2h_R2 **Scan** 31371
**Peptide** AANTAKAFNEALPLTGVVLTKVDGDARG **Charge** 4 **Spectral Angle** 0.8

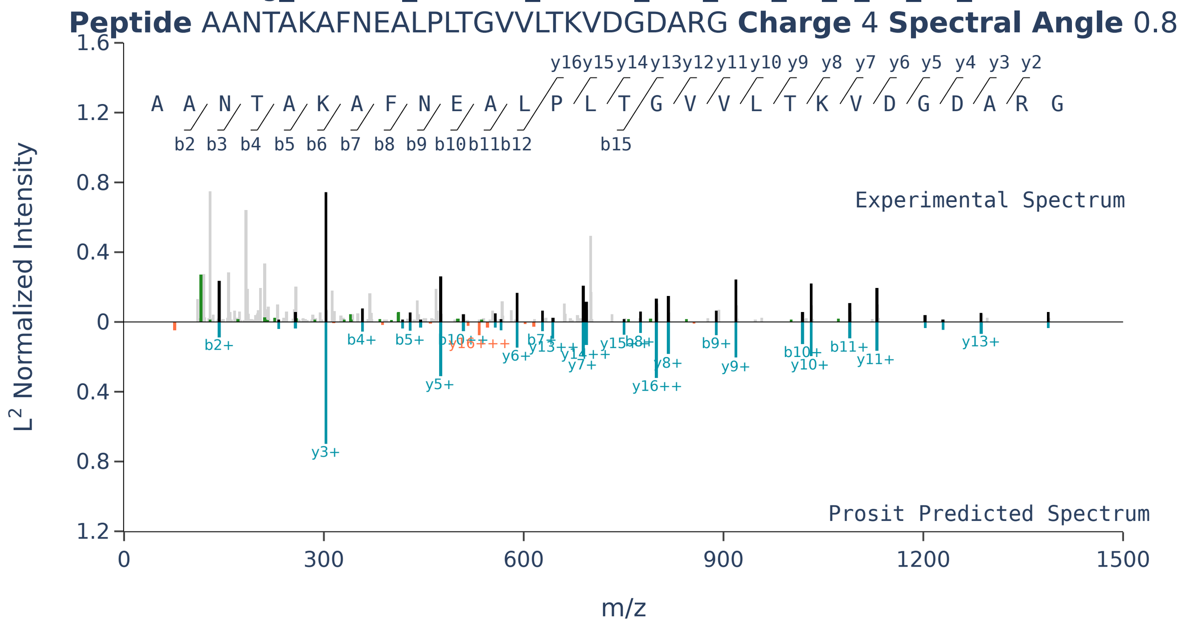


**Source** Ncheng_210623_230623_HFGoe_FFH_20S_25_1_A1_4h_R1 **Scan** 20401
**Peptide** IAAGCGMQVQDVNRL **Charge** 2 **Spectral Angle** 0.7

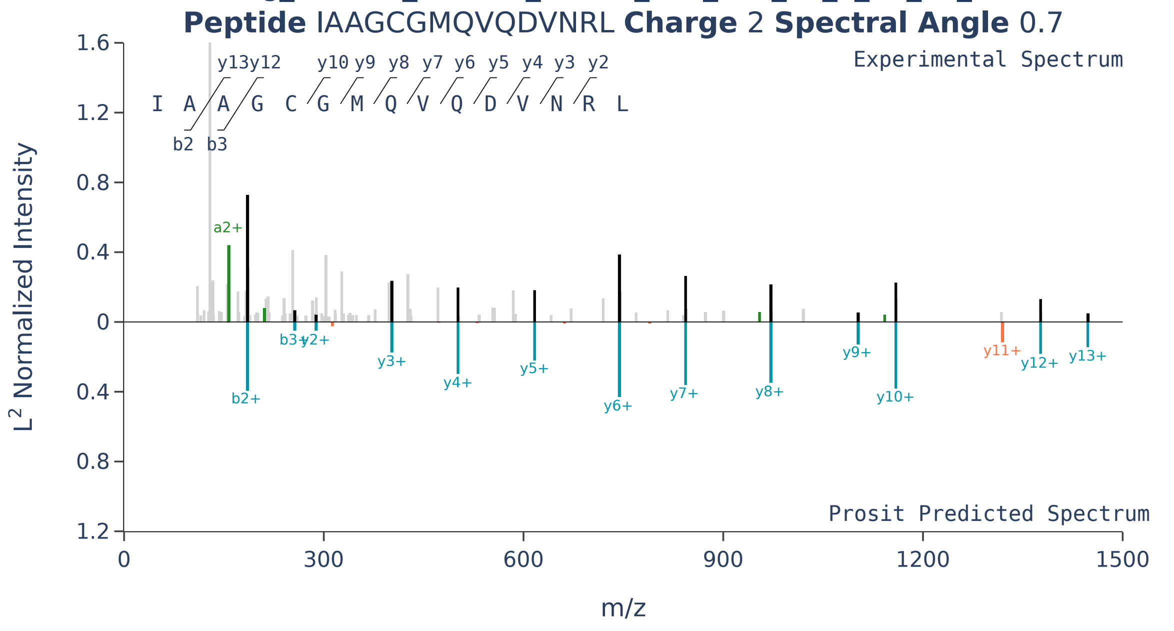


**Source** Ncheng_210623_230623_HFGoe_FFH_20S_25_1_A2_24h_R2 **Scan** 6153
**Peptide** RTLRNISGR **Charge** 3 **Spectral Angle** 0.8

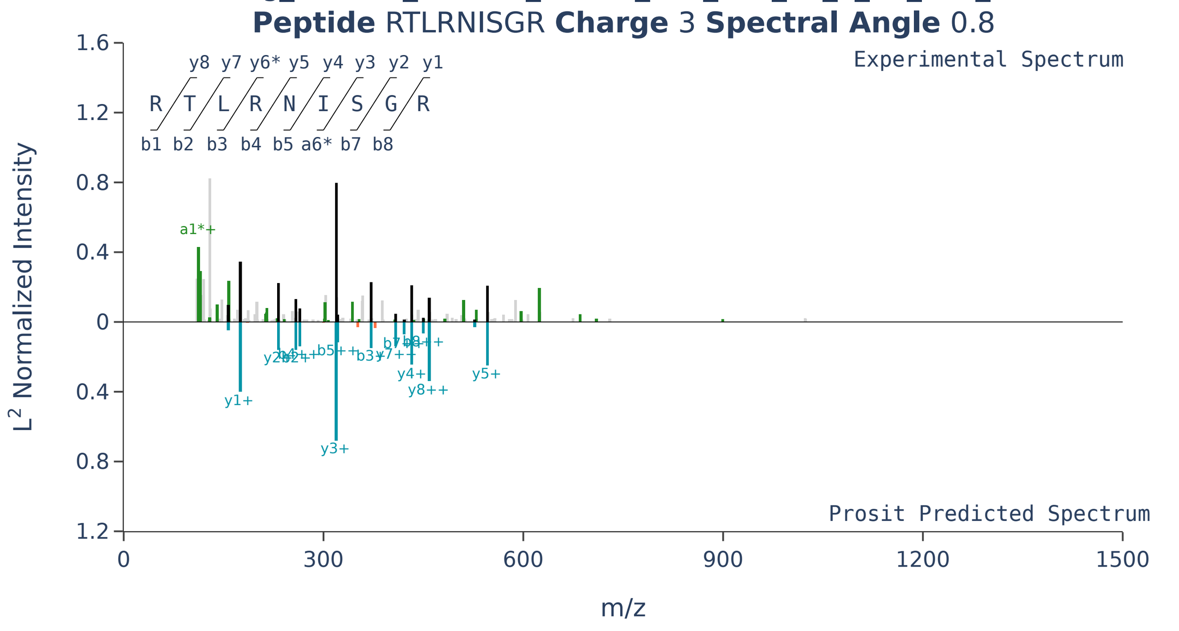

**Source** Ncheng_210623_230623_HFGoe_FFH_20S_25_1_A2_4h_R1 **Scan** 23346
**Peptide** TAGRLHVDEAMMDEIKQVHA **Charge** 3 **Spectral Angle** 0.87

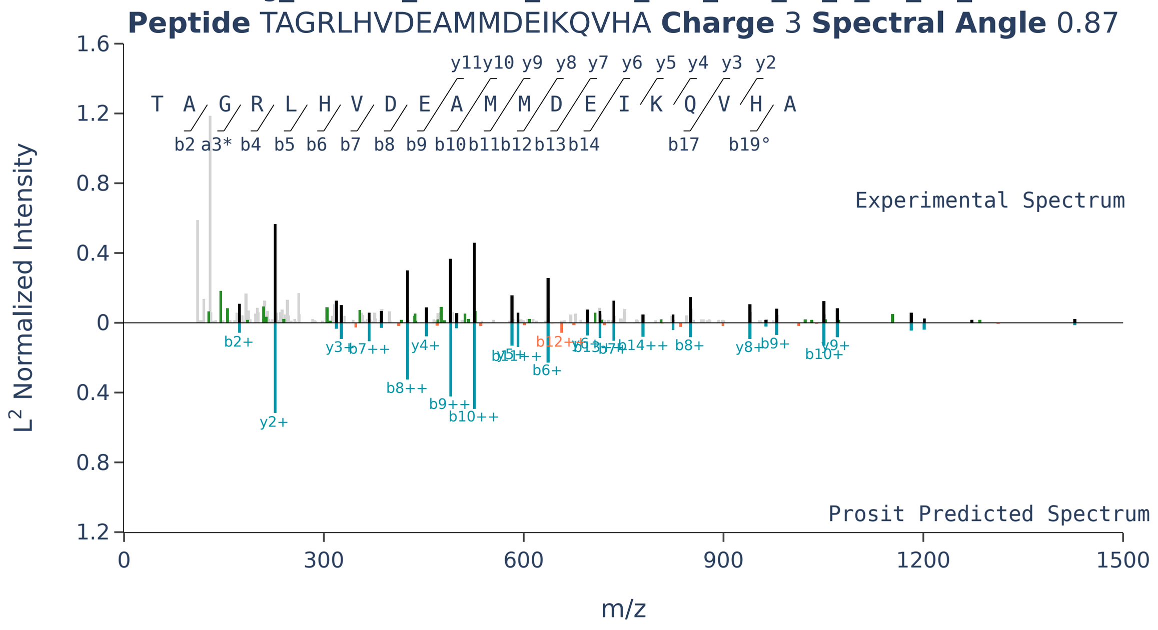


**Source** Ncheng_210623_230623_HFGoe_FFH_20S_25_1_A1_4h_R2 **Scan** 22456
**Peptide** ITGKPIKFLGVGE **Charge** 3 **Spectral Angle** 0.9

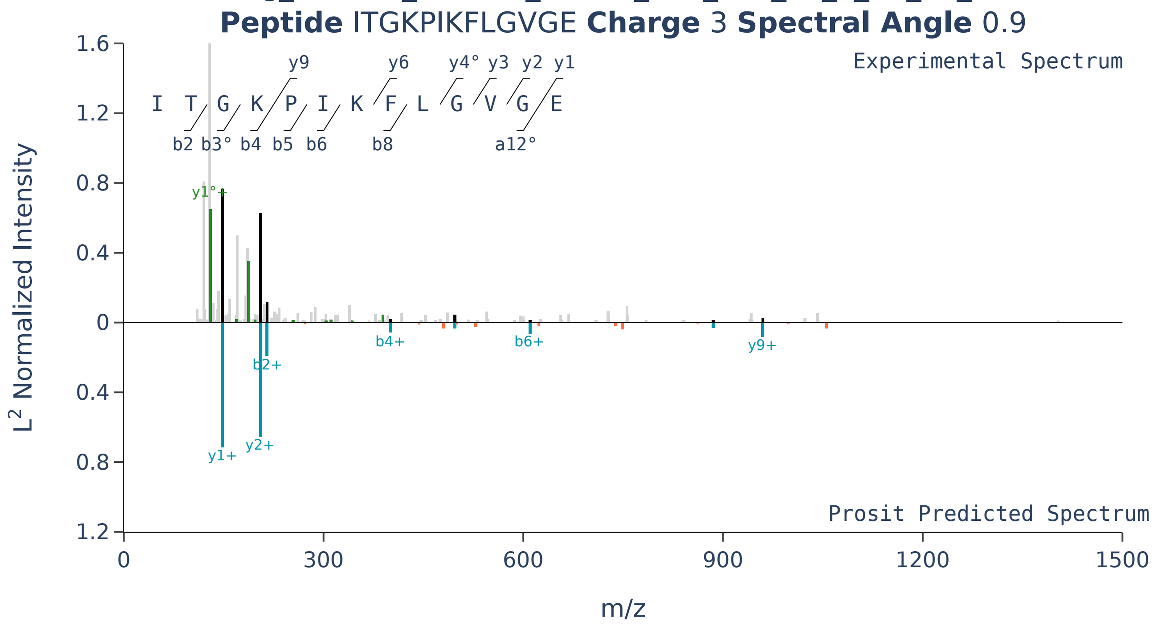


**Source** Ncheng_210623_230623_HFGoe_FFH_20S_25_1_A2_4h_R2 **Scan** 25382
**Peptide** GMQVQDVNRLL **Charge** 2 **Spectral Angle** 0.75

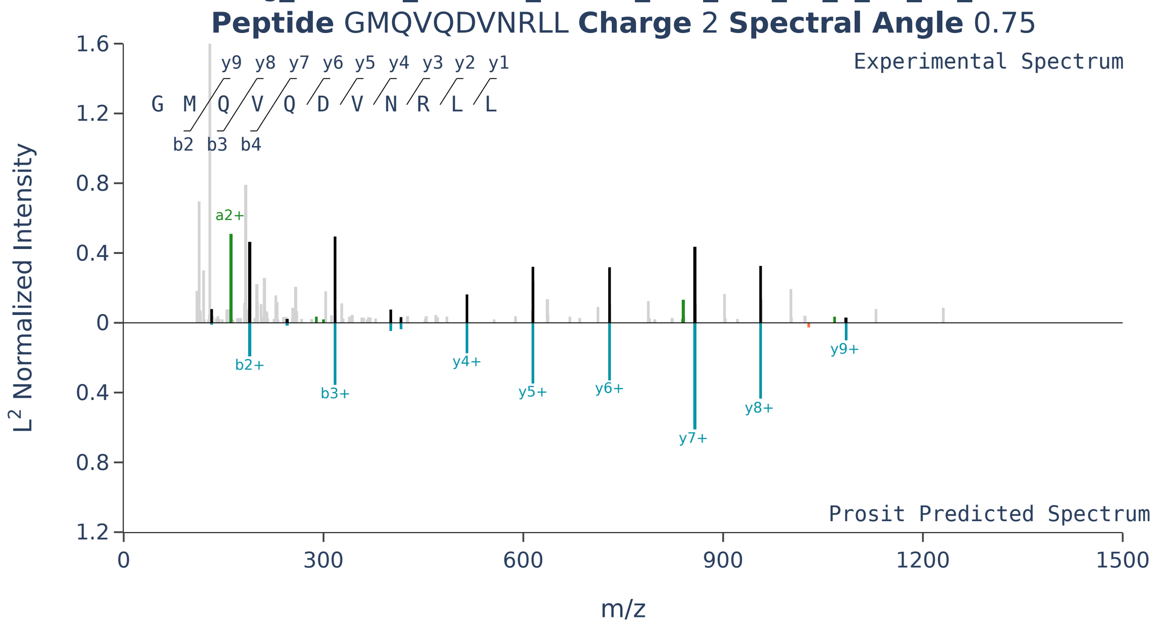


**Source** Ncheng_210623_230623_HFGoe_FFH_20S_25_1_A1_1h_R1 **Scan** 26307
**Peptide** RNELVAAMGEENQTLNL **Charge** 2 **Spectral Angle** 0.81

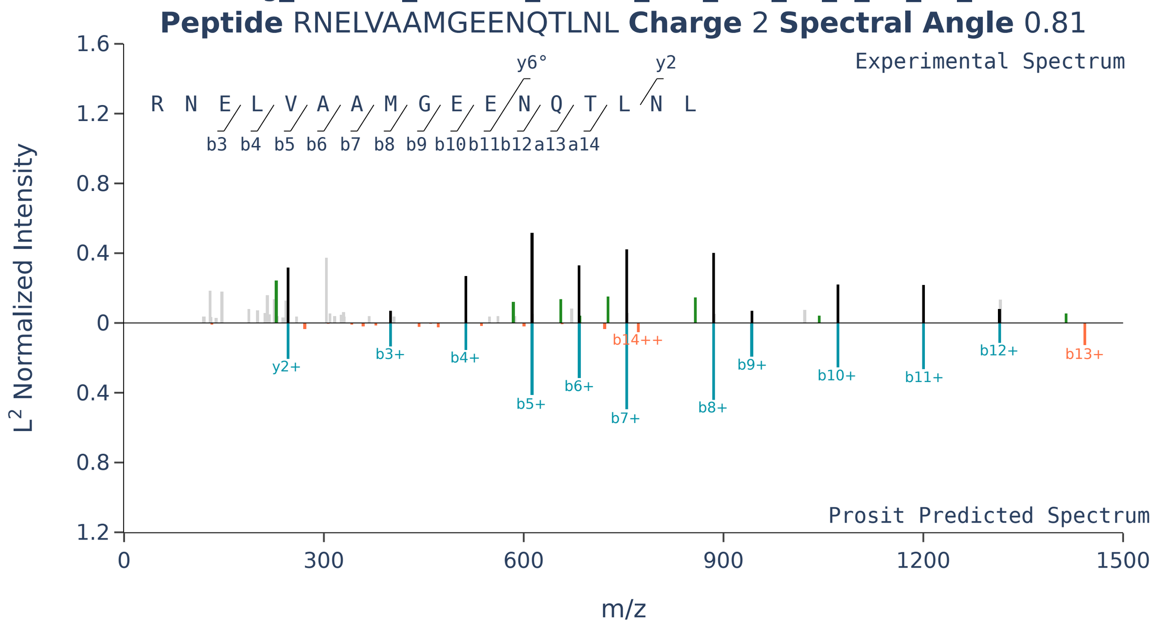


**Source** Ncheng_210623_230623_HFGoe_FFH_20S_25_1_A1_24h_R1 **Scan** 16225
**Peptide** IEDIESKVDRAQAEKL **Charge** 2 **Spectral Angle** 0.81

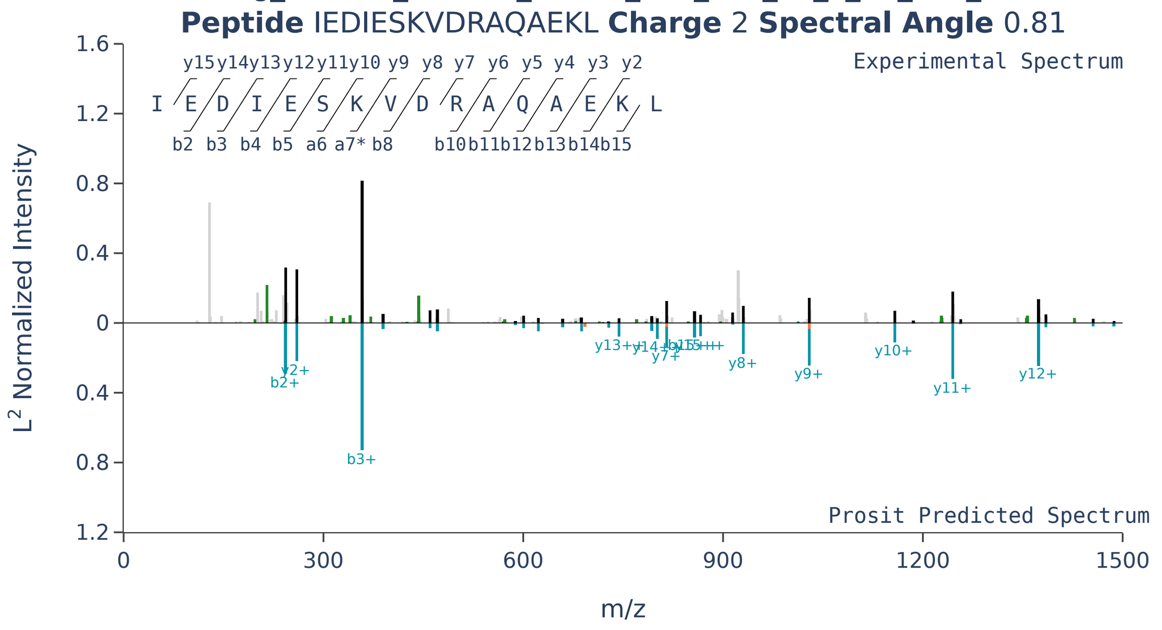


**Source** Ncheng_210623_230623_HFGoe_FFH_20S_25_1_A2_2h_R1 **Scan** 9840
**Peptide** GQDAANTAKAFNE **Charge** 2 **Spectral Angle** 0.81

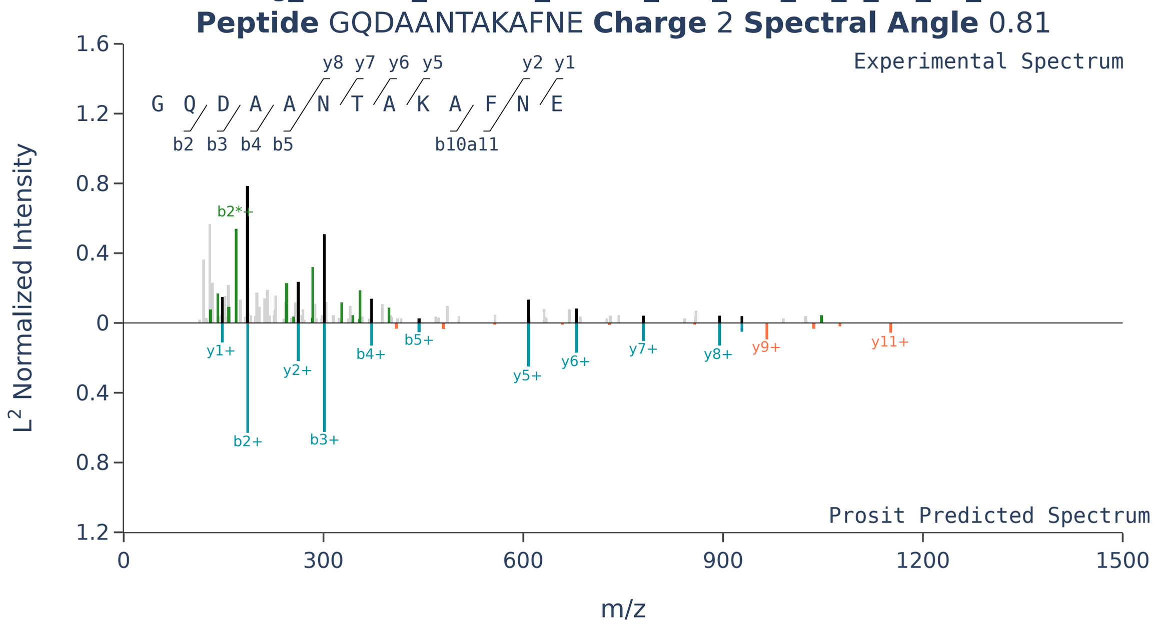


**Source** Ncheng_210623_230623_HFGoe_FFH_20S_25_1_A1_2h_R1 **Scan** 16999
**Peptide** EPFHPDRIASRI **Charge** 4 **Spectral Angle** 0.83

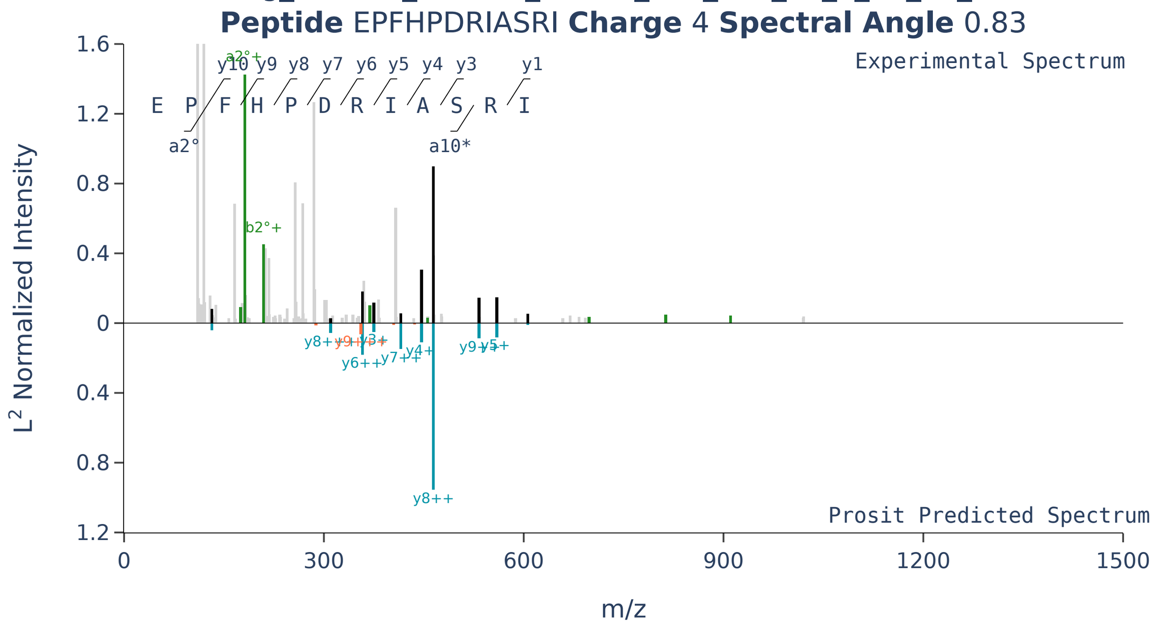


**Source** Ncheng_210623_230623_HFGoe_FFH_20S_25_1_A2_4h_R1 **Scan** 15287
**Peptide** TKVDGDARGGAALSL **Charge** 2 **Spectral Angle** 0.8

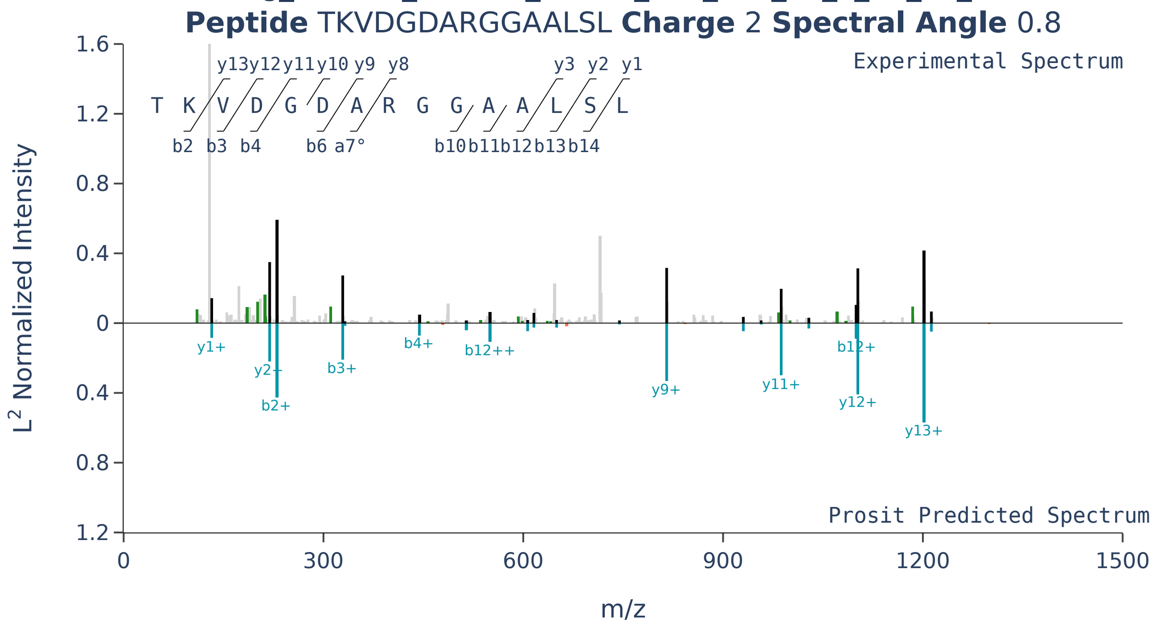


**Source** Ncheng_210623_230623_HFGoe_FFH_20S_25_1_A2_4h_R1 **Scan** 20786
**Peptide** KQLETLAEQVGVD **Charge** 2 **Spectral Angle** 0.66

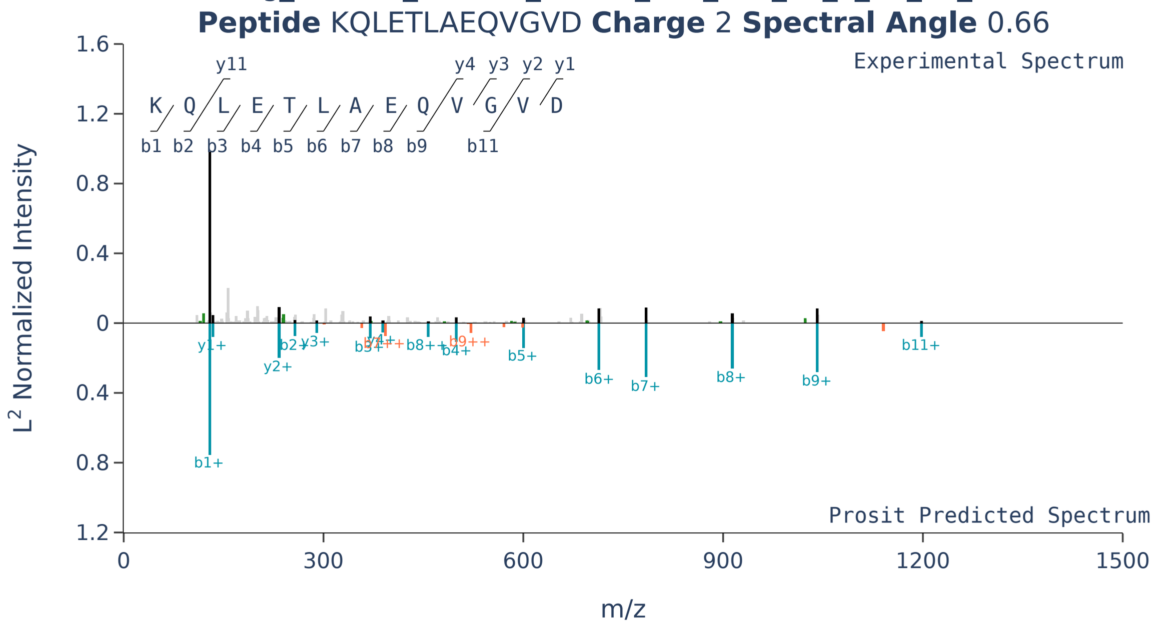


**Source** Ncheng_210623_230623_HFGoe_FFH_20S_25_1_A2_1h_R2 **Scan** 5900
**Peptide** VDTAGRLH **Charge** 2 **Spectral Angle** 0.84

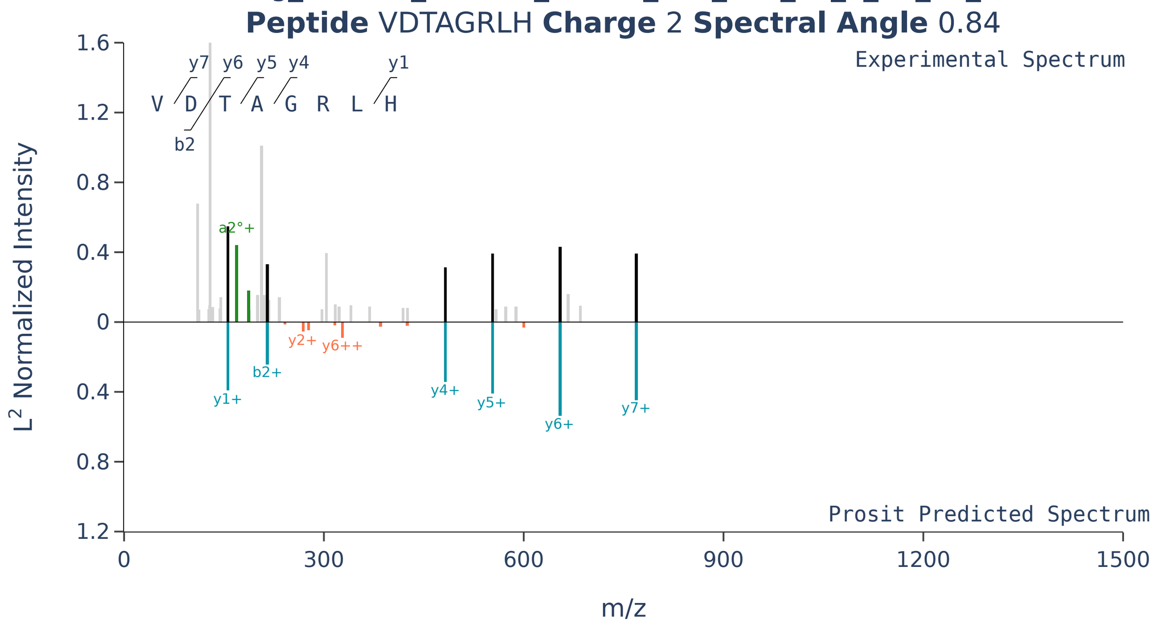


**Source** Ncheng_210623_230623_HFGoe_FFH_20S_25_1_A2_24h_R2 **Scan** 6982
**Peptide** IPDNVKS **Charge** 1 **Spectral Angle** 0.79

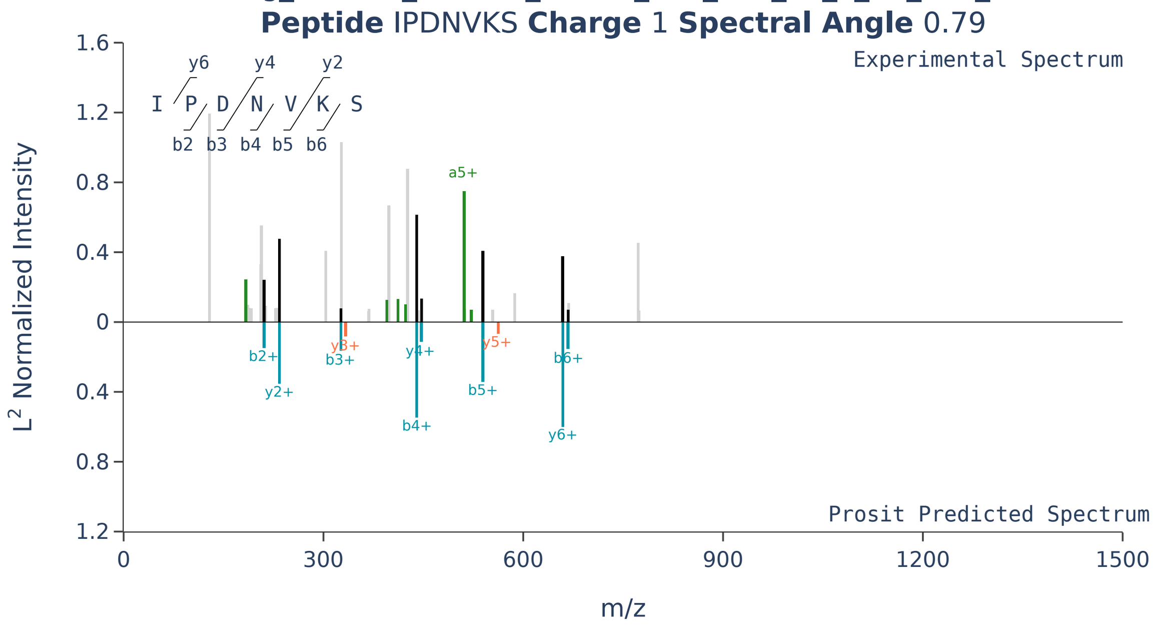


**Source** Ncheng_210623_230623_HFGoe_FFH_20S_25_1_A1_2h_R2 **Scan** 23703
**Peptide** VRNELVAAMGEENQTL **Charge** 2 **Spectral Angle** 0.82

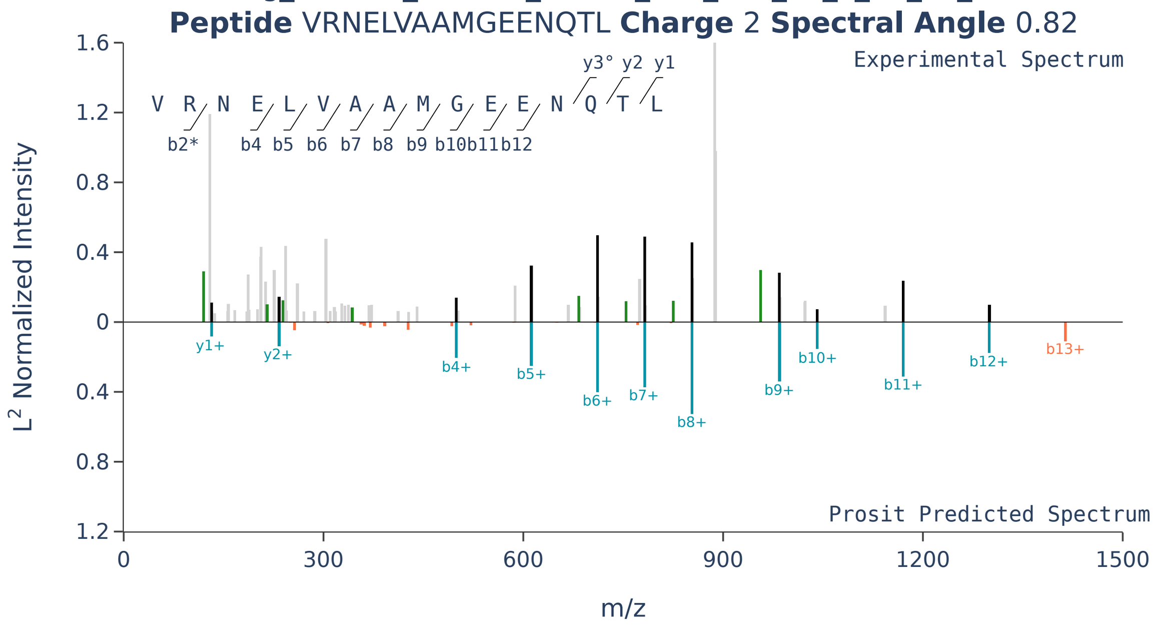


**Source** Ncheng_210623_230623_HFGoe_FFH_20S_25_1_A1_1h_R2 **Scan** 34142
**Peptide** LETLAEQVGVDFFPSDVGQKPVD **Charge** 3 **Spectral Angle** 0.82

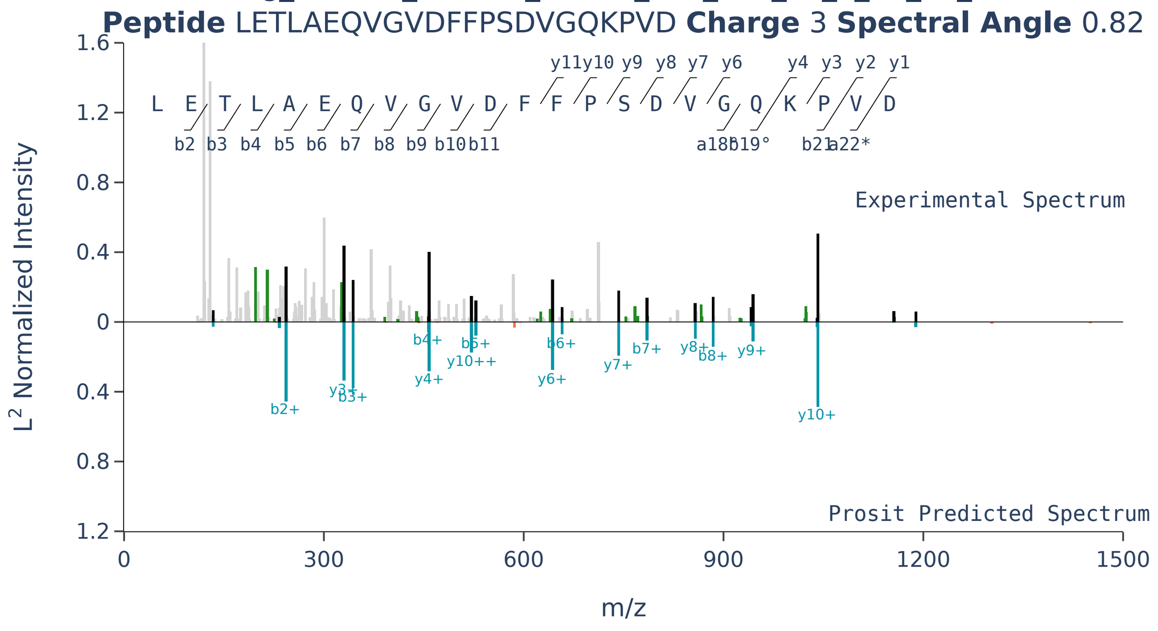


**Source** Ncheng_210623_230623_HFGoe_FFH_20S_25_1_A1_24h_R1 **Scan** 13114
**Peptide** VGQKPVDIVN **Charge** 1 **Spectral Angle** 0.82

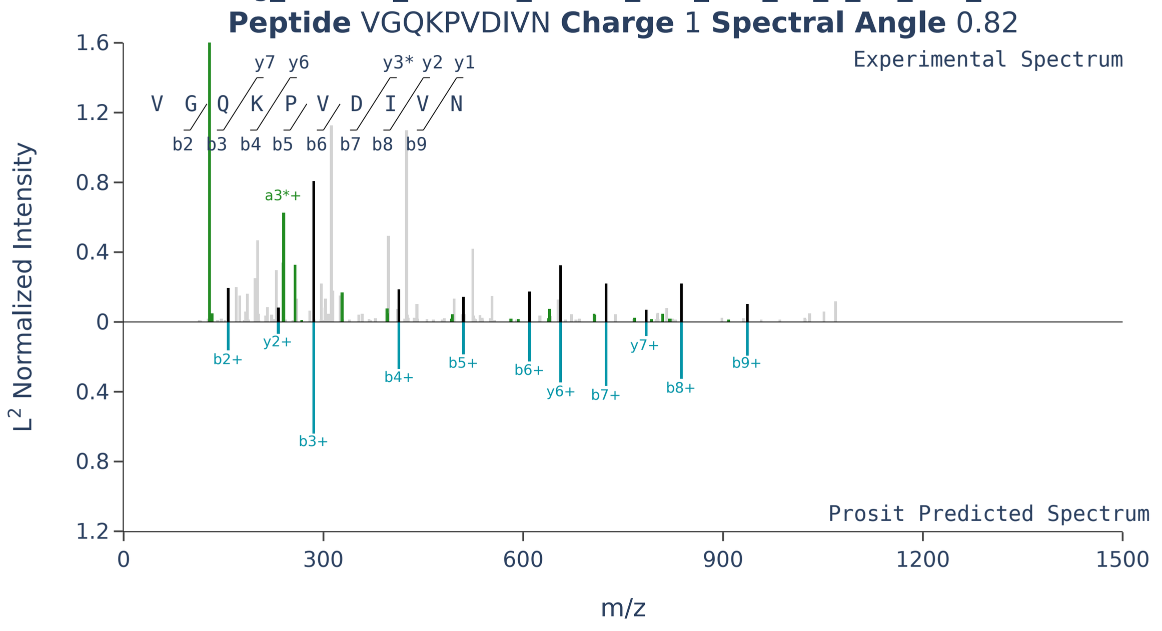


**Source** Ncheng_210623_230623_HFGoe_FFH_20S_25_1_A2_4h_R2 **Scan** 20858
**Peptide** GEKTEALEPFHPDRIASRL **Charge** 3 **Spectral Angle** 0.76

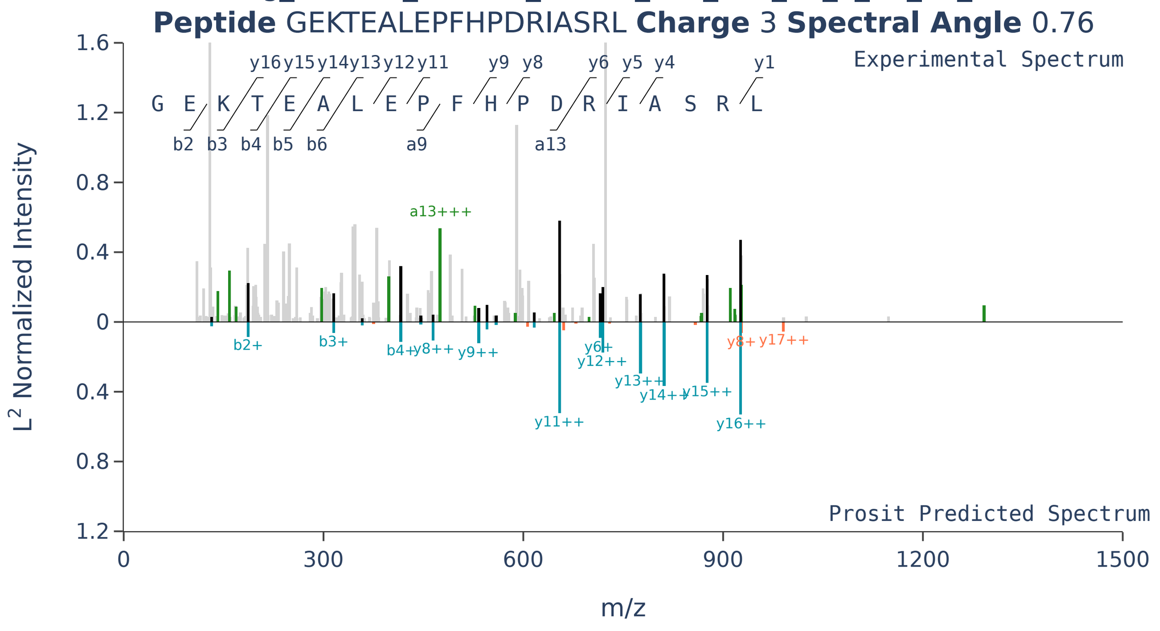

**Source** Ncheng_210623_230623_HFGoe_FFH_20S_25_1_A1_2h_R1 **Scan** 32138
**Peptide** EVNKSLTPGQEFVKIVRNELVA **Charge** 3 **Spectral Angle** 0.71

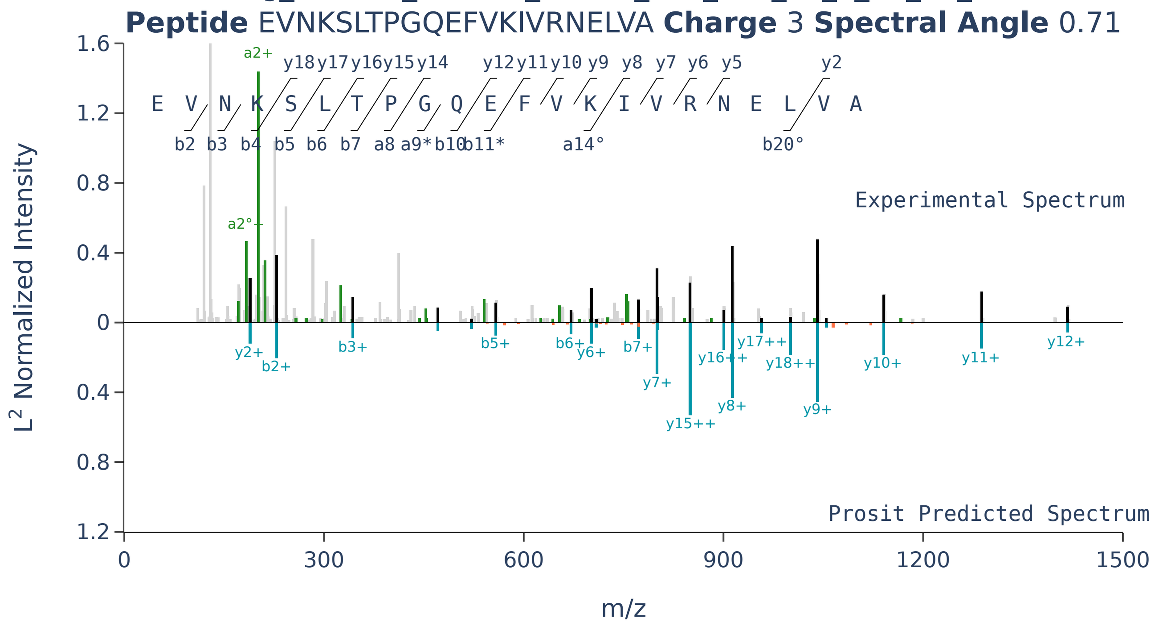


**Source** Ncheng_210623_230623_HFGoe_FFH_20S_25_1_A1_1h_R2 **Scan** 9515
**Peptide** QAEKLASKL **Charge** 2 **Spectral Angle** 0.68

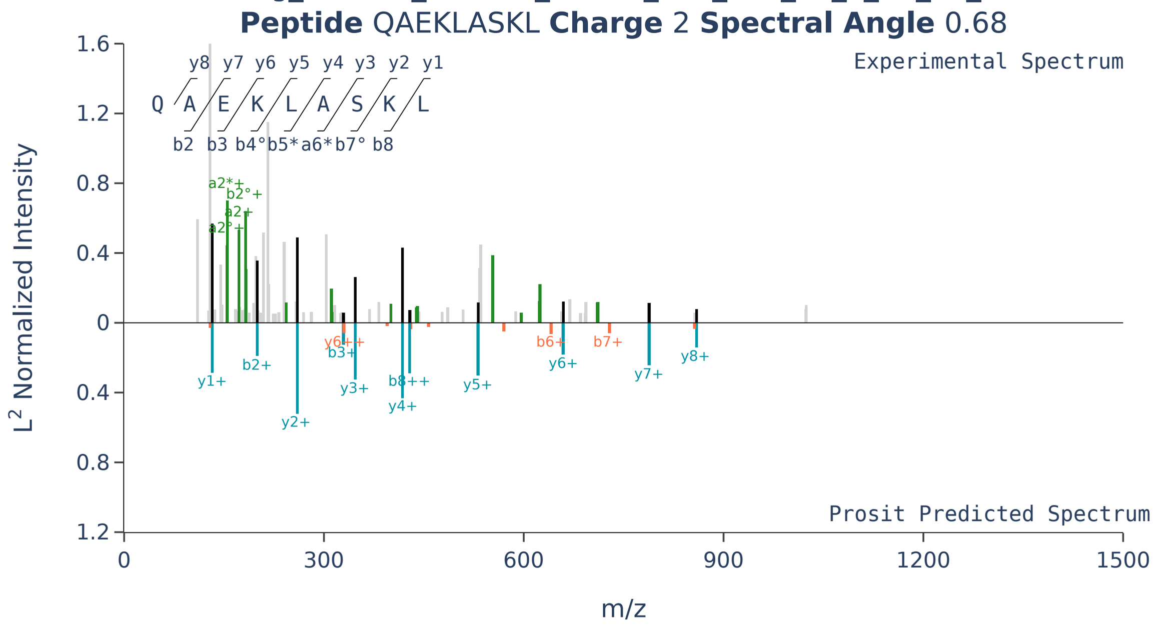


**Source** Ncheng_210623_230623_HFGoe_FFH_20S_25_1_A2_24h_R1 **Scan** 7265
**Peptide** KLGKFLREKH **Charge** 2 **Spectral Angle** 0.78

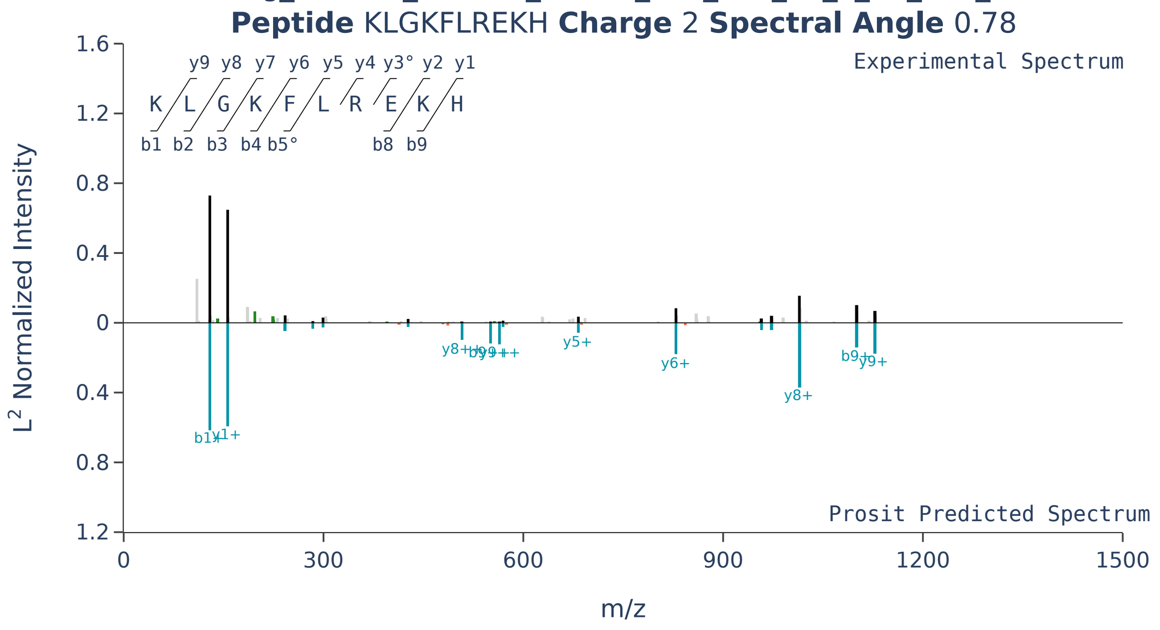


**Source** Ncheng_210623_230623_HFGoe_FFH_20S_25_1_A2_4h_R2 **Scan** 15934
**Peptide** EALINSMTMKER **Charge** 3 **Spectral Angle** 0.81

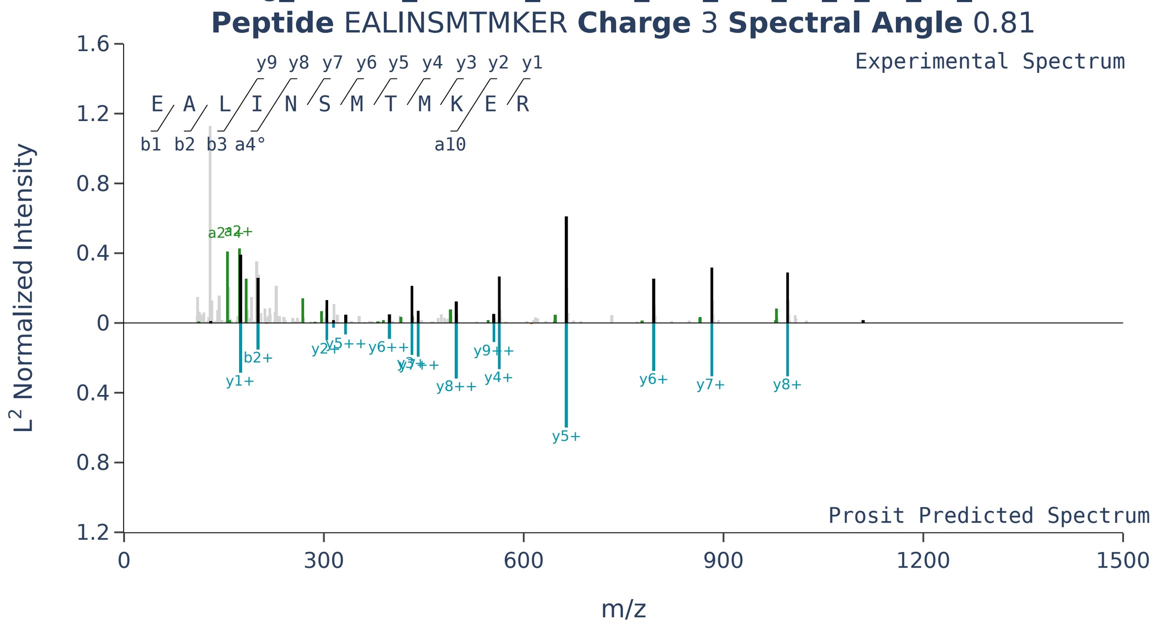


**Source** Ncheng_210623_230623_HFGoe_FFH_20S_25_1_A1_2h_R2 **Scan** 30463
**Peptide** EVNKSLTPGQEFVKIVRNEL **Charge** 3 **Spectral Angle** 0.73

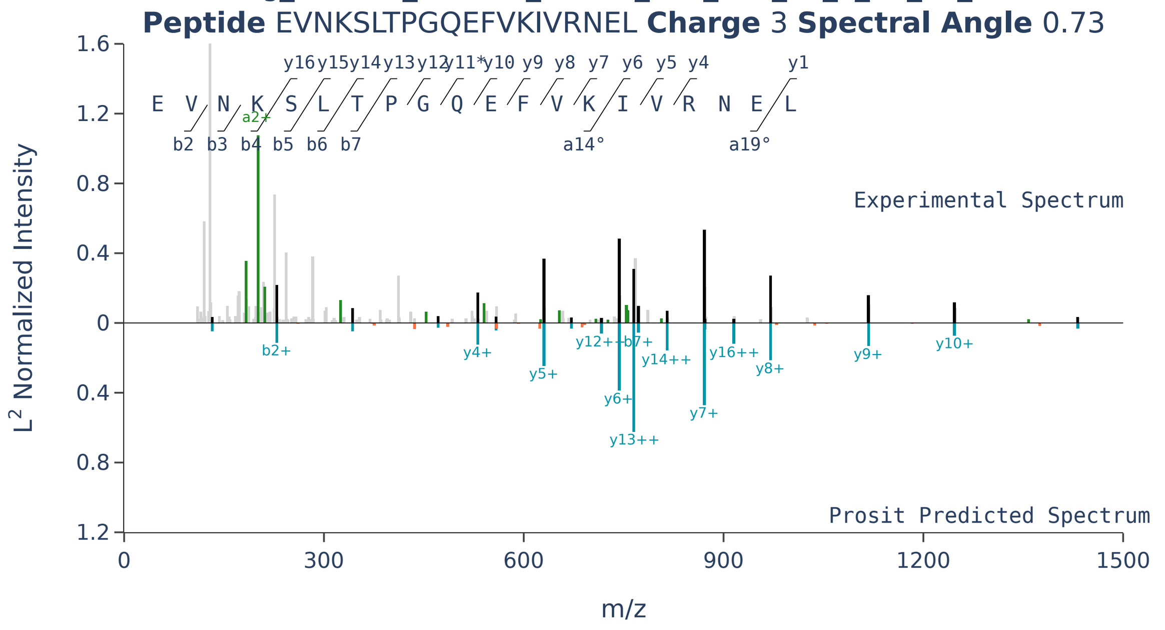


**Source** Ncheng_210623_230623_HFGoe_FFH_20S_25_1_A2_4h_R2 **Scan** 7220
**Peptide** EDIESKVDR **Charge** 2 **Spectral Angle** 0.75

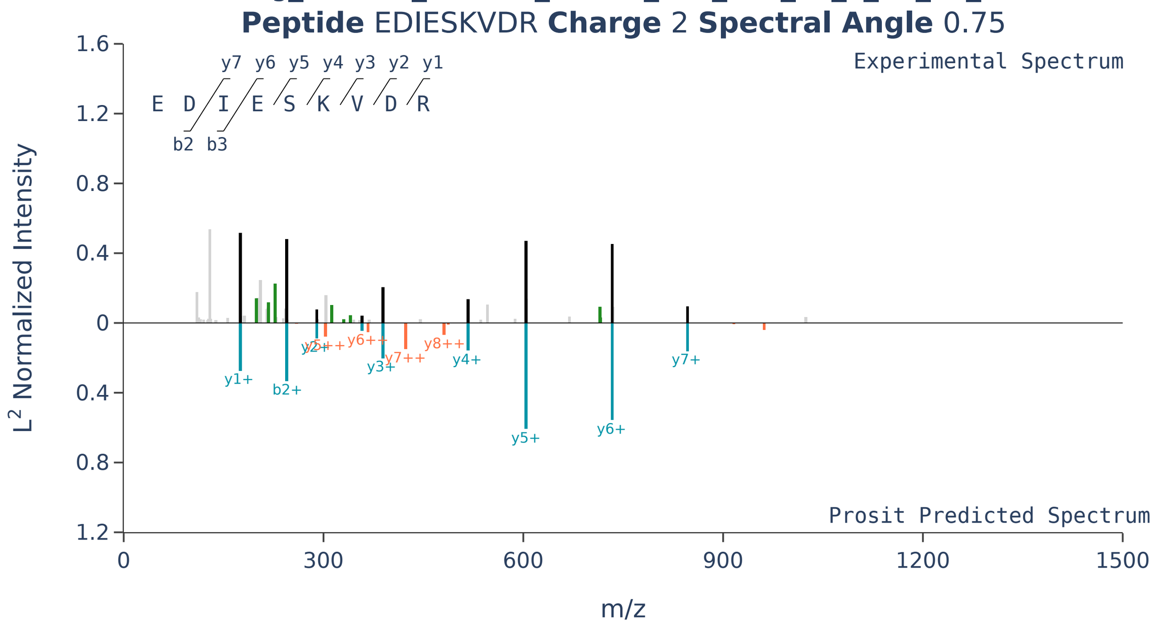


**Source** Ncheng_210623_230623_HFGoe_FFH_20S_25_1_A2_2h_R1 **Scan** 11304
**Peptide** SADVYRPAAIKQ **Charge** 2 **Spectral Angle** 0.72

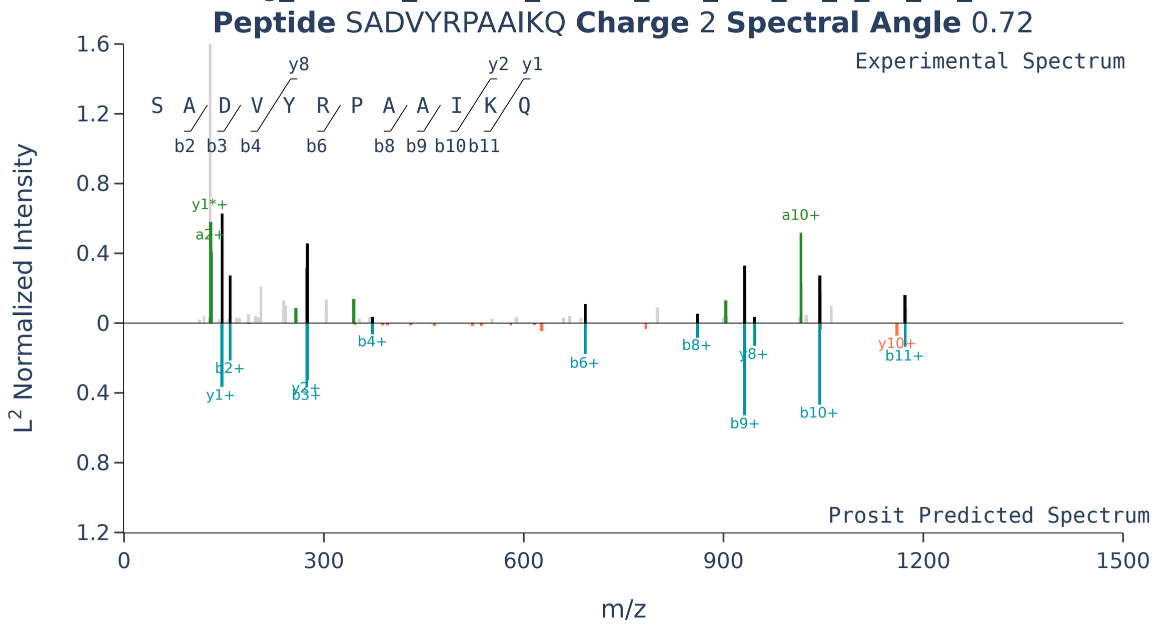


**Source** Ncheng_210623_230623_HFGoe_FFH_20S_25_1_A1_2h_R2 **Scan** 22590
**Peptide** GHEVNKSLTPGQEFVKIV **Charge** 2 **Spectral Angle** 0.86

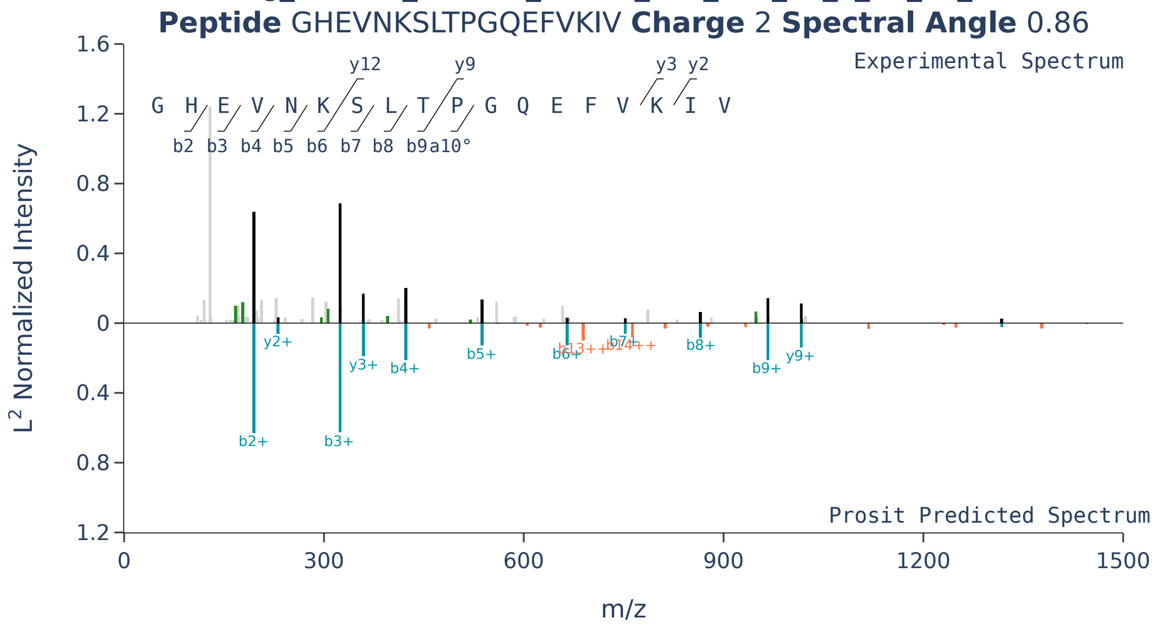


**Source** Ncheng_210623_230623_HFGoe_FFH_20S_25_1_A1_2h_R2 **Scan** 17899
**Peptide** GVVLTKVDGDARGGAAL **Charge** 2 **Spectral Angle** 0.73

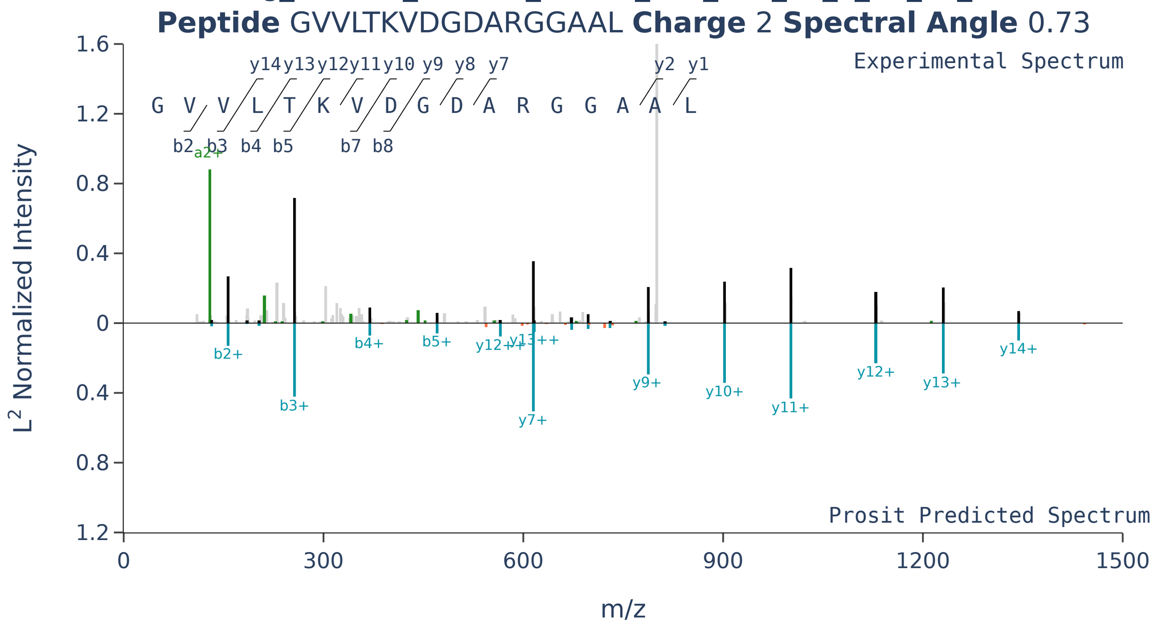


**Source** Ncheng_210623_230623_HFGoe_FFH_20S_25_1_A2_4h_R1 **Scan** 12841
**Peptide** GGMAKMMRSM **Charge** 3 **Spectral Angle** 0.85

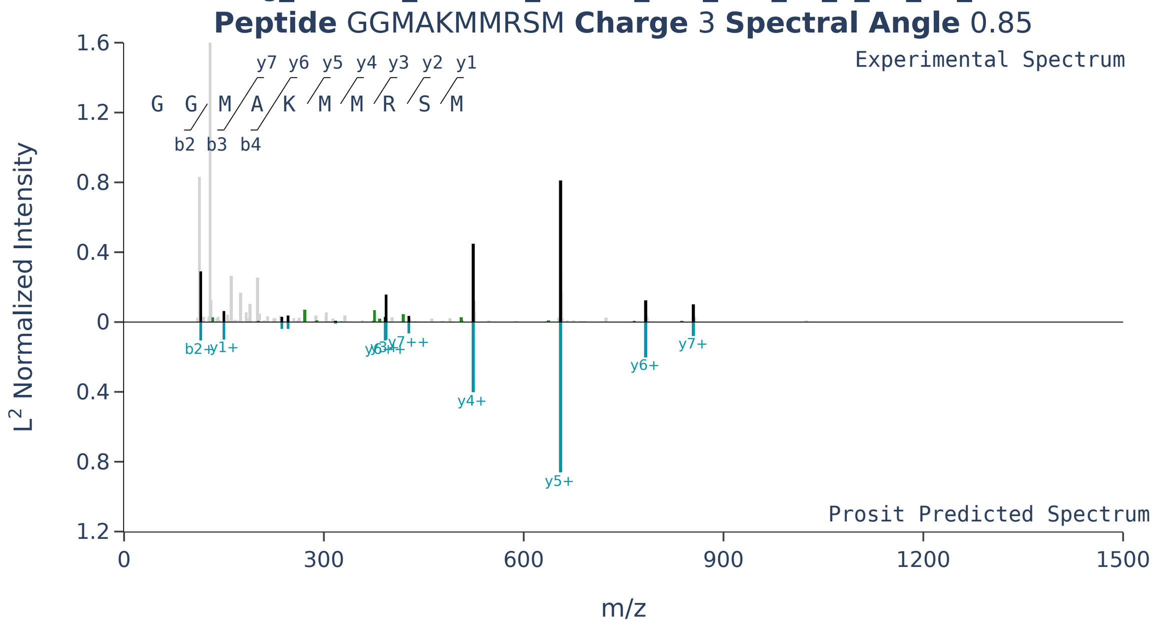


**Source** Ncheng_210623_230623_HFGoe_FFH_20S_25_1_A2_24h_R1 **Scan** 11277
**Peptide** LTKVDGDARGGAAL **Charge** 2 **Spectral Angle** 0.82

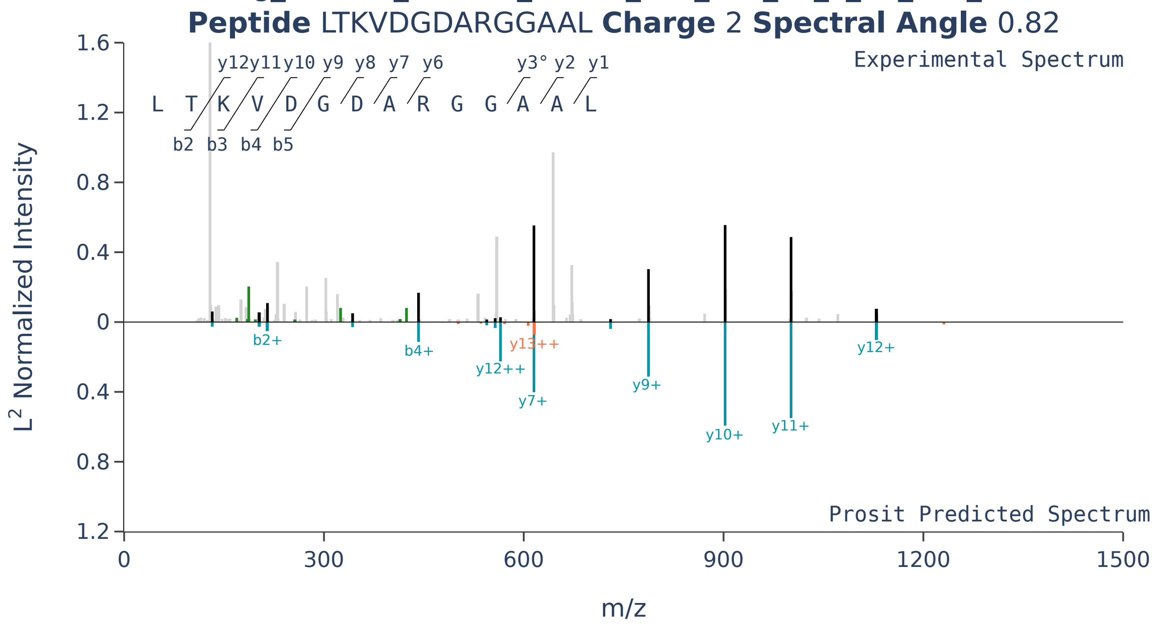


**Source** Ncheng_210623_230623_HFGoe_FFH_20S_25_1_A2_4h_R1 **Scan** 20902
**Peptide** AVGHEVNKSLTPGQEFVKI **Charge** 2 **Spectral Angle** 0.85

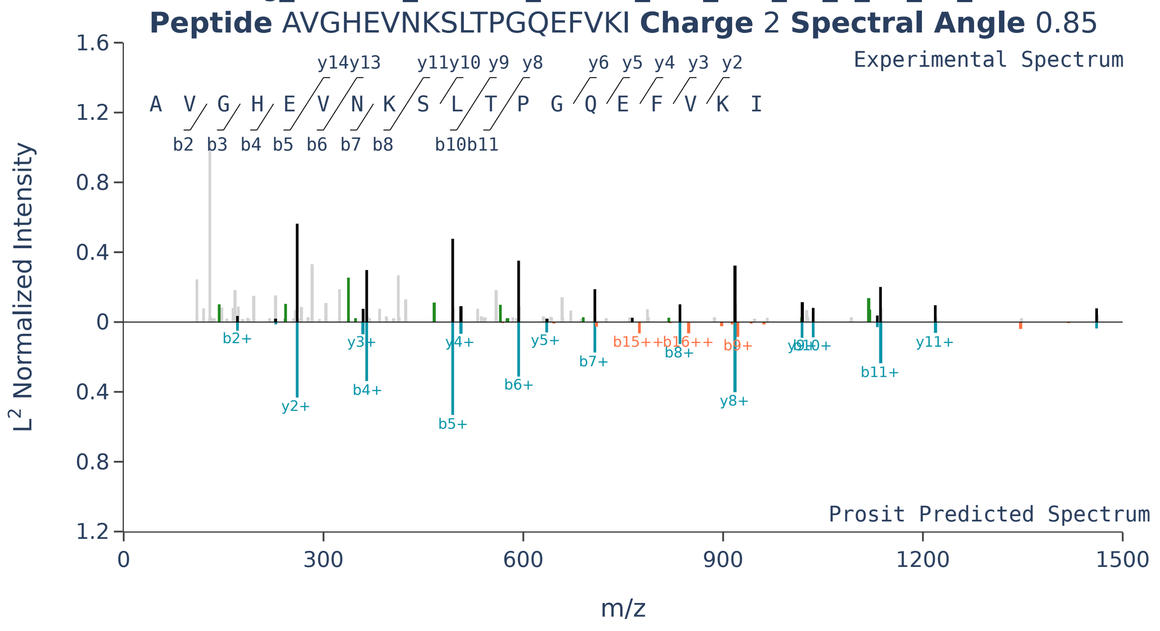


**Source** Ncheng_210623_230623_HFGoe_FFH_20S_25_1_A2_2h_R1 **Scan** 30727
**Peptide** DDKVLVRMEALL **Charge** 2 **Spectral Angle** 0.84

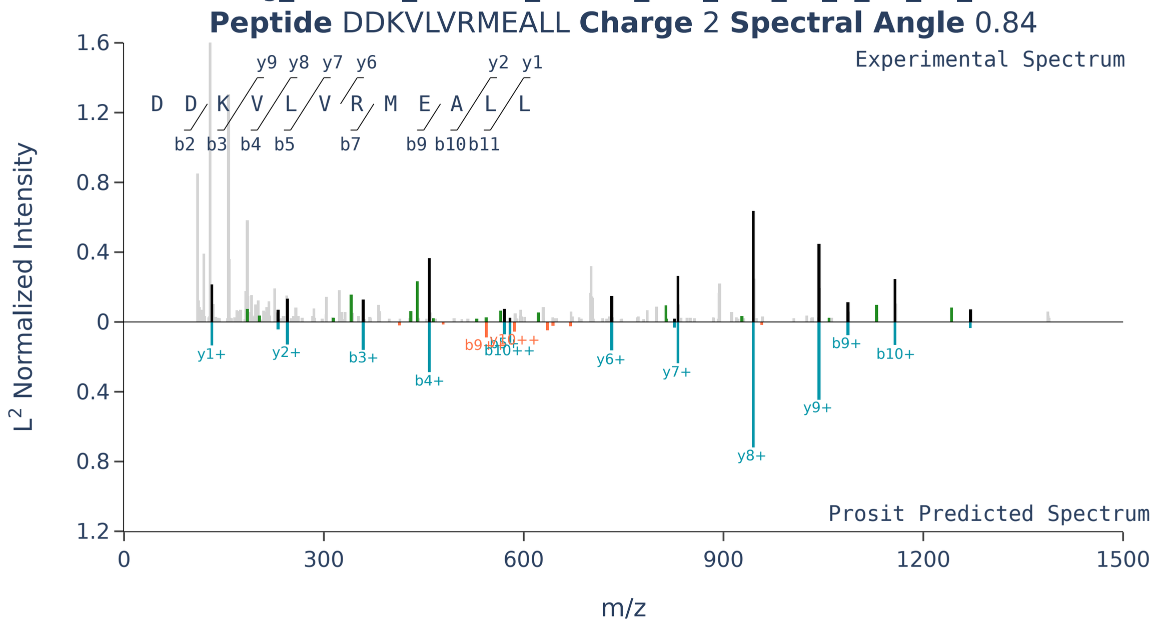


**Source** Ncheng_210623_230623_HFGoe_FFH_20S_25_1_A1_2h_R2 **Scan** 30374
**Peptide** TEDNVKDTLREVRMALLE **Charge** 3 **Spectral Angle** 0.78

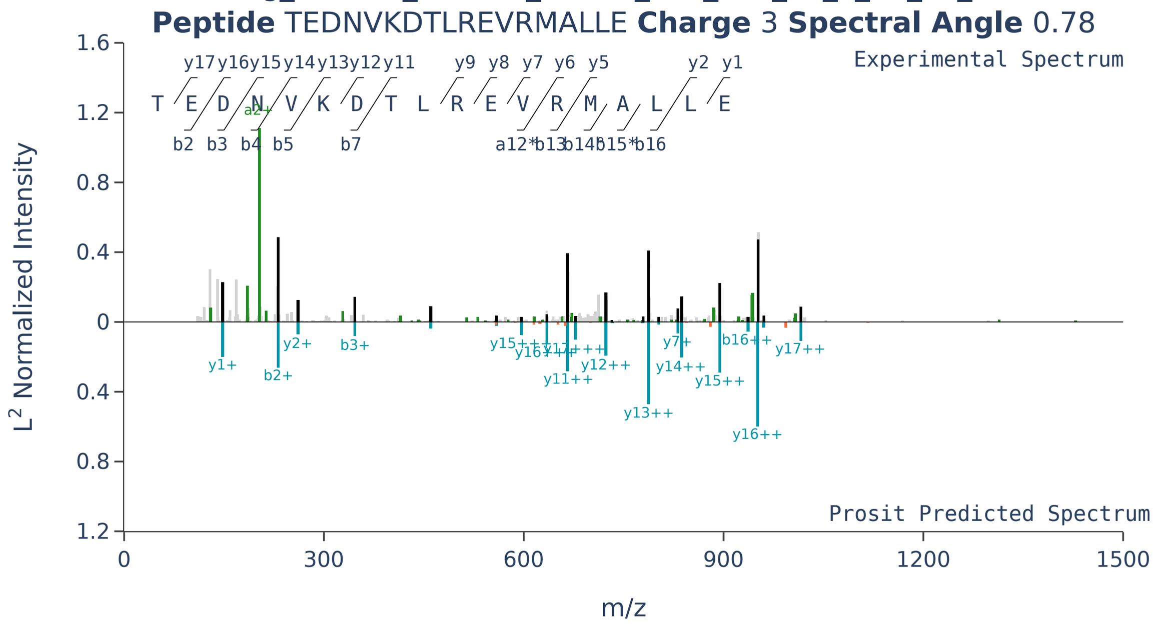


**Source** Ncheng_210623_230623_HFGoe_FFH_20S_25_1_A1_4h_R1 **Scan** 20447
**Peptide** LLVDTAGRLHVD **Charge** 3 **Spectral Angle** 0.77

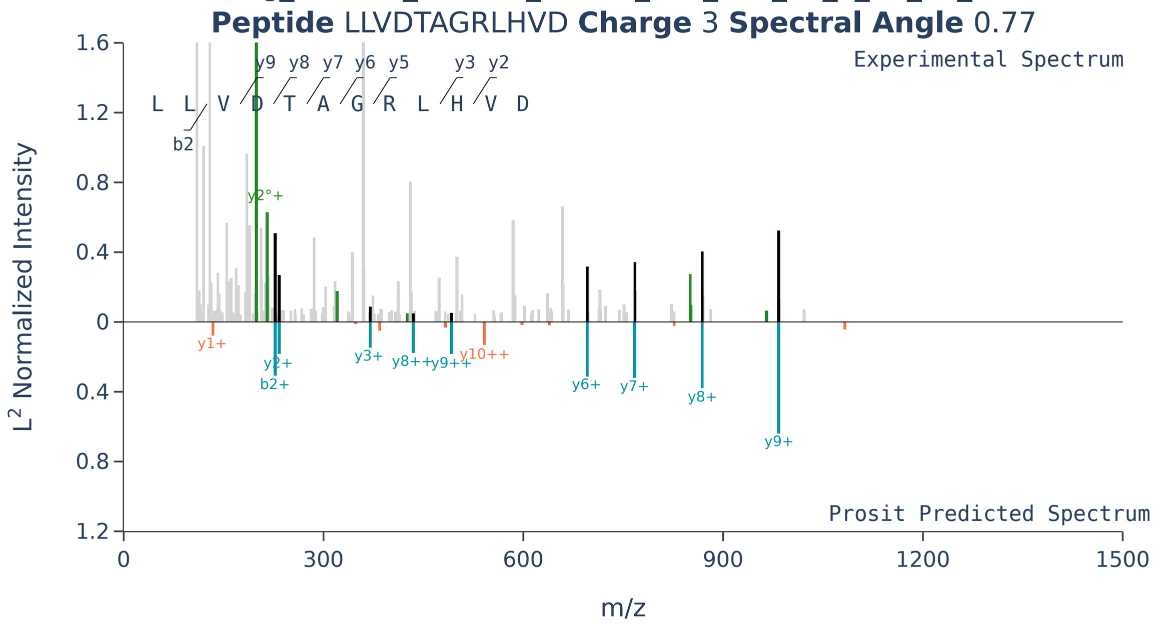

**Source** Ncheng_210623_230623_HFGoe_FFH_20S_25_1_A1_4h_R1 **Scan** 19285
**Peptide** EALEPFHPDRIAS **Charge** 3 **Spectral Angle** 0.79

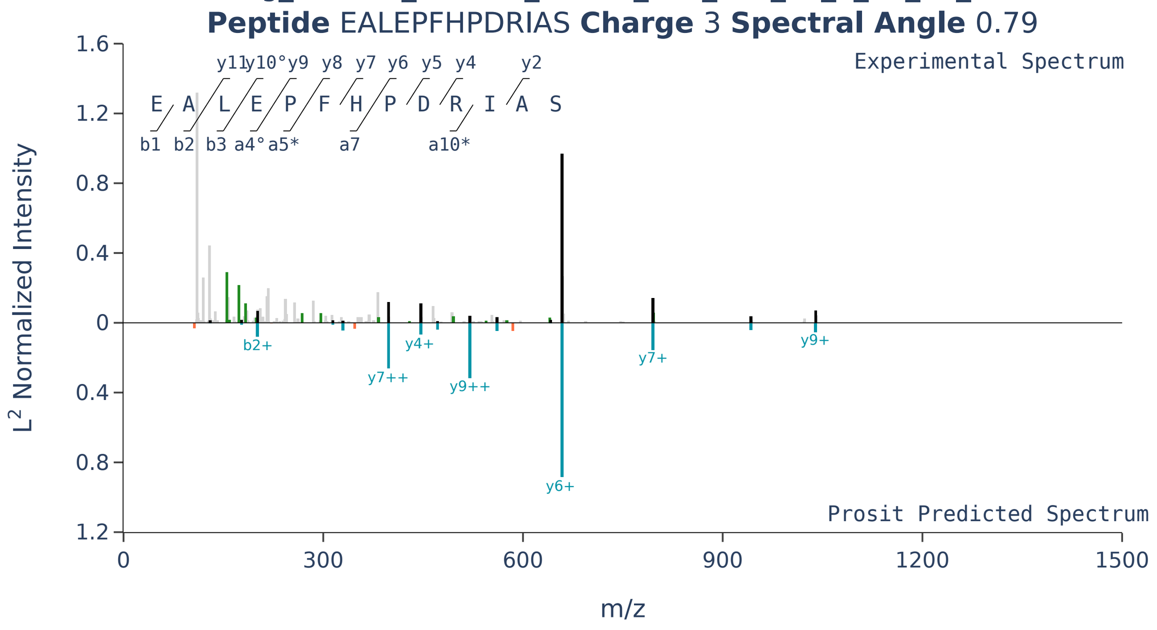

**Source** Ncheng_210623_230623_HFGoe_FFH_20S_25_1_A2_4h_R2 **Scan** 25074
**Peptide** LVVSADVYRPAAIKQLE **Charge** 2 **Spectral Angle** 0.8

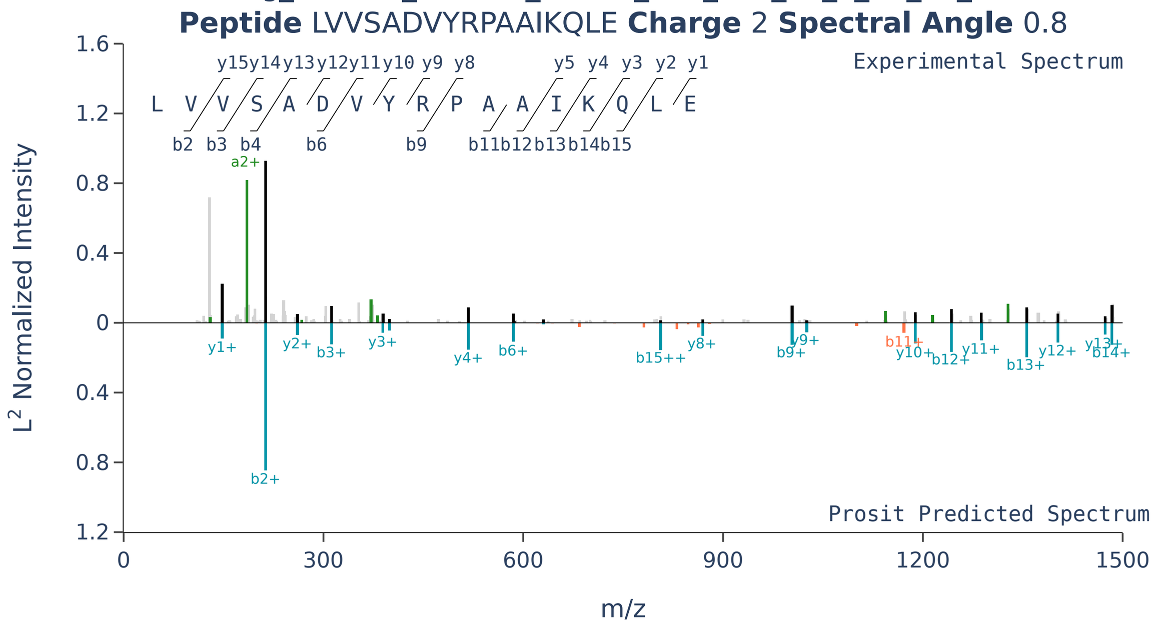

**Source** Ncheng_210623_230623_HFGoe_FFH_20S_25_1_A2_2h_R2 **Scan** 20790
**Peptide** KLPGMGQIPDNVKSQM **Charge** 2 **Spectral Angle** 0.8

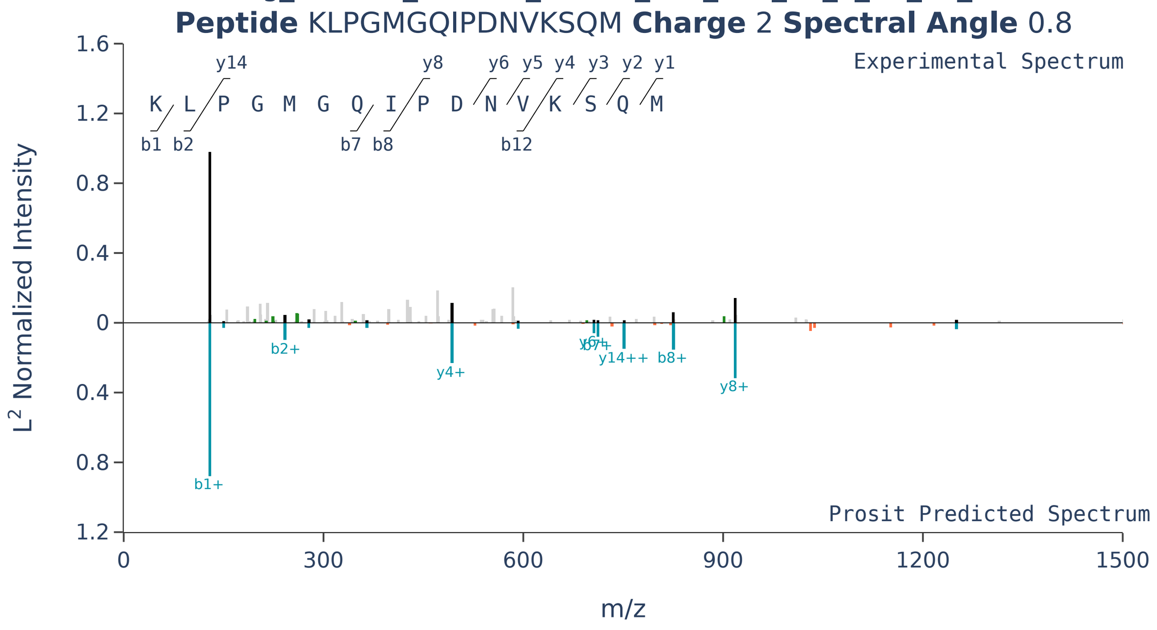

**Source** Ncheng_210623_230623_HFGoe_FFH_20S_25_1_A2_24h_R2 **Scan** 12259
**Peptide** GMAKMMRSM **Charge** 2 **Spectral Angle** 0.75

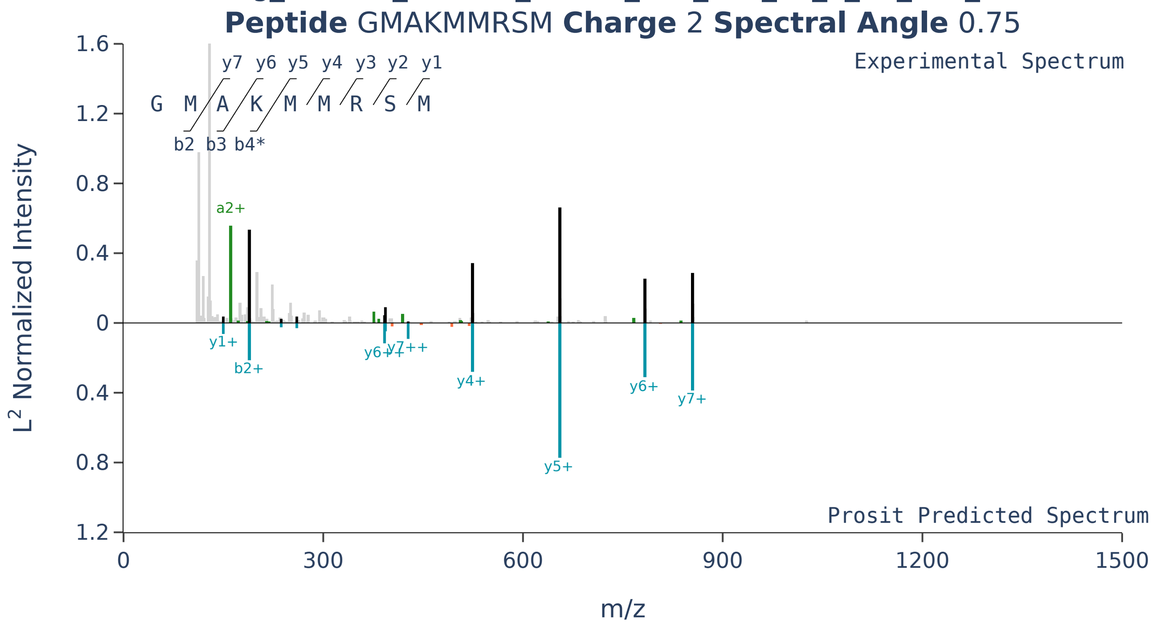

**Source** Ncheng_210623_230623_HFGoe_FFH_20S_25_1_A1_4h_R1 **Scan** 28956
**Peptide** VRNELVAAMGEENQTLNL **Charge** 2 **Spectral Angle** 0.81

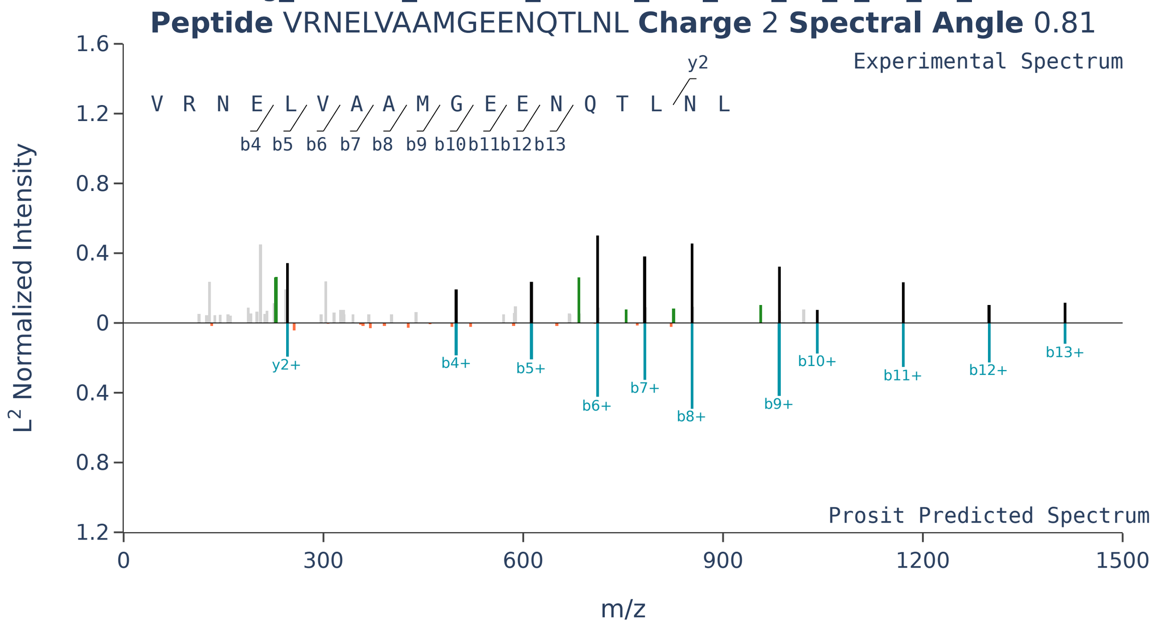

**Source** Ncheng_210623_230623_HFGoe_FFH_20S_25_1_A2_4h_R1 **Scan** 31323
**Peptide** NKSLTPGQEFVKIVRNELVA **Charge** 2 **Spectral Angle** 0.79

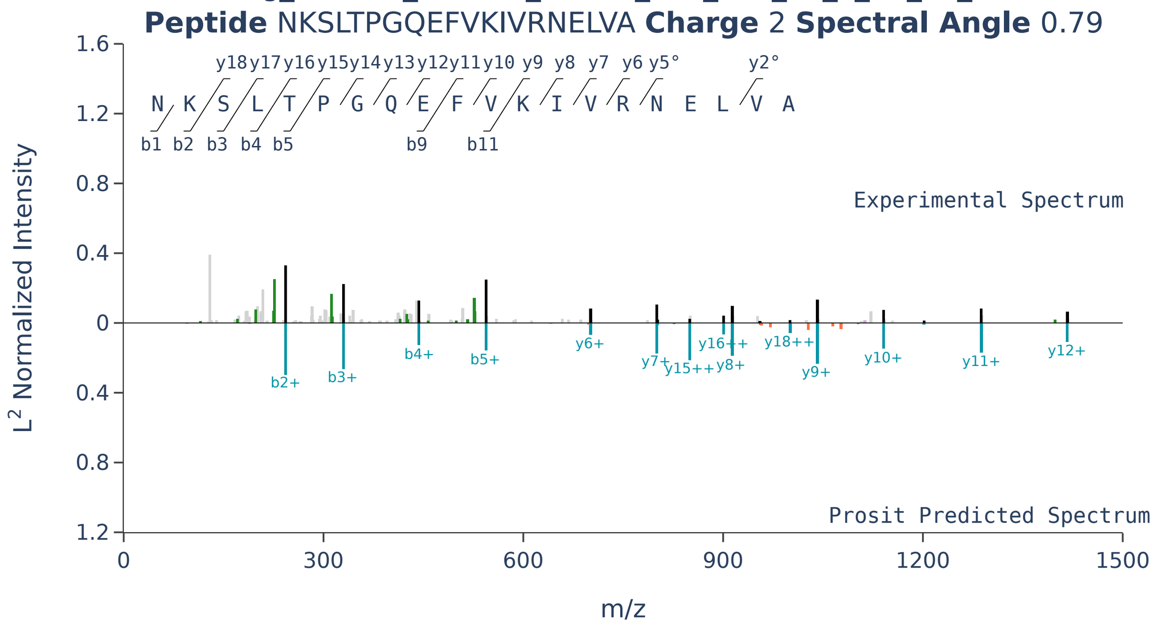

**Source** Ncheng_210623_230623_HFGoe_FFH_20S_25_1_A1_2h_R2 **Scan** 21751
**Peptide** LLVDTAGRLHV **Charge** 3 **Spectral Angle** 0.84

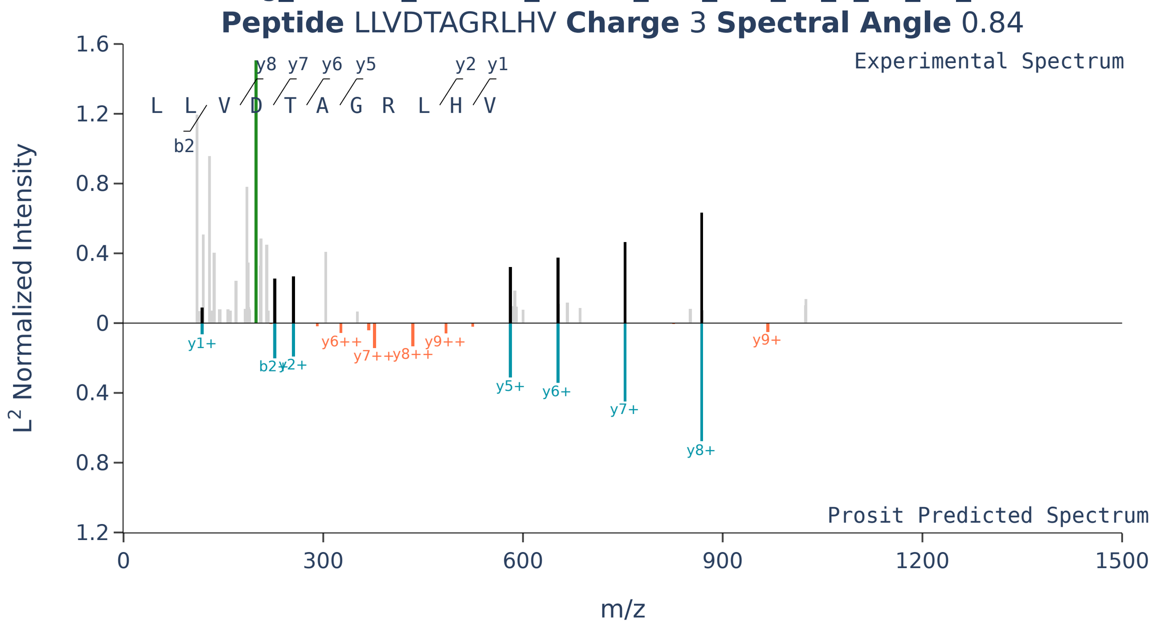

**Source** Ncheng_210623_230623_HFGoe_FFH_20S_25_1_A1_4h_R1 **Scan** 10290
**Peptide** TLREVRM **Charge** 2 **Spectral Angle** 0.77

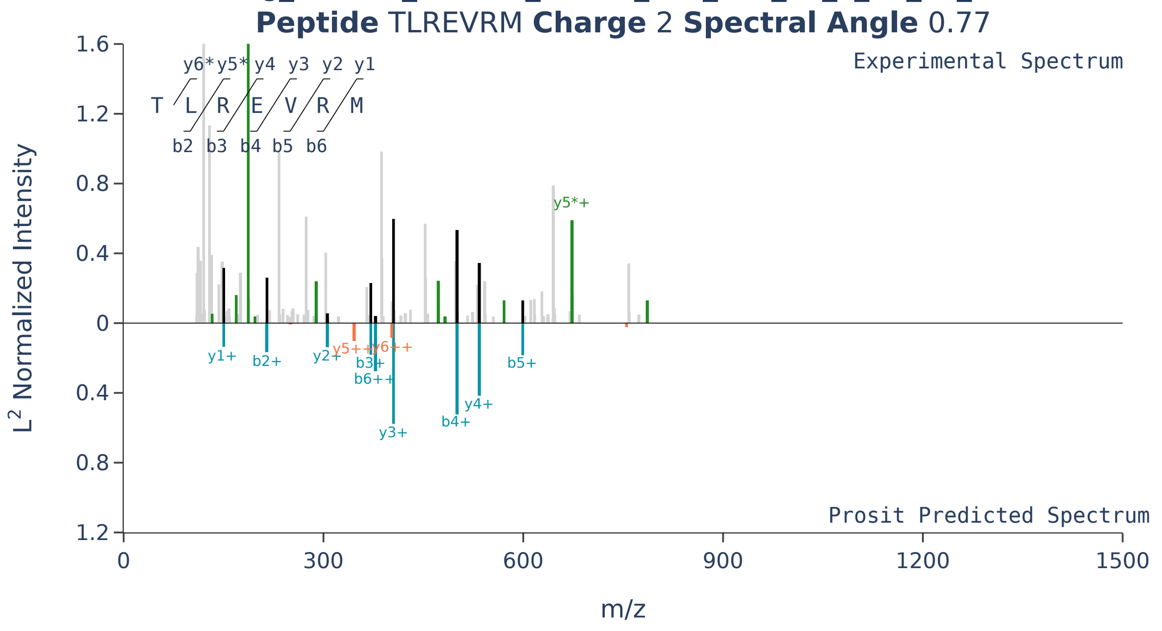

**Source** Ncheng_210623_230623_HFGoe_FFH_20S_25_1_A2_4h_R2 **Scan** 20106
**Peptide** NKSLTPGQEFVKIVR **Charge** 2 **Spectral Angle** 0.84

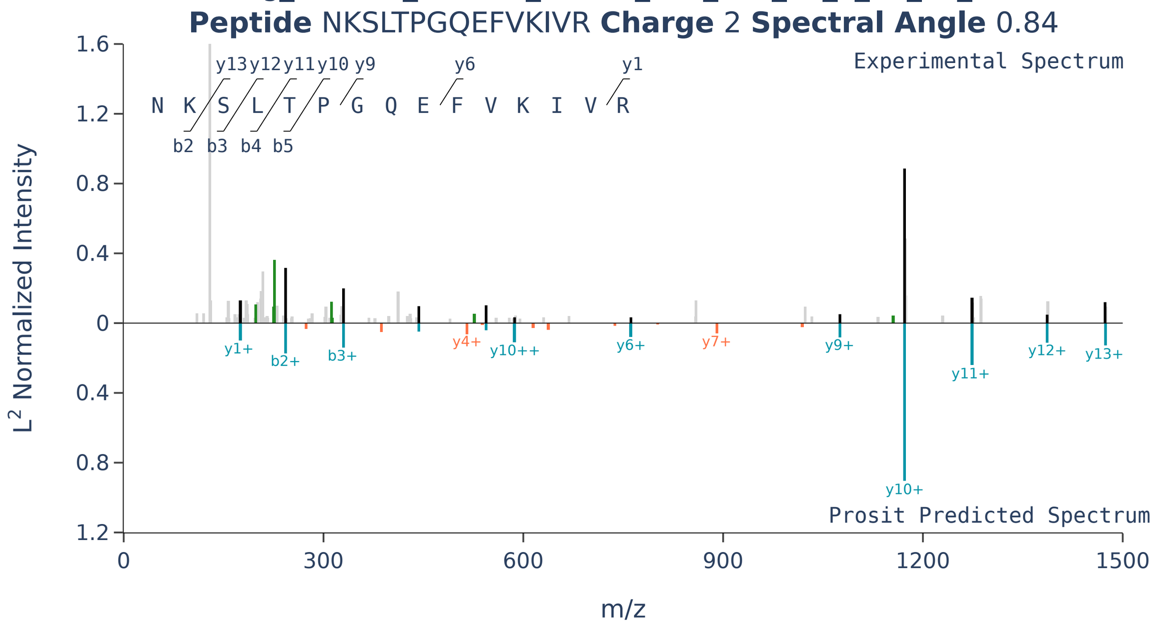

**Source** Ncheng_210623_230623_HFGoe_FFH_20S_25_1_A1_24h_R1 **Scan** 15405
**Peptide** DGDARGGAALSIRHI **Charge** 2 **Spectral Angle** 0.78

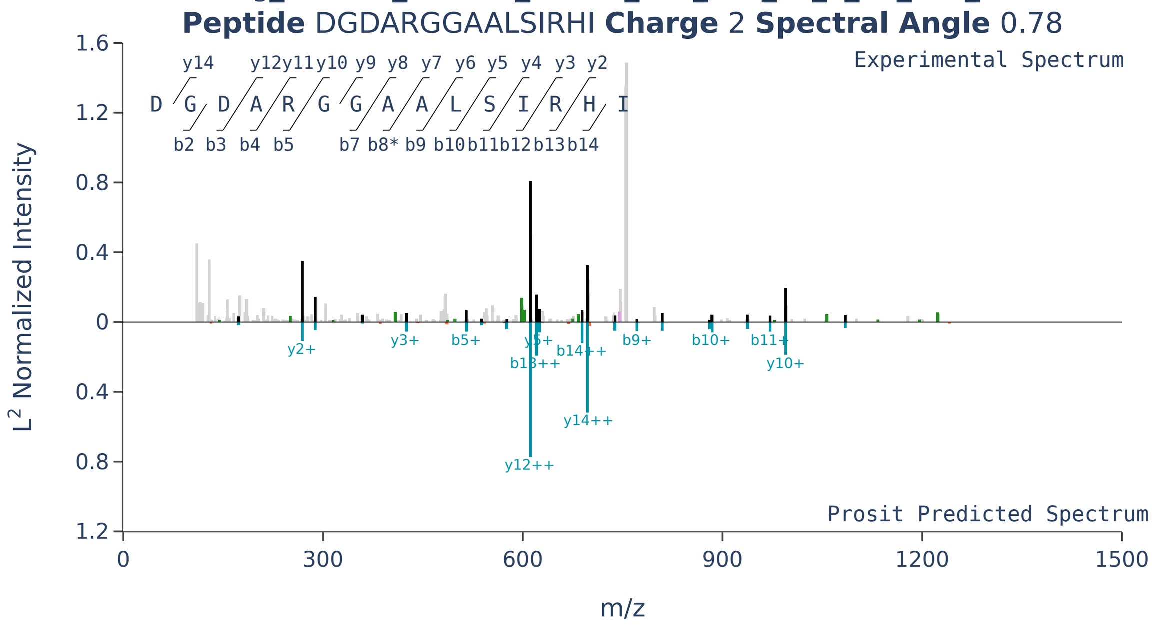

**Source** Ncheng_210623_230623_HFGoe_FFH_20S_25_1_A1_24h_R1 **Scan** 10488
**Peptide** AAIKQLE **Charge** 2 **Spectral Angle** 0.83

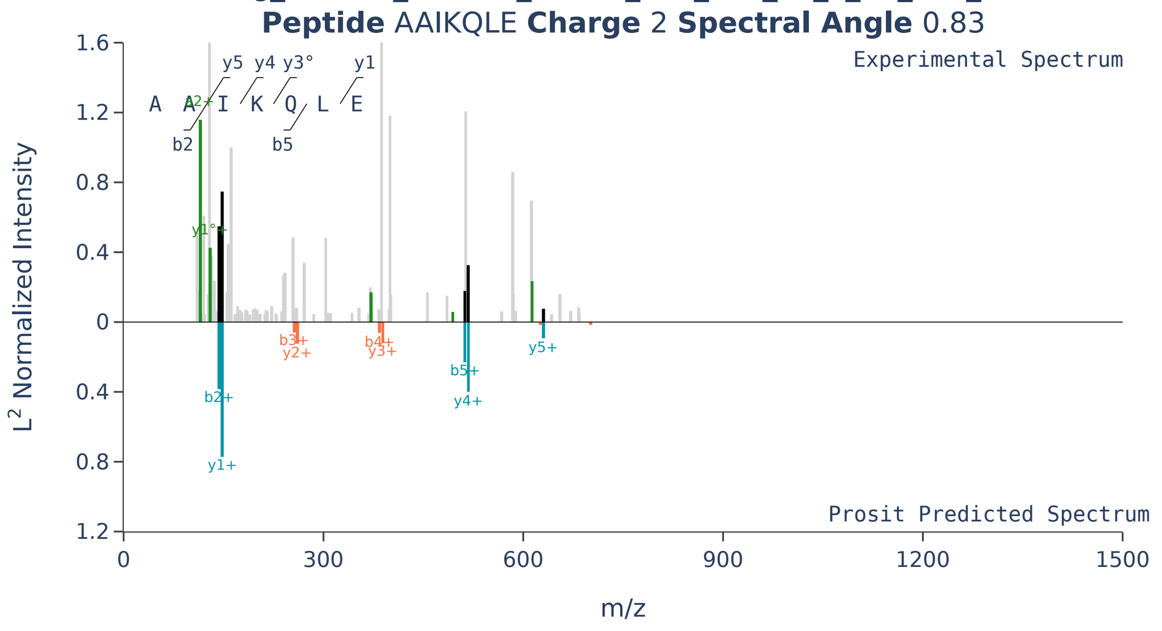

**Source** Ncheng_210623_230623_HFGoe_FFH_20S_25_1_A2_24h_R1 **Scan** 17956
**Peptide** VDGDARGGAALSI **Charge** 2 **Spectral Angle** 0.82

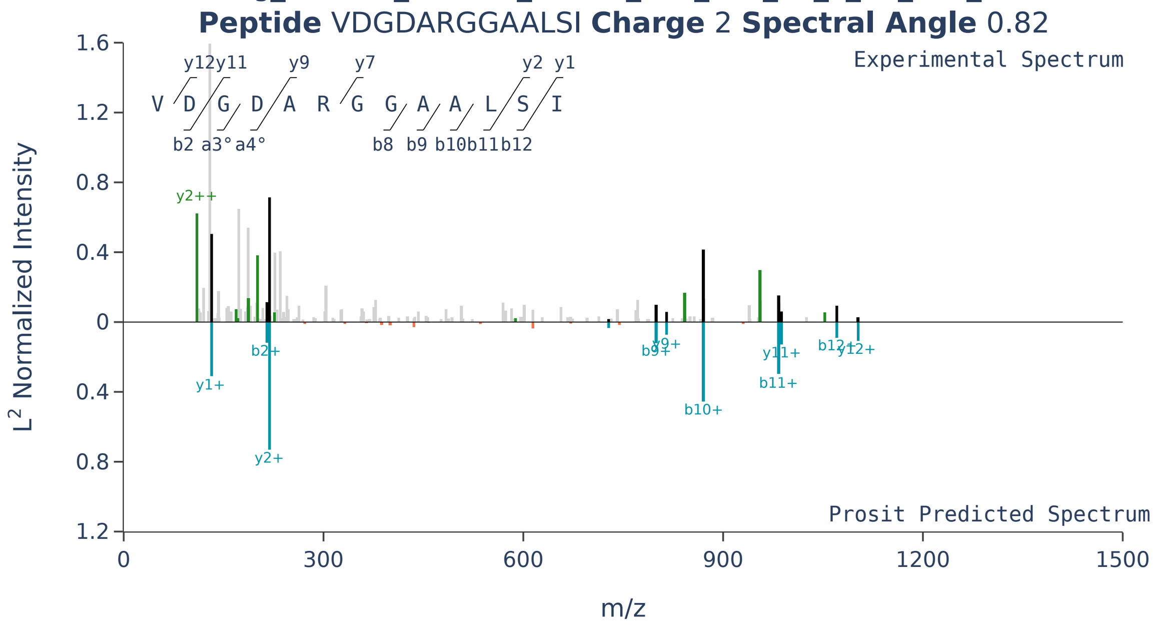

**Source** Ncheng_210623_230623_HFGoe_FFH_20S_25_1_A2_2h_R1 **Scan** 27361
**Peptide** KAVGHEVNKSLTPGQEFVKIVRNELVA **Charge** 3 **Spectral Angle** 0.77

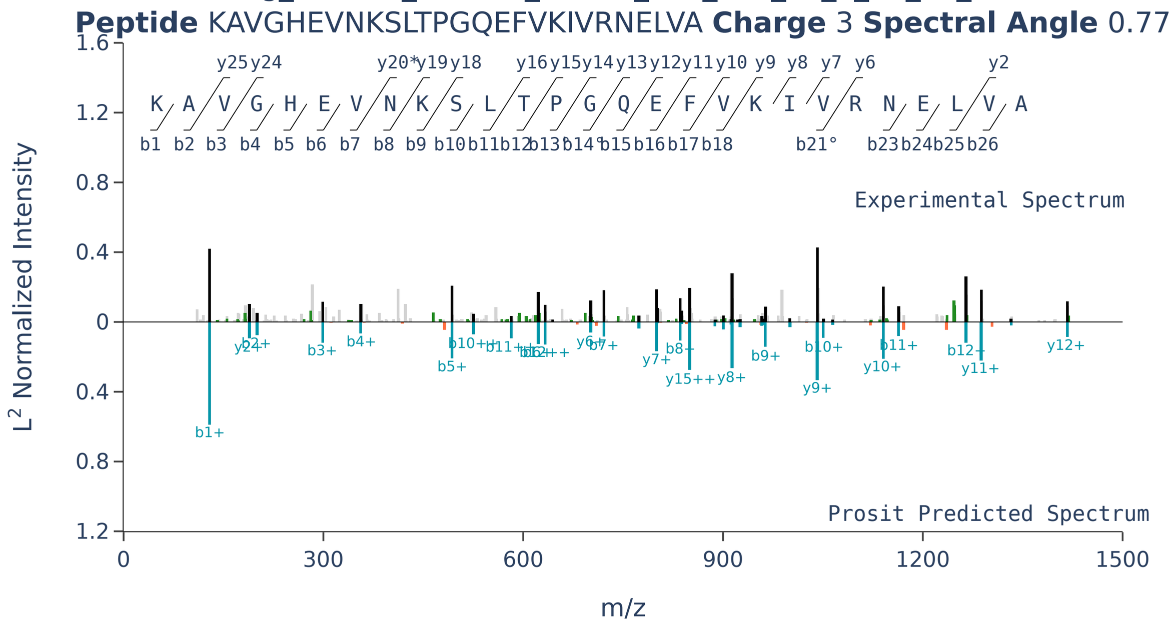

**Source** Ncheng_210623_230623_HFGoe_FFH_20S_25_1_A1_24h_R1 **Scan** 6162
**Peptide** HEVNKSL **Charge** 1 **Spectral Angle** 0.83

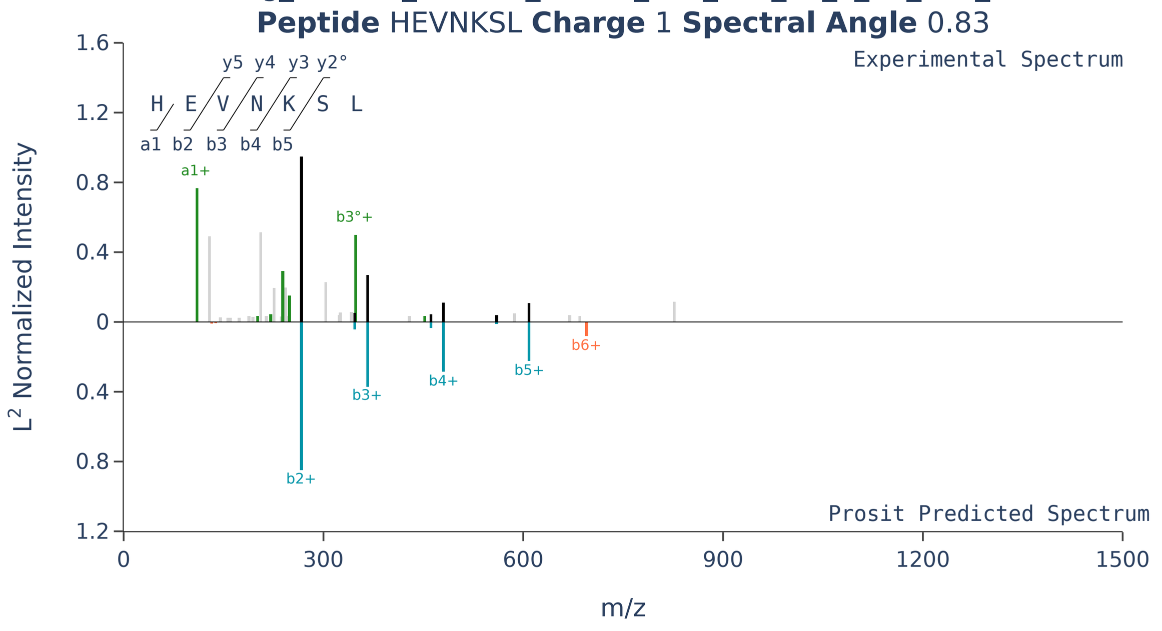

**Source** Ncheng_210623_230623_HFGoe_FFH_20S_25_1_A2_24h_R2 **Scan** 6455
**Peptide** ASKLKKGDGF **Charge** 3 **Spectral Angle** 0.79

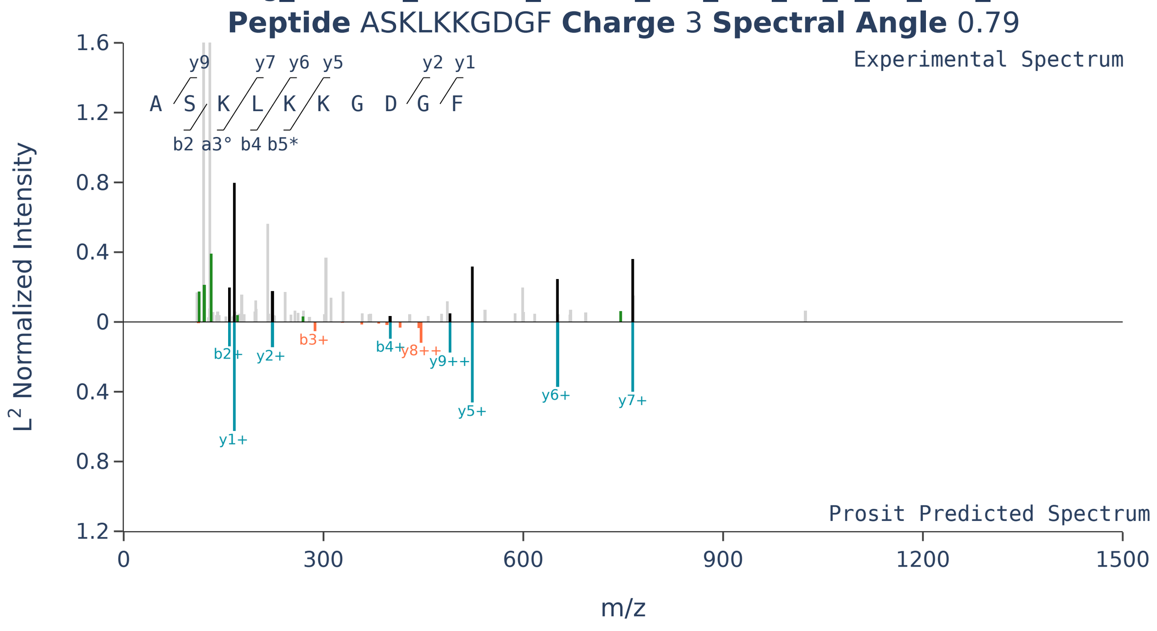

**Source** Ncheng_210623_230623_HFGoe_FFH_20S_25_1_A1_4h_R1 **Scan** 12369
**Peptide** TAGRLHVDEAMMD **Charge** 3 **Spectral Angle** 0.73

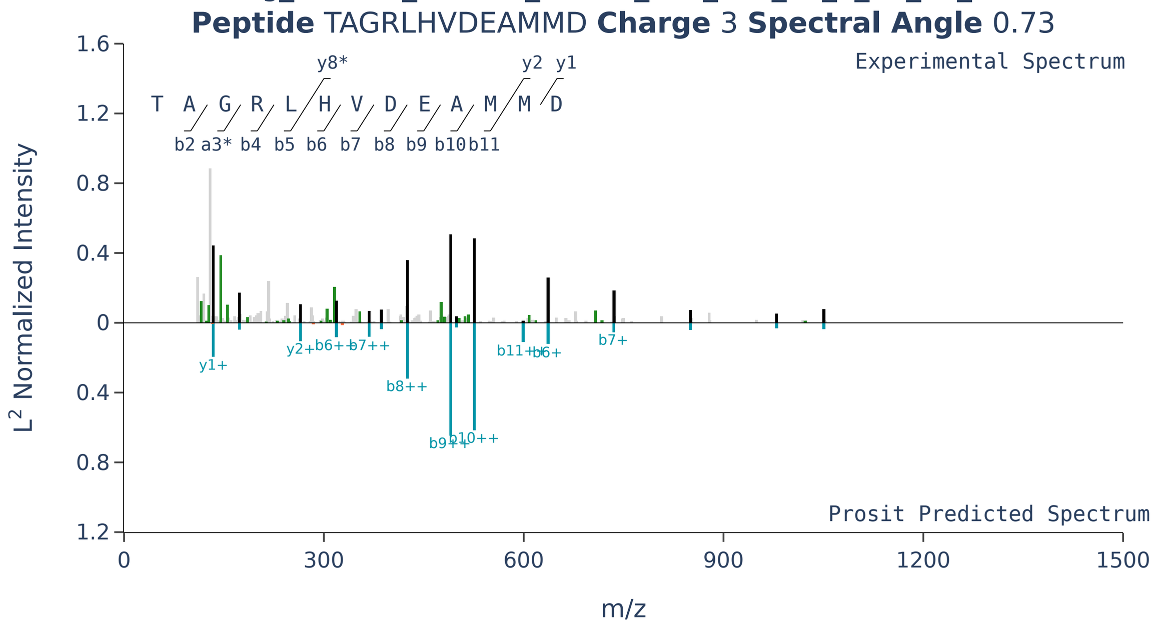


**Source** Ncheng_210623_230623_HFGoe_FFH_20S_25_1_A1_24h_R1 **Scan** 11525
**Peptide** RIAAGCGMQVQ **Charge** 2 **Spectral Angle** 0.71

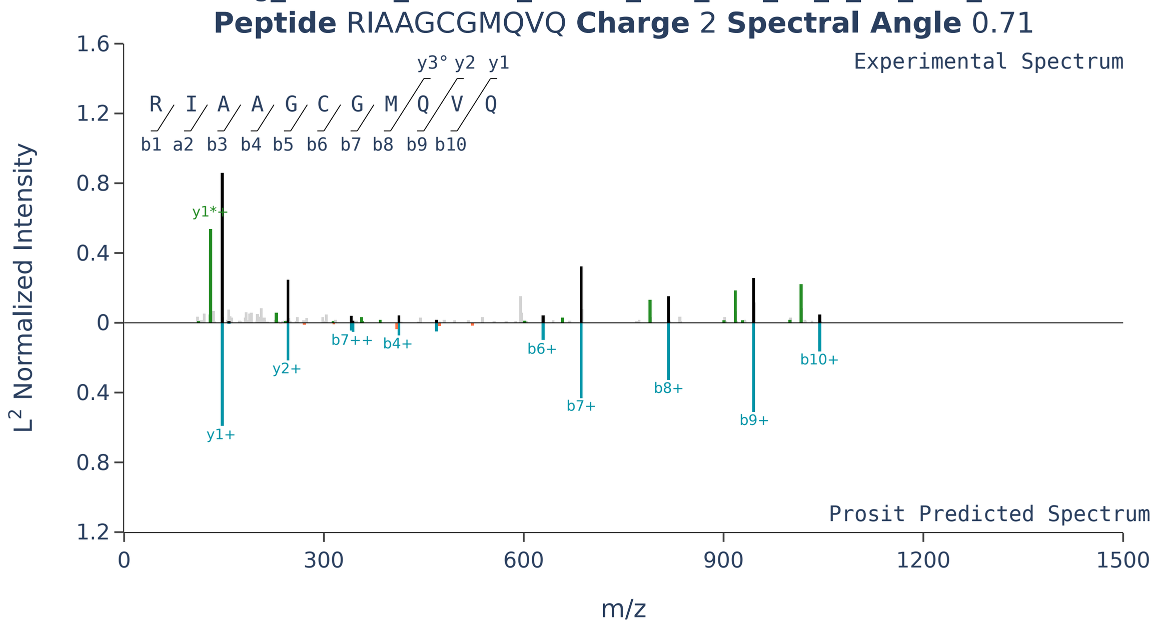


**Source** Ncheng_210623_230623_HFGoe_FFH_20S_25_1_A1_4h_R1 **Scan** 14949
**Peptide** LGVGEKTEAL **Charge** 2 **Spectral Angle** 0.69

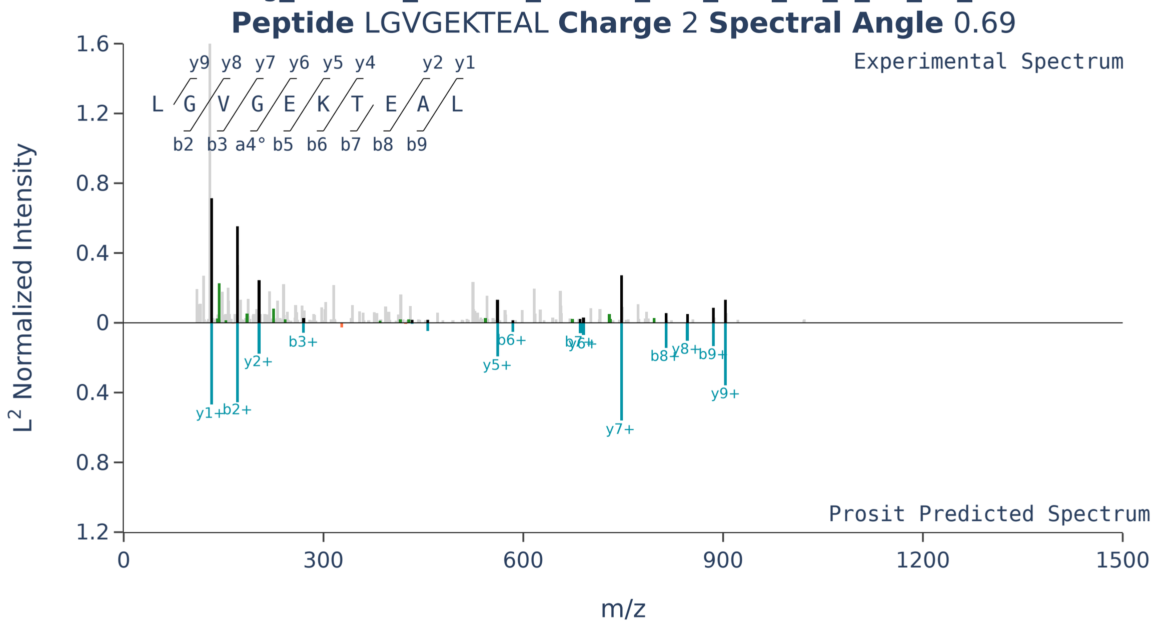


**Source** Ncheng_210623_230623_HFGoe_FFH_20S_25_1_A2_24h_R2 **Scan** 10127
**Peptide** TLREVRMA **Charge** 2 **Spectral Angle** 0.71

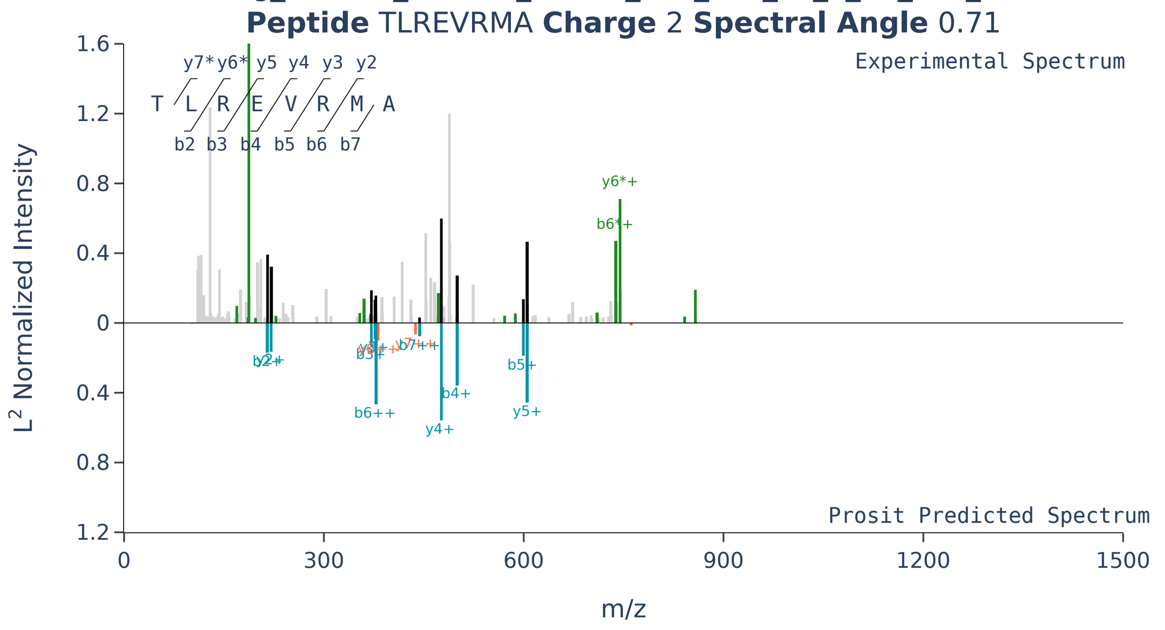


**Source** Ncheng_210623_230623_HFGoe_FFH_20S_25_1_A2_24h_R2 **Scan** 22034
**Peptide** MGEENQTLNLAA **Charge** 1 **Spectral Angle** 0.76

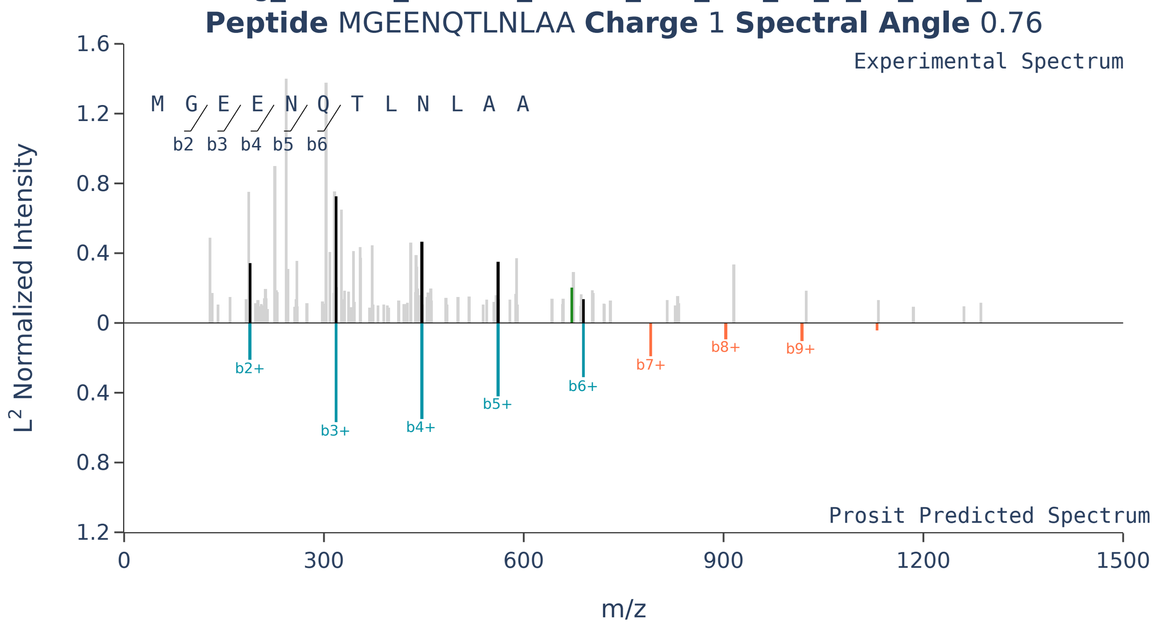


**Source** Ncheng_210623_230623_HFGoe_FFH_20S_25_1_A1_2h_R1 **Scan** 26903
**Peptide** ASLMGKLPGMGQIPDNVKSQMDD **Charge** 3 **Spectral Angle** 0.74

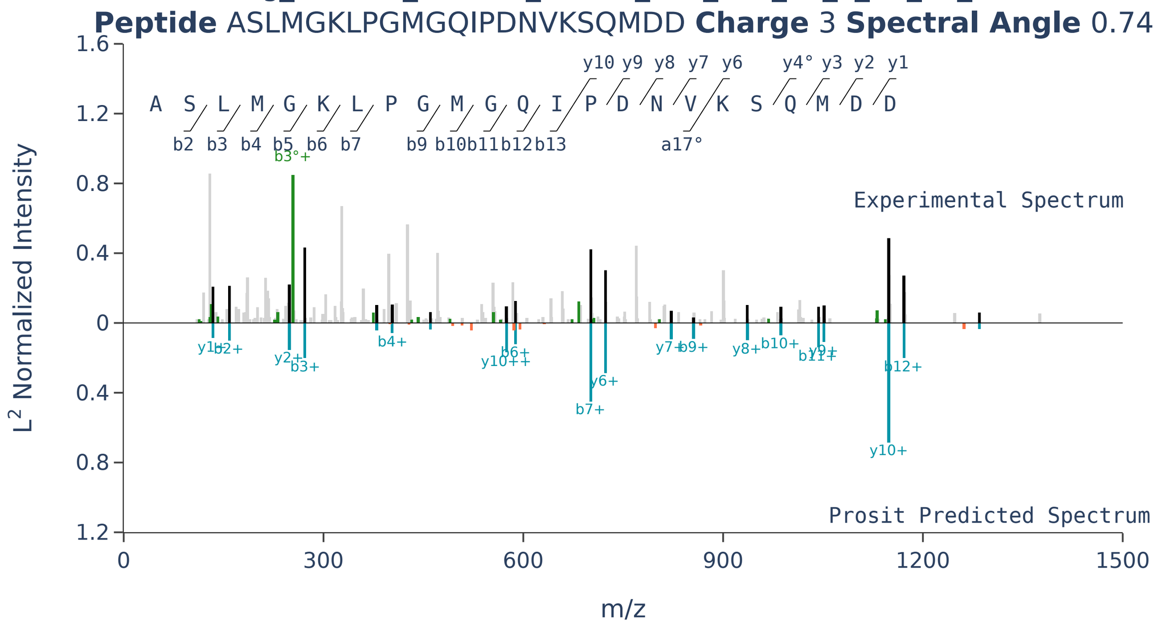


**Source** Ncheng_210623_230623_HFGoe_FFH_20S_25_1_A1_4h_R1 **Scan** 19681
**Peptide** KPIKFLGVGEKTEAL **Charge** 2 **Spectral Angle** 0.8

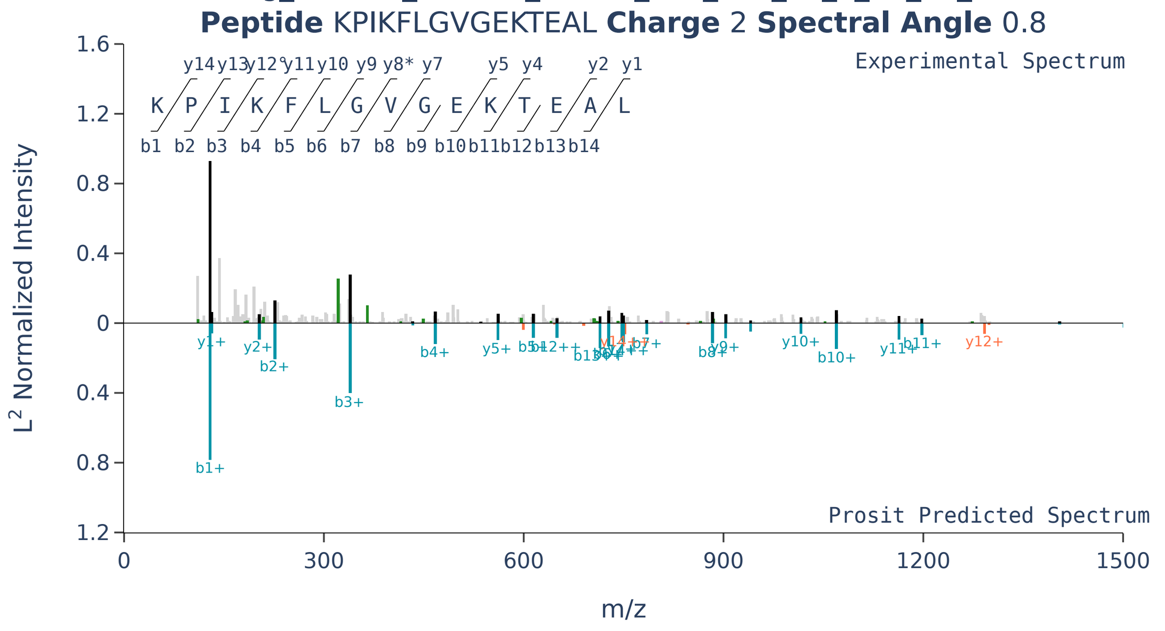


**Source** Ncheng_210623_230623_HFGoe_FFH_20S_25_1_A1_2h_R1 **Scan** 32805
**Peptide** EENQTLNLAAQPPAVVL **Charge** 2 **Spectral Angle** 0.91

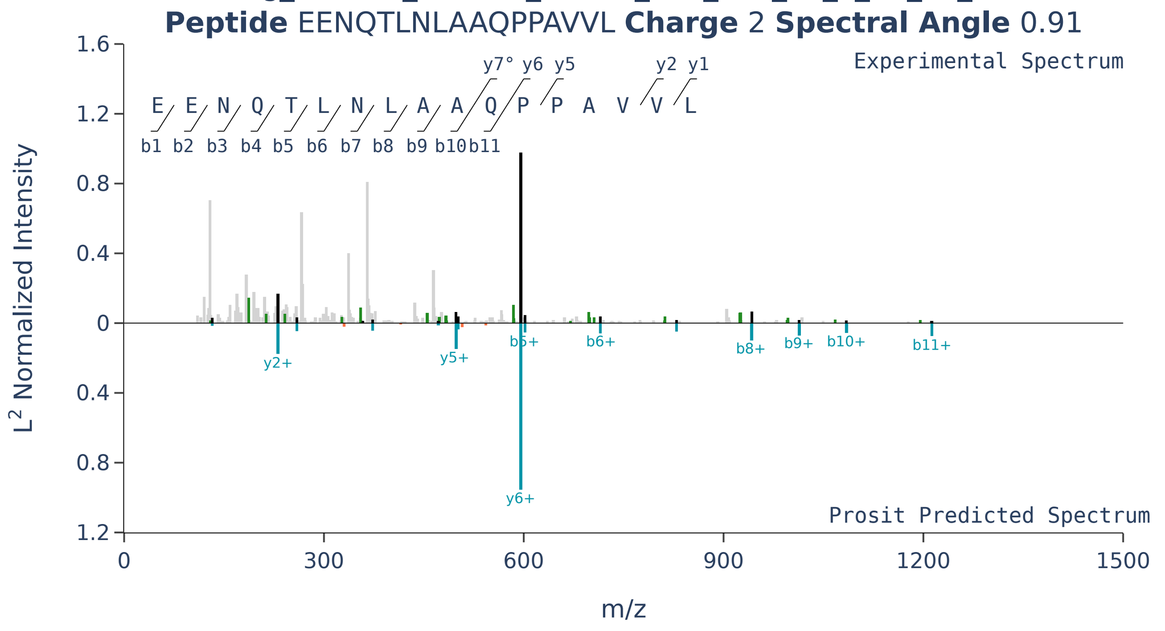


**Source** Ncheng_210623_230623_HFGoe_FFH_20S_25_1_A2_4h_R1 **Scan** 6706
**Peptide** DRAQAEKLA **Charge** 2 **Spectral Angle** 0.75

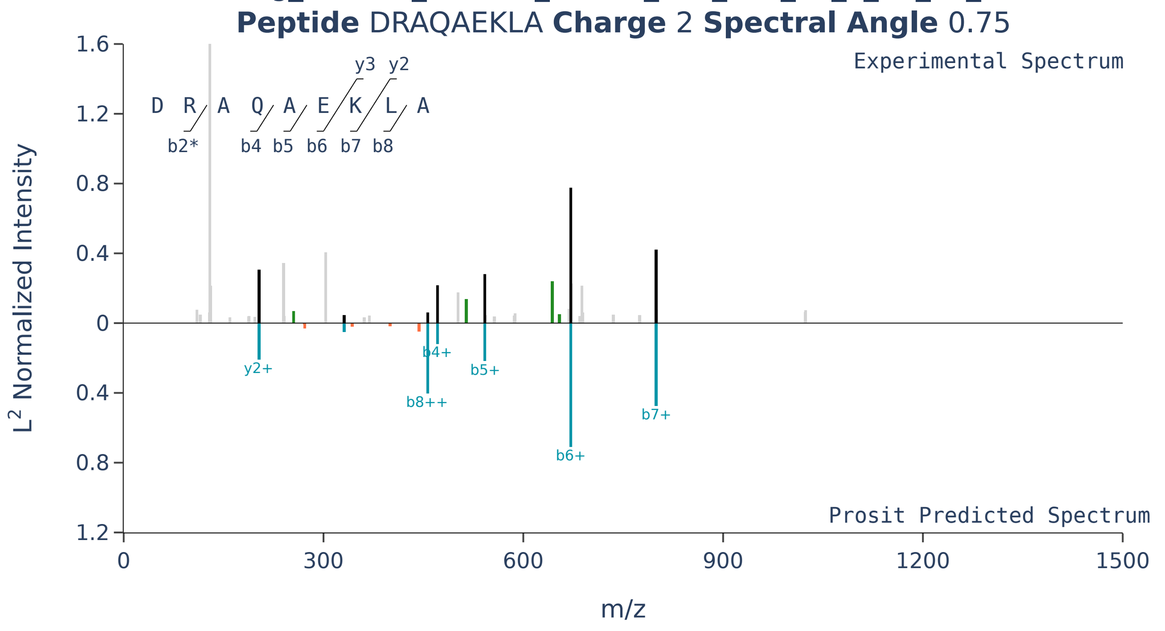


**Source** Ncheng_210623_230623_HFGoe_FFH_20S_25_1_A1_2h_R1 **Scan** 27408
**Peptide** KAVGHEVNKSLTPGQEFVKIVRNELV **Charge** 3 **Spectral Angle** 0.76

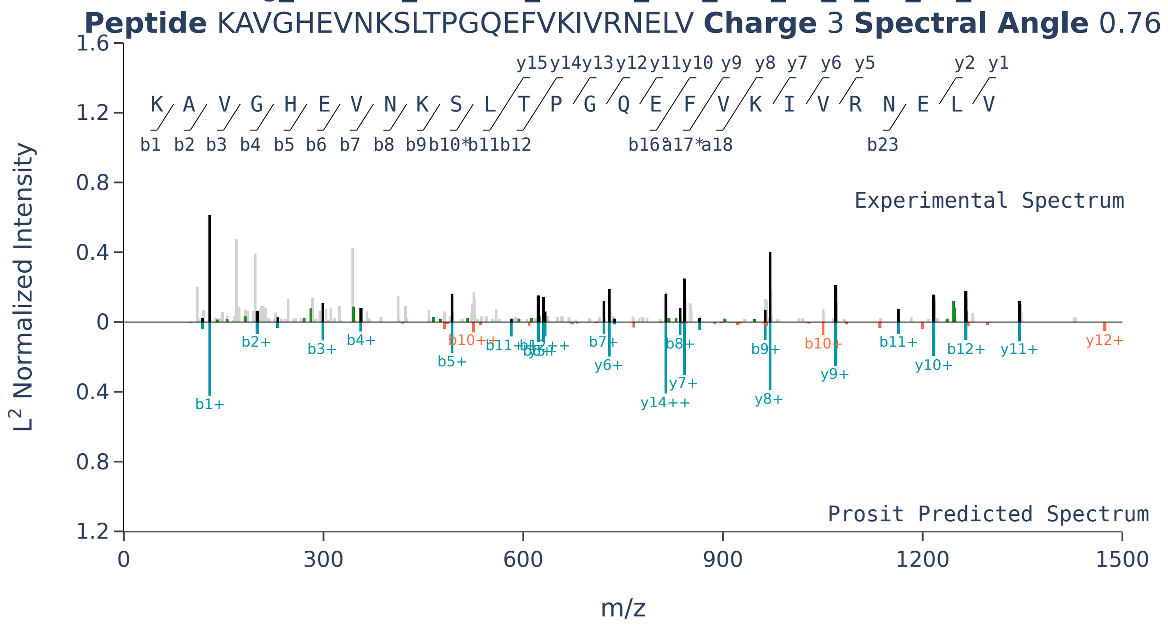


**Source** Ncheng_210623_230623_HFGoe_FFH_20S_25_1_A2_4h_R1 **Scan** 15028
**Peptide** AIINSMTMKERA **Charge** 2 **Spectral Angle** 0.64

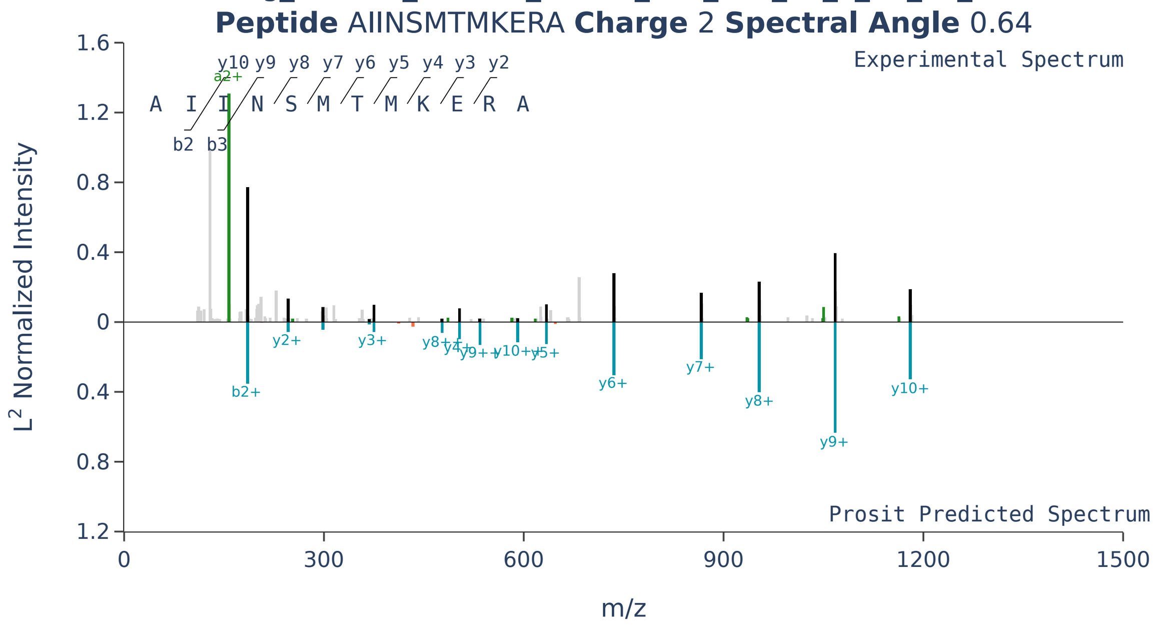


**Source** Ncheng_210623_230623_HFGoe_FFH_20S_25_1_A2_4h_R1 **Scan** 9806
**Peptide** CGMQVQDVNR **Charge** 2 **Spectral Angle** 0.73

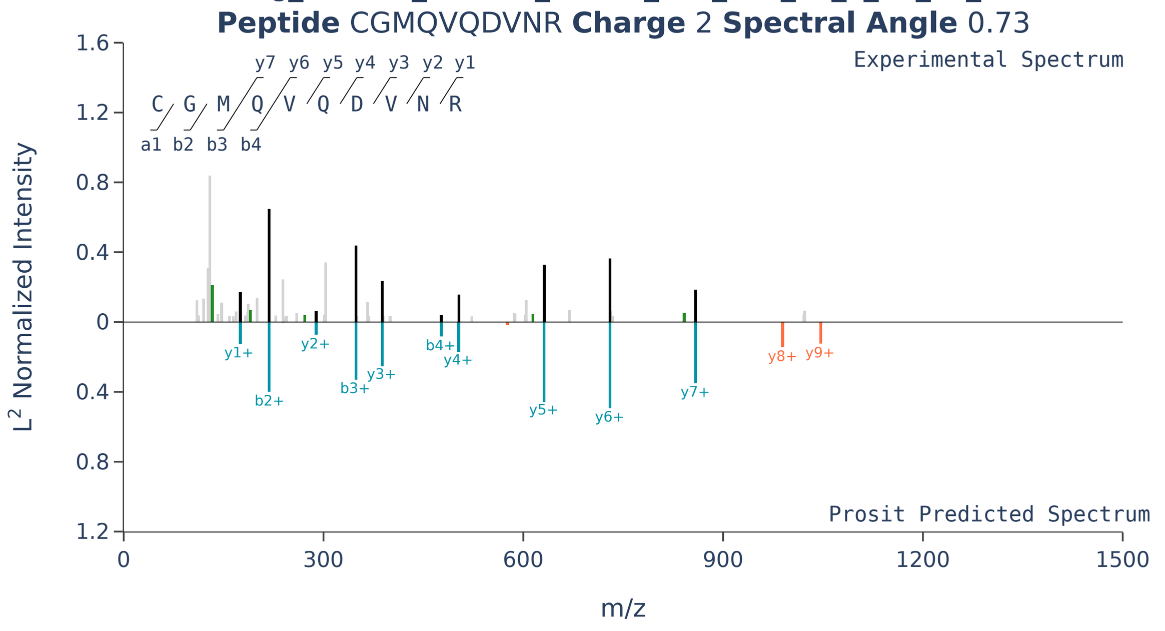


**Source** Ncheng_210623_230623_HFGoe_FFH_20S_25_1_A1_2h_R1 **Scan** 21994
**Peptide** VLVVSADVYRPAAIKQ **Charge** 2 **Spectral Angle** 0.86

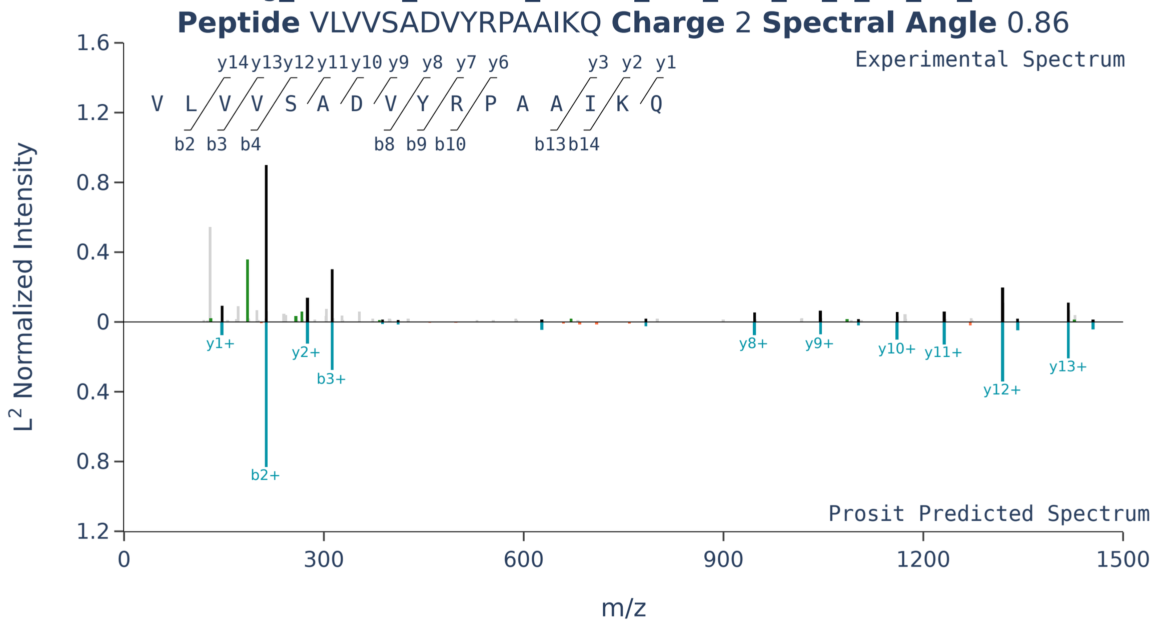


**Source** Ncheng_210623_230623_HFGoe_FFH_20S_25_1_A1_2h_R1 **Scan** 33806
**Peptide** AANTAKAFNEALPLTGVVLT **Charge** 2 **Spectral Angle** 0.8

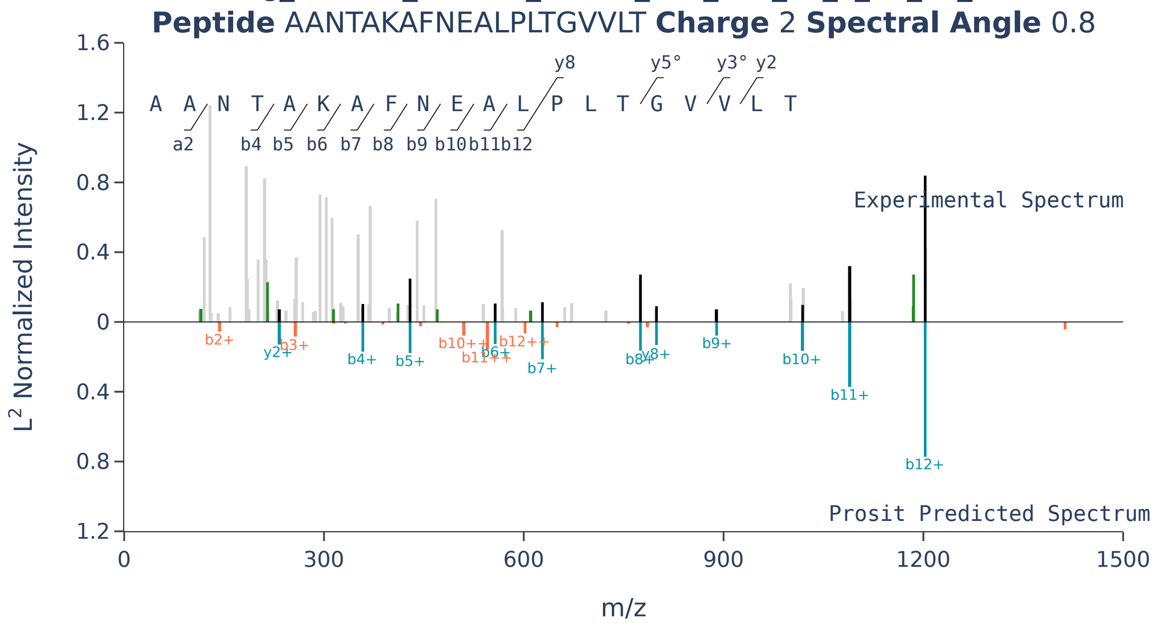


**Source** Ncheng_210623_230623_HFGoe_FFH_20S_25_1_A2_24h_R2 **Scan** 6099
**Peptide** SKLKKGDGFD **Charge** 1 **Spectral Angle** 0.87

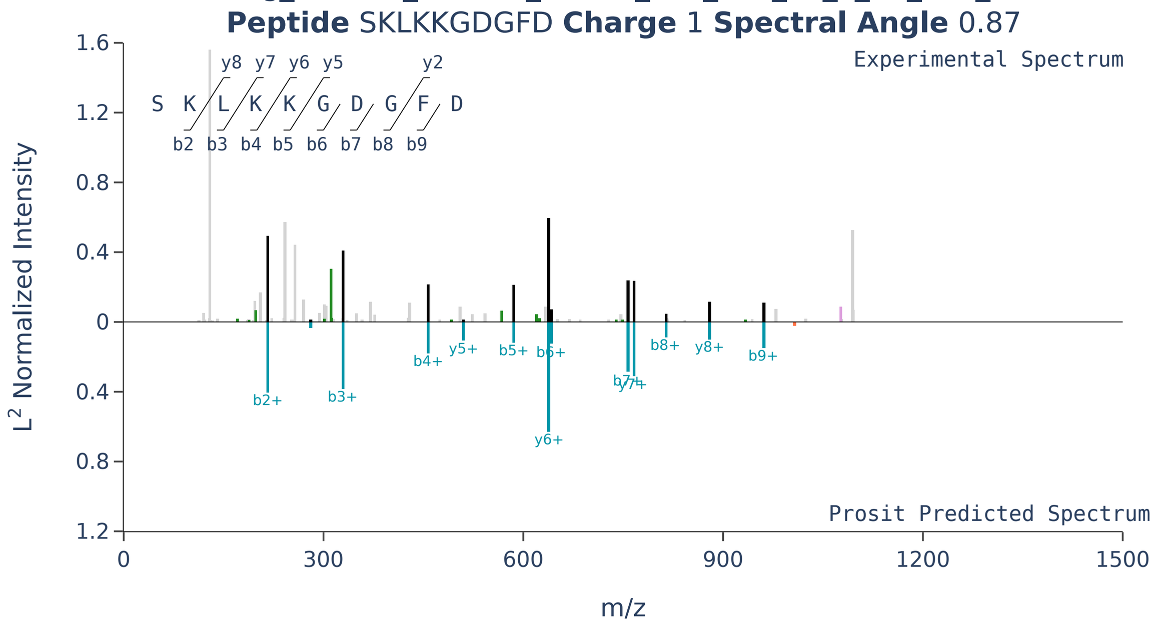

**Source** Ncheng_210623_230623_HFGoe_FFH_20S_25_1_A1_2h_R2 **Scan** 18760
**Peptide** SLRHITGKPIKFLGVGE **Charge** 3 **Spectral Angle** 0.83

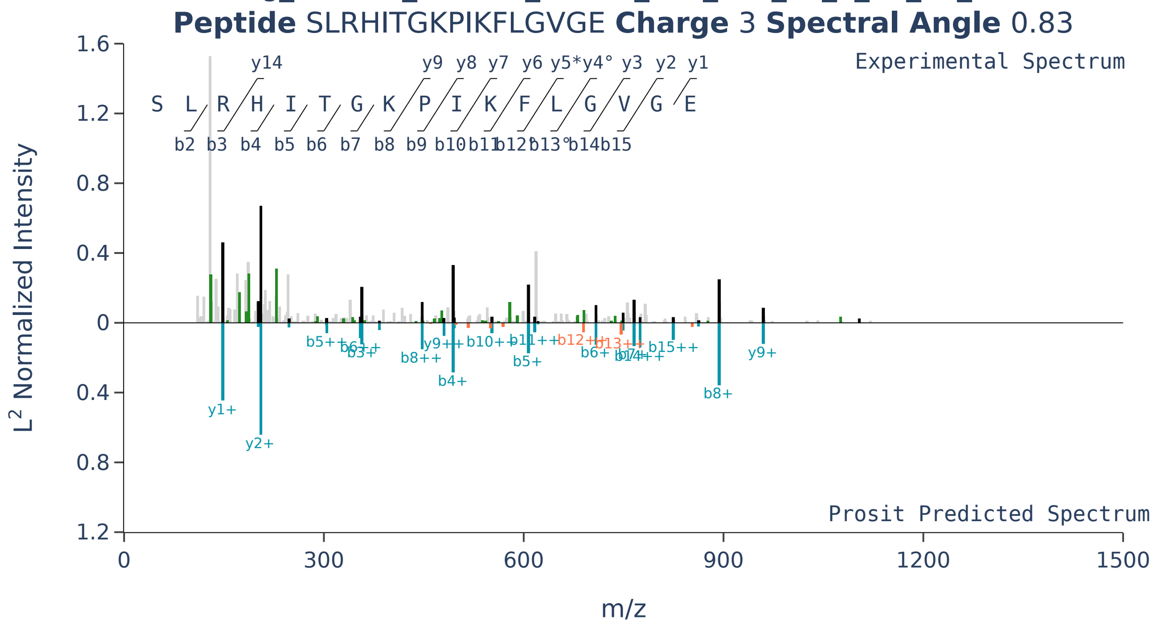


**Source** Ncheng_210623_230623_HFGoe_FFH_20S_25_1_A1_24h_R2 **Scan** 17201
**Peptide** LEPFHPDRIAS **Charge** 3 **Spectral Angle** 0.87

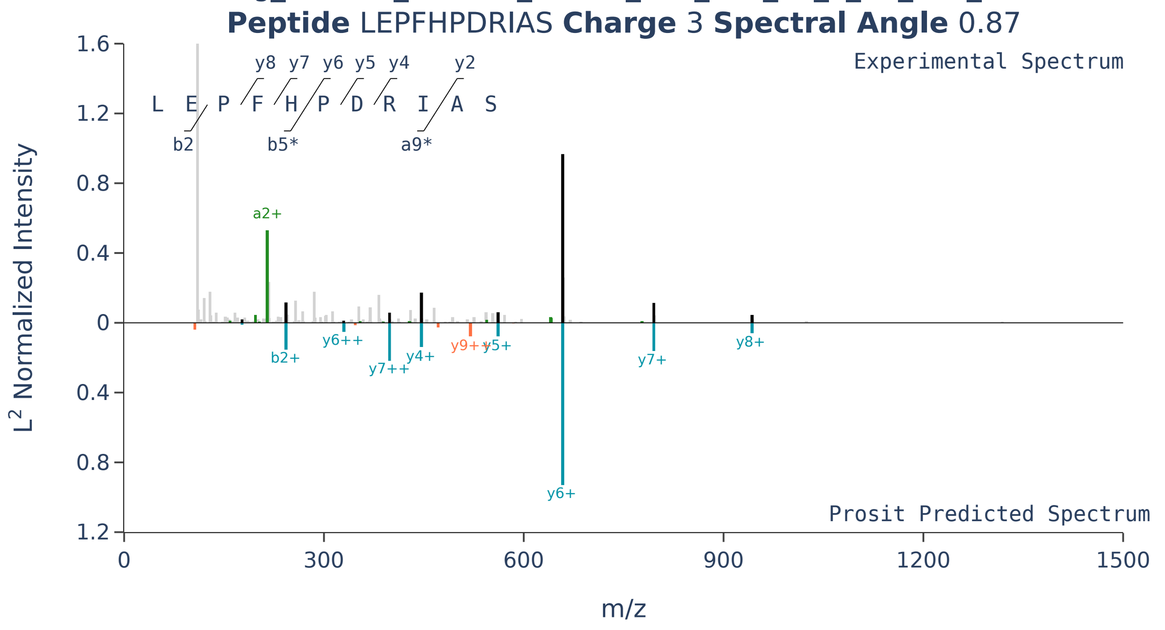


**Source** Ncheng_210623_230623_HFGoe_FFH_20S_25_1_A1_1h_R2 **Scan** 30633
**Peptide** AKLKFYDVLLVDTAGRLHVD **Charge** 3 **Spectral Angle** 0.86

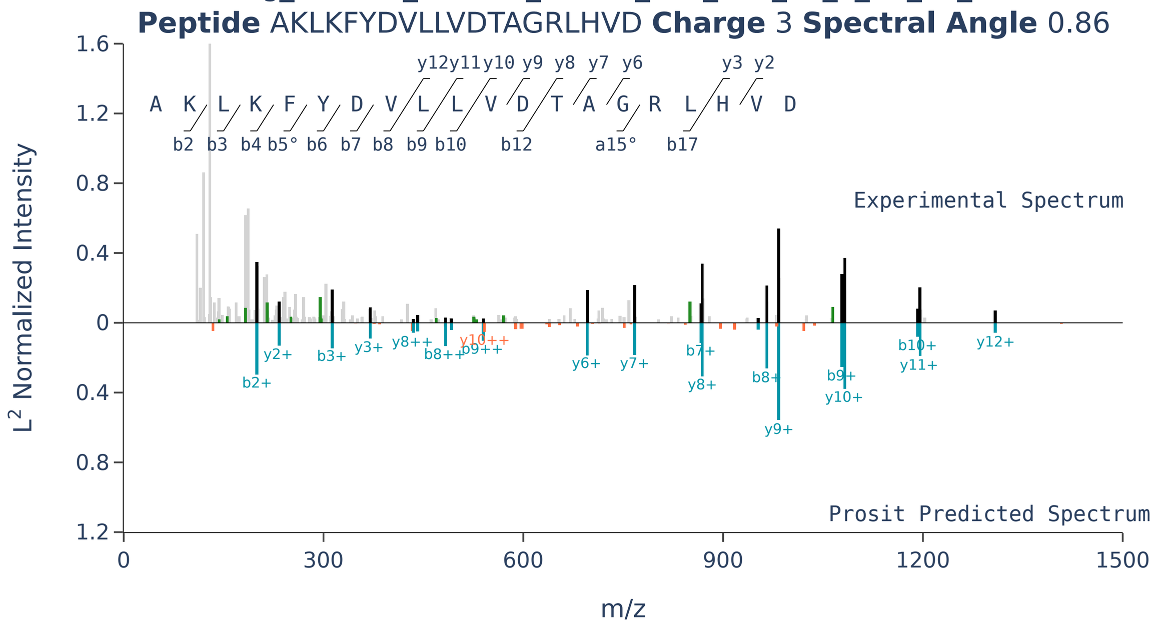


**Source** Ncheng_210623_230623_HFGoe_FFH_20S_25_1_A2_4h_R2 **Scan** 25158
**Peptide** LMGKLPGMGQIPDNVKSQMDD **Charge** 3 **Spectral Angle** 0.74

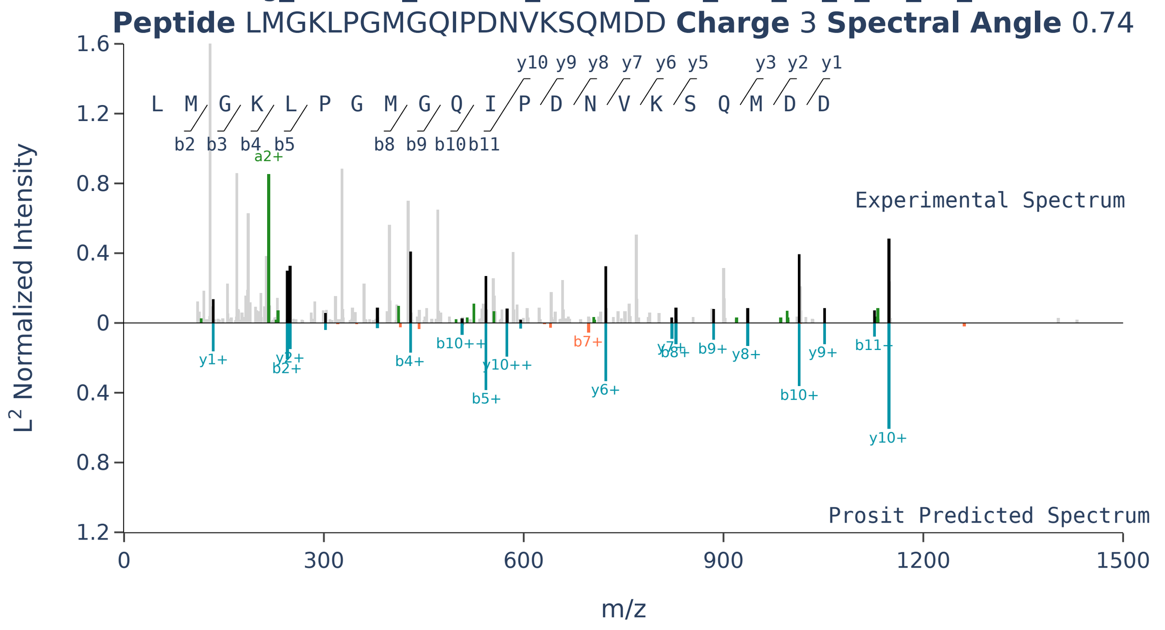


**Source** Ncheng_210623_230623_HFGoe_FFH_20S_25_1_A2_4h_R1 **Scan** 29455
**Peptide** NKSLTPGQEFVKIVRNEL **Charge** 2 **Spectral Angle** 0.8

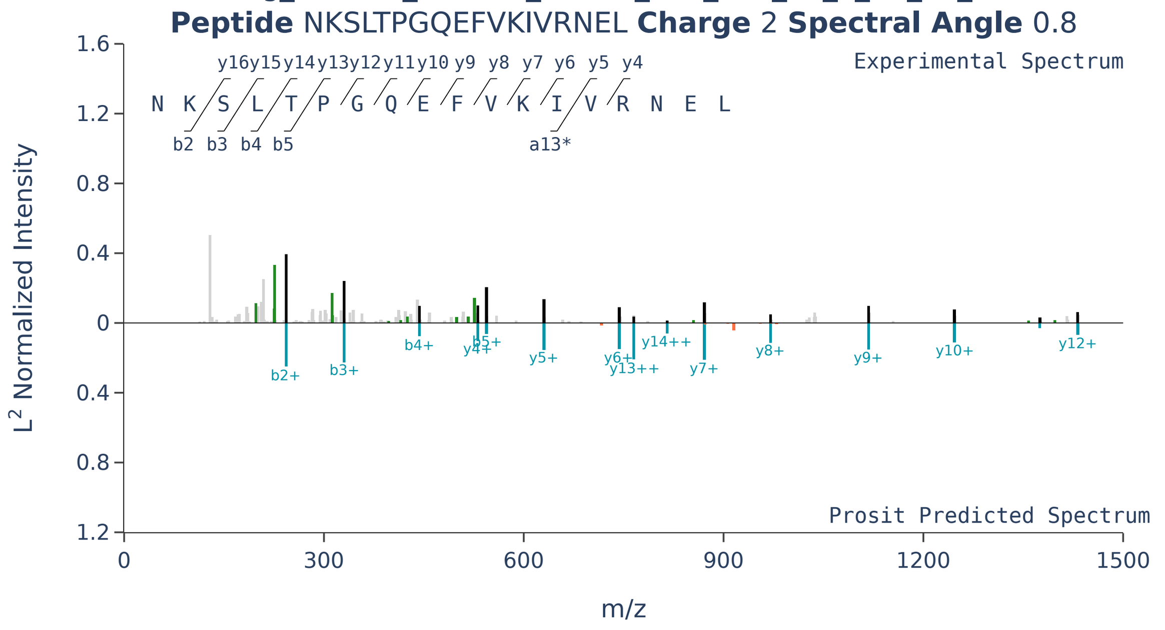


**Source** Ncheng_210623_230623_HFGoe_FFH_20S_25_1_A1_24h_R2 **Scan** 11355
**Peptide** GKTTSVGKLGKF **Charge** 2 **Spectral Angle** 0.64

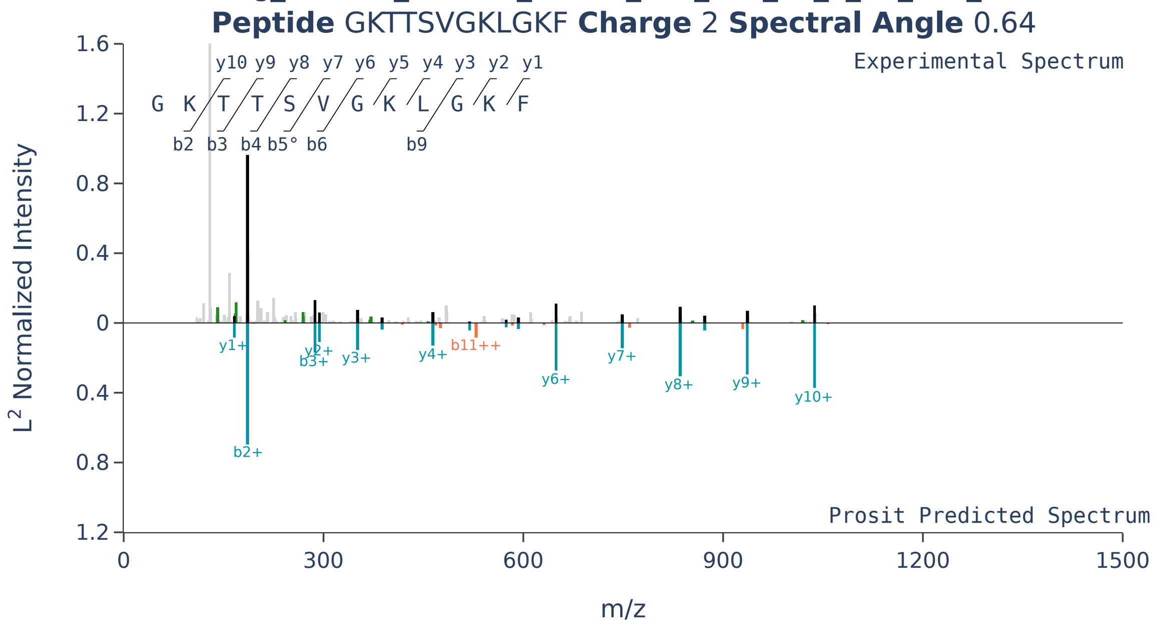


**Source** Ncheng_210623_230623_HFGoe_FFH_20S_25_1_A2_4h_R2 **Scan** 33686
**Peptide** EALPLTGVVLTKV **Charge** 2 **Spectral Angle** 0.74

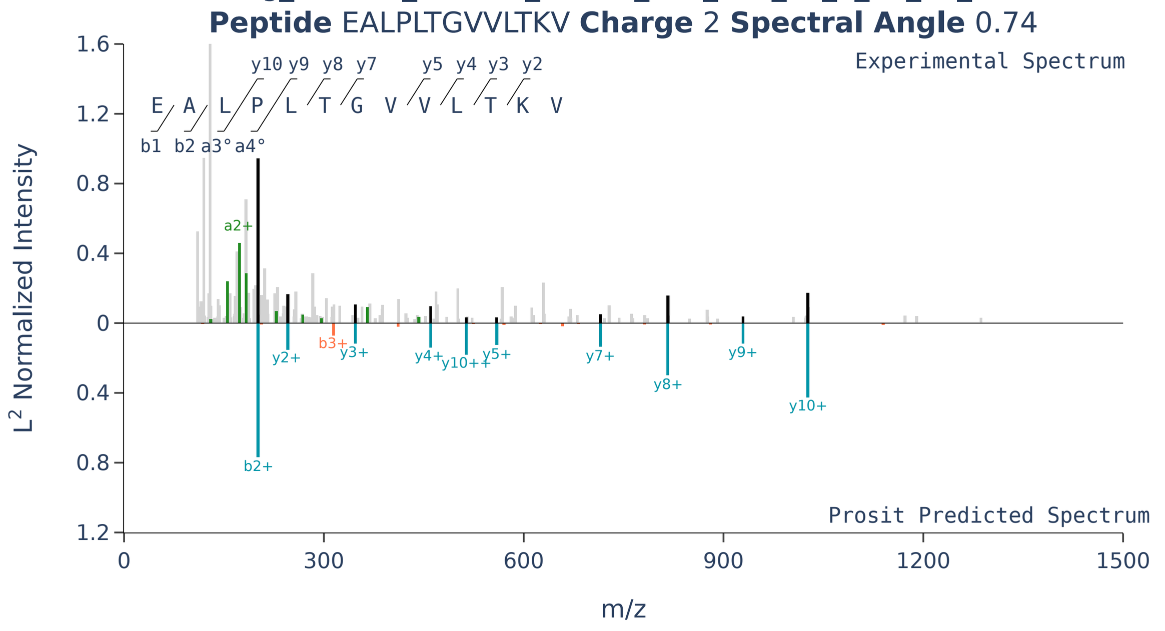


**Source** Ncheng_210623_230623_HFGoe_FFH_20S_25_1_A2_24h_R2 **Scan** 15601
**Peptide** VDGDARGGAALSIRHL **Charge** 2 **Spectral Angle** 0.81

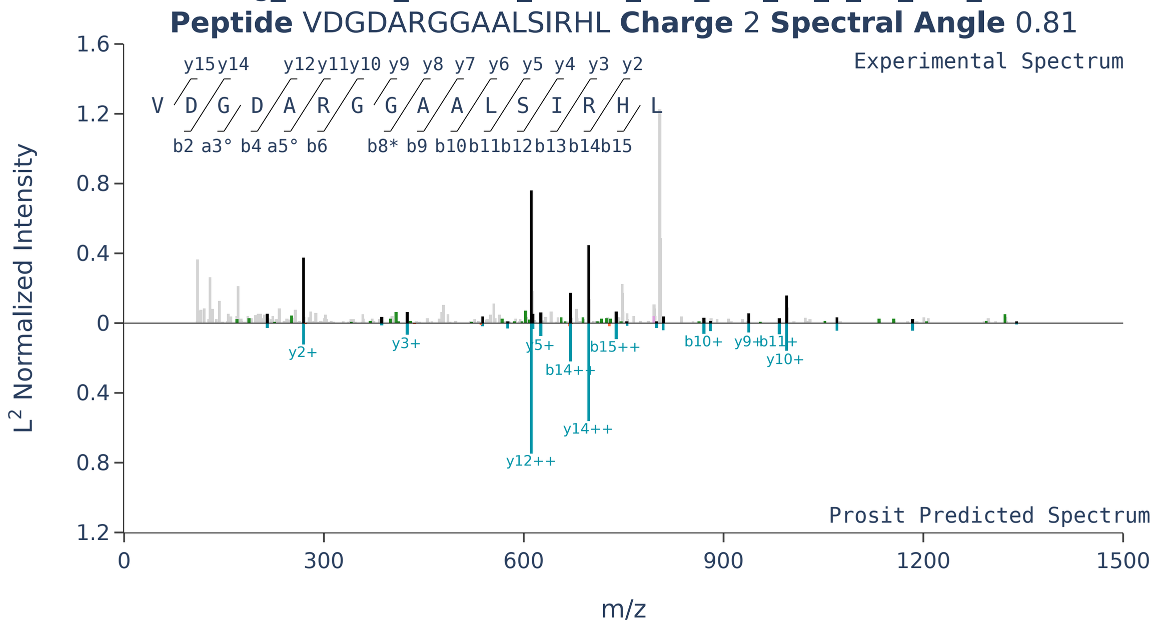


**Source** Ncheng_210623_230623_HFGoe_FFH_20S_25_1_A2_4h_R2 **Scan** 33593
**Peptide** DVLSLLE **Charge** 1 **Spectral Angle** 0.81

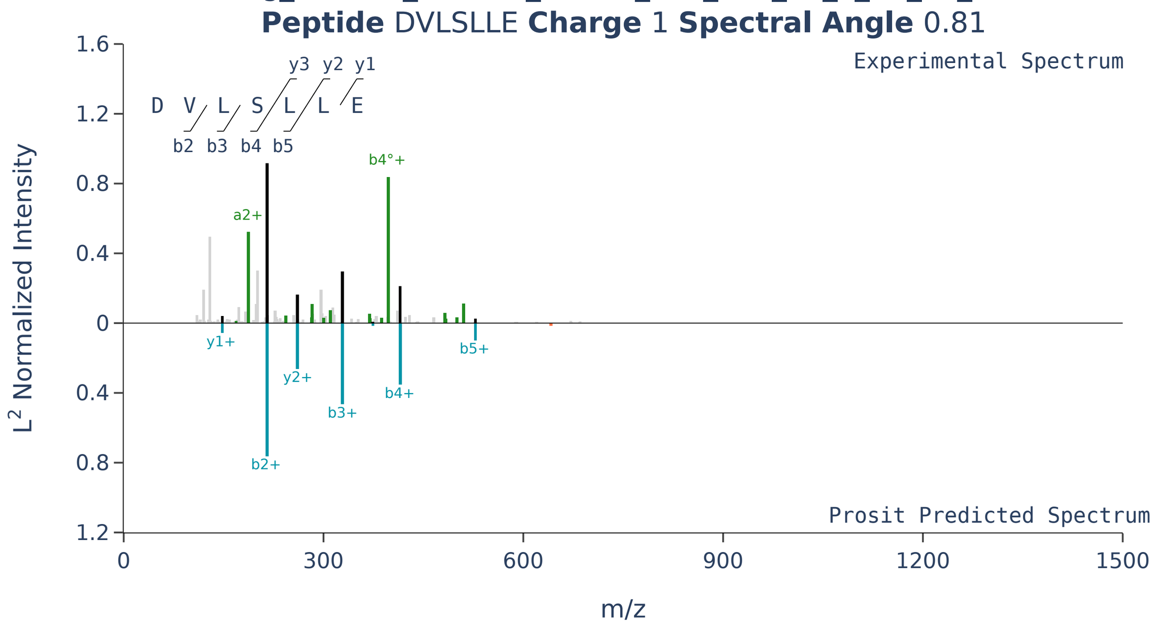


**Source** Ncheng_210623_230623_HFGoe_FFH_20S_25_1_A2_4h_R1 **Scan** 20432
**Peptide** LEPFHPDRIASRI **Charge** 3 **Spectral Angle** 0.77

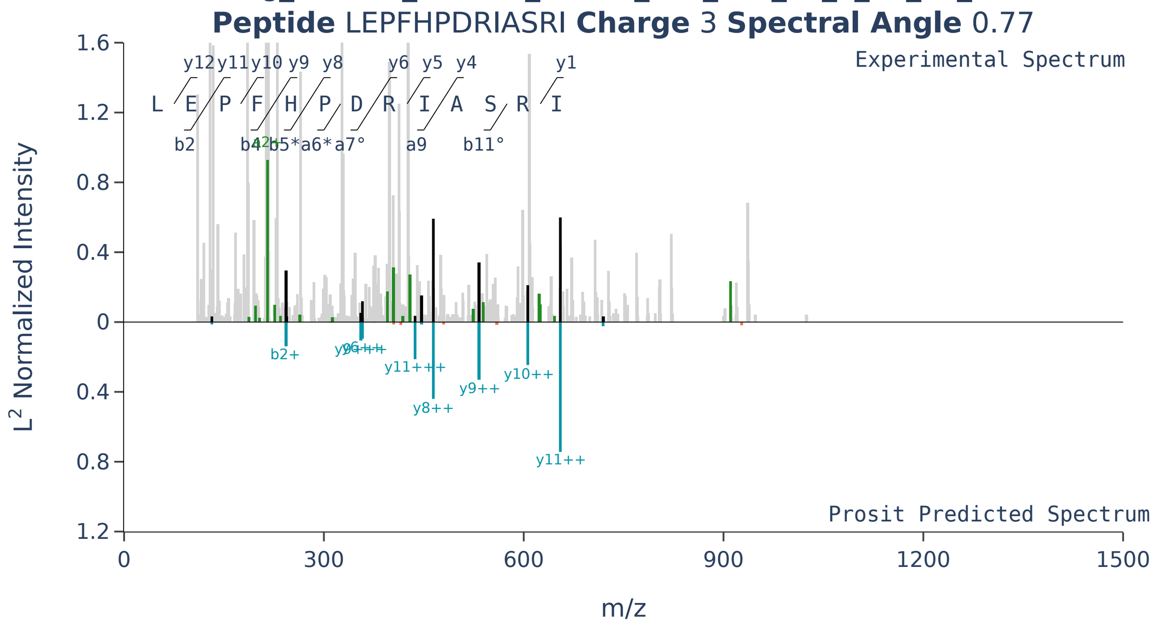


**Source** Ncheng_210623_230623_HFGoe_FFH_20S_25_1_A1_24h_R2 **Scan** 9095
**Peptide** GMAKMMRSMK **Charge** 3 **Spectral Angle** 0.79

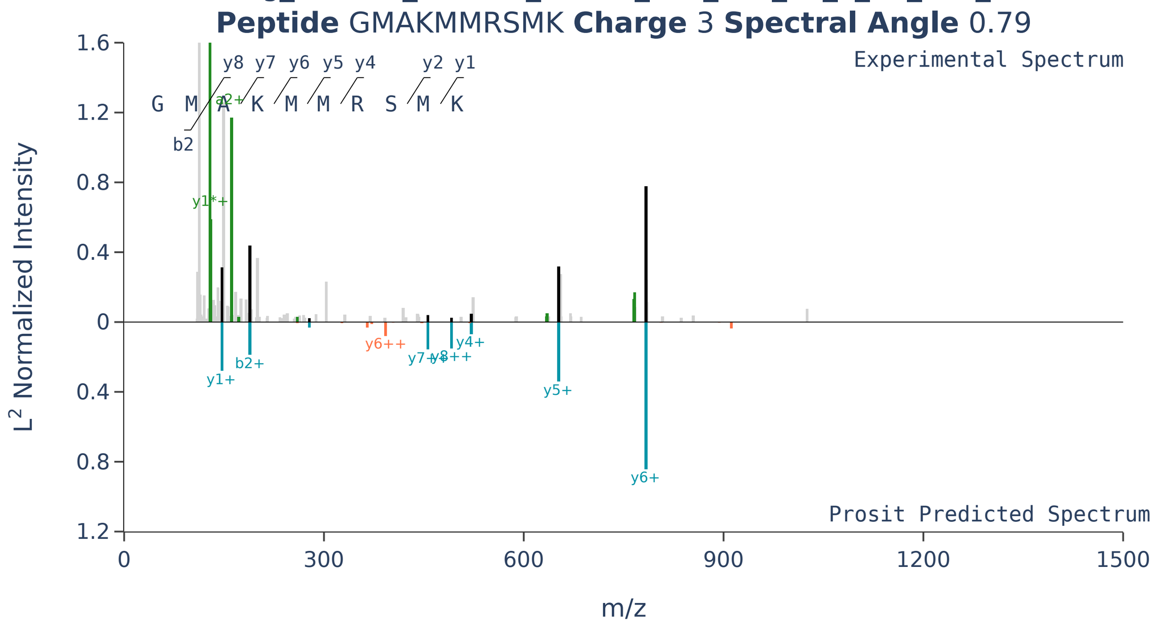


**Source** Ncheng_210623_230623_HFGoe_FFH_20S_25_1_A1_1h_R1 **Scan** 35579
**Peptide** AEQVGVDFFPSDVGQKPVDIVNAALKEA **Charge** 3 **Spectral Angle** 0.9

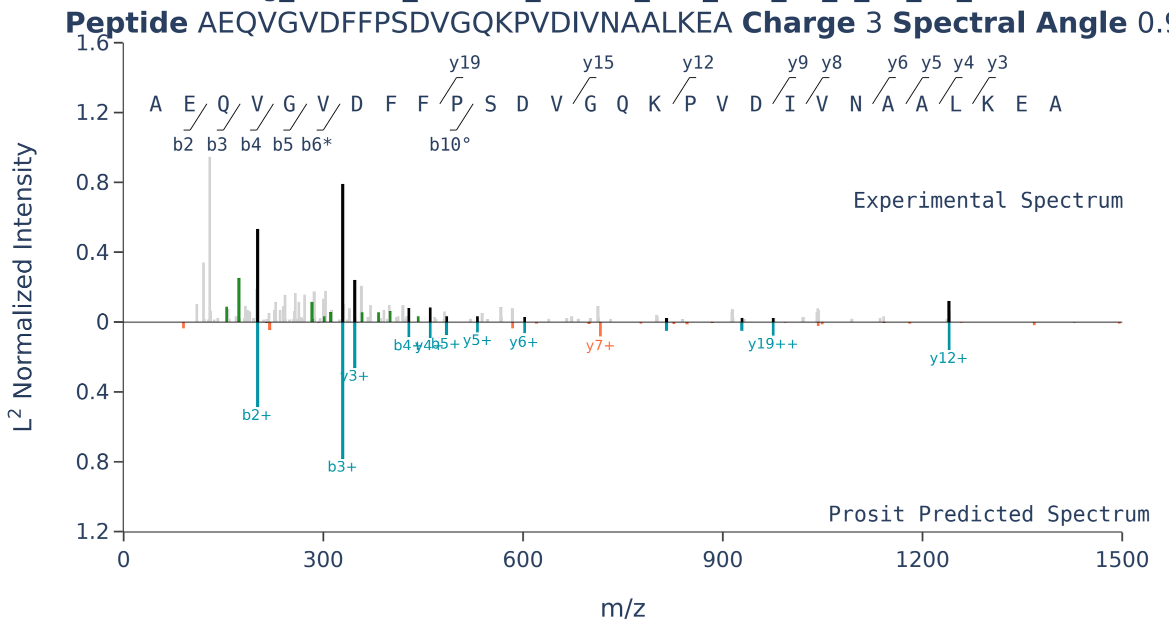


**Source** Ncheng_210623_230623_HFGoe_FFH_20S_25_1_A2_24h_R1 **Scan** 14906
**Peptide** LTEDNVKDTL **Charge** 2 **Spectral Angle** 0.76

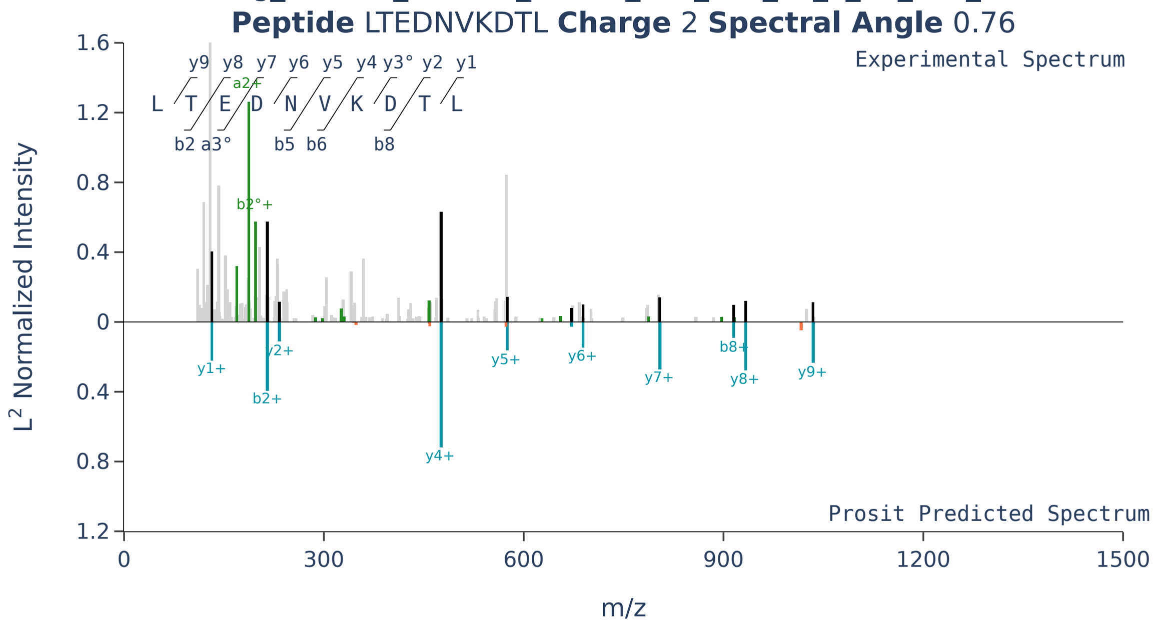


**Source** Ncheng_210623_230623_HFGoe_FFH_20S_25_1_A2_4h_R1 **Scan** 24729
**Peptide** RLAAGCGMQVQDVNRLL **Charge** 3 **Spectral Angle** 0.85

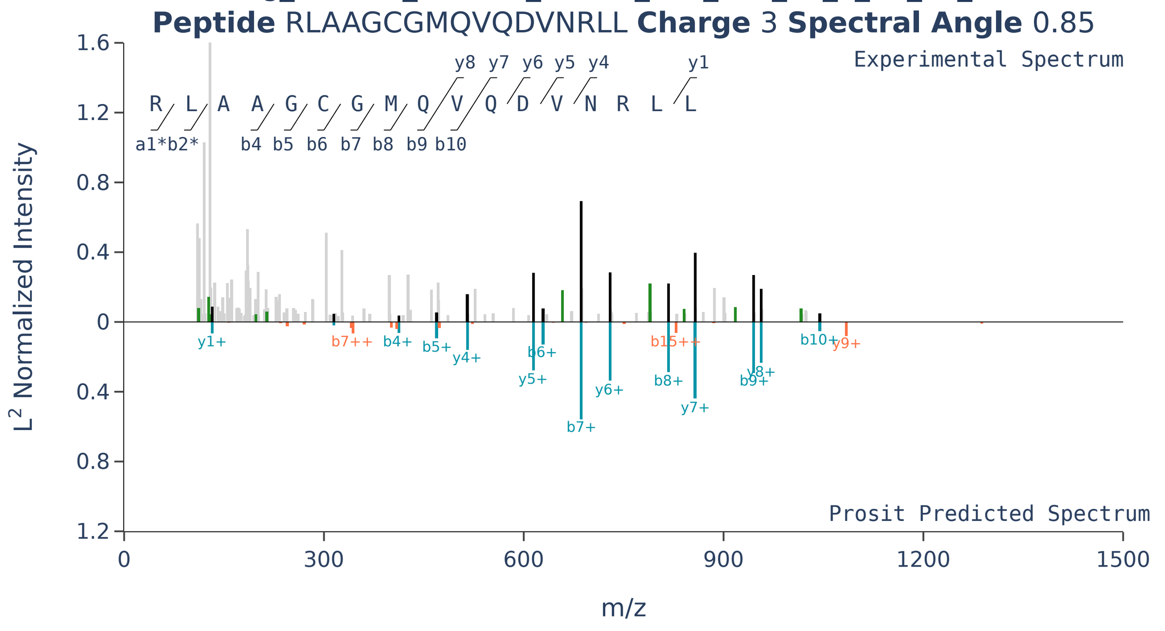


**Source** Ncheng_210623_230623_HFGoe_FFH_20S_25_1_A2_4h_R2 **Scan** 25077
**Peptide** MEAIINSMTMKERAKPELL **Charge** 2 **Spectral Angle** 0.88

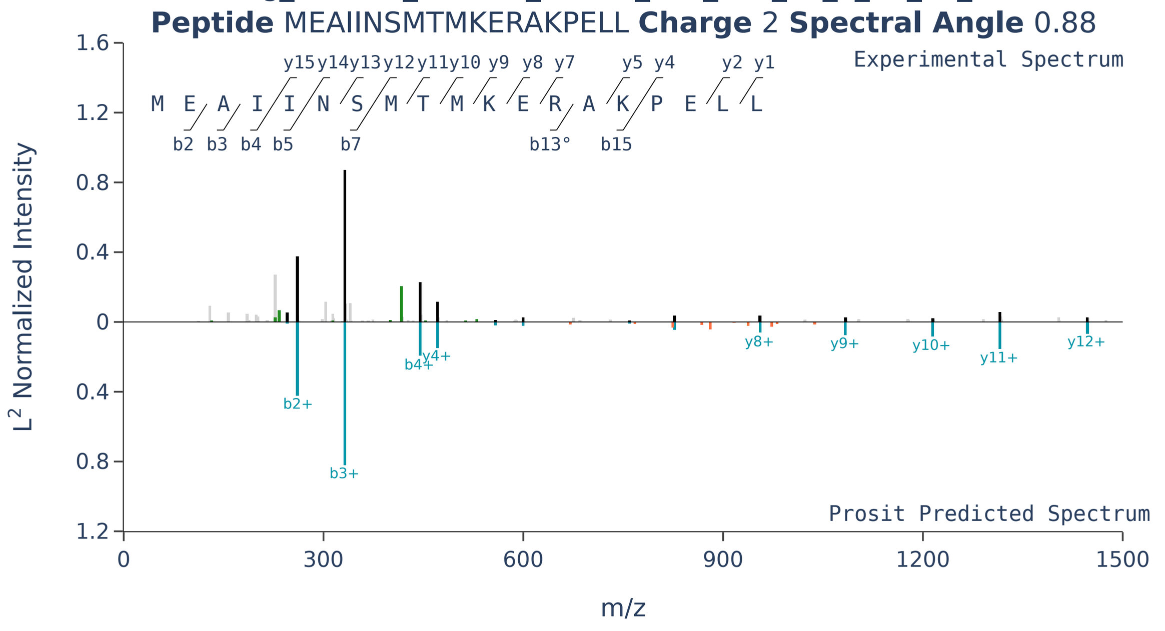

**Source** Ncheng_210623_230623_HFGoe_FFH_20S_25_1_A2_24h_R2 **Scan** 8026
**Peptide** GGMAKMMRS **Charge** 2 **Spectral Angle** 0.85

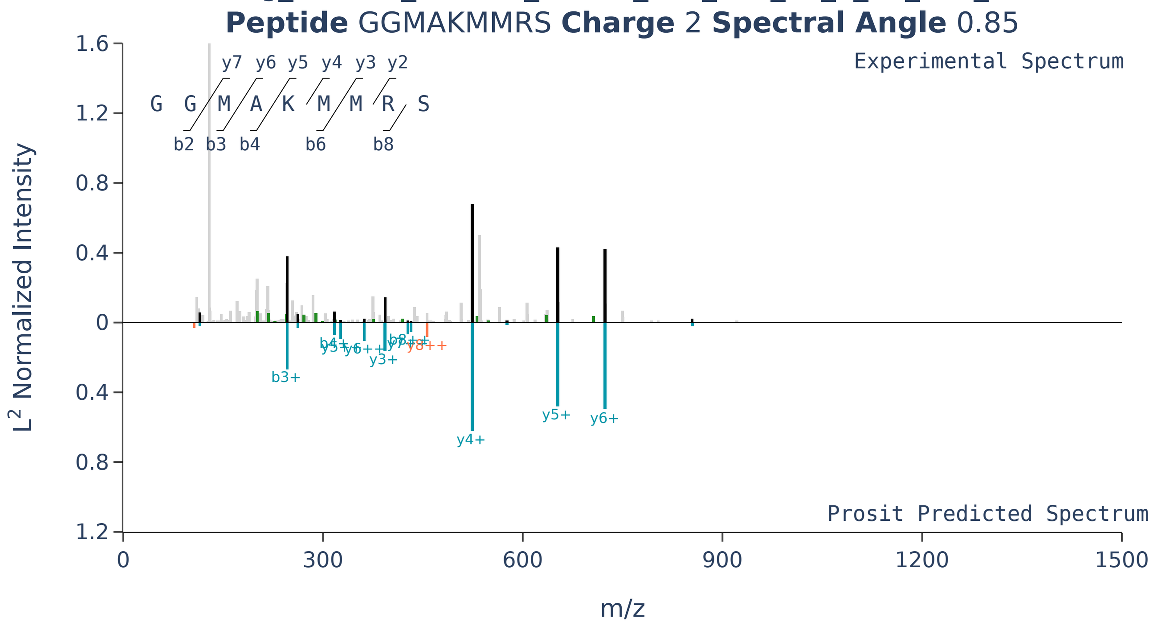


**Source** Ncheng_210623_230623_HFGoe_FFH_20S_25_1_A1_1h_R1 **Scan** 12222
**Peptide** TAGRLHVDEAMM **Charge** 3 **Spectral Angle** 0.69

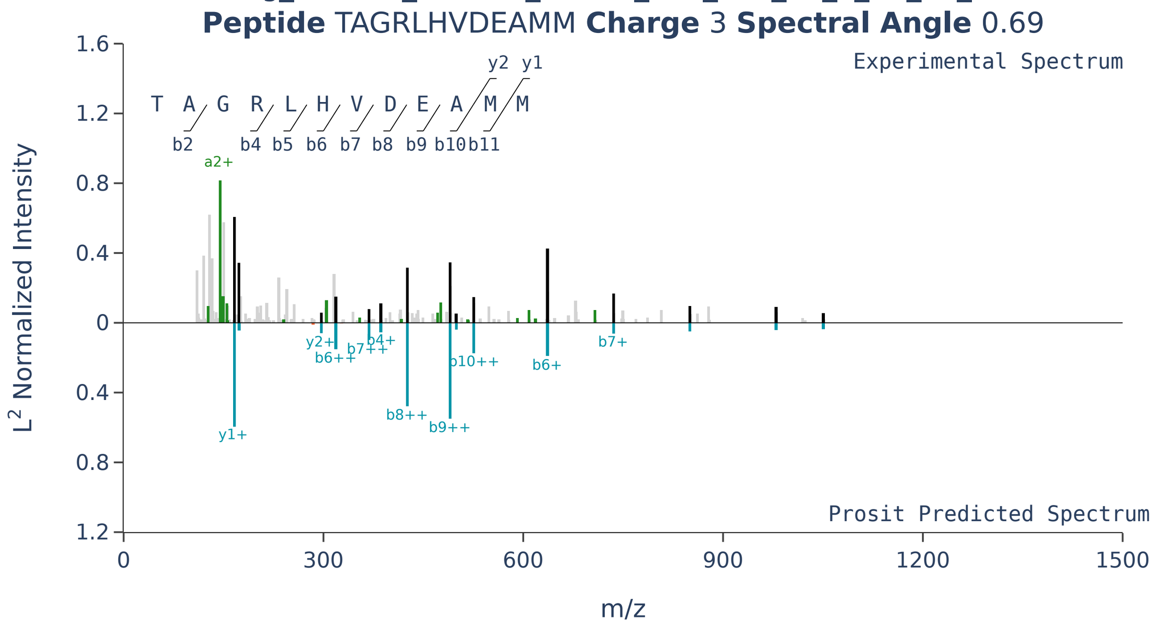


**Source** Ncheng_210623_230623_HFGoe_FFH_20S_25_1_A1_4h_R1 **Scan** 18379
**Peptide** EKAVGHEVNKSLTPGQEFVKIVRN **Charge** 3 **Spectral Angle** 0.77

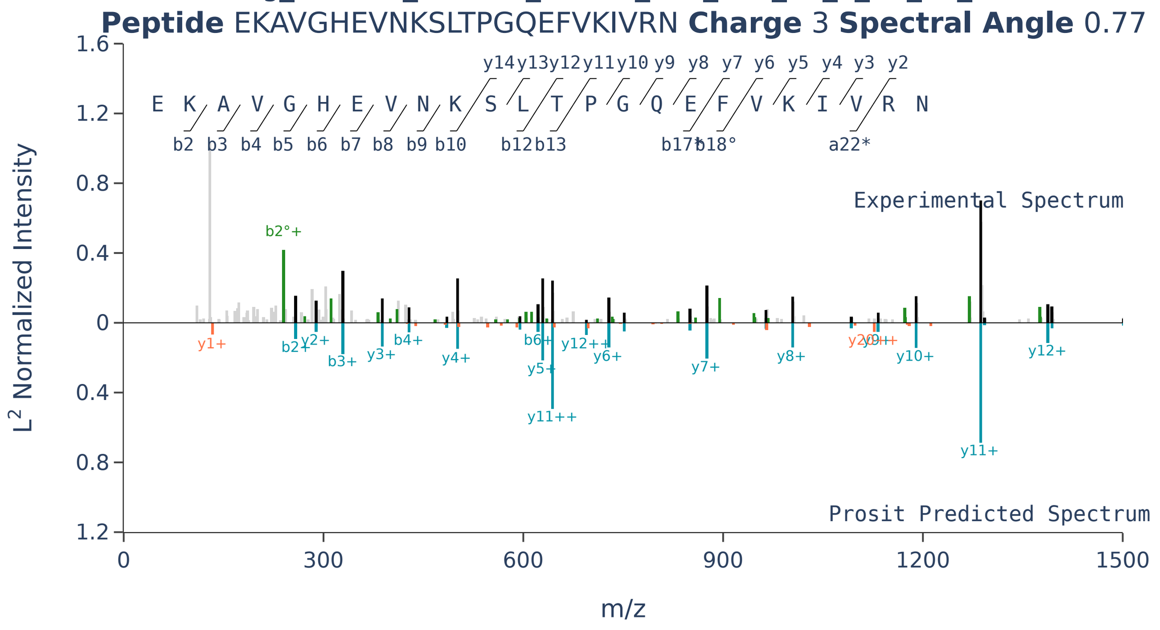


**Source** Ncheng_210623_230623_HFGoe_FFH_20S_25_1_A1_24h_R1 **Scan** 10050
**Peptide** RPAAIKQLE **Charge** 2 **Spectral Angle** 0.7

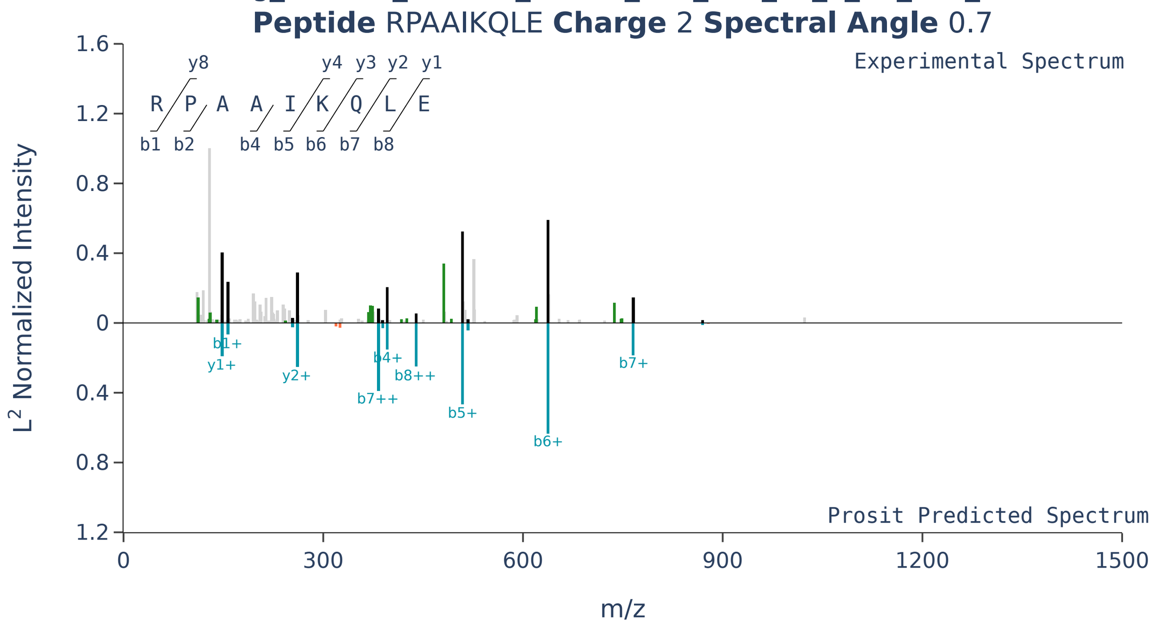


**Source** Ncheng_210623_230623_HFGoe_FFH_20S_25_1_A2_24h_R1 **Scan** 8861
**Peptide** DMQRMMKKMKKGGMAK **Charge** 5 **Spectral Angle** 0.83

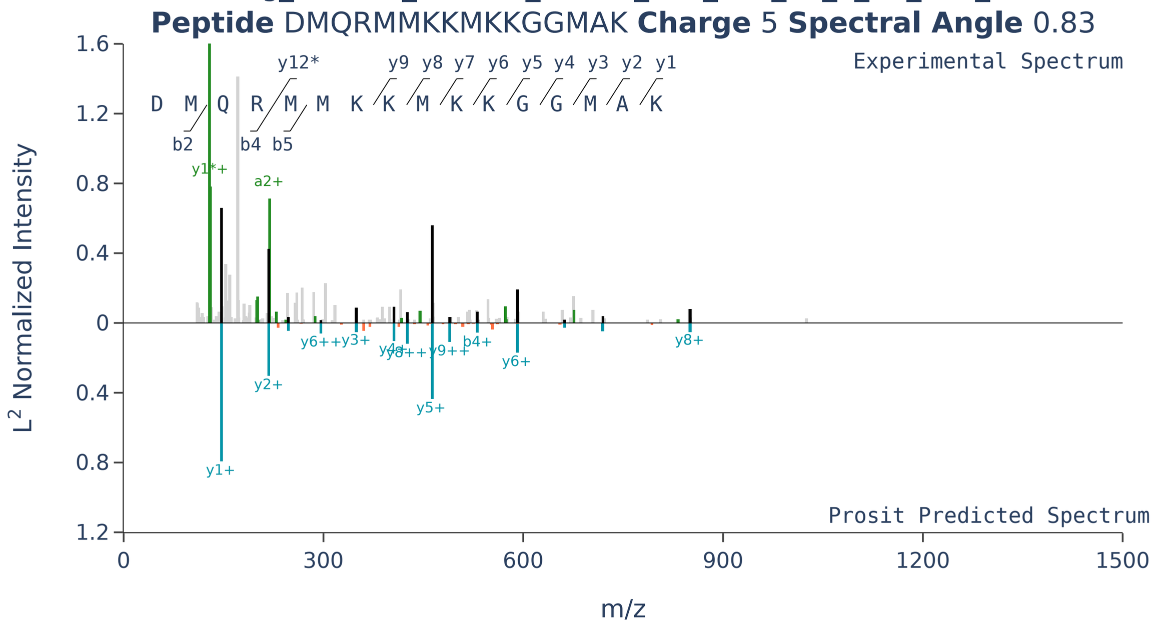


**Source** Ncheng_210623_230623_HFGoe_FFH_20S_25_1_A2_4h_R1 **Scan** 24388
**Peptide** TGKPIKFLGVGEKTEALEPFHPDRL **Charge** 5 **Spectral Angle** 0.87

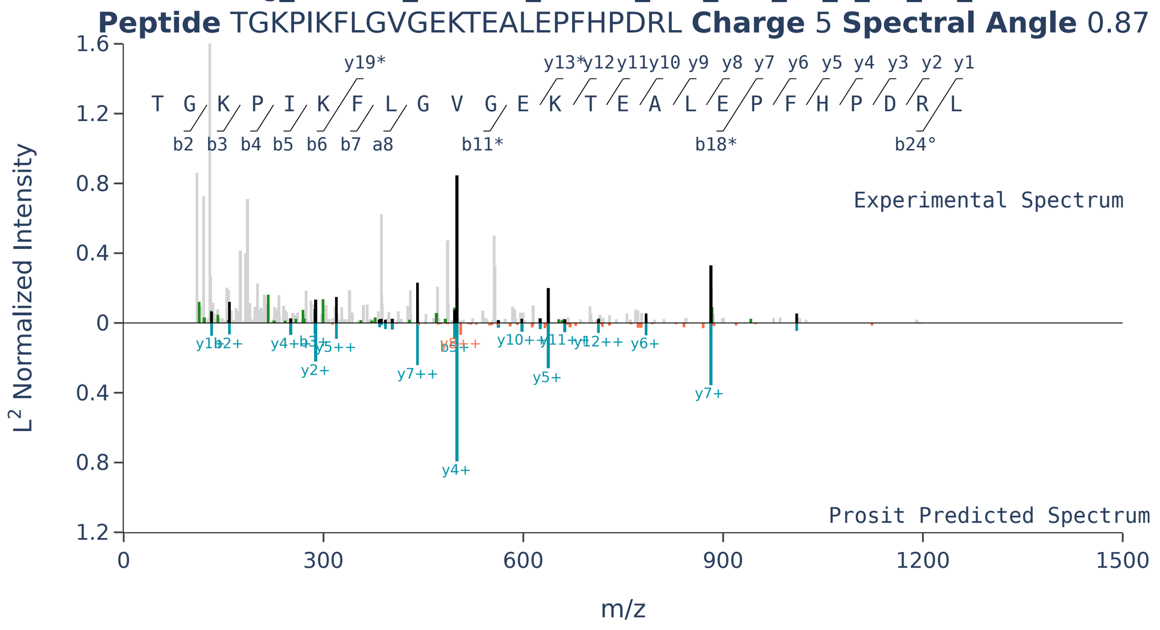


**Source** Ncheng_210623_230623_HFGoe_FFH_20S_25_1_A2_4h_R1 **Scan** 20528
**Peptide** GKLPGMGQIPDNVKSQMD **Charge** 2 **Spectral Angle** 0.8

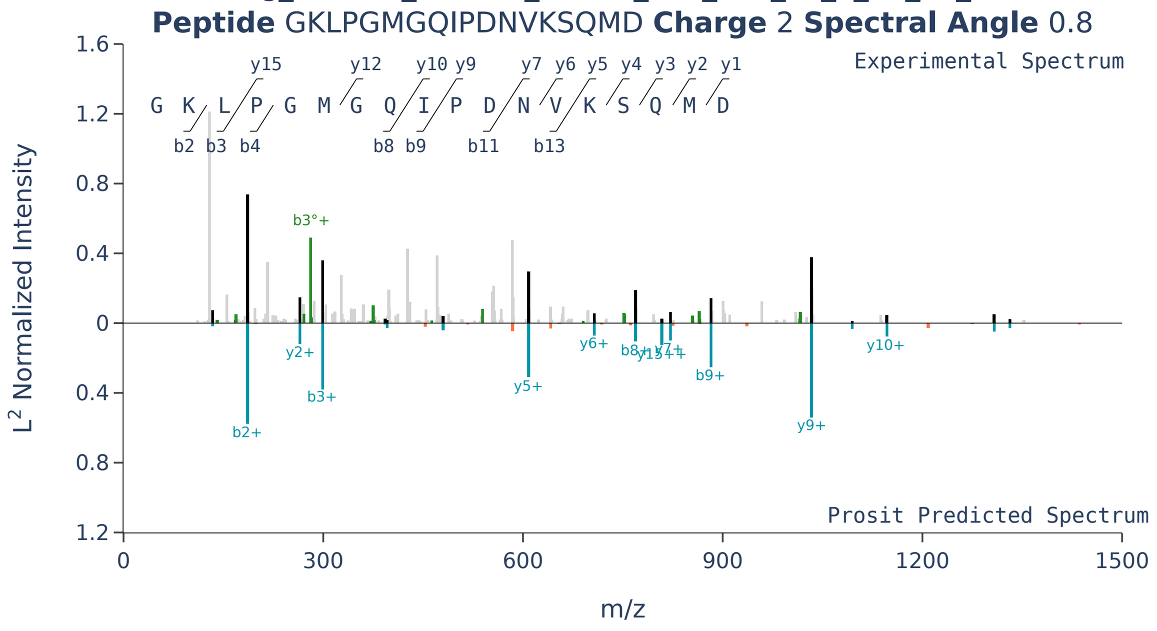


**Source** Ncheng_210623_230623_HFGoe_FFH_20S_25_1_A2_24h_R1 **Scan** 10684
**Peptide** EFINRVKEKAVGHE **Charge** 2 **Spectral Angle** 0.76

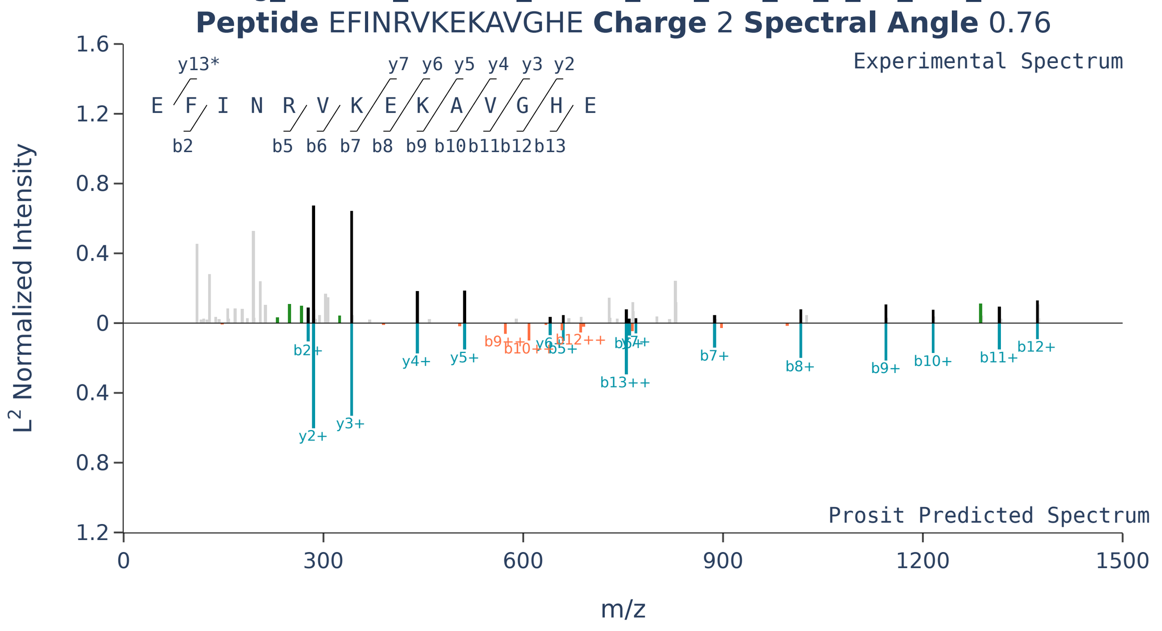


**Source** Ncheng_210623_230623_HFGoe_FFH_20S_25_1_A2_24h_R1 **Scan** 9148
**Peptide** AGLQGAGKTTSVG **Charge** 1 **Spectral Angle** 0.67

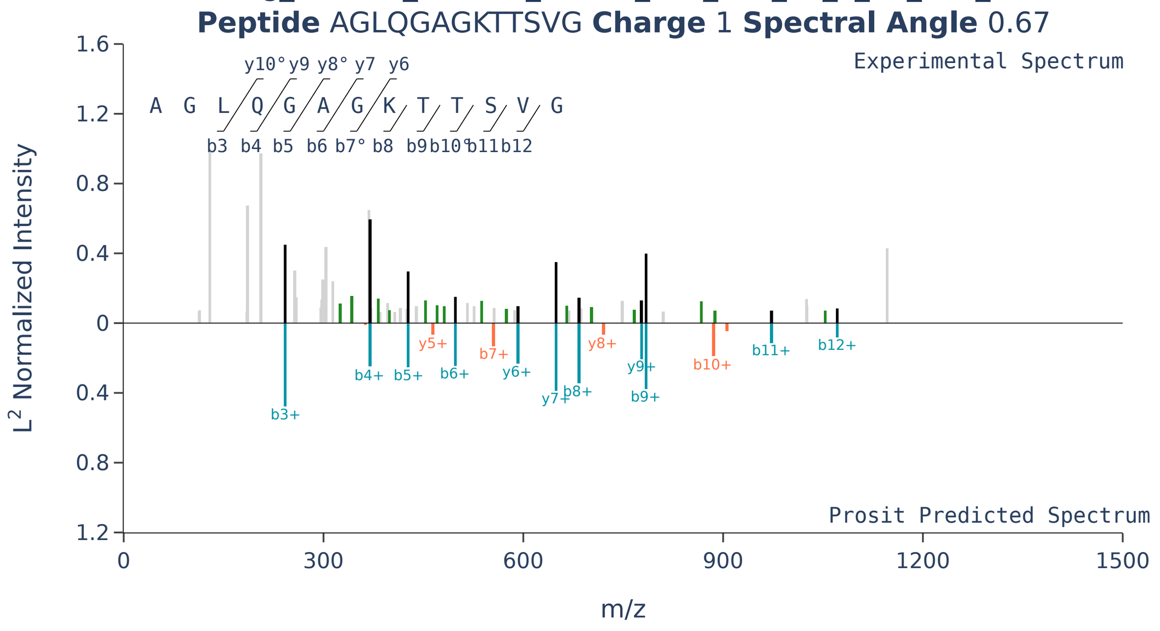


**Source** Ncheng_210623_230623_HFGoe_FFH_20S_25_1_A2_24h_R1 **Scan** 12010
**Peptide** GRLTEDNVKDTLRE **Charge** 4 **Spectral Angle** 0.79

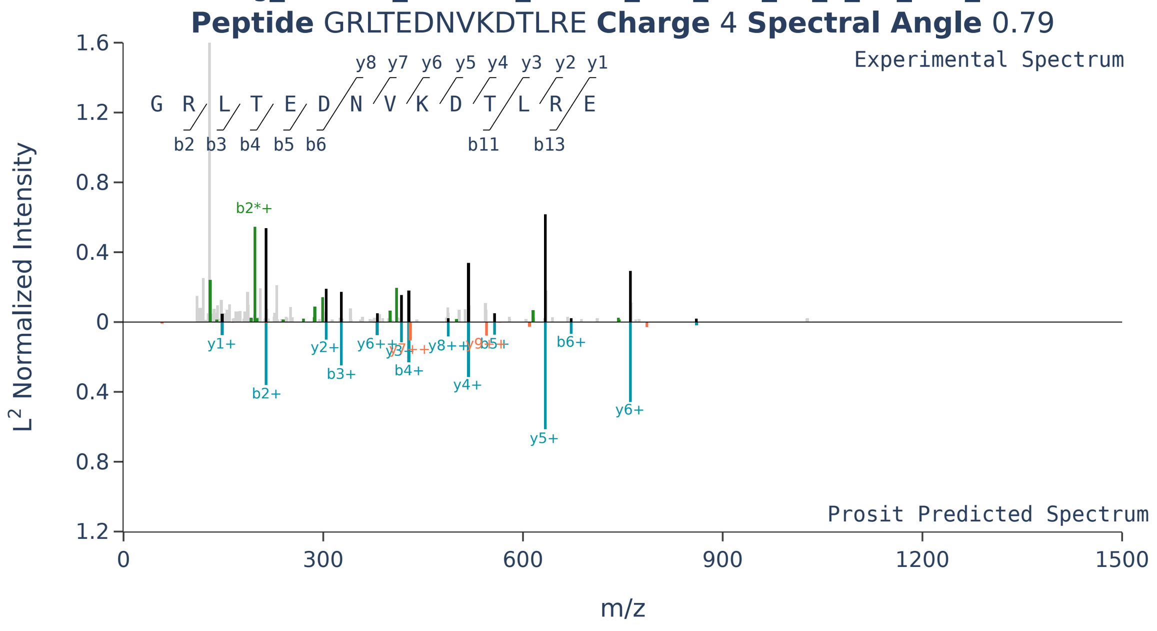


**Source** Ncheng_210623_230623_HFGoe_FFH_20S_25_1_A1_1h_R1 **Scan** 12945
**Peptide** KKGDGFDL **Charge** 2 **Spectral Angle** 0.84

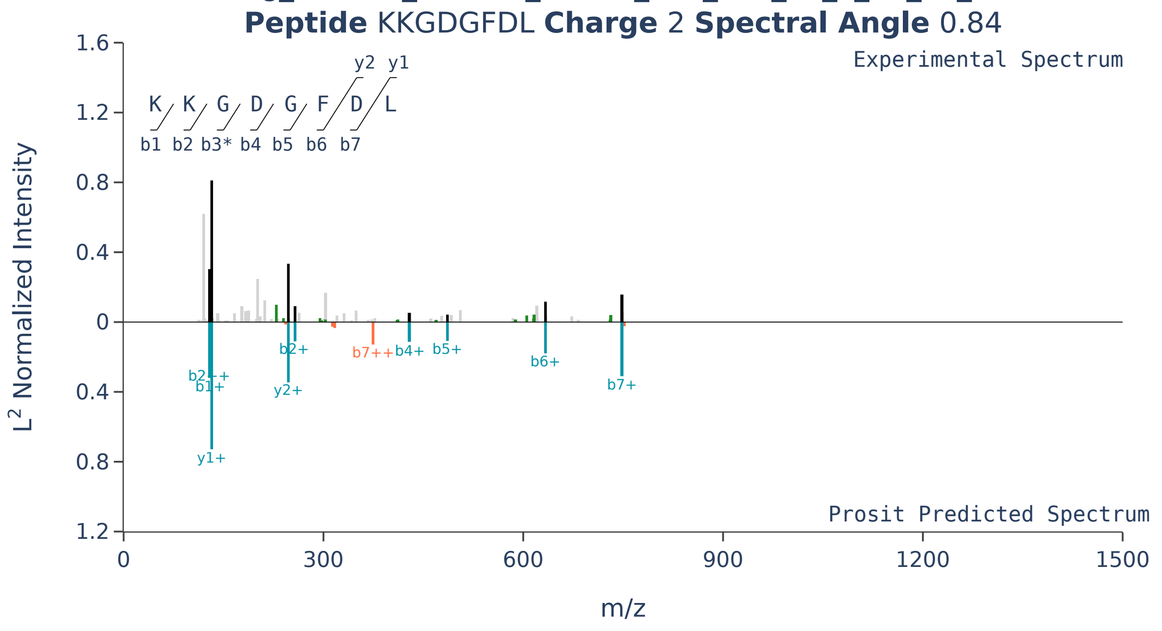


**Source** Ncheng_210623_230623_HFGoe_FFH_20S_25_1_A2_4h_R1 **Scan** 20320
**Peptide** TPGQEFVKIVRNE **Charge** 2 **Spectral Angle** 0.69

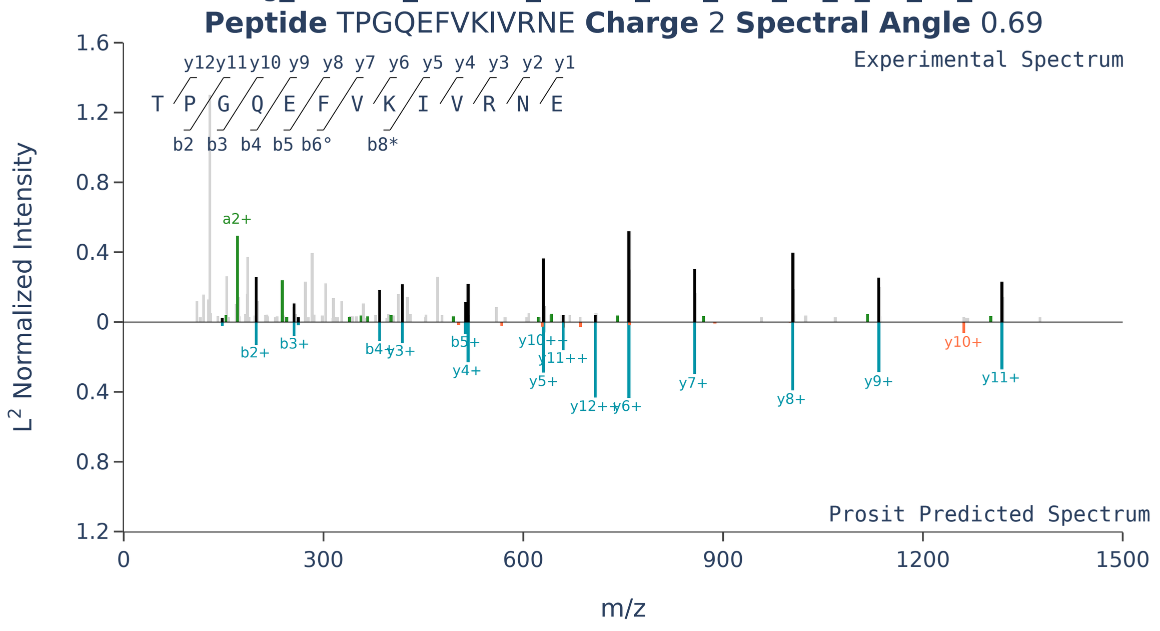


**Source** Ncheng_210623_230623_HFGoe_FFH_20S_25_1_A2_24h_R2 **Scan** 10035
**Peptide** NSMTMKERAKPEIIKG **Charge** 2 **Spectral Angle** 0.76

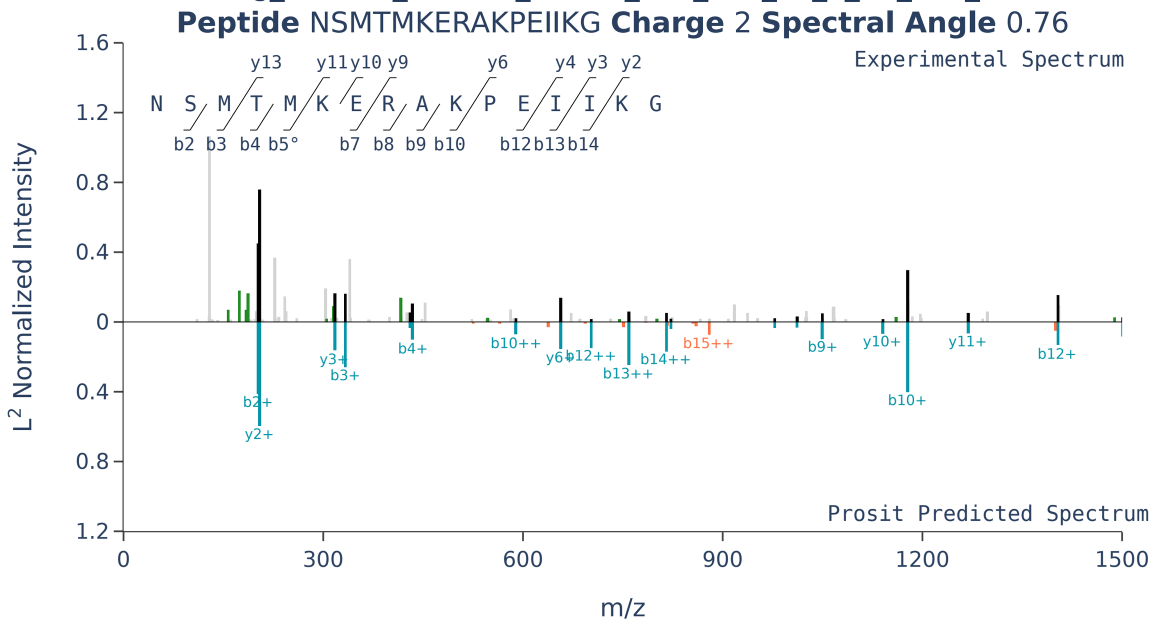


**Source** Ncheng_210623_230623_HFGoe_FFH_20S_25_1_A2_4h_R1 **Scan** 24127
**Peptide** DVNRLLKQFD **Charge** 2 **Spectral Angle** 0.7

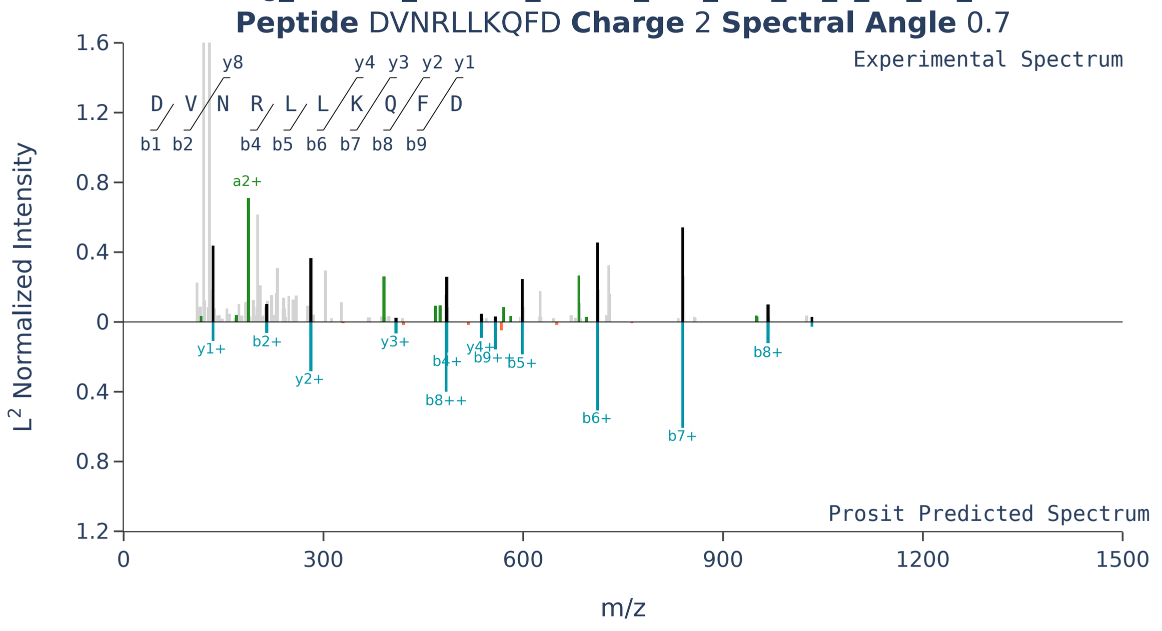


**Source** Ncheng_210623_230623_HFGoe_FFH_20S_25_1_A1_2h_R2 **Scan** 7182
**Peptide** NISGRGRLTE **Charge** 3 **Spectral Angle** 0.76

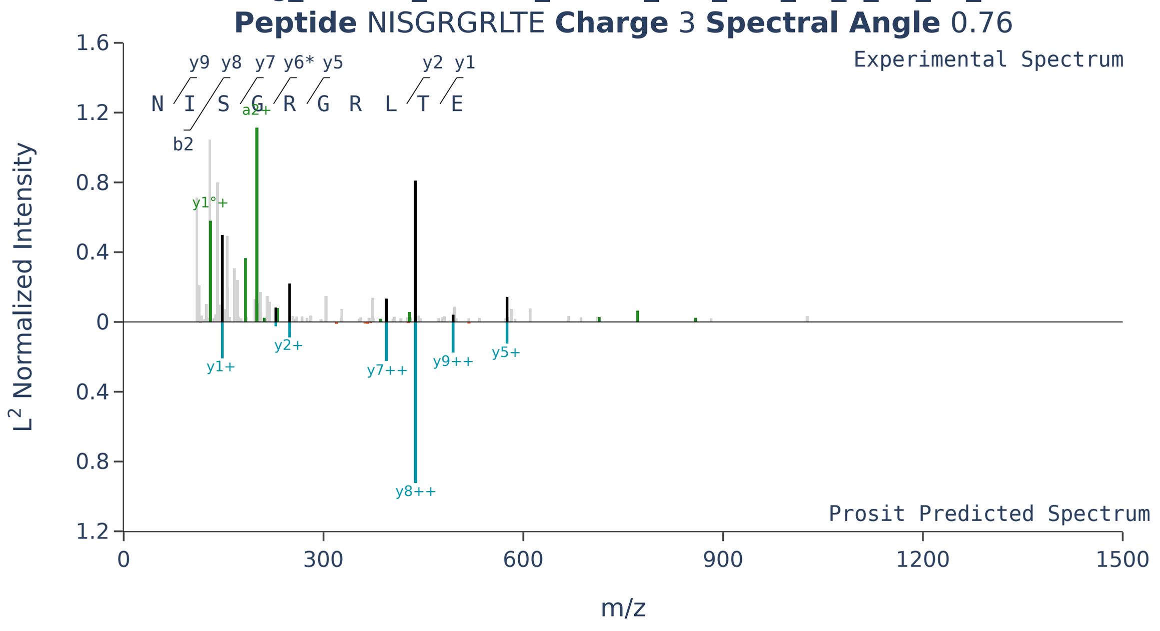

**Source** Ncheng_210623_230623_HFGoe_FFH_20S_25_1_A2_4h_R2 **Scan** 21647
**Peptide** KSLTPGQEFVKIVRNE **Charge** 2 **Spectral Angle** 0.68

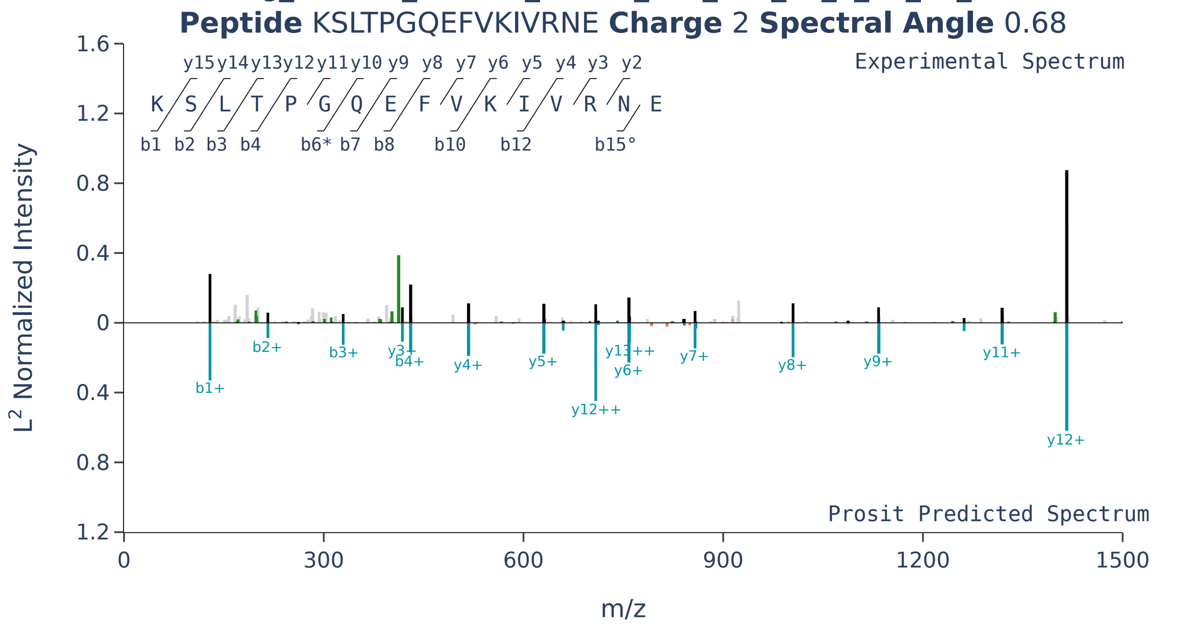


**Source** Ncheng_210623_230623_HFGoe_FFH_20S_25_1_A2_2h_R1 **Scan** 30776
**Peptide** GGMASLMGKLPGMGQIPDNVKSQMDD **Charge** 3 **Spectral Angle** 0.85

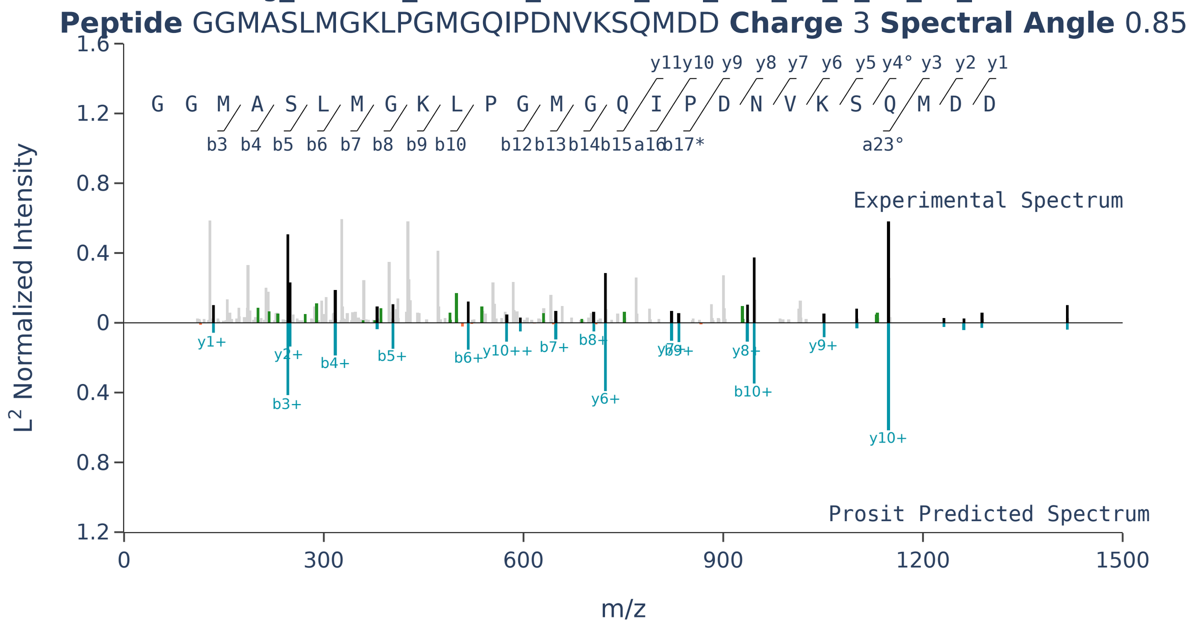


**Source** Ncheng_210623_230623_HFGoe_FFH_20S_25_1_A2_1h_R1 **Scan** 18396
**Peptide** PIKFLGVGEKTE **Charge** 3 **Spectral Angle** 0.81

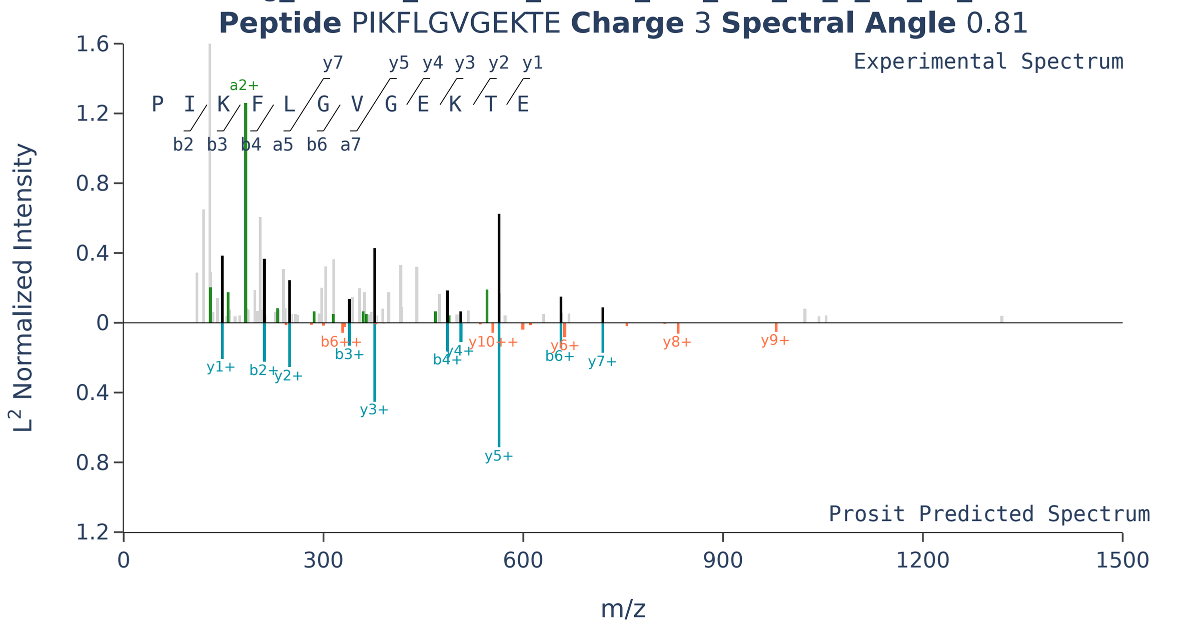


**Source** Ncheng_210623_230623_HFGoe_FFH_20S_25_1_A1_24h_R2 **Scan** 5878
**Peptide** KKGGMAKMMR **Charge** 3 **Spectral Angle** 0.75

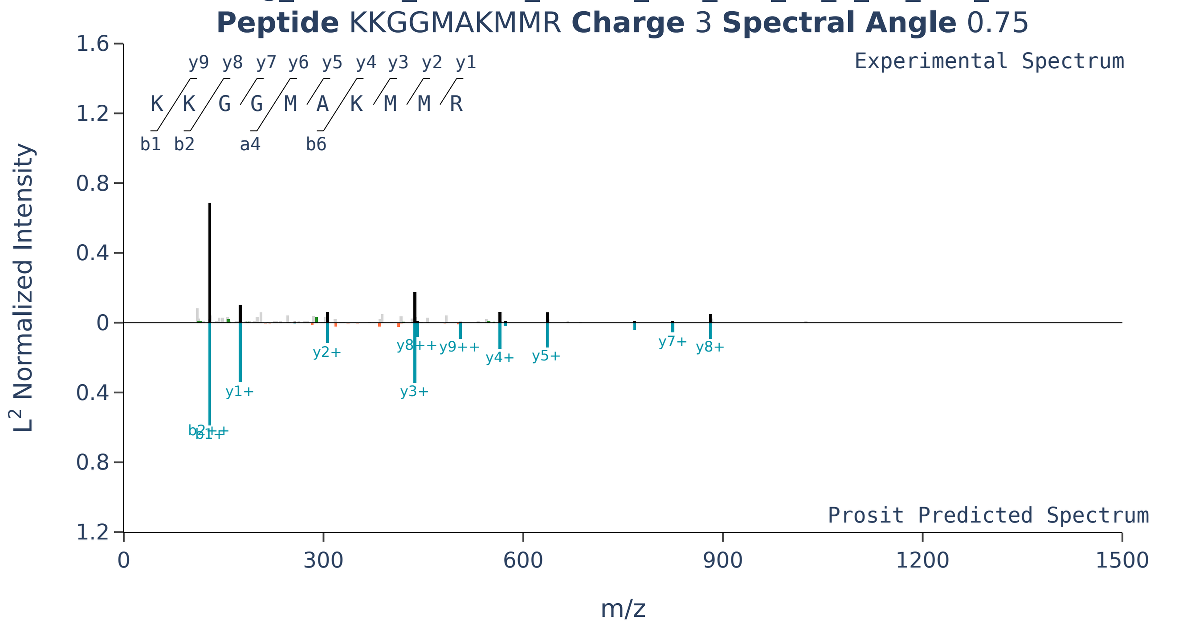


**Source** Ncheng_210623_230623_HFGoe_FFH_20S_25_1_A1_4h_R1 **Scan** 20188
**Peptide** DAANTAKAFNEAL **Charge** 2 **Spectral Angle** 0.83

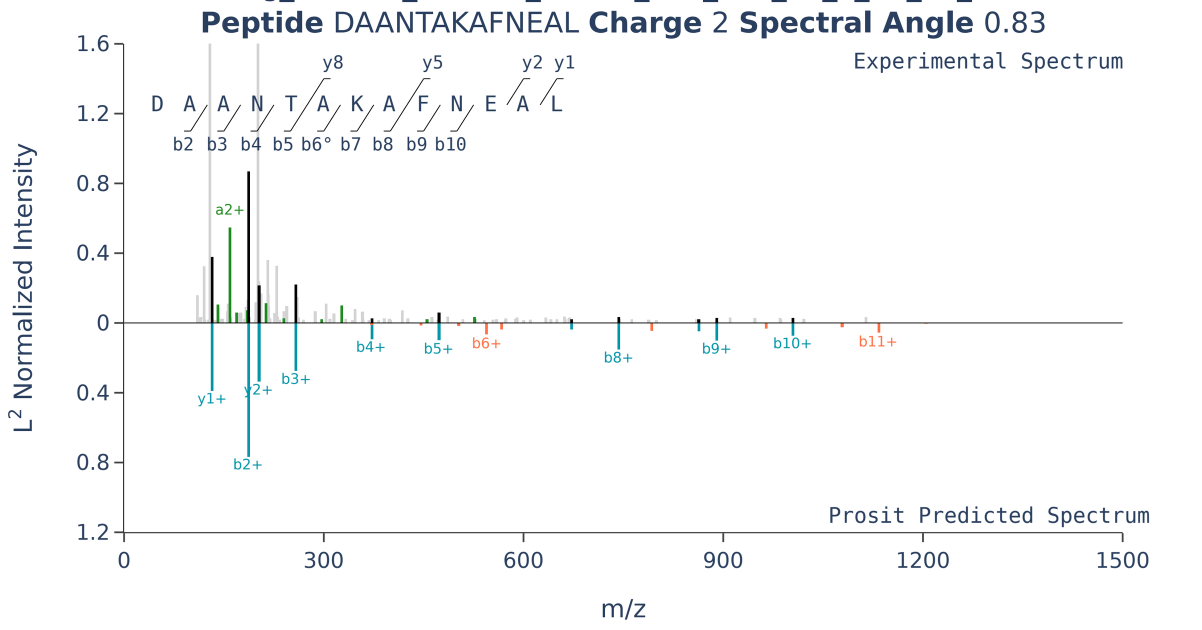


**Source** Ncheng_210623_230623_HFGoe_FFH_20S_25_1_A2_2h_R1 **Scan** 22413
**Peptide** QDVNRLLKQFD **Charge** 2 **Spectral Angle** 0.68

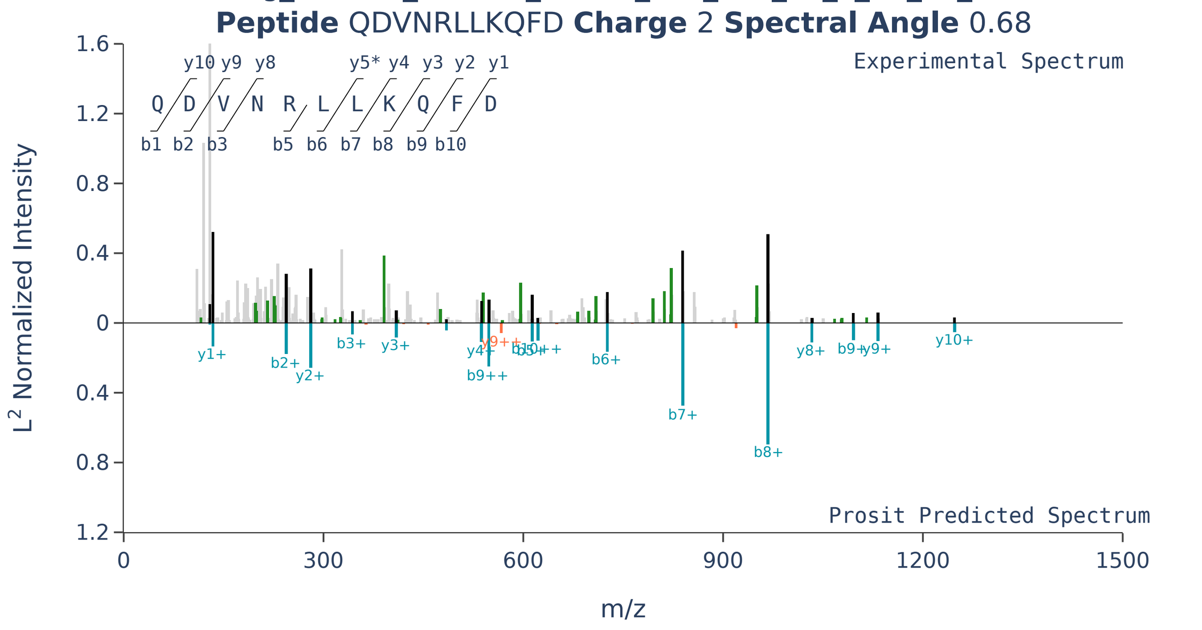


**Source** Ncheng_210623_230623_HFGoe_FFH_20S_25_1_A2_24h_R1 **Scan** 8083
**Peptide** RSMKGMM **Charge** 2 **Spectral Angle** 0.7

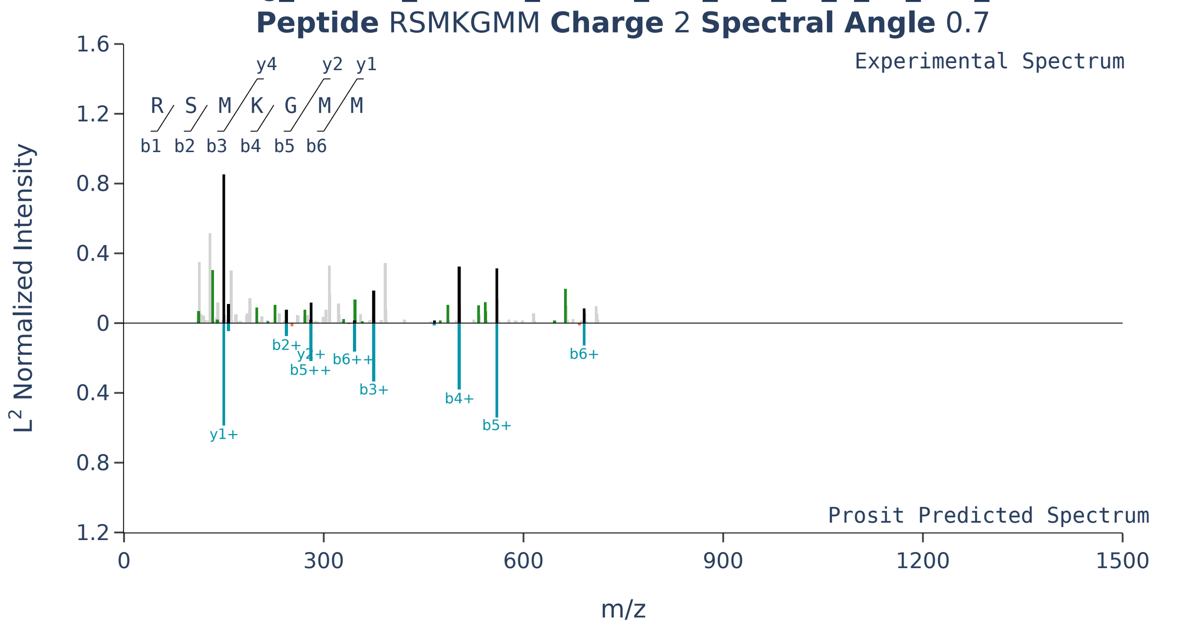


**Source** Ncheng_210623_230623_HFGoe_FFH_20S_25_1_A2_24h_R2 **Scan** 17240
**Peptide** VKEKAVGHEVNKSLTPGQEFVKIVRN **Charge** 3 **Spectral Angle** 0.79

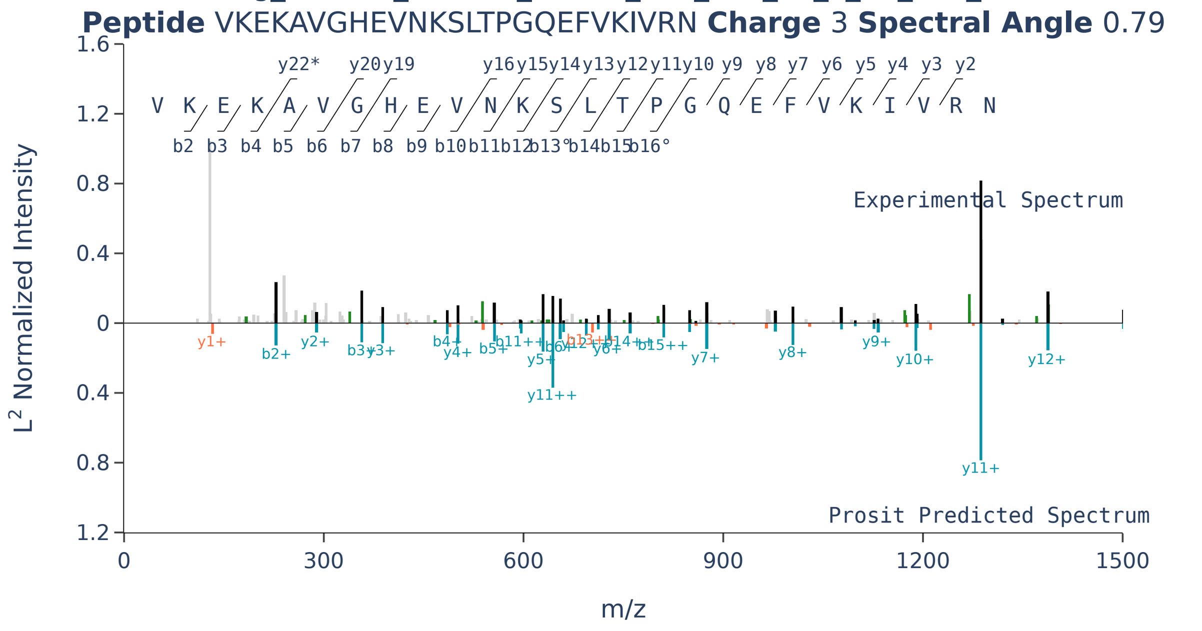


**Source** Ncheng_210623_230623_HFGoe_FFH_20S_25_1_A2_4h_R1 **Scan** 32097
**Peptide** PAAIKQLETLAEQV **Charge** 2 **Spectral Angle** 0.8

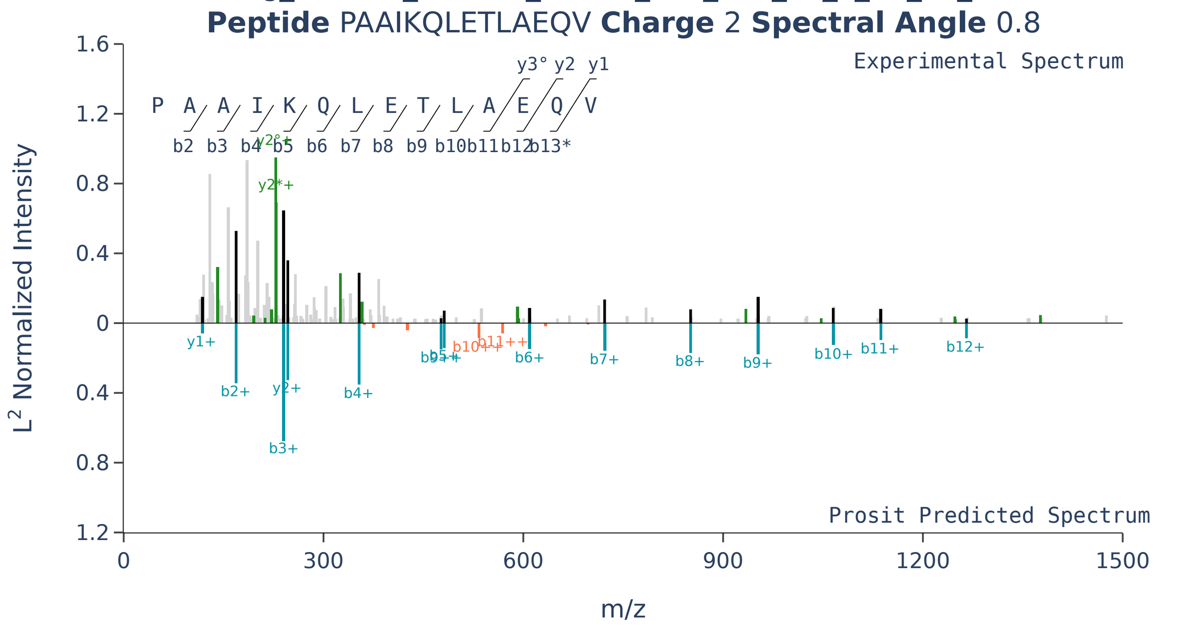


**Source** Ncheng_210623_230623_HFGoe_FFH_20S_25_1_A2_4h_R1 **Scan** 16200
**Peptide** GKLGKFL **Charge** 2 **Spectral Angle** 0.74

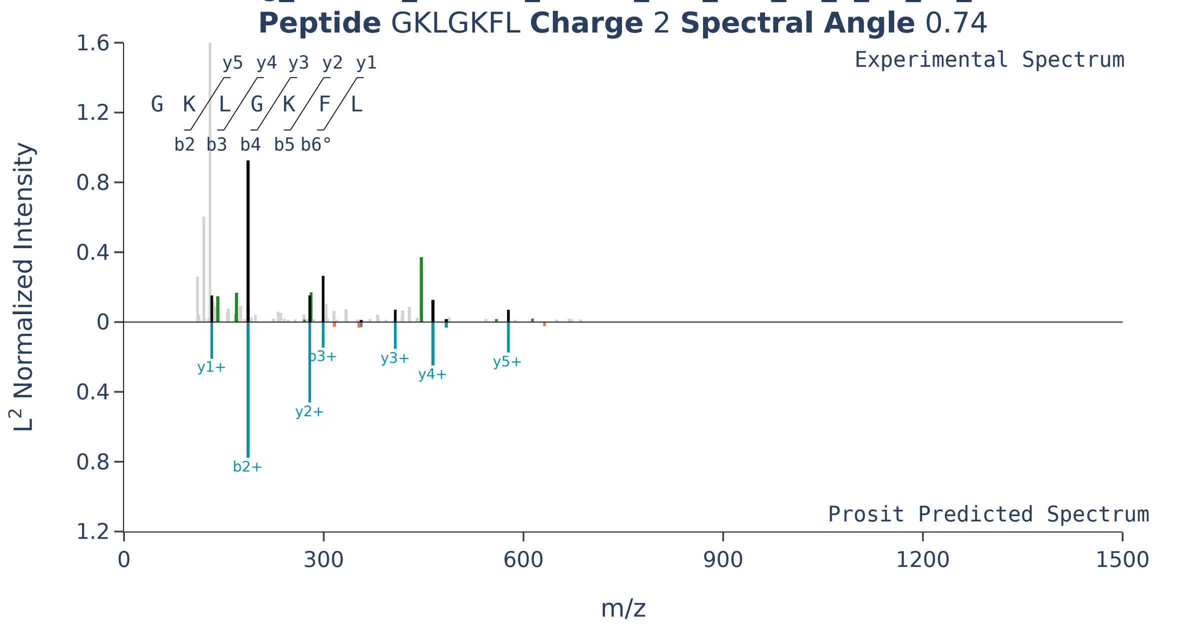


**Source** Ncheng_210623_230623_HFGoe_FFH_20S_25_1_A1_24h_R2 **Scan** 21507
**Peptide** NLAAQPPAVV **Charge** 2 **Spectral Angle** 0.77

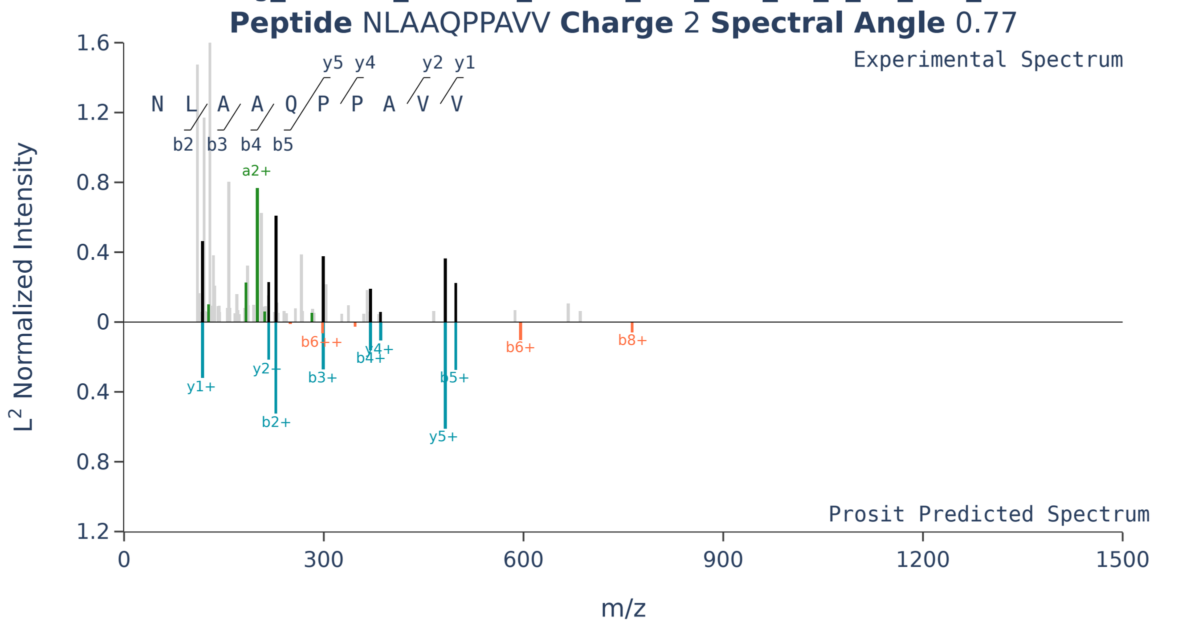


**Source** Ncheng_210623_230623_HFGoe_FFH_20S_25_1_A2_4h_R1 **Scan** 29997
**Peptide** VNKSLTPGQEFVKIVRNEL **Charge** 3 **Spectral Angle** 0.69

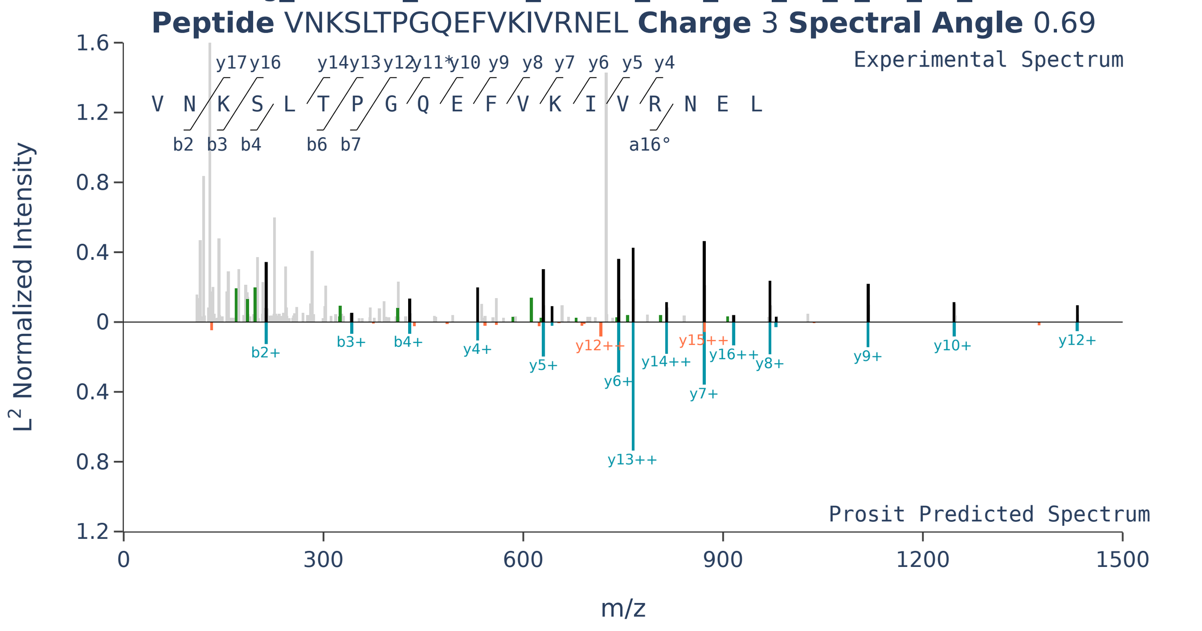


**Source** Ncheng_210623_230623_HFGoe_FFH_20S_25_1_A1_4h_R2 **Scan** 17983
**Peptide** GLQGAGKTTSVGKLGKFLRE **Charge** 3 **Spectral Angle** 0.76

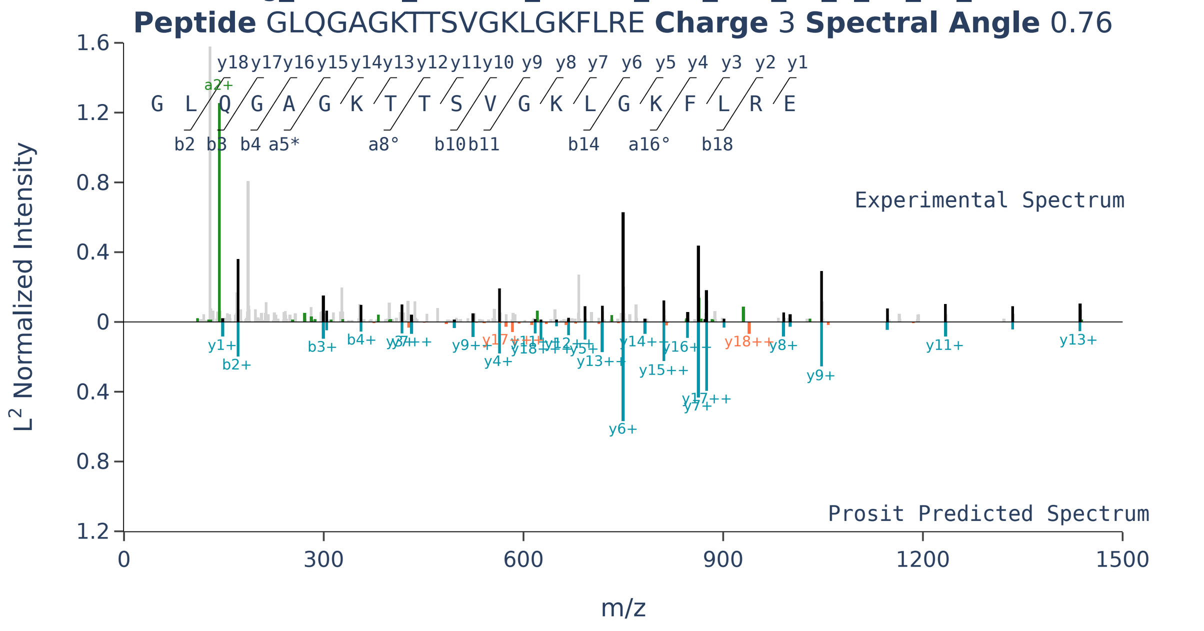


**Source** Ncheng_210623_230623_HFGoe_FFH_20S_25_1_A2_24h_R1 **Scan** 6617
**Peptide** FINRVKEK **Charge** 2 **Spectral Angle** 0.76

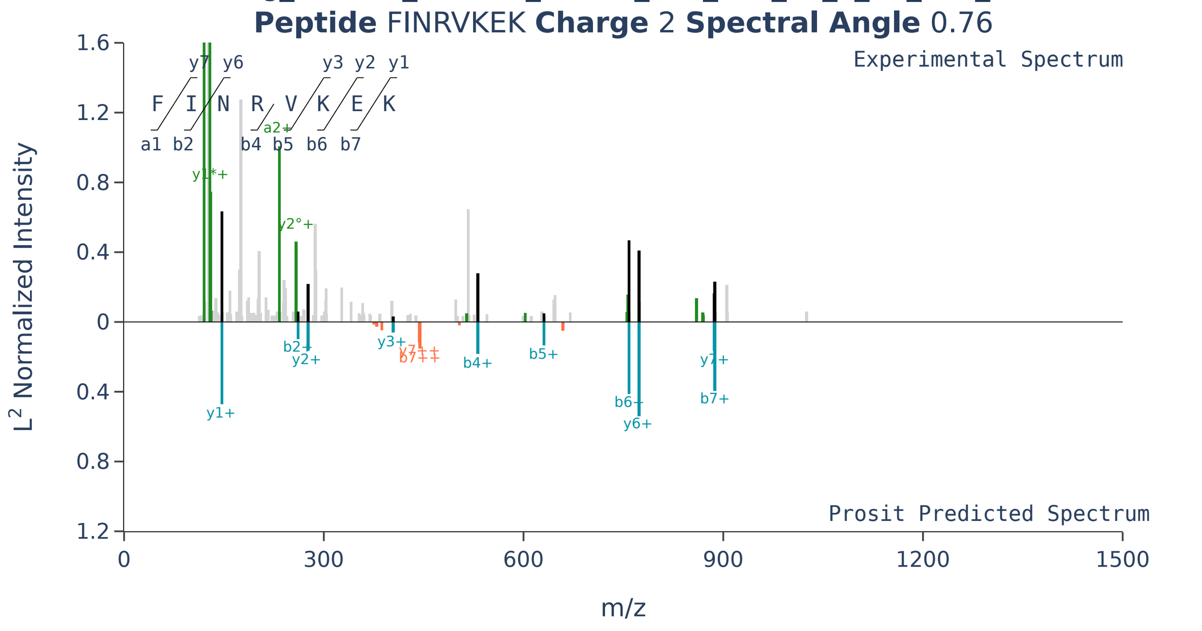


**Source** Ncheng_210623_230623_HFGoe_FFH_20S_25_1_A2_24h_R1 **Scan** 6816
**Peptide** REKHKKKVLVV **Charge** 2 **Spectral Angle** 0.67

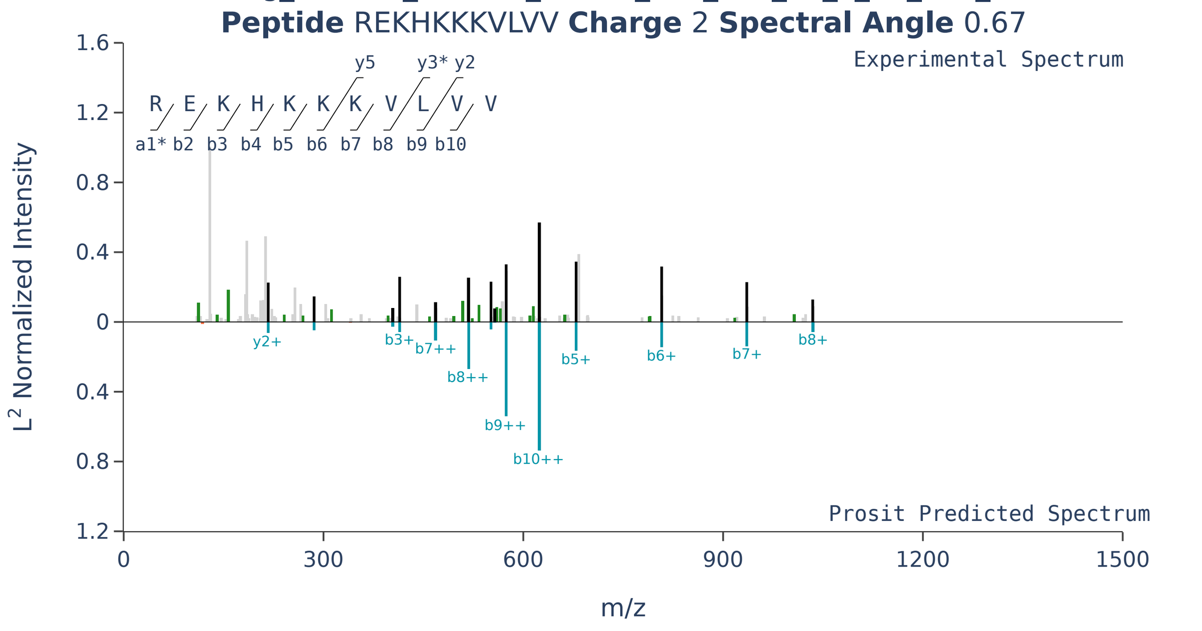

**Source** Ncheng_210623_230623_HFGoe_FFH_20S_25_1_A1_2h_R1 **Scan** 19714
**Peptide** GRLTEDNVKDTLREVRMA **Charge** 3 **Spectral Angle** 0.79

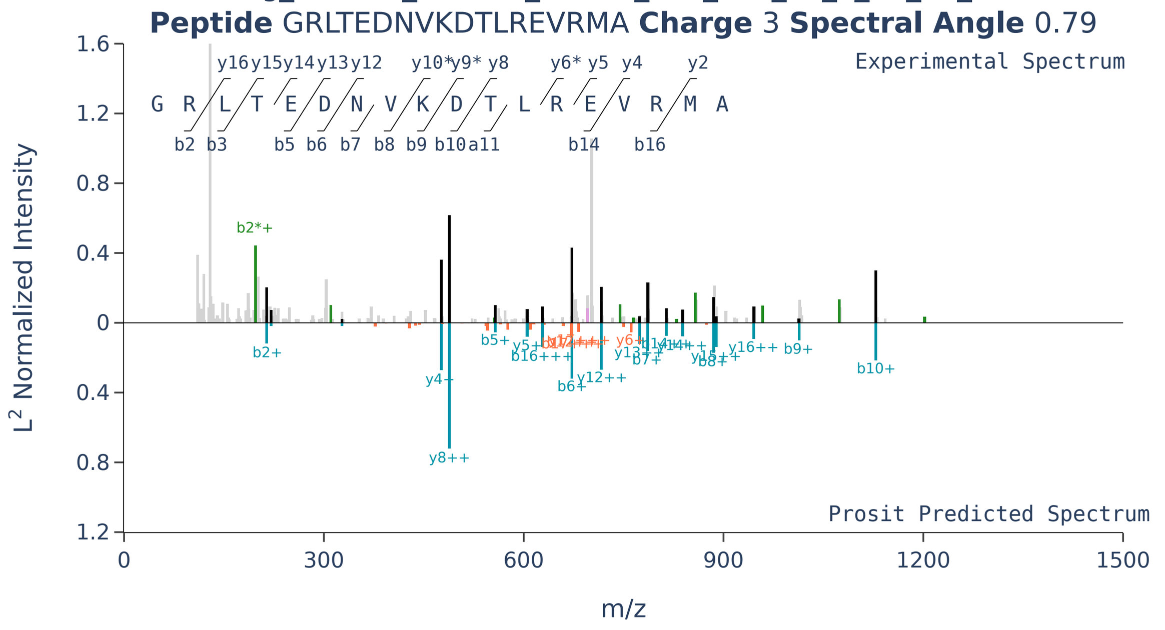


**Source** Ncheng_210623_230623_HFGoe_FFH_20S_25_1_A1_4h_R1 **Scan** 19365
**Peptide** ALEPFHPDRLA **Charge** 3 **Spectral Angle** 0.85

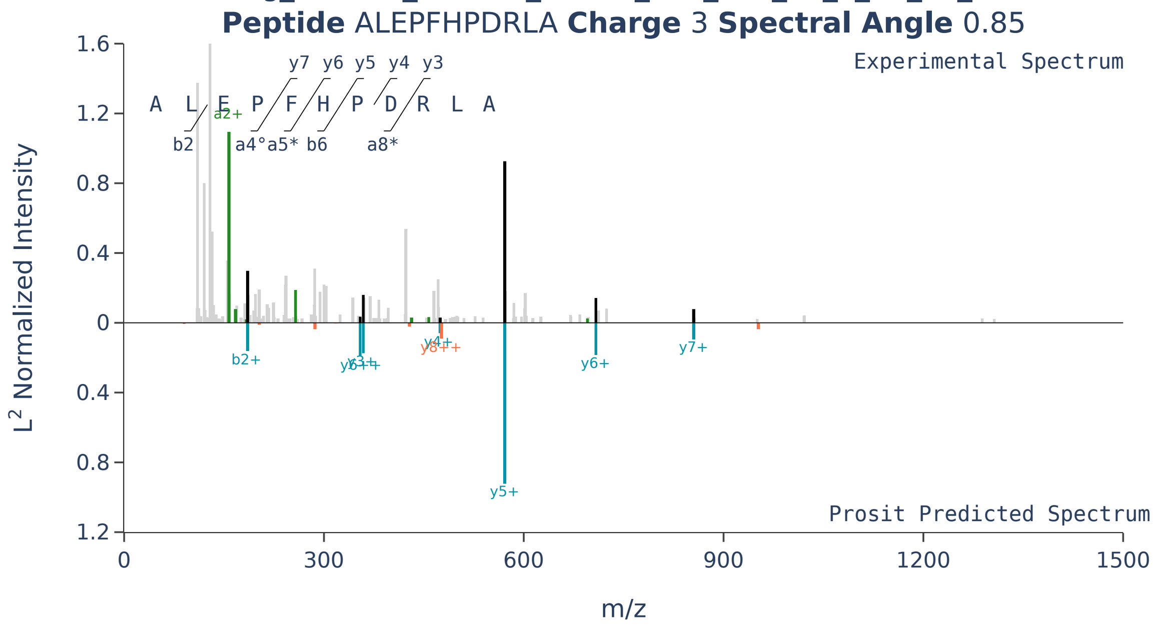


**Source** Ncheng_210623_230623_HFGoe_FFH_20S_25_1_A2_24h_R1 **Scan** 6385
**Peptide** TEDNVKD **Charge** 1 **Spectral Angle** 0.9

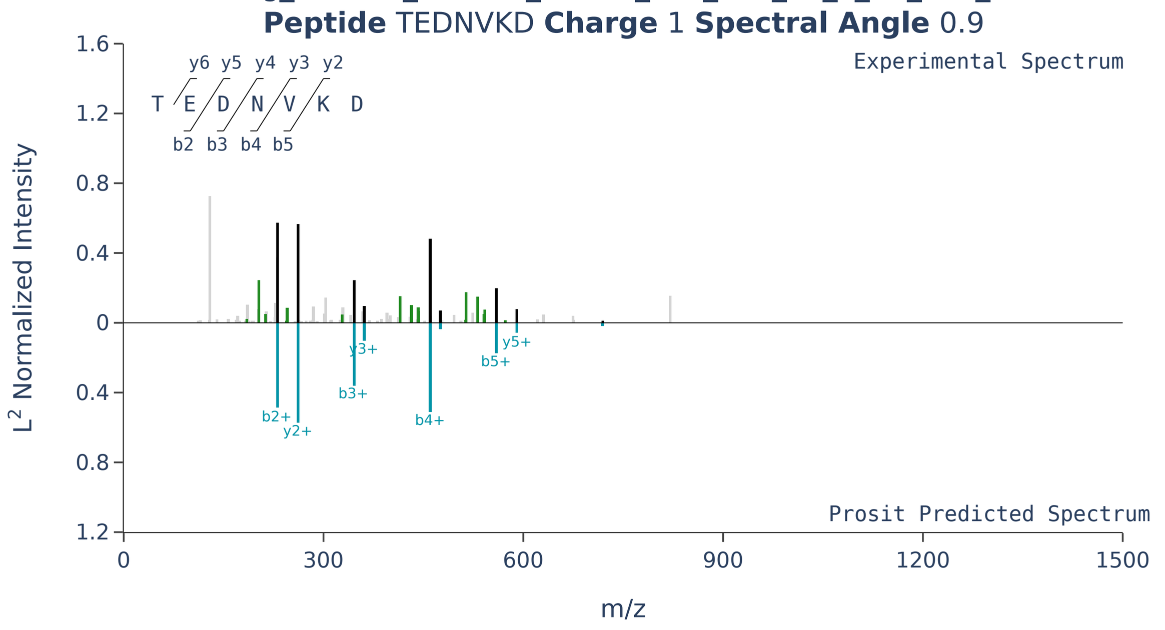


**Source** Ncheng_210623_230623_HFGoe_FFH_20S_25_1_A2_4h_R2 **Scan** 23867
**Peptide** LGVGEKTEALEPFHPDRIASRL **Charge** 3 **Spectral Angle** 0.82

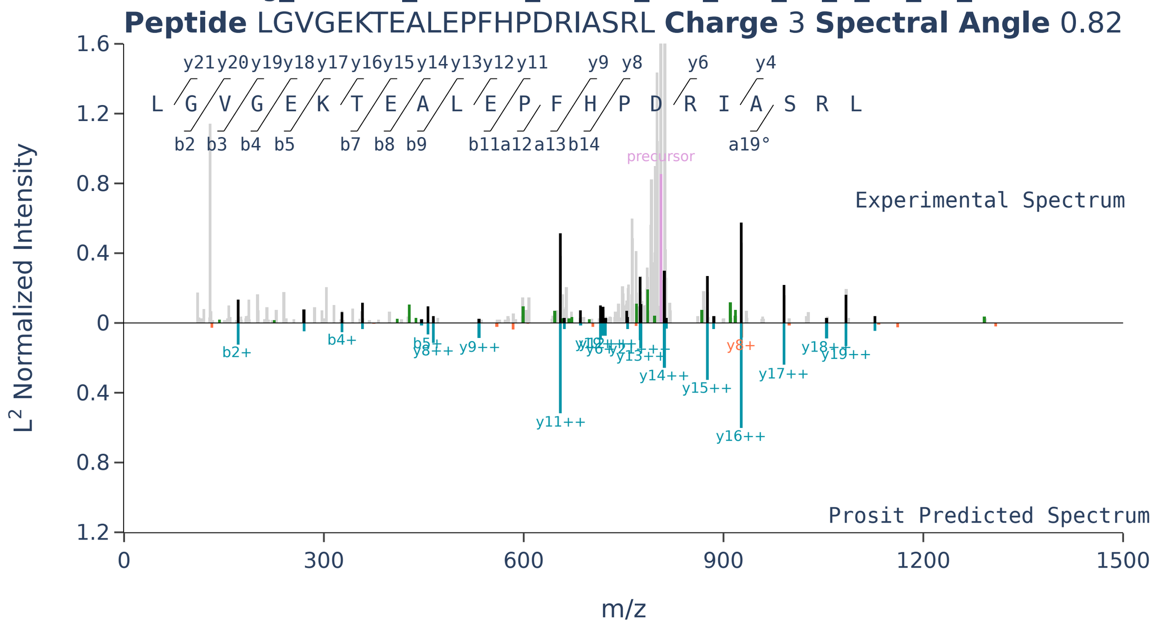


**Source** Ncheng_210623_230623_HFGoe_FFH_20S_25_1_A2_2h_R1 **Scan** 29195
**Peptide** GHEVNKSLTPGQEFVKIVRNELVA **Charge** 3 **Spectral Angle** 0.87

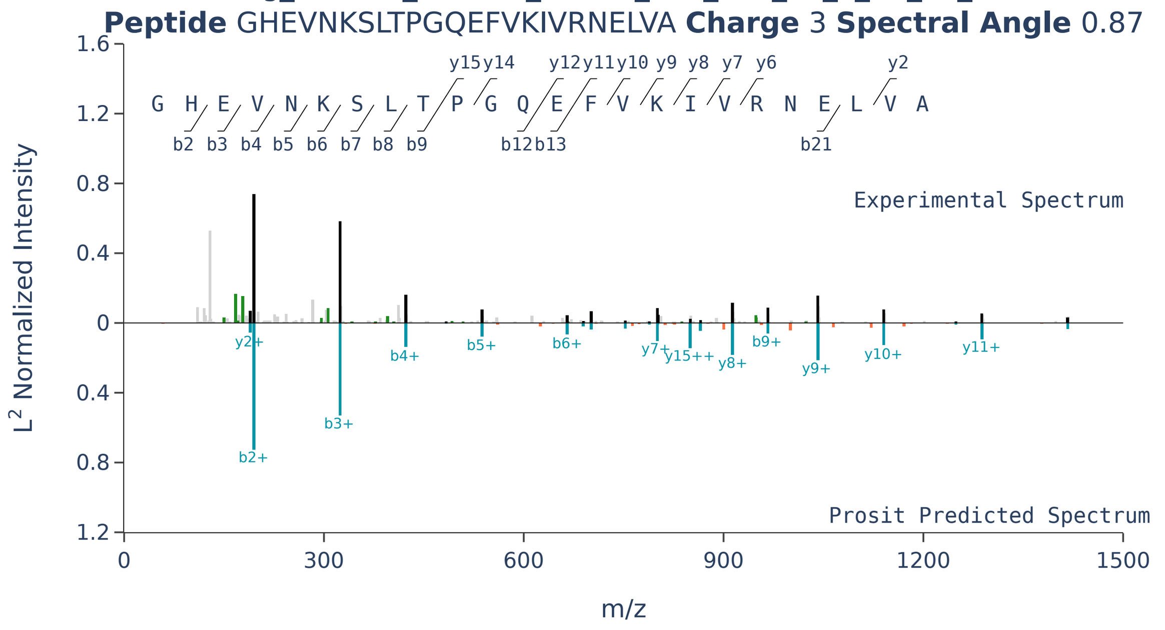


**Source** Ncheng_210623_230623_HFGoe_FFH_20S_25_1_A1_24h_R1 **Scan** 15509
**Peptide** KSQMDDKVLVRM **Charge** 2 **Spectral Angle** 0.62

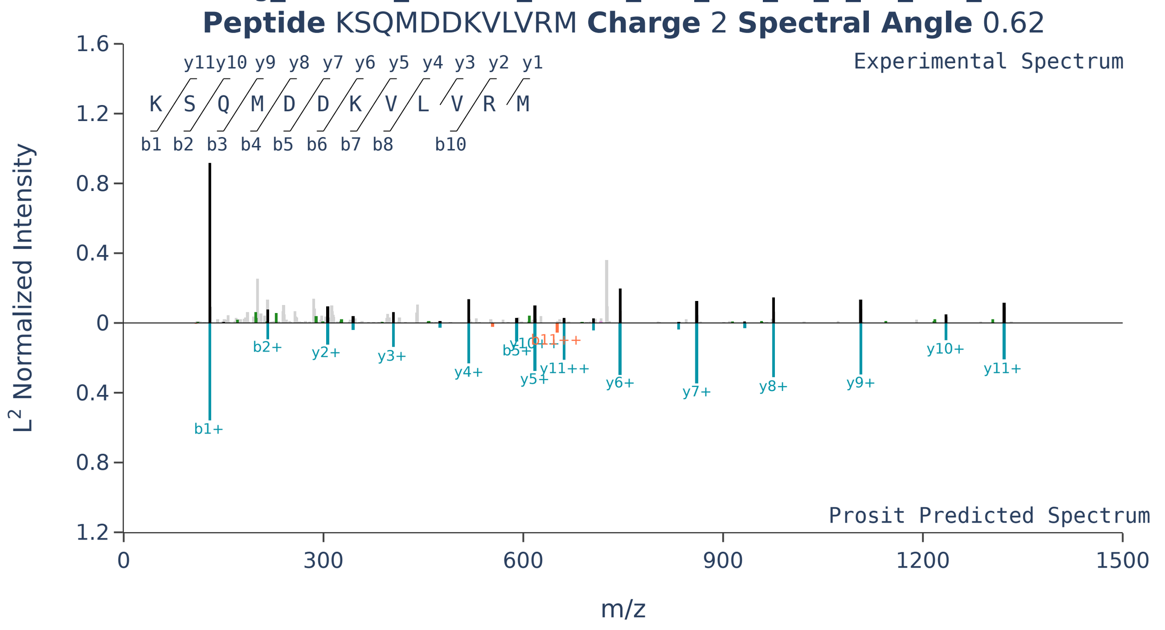


**Source** Ncheng_210623_230623_HFGoe_FFH_20S_25_1_A2_24h_R2 **Scan** 7284
**Peptide** RQMKNMGGM **Charge** 2 **Spectral Angle** 0.75

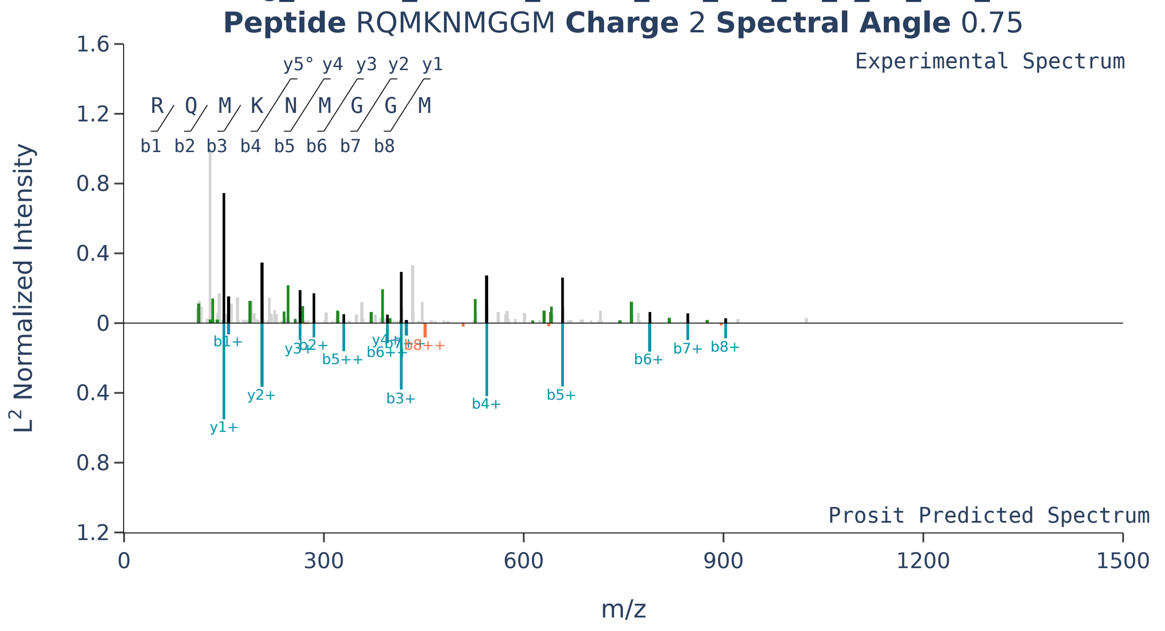


**Source** Ncheng_210623_230623_HFGoe_FFH_20S_25_1_A1_2h_R1 **Scan** 10171
**Peptide** VVLTKVDGDAR **Charge** 2 **Spectral Angle** 0.73

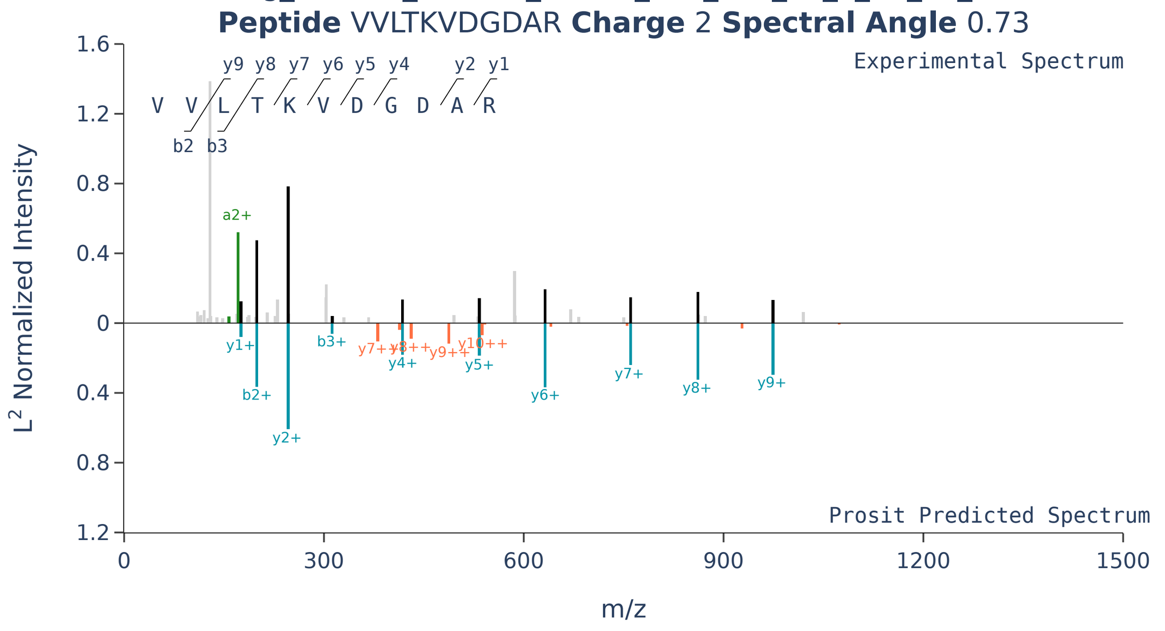


**Source** Ncheng_210623_230623_HFGoe_FFH_20S_25_1_A1_24h_R1 **Scan** 14931
**Peptide** KSLTPGQEF **Charge** 1 **Spectral Angle** 0.89

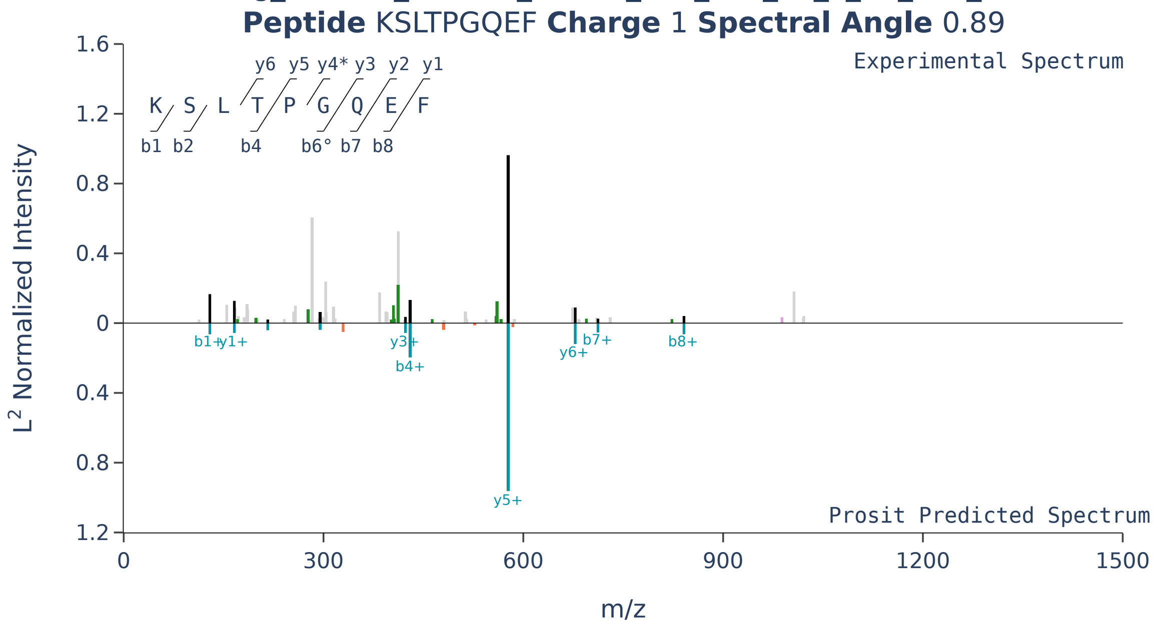


**Source** Ncheng_210623_230623_HFGoe_FFH_20S_25_1_A2_2h_R1 **Scan** 35459
**Peptide** VDFFPSDVGQKPVDIVNAALKEA **Charge** 3 **Spectral Angle** 0.87

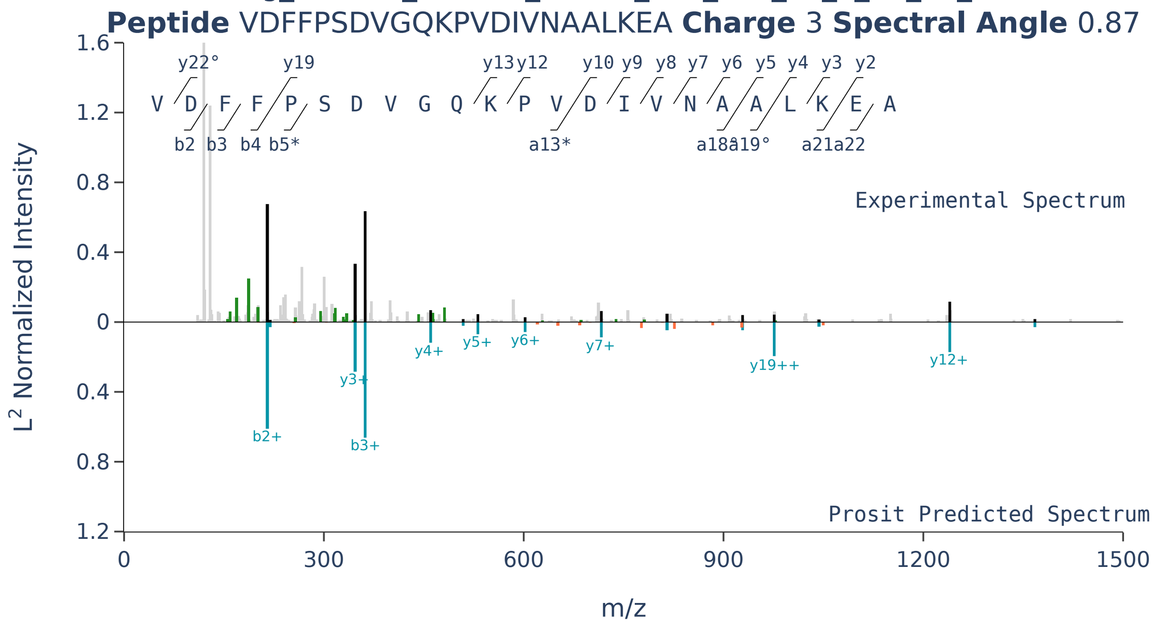


**Source** Ncheng_210623_230623_HFGoe_FFH_20S_25_1_A1_2h_R2 **Scan** 32330
**Peptide** AKLKFYDVLLVDTAGRLHV **Charge** 3 **Spectral Angle** 0.85

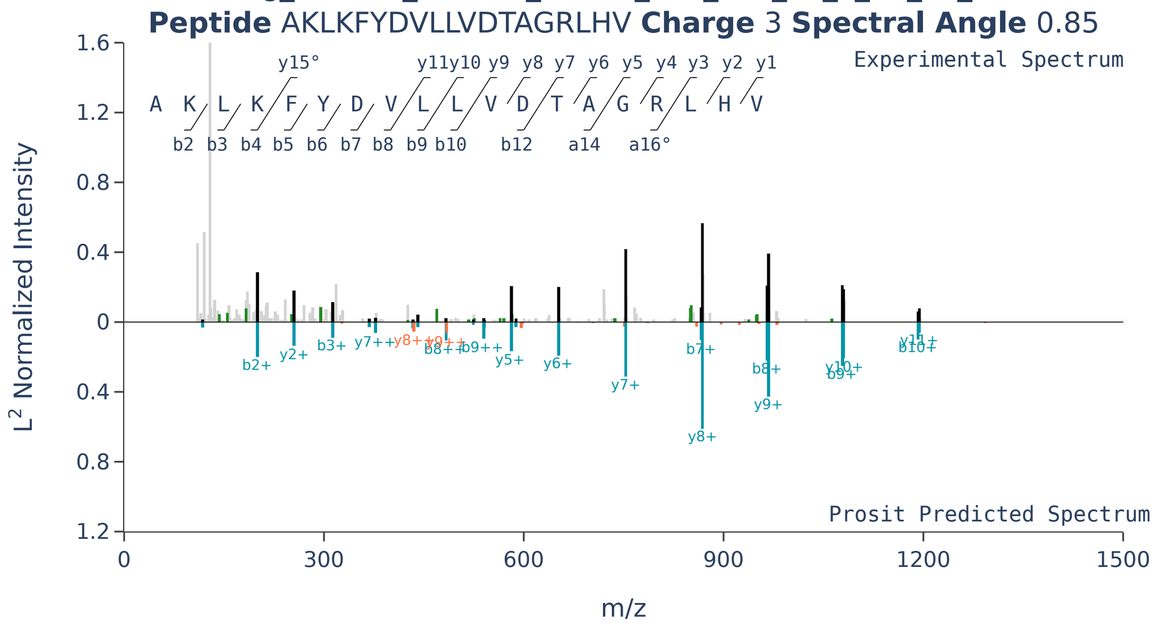


**Source** Ncheng_210623_230623_HFGoe_FFH_20S_25_1_A2_4h_R1 **Scan** 30856
**Peptide** AAGCGMQVQDVNRLLKQF **Charge** 3 **Spectral Angle** 0.71

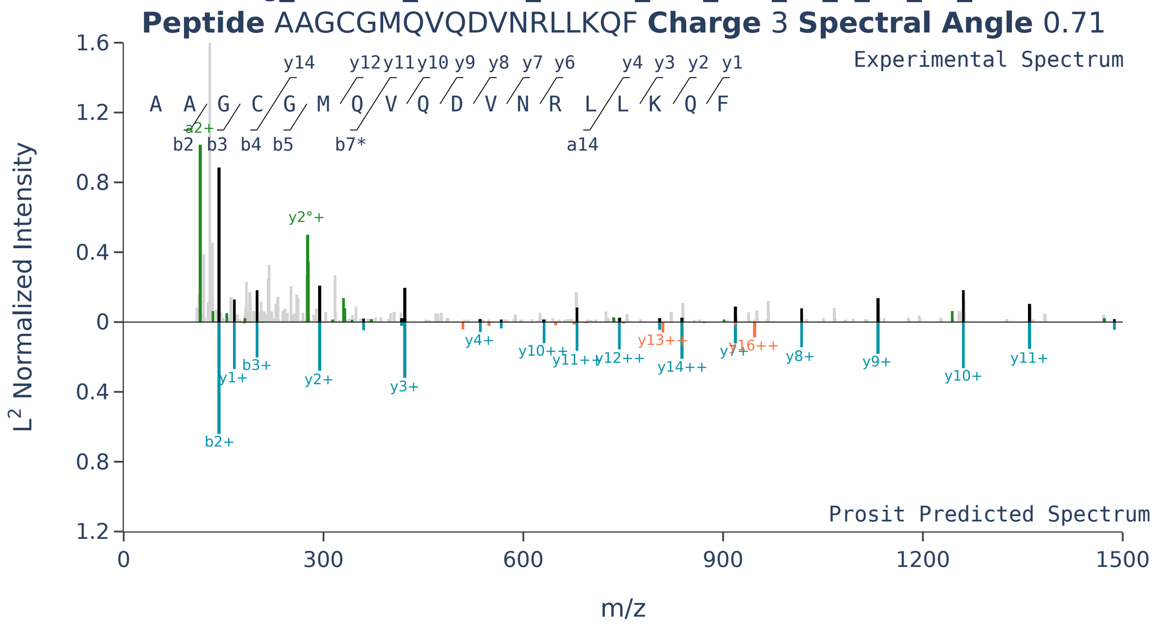


**Source** Ncheng_210623_230623_HFGoe_FFH_20S_25_1_A2_24h_R2 **Scan** 16268
**Peptide** AQAEKLASKLKKGDGFDLND **Charge** 3 **Spectral Angle** 0.87

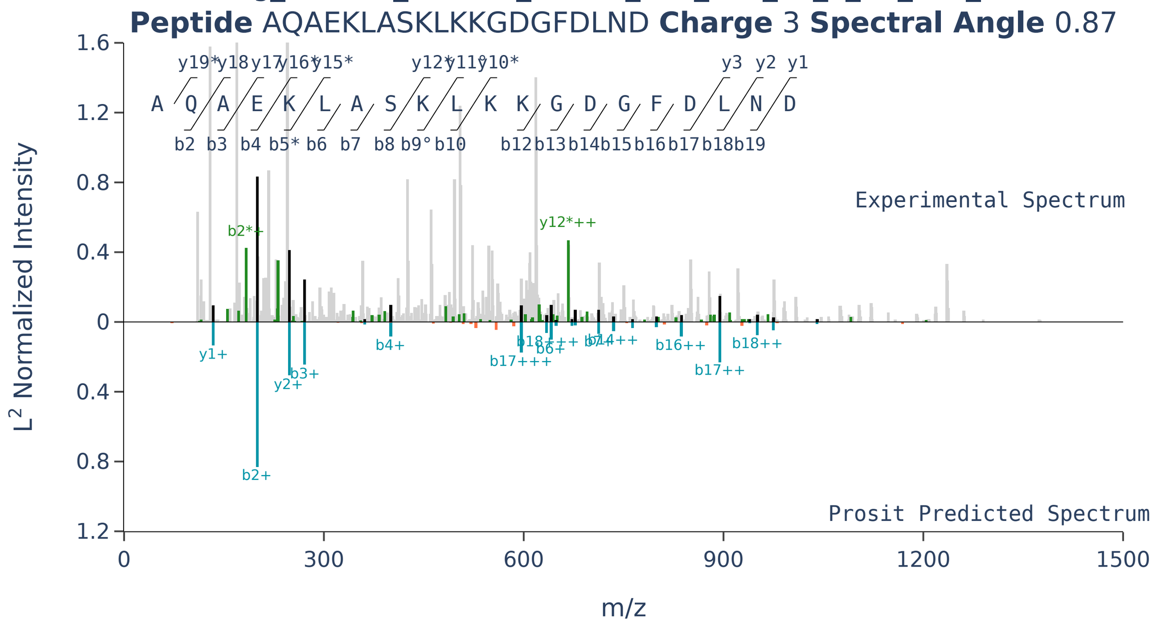


**Source** Ncheng_210623_230623_HFGoe_FFH_20S_25_1_A2_4h_R2 **Scan** 13695
**Peptide** LMAGLQGAGKTT **Charge** 2 **Spectral Angle** 0.71

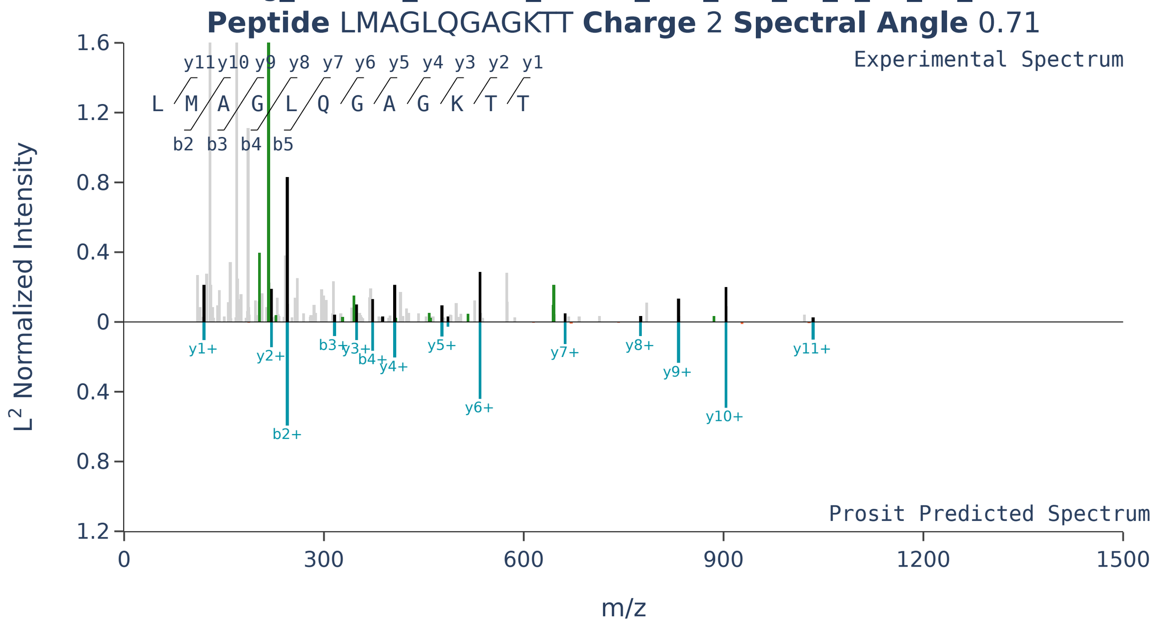


**Source** Ncheng_210623_230623_HFGoe_FFH_20S_25_1_A2_4h_R1 **Scan** 10564
**Peptide** HVDEAMM **Charge** 2 **Spectral Angle** 0.84

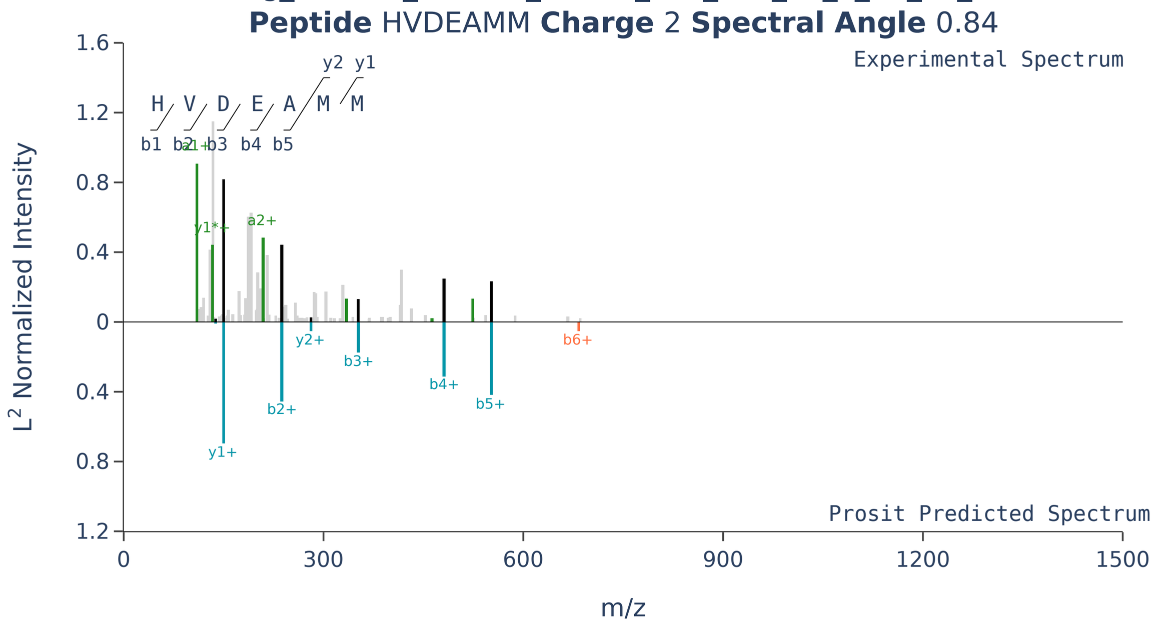

**Source** Ncheng_210623_230623_HFGoe_FFH_20S_25_1_A2_2h_R2 **Scan** 11475
**Peptide** KKGDGFDLND **Charge** 3 **Spectral Angle** 0.72

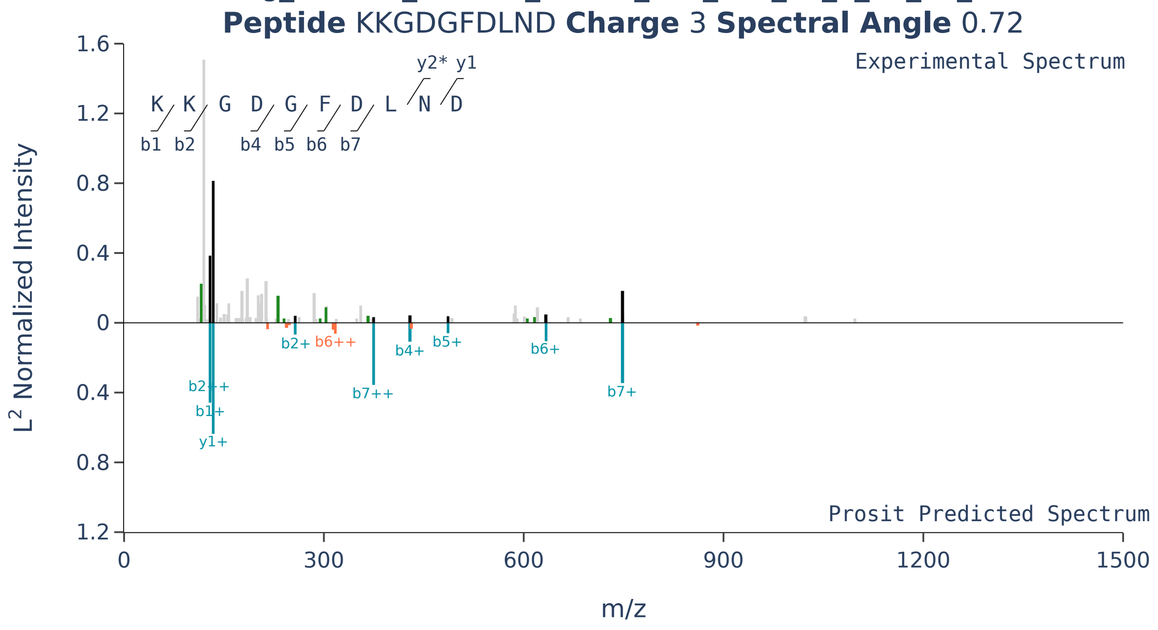


**Source** Ncheng_210623_230623_HFGoe_FFH_20S_25_1_A2_4h_R2 **Scan** 9832
**Peptide** VDTAGRLHVD **Charge** 1 **Spectral Angle** 0.78

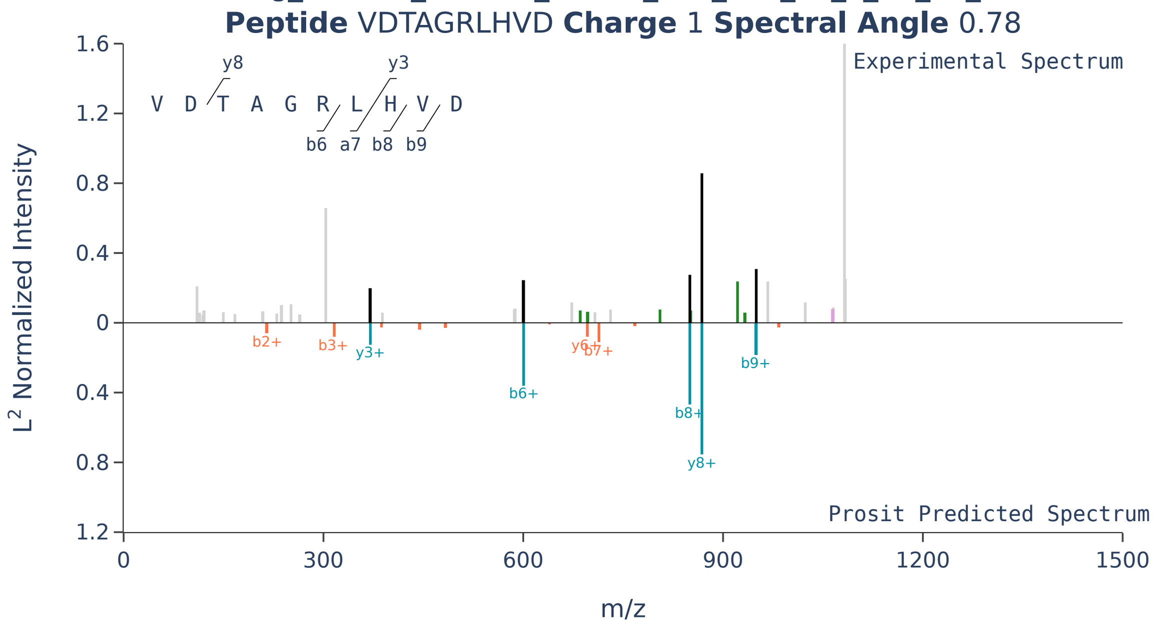


**Source** Ncheng_210623_230623_HFGoe_FFH_20S_25_1_A2_24h_R1 **Scan** 16216
**Peptide** SRILGMGD **Charge** 2 **Spectral Angle** 0.68

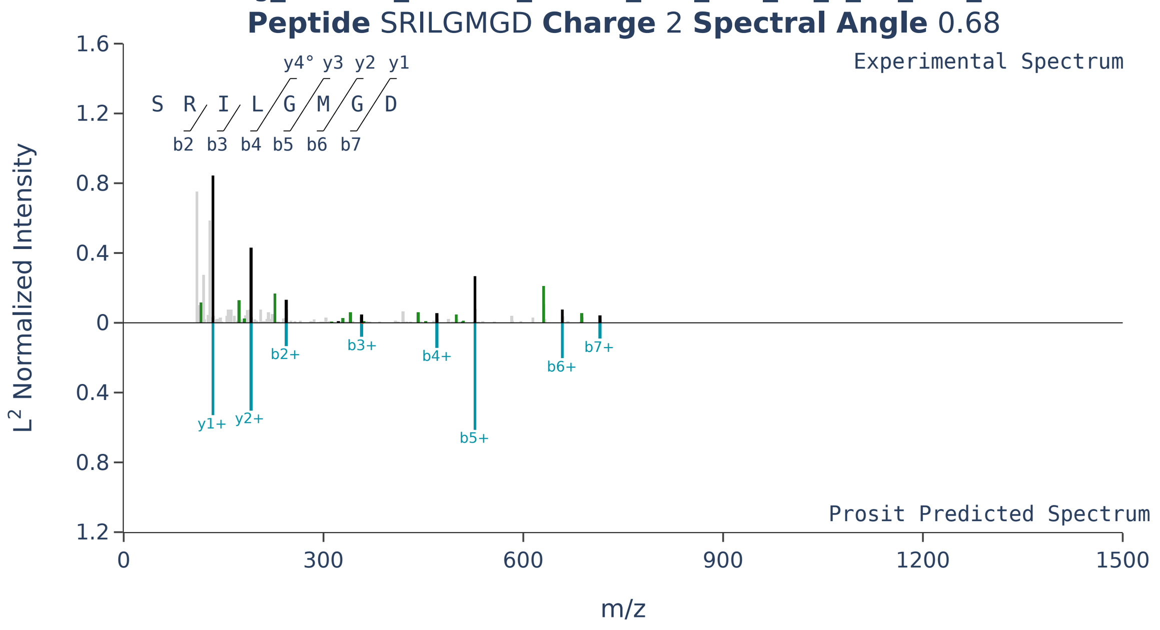


**Source** Ncheng_210623_230623_HFGoe_FFH_20S_25_1_A2_24h_R2 **Scan** 12426
**Peptide** VGKLGKF **Charge** 2 **Spectral Angle** 0.8

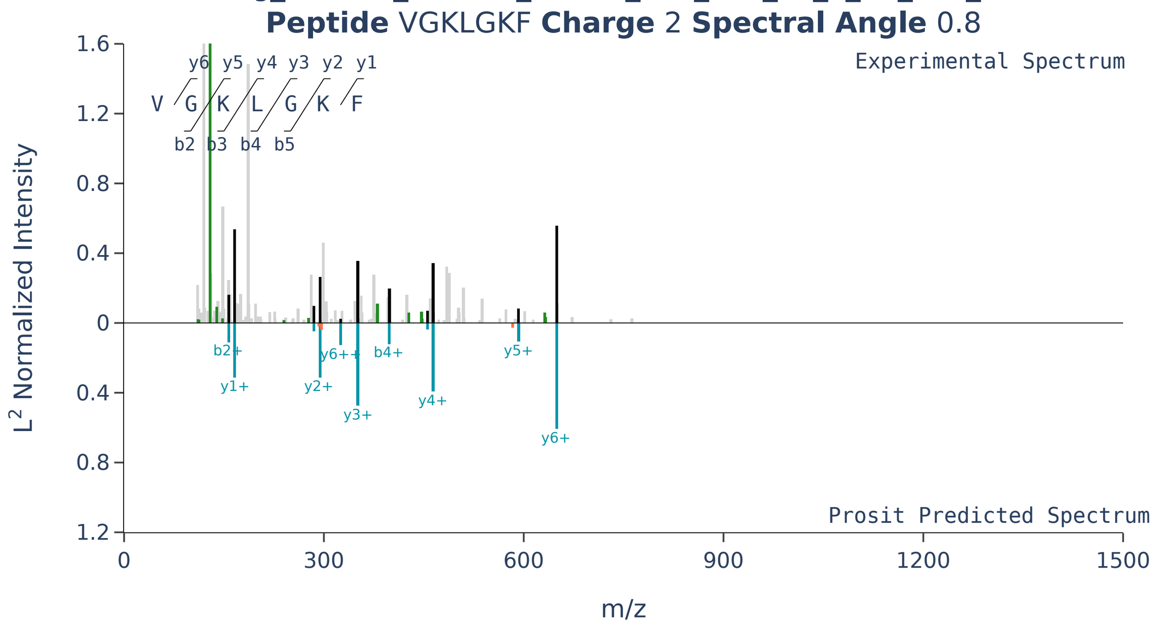


**Source** Ncheng_210623_230623_HFGoe_FFH_20S_25_1_A2_2h_R1 **Scan** 25462
**Peptide** VGQKPVDIVNAALKEA **Charge** 3 **Spectral Angle** 0.8

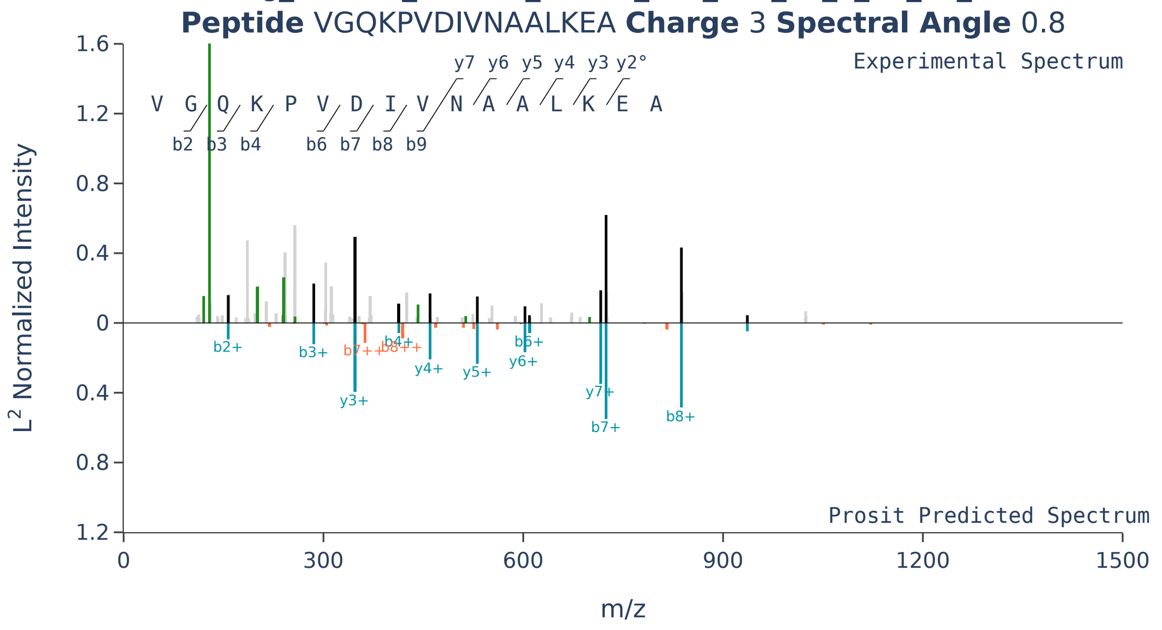


**Source** Ncheng_210623_230623_HFGoe_FFH_20S_25_1_A1_1h_R2 **Scan** 33058
**Peptide** ADVALPVVREFINRVKEKAVGH **Charge** 3 **Spectral Angle** 0.84

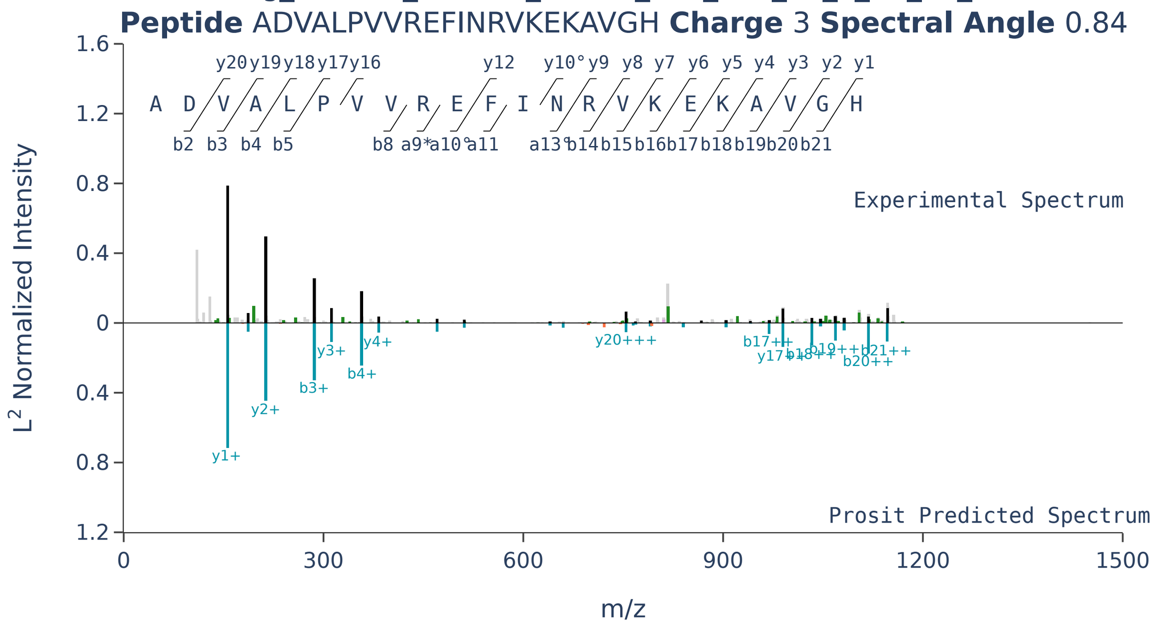


**Source** Ncheng_210623_230623_HFGoe_FFH_20S_25_1_A2_24h_R2 **Scan** 22325
**Peptide** AGCGMQVQDVNRLLKQ **Charge** 2 **Spectral Angle** 0.68

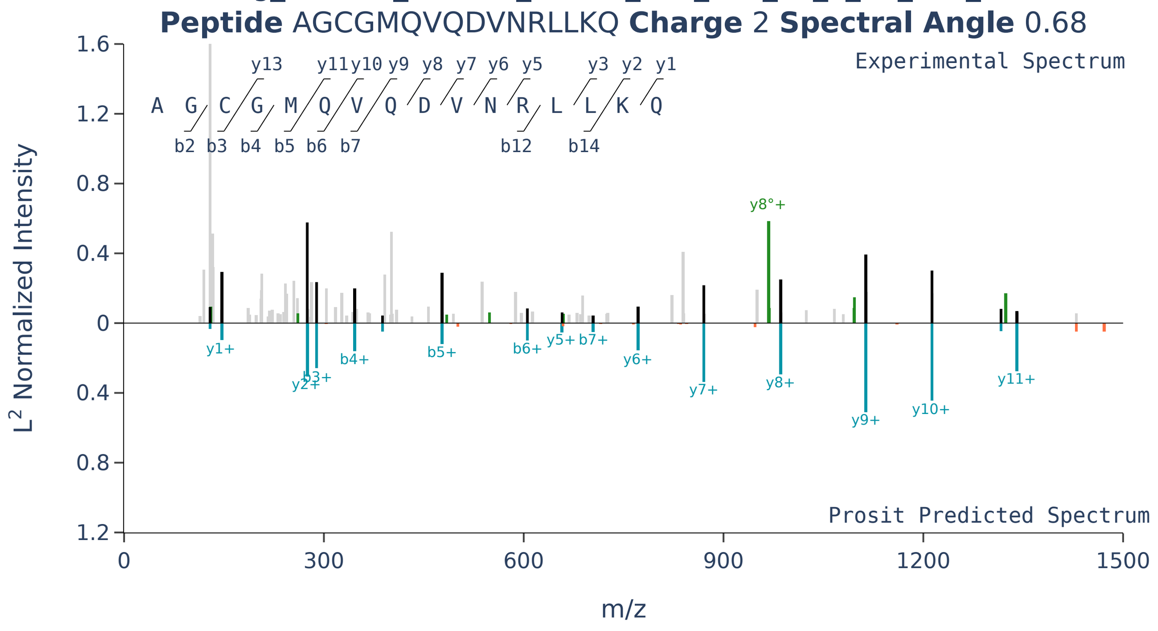


**Source** Ncheng_210623_230623_HFGoe_FFH_20S_25_1_A1_4h_R2 **Scan** 26282
**Peptide** GQDAANTAKAFNEALPLT **Charge** 2 **Spectral Angle** 0.89

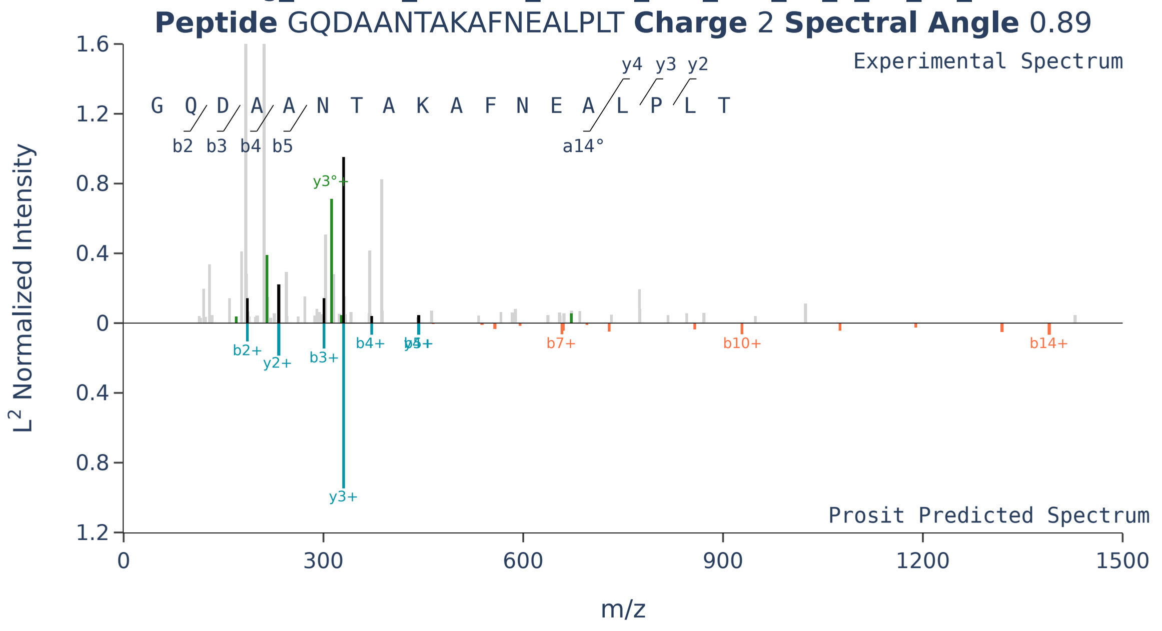


**Source** Ncheng_210623_230623_HFGoe_FFH_20S_25_1_A2_4h_R2 **Scan** 27868
**Peptide** ASRILGMGDVL **Charge** 2 **Spectral Angle** 0.89

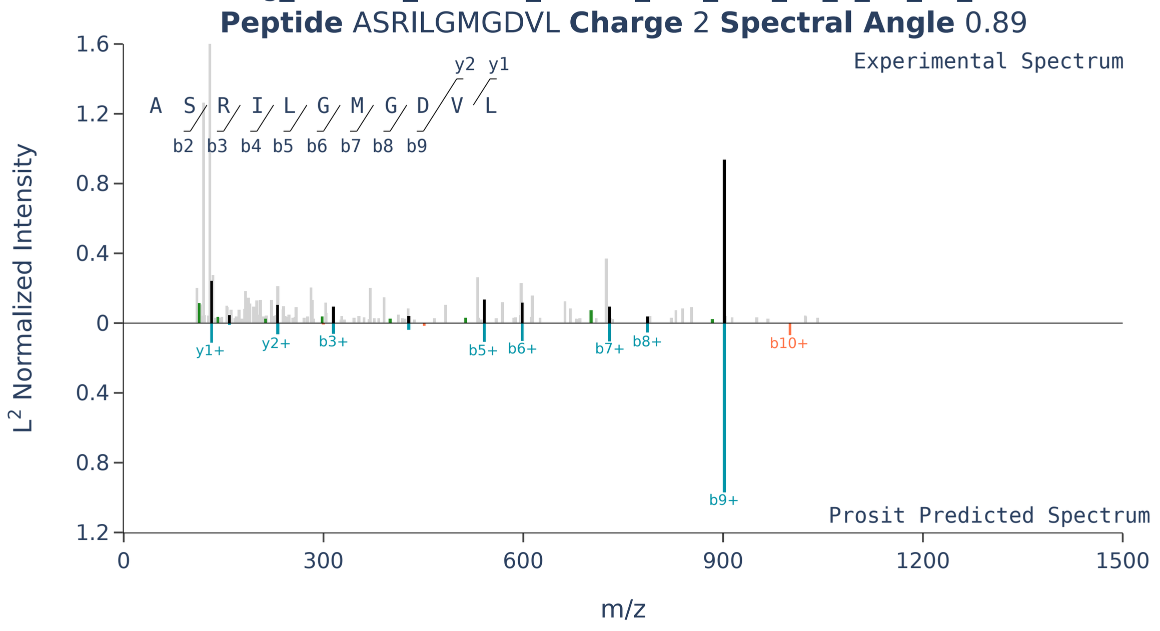


**Source** Ncheng_210623_230623_HFGoe_FFH_20S_25_1_A1_24h_R2 **Scan** 16091
**Peptide** SIRHITGKPIKFLGVGEKT **Charge** 5 **Spectral Angle** 0.77

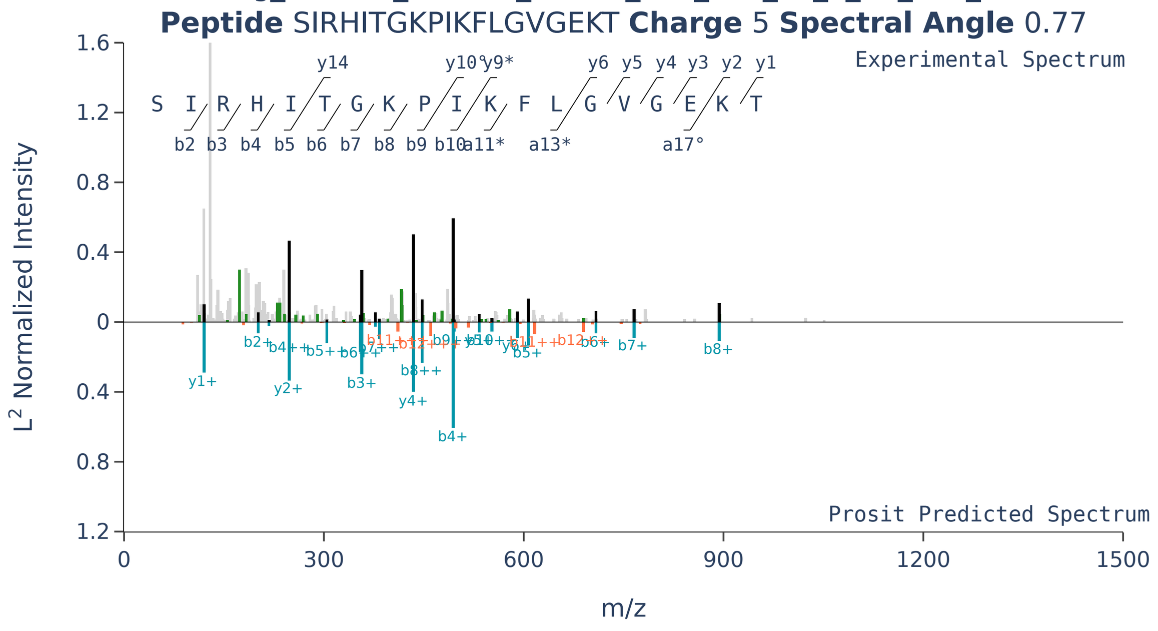


**Source** Ncheng_210623_230623_HFGoe_FFH_20S_25_1_A1_4h_R1 **Scan** 23643
**Peptide** VQDVNRLLKQFD **Charge** 2 **Spectral Angle** 0.7

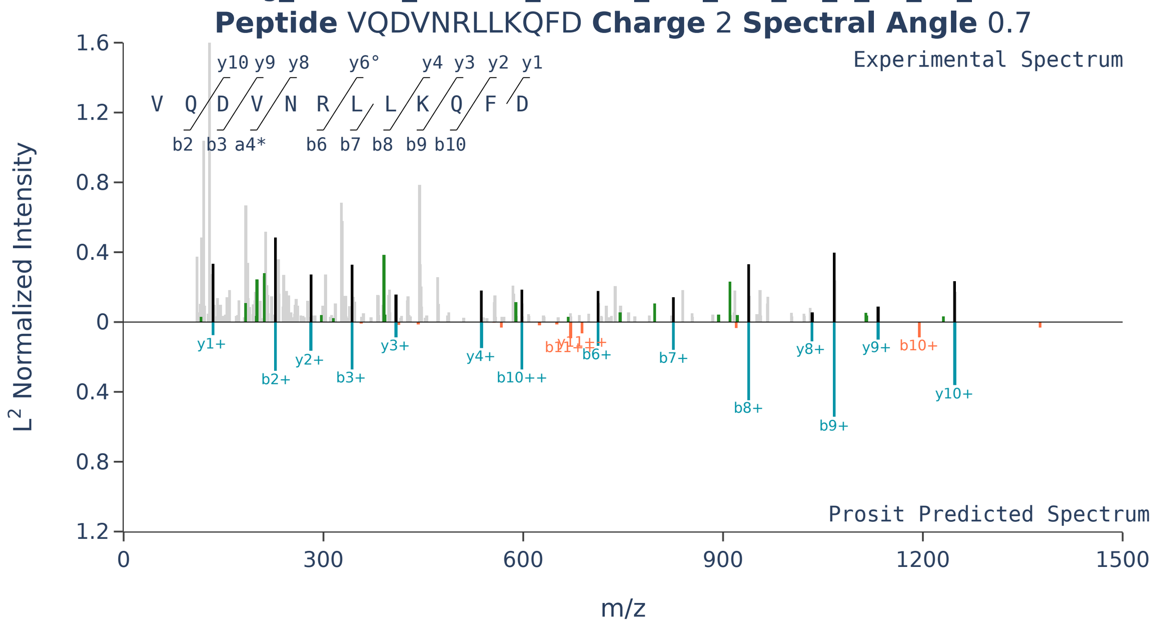


**Source** Ncheng_210623_230623_HFGoe_FFH_20S_25_1_A1_1h_R2 **Scan** 35837
**Peptide** QVGVDFFPSDVGQKPVDIVNAALKEA **Charge** 3 **Spectral Angle** 0.81

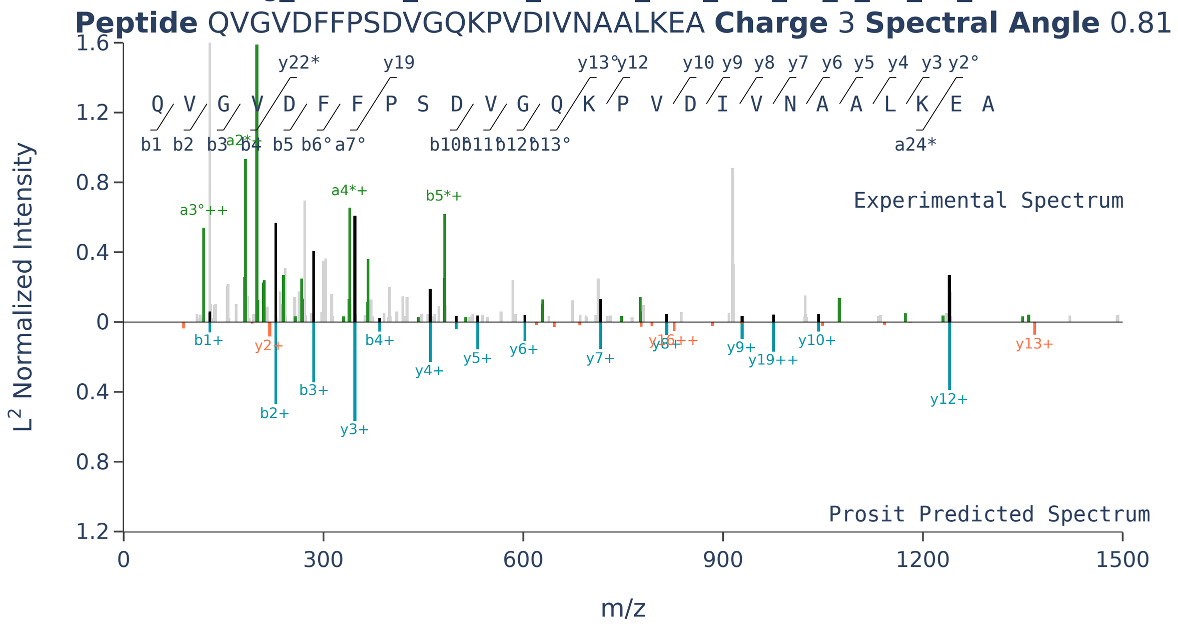


**Source** Ncheng_210623_230623_HFGoe_FFH_20S_25_1_A1_24h_R2 **Scan** 13664
**Peptide** LVDTAGRLHVDE **Charge** 2 **Spectral Angle** 0.75

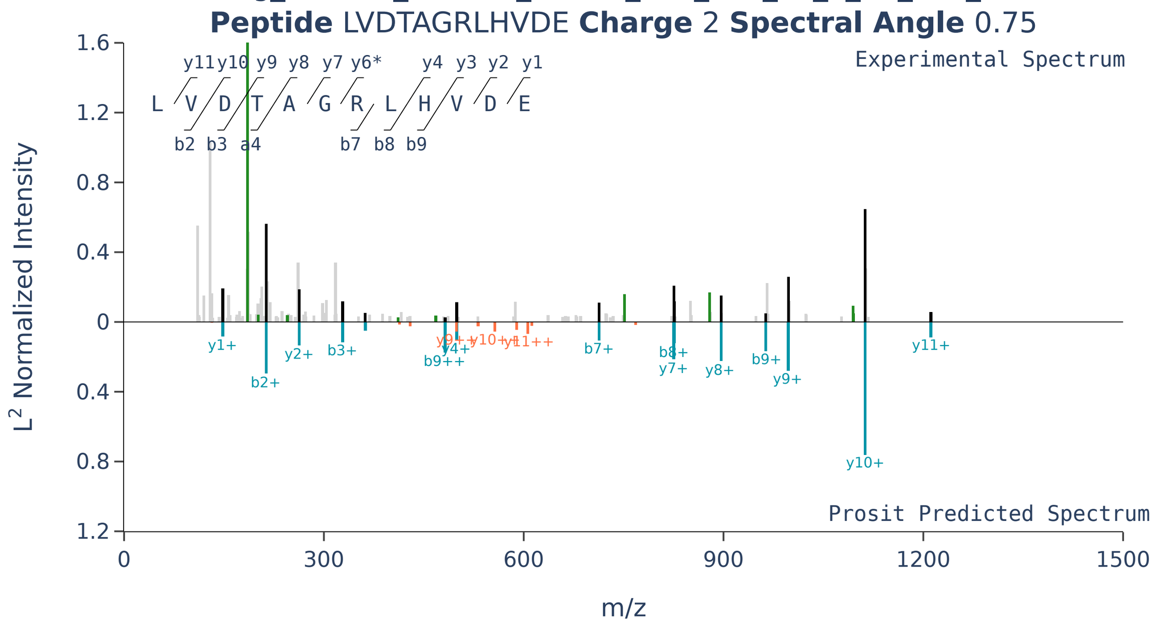


**Source** Ncheng_210623_230623_HFGoe_FFH_20S_25_1_A2_4h_R2 **Scan** 21059
**Peptide** KAVGHEVNKSLTPGQEFVKIV **Charge** 2 **Spectral Angle** 0.65

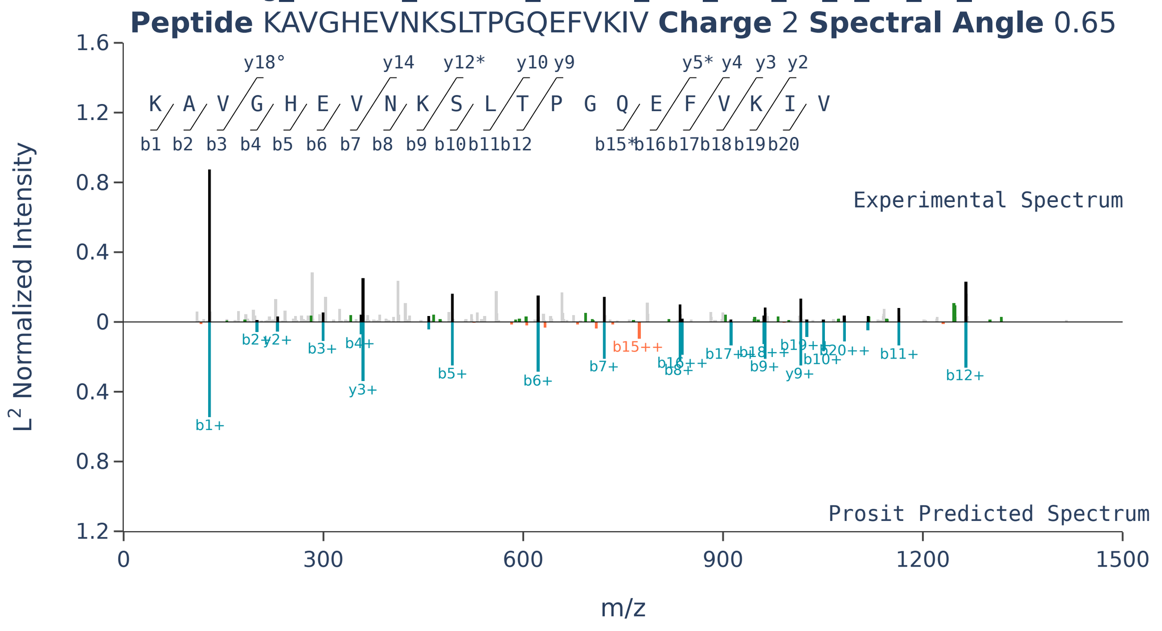


**Source** Ncheng_210623_230623_HFGoe_FFH_20S_25_1_A1_4h_R2 **Scan** 20663
**Peptide** VNKSLTPGQEFVKIVRN **Charge** 2 **Spectral Angle** 0.88

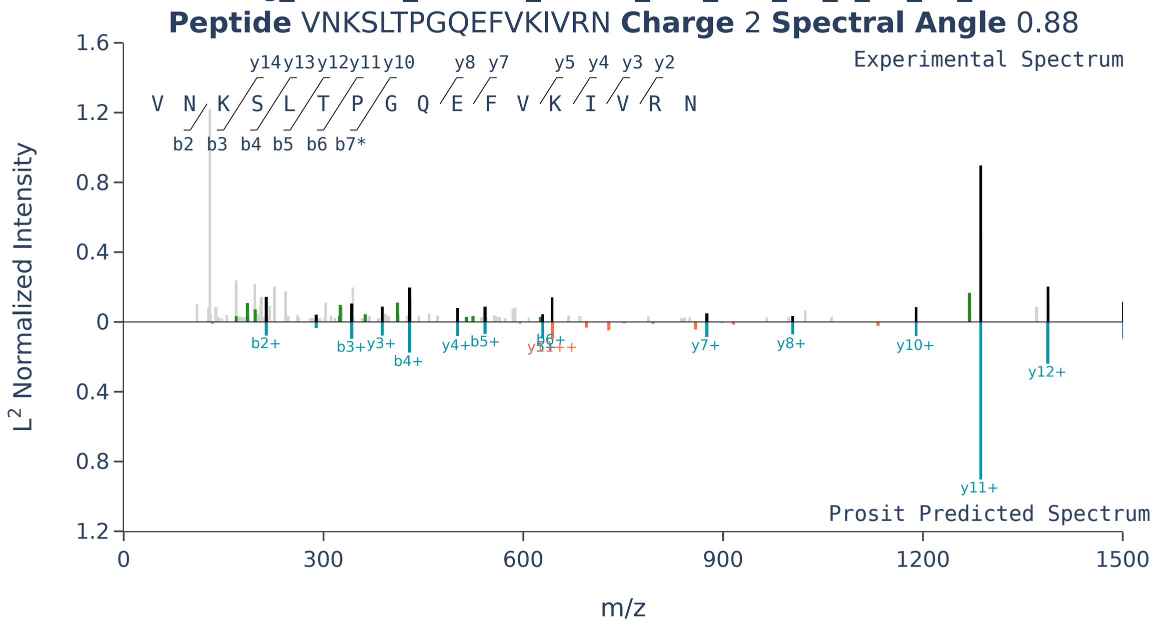

**Source** Ncheng_210623_230623_HFGoe_FFH_20S_25_1_A2_24h_R2 **Scan** 15923
**Peptide** AGLQGAGKTTSVGKLGKF **Charge** 2 **Spectral Angle** 0.79

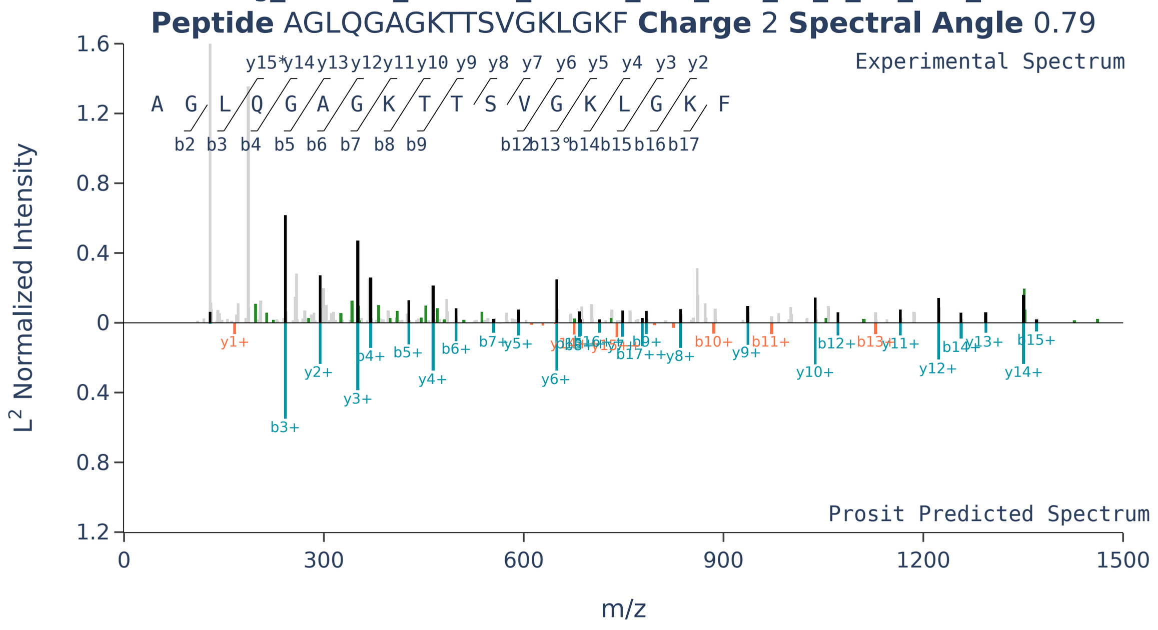


**Source** Ncheng_210623_230623_HFGoe_FFH_20S_25_1_A1_24h_R2 **Scan** 12500
**Peptide** NISGRGRLTEDNVKDTLRE **Charge** 4 **Spectral Angle** 0.76

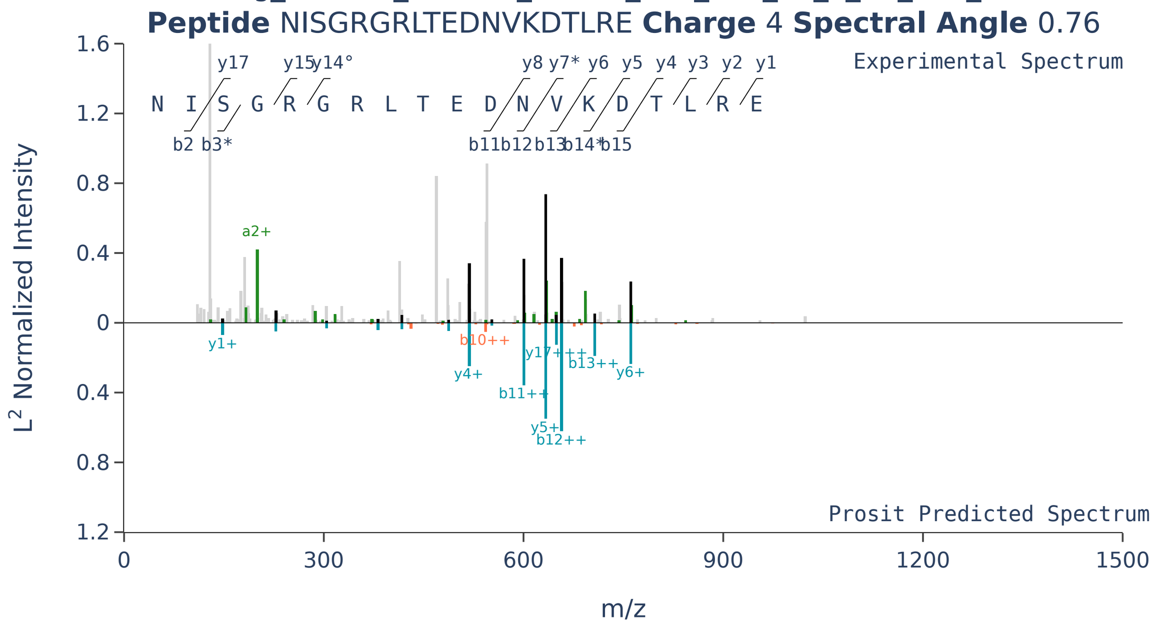


**Source** Ncheng_210623_230623_HFGoe_FFH_20S_25_1_A2_4h_R1 **Scan** 29151
**Peptide** IAAGCGMQVQDVNRLLKQFD **Charge** 3 **Spectral Angle** 0.81

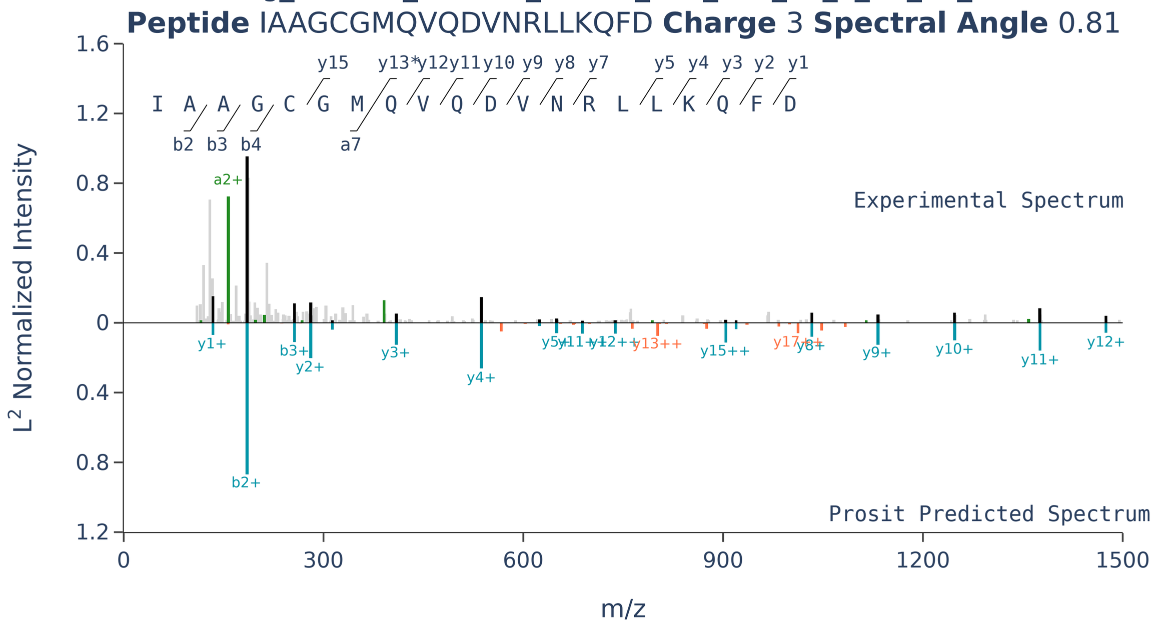


**Source** Ncheng_210623_230623_HFGoe_FFH_20S_25_1_A1_2h_R1 **Scan** 8140
**Peptide** EDNVKDTLRE **Charge** 3 **Spectral Angle** 0.82

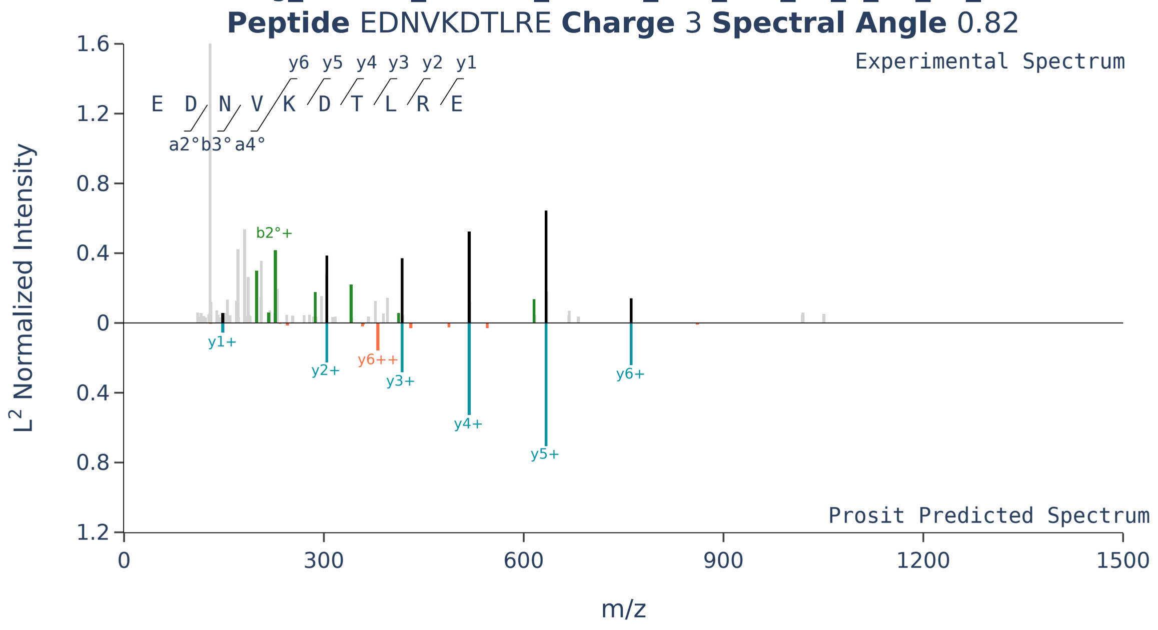


**Source** Ncheng_210623_230623_HFGoe_FFH_20S_25_1_A2_1h_R2 **Scan** 9182
**Peptide** RMEAIIN **Charge** 2 **Spectral Angle** 0.69

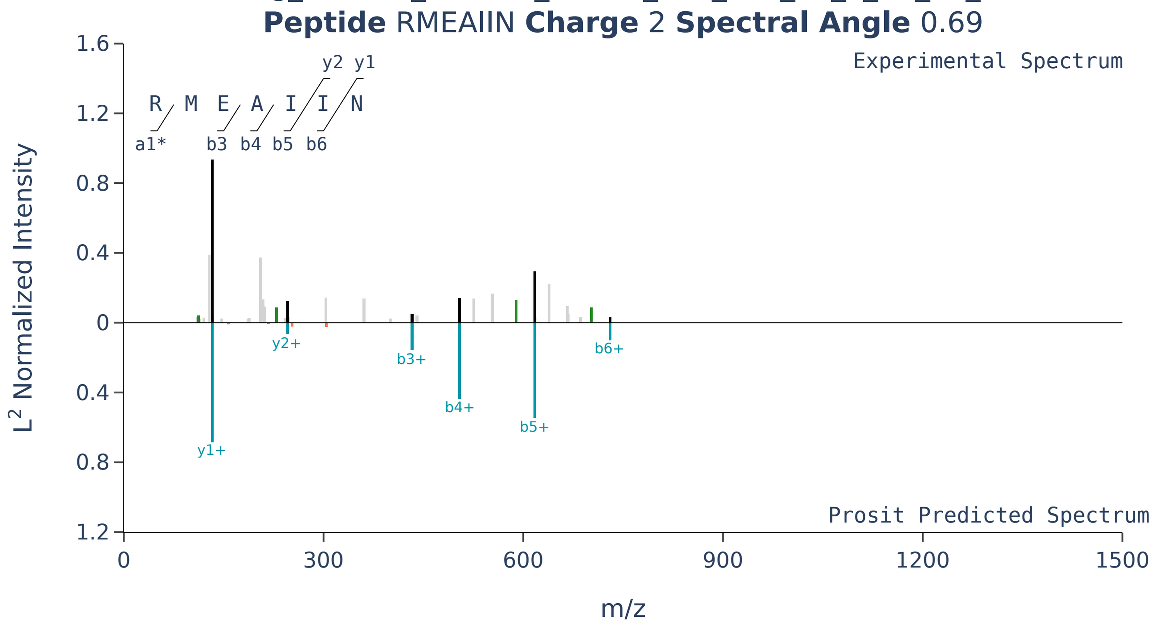


**Source** Ncheng_210623_230623_HFGoe_FFH_20S_25_1_A1_2h_R2 **Scan** 35139
**Peptide** AMTGQDAANTAKAFNEALPLTGVVLTKVD **Charge** 3 **Spectral Angle** 0.79

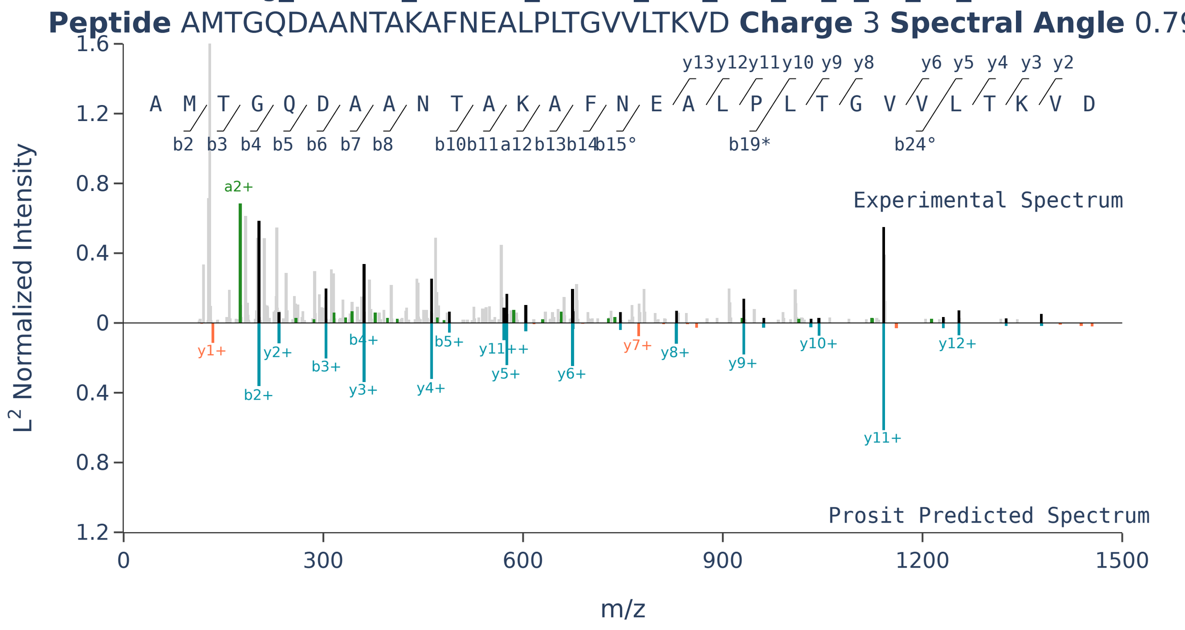


**Source** Ncheng_210623_230623_HFGoe_FFH_20S_25_1_A2_2h_R1 **Scan** 10325
**Peptide** PFHPDRLAS **Charge** 2 **Spectral Angle** 0.83

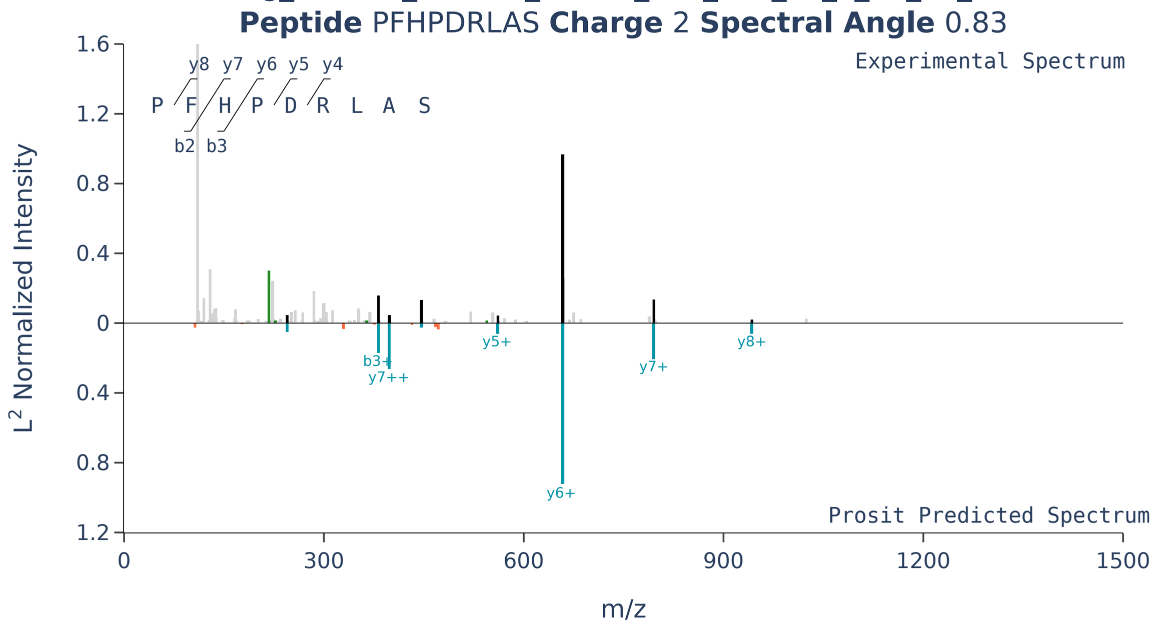


**Source** Ncheng_210623_230623_HFGoe_FFH_20S_25_1_A1_4h_R2 **Scan** 28444
**Peptide** GHEVNKSLTPGQEFVKIVRNEL **Charge** 3 **Spectral Angle** 0.84

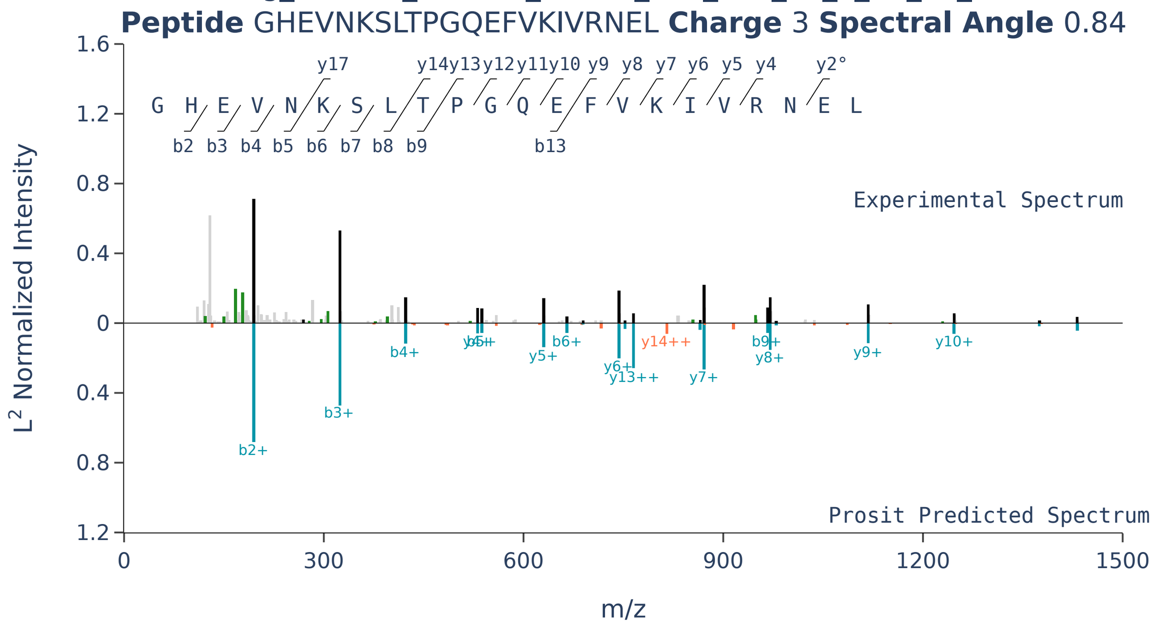


**Source** Ncheng_210623_230623_HFGoe_FFH_20S_25_1_A2_24h_R2 **Scan** 15341
**Peptide** TEDNVKDTLREV **Charge** 2 **Spectral Angle** 0.77

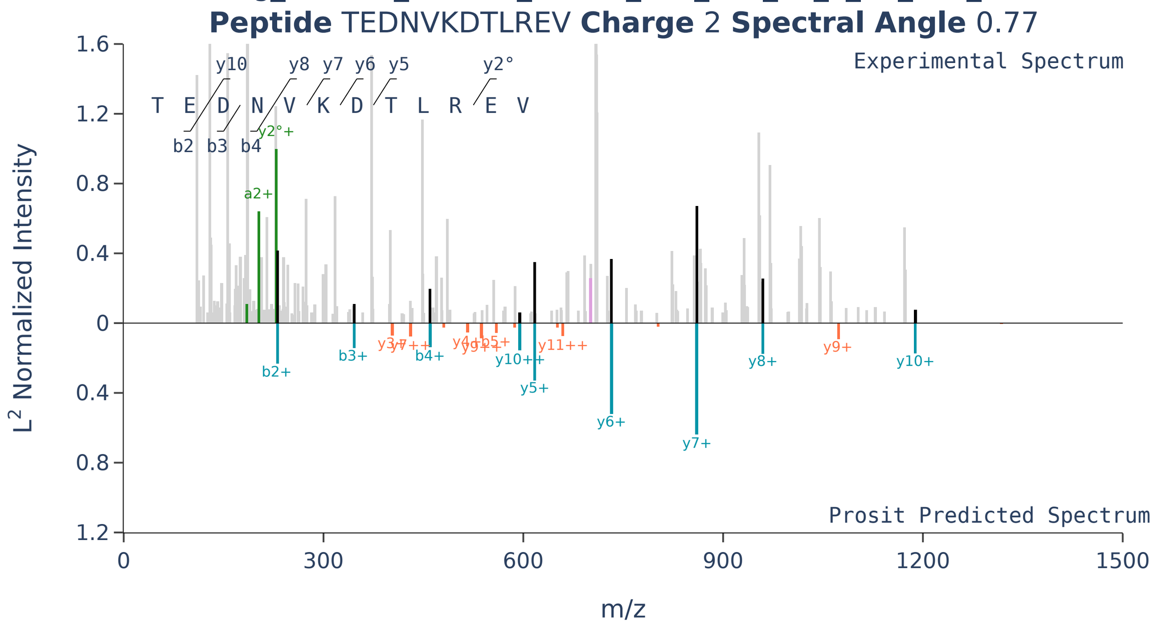


**Source** Ncheng_210623_230623_HFGoe_FFH_20S_25_1_A2_24h_R1 **Scan** 6770
**Peptide** SRTLRNISGR **Charge** 3 **Spectral Angle** 0.75

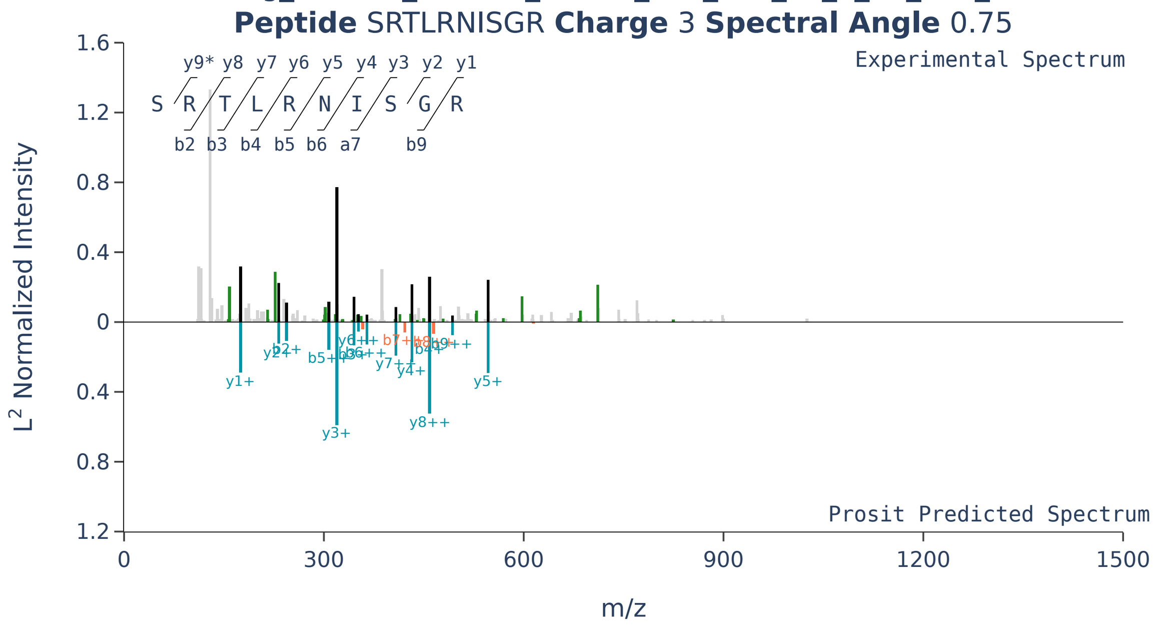


**Source** Ncheng_210623_230623_HFGoe_FFH_20S_25_1_A2_2h_R2 **Scan** 22195
**Peptide** EPFHPDRIASRLL **Charge** 3 **Spectral Angle** 0.9

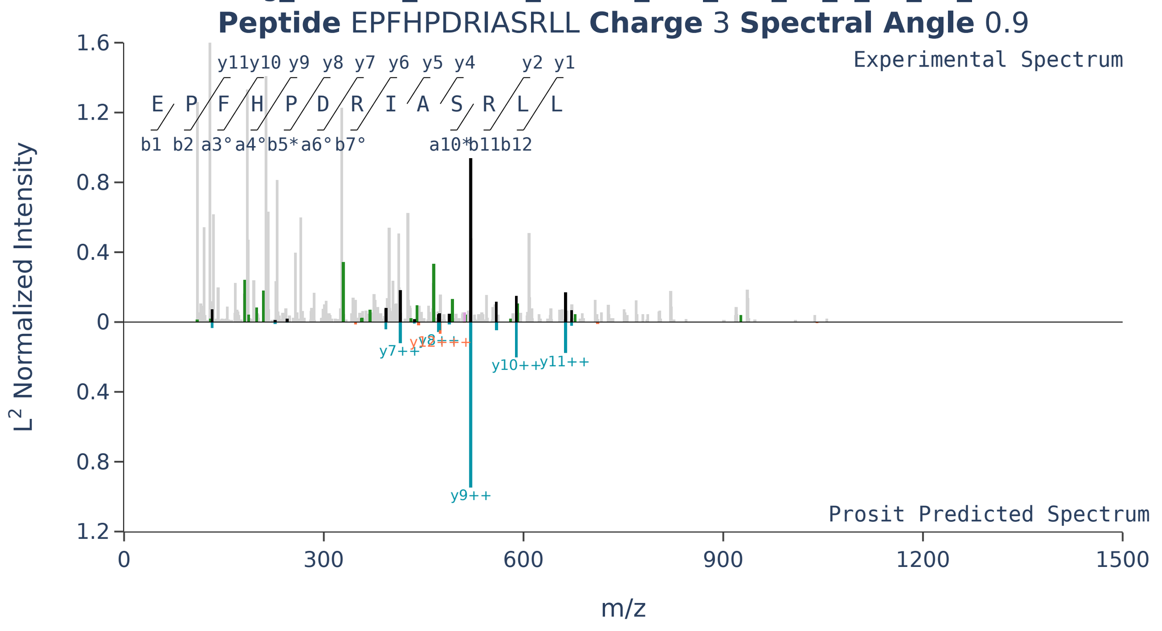


**Source** Ncheng_210623_230623_HFGoe_FFH_20S_25_1_A1_4h_R1 **Scan** 7321
**Peptide** TAGRLHVDE **Charge** 2 **Spectral Angle** 0.73

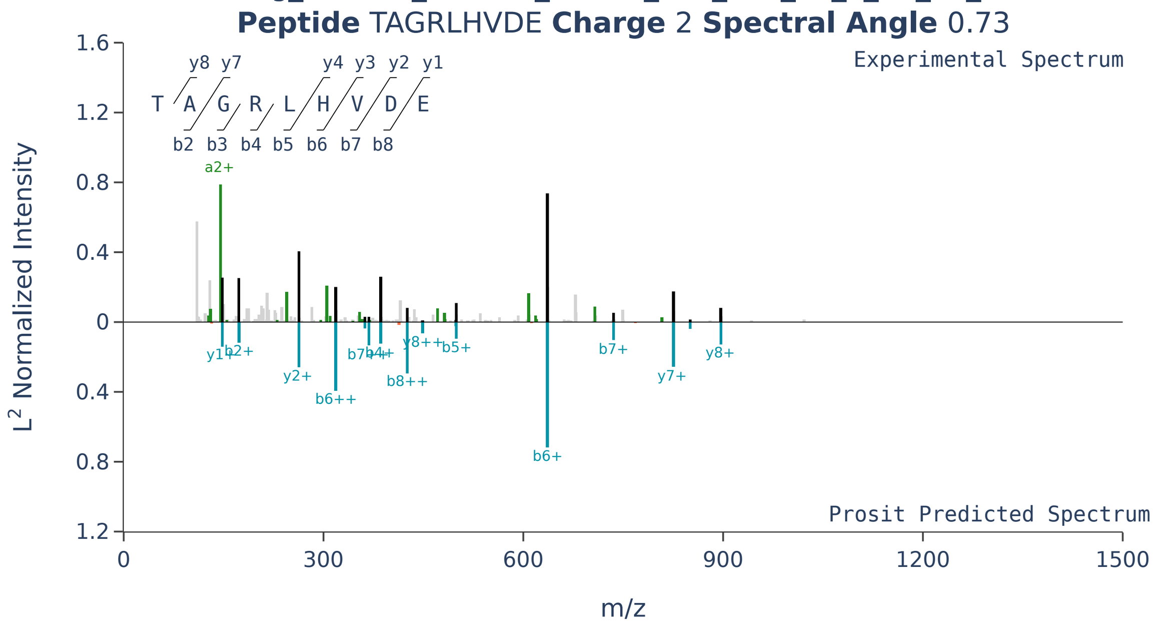


**Source** Ncheng_210623_230623_HFGoe_FFH_20S_25_1_A1_2h_R1 **Scan** 8637
**Peptide** AGCGMQVQD **Charge** 1 **Spectral Angle** 0.67

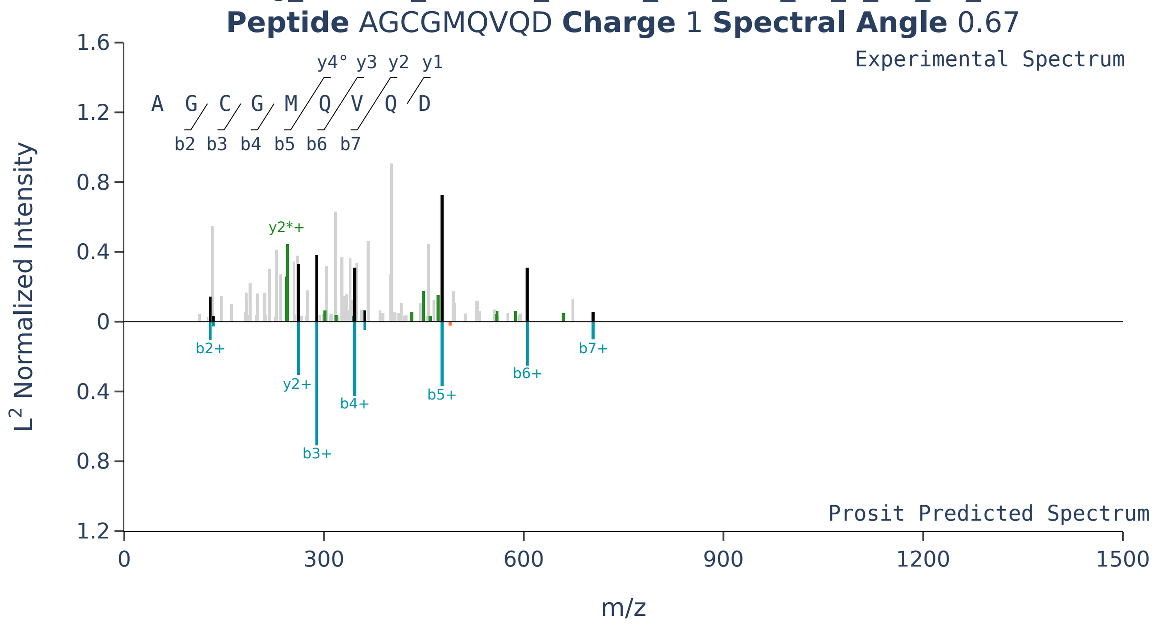


**Source** Ncheng_210623_230623_HFGoe_FFH_20S_25_1_A2_24h_R2 **Scan** 15305
**Peptide** AAGCGMQVQDV **Charge** 2 **Spectral Angle** 0.81

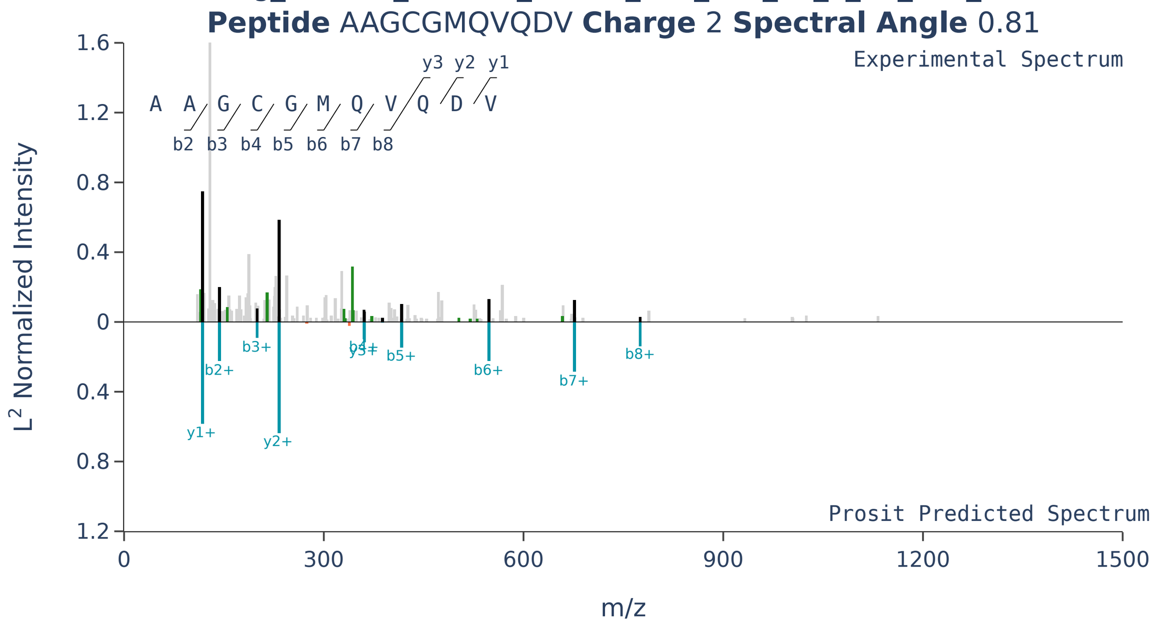


**Source** Ncheng_210623_230623_HFGoe_FFH_20S_25_1_A2_24h_R2 **Scan** 18079
**Peptide** GKTTSVGKLGKFL **Charge** 2 **Spectral Angle** 0.75

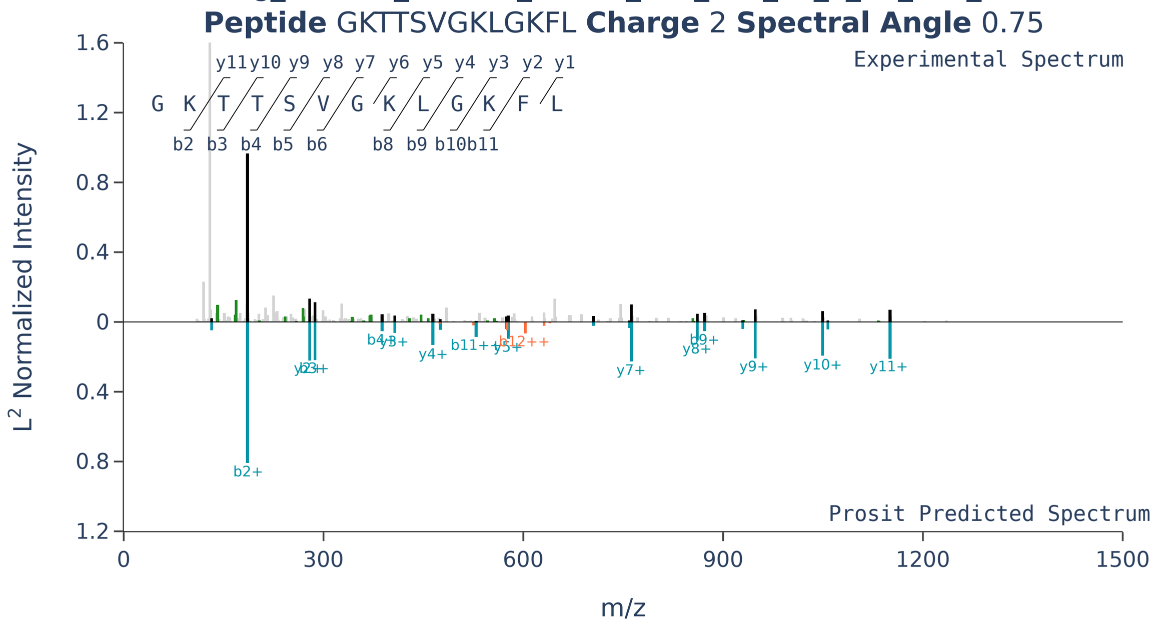

**Source** Ncheng_210623_230623_HFGoe_FFH_20S_25_1_A1_1h_R2 **Scan** 13265
**Peptide** KAFNEAL **Charge** 2 **Spectral Angle** 0.77

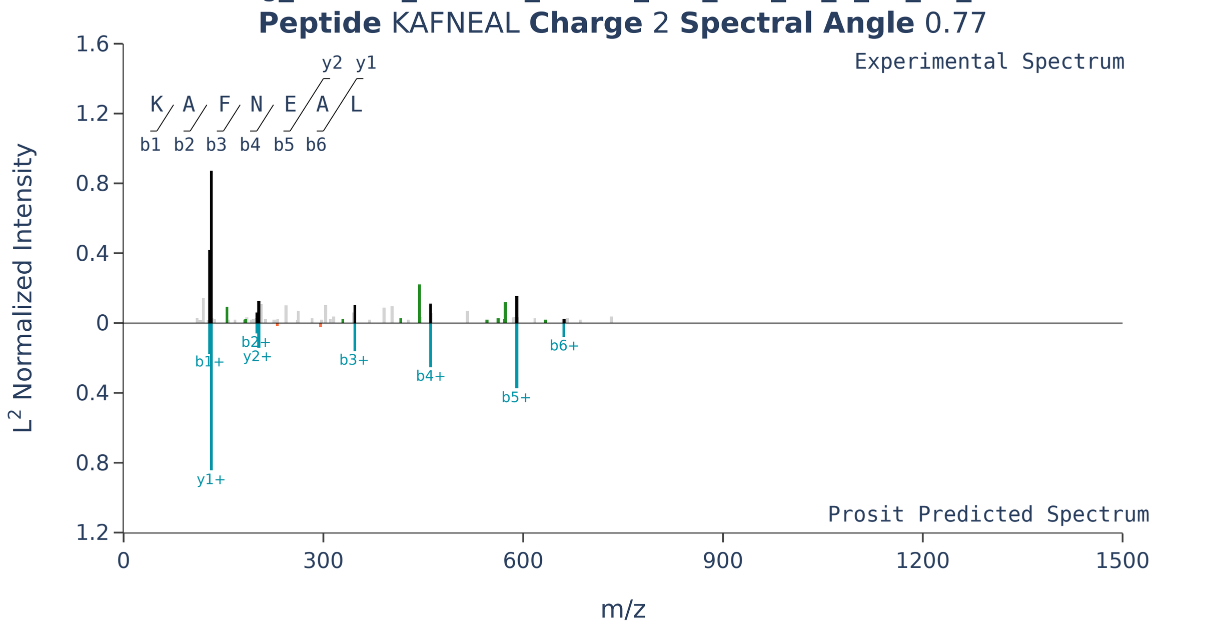


**Source** Ncheng_210623_230623_HFGoe_FFH_20S_25_1_A2_4h_R1 **Scan** 18519
**Peptide** KEKAVGHEVNKSLTPGQEFVKIVRNE **Charge** 3 **Spectral Angle** 0.81

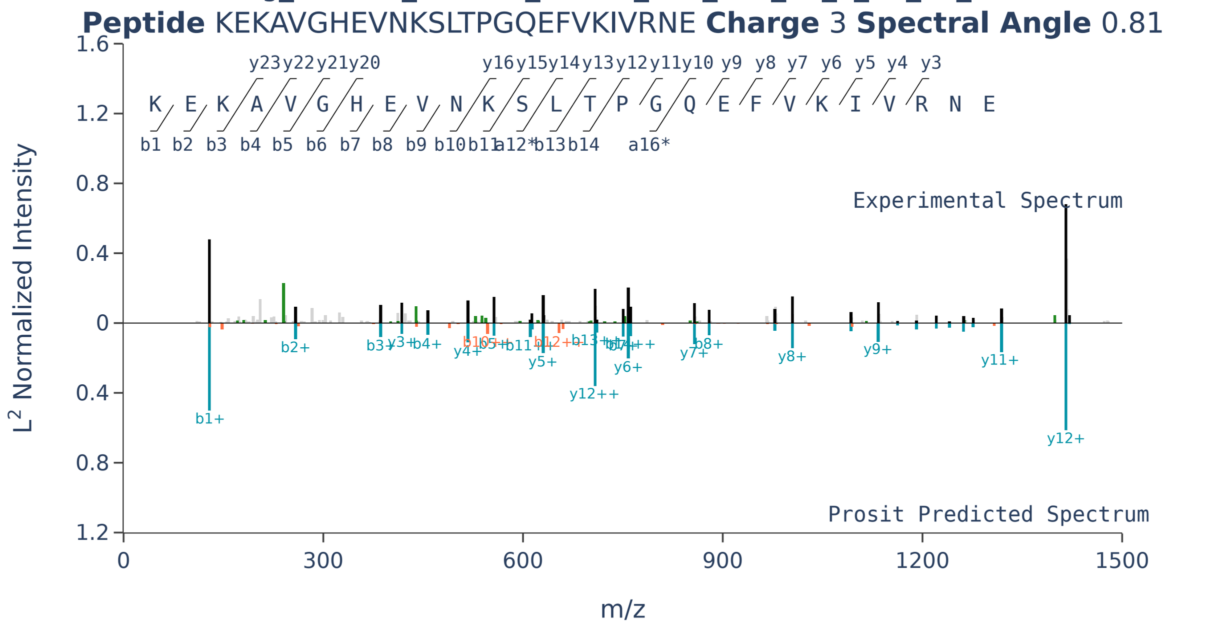


**Source** Ncheng_210623_230623_HFGoe_FFH_20S_25_1_A1_24h_R1 **Scan** 10553
**Peptide** IEDIESKVDR **Charge** 2 **Spectral Angle** 0.72

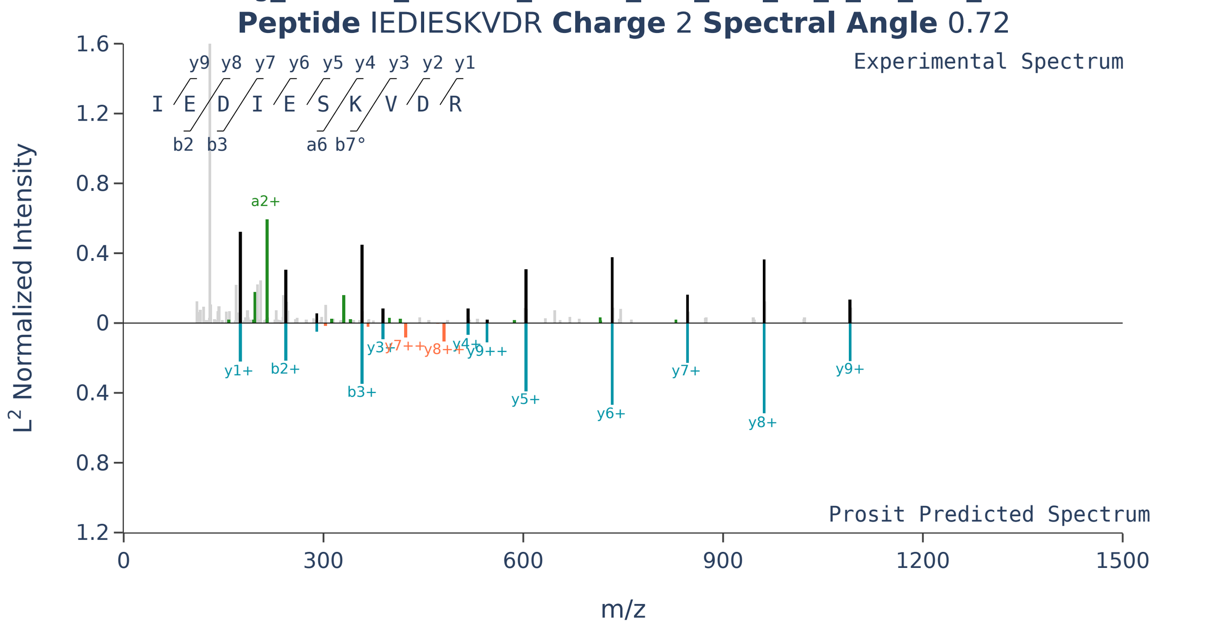


**Source** Ncheng_210623_230623_HFGoe_FFH_20S_25_1_A1_4h_R1 **Scan** 11837
**Peptide** QIPDNVKSQMD **Charge** 1 **Spectral Angle** 0.7

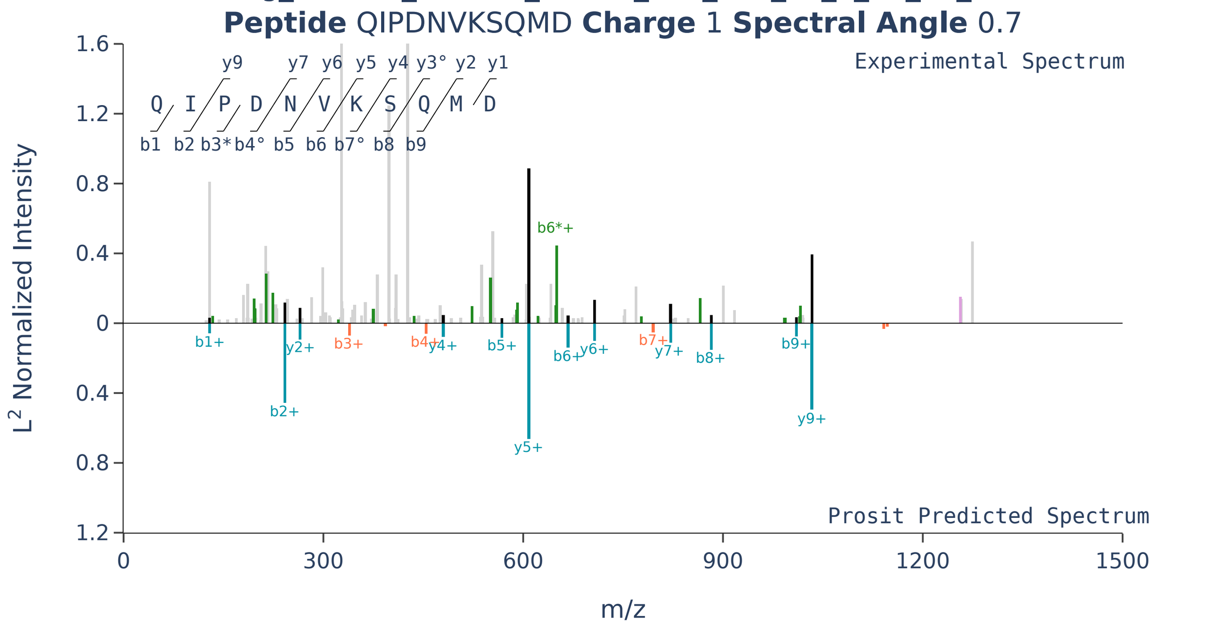


**Source** Ncheng_210623_230623_HFGoe_FFH_20S_25_1_A2_24h_R1 **Scan** 13219
**Peptide** DNLTDRLSRT **Charge** 3 **Spectral Angle** 0.61

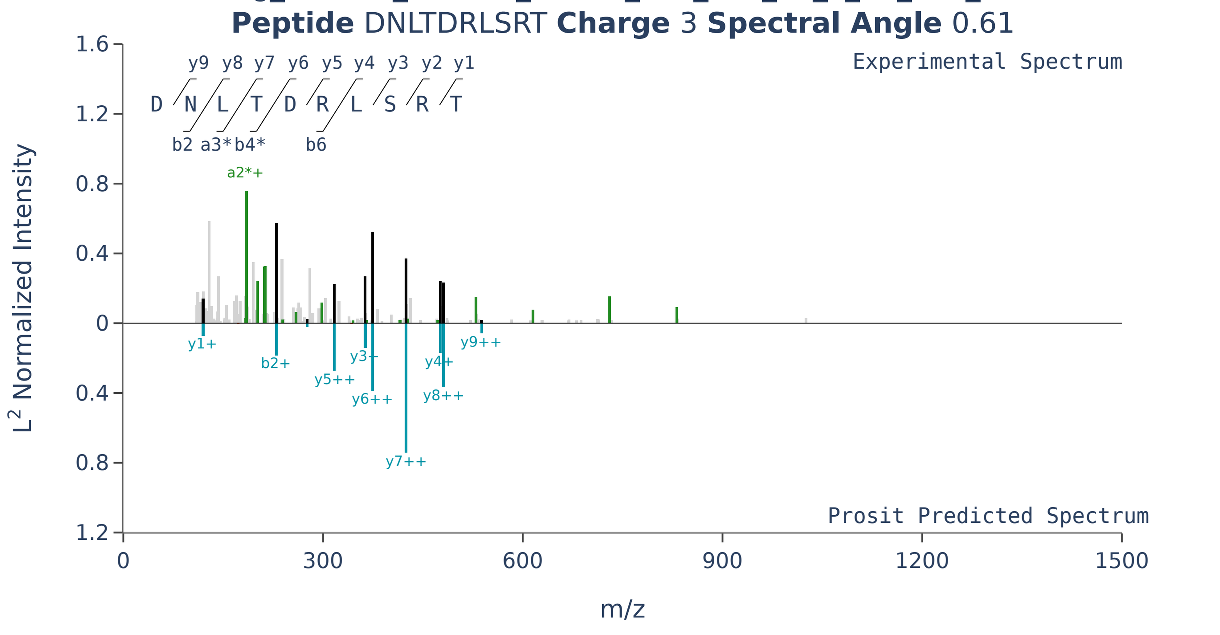


**Source** Ncheng_210623_230623_HFGoe_FFH_20S_25_1_A1_4h_R1 **Scan** 11677
**Peptide** VKDTLREVRM **Charge** 3 **Spectral Angle** 0.62

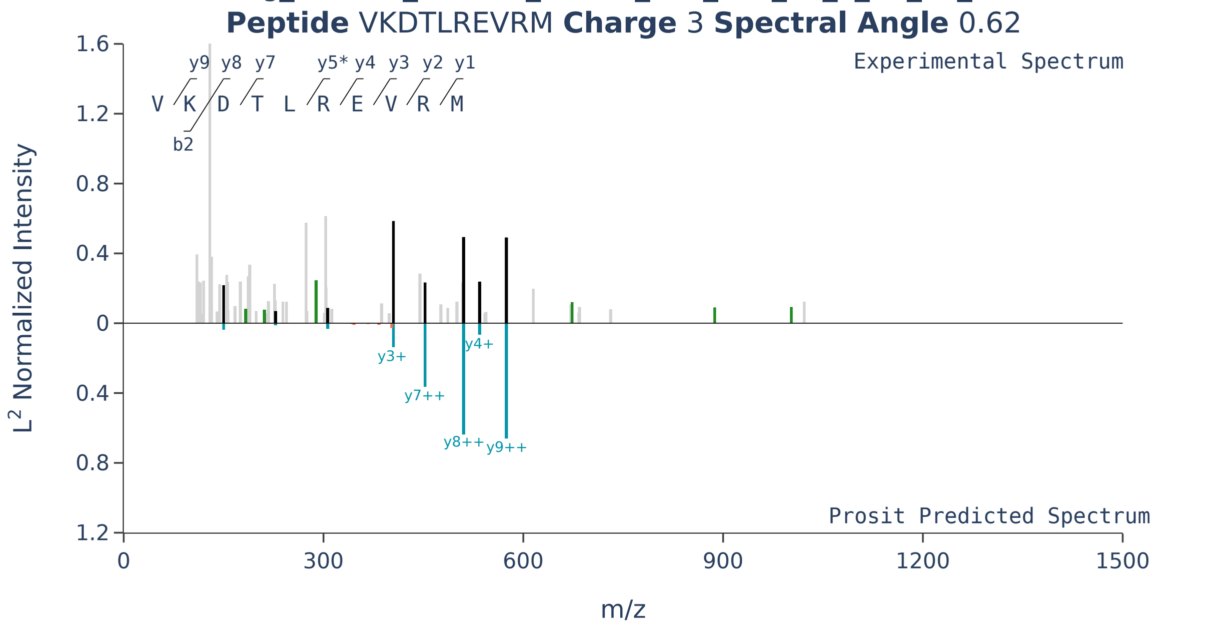


**Source** Ncheng_210623_230623_HFGoe_FFH_20S_25_1_A1_24h_R2 **Scan** 7305
**Peptide** GHEVNKSLTPGQ **Charge** 2 **Spectral Angle** 0.78

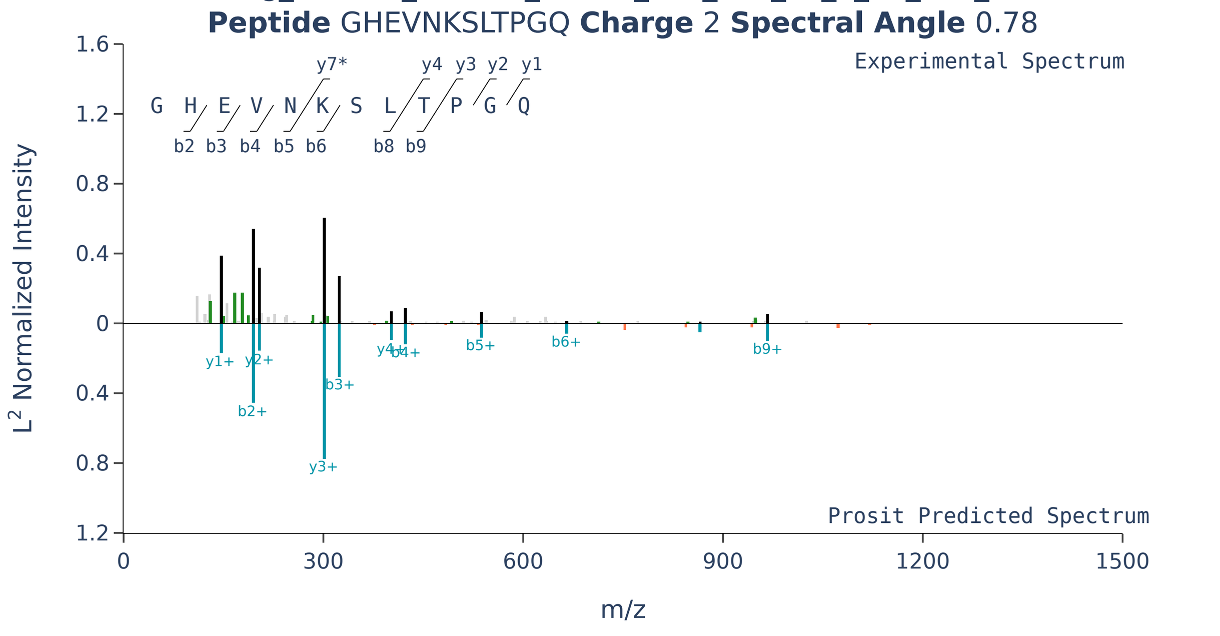


**Source** Ncheng_210623_230623_HFGoe_FFH_20S_25_1_A2_24h_R2 **Scan** 13577
**Peptide** LVDTAGRLHVD **Charge** 3 **Spectral Angle** 0.78

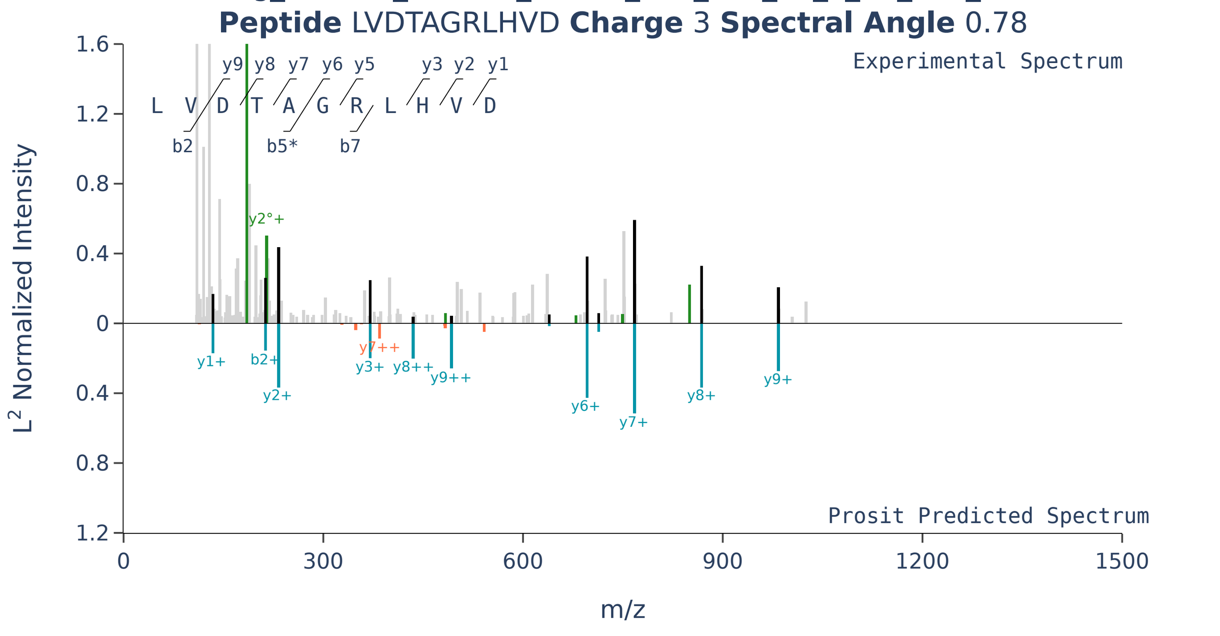


**Source** Ncheng_210623_230623_HFGoe_FFH_20S_25_1_A2_24h_R2 **Scan** 5326
**Peptide** RPAAIKQ **Charge** 2 **Spectral Angle** 0.65

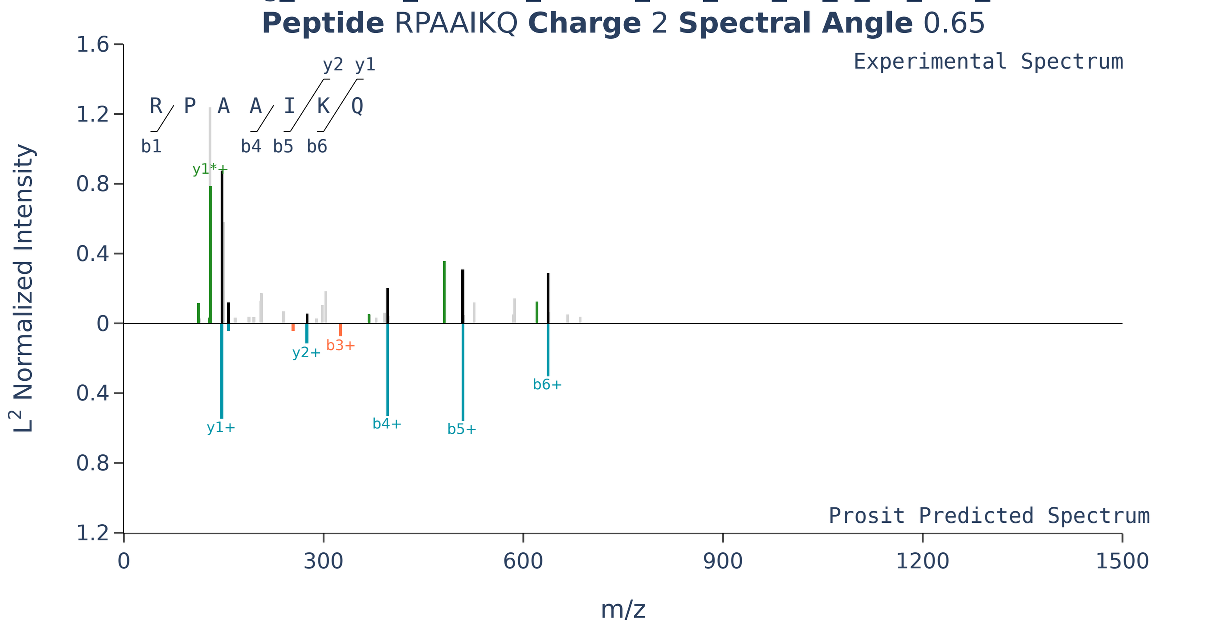


**Source** Ncheng_210623_230623_HFGoe_FFH_20S_25_1_A1_24h_R1 **Scan** 17268
**Peptide** RHITGKPIKFLGV **Charge** 4 **Spectral Angle** 0.69

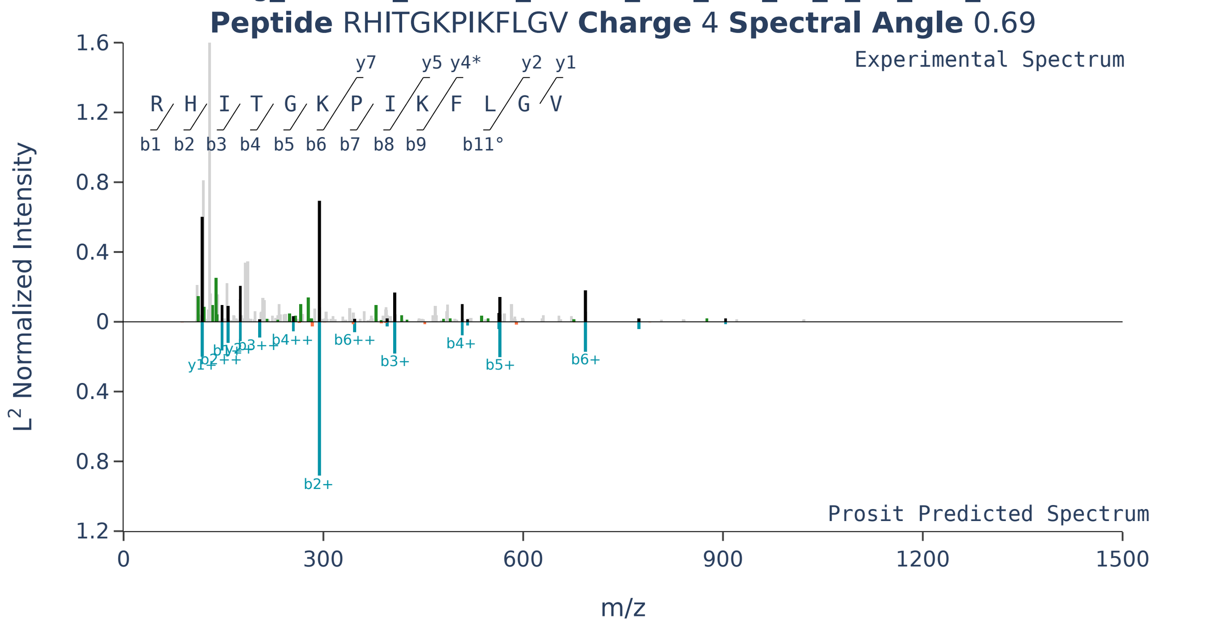


**Source** Ncheng_210623_230623_HFGoe_FFH_20S_25_1_A1_4h_R1 **Scan** 21419
**Peptide** GAALSIRHITGKPIKFLGVGEKTE **Charge** 3 **Spectral Angle** 0.82

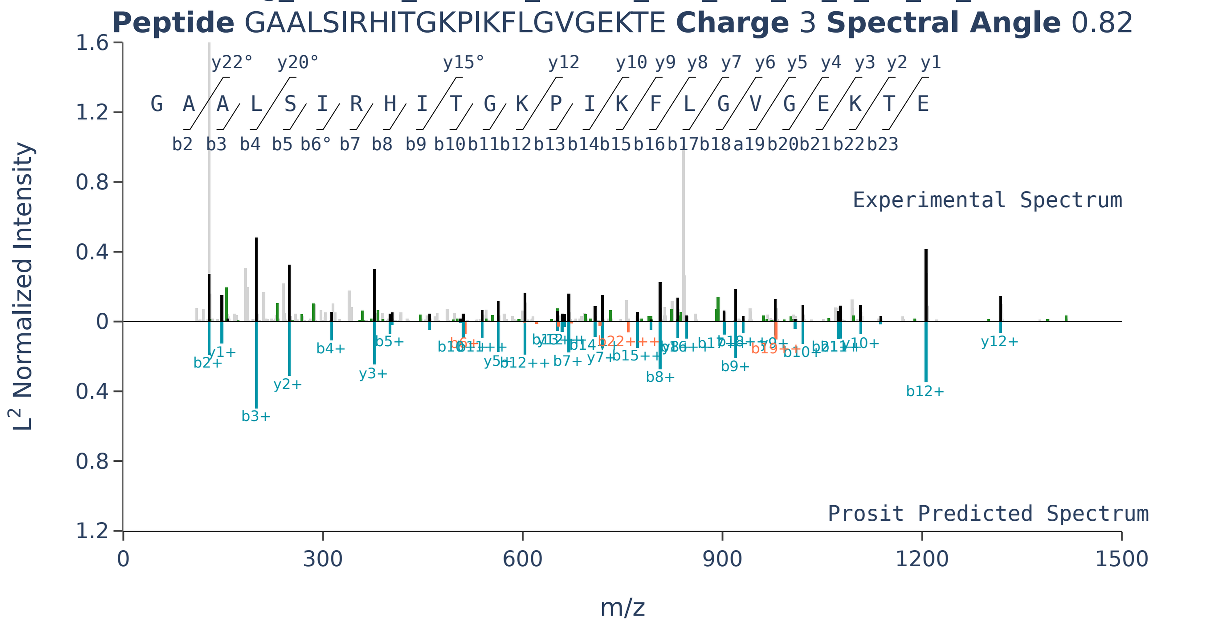


**Source** Ncheng_210623_230623_HFGoe_FFH_20S_25_1_A1_4h_R2 **Scan** 13921
**Peptide** RLHVDEAMMD **Charge** 2 **Spectral Angle** 0.75

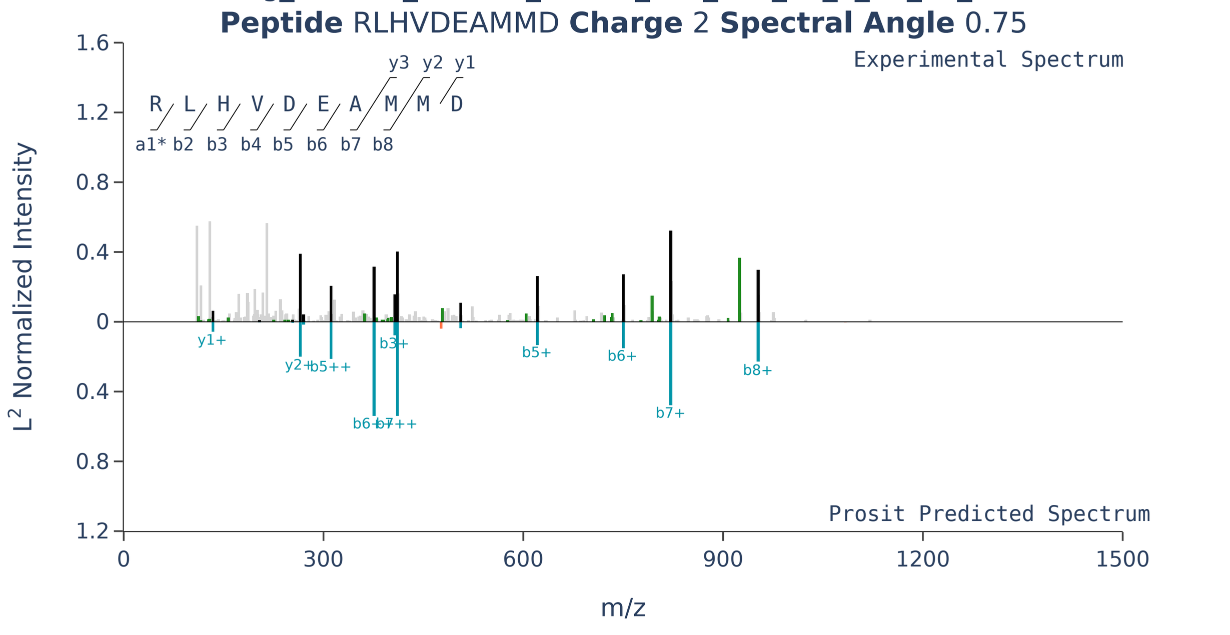


**Source** Ncheng_210623_230623_HFGoe_FFH_20S_25_1_A2_4h_R1 **Scan** 17496
**Peptide** KAVGHEVNKSLTPGQEFVKIVRN **Charge** 3 **Spectral Angle** 0.73

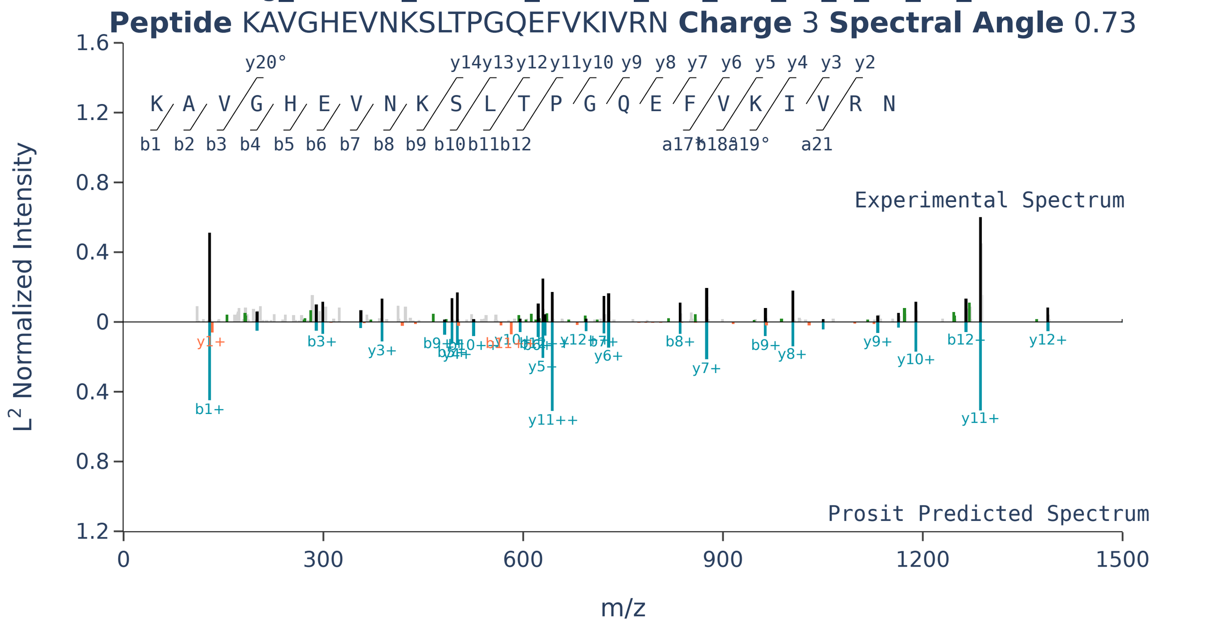


**Source** Ncheng_210623_230623_HFGoe_FFH_20S_25_1_A1_4h_R1 **Scan** 21651
**Peptide** RLAAGCGMQVQDVNRLLKQ **Charge** 4 **Spectral Angle** 0.79

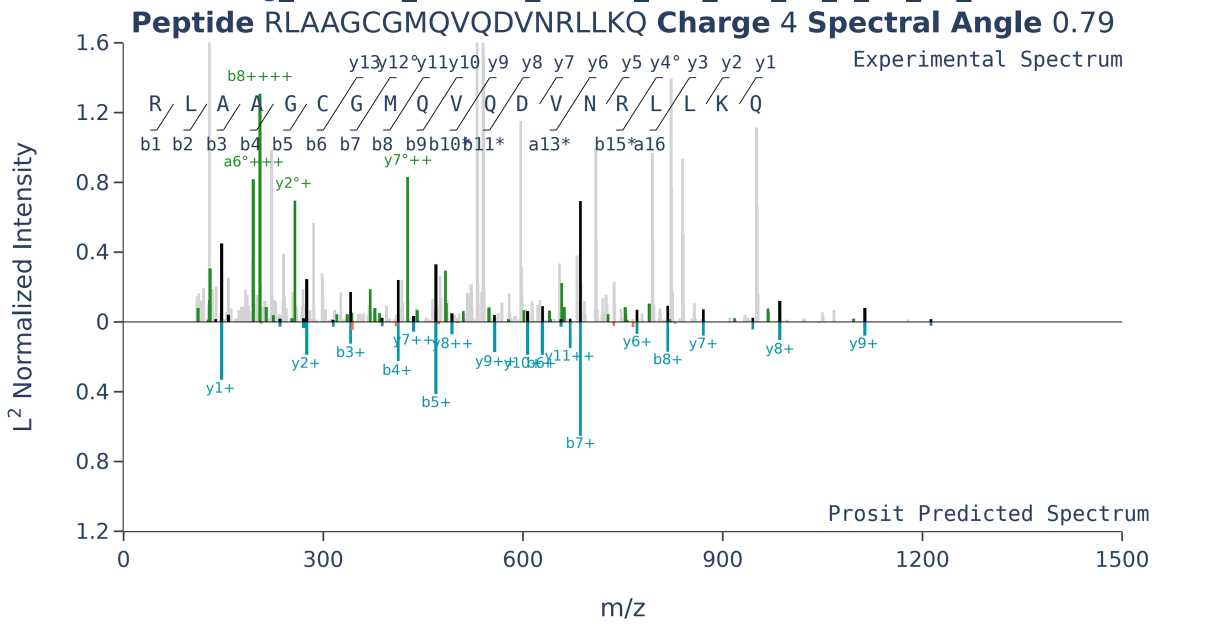


**Source** Ncheng_210623_230623_HFGoe_FFH_20S_25_1_A2_2h_R1 **Scan** 27049
**Peptide** LPGMGQIPDNVKSQMDDKVLVRME **Charge** 3 **Spectral Angle** 0.71

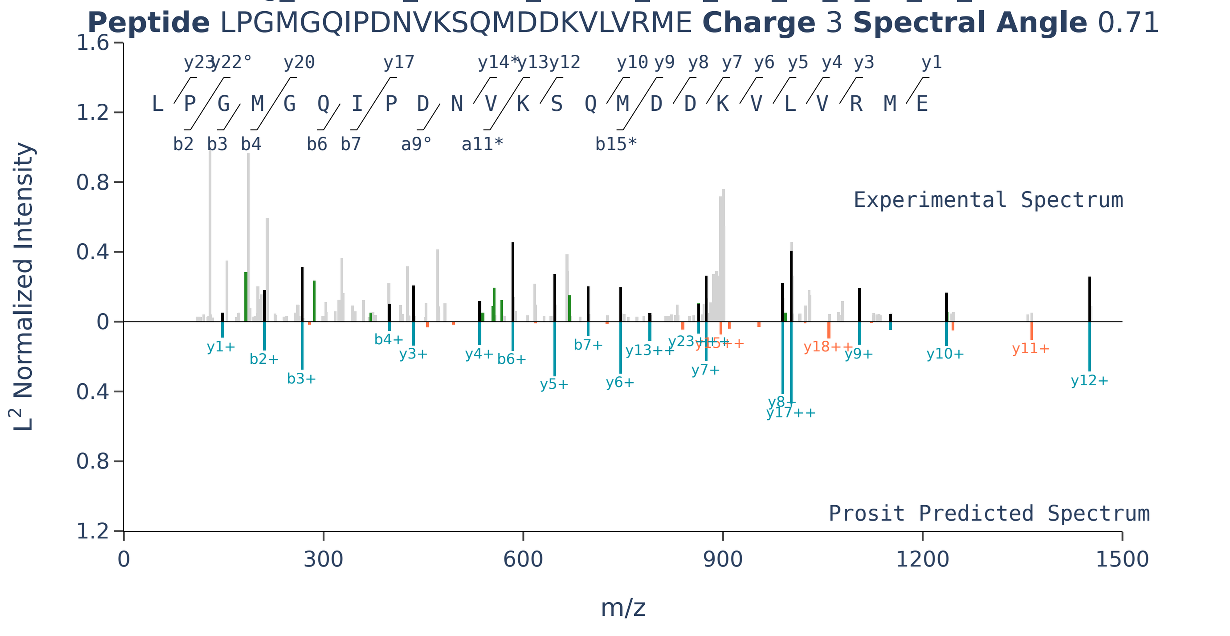

**Source** Ncheng_210623_230623_HFGoe_FFH_20S_25_1_A2_4h_R2 **Scan** 34034
**Peptide** GEENQTLNLAAQPPAVVLMAGLQGAGKTT **Charge** 3 **Spectral Angle** 0.78

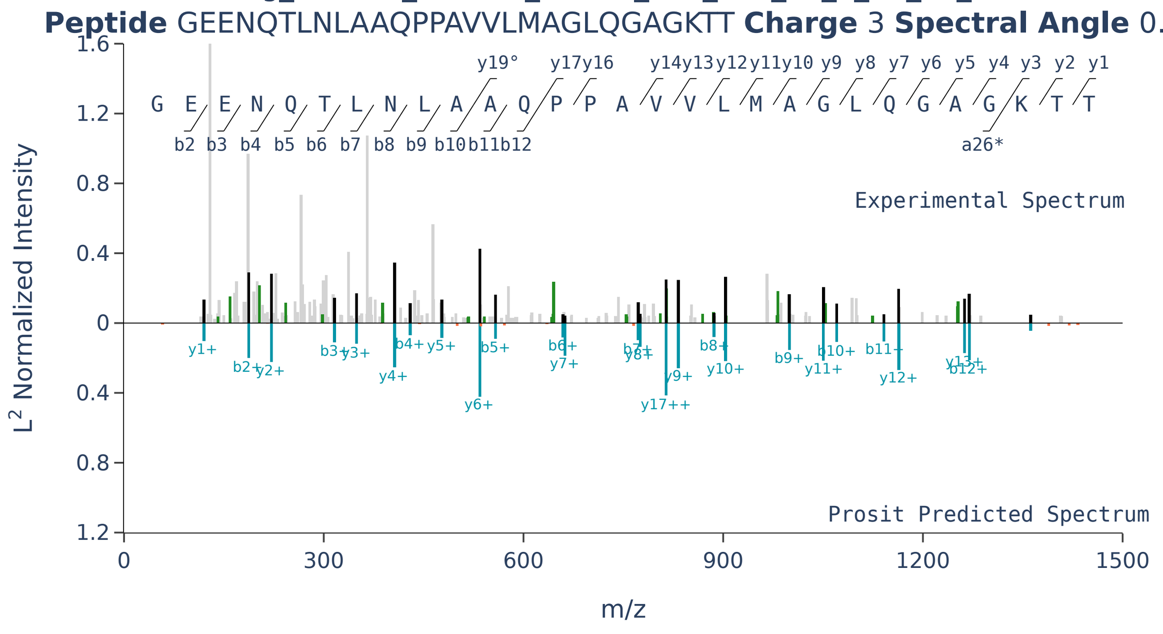


**Source** Ncheng_210623_230623_HFGoe_FFH_20S_25_1_A2_2h_R1 **Scan** 13893
**Peptide** PSDVGQKPVDI **Charge** 2 **Spectral Angle** 0.78

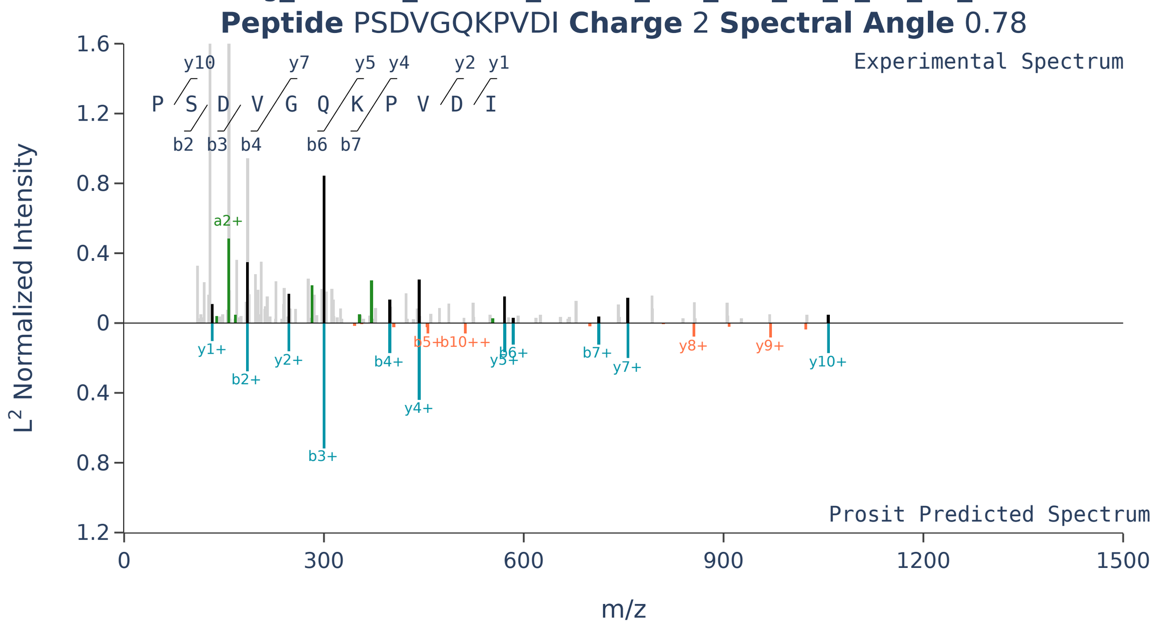


**Source** Ncheng_210623_230623_HFGoe_FFH_20S_25_1_A2_4h_R2 **Scan** 17689
**Peptide** RGRLTEDNVKDTLREVRM **Charge** 5 **Spectral Angle** 0.77

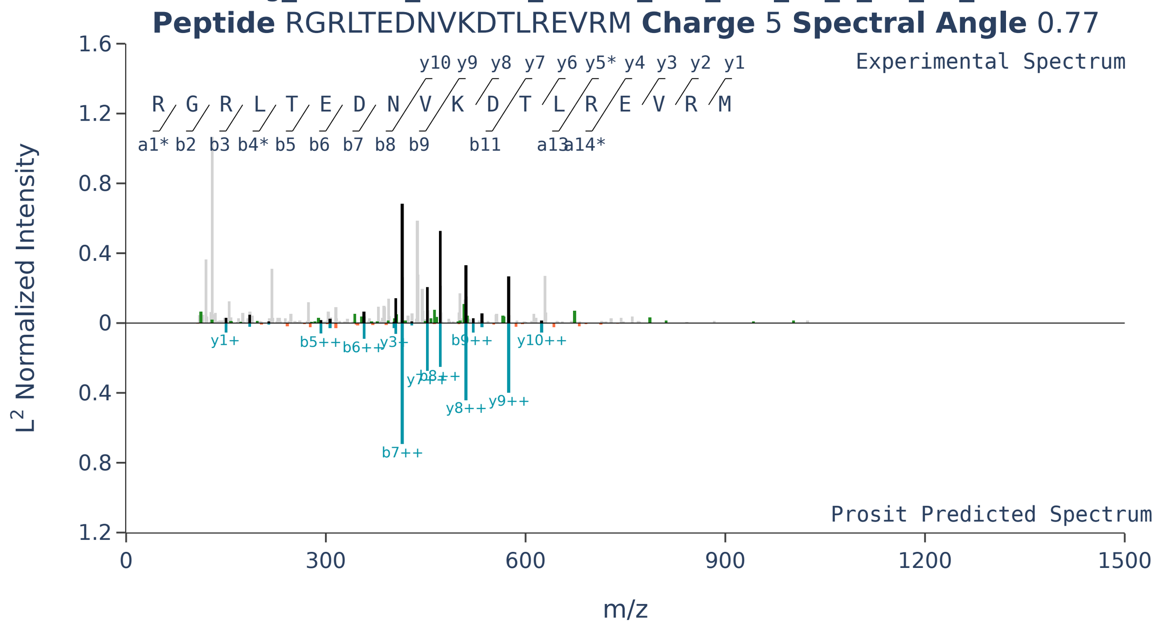


**Source** Ncheng_210623_230623_HFGoe_FFH_20S_25_1_A2_24h_R2 **Scan** 9992
**Peptide** SMTMKERAKPEIIKG **Charge** 2 **Spectral Angle** 0.75

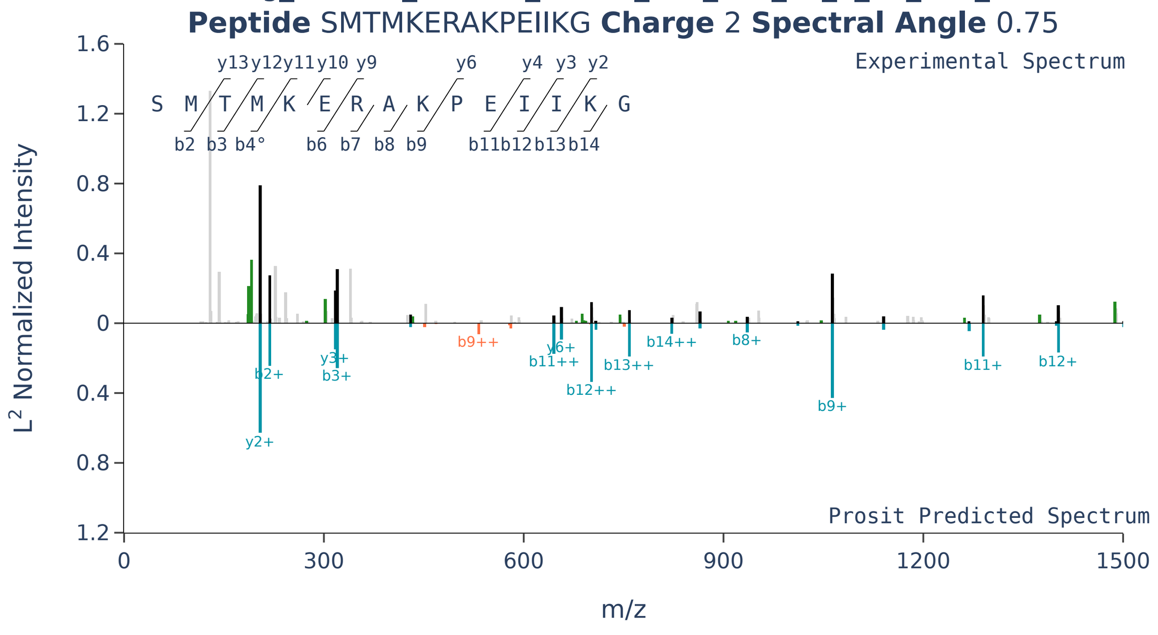


**Source** Ncheng_210623_230623_HFGoe_FFH_20S_25_1_A2_4h_R1 **Scan** 21782
**Peptide** PGMGQIPDNVKSQMDDKVLVR **Charge** 3 **Spectral Angle** 0.71

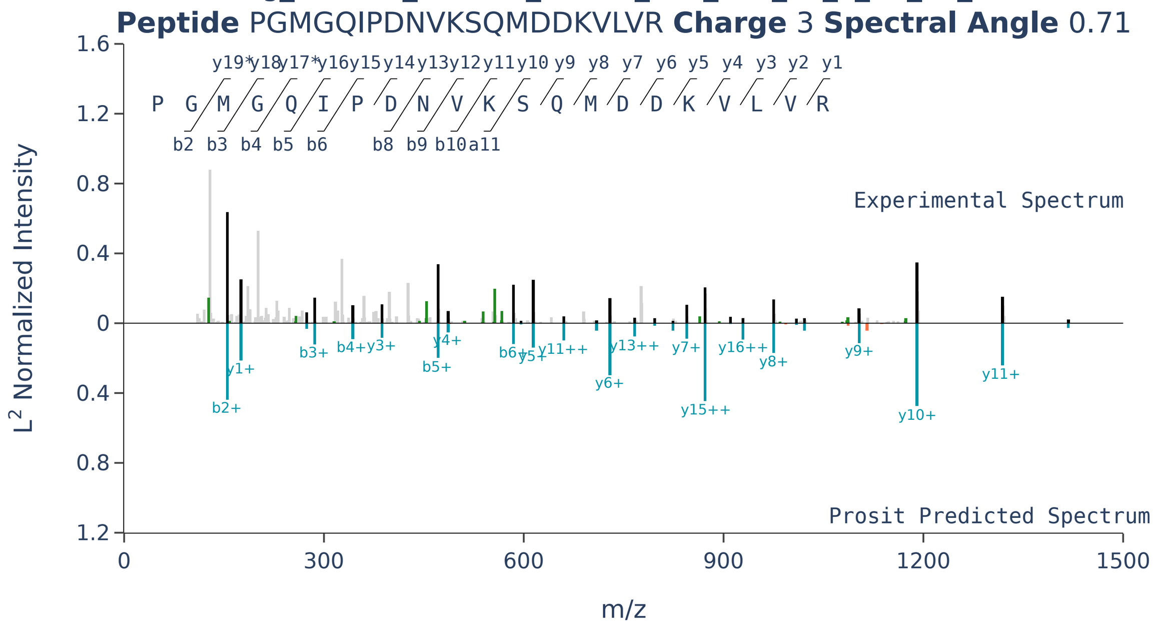


**Source** Ncheng_210623_230623_HFGoe_FFH_20S_25_1_A1_4h_R2 **Scan** 10120
**Peptide** KQVHASINPVE **Charge** 1 **Spectral Angle** 0.82

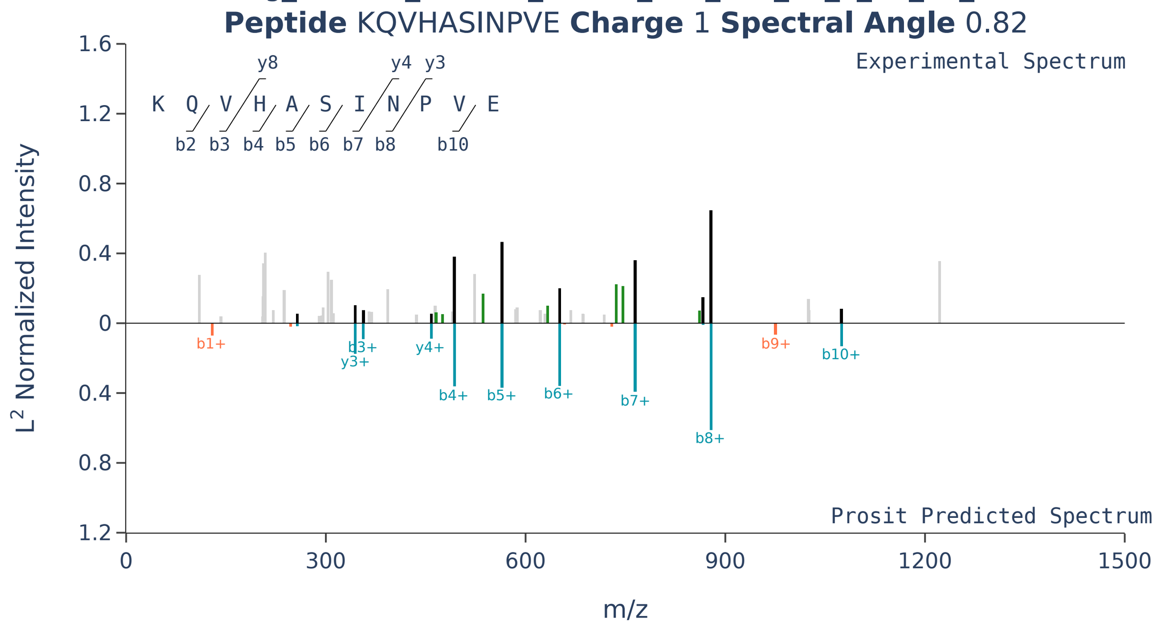


**Source** Ncheng_210623_230623_HFGoe_FFH_20S_25_1_A2_24h_R1 **Scan** 7546
**Peptide** AGRLHVDEA **Charge** 2 **Spectral Angle** 0.78

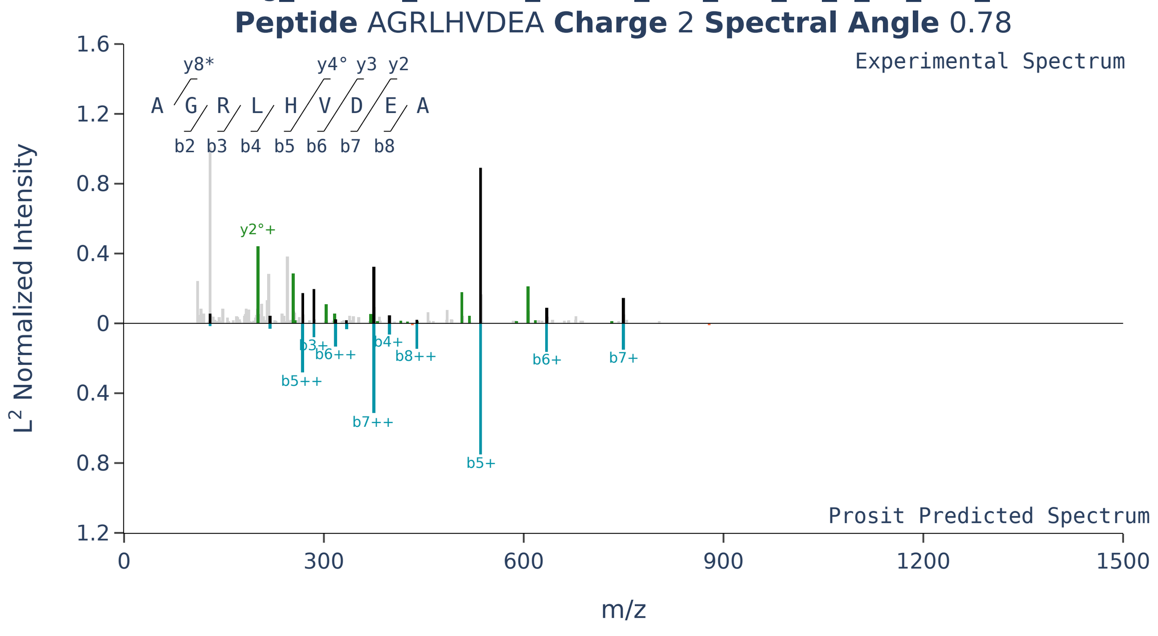


**Source** Ncheng_210623_230623_HFGoe_FFH_20S_25_1_A1_2h_R1 **Scan** 34206
**Peptide** FNEALPLTGVVLTKVDGDARGGAAL **Charge** 3 **Spectral Angle** 0.83

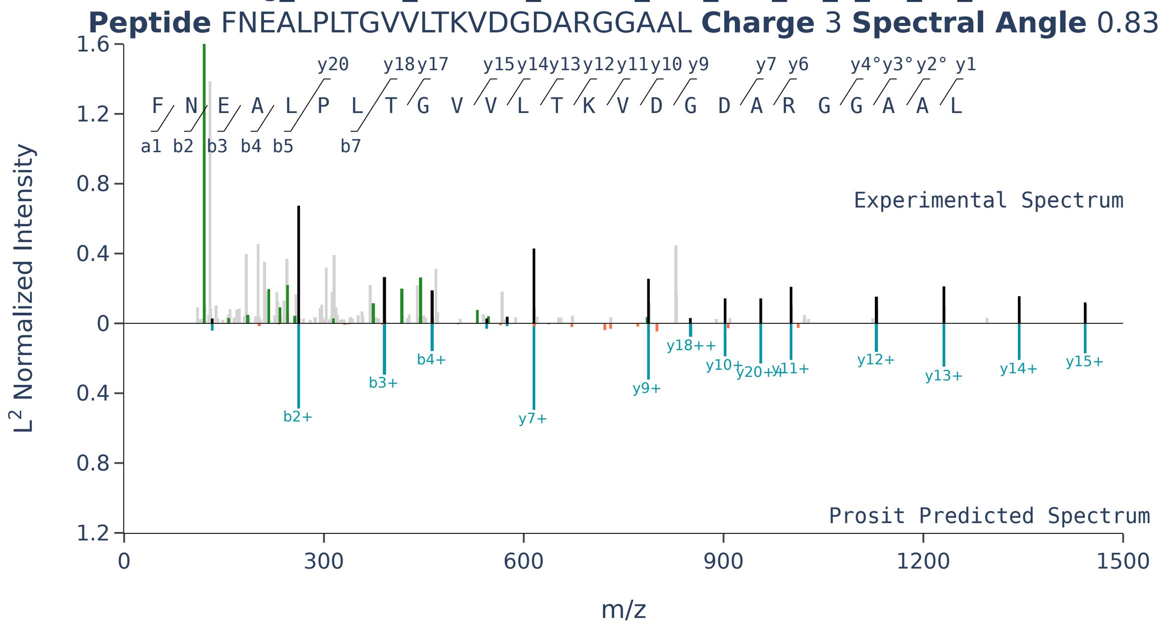


**Source** Ncheng_210623_230623_HFGoe_FFH_20S_25_1_A2_4h_R2 **Scan** 21288
**Peptide** VGQKPVDIVNAALK **Charge** 3 **Spectral Angle** 0.73

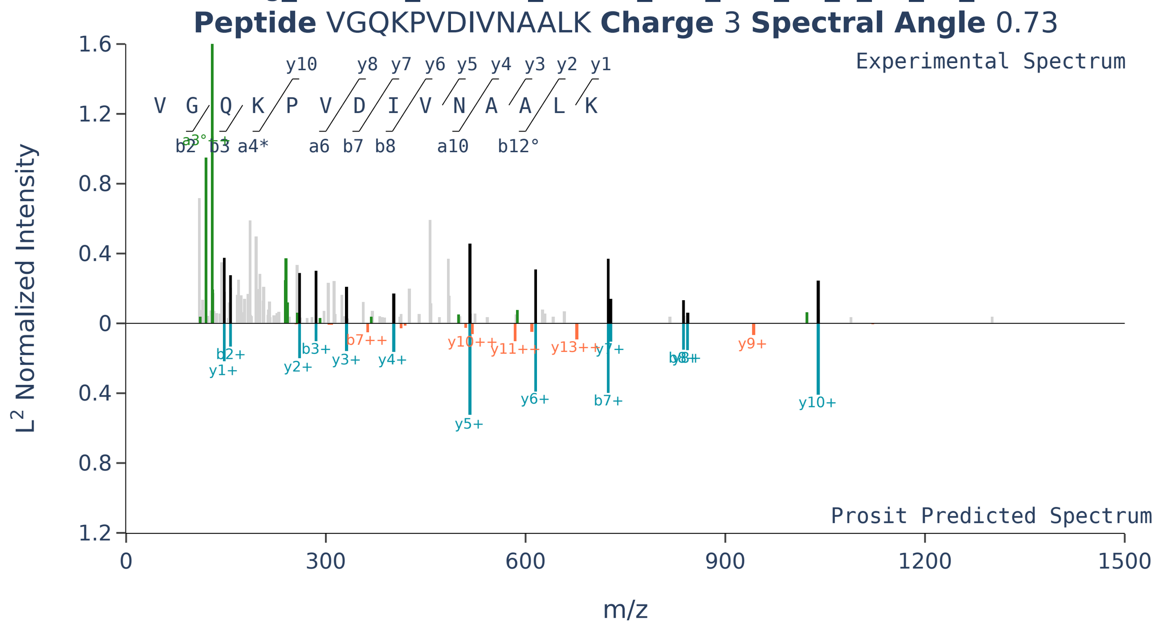


**Source** Ncheng_210623_230623_HFGoe_FFH_20S_25_1_A1_1h_R1 **Scan** 5935
**Peptide** ALKEAKLK **Charge** 2 **Spectral Angle** 0.77

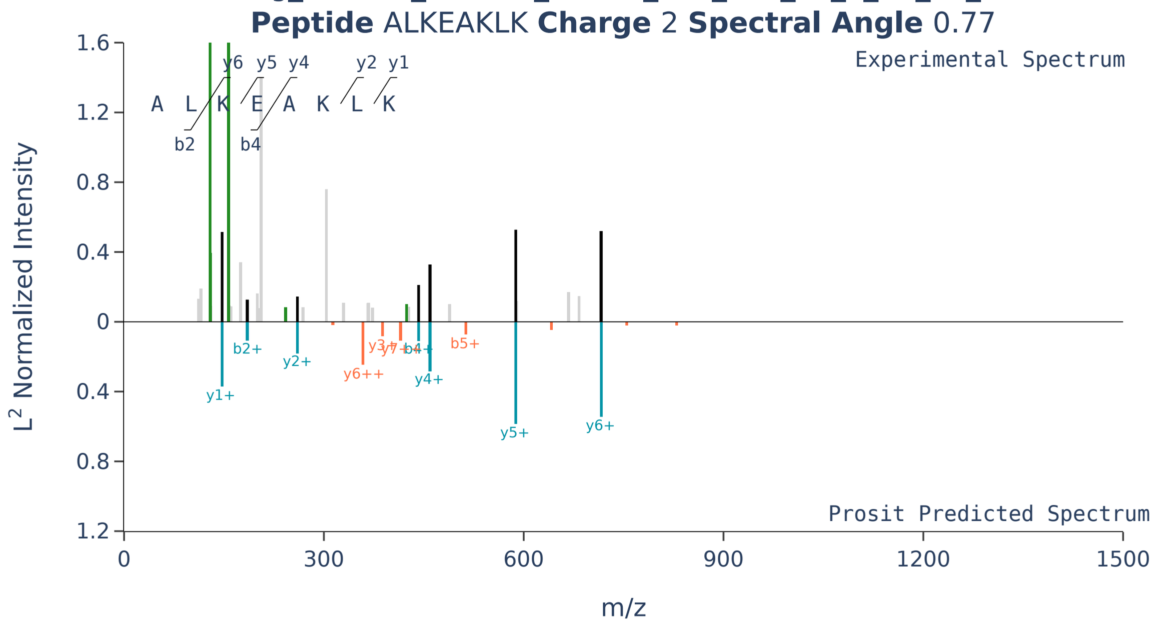


**Source** Ncheng_210623_230623_HFGoe_FFH_20S_25_1_A2_4h_R2 **Scan** 21837
**Peptide** KPIKFLGVGEKTEALEPFHPDRIASR **Charge** 4 **Spectral Angle** 0.8

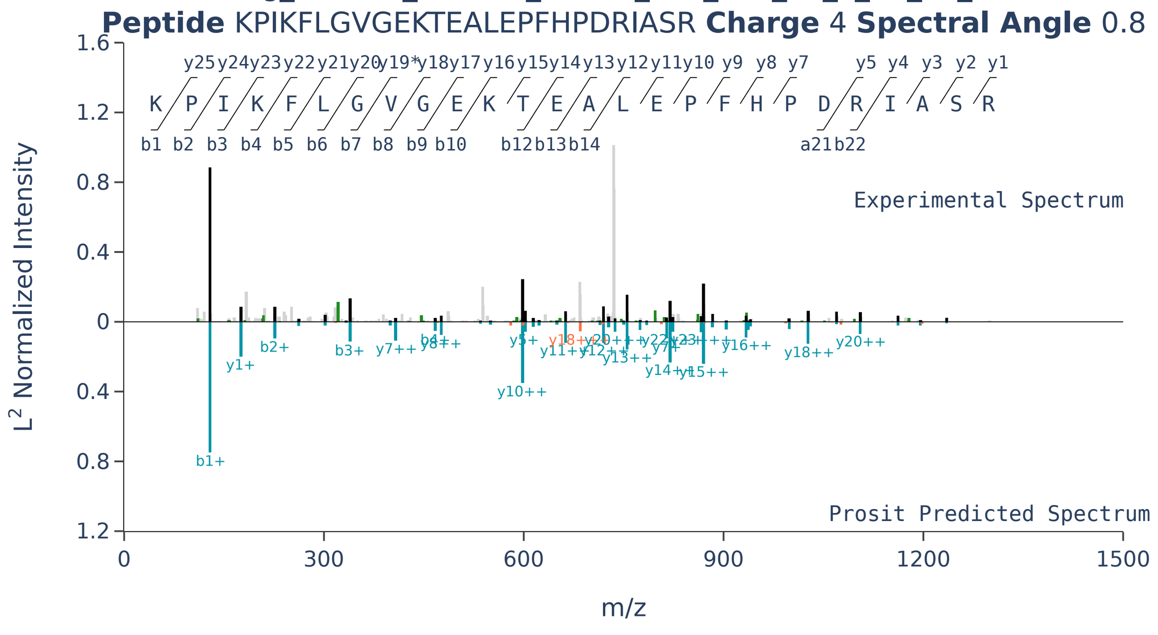


**Source** Ncheng_210623_230623_HFGoe_FFH_20S_25_1_A1_24h_R1 **Scan** 19828
**Peptide** SVGKLGKFL **Charge** 2 **Spectral Angle** 0.71

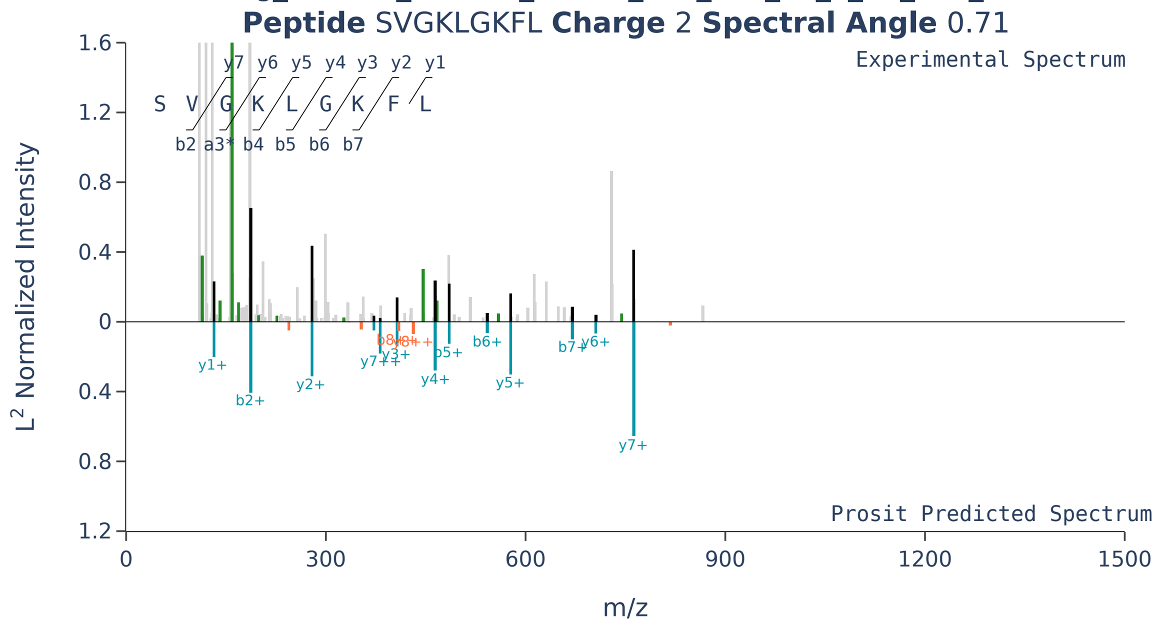


**Source** Ncheng_210623_230623_HFGoe_FFH_20S_25_1_A1_2h_R2 **Scan** 29105
**Peptide** VDAMTGQDAANTAKAFNEALPLTG **Charge** 2 **Spectral Angle** 0.79

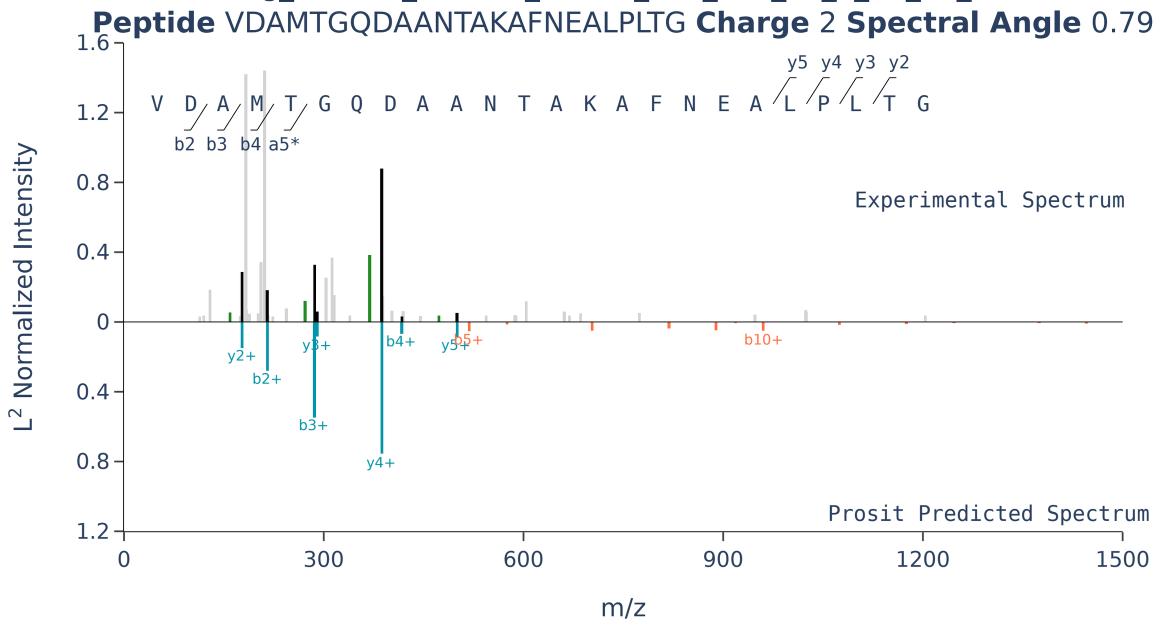


**Source** Ncheng_210623_230623_HFGoe_FFH_20S_25_1_A2_24h_R1 **Scan** 18199
**Peptide** ALLNSMTMKE **Charge** 2 **Spectral Angle** 0.75

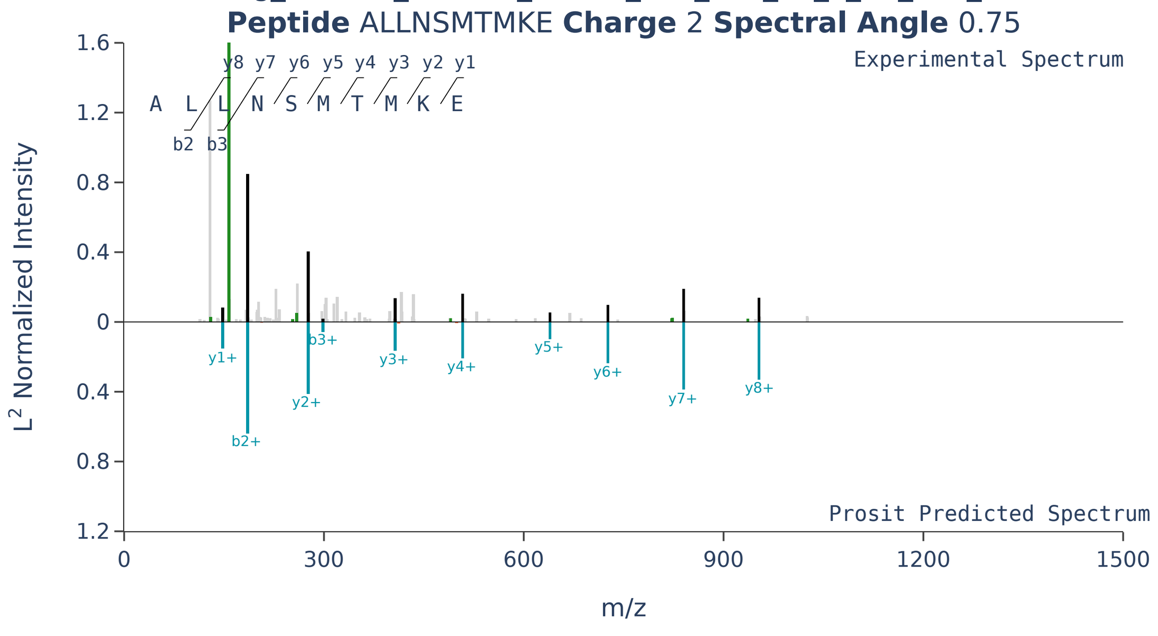


**Source** Ncheng_210623_230623_HFGoe_FFH_20S_25_1_A2_4h_R2 **Scan** 18096
**Peptide** QGAGKTTSVGKLGKFL **Charge** 2 **Spectral Angle** 0.75

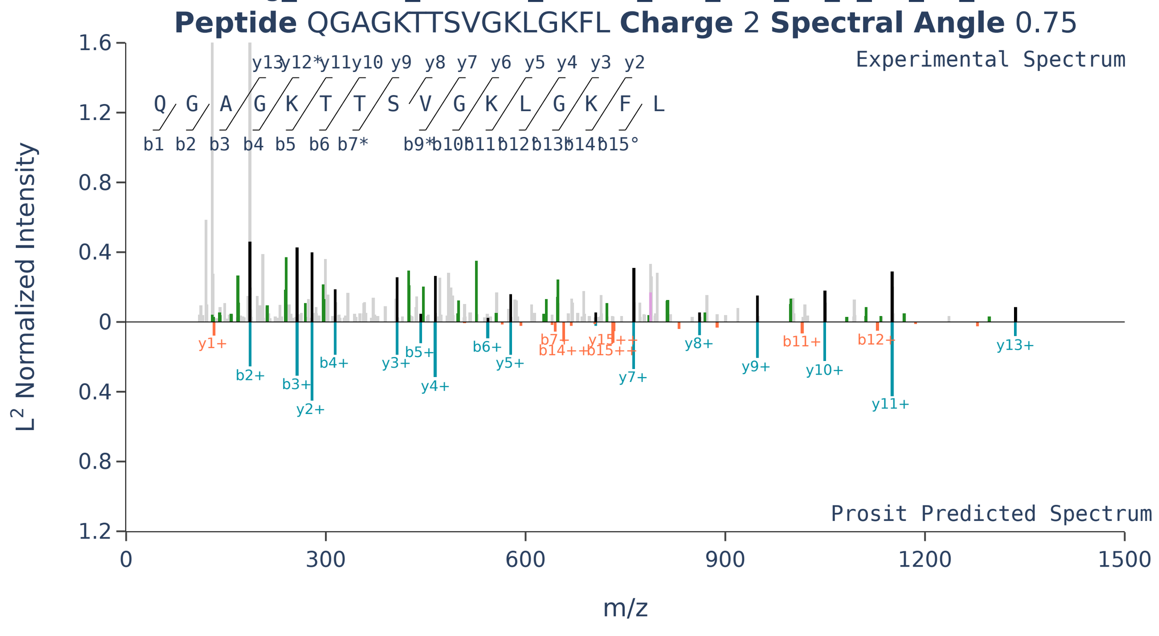

**Source** Ncheng_210623_230623_HFGoe_FFH_20S_25_1_A1_4h_R2 **Scan** 15067
**Peptide** DGDARGGAALSIRHL **Charge** 3 **Spectral Angle** 0.88

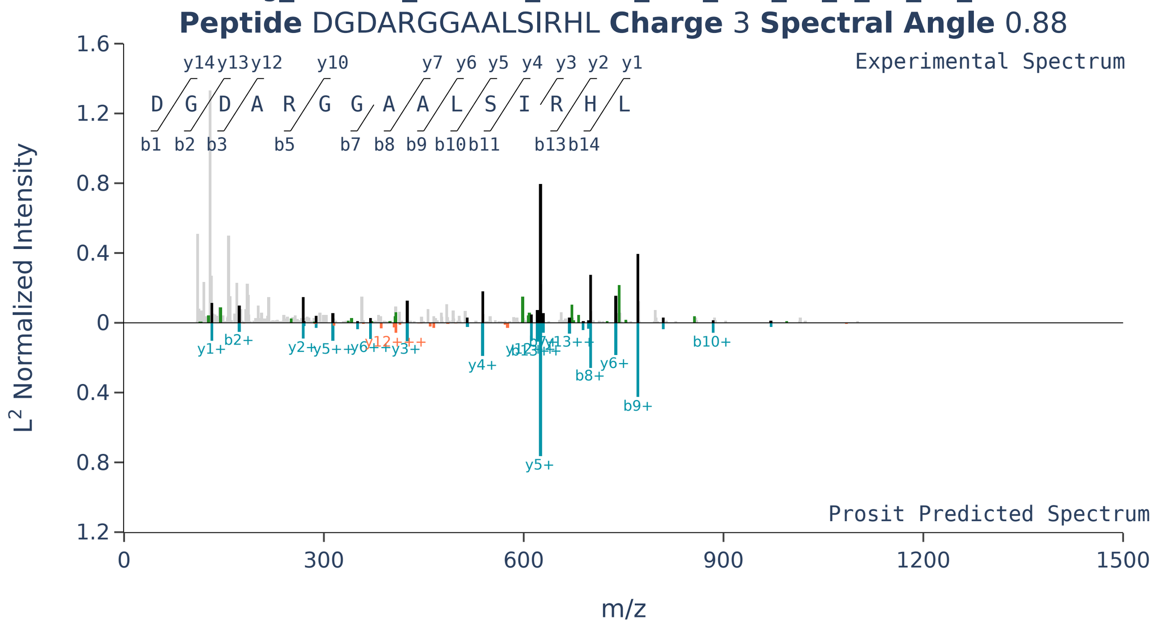


**Source** Ncheng_210623_230623_HFGoe_FFH_20S_25_1_A1_24h_R1 **Scan** 11927
**Peptide** GKLGKFLRE **Charge** 3 **Spectral Angle** 0.69

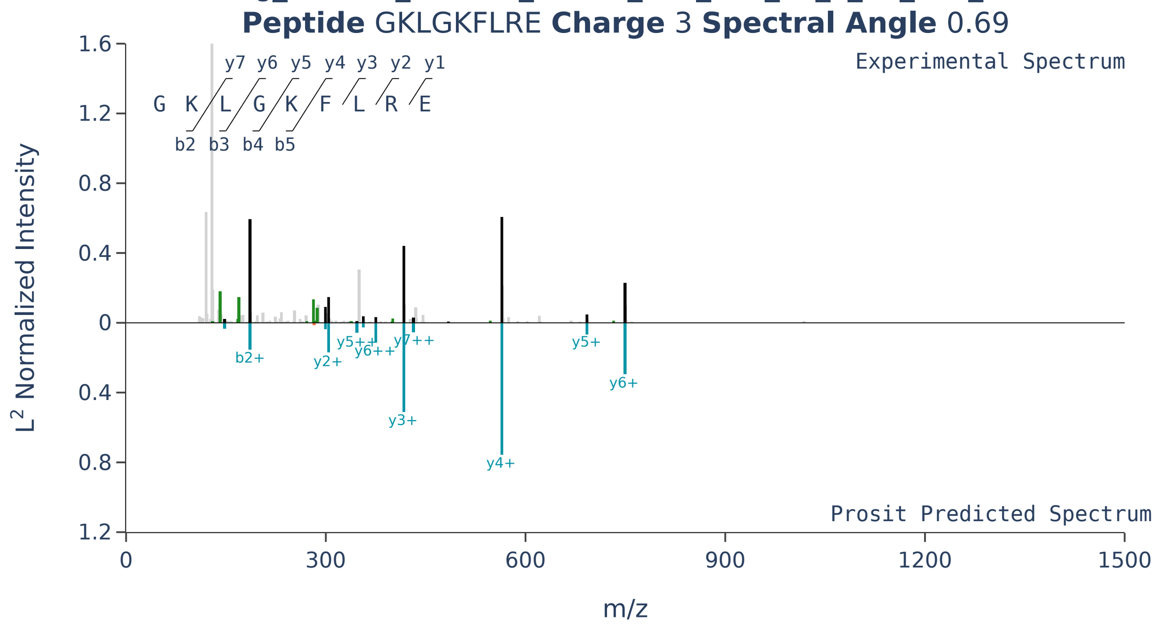


**Source** Ncheng_210623_230623_HFGoe_FFH_20S_25_1_A2_4h_R1 **Scan** 14350
**Peptide** AGRLHVDEAMMD **Charge** 2 **Spectral Angle** 0.71

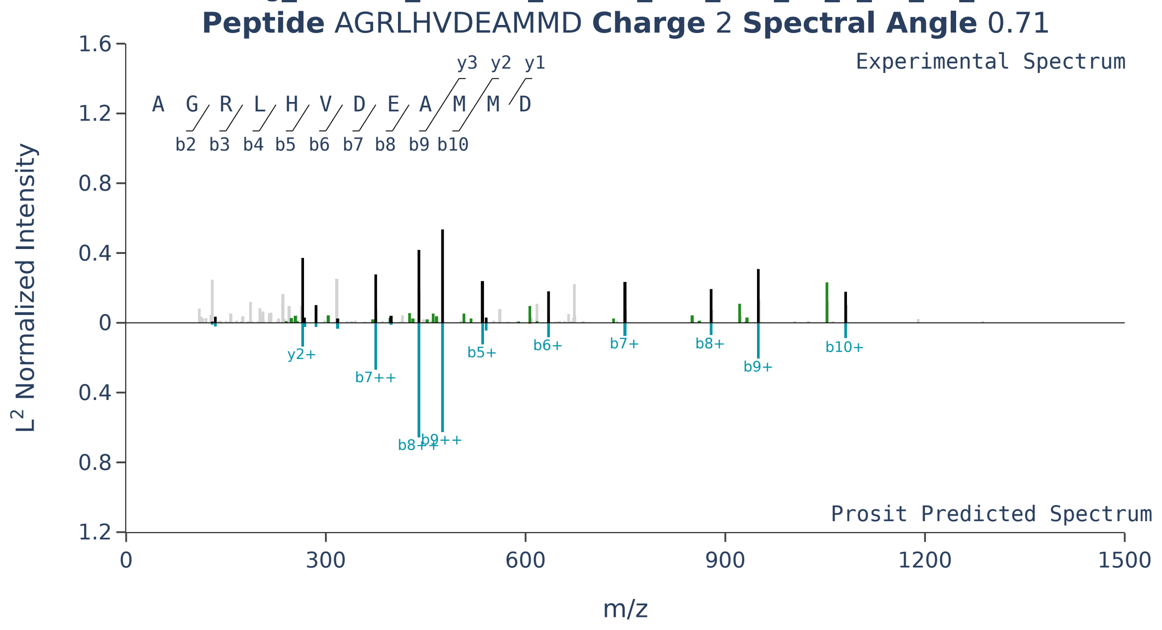


**Source** Ncheng_210623_230623_HFGoe_FFH_20S_25_1_A2_4h_R1 **Scan** 19386
**Peptide** ALEPFHPDRIA **Charge** 3 **Spectral Angle** 0.86

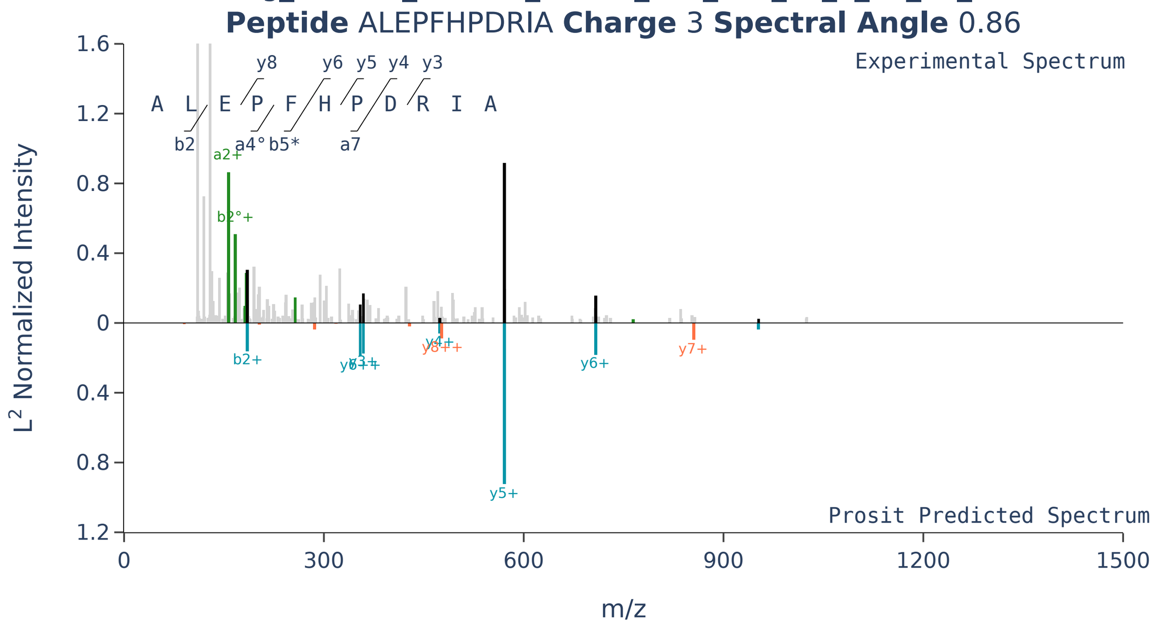


**Source** Ncheng_210623_230623_HFGoe_FFH_20S_25_1_A1_4h_R1 **Scan** 31997
**Peptide** EALPLTGVVLTKVD **Charge** 2 **Spectral Angle** 0.79

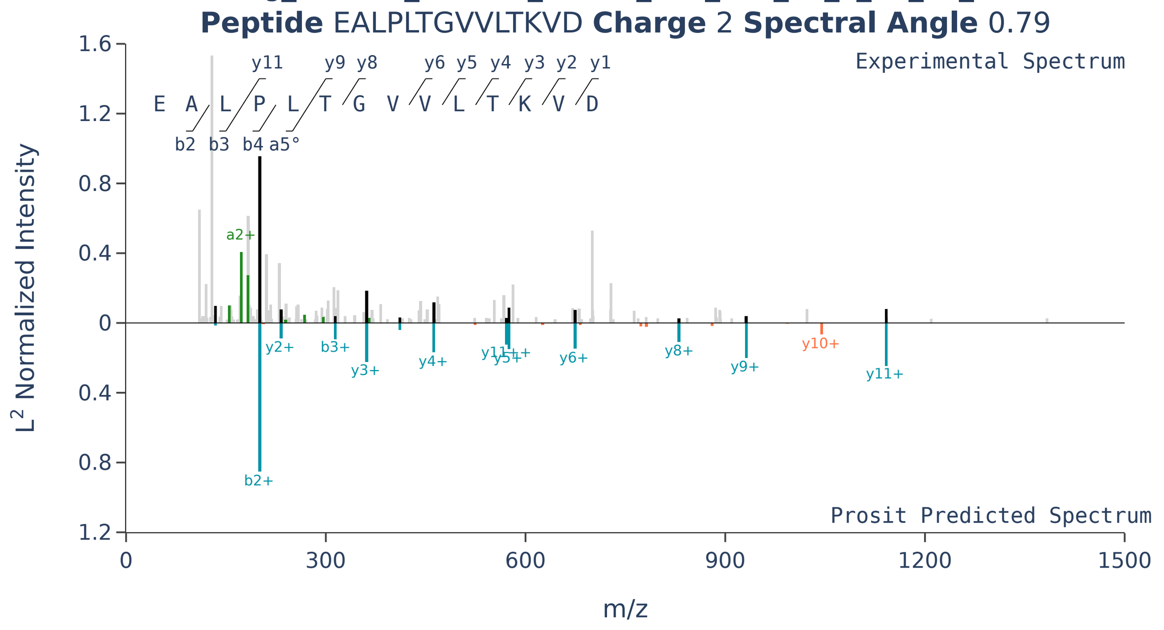


**Source** Ncheng_210623_230623_HFGoe_FFH_20S_25_1_A1_24h_R1 **Scan** 10648
**Peptide** GQDAANTAKAF **Charge** 2 **Spectral Angle** 0.71

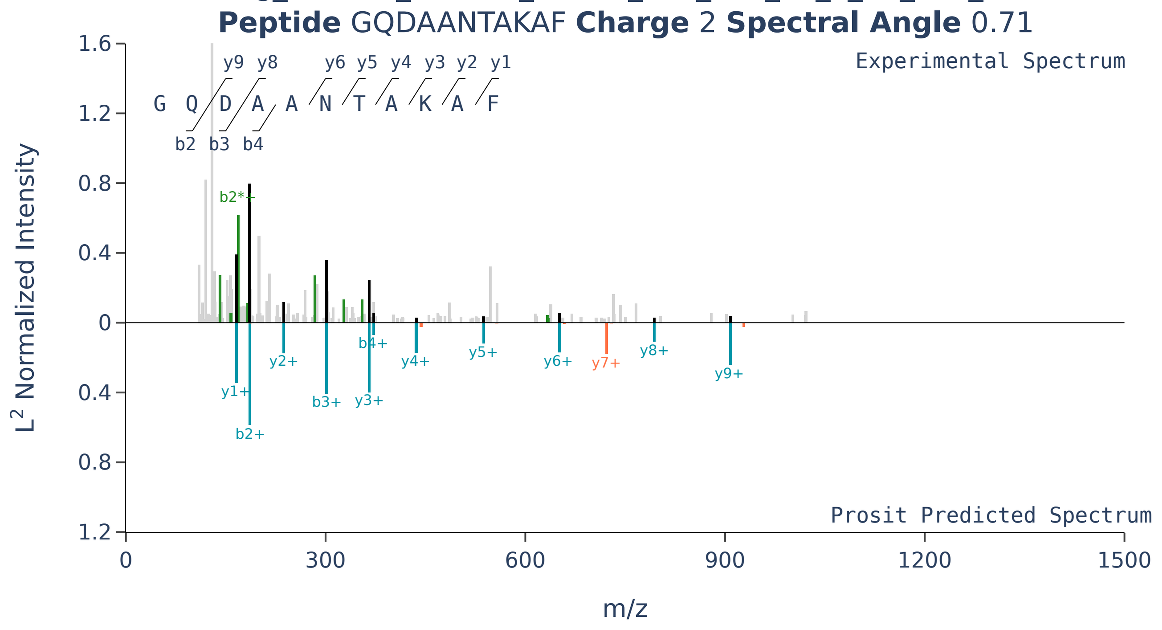


**Source** Ncheng_210623_230623_HFGoe_FFH_20S_25_1_A1_24h_R1 **Scan** 8496
**Peptide** ADVYRPA **Charge** 2 **Spectral Angle** 0.73

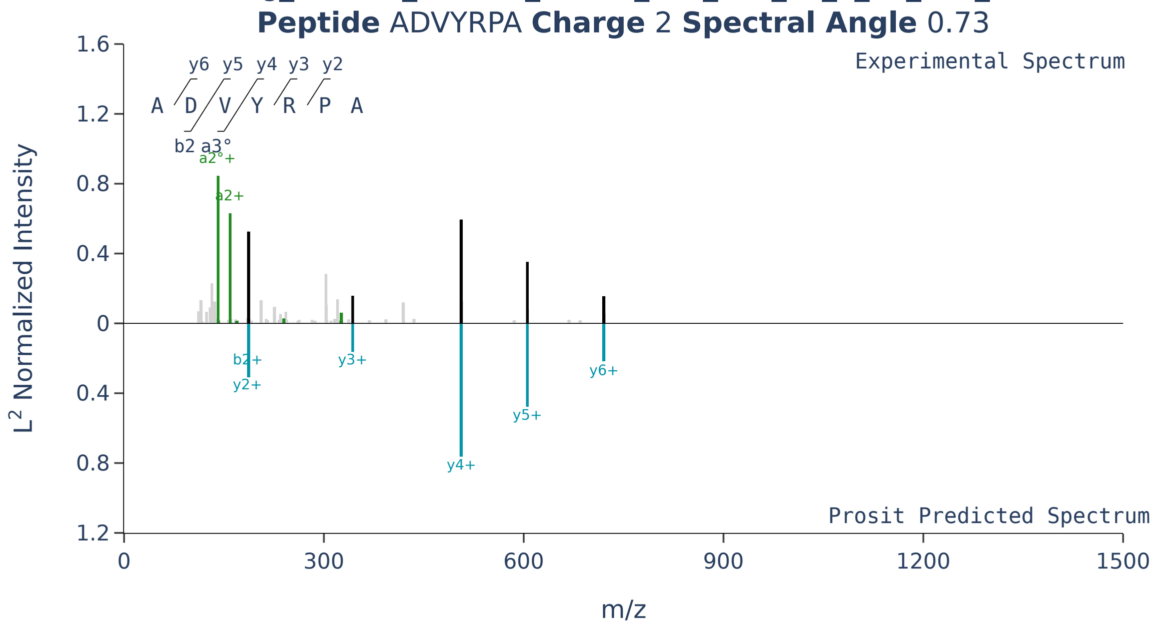


**Source** Ncheng_210623_230623_HFGoe_FFH_20S_25_1_A2_2h_R1 **Scan** 24431
**Peptide** FVKIVRNELVAAM **Charge** 3 **Spectral Angle** 0.78

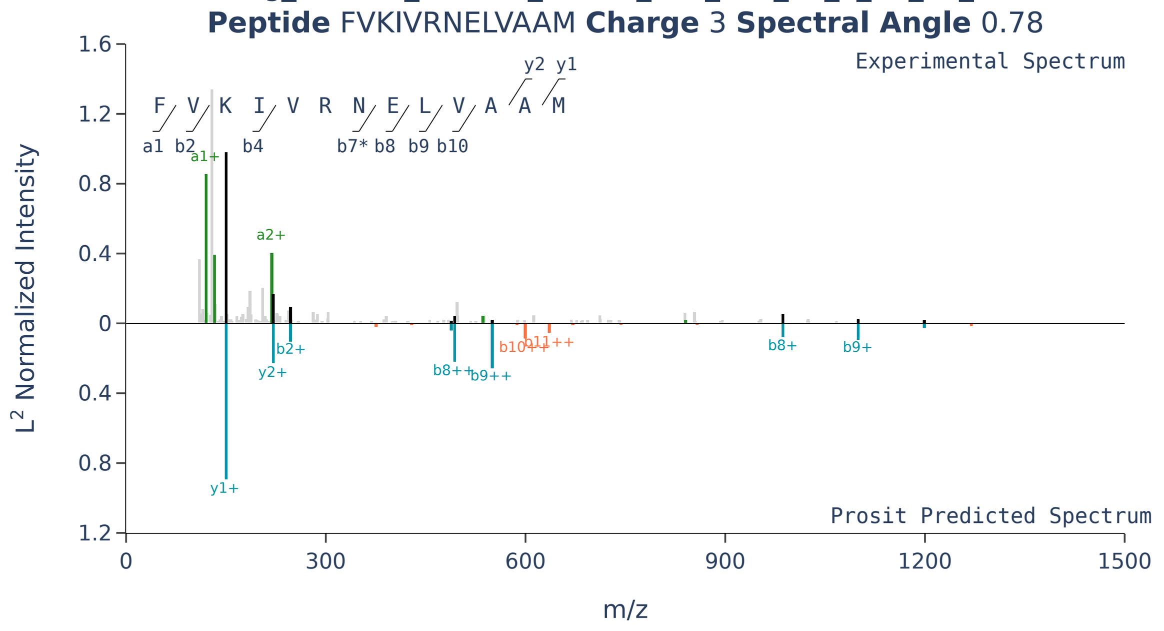


**Source** Ncheng_210623_230623_HFGoe_FFH_20S_25_1_A1_2h_R2 **Scan** 19434
**Peptide** GRLTEDNVKDTLREVRM **Charge** 3 **Spectral Angle** 0.71

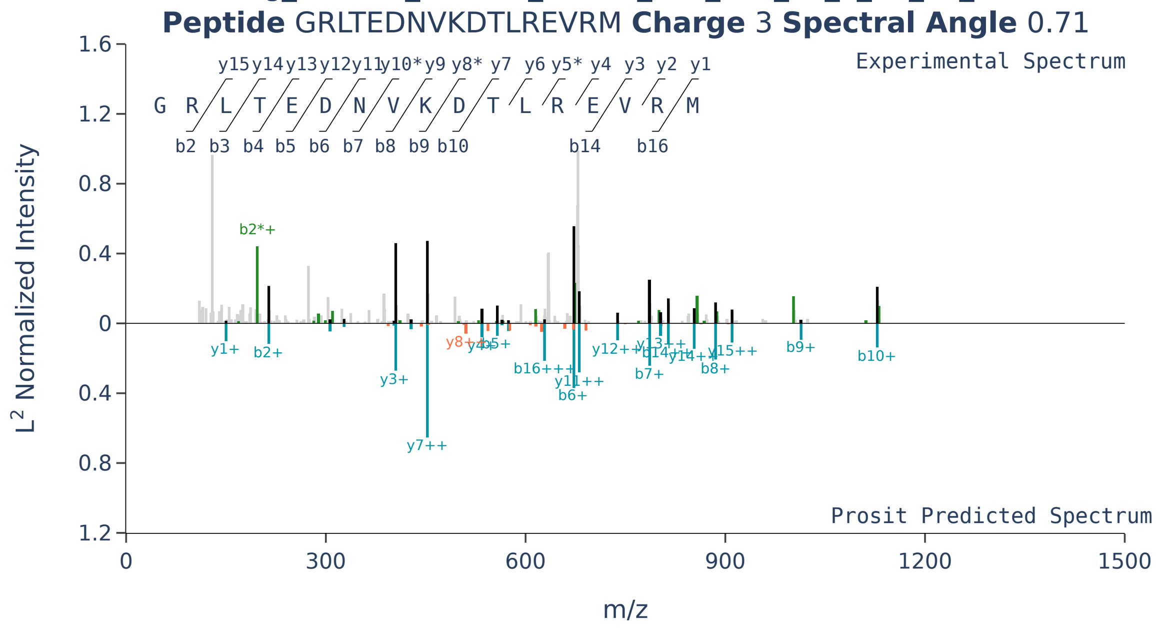


**Source** Ncheng_210623_230623_HFGoe_FFH_20S_25_1_A1_24h_R2 **Scan** 16287
**Peptide** RIAAGCGMQVQDV **Charge** 2 **Spectral Angle** 0.74

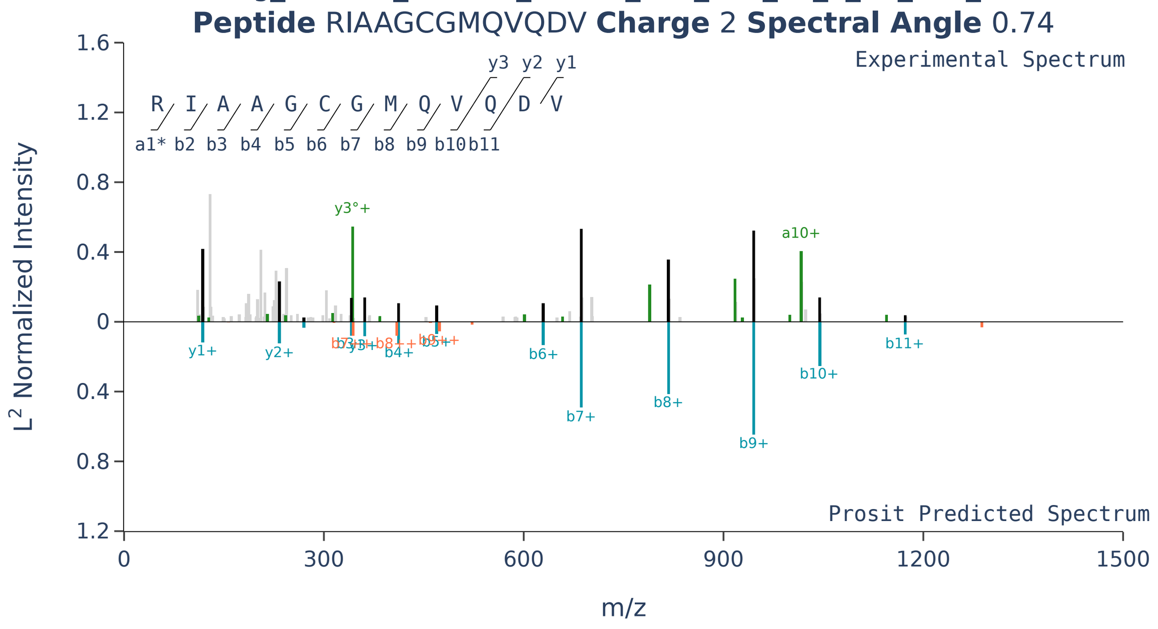


**Source** Ncheng_210623_230623_HFGoe_FFH_20S_25_1_A2_24h_R1 **Scan** 8424
**Peptide** VGHEVNKSLTPGQ **Charge** 2 **Spectral Angle** 0.8

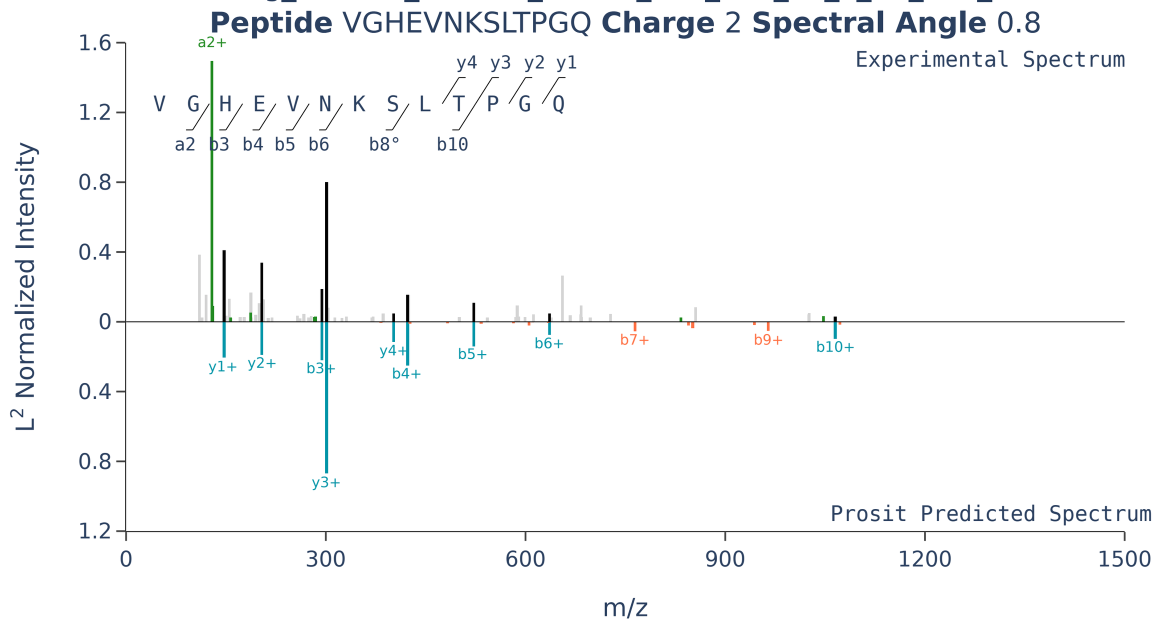


**Source** Ncheng_210623_230623_HFGoe_FFH_20S_25_1_A1_24h_R2 **Scan** 29197
**Peptide** DVALPVV **Charge** 1 **Spectral Angle** 0.77

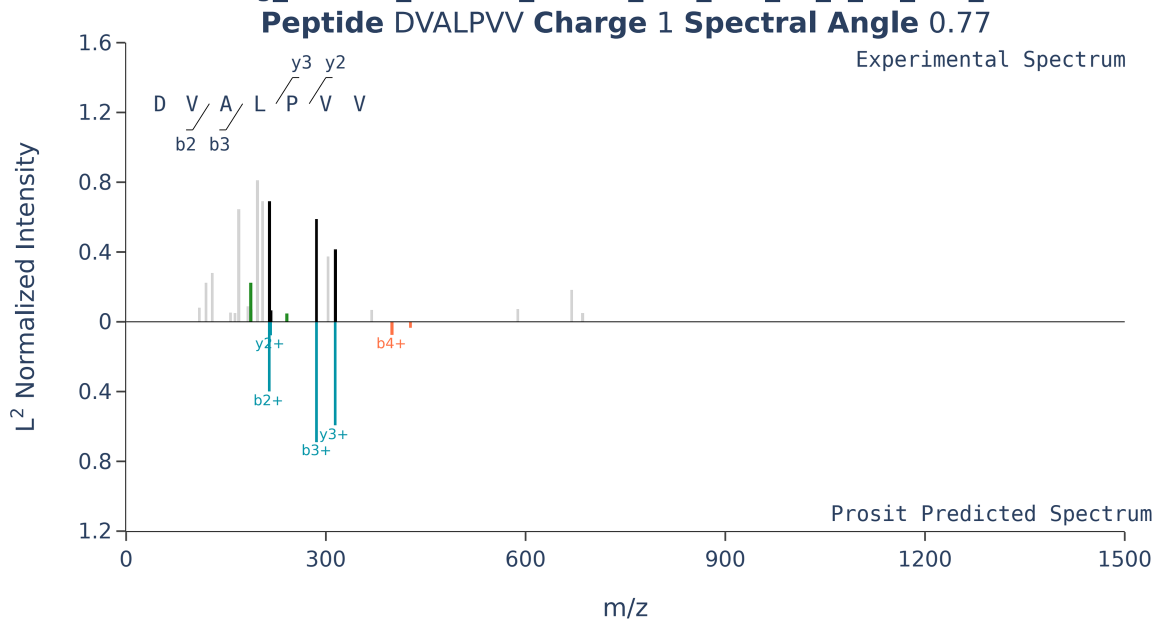


**Source** Ncheng_210623_230623_HFGoe_FFH_20S_25_1_A2_4h_R2 **Scan** 17654
**Peptide** TLRNISGRGRLTEDNVKDTLREV **Charge** 5 **Spectral Angle** 0.8

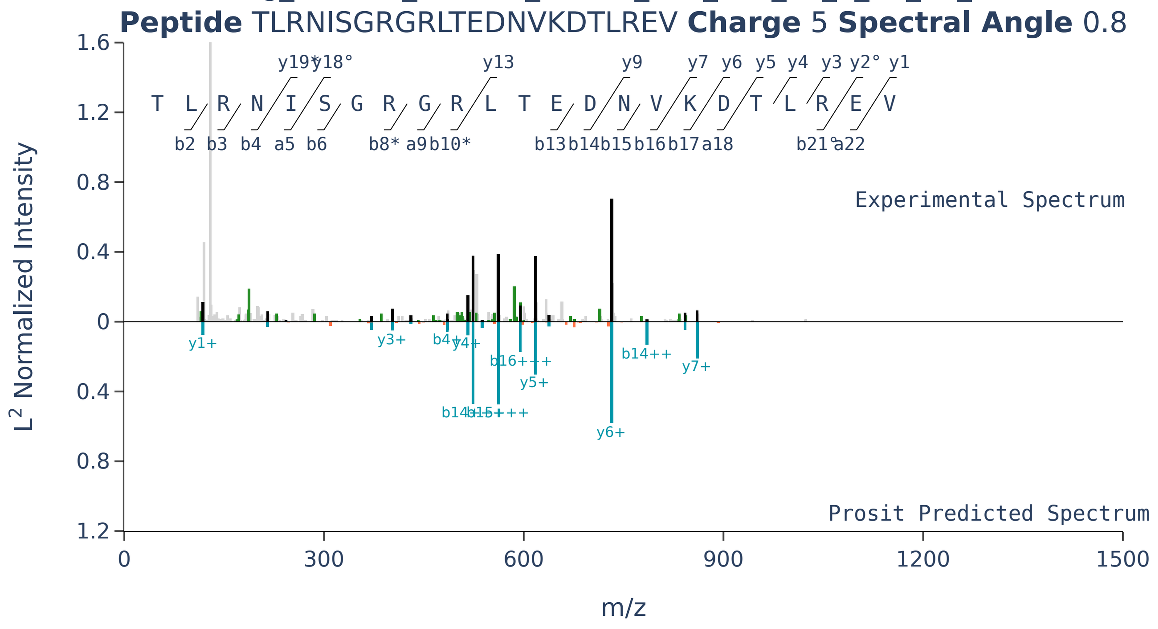


**Source** Ncheng_210623_230623_HFGoe_FFH_20S_25_1_A2_4h_R2 **Scan** 23690
**Peptide** SLTPGQEFVKIVRN **Charge** 2 **Spectral Angle** 0.56

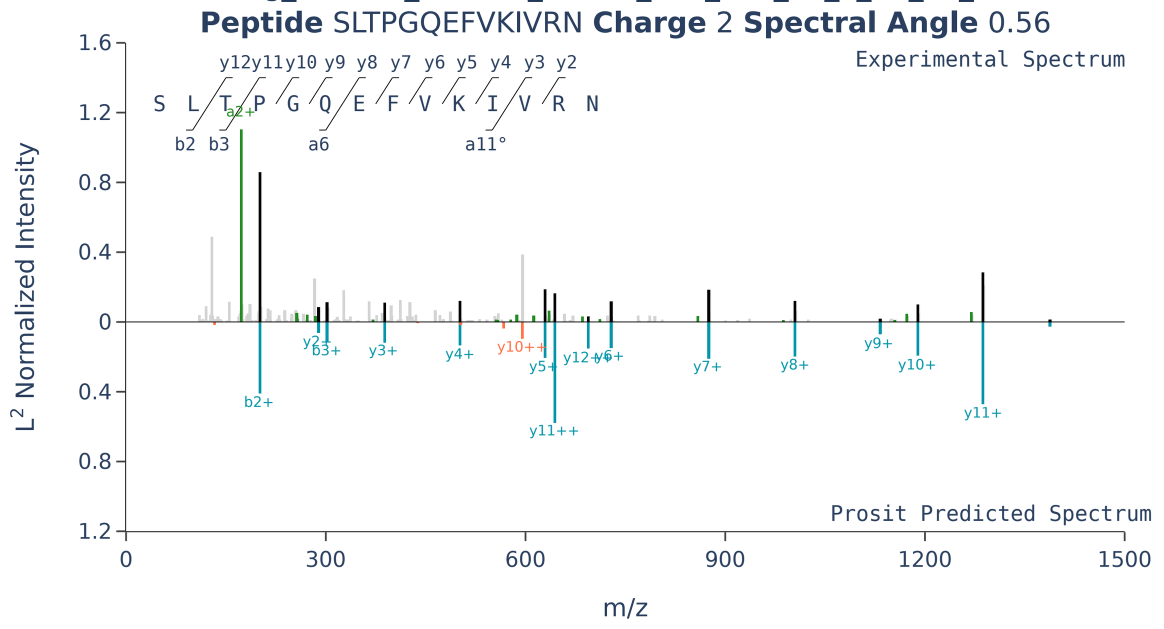


**Source** Ncheng_210623_230623_HFGoe_FFH_20S_25_1_A2_24h_R2 **Scan** 24942
**Peptide** KSLTPGQEFVKIV **Charge** 2 **Spectral Angle** 0.89

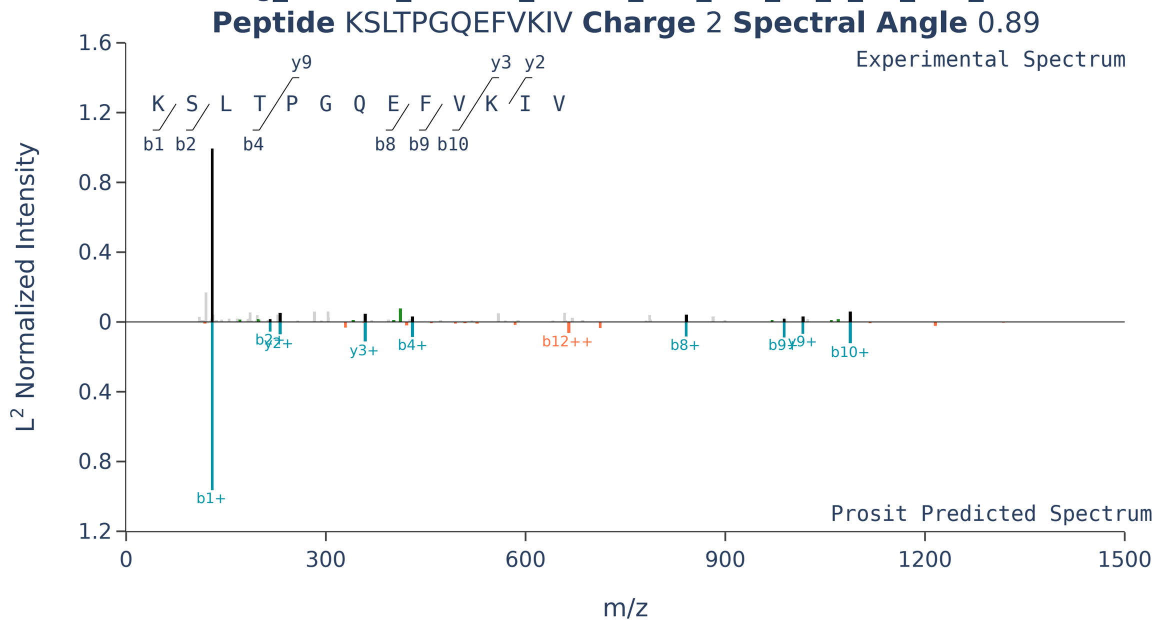

**Source** Ncheng_210623_230623_HFGoe_FFH_20S_25_1_A2_4h_R1 **Scan** 32601
**Peptide** AKLKFYDVLLV **Charge** 2 **Spectral Angle** 0.71

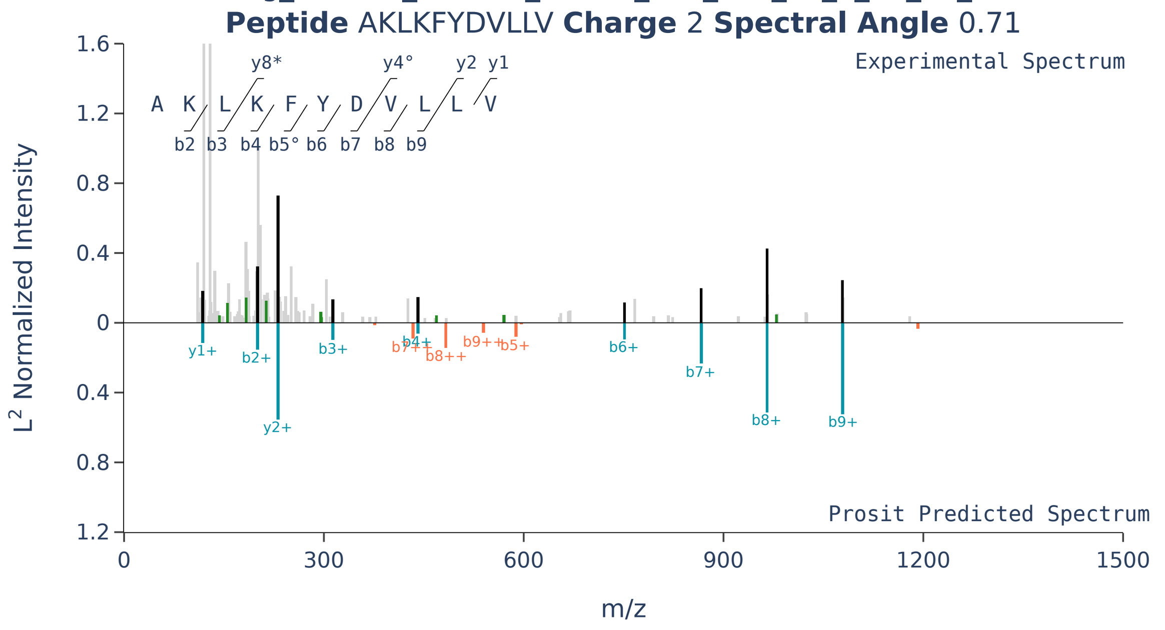


**Source** Ncheng_210623_230623_HFGoe_FFH_20S_25_1_A1_2h_R2 **Scan** 19654
**Peptide** LVNAALKEAKLKF **Charge** 4 **Spectral Angle** 0.77

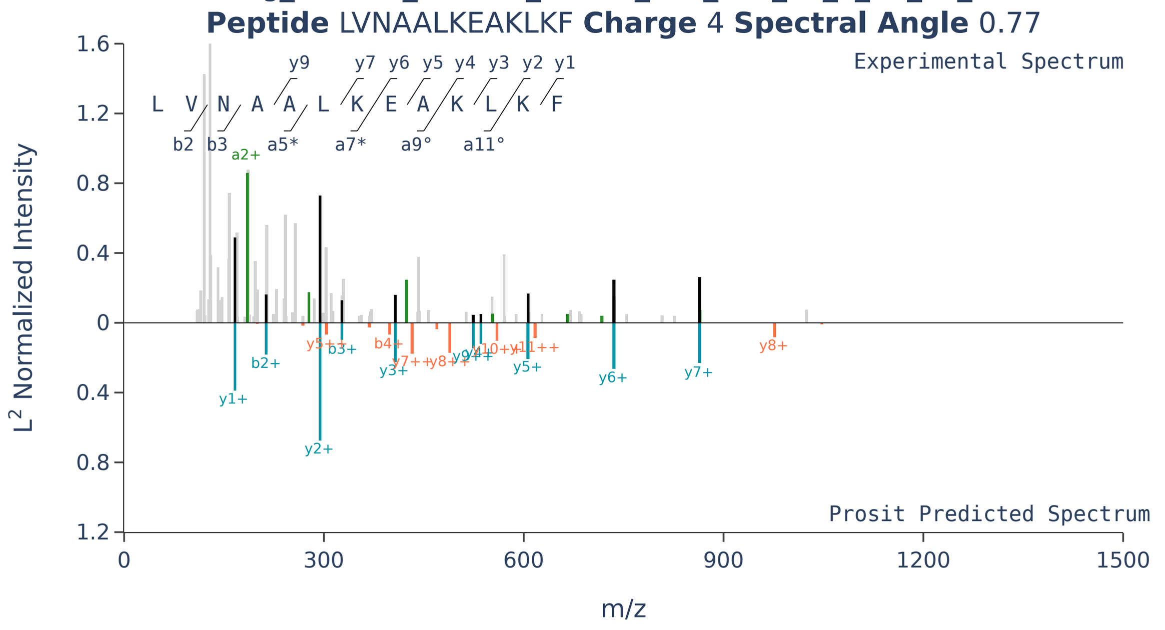


**Source** Ncheng_210623_230623_HFGoe_FFH_20S_25_1_A1_4h_R1 **Scan** 11702
**Peptide** IEDIESKVDRA **Charge** 2 **Spectral Angle** 0.72

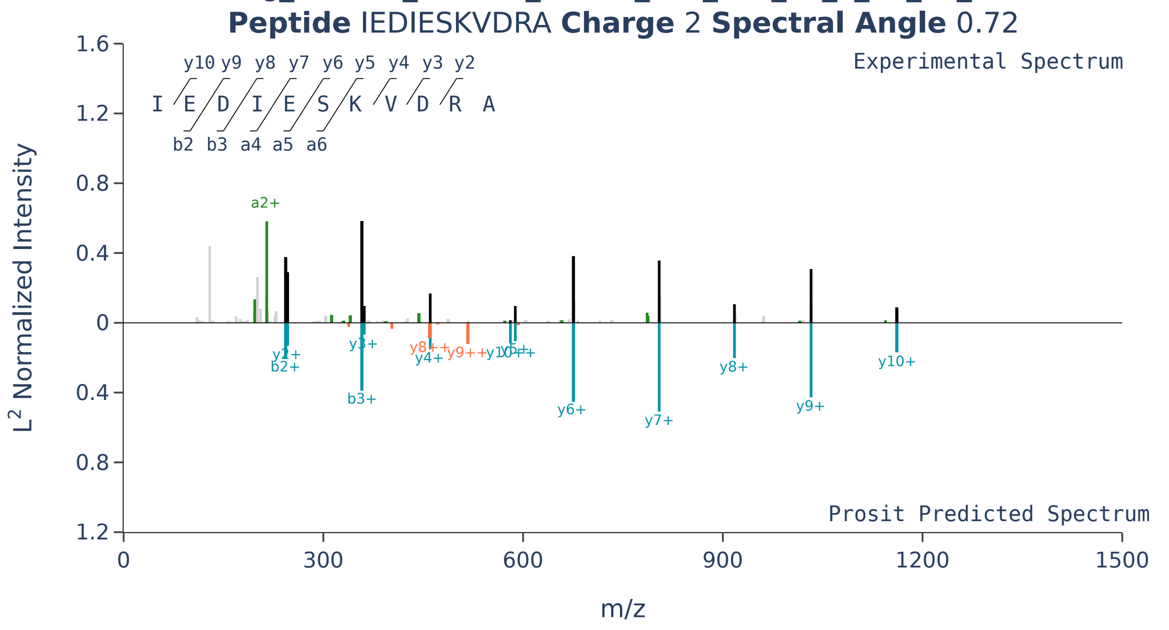


**Source** Ncheng_210623_230623_HFGoe_FFH_20S_25_1_A2_1h_R1 **Scan** 25596
**Peptide** DEAMMDEIKQVHASINPVE **Charge** 3 **Spectral Angle** 0.77

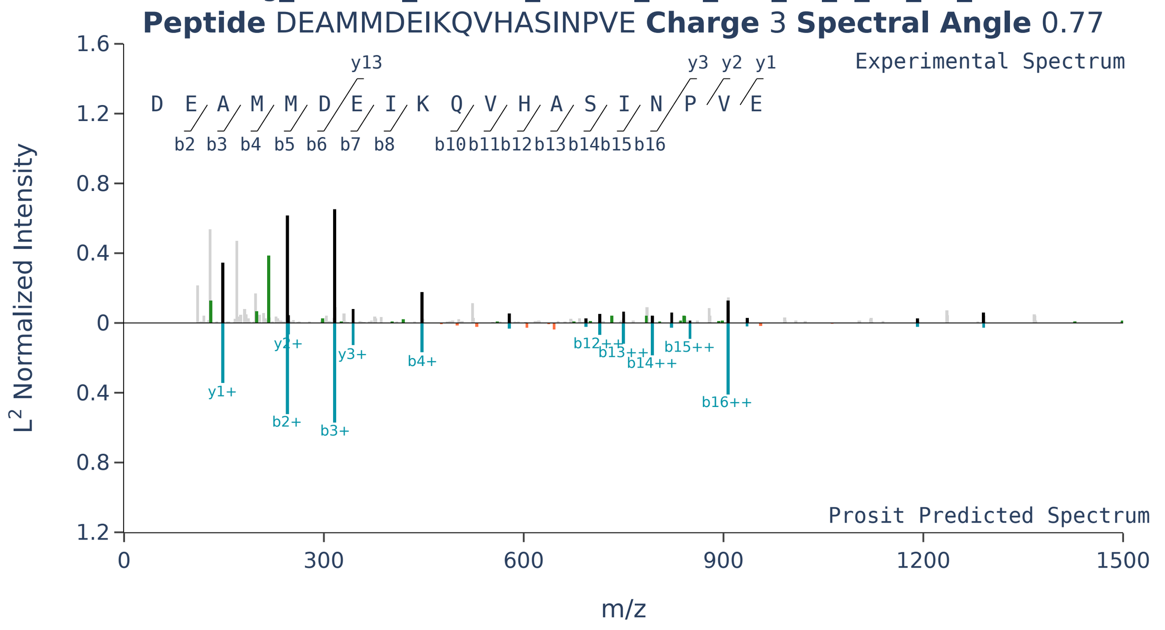


**Source** Ncheng_210623_230623_HFGoe_FFH_20S_25_1_A2_4h_R1 **Scan** 23849
**Peptide** LPGMGQIPDNVKSQM **Charge** 2 **Spectral Angle** 0.74

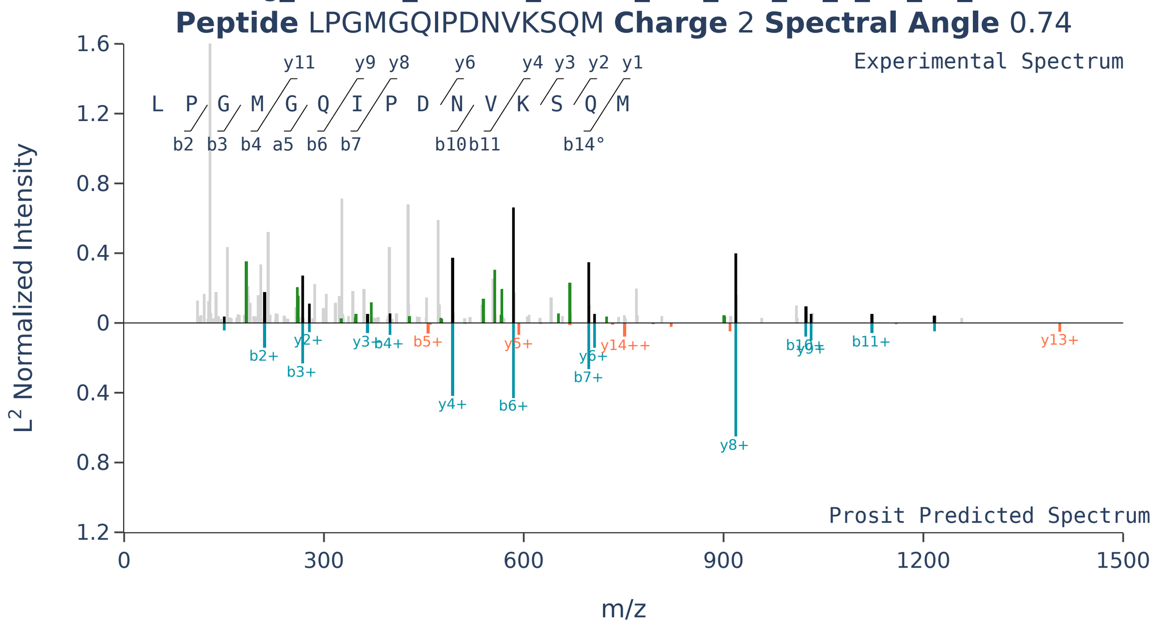


**Source** Ncheng_210623_230623_HFGoe_FFH_20S_25_1_A1_2h_R2 **Scan** 30625
**Peptide** LNDFLEQLRQMKNMG **Charge** 2 **Spectral Angle** 0.77

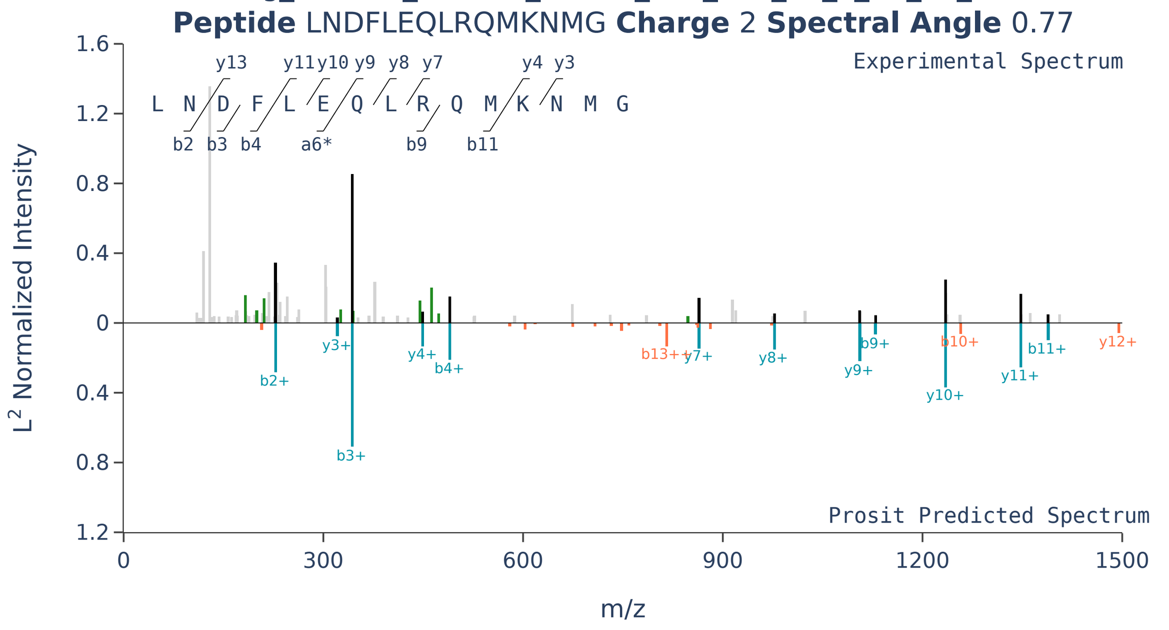


**Source** Ncheng_210623_230623_HFGoe_FFH_20S_25_1_A2_24h_R1 **Scan** 21607
**Peptide** GMQVQDVNRLLKQ **Charge** 2 **Spectral Angle** 0.73

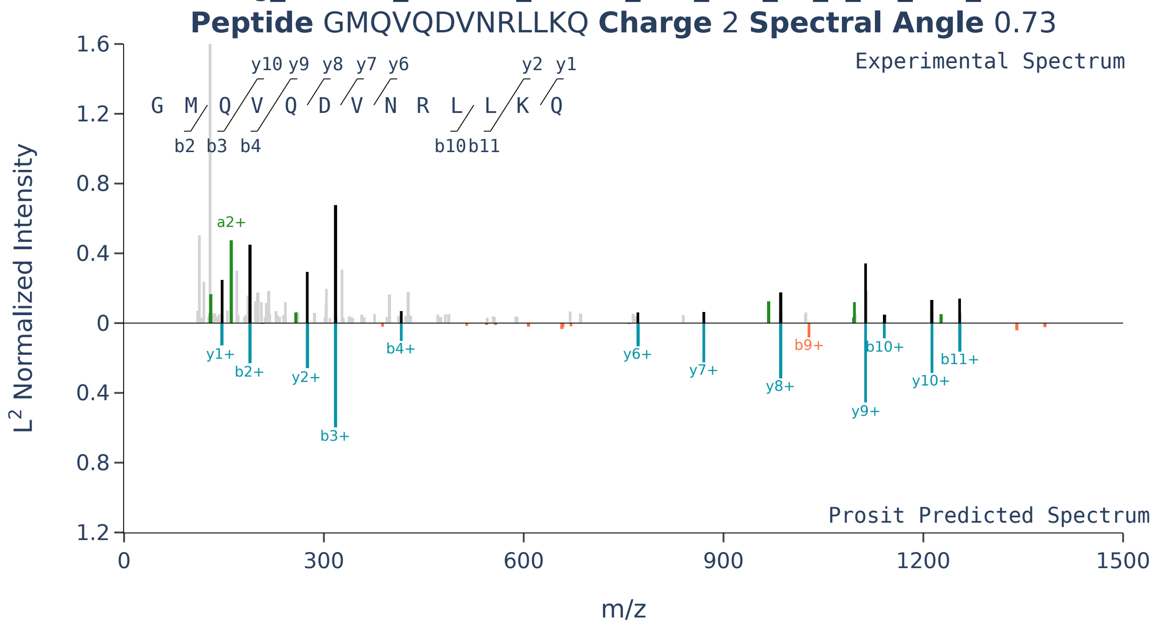


**Source** Ncheng_210623_230623_HFGoe_FFH_20S_25_1_A2_4h_R2 **Scan** 22286
**Peptide** GQIPDNVKSQMDDKVLVRMEA **Charge** 3 **Spectral Angle** 0.81

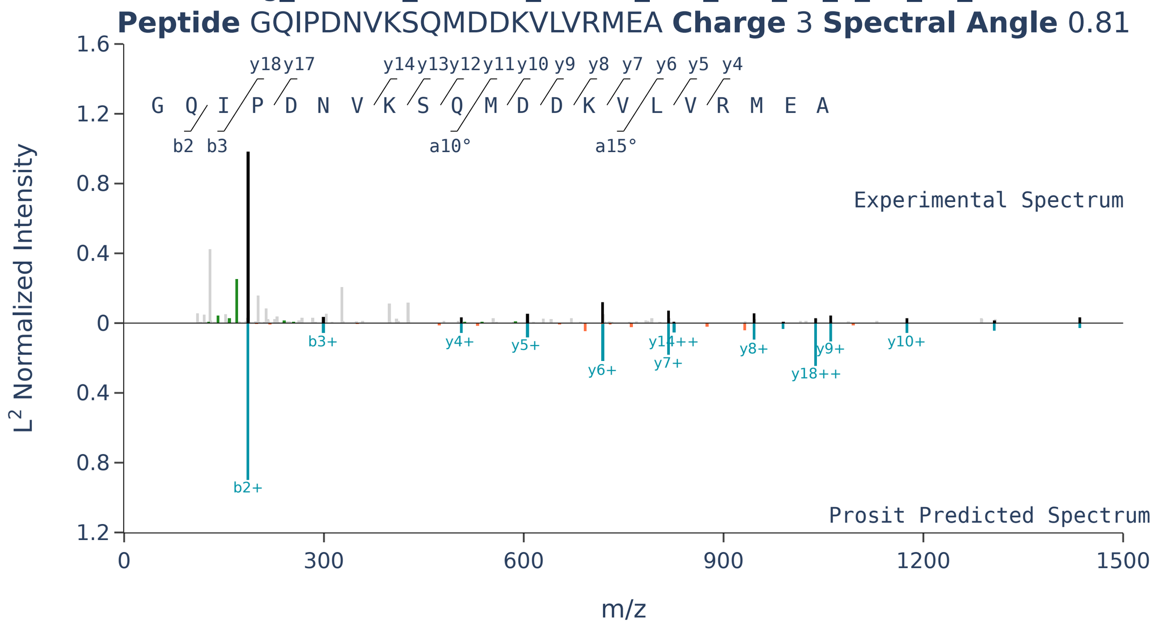


**Source** Ncheng_210623_230623_HFGoe_FFH_20S_25_1_A2_4h_R2 **Scan** 21365
**Peptide** TGKPIKFLGV **Charge** 2 **Spectral Angle** 0.7

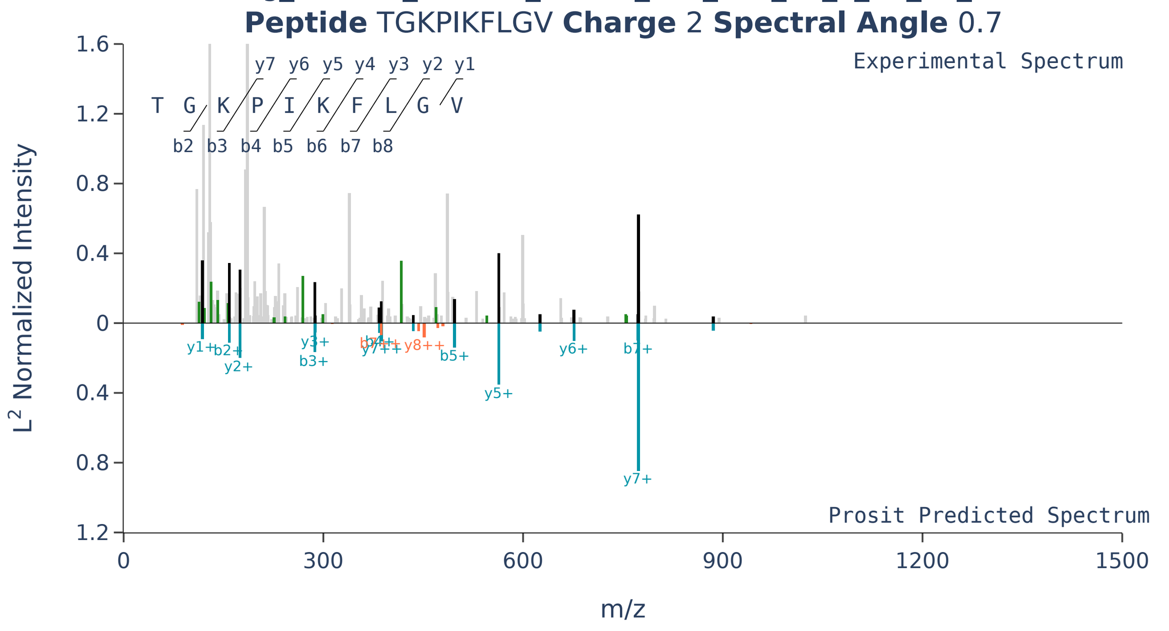


**Source** Ncheng_210623_230623_HFGoe_FFH_20S_25_1_A1_24h_R1 **Scan** 13896
**Peptide** EDIESKVDRAQAEKL **Charge** 2 **Spectral Angle** 0.78

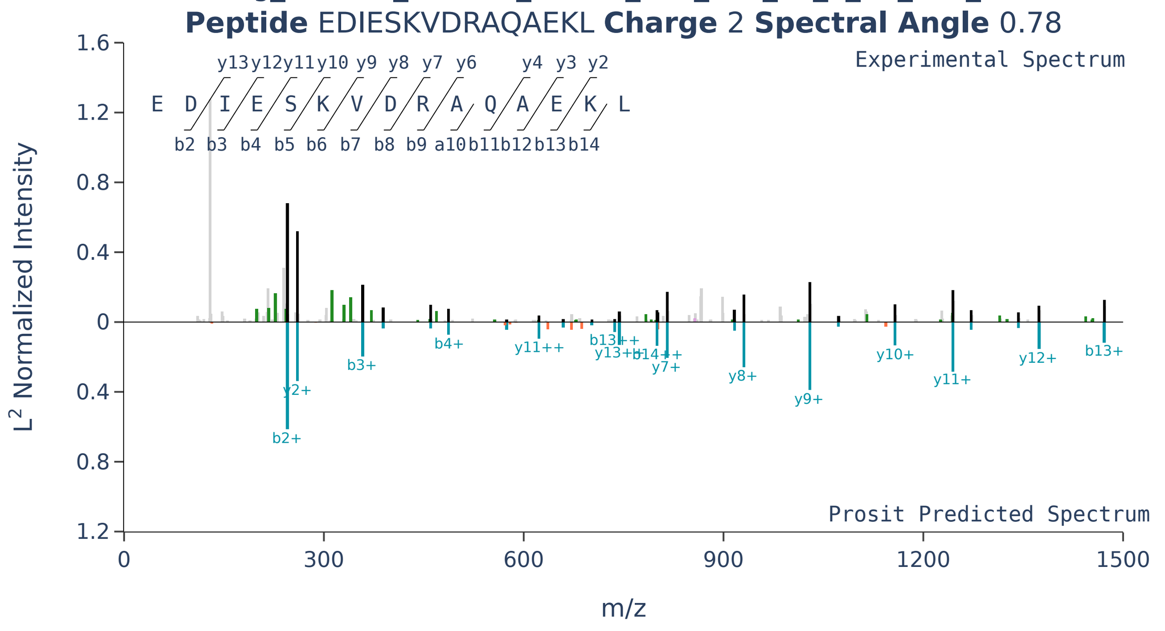


**Source** Ncheng_210623_230623_HFGoe_FFH_20S_25_1_A1_24h_R1 **Scan** 15588
**Peptide** GKPIKFLGVGEKTE **Charge** 2 **Spectral Angle** 0.74

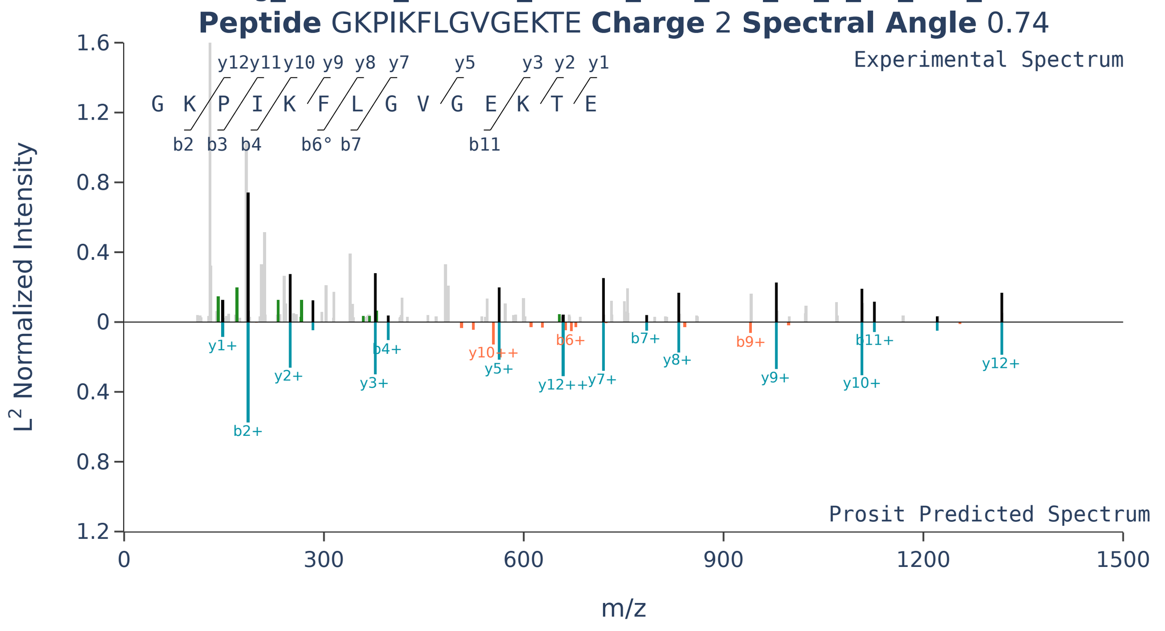


**Source** Ncheng_210623_230623_HFGoe_FFH_20S_25_1_A1_2h_R2 **Scan** 31177
**Peptide** GGMASLMGKLPGMGQIPDNVKSQMD **Charge** 2 **Spectral Angle** 0.8

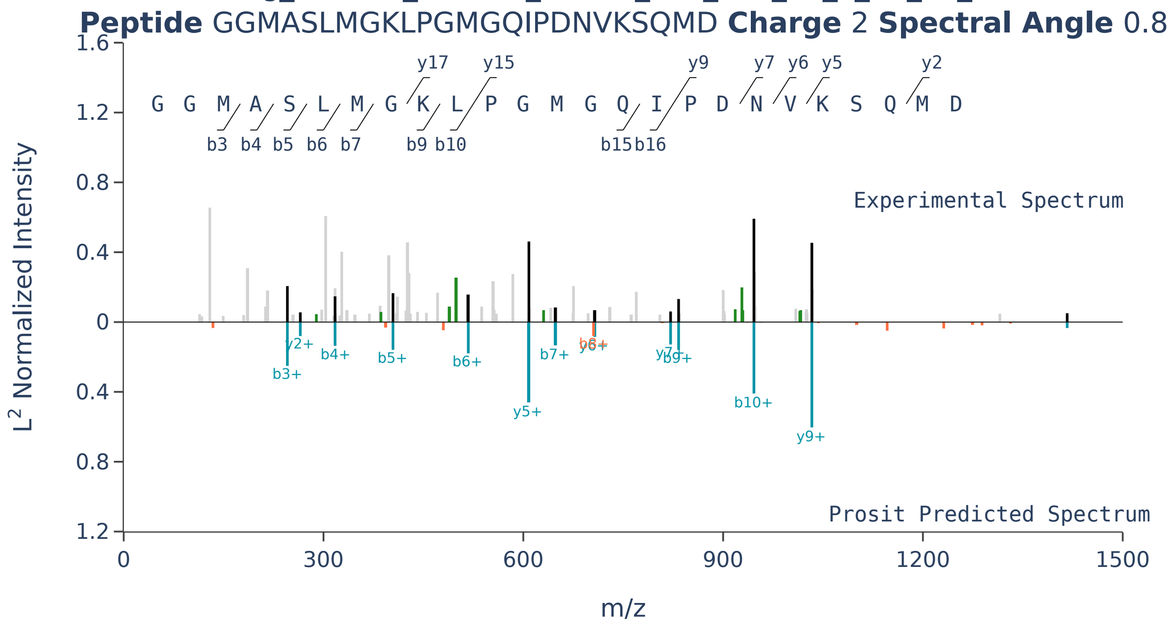


**Source** Ncheng_210623_230623_HFGoe_FFH_20S_25_1_A2_24h_R2 **Scan** 16520
**Peptide** INRVKEKAVGHEVNKSLTPGQEFVKIVRN **Charge** 3 **Spectral Angle** 0.79

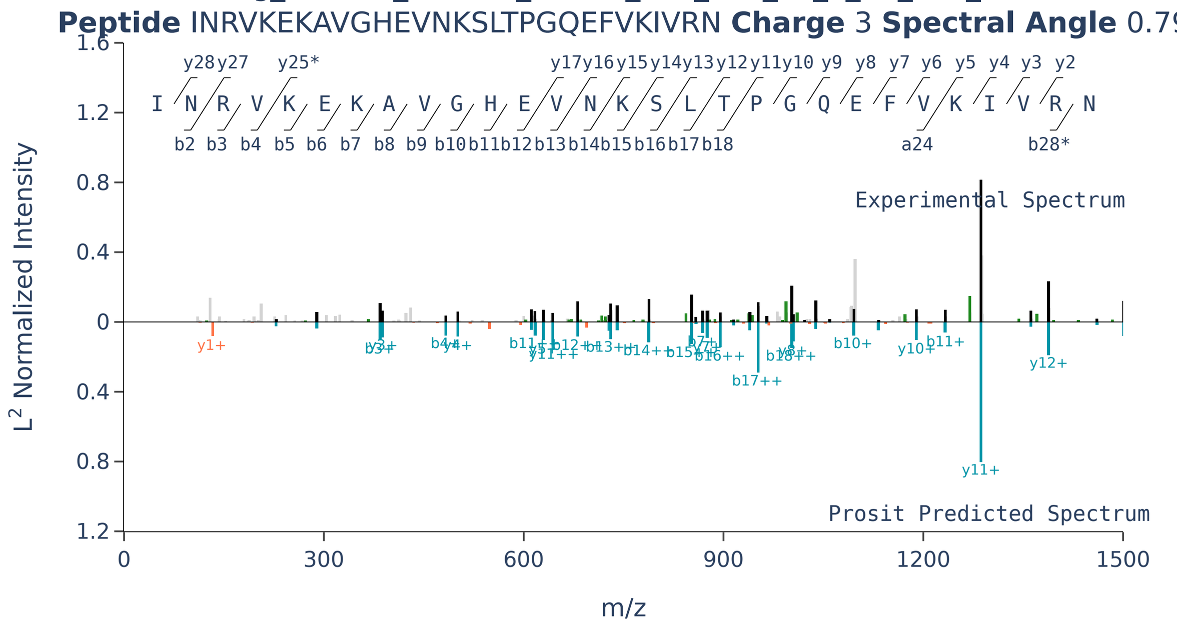


**Source** Ncheng_210623_230623_HFGoe_FFH_20S_25_1_A1_24h_R2 **Scan** 16583
**Peptide** KQLETLAEQVG **Charge** 2 **Spectral Angle** 0.73

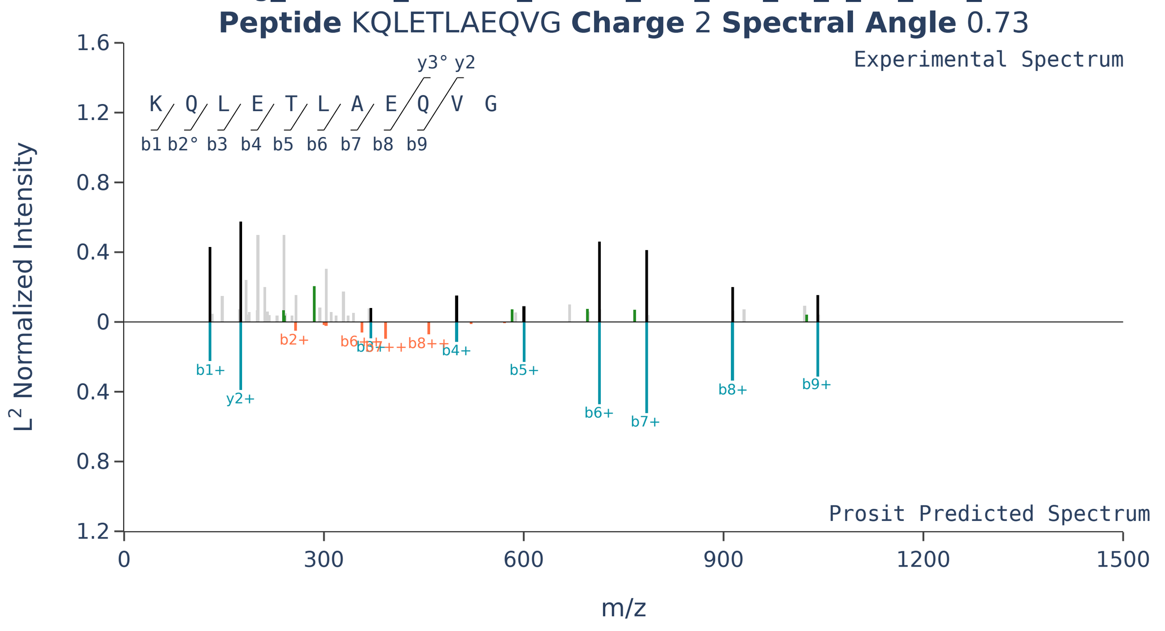


**Source** Ncheng_210623_230623_HFGoe_FFH_20S_25_1_A2_24h_R2 **Scan** 17976
**Peptide** TTSVGKLGKFLRE **Charge** 3 **Spectral Angle** 0.6

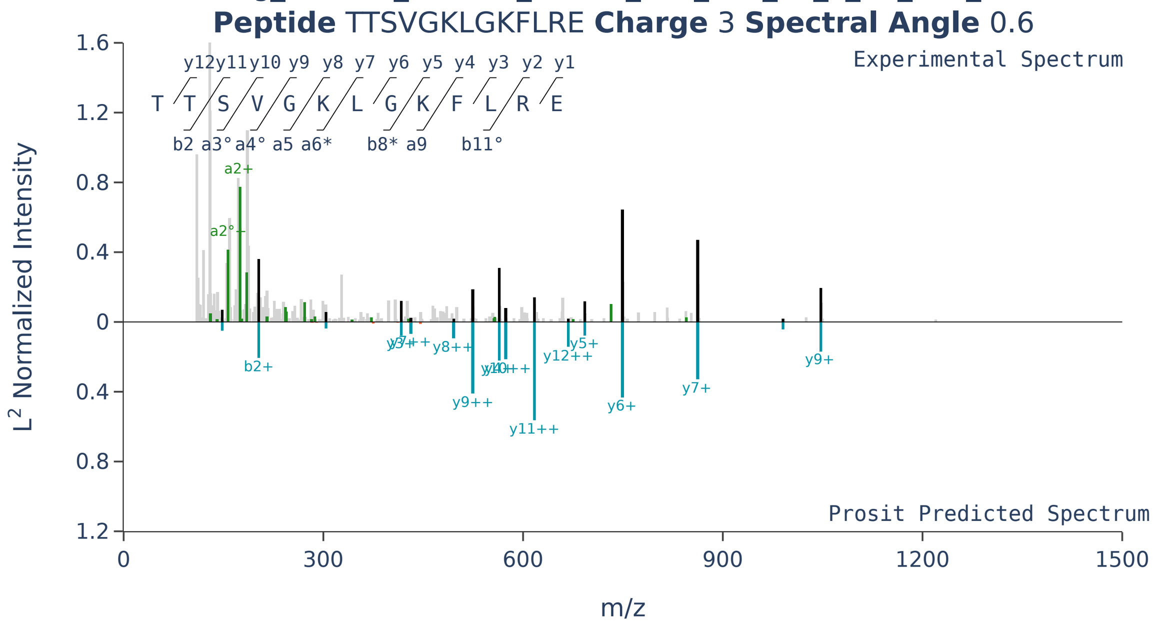

**Source** Ncheng_210623_230623_HFGoe_FFH_20S_25_1_A1_4h_R2 **Scan** 19086
**Peptide** KPIKFLGVGEKTEALE **Charge** 2 **Spectral Angle** 0.74

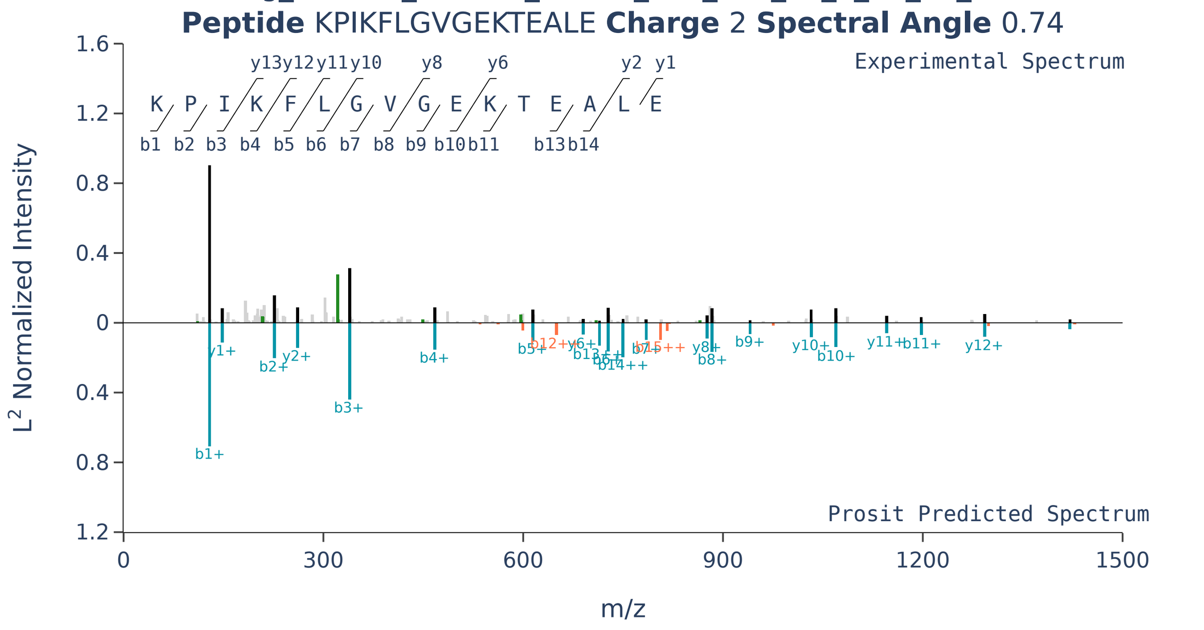


**Source** Ncheng_210623_230623_HFGoe_FFH_20S_25_1_A1_4h_R2 **Scan** 19597
**Peptide** VSADVYRPAAIKQLE **Charge** 2 **Spectral Angle** 0.67

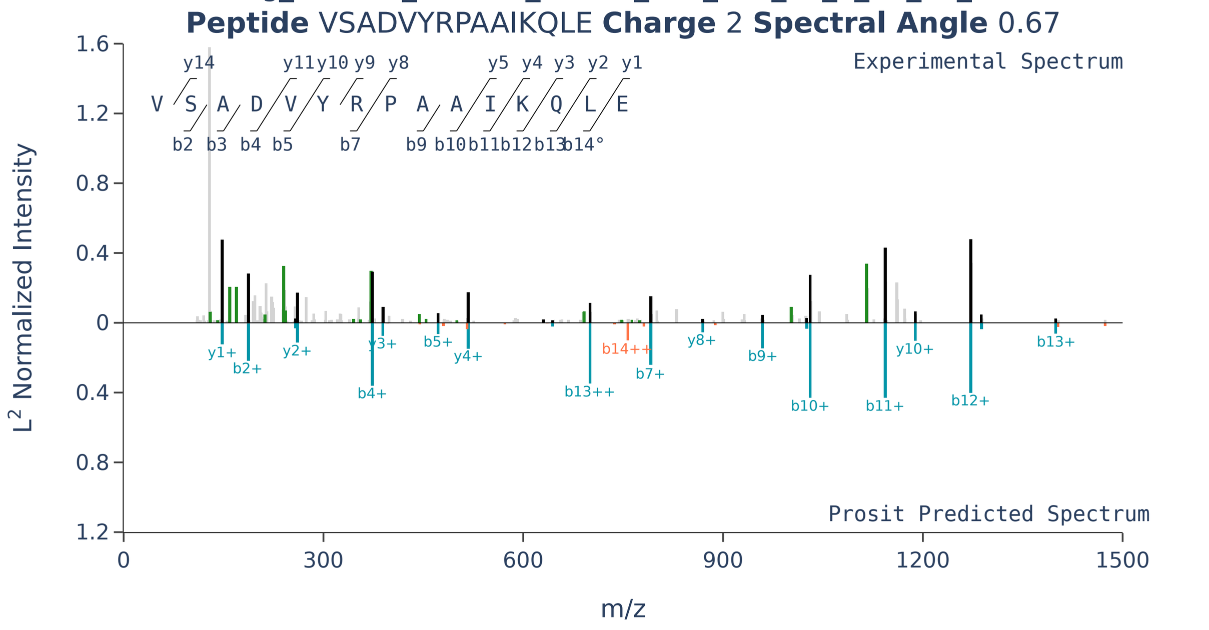


**Source** Ncheng_210623_230623_HFGoe_FFH_20S_25_1_A2_4h_R2 **Scan** 21430
**Peptide** VGHEVNKSLTPGQEFVKIVRNE **Charge** 3 **Spectral Angle** 0.7

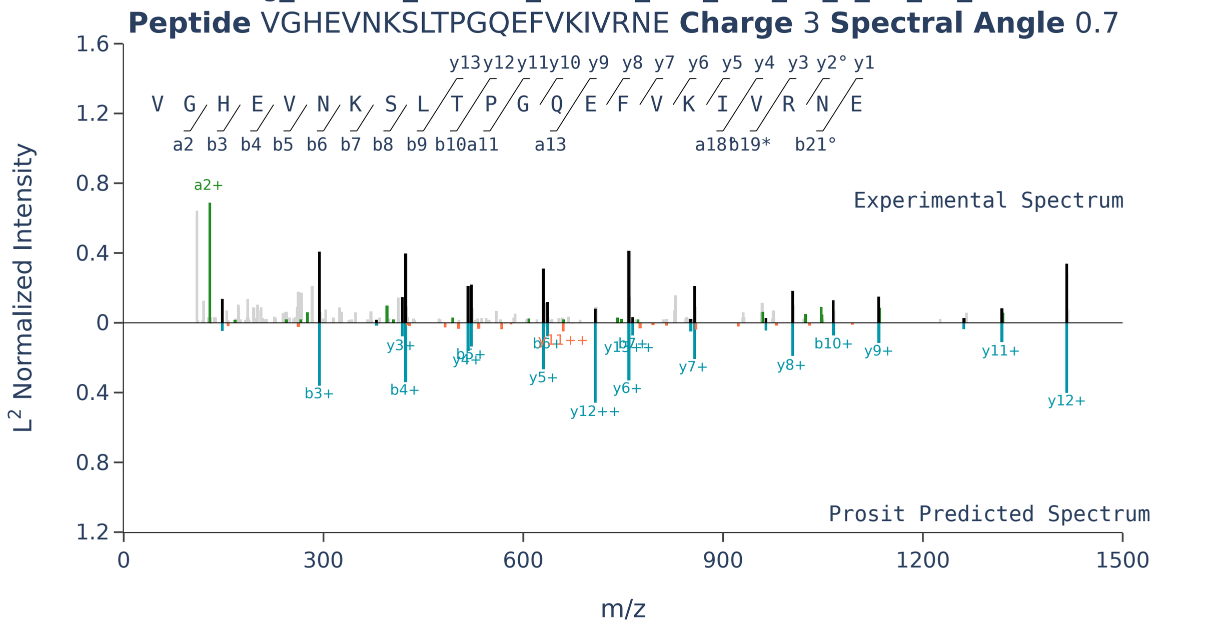


**Source** Ncheng_210623_230623_HFGoe_FFH_20S_25_1_A2_2h_R2 **Scan** 28723
**Peptide** GMASLMGKLPGMGQIPDNVKSQ **Charge** 3 **Spectral Angle** 0.79

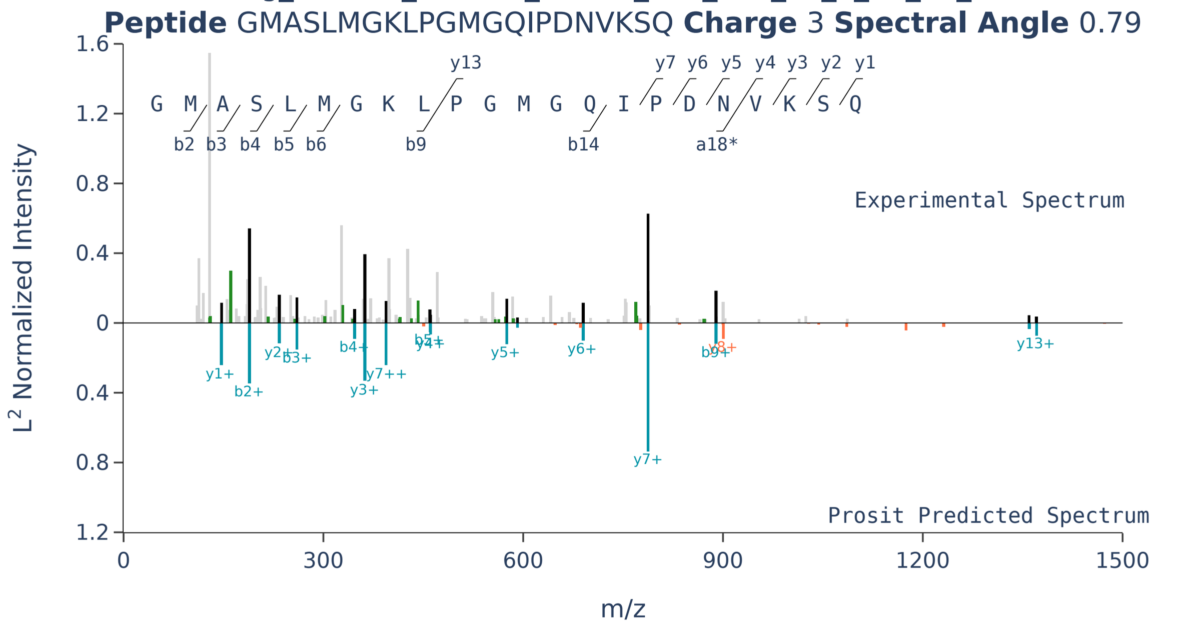


**Source** Ncheng_210623_230623_HFGoe_FFH_20S_25_1_A2_24h_R2 **Scan** 14091
**Peptide** NISGRGRLTEDNVKDTL **Charge** 3 **Spectral Angle** 0.66

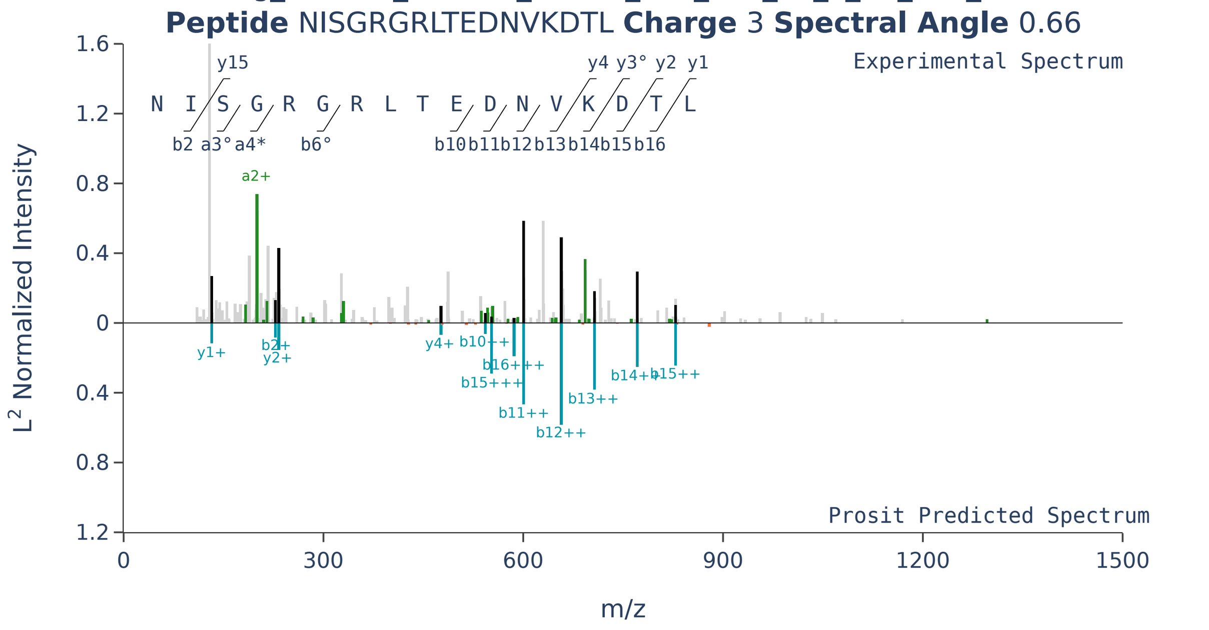


**Source** Ncheng_210623_230623_HFGoe_FFH_20S_25_1_A2_24h_R1 **Scan** 12296
**Peptide** KEAKLKFY **Charge** 2 **Spectral Angle** 0.79

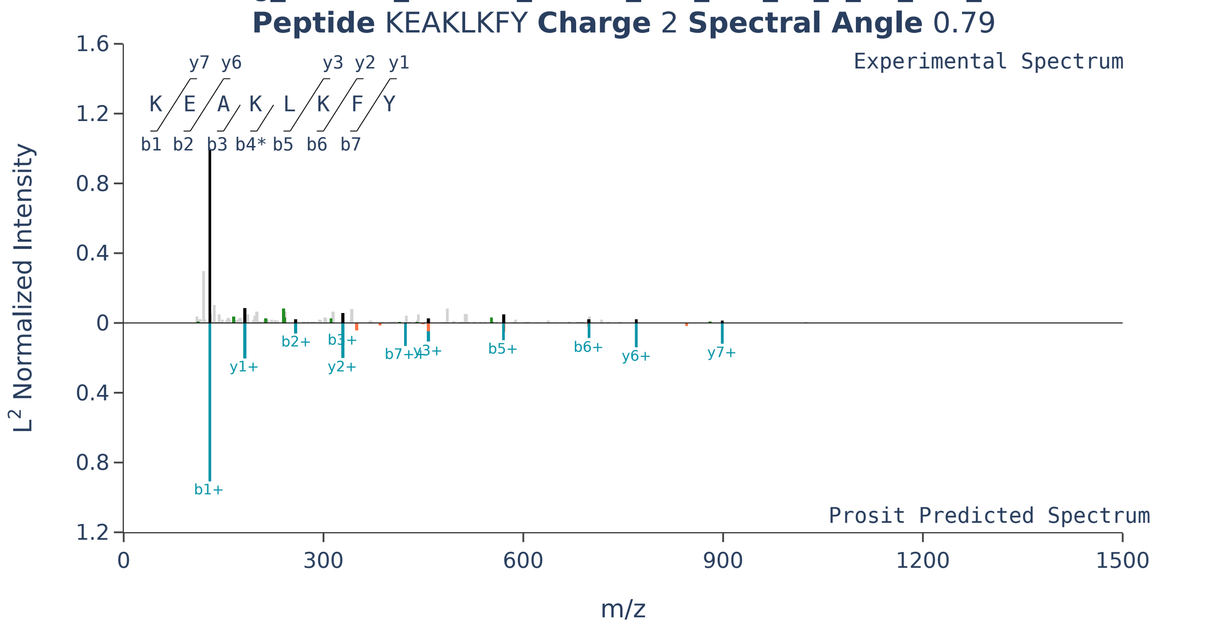


**Source** Ncheng_210623_230623_HFGoe_FFH_20S_25_1_A1_4h_R2 **Scan** 18688
**Peptide** LRHITGKPIKFLGVGE **Charge** 4 **Spectral Angle** 0.79

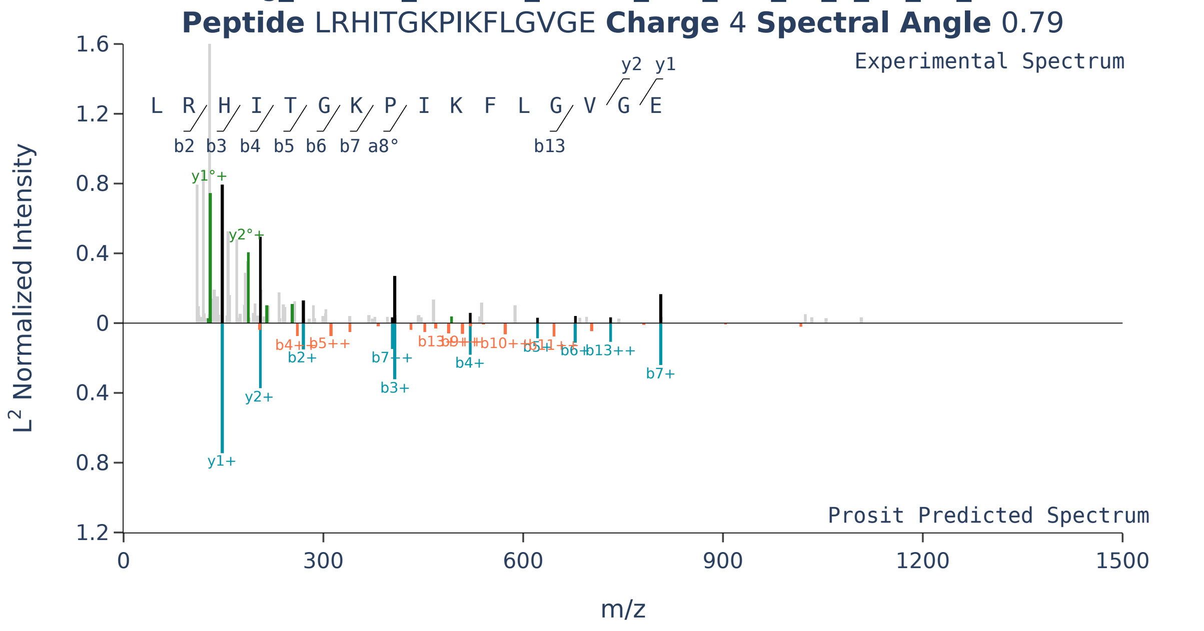


**Source** Ncheng_210623_230623_HFGoe_FFH_20S_25_1_A2_4h_R2 **Scan** 20583
**Peptide** KPIKFLGV **Charge** 2 **Spectral Angle** 0.58

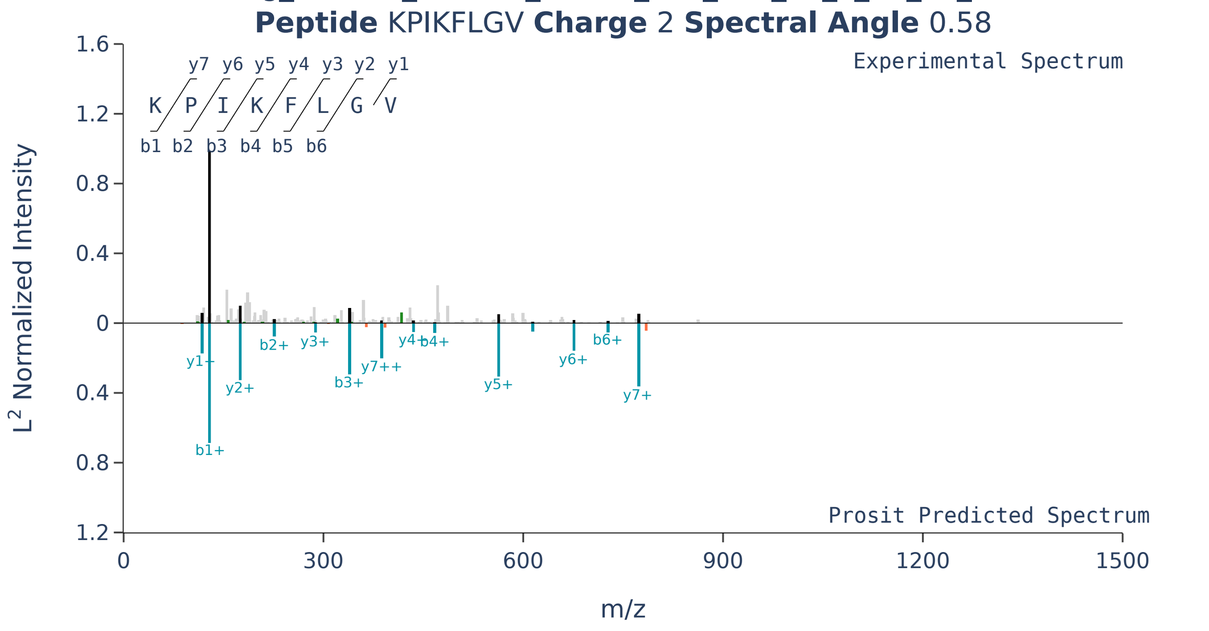


**Source** Ncheng_210623_230623_HFGoe_FFH_20S_25_1_A1_2h_R2 **Scan** 31109
**Peptide** AVGHEVNKSLTPGQEFVKIVRNELVAA **Charge** 3 **Spectral Angle** 0.83

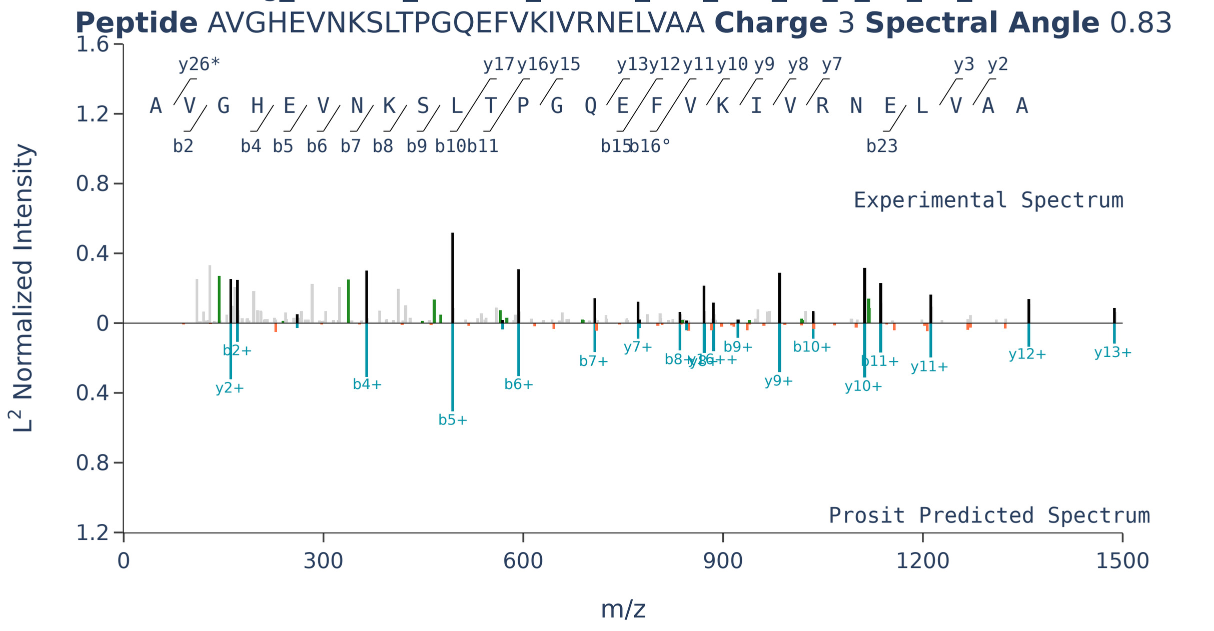


**Source** Ncheng_210623_230623_HFGoe_FFH_20S_25_1_A1_2h_R2 **Scan** 27102
**Peptide** SLMGKLPGMGQIPDNVKSQM **Charge** 3 **Spectral Angle** 0.69

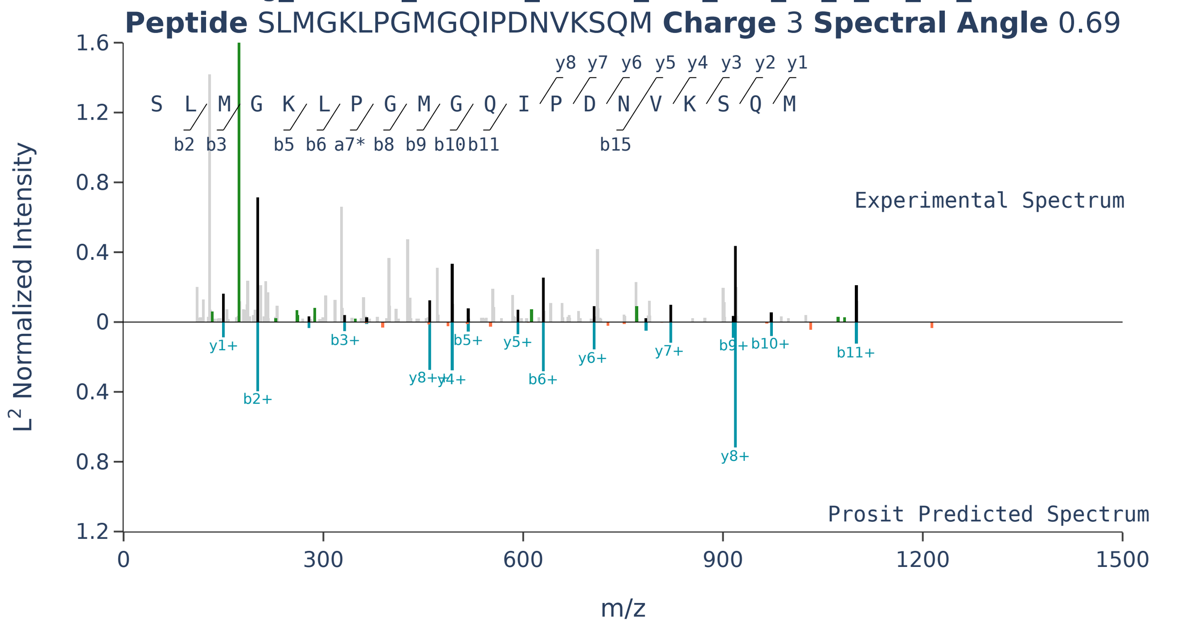


**Source** Ncheng_210623_230623_HFGoe_FFH_20S_25_1_A2_4h_R2 **Scan** 18370
**Peptide** KVDGDARGGAALSIRHITGKPLKF **Charge** 6 **Spectral Angle** 0.84

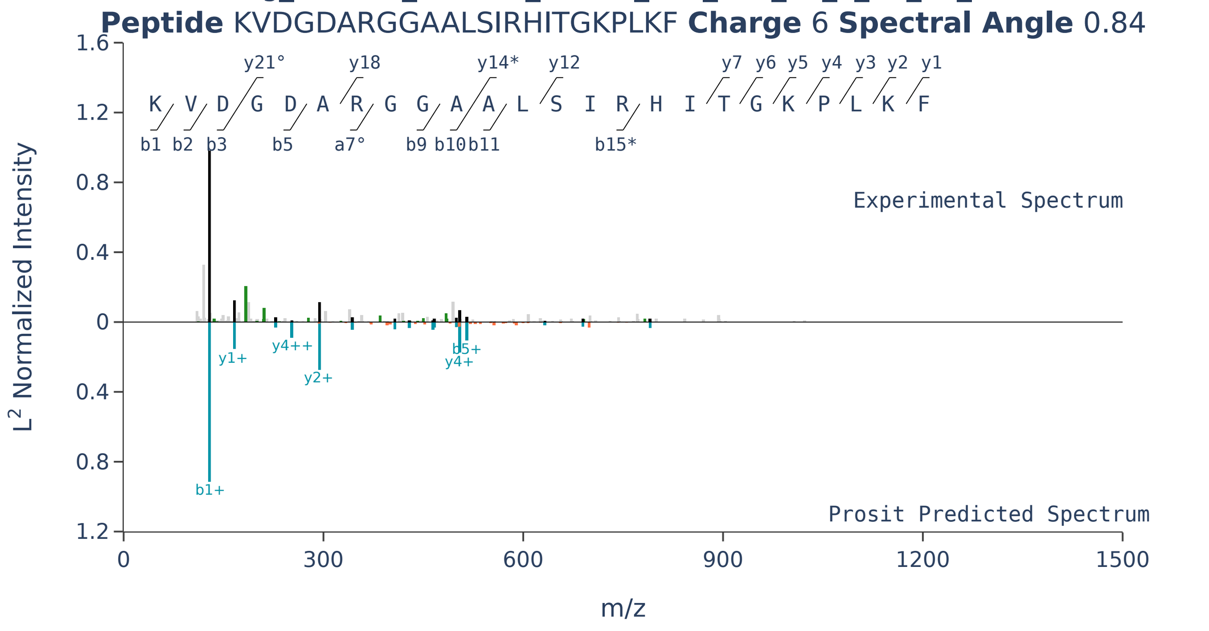


**Source** Ncheng_210623_230623_HFGoe_FFH_20S_25_1_A2_4h_R1 **Scan** 24845
**Peptide** MEAIINSMTMKERAKPEII **Charge** 2 **Spectral Angle** 0.89

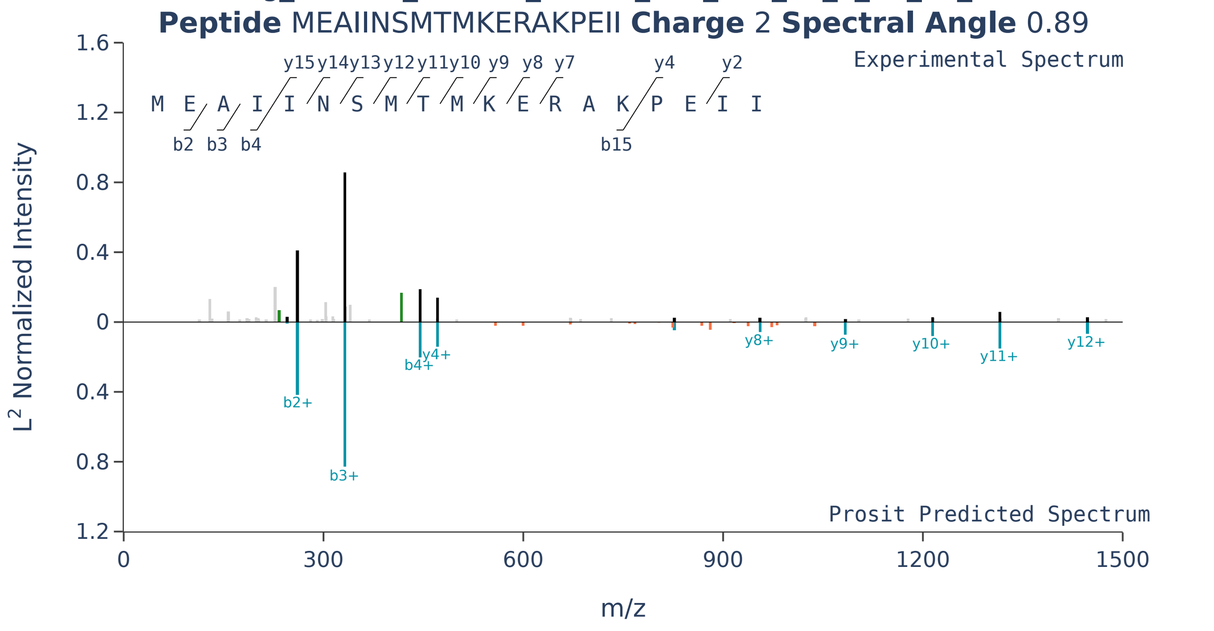


**Source** Ncheng_210623_230623_HFGoe_FFH_20S_25_1_A1_2h_R2 **Scan** 22682
**Peptide** KLPGMGQIPDNVKSQMDDKVLVR **Charge** 3 **Spectral Angle** 0.73

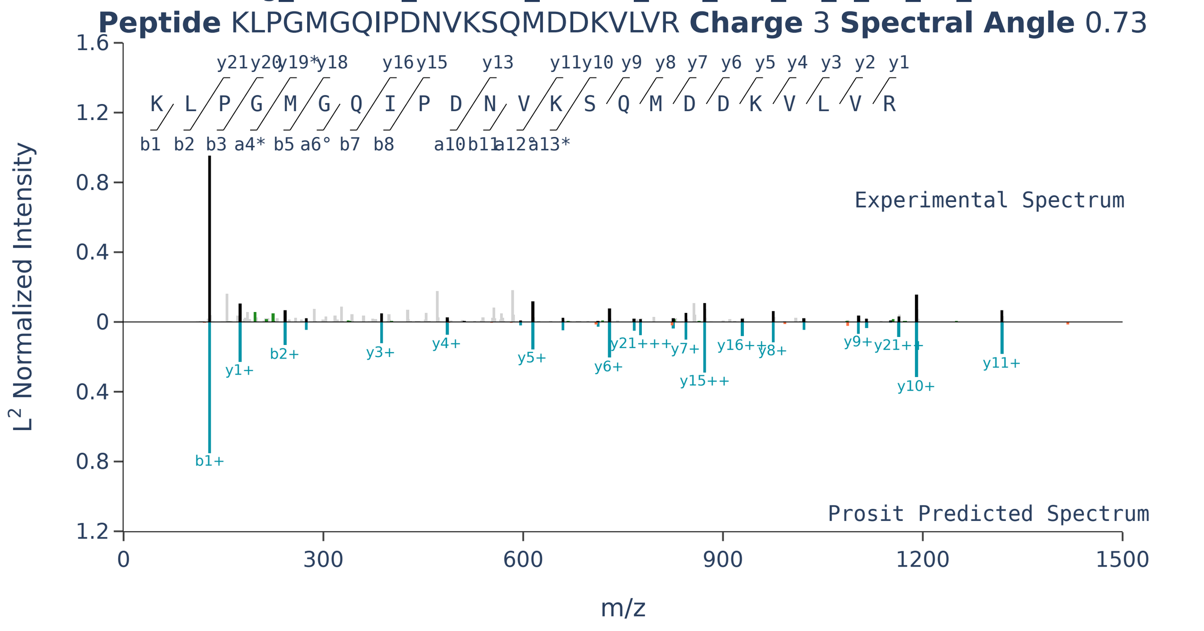


**Source** Ncheng_210623_230623_HFGoe_FFH_20S_25_1_A2_4h_R1 **Scan** 25987
**Peptide** ASLMGKLPGMGQIPDNVKS **Charge** 3 **Spectral Angle** 0.81

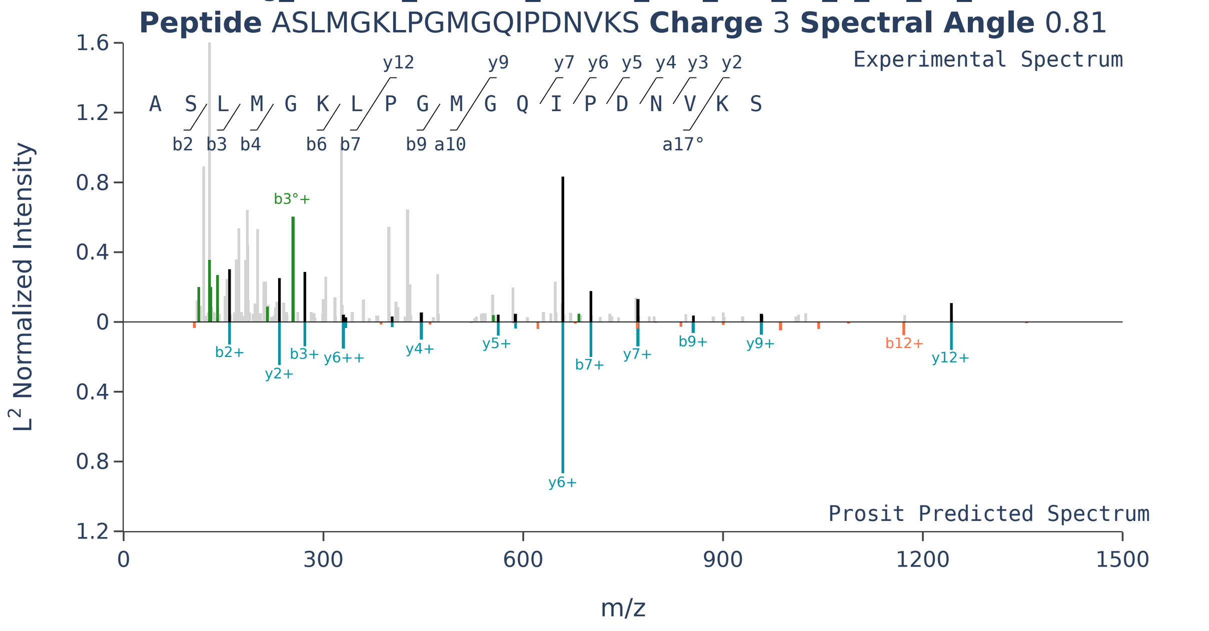


**Source** Ncheng_210623_230623_HFGoe_FFH_20S_25_1_A2_4h_R1 **Scan** 19191
**Peptide** SIRHITGKPIKFLGVGE **Charge** 4 **Spectral Angle** 0.81

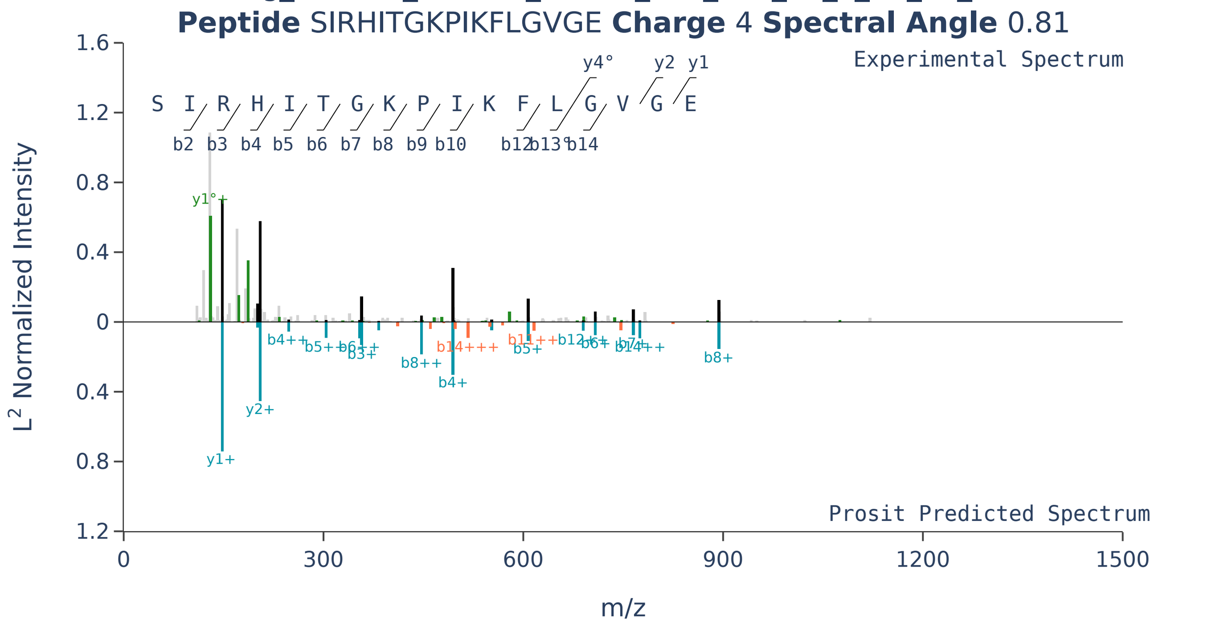

**Source** Ncheng_210623_230623_HFGoe_FFH_20S_25_1_A1_2h_R2 **Scan** 19453
**Peptide** KNMGGMASLMG **Charge** 2 **Spectral Angle** 0.62

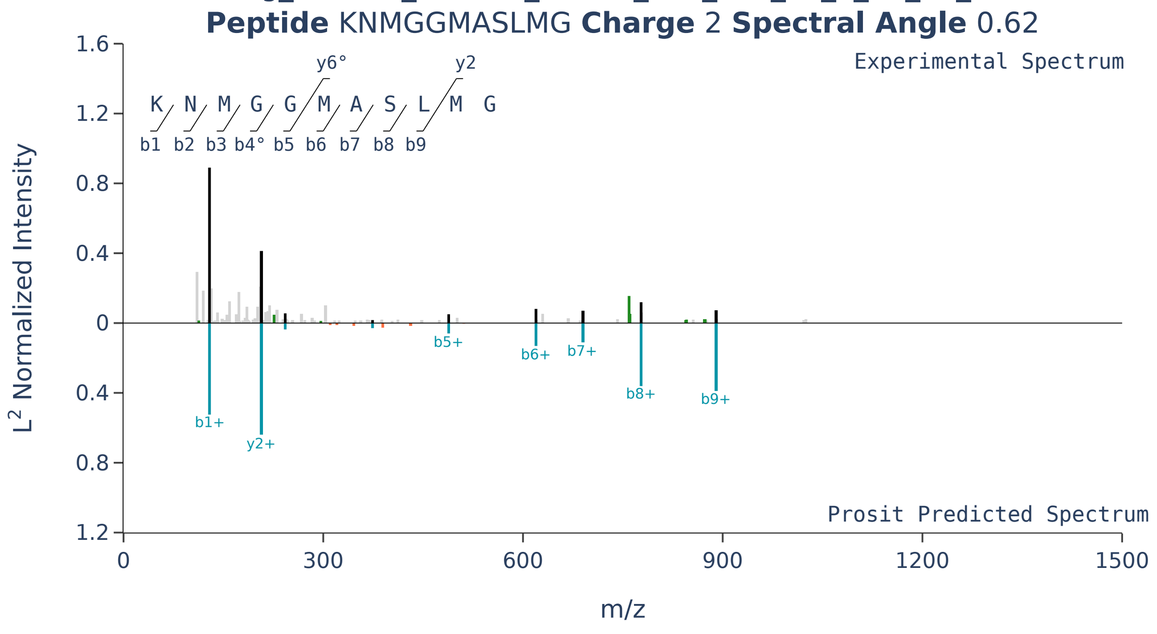


**Source** Ncheng_210623_230623_HFGoe_FFH_20S_25_1_A2_24h_R2 **Scan** 8466
**Peptide** SRTLRNI **Charge** 2 **Spectral Angle** 0.75

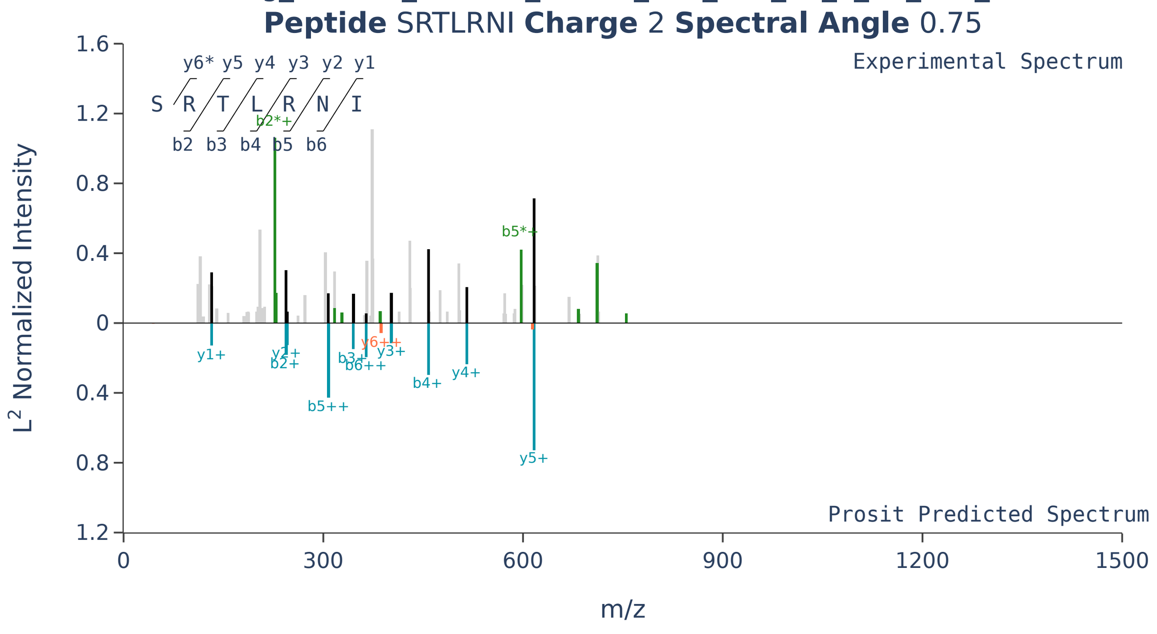


**Source** Ncheng_210623_230623_HFGoe_FFH_20S_25_1_A2_4h_R2 **Scan** 18940
**Peptide** DVYRPAAIKQLET **Charge** 2 **Spectral Angle** 0.76

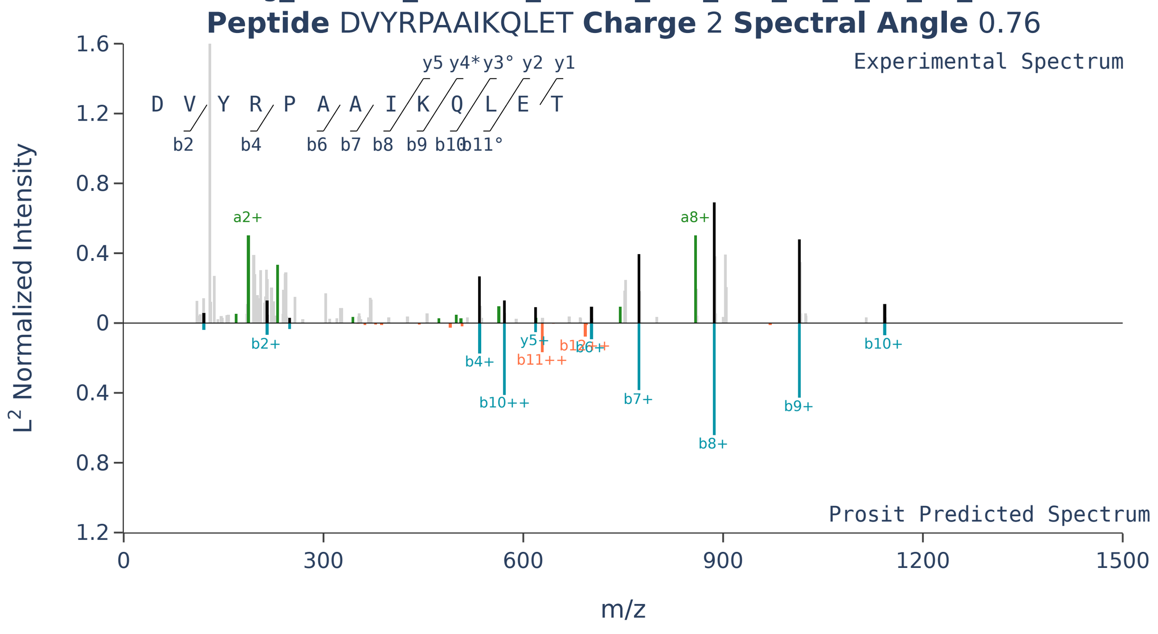


**Source** Ncheng_210623_230623_HFGoe_FFH_20S_25_1_A2_24h_R1 **Scan** 12884
**Peptide** QIPDNVKSQM **Charge** 2 **Spectral Angle** 0.62

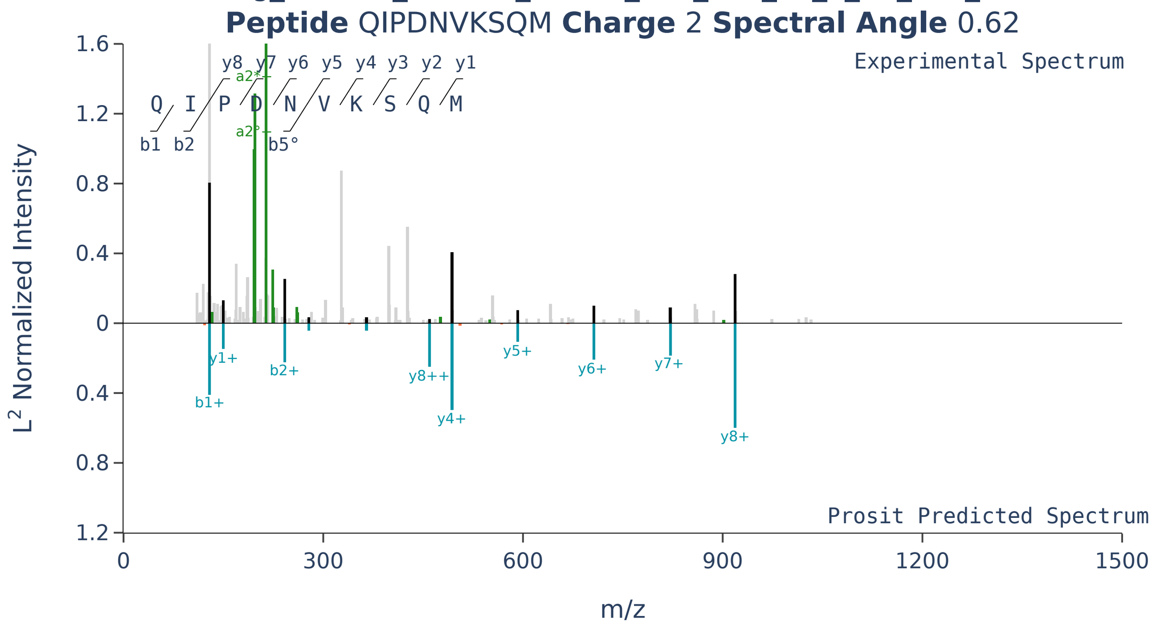


**Source** Ncheng_210623_230623_HFGoe_FFH_20S_25_1_A2_2h_R1 **Scan** 25942
**Peptide** AMGEENQTLNLAAQPPAV **Charge** 2 **Spectral Angle** 0.97

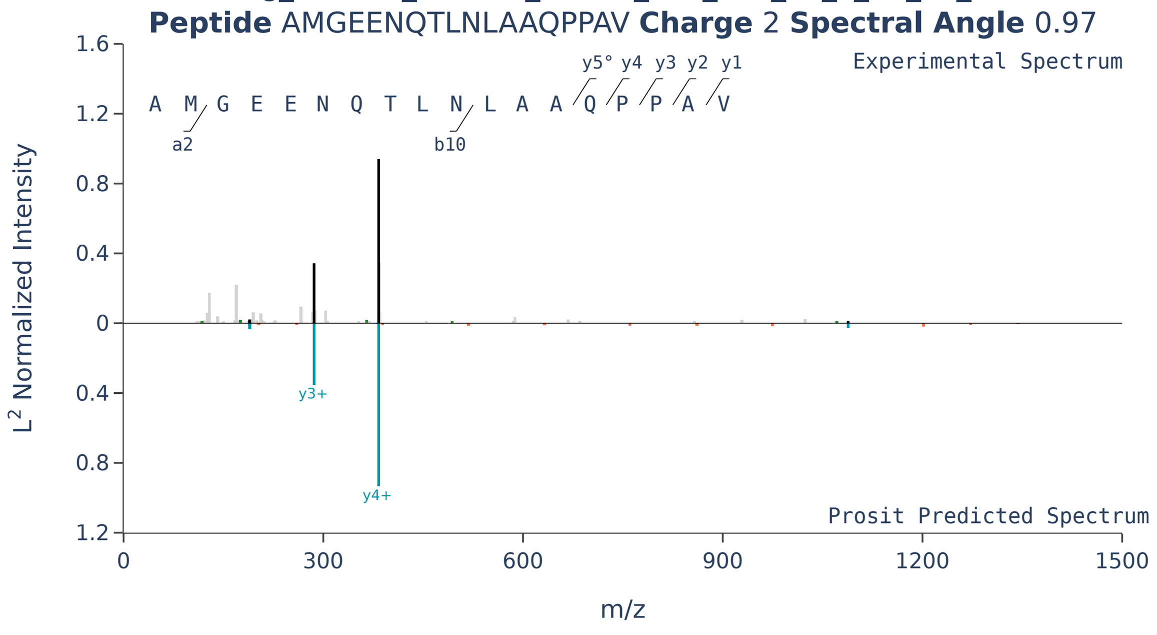


**Source** Ncheng_210623_230623_HFGoe_FFH_20S_25_1_A2_4h_R1 **Scan** 22122
**Peptide** VVLTKVDGDARGGAALSI **Charge** 2 **Spectral Angle** 0.71

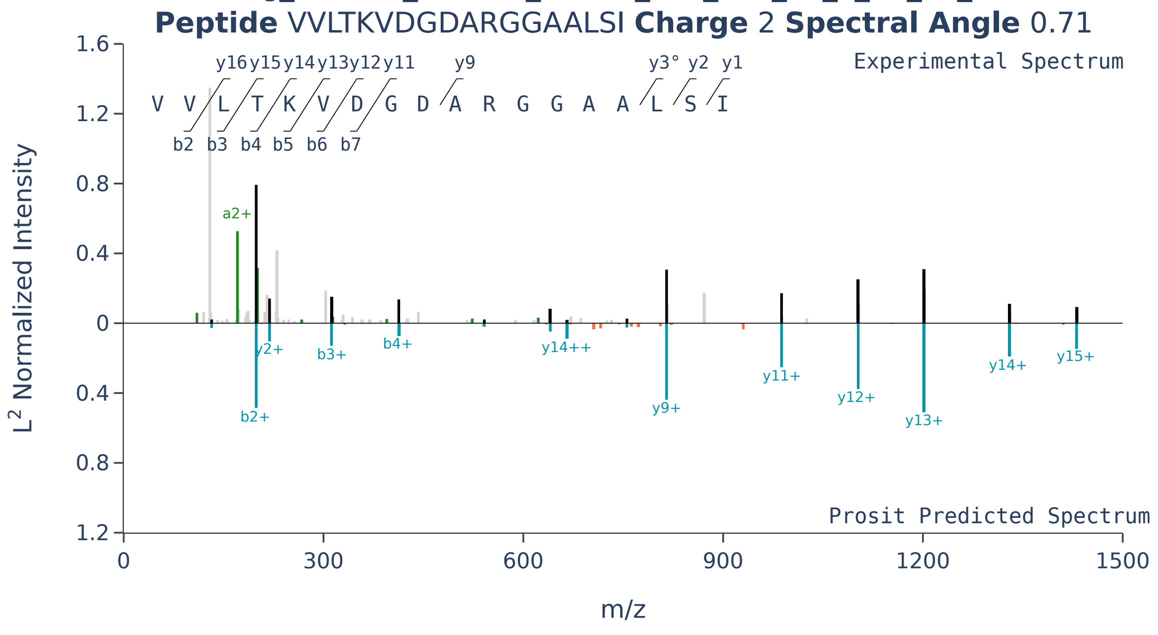


**Source** Ncheng_210623_230623_HFGoe_FFH_20S_25_1_A1_4h_R1 **Scan** 26856
**Peptide** DAANTAKAFNEALPLTG **Charge** 2 **Spectral Angle** 0.77

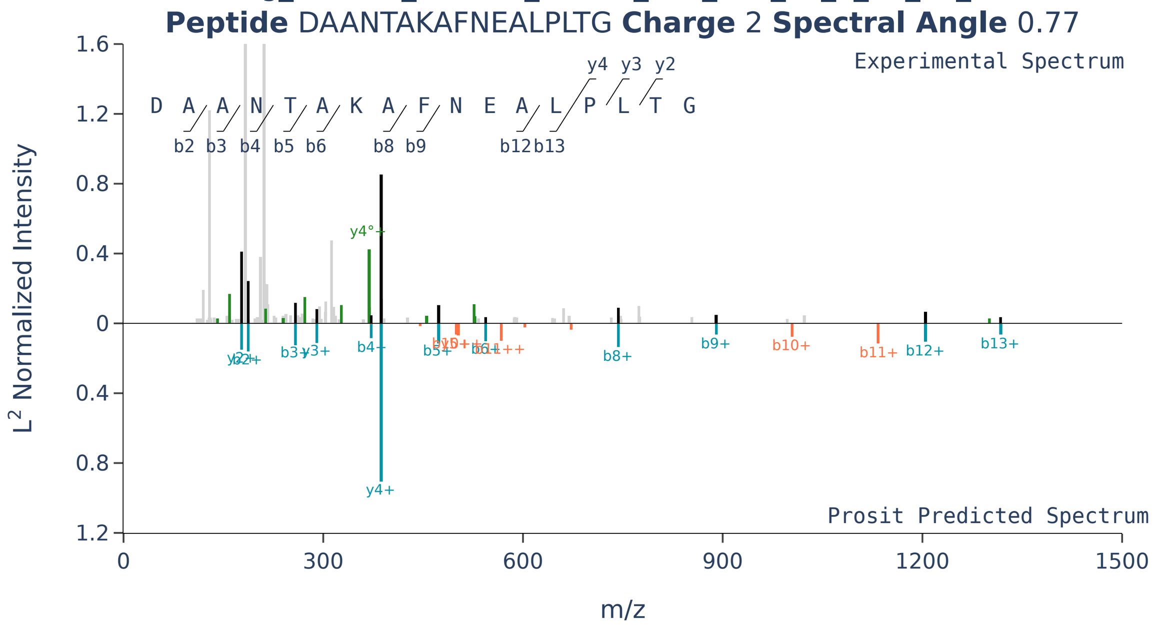


**Source** Ncheng_210623_230623_HFGoe_FFH_20S_25_1_A2_4h_R1 **Scan** 35085
**Peptide** EALPLTGVVLTKVDGDARGGAALSL **Charge** 3 **Spectral Angle** 0.76

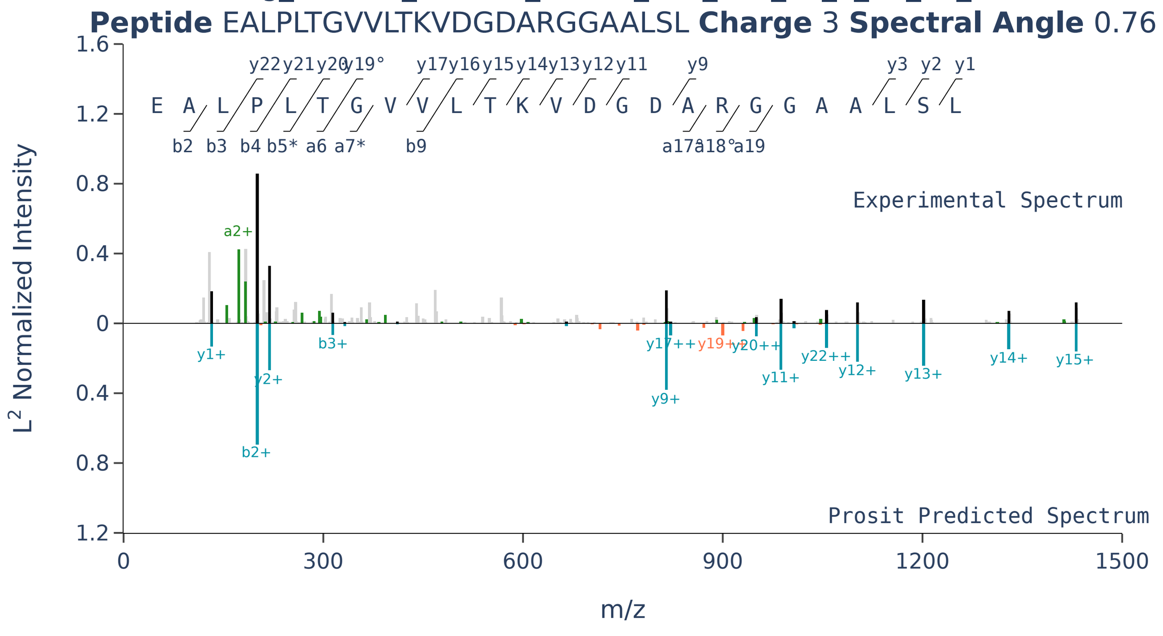


**Source** Ncheng_210623_230623_HFGoe_FFH_20S_25_1_A2_4h_R2 **Scan** 14761
**Peptide** MMDEIKQVH **Charge** 2 **Spectral Angle** 0.84

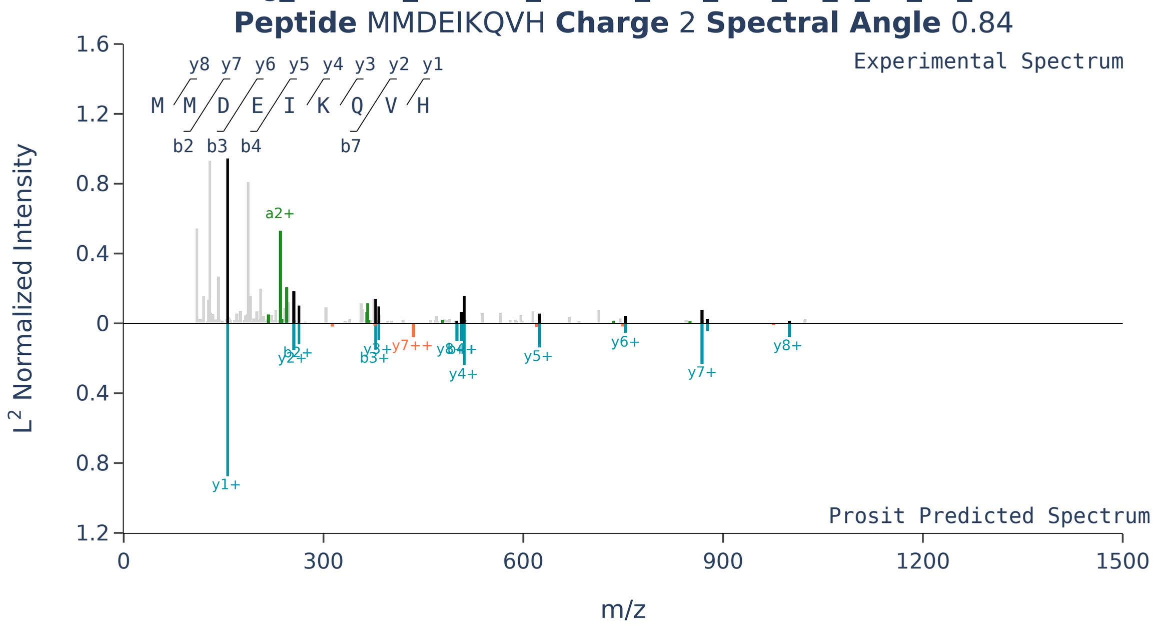


**Source** Ncheng_210623_230623_HFGoe_FFH_20S_25_1_A1_2h_R1 **Scan** 30201
**Peptide** KFYDVLLVDTAGRLHVD **Charge** 3 **Spectral Angle** 0.82

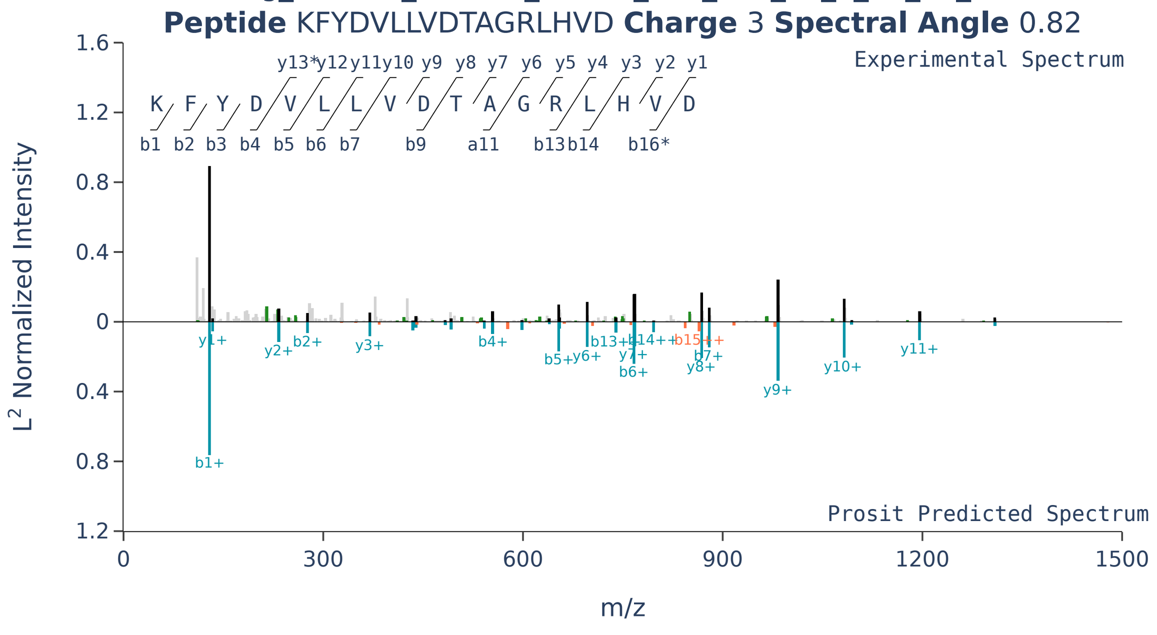


**Source** Ncheng_210623_230623_HFGoe_FFH_20S_25_1_A1_24h_R1 **Scan** 15630
**Peptide** KVDGDARGGAALSL **Charge** 2 **Spectral Angle** 0.72

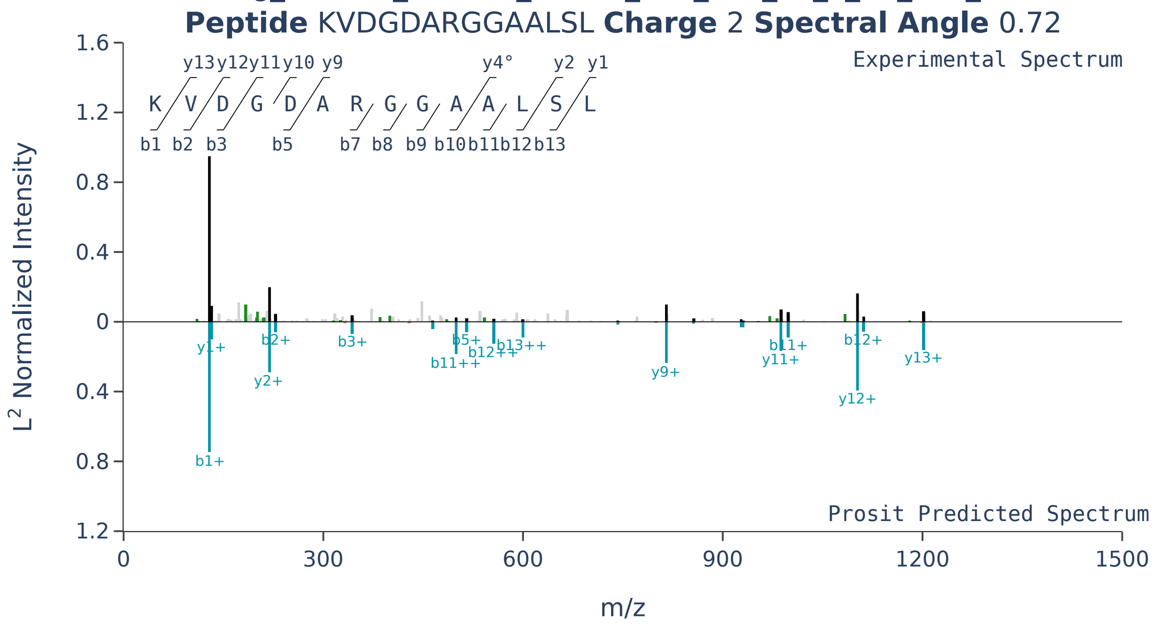


**Source** Ncheng_210623_230623_HFGoe_FFH_20S_25_1_A2_4h_R1 **Scan** 8009
**Peptide** DMQRMMK **Charge** 2 **Spectral Angle** 0.79

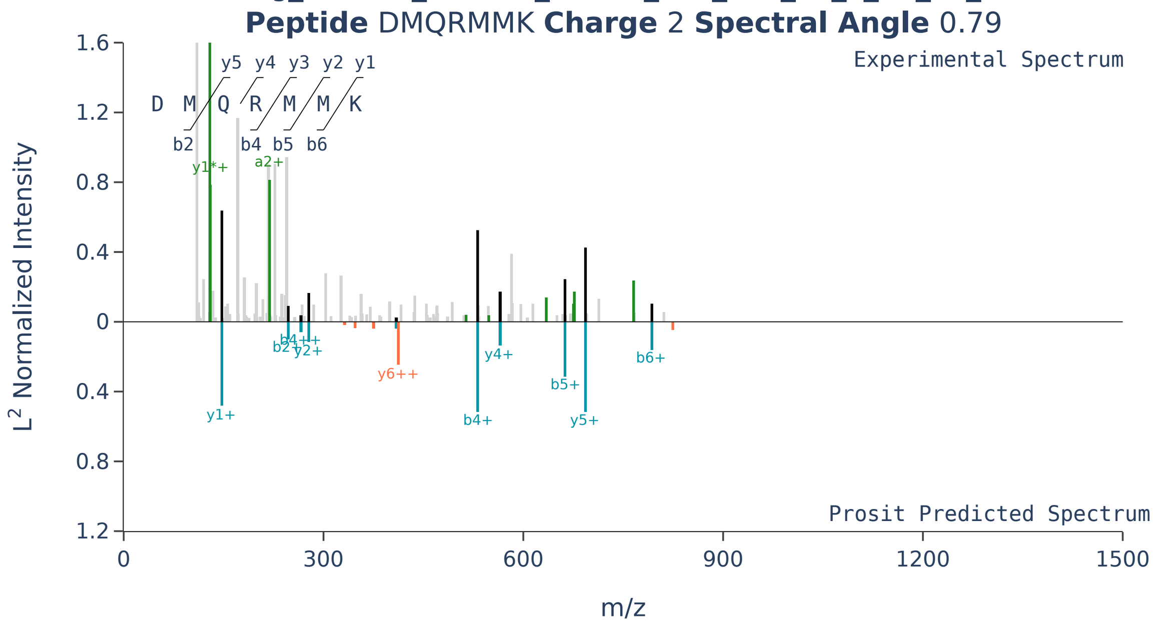


**Source** Ncheng_210623_230623_HFGoe_FFH_20S_25_1_A2_2h_R2 **Scan** 25633
**Peptide** QDAANTAKAFNEALPLTG **Charge** 2 **Spectral Angle** 0.82

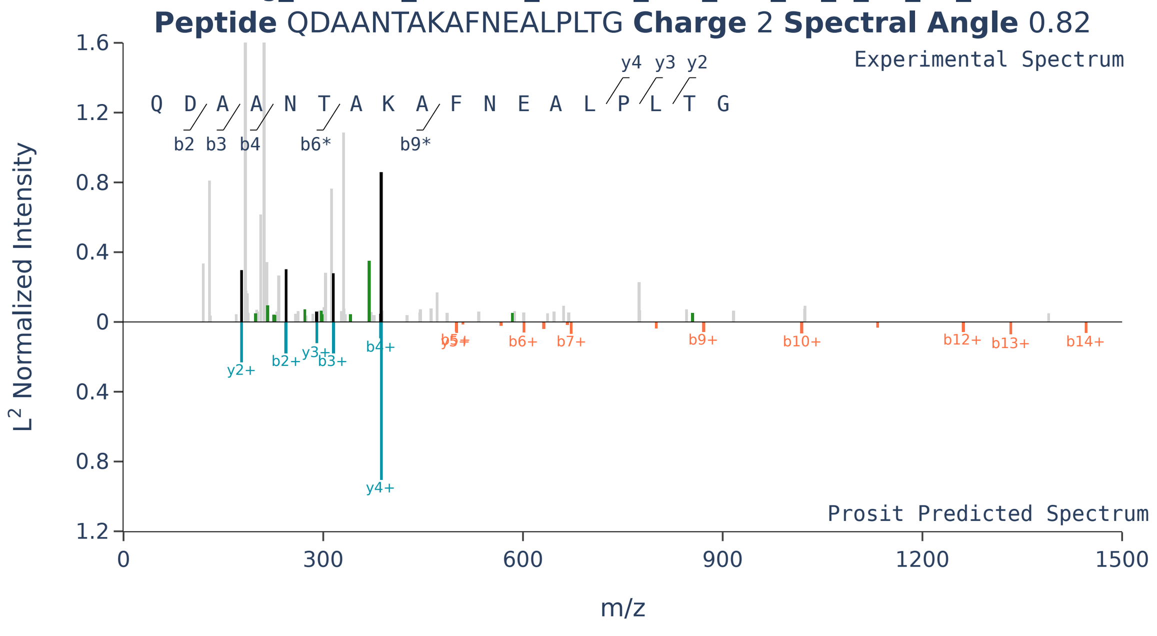


**Source** Ncheng_210623_230623_HFGoe_FFH_20S_25_1_A1_24h_R2 **Scan** 14851
**Peptide** AGCGMQVQDV **Charge** 2 **Spectral Angle** 0.85

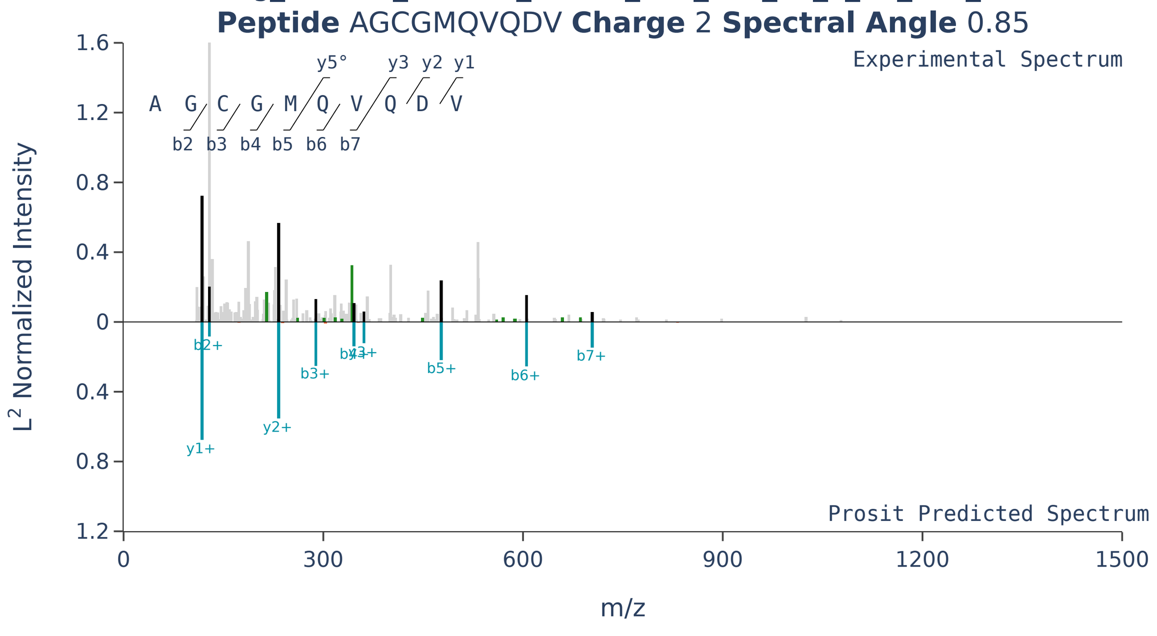


**Source** Ncheng_210623_230623_HFGoe_FFH_20S_25_1_A2_24h_R1 **Scan** 6132
**Peptide** AGKTTSVGKLGK **Charge** 3 **Spectral Angle** 0.86

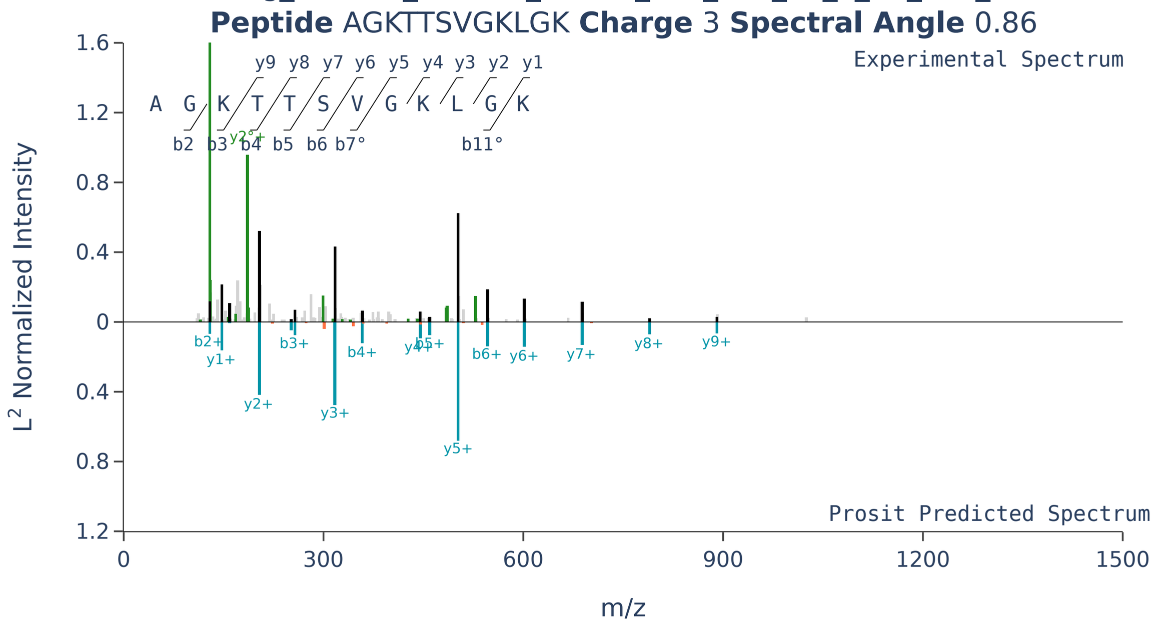

**Source** Ncheng_210623_230623_HFGoe_FFH_20S_25_1_A2_4h_R1 **Scan** 29396
**Peptide** RLAAGCGMQVQDVNRLLKQF **Charge** 3 **Spectral Angle** 0.78

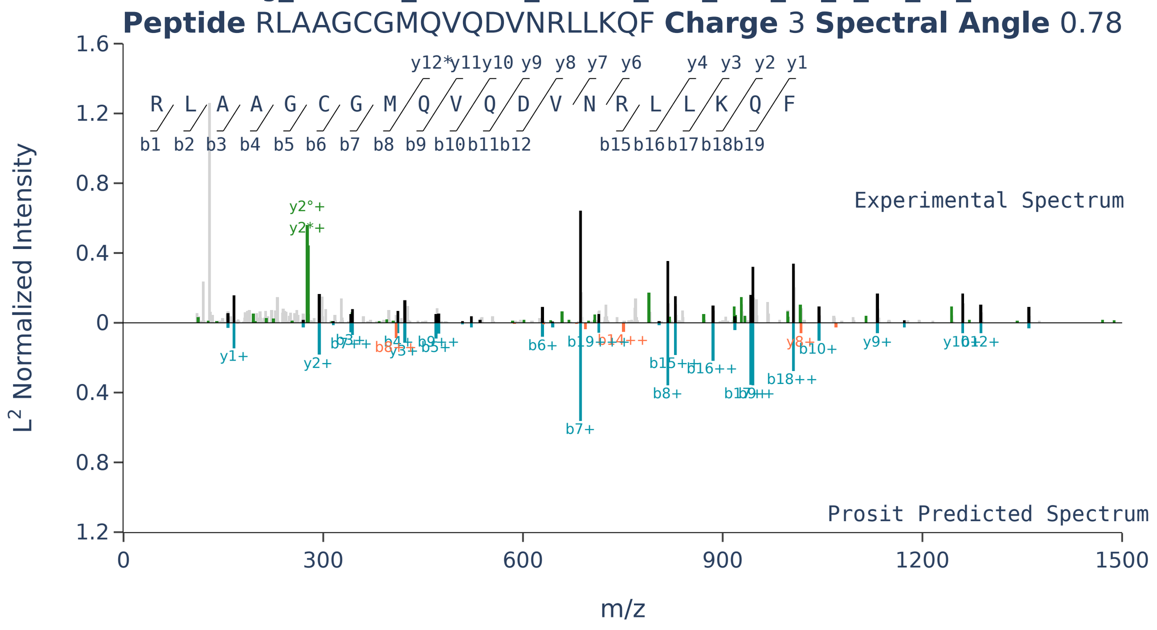


**Source** Ncheng_210623_230623_HFGoe_FFH_20S_25_1_A2_4h_R1 **Scan** 19607
**Peptide** EFINRVKEKAVGHEVNKSLTPGQEFVKIVR **Charge** 4 **Spectral Angle** 0.86

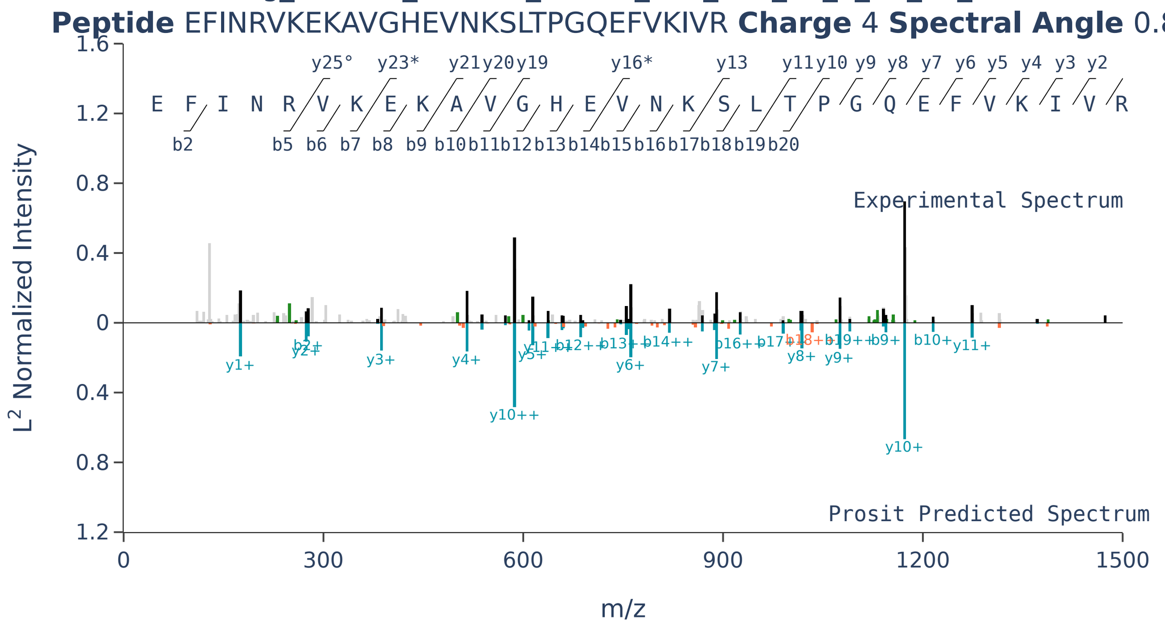


**Source** Ncheng_210623_230623_HFGoe_FFH_20S_25_1_A2_4h_R2 **Scan** 9666
**Peptide** VDAMTGQDAANTA **Charge** 2 **Spectral Angle** 0.87

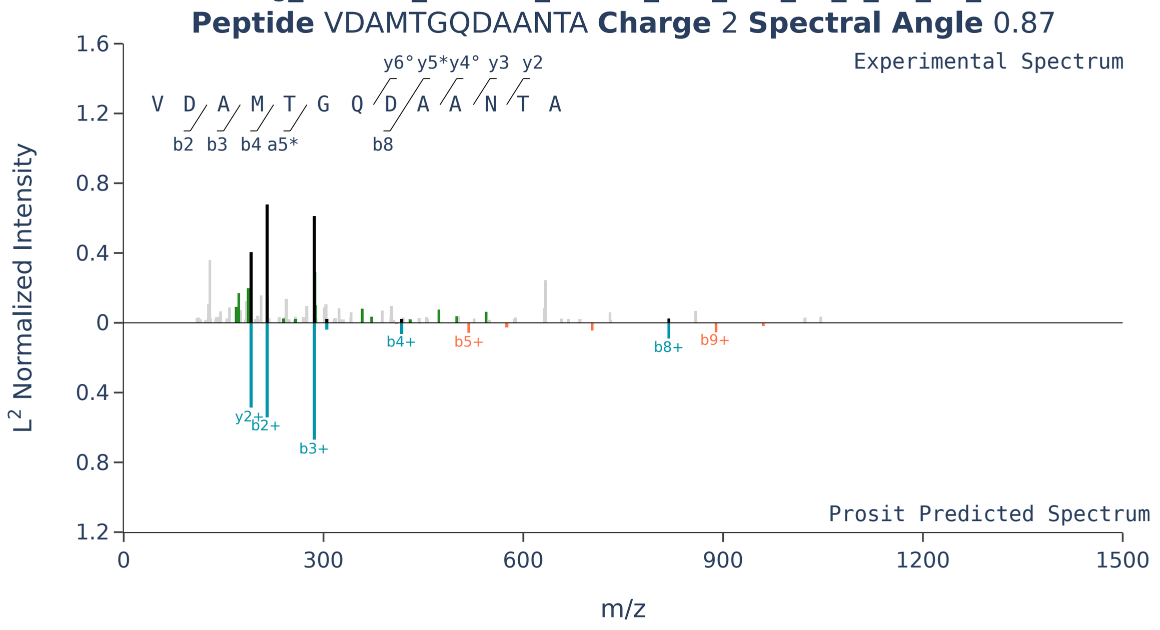


**Source** Ncheng_210623_230623_HFGoe_FFH_20S_25_1_A2_24h_R1 **Scan** 15164
**Peptide** GKTTSVGKLGKFLRE **Charge** 2 **Spectral Angle** 0.77

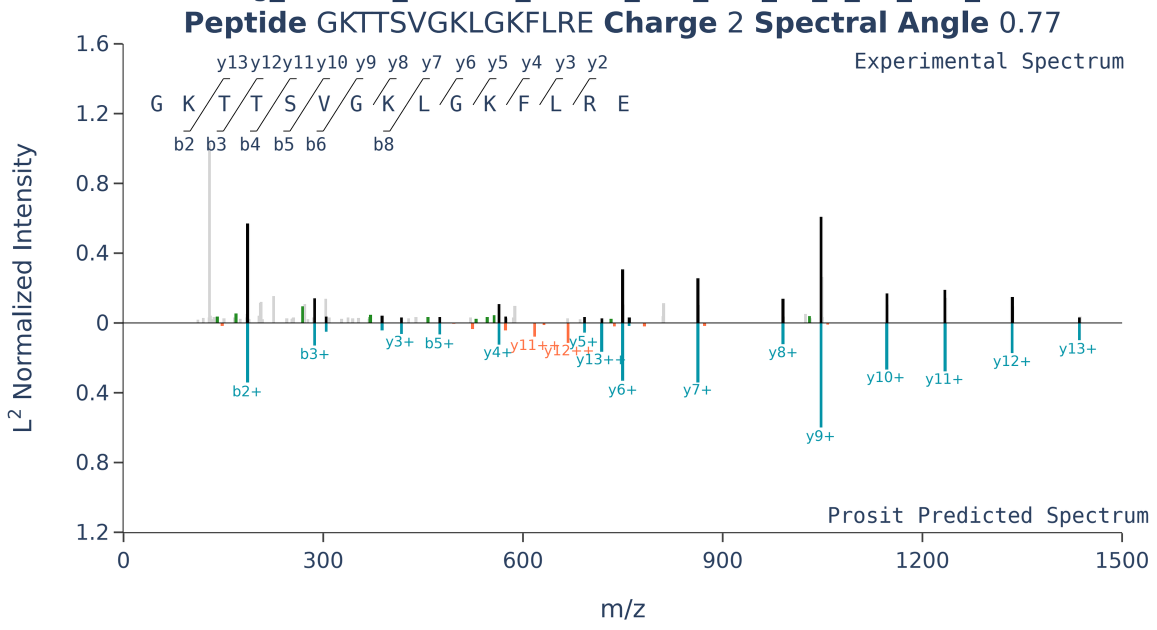


**Source** Ncheng_210623_230623_HFGoe_FFH_20S_25_1_A2_24h_R2 **Scan** 10242
**Peptide** VDTAGRLHVDE **Charge** 1 **Spectral Angle** 0.72

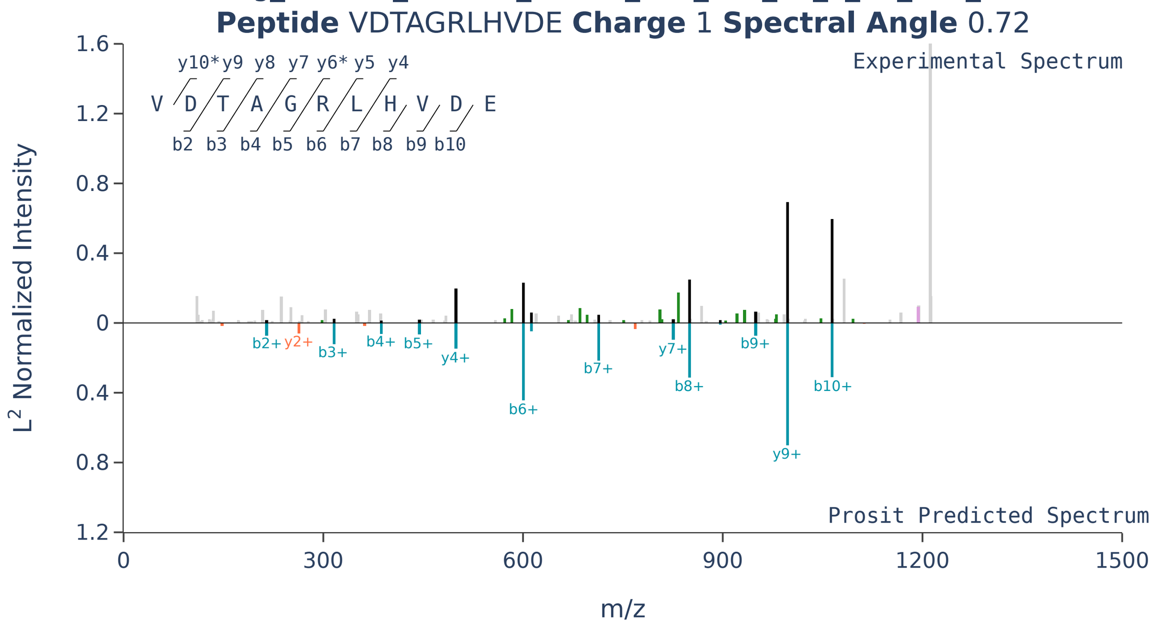


**Source** Ncheng_210623_230623_HFGoe_FFH_20S_25_1_A1_2h_R2 **Scan** 17424
**Peptide** GEKTEALEPFHPDRLAS **Charge** 3 **Spectral Angle** 0.85

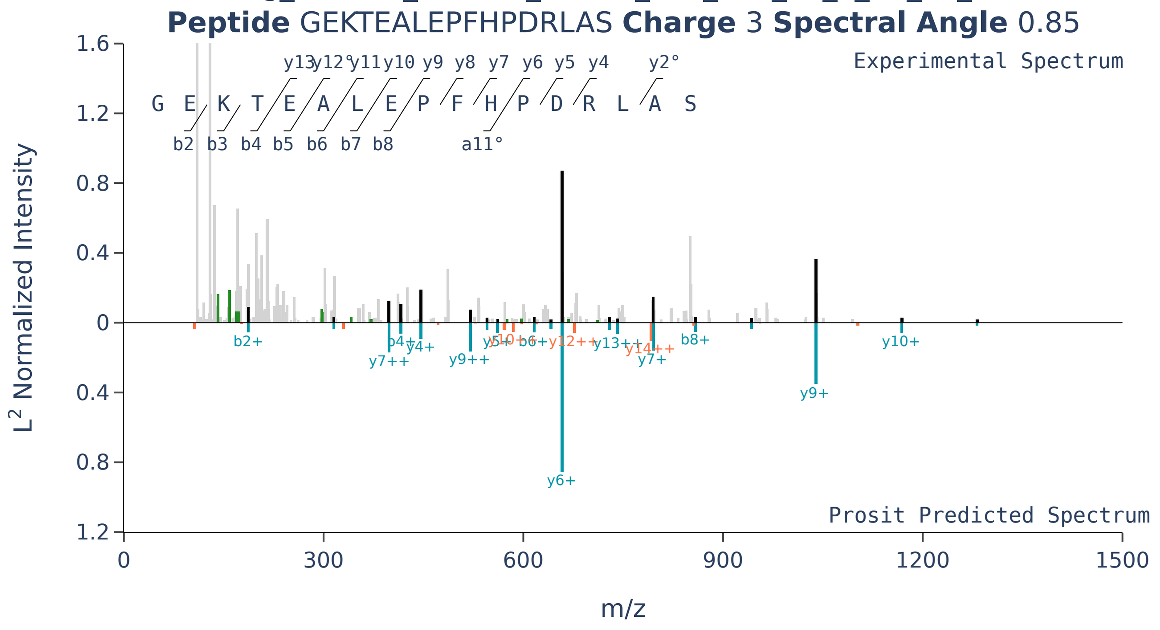


**Source** Ncheng_210623_230623_HFGoe_FFH_20S_25_1_A1_1h_R1 **Scan** 30112
**Peptide** KLKFYDVLLVDTAGRLHVD **Charge** 3 **Spectral Angle** 0.8

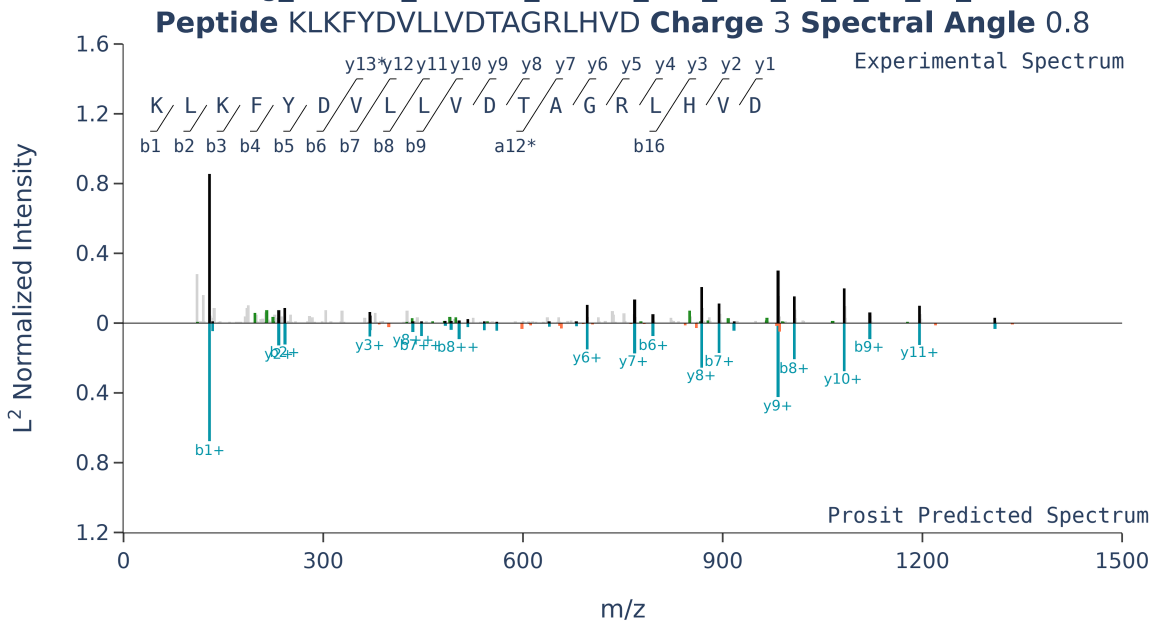


**Source** Ncheng_210623_230623_HFGoe_FFH_20S_25_1_A1_2h_R1 **Scan** 26690
**Peptide** SADVYRPAAIKQLETLAE **Charge** 3 **Spectral Angle** 0.78

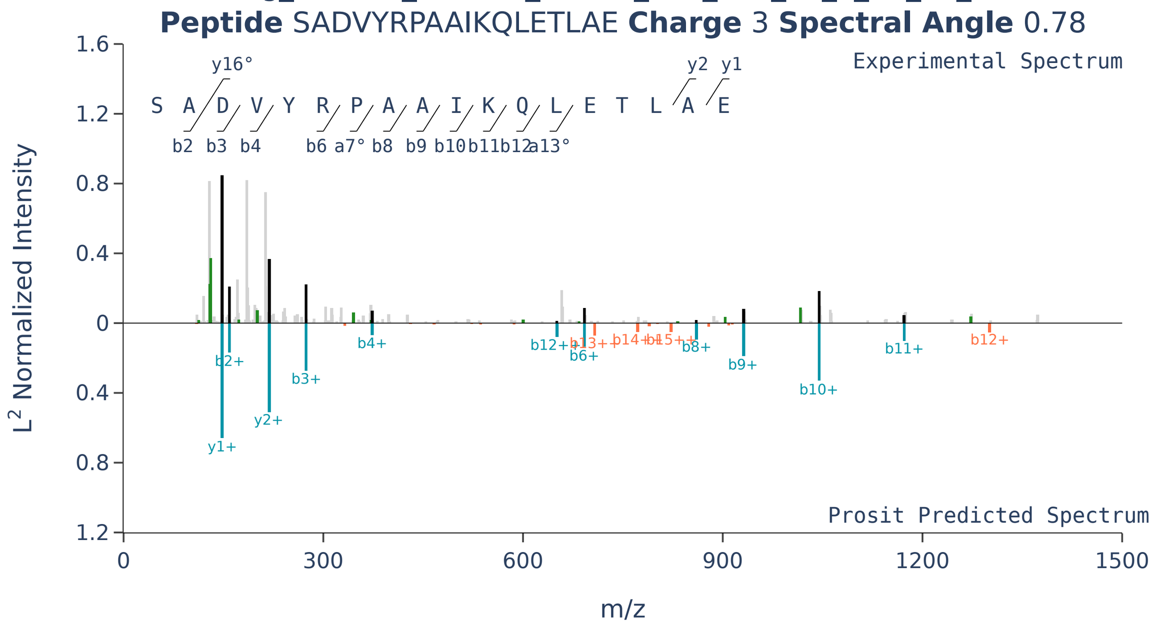


**Source** Ncheng_210623_230623_HFGoe_FFH_20S_25_1_A2_24h_R2 **Scan** 12546
**Peptide** NLSGRGRLTEDNVKDTLRE **Charge** 4 **Spectral Angle** 0.81

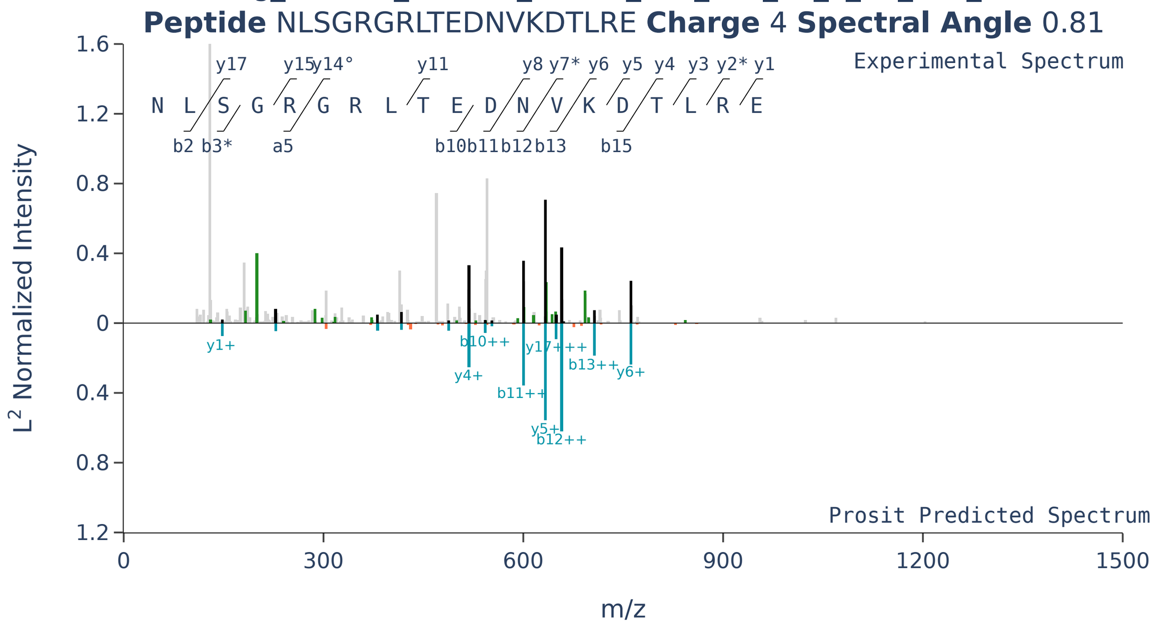


**Source** Ncheng_210623_230623_HFGoe_FFH_20S_25_1_A2_1h_R1 **Scan** 12821
**Peptide** RIAAGCGMQV **Charge** 2 **Spectral Angle** 0.83

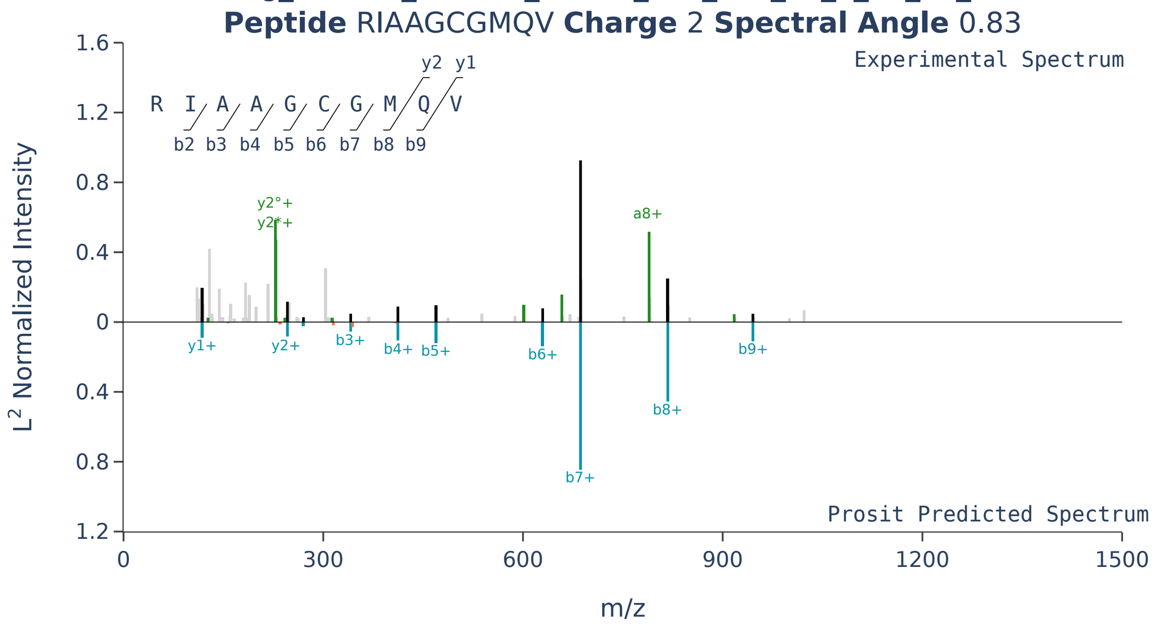


**Source** Ncheng_210623_230623_HFGoe_FFH_20S_25_1_A2_24h_R2 **Scan** 10265
**Peptide** KGGMAKMMRSM **Charge** 3 **Spectral Angle** 0.79

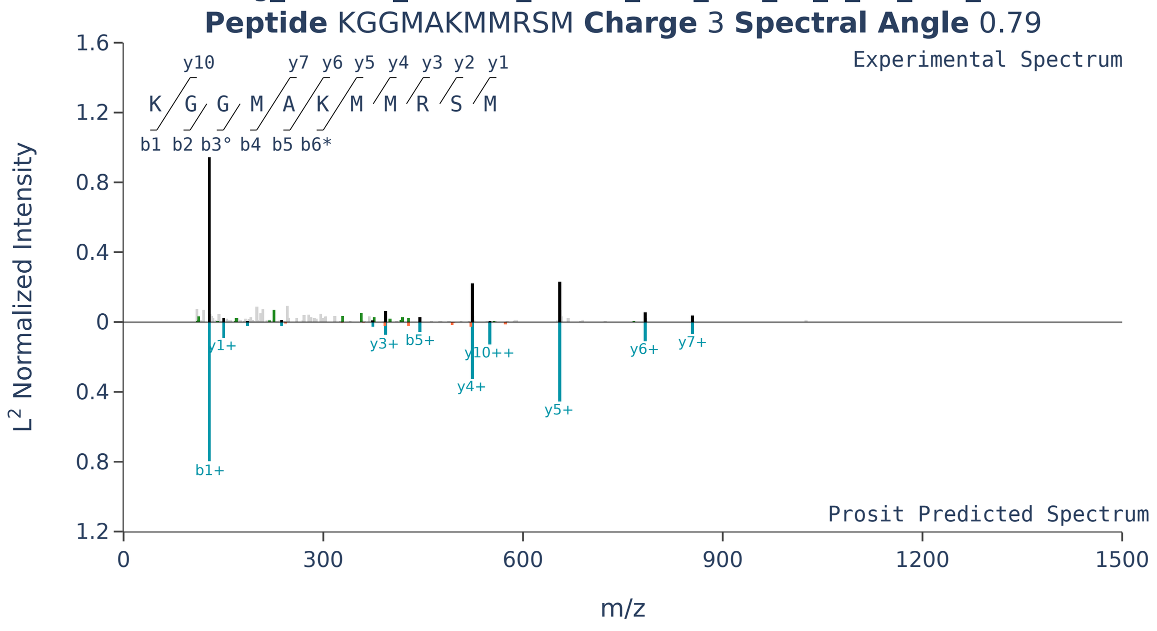


**Source** Ncheng_210623_230623_HFGoe_FFH_20S_25_1_A1_2h_R1 **Scan** 28664
**Peptide** AALKEAKLKFYDVLL **Charge** 3 **Spectral Angle** 0.68

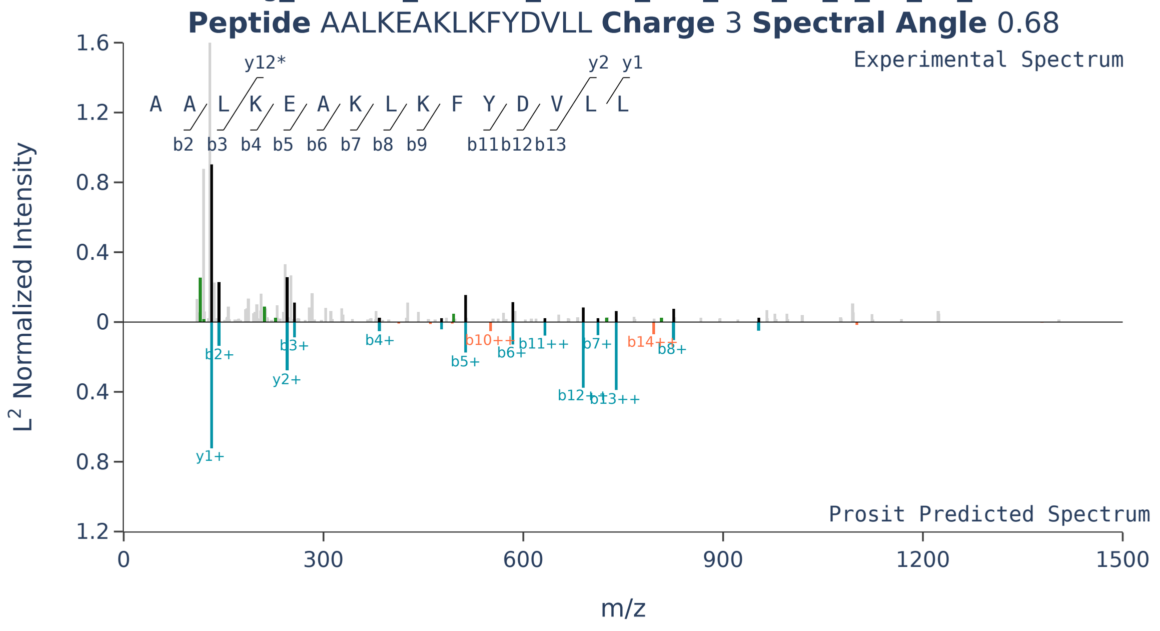


**Source** Ncheng_210623_230623_HFGoe_FFH_20S_25_1_A2_4h_R1 **Scan** 34386
**Peptide** AANTAKAFNEALPLTGVVLTKV **Charge** 2 **Spectral Angle** 0.79

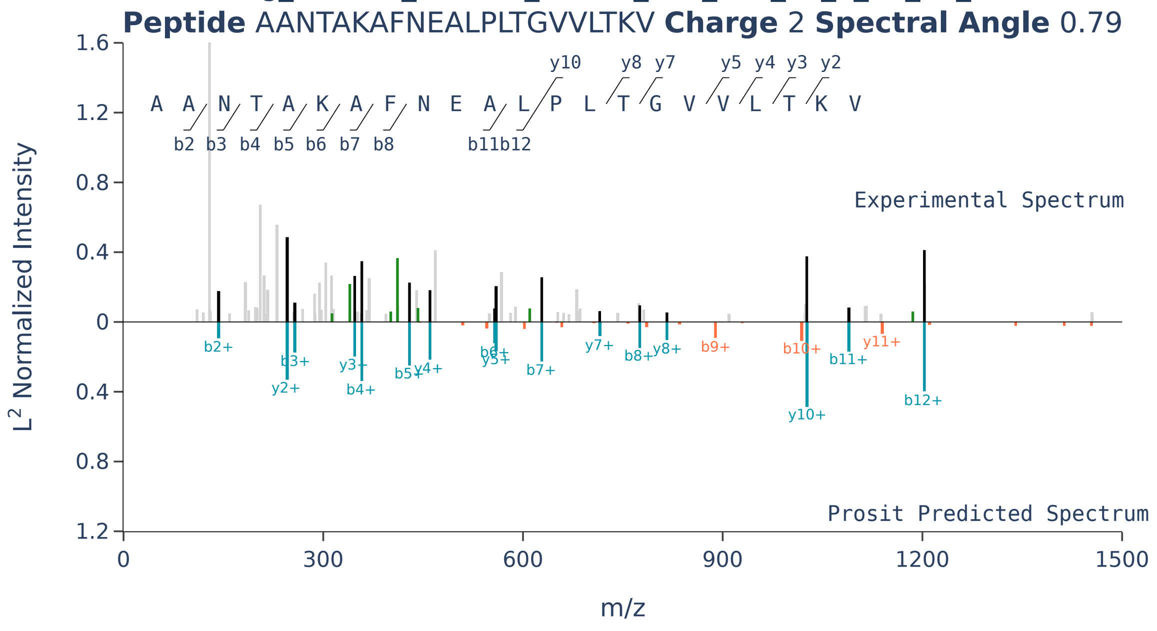


**Source** Ncheng_210623_230623_HFGoe_FFH_20S_25_1_A2_2h_R1 **Scan** 34982
**Peptide** TGQDAANTAKAFNEALPLTGVVLTKV **Charge** 3 **Spectral Angle** 0.8

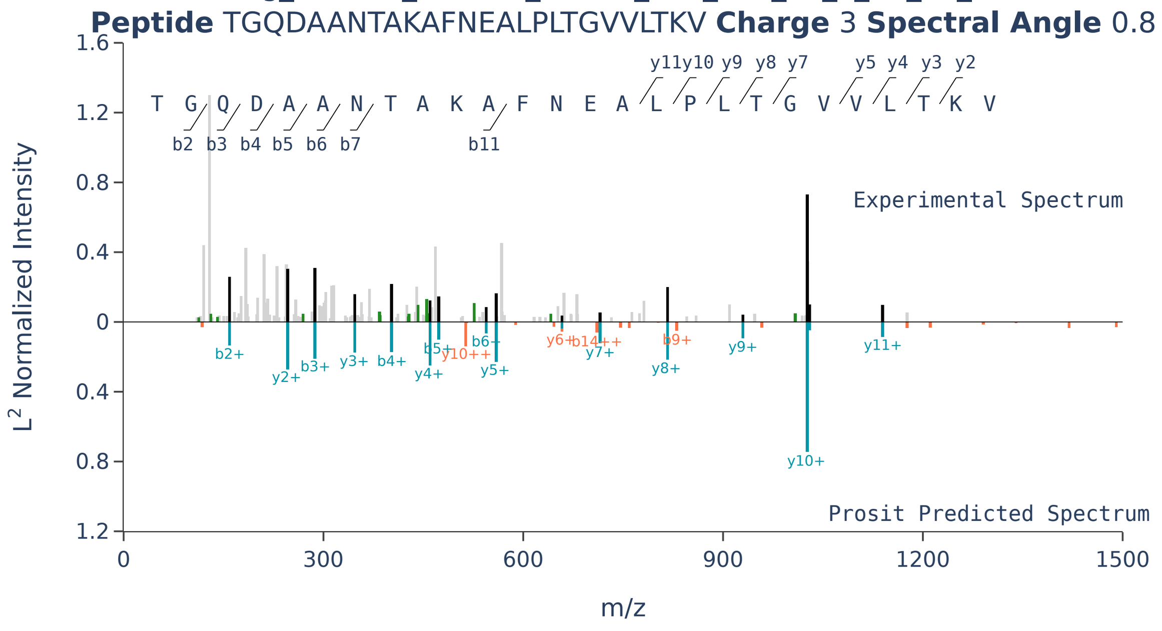


**Source** Ncheng_210623_230623_HFGoe_FFH_20S_25_1_A1_4h_R1 **Scan** 15170
**Peptide** SGRGRLTEDNVKDTLREV **Charge** 3 **Spectral Angle** 0.73

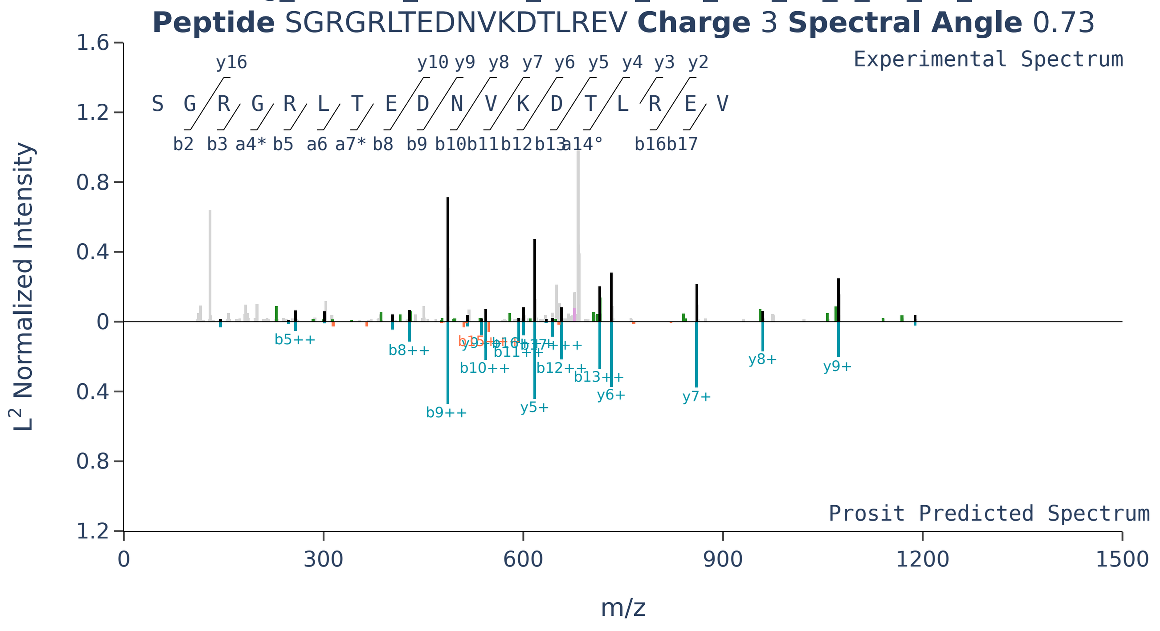

**Source** Ncheng_210623_230623_HFGoe_FFH_20S_25_1_A1_4h_R2 **Scan** 14470
**Peptide** KEKAVGHEVNKSLTPGQEFV **Charge** 2 **Spectral Angle** 0.77

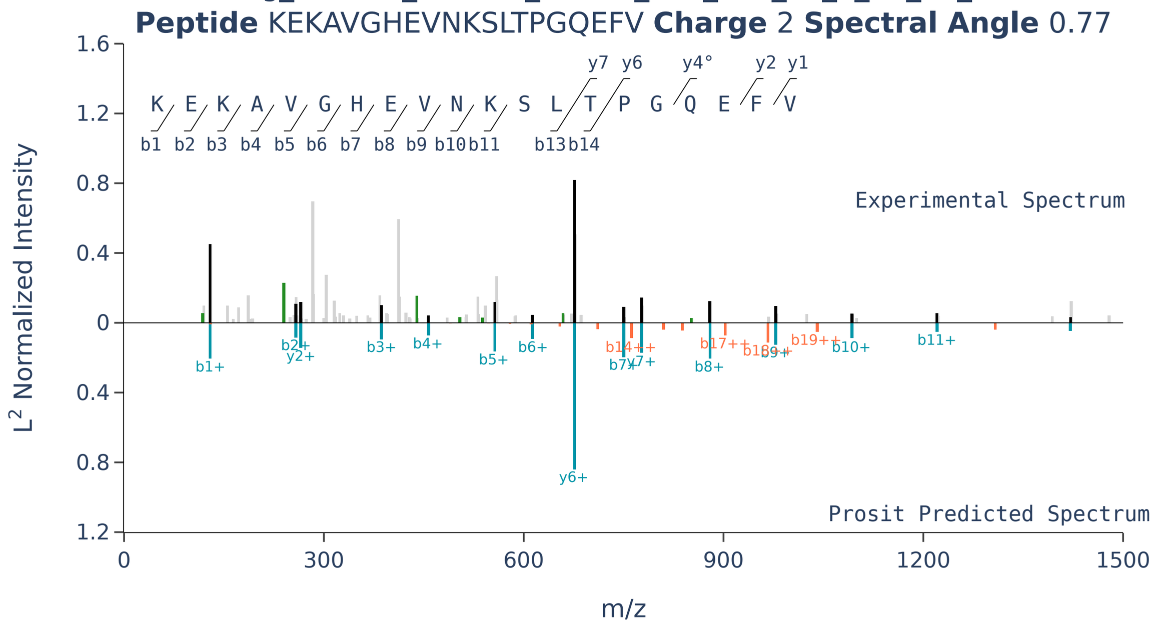


**Source** Ncheng_210623_230623_HFGoe_FFH_20S_25_1_A2_24h_R1 **Scan** 16954
**Peptide** PDNVKSQMDDKVLV **Charge** 2 **Spectral Angle** 0.79

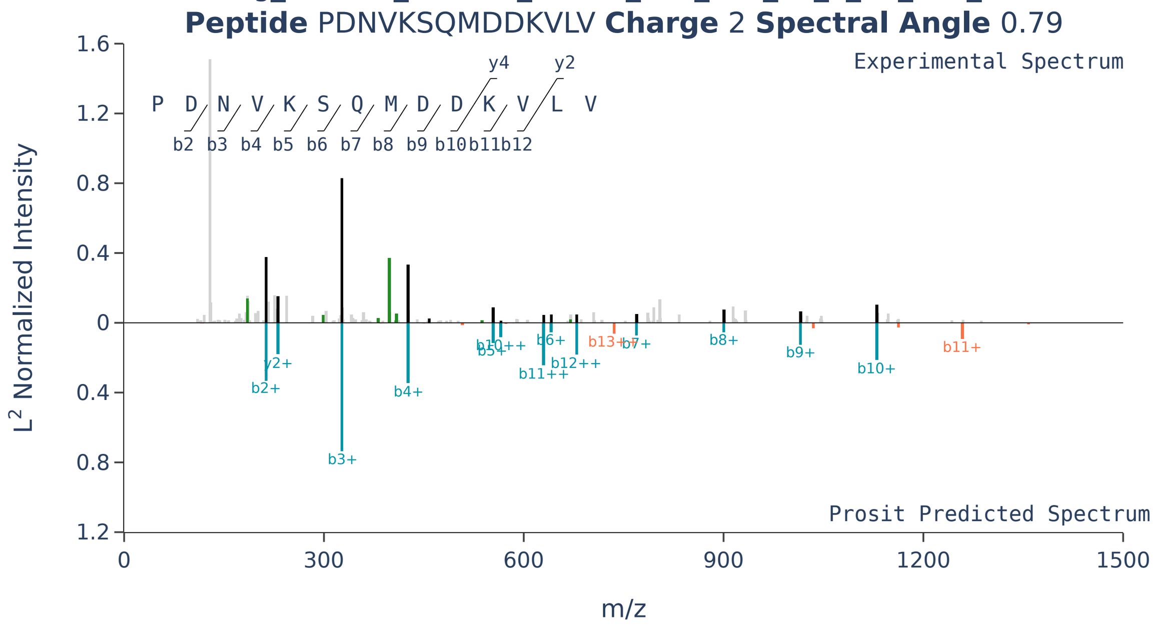


**Source** Ncheng_210623_230623_HFGoe_FFH_20S_25_1_A1_4h_R2 **Scan** 15234
**Peptide** GRGRLTEDNVKDTLREV **Charge** 4 **Spectral Angle** 0.83

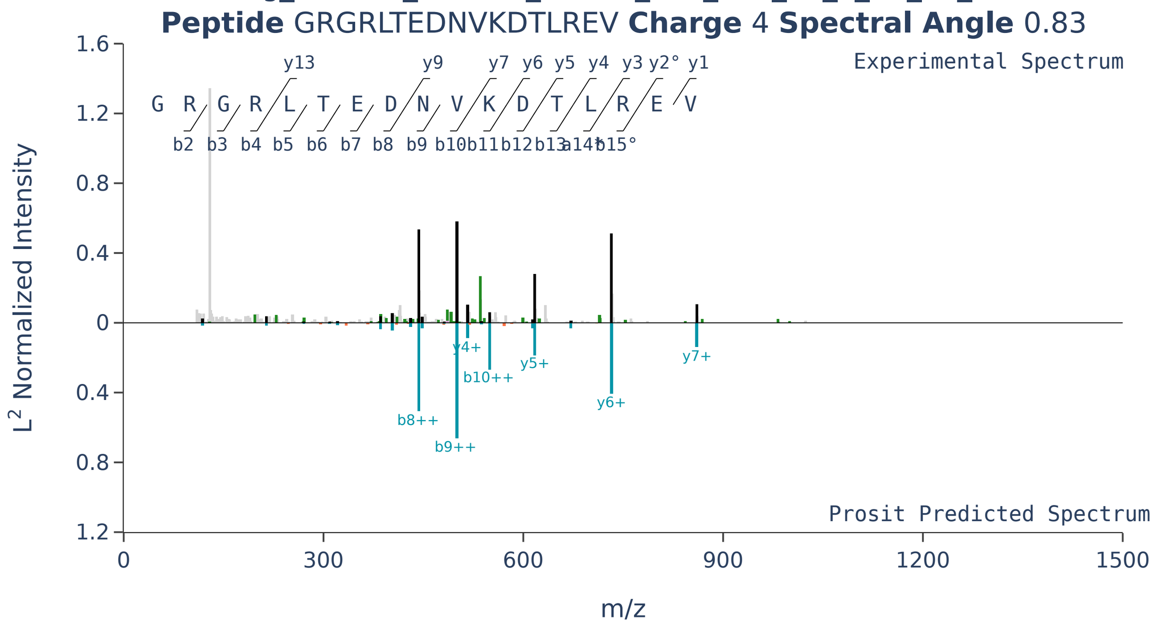


**Source** Ncheng_210623_230623_HFGoe_FFH_20S_25_1_A2_24h_R2 **Scan** 21699
**Peptide** KAVGHEVNKSLTPGQEFVKLV **Charge** 3 **Spectral Angle** 0.87

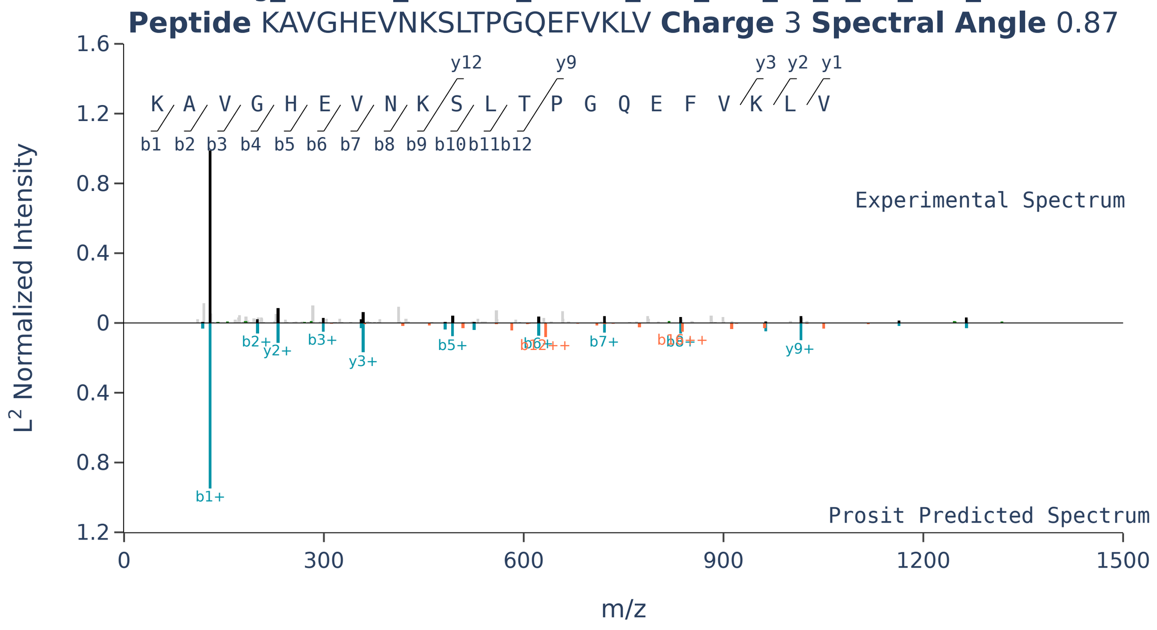


**Source** Ncheng_210623_230623_HFGoe_FFH_20S_25_1_A1_2h_R2 **Scan** 20294
**Peptide** VKIVRNELVAAM **Charge** 3 **Spectral Angle** 0.81

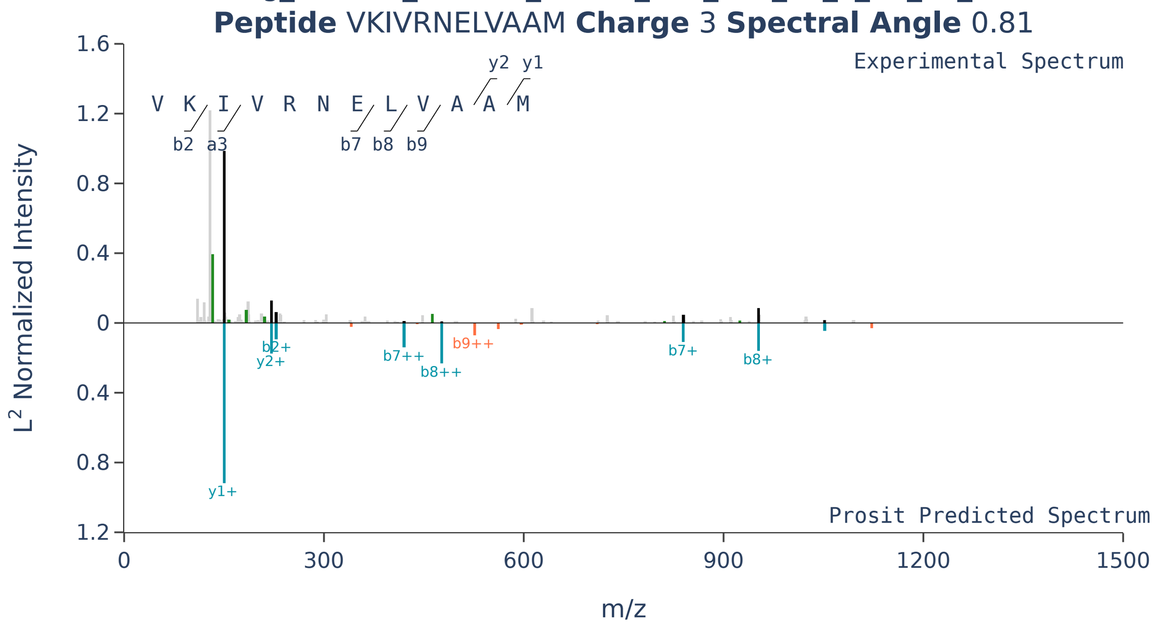


**Source** Ncheng_210623_230623_HFGoe_FFH_20S_25_1_A1_2h_R2 **Scan** 6881
**Peptide** NSMTMKE **Charge** 1 **Spectral Angle** 0.88

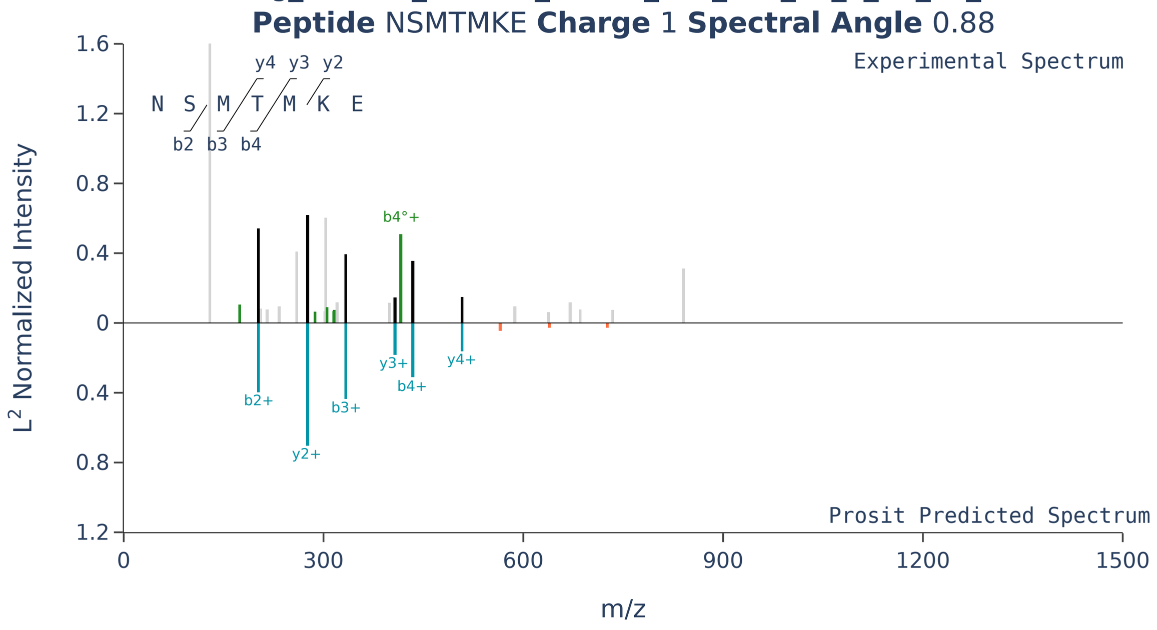


**Source** Ncheng_210623_230623_HFGoe_FFH_20S_25_1_A1_24h_R2 **Scan** 19593
**Peptide** PVVREFINRV **Charge** 3 **Spectral Angle** 0.64

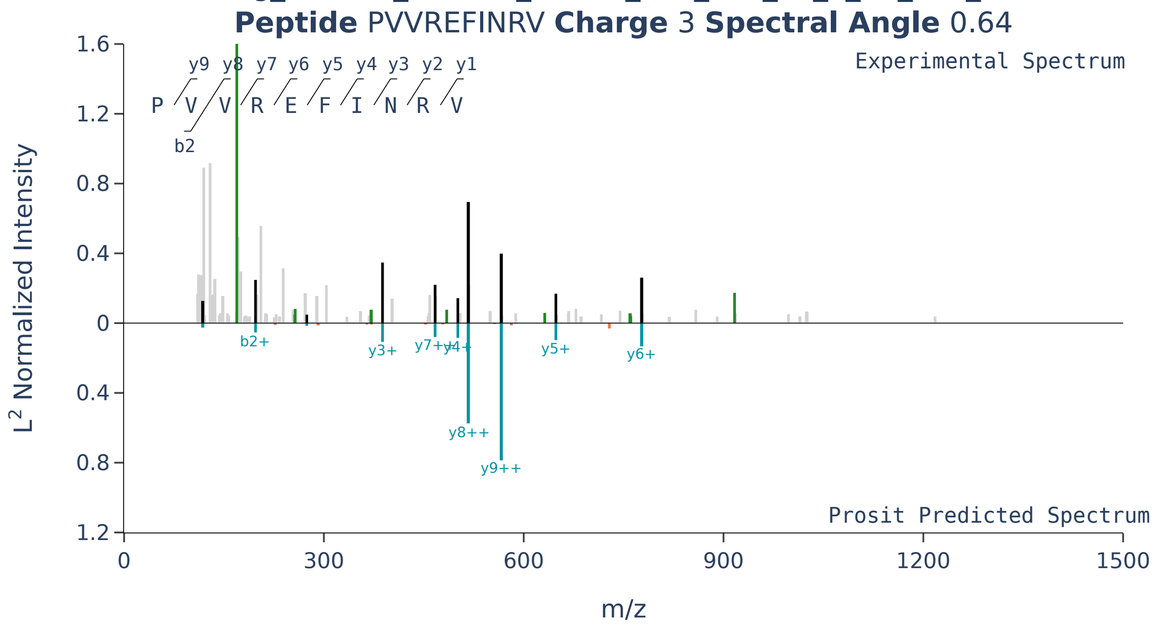


**Source** Ncheng_210623_230623_HFGoe_FFH_20S_25_1_A2_24h_R1 **Scan** 9547
**Peptide** TKVDGDARGGAALSIRH **Charge** 2 **Spectral Angle** 0.74

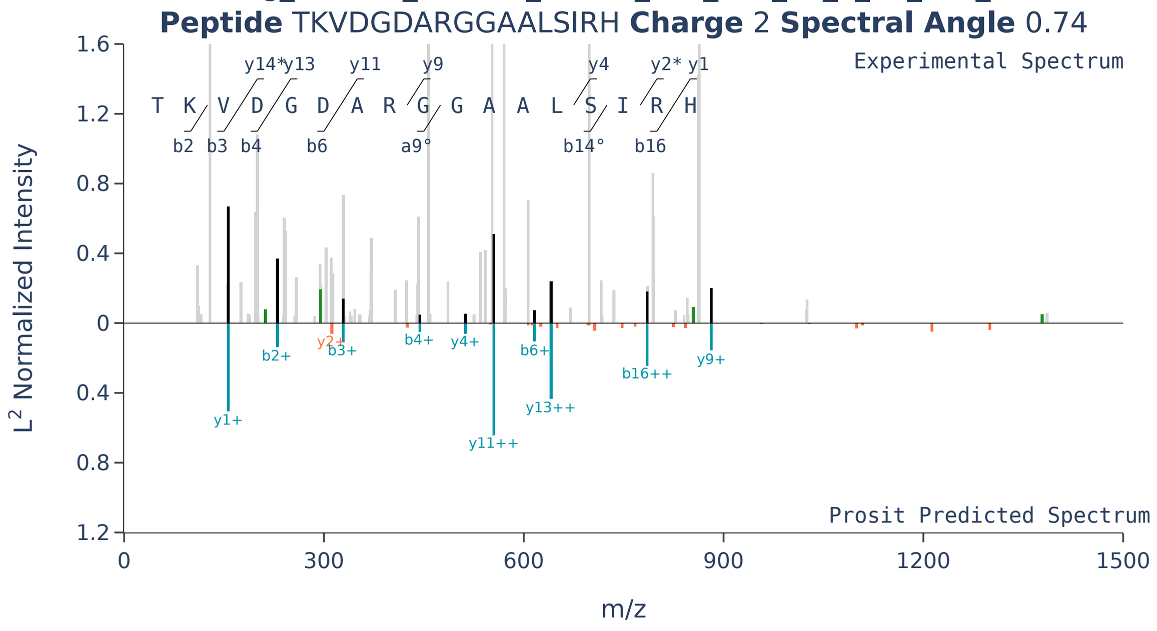


**Source** Ncheng_210623_230623_HFGoe_FFH_20S_25_1_A1_2h_R2 **Scan** 28918
**Peptide** RIAAGCGMQVQDVNRLLKQF **Charge** 3 **Spectral Angle** 0.71

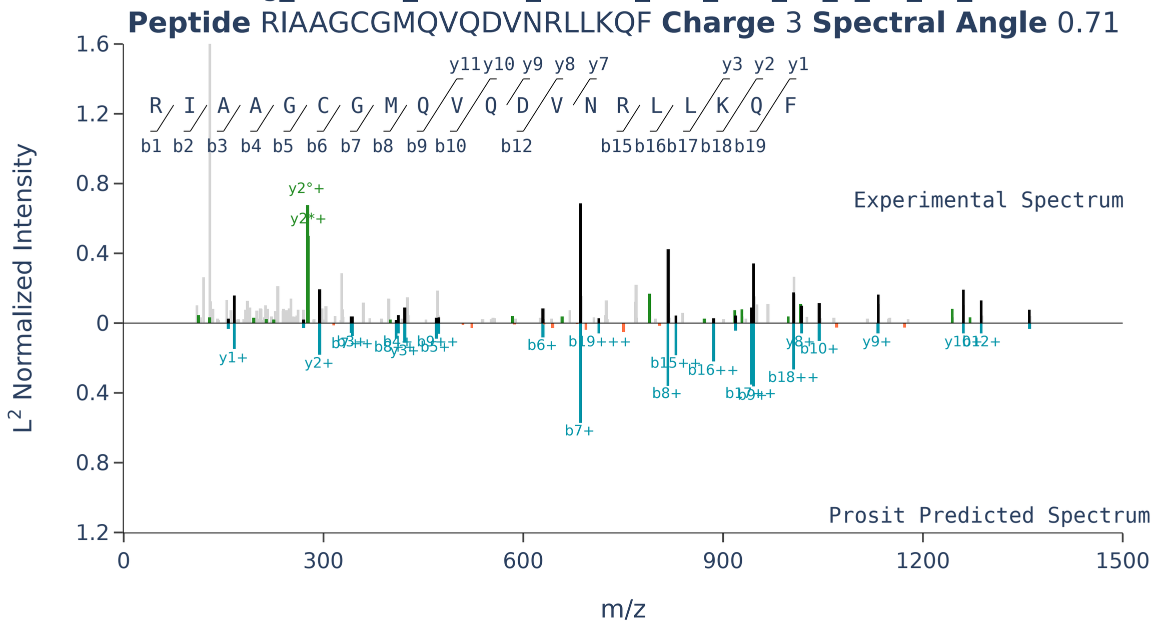


**Source** Ncheng_210623_230623_HFGoe_FFH_20S_25_1_A1_24h_R1 **Scan** 13141
**Peptide** TKVDGDARGGAALSIRHITGK **Charge** 5 **Spectral Angle** 0.75

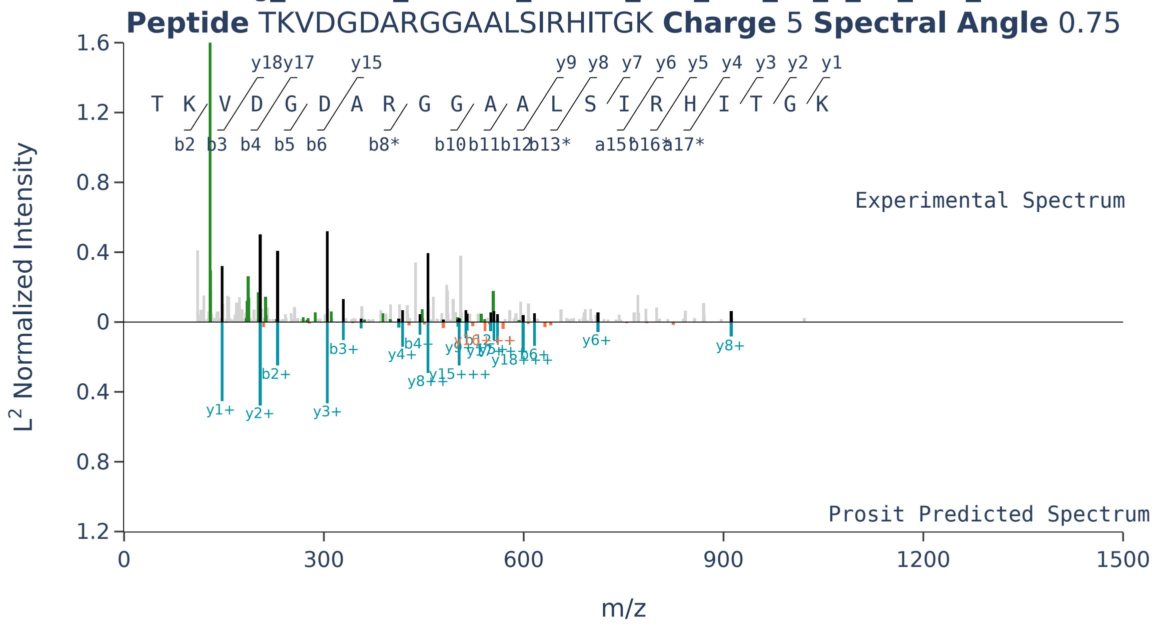


**Source** Ncheng_210623_230623_HFGoe_FFH_20S_25_1_A2_4h_R1 **Scan** 23105
**Peptide** MAGLQGAGKTTSVGKLGKFL **Charge** 3 **Spectral Angle** 0.78

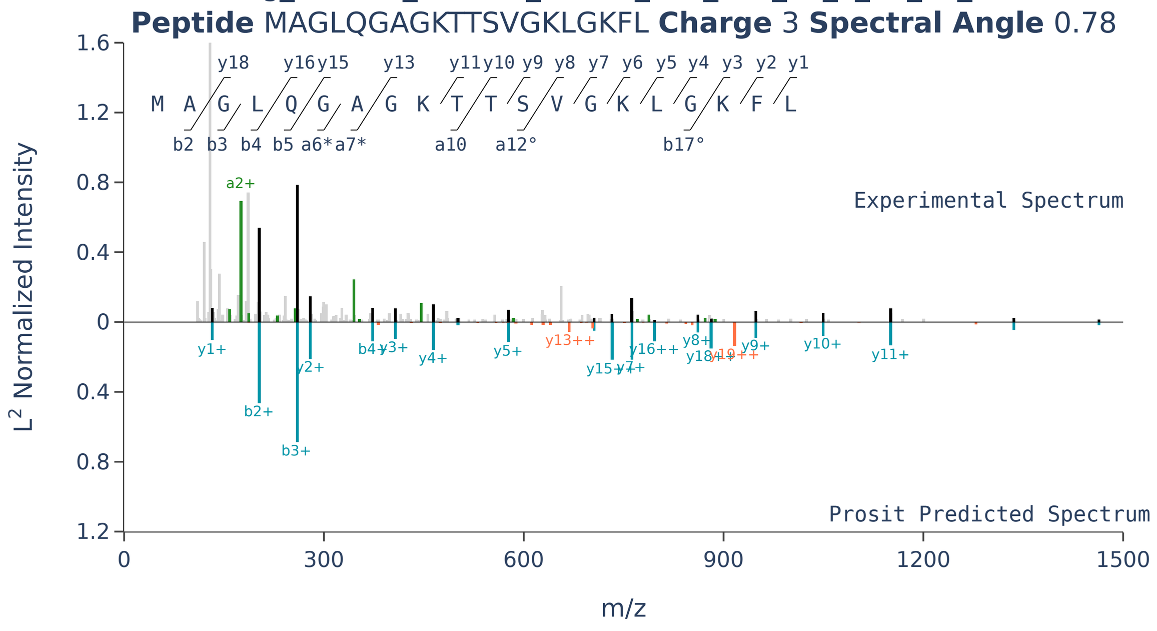


**Source** Ncheng_210623_230623_HFGoe_FFH_20S_25_1_A1_24h_R1 **Scan** 17446
**Peptide** EDNVKDTLREVRM **Charge** 3 **Spectral Angle** 0.8

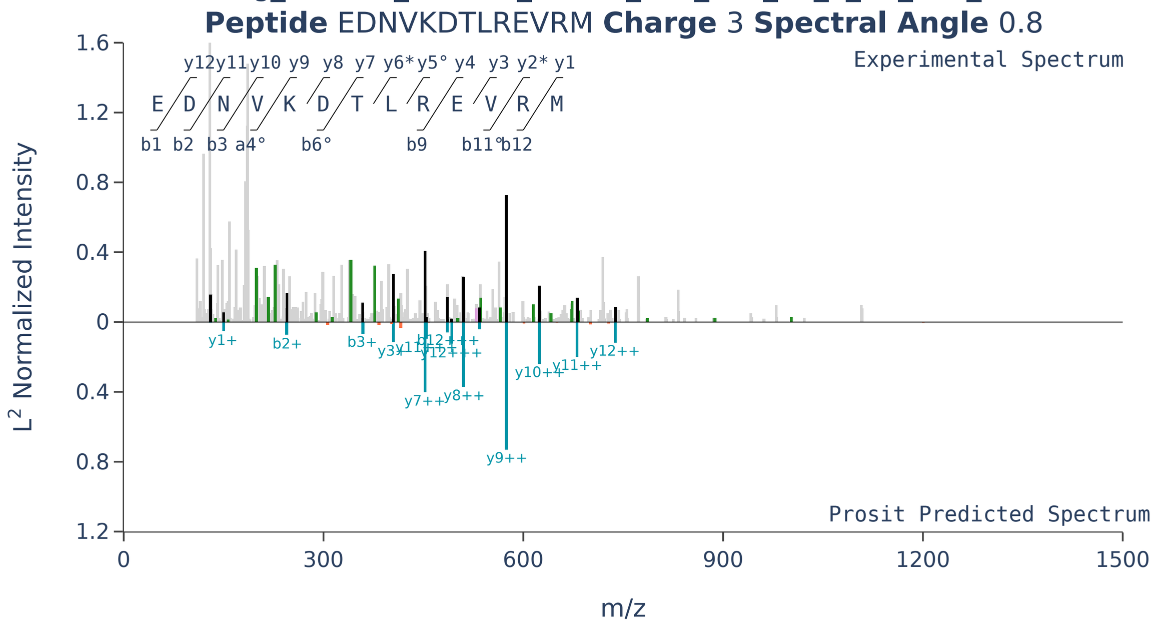


**Source** Ncheng_210623_230623_HFGoe_FFH_20S_25_1_A2_24h_R2 **Scan** 6847
**Peptide** RAKPEIIK **Charge** 2 **Spectral Angle** 0.69

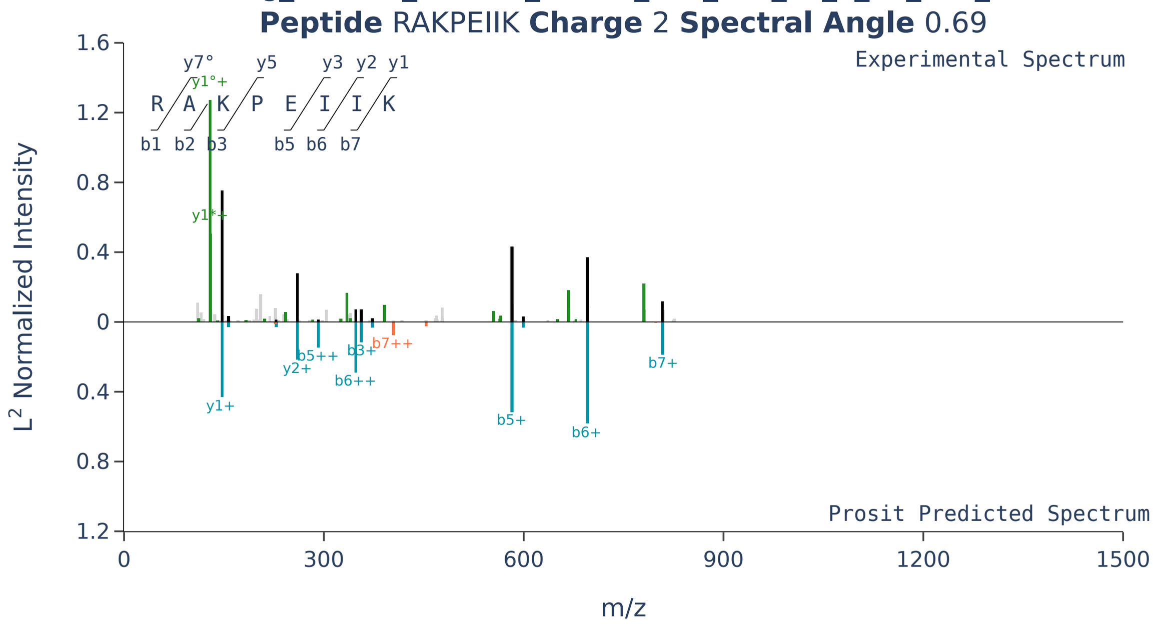


**Source** Ncheng_210623_230623_HFGoe_FFH_20S_25_1_A2_4h_R1 **Scan** 10485
**Peptide** TEDNVKDTL **Charge** 1 **Spectral Angle** 0.78

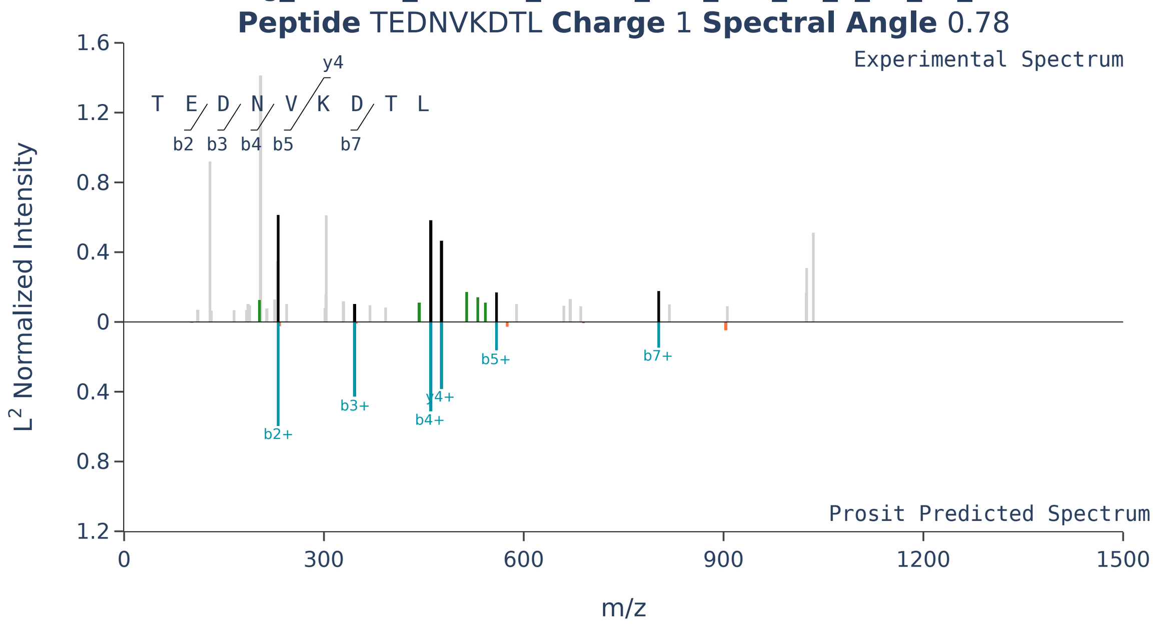


**Source** Ncheng_210623_230623_HFGoe_FFH_20S_25_1_A1_4h_R2 **Scan** 12371
**Peptide** EPFHPDRLAS **Charge** 2 **Spectral Angle** 0.92

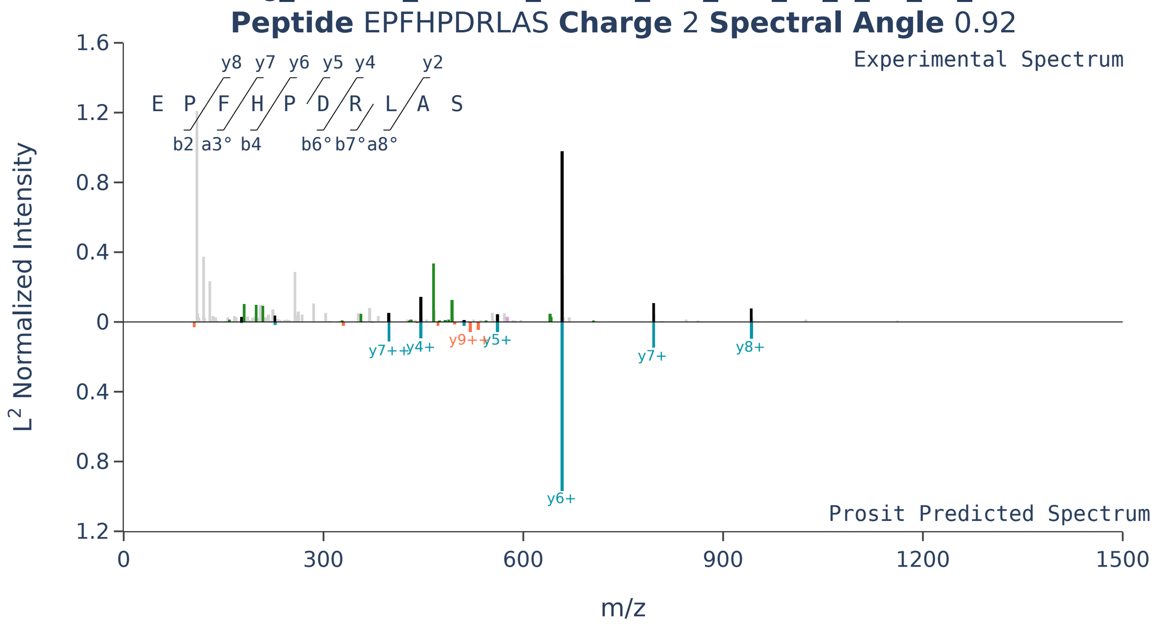

**Source** Ncheng_210623_230623_HFGoe_FFH_20S_25_1_A2_24h_R2 **Scan** 8462
**Peptide** DMQRMMKKMK **Charge** 2 **Spectral Angle** 0.72

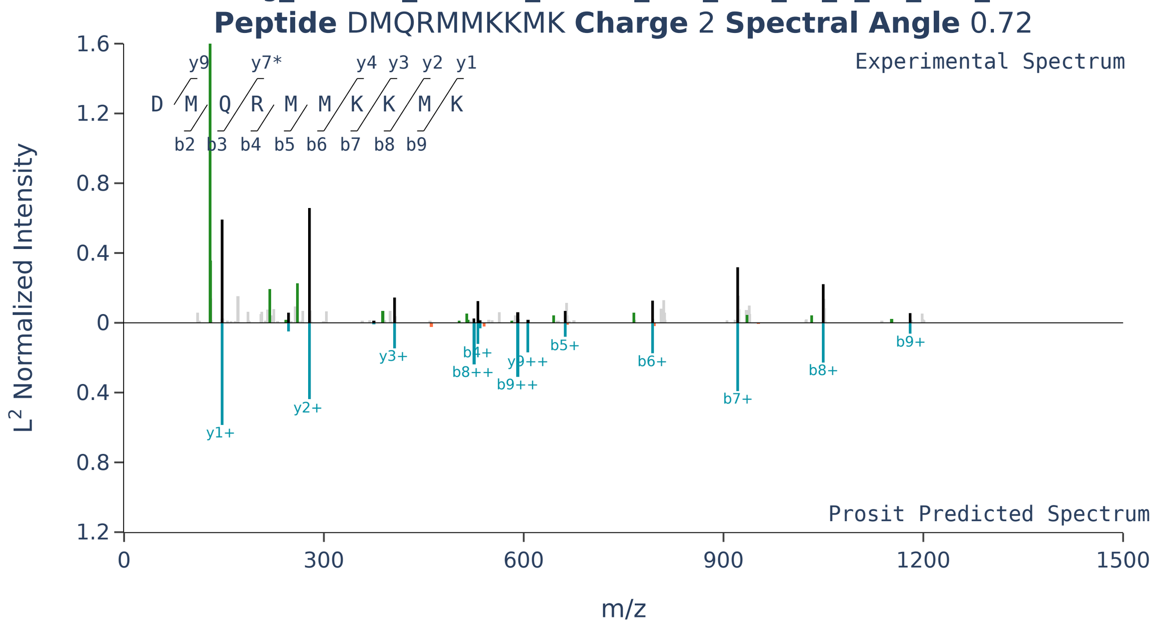


**Source** Ncheng_210623_230623_HFGoe_FFH_20S_25_1_A1_4h_R2 **Scan** 18874
**Peptide** VSADVYRPAAL **Charge** 2 **Spectral Angle** 0.76

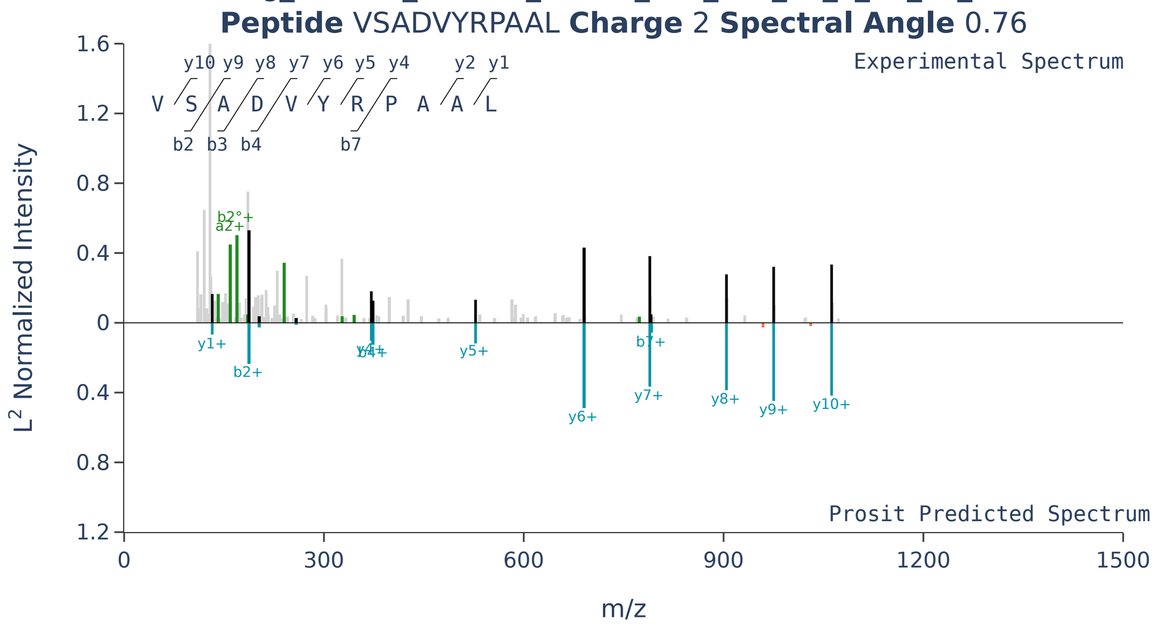


**Source** Ncheng_210623_230623_HFGoe_FFH_20S_25_1_A1_24h_R1 **Scan** 23466
**Peptide** SLTPGQEFV **Charge** 2 **Spectral Angle** 0.82

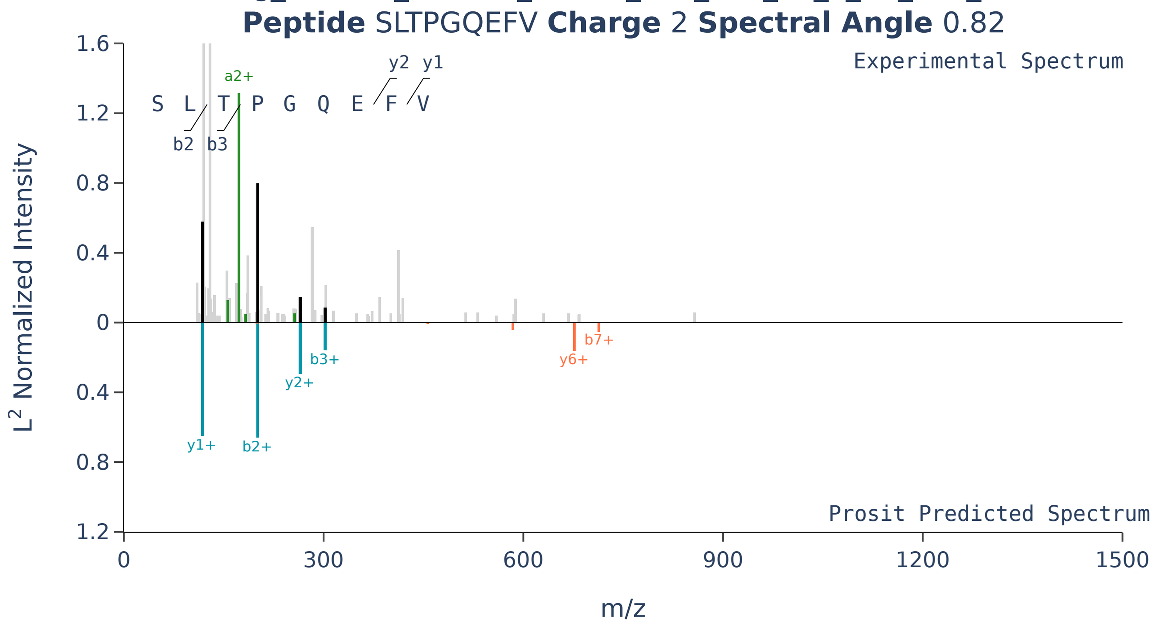


**Source** Ncheng_210623_230623_HFGoe_FFH_20S_25_1_A2_2h_R1 **Scan** 20706
**Peptide** QIPDNVKSQMDDKVLVRME **Charge** 3 **Spectral Angle** 0.61

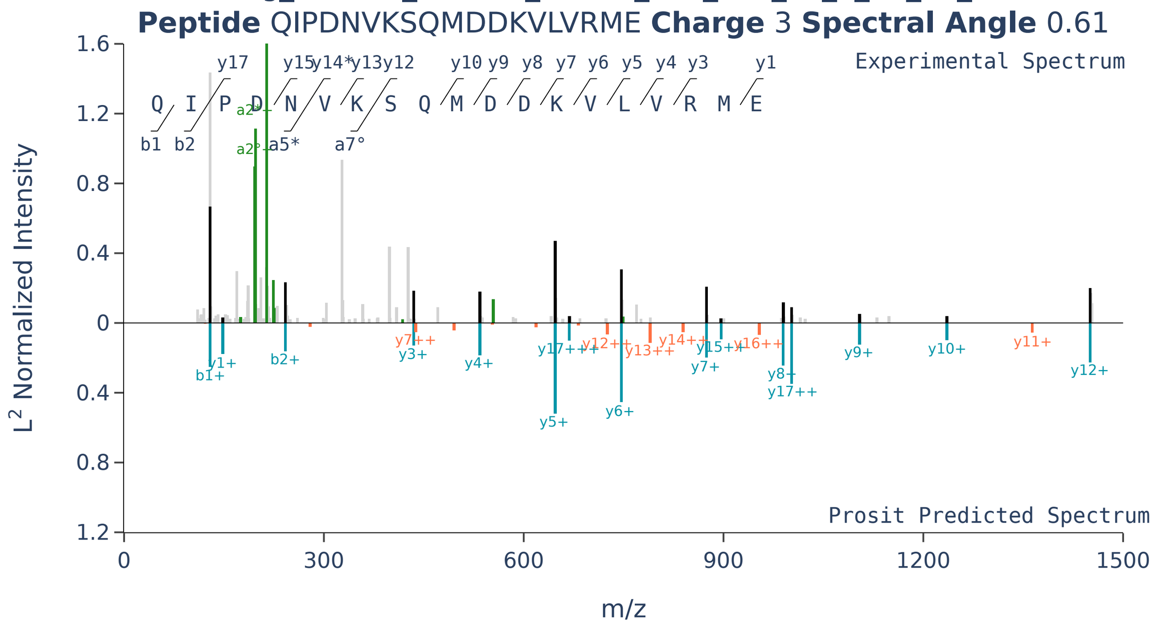


**Source** Ncheng_210623_230623_HFGoe_FFH_20S_25_1_A1_4h_R1 **Scan** 18194
**Peptide** GEENQTLNLAA **Charge** 1 **Spectral Angle** 0.7

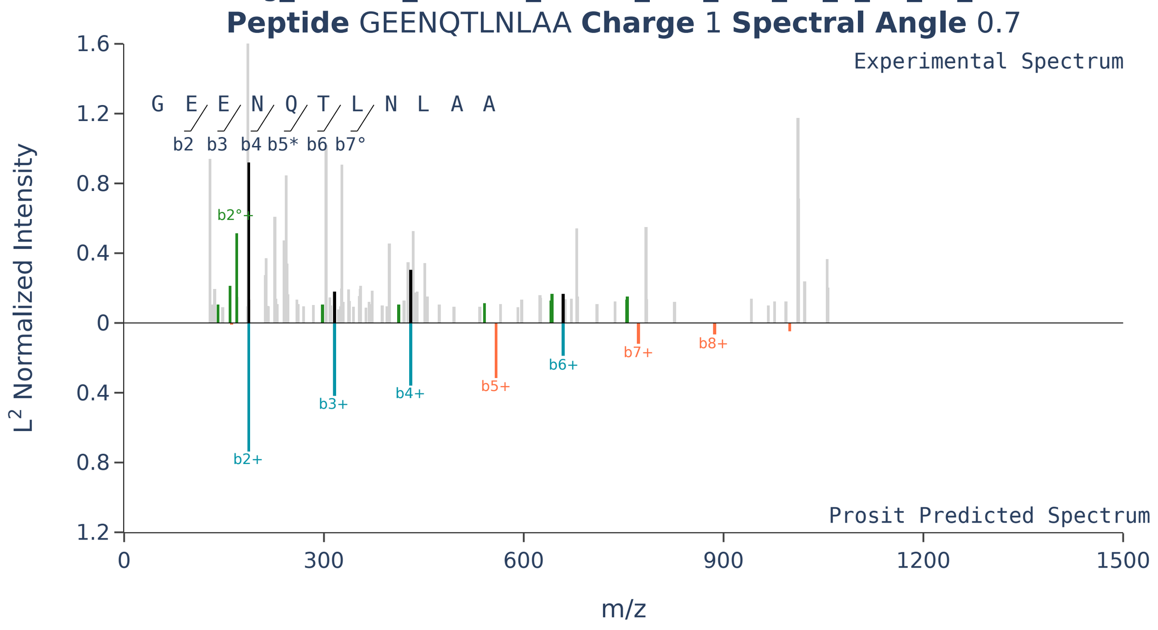


**Source** Ncheng_210623_230623_HFGoe_FFH_20S_25_1_A1_24h_R1 **Scan** 11048
**Peptide** RNISGRGRLTEDNVKDTLRE **Charge** 5 **Spectral Angle** 0.75

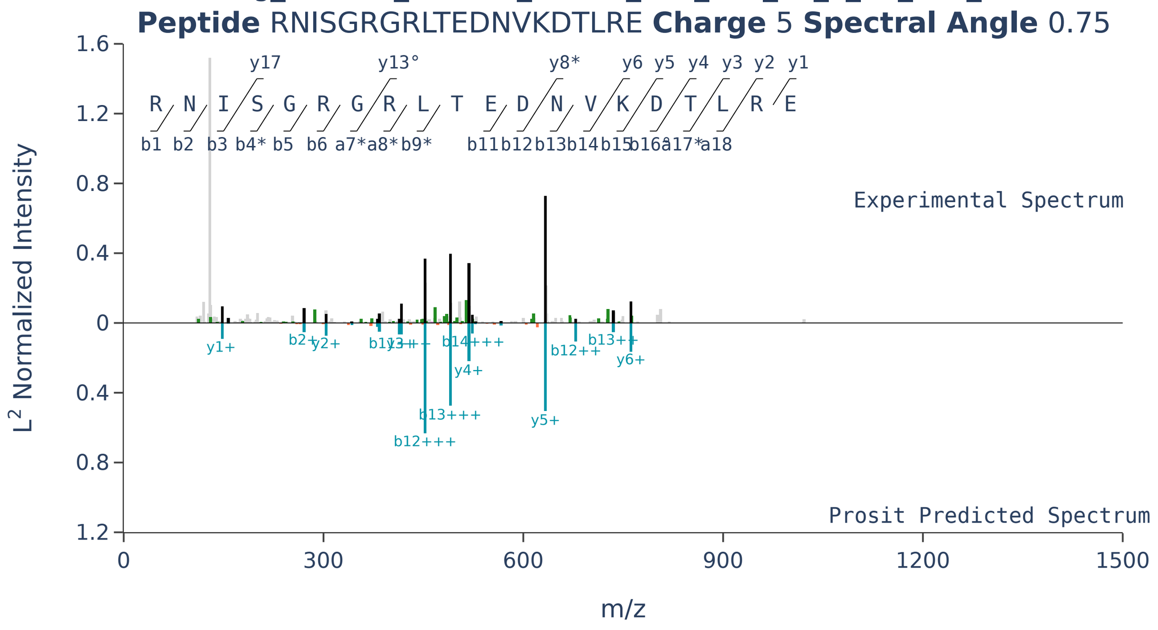


**Source** Ncheng_210623_230623_HFGoe_FFH_20S_25_1_A1_24h_R2 **Scan** 9705
**Peptide** KSQMDDKVLVR **Charge** 3 **Spectral Angle** 0.61

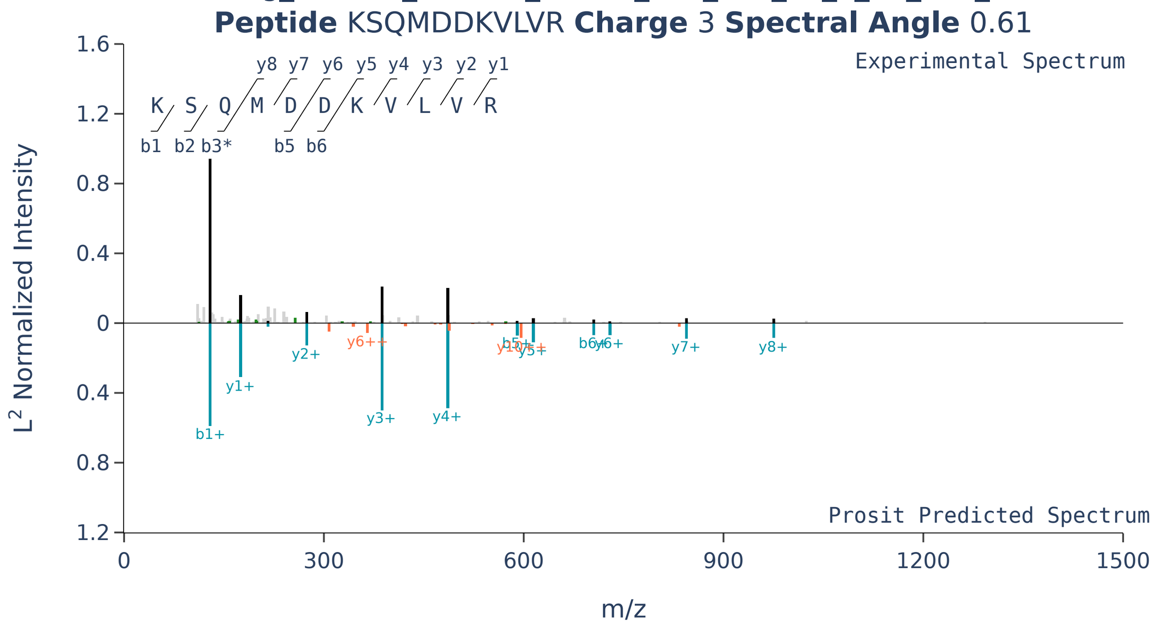


**Source** Ncheng_210623_230623_HFGoe_FFH_20S_25_1_A2_24h_R2 **Scan** 14706
**Peptide** AVGHEVNKSLTPGQEFVK **Charge** 3 **Spectral Angle** 0.74

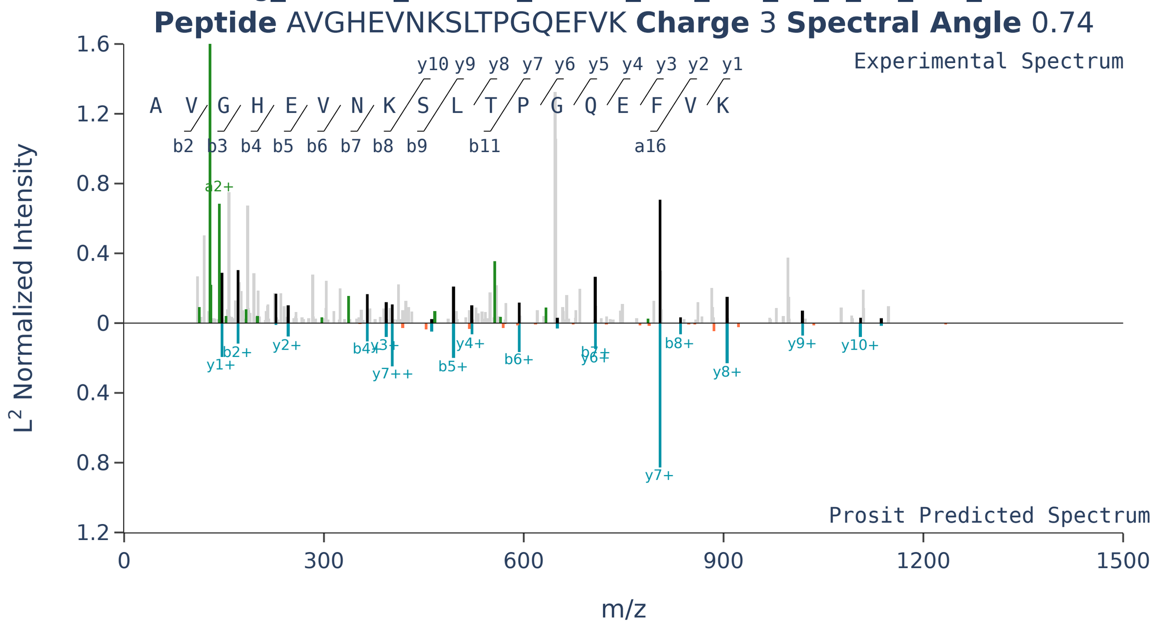


**Source** Ncheng_210623_230623_HFGoe_FFH_20S_25_1_A2_4h_R1 **Scan** 7183
**Peptide** VAAMGEENQ **Charge** 1 **Spectral Angle** 0.79

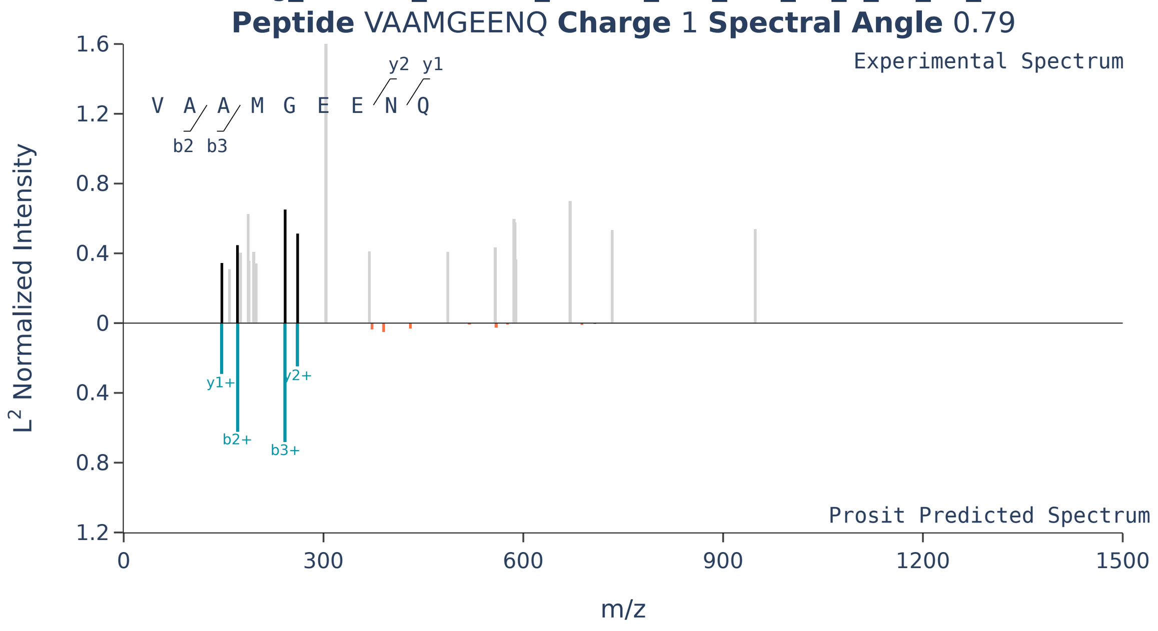


**Source** Ncheng_210623_230623_HFGoe_FFH_20S_25_1_A2_24h_R1 **Scan** 7256
**Peptide** QAEKLASKLK **Charge** 3 **Spectral Angle** 0.92

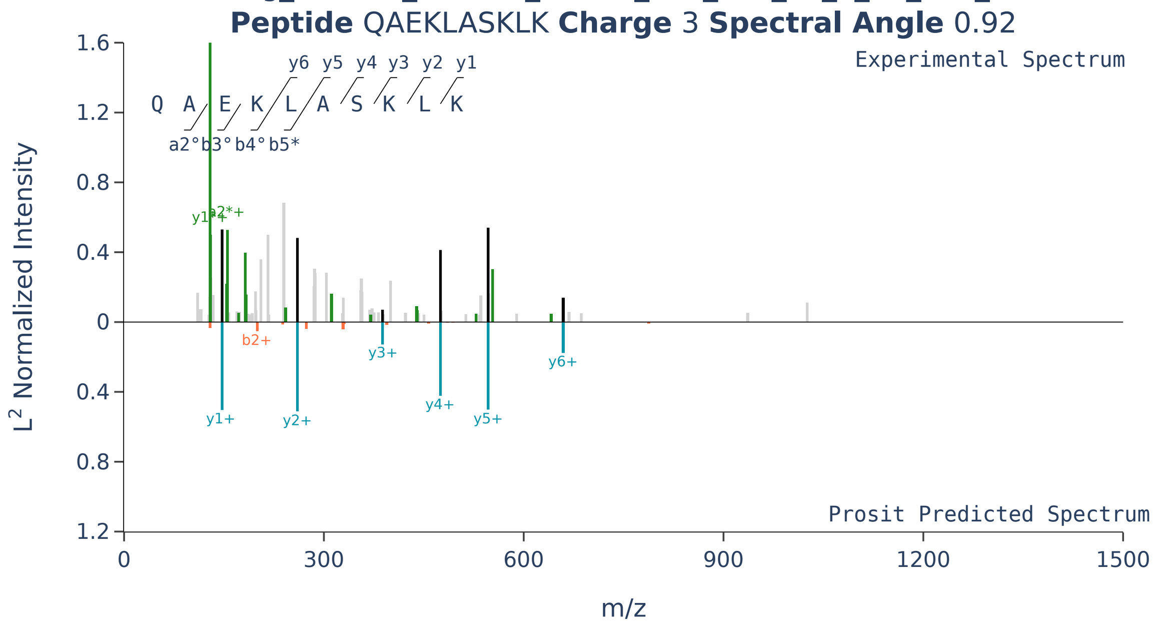


**Source** Ncheng_210623_230623_HFGoe_FFH_20S_25_1_A2_4h_R2 **Scan** 10735
**Peptide** DIESKVDRAQAE **Charge** 2 **Spectral Angle** 0.74

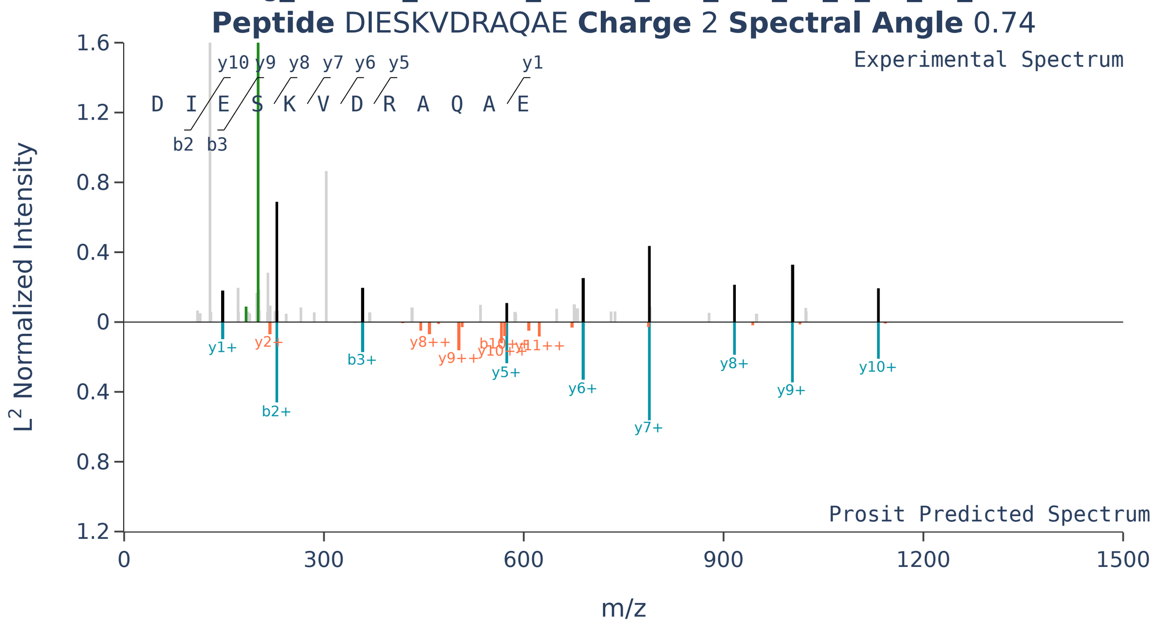


**Source** Ncheng_210623_230623_HFGoe_FFH_20S_25_1_A2_4h_R2 **Scan** 25815
**Peptide** SADVYRPAAIKQLETL **Charge** 2 **Spectral Angle** 0.76

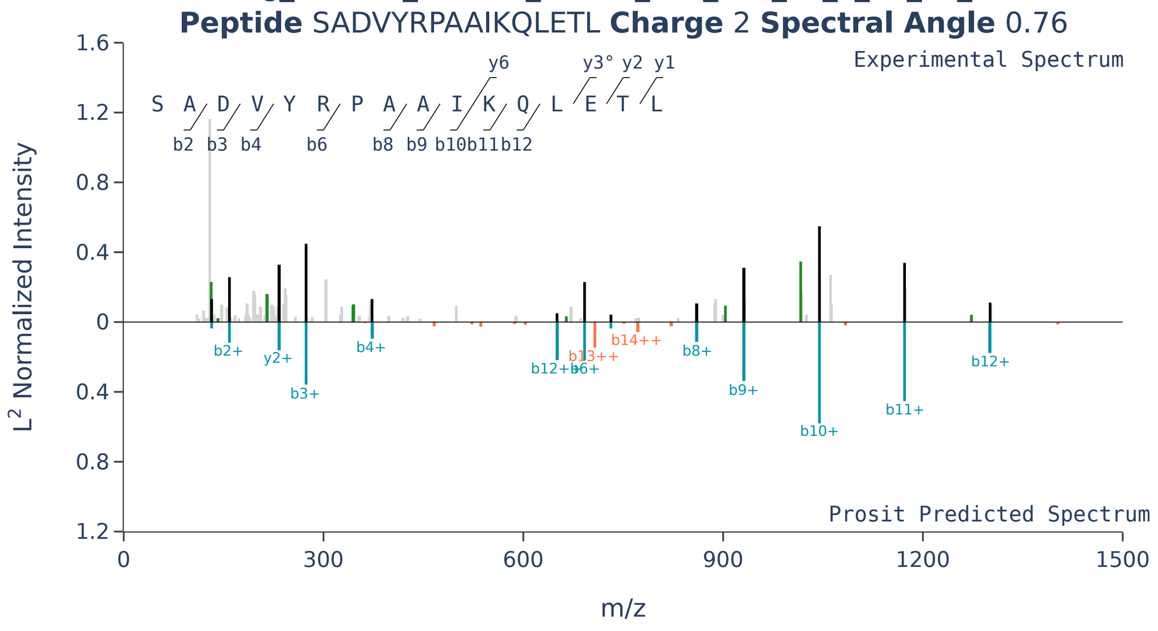


**Source** Ncheng_210623_230623_HFGoe_FFH_20S_25_1_A1_24h_R1 **Scan** 11045
**Peptide** EPFHPDRIASR **Charge** 3 **Spectral Angle** 0.85

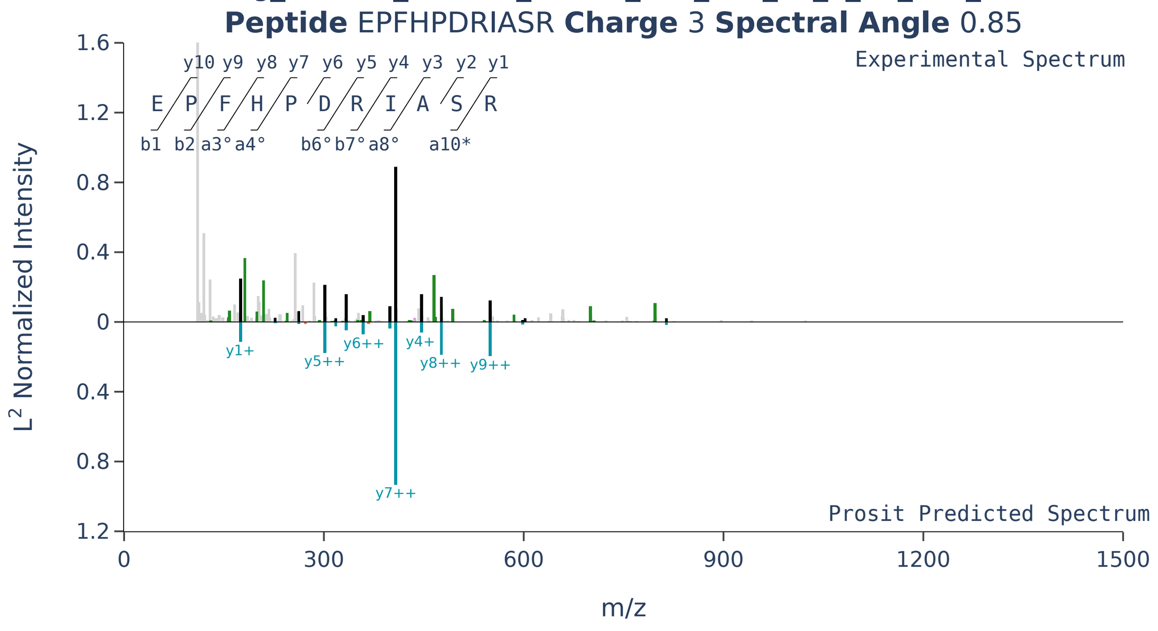


**Source** Ncheng_210623_230623_HFGoe_FFH_20S_25_1_A1_4h_R1 **Scan** 8692
**Peptide** KLVRNEL **Charge** 2 **Spectral Angle** 0.74

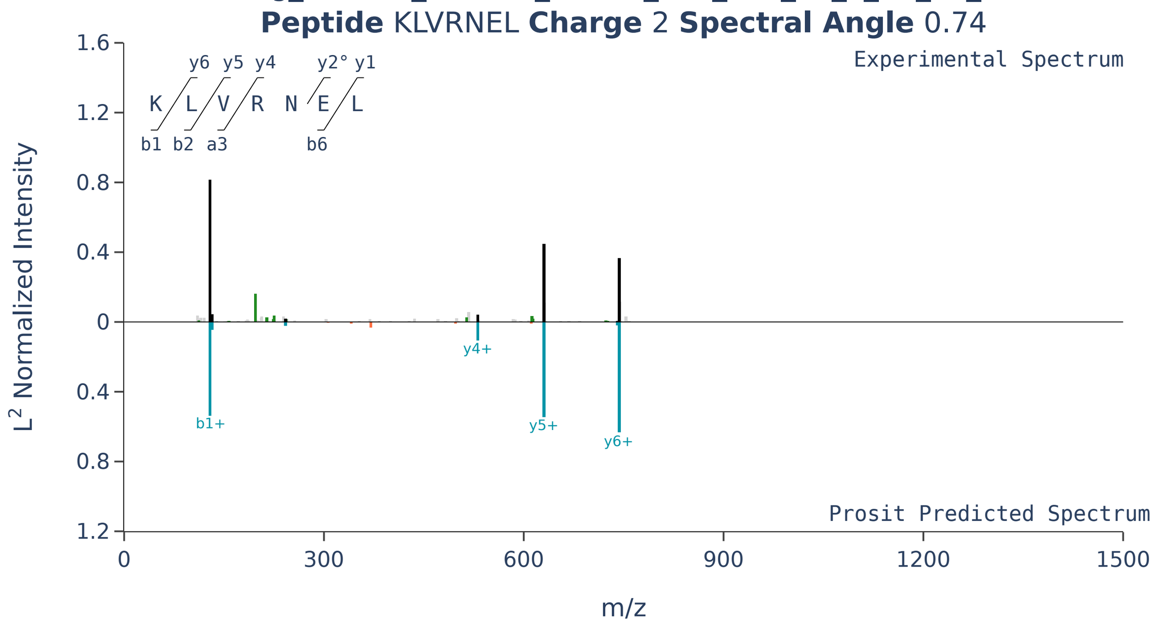


**Source** Ncheng_210623_230623_HFGoe_FFH_20S_25_1_A2_4h_R1 **Scan** 17835
**Peptide** SGRGRLTEDNVKDTLREVRM **Charge** 5 **Spectral Angle** 0.77

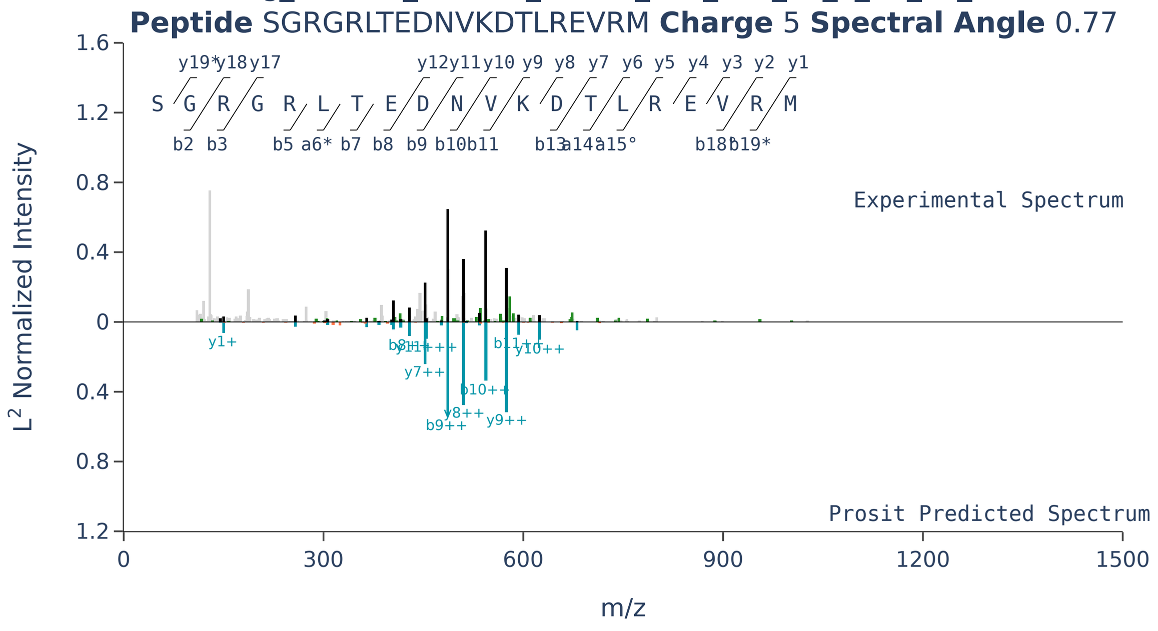

**Source** Ncheng_210623_230623_HFGoe_FFH_20S_25_1_A1_2h_R2 **Scan** 30523
**Peptide** ALKEAKLKFYDVLLV **Charge** 2 **Spectral Angle** 0.69

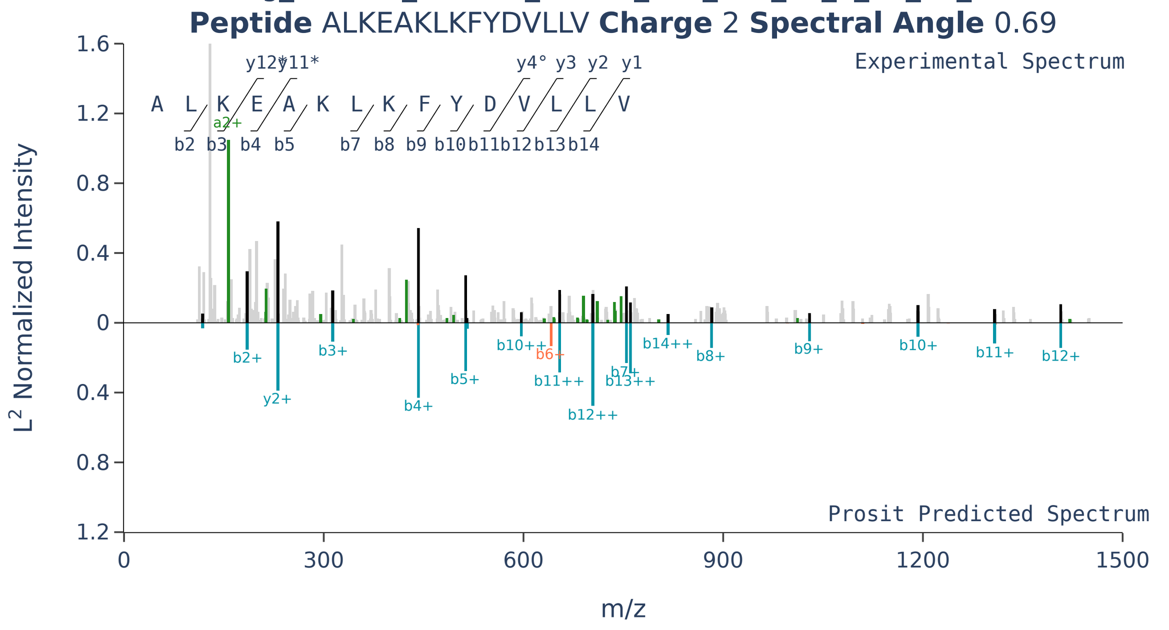


**Source** Ncheng_210623_230623_HFGoe_FFH_20S_25_1_A2_1h_R2 **Scan** 25236
**Peptide** KAVGHEVNKSLTPGQEFVKIVRNEL **Charge** 4 **Spectral Angle** 0.78

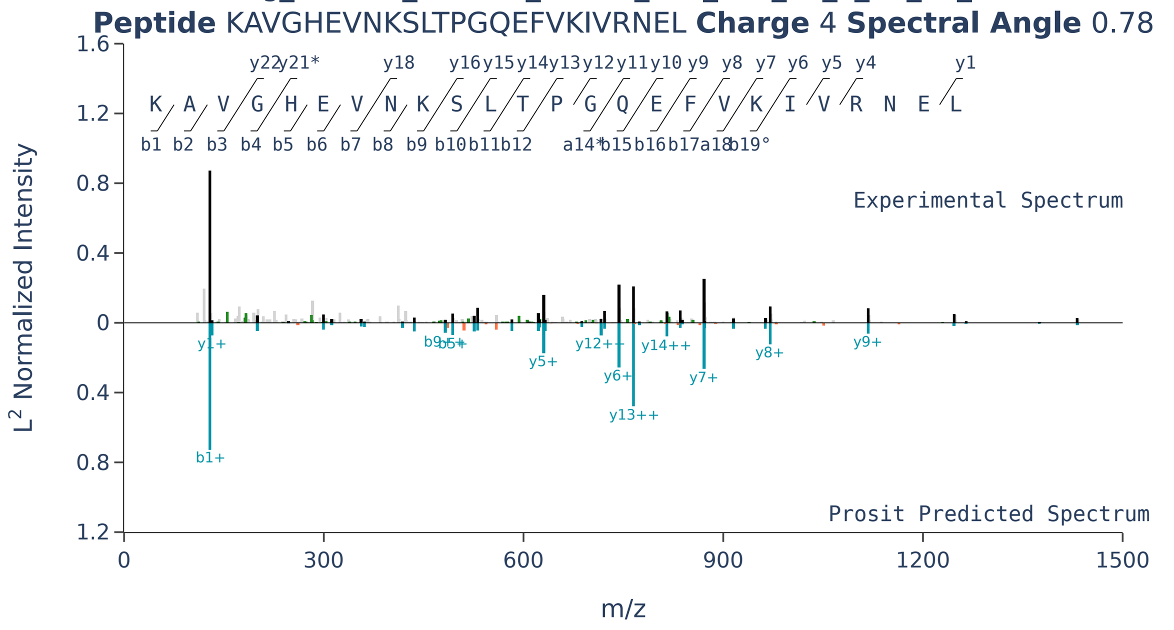


**Source** Ncheng_210623_230623_HFGoe_FFH_20S_25_1_A1_24h_R1 **Scan** 5334
**Peptide** SMTMKERAK **Charge** 3 **Spectral Angle** 0.78

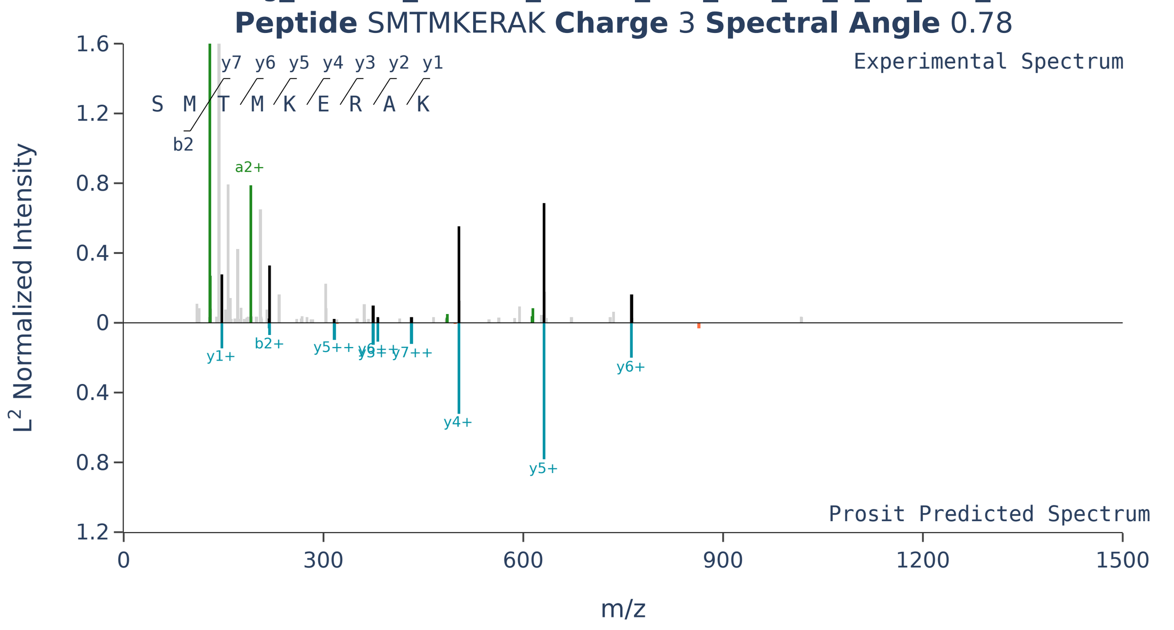


**Source** Ncheng_210623_230623_HFGoe_FFH_20S_25_1_A1_4h_R1 **Scan** 19477
**Peptide** TGKPIKFLGVGEKTEALE **Charge** 2 **Spectral Angle** 0.77

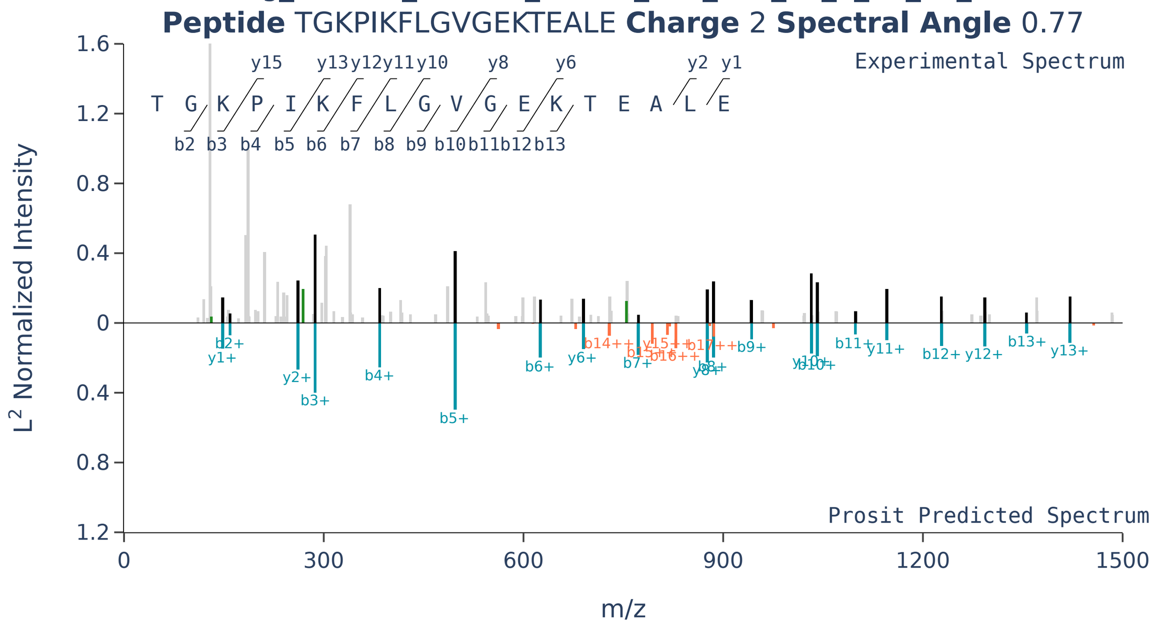


**Source** Ncheng_210623_230623_HFGoe_FFH_20S_25_1_A1_24h_R1 **Scan** 5775
**Peptide** AMTGQDAANTAK **Charge** 2 **Spectral Angle** 0.67

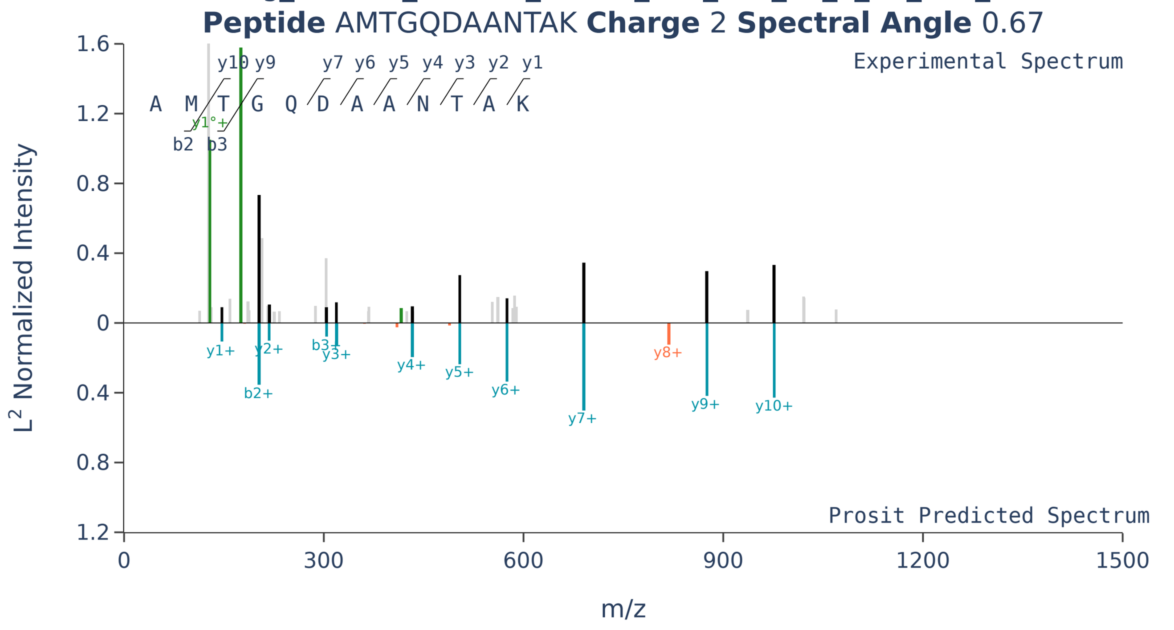


**Source** Ncheng_210623_230623_HFGoe_FFH_20S_25_1_A1_4h_R2 **Scan** 26144
**Peptide** QDAANTAKAFNEALPLT **Charge** 2 **Spectral Angle** 0.86

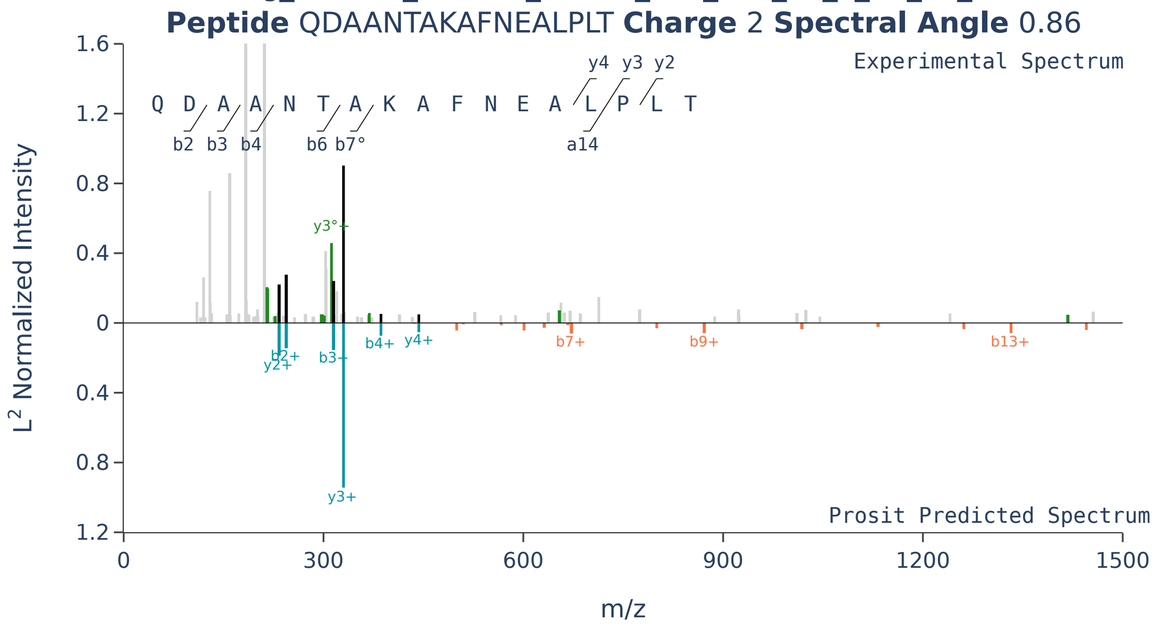


**Source** Ncheng_210623_230623_HFGoe_FFH_20S_25_1_A1_1h_R1 **Scan** 28868
**Peptide** SLIEDIESKVDRAQAEKL **Charge** 3 **Spectral Angle** 0.64

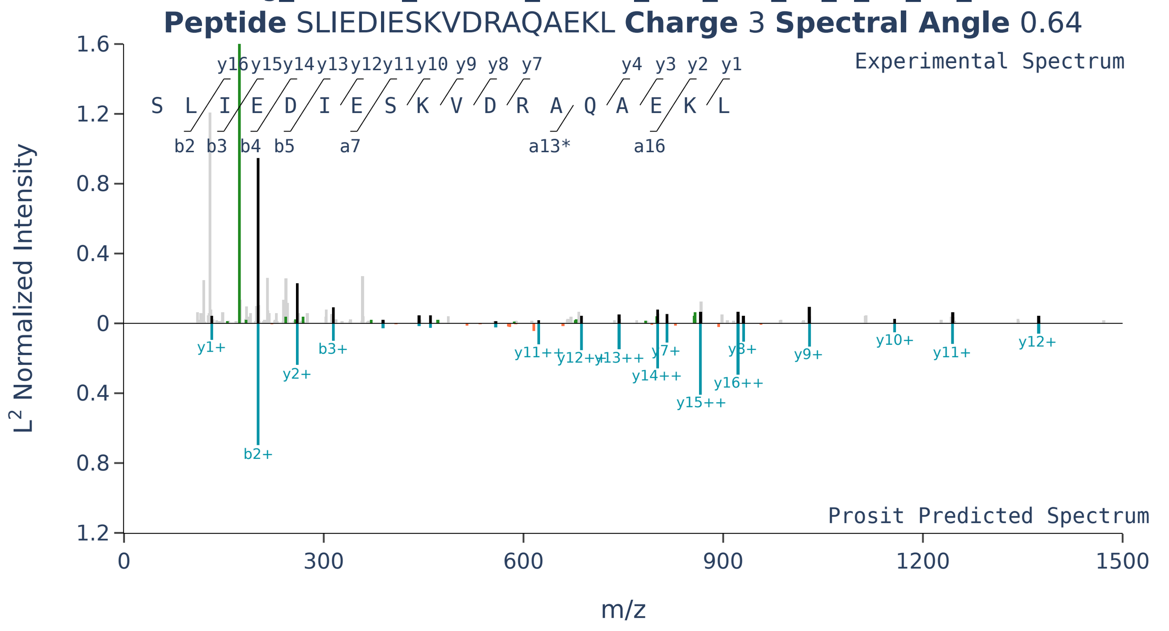


**Source** Ncheng_210623_230623_HFGoe_FFH_20S_25_1_A1_4h_R1 **Scan** 23371
**Peptide** VRMEAIINSMTMKER **Charge** 3 **Spectral Angle** 0.82

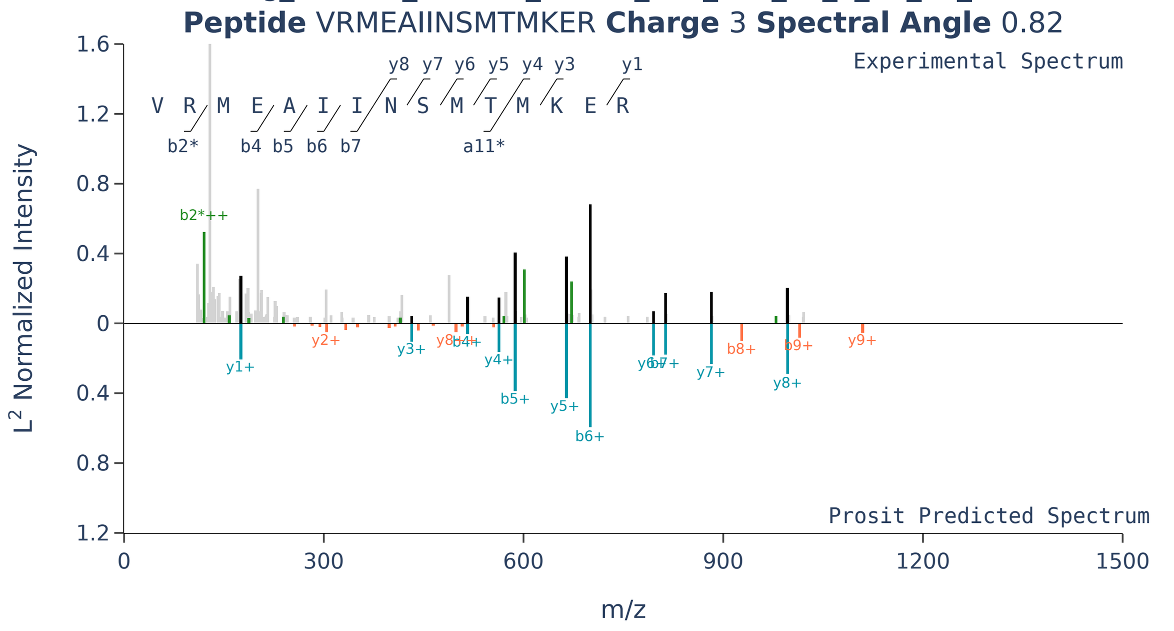


**Source** Ncheng_210623_230623_HFGoe_FFH_20S_25_1_A1_24h_R1 **Scan** 11419
**Peptide** VVLTKVDGDARGGAA **Charge** 2 **Spectral Angle** 0.72

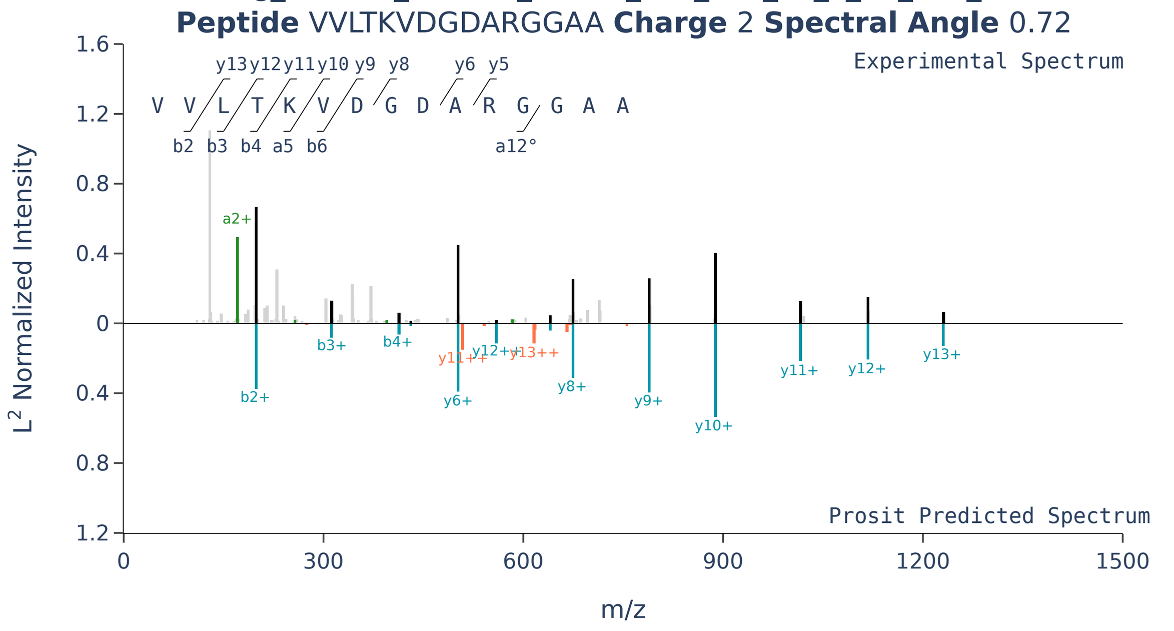


**Source** Ncheng_210623_230623_HFGoe_FFH_20S_25_1_A1_4h_R2 **Scan** 12661
**Peptide** LVAAMGEENQ **Charge** 2 **Spectral Angle** 0.77

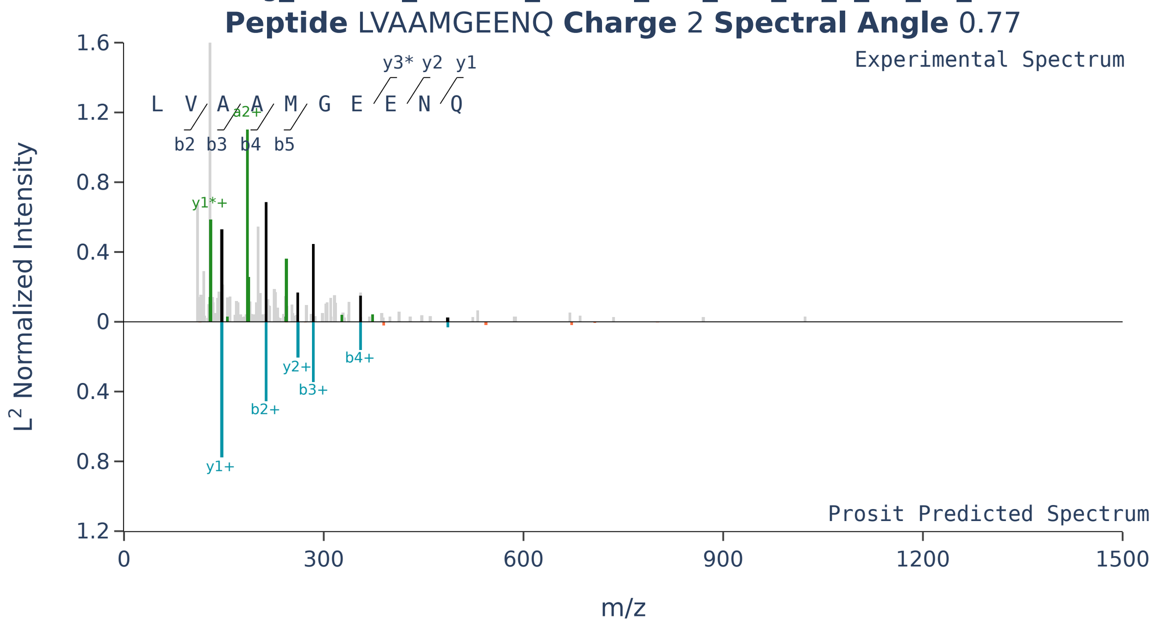


**Source** Ncheng_210623_230623_HFGoe_FFH_20S_25_1_A1_2h_R2 **Scan** 11823
**Peptide** RIAAGCGMQVQD **Charge** 2 **Spectral Angle** 0.79

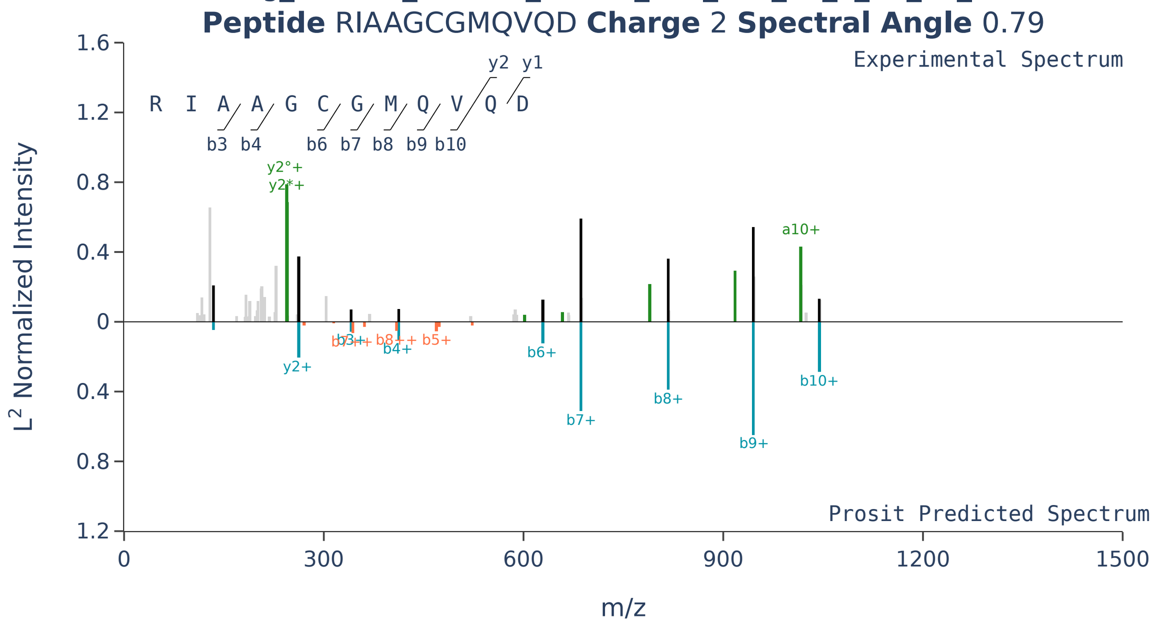


**Source** Ncheng_210623_230623_HFGoe_FFH_20S_25_1_A1_4h_R1 **Scan** 29838
**Peptide** KLKFYDVLL **Charge** 3 **Spectral Angle** 0.8

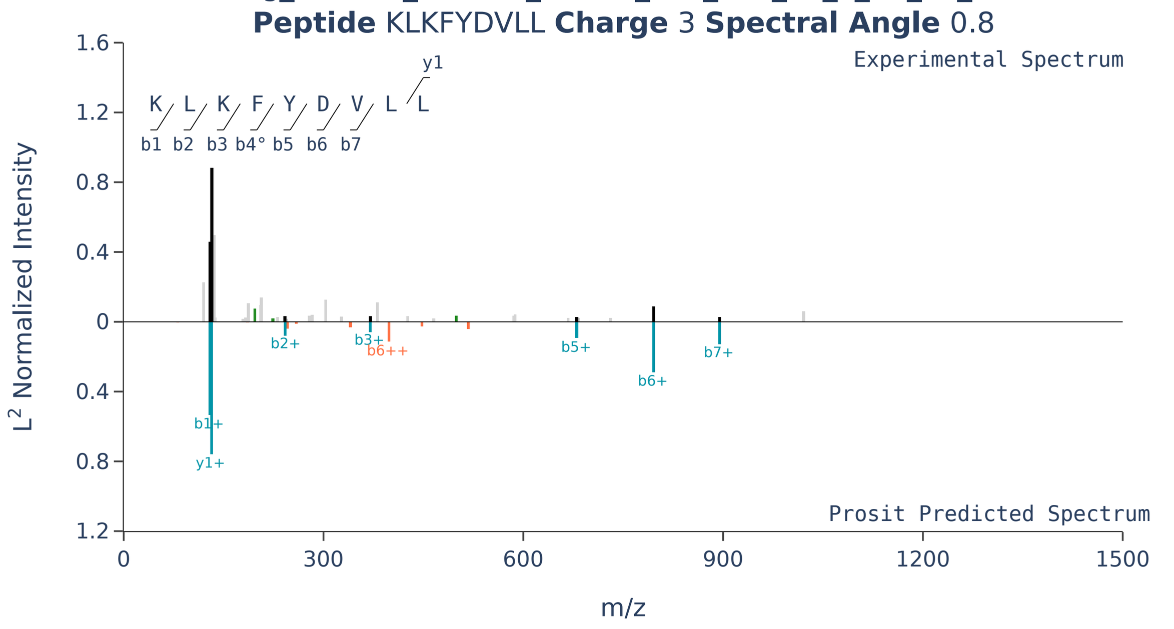


**Source** Ncheng_210623_230623_HFGoe_FFH_20S_25_1_A2_4h_R1 **Scan** 30228
**Peptide** VGHEVNKSLTPGQEFVKIVRNELVA **Charge** 3 **Spectral Angle** 0.81

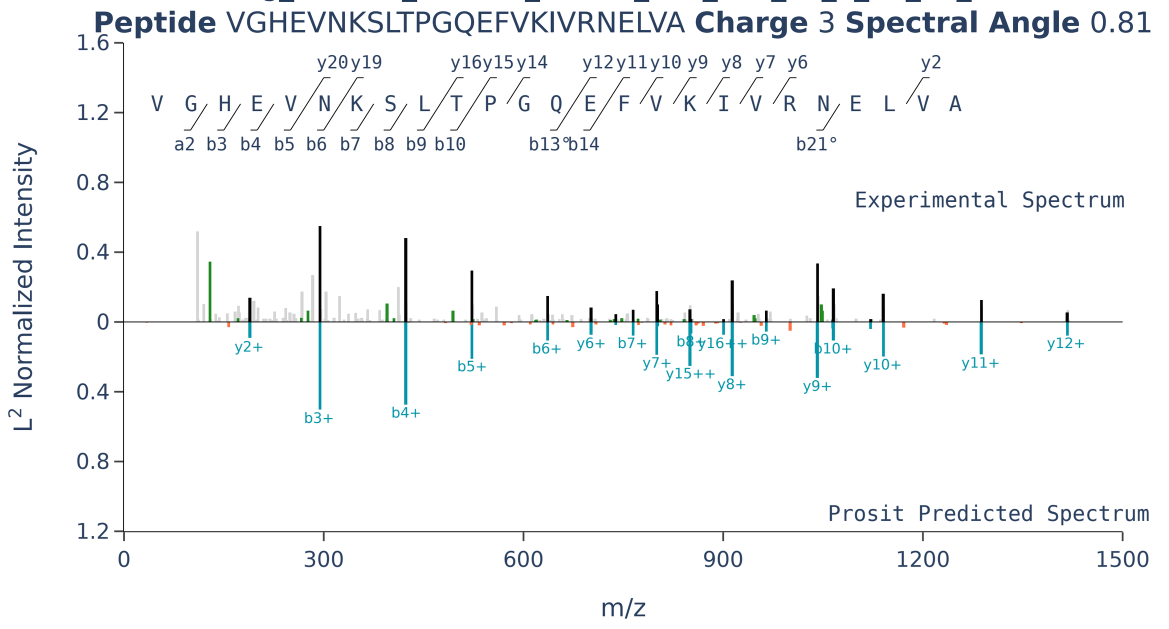


**Source** Ncheng_210623_230623_HFGoe_FFH_20S_25_1_A2_4h_R1 **Scan** 29918
**Peptide** GHEVNKSLTPGQEFVKIVRNELV **Charge** 3 **Spectral Angle** 0.84

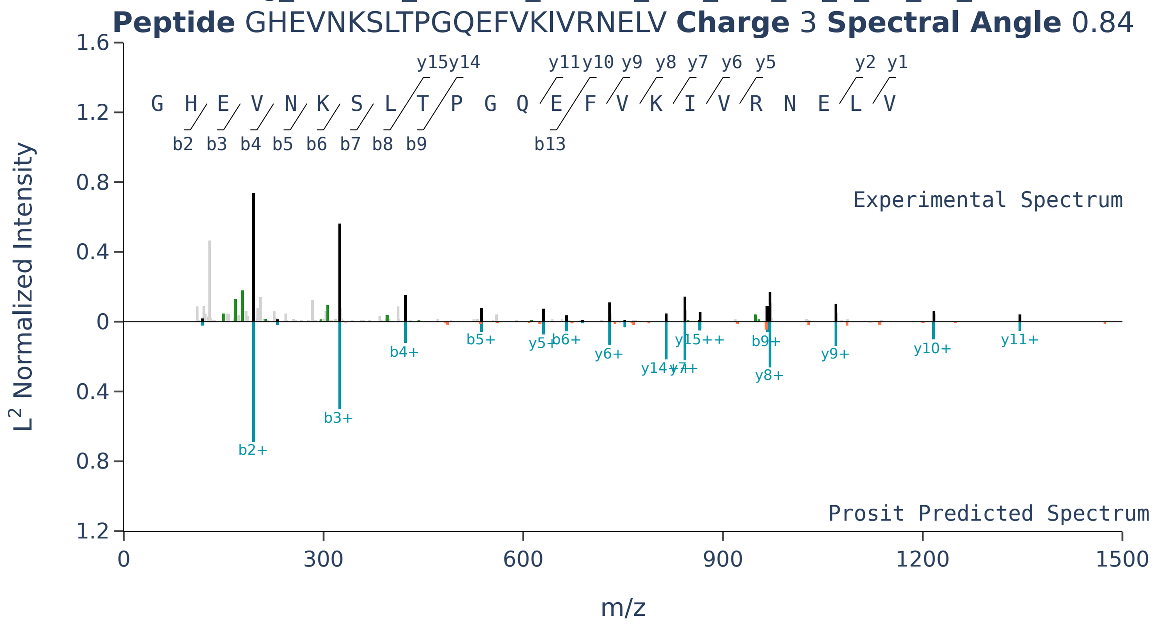


**Source** Ncheng_210623_230623_HFGoe_FFH_20S_25_1_A1_4h_R2 **Scan** 16819
**Peptide** PSDVGQKPVDIVNA **Charge** 2 **Spectral Angle** 0.72

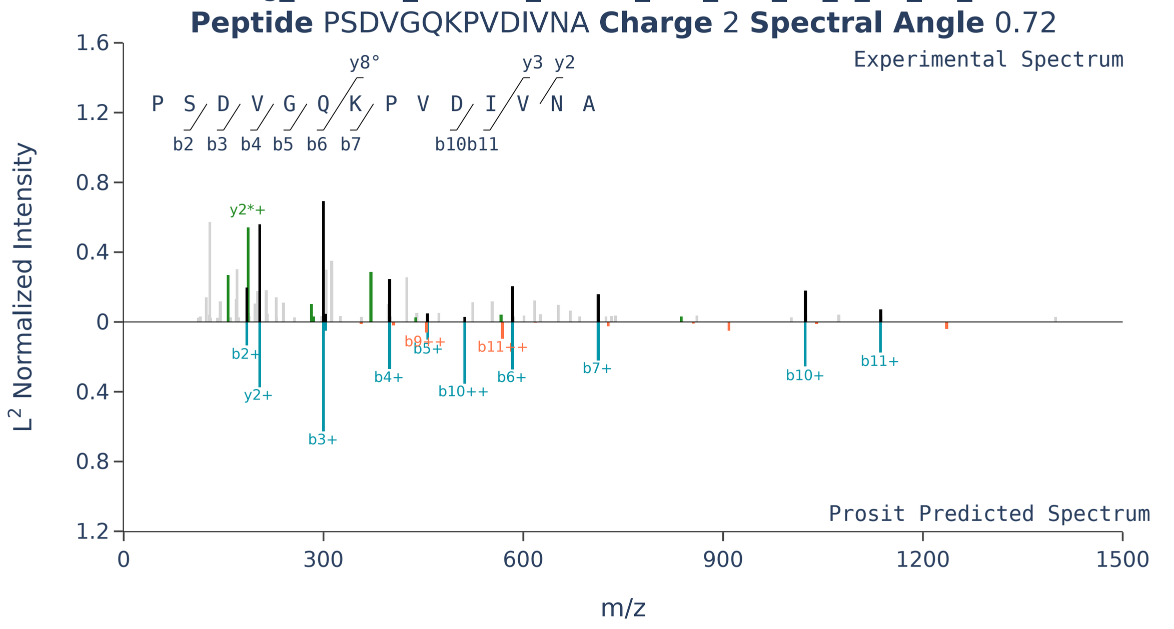

**Source** Ncheng_210623_230623_HFGoe_FFH_20S_25_1_A1_2h_R1 **Scan** 18337
**Peptide** DNVKDTLREVRM **Charge** 3 **Spectral Angle** 0.65

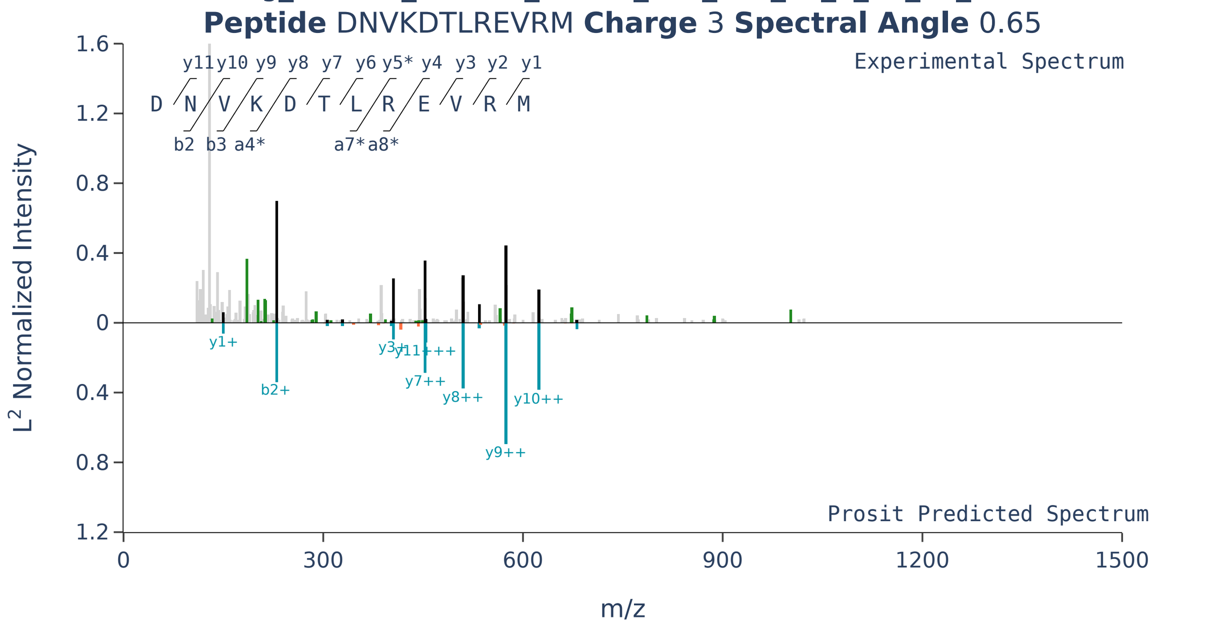


**Source** Ncheng_210623_230623_HFGoe_FFH_20S_25_1_A1_4h_R1 **Scan** 34518
**Peptide** AMGEENQTLNLAAQPPAVVL **Charge** 2 **Spectral Angle** 0.92

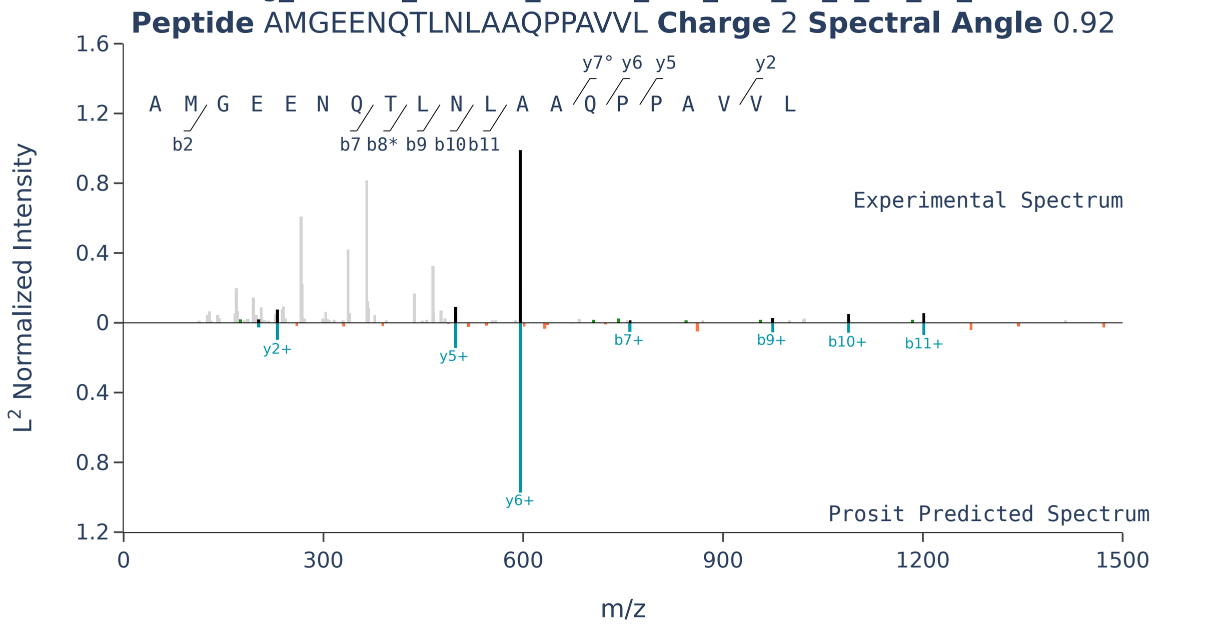


**Source** Ncheng_210623_230623_HFGoe_FFH_20S_25_1_A1_2h_R1 **Scan** 19509
**Peptide** RLTEDNVKDTLREVRM **Charge** 4 **Spectral Angle** 0.75

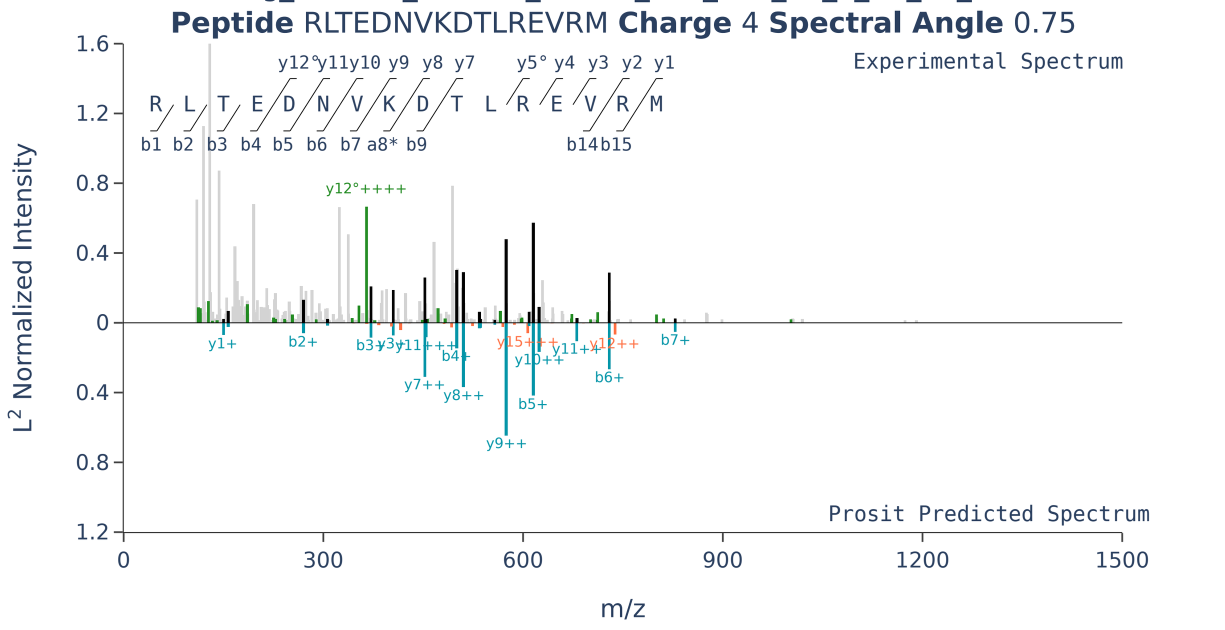


**Source** Ncheng_210623_230623_HFGoe_FFH_20S_25_1_A2_24h_R2 **Scan** 15425
**Peptide** QVQDVNRLLKQ **Charge** 2 **Spectral Angle** 0.69

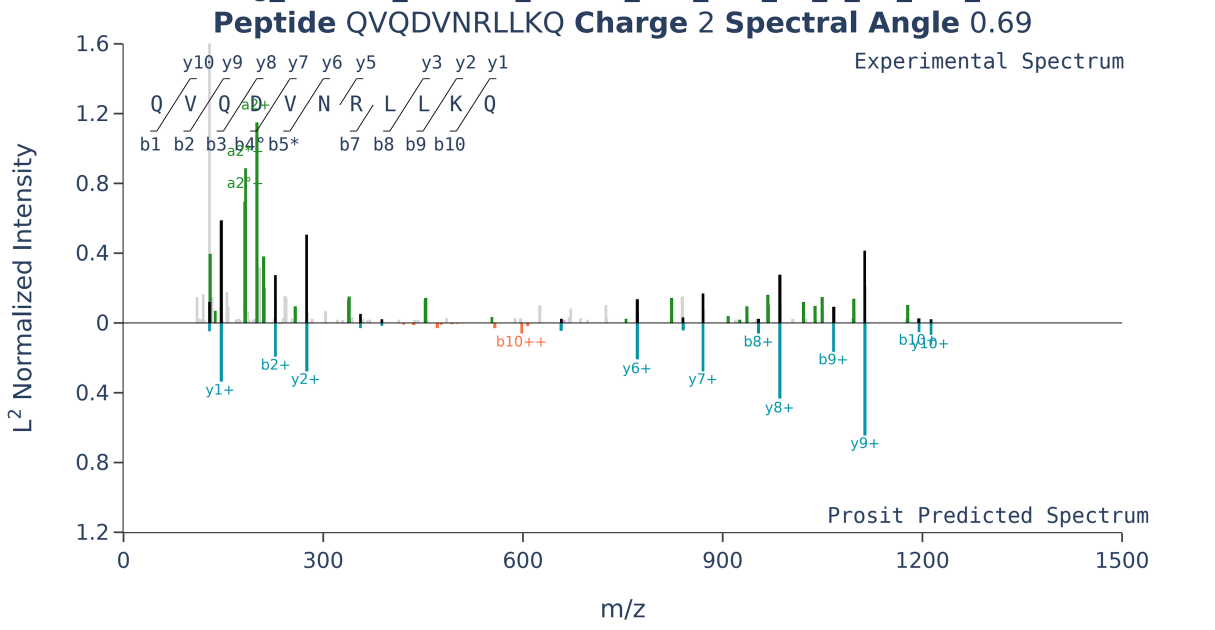


**Source** Ncheng_210623_230623_HFGoe_FFH_20S_25_1_A1_2h_R1 **Scan** 21034
**Peptide** GKLPGMGQIPDNVKSQM **Charge** 2 **Spectral Angle** 0.78

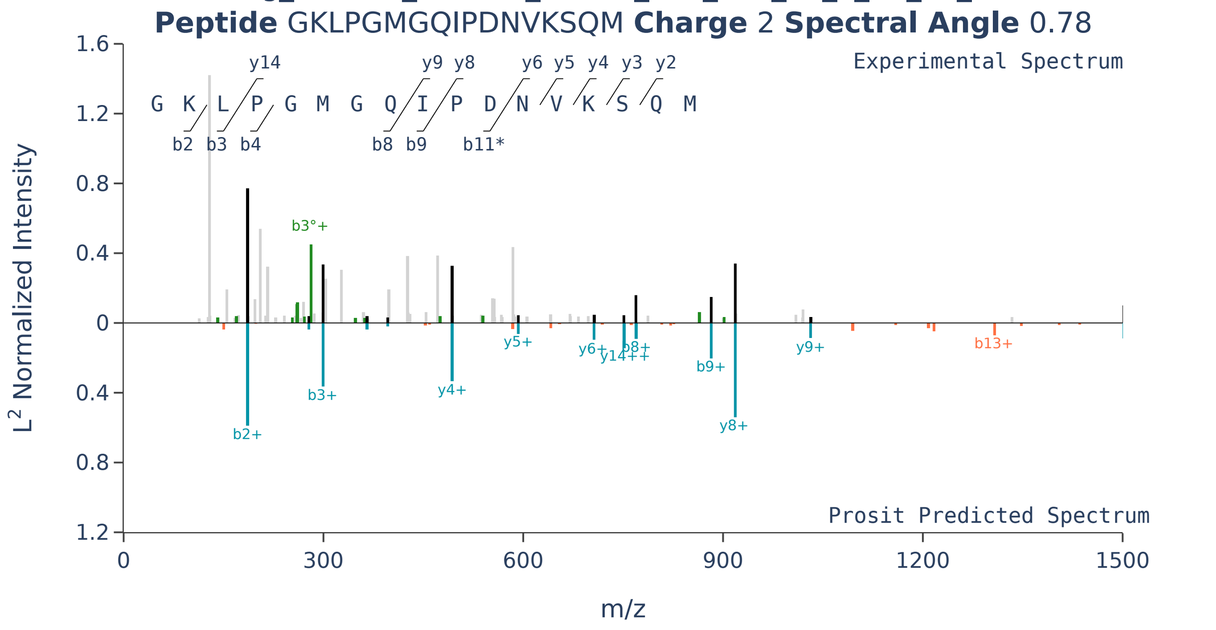


**Source** Ncheng_210623_230623_HFGoe_FFH_20S_25_1_A2_4h_R2 **Scan** 33171
**Peptide** MGGMASLMGKLPGMGQIPDNVKSQMDD **Charge** 3 **Spectral Angle** 0.83

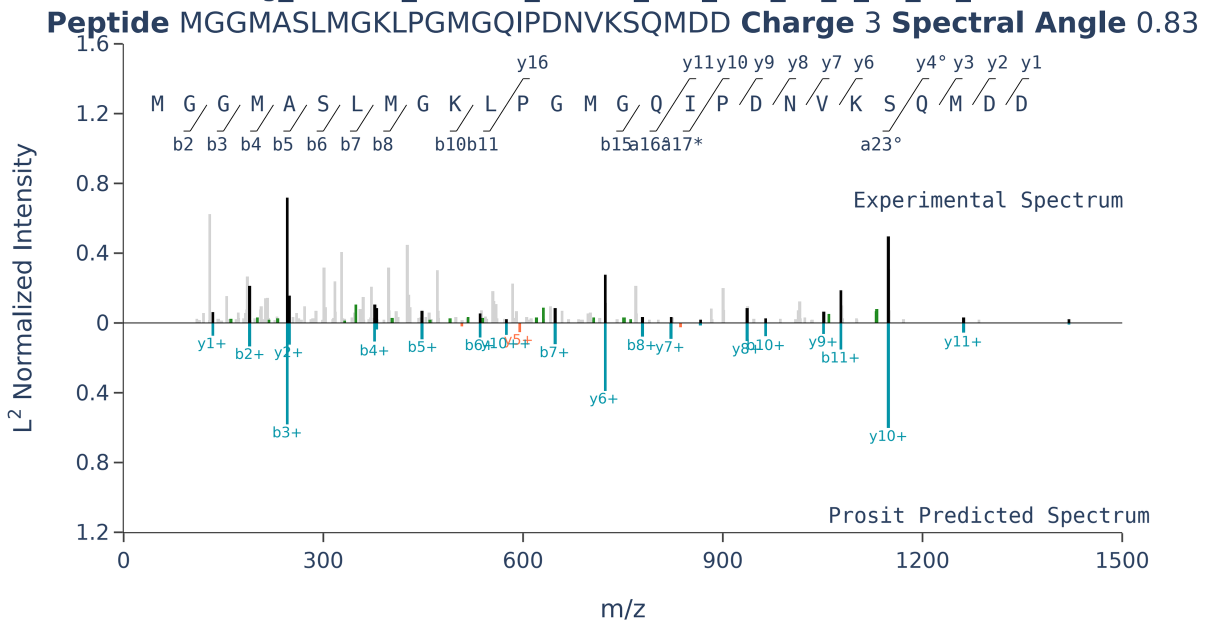


**Source** Ncheng_210623_230623_HFGoe_FFH_20S_25_1_A2_24h_R1 **Scan** 6027
**Peptide** KKGGMAKMMRS **Charge** 2 **Spectral Angle** 0.74

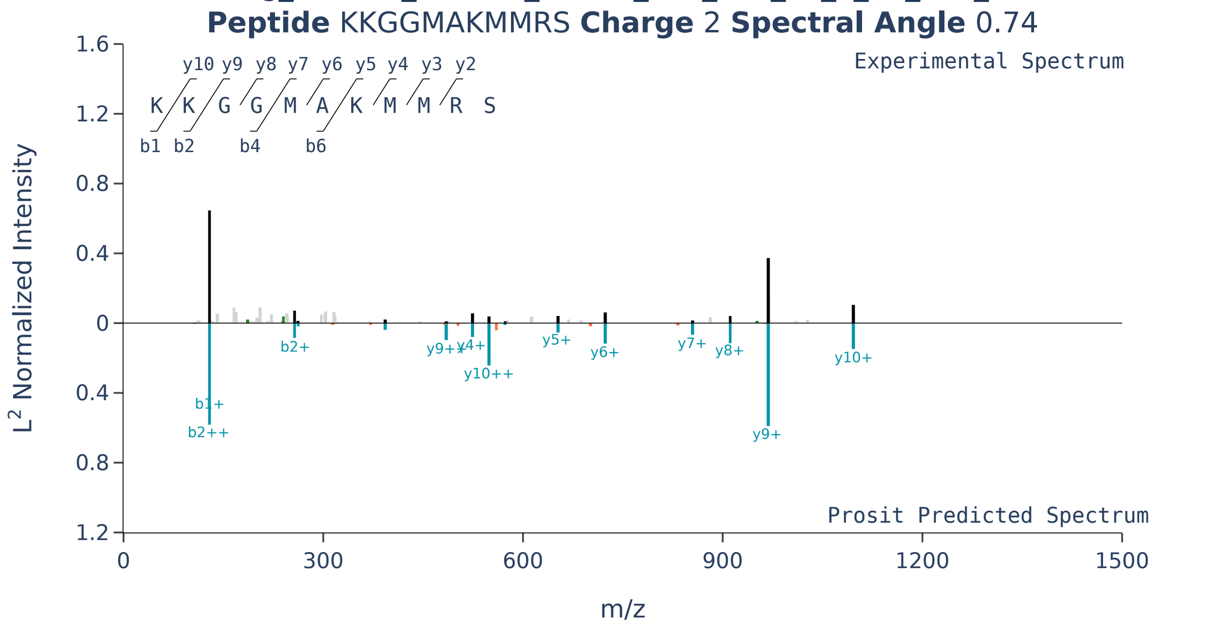


**Source** Ncheng_210623_230623_HFGoe_FFH_20S_25_1_A1_4h_R1 **Scan** 19414
**Peptide** PLKFLGVGEKT **Charge** 3 **Spectral Angle** 0.68

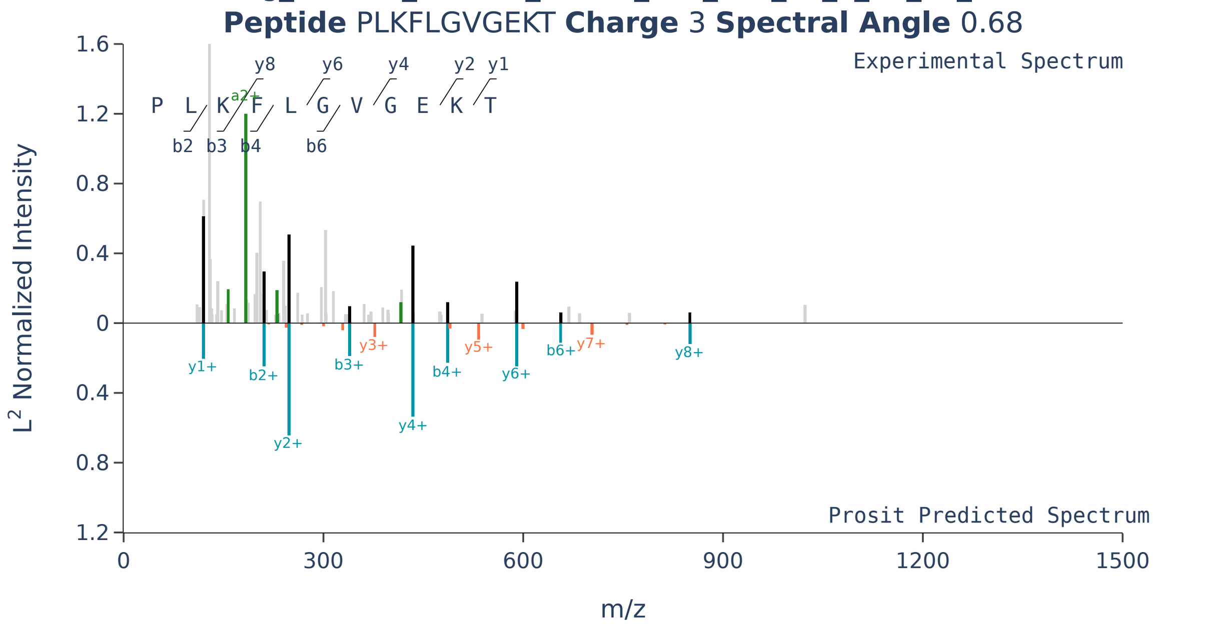


**Source** Ncheng_210623_230623_HFGoe_FFH_20S_25_1_A2_4h_R2 **Scan** 29910
**Peptide** GMASLMGKLPGMGQIPDNVKS **Charge** 2 **Spectral Angle** 0.77

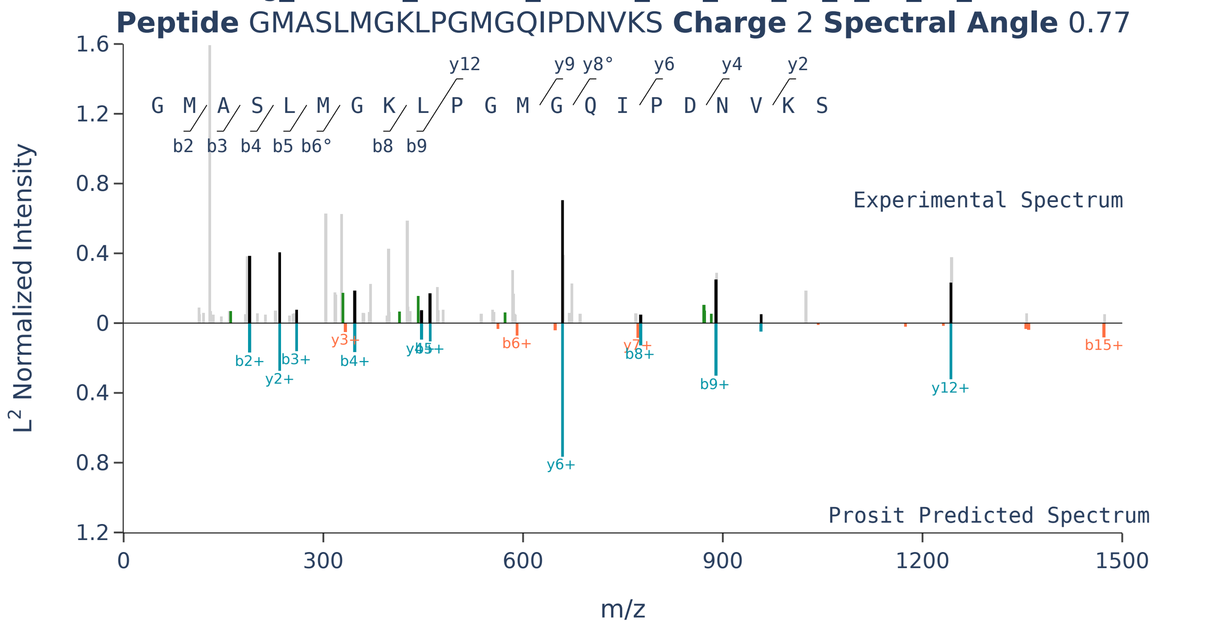


**Source** Ncheng_210623_230623_HFGoe_FFH_20S_25_1_A1_2h_R1 **Scan** 30769
**Peptide** AMTGQDAANTAKAFNEALPLTGV **Charge** 2 **Spectral Angle** 0.9

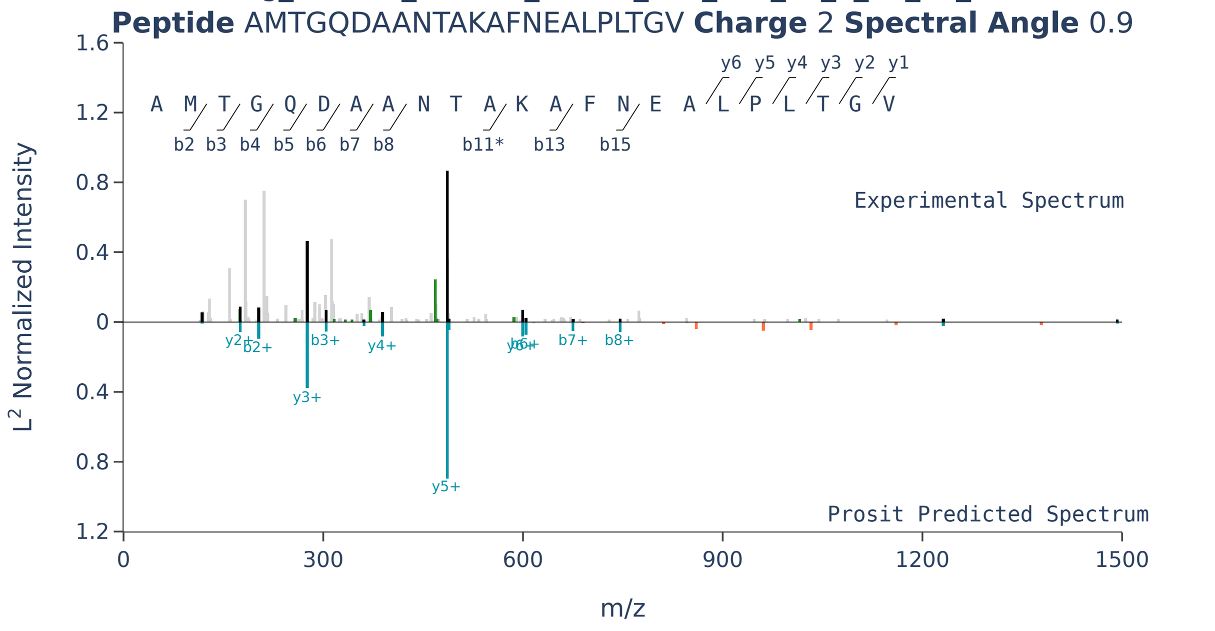


**Source** Ncheng_210623_230623_HFGoe_FFH_20S_25_1_A1_1h_R2 **Scan** 20335
**Peptide** VRMALLE **Charge** 2 **Spectral Angle** 0.78

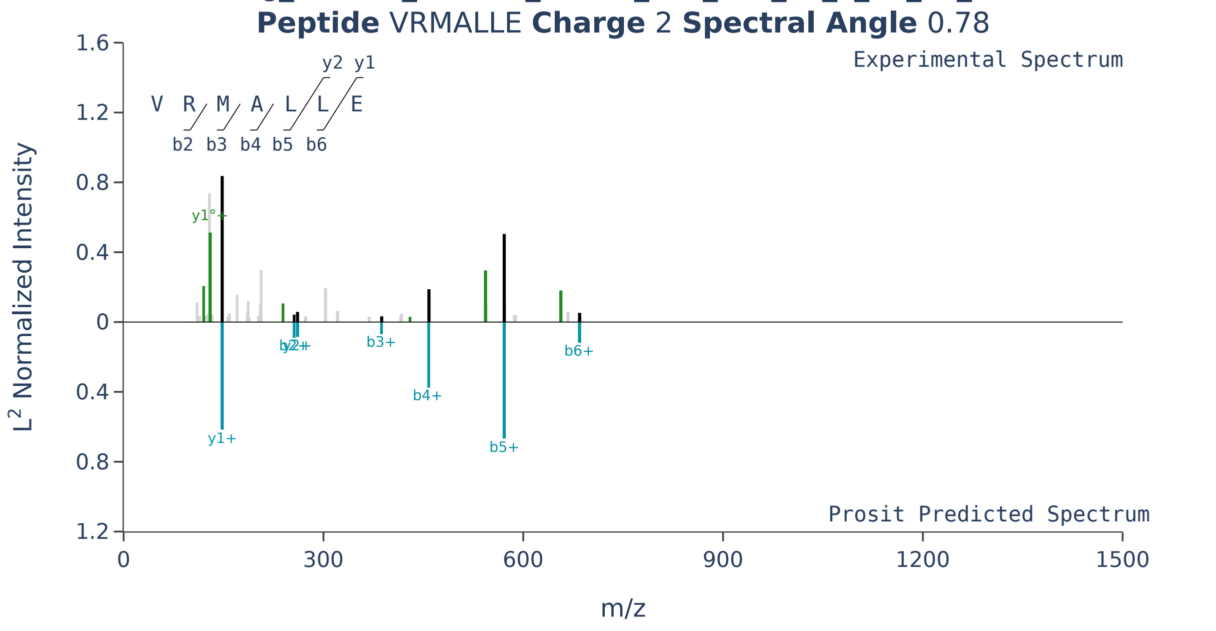


**Source** Ncheng_210623_230623_HFGoe_FFH_20S_25_1_A2_24h_R1 **Scan** 16251
**Peptide** LEDIESKVDRAQAEKL **Charge** 2 **Spectral Angle** 0.82

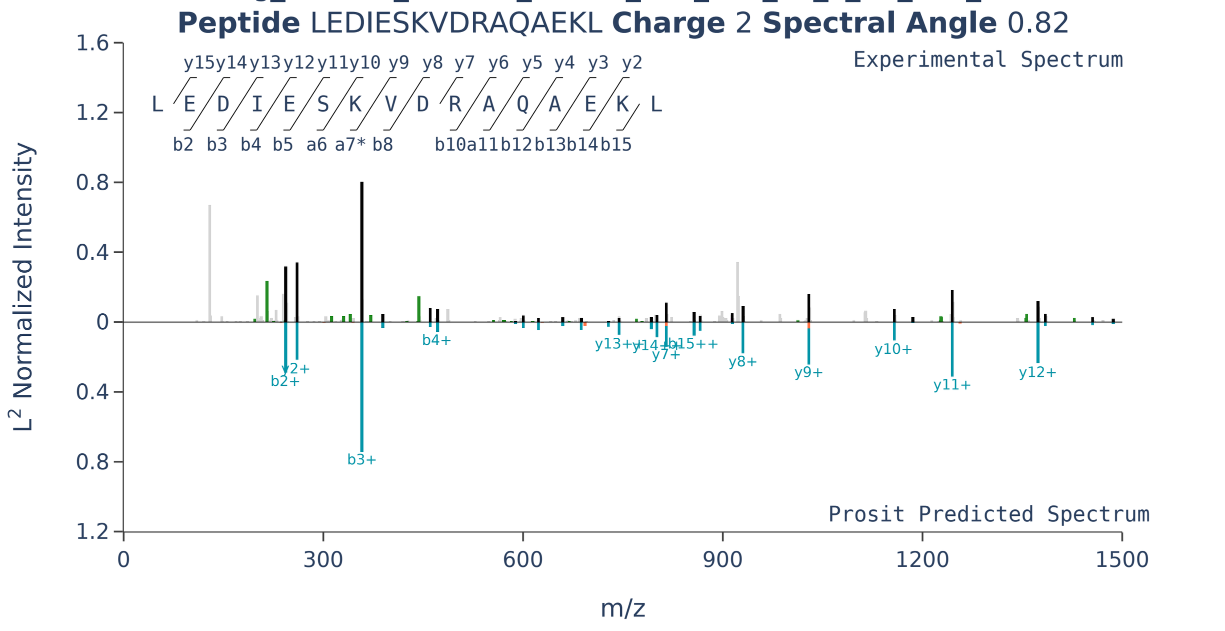


**Source** Ncheng_210623_230623_HFGoe_FFH_20S_25_1_A2_1h_R1 **Scan** 13794
**Peptide** CGMQVQDV **Charge** 2 **Spectral Angle** 0.81

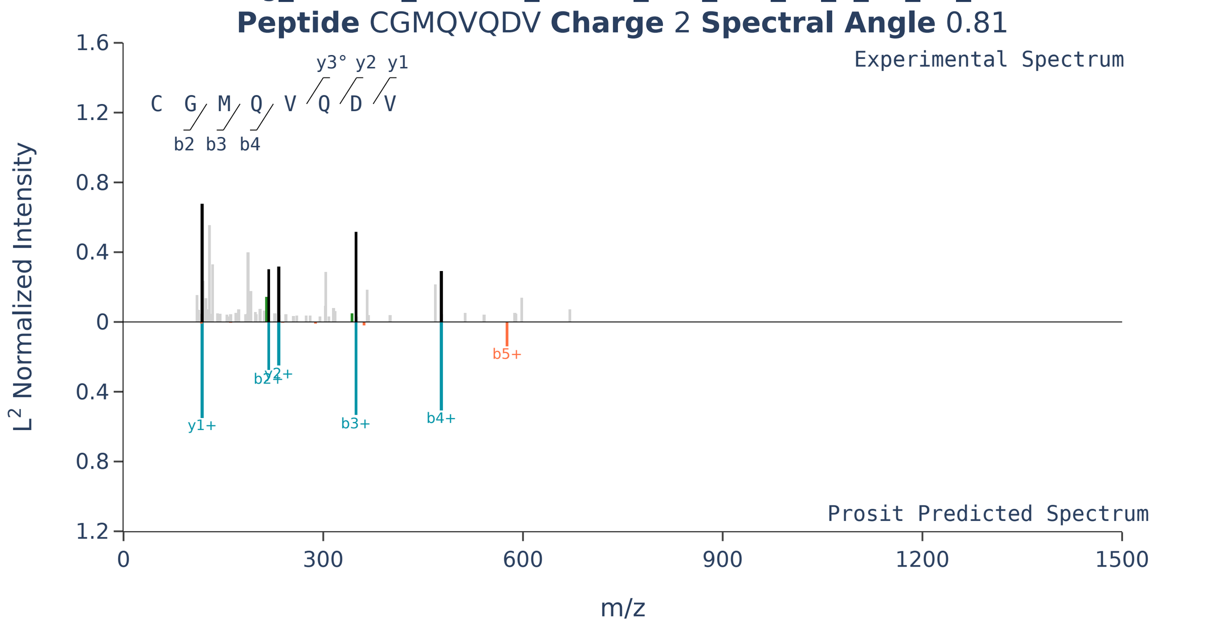


**Source** Ncheng_210623_230623_HFGoe_FFH_20S_25_1_A1_24h_R1 **Scan** 5148
**Peptide** MTMKERAK **Charge** 2 **Spectral Angle** 0.72

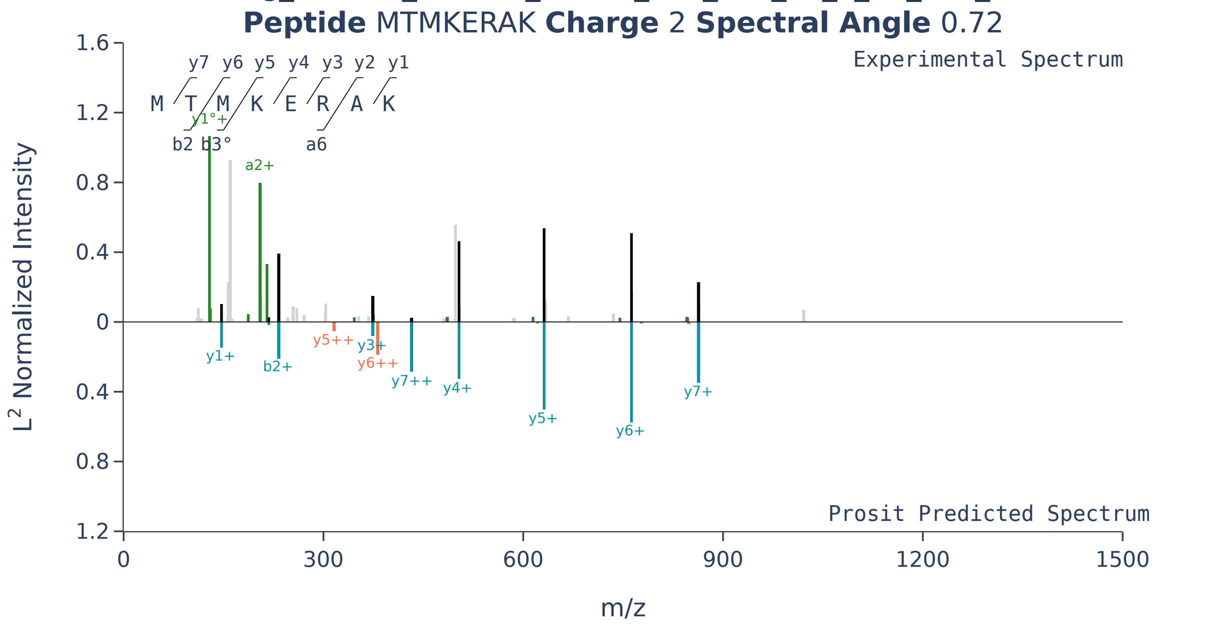


**Source** Ncheng_210623_230623_HFGoe_FFH_20S_25_1_A2_4h_R1 **Scan** 26002
**Peptide** KLPGMGQIPDNVKSQMDDKVLVRM **Charge** 3 **Spectral Angle** 0.74

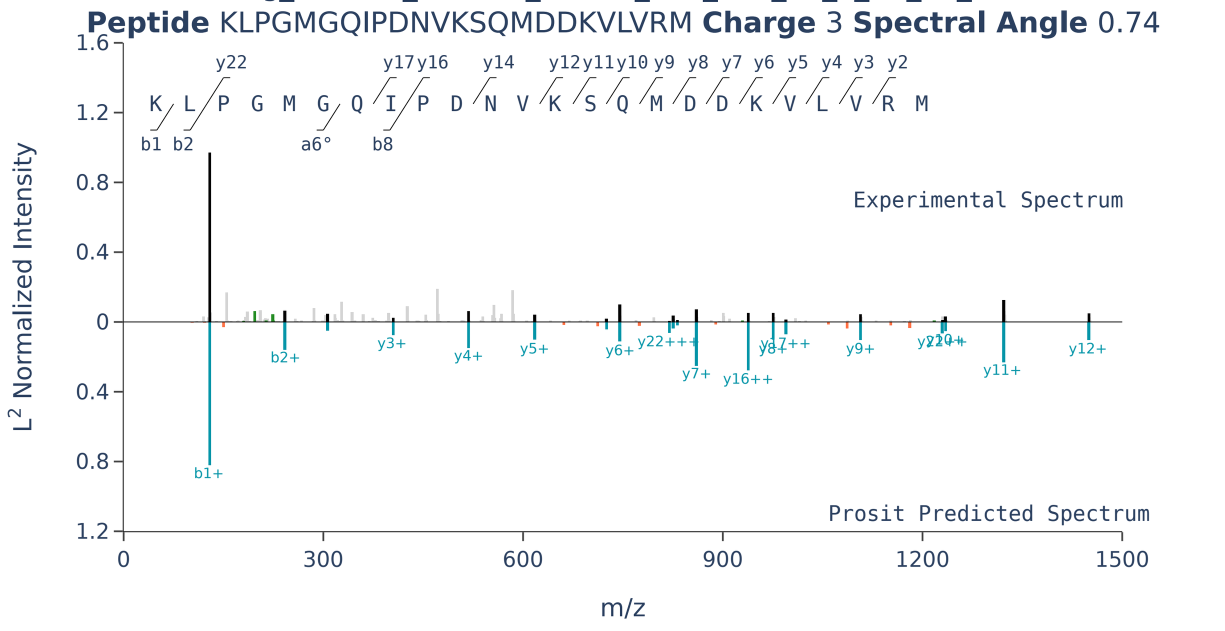

**Source** Ncheng_210623_230623_HFGoe_FFH_20S_25_1_A2_24h_R2 **Scan** 19914
**Peptide** PIKFLGVGEKT **Charge** 3 **Spectral Angle** 0.74

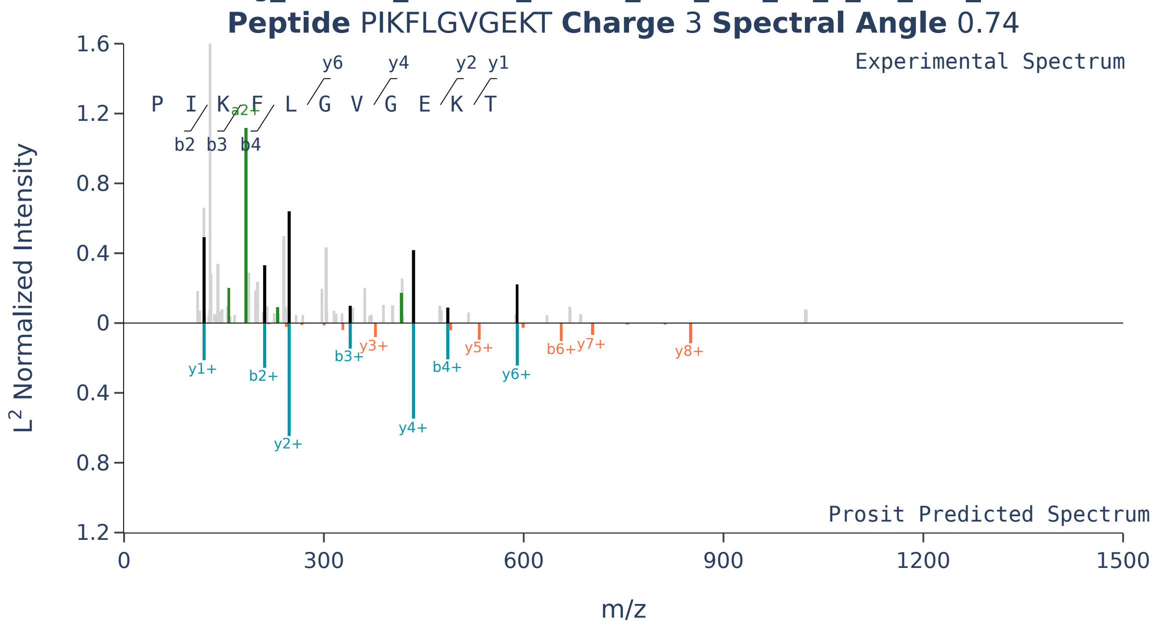


**Source** Ncheng_210623_230623_HFGoe_FFH_20S_25_1_A2_4h_R1 **Scan** 5333
**Peptide** KAVGHEV **Charge** 2 **Spectral Angle** 0.87

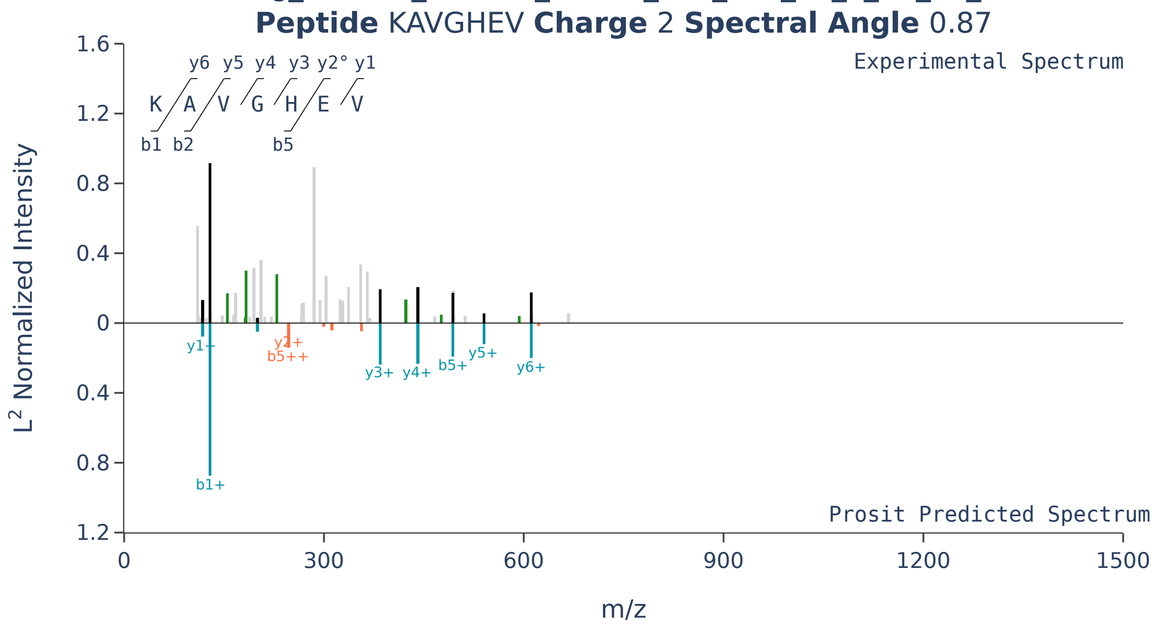


**Source** Ncheng_210623_230623_HFGoe_FFH_20S_25_1_A2_2h_R2 **Scan** 29744
**Peptide** TEDNVKDTLREVRMALL **Charge** 3 **Spectral Angle** 0.67

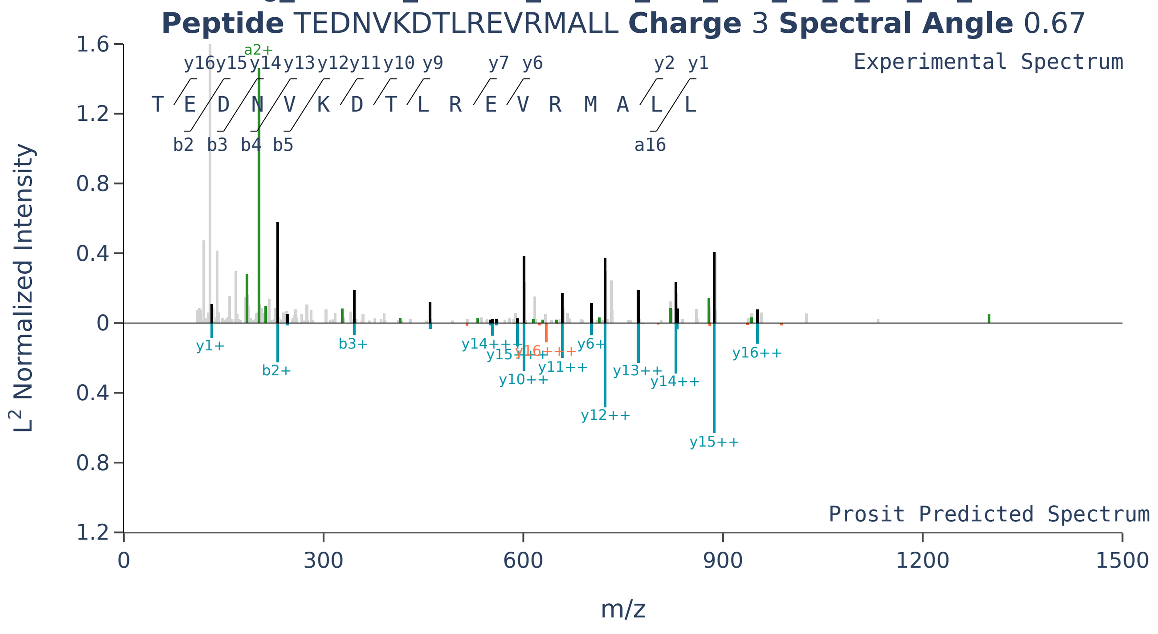


**Source** Ncheng_210623_230623_HFGoe_FFH_20S_25_1_A1_2h_R2 **Scan** 33948
**Peptide** QDAANTAKAFNEALPLTGVVLTKVD **Charge** 3 **Spectral Angle** 0.75

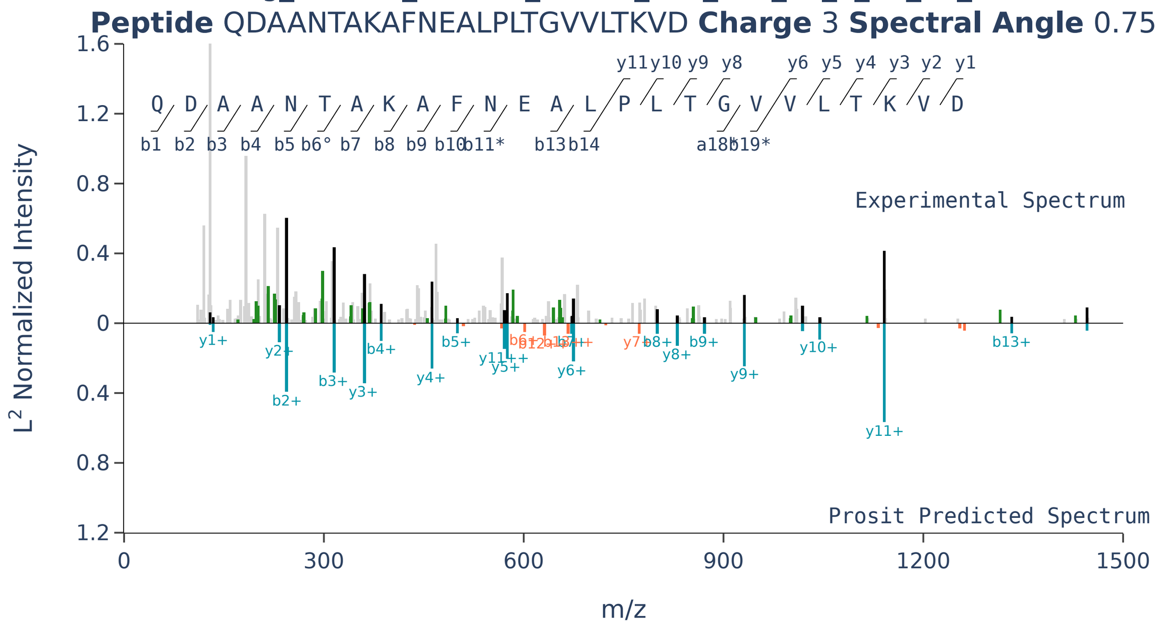


**Source** Ncheng_210623_230623_HFGoe_FFH_20S_25_1_A1_24h_R1 **Scan** 12869
**Peptide** DGDARGGAALSLR **Charge** 2 **Spectral Angle** 0.71

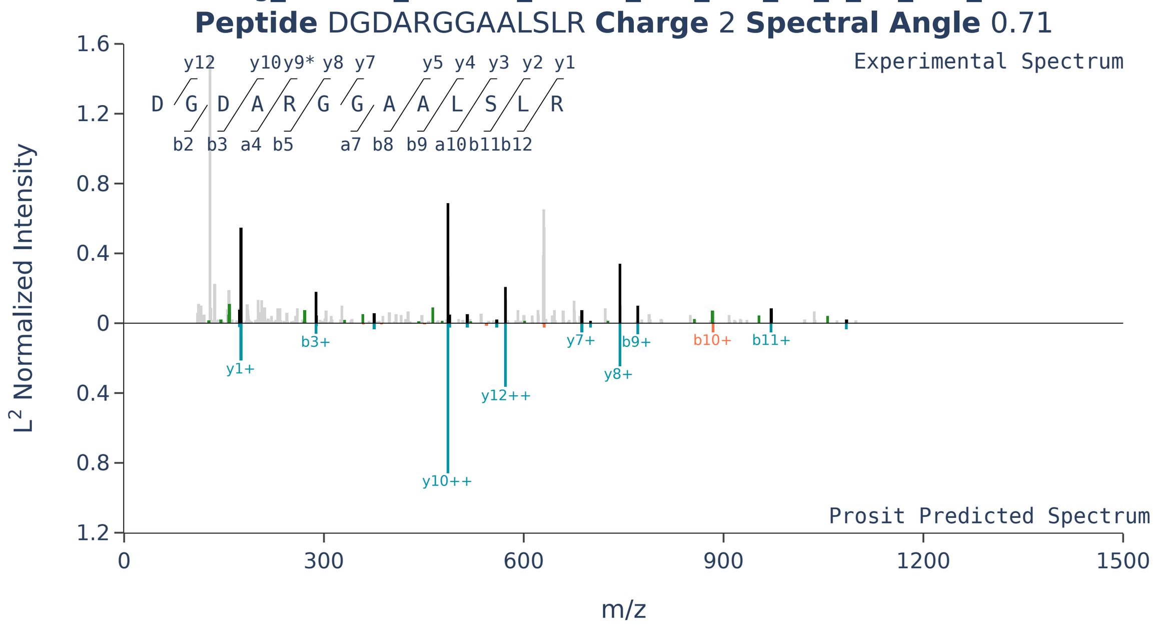


**Source** Ncheng_210623_230623_HFGoe_FFH_20S_25_1_A1_24h_R2 **Scan** 13564
**Peptide** VSADVYRPAALKQ **Charge** 2 **Spectral Angle** 0.7

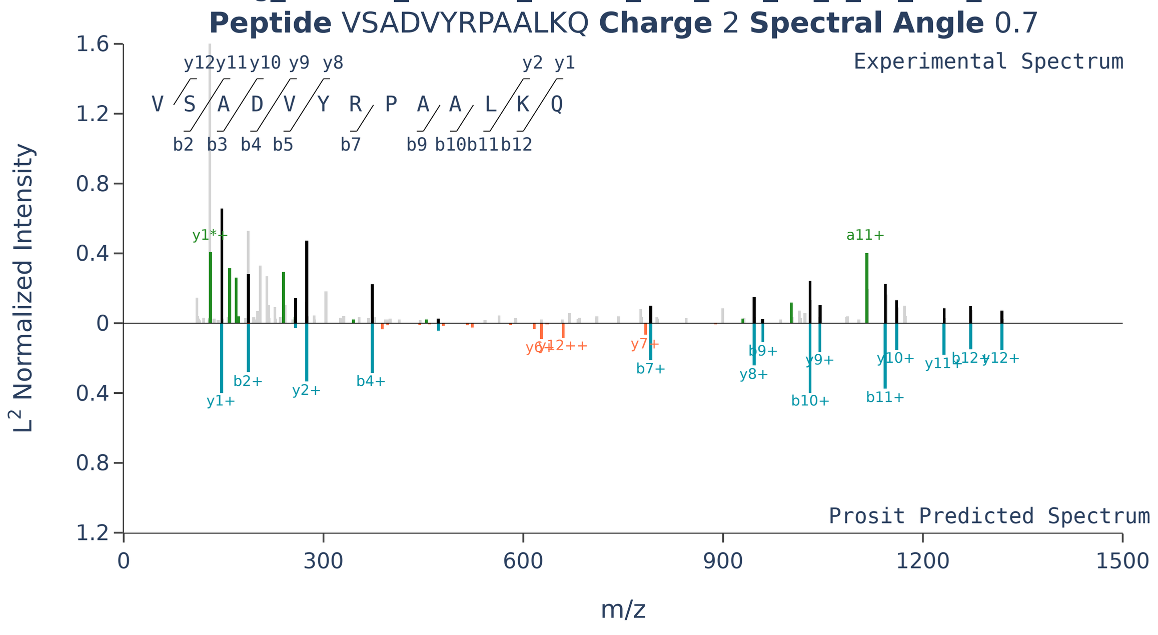


**Source** Ncheng_210623_230623_HFGoe_FFH_20S_25_1_A1_2h_R1 **Scan** 17948
**Peptide** REVRMALLE **Charge** 2 **Spectral Angle** 0.75

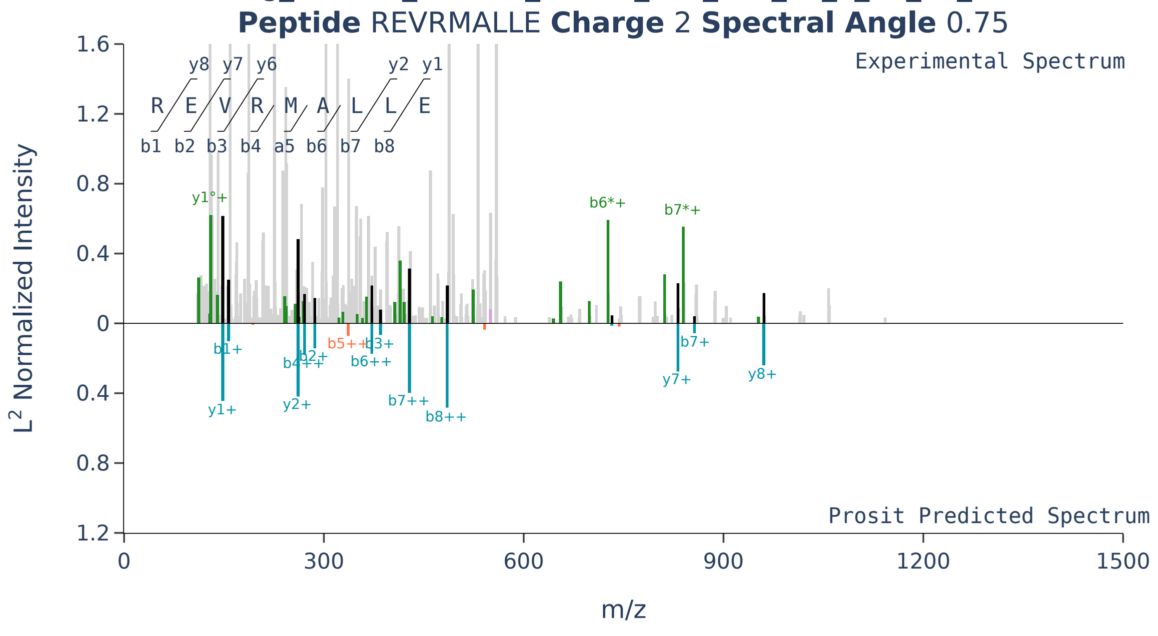


**Source** Ncheng_210623_230623_HFGoe_FFH_20S_25_1_A1_1h_R1 **Scan** 21961
**Peptide** HEVNKSLTPGQEFVKIV **Charge** 4 **Spectral Angle** 0.72

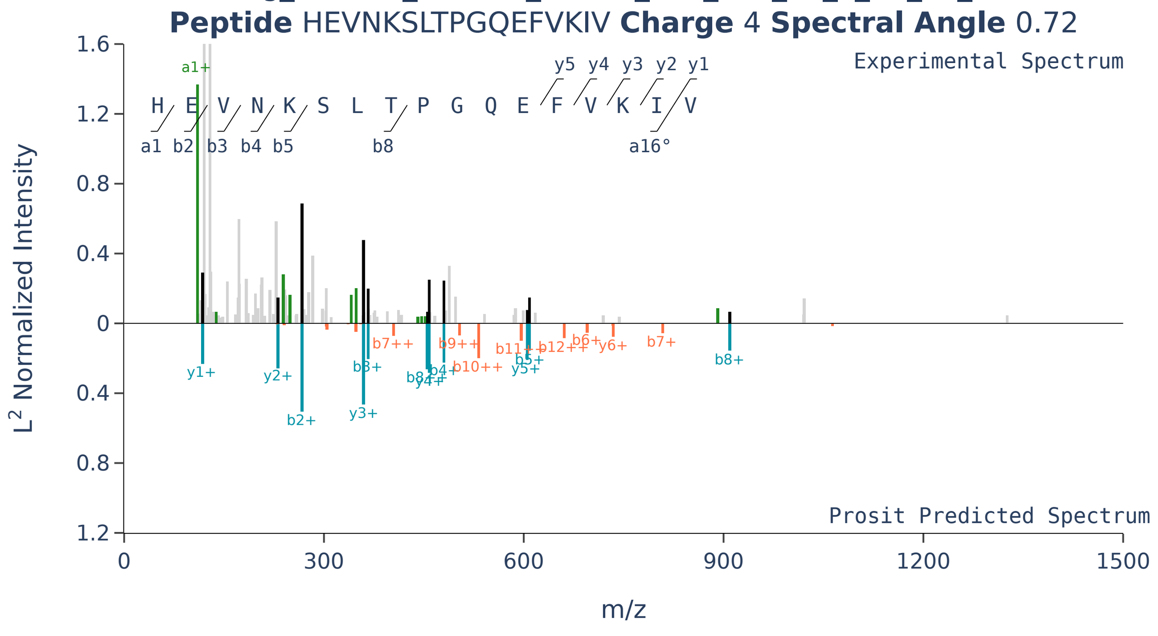


**Source** Ncheng_210623_230623_HFGoe_FFH_20S_25_1_A2_24h_R1 **Scan** 6158
**Peptide** MTGQDAANTAKA **Charge** 2 **Spectral Angle** 0.68

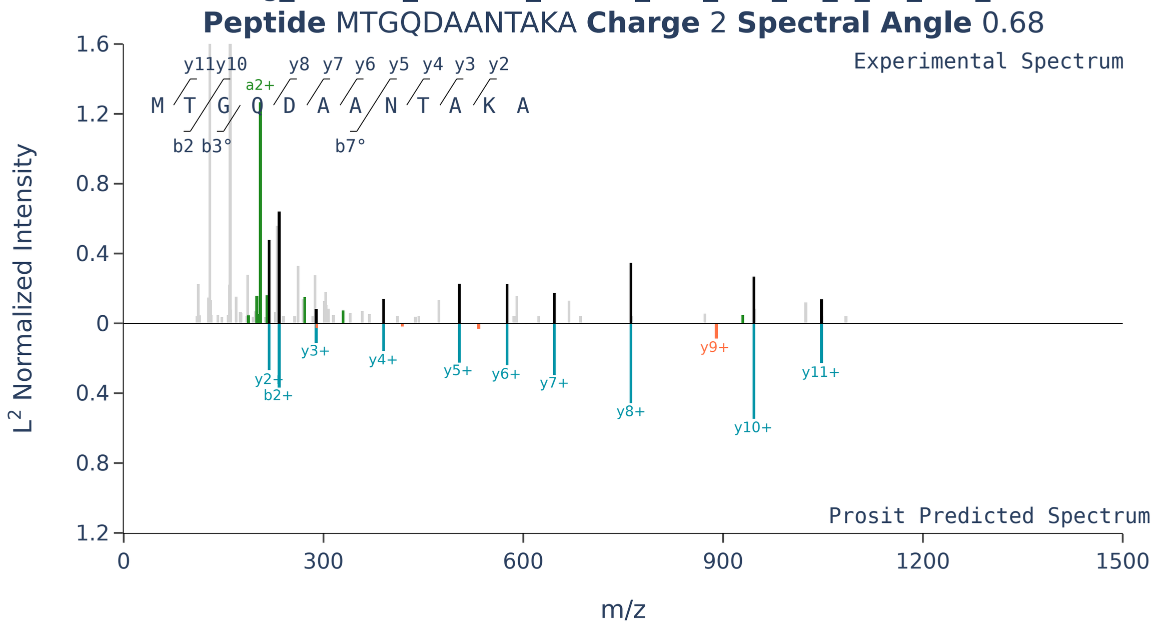


**Source** Ncheng_210623_230623_HFGoe_FFH_20S_25_1_A2_4h_R2 **Scan** 15050
**Peptide** ALEPFHPDRIASR **Charge** 3 **Spectral Angle** 0.63

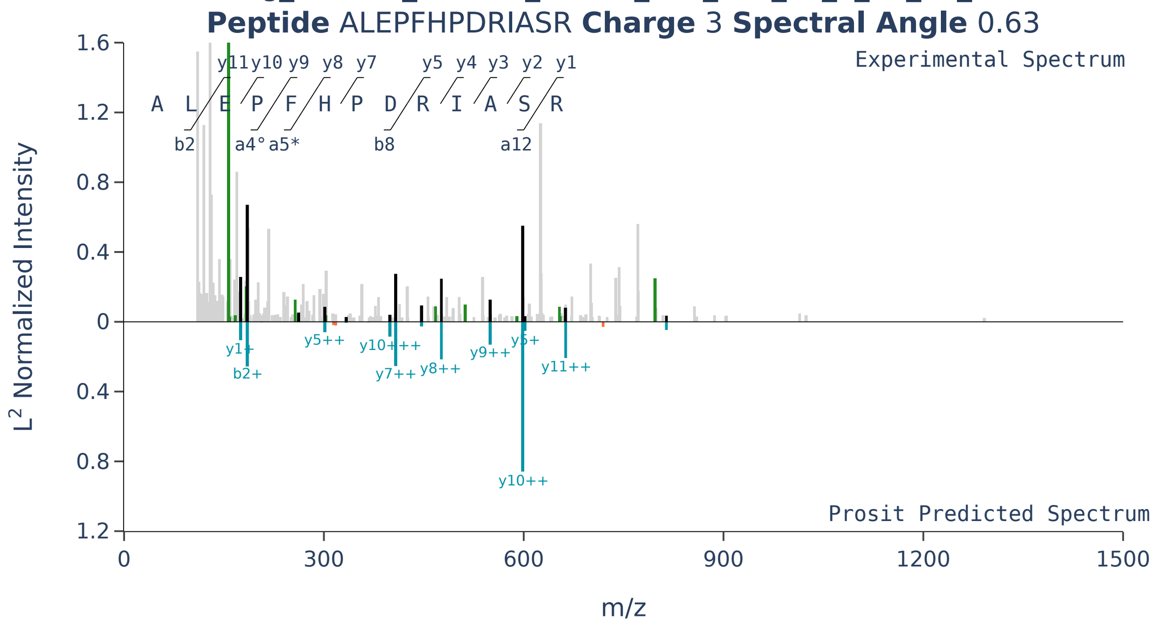


**Source** Ncheng_210623_230623_HFGoe_FFH_20S_25_1_A2_24h_R1 **Scan** 15284
**Peptide** GLQGAGKTTSVGKLGKF **Charge** 2 **Spectral Angle** 0.76

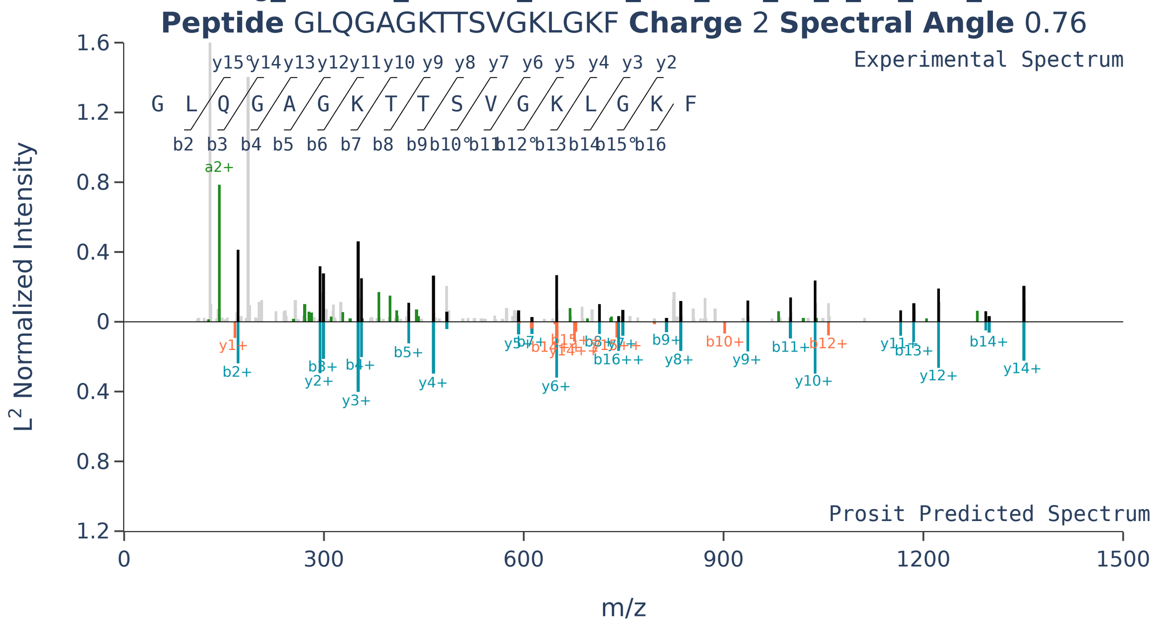


**Source** Ncheng_210623_230623_HFGoe_FFH_20S_25_1_A1_2h_R1 **Scan** 29287
**Peptide** ALPLTGVVLTKVDGDARGGAALSIRHL **Charge** 4 **Spectral Angle** 0.73

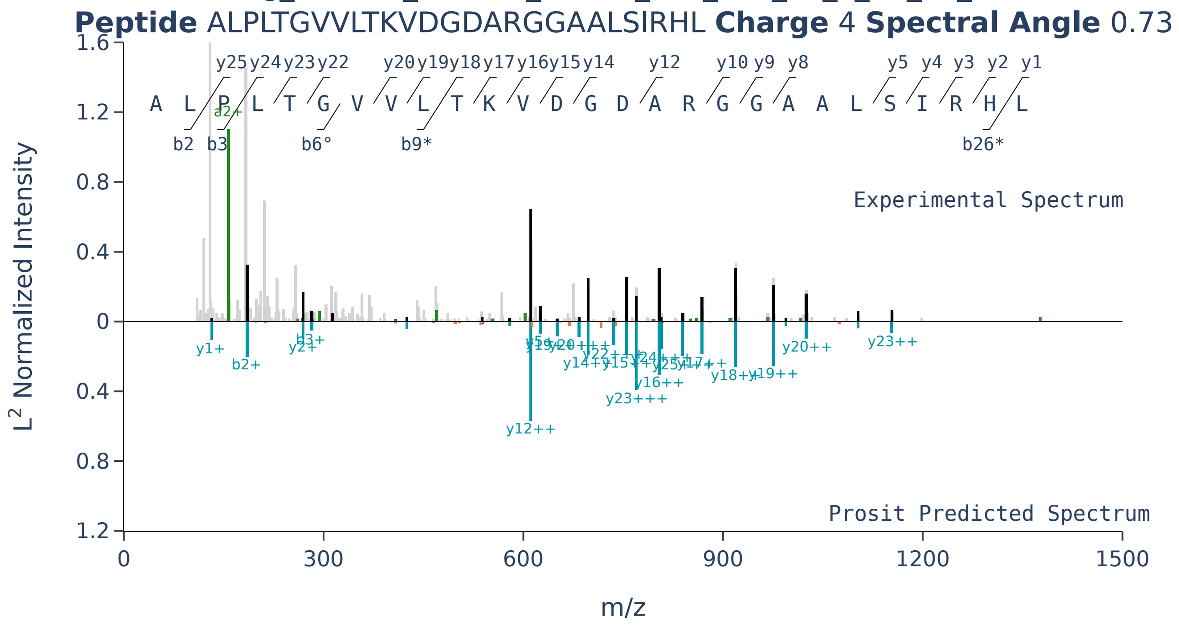


**Source** Ncheng_210623_230623_HFGoe_FFH_20S_25_1_A1_2h_R2 **Scan** 20070
**Peptide** HVDEAMMDEIKQVHA **Charge** 3 **Spectral Angle** 0.79

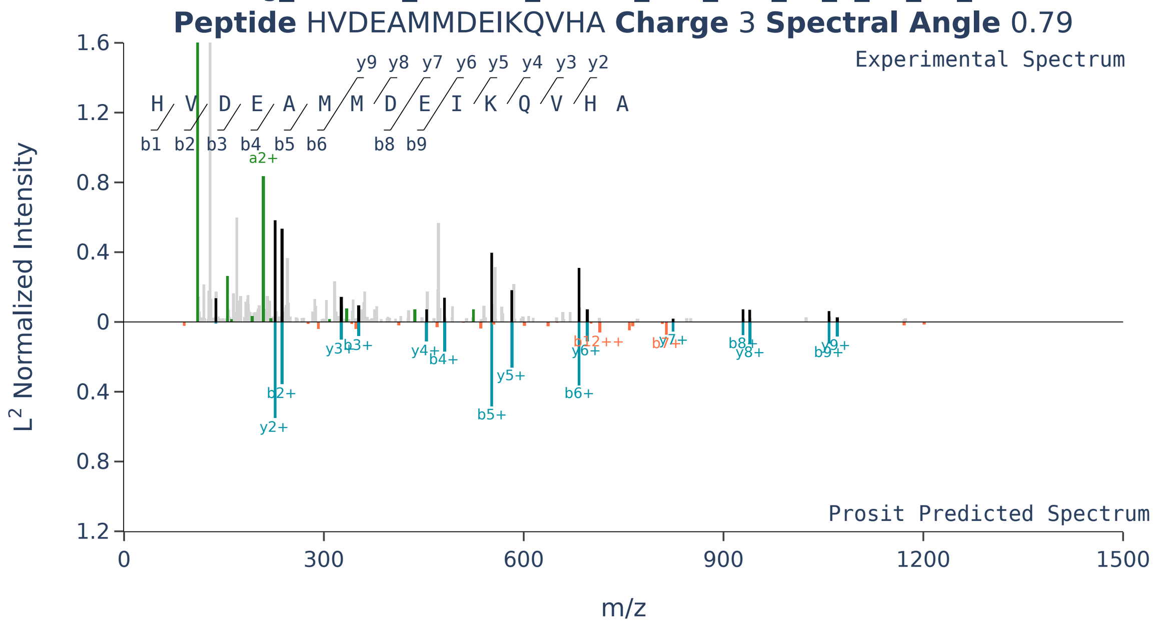


**Source** Ncheng_210623_230623_HFGoe_FFH_20S_25_1_A2_4h_R1 **Scan** 24342
**Peptide** NRLLKQFDDMQRMMKKM **Charge** 5 **Spectral Angle** 0.83

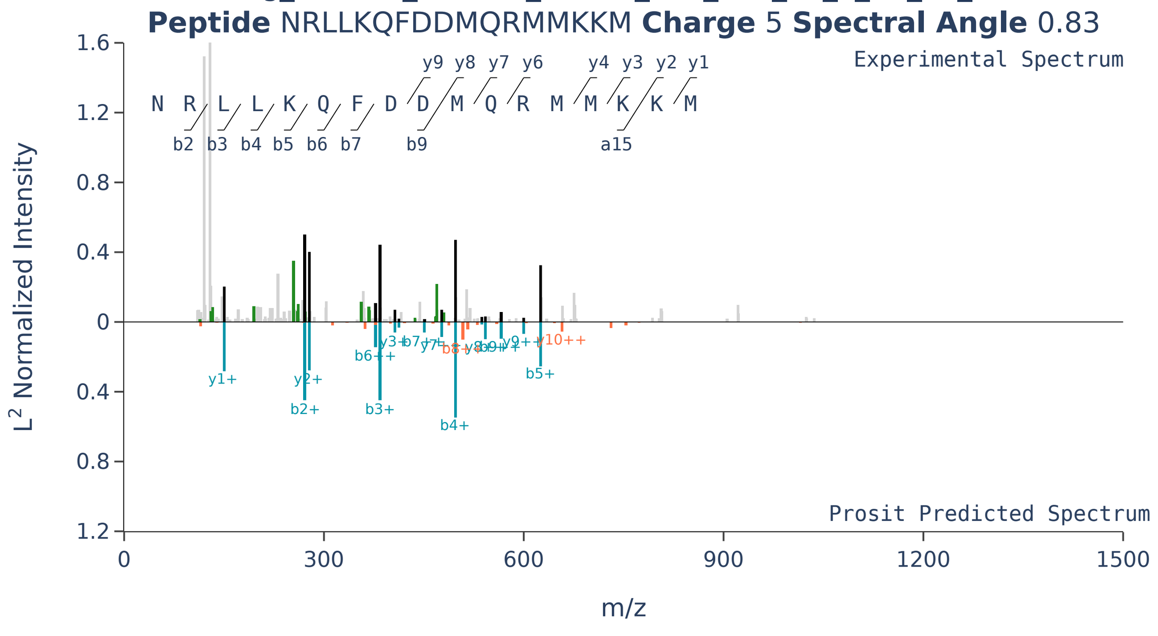


**Source** Ncheng_210623_230623_HFGoe_FFH_20S_25_1_A2_2h_R1 **Scan** 11475
**Peptide** IRHITGKPIKF **Charge** 3 **Spectral Angle** 0.79

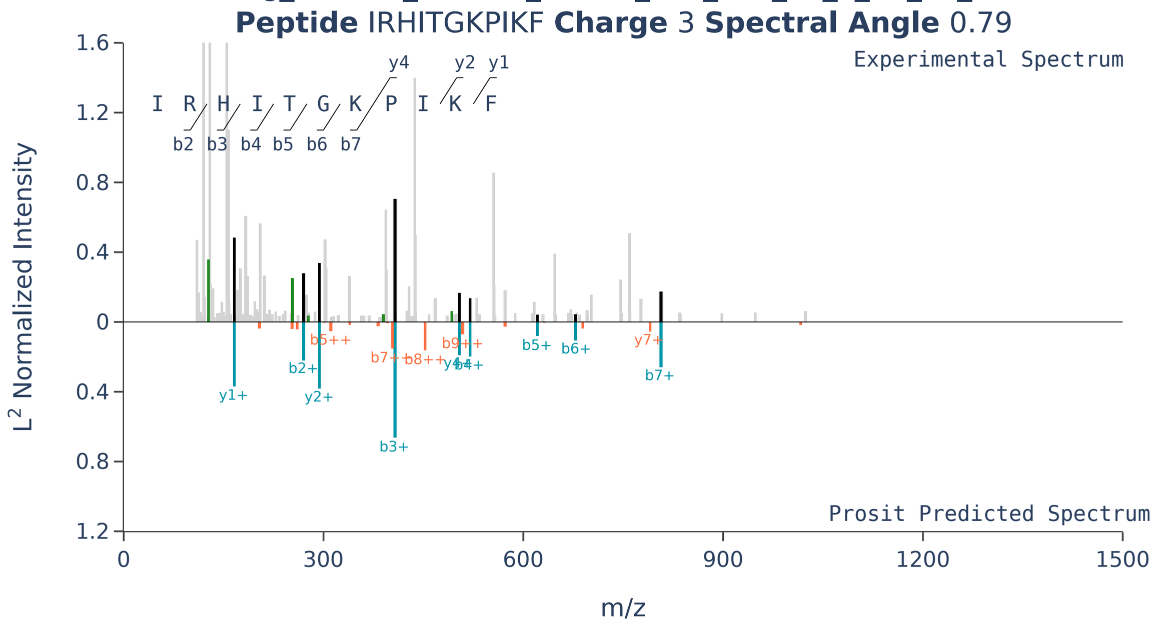

**Source** Ncheng_210623_230623_HFGoe_FFH_20S_25_1_A2_24h_R2 **Scan** 17741
**Peptide** TEDNVKDTLREVRM **Charge** 4 **Spectral Angle** 0.8

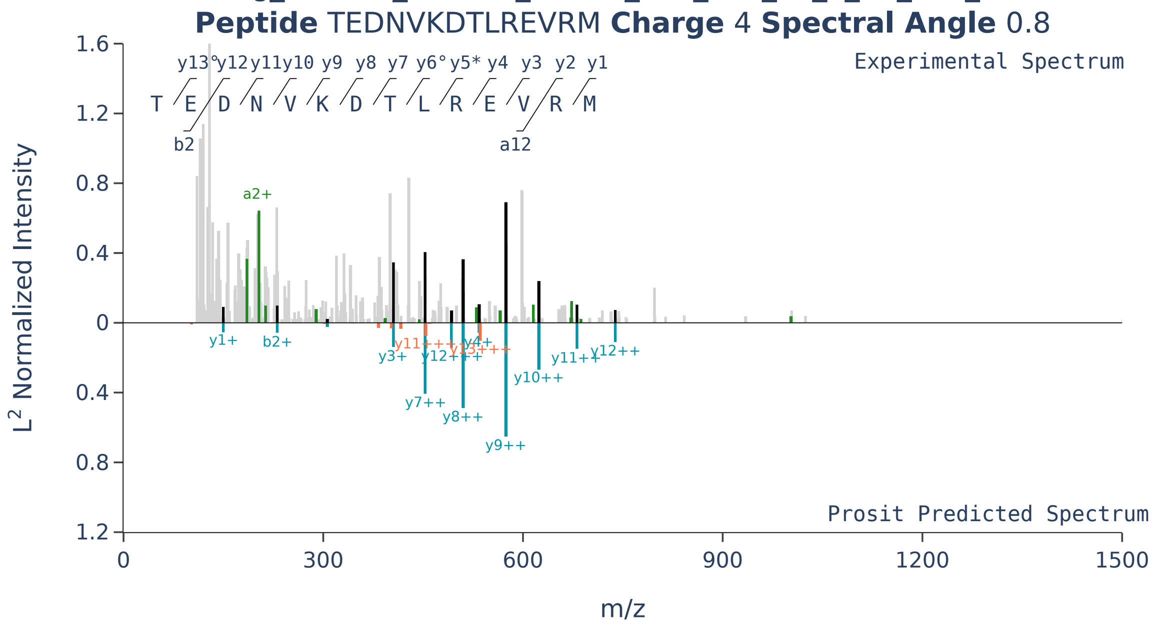


**Source** Ncheng_210623_230623_HFGoe_FFH_20S_25_1_A2_4h_R1 **Scan** 18509
**Peptide** SADVYRPAAIKQLET **Charge** 2 **Spectral Angle** 0.72

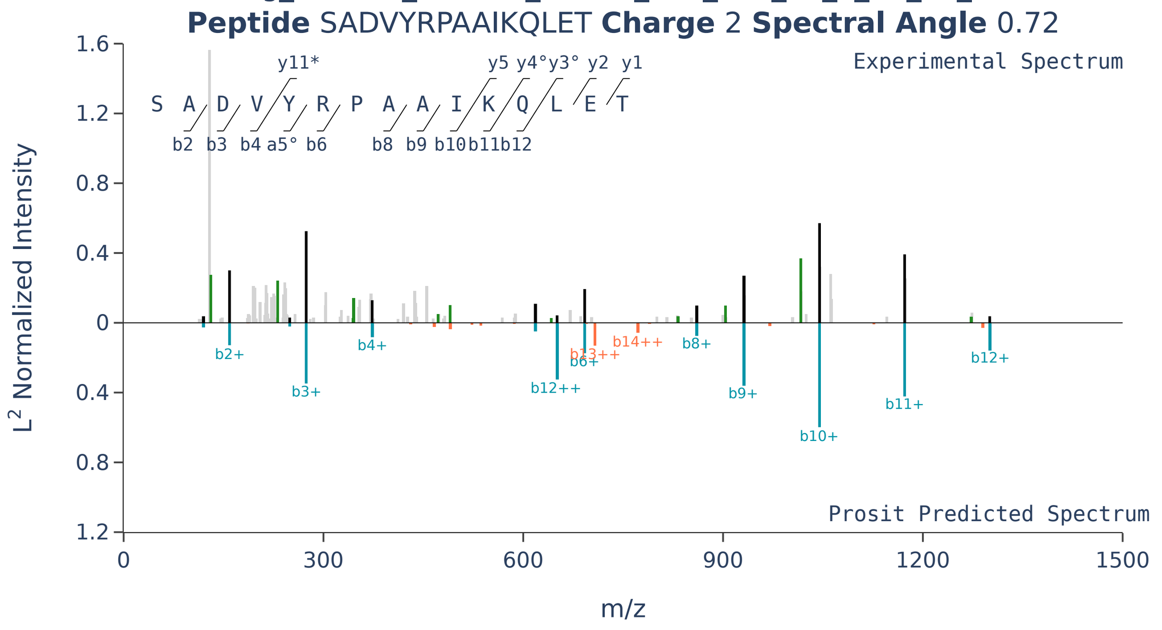


**Source** Ncheng_210623_230623_HFGoe_FFH_20S_25_1_A2_24h_R1 **Scan** 9929
**Peptide** QMKNMGGMA **Charge** 2 **Spectral Angle** 0.73

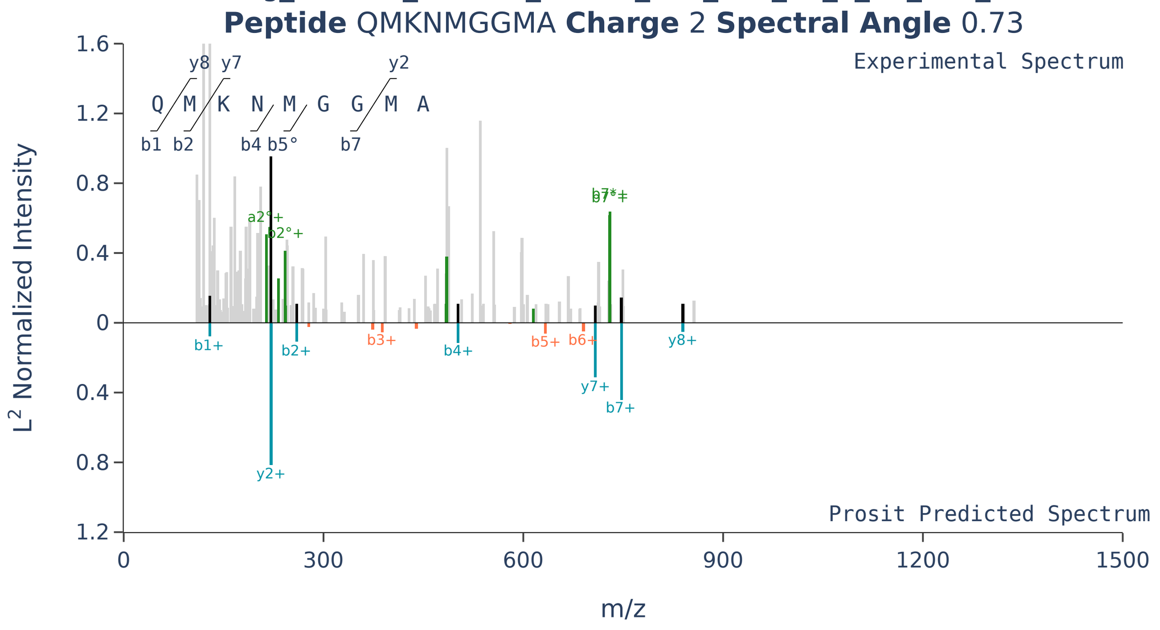


**Source** Ncheng_210623_230623_HFGoe_FFH_20S_25_1_A1_24h_R2 **Scan** 14345
**Peptide** EDNVKDTLREV **Charge** 2 **Spectral Angle** 0.81

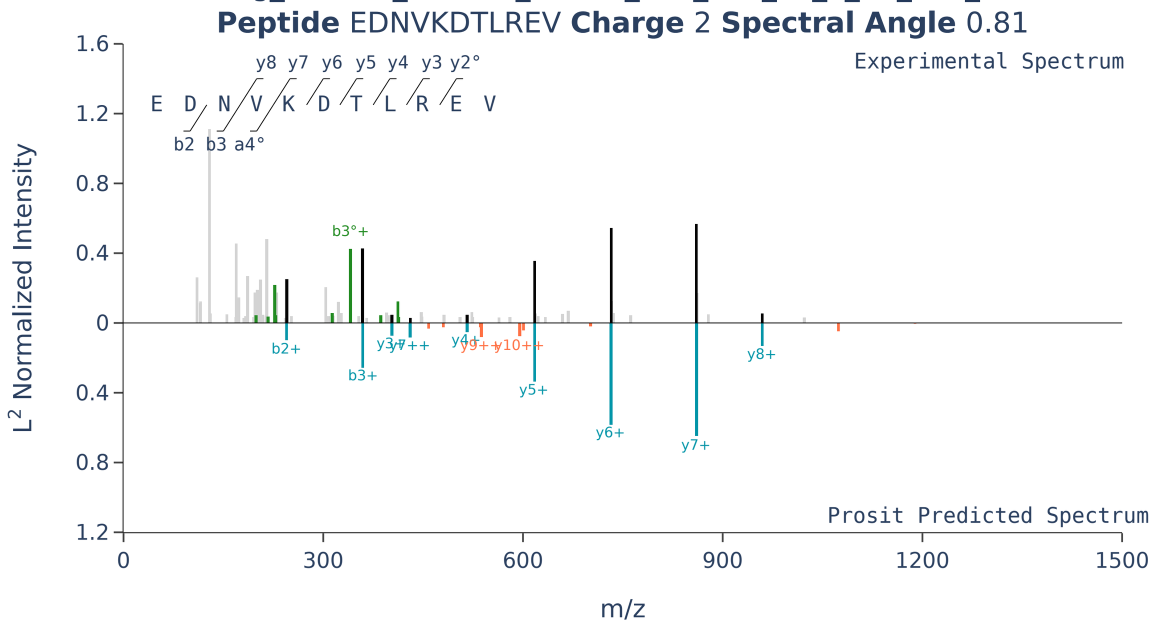


**Source** Ncheng_210623_230623_HFGoe_FFH_20S_25_1_A2_24h_R2 **Scan** 10623
**Peptide** KLKKGDGFDLND **Charge** 2 **Spectral Angle** 0.71

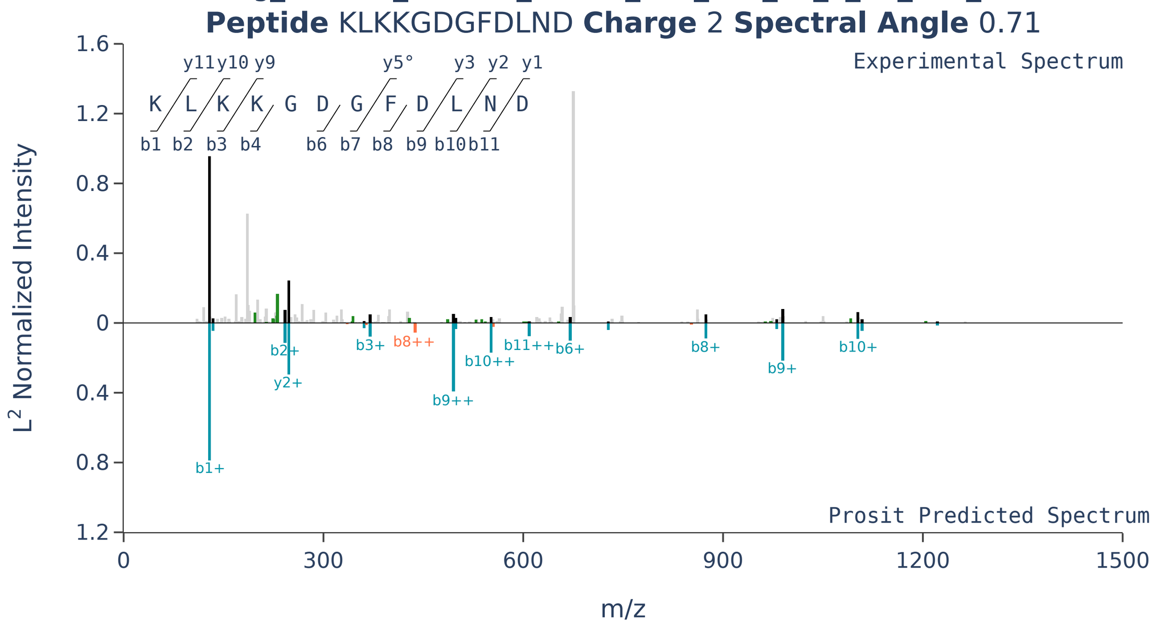


**Source** Ncheng_210623_230623_HFGoe_FFH_20S_25_1_A1_2h_R1 **Scan** 22658
**Peptide** DEIKQVHASINPVETL **Charge** 3 **Spectral Angle** 0.68

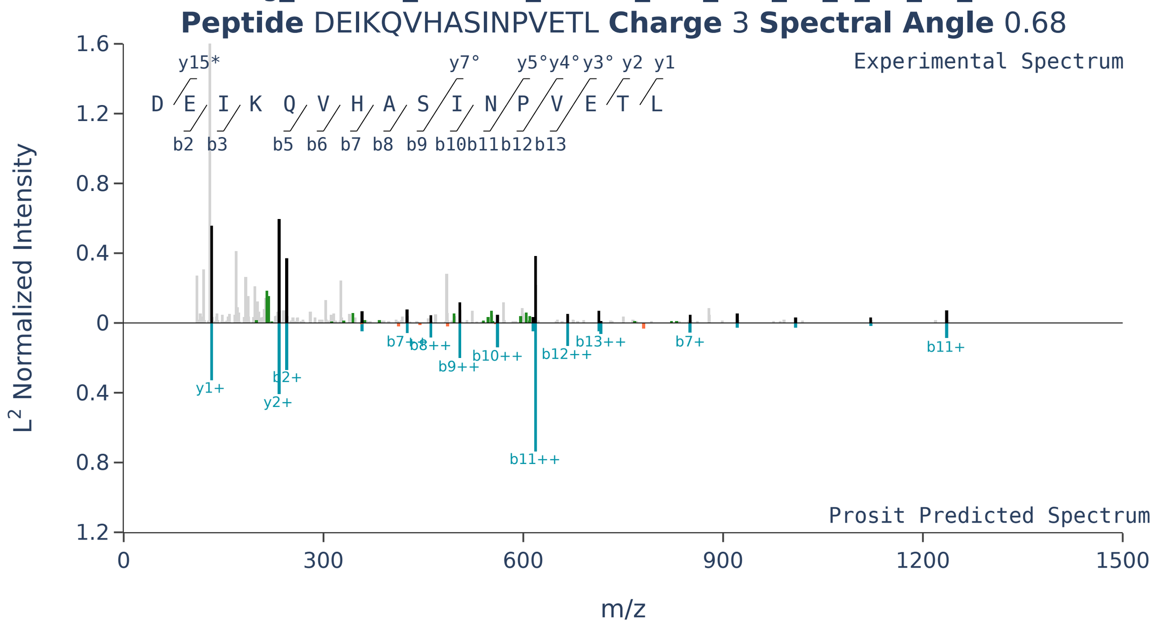


**Source** Ncheng_210623_230623_HFGoe_FFH_20S_25_1_A2_24h_R1 **Scan** 16563
**Peptide** KQFDDMQRMM **Charge** 2 **Spectral Angle** 0.66

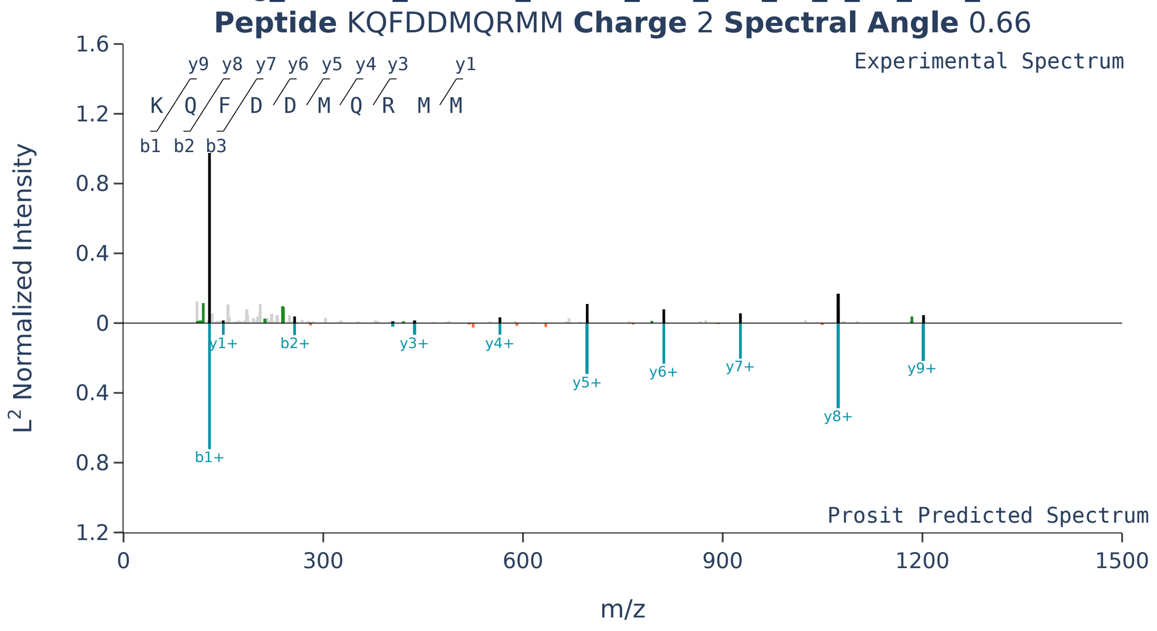


**Source** Ncheng_210623_230623_HFGoe_FFH_20S_25_1_A2_24h_R1 **Scan** 7732
**Peptide** DMQRMMKKMKK **Charge** 2 **Spectral Angle** 0.64

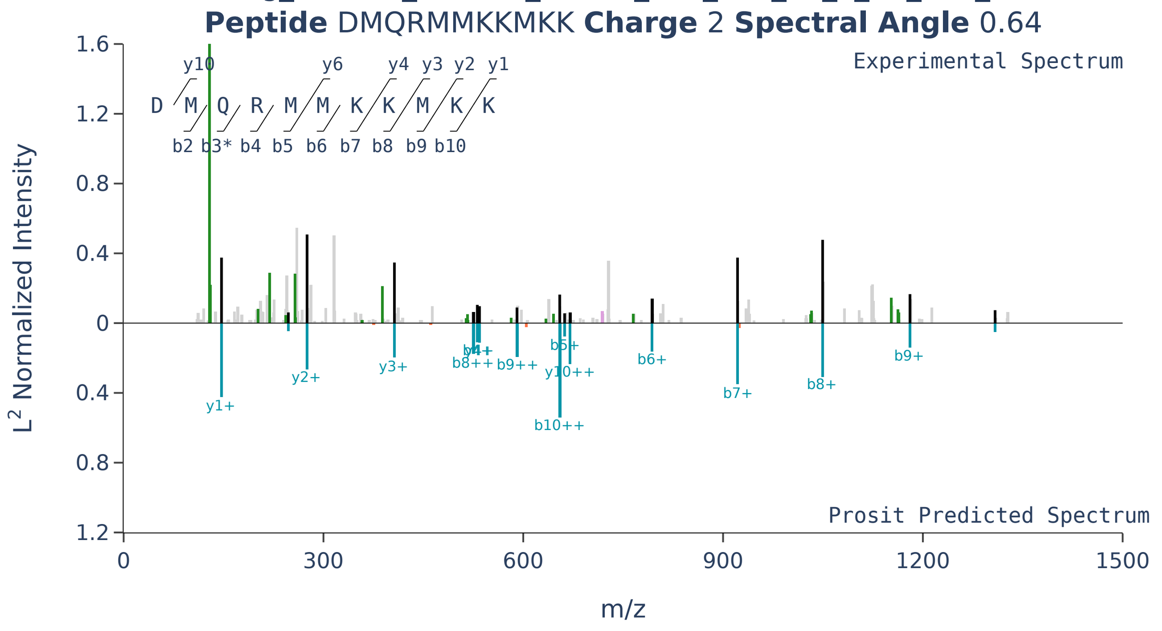


**Source** Ncheng_210623_230623_HFGoe_FFH_20S_25_1_A1_1h_R2 **Scan** 32460
**Peptide** LTEDNVKDTLREVRMALLE **Charge** 3 **Spectral Angle** 0.7

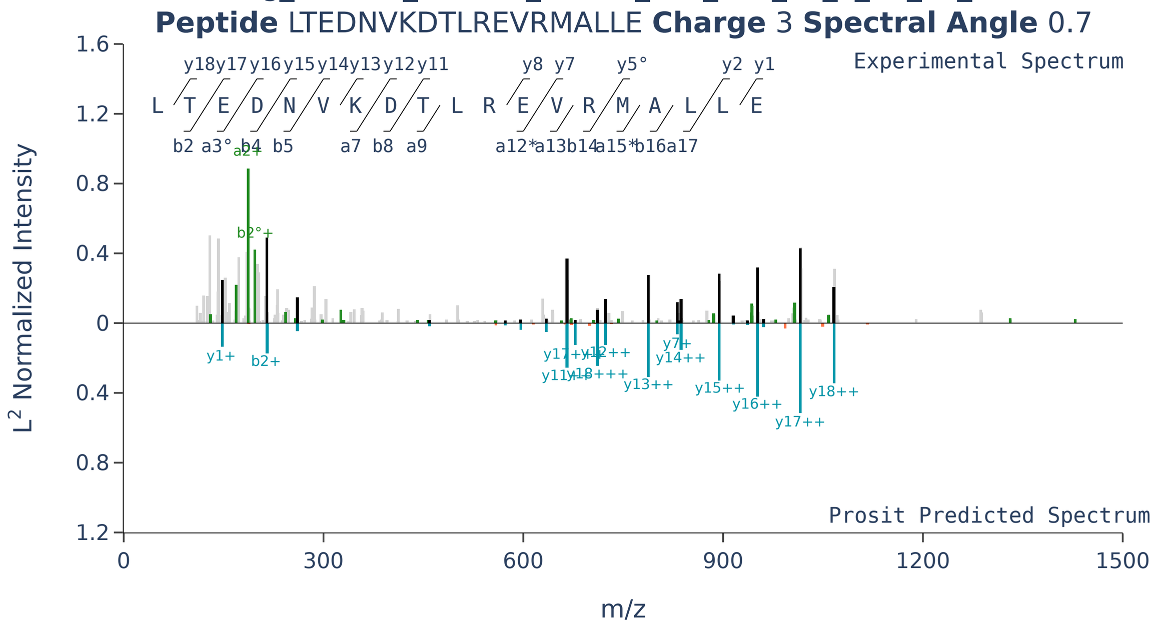


**Source** Ncheng_210623_230623_HFGoe_FFH_20S_25_1_A1_2h_R1 **Scan** 25594
**Peptide** GEKTEALEPFHPDRIASRILG **Charge** 3 **Spectral Angle** 0.76

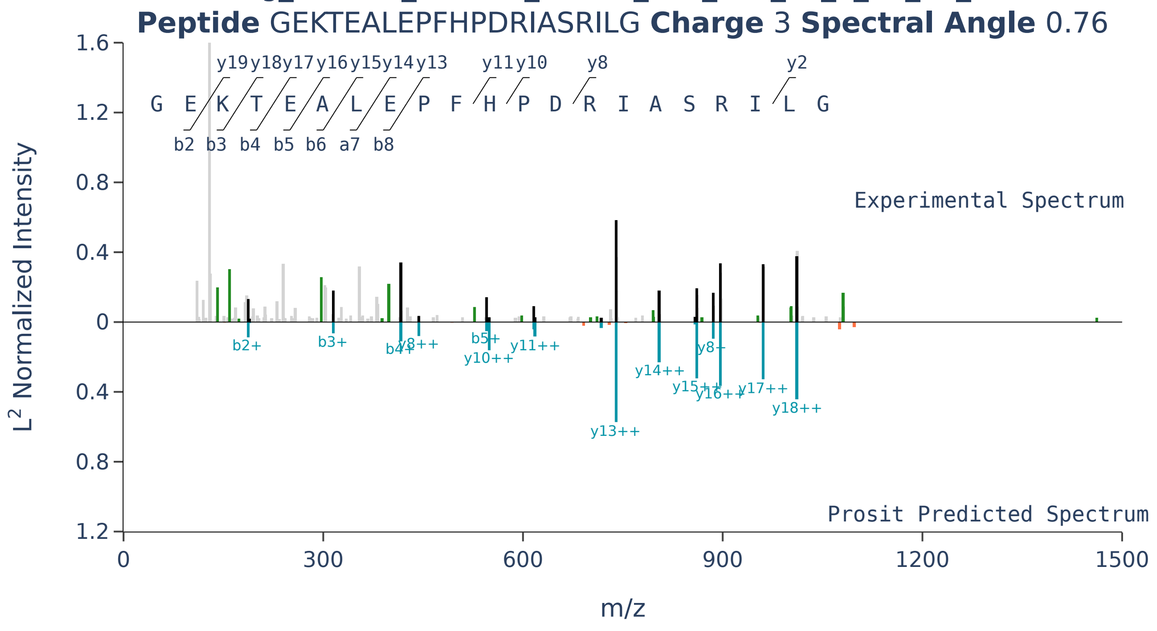


**Source** Ncheng_210623_230623_HFGoe_FFH_20S_25_1_A1_4h_R2 **Scan** 19331
**Peptide** NLSGRGRLTEDNVKDTLREVRM **Charge** 5 **Spectral Angle** 0.76

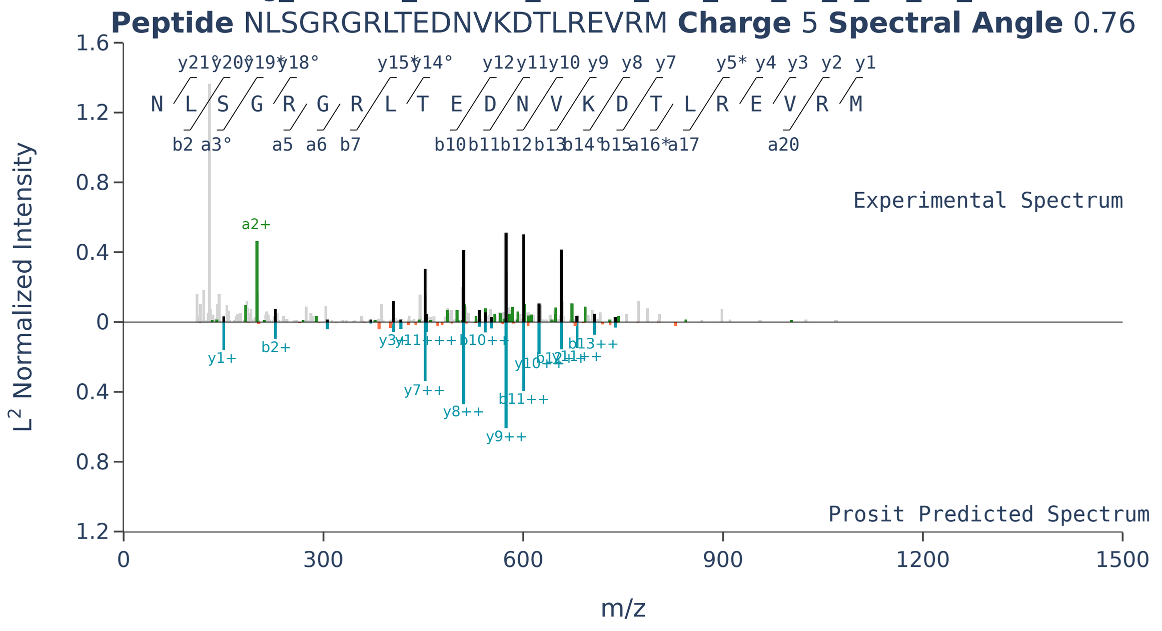


**Source** Ncheng_210623_230623_HFGoe_FFH_20S_25_1_A2_24h_R2 **Scan** 12247
**Peptide** IVNAALKEAKLK **Charge** 2 **Spectral Angle** 0.68

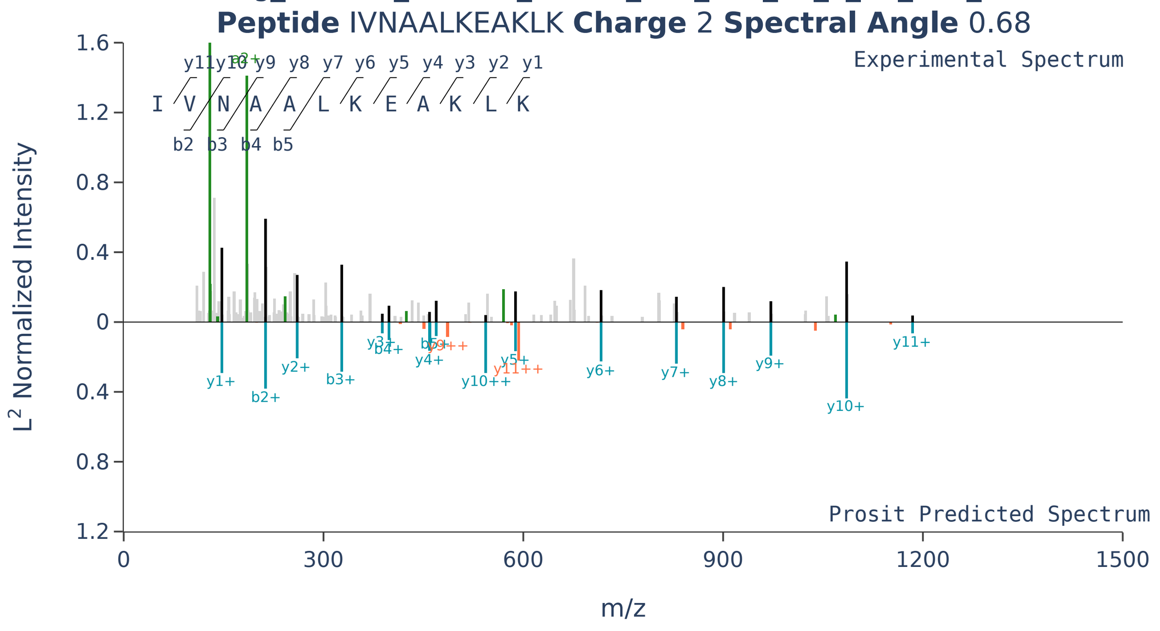


**Source** Ncheng_210623_230623_HFGoe_FFH_20S_25_1_A1_24h_R1 **Scan** 14146
**Peptide** NLSGRGRLTEDNVKDTL **Charge** 3 **Spectral Angle** 0.69

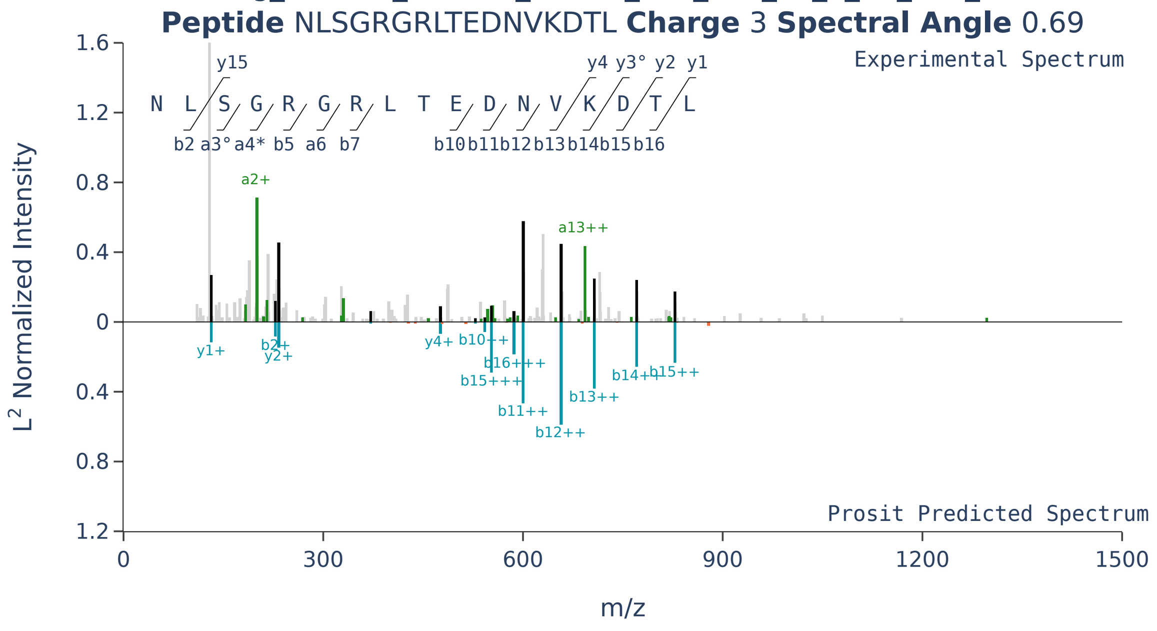


**Source** Ncheng_210623_230623_HFGoe_FFH_20S_25_1_A2_4h_R2 **Scan** 22015
**Peptide** FLEQLRQMKNMG **Charge** 2 **Spectral Angle** 0.65

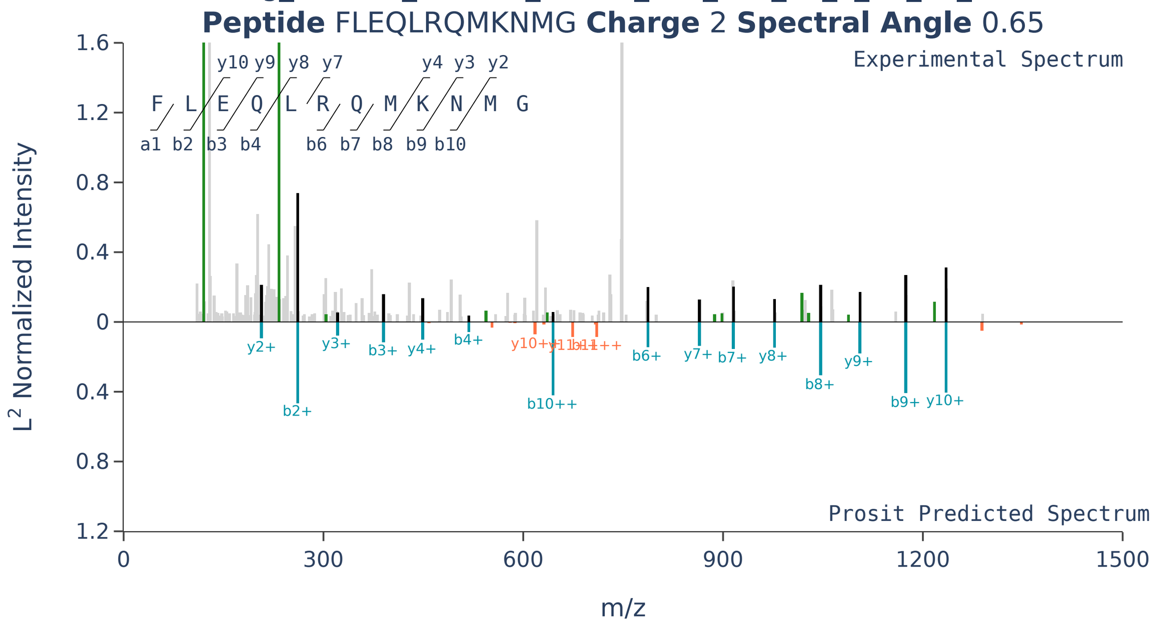


**Source** Ncheng_210623_230623_HFGoe_FFH_20S_25_1_A1_24h_R2 **Scan** 11149
**Peptide** SVGKLGKFLREKH **Charge** 2 **Spectral Angle** 0.65

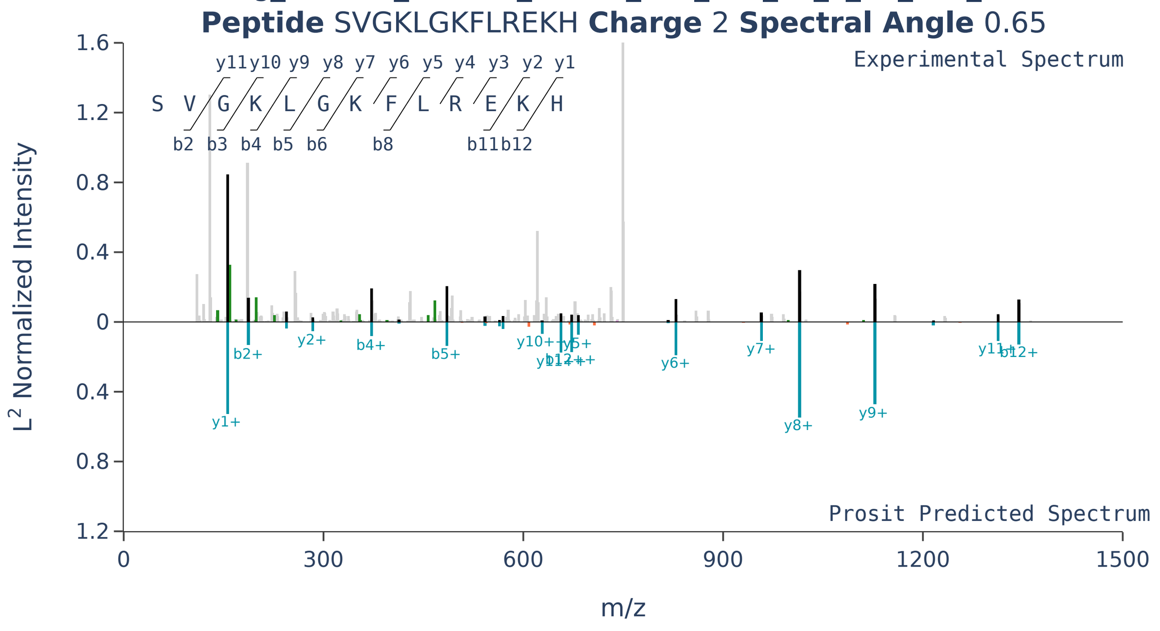

**Source** Ncheng_210623_230623_HFGoe_FFH_20S_25_1_A1_4h_R2 **Scan** 20953
**Peptide** VVSADVYRPAAIKQLE **Charge** 2 **Spectral Angle** 0.67

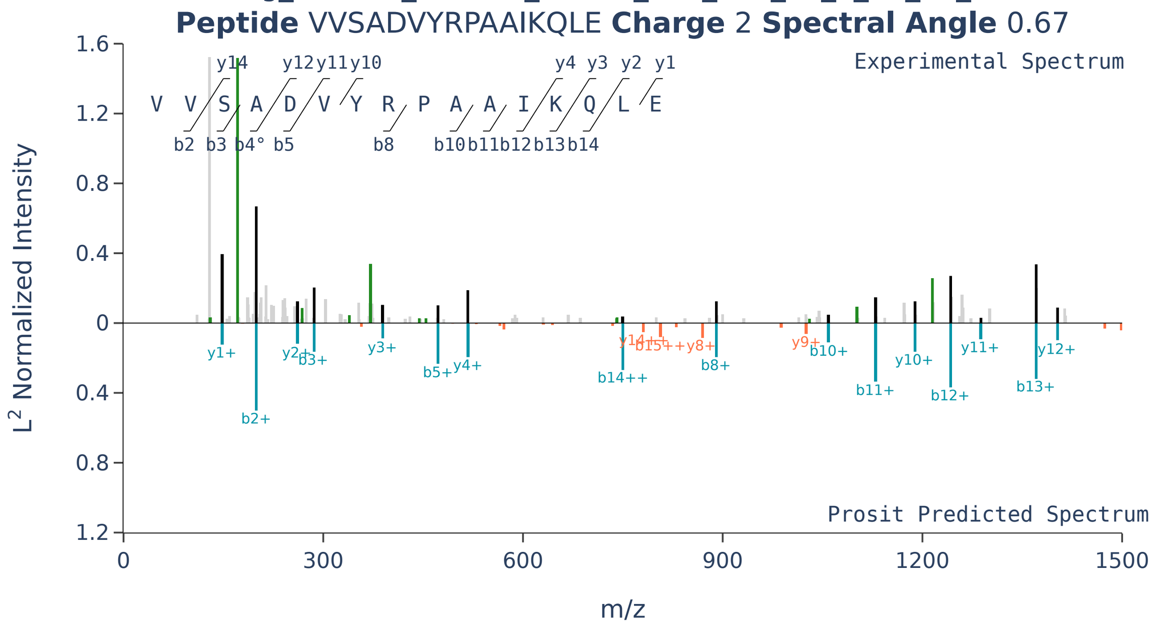


**Source** Ncheng_210623_230623_HFGoe_FFH_20S_25_1_A2_4h_R2 **Scan** 20346
**Peptide** HEVNKSLTPGQEFVKI **Charge** 2 **Spectral Angle** 0.82

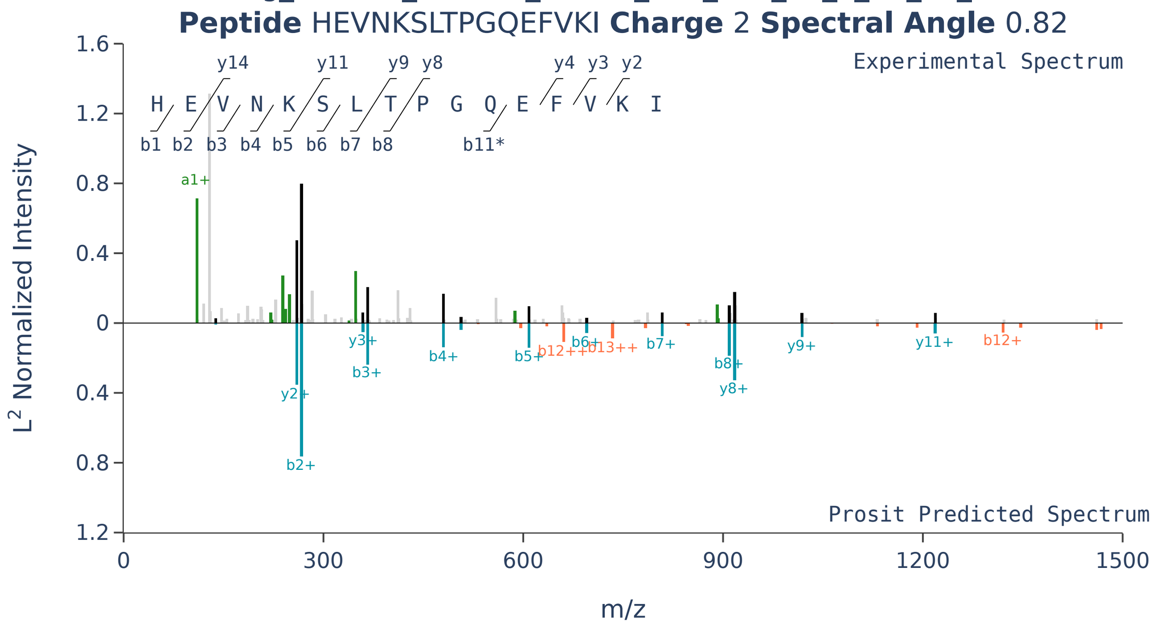


**Source** Ncheng_210623_230623_HFGoe_FFH_20S_25_1_A1_4h_R2 **Scan** 14029
**Peptide** KQFDDMQRMMKKMK **Charge** 4 **Spectral Angle** 0.8

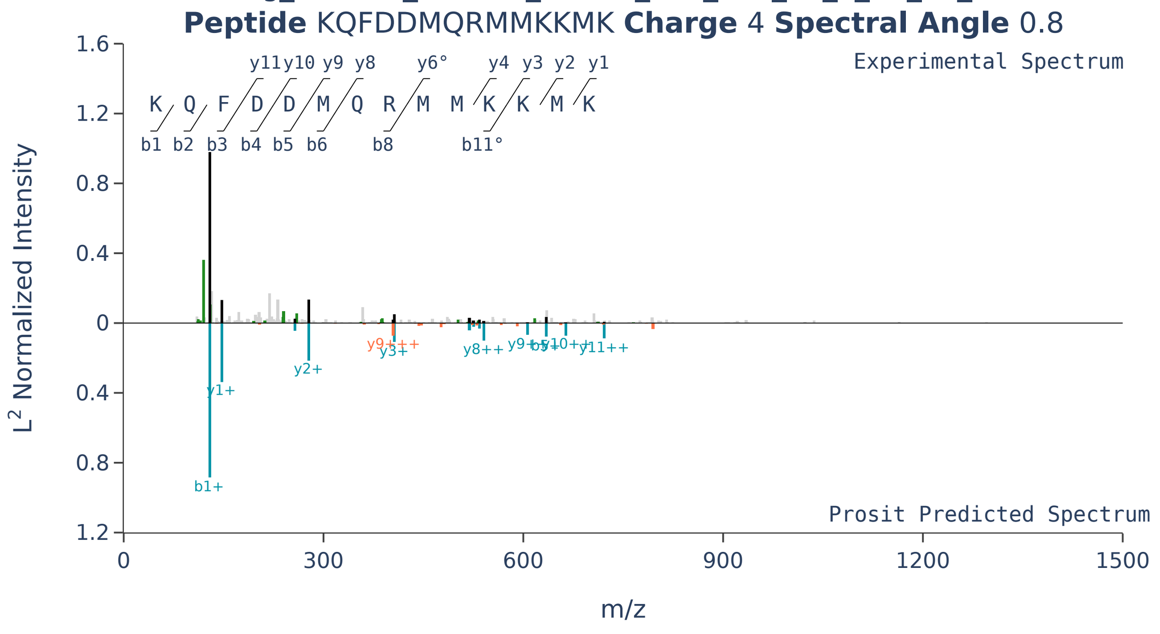


**Source** Ncheng_210623_230623_HFGoe_FFH_20S_25_1_A2_24h_R2 **Scan** 7845
**Peptide** EFINRVKEK **Charge** 2 **Spectral Angle** 0.75

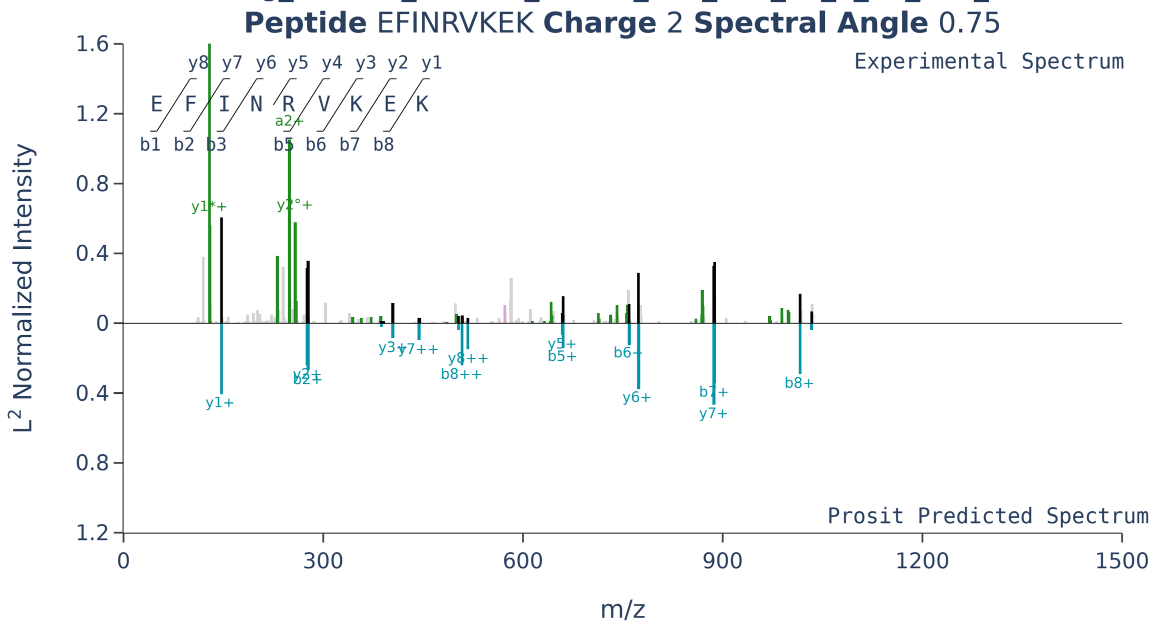


**Source** Ncheng_210623_230623_HFGoe_FFH_20S_25_1_A1_2h_R1 **Scan** 37486
**Peptide** ELVAAMGEENQTLNLAAQPPAVVL **Charge** 2 **Spectral Angle** 0.92

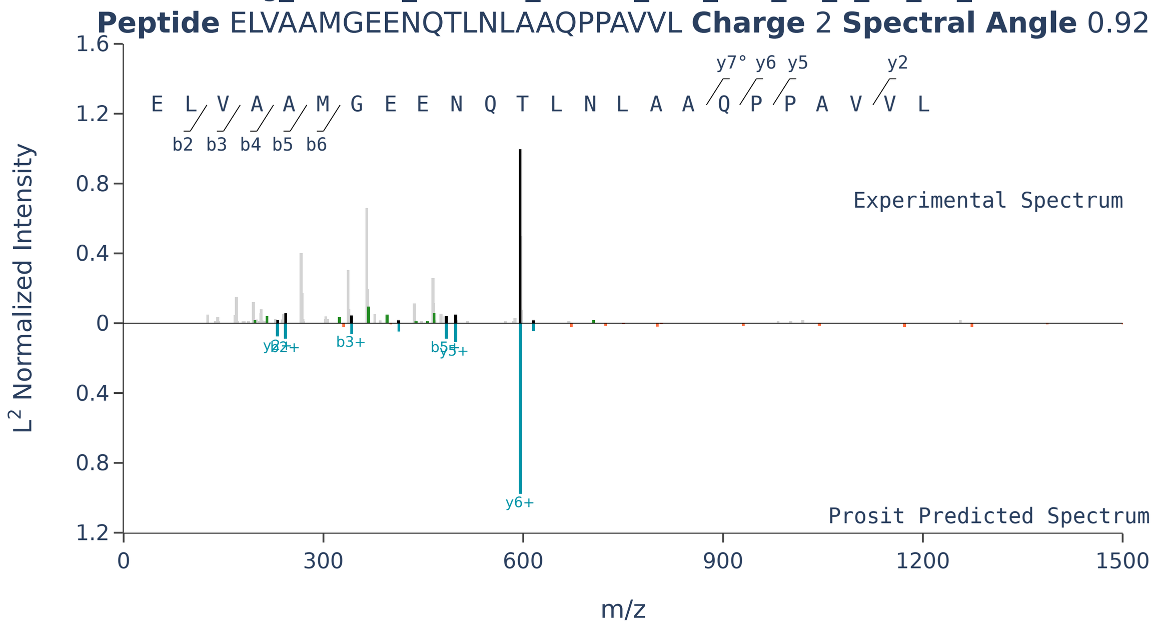


**Source** Ncheng_210623_230623_HFGoe_FFH_20S_25_1_A1_2h_R2 **Scan** 16651
**Peptide** GVGEKTEALEPFH **Charge** 3 **Spectral Angle** 0.86

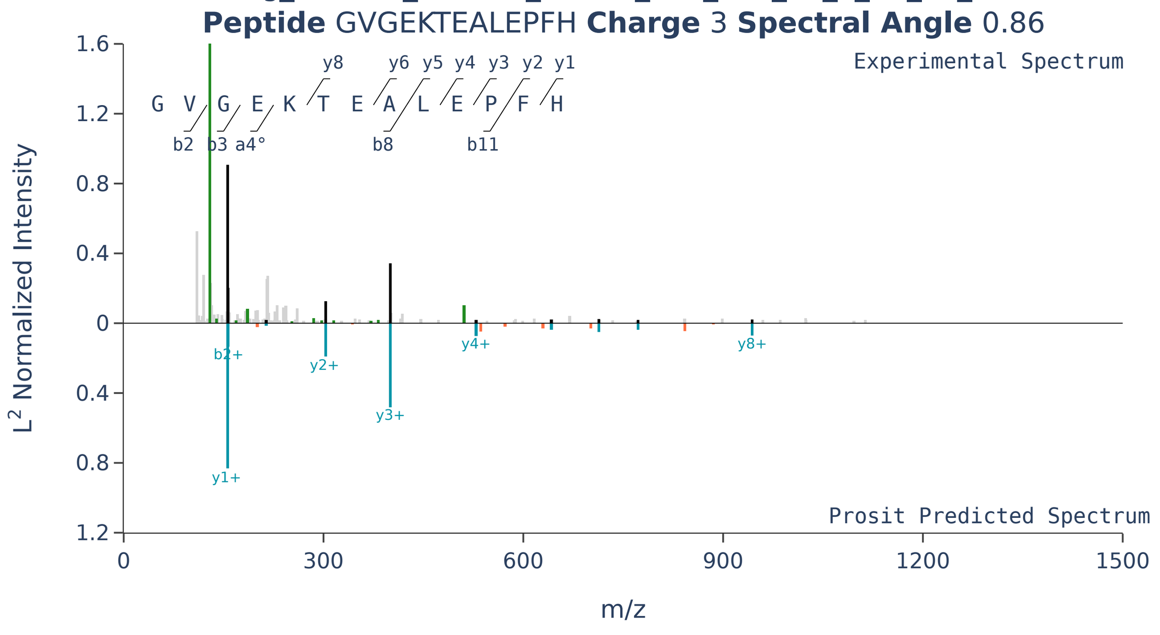


**Source** Ncheng_210623_230623_HFGoe_FFH_20S_25_1_A1_24h_R1 **Scan** 11382
**Peptide** TKVDGDARGGAALSIR **Charge** 2 **Spectral Angle** 0.72

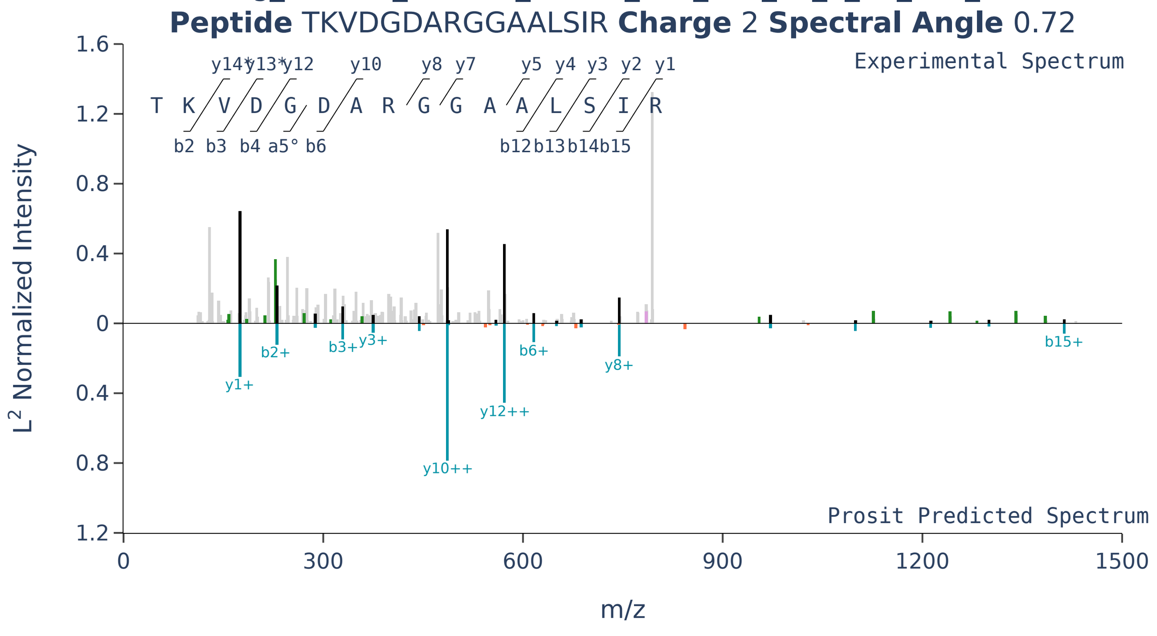


**Source** Ncheng_210623_230623_HFGoe_FFH_20S_25_1_A2_24h_R2 **Scan** 8943
**Peptide** VYRPAALKQ **Charge** 2 **Spectral Angle** 0.66

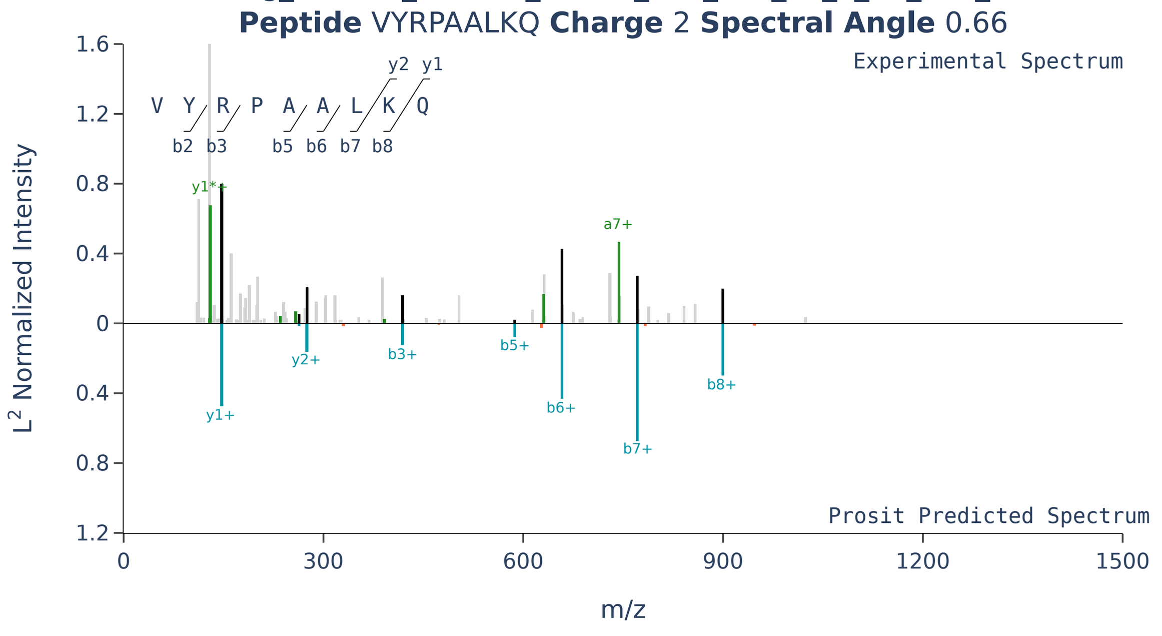


**Source** Ncheng_210623_230623_HFGoe_FFH_20S_25_1_A2_1h_R1 **Scan** 28913
**Peptide** AAGCGMQVQDVNRLLKQFD **Charge** 3 **Spectral Angle** 0.71

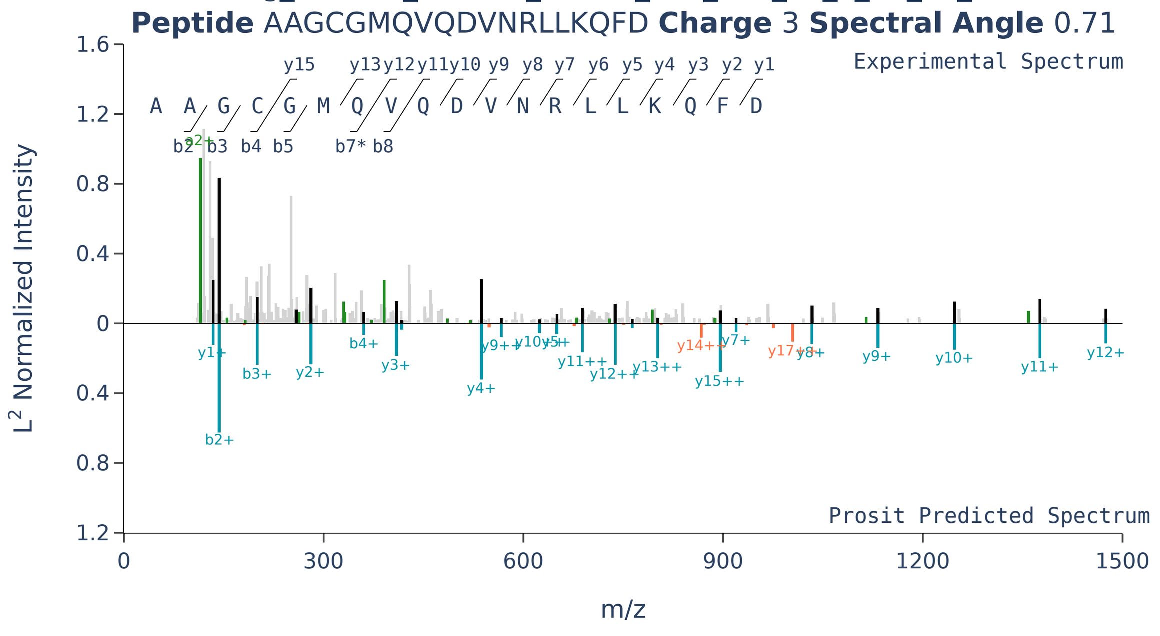


**Source** Ncheng_210623_230623_HFGoe_FFH_20S_25_1_A2_2h_R1 **Scan** 30655
**Peptide** HEVNKSLTPGQEFVKIVRNELVAA **Charge** 3 **Spectral Angle** 0.81

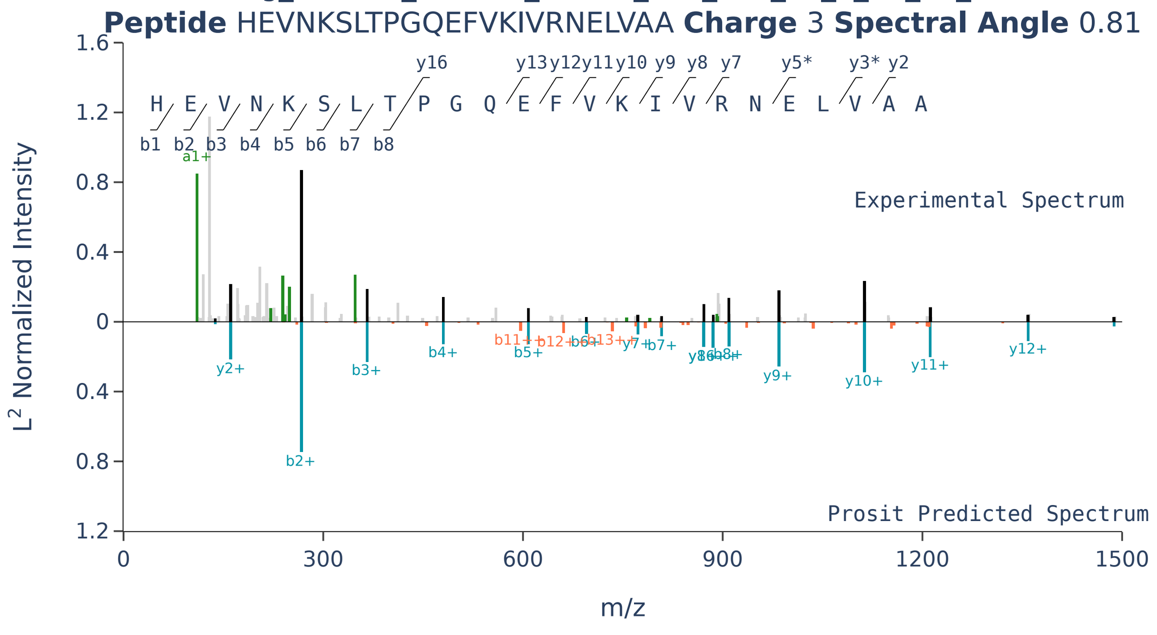


**Source** Ncheng_210623_230623_HFGoe_FFH_20S_25_1_A1_4h_R1 **Scan** 19489
**Peptide** DLESKVDRAQAEKLASKLKKGDGF **Charge** 3 **Spectral Angle** 0.78

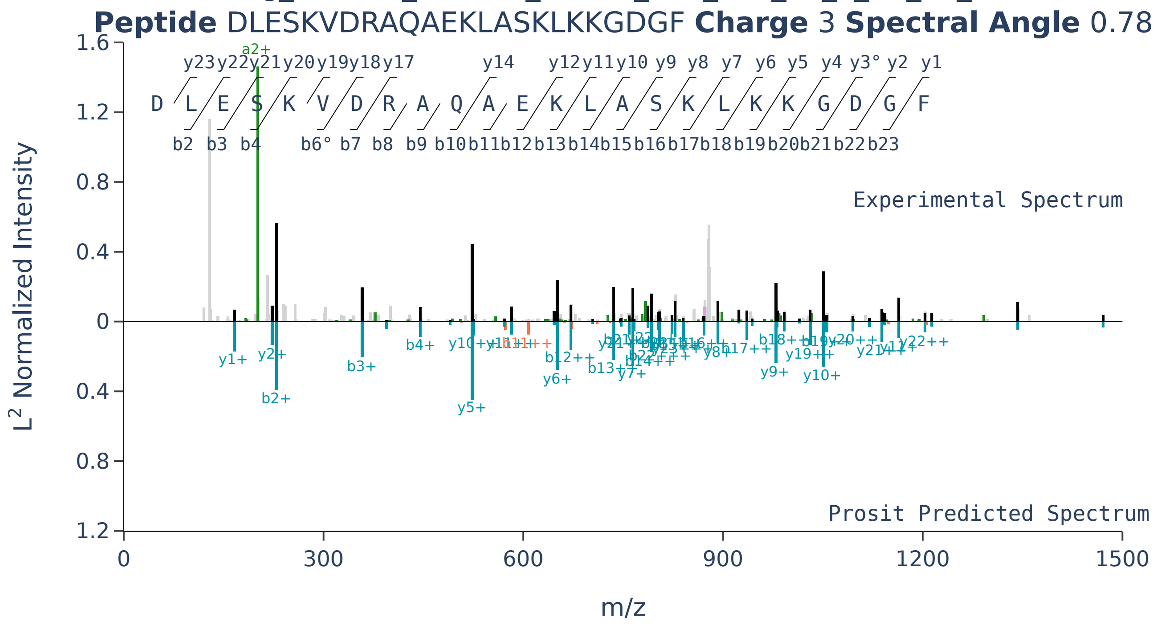


**Source** Ncheng_210623_230623_HFGoe_FFH_20S_25_1_A1_2h_R1 **Scan** 17554
**Peptide** FLEQLRQMKN **Charge** 2 **Spectral Angle** 0.6

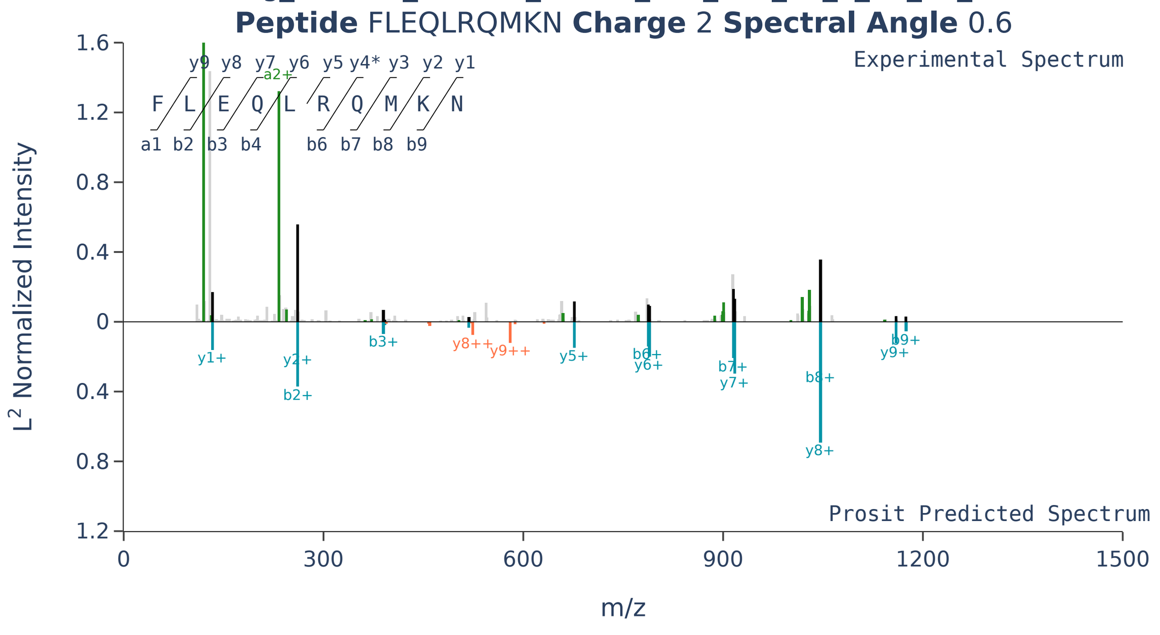


**Source** Ncheng_210623_230623_HFGoe_FFH_20S_25_1_A2_24h_R2 **Scan** 9074
**Peptide** IINSMTMKERAK **Charge** 4 **Spectral Angle** 0.76

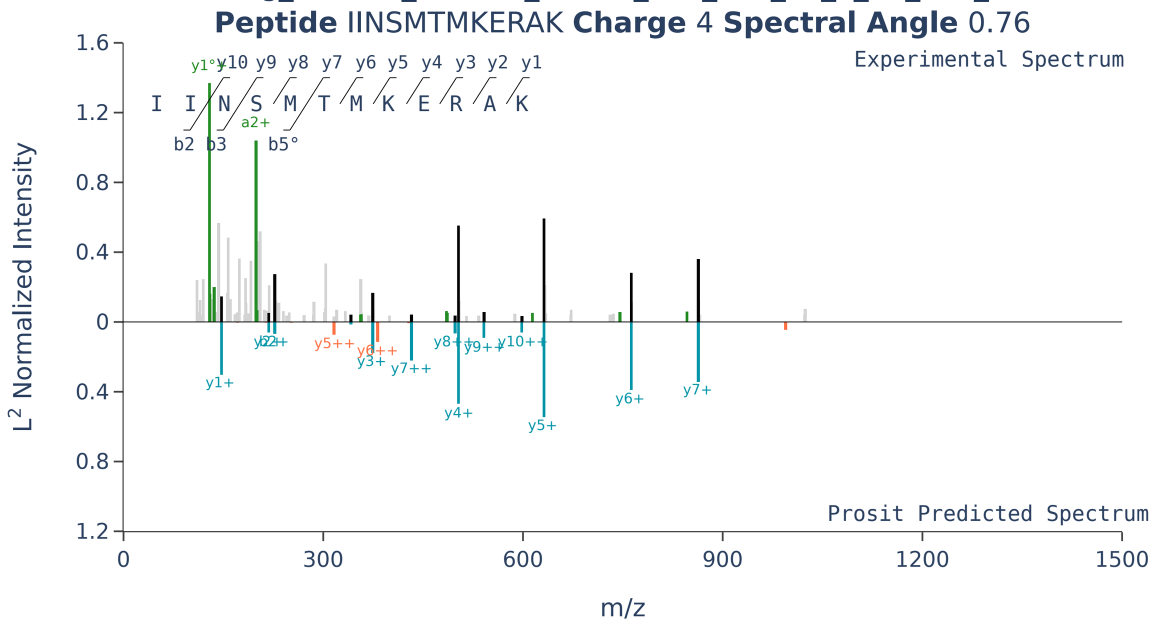


**Source** Ncheng_210623_230623_HFGoe_FFH_20S_25_1_A2_24h_R1 **Scan** 20047
**Peptide** KSLTPGQEFVKIVR **Charge** 2 **Spectral Angle** 0.76

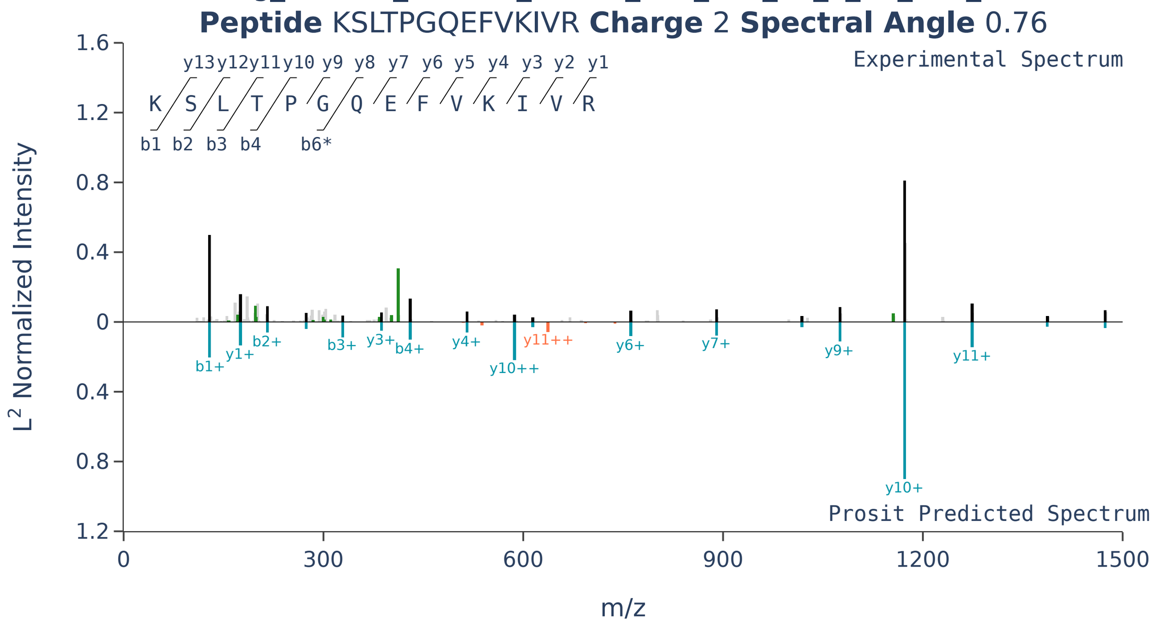


**Source** Ncheng_210623_230623_HFGoe_FFH_20S_25_1_A2_4h_R1 **Scan** 29484
**Peptide** SLMGKLPGMGQIPDNVKSQMDDKVLVRME **Charge** 4 **Spectral Angle** 0.69

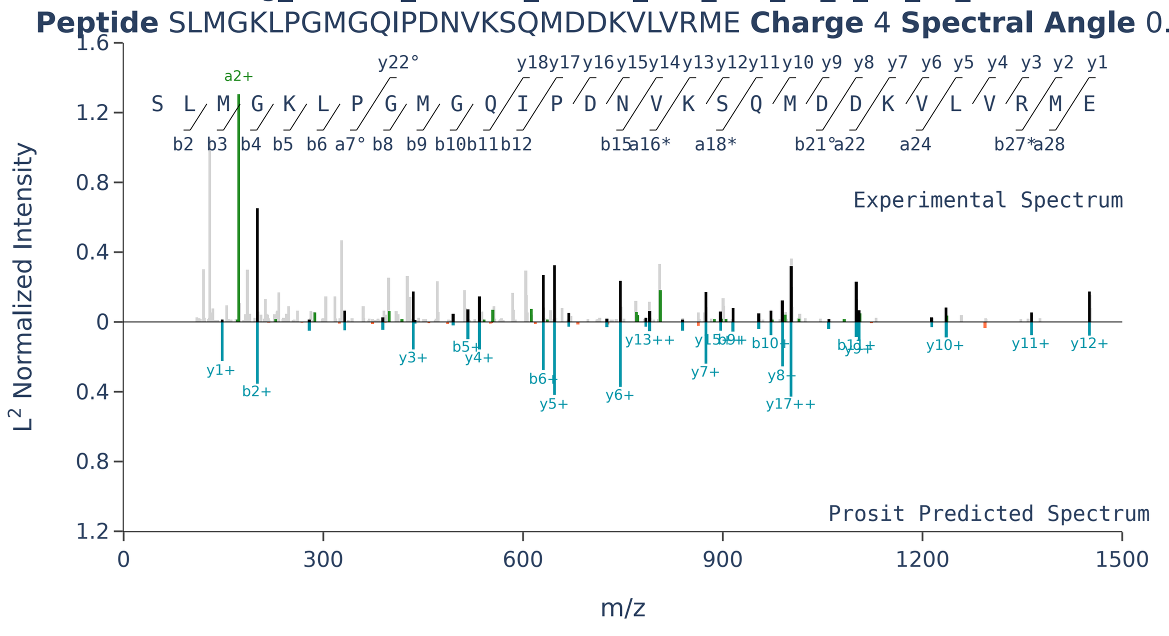

**Source** Ncheng_210623_230623_HFGoe_FFH_20S_25_1_A2_24h_R1 **Scan** 27166
**Peptide** LPGMGQIPDNV **Charge** 2 **Spectral Angle** 0.82

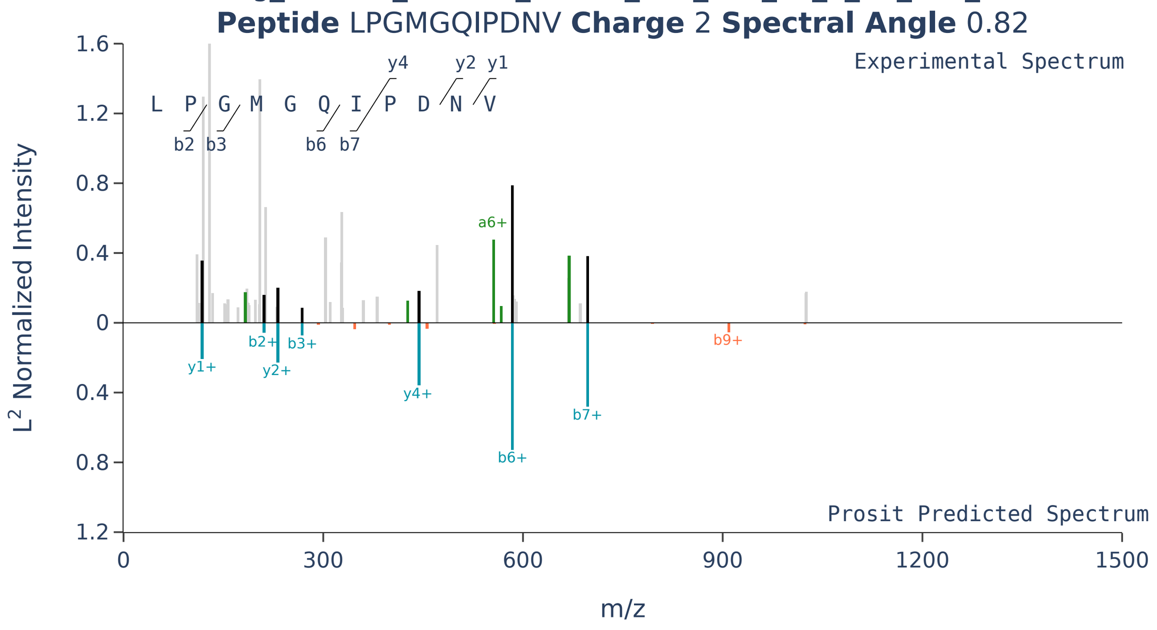


**Source** Ncheng_210623_230623_HFGoe_FFH_20S_25_1_A1_2h_R2 **Scan** 23330
**Peptide** LGVGEKTEALEPFHPDRIASRI **Charge** 3 **Spectral Angle** 0.79

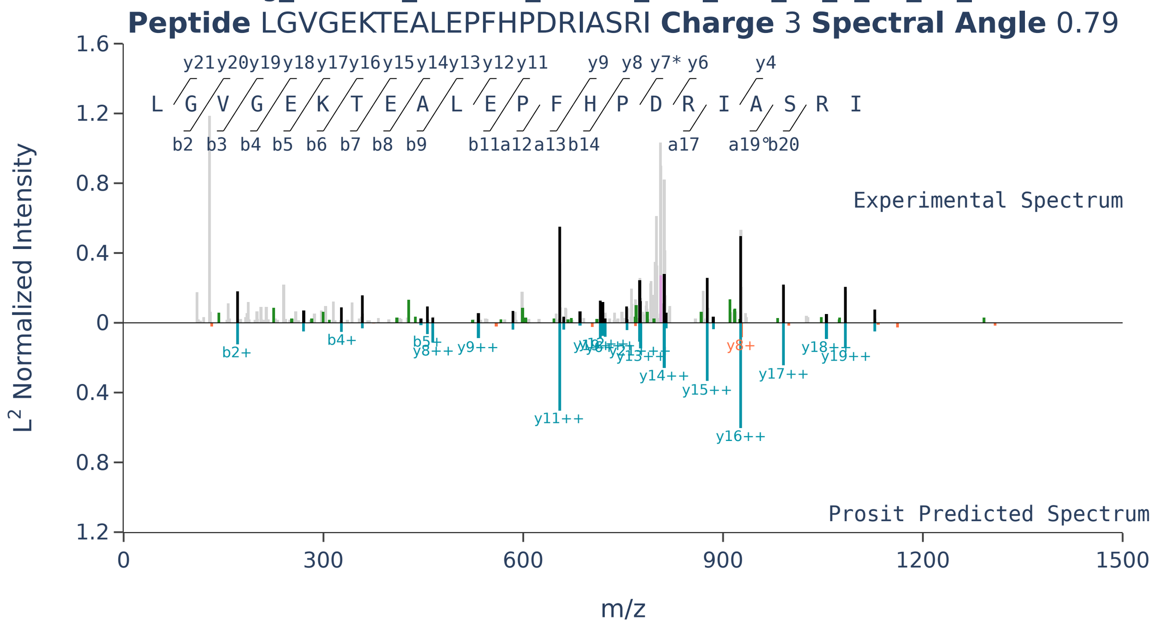


**Source** Ncheng_210623_230623_HFGoe_FFH_20S_25_1_A1_24h_R2 **Scan** 25231
**Peptide** AKLKFYDVL **Charge** 2 **Spectral Angle** 0.65

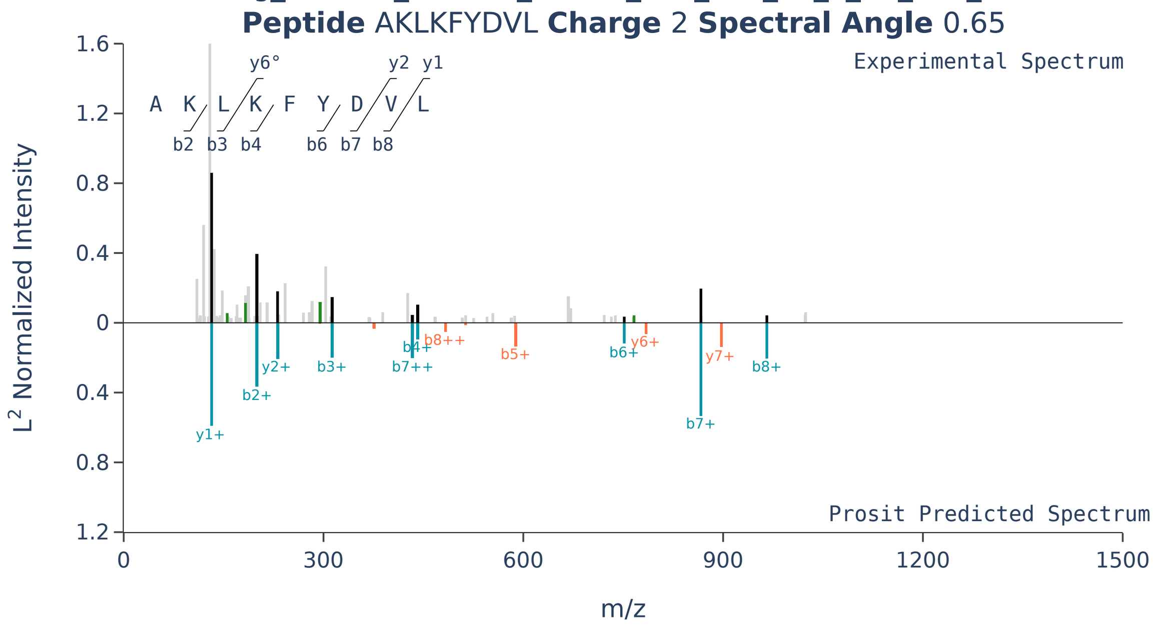


**Source** Ncheng_210623_230623_HFGoe_FFH_20S_25_1_A2_2h_R2 **Scan** 13340
**Peptide** SMTMKERAKPEII **Charge** 2 **Spectral Angle** 0.68

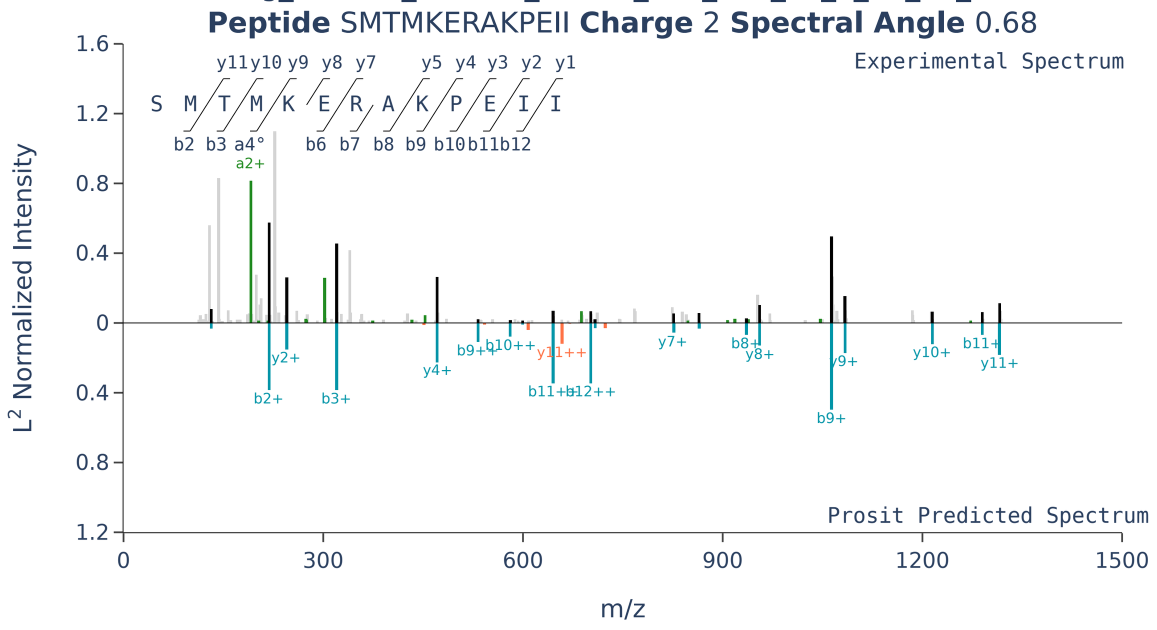


**Source** Ncheng_210623_230623_HFGoe_FFH_20S_25_1_A1_24h_R1 **Scan** 9931
**Peptide** AGLQGAGKTTSVGKLGK **Charge** 2 **Spectral Angle** 0.74

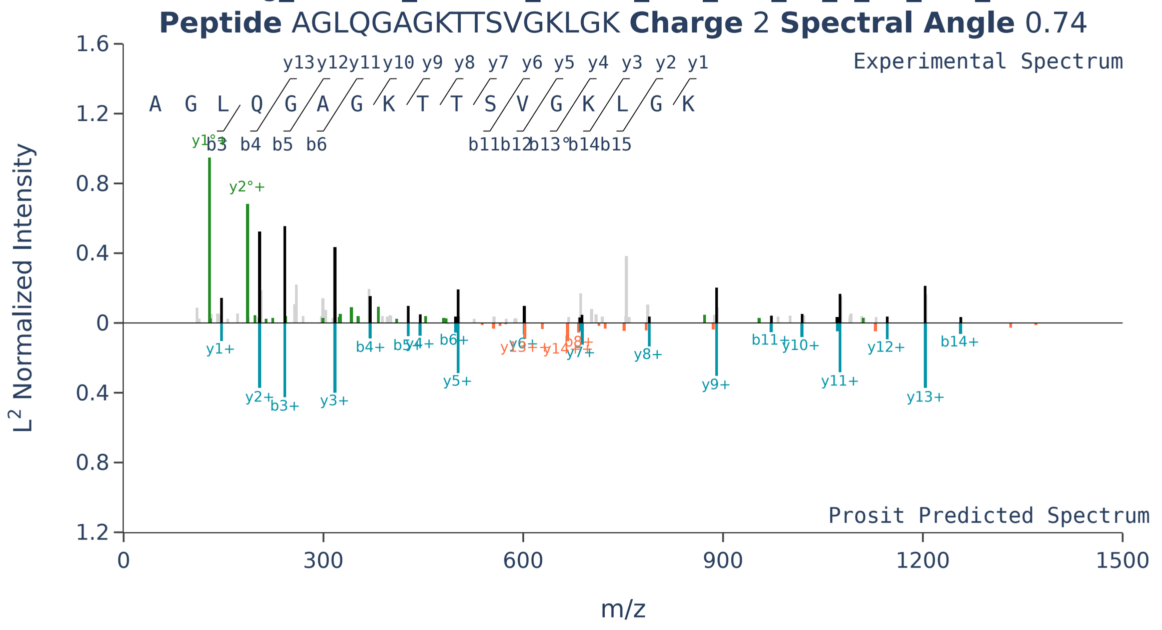


**Source** Ncheng_210623_230623_HFGoe_FFH_20S_25_1_A2_24h_R2 **Scan** 21029
**Peptide** LPGMGQIPDNVKS **Charge** 2 **Spectral Angle** 0.75

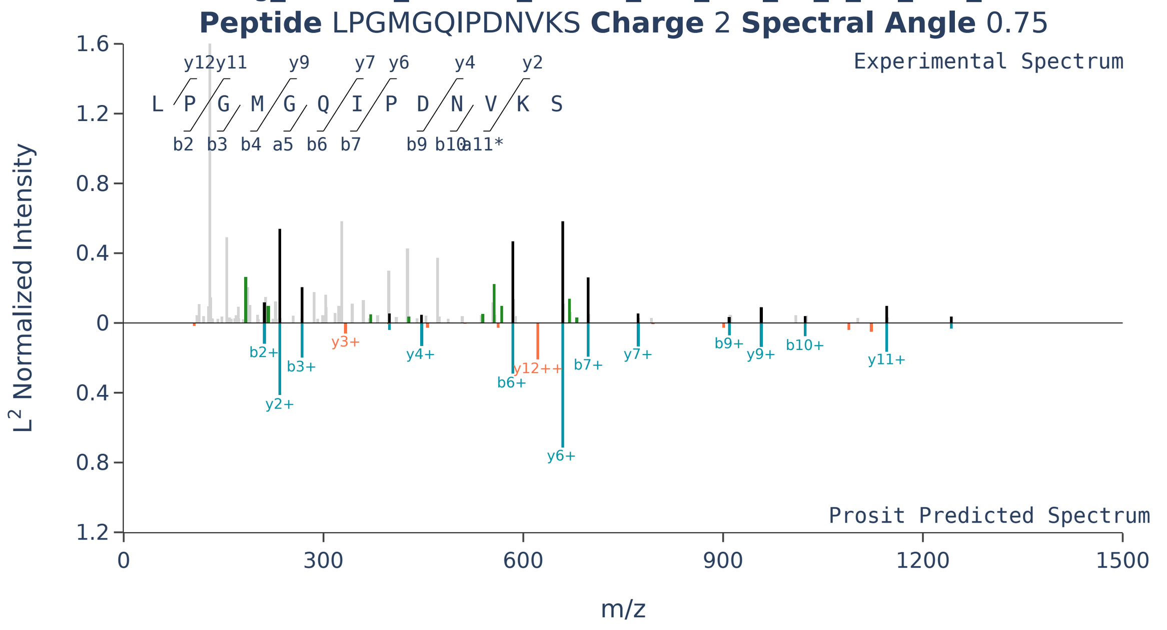


**Source** Ncheng_210623_230623_HFGoe_FFH_20S_25_1_A1_1h_R2 **Scan** 10792
**Peptide** REFINRV **Charge** 2 **Spectral Angle** 0.73

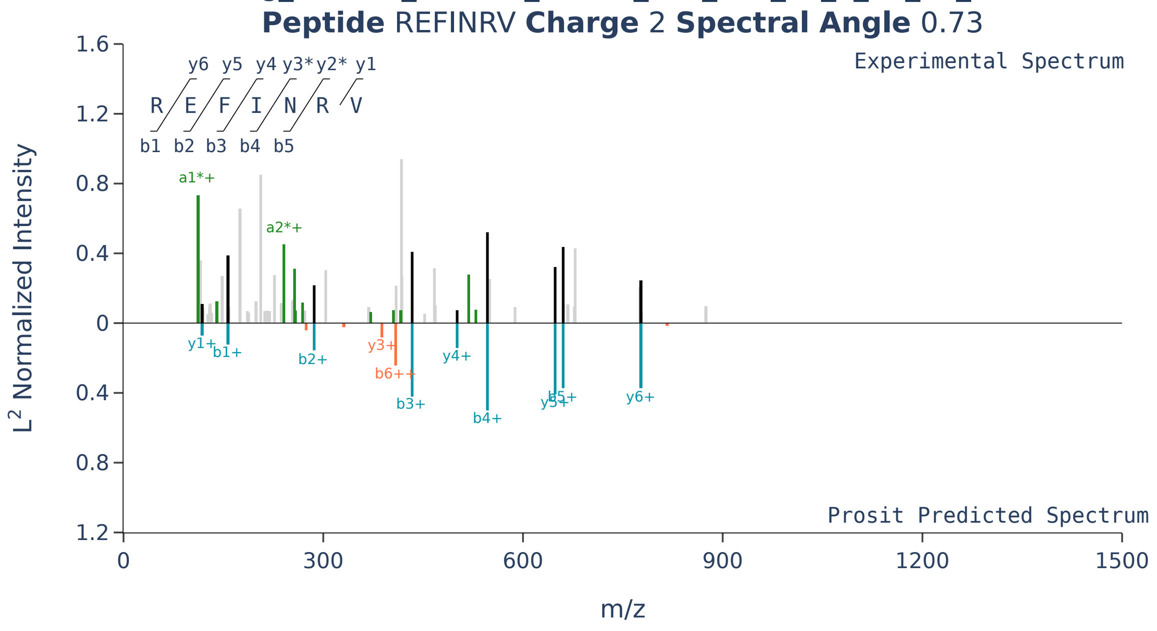


**Source** Ncheng_210623_230623_HFGoe_FFH_20S_25_1_A2_2h_R2 **Scan** 31899
**Peptide** NEALPLTGVVLTKVDGDARGGAAL **Charge** 3 **Spectral Angle** 0.82

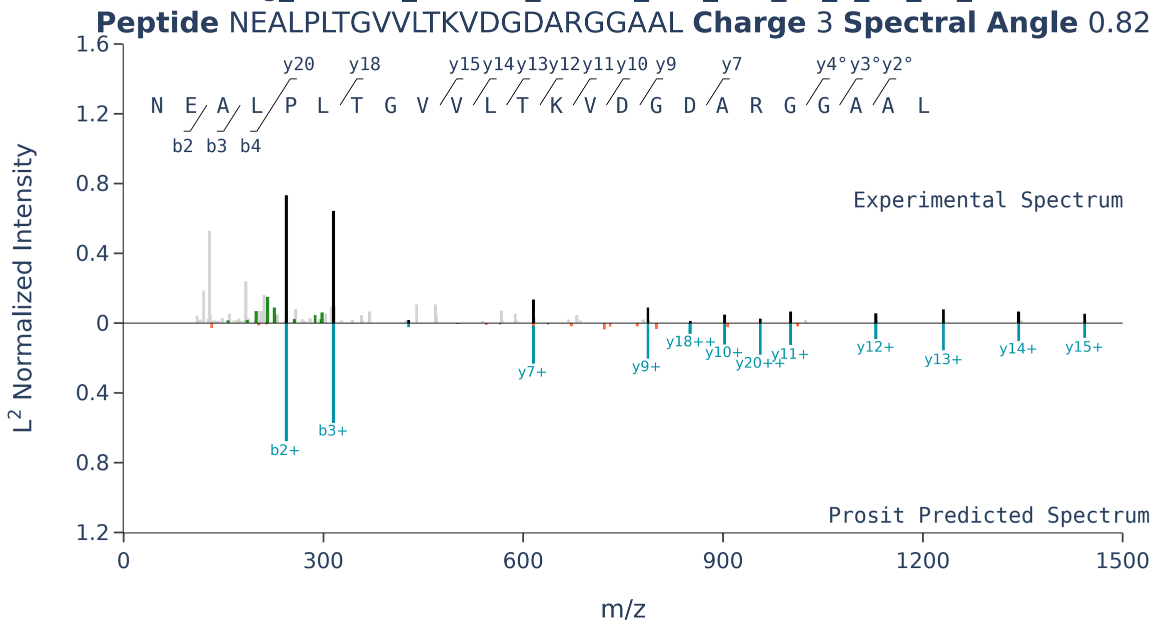


**Source** Ncheng_210623_230623_HFGoe_FFH_20S_25_1_A2_2h_R2 **Scan** 8589
**Peptide** DIESKVDRAQA **Charge** 2 **Spectral Angle** 0.79

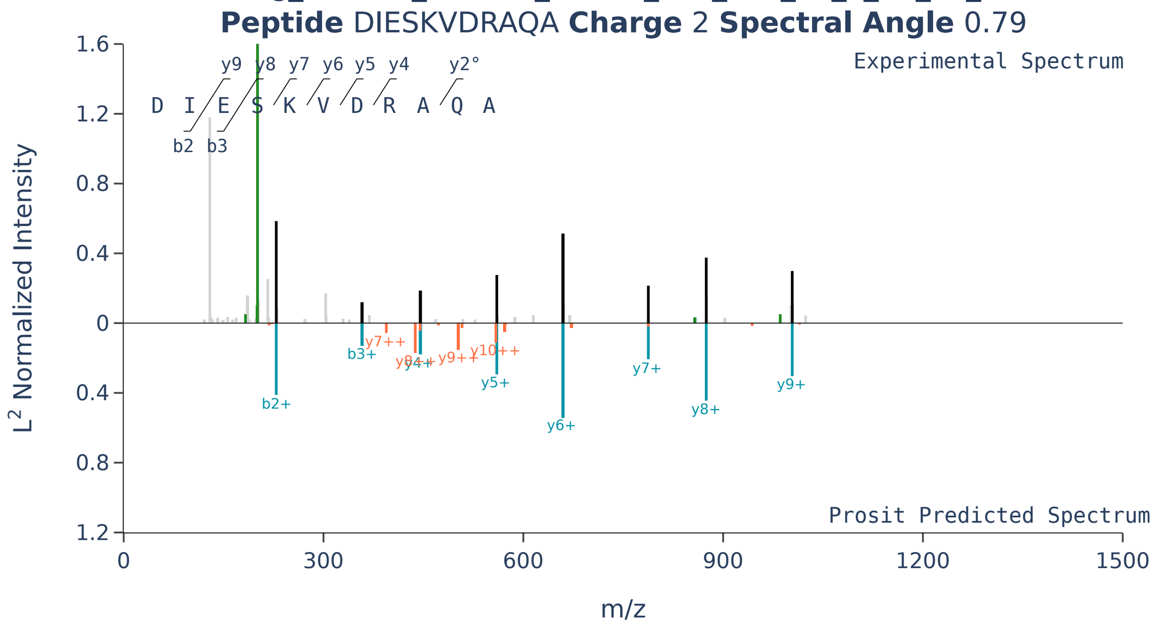


**Source** Ncheng_210623_230623_HFGoe_FFH_20S_25_1_A2_24h_R1 **Scan** 11799
**Peptide** AGKTTSVGKLGKF **Charge** 3 **Spectral Angle** 0.77

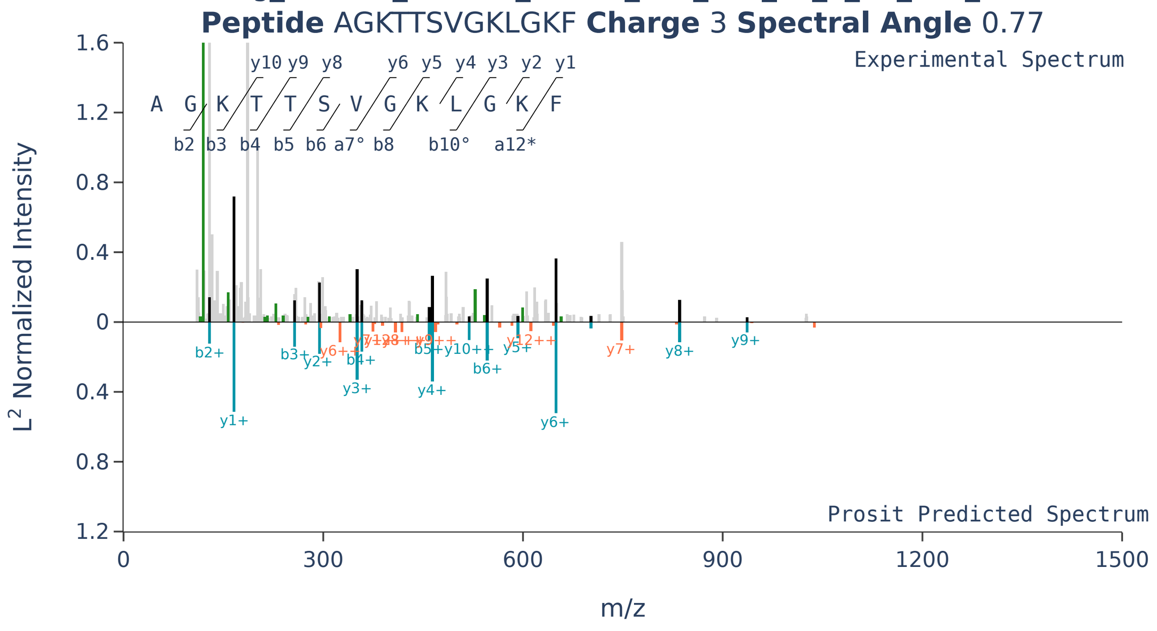


**Source** Ncheng_210623_230623_HFGoe_FFH_20S_25_1_A2_1h_R1 **Scan** 27932
**Peptide** RIAAGCGMQVQDVNRLLKQFD **Charge** 3 **Spectral Angle** 0.77

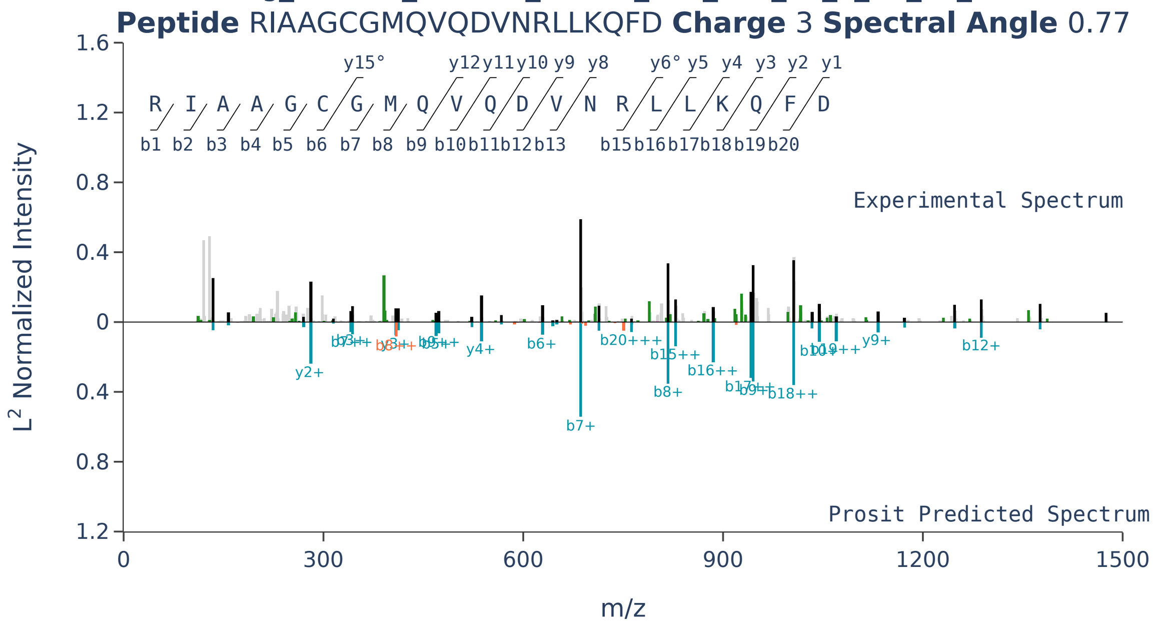


**Source** Ncheng_210623_230623_HFGoe_FFH_20S_25_1_A2_2h_R1 **Scan** 6086
**Peptide** FLNRVKEK **Charge** 2 **Spectral Angle** 0.77

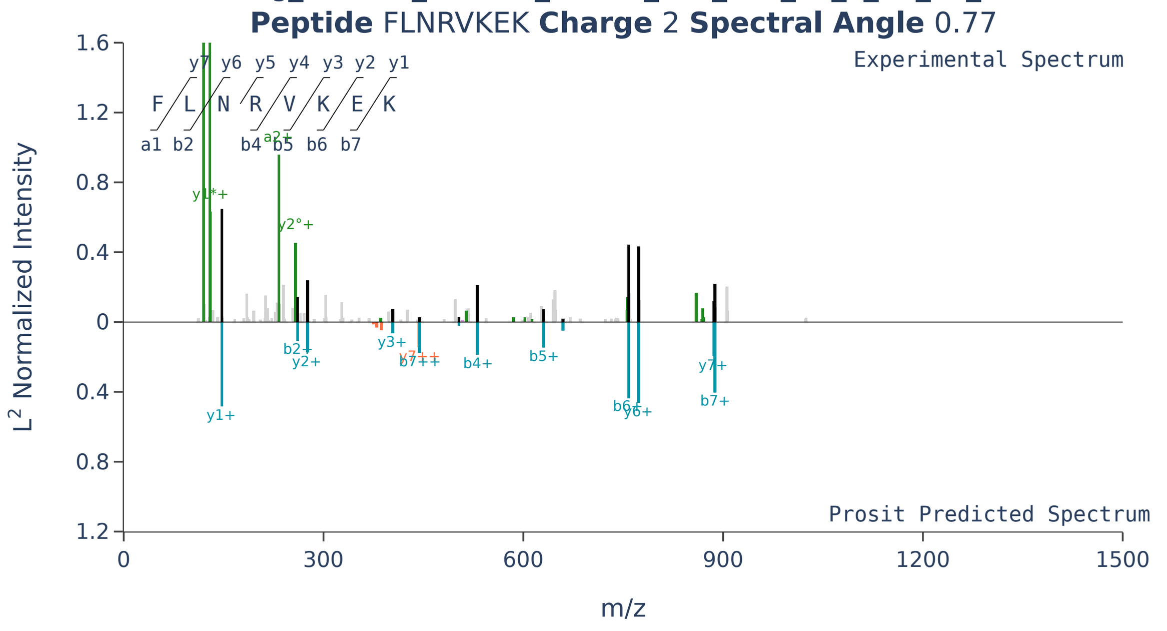


**Source** Ncheng_210623_230623_HFGoe_FFH_20S_25_1_A1_2h_R2 **Scan** 25242
**Peptide** KLPGMGQIPDNVKSQMDDKVLVRME **Charge** 3 **Spectral Angle** 0.76

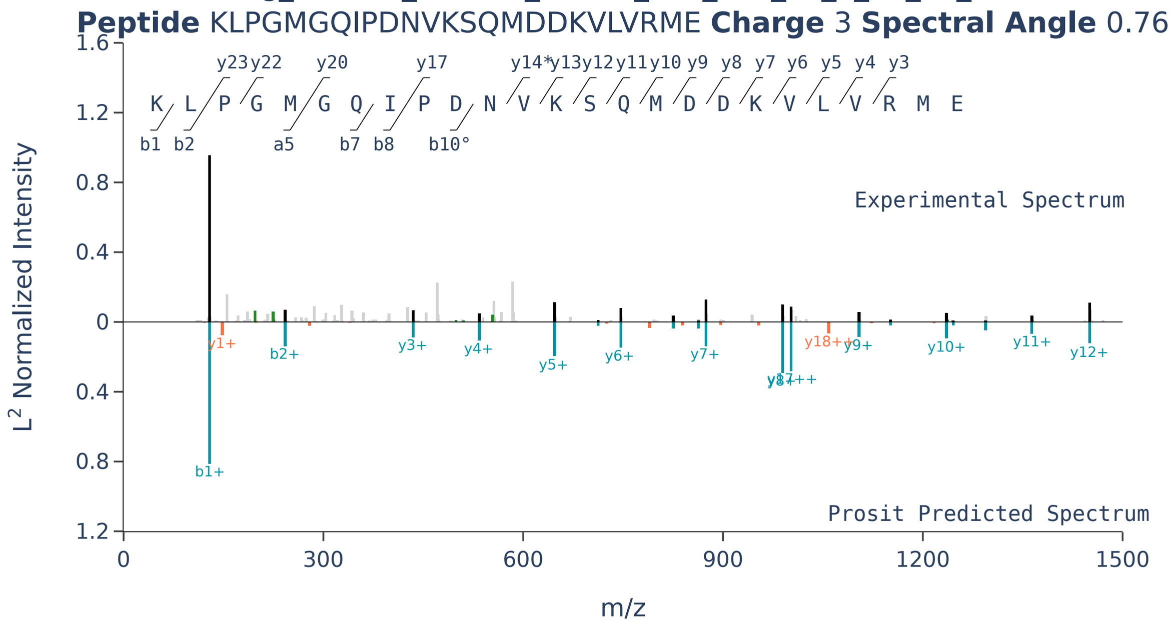


**Source** Ncheng_210623_230623_HFGoe_FFH_20S_25_1_A1_2h_R2 **Scan** 30068
**Peptide** GGMASLMGKLPGMGQIPDNVKS **Charge** 3 **Spectral Angle** 0.88

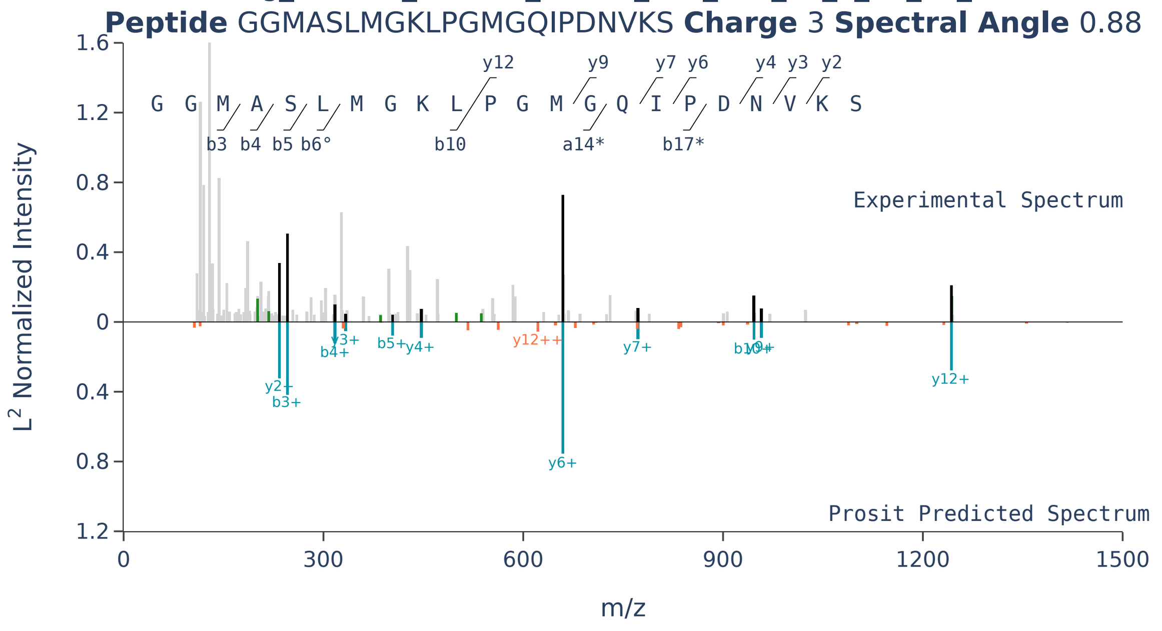


**Source** Ncheng_210623_230623_HFGoe_FFH_20S_25_1_A2_4h_R1 **Scan** 25368
**Peptide** GQKPVDIVNAALKEA **Charge** 3 **Spectral Angle** 0.65

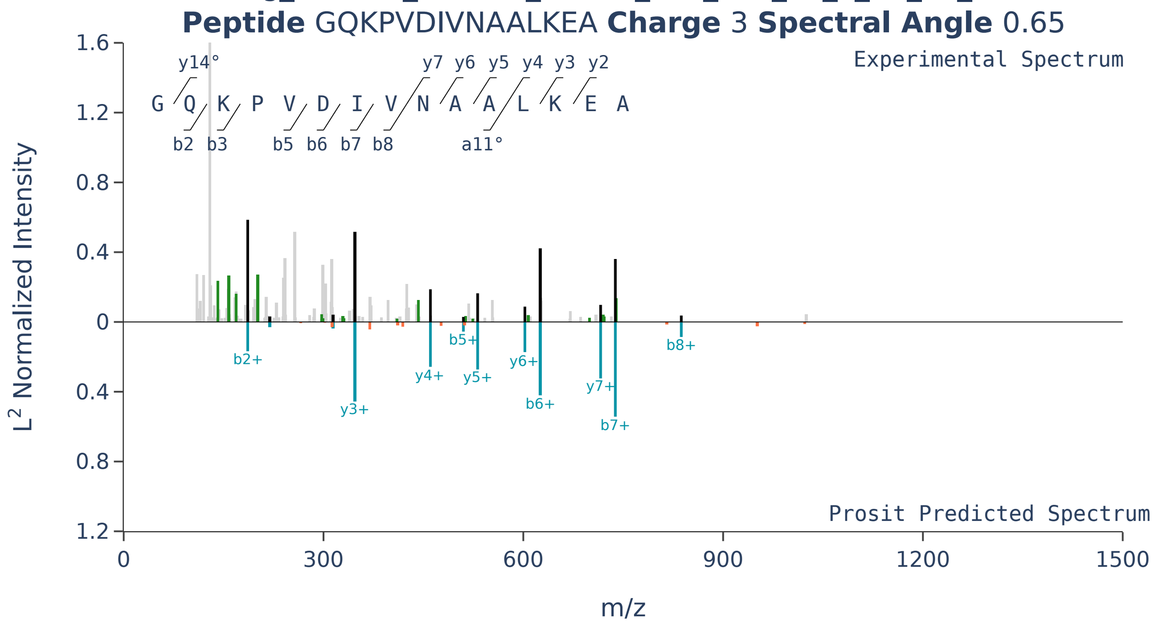

**Source** Ncheng_210623_230623_HFGoe_FFH_20S_25_1_A2_24h_R2 **Scan** 17414
**Peptide** DTAGRLHVDEAMMD **Charge** 3 **Spectral Angle** 0.64

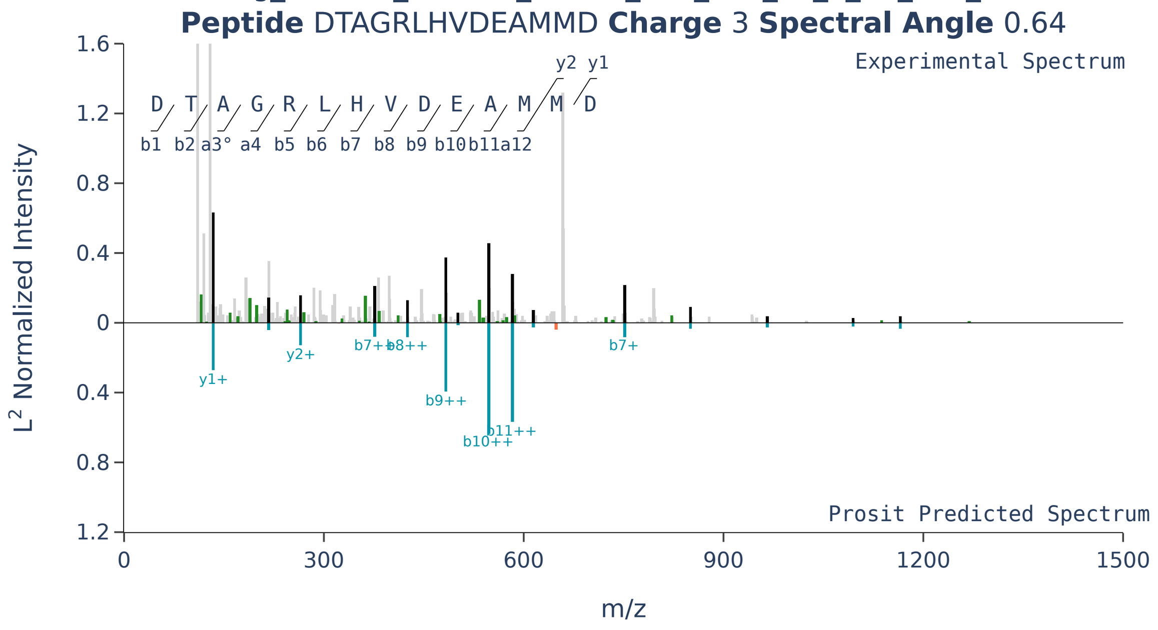


**Source** Ncheng_210623_230623_HFGoe_FFH_20S_25_1_A2_24h_R2 **Scan** 10503
**Peptide** PFHPDRIAS **Charge** 2 **Spectral Angle** 0.82

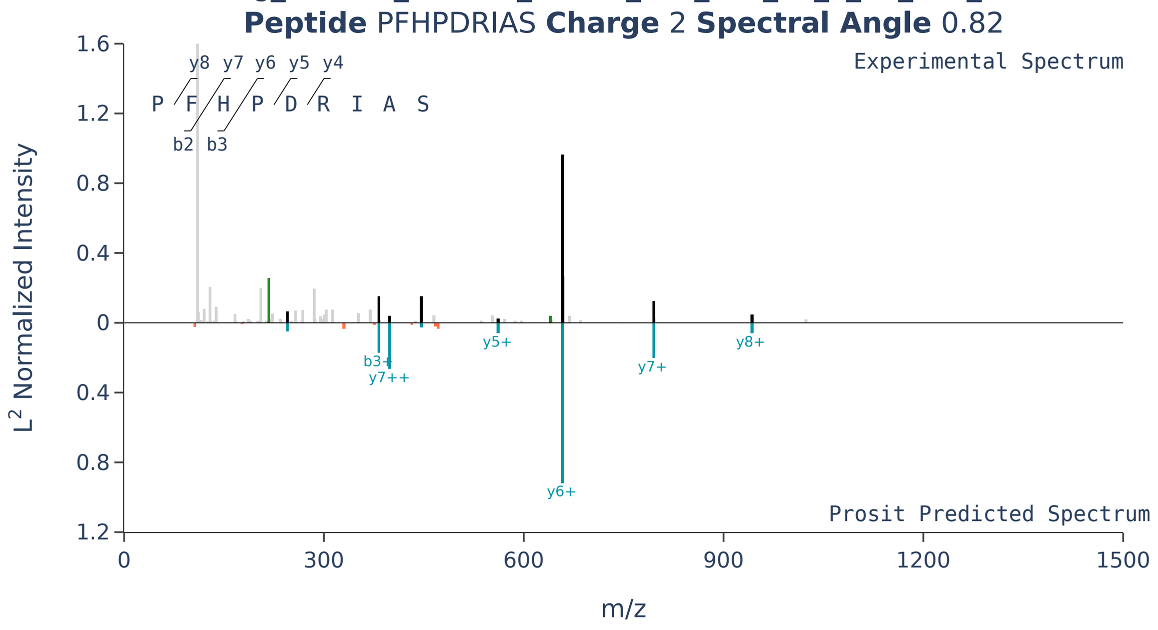


**Source** Ncheng_210623_230623_HFGoe_FFH_20S_25_1_A2_24h_R1 **Scan** 18007
**Peptide** ALLNSMTMKERAKPEI **Charge** 3 **Spectral Angle** 0.61

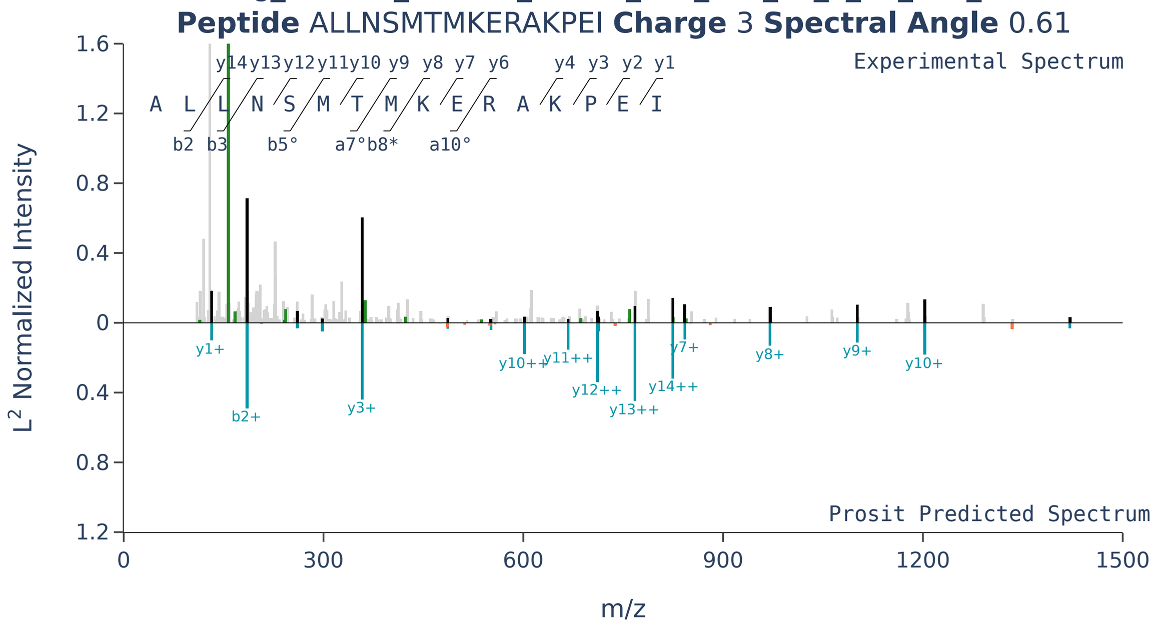


**Source** Ncheng_210623_230623_HFGoe_FFH_20S_25_1_A1_2h_R1 **Scan** 27939
**Peptide** DKVLVRMEALL **Charge** 2 **Spectral Angle** 0.82

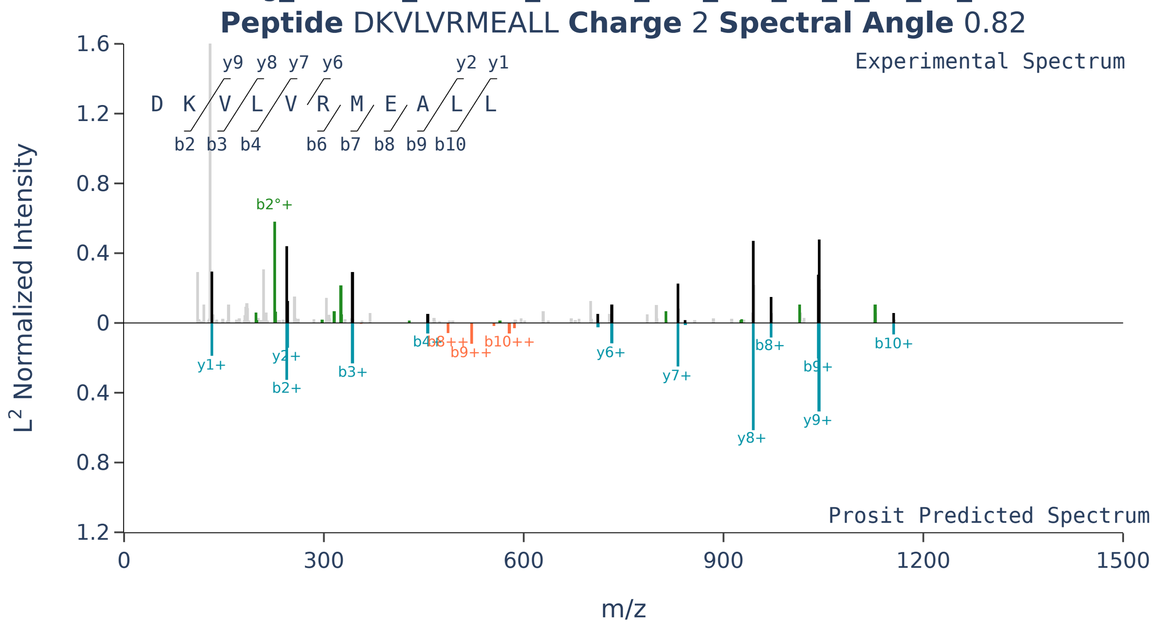


**Source** Ncheng_210623_230623_HFGoe_FFH_20S_25_1_A2_24h_R1 **Scan** 13143
**Peptide** AAMGEENQTL **Charge** 2 **Spectral Angle** 0.77

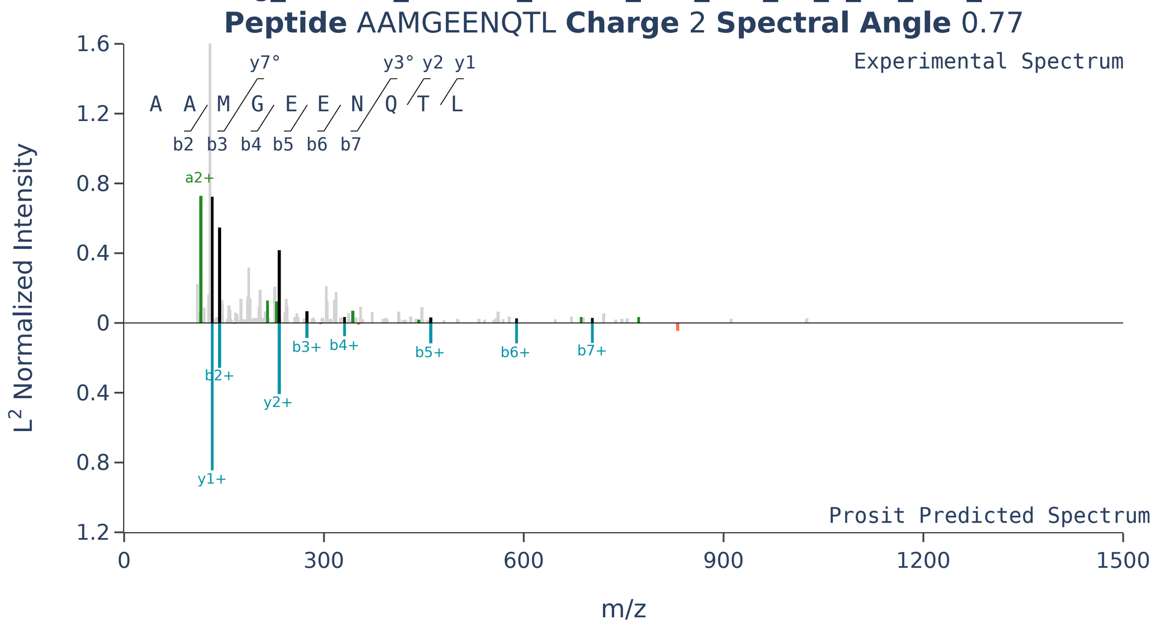


**Source** Ncheng_210623_230623_HFGoe_FFH_20S_25_1_A2_1h_R1 **Scan** 32272
**Peptide** KAVGHEVNKSLTPGQEFVKIVRNELVAAM **Charge** 3 **Spectral Angle** 0.65

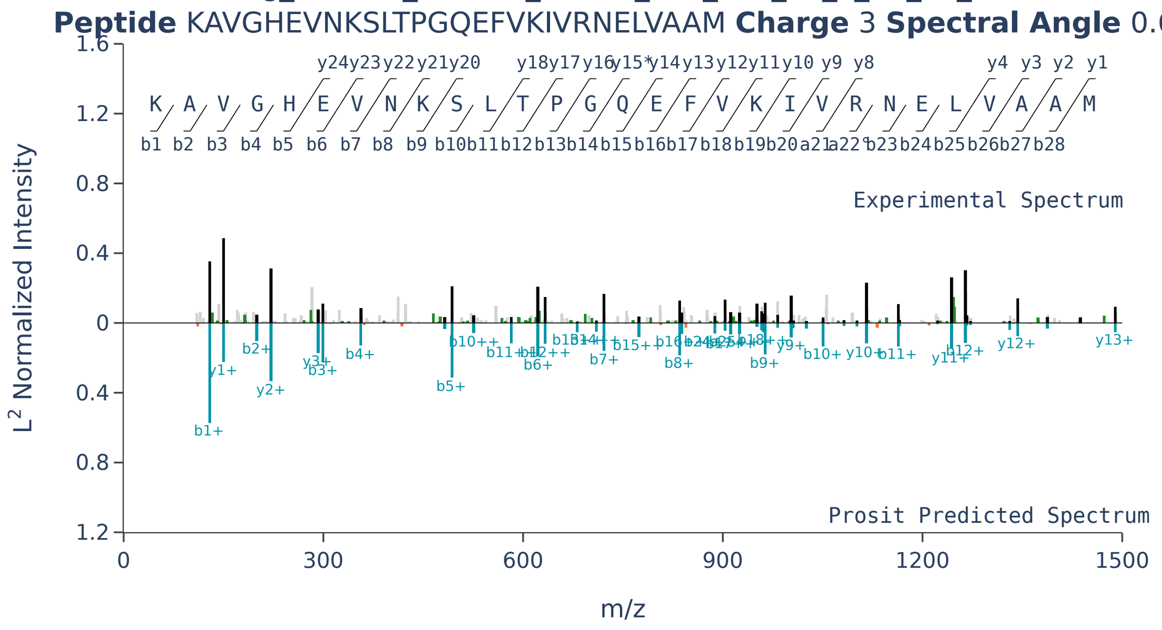


**Source** Ncheng_210623_230623_HFGoe_FFH_20S_25_1_A2_4h_R1 **Scan** 29104
**Peptide** MGEENQTLNLAAQPPAVV **Charge** 2 **Spectral Angle** 0.94

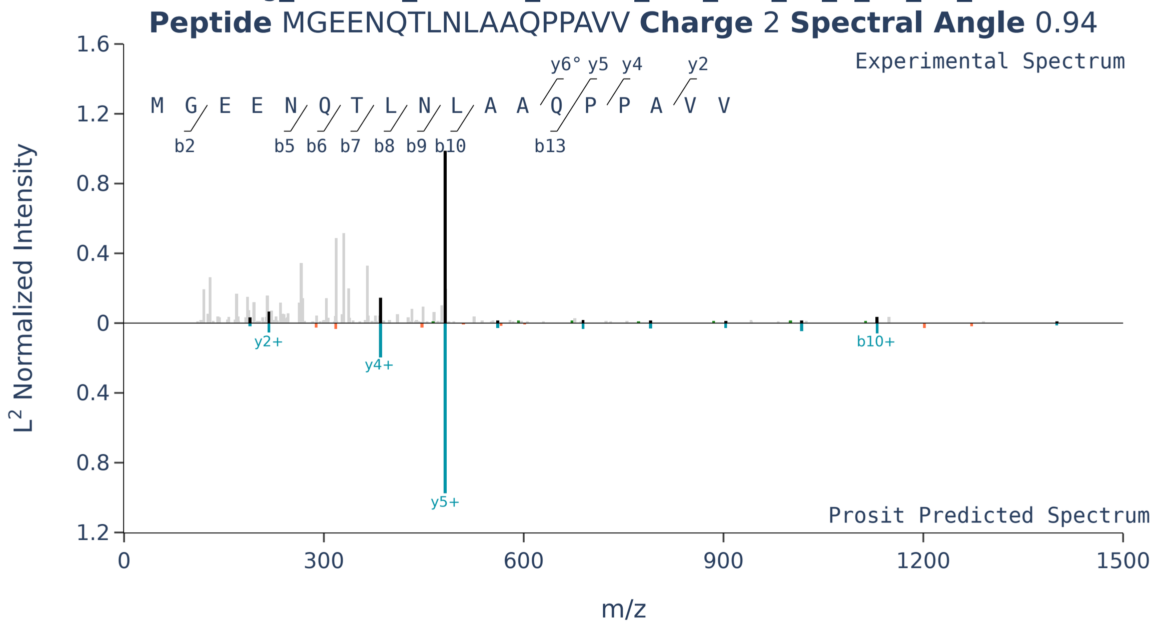


**Source** Ncheng_210623_230623_HFGoe_FFH_20S_25_1_A1_24h_R1 **Scan** 12119
**Peptide** QGAGKTTSVGKLGKF **Charge** 2 **Spectral Angle** 0.71

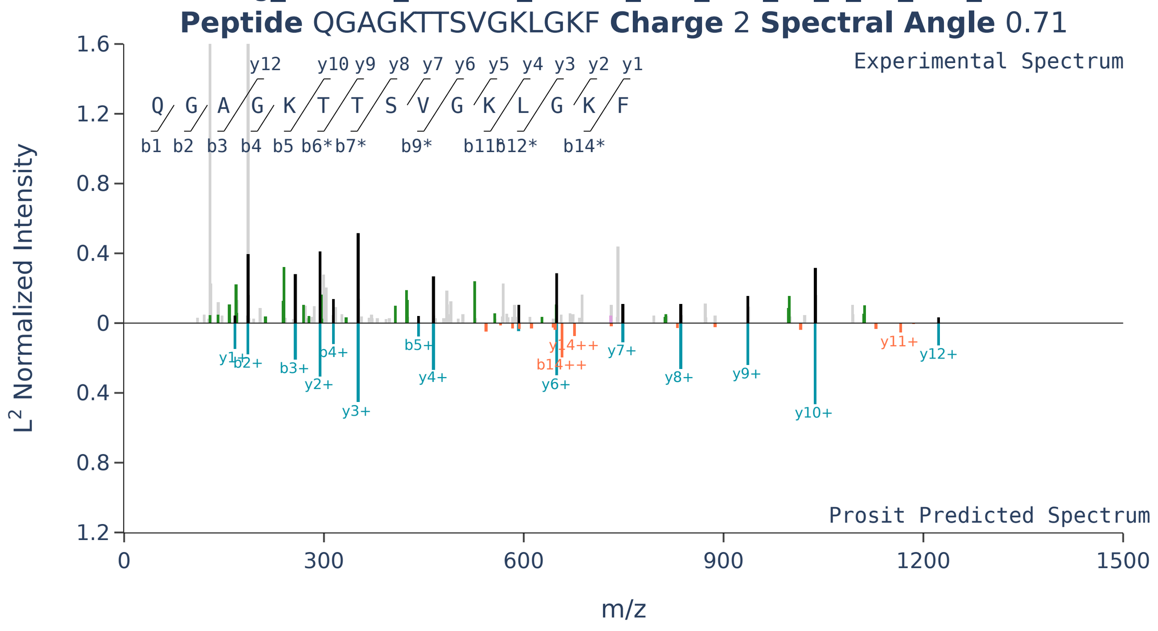


**Source** Ncheng_210623_230623_HFGoe_FFH_20S_25_1_A1_24h_R1 **Scan** 6829
**Peptide** AKPEIIKGSR **Charge** 2 **Spectral Angle** 0.7

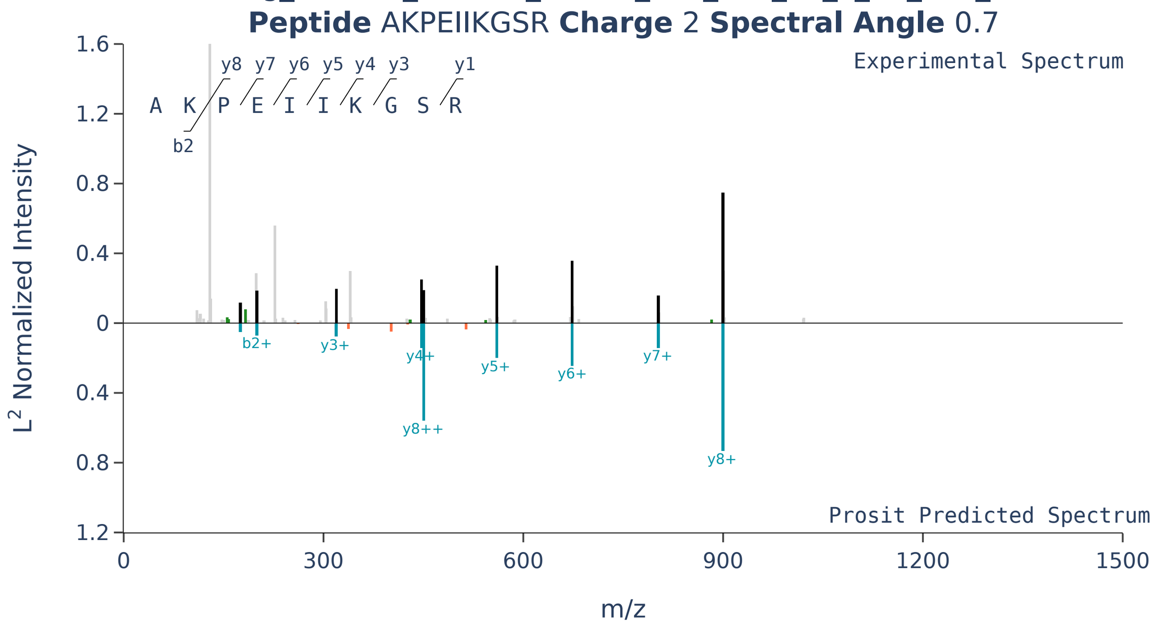


**Source** Ncheng_210623_230623_HFGoe_FFH_20S_25_1_A2_24h_R1 **Scan** 4793
**Peptide** KVDRAQAE **Charge** 2 **Spectral Angle** 0.68

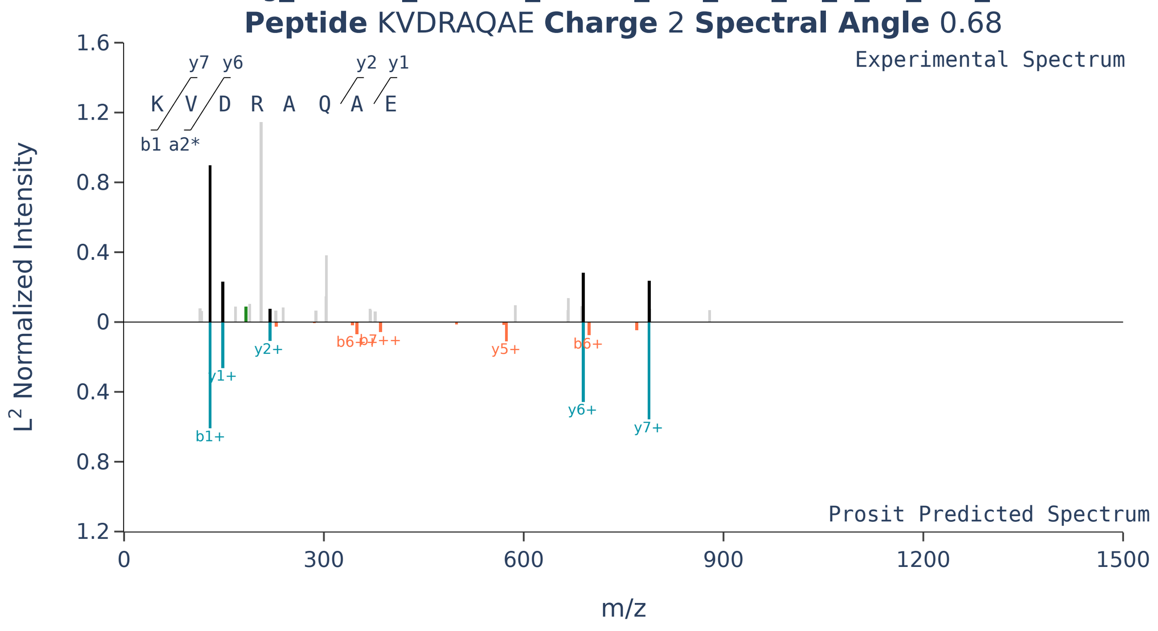


**Source** Ncheng_210623_230623_HFGoe_FFH_20S_25_1_A1_2h_R2 **Scan** 10361
**Peptide** LRQMKNMGGMA **Charge** 2 **Spectral Angle** 0.75

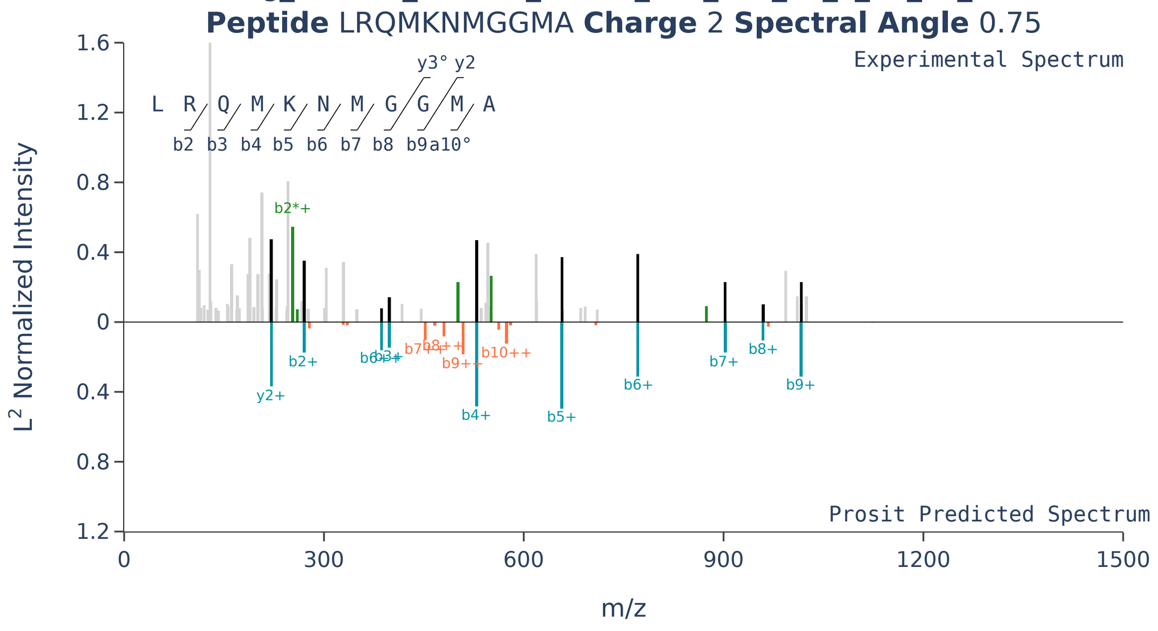


**Source** Ncheng_210623_230623_HFGoe_FFH_20S_25_1_A1_1h_R2 **Scan** 35099
**Peptide** GVDFFPSDVGQKPVDIVNAALKEA **Charge** 3 **Spectral Angle** 0.84

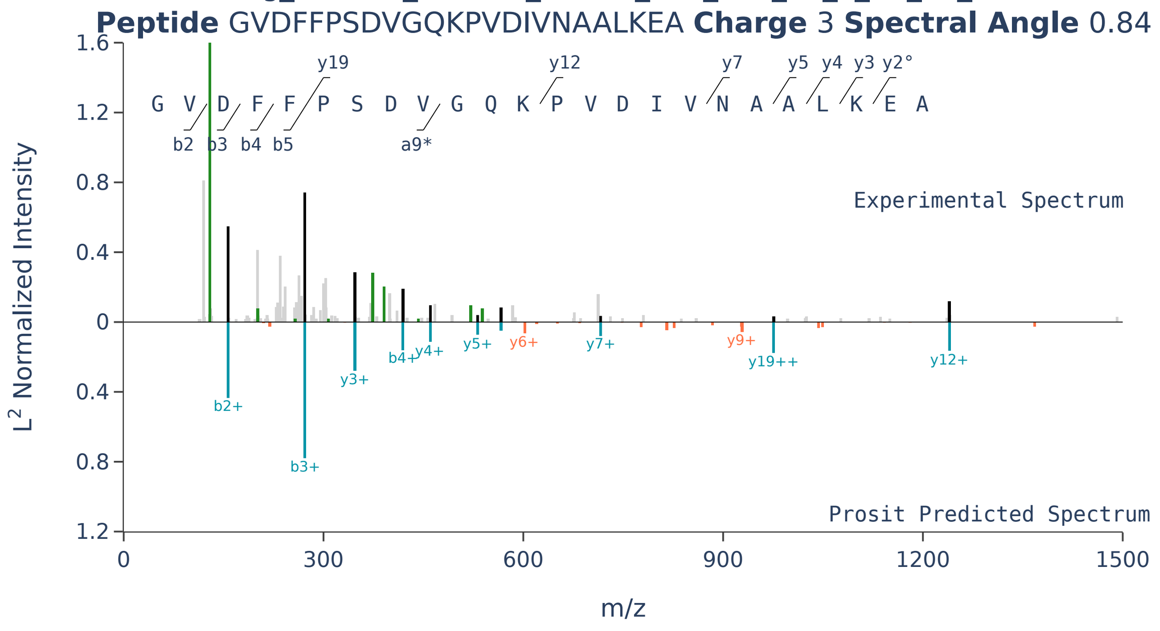


**Source** Ncheng_210623_230623_HFGoe_FFH_20S_25_1_A2_4h_R1 **Scan** 5383
**Peptide** VGQKPVD **Charge** 2 **Spectral Angle** 0.7

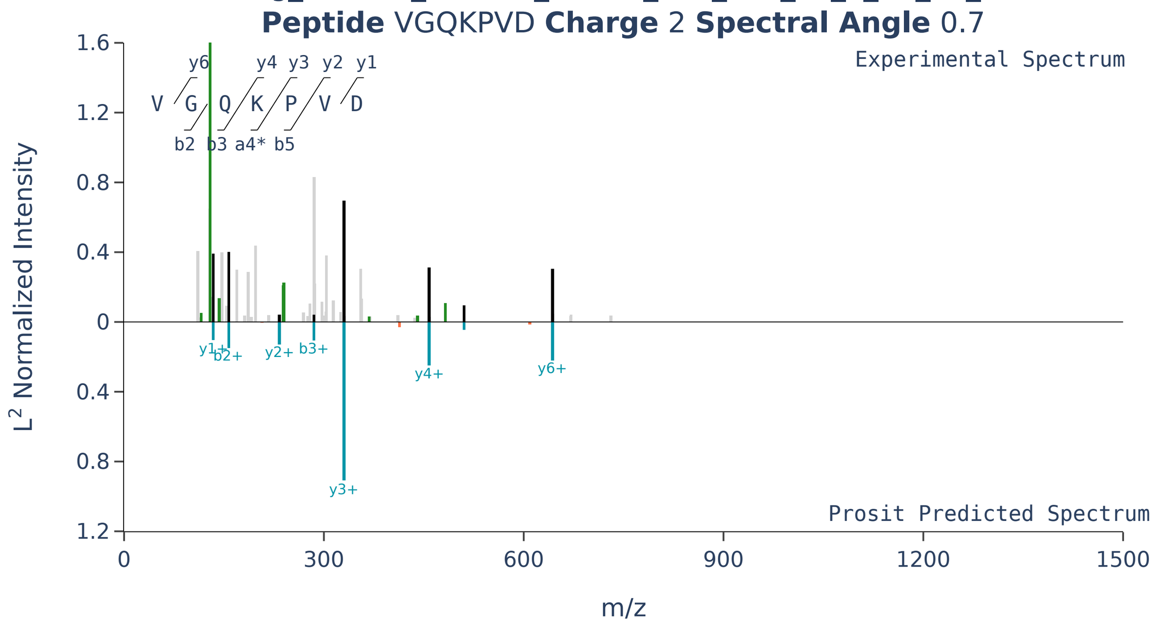


**Source** Ncheng_210623_230623_HFGoe_FFH_20S_25_1_A1_1h_R1 **Scan** 18282
**Peptide** GVGEKTEALEPFHPDRIAS **Charge** 3 **Spectral Angle** 0.82

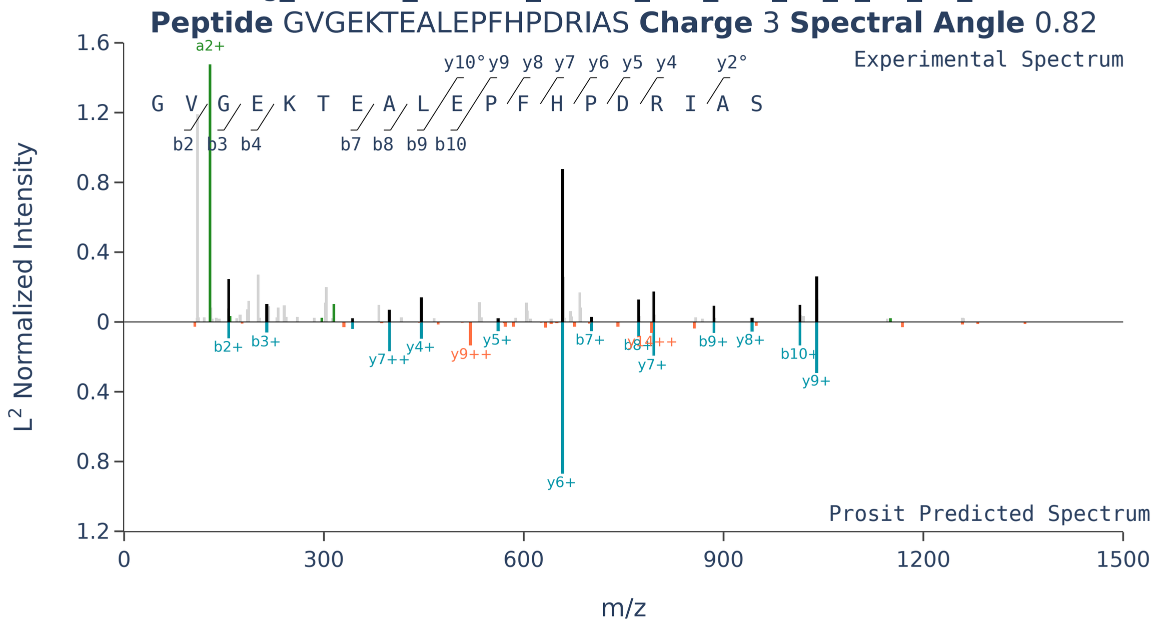


**Source** Ncheng_210623_230623_HFGoe_FFH_20S_25_1_A2_4h_R2 **Scan** 29684
**Peptide** DAMTGQDAANTAKAFNEALPLTG **Charge** 2 **Spectral Angle** 0.82

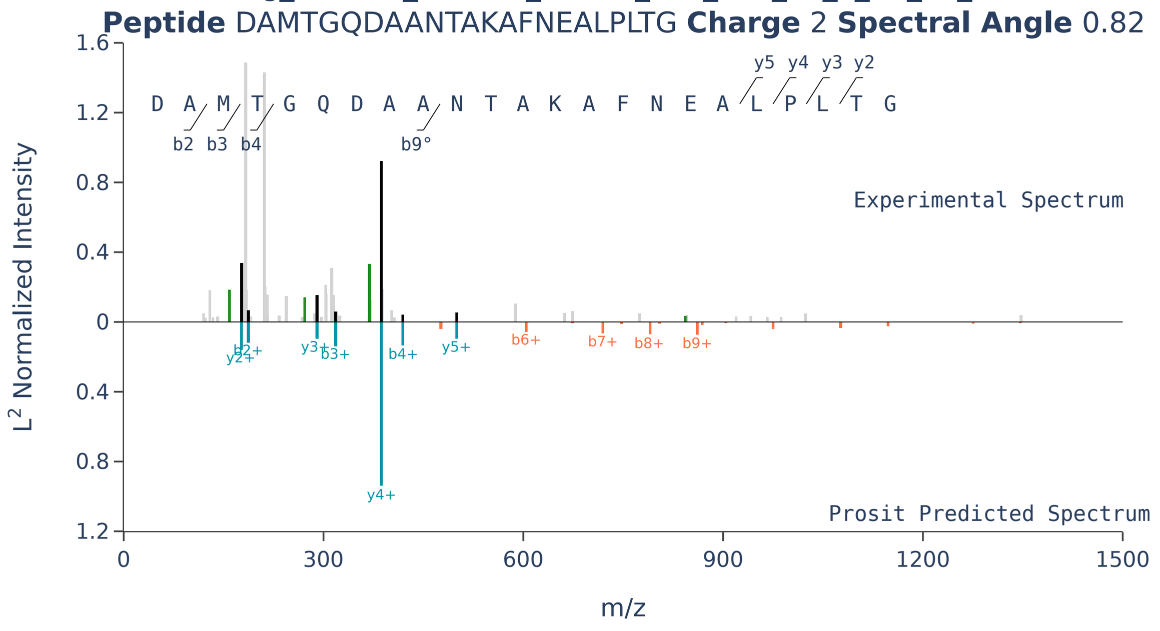

**Source** Ncheng_210623_230623_HFGoe_FFH_20S_25_1_A1_2h_R2 **Scan** 28698
**Peptide** KSLTPGQEFVKIVRNEL **Charge** 2 **Spectral Angle** 0.54

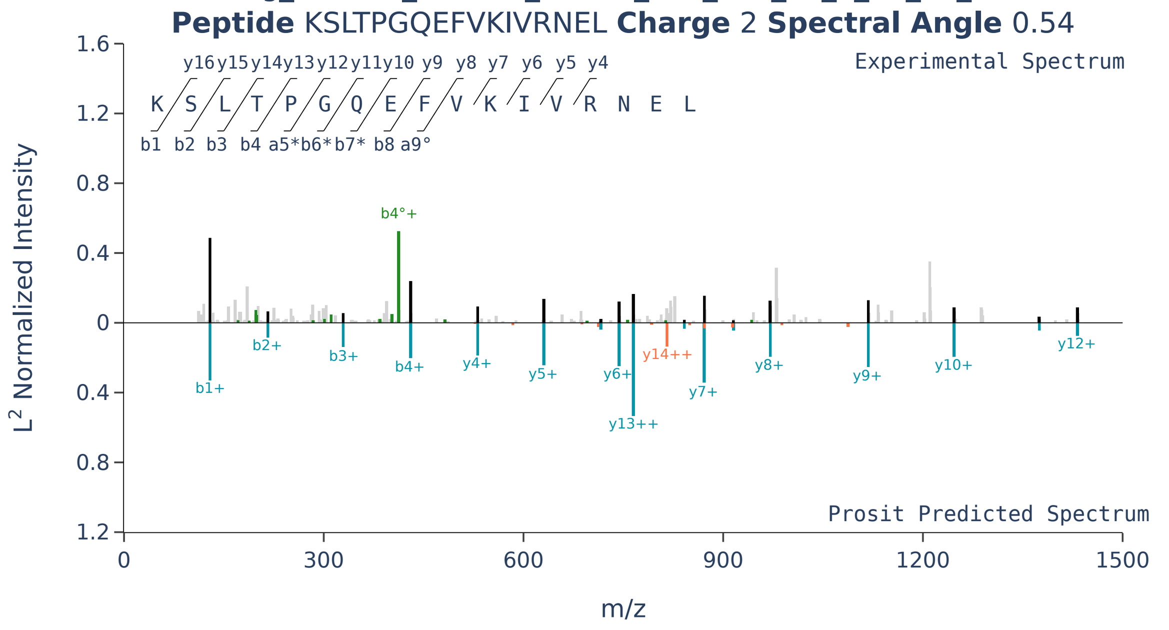


**Source** Ncheng_210623_230623_HFGoe_FFH_20S_25_1_A2_24h_R2 **Scan** 19840
**Peptide** KEKAVGHEVNKSLTPGQEFVKIV **Charge** 2 **Spectral Angle** 0.71

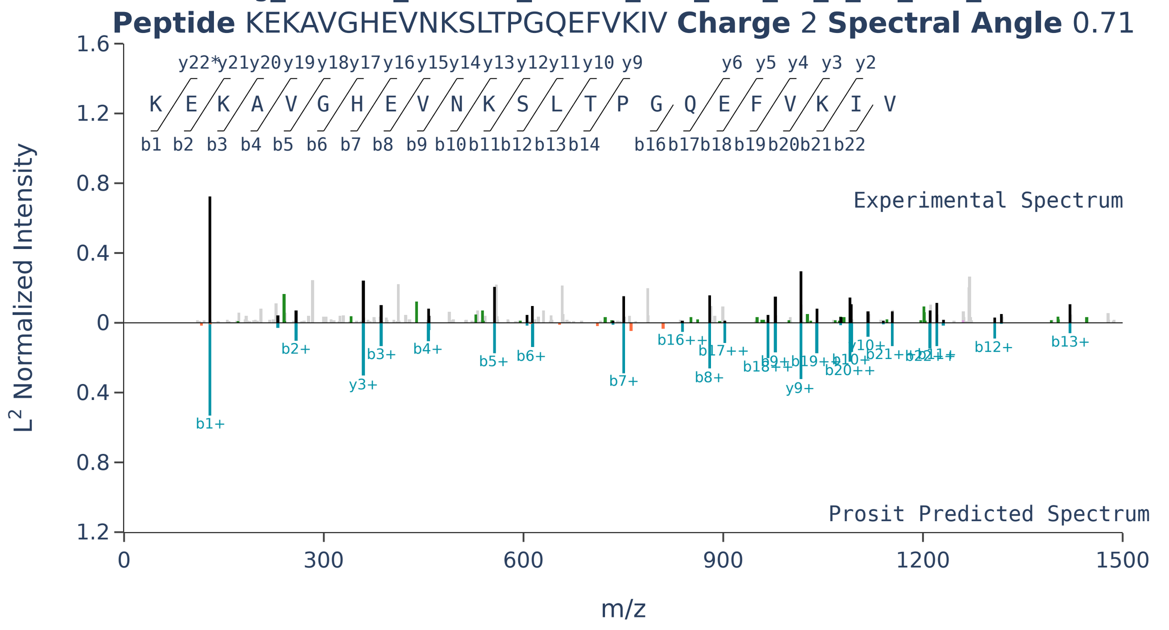


**Source** Ncheng_210623_230623_HFGoe_FFH_20S_25_1_A1_24h_R2 **Scan** 8308
**Peptide** KLGKFLREK **Charge** 2 **Spectral Angle** 0.59

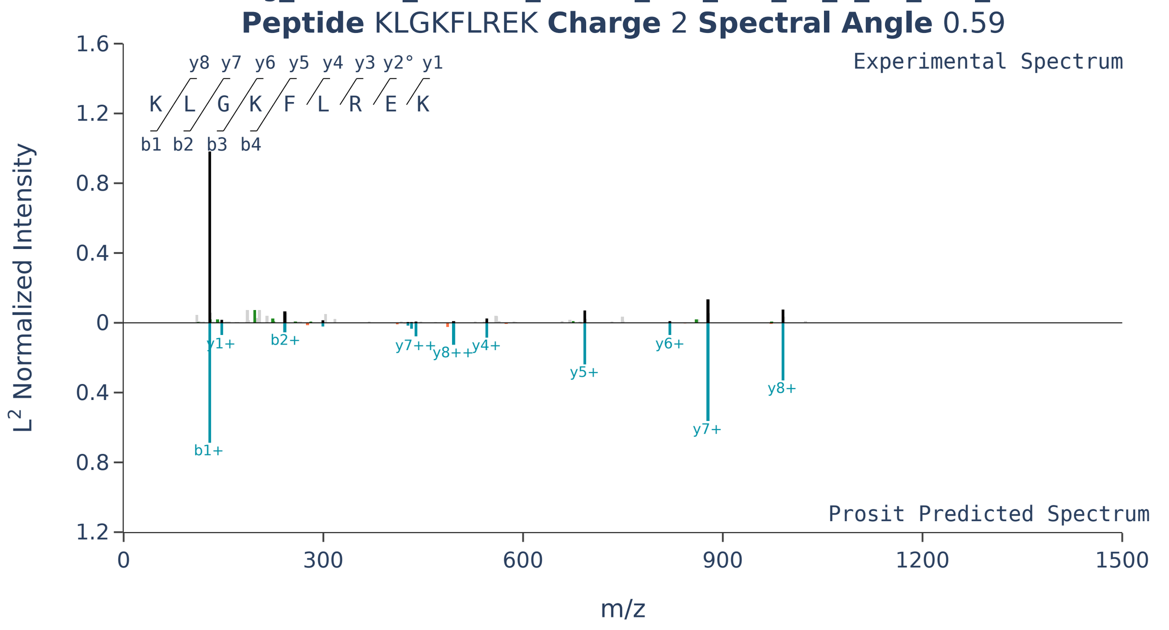


**Source** Ncheng_210623_230623_HFGoe_FFH_20S_25_1_A1_24h_R2 **Scan** 16038
**Peptide** DLESKVDRAQAEKLASKLKKG **Charge** 3 **Spectral Angle** 0.81

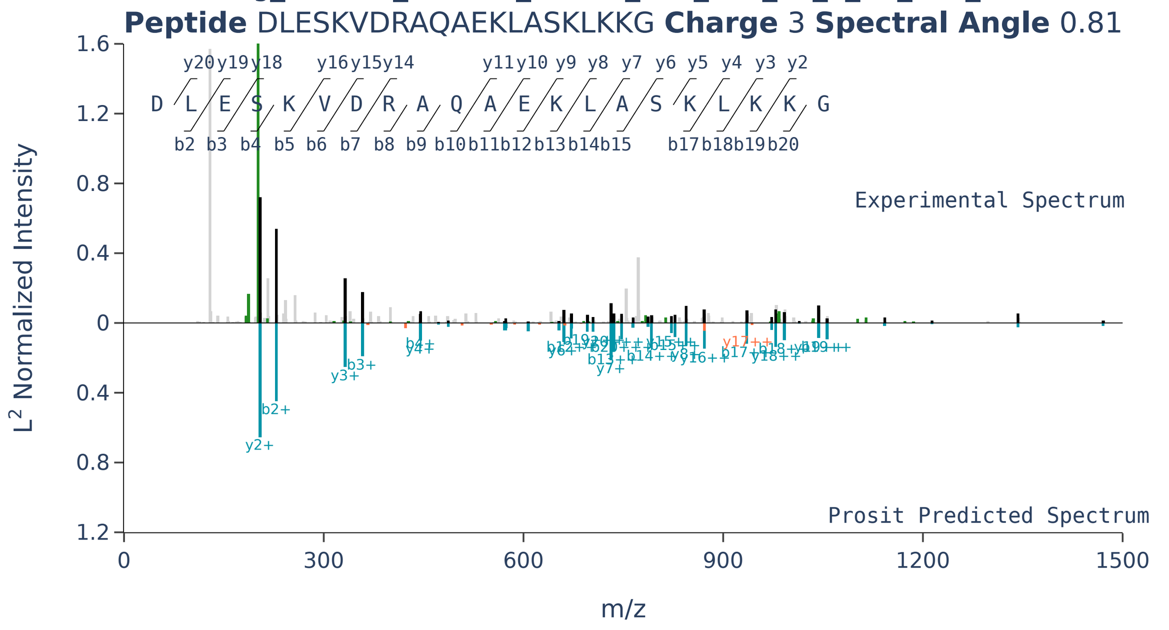


**Source** Ncheng_210623_230623_HFGoe_FFH_20S_25_1_A2_24h_R2 **Scan** 9641
**Peptide** DTAGRLHVDE **Charge** 2 **Spectral Angle** 0.6

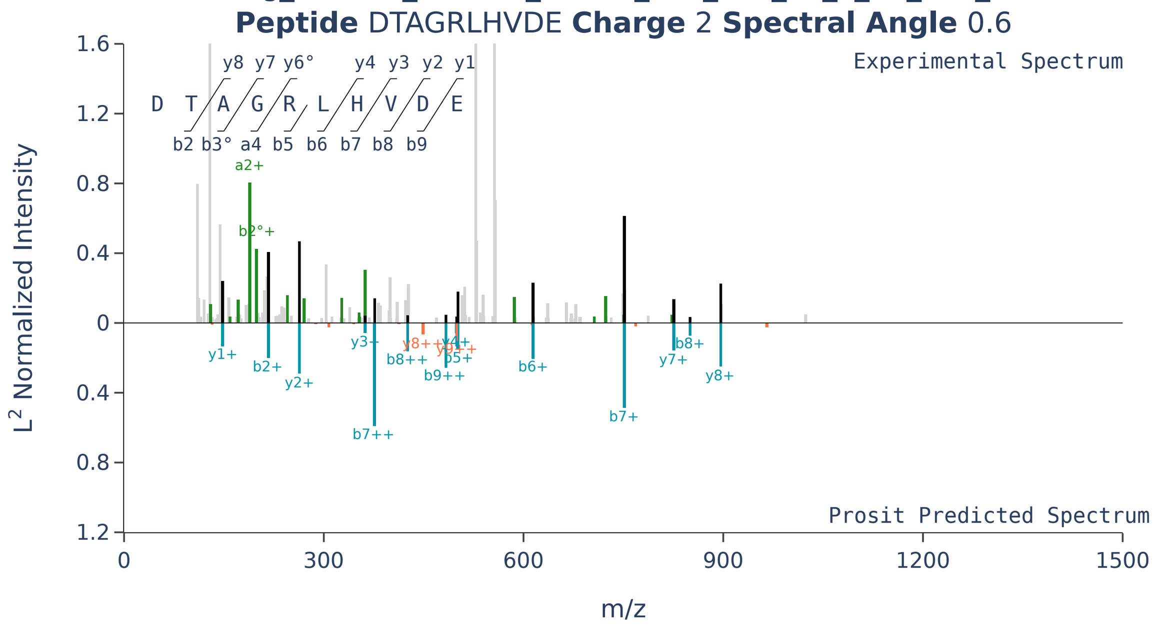


**Source** Ncheng_210623_230623_HFGoe_FFH_20S_25_1_A2_4h_R2 **Scan** 11289
**Peptide** ADVYRPAAIK **Charge** 2 **Spectral Angle** 0.66

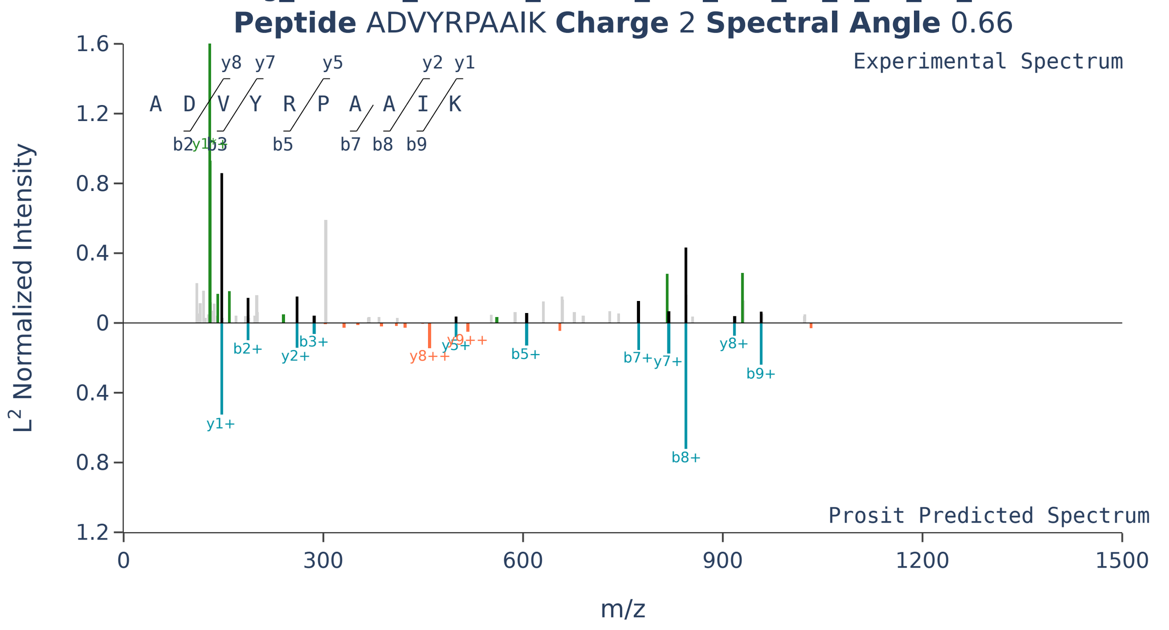


**Source** Ncheng_210623_230623_HFGoe_FFH_20S_25_1_A2_2h_R1 **Scan** 25337
**Peptide** PSDVGQKPVDIVNAAL **Charge** 2 **Spectral Angle** 0.7

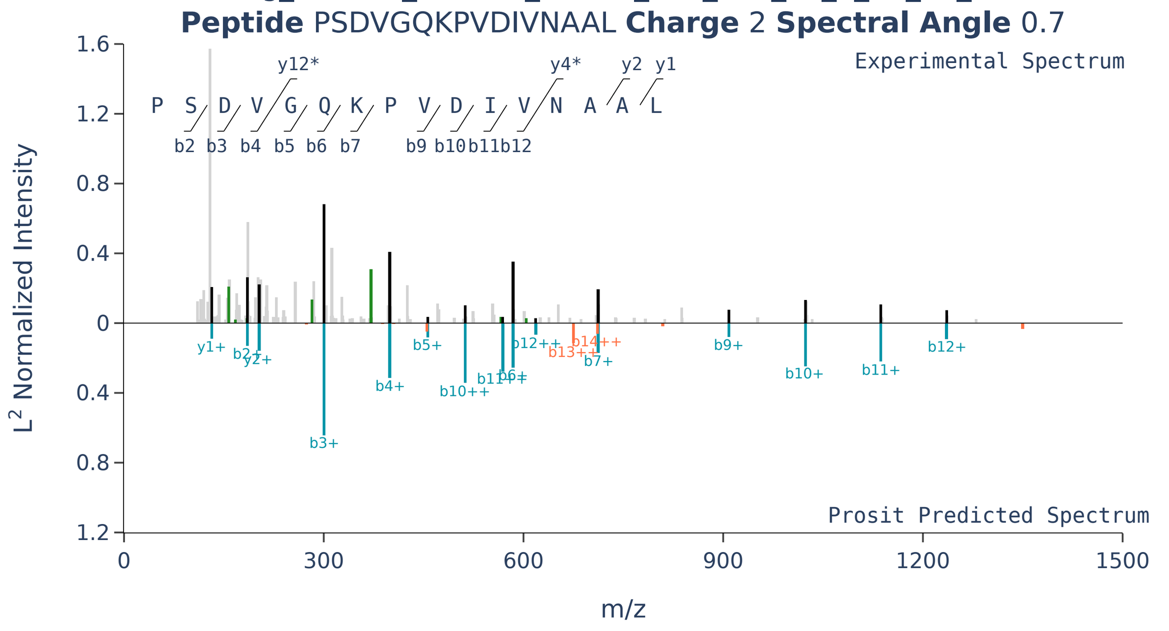


**Source** Ncheng_210623_230623_HFGoe_FFH_20S_25_1_A2_24h_R1 **Scan** 5635
**Peptide** EIIKGSRKR **Charge** 2 **Spectral Angle** 0.76

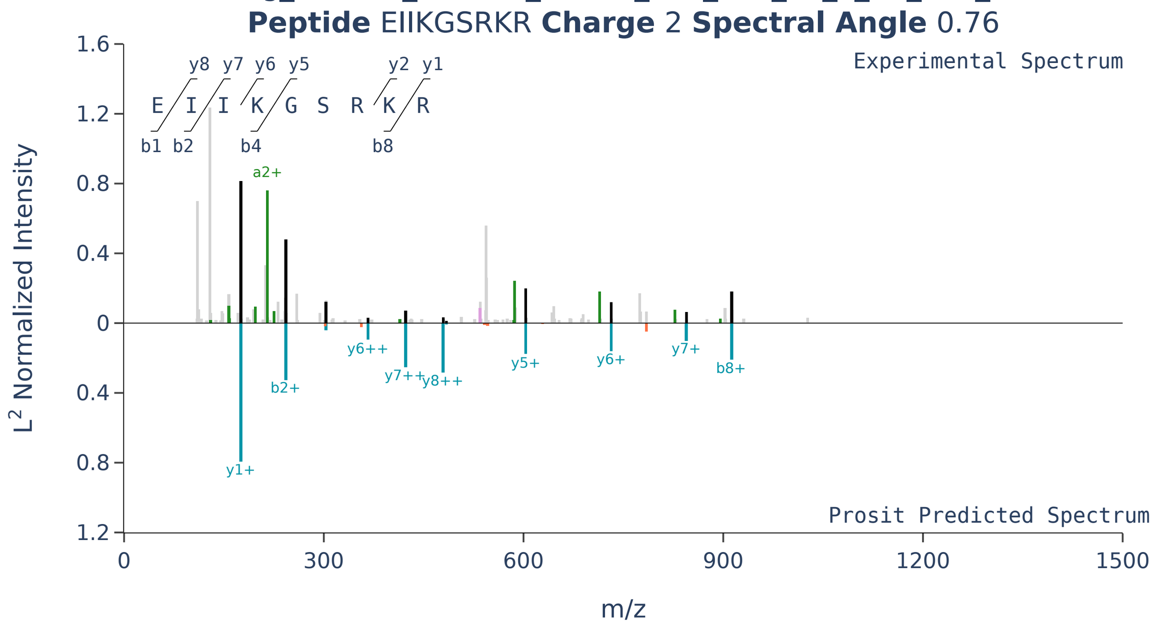


**Source** Ncheng_210623_230623_HFGoe_FFH_20S_25_1_A2_24h_R2 **Scan** 22907
**Peptide** PGQEFVKIV **Charge** 2 **Spectral Angle** 0.67

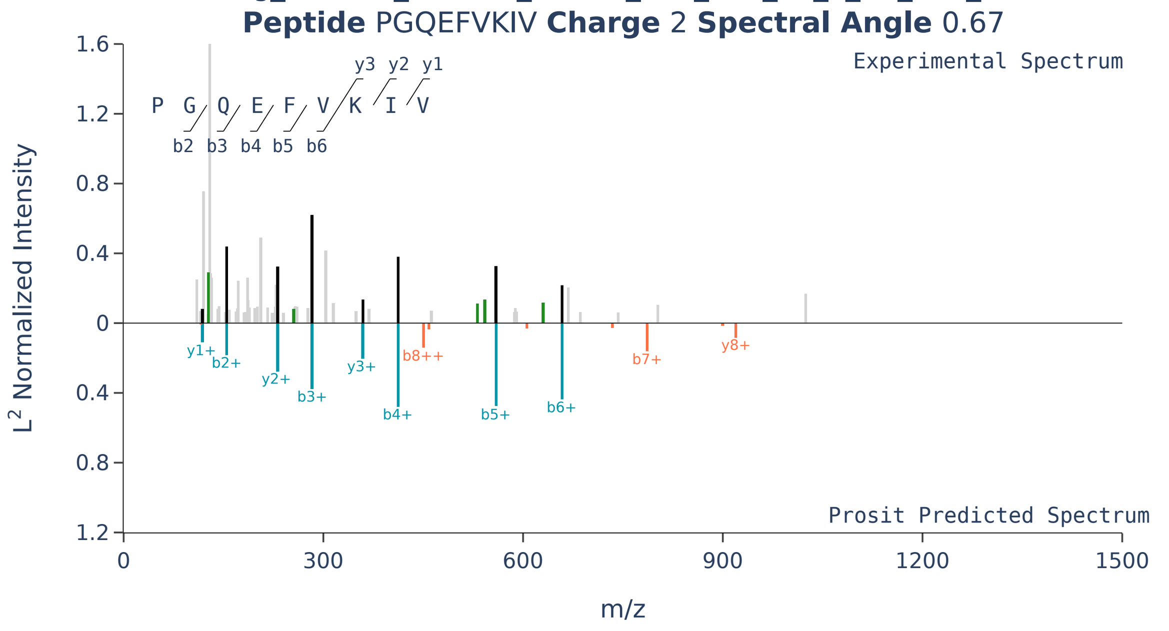


**Source** Ncheng_210623_230623_HFGoe_FFH_20S_25_1_A2_24h_R1 **Scan** 9610
**Peptide** KERAKPEII **Charge** 3 **Spectral Angle** 0.67

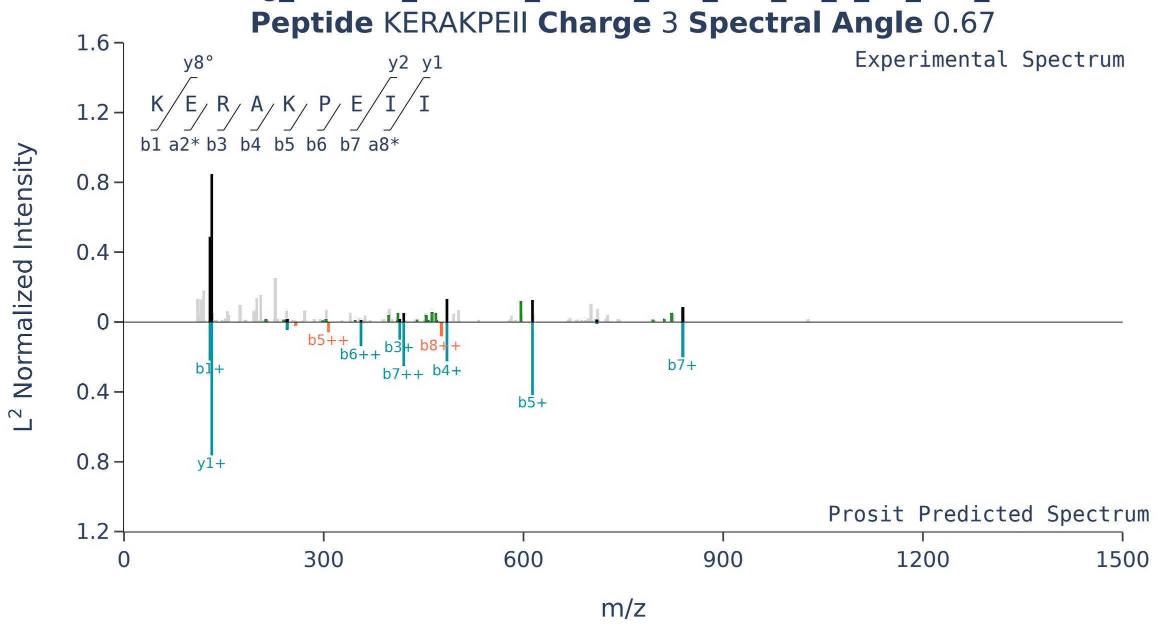


**Source** Ncheng_210623_230623_HFGoe_FFH_20S_25_1_A1_2h_R2 **Scan** 24583
**Peptide** PGMGQIPDNVKSQMDDKVLVRME **Charge** 3 **Spectral Angle** 0.68

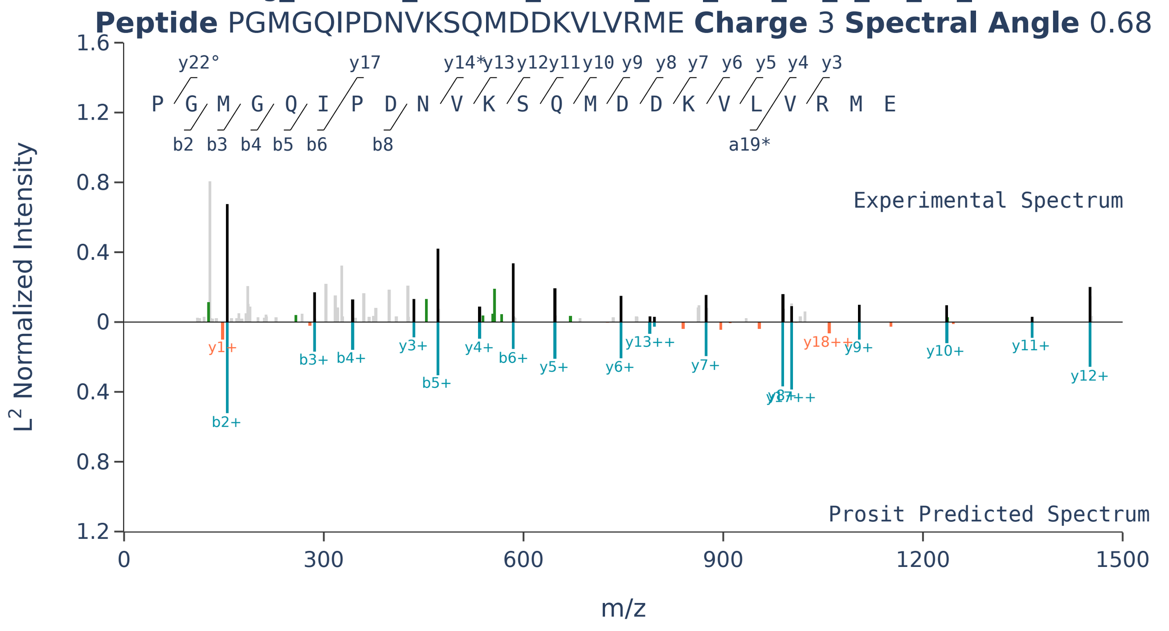


**Source** Ncheng_210623_230623_HFGoe_FFH_20S_25_1_A2_4h_R1 **Scan** 20875
**Peptide** MFDNLTDRLSR **Charge** 3 **Spectral Angle** 0.57

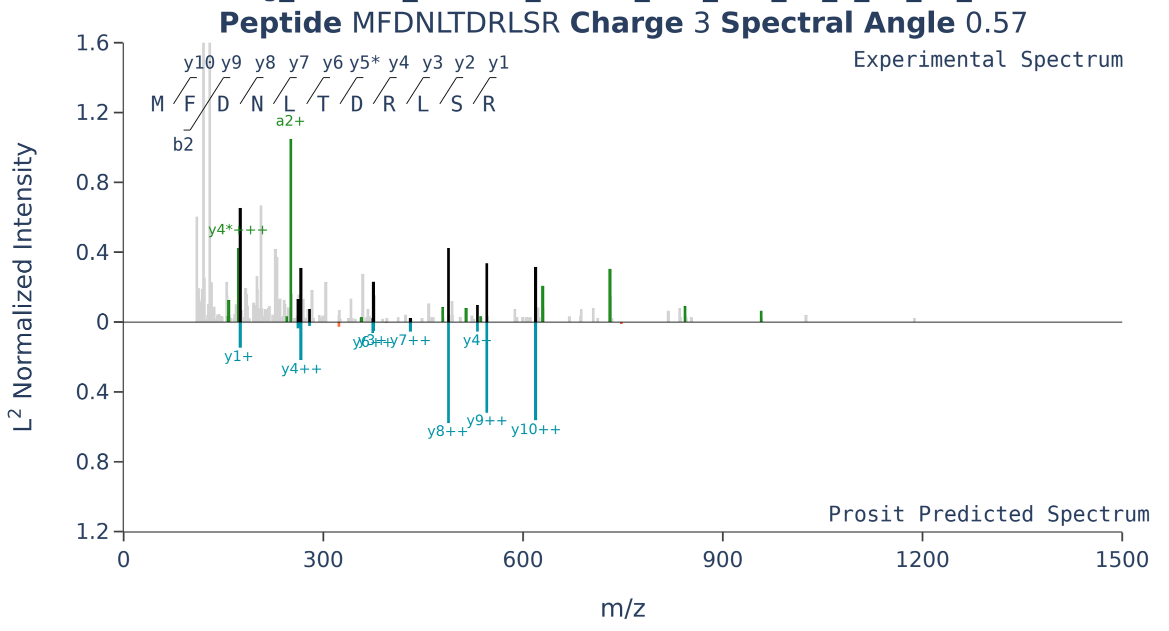


**Source** Ncheng_210623_230623_HFGoe_FFH_20S_25_1_A2_24h_R2 **Scan** 18040
**Peptide** VVLTKVDGDARGGAALSLR **Charge** 3 **Spectral Angle** 0.66

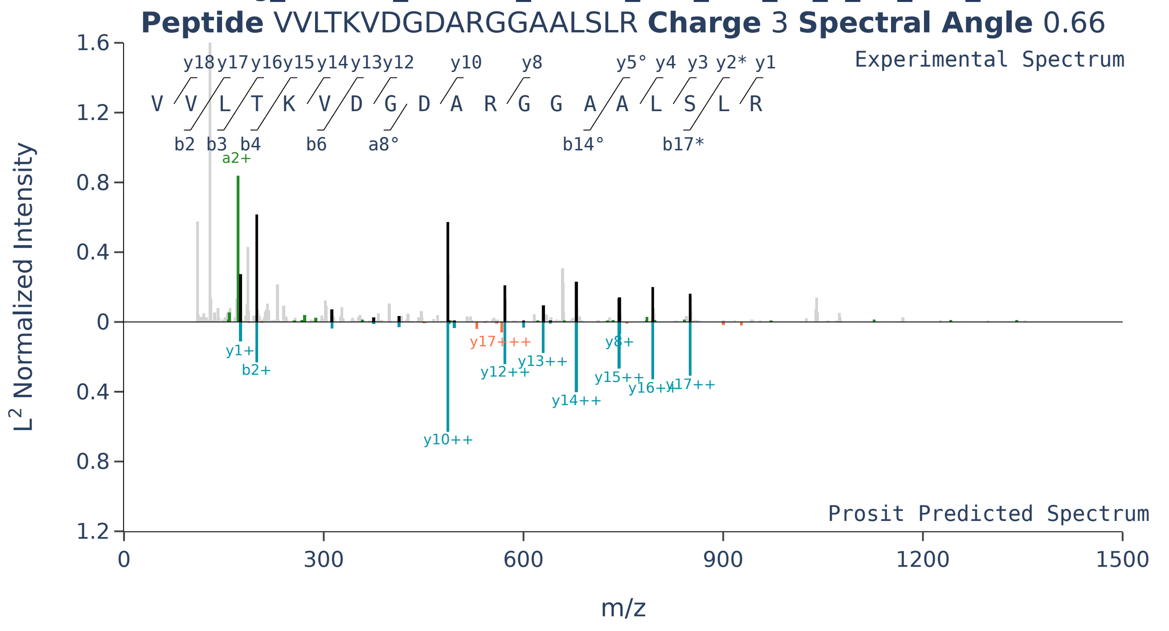


**Source** Ncheng_210623_230623_HFGoe_FFH_20S_25_1_A2_24h_R2 **Scan** 6902
**Peptide** KEKAVGHEVNKSLTPGQ **Charge** 2 **Spectral Angle** 0.8

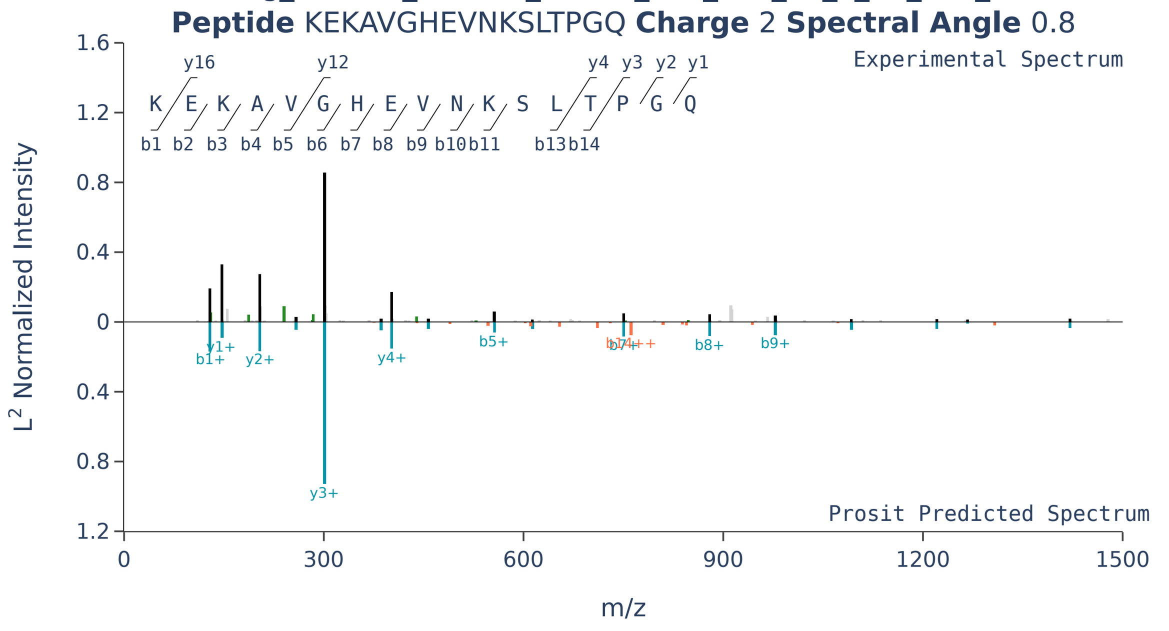


**Source** Ncheng_210623_230623_HFGoe_FFH_20S_25_1_A1_2h_R2 **Scan** 31671
**Peptide** GQIPDNVKSQMDDKVLVRMEAII **Charge** 3 **Spectral Angle** 0.84

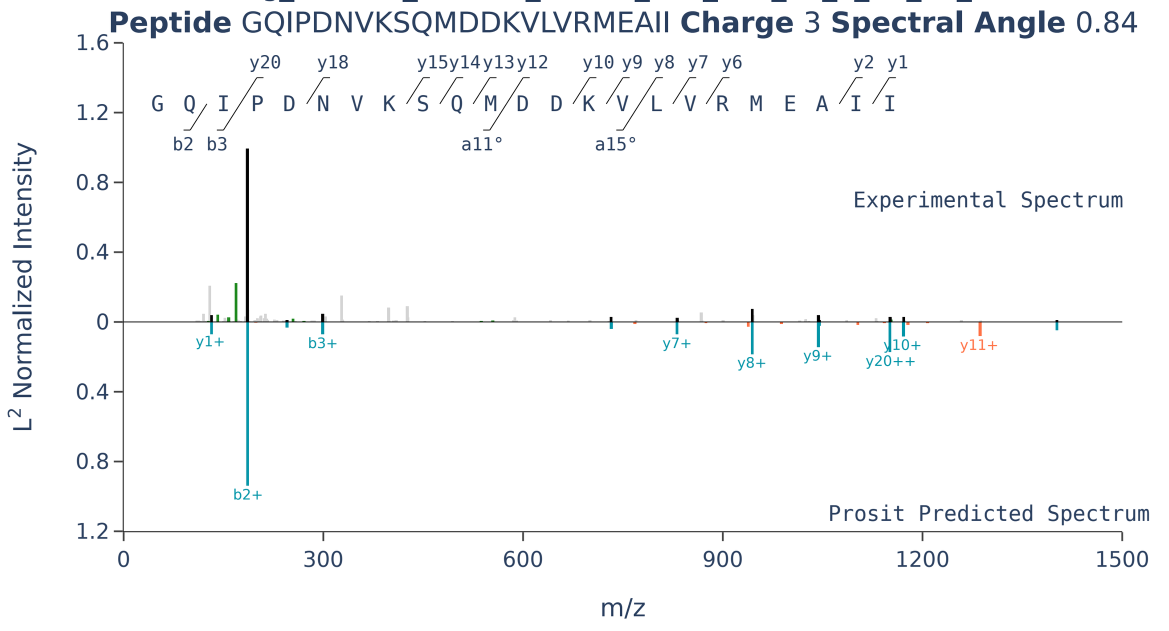

**Source** Ncheng_210623_230623_HFGoe_FFH_20S_25_1_A2_4h_R2 **Scan** 18820
**Peptide** KAVGHEVNKSLTPGQEFVKI **Charge** 3 **Spectral Angle** 0.83

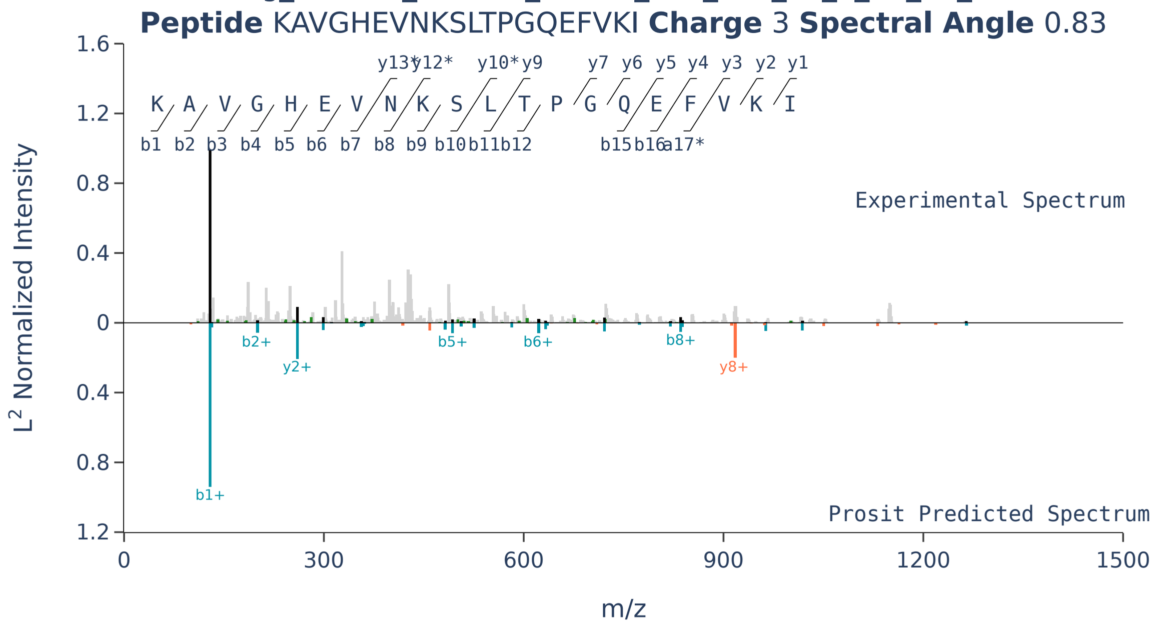


**Source** Ncheng_210623_230623_HFGoe_FFH_20S_25_1_A2_4h_R2 **Scan** 11926
**Peptide** AIINSMTMKERAK **Charge** 4 **Spectral Angle** 0.68

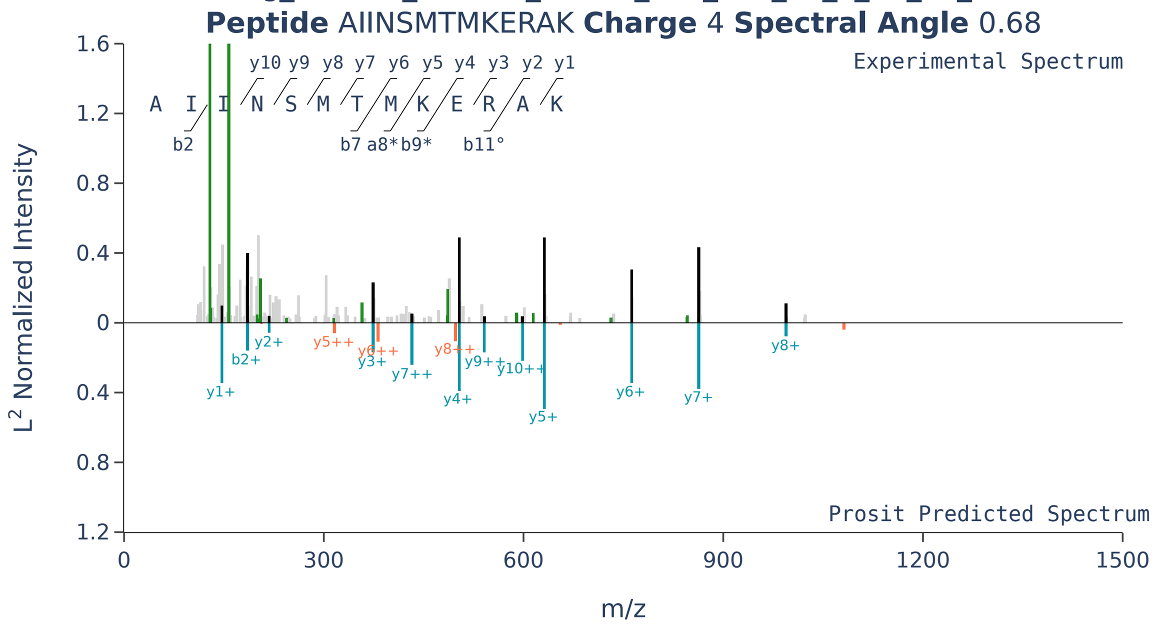


**Source** Ncheng_210623_230623_HFGoe_FFH_20S_25_1_A2_4h_R1 **Scan** 19488
**Peptide** GKLPGMGQI **Charge** 2 **Spectral Angle** 0.79

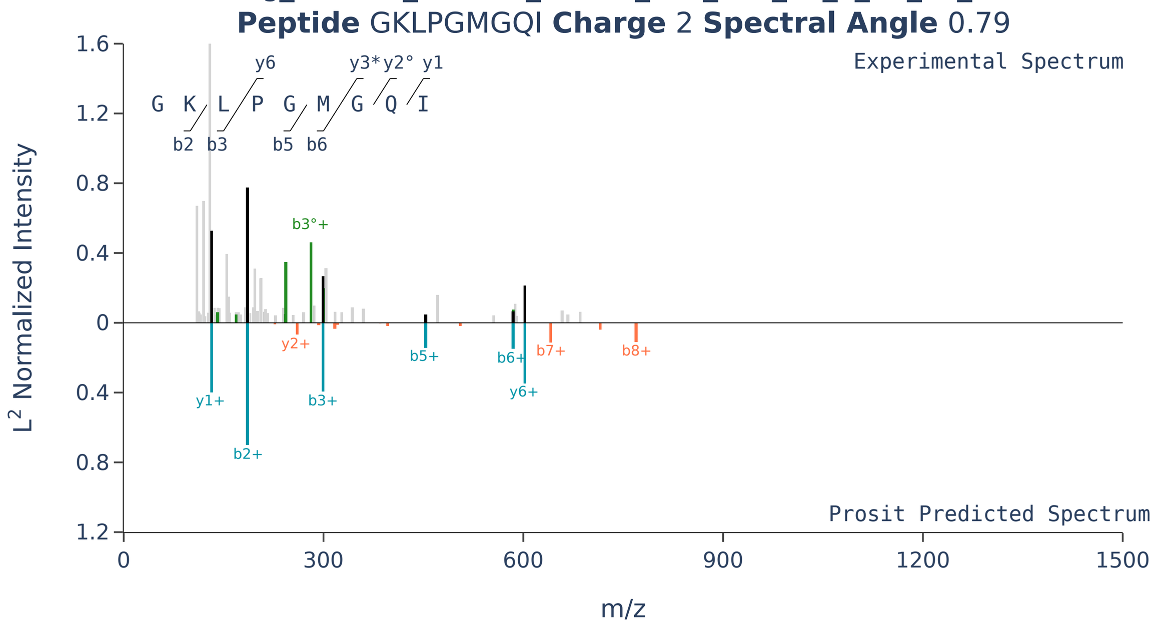


**Source** Ncheng_210623_230623_HFGoe_FFH_20S_25_1_A1_2h_R2 **Scan** 7287
**Peptide** SRTLRNISG **Charge** 2 **Spectral Angle** 0.68

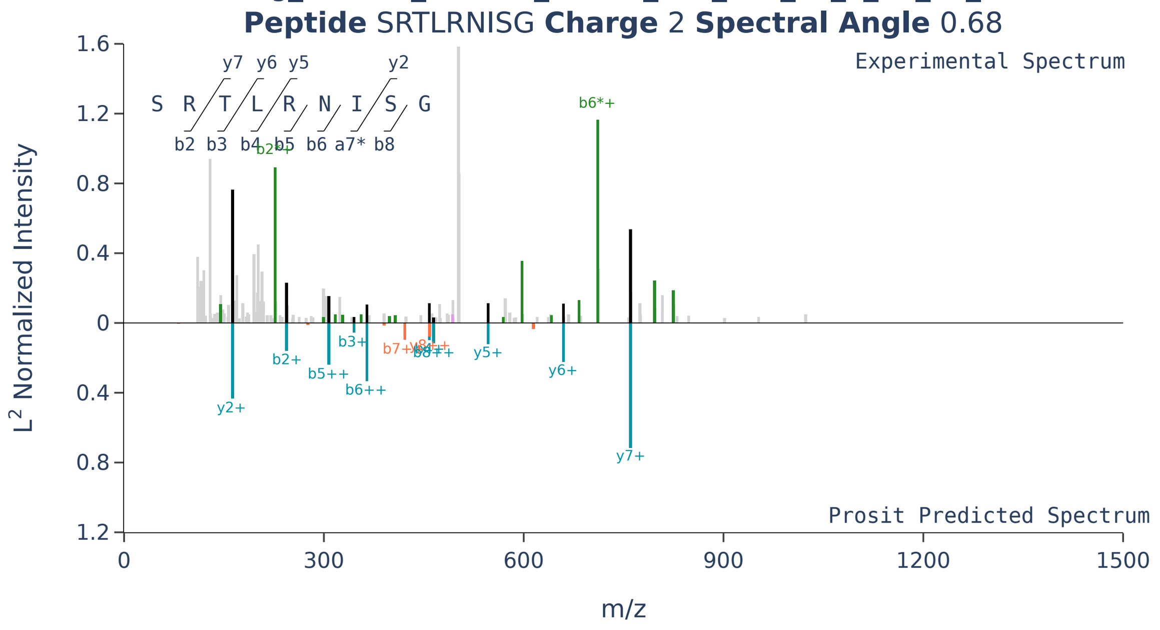


**Source** Ncheng_210623_230623_HFGoe_FFH_20S_25_1_A2_4h_R2 **Scan** 30985
**Peptide** CGMQVQDVNRLLKQF **Charge** 3 **Spectral Angle** 0.81

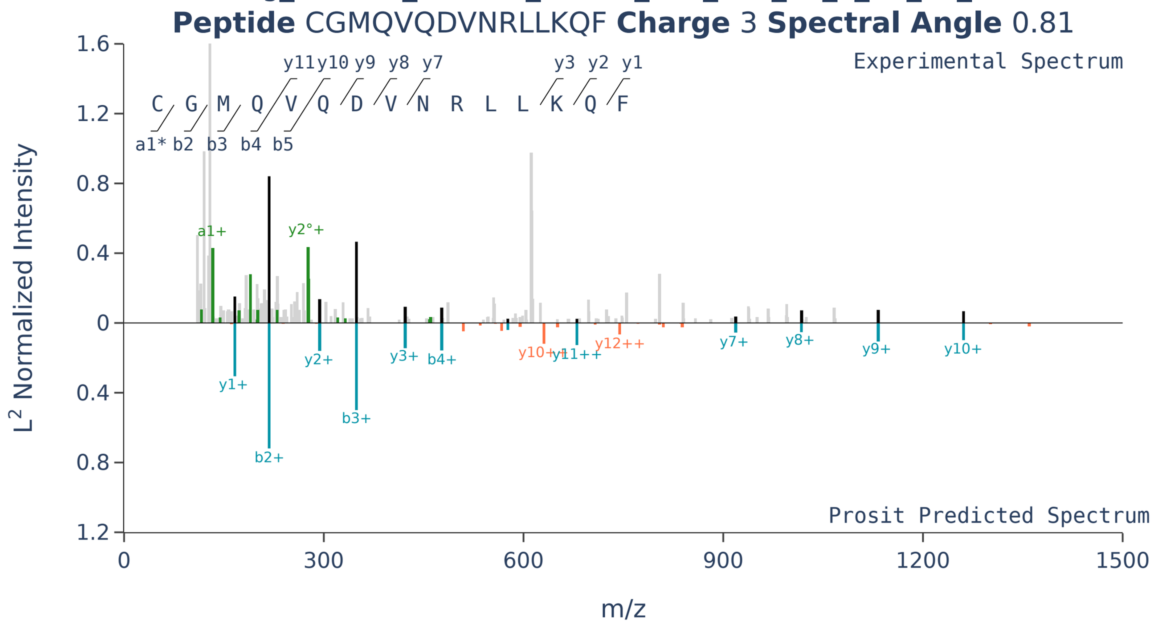


**Source** Ncheng_210623_230623_HFGoe_FFH_20S_25_1_A2_24h_R1 **Scan** 15689
**Peptide** VDGDARGGAALSIRHI **Charge** 2 **Spectral Angle** 0.77

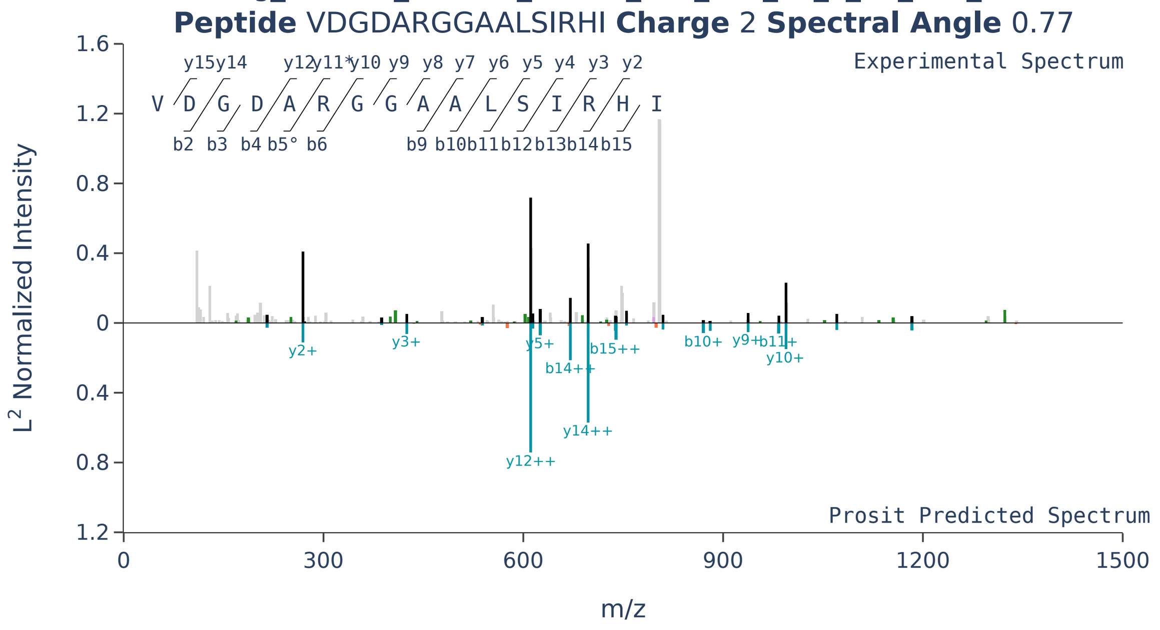


**Source** Ncheng_210623_230623_HFGoe_FFH_20S_25_1_A1_2h_R1 **Scan** 10670
**Peptide** ADVYRPAAIKQ **Charge** 2 **Spectral Angle** 0.65

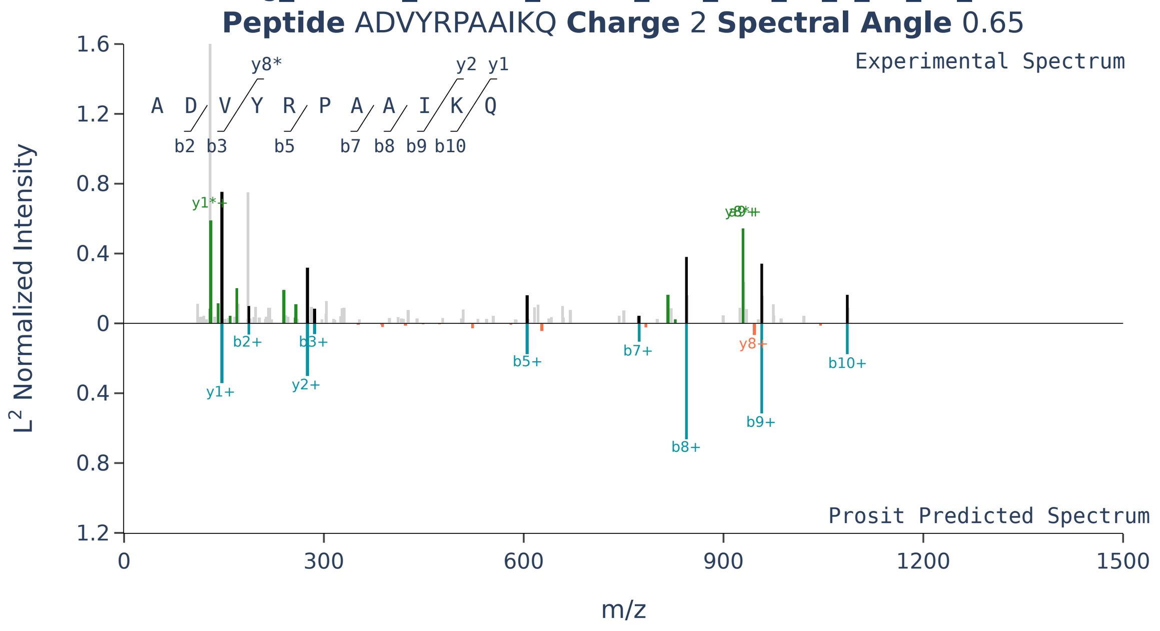


**Source** Ncheng_210623_230623_HFGoe_FFH_20S_25_1_A1_2h_R2 **Scan** 28734
**Peptide** KAVGHEVNKSLTPGQEFVKIVRNELVAA **Charge** 3 **Spectral Angle** 0.77

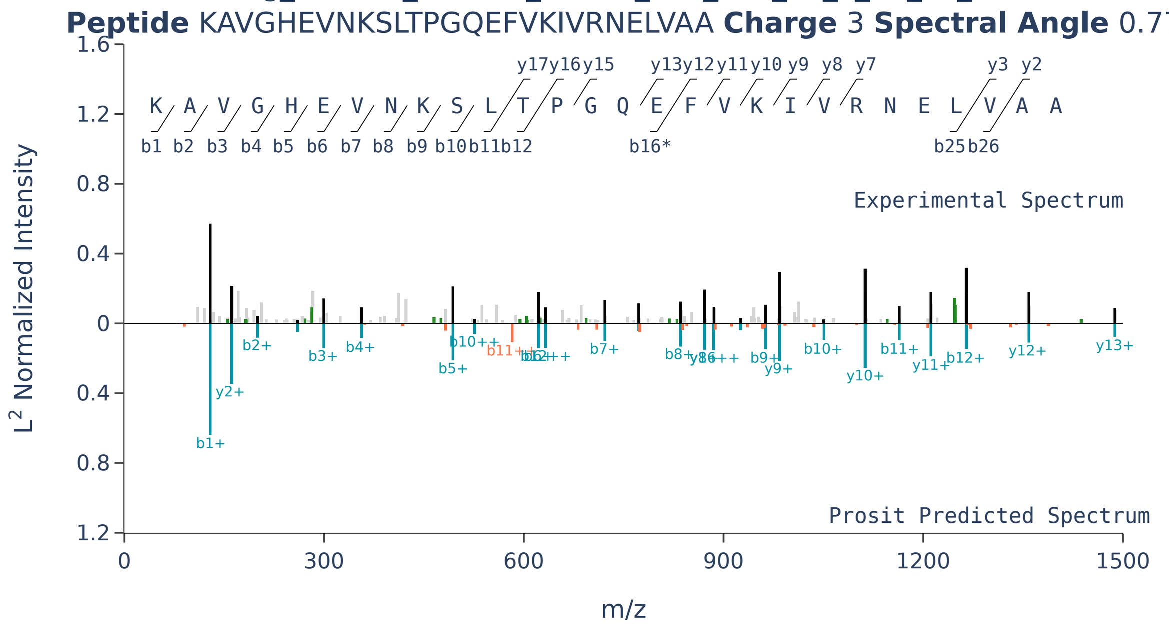


**Source** Ncheng_210623_230623_HFGoe_FFH_20S_25_1_A2_24h_R1 **Scan** 19282
**Peptide** VVLTKVDGDARGGAALSIRHL **Charge** 3 **Spectral Angle** 0.74

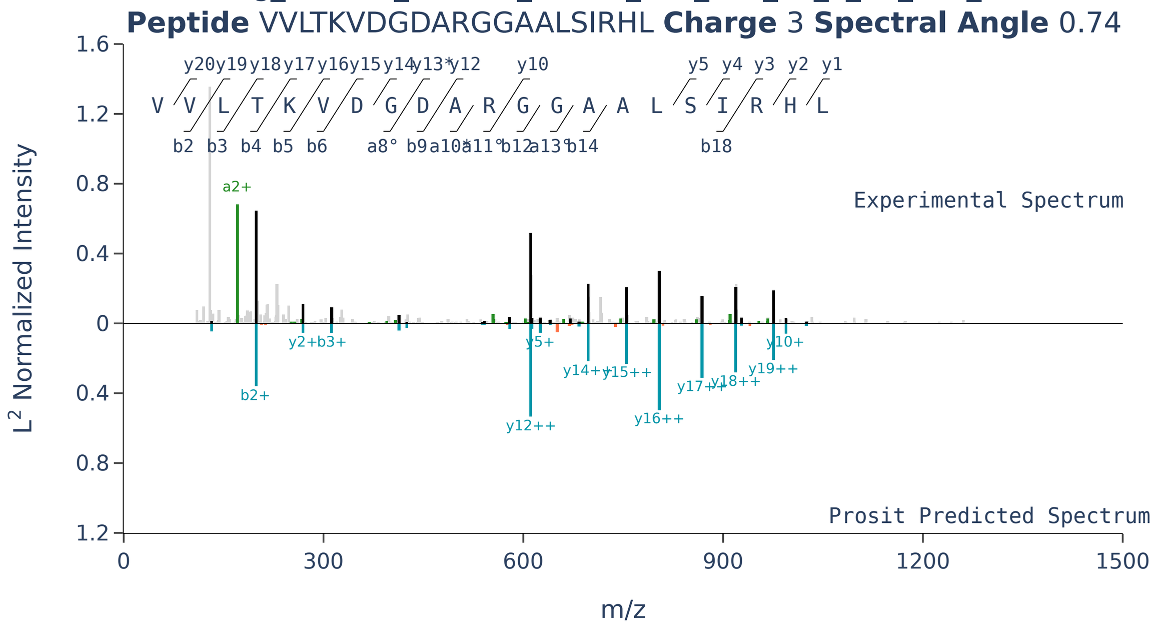


**Source** Ncheng_210623_230623_HFGoe_FFH_20S_25_1_A2_24h_R1 **Scan** 16386
**Peptide** HITGKPIKFLGVGEKTE **Charge** 2 **Spectral Angle** 0.85

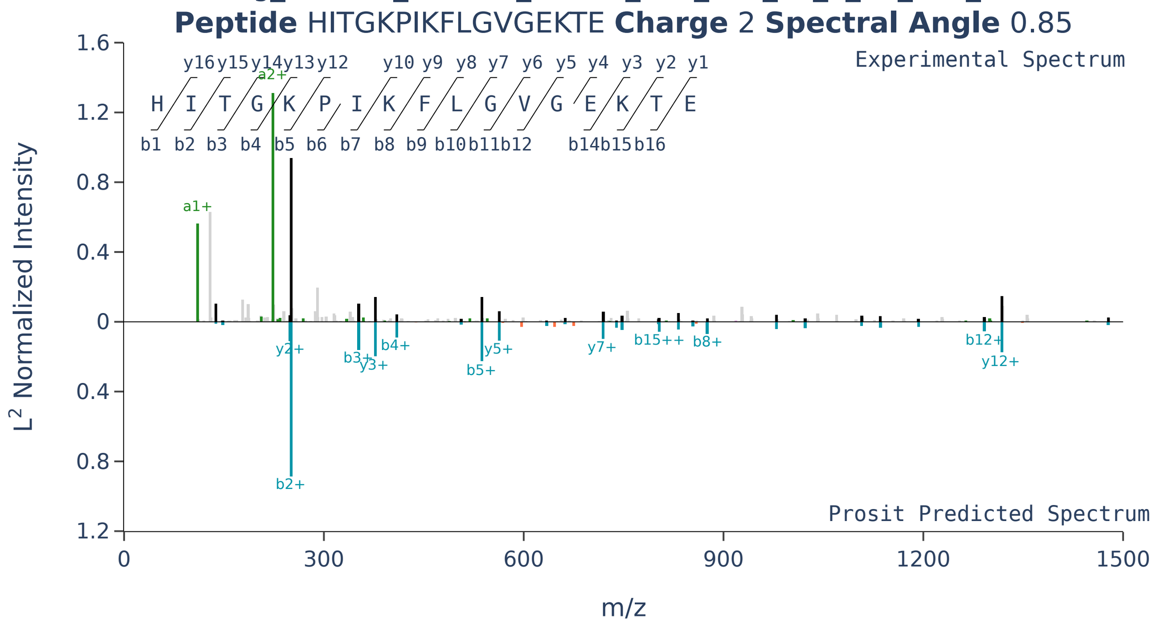


**Source** Ncheng_210623_230623_HFGoe_FFH_20S_25_1_A1_4h_R2 **Scan** 20351
**Peptide** TGKPIKFLGVGEKTEAL **Charge** 2 **Spectral Angle** 0.73

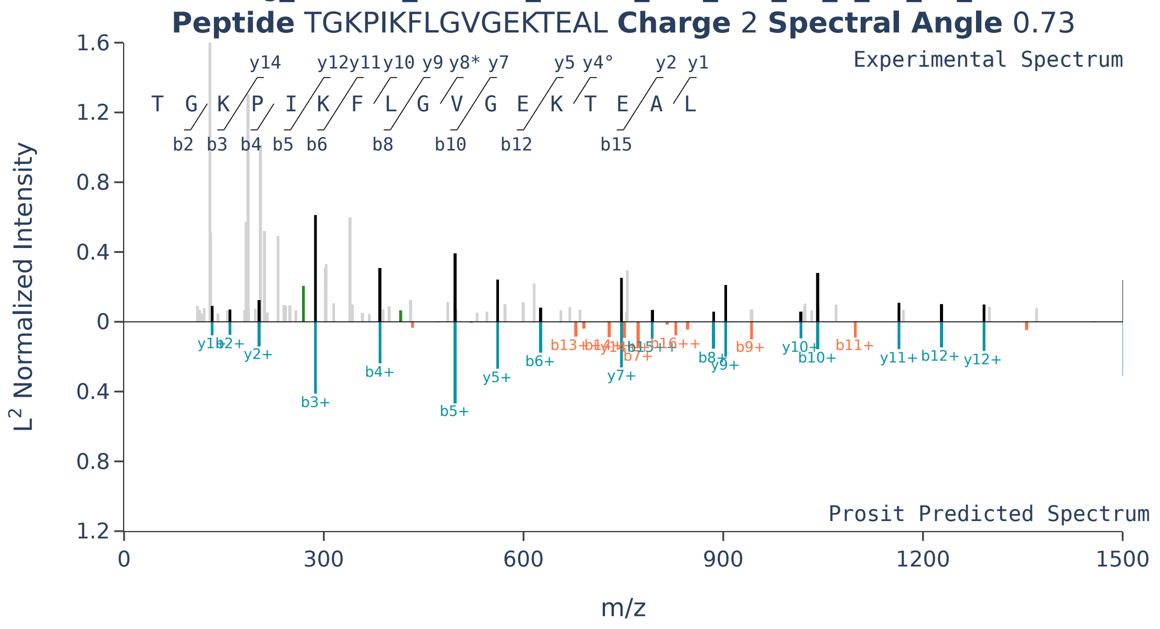


**Source** Ncheng_210623_230623_HFGoe_FFH_20S_25_1_A2_4h_R1 **Scan** 26486
**Peptide** MGEENQTLNLAAQPPAV **Charge** 2 **Spectral Angle** 0.96

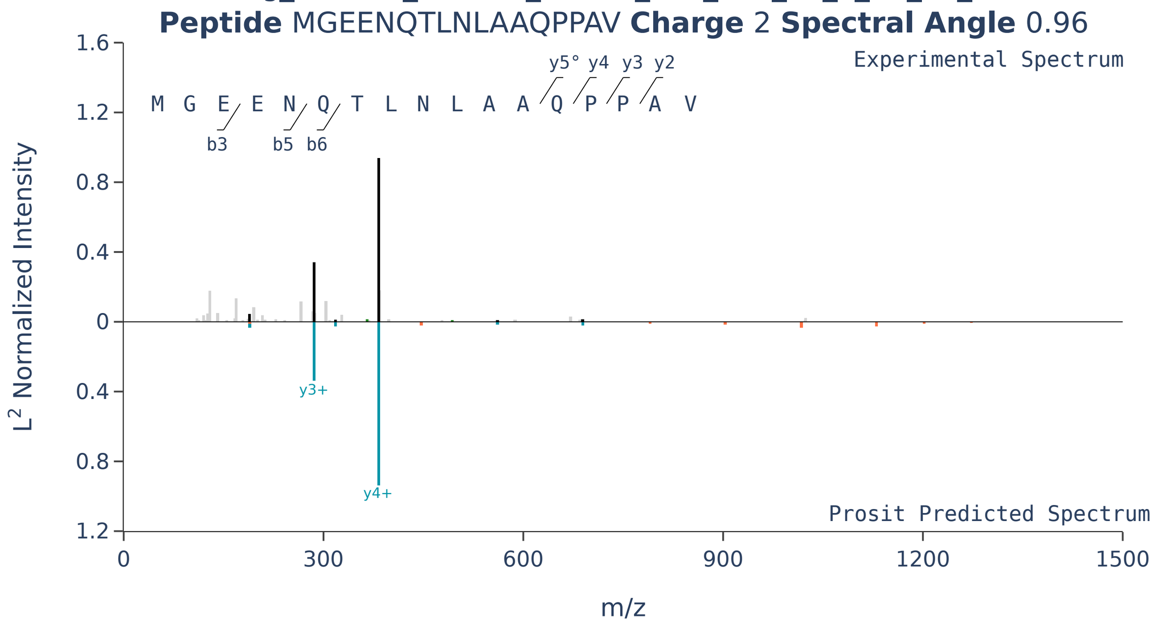


**Source** Ncheng_210623_230623_HFGoe_FFH_20S_25_1_A1_2h_R1 **Scan** 31158
**Peptide** VNKSLTPGQEFVKIVRNELVA **Charge** 3 **Spectral Angle** 0.67

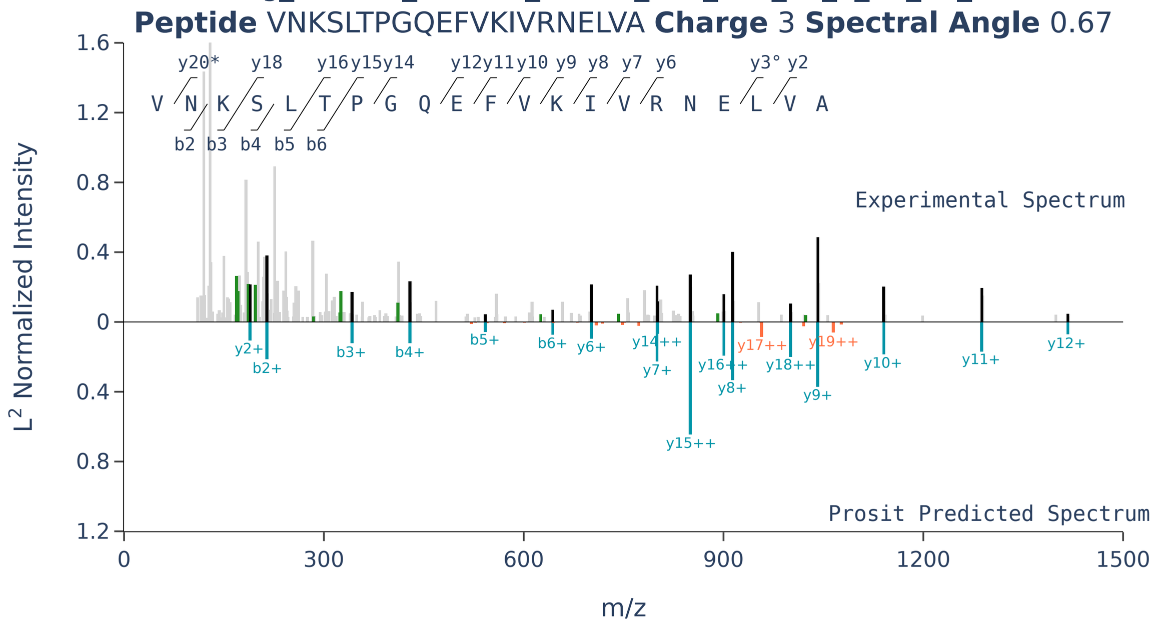


**Source** Ncheng_210623_230623_HFGoe_FFH_20S_25_1_A2_2h_R2 **Scan** 10604
**Peptide** IINSMTMKER **Charge** 3 **Spectral Angle** 0.59

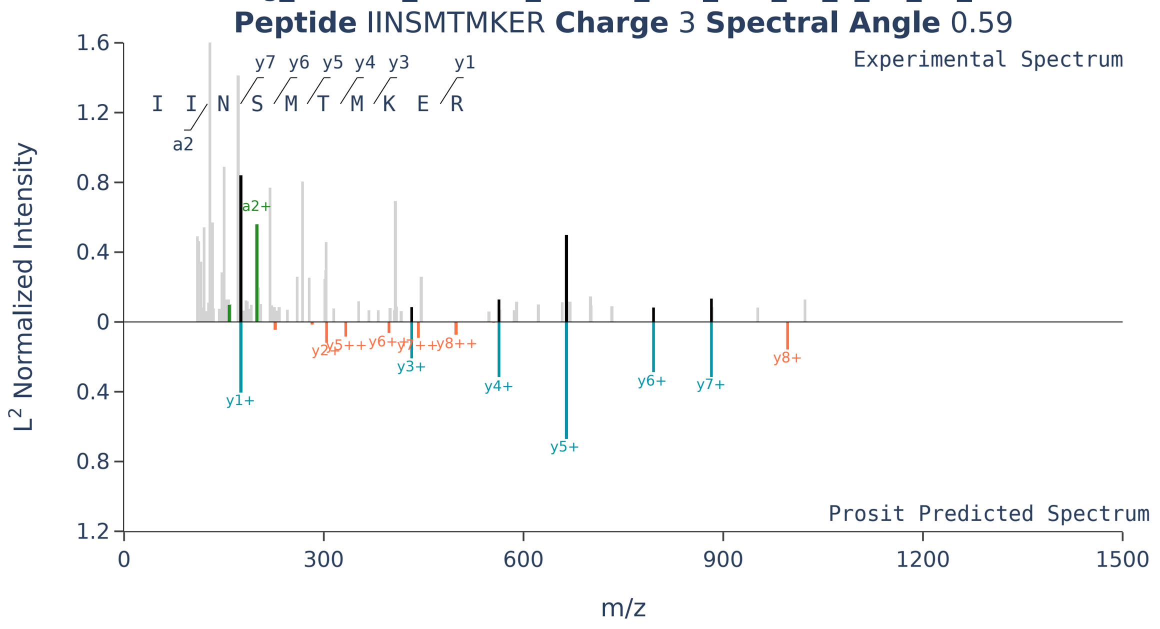


**Source** Ncheng_210623_230623_HFGoe_FFH_20S_25_1_A1_2h_R1 **Scan** 32952
**Peptide** SADVYRPAAIKQLETLAEQV **Charge** 2 **Spectral Angle** 0.79

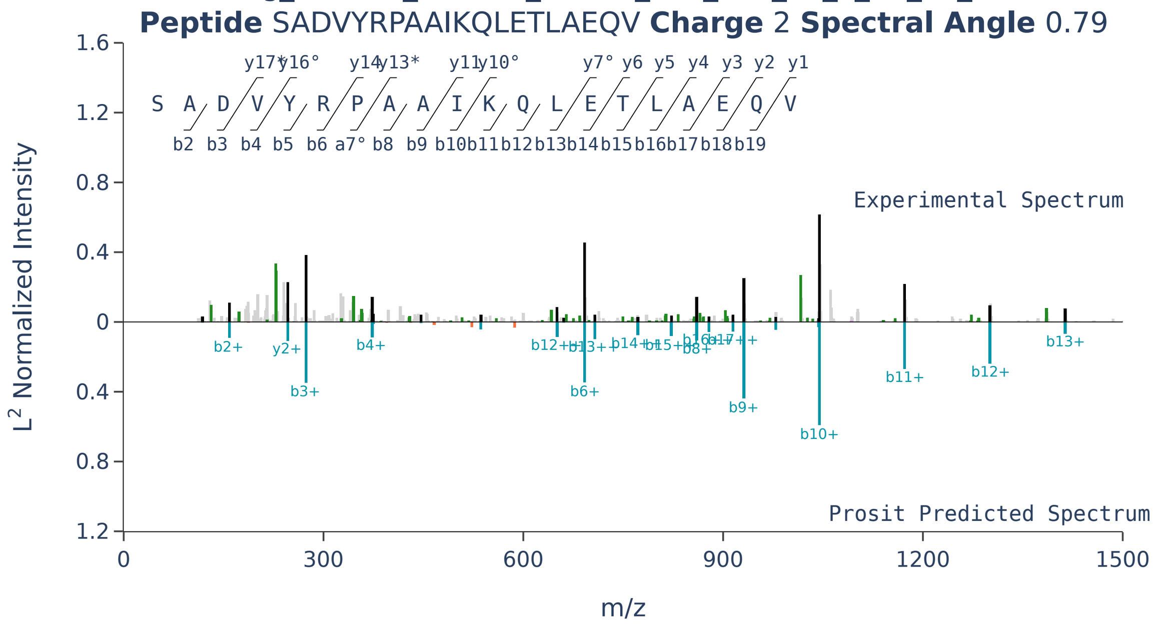

**Source** Ncheng_210623_230623_HFGoe_FFH_20S_25_1_A2_24h_R2 **Scan** 11675
**Peptide** ALLNSMTMKERAKPEIIKGSRKR **Charge** 6 **Spectral Angle** 0.67

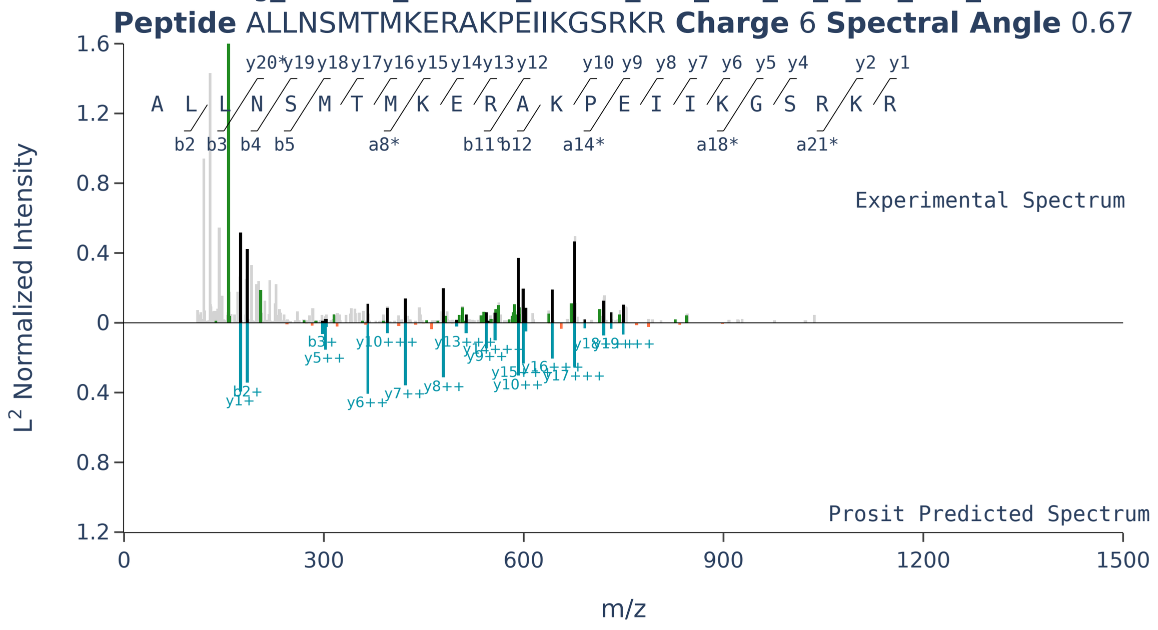

**Source** Ncheng_210623_230623_HFGoe_FFH_20S_25_1_A1_2h_R2 **Scan** 31106
**Peptide** AKLKFYDVLLVDTAGRLHVDE **Charge** 3 **Spectral Angle** 0.85

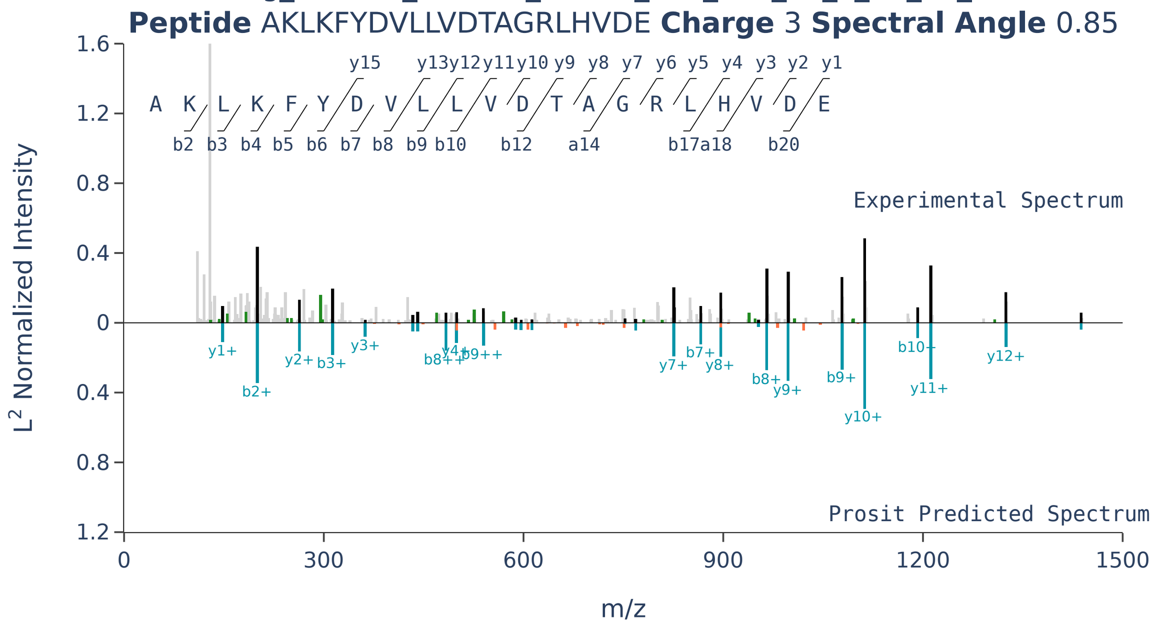

**Source** Ncheng_210623_230623_HFGoe_FFH_20S_25_1_A2_2h_R2 **Scan** 30680
**Peptide** GMASLMGKLPGMGQIPDNVKSQM **Charge** 3 **Spectral Angle** 0.79

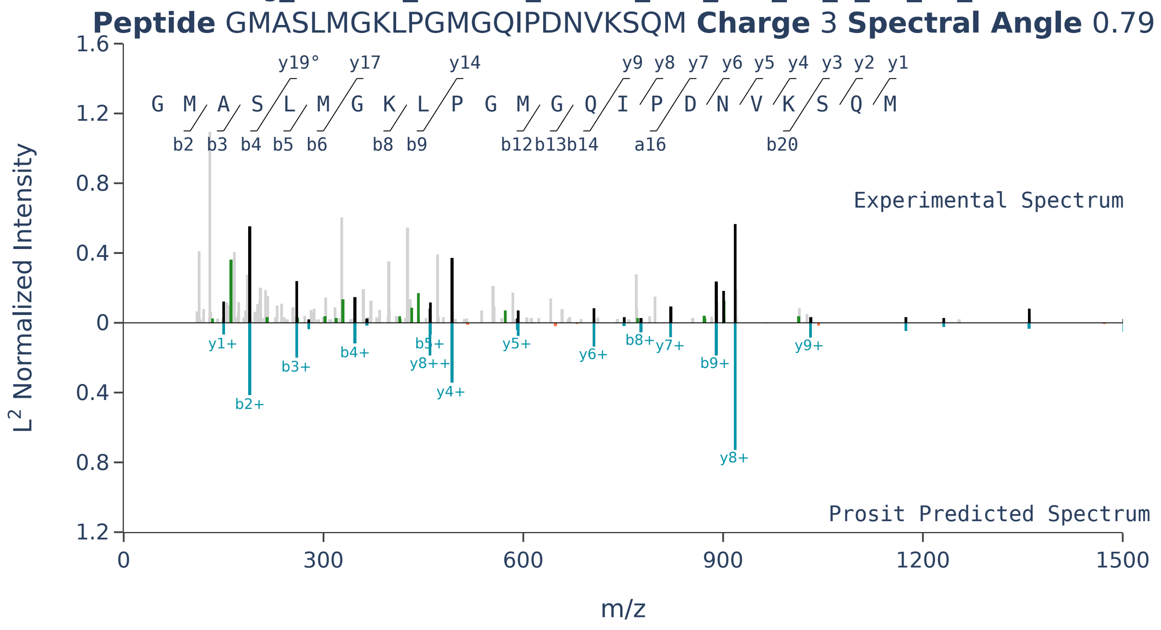

**Source** Ncheng_210623_230623_HFGoe_FFH_20S_25_1_A1_4h_R2 **Scan** 17553
**Peptide** TEALEPFHPDRIASR **Charge** 4 **Spectral Angle** 0.65

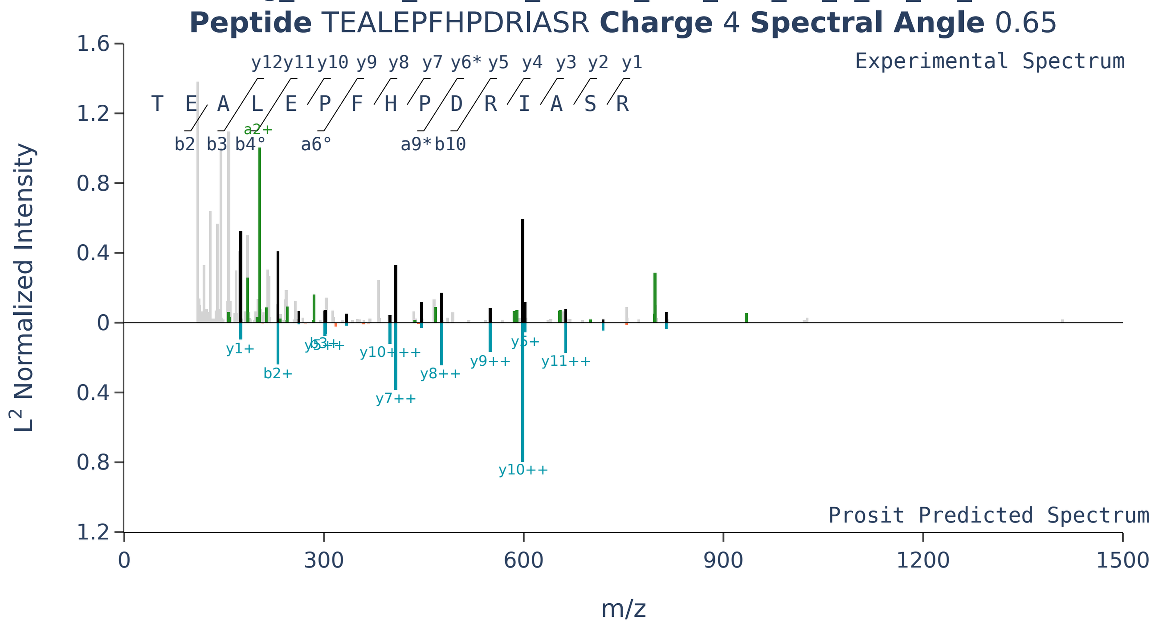

**Source** Ncheng_210623_230623_HFGoe_FFH_20S_25_1_A1_24h_R2 **Scan** 5288
**Peptide** KEKAVGHEV **Charge** 1 **Spectral Angle** 0.69

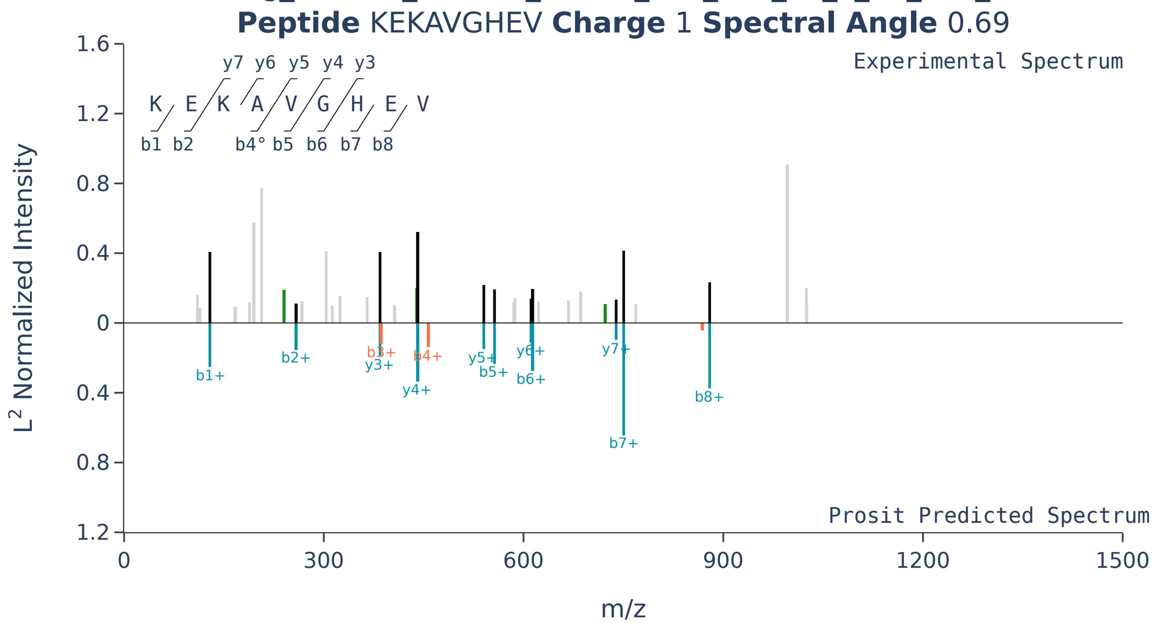

**Source** Ncheng_210623_230623_HFGoe_FFH_20S_25_1_A1_24h_R1 **Scan** 6826
**Peptide** IPDNVKSQ **Charge** 2 **Spectral Angle** 0.59

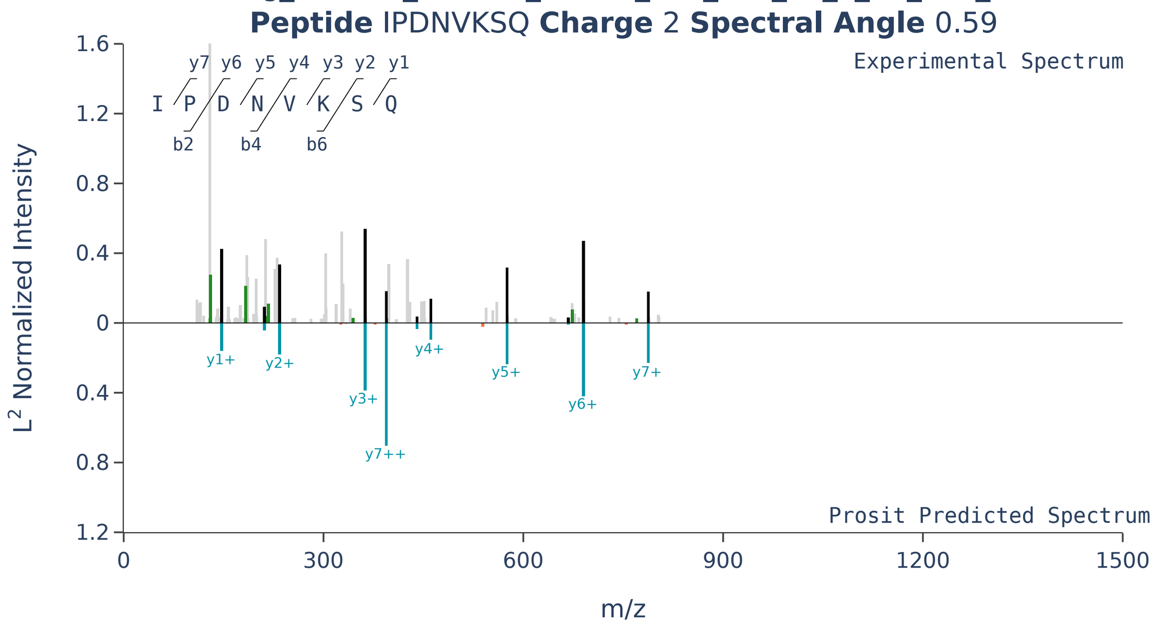

**Source** Ncheng_210623_230623_HFGoe_FFH_20S_25_1_A1_2h_R2 **Scan** 21558
**Peptide** KEAKLKFYDVL **Charge** 3 **Spectral Angle** 0.83

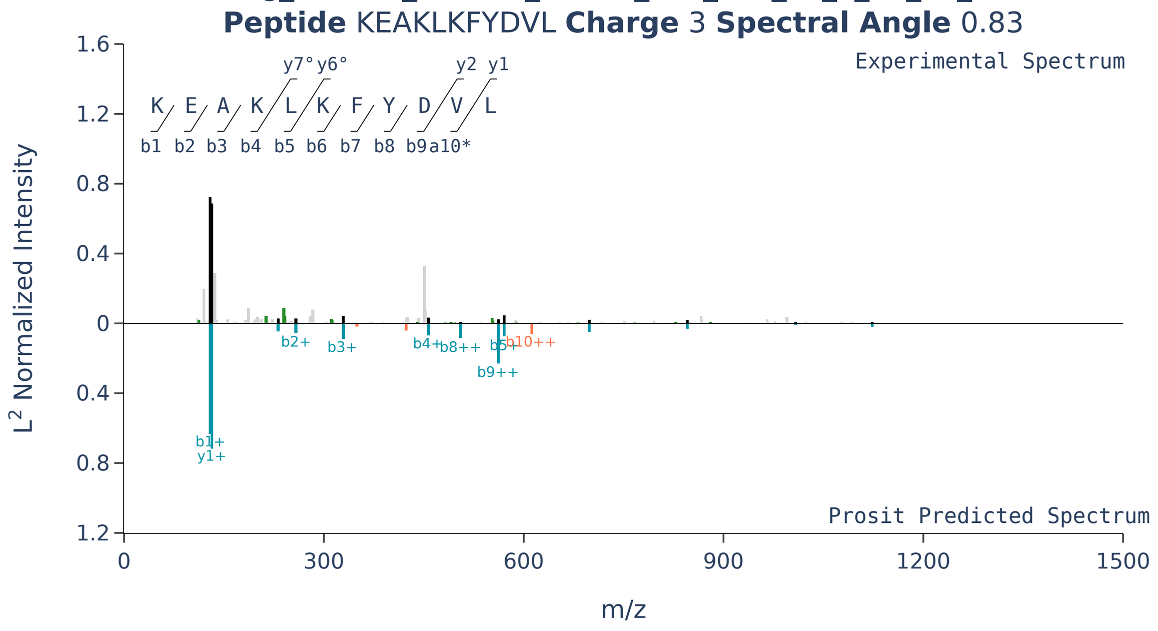

**Source** Ncheng_210623_230623_HFGoe_FFH_20S_25_1_A1_24h_R2 **Scan** 10830
**Peptide** KVDGDARGGAALSIR **Charge** 4 **Spectral Angle** 0.8

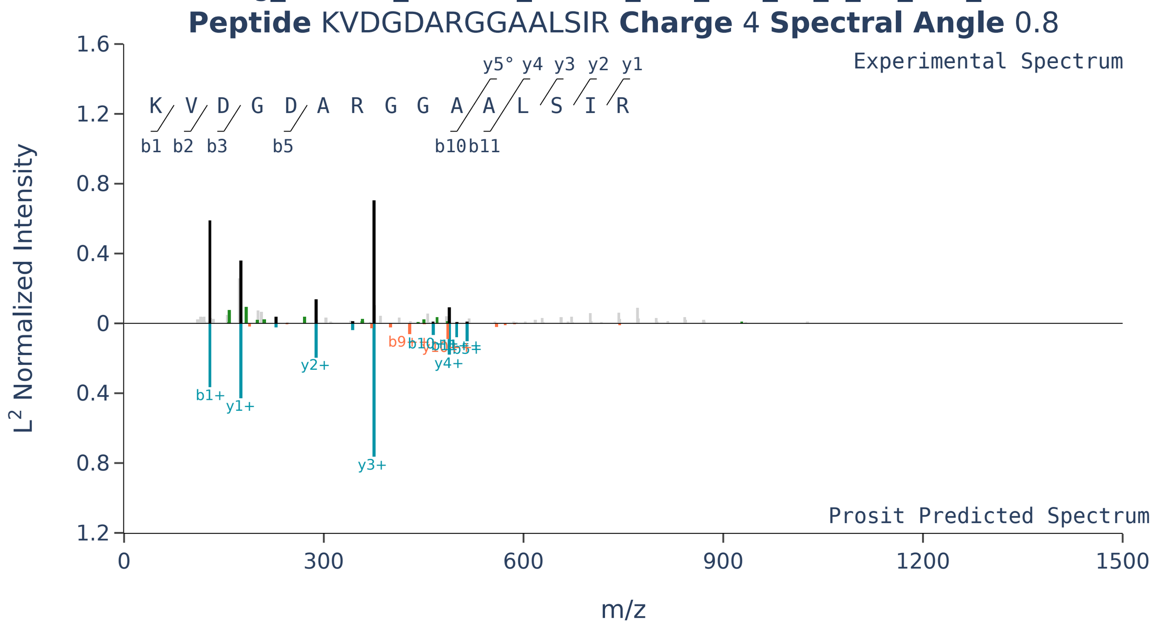

**Source** Ncheng_210623_230623_HFGoe_FFH_20S_25_1_A1_2h_R1 **Scan** 5727
**Peptide** SMTMKER **Charge** 2 **Spectral Angle** 0.85

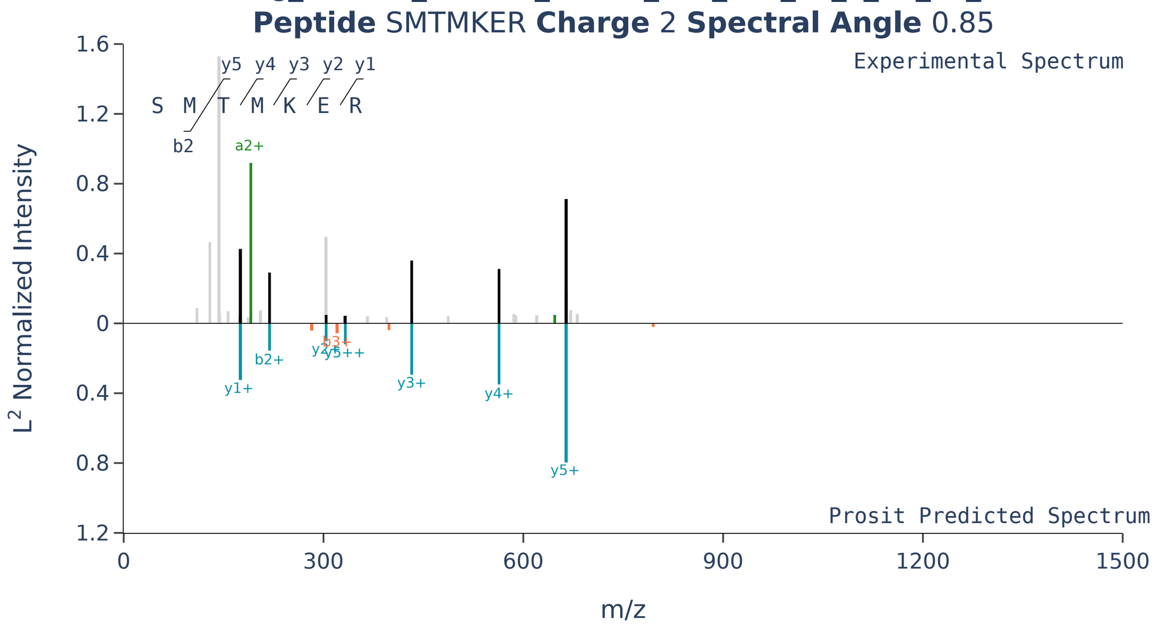

**Source** Ncheng_210623_230623_HFGoe_FFH_20S_25_1_A1_2h_R2 **Scan** 29474
**Peptide** ALPLTGVVLTKVDGDARGGAALSIRHI **Charge** 4 **Spectral Angle** 0.7

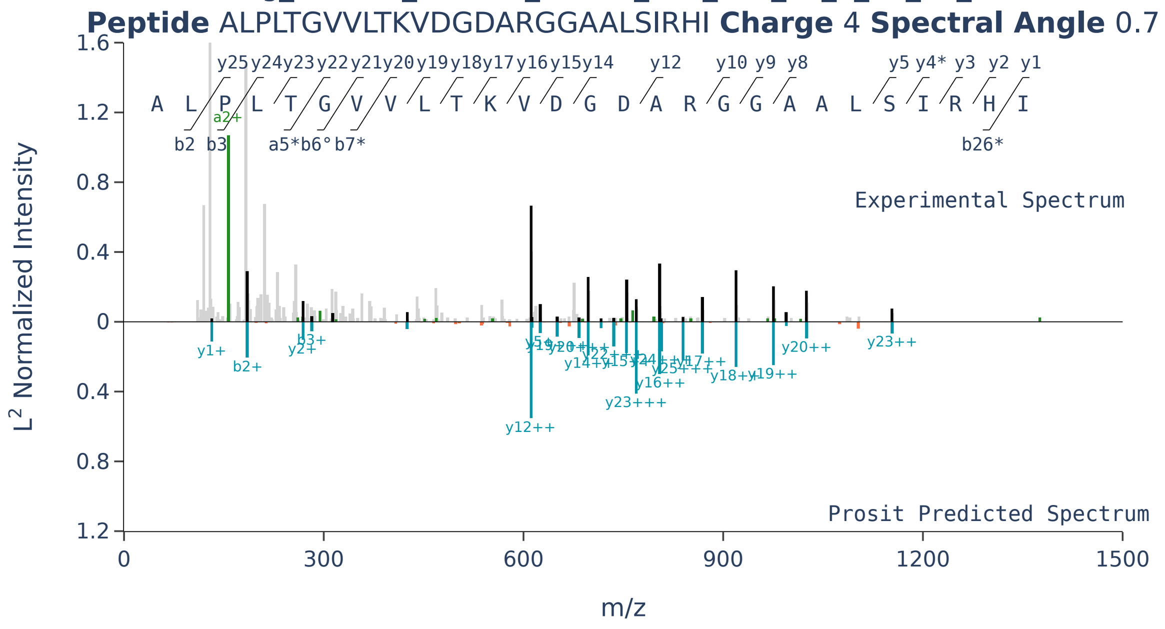

**Source** Ncheng_210623_230623_HFGoe_FFH_20S_25_1_A2_4h_R2 **Scan** 33392
**Peptide** NKSLTPGQEFVKIVRNELVAAM **Charge** 3 **Spectral Angle** 0.86

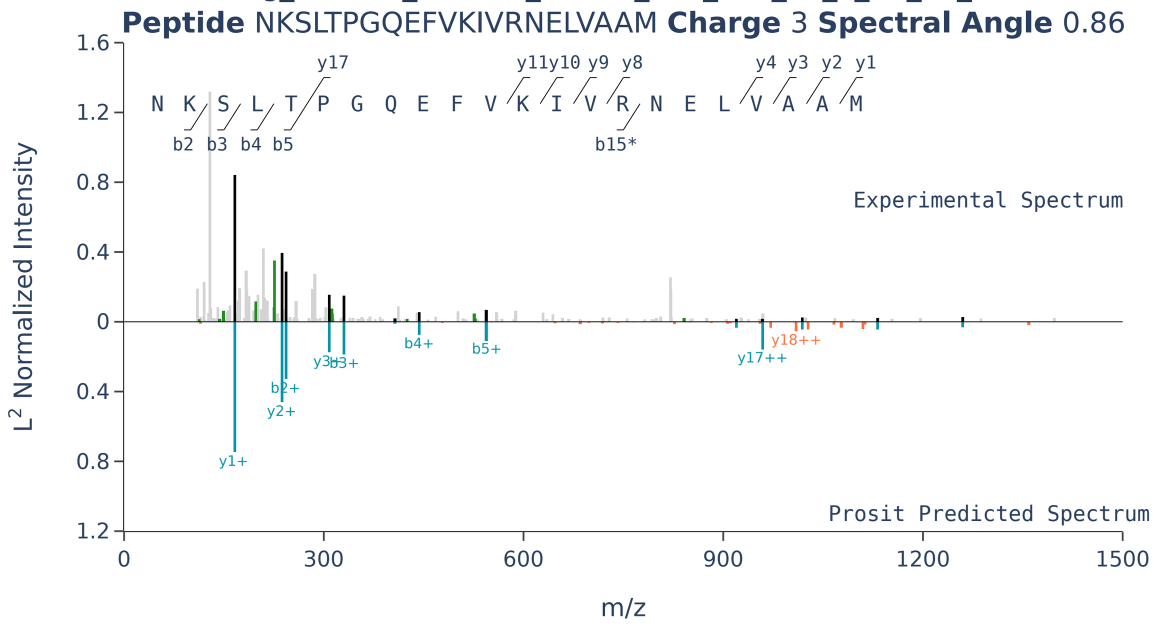

**Source** Ncheng_210623_230623_HFGoe_FFH_20S_25_1_A1_24h_R2 **Scan** 8980
**Peptide** VYRPAAIKQ **Charge** 2 **Spectral Angle** 0.69

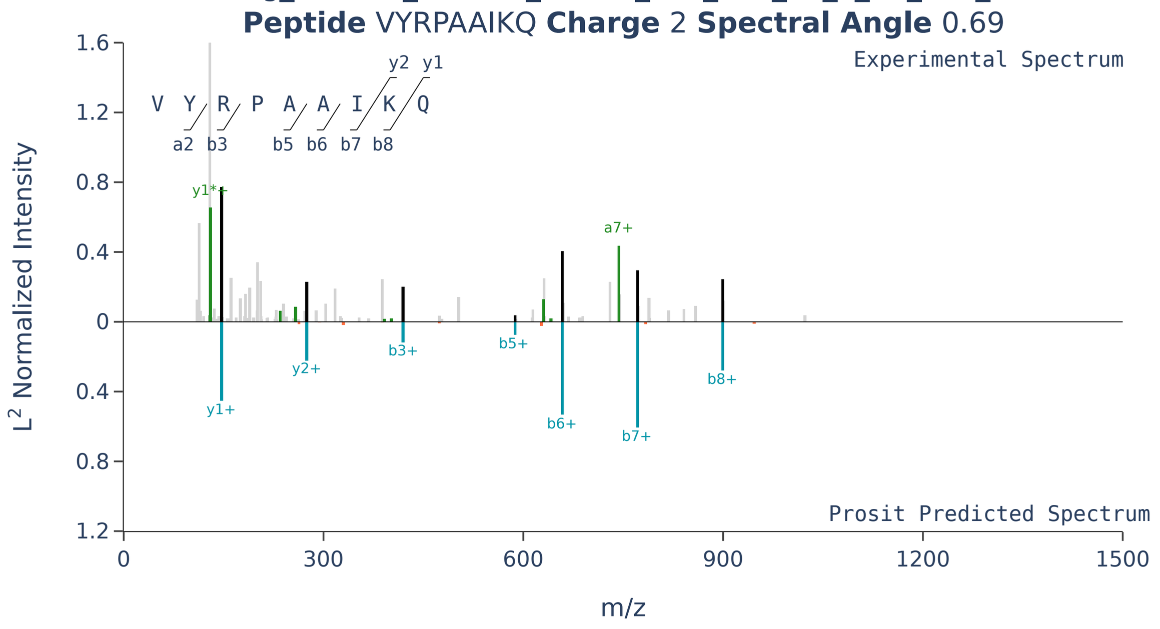

**Source** Ncheng_210623_230623_HFGoe_FFH_20S_25_1_A1_2h_R2 **Scan** 29944
**Peptide** KEAKLKFYDVLLVDTAGRLHVD **Charge** 3 **Spectral Angle** 0.74

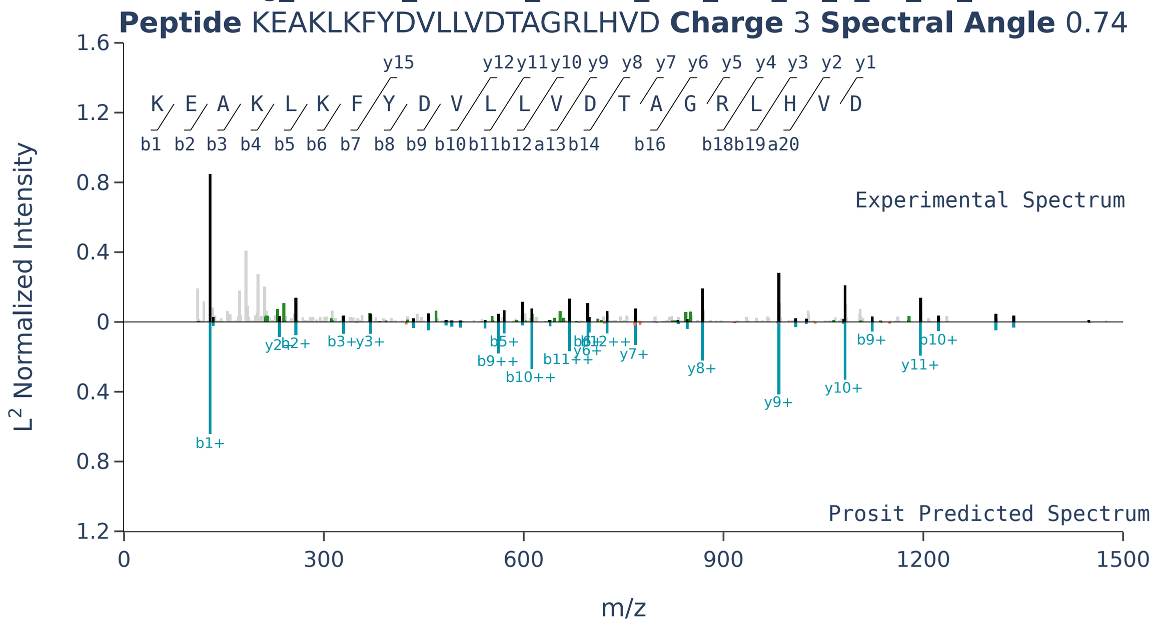

**Source** Ncheng_210623_230623_HFGoe_FFH_20S_25_1_A1_2h_R1 **Scan** 25223
**Peptide** LMGKLPGMGQIPDNVKSQM **Charge** 3 **Spectral Angle** 0.66

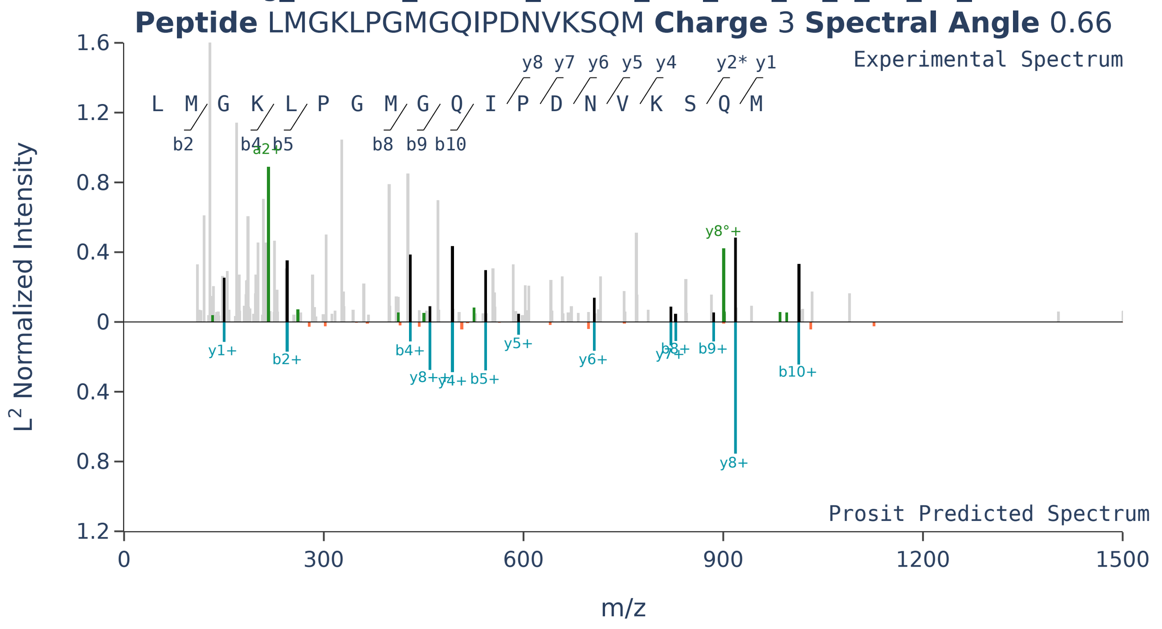

**Source** Ncheng_210623_230623_HFGoe_FFH_20S_25_1_A1_24h_R1 **Scan** 13713
**Peptide** VSADVYRPAAIKQ **Charge** 2 **Spectral Angle** 0.69

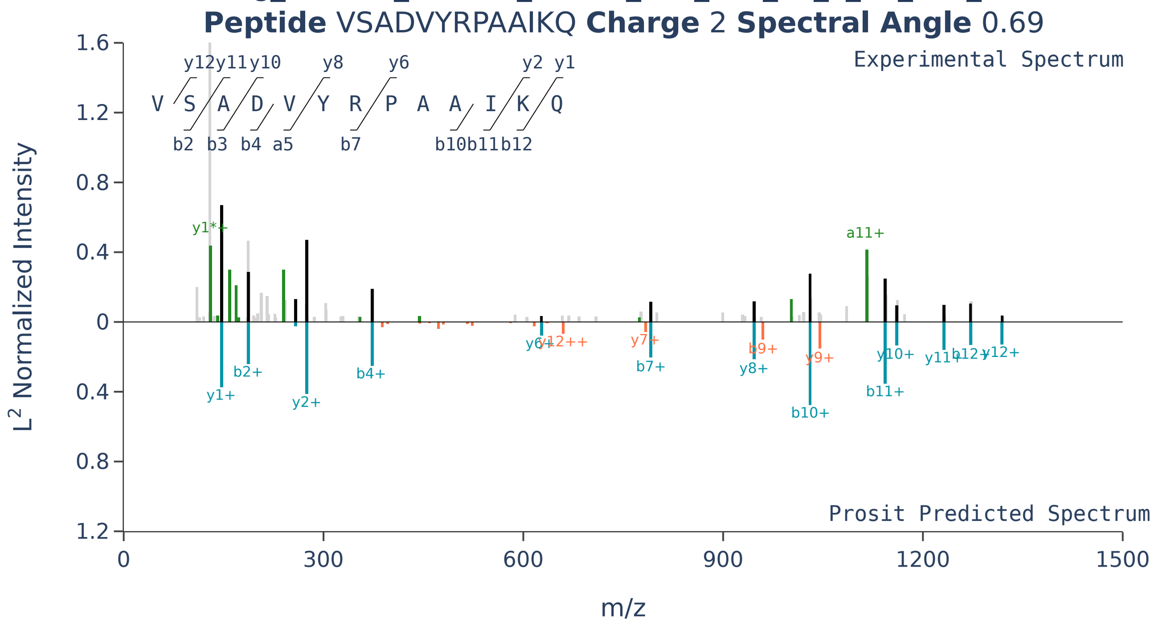

**Source** Ncheng_210623_230623_HFGoe_FFH_20S_25_1_A1_4h_R1 **Scan** 20686
**Peptide** VGHEVNKSLTPGQEFVKL **Charge** 4 **Spectral Angle** 0.77

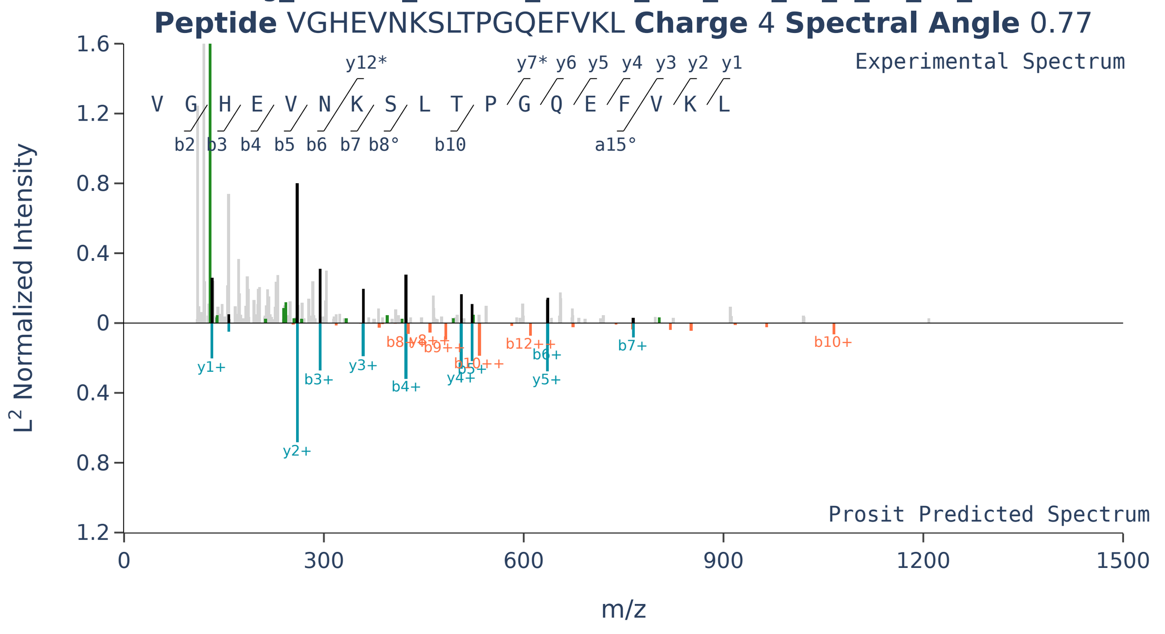


**Source** Ncheng_210623_230623_HFGoe_FFH_20S_25_1_A1_2h_R1 **Scan** 21582
**Peptide** GVGEKTEALEPFHPDRIASRL **Charge** 4 **Spectral Angle** 0.66

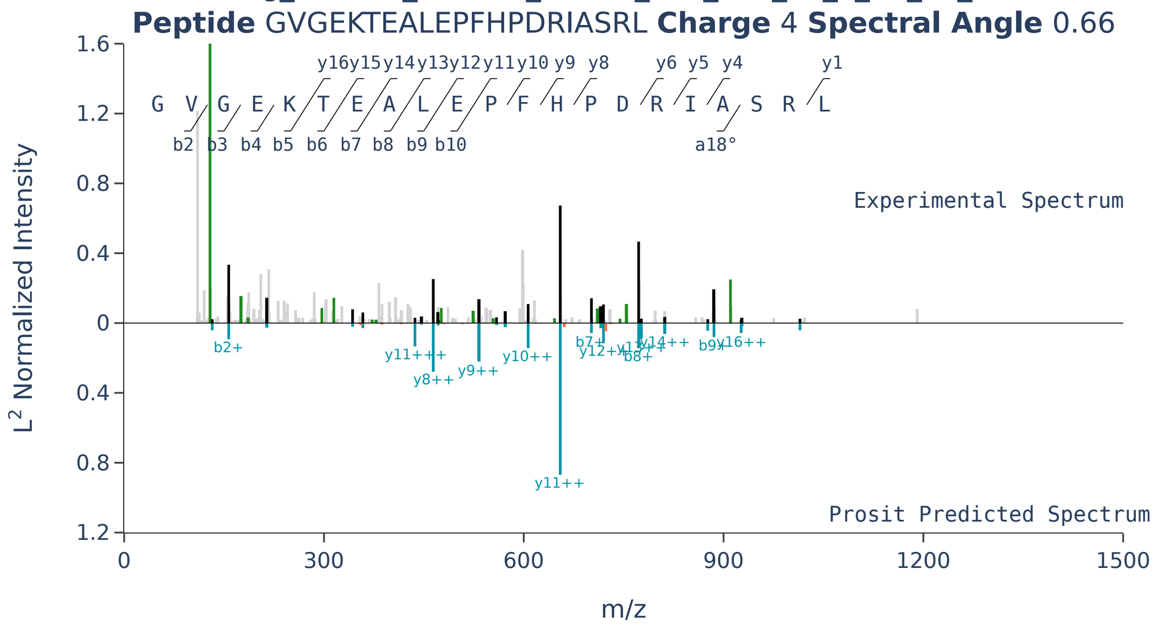


**Source** Ncheng_210623_230623_HFGoe_FFH_20S_25_1_A2_24h_R1 **Scan** 7302
**Peptide** GKTTSVGKLG **Charge** 2 **Spectral Angle** 0.72

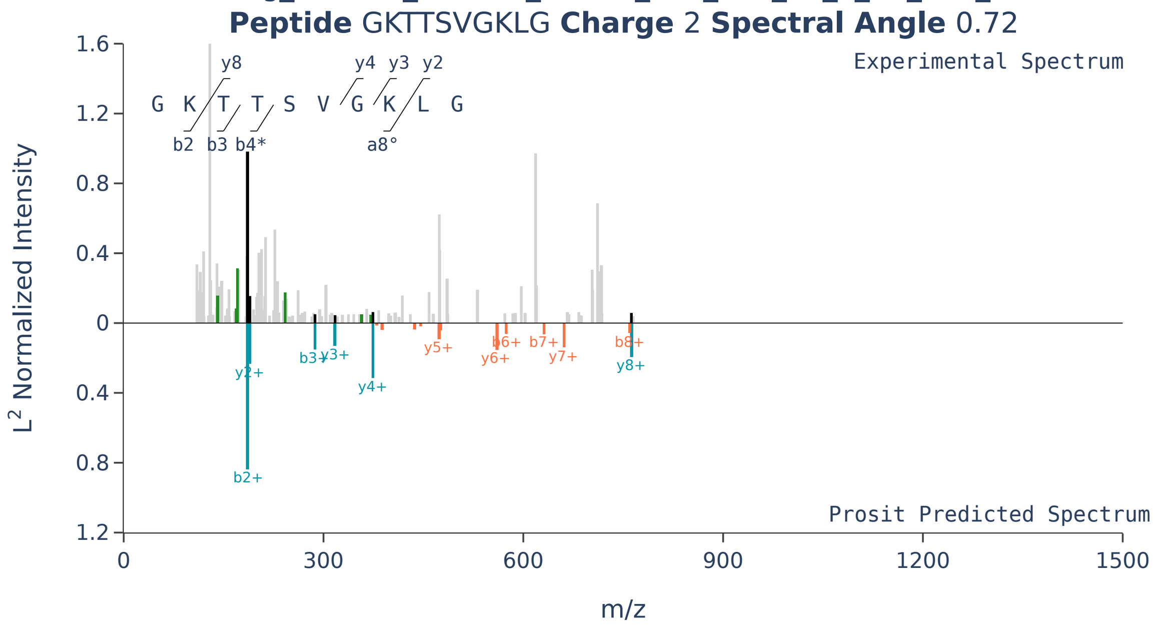


**Source** Ncheng_210623_230623_HFGoe_FFH_20S_25_1_A1_2h_R2 **Scan** 10564
**Peptide** DEIKQVHASIN **Charge** 3 **Spectral Angle** 0.76

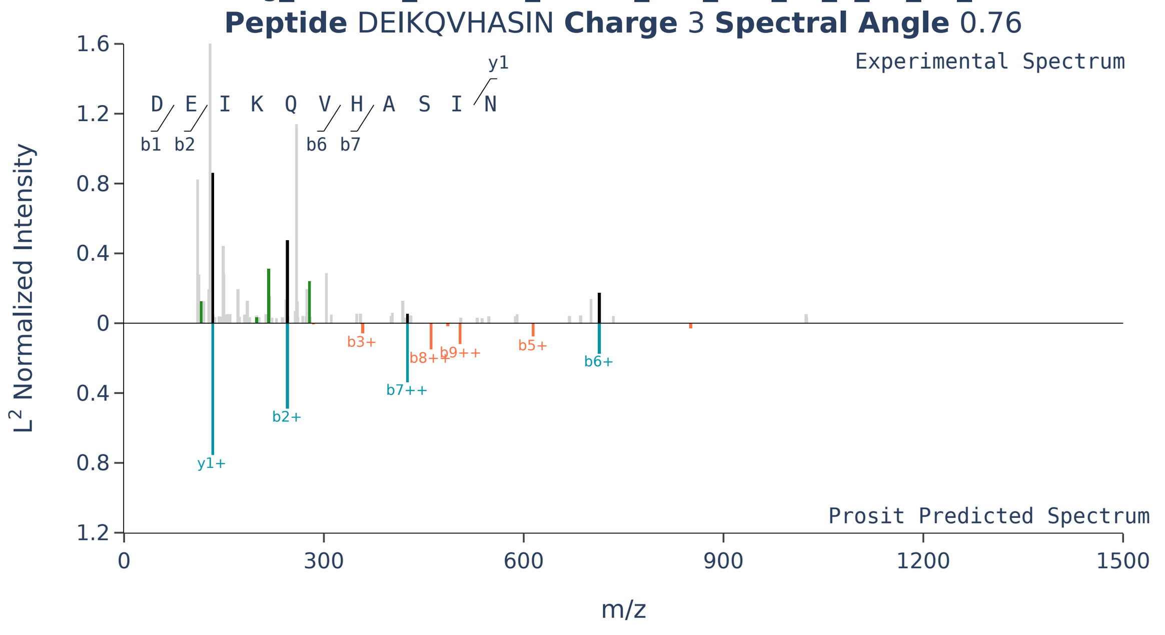


**Source** Ncheng_210623_230623_HFGoe_FFH_20S_25_1_A1_24h_R1 **Scan** 15525
**Peptide** VLTKVDGDARGGAALSLR **Charge** 4 **Spectral Angle** 0.71

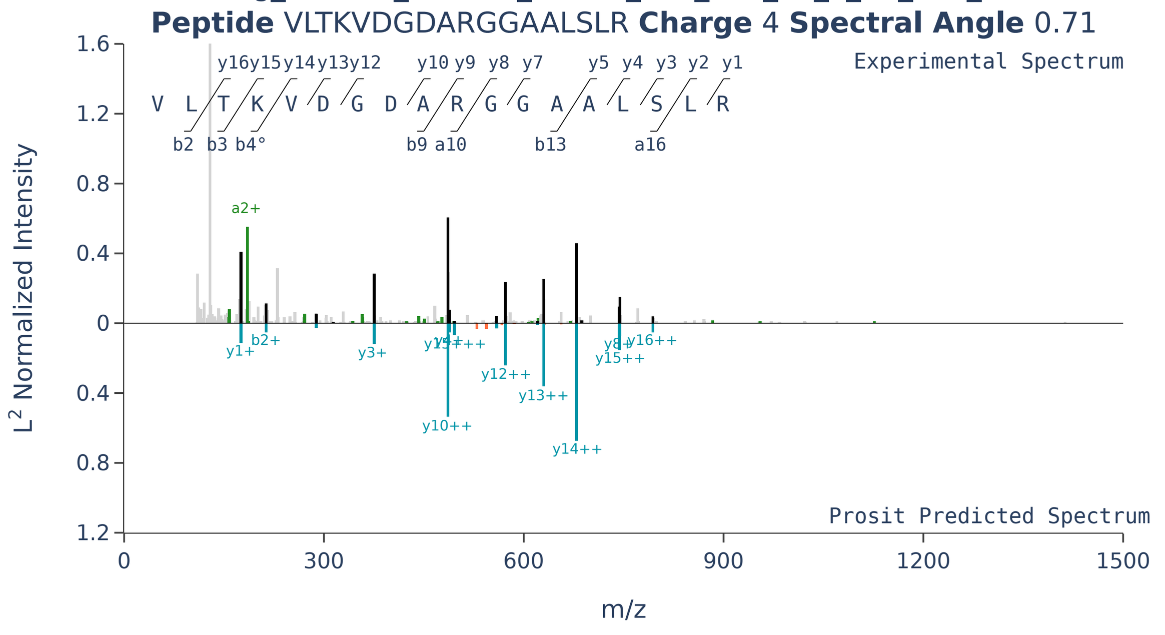


**Source** Ncheng_210623_230623_HFGoe_FFH_20S_25_1_A1_4h_R2 **Scan** 18279
**Peptide** REFINRVKEKAVGHEVNKSLTPGQEFVKL **Charge** 5 **Spectral Angle** 0.69

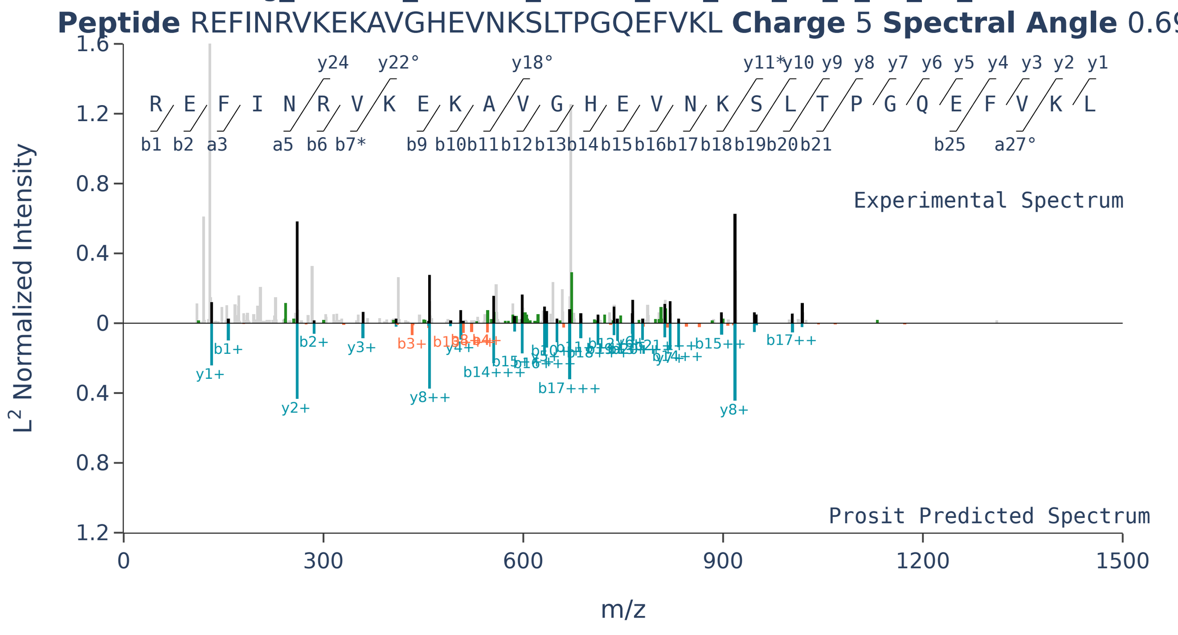


**Source** Ncheng_210623_230623_HFGoe_FFH_20S_25_1_A2_4h_R2 **Scan** 13682
**Peptide** TKVDGDARGGAALSIRHL **Charge** 5 **Spectral Angle** 0.85

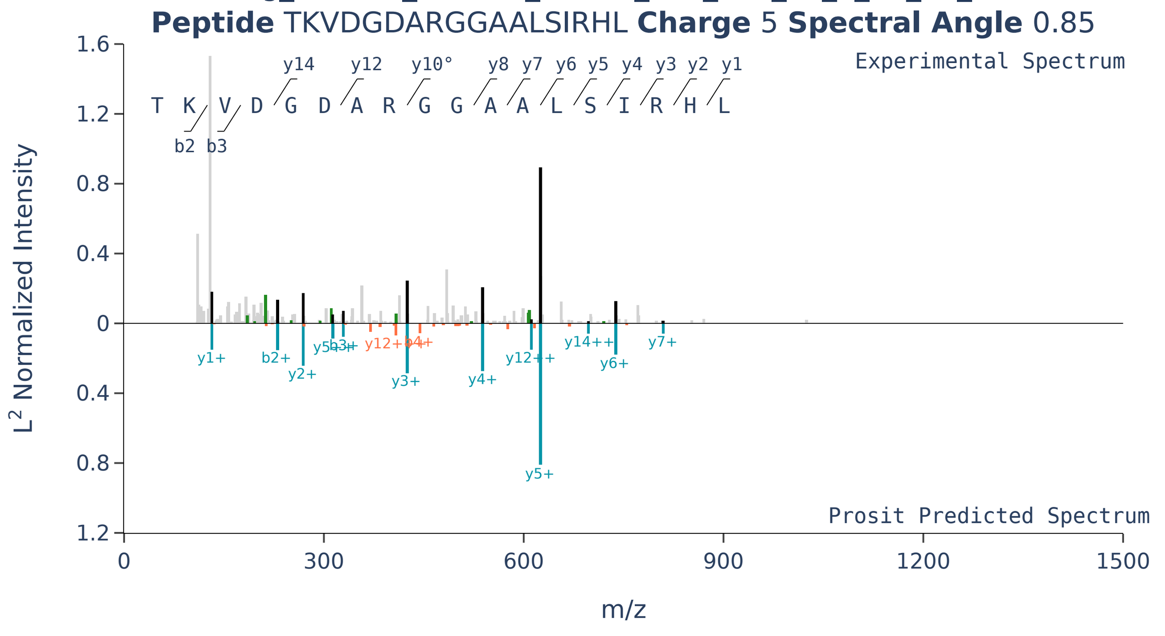


**Source** Ncheng_210623_230623_HFGoe_FFH_20S_25_1_A1_2h_R2 **Scan** 34012
**Peptide** EADVALPVVREFINRVKE **Charge** 3 **Spectral Angle** 0.71

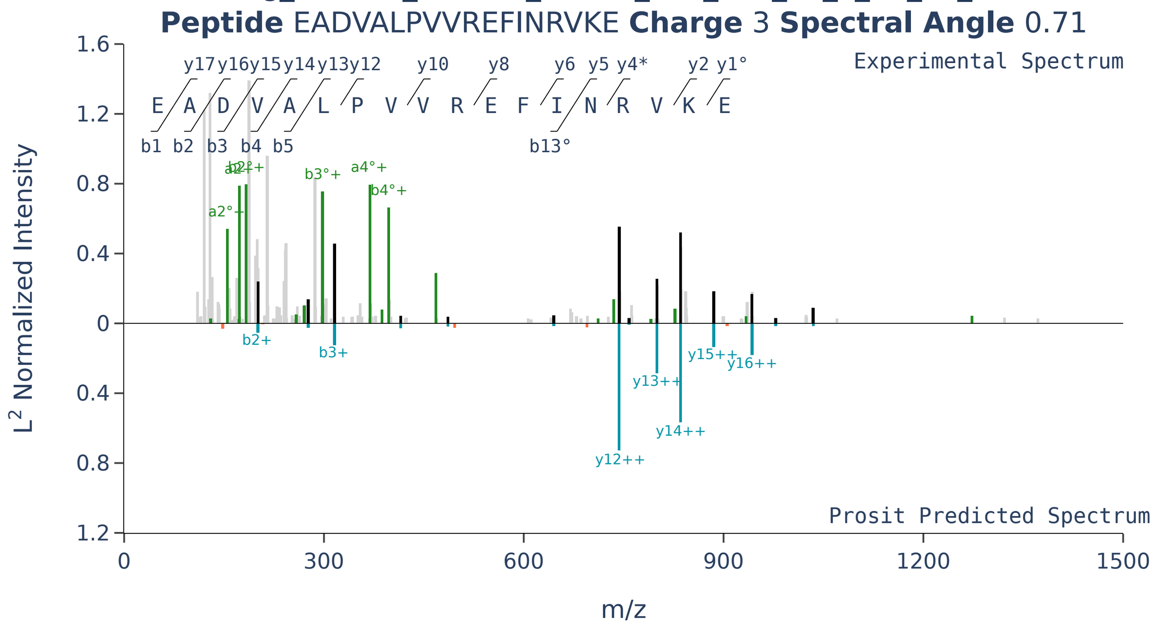


**Source** Ncheng_210623_230623_HFGoe_FFH_20S_25_1_A1_2h_R2 **Scan** 30957
**Peptide** VGQKPVDIVNAALKEAKL **Charge** 2 **Spectral Angle** 0.71

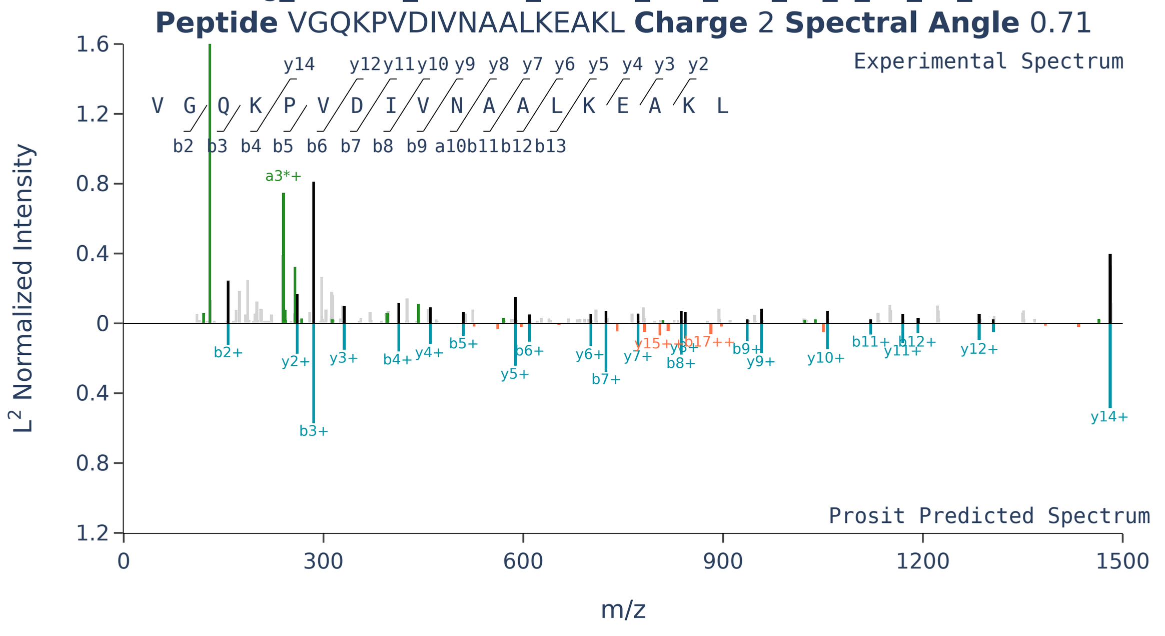


**Source** Ncheng_210623_230623_HFGoe_FFH_20S_25_1_A2_2h_R1 **Scan** 23838
**Peptide** TGKPIKFLGVGEKTEALEPFHPDRI **Charge** 3 **Spectral Angle** 0.85

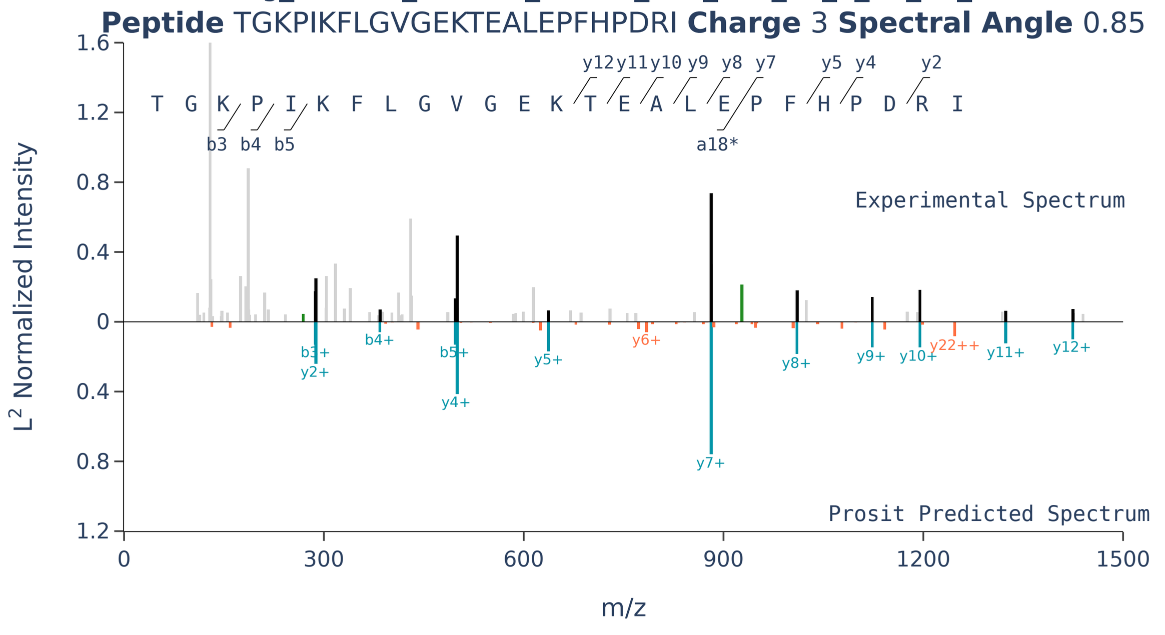


**Source** Ncheng_210623_230623_HFGoe_FFH_20S_25_1_A2_2h_R2 **Scan** 7734
**Peptide** KVDGDARGGAAL **Charge** 1 **Spectral Angle** 0.92

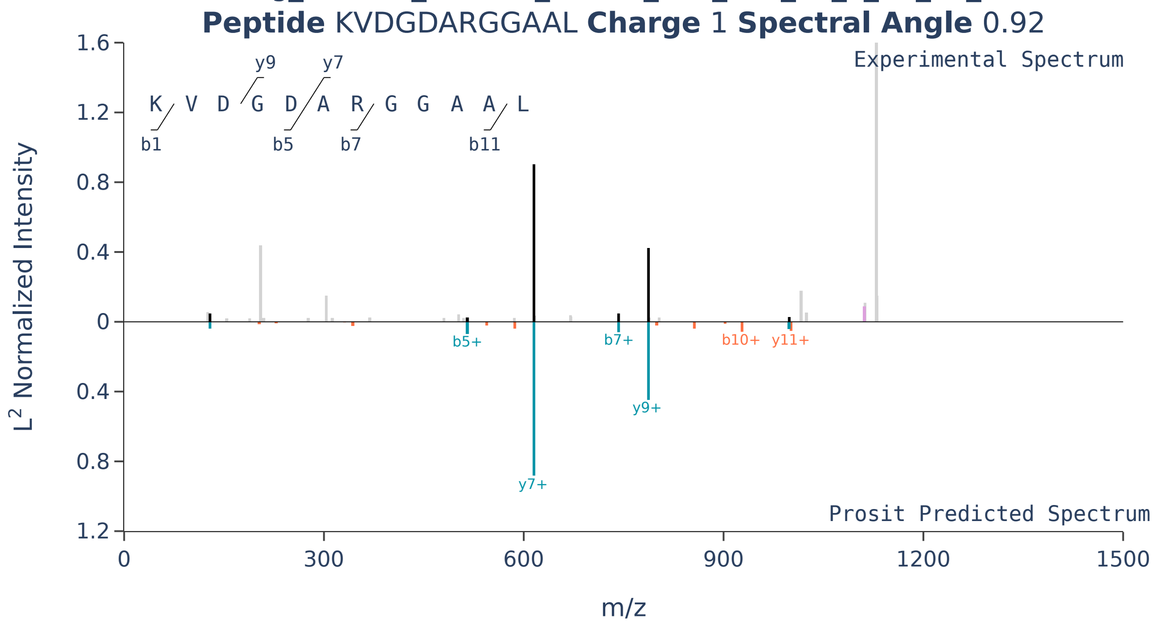


**Source** Ncheng_210623_230623_HFGoe_FFH_20S_25_1_A2_4h_R1 **Scan** 14353
**Peptide** AALKEAKLKF **Charge** 3 **Spectral Angle** 0.7

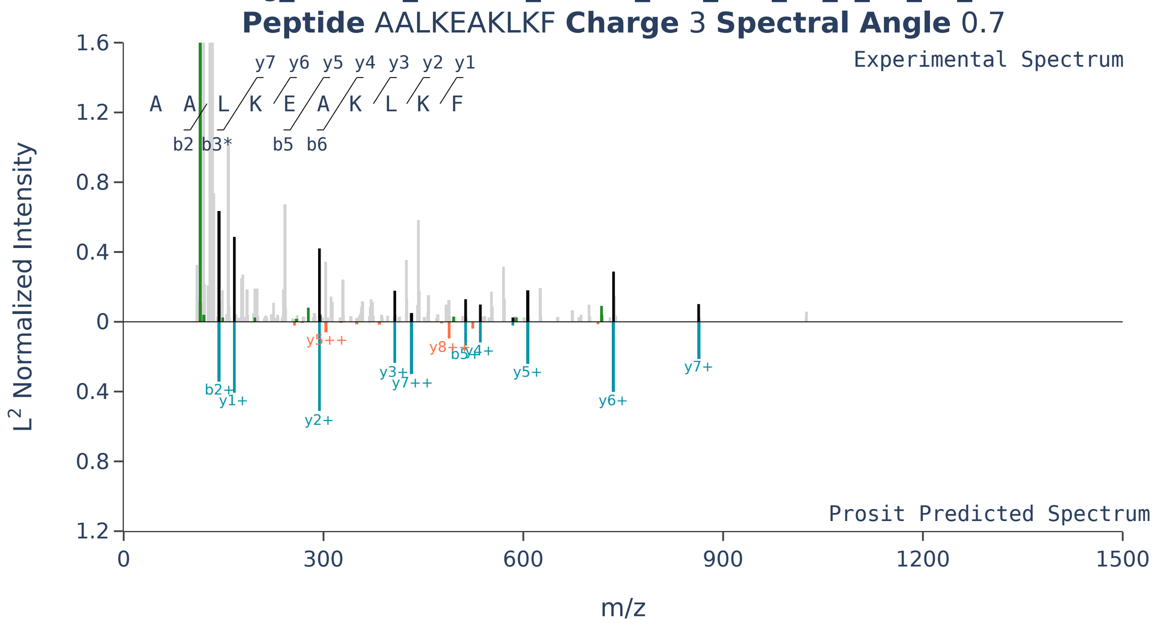


**Source** Ncheng_210623_230623_HFGoe_FFH_20S_25_1_A1_24h_R2 **Scan** 7231
**Peptide** KEKAVGHEVNKSLTPGQE **Charge** 2 **Spectral Angle** 0.87

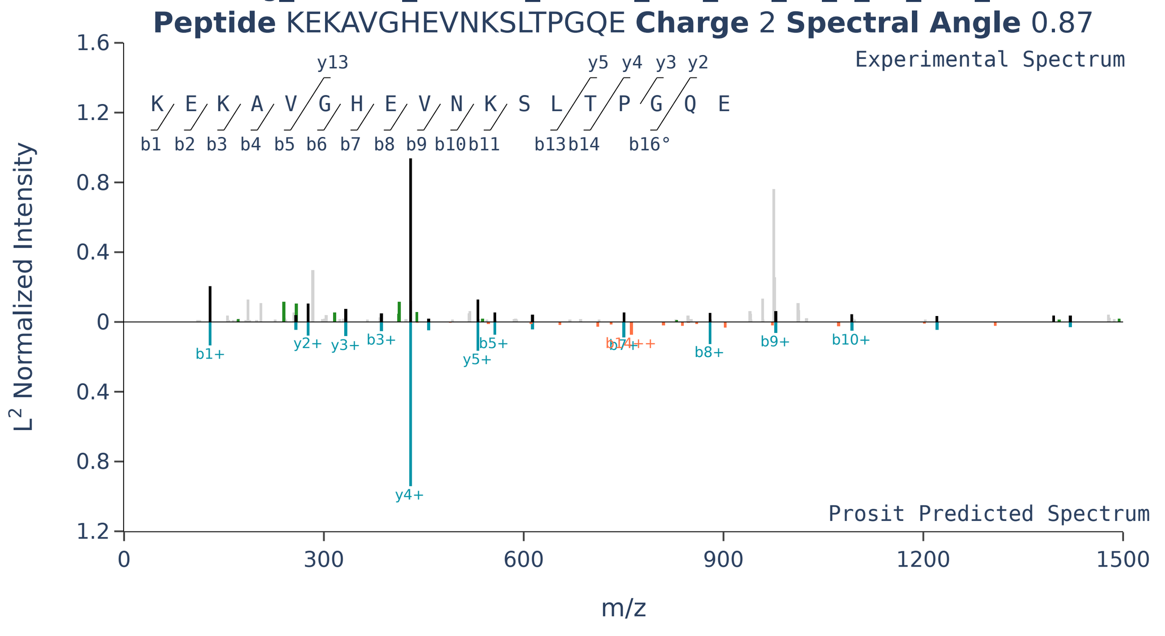


**Source** Ncheng_210623_230623_HFGoe_FFH_20S_25_1_A2_2h_R2 **Scan** 11433
**Peptide** SADVYRPAAIK **Charge** 2 **Spectral Angle** 0.68

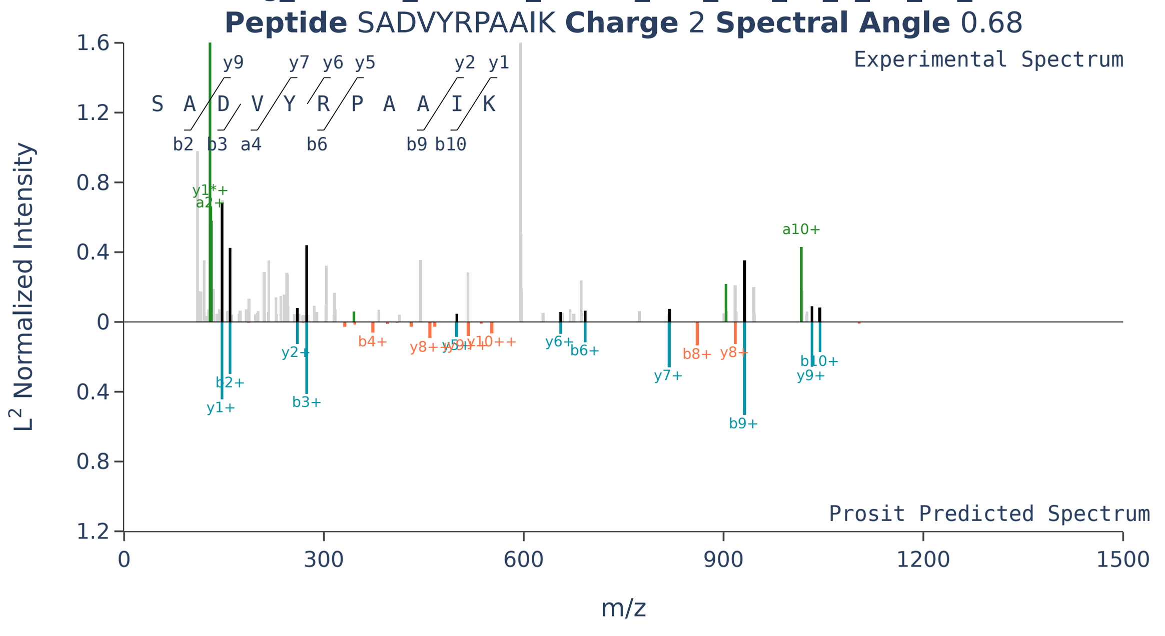


**Source** Ncheng_210623_230623_HFGoe_FFH_20S_25_1_A2_4h_R1 **Scan** 6297
**Peptide** SMTMKERA **Charge** 2 **Spectral Angle** 0.79

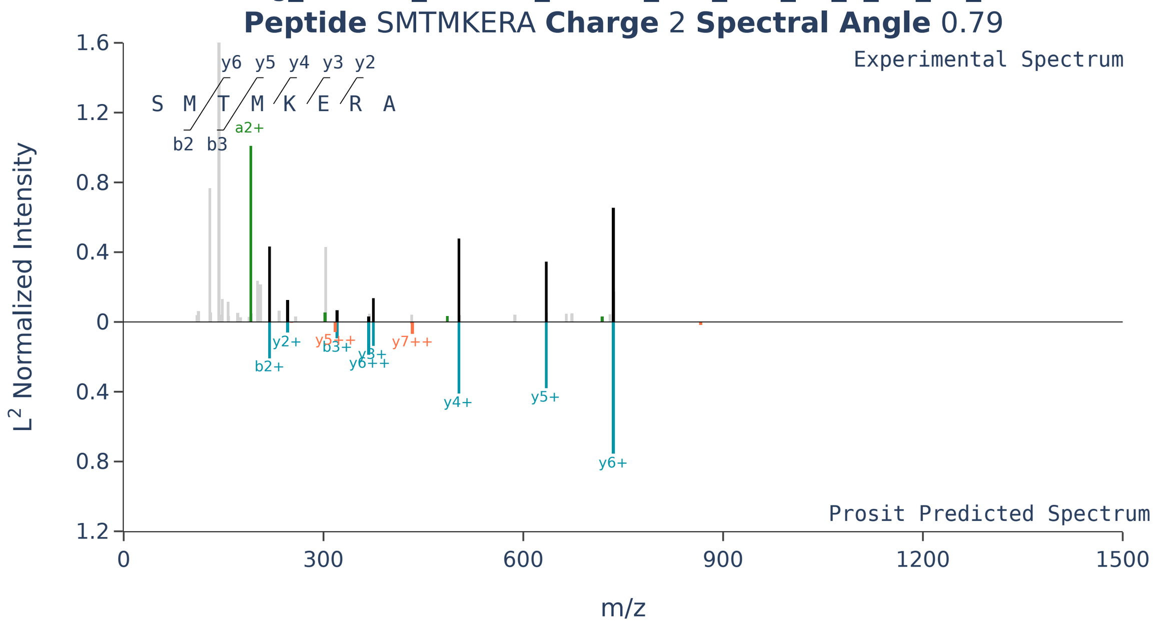

**Source** Ncheng_210623_230623_HFGoe_FFH_20S_25_1_A2_2h_R1 **Scan** 26570
**Peptide** DEAMMDEIKQVHASINPVET **Charge** 3 **Spectral Angle** 0.74

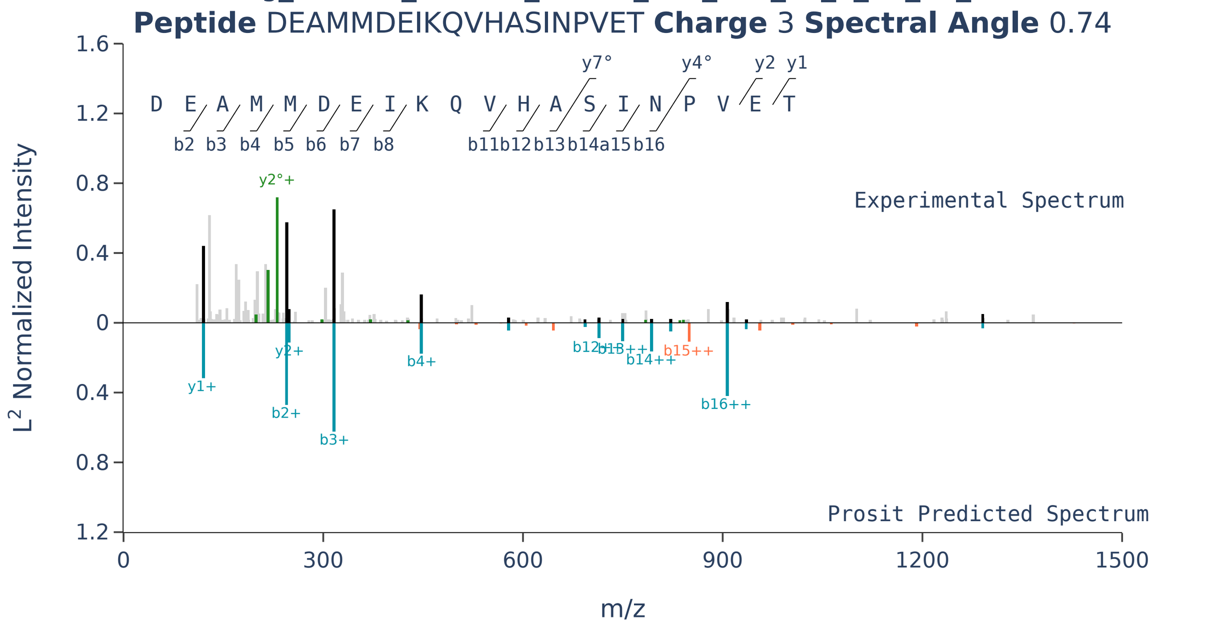


**Source** Ncheng_210623_230623_HFGoe_FFH_20S_25_1_A1_24h_R2 **Scan** 5723
**Peptide** AKPEIIKGSRK **Charge** 2 **Spectral Angle** 0.57

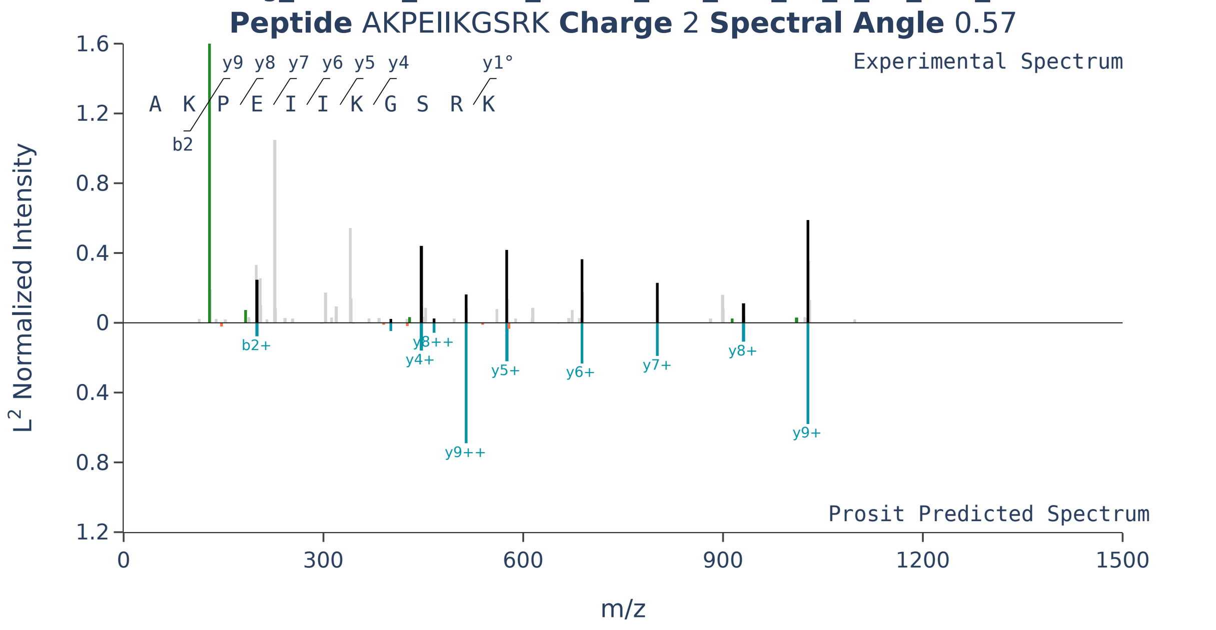


**Source** Ncheng_210623_230623_HFGoe_FFH_20S_25_1_A2_4h_R1 **Scan** 11788
**Peptide** DVNRLLKQ **Charge** 2 **Spectral Angle** 0.68

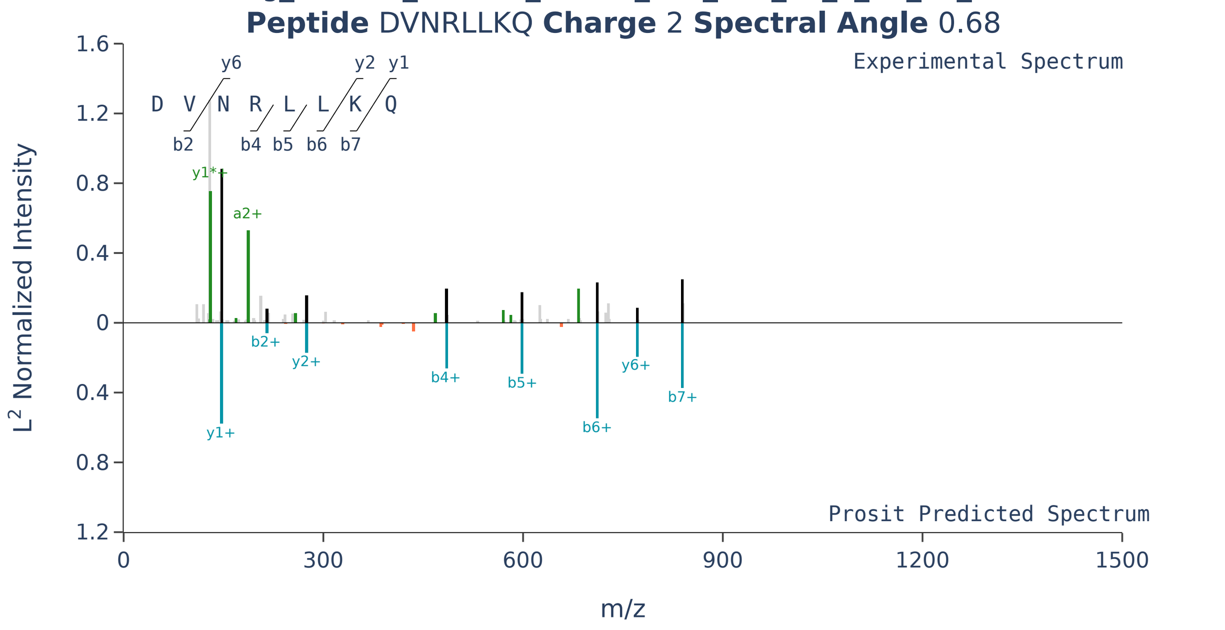


**Source** Ncheng_210623_230623_HFGoe_FFH_20S_25_1_A1_4h_R1 **Scan** 26787
**Peptide** GVGEKTEALEPFHPDRIASRILG **Charge** 4 **Spectral Angle** 0.88

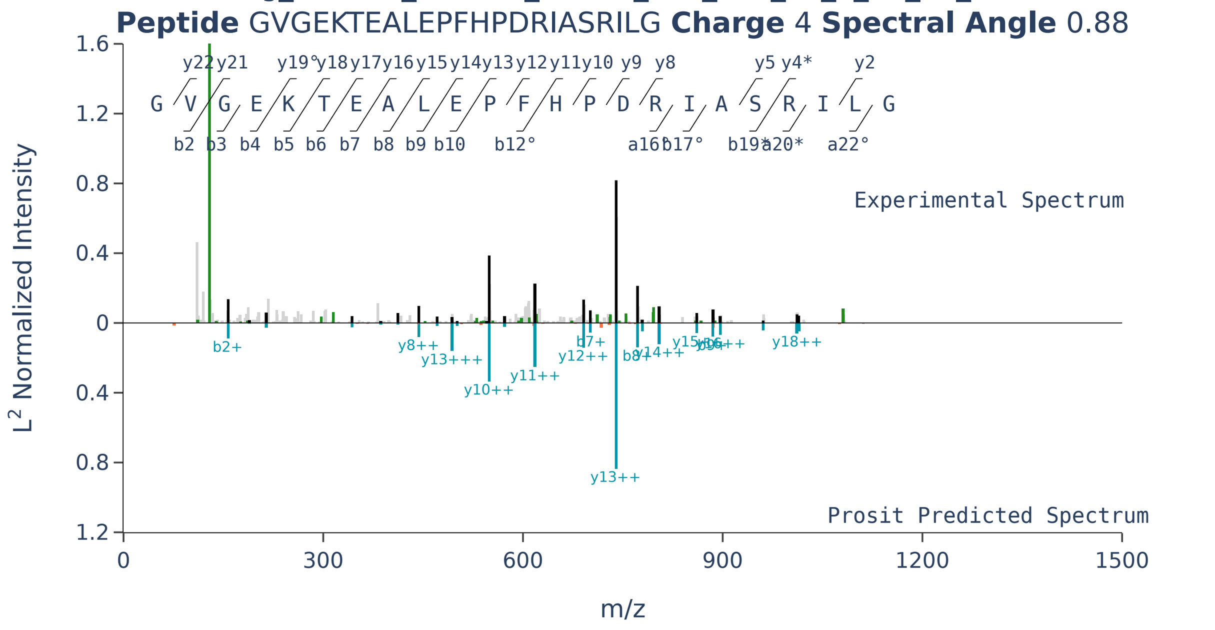


**Source** Ncheng_210623_230623_HFGoe_FFH_20S_25_1_A2_24h_R2 **Scan** 19922
**Peptide** LGVGEKTEALEPFHPDRIASR **Charge** 5 **Spectral Angle** 0.8

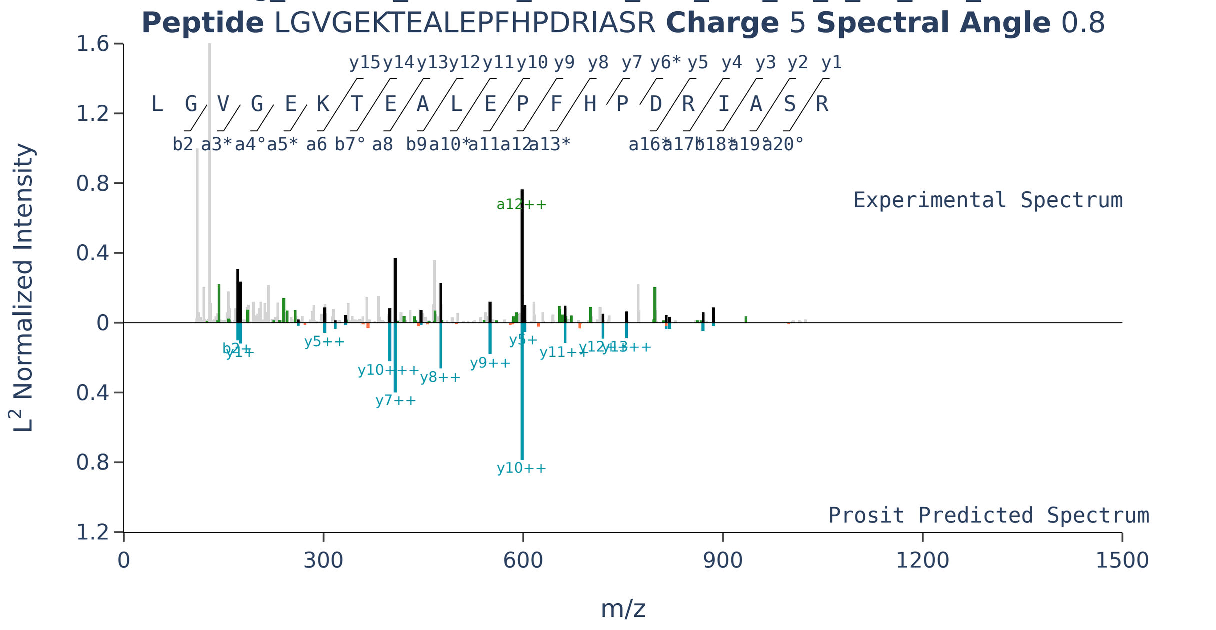


**Source** Ncheng_210623_230623_HFGoe_FFH_20S_25_1_A2_4h_R2 **Scan** 28877
**Peptide** EFVKIVRNELVAAM **Charge** 2 **Spectral Angle** 0.68

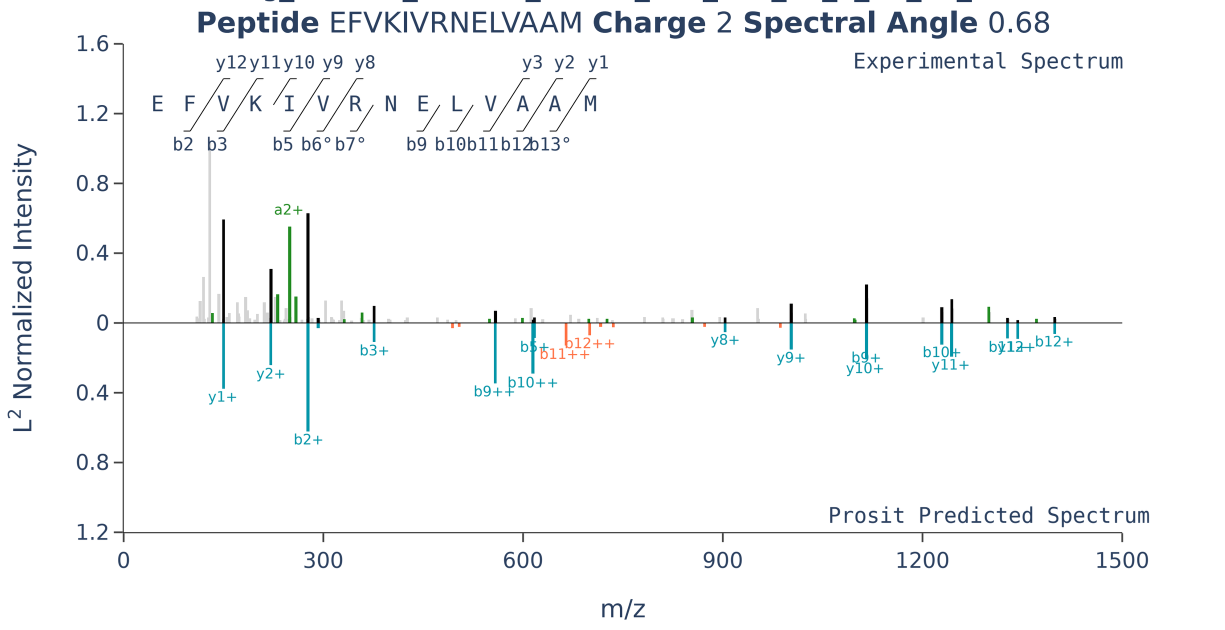


**Source** Ncheng_210623_230623_HFGoe_FFH_20S_25_1_A1_4h_R1 **Scan** 10066
**Peptide** EFINRVKEKAVGH **Charge** 2 **Spectral Angle** 0.83

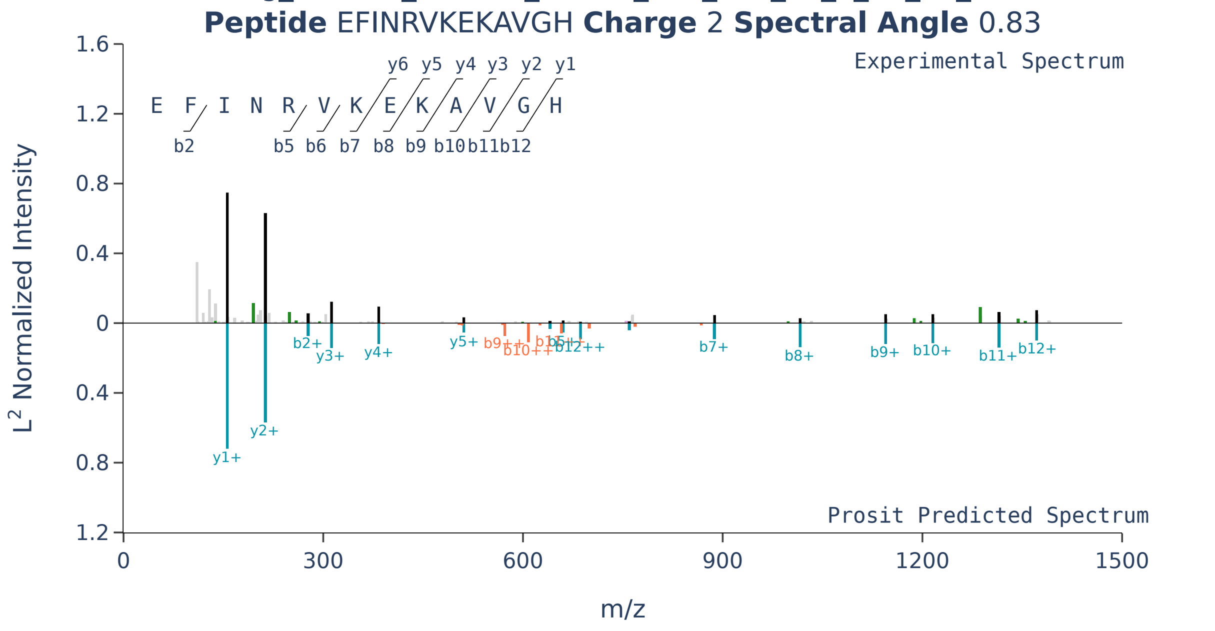


**Source** Ncheng_210623_230623_HFGoe_FFH_20S_25_1_A1_4h_R1 **Scan** 8403
**Peptide** KKGGMAKMMRSM **Charge** 2 **Spectral Angle** 0.7

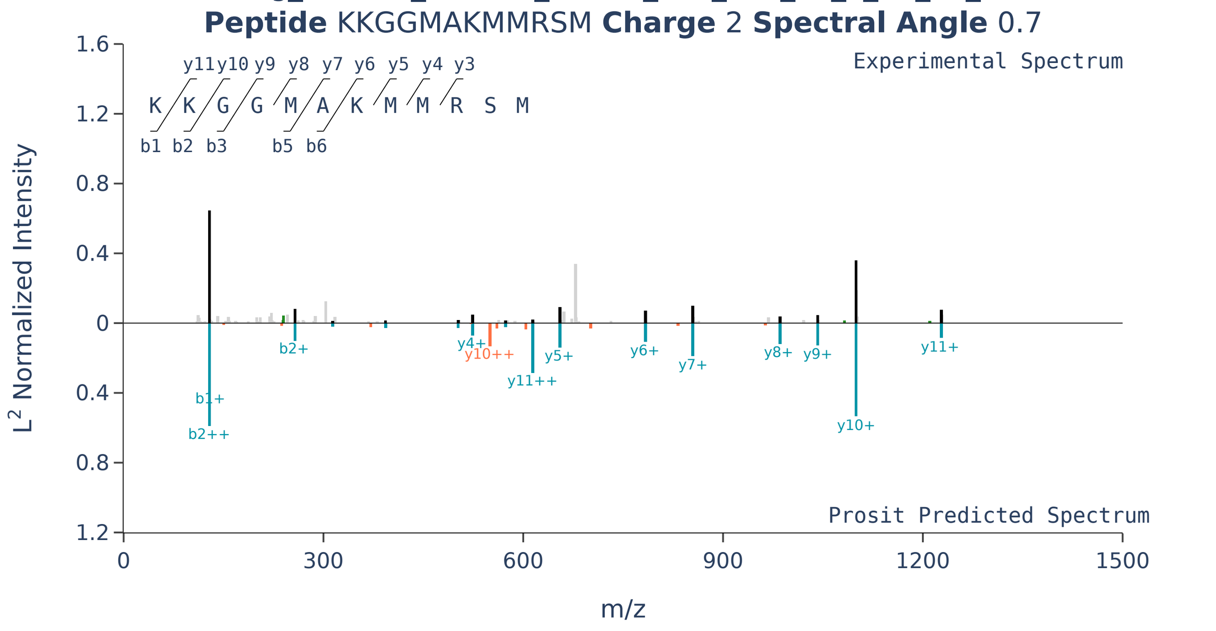


**Source** Ncheng_210623_230623_HFGoe_FFH_20S_25_1_A1_4h_R1 **Scan** 24866
**Peptide** KTEALEPFHPDRIASRLL **Charge** 3 **Spectral Angle** 0.64

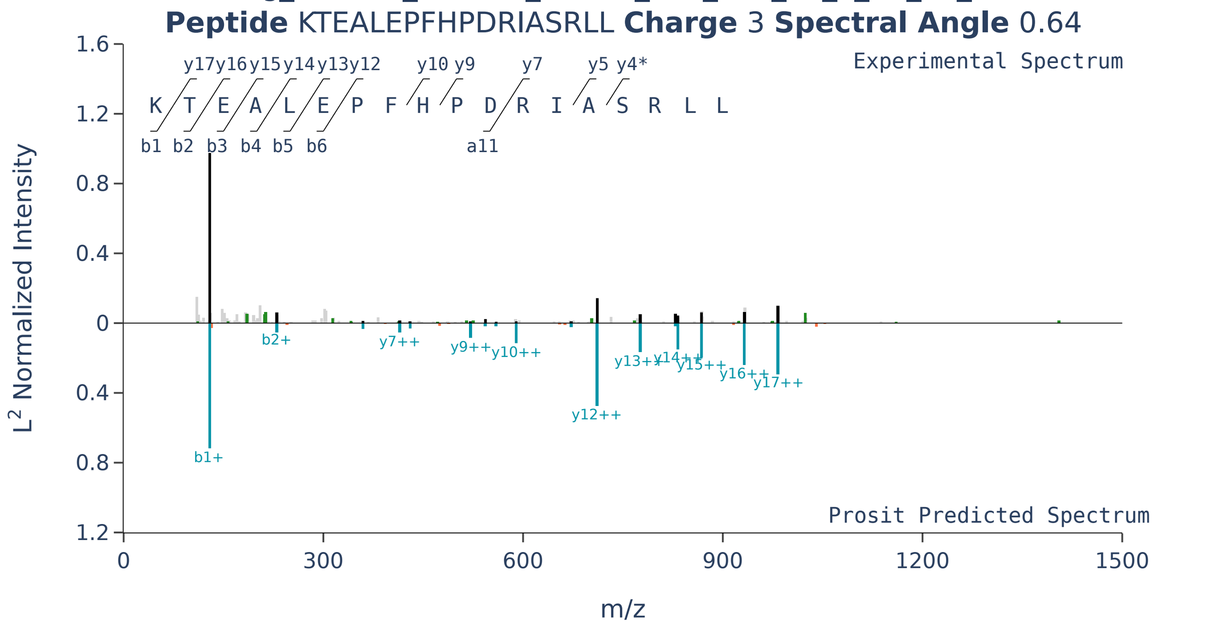


**Source** Ncheng_210623_230623_HFGoe_FFH_20S_25_1_A2_24h_R1 **Scan** 16550
**Peptide** INRVKEKAVGHEVNKSLTPGQEFVKIVR **Charge** 4 **Spectral Angle** 0.77

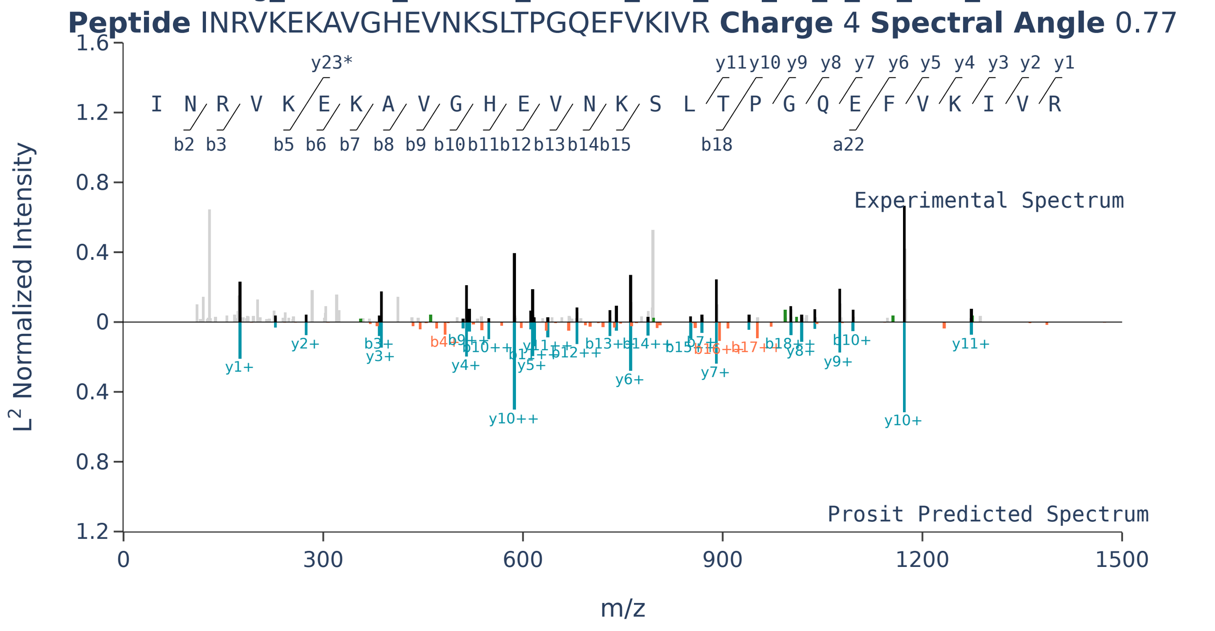


**Source** Ncheng_210623_230623_HFGoe_FFH_20S_25_1_A1_24h_R2 **Scan** 7539
**Peptide** AQAEKLASKLKKG **Charge** 2 **Spectral Angle** 0.8

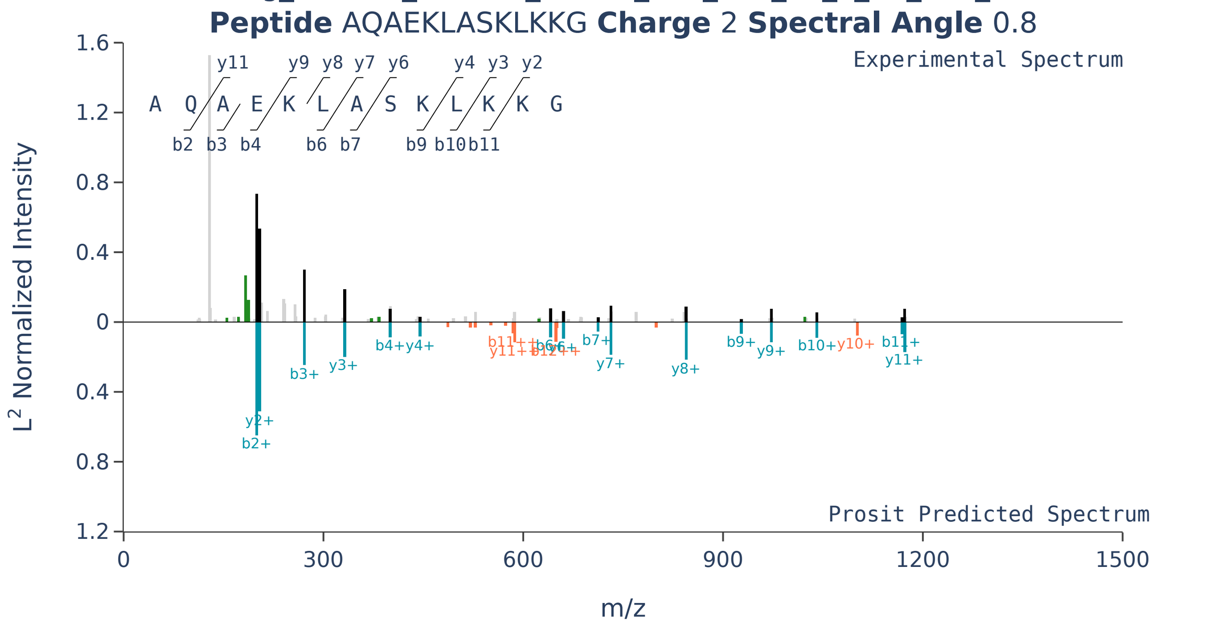


**Source** Ncheng_210623_230623_HFGoe_FFH_20S_25_1_A2_24h_R1 **Scan** 7519
**Peptide** RAQAEKLASKLK **Charge** 3 **Spectral Angle** 0.77

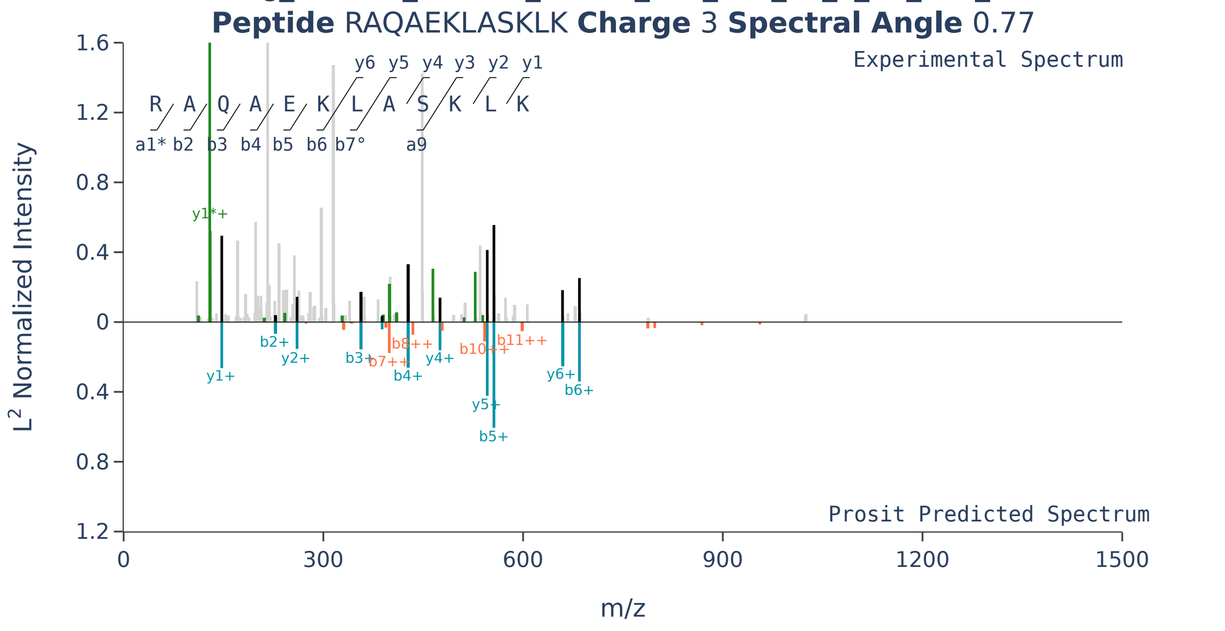


**Source** Ncheng_210623_230623_HFGoe_FFH_20S_25_1_A1_2h_R1 **Scan** 26809
**Peptide** VLVVSADVYRPAAIKQL **Charge** 2 **Spectral Angle** 0.81

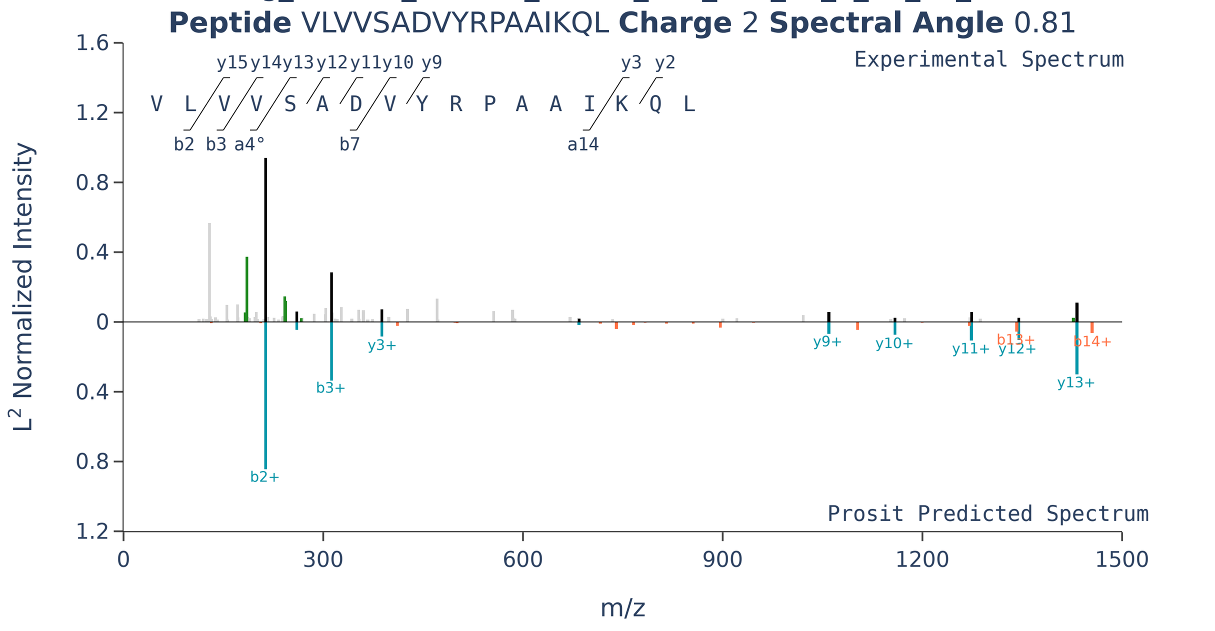


**Source** Ncheng_210623_230623_HFGoe_FFH_20S_25_1_A1_2h_R2 **Scan** 30192
**Peptide** GQIPDNVKSQMDDKVLVRMEAILN **Charge** 3 **Spectral Angle** 0.88

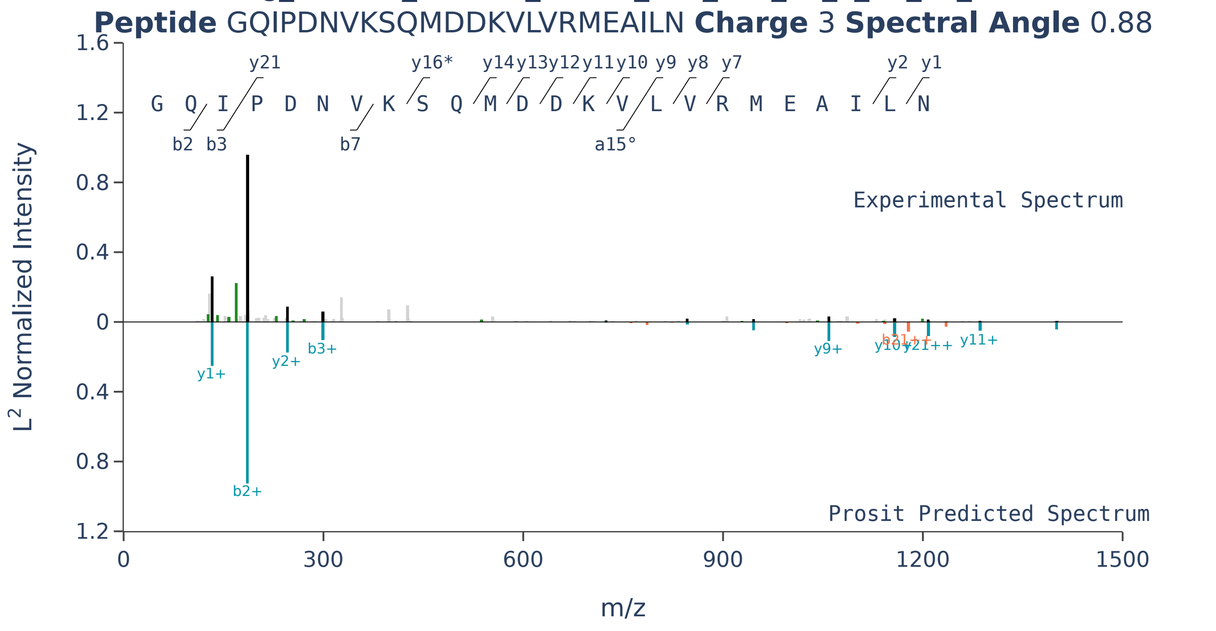


**Source** Ncheng_210623_230623_HFGoe_FFH_20S_25_1_A1_2h_R1 **Scan** 12688
**Peptide** IAAGCGMQVQD **Charge** 2 **Spectral Angle** 0.79

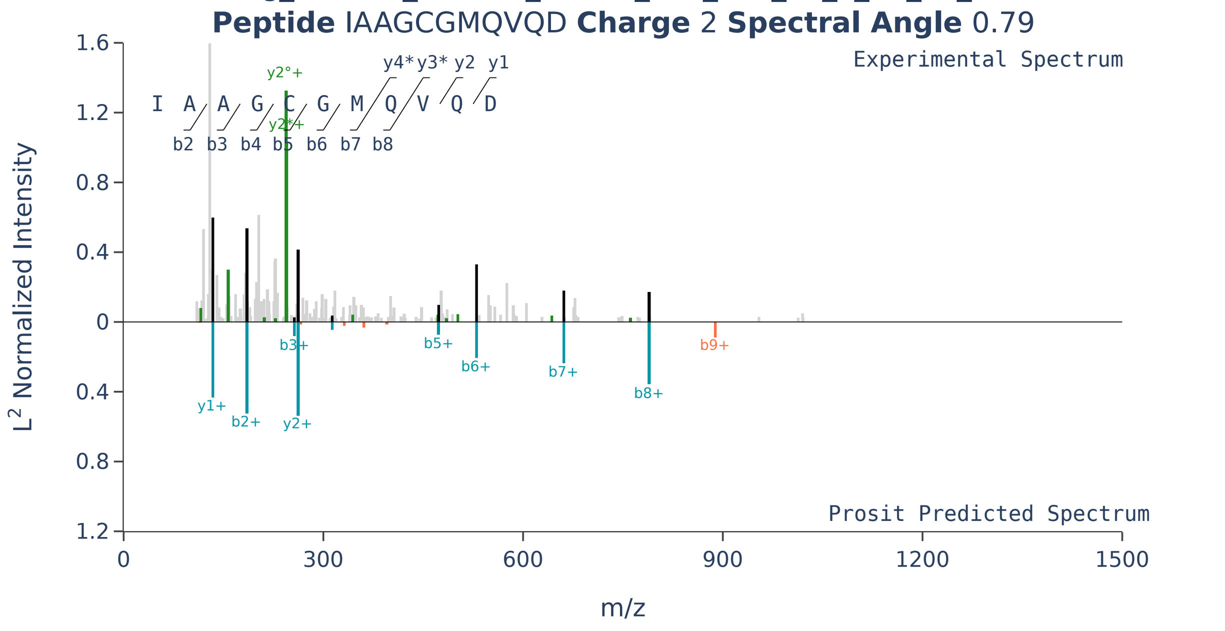

**Source** Ncheng_210623_230623_HFGoe_FFH_20S_25_1_A2_24h_R2 **Scan** 13107
**Peptide** AGKTTSVGKLGKFLREK **Charge** 4 **Spectral Angle** 0.67

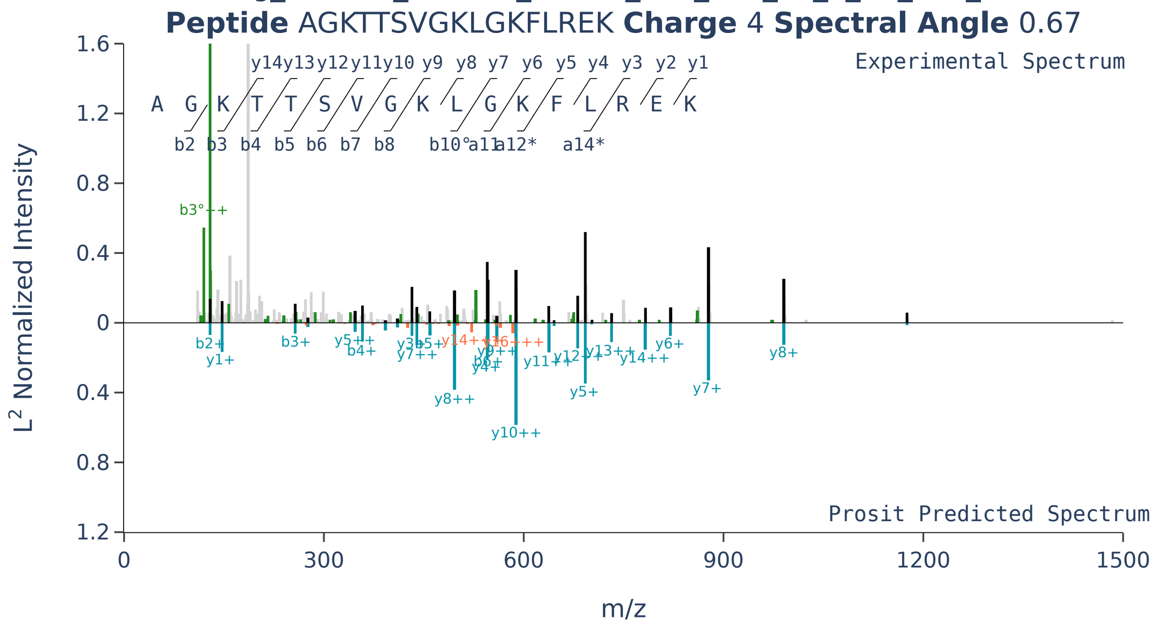


**Source** Ncheng_210623_230623_HFGoe_FFH_20S_25_1_A2_24h_R2 **Scan** 11833
**Peptide** AQAEKLASKLKKGDGFD **Charge** 2 **Spectral Angle** 0.73

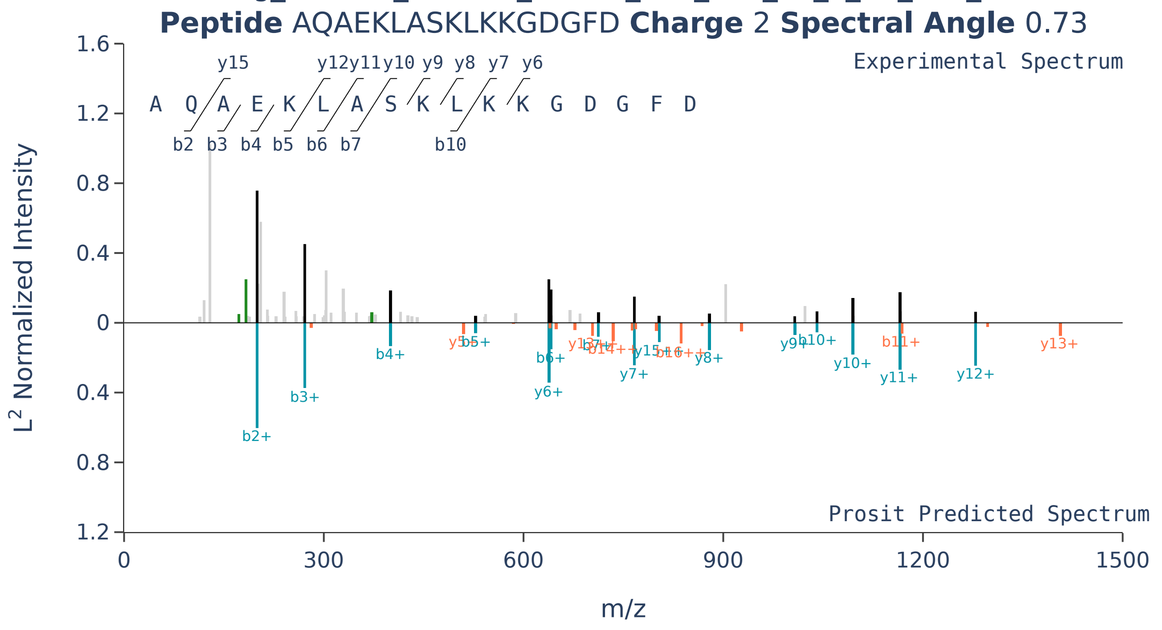


**Source** Ncheng_210623_230623_HFGoe_FFH_20S_25_1_A2_24h_R1 **Scan** 7914
**Peptide** DMQRMMKKM **Charge** 2 **Spectral Angle** 0.65

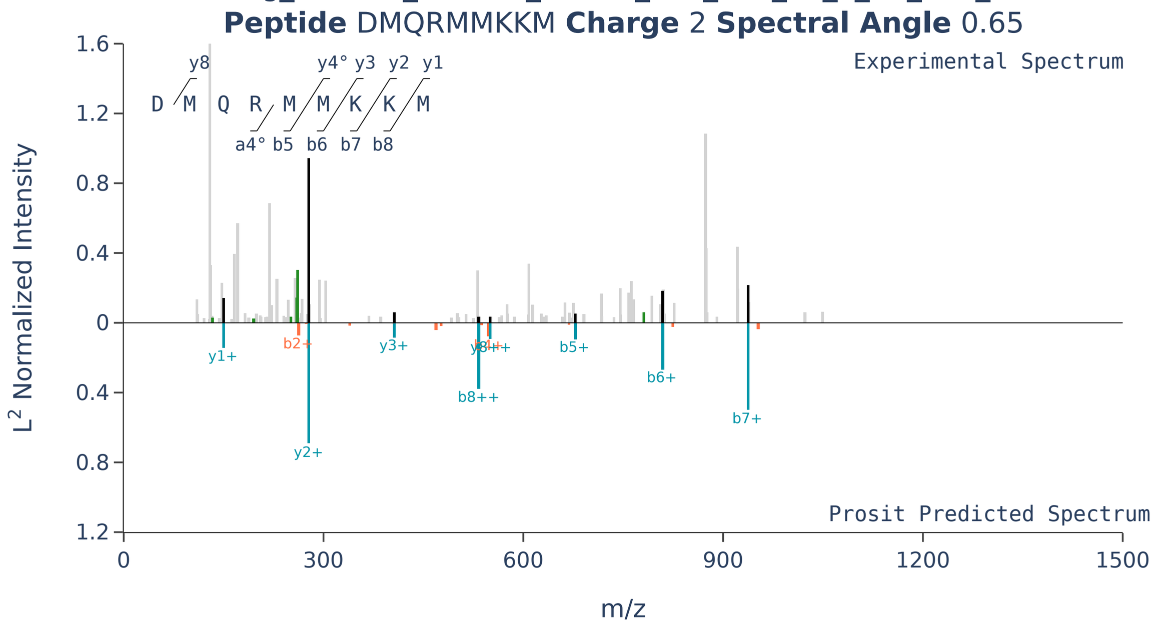


**Source** Ncheng_210623_230623_HFGoe_FFH_20S_25_1_A1_2h_R1 **Scan** 31334
**Peptide** IVNAALKEAKLKFYDVLL **Charge** 3 **Spectral Angle** 0.81

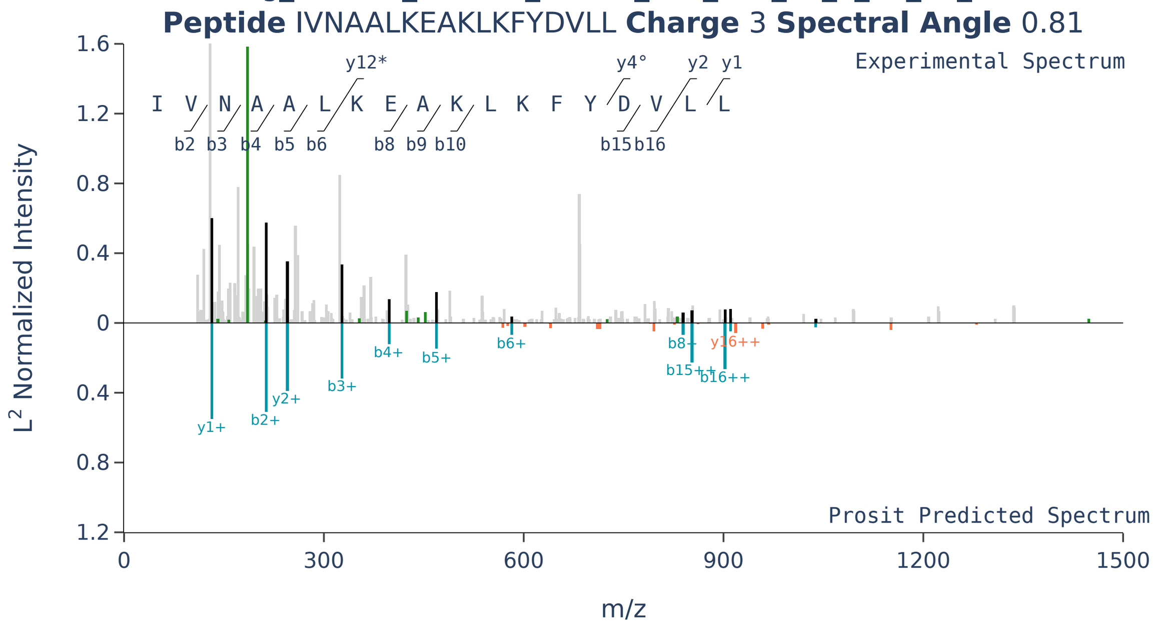


**Source** Ncheng_210623_230623_HFGoe_FFH_20S_25_1_A2_24h_R1 **Scan** 8919
**Peptide** TEDNVKDTLRE **Charge** 2 **Spectral Angle** 0.78

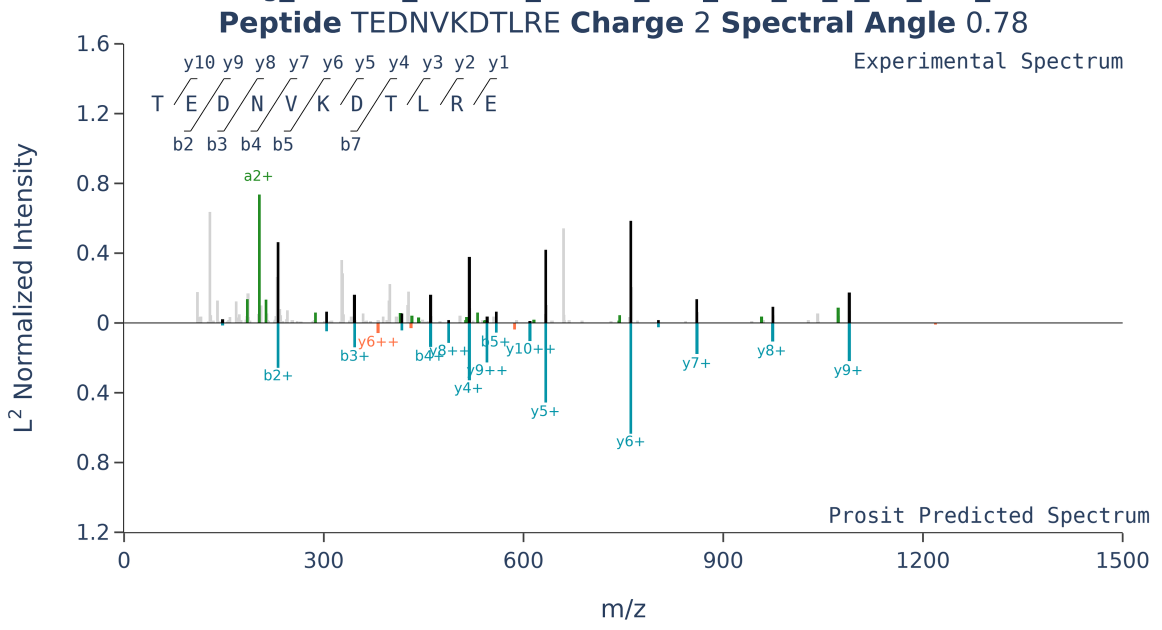


**Source** Ncheng_210623_230623_HFGoe_FFH_20S_25_1_A2_24h_R2 **Scan** 11655
**Peptide** QDVNRLLKQ **Charge** 1 **Spectral Angle** 0.82

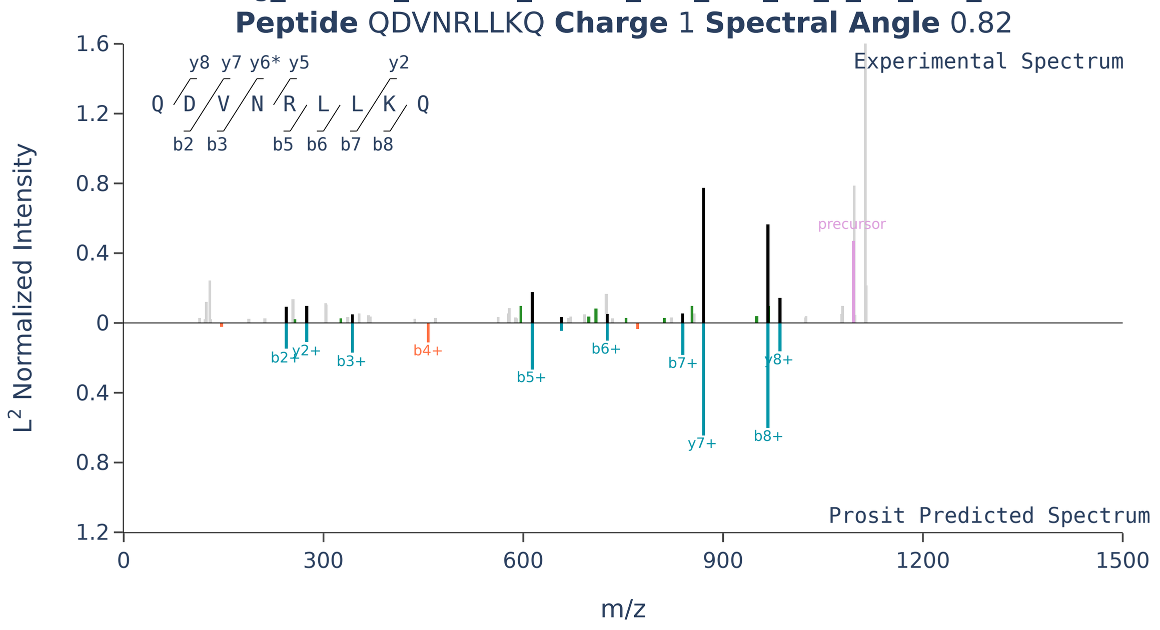


**Source** Ncheng_210623_230623_HFGoe_FFH_20S_25_1_A1_24h_R2 **Scan** 23077
**Peptide** MGKLPGMGQI **Charge** 2 **Spectral Angle** 0.77

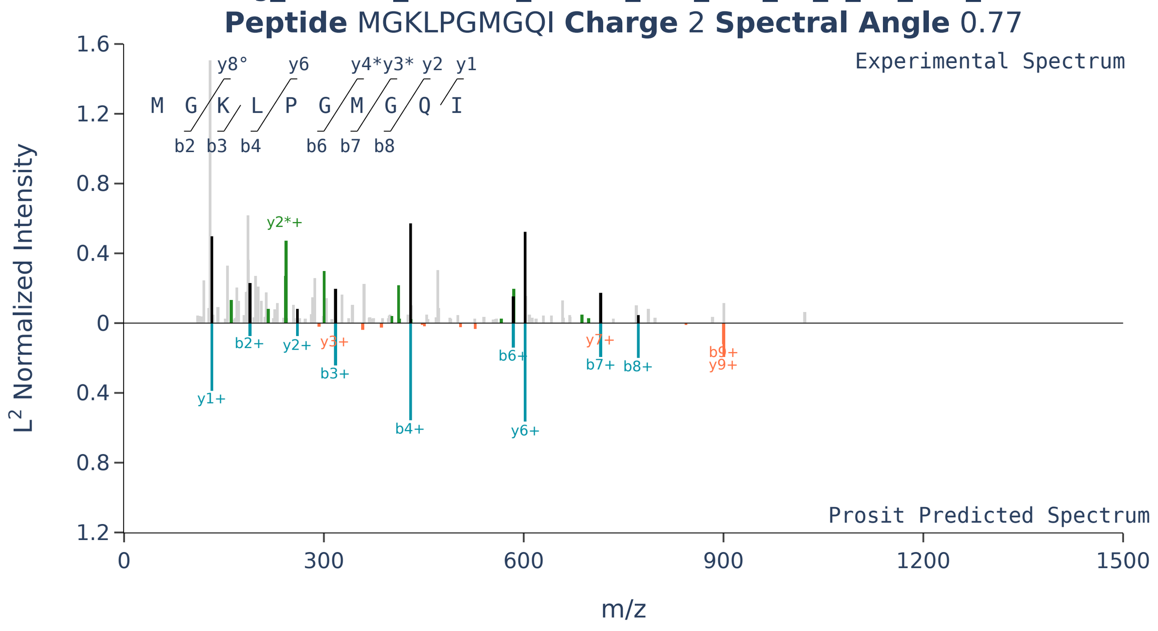


**Source** Ncheng_210623_230623_HFGoe_FFH_20S_25_1_A2_24h_R2 **Scan** 19005
**Peptide** AGLQGAGKTTSVGKLGKFLRE **Charge** 2 **Spectral Angle** 0.73

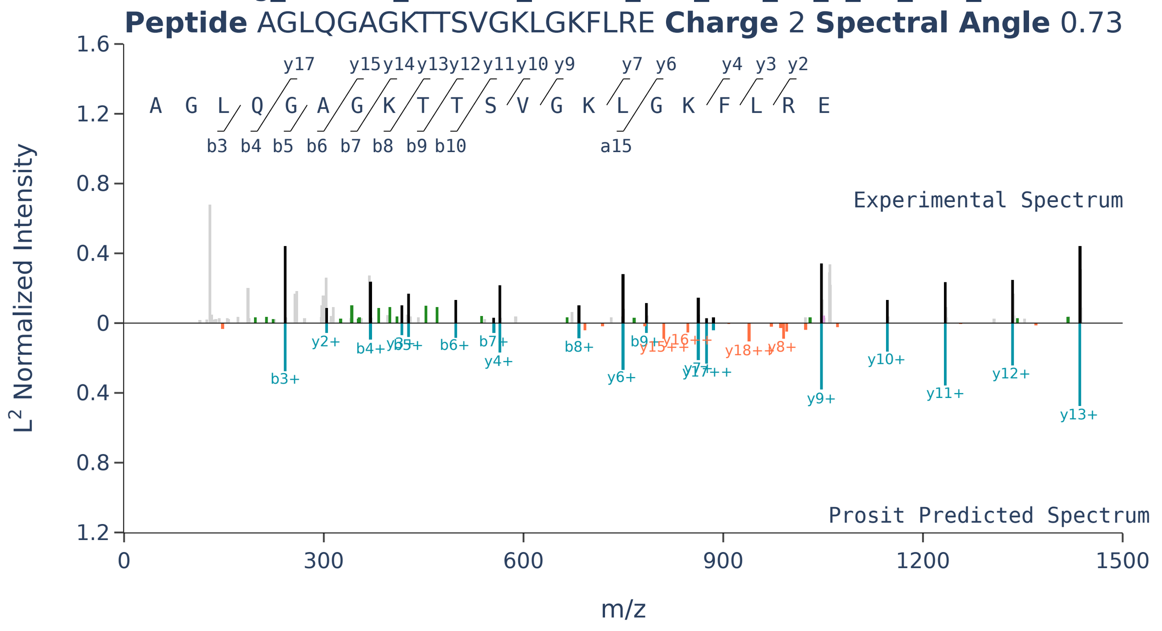


**Source** Ncheng_210623_230623_HFGoe_FFH_20S_25_1_A1_24h_R1 **Scan** 13972
**Peptide** DGDARGGAALSIRHITGK **Charge** 5 **Spectral Angle** 0.81

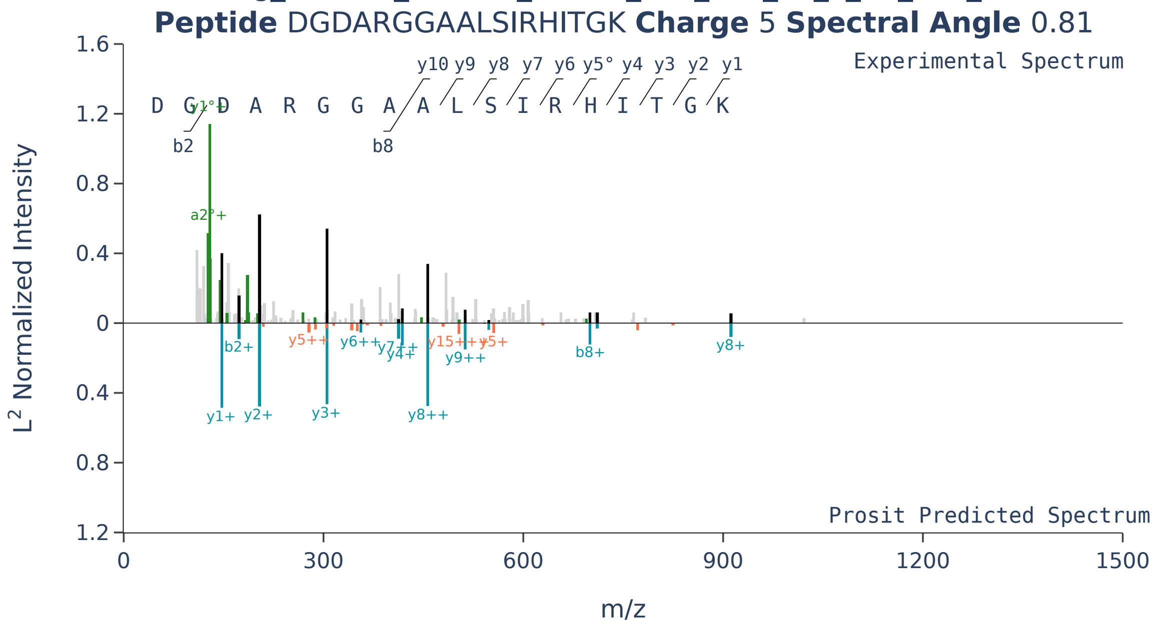


**Source** Ncheng_210623_230623_HFGoe_FFH_20S_25_1_A2_24h_R2 **Scan** 15376
**Peptide** GAALSIRHI **Charge** 2 **Spectral Angle** 0.71

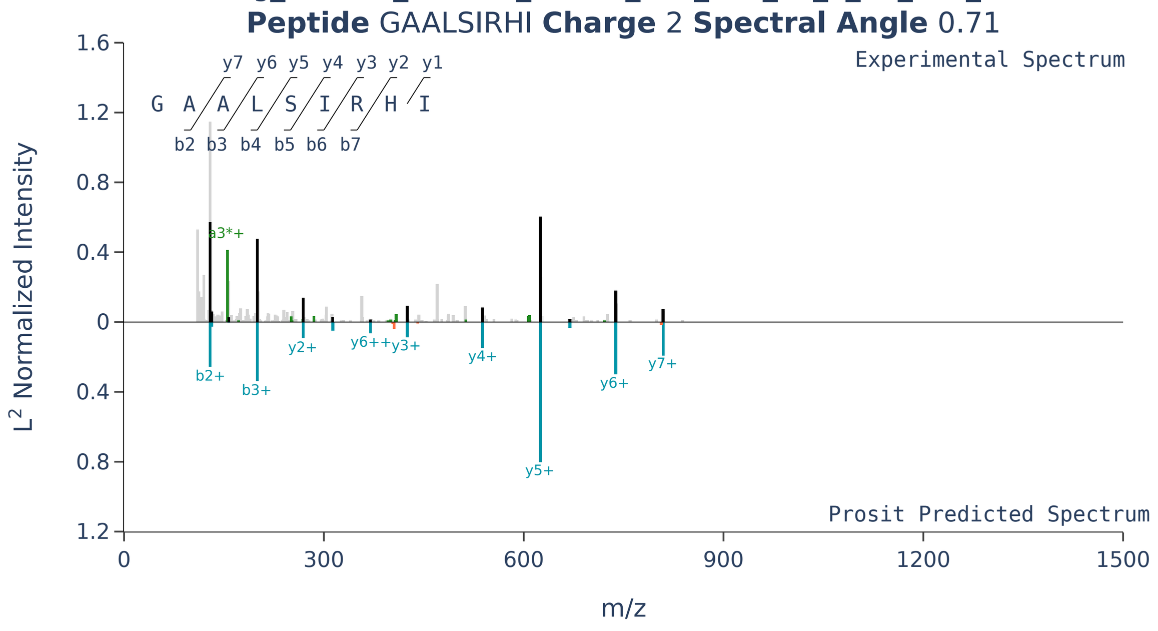


**Source** Ncheng_210623_230623_HFGoe_FFH_20S_25_1_A1_2h_R2 **Scan** 32154
**Peptide** QMDDKVLVRMEAII **Charge** 2 **Spectral Angle** 0.72

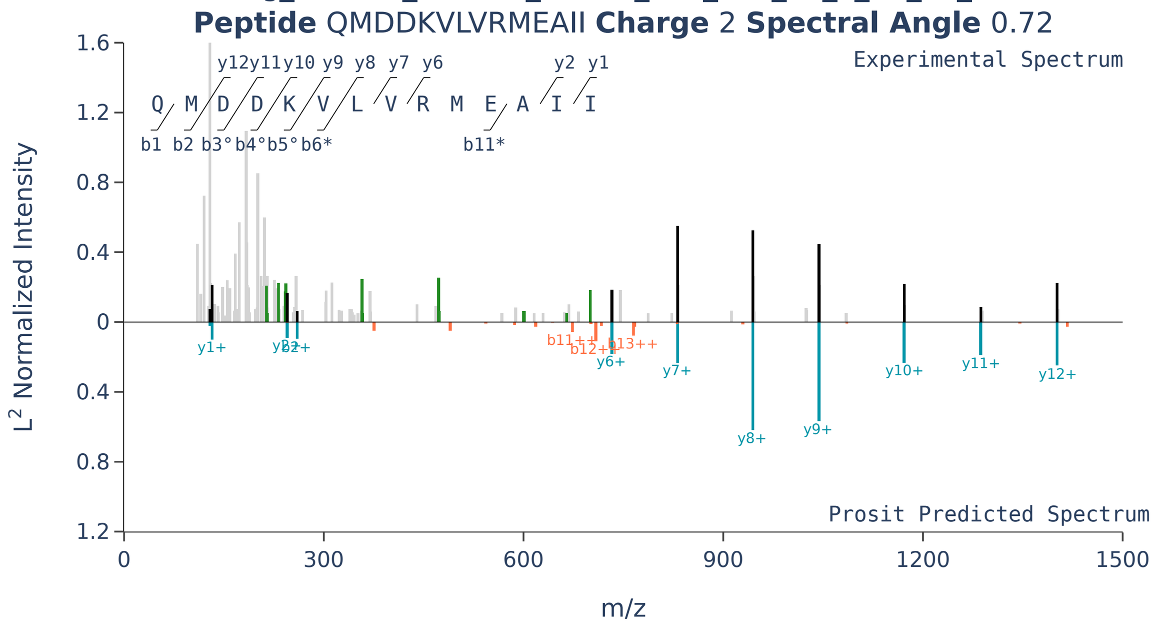


**Source** Ncheng_210623_230623_HFGoe_FFH_20S_25_1_A1_4h_R1 **Scan** 15278
**Peptide** VVLTKVDGDARGGAALSIRH **Charge** 3 **Spectral Angle** 0.54

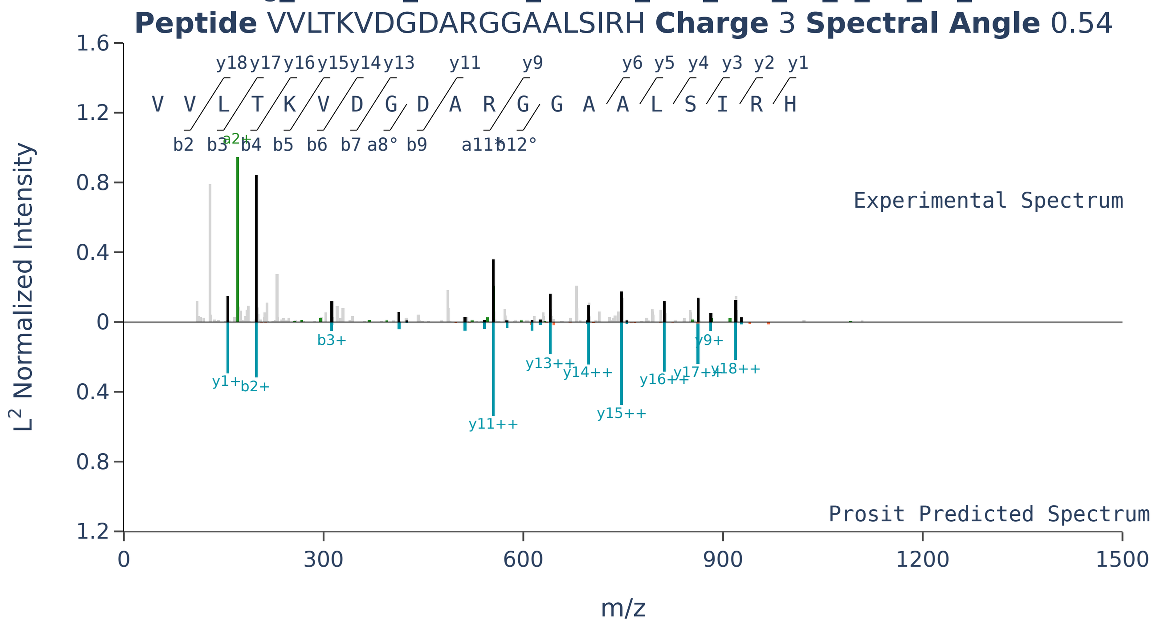


**Source** Ncheng_210623_230623_HFGoe_FFH_20S_25_1_A1_2h_R2 **Scan** 27540
**Peptide** DVYRPAAIKQLETLAE **Charge** 3 **Spectral Angle** 0.78

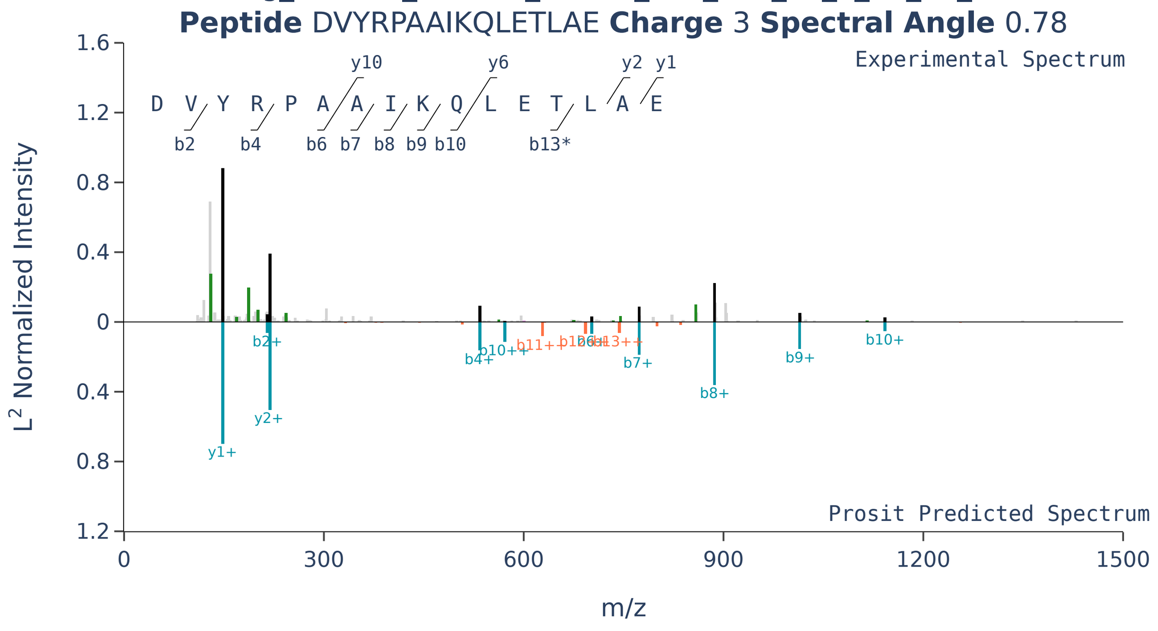


**Source** Ncheng_210623_230623_HFGoe_FFH_20S_25_1_A1_2h_R2 **Scan** 30789
**Peptide** VNAALKEAKLKFYDVLLVD **Charge** 2 **Spectral Angle** 0.66

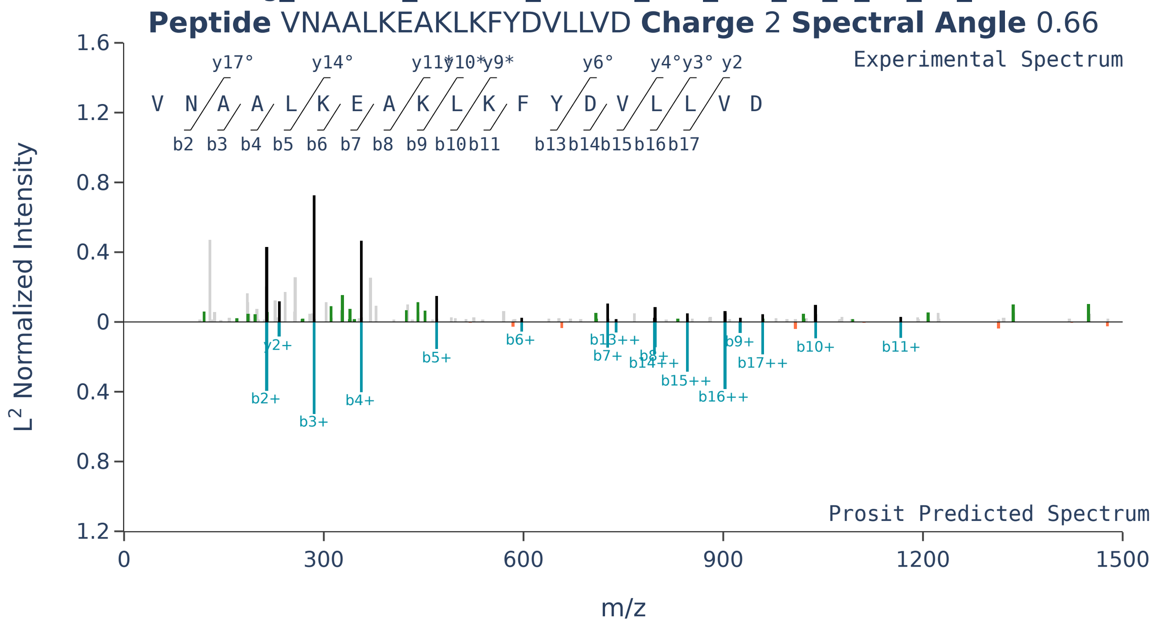


**Source** Ncheng_210623_230623_HFGoe_FFH_20S_25_1_A2_1h_R1 **Scan** 29210
**Peptide** EALPLTGVVLTKVDGDARGGAALSIRHL **Charge** 5 **Spectral Angle** 0.75

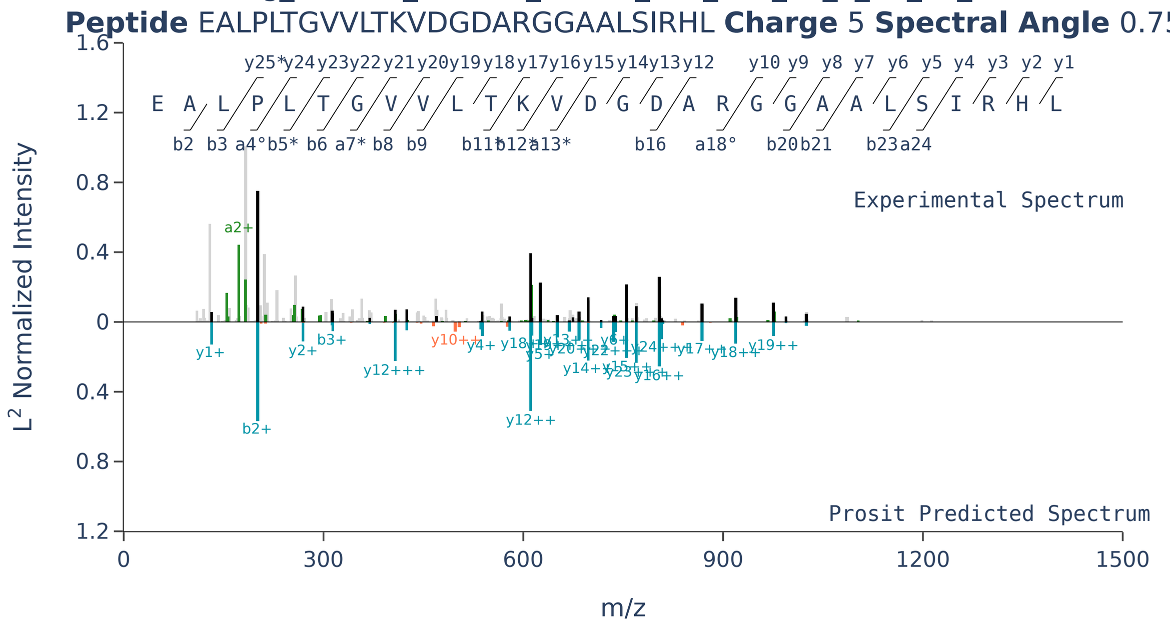

**Source** Ncheng_210623_230623_HFGoe_FFH_20S_25_1_A2_2h_R2 **Scan** 39122
**Peptide** RILGMGDVLSLIE **Charge** 2 **Spectral Angle** 0.72

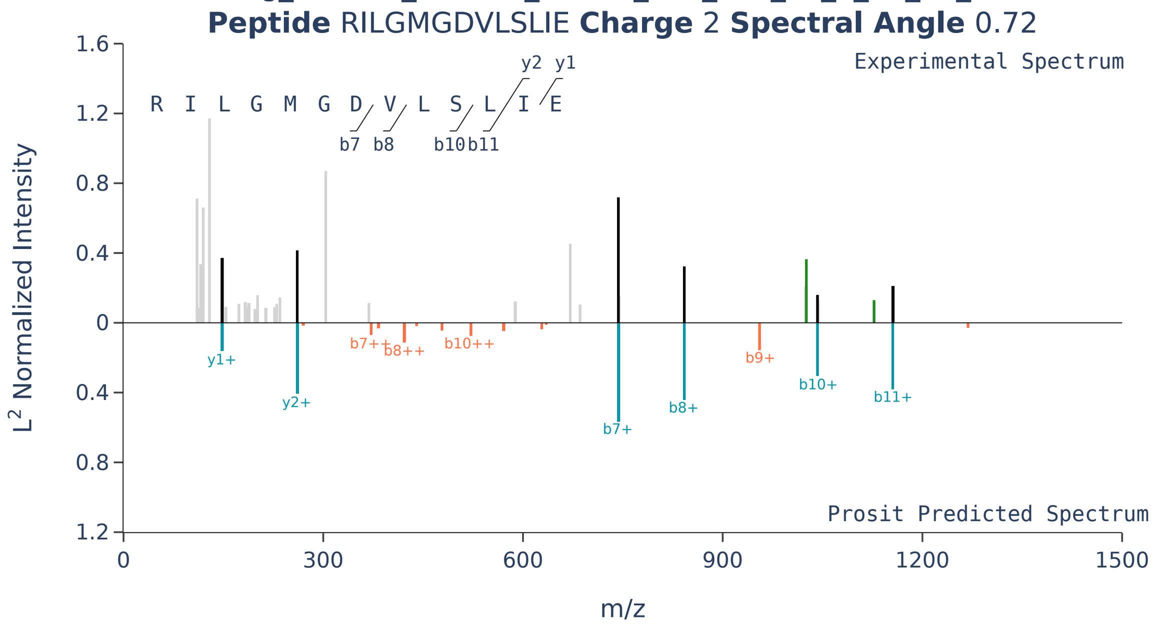


**Source** Ncheng_210623_230623_HFGoe_FFH_20S_25_1_A1_4h_R1 **Scan** 12627
**Peptide** AMMDELKQ **Charge** 2 **Spectral Angle** 0.79

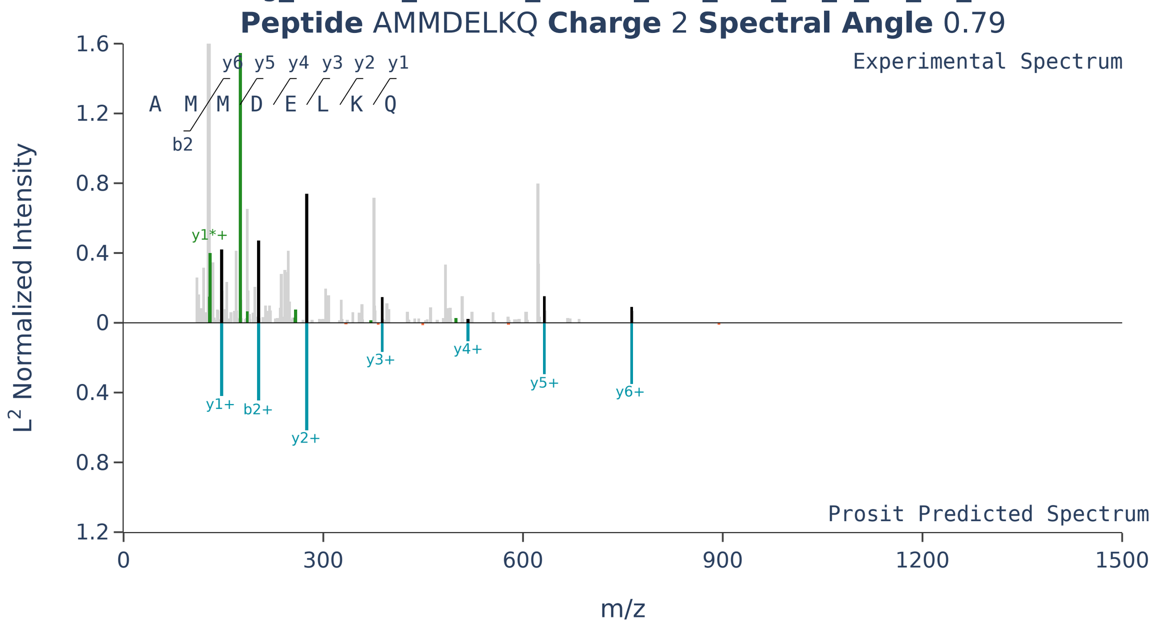


**Source** Ncheng_210623_230623_HFGoe_FFH_20S_25_1_A2_2h_R2 **Scan** 26469
**Peptide** ADVYRPAAIKQLETLAE **Charge** 3 **Spectral Angle** 0.79

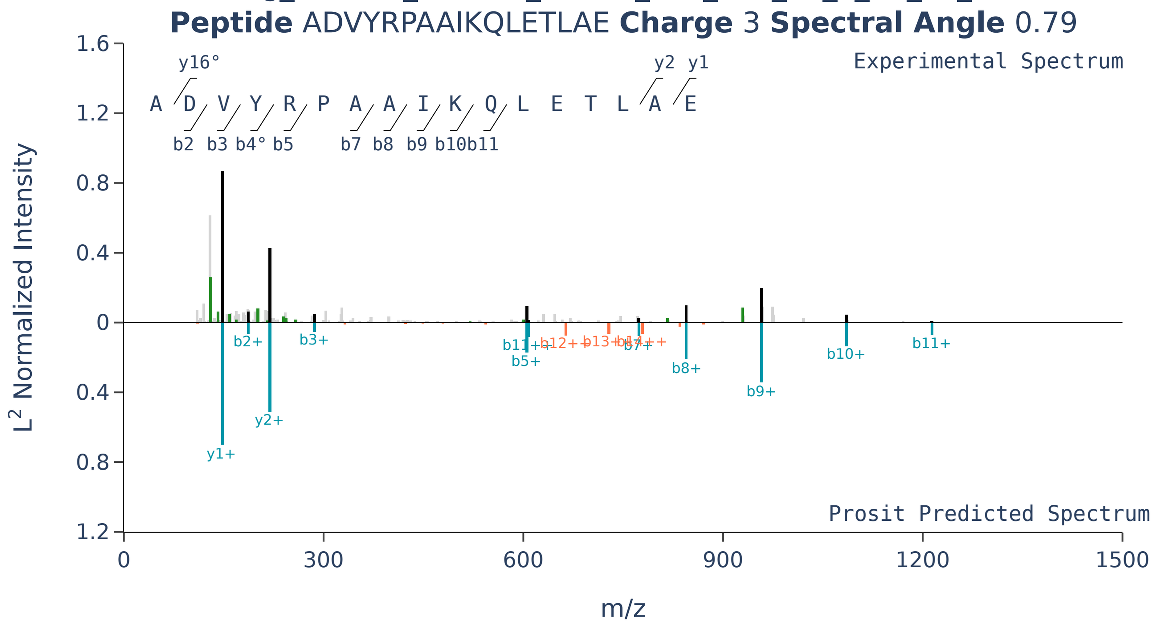


**Source** Ncheng_210623_230623_HFGoe_FFH_20S_25_1_A1_4h_R2 **Scan** 19335
**Peptide** EQLRQMKNMGGMASL **Charge** 2 **Spectral Angle** 0.76

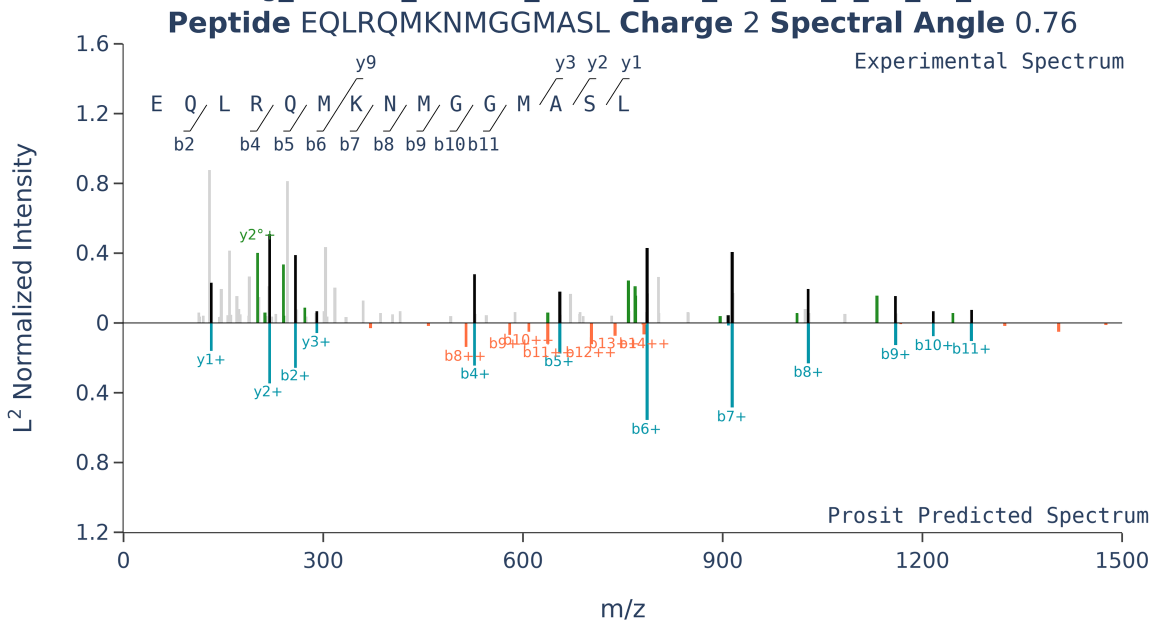


**Source** Ncheng_210623_230623_HFGoe_FFH_20S_25_1_A2_4h_R1 **Scan** 32138
**Peptide** GQIPDNVKSQMDDKVLVRMEALL **Charge** 3 **Spectral Angle** 0.83

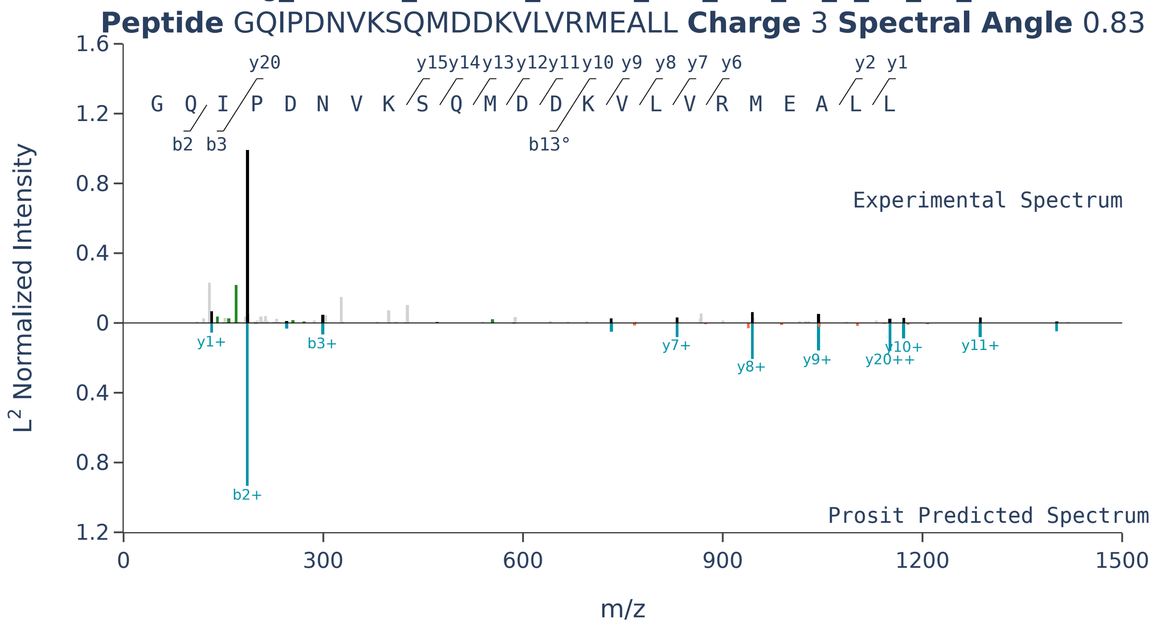


**Source** Ncheng_210623_230623_HFGoe_FFH_20S_25_1_A1_2h_R2 **Scan** 34100
**Peptide** DKVLVRMEAIINSMTMKER **Charge** 3 **Spectral Angle** 0.83

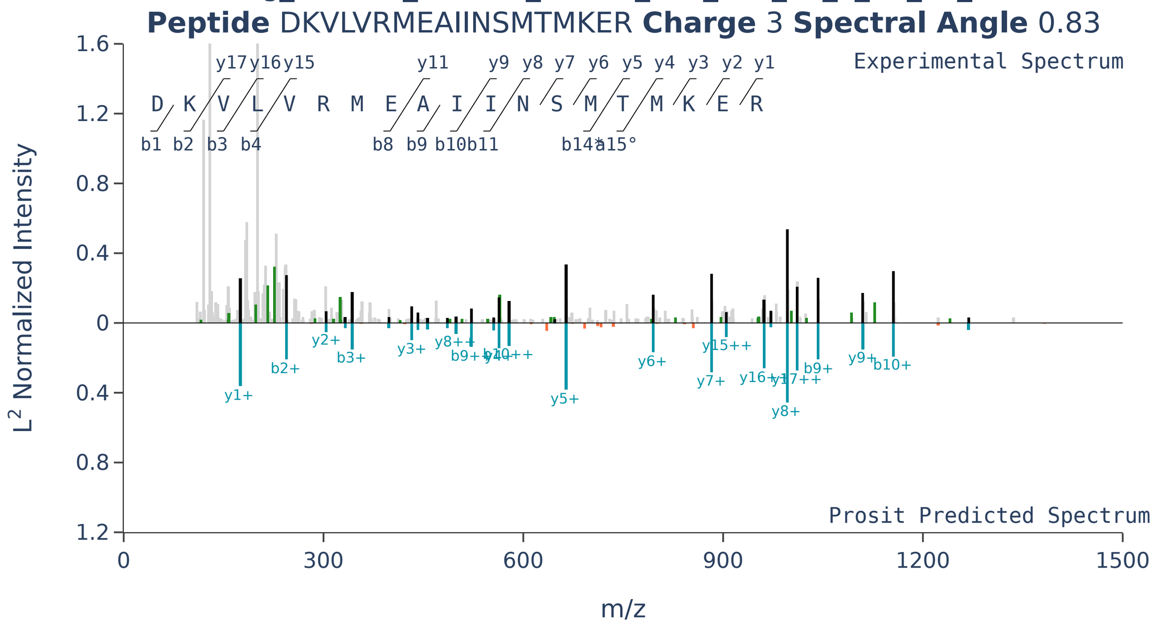


**Source** Ncheng_210623_230623_HFGoe_FFH_20S_25_1_A1_2h_R2 **Scan** 27654
**Peptide** KVLVRMEAIINSM **Charge** 2 **Spectral Angle** 0.6

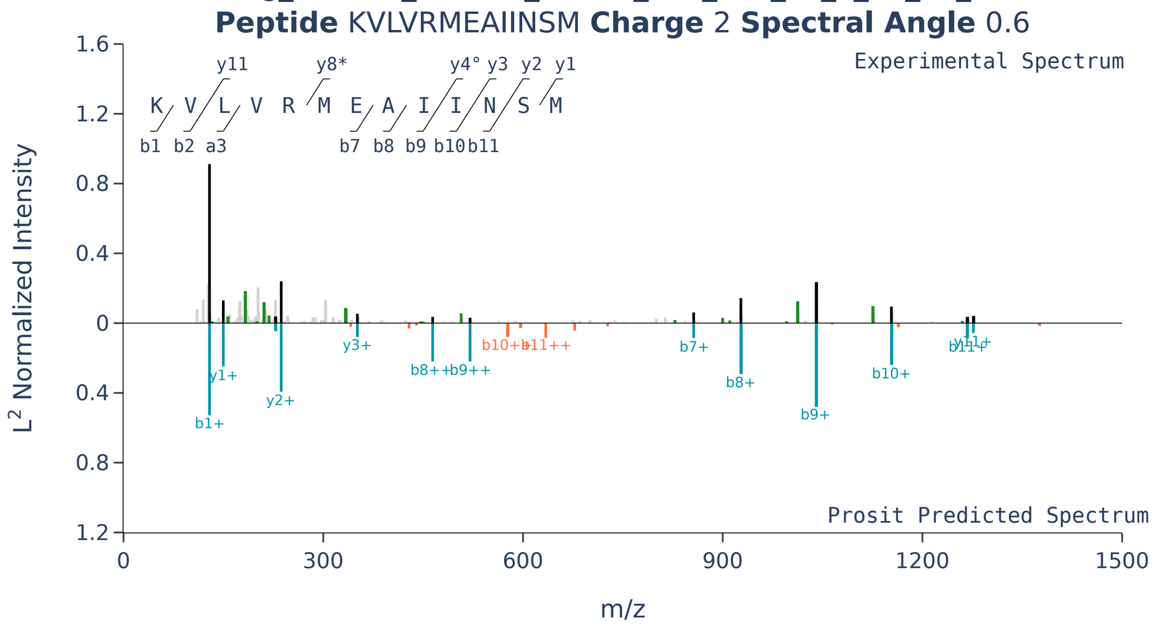


**Source** Ncheng_210623_230623_HFGoe_FFH_20S_25_1_A1_24h_R2 **Scan** 16846
**Peptide** QMDDKVLV **Charge** 2 **Spectral Angle** 0.88

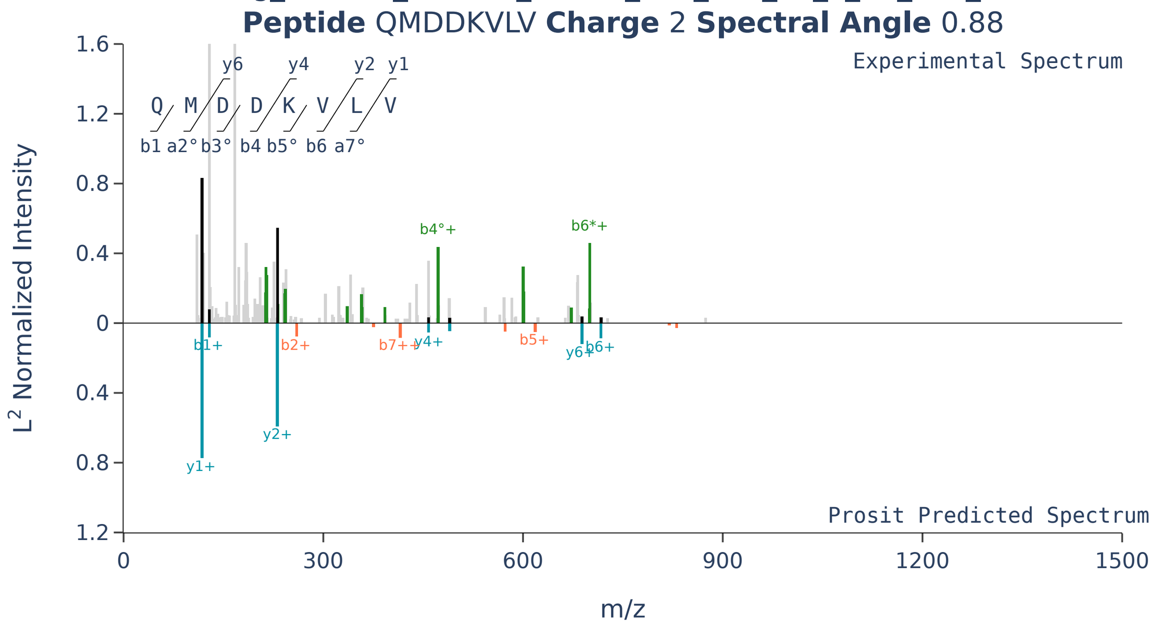


**Source** Ncheng_210623_230623_HFGoe_FFH_20S_25_1_A1_24h_R1 **Scan** 12093
**Peptide** KEKAVGHEVNKSLTPGQEF **Charge** 2 **Spectral Angle** 0.8

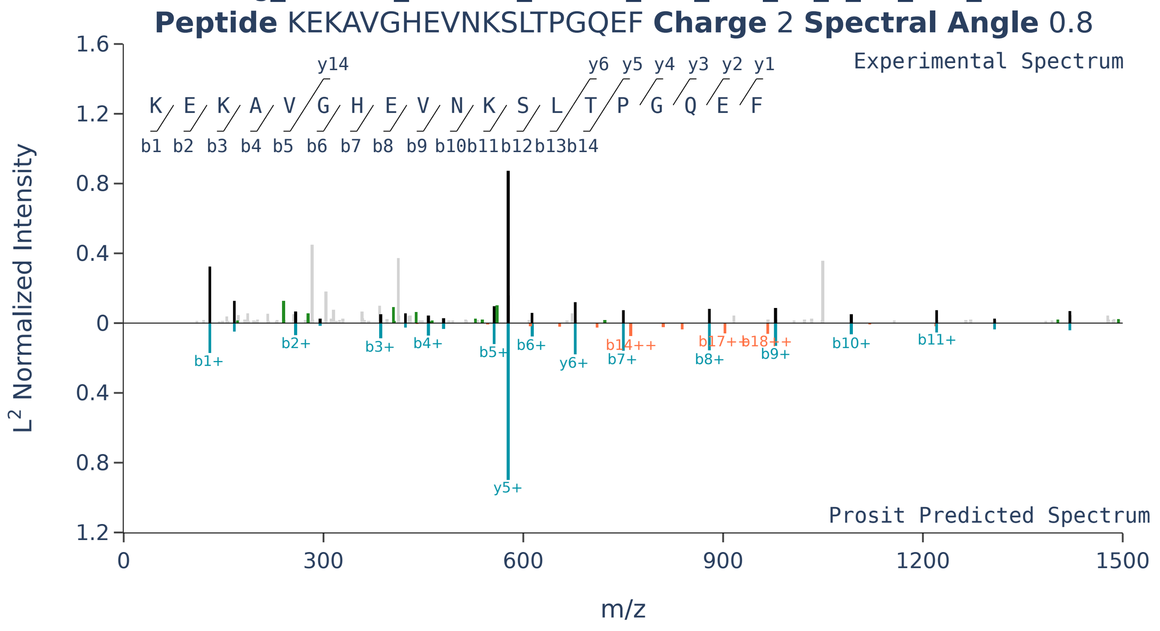


**Source** Ncheng_210623_230623_HFGoe_FFH_20S_25_1_A2_1h_R2 **Scan** 8228
**Peptide** DLESKVDRAQA **Charge** 3 **Spectral Angle** 0.87

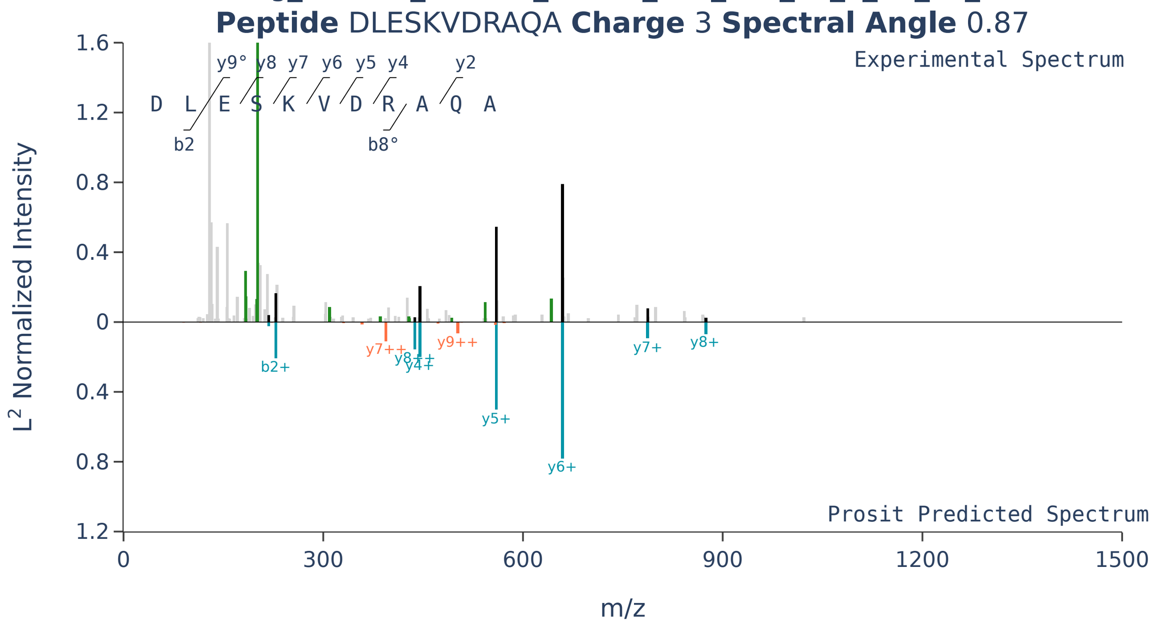


**Source** Ncheng_210623_230623_HFGoe_FFH_20S_25_1_A1_24h_R2 **Scan** 11380
**Peptide** MDDKVLVR **Charge** 2 **Spectral Angle** 0.69

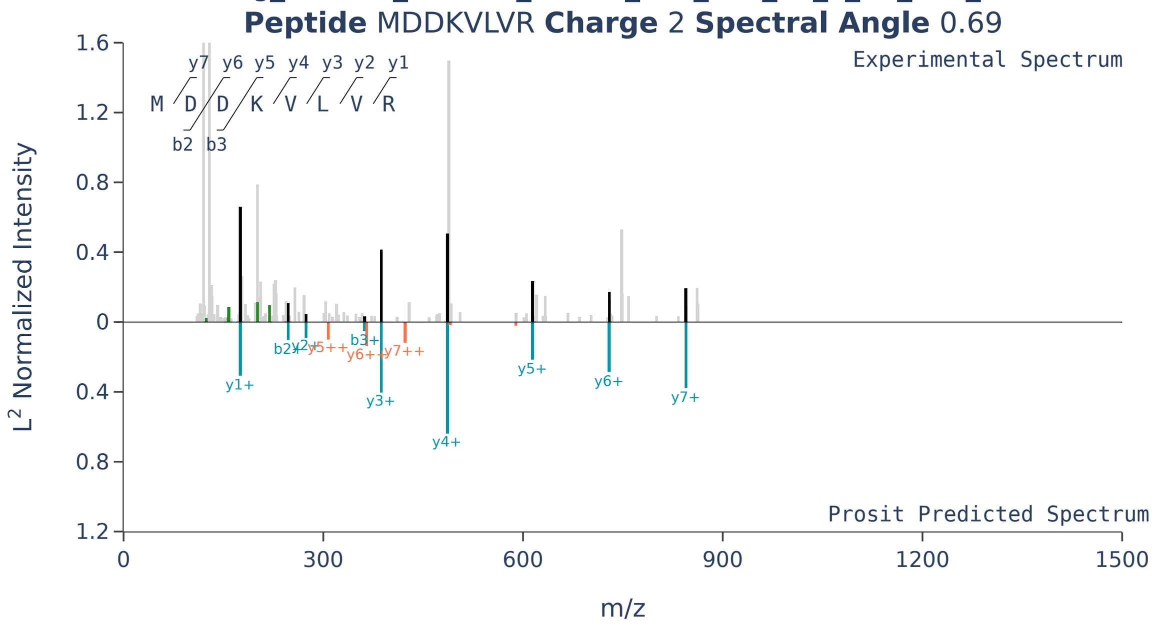


**Source** Ncheng_210623_230623_HFGoe_FFH_20S_25_1_A1_24h_R1 **Scan** 25763
**Peptide** NELVAAMGEENQTL **Charge** 2 **Spectral Angle** 0.89

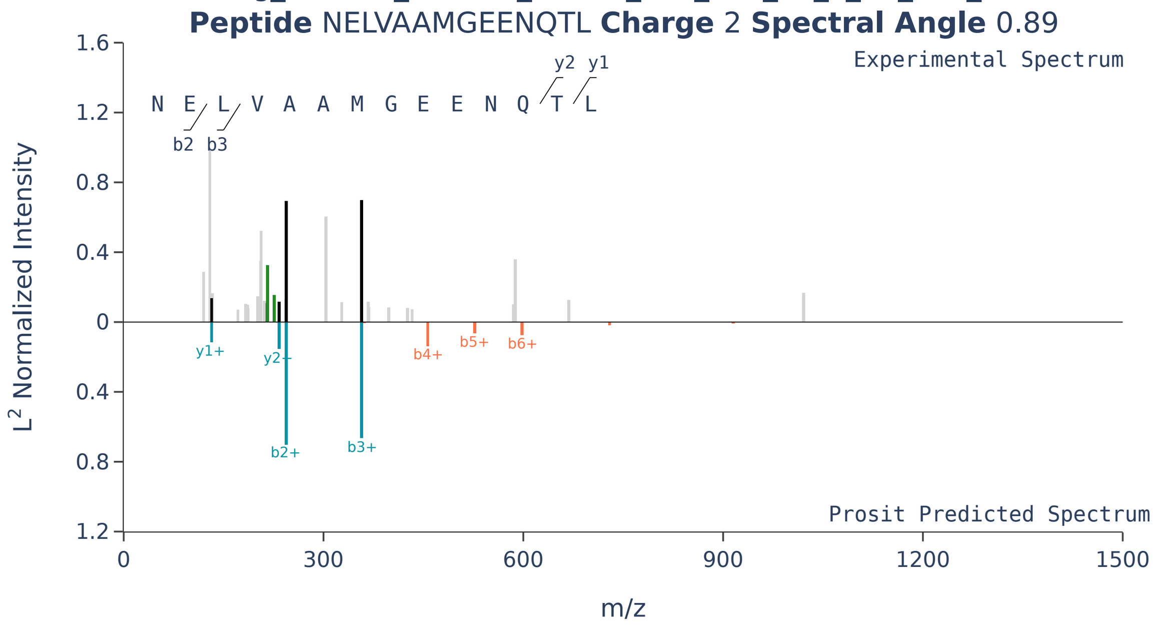


**Source** Ncheng_210623_230623_HFGoe_FFH_20S_25_1_A2_4h_R1 **Scan** 27366
**Peptide** KEAKLKFYDVLL **Charge** 2 **Spectral Angle** 0.65

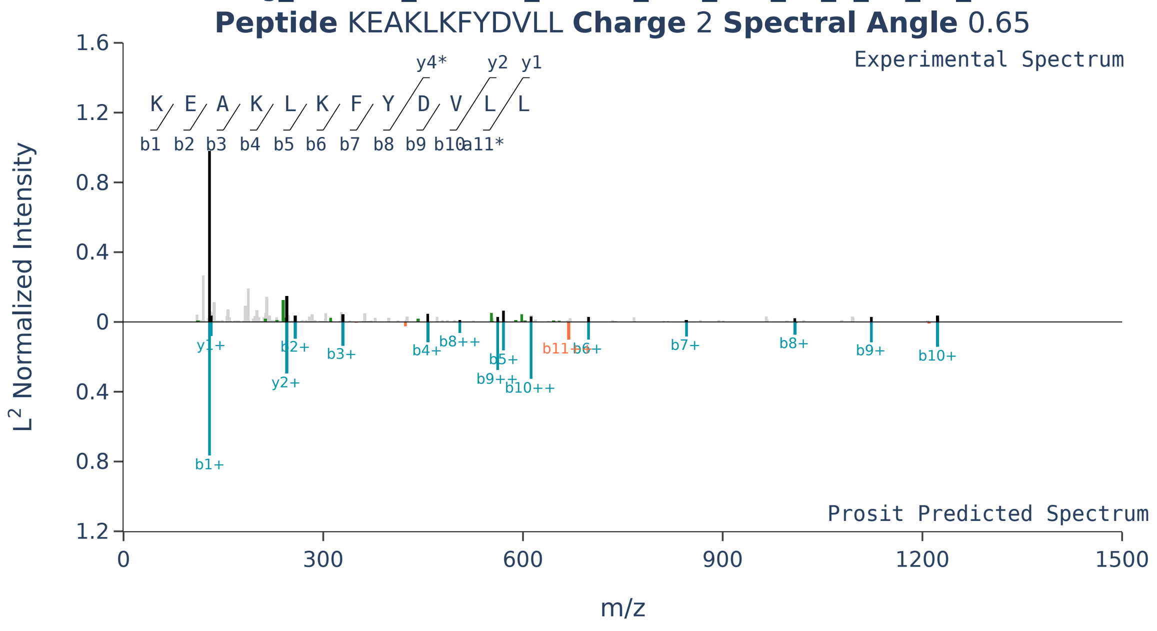


**Source** Ncheng_210623_230623_HFGoe_FFH_20S_25_1_A2_24h_R2 **Scan** 9415
**Peptide** AEKLASKLKKGDGFD **Charge** 2 **Spectral Angle** 0.71

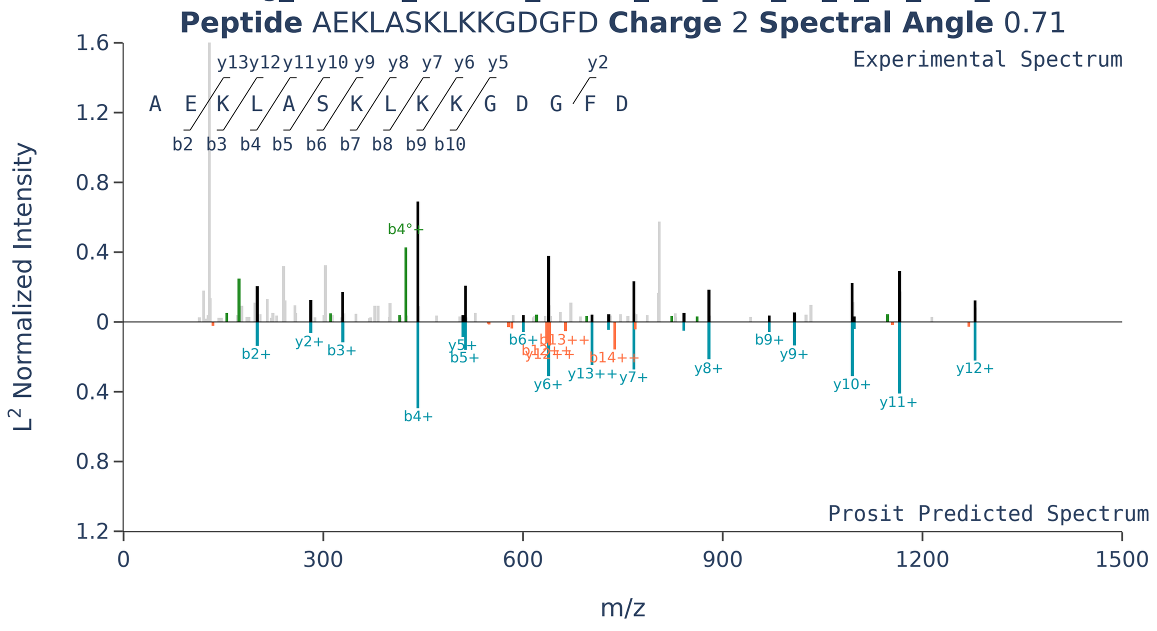


**Source** Ncheng_210623_230623_HFGoe_FFH_20S_25_1_A2_24h_R1 **Scan** 11479
**Peptide** TKVDGDARGGAALSLR **Charge** 4 **Spectral Angle** 0.85

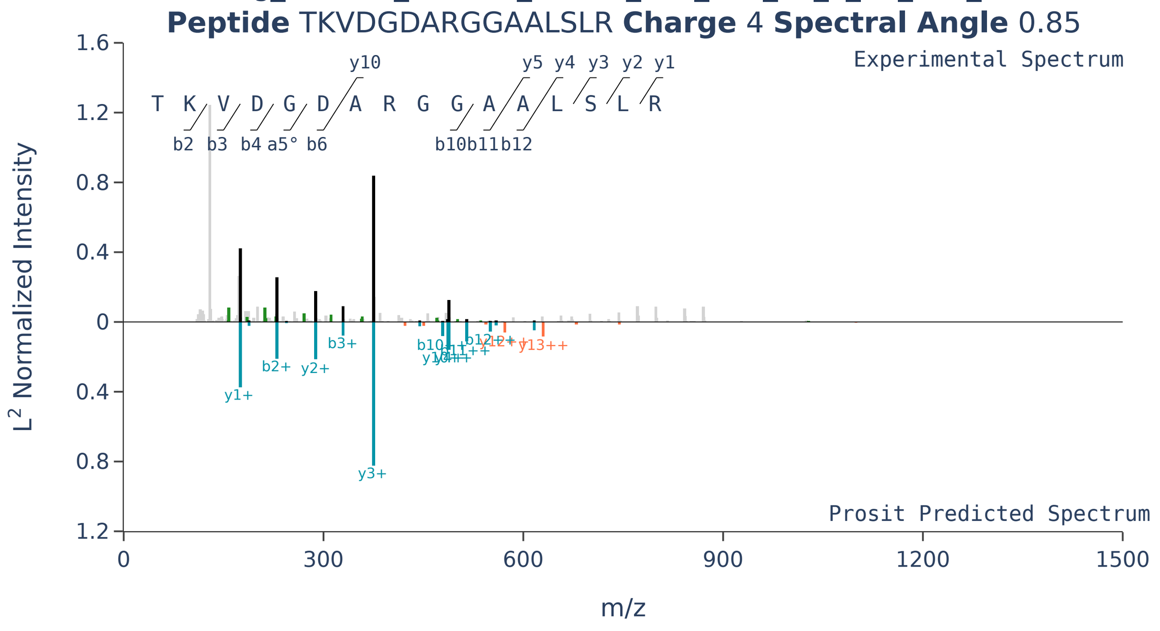

**Source** Ncheng_210623_230623_HFGoe_FFH_20S_25_1_A2_24h_R1 **Scan** 12474
**Peptide** SIRHITGKPIKF **Charge** 2 **Spectral Angle** 0.77

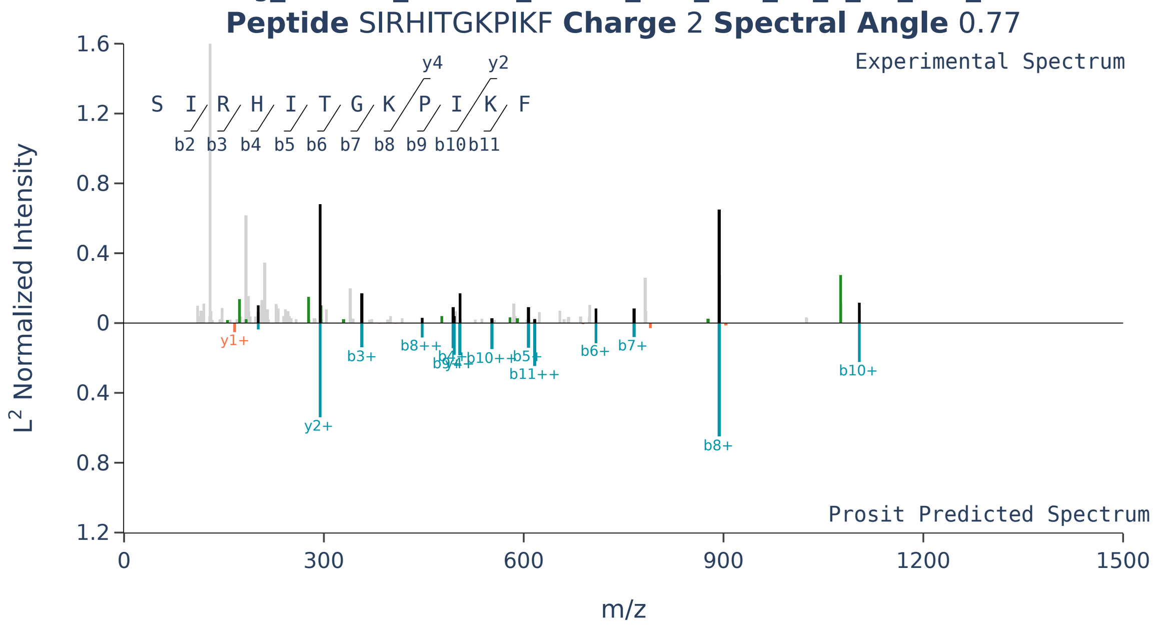

**Source** Ncheng_210623_230623_HFGoe_FFH_20S_25_1_A1_2h_R2 **Scan** 20905
**Peptide** KVLVRMEAIIN **Charge** 3 **Spectral Angle** 0.81

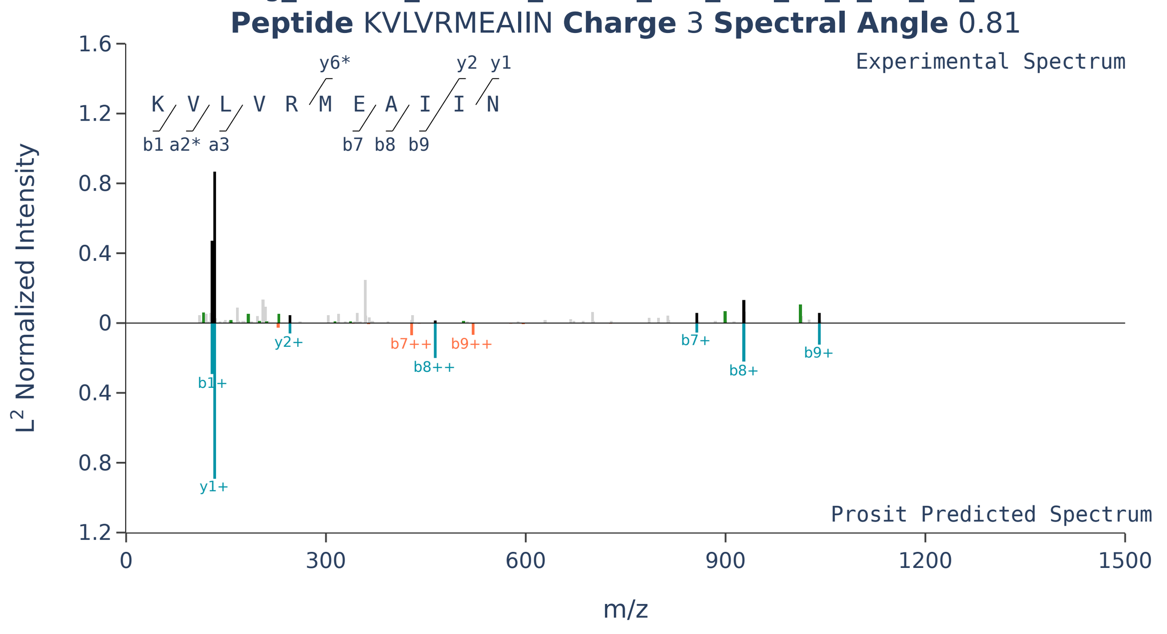

**Source** Ncheng_210623_230623_HFGoe_FFH_20S_25_1_A2_4h_R1 **Scan** 26419
**Peptide** DVYRPAAIKQLETL **Charge** 2 **Spectral Angle** 0.75

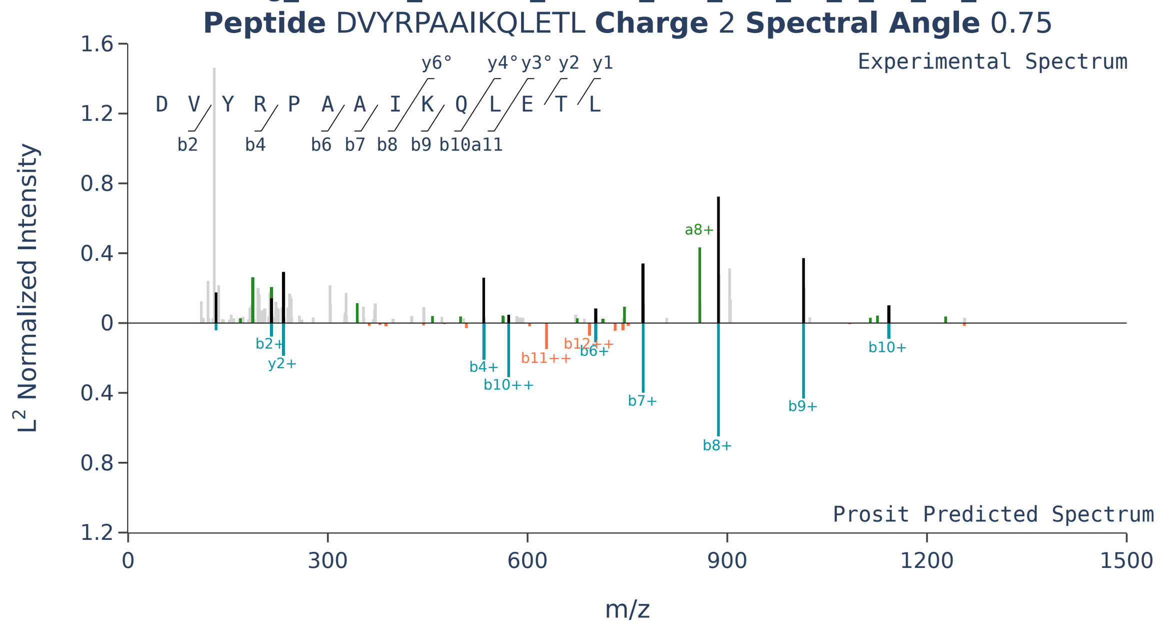

**Source** Ncheng_210623_230623_HFGoe_FFH_20S_25_1_A2_24h_R1 **Scan** 6453
**Peptide** GLQGAGKTTSVGK **Charge** 2 **Spectral Angle** 0.67

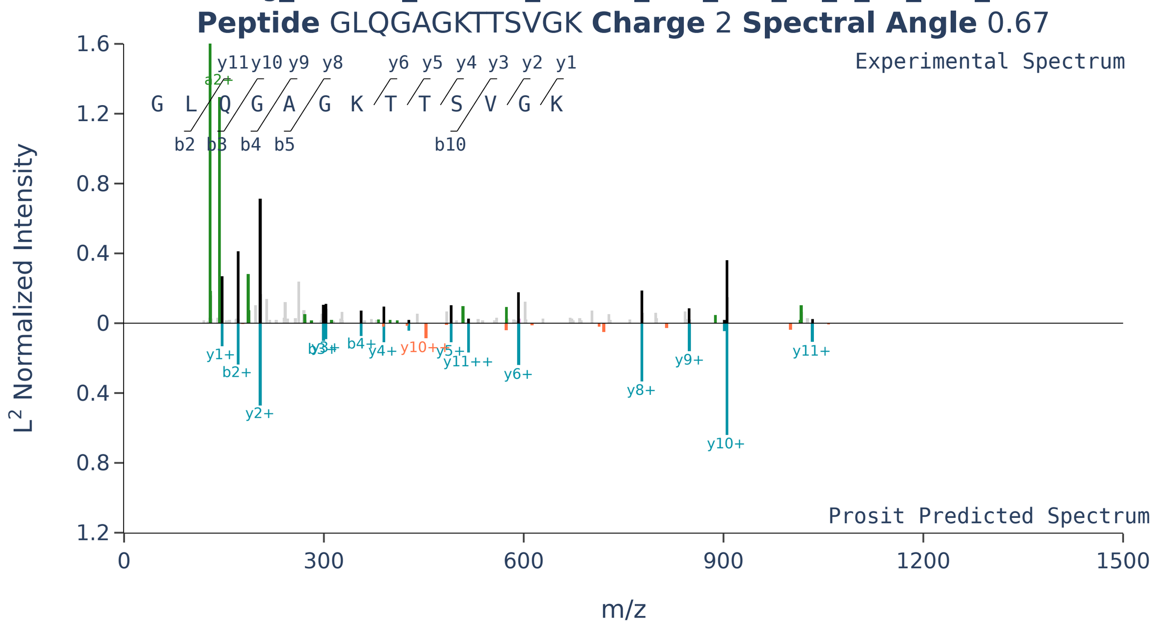

**Source** Ncheng_210623_230623_HFGoe_FFH_20S_25_1_A1_2h_R2 **Scan** 35445
**Peptide** VGHEVNKSLTPGQEFVKIVRNELVAAM **Charge** 3 **Spectral Angle** 0.77

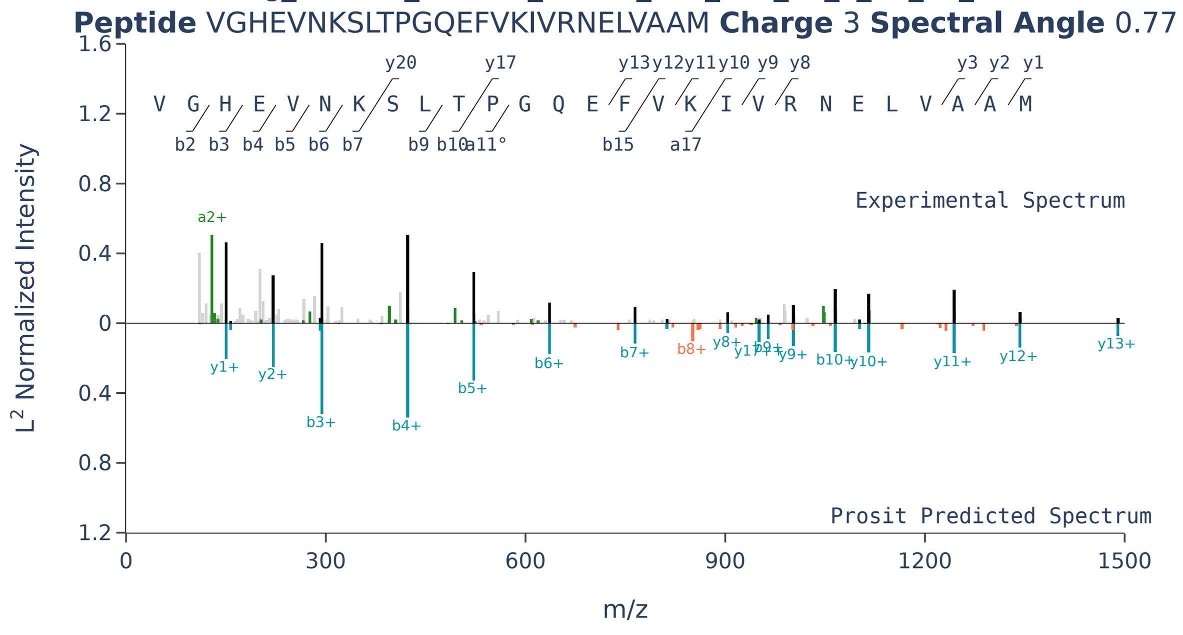

**Source** Ncheng_210623_230623_HFGoe_FFH_20S_25_1_A2_24h_R2 **Scan** 23887
**Peptide** AKAFNEALPLT **Charge** 2 **Spectral Angle** 0.66

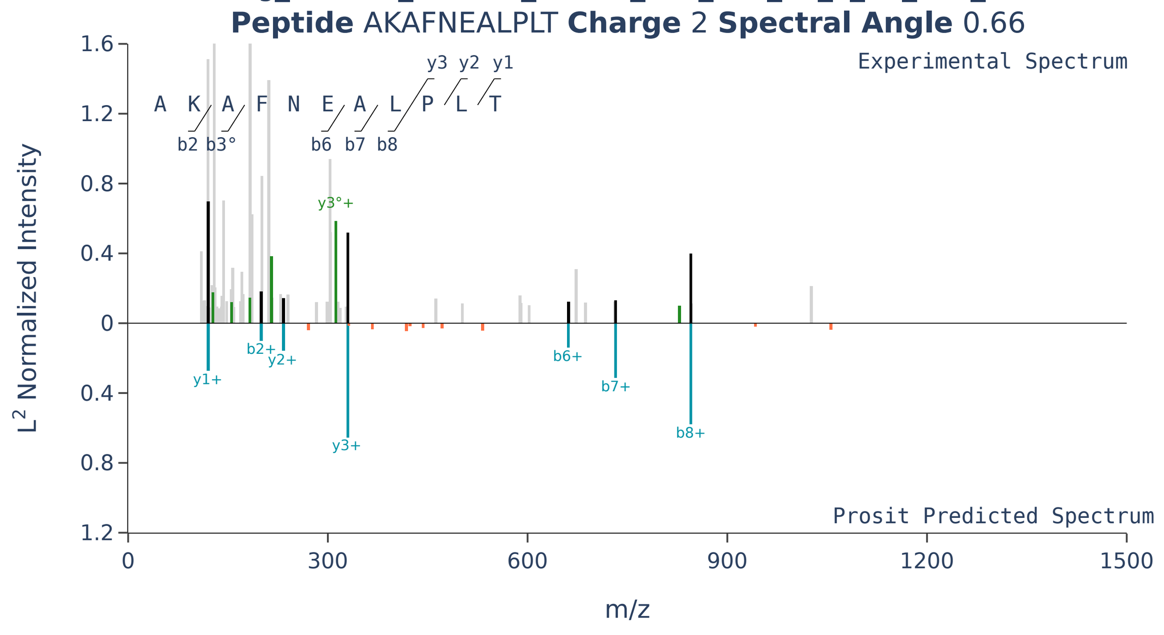

**Source** Ncheng_210623_230623_HFGoe_FFH_20S_25_1_A2_24h_R1 **Scan** 10635
**Peptide** RAQAEKLASKLKKGDGFD **Charge** 2 **Spectral Angle** 0.68

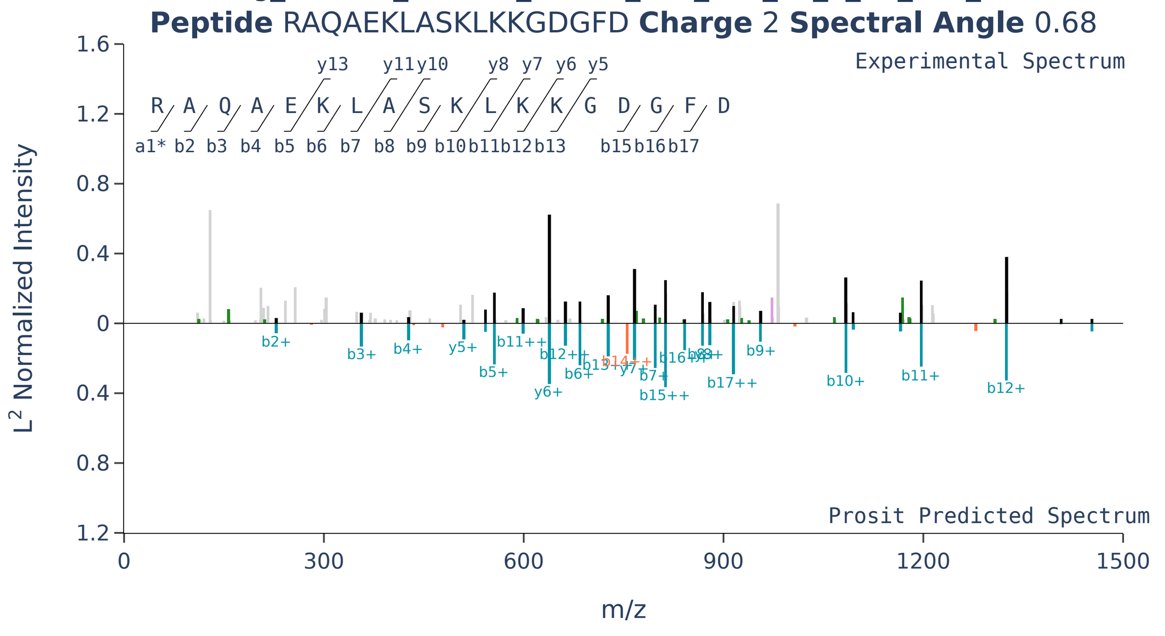

**Source** Ncheng_210623_230623_HFGoe_FFH_20S_25_1_A1_2h_R1 **Scan** 27153
**Peptide** MTGQDAANTAKAFNEALPLTG **Charge** 2 **Spectral Angle** 0.84

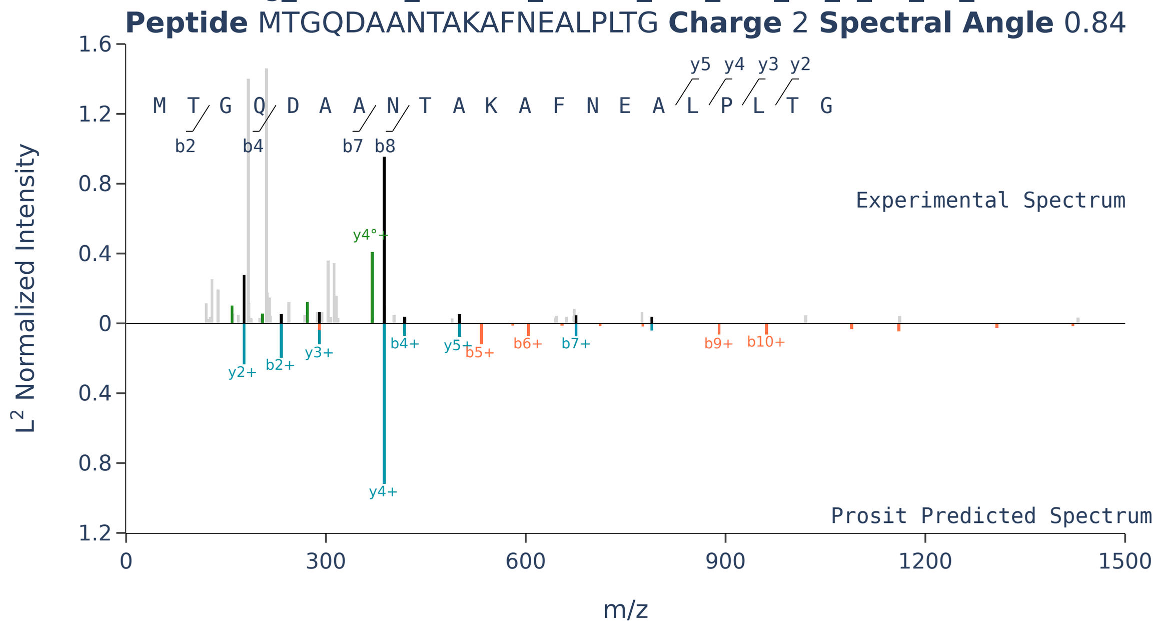

**Source** Ncheng_210623_230623_HFGoe_FFH_20S_25_1_A1_2h_R2 **Scan** 28879
**Peptide** EALPLTGVVLTKVDGDARGG **Charge** 3 **Spectral Angle** 0.67

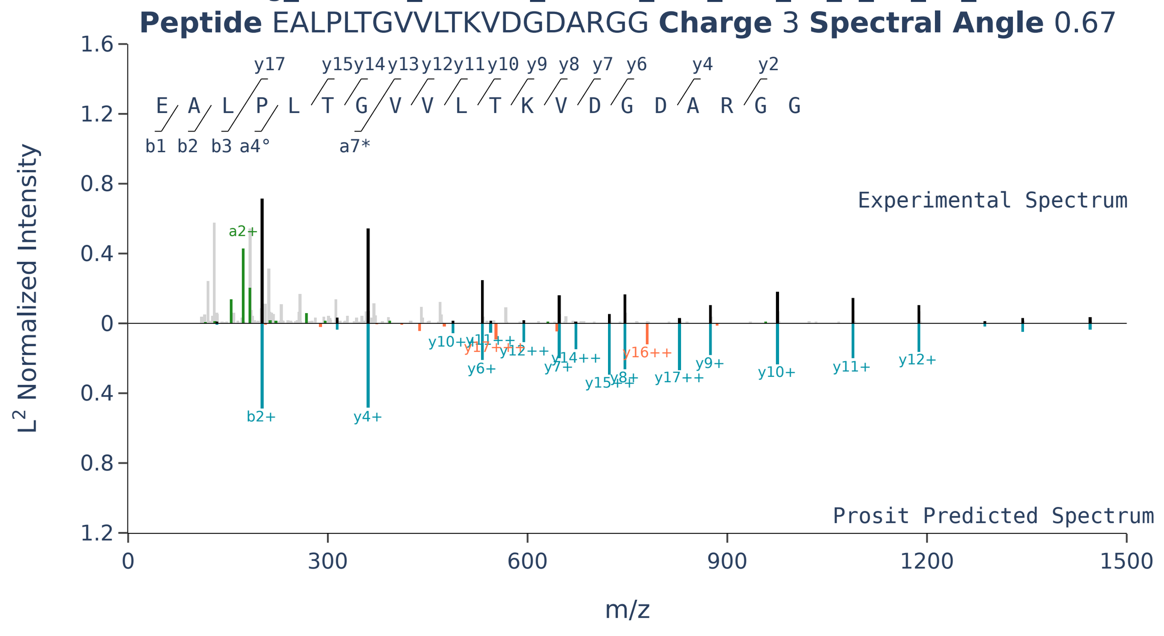

**Source** Ncheng_210623_230623_HFGoe_FFH_20S_25_1_A2_2h_R2 **Scan** 35314
**Peptide** ENQTLNLAAQPPAVVLM **Charge** 2 **Spectral Angle** 0.82

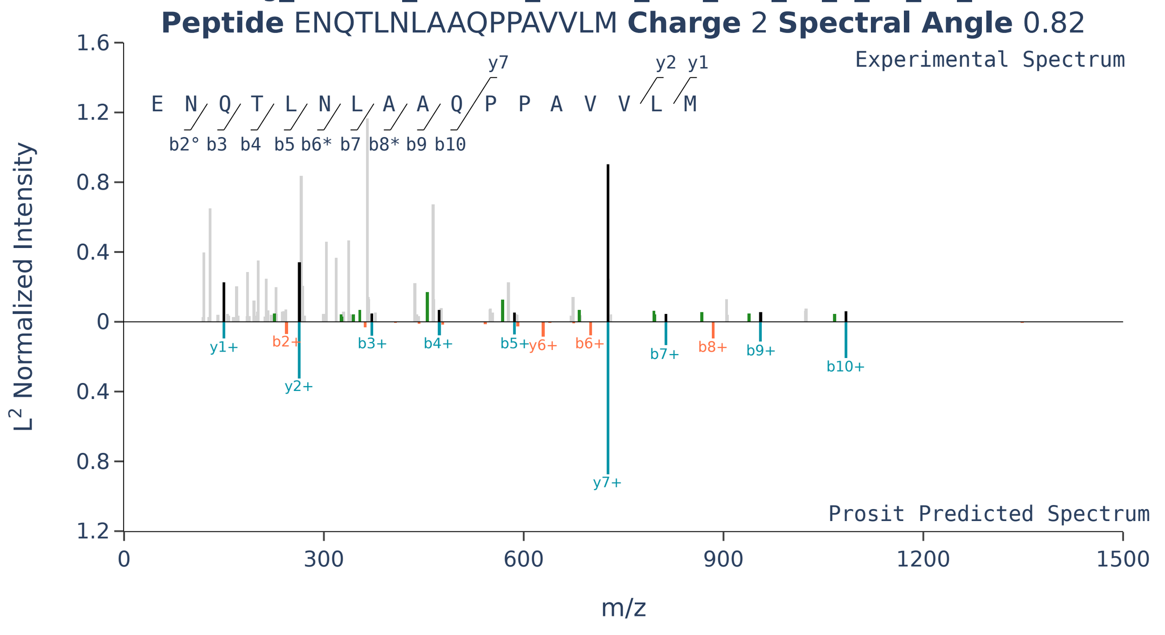

**Source** Ncheng_210623_230623_HFGoe_FFH_20S_25_1_A2_2h_R2 **Scan** 29599
**Peptide** KNMGGMASLMGKLPGMGQIPDNVKSQMD **Charge** 3 **Spectral Angle** 0.81

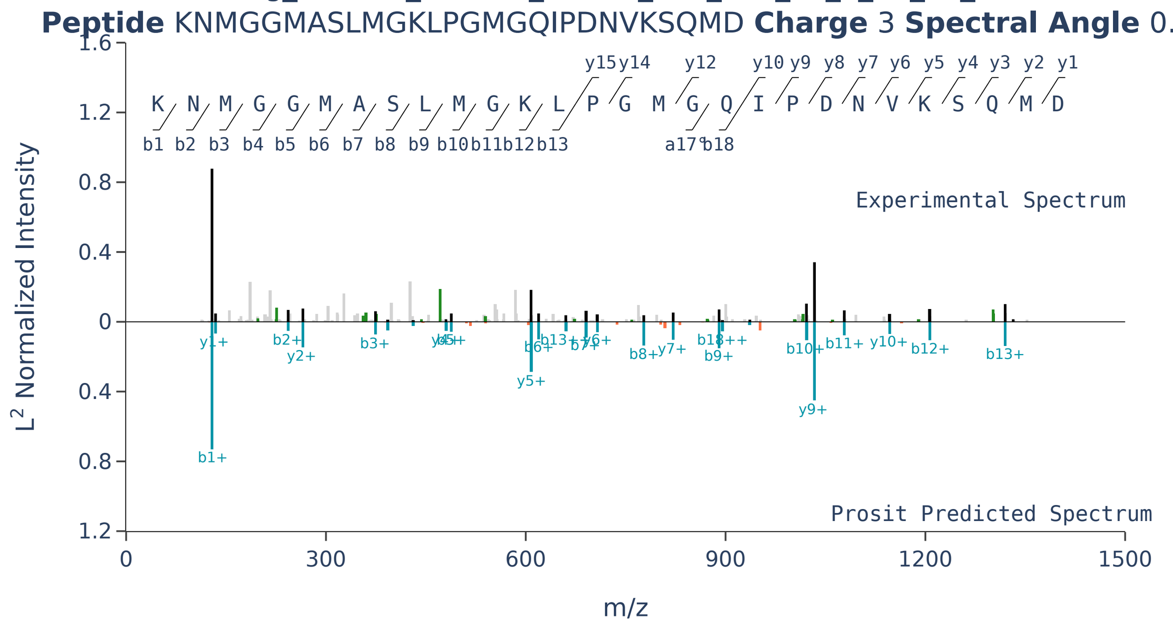

**Source** Ncheng_210623_230623_HFGoe_FFH_20S_25_1_A1_2h_R2 **Scan** 33763
**Peptide** ADVALPVVREFINRVKEKAVGHE **Charge** 3 **Spectral Angle** 0.83

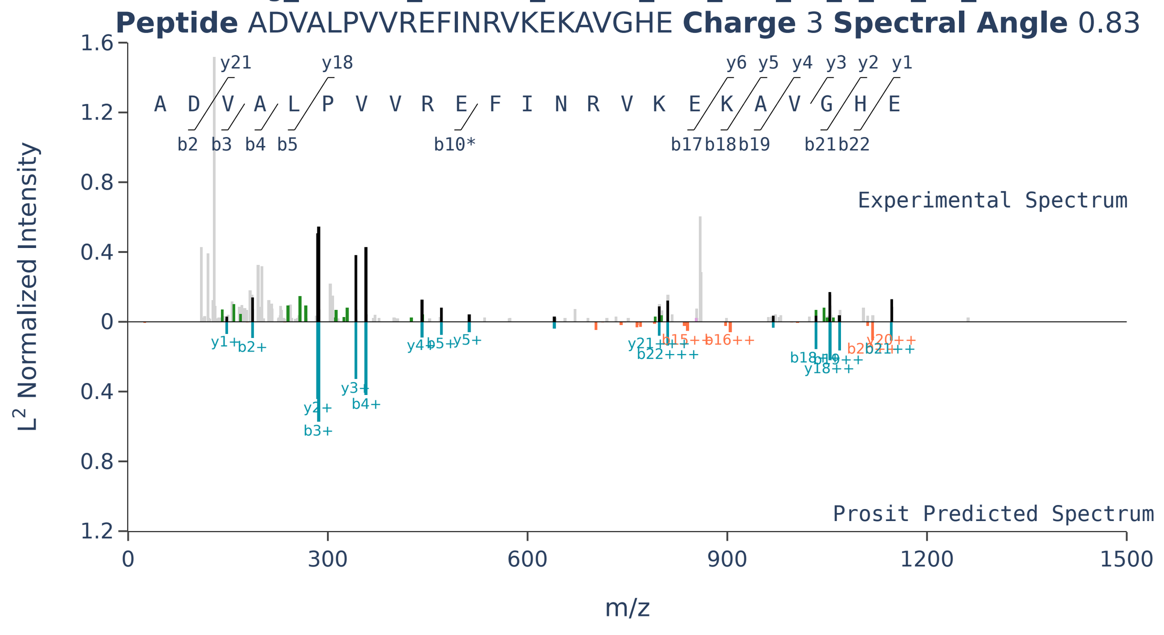

**Source** Ncheng_210623_230623_HFGoe_FFH_20S_25_1_A2_4h_R1 **Scan** 29516
**Peptide** KNMGGMASLMGKLPGM **Charge** 3 **Spectral Angle** 0.68

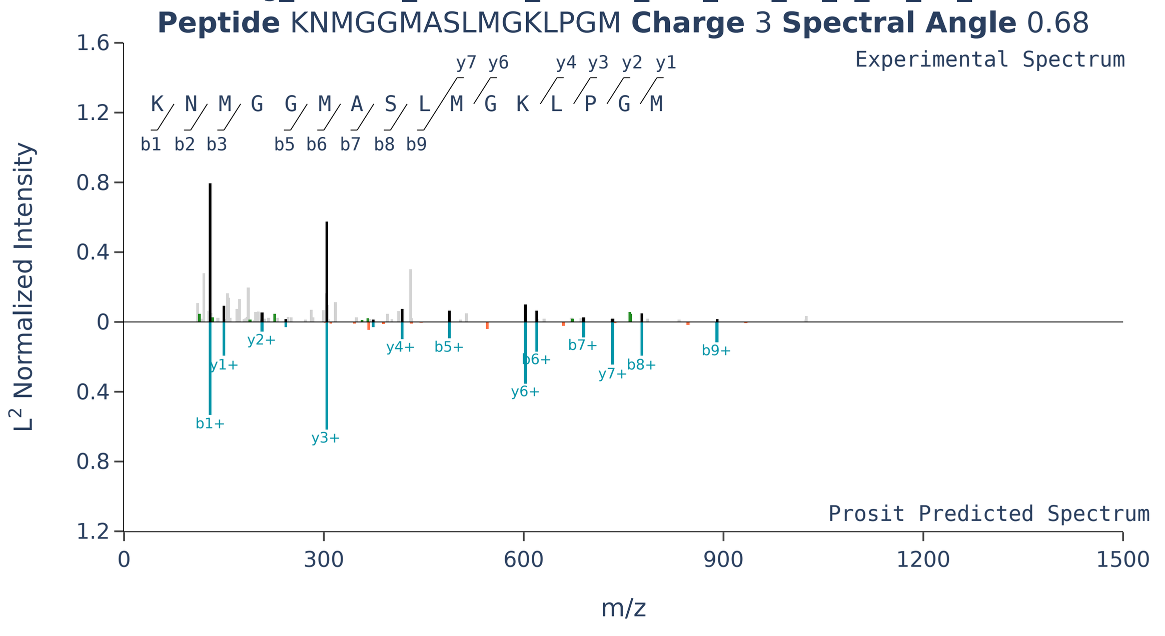

**Source** Ncheng_210623_230623_HFGoe_FFH_20S_25_1_A1_2h_R1 **Scan** 19681
**Peptide** FVKIVRNELVA **Charge** 3 **Spectral Angle** 0.6

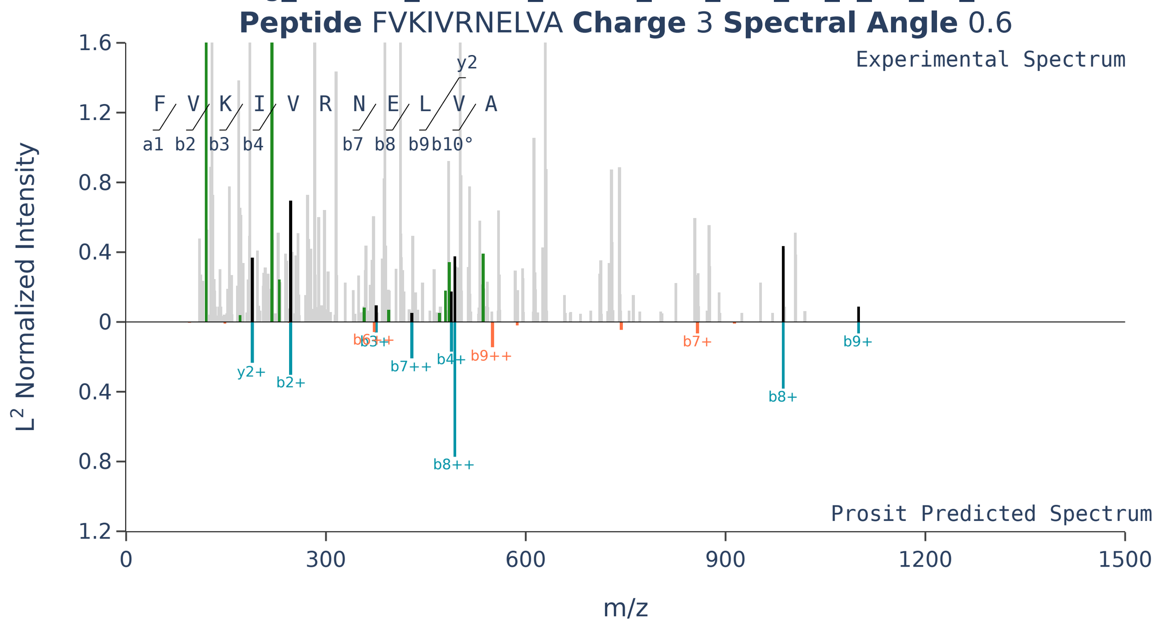

**Source** Ncheng_210623_230623_HFGoe_FFH_20S_25_1_A2_24h_R1 **Scan** 6824
**Peptide** RAQAEKLASKLKK **Charge** 3 **Spectral Angle** 0.81

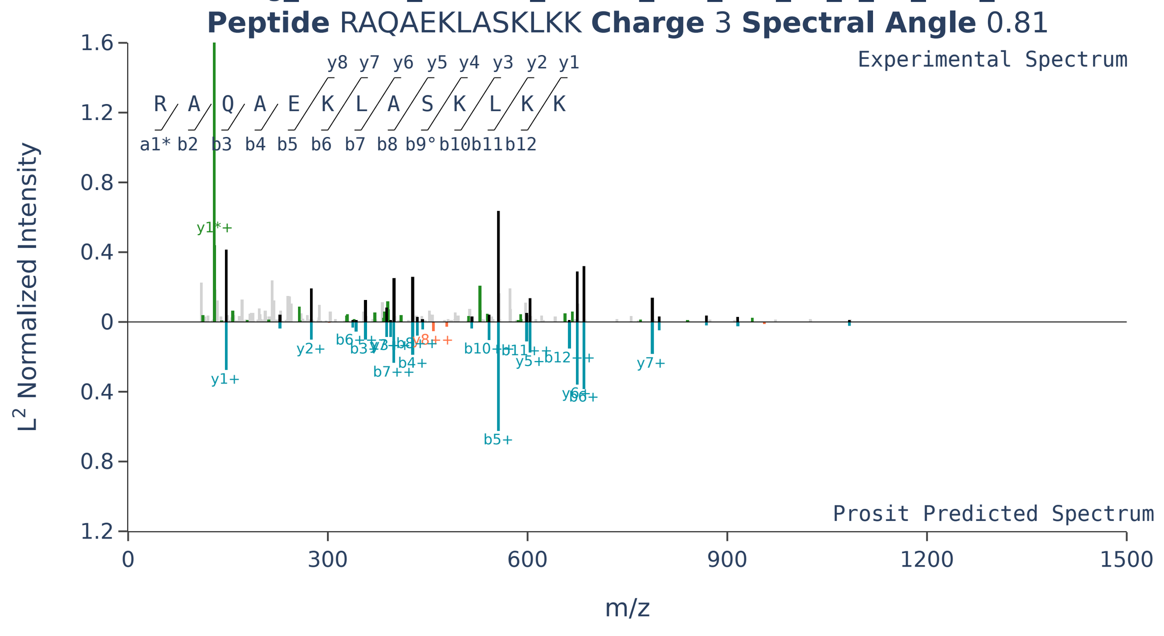

**Source** Ncheng_210623_230623_HFGoe_FFH_20S_25_1_A1_24h_R1 **Scan** 17088
**Peptide** EAIINSMTMKERA **Charge** 2 **Spectral Angle** 0.68

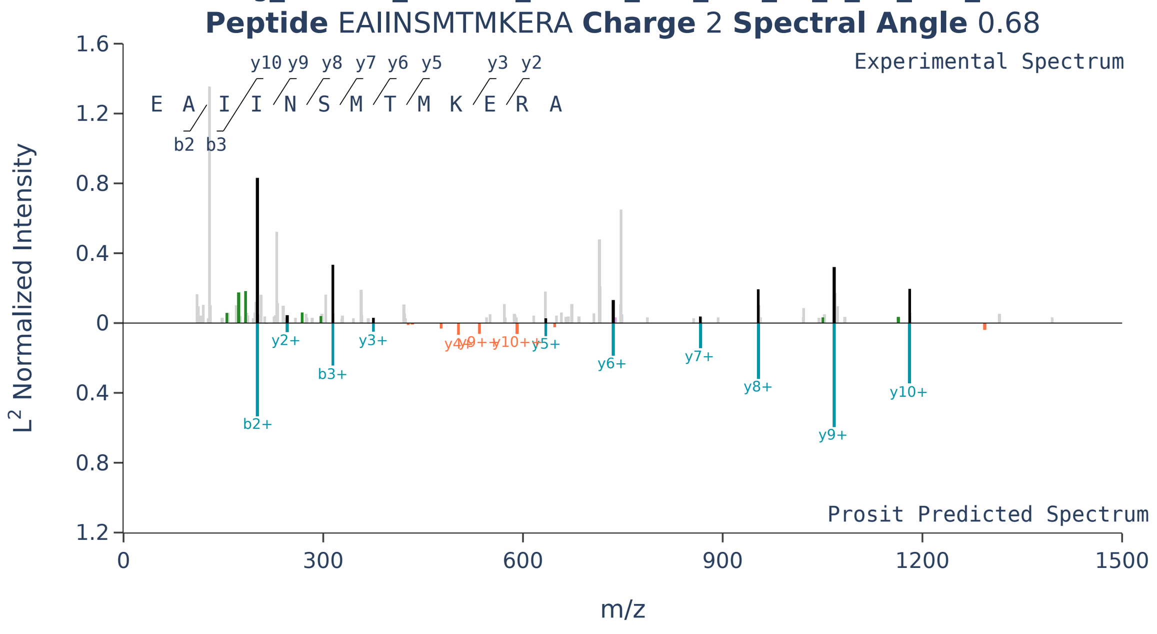

**Source** Ncheng_210623_230623_HFGoe_FFH_20S_25_1_A2_4h_R1 **Scan** 25279
**Peptide** NDFLEQLRQMKN **Charge** 2 **Spectral Angle** 0.73

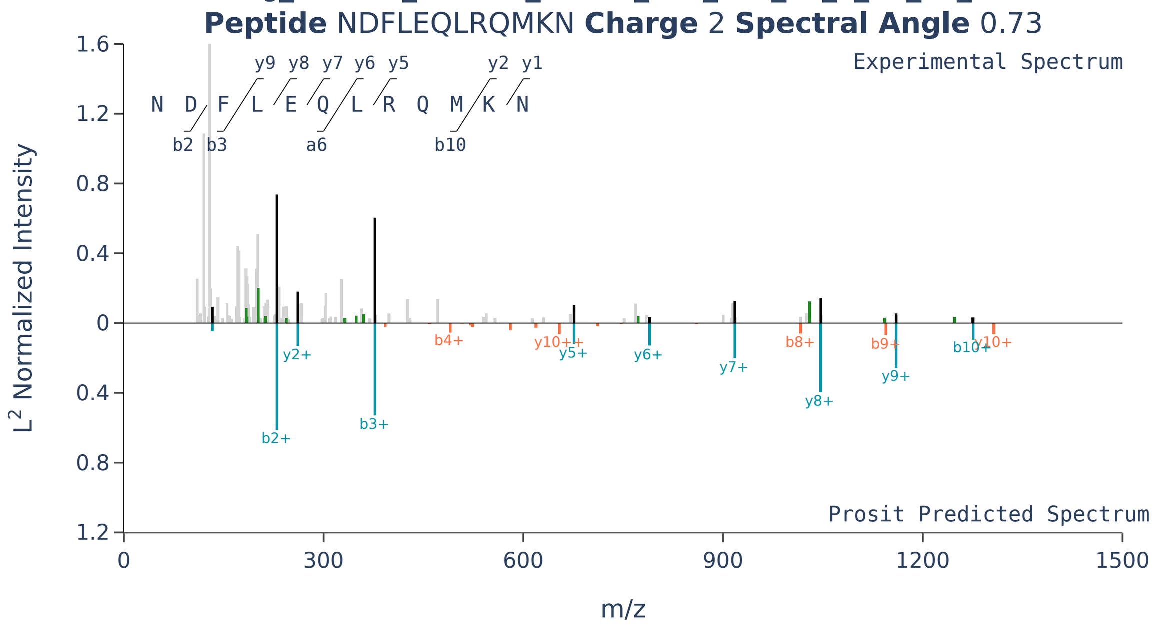

**Source** Ncheng_210623_230623_HFGoe_FFH_20S_25_1_A2_24h_R1 **Scan** 6712
**Peptide** ISGRGRL **Charge** 2 **Spectral Angle** 0.86

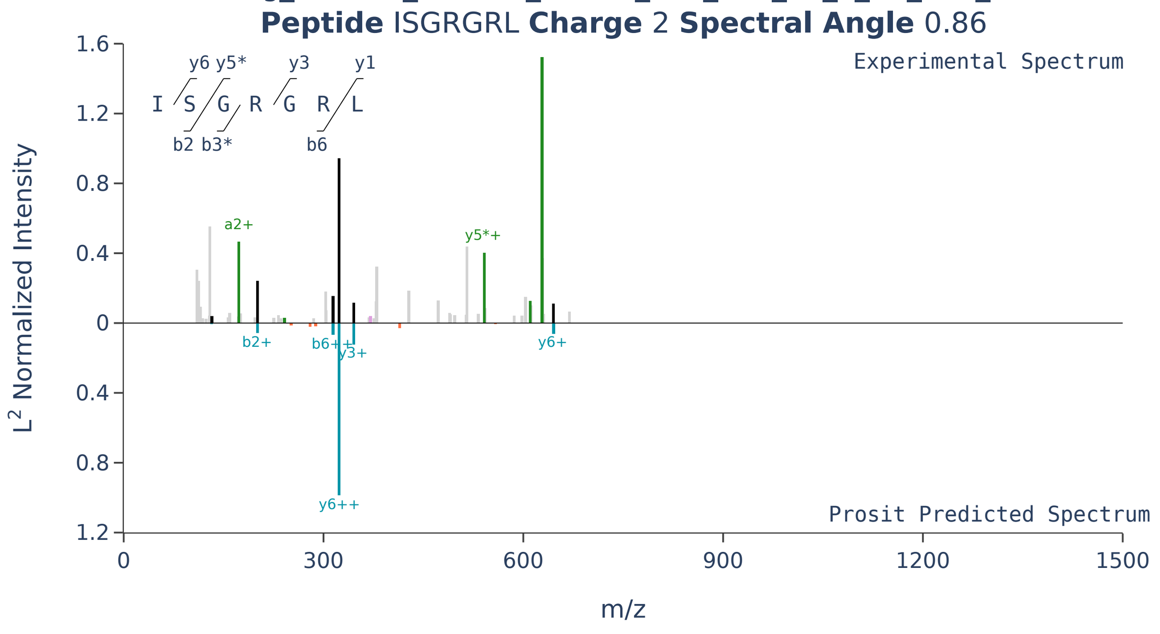

**Source** Ncheng_210623_230623_HFGoe_FFH_20S_25_1_A2_2h_R2 **Scan** 18572
**Peptide** MDDKVLVRMEA **Charge** 3 **Spectral Angle** 0.85

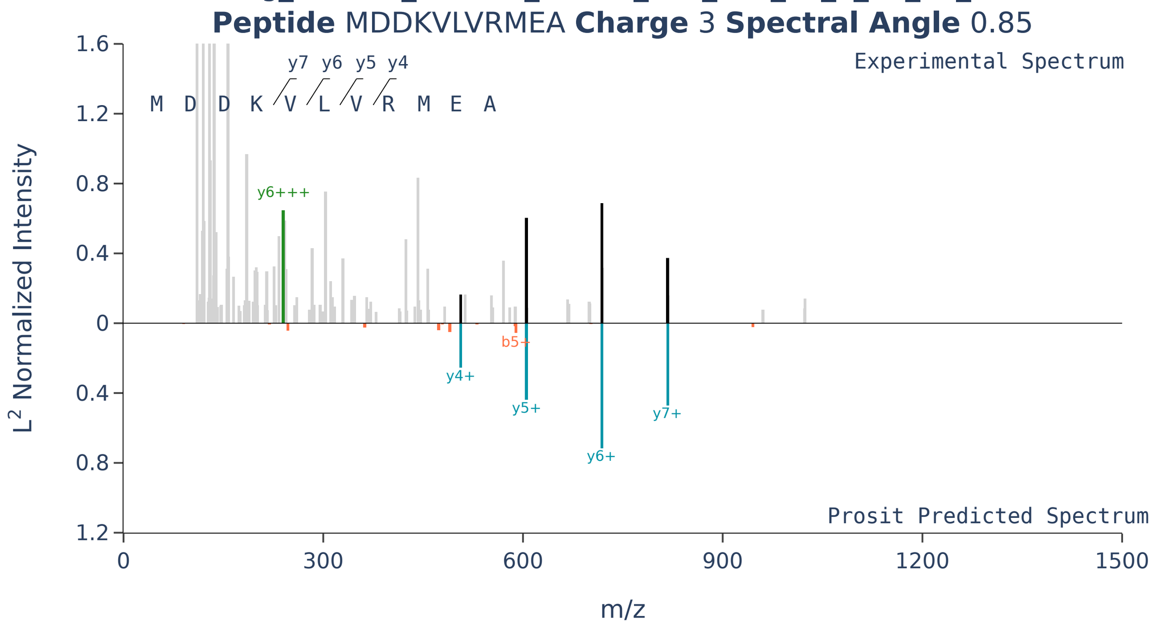

**Source** Ncheng_210623_230623_HFGoe_FFH_20S_25_1_A1_24h_R2 **Scan** 11225
**Peptide** GLQGAGKTTSVGKLG **Charge** 2 **Spectral Angle** 0.7

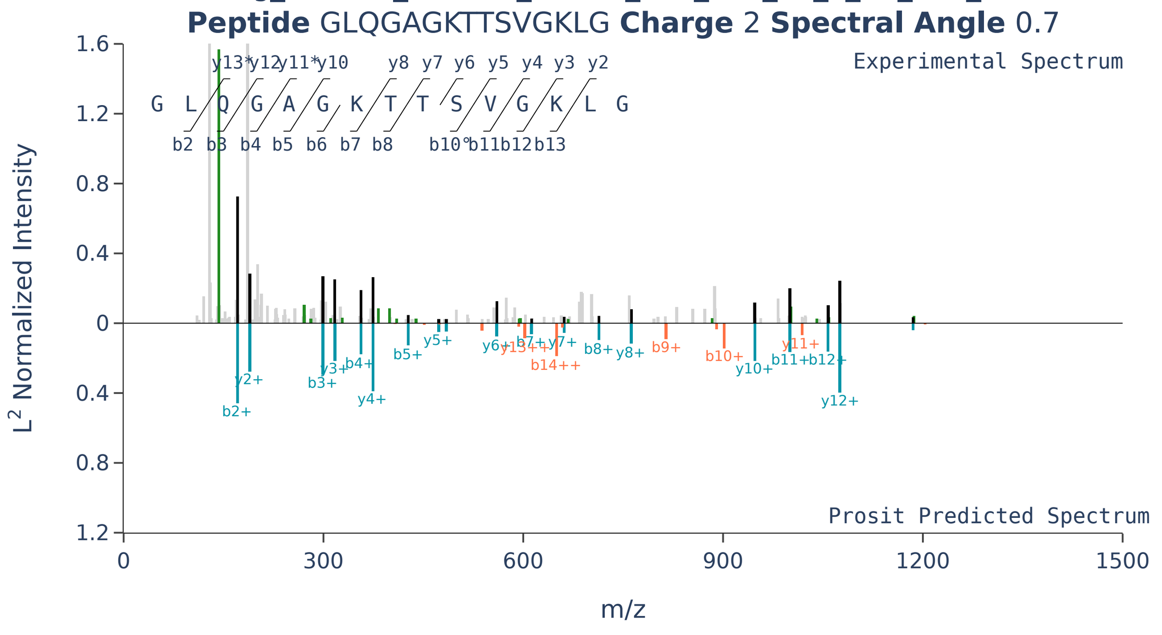

**Source** Ncheng_210623_230623_HFGoe_FFH_20S_25_1_A2_4h_R2 **Scan** 7702
**Peptide** FLNRVKE **Charge** 2 **Spectral Angle** 0.75

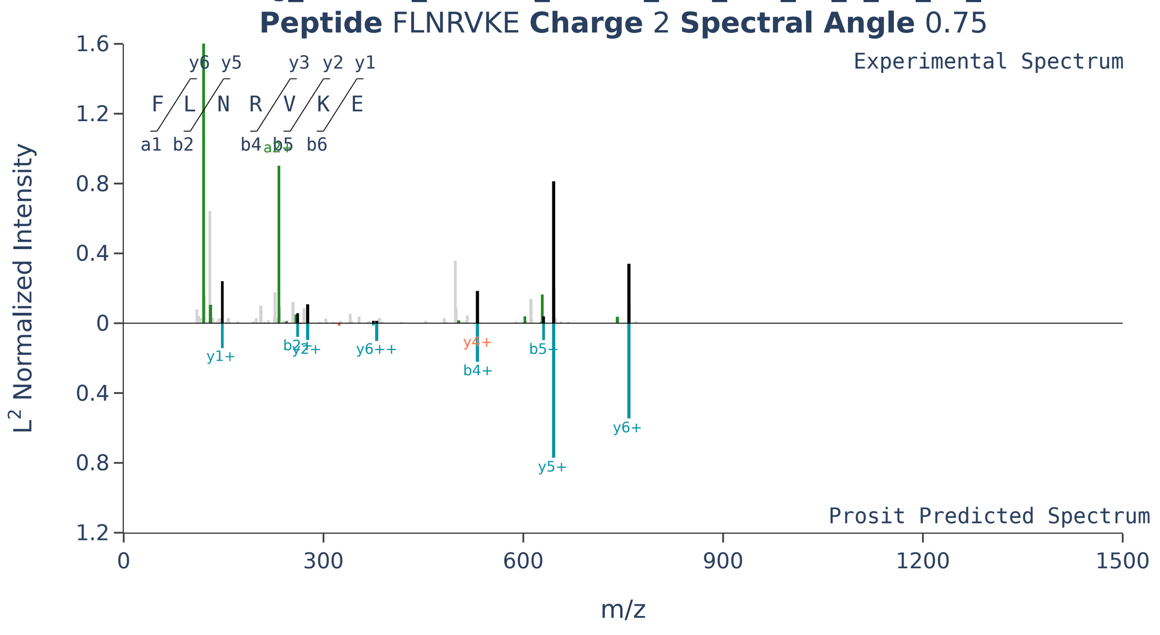

**Source** Ncheng_210623_230623_HFGoe_FFH_20S_25_1_A1_2h_R1 **Scan** 24321
**Peptide** KVLVRMEAII **Charge** 3 **Spectral Angle** 0.88

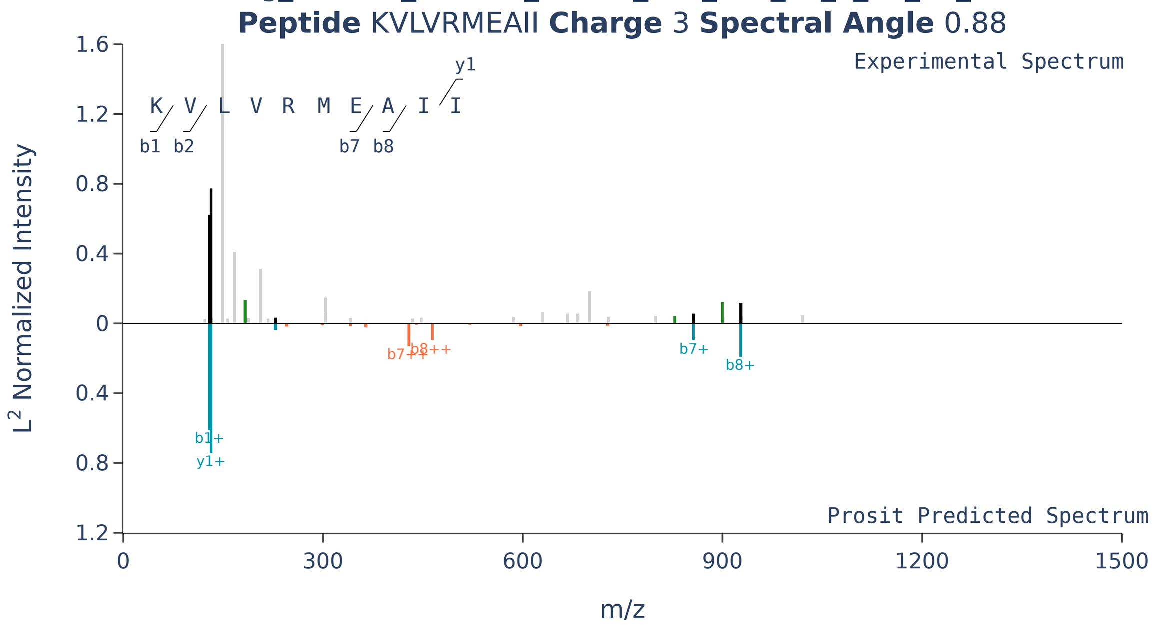

**Source** Ncheng_210623_230623_HFGoe_FFH_20S_25_1_A2_24h_R1 **Scan** 13723
**Peptide** QGAGKTTSVGKLGKFLREK **Charge** 5 **Spectral Angle** 0.74

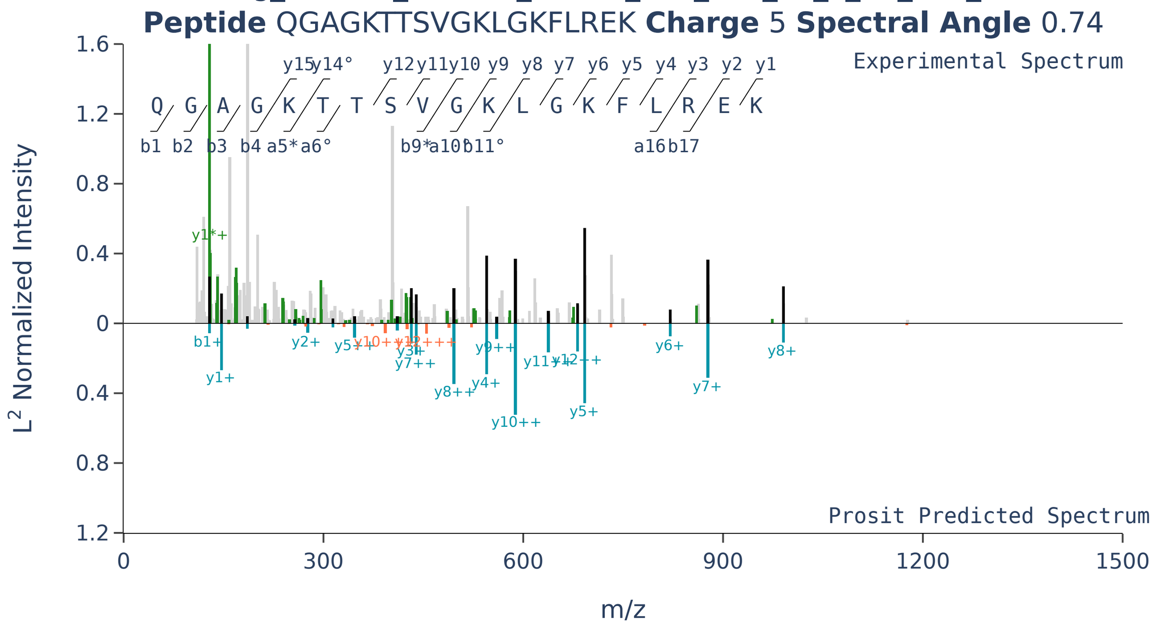

**Source** Ncheng_210623_230623_HFGoe_FFH_20S_25_1_A1_4h_R1 **Scan** 20032
**Peptide** KTEALEPFHPDRIASRL **Charge** 3 **Spectral Angle** 0.62

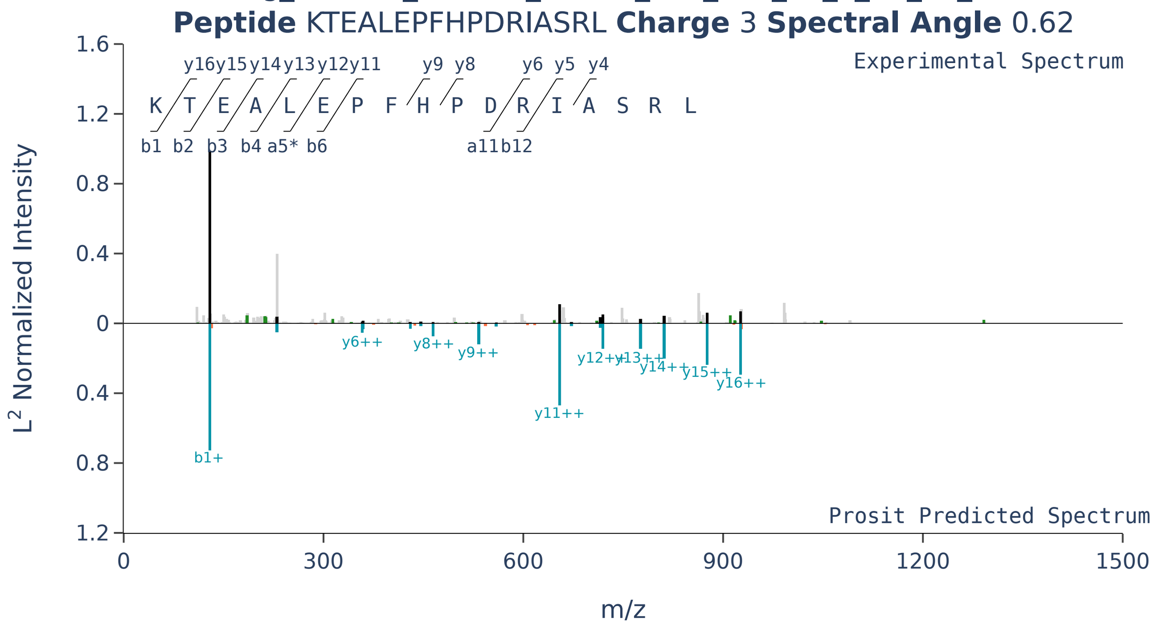

**Source** Ncheng_210623_230623_HFGoe_FFH_20S_25_1_A1_4h_R2 **Scan** 12163
**Peptide** AALSIRHITGK **Charge** 3 **Spectral Angle** 0.77

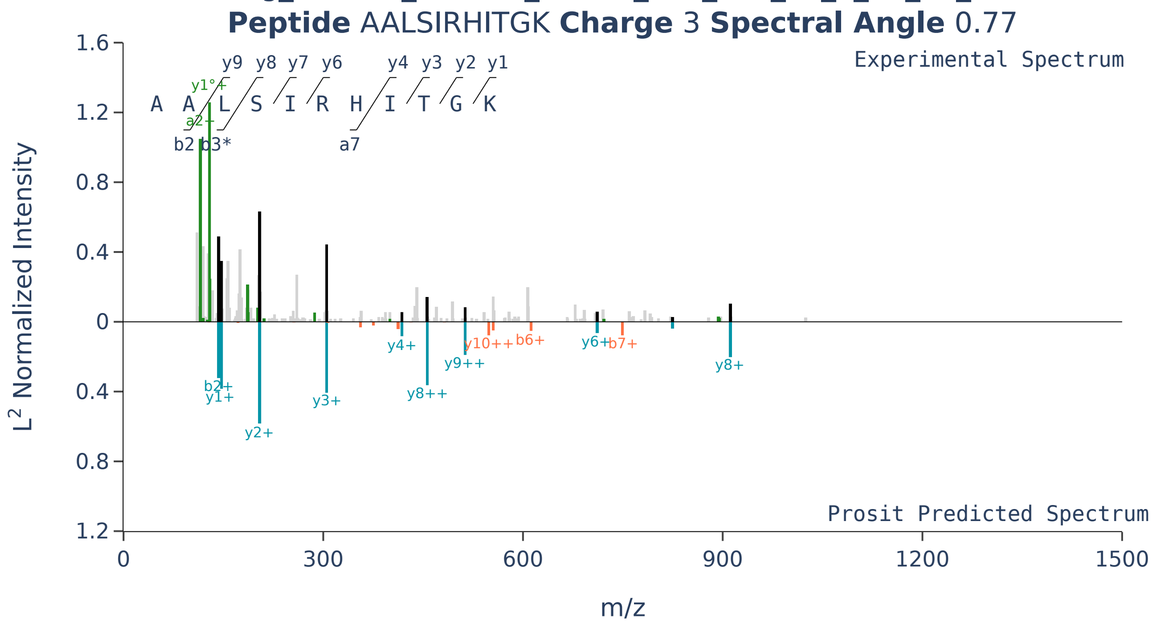

**Source** Ncheng_210623_230623_HFGoe_FFH_20S_25_1_A1_2h_R2 **Scan** 32109
**Peptide** LVAAMGEENQTLNLAAQPPAVV **Charge** 2 **Spectral Angle** 0.92

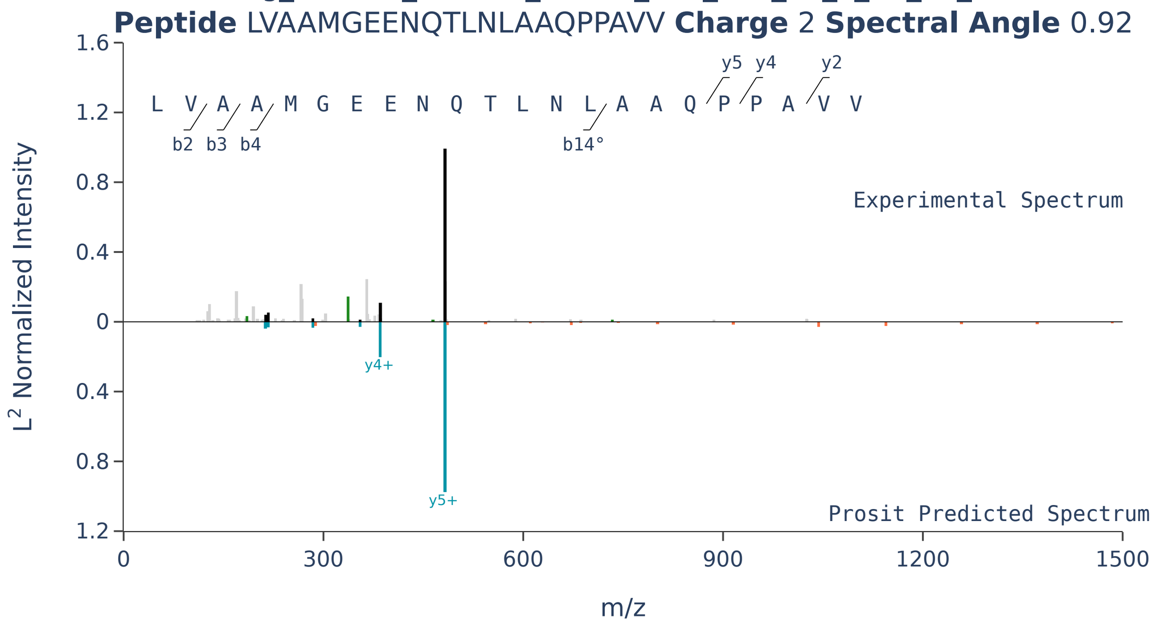

**Source** Ncheng_210623_230623_HFGoe_FFH_20S_25_1_A1_2h_R2 **Scan** 37998
**Peptide** GQIPDNVKSQMDDKVLVRMEAIINSM **Charge** 4 **Spectral Angle** 0.91

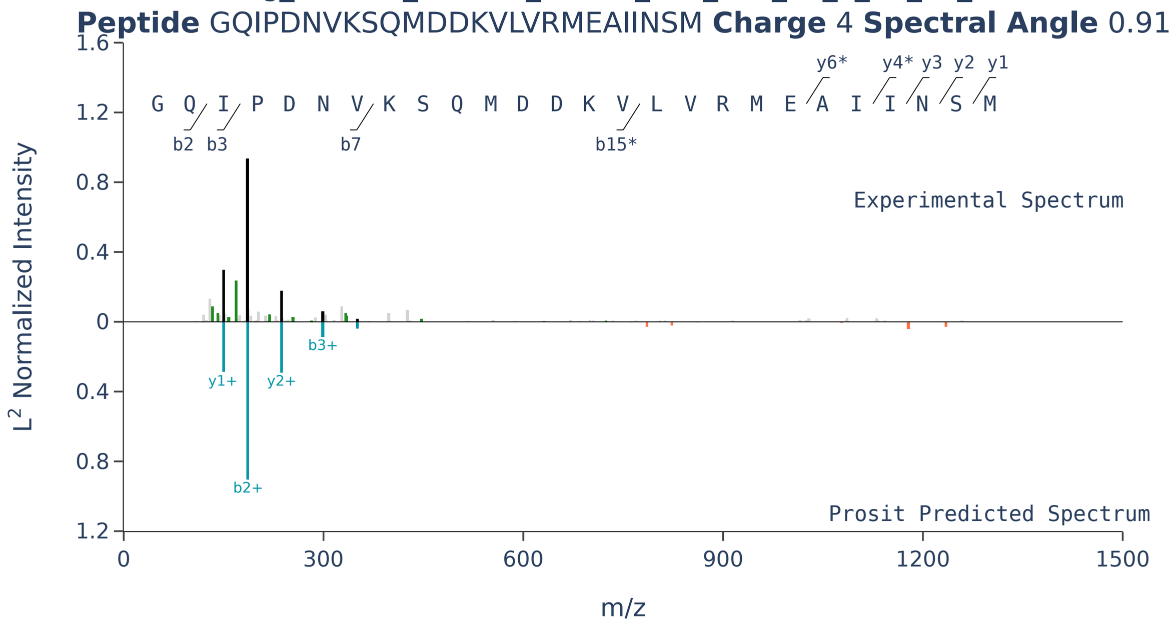

**Source** Ncheng_210623_230623_HFGoe_FFH_20S_25_1_A2_2h_R2 **Scan** 18583
**Peptide** DLESKVDRAQAEKLASKLKKGDGFD **Charge** 3 **Spectral Angle** 0.76

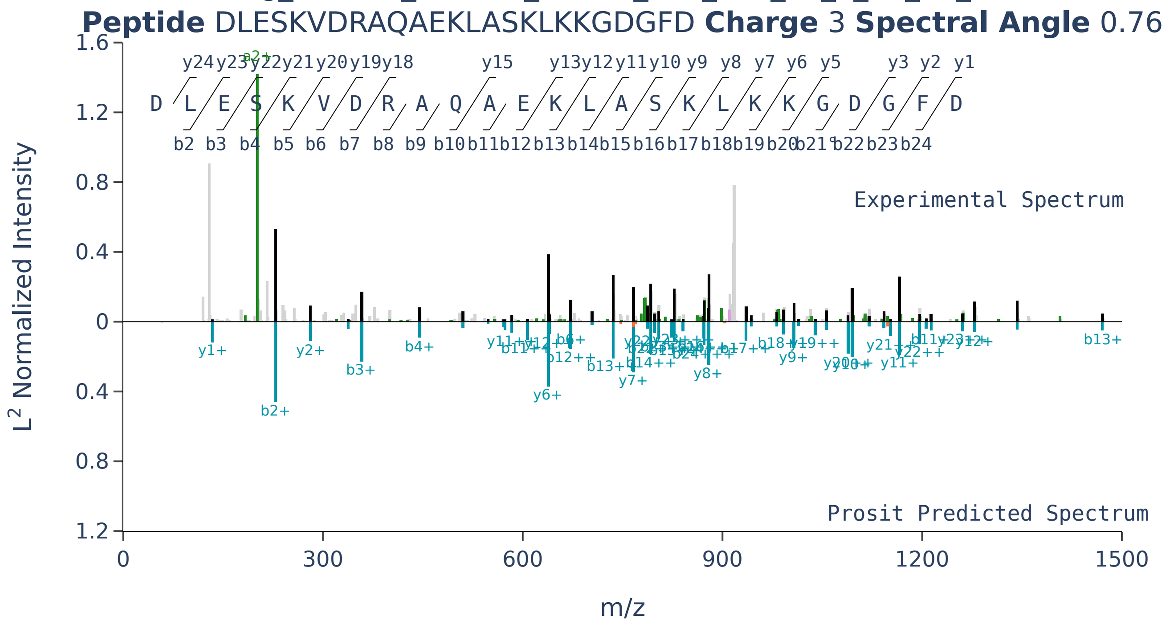

**Source** Ncheng_210623_230623_HFGoe_FFH_20S_25_1_A1_24h_R1 **Scan** 9930
**Peptide** NLTDRLSR **Charge** 2 **Spectral Angle** 0.65

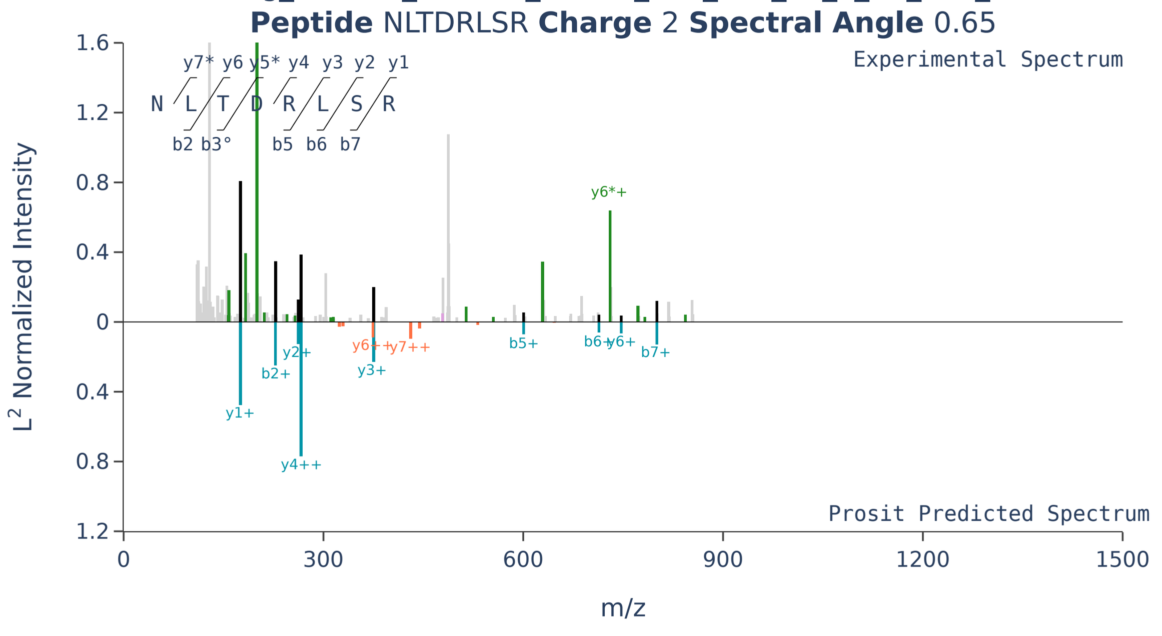

**Source** Ncheng_210623_230623_HFGoe_FFH_20S_25_1_A1_4h_R2 **Scan** 20999
**Peptide** AVGHEVNKSLTPGQEFVKL **Charge** 3 **Spectral Angle** 0.76

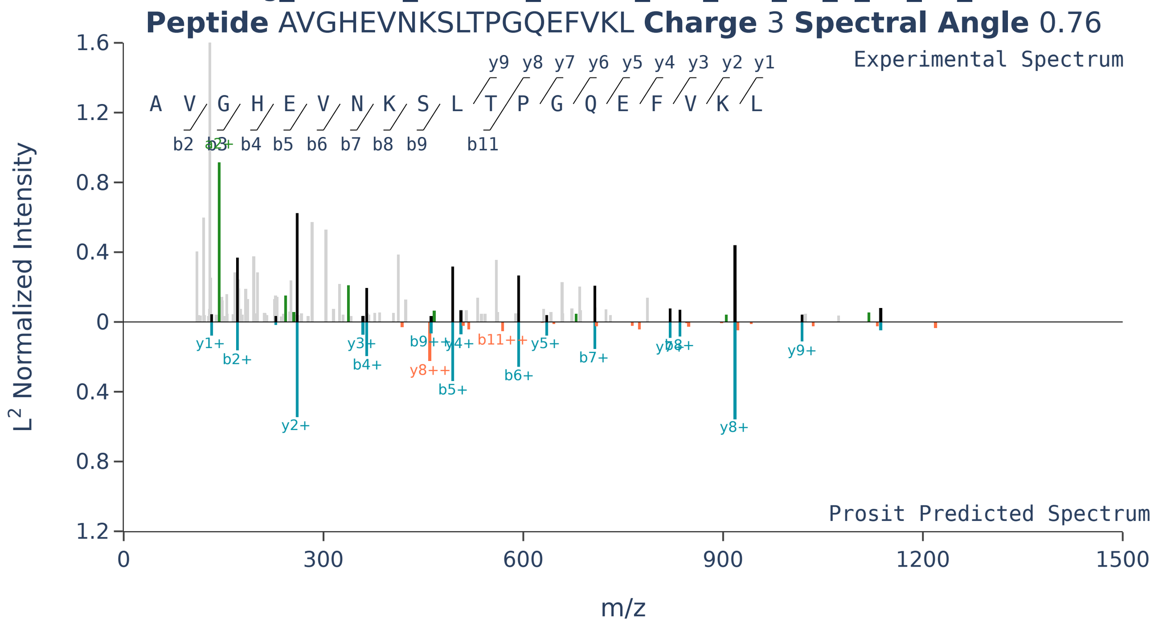

**Source** Ncheng_210623_230623_HFGoe_FFH_20S_25_1_A2_24h_R1 **Scan** 23552
**Peptide** HEVNKSLTPGQEFVKLV **Charge** 4 **Spectral Angle** 0.71

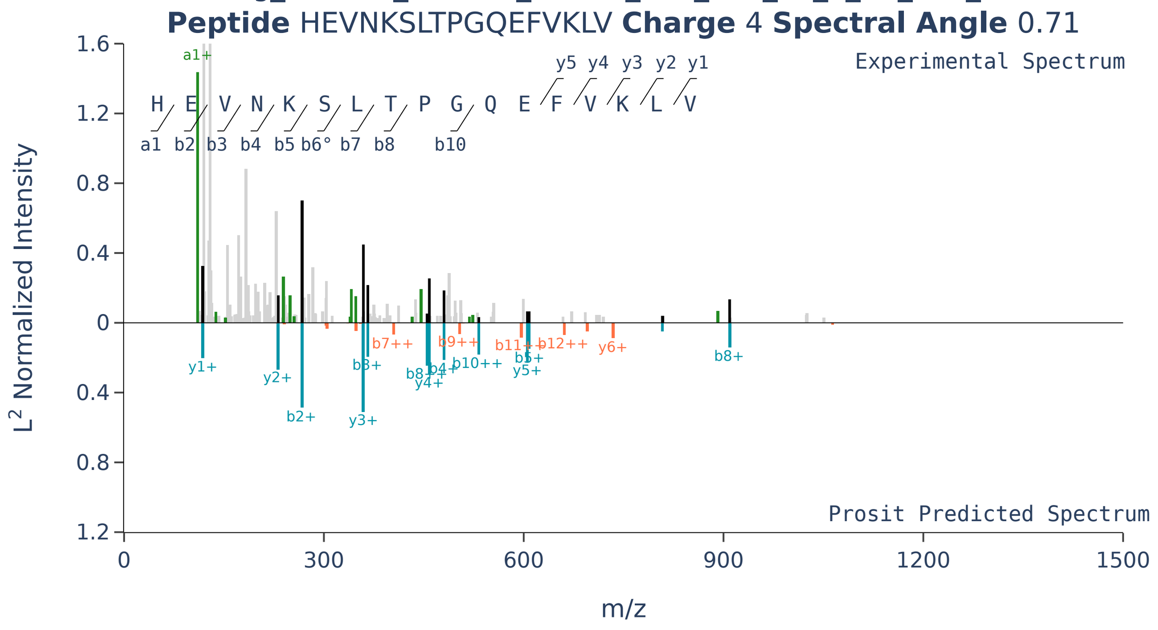


**Source** Ncheng_210623_230623_HFGoe_FFH_20S_25_1_A1_24h_R2 **Scan** 7942
**Peptide** TAGRLHVDEA **Charge** 3 **Spectral Angle** 0.63

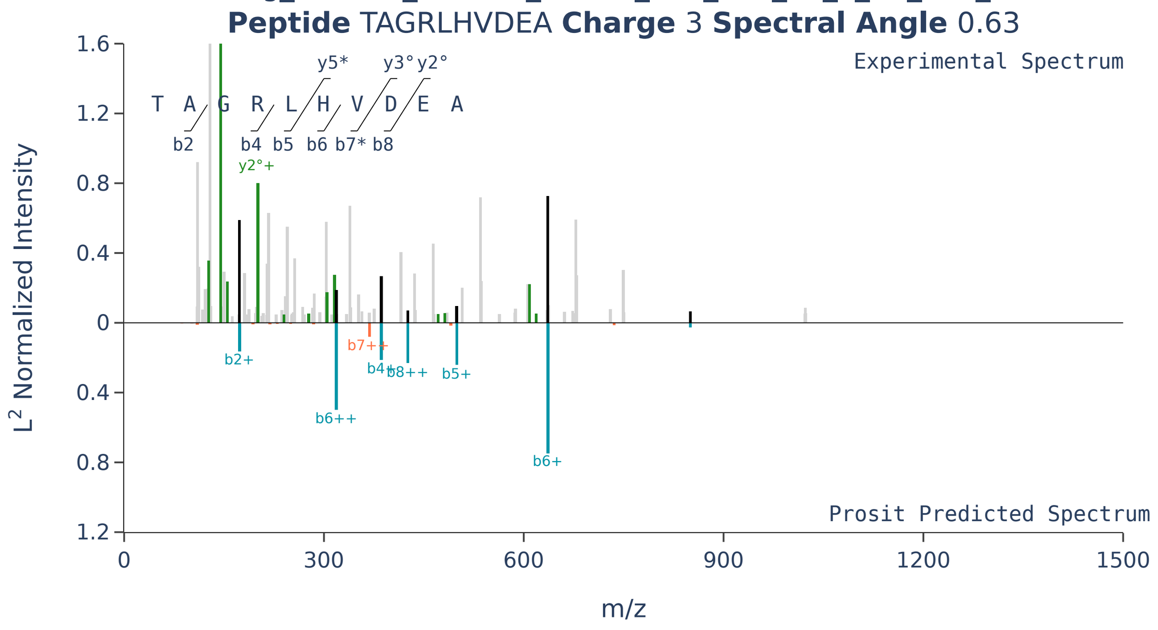


**Source** Ncheng_210623_230623_HFGoe_FFH_20S_25_1_A1_4h_R2 **Scan** 10126
**Peptide** RGRLTEDNVKDTLRE **Charge** 4 **Spectral Angle** 0.66

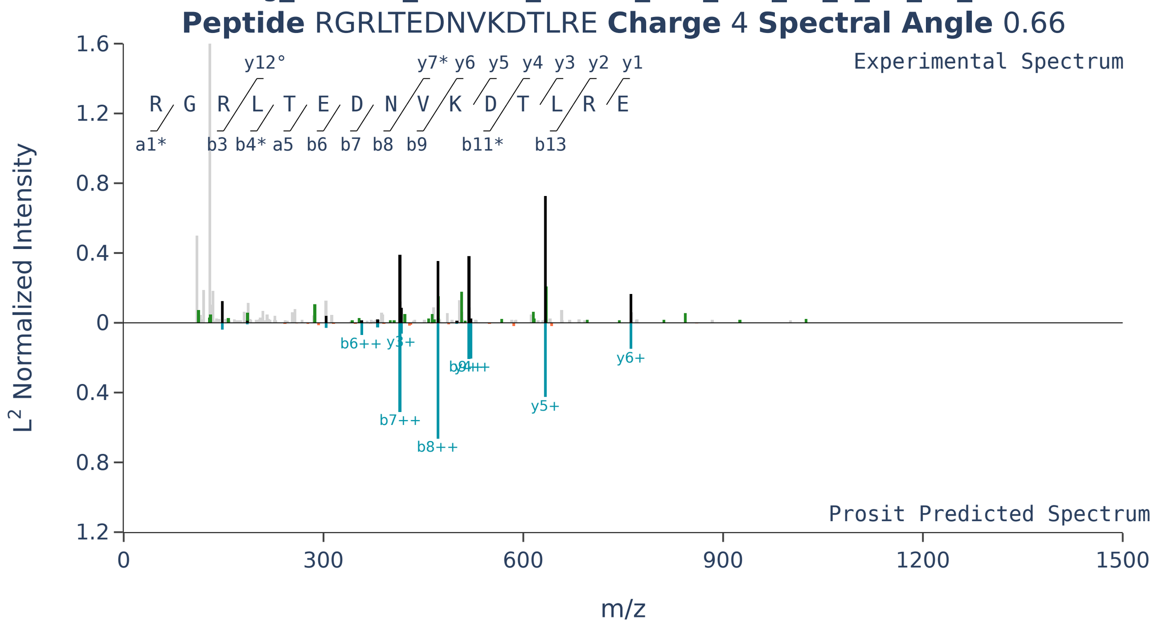


**Source** Ncheng_210623_230623_HFGoe_FFH_20S_25_1_A1_2h_R1 **Scan** 10282
**Peptide** MKERAKPEII **Charge** 2 **Spectral Angle** 0.53

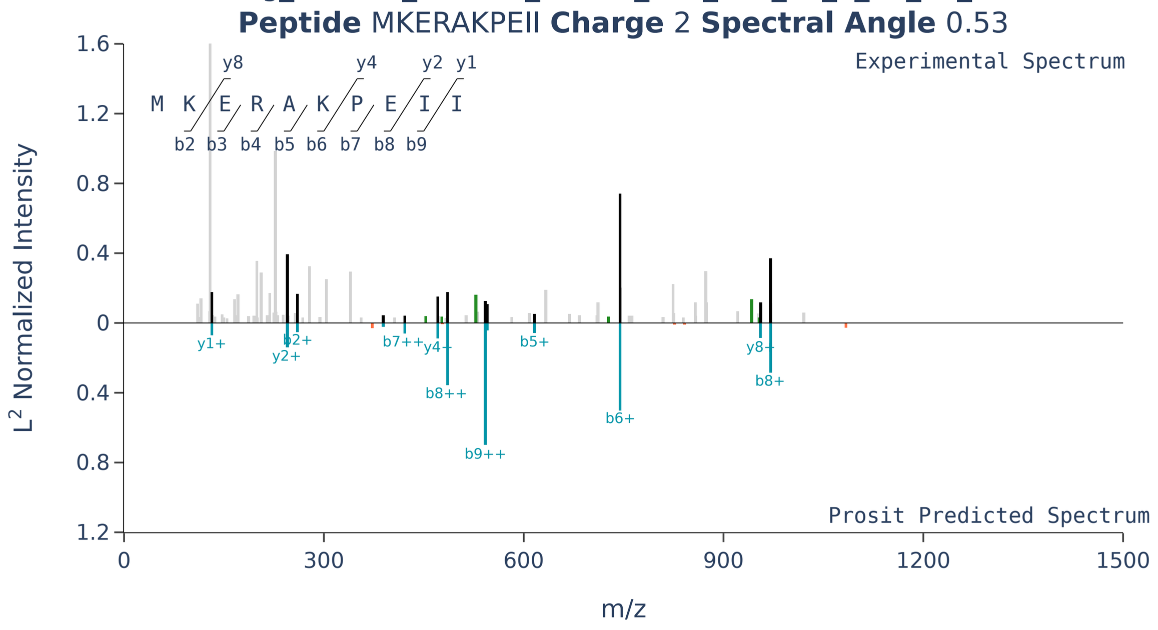


**Source** Ncheng_210623_230623_HFGoe_FFH_20S_25_1_A2_4h_R1 **Scan** 34452
**Peptide** GQDAANTAKAFNEALPLTGVVLTKVD **Charge** 3 **Spectral Angle** 0.75

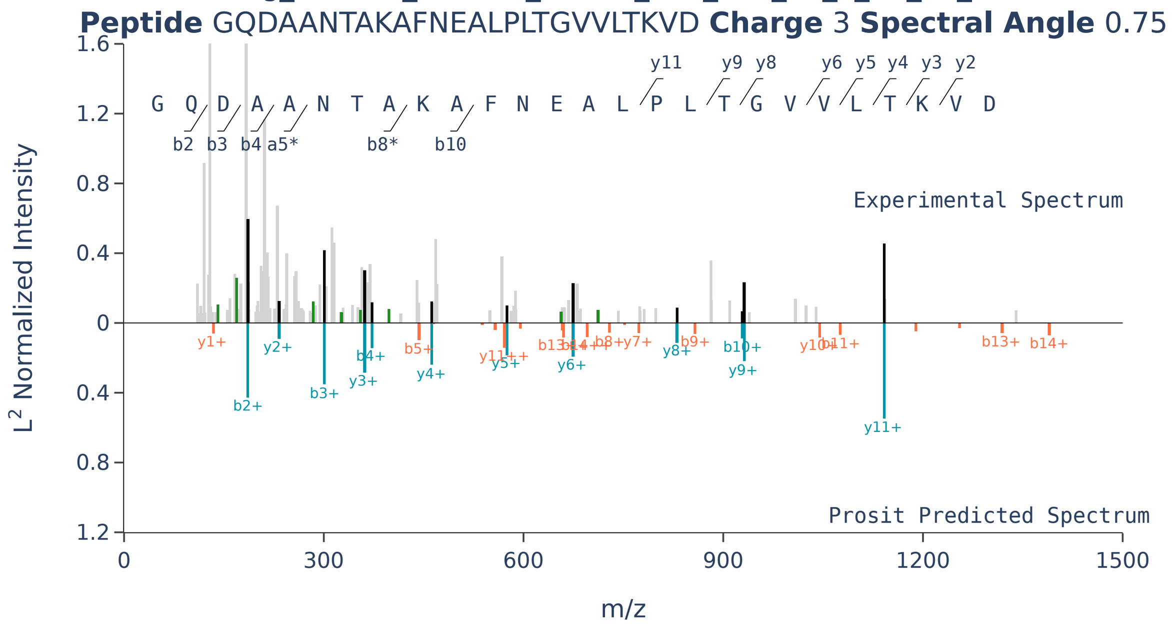


**Source** Ncheng_210623_230623_HFGoe_FFH_20S_25_1_A1_4h_R1 **Scan** 22508
**Peptide** EPFHPDRIASRIL **Charge** 3 **Spectral Angle** 0.9

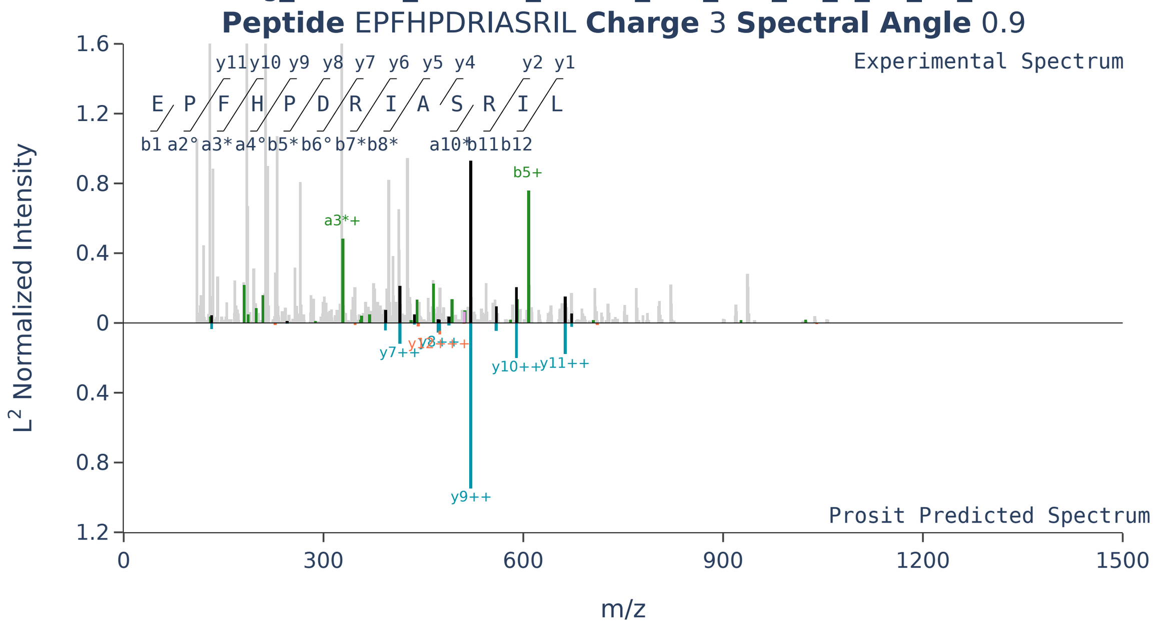


**Source** Ncheng_210623_230623_HFGoe_FFH_20S_25_1_A1_24h_R1 **Scan** 5715
**Peptide** GLQGAGKTT **Charge** 2 **Spectral Angle** 0.79

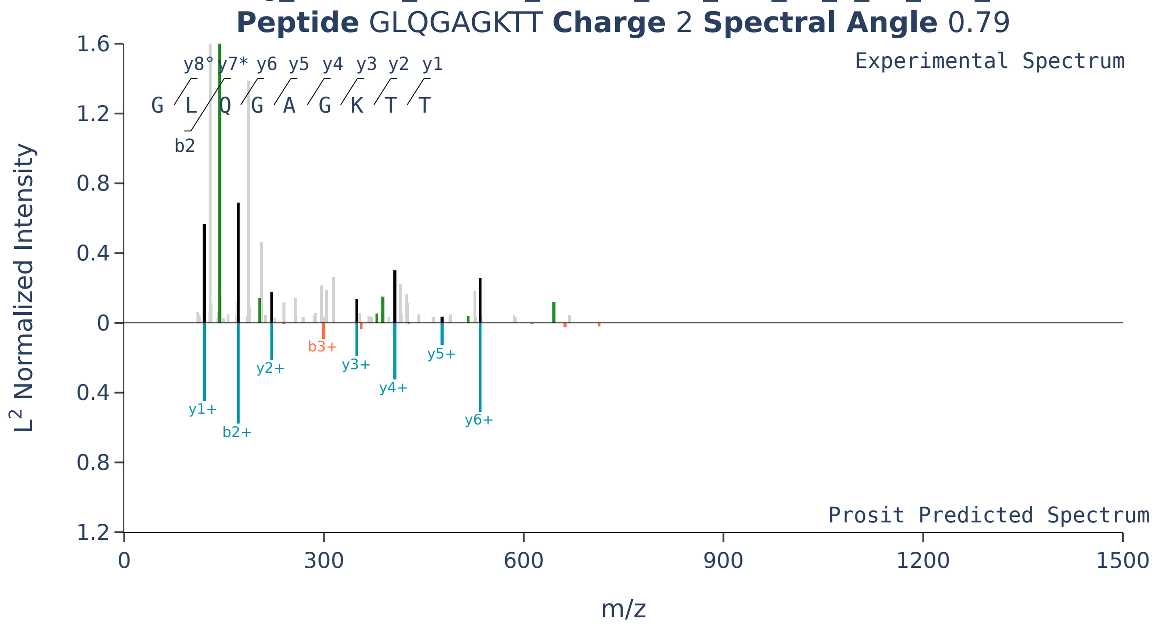


**Source** Ncheng_210623_230623_HFGoe_FFH_20S_25_1_A1_2h_R2 **Scan** 37450
**Peptide** ASKLKKGDGFDLNDFLEQLRQM **Charge** 3 **Spectral Angle** 0.8

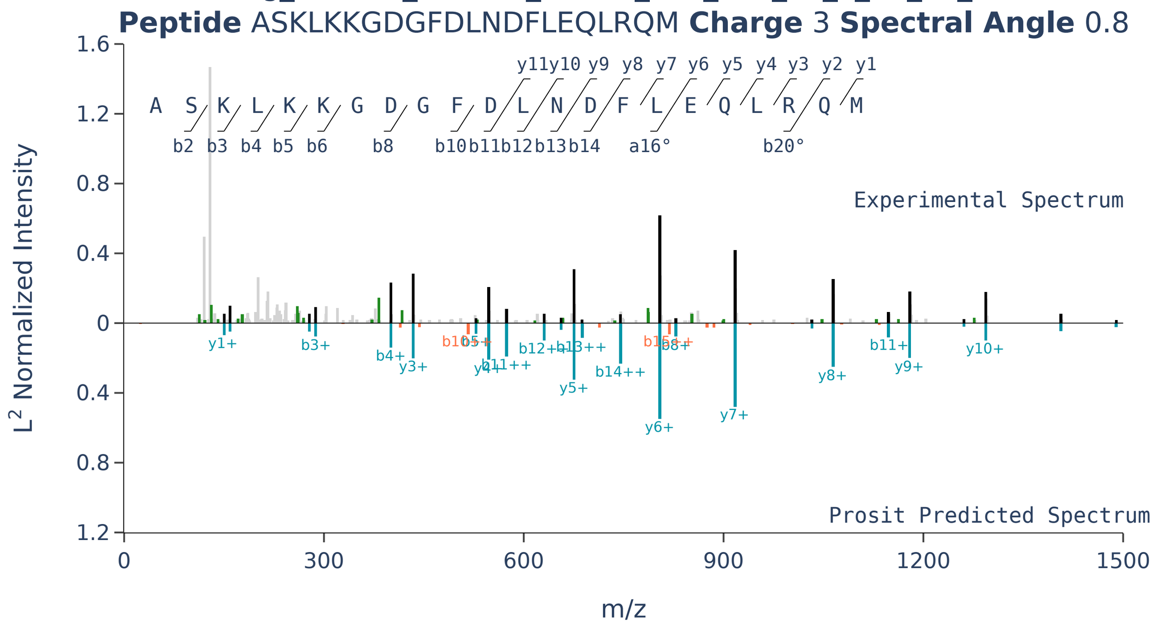


**Source** Ncheng_210623_230623_HFGoe_FFH_20S_25_1_A1_1h_R1 **Scan** 29556
**Peptide** KLKFYDVLLVD **Charge** 3 **Spectral Angle** 0.71

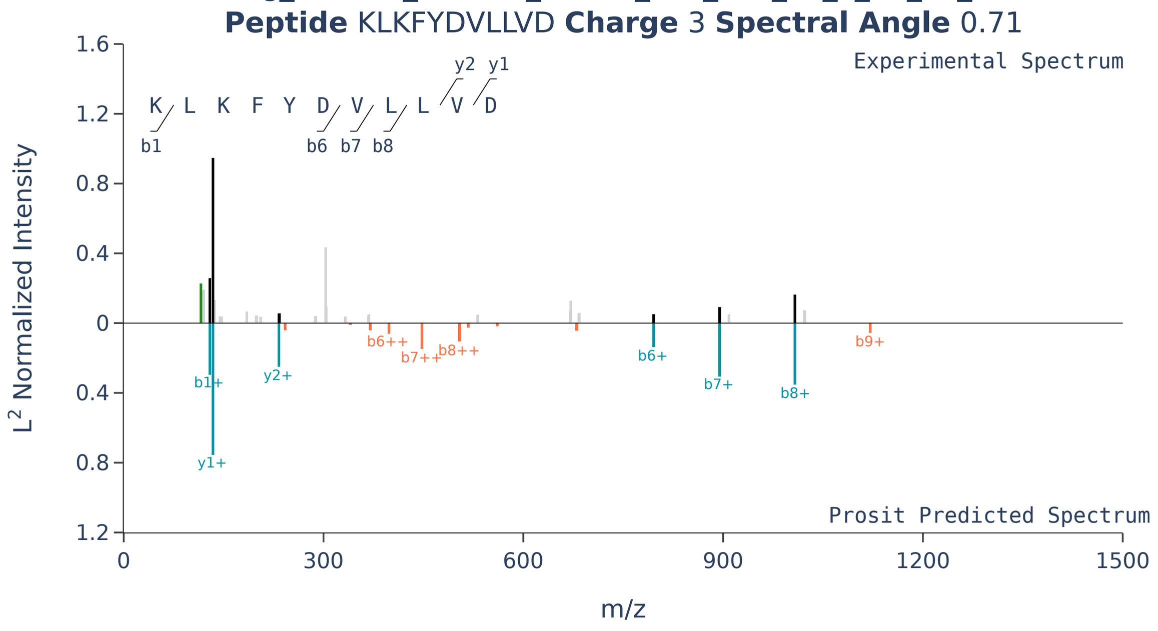


**Source** Ncheng_210623_230623_HFGoe_FFH_20S_25_1_A1_24h_R1 **Scan** 17024
**Peptide** KEKAVGHEVNKSLTPGQEFVKIVR **Charge** 3 **Spectral Angle** 0.82

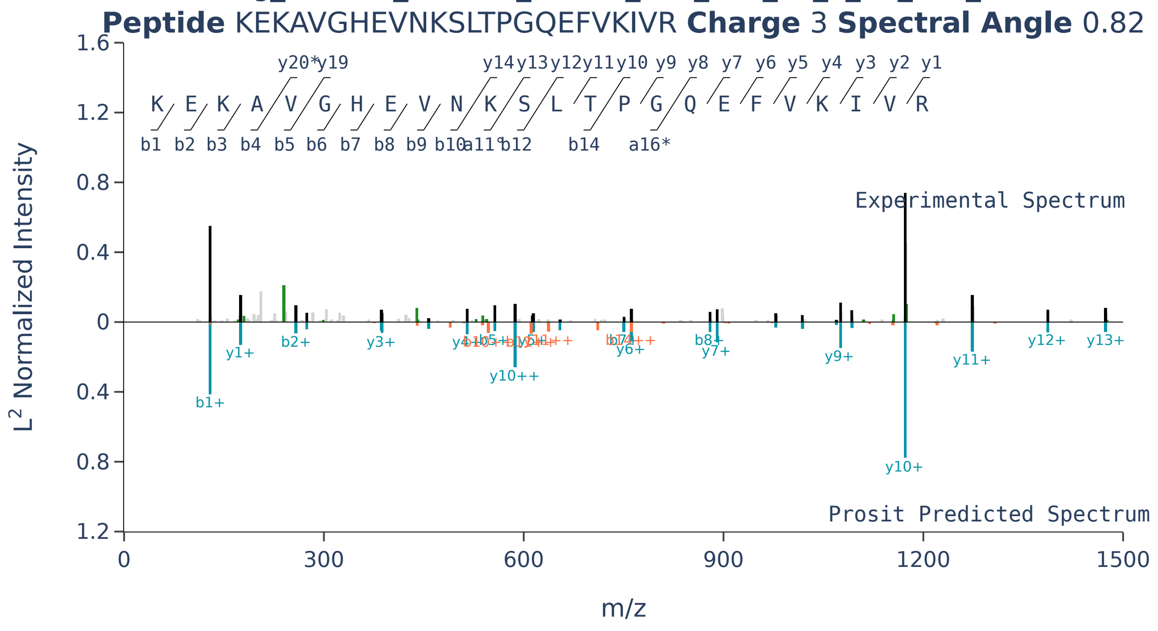


**Source** Ncheng_210623_230623_HFGoe_FFH_20S_25_1_A2_2h_R1 **Scan** 32792
**Peptide** ELKQVHASINPVETLFVVD **Charge** 2 **Spectral Angle** 0.67

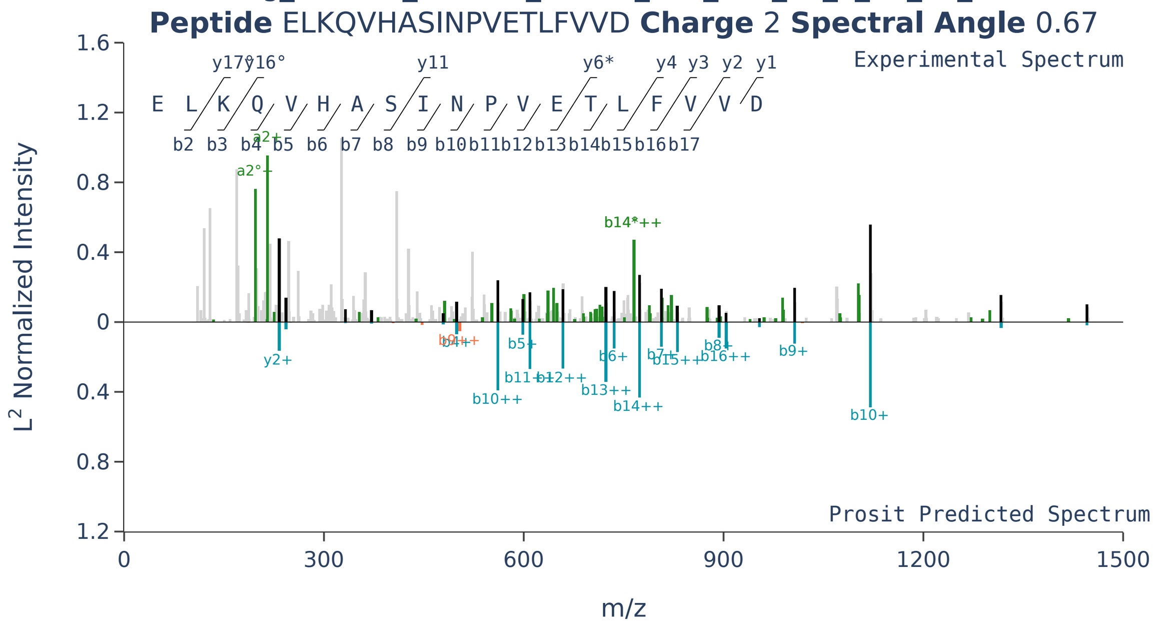


**Source** Ncheng_210623_230623_HFGoe_FFH_20S_25_1_A1_24h_R1 **Scan** 6116
**Peptide** DVGQKPVD **Charge** 2 **Spectral Angle** 0.71

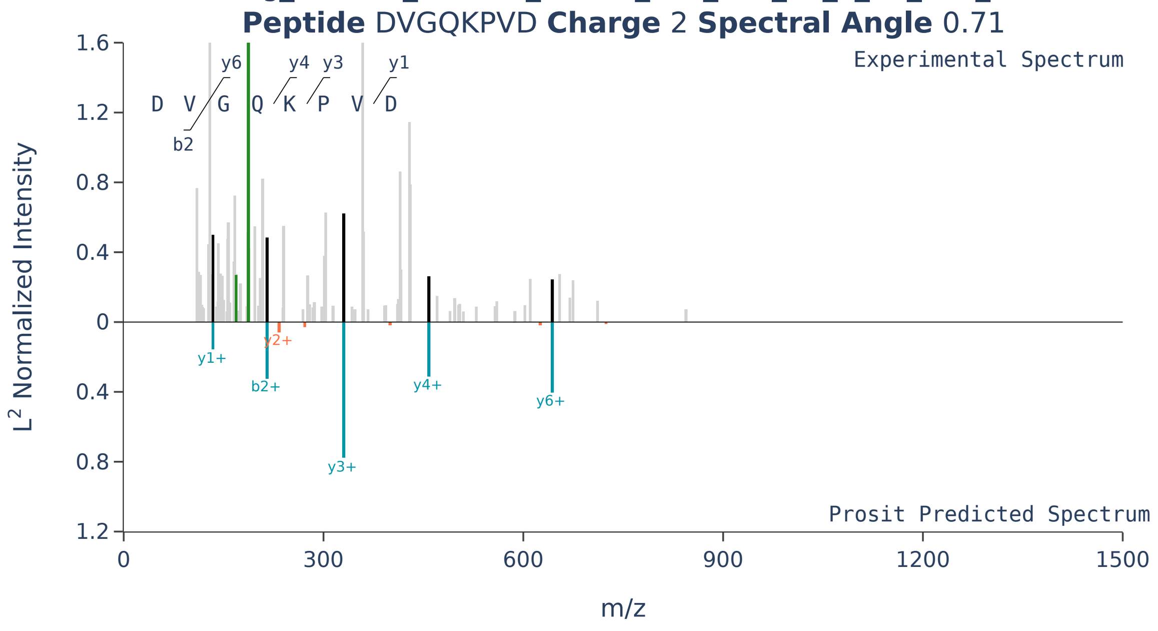


**Source** Ncheng_210623_230623_HFGoe_FFH_20S_25_1_A2_4h_R2 **Scan** 19921
**Peptide** IVNAALKEAKLKF **Charge** 4 **Spectral Angle** 0.74

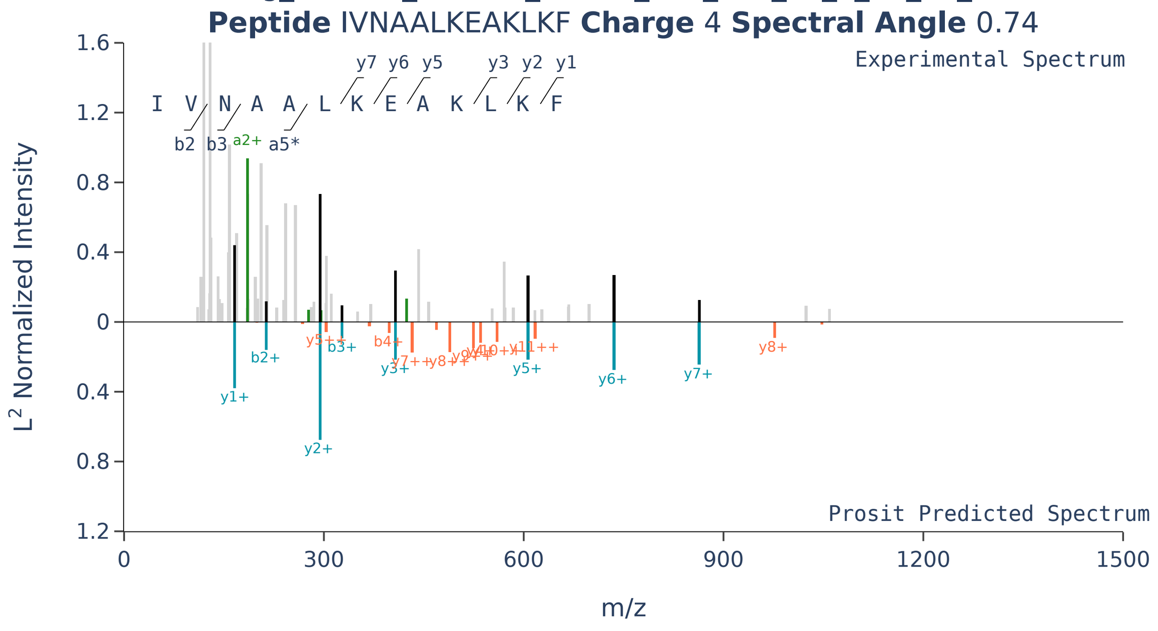


**Source** Ncheng_210623_230623_HFGoe_FFH_20S_25_1_A1_24h_R1 **Scan** 20315
**Peptide** AMGEENQTLNLA **Charge** 2 **Spectral Angle** 0.69

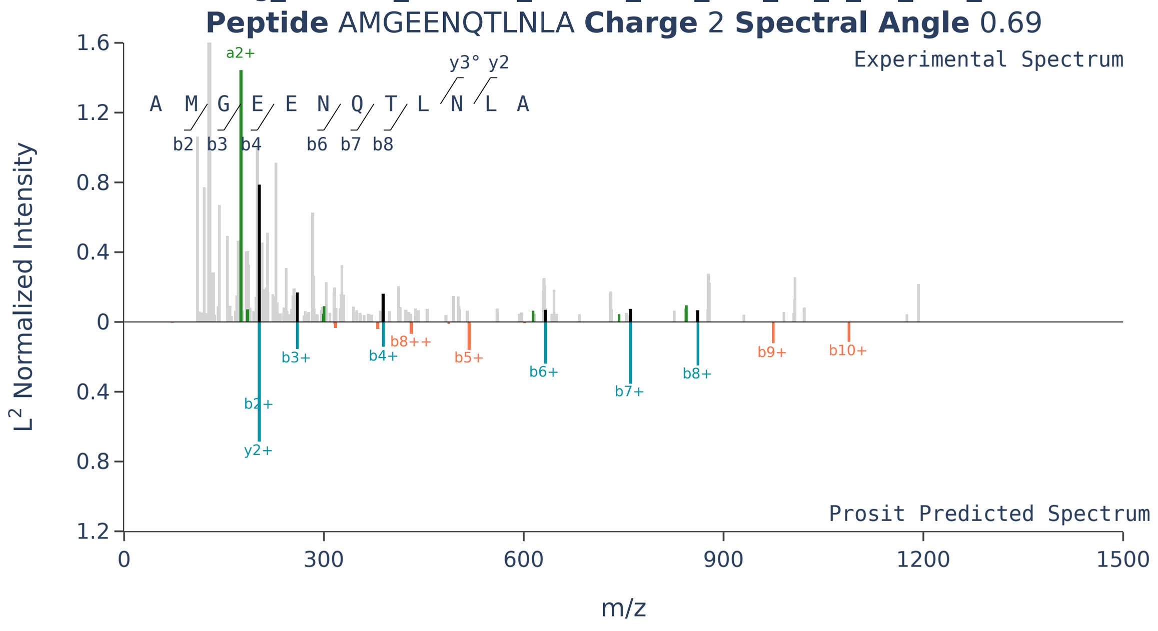


**Source** Ncheng_210623_230623_HFGoe_FFH_20S_25_1_A2_4h_R1 **Scan** 29875
**Peptide** HEVNKSLTPGQEFVKIVRNELV **Charge** 3 **Spectral Angle** 0.76

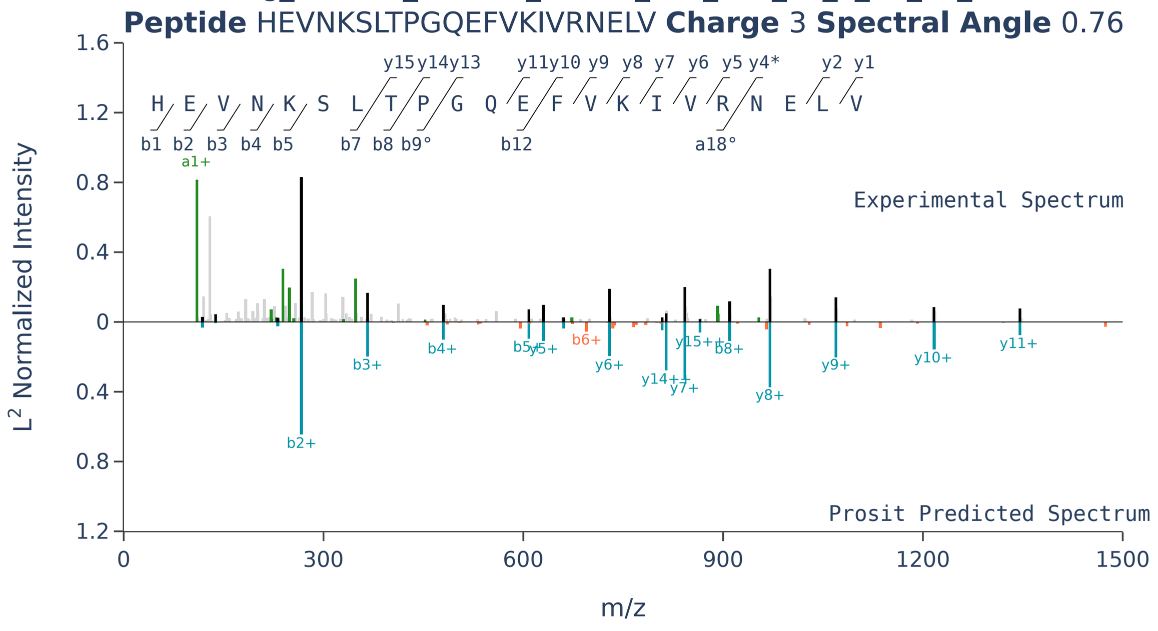

**Source** Ncheng_210623_230623_HFGoe_FFH_20S_25_1_A2_24h_R2 **Scan** 8532
**Peptide** QRMMKKMK **Charge** 3 **Spectral Angle** 0.85

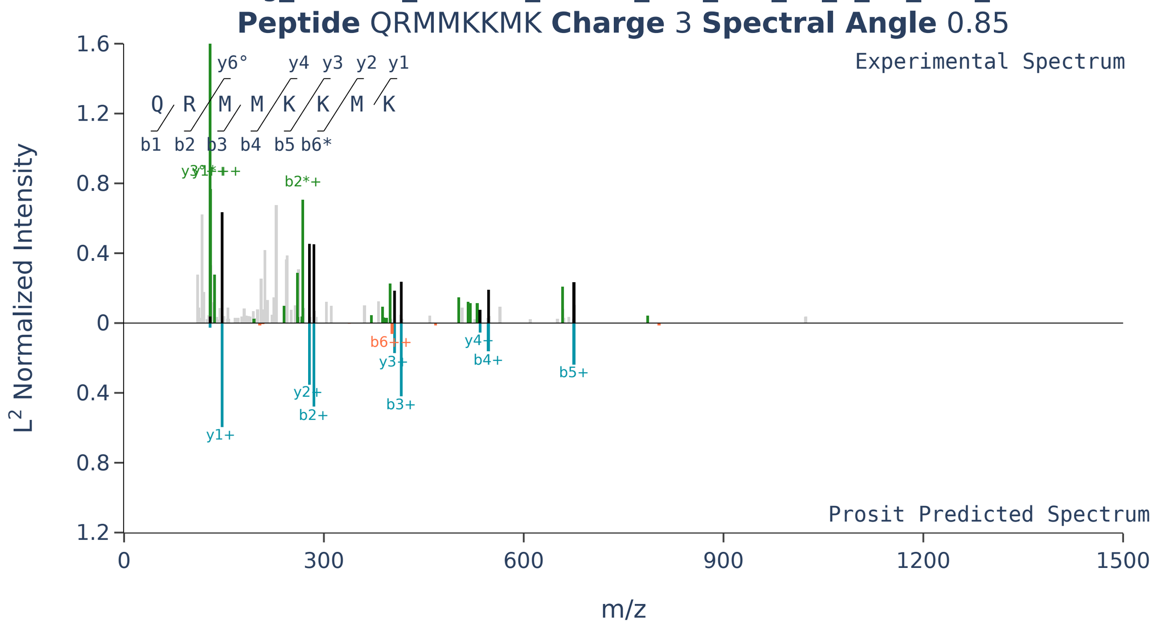


**Source** Ncheng_210623_230623_HFGoe_FFH_20S_25_1_A1_4h_R2 **Scan** 21409
**Peptide** PFHPDRIASRILG **Charge** 3 **Spectral Angle** 0.8

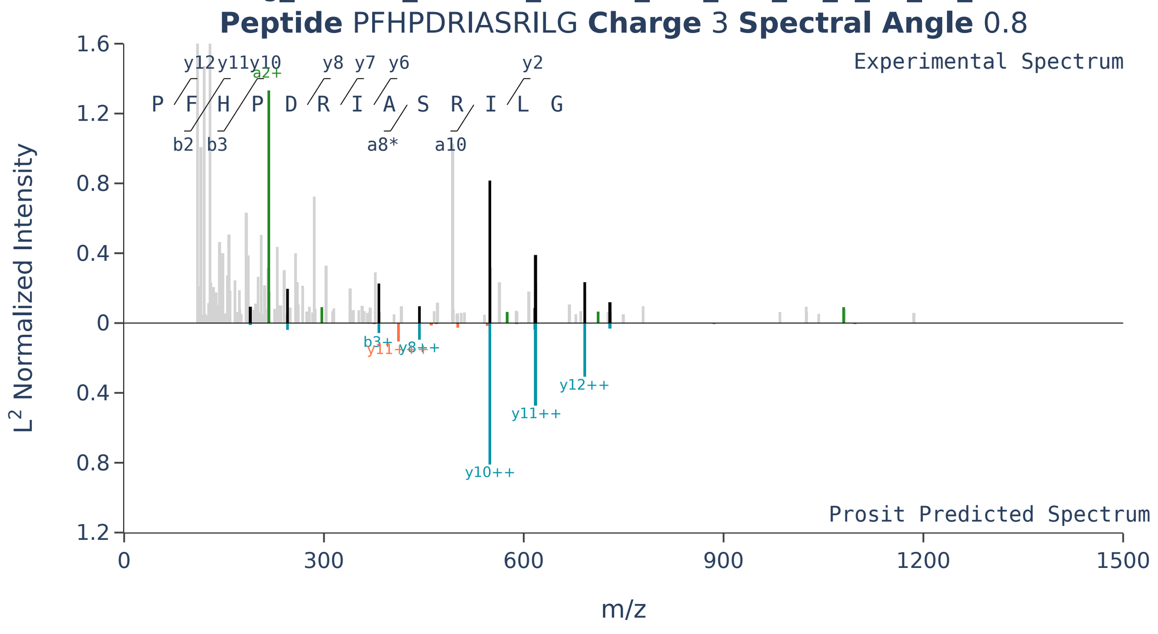


**Source** Ncheng_210623_230623_HFGoe_FFH_20S_25_1_A1_4h_R1 **Scan** 17874
**Peptide** GRGRLTEDNVKDTLREVRM **Charge** 5 **Spectral Angle** 0.77

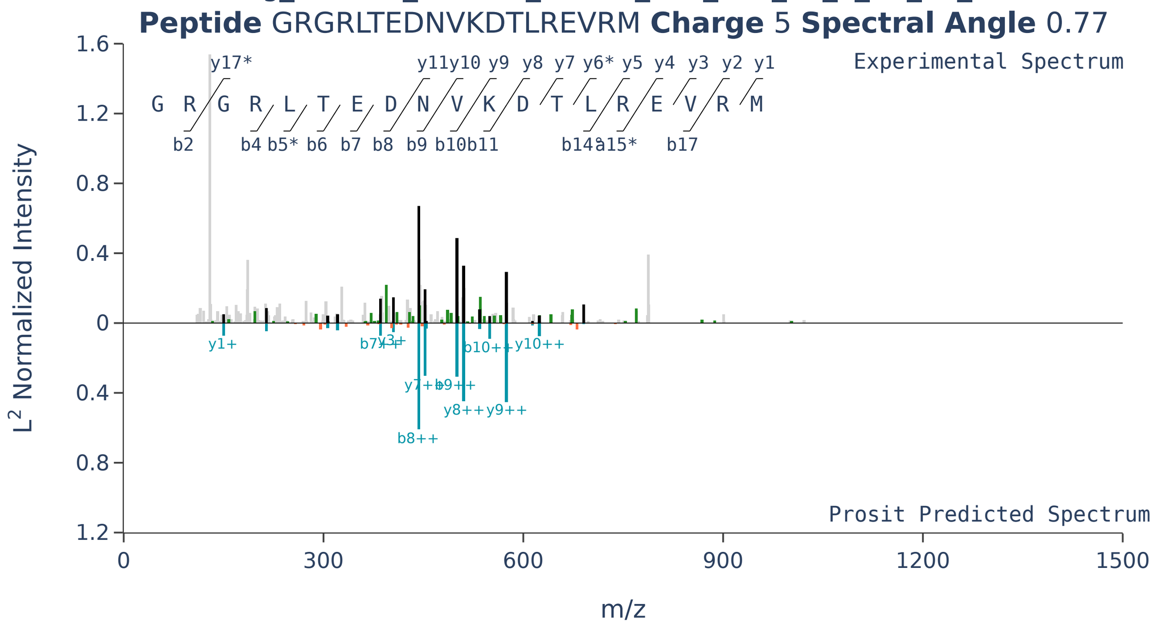


**Source** Ncheng_210623_230623_HFGoe_FFH_20S_25_1_A2_24h_R2 **Scan** 23147
**Peptide** KAFNEALPLTG **Charge** 1 **Spectral Angle** 0.61

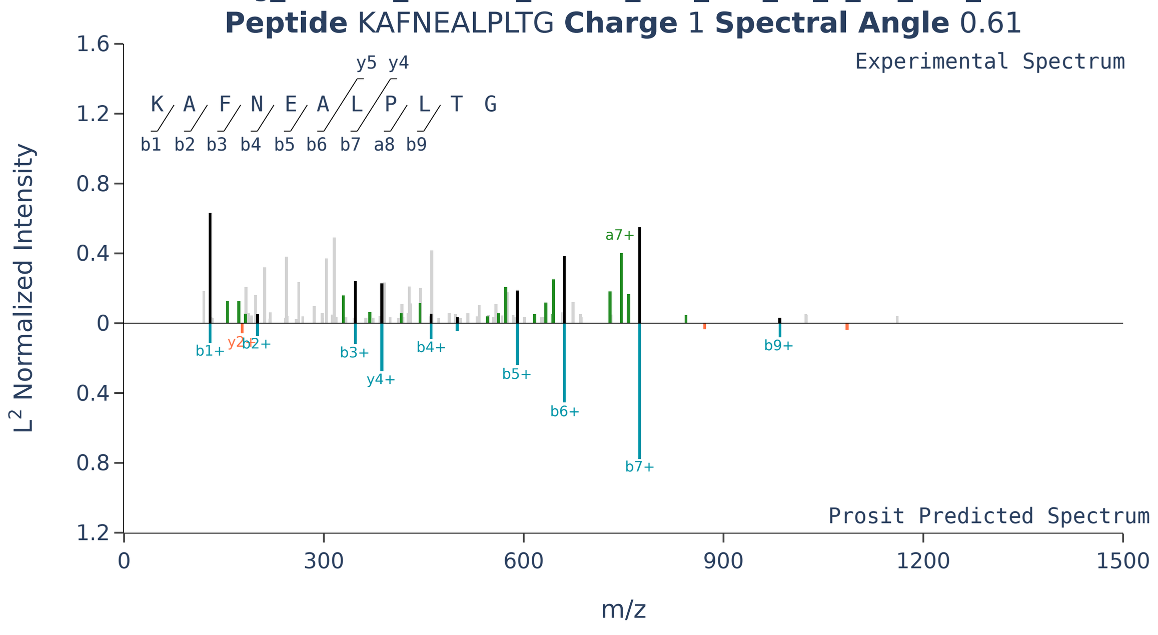


**Source** Ncheng_210623_230623_HFGoe_FFH_20S_25_1_A1_24h_R2 **Scan** 22434
**Peptide** PAAIKQLETL **Charge** 2 **Spectral Angle** 0.71

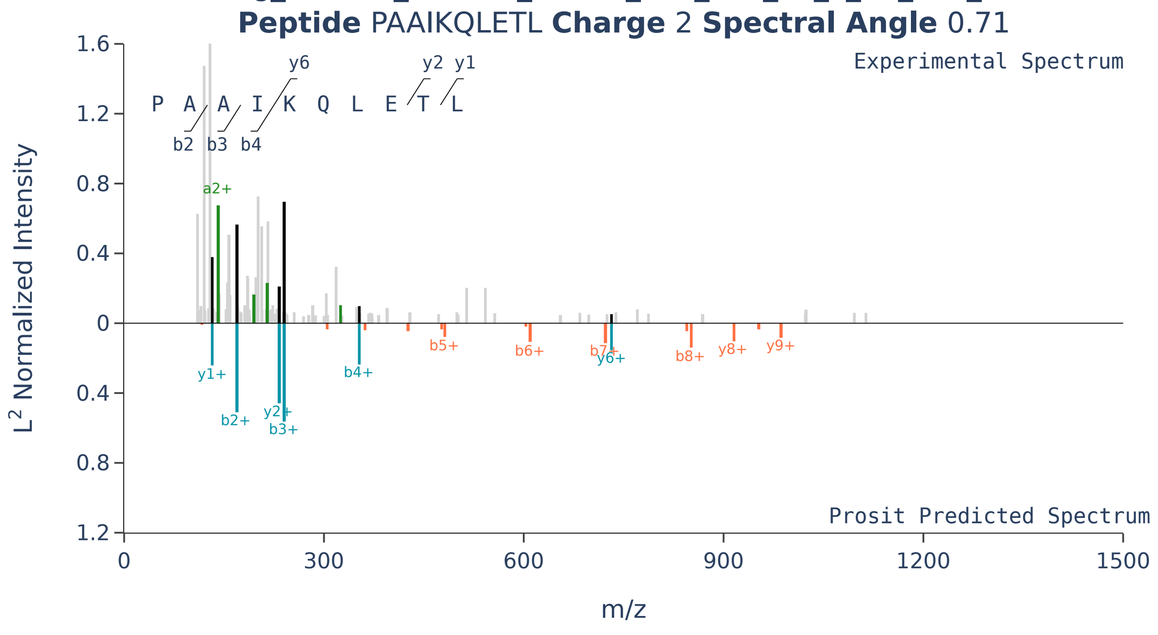


**Source** Ncheng_210623_230623_HFGoe_FFH_20S_25_1_A1_4h_R2 **Scan** 10068
**Peptide** GGMAKMMRSMKG **Charge** 2 **Spectral Angle** 0.74

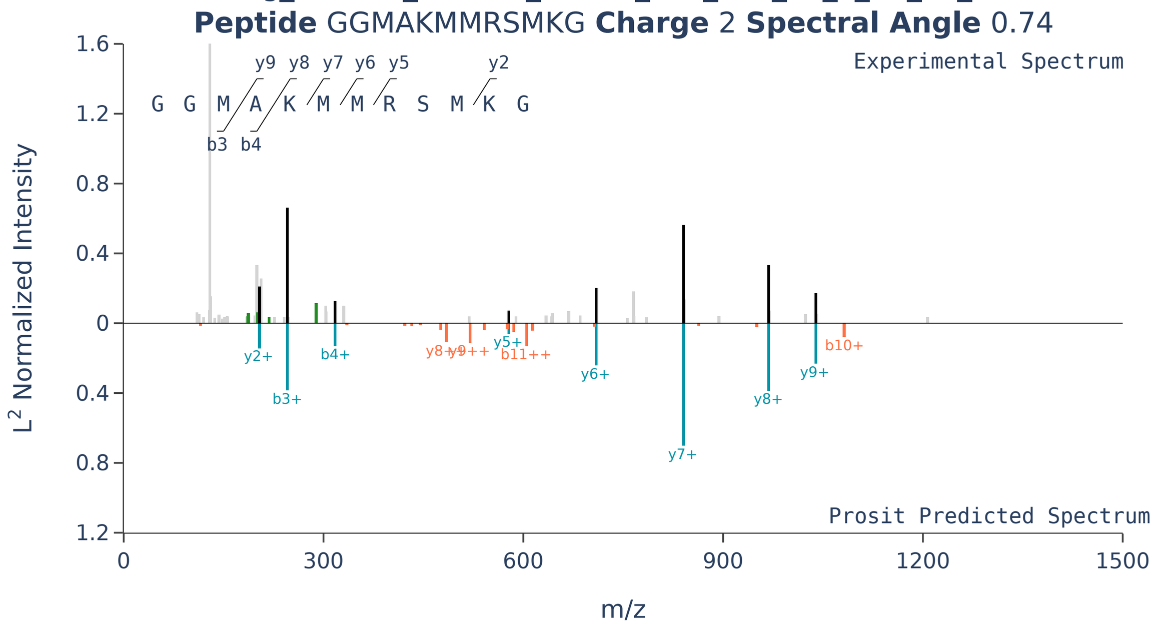


**Source** Ncheng_210623_230623_HFGoe_FFH_20S_25_1_A2_4h_R1 **Scan** 12836
**Peptide** LAAGCGMQVQD **Charge** 2 **Spectral Angle** 0.77

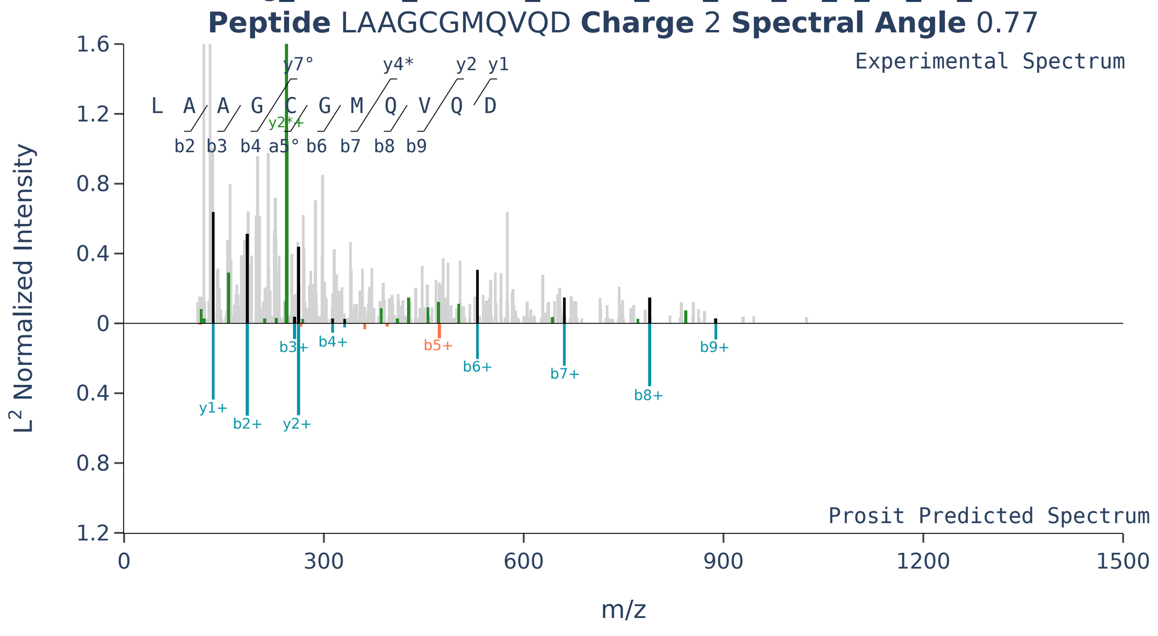


**Source** Ncheng_210623_230623_HFGoe_FFH_20S_25_1_A1_1h_R2 **Scan** 22453
**Peptide** AVGHEVNKSLTPGQEFVKLV **Charge** 3 **Spectral Angle** 0.76

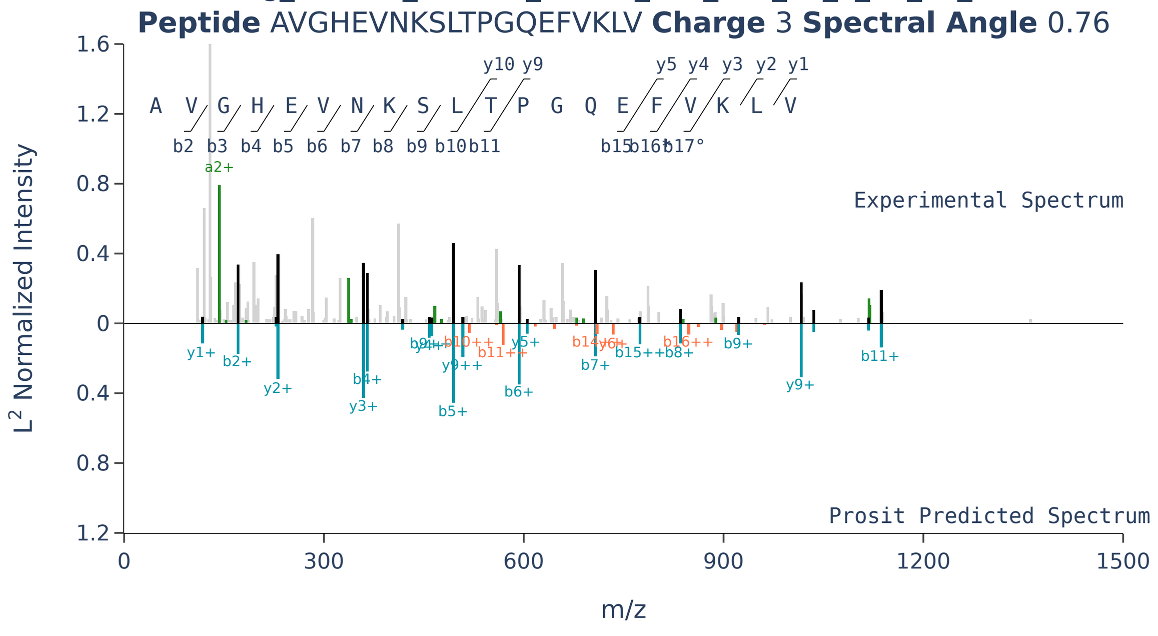


**Source** Ncheng_210623_230623_HFGoe_FFH_20S_25_1_A2_24h_R1 **Scan** 27630
**Peptide** DAANTAKAFNEALPLT **Charge** 2 **Spectral Angle** 0.87

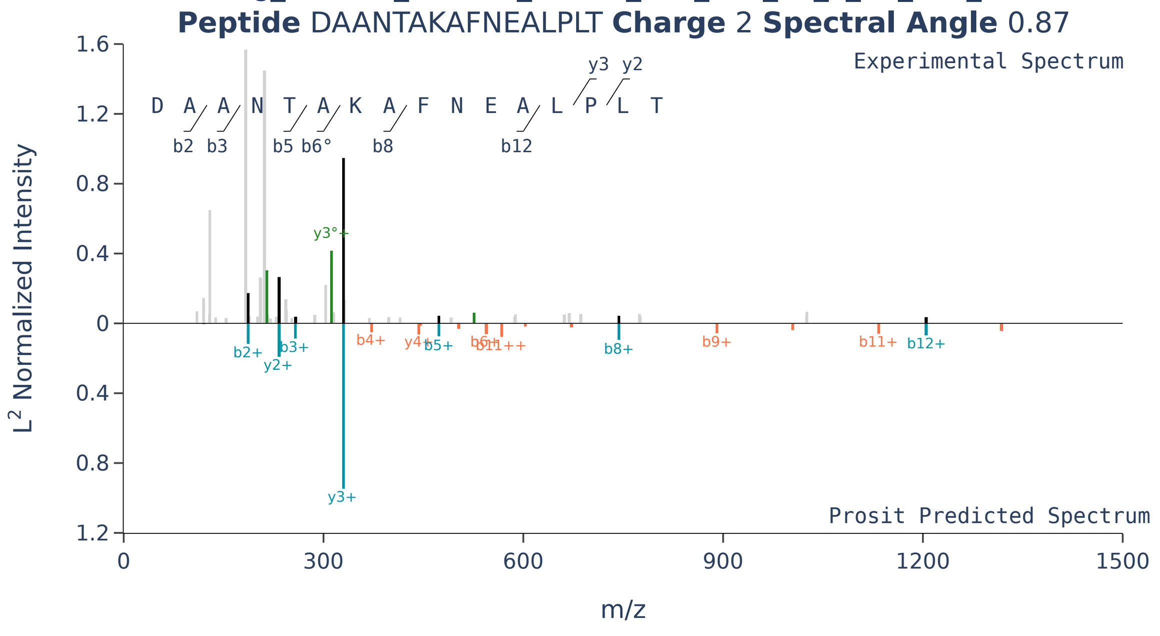


**Source** Ncheng_210623_230623_HFGoe_FFH_20S_25_1_A1_4h_R1 **Scan** 20663
**Peptide** GEKTEALEPFHPDRIASRI **Charge** 4 **Spectral Angle** 0.75

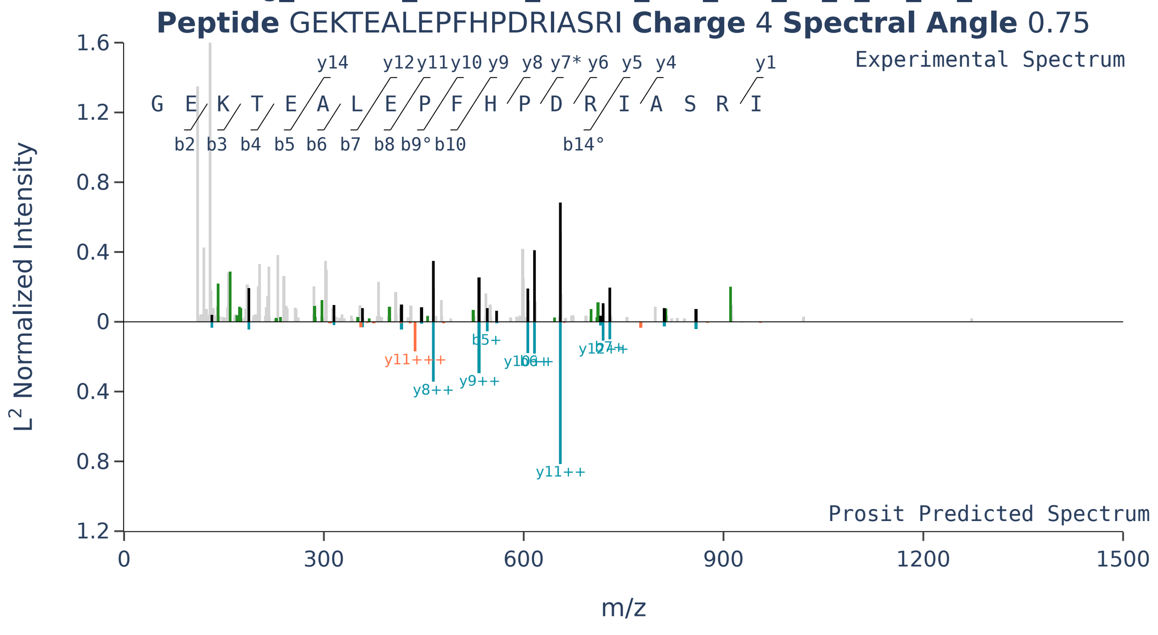


**Source** Ncheng_210623_230623_HFGoe_FFH_20S_25_1_A2_24h_R1 **Scan** 11800
**Peptide** ALLNSMTMKER **Charge** 3 **Spectral Angle** 0.6

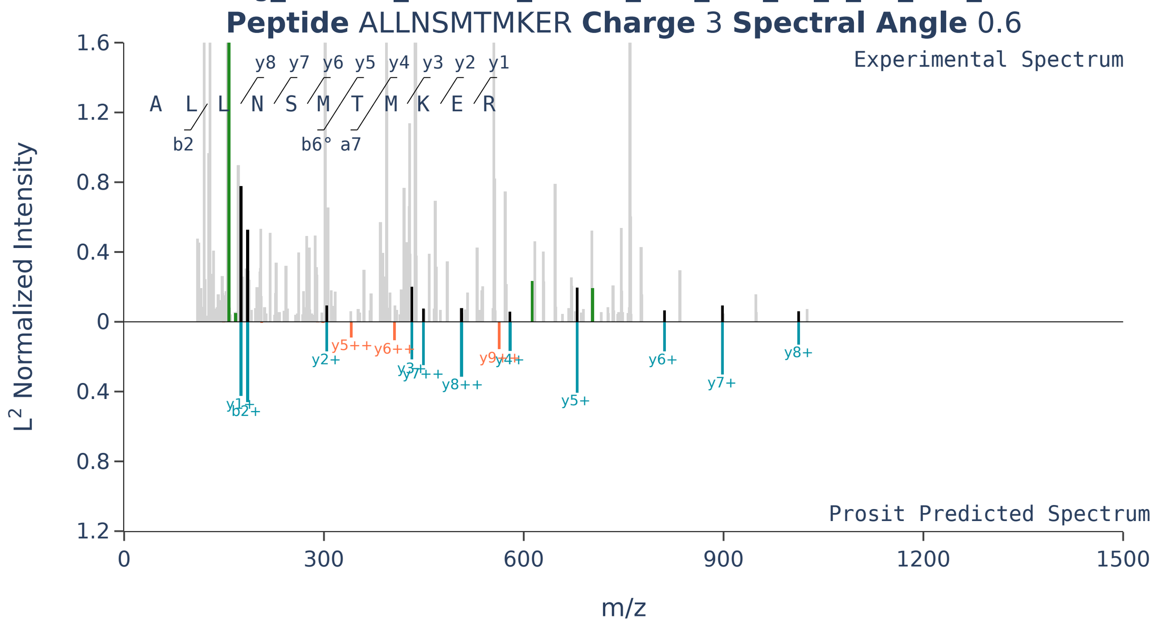


**Source** Ncheng_210623_230623_HFGoe_FFH_20S_25_1_A2_4h_R1 **Scan** 25474
**Peptide** DKVLVRMEAIIN **Charge** 2 **Spectral Angle** 0.66

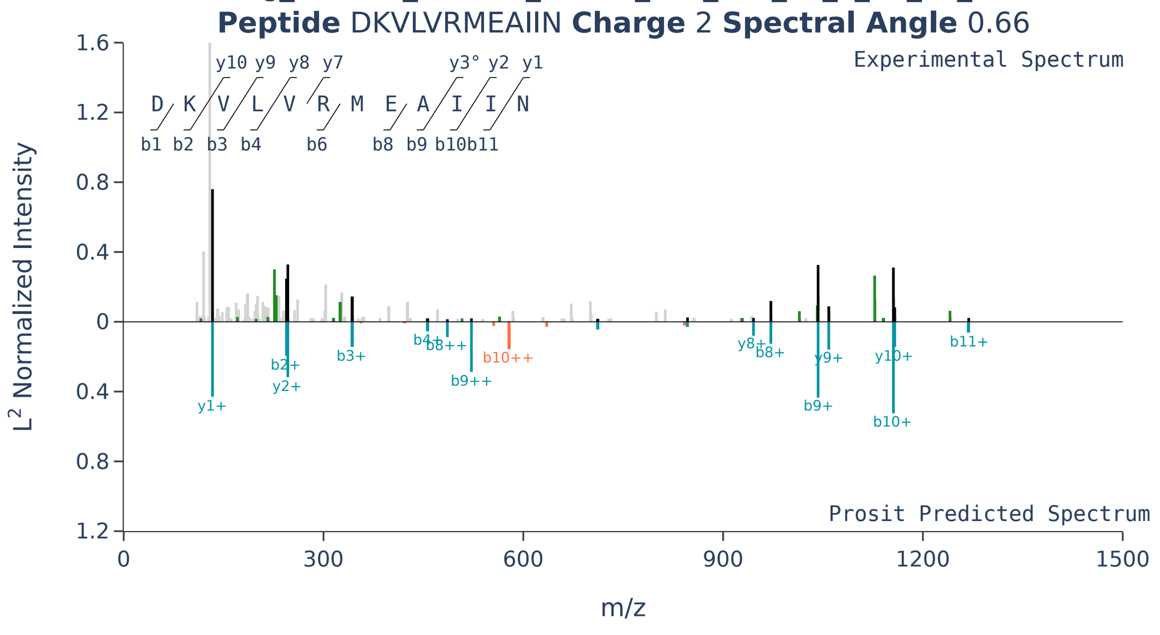


**Source** Ncheng_210623_230623_HFGoe_FFH_20S_25_1_A2_4h_R1 **Scan** 22260
**Peptide** EALEPFHPDRIASRI **Charge** 4 **Spectral Angle** 0.81

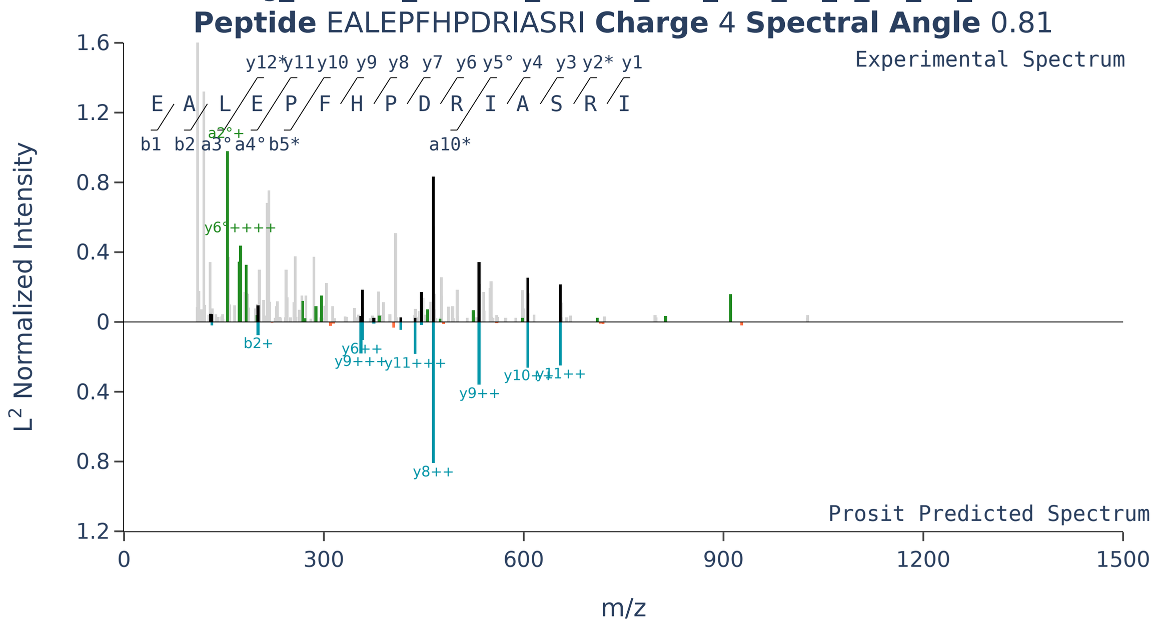


**Source** Ncheng_210623_230623_HFGoe_FFH_20S_25_1_A2_4h_R2 **Scan** 20291
**Peptide** RPAAIKQLETLAE **Charge** 2 **Spectral Angle** 0.72

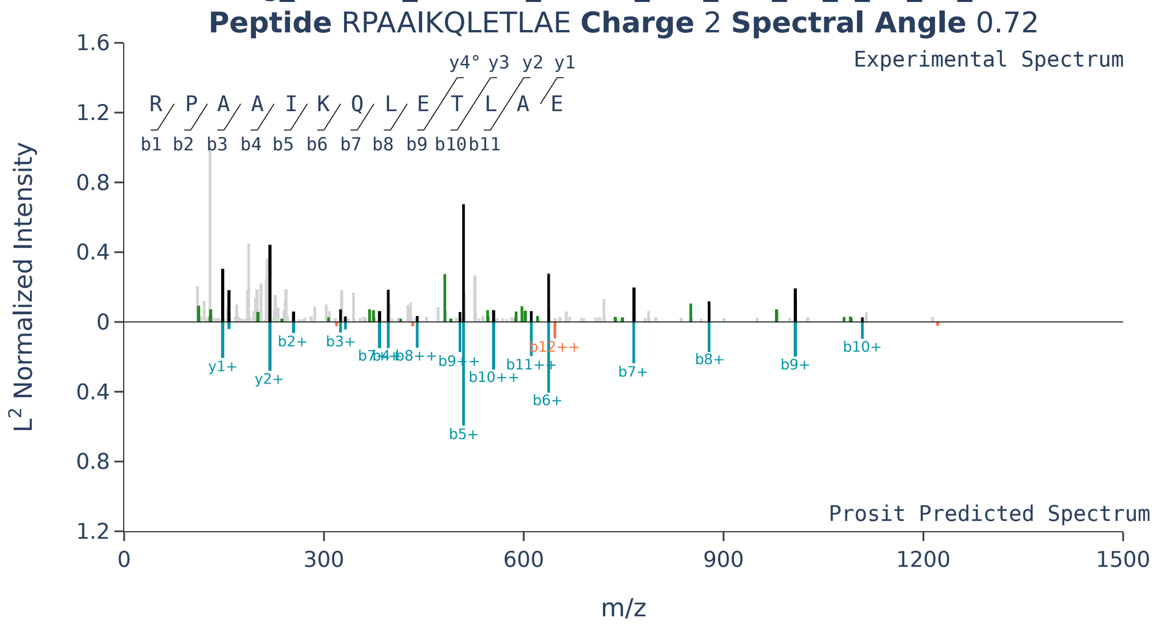


**Source** Ncheng_210623_230623_HFGoe_FFH_20S_25_1_A2_4h_R2 **Scan** 21571
**Peptide** LTEDNVKDTLREVRM **Charge** 2 **Spectral Angle** 0.69

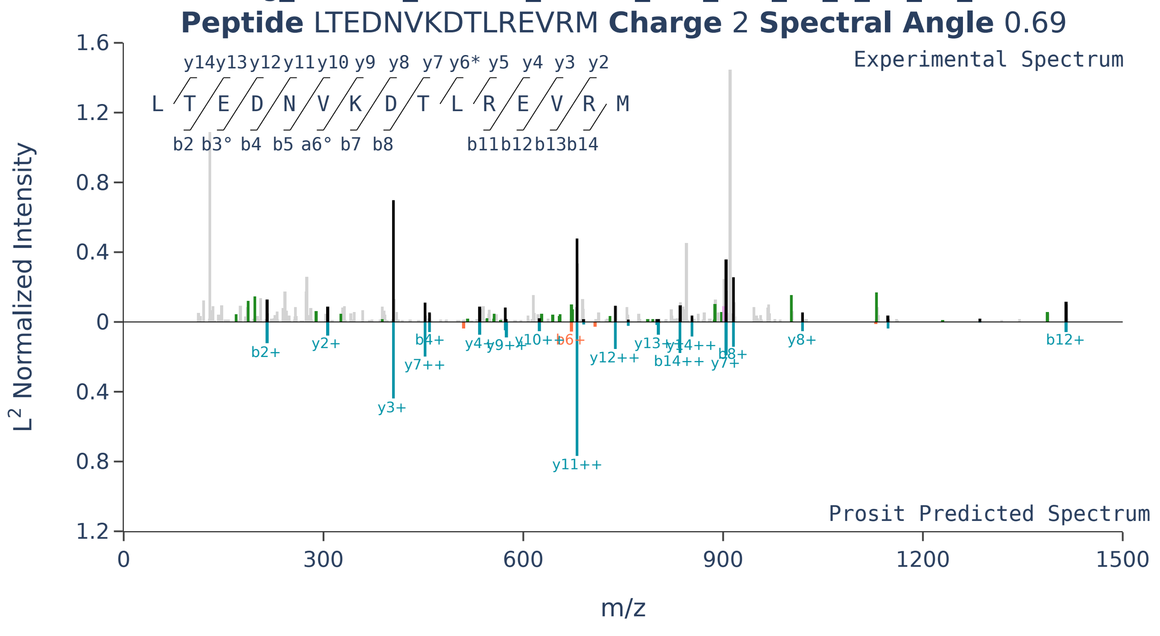

**Source** Ncheng_210623_230623_HFGoe_FFH_20S_25_1_A1_2h_R2 **Scan** 33134
**Peptide** LVNAALKEAKLKFYDVLLV **Charge** 2 **Spectral Angle** 0.72

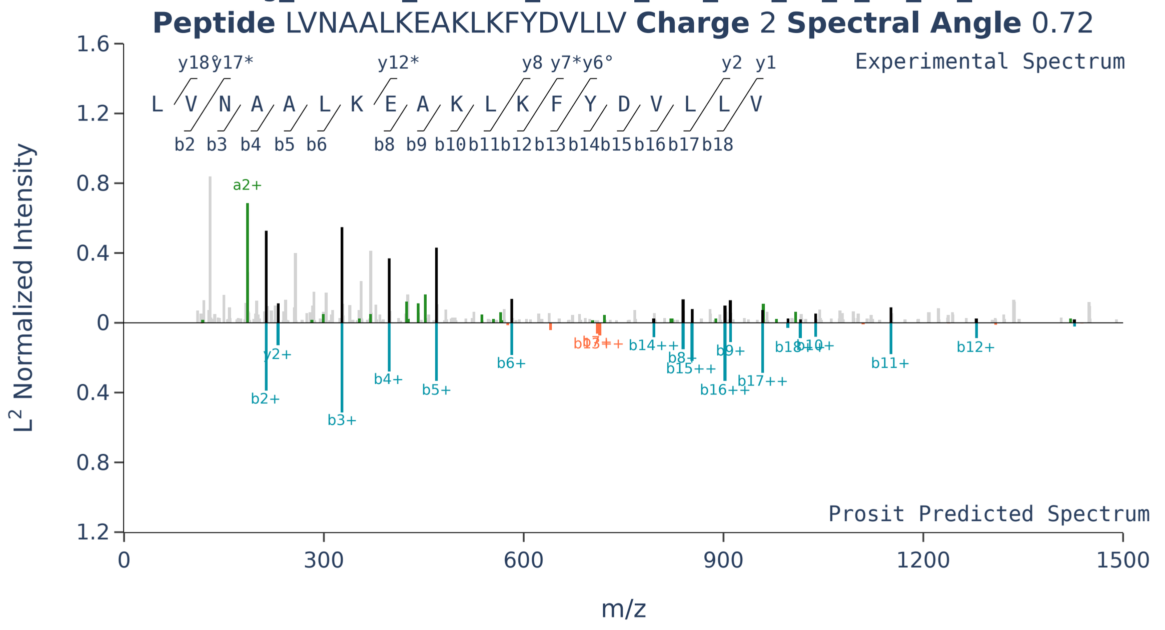


**Source** Ncheng_210623_230623_HFGoe_FFH_20S_25_1_A1_4h_R2 **Scan** 23747
**Peptide** AVGHEVNKSLTPGQEFVKIV **Charge** 2 **Spectral Angle** 0.78

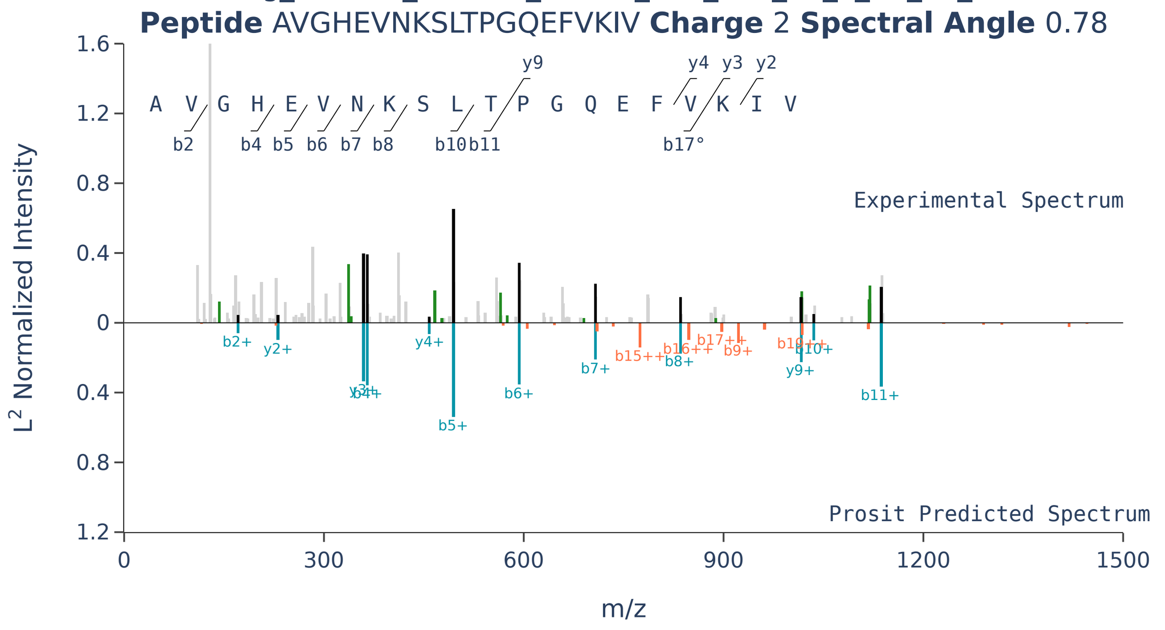


**Source** Ncheng_210623_230623_HFGoe_FFH_20S_25_1_A2_4h_R1 **Scan** 18636
**Peptide** IRHITGKPIKFLGVGE **Charge** 4 **Spectral Angle** 0.76

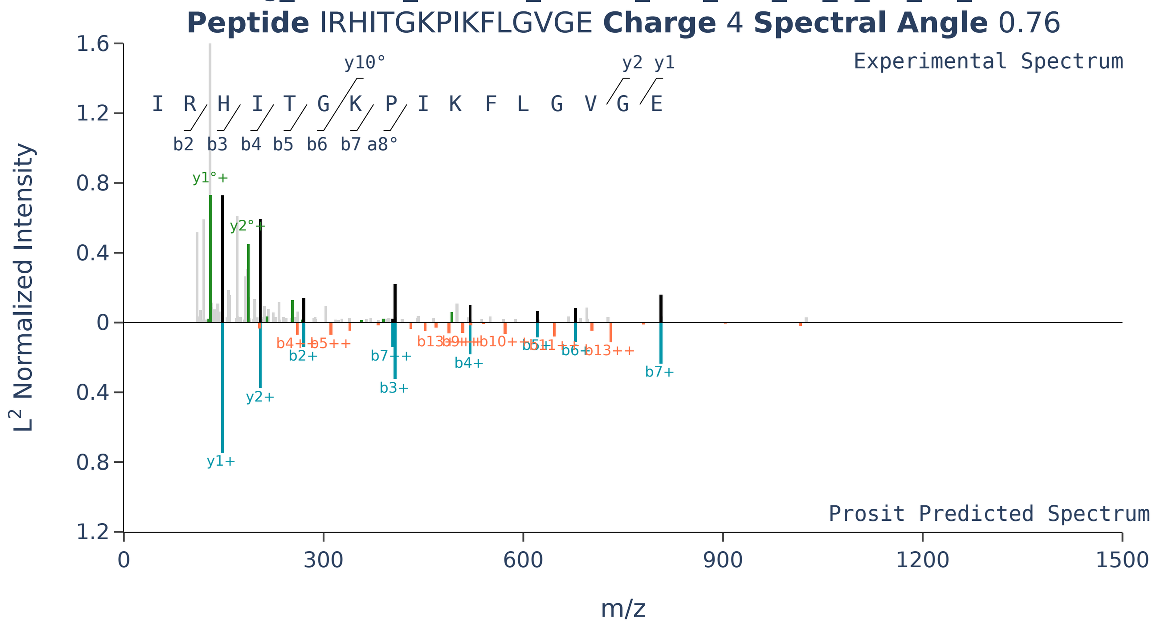


**Source** Ncheng_210623_230623_HFGoe_FFH_20S_25_1_A2_2h_R1 **Scan** 8774
**Peptide** QIPDNVKSQMDD **Charge** 2 **Spectral Angle** 0.61

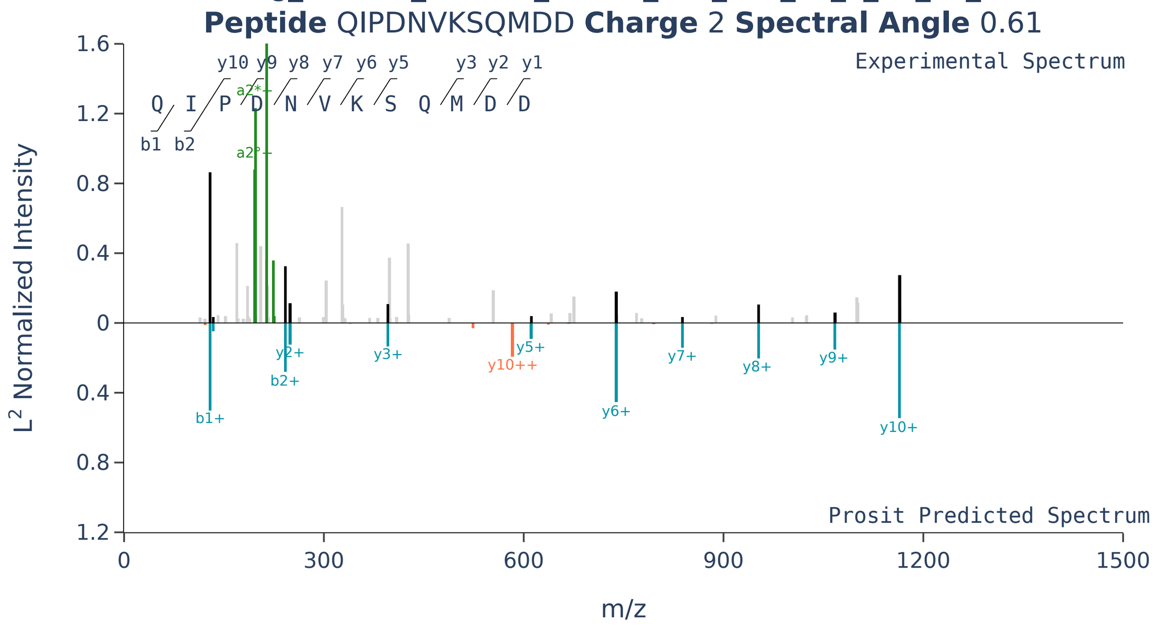


**Source** Ncheng_210623_230623_HFGoe_FFH_20S_25_1_A1_4h_R2 **Scan** 18892
**Peptide** DIESKVDRAQAEKLASKL **Charge** 5 **Spectral Angle** 0.77

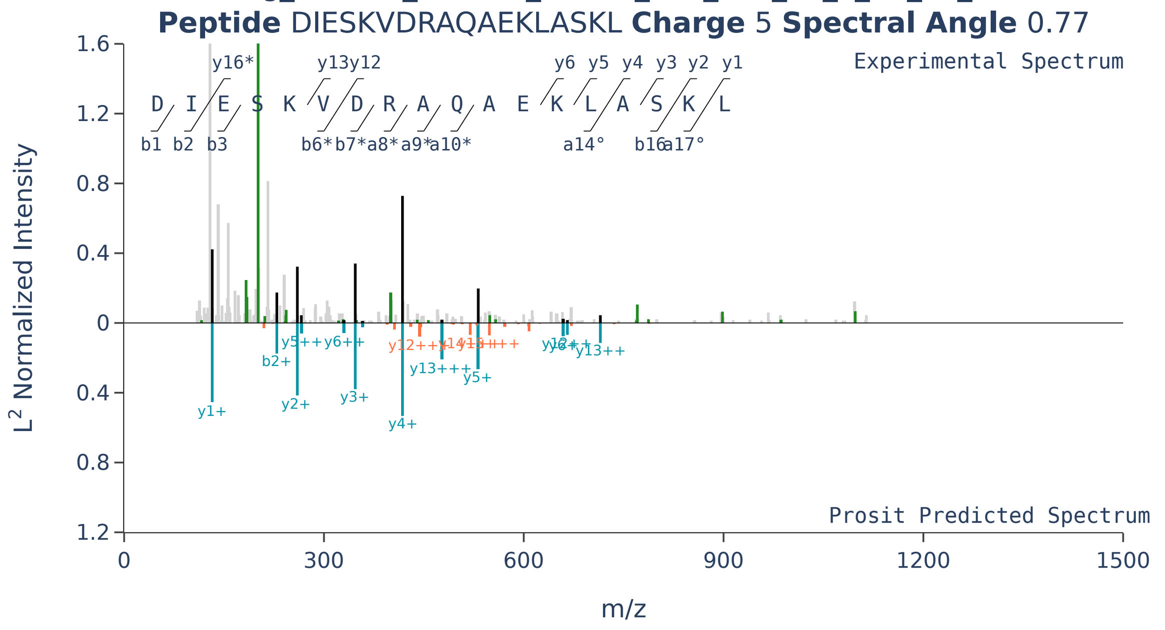


**Source** Ncheng_210623_230623_HFGoe_FFH_20S_25_1_A1_2h_R2 **Scan** 27313
**Peptide** PSDVGQKPVDVINAALKEA **Charge** 3 **Spectral Angle** 0.7

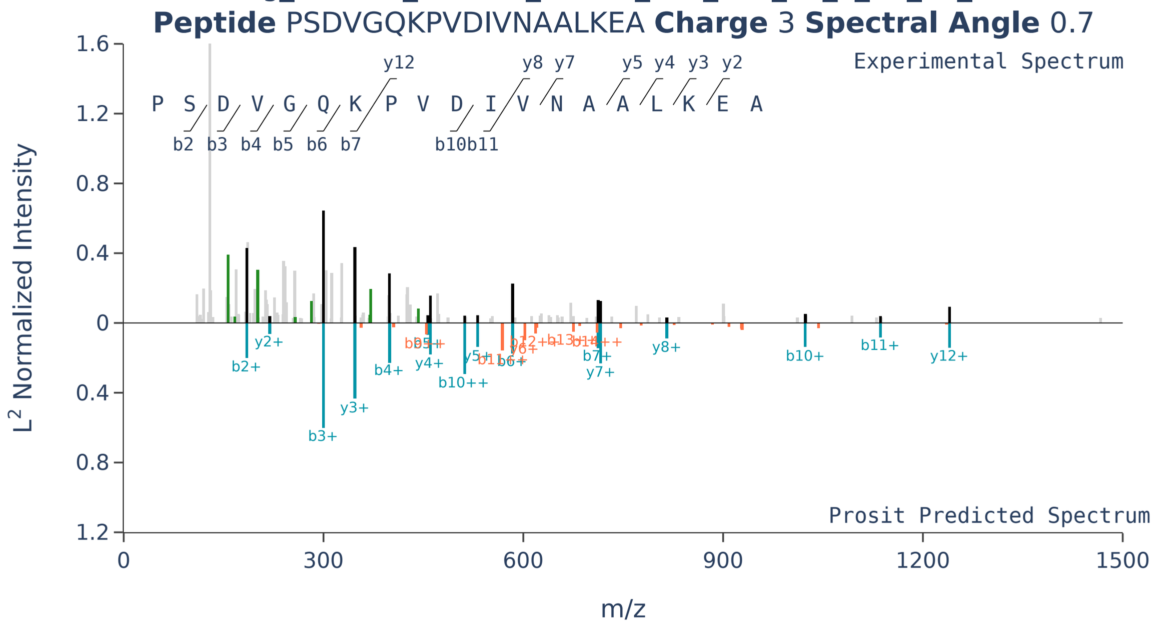


**Source** Ncheng_210623_230623_HFGoe_FFH_20S_25_1_A2_4h_R1 **Scan** 29386
**Peptide** KTEALEPFHPDRIASRILGM **Charge** 3 **Spectral Angle** 0.64

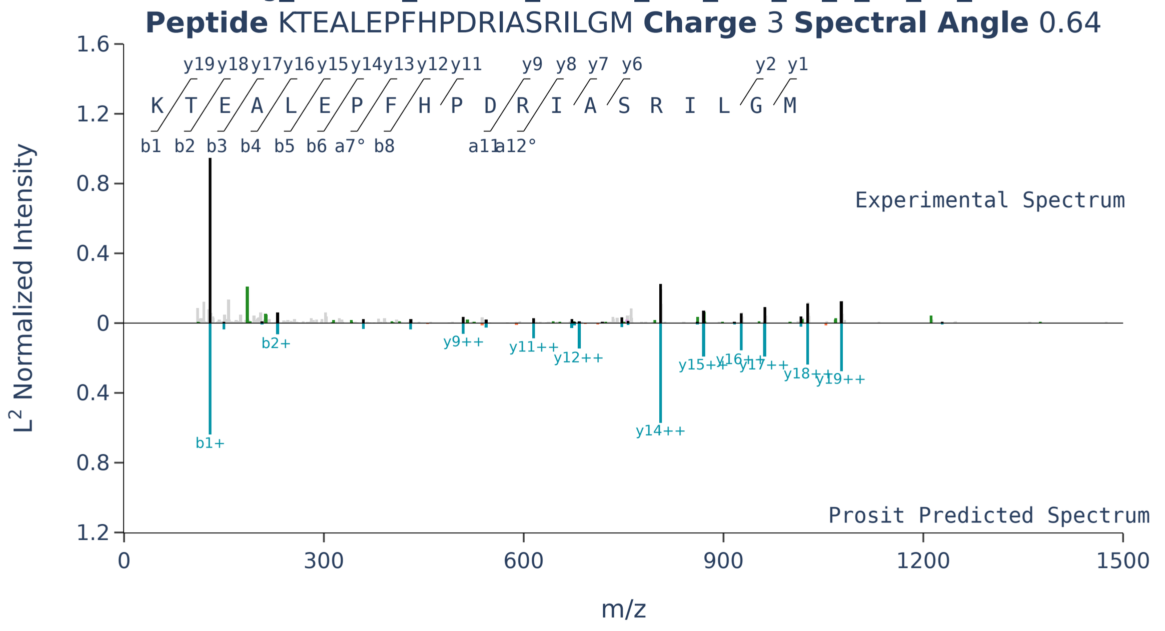


**Source** Ncheng_210623_230623_HFGoe_FFH_20S_25_1_A1_2h_R2 **Scan** 30064
**Peptide** VVDAMTGQDAANTAKAFNEALPLTG **Charge** 3 **Spectral Angle** 0.79

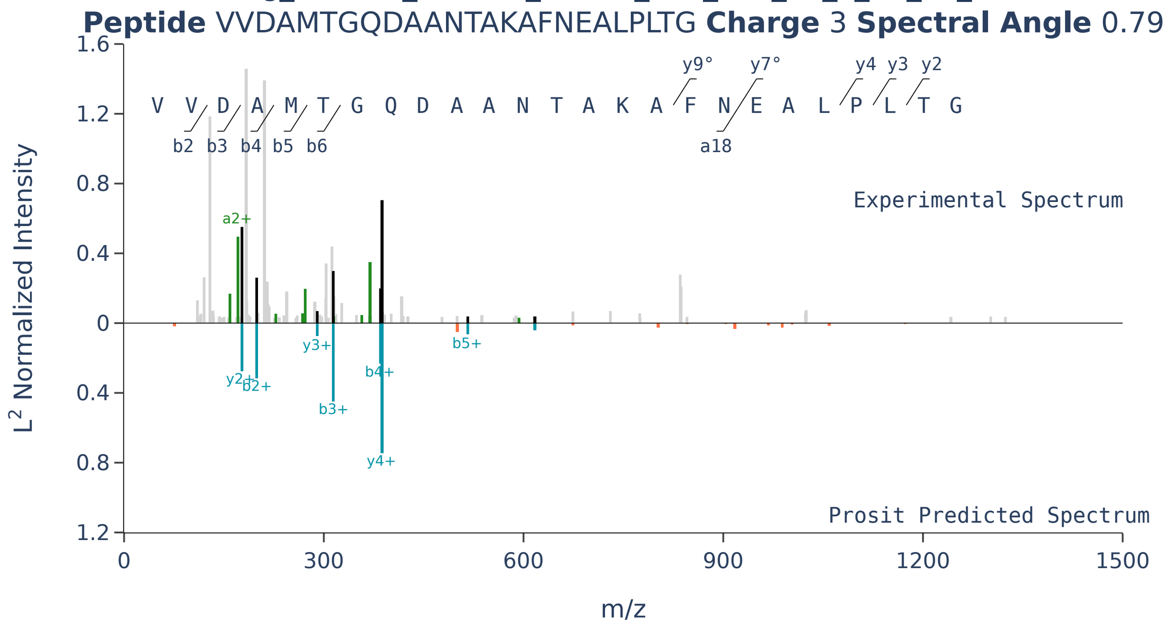


**Source** Ncheng_210623_230623_HFGoe_FFH_20S_25_1_A2_24h_R1 **Scan** 5181
**Peptide** FLREKHK **Charge** 2 **Spectral Angle** 0.7

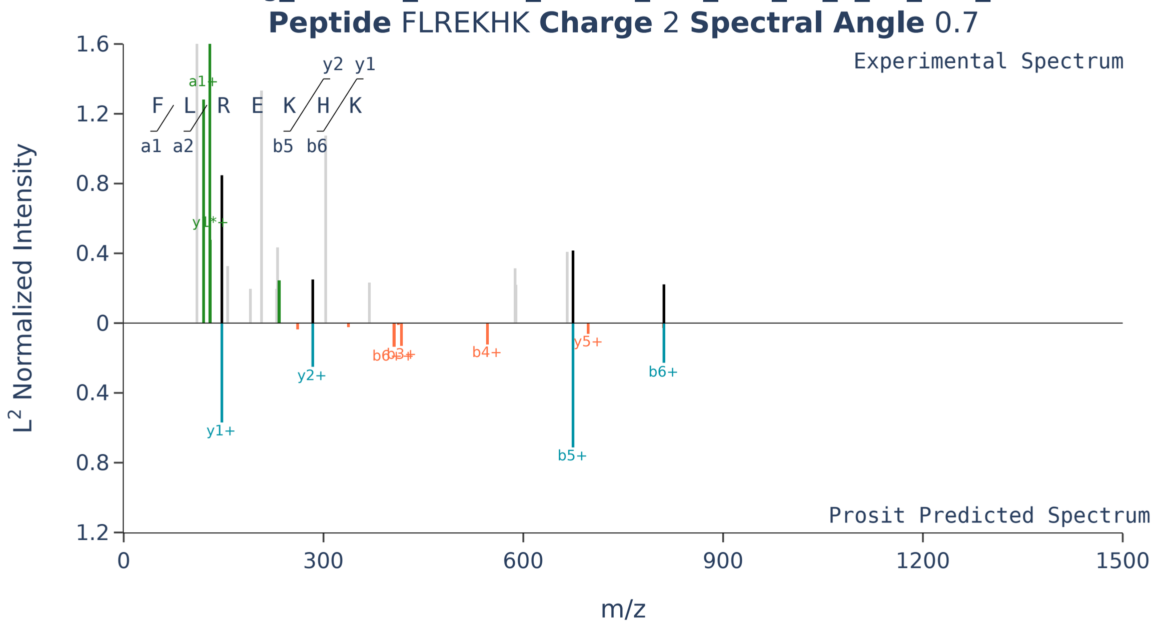


**Source** Ncheng_210623_230623_HFGoe_FFH_20S_25_1_A2_4h_R1 **Scan** 31322
**Peptide** KEAKLKFYDVLLVDTAGRLHV **Charge** 3 **Spectral Angle** 0.79

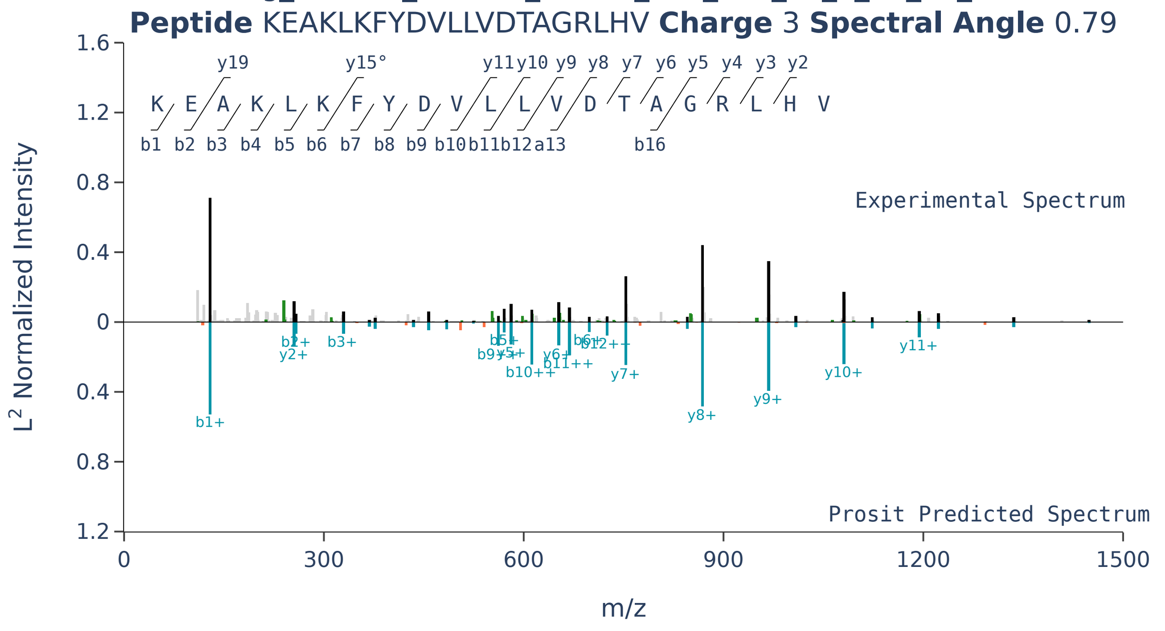


**Source** Ncheng_210623_230623_HFGoe_FFH_20S_25_1_A2_2h_R1 **Scan** 15543
**Peptide** IESKVDRAQAEKLASKL **Charge** 2 **Spectral Angle** 0.67

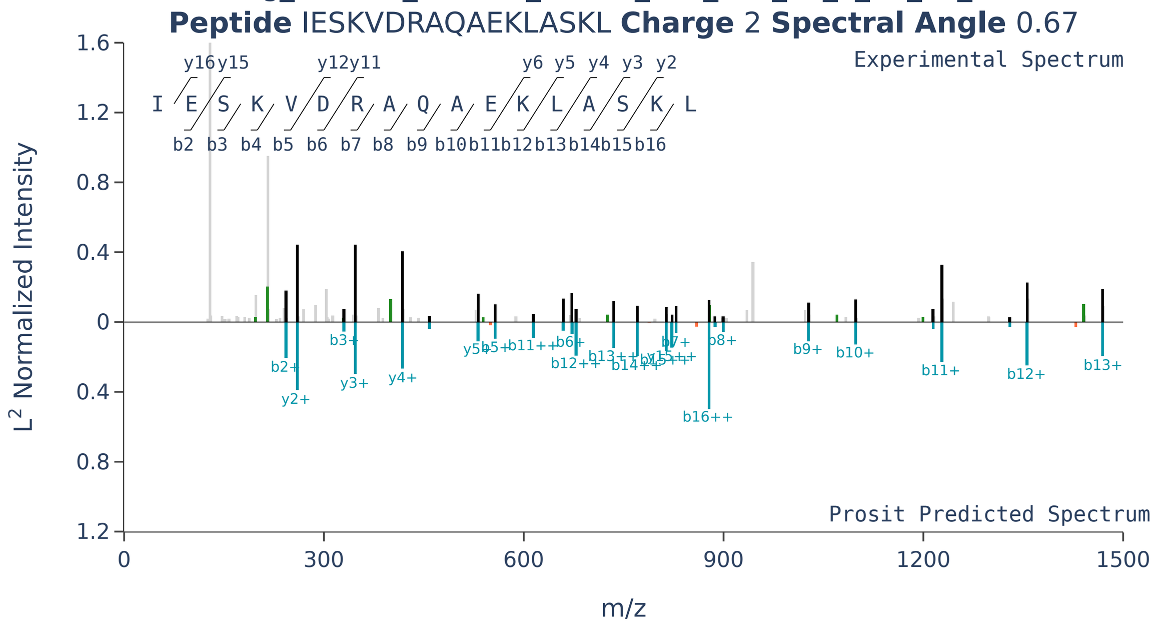


**Source** Ncheng_210623_230623_HFGoe_FFH_20S_25_1_A1_24h_R1 **Scan** 8117
**Peptide** AKPEIIKGS **Charge** 2 **Spectral Angle** 0.61

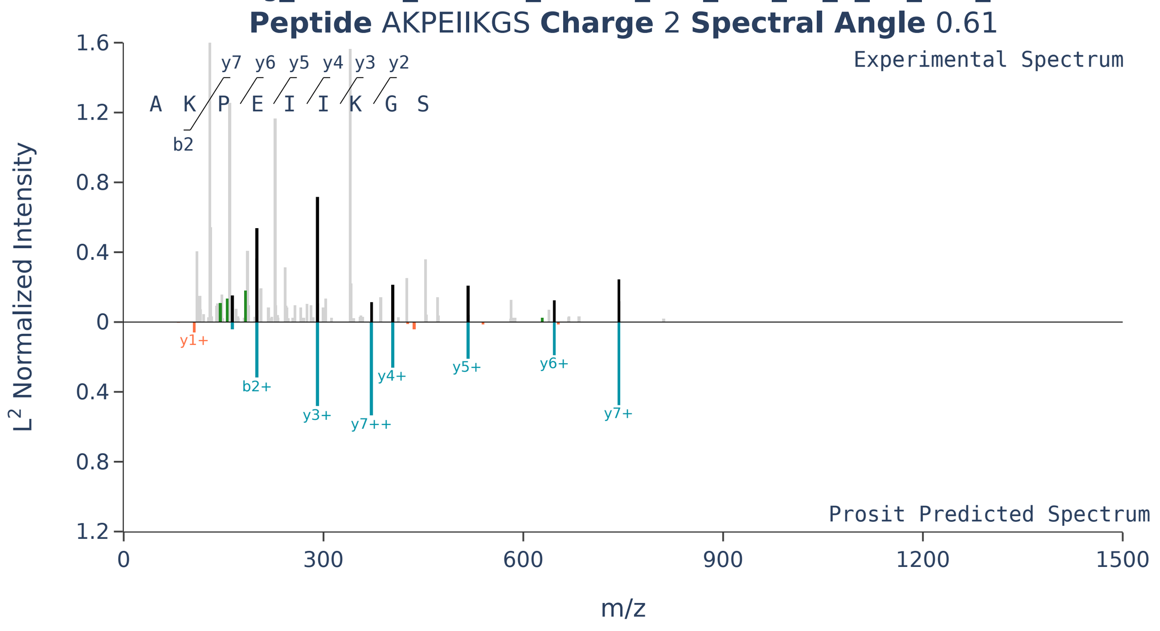


**Source** Ncheng_210623_230623_HFGoe_FFH_20S_25_1_A1_4h_R2 **Scan** 14861
**Peptide** GRLHVDEAMM **Charge** 3 **Spectral Angle** 0.64

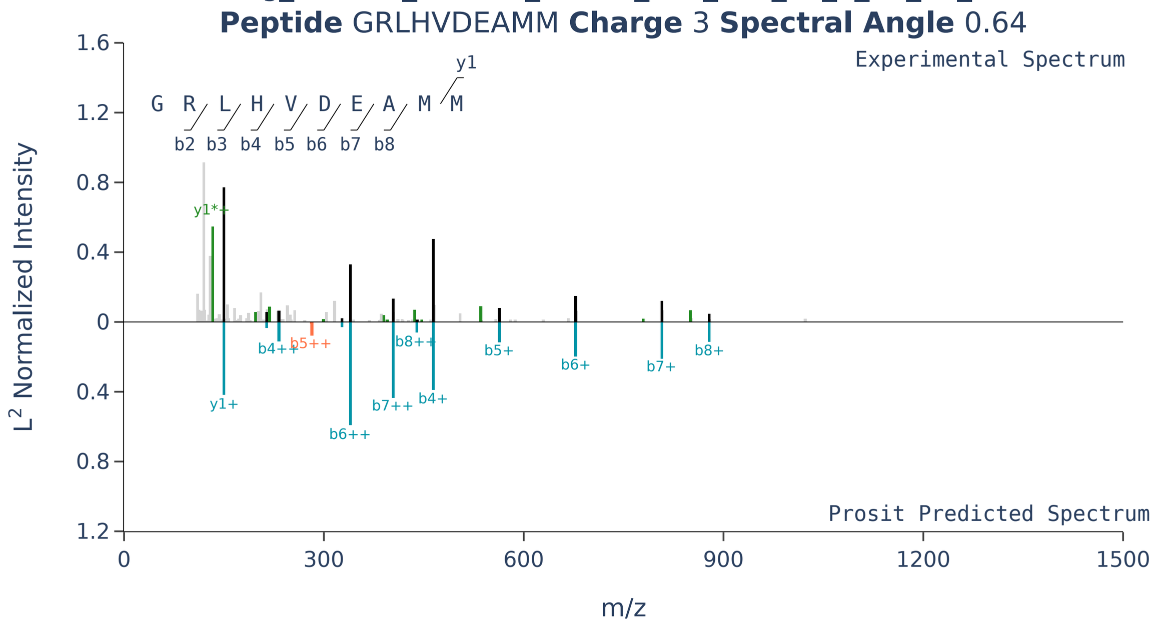


**Source** Ncheng_210623_230623_HFGoe_FFH_20S_25_1_A1_4h_R1 **Scan** 25412
**Peptide** QVQDVNRLLKQFD **Charge** 2 **Spectral Angle** 0.6

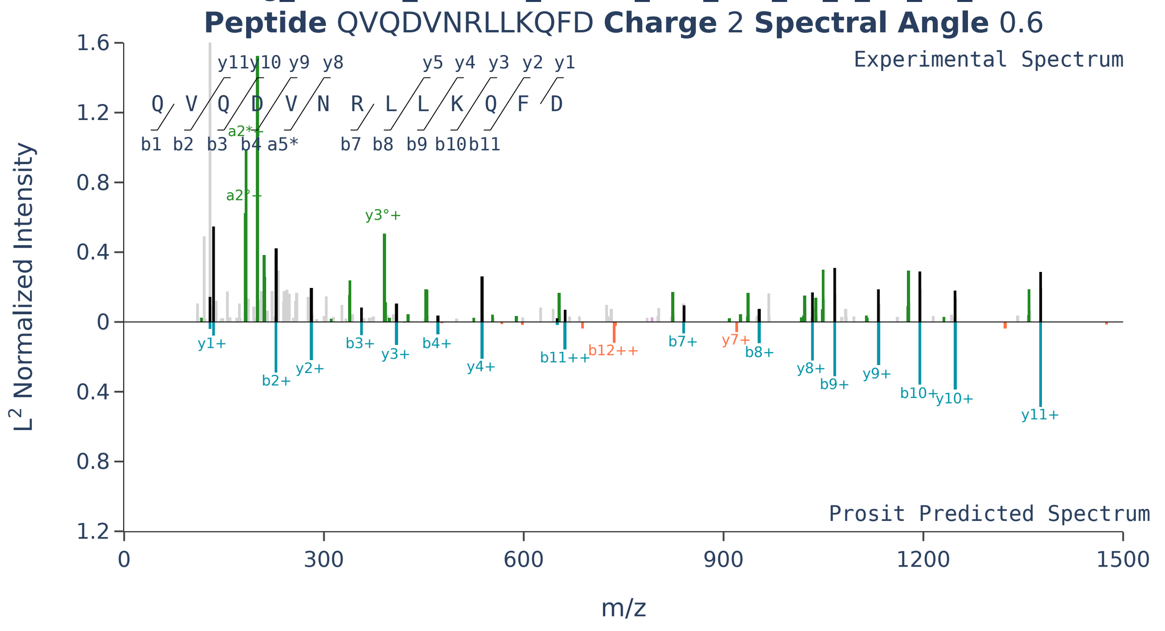


**Source** Ncheng_210623_230623_HFGoe_FFH_20S_25_1_A2_4h_R1 **Scan** 31977
**Peptide** QDAANTAKAFNEALPLTGVVLTK **Charge** 3 **Spectral Angle** 0.8

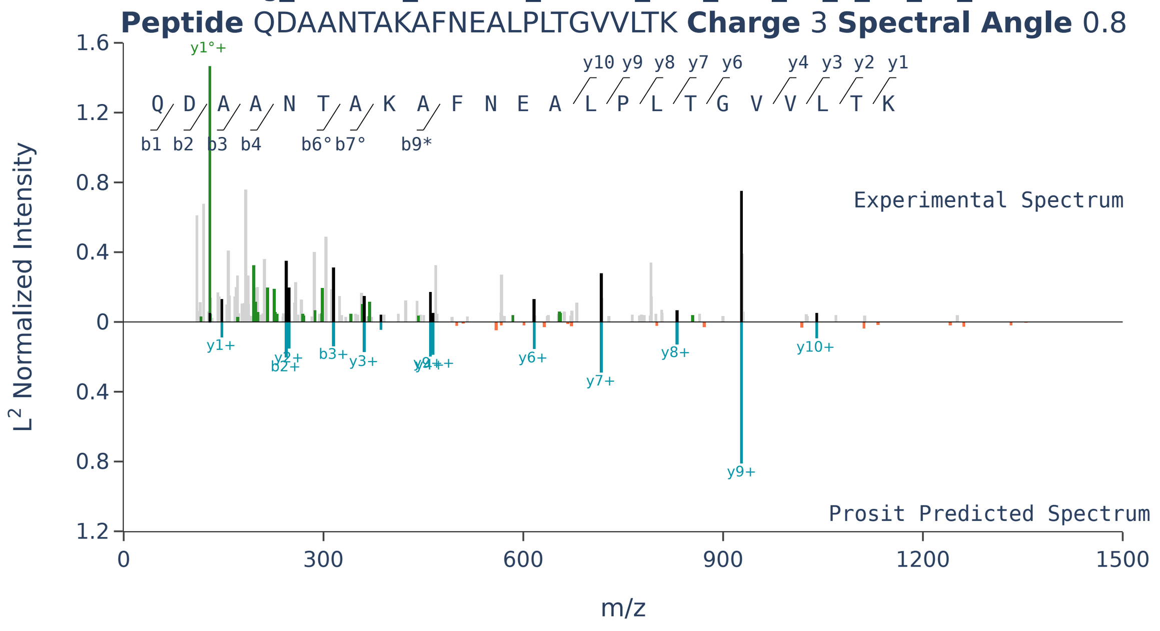

**Source** Ncheng_210623_230623_HFGoe_FFH_20S_25_1_A1_4h_R1 **Scan** 15327
**Peptide** RQMKNMGGMASLMGK **Charge** 3 **Spectral Angle** 0.71

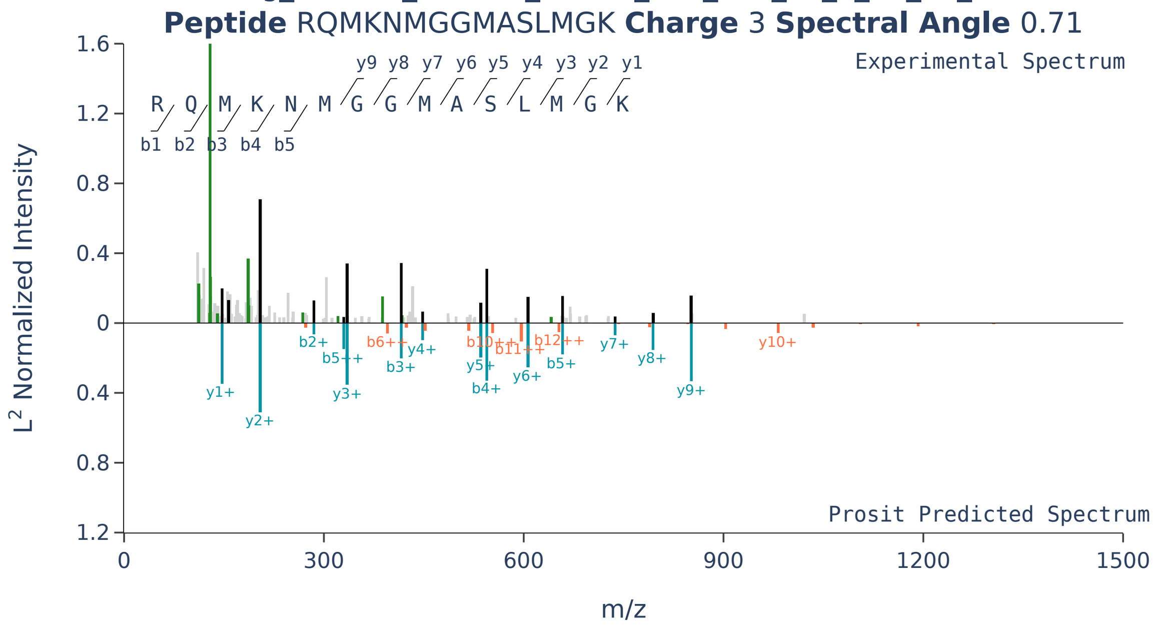


**Source** Ncheng_210623_230623_HFGoe_FFH_20S_25_1_A2_4h_R2 **Scan** 19469
**Peptide** SLRHITGKPIKFLGV **Charge** 4 **Spectral Angle** 0.69

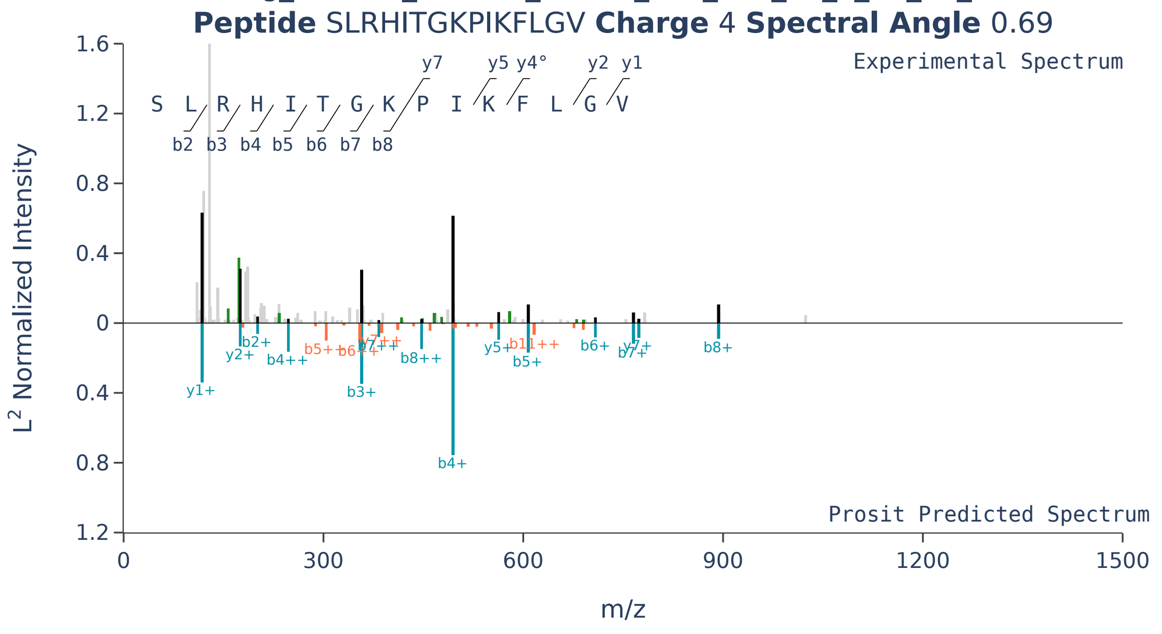


**Source** Ncheng_210623_230623_HFGoe_FFH_20S_25_1_A1_4h_R1 **Scan** 11861
**Peptide** VVLTKVDGD **Charge** 2 **Spectral Angle** 0.77

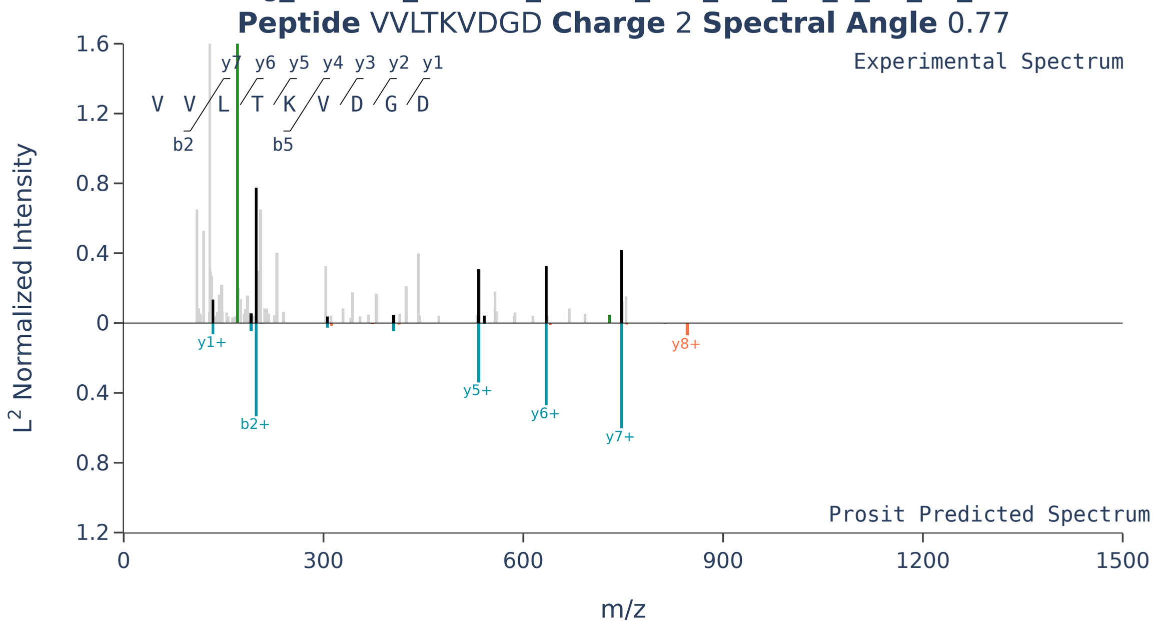


**Source** Ncheng_210623_230623_HFGoe_FFH_20S_25_1_A1_2h_R2 **Scan** 31873
**Peptide** KLKFYDVLLV **Charge** 3 **Spectral Angle** 0.71

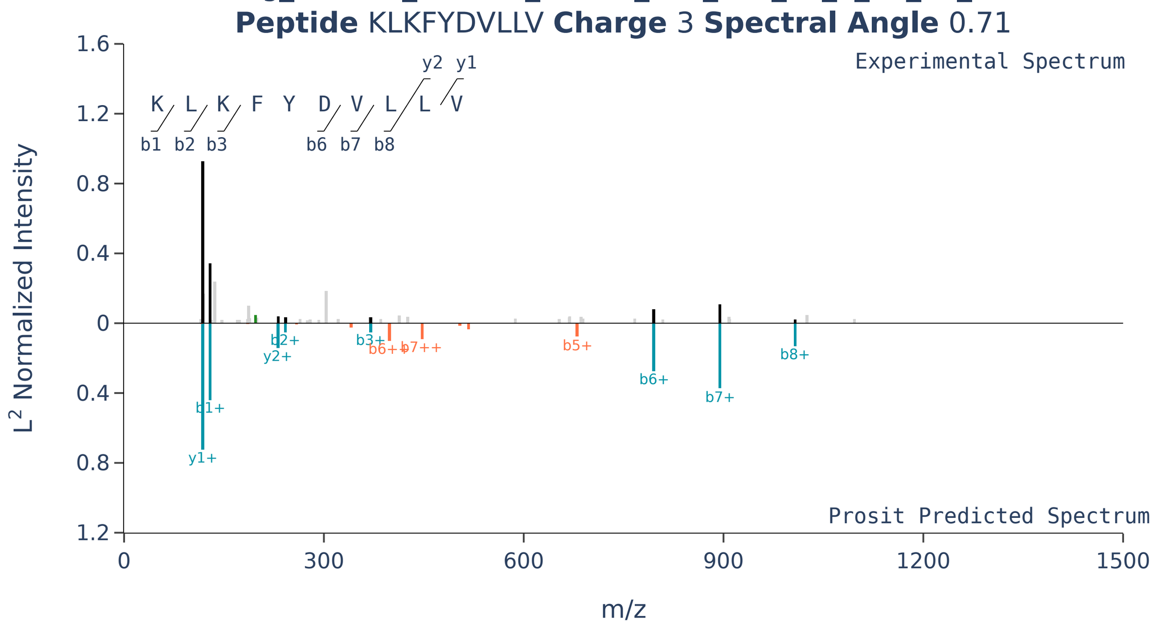


**Source** Ncheng_210623_230623_HFGoe_FFH_20S_25_1_A1_2h_R1 **Scan** 39742
**Peptide** ELVAAMGEENQTLNLAAQPPAVVLM **Charge** 2 **Spectral Angle** 0.9

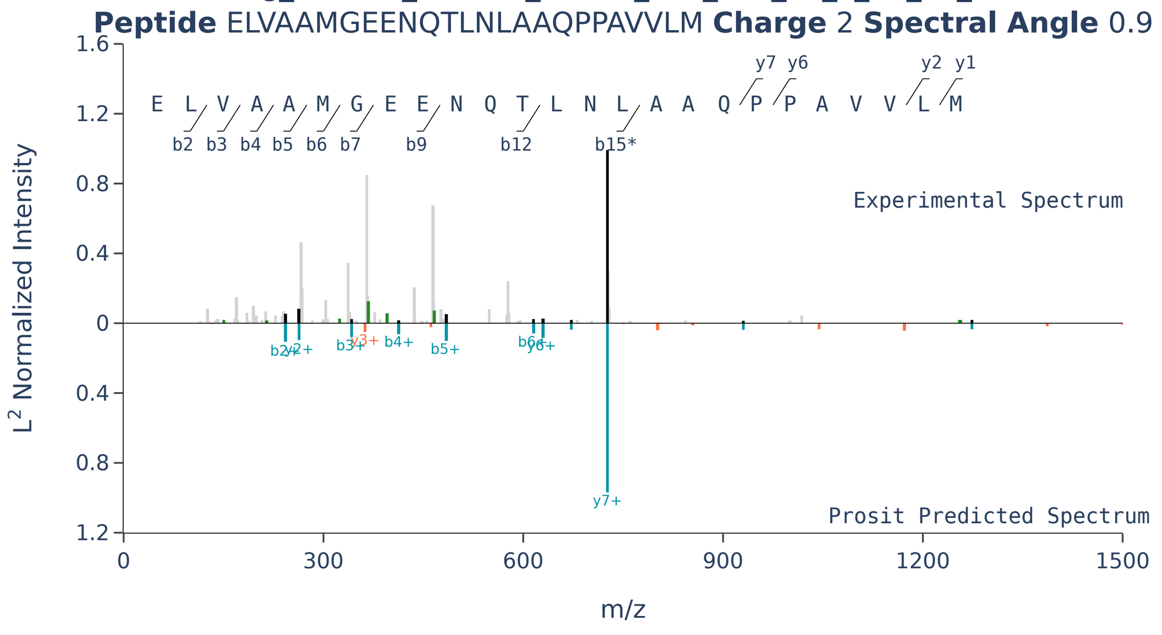


**Source** Ncheng_210623_230623_HFGoe_FFH_20S_25_1_A2_24h_R2 **Scan** 13718
**Peptide** EFINRVKEKAVGHEVNKSLTPGQ **Charge** 3 **Spectral Angle** 0.84

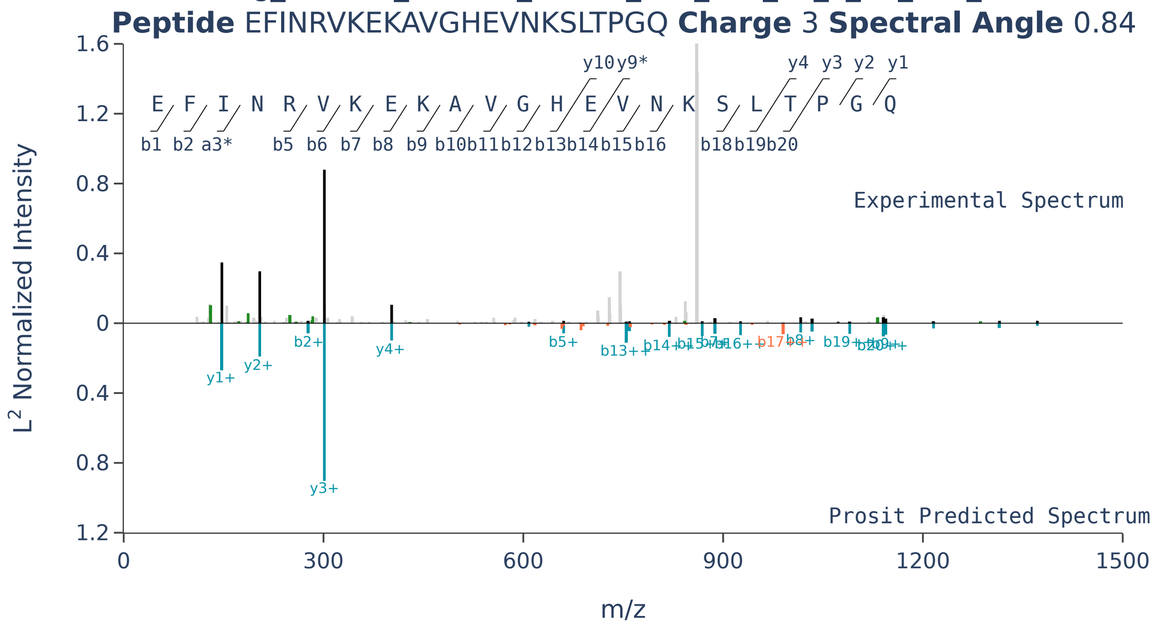


**Source** Ncheng_210623_230623_HFGoe_FFH_20S_25_1_A1_24h_R2 **Scan** 13174
**Peptide** DIESKVDRAQAEKLA **Charge** 2 **Spectral Angle** 0.74

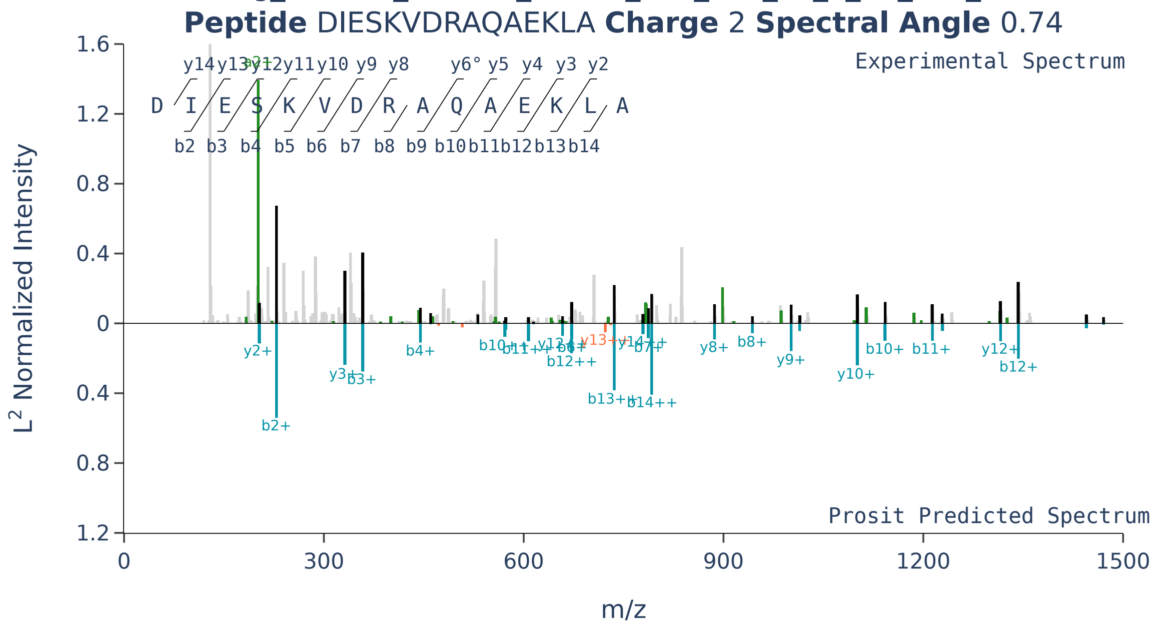


**Source** Ncheng_210623_230623_HFGoe_FFH_20S_25_1_A1_2h_R2 **Scan** 27986
**Peptide** KLKFYDVLLVDTAGR **Charge** 3 **Spectral Angle** 0.51

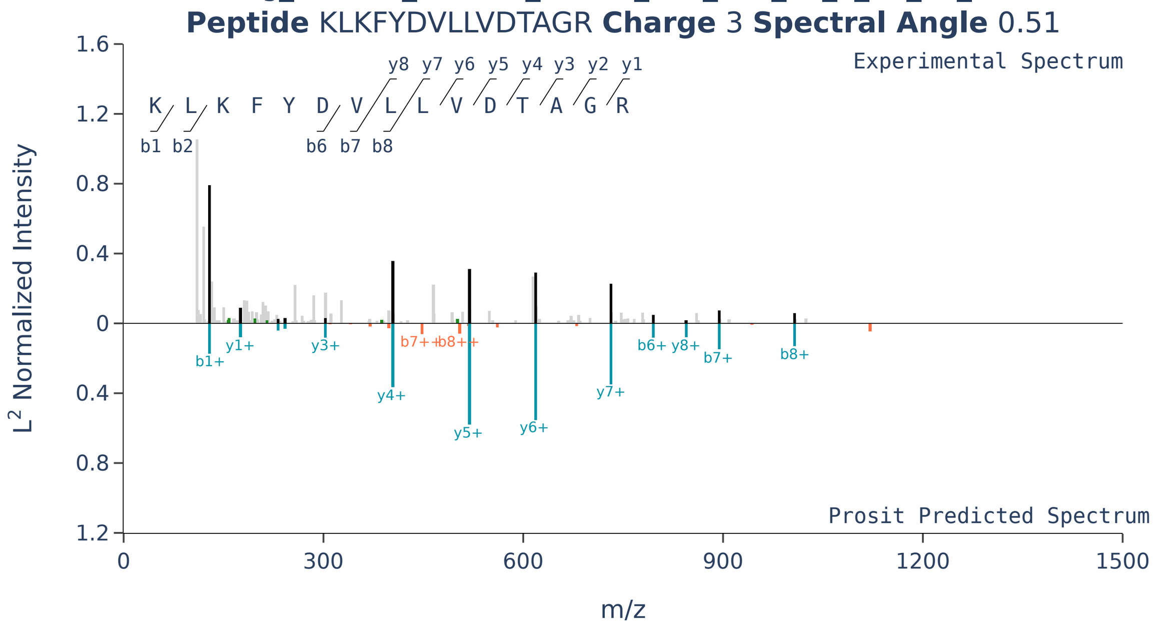


**Source** Ncheng_210623_230623_HFGoe_FFH_20S_25_1_A2_24h_R1 **Scan** 12339
**Peptide** EFINRVKEKAVGHEV **Charge** 2 **Spectral Angle** 0.59

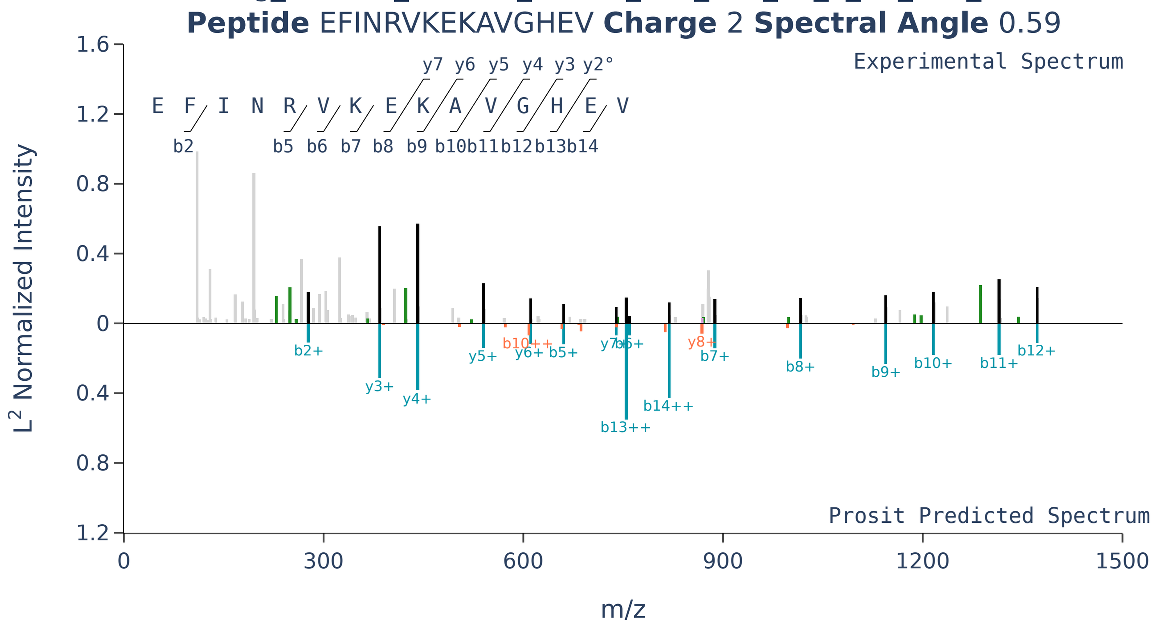


**Source** Ncheng_210623_230623_HFGoe_FFH_20S_25_1_A1_2h_R1 **Scan** 32601
**Peptide** TLFVVDAMTGQDAANTAKAF **Charge** 2 **Spectral Angle** 0.89

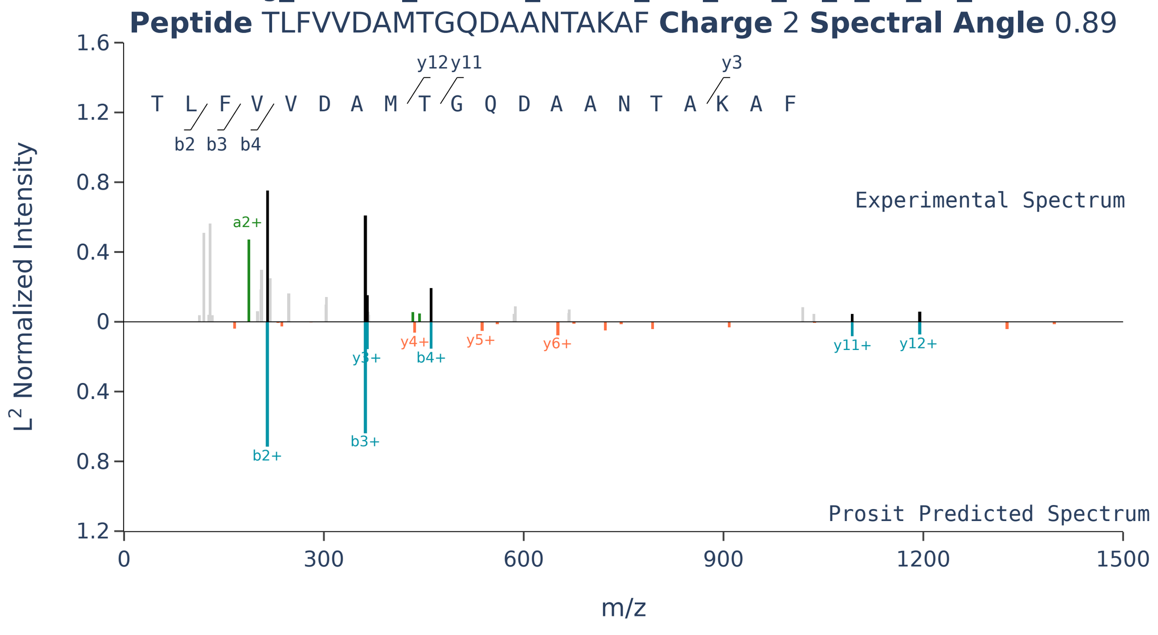


**Source** Ncheng_210623_230623_HFGoe_FFH_20S_25_1_A2_4h_R1 **Scan** 18821
**Peptide** VVDAMTGQDAANTAKAFNE **Charge** 2 **Spectral Angle** 0.82

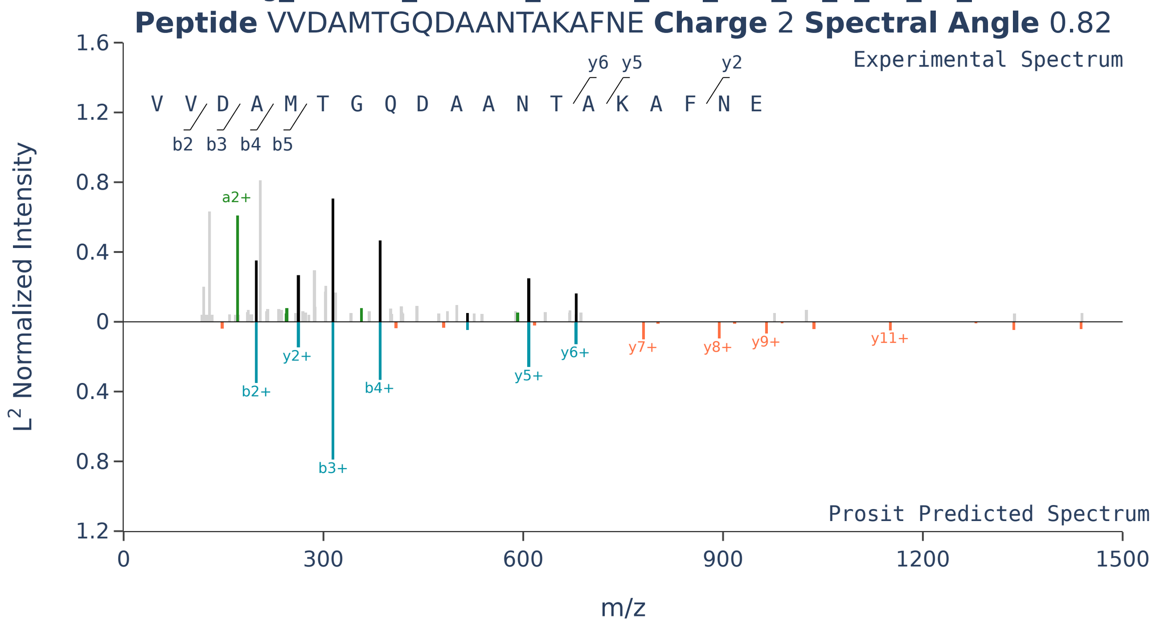


**Source** Ncheng_210623_230623_HFGoe_FFH_20S_25_1_A2_4h_R1 **Scan** 21590
**Peptide** KVDGDARGGAALSIRHITGKPIKFL **Charge** 3 **Spectral Angle** 0.64

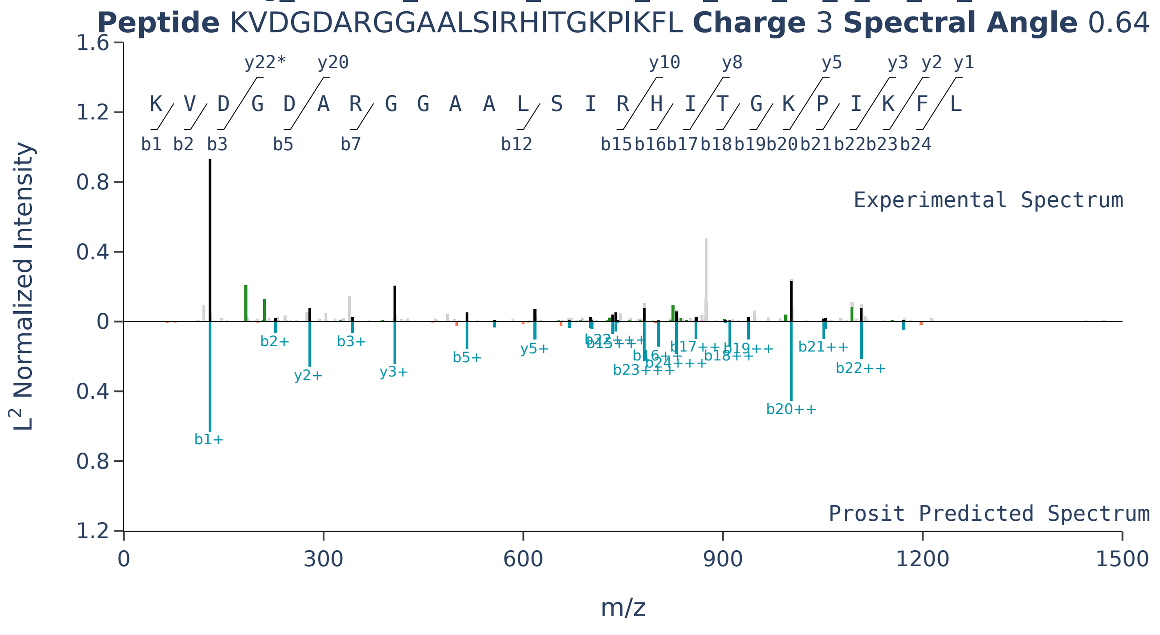


**Source** Ncheng_210623_230623_HFGoe_FFH_20S_25_1_A1_2h_R2 **Scan** 30101
**Peptide** KFYDVLLVDTAGRLHVDE **Charge** 2 **Spectral Angle** 0.64

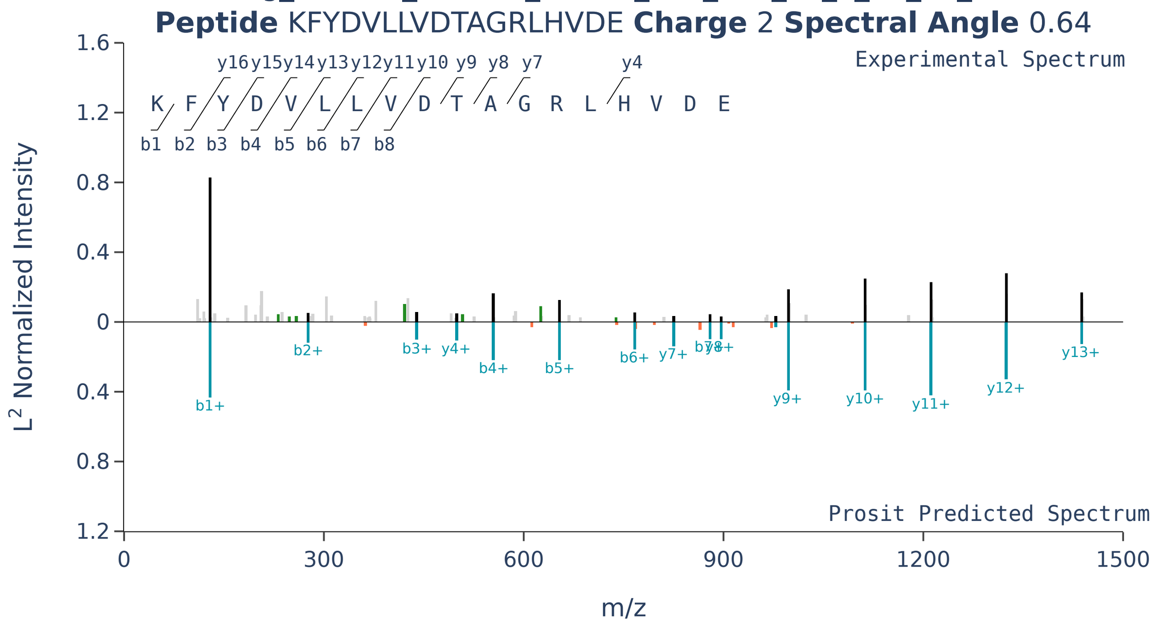


**Source** Ncheng_210623_230623_HFGoe_FFH_20S_25_1_A1_2h_R2 **Scan** 26676
**Peptide** KVLVRMEAIINSMTMKER **Charge** 3 **Spectral Angle** 0.77

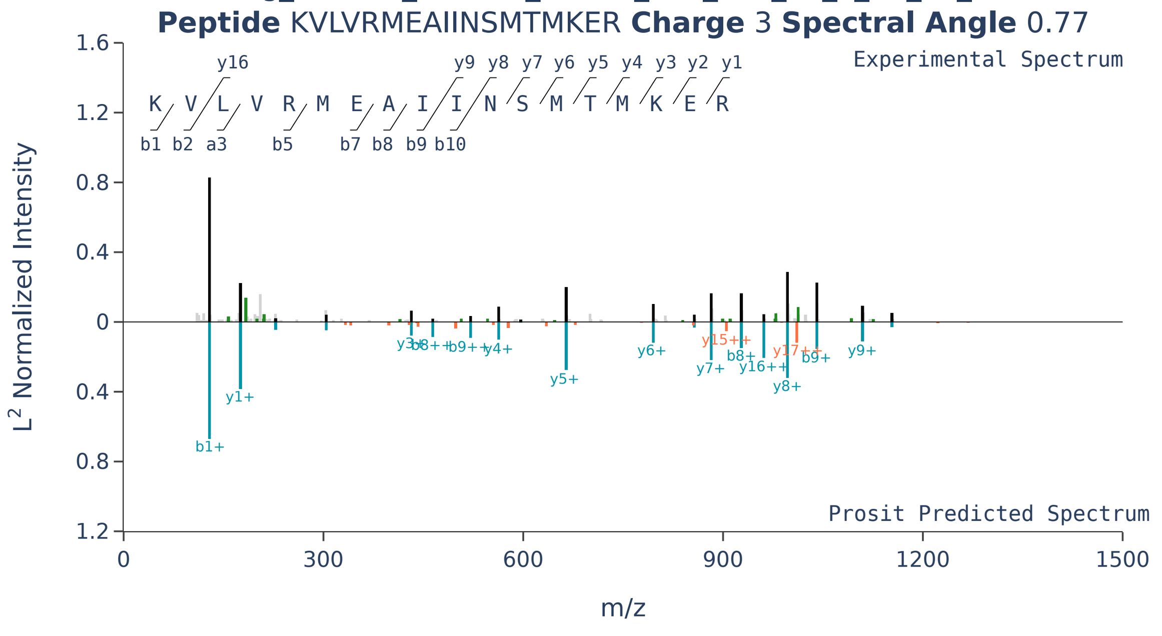


**Source** Ncheng_210623_230623_HFGoe_FFH_20S_25_1_A1_24h_R1 **Scan** 6013
**Peptide** GKTTSVGKLGK **Charge** 2 **Spectral Angle** 0.6

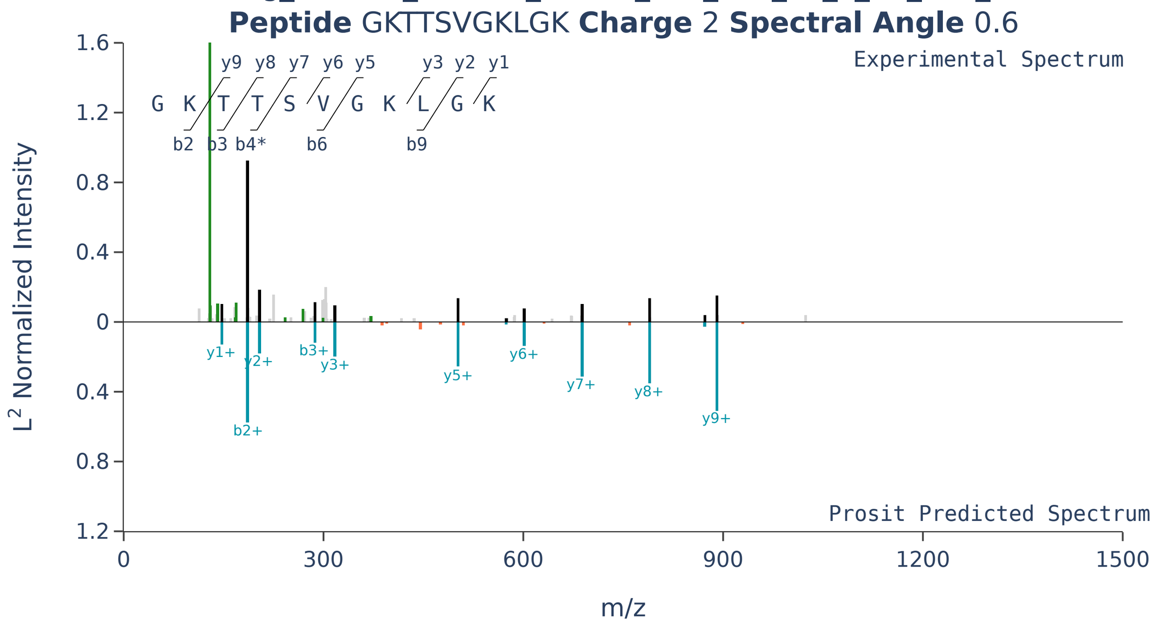

**Source** Ncheng_210623_230623_HFGoe_FFH_20S_25_1_A2_2h_R1 **Scan** 20394
**Peptide** IVNAALKEAKLKFYD **Charge** 2 **Spectral Angle** 0.73

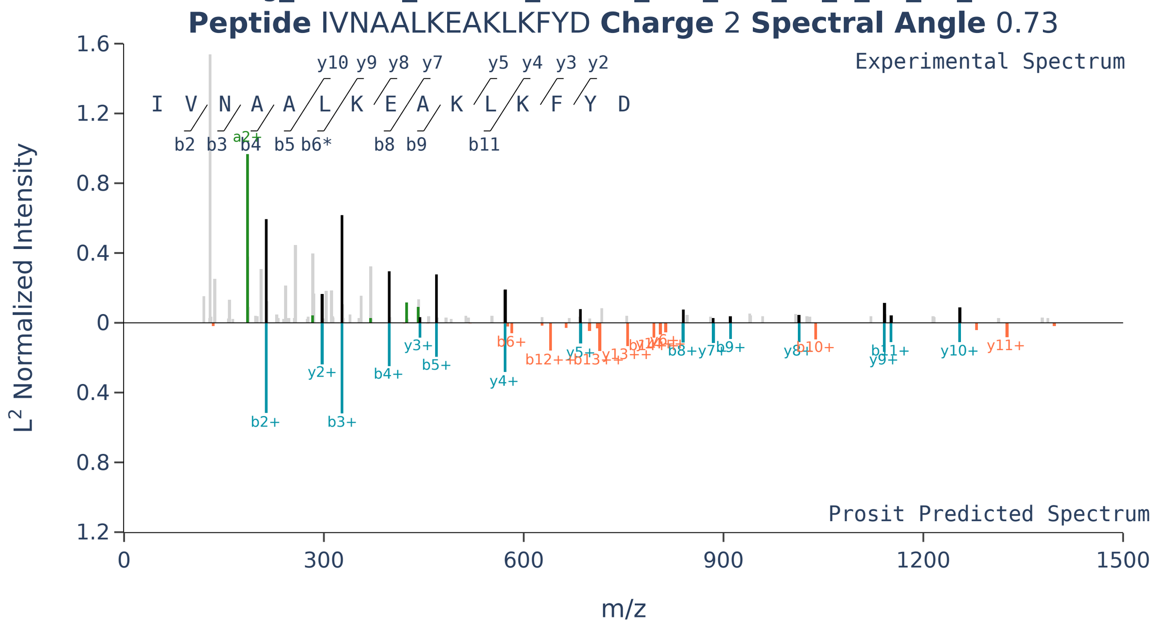


**Source** Ncheng_210623_230623_HFGoe_FFH_20S_25_1_A2_24h_R1 **Scan** 6358
**Peptide** QGAGKTTSVGKLGK **Charge** 2 **Spectral Angle** 0.77

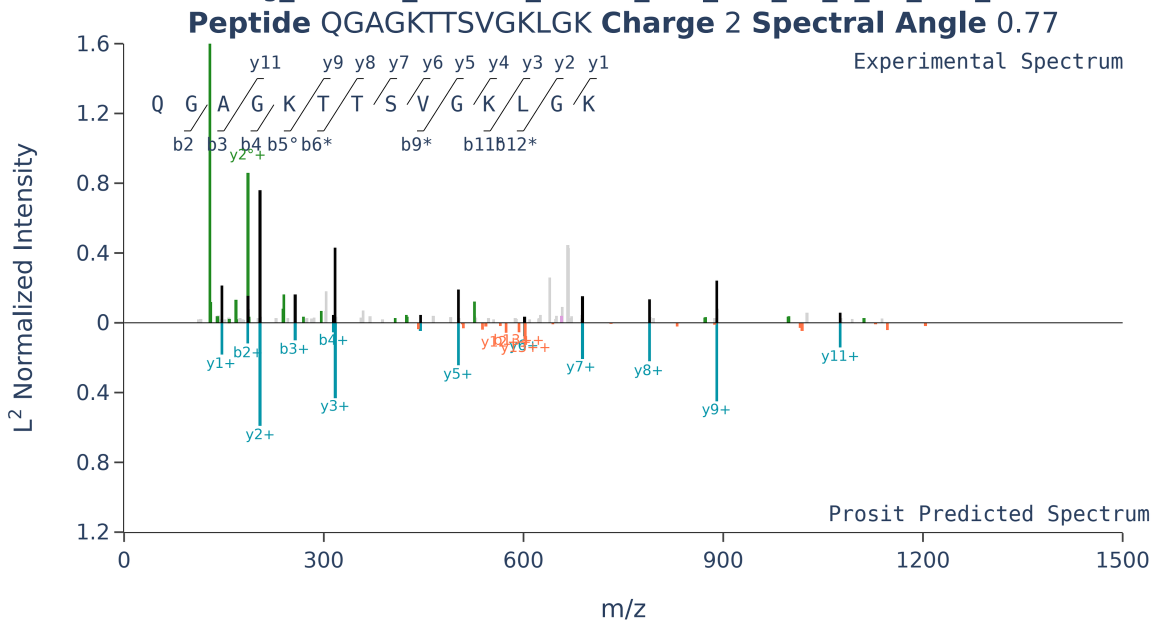


**Source** Ncheng_210623_230623_HFGoe_FFH_20S_25_1_A1_2h_R2 **Scan** 21956
**Peptide** VDEAMMDEIKQVHA **Charge** 3 **Spectral Angle** 0.72

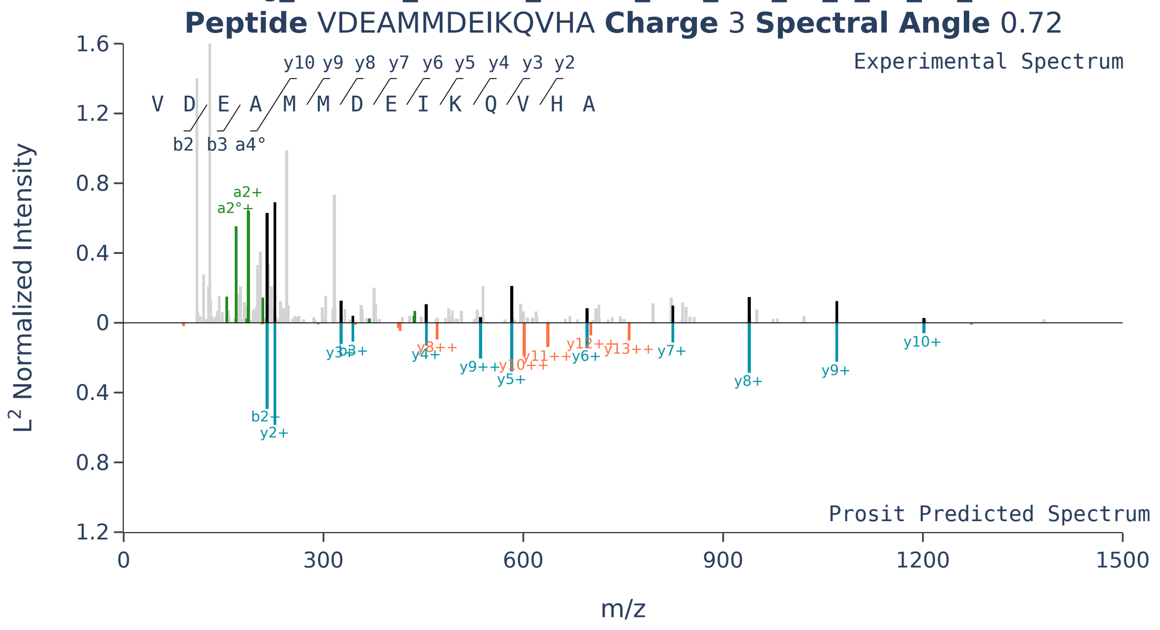


**Source** Ncheng_210623_230623_HFGoe_FFH_20S_25_1_A2_4h_R2 **Scan** 6515
**Peptide** DMQRMMKK **Charge** 2 **Spectral Angle** 0.75

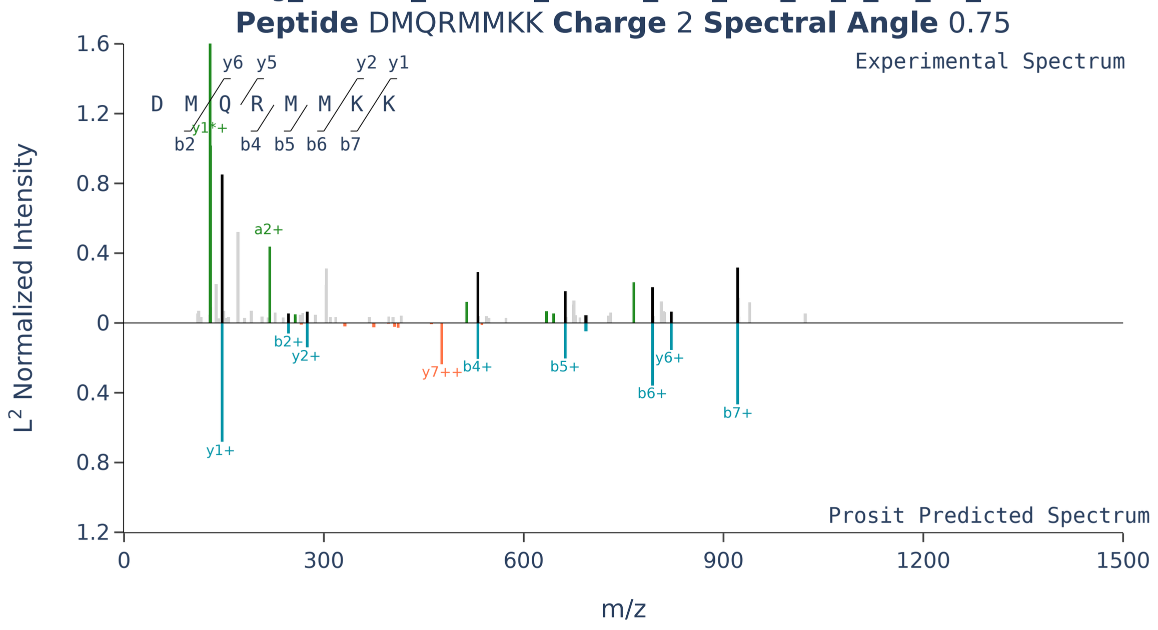


**Source** Ncheng_210623_230623_HFGoe_FFH_20S_25_1_A2_4h_R1 **Scan** 21245
**Peptide** QLPDNVKSQMDDKVLVRME **Charge** 3 **Spectral Angle** 0.6

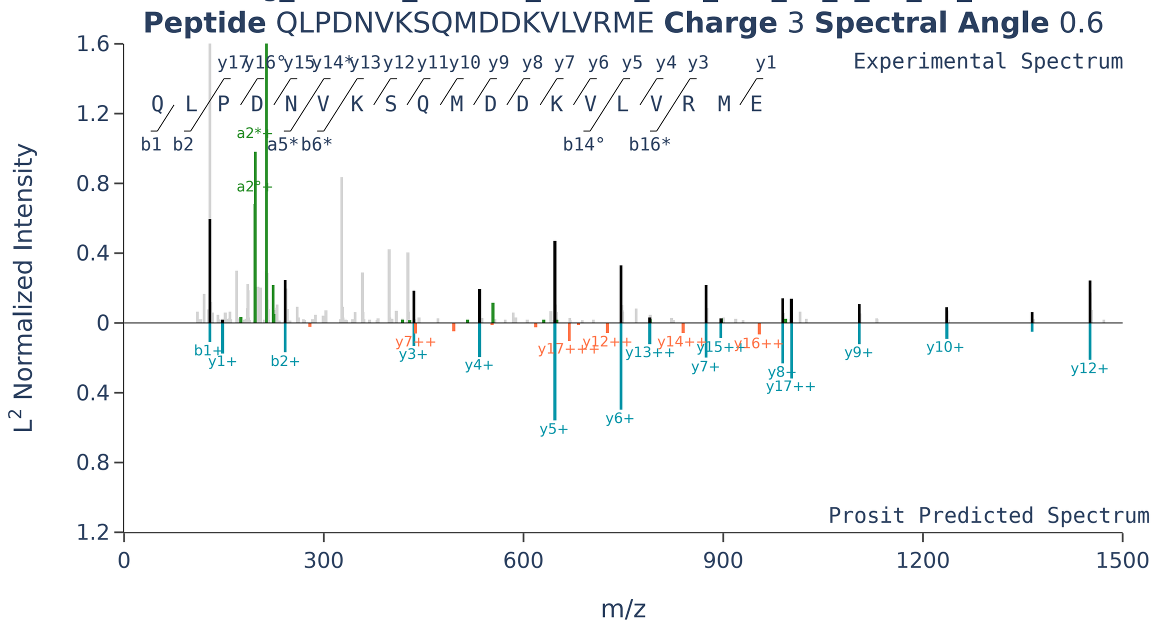


**Source** Ncheng_210623_230623_HFGoe_FFH_20S_25_1_A2_4h_R2 **Scan** 32291
**Peptide** GGMASLMGKLPGMGQIPDNVKSQM **Charge** 3 **Spectral Angle** 0.82

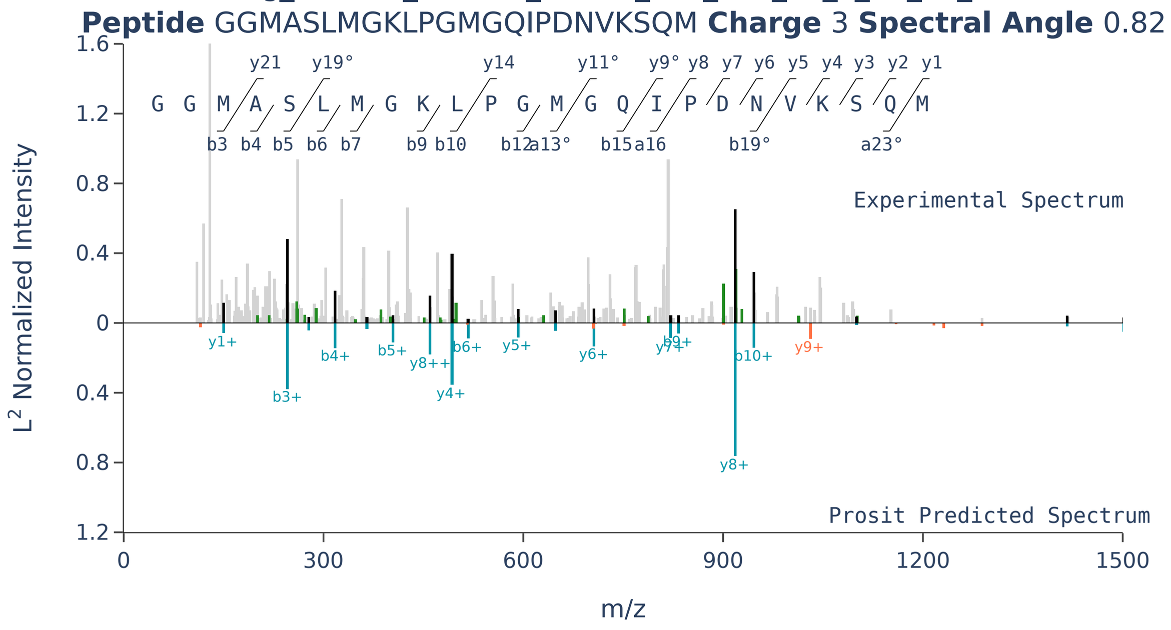


**Source** Ncheng_210623_230623_HFGoe_FFH_20S_25_1_A2_24h_R1 **Scan** 10783
**Peptide** LTDRLSRT **Charge** 2 **Spectral Angle** 0.75

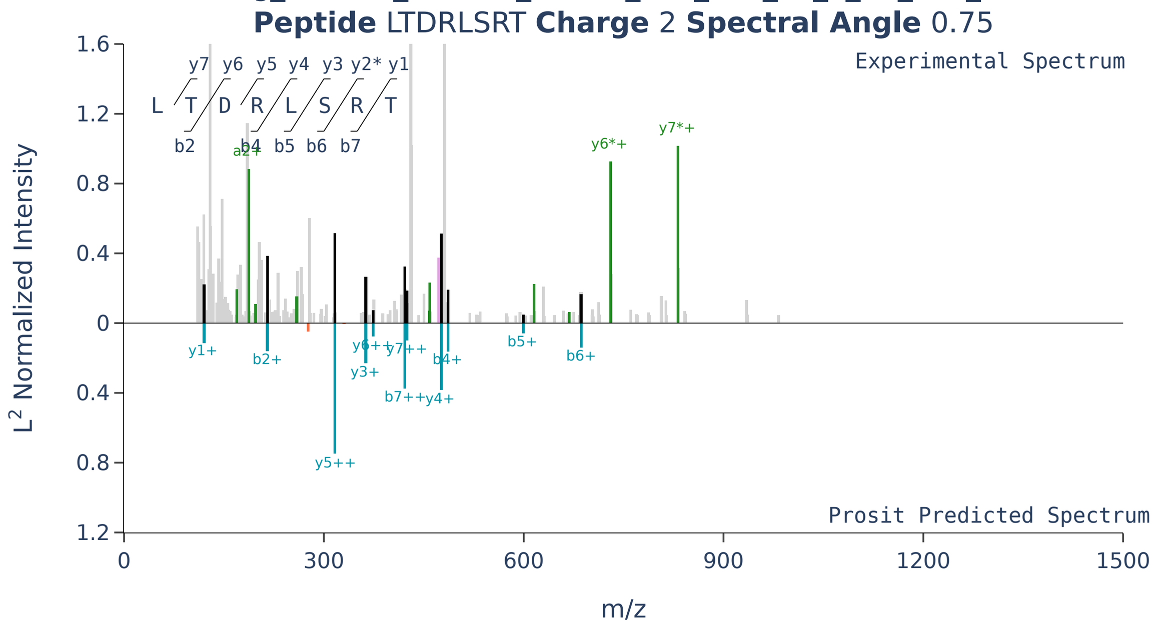


**Source** Ncheng_210623_230623_HFGoe_FFH_20S_25_1_A2_4h_R2 **Scan** 20366
**Peptide** MAGLQGAGKTTSVGKLGKFLRE **Charge** 5 **Spectral Angle** 0.7

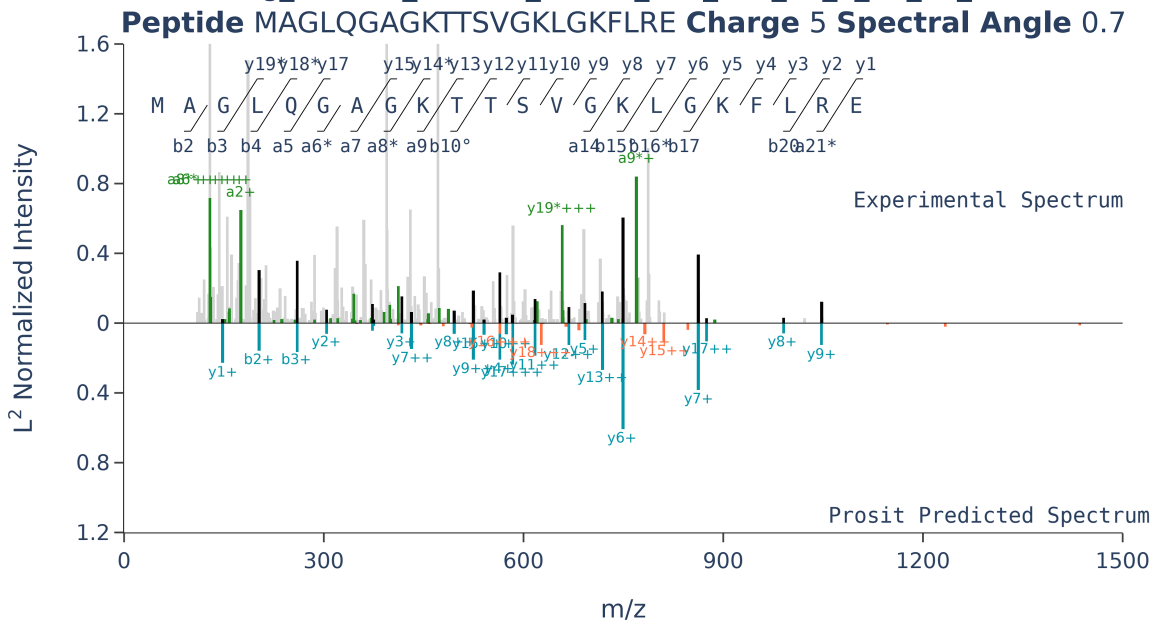


**Source** Ncheng_210623_230623_HFGoe_FFH_20S_25_1_A1_2h_R2 **Scan** 32572
**Peptide** DNVKDTLREVRMALLEAD **Charge** 3 **Spectral Angle** 0.64

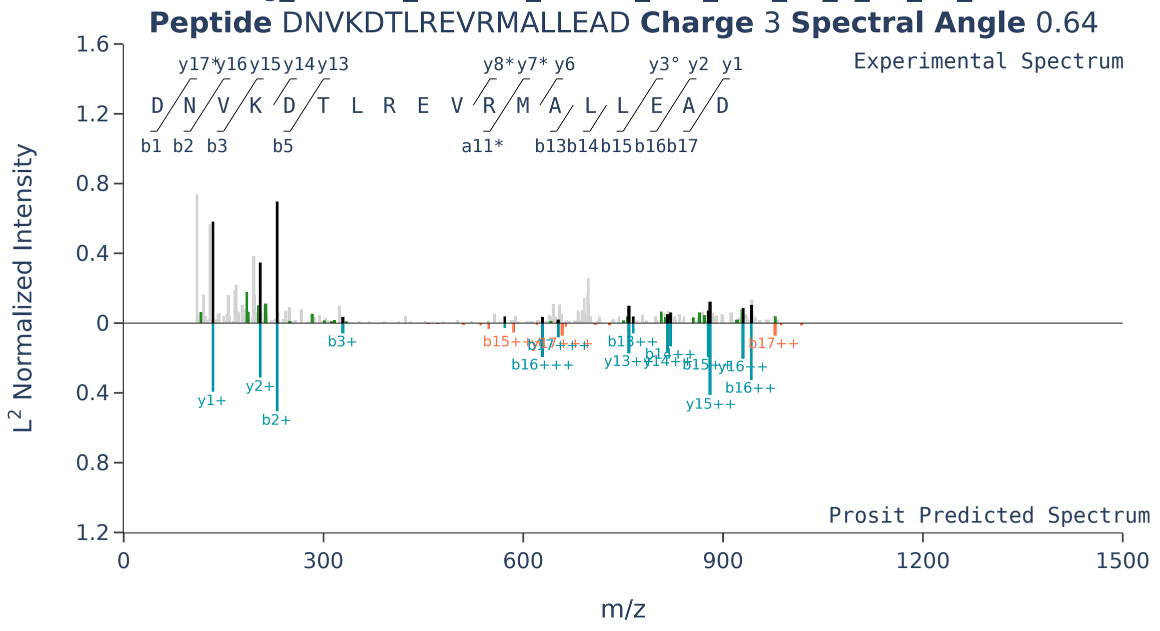


**Source** Ncheng_210623_230623_HFGoe_FFH_20S_25_1_A2_24h_R2 **Scan** 9850
**Peptide** MAGLQGAGKTTSVGK **Charge** 2 **Spectral Angle** 0.72

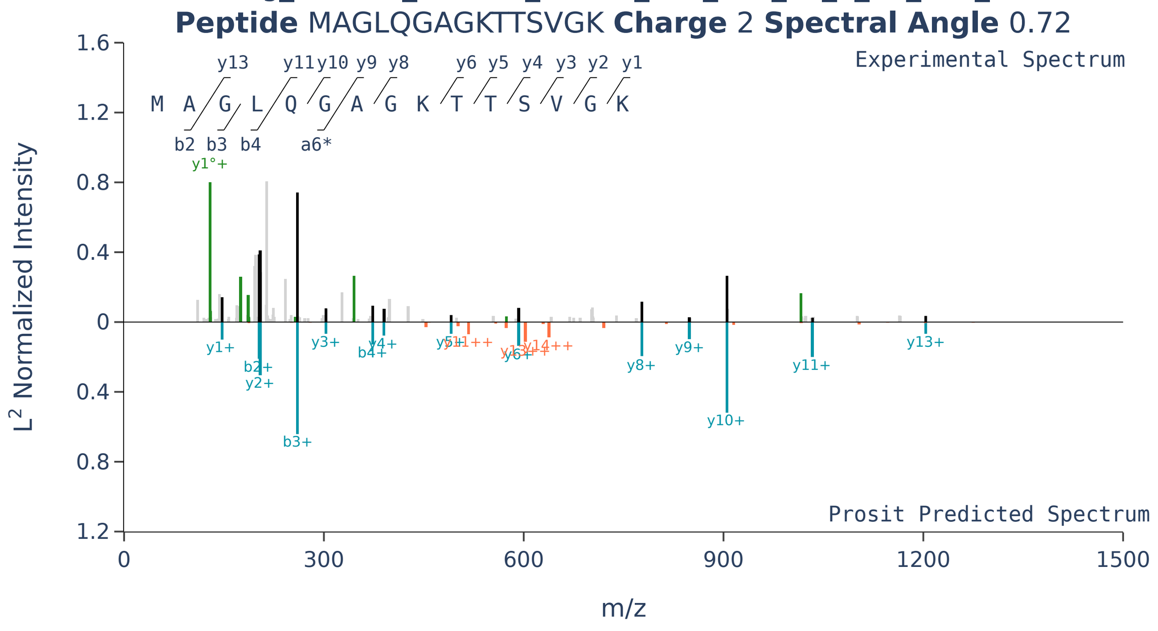


**Source** Ncheng_210623_230623_HFGoe_FFH_20S_25_1_A1_4h_R1 **Scan** 13737
**Peptide** RIAAGCGMQVQDVN **Charge** 2 **Spectral Angle** 0.74

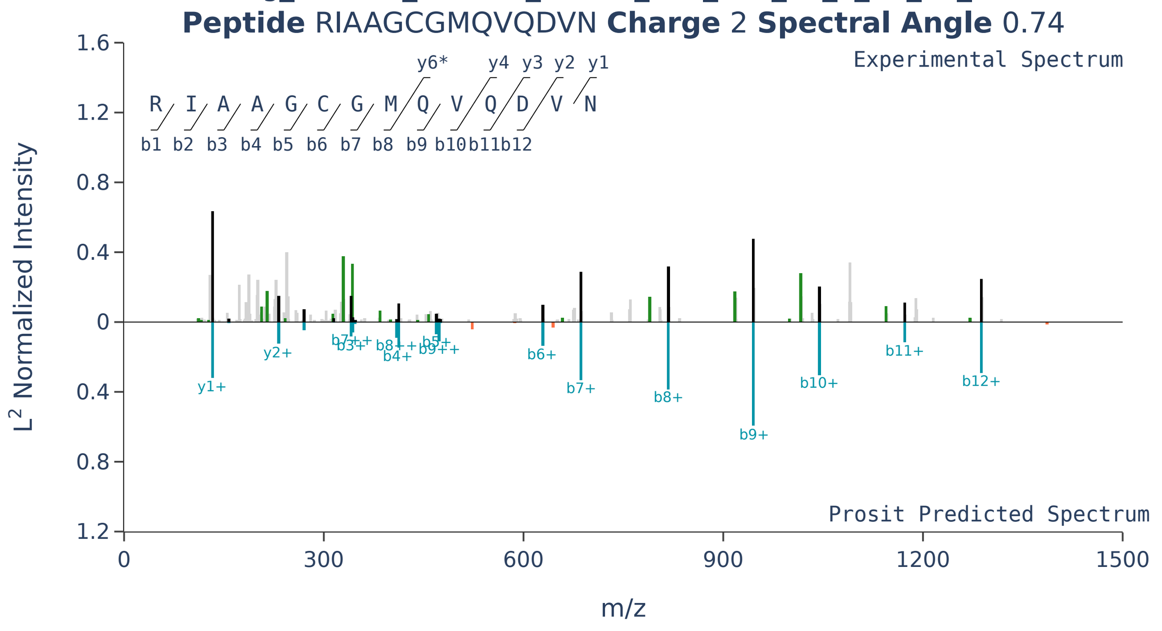


**Source** Ncheng_210623_230623_HFGoe_FFH_20S_25_1_A2_24h_R2 **Scan** 5976
**Peptide** KLKKGDGFD **Charge** 2 **Spectral Angle** 0.74

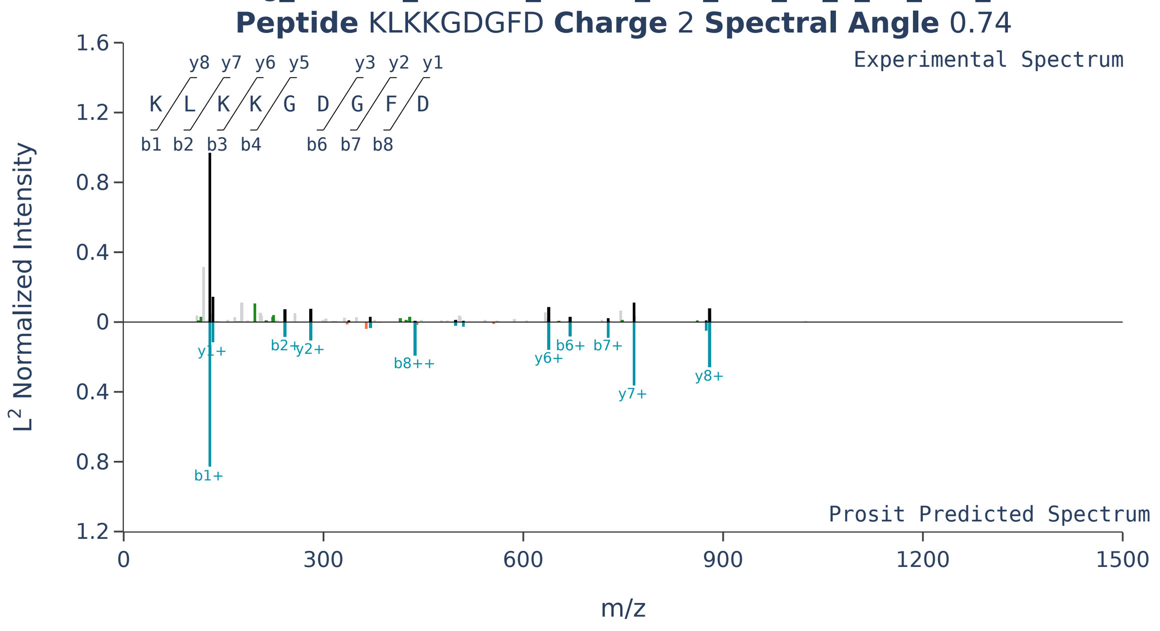


**Source** Ncheng_210623_230623_HFGoe_FFH_20S_25_1_A1_2h_R1 **Scan** 31110
**Peptide** AANTAKAFNEALPLTGVVLTKVDGDARGG **Charge** 3 **Spectral Angle** 0.8

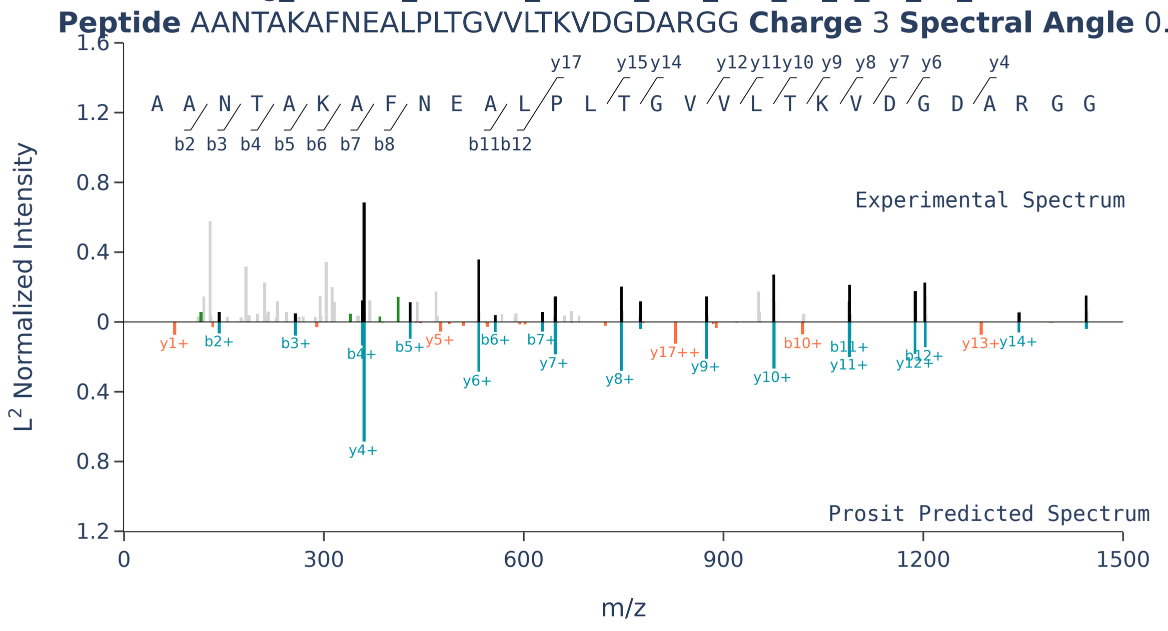


**Source** Ncheng_210623_230623_HFGoe_FFH_20S_25_1_A2_4h_R1 **Scan** 25410
**Peptide** DTAGRLHVDEAMMDEIKQVHA **Charge** 3 **Spectral Angle** 0.77

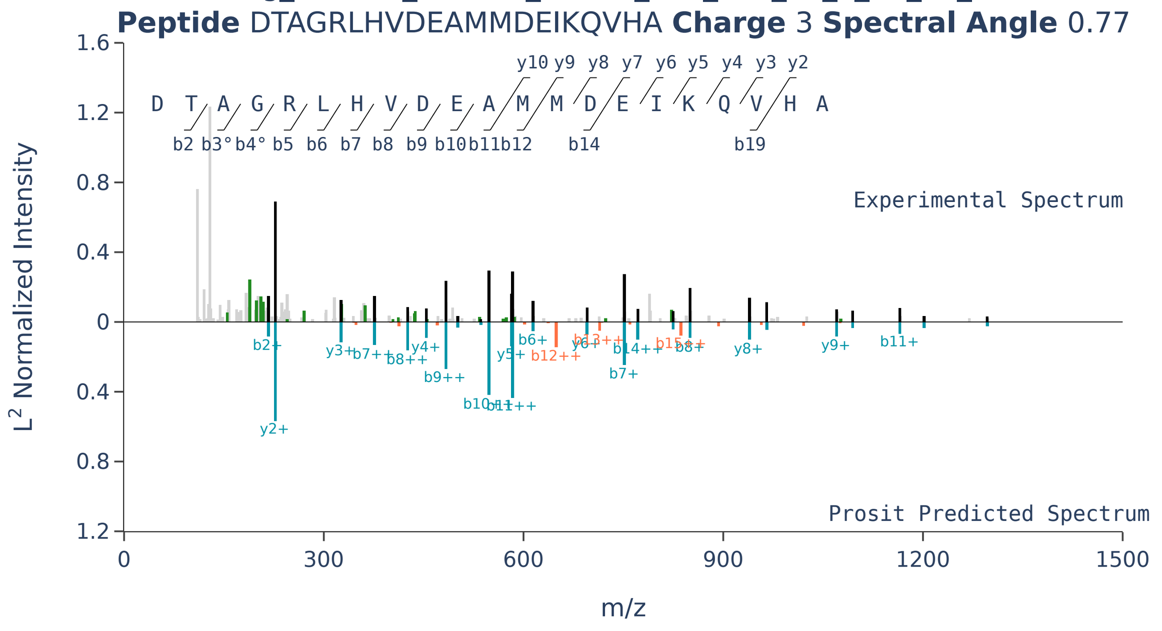


**Source** Ncheng_210623_230623_HFGoe_FFH_20S_25_1_A2_2h_R2 **Scan** 14644
**Peptide** KTEALEPFHPDRIASR **Charge** 5 **Spectral Angle** 0.68

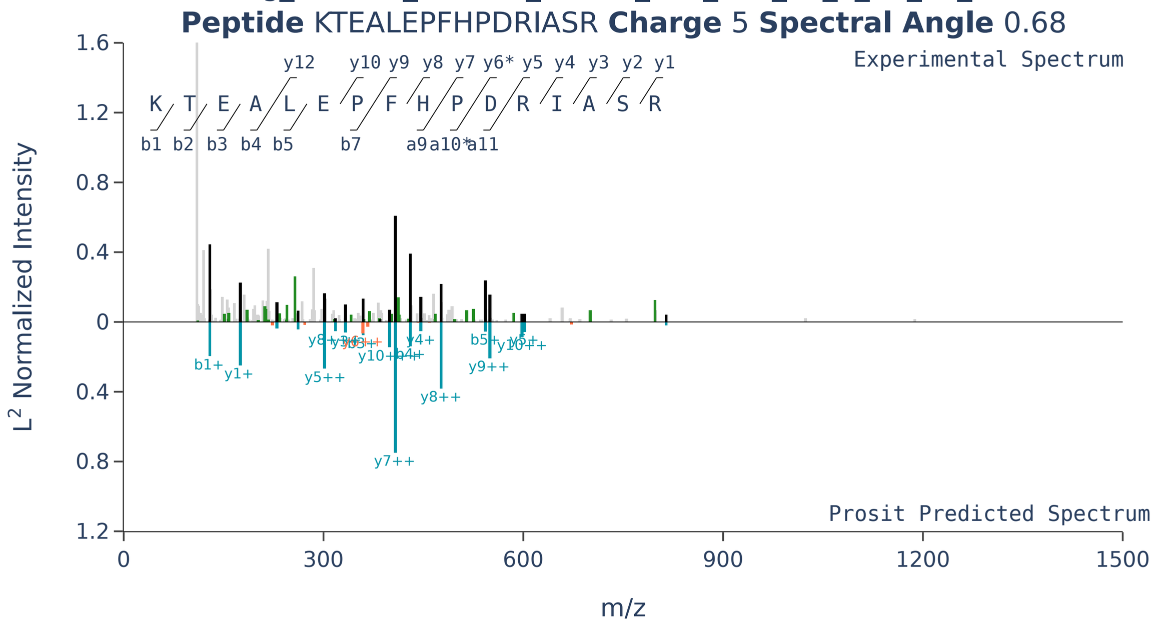

**Source** Ncheng_210623_230623_HFGoe_FFH_20S_25_1_A1_2h_R2 **Scan** 31928
**Peptide** KSLTPGQEFVKIVRNELVAA **Charge** 3 **Spectral Angle** 0.73

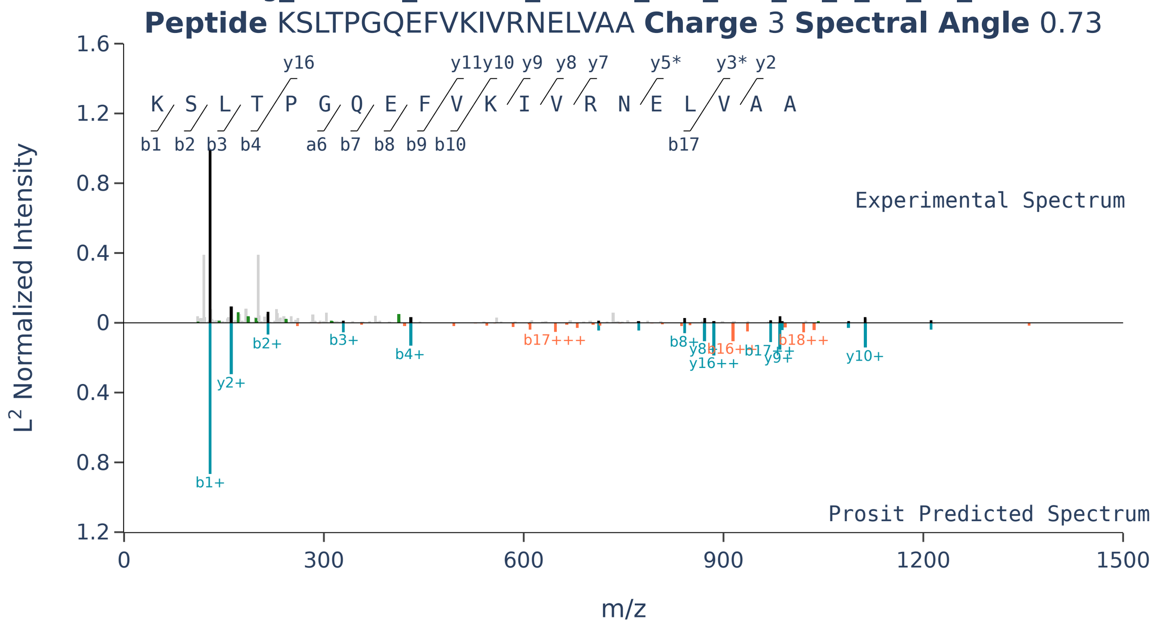


**Source** Ncheng_210623_230623_HFGoe_FFH_20S_25_1_A2_4h_R1 **Scan** 33088
**Peptide** AAQPPAVVLMAGLQGAGKTT **Charge** 3 **Spectral Angle** 0.78

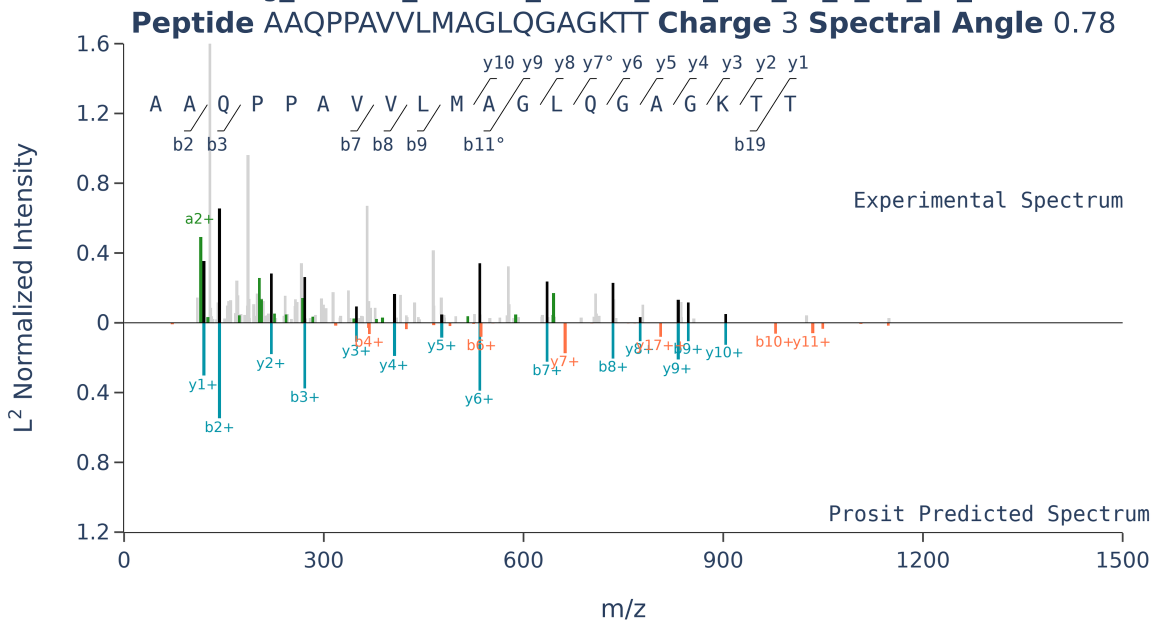


**Source** Ncheng_210623_230623_HFGoe_FFH_20S_25_1_A1_2h_R2 **Scan** 27052
**Peptide** MGKLPGMGQIPDNVKSQMDDKVLV **Charge** 3 **Spectral Angle** 0.81

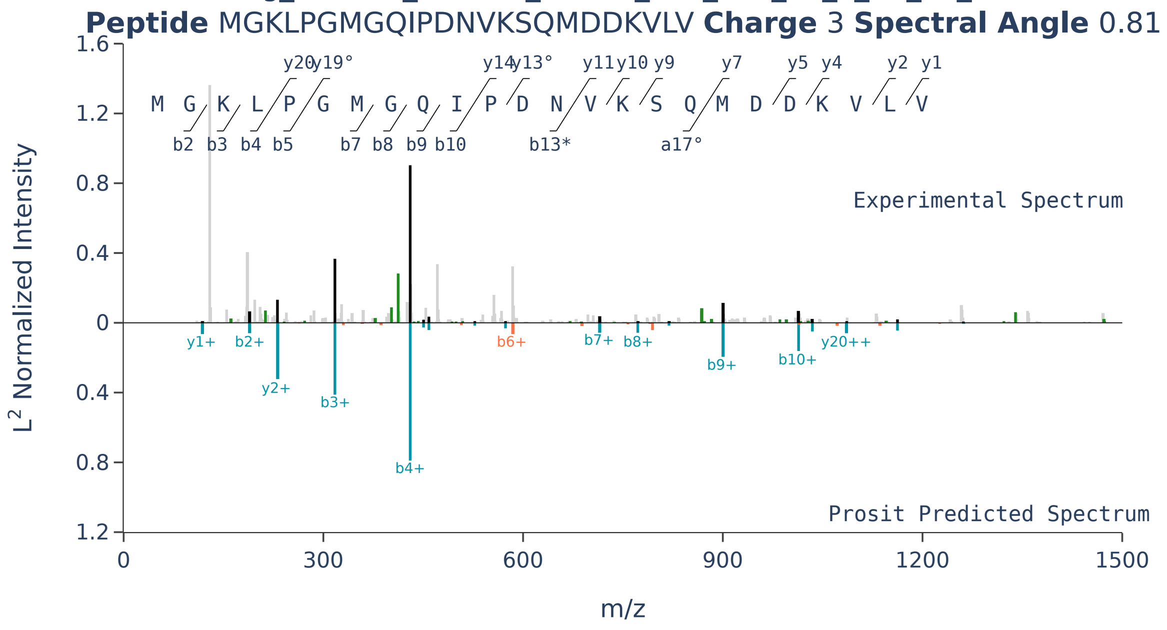


**Source** Ncheng_210623_230623_HFGoe_FFH_20S_25_1_A2_2h_R1 **Scan** 21122
**Peptide** LGVGEKTEALEPFHPDRIAS **Charge** 3 **Spectral Angle** 0.84

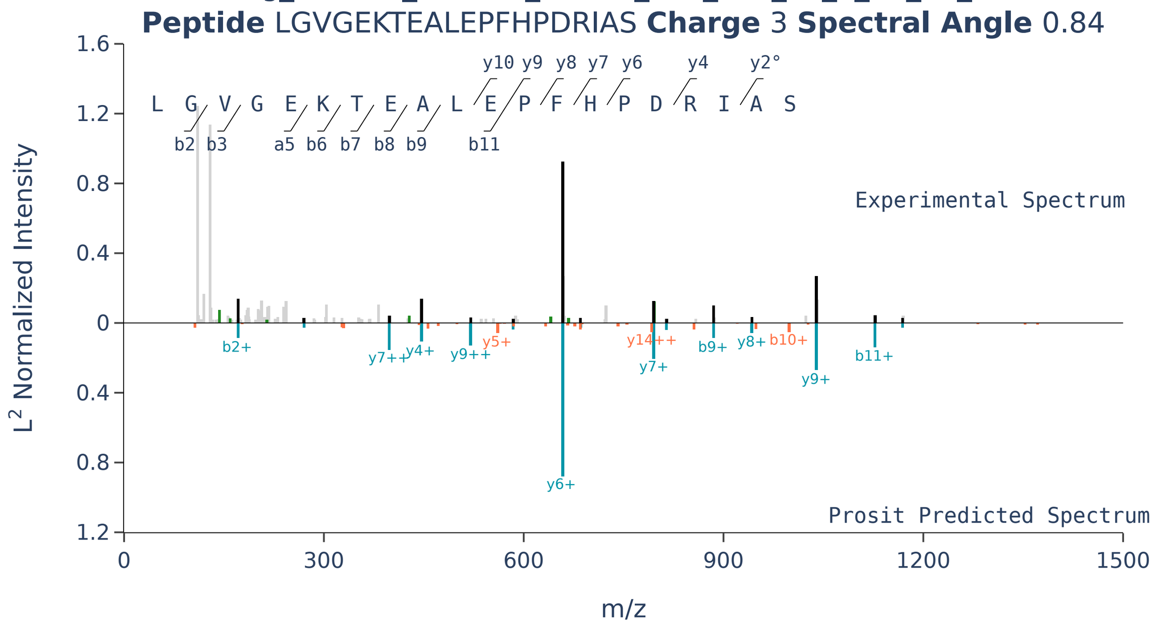


**Source** Ncheng_210623_230623_HFGoe_FFH_20S_25_1_A2_4h_R1 **Scan** 29049
**Peptide** NDFLEQLRQMKNMG **Charge** 2 **Spectral Angle** 0.78

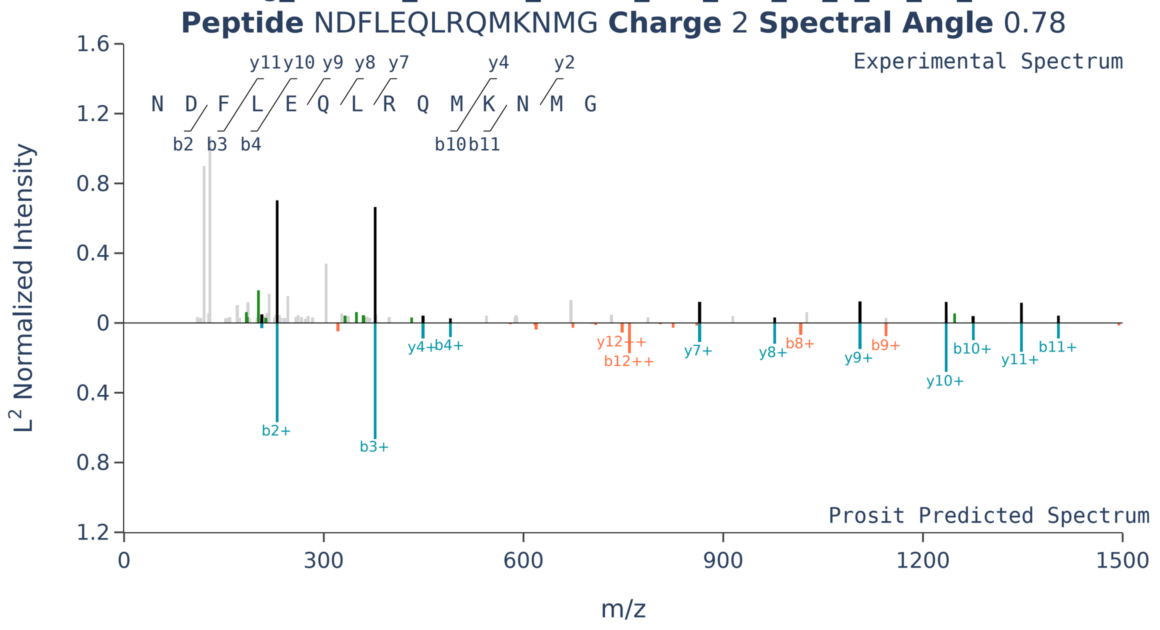


**Source** Ncheng_210623_230623_HFGoe_FFH_20S_25_1_A2_4h_R2 **Scan** 23662
**Peptide** RQMKNMGGMASLMGKL **Charge** 4 **Spectral Angle** 0.74

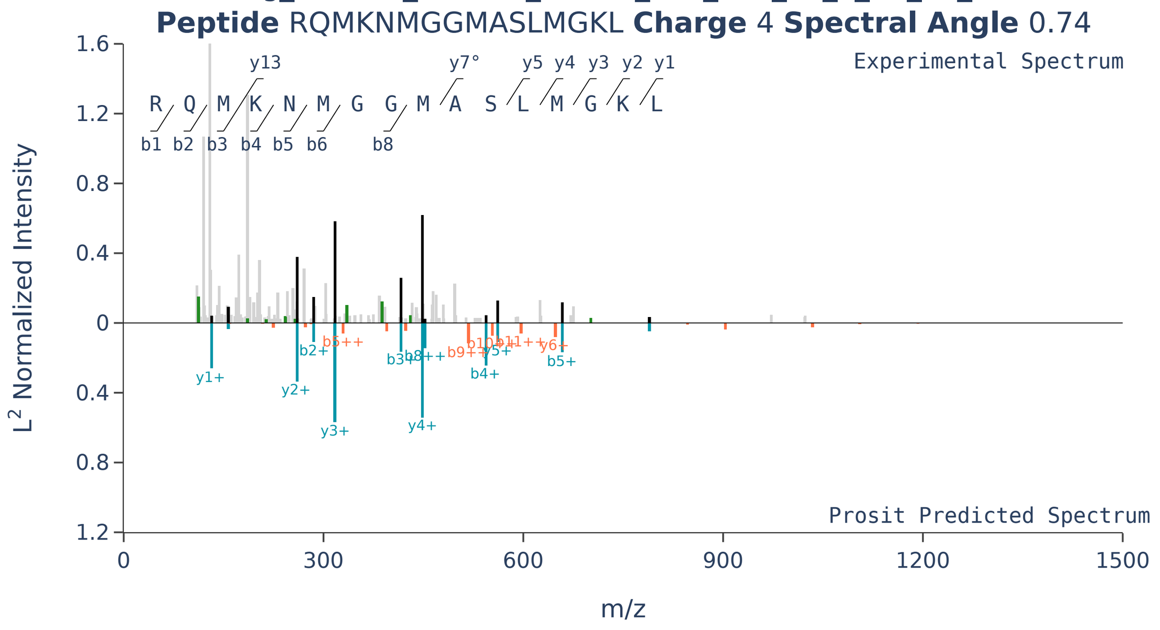


**Source** Ncheng_210623_230623_HFGoe_FFH_20S_25_1_A1_24h_R1 **Scan** 29357
**Peptide** NLAAQPPAVVL **Charge** 2 **Spectral Angle** 0.72

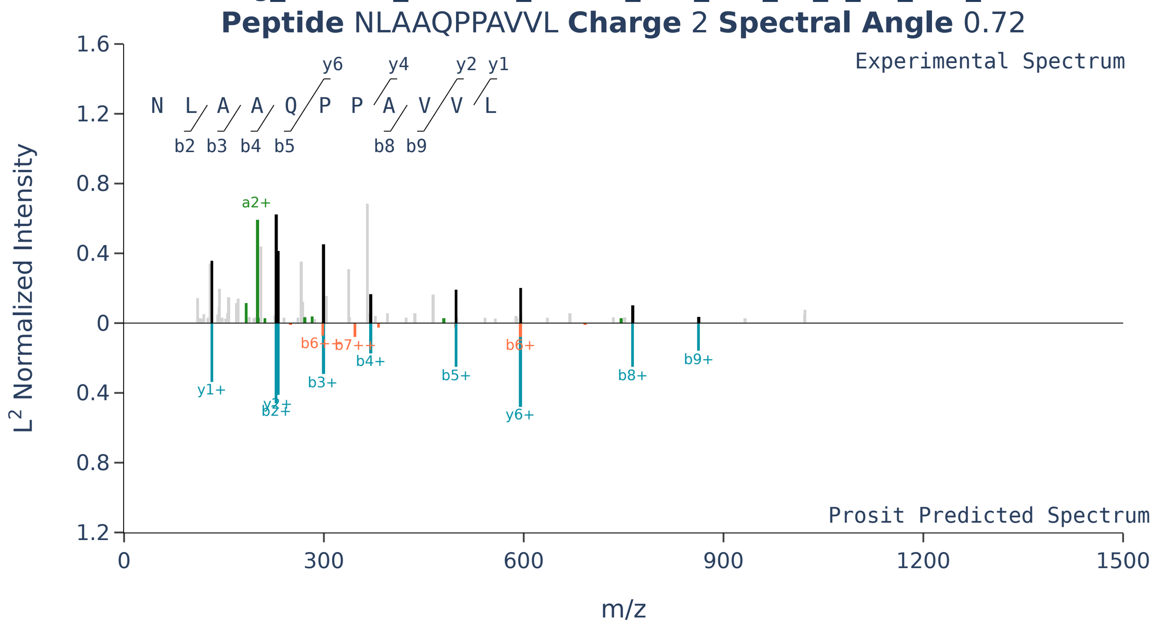


**Source** Ncheng_210623_230623_HFGoe_FFH_20S_25_1_A1_2h_R1 **Scan** 18281
**Peptide** VNAALKEAKLKFYD **Charge** 2 **Spectral Angle** 0.78

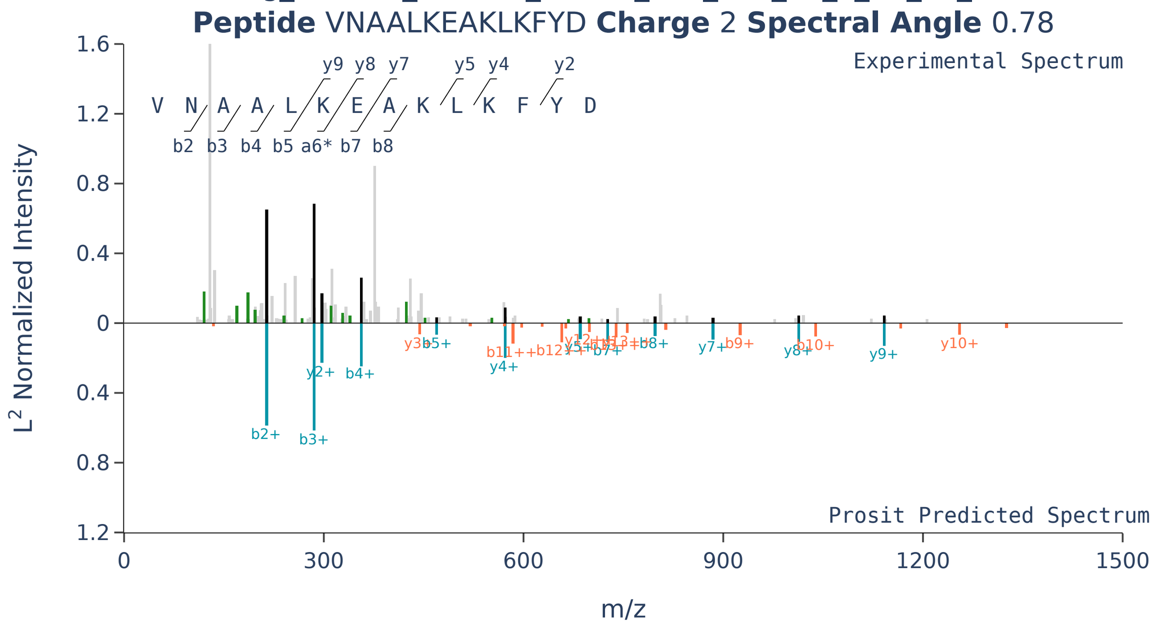


**Source** Ncheng_210623_230623_HFGoe_FFH_20S_25_1_A1_2h_R2 **Scan** 34974
**Peptide** SLTPGQEFVKIVRNELVA **Charge** 2 **Spectral Angle** 0.68

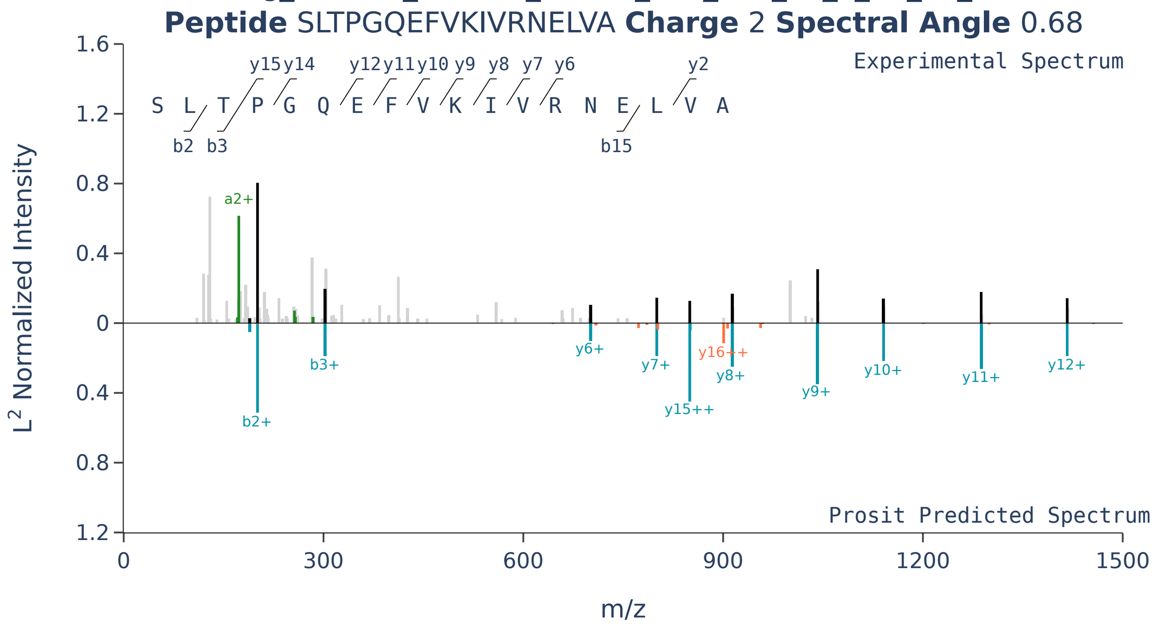


**Source** Ncheng_210623_230623_HFGoe_FFH_20S_25_1_A1_24h_R2 **Scan** 22684
**Peptide** PGQEFVKLV **Charge** 2 **Spectral Angle** 0.67

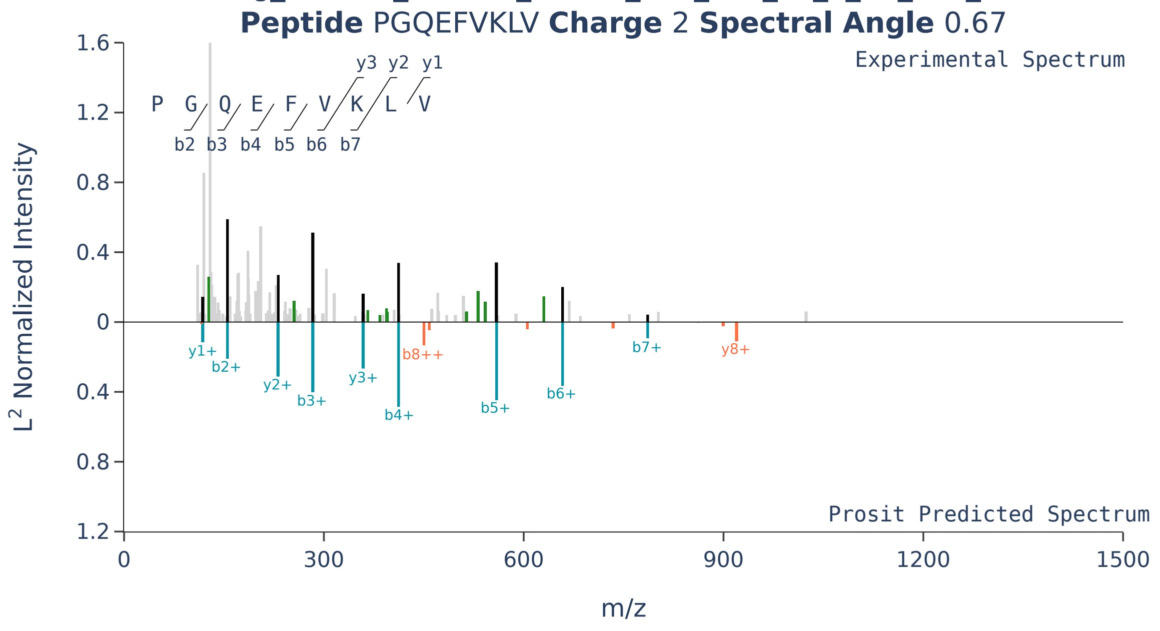


**Source** Ncheng_210623_230623_HFGoe_FFH_20S_25_1_A2_24h_R2 **Scan** 12897
**Peptide** LKKGDGFDLND **Charge** 2 **Spectral Angle** 0.74

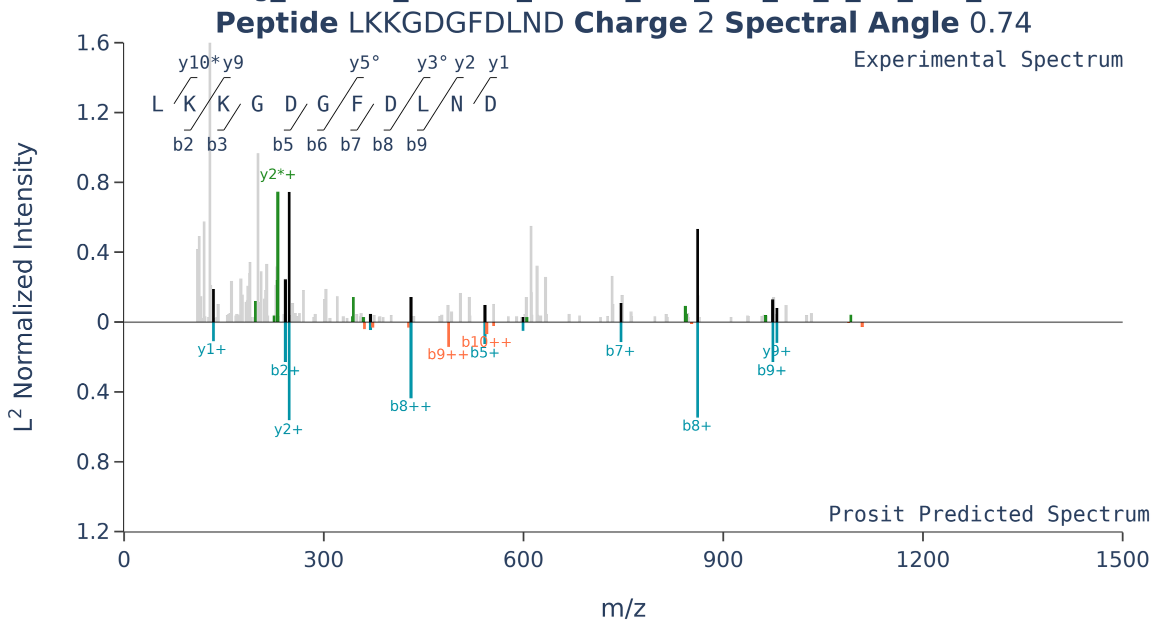


**Source** Ncheng_210623_230623_HFGoe_FFH_20S_25_1_A2_4h_R1 **Scan** 31080
**Peptide** AKLKFYDVLLVD **Charge** 3 **Spectral Angle** 0.69

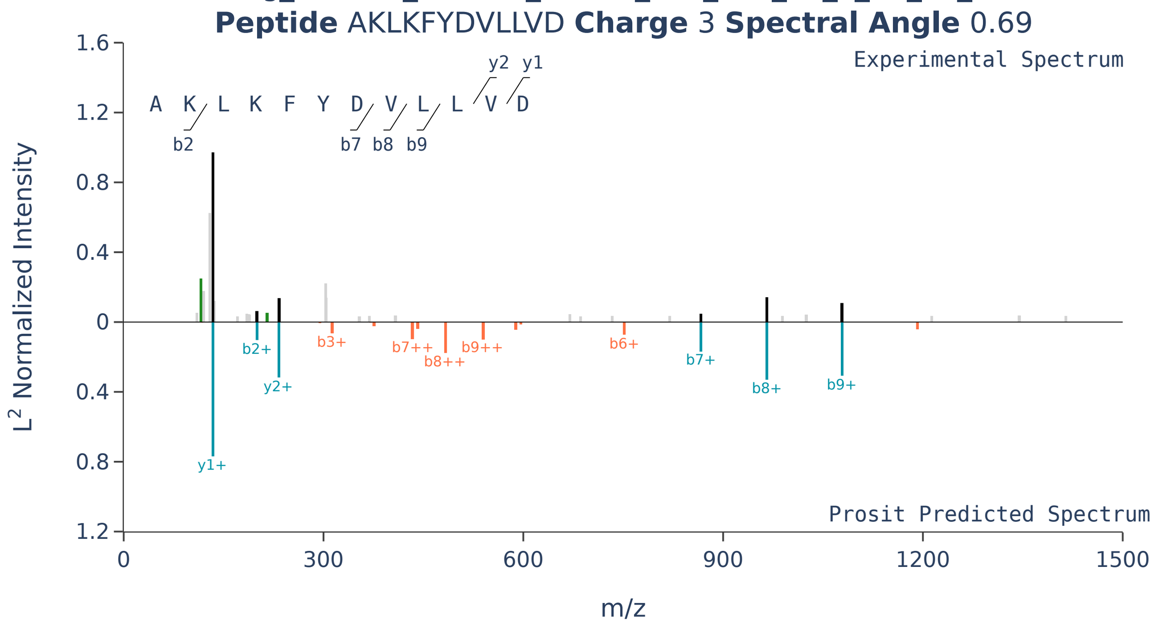


**Source** Ncheng_210623_230623_HFGoe_FFH_20S_25_1_A2_4h_R1 **Scan** 29494
**Peptide** NEALPLTGVVLTKVDGDARGG **Charge** 3 **Spectral Angle** 0.8

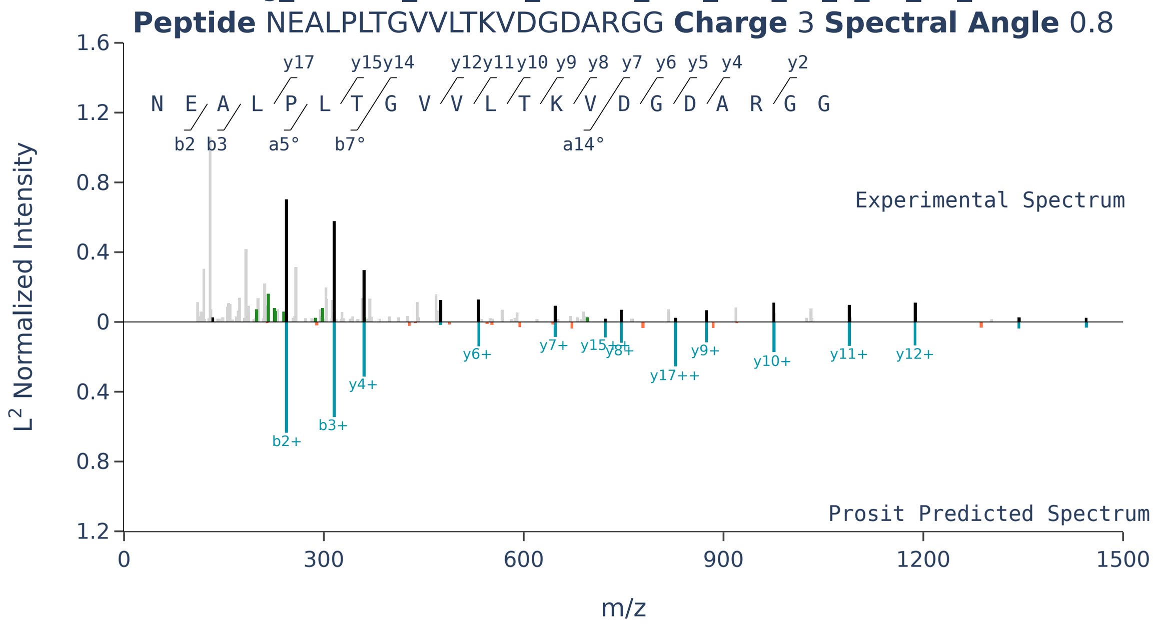


**Source** Ncheng_210623_230623_HFGoe_FFH_20S_25_1_A2_24h_R1 **Scan** 5254
**Peptide** LASKLKK **Charge** 2 **Spectral Angle** 0.84

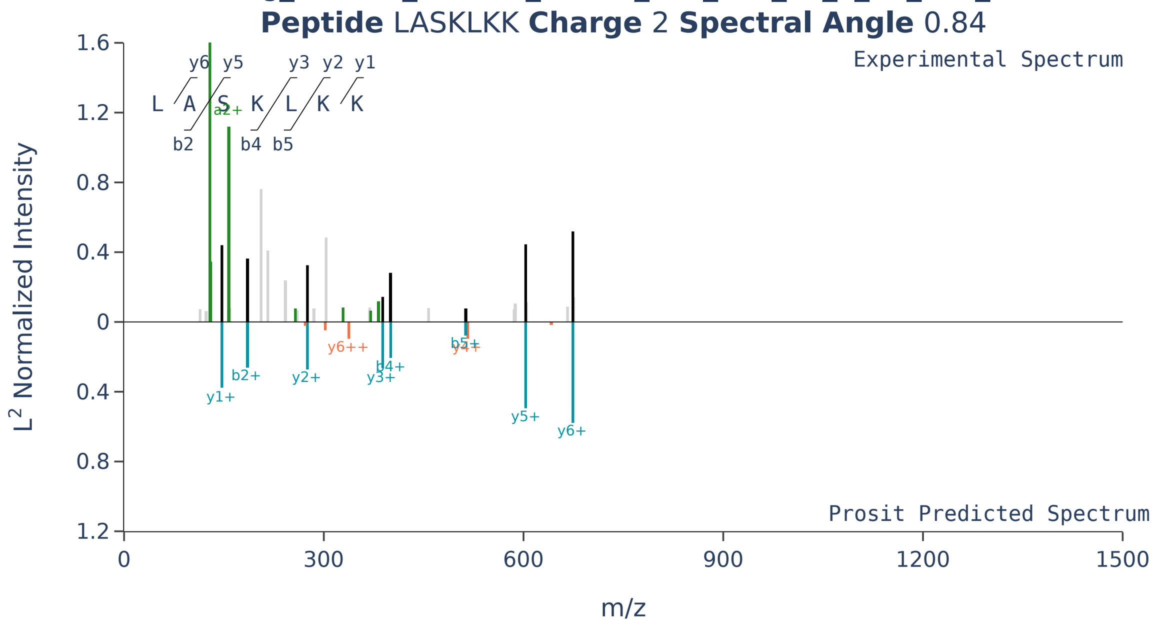


**Source** Ncheng_210623_230623_HFGoe_FFH_20S_25_1_A1_24h_R2 **Scan** 14304
**Peptide** ETLAEQV **Charge** 1 **Spectral Angle** 0.79

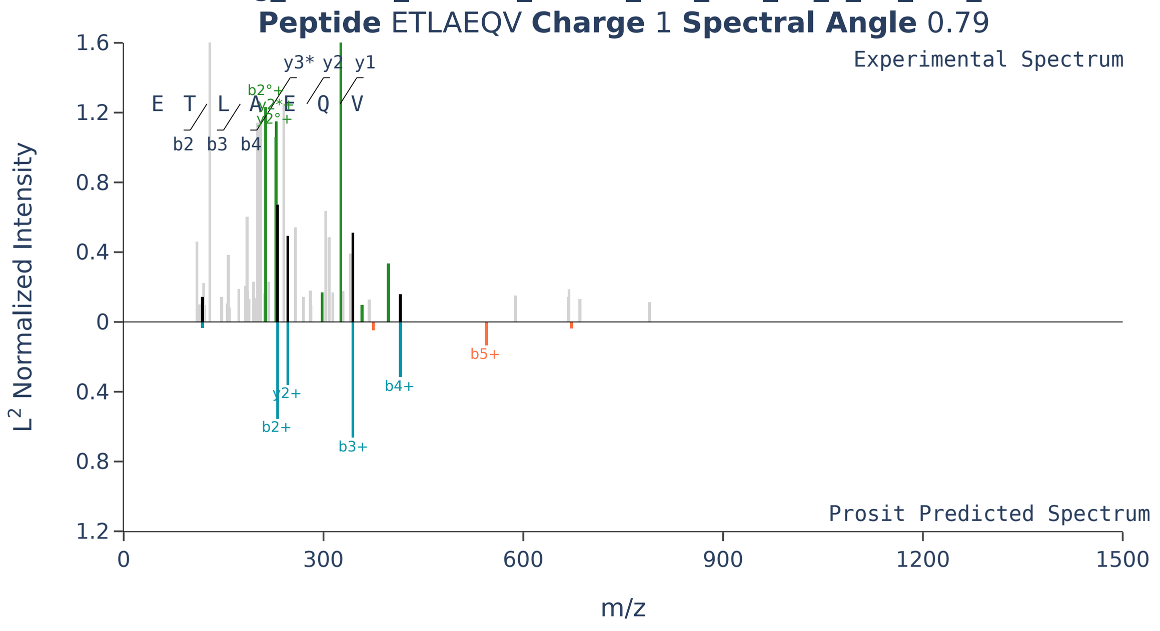

**Source** Ncheng_210623_230623_HFGoe_FFH_20S_25_1_A1_4h_R1 **Scan** 15938
**Peptide** RMEAIINSMTMKERAKPEIIKGSRK **Charge** 5 **Spectral Angle** 0.74

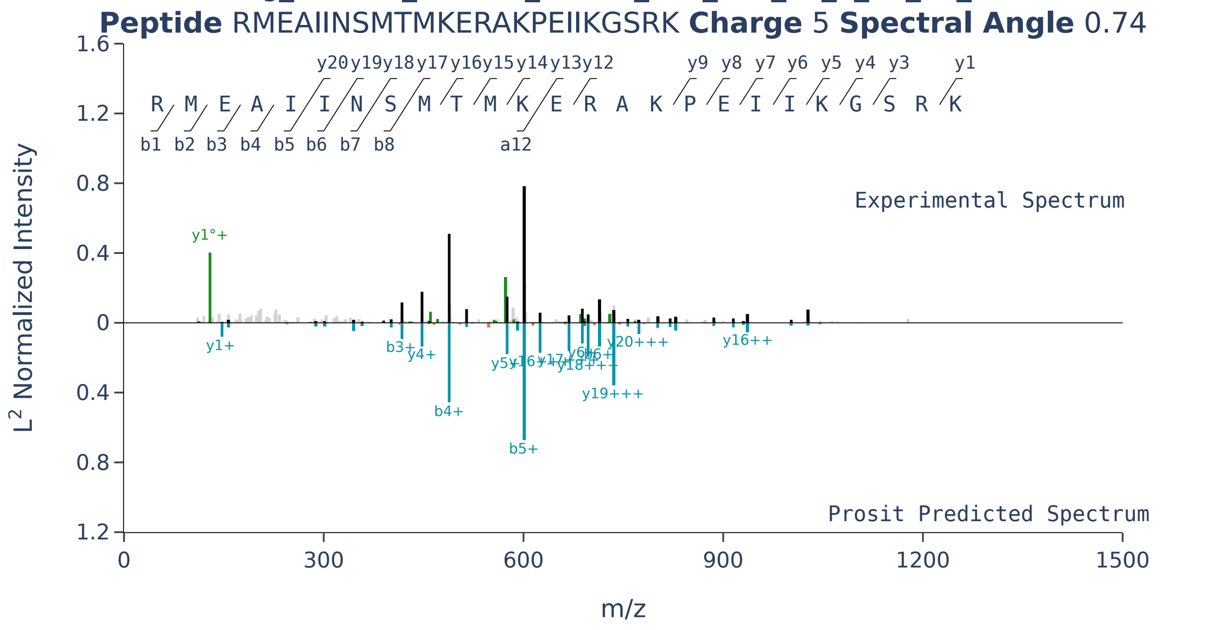


**Source** Ncheng_210623_230623_HFGoe_FFH_20S_25_1_A1_2h_R2 **Scan** 22658
**Peptide** GRLHVDEAMMDEIKQVHA **Charge** 3 **Spectral Angle** 0.65

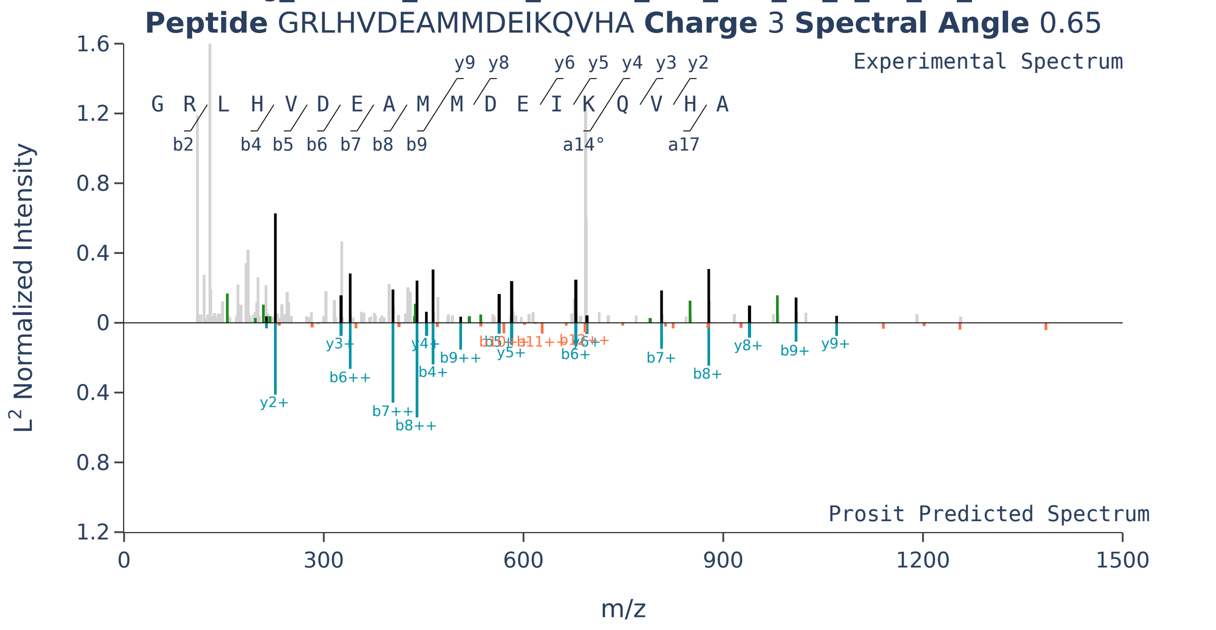


**Source** Ncheng_210623_230623_HFGoe_FFH_20S_25_1_A2_2h_R1 **Scan** 31022
**Peptide** LAAGCGMQVQDVNRLLKQFD **Charge** 3 **Spectral Angle** 0.8

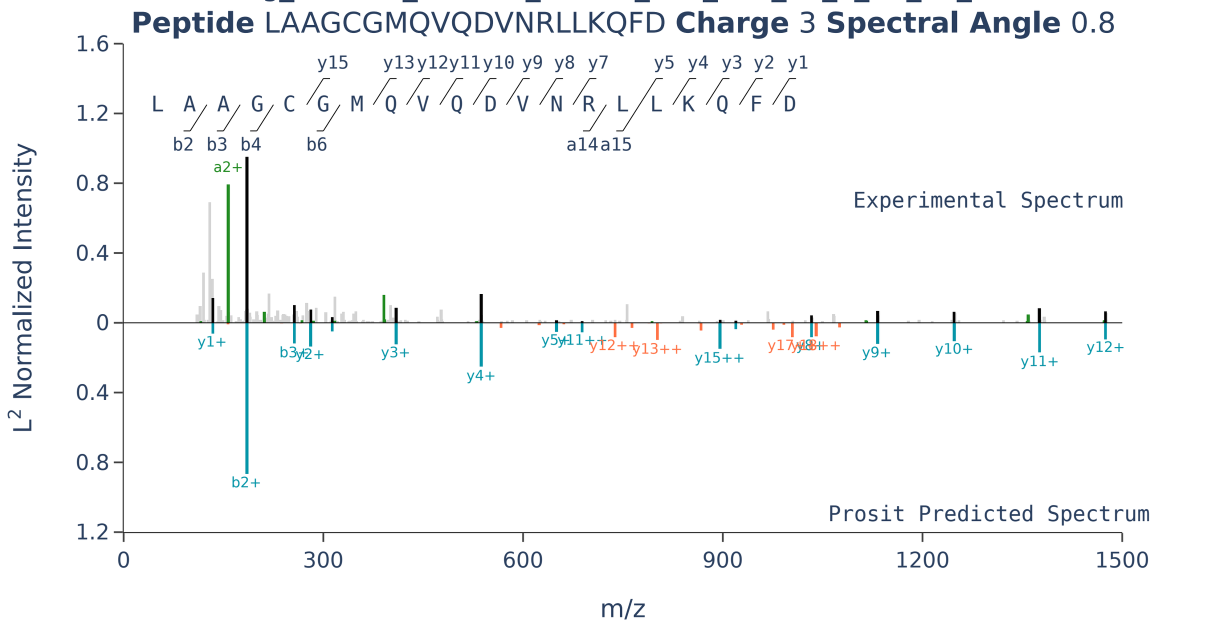


**Source** Ncheng_210623_230623_HFGoe_FFH_20S_25_1_A1_4h_R1 **Scan** 14566
**Peptide** EIKQVHASINPVET **Charge** 2 **Spectral Angle** 0.72

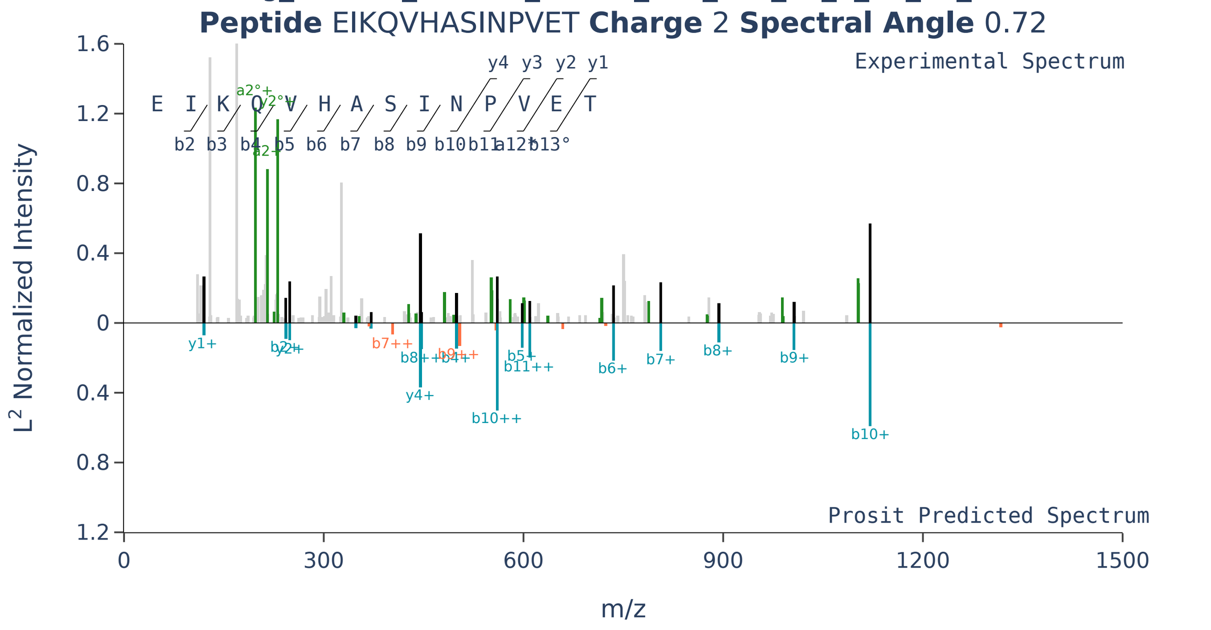


**Source** Ncheng_210623_230623_HFGoe_FFH_20S_25_1_A1_4h_R1 **Scan** 13014
**Peptide** FPSDVGQKPVD **Charge** 2 **Spectral Angle** 0.68

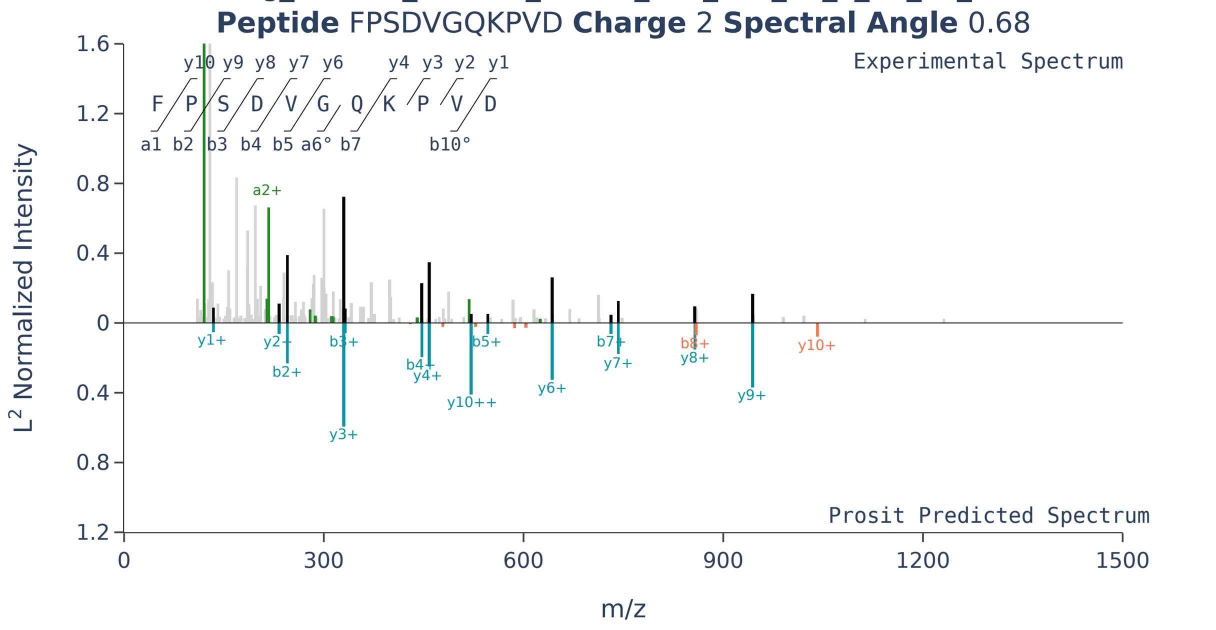


**Source** Ncheng_210623_230623_HFGoe_FFH_20S_25_1_A1_24h_R1 **Scan** 21026
**Peptide** TSVGKLGKFL **Charge** 3 **Spectral Angle** 0.72

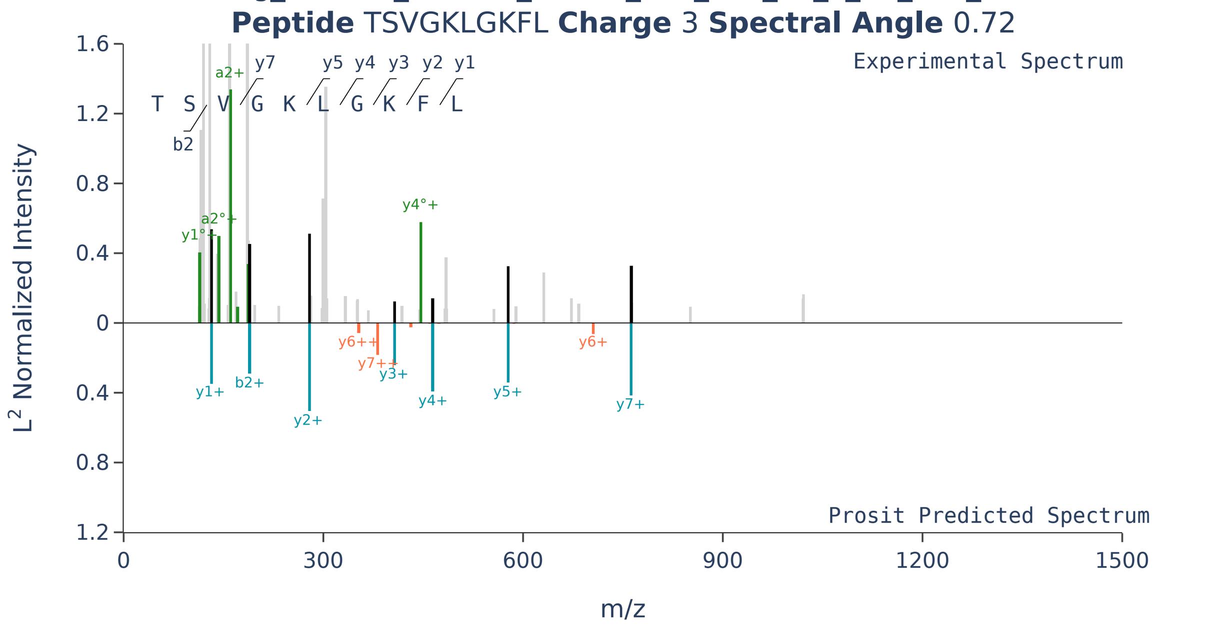


**Source** Ncheng_210623_230623_HFGoe_FFH_20S_25_1_A2_4h_R1 **Scan** 28492
**Peptide** VGHEVNKSLTPGQEFVKIVRNEL **Charge** 3 **Spectral Angle** 0.73

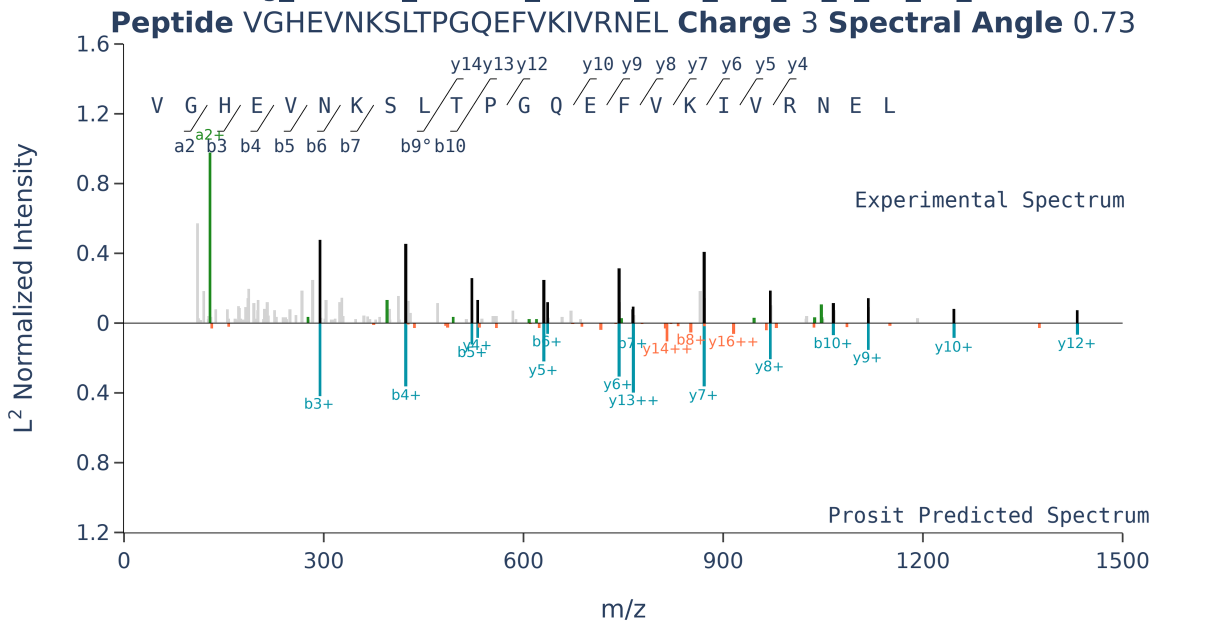


**Source** Ncheng_210623_230623_HFGoe_FFH_20S_25_1_A1_24h_R2 **Scan** 9126
**Peptide** FINRVKEKAVGHEV **Charge** 2 **Spectral Angle** 0.53

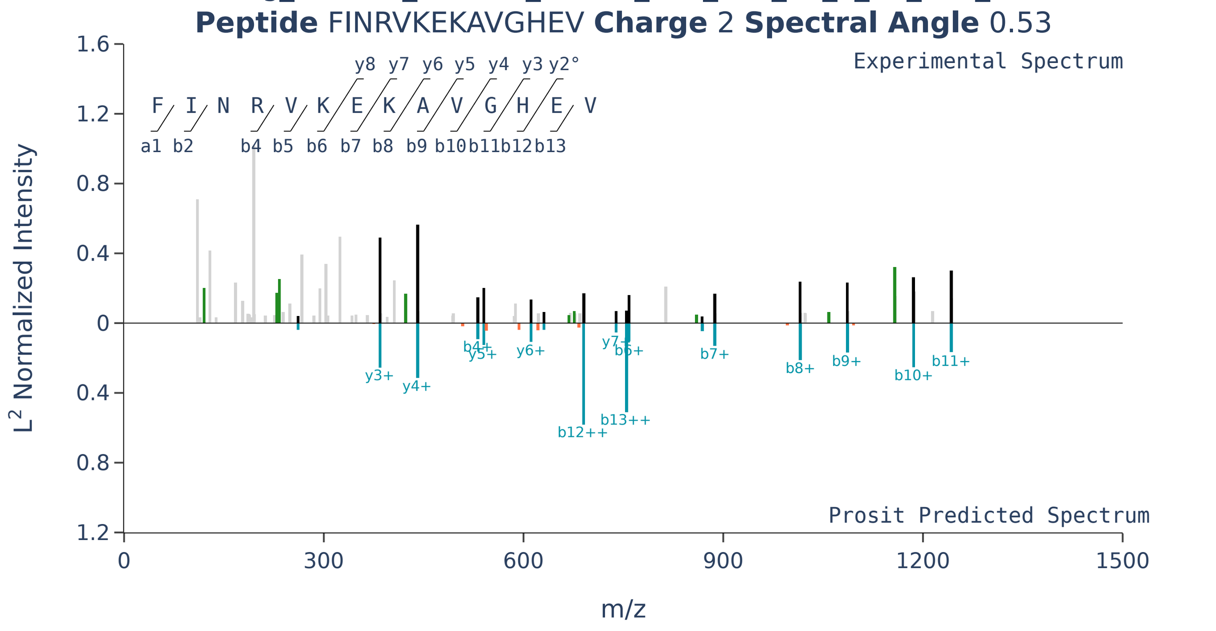


**Source** Ncheng_210623_230623_HFGoe_FFH_20S_25_1_A2_4h_R2 **Scan** 29350
**Peptide** SLTPGQEFVKIV **Charge** 2 **Spectral Angle** 0.8

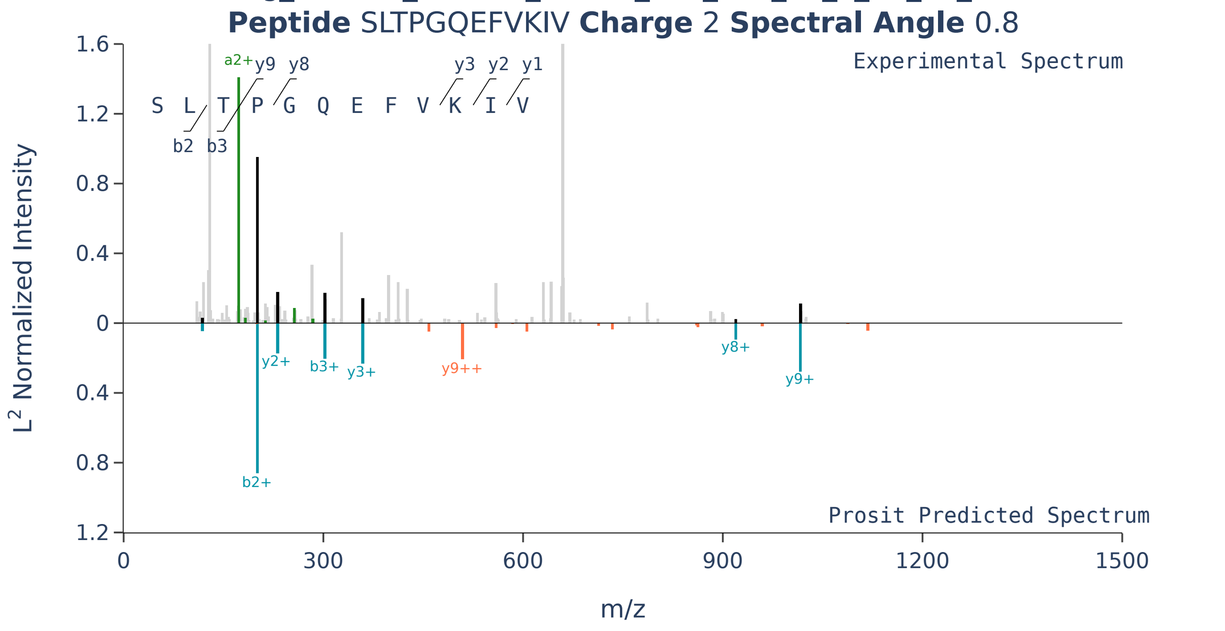


**Source** Ncheng_210623_230623_HFGoe_FFH_20S_25_1_A2_24h_R1 **Scan** 9502
**Peptide** AMGEENQTLN **Charge** 1 **Spectral Angle** 0.73

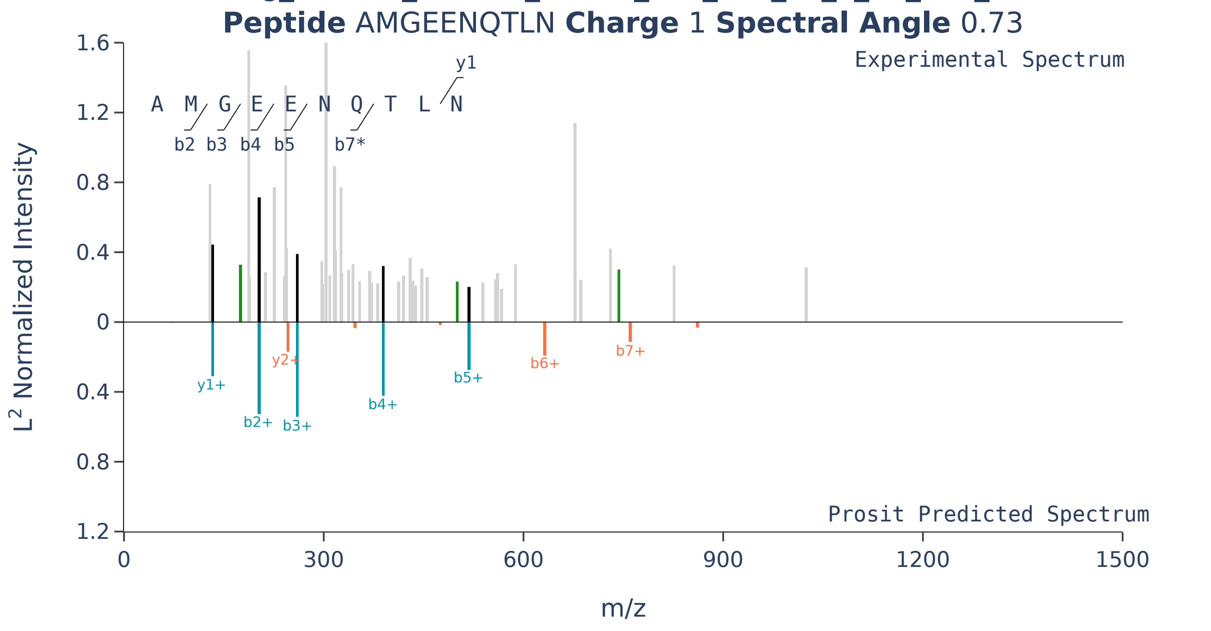


**Source** Ncheng_210623_230623_HFGoe_FFH_20S_25_1_A2_4h_R2 **Scan** 32277
**Peptide** EALPLTGVVLTKVDGD **Charge** 2 **Spectral Angle** 0.69

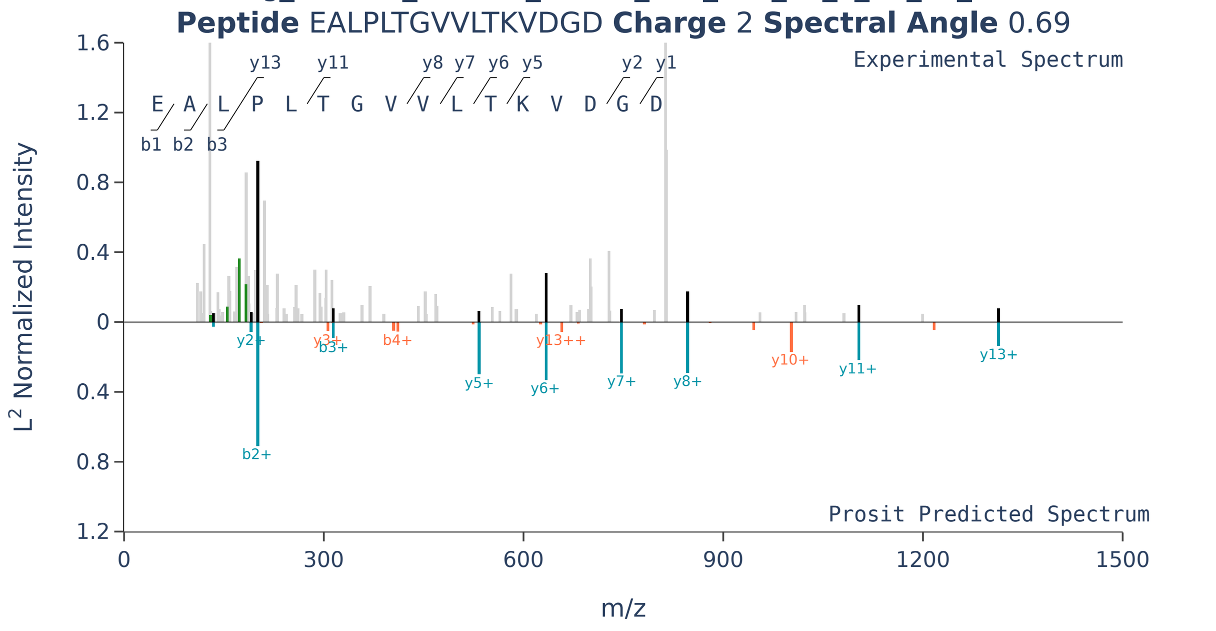


**Source** Ncheng_210623_230623_HFGoe_FFH_20S_25_1_A2_2h_R1 **Scan** 34212
**Peptide** ADVALPVVREFINRVKEKAVG **Charge** 3 **Spectral Angle** 0.89

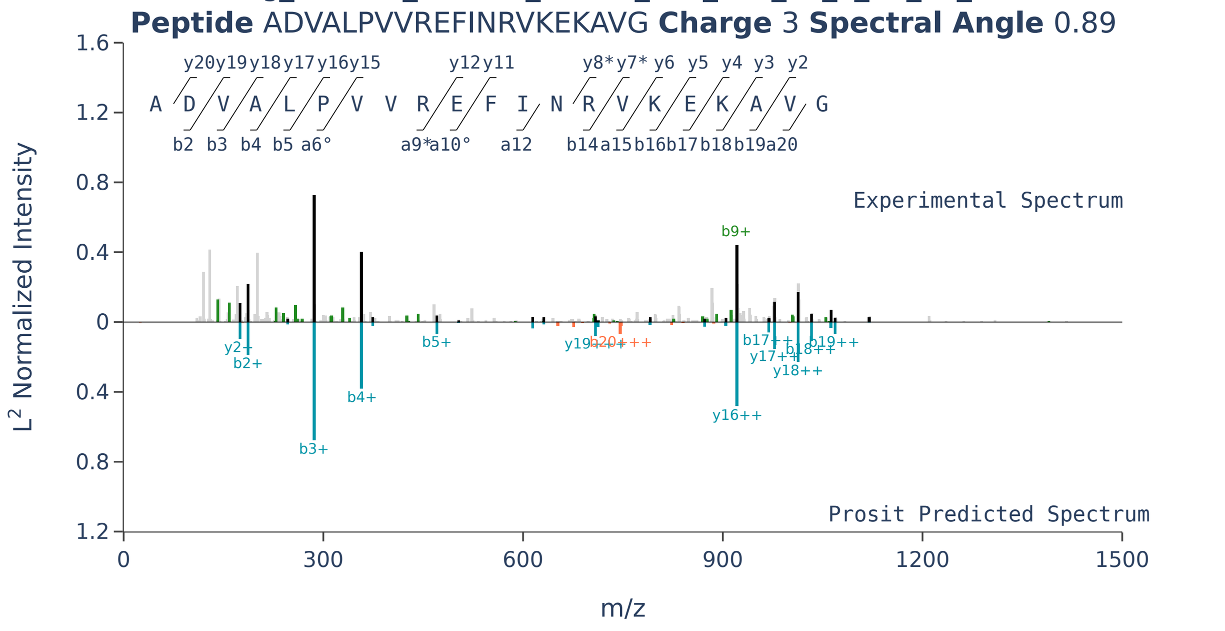


**Source** Ncheng_210623_230623_HFGoe_FFH_20S_25_1_A1_24h_R2 **Scan** 20600
**Peptide** HITGKPIKFLGV **Charge** 3 **Spectral Angle** 0.8

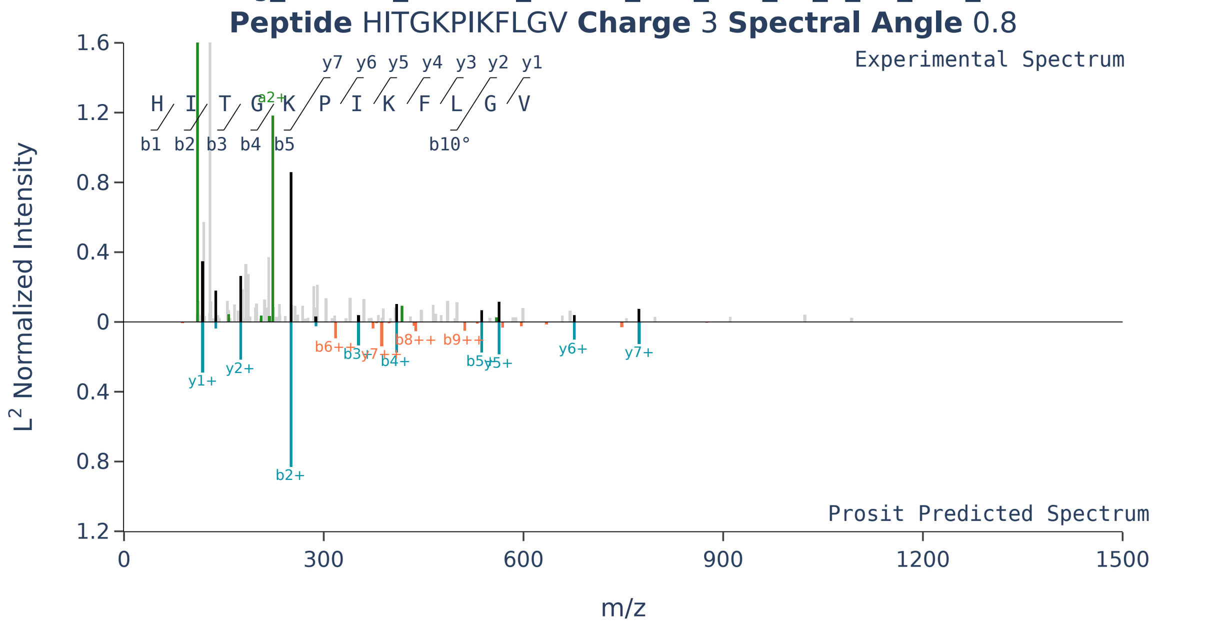


**Source** Ncheng_210623_230623_HFGoe_FFH_20S_25_1_A1_24h_R2 **Scan** 24083
**Peptide** LNLAAQPPAV **Charge** 2 **Spectral Angle** 0.84

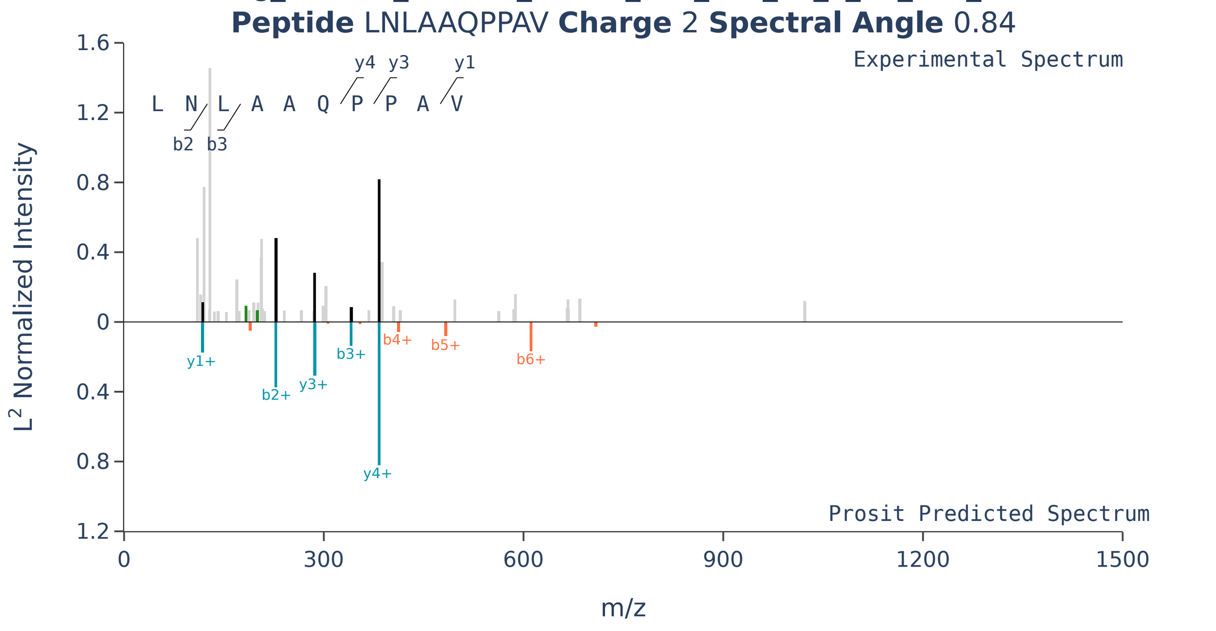


**Source** Ncheng_210623_230623_HFGoe_FFH_20S_25_1_A1_4h_R2 **Scan** 18332
**Peptide** RMALLEAD **Charge** 2 **Spectral Angle** 0.68

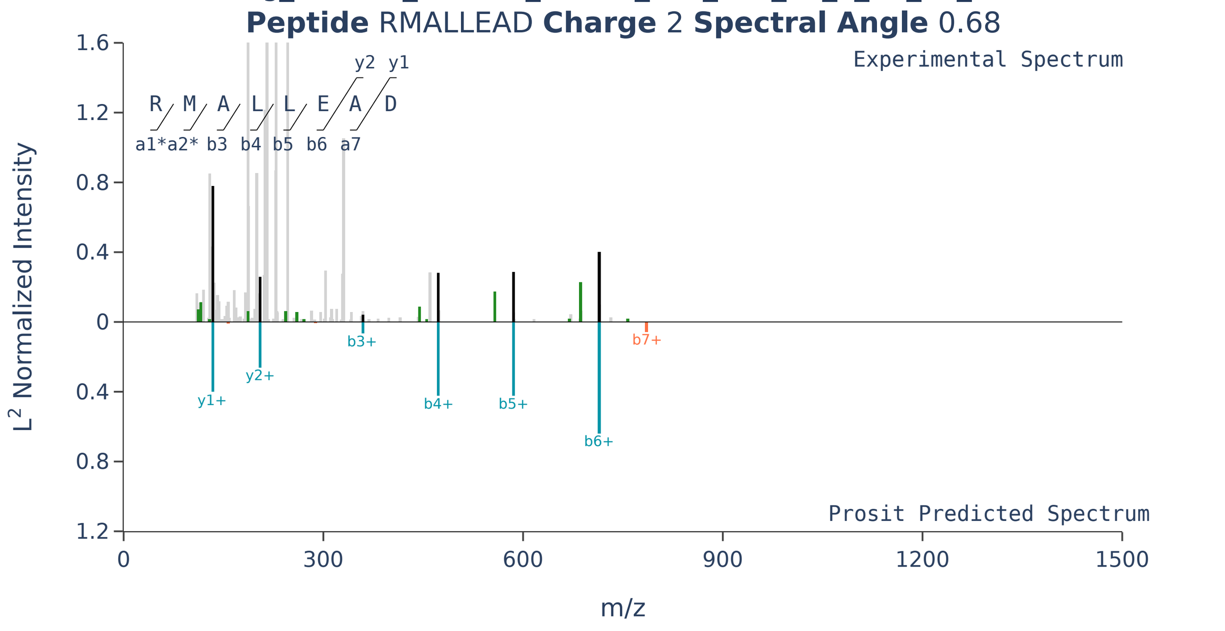

**Source** Ncheng_210623_230623_HFGoe_FFH_20S_25_1_A2_2h_R1 **Scan** 20591
**Peptide** KKVLVVSADVYRPAAIKQLE **Charge** 3 **Spectral Angle** 0.9

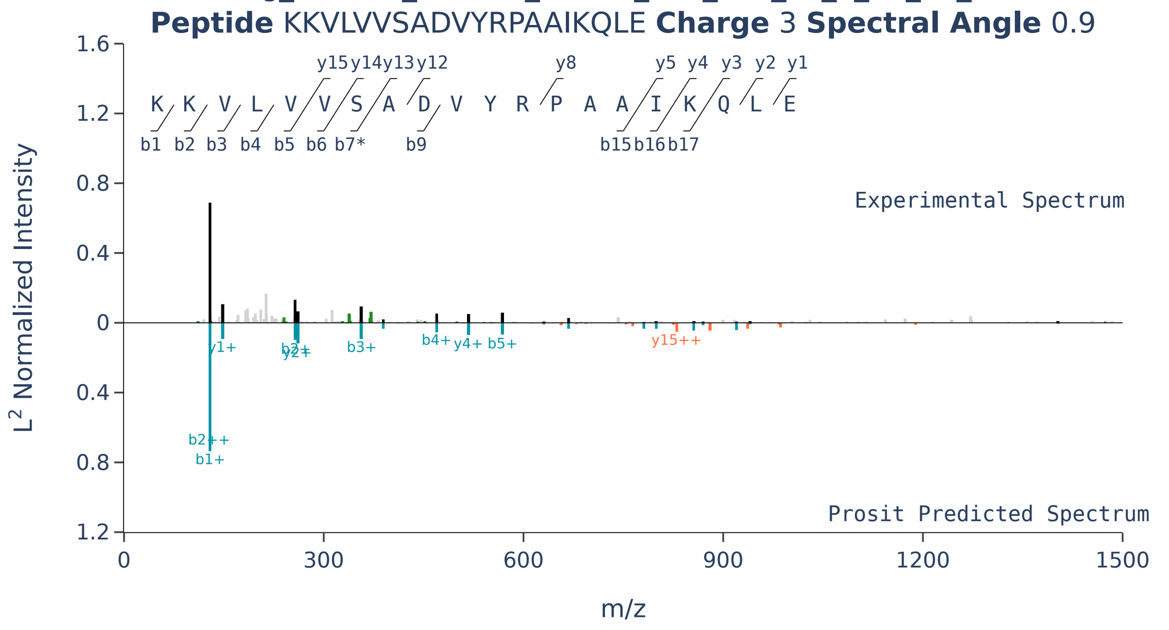


**Source** Ncheng_210623_230623_HFGoe_FFH_20S_25_1_A2_4h_R2 **Scan** 23191
**Peptide** GKLPGMGQIPDNVKSQMDDKVLVR **Charge** 4 **Spectral Angle** 0.72

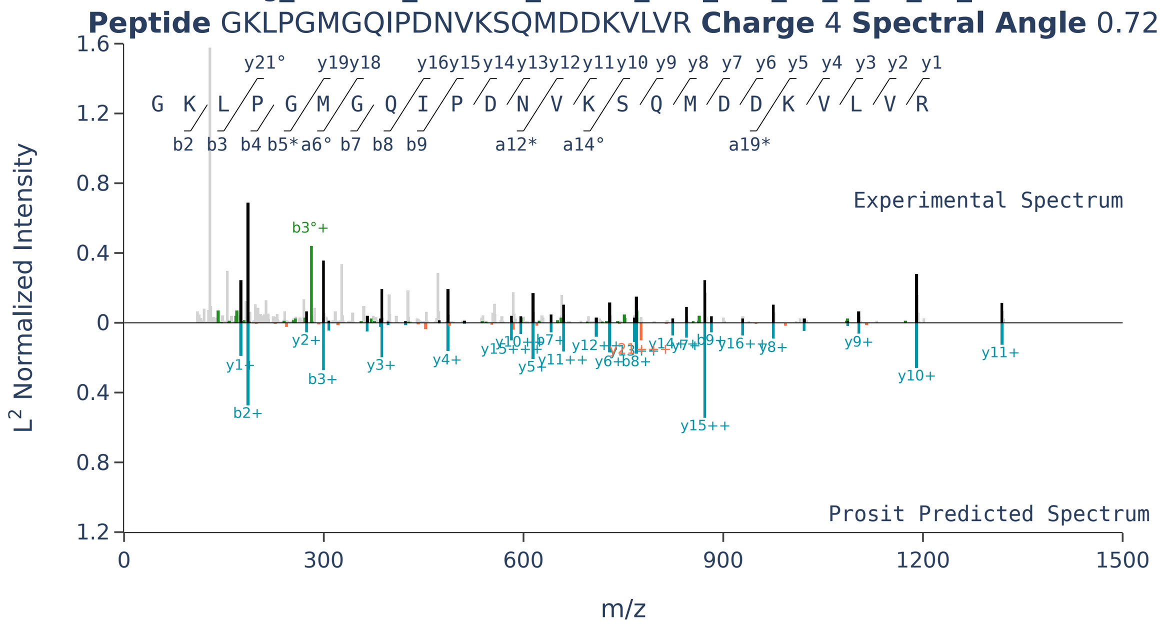


**Source** Ncheng_210623_230623_HFGoe_FFH_20S_25_1_A1_4h_R2 **Scan** 5953
**Peptide** RAKPEIIKGSR **Charge** 3 **Spectral Angle** 0.85

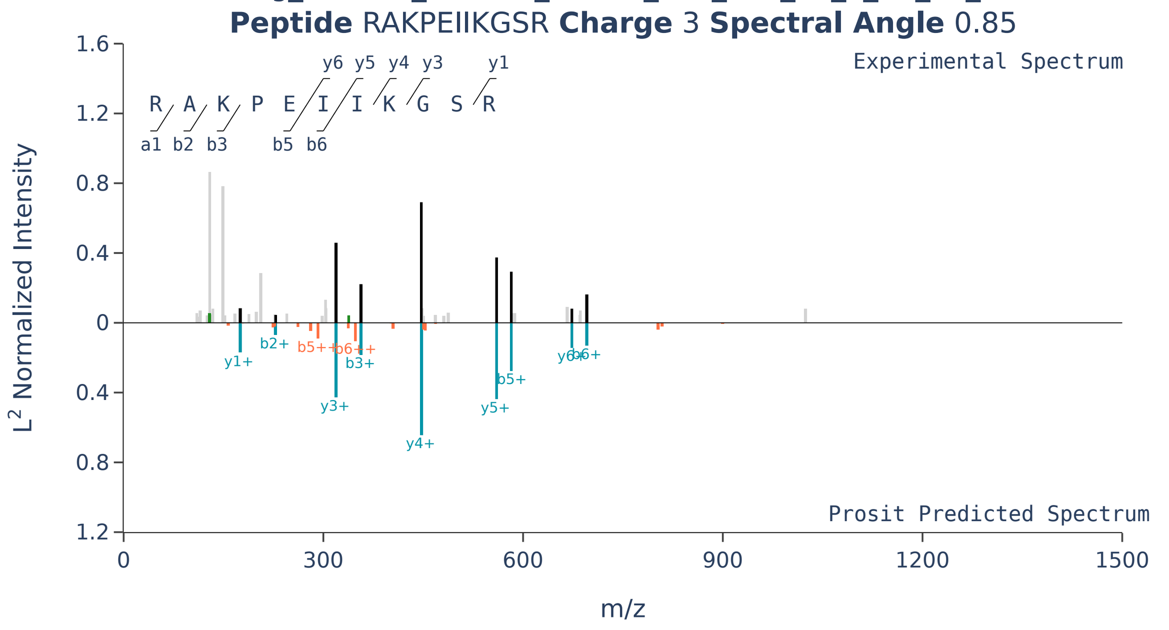


**Source** Ncheng_210623_230623_HFGoe_FFH_20S_25_1_A1_24h_R2 **Scan** 12272
**Peptide** MAGLQGAGKTTSVGKLGK **Charge** 2 **Spectral Angle** 0.73

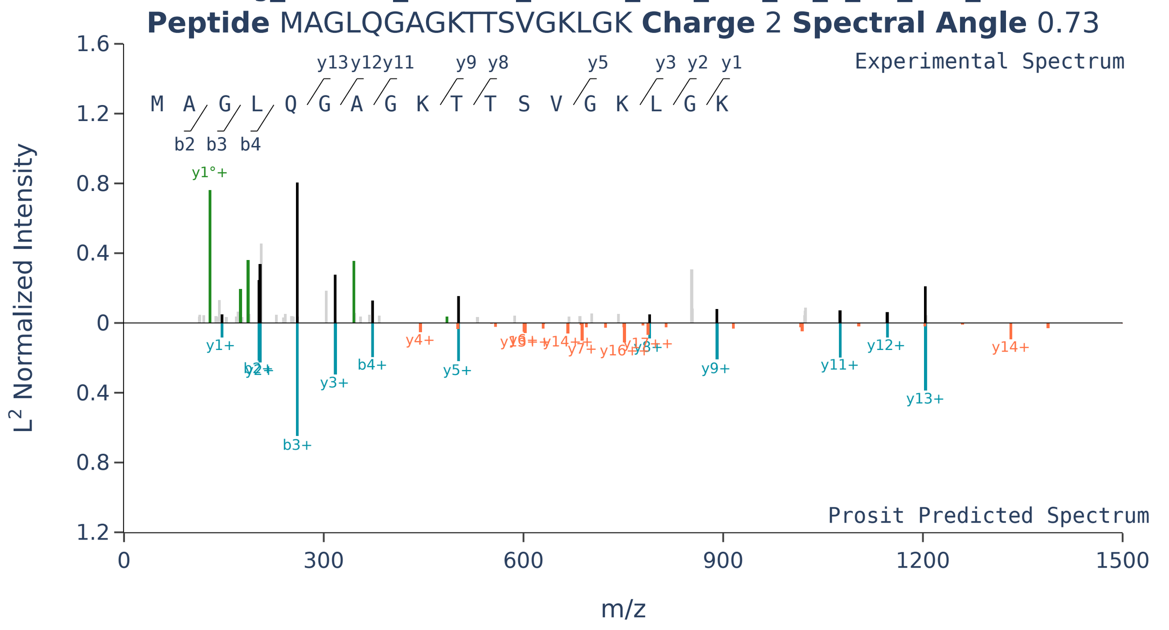


**Source** Ncheng_210623_230623_HFGoe_FFH_20S_25_1_A2_4h_R2 **Scan** 31750
**Peptide** VGHEVNKSLTPGQEFVKIVRNELVAA **Charge** 3 **Spectral Angle** 0.77

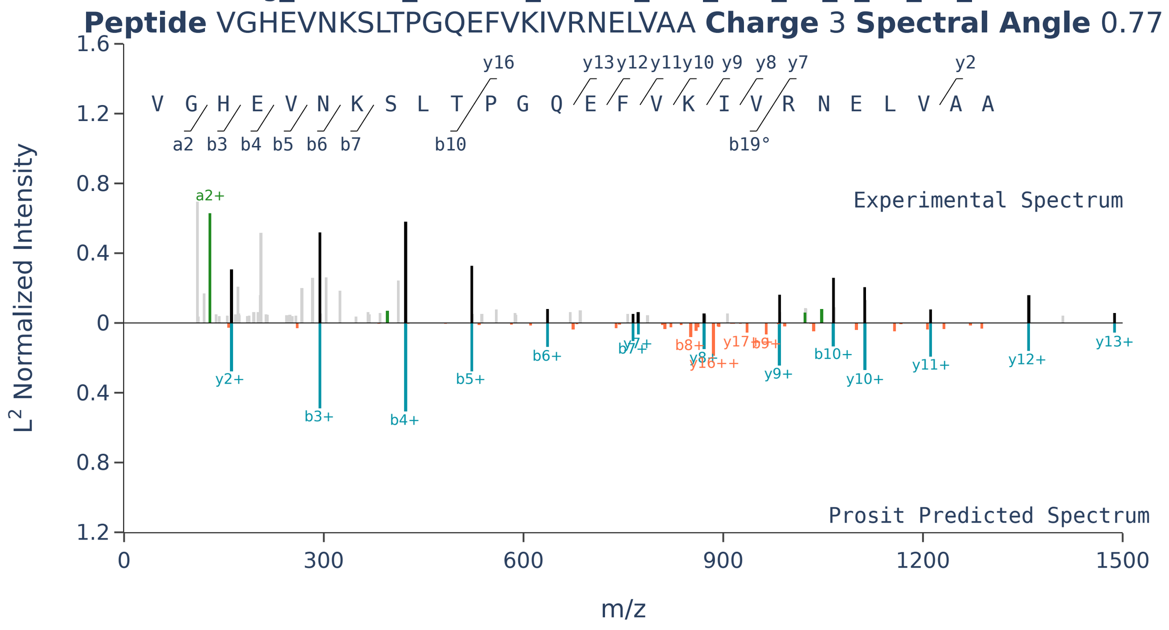


**Source** Ncheng_210623_230623_HFGoe_FFH_20S_25_1_A1_2h_R1 **Scan** 18585
**Peptide** ISGRGRLTEDNVKDTLREVRM **Charge** 5 **Spectral Angle** 0.76

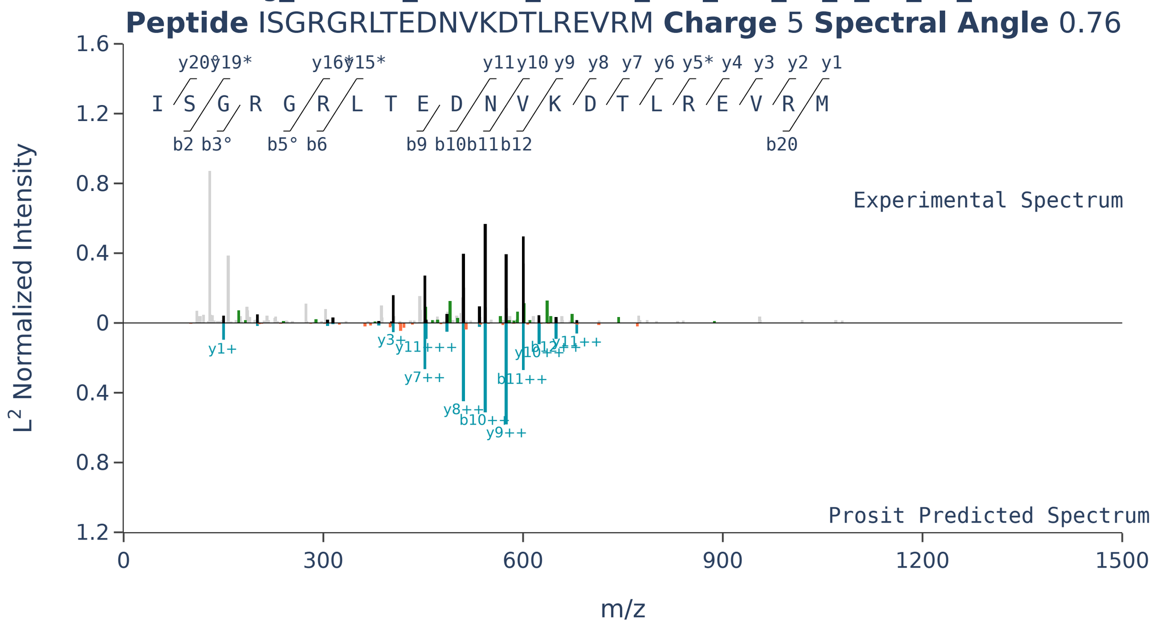


**Source** Ncheng_210623_230623_HFGoe_FFH_20S_25_1_A2_24h_R1 **Scan** 19224
**Peptide** DLESKVDRAQAEKLASKL **Charge** 3 **Spectral Angle** 0.7

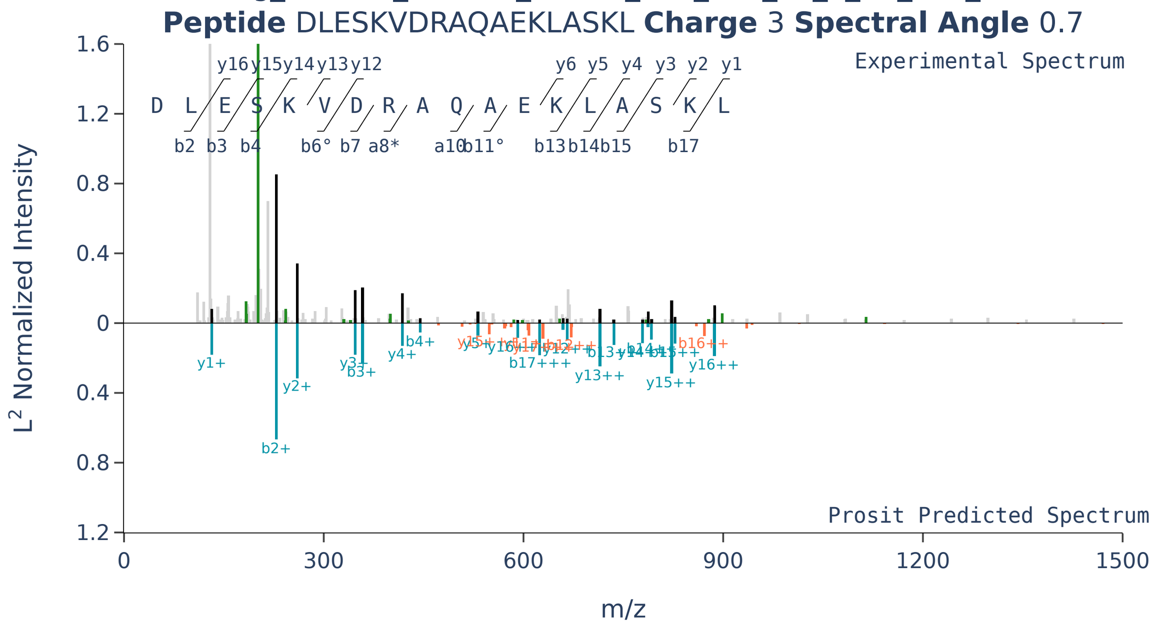


**Source** Ncheng_210623_230623_HFGoe_FFH_20S_25_1_A1_2h_R2 **Scan** 17549
**Peptide** PDNVKSQMDDKVLVRM **Charge** 4 **Spectral Angle** 0.87

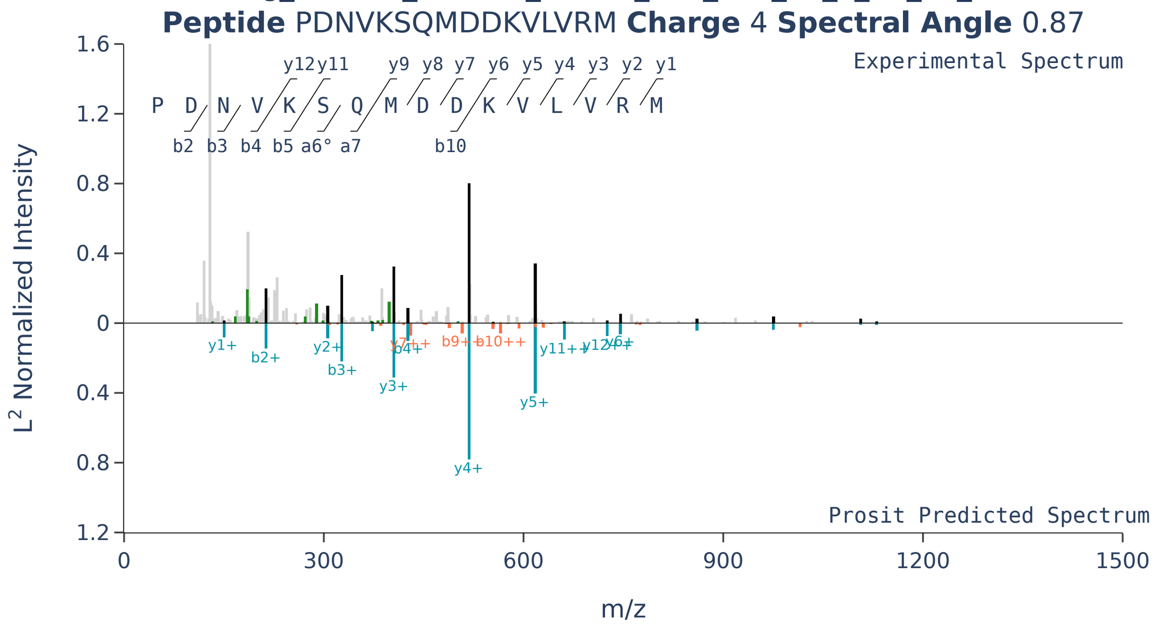


**Source** Ncheng_210623_230623_HFGoe_FFH_20S_25_1_A2_24h_R2 **Scan** 18094
**Peptide** PDNVKSQ **Charge** 1 **Spectral Angle** 0.88

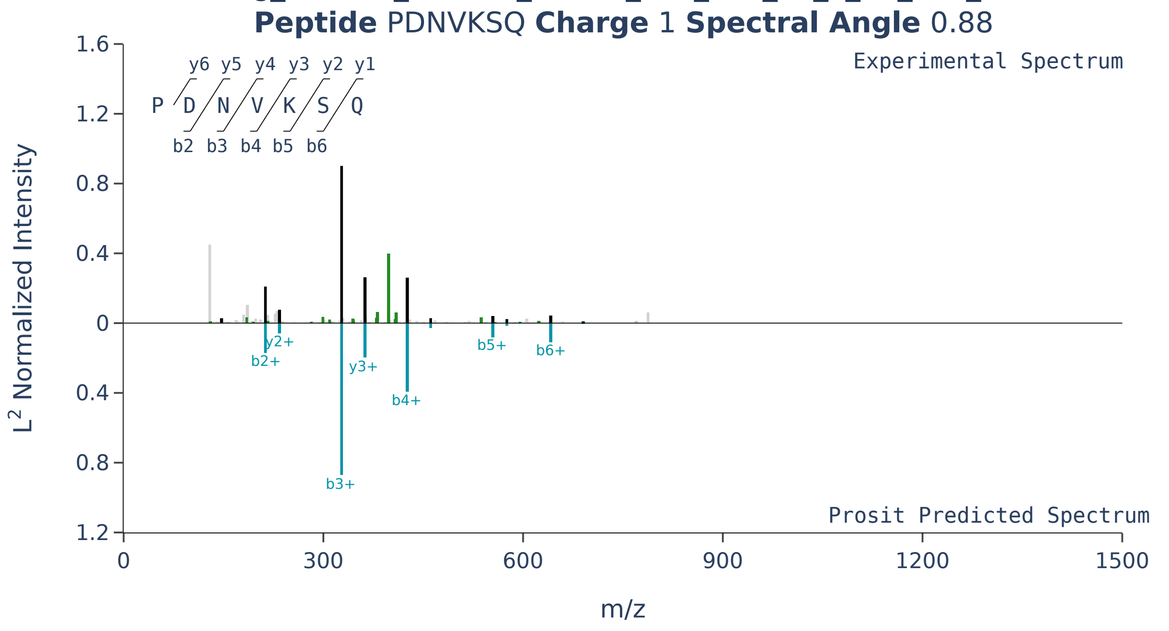


**Source** Ncheng_210623_230623_HFGoe_FFH_20S_25_1_A2_24h_R2 **Scan** 9093
**Peptide** LLNSMTMKERAK **Charge** 2 **Spectral Angle** 0.73

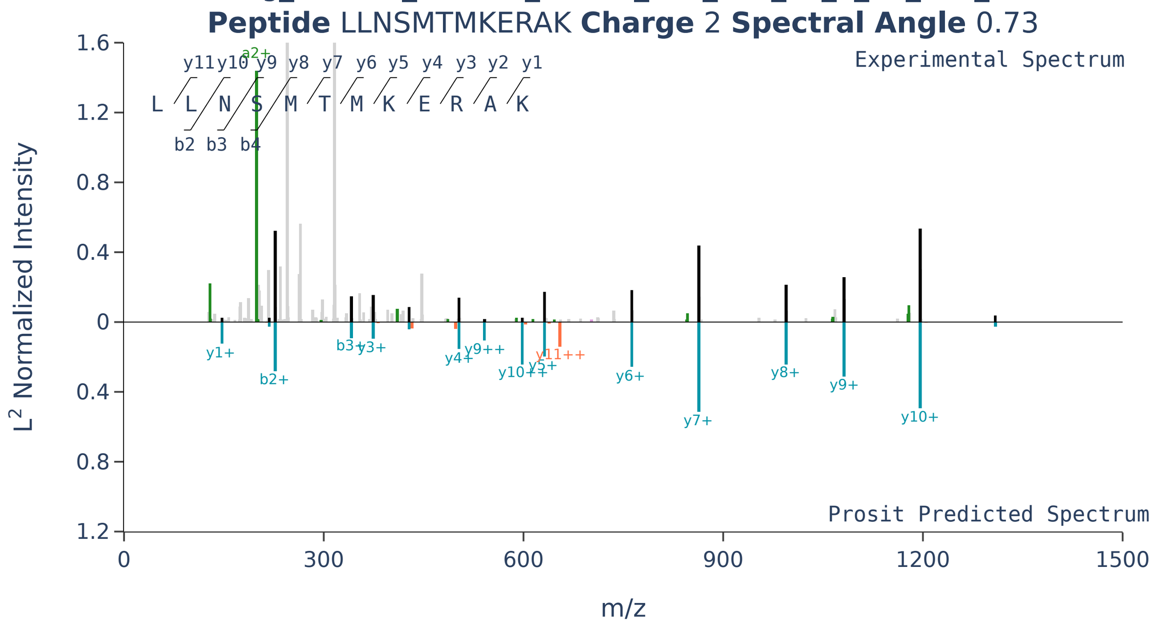


**Source** Ncheng_210623_230623_HFGoe_FFH_20S_25_1_A1_4h_R1 **Scan** 15204
**Peptide** LRHITGKPIKFLGVGEKTE **Charge** 4 **Spectral Angle** 0.71

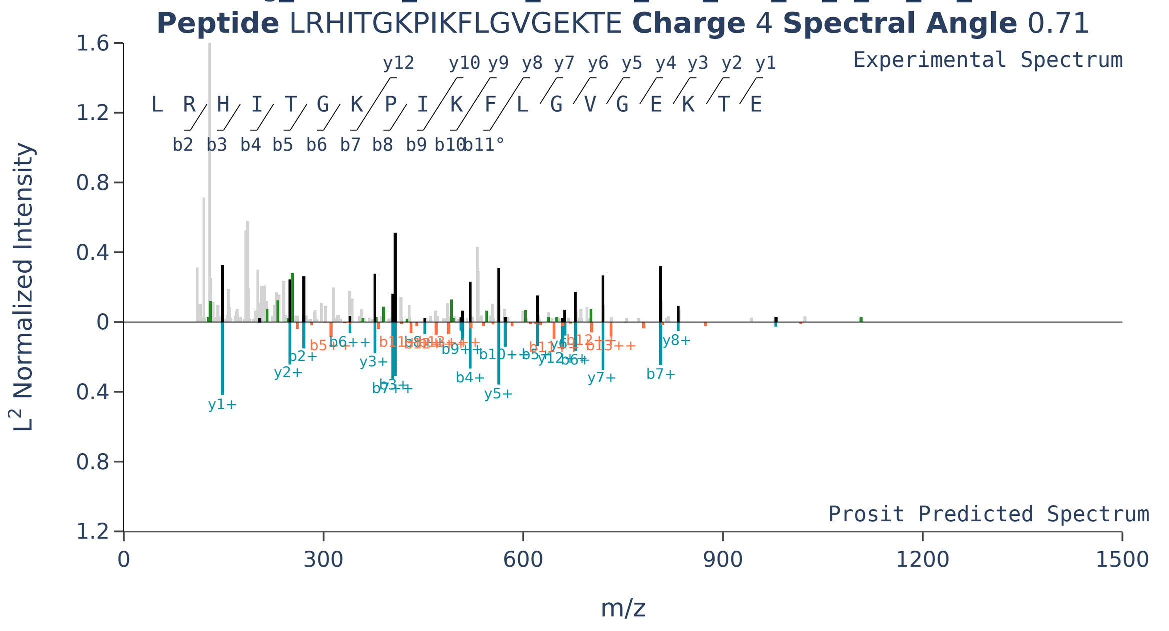


**Source** Ncheng_210623_230623_HFGoe_FFH_20S_25_1_A2_24h_R1 **Scan** 7225
**Peptide** FINRVKEKAVGH **Charge** 2 **Spectral Angle** 0.79

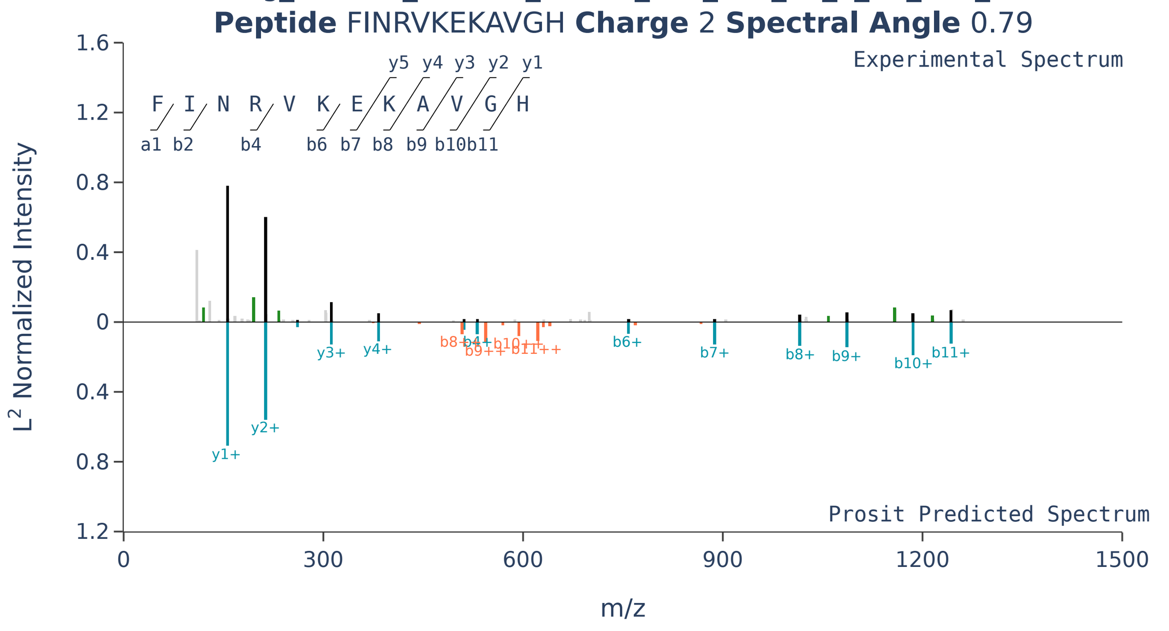


**Source** Ncheng_210623_230623_HFGoe_FFH_20S_25_1_A1_4h_R1 **Scan** 32102
**Peptide** TGQDAANTAKAFNEALPLTGVVLTK **Charge** 3 **Spectral Angle** 0.84

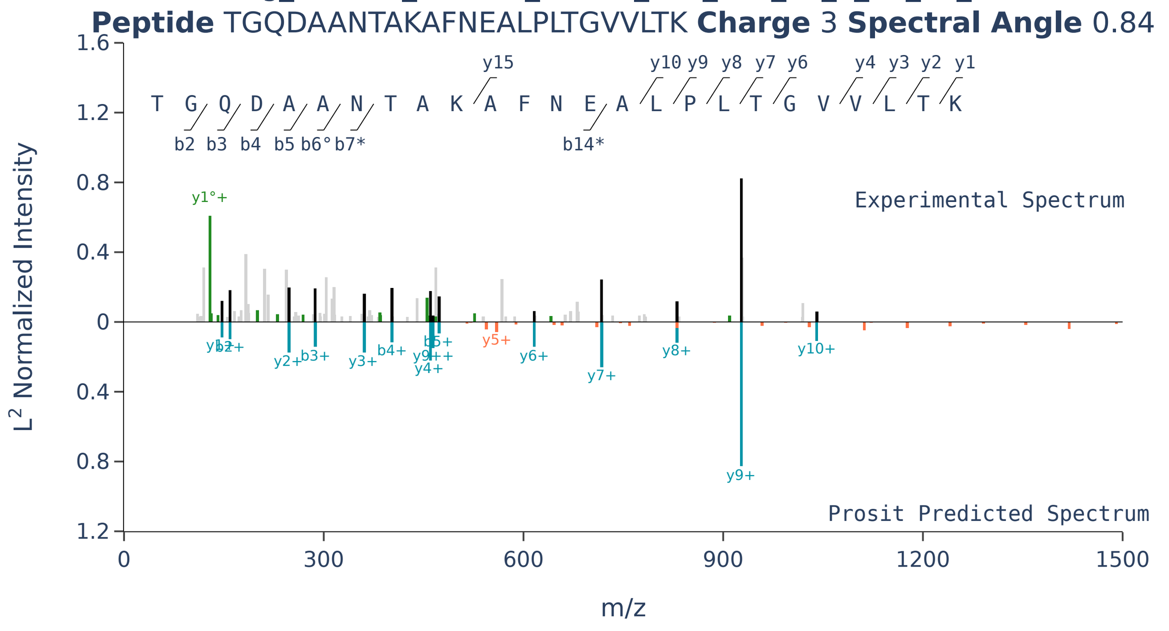


**Source** Ncheng_210623_230623_HFGoe_FFH_20S_25_1_A2_4h_R1 **Scan** 20808
**Peptide** PFHPDRIASRLL **Charge** 3 **Spectral Angle** 0.71

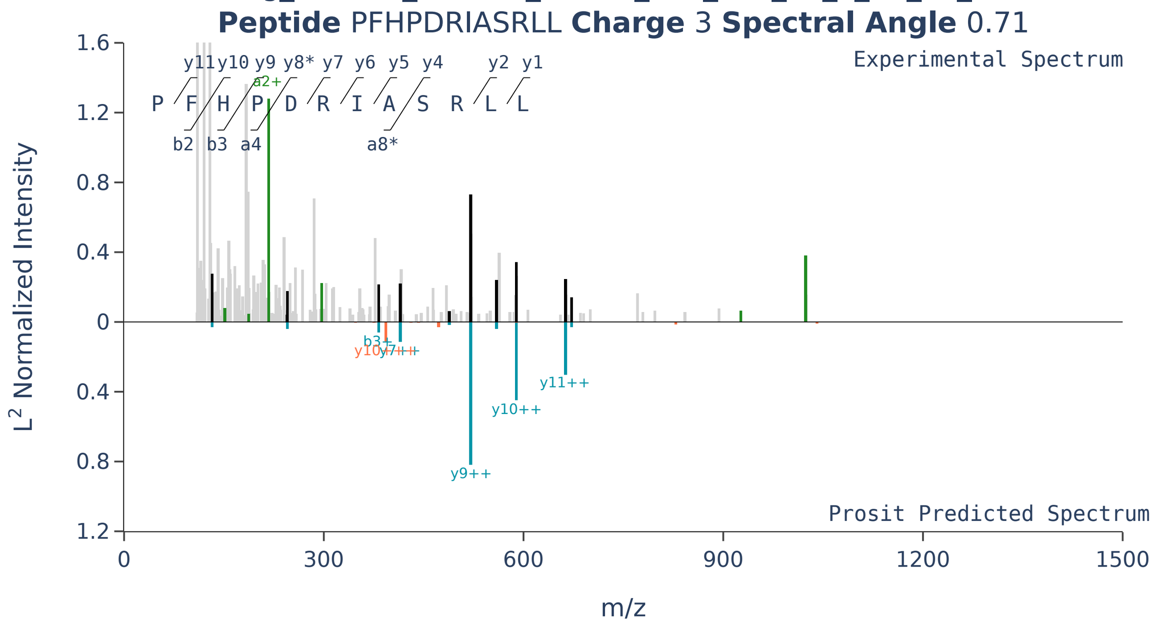


**Source** Ncheng_210623_230623_HFGoe_FFH_20S_25_1_A1_24h_R1 **Scan** 8015
**Peptide** DMQRMMKKMKKG **Charge** 2 **Spectral Angle** 0.67

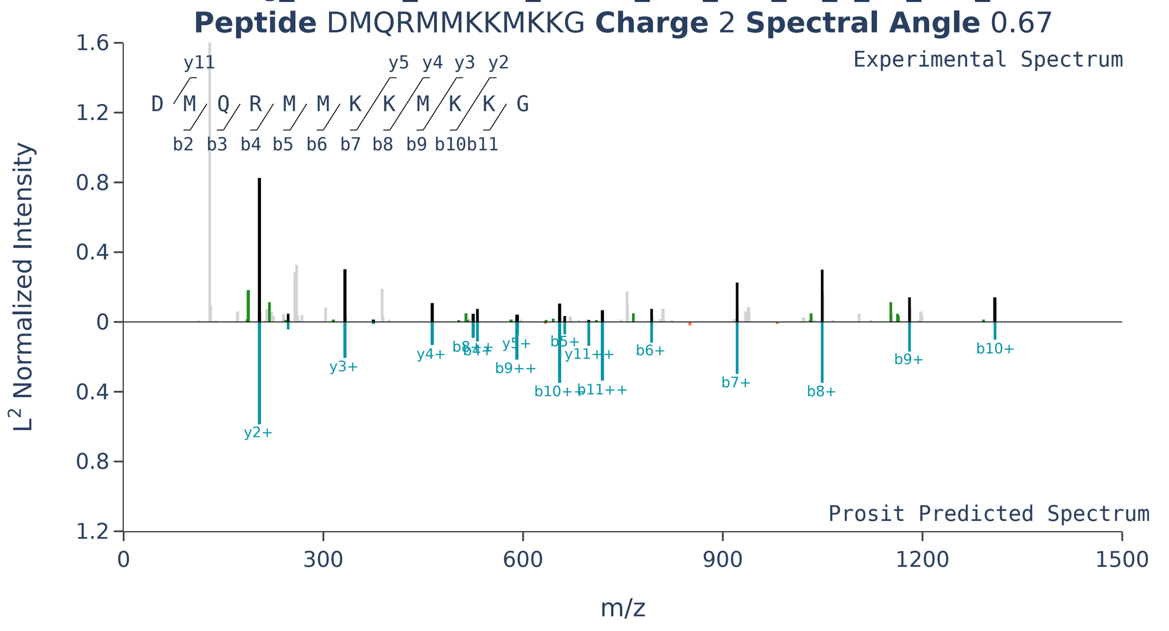

**Source** Ncheng_210623_230623_HFGoe_FFH_20S_25_1_A1_2h_R2 **Scan** 30100
**Peptide** KNMGGMASLMGKLPGMGQIPDNVKSQM **Charge** 3 **Spectral Angle** 0.75

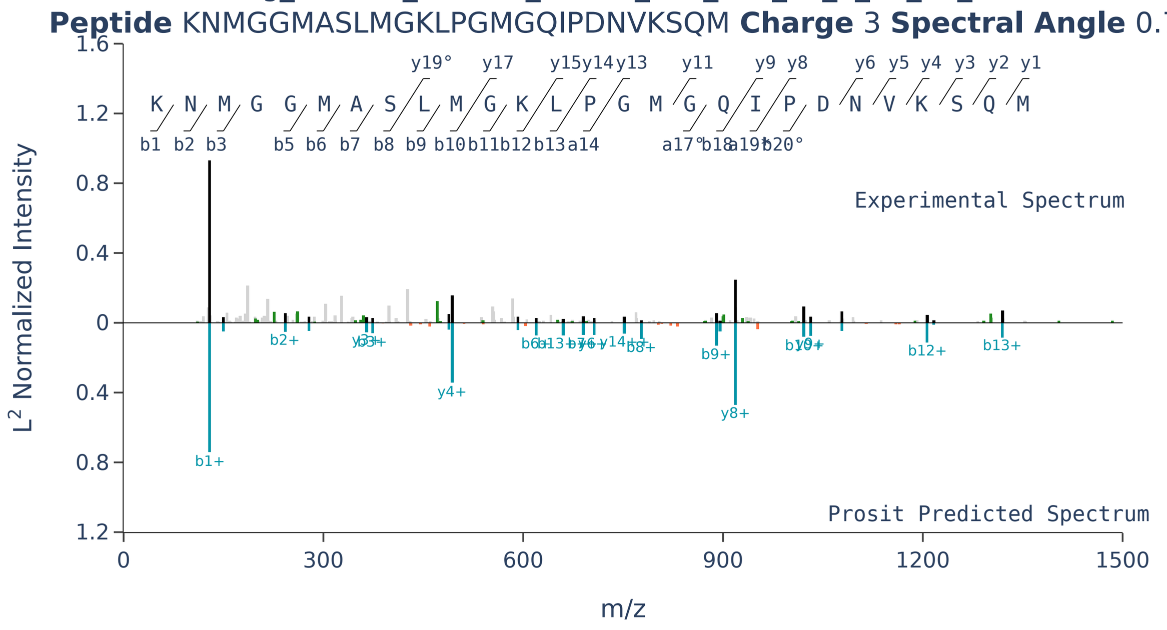


**Source** Ncheng_210623_230623_HFGoe_FFH_20S_25_1_A2_24h_R1 **Scan** 6500
**Peptide** QAEKLASKLKKG **Charge** 3 **Spectral Angle** 0.76

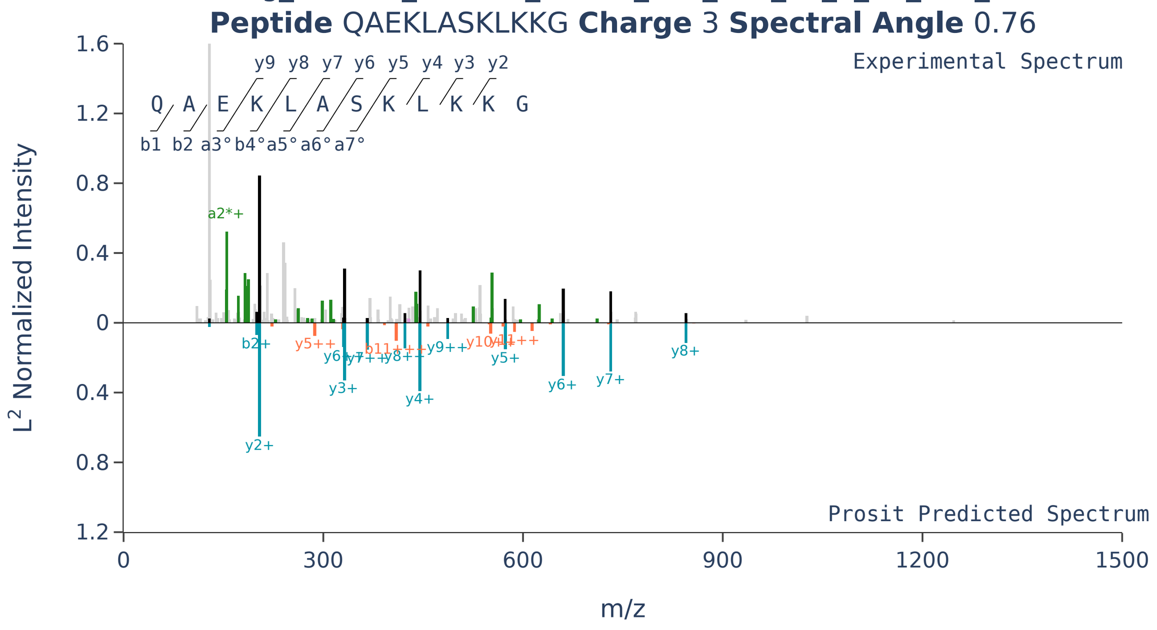


**Source** Ncheng_210623_230623_HFGoe_FFH_20S_25_1_A2_24h_R2 **Scan** 16488
**Peptide** KQFDDMQRMMKKM **Charge** 3 **Spectral Angle** 0.87

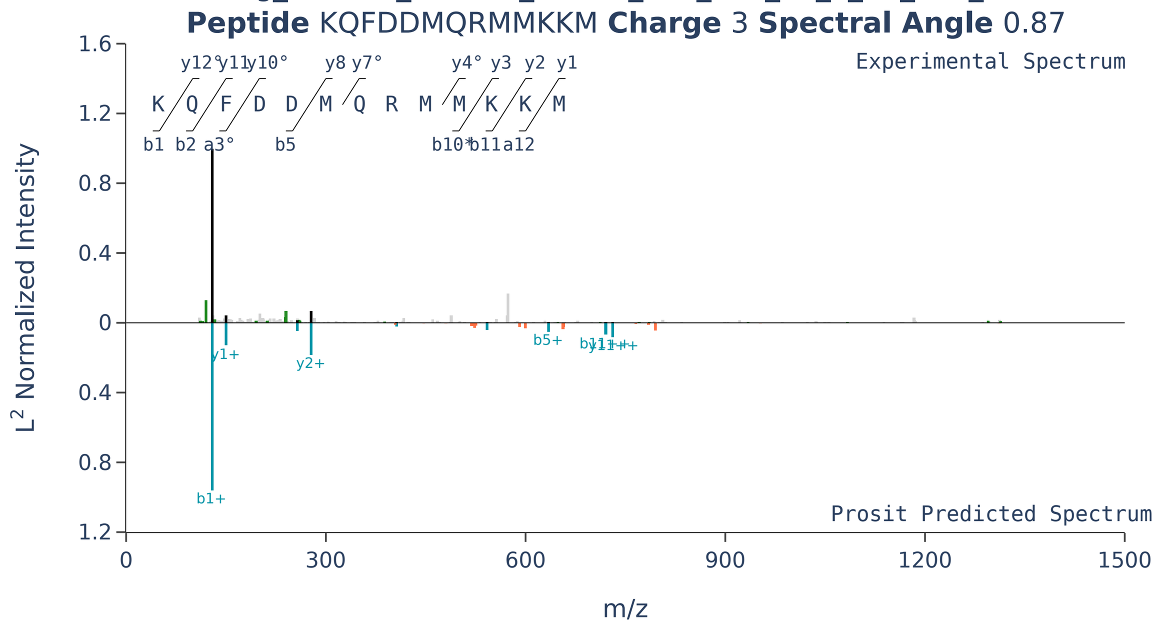


**Source** Ncheng_210623_230623_HFGoe_FFH_20S_25_1_A2_24h_R1 **Scan** 18671
**Peptide** SGRGRLTEDNVKDTLREVRMA **Charge** 4 **Spectral Angle** 0.75

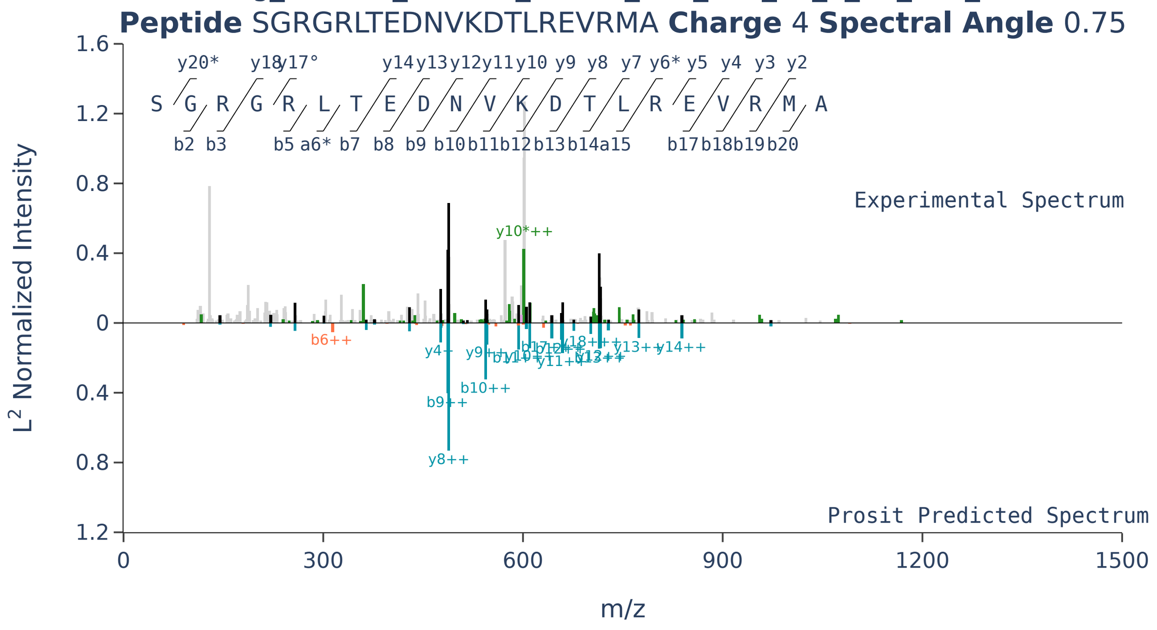


**Source** Ncheng_210623_230623_HFGoe_FFH_20S_25_1_A1_4h_R1 **Scan** 20763
**Peptide** PFHPDRIASRIL **Charge** 3 **Spectral Angle** 0.73

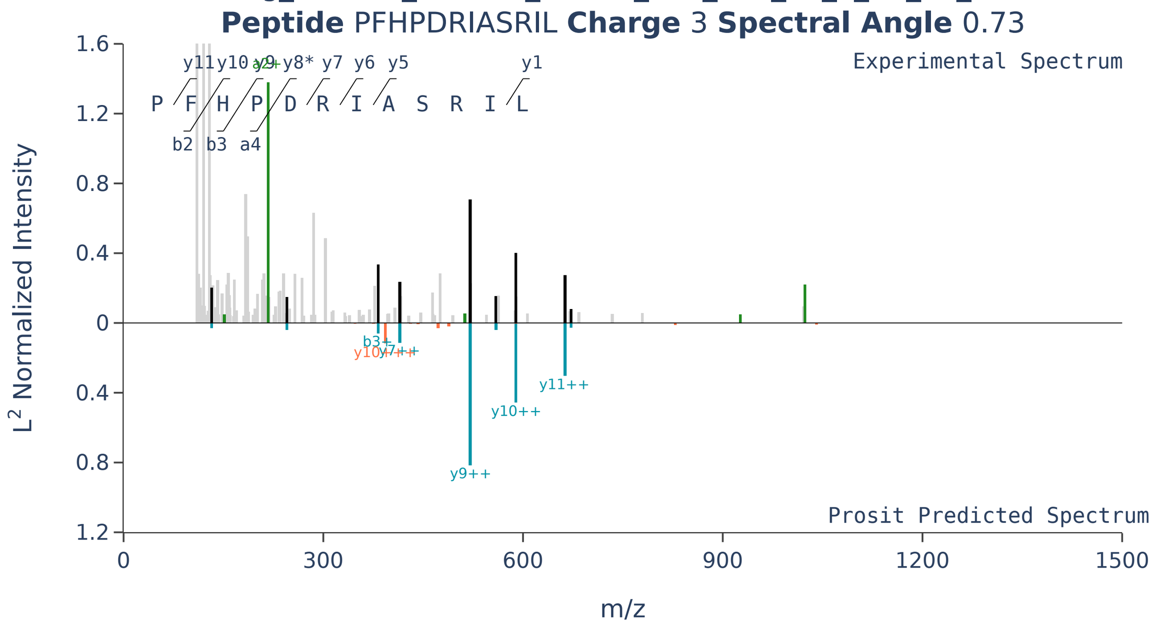


**Source** Ncheng_210623_230623_HFGoe_FFH_20S_25_1_A2_24h_R2 **Scan** 10476
**Peptide** QAEKLASKLKKGDGF **Charge** 2 **Spectral Angle** 0.7

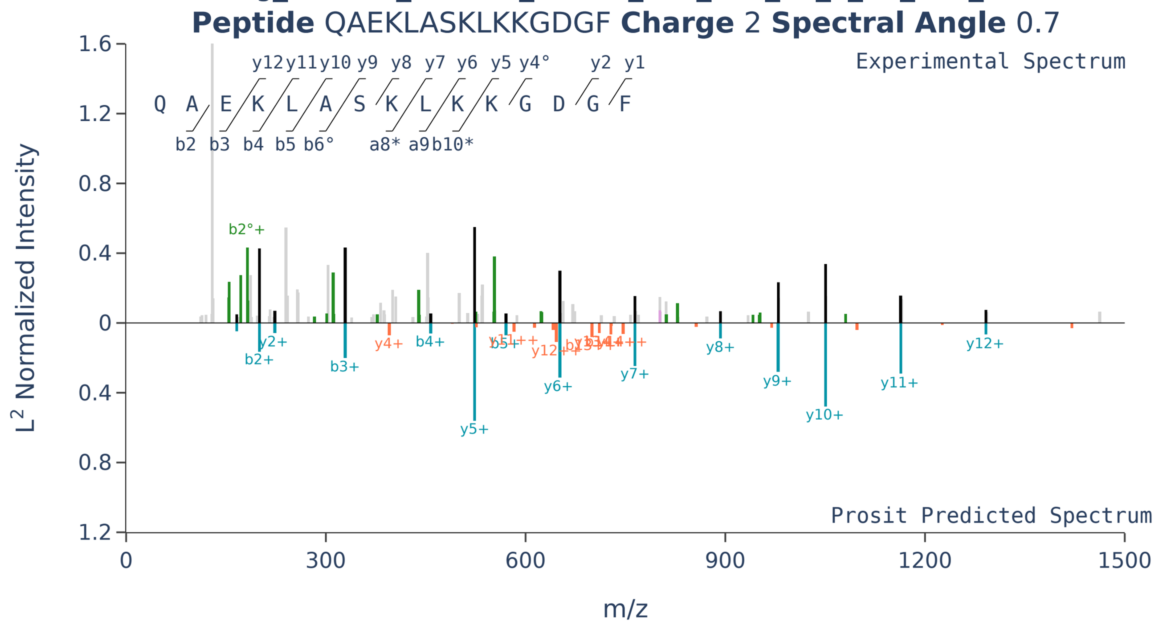


**Source** Ncheng_210623_230623_HFGoe_FFH_20S_25_1_A1_24h_R2 **Scan** 9448
**Peptide** RHITGKPLKF **Charge** 2 **Spectral Angle** 0.7

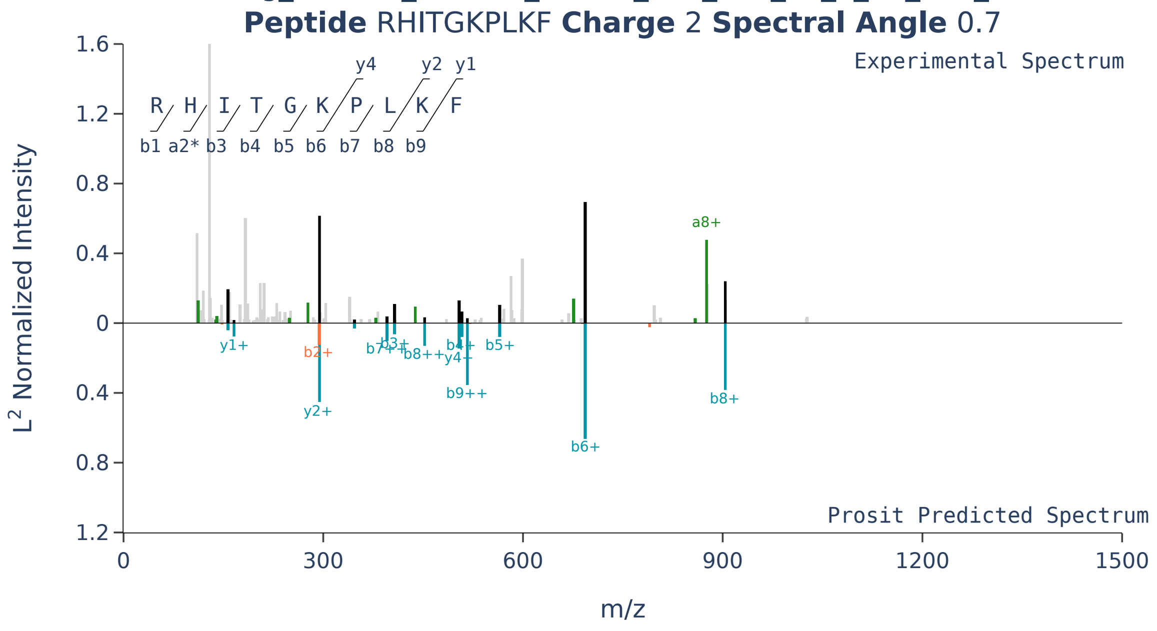


**Source** Ncheng_210623_230623_HFGoe_FFH_20S_25_1_A2_24h_R2 **Scan** 7522
**Peptide** DDMQRMMKK **Charge** 2 **Spectral Angle** 0.73

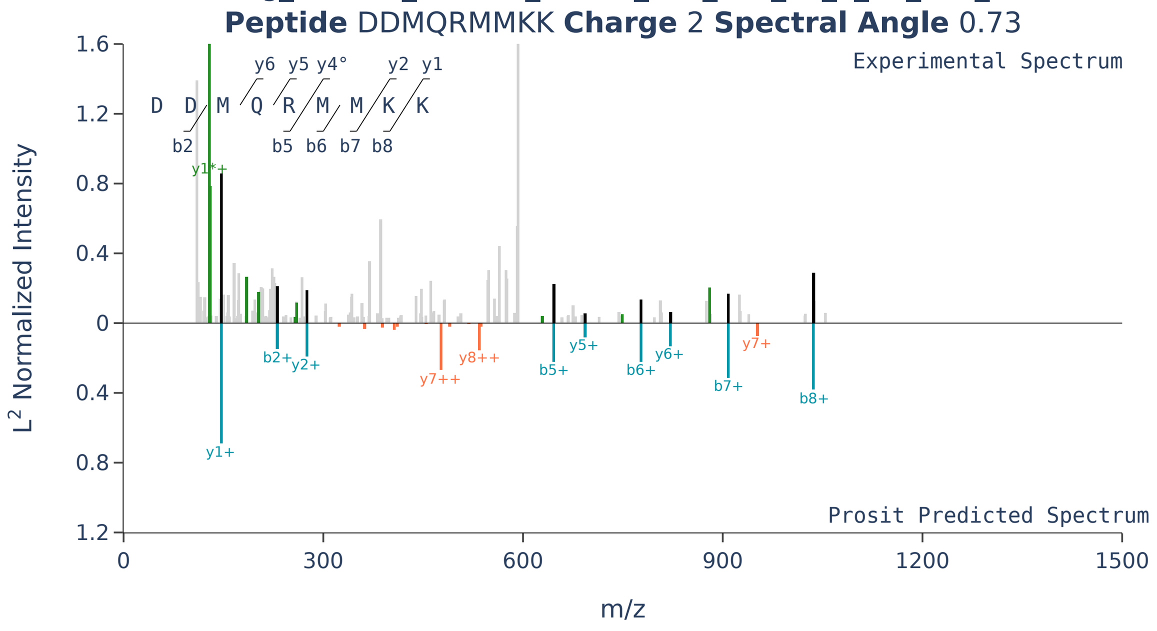


**Source** Ncheng_210623_230623_HFGoe_FFH_20S_25_1_A1_2h_R1 **Scan** 15083
**Peptide** TKVDGDARGGAALSI **Charge** 2 **Spectral Angle** 0.73

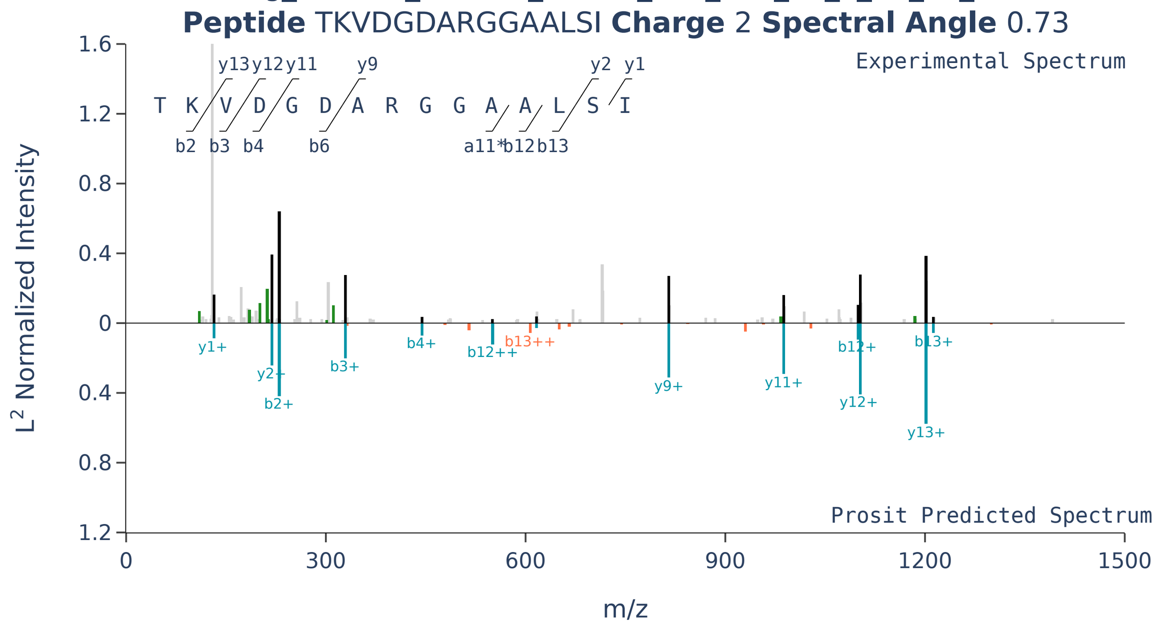


**Source** Ncheng_210623_230623_HFGoe_FFH_20S_25_1_A1_4h_R1 **Scan** 24256
**Peptide** MGKLPGMGQIPDNVKSQMDDKVLVR **Charge** 4 **Spectral Angle** 0.66

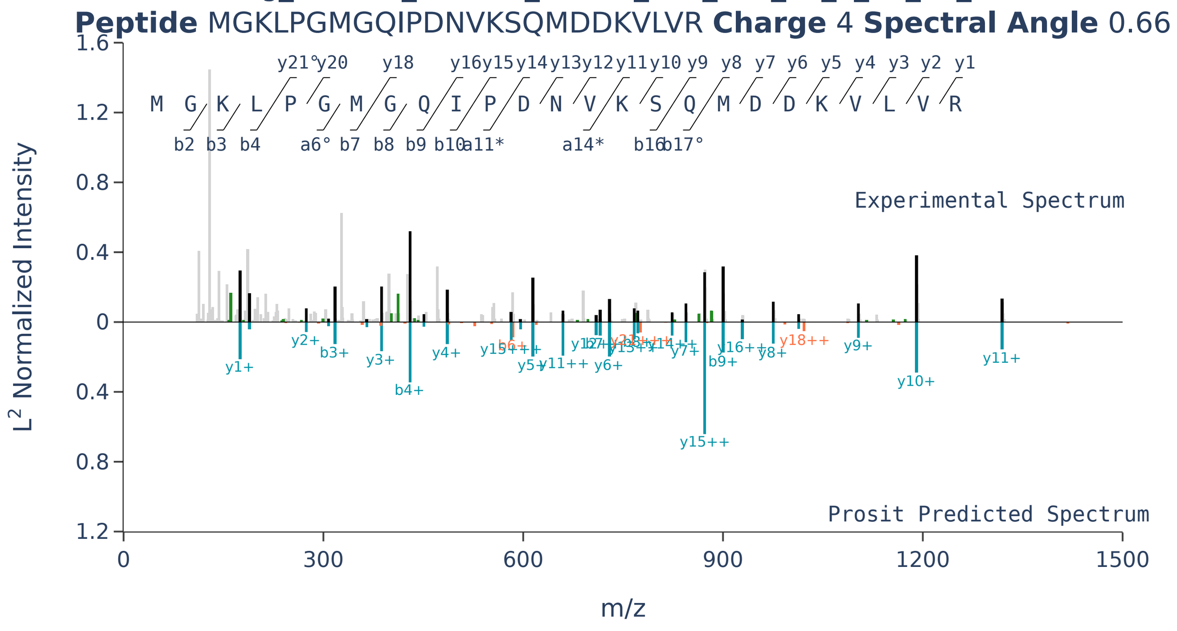


**Source** Ncheng_210623_230623_HFGoe_FFH_20S_25_1_A2_4h_R1 **Scan** 18244
**Peptide** KVDGDARGGAALSIRHITGKPIKF **Charge** 6 **Spectral Angle** 0.82

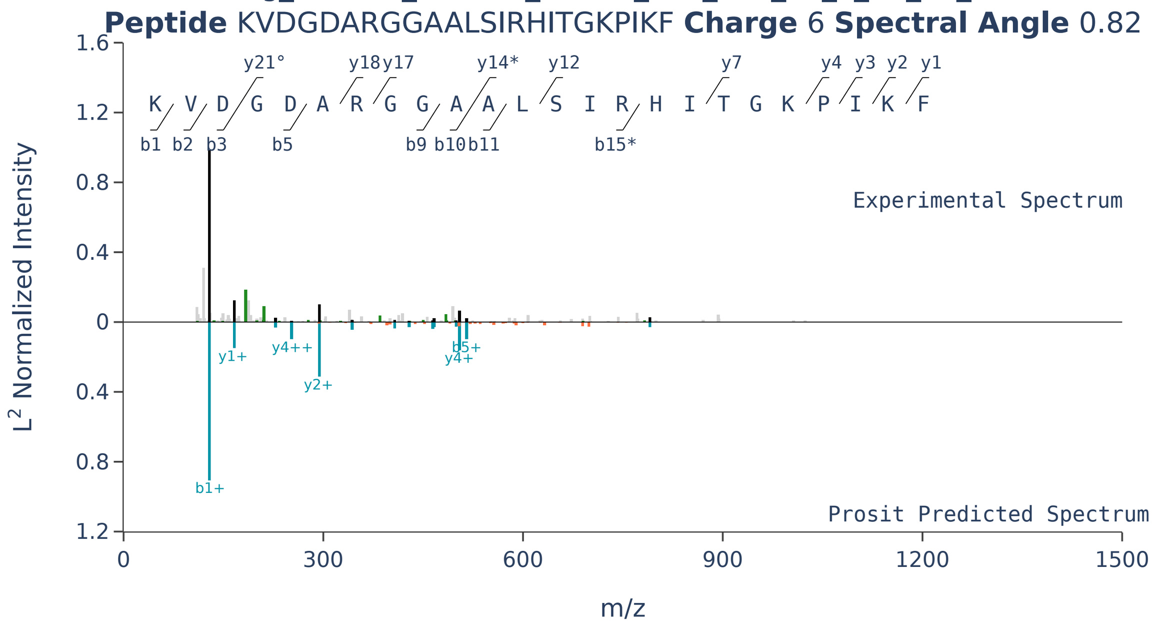


**Source** Ncheng_210623_230623_HFGoe_FFH_20S_25_1_A1_24h_R2 **Scan** 10437
**Peptide** SGRGRLTEDNVKDTLRE **Charge** 3 **Spectral Angle** 0.75

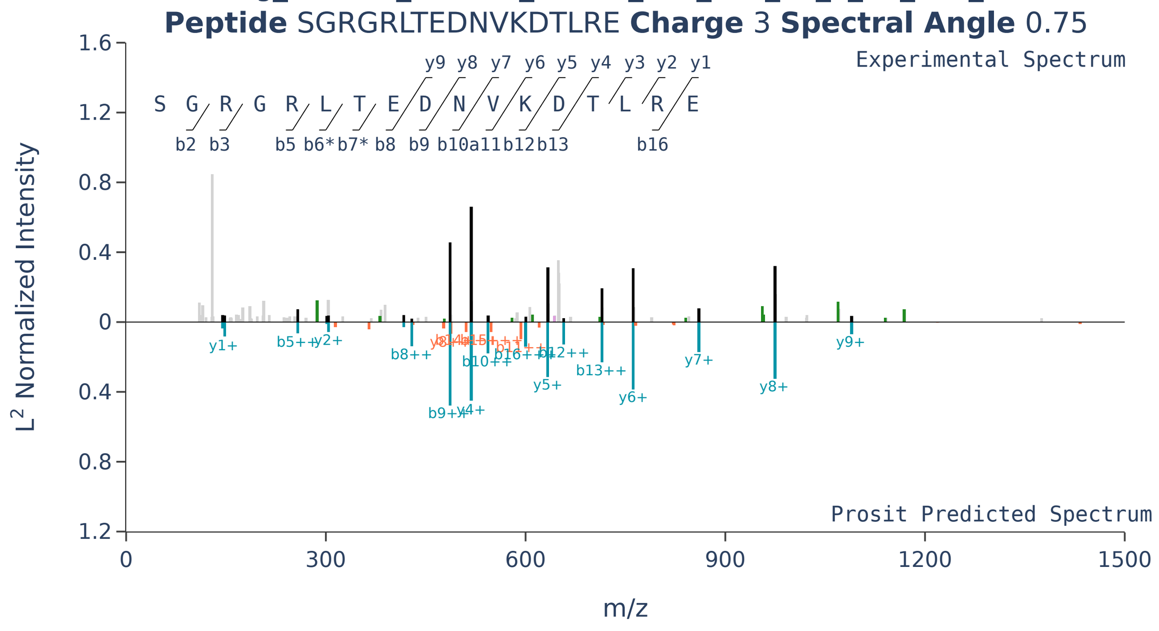


**Source** Ncheng_210623_230623_HFGoe_FFH_20S_25_1_A2_24h_R2 **Scan** 12882
**Peptide** DRLSRTLRNISGR **Charge** 4 **Spectral Angle** 0.75

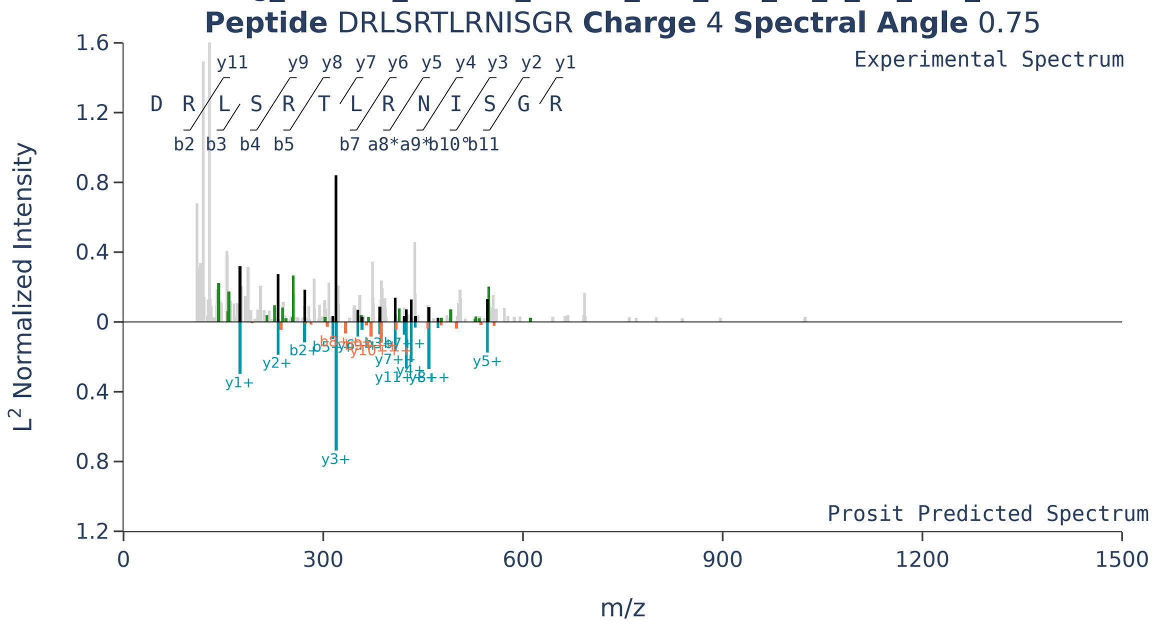


**Source** Ncheng_210623_230623_HFGoe_FFH_20S_25_1_A1_24h_R1 **Scan** 14700
**Peptide** GDARGGAALSIRHLTG **Charge** 3 **Spectral Angle** 0.74

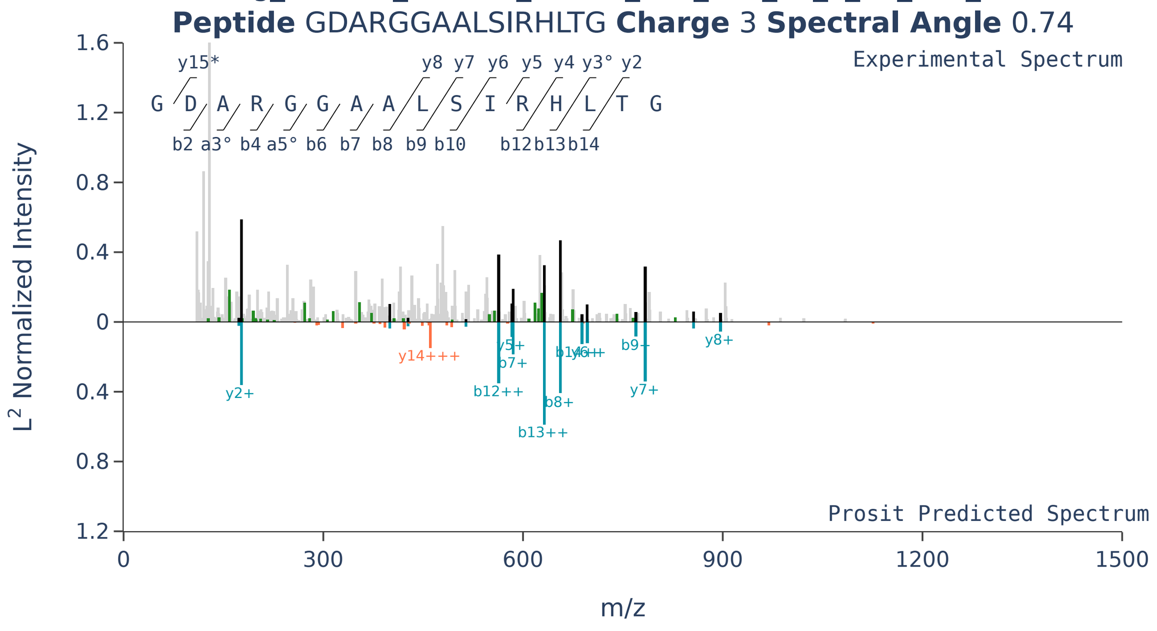


**Source** Ncheng_210623_230623_HFGoe_FFH_20S_25_1_A1_2h_R2 **Scan** 31135
**Peptide** KVLVRMEAIINSMTM **Charge** 2 **Spectral Angle** 0.55

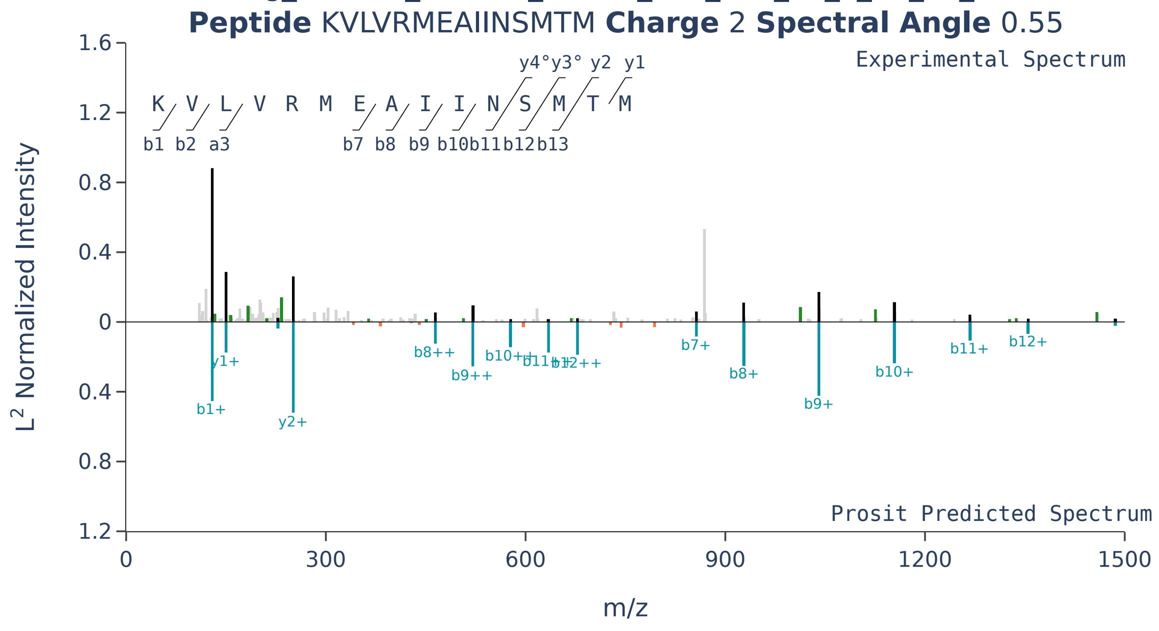

**Source** Ncheng_210623_230623_HFGoe_FFH_20S_25_1_A2_24h_R1 **Scan** 6948
**Peptide** MQRMMKKM **Charge** 3 **Spectral Angle** 0.78

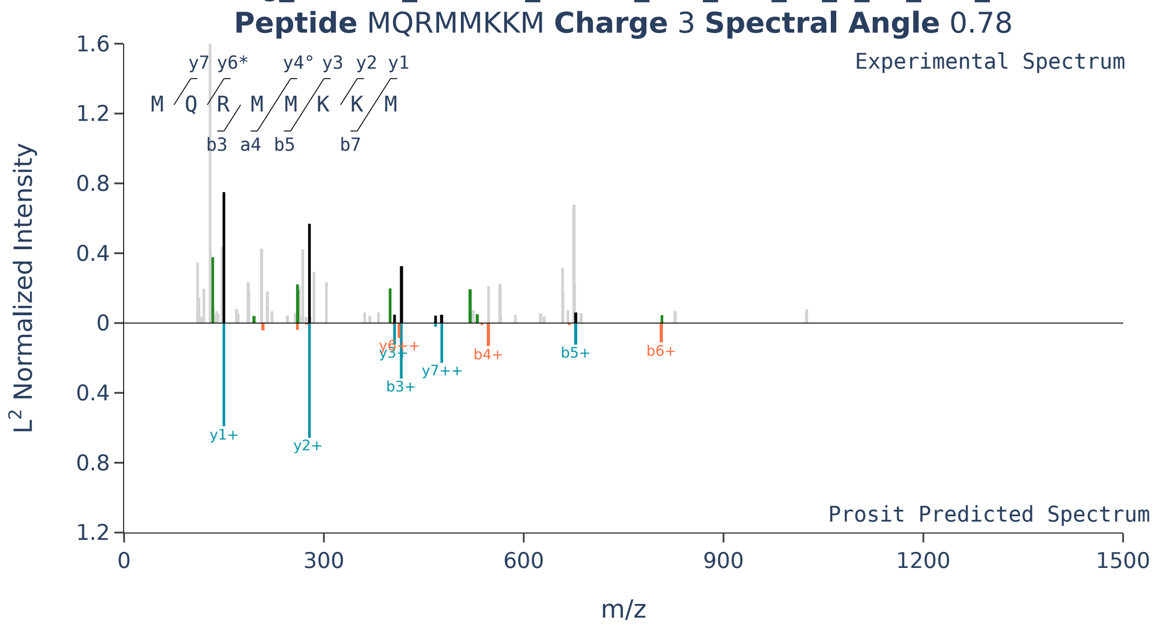


**Source** Ncheng_210623_230623_HFGoe_FFH_20S_25_1_A1_4h_R1 **Scan** 20094
**Peptide** AMMDEIKQVHASINPVE **Charge** 3 **Spectral Angle** 0.59

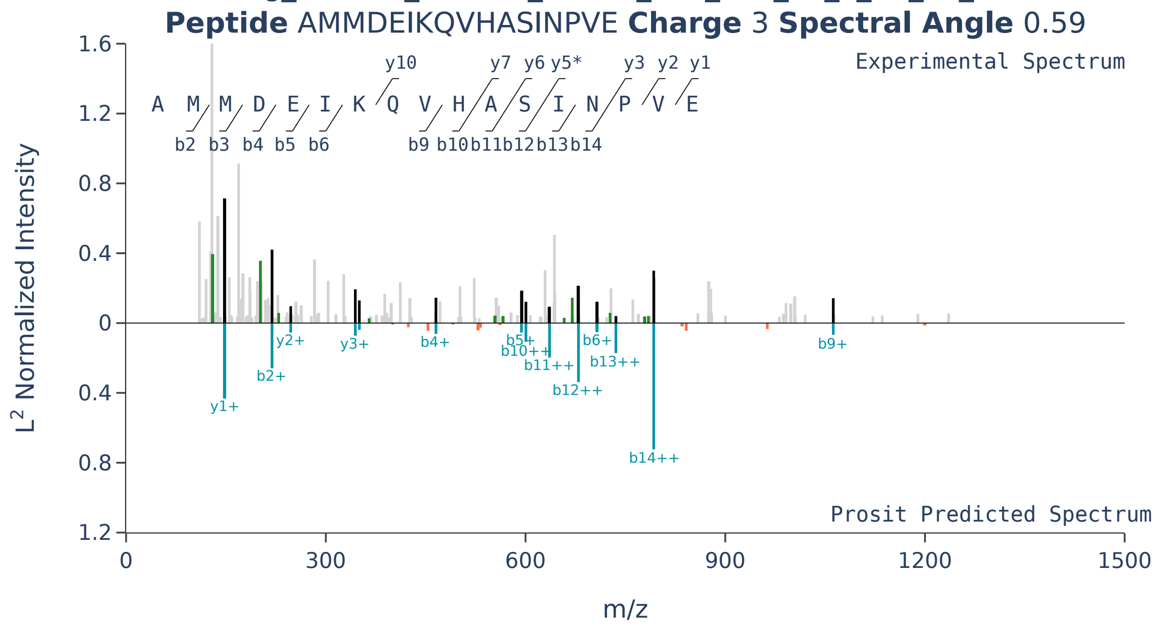


**Source** Ncheng_210623_230623_HFGoe_FFH_20S_25_1_A1_24h_R1 **Scan** 25148
**Peptide** KLKFYDVL **Charge** 2 **Spectral Angle** 0.59

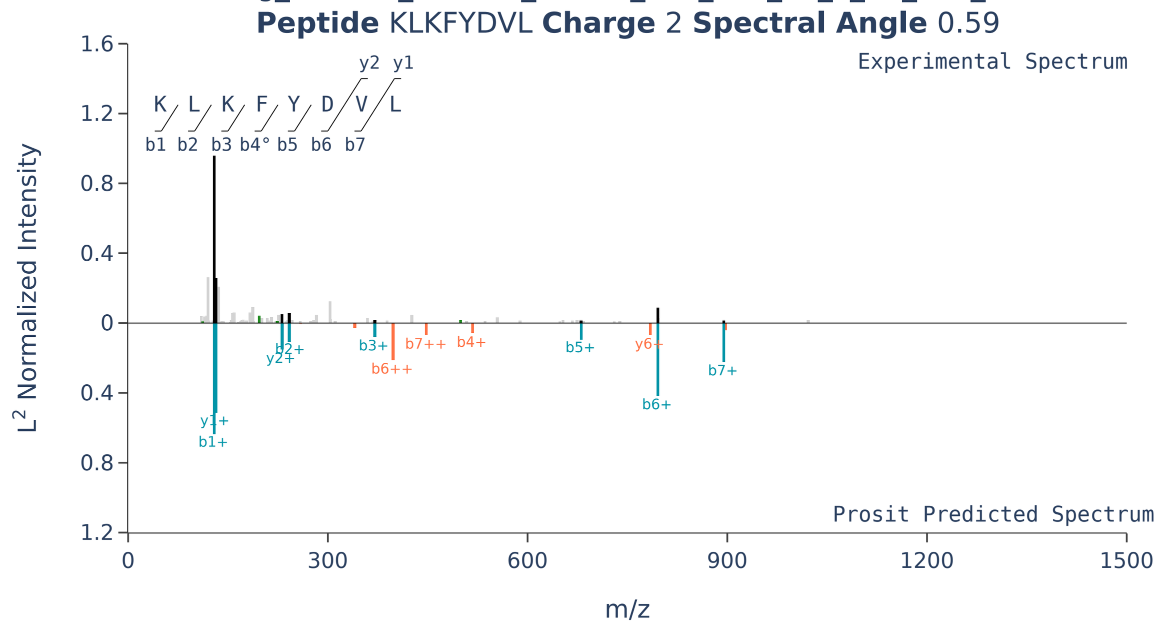


**Source** Ncheng_210623_230623_HFGoe_FFH_20S_25_1_A1_24h_R1 **Scan** 15796
**Peptide** NLTDRLSRTLR **Charge** 2 **Spectral Angle** 0.66

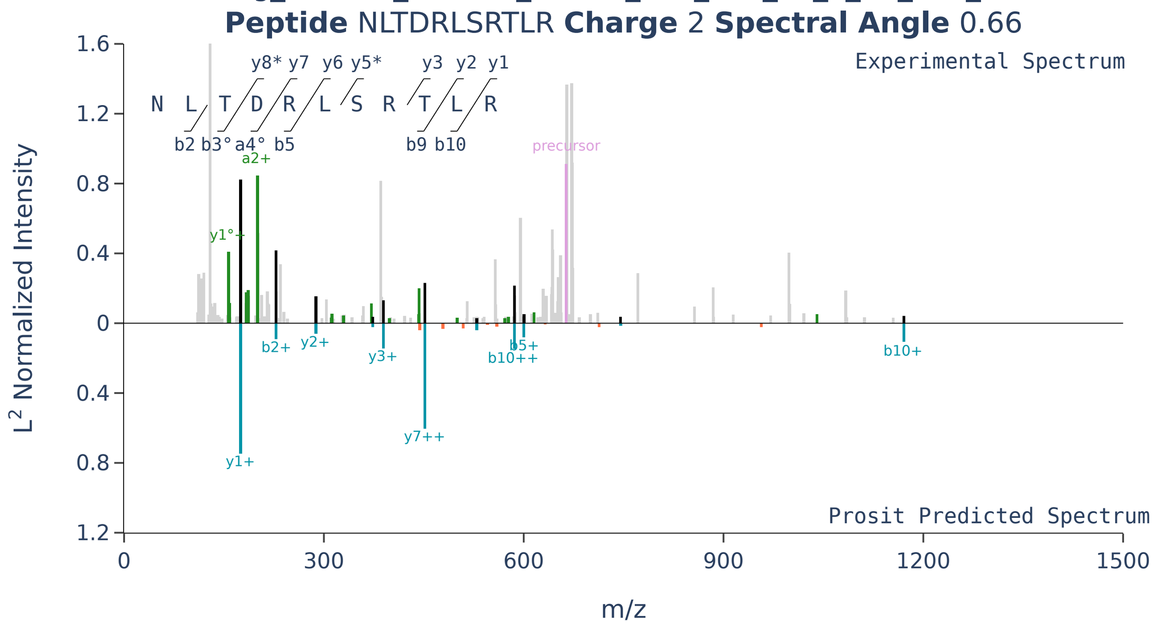


**Source** Ncheng_210623_230623_HFGoe_FFH_20S_25_1_A2_24h_R2 **Scan** 12692
**Peptide** SKLKKGDGFDL **Charge** 3 **Spectral Angle** 0.82

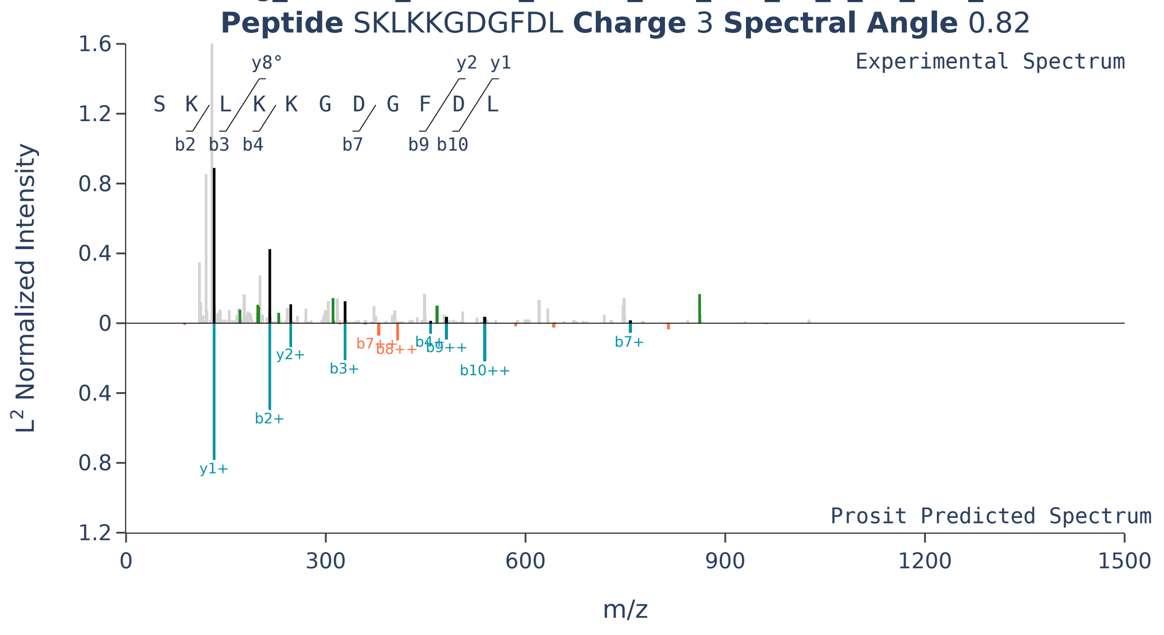


**Source** Ncheng_210623_230623_HFGoe_FFH_20S_25_1_A2_4h_R1 **Scan** 15703
**Peptide** AALKEAKLKFYD **Charge** 3 **Spectral Angle** 0.61

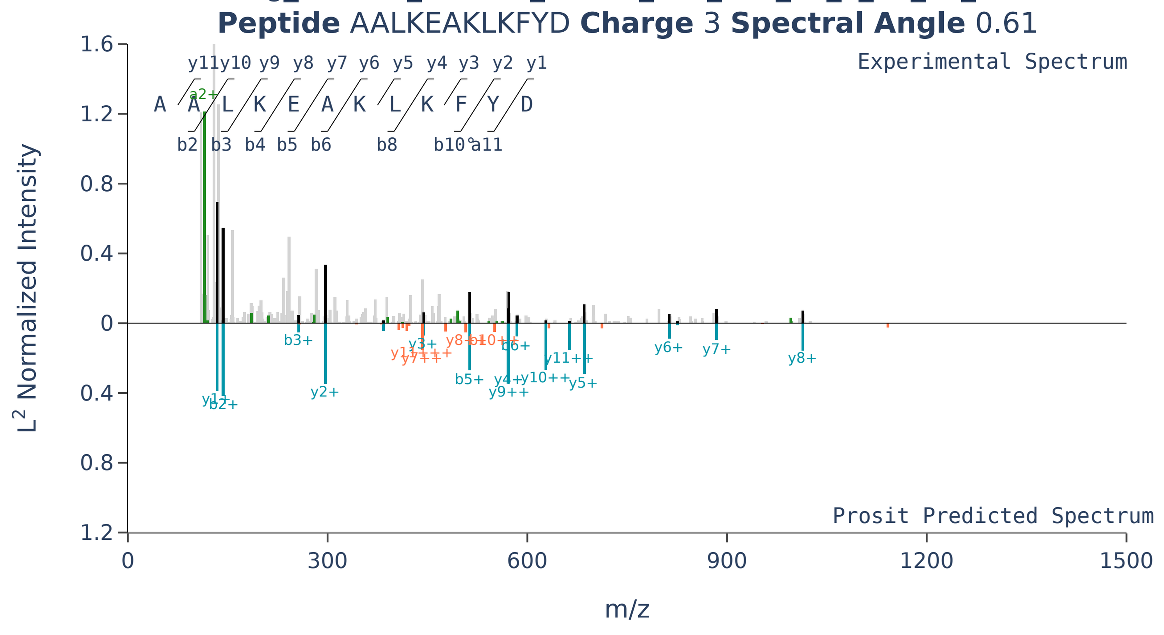


**Source** Ncheng_210623_230623_HFGoe_FFH_20S_25_1_A1_4h_R1 **Scan** 19221
**Peptide** VVLTKVDGDARGGAALSIRHITG **Charge** 3 **Spectral Angle** 0.72

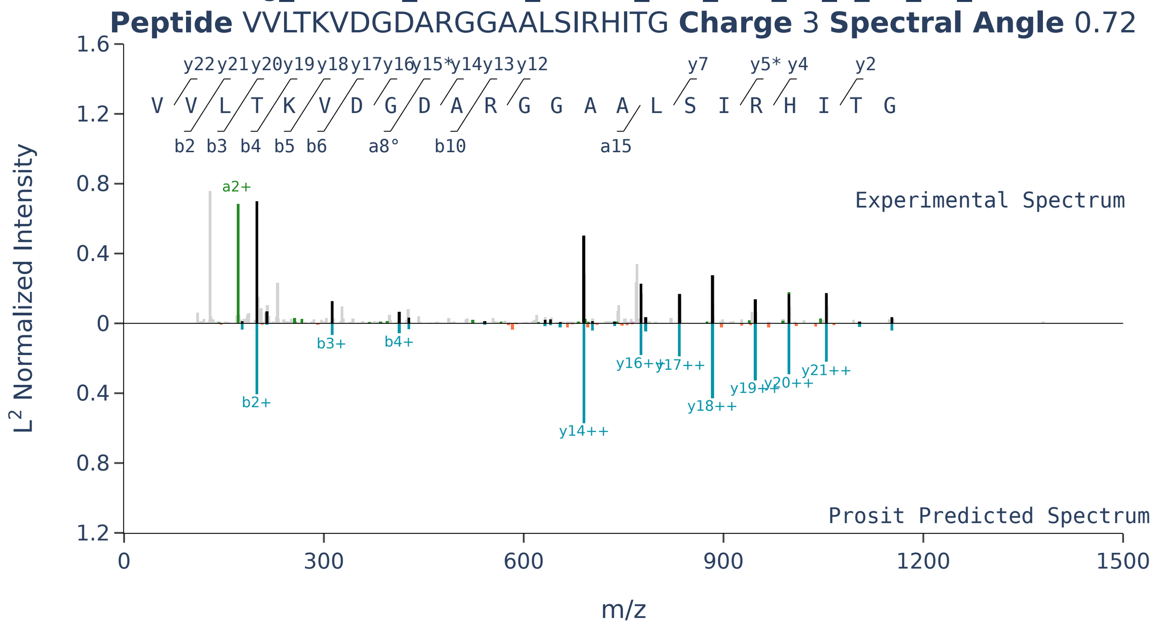


**Source** Ncheng_210623_230623_HFGoe_FFH_20S_25_1_A1_24h_R2 **Scan** 19332
**Peptide** TGVVLTKVDGDARGGAAL **Charge** 3 **Spectral Angle** 0.68

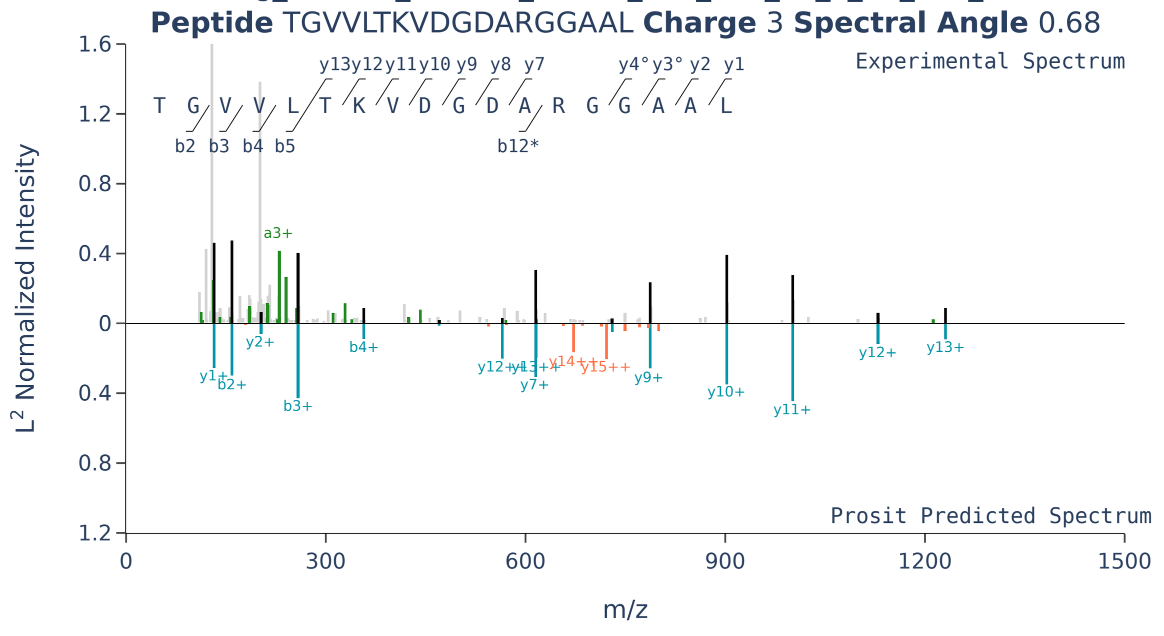


**Source** Ncheng_210623_230623_HFGoe_FFH_20S_25_1_A1_1h_R2 **Scan** 31872
**Peptide** SADVYRPAAIKQLETLAEQVG **Charge** 3 **Spectral Angle** 0.74

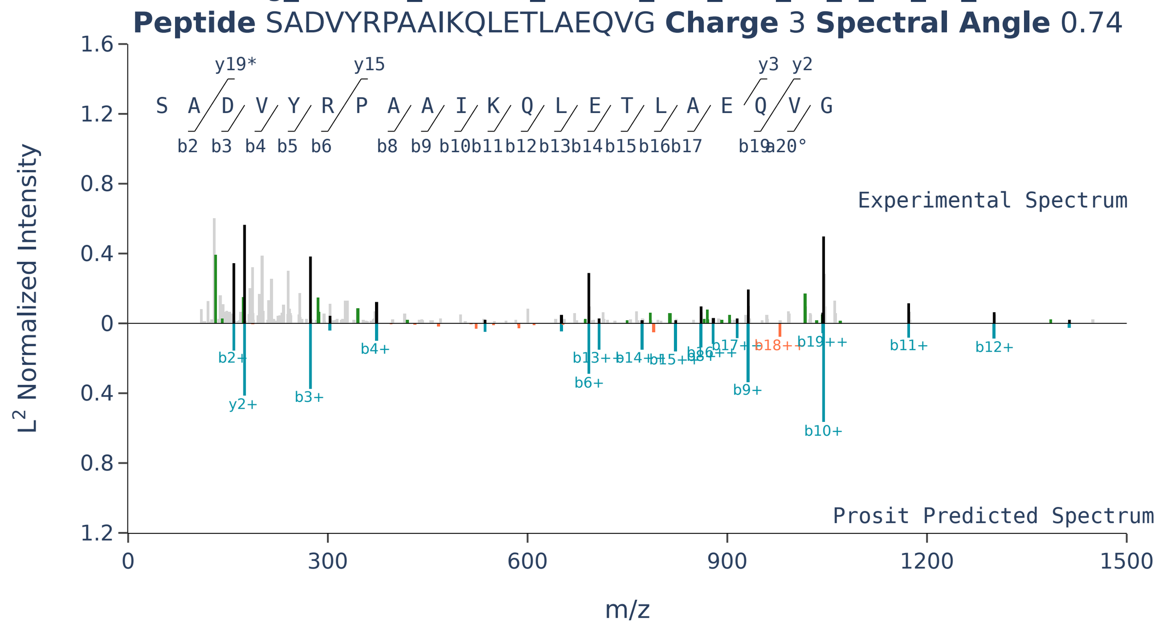


**Source** Ncheng_210623_230623_HFGoe_FFH_20S_25_1_A2_4h_R1 **Scan** 21883
**Peptide** GQIPDNVKSQMDDKVLVRM **Charge** 2 **Spectral Angle** 0.64

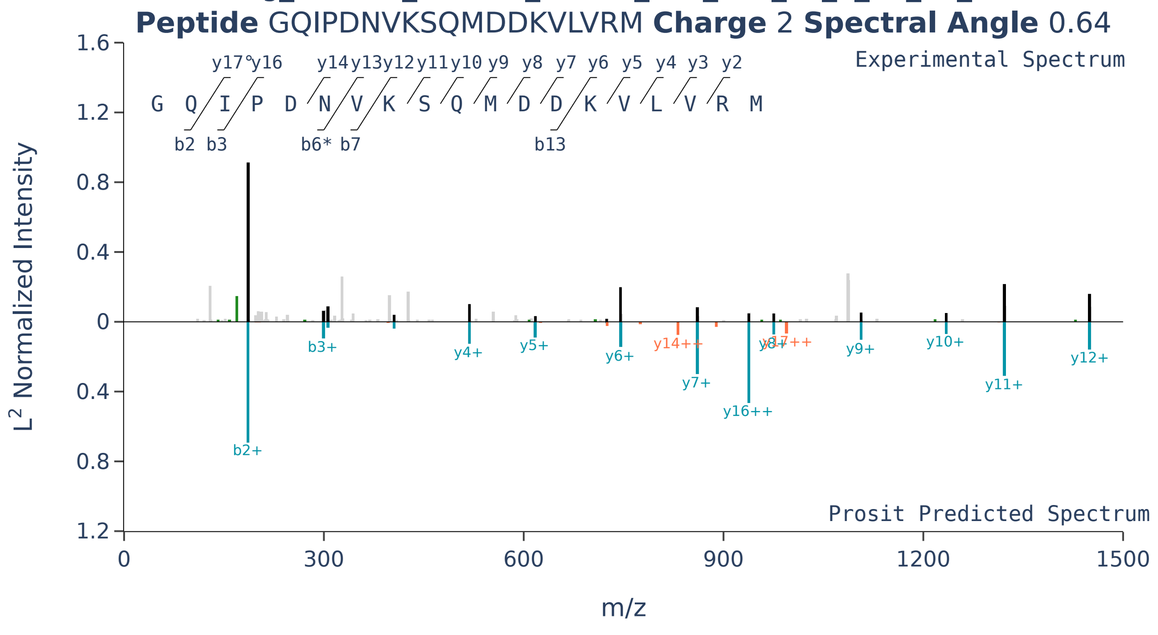


**Source** Ncheng_210623_230623_HFGoe_FFH_20S_25_1_A2_2h_R1 **Scan** 29586
**Peptide** KNMGGMASLMGKLPGMGQIPDNVKSQMDD **Charge** 3 **Spectral Angle** 0.75

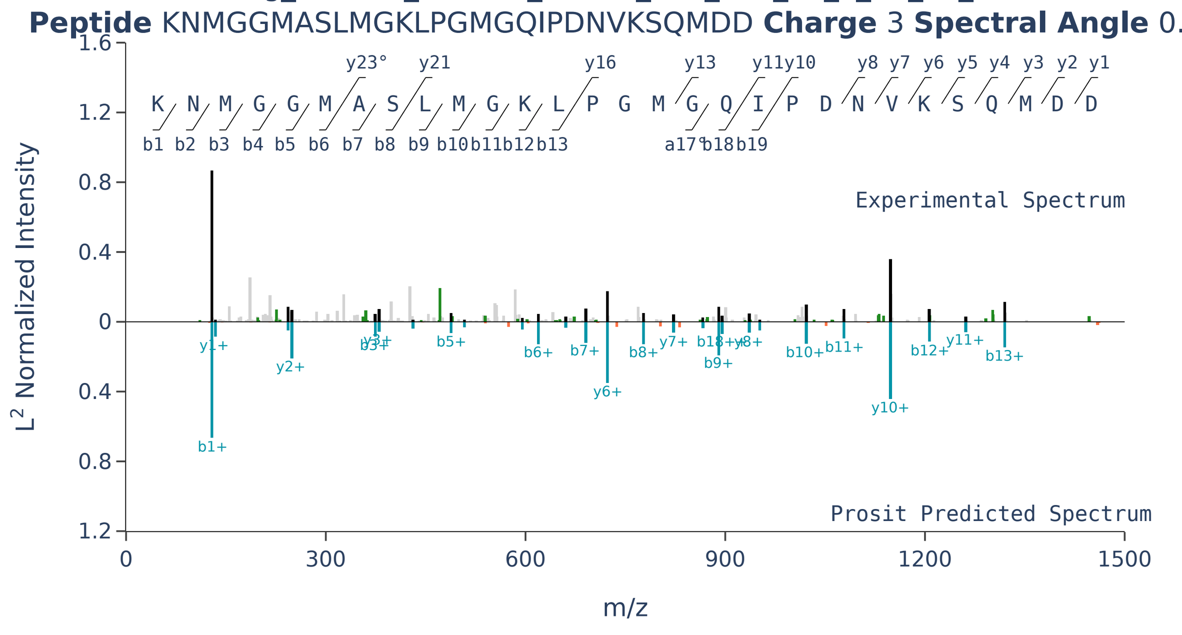


**Source** Ncheng_210623_230623_HFGoe_FFH_20S_25_1_A2_4h_R1 **Scan** 30097
**Peptide** GCGMQVQDVNRLLKQFD **Charge** 2 **Spectral Angle** 0.74

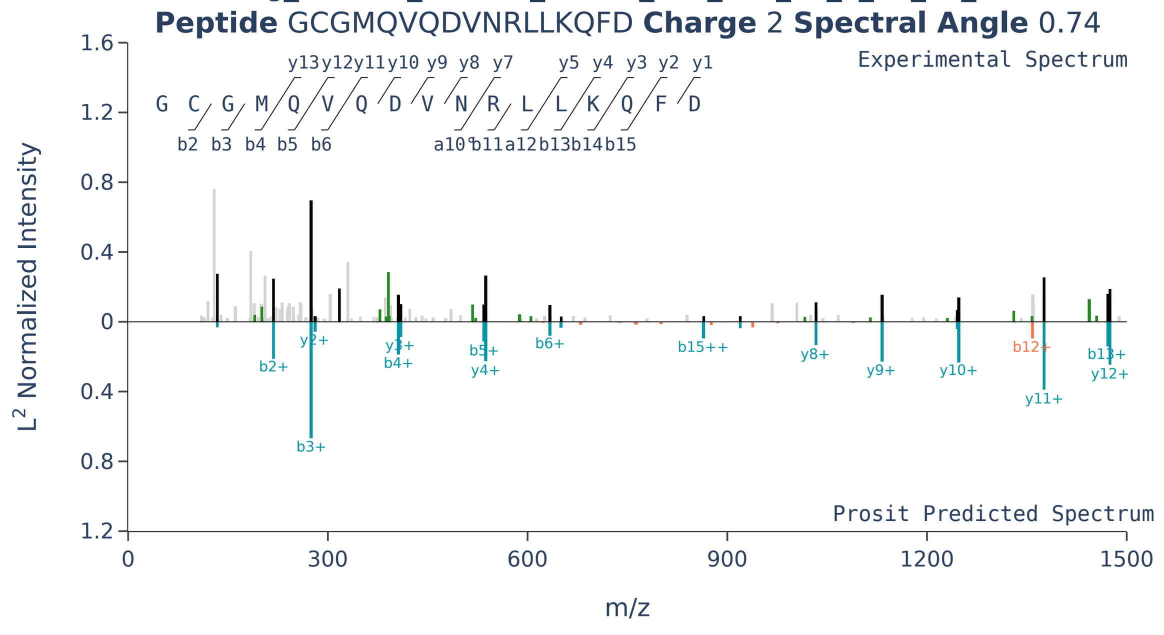


**Source** Ncheng_210623_230623_HFGoe_FFH_20S_25_1_A1_24h_R1 **Scan** 14138
**Peptide** TKVDGDARGGAALSIRHI **Charge** 5 **Spectral Angle** 0.83

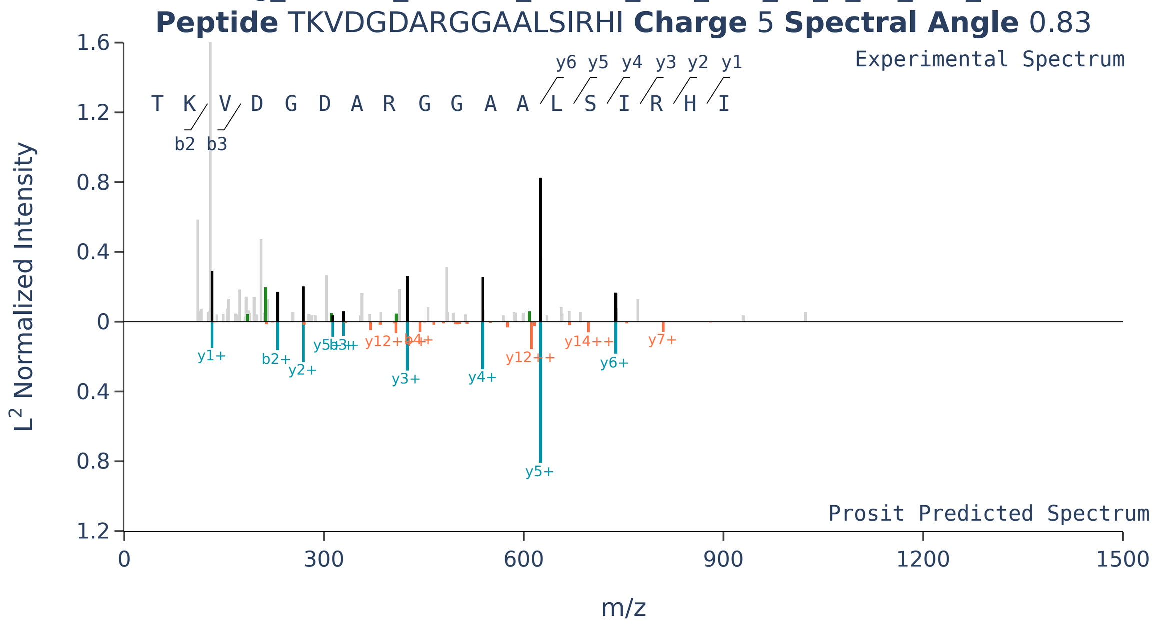


**Source** Ncheng_210623_230623_HFGoe_FFH_20S_25_1_A2_24h_R1 **Scan** 13654
**Peptide** KVDGDARGGAALSIRHI **Charge** 2 **Spectral Angle** 0.75

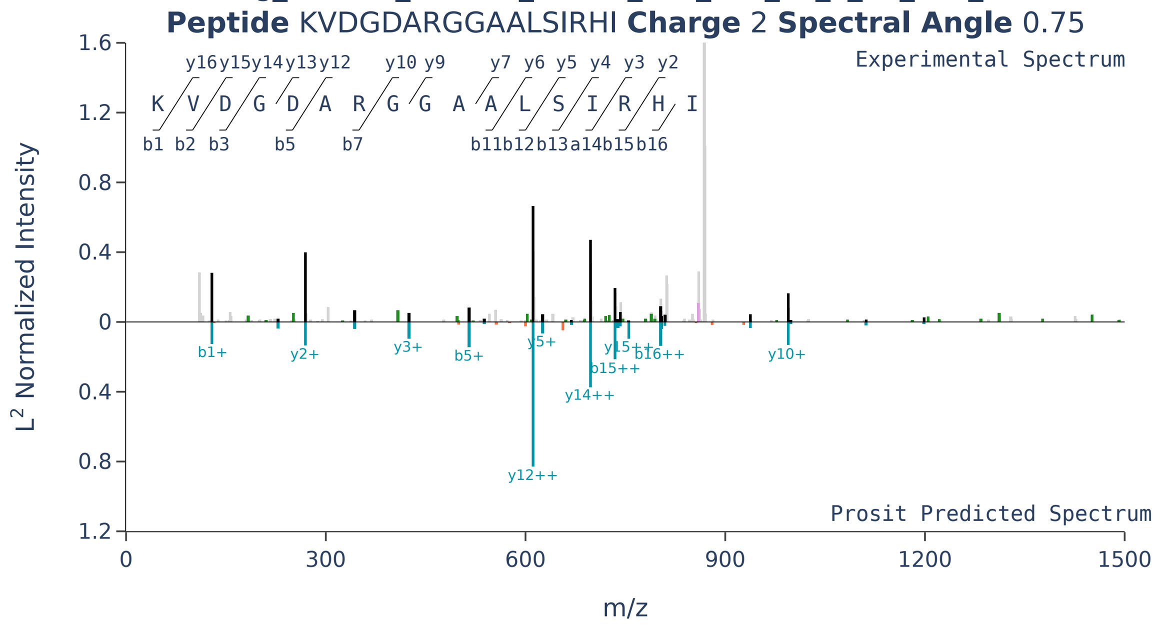


**Source** Ncheng_210623_230623_HFGoe_FFH_20S_25_1_A1_4h_R1 **Scan** 12732
**Peptide** EAIINSMTMKERAKPEIIKGSRKR **Charge** 6 **Spectral Angle** 0.74

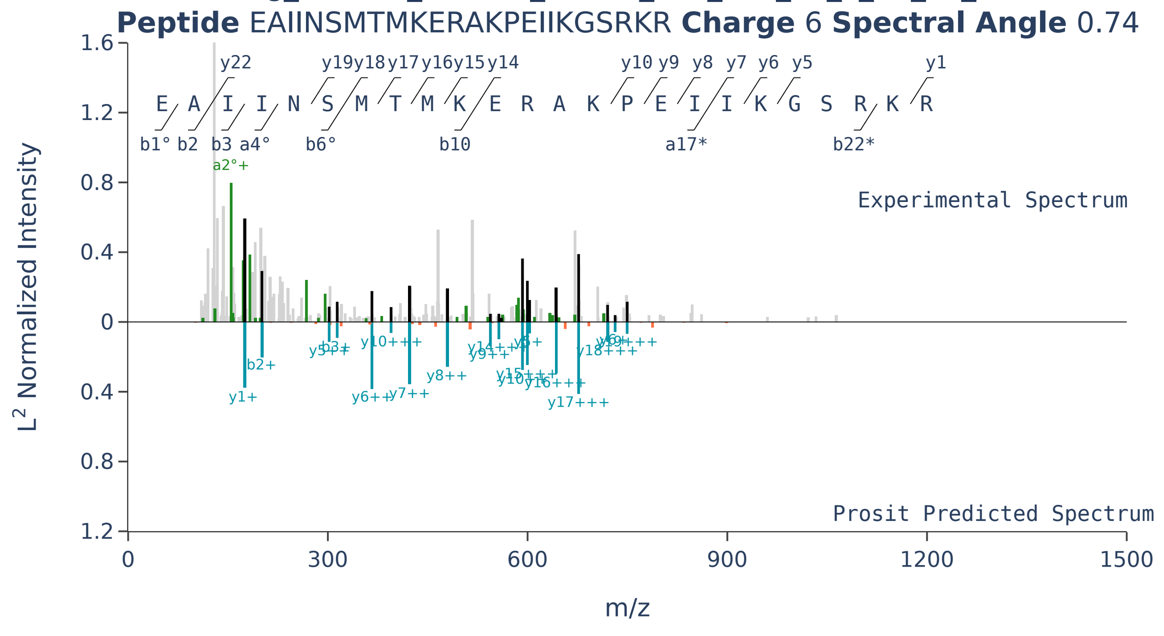

**Source** Ncheng_210623_230623_HFGoe_FFH_20S_25_1_A2_24h_R2 **Scan** 14272
**Peptide** VDGDARGGAALSIRHITGK **Charge** 5 **Spectral Angle** 0.82

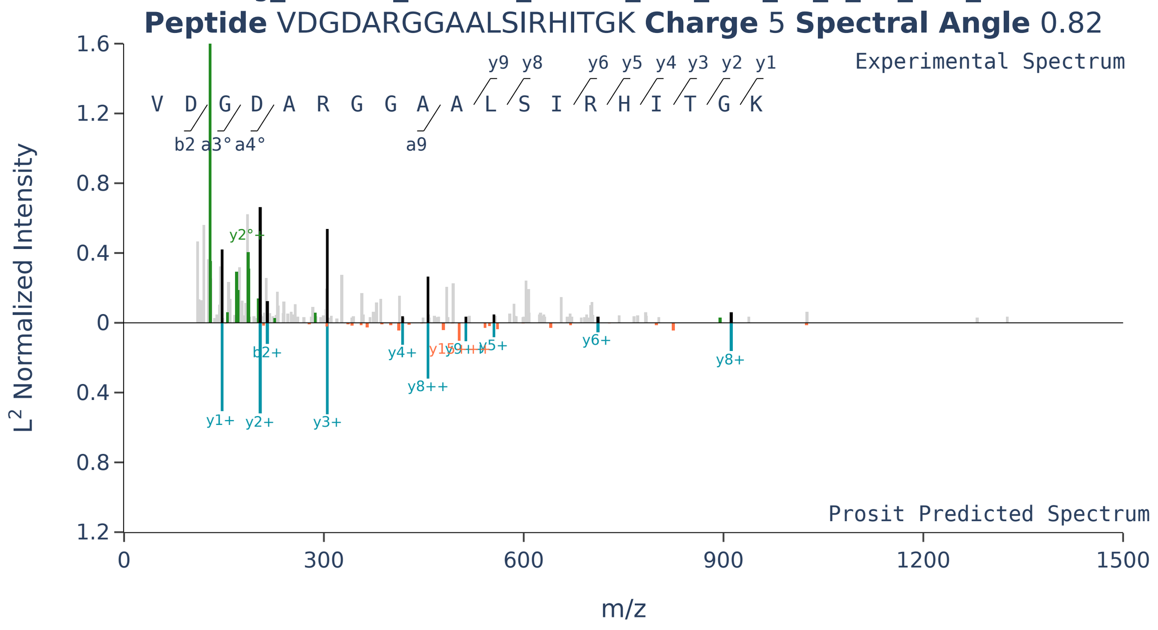


**Source** Ncheng_210623_230623_HFGoe_FFH_20S_25_1_A1_2h_R1 **Scan** 20074
**Peptide** KPVDIVNAALKE **Charge** 3 **Spectral Angle** 0.55

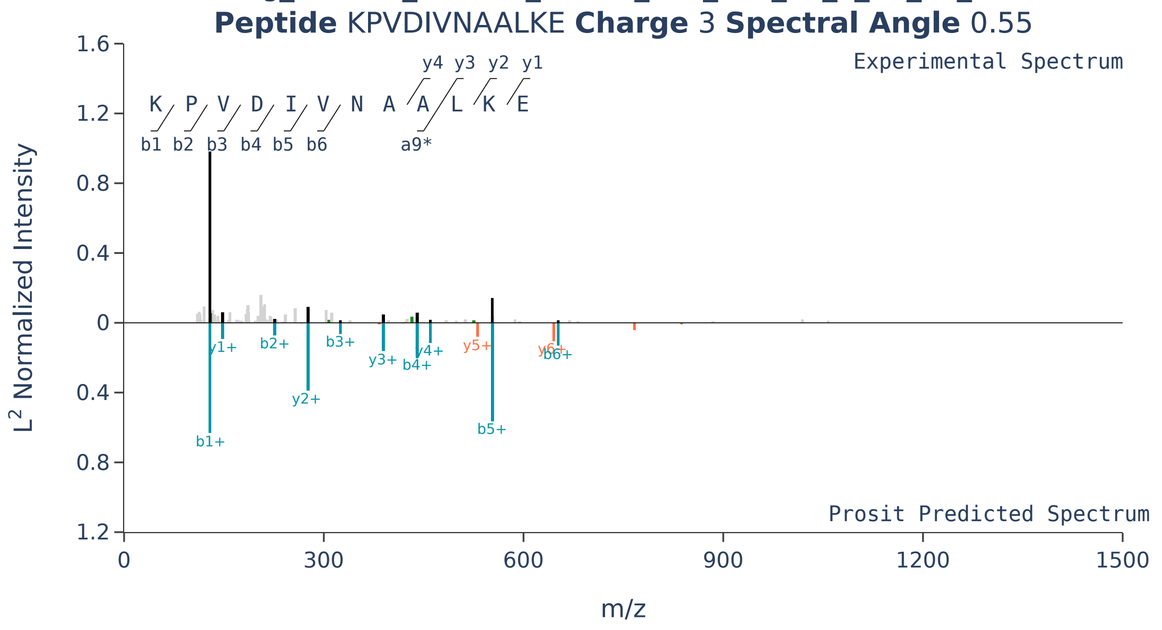


**Source** Ncheng_210623_230623_HFGoe_FFH_20S_25_1_A2_24h_R1 **Scan** 13148
**Peptide** DLESKVDRAQAEKLAS **Charge** 2 **Spectral Angle** 0.71

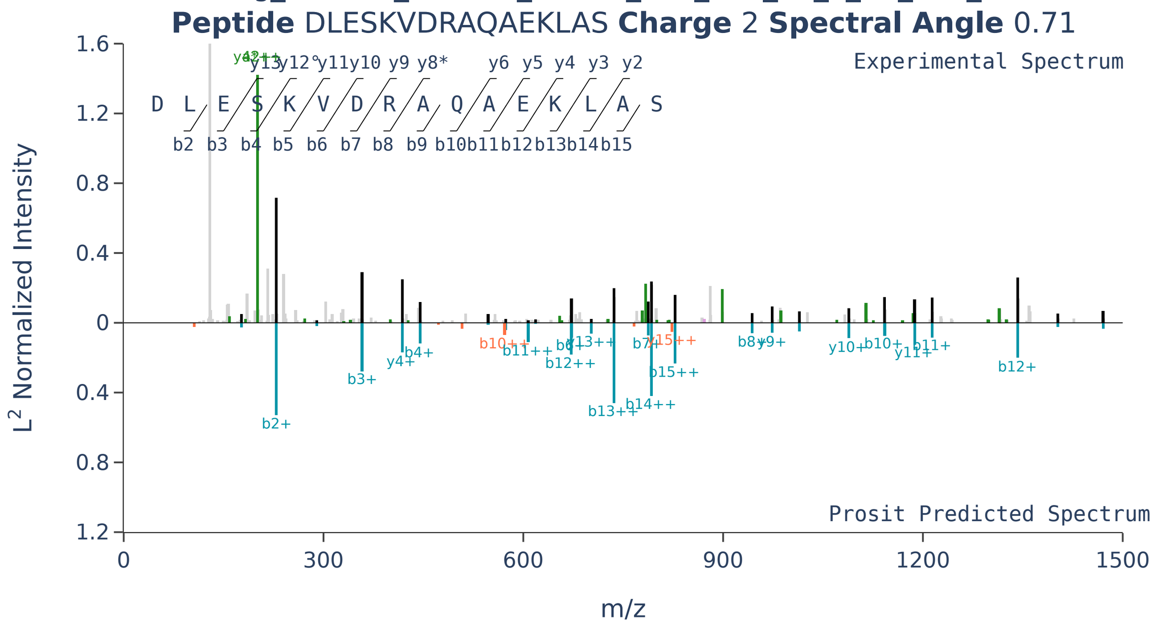


**Source** Ncheng_210623_230623_HFGoe_FFH_20S_25_1_A2_24h_R1 **Scan** 13751
**Peptide** KQFDDMQRMMKKMKKG **Charge** 3 **Spectral Angle** 0.91

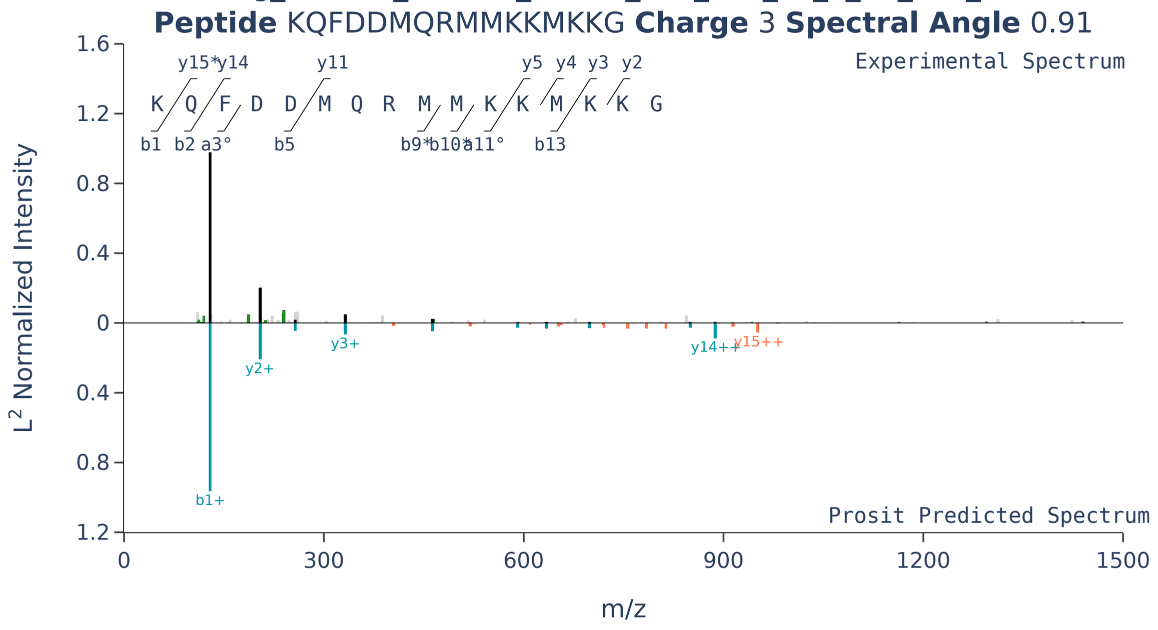


**Source** Ncheng_210623_230623_HFGoe_FFH_20S_25_1_A1_2h_R2 **Scan** 30282
**Peptide** PDNVKSQMDDKVLVRMEAII **Charge** 3 **Spectral Angle** 0.75

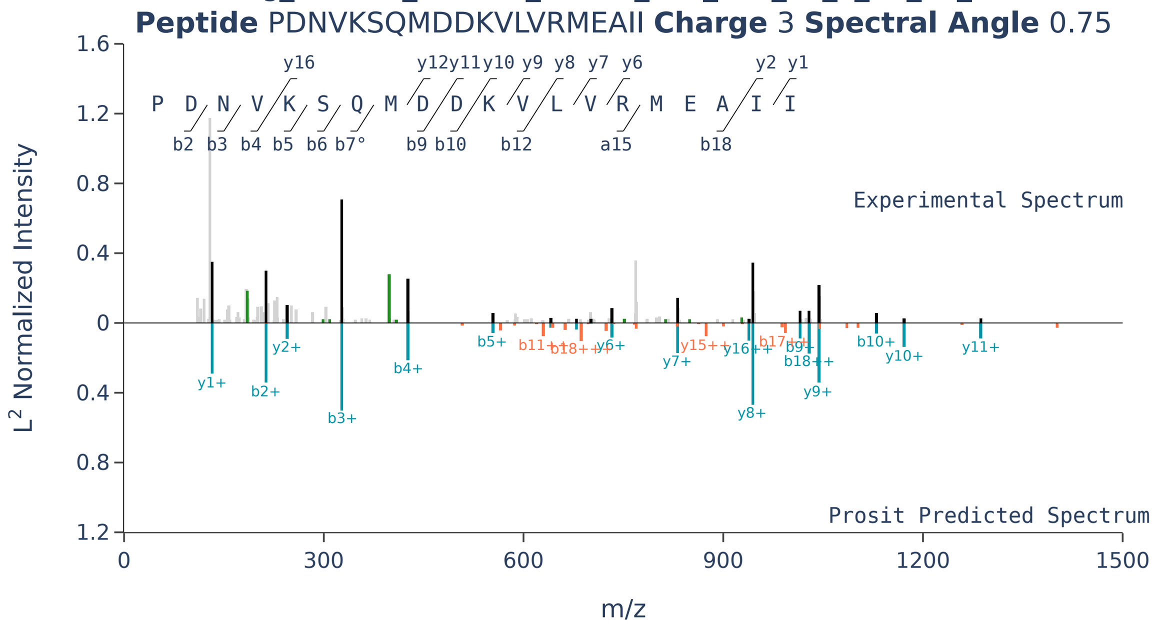


**Source** Ncheng_210623_230623_HFGoe_FFH_20S_25_1_A2_24h_R1 **Scan** 7575
**Peptide** RQMKNMGGMA **Charge** 2 **Spectral Angle** 0.72

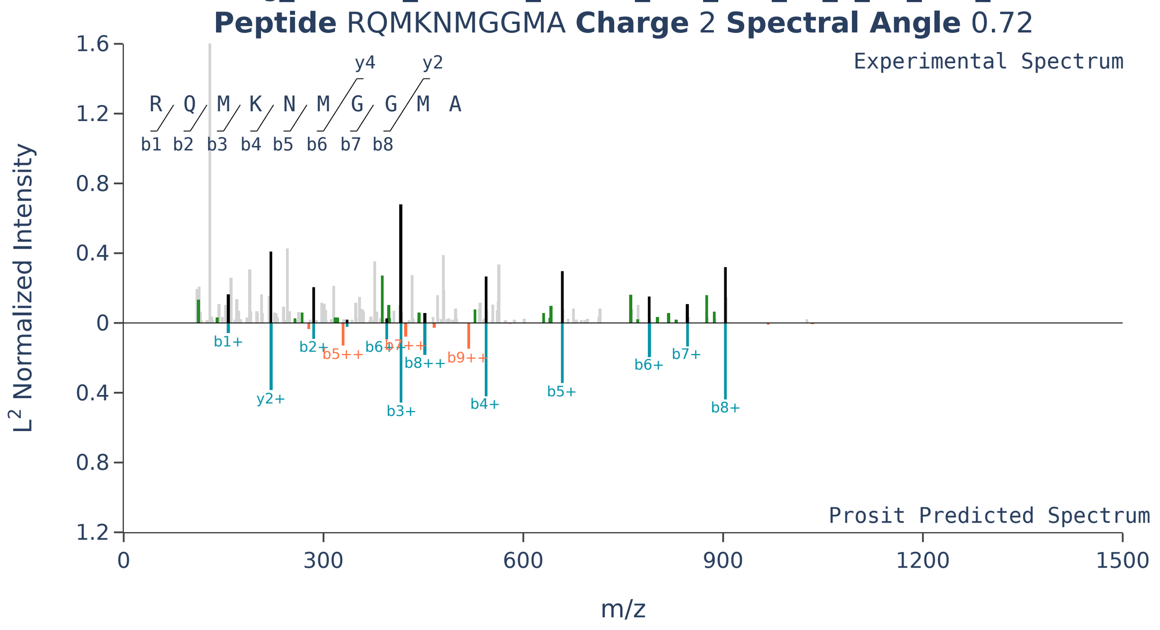


**Source** Ncheng_210623_230623_HFGoe_FFH_20S_25_1_A2_4h_R1 **Scan** 21363
**Peptide** PDRIASRILGMGD **Charge** 3 **Spectral Angle** 0.59

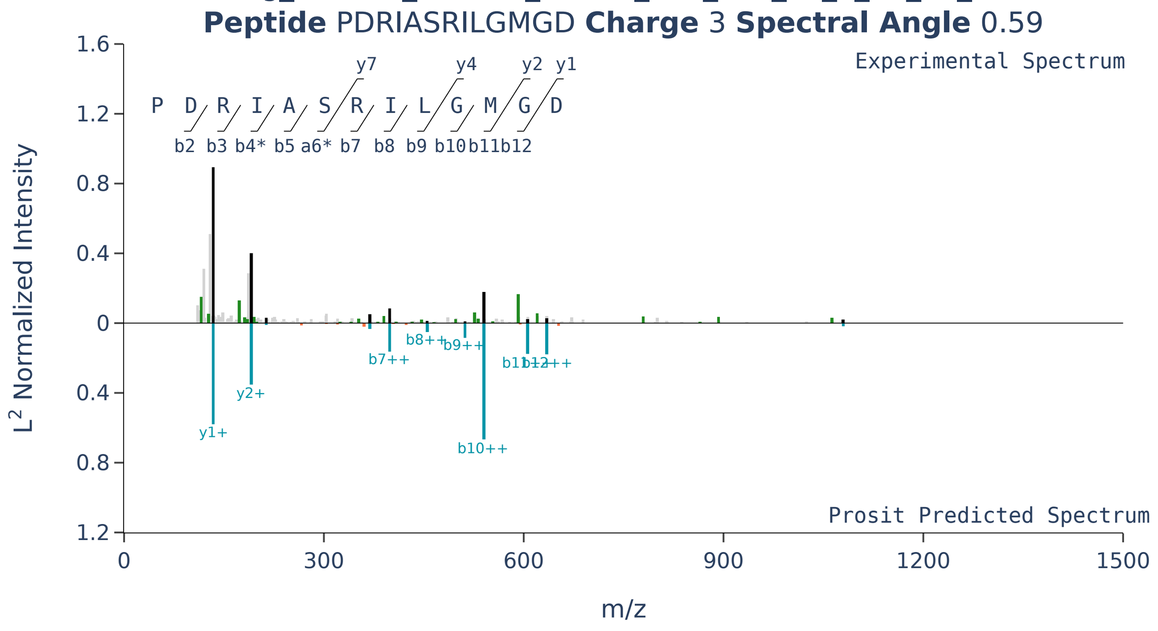


**Source** Ncheng_210623_230623_HFGoe_FFH_20S_25_1_A1_24h_R1 **Scan** 7806
**Peptide** VDRAQAEKL **Charge** 2 **Spectral Angle** 0.84

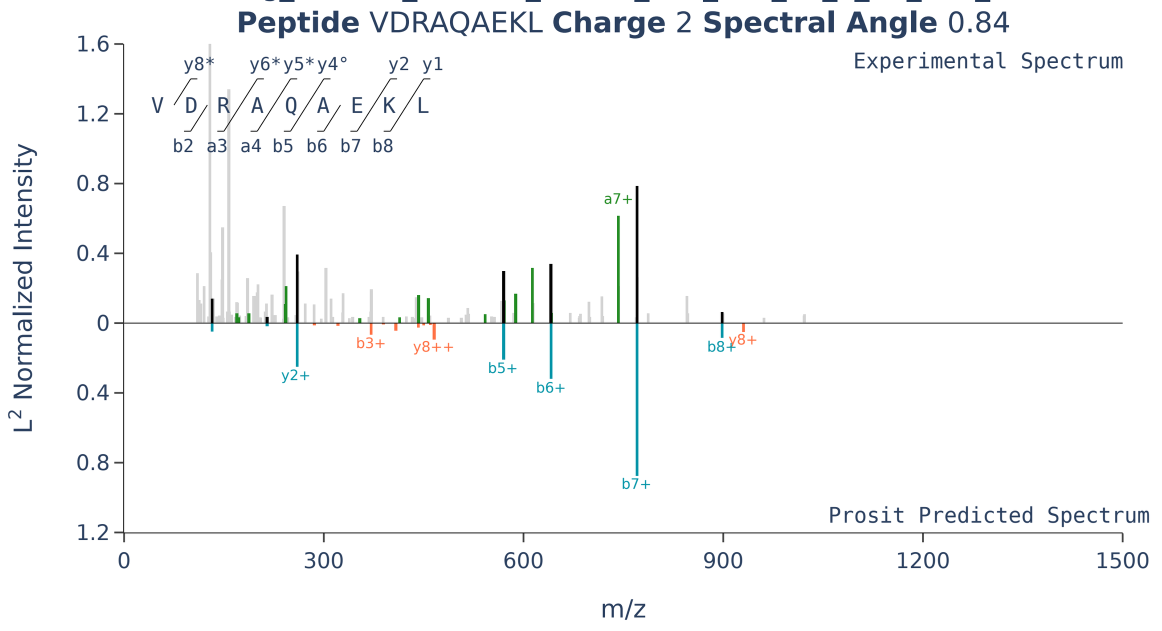


**Source** Ncheng_210623_230623_HFGoe_FFH_20S_25_1_A1_24h_R1 **Scan** 16538
**Peptide** FDNLTDRLSRT **Charge** 3 **Spectral Angle** 0.64

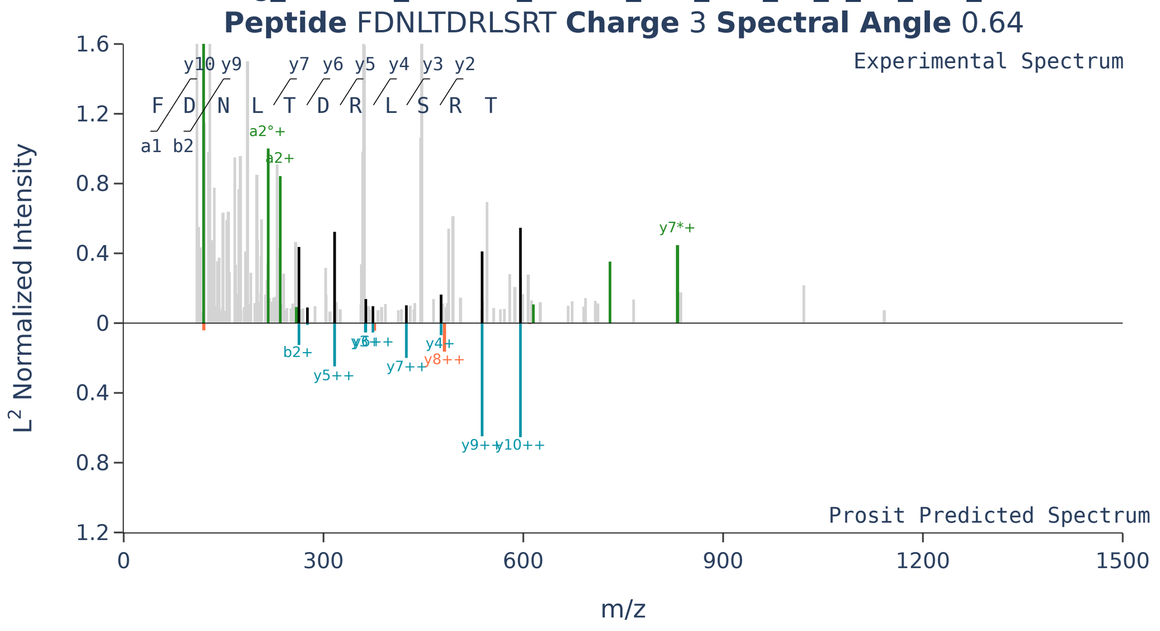


**Source** Ncheng_210623_230623_HFGoe_FFH_20S_25_1_A1_24h_R2 **Scan** 8695
**Peptide** DLESKVDRAQAE **Charge** 1 **Spectral Angle** 0.72

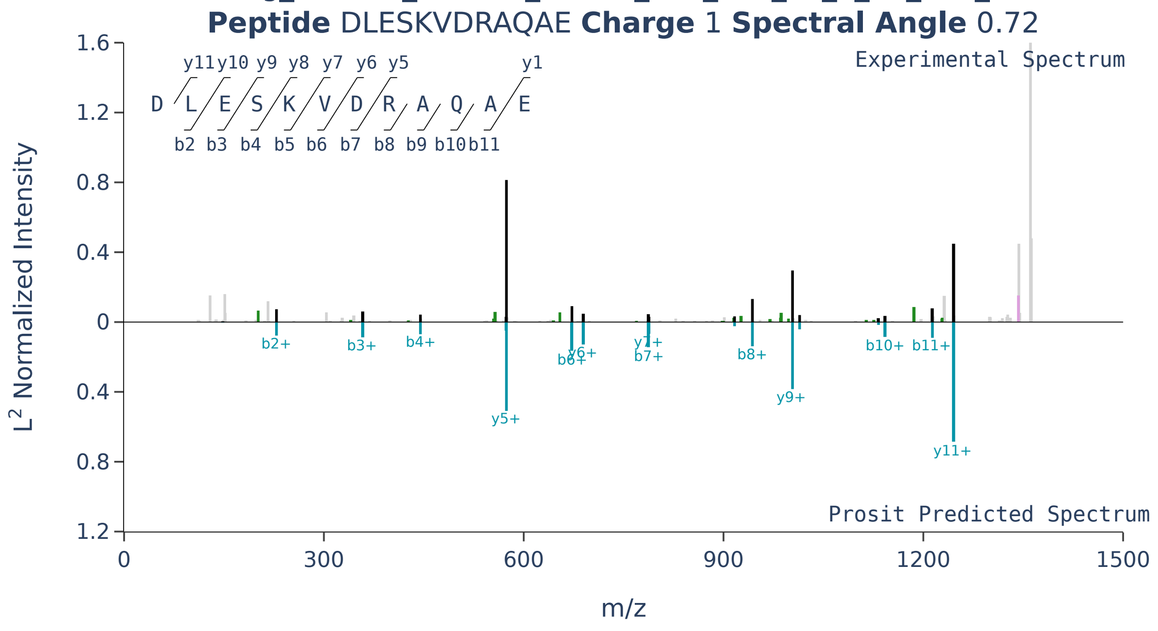


**Source** Ncheng_210623_230623_HFGoe_FFH_20S_25_1_A1_2h_R2 **Scan** 32276
**Peptide** FNEALPLTGVVLTKVDGDARGGAALSIRHL **Charge** 4 **Spectral Angle** 0.71

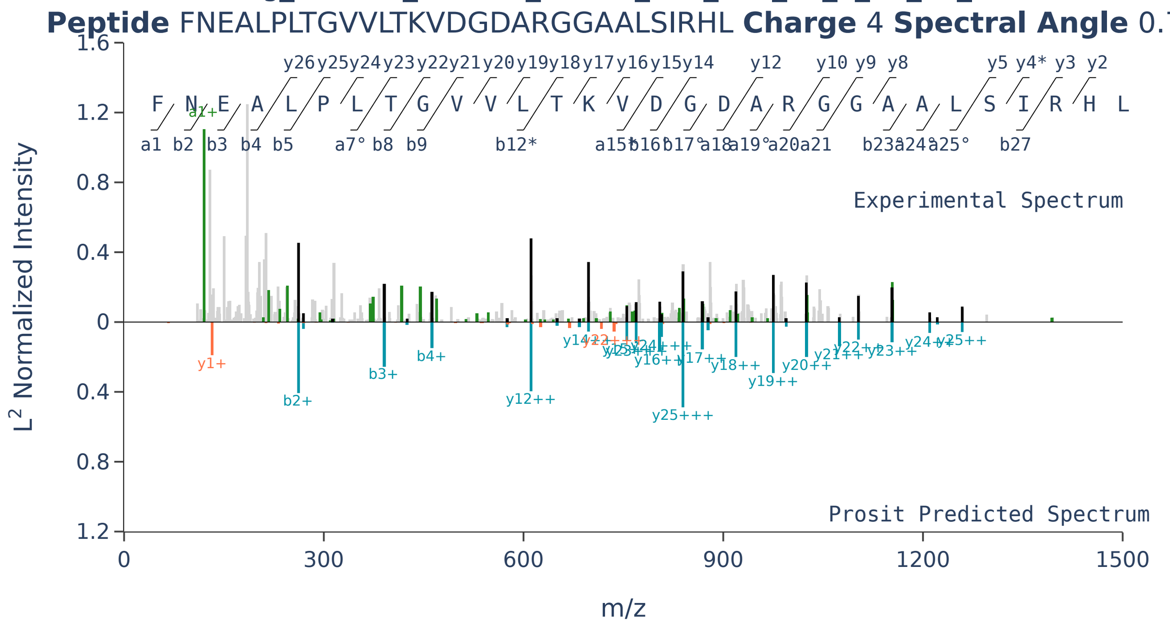


**Source** Ncheng_210623_230623_HFGoe_FFH_20S_25_1_A1_24h_R1 **Scan** 26286
**Peptide** LEADVALPVVRE **Charge** 2 **Spectral Angle** 0.81

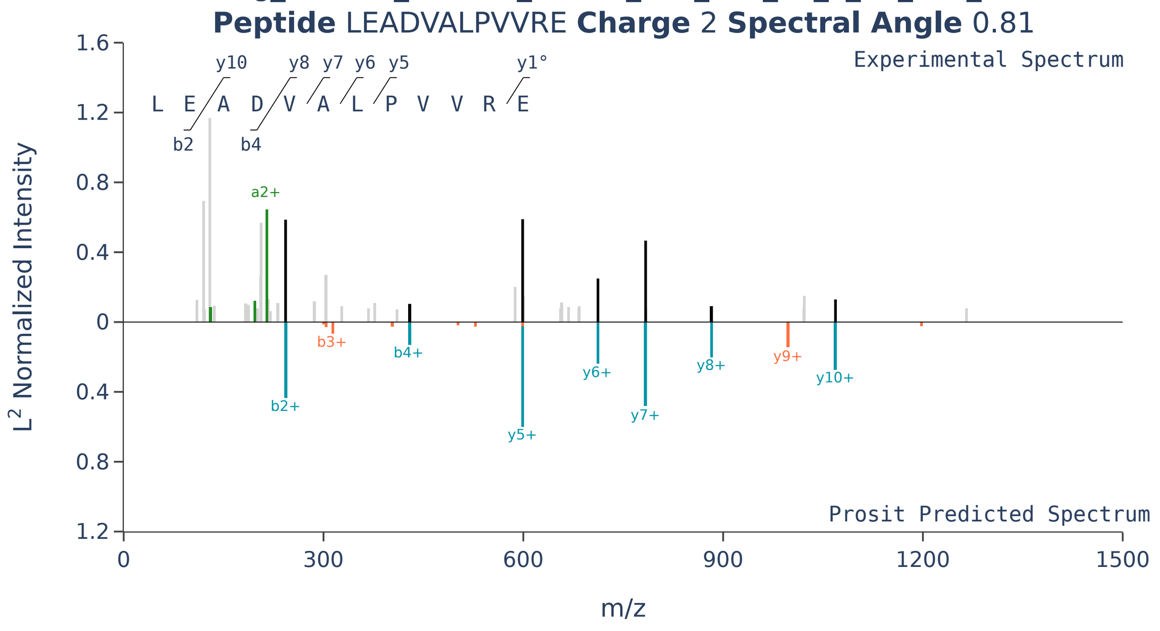


**Source** Ncheng_210623_230623_HFGoe_FFH_20S_25_1_A2_4h_R1 **Scan** 25468
**Peptide** PGMGQIPDNVKSQMDDKVLVRM **Charge** 3 **Spectral Angle** 0.66

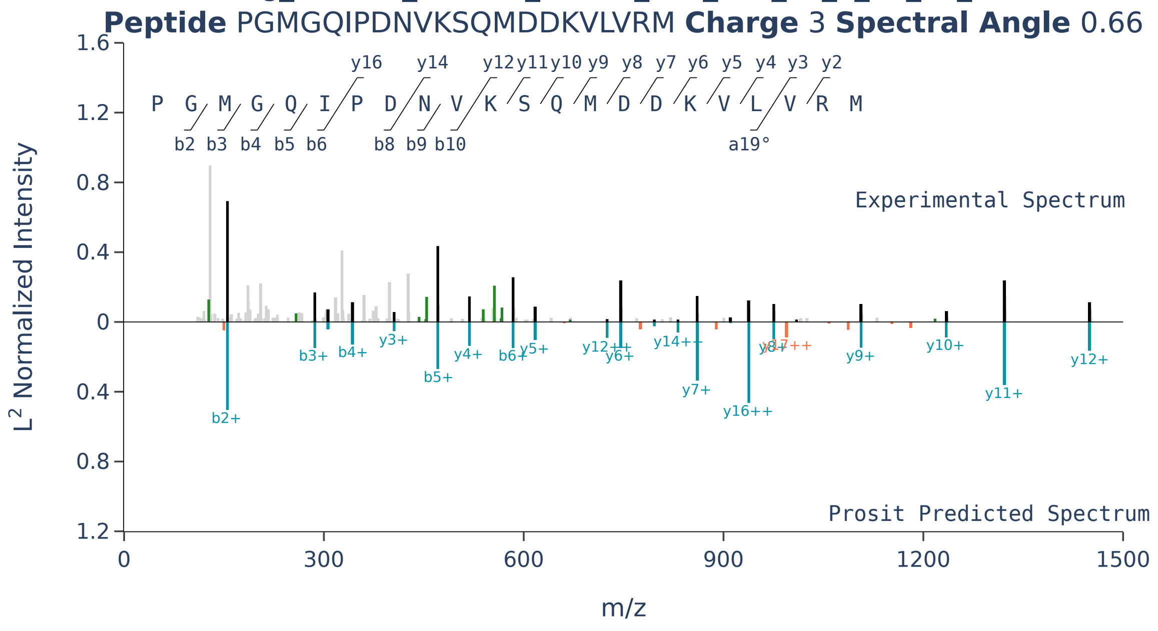


**Source** Ncheng_210623_230623_HFGoe_FFH_20S_25_1_A2_4h_R1 **Scan** 15010
**Peptide** LKEAKLKFYD **Charge** 3 **Spectral Angle** 0.7

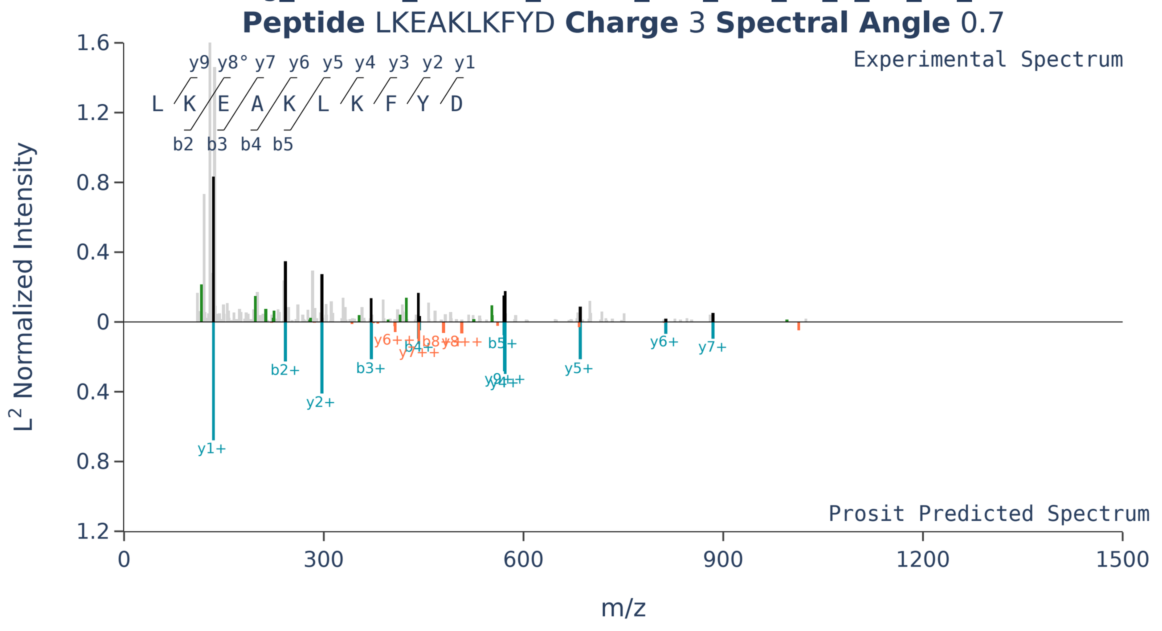


**Source** Ncheng_210623_230623_HFGoe_FFH_20S_25_1_A1_2h_R2 **Scan** 26320
**Peptide** MGKLPGMGQIPDNVKSQMDDKVLVRME **Charge** 3 **Spectral Angle** 0.67

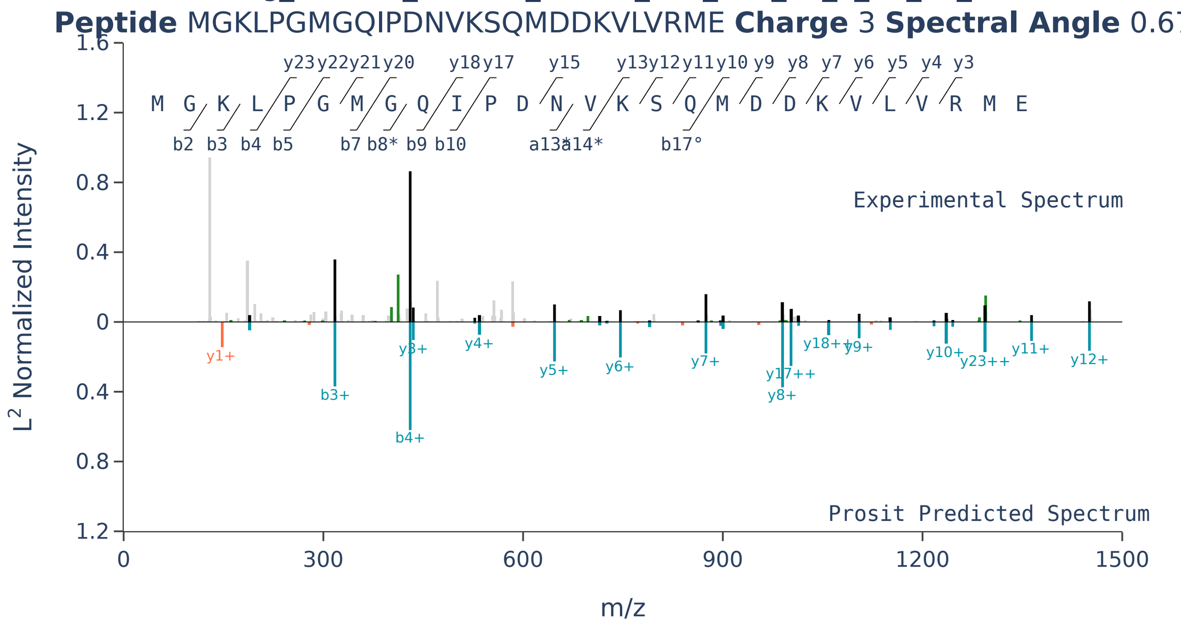

**Source** Ncheng_210623_230623_HFGoe_FFH_20S_25_1_A2_2h_R2 **Scan** 30488
**Peptide** KLKFYDVLLVDT **Charge** 3 **Spectral Angle** 0.67

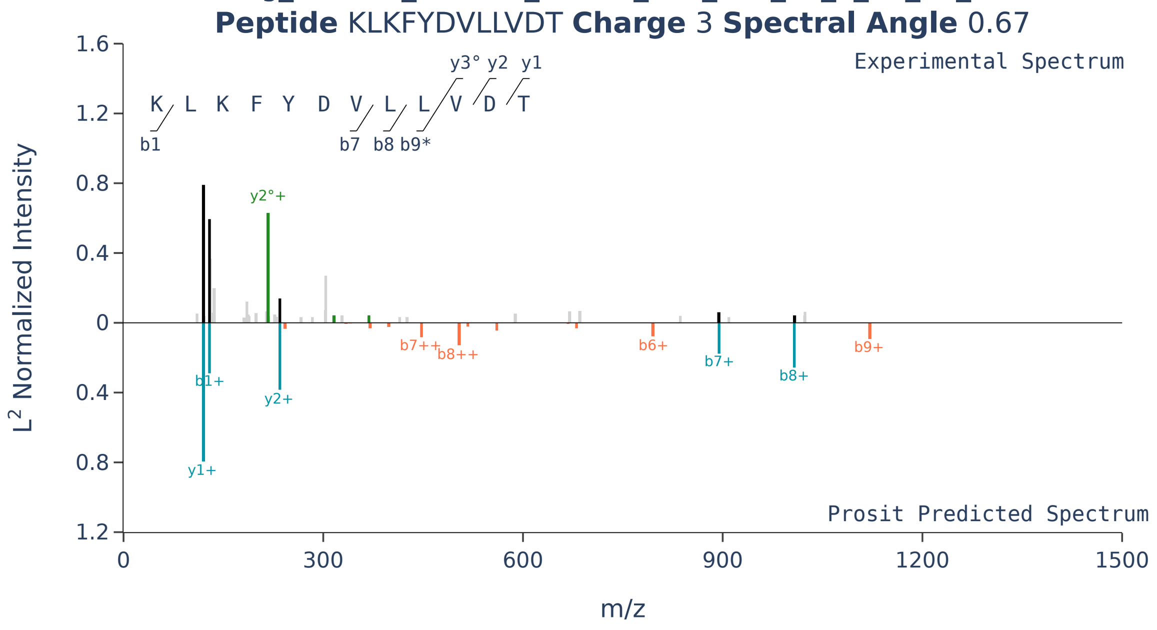


**Source** Ncheng_210623_230623_HFGoe_FFH_20S_25_1_A1_2h_R1 **Scan** 20539
**Peptide** KKVLVVSADVYRPAAI **Charge** 2 **Spectral Angle** 0.49

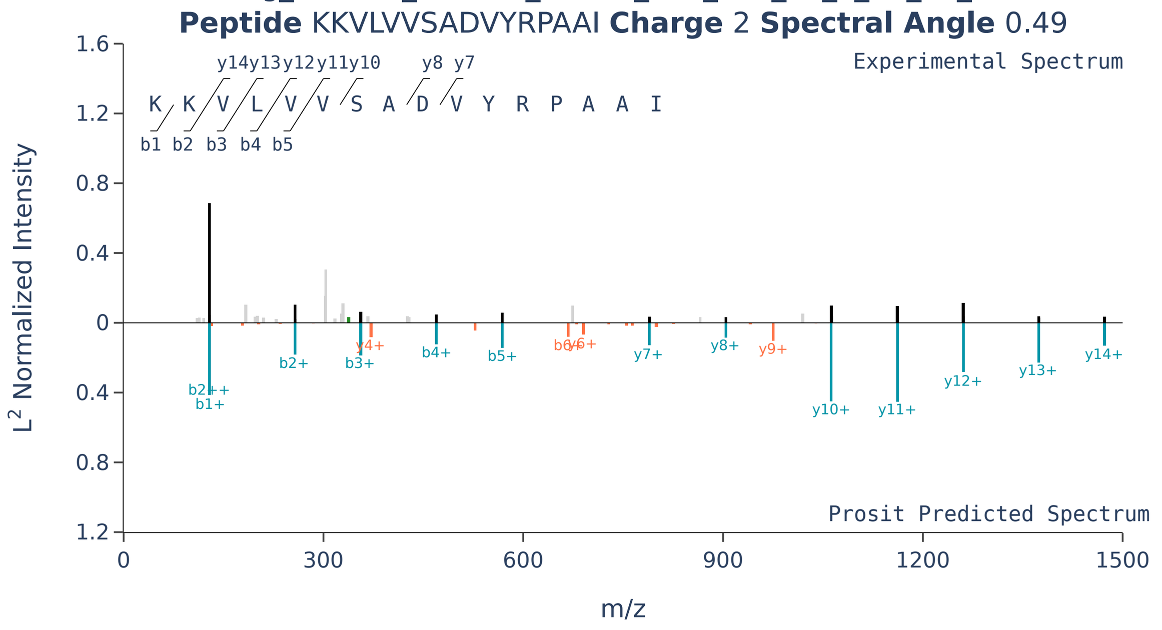


**Source** Ncheng_210623_230623_HFGoe_FFH_20S_25_1_A1_1h_R1 **Scan** 18124
**Peptide** LTKVDGDARGGAALSIRHL **Charge** 4 **Spectral Angle** 0.71

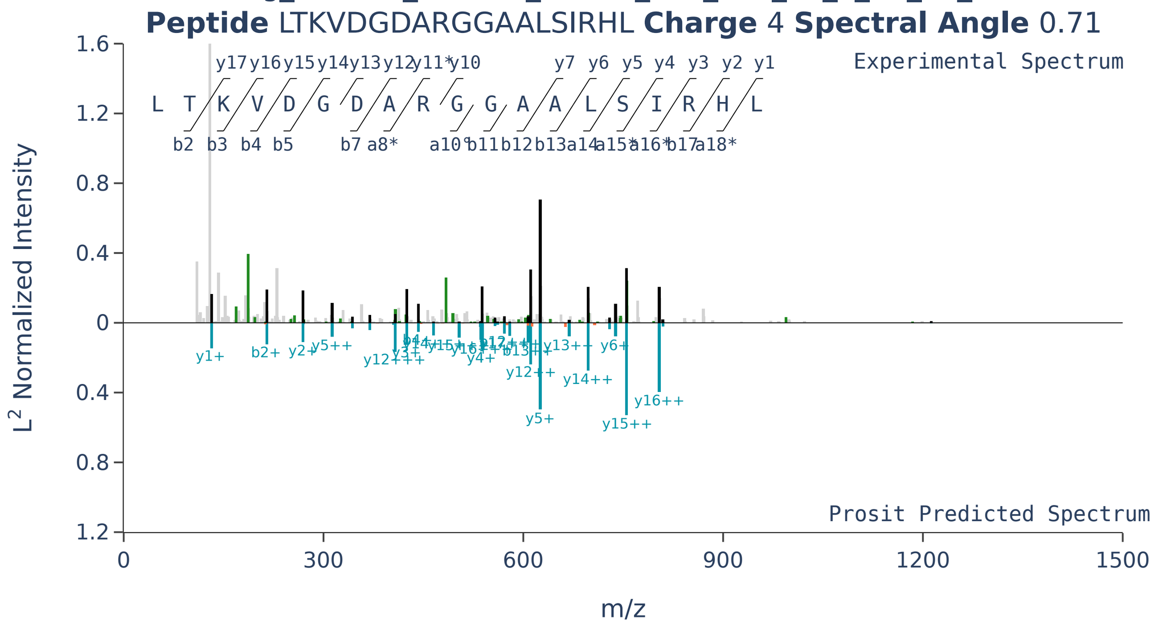


**Source** Ncheng_210623_230623_HFGoe_FFH_20S_25_1_A1_24h_R1 **Scan** 18073
**Peptide** LNRVKEKAVGHEVNKSLTPGQEFVKIVRNE **Charge** 5 **Spectral Angle** 0.72

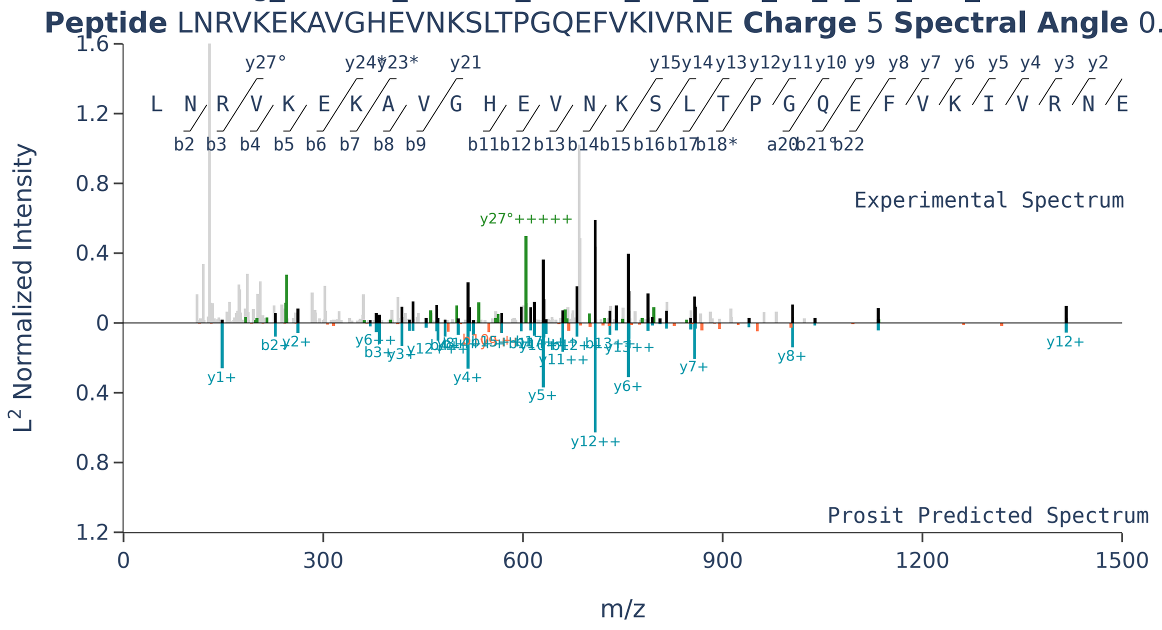


**Source** Ncheng_210623_230623_HFGoe_FFH_20S_25_1_A1_2h_R2 **Scan** 20479
**Peptide** VGHEVNKSLTPGQEFVKI **Charge** 4 **Spectral Angle** 0.74

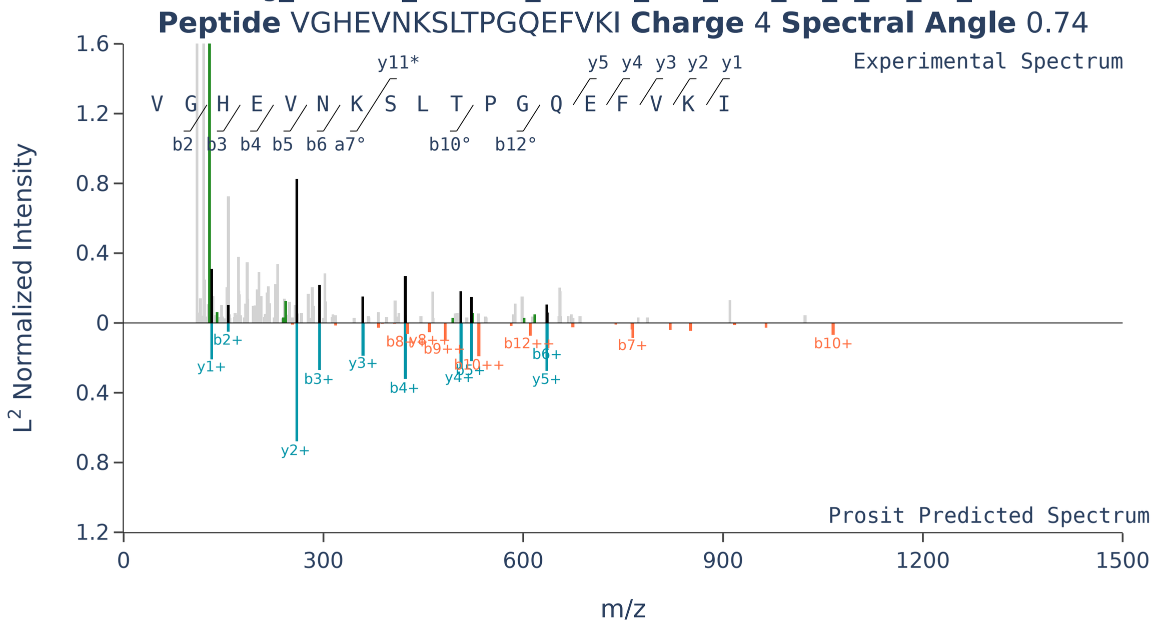


**Source** Ncheng_210623_230623_HFGoe_FFH_20S_25_1_A2_4h_R1 **Scan** 17644
**Peptide** VVLTKVDGDARGGAALSIR **Charge** 2 **Spectral Angle** 0.62

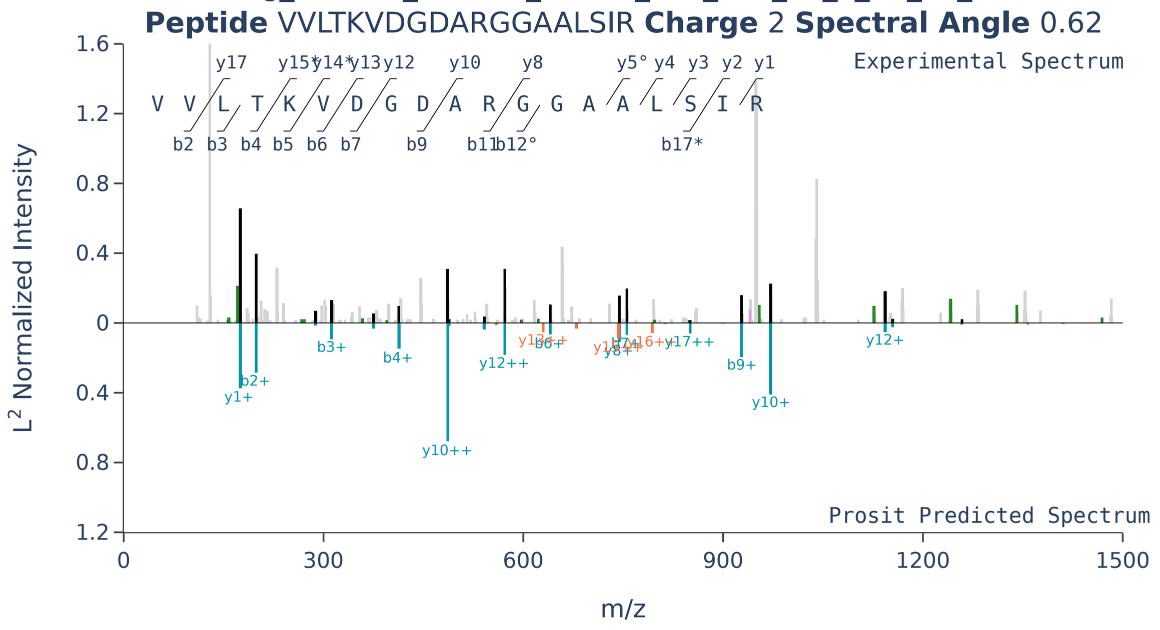


**Source** Ncheng_210623_230623_HFGoe_FFH_20S_25_1_A1_2h_R1 **Scan** 28577
**Peptide** KLKFYDVLLVDTAGRLH **Charge** 3 **Spectral Angle** 0.64

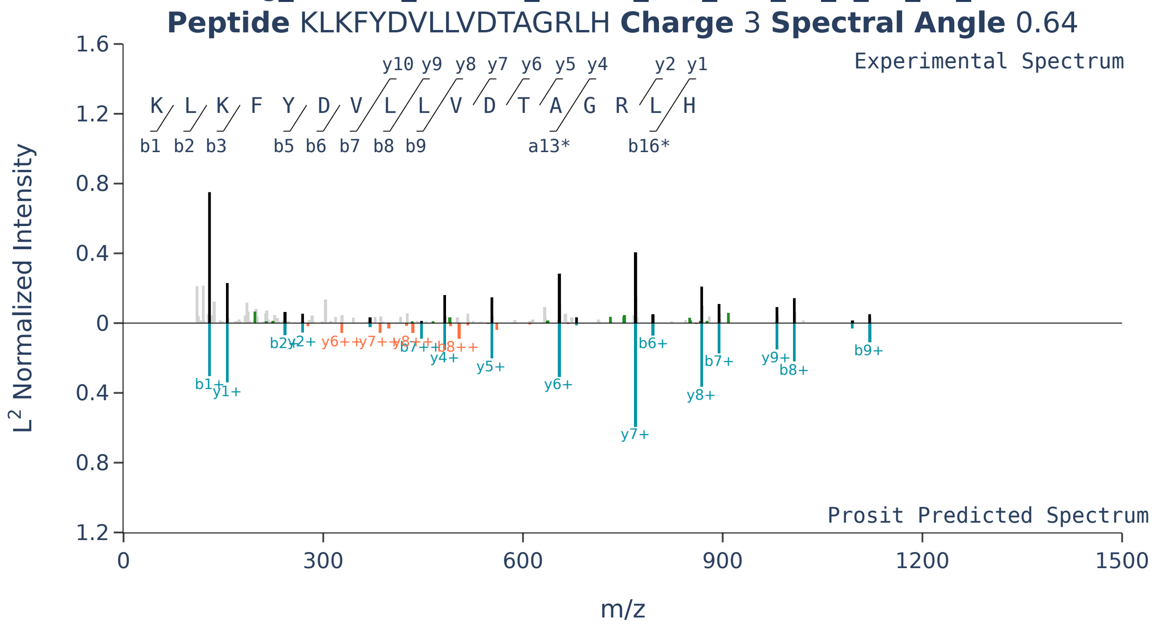


**Source** Ncheng_210623_230623_HFGoe_FFH_20S_25_1_A1_4h_R2 **Scan** 9205
**Peptide** VNAALKEAKLK **Charge** 3 **Spectral Angle** 0.71

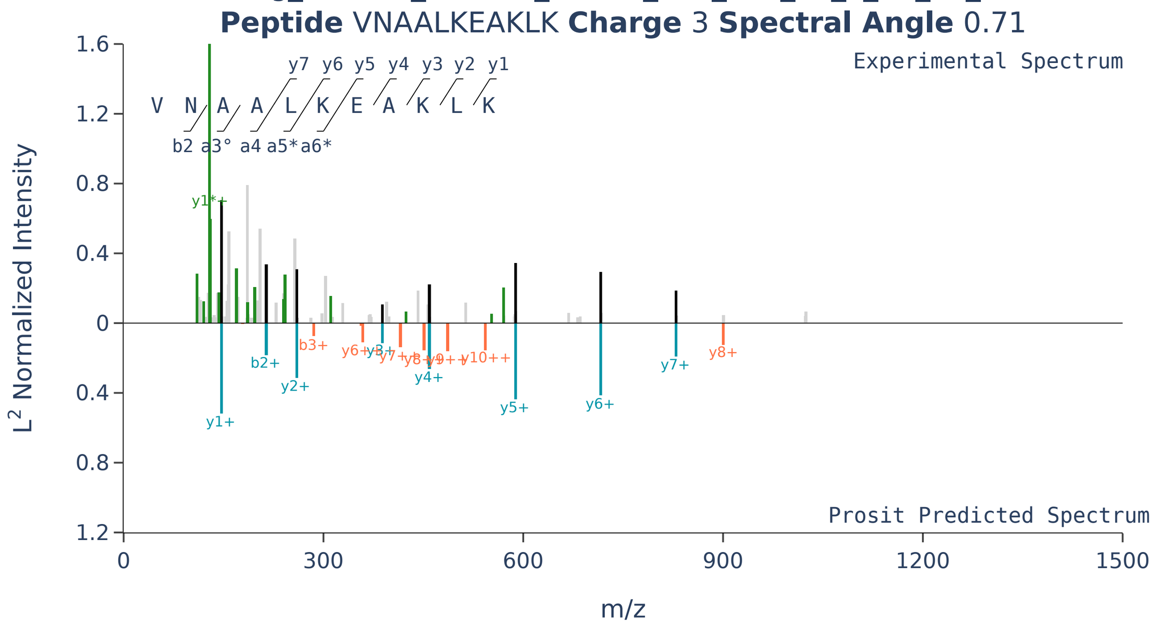


**Source** Ncheng_210623_230623_HFGoe_FFH_20S_25_1_A1_24h_R2 **Scan** 7296
**Peptide** AQAEKLASKLKK **Charge** 3 **Spectral Angle** 0.7

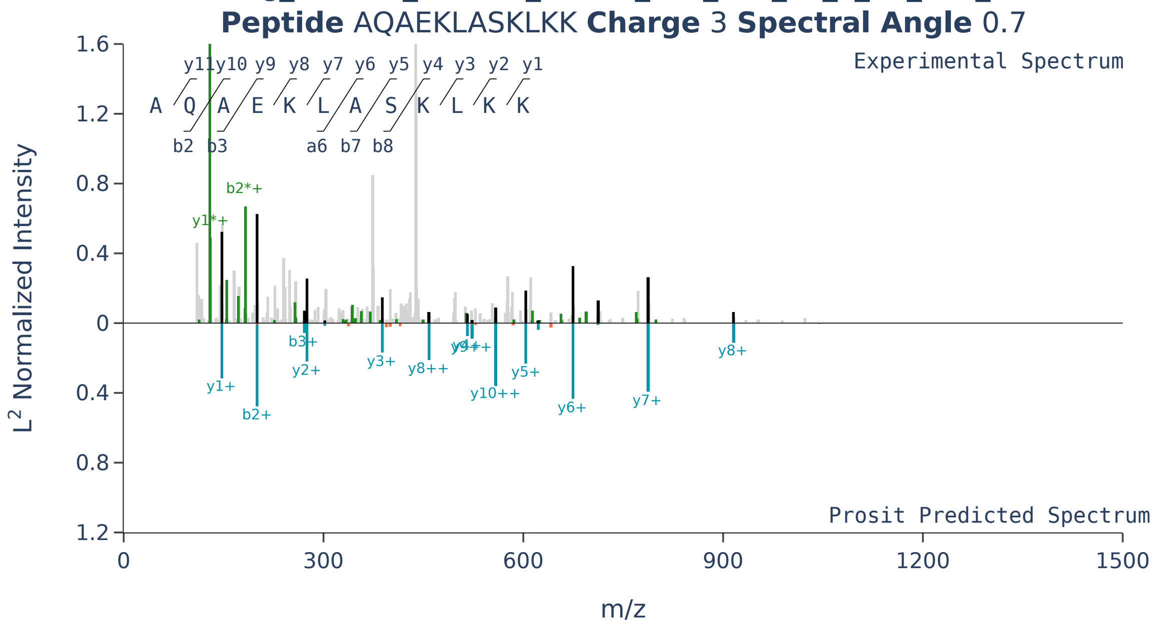


**Source** Ncheng_210623_230623_HFGoe_FFH_20S_25_1_A1_2h_R1 **Scan** 19005
**Peptide** TAGRLHVDEAMMDEIKQ **Charge** 4 **Spectral Angle** 0.53

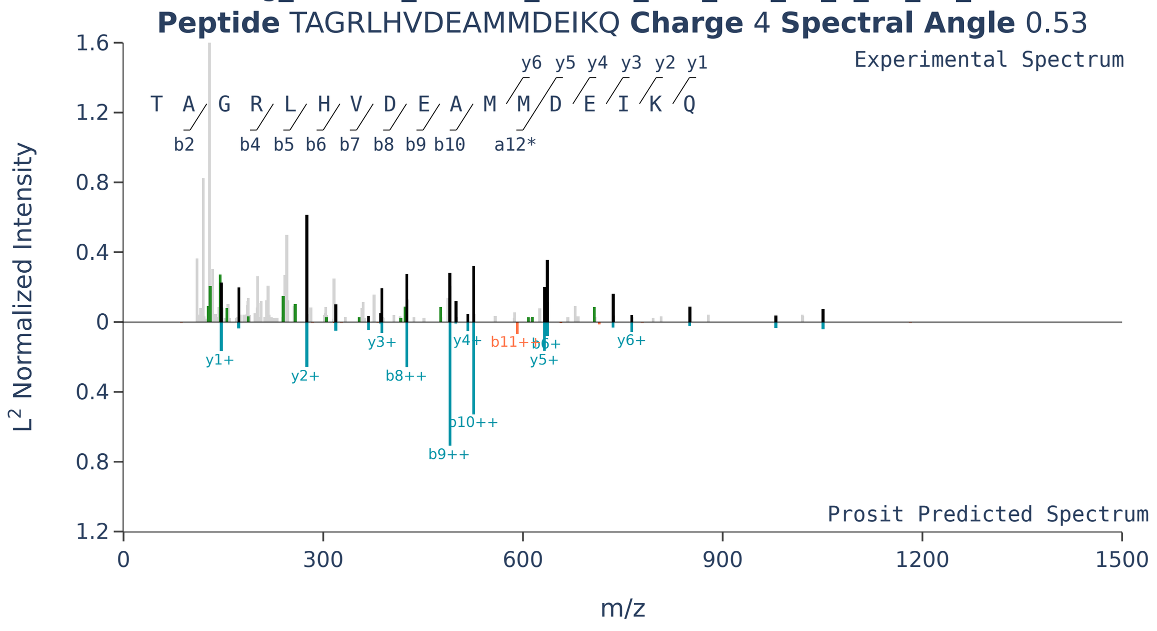


**Source** Ncheng_210623_230623_HFGoe_FFH_20S_25_1_A2_4h_R1 **Scan** 30891
**Peptide** GRLTEDNVKDTLREVRMALL **Charge** 3 **Spectral Angle** 0.78

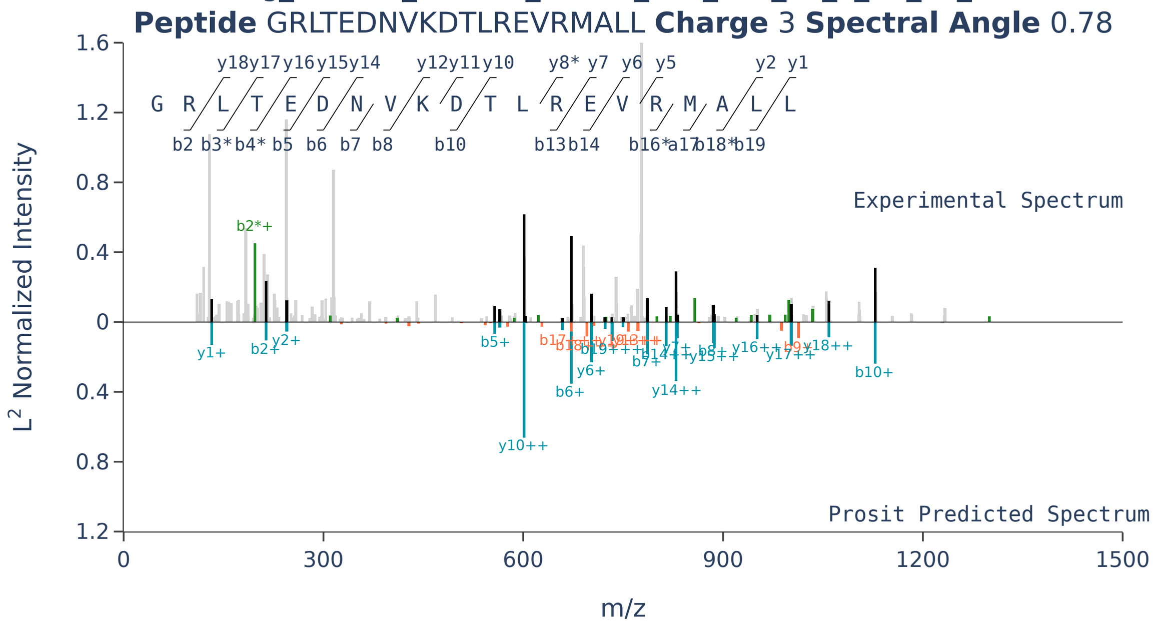


**Source** Ncheng_210623_230623_HFGoe_FFH_20S_25_1_A1_2h_R2 **Scan** 17741
**Peptide** LIEDIESKVDRAQAE **Charge** 2 **Spectral Angle** 0.78

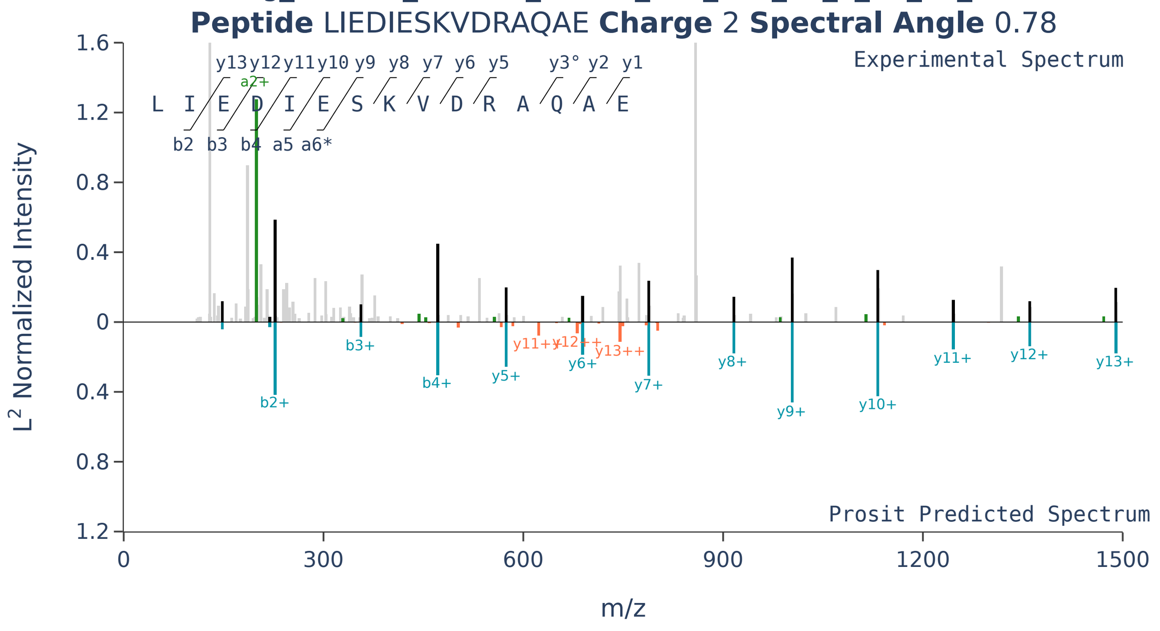


**Source** Ncheng_210623_230623_HFGoe_FFH_20S_25_1_A2_4h_R1 **Scan** 32709
**Peptide** MDDKVLVRMEAII **Charge** 2 **Spectral Angle** 0.76

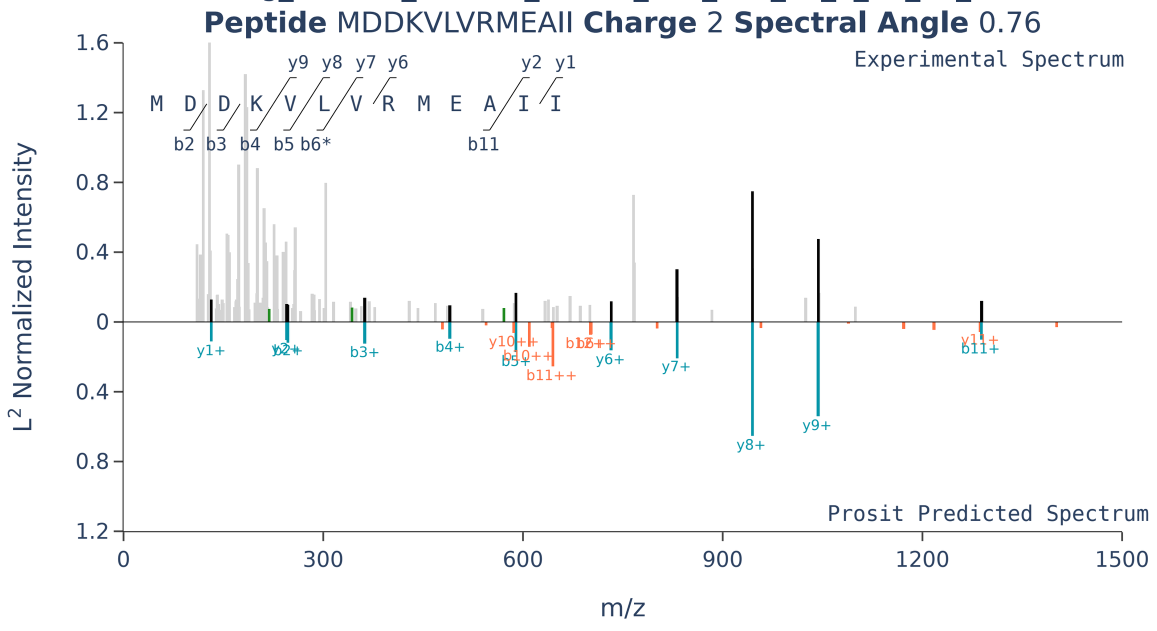


**Source** Ncheng_210623_230623_HFGoe_FFH_20S_25_1_A2_4h_R1 **Scan** 26297
**Peptide** GVGEKTEALEPFHPDRIASRLL **Charge** 4 **Spectral Angle** 0.69

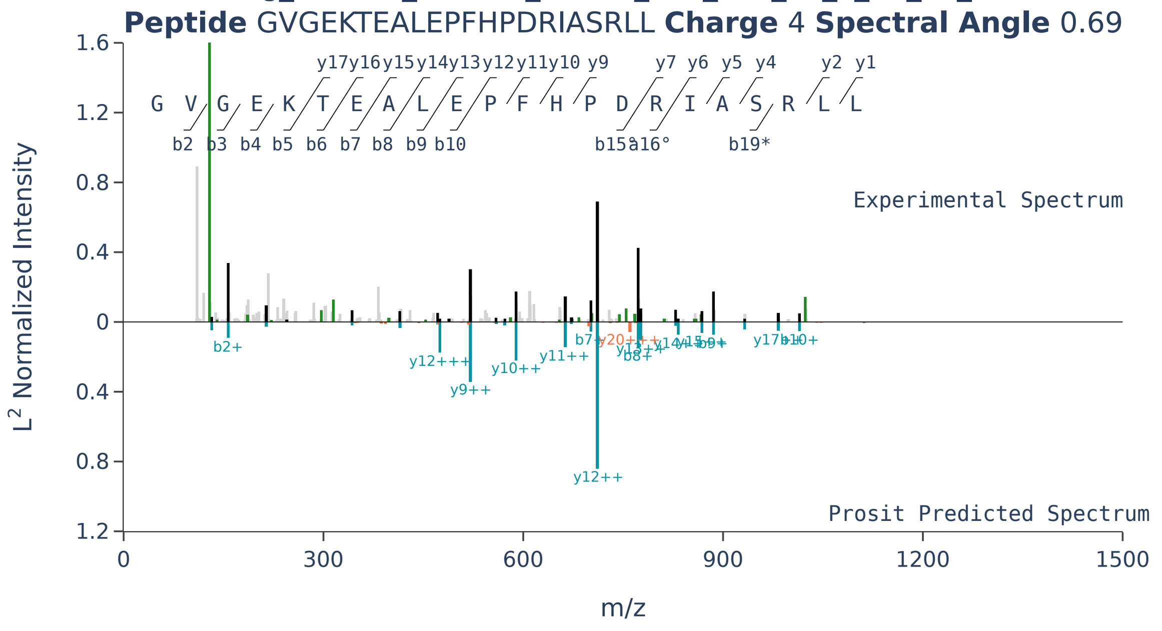


**Source** Ncheng_210623_230623_HFGoe_FFH_20S_25_1_A1_2h_R2 **Scan** 20574
**Peptide** LVNAALKEAKLKFYD **Charge** 2 **Spectral Angle** 0.75

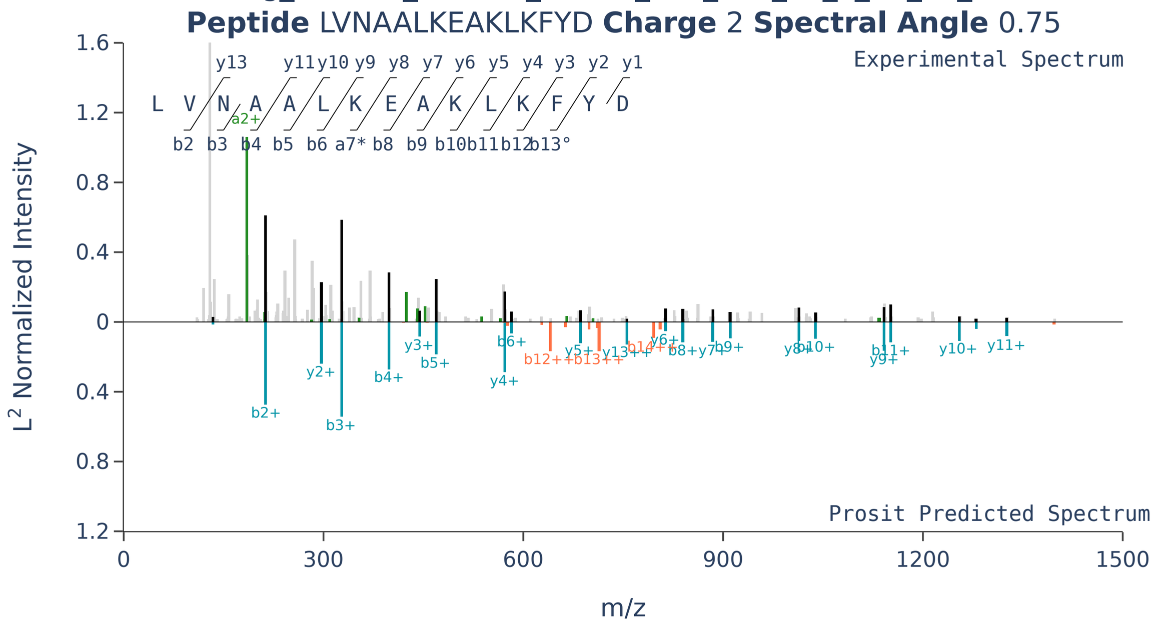

**Source** Ncheng_210623_230623_HFGoe_FFH_20S_25_1_A2_24h_R2 **Scan** 13811
**Peptide** EKLASKLKKGDGFDLND **Charge** 2 **Spectral Angle** 0.74

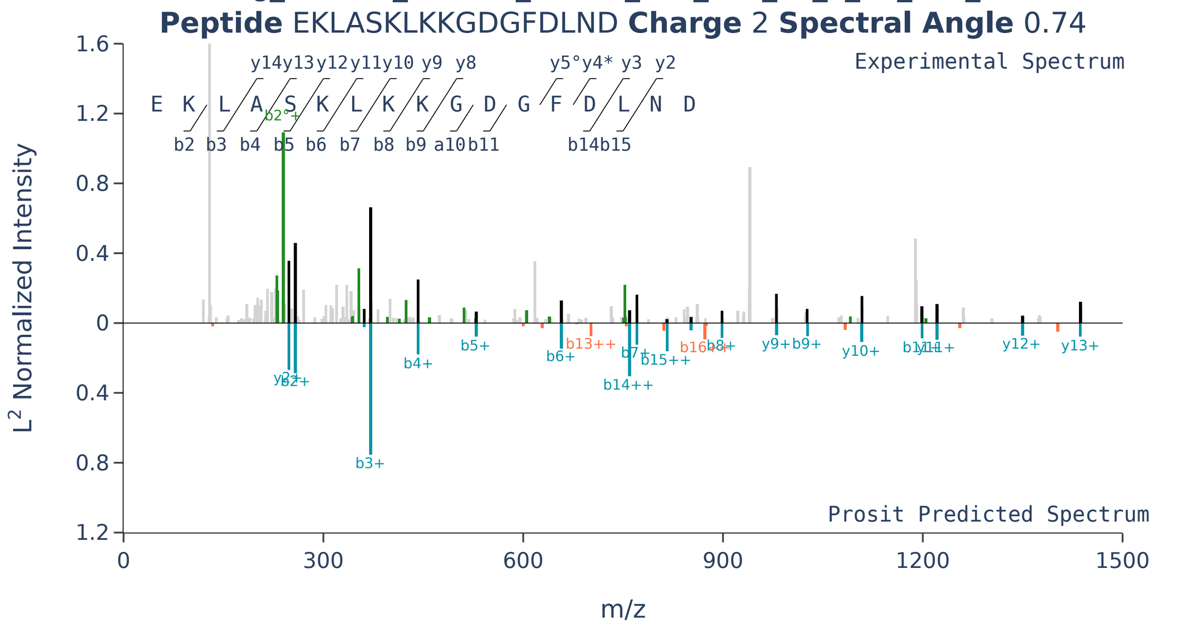


**Source** Ncheng_210623_230623_HFGoe_FFH_20S_25_1_A1_2h_R1 **Scan** 5107
**Peptide** KLASKLKK **Charge** 2 **Spectral Angle** 0.84

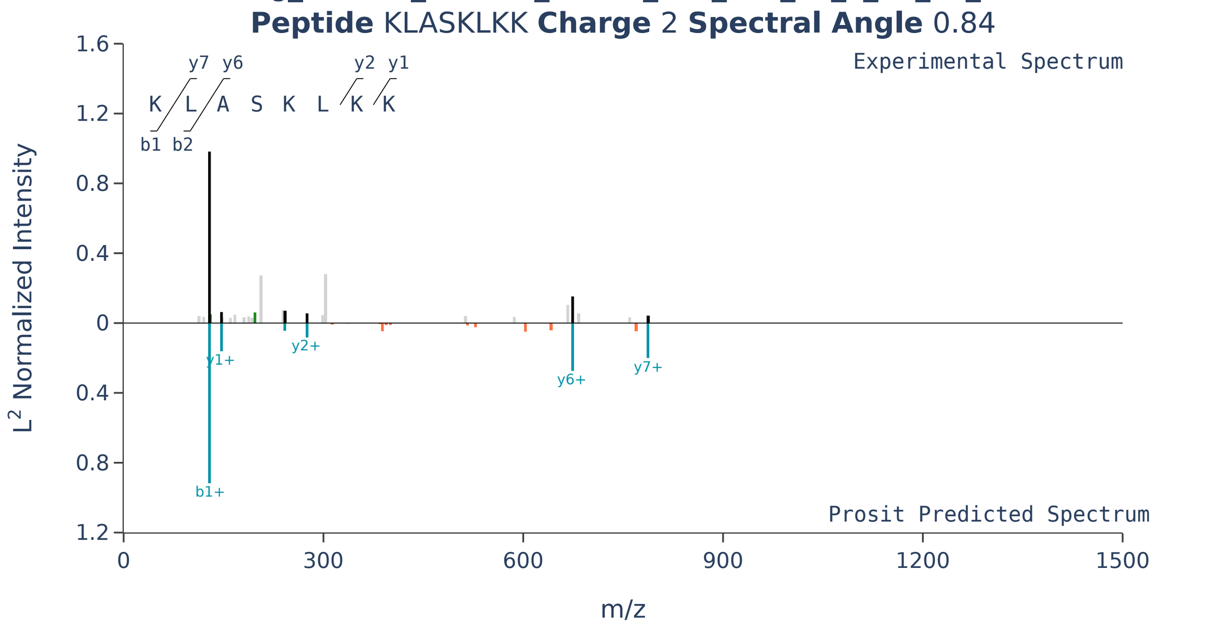


**Source** Ncheng_210623_230623_HFGoe_FFH_20S_25_1_A2_4h_R1 **Scan** 38305
**Peptide** DAMTGQDAANTAKAFNEALPLTGVVLTKV **Charge** 3 **Spectral Angle** 0.79

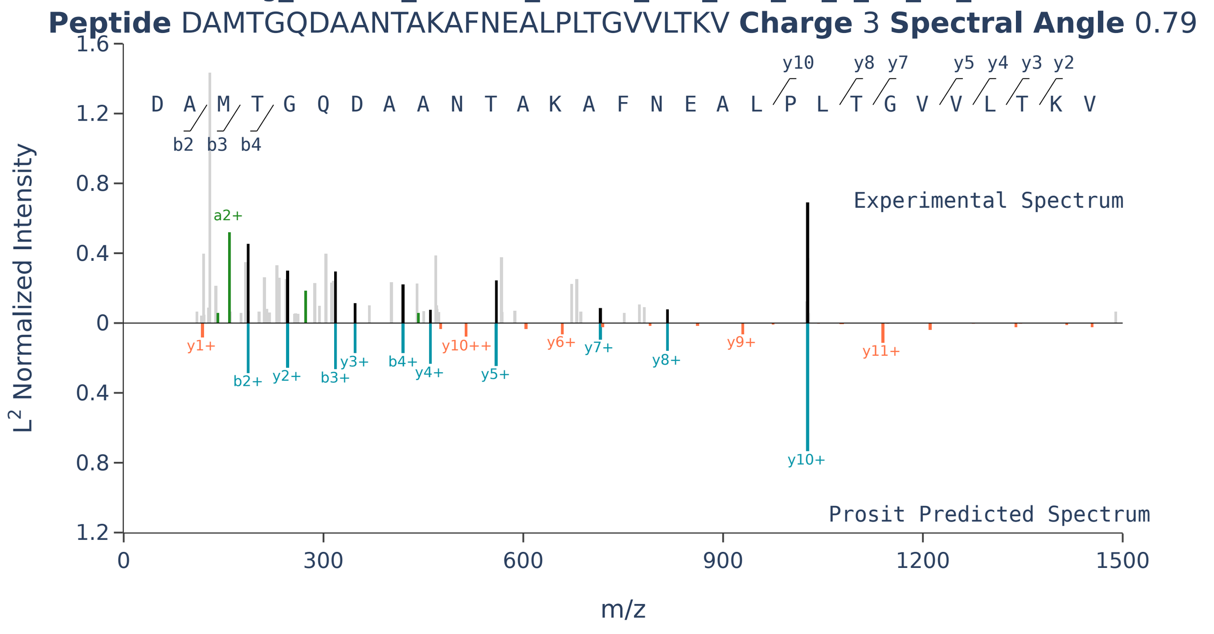


**Source** Ncheng_210623_230623_HFGoe_FFH_20S_25_1_A2_24h_R2 **Scan** 8261
**Peptide** EDNVKDTLR **Charge** 2 **Spectral Angle** 0.77

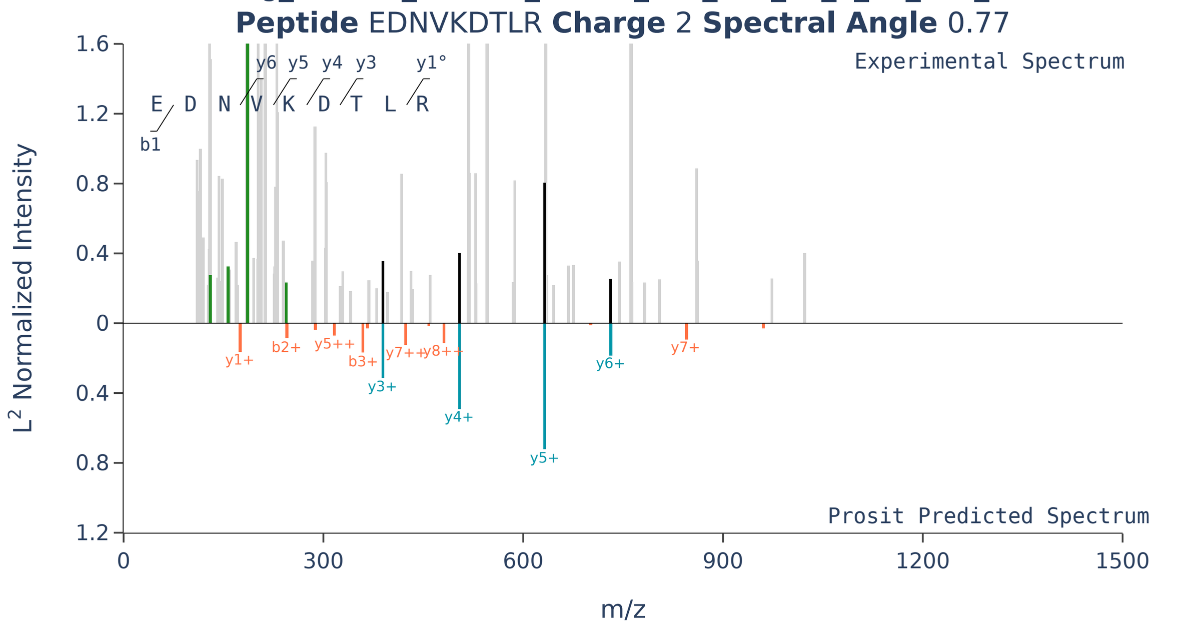


**Source** Ncheng_210623_230623_HFGoe_FFH_20S_25_1_A2_4h_R1 **Scan** 28416
**Peptide** DVGQKPVDIVNAALKEA **Charge** 3 **Spectral Angle** 0.75

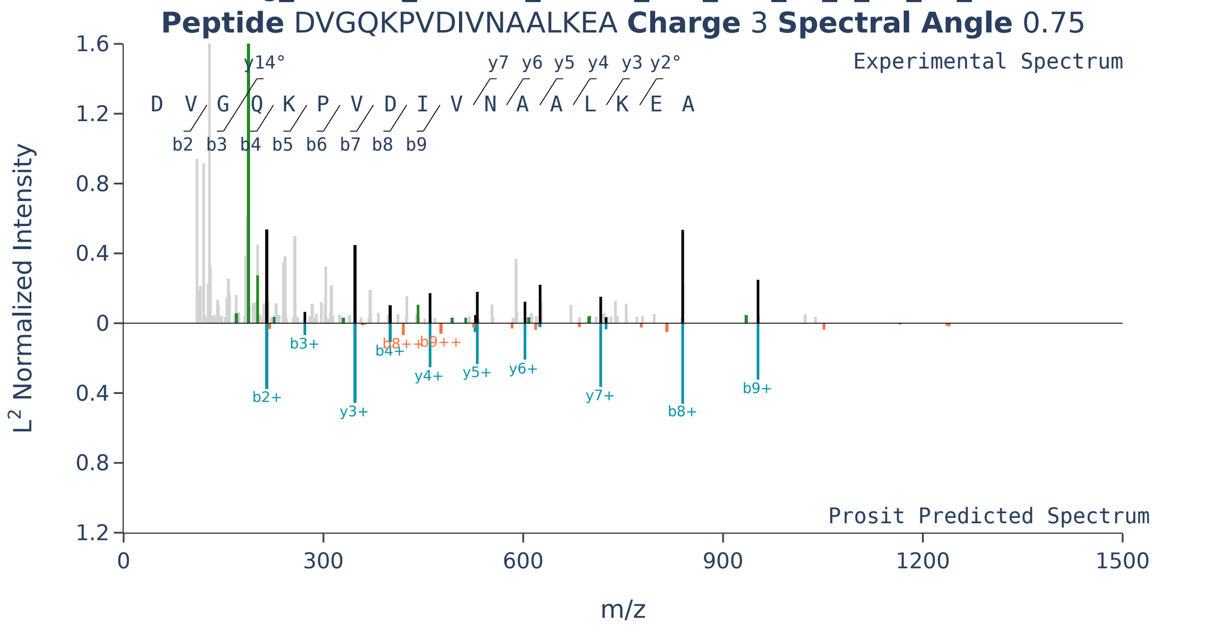


**Source** Ncheng_210623_230623_HFGoe_FFH_20S_25_1_A1_1h_R2 **Scan** 18261
**Peptide** QMDDKVLVRMEA **Charge** 3 **Spectral Angle** 0.9

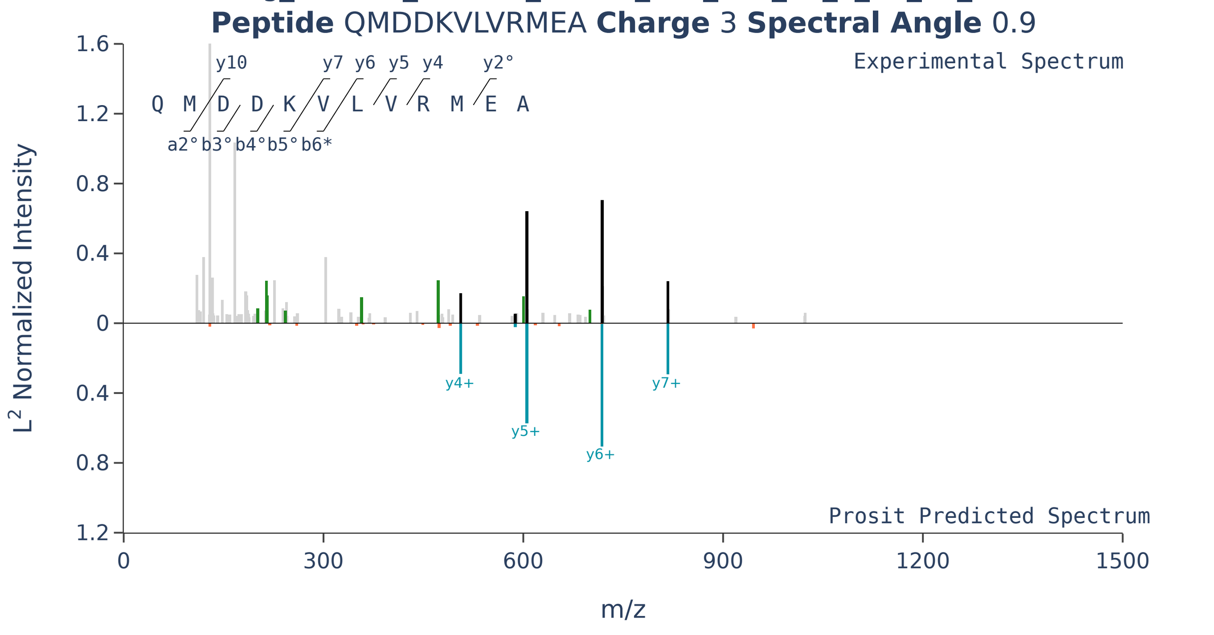


**Source** Ncheng_210623_230623_HFGoe_FFH_20S_25_1_A1_24h_R2 **Scan** 10275
**Peptide** DRAQAEKLASKLK **Charge** 4 **Spectral Angle** 0.75

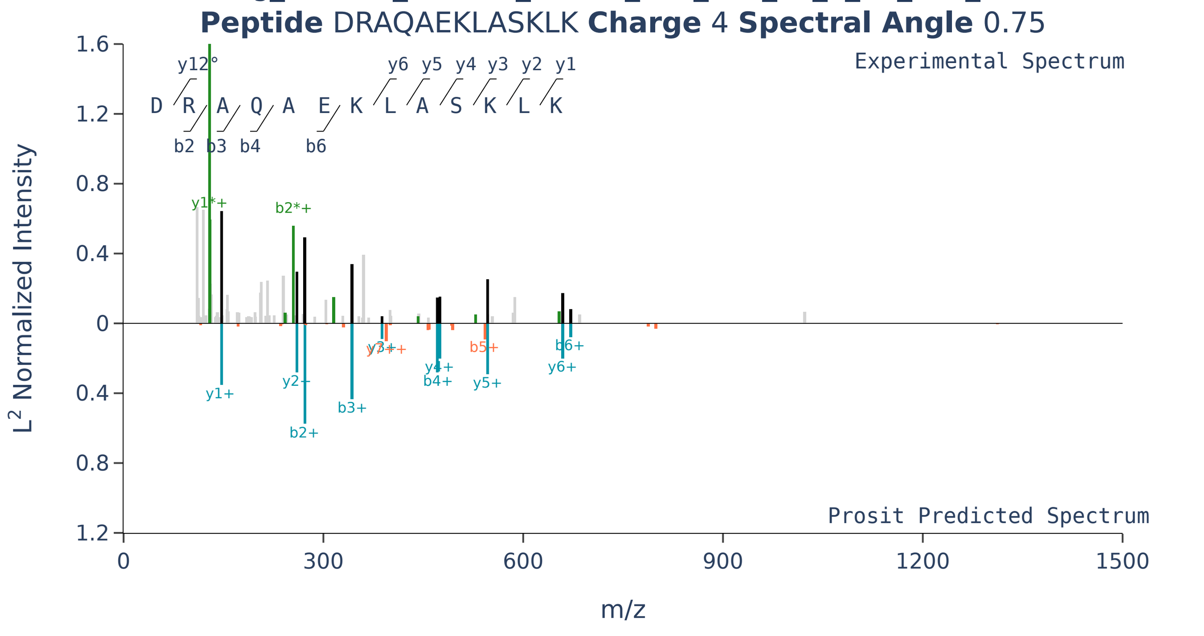


**Source** Ncheng_210623_230623_HFGoe_FFH_20S_25_1_A1_1h_R1 **Scan** 32302
**Peptide** ETLAEQVGVDFFPSDVGQKPVD **Charge** 2 **Spectral Angle** 0.79

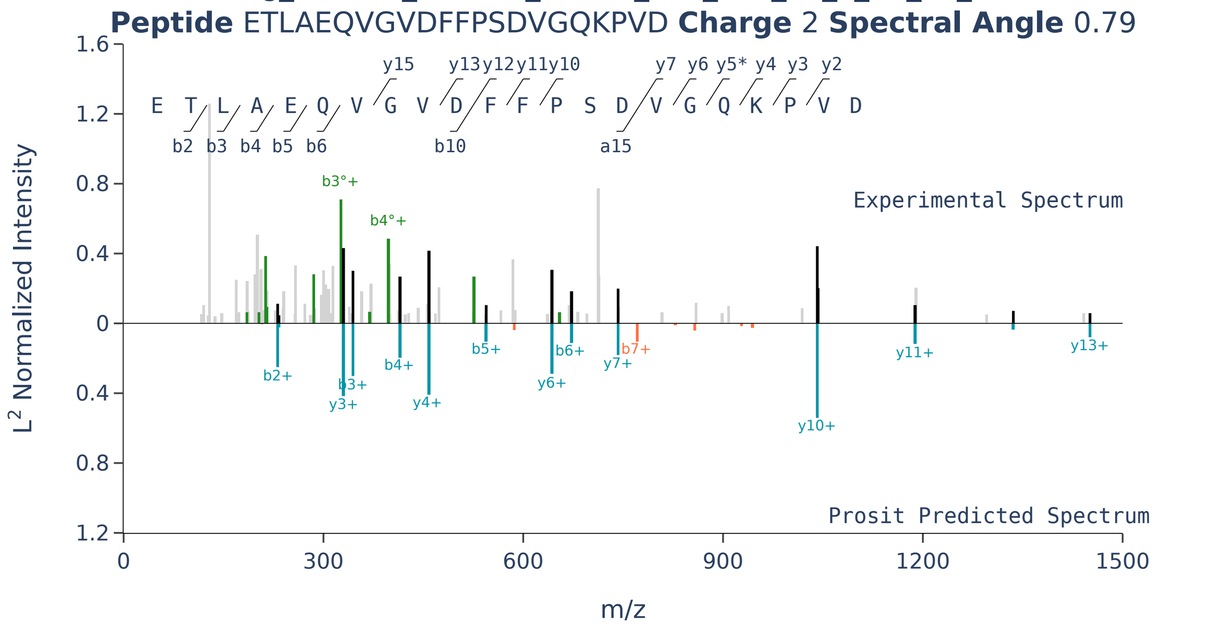


**Source** Ncheng_210623_230623_HFGoe_FFH_20S_25_1_A2_4h_R2 **Scan** 14305
**Peptide** ESKVDRAQAEKLASKL **Charge** 2 **Spectral Angle** 0.68

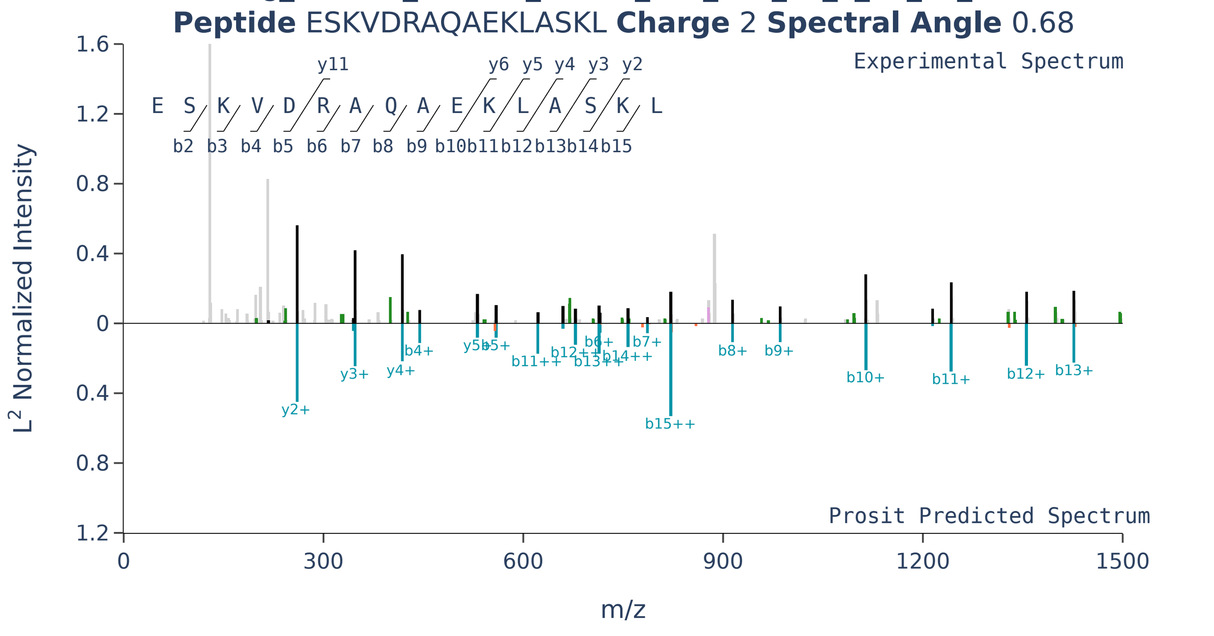


**Source** Ncheng_210623_230623_HFGoe_FFH_20S_25_1_A2_4h_R2 **Scan** 10602
**Peptide** MRSMKGMM **Charge** 2 **Spectral Angle** 0.62

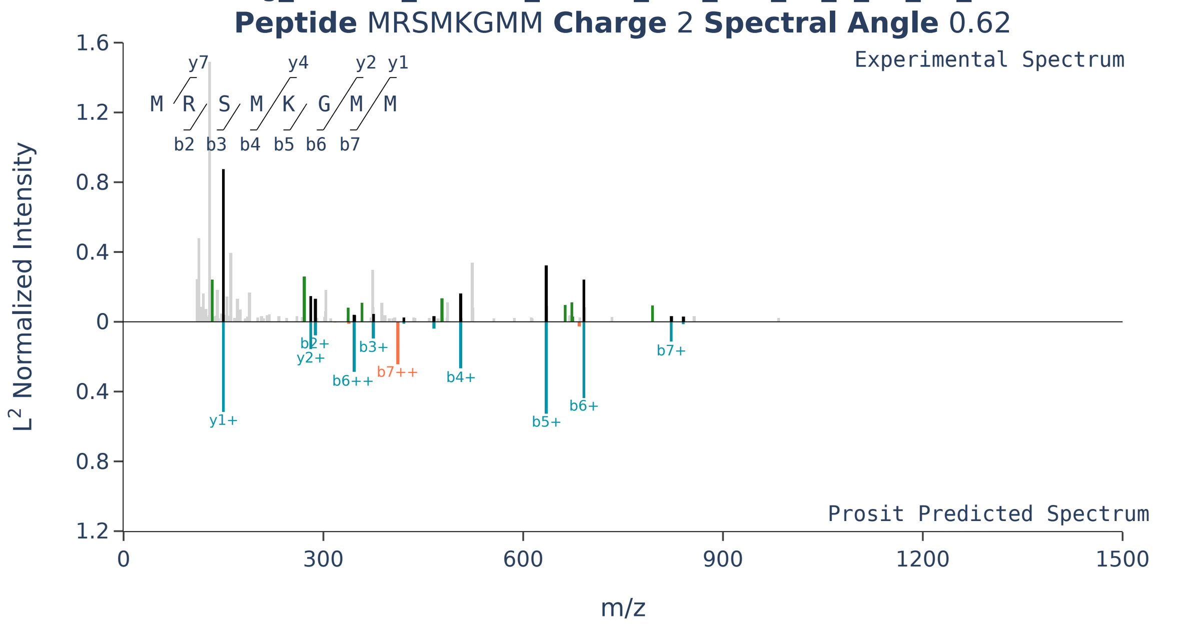


**Source** Ncheng_210623_230623_HFGoe_FFH_20S_25_1_A1_24h_R2 **Scan** 22078
**Peptide** EKAVGHEVNKSLTPGQEFVKLV **Charge** 3 **Spectral Angle** 0.77

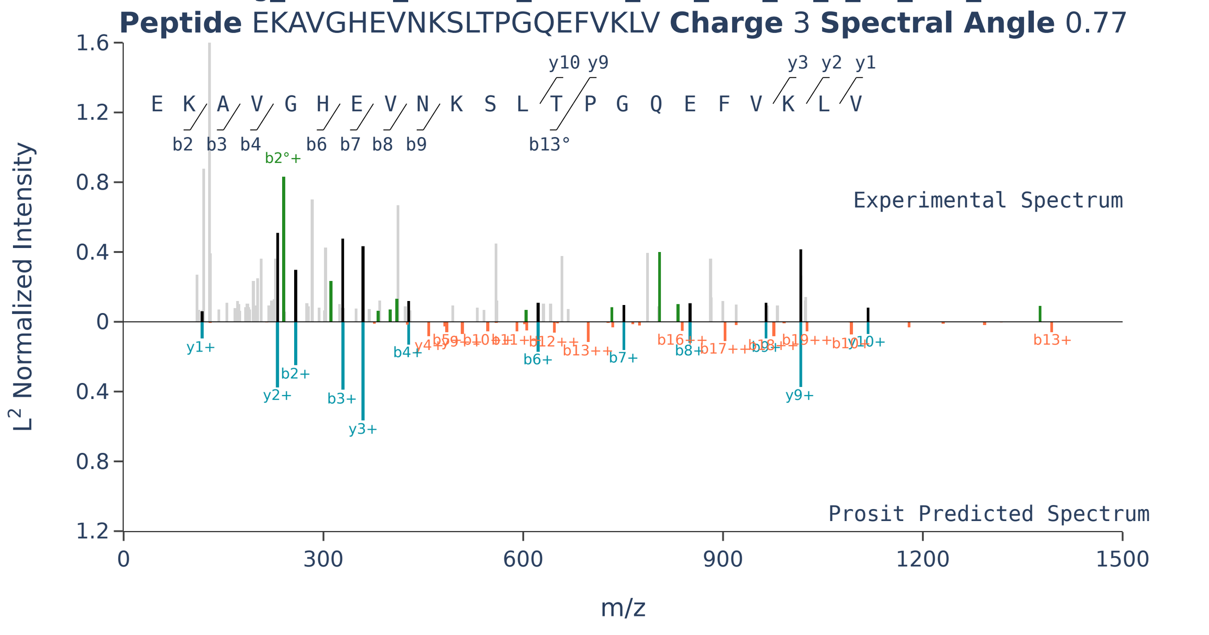


**Source** Ncheng_210623_230623_HFGoe_FFH_20S_25_1_A2_4h_R1 **Scan** 13130
**Peptide** PDNVKSQM **Charge** 1 **Spectral Angle** 0.9

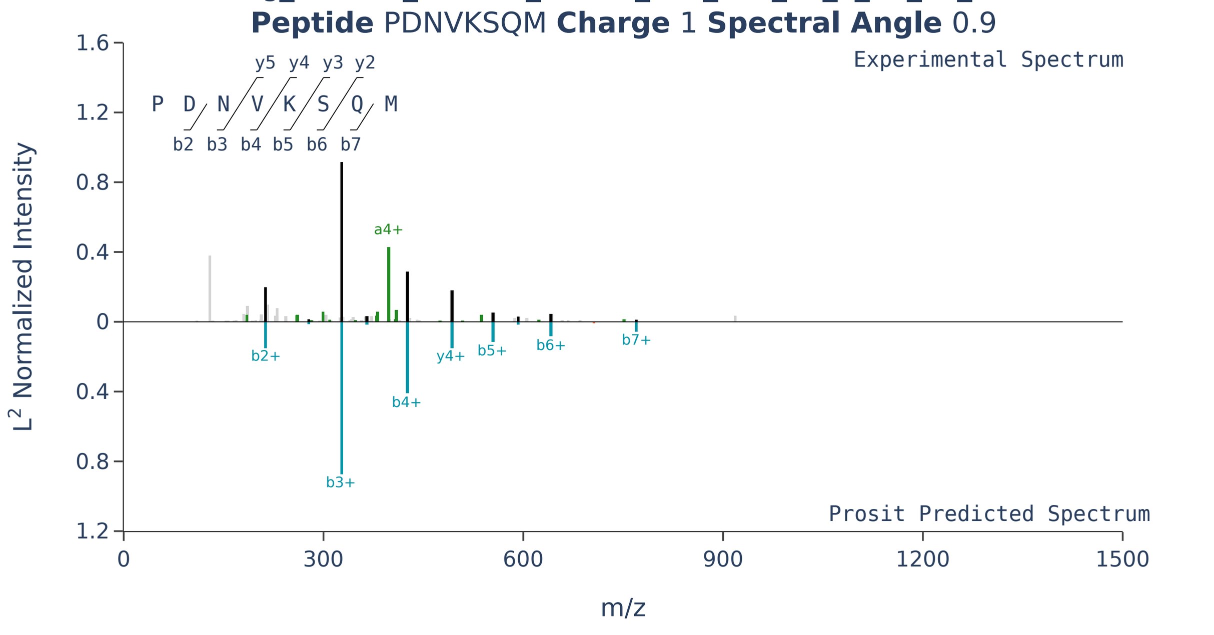


**Source** Ncheng_210623_230623_HFGoe_FFH_20S_25_1_A2_2h_R2 **Scan** 23225
**Peptide** DTAGRLHVDEAMMDEIKQVH **Charge** 4 **Spectral Angle** 0.77

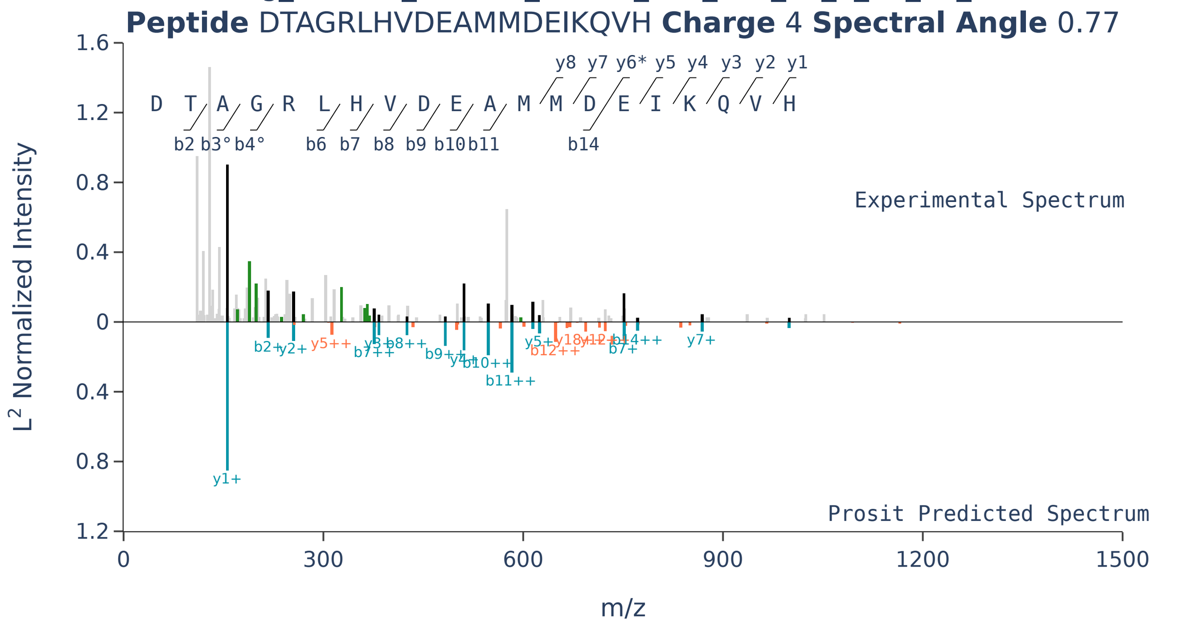


**Source** Ncheng_210623_230623_HFGoe_FFH_20S_25_1_A1_4h_R2 **Scan** 10859
**Peptide** DNVKDTLREVR **Charge** 2 **Spectral Angle** 0.65

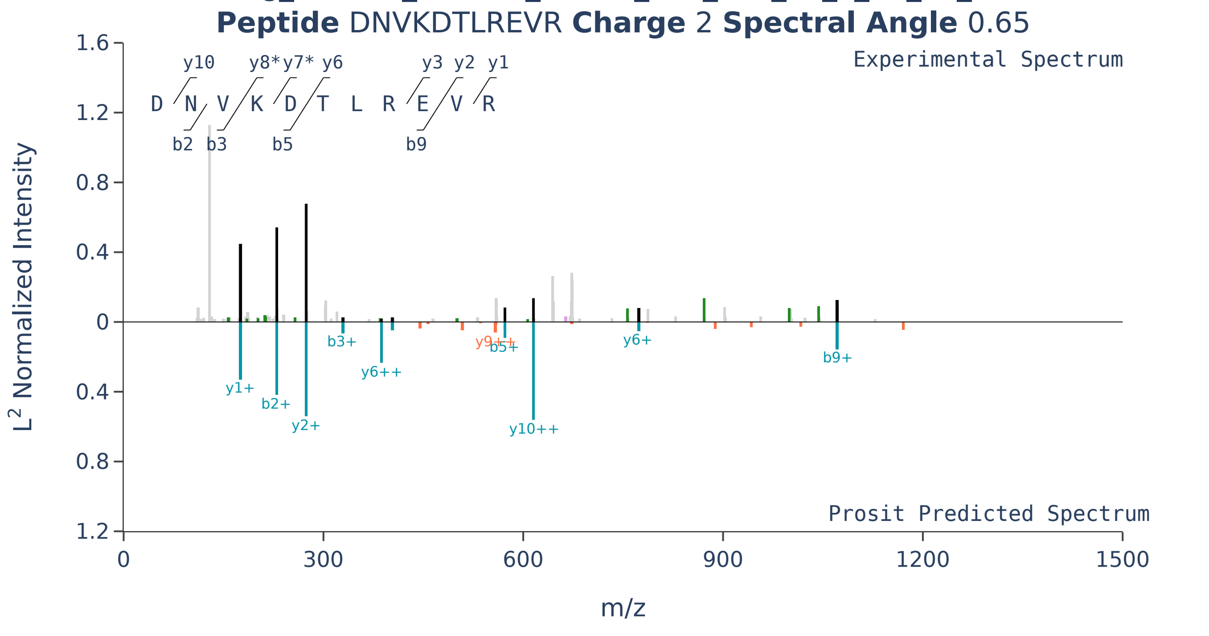


**Source** Ncheng_210623_230623_HFGoe_FFH_20S_25_1_A1_2h_R2 **Scan** 27076
**Peptide** SLMGKLPGMGQIPDNVKSQMDDKVLVR **Charge** 3 **Spectral Angle** 0.65

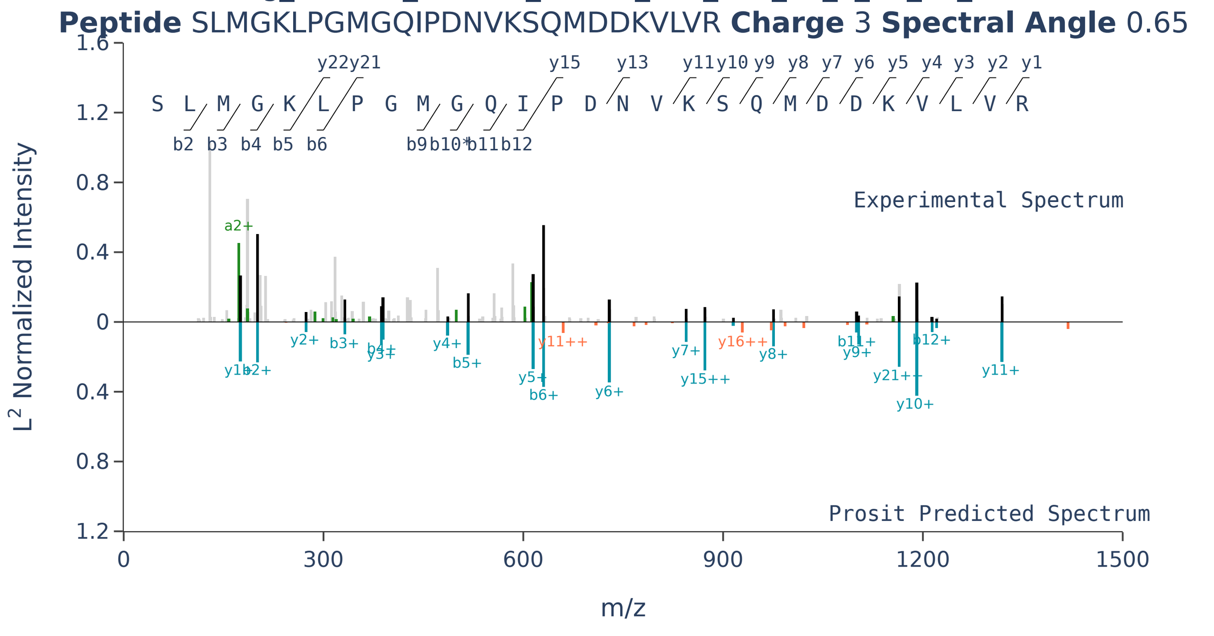

**Source** Ncheng_210623_230623_HFGoe_FFH_20S_25_1_A1_1h_R2 **Scan** 31634
**Peptide** QVGVDFFPSDVGQKPVDIVNA **Charge** 2 **Spectral Angle** 0.68

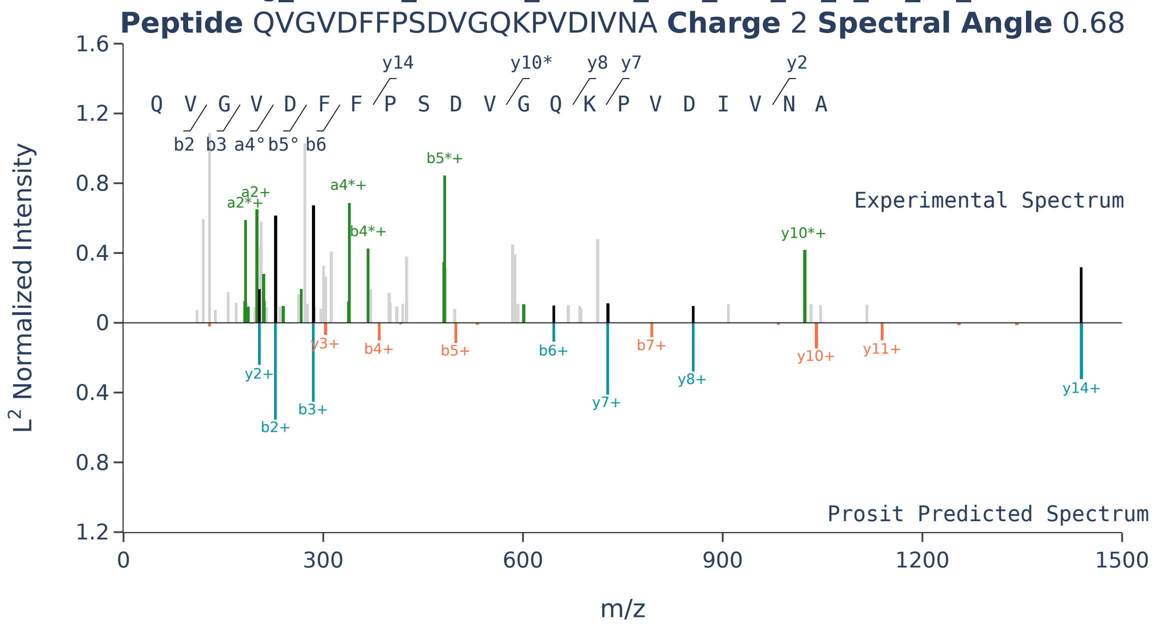


**Source** Ncheng_210623_230623_HFGoe_FFH_20S_25_1_A1_2h_R1 **Scan** 28474
**Peptide** DDKVLVRMEAIIN **Charge** 2 **Spectral Angle** 0.64

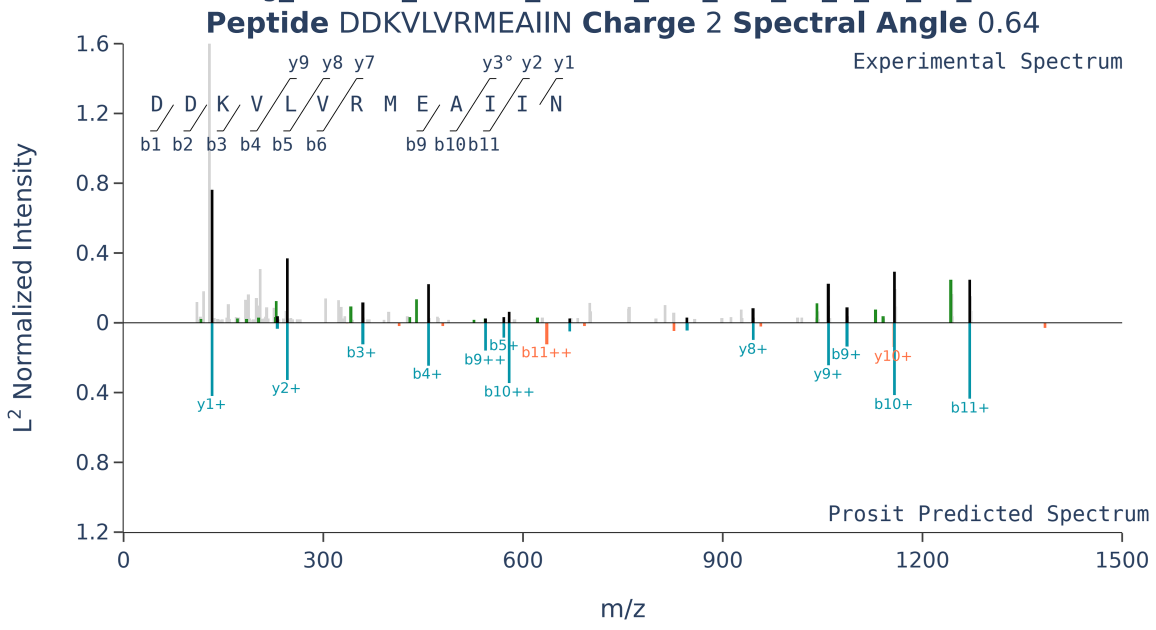


**Source** Ncheng_210623_230623_HFGoe_FFH_20S_25_1_A2_24h_R1 **Scan** 12388
**Peptide** LVNAALKEAKLK **Charge** 3 **Spectral Angle** 0.6

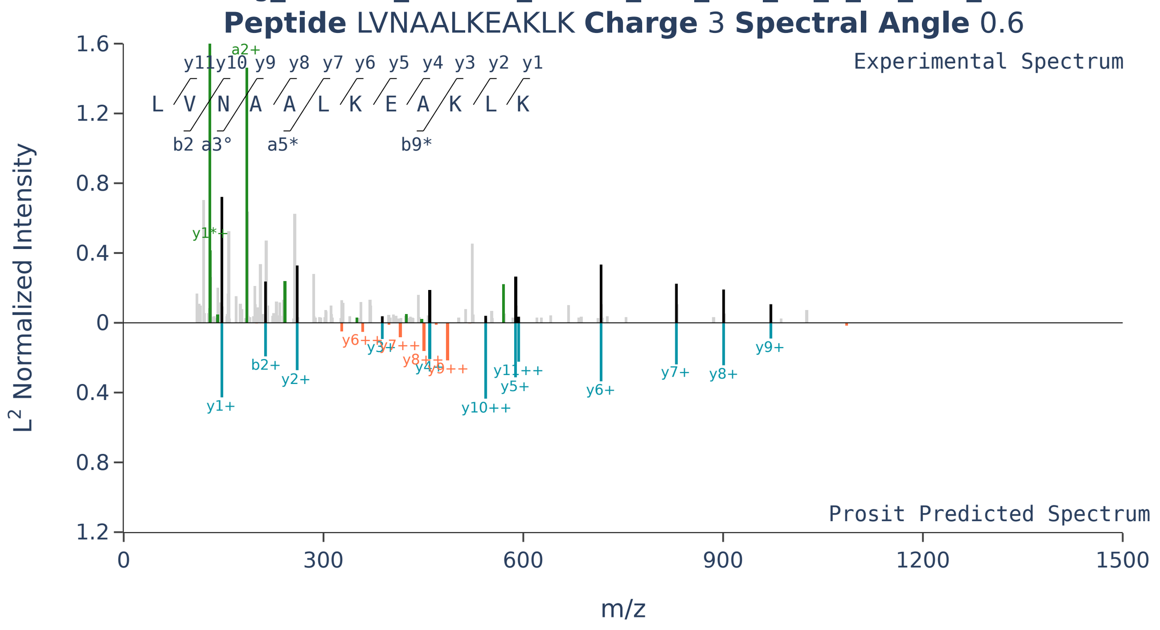


**Source** Ncheng_210623_230623_HFGoe_FFH_20S_25_1_A1_4h_R1 **Scan** 29151
**Peptide** SLTPGQEFVKLV **Charge** 2 **Spectral Angle** 0.76

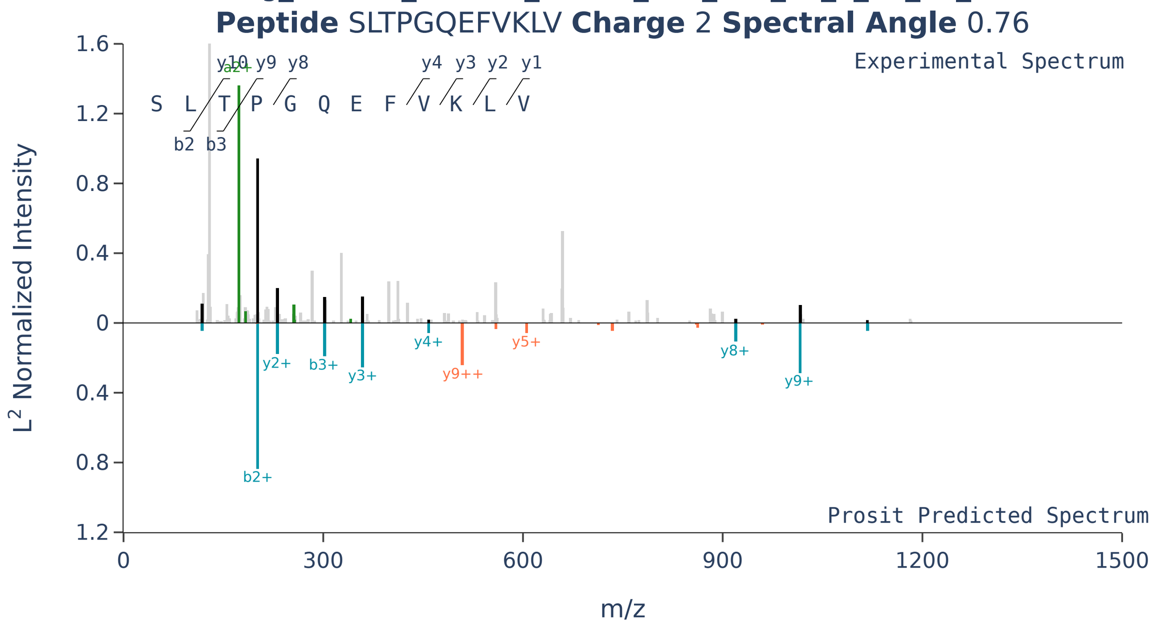


**Source** Ncheng_210623_230623_HFGoe_FFH_20S_25_1_A1_24h_R1 **Scan** 11988
**Peptide** HITGKPIKF **Charge** 1 **Spectral Angle** 0.5

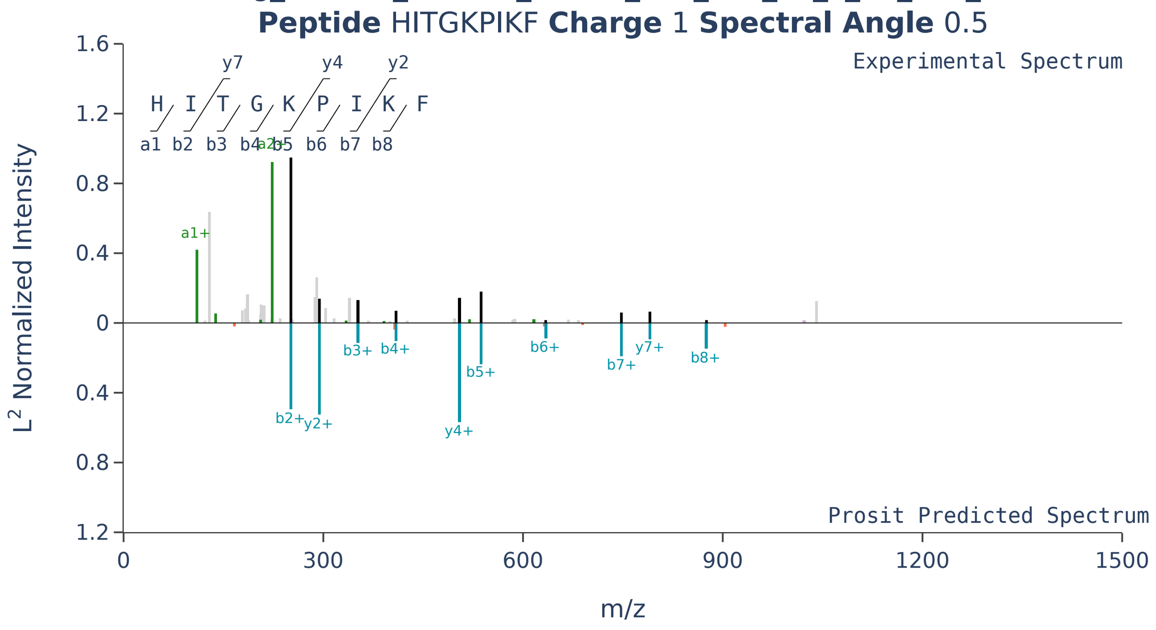


**Source** Ncheng_210623_230623_HFGoe_FFH_20S_25_1_A1_24h_R2 **Scan** 6974
**Peptide** AGLQGAGKTTSVGK **Charge** 2 **Spectral Angle** 0.72

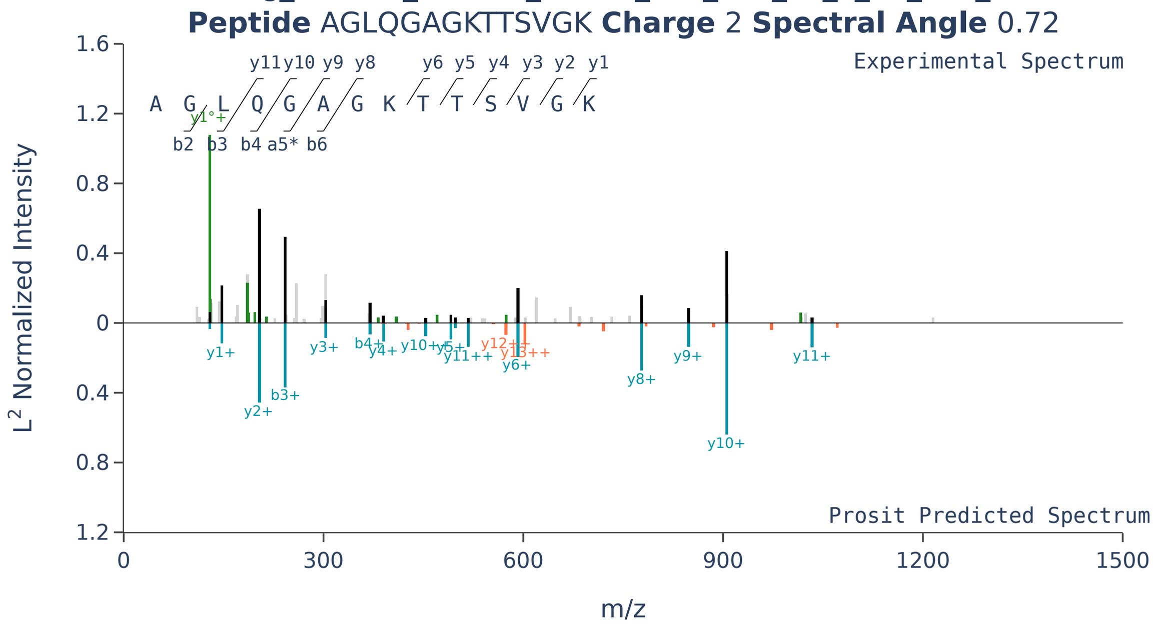


**Source** Ncheng_210623_230623_HFGoe_FFH_20S_25_1_A2_24h_R1 **Scan** 12808
**Peptide** TSVGKLGKFLREKH **Charge** 3 **Spectral Angle** 0.71

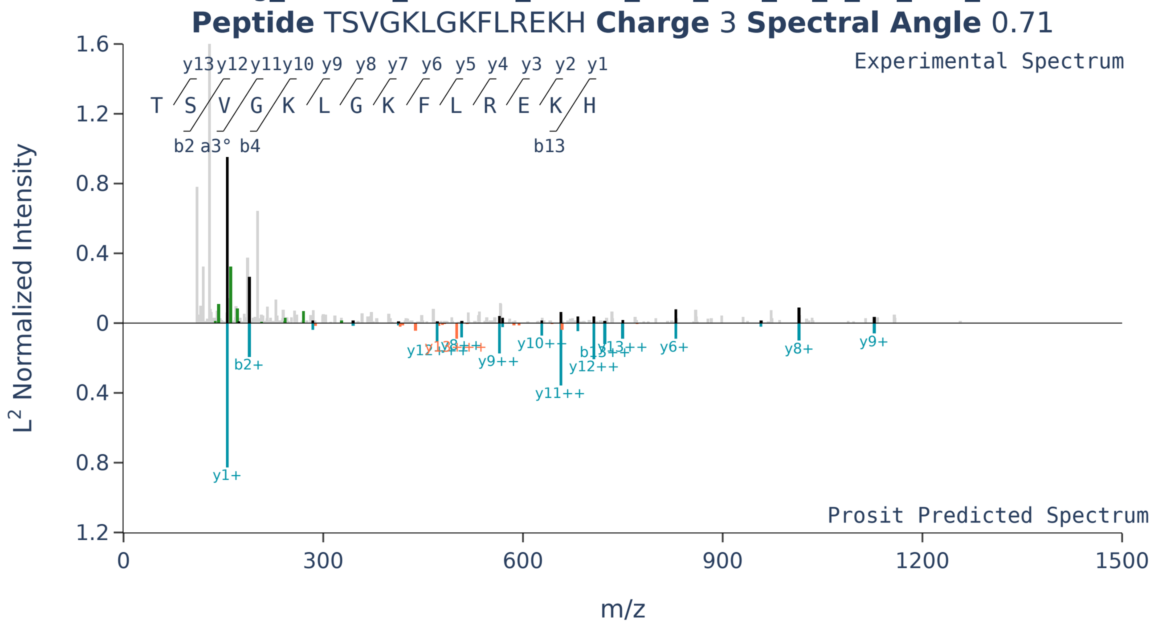


**Source** Ncheng_210623_230623_HFGoe_FFH_20S_25_1_A2_2h_R2 **Scan** 19758
**Peptide** EKAVGHEVNKSLTPGQEFVKIVRNE **Charge** 4 **Spectral Angle** 0.69

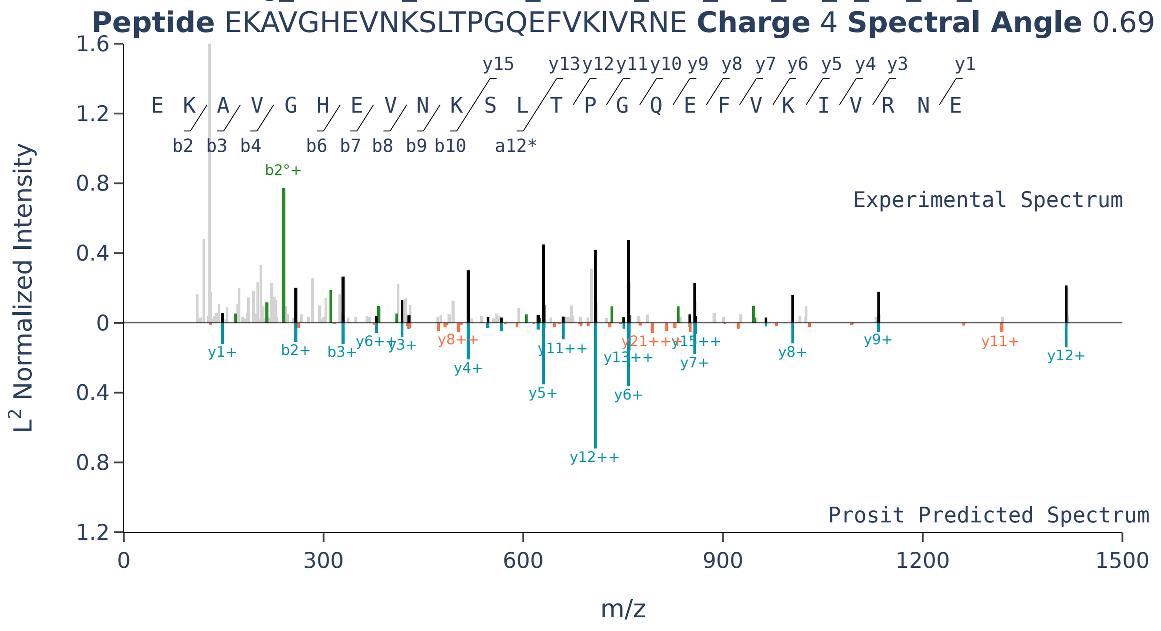


**Source** Ncheng_210623_230623_HFGoe_FFH_20S_25_1_A1_24h_R1 **Scan** 7449
**Peptide** MKERAKPEIIKG **Charge** 2 **Spectral Angle** 0.65

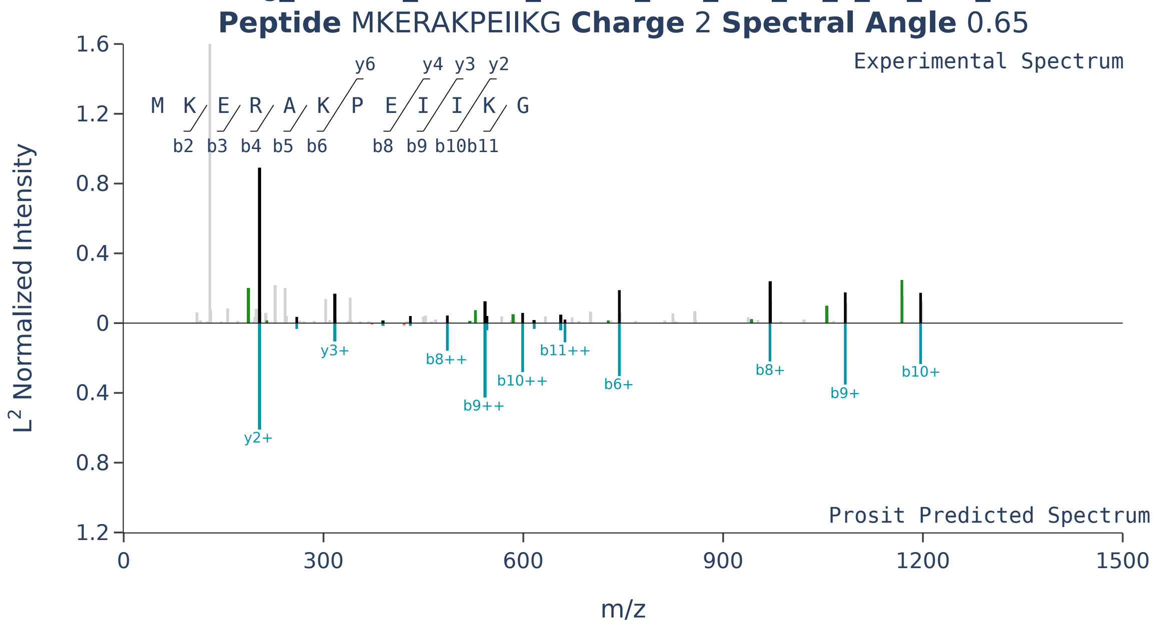


**Source** Ncheng_210623_230623_HFGoe_FFH_20S_25_1_A1_4h_R2 **Scan** 6611
**Peptide** KVDRAQAEKL **Charge** 3 **Spectral Angle** 0.92

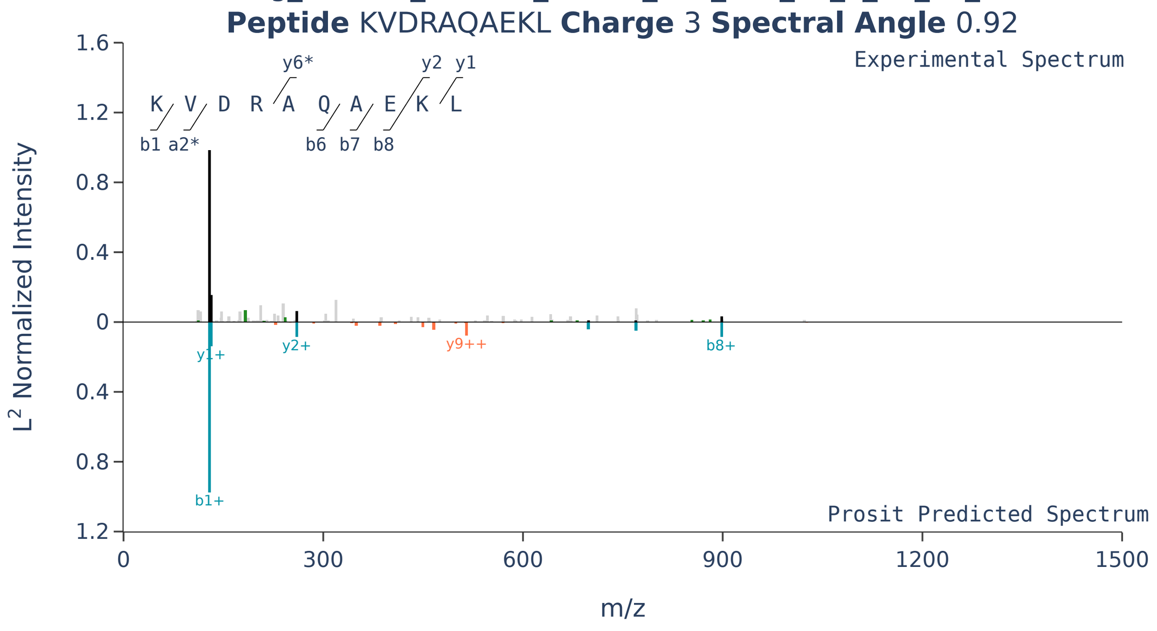


**Source** Ncheng_210623_230623_HFGoe_FFH_20S_25_1_A1_4h_R1 **Scan** 20163
**Peptide** EFINRVKEKAVGHEVNKSLTPGQEFVKL **Charge** 4 **Spectral Angle** 0.77

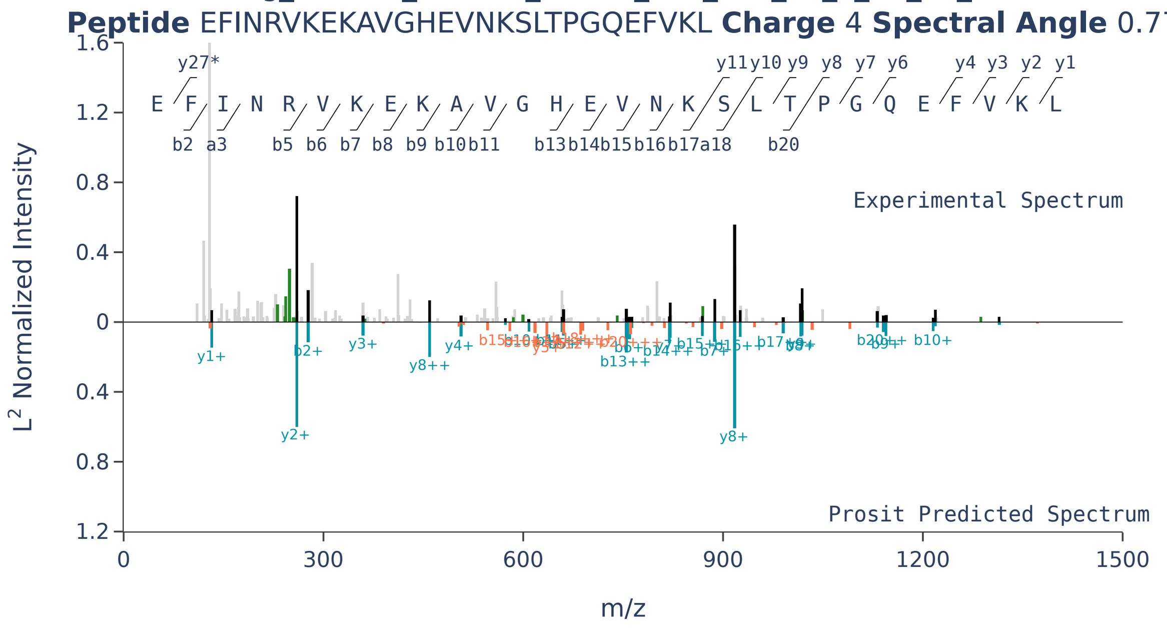


**Source** Ncheng_210623_230623_HFGoe_FFH_20S_25_1_A2_4h_R2 **Scan** 17941
**Peptide** DTAGRLHVDEAMM **Charge** 2 **Spectral Angle** 0.7

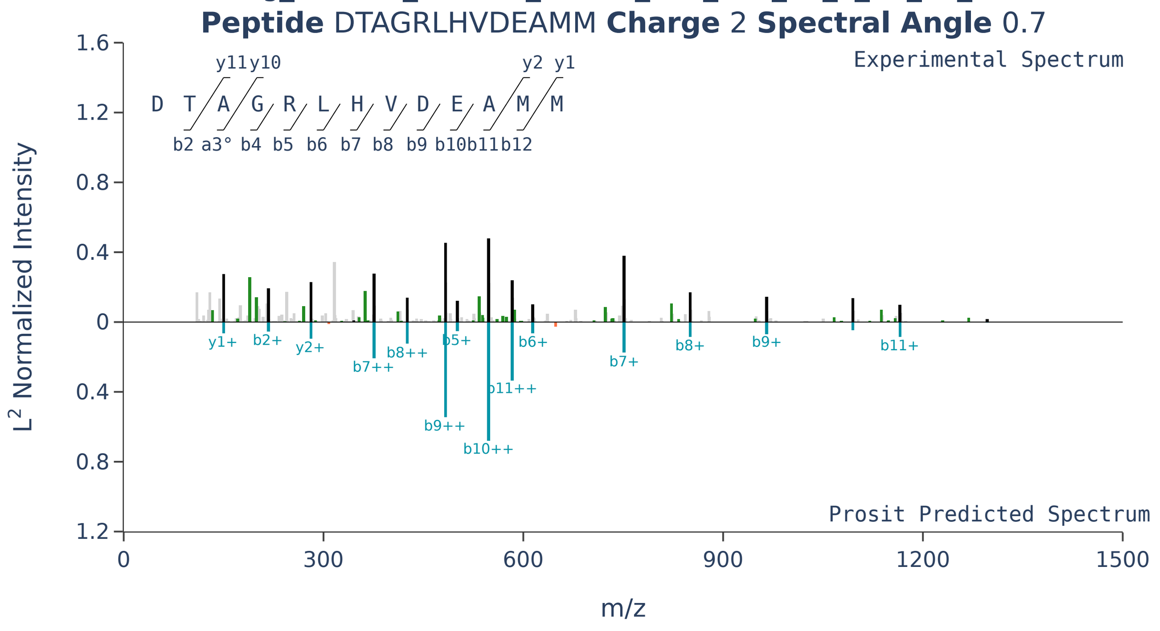


**Source** Ncheng_210623_230623_HFGoe_FFH_20S_25_1_A2_4h_R2 **Scan** 23428
**Peptide** PIKFLGVGEKTEALE **Charge** 3 **Spectral Angle** 0.84

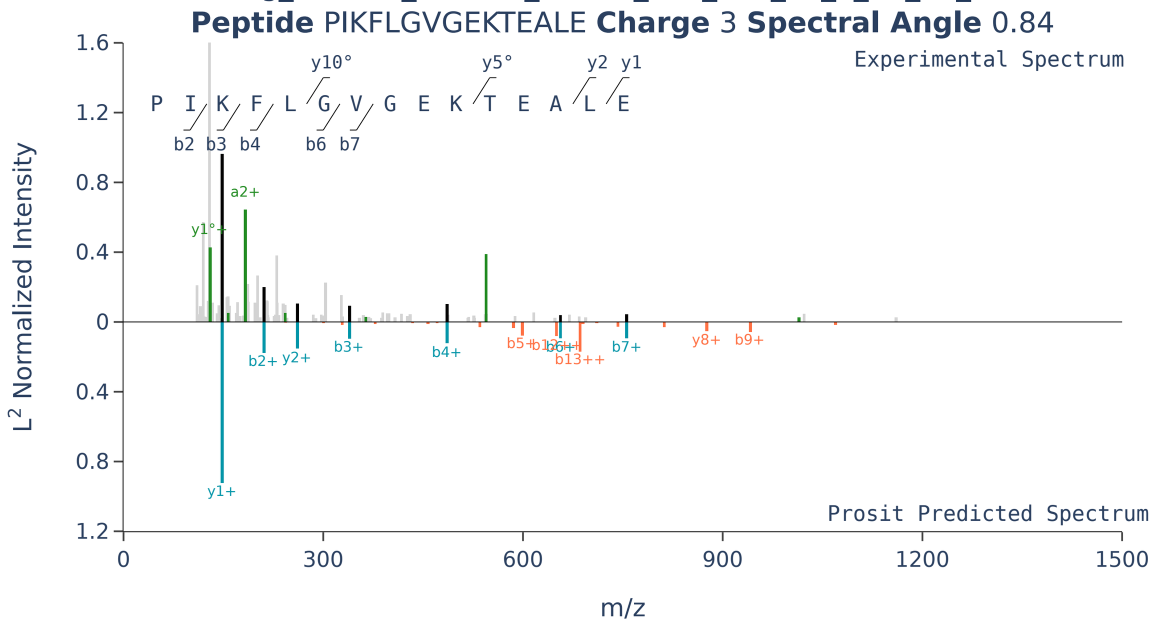


**Source** Ncheng_210623_230623_HFGoe_FFH_20S_25_1_A2_4h_R1 **Scan** 30118
**Peptide** GGMASLMGKLPGMGQIPDNVKSQ **Charge** 3 **Spectral Angle** 0.81

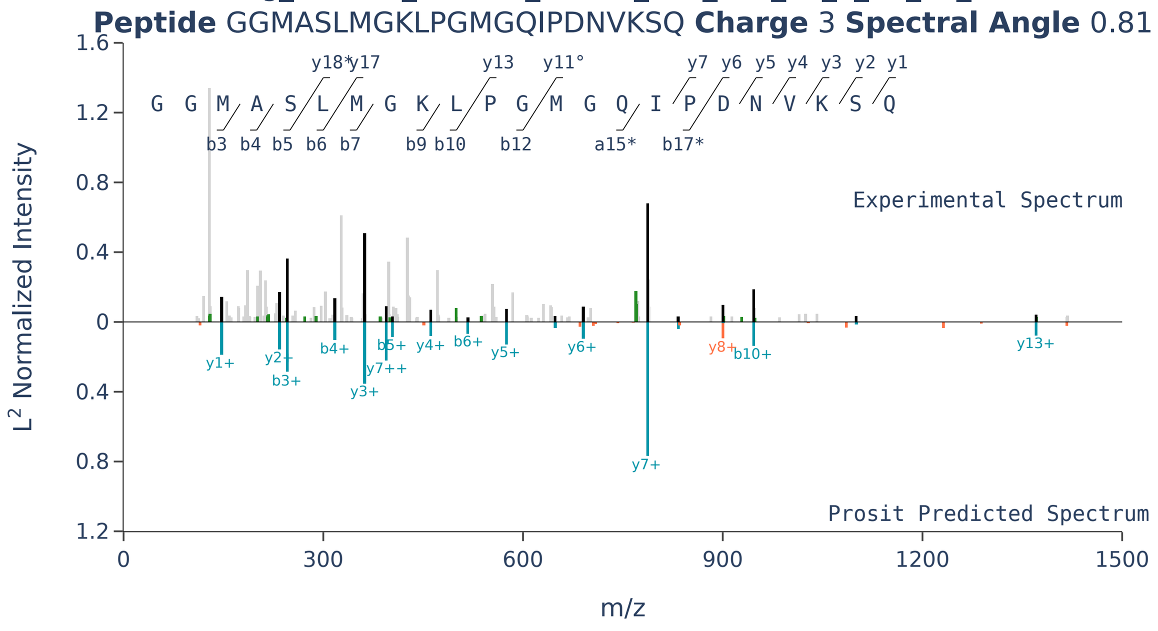


**Source** Ncheng_210623_230623_HFGoe_FFH_20S_25_1_A2_2h_R2 **Scan** 13409
**Peptide** LVAAMGE **Charge** 1 **Spectral Angle** 0.81

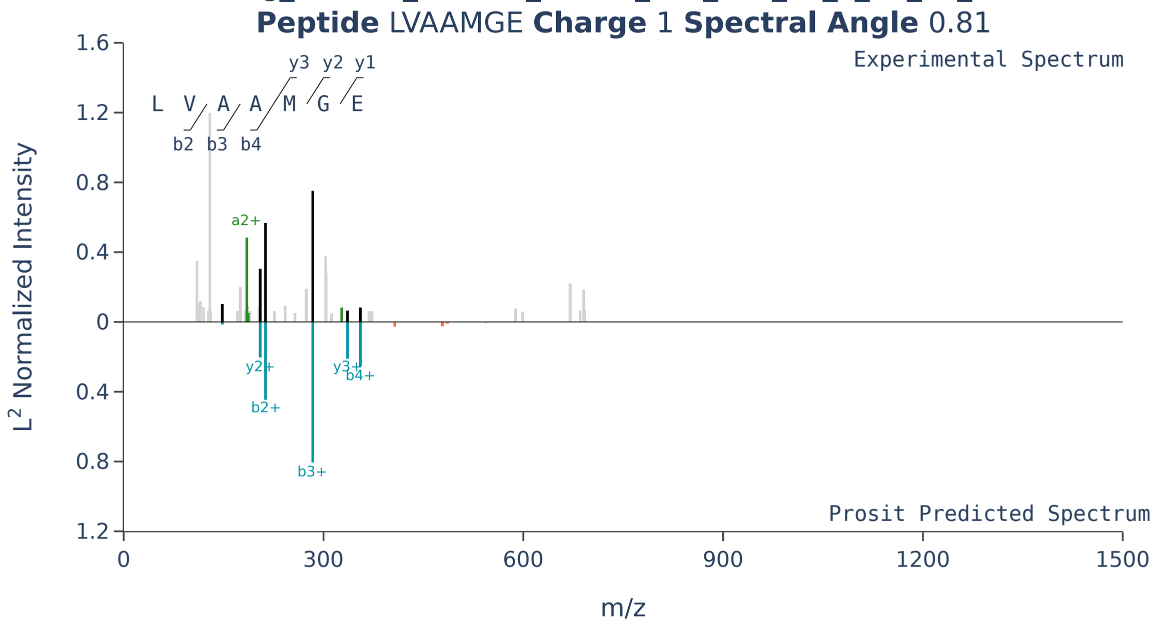

**Source** Ncheng_210623_230623_HFGoe_FFH_20S_25_1_A2_4h_R2 **Scan** 26695
**Peptide** LVAAMGEENQTLNLA **Charge** 2 **Spectral Angle** 0.8

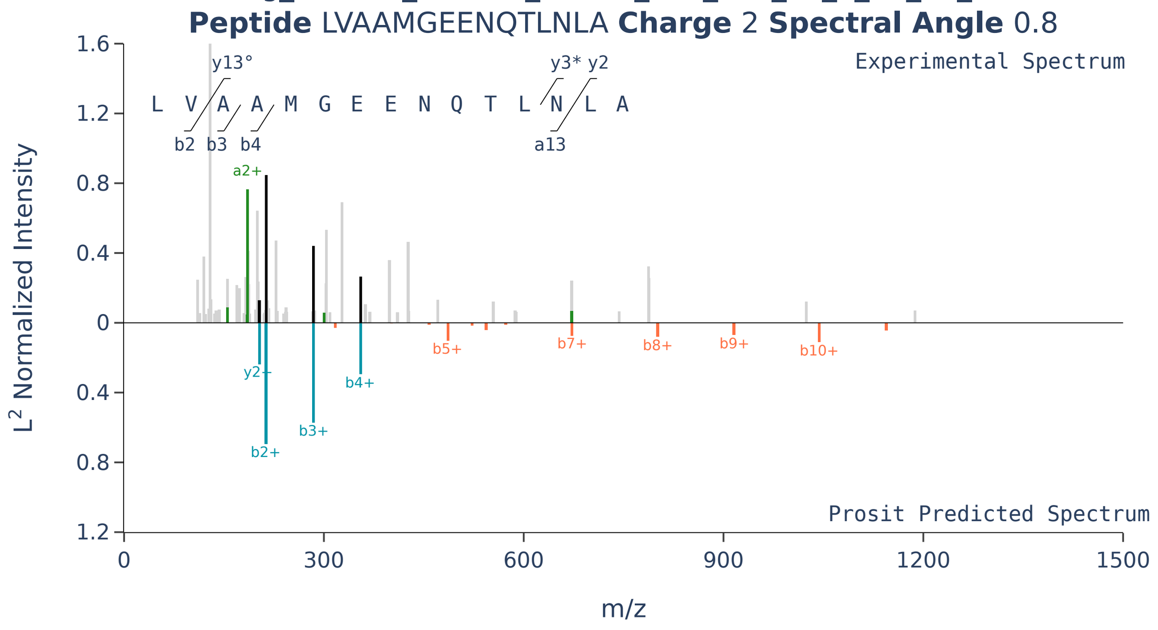


**Source** Ncheng_210623_230623_HFGoe_FFH_20S_25_1_A2_4h_R2 **Scan** 30882
**Peptide** GQIPDNVKSQMDDKVLVRMEAIIN **Charge** 4 **Spectral Angle** 0.95

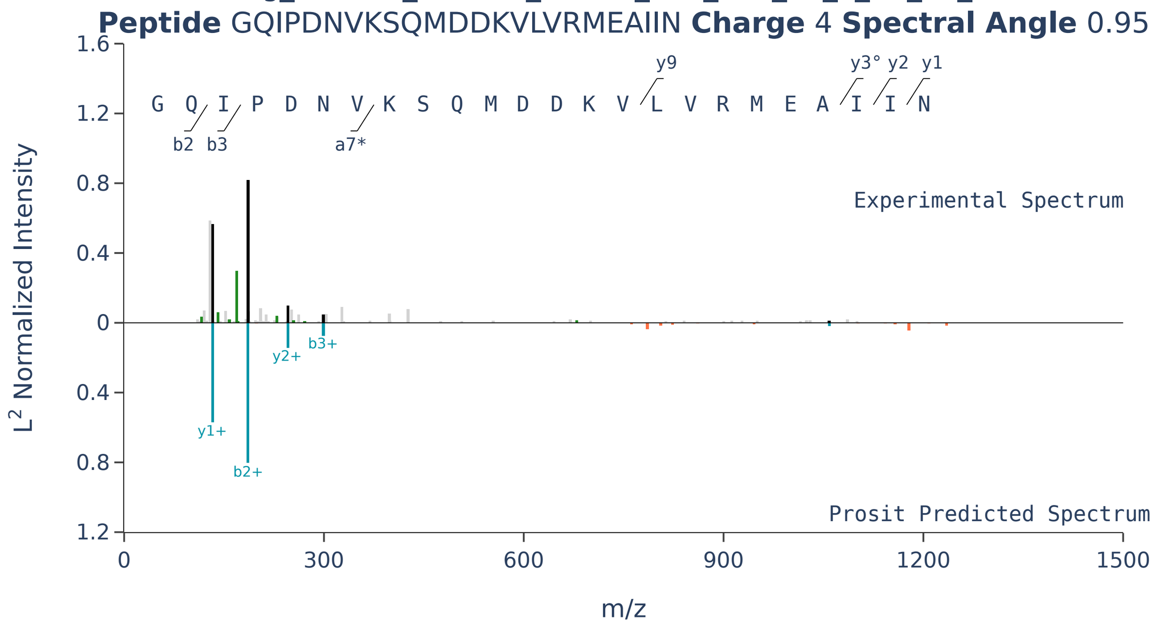


**Source** Ncheng_210623_230623_HFGoe_FFH_20S_25_1_A1_24h_R2 **Scan** 21394
**Peptide** TPGQEFVKL **Charge** 2 **Spectral Angle** 0.61

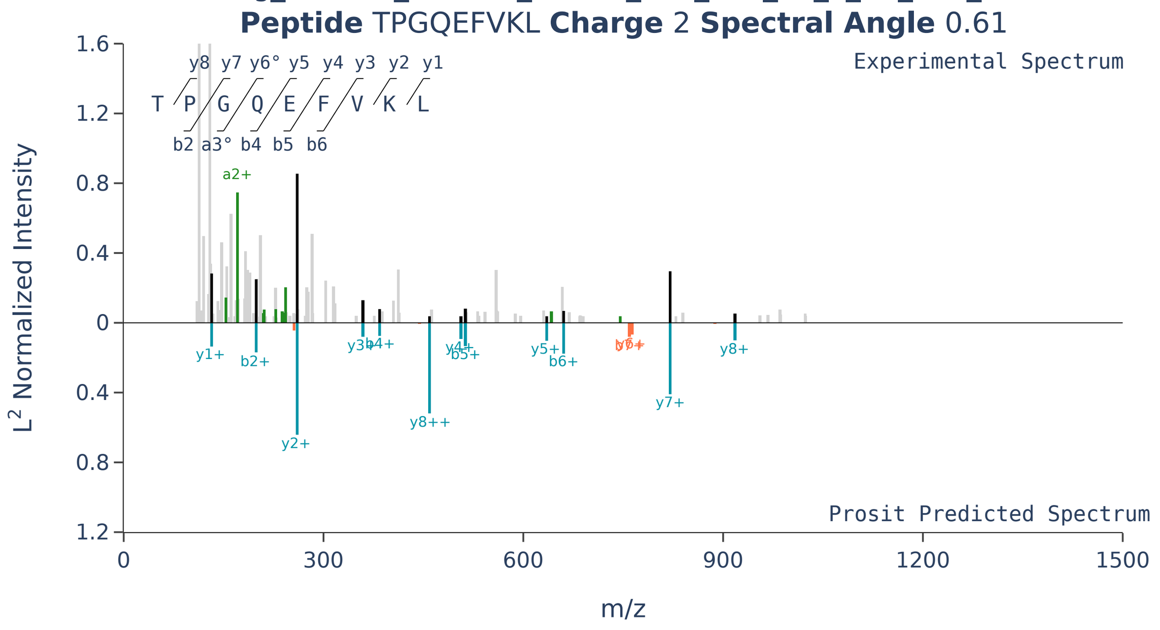


**Source** Ncheng_210623_230623_HFGoe_FFH_20S_25_1_A2_4h_R1 **Scan** 7242
**Peptide** RGRLTEDNV **Charge** 3 **Spectral Angle** 0.61

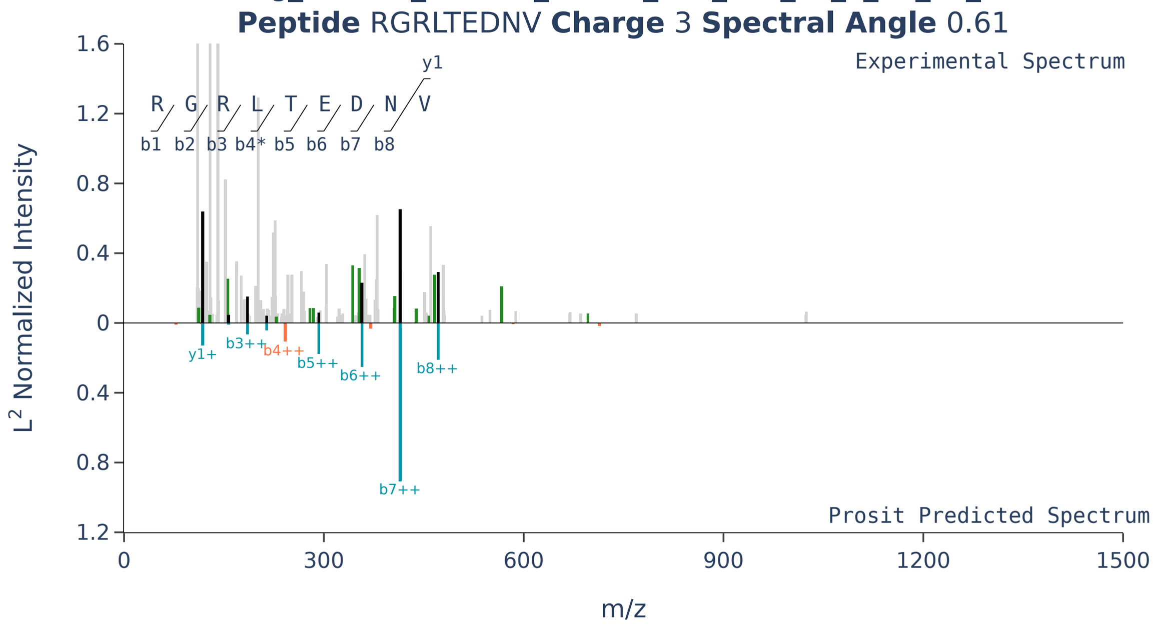


**Source** Ncheng_210623_230623_HFGoe_FFH_20S_25_1_A1_1h_R2 **Scan** 34399
**Peptide** VHASINPVETLFVVD **Charge** 2 **Spectral Angle** 0.67

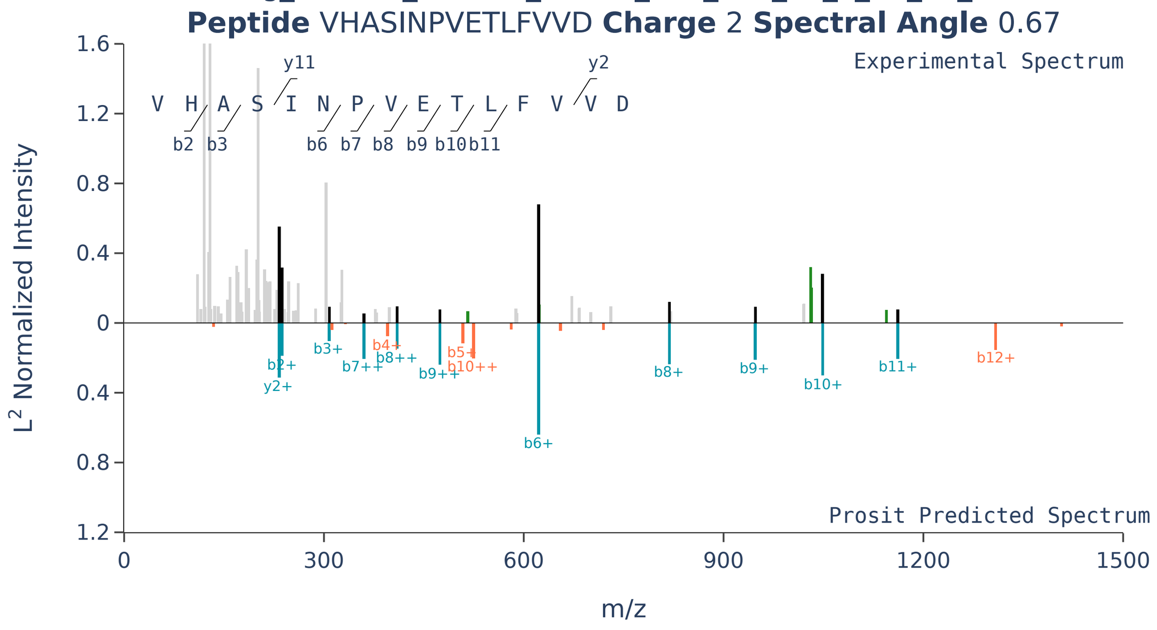


**Source** Ncheng_210623_230623_HFGoe_FFH_20S_25_1_A2_4h_R1 **Scan** 12120
**Peptide** IEDIESKVDRAQAE **Charge** 2 **Spectral Angle** 0.78

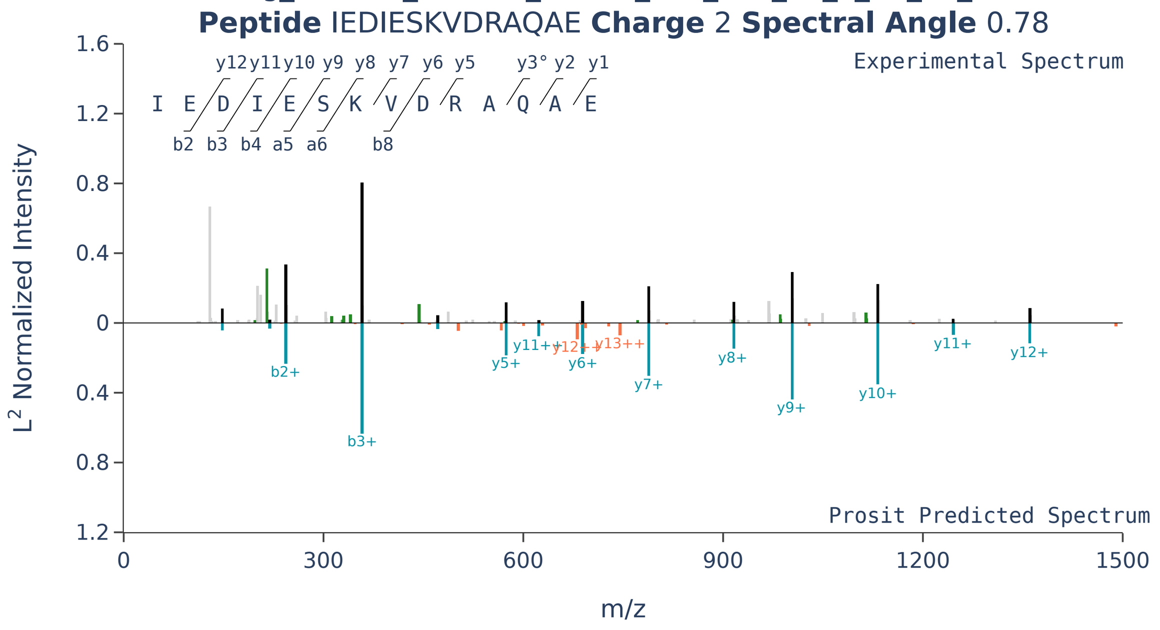


**Source** Ncheng_210623_230623_HFGoe_FFH_20S_25_1_A1_24h_R2 **Scan** 15920
**Peptide** VDGDARGGAALSIRHLTG **Charge** 3 **Spectral Angle** 0.69

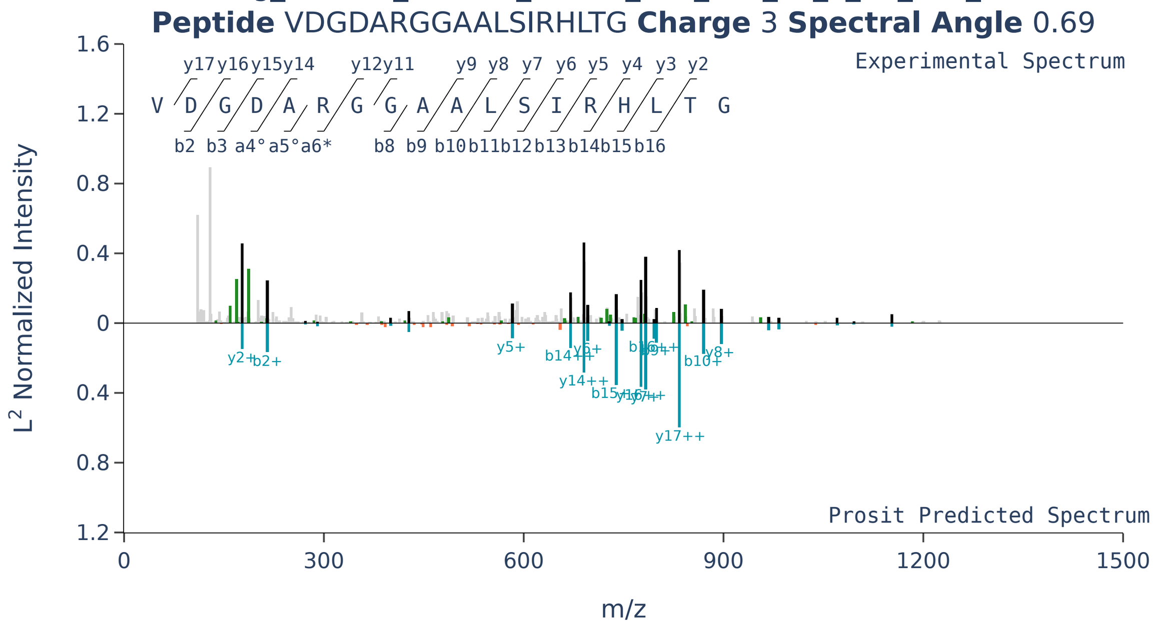


**Source** Ncheng_210623_230623_HFGoe_FFH_20S_25_1_A1_24h_R1 **Scan** 12866
**Peptide** KLKKGDGFDL **Charge** 2 **Spectral Angle** 0.68

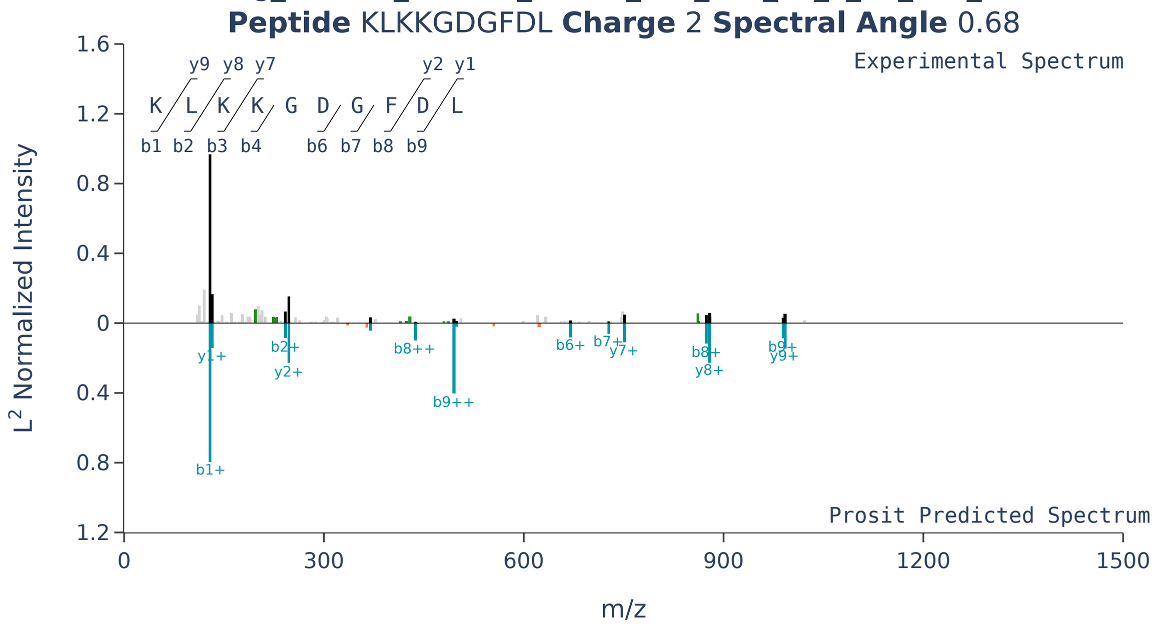


**Source** Ncheng_210623_230623_HFGoe_FFH_20S_25_1_A1_4h_R2 **Scan** 9788
**Peptide** RLSRTLRNL **Charge** 3 **Spectral Angle** 0.64

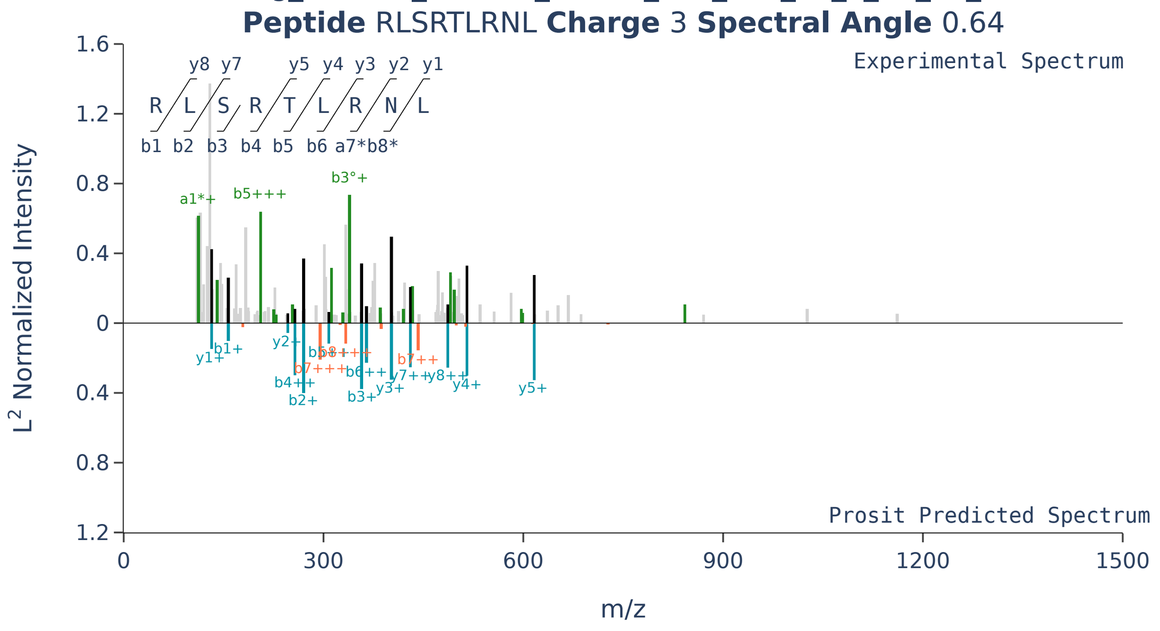


**Source** Ncheng_210623_230623_HFGoe_FFH_20S_25_1_A1_4h_R1 **Scan** 14442
**Peptide** VDTAGRLHVDEAMMD **Charge** 3 **Spectral Angle** 0.59

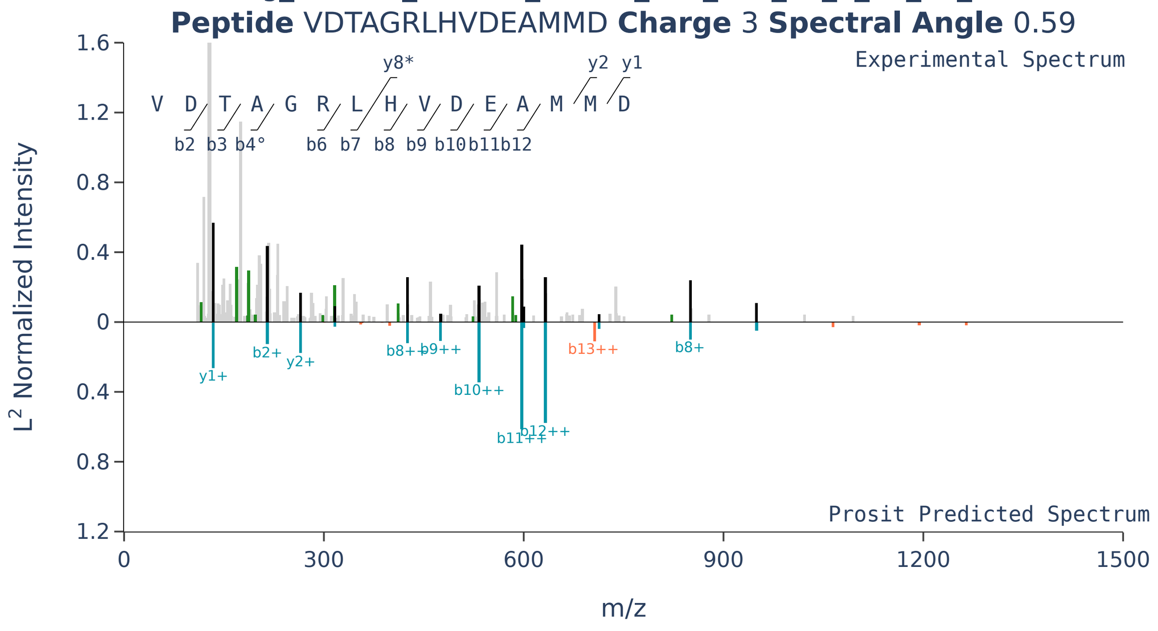


**Source** Ncheng_210623_230623_HFGoe_FFH_20S_25_1_A2_2h_R2 **Scan** 33953
**Peptide** VLSLIEDIESKVDRAQAE **Charge** 3 **Spectral Angle** 0.6

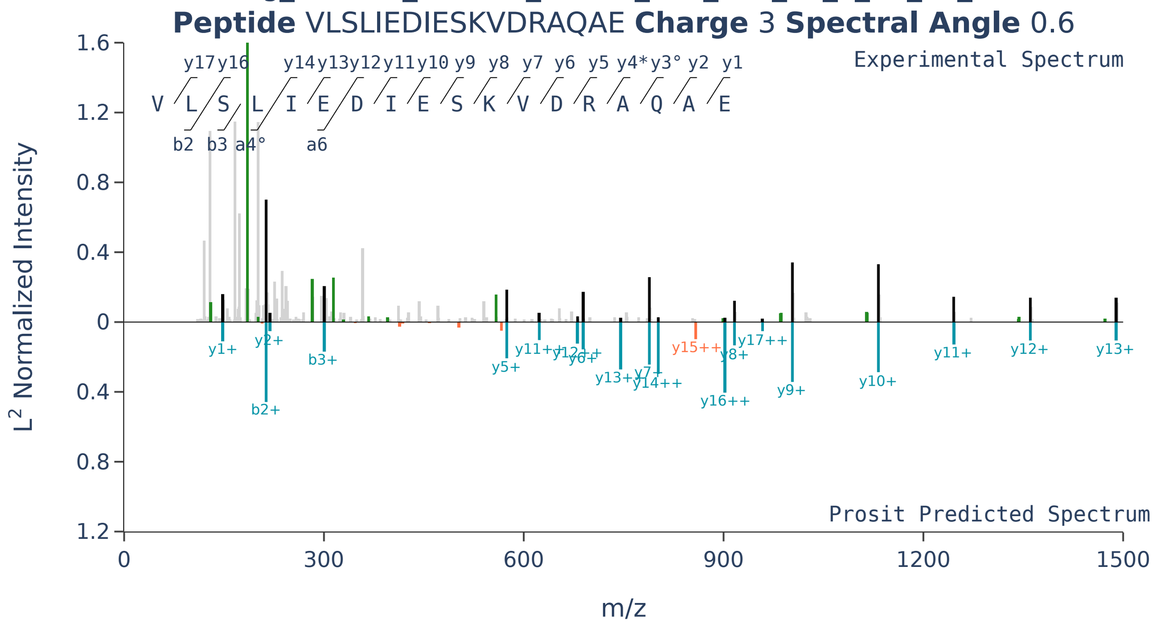


**Source** Ncheng_210623_230623_HFGoe_FFH_20S_25_1_A1_2h_R2 **Scan** 26671
**Peptide** MGKLPGMGQIPDNVKSQMDDKVLVRM **Charge** 3 **Spectral Angle** 0.66

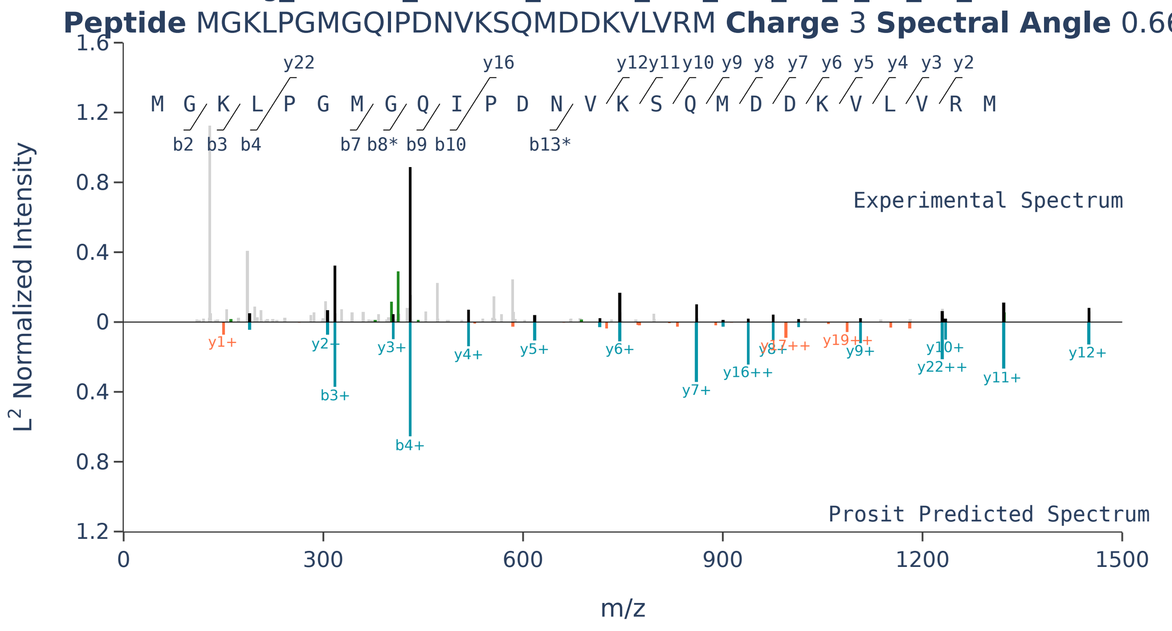


**Source** Ncheng_210623_230623_HFGoe_FFH_20S_25_1_A1_4h_R2 **Scan** 22318
**Peptide** EAIINSMTMKERAKPEII **Charge** 2 **Spectral Angle** 0.78

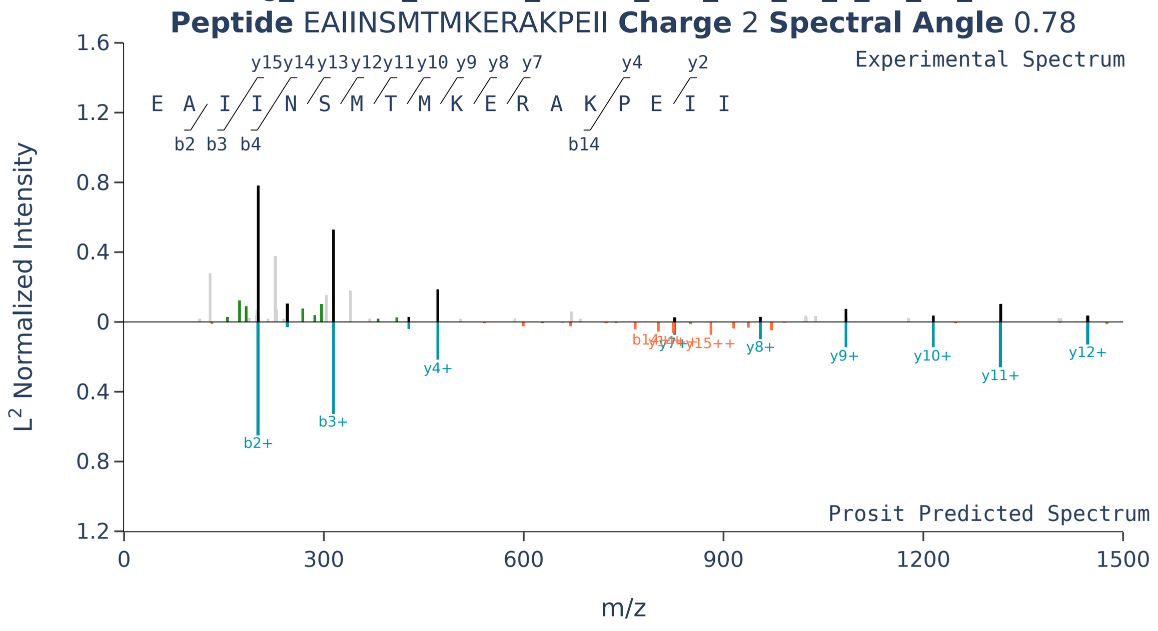


**Source** Ncheng_210623_230623_HFGoe_FFH_20S_25_1_A2_4h_R1 **Scan** 23257
**Peptide** PLKFLGVGEKTEALE **Charge** 3 **Spectral Angle** 0.84

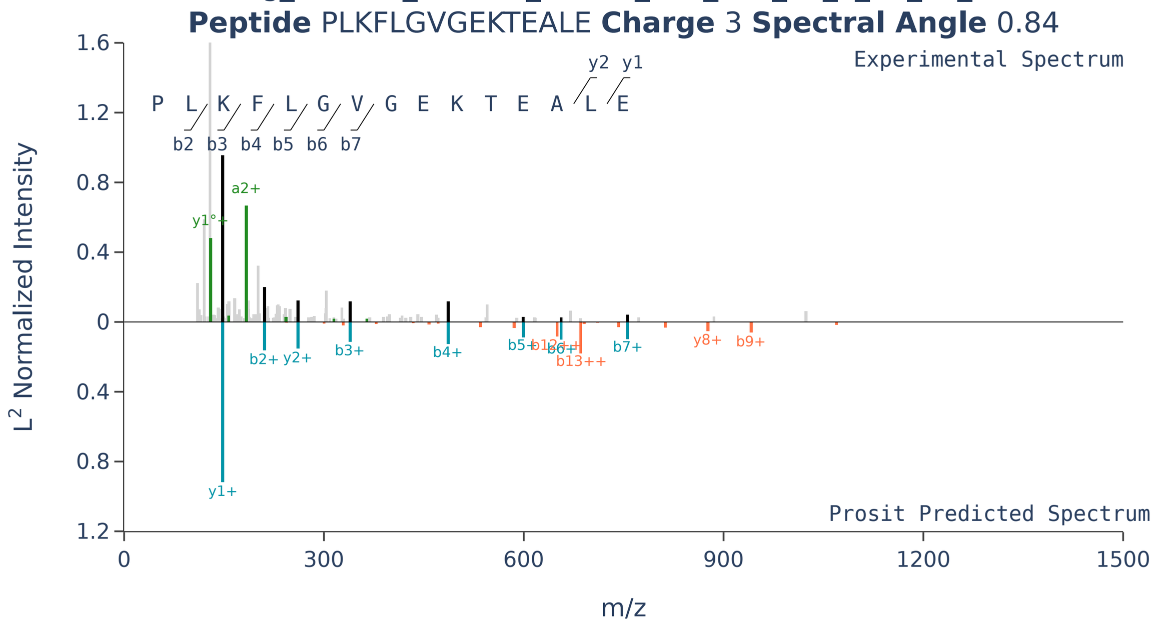


**Source** Ncheng_210623_230623_HFGoe_FFH_20S_25_1_A2_1h_R1 **Scan** 31747
**Peptide** ENQTLNLAAQPPAVVL **Charge** 2 **Spectral Angle** 0.86

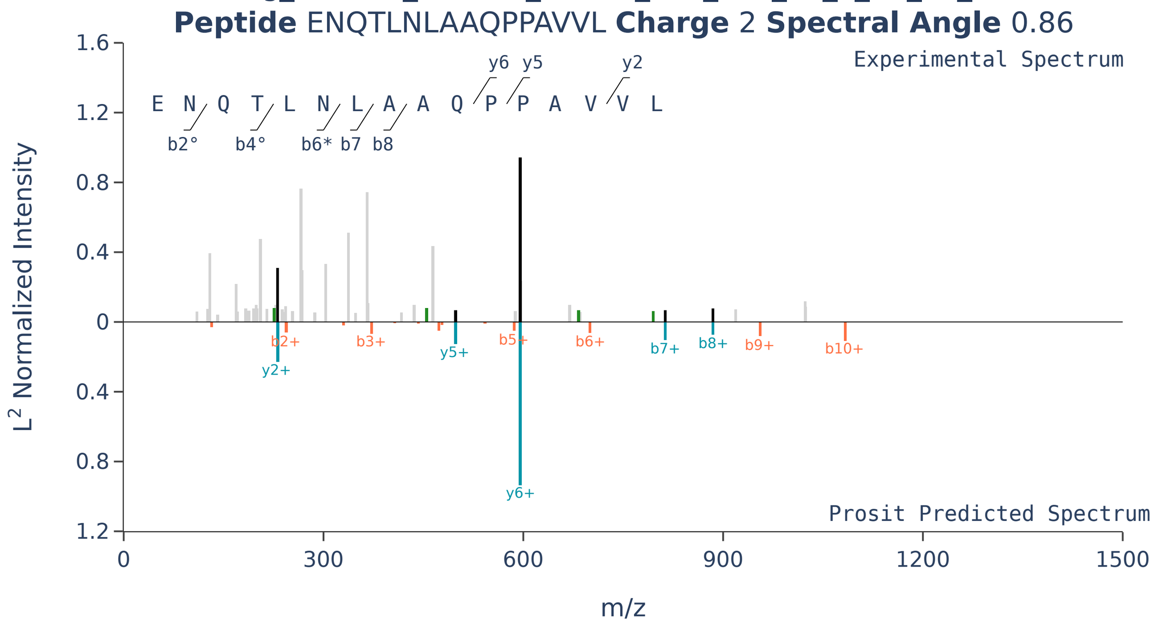

**Source** Ncheng_210623_230623_HFGoe_FFH_20S_25_1_A1_2h_R1 **Scan** 22661
**Peptide** LTPGQEFVKIVRN **Charge** 3 **Spectral Angle** 0.55

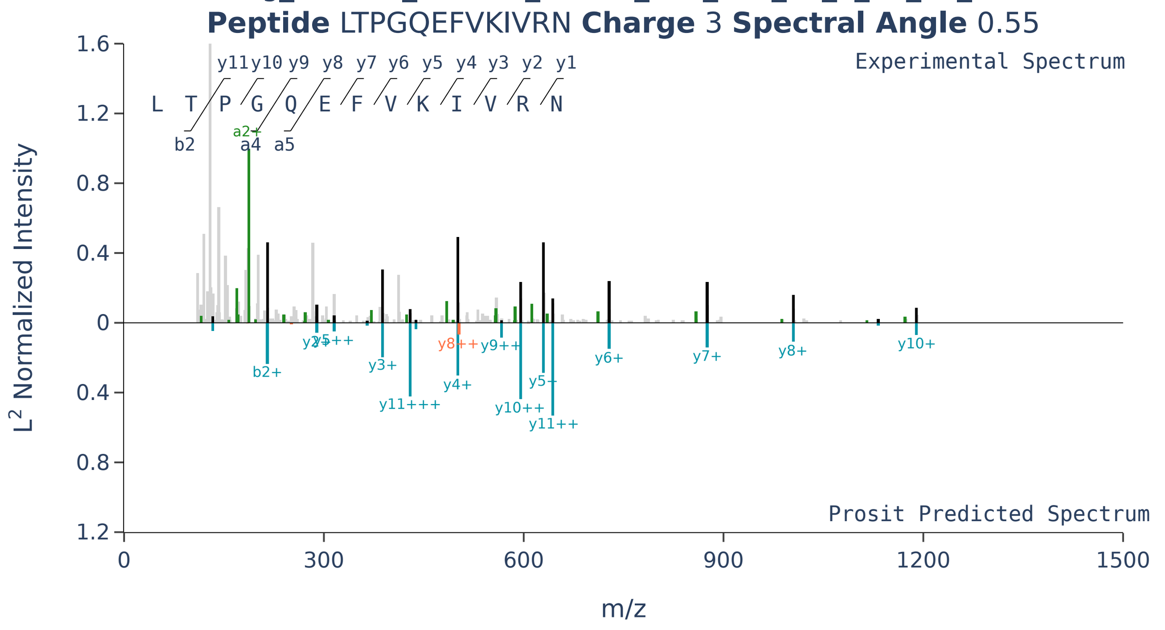


**Source** Ncheng_210623_230623_HFGoe_FFH_20S_25_1_A1_24h_R2 **Scan** 11363
**Peptide** GQDAANTAKAFNEA **Charge** 2 **Spectral Angle** 0.8

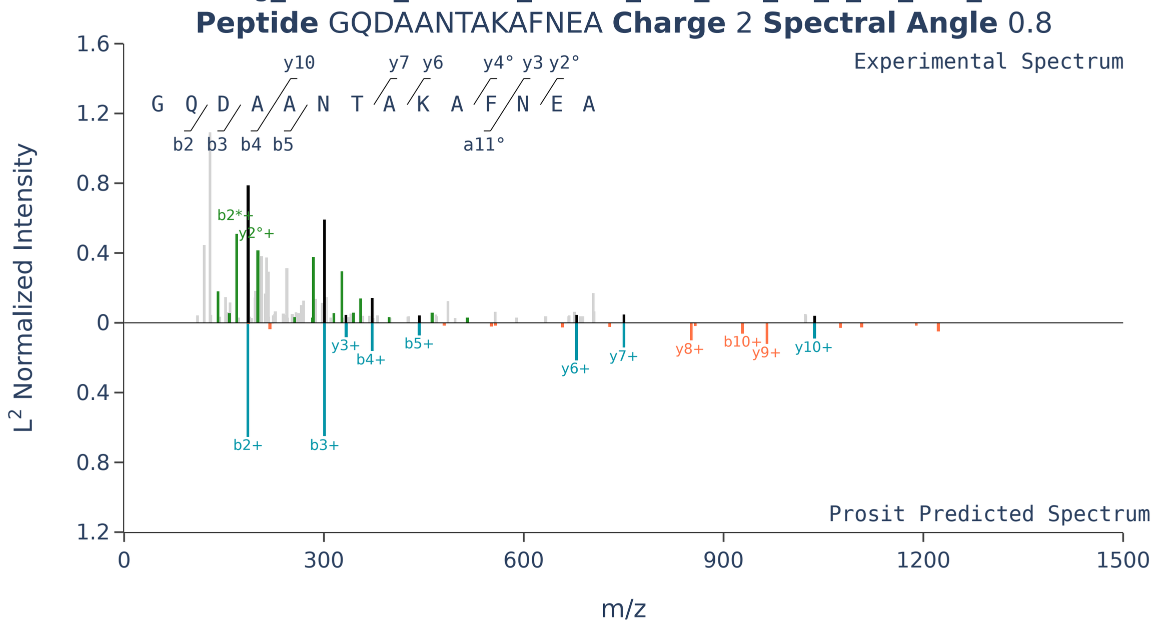


**Source** Ncheng_210623_230623_HFGoe_FFH_20S_25_1_A2_4h_R1 **Scan** 27620
**Peptide** ELVAAMGEENQTLNLAAQ **Charge** 2 **Spectral Angle** 0.87

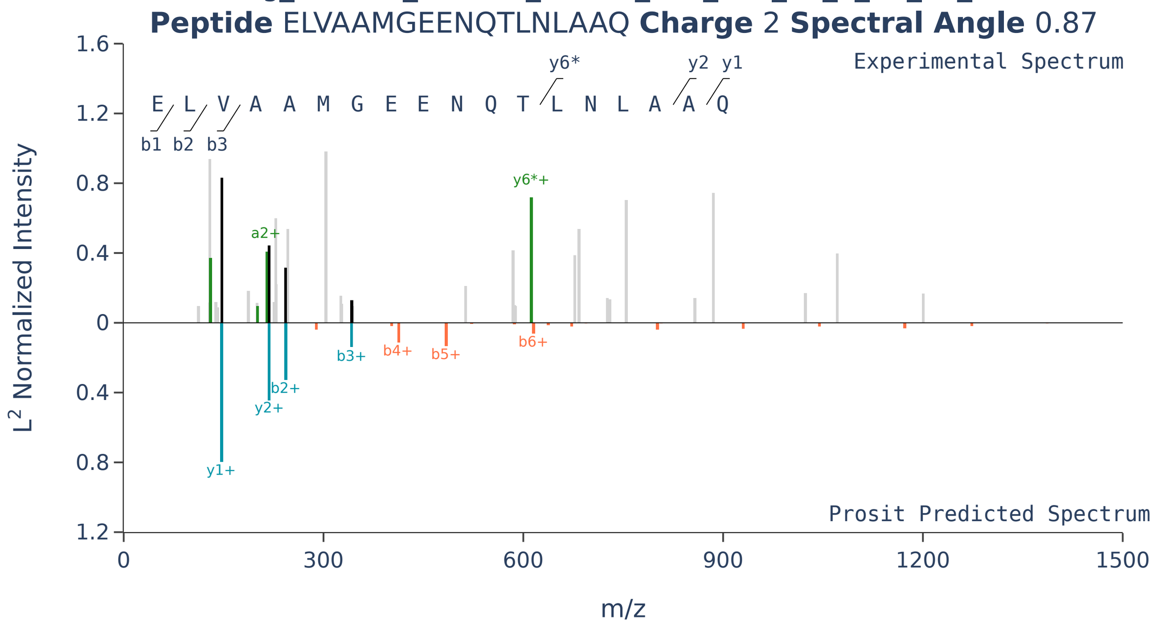


**Source** Ncheng_210623_230623_HFGoe_FFH_20S_25_1_A2_24h_R2 **Scan** 13407
**Peptide** KVDRAQAEKLASKL **Charge** 2 **Spectral Angle** 0.76

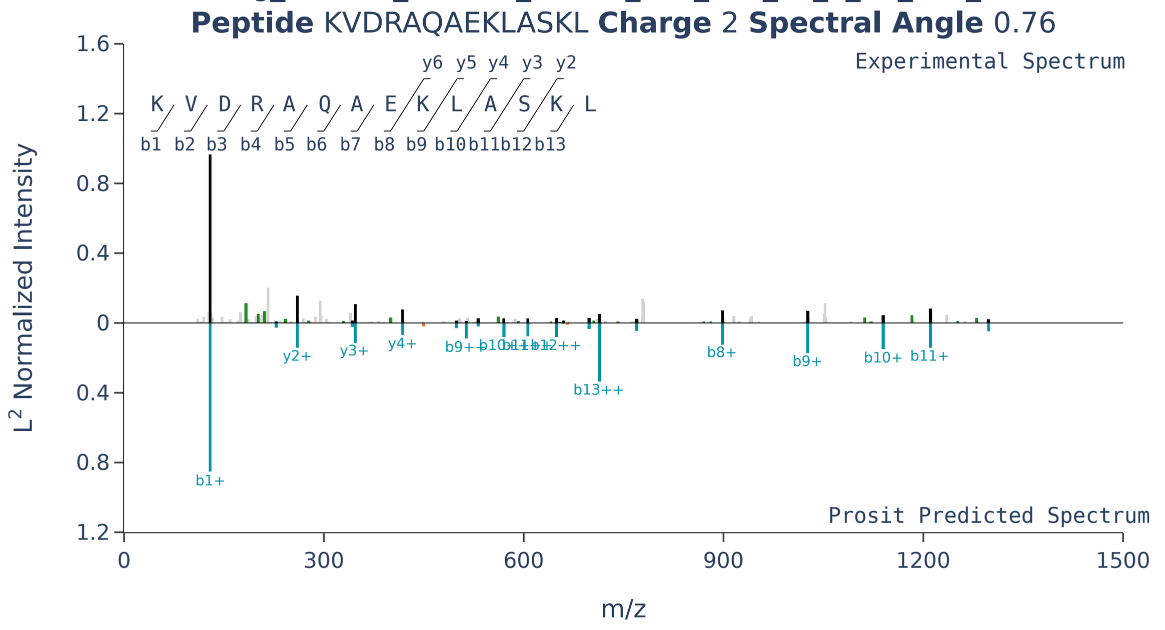


**Source** Ncheng_210623_230623_HFGoe_FFH_20S_25_1_A1_2h_R1 **Scan** 23455
**Peptide** AGRLHVDEAMMDEIKQV **Charge** 3 **Spectral Angle** 0.68

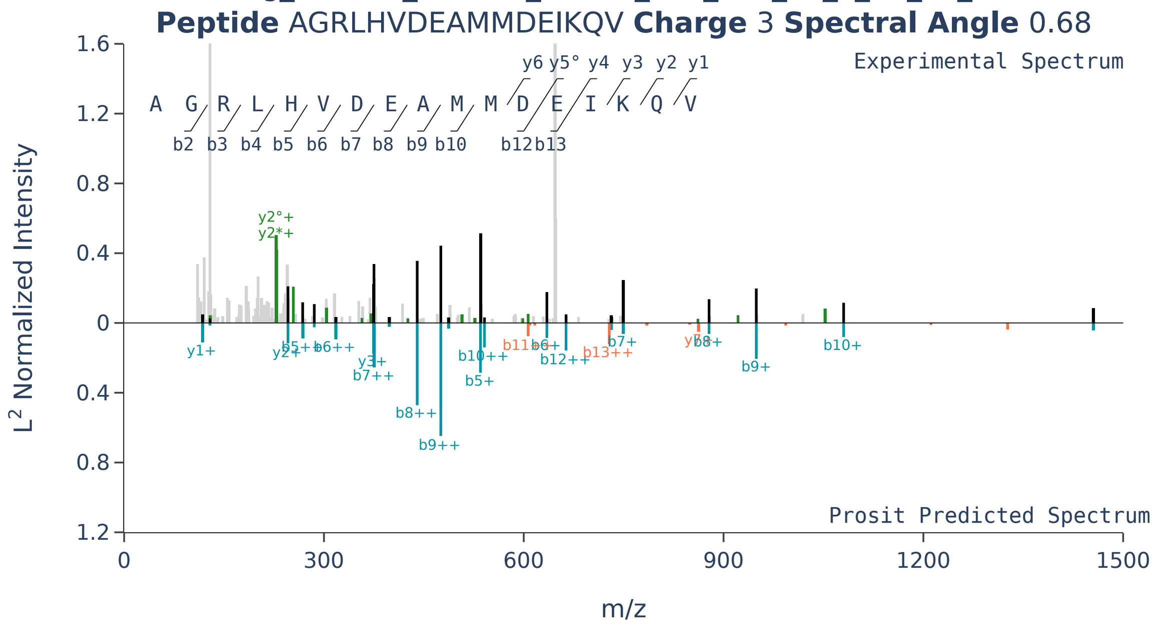


**Source** Ncheng_210623_230623_HFGoe_FFH_20S_25_1_A2_4h_R1 **Scan** 24609
**Peptide** VGQKPVDIVNAAL **Charge** 2 **Spectral Angle** 0.73

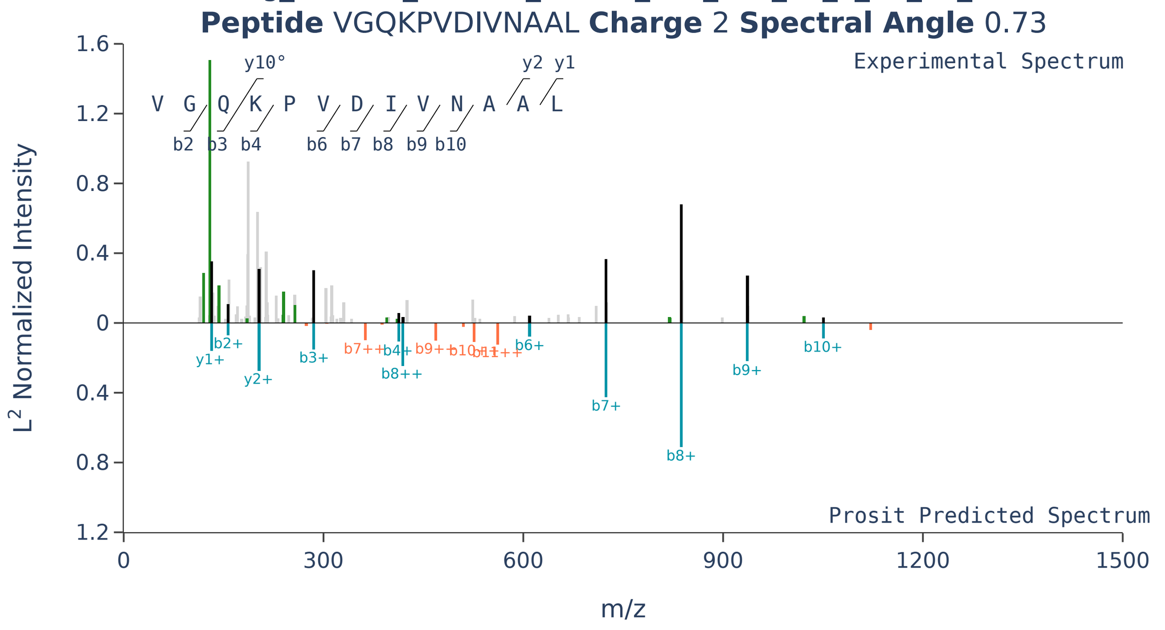


**Source** Ncheng_210623_230623_HFGoe_FFH_20S_25_1_A1_2h_R1 **Scan** 32483
**Peptide** AANTAKAFNEALPLTGVVLTKVD **Charge** 3 **Spectral Angle** 0.75

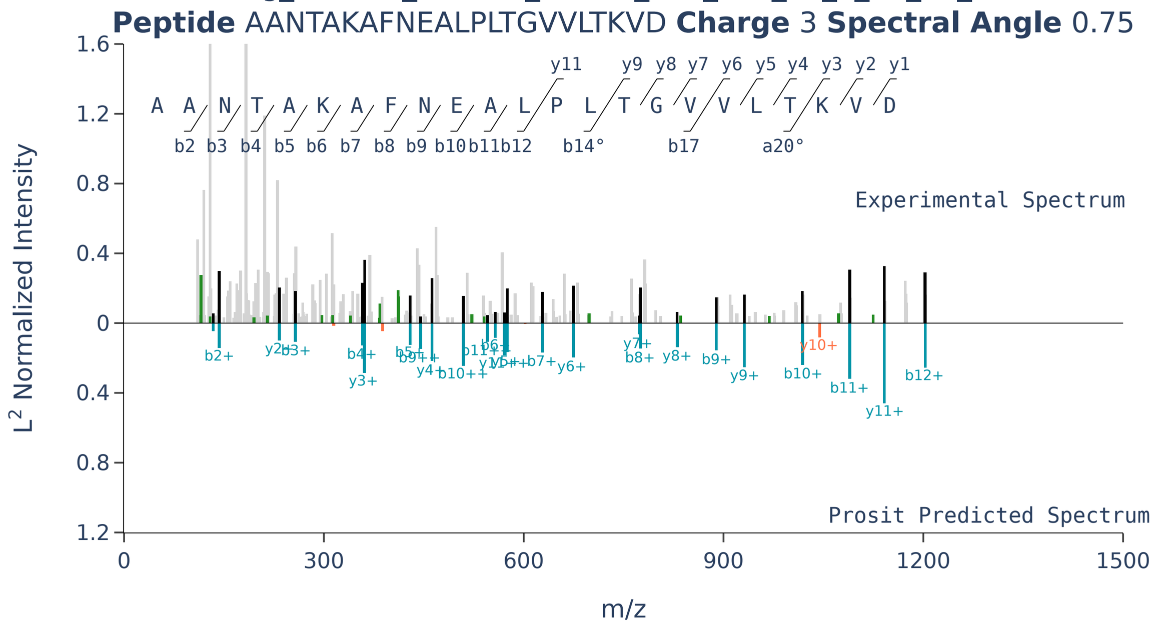


**Source** Ncheng_210623_230623_HFGoe_FFH_20S_25_1_A2_24h_R2 **Scan** 11952
**Peptide** TGQDAANTAKAFNEA **Charge** 2 **Spectral Angle** 0.7

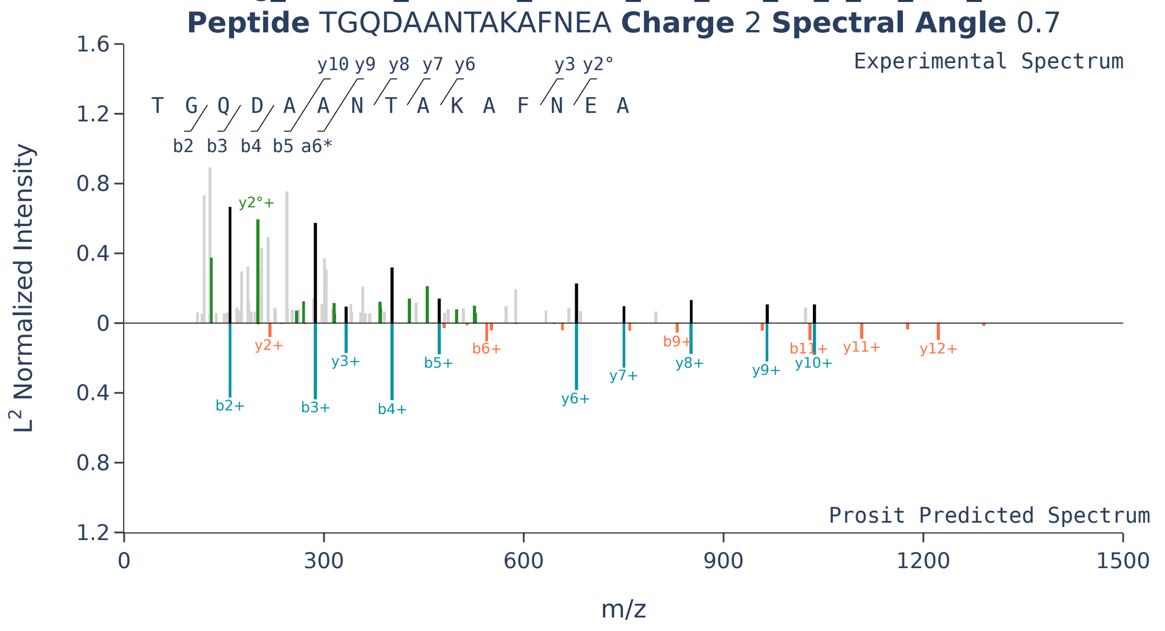


**Source** Ncheng_210623_230623_HFGoe_FFH_20S_25_1_A2_4h_R1 **Scan** 31081
**Peptide** NKSLTPGQEFVKIVRNELV **Charge** 3 **Spectral Angle** 0.66

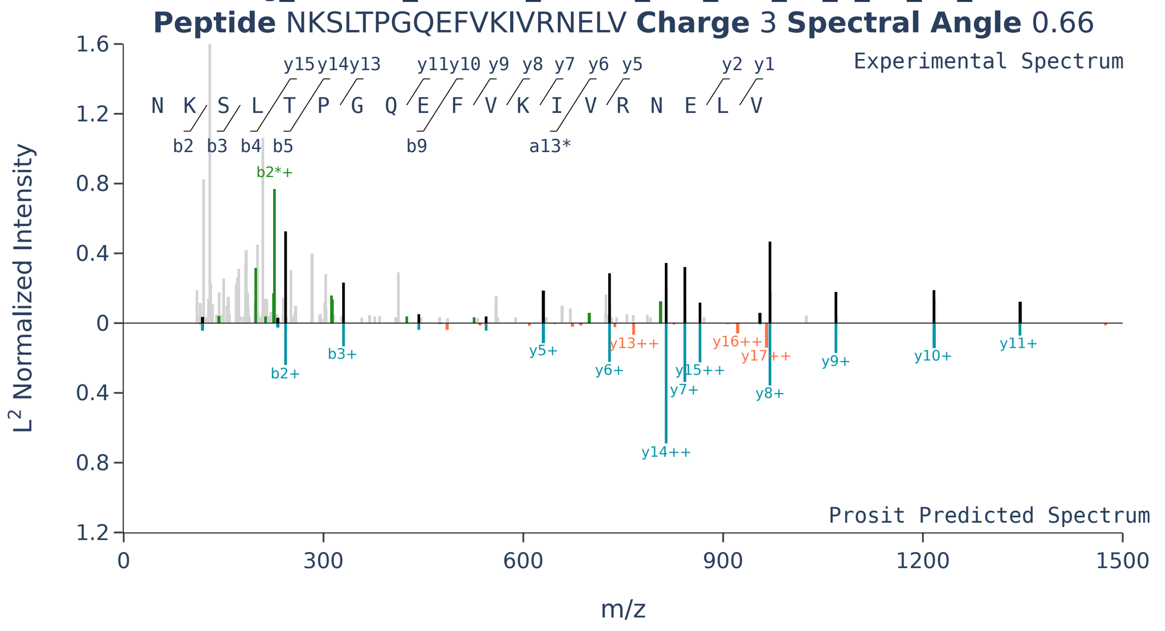


**Source** Ncheng_210623_230623_HFGoe_FFH_20S_25_1_A2_4h_R2 **Scan** 20899
**Peptide** GLQGAGKTTSVGKLGKFL **Charge** 2 **Spectral Angle** 0.64

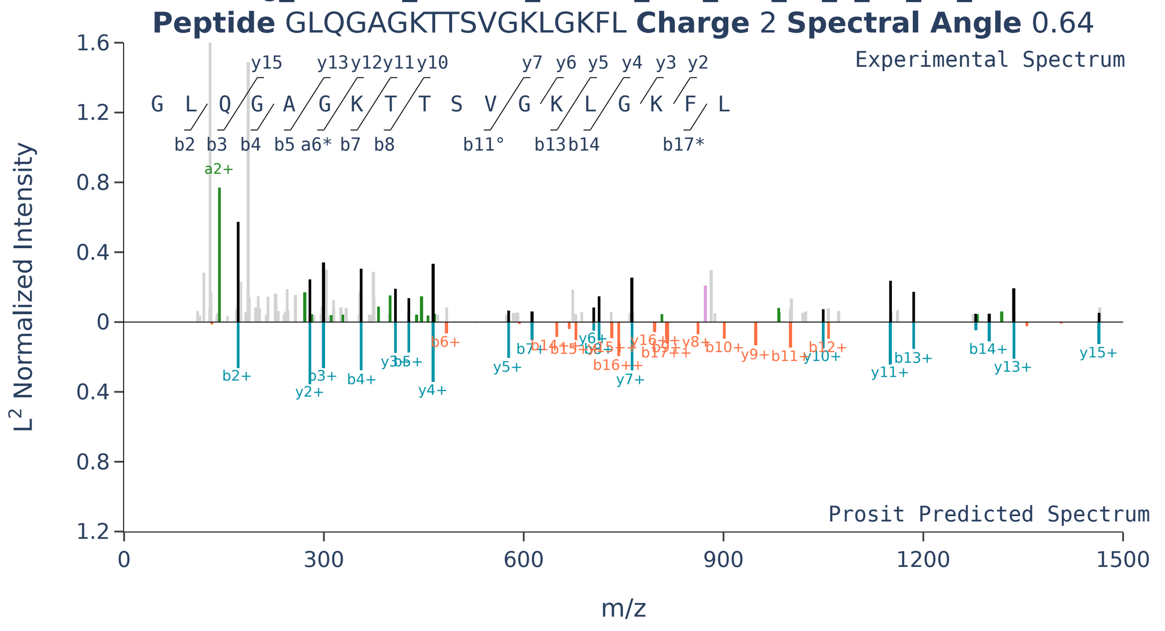


**Source** Ncheng_210623_230623_HFGoe_FFH_20S_25_1_A2_4h_R1 **Scan** 8606
**Peptide** ESKVDRAQAE **Charge** 2 **Spectral Angle** 0.81

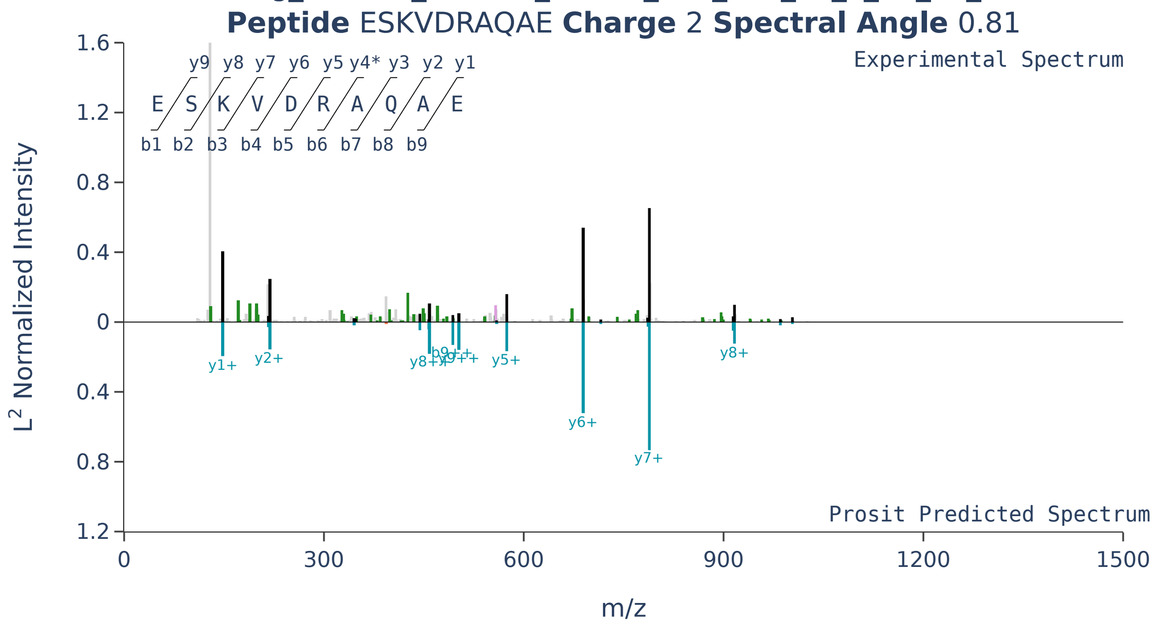


**Source** Ncheng_210623_230623_HFGoe_FFH_20S_25_1_A2_2h_R1 **Scan** 38303
**Peptide** ENQTLNLAAQPPAVVLMAGLQGAGKTT **Charge** 3 **Spectral Angle** 0.68

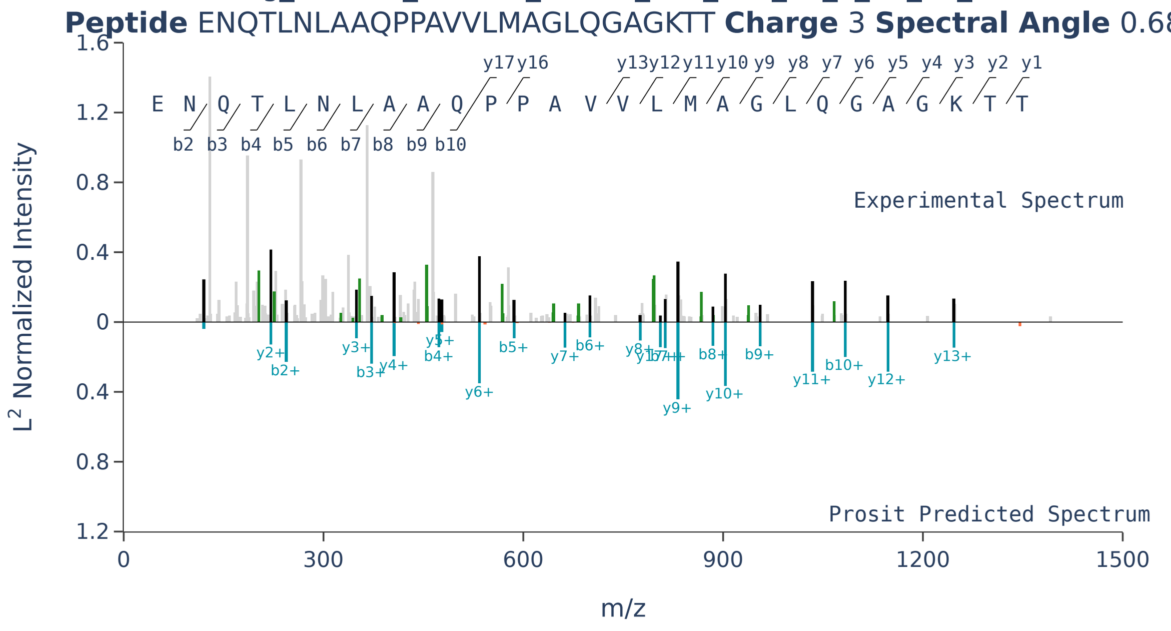


**Source** Ncheng_210623_230623_HFGoe_FFH_20S_25_1_A2_4h_R2 **Scan** 31611
**Peptide** GHEVNKSLTPGQEFVKIVRNELVAA **Charge** 3 **Spectral Angle** 0.83

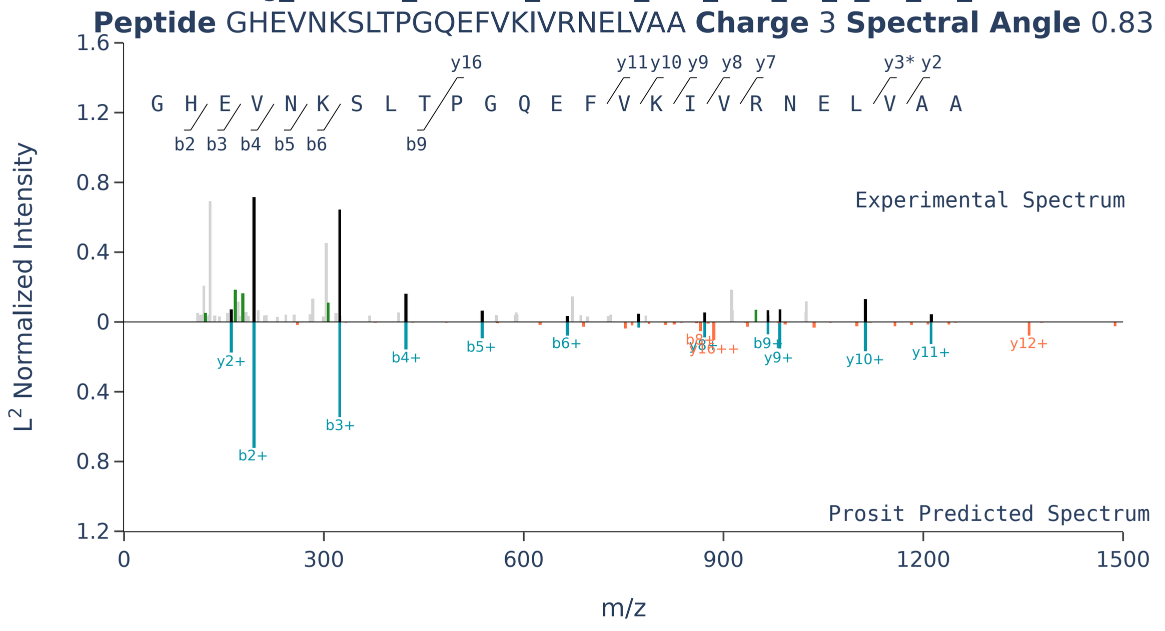


**Source** Ncheng_210623_230623_HFGoe_FFH_20S_25_1_A1_2h_R2 **Scan** 18986
**Peptide** ETLAEQVGVD **Charge** 2 **Spectral Angle** 0.7

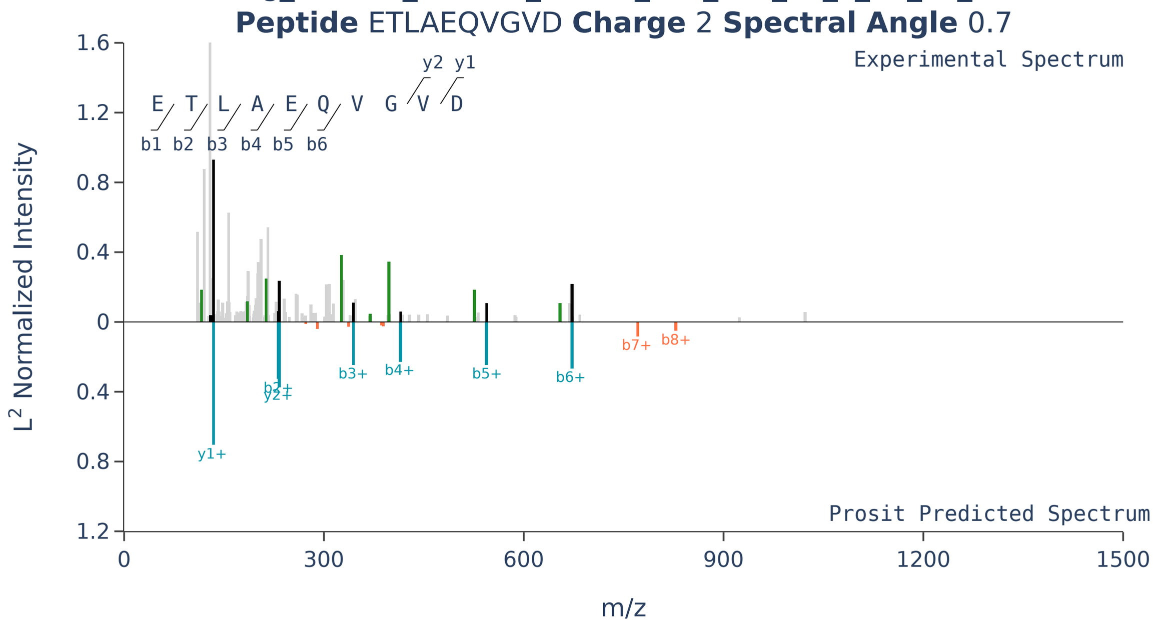


**Source** Ncheng_210623_230623_HFGoe_FFH_20S_25_1_A2_24h_R1 **Scan** 19946
**Peptide** VGHEVNKSLTPGQEFVKIVR **Charge** 3 **Spectral Angle** 0.68

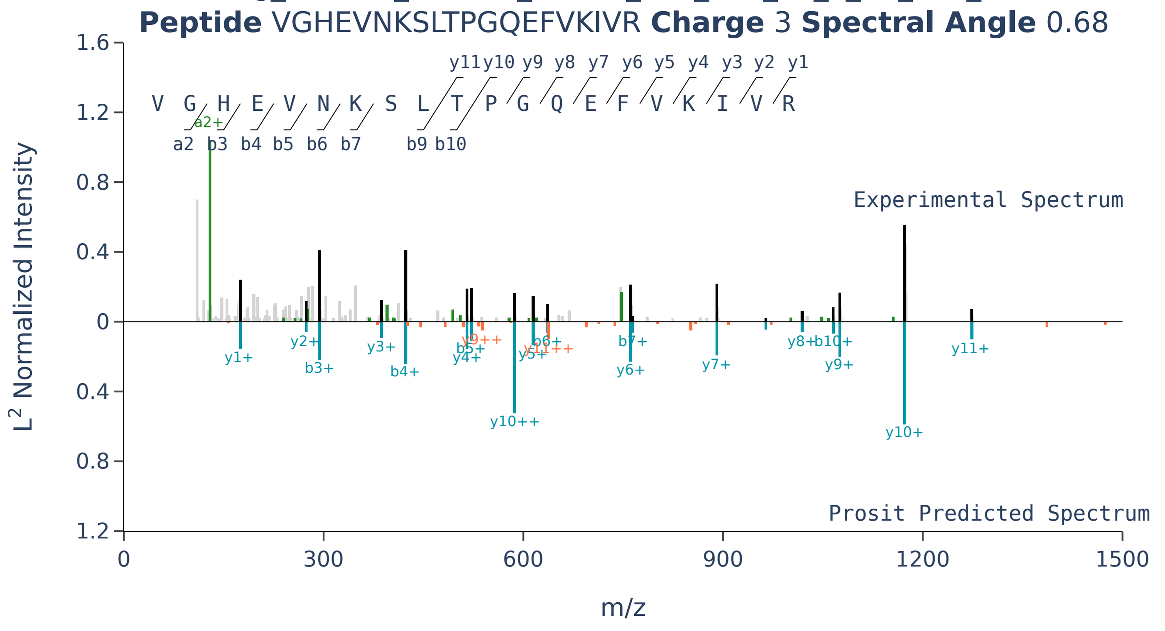

**Source** Ncheng_210623_230623_HFGoe_FFH_20S_25_1_A2_24h_R2 **Scan** 9228
**Peptide** EQLRQMKNM **Charge** 2 **Spectral Angle** 0.5

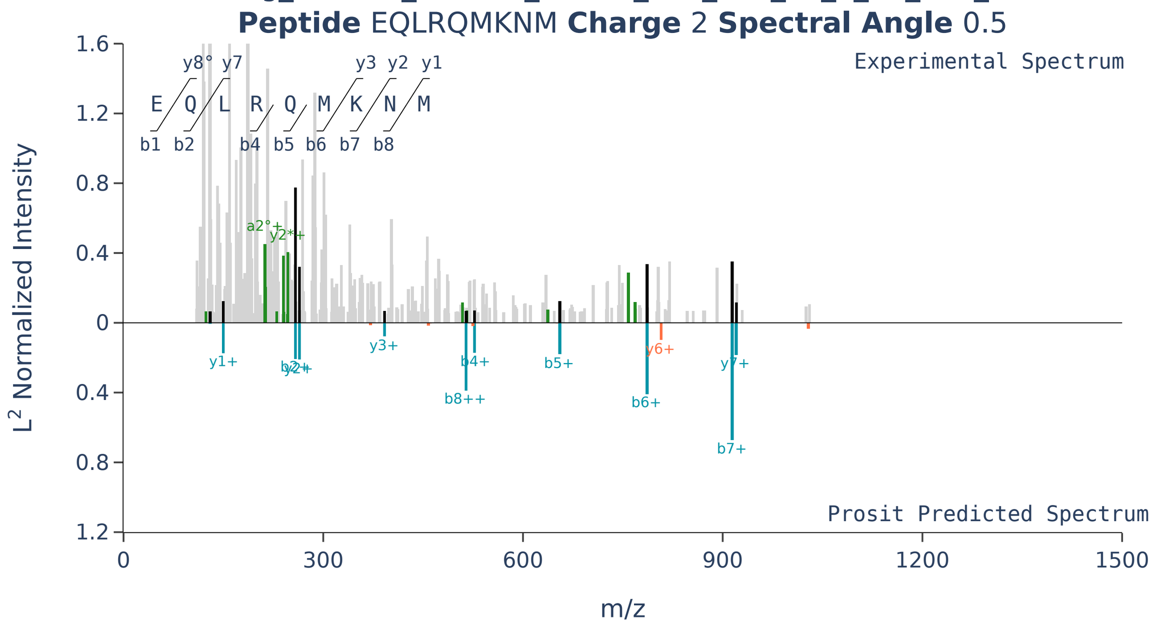


**Source** Ncheng_210623_230623_HFGoe_FFH_20S_25_1_A1_24h_R1 **Scan** 10384
**Peptide** QAEKLASKLKKGDGFD **Charge** 2 **Spectral Angle** 0.67

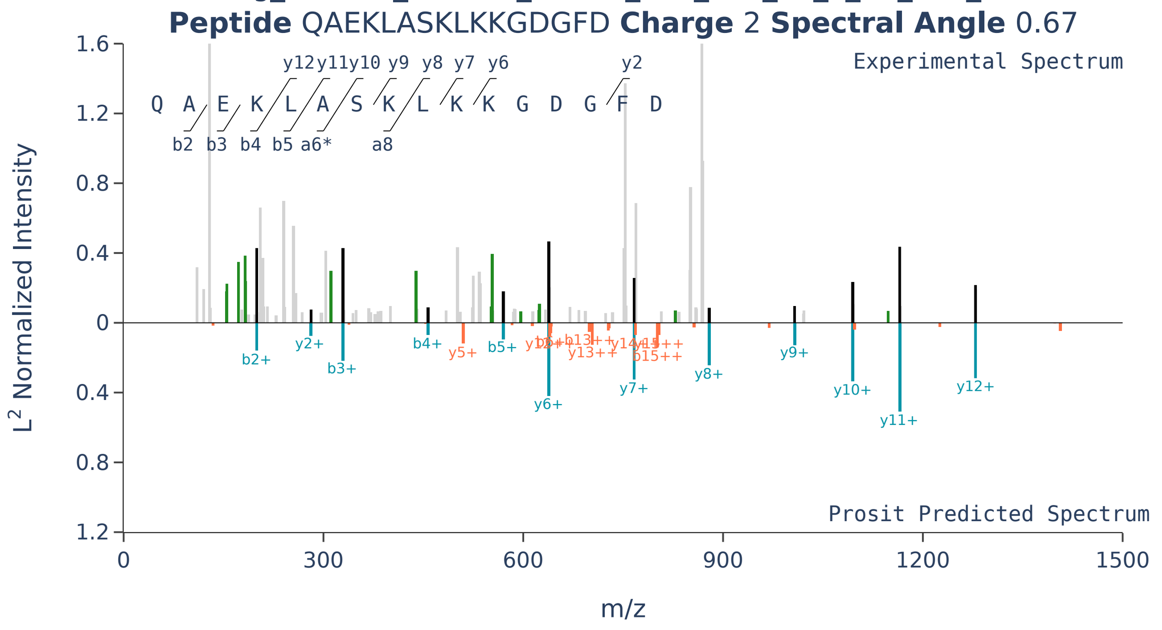


**Source** Ncheng_210623_230623_HFGoe_FFH_20S_25_1_A2_24h_R2 **Scan** 13641
**Peptide** NSMTMKERAKPEII **Charge** 2 **Spectral Angle** 0.68

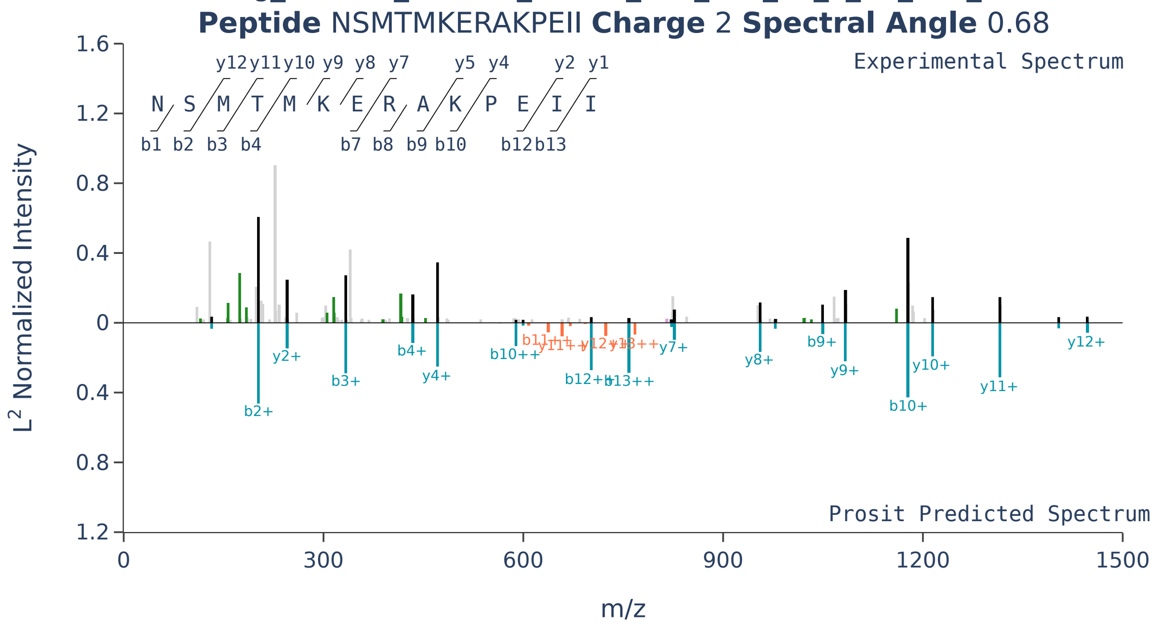


**Source** Ncheng_210623_230623_HFGoe_FFH_20S_25_1_A1_4h_R1 **Scan** 16955
**Peptide** RHITGKPIKFLGVGE **Charge** 3 **Spectral Angle** 0.82

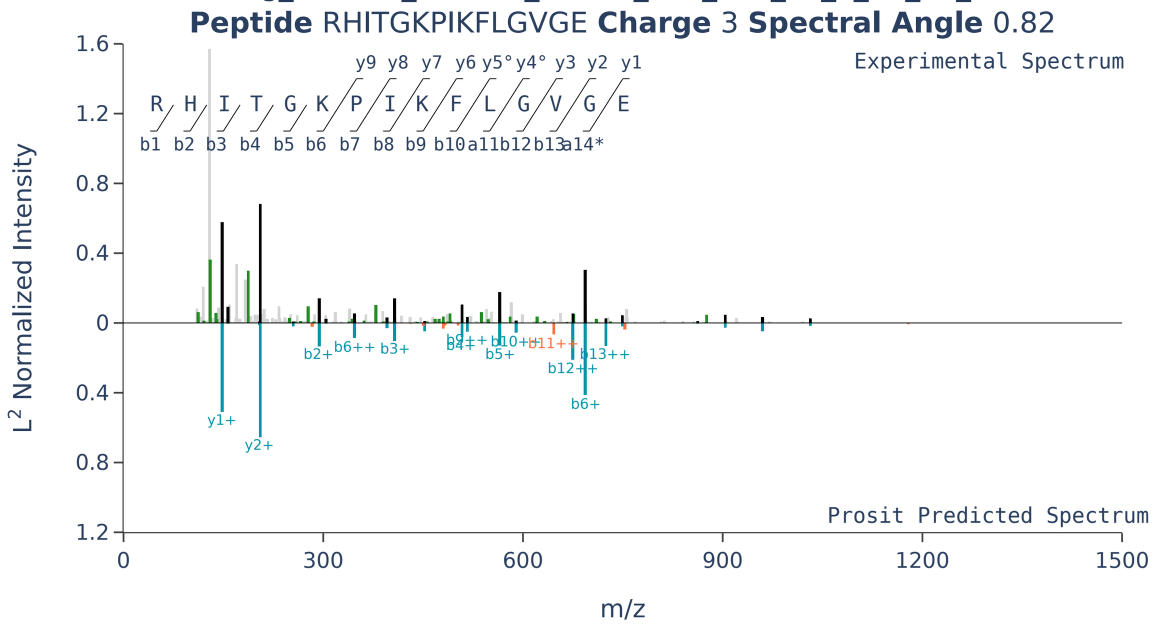


**Source** Ncheng_210623_230623_HFGoe_FFH_20S_25_1_A2_24h_R1 **Scan** 19332
**Peptide** ALKEAKLKFYDV **Charge** 3 **Spectral Angle** 0.73

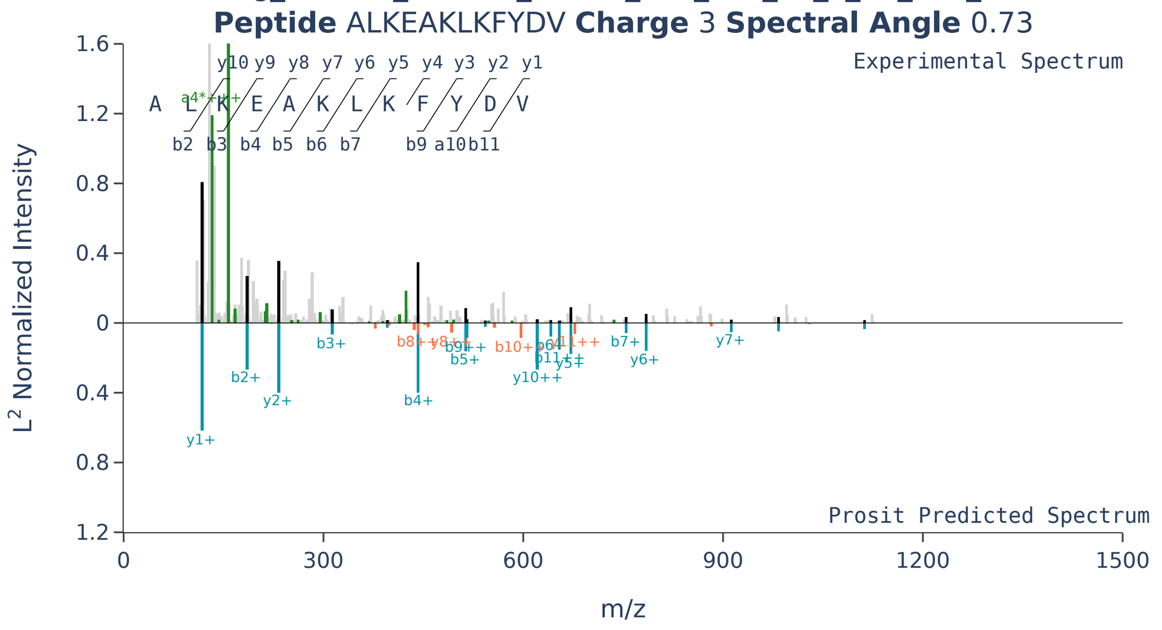


**Source** Ncheng_210623_230623_HFGoe_FFH_20S_25_1_A2_2h_R1 **Scan** 31747
**Peptide** IVNAALKEAKLKFYDVLLVD **Charge** 3 **Spectral Angle** 0.64

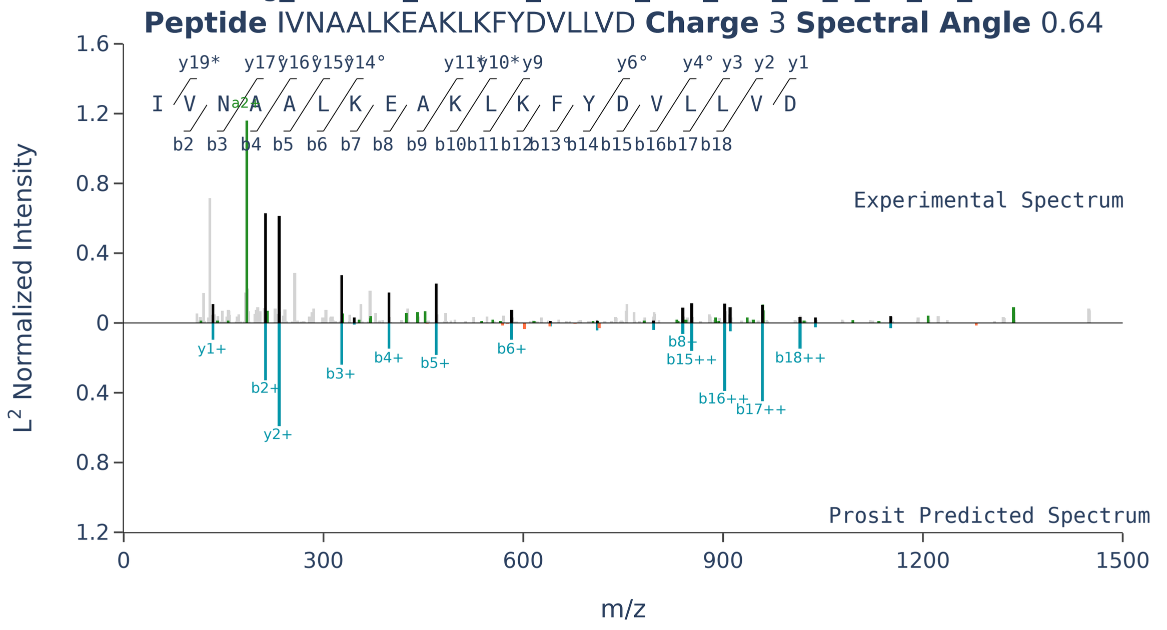


**Source** Ncheng_210623_230623_HFGoe_FFH_20S_25_1_A1_24h_R2 **Scan** 6803
**Peptide** AALKEAKLK **Charge** 2 **Spectral Angle** 0.66

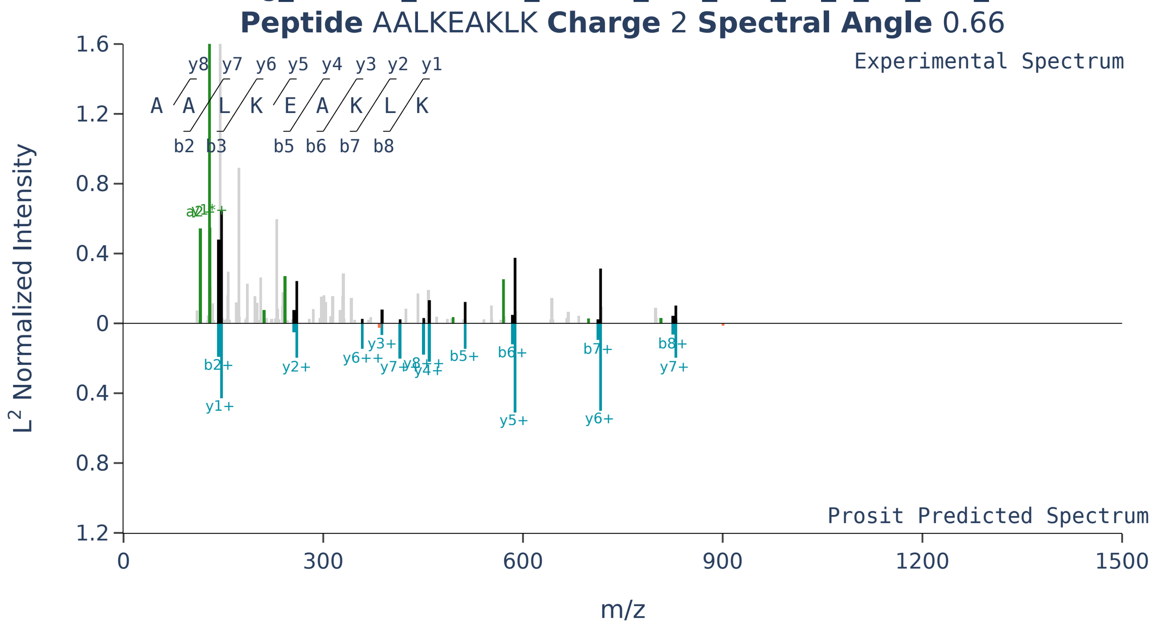


**Source** Ncheng_210623_230623_HFGoe_FFH_20S_25_1_A1_24h_R2 **Scan** 10140
**Peptide** SMTMKERAKPEI **Charge** 2 **Spectral Angle** 0.7

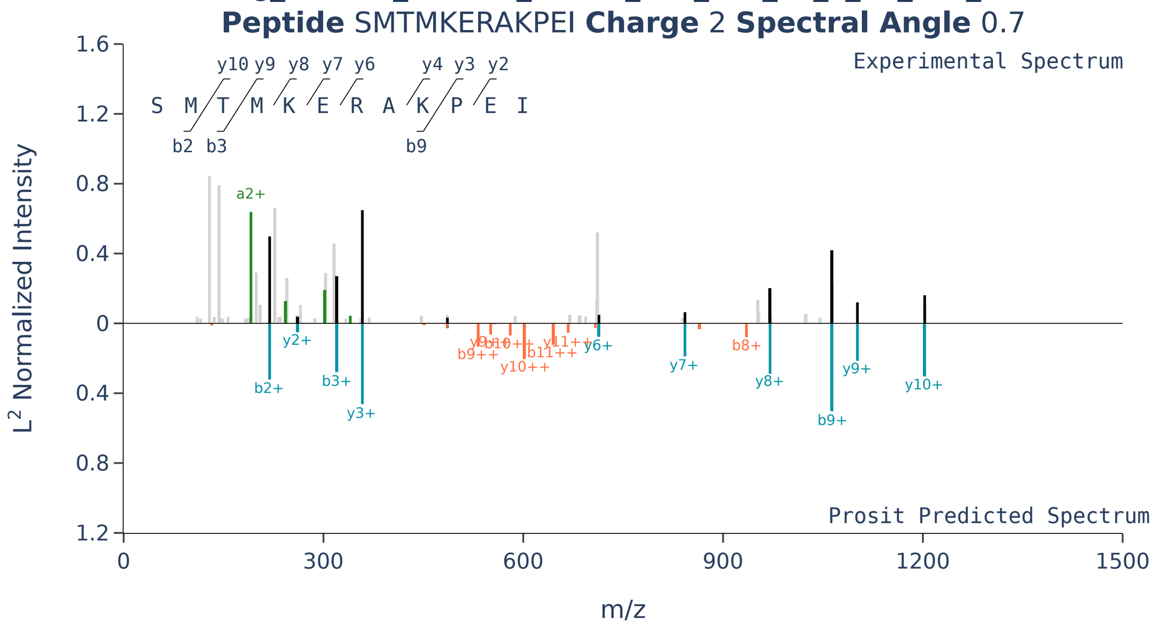


**Source** Ncheng_210623_230623_HFGoe_FFH_20S_25_1_A1_24h_R2 **Scan** 9021
**Peptide** GLQGAGKTTSVGKLGK **Charge** 2 **Spectral Angle** 0.68

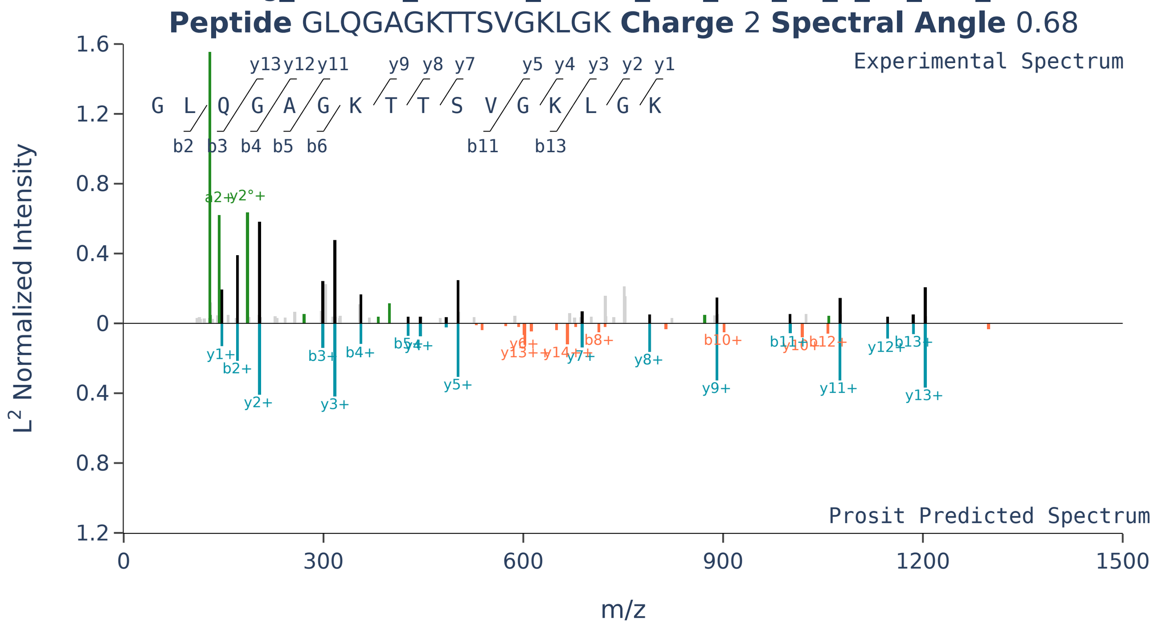


**Source** Ncheng_210623_230623_HFGoe_FFH_20S_25_1_A2_24h_R1 **Scan** 8210
**Peptide** MKKGGMAKMM **Charge** 3 **Spectral Angle** 0.81

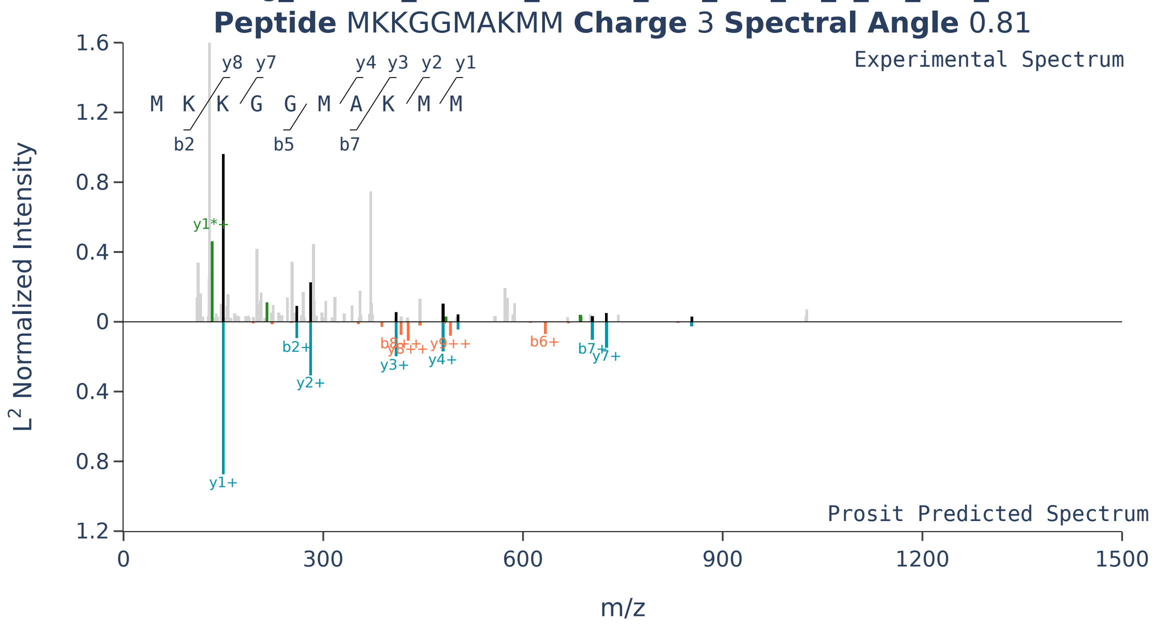


**Source** Ncheng_210623_230623_HFGoe_FFH_20S_25_1_A1_24h_R2 **Scan** 9475
**Peptide** TLRNISGRGRL **Charge** 2 **Spectral Angle** 0.74

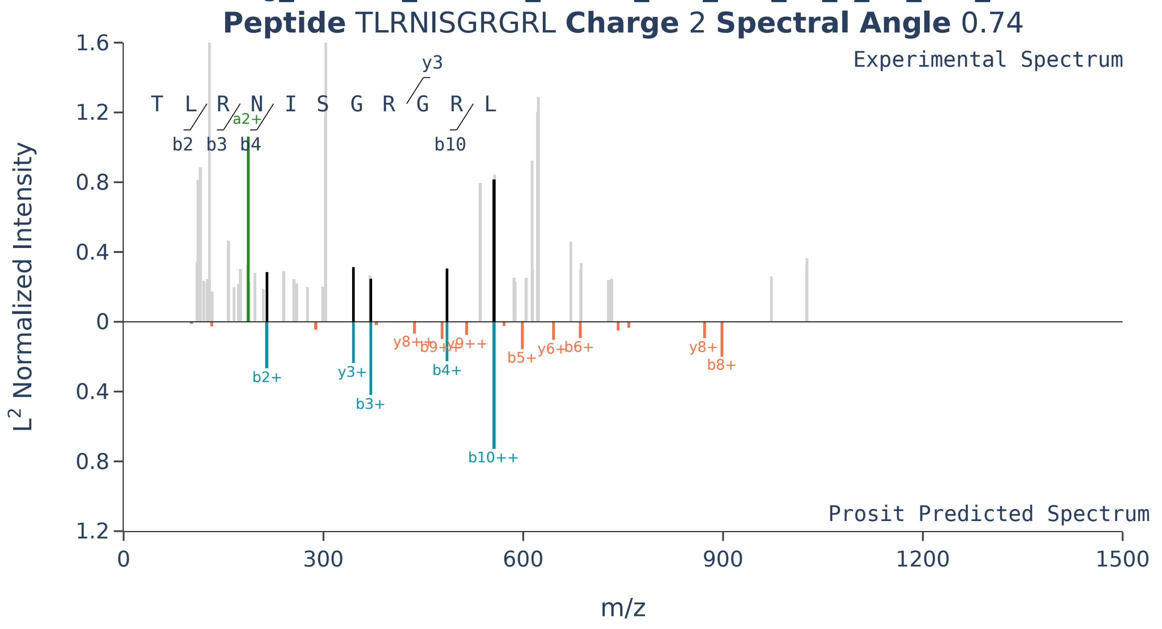


**Source** Ncheng_210623_230623_HFGoe_FFH_20S_25_1_A2_24h_R2 **Scan** 9619
**Peptide** MTMKERAKPEIIKG **Charge** 2 **Spectral Angle** 0.66

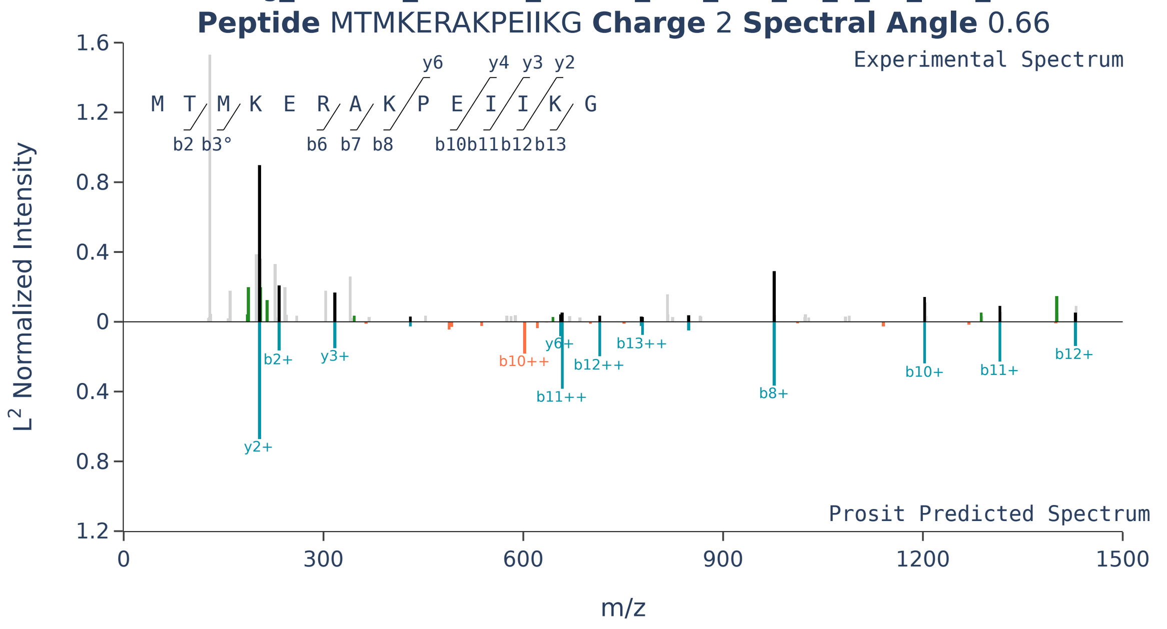


**Source** Ncheng_210623_230623_HFGoe_FFH_20S_25_1_A2_2h_R2 **Scan** 12137
**Peptide** VVDAMTGQDAANTA **Charge** 2 **Spectral Angle** 0.81

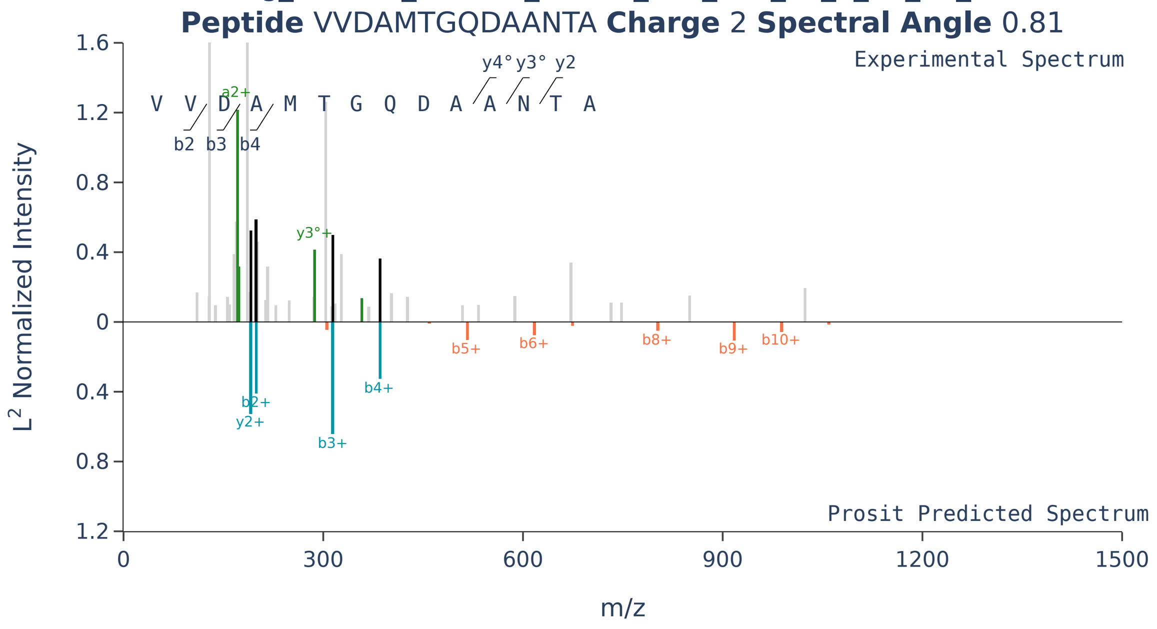


**Source** Ncheng_210623_230623_HFGoe_FFH_20S_25_1_A2_1h_R2 **Scan** 15110
**Peptide** VNRLLKQF **Charge** 2 **Spectral Angle** 0.71

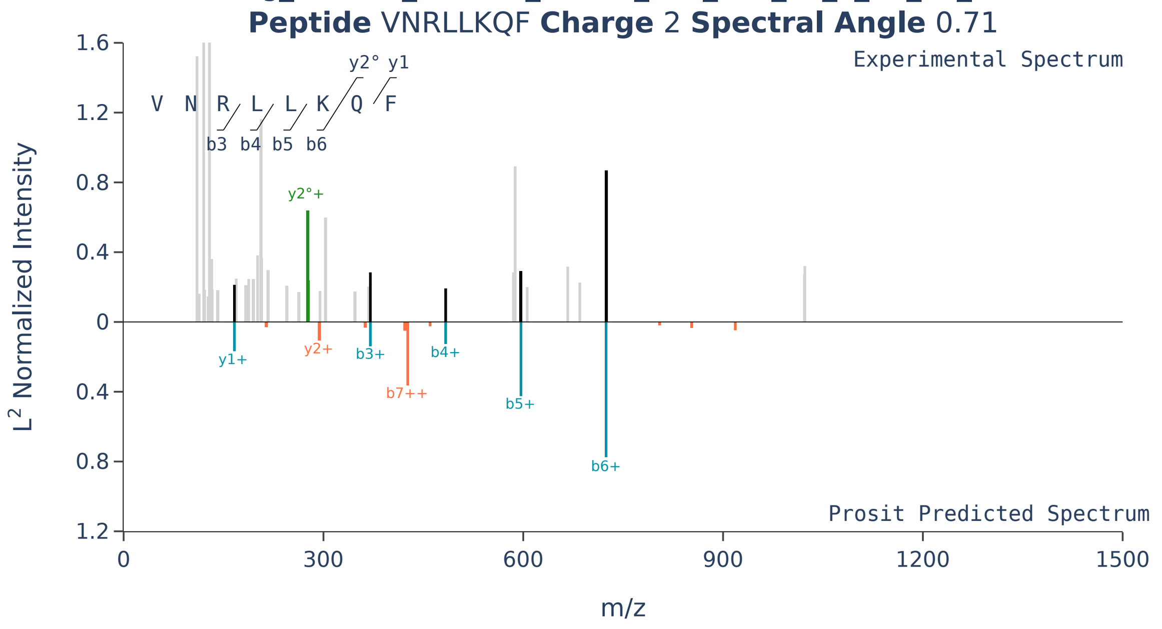


**Source** Ncheng_210623_230623_HFGoe_FFH_20S_25_1_A2_4h_R2 **Scan** 10543
**Peptide** KLGKFLRE **Charge** 2 **Spectral Angle** 0.72

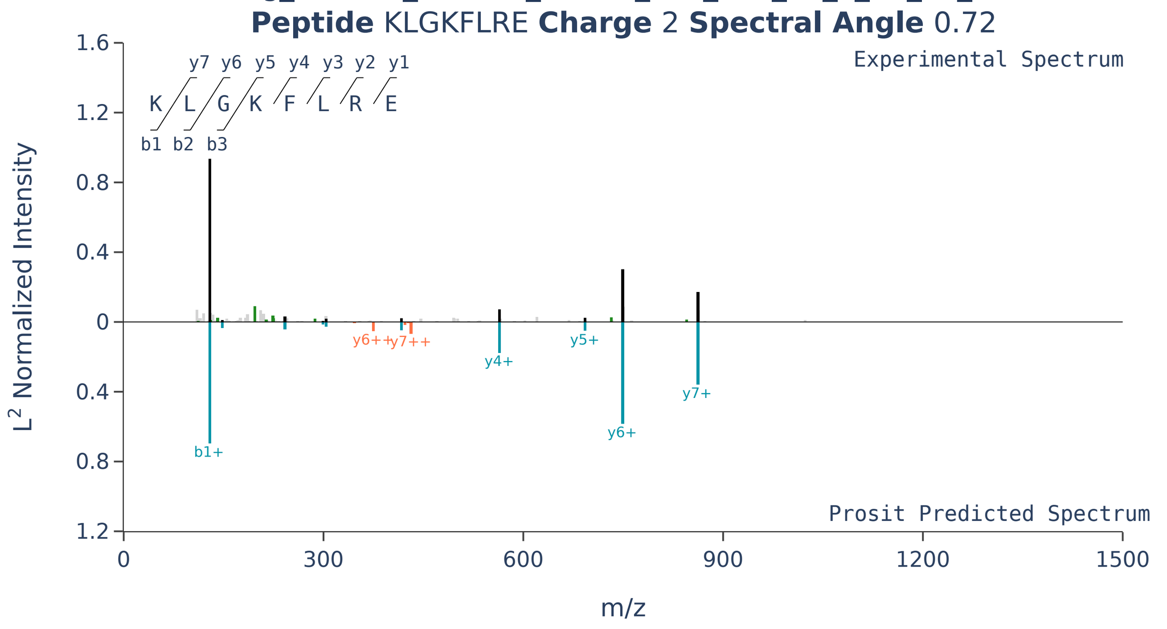

**Source** Ncheng_210623_230623_HFGoe_FFH_20S_25_1_A1_2h_R1 **Scan** 31177
**Peptide** ADVALPVVREFINRVKEKAVGHEVNK **Charge** 5 **Spectral Angle** 0.83

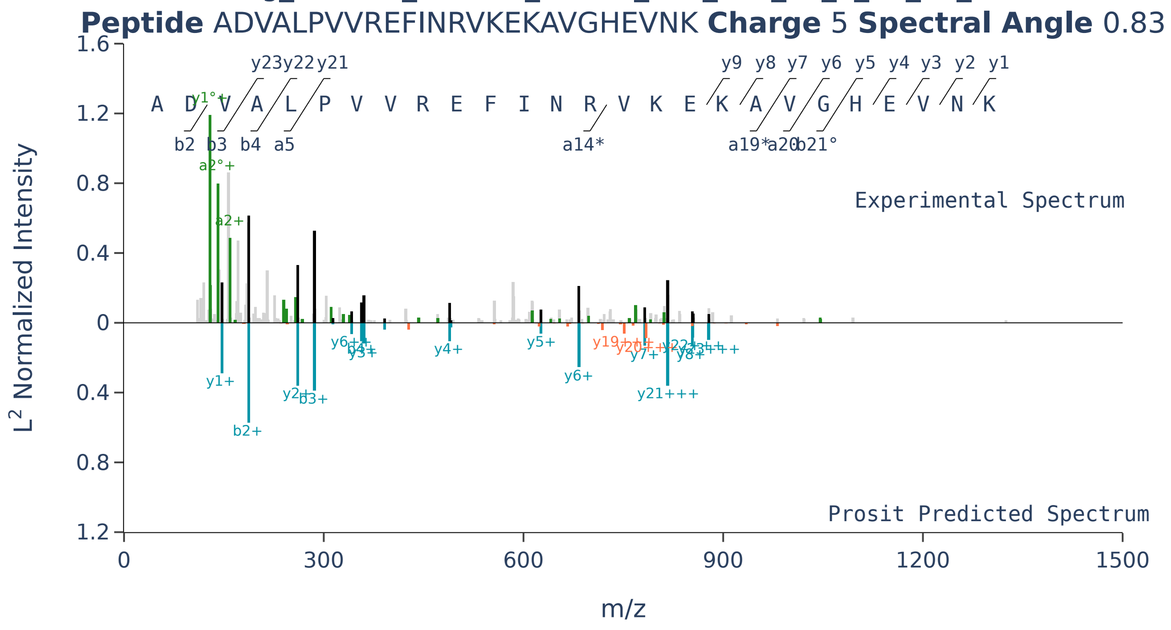


**Source** Ncheng_210623_230623_HFGoe_FFH_20S_25_1_A1_24h_R2 **Scan** 19663
**Peptide** EAKLKFYDV **Charge** 2 **Spectral Angle** 0.57

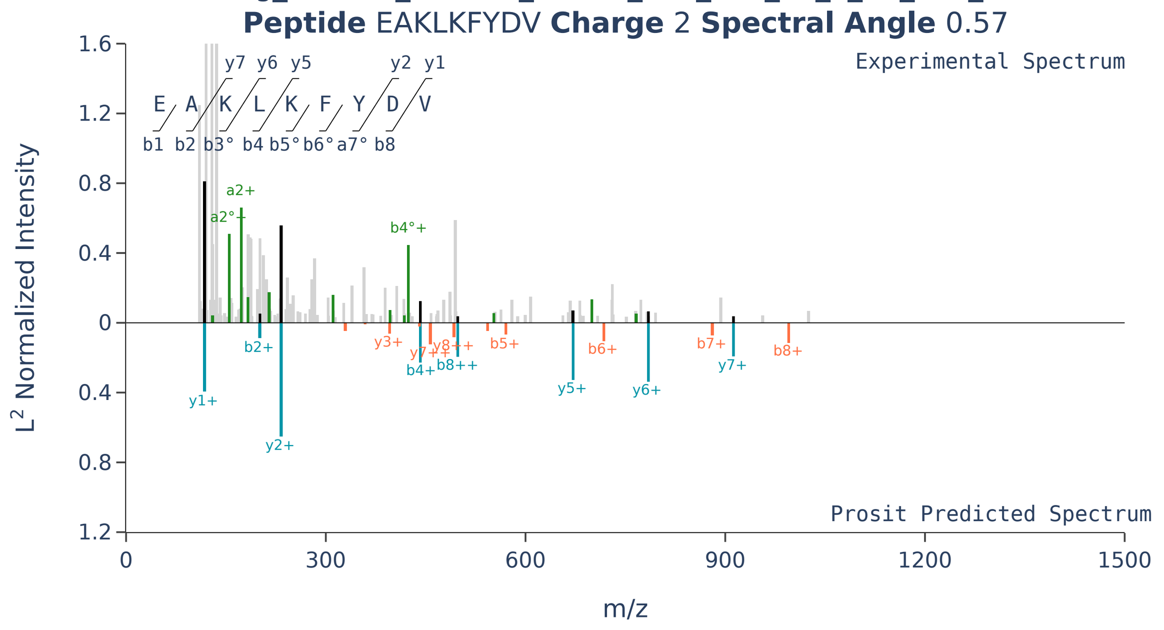


**Source** Ncheng_210623_230623_HFGoe_FFH_20S_25_1_A2_4h_R2 **Scan** 40837
**Peptide** DLNDFLEQL **Charge** 2 **Spectral Angle** 0.79

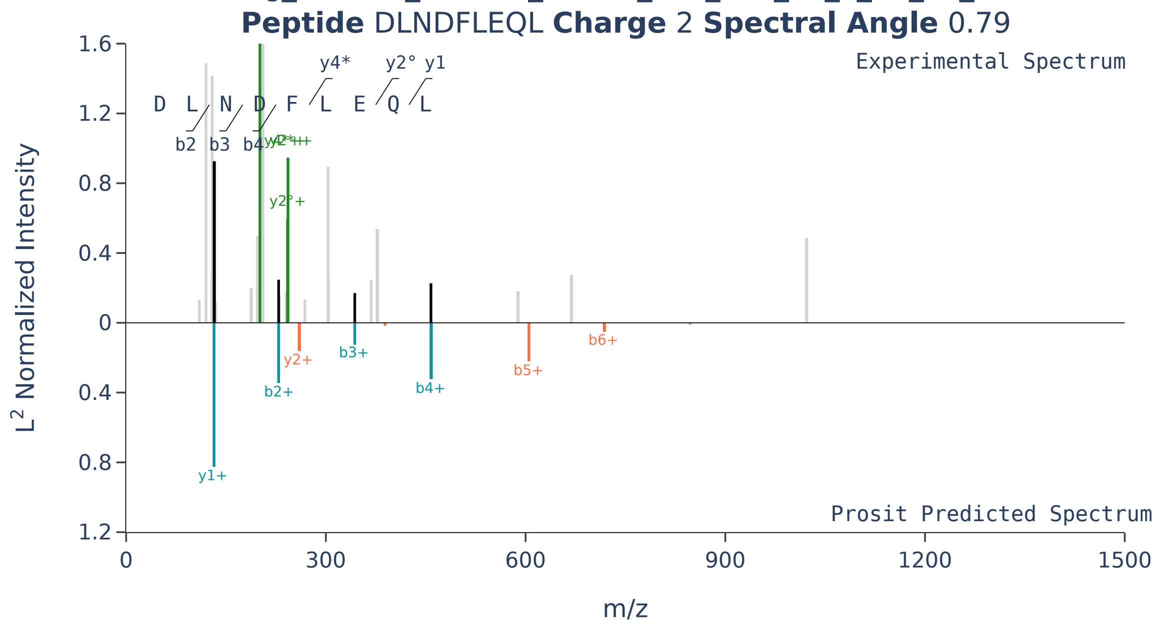


**Source** Ncheng_210623_230623_HFGoe_FFH_20S_25_1_A2_24h_R1 **Scan** 10246
**Peptide** VDGDARGGAALSIRH **Charge** 4 **Spectral Angle** 0.84

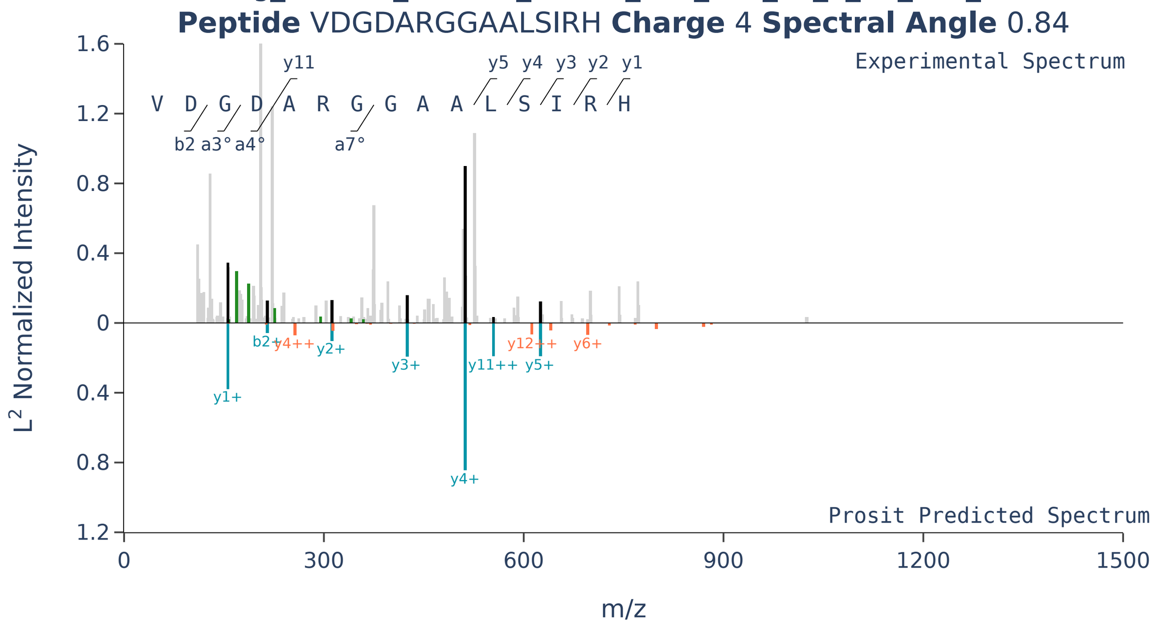


**Source** Ncheng_210623_230623_HFGoe_FFH_20S_25_1_A1_1h_R2 **Scan** 35559
**Peptide** LVAAMGEENQTLNLAAQPPAVVL **Charge** 2 **Spectral Angle** 0.91

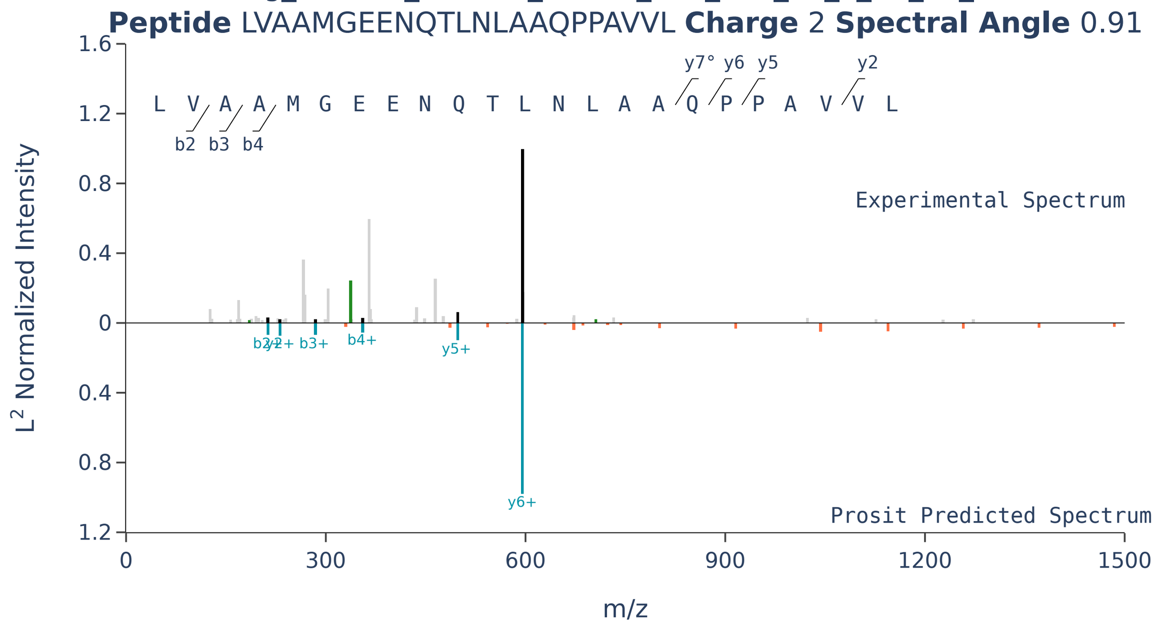


**Source** Ncheng_210623_230623_HFGoe_FFH_20S_25_1_A1_24h_R2 **Scan** 6985
**Peptide** KMKKGGMAKMM **Charge** 3 **Spectral Angle** 0.95

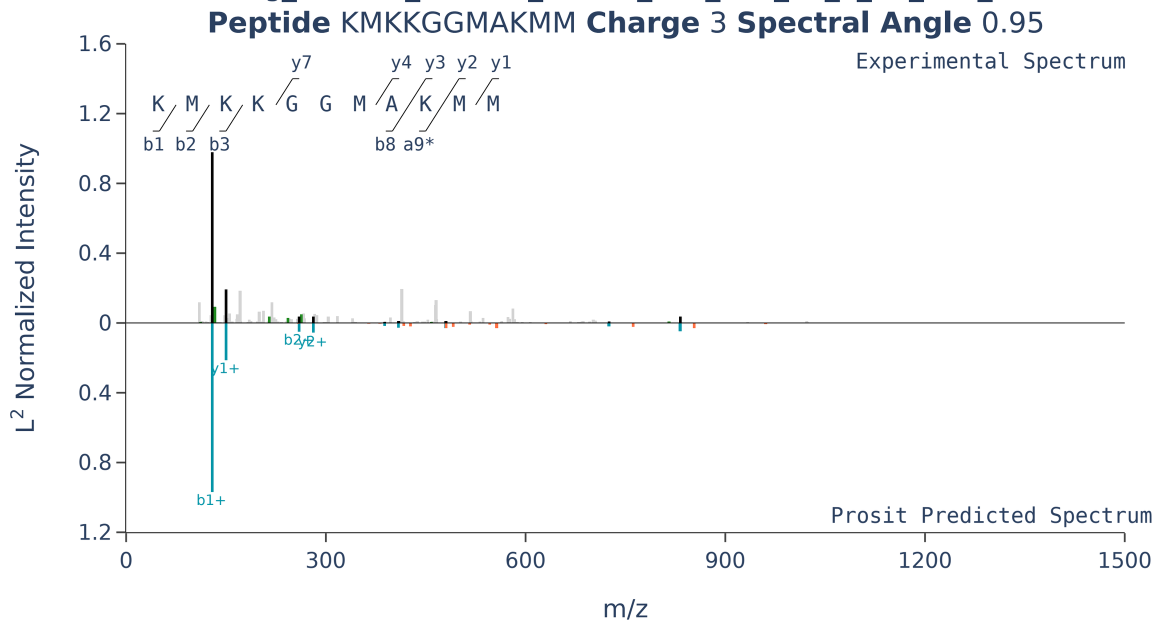


**Source** Ncheng_210623_230623_HFGoe_FFH_20S_25_1_A1_4h_R1 **Scan** 12471
**Peptide** GDARGGAALSIRHITGK **Charge** 4 **Spectral Angle** 0.7

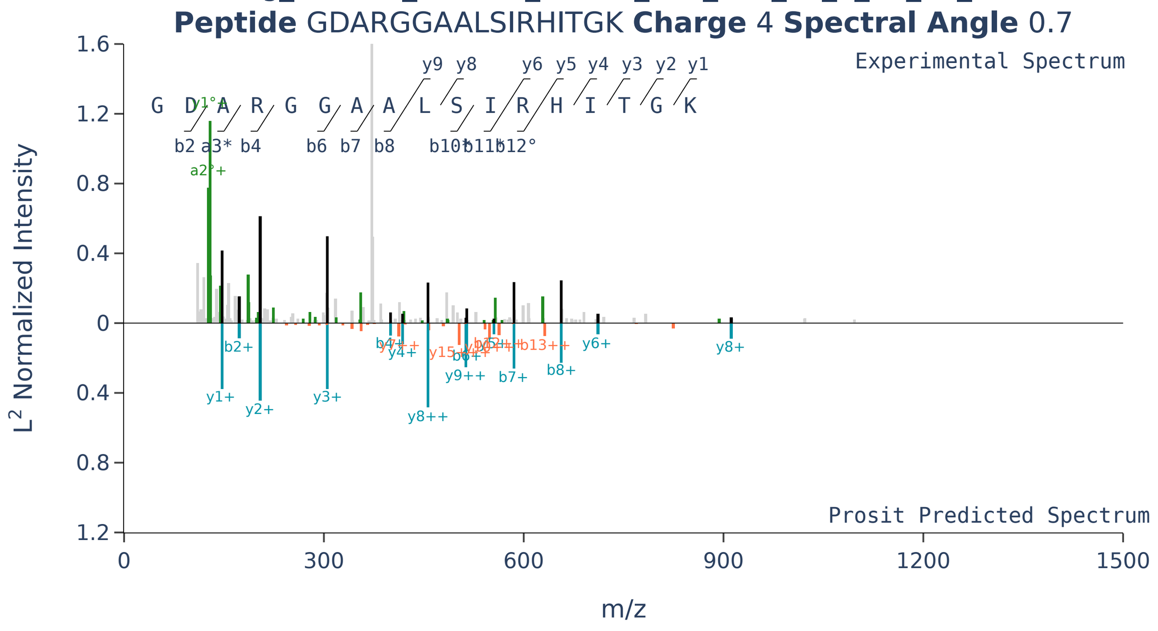


**Source** Ncheng_210623_230623_HFGoe_FFH_20S_25_1_A2_4h_R2 **Scan** 34271
**Peptide** EVNKSLTPGQEFVKIVRNELVAA **Charge** 3 **Spectral Angle** 0.68

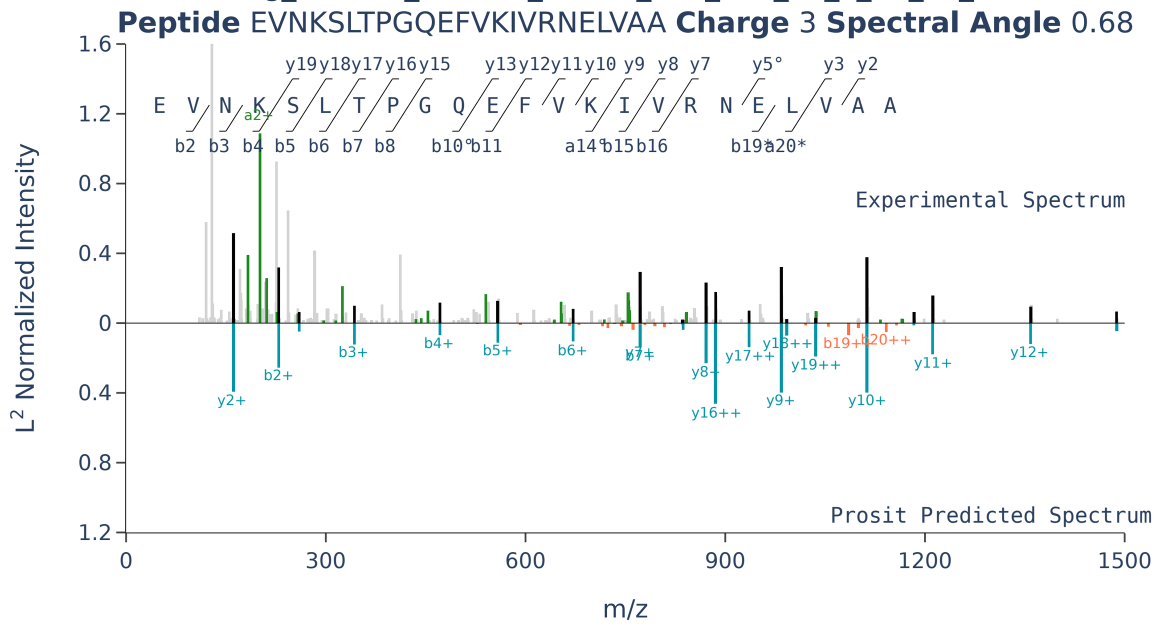


**Source** Ncheng_210623_230623_HFGoe_FFH_20S_25_1_A2_4h_R2 **Scan** 39699
**Peptide** EENQTLNLAAQPPAVVLMAGLQGAGKTT **Charge** 3 **Spectral Angle** 0.66

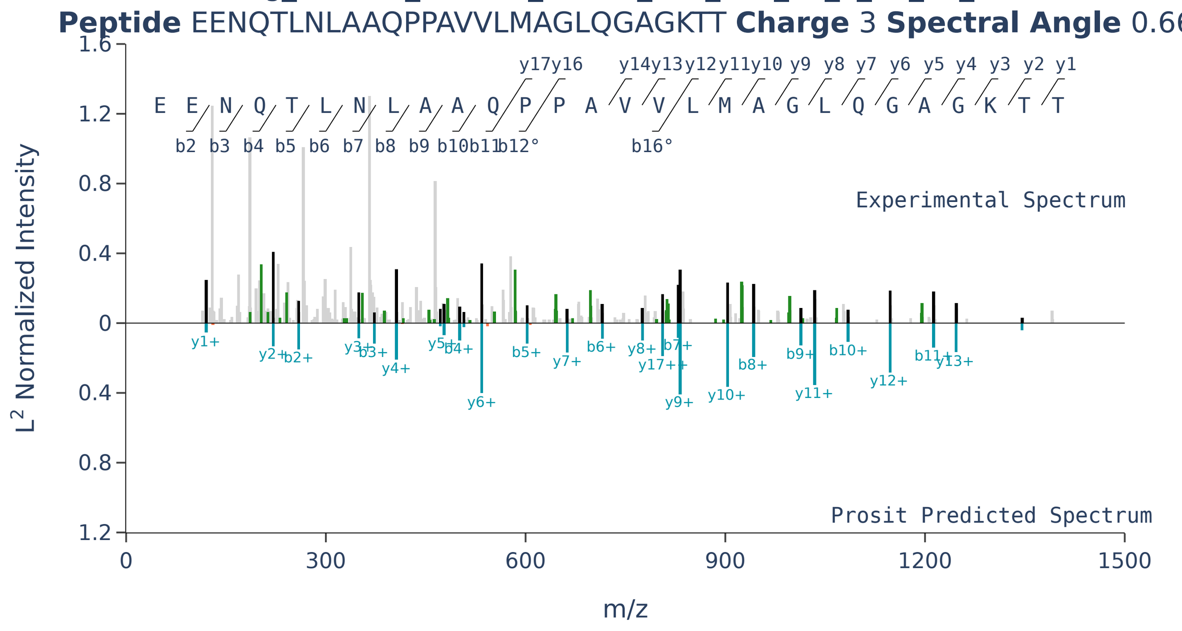


**Source** Ncheng_210623_230623_HFGoe_FFH_20S_25_1_A2_24h_R1 **Scan** 15012
**Peptide** RQMKNMGGMASL **Charge** 2 **Spectral Angle** 0.73

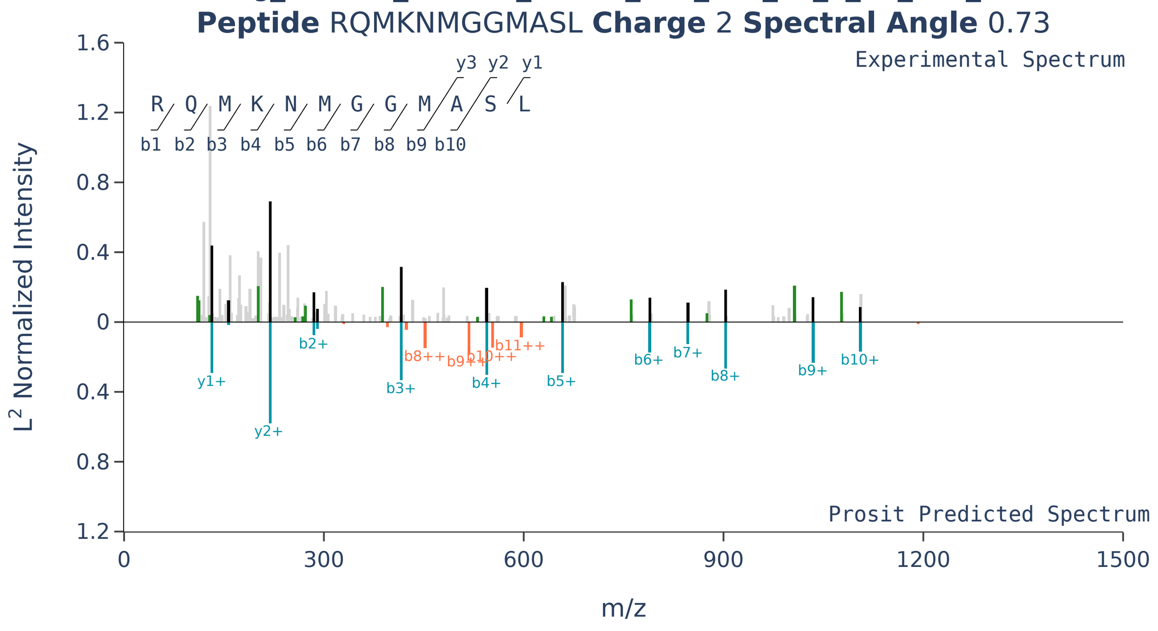


**Source** Ncheng_210623_230623_HFGoe_FFH_20S_25_1_A1_4h_R2 **Scan** 17278
**Peptide** NISGRGRLTEDNVKDTLREV **Charge** 2 **Spectral Angle** 0.82

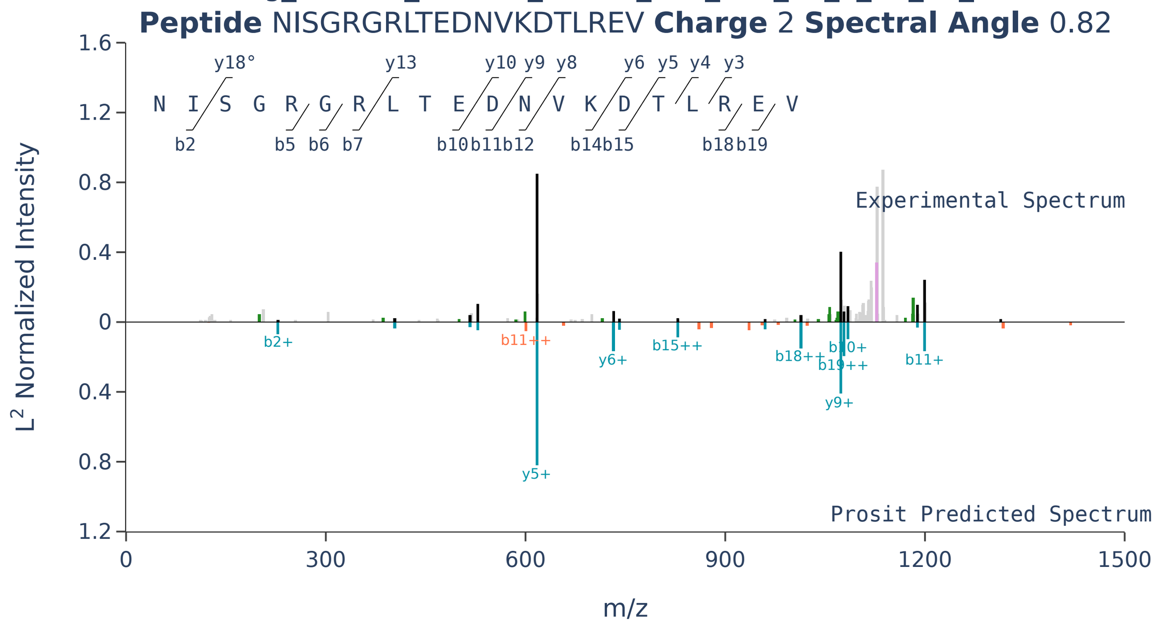


**Source** Ncheng_210623_230623_HFGoe_FFH_20S_25_1_A2_24h_R2 **Scan** 7370
**Peptide** SMTMKERAKPEIIKGSRK **Charge** 5 **Spectral Angle** 0.76

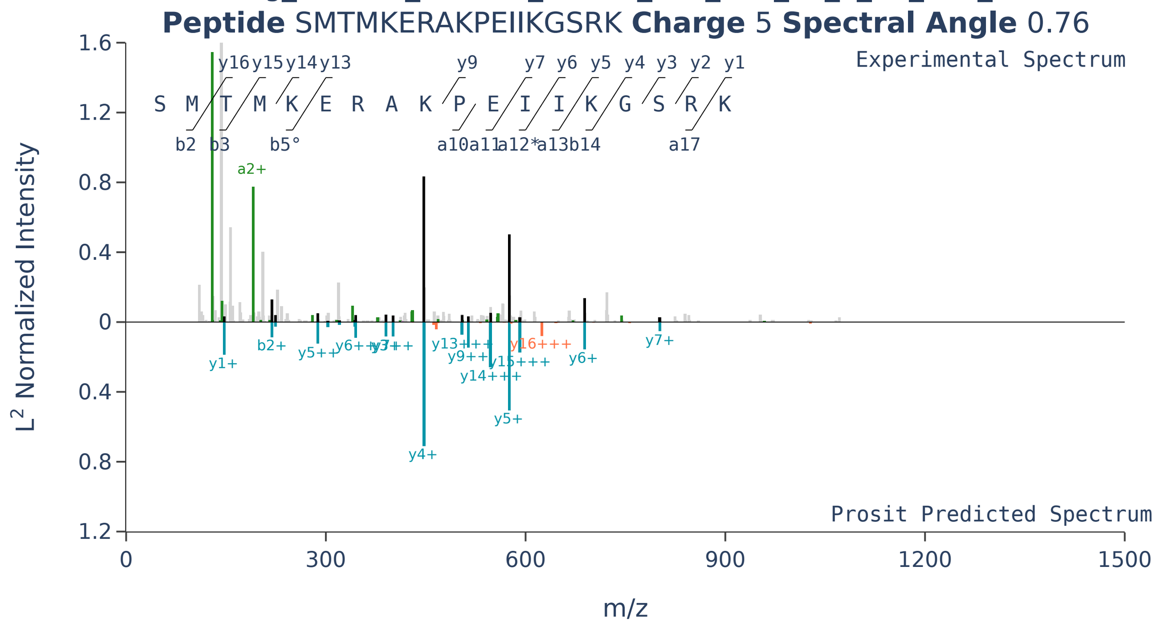


**Source** Ncheng_210623_230623_HFGoe_FFH_20S_25_1_A1_1h_R1 **Scan** 17113
**Peptide** LKEAKLKFYDV **Charge** 3 **Spectral Angle** 0.78

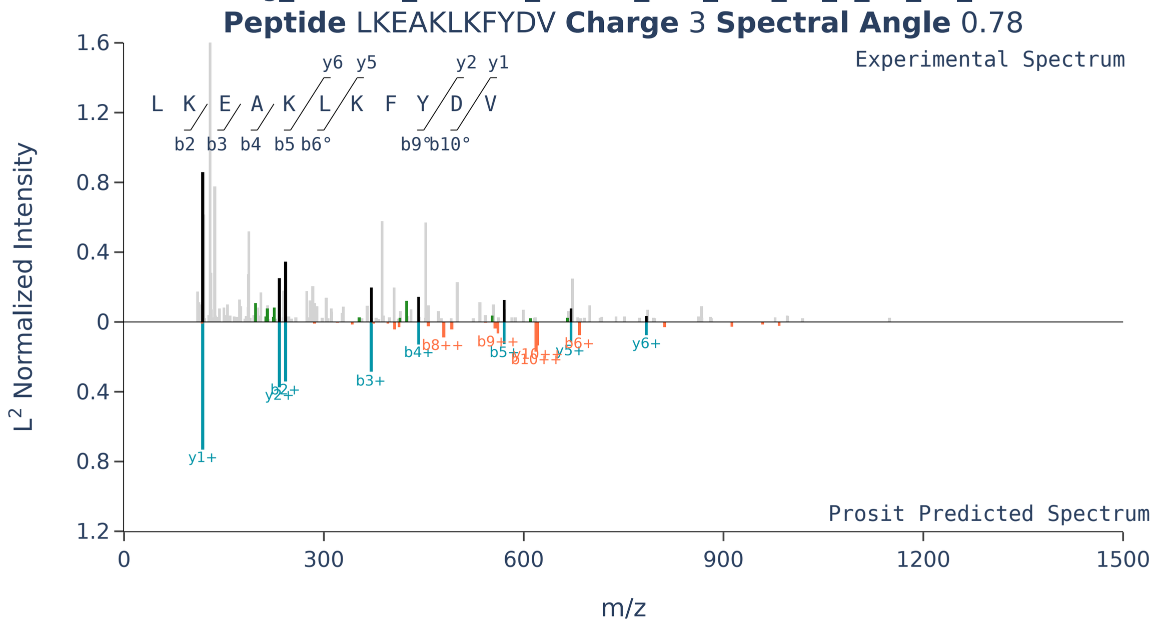


**Source** Ncheng_210623_230623_HFGoe_FFH_20S_25_1_A1_24h_R1 **Scan** 6648
**Peptide** KERAKPEIIKG **Charge** 2 **Spectral Angle** 0.71

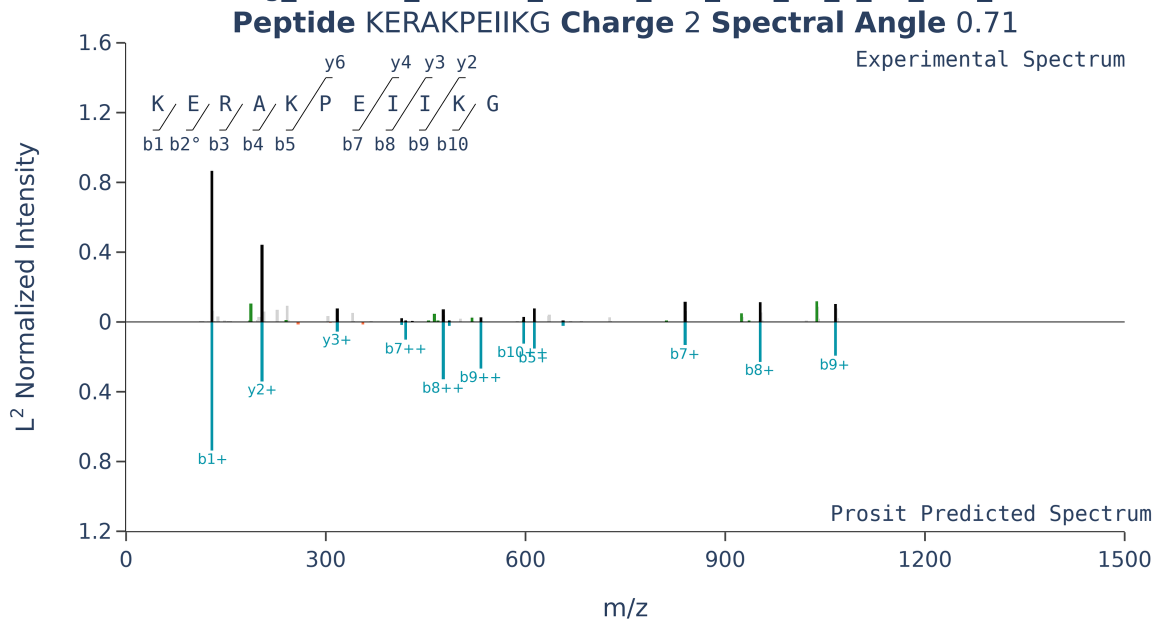


**Source** Ncheng_210623_230623_HFGoe_FFH_20S_25_1_A1_4h_R1 **Scan** 14481
**Peptide** GDARGGAALSIRHITG **Charge** 2 **Spectral Angle** 0.87

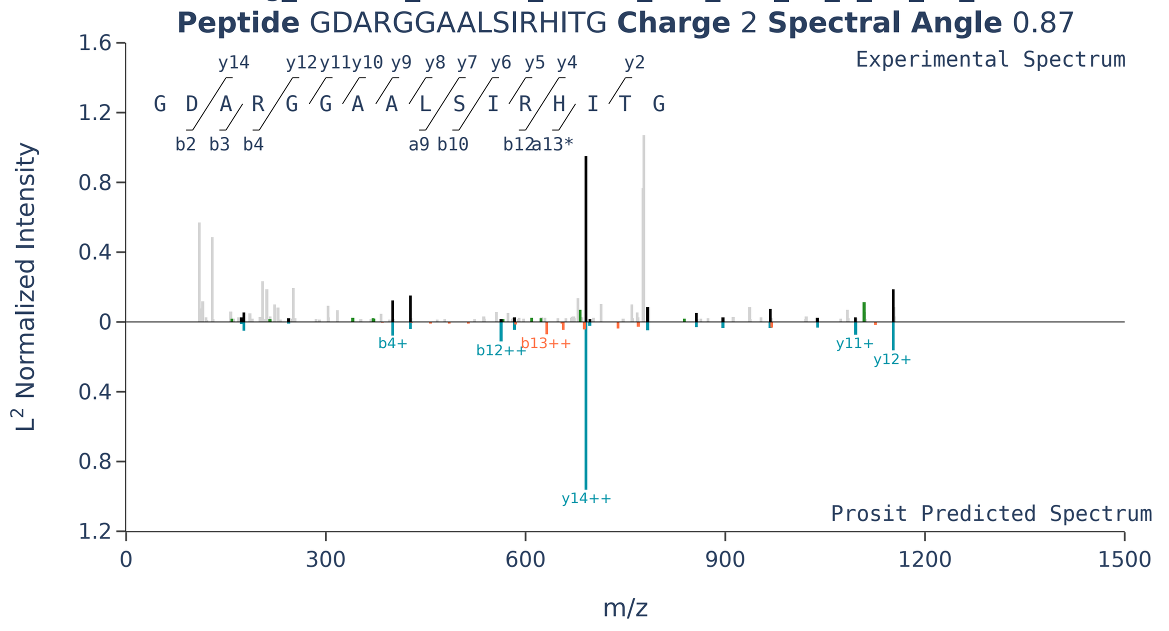

**Source** Ncheng_210623_230623_HFGoe_FFH_20S_25_1_A1_1h_R2 **Scan** 27575
**Peptide** YDVLLVDTAGRLHVD **Charge** 3 **Spectral Angle** 0.78

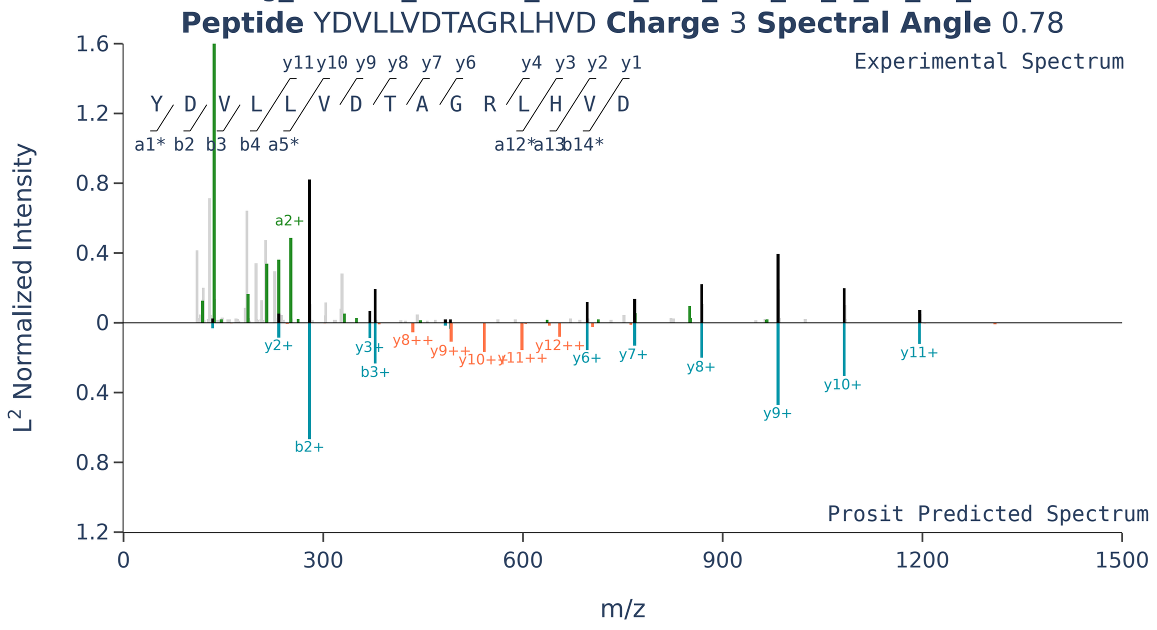


**Source** Ncheng_210623_230623_HFGoe_FFH_20S_25_1_A2_24h_R2 **Scan** 23537
**Peptide** MGKLPGMGQIPD **Charge** 2 **Spectral Angle** 0.92

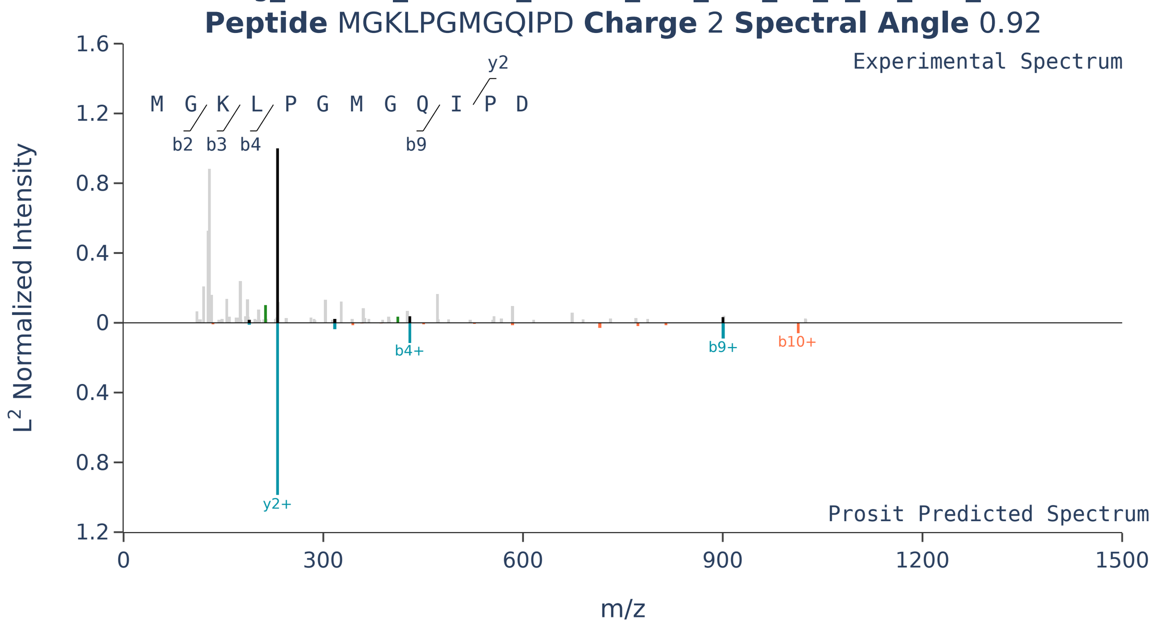


**Source** Ncheng_210623_230623_HFGoe_FFH_20S_25_1_A2_24h_R2 **Scan** 18500
**Peptide** MAGLQGAGKTTSVGKLGKF **Charge** 3 **Spectral Angle** 0.68

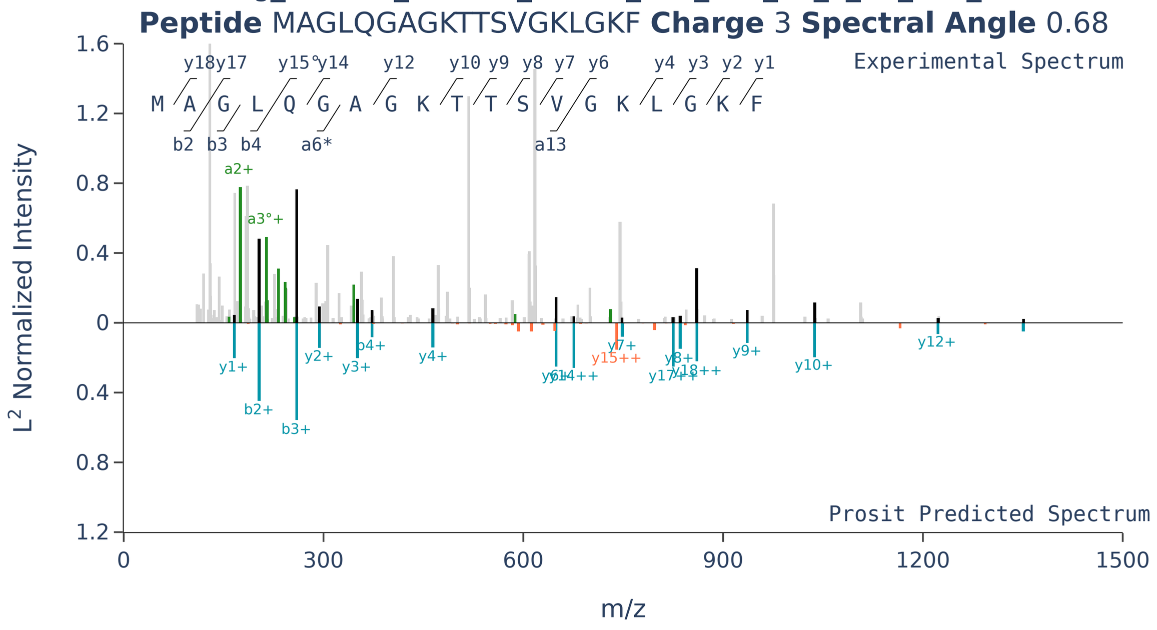


**Source** Ncheng_210623_230623_HFGoe_FFH_20S_25_1_A1_24h_R2 **Scan** 7623
**Peptide** TMKERAKPEIIKG **Charge** 2 **Spectral Angle** 0.61

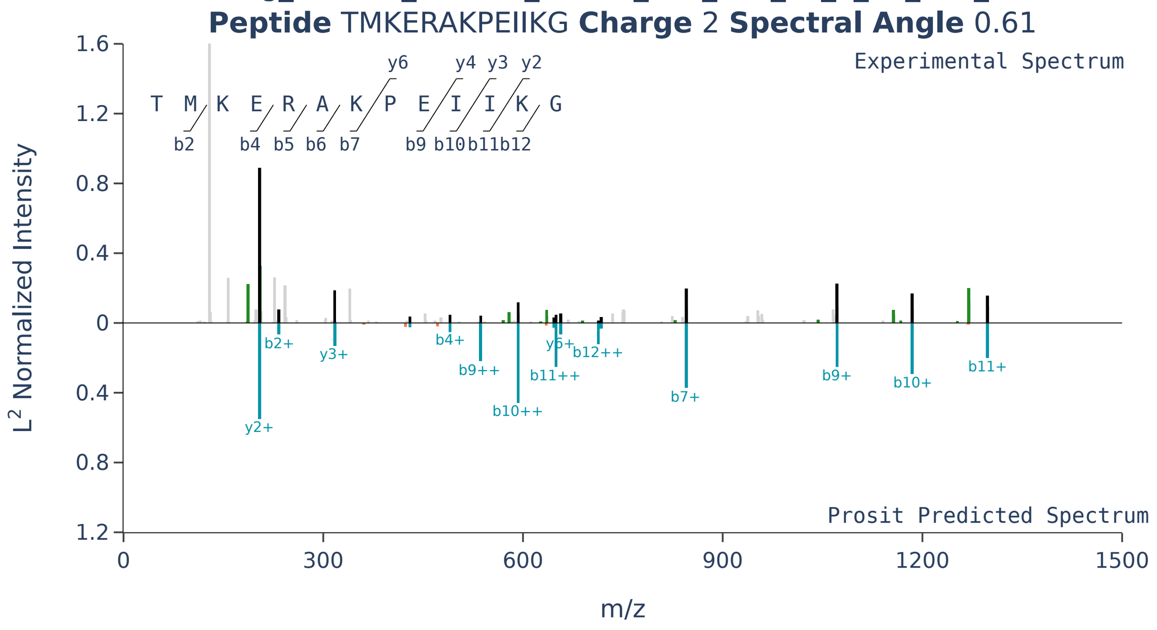


**Source** Ncheng_210623_230623_HFGoe_FFH_20S_25_1_A2_2h_R1 **Scan** 15202
**Peptide** ALKEAKLKFY **Charge** 3 **Spectral Angle** 0.67

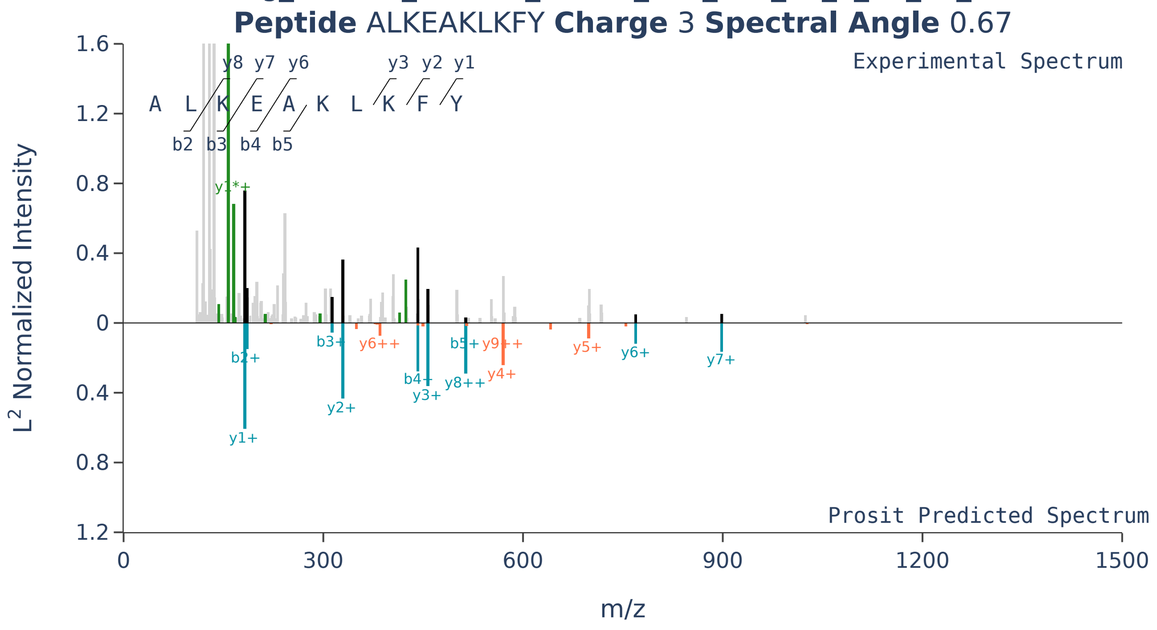


**Source** Ncheng_210623_230623_HFGoe_FFH_20S_25_1_A2_24h_R2 **Scan** 5602
**Peptide** RAQAEKLAS **Charge** 2 **Spectral Angle** 0.65

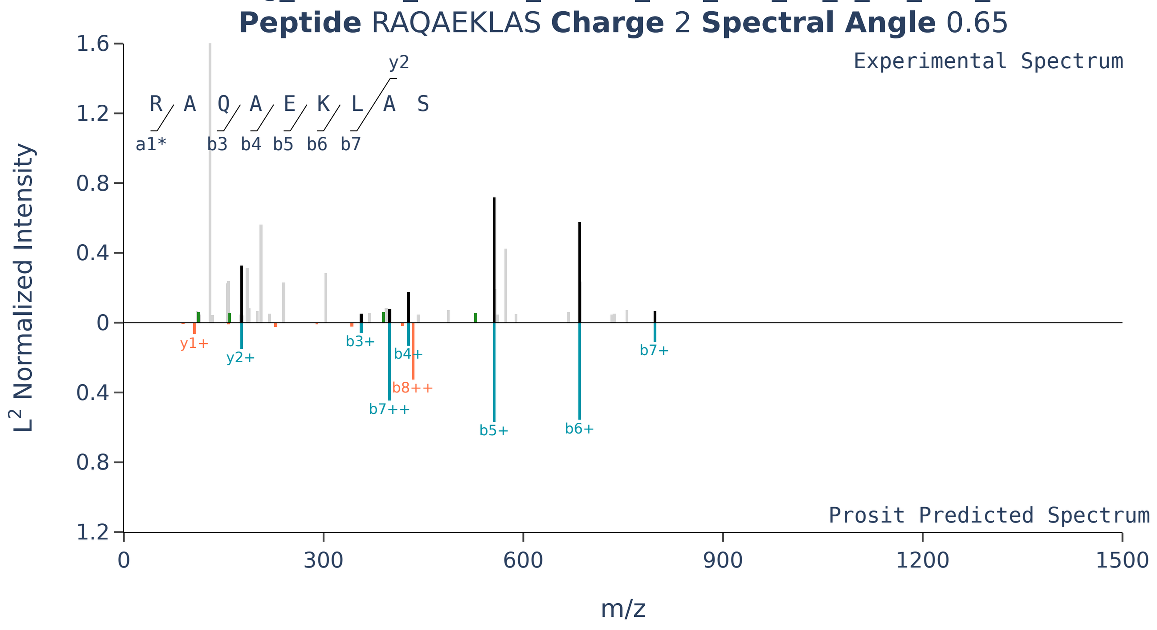


**Source** Ncheng_210623_230623_HFGoe_FFH_20S_25_1_A2_24h_R2 **Scan** 10253
**Peptide** NVKDTLREVRM **Charge** 2 **Spectral Angle** 0.6

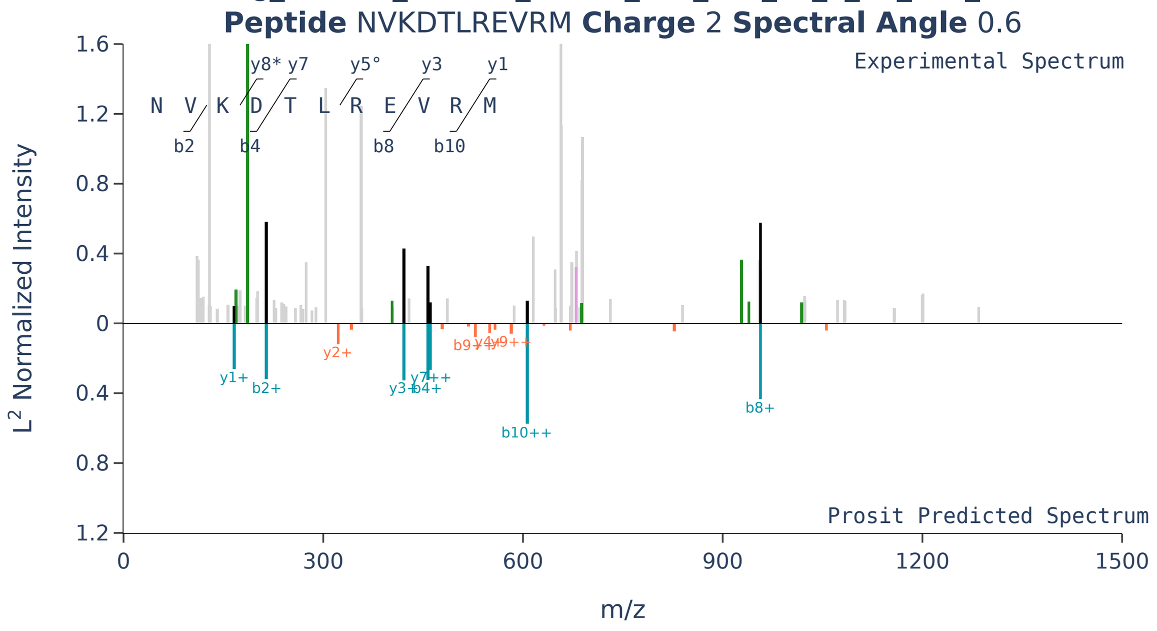


**Source** Ncheng_210623_230623_HFGoe_FFH_20S_25_1_A1_24h_R1 **Scan** 12303
**Peptide** QLPDNVKSQMDD **Charge** 1 **Spectral Angle** 0.84

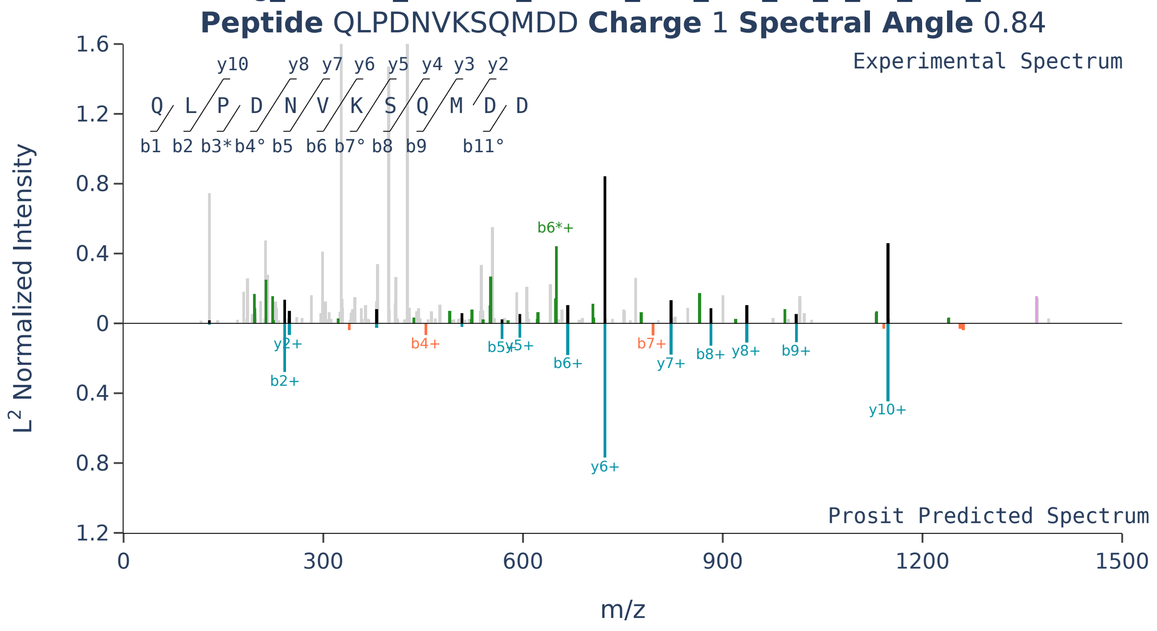


**Source** Ncheng_210623_230623_HFGoe_FFH_20S_25_1_A1_2h_R2 **Scan** 30705
**Peptide** KLKFYDVLLVDTAGRLHVDE **Charge** 3 **Spectral Angle** 0.8

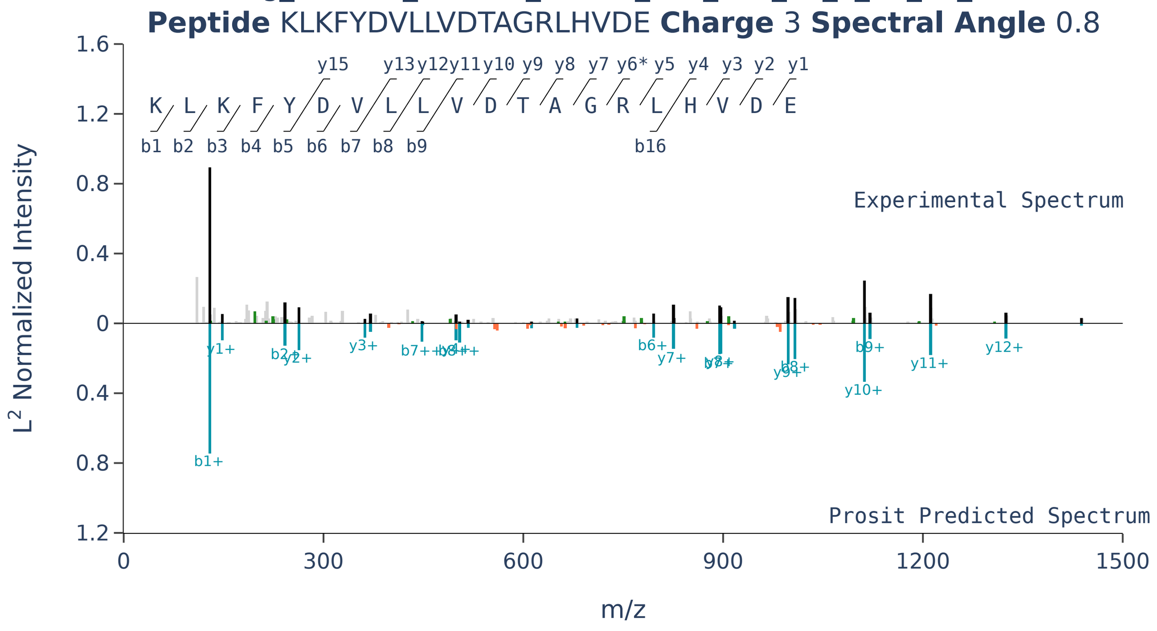


**Source** Ncheng_210623_230623_HFGoe_FFH_20S_25_1_A1_2h_R1 **Scan** 26467
**Peptide** DVLLVDTAGRLHVD **Charge** 3 **Spectral Angle** 0.69

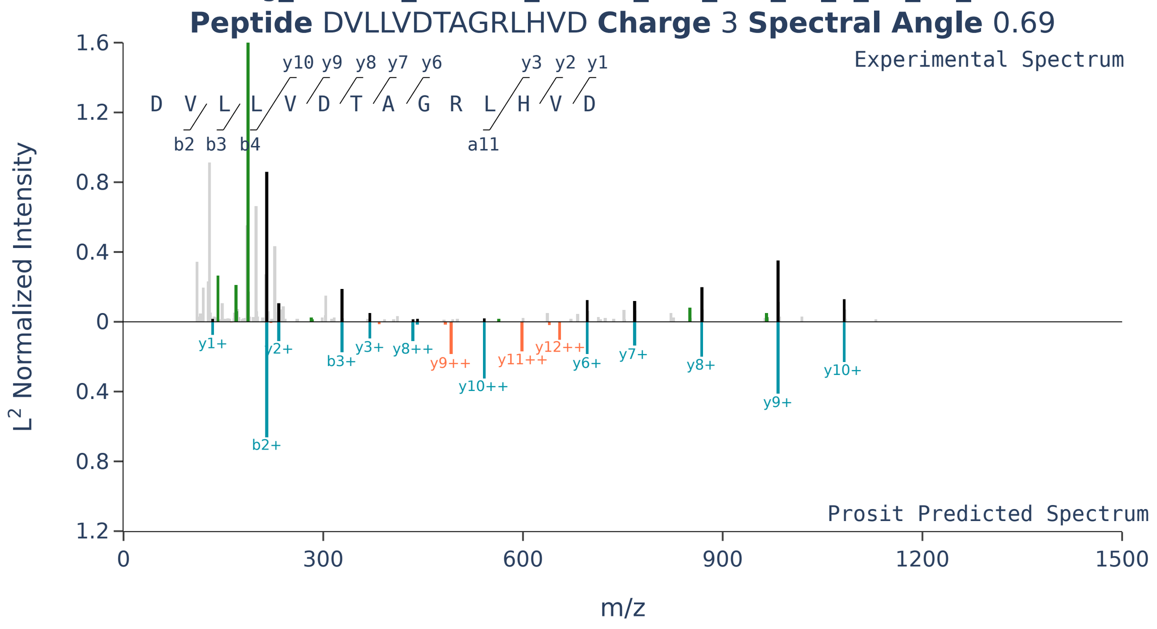


**Source** Ncheng_210623_230623_HFGoe_FFH_20S_25_1_A1_24h_R2 **Scan** 6852
**Peptide** SKVDRAQAEKL **Charge** 2 **Spectral Angle** 0.69

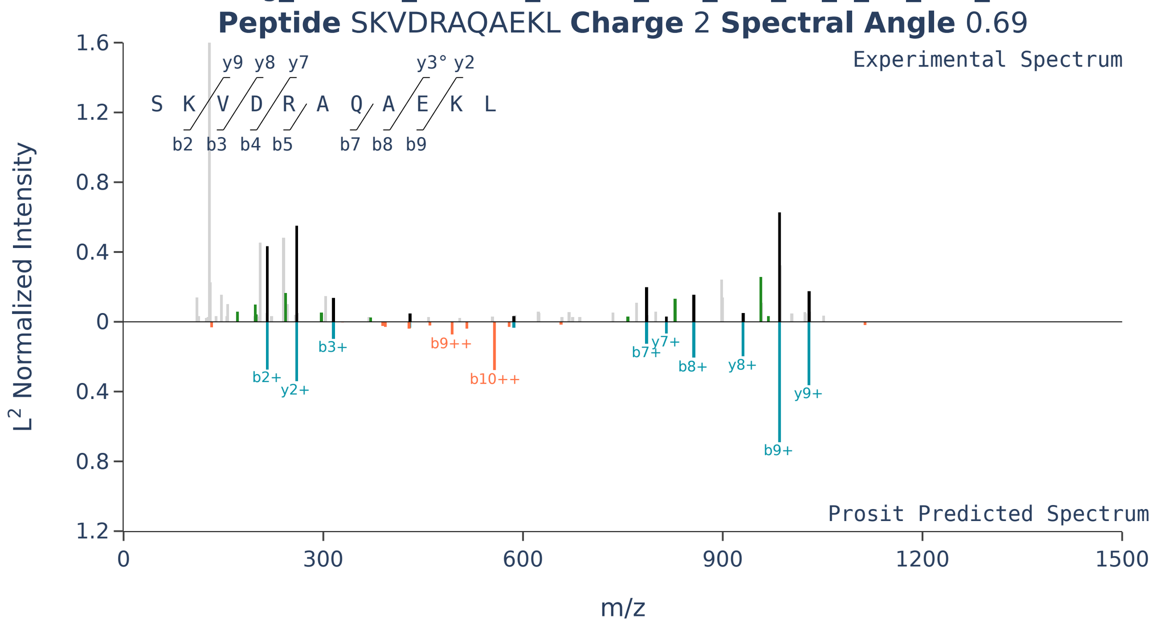


**Source** Ncheng_210623_230623_HFGoe_FFH_20S_25_1_A1_24h_R1 **Scan** 19723
**Peptide** SIRHITGKPIKFLGV **Charge** 4 **Spectral Angle** 0.69

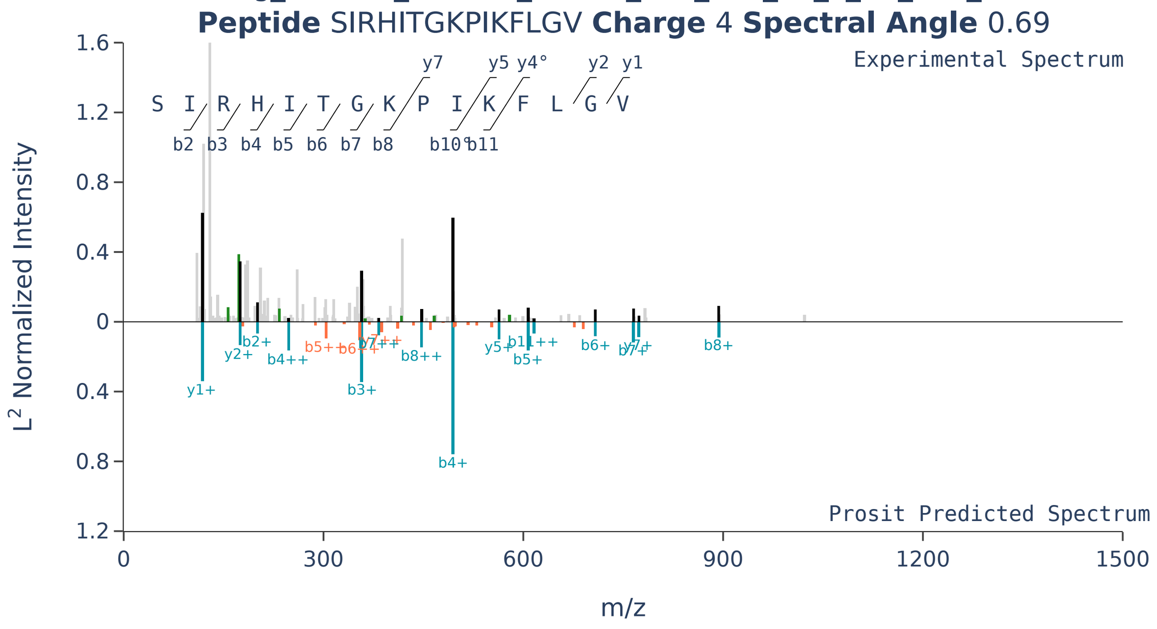


**Source** Ncheng_210623_230623_HFGoe_FFH_20S_25_1_A1_4h_R2 **Scan** 21851
**Peptide** EFINRVKEKAVGHEVNKSLTPGQEFVKLV **Charge** 3 **Spectral Angle** 0.68

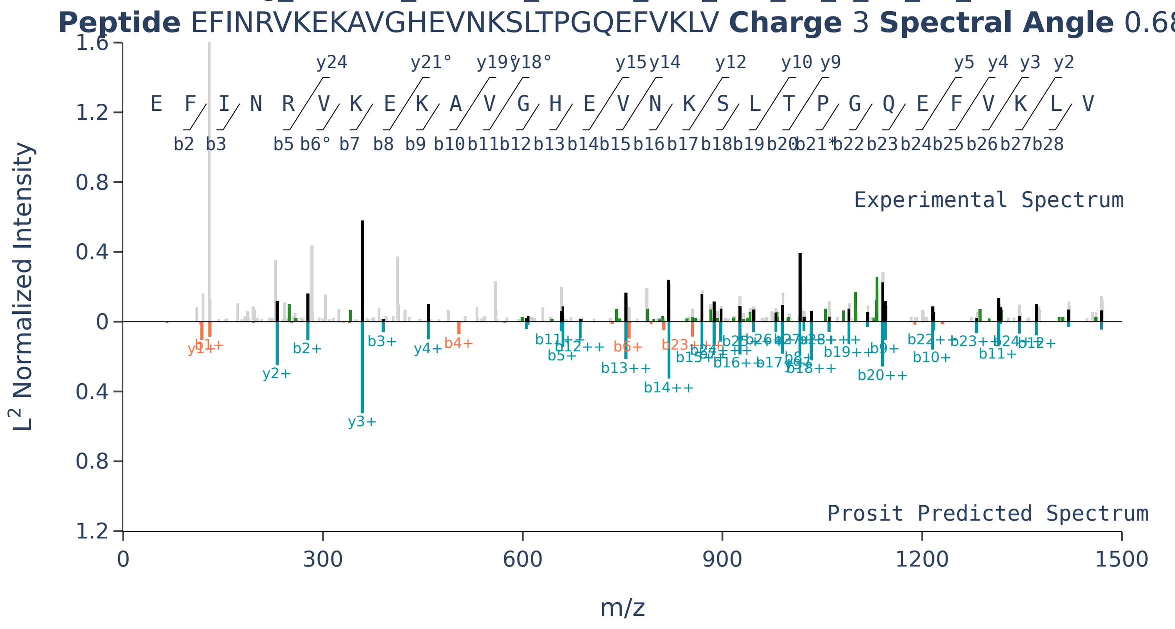


**Source** Ncheng_210623_230623_HFGoe_FFH_20S_25_1_A2_2h_R1 **Scan** 7643
**Peptide** FINRVKE **Charge** 2 **Spectral Angle** 0.75

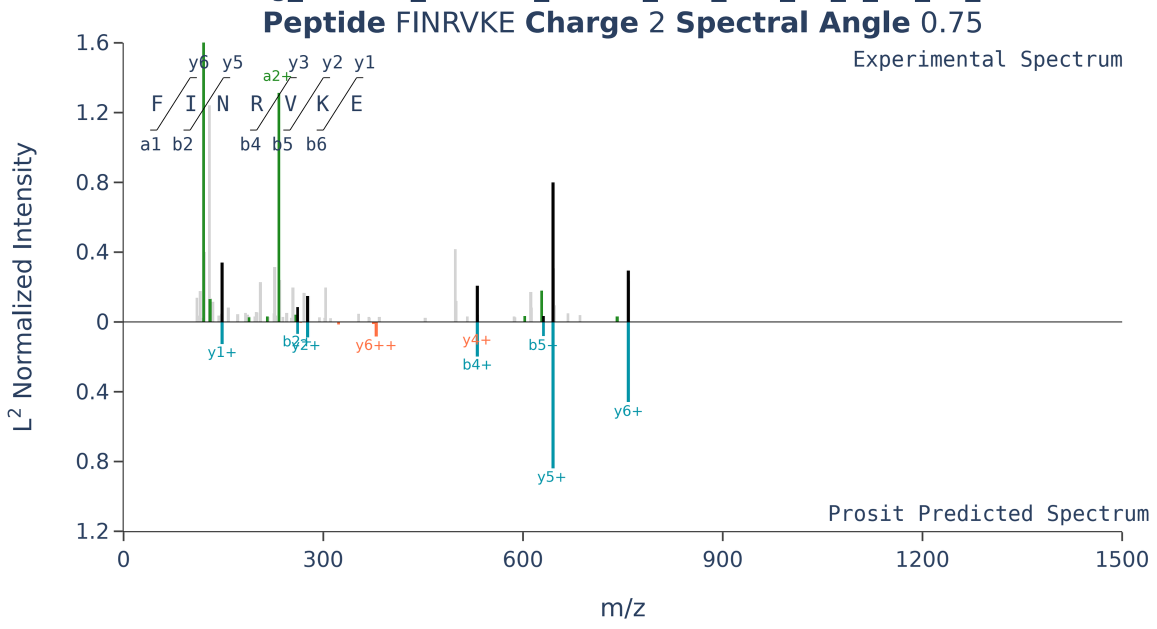


**Source** Ncheng_210623_230623_HFGoe_FFH_20S_25_1_A1_24h_R1 **Scan** 15221
**Peptide** MTGQDAANTAKAFNEA **Charge** 2 **Spectral Angle** 0.69

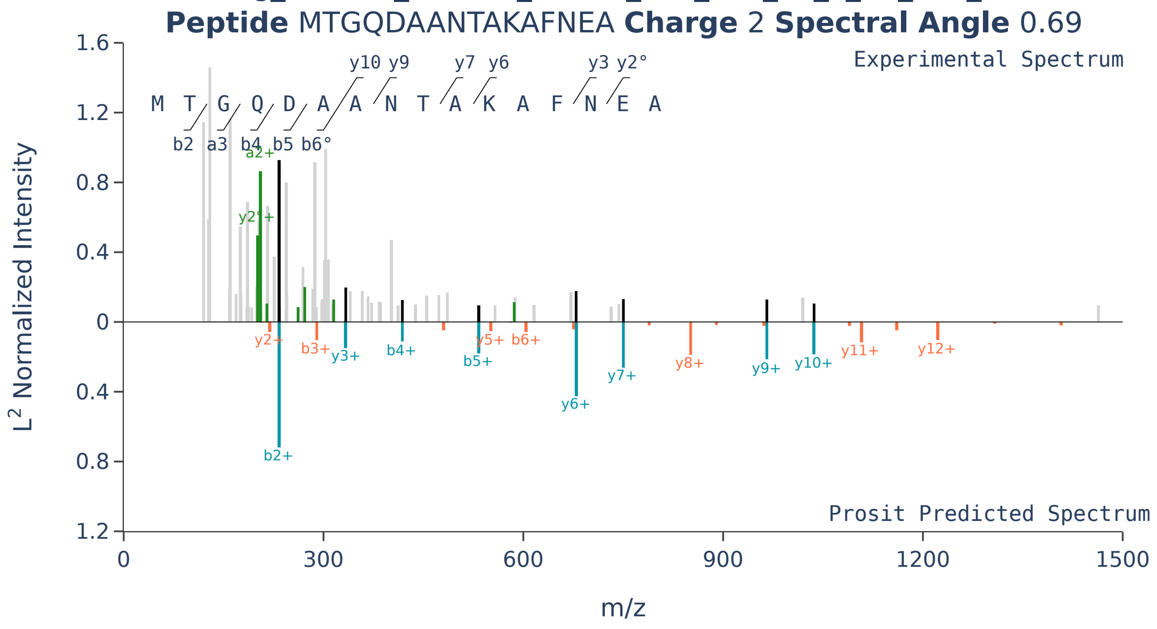

**Source** Ncheng_210623_230623_HFGoe_FFH_20S_25_1_A2_1h_R1 **Scan** 15075
**Peptide** SIRHITGKPIKFLGVGEKTE **Charge** 3 **Spectral Angle** 0.68

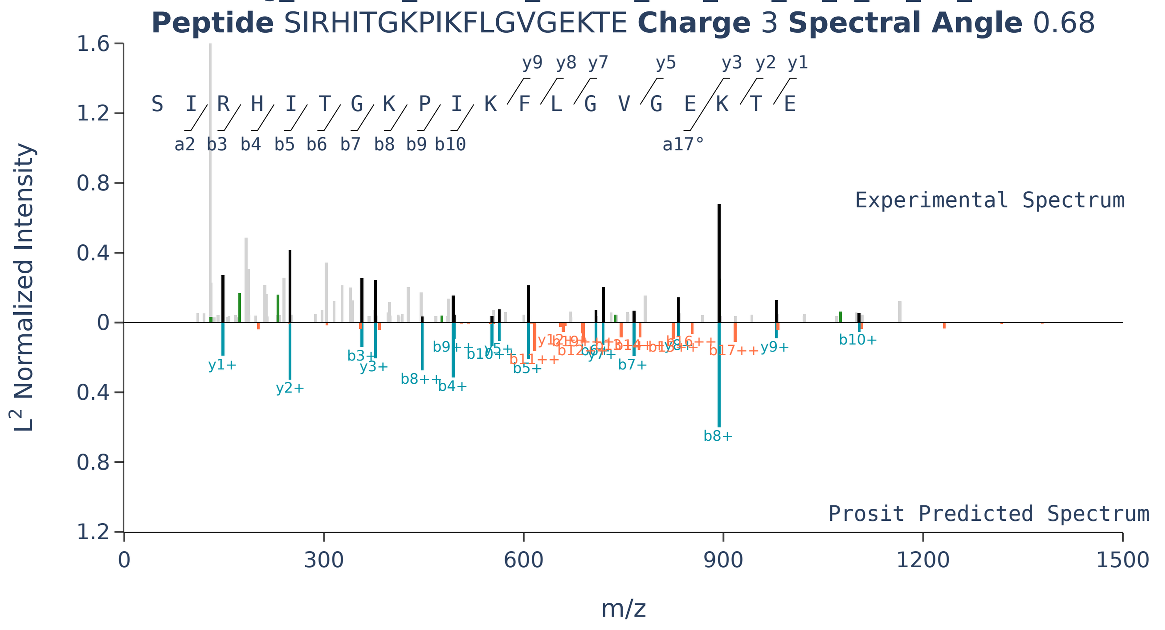


**Source** Ncheng_210623_230623_HFGoe_FFH_20S_25_1_A2_4h_R2 **Scan** 30350
**Peptide** AKLKFYDVLL **Charge** 3 **Spectral Angle** 0.76

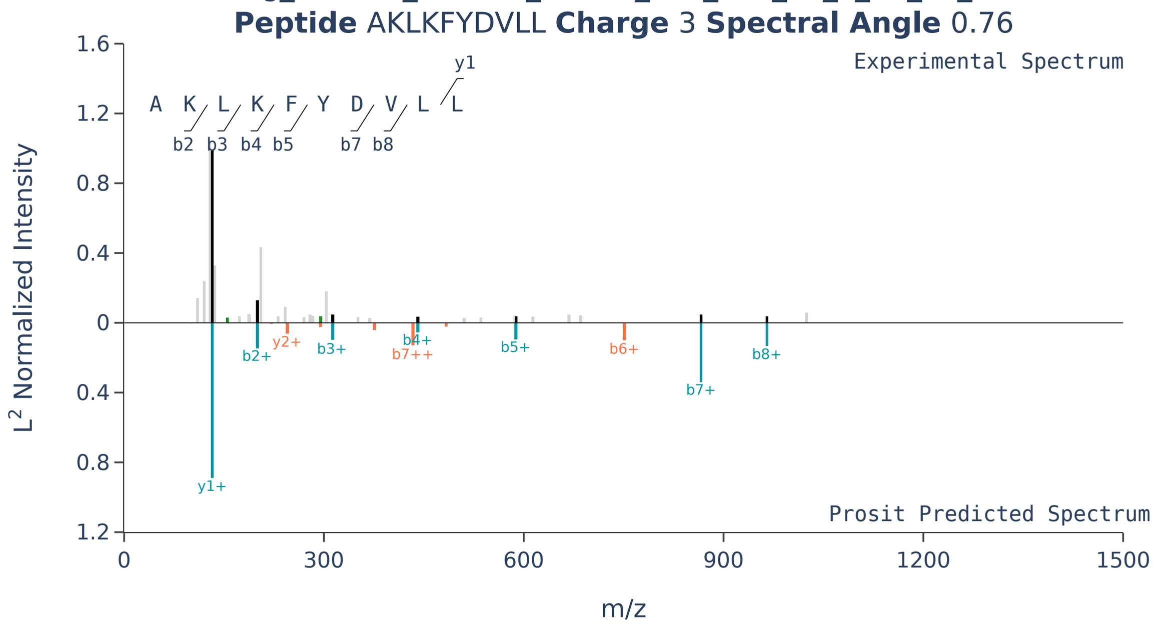


**Source** Ncheng_210623_230623_HFGoe_FFH_20S_25_1_A1_4h_R2 **Scan** 32005
**Peptide** RNELVAAMGEENQTLNLAAQPPAVV **Charge** 3 **Spectral Angle** 0.88

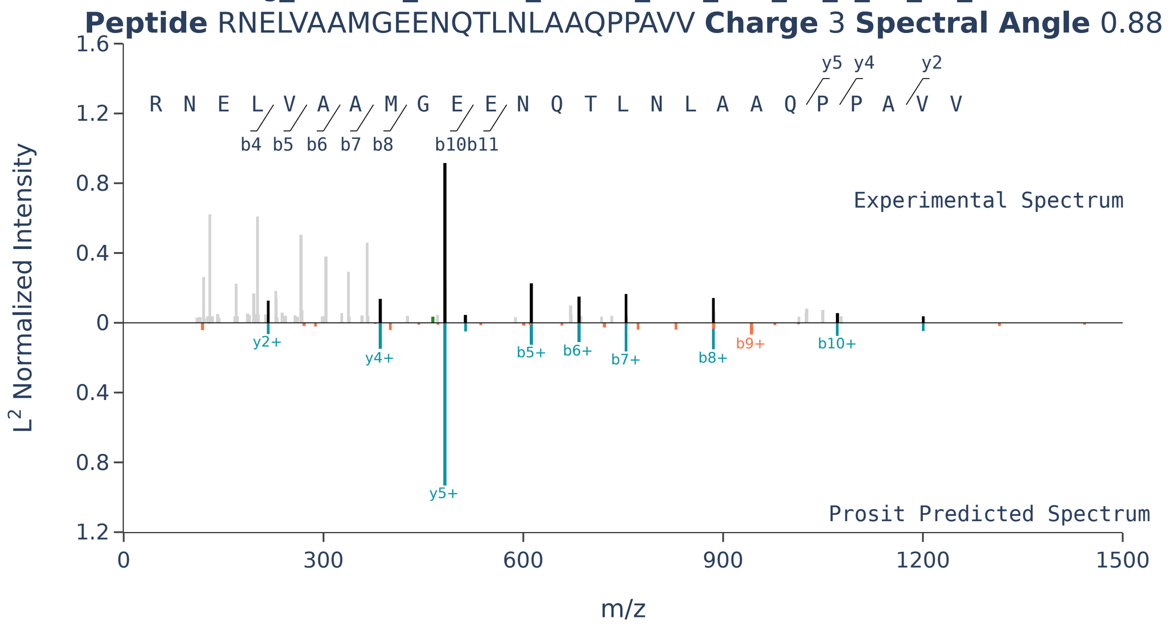


**Source** Ncheng_210623_230623_HFGoe_FFH_20S_25_1_A1_2h_R2 **Scan** 32450
**Peptide** EADVALPVVREFINRVKEKAVGHEVNK **Charge** 4 **Spectral Angle** 0.74

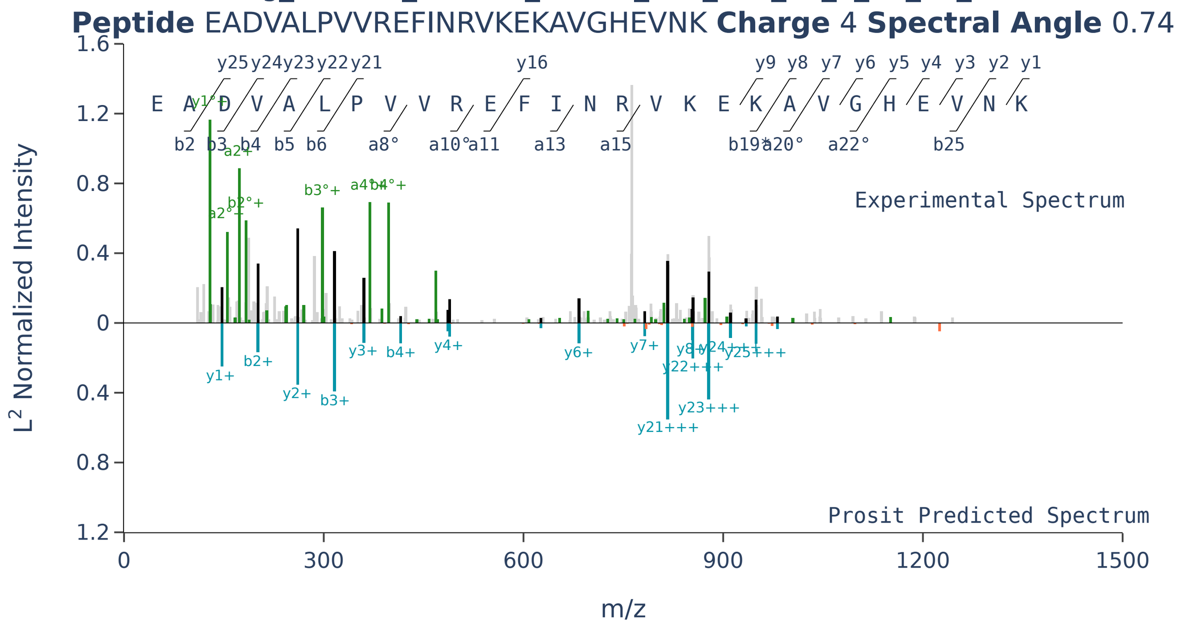


**Source** Ncheng_210623_230623_HFGoe_FFH_20S_25_1_A2_24h_R1 **Scan** 9653
**Peptide** GMAKMMRSMKG **Charge** 2 **Spectral Angle** 0.67

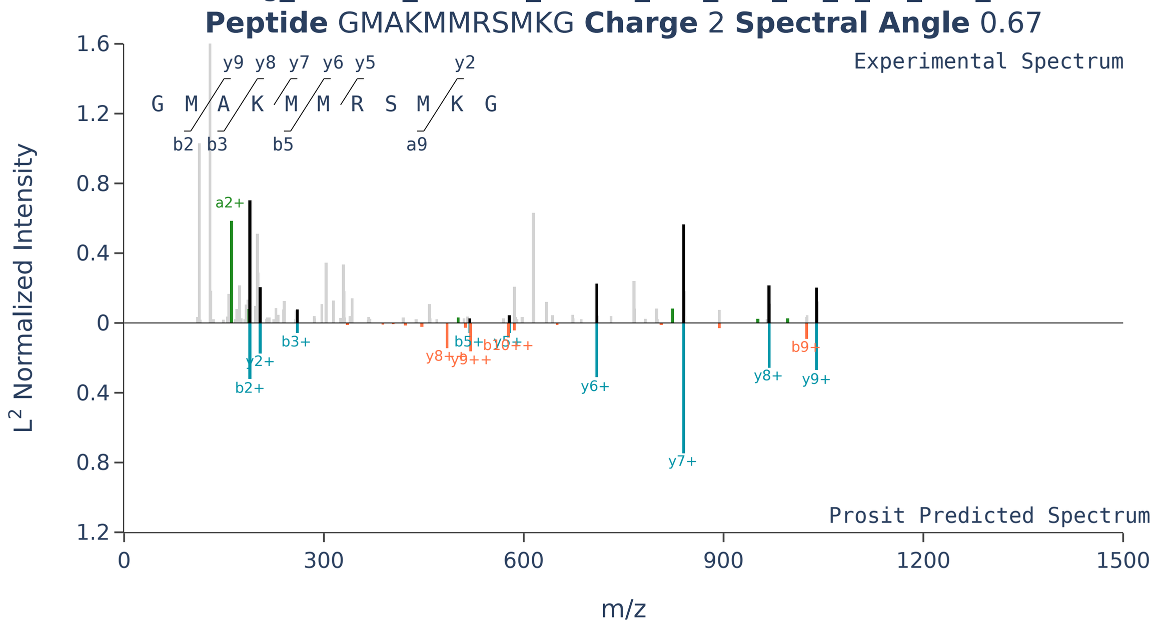


**Source** Ncheng_210623_230623_HFGoe_FFH_20S_25_1_A2_24h_R2 **Scan** 17325
**Peptide** KEKAVGHEVNKSLTPGQEFVKLVR **Charge** 5 **Spectral Angle** 0.61

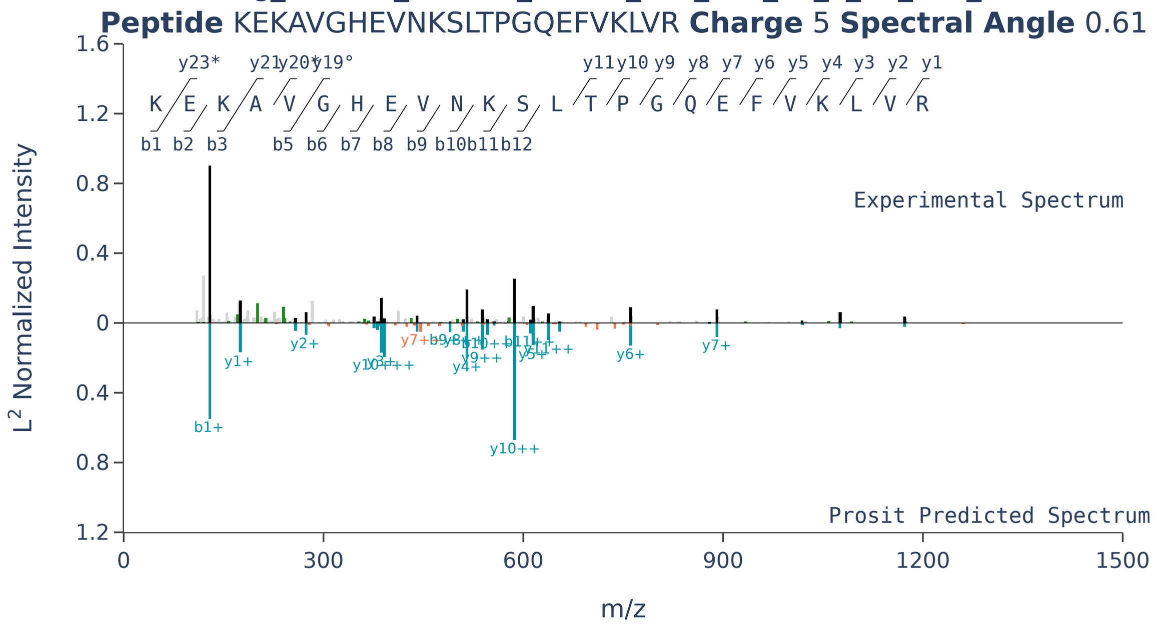


**Source** Ncheng_210623_230623_HFGoe_FFH_20S_25_1_A2_2h_R1 **Scan** 26120
**Peptide** KEKAVGHEVNKSLTPGQEFVKIVRNELV **Charge** 4 **Spectral Angle** 0.76

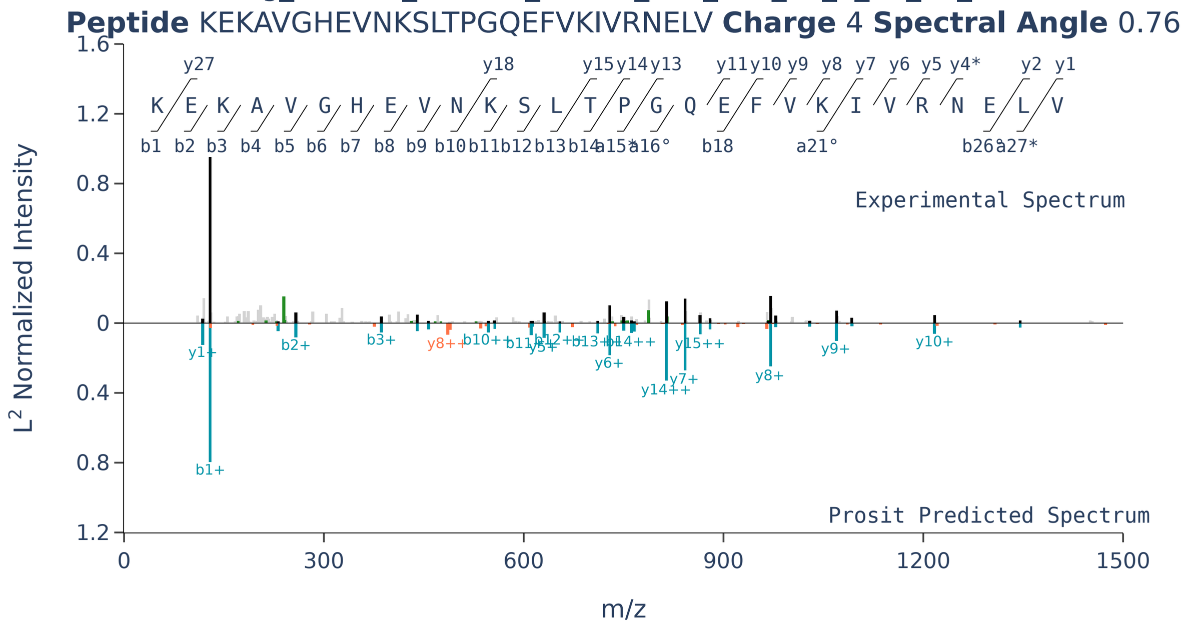


**Source** Ncheng_210623_230623_HFGoe_FFH_20S_25_1_A1_4h_R1 **Scan** 10848
**Peptide** AGCGMQV **Charge** 1 **Spectral Angle** 0.68

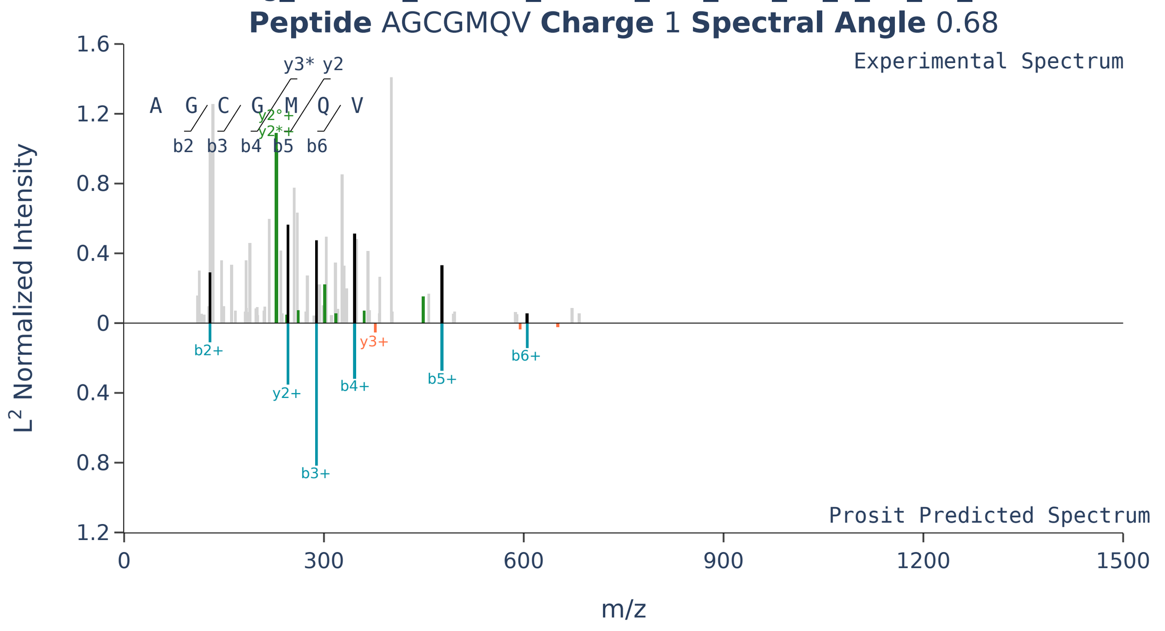


**Source** Ncheng_210623_230623_HFGoe_FFH_20S_25_1_A2_4h_R2 **Scan** 20843
**Peptide** AIINSMTMKERAKPEII **Charge** 2 **Spectral Angle** 0.71

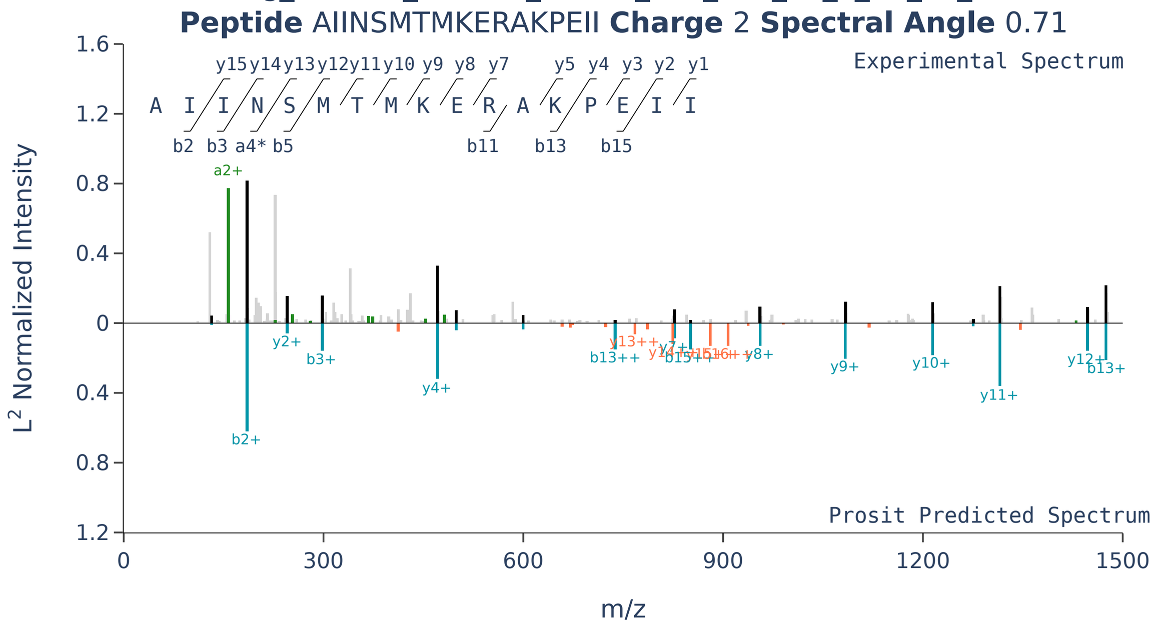


**Source** Ncheng_210623_230623_HFGoe_FFH_20S_25_1_A1_24h_R1 **Scan** 7380
**Peptide** TDRLSRTLR **Charge** 2 **Spectral Angle** 0.61

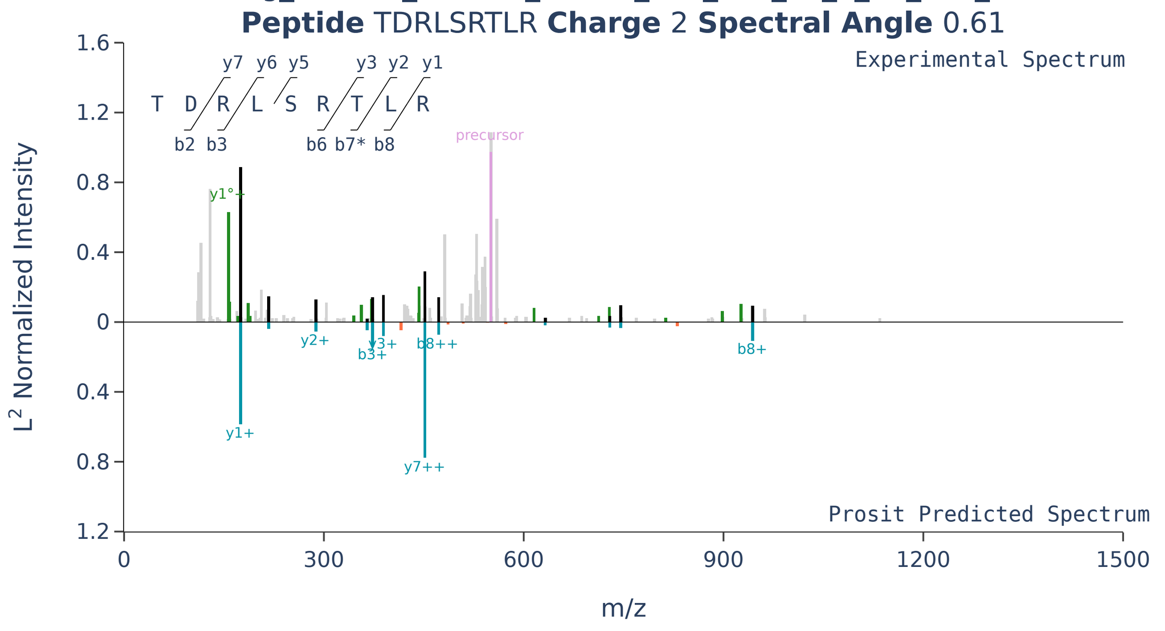


**Source** Ncheng_210623_230623_HFGoe_FFH_20S_25_1_A2_2h_R2 **Scan** 31296
**Peptide** ADVALPVVREFINRVKEK **Charge** 4 **Spectral Angle** 0.76

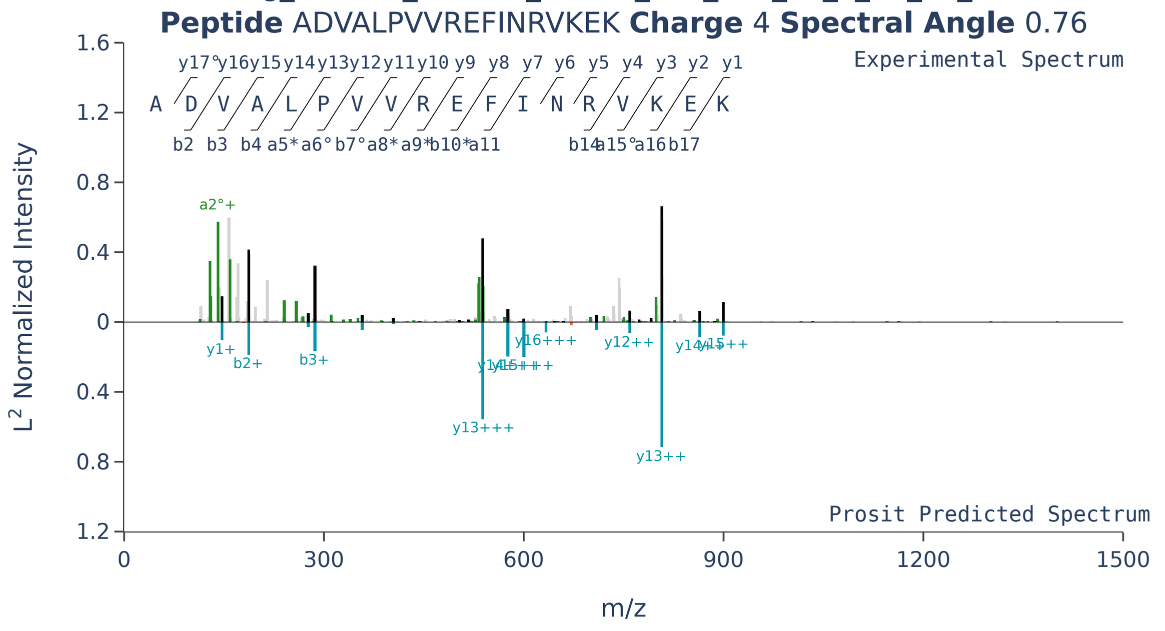


**Source** Ncheng_210623_230623_HFGoe_FFH_20S_25_1_A2_1h_R2 **Scan** 10688
**Peptide** VDEAMMD **Charge** 1 **Spectral Angle** 0.72

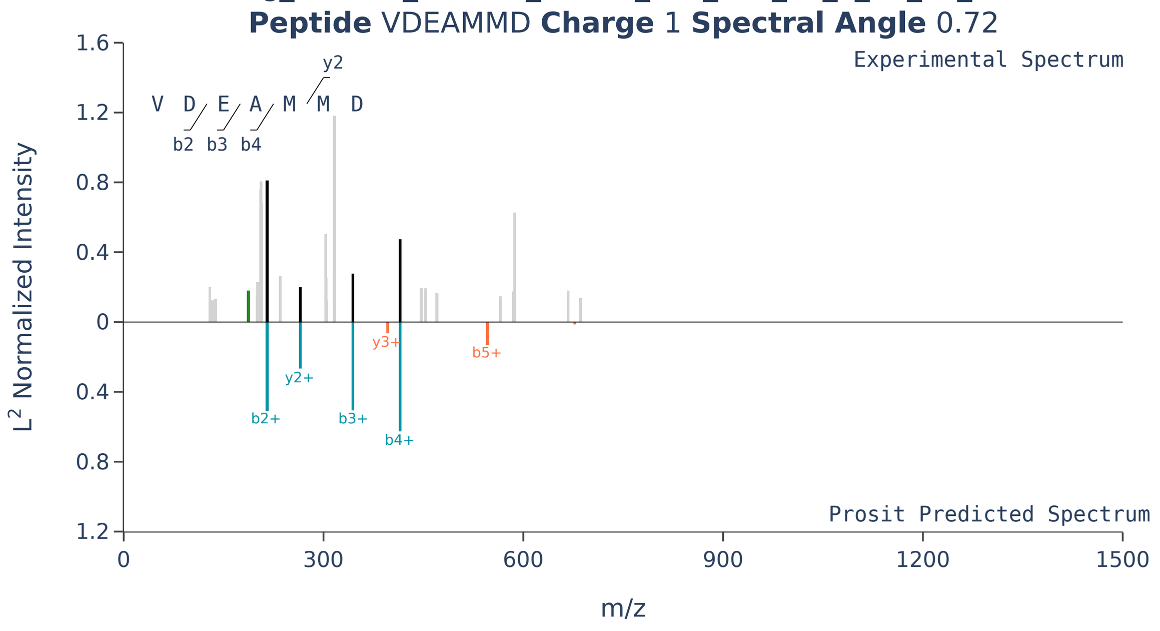


**Source** Ncheng_210623_230623_HFGoe_FFH_20S_25_1_A1_24h_R2 **Scan** 5904
**Peptide** GRGRLTEDNVKD **Charge** 3 **Spectral Angle** 0.65

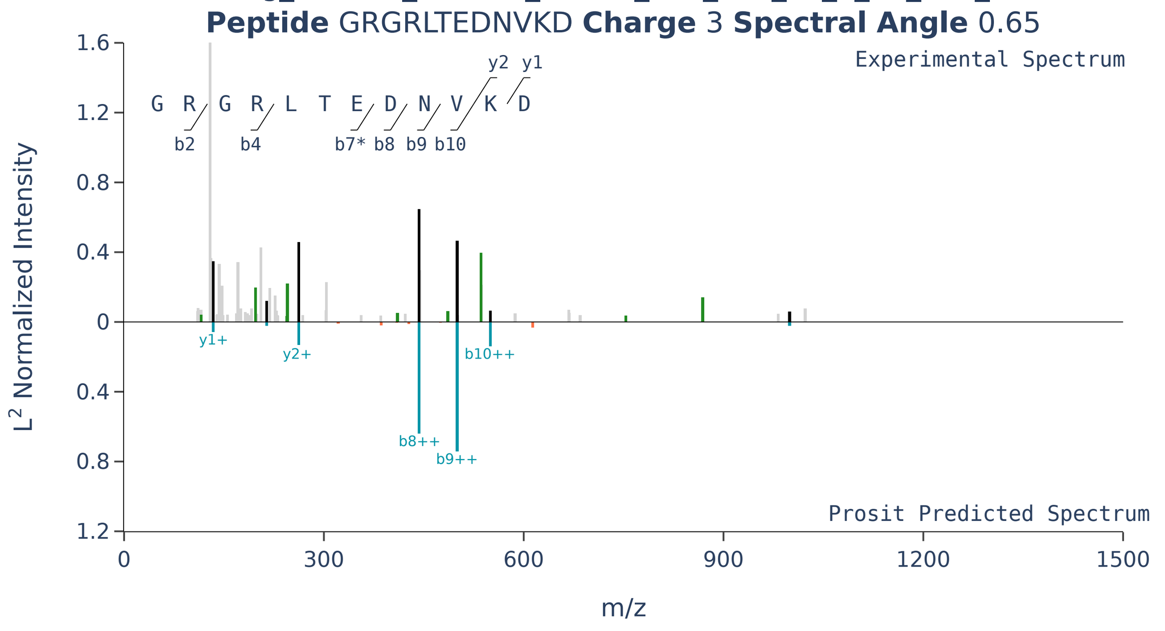


**Source** Ncheng_210623_230623_HFGoe_FFH_20S_25_1_A1_1h_R2 **Scan** 28945
**Peptide** GEKTEALEPFHPDRIASRILGMGD **Charge** 3 **Spectral Angle** 0.76

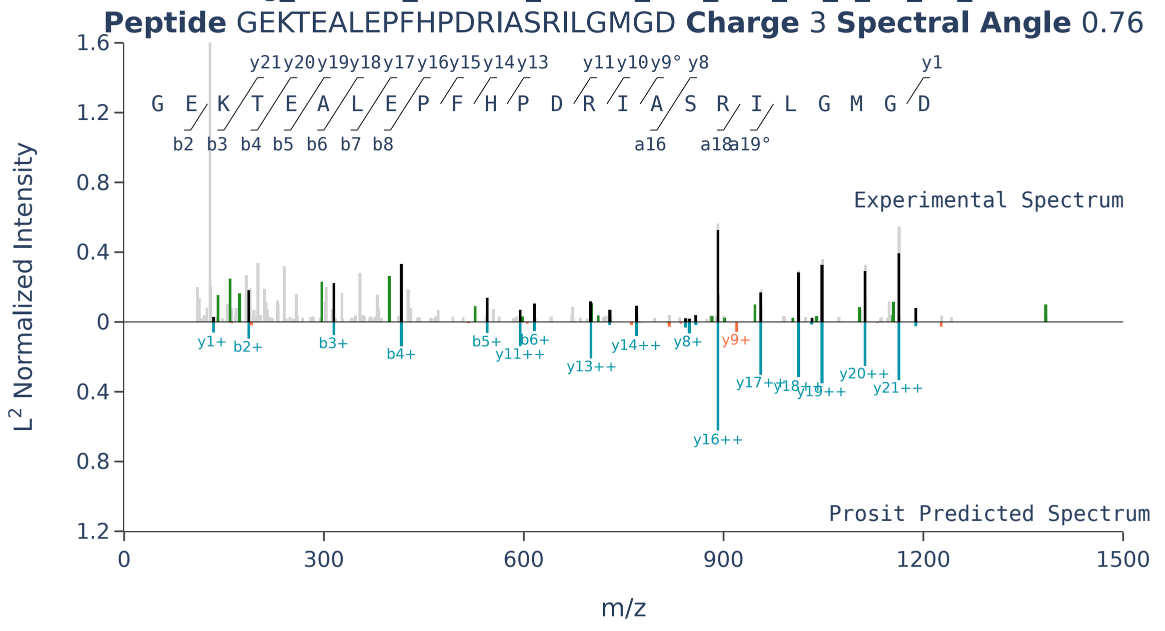


**Source** Ncheng_210623_230623_HFGoe_FFH_20S_25_1_A2_1h_R1 **Scan** 6991
**Peptide** GRLHVDEA **Charge** 2 **Spectral Angle** 0.69

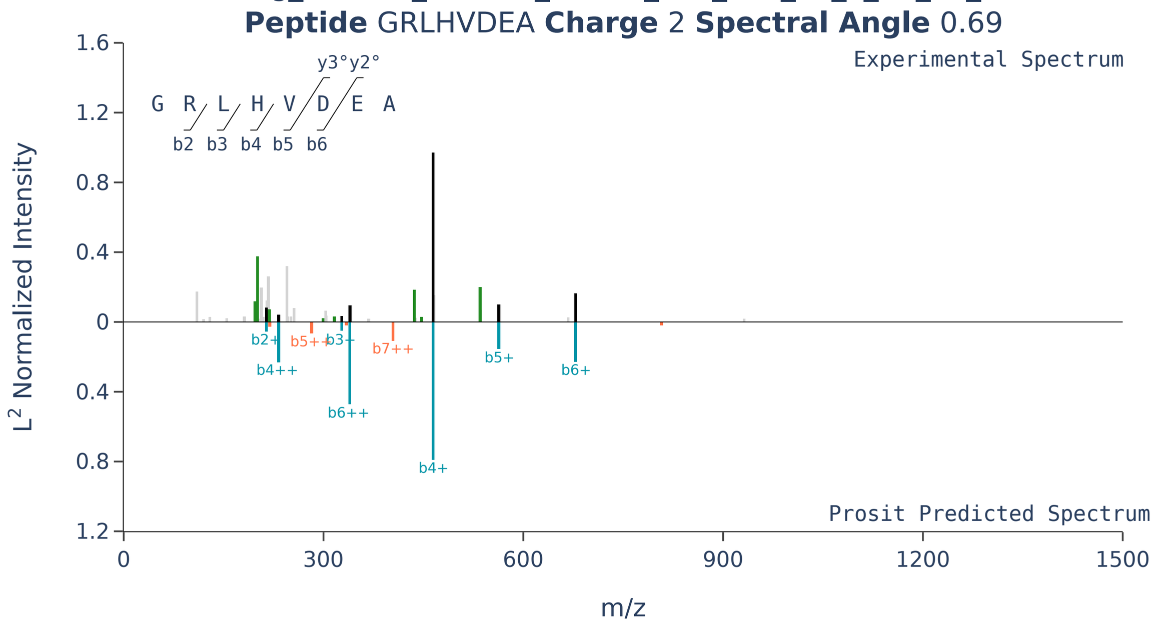

**Source** Ncheng_210623_230623_HFGoe_FFH_20S_25_1_A1_24h_R2 **Scan** 18938
**Peptide** VGEKTEALEPFHPDRI **Charge** 4 **Spectral Angle** 0.79

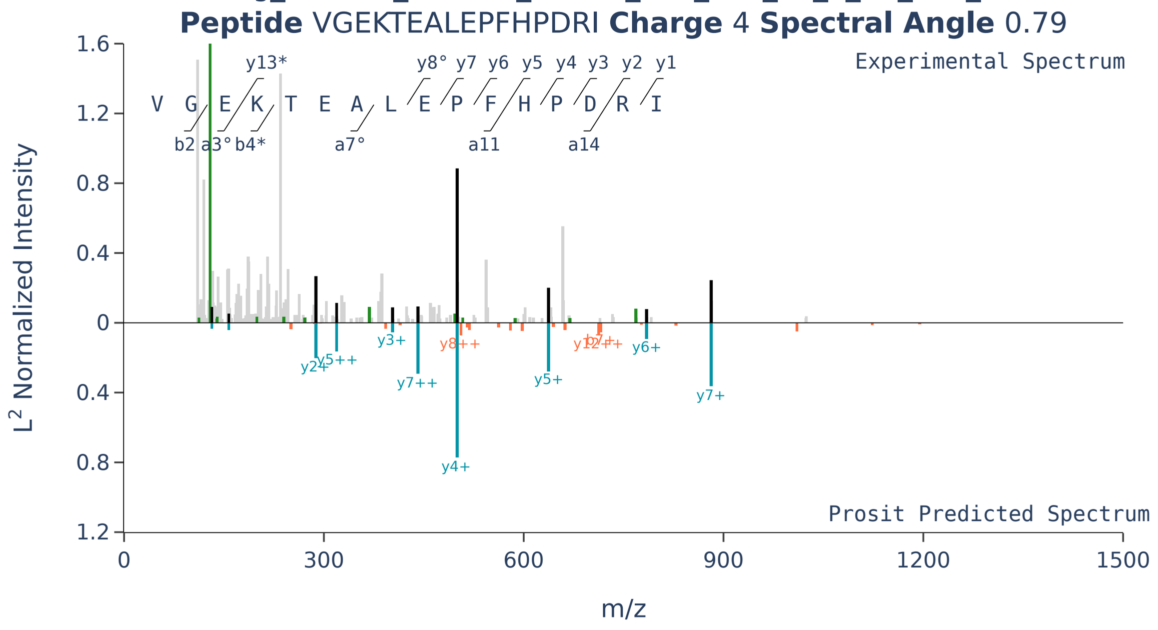


**Source** Ncheng_210623_230623_HFGoe_FFH_20S_25_1_A2_24h_R1 **Scan** 17069
**Peptide** TSVGKLGKFLRE **Charge** 3 **Spectral Angle** 0.65

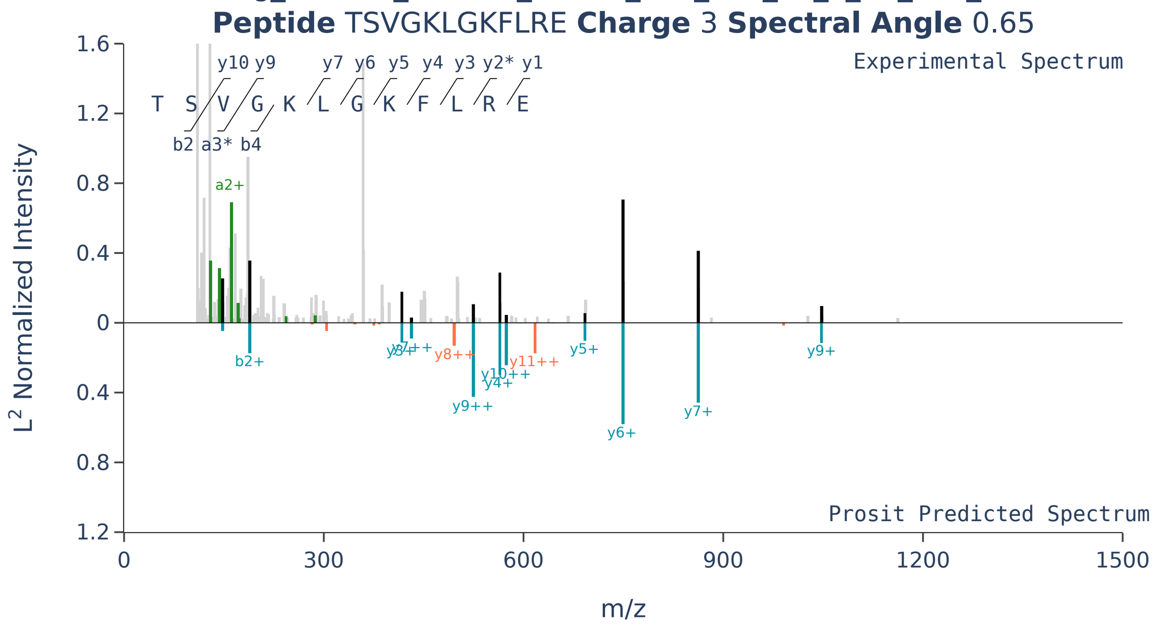


**Source** Ncheng_210623_230623_HFGoe_FFH_20S_25_1_A2_4h_R2 **Scan** 16860
**Peptide** LRNISGRGRLTEDNVKDTLREV **Charge** 5 **Spectral Angle** 0.61

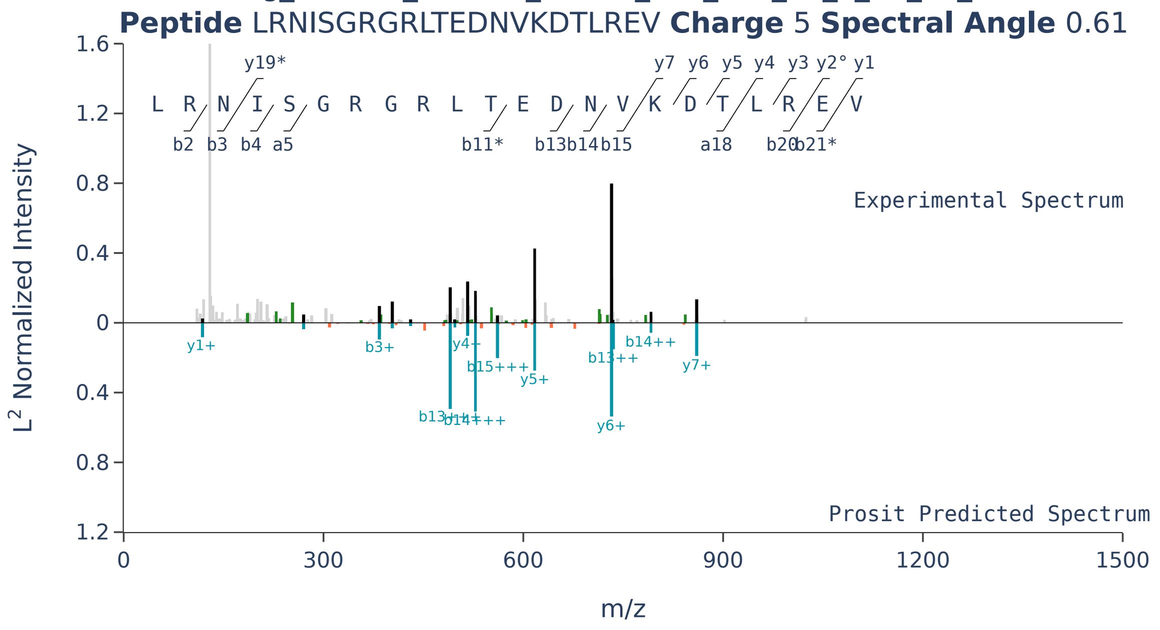


**Source** Ncheng_210623_230623_HFGoe_FFH_20S_25_1_A1_24h_R1 **Scan** 9178
**Peptide** PFHPDRIASR **Charge** 3 **Spectral Angle** 0.63

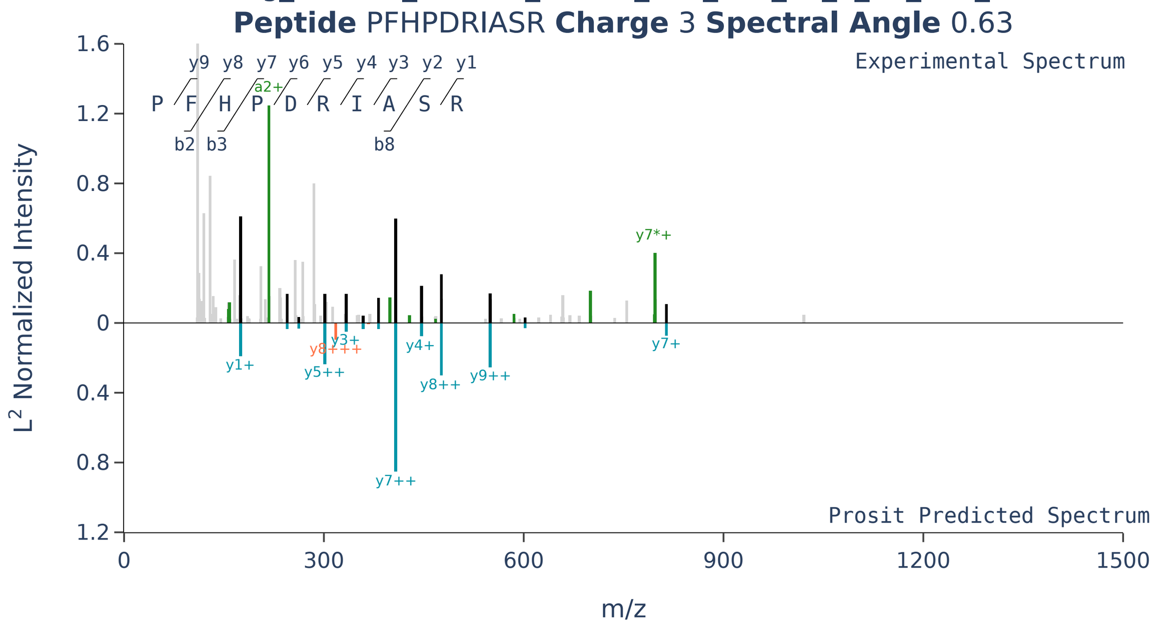


**Source** Ncheng_210623_230623_HFGoe_FFH_20S_25_1_A1_24h_R2 **Scan** 11695
**Peptide** AGCGMQVQDVN **Charge** 2 **Spectral Angle** 0.84

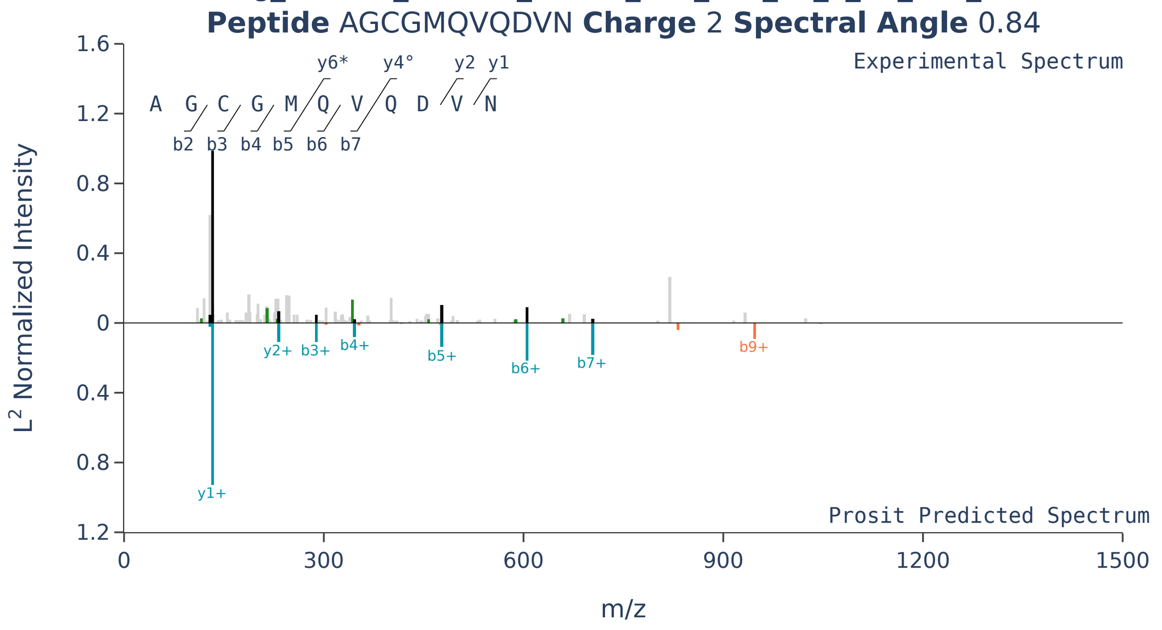


**Source** Ncheng_210623_230623_HFGoe_FFH_20S_25_1_A2_4h_R1 **Scan** 25304
**Peptide** MFDNLTDRLSRTLR **Charge** 3 **Spectral Angle** 0.63

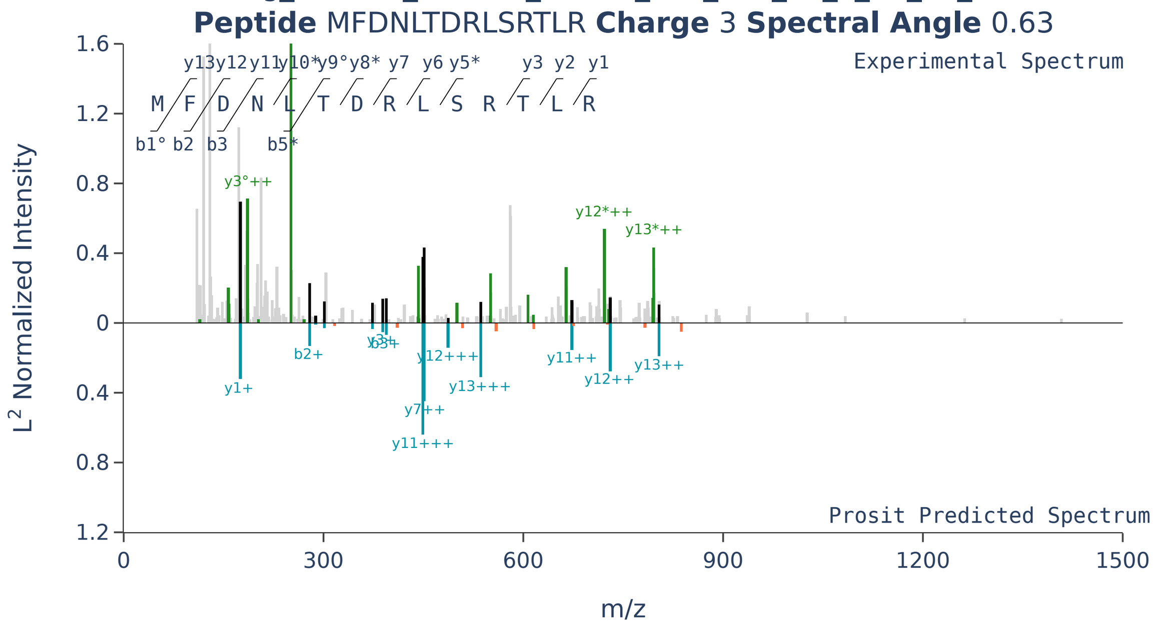


**Source** Ncheng_210623_230623_HFGoe_FFH_20S_25_1_A2_4h_R2 **Scan** 23163
**Peptide** KLPGMGQIPDNVKSQMDDKVLV **Charge** 3 **Spectral Angle** 0.89

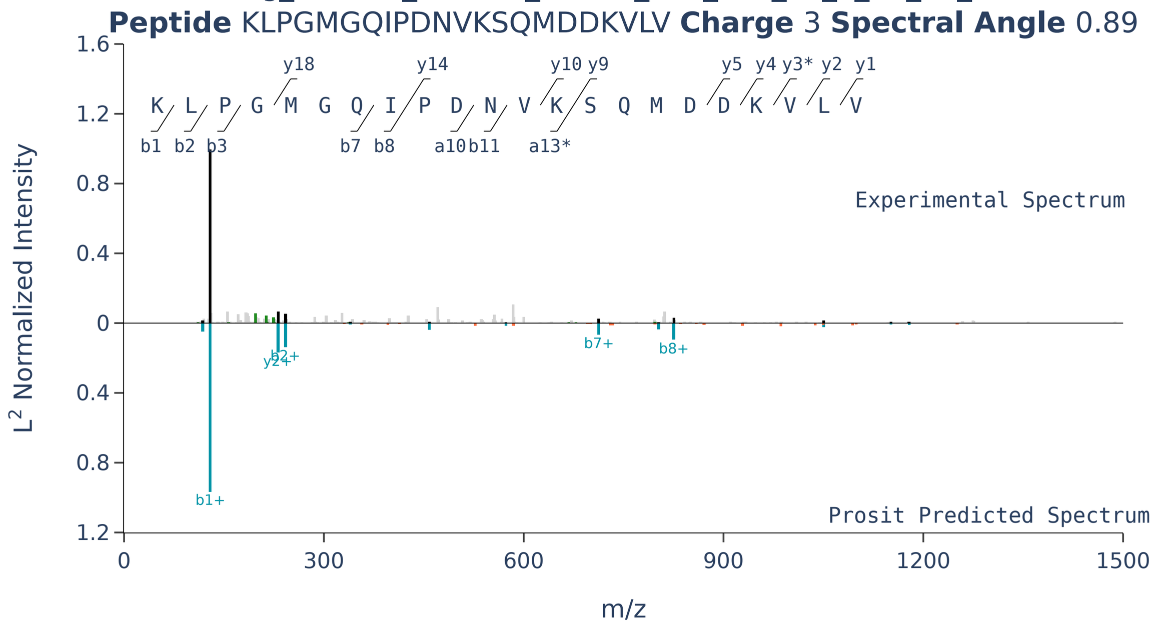


**Source** Ncheng_210623_230623_HFGoe_FFH_20S_25_1_A1_24h_R2 **Scan** 16309
**Peptide** HITGKPIKFLGVGEKT **Charge** 3 **Spectral Angle** 0.79

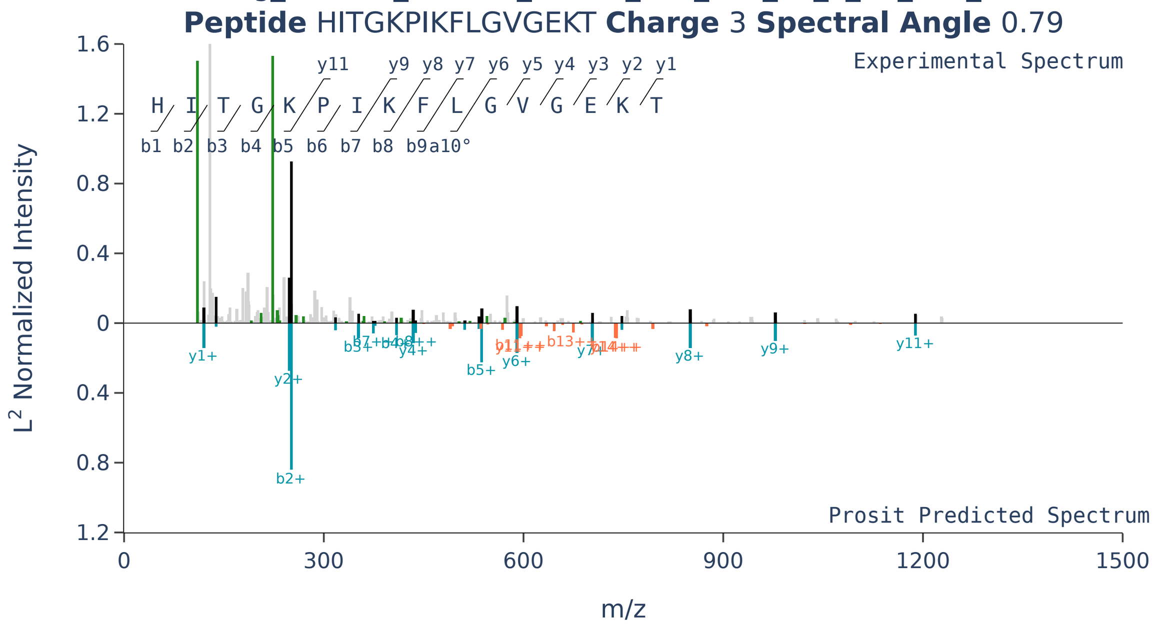


**Source** Ncheng_210623_230623_HFGoe_FFH_20S_25_1_A2_24h_R2 **Scan** 8104
**Peptide** DMQRMMKKMKKGG **Charge** 2 **Spectral Angle** 0.63

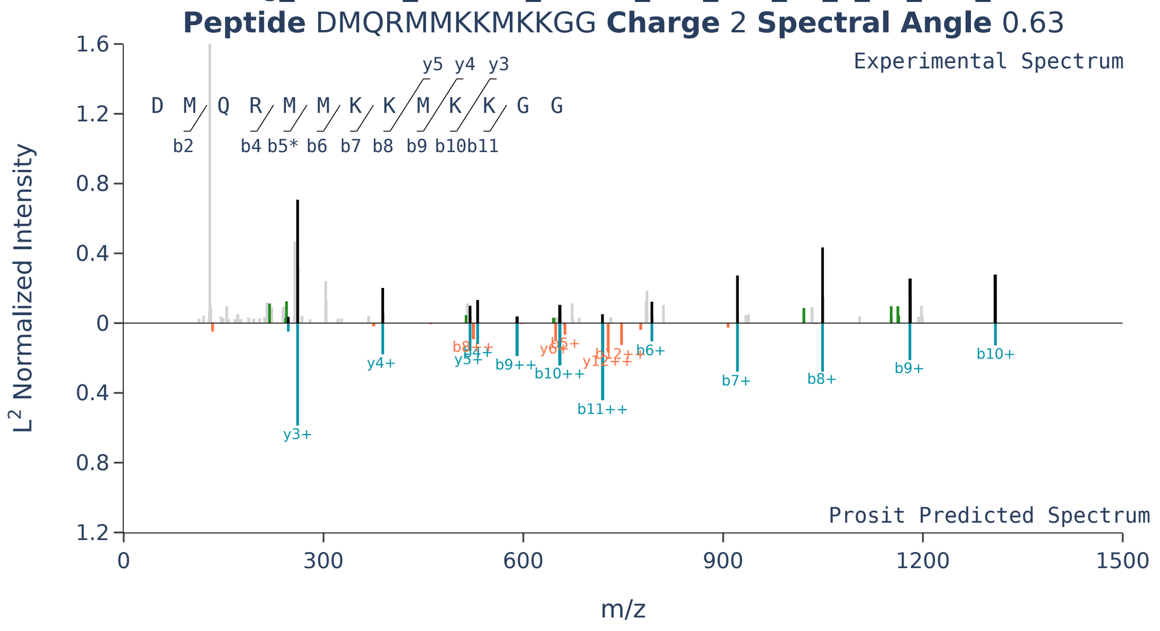


**Source** Ncheng_210623_230623_HFGoe_FFH_20S_25_1_A1_2h_R2 **Scan** 24996
**Peptide** EAMMDEIKQVHASINPVET **Charge** 3 **Spectral Angle** 0.58

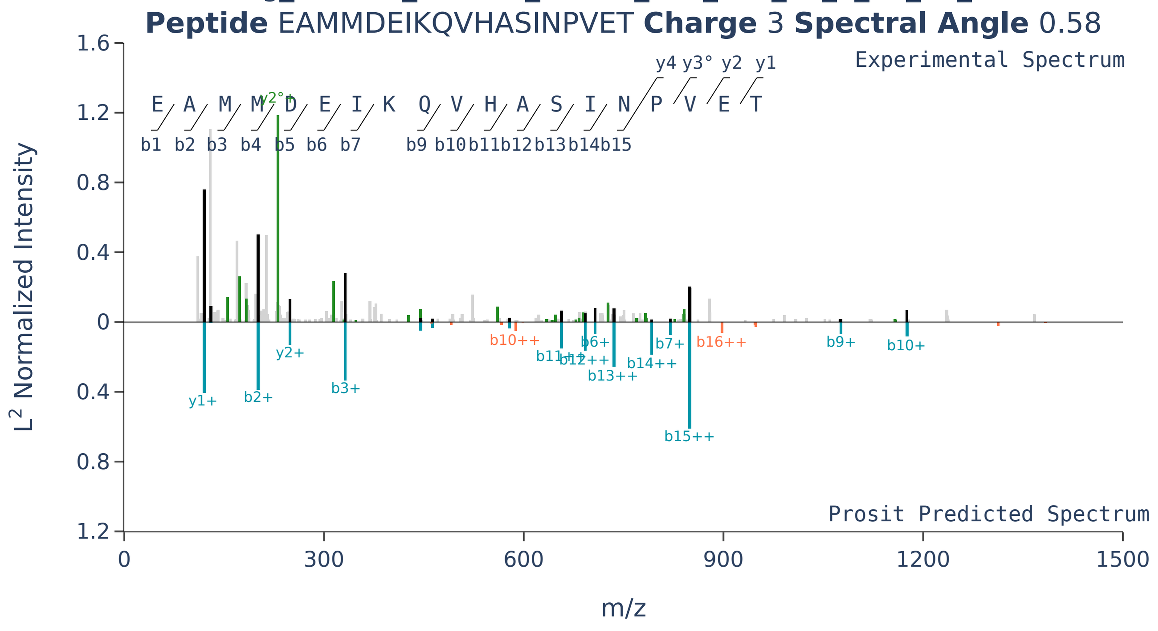


**Source** Ncheng_210623_230623_HFGoe_FFH_20S_25_1_A2_4h_R1 **Scan** 9784
**Peptide** GGMAKMMRSMK **Charge** 3 **Spectral Angle** 0.68

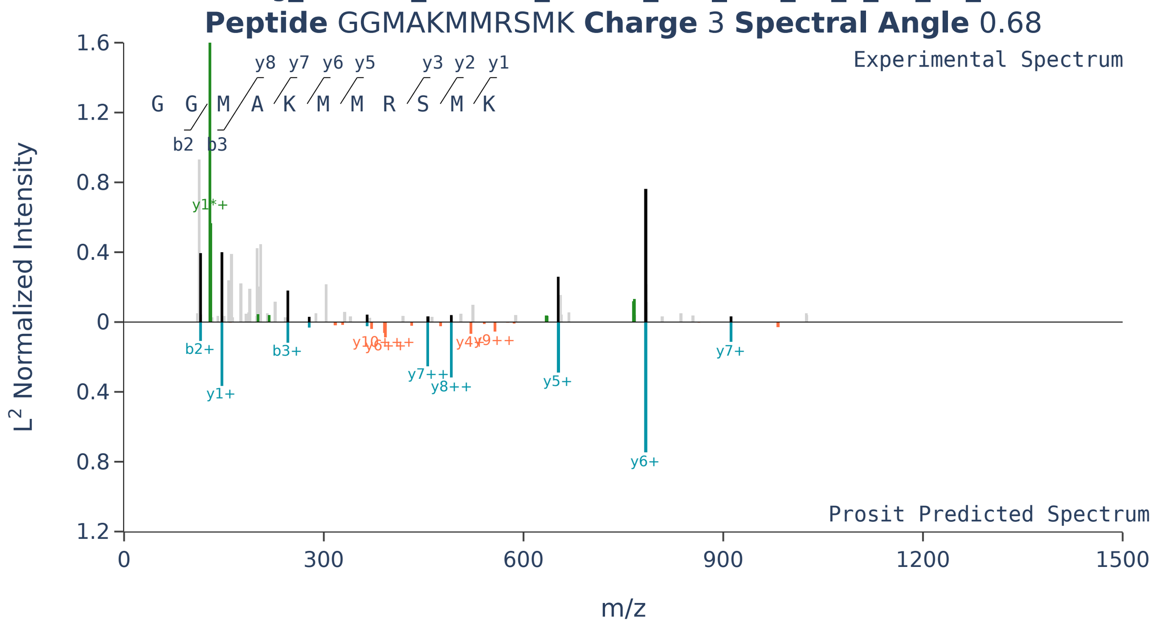


**Source** Ncheng_210623_230623_HFGoe_FFH_20S_25_1_A1_24h_R2 **Scan** 13149
**Peptide** KQFDDMQRMMKKMKK **Charge** 5 **Spectral Angle** 0.89

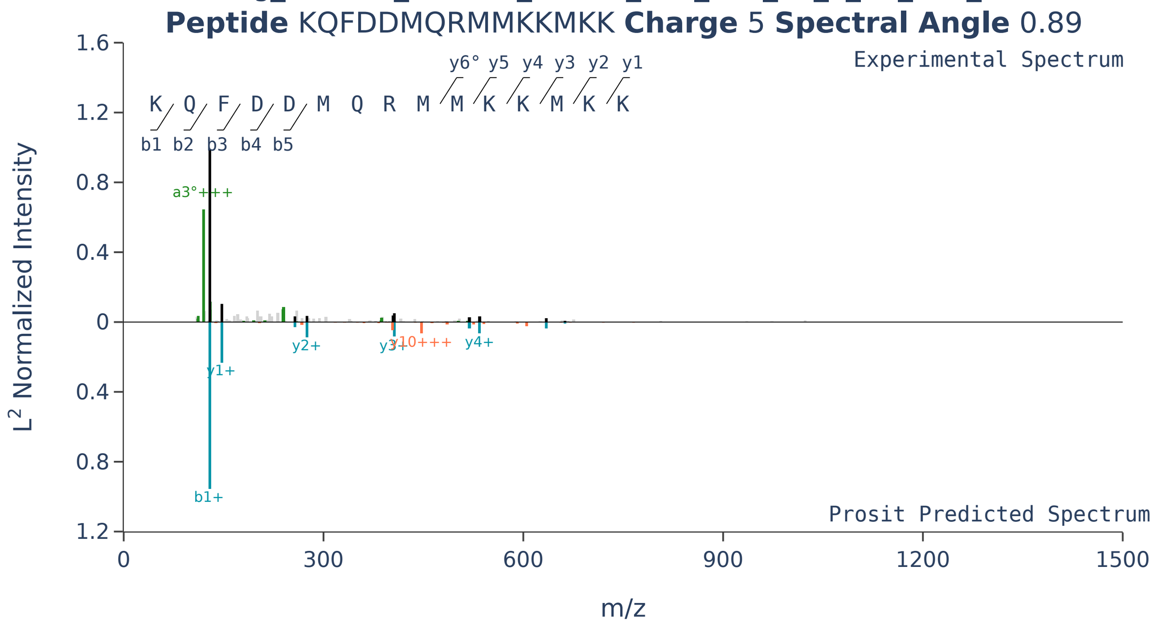


**Source** Ncheng_210623_230623_HFGoe_FFH_20S_25_1_A1_4h_R2 **Scan** 14823
**Peptide** GQKPVDIVNAA **Charge** 2 **Spectral Angle** 0.58

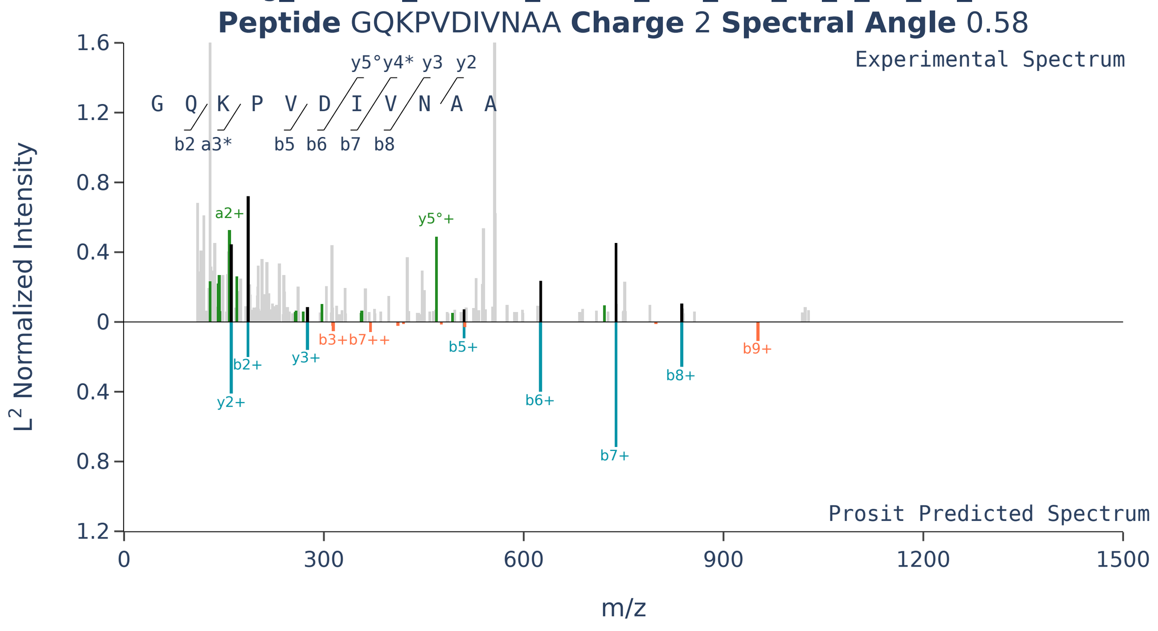


**Source** Ncheng_210623_230623_HFGoe_FFH_20S_25_1_A2_4h_R1 **Scan** 23292
**Peptide** TGKPIKFLGVGEKTEALEPFH **Charge** 4 **Spectral Angle** 0.85

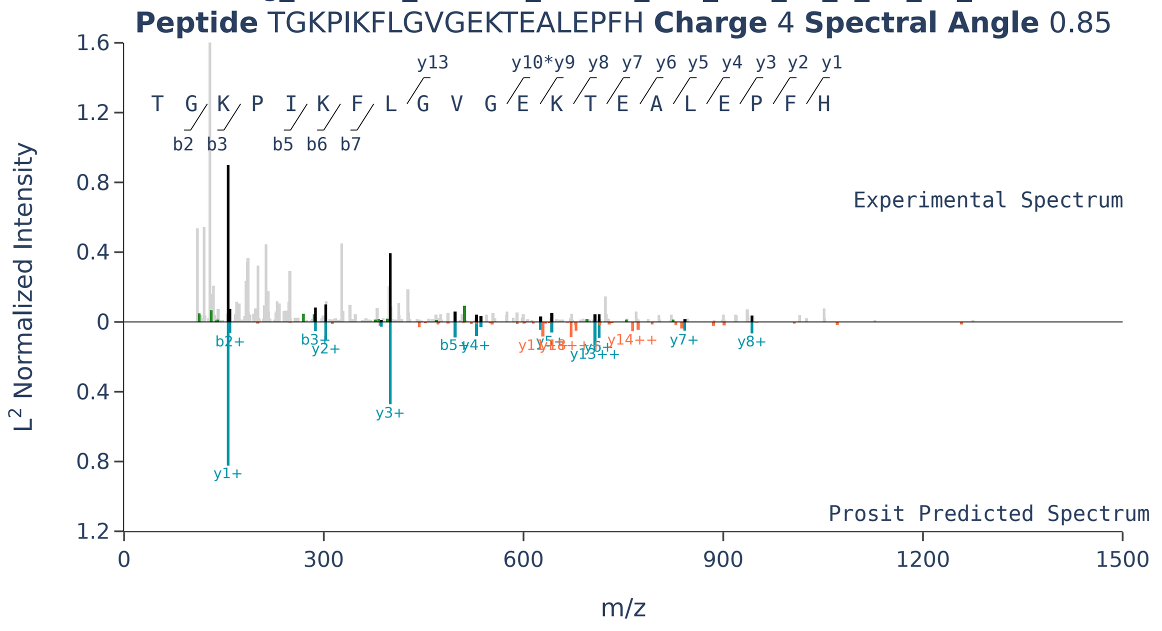


**Source** Ncheng_210623_230623_HFGoe_FFH_20S_25_1_A2_24h_R1 **Scan** 11814
**Peptide** AIINSMTMKERAKPEIIKGSRKR **Charge** 6 **Spectral Angle** 0.65

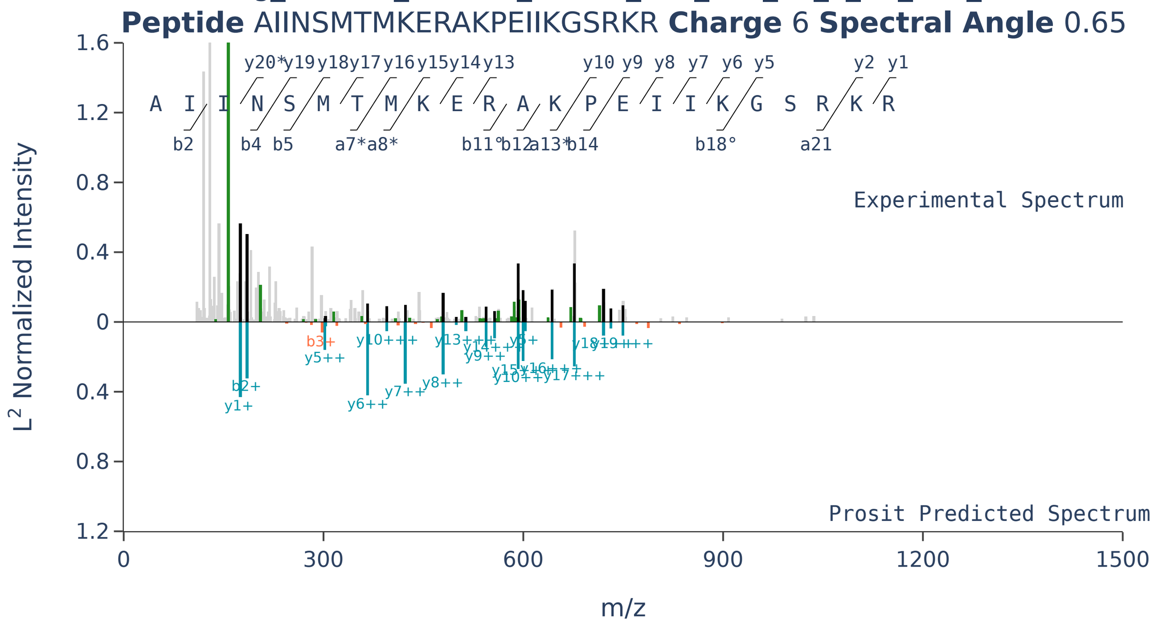

**Source** Ncheng_210623_230623_HFGoe_FFH_20S_25_1_A1_24h_R1 **Scan** 7003
**Peptide** RAQAEKLASKLKKG **Charge** 4 **Spectral Angle** 0.78

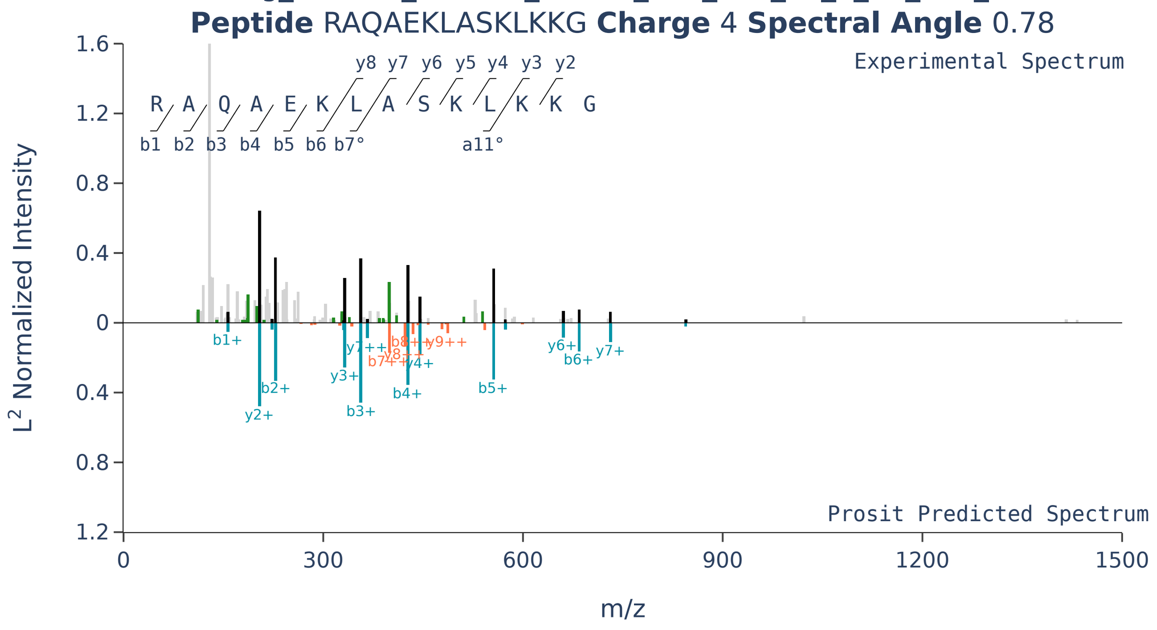


**Source** Ncheng_210623_230623_HFGoe_FFH_20S_25_1_A1_4h_R2 **Scan** 23306
**Peptide** EPFHPDRIASRILG **Charge** 4 **Spectral Angle** 0.79

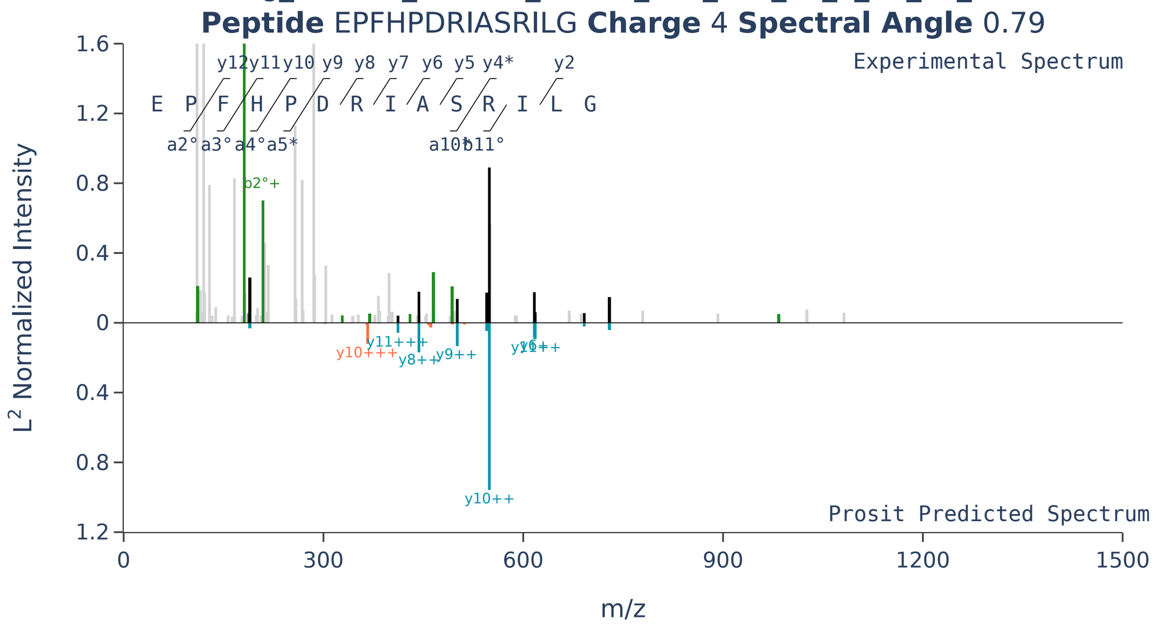


**Source** Ncheng_210623_230623_HFGoe_FFH_20S_25_1_A2_24h_R2 **Scan** 14400
**Peptide** AGKTTSVGKLGKFLR **Charge** 4 **Spectral Angle** 0.72

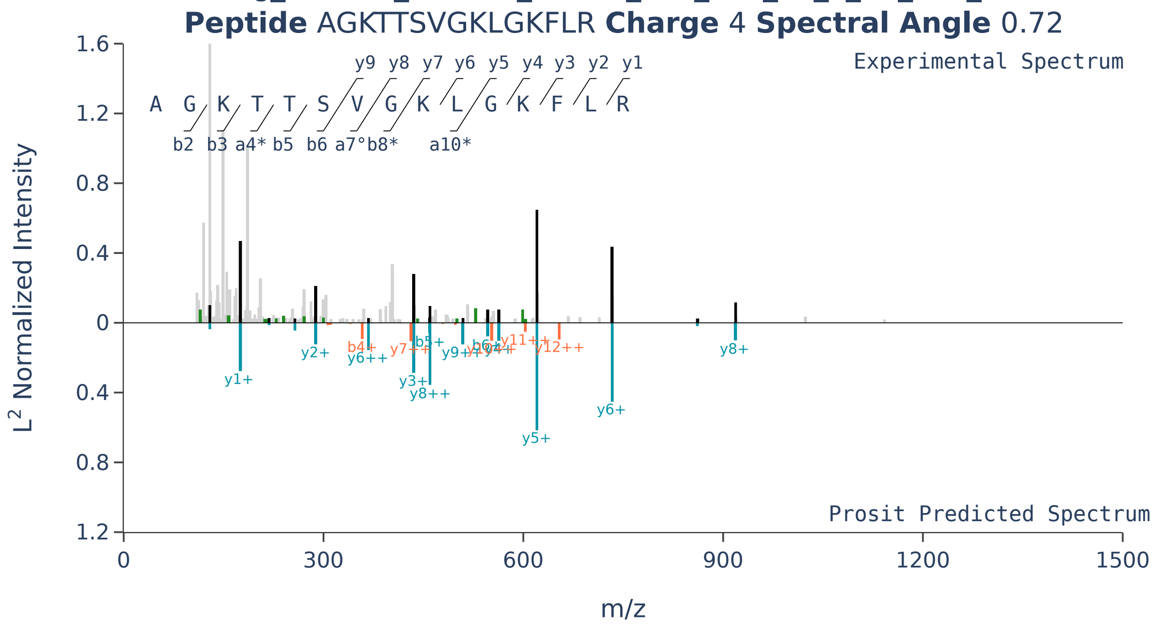


**Source** Ncheng_210623_230623_HFGoe_FFH_20S_25_1_A1_2h_R2 **Scan** 30459
**Peptide** NEALPLTGVVLTKVDGDARGGAALSIRHI **Charge** 4 **Spectral Angle** 0.78

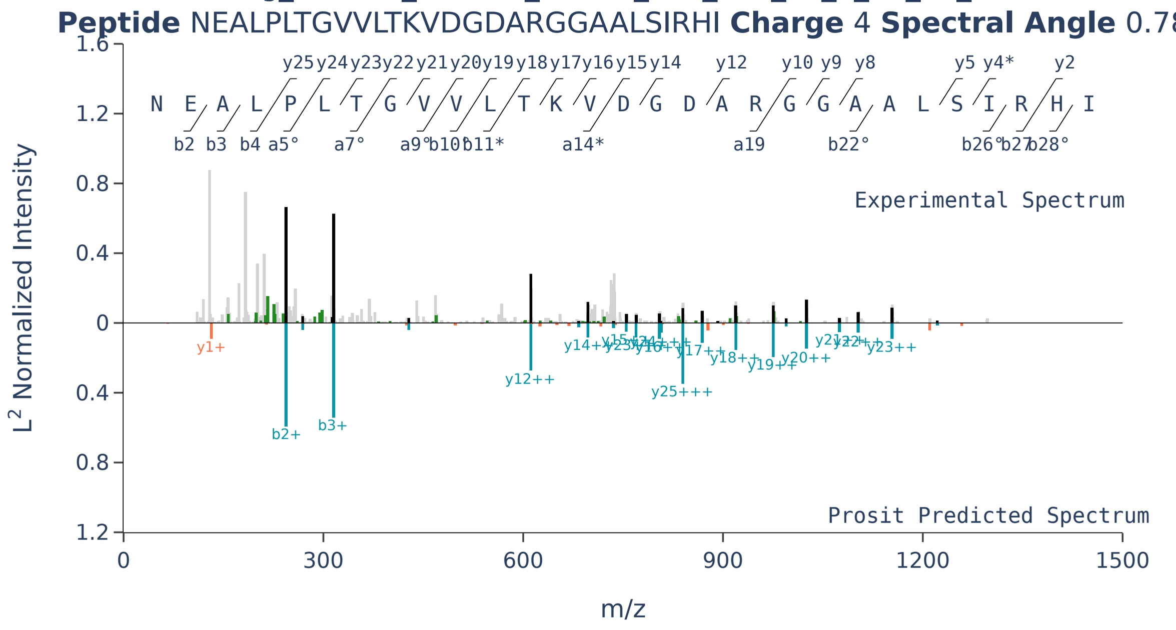


**Source** Ncheng_210623_230623_HFGoe_FFH_20S_25_1_A2_4h_R1 **Scan** 21440
**Peptide** ALEPFHPDRIASRI **Charge** 2 **Spectral Angle** 0.67

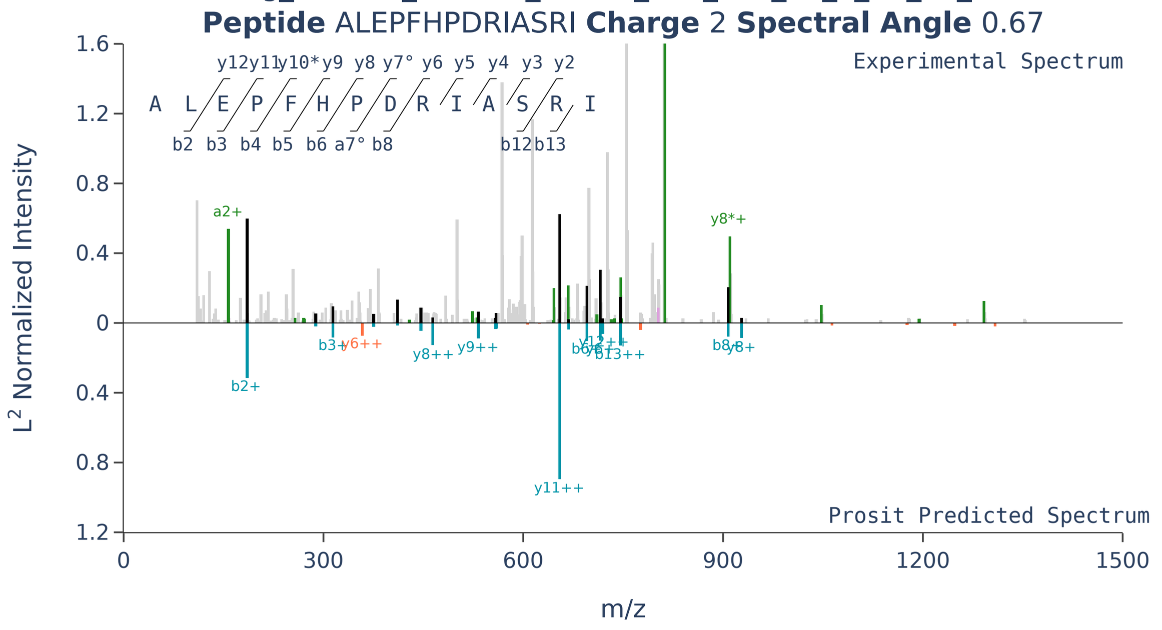


**Source** Ncheng_210623_230623_HFGoe_FFH_20S_25_1_A1_4h_R1 **Scan** 14162
**Peptide** IINSMTMKERAKPEIIKG **Charge** 5 **Spectral Angle** 0.85

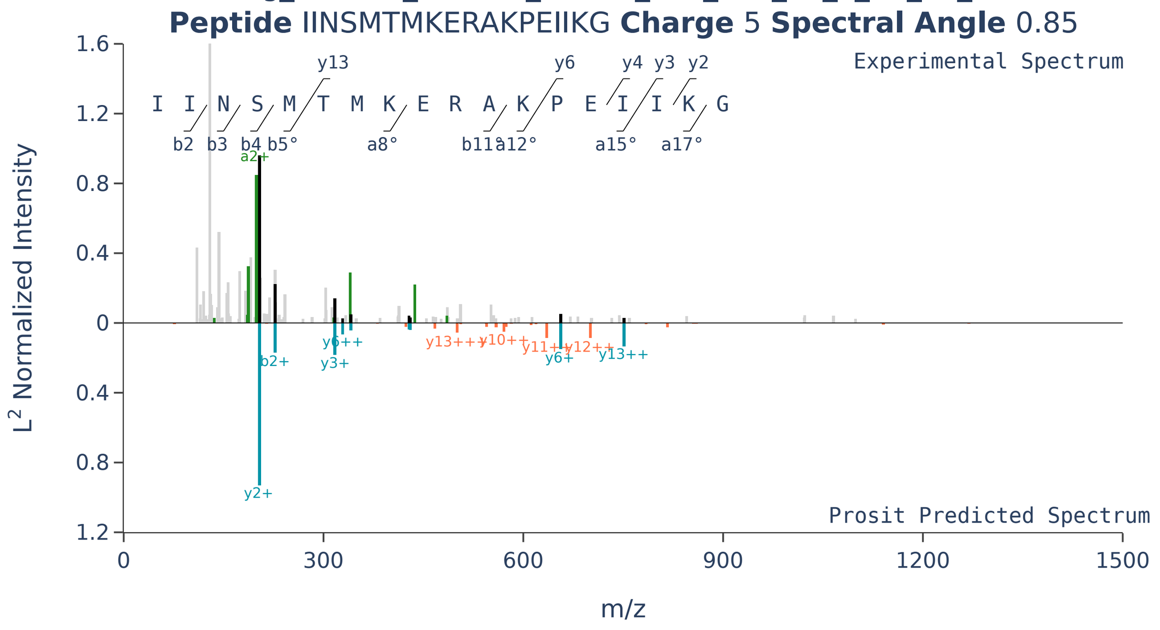


**Source** Ncheng_210623_230623_HFGoe_FFH_20S_25_1_A1_24h_R1 **Scan** 15606
**Peptide** GAGKTTSVGKLGKFLRE **Charge** 4 **Spectral Angle** 0.62

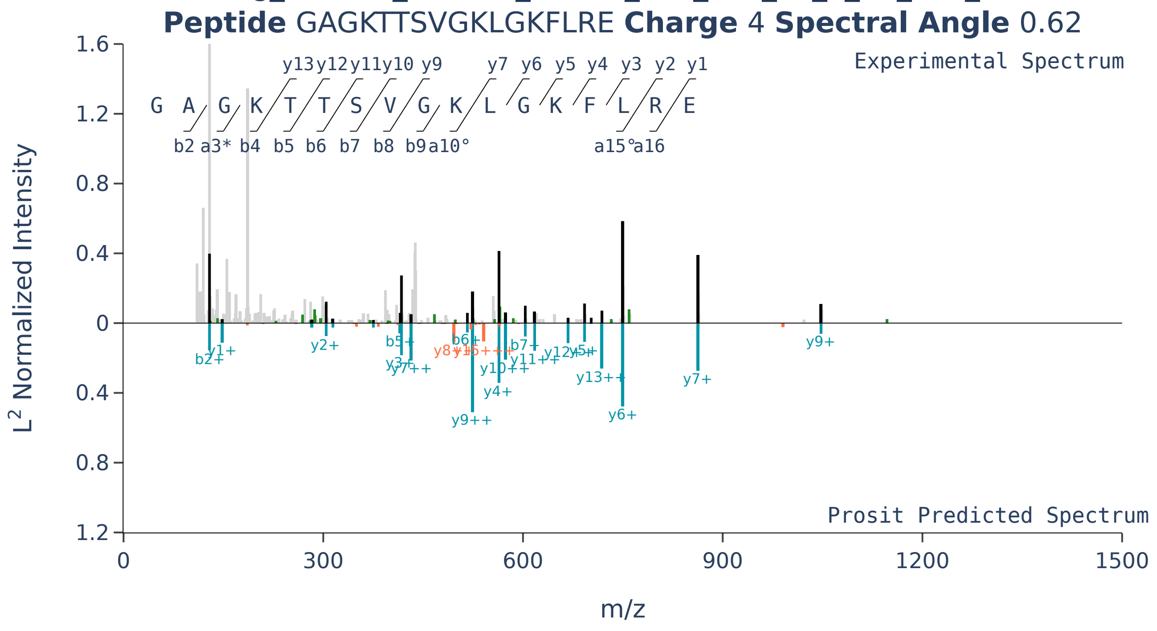


**Source** Ncheng_210623_230623_HFGoe_FFH_20S_25_1_A2_2h_R1 **Scan** 16455
**Peptide** SIRHITGKPIKFL **Charge** 3 **Spectral Angle** 0.71

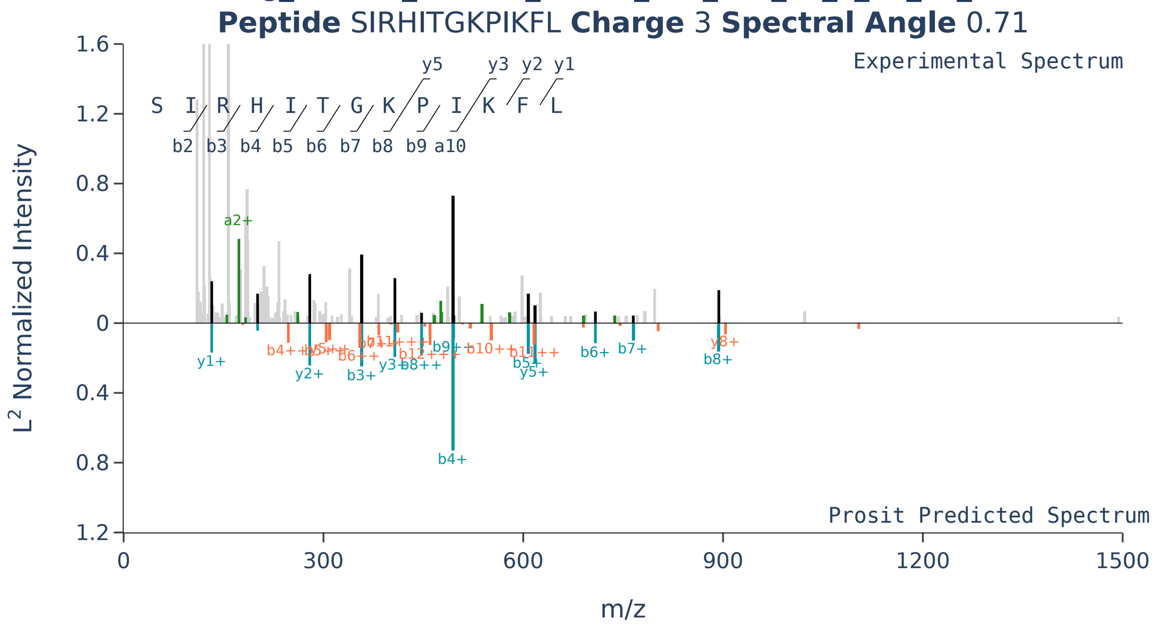


**Source** Ncheng_210623_230623_HFGoe_FFH_20S_25_1_A2_24h_R1 **Scan** 10758
**Peptide** TMKERAKPELL **Charge** 2 **Spectral Angle** 0.63

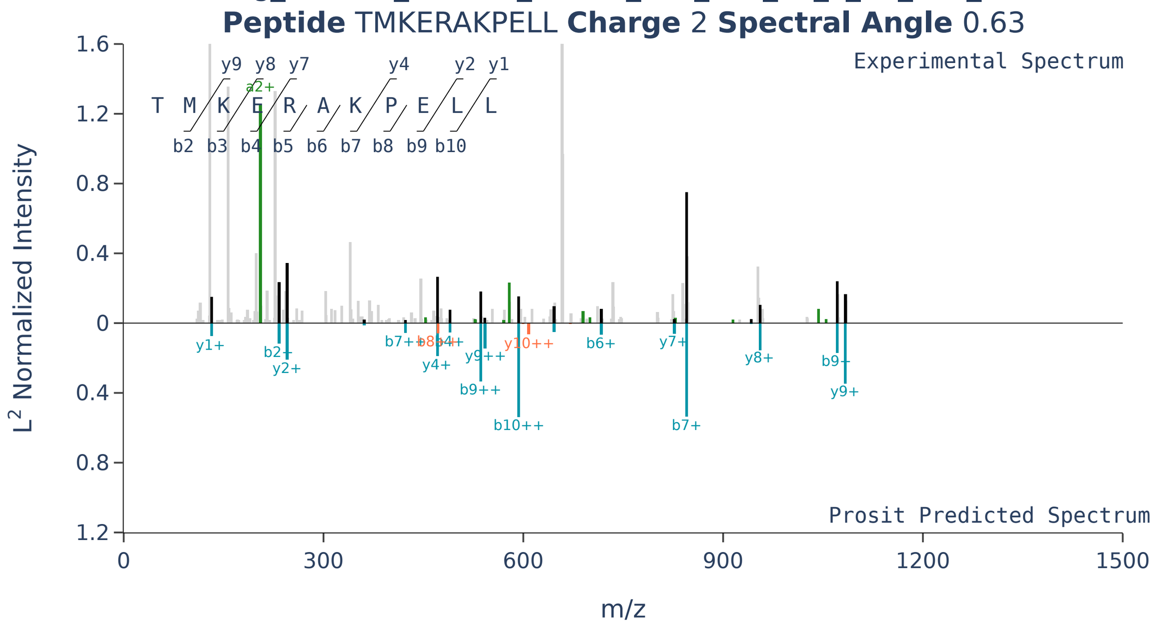


**Source** Ncheng_210623_230623_HFGoe_FFH_20S_25_1_A1_2h_R2 **Scan** 27436
**Peptide** MTGQDAANTAKAFNEALPLT **Charge** 2 **Spectral Angle** 0.89

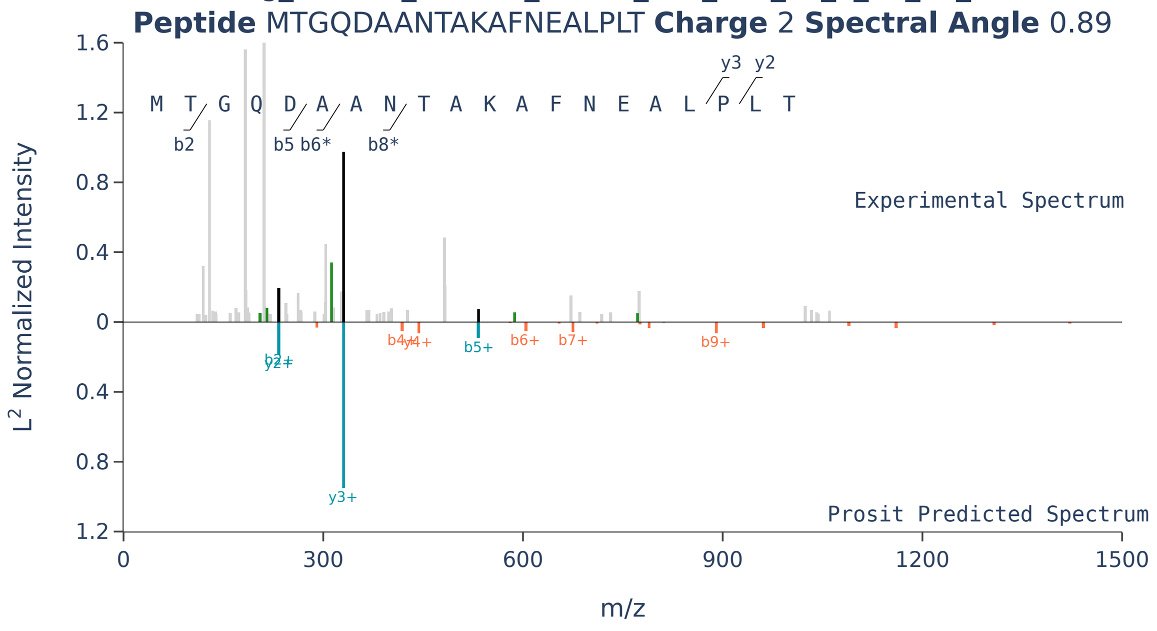


**Source** Ncheng_210623_230623_HFGoe_FFH_20S_25_1_A1_4h_R1 **Scan** 31161
**Peptide** EALPLTGVVLTKVDGDARGGAALSIR **Charge** 3 **Spectral Angle** 0.61

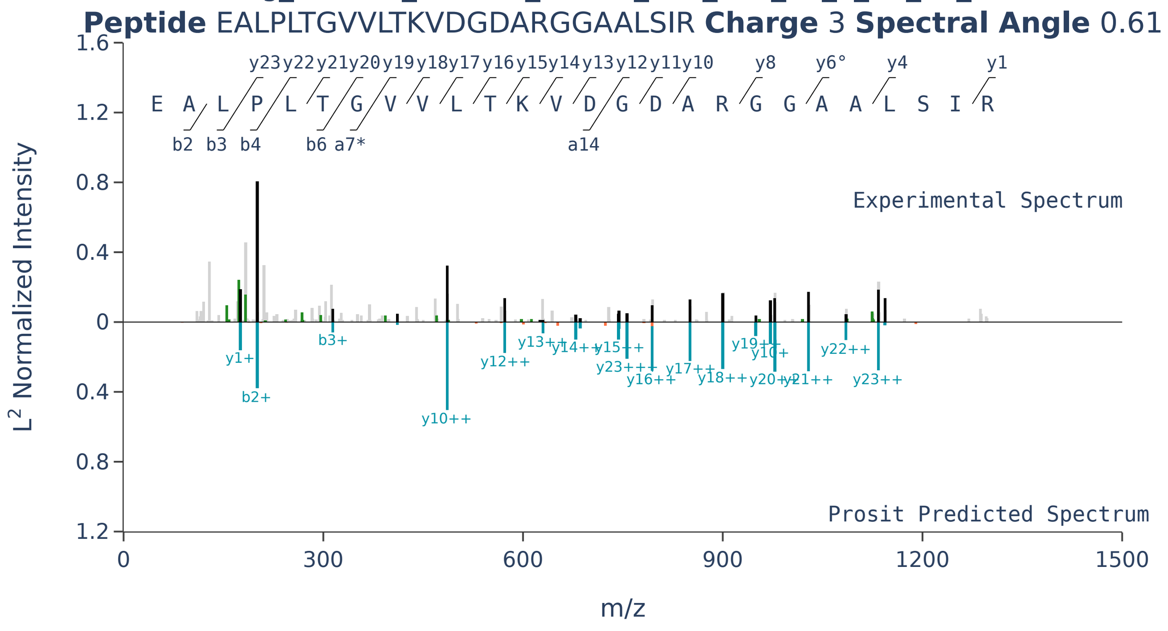


**Source** Ncheng_210623_230623_HFGoe_FFH_20S_25_1_A1_2h_R2 **Scan** 19008
**Peptide** TAGRLHVDEAMMDELKQ **Charge** 4 **Spectral Angle** 0.53

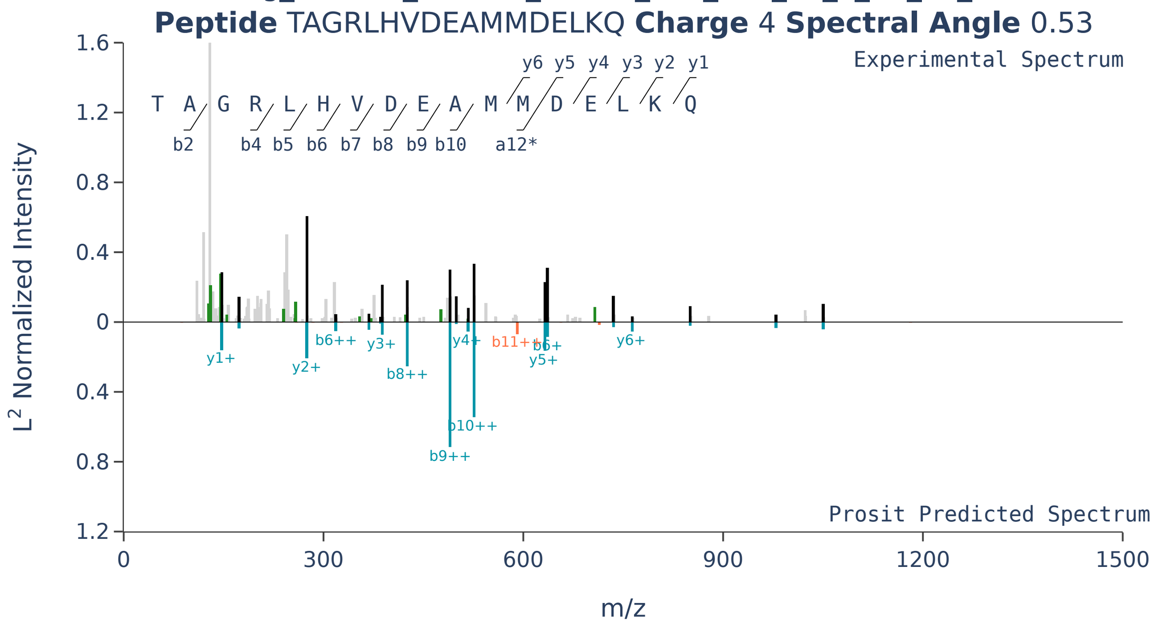


**Source** Ncheng_210623_230623_HFGoe_FFH_20S_25_1_A2_2h_R2 **Scan** 13160
**Peptide** RHITGKPIKFLGVGEKT **Charge** 5 **Spectral Angle** 0.77

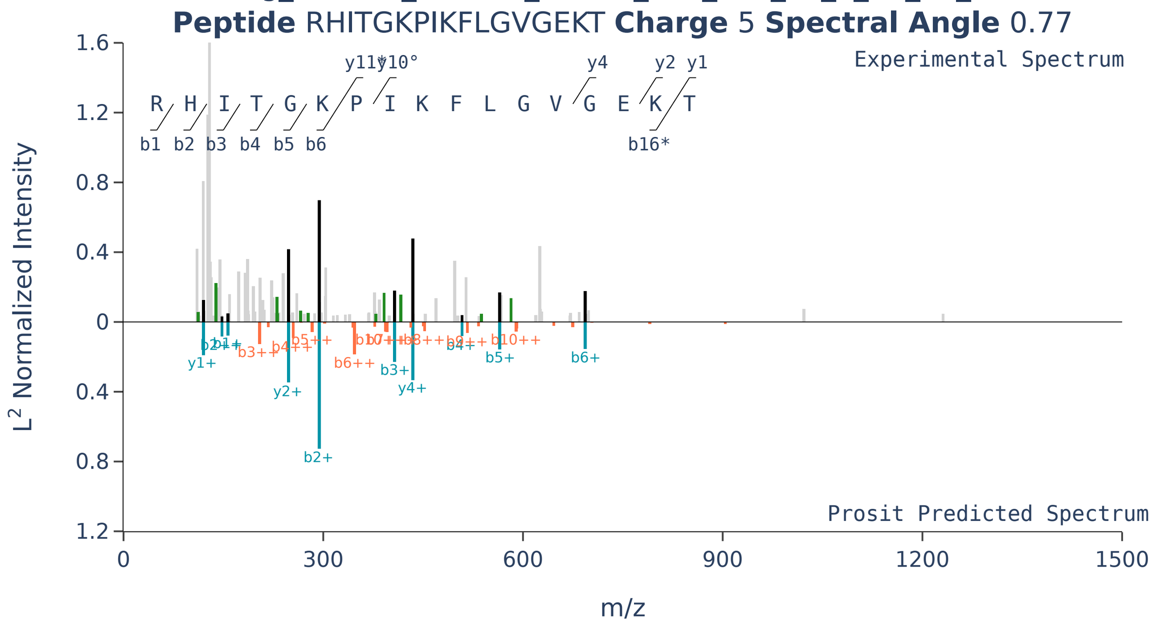


**Source** Ncheng_210623_230623_HFGoe_FFH_20S_25_1_A1_4h_R2 **Scan** 19595
**Peptide** VLTKVDGDARGGAALSI **Charge** 2 **Spectral Angle** 0.72

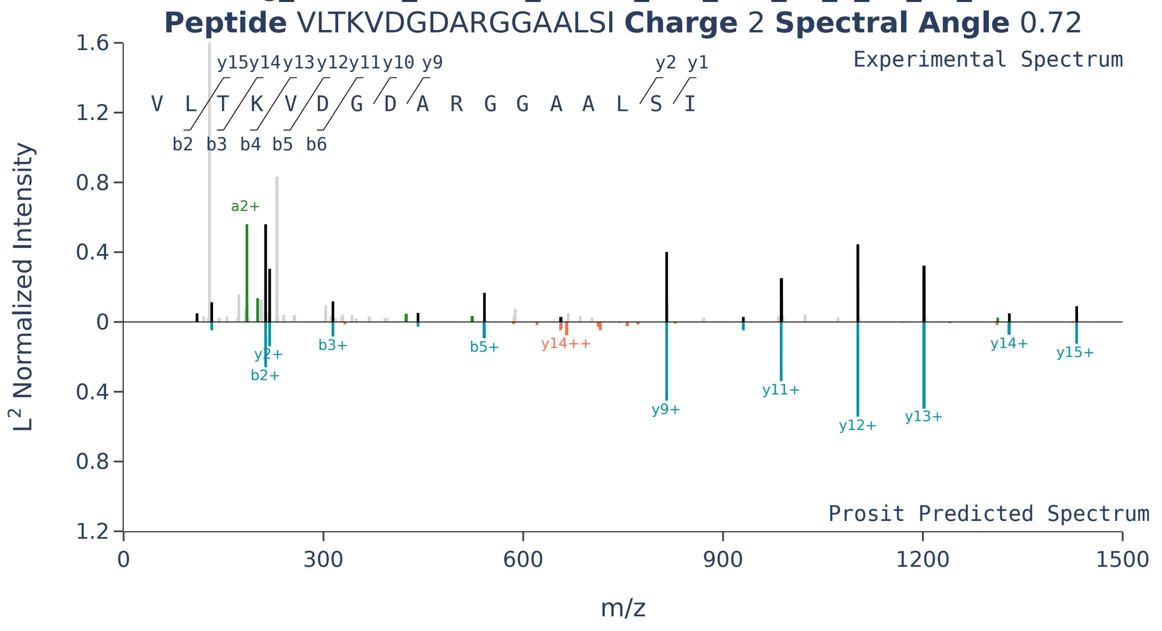


**Source** Ncheng_210623_230623_HFGoe_FFH_20S_25_1_A1_2h_R2 **Scan** 35006
**Peptide** RNELVAAMGEENQTLNLAAQPPAVVL **Charge** 3 **Spectral Angle** 0.7

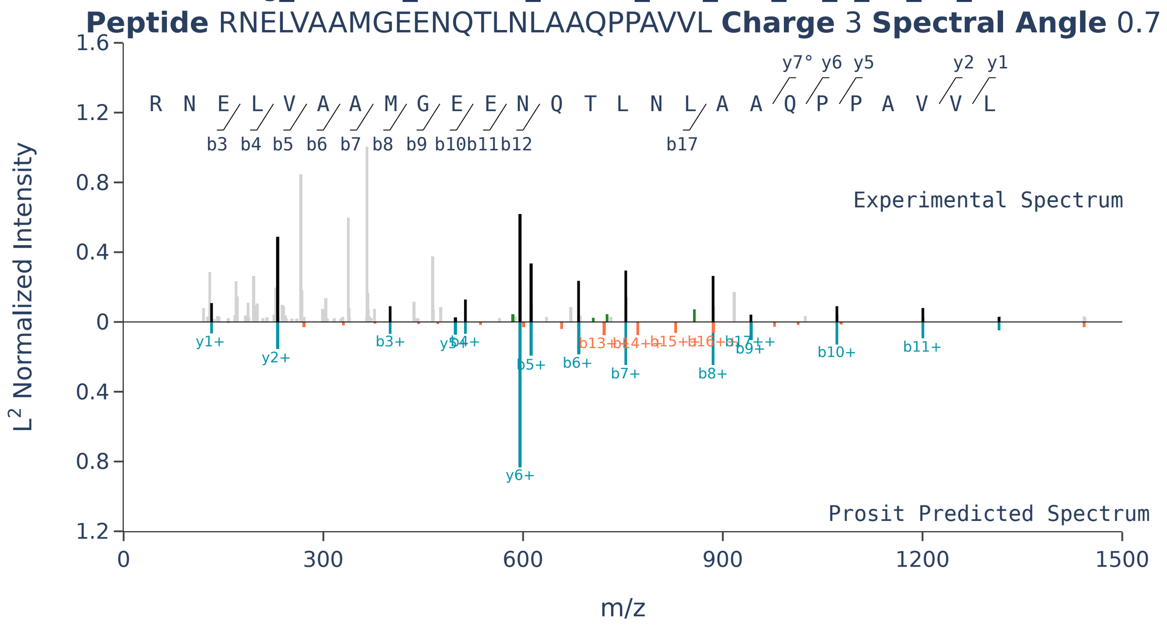

**Source** Ncheng_210623_230623_HFGoe_FFH_20S_25_1_A2_4h_R1 **Scan** 19997
**Peptide** GVVLTKVDGDARGGAALSIRHI **Charge** 3 **Spectral Angle** 0.65

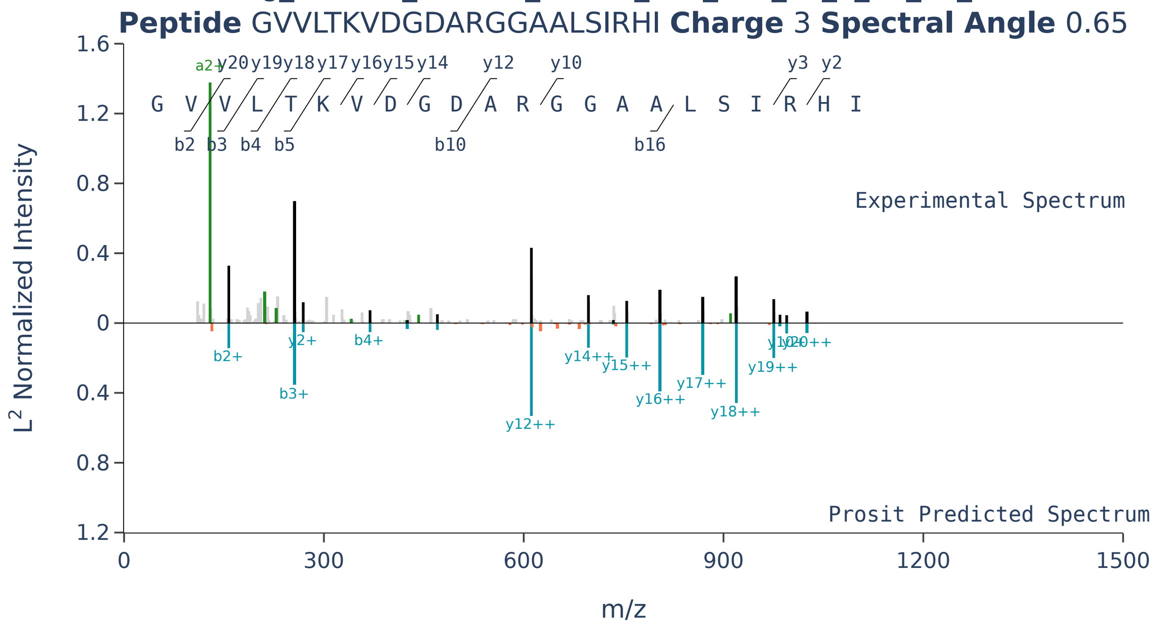

**Source** Ncheng_210623_230623_HFGoe_FFH_20S_25_1_A2_2h_R1 **Scan** 31571
**Peptide** ALKEAKLKFYDVLLVDTAGRL **Charge** 3 **Spectral Angle** 0.85

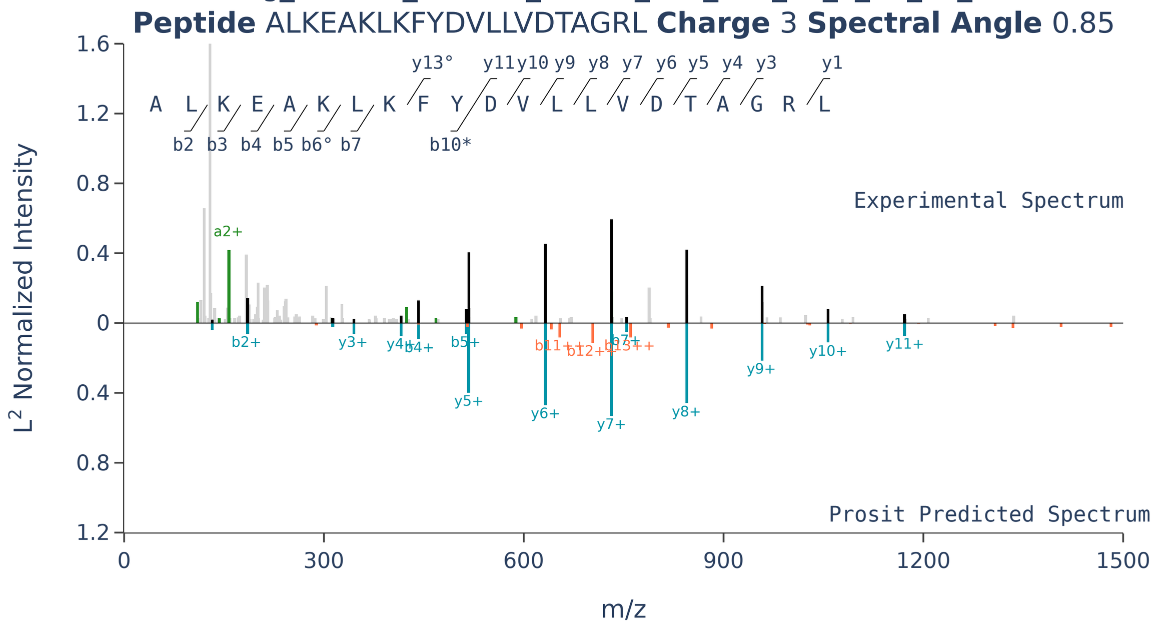

**Source** Ncheng_210623_230623_HFGoe_FFH_20S_25_1_A2_24h_R1 **Scan** 7771
**Peptide** DAMTGQDAANTA **Charge** 2 **Spectral Angle** 0.79

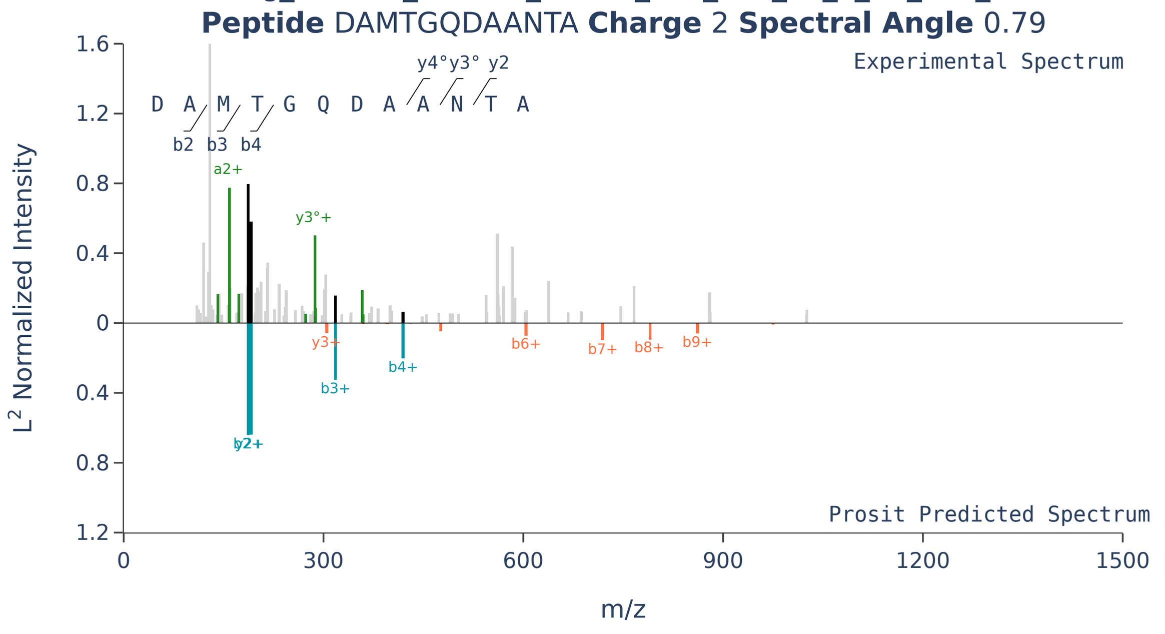

**Source** Ncheng_210623_230623_HFGoe_FFH_20S_25_1_A1_24h_R1 **Scan** 7713
**Peptide** TMKERAKPEIIKGS **Charge** 2 **Spectral Angle** 0.65

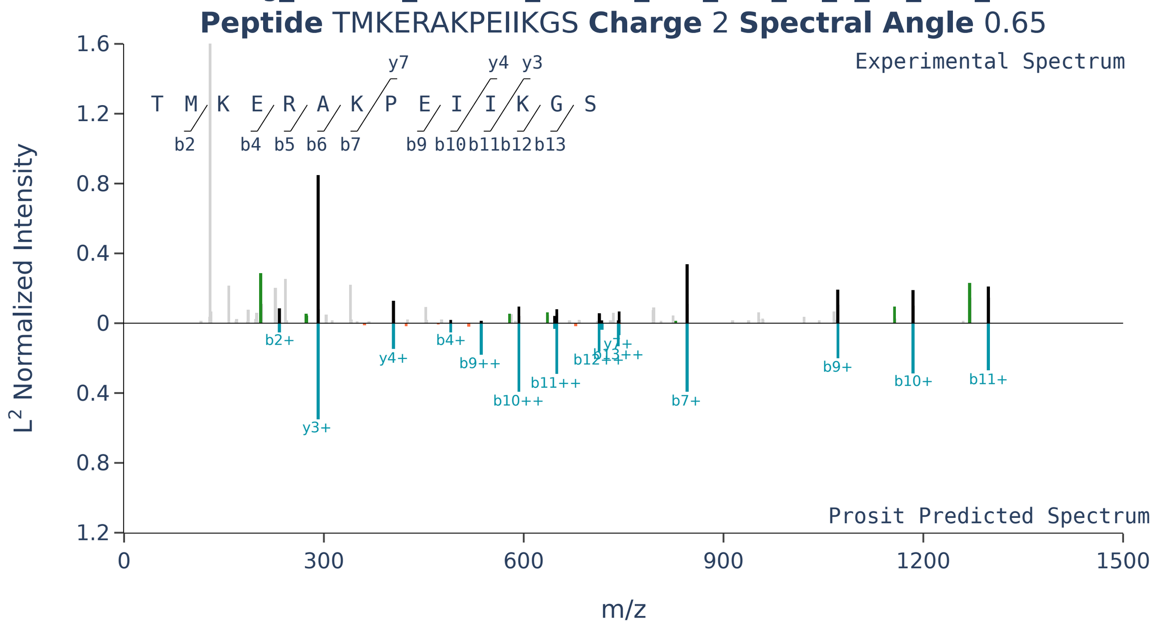

**Source** Ncheng_210623_230623_HFGoe_FFH_20S_25_1_A1_24h_R1 **Scan** 7857
**Peptide** NLSGRGRLTEDNVKD **Charge** 3 **Spectral Angle** 0.69

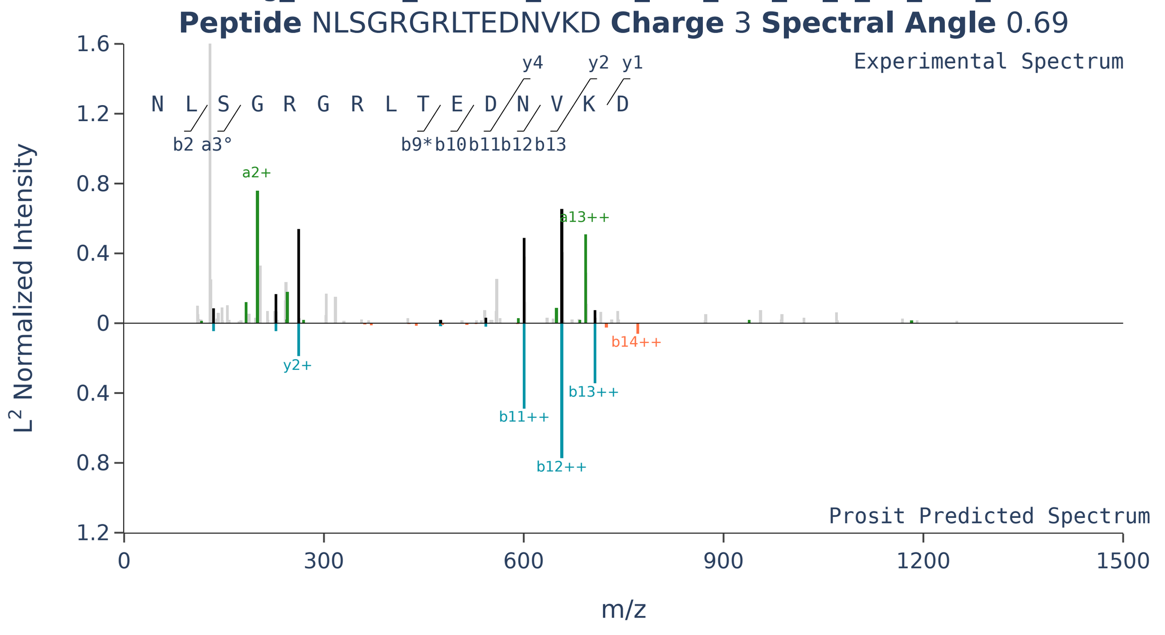

**Source** Ncheng_210623_230623_HFGoe_FFH_20S_25_1_A2_4h_R2 **Scan** 24790
**Peptide** FVVDAMTGQDAANTAKAFN **Charge** 2 **Spectral Angle** 0.83

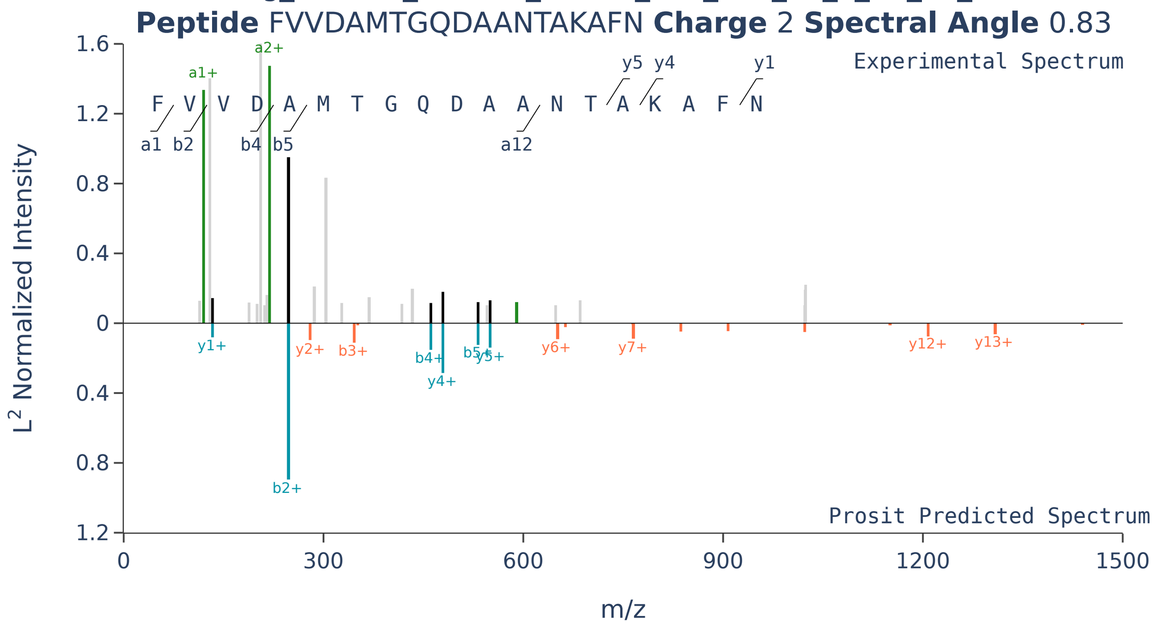

**Source** Ncheng_210623_230623_HFGoe_FFH_20S_25_1_A2_4h_R2 **Scan** 29088
**Peptide** KNMGGMASLMGKLPGMGQIPDNVKSQ **Charge** 3 **Spectral Angle** 0.77

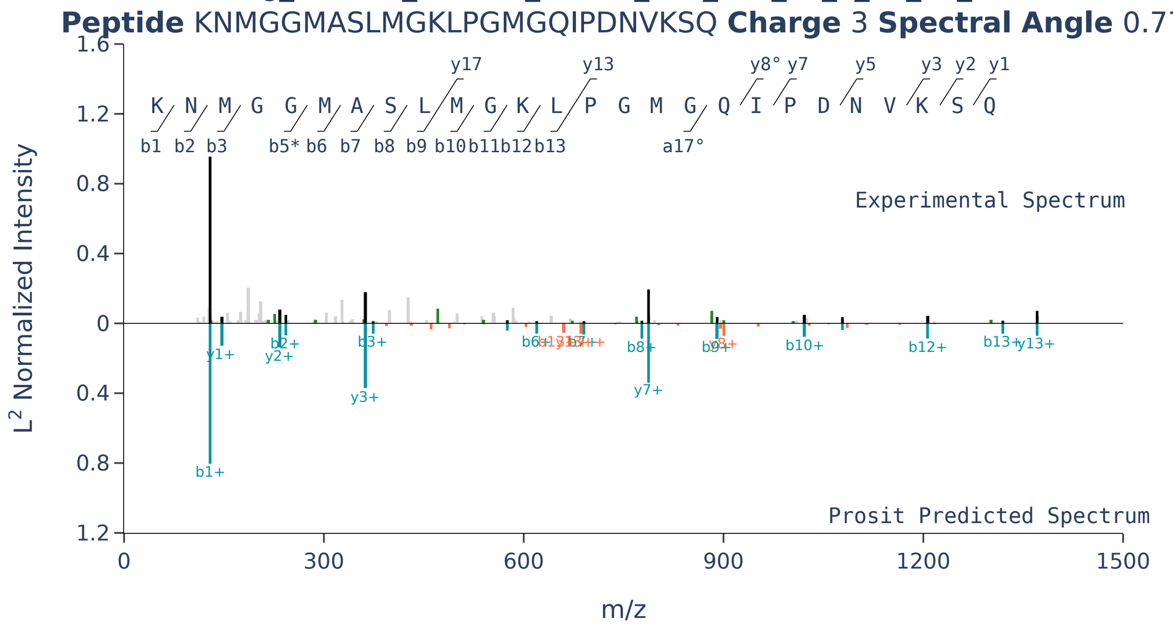

**Source** Ncheng_210623_230623_HFGoe_FFH_20S_25_1_A1_24h_R2 **Scan** 17544
**Peptide** KEKAVGHEVNKSLTPGQEFVKI **Charge** 2 **Spectral Angle** 0.69

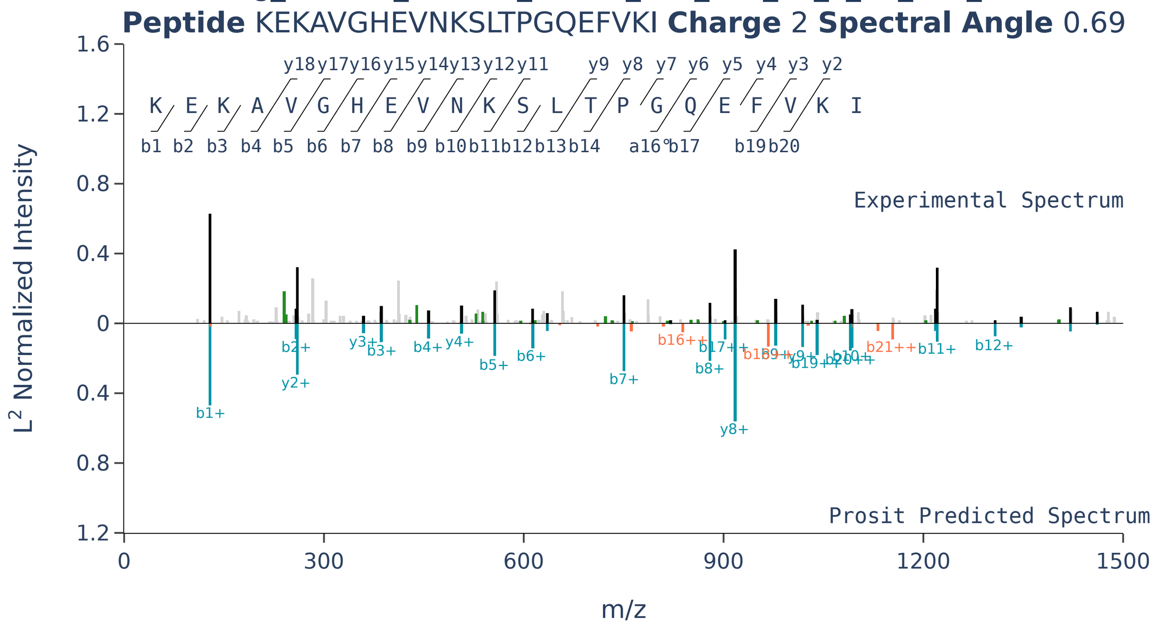

**Source** Ncheng_210623_230623_HFGoe_FFH_20S_25_1_A1_24h_R1 **Scan** 10708
**Peptide** TMKERAKPEII **Charge** 2 **Spectral Angle** 0.59

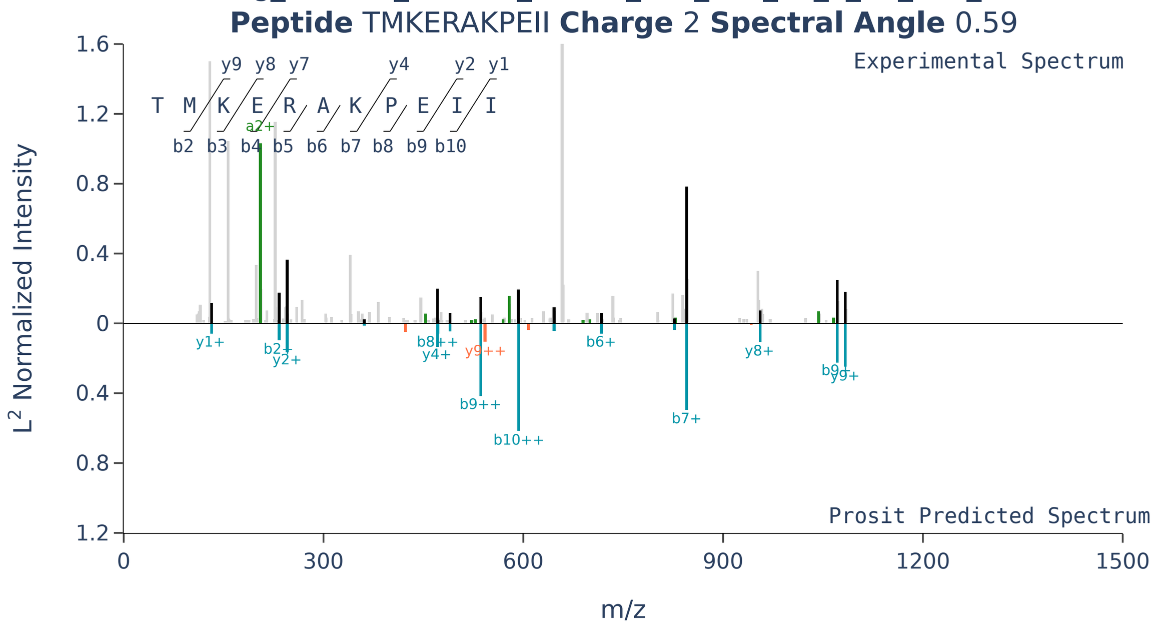

**Source** Ncheng_210623_230623_HFGoe_FFH_20S_25_1_A1_24h_R1 **Scan** 5137
**Peptide** RAQAEKLASK **Charge** 2 **Spectral Angle** 0.68

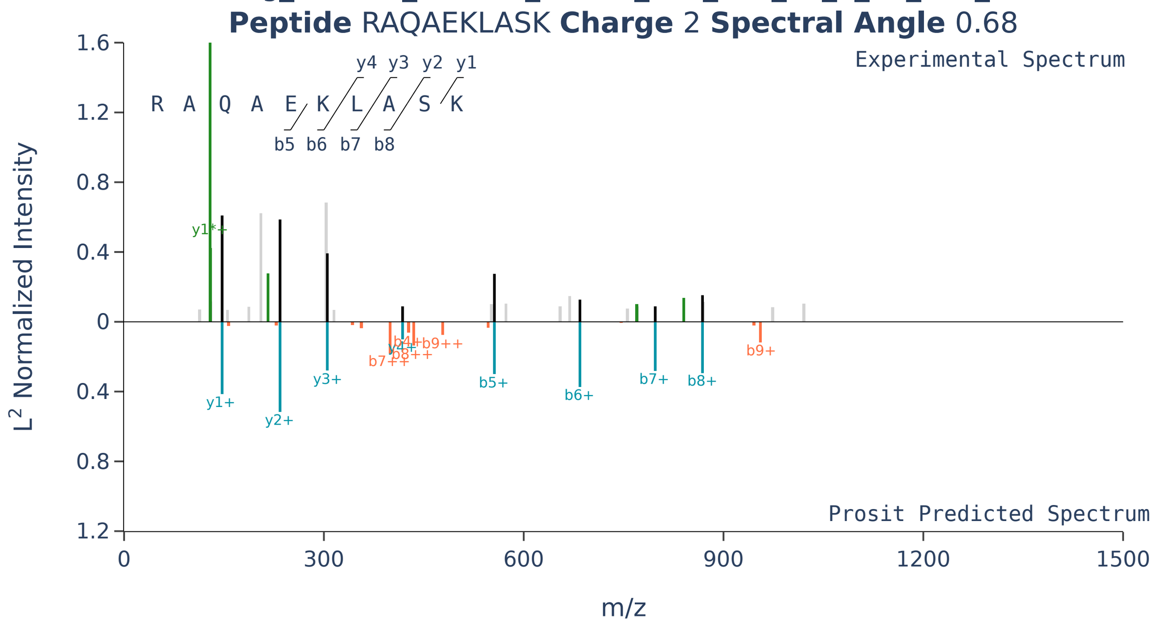

**Source** Ncheng_210623_230623_HFGoe_FFH_20S_25_1_A2_24h_R2 **Scan** 18389
**Peptide** EALINSMTMKERAKPEIIKG **Charge** 3 **Spectral Angle** 0.77

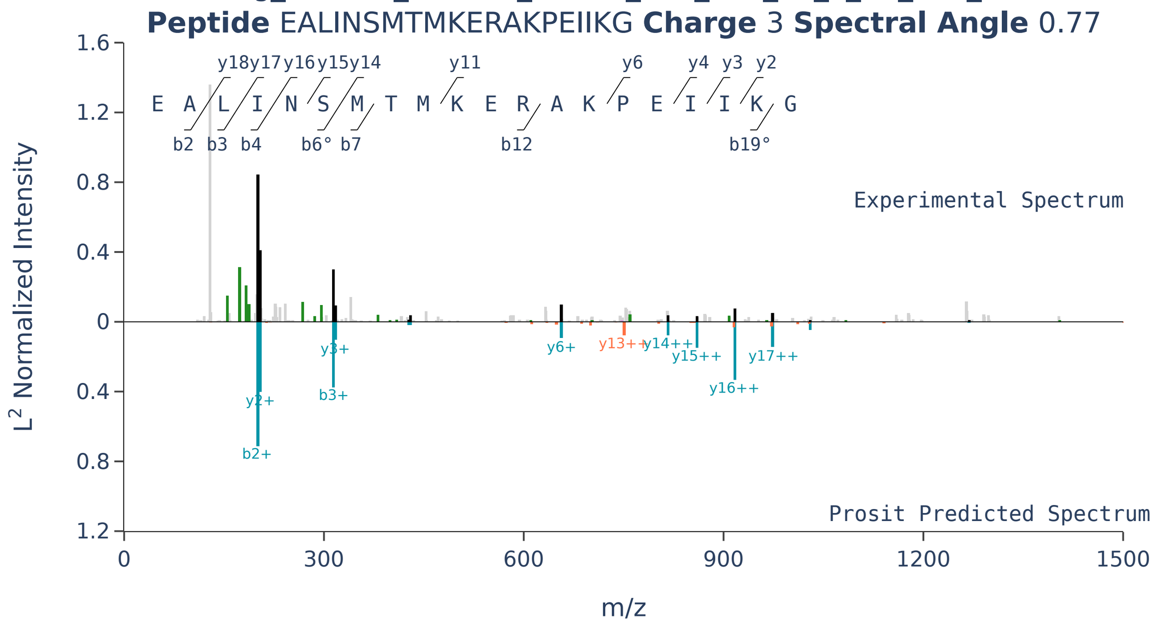

**Source** Ncheng_210623_230623_HFGoe_FFH_20S_25_1_A2_24h_R2 **Scan** 9347
**Peptide** IESKVDRAQAEKL **Charge** 2 **Spectral Angle** 0.65

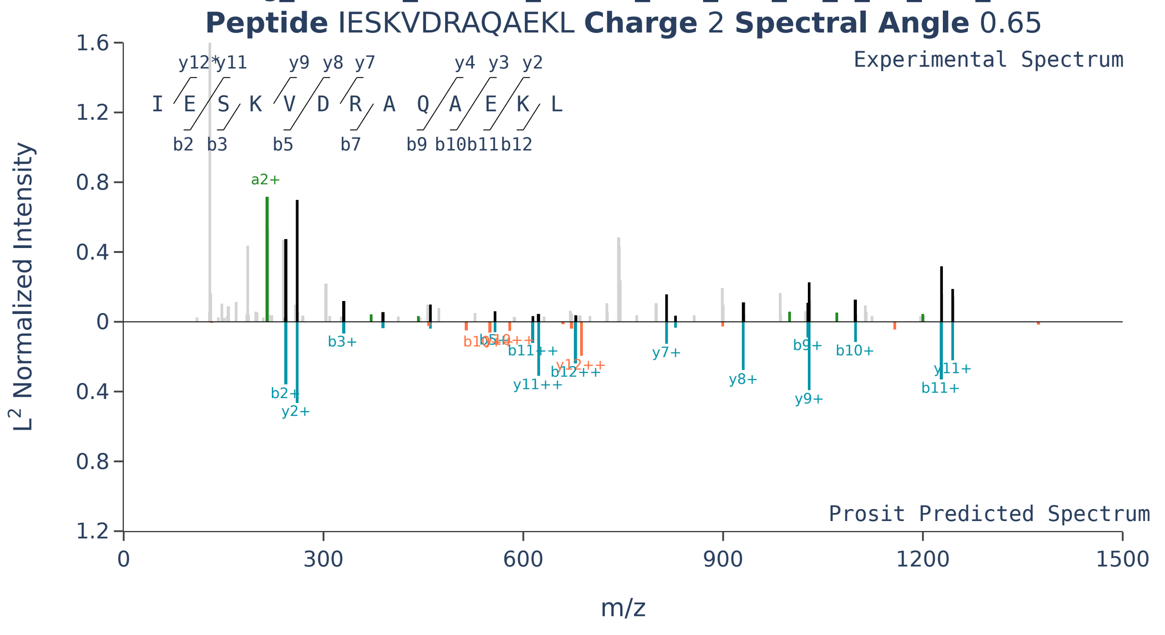

**Source** Ncheng_210623_230623_HFGoe_FFH_20S_25_1_A1_24h_R1 **Scan** 5728
**Peptide** EKLASKLKK **Charge** 2 **Spectral Angle** 0.78

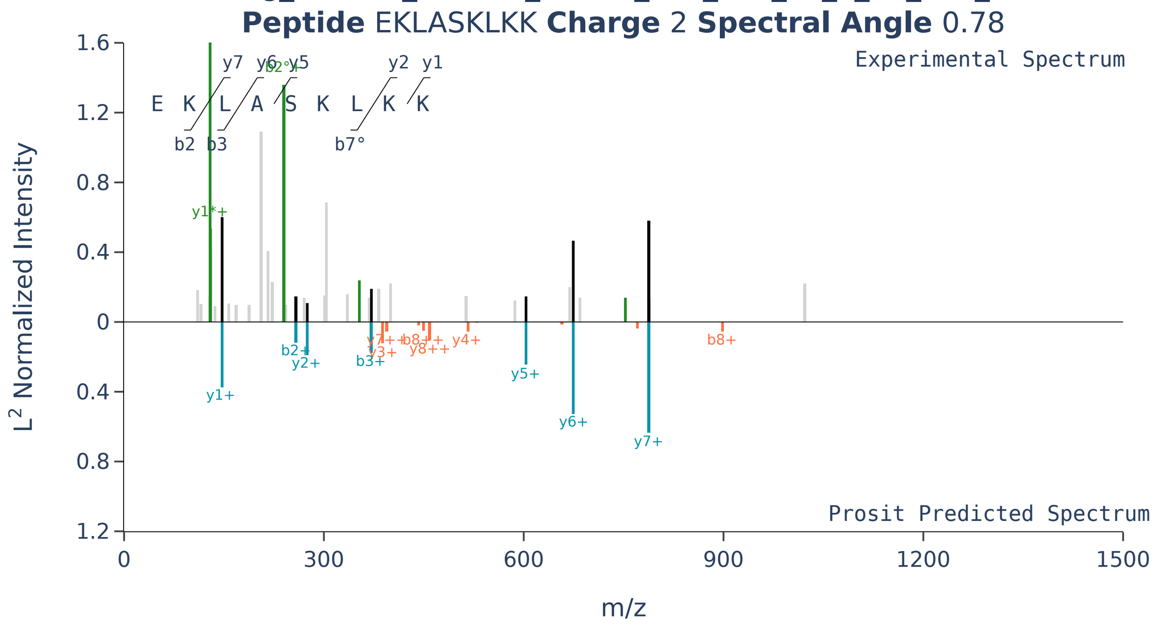

**Source** Ncheng_210623_230623_HFGoe_FFH_20S_25_1_A1_4h_R1 **Scan** 5692
**Peptide** IESKVDRAQAE **Charge** 3 **Spectral Angle** 0.7

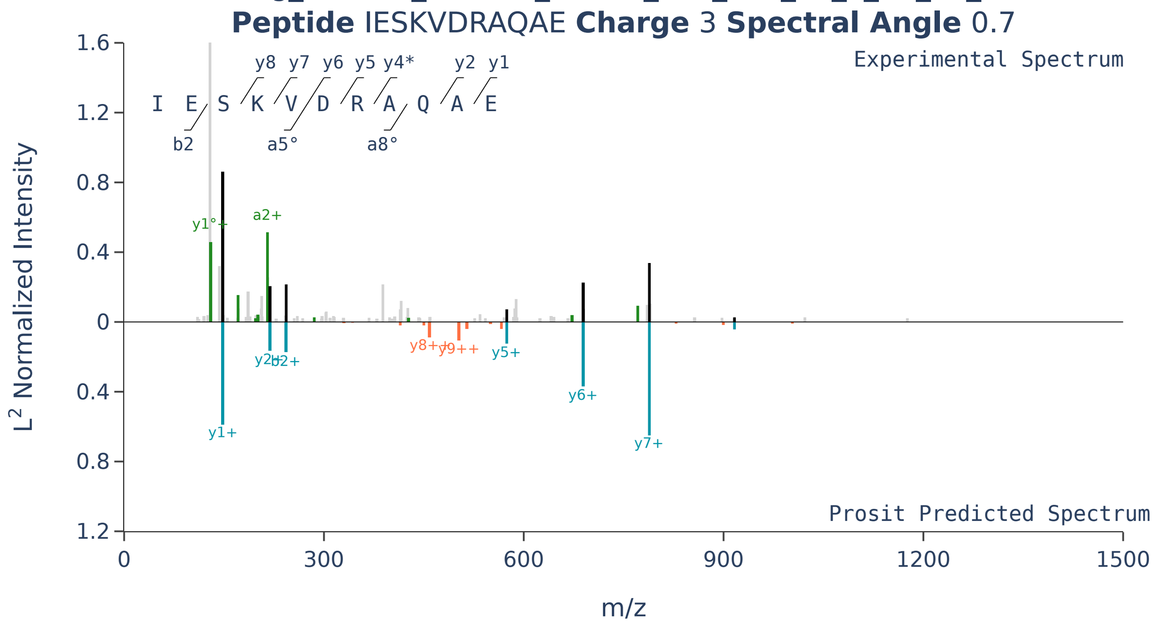

**Source** Ncheng_210623_230623_HFGoe_FFH_20S_25_1_A2_2h_R1 **Scan** 26069
**Peptide** PFHPDRIASRILGMGD **Charge** 3 **Spectral Angle** 0.71

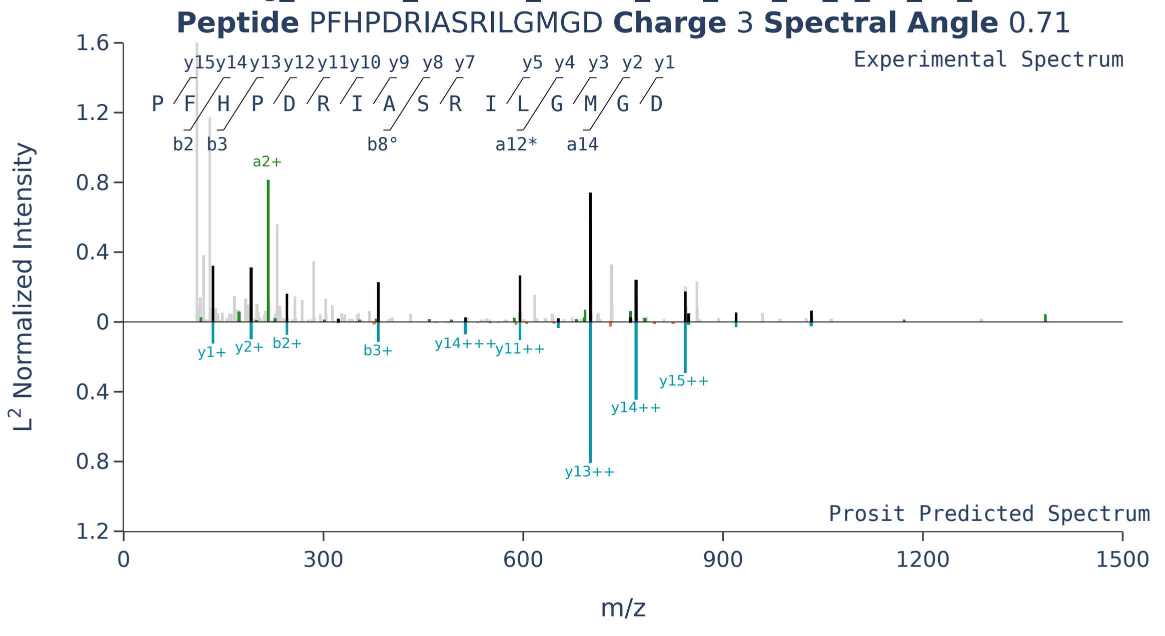

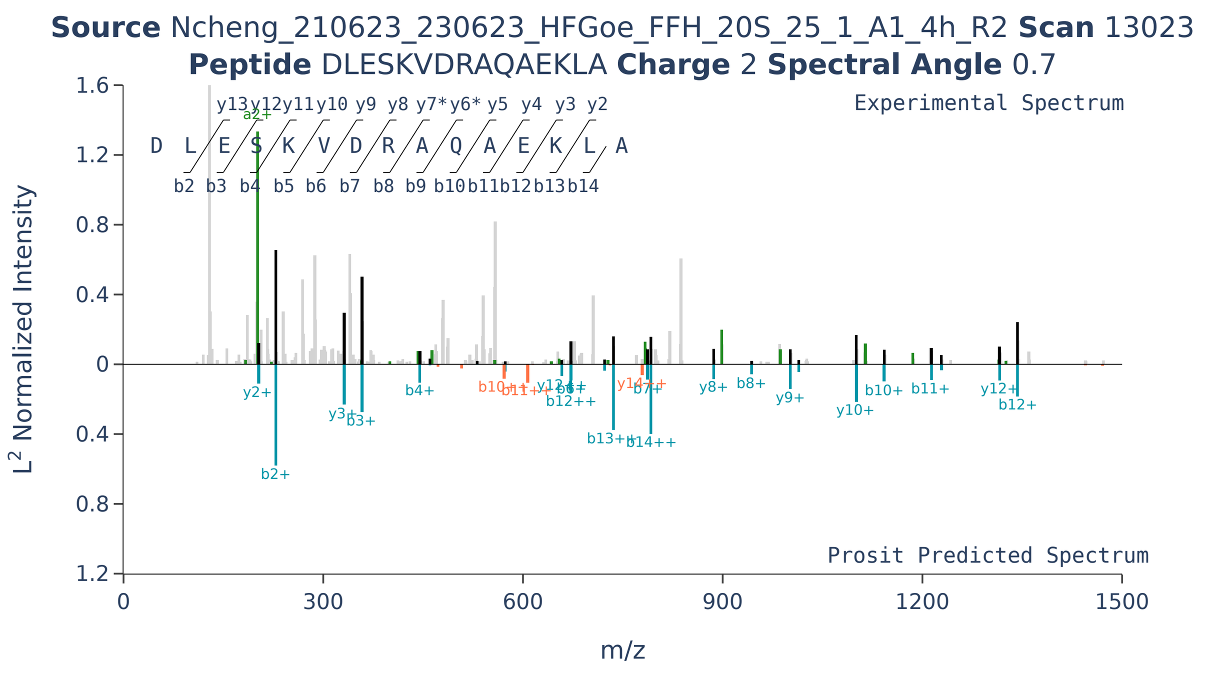
Source Ncheng_210623_230623_HFGoe_FFH_20S_25_1_A1_4h_R2 Scan 13023
Peptide DLESKVDRAQAEKLA Charge 2 Spectral Angle 0.7
Experimental Spectrum
Prosit Predicted Spectrum
L² Normalized Intensity
m/z

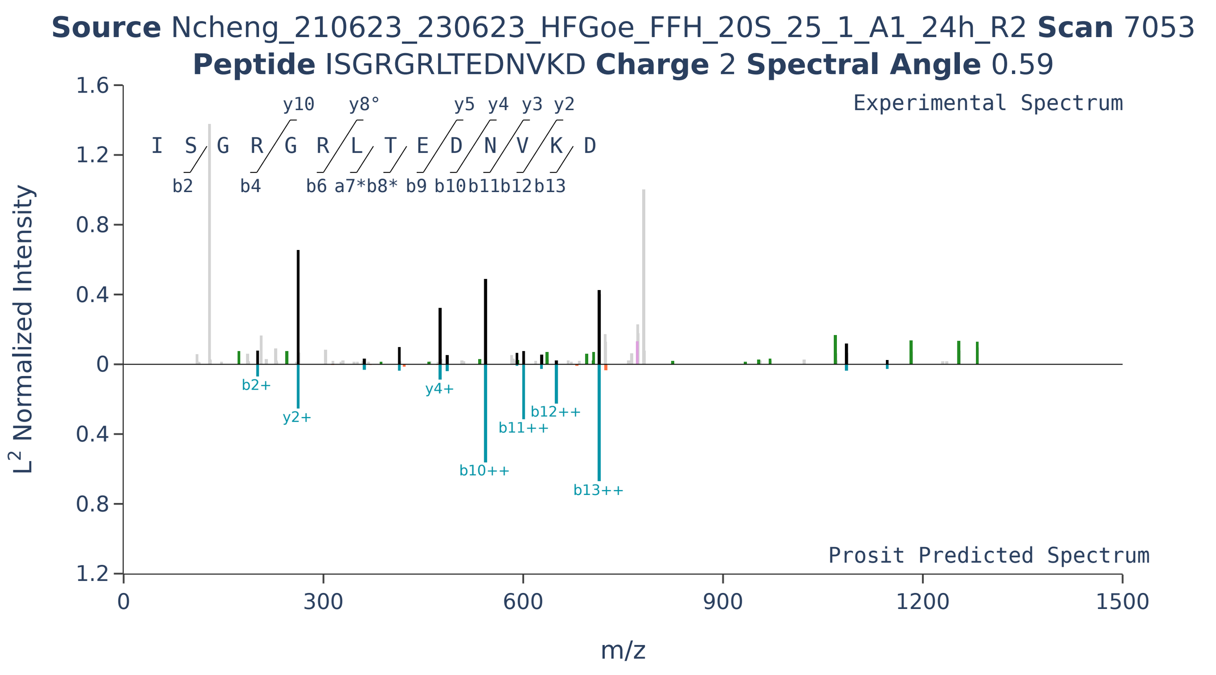
Source Ncheng_210623_230623_HFGoe_FFH_20S_25_1_A1_24h_R2 Scan 7053
Peptide ISGRGRLTEDNVKD Charge 2 Spectral Angle 0.59
Experimental Spectrum
Prosit Predicted Spectrum
L² Normalized Intensity
m/z

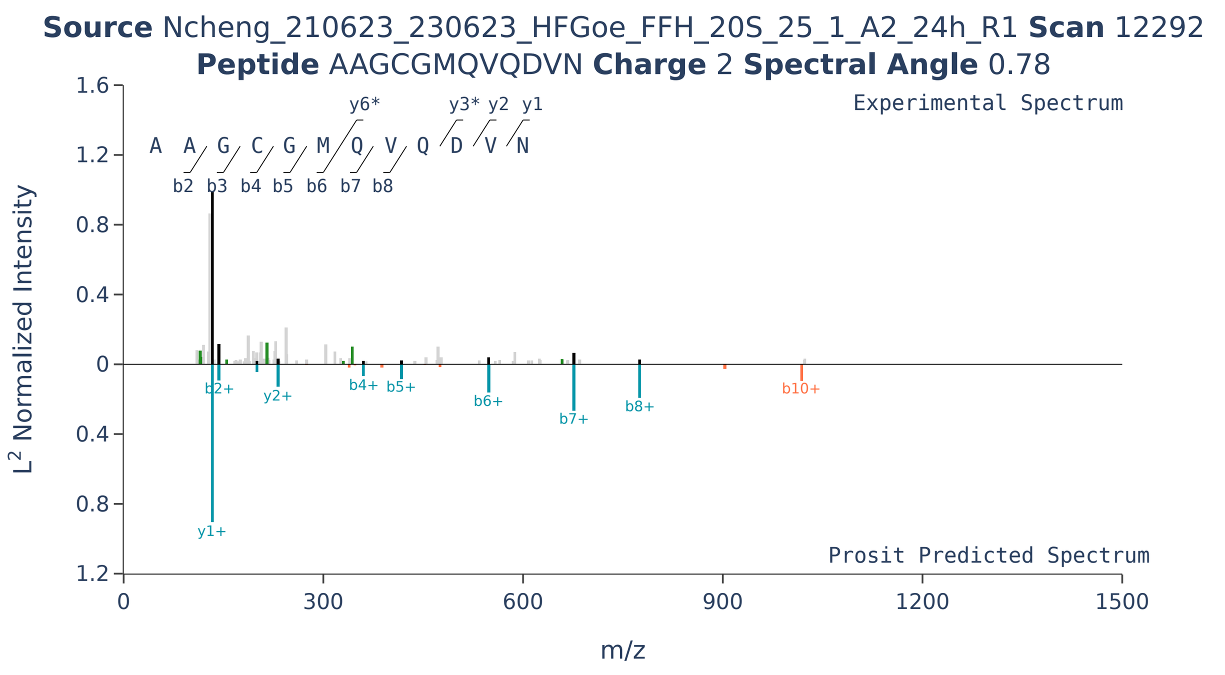
Source Ncheng_210623_230623_HFGoe_FFH_20S_25_1_A2_24h_R1 Scan 12292
Peptide AAGCGMQVQDVN Charge 2 Spectral Angle 0.78
Experimental Spectrum
Prosit Predicted Spectrum
L² Normalized Intensity
m/z

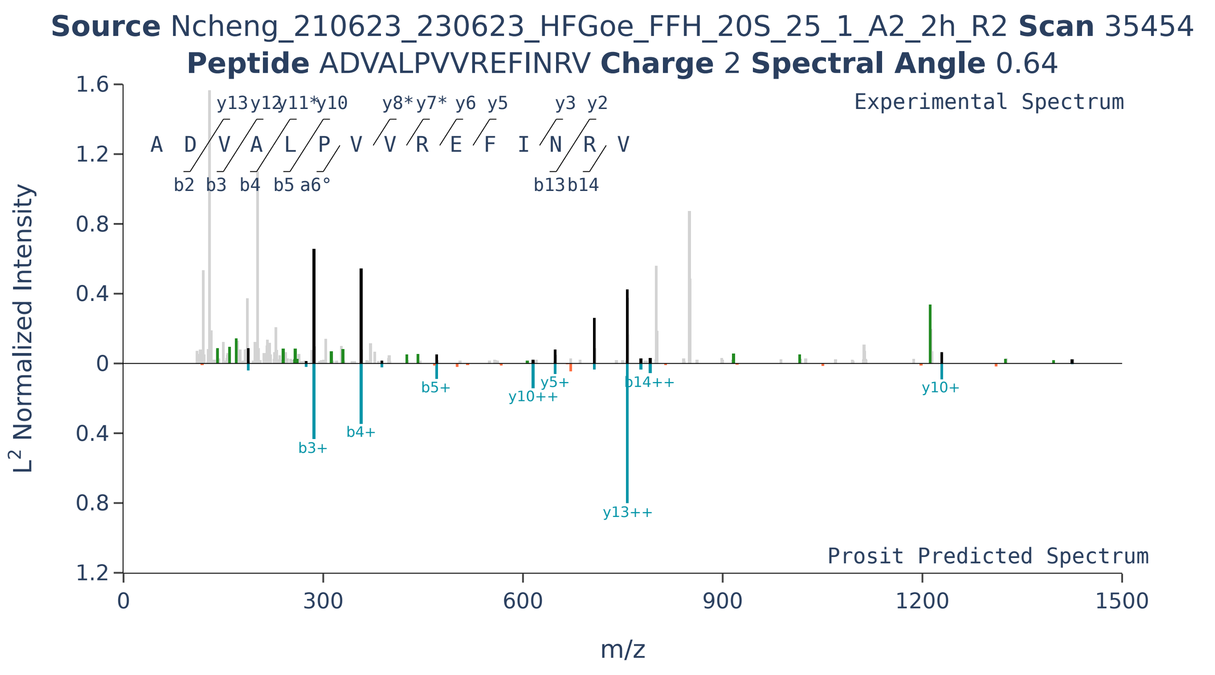
Source Ncheng_210623_230623_HFGoe_FFH_20S_25_1_A2_2h_R2 Scan 35454
Peptide ADVALPVVREFINRV Charge 2 Spectral Angle 0.64
Experimental Spectrum
Prosit Predicted Spectrum
L² Normalized Intensity
m/z

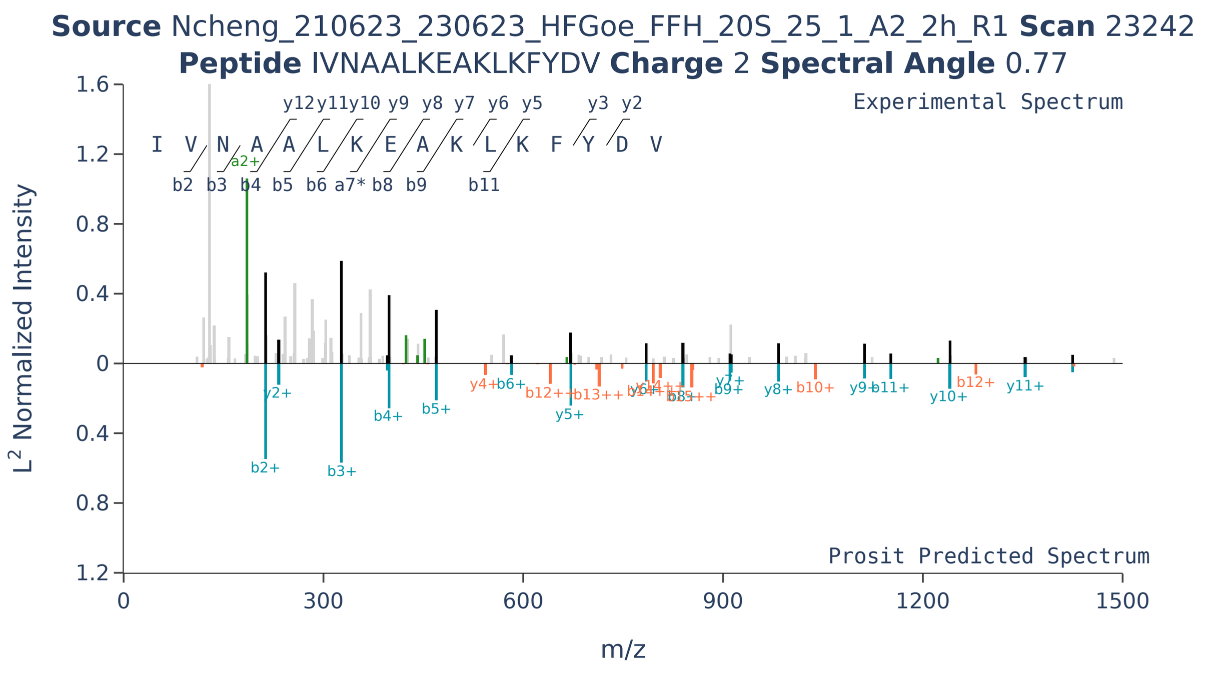
Source Ncheng_210623_230623_HFGoe_FFH_20S_25_1_A2_2h_R1 Scan 23242
Peptide IVNAALKEAKLKFYDV Charge 2 Spectral Angle 0.77
Experimental Spectrum
Prosit Predicted Spectrum
L² Normalized Intensity
m/z

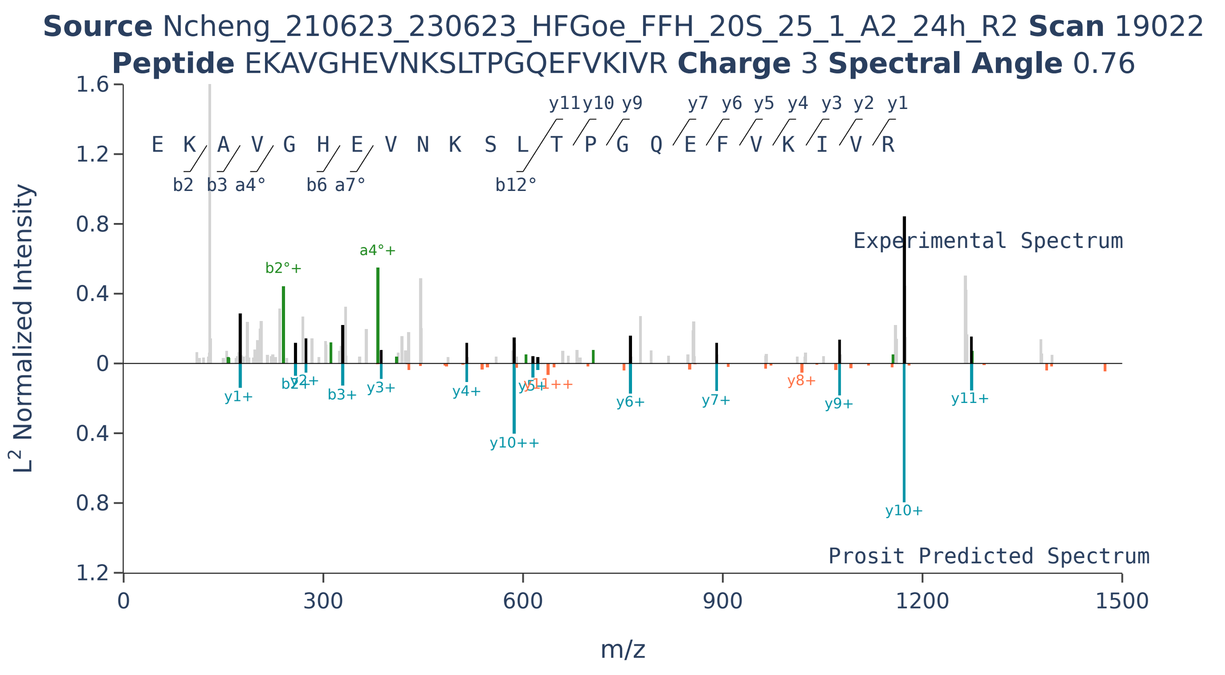
Source Ncheng_210623_230623_HFGoe_FFH_20S_25_1_A2_24h_R2 Scan 19022
Peptide EKAVGHEVNKSLTPGQEFVKIVR Charge 3 Spectral Angle 0.76
Experimental Spectrum
Prosit Predicted Spectrum
L² Normalized Intensity
m/z

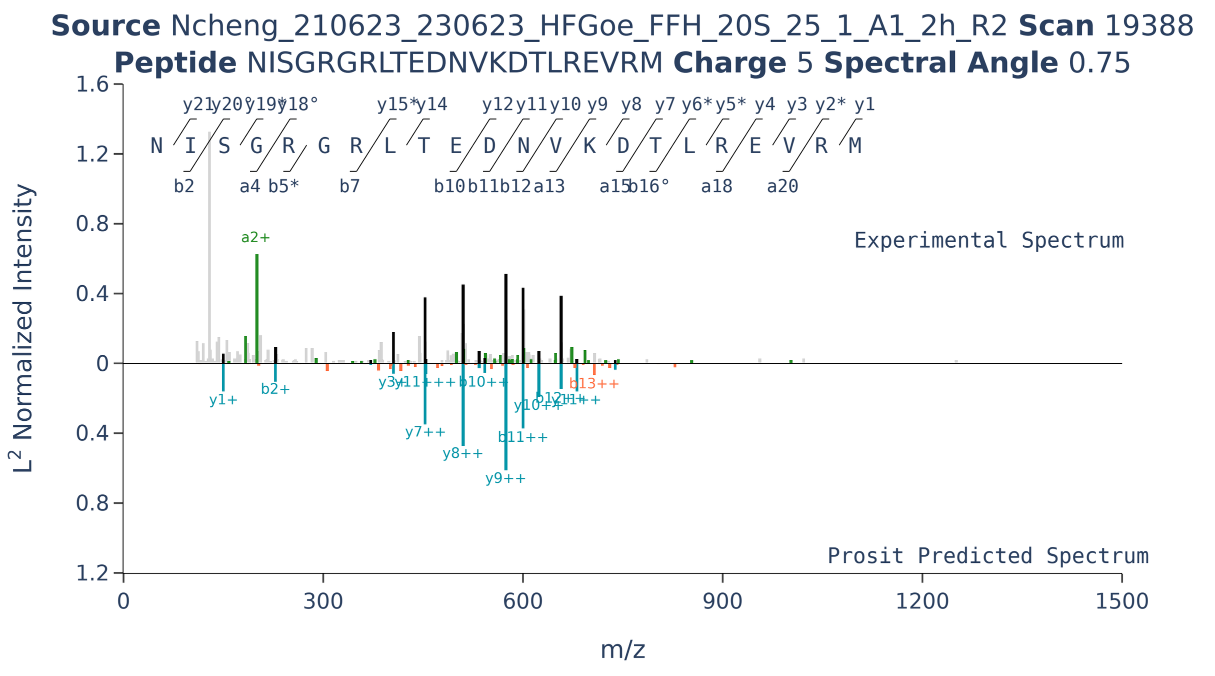
Source Ncheng_210623_230623_HFGoe_FFH_20S_25_1_A1_2h_R2 Scan 19388
Peptide NISGRGRLTEDNVKDTLREVRM Charge 5 Spectral Angle 0.75
Experimental Spectrum
Prosit Predicted Spectrum
L² Normalized Intensity
m/z

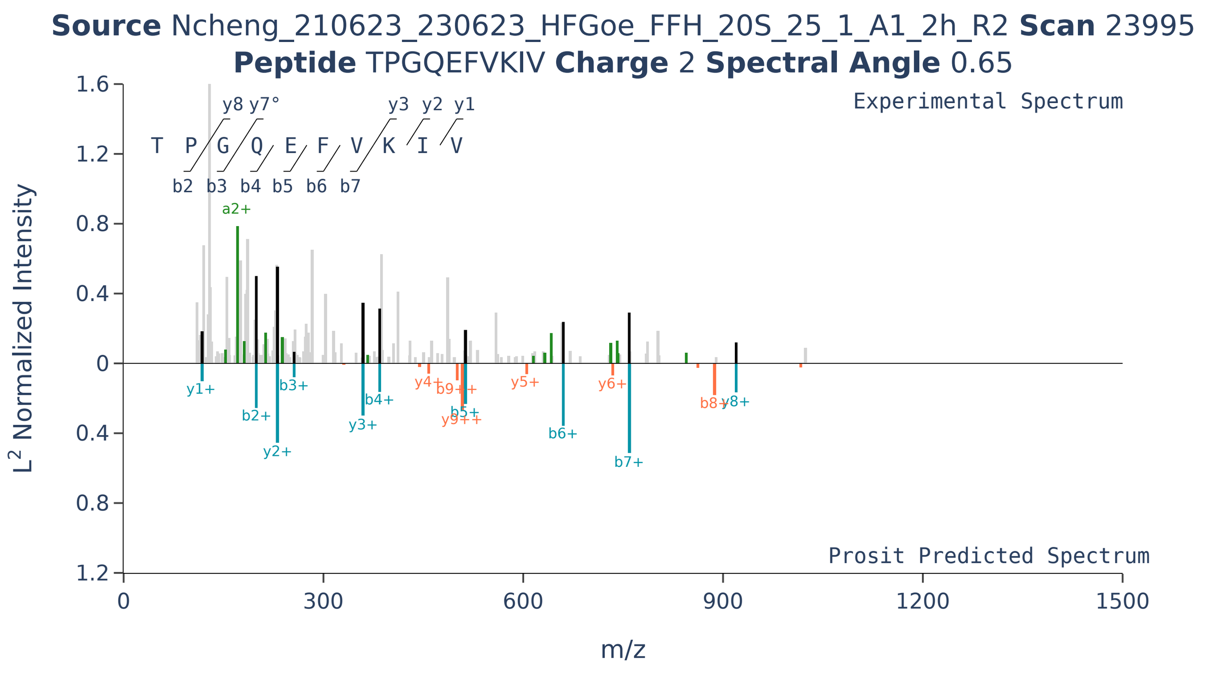
Source Ncheng_210623_230623_HFGoe_FFH_20S_25_1_A1_2h_R2 Scan 23995
Peptide TPGQEFVKIV Charge 2 Spectral Angle 0.65
Experimental Spectrum
Prosit Predicted Spectrum
L² Normalized Intensity
m/z

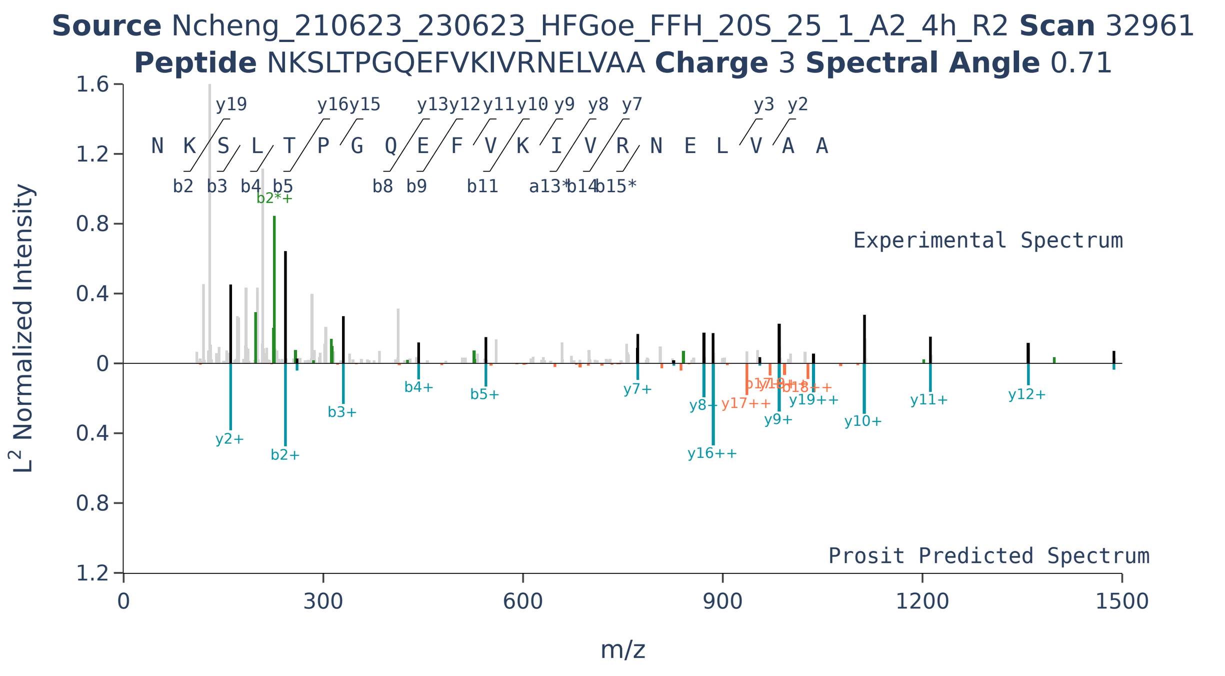
Source Ncheng_210623_230623_HFGoe_FFH_20S_25_1_A2_4h_R2 Scan 32961
Peptide NKSLTPGQEFVKIVRNELVAA Charge 3 Spectral Angle 0.71
Experimental Spectrum
Prosit Predicted Spectrum
L² Normalized Intensity
m/z

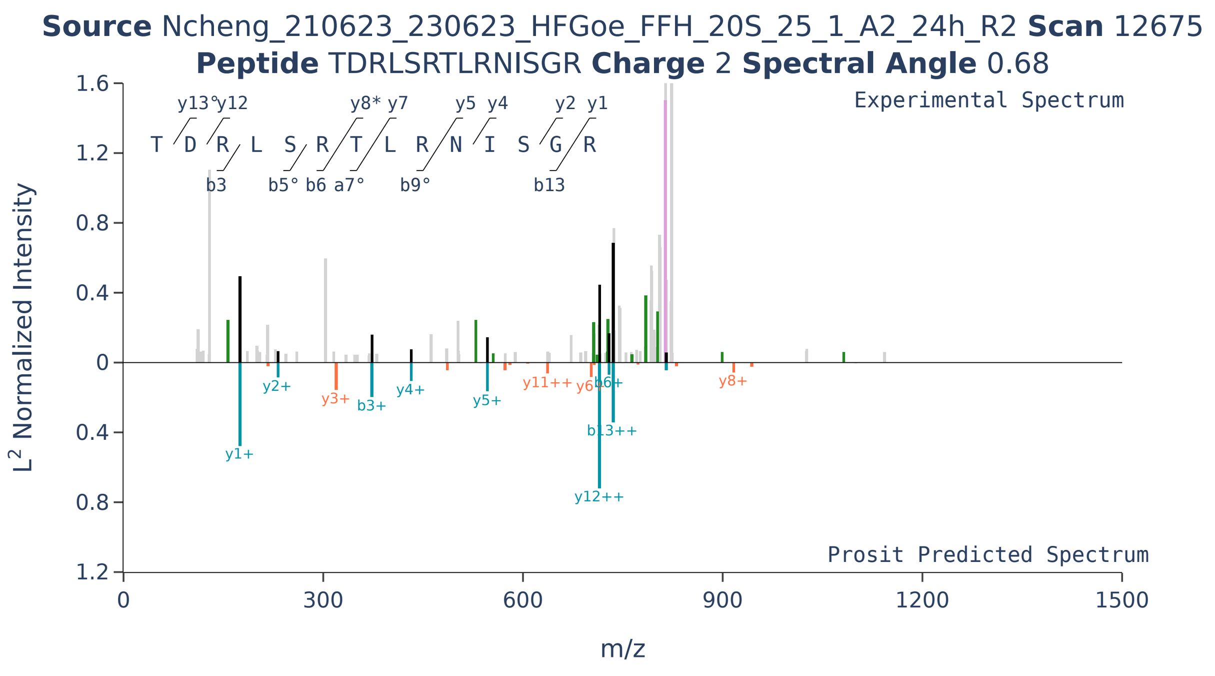
Source Ncheng_210623_230623_HFGoe_FFH_20S_25_1_A2_24h_R2 Scan 12675
Peptide TDRLSRTLRNISGR Charge 2 Spectral Angle 0.68
Experimental Spectrum
Prosit Predicted Spectrum
L² Normalized Intensity
m/z

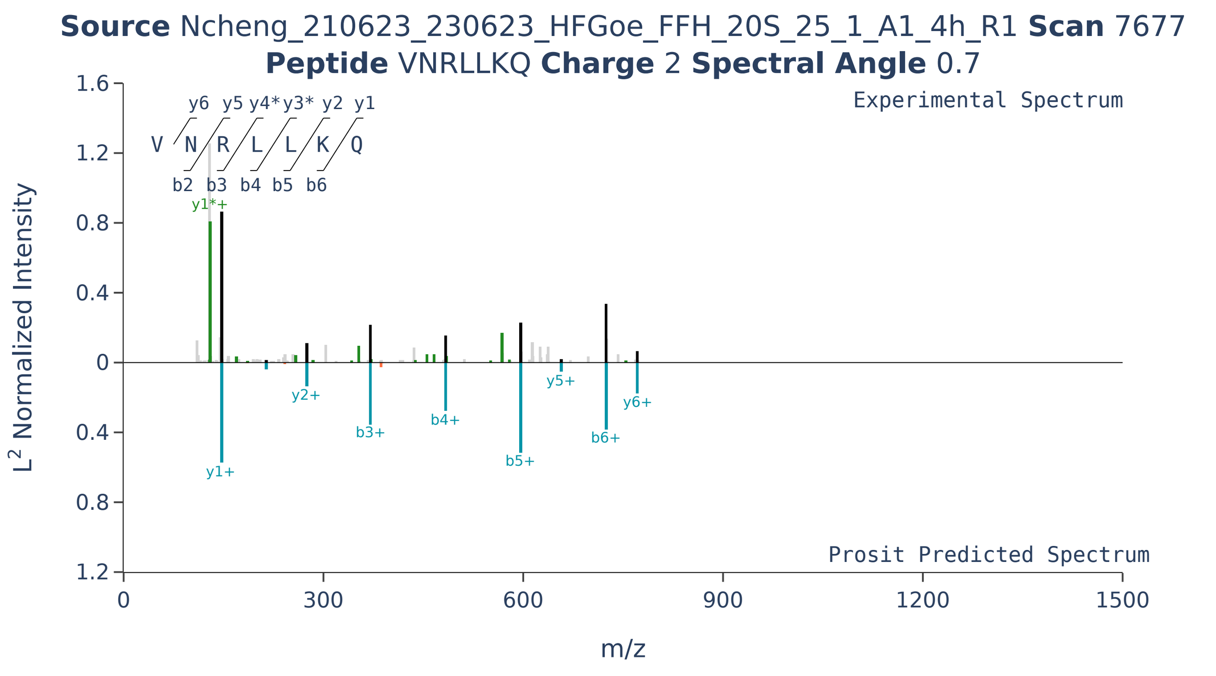
Source Ncheng_210623_230623_HFGoe_FFH_20S_25_1_A1_4h_R1 Scan 7677
Peptide VNRLLKQ Charge 2 Spectral Angle 0.7
Experimental Spectrum
Prosit Predicted Spectrum
L² Normalized Intensity
m/z

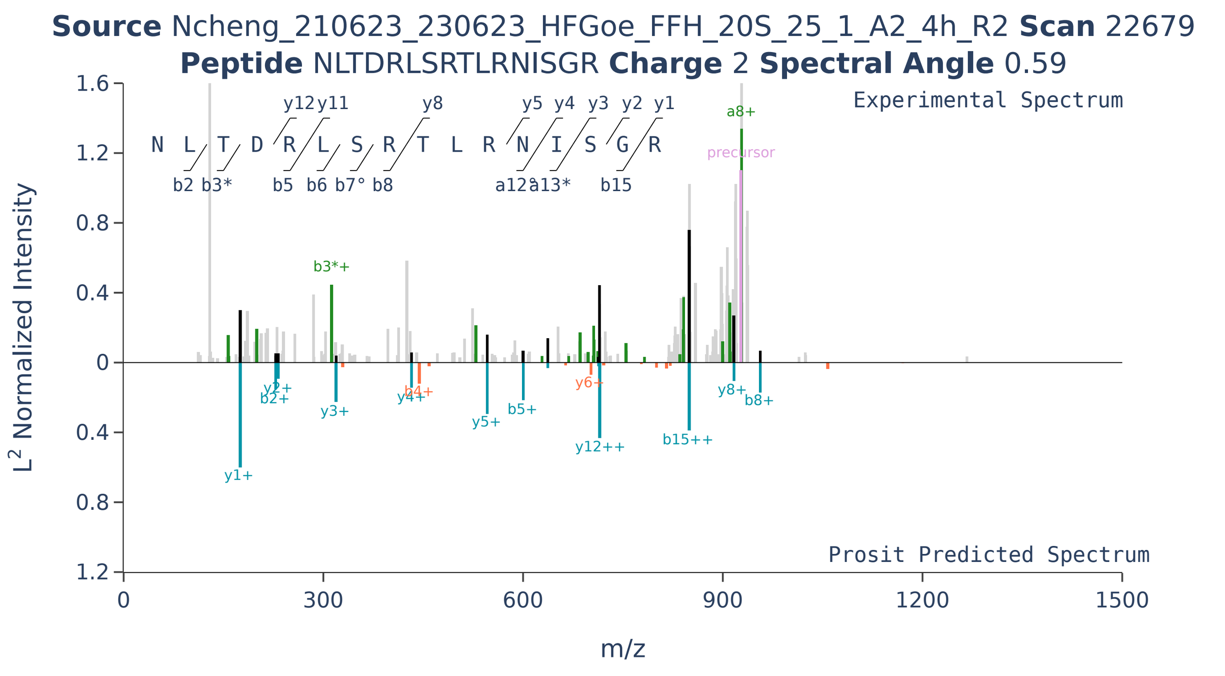
Source Ncheng_210623_230623_HFGoe_FFH_20S_25_1_A2_4h_R2 Scan 22679
Peptide NLTDRLSRTLRNISGR Charge 2 Spectral Angle 0.59
Experimental Spectrum
Prosit Predicted Spectrum
L² Normalized Intensity
m/z

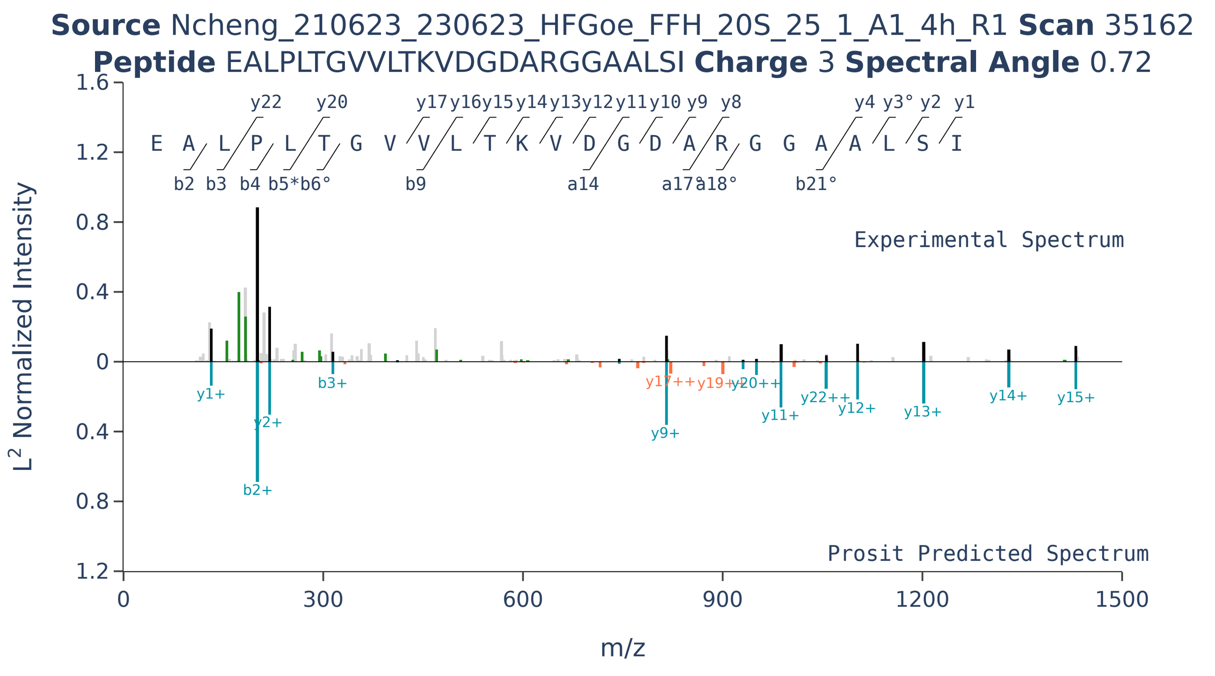
Source Ncheng_210623_230623_HFGoe_FFH_20S_25_1_A1_4h_R1 Scan 35162
Peptide EALPLTGVVLTKVDGDARGGAALSI Charge 3 Spectral Angle 0.72
Experimental Spectrum
Prosit Predicted Spectrum
L² Normalized Intensity
m/z

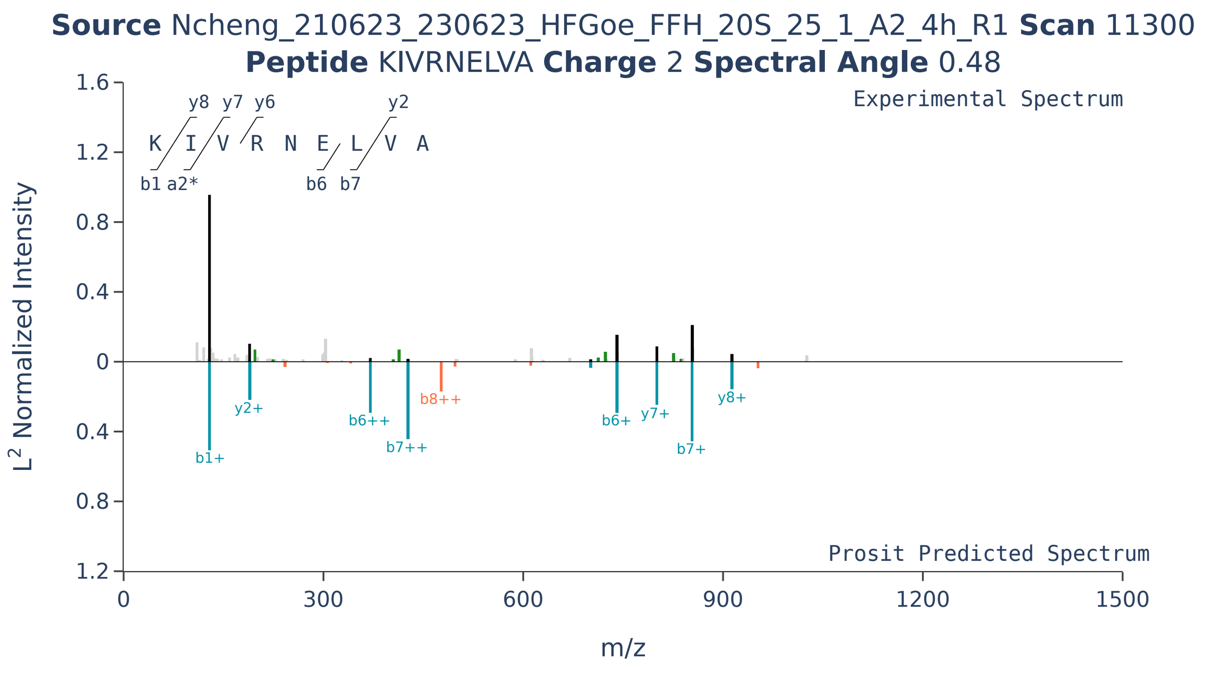
Source Ncheng_210623_230623_HFGoe_FFH_20S_25_1_A2_4h_R1 Scan 11300
Peptide KIVRNELVA Charge 2 Spectral Angle 0.48
Experimental Spectrum
Prosit Predicted Spectrum
L² Normalized Intensity
m/z

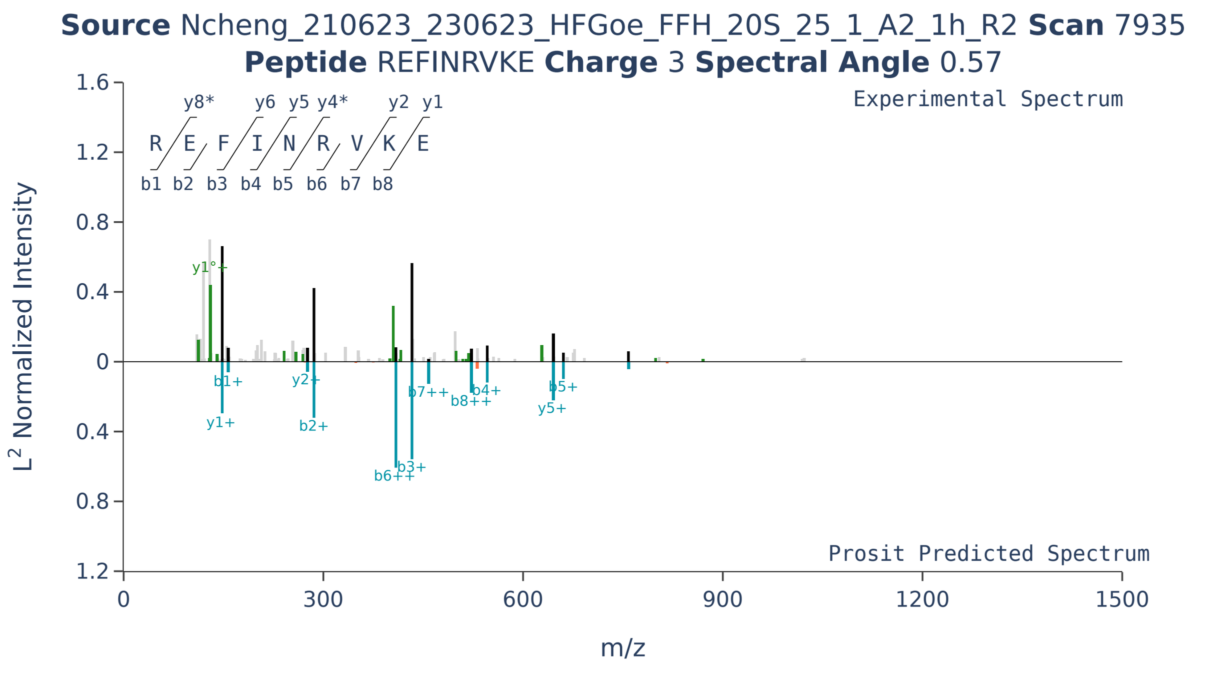
Source Ncheng_210623_230623_HFGoe_FFH_20S_25_1_A2_1h_R2 Scan 7935
Peptide REFINRVKE Charge 3 Spectral Angle 0.57
Experimental Spectrum
Prosit Predicted Spectrum
L² Normalized Intensity
m/z

**Source** Ncheng_210623_230623_HFGoe_FFH_20S_25_1_A2_24h_R1 **Scan** 14127
**Peptide** DGDARGGAALSIRHITGKPLK **Charge** 4 **Spectral Angle** 0.65

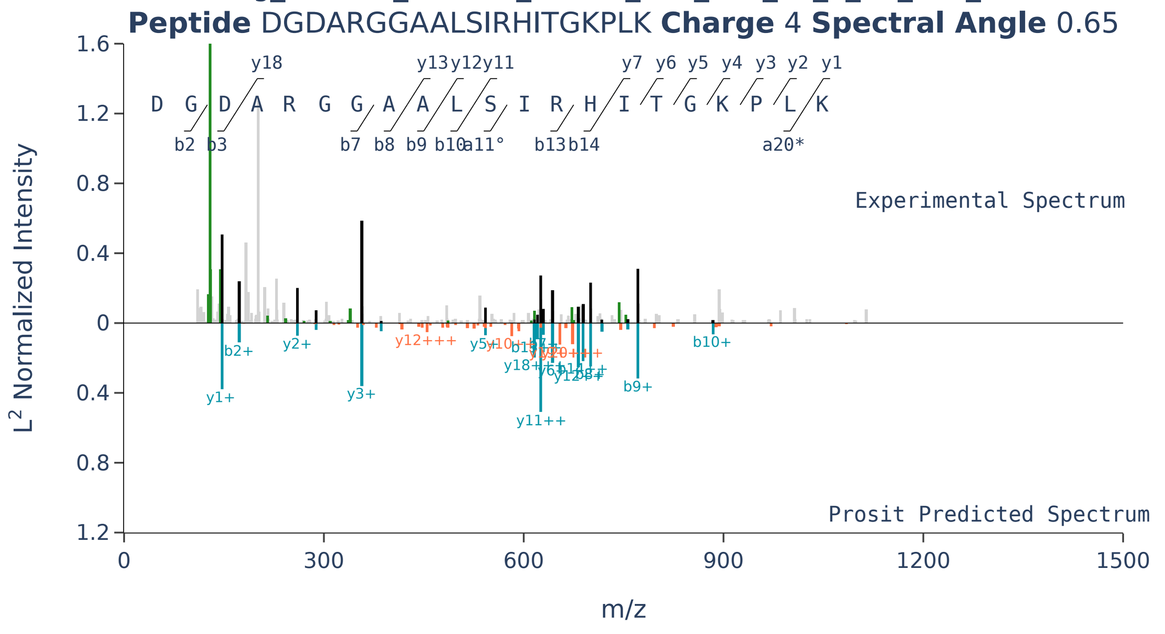


**Source** Ncheng_210623_230623_HFGoe_FFH_20S_25_1_A1_24h_R1 **Scan** 17294
**Peptide** VNAALKEAKLKF **Charge** 3 **Spectral Angle** 0.7

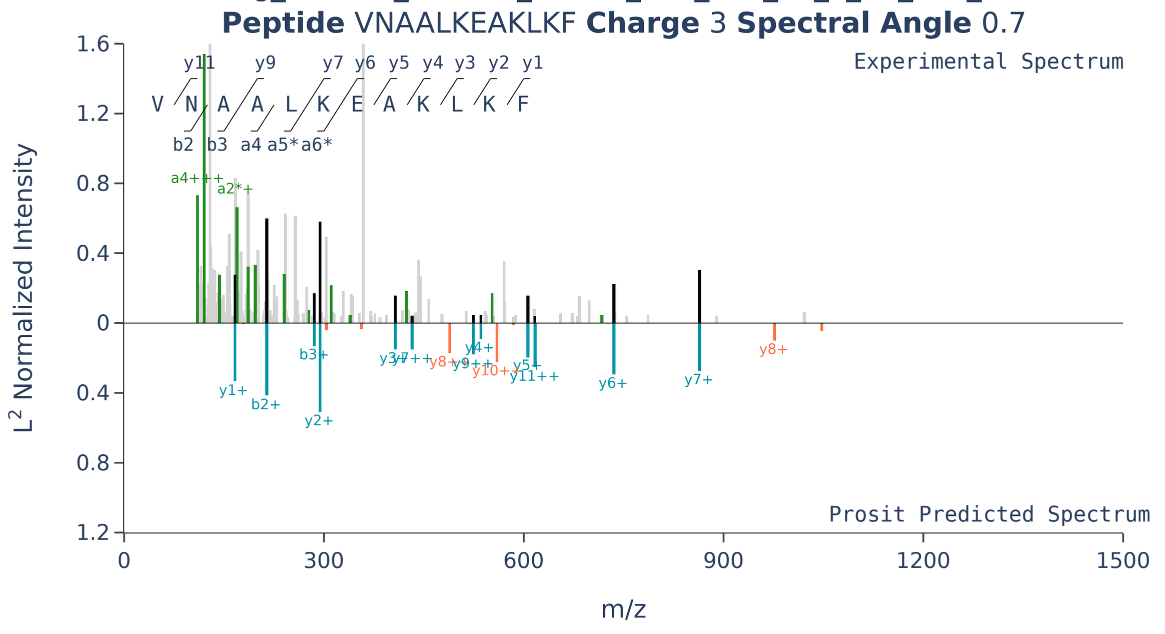


**Source** Ncheng_210623_230623_HFGoe_FFH_20S_25_1_A2_4h_R2 **Scan** 29810
**Peptide** FLEQLRQMKNMGGMASL **Charge** 2 **Spectral Angle** 0.74

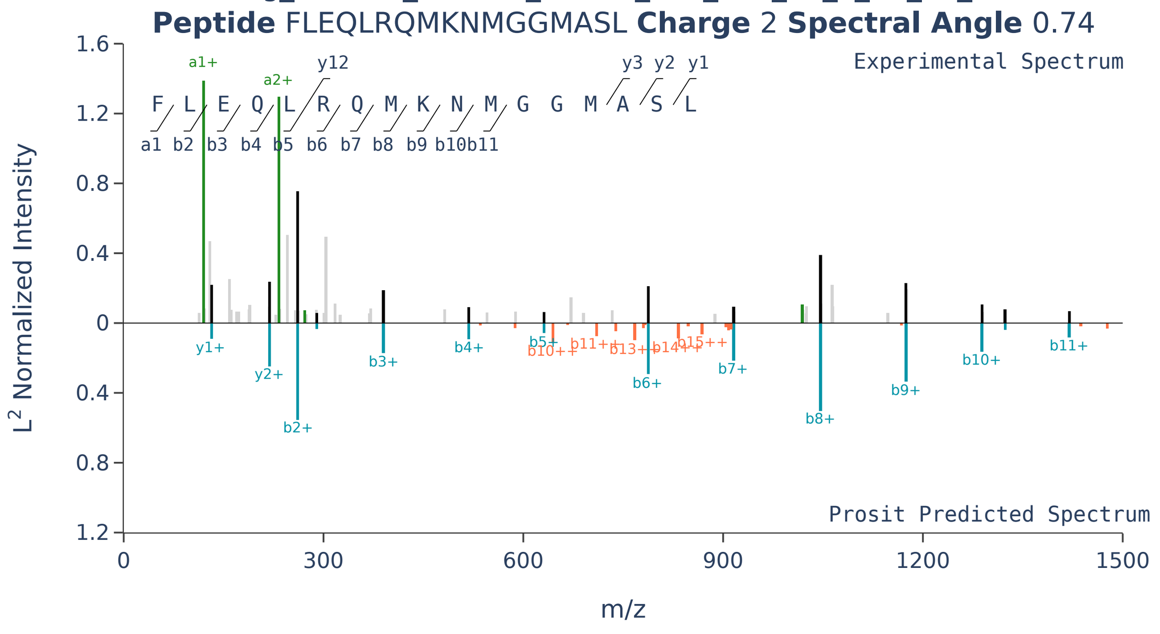


**Source** Ncheng_210623_230623_HFGoe_FFH_20S_25_1_A2_4h_R1 **Scan** 11608
**Peptide** QLRQMKNMGGMA **Charge** 2 **Spectral Angle** 0.71

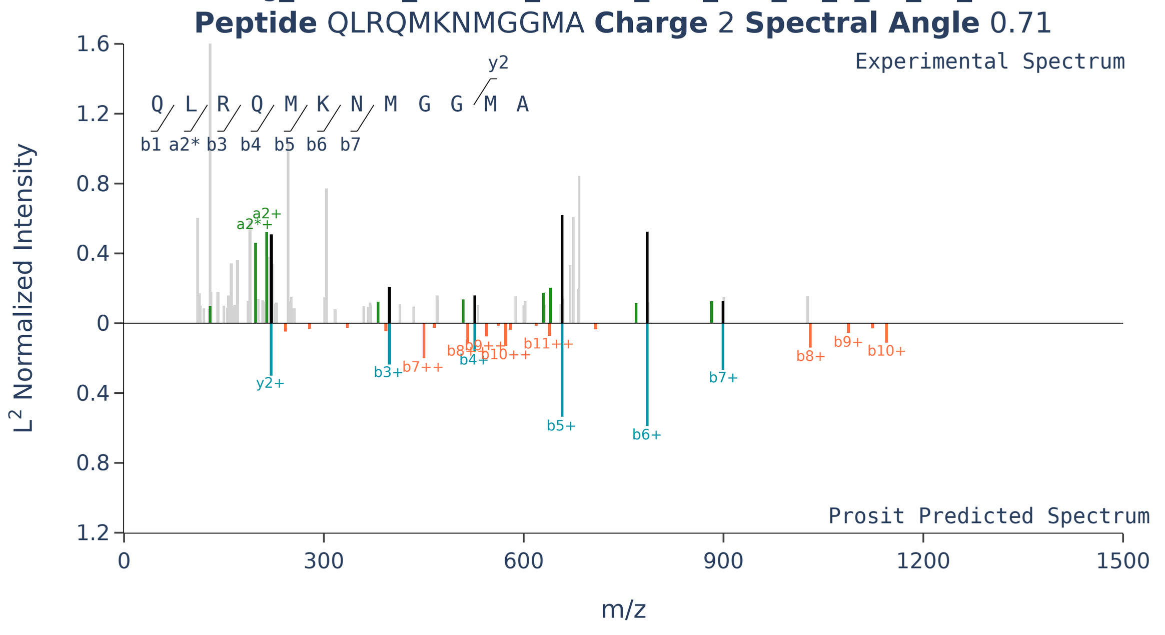


**Source** Ncheng_210623_230623_HFGoe_FFH_20S_25_1_A2_24h_R1 **Scan** 10944
**Peptide** RAQAEKLASKLKKGDGF **Charge** 4 **Spectral Angle** 0.66

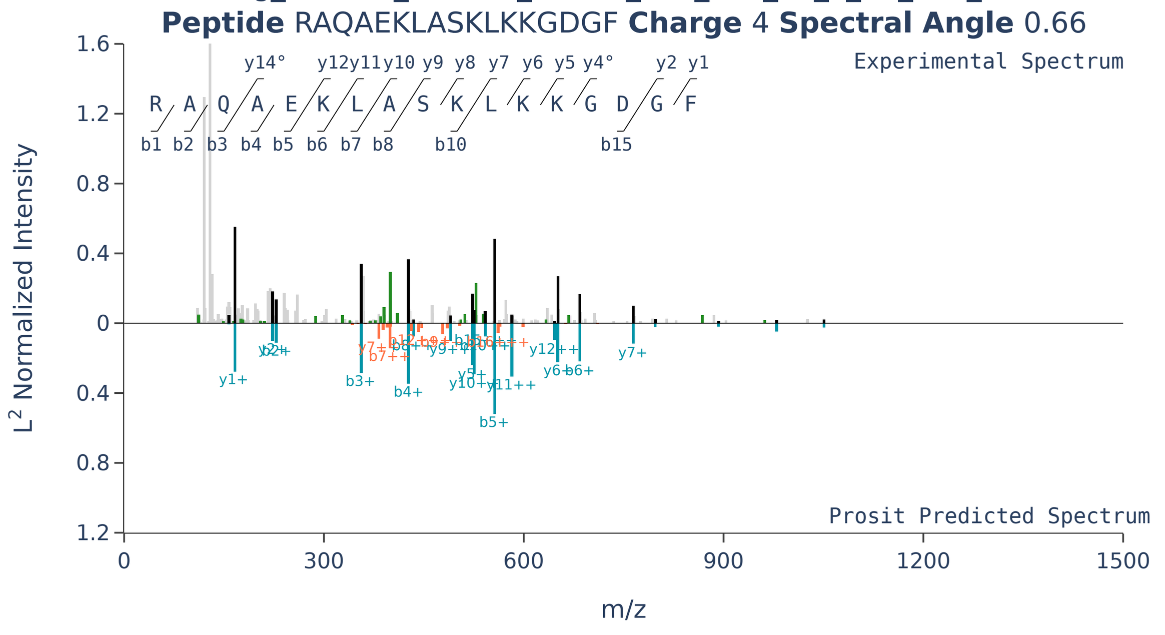


**Source** Ncheng_210623_230623_HFGoe_FFH_20S_25_1_A1_2h_R2 **Scan** 30648
**Peptide** TPGQEFVKIVRNELVA **Charge** 2 **Spectral Angle** 0.71

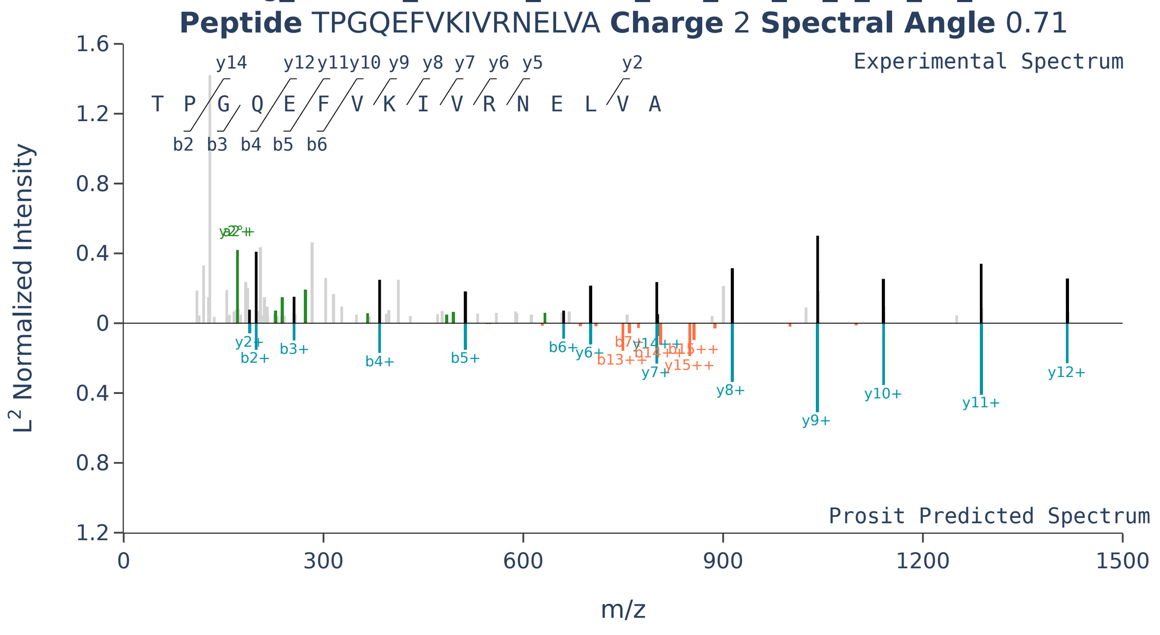


**Source** Ncheng_210623_230623_HFGoe_FFH_20S_25_1_A2_4h_R1 **Scan** 17128
**Peptide** LKQFDDMQRMMKKMKKG **Charge** 4 **Spectral Angle** 0.68

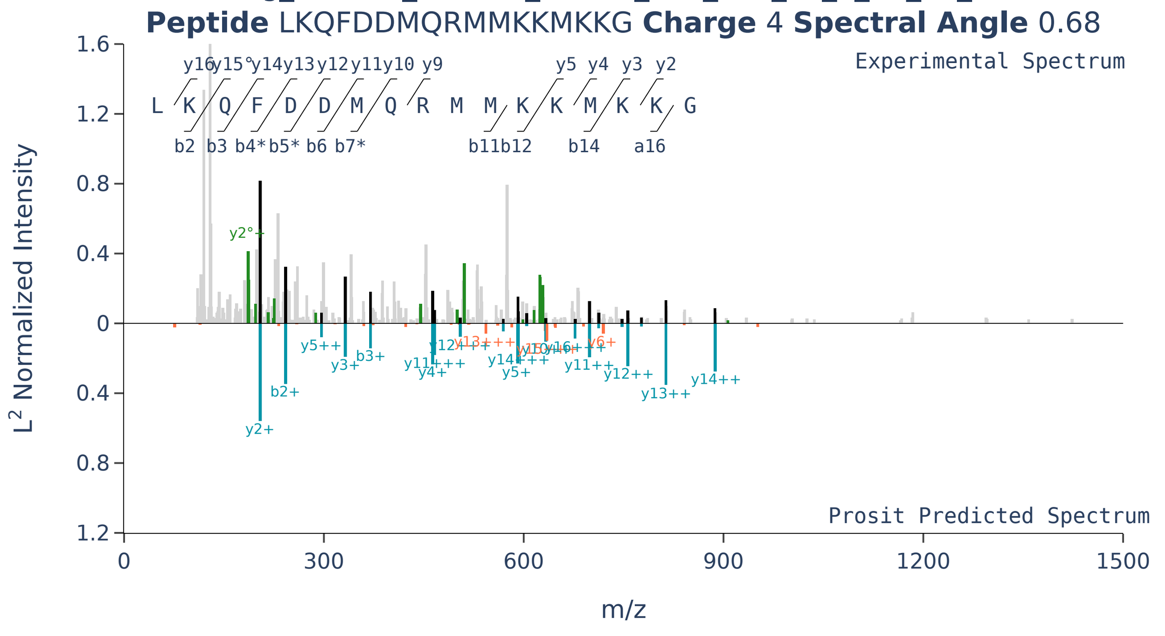


**Source** Ncheng_210623_230623_HFGoe_FFH_20S_25_1_A2_24h_R1 **Scan** 12044
**Peptide** HITGKPLKF **Charge** 2 **Spectral Angle** 0.72

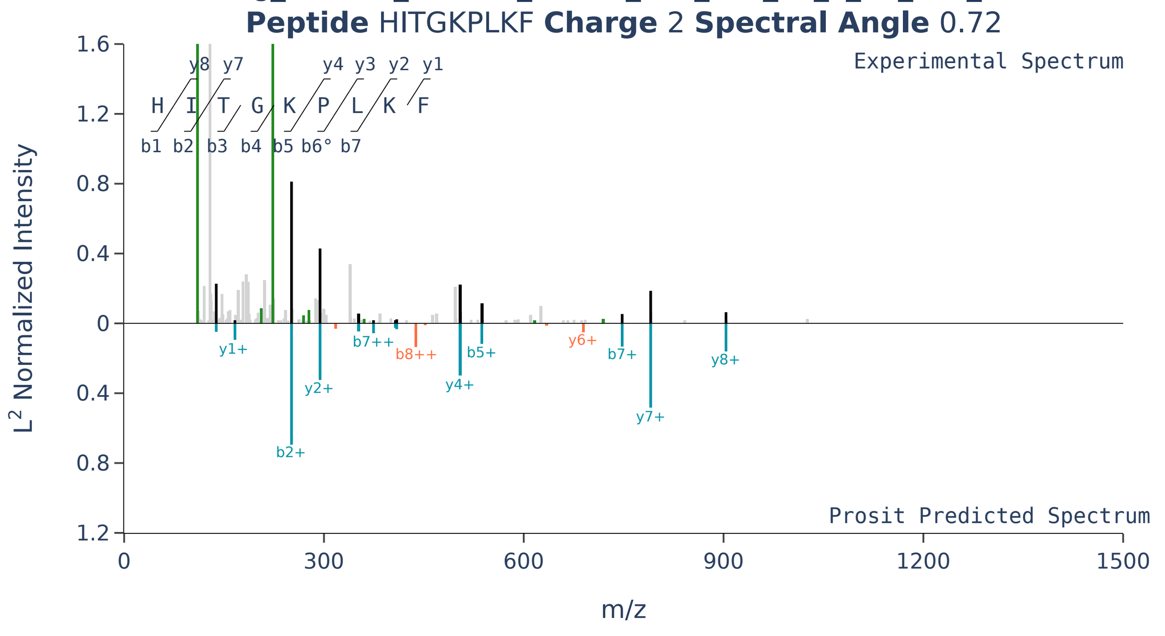


**Source** Ncheng_210623_230623_HFGoe_FFH_20S_25_1_A1_24h_R1 **Scan** 7876
**Peptide** NISGRGRLTEDNVKD **Charge** 2 **Spectral Angle** 0.76

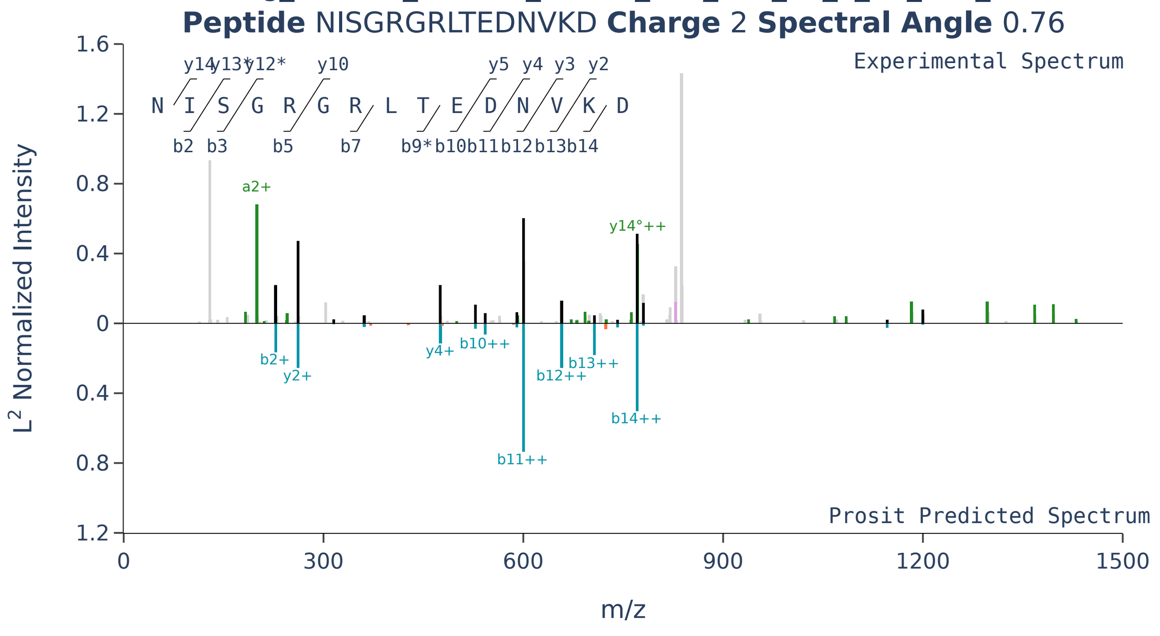


**Source** Ncheng_210623_230623_HFGoe_FFH_20S_25_1_A2_2h_R2 **Scan** 35115
**Peptide** KKGDGFDLNDFLEQLRQMKN **Charge** 3 **Spectral Angle** 0.84

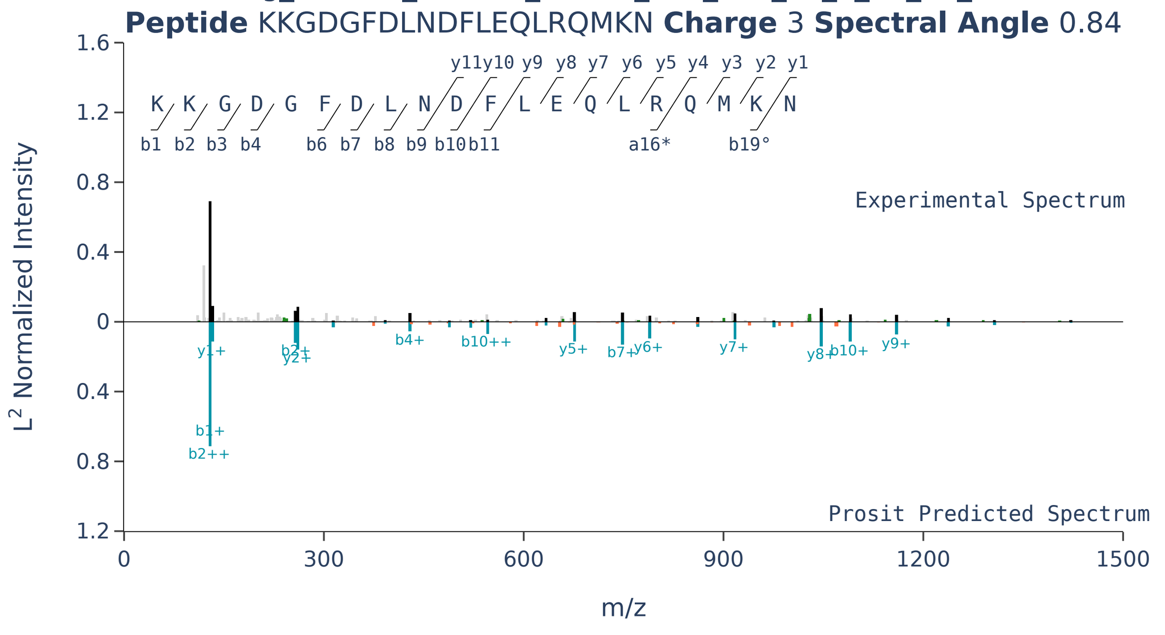


**Source** Ncheng_210623_230623_HFGoe_FFH_20S_25_1_A1_24h_R1 **Scan** 8412
**Peptide** GKLGKFLREKH **Charge** 3 **Spectral Angle** 0.79

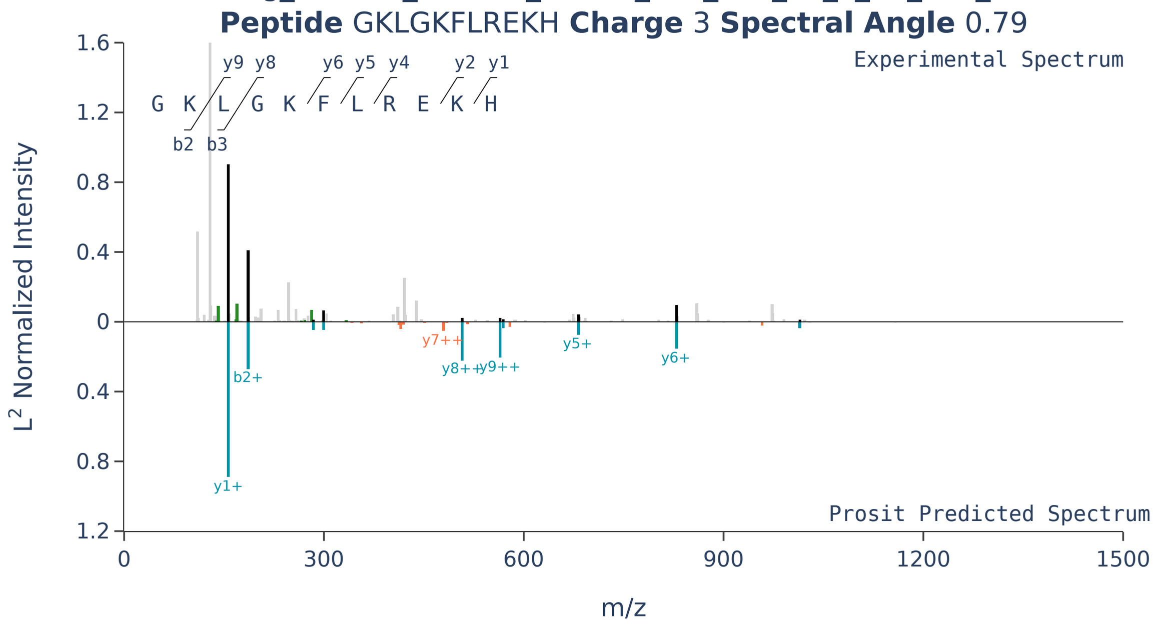


**Source** Ncheng_210623_230623_HFGoe_FFH_20S_25_1_A1_2h_R2 **Scan** 26184
**Peptide** ALEPFHPDRIASRIL **Charge** 3 **Spectral Angle** 0.56

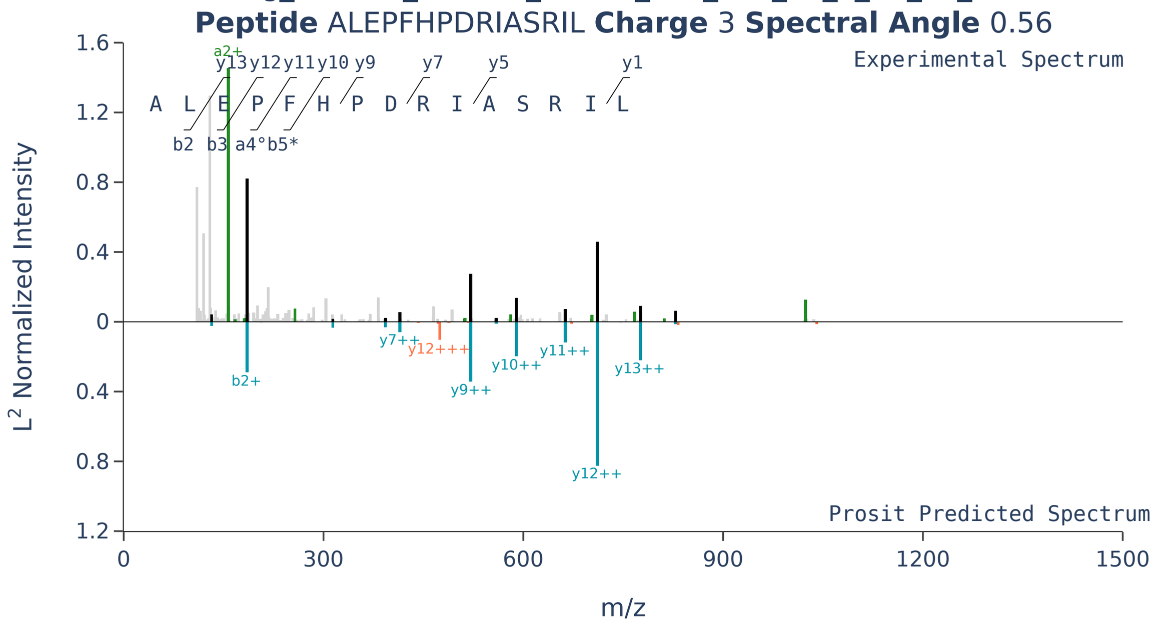


**Source** Ncheng_210623_230623_HFGoe_FFH_20S_25_1_A1_1h_R1 **Scan** 19000
**Peptide** HITGKPIKFLGVGE **Charge** 3 **Spectral Angle** 0.87

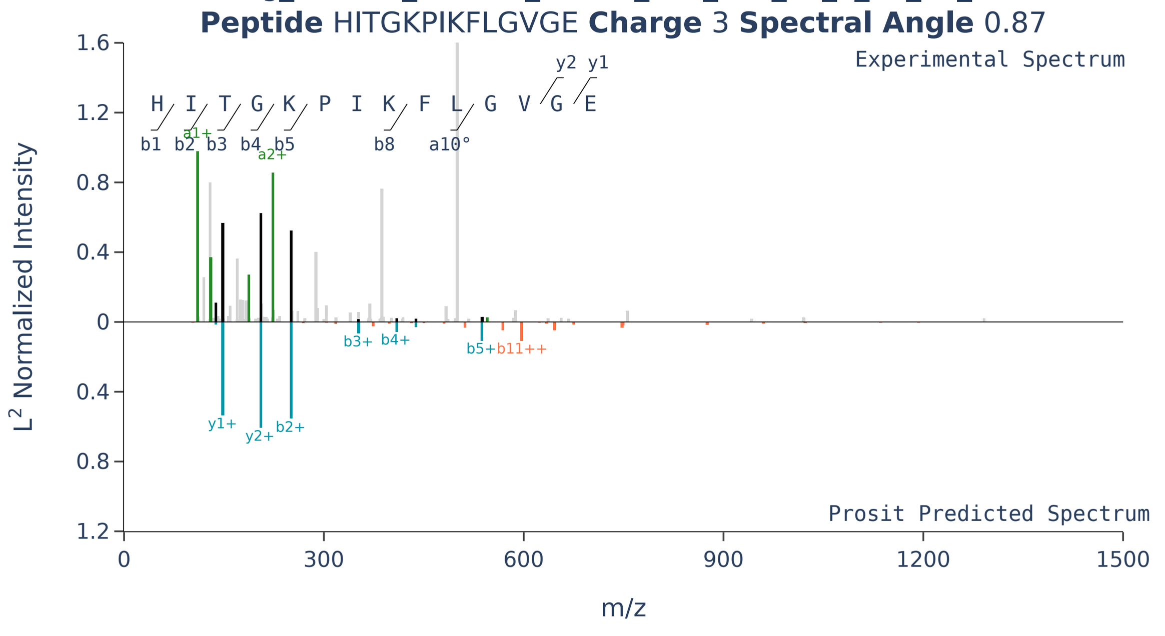


**Source** Ncheng_210623_230623_HFGoe_FFH_20S_25_1_A1_4h_R1 **Scan** 19531
**Peptide** QDAANTAKAFNEAL **Charge** 2 **Spectral Angle** 0.89

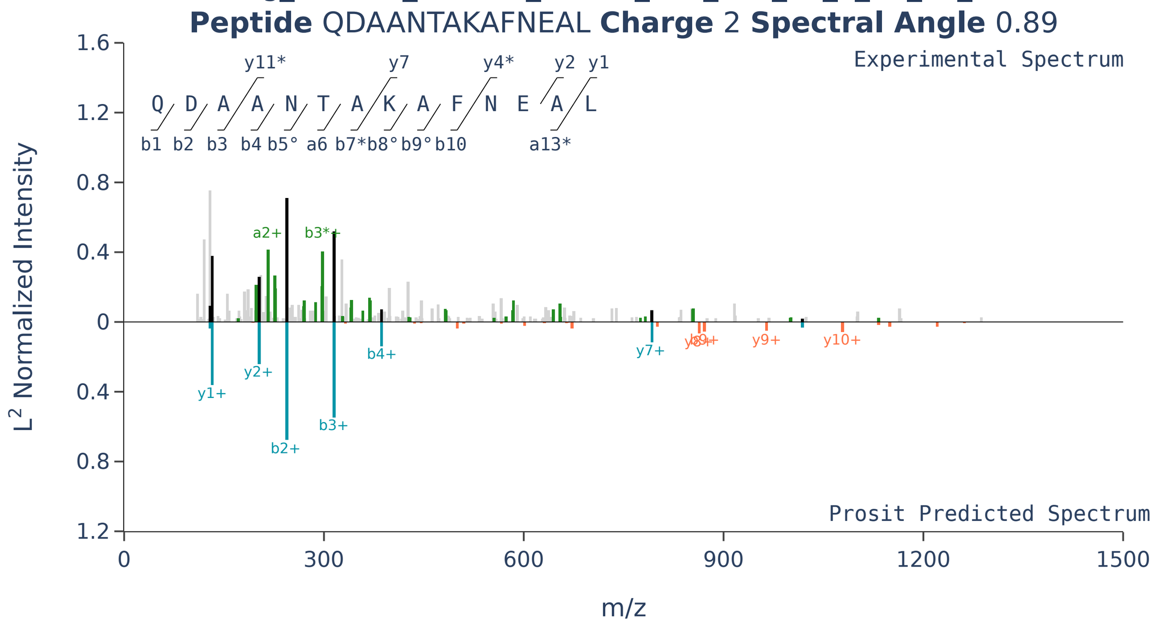


**Source** Ncheng_210623_230623_HFGoe_FFH_20S_25_1_A2_4h_R1 **Scan** 32418
**Peptide** KAFNEALPLTGVVLTKVDGDARGGAAL **Charge** 3 **Spectral Angle** 0.64

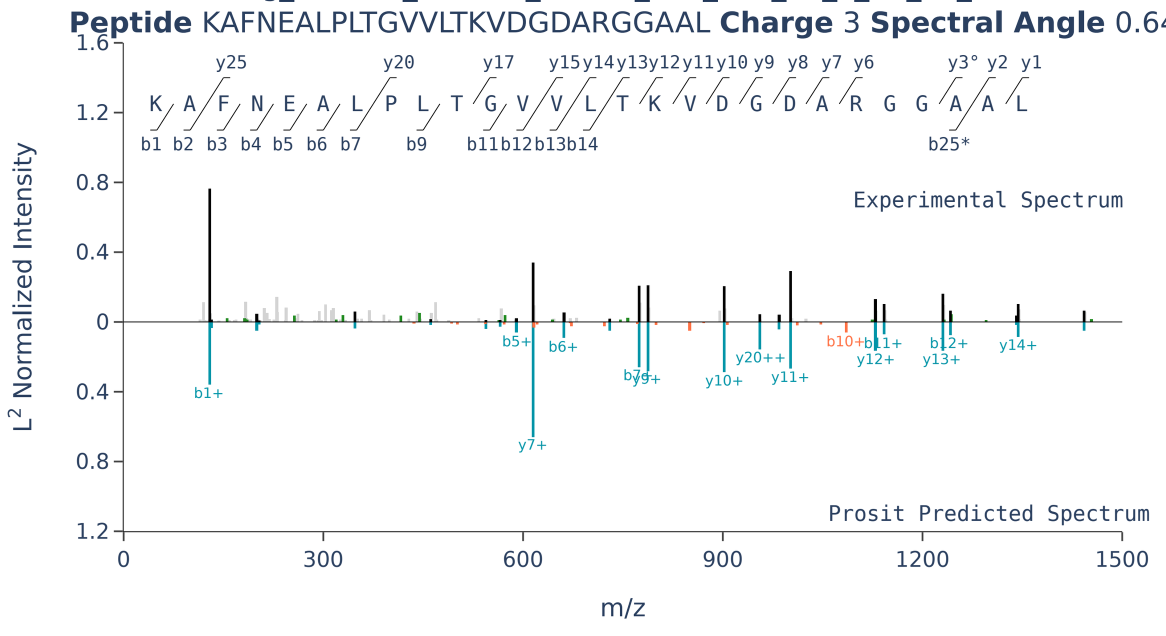

**Source** Ncheng_210623_230623_HFGoe_FFH_20S_25_1_A1_4h_R1 **Scan** 11870
**Peptide** DMQRMMKKMKKGGMAKMMR **Charge** 3 **Spectral Angle** 0.78

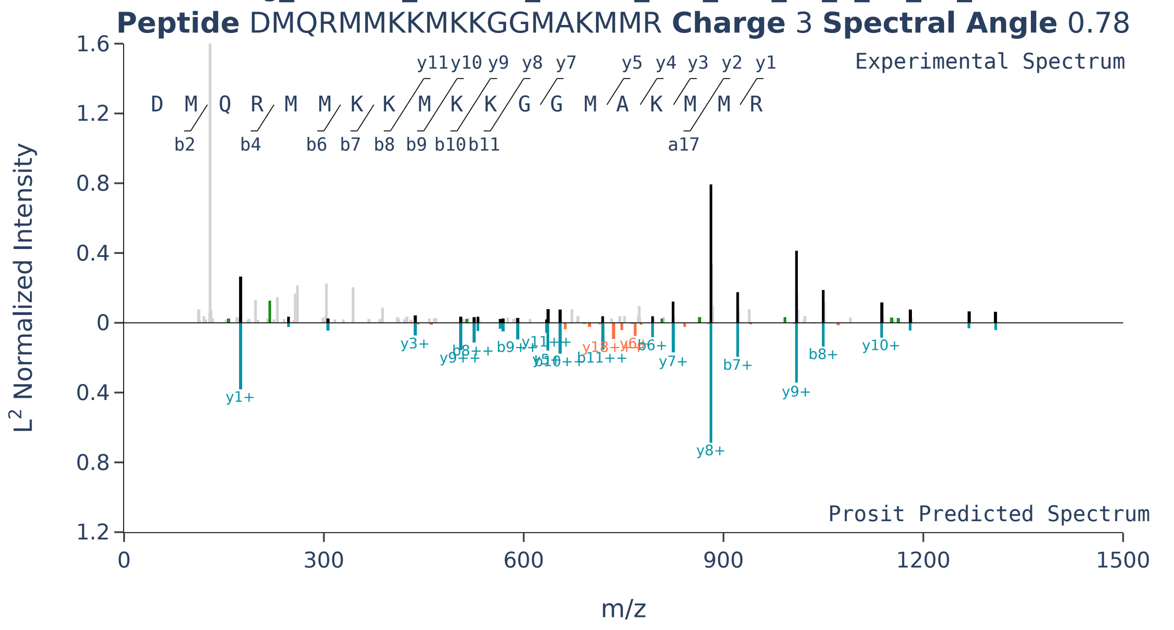


**Source** Ncheng_210623_230623_HFGoe_FFH_20S_25_1_A2_2h_R1 **Scan** 30602
**Peptide** ALKEAKLKFYDVLLVDTAGRLHVD **Charge** 4 **Spectral Angle** 0.77

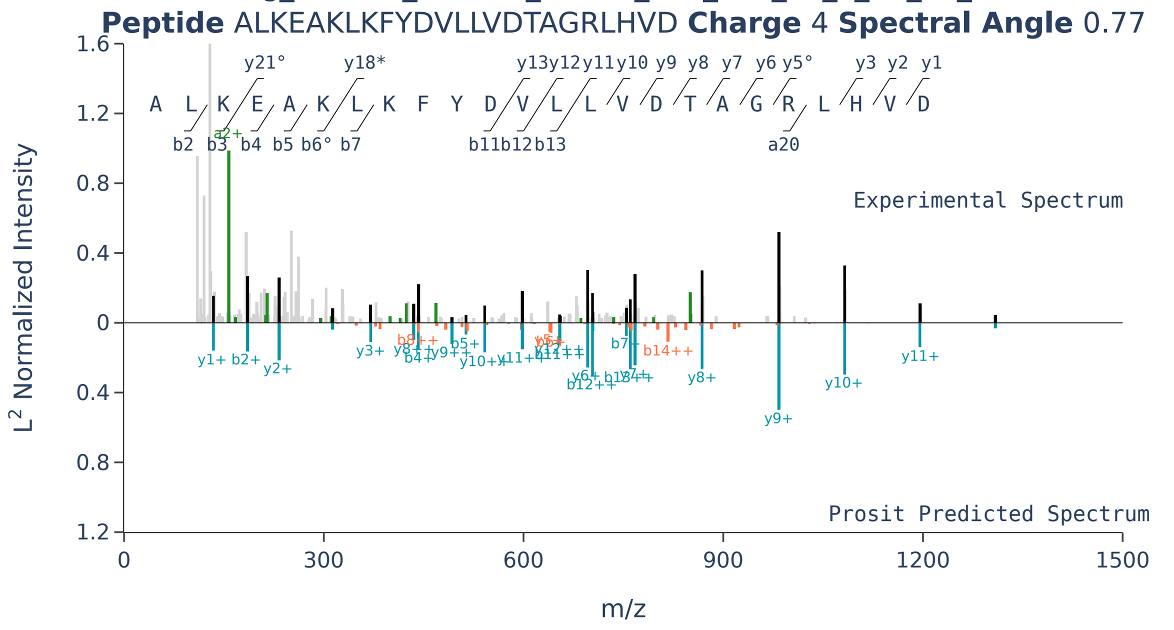


**Source** Ncheng_210623_230623_HFGoe_FFH_20S_25_1_A2_4h_R1 **Scan** 17483
**Peptide** INRVKEKAVGHEVNKSLTPGQEFVKIVRNE **Charge** 4 **Spectral Angle** 0.7

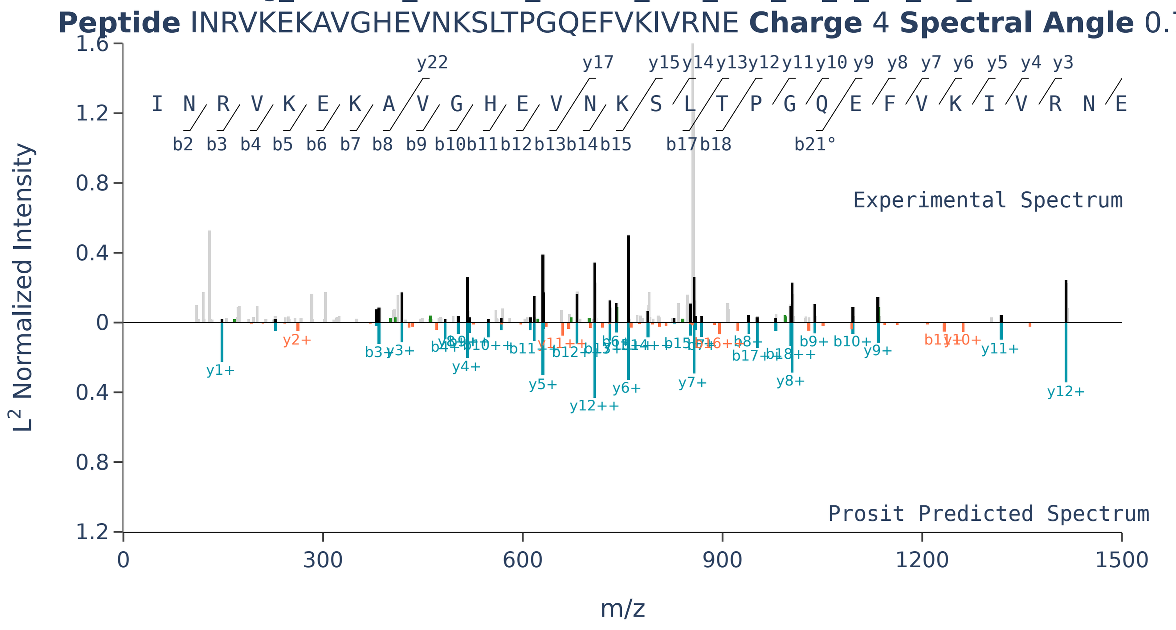


**Source** Ncheng_210623_230623_HFGoe_FFH_20S_25_1_A1_24h_R2 **Scan** 12782
**Peptide** AIINSMTMKERAKPEIIKGSRK **Charge** 6 **Spectral Angle** 0.78

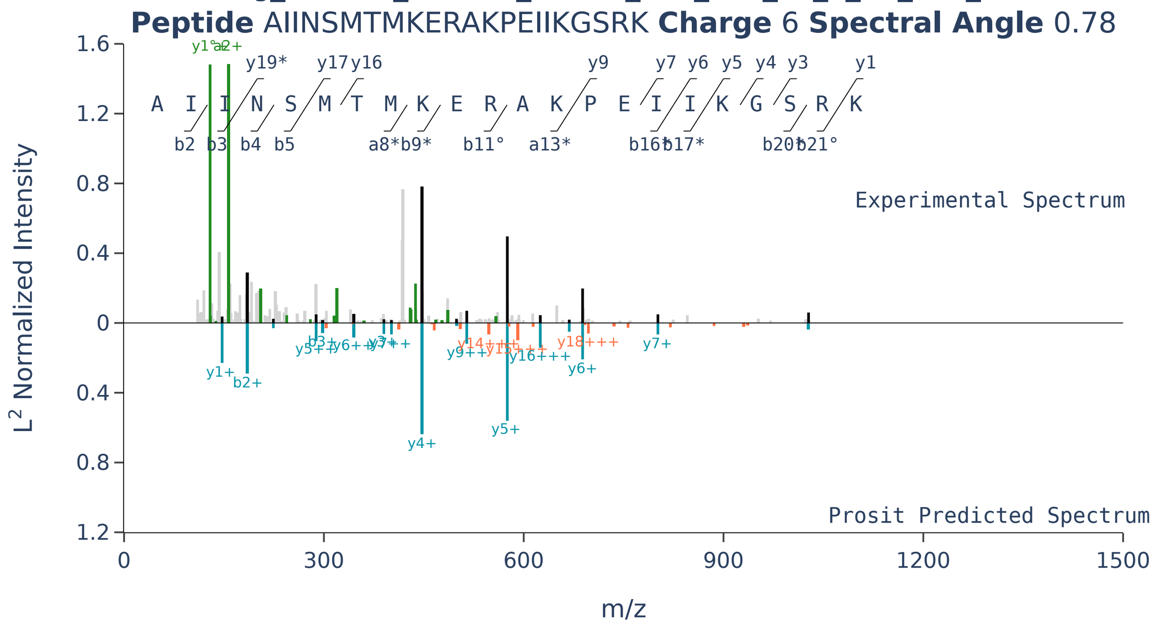


**Source** Ncheng_210623_230623_HFGoe_FFH_20S_25_1_A2_24h_R1 **Scan** 8024
**Peptide** KLASKLKKGDGF **Charge** 3 **Spectral Angle** 0.67

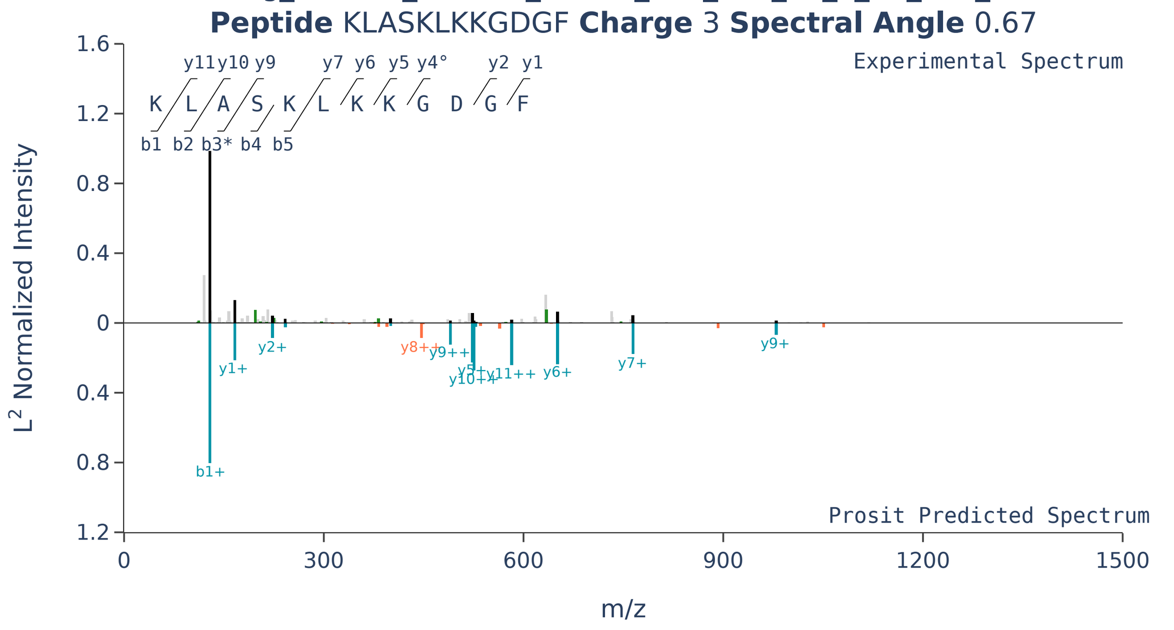


**Source** Ncheng_210623_230623_HFGoe_FFH_20S_25_1_A1_24h_R1 **Scan** 27036
**Peptide** LGMGDVL **Charge** 1 **Spectral Angle** 0.77

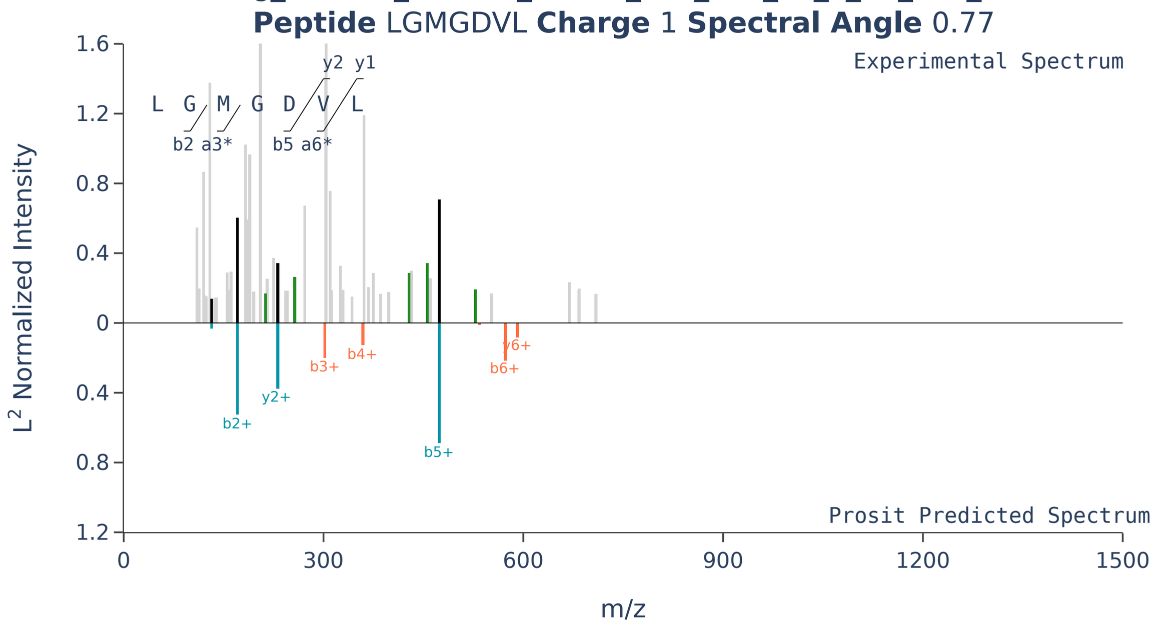


**Source** Ncheng_210623_230623_HFGoe_FFH_20S_25_1_A2_4h_R2 **Scan** 20486
**Peptide** EFVKIVRNEL **Charge** 2 **Spectral Angle** 0.82

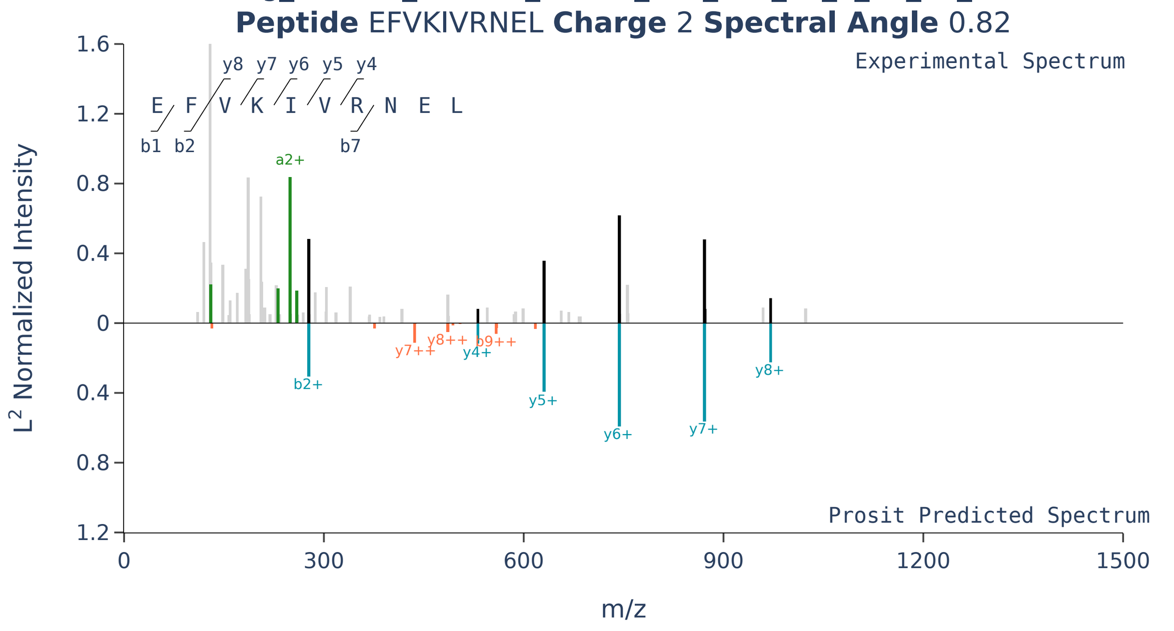


**Source** Ncheng_210623_230623_HFGoe_FFH_20S_25_1_A1_24h_R2 **Scan** 21229
**Peptide** MGKLPGMGQIPDNVK **Charge** 3 **Spectral Angle** 0.87

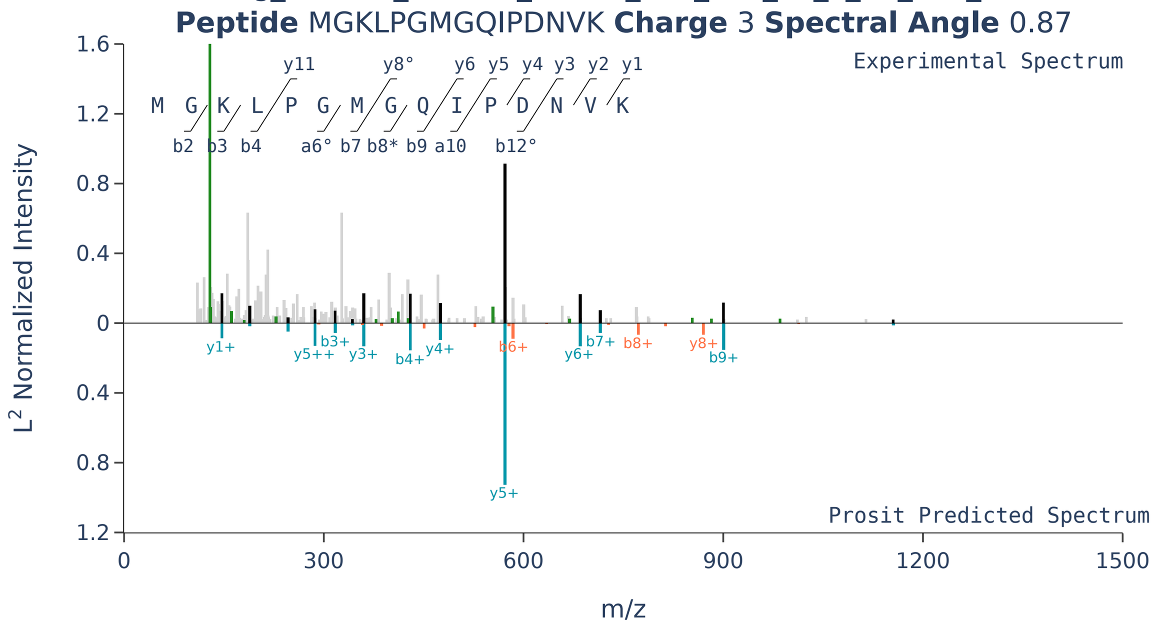


**Source** Ncheng_210623_230623_HFGoe_FFH_20S_25_1_A2_4h_R2 **Scan** 13753
**Peptide** EDIESKVDRAQAEKLA **Charge** 2 **Spectral Angle** 0.67

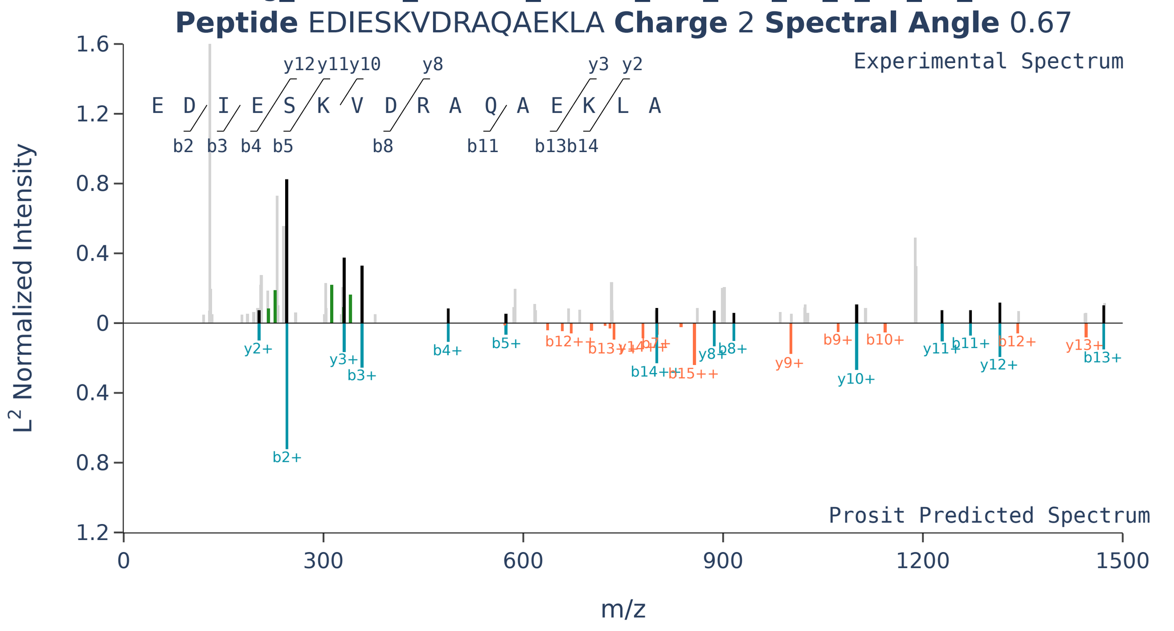


**Source** Ncheng_210623_230623_HFGoe_FFH_20S_25_1_A1_24h_R2 **Scan** 11496
**Peptide** KQFDDMQRMMKK **Charge** 4 **Spectral Angle** 0.86

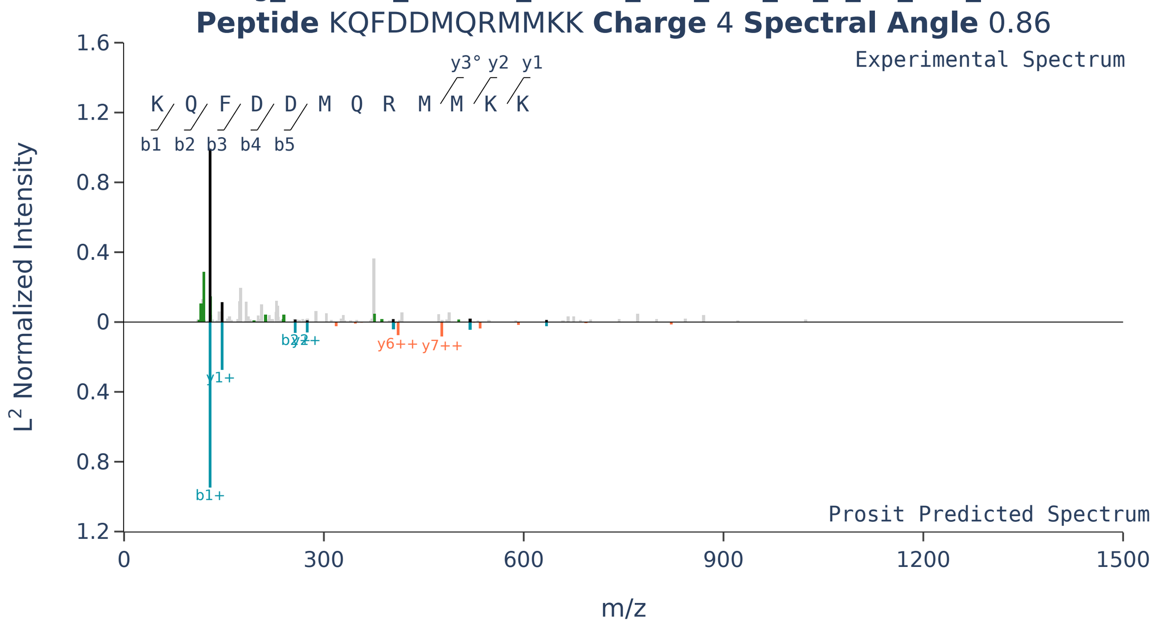


**Source** Ncheng_210623_230623_HFGoe_FFH_20S_25_1_A1_2h_R1 **Scan** 30788
**Peptide** MKNMGGMASLMGKLPGMGQIPDNVKSQMD **Charge** 4 **Spectral Angle** 0.85

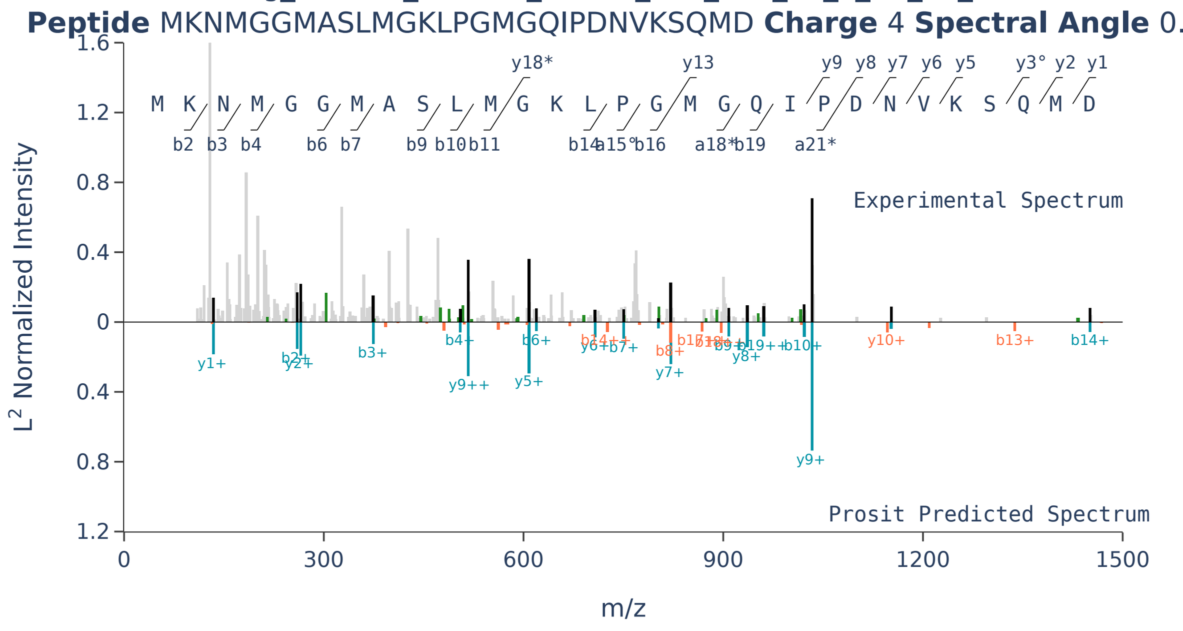


**Source** Ncheng_210623_230623_HFGoe_FFH_20S_25_1_A2_4h_R1 **Scan** 37112
**Peptide** AMGEENQTLNLAAQPPAVVLM **Charge** 2 **Spectral Angle** 0.87

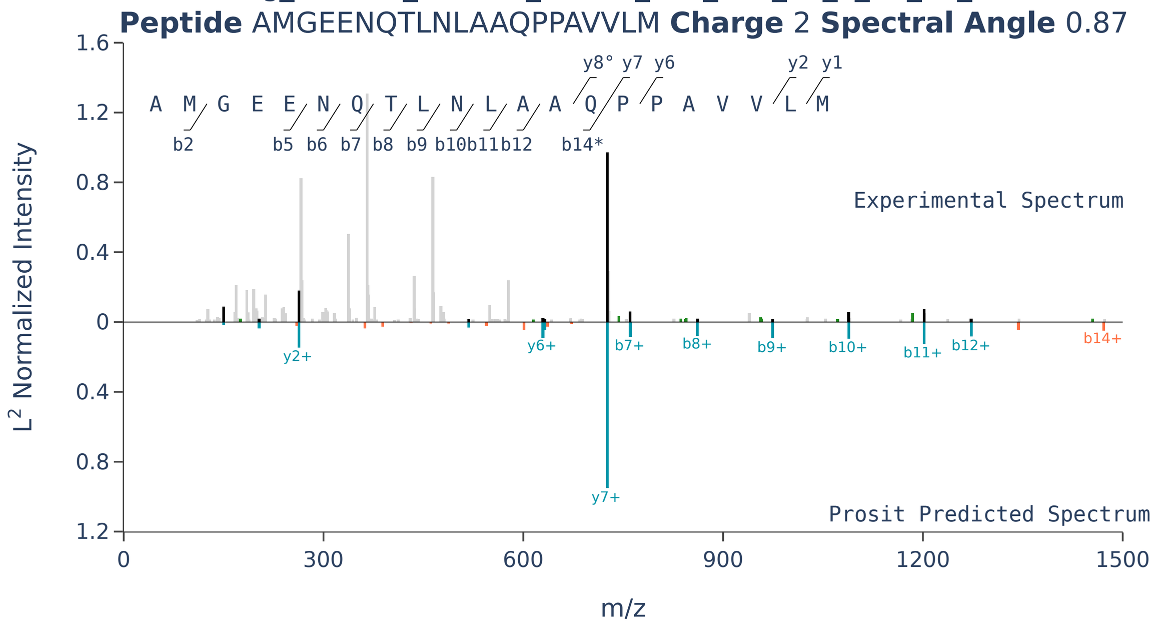


**Source** Ncheng_210623_230623_HFGoe_FFH_20S_25_1_A2_4h_R2 **Scan** 33739
**Peptide** NMGGMASLMGKLPGMGQIPDNVKSQMD **Charge** 3 **Spectral Angle** 0.82

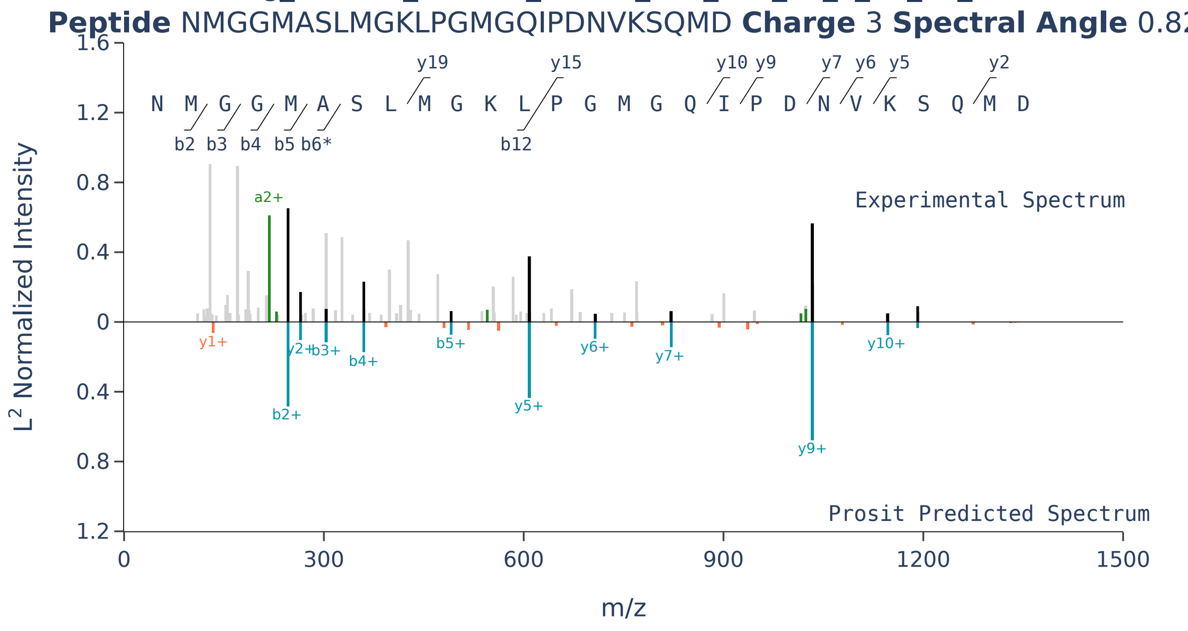


**Source** Ncheng_210623_230623_HFGoe_FFH_20S_25_1_A2_24h_R1 **Scan** 8630
**Peptide** FLNRVKEKAV **Charge** 3 **Spectral Angle** 0.53

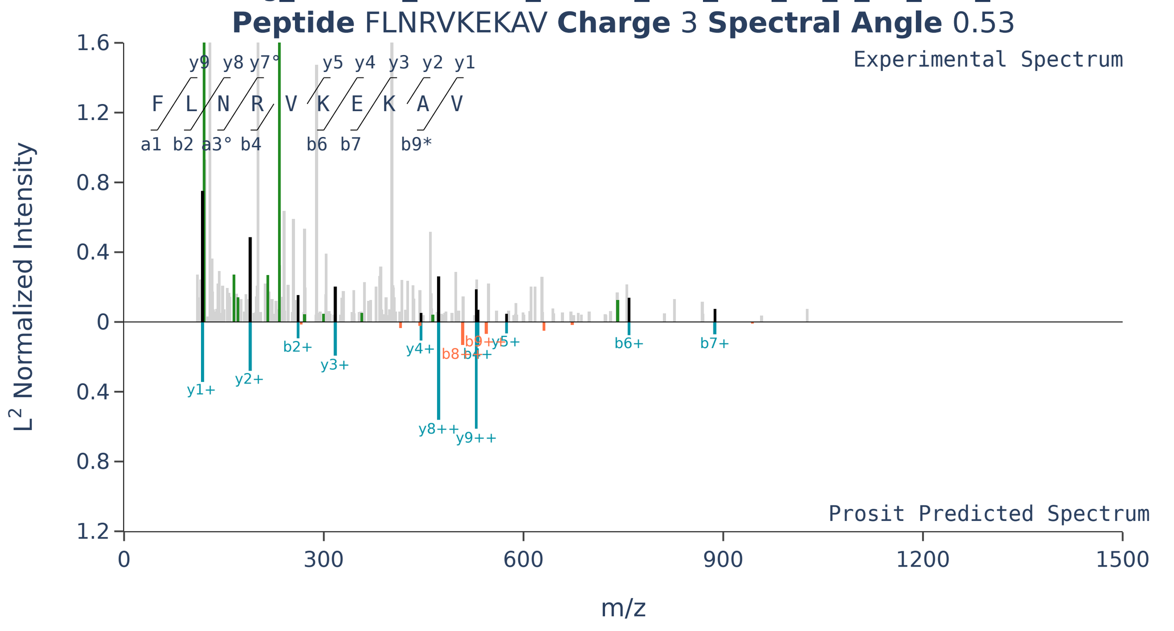


**Source** Ncheng_210623_230623_HFGoe_FFH_20S_25_1_A2_4h_R2 **Scan** 11974
**Peptide** TAGRLHVDEAM **Charge** 3 **Spectral Angle** 0.52

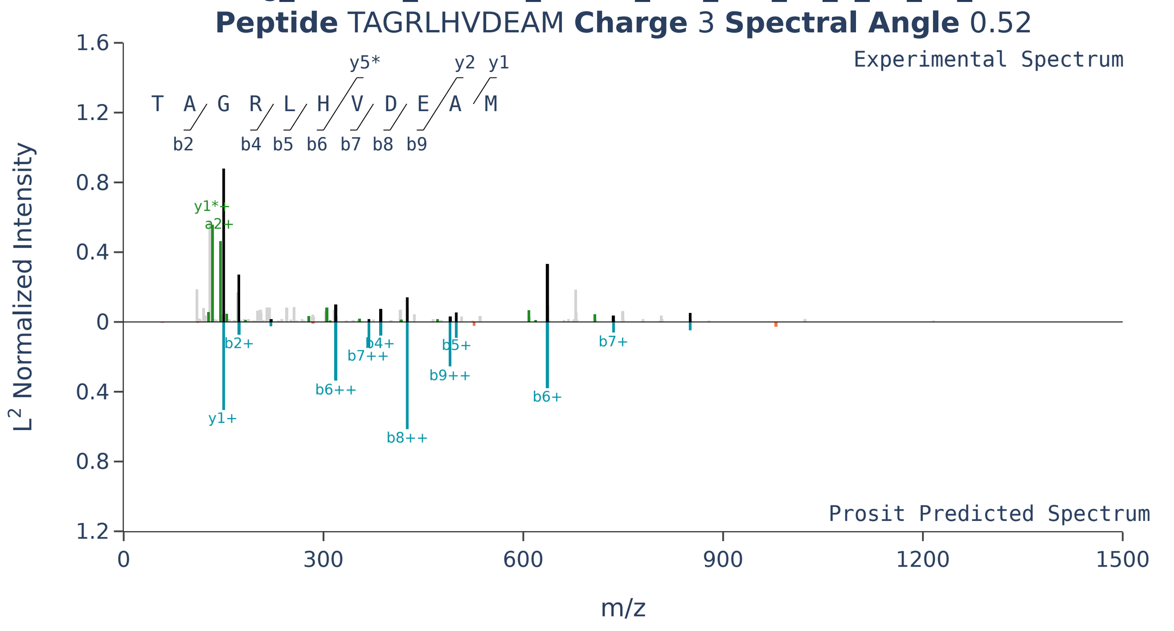

**Source** Ncheng_210623_230623_HFGoe_FFH_20S_25_1_A2_24h_R1 **Scan** 6012
**Peptide** MKKGGMAKM **Charge** 2 **Spectral Angle** 0.73

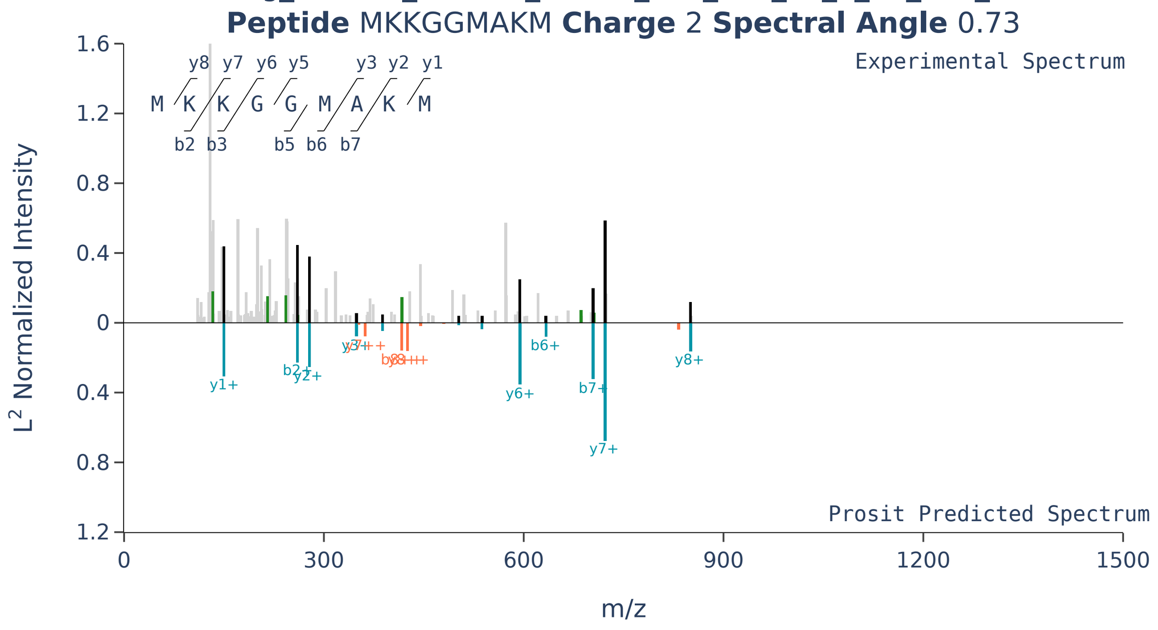


**Source** Ncheng_210623_230623_HFGoe_FFH_20S_25_1_A2_4h_R1 **Scan** 34394
**Peptide** ALPLTGVVLTKVDGDARGGAALSI **Charge** 3 **Spectral Angle** 0.73

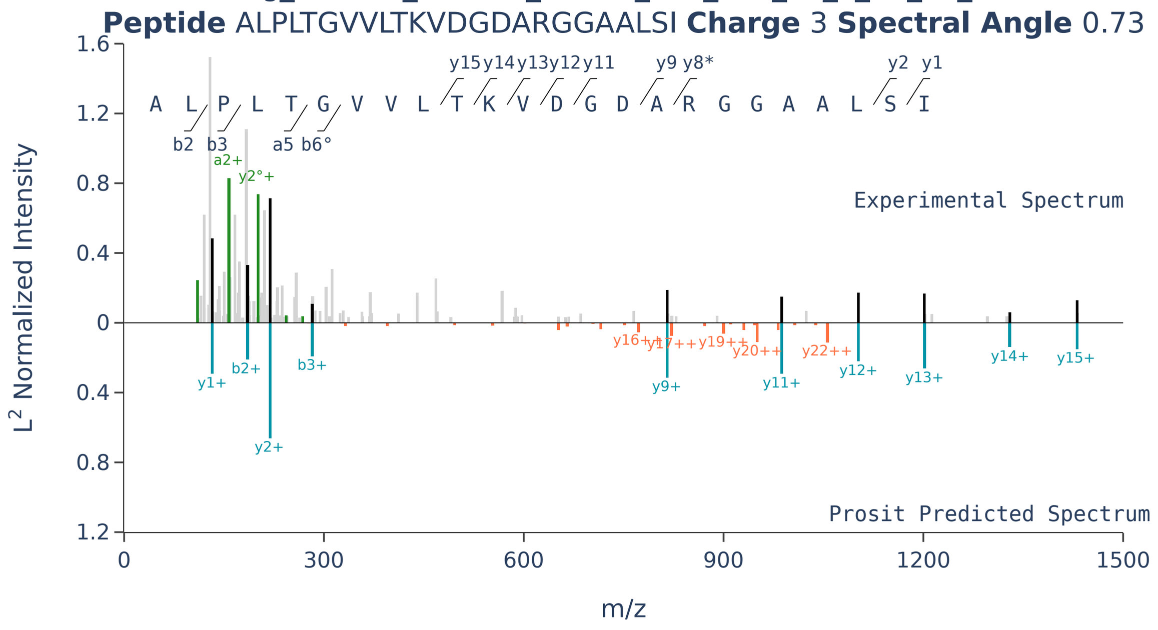


**Source** Ncheng_210623_230623_HFGoe_FFH_20S_25_1_A1_24h_R1 **Scan** 7151
**Peptide** LREKHKKKVLVV **Charge** 2 **Spectral Angle** 0.59

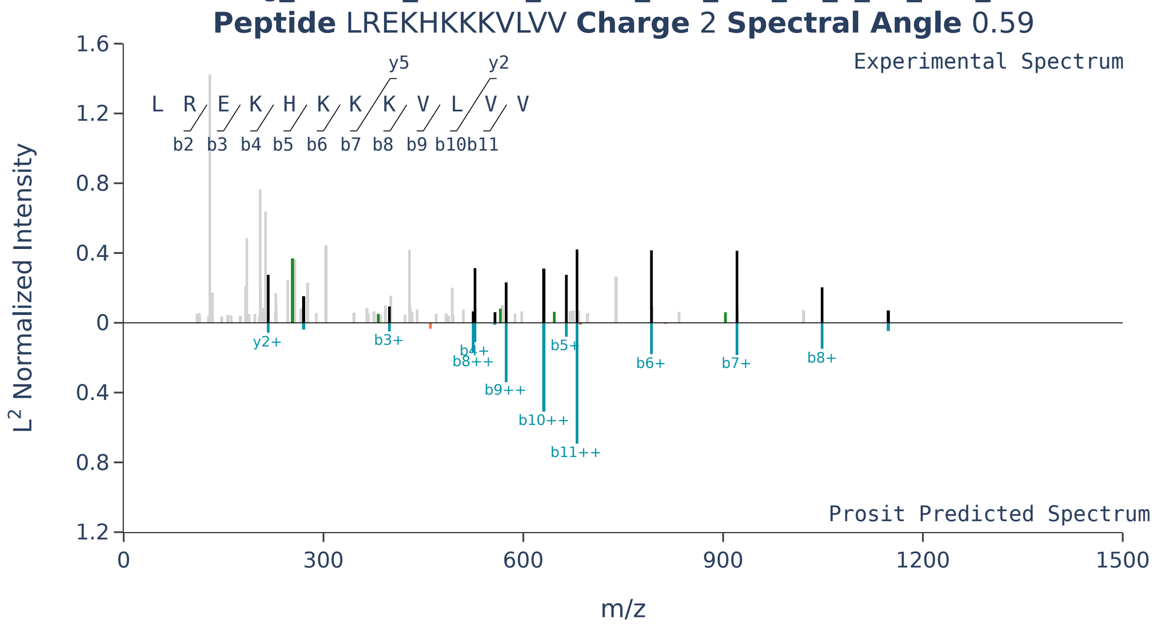


**Source** Ncheng_210623_230623_HFGoe_FFH_20S_25_1_A2_2h_R1 **Scan** 31380
**Peptide** QIPDNVKSQMDDKVLVRMEAII **Charge** 4 **Spectral Angle** 0.7

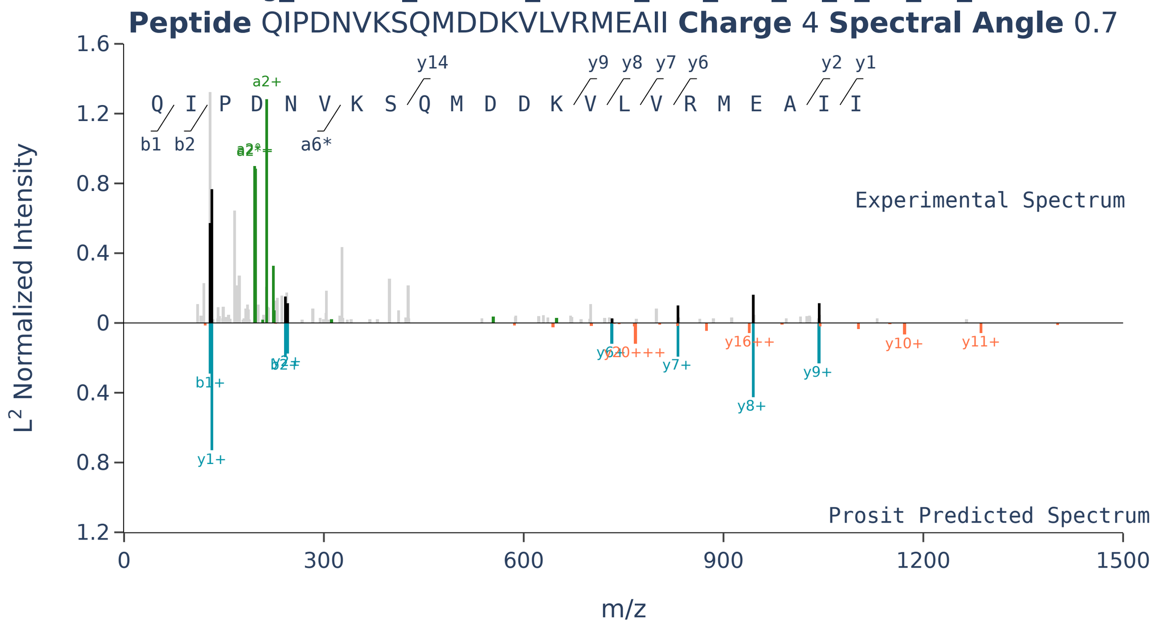


**Source** Ncheng_210623_230623_HFGoe_FFH_20S_25_1_A1_4h_R1 **Scan** 25078
**Peptide** PLTGVVLTKVDGDARGGAALSIRHI **Charge** 3 **Spectral Angle** 0.68

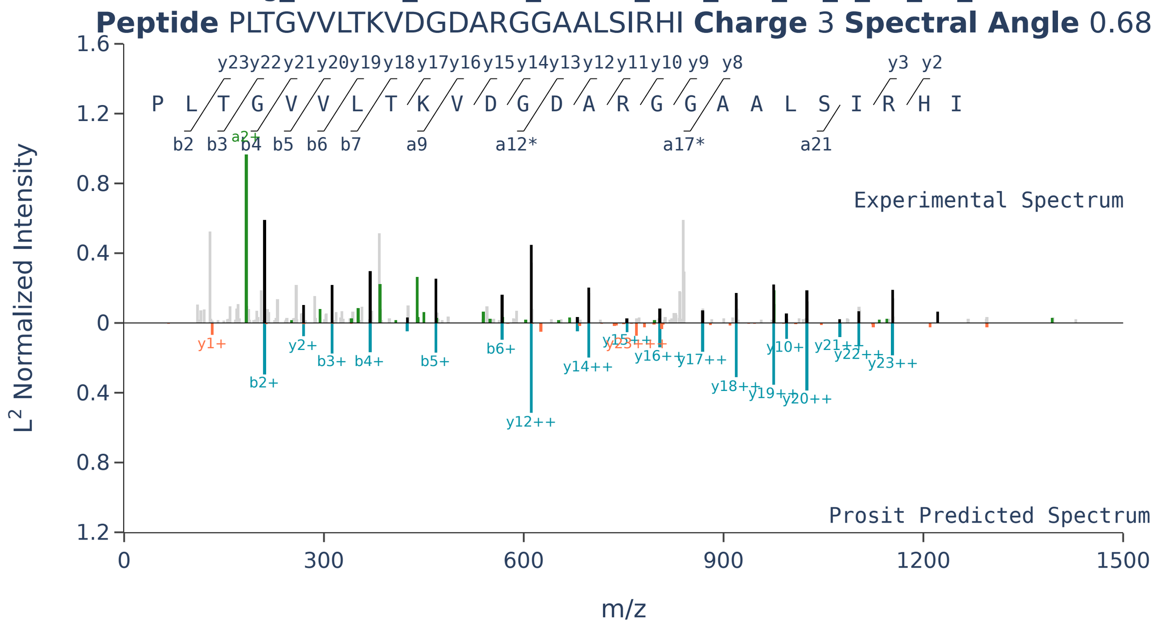


**Source** Ncheng_210623_230623_HFGoe_FFH_20S_25_1_A2_2h_R2 **Scan** 31715
**Peptide** ALKEAKLKFYDVLLVDTAGRLHV **Charge** 3 **Spectral Angle** 0.84

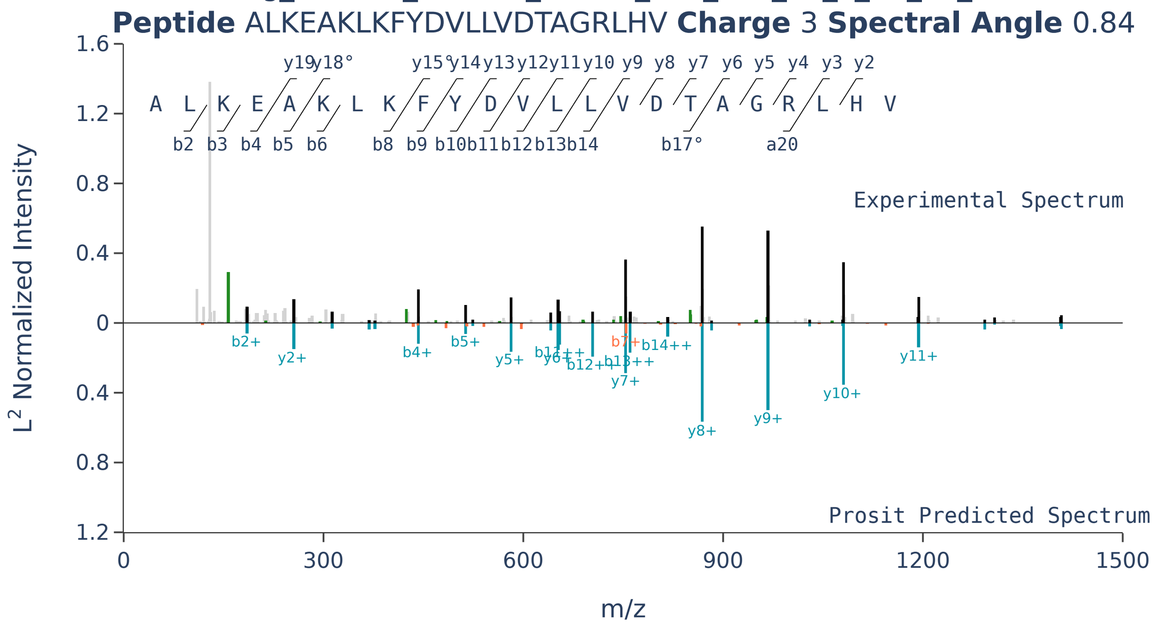


**Source** Ncheng_210623_230623_HFGoe_FFH_20S_25_1_A1_1h_R1 **Scan** 14365
**Peptide** ALKEAKLKFYD **Charge** 2 **Spectral Angle** 0.62

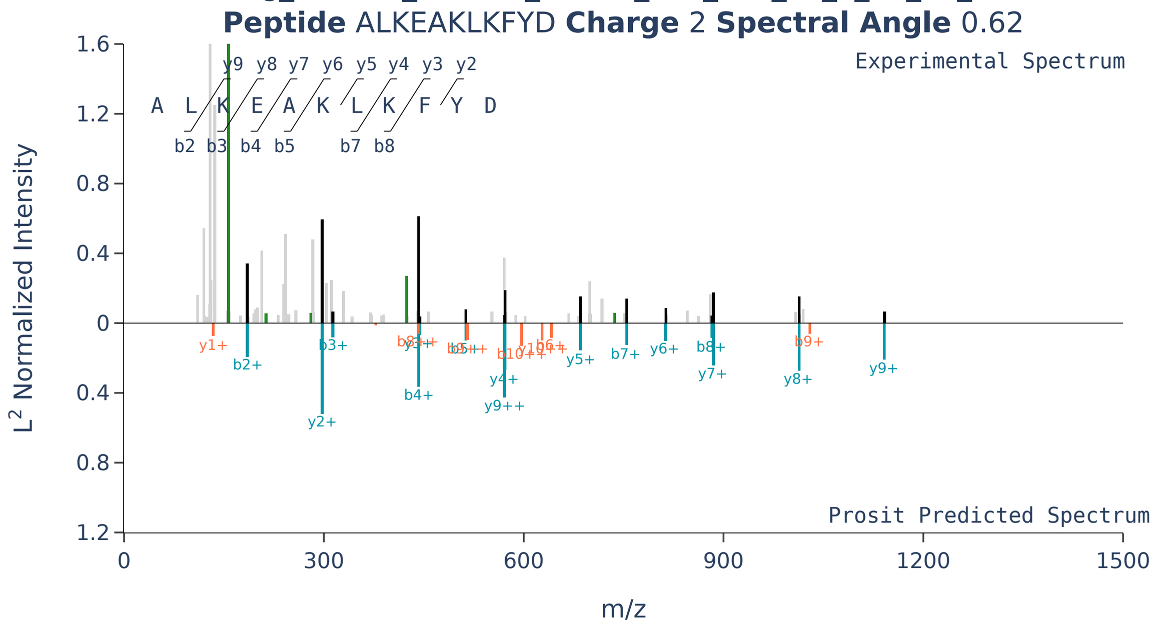


**Source** Ncheng_210623_230623_HFGoe_FFH_20S_25_1_A1_2h_R2 **Scan** 24849
**Peptide** PGMGQIPDNVKSQMDDKVLV **Charge** 3 **Spectral Angle** 0.84

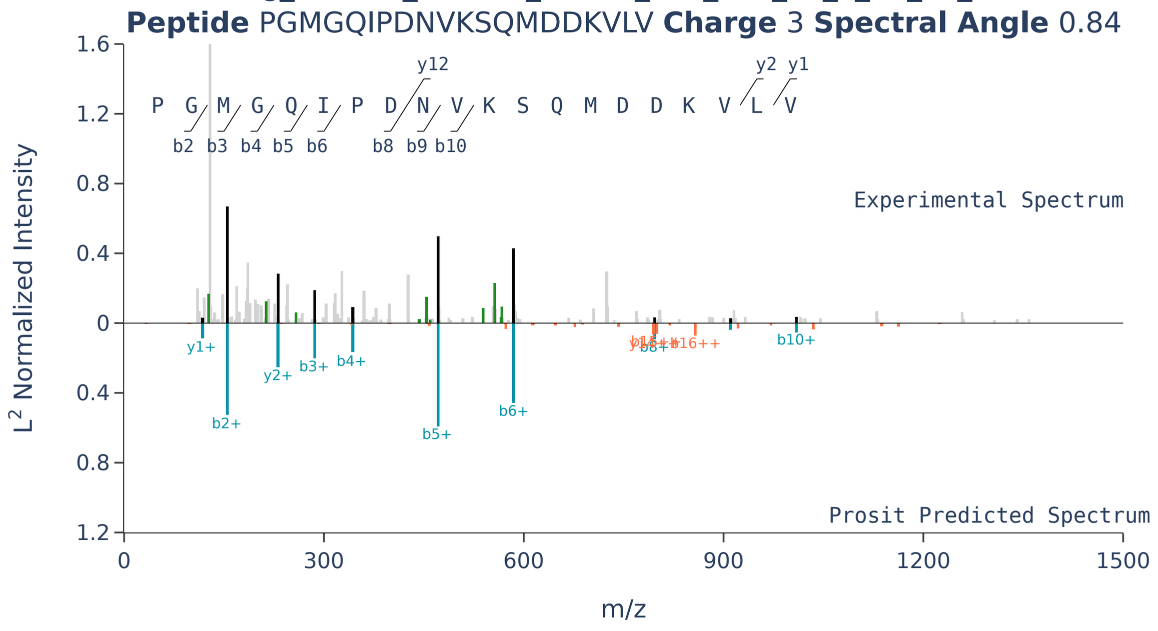


**Source** Ncheng_210623_230623_HFGoe_FFH_20S_25_1_A1_2h_R2 **Scan** 13978
**Peptide** LVDTAGRLHVDEA **Charge** 2 **Spectral Angle** 0.71

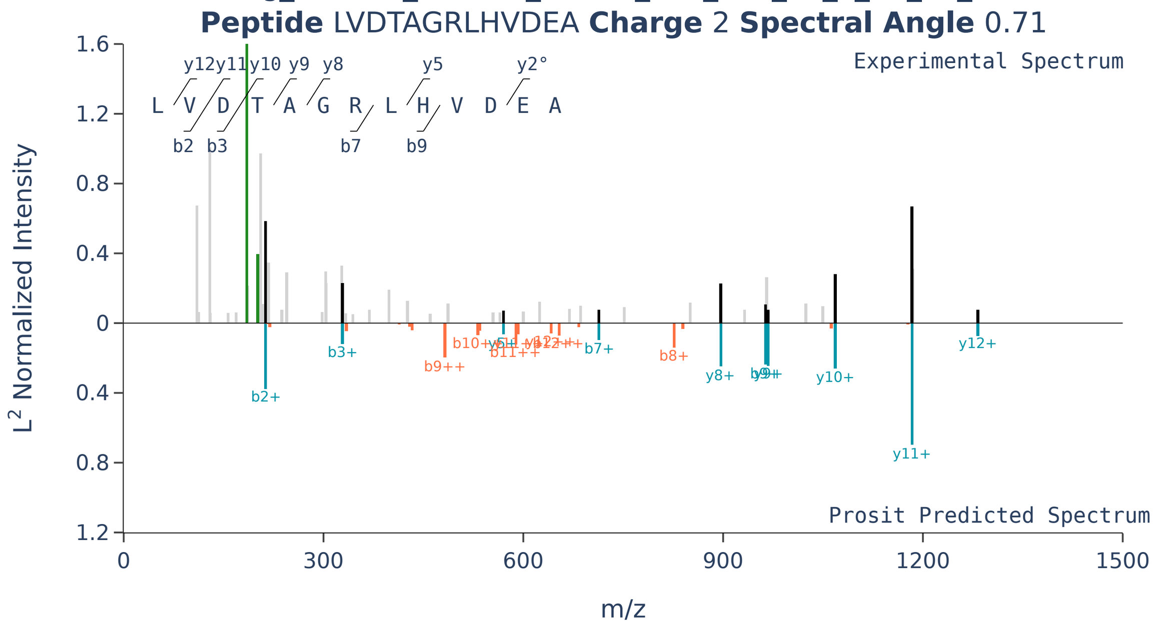


**Source** Ncheng_210623_230623_HFGoe_FFH_20S_25_1_A1_4h_R1 **Scan** 12456
**Peptide** LTEDNVKDTLRE **Charge** 2 **Spectral Angle** 0.72

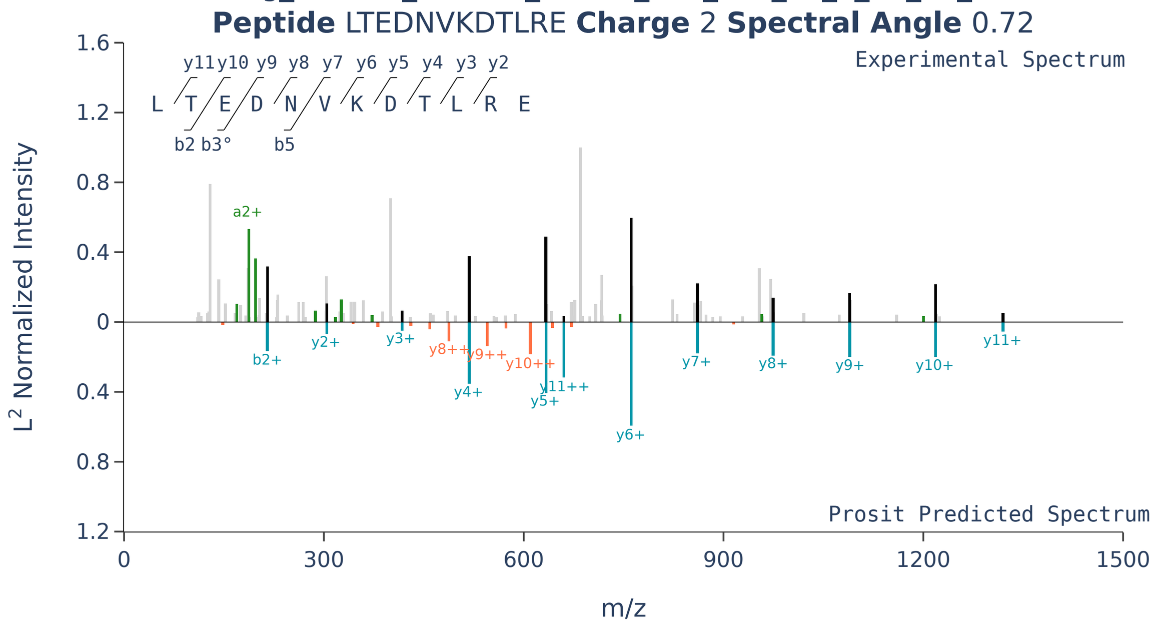


**Source** Ncheng_210623_230623_HFGoe_FFH_20S_25_1_A2_2h_R2 **Scan** 34159
**Peptide** VAAMGEENQTLNLAAQPPAVVL **Charge** 2 **Spectral Angle** 0.9

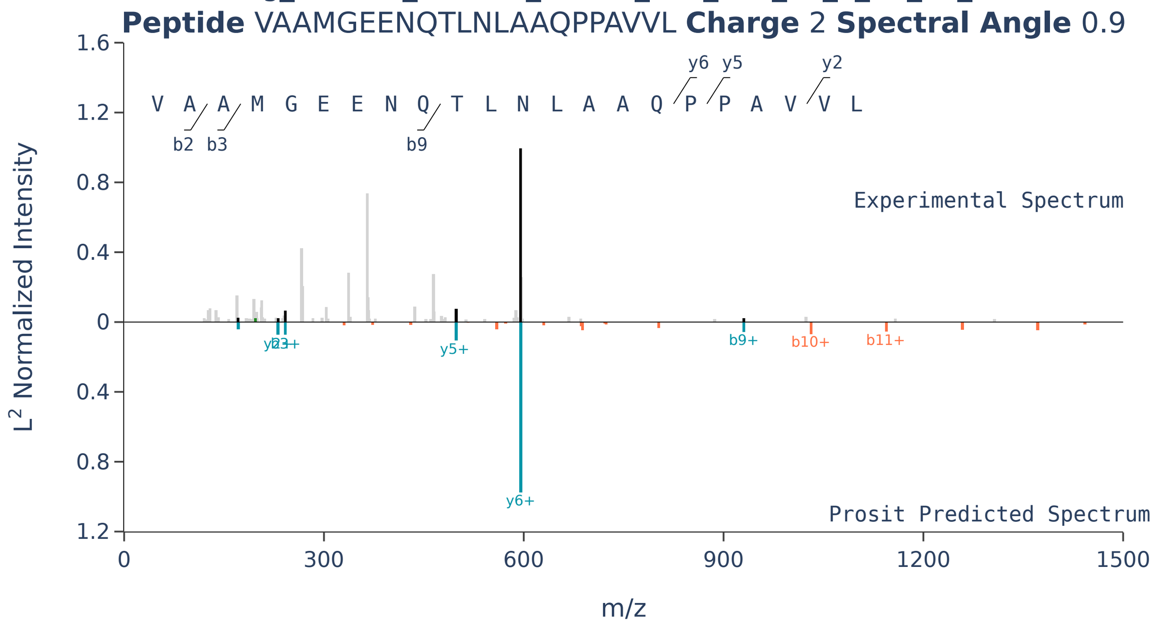


**Source** Ncheng_210623_230623_HFGoe_FFH_20S_25_1_A2_24h_R1 **Scan** 25298
**Peptide** QEFVKIV **Charge** 2 **Spectral Angle** 0.82

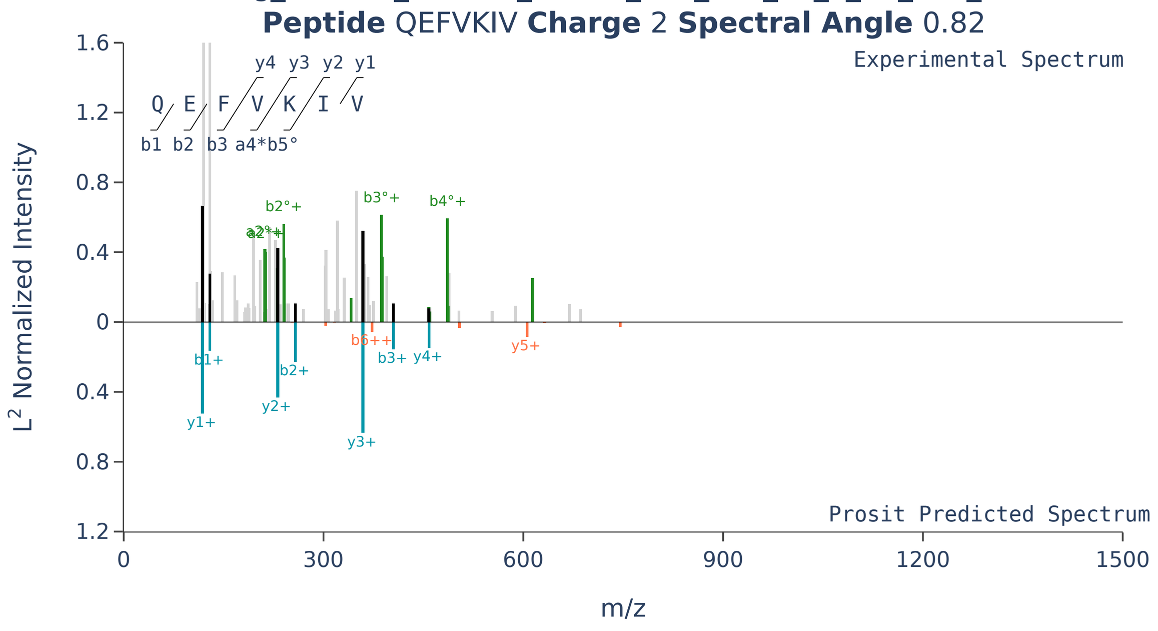


**Source** Ncheng_210623_230623_HFGoe_FFH_20S_25_1_A2_24h_R2 **Scan** 10992
**Peptide** VDTAGRLHVDEA **Charge** 2 **Spectral Angle** 0.63

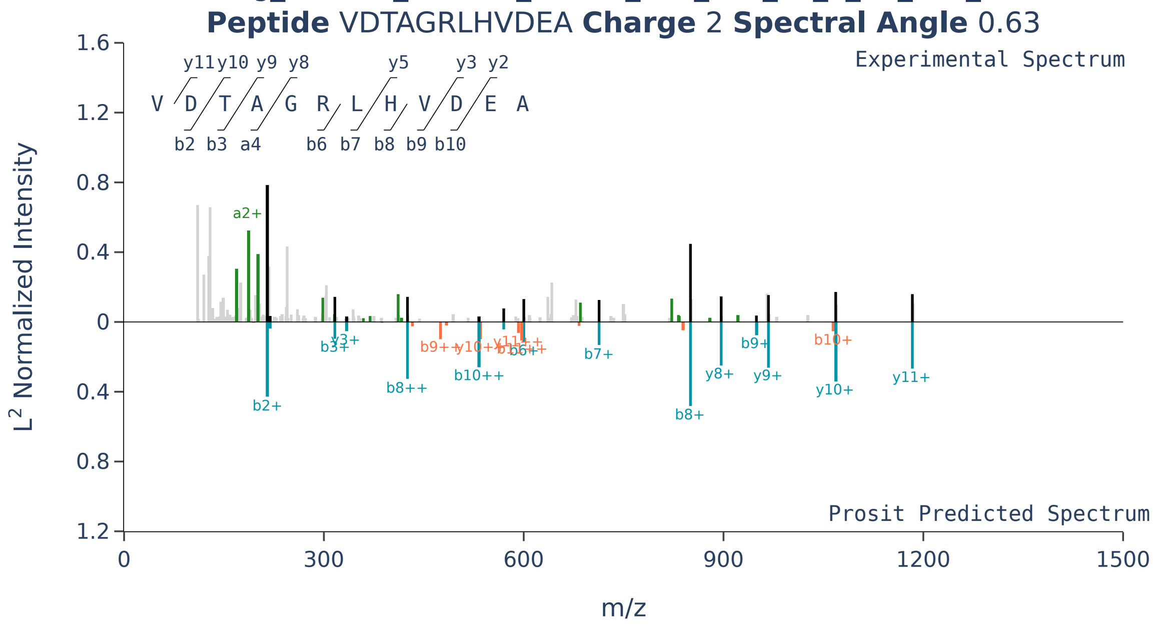


**Source** Ncheng_210623_230623_HFGoe_FFH_20S_25_1_A2_2h_R2 **Scan** 6169
**Peptide** DEIKQVHAS **Charge** 2 **Spectral Angle** 0.73

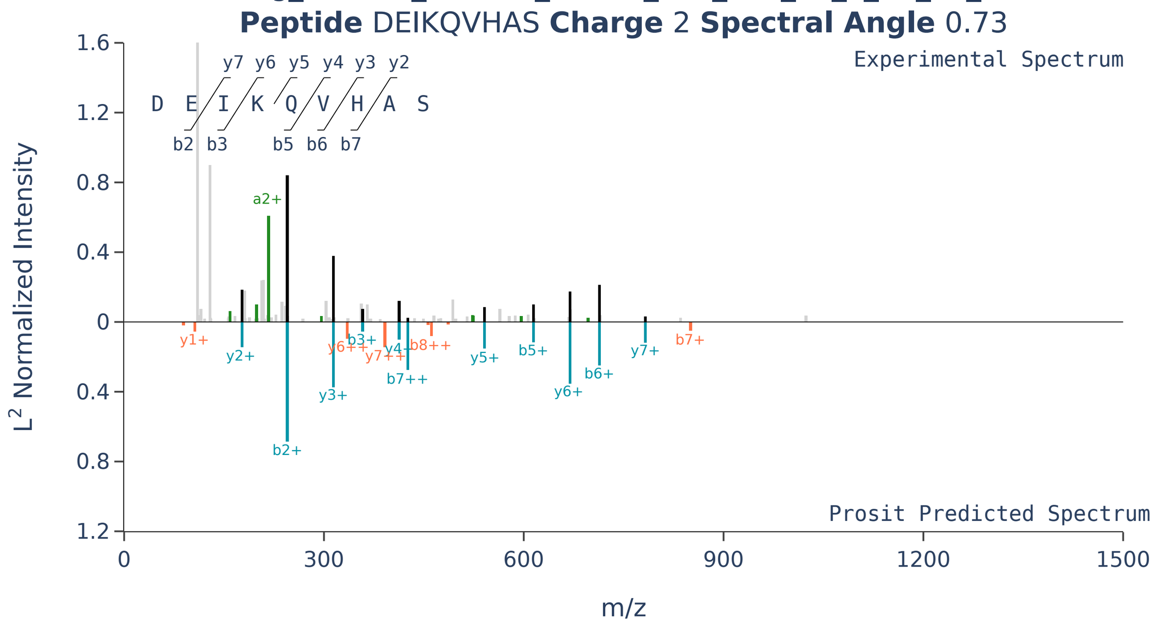


**Source** Ncheng_210623_230623_HFGoe_FFH_20S_25_1_A1_4h_R2 **Scan** 24423
**Peptide** TPGQEFVKLV **Charge** 2 **Spectral Angle** 0.65

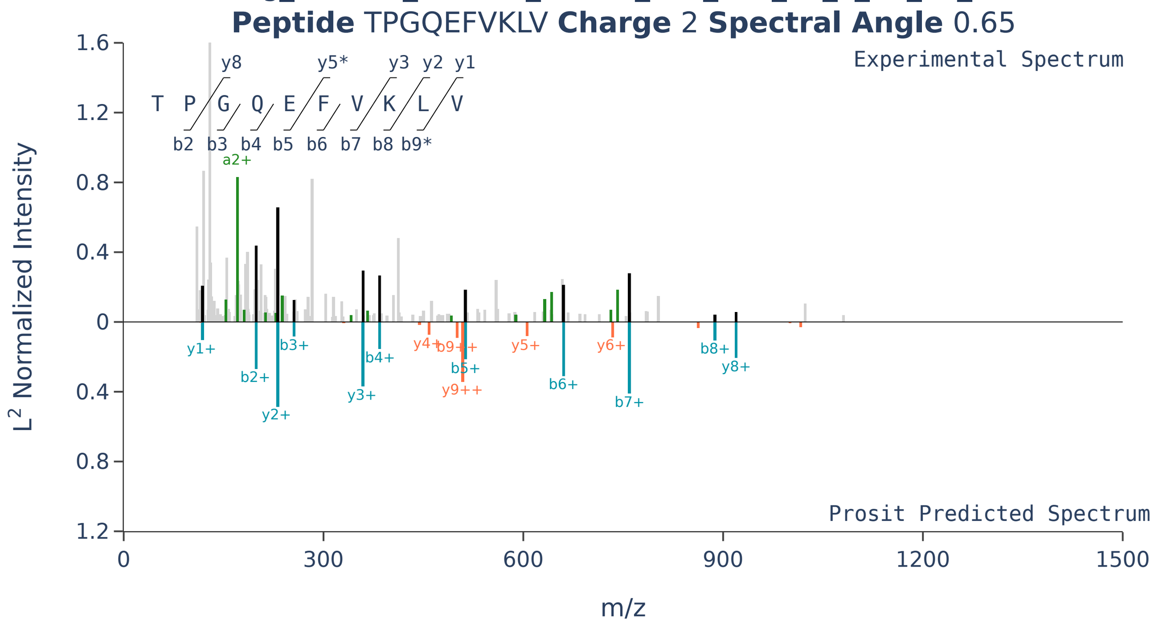

**Source** Ncheng_210623_230623_HFGoe_FFH_20S_25_1_A1_2h_R1 **Scan** 28372
**Peptide** SLIEDIESKVDRA **Charge** 3 **Spectral Angle** 0.62

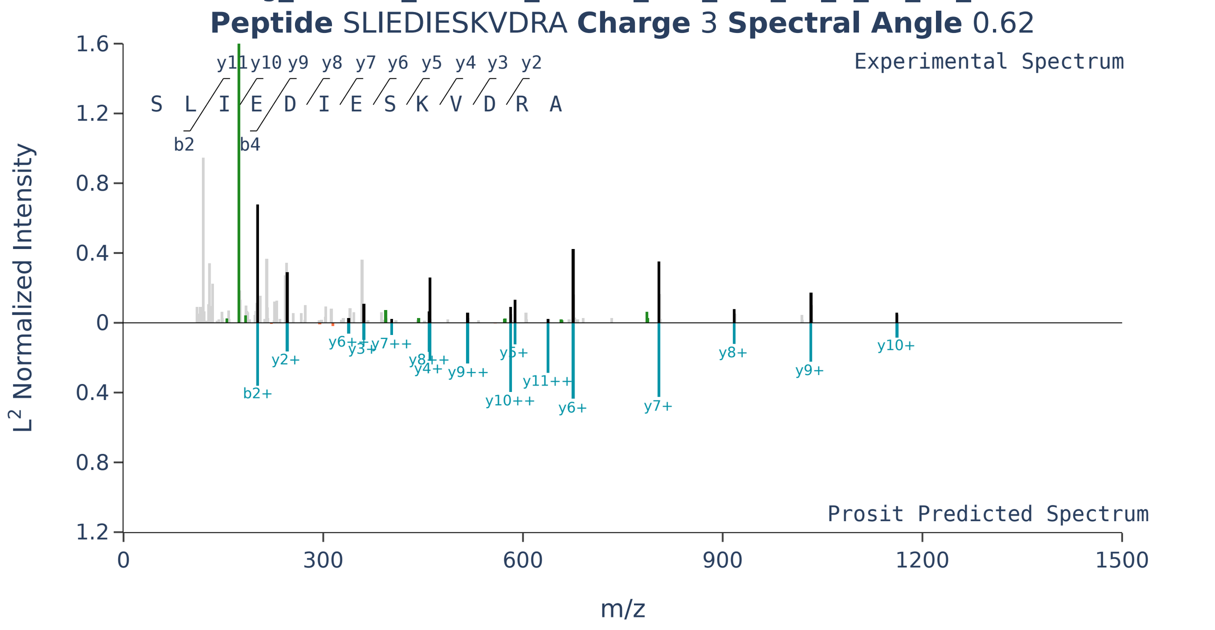


**Source** Ncheng_210623_230623_HFGoe_FFH_20S_25_1_A2_2h_R1 **Scan** 23159
**Peptide** TGKPIKFLGVGEKTEALEPFHPDRIAS **Charge** 3 **Spectral Angle** 0.78

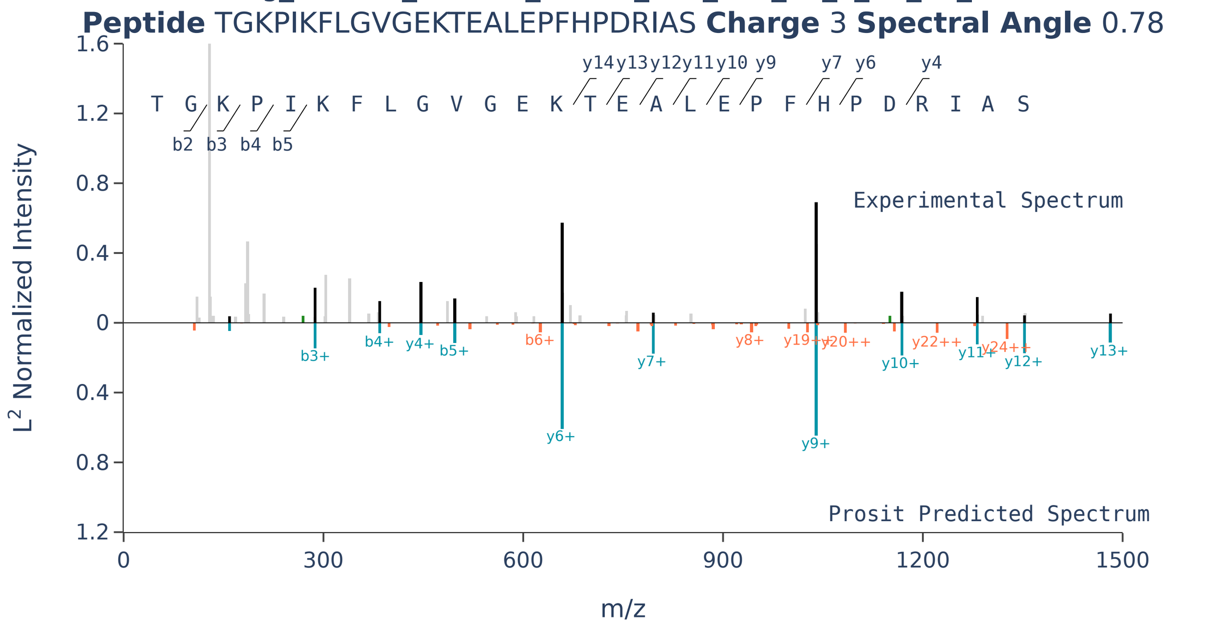


**Source** Ncheng_210623_230623_HFGoe_FFH_20S_25_1_A2_24h_R2 **Scan** 14791
**Peptide** QGAGKTTSVGKLGKFLR **Charge** 3 **Spectral Angle** 0.81

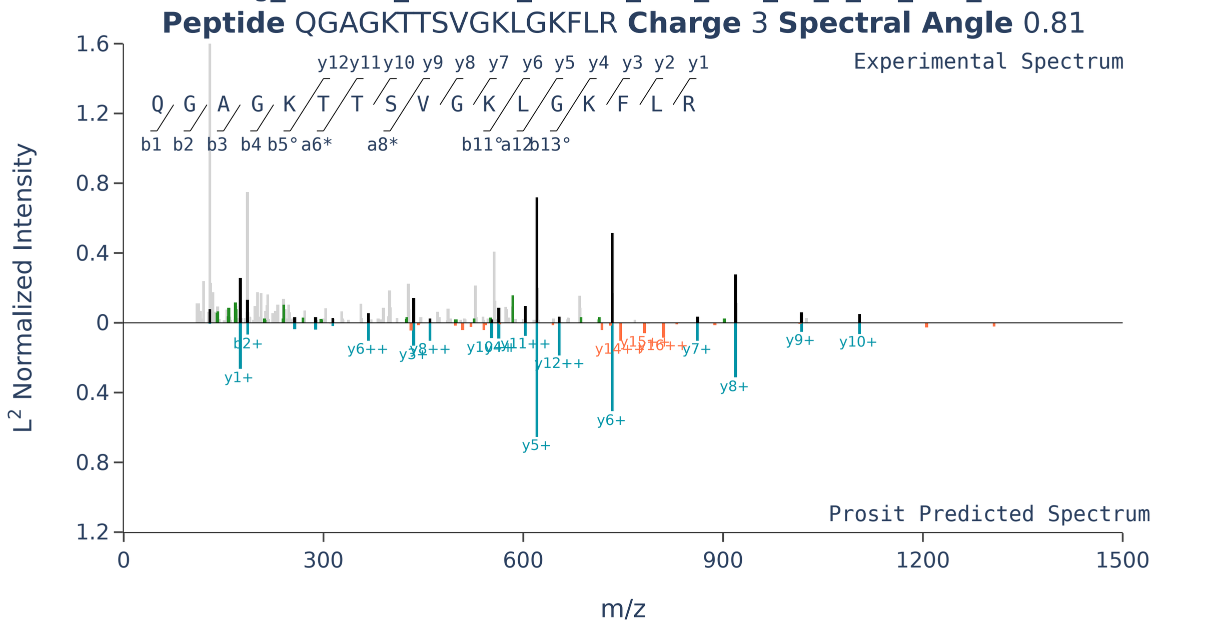


**Source** Ncheng_210623_230623_HFGoe_FFH_20S_25_1_A2_24h_R2 **Scan** 6798
**Peptide** RAKPEIIKGS **Charge** 2 **Spectral Angle** 0.67

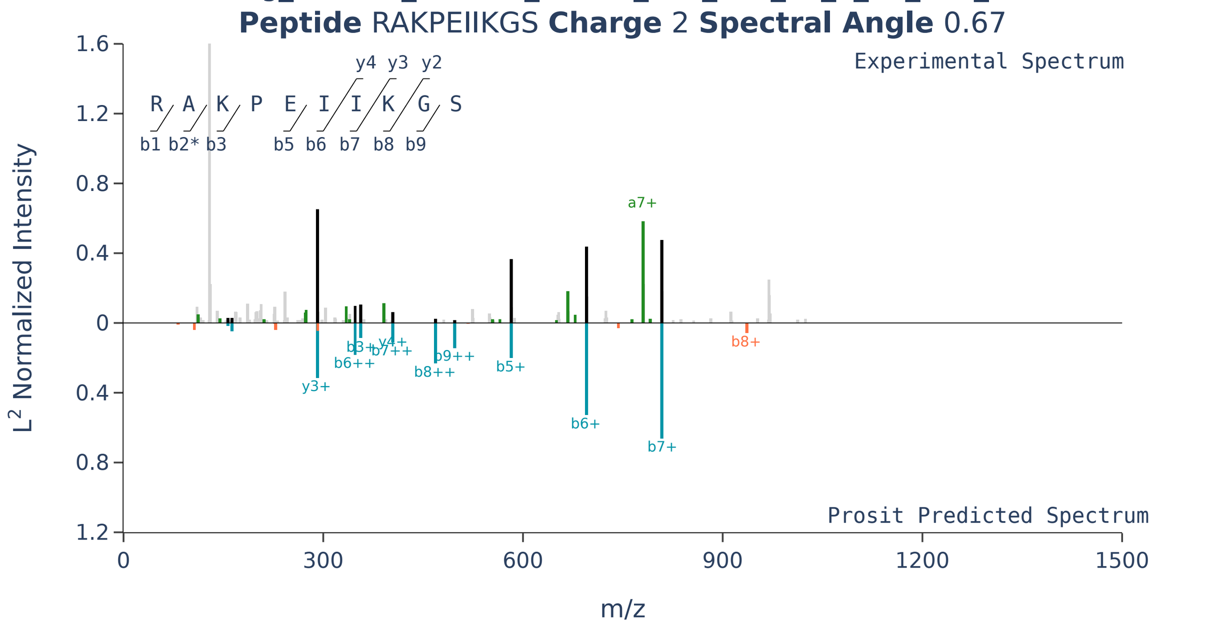


**Source** Ncheng_210623_230623_HFGoe_FFH_20S_25_1_A1_4h_R2 **Scan** 19079
**Peptide** DIESKVDRAQAEKLASKLKKGDGFD **Charge** 3 **Spectral Angle** 0.73

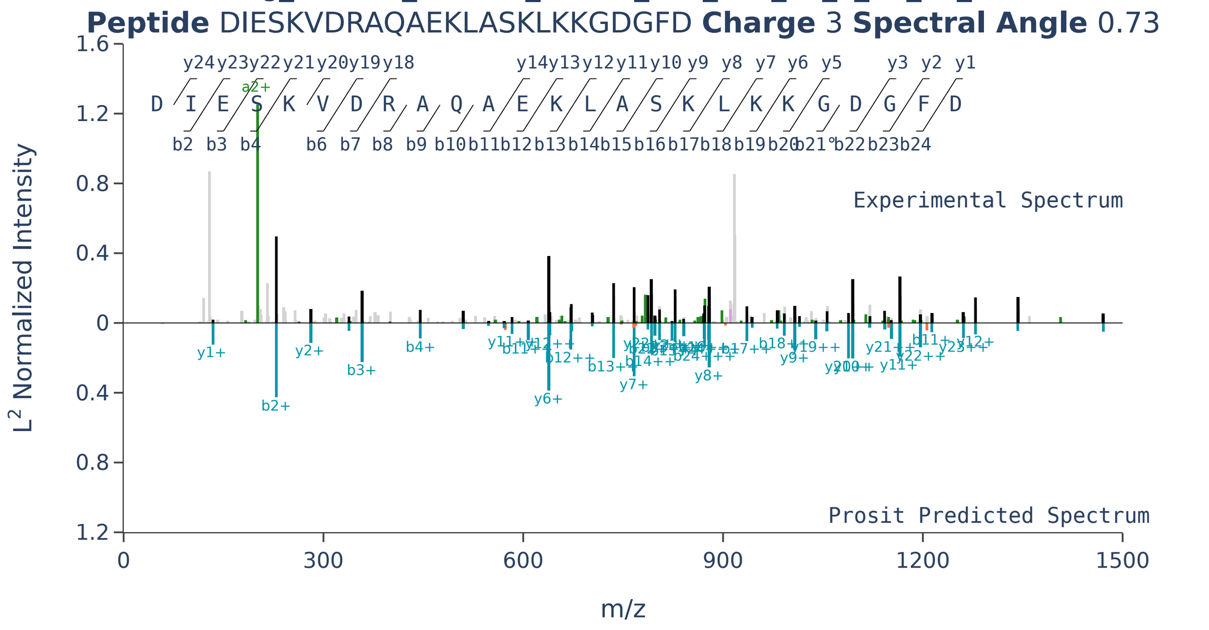


**Source** Ncheng_210623_230623_HFGoe_FFH_20S_25_1_A1_24h_R2 **Scan** 19350
**Peptide** EKAVGHEVNKSLTPGQEFVKL **Charge** 4 **Spectral Angle** 0.73

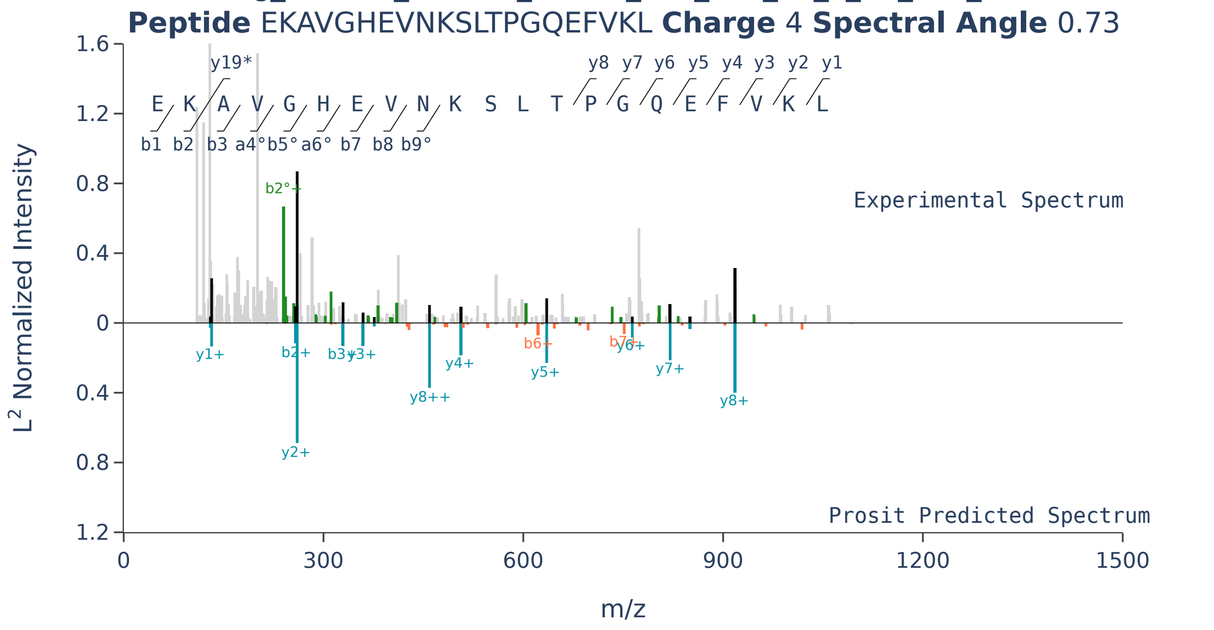


**Source** Ncheng_210623_230623_HFGoe_FFH_20S_25_1_A2_2h_R1 **Scan** 21282
**Peptide** CGMQVQDVNRLLKQ **Charge** 3 **Spectral Angle** 0.74

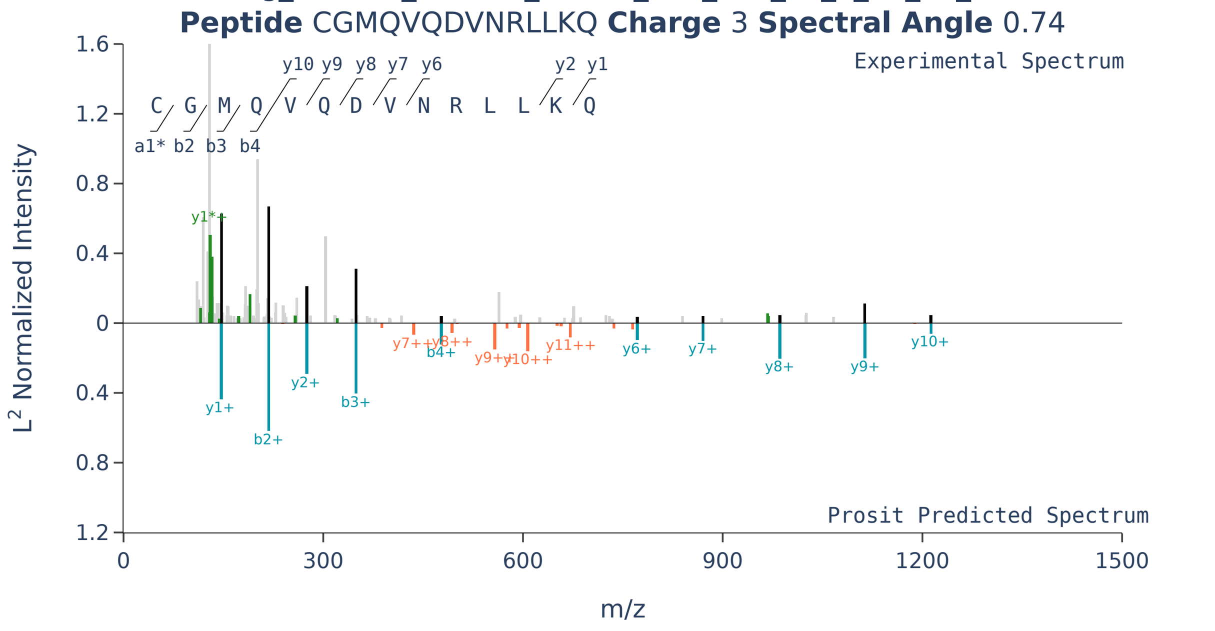


**Source** Ncheng_210623_230623_HFGoe_FFH_20S_25_1_A1_2h_R2 **Scan** 24846
**Peptide** KVLVRMEAIINSMTMKERAK **Charge** 4 **Spectral Angle** 0.79

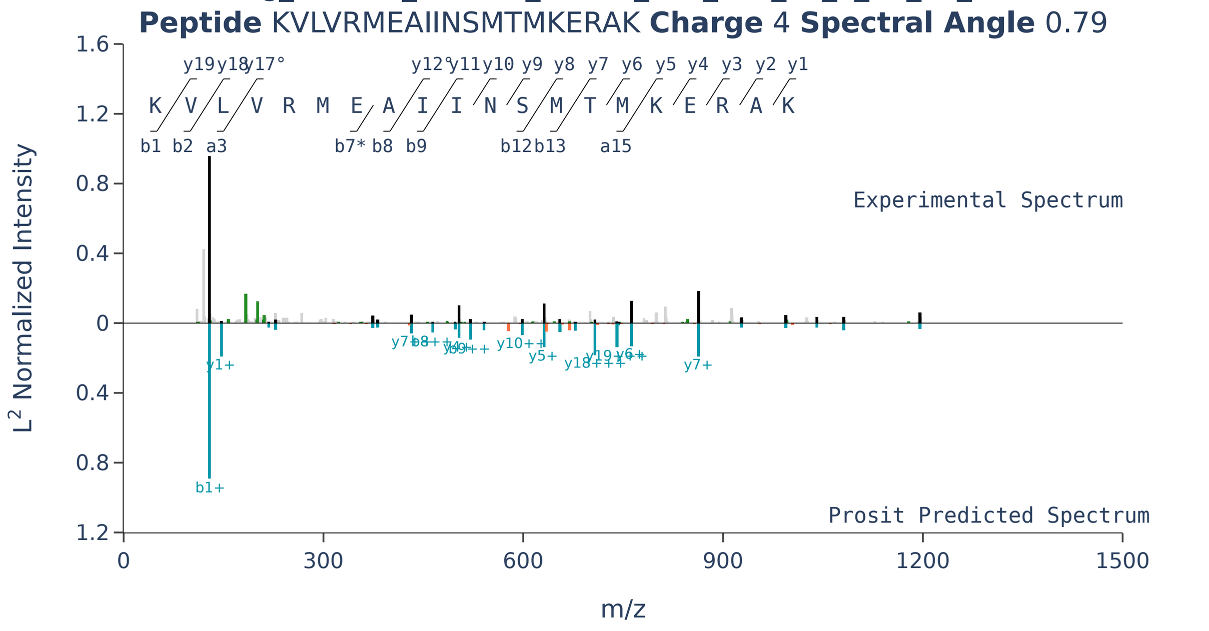


**Source** Ncheng_210623_230623_HFGoe_FFH_20S_25_1_A1_1h_R2 **Scan** 35897
**Peptide** AEQVGVDFFPSDVGQKPVDIVNAAL **Charge** 3 **Spectral Angle** 0.76

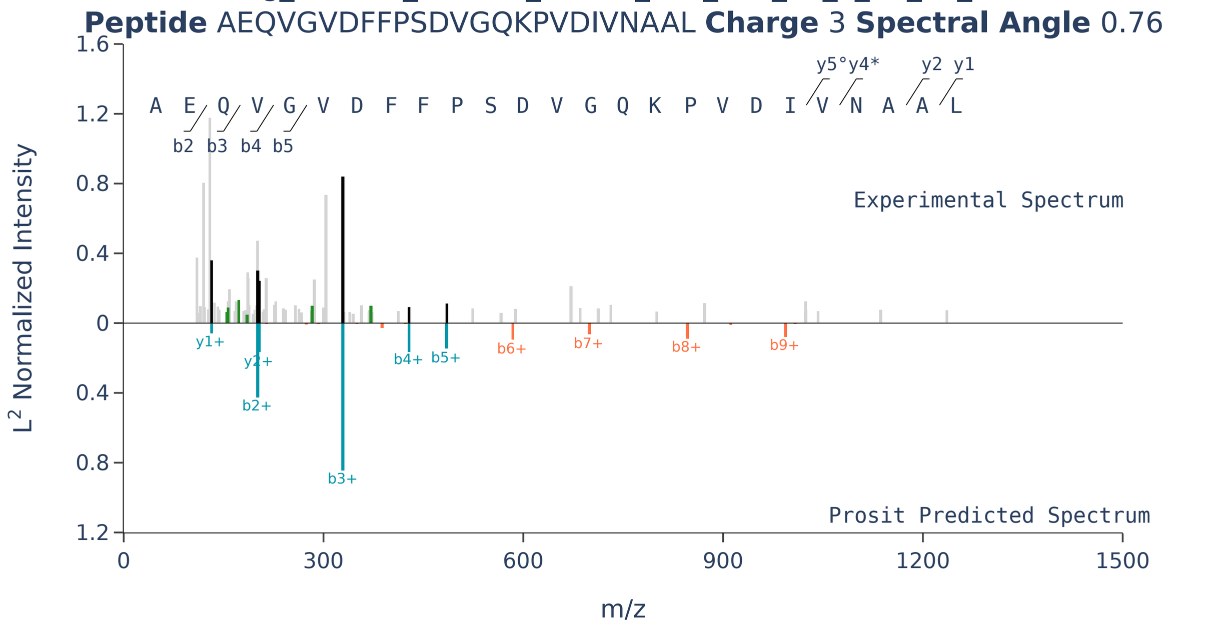


**Source** Ncheng_210623_230623_HFGoe_FFH_20S_25_1_A1_4h_R2 **Scan** 24499
**Peptide** KVLVRMEALL **Charge** 2 **Spectral Angle** 0.55

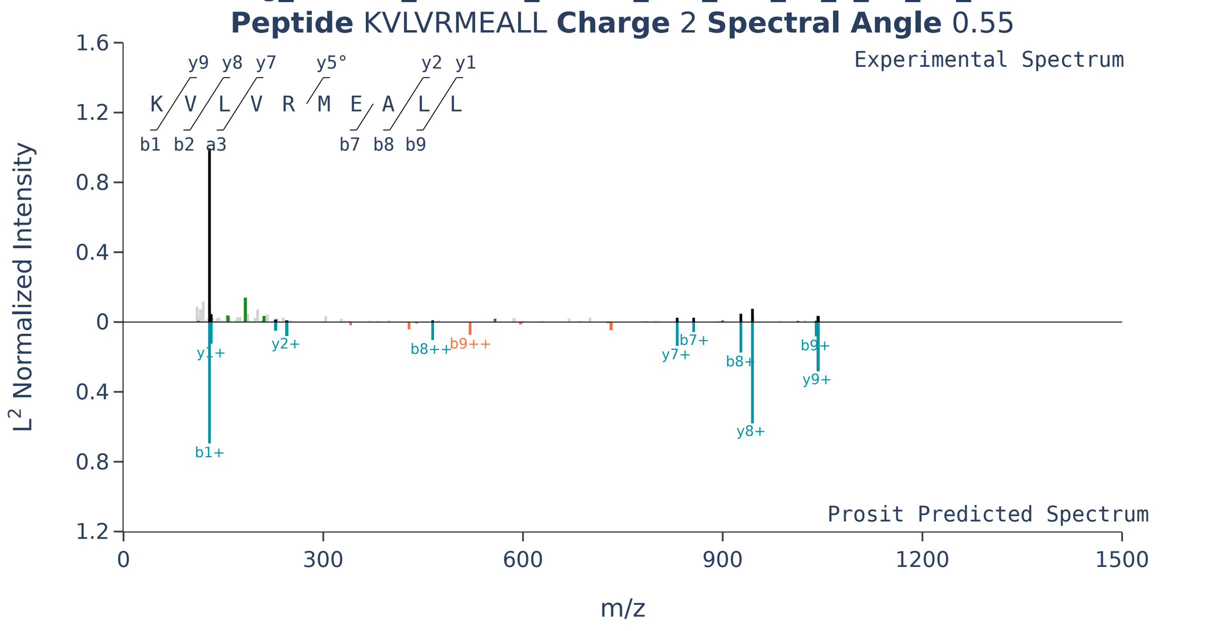


**Source** Ncheng_210623_230623_HFGoe_FFH_20S_25_1_A1_4h_R2 **Scan** 22234
**Peptide** VVLTKVDGDARGGAALSL **Charge** 3 **Spectral Angle** 0.79

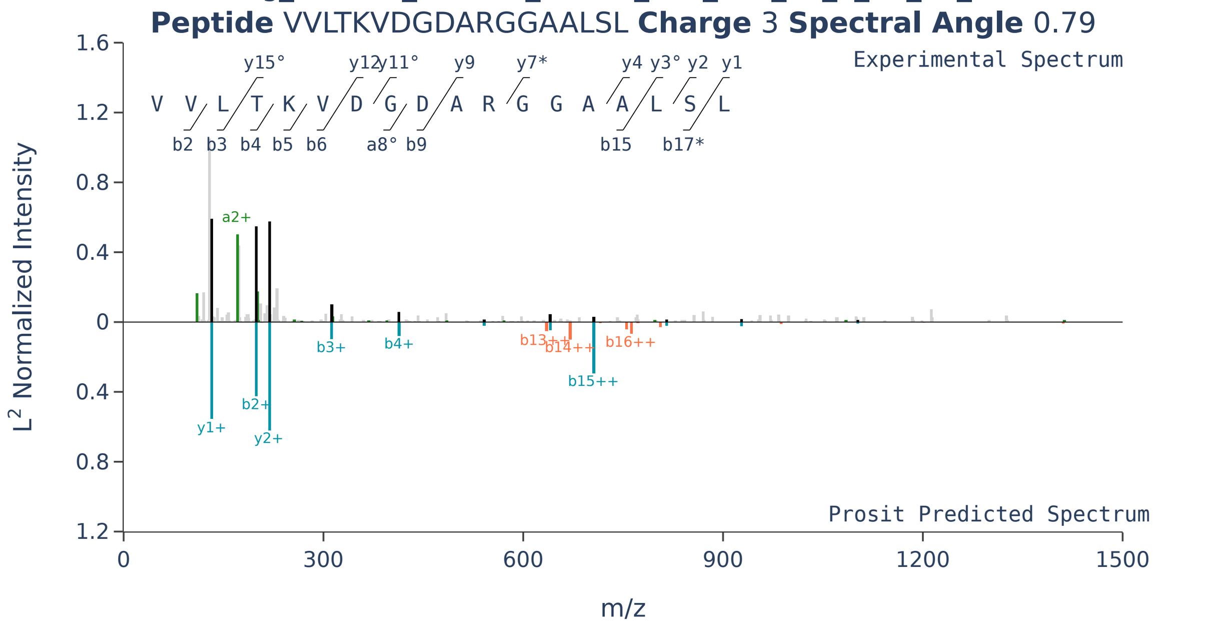


**Source** Ncheng_210623_230623_HFGoe_FFH_20S_25_1_A1_24h_R2 **Scan** 11207
**Peptide** VGKLGKFLREK **Charge** 3 **Spectral Angle** 0.67

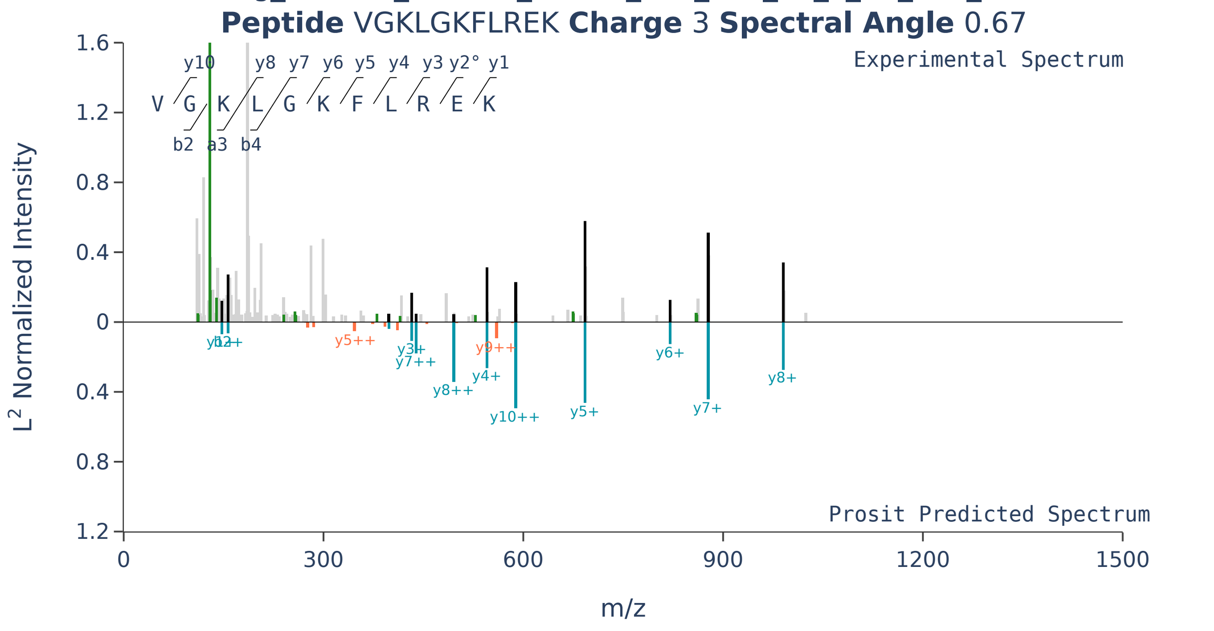


**Source** Ncheng_210623_230623_HFGoe_FFH_20S_25_1_A2_4h_R1 **Scan** 38551
**Peptide** KKGDGFDLNDFLEQL **Charge** 2 **Spectral Angle** 0.65

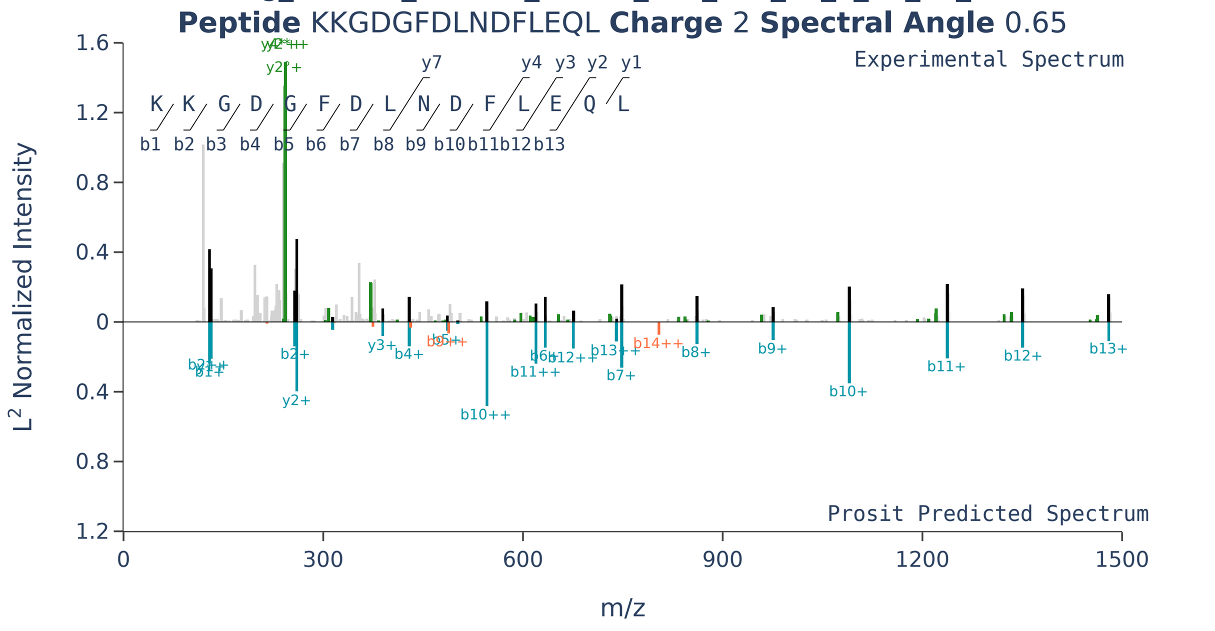


**Source** Ncheng_210623_230623_HFGoe_FFH_20S_25_1_A1_24h_R2 **Scan** 6309
**Peptide** QAEKLASKLKK **Charge** 2 **Spectral Angle** 0.67

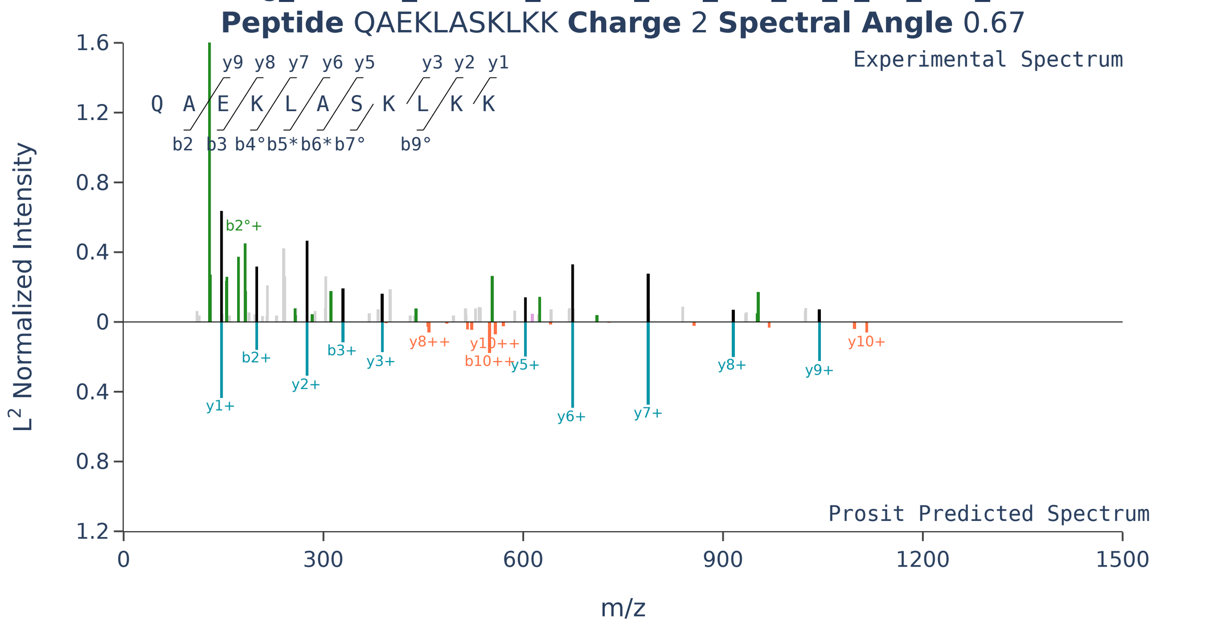


**Source** Ncheng_210623_230623_HFGoe_FFH_20S_25_1_A1_24h_R1 **Scan** 17906
**Peptide** TEDNVKDTLREVRMA **Charge** 3 **Spectral Angle** 0.57

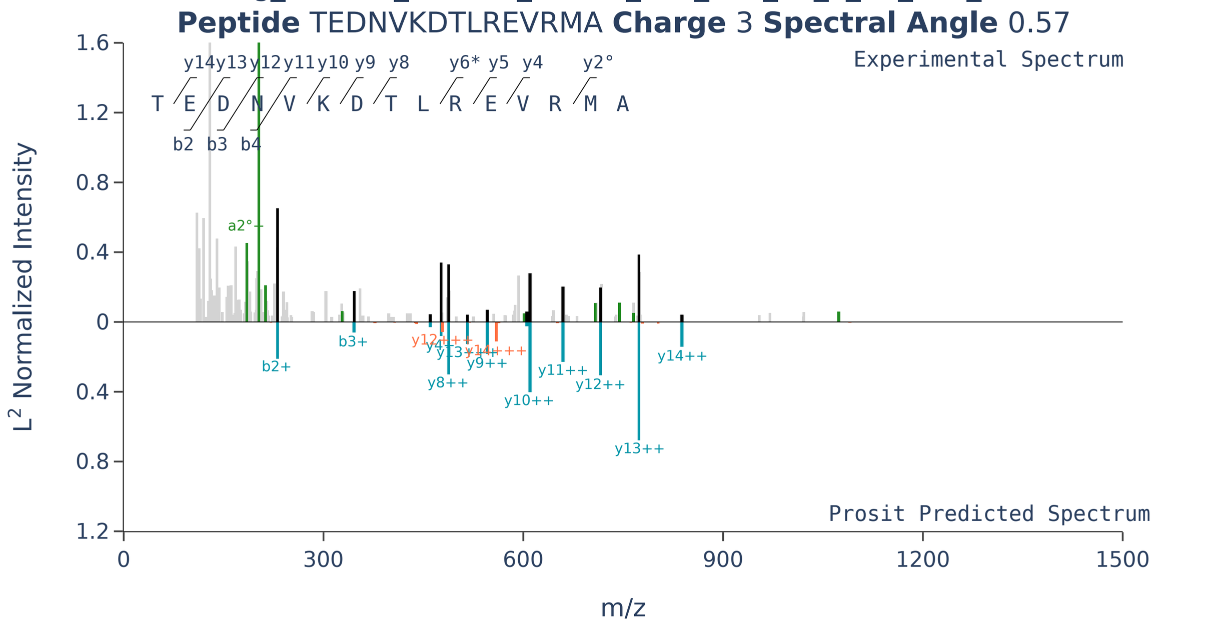

**Source** Ncheng_210623_230623_HFGoe_FFH_20S_25_1_A1_2h_R2 **Scan** 28415
**Peptide** DVYRPAAIKQLETLAEQ **Charge** 3 **Spectral Angle** 0.84

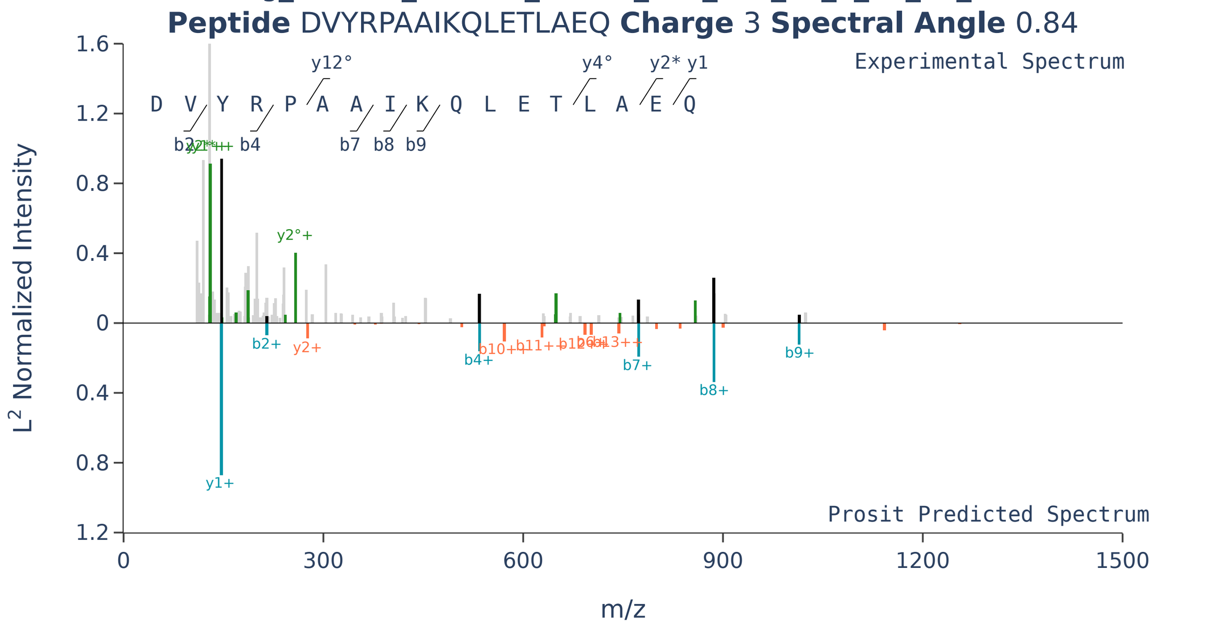


**Source** Ncheng_210623_230623_HFGoe_FFH_20S_25_1_A2_24h_R1 **Scan** 11451
**Peptide** EFINRVKEKAV **Charge** 2 **Spectral Angle** 0.51

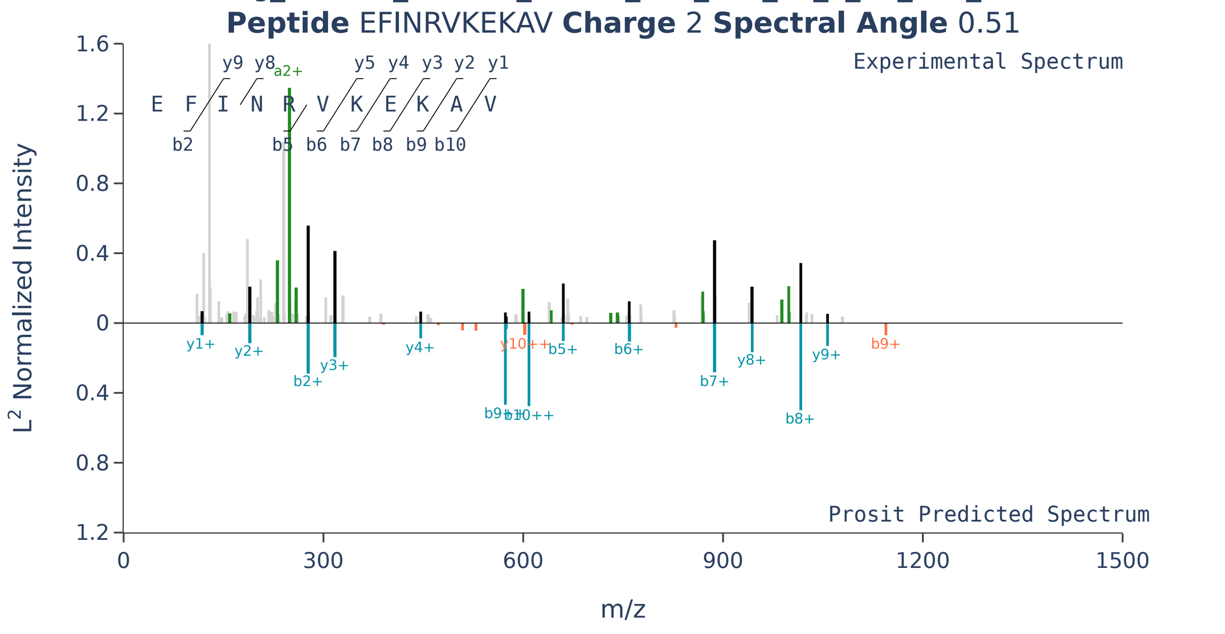


**Source** Ncheng_210623_230623_HFGoe_FFH_20S_25_1_A2_4h_R1 **Scan** 18413
**Peptide** TKVDGDARGGAALSIRHITGKPLKF **Charge** 6 **Spectral Angle** 0.78

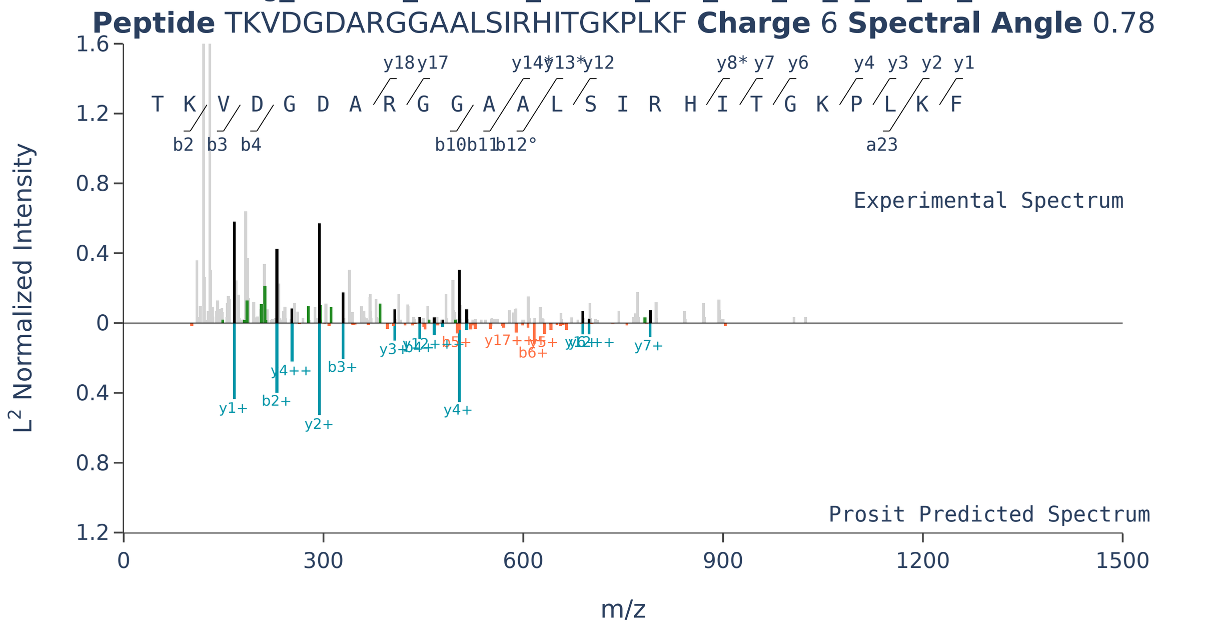


**Source** Ncheng_210623_230623_HFGoe_FFH_20S_25_1_A2_4h_R2 **Scan** 12843
**Peptide** EALINSMTMKERAKPEIIKGSRKR **Charge** 3 **Spectral Angle** 0.77

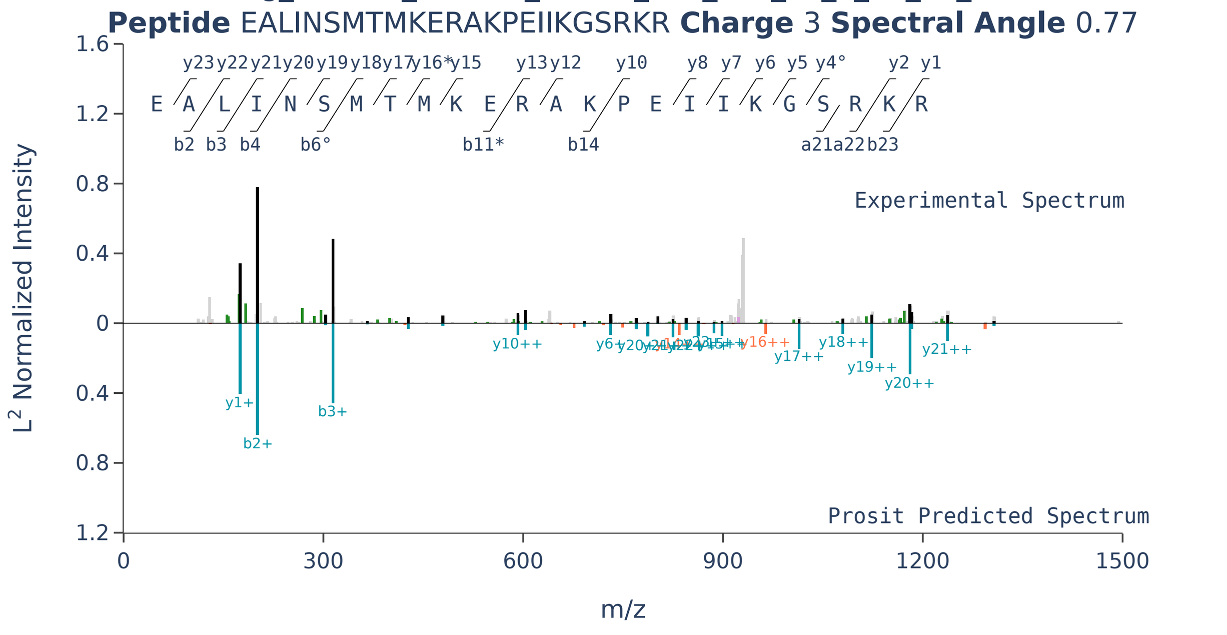


**Source** Ncheng_210623_230623_HFGoe_FFH_20S_25_1_A2_2h_R1 **Scan** 30934
**Peptide** AKLKFYDVLLVDT **Charge** 3 **Spectral Angle** 0.73

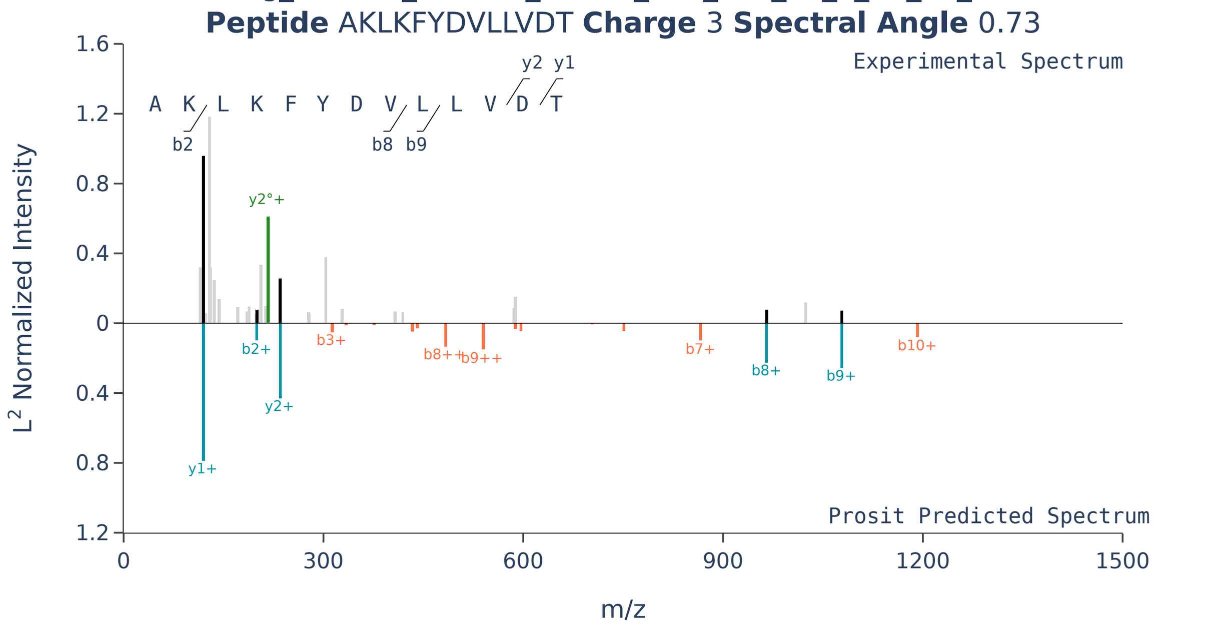


**Source** Ncheng_210623_230623_HFGoe_FFH_20S_25_1_A2_24h_R1 **Scan** 16049
**Peptide** VDGDARGGAALSIRHITG **Charge** 3 **Spectral Angle** 0.61

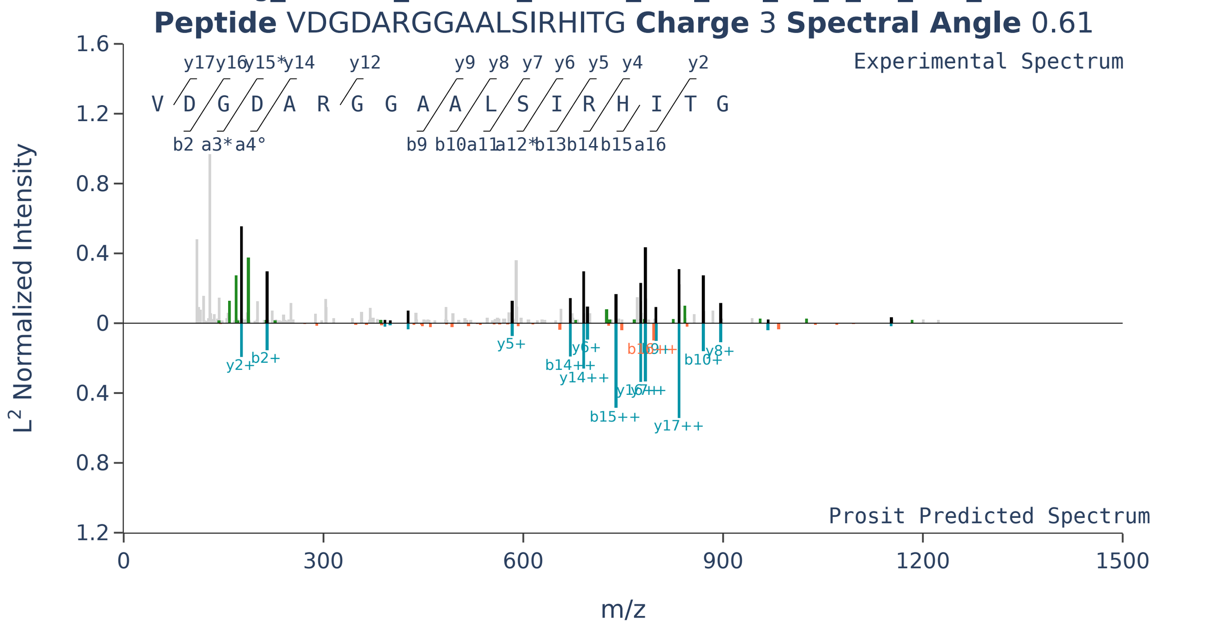


**Source** Ncheng_210623_230623_HFGoe_FFH_20S_25_1_A1_24h_R2 **Scan** 24205
**Peptide** NTAKAFNEALPLTG **Charge** 2 **Spectral Angle** 0.66

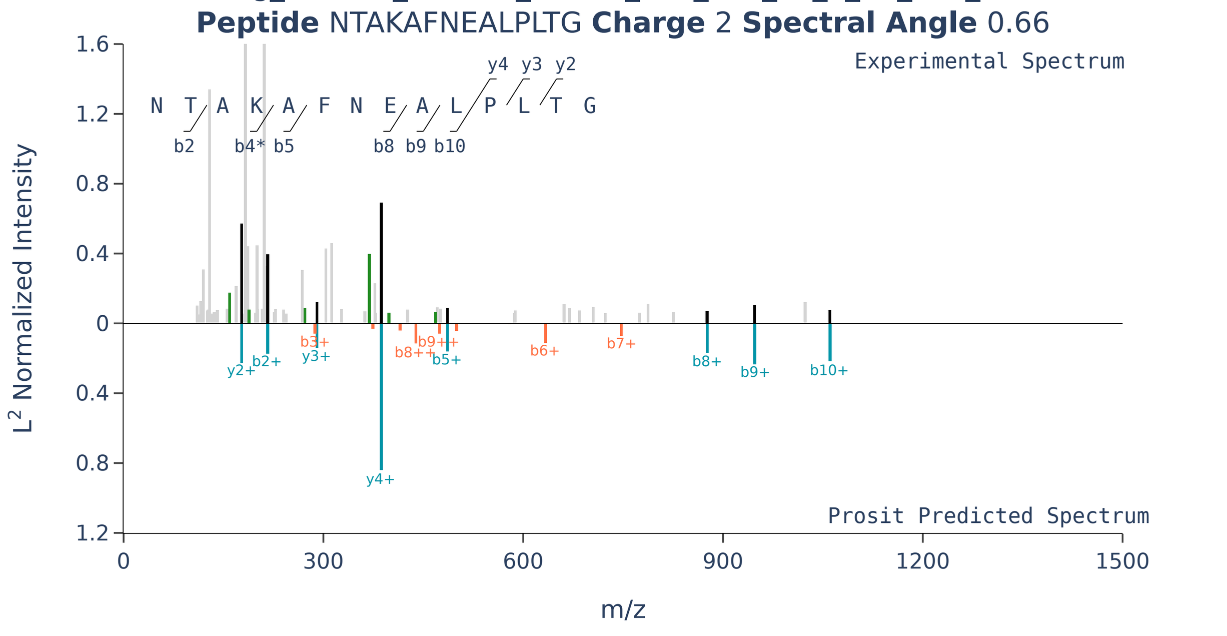


**Source** Ncheng_210623_230623_HFGoe_FFH_20S_25_1_A2_24h_R1 **Scan** 16944
**Peptide** RNELVAAMGEEN **Charge** 2 **Spectral Angle** 0.56

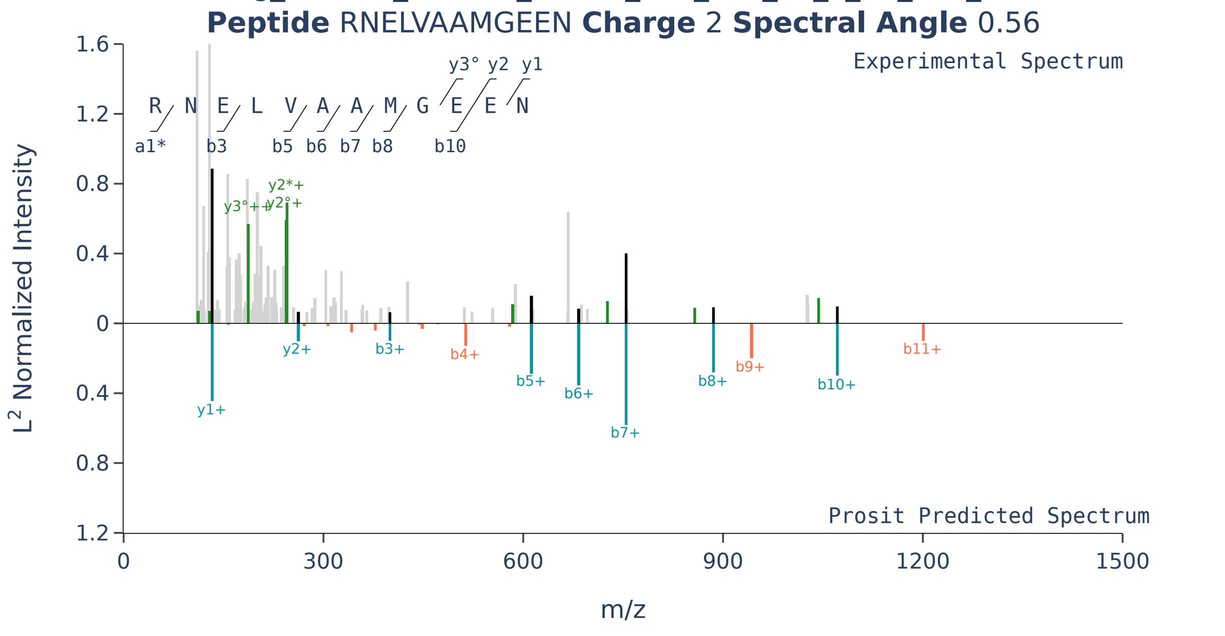


**Source** Ncheng_210623_230623_HFGoe_FFH_20S_25_1_A1_2h_R1 **Scan** 23329
**Peptide** KVLVVSADVYRPAAIKQLE **Charge** 3 **Spectral Angle** 0.88

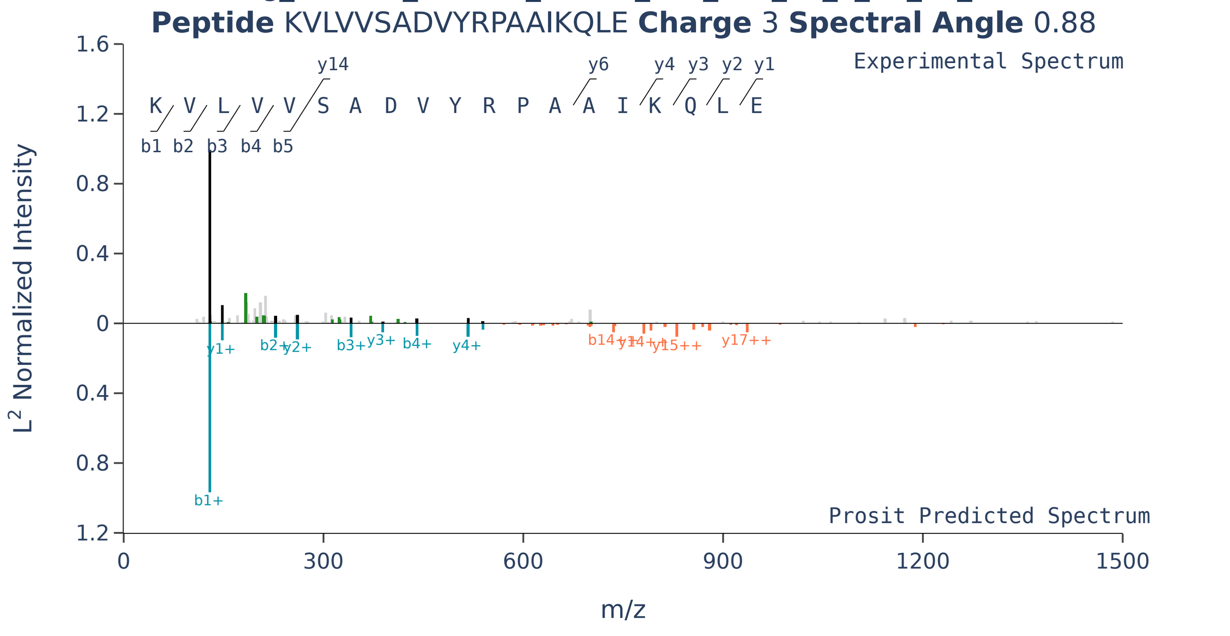


**Source** Ncheng_210623_230623_HFGoe_FFH_20S_25_1_A2_24h_R2 **Scan** 13897
**Peptide** EFINRVKEKAVGHEVNKSLTPGQE **Charge** 3 **Spectral Angle** 0.76

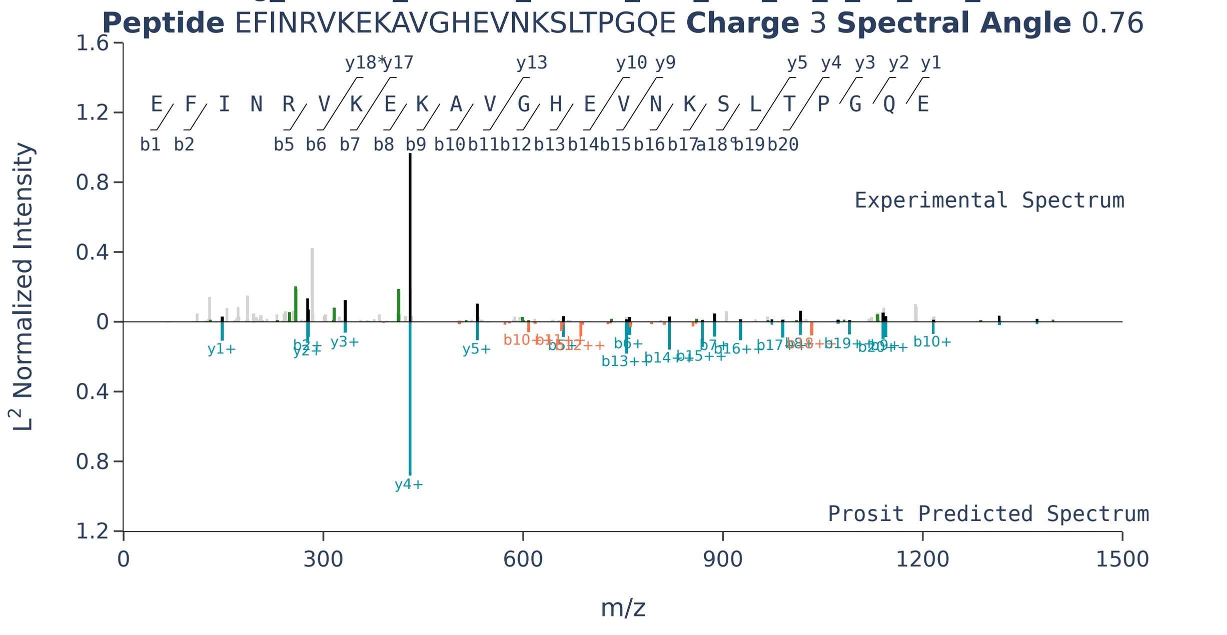


**Source** Ncheng_210623_230623_HFGoe_FFH_20S_25_1_A1_24h_R2 **Scan** 19347
**Peptide** TPGQEFVKIVR **Charge** 2 **Spectral Angle** 0.64

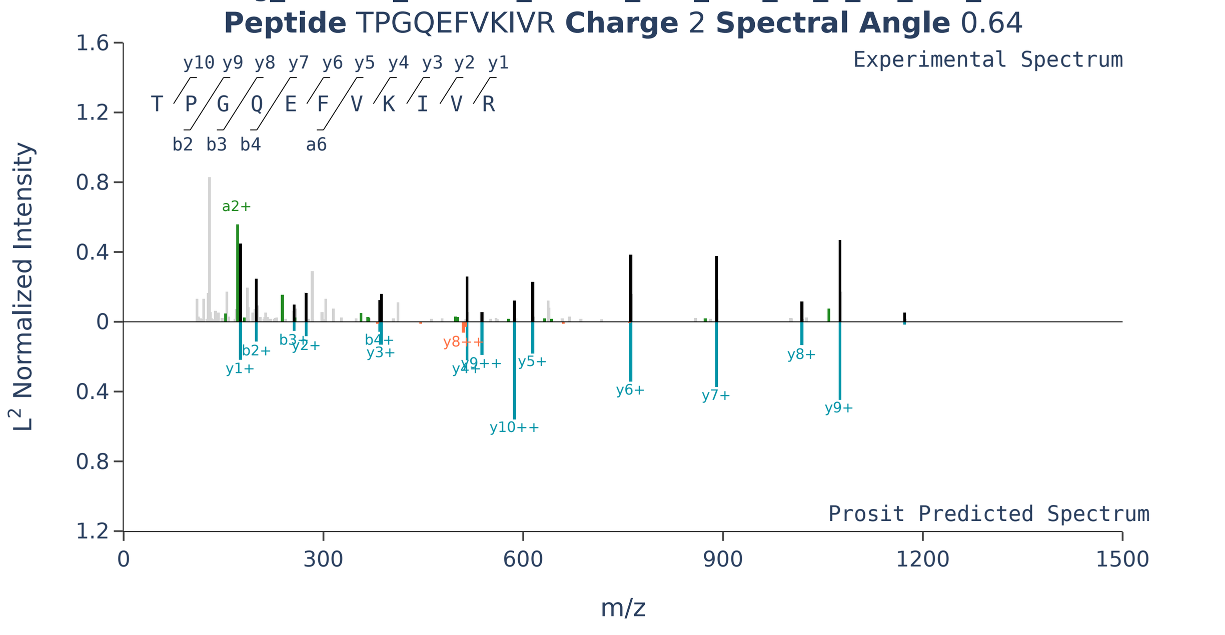


**Source** Ncheng_210623_230623_HFGoe_FFH_20S_25_1_A2_24h_R1 **Scan** 13057
**Peptide** EQLRQMKNMGGMA **Charge** 2 **Spectral Angle** 0.71

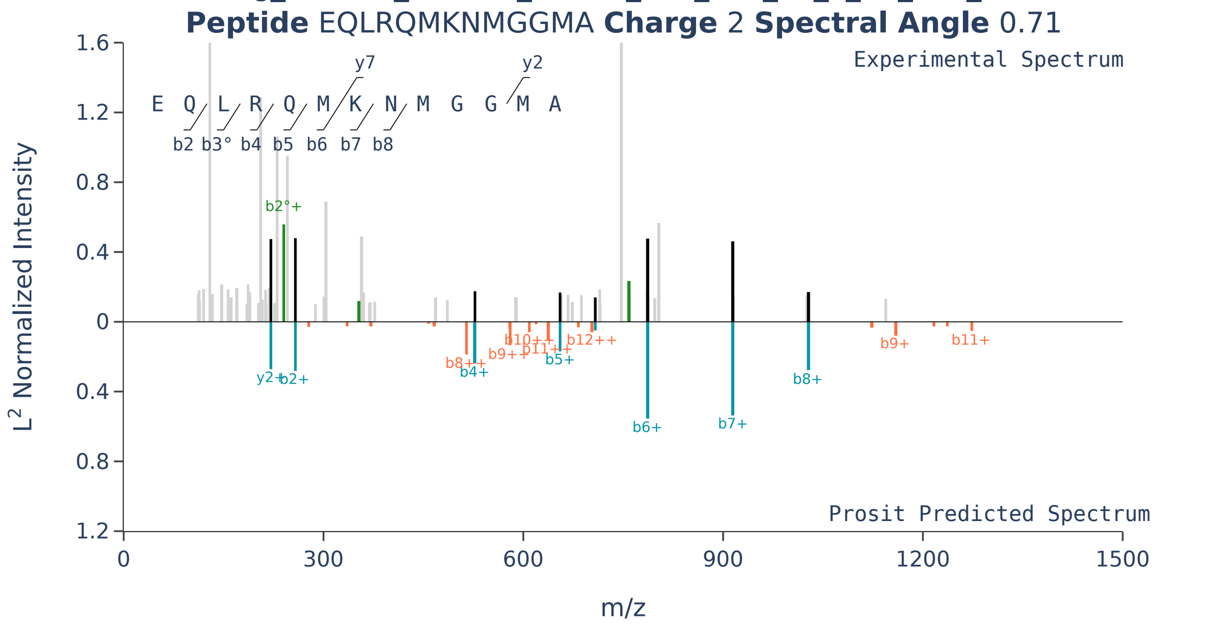


**Source** Ncheng_210623_230623_HFGoe_FFH_20S_25_1_A1_2h_R2 **Scan** 17107
**Peptide** QIPDNVKSQMDDKVLVR **Charge** 4 **Spectral Angle** 0.64

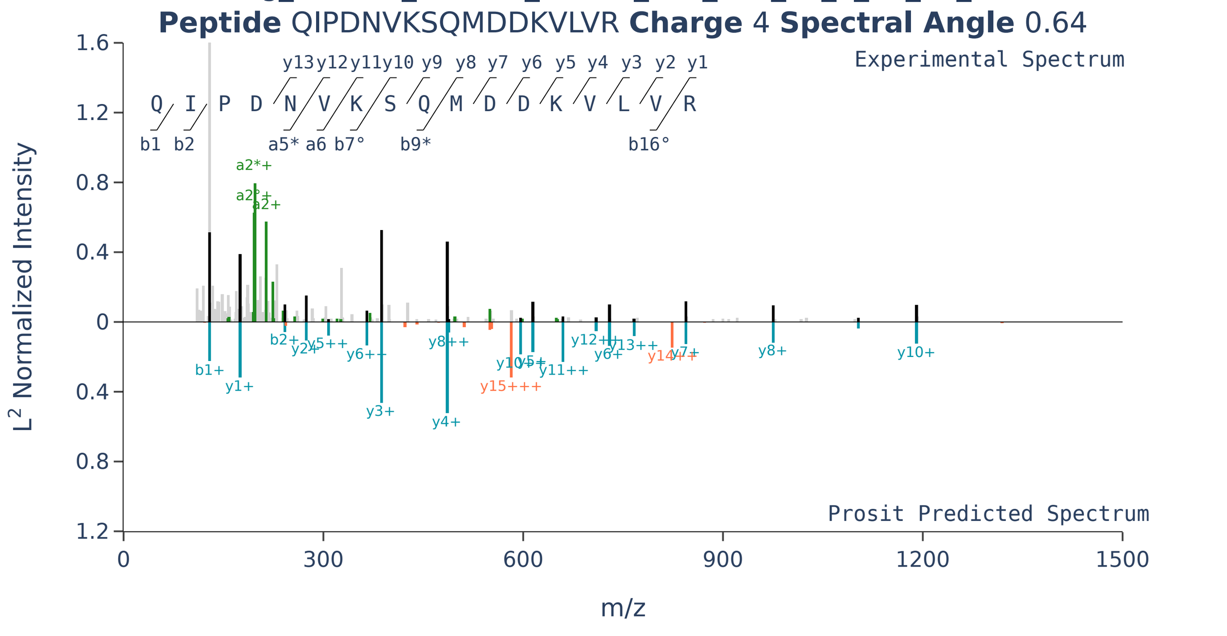


**Source** Ncheng_210623_230623_HFGoe_FFH_20S_25_1_A1_1h_R1 **Scan** 24466
**Peptide** KTEALEPFHPDRIASRILG **Charge** 3 **Spectral Angle** 0.59

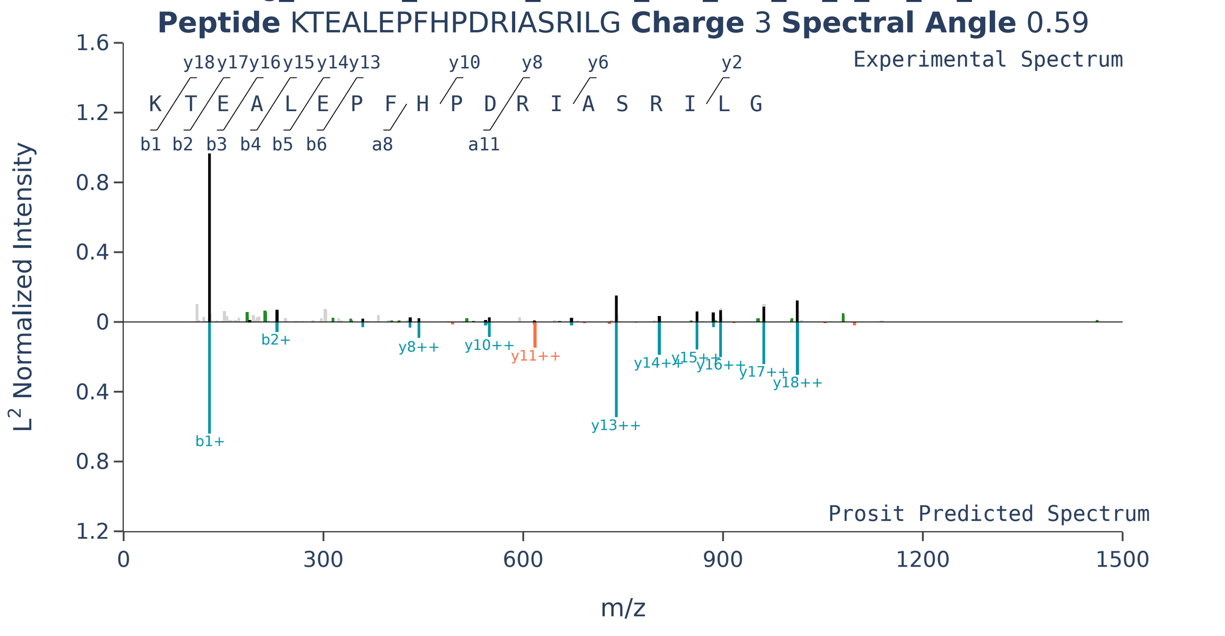


**Source** Ncheng_210623_230623_HFGoe_FFH_20S_25_1_A2_4h_R1 **Scan** 30804
**Peptide** KSLTPGQEFVKIVRNELV **Charge** 3 **Spectral Angle** 0.66

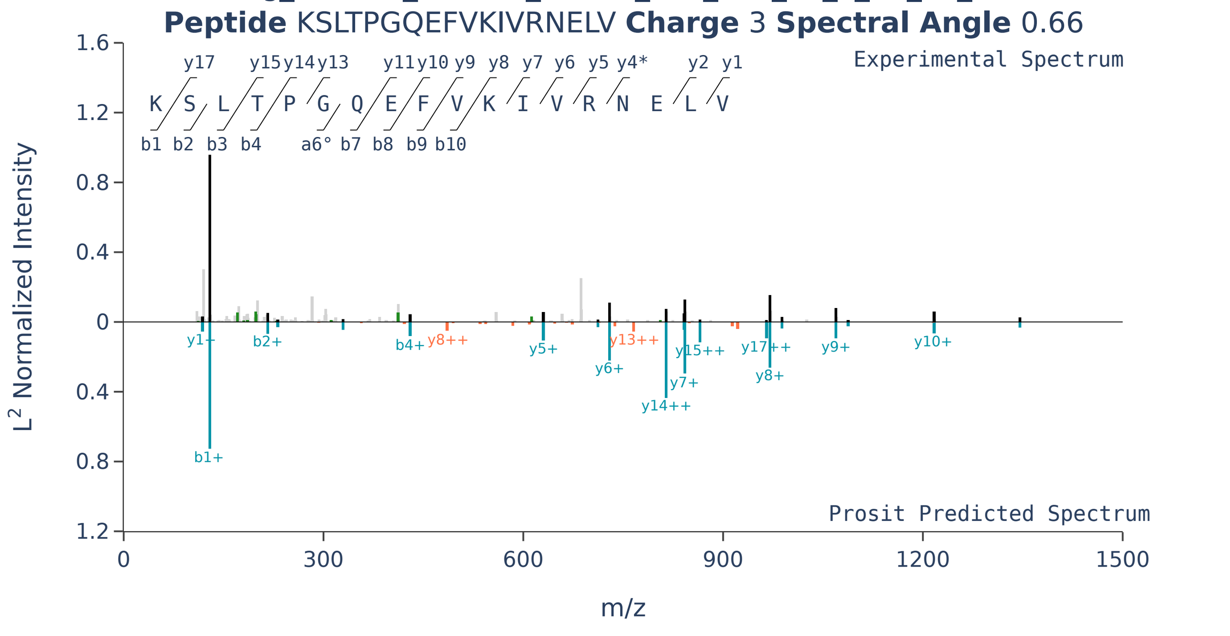

**Source** Ncheng_210623_230623_HFGoe_FFH_20S_25_1_A1_24h_R1 **Scan** 5842
**Peptide** EKHKKKVLV **Charge** 2 **Spectral Angle** 0.6

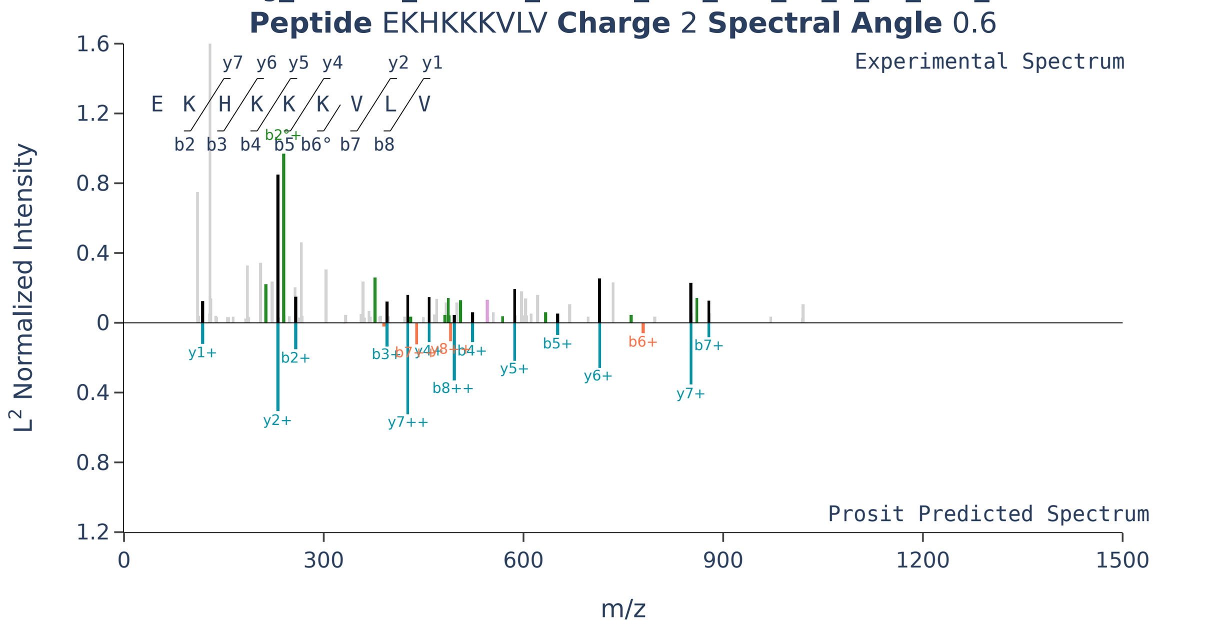


**Source** Ncheng_210623_230623_HFGoe_FFH_20S_25_1_A1_2h_R2 **Scan** 26091
**Peptide** DEAMMDEIKQVHASI **Charge** 2 **Spectral Angle** 0.89

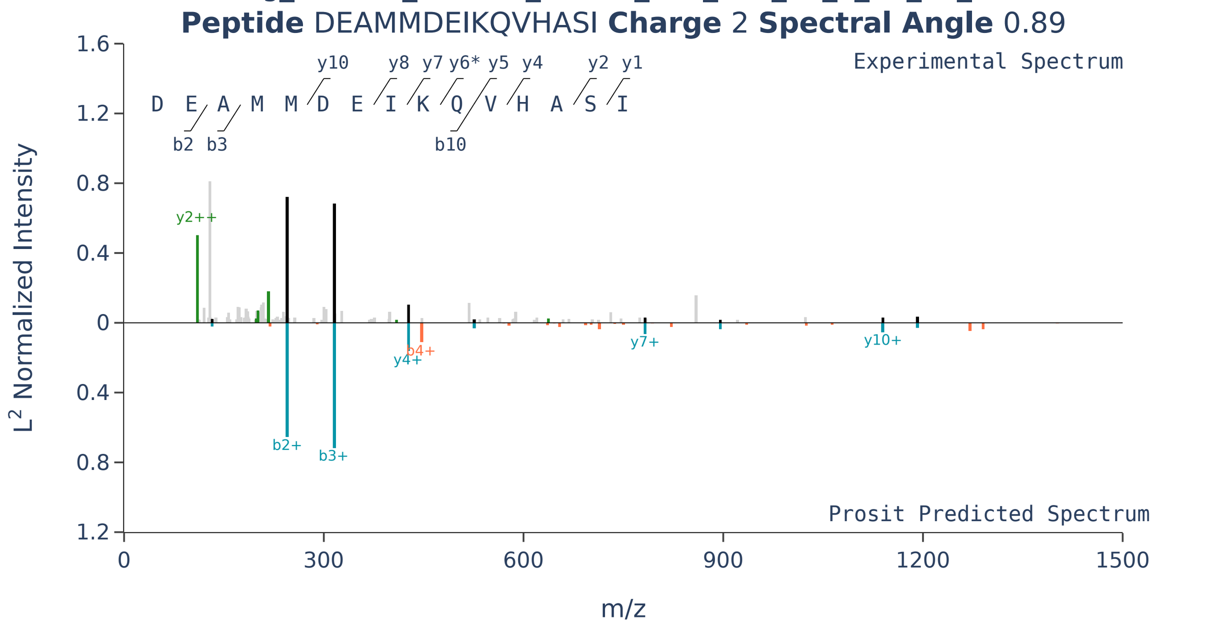


**Source** Ncheng_210623_230623_HFGoe_FFH_20S_25_1_A2_4h_R1 **Scan** 17930
**Peptide** RGRLTEDNVKDTLREVRMA **Charge** 5 **Spectral Angle** 0.81

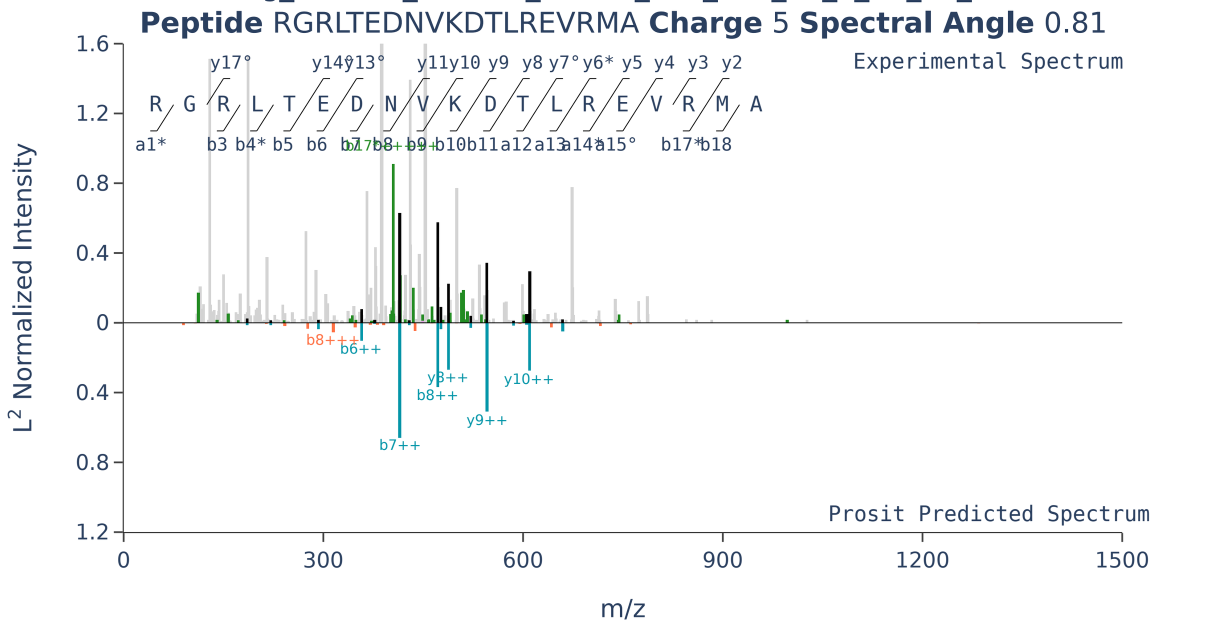


**Source** Ncheng_210623_230623_HFGoe_FFH_20S_25_1_A2_2h_R2 **Scan** 29614
**Peptide** YDVLLVDTAGRLHV **Charge** 3 **Spectral Angle** 0.77

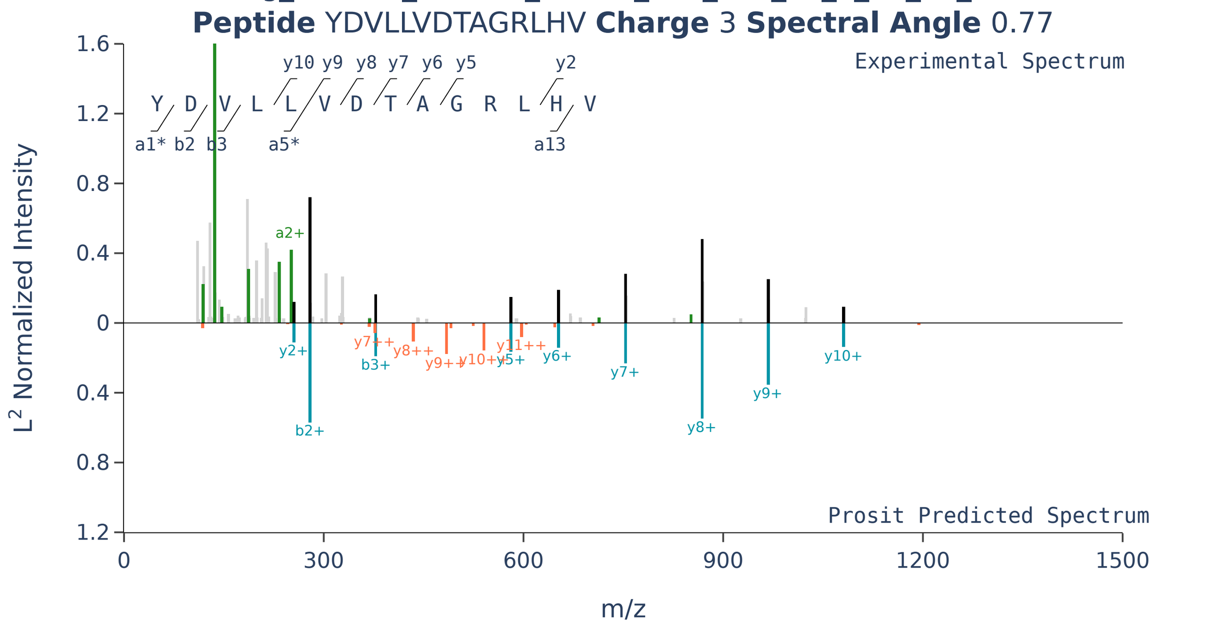


**Source** Ncheng_210623_230623_HFGoe_FFH_20S_25_1_A1_24h_R2 **Scan** 9252
**Peptide** DRAQAEKLASKLKK **Charge** 4 **Spectral Angle** 0.74

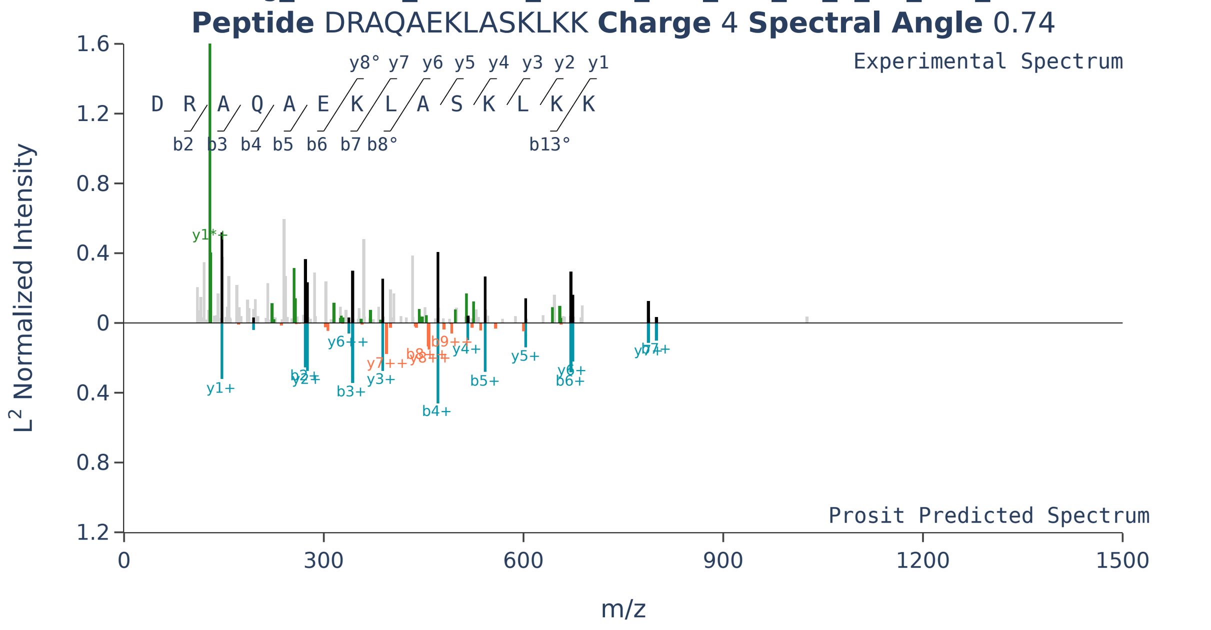


**Source** Ncheng_210623_230623_HFGoe_FFH_20S_25_1_A2_24h_R1 **Scan** 6702
**Peptide** KAVGHEVNKSL **Charge** 1 **Spectral Angle** 0.83

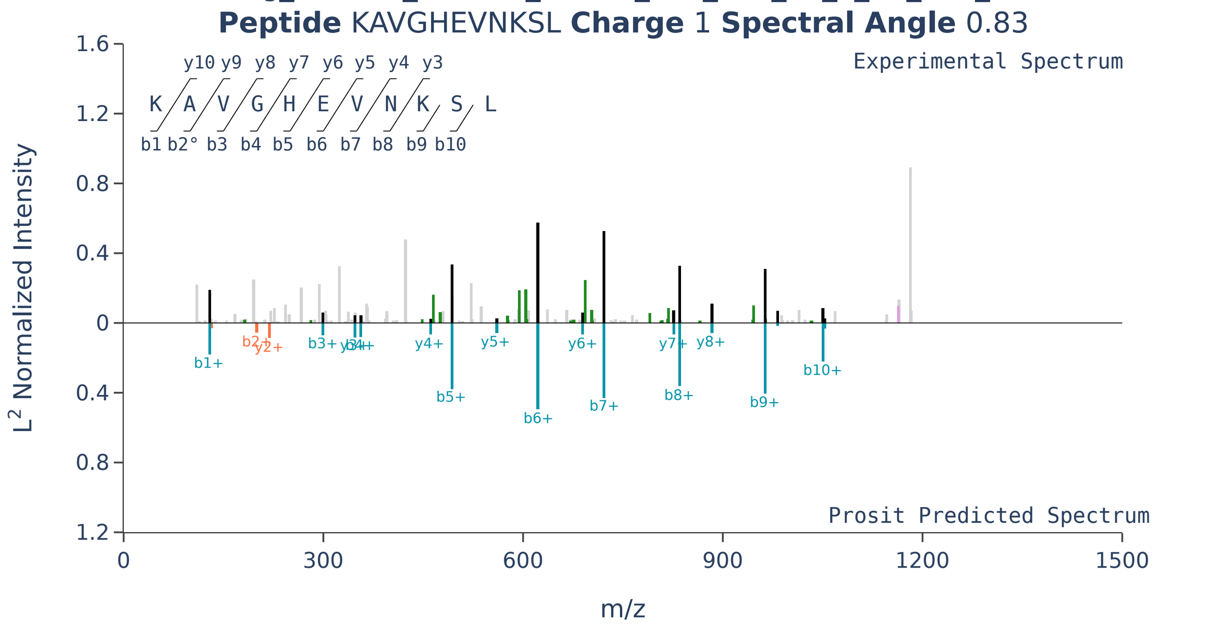


**Source** Ncheng_210623_230623_HFGoe_FFH_20S_25_1_A2_2h_R1 **Scan** 30325
**Peptide** ALKEAKLKFYDVLLVDTAGRLHVDE **Charge** 3 **Spectral Angle** 0.79

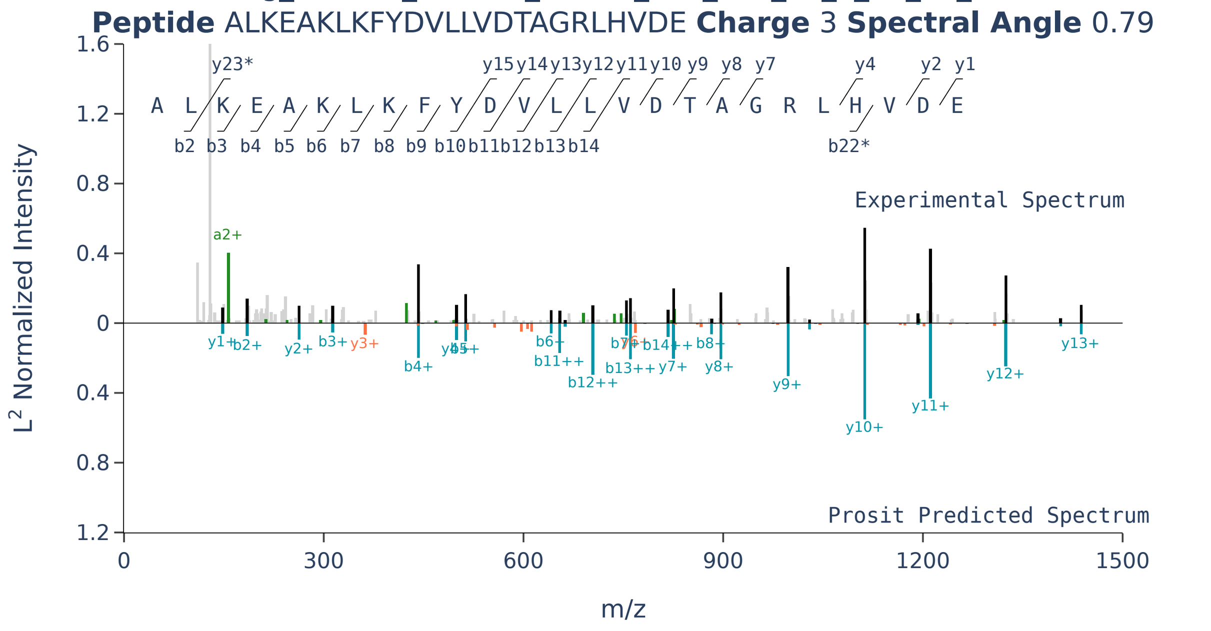


**Source** Ncheng_210623_230623_HFGoe_FFH_20S_25_1_A1_4h_R1 **Scan** 11102
**Peptide** DMQRMMKKMKKGGMAKM **Charge** 5 **Spectral Angle** 0.87

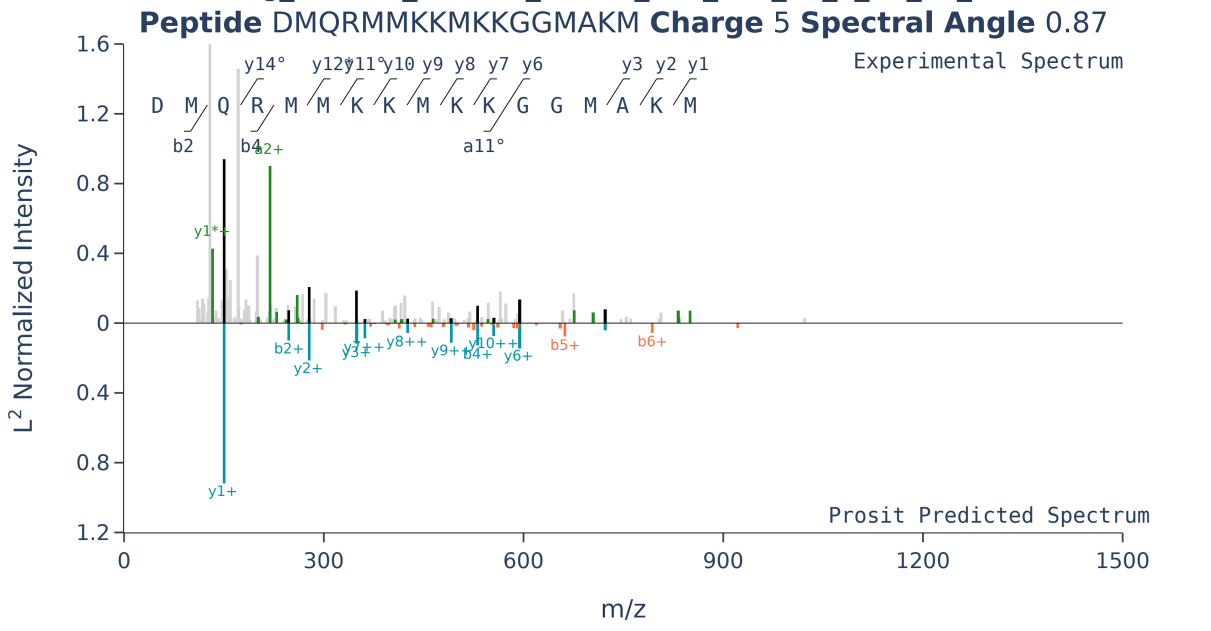


**Source** Ncheng_210623_230623_HFGoe_FFH_20S_25_1_A1_4h_R1 **Scan** 21843
**Peptide** DGDARGGAALSIRHITGKPIKFLGVGEKT **Charge** 4 **Spectral Angle** 0.67

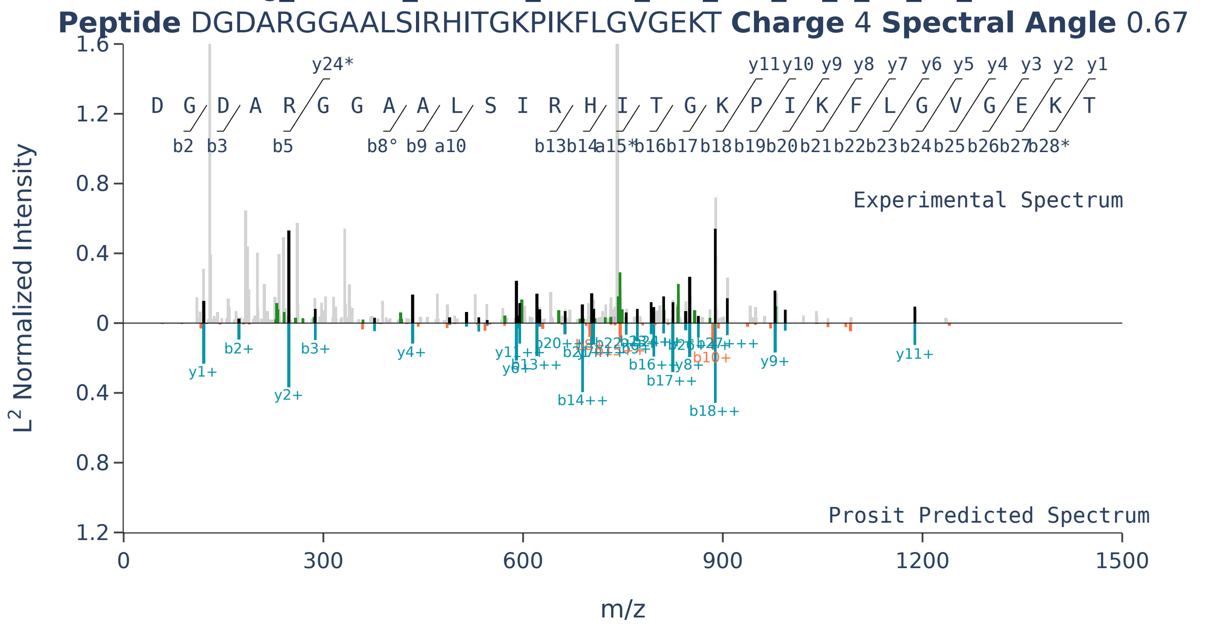


**Source** Ncheng_210623_230623_HFGoe_FFH_20S_25_1_A2_4h_R2 **Scan** 17336
**Peptide** DNVKDTLREVRMA **Charge** 4 **Spectral Angle** 0.67

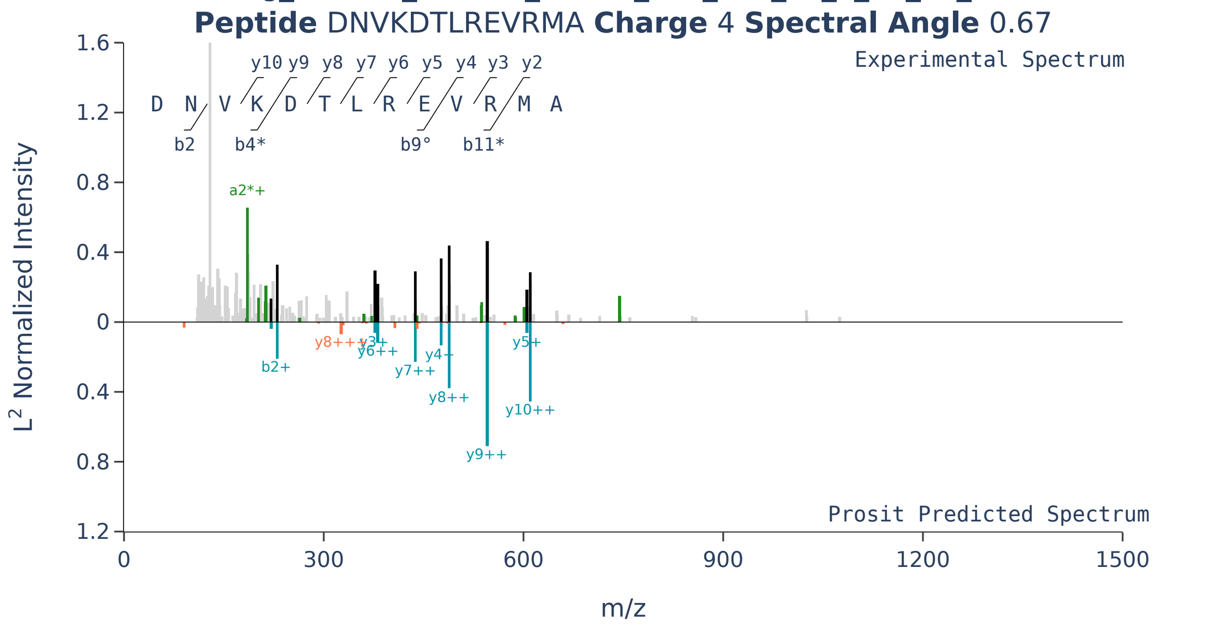


**Source** Ncheng_210623_230623_HFGoe_FFH_20S_25_1_A1_24h_R1 **Scan** 12069
**Peptide** KLASKLKKGDGFDLND **Charge** 2 **Spectral Angle** 0.72

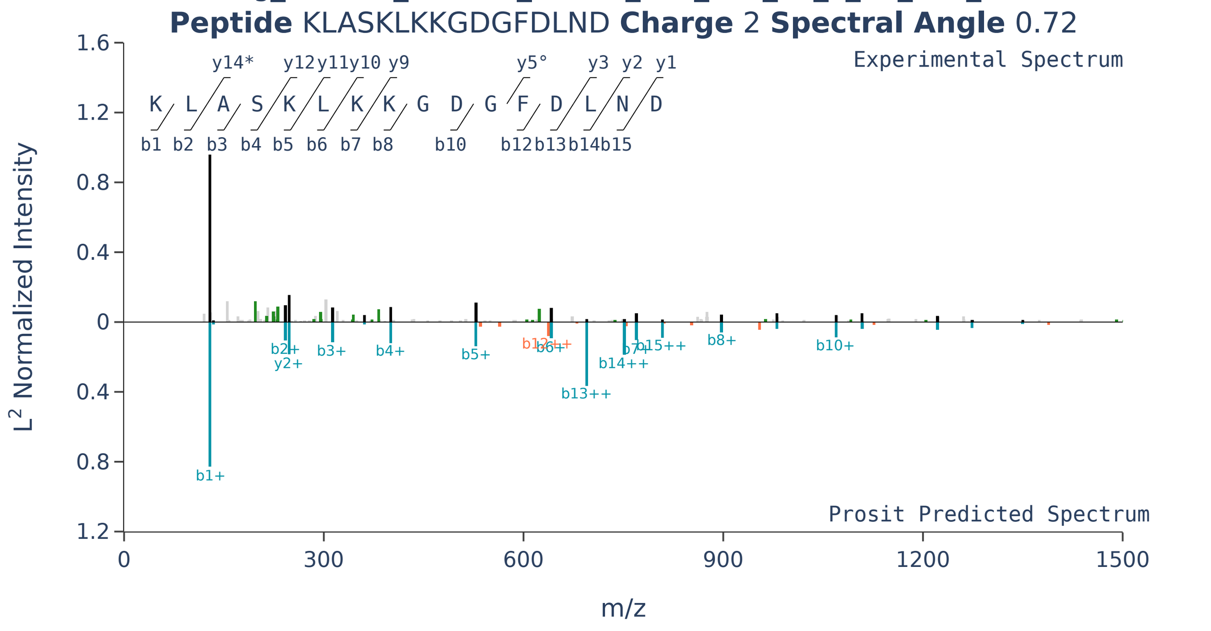


**Source** Ncheng_210623_230623_HFGoe_FFH_20S_25_1_A1_2h_R2 **Scan** 28606
**Peptide** DDKVLVRMEAILN **Charge** 2 **Spectral Angle** 0.66

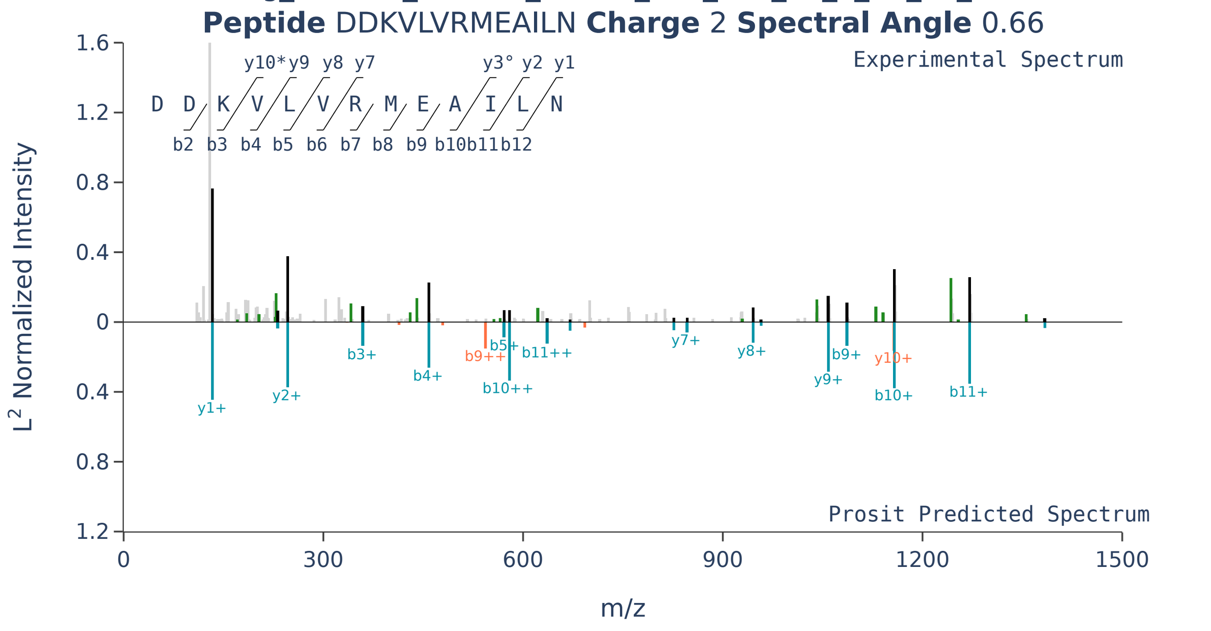


**Source** Ncheng_210623_230623_HFGoe_FFH_20S_25_1_A2_24h_R2 **Scan** 17684
**Peptide** NLSGRGRLTEDNVKDTLREV **Charge** 2 **Spectral Angle** 0.8

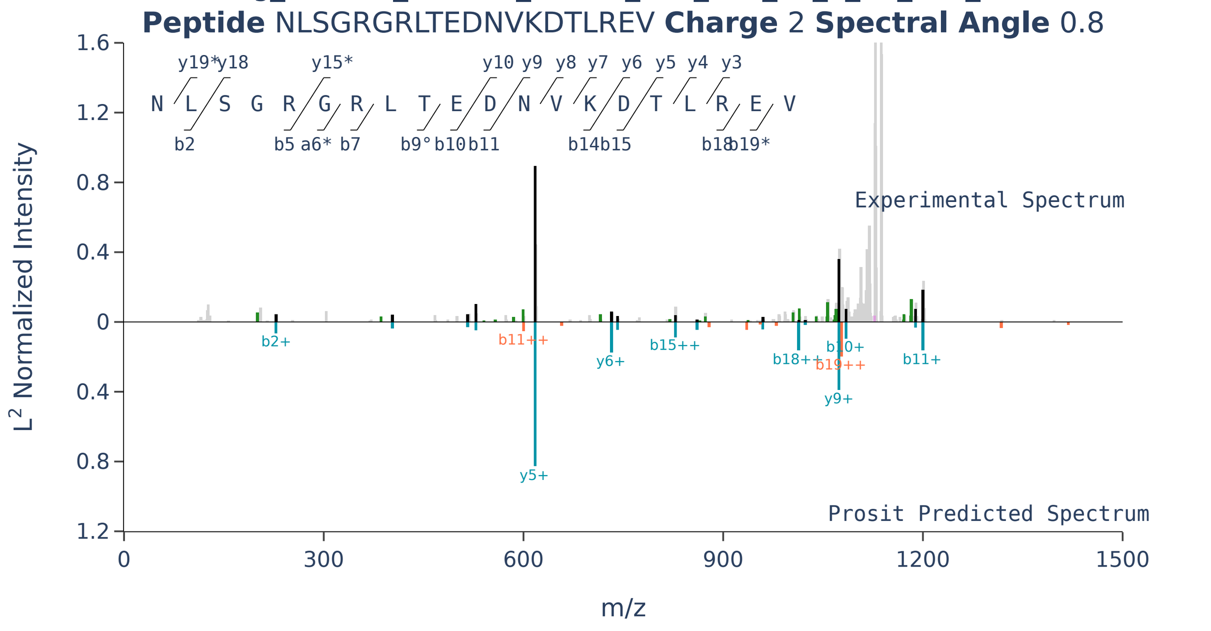


**Source** Ncheng_210623_230623_HFGoe_FFH_20S_25_1_A1_2h_R2 **Scan** 27237
**Peptide** LGVGEKTEALEPFHPDRIASRLL **Charge** 4 **Spectral Angle** 0.72

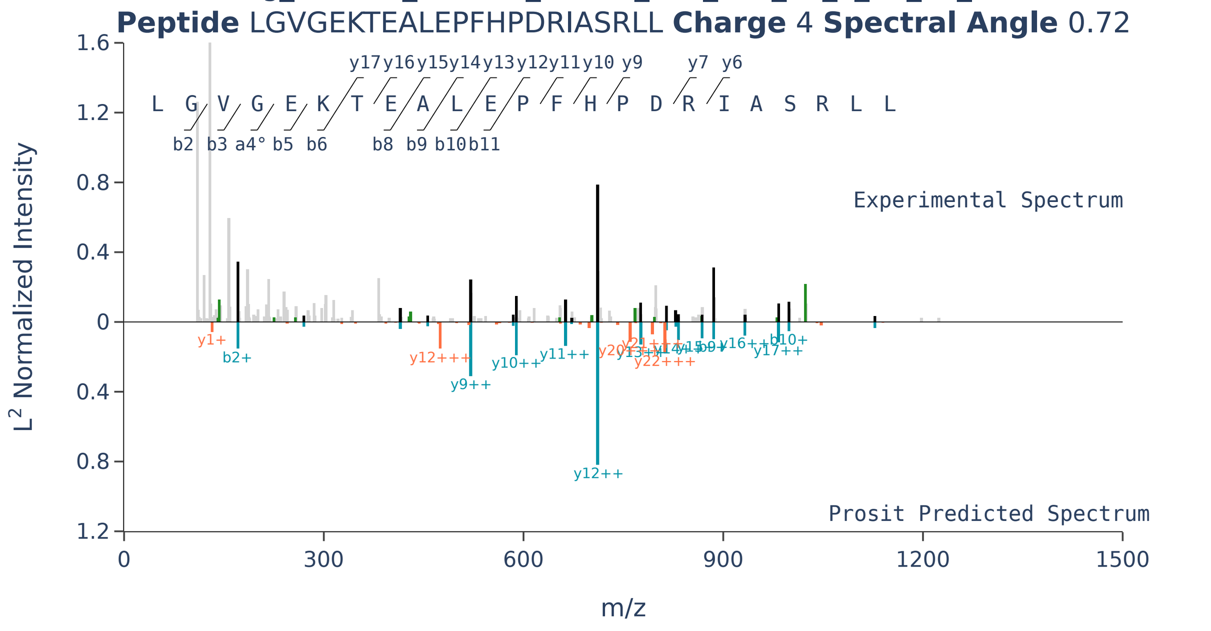


**Source** Ncheng_210623_230623_HFGoe_FFH_20S_25_1_A1_4h_R1 **Scan** 25157
**Peptide** KEKAVGHEVNKSLTPGQEFVKIVRNEL **Charge** 4 **Spectral Angle** 0.73

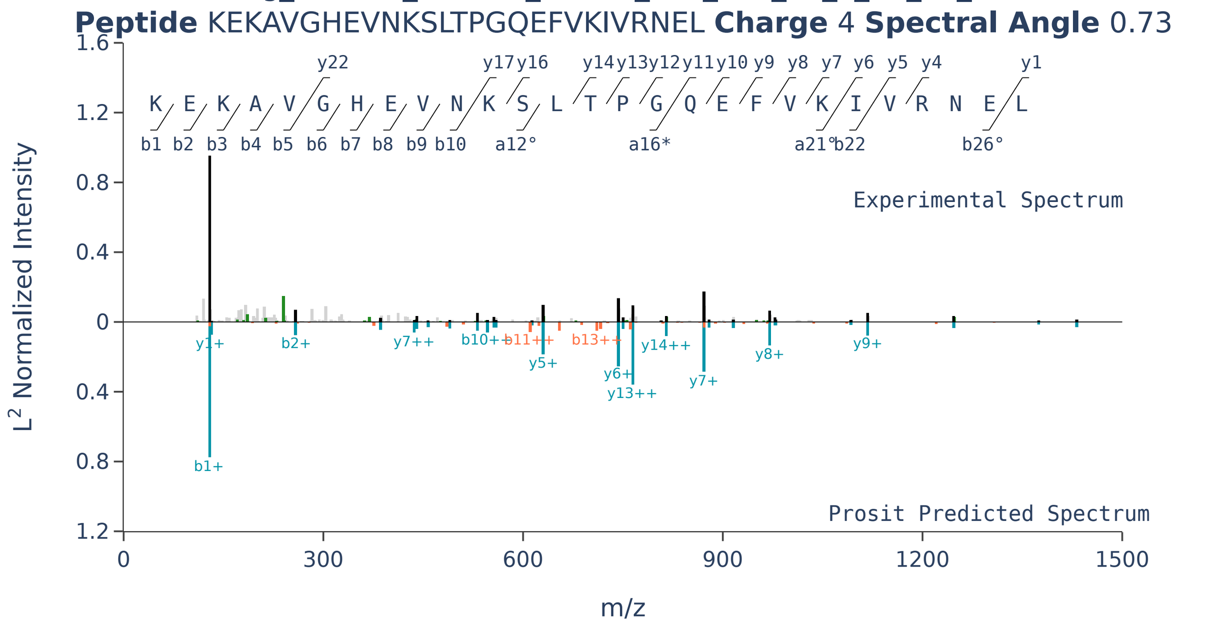

**Source** Ncheng_210623_230623_HFGoe_FFH_20S_25_1_A1_24h_R2 **Scan** 10794
**Peptide** QDVNRLLK **Charge** 2 **Spectral Angle** 0.65

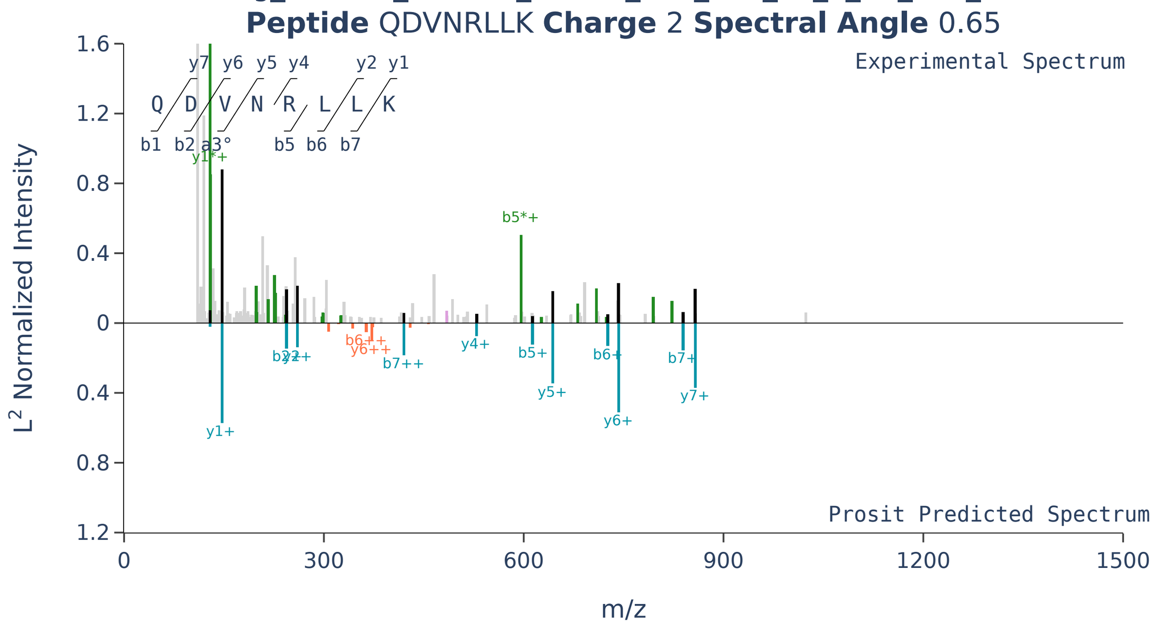


**Source** Ncheng_210623_230623_HFGoe_FFH_20S_25_1_A1_2h_R2 **Scan** 29485
**Peptide** GEKTEALEPFHPDRIASRILGM **Charge** 3 **Spectral Angle** 0.67

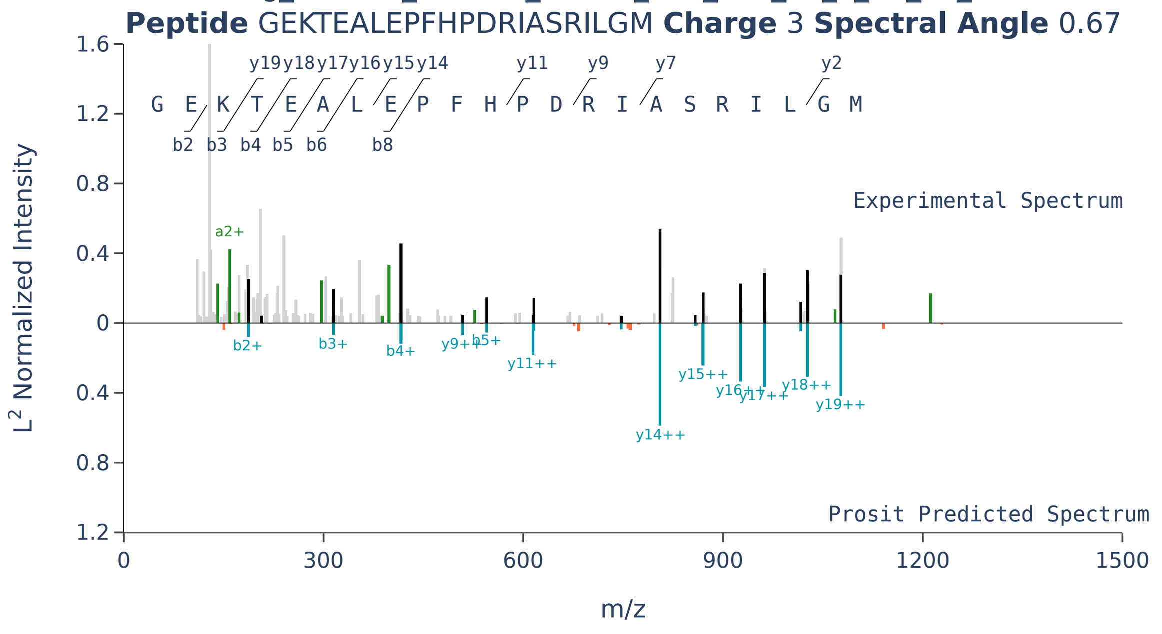


**Source** Ncheng_210623_230623_HFGoe_FFH_20S_25_1_A1_24h_R1 **Scan** 7076
**Peptide** RAKPEIIKG **Charge** 2 **Spectral Angle** 0.65

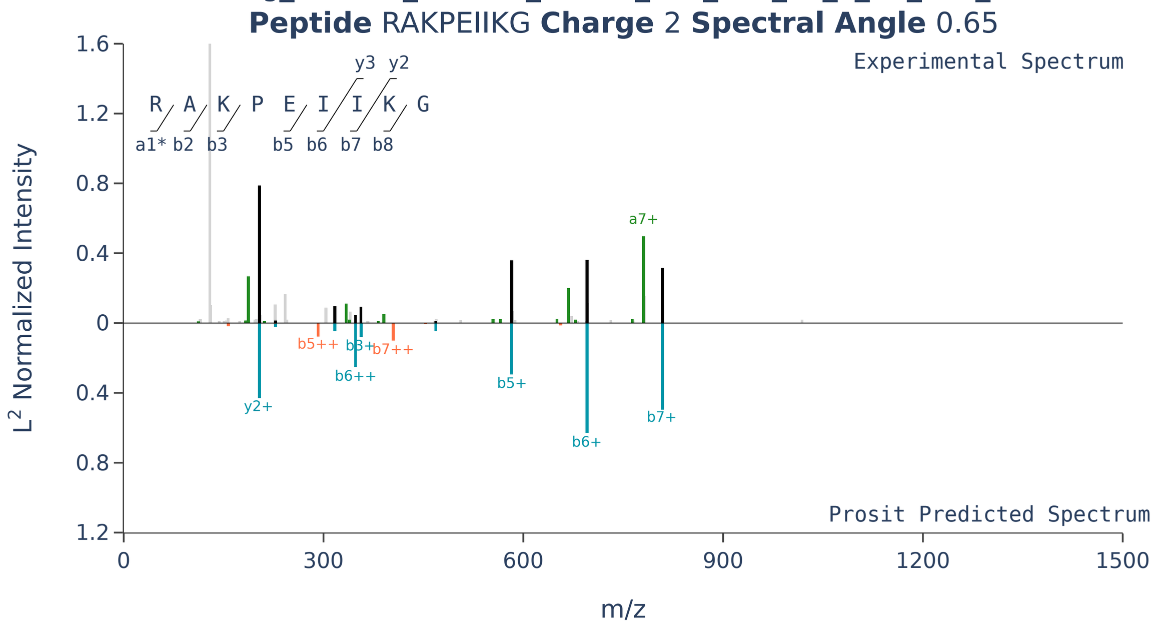


**Source** Ncheng_210623_230623_HFGoe_FFH_20S_25_1_A1_2h_R2 **Scan** 29937
**Peptide** GMASLMGKLPGMGQIPDNVKSQMDDKVLVR **Charge** 5 **Spectral Angle** 0.64

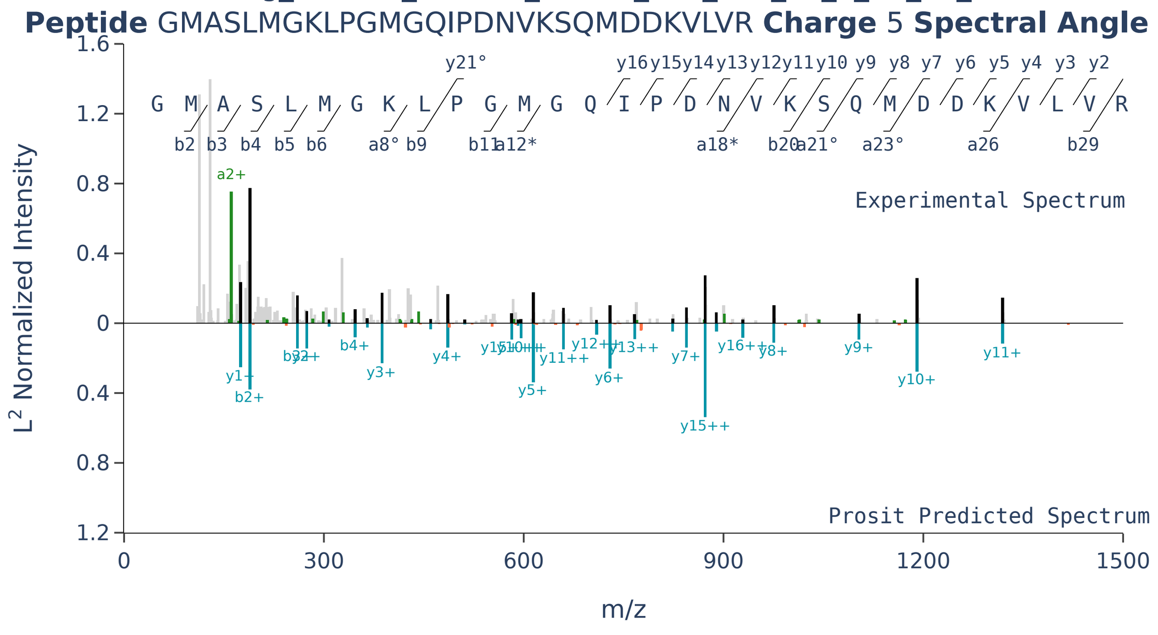


**Source** Ncheng_210623_230623_HFGoe_FFH_20S_25_1_A2_4h_R1 **Scan** 14378
**Peptide** PVVREFINRVKEK **Charge** 3 **Spectral Angle** 0.63

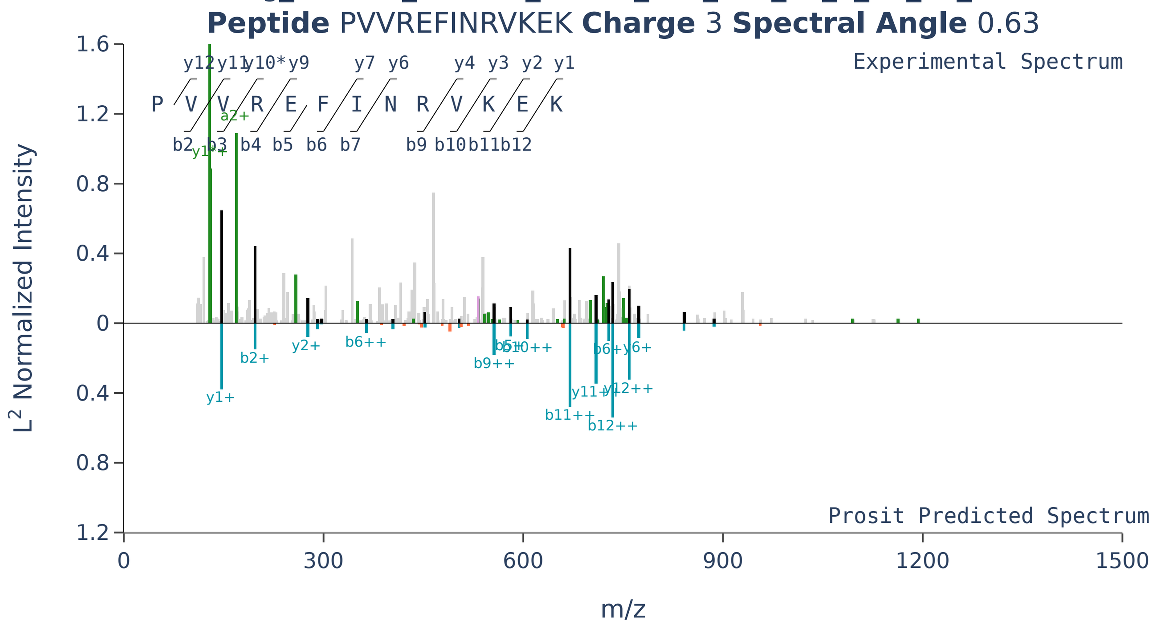


**Source** Ncheng_210623_230623_HFGoe_FFH_20S_25_1_A1_2h_R2 **Scan** 24070
**Peptide** PIKFLGVGEKTEAL **Charge** 3 **Spectral Angle** 0.76

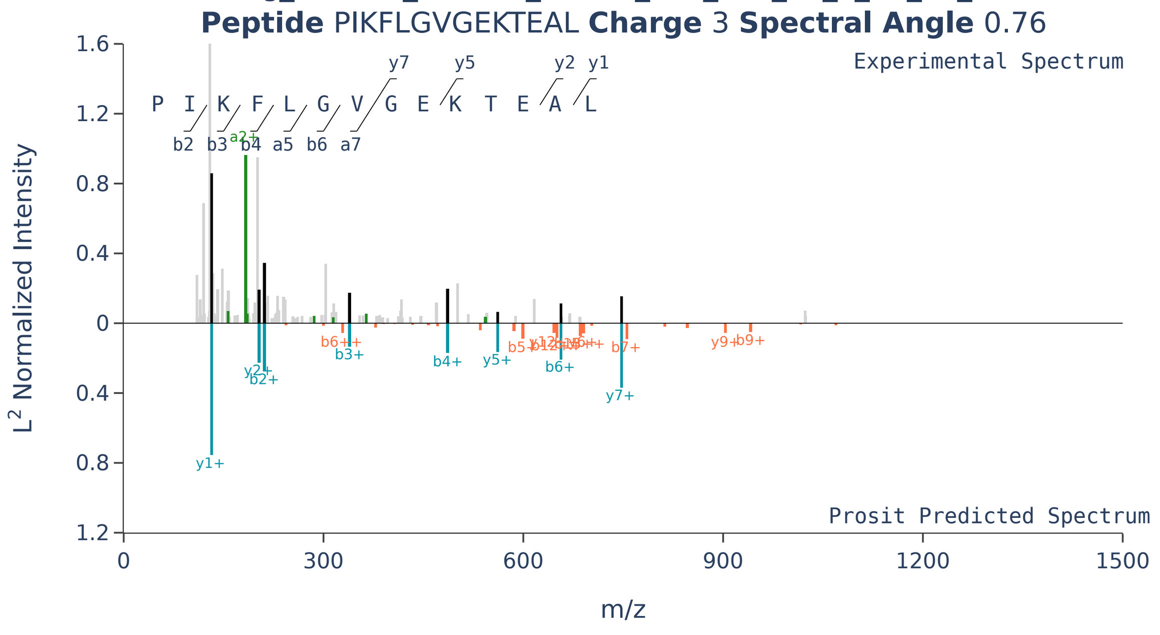


**Source** Ncheng_210623_230623_HFGoe_FFH_20S_25_1_A1_24h_R2 **Scan** 16072
**Peptide** GLQGAGKTTSVGKLGKFLREK **Charge** 3 **Spectral Angle** 0.65

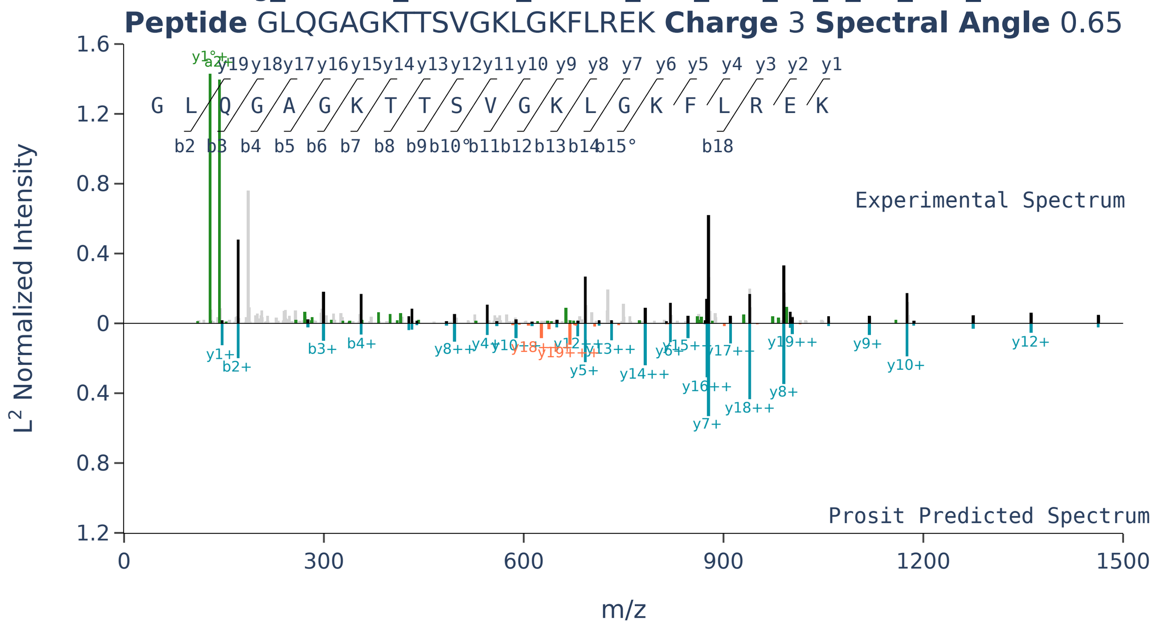


**Source** Ncheng_210623_230623_HFGoe_FFH_20S_25_1_A2_4h_R1 **Scan** 25688
**Peptide** GKLPGMGQIPDNVKSQMDDKVLVRME **Charge** 3 **Spectral Angle** 0.68

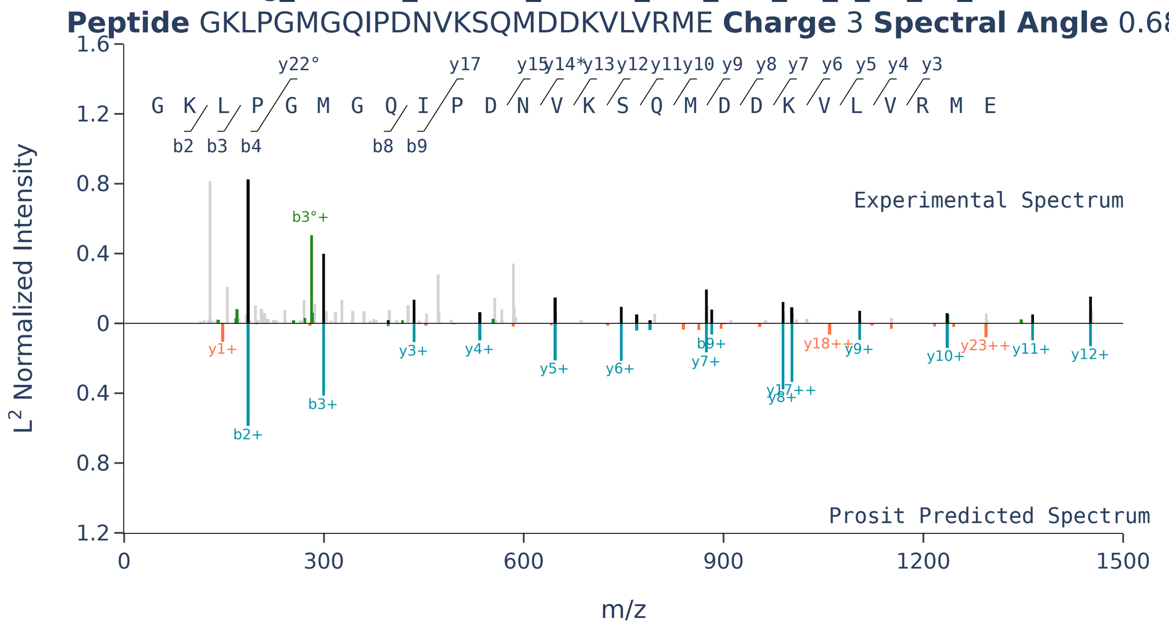


**Source** Ncheng_210623_230623_HFGoe_FFH_20S_25_1_A2_24h_R2 **Scan** 13015
**Peptide** DIESKVDRAQAEKLAS **Charge** 2 **Spectral Angle** 0.65

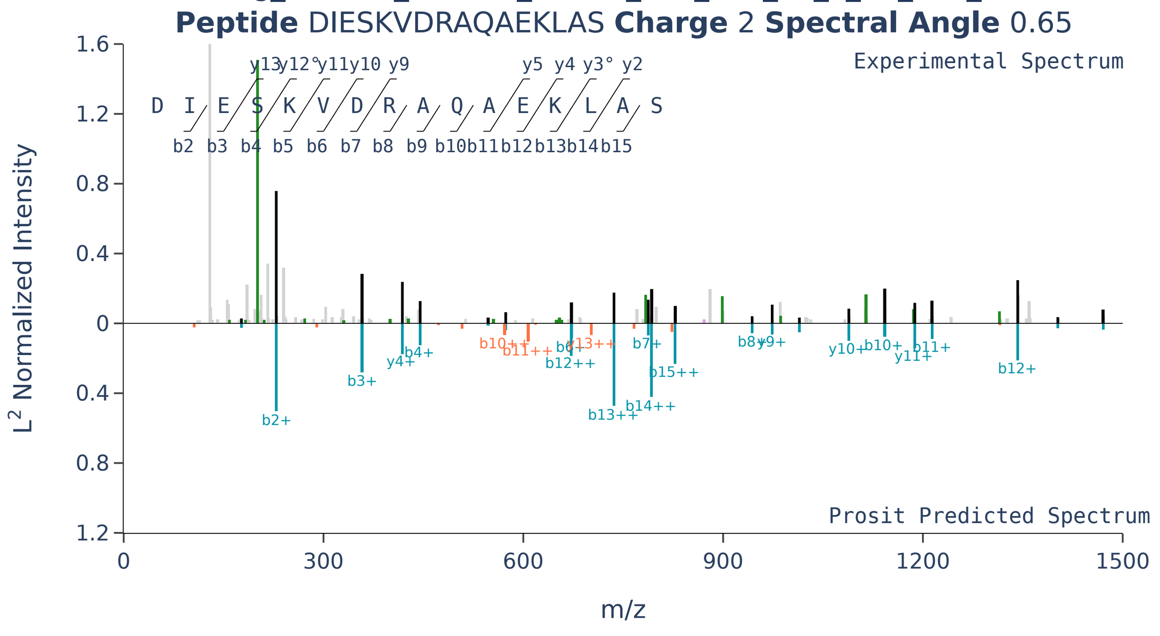


**Source** Ncheng_210623_230623_HFGoe_FFH_20S_25_1_A2_4h_R2 **Scan** 28073
**Peptide** RQMKNMGGMASLMGKLPGM **Charge** 3 **Spectral Angle** 0.84

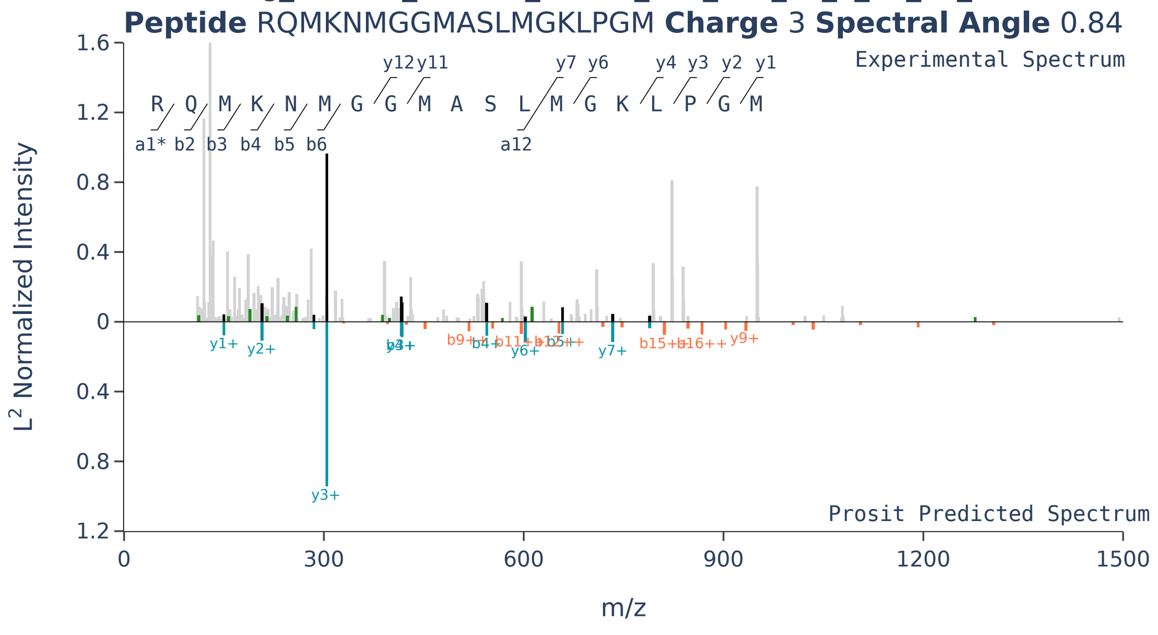


**Source** Ncheng_210623_230623_HFGoe_FFH_20S_25_1_A2_4h_R2 **Scan** 7571
**Peptide** QLRQMKNMG **Charge** 2 **Spectral Angle** 0.7

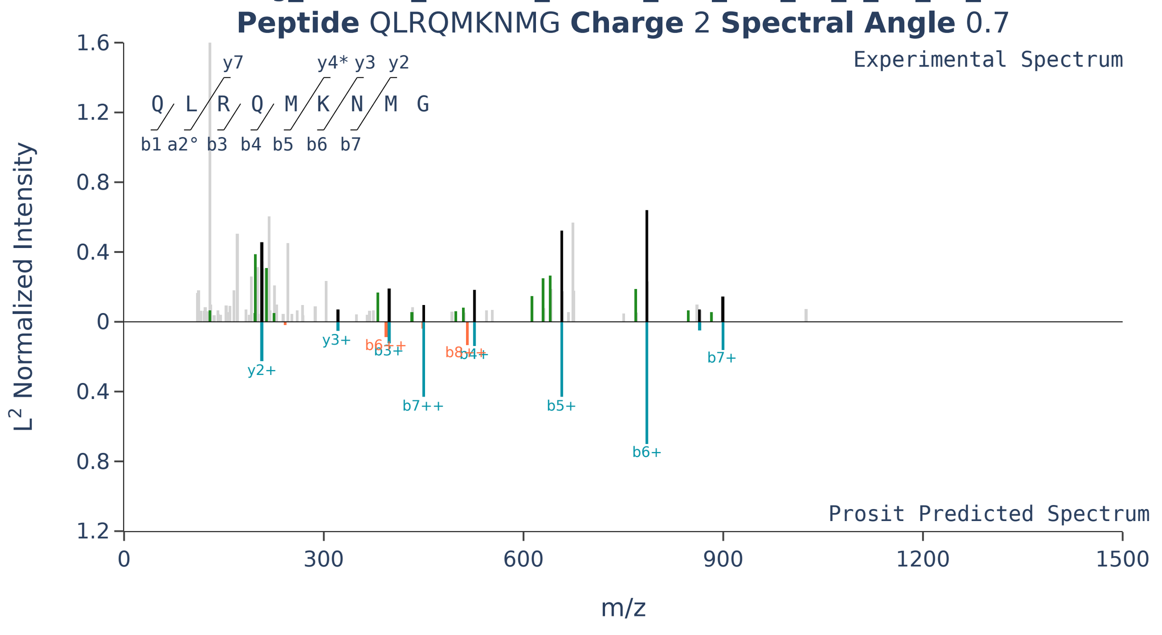


**Source** Ncheng_210623_230623_HFGoe_FFH_20S_25_1_A1_1h_R1 **Scan** 35774
**Peptide** SLTPGQEFVKIVRNELVAAM **Charge** 3 **Spectral Angle** 0.85

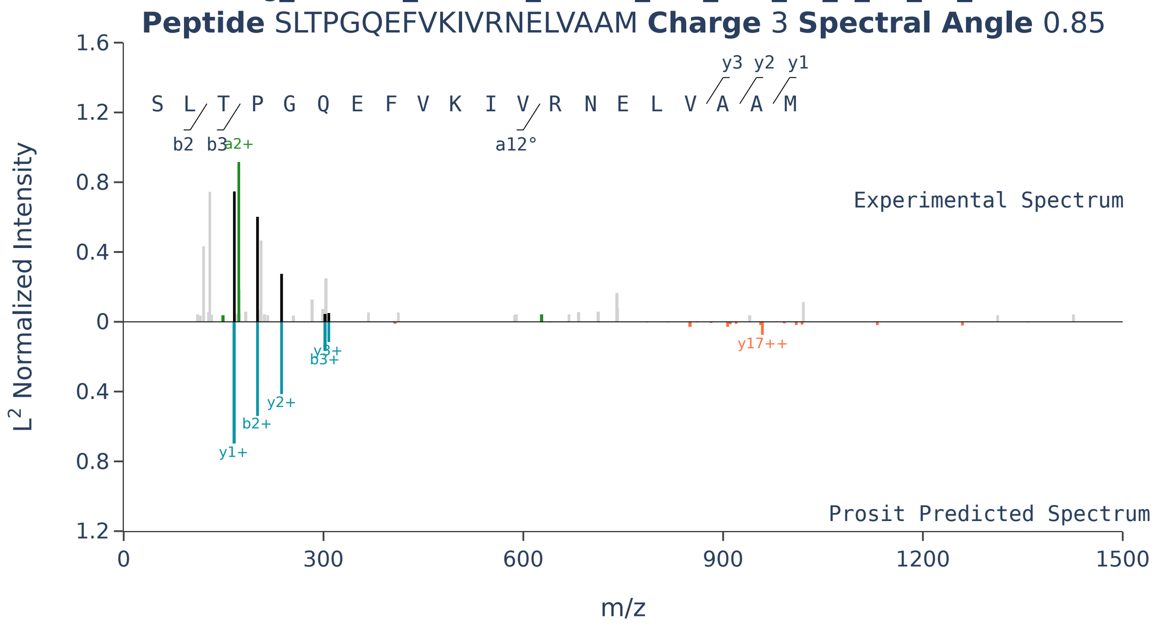


**Source** Ncheng_210623_230623_HFGoe_FFH_20S_25_1_A1_1h_R1 **Scan** 14595
**Peptide** IRHITGKPIKFLGVGEKTE **Charge** 4 **Spectral Angle** 0.67

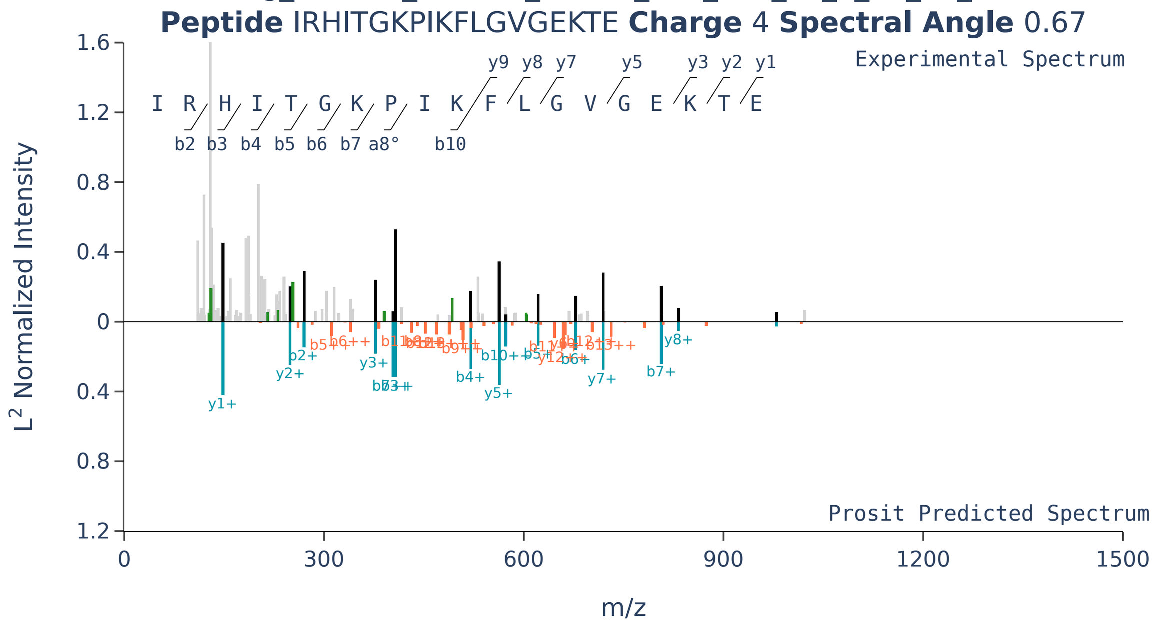


**Source** Ncheng_210623_230623_HFGoe_FFH_20S_25_1_A1_1h_R2 **Scan** 35097
**Peptide** ADVALPVVREFI **Charge** 2 **Spectral Angle** 0.83

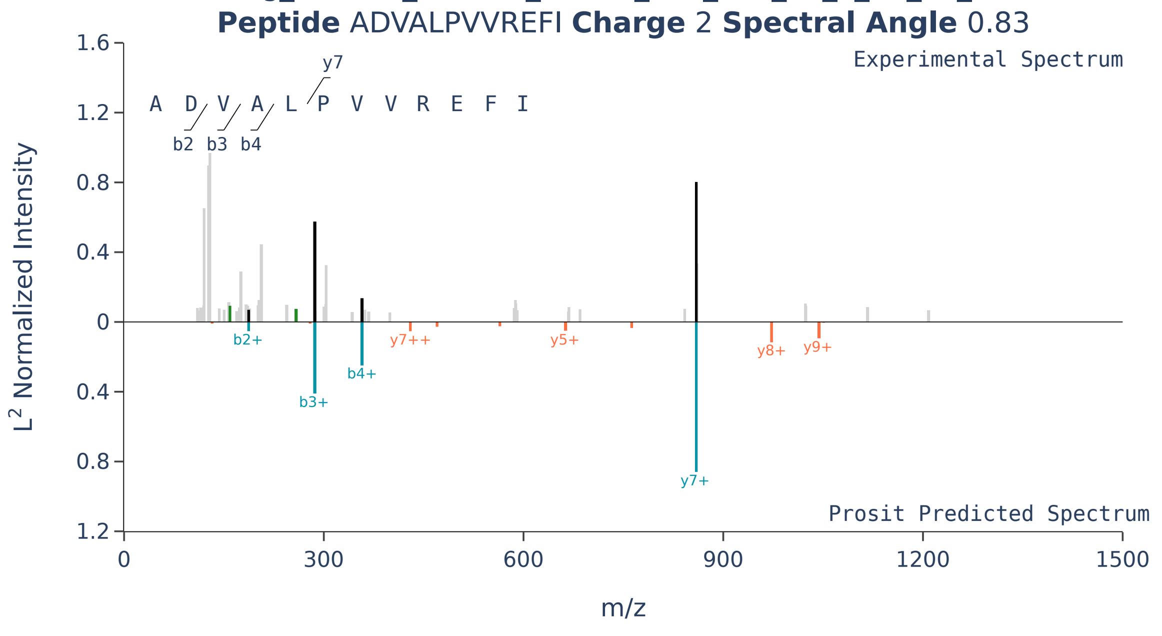


**Source** Ncheng_210623_230623_HFGoe_FFH_20S_25_1_A2_24h_R2 **Scan** 12007
**Peptide** QLPDNVKSQMD **Charge** 1 **Spectral Angle** 0.76

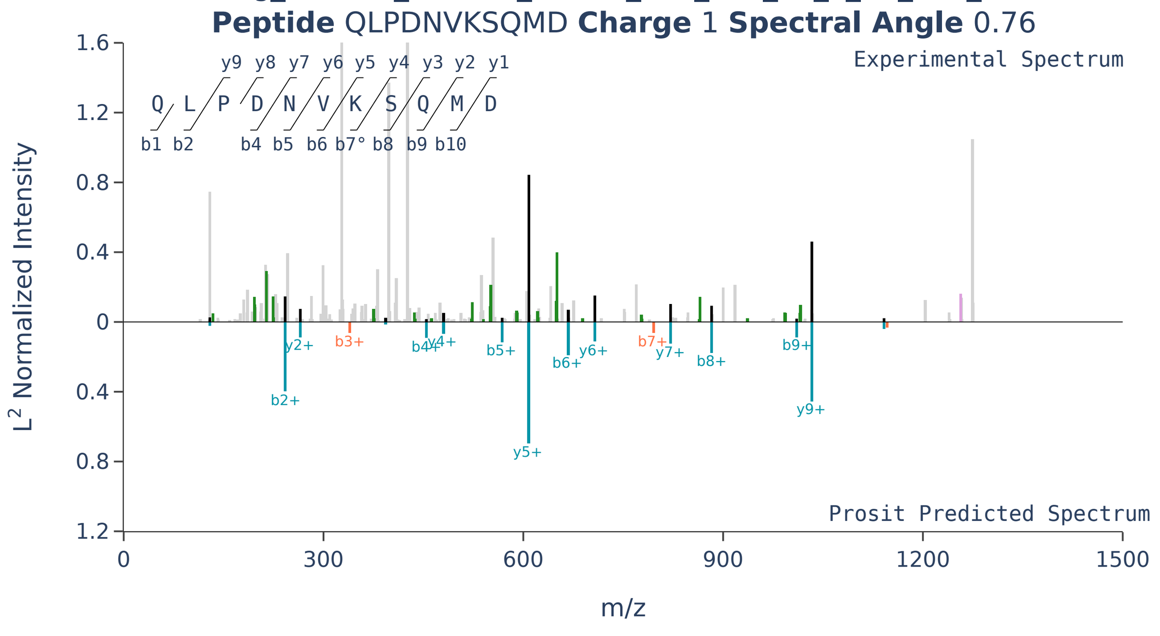

**Source** Ncheng_210623_230623_HFGoe_FFH_20S_25_1_A1_4h_R1 **Scan** 28481
**Peptide** LMGKLPGMGQIPDNV **Charge** 2 **Spectral Angle** 0.8

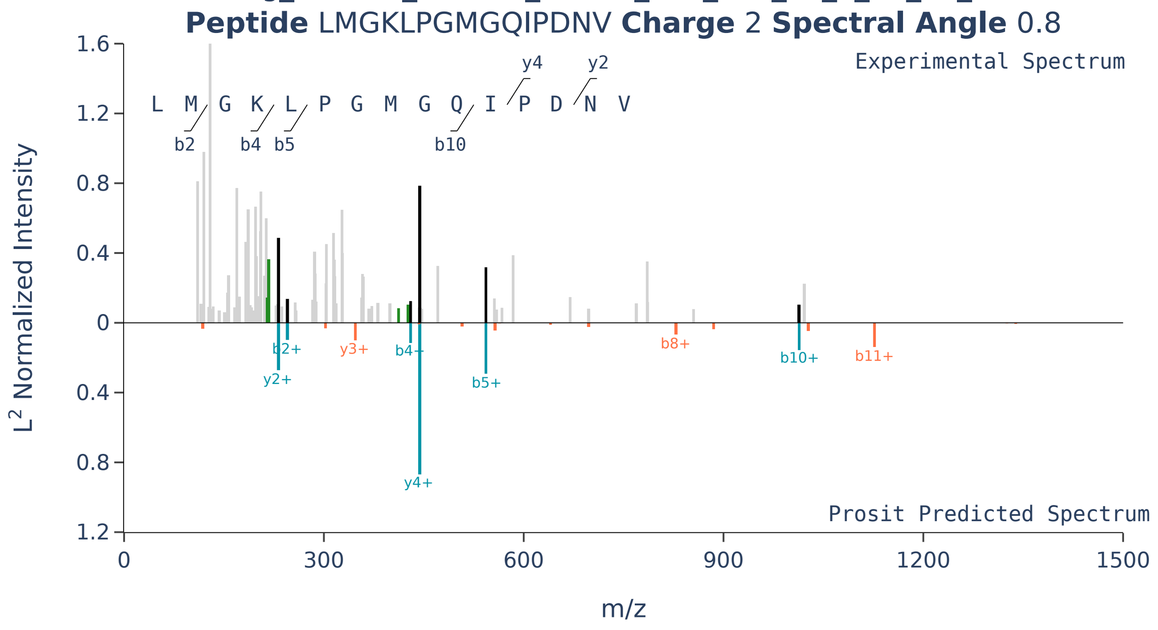


**Source** Ncheng_210623_230623_HFGoe_FFH_20S_25_1_A1_2h_R2 **Scan** 20536
**Peptide** IEDIESKVDRAQAEKLASKL **Charge** 3 **Spectral Angle** 0.77

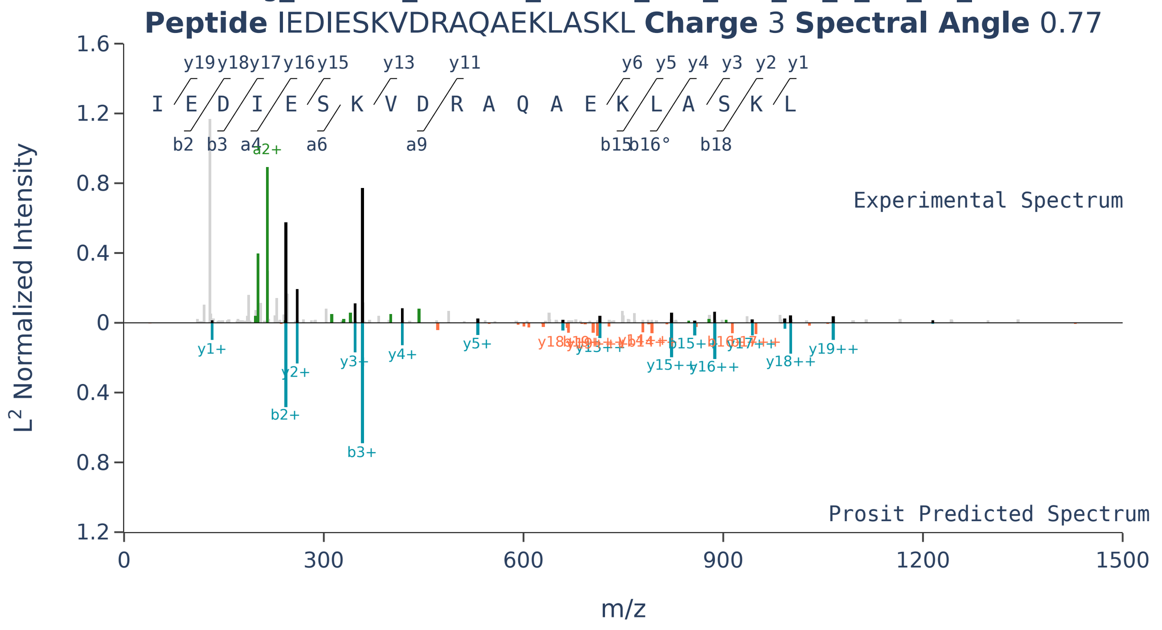


**Source** Ncheng_210623_230623_HFGoe_FFH_20S_25_1_A1_2h_R2 **Scan** 34891
**Peptide** TLAEQVGVDFFPSDVGQKPVDIVNA **Charge** 3 **Spectral Angle** 0.63

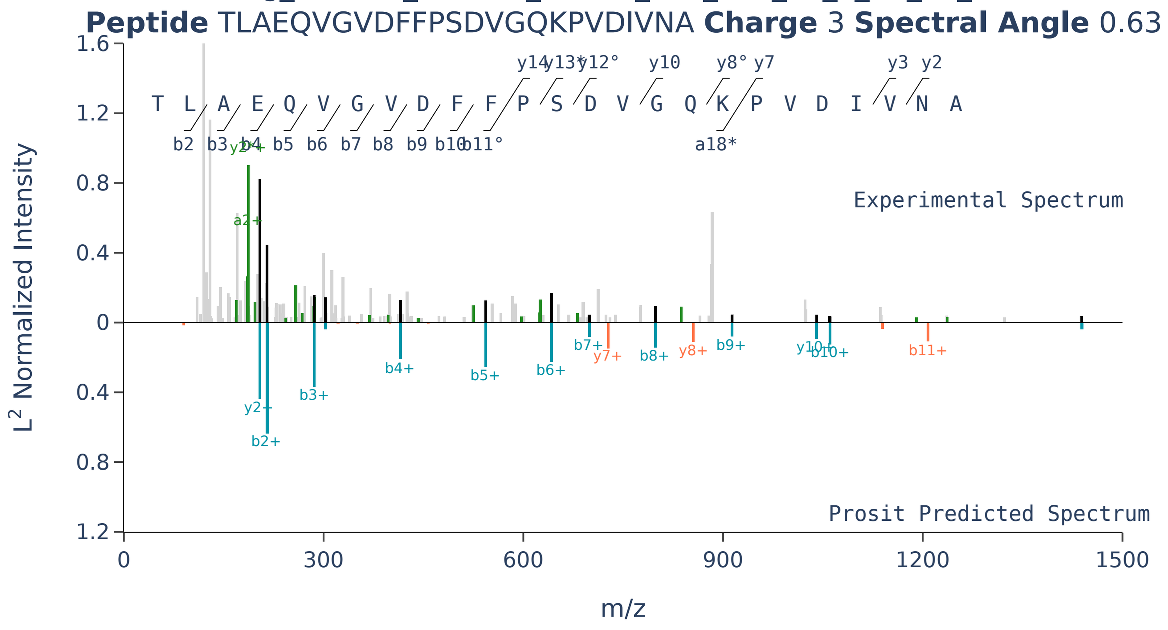


**Source** Ncheng_210623_230623_HFGoe_FFH_20S_25_1_A2_4h_R1 **Scan** 6596
**Peptide** LTEDNVKDT **Charge** 2 **Spectral Angle** 0.71

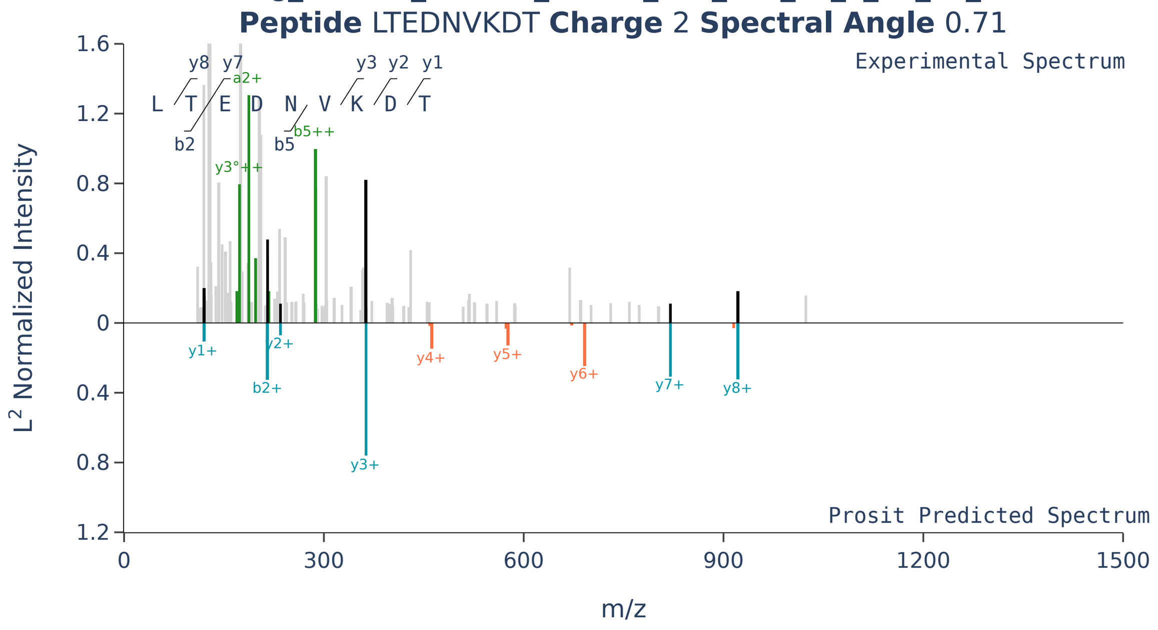


**Source** Ncheng_210623_230623_HFGoe_FFH_20S_25_1_A1_2h_R1 **Scan** 31008
**Peptide** SLIEDIESKVDRAQAEKLASKL **Charge** 3 **Spectral Angle** 0.72

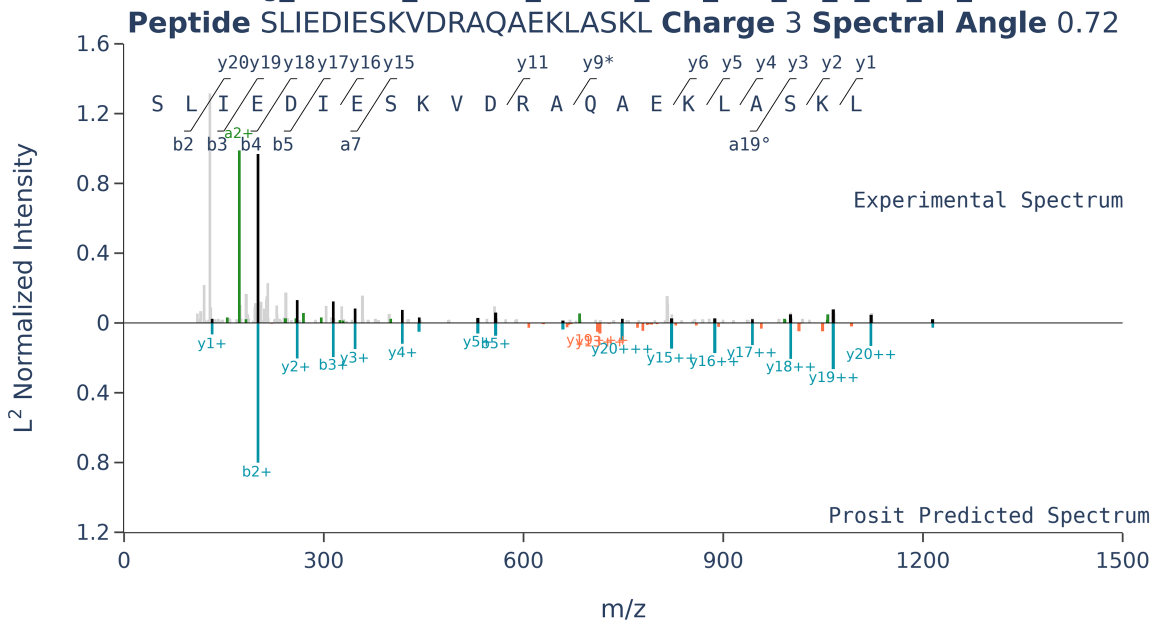


**Source** Ncheng_210623_230623_HFGoe_FFH_20S_25_1_A1_2h_R1 **Scan** 13751
**Peptide** GRLHVDEAMMD **Charge** 3 **Spectral Angle** 0.46

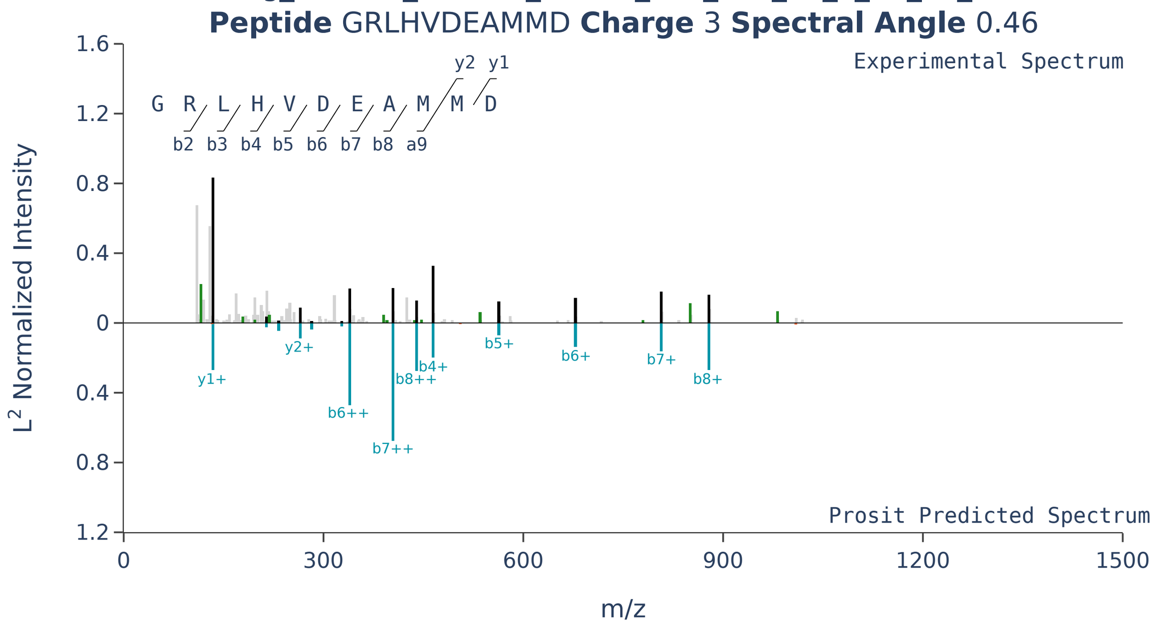


**Source** Ncheng_210623_230623_HFGoe_FFH_20S_25_1_A2_4h_R1 **Scan** 23514
**Peptide** LLEDIESKVDRAQAEKLASKLKKGDGFD **Charge** 4 **Spectral Angle** 0.71

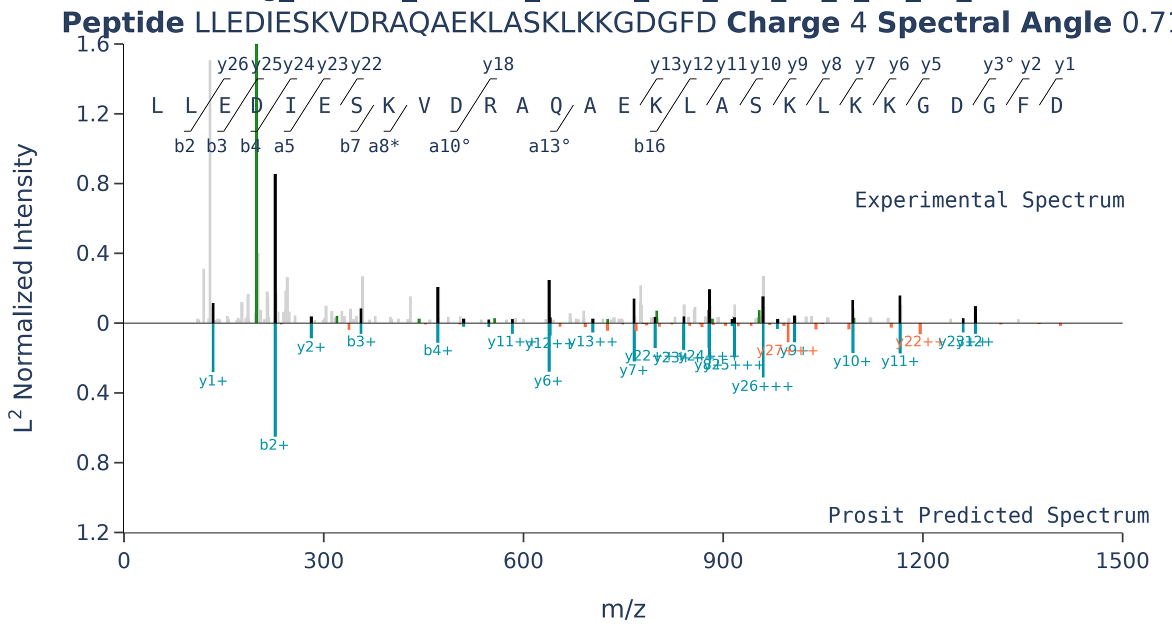


**Source** Ncheng_210623_230623_HFGoe_FFH_20S_25_1_A1_2h_R2 **Scan** 29286
**Peptide** ALKEAKLKFYDVLLVD **Charge** 2 **Spectral Angle** 0.59

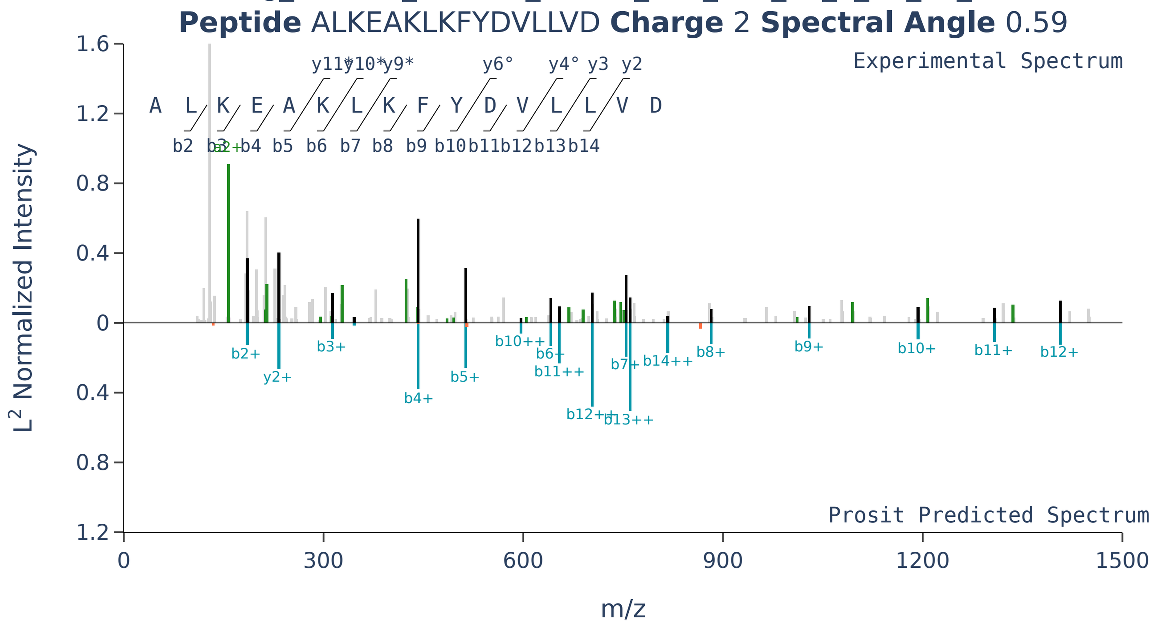


**Source** Ncheng_210623_230623_HFGoe_FFH_20S_25_1_A2_24h_R2 **Scan** 9450
**Peptide** RHITGKPIKF **Charge** 3 **Spectral Angle** 0.69

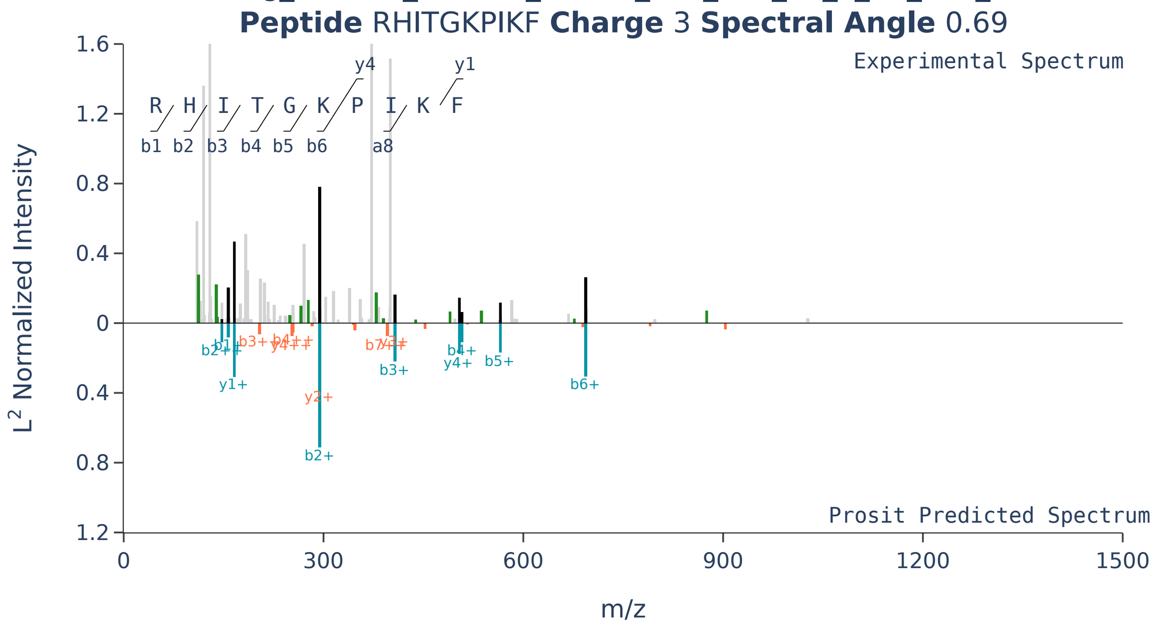


**Source** Ncheng_210623_230623_HFGoe_FFH_20S_25_1_A2_24h_R1 **Scan** 10617
**Peptide** DDMQRMMKKMK **Charge** 2 **Spectral Angle** 0.72

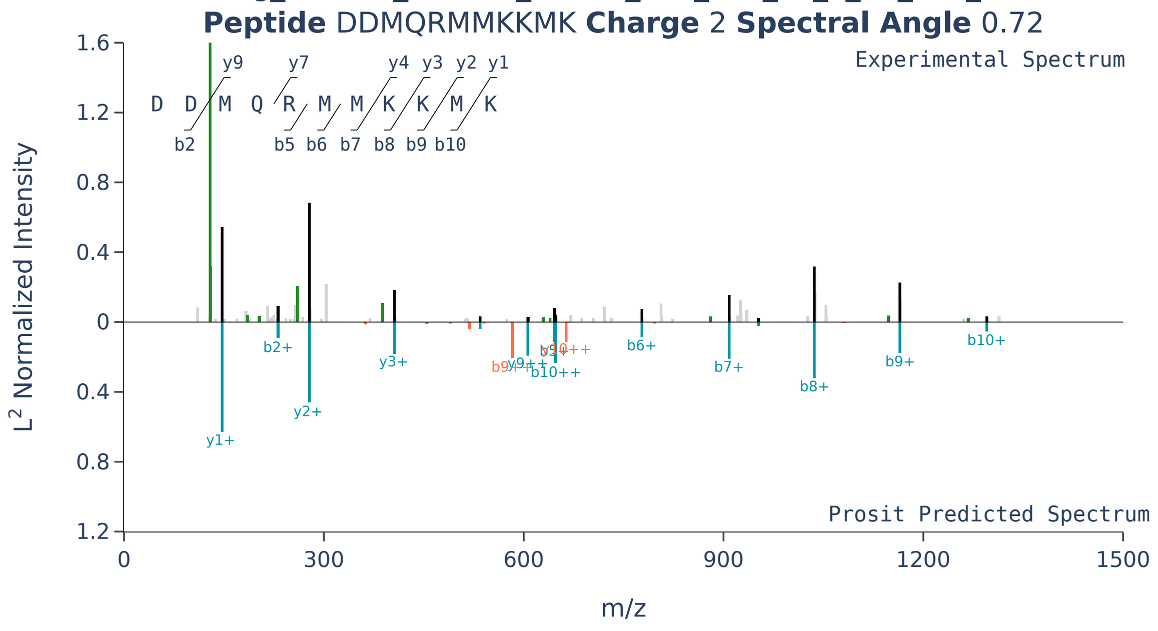


**Source** Ncheng_210623_230623_HFGoe_FFH_20S_25_1_A1_2h_R1 **Scan** 28152
**Peptide** VKIVRNELVAAMGEENQTLNL **Charge** 3 **Spectral Angle** 0.63

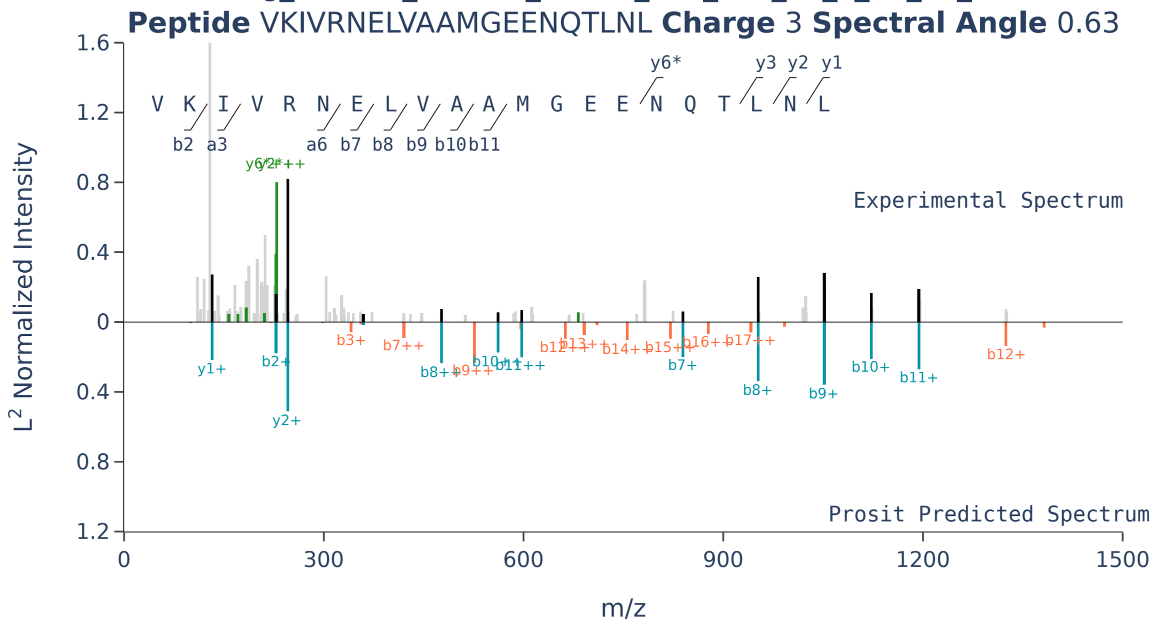


**Source** Ncheng_210623_230623_HFGoe_FFH_20S_25_1_A1_2h_R1 **Scan** 32111
**Peptide** MDDKVLVRMEALL **Charge** 2 **Spectral Angle** 0.76

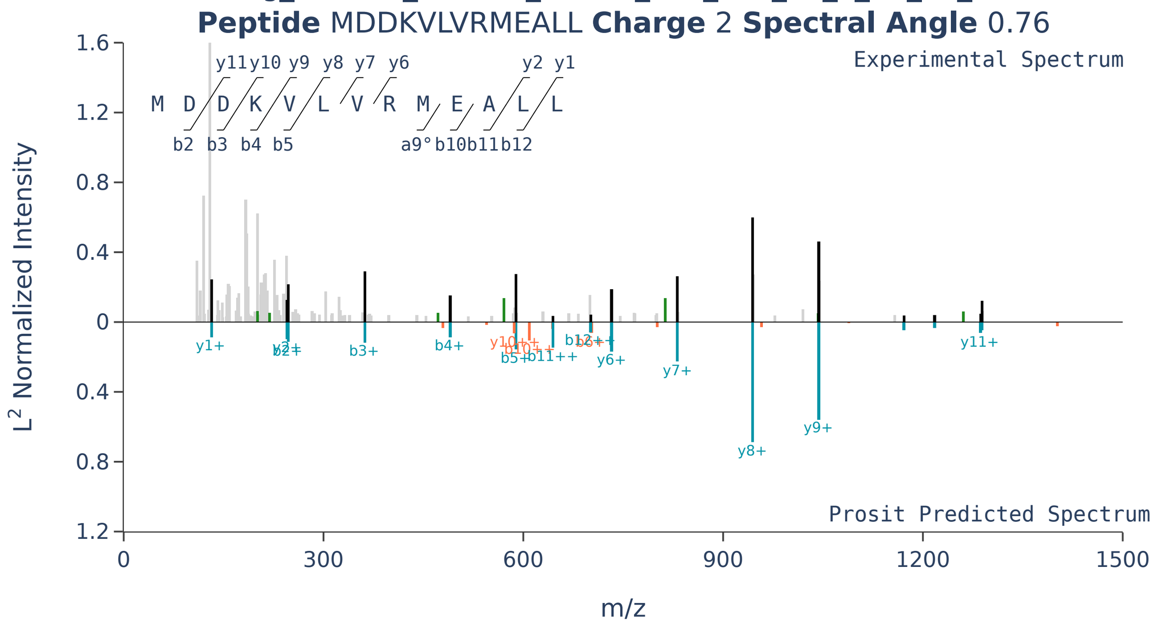


**Source** Ncheng_210623_230623_HFGoe_FFH_20S_25_1_A2_24h_R2 **Scan** 5704
**Peptide** LESKVDRAQAE **Charge** 3 **Spectral Angle** 0.77

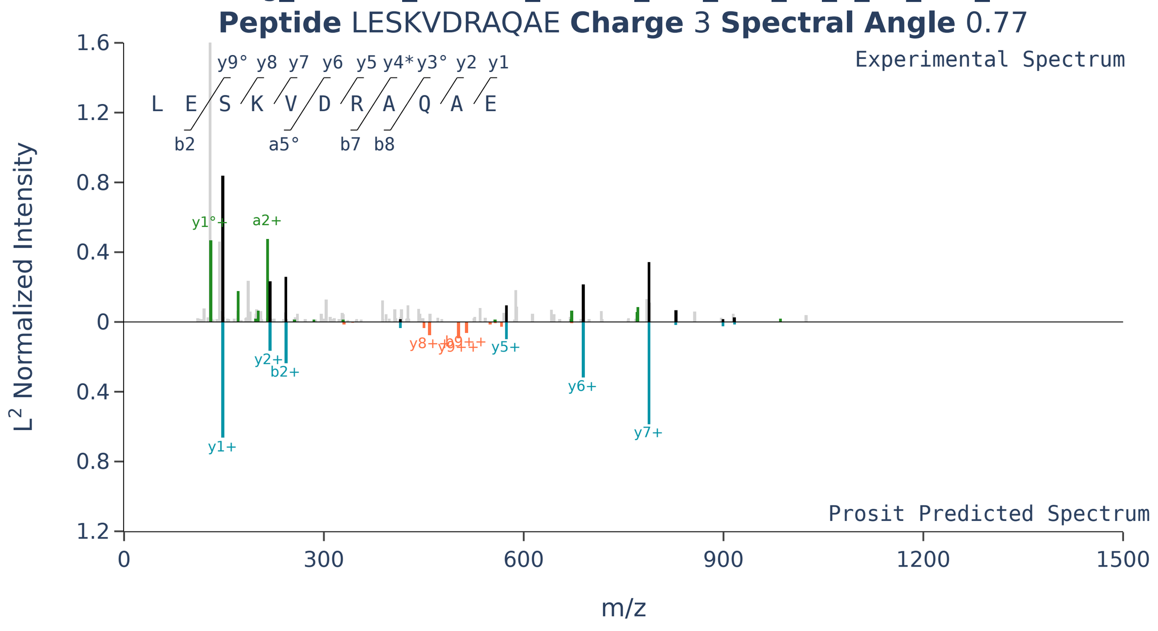


**Source** Ncheng_210623_230623_HFGoe_FFH_20S_25_1_A2_4h_R2 **Scan** 25293
**Peptide** LPGMGQIPDNVKSQMDDKVLVR **Charge** 4 **Spectral Angle** 0.61

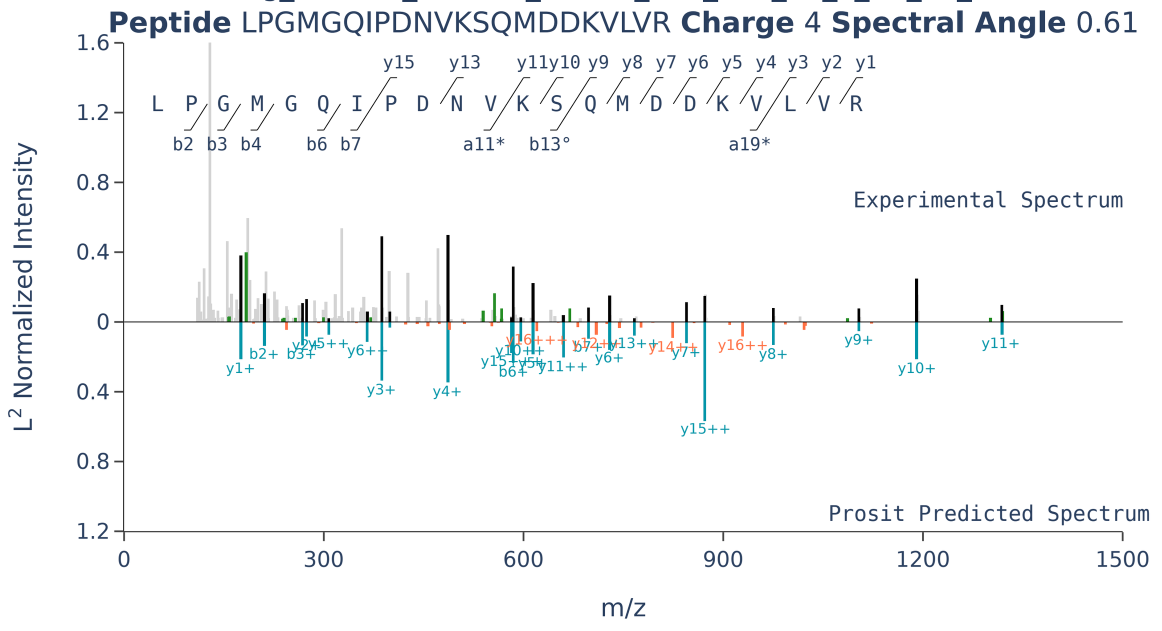


**Source** Ncheng_210623_230623_HFGoe_FFH_20S_25_1_A2_24h_R2 **Scan** 16463
**Peptide** GDARGGAALSI **Charge** 1 **Spectral Angle** 0.93

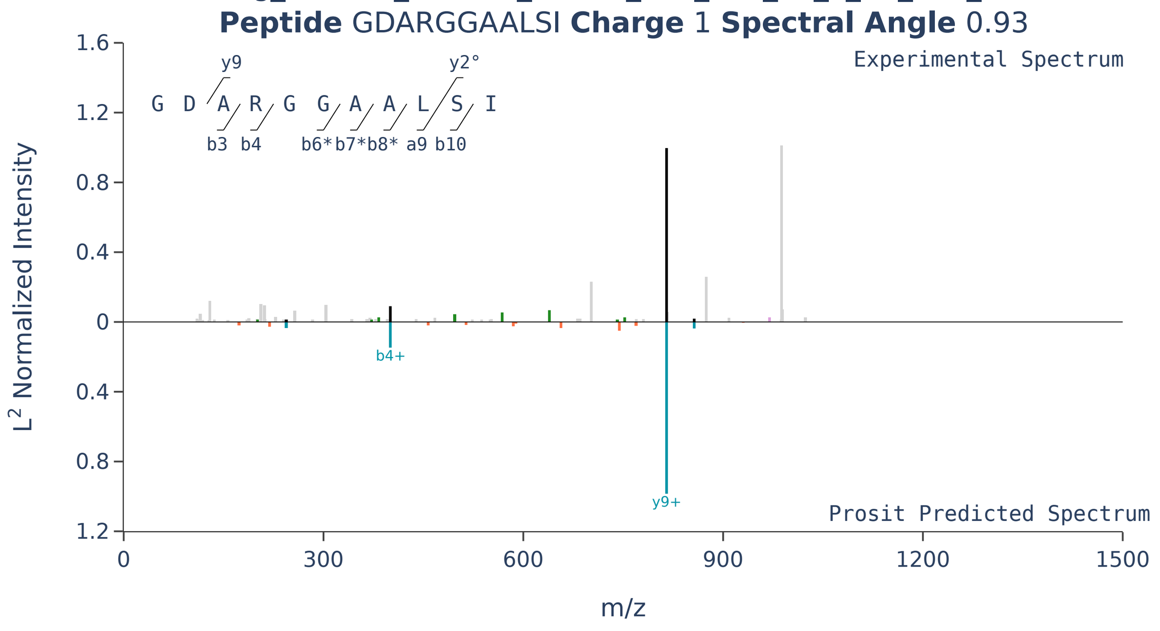

**Source** Ncheng_210623_230623_HFGoe_FFH_20S_25_1_A1_4h_R1 **Scan** 31696
**Peptide** ALPLTGVVLTKVDGDARGGAAL **Charge** 3 **Spectral Angle** 0.5

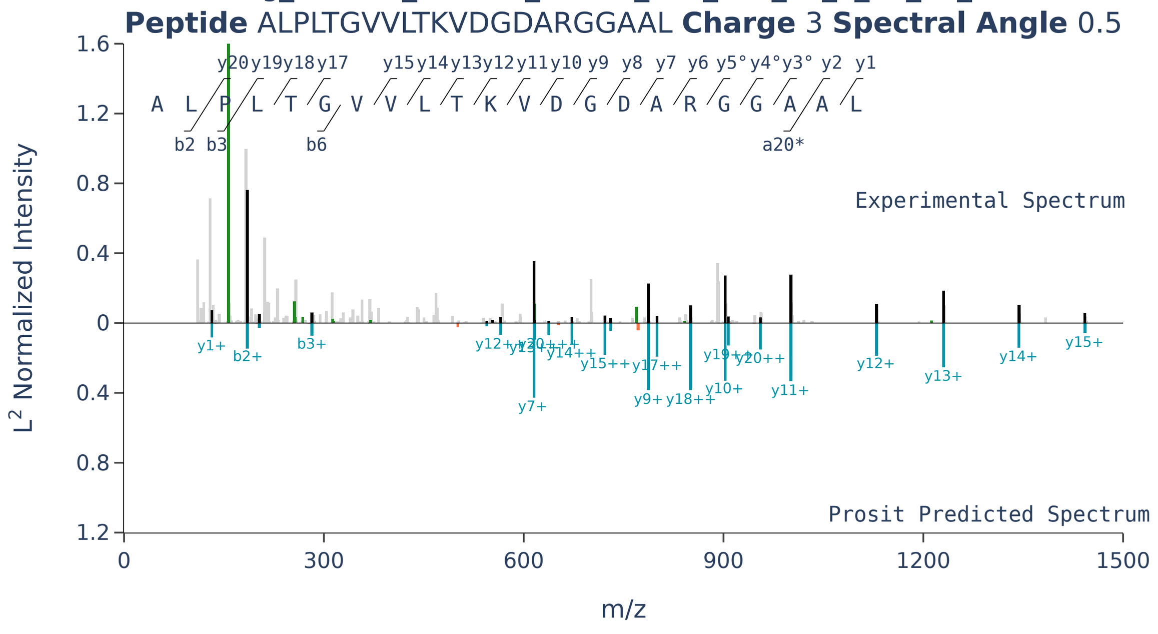


**Source** Ncheng_210623_230623_HFGoe_FFH_20S_25_1_A2_24h_R1 **Scan** 18020
**Peptide** AIINSMTMKERAKPEI **Charge** 4 **Spectral Angle** 0.56

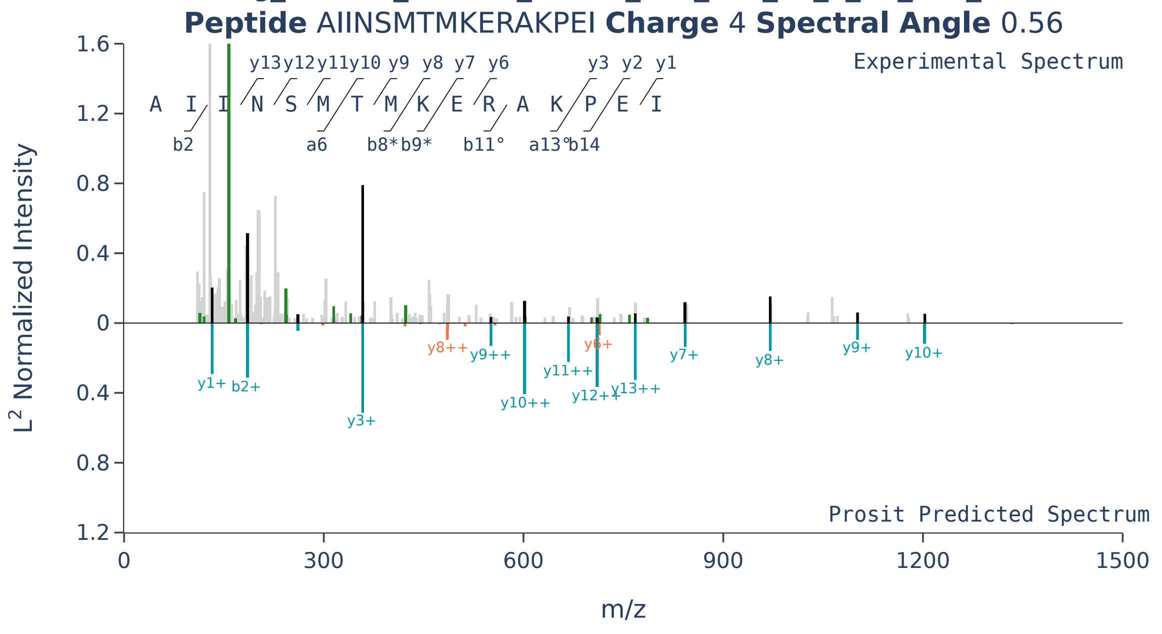


**Source** Ncheng_210623_230623_HFGoe_FFH_20S_25_1_A1_2h_R1 **Scan** 17600
**Peptide** KTEALEPFHPDRLA **Charge** 3 **Spectral Angle** 0.67

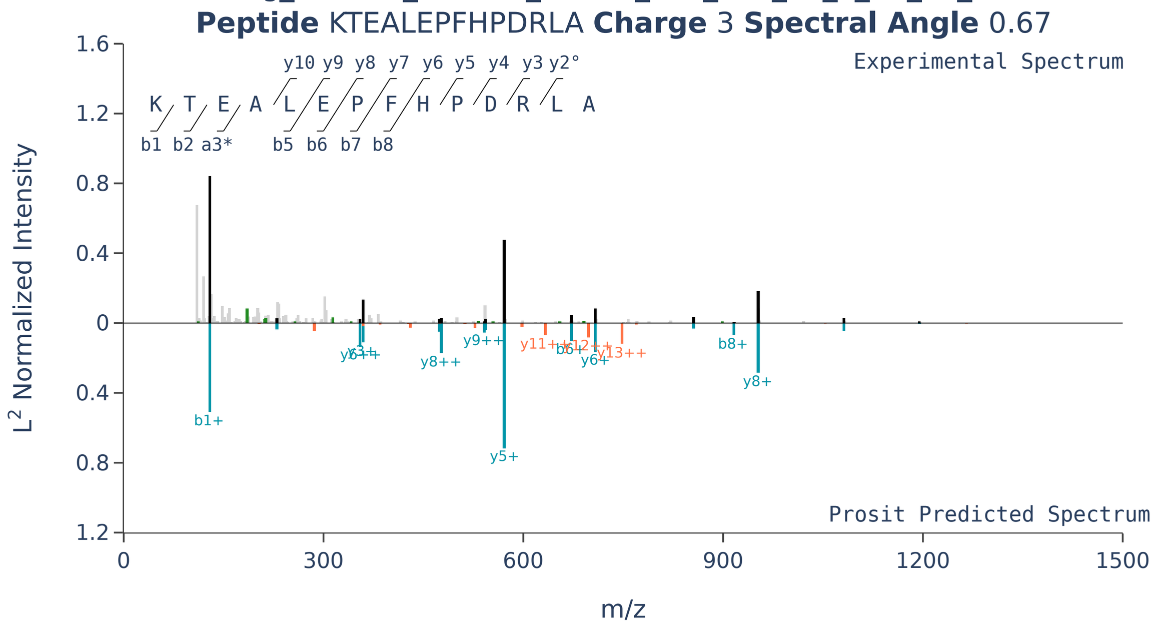


**Source** Ncheng_210623_230623_HFGoe_FFH_20S_25_1_A2_24h_R1 **Scan** 15992
**Peptide** RLLGMGD **Charge** 2 **Spectral Angle** 0.78

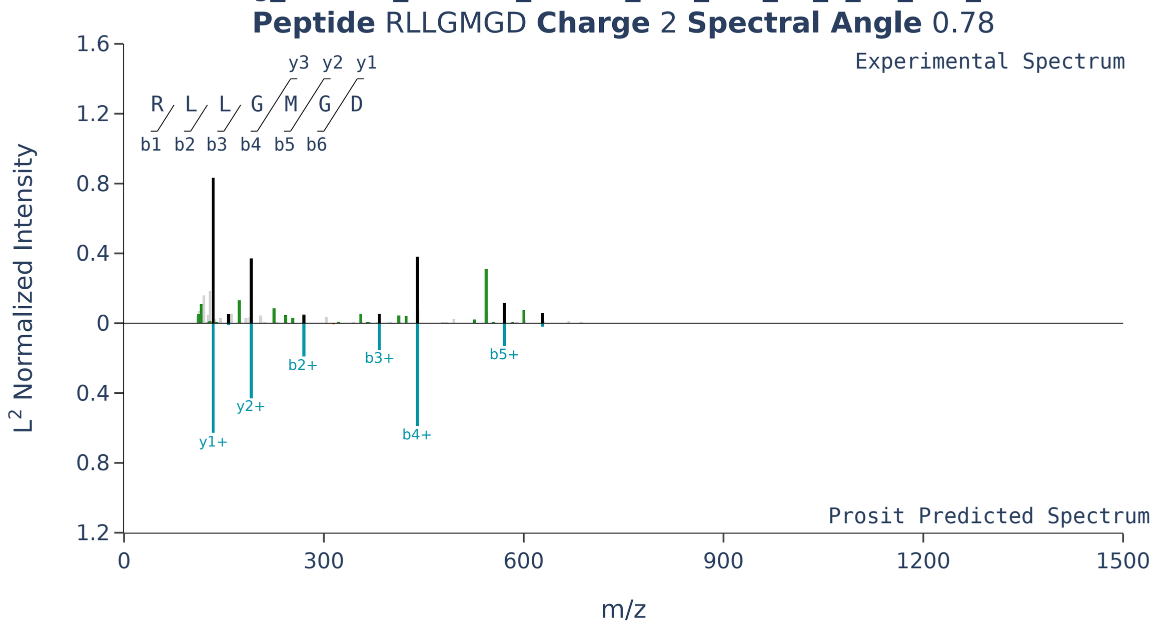


**Source** Ncheng_210623_230623_HFGoe_FFH_20S_25_1_A1_2h_R2 **Scan** 13553
**Peptide** LRNISGRGRLTEDNVKDTL **Charge** 4 **Spectral Angle** 0.63

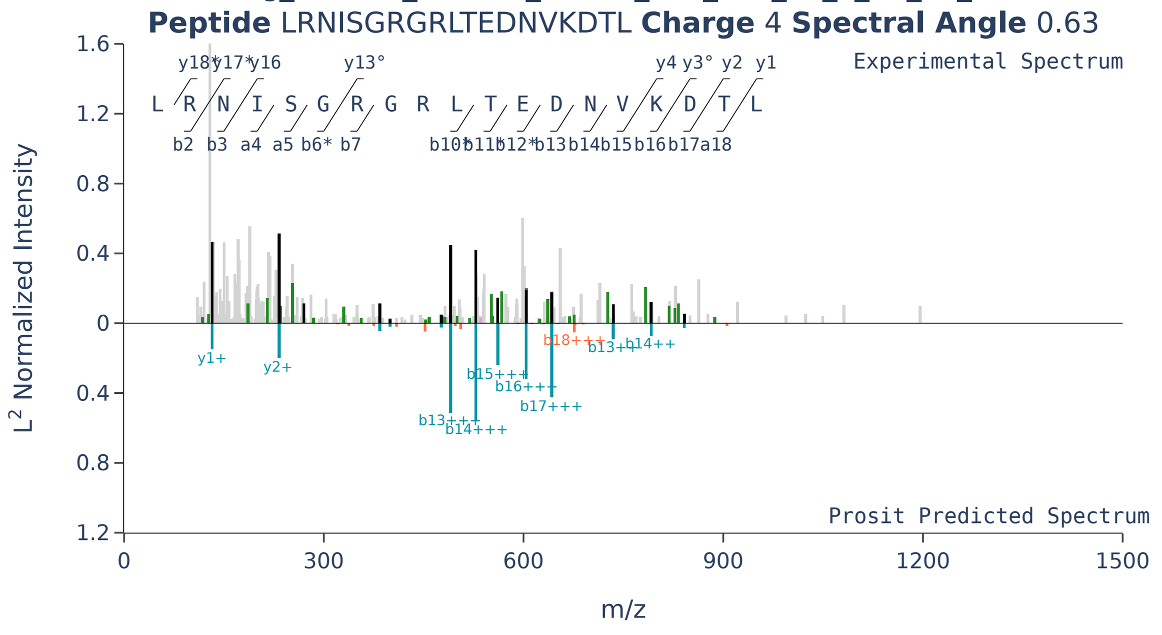


**Source** Ncheng_210623_230623_HFGoe_FFH_20S_25_1_A2_4h_R2 **Scan** 37009
**Peptide** KKGDGFDLNDFLEQLRQM **Charge** 3 **Spectral Angle** 0.74

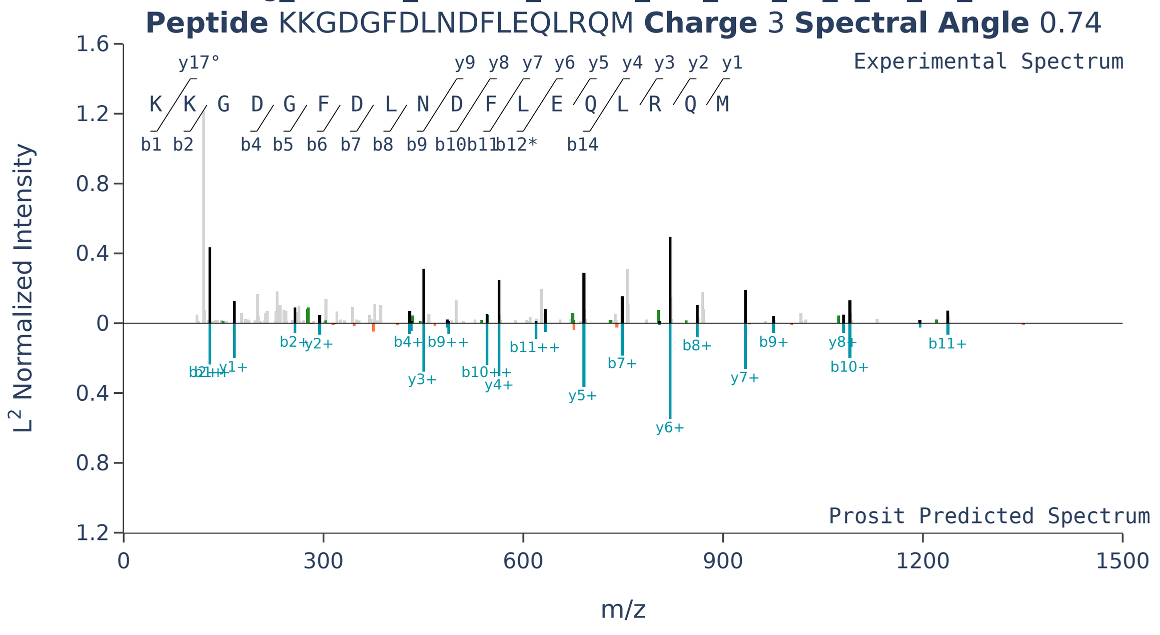


**Source** Ncheng_210623_230623_HFGoe_FFH_20S_25_1_A2_4h_R1 **Scan** 24301
**Peptide** ALKEAKLKFYDVL **Charge** 2 **Spectral Angle** 0.67

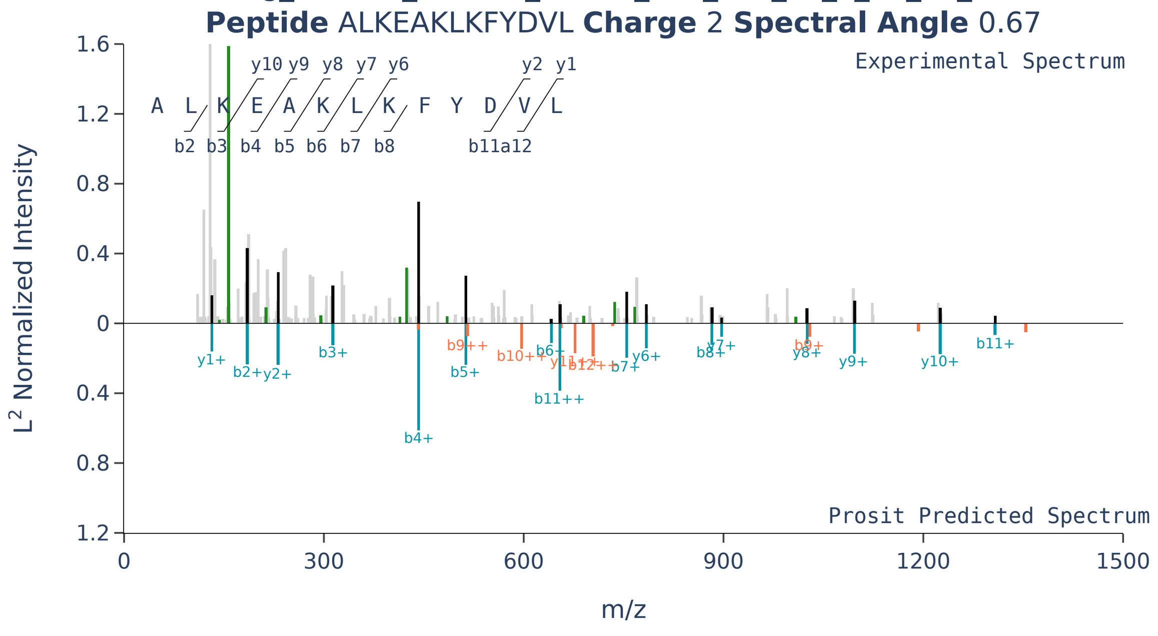


**Source** Ncheng_210623_230623_HFGoe_FFH_20S_25_1_A1_24h_R2 **Scan** 9007
**Peptide** LESKVDRAQAEKL **Charge** 2 **Spectral Angle** 0.69

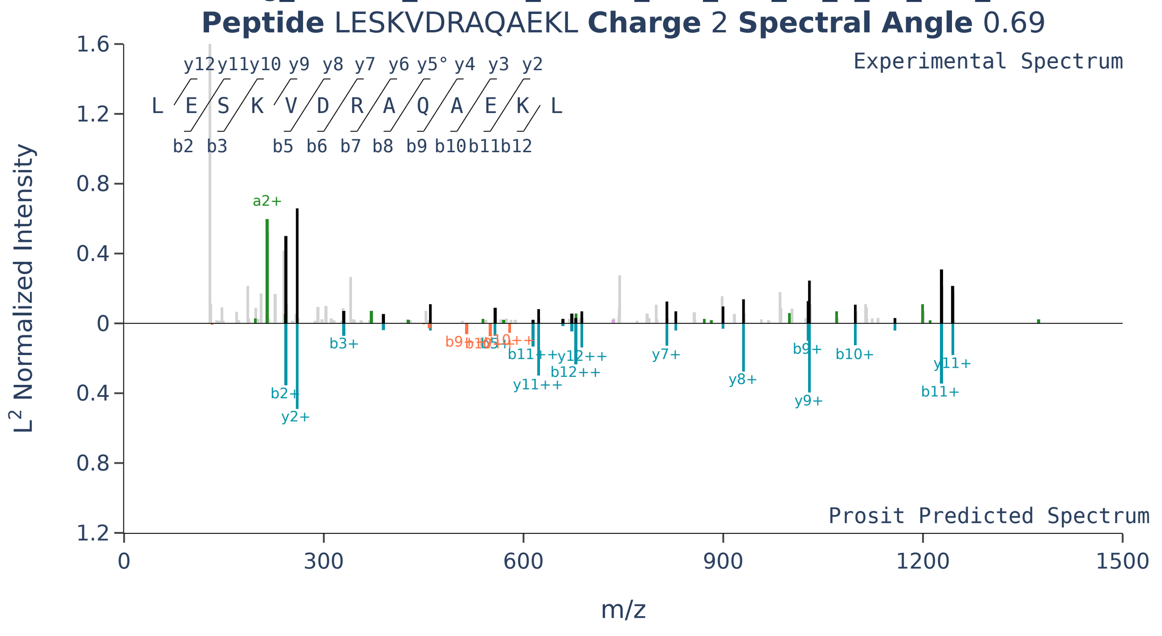


**Source** Ncheng_210623_230623_HFGoe_FFH_20S_25_1_A2_1h_R2 **Scan** 28383
**Peptide** KFYDVLLVD **Charge** 2 **Spectral Angle** 0.56

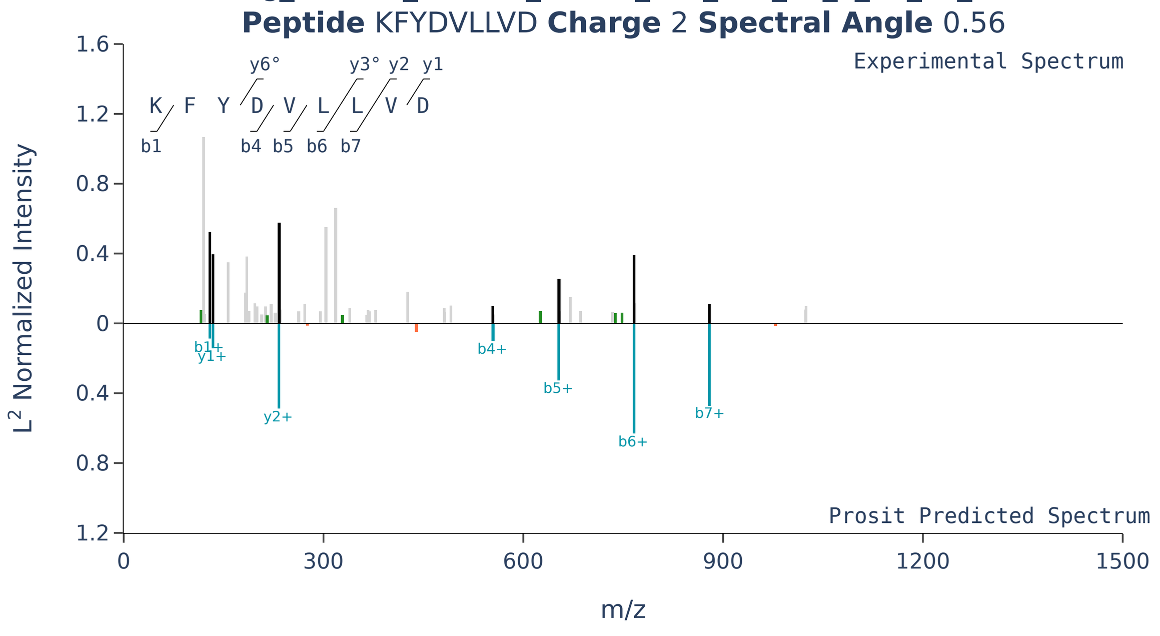


**Source** Ncheng_210623_230623_HFGoe_FFH_20S_25_1_A1_4h_R1 **Scan** 35848
**Peptide** GQDAANTAKAFNEALPLTGVVLTKV **Charge** 3 **Spectral Angle** 0.79

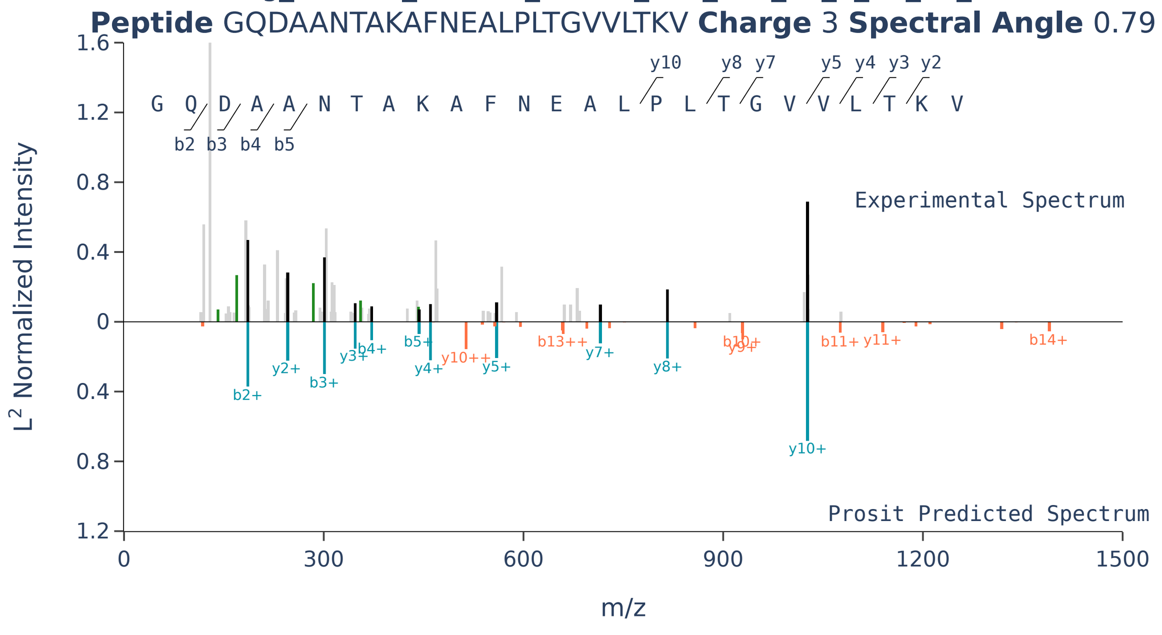


**Source** Ncheng_210623_230623_HFGoe_FFH_20S_25_1_A1_4h_R1 **Scan** 25283
**Peptide** VDAMTGQDAANTAKAFNEAL **Charge** 2 **Spectral Angle** 0.9

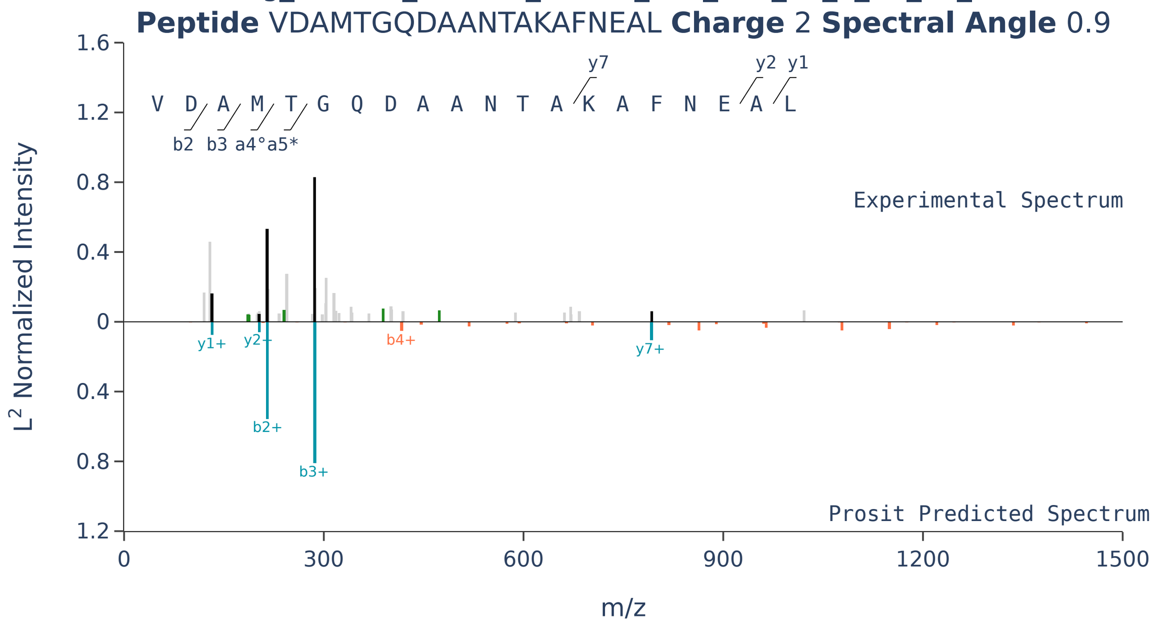


**Source** Ncheng_210623_230623_HFGoe_FFH_20S_25_1_A1_1h_R1 **Scan** 31474
**Peptide** FYDVLLVDTAGRLHVDE **Charge** 3 **Spectral Angle** 0.79

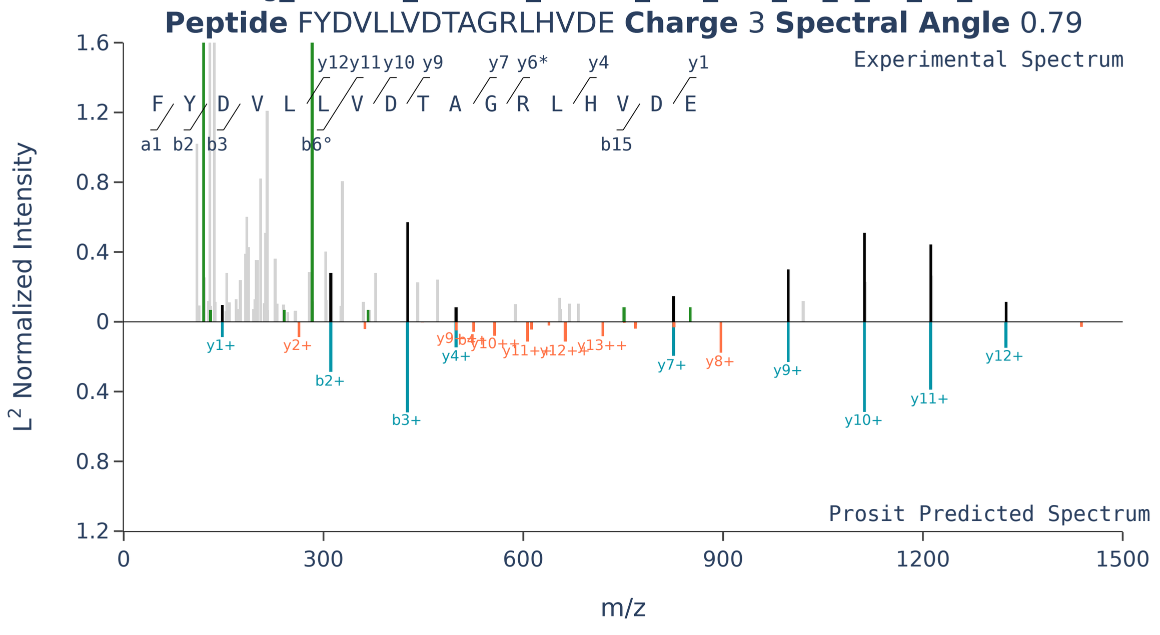


**Source** Ncheng_210623_230623_HFGoe_FFH_20S_25_1_A1_4h_R2 **Scan** 16345
**Peptide** DEIKQVHASINPVET **Charge** 3 **Spectral Angle** 0.46

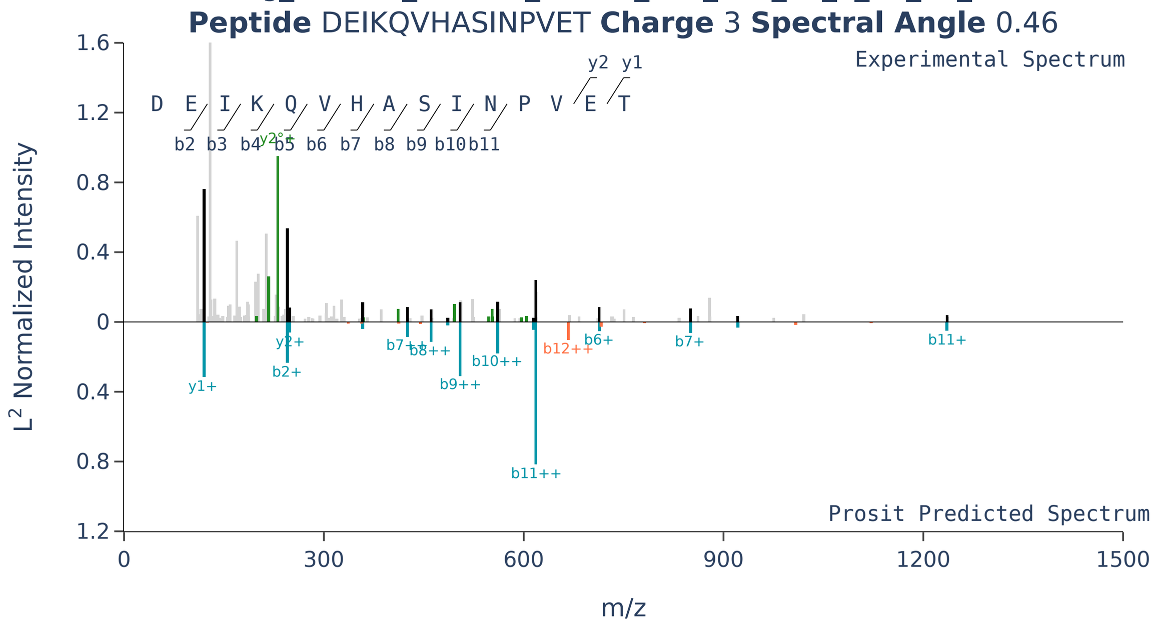


**Source** Ncheng_210623_230623_HFGoe_FFH_20S_25_1_A1_1h_R1 **Scan** 31136
**Peptide** LVNAALKEAKLKFYDVLLVD **Charge** 3 **Spectral Angle** 0.59

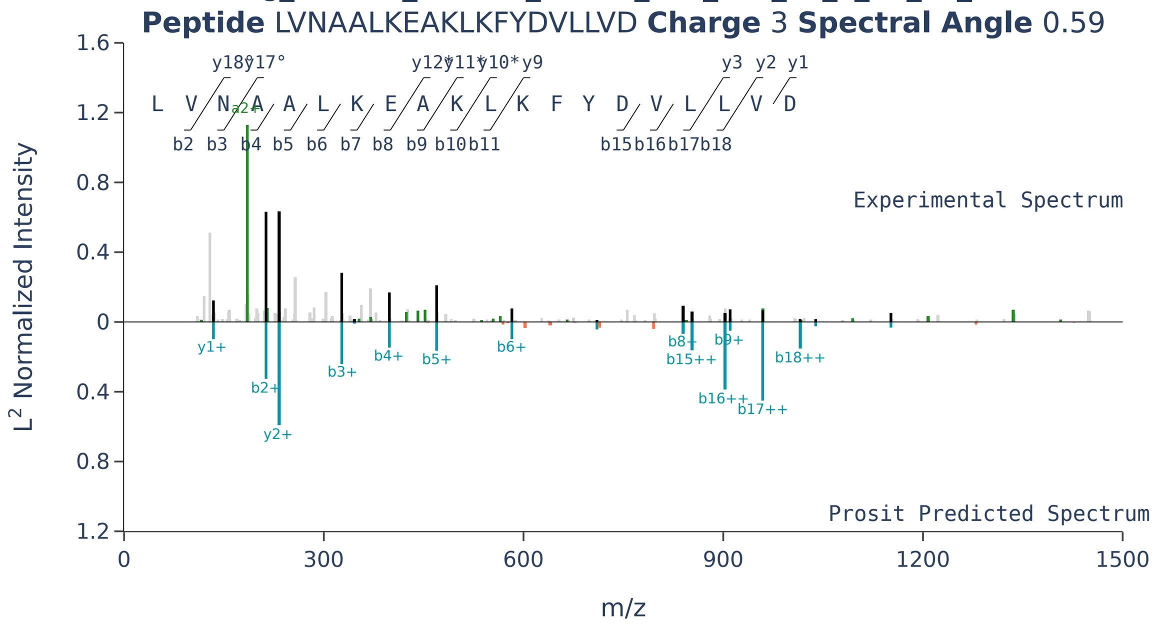


**Source** Ncheng_210623_230623_HFGoe_FFH_20S_25_1_A2_2h_R2 **Scan** 17057
**Peptide** QLPDNVKSQMDDKVLVR **Charge** 4 **Spectral Angle** 0.62

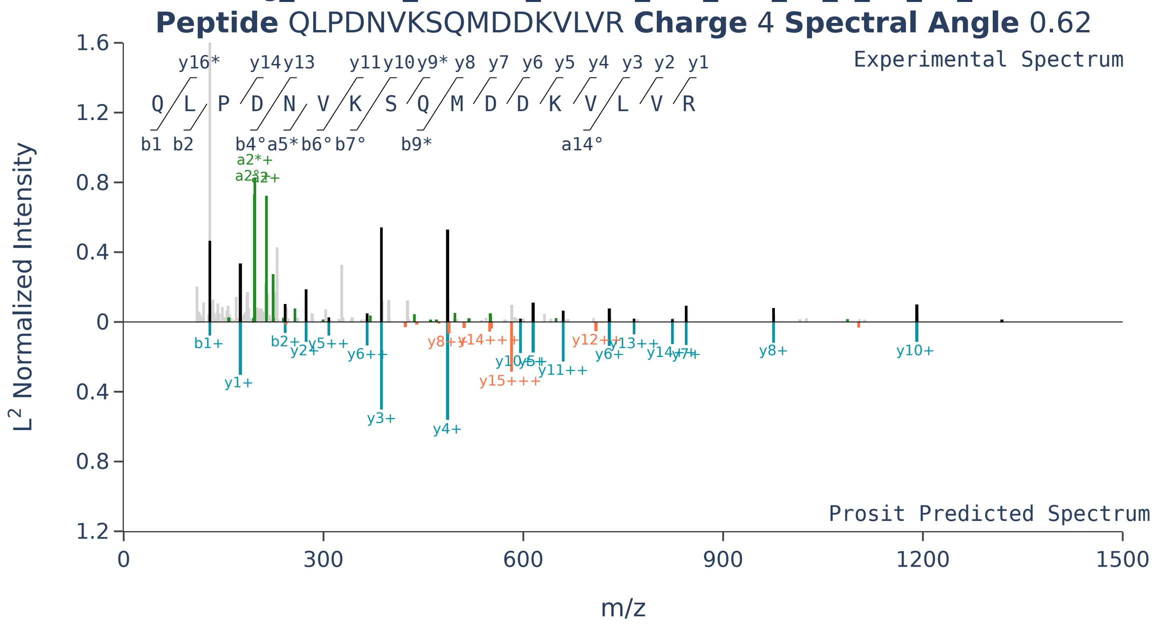

**Source** Ncheng_210623_230623_HFGoe_FFH_20S_25_1_A2_24h_R2 **Scan** 9077
**Peptide** EKLASKLKKGDGFD **Charge** 2 **Spectral Angle** 0.72

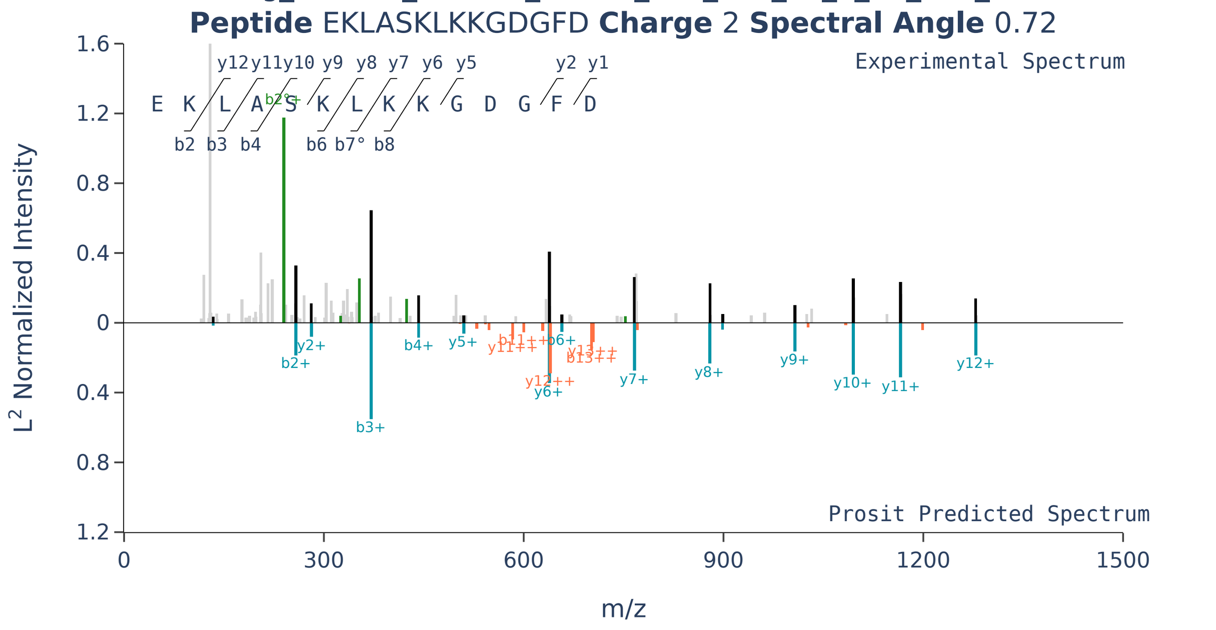


**Source** Ncheng_210623_230623_HFGoe_FFH_20S_25_1_A1_2h_R1 **Scan** 28465
**Peptide** SLIEDIESKVDRAQAE **Charge** 3 **Spectral Angle** 0.63

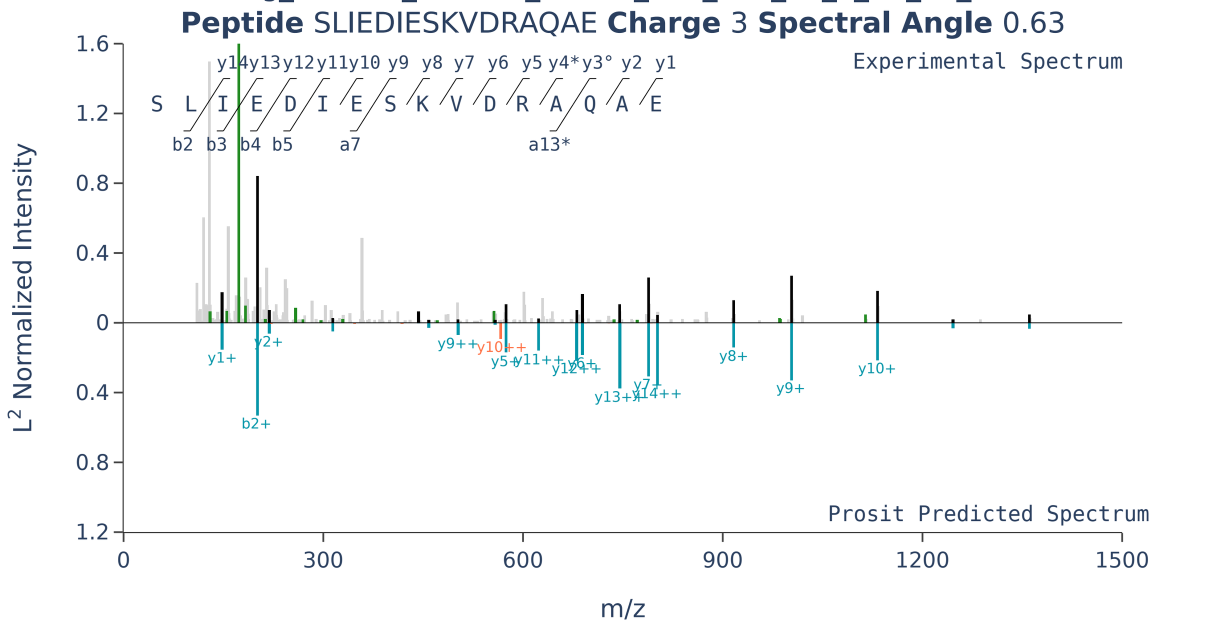


**Source** Ncheng_210623_230623_HFGoe_FFH_20S_25_1_A1_2h_R1 **Scan** 31180
**Peptide** RLTEDNVKDTLREVRMALLE **Charge** 4 **Spectral Angle** 0.7

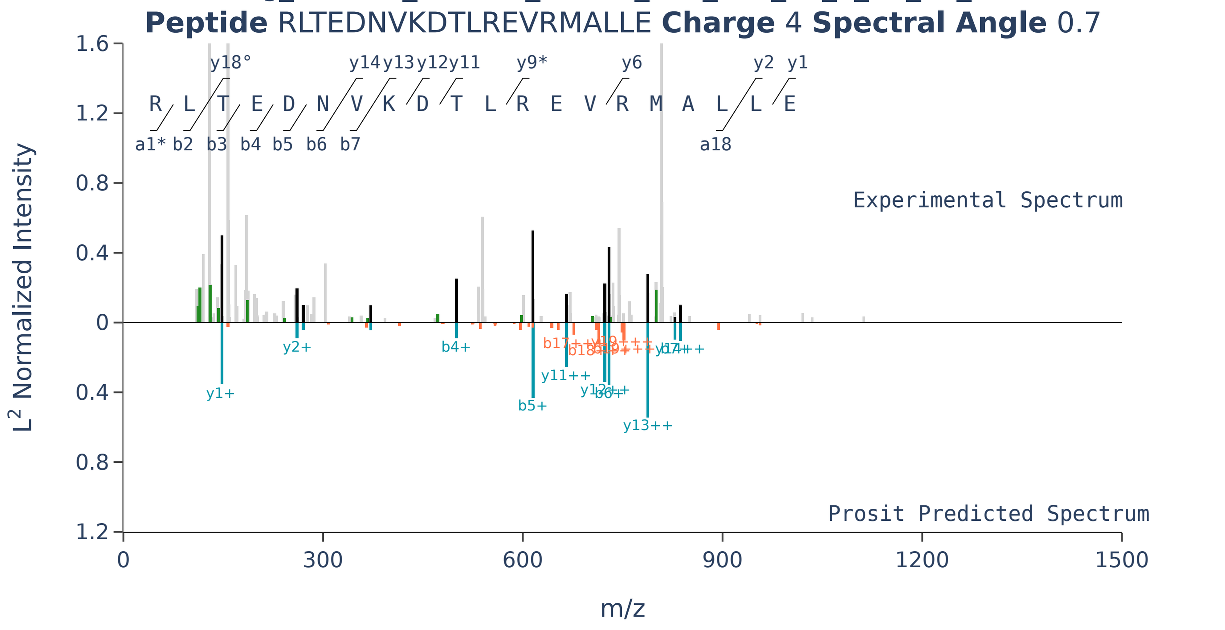


**Source** Ncheng_210623_230623_HFGoe_FFH_20S_25_1_A1_2h_R2 **Scan** 28496
**Peptide** ALKEAKLKFYDVLL **Charge** 3 **Spectral Angle** 0.69

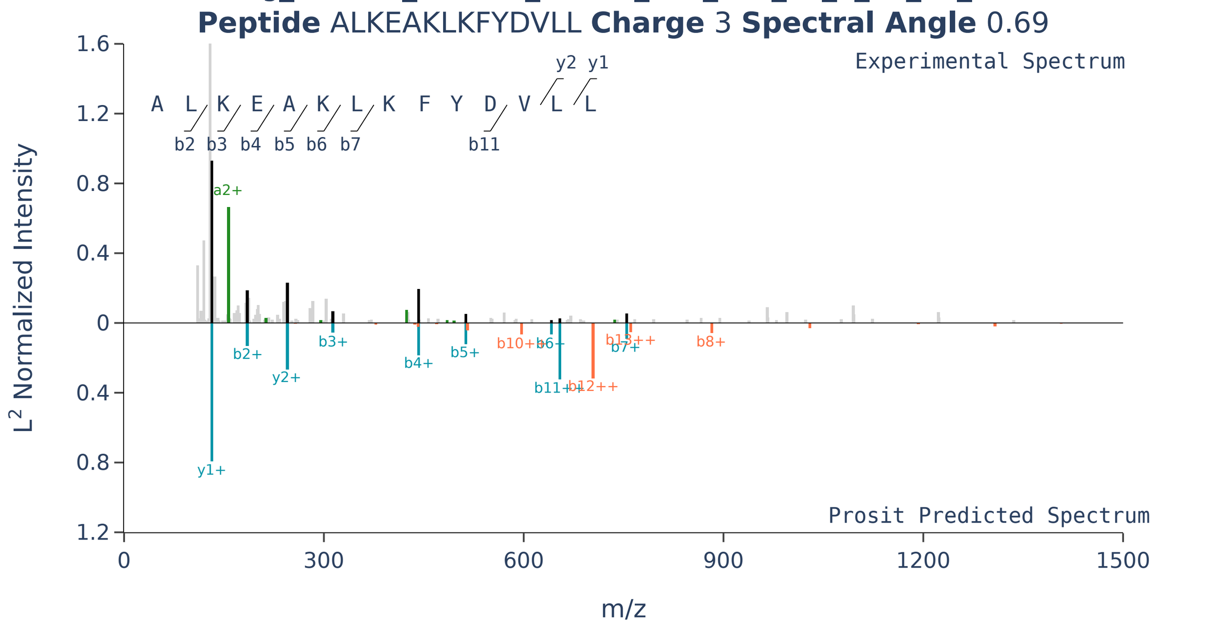


**Source** Ncheng_210623_230623_HFGoe_FFH_20S_25_1_A2_24h_R1 **Scan** 7018
**Peptide** KVDRAQAEKLA **Charge** 2 **Spectral Angle** 0.62

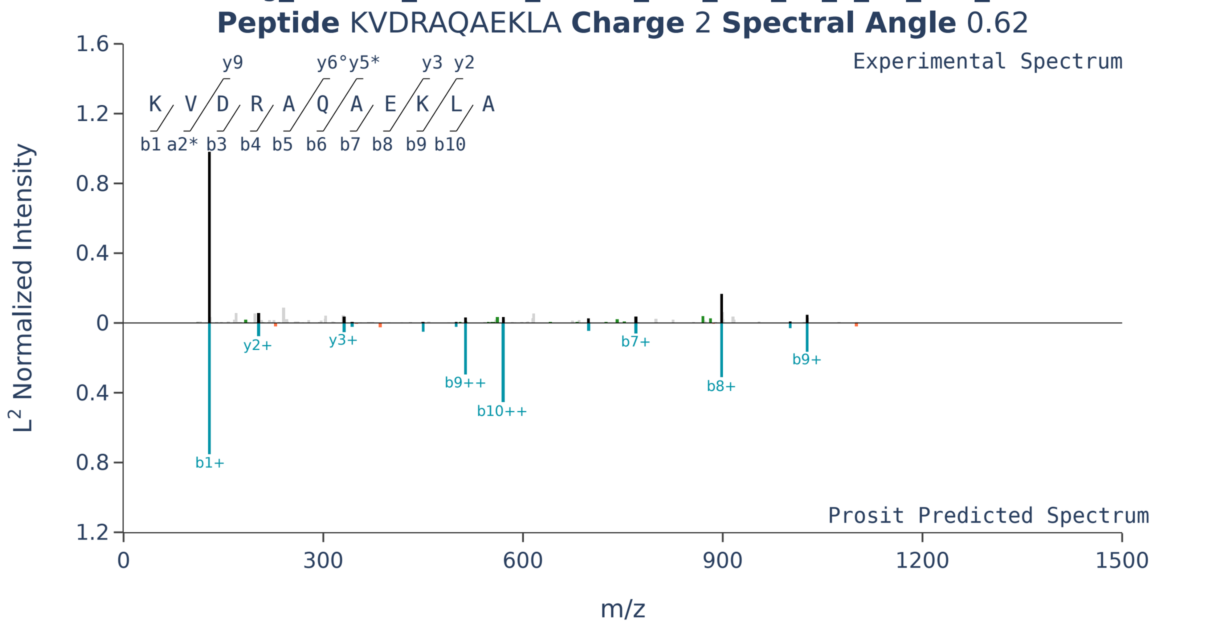


**Source** Ncheng_210623_230623_HFGoe_FFH_20S_25_1_A1_1h_R2 **Scan** 17159
**Peptide** ITGKPIKFLGVGEKTE **Charge** 2 **Spectral Angle** 0.71

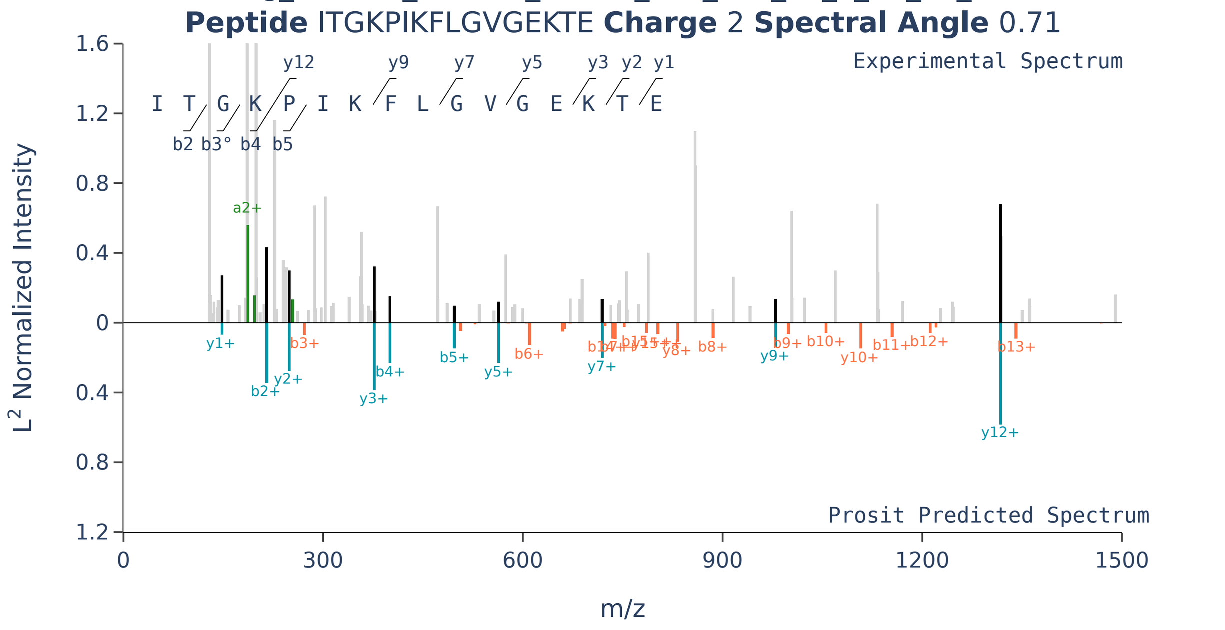


**Source** Ncheng_210623_230623_HFGoe_FFH_20S_25_1_A1_24h_R1 **Scan** 15247
**Peptide** MEAIINSMTMKERAKPEIIKGSRKR **Charge** 6 **Spectral Angle** 0.64

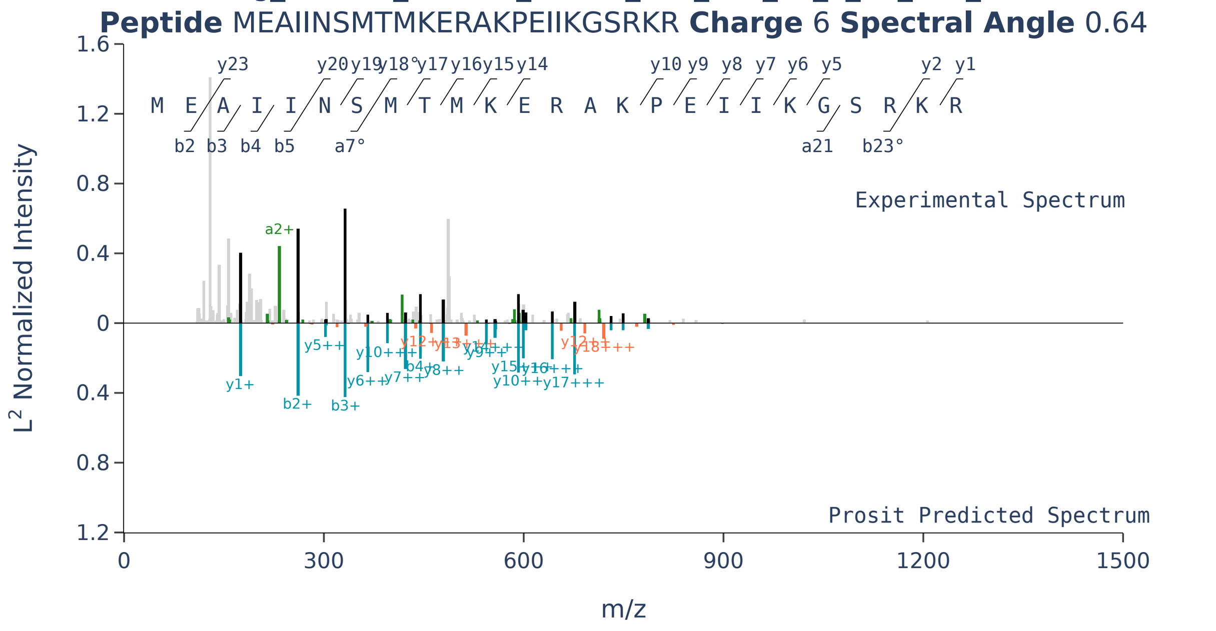


**Source** Ncheng_210623_230623_HFGoe_FFH_20S_25_1_A1_2h_R2 **Scan** 32402
**Peptide** ADVALPVVREFINRVKEKAVGHEVNKSL **Charge** 4 **Spectral Angle** 0.88

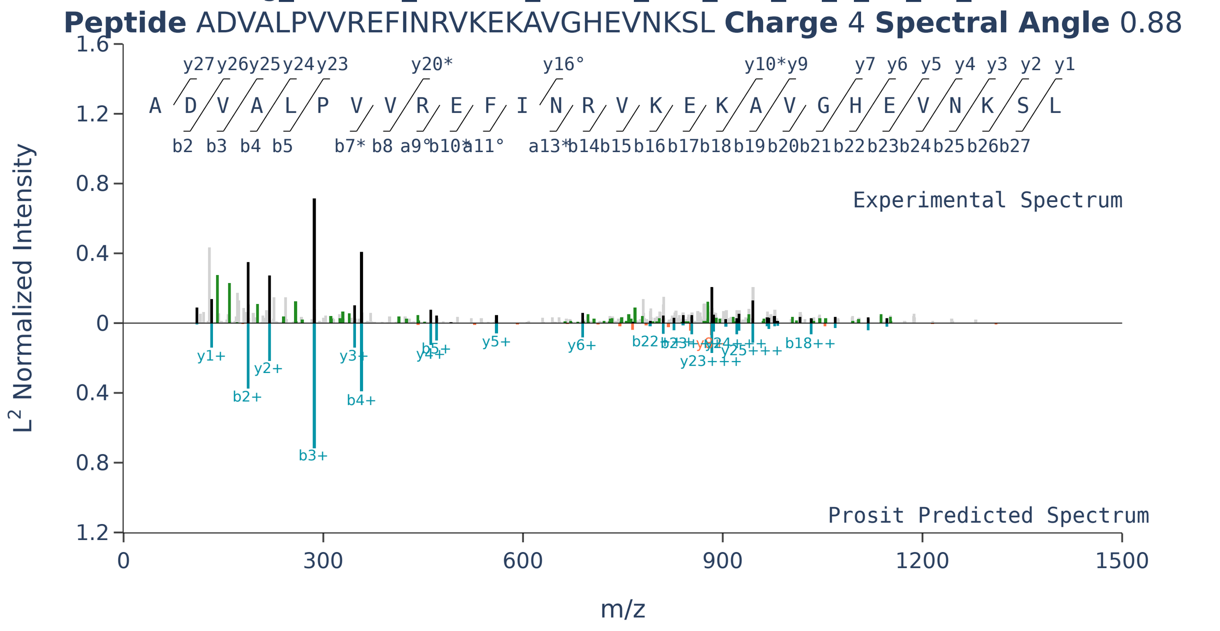


**Source** Ncheng_210623_230623_HFGoe_FFH_20S_25_1_A1_2h_R2 **Scan** 27134
**Peptide** VGQKPVDIVNAALKEAKLK **Charge** 3 **Spectral Angle** 0.71

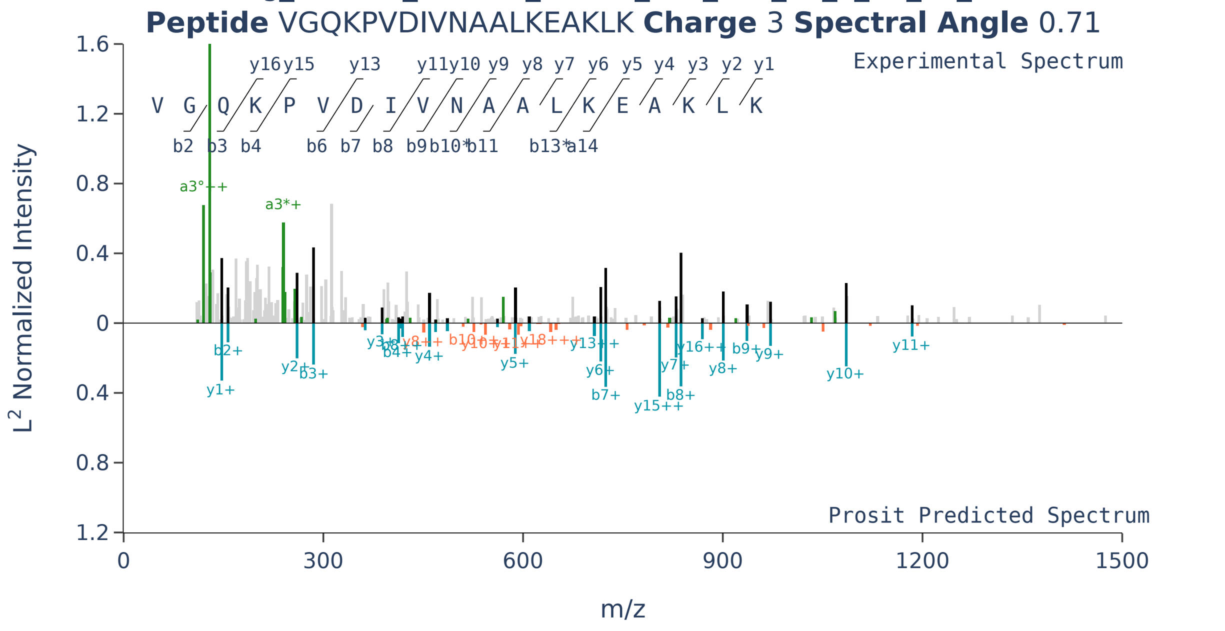


**Source** Ncheng_210623_230623_HFGoe_FFH_20S_25_1_A2_4h_R1 **Scan** 18554
**Peptide** VKEKAVGHEVNKSLTPGQEFVKIVRNE **Charge** 4 **Spectral Angle** 0.72

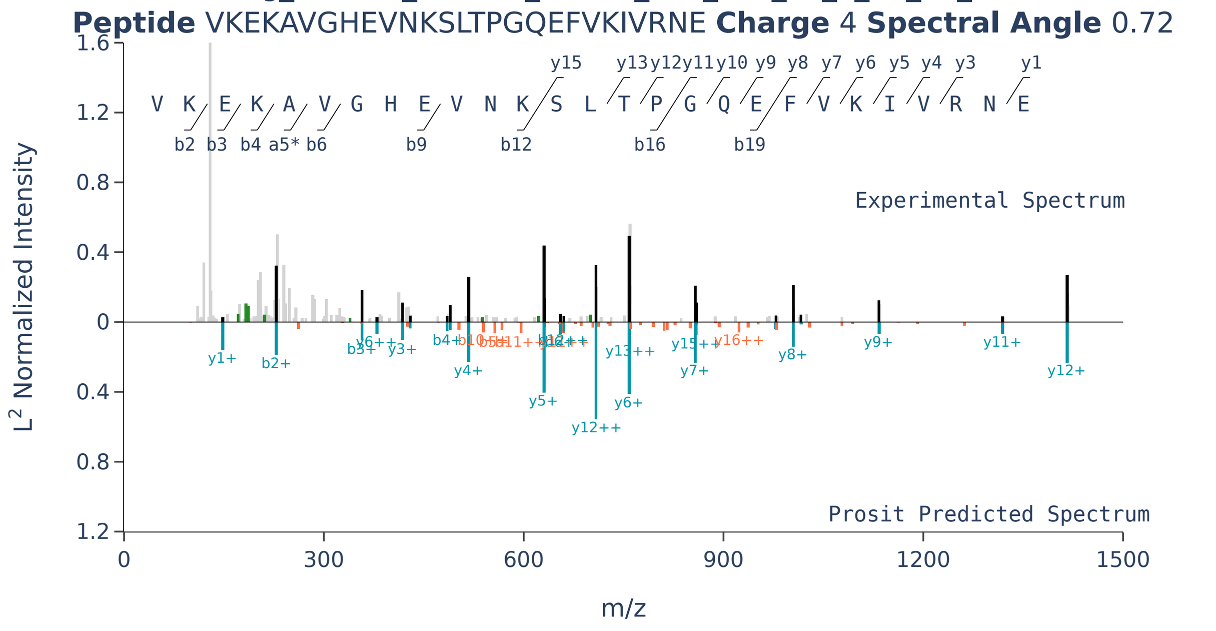


**Source** Ncheng_210623_230623_HFGoe_FFH_20S_25_1_A1_4h_R1 **Scan** 22165
**Peptide** GDARGGAALSIRHITGKPIKFL **Charge** 3 **Spectral Angle** 0.66

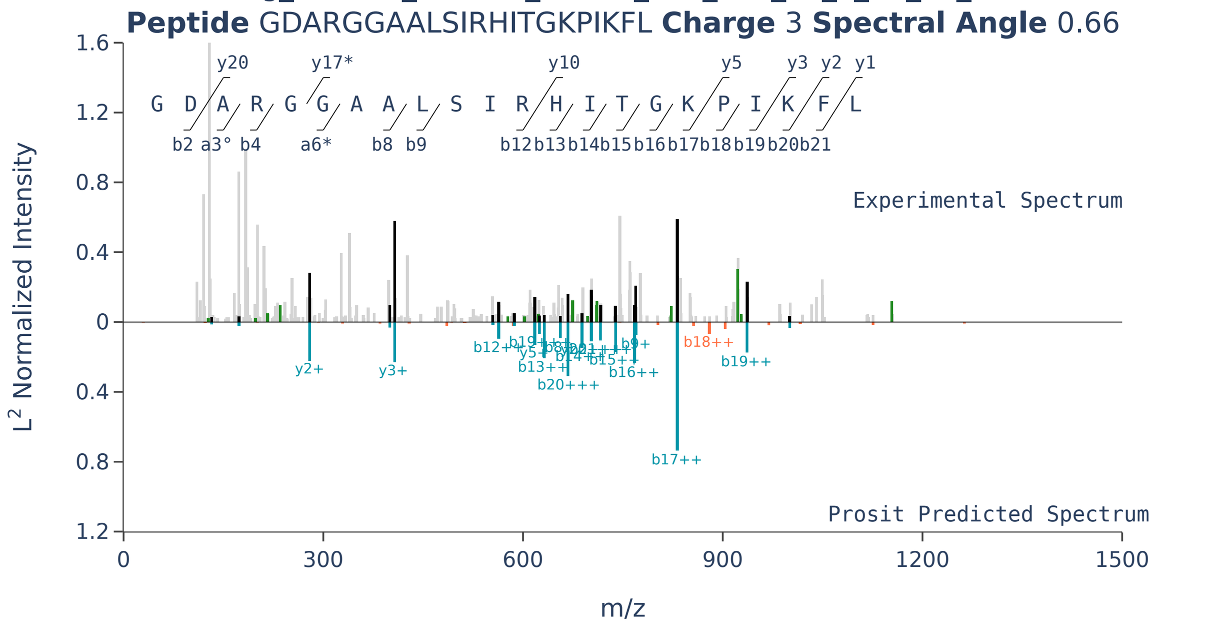


**Source** Ncheng_210623_230623_HFGoe_FFH_20S_25_1_A1_24h_R1 **Scan** 13186
**Peptide** TDRLSRTLRNISG **Charge** 2 **Spectral Angle** 0.78

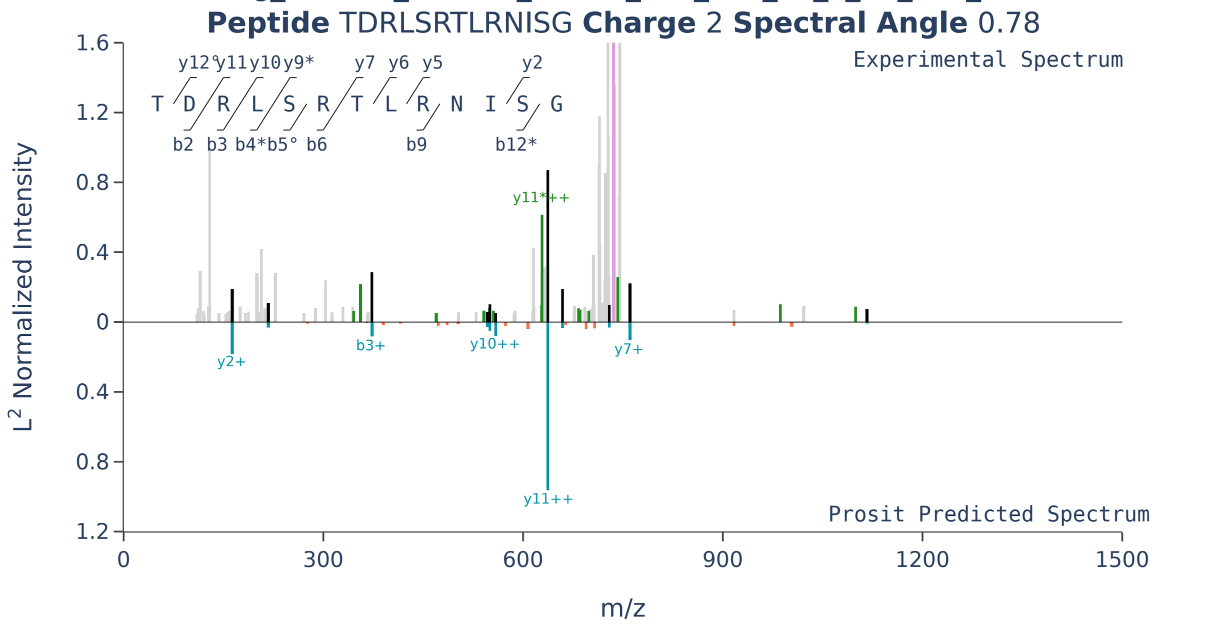


**Source** Ncheng_210623_230623_HFGoe_FFH_20S_25_1_A2_4h_R2 **Scan** 14520
**Peptide** AIINSMTMKERAKPEIIKGSR **Charge** 5 **Spectral Angle** 0.57

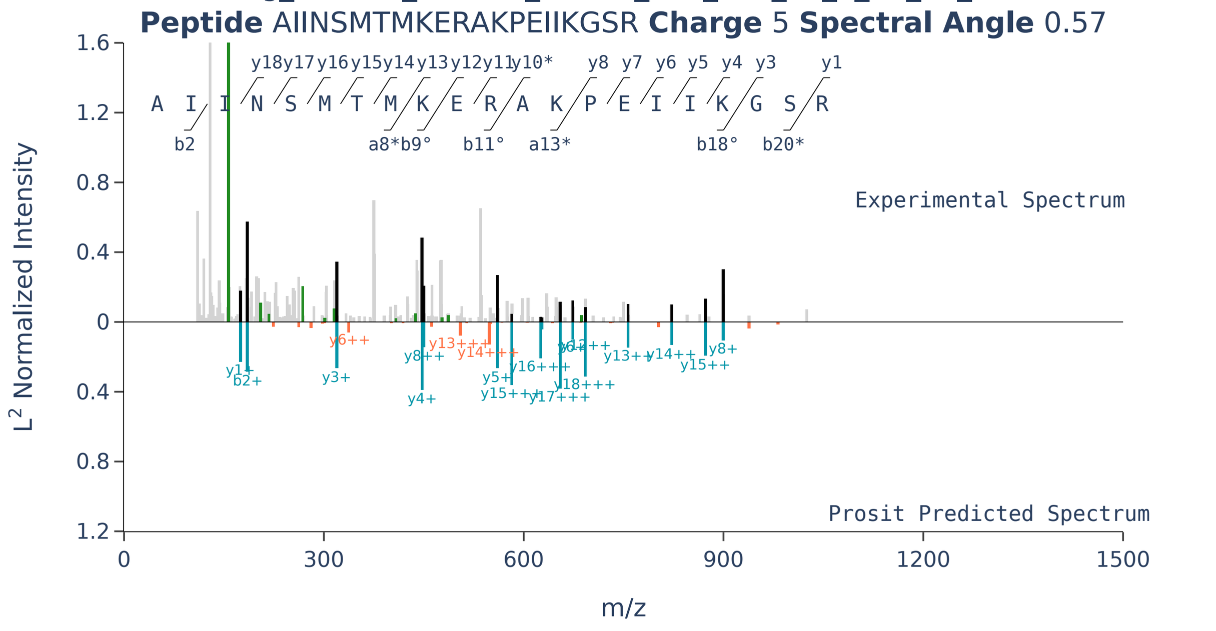


**Source** Ncheng_210623_230623_HFGoe_FFH_20S_25_1_A1_24h_R2 **Scan** 13388
**Peptide** LTKVDGDARGGAALSLR **Charge** 3 **Spectral Angle** 0.65

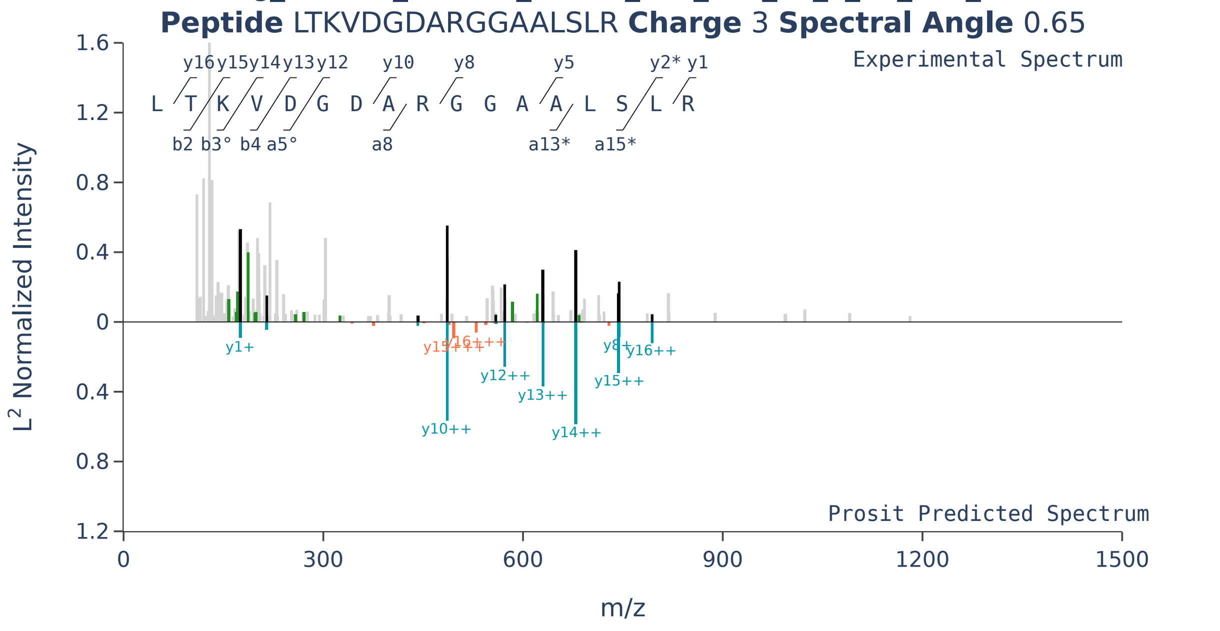


**Source** Ncheng_210623_230623_HFGoe_FFH_20S_25_1_A1_2h_R2 **Scan** 13689
**Peptide** RLAAGCGMQV **Charge** 2 **Spectral Angle** 0.84

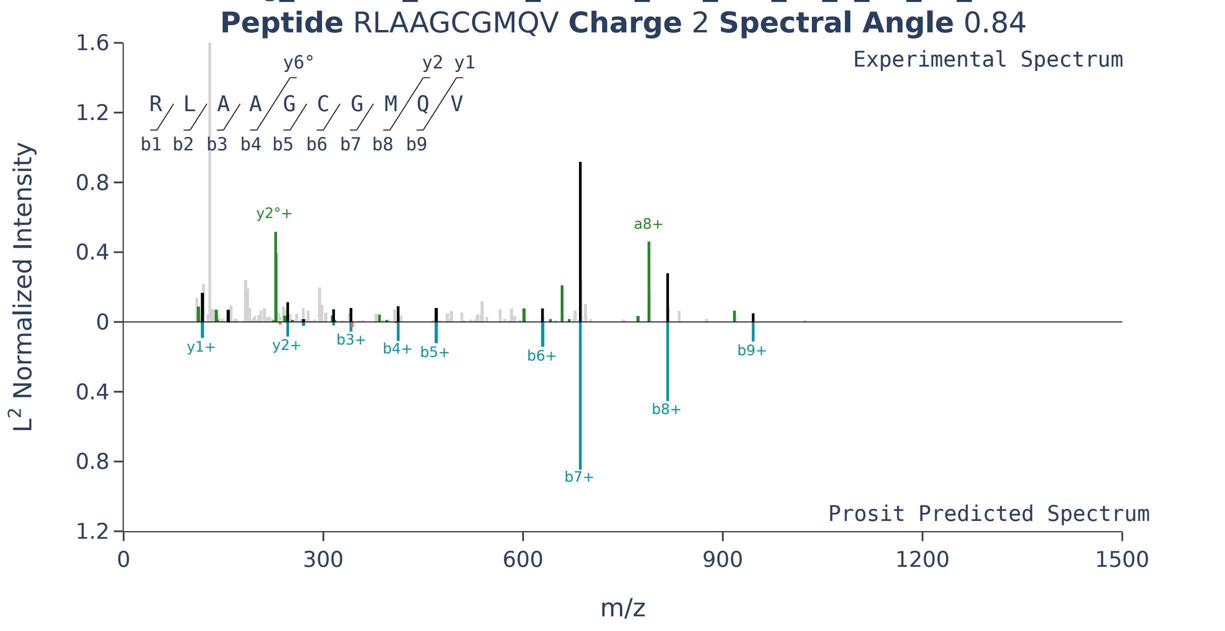

**Source** Ncheng_210623_230623_HFGoe_FFH_20S_25_1_A1_4h_R1 **Scan** 20883
**Peptide** QIPDNVKSQMDDKVLV **Charge** 2 **Spectral Angle** 0.79

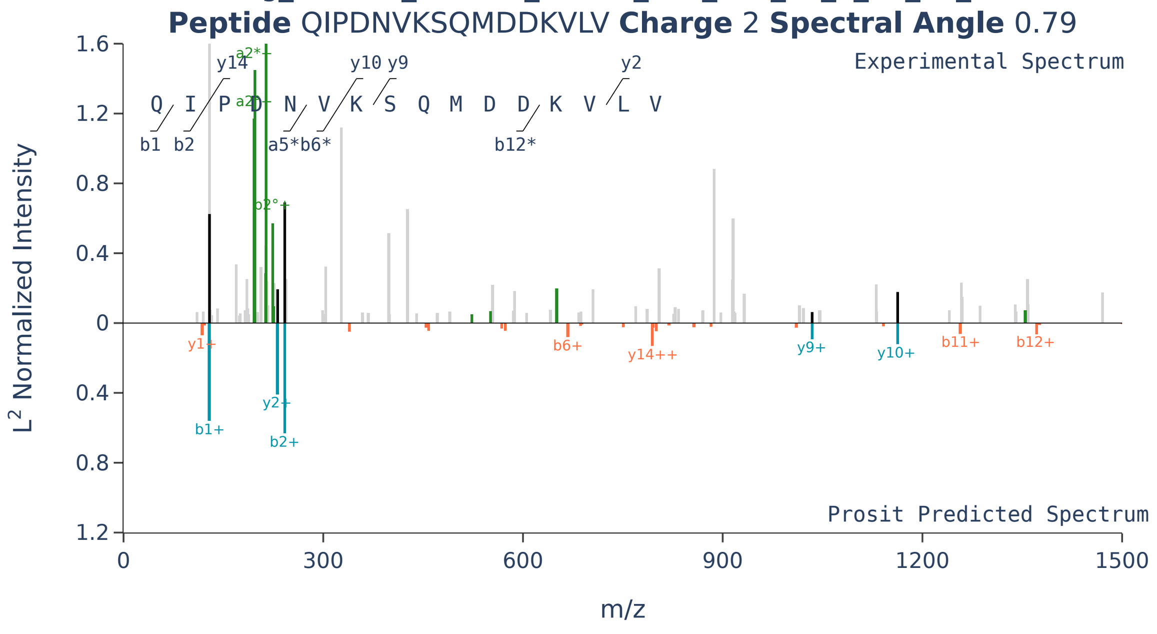


**Source** Ncheng_210623_230623_HFGoe_FFH_20S_25_1_A2_24h_R1 **Scan** 12944
**Peptide** LEQLRQMKNMG **Charge** 2 **Spectral Angle** 0.58

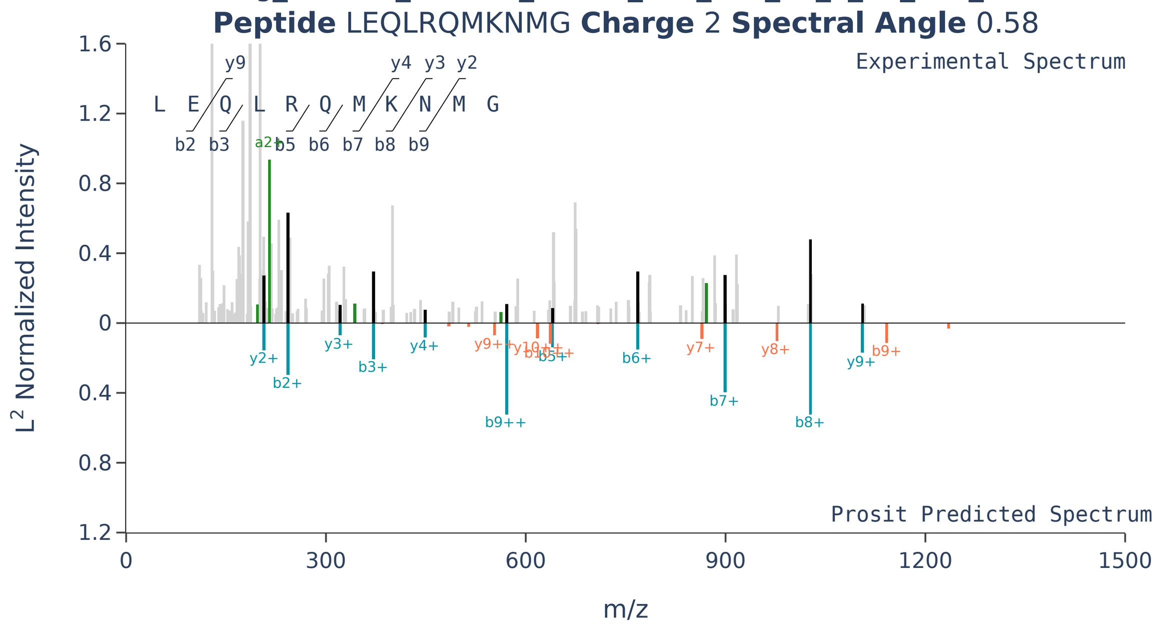


**Source** Ncheng_210623_230623_HFGoe_FFH_20S_25_1_A2_4h_R2 **Scan** 15772
**Peptide** NRVKEKAVGHEVNKSLTPGQEFVKIVRN **Charge** 4 **Spectral Angle** 0.63

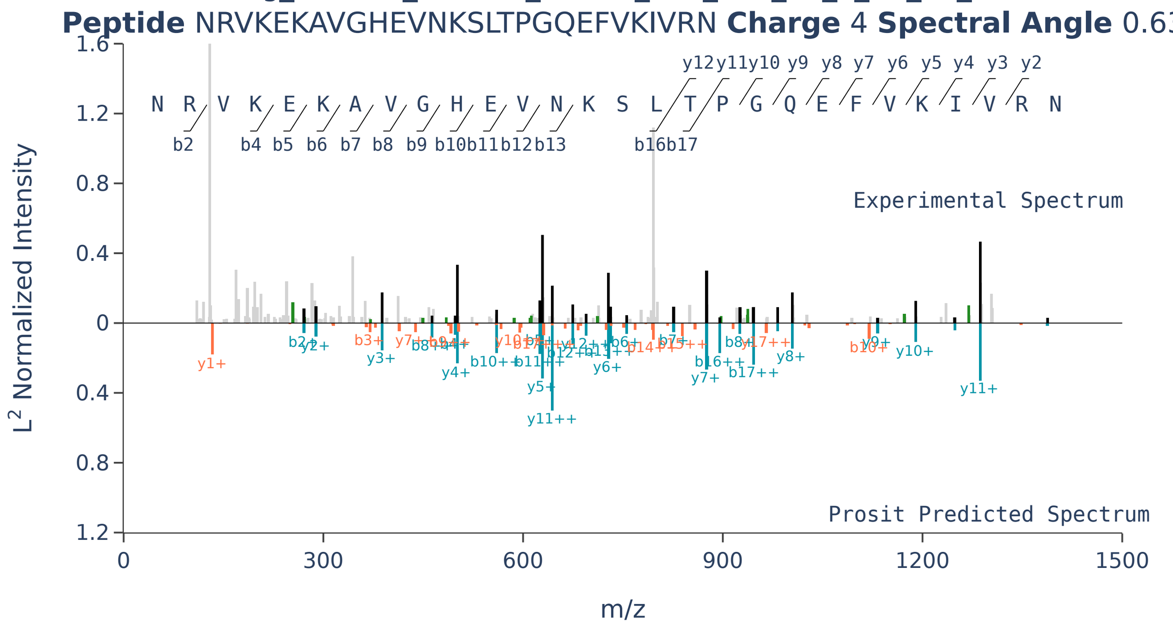


**Source** Ncheng_210623_230623_HFGoe_FFH_20S_25_1_A1_2h_R1 **Scan** 13140
**Peptide** KLASKLKKGDGFDL **Charge** 4 **Spectral Angle** 0.94

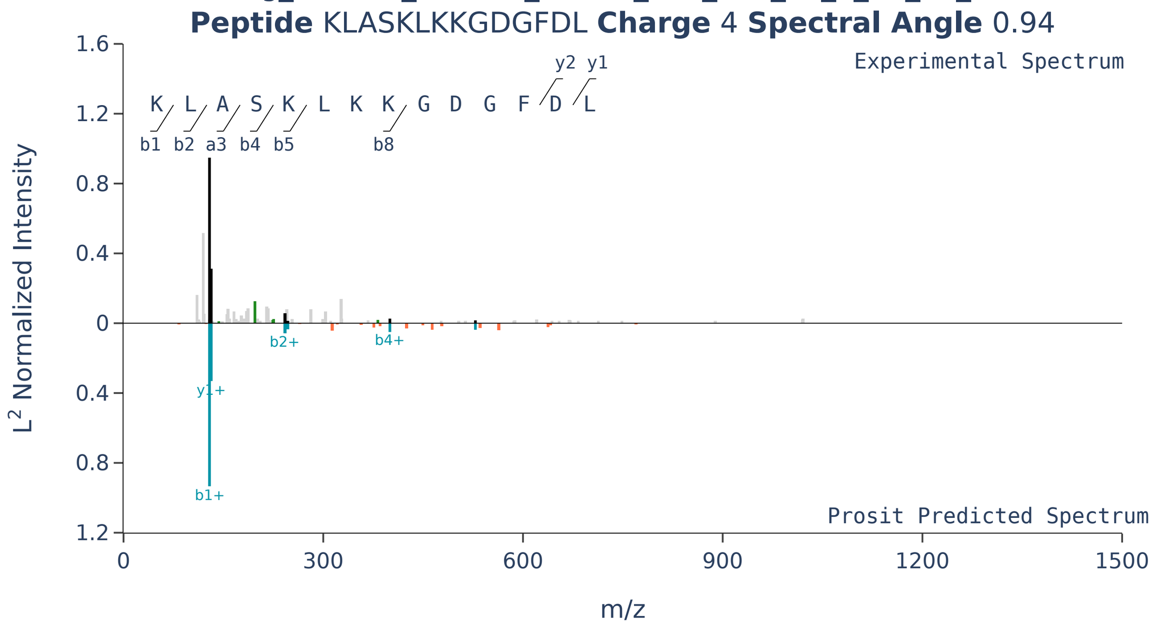


**Source** Ncheng_210623_230623_HFGoe_FFH_20S_25_1_A2_4h_R2 **Scan** 14366
**Peptide** RAQAEKLASKLKKGDGFDLND **Charge** 2 **Spectral Angle** 0.6

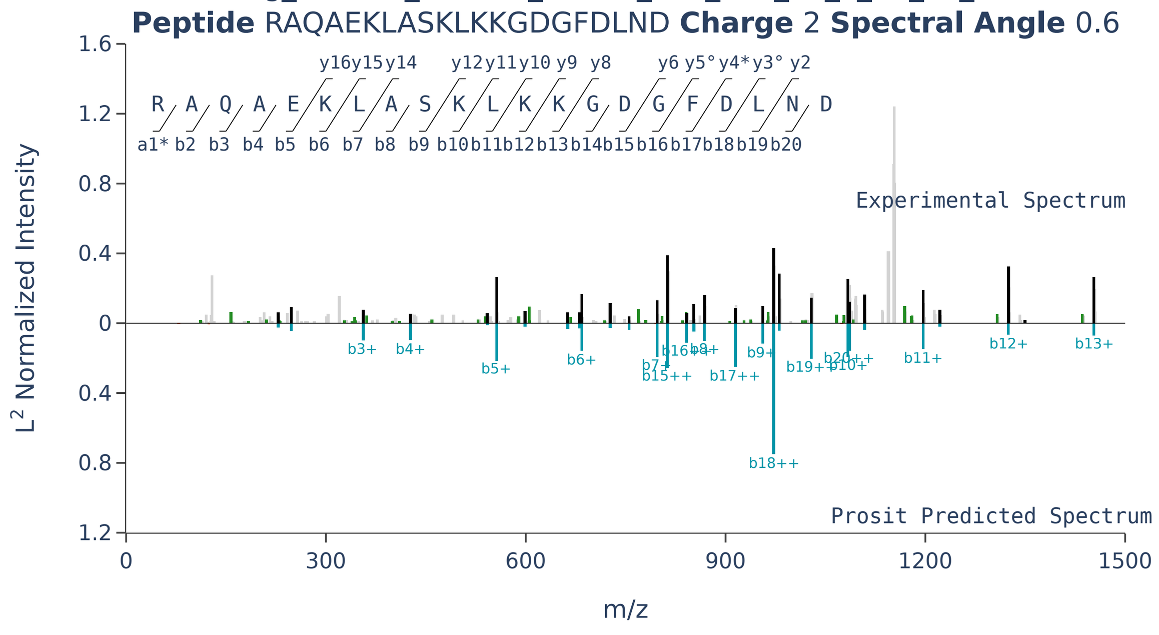


**Source** Ncheng_210623_230623_HFGoe_FFH_20S_25_1_A1_4h_R2 **Scan** 28257
**Peptide** SADVYRPAAIKQLETLAEQ **Charge** 3 **Spectral Angle** 0.78

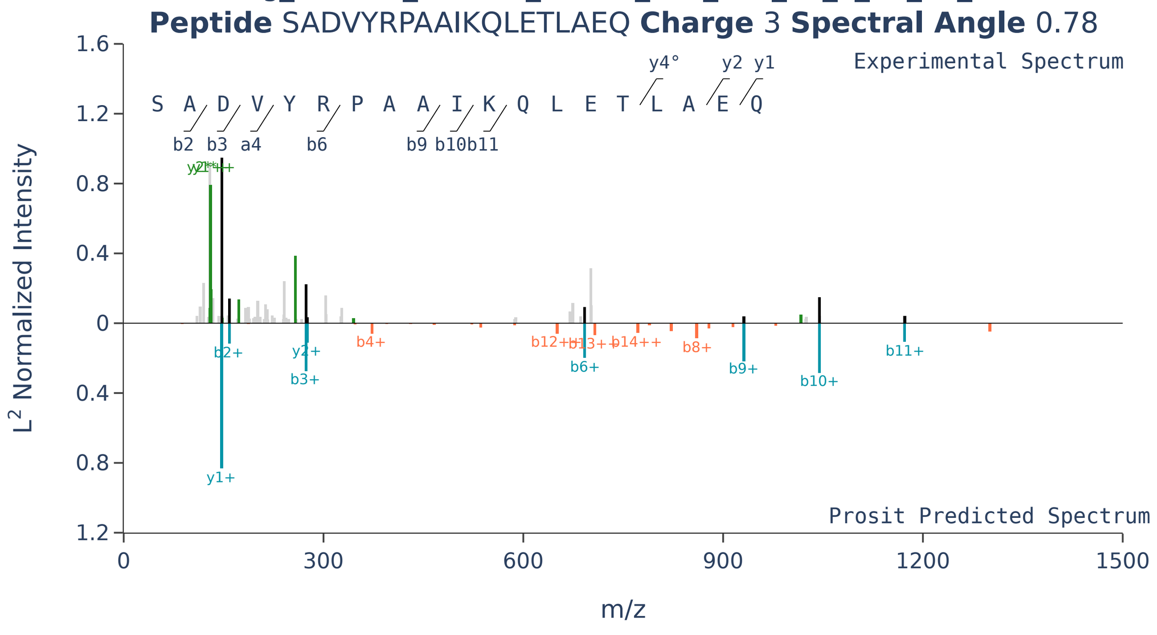


**Source** Ncheng_210623_230623_HFGoe_FFH_20S_25_1_A2_2h_R1 **Scan** 29141
**Peptide** KKGDGFDLNDFLE **Charge** 2 **Spectral Angle** 0.48

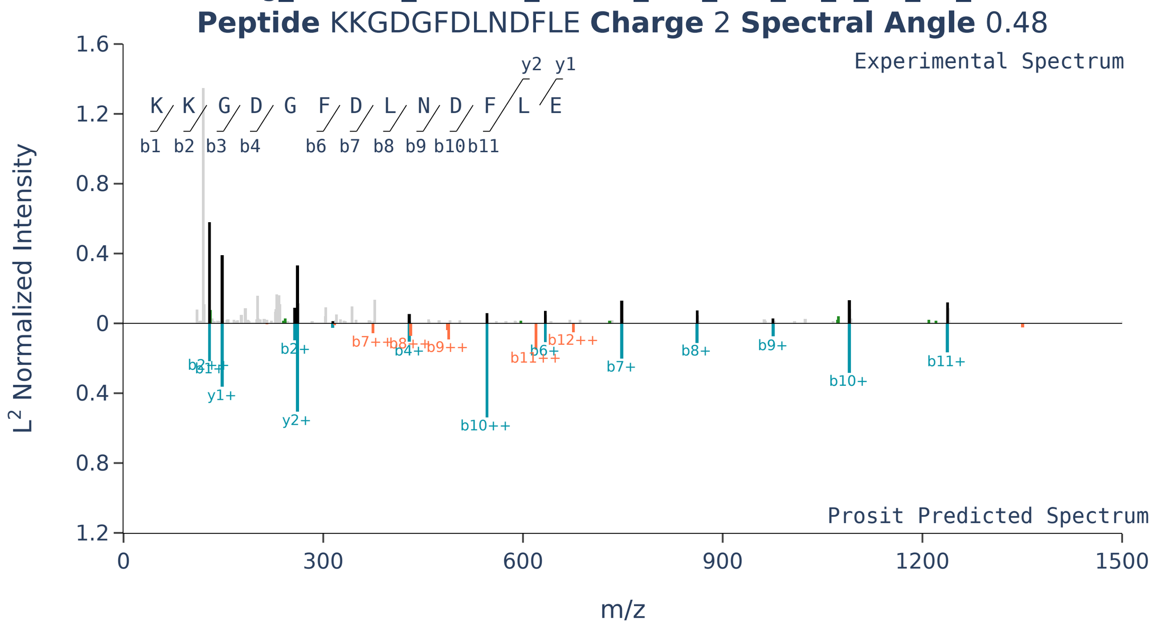


**Source** Ncheng_210623_230623_HFGoe_FFH_20S_25_1_A2_4h_R1 **Scan** 30644
**Peptide** EALPLTGVVLTKVDGDARGGAALSIRHI **Charge** 3 **Spectral Angle** 0.58

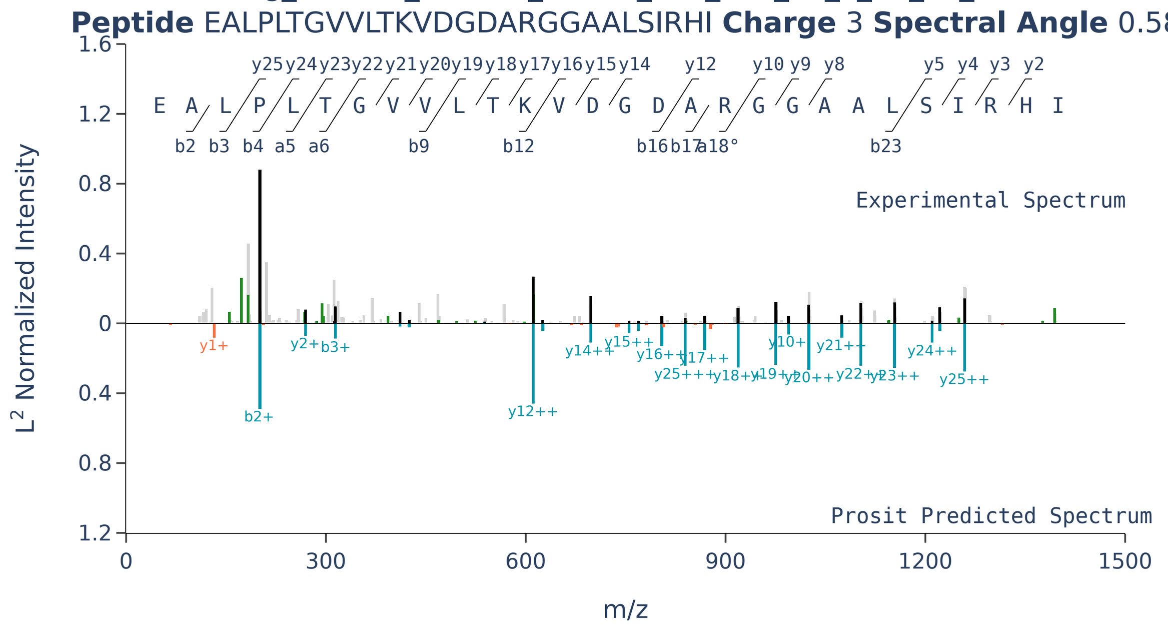


**Source** Ncheng_210623_230623_HFGoe_FFH_20S_25_1_A2_24h_R1 **Scan** 8829
**Peptide** LRNISGRGRLTEDNVKD **Charge** 4 **Spectral Angle** 0.63

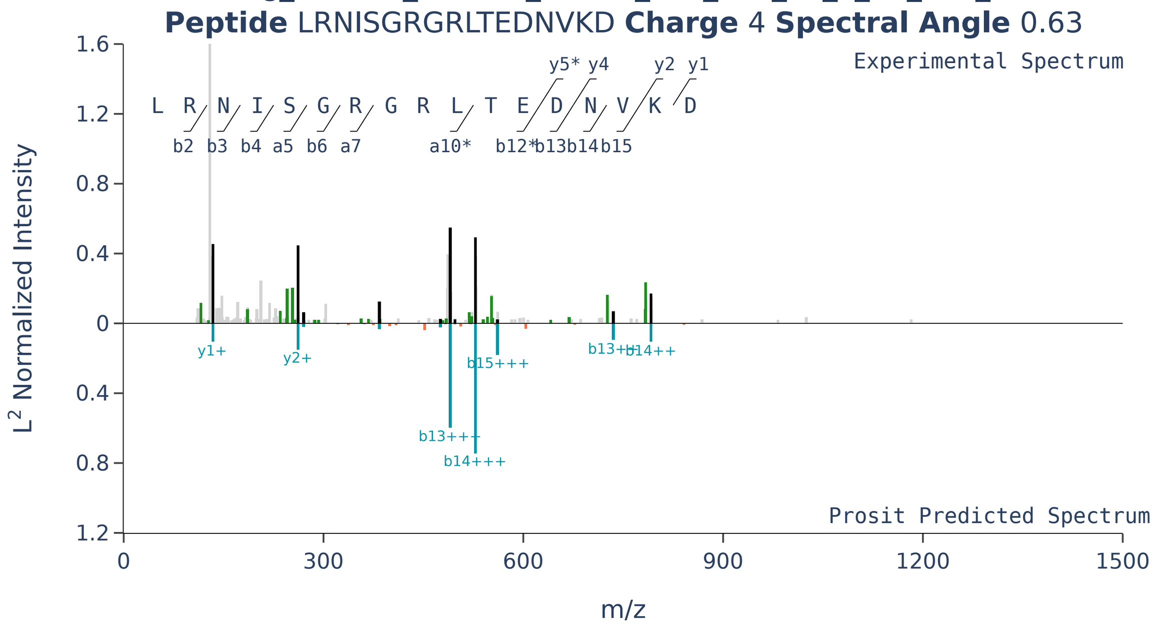


**Source** Ncheng_210623_230623_HFGoe_FFH_20S_25_1_A1_1h_R2 **Scan** 20143
**Peptide** AALSIRHITGKPIKFLGVGEKTE **Charge** 3 **Spectral Angle** 0.68

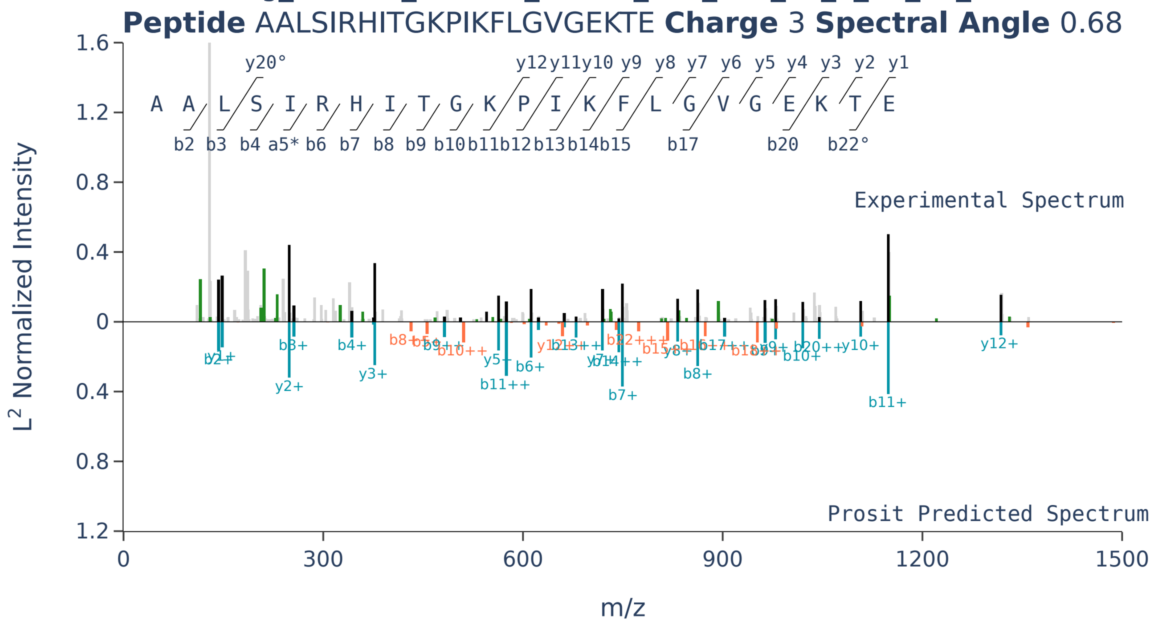


**Source** Ncheng_210623_230623_HFGoe_FFH_20S_25_1_A1_4h_R1 **Scan** 26052
**Peptide** EVNKSLTPGQEFVKIV **Charge** 2 **Spectral Angle** 0.7

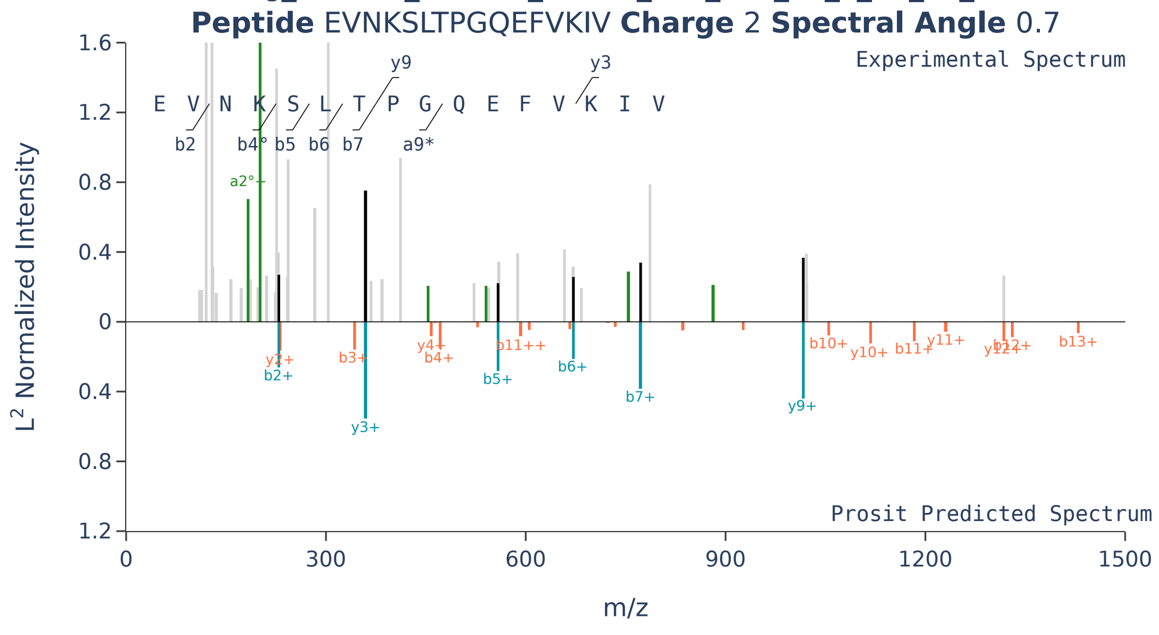


**Source** Ncheng_210623_230623_HFGoe_FFH_20S_25_1_A2_4h_R2 **Scan** 24167
**Peptide** SLTPGQEFVKIVR **Charge** 2 **Spectral Angle** 0.54

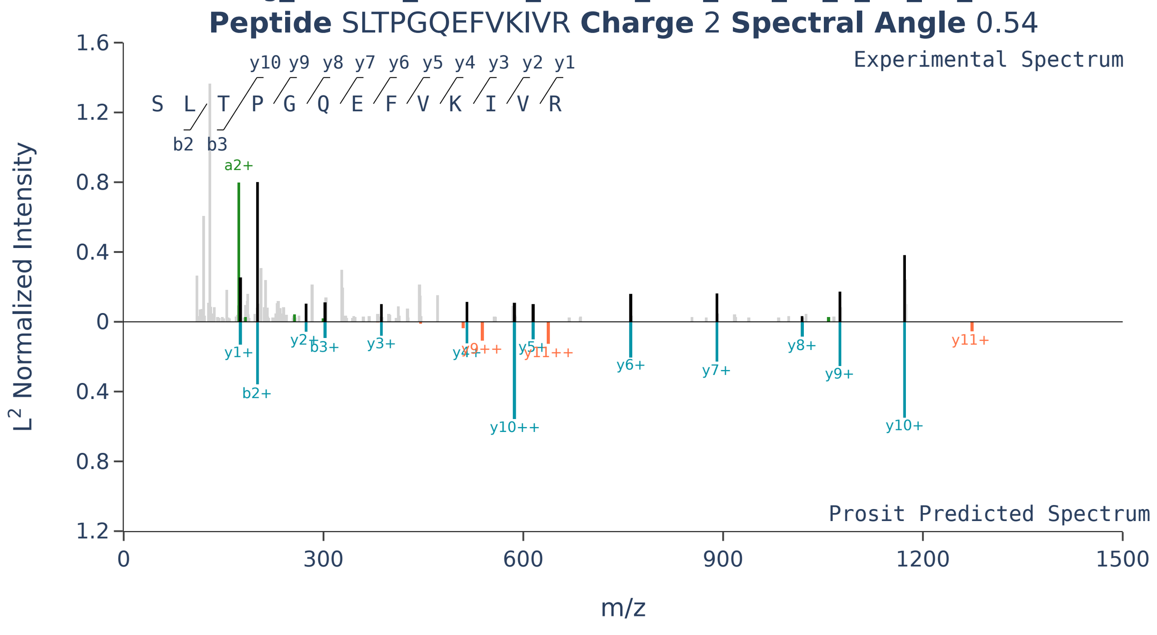


**Source** Ncheng_210623_230623_HFGoe_FFH_20S_25_1_A2_24h_R2 **Scan** 16402
**Peptide** PVVREFINRVKE **Charge** 2 **Spectral Angle** 0.61

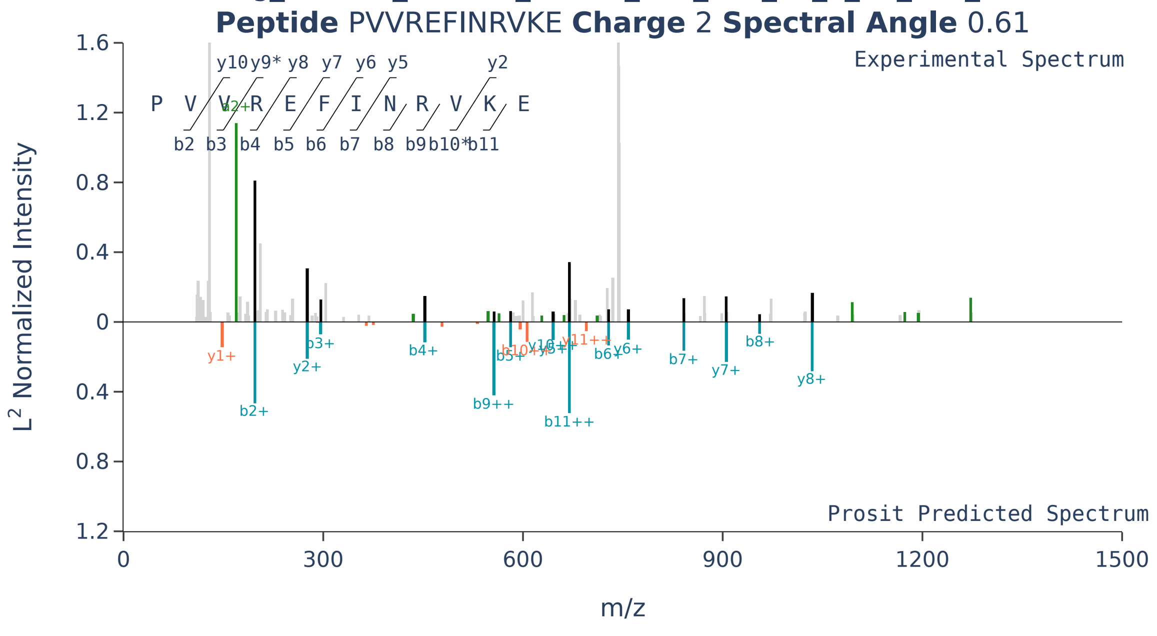


**Source** Ncheng_210623_230623_HFGoe_FFH_20S_25_1_A1_24h_R2 **Scan** 9069
**Peptide** EFINRVKEKA **Charge** 2 **Spectral Angle** 0.61

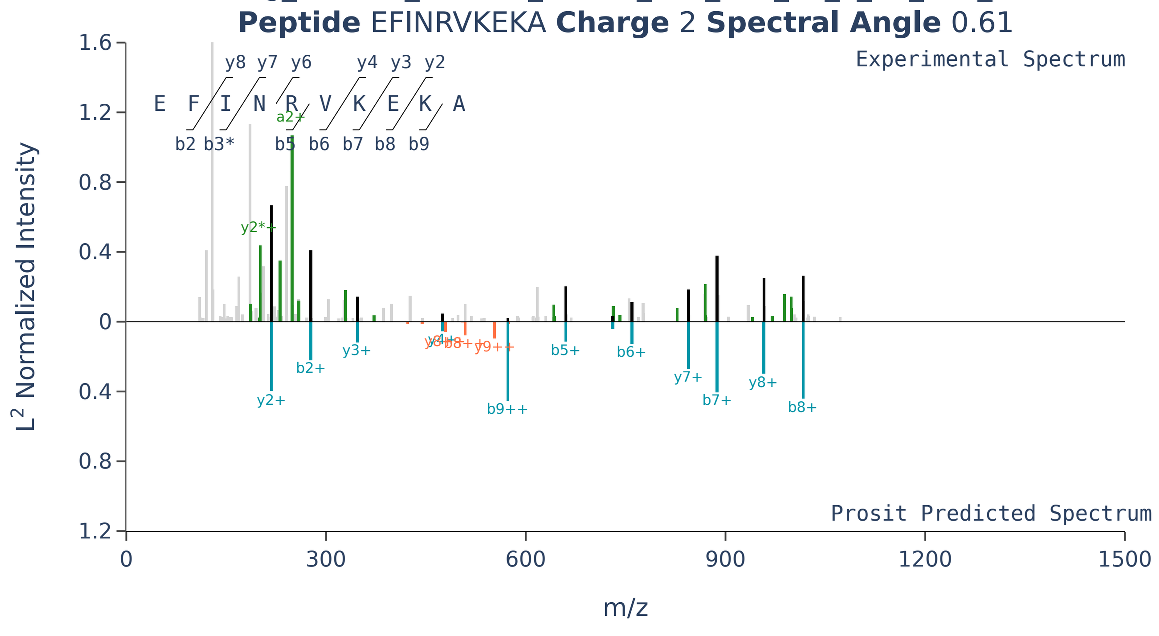


**Source** Ncheng_210623_230623_HFGoe_FFH_20S_25_1_A2_24h_R2 **Scan** 26559
**Peptide** SLTPGQEFVKI **Charge** 2 **Spectral Angle** 0.65

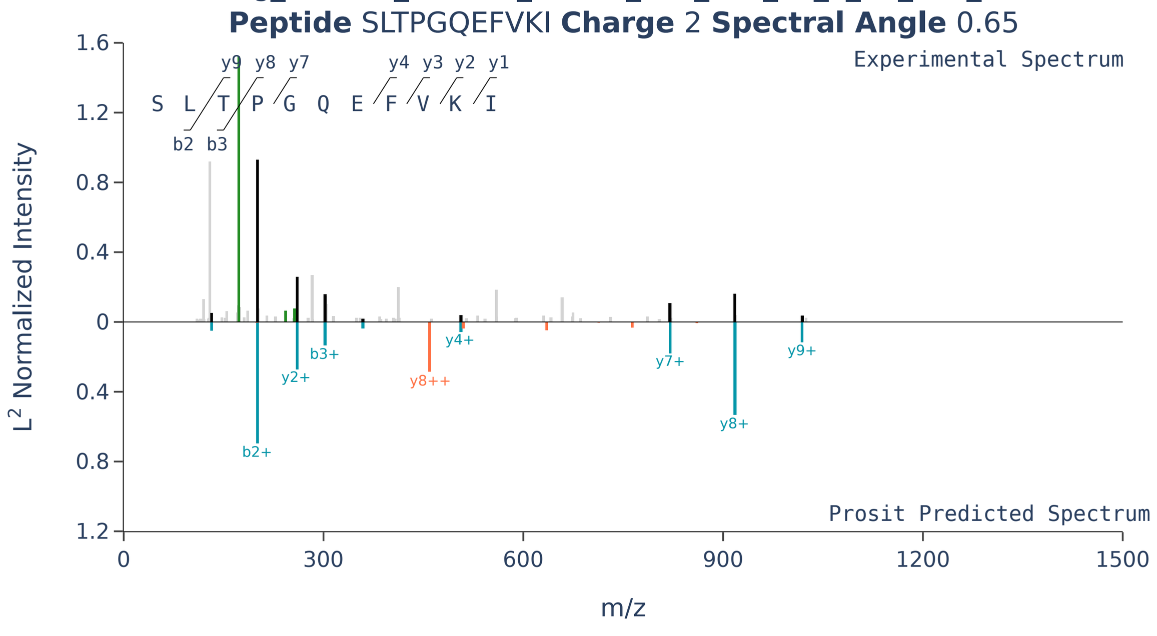

**Source** Ncheng_210623_230623_HFGoe_FFH_20S_25_1_A1_24h_R2 **Scan** 13922
**Peptide** KQFDDMQRMMKKMKKGGMAK **Charge** 5 **Spectral Angle** 0.88

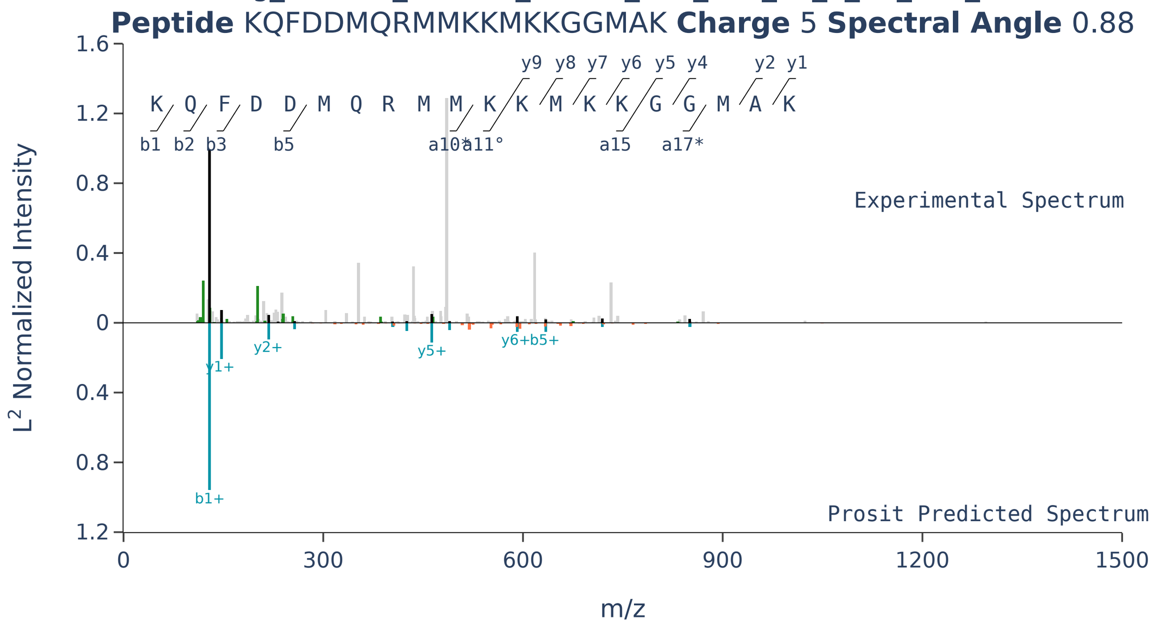


**Source** Ncheng_210623_230623_HFGoe_FFH_20S_25_1_A1_2h_R1 **Scan** 27897
**Peptide** IVNAALKEAKLKFYDVL **Charge** 4 **Spectral Angle** 0.88

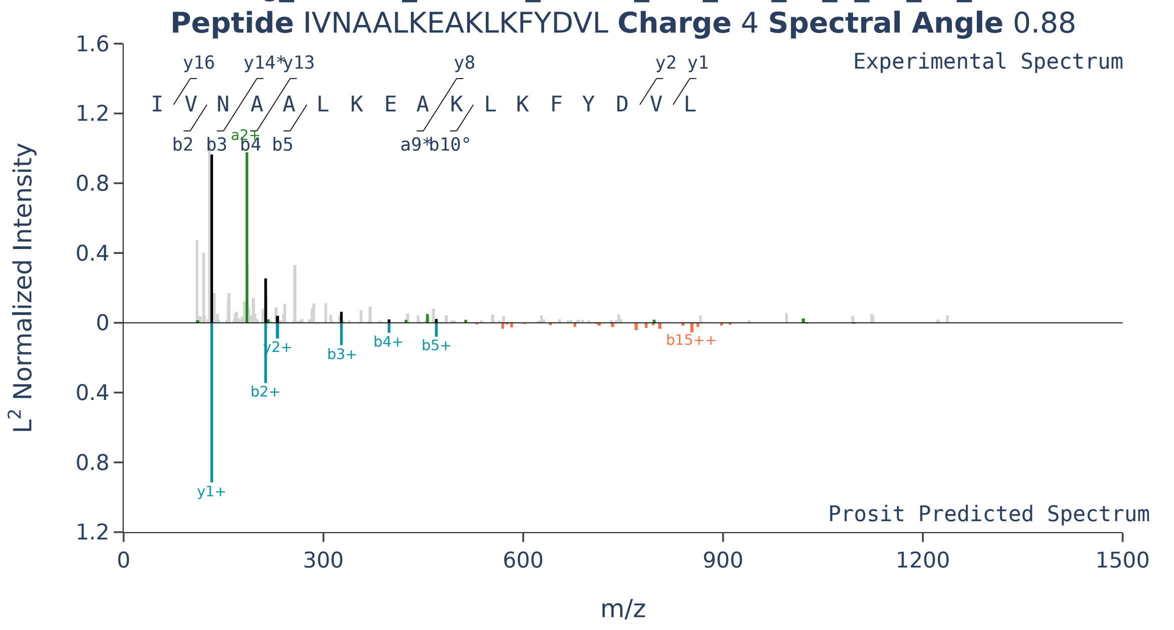


**Source** Ncheng_210623_230623_HFGoe_FFH_20S_25_1_A1_2h_R2 **Scan** 30151
**Peptide** GVGEKTEALEPFHPDRIASRILGMGD **Charge** 4 **Spectral Angle** 0.8

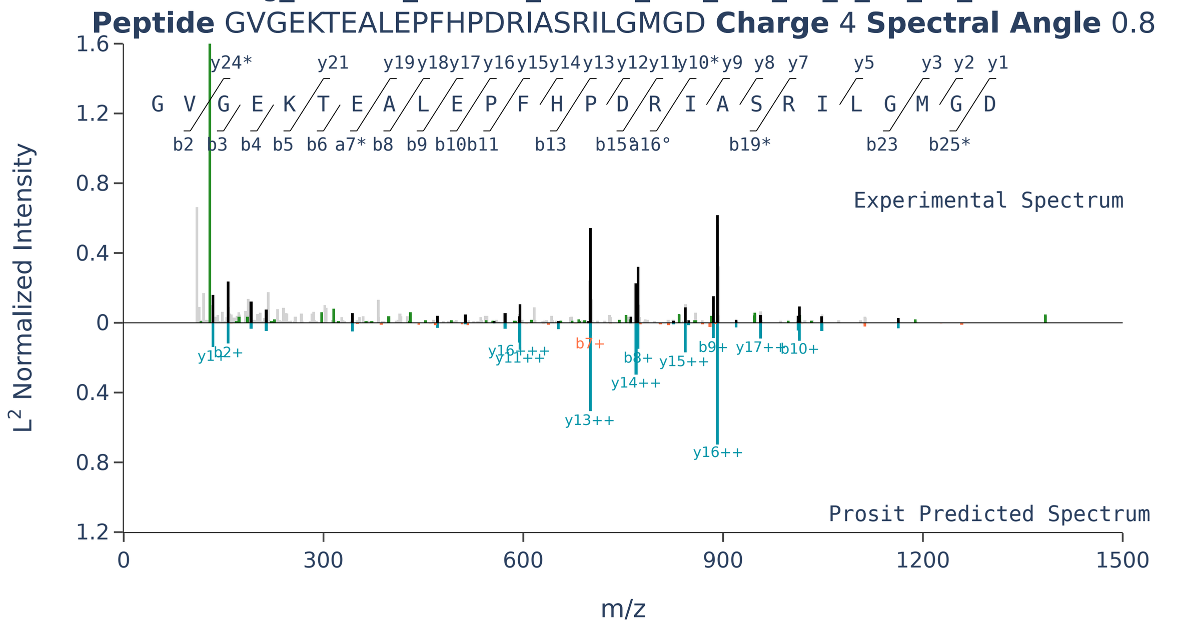


**Source** Ncheng_210623_230623_HFGoe_FFH_20S_25_1_A1_2h_R1 **Scan** 19415
**Peptide** DIESKVDRAQAEKLASKLKKGDGF **Charge** 3 **Spectral Angle** 0.72

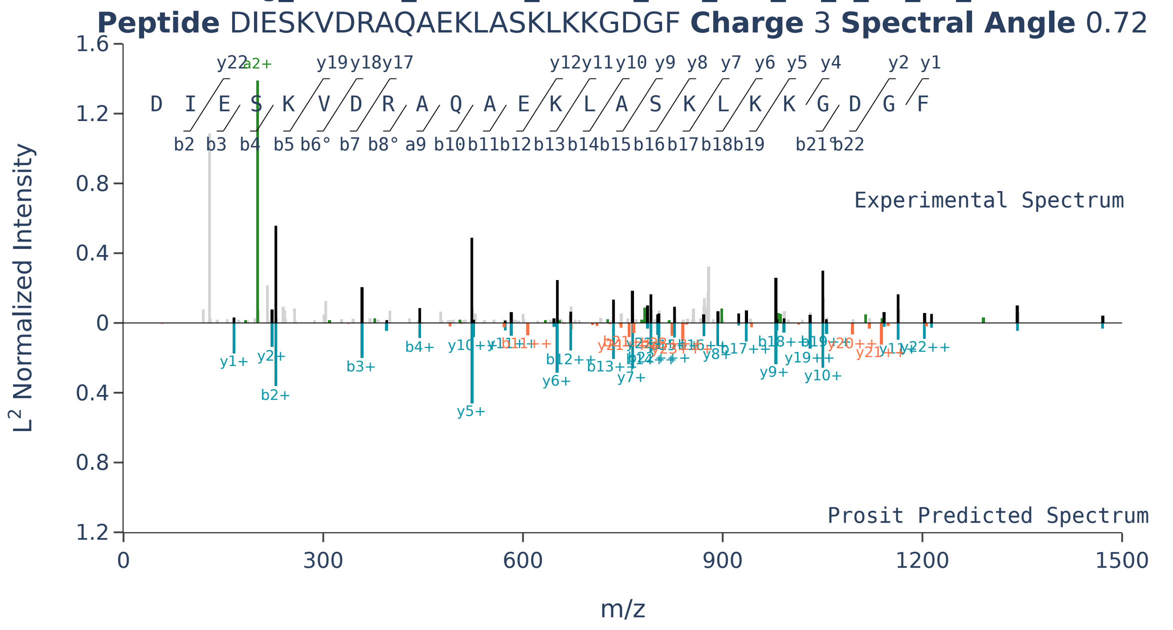


**Source** Ncheng_210623_230623_HFGoe_FFH_20S_25_1_A2_4h_R1 **Scan** 35656
**Peptide** QDAANTAKAFNEALPLTGVVLTKV **Charge** 3 **Spectral Angle** 0.71

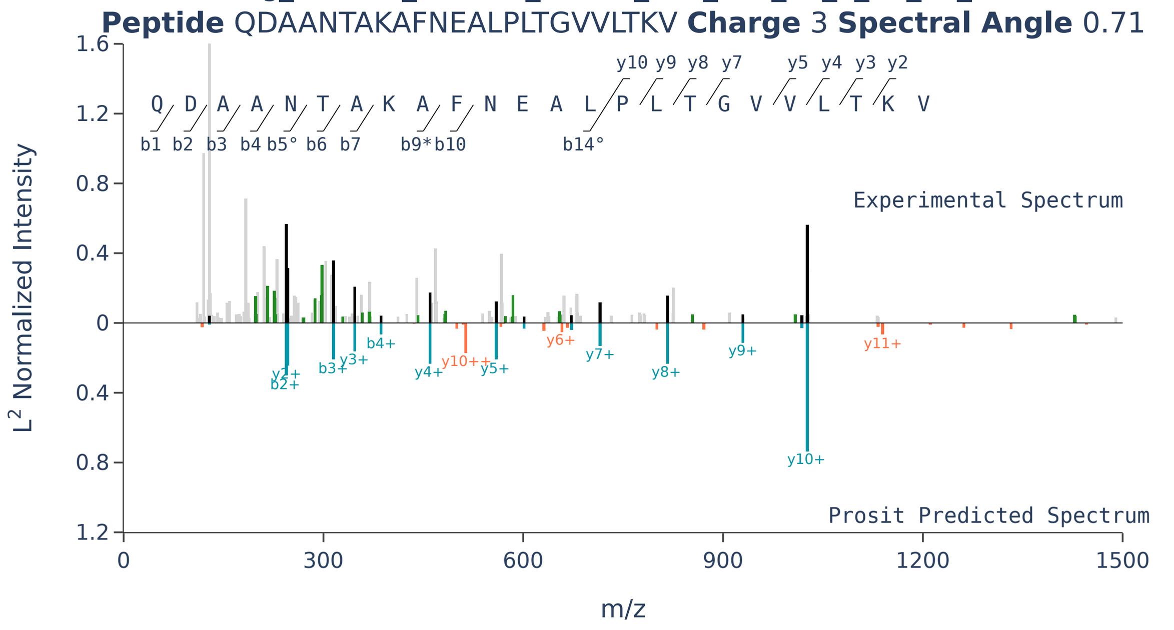


**Source** Ncheng_210623_230623_HFGoe_FFH_20S_25_1_A2_24h_R2 **Scan** 17175
**Peptide** KTEALEPFHPDRLAS **Charge** 3 **Spectral Angle** 0.69

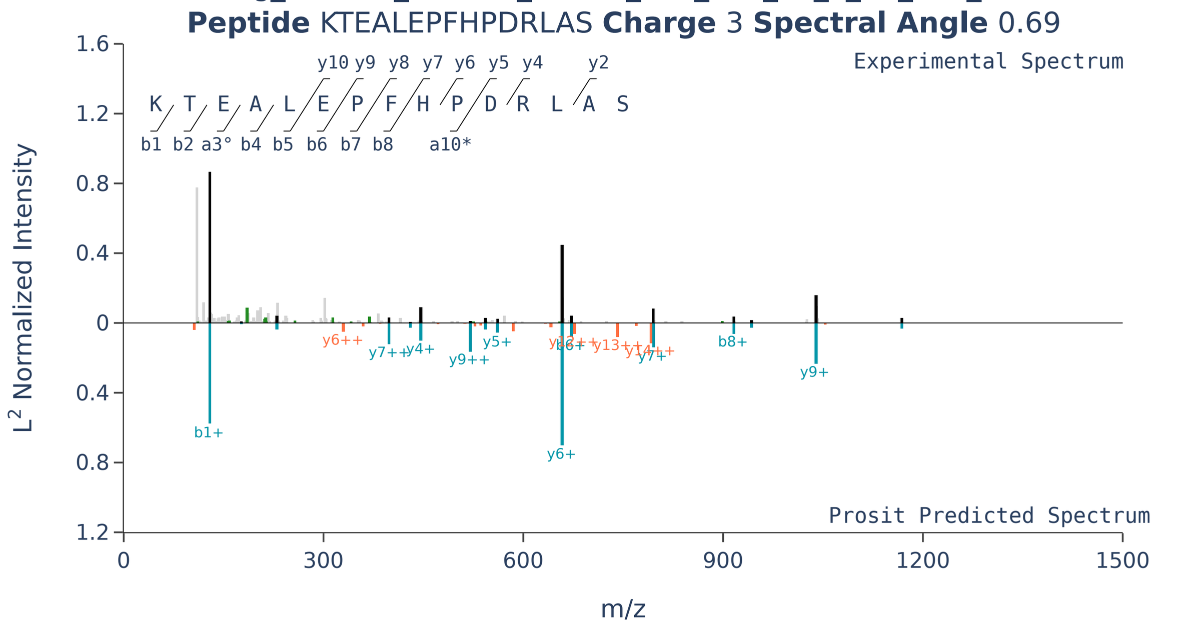


**Source** Ncheng_210623_230623_HFGoe_FFH_20S_25_1_A1_24h_R2 **Scan** 16977
**Peptide** LESKVDRAQAEKLASKLKKGDGFD **Charge** 3 **Spectral Angle** 0.69

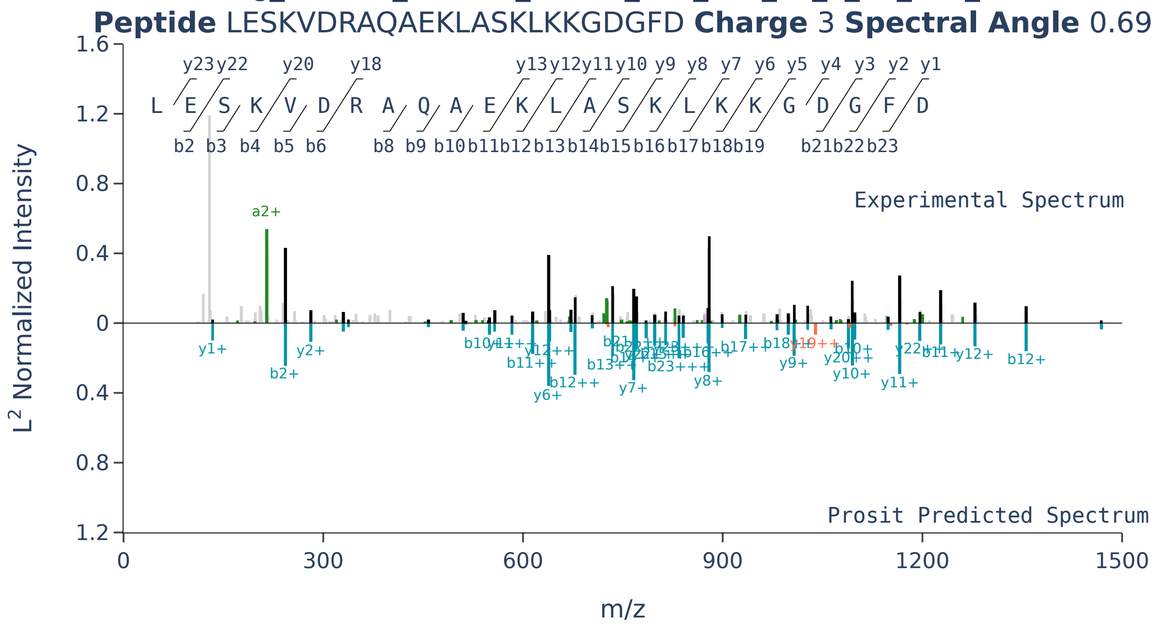


**Source** Ncheng_210623_230623_HFGoe_FFH_20S_25_1_A1_24h_R2 **Scan** 11436
**Peptide** KVDGDARGGAALSLR **Charge** 3 **Spectral Angle** 0.4

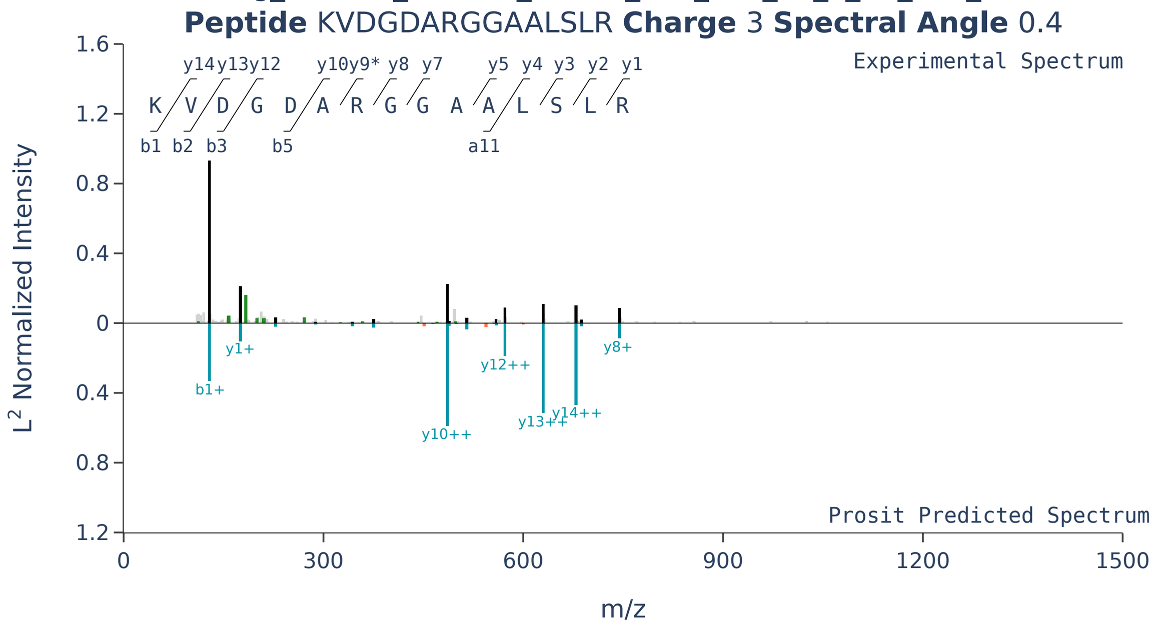


**Source** Ncheng_210623_230623_HFGoe_FFH_20S_25_1_A2_2h_R2 **Scan** 18601
**Peptide** SIRHITGKPIKFLGVGEKTEALE **Charge** 5 **Spectral Angle** 0.83

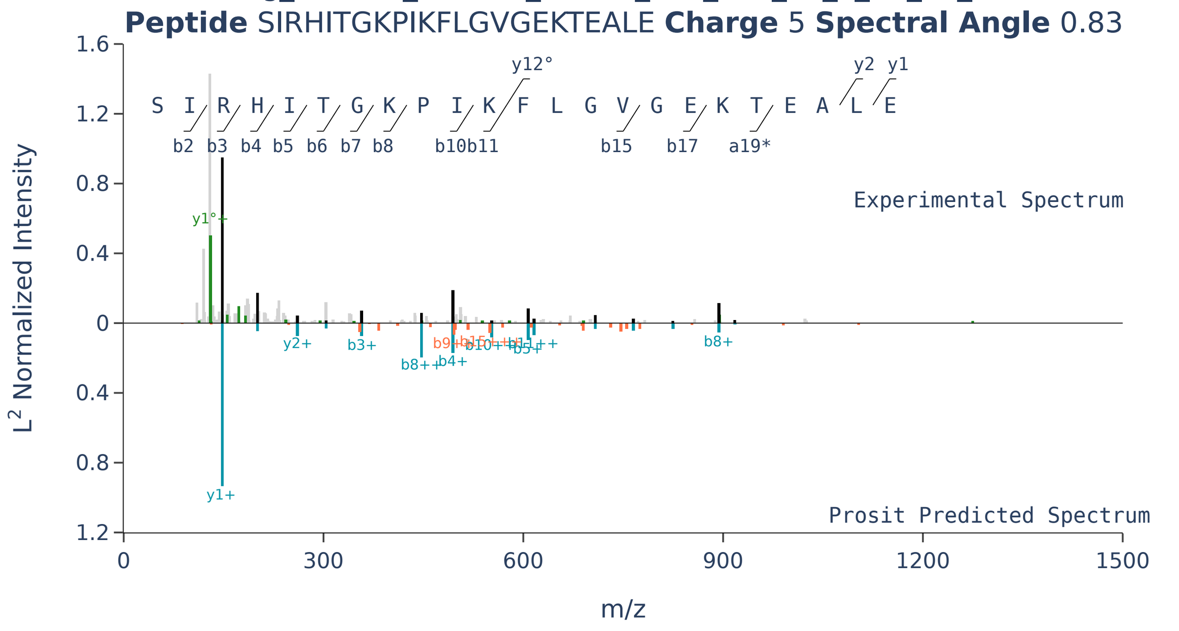


**Source** Ncheng_210623_230623_HFGoe_FFH_20S_25_1_A1_2h_R2 **Scan** 38090
**Peptide** KKGDGFDLNDFLEQLRQMKNM **Charge** 3 **Spectral Angle** 0.85

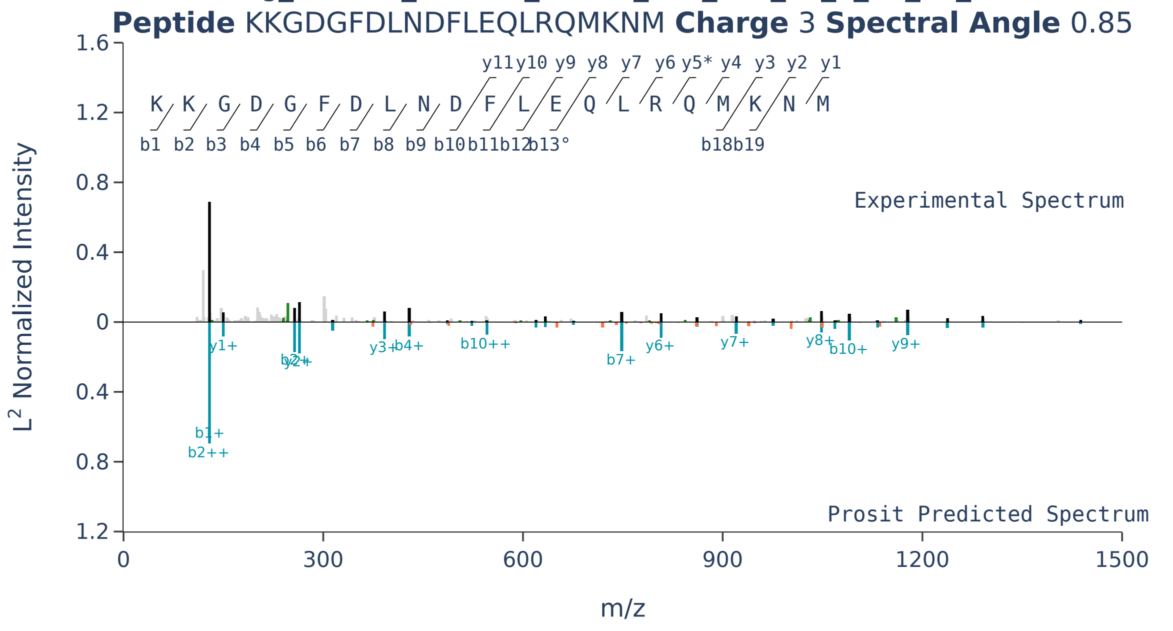


**Source** Ncheng_210623_230623_HFGoe_FFH_20S_25_1_A2_1h_R1 **Scan** 22591
**Peptide** LKEAKLKFYDVL **Charge** 3 **Spectral Angle** 0.77

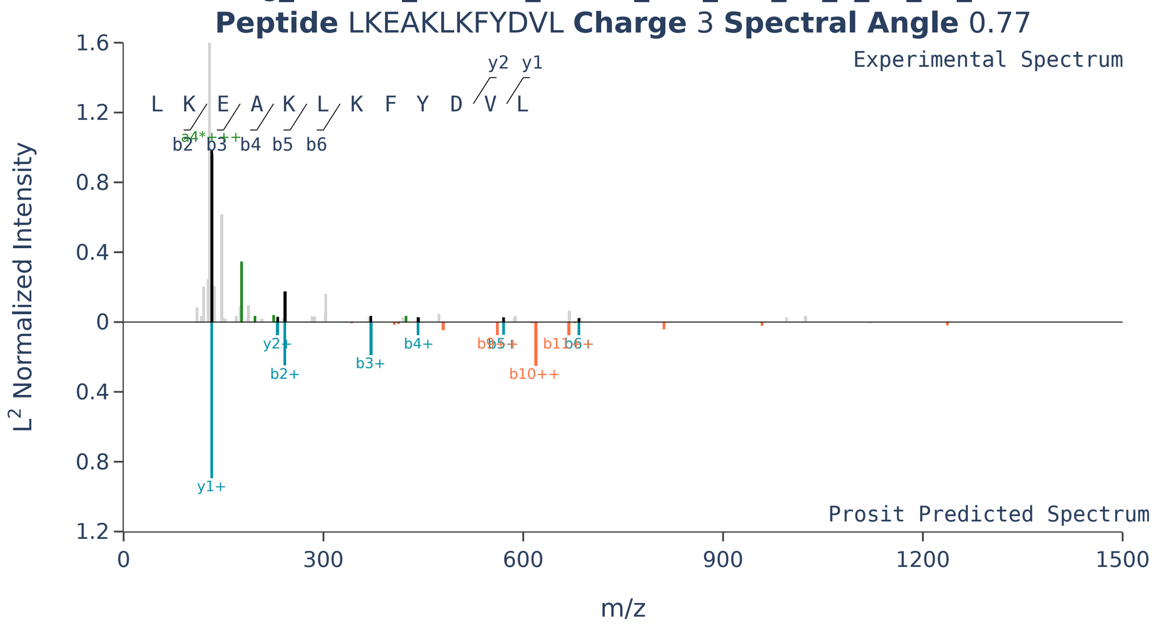


**Source** Ncheng_210623_230623_HFGoe_FFH_20S_25_1_A2_24h_R2 **Scan** 7096
**Peptide** MTMKERAKPEIIKGSRK **Charge** 5 **Spectral Angle** 0.8

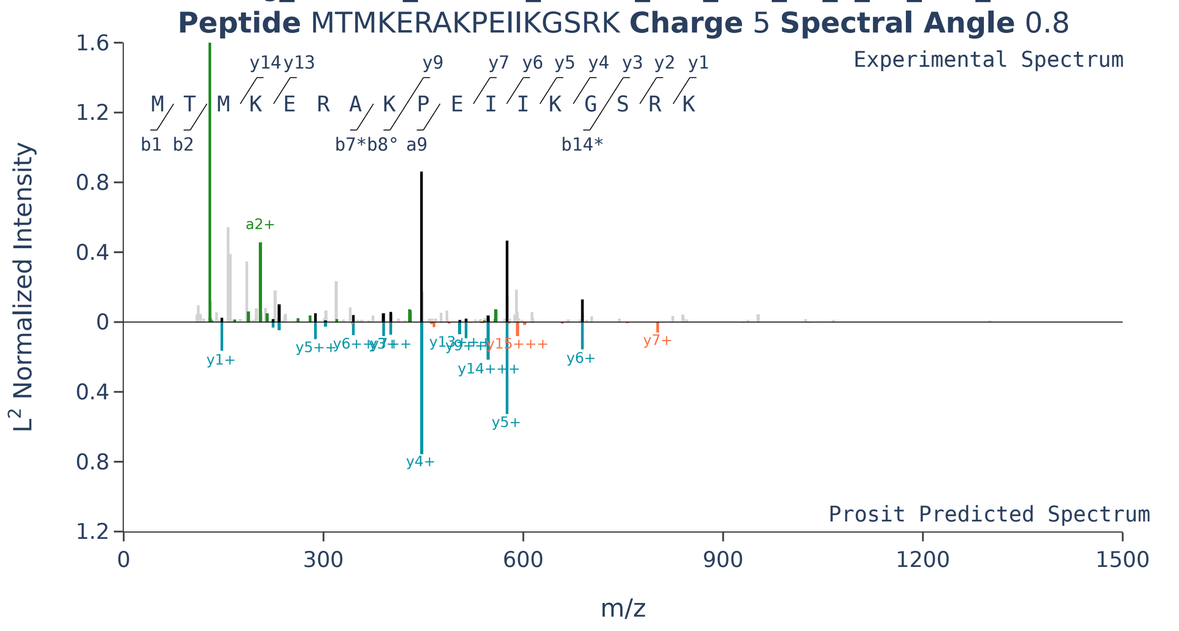


**Source** Ncheng_210623_230623_HFGoe_FFH_20S_25_1_A2_24h_R1 **Scan** 6474
**Peptide** INRVKEKAVGHEV **Charge** 2 **Spectral Angle** 0.56

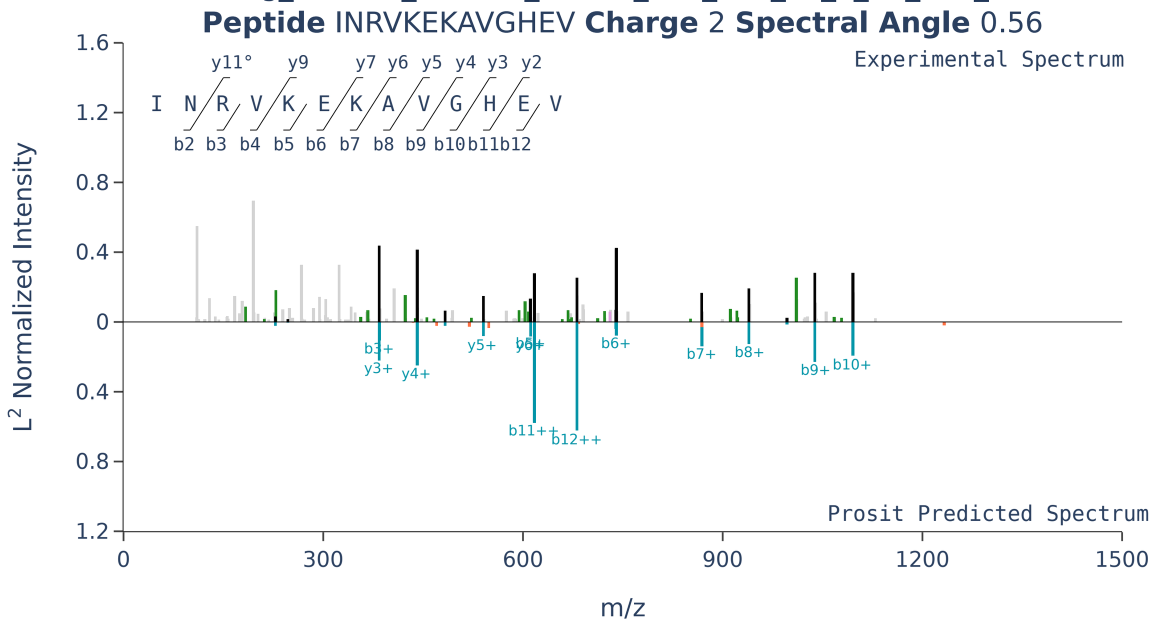


**Source** Ncheng_210623_230623_HFGoe_FFH_20S_25_1_A2_2h_R1 **Scan** 13663
**Peptide** EAMMDEIKQ **Charge** 2 **Spectral Angle** 0.73

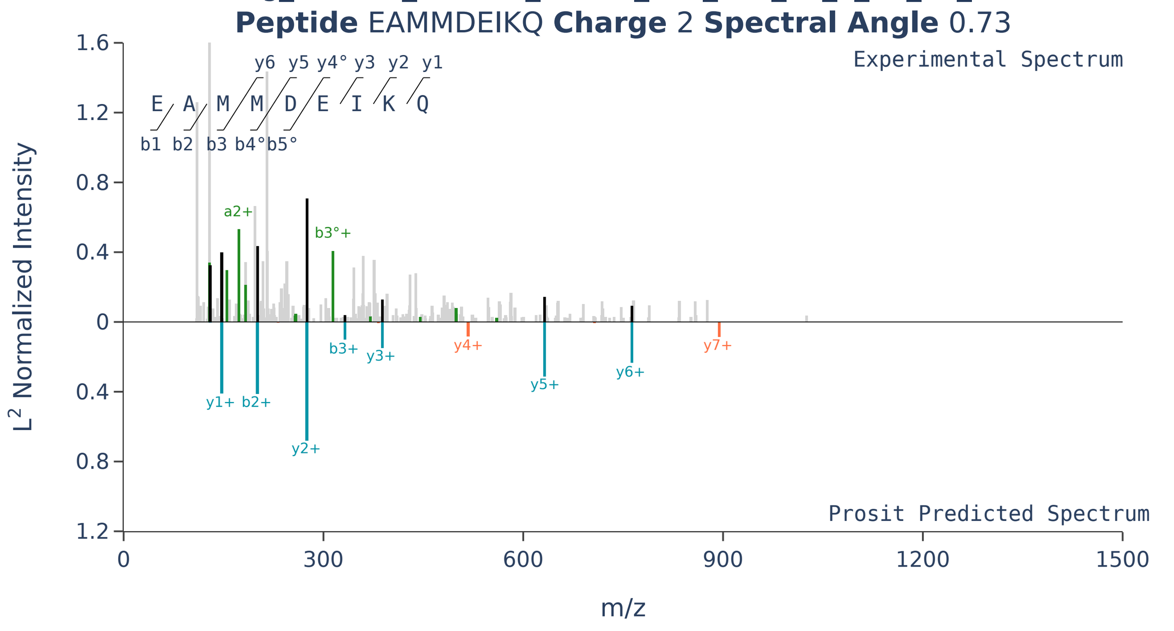


**Source** Ncheng_210623_230623_HFGoe_FFH_20S_25_1_A1_4h_R1 **Scan** 15025
**Peptide** RNISGRGRLTEDNVKDTLREV **Charge** 4 **Spectral Angle** 0.64

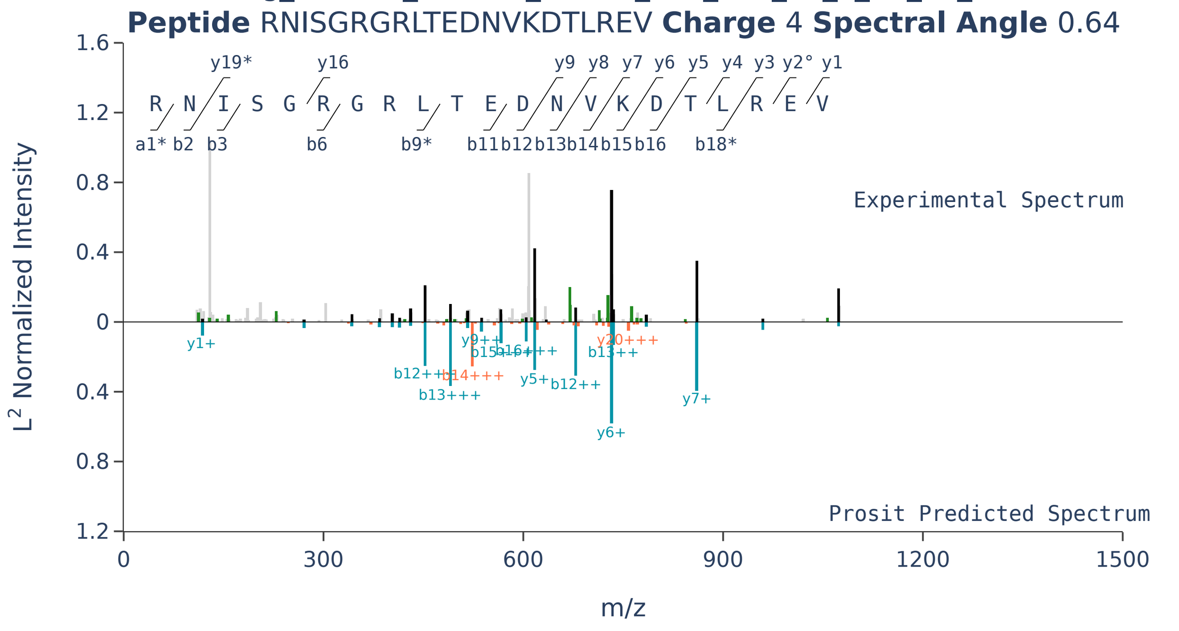

**Source** Ncheng_210623_230623_HFGoe_FFH_20S_25_1_A2_4h_R1 **Scan** 28813
**Peptide** MFDNLTDRLSRTLRNISGR **Charge** 3 **Spectral Angle** 0.63

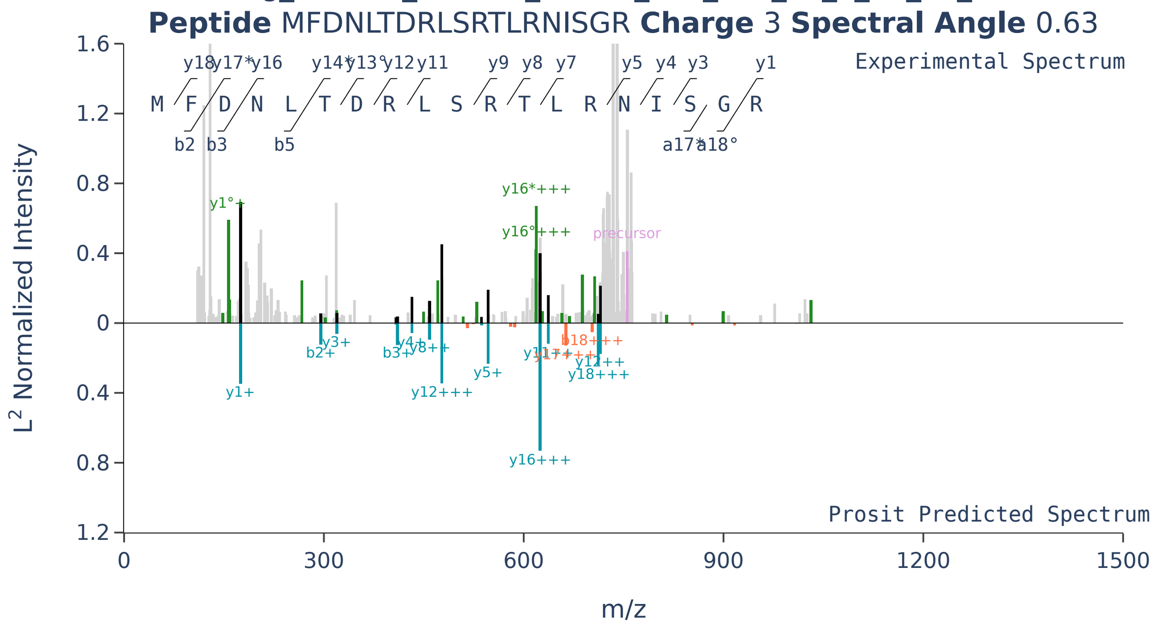


**Source** Ncheng_210623_230623_HFGoe_FFH_20S_25_1_A2_24h_R2 **Scan** 7156
**Peptide** FLNRVKEKAVGHEVNK **Charge** 5 **Spectral Angle** 0.65

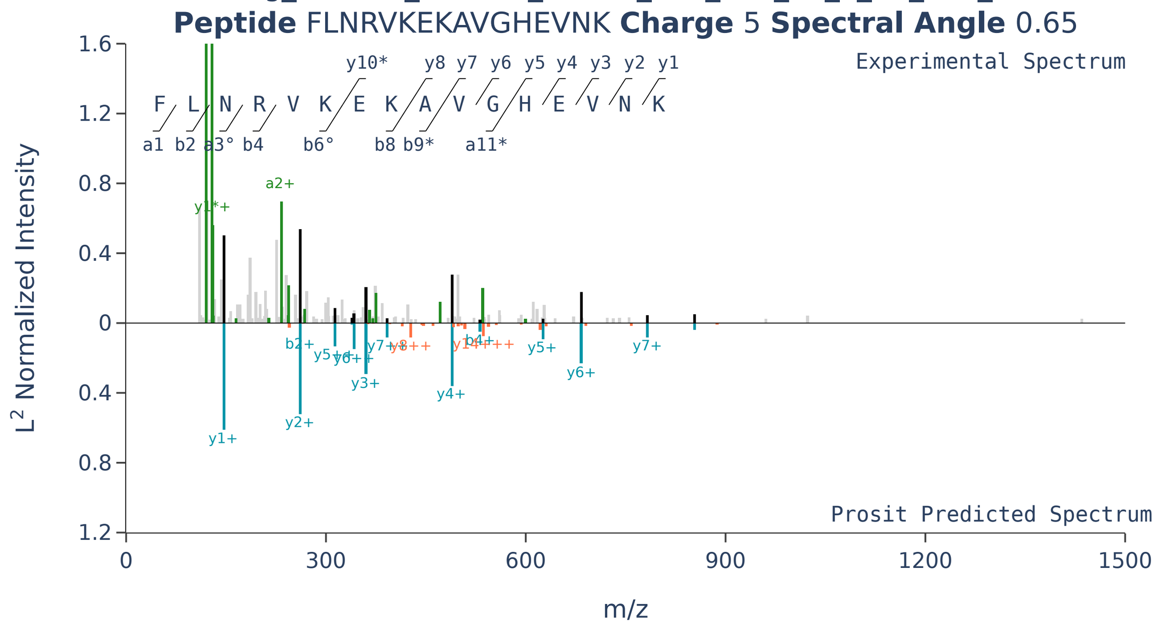


**Source** Ncheng_210623_230623_HFGoe_FFH_20S_25_1_A2_4h_R2 **Scan** 17310
**Peptide** KQLETLAEQV **Charge** 1 **Spectral Angle** 0.75

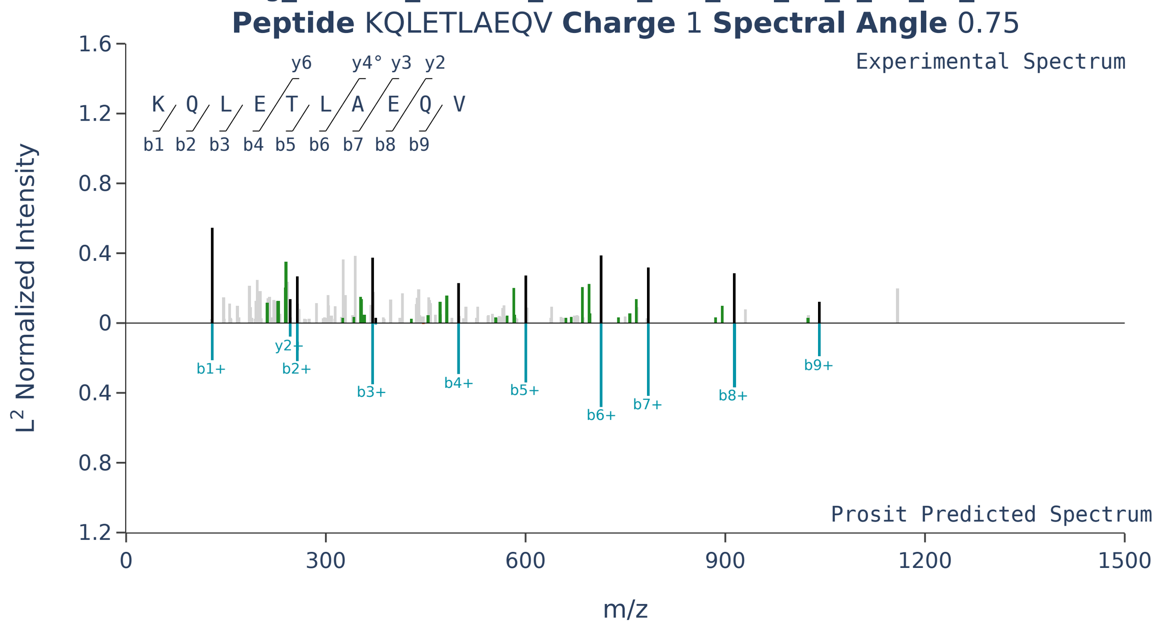


**Source** Ncheng_210623_230623_HFGoe_FFH_20S_25_1_A2_24h_R1 **Scan** 10305
**Peptide** PDRIASRI **Charge** 2 **Spectral Angle** 0.44

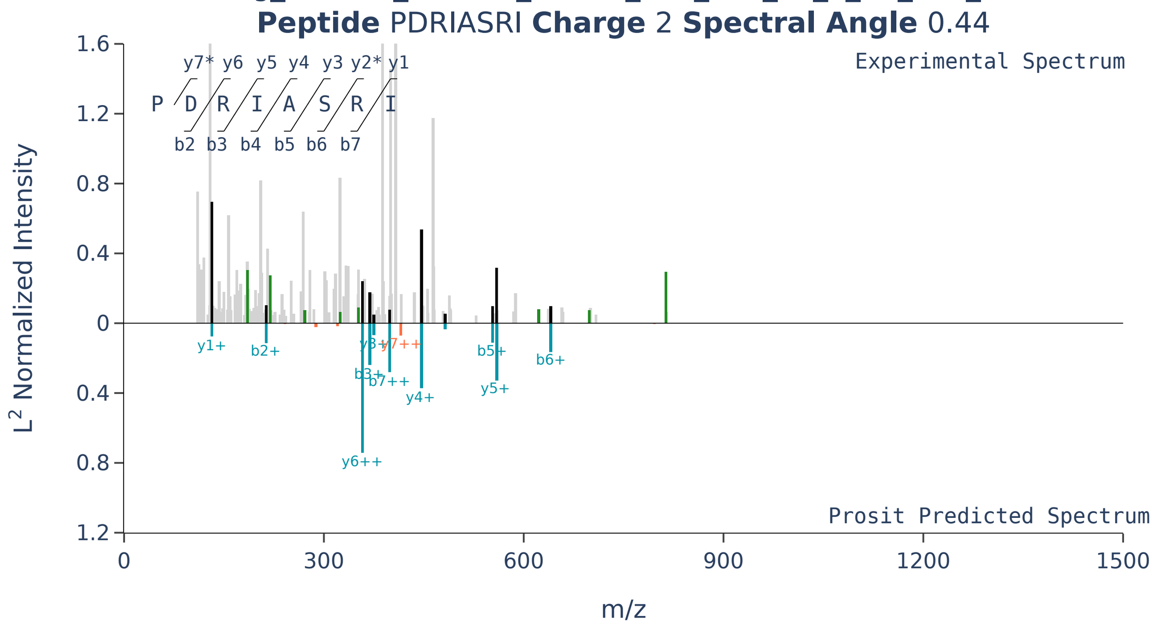


**Source** Ncheng_210623_230623_HFGoe_FFH_20S_25_1_A1_2h_R2 **Scan** 36741
**Peptide** TLAEQVGVDFFPSDVGQKPVDIVNAALKE **Charge** 3 **Spectral Angle** 0.79

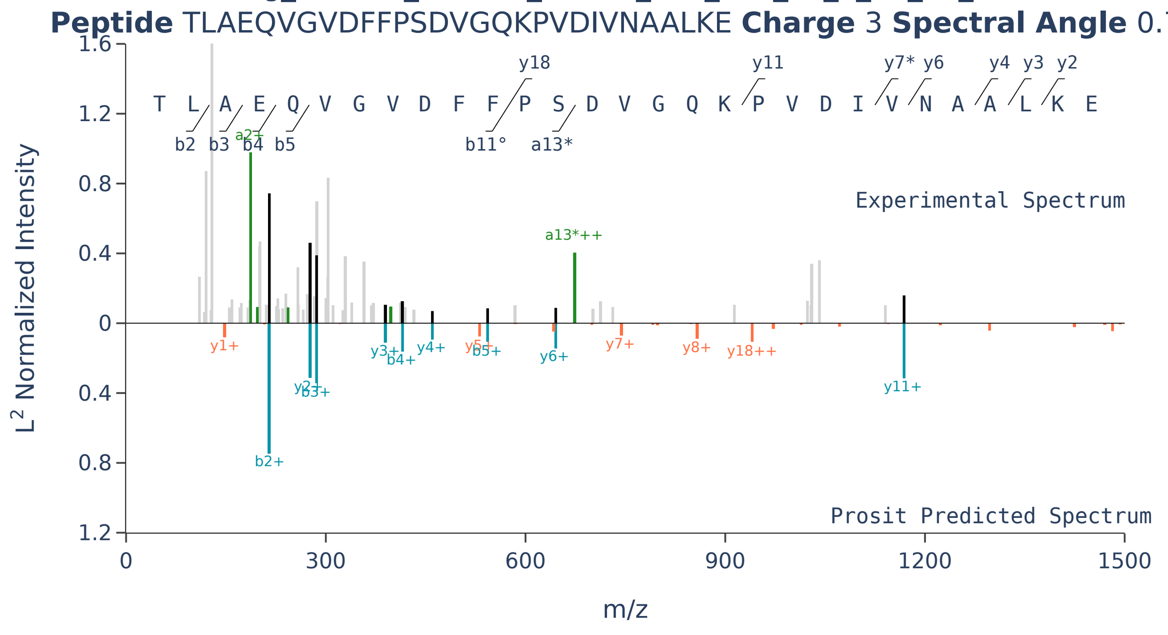


**Source** Ncheng_210623_230623_HFGoe_FFH_20S_25_1_A1_24h_R2 **Scan** 8951
**Peptide** ARGGAALSIRH **Charge** 3 **Spectral Angle** 0.86

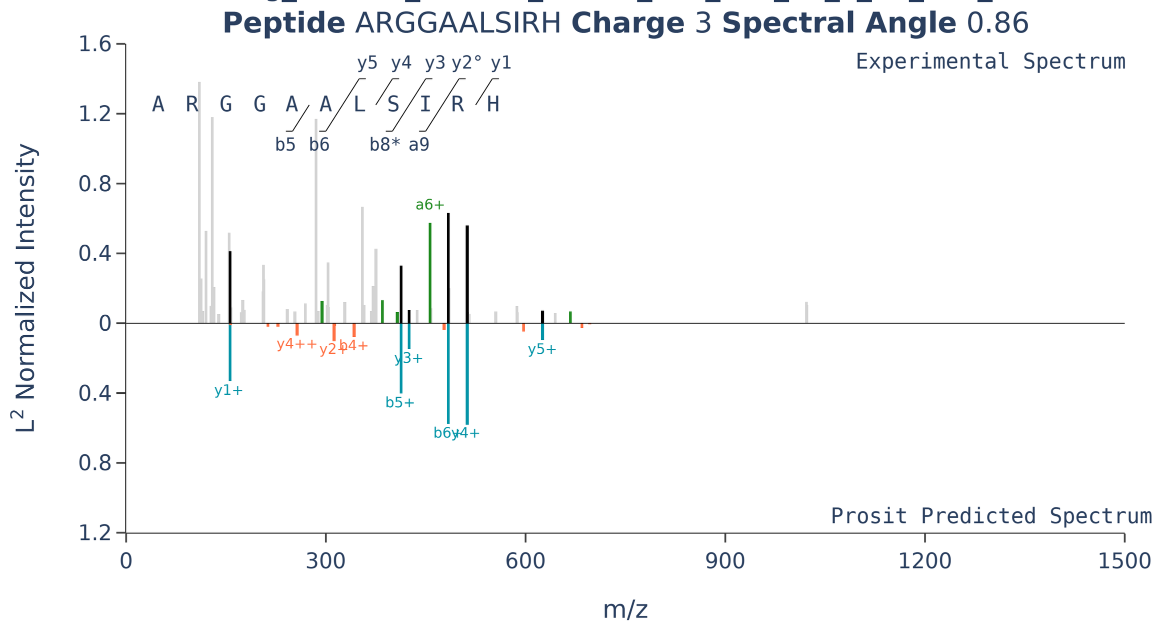


**Source** Ncheng_210623_230623_HFGoe_FFH_20S_25_1_A1_24h_R2 **Scan** 6370
**Peptide** DRAQAEKLAS **Charge** 2 **Spectral Angle** 0.54

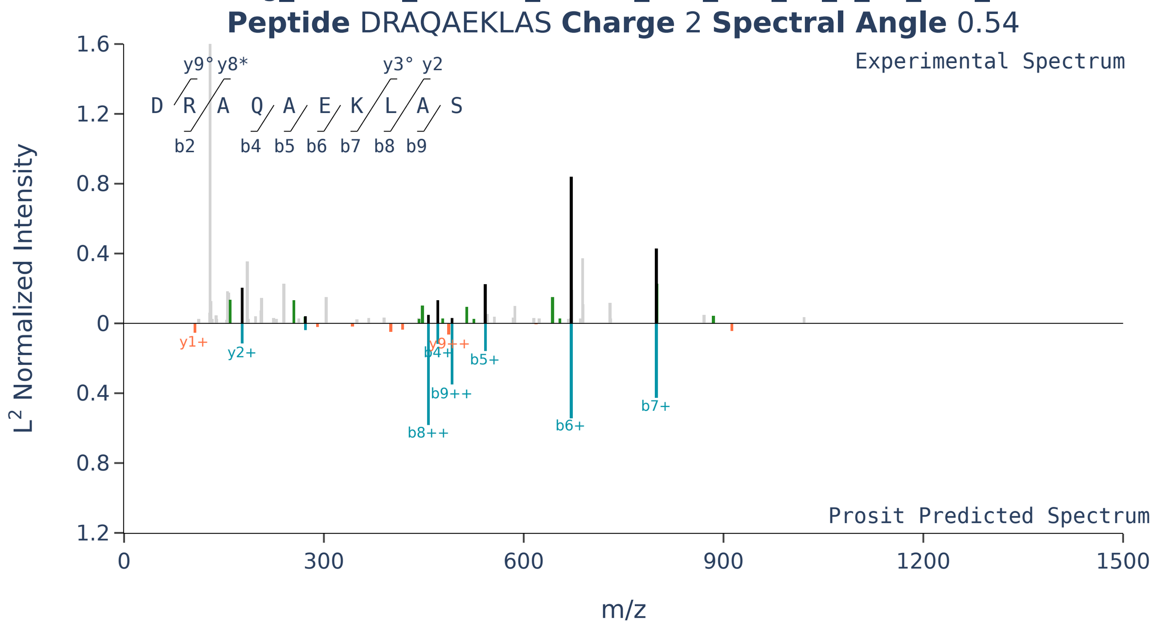


**Source** Ncheng_210623_230623_HFGoe_FFH_20S_25_1_A1_2h_R1 **Scan** 20301
**Peptide** LLVDTAGRLHVDEA **Charge** 2 **Spectral Angle** 0.72

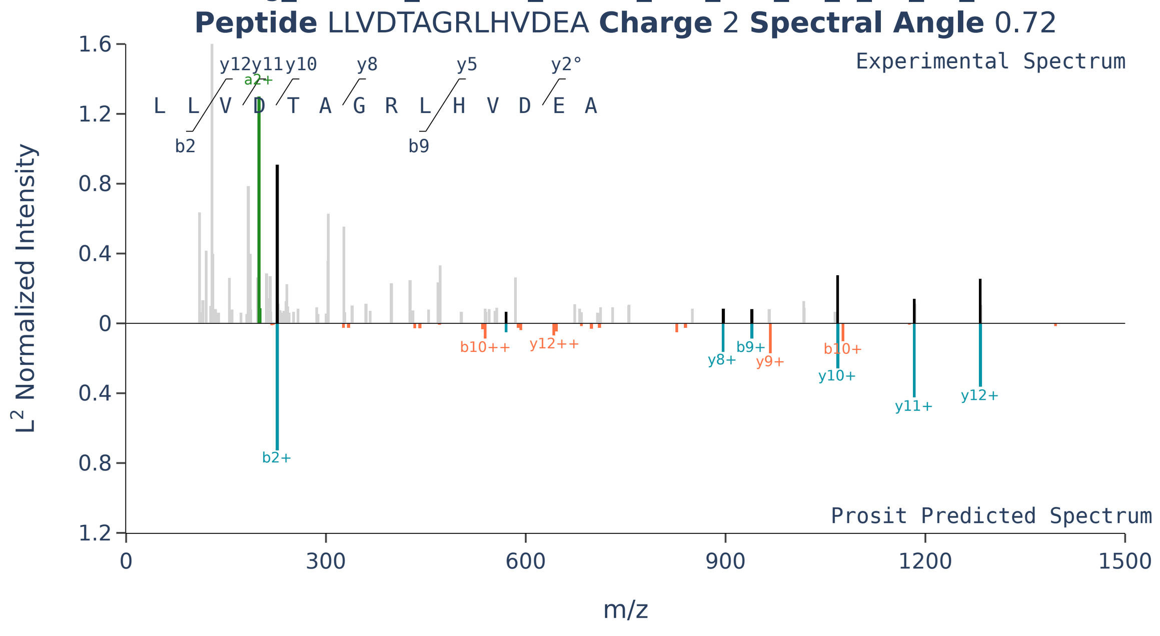


**Source** Ncheng_210623_230623_HFGoe_FFH_20S_25_1_A1_24h_R2 **Scan** 17320
**Peptide** KGDGFDL **Charge** 2 **Spectral Angle** 0.66

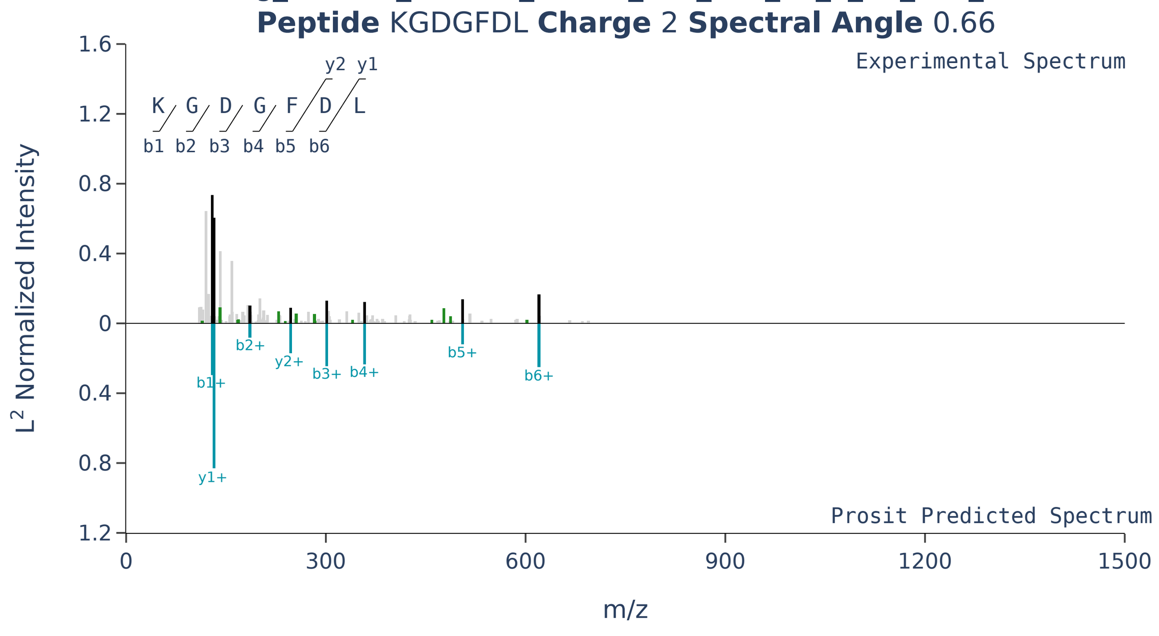


**Source** Ncheng_210623_230623_HFGoe_FFH_20S_25_1_A2_2h_R2 **Scan** 33440
**Peptide** AAMGEENQTLNLAAQPPAVVL **Charge** 2 **Spectral Angle** 0.89

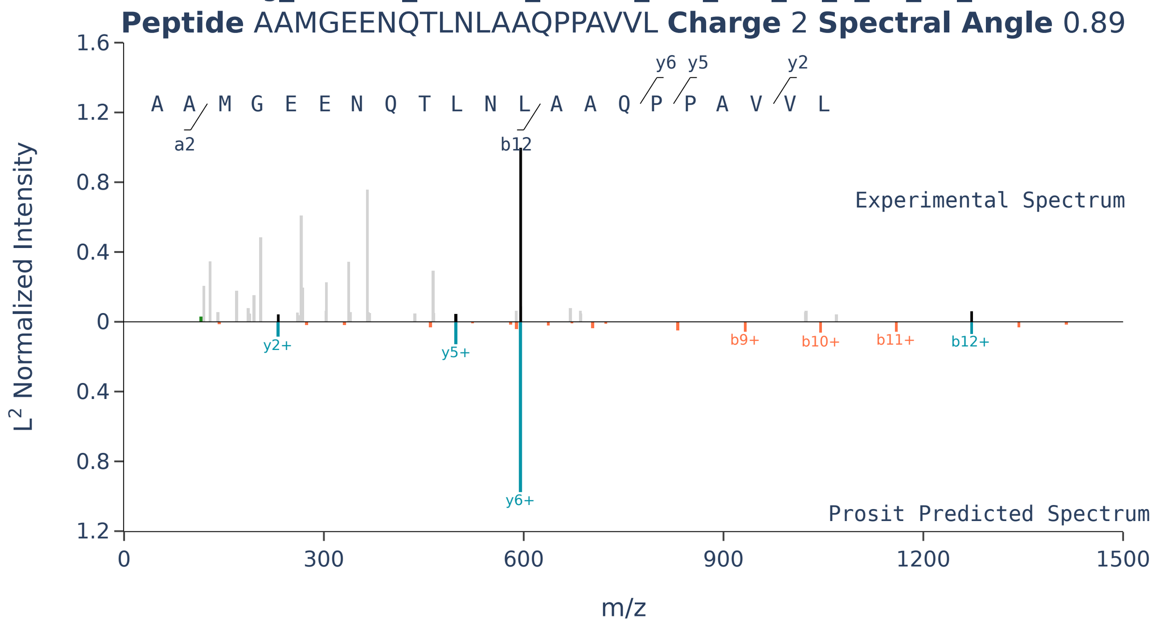


**Source** Ncheng_210623_230623_HFGoe_FFH_20S_25_1_A1_4h_R1 **Scan** 25129
**Peptide** EAMMDEIKQVHASINPVE **Charge** 2 **Spectral Angle** 0.73

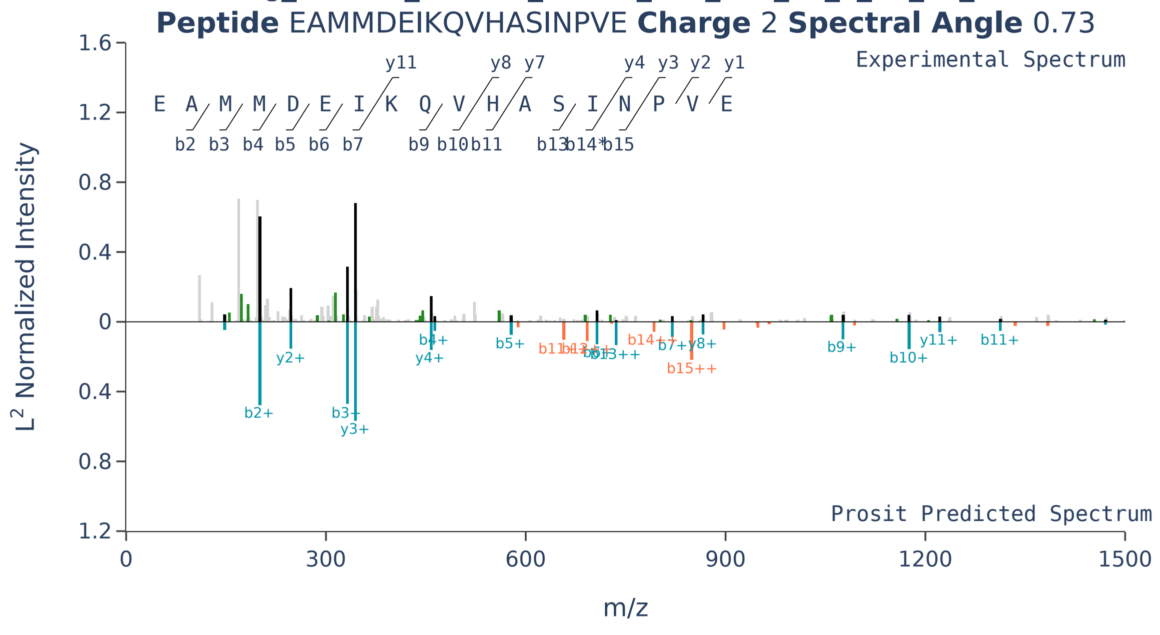


**Source** Ncheng_210623_230623_HFGoe_FFH_20S_25_1_A2_24h_R2 **Scan** 11433
**Peptide** GQKPVDIVN **Charge** 2 **Spectral Angle** 0.78

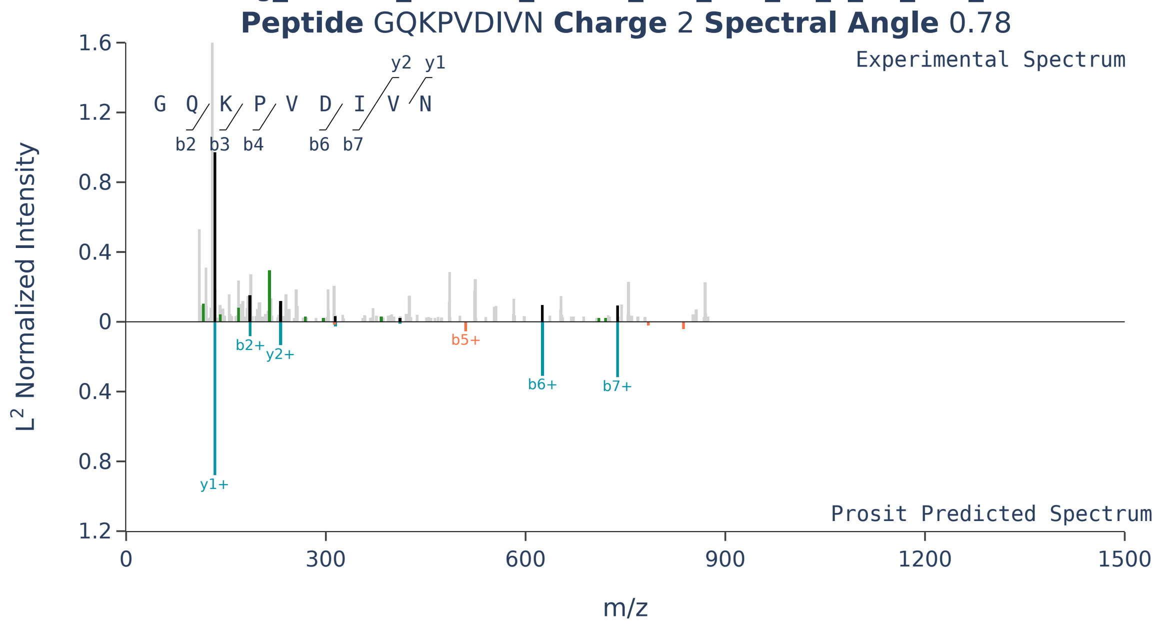


**Source** Ncheng_210623_230623_HFGoe_FFH_20S_25_1_A1_2h_R1 **Scan** 28853
**Peptide** DTAGRLHVDEAMMDEIKQVHASINPVE **Charge** 4 **Spectral Angle** 0.74

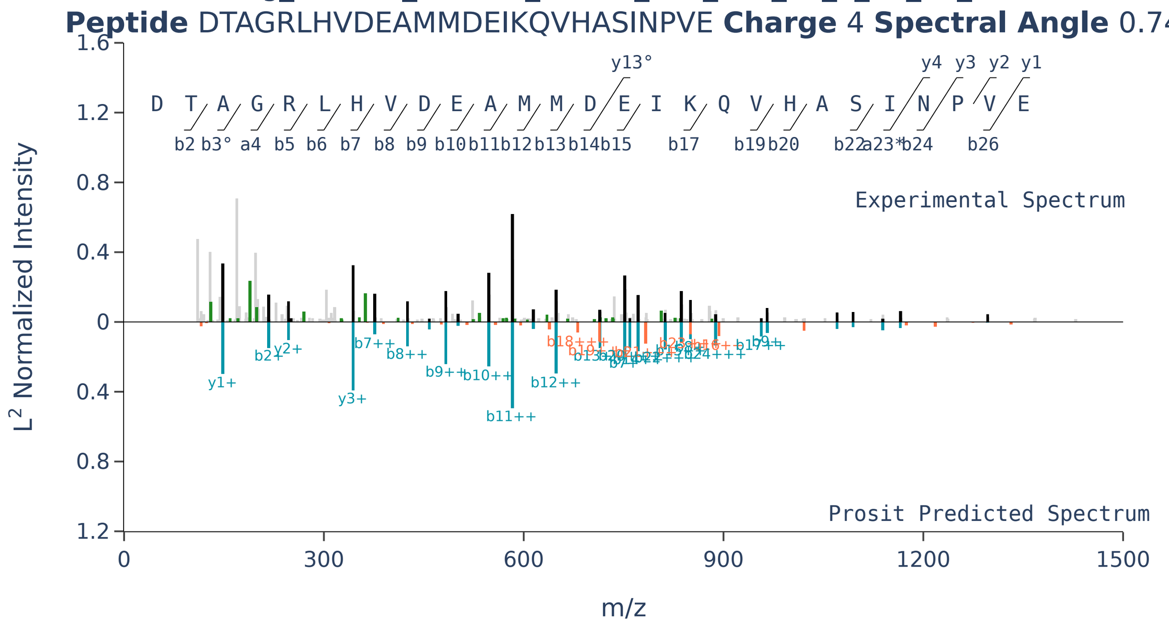


**Source** Ncheng_210623_230623_HFGoe_FFH_20S_25_1_A2_2h_R2 **Scan** 16309
**Peptide** ISGRGRLTEDNVKDTLREV **Charge** 4 **Spectral Angle** 0.71

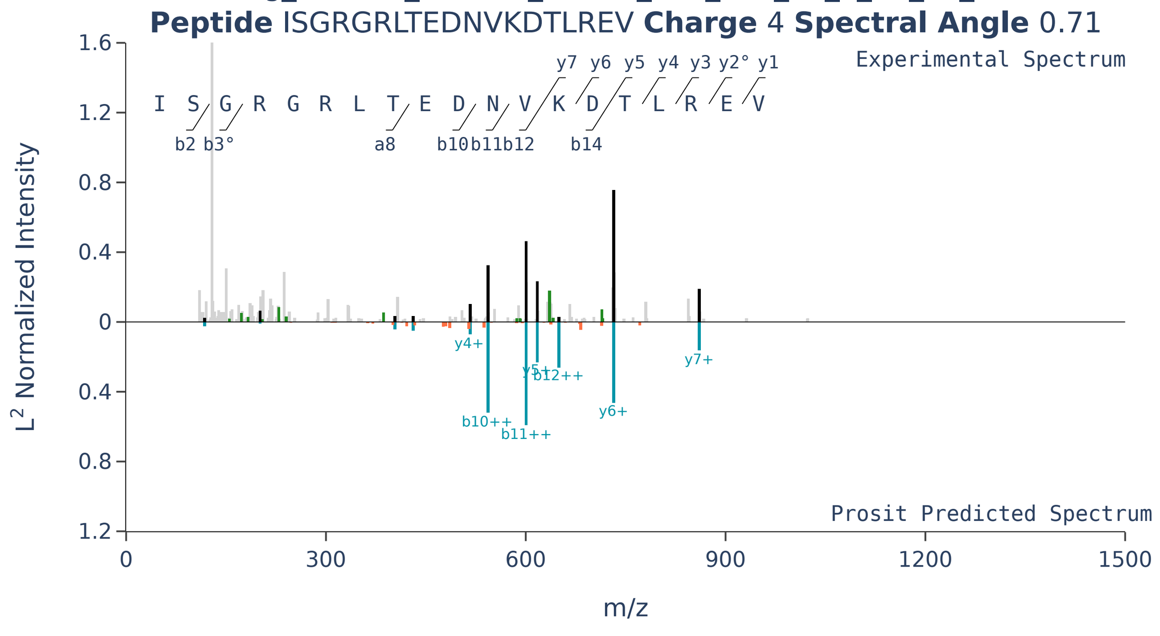


**Source** Ncheng_210623_230623_HFGoe_FFH_20S_25_1_A2_2h_R1 **Scan** 31632
**Peptide** KLKFYDVLLVDTAGRLHV **Charge** 3 **Spectral Angle** 0.67

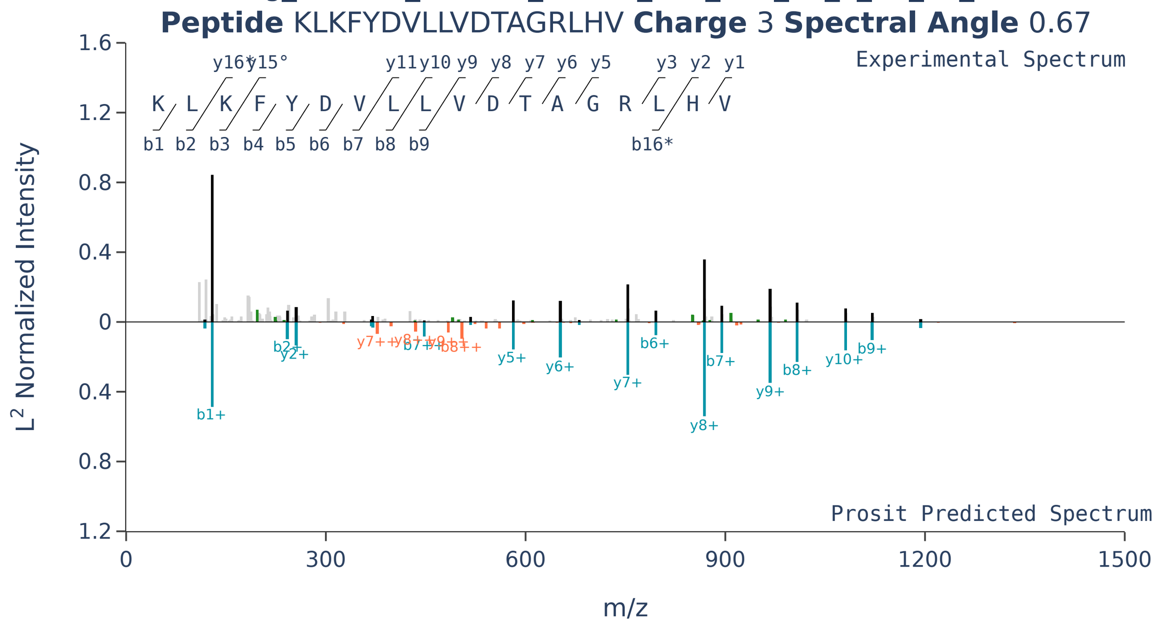

**Source** Ncheng_210623_230623_HFGoe_FFH_20S_25_1_A2_4h_R2 **Scan** 18407
**Peptide** ALLNSMTMKERAKPEII **Charge** 3 **Spectral Angle** 0.7

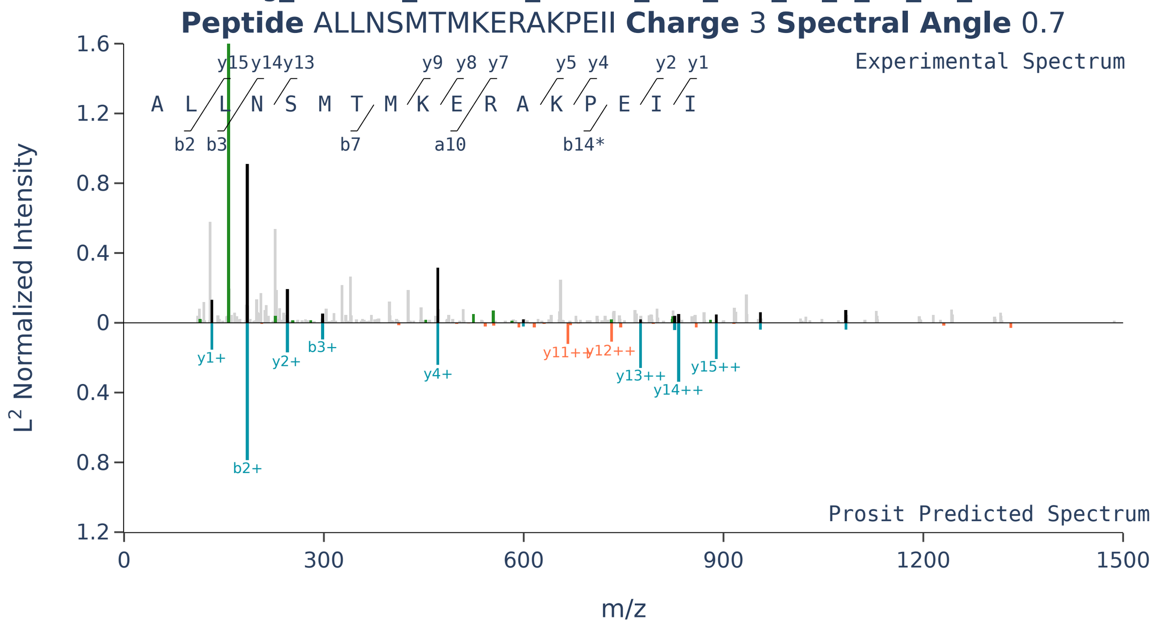


**Source** Ncheng_210623_230623_HFGoe_FFH_20S_25_1_A1_2h_R1 **Scan** 28583
**Peptide** ALKEAKLKFYDVLLVDTAGRLH **Charge** 4 **Spectral Angle** 0.67

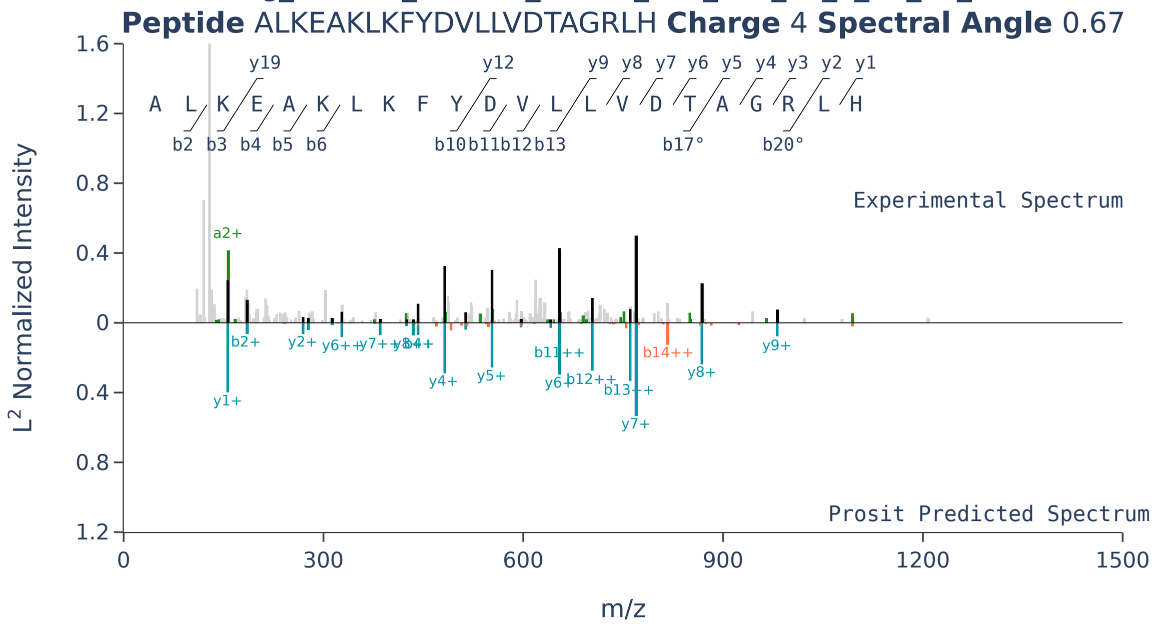


**Source** Ncheng_210623_230623_HFGoe_FFH_20S_25_1_A2_1h_R1 **Scan** 31356
**Peptide** KSLTPGQEFVKIVRNELVAAM **Charge** 3 **Spectral Angle** 0.88

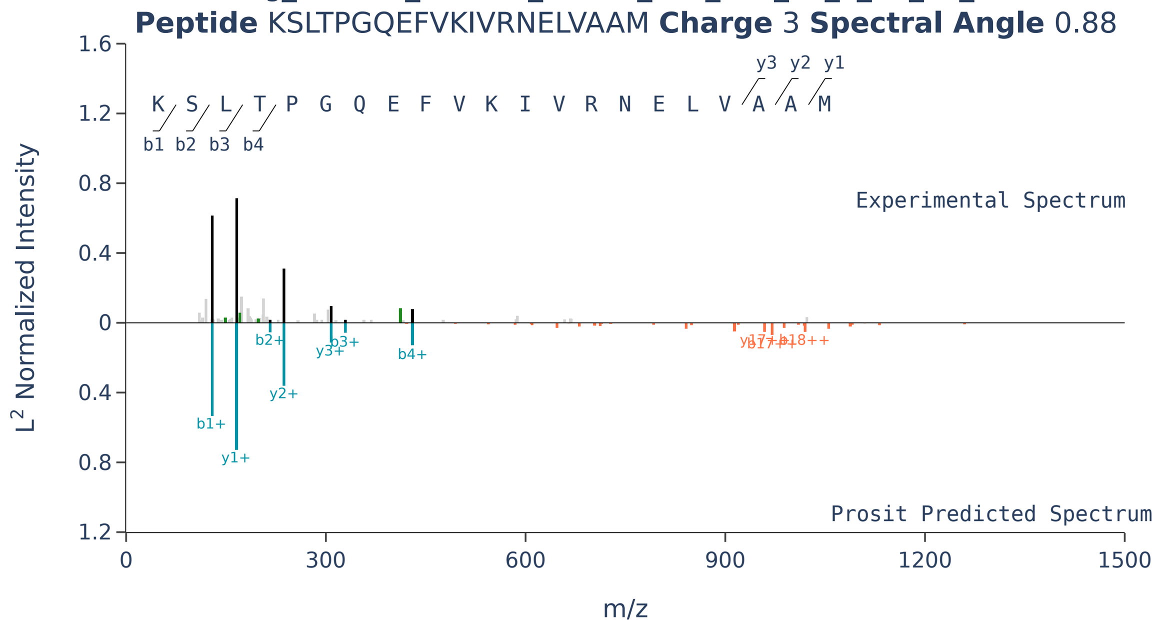


**Source** Ncheng_210623_230623_HFGoe_FFH_20S_25_1_A2_4h_R1 **Scan** 11363
**Peptide** RGRLTEDNVKDTL **Charge** 4 **Spectral Angle** 0.56

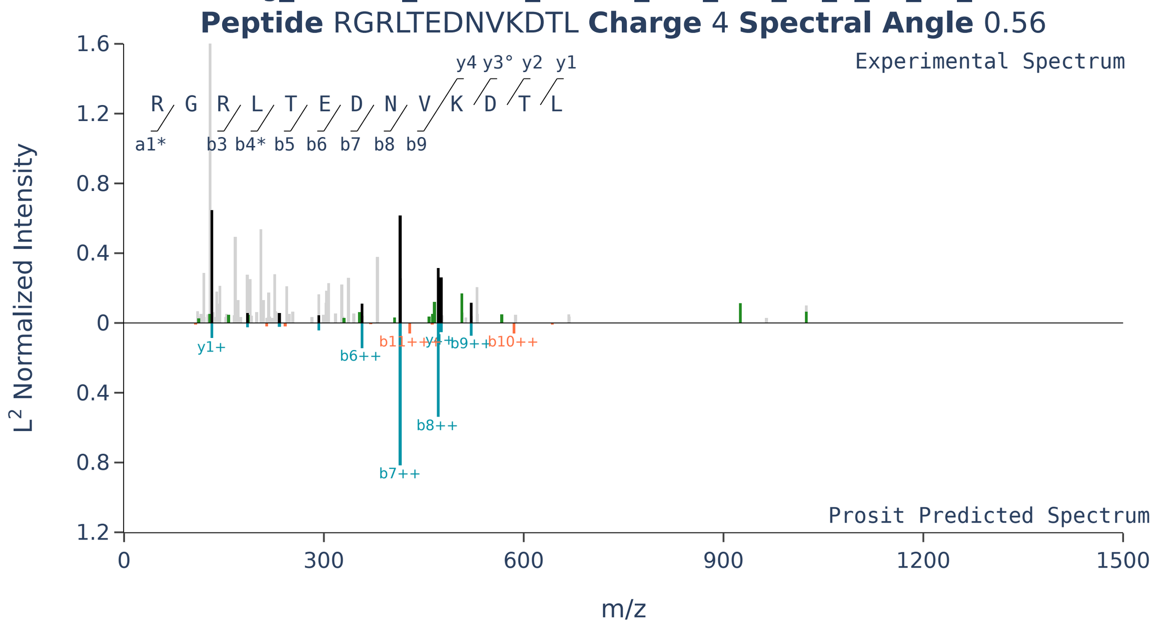


**Source** Ncheng_210623_230623_HFGoe_FFH_20S_25_1_A1_4h_R1 **Scan** 12939
**Peptide** MTMKERAKPELL **Charge** 2 **Spectral Angle** 0.67

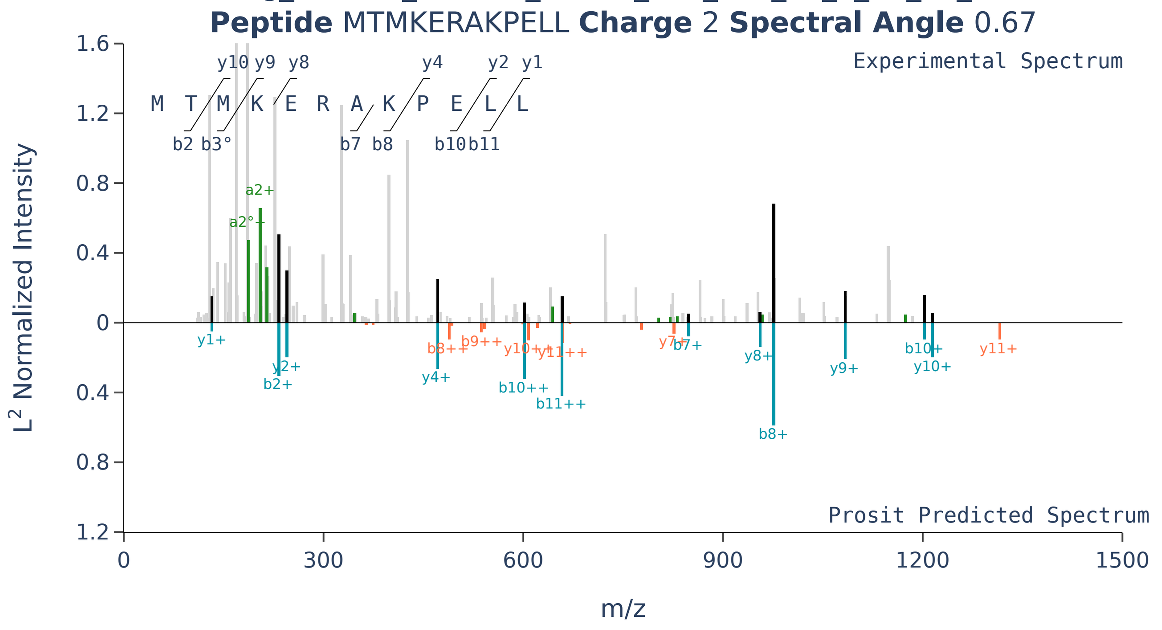


**Source** Ncheng_210623_230623_HFGoe_FFH_20S_25_1_A2_24h_R1 **Scan** 13628
**Peptide** DIESKVDRAQAEKL **Charge** 2 **Spectral Angle** 0.71

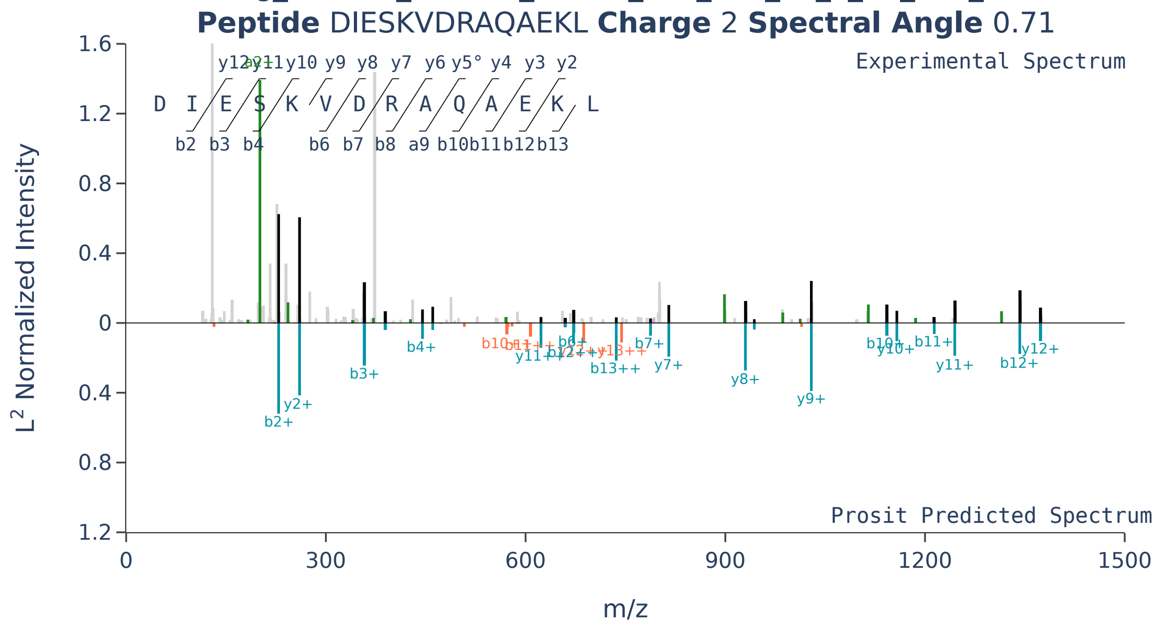


**Source** Ncheng_210623_230623_HFGoe_FFH_20S_25_1_A1_2h_R2 **Scan** 30261
**Peptide** VLSLIEDIESKVDRAQAEKLASKLKK **Charge** 5 **Spectral Angle** 0.69

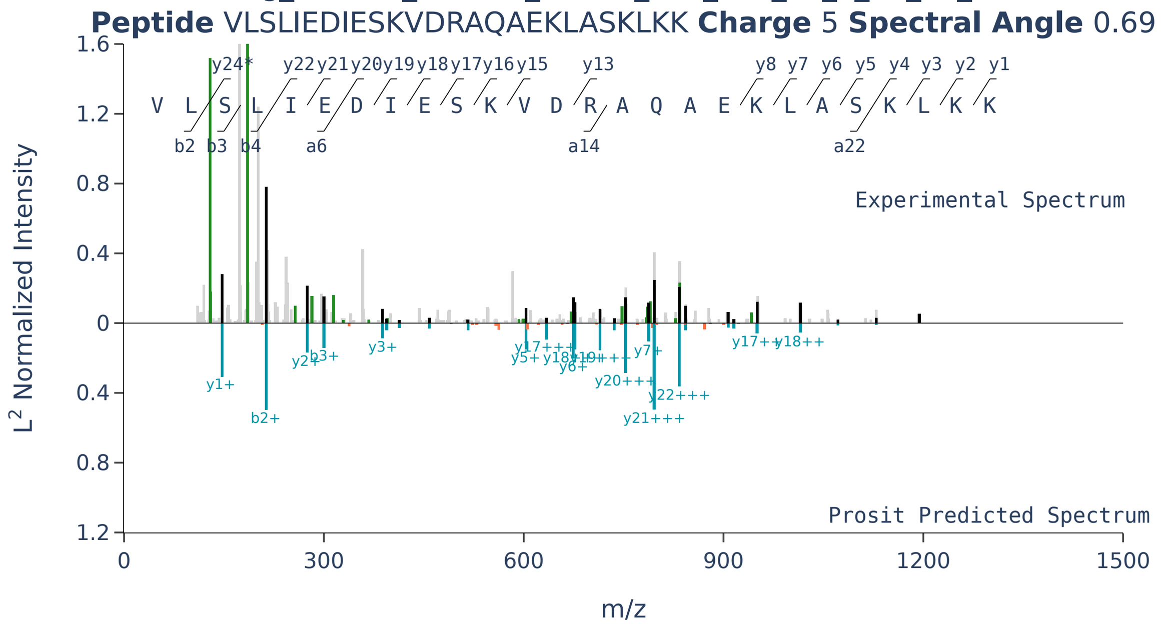


**Source** Ncheng_210623_230623_HFGoe_FFH_20S_25_1_A1_4h_R2 **Scan** 19327
**Peptide** SLRHITGKPIKFLGVGEKTEAL **Charge** 5 **Spectral Angle** 0.76

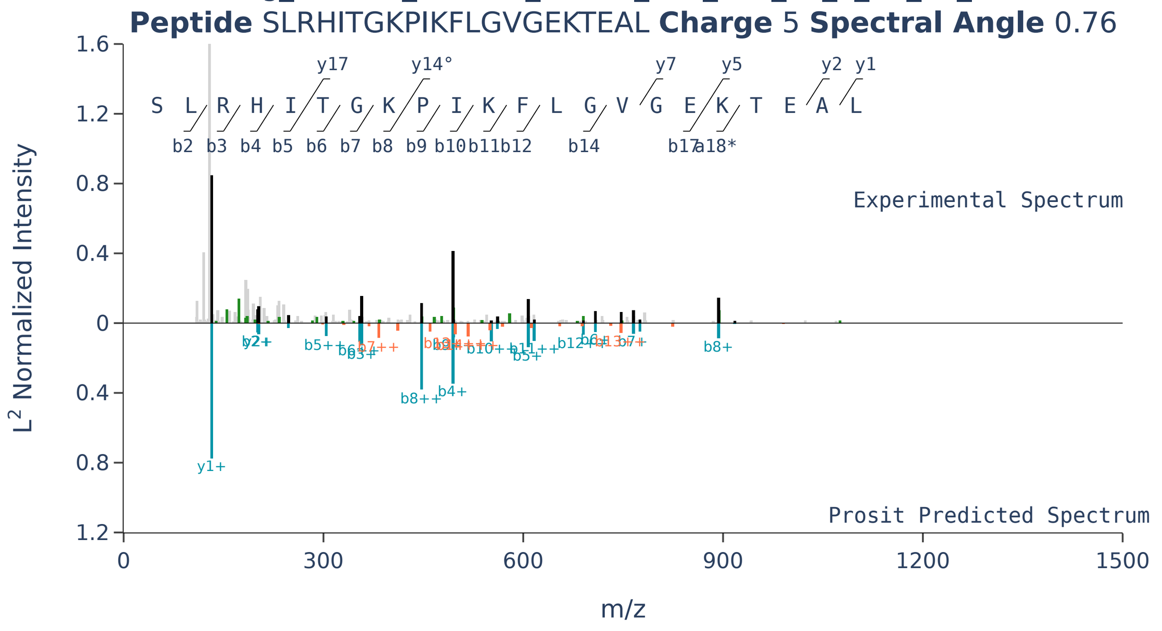


**Source** Ncheng_210623_230623_HFGoe_FFH_20S_25_1_A1_24h_R1 **Scan** 10746
**Peptide** SKLKKGDGFDLND **Charge** 3 **Spectral Angle** 0.53

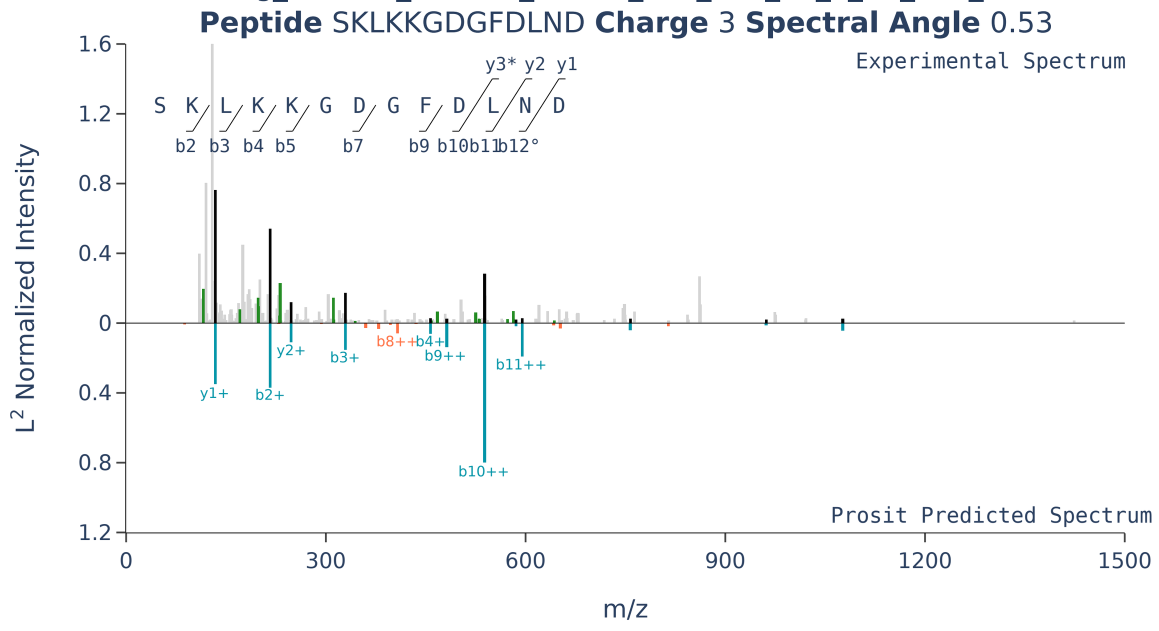


**Source** Ncheng_210623_230623_HFGoe_FFH_20S_25_1_A2_24h_R1 **Scan** 19523
**Peptide** EKAVGHEVNKSLTPGQEFVKI **Charge** 4 **Spectral Angle** 0.72

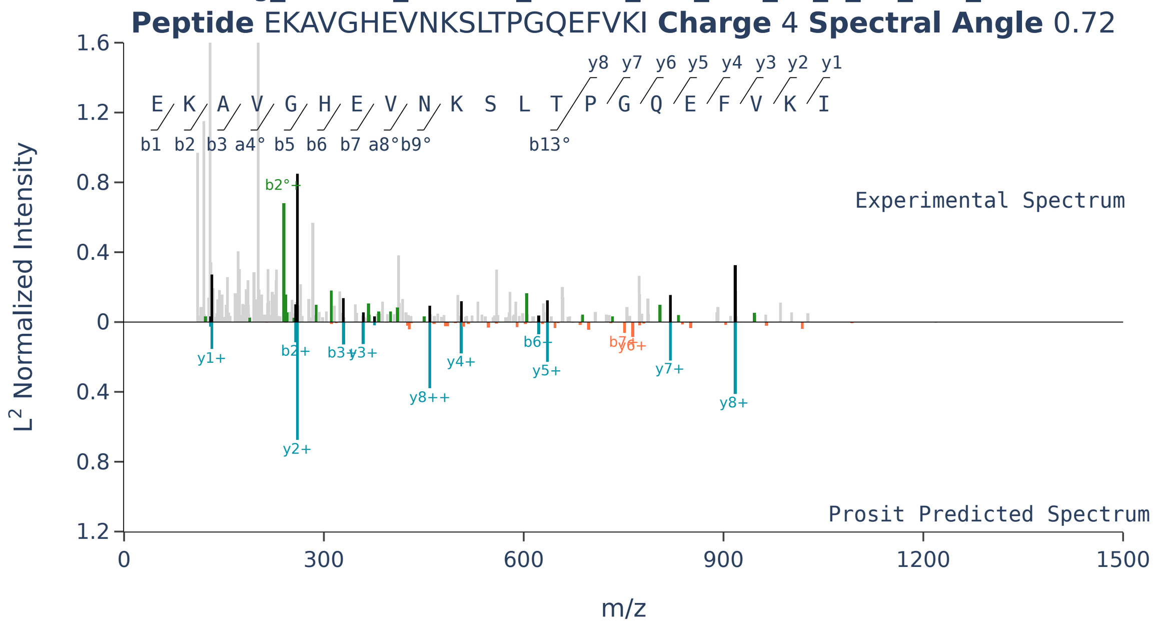


**Source** Ncheng_210623_230623_HFGoe_FFH_20S_25_1_A2_4h_R2 **Scan** 15827
**Peptide** DLESKVDRAQAEKLASKLKK **Charge** 5 **Spectral Angle** 0.73

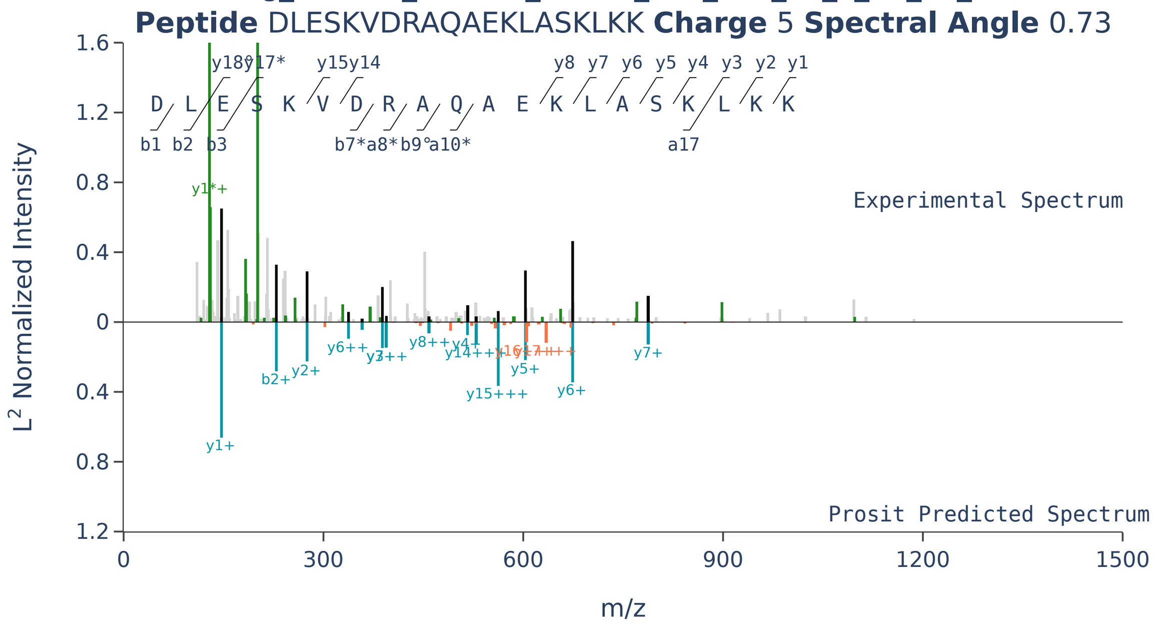


**Source** Ncheng_210623_230623_HFGoe_FFH_20S_25_1_A2_2h_R2 **Scan** 37119
**Peptide** KKGDGFDLNDFLEQLRQMKNMG **Charge** 3 **Spectral Angle** 0.87

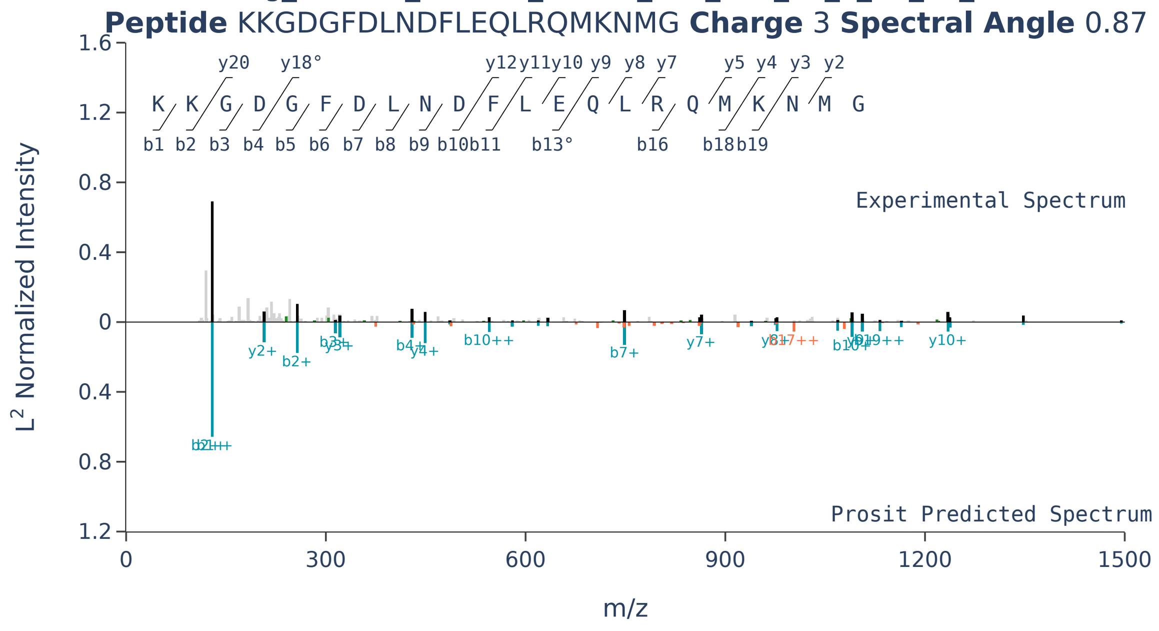


**Source** Ncheng_210623_230623_HFGoe_FFH_20S_25_1_A2_24h_R1 **Scan** 9607
**Peptide** EDIESKVDRAQAE **Charge** 2 **Spectral Angle** 0.76

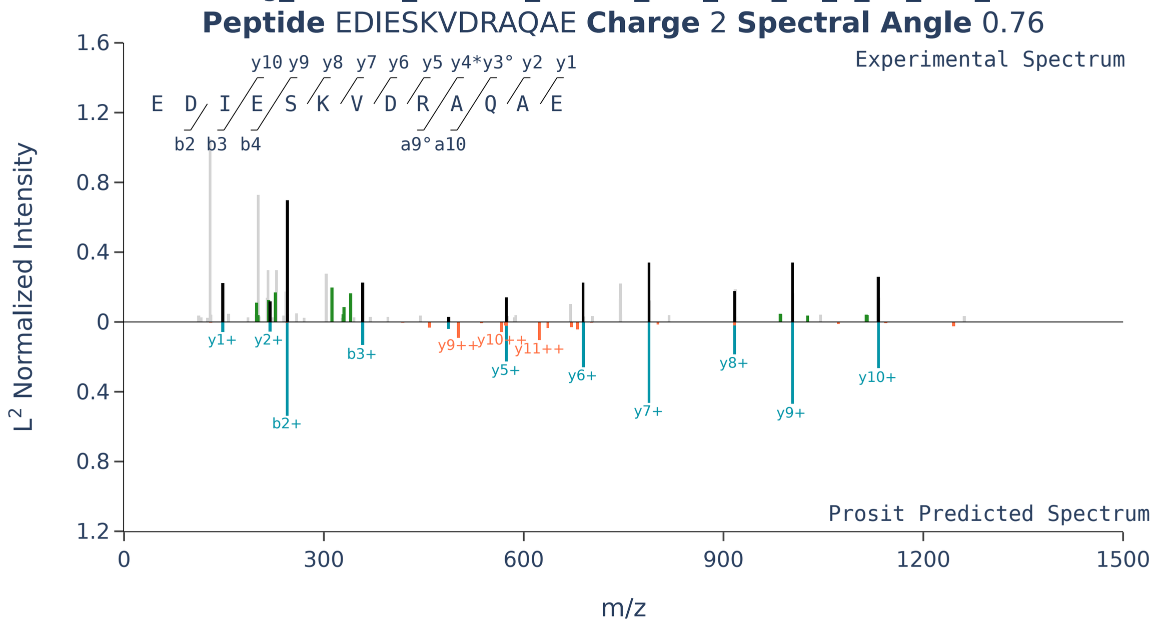


**Source** Ncheng_210623_230623_HFGoe_FFH_20S_25_1_A2_4h_R2 **Scan** 8792
**Peptide** EKLASKL **Charge** 2 **Spectral Angle** 0.8

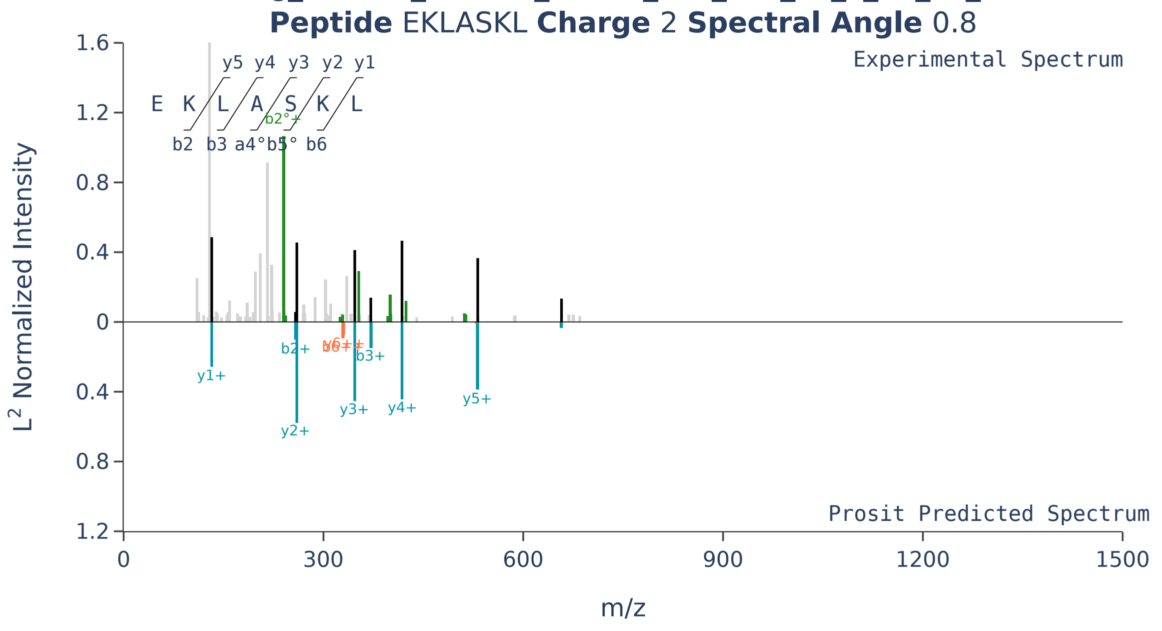


**Source** Ncheng_210623_230623_HFGoe_FFH_20S_25_1_A2_2h_R1 **Scan** 36598
**Peptide** VAAMGEENQTLNLAAQPPAVVLM **Charge** 2 **Spectral Angle** 0.85

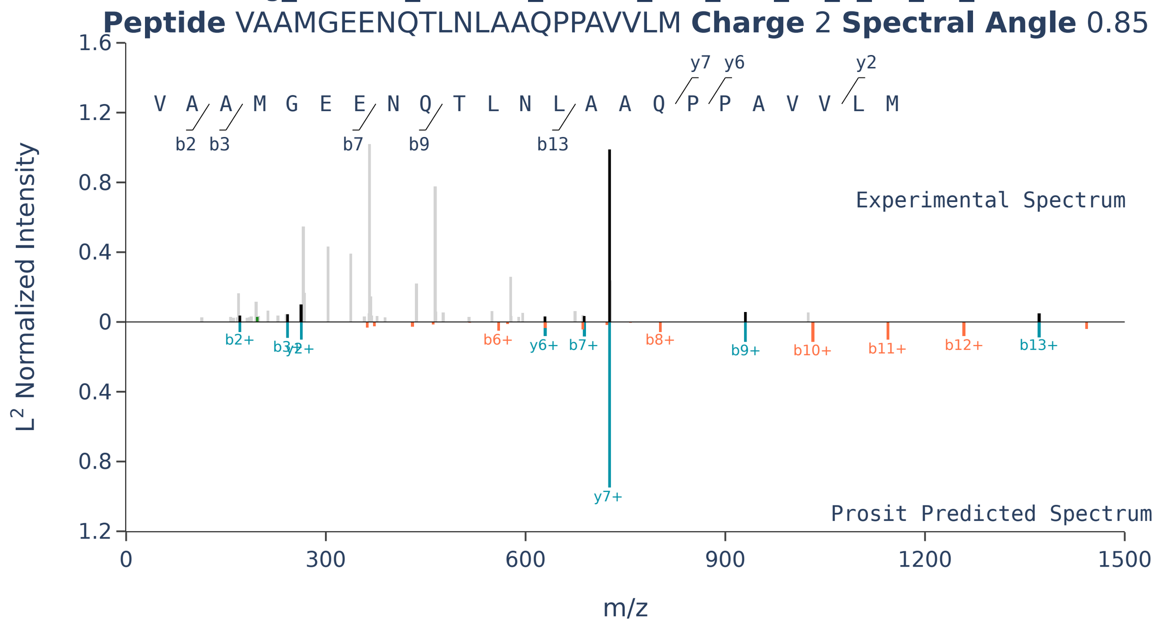

**Source** Ncheng_210623_230623_HFGoe_FFH_20S_25_1_A1_2h_R2 **Scan** 25605
**Peptide** GKLPGMGQIPDNVKSQMDDKVLVRM **Charge** 4 **Spectral Angle** 0.71

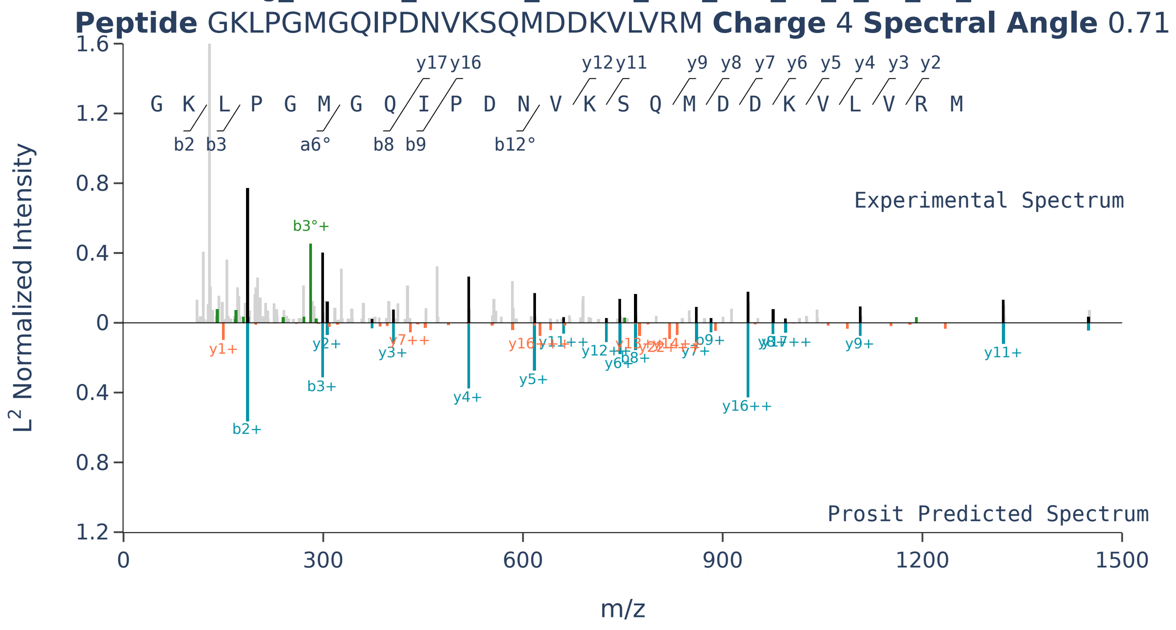


**Source** Ncheng_210623_230623_HFGoe_FFH_20S_25_1_A1_4h_R1 **Scan** 16303
**Peptide** DVYRPAAL **Charge** 2 **Spectral Angle** 0.8

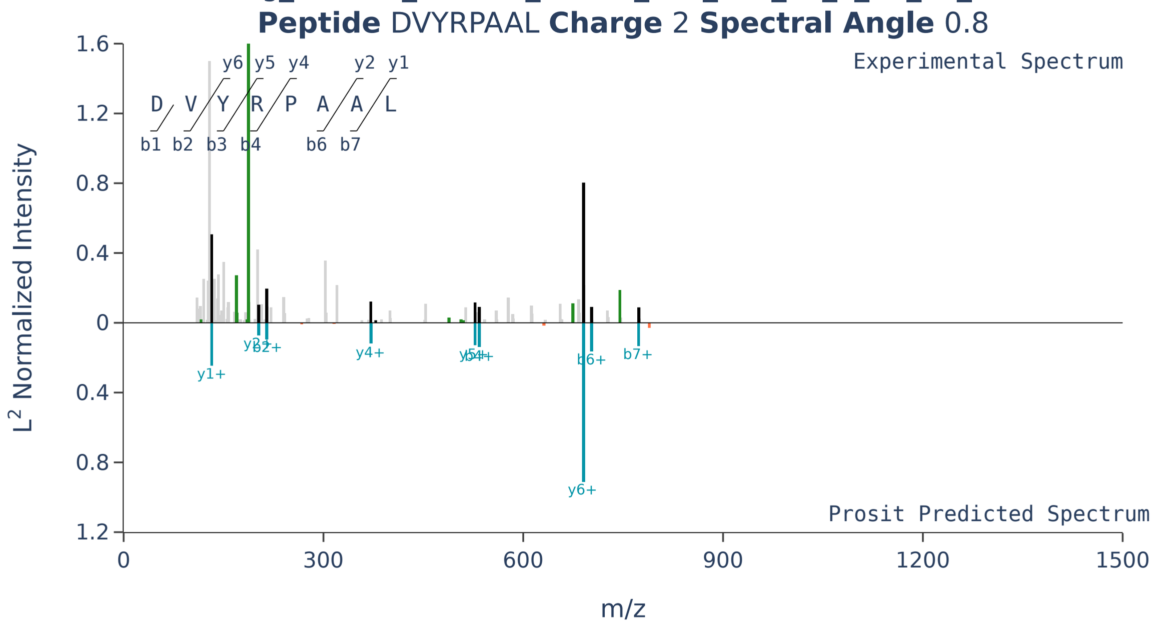


**Source** Ncheng_210623_230623_HFGoe_FFH_20S_25_1_A1_1h_R1 **Scan** 32321
**Peptide** ADVALPVVREFINRVKEKAVGHEVN **Charge** 5 **Spectral Angle** 0.82

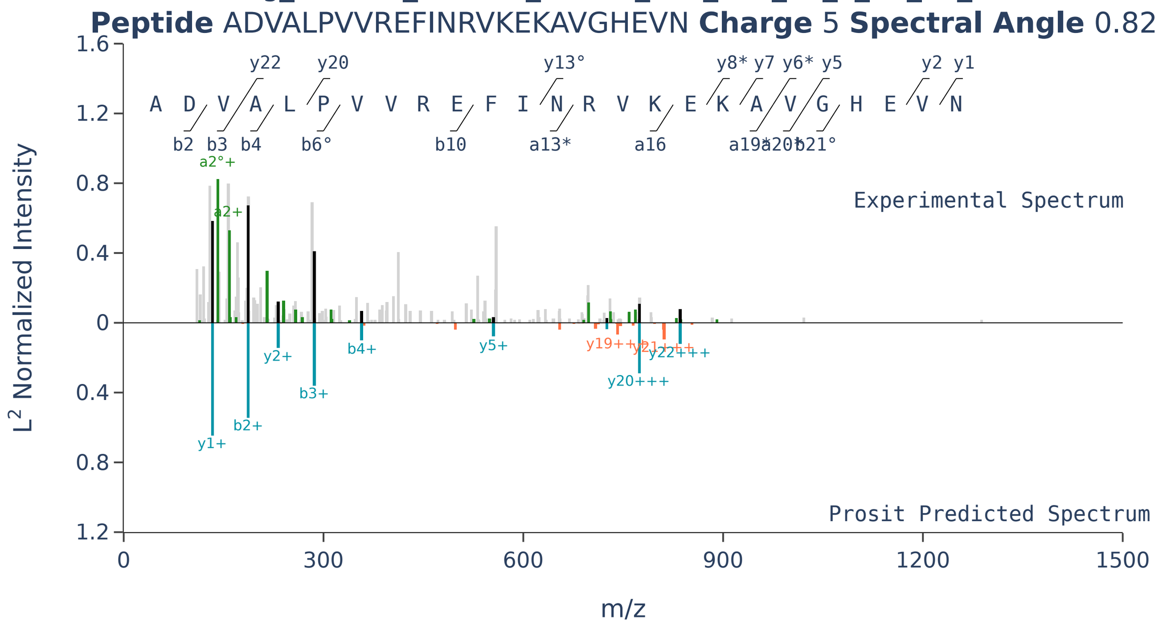


**Source** Ncheng_210623_230623_HFGoe_FFH_20S_25_1_A1_24h_R1 **Scan** 6056
**Peptide** RNISGRGRLT **Charge** 3 **Spectral Angle** 0.68

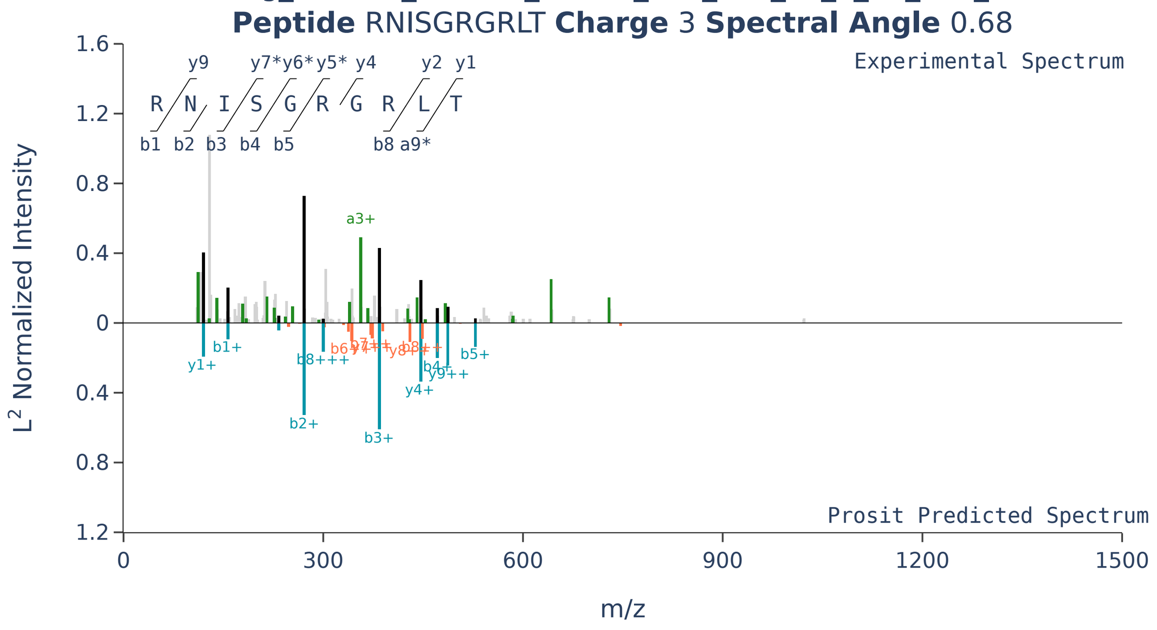


**Source** Ncheng_210623_230623_HFGoe_FFH_20S_25_1_A2_4h_R1 **Scan** 29661
**Peptide** ALPVVREFINRV **Charge** 3 **Spectral Angle** 0.56

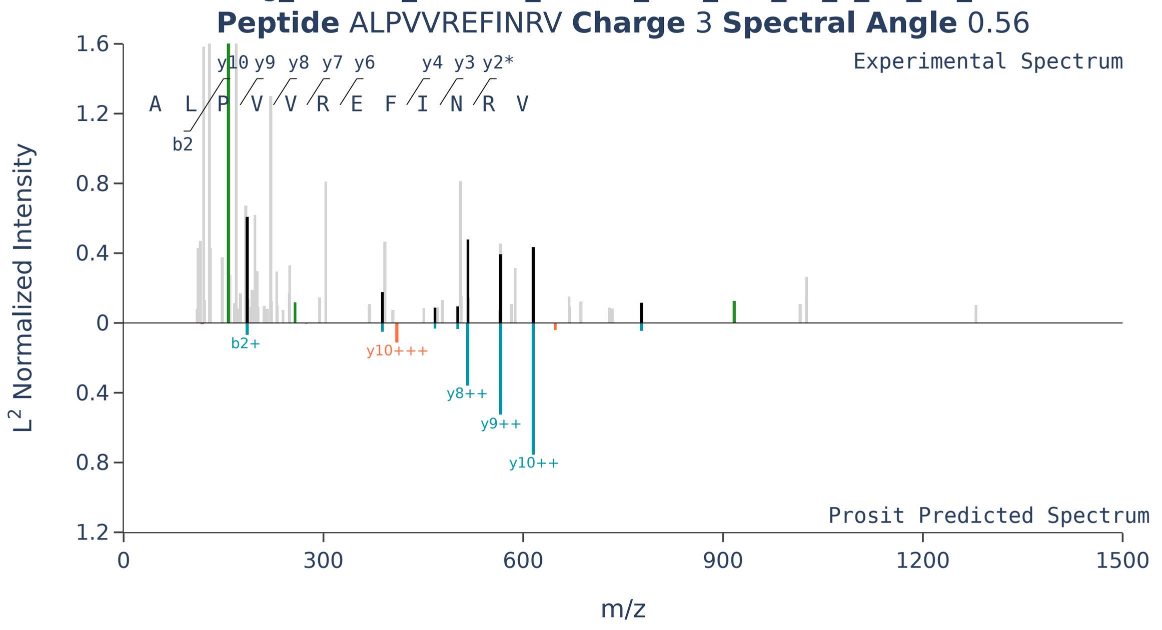


**Source** Ncheng_210623_230623_HFGoe_FFH_20S_25_1_A2_24h_R2 **Scan** 8235
**Peptide** FINRVKEKAVGHEVN **Charge** 2 **Spectral Angle** 0.49

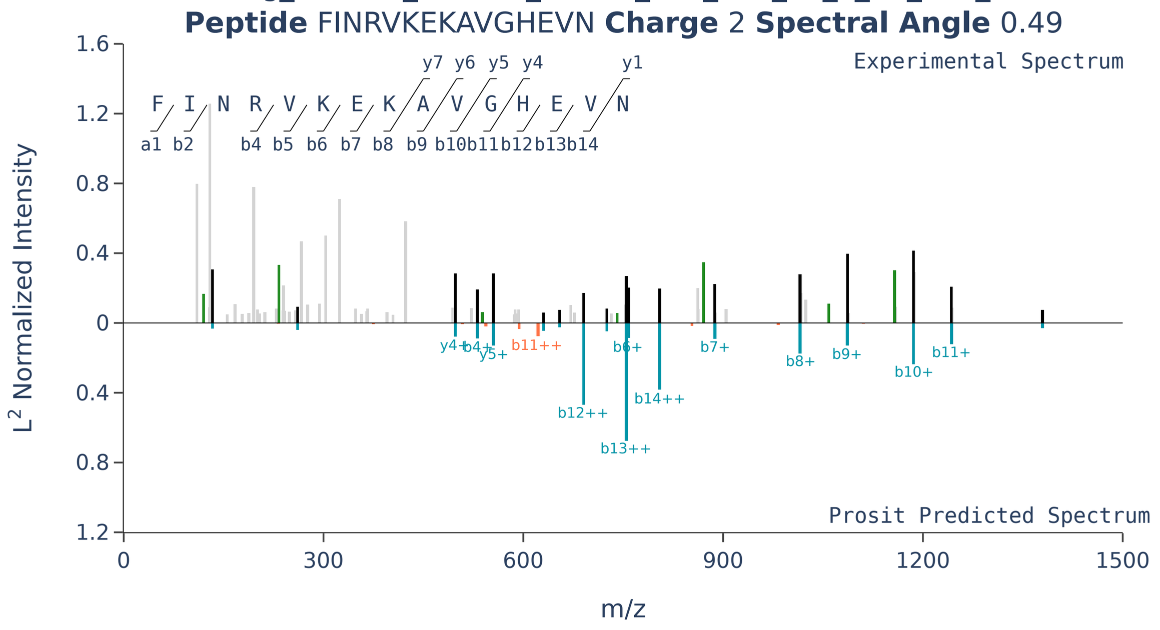


**Source** Ncheng_210623_230623_HFGoe_FFH_20S_25_1_A2_24h_R2 **Scan** 27879
**Peptide** KAFNEALPLTGV **Charge** 2 **Spectral Angle** 0.57

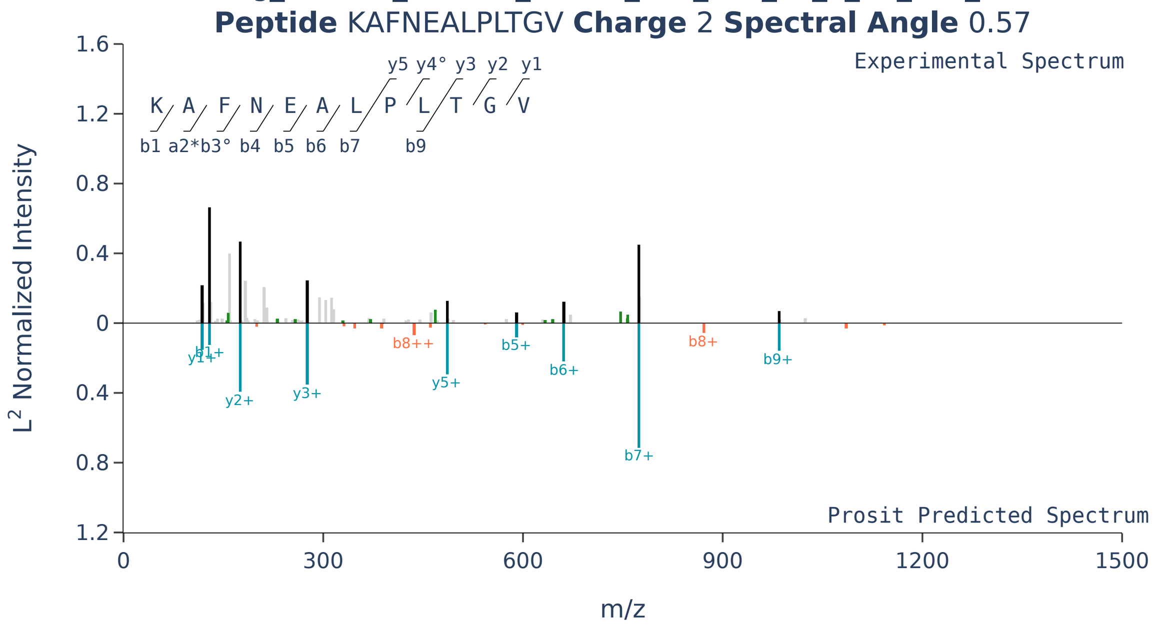


**Source** Ncheng_210623_230623_HFGoe_FFH_20S_25_1_A2_24h_R1 **Scan** 5603
**Peptide** INRVKEKAVG **Charge** 1 **Spectral Angle** 0.65

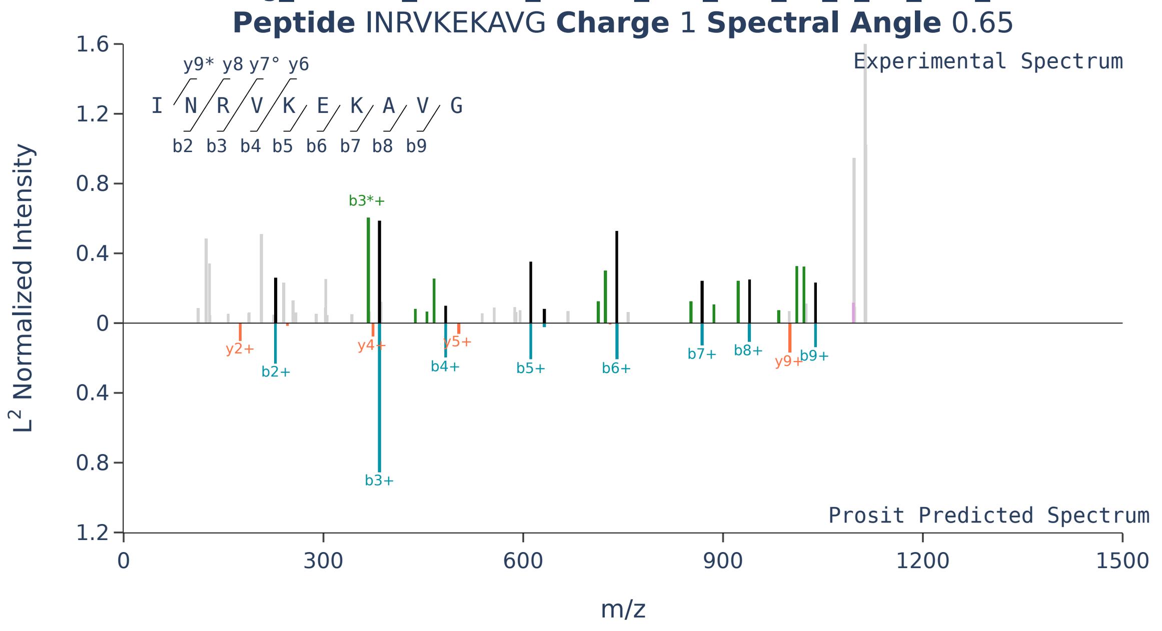


**Source** Ncheng_210623_230623_HFGoe_FFH_20S_25_1_A1_1h_R2 **Scan** 14183
**Peptide** KVDGDARGGAALSI **Charge** 2 **Spectral Angle** 0.71

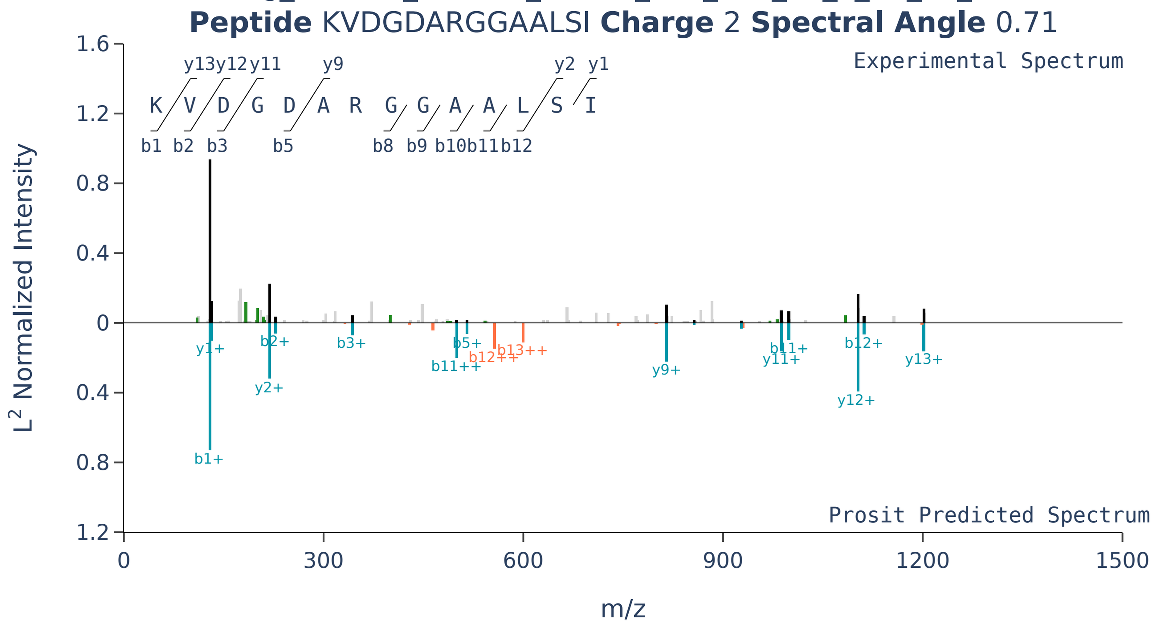


**Source** Ncheng_210623_230623_HFGoe_FFH_20S_25_1_A2_24h_R2 **Scan** 19813
**Peptide** VGHEVNKSLTPGQEFVKLVR **Charge** 5 **Spectral Angle** 0.58

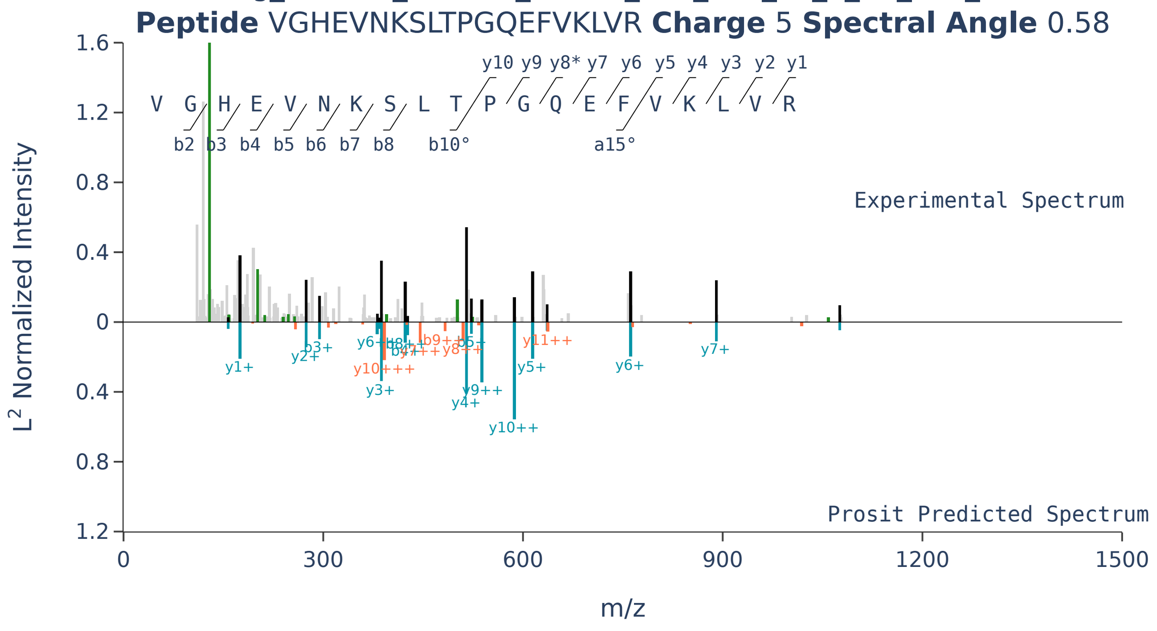


**Source** Ncheng_210623_230623_HFGoe_FFH_20S_25_1_A1_24h_R1 **Scan** 11496
**Peptide** DAANTAKAFNEA **Charge** 2 **Spectral Angle** 0.81

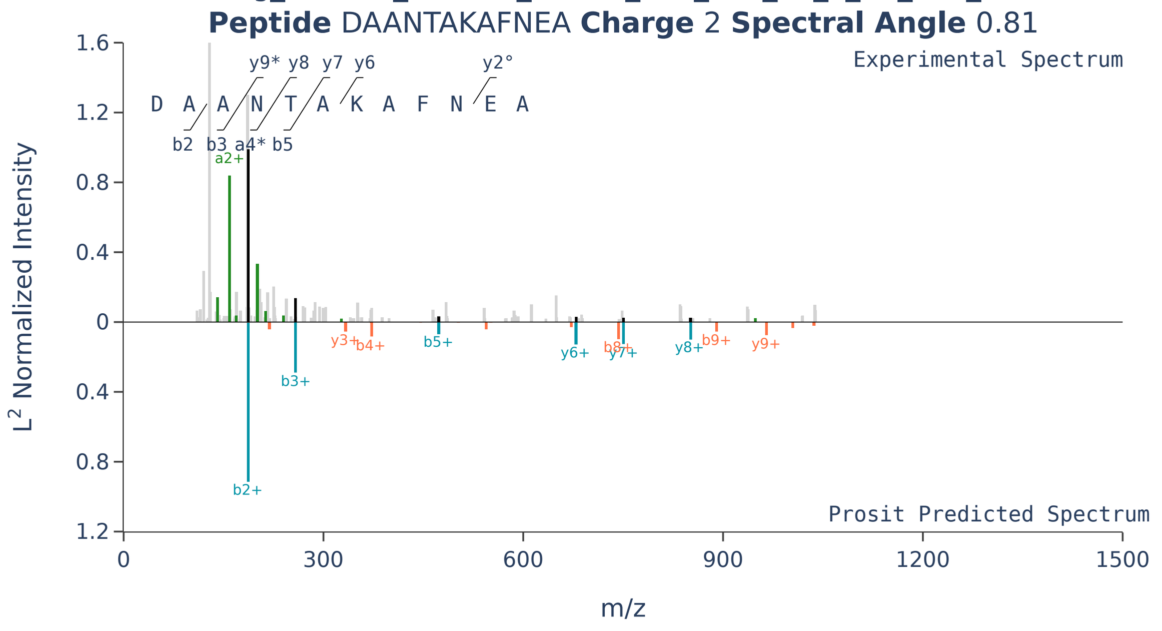


**Source** Ncheng_210623_230623_HFGoe_FFH_20S_25_1_A1_24h_R2 **Scan** 29249
**Peptide** AAQPPAVVLMA **Charge** 2 **Spectral Angle** 0.7

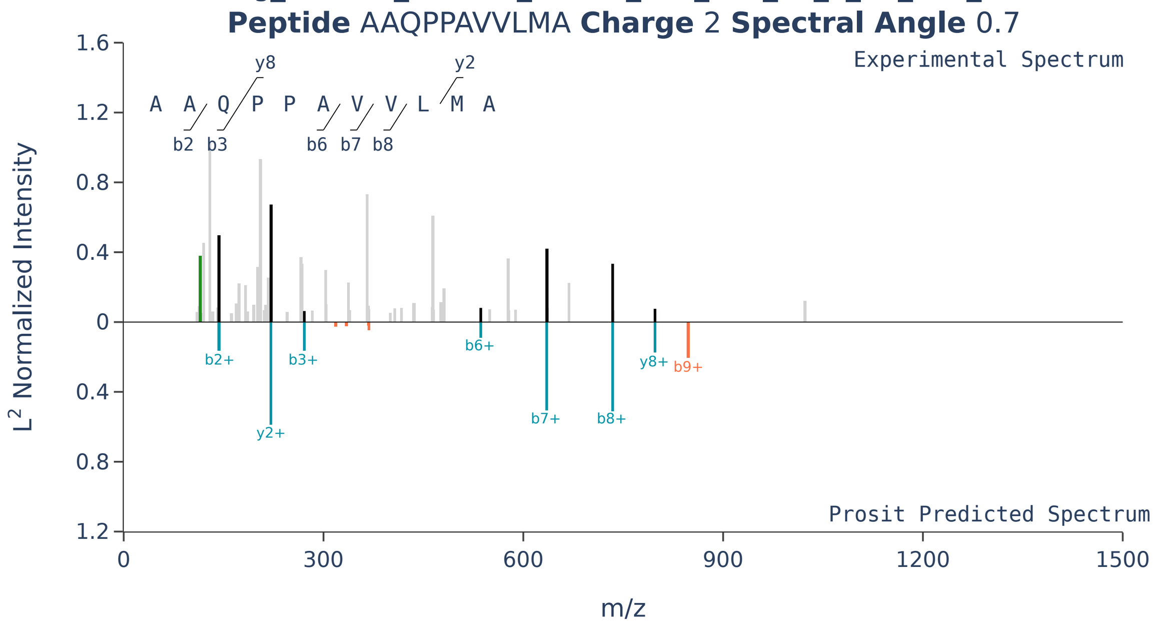


**Source** Ncheng_210623_230623_HFGoe_FFH_20S_25_1_A2_24h_R1 **Scan** 16172
**Peptide** DIESKVDRAQAEKLASKLKKG **Charge** 6 **Spectral Angle** 0.86

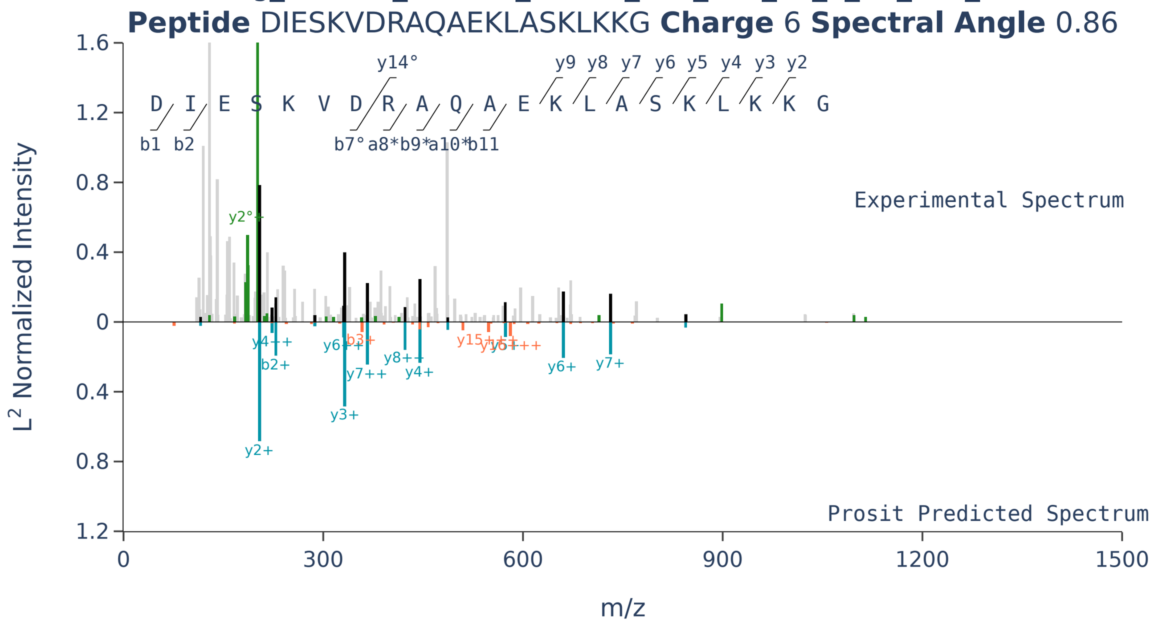


**Source** Ncheng_210623_230623_HFGoe_FFH_20S_25_1_A1_2h_R2 **Scan** 23017
**Peptide** GVVLTKVDGDARGGAALSI **Charge** 2 **Spectral Angle** 0.64

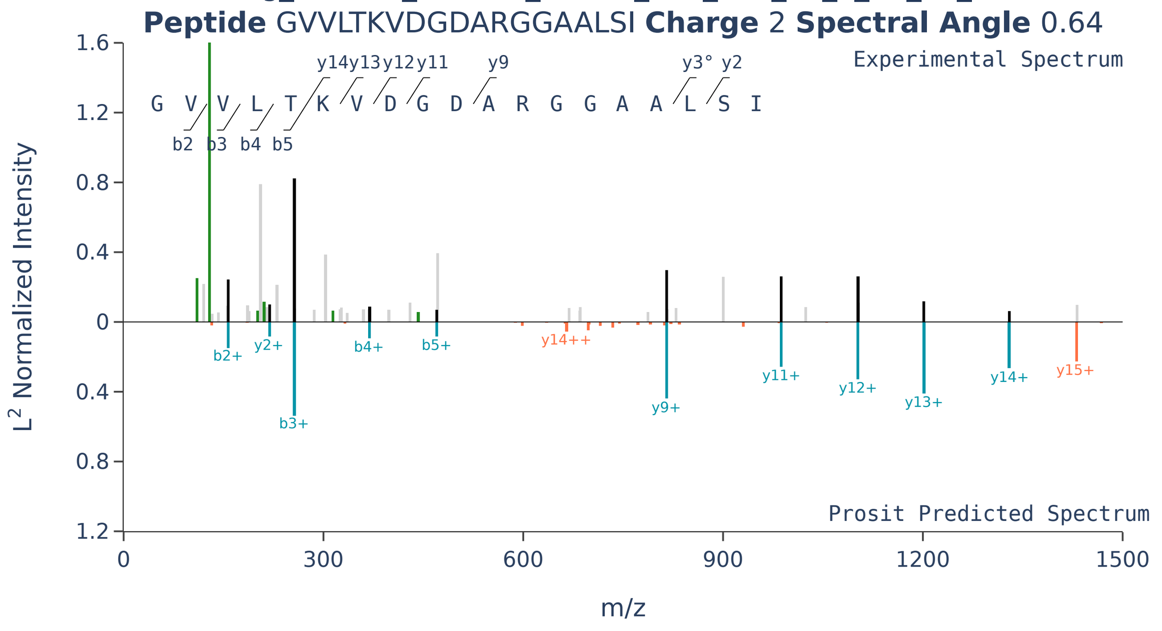


**Source** Ncheng_210623_230623_HFGoe_FFH_20S_25_1_A1_2h_R1 **Scan** 31474
**Peptide** KFYDVLLVDTAGRLHV **Charge** 3 **Spectral Angle** 0.67

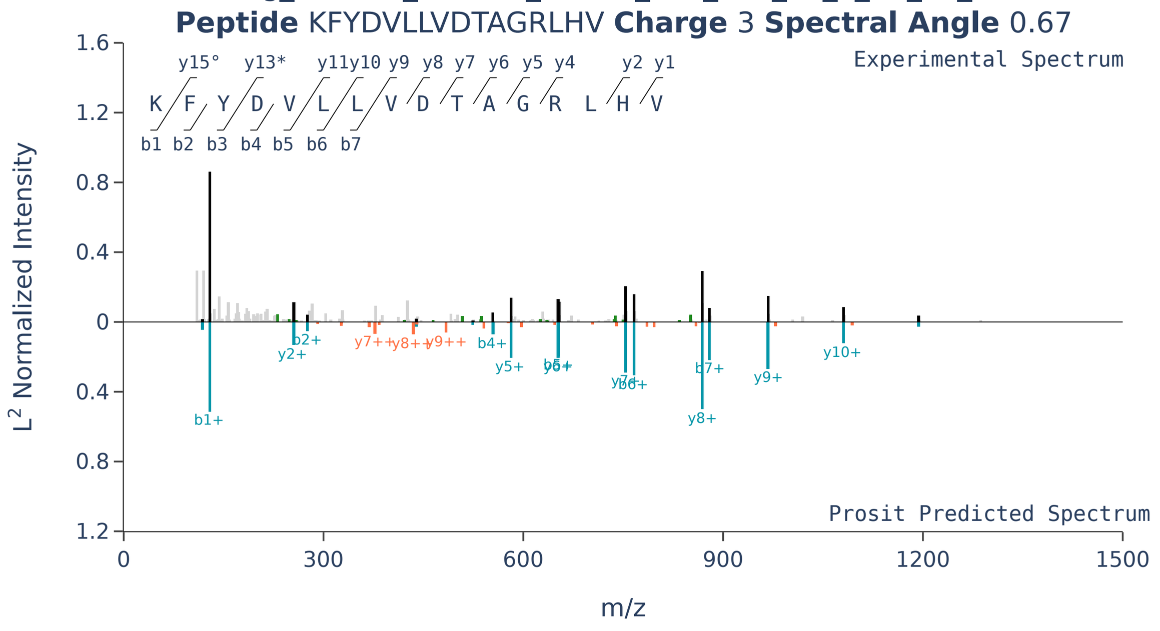

**Source** Ncheng_210623_230623_HFGoe_FFH_20S_25_1_A1_2h_R2 **Scan** 29908
**Peptide** AALKEAKLKFYDVLLVD **Charge** 3 **Spectral Angle** 0.59

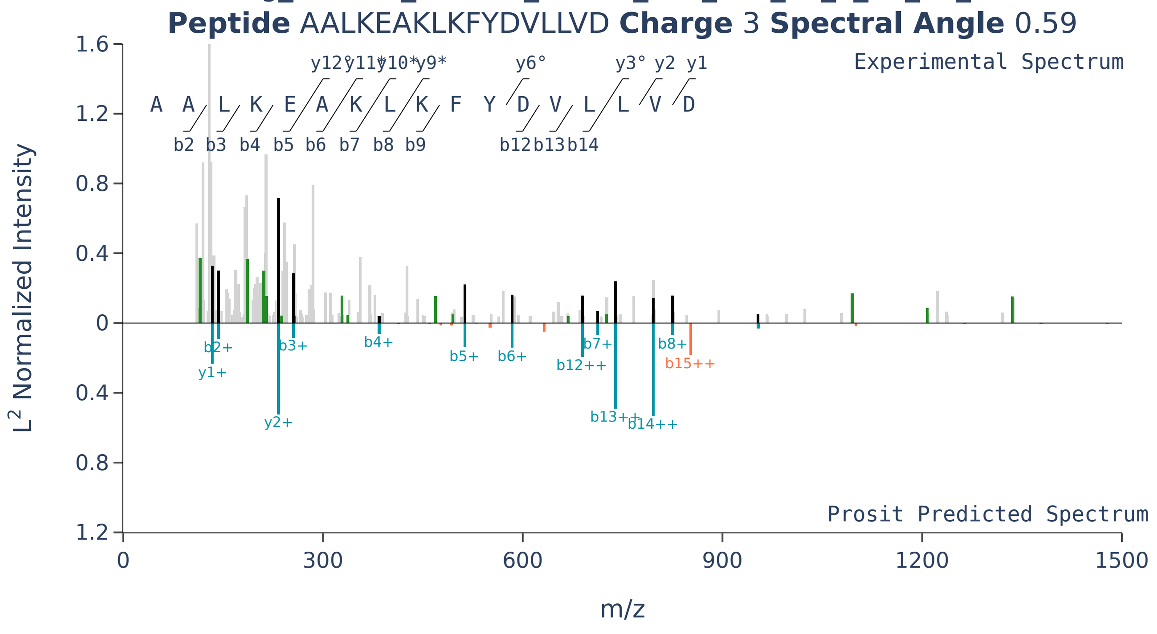


**Source** Ncheng_210623_230623_HFGoe_FFH_20S_25_1_A2_24h_R2 **Scan** 15724
**Peptide** DGDARGGAALSIRHITG **Charge** 4 **Spectral Angle** 0.74

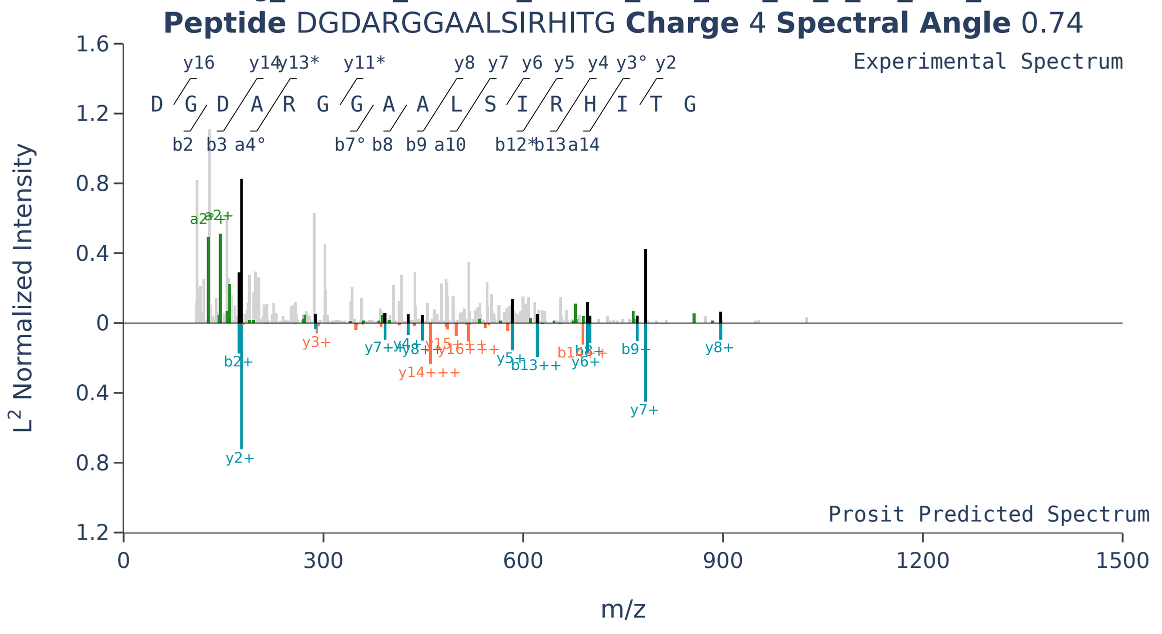


**Source** Ncheng_210623_230623_HFGoe_FFH_20S_25_1_A1_2h_R2 **Scan** 17995
**Peptide** REFINRVKEKAVGHEVNKSLTPGQEFVKI **Charge** 4 **Spectral Angle** 0.64

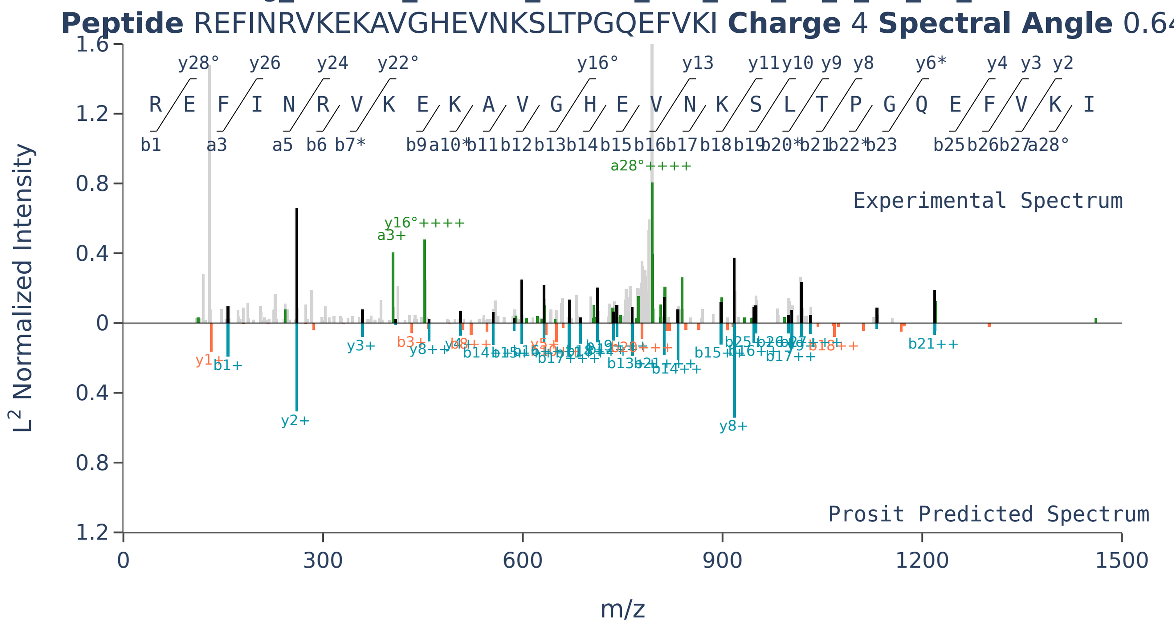


**Source** Ncheng_210623_230623_HFGoe_FFH_20S_25_1_A2_24h_R1 **Scan** 13007
**Peptide** ALLNSMTMKERAKPEIIKGSRK **Charge** 3 **Spectral Angle** 0.58

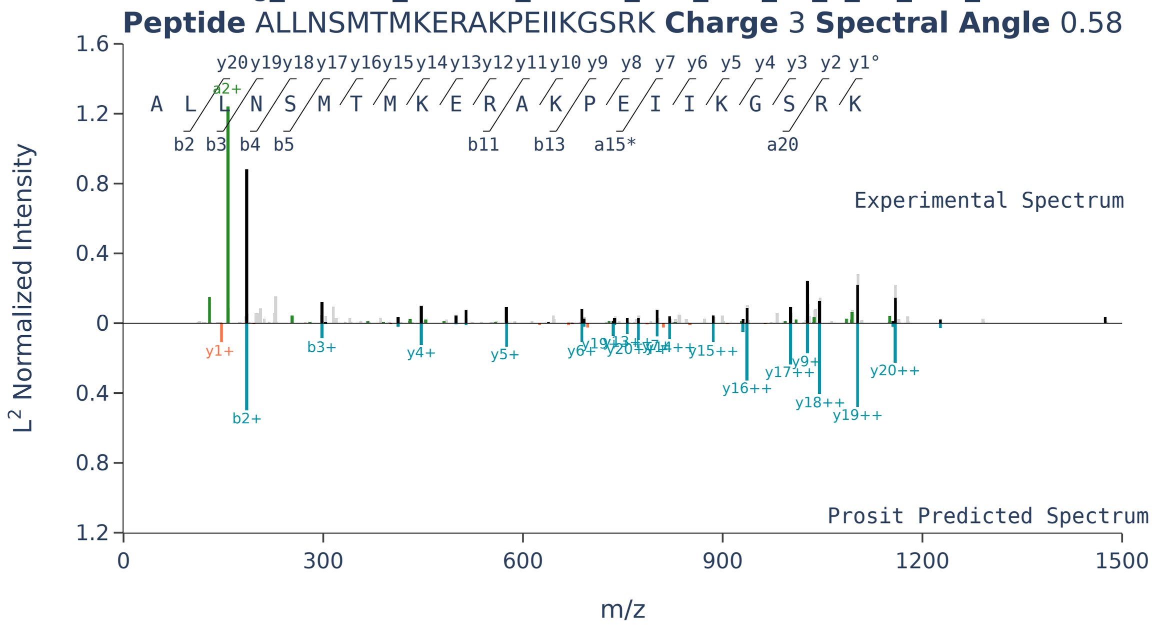


**Source** Ncheng_210623_230623_HFGoe_FFH_20S_25_1_A1_4h_R2 **Scan** 21711
**Peptide** IVNAALKEAKLKFY **Charge** 3 **Spectral Angle** 0.61

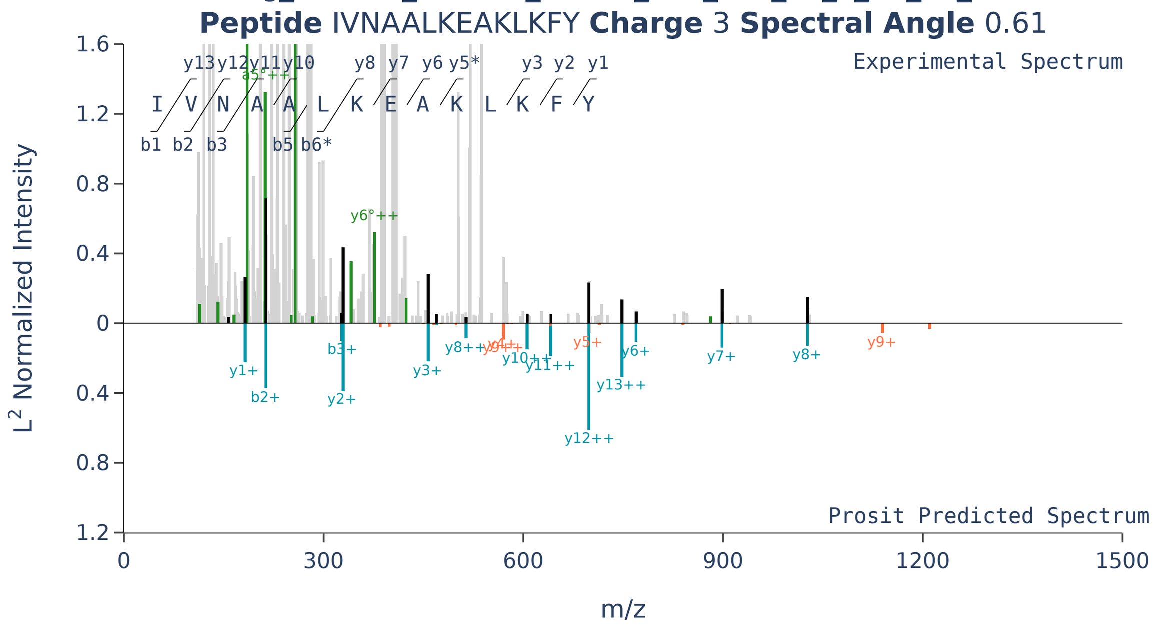


**Source** Ncheng_210623_230623_HFGoe_FFH_20S_25_1_A2_4h_R2 **Scan** 26396
**Peptide** TGQDAANTAKAFNEALPLT **Charge** 2 **Spectral Angle** 0.87

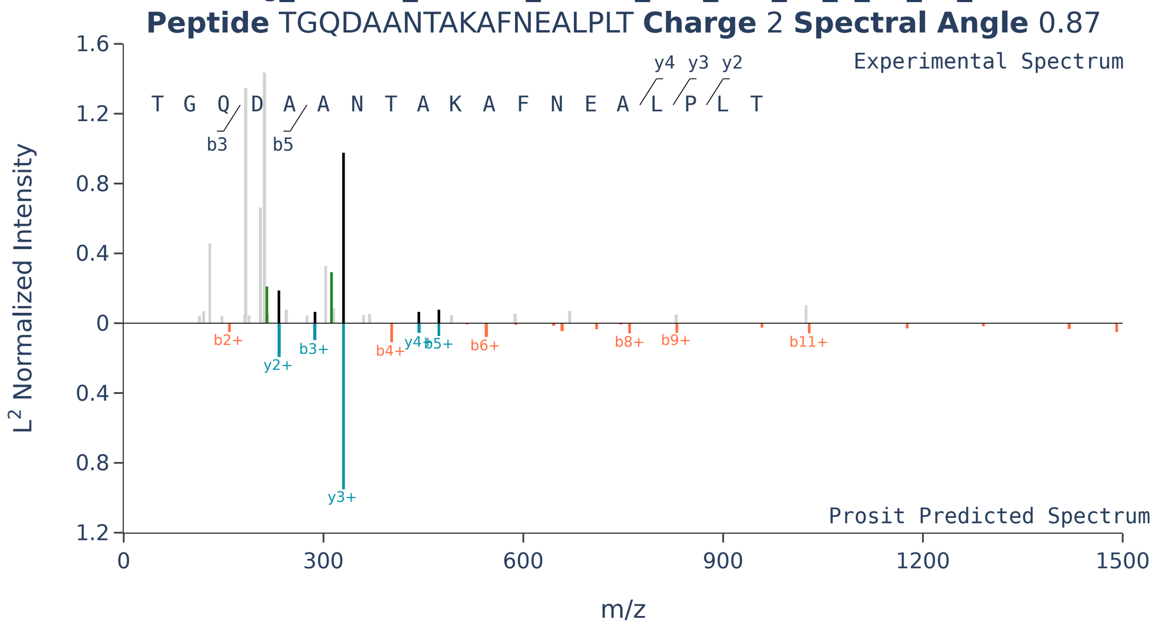


**Source** Ncheng_210623_230623_HFGoe_FFH_20S_25_1_A1_4h_R1 **Scan** 23628
**Peptide** TPGQEFVKIVRN **Charge** 2 **Spectral Angle** 0.8

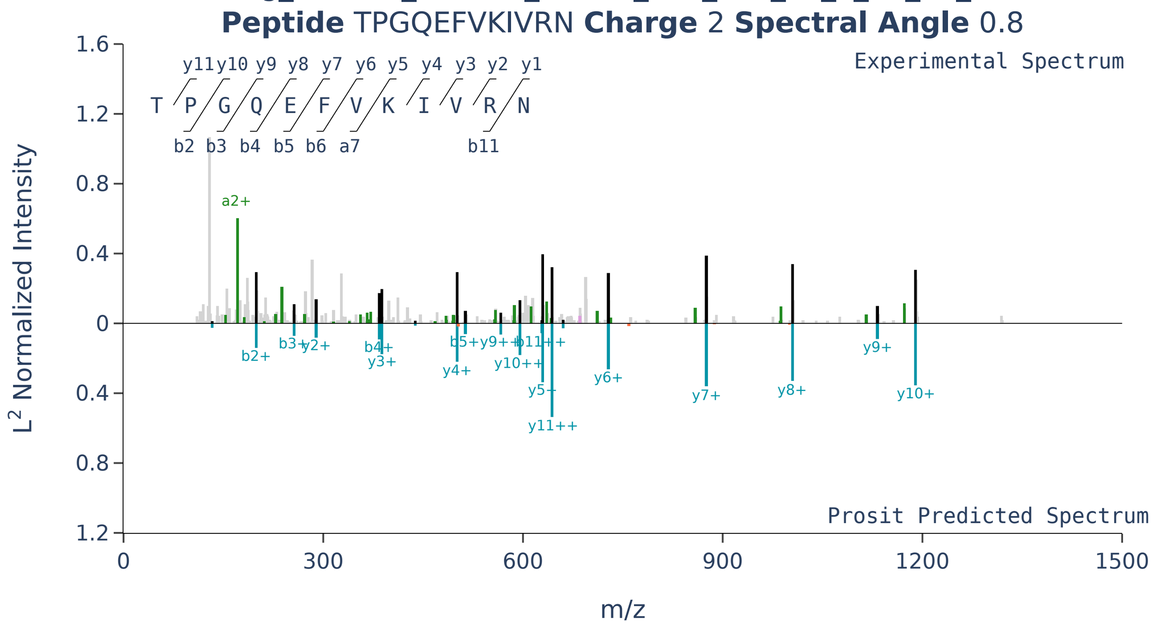


**Source** Ncheng_210623_230623_HFGoe_FFH_20S_25_1_A2_4h_R2 **Scan** 21480
**Peptide** KKVLVVSADVYRPAAIKQL **Charge** 3 **Spectral Angle** 0.88

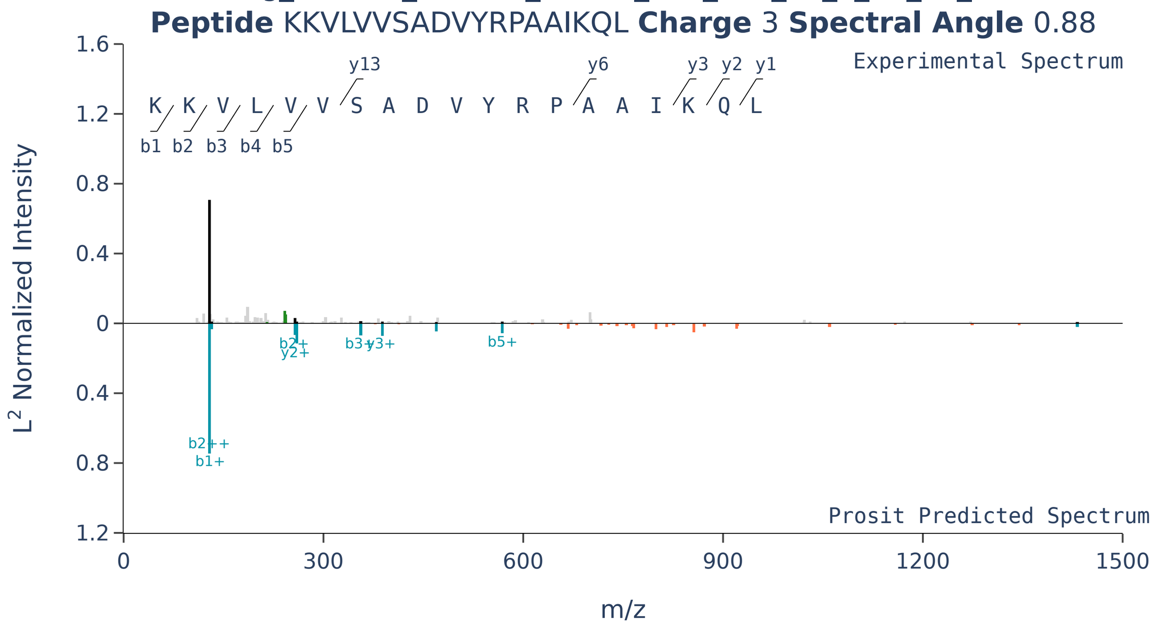


**Source** Ncheng_210623_230623_HFGoe_FFH_20S_25_1_A1_2h_R2 **Scan** 22562
**Peptide** RLHVDEAMMDEIKQVHA **Charge** 4 **Spectral Angle** 0.72

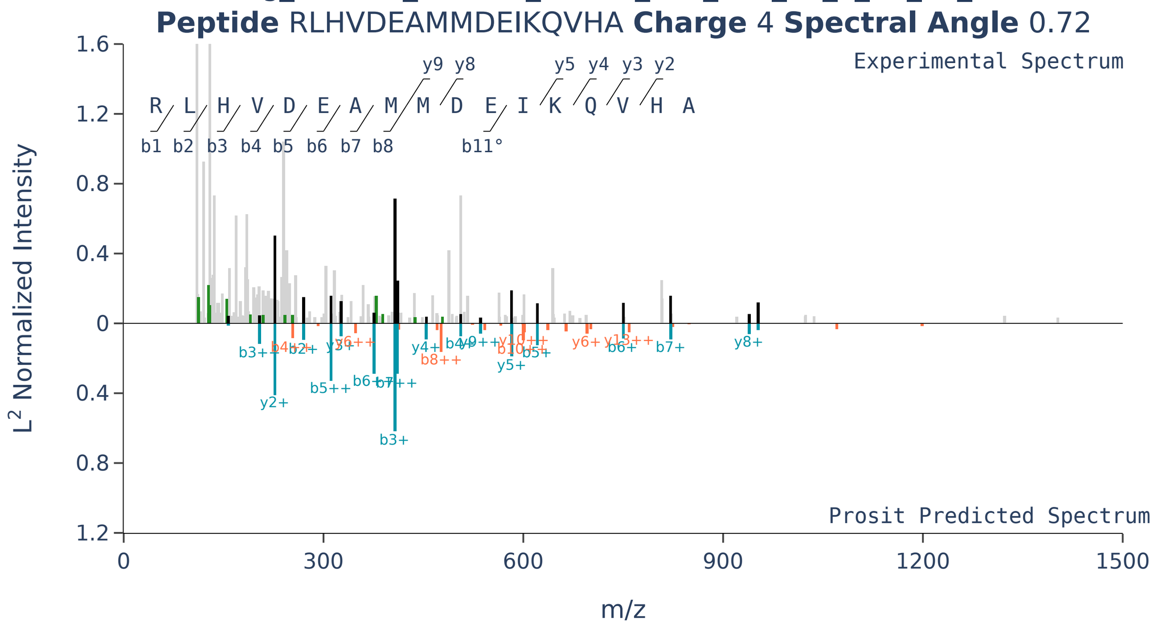


**Source** Ncheng_210623_230623_HFGoe_FFH_20S_25_1_A2_4h_R1 **Scan** 20828
**Peptide** MMDEIKQVHASINPVE **Charge** 3 **Spectral Angle** 0.51

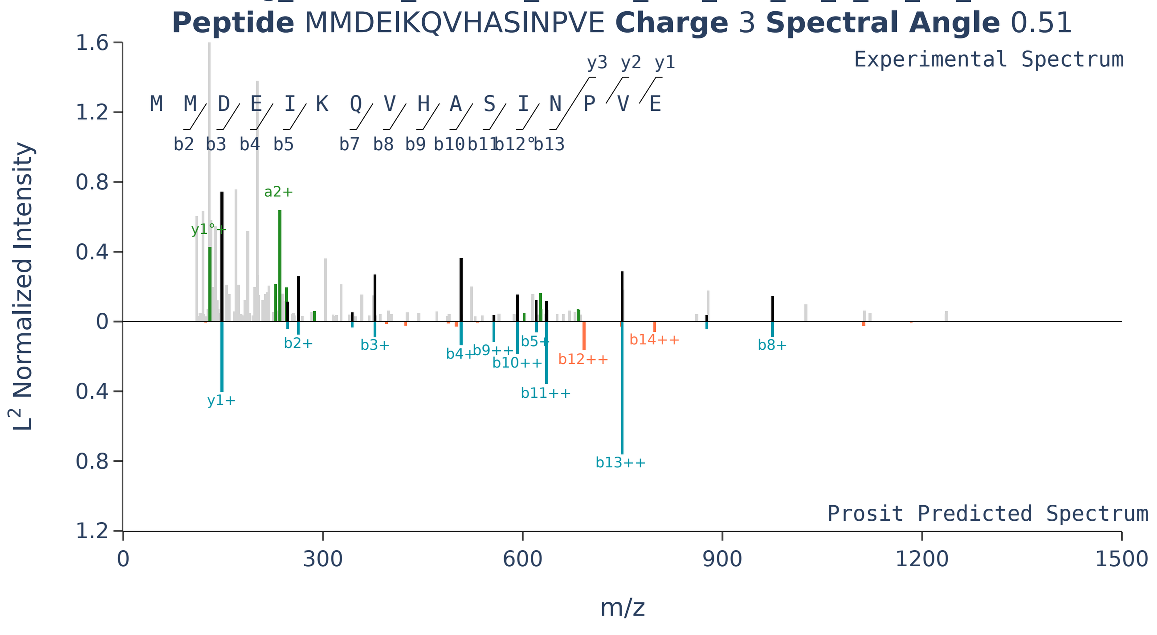


**Source** Ncheng_210623_230623_HFGoe_FFH_20S_25_1_A1_2h_R2 **Scan** 23409
**Peptide** TGKPIKFLGVGEKTEALEPFHPDRLAS **Charge** 4 **Spectral Angle** 0.84

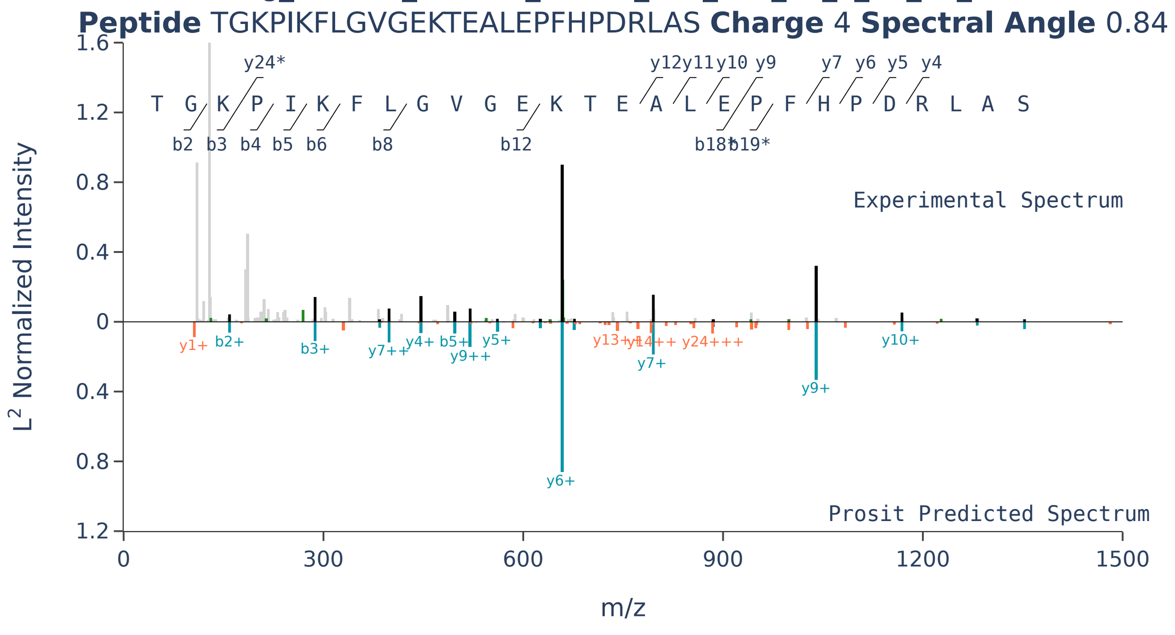


**Source** Ncheng_210623_230623_HFGoe_FFH_20S_25_1_A1_24h_R2 **Scan** 16379
**Peptide** HITGKPIKFLGVGEKTEA **Charge** 4 **Spectral Angle** 0.77

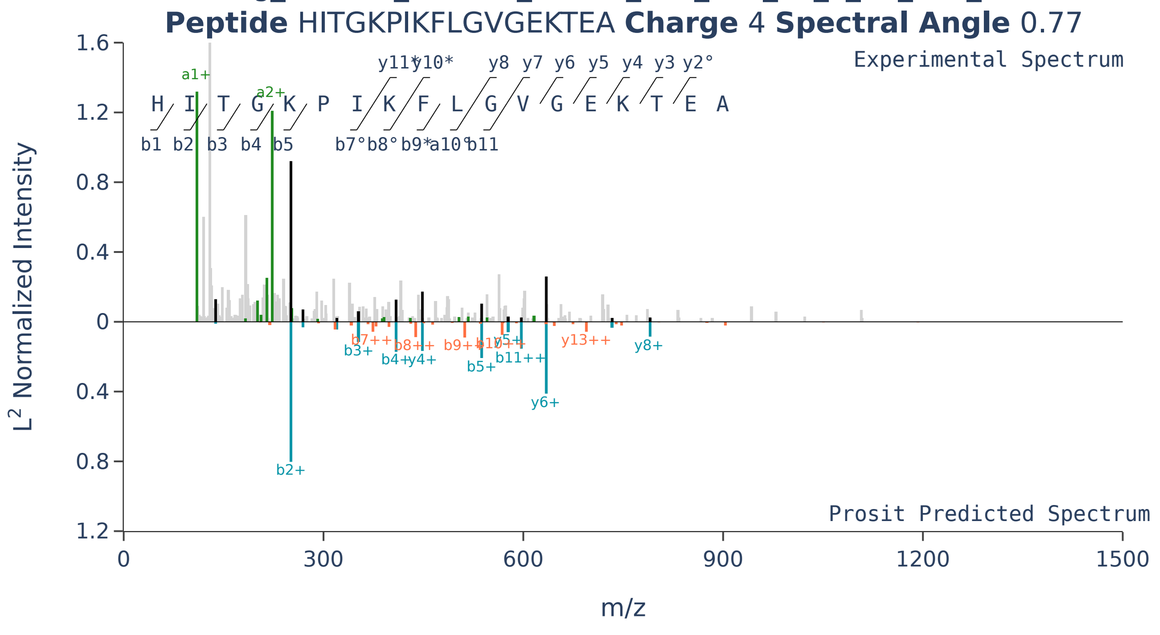


**Source** Ncheng_210623_230623_HFGoe_FFH_20S_25_1_A2_4h_R2 **Scan** 33507
**Peptide** TEALEPFHPDRIASRILGMGDVLSL **Charge** 4 **Spectral Angle** 0.85

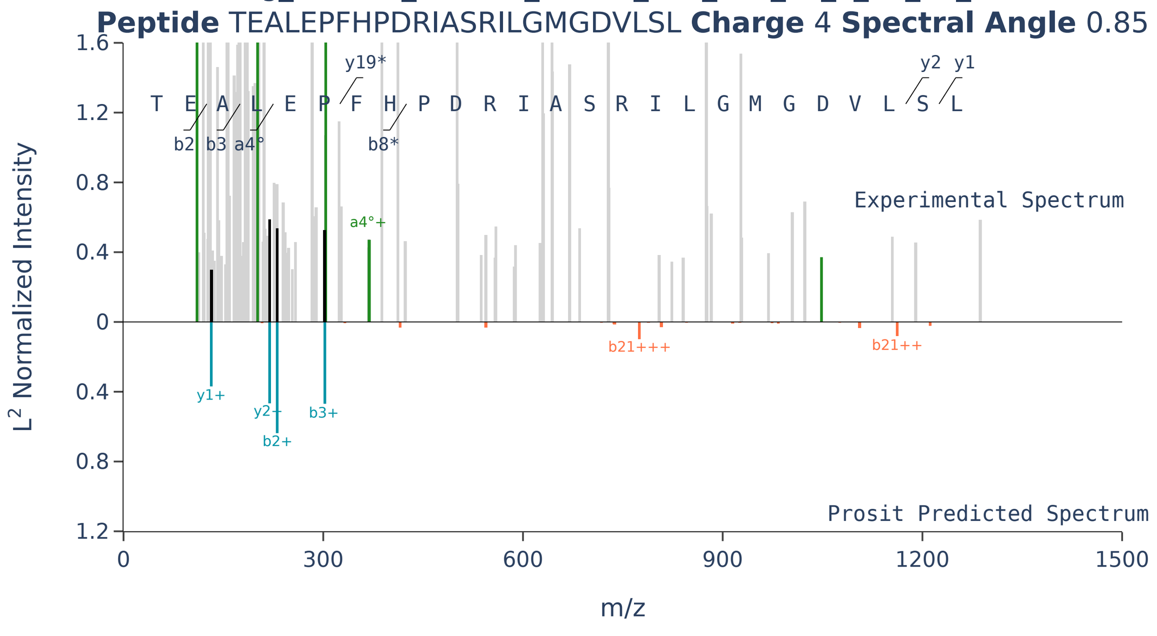


**Source** Ncheng_210623_230623_HFGoe_FFH_20S_25_1_A1_2h_R2 **Scan** 38639
**Peptide** TLFVVDAMTGQDAANTAKAFNEALPLTG **Charge** 3 **Spectral Angle** 0.84

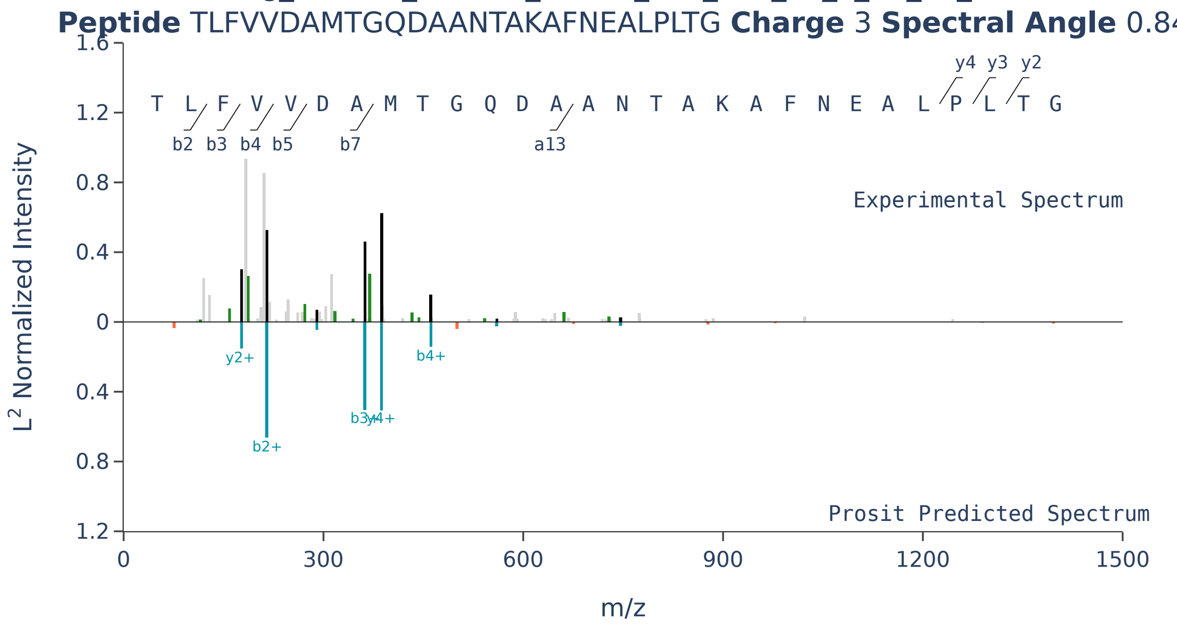


**Source** Ncheng_210623_230623_HFGoe_FFH_20S_25_1_A1_1h_R2 **Scan** 21572
**Peptide** LGVGEKTEALEPFHPDRI **Charge** 3 **Spectral Angle** 0.76

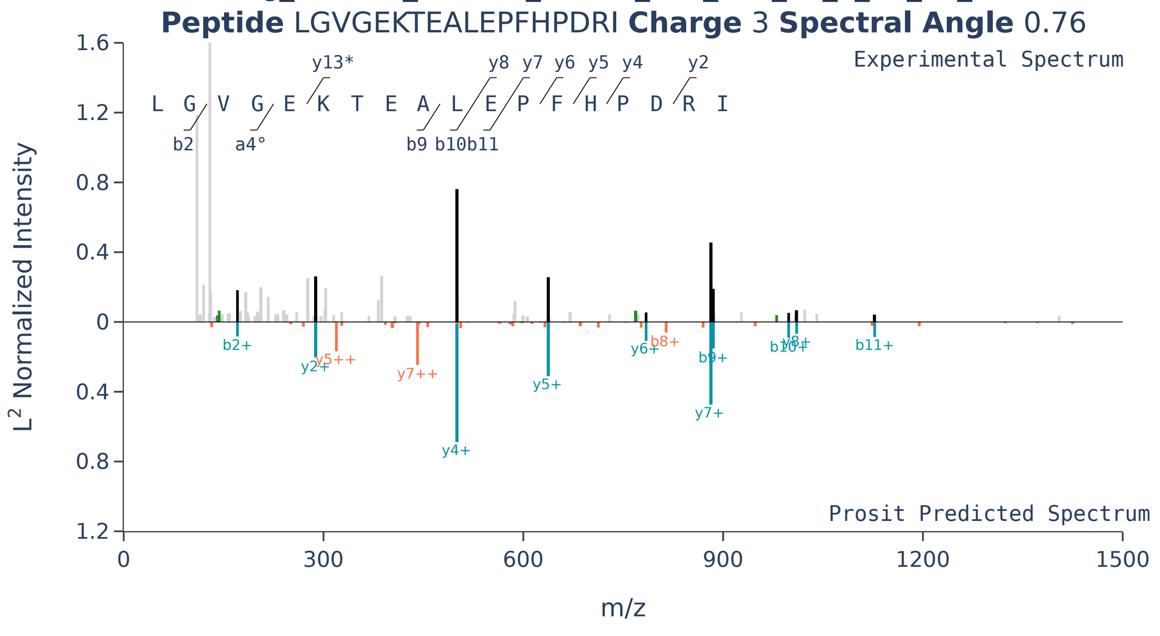

**Source** Ncheng_210623_230623_HFGoe_FFH_20S_25_1_A1_2h_R2 **Scan** 27754
**Peptide** LMGKLPGMGQIPDNVKSQMDDKVLVRME **Charge** 5 **Spectral Angle** 0.77

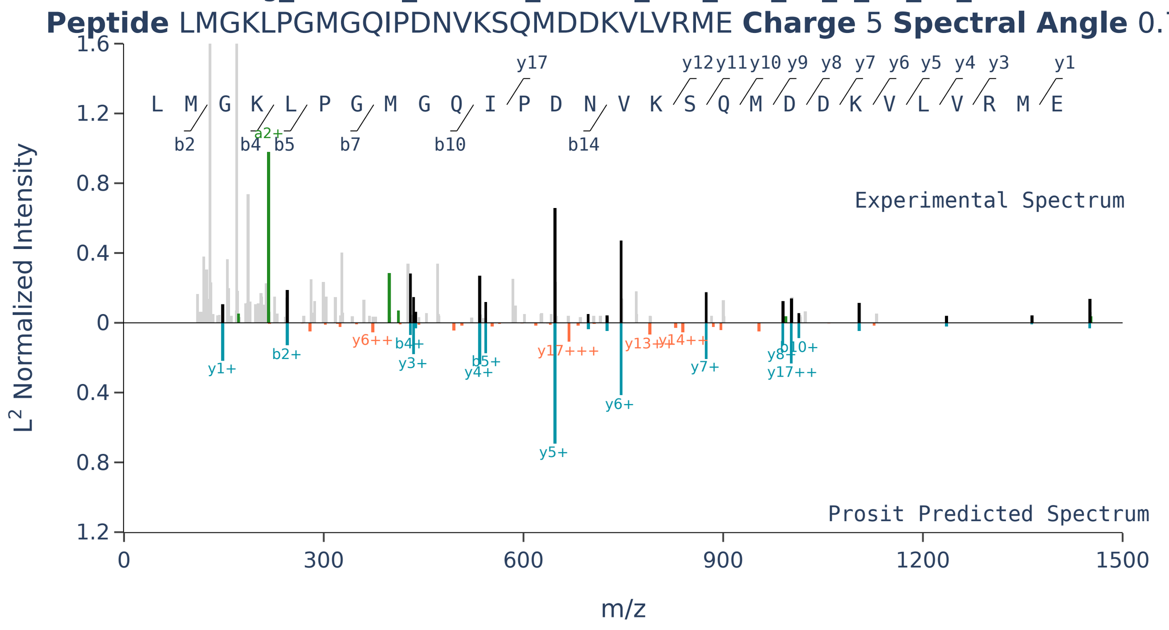


**Source** Ncheng_210623_230623_HFGoe_FFH_20S_25_1_A2_24h_R1 **Scan** 9957
**Peptide** TDRLSRTL **Charge** 2 **Spectral Angle** 0.61

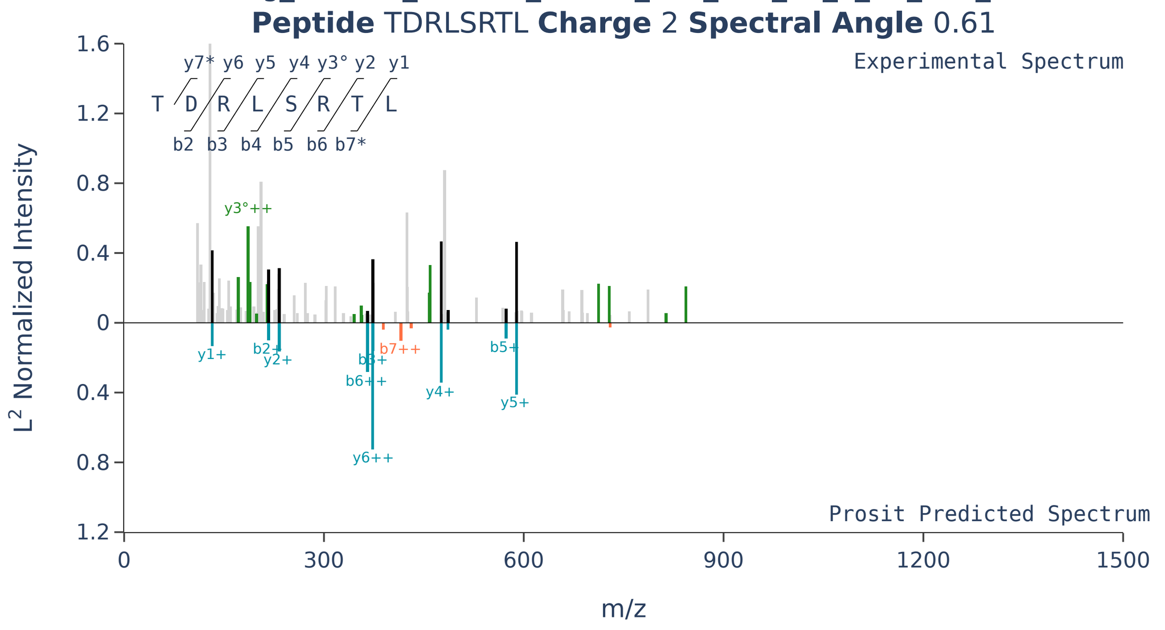


**Source** Ncheng_210623_230623_HFGoe_FFH_20S_25_1_A2_4h_R1 **Scan** 24387
**Peptide** PLKFLGVGEKTEAL **Charge** 3 **Spectral Angle** 0.74

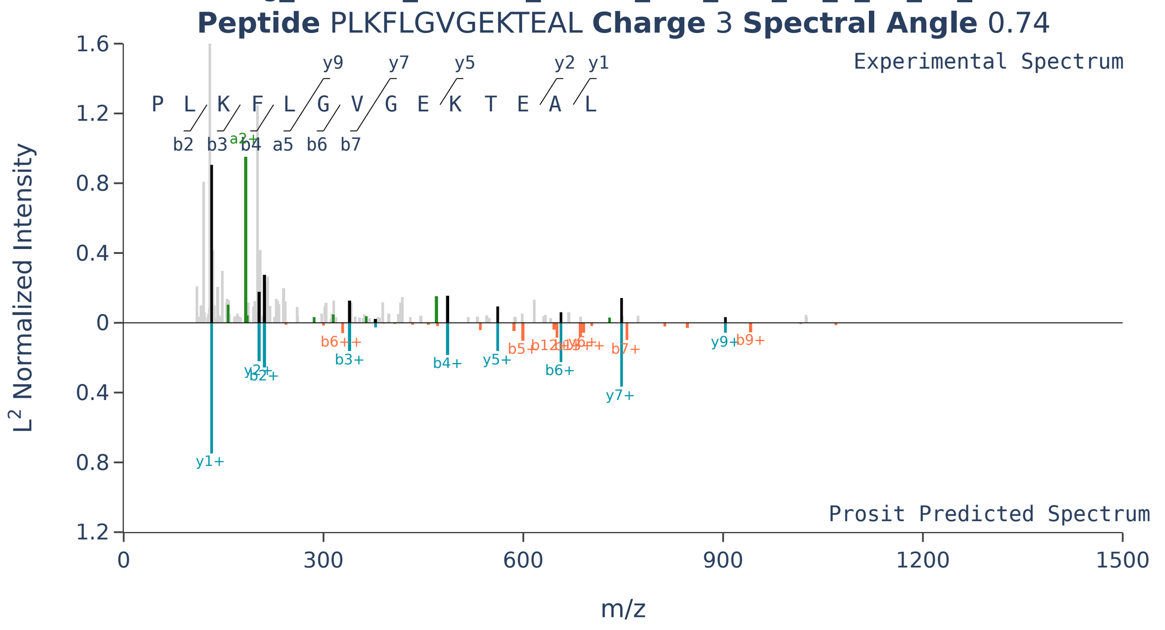


**Source** Ncheng_210623_230623_HFGoe_FFH_20S_25_1_A2_2h_R2 **Scan** 31065
**Peptide** MGGMASLMGKLPGMGQIPDNVKSQ **Charge** 3 **Spectral Angle** 0.74

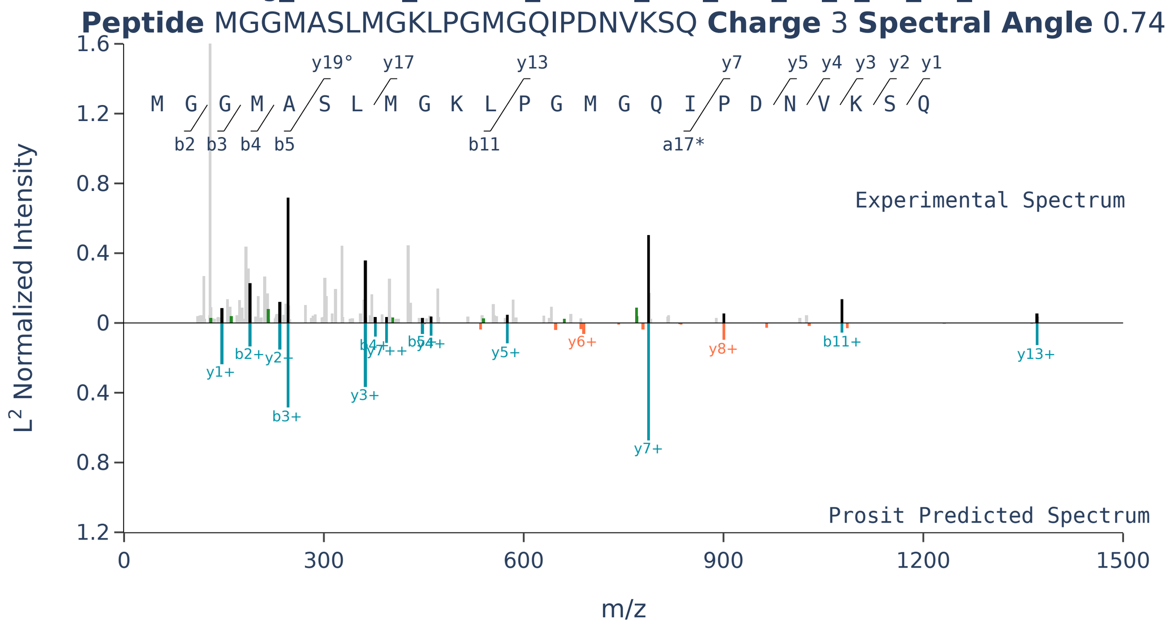


**Source** Ncheng_210623_230623_HFGoe_FFH_20S_25_1_A2_4h_R1 **Scan** 33654
**Peptide** IVNAALKEAKLKFYDVLLV **Charge** 3 **Spectral Angle** 0.66

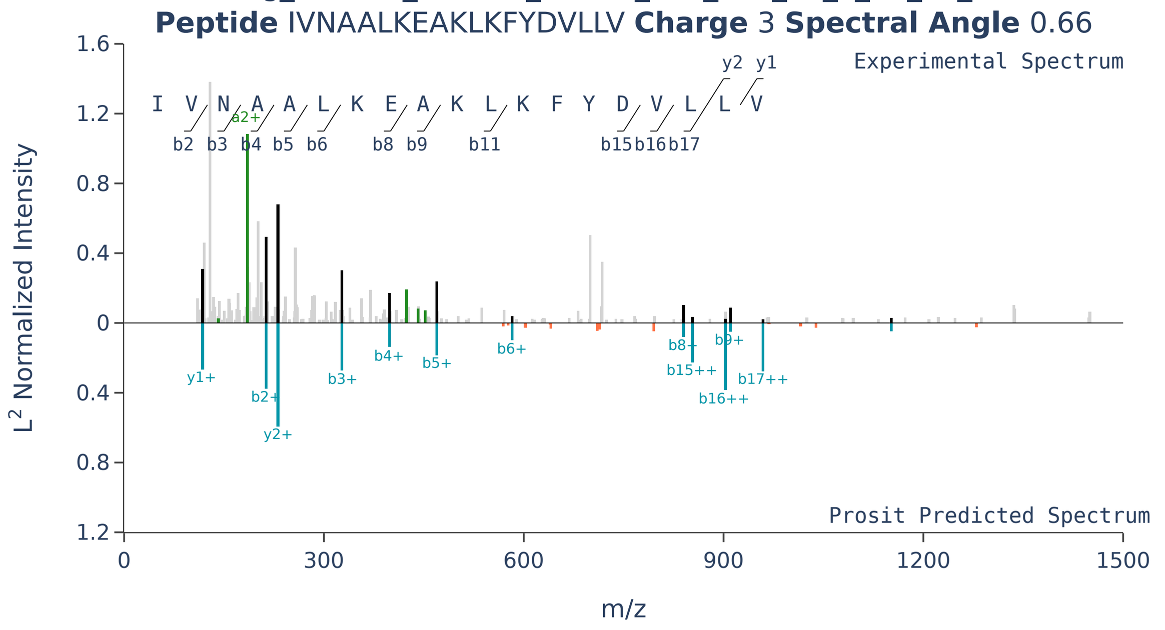


**Source** Ncheng_210623_230623_HFGoe_FFH_20S_25_1_A2_4h_R1 **Scan** 12198
**Peptide** AKMMRSM **Charge** 2 **Spectral Angle** 0.92

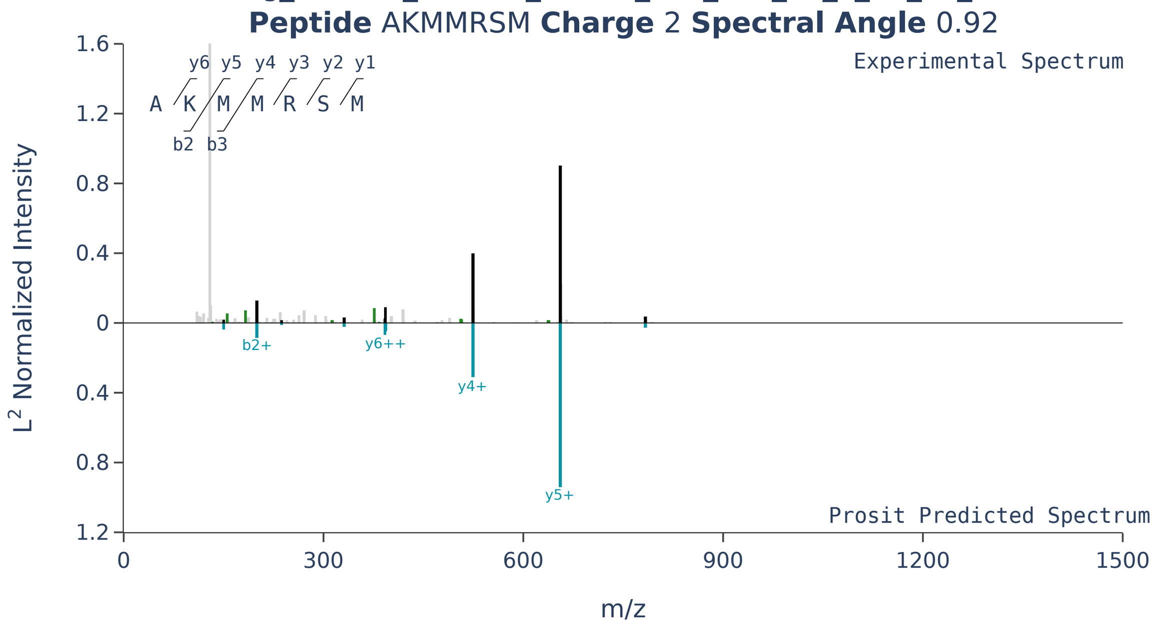


**Source** Ncheng_210623_230623_HFGoe_FFH_20S_25_1_A2_2h_R2 **Scan** 21958
**Peptide** DNLTDRLSRTL **Charge** 2 **Spectral Angle** 0.49

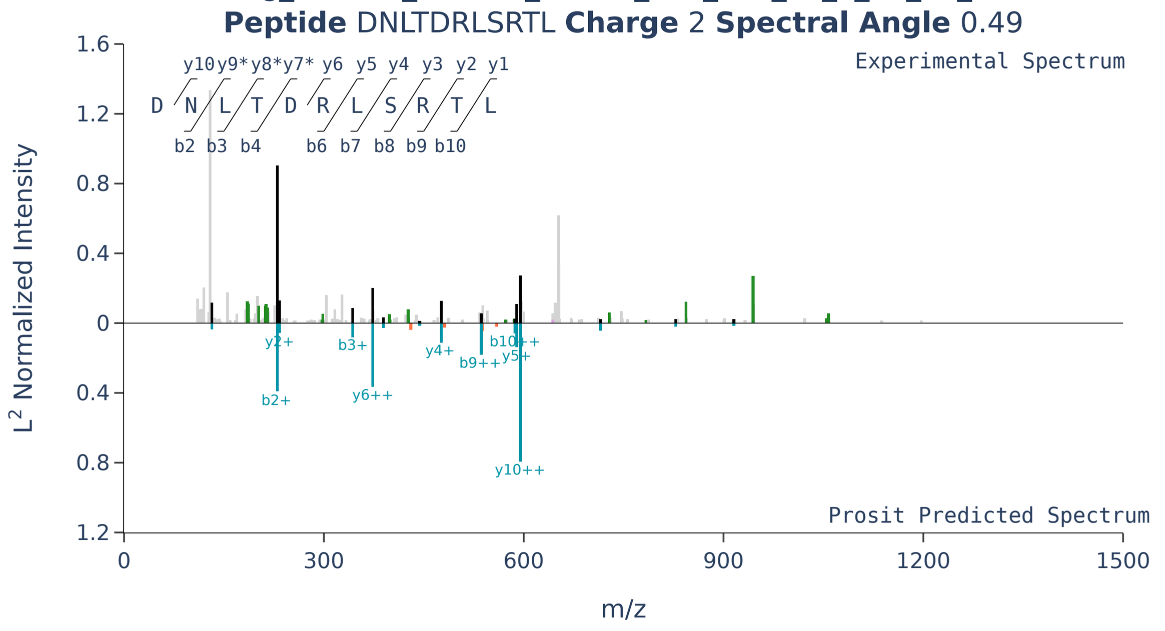


**Source** Ncheng_210623_230623_HFGoe_FFH_20S_25_1_A2_24h_R2 **Scan** 14748
**Peptide** QAEKLASKLKKGDGFDLND **Charge** 2 **Spectral Angle** 0.66

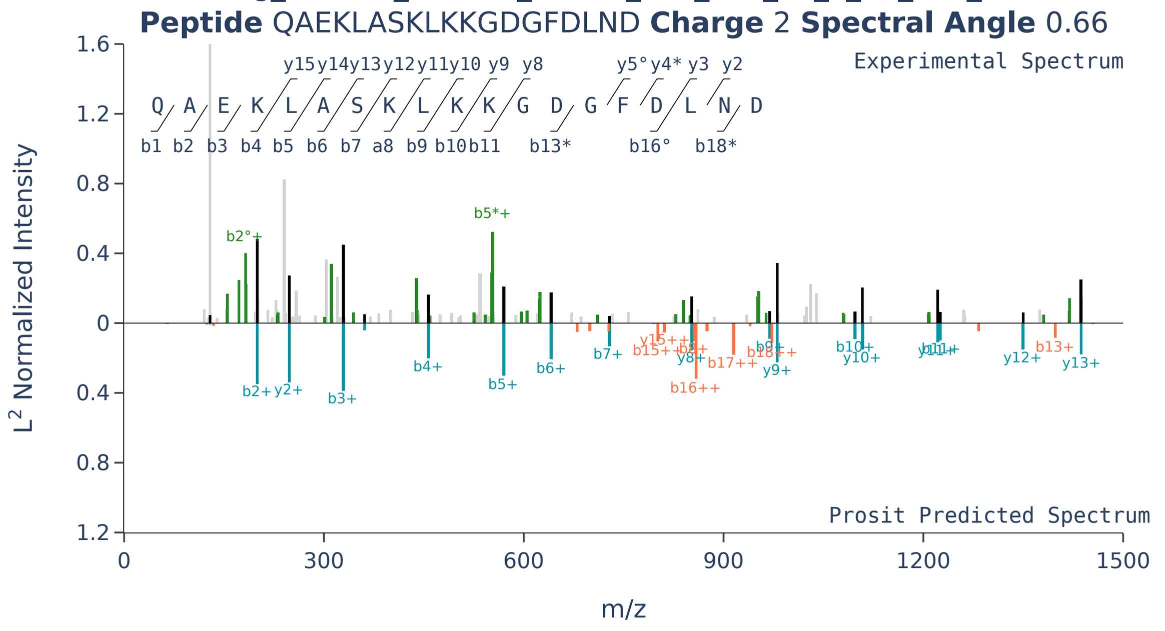


**Source** Ncheng_210623_230623_HFGoe_FFH_20S_25_1_A1_24h_R1 **Scan** 18030
**Peptide** KAVGHEVNKSLTPGQEFVKLVR **Charge** 4 **Spectral Angle** 0.58

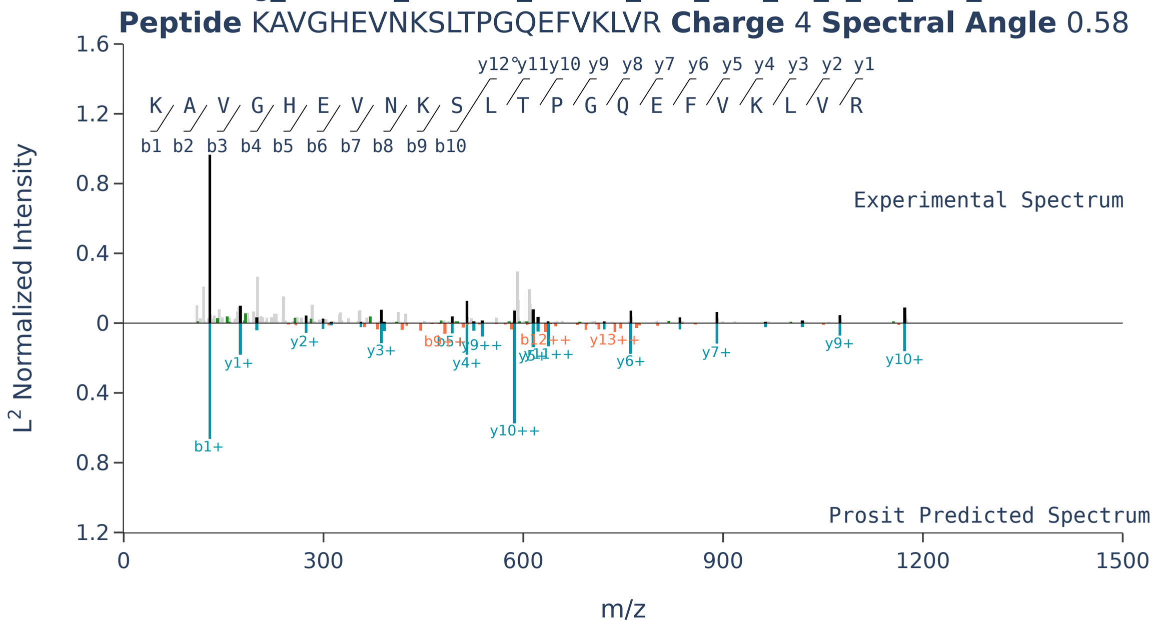


**Source** Ncheng_210623_230623_HFGoe_FFH_20S_25_1_A1_2h_R2 **Scan** 5684
**Peptide** RGRLTEDNVKD **Charge** 3 **Spectral Angle** 0.61

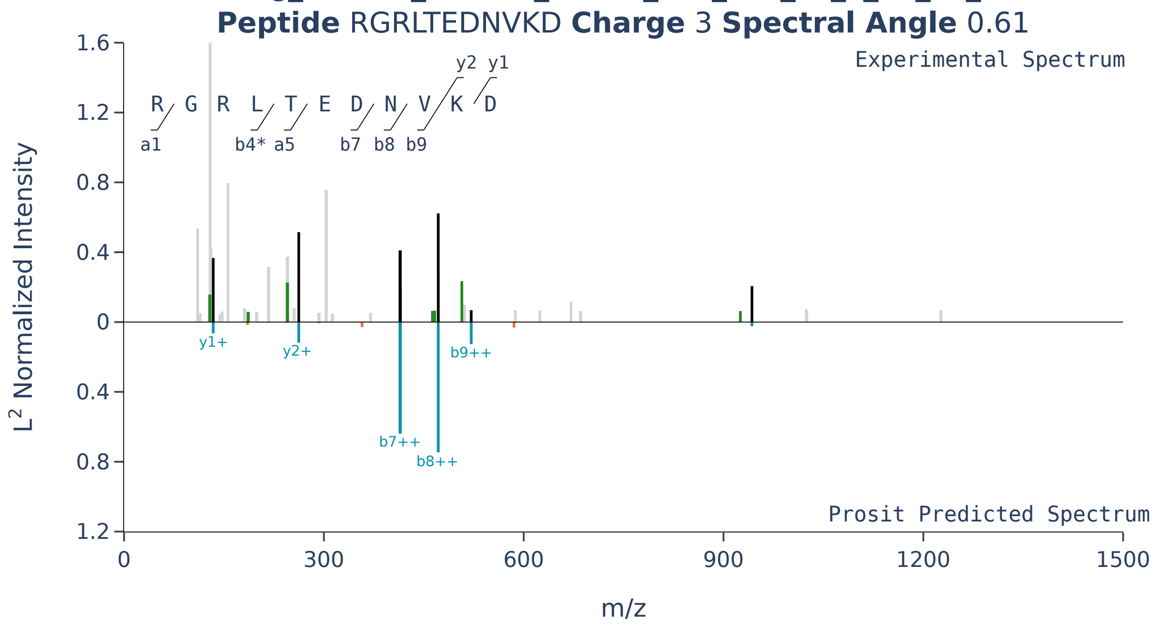


**Source** Ncheng_210623_230623_HFGoe_FFH_20S_25_1_A1_1h_R1 **Scan** 20705
**Peptide** LTEDNVKDTLREVRMA **Charge** 4 **Spectral Angle** 0.67

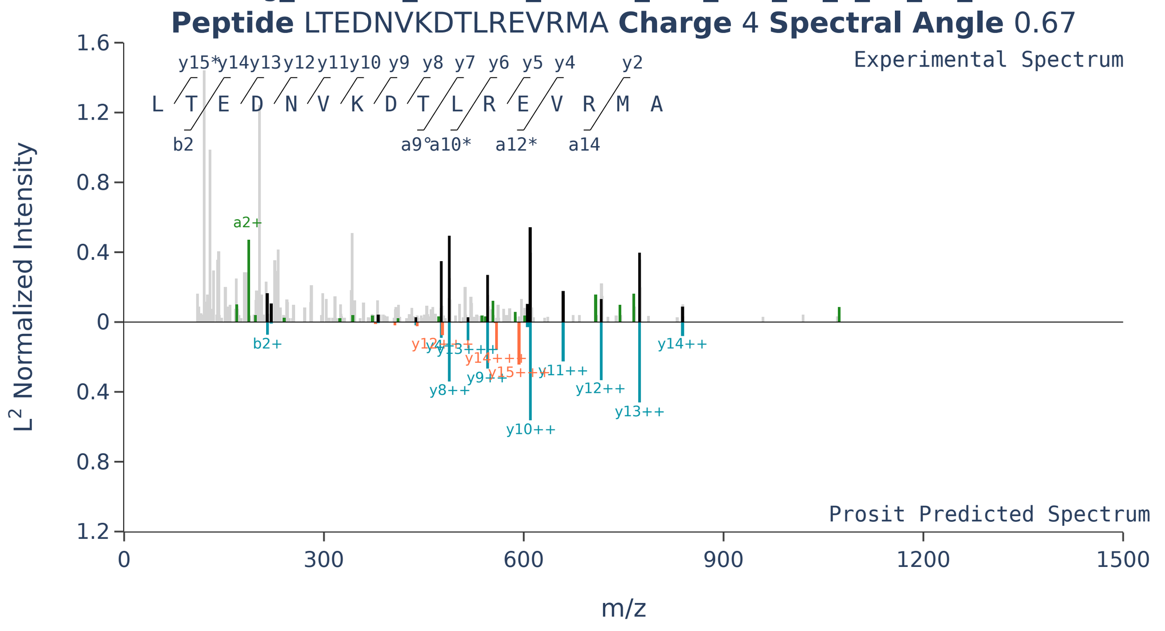


**Source** Ncheng_210623_230623_HFGoe_FFH_20S_25_1_A1_1h_R2 **Scan** 24173
**Peptide** VDTAGRLHVDEAMMDEIKQVHA **Charge** 3 **Spectral Angle** 0.68

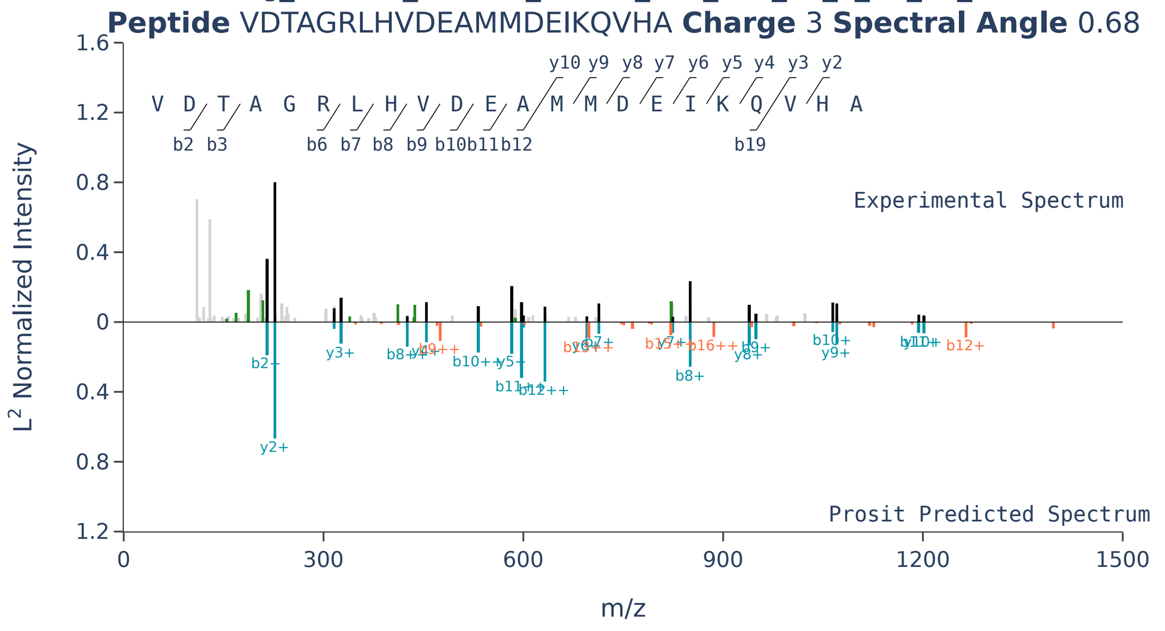


**Source** Ncheng_210623_230623_HFGoe_FFH_20S_25_1_A2_24h_R2 **Scan** 12845
**Peptide** KVDGDARGGAALSIRHITGKPLK **Charge** 5 **Spectral Angle** 0.7

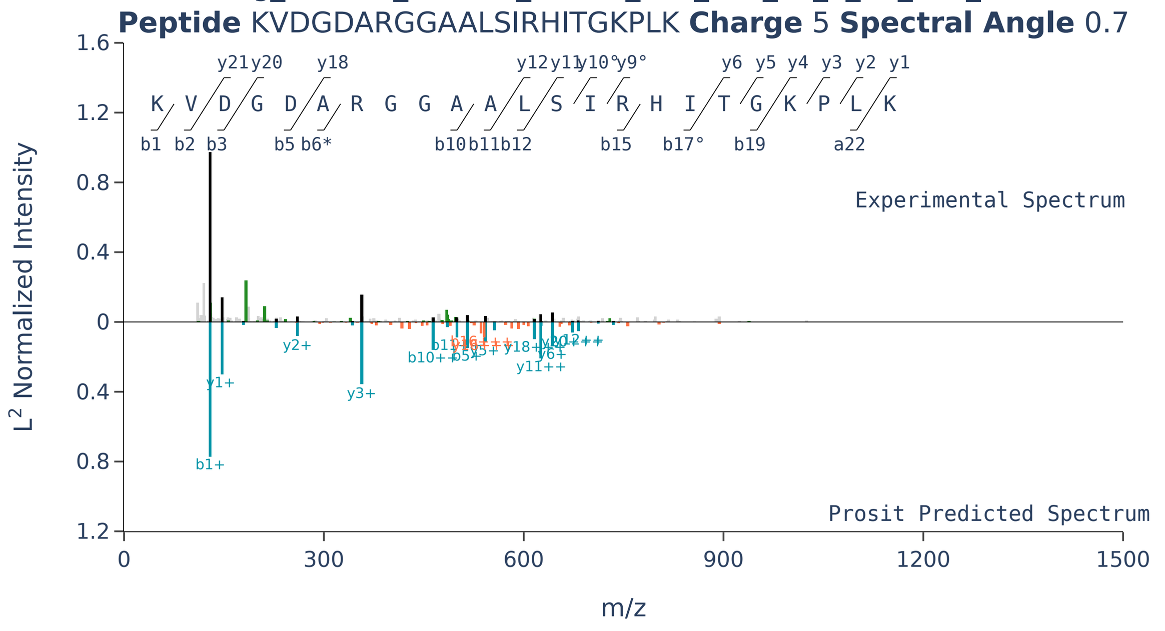


**Source** Ncheng_210623_230623_HFGoe_FFH_20S_25_1_A2_4h_R2 **Scan** 9654
**Peptide** DRLSRTL **Charge** 2 **Spectral Angle** 0.39

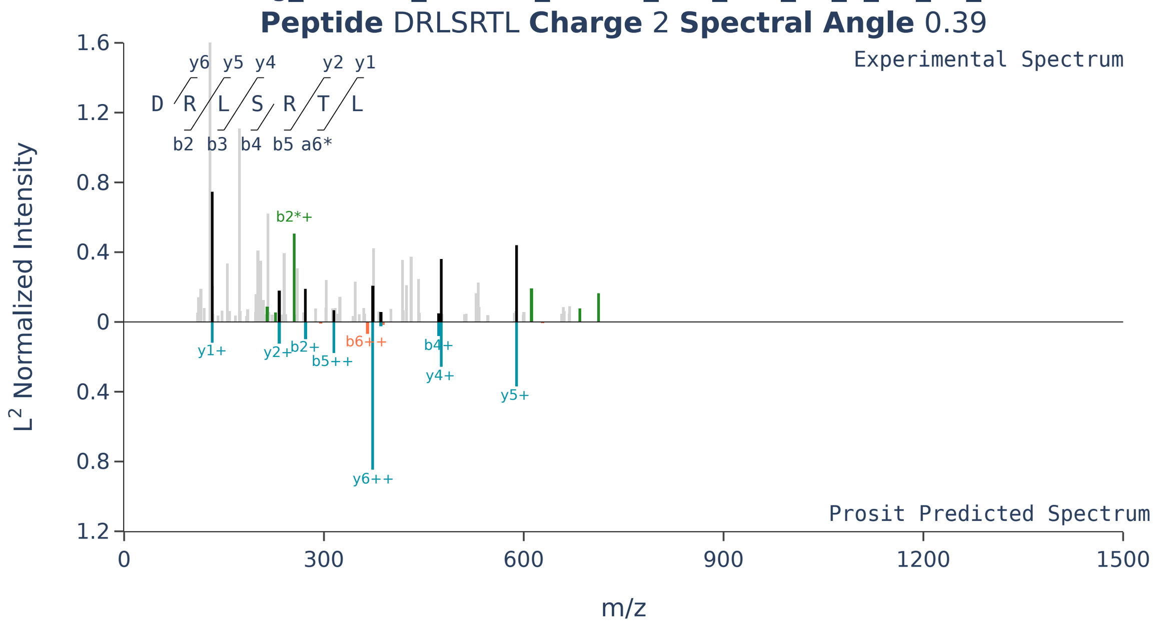


**Source** Ncheng_210623_230623_HFGoe_FFH_20S_25_1_A1_1h_R2 **Scan** 8700
**Peptide** REFINRVKEKAV **Charge** 4 **Spectral Angle** 0.79

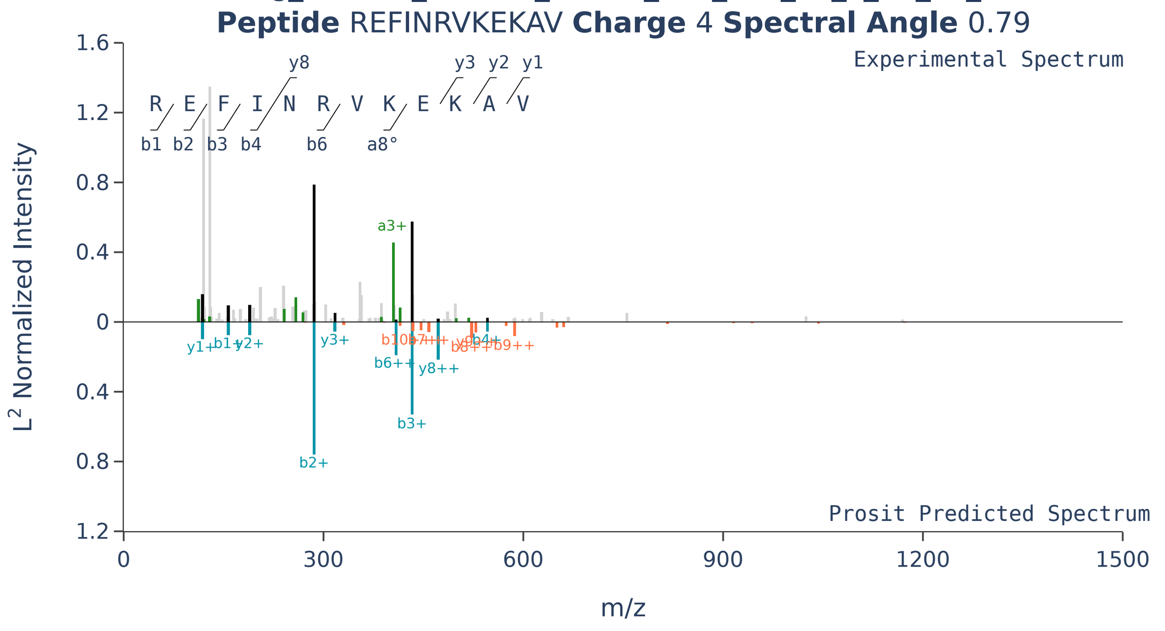

**Source** Ncheng_210623_230623_HFGoe_FFH_20S_25_1_A1_2h_R1 **Scan** 12178
**Peptide** KVDGDARGGAALSIRHITGK **Charge** 5 **Spectral Angle** 0.74

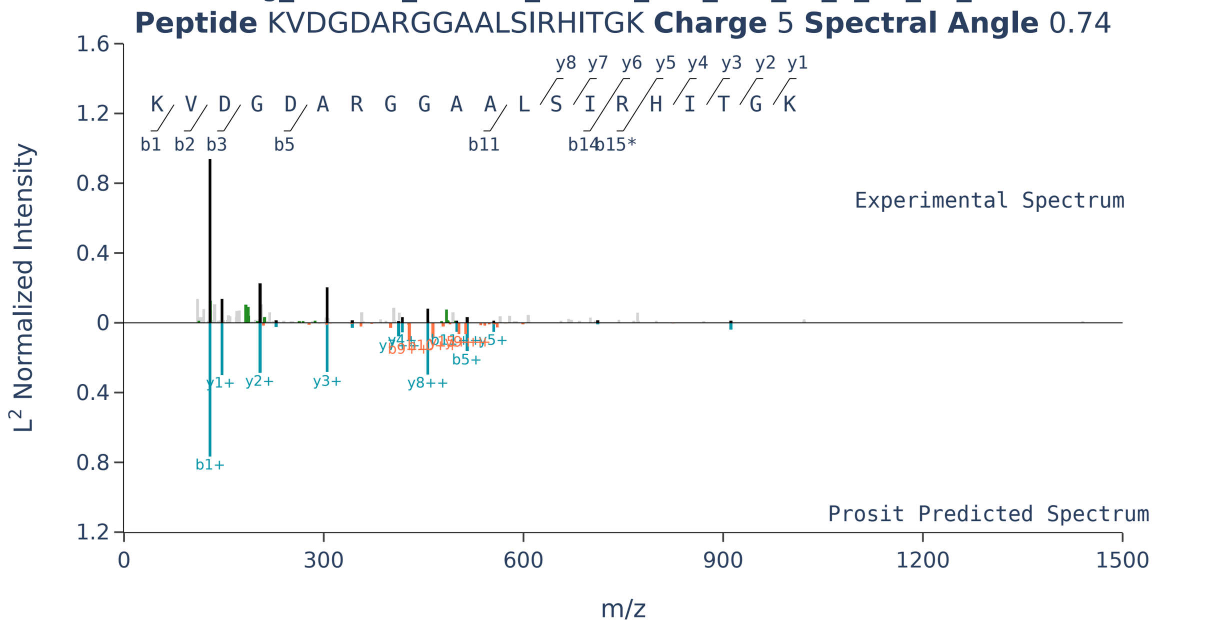


**Source** Ncheng_210623_230623_HFGoe_FFH_20S_25_1_A1_24h_R2 **Scan** 9509
**Peptide** GDARGGAALSIRH **Charge** 3 **Spectral Angle** 0.86

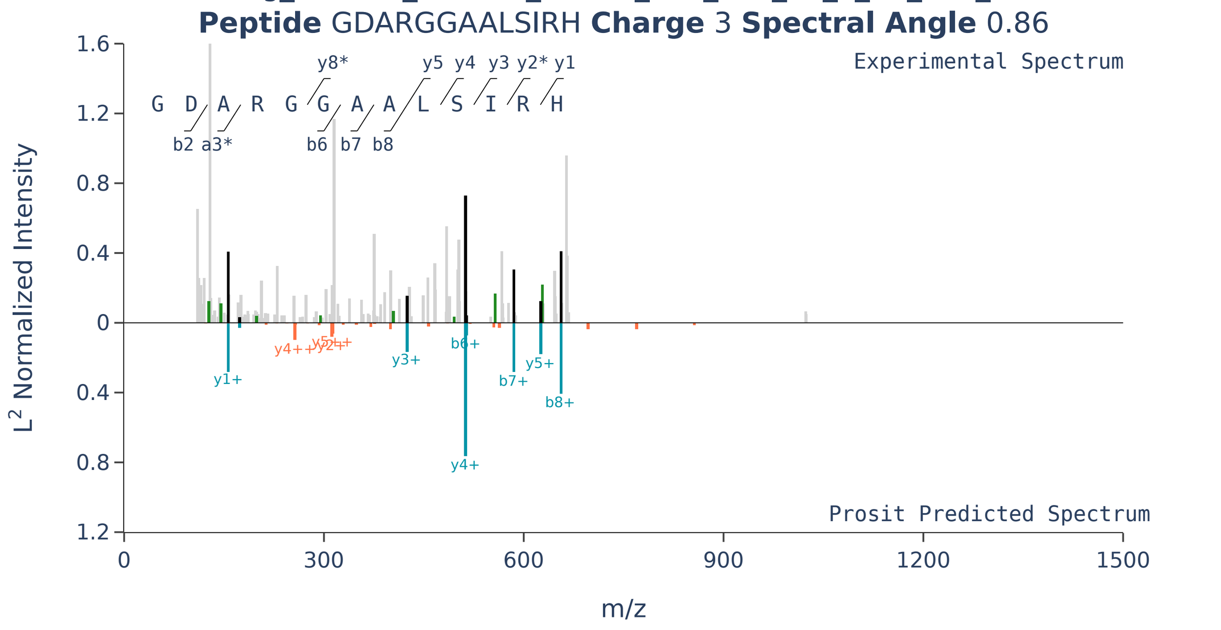


**Source** Ncheng_210623_230623_HFGoe_FFH_20S_25_1_A1_2h_R2 **Scan** 36340
**Peptide** TLFVVDAMTGQDAANTAKAFNEAL **Charge** 3 **Spectral Angle** 0.79

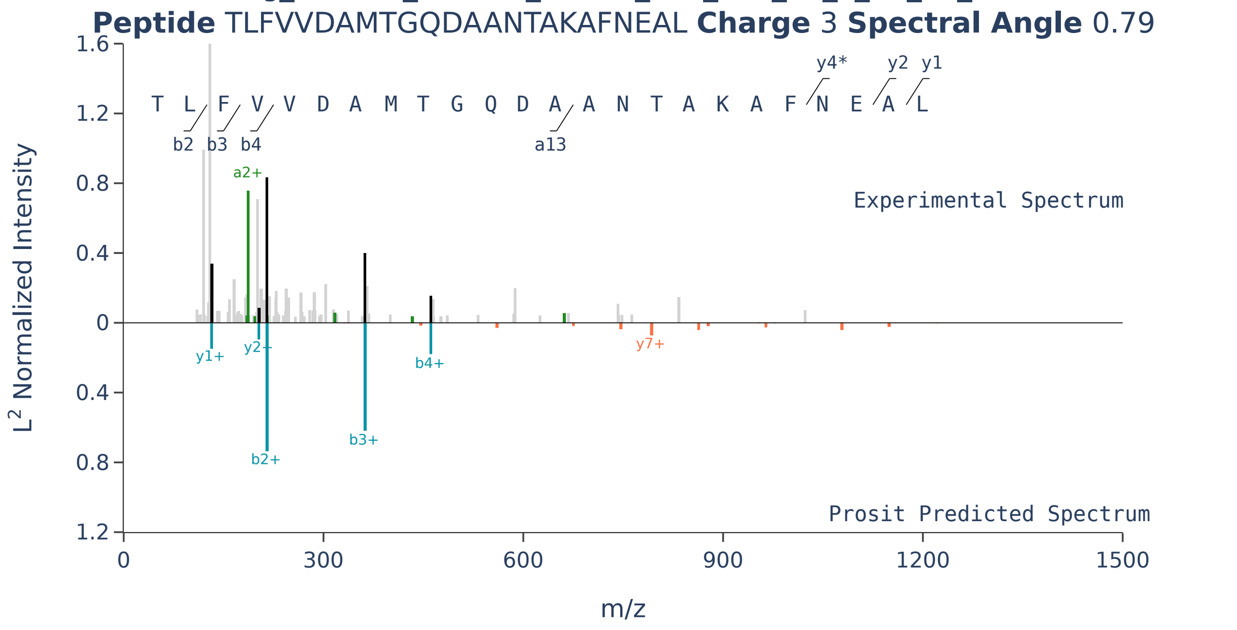


**Source** Ncheng_210623_230623_HFGoe_FFH_20S_25_1_A1_2h_R2 **Scan** 31045
**Peptide** GRLTEDNVKDTLREVRMALLE **Charge** 4 **Spectral Angle** 0.66

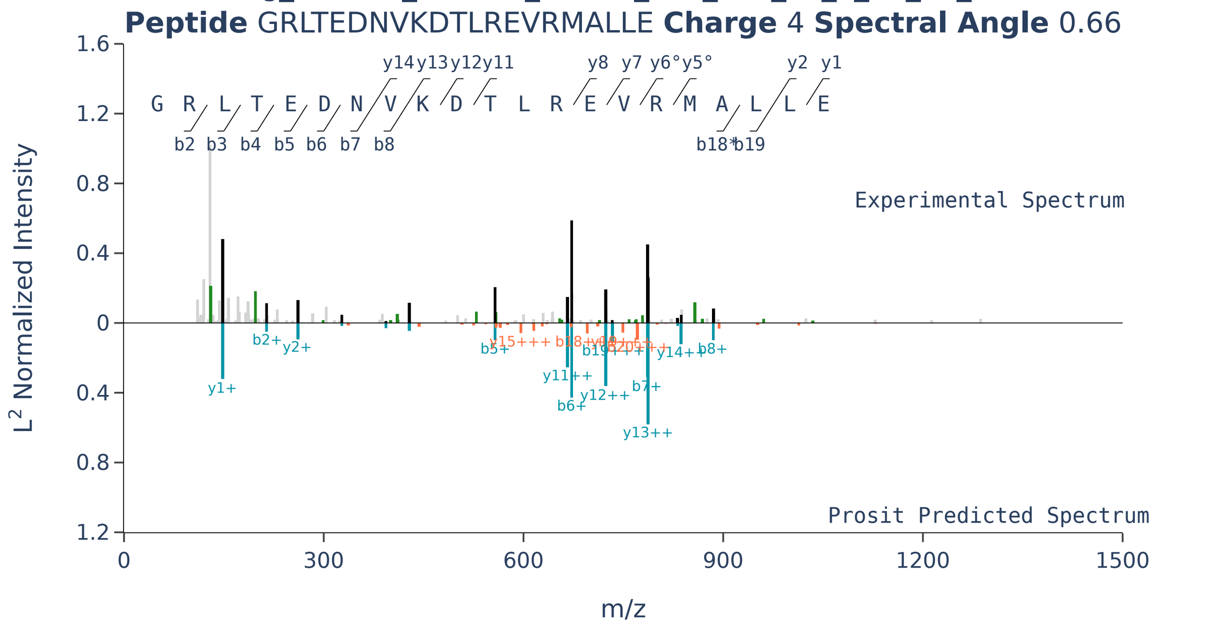


**Source** Ncheng_210623_230623_HFGoe_FFH_20S_25_1_A1_2h_R1 **Scan** 29753
**Peptide** GMQVQDVNRLLKQF **Charge** 3 **Spectral Angle** 0.64

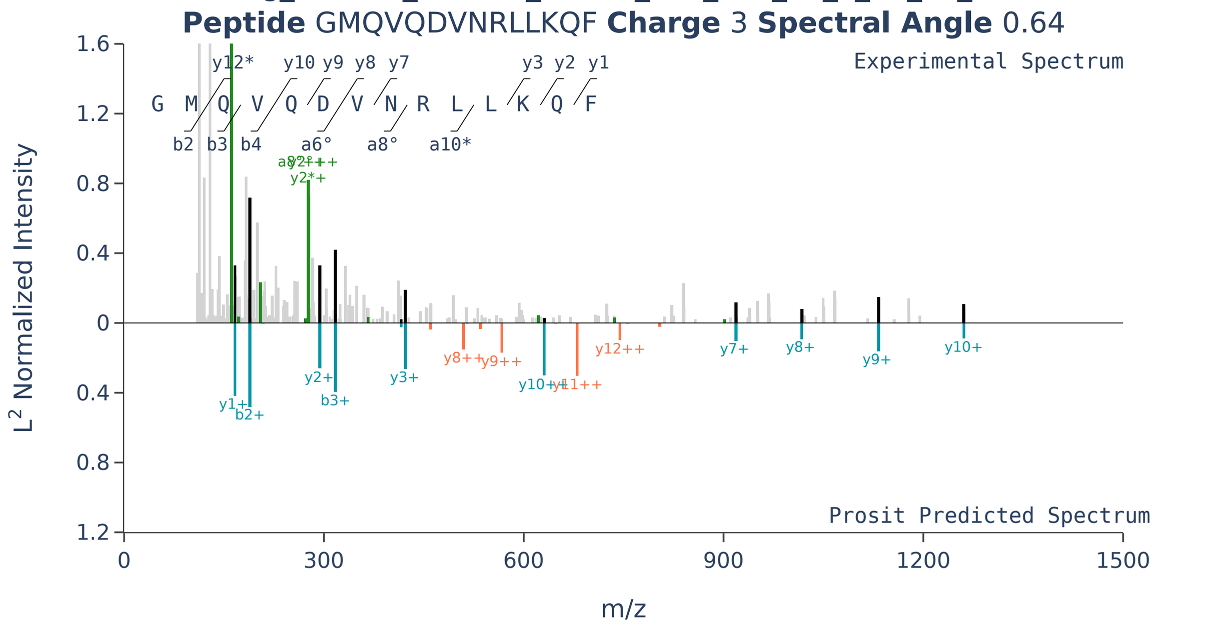


**Source** Ncheng_210623_230623_HFGoe_FFH_20S_25_1_A1_2h_R2 **Scan** 18897
**Peptide** LSGRGRLTEDNVKDTLREVRMA **Charge** 5 **Spectral Angle** 0.71

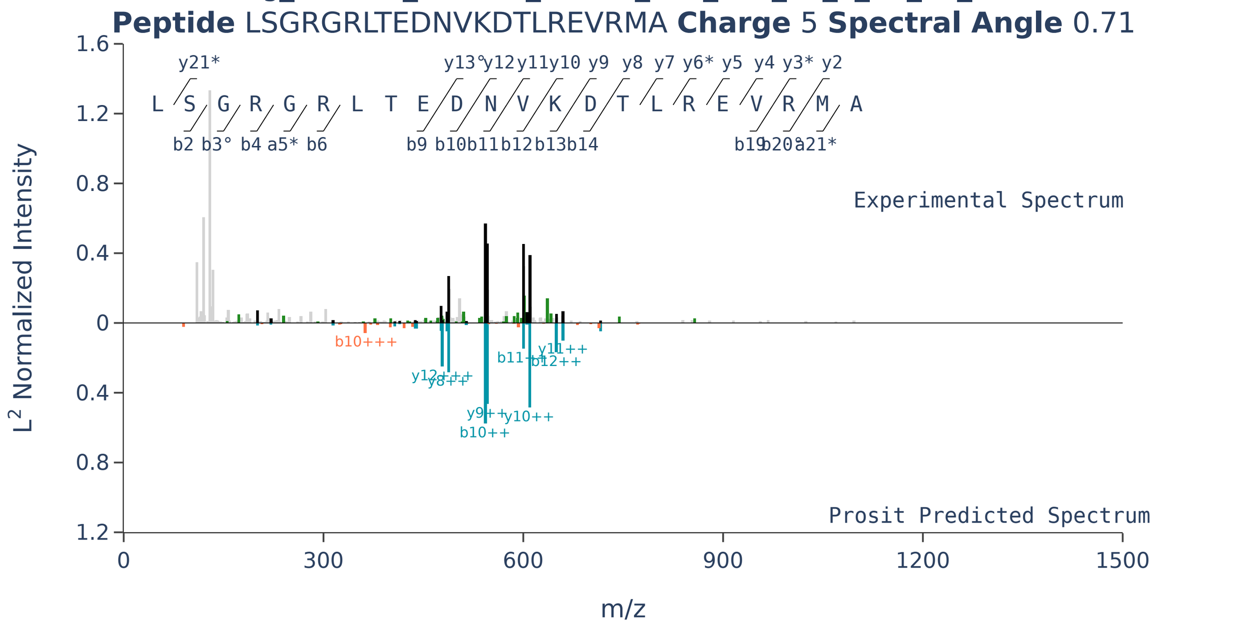


**Source** Ncheng_210623_230623_HFGoe_FFH_20S_25_1_A2_4h_R2 **Scan** 30312
**Peptide** TGQDAANTAKAFNEALPLTGV **Charge** 2 **Spectral Angle** 0.76

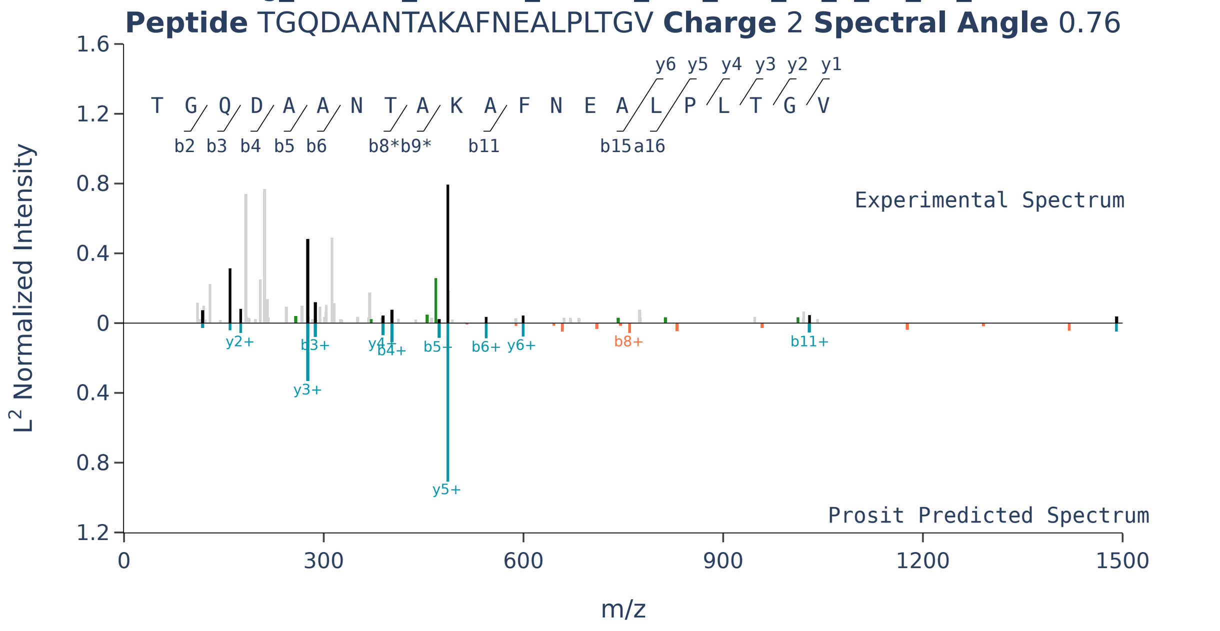


**Source** Ncheng_210623_230623_HFGoe_FFH_20S_25_1_A1_2h_R1 **Scan** 32412
**Peptide** ADVYRPAAIKQLETLAEQVG **Charge** 3 **Spectral Angle** 0.72

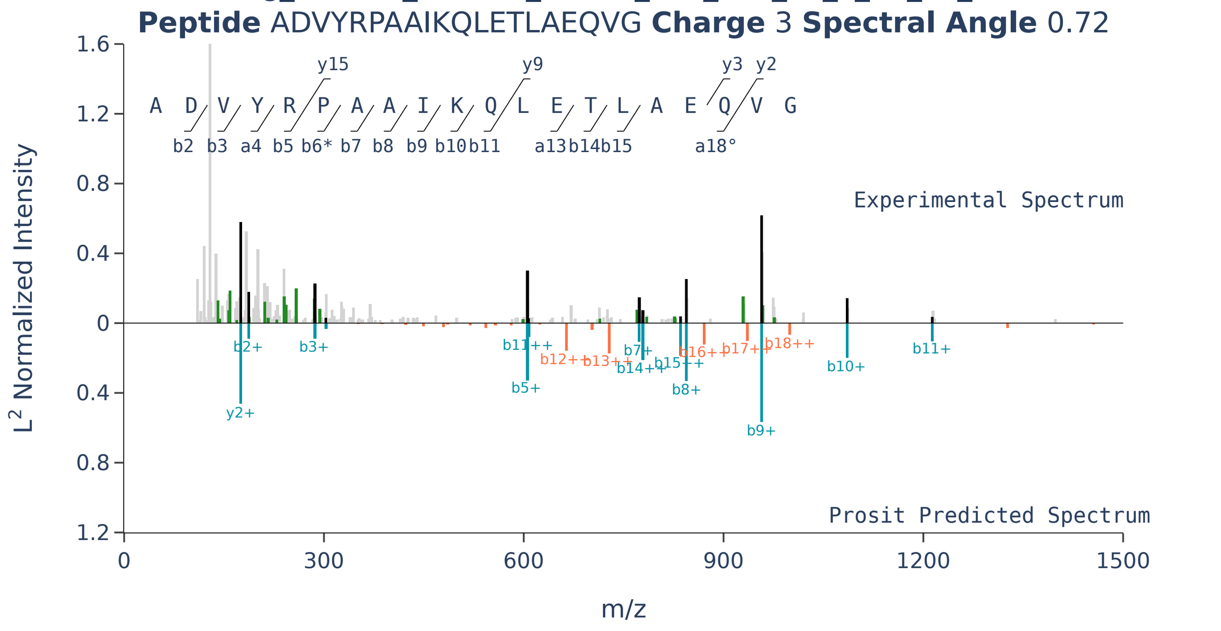


**Source** Ncheng_210623_230623_HFGoe_FFH_20S_25_1_A1_4h_R2 **Scan** 13874
**Peptide** AEKLASKLKKGDGFDLND **Charge** 2 **Spectral Angle** 0.7

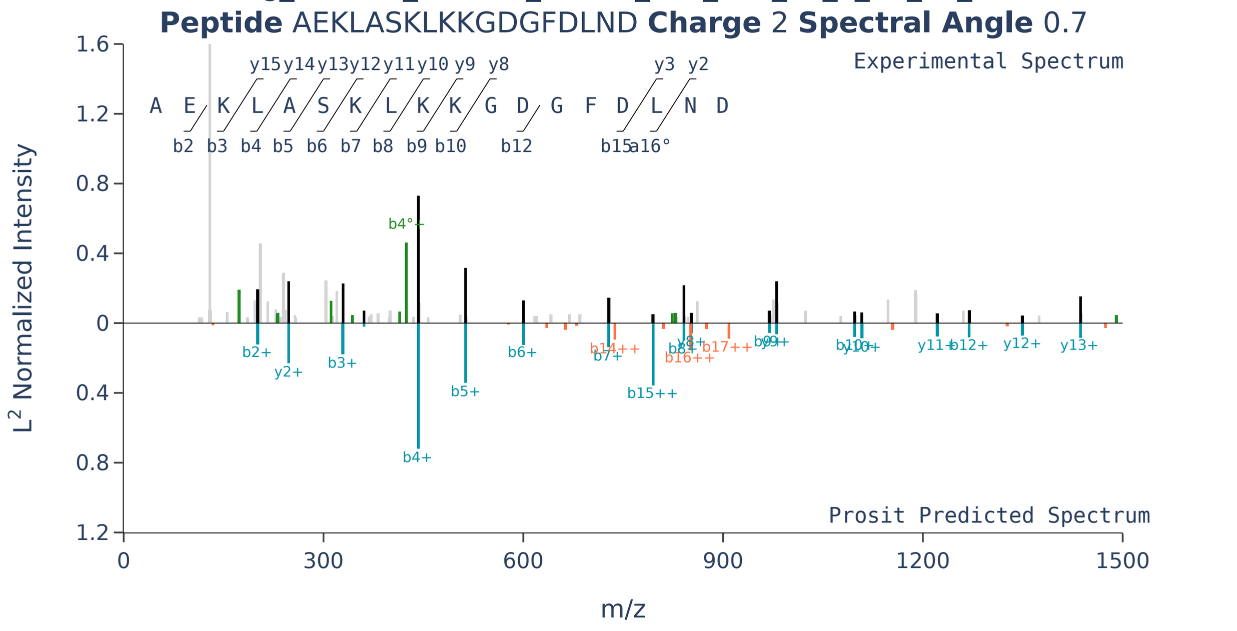


**Source** Ncheng_210623_230623_HFGoe_FFH_20S_25_1_A2_1h_R2 **Scan** 11822
**Peptide** VAAMGEENQTLN **Charge** 2 **Spectral Angle** 0.86

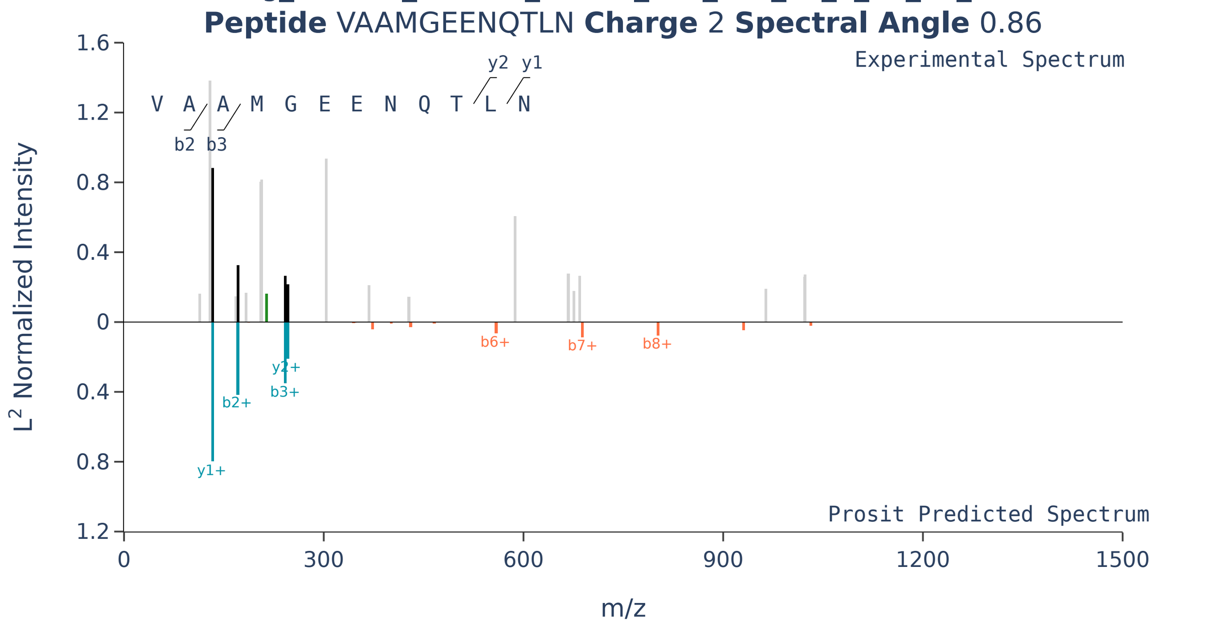


**Source** Ncheng_210623_230623_HFGoe_FFH_20S_25_1_A1_4h_R1 **Scan** 22204
**Peptide** LMAGLQGAGKTTSVGKLGKFLRE **Charge** 4 **Spectral Angle** 0.62

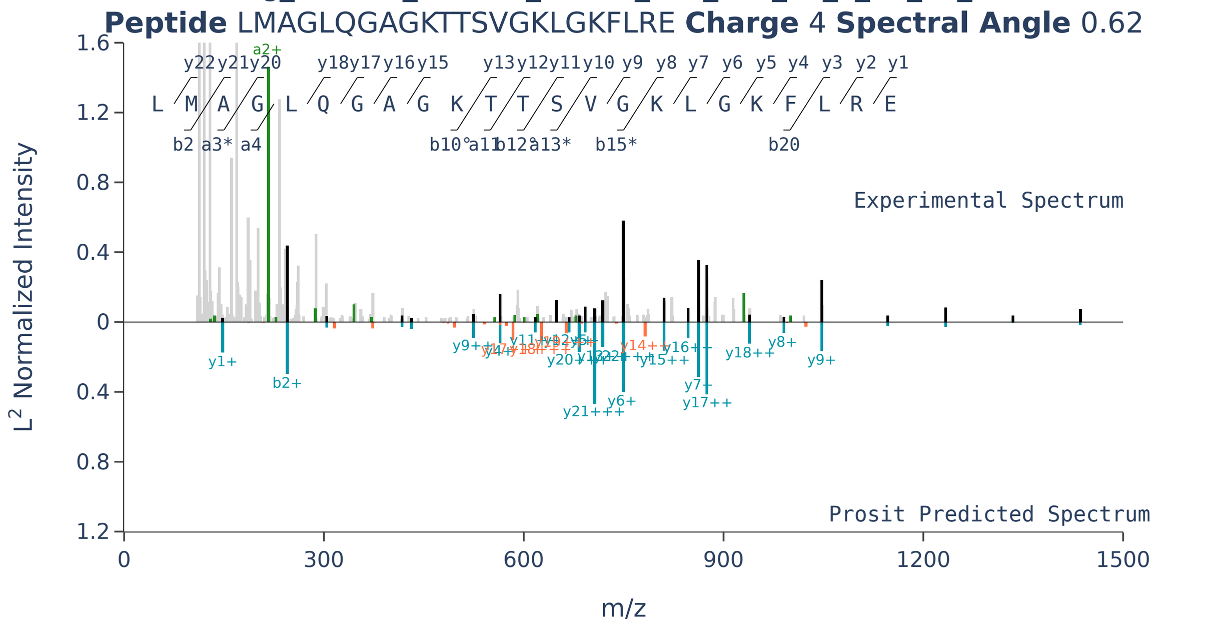


**Source** Ncheng_210623_230623_HFGoe_FFH_20S_25_1_A1_2h_R1 **Scan** 14082
**Peptide** TKVDGDARGGAALSIRHITG **Charge** 5 **Spectral Angle** 0.81

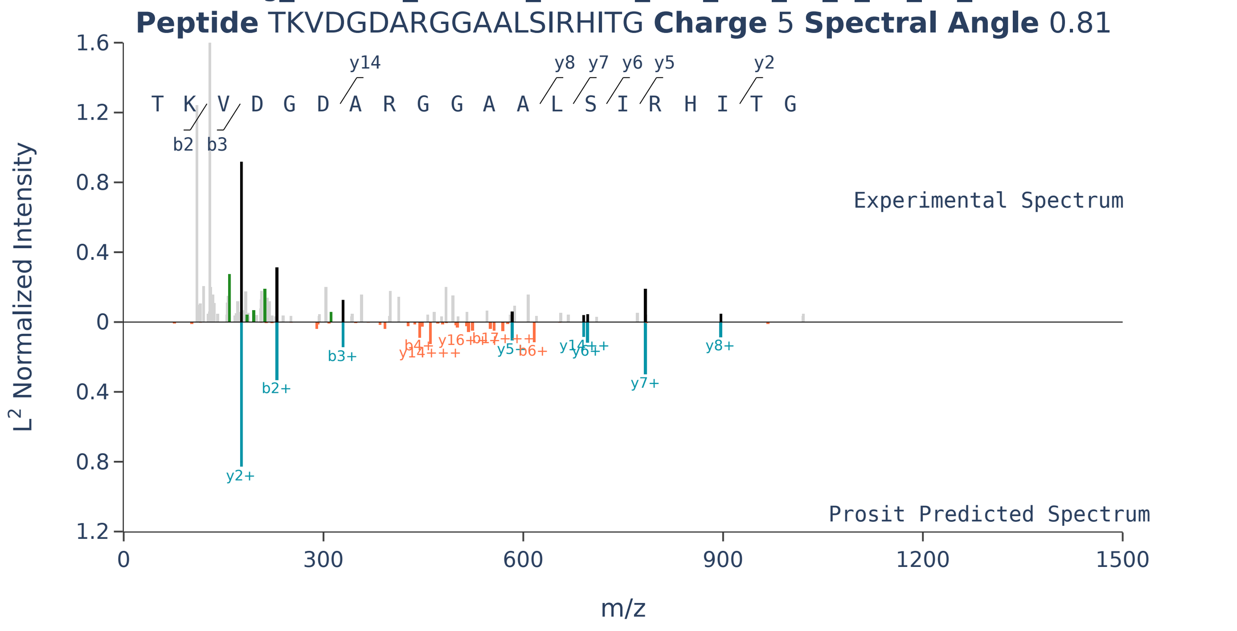


**Source** Ncheng_210623_230623_HFGoe_FFH_20S_25_1_A2_4h_R1 **Scan** 23291
**Peptide** RMEAIINSMTMKERAKPEII **Charge** 3 **Spectral Angle** 0.66

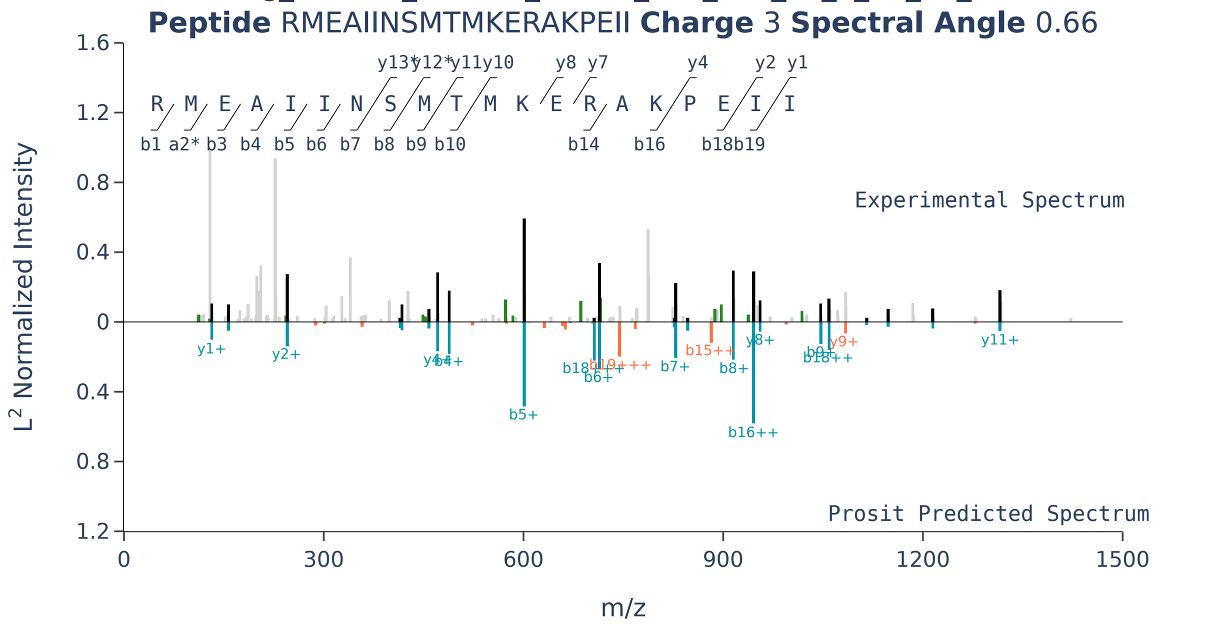


**Source** Ncheng_210623_230623_HFGoe_FFH_20S_25_1_A1_24h_R1 **Scan** 7754
**Peptide** TMKERAKPEIIK **Charge** 2 **Spectral Angle** 0.57

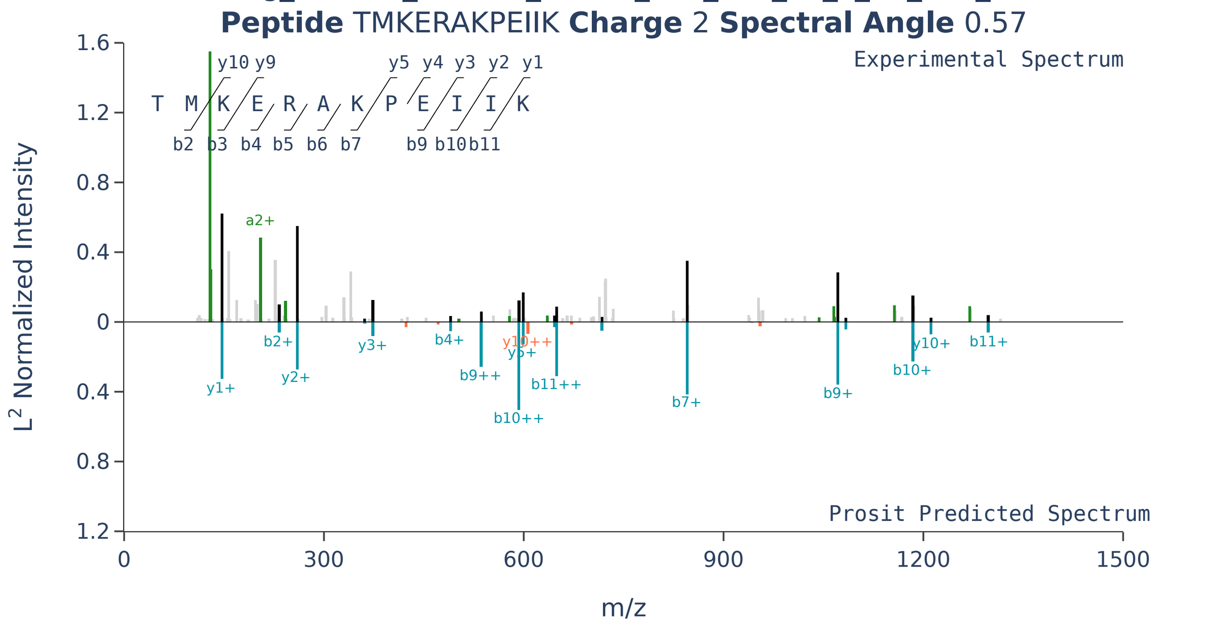


**Source** Ncheng_210623_230623_HFGoe_FFH_20S_25_1_A1_2h_R2 **Scan** 22982
**Peptide** VGHEVNKSLTPGQEFVKIV **Charge** 4 **Spectral Angle** 0.63

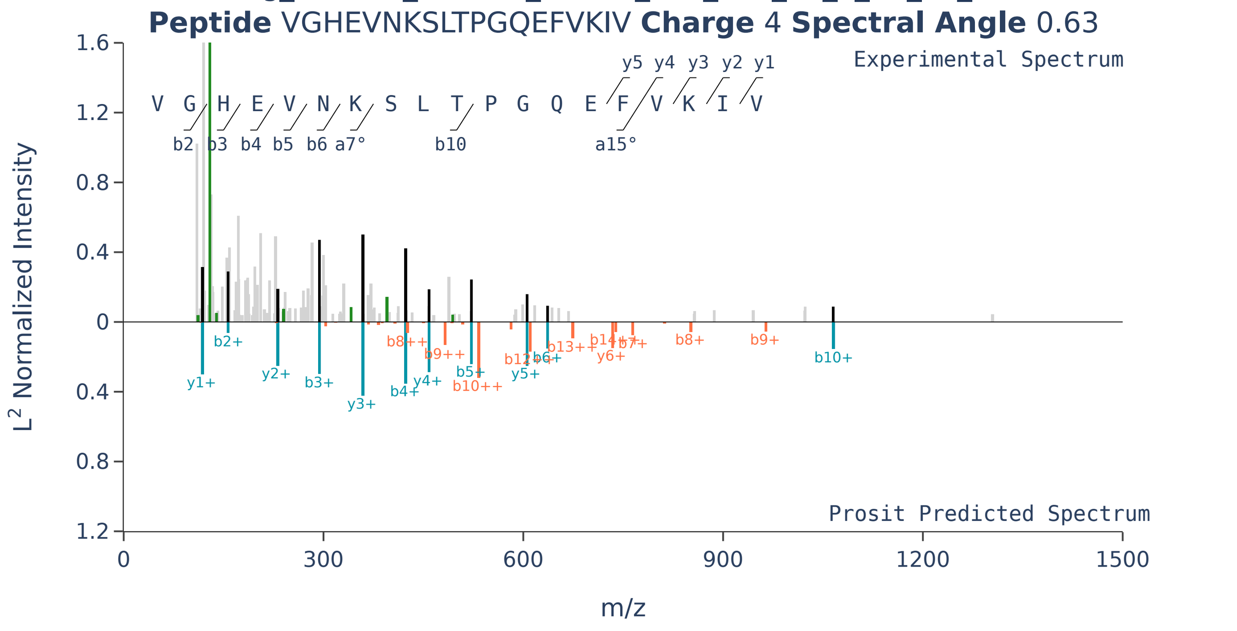

**Source** Ncheng_210623_230623_HFGoe_FFH_20S_25_1_A2_2h_R1 **Scan** 31178
**Peptide** LVNAALKEAKLKFYDVLL **Charge** 4 **Spectral Angle** 0.84

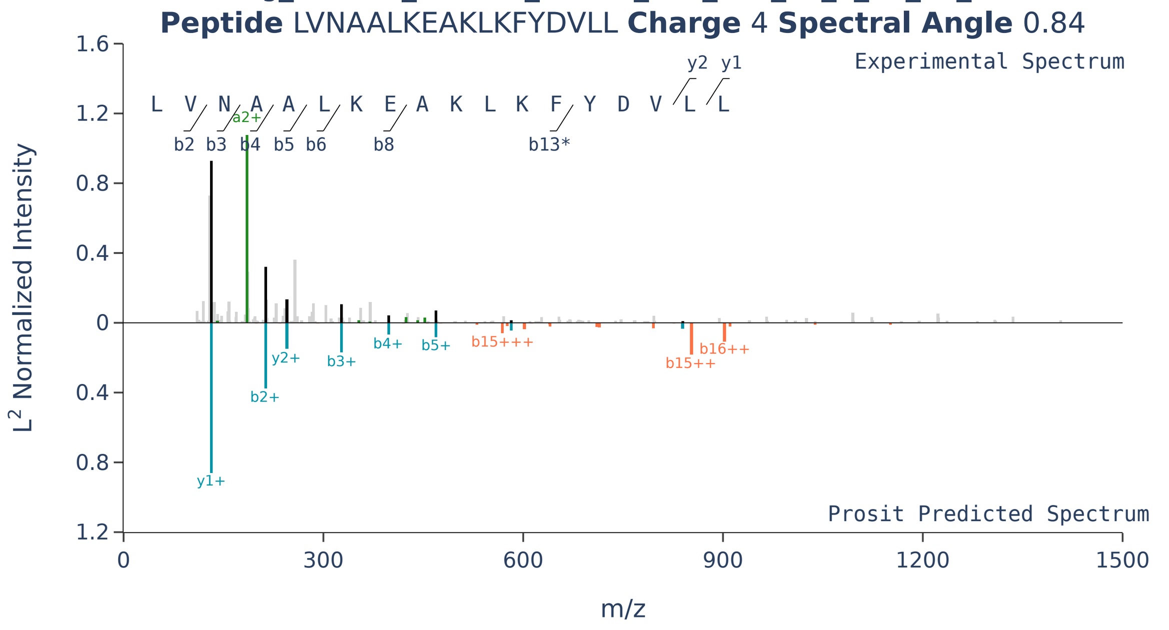


**Source** Ncheng_210623_230623_HFGoe_FFH_20S_25_1_A2_4h_R2 **Scan** 23368
**Peptide** VGHEVNKSLTPGQEFVKLV **Charge** 4 **Spectral Angle** 0.67

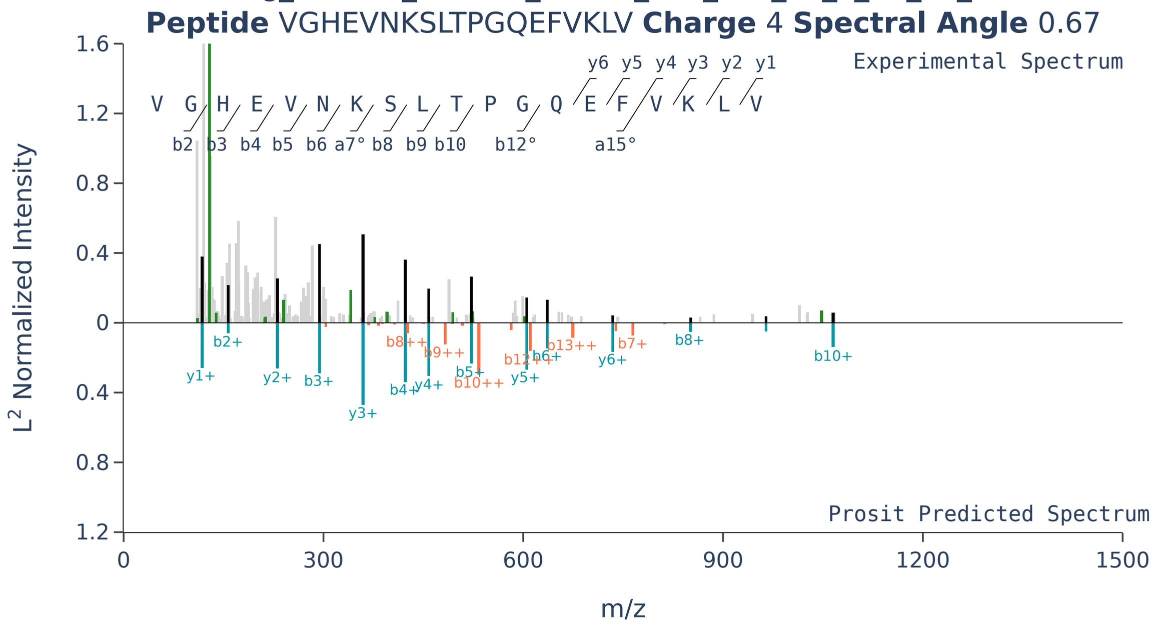


**Source** Ncheng_210623_230623_HFGoe_FFH_20S_25_1_A1_1h_R2 **Scan** 33226
**Peptide** ADVALPVVREFINRVKEKAVGHEV **Charge** 5 **Spectral Angle** 0.54

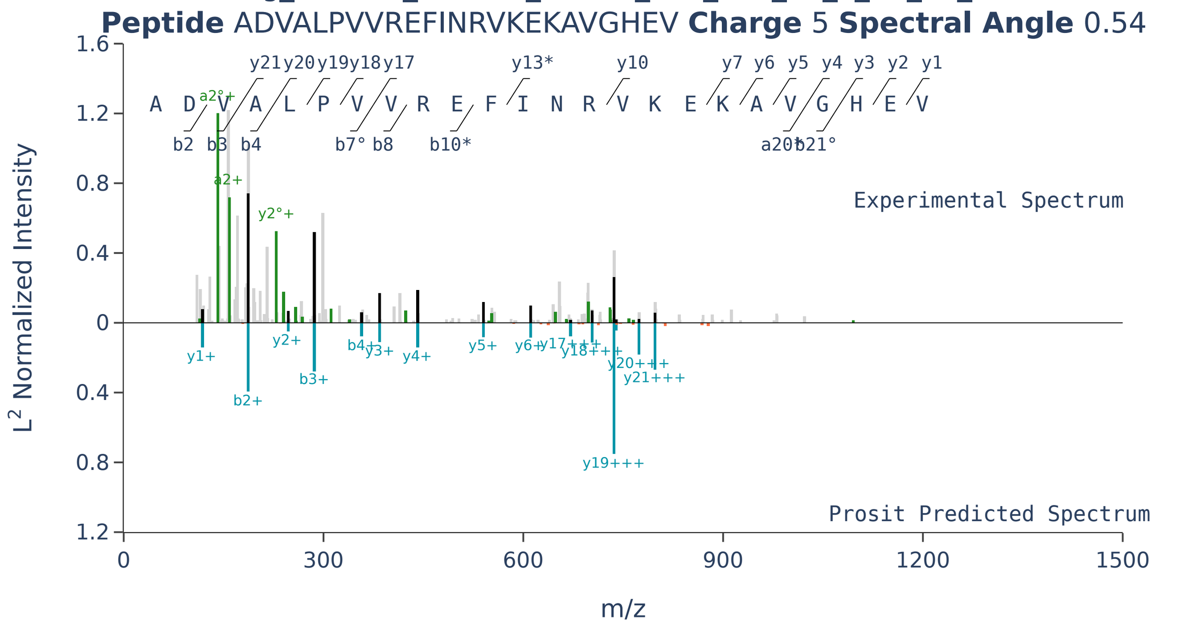


**Source** Ncheng_210623_230623_HFGoe_FFH_20S_25_1_A1_2h_R2 **Scan** 36945
**Peptide** KSQMDDKVLVRMEAIINSMTMKE **Charge** 4 **Spectral Angle** 0.73

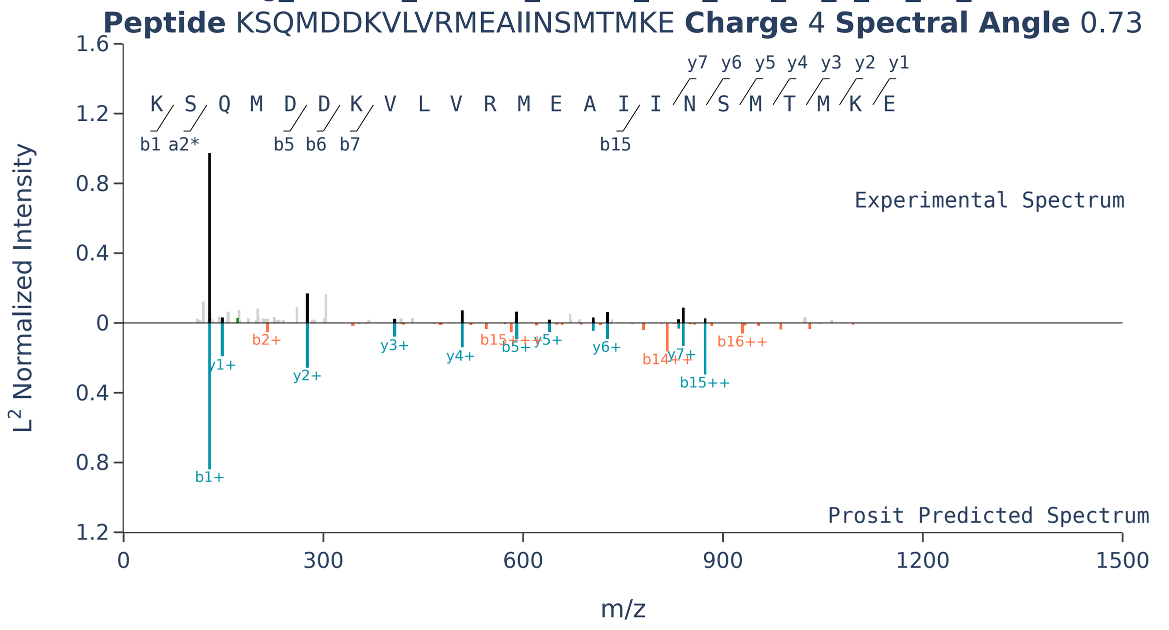


**Source** Ncheng_210623_230623_HFGoe_FFH_20S_25_1_A1_4h_R1 **Scan** 23173
**Peptide** VLLVDTAGRLHVDE **Charge** 2 **Spectral Angle** 0.51

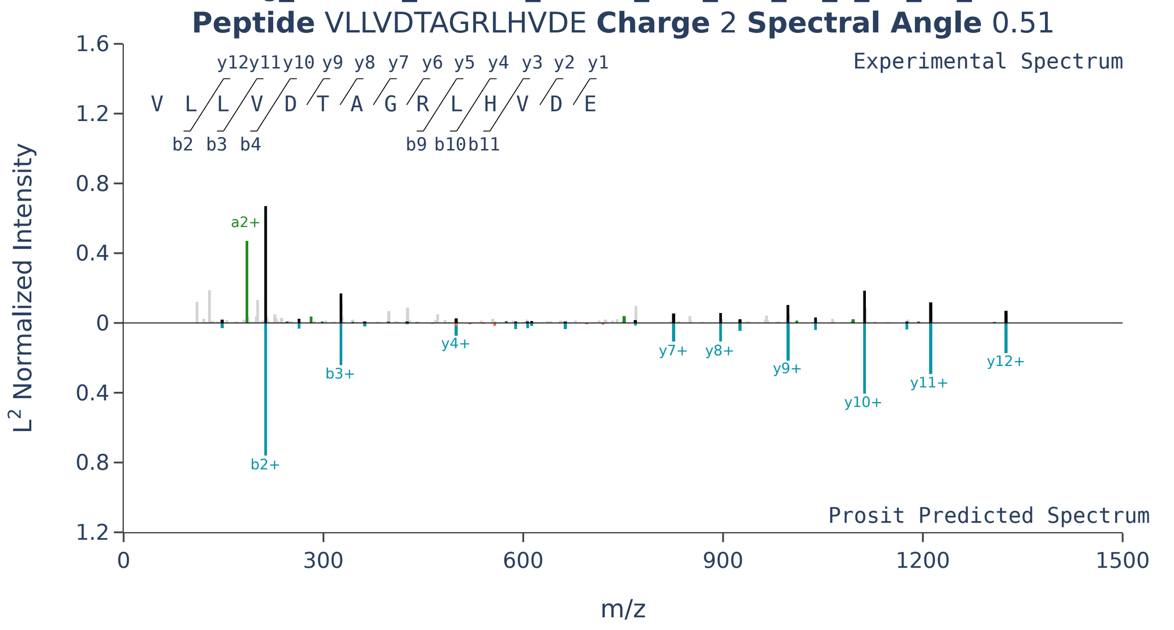


**Source** Ncheng_210623_230623_HFGoe_FFH_20S_25_1_A1_24h_R2 **Scan** 14726
**Peptide** KTTSVGKLGKFLRE **Charge** 4 **Spectral Angle** 0.66

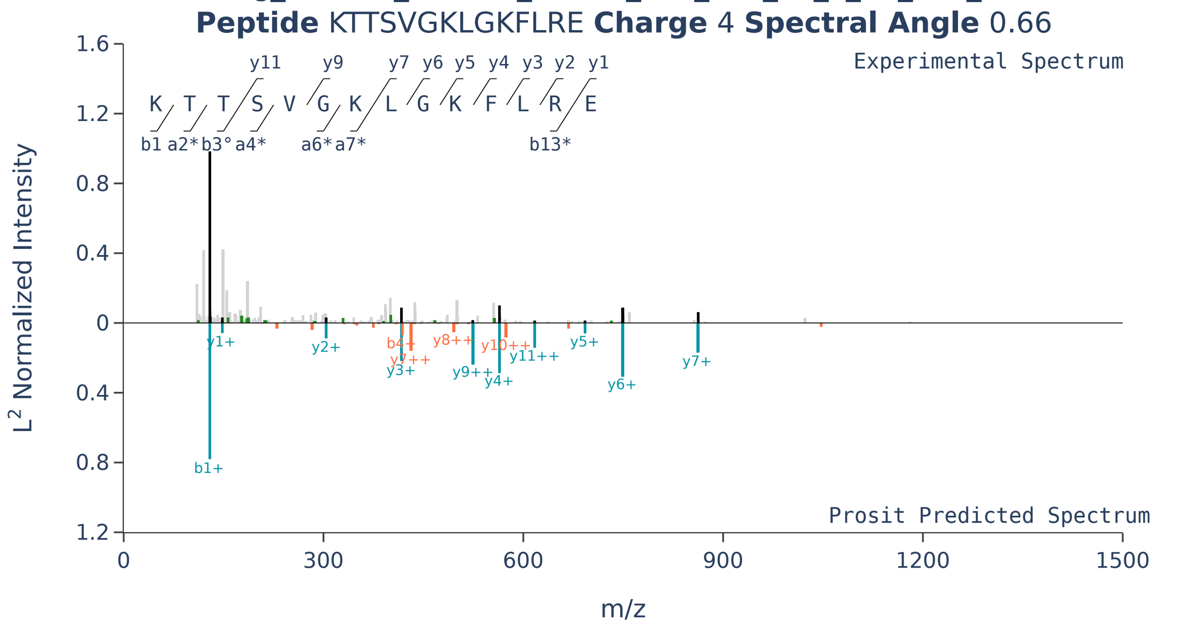


**Source** Ncheng_210623_230623_HFGoe_FFH_20S_25_1_A1_24h_R1 **Scan** 13032
**Peptide** DDMQRMMKKM **Charge** 2 **Spectral Angle** 0.72

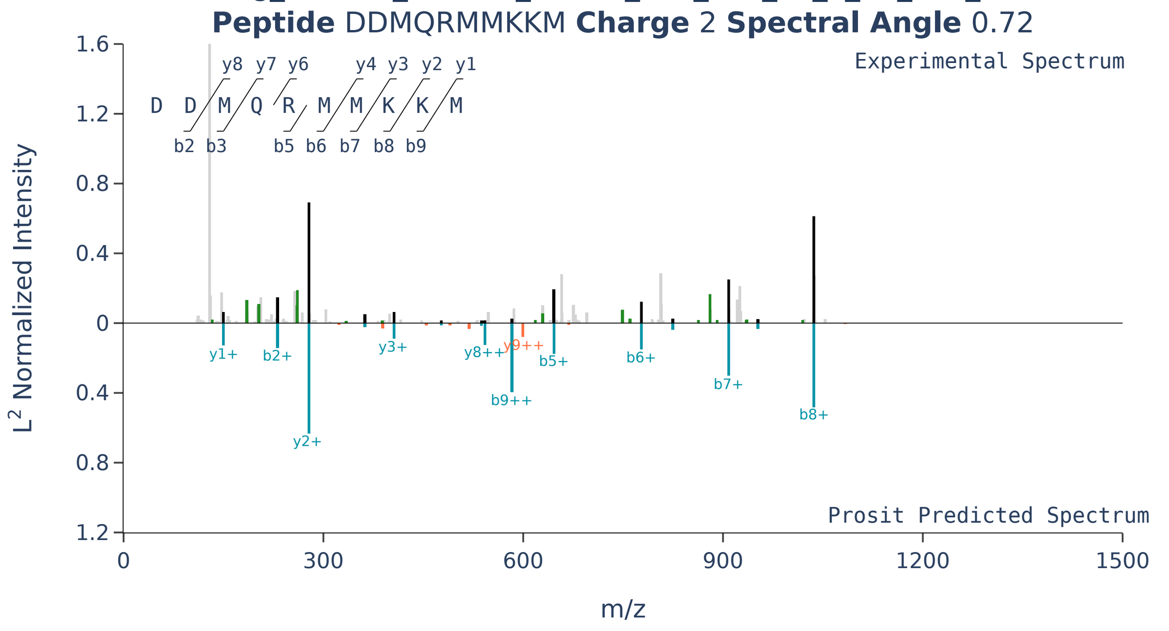


**Source** Ncheng_210623_230623_HFGoe_FFH_20S_25_1_A1_4h_R2 **Scan** 20485
**Peptide** MEAIINSMTMKERAKPEIIKG **Charge** 3 **Spectral Angle** 0.85

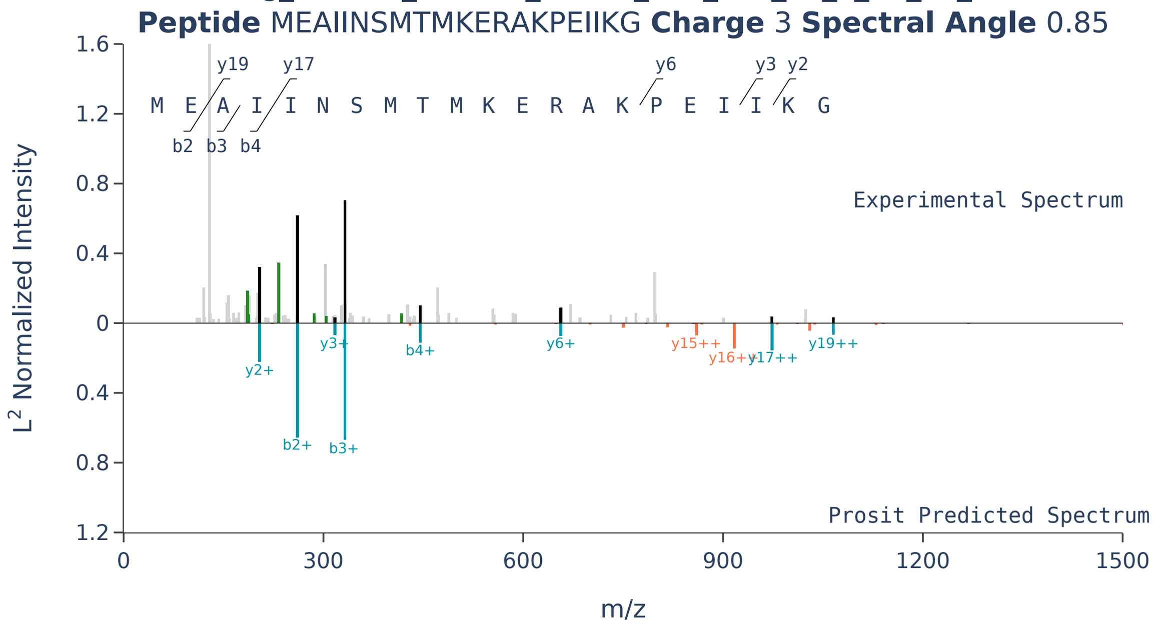


**Source** Ncheng_210623_230623_HFGoe_FFH_20S_25_1_A1_24h_R2 **Scan** 5721
**Peptide** MQRMMKKMK **Charge** 2 **Spectral Angle** 0.67

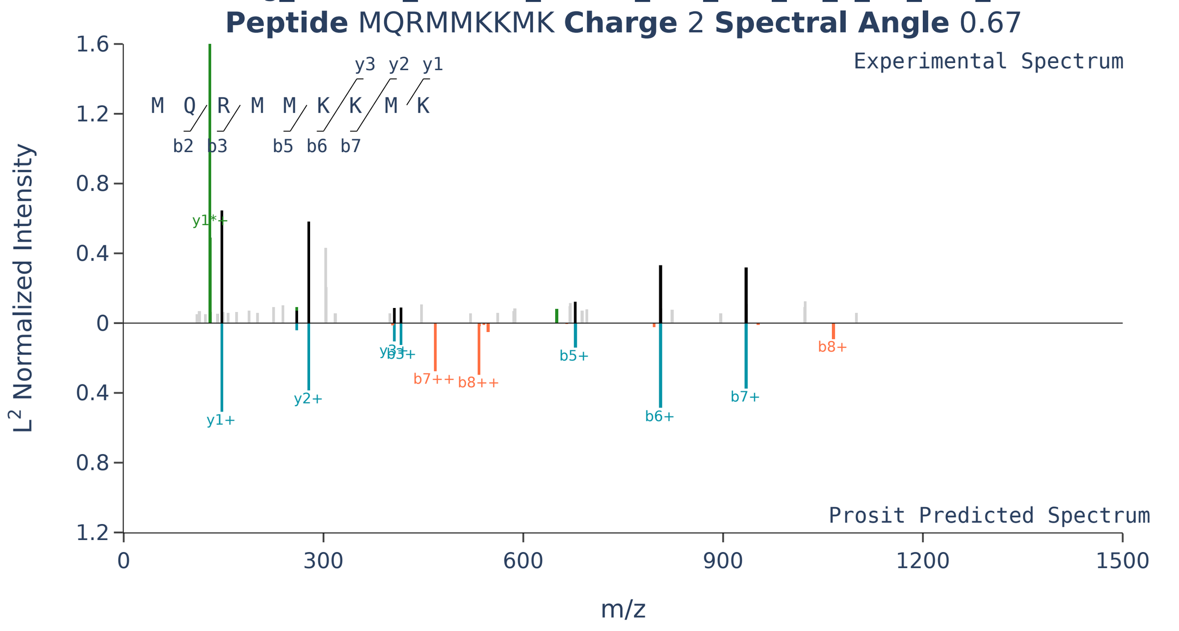


**Source** Ncheng_210623_230623_HFGoe_FFH_20S_25_1_A1_4h_R2 **Scan** 17689
**Peptide** FINRVKEKAVGHEVNKSLTPGQEFVKIVRN **Charge** 3 **Spectral Angle** 0.76

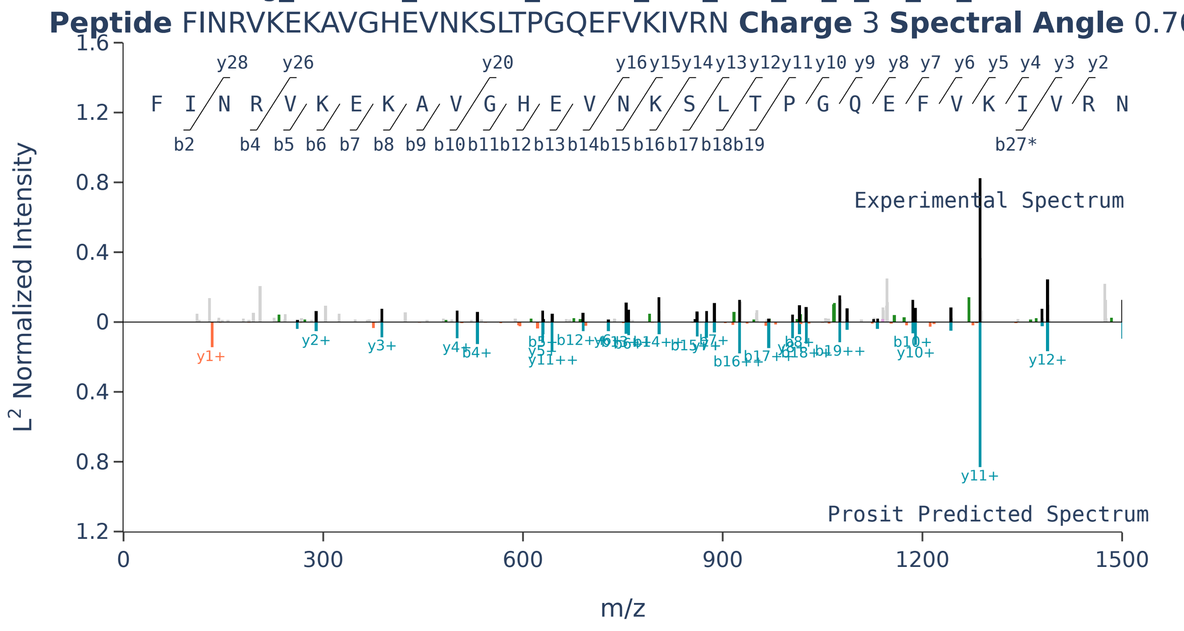


**Source** Ncheng_210623_230623_HFGoe_FFH_20S_25_1_A2_24h_R2 **Scan** 20700
**Peptide** PLTGVVLTKVDGDARGG **Charge** 3 **Spectral Angle** 0.72

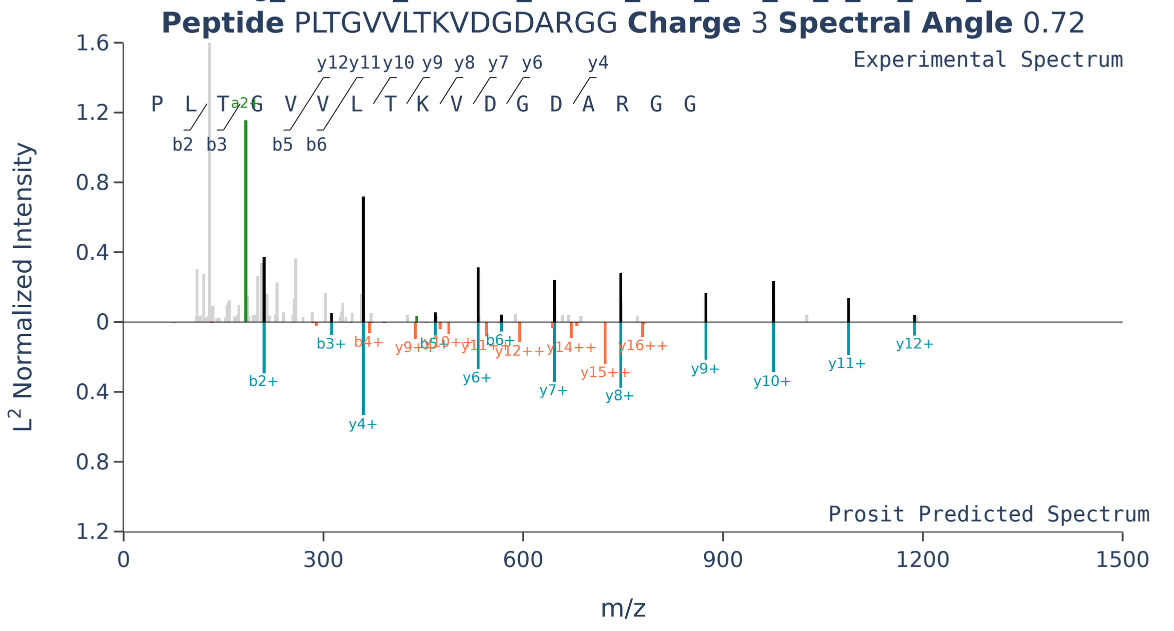


**Source** Ncheng_210623_230623_HFGoe_FFH_20S_25_1_A1_2h_R2 **Scan** 37211
**Peptide** VNKSLTPGQEFVKIVRNELVAAM **Charge** 4 **Spectral Angle** 0.83

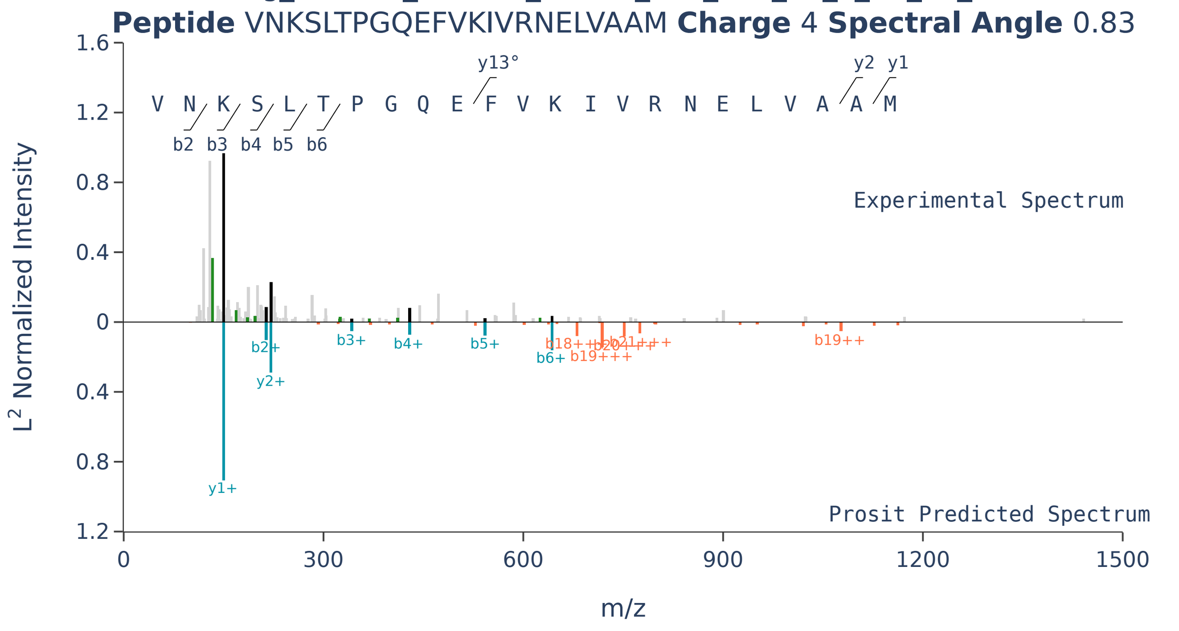


**Source** Ncheng_210623_230623_HFGoe_FFH_20S_25_1_A1_2h_R1 **Scan** 20068
**Peptide** LVDTAGRLHVDEAMMD **Charge** 3 **Spectral Angle** 0.46

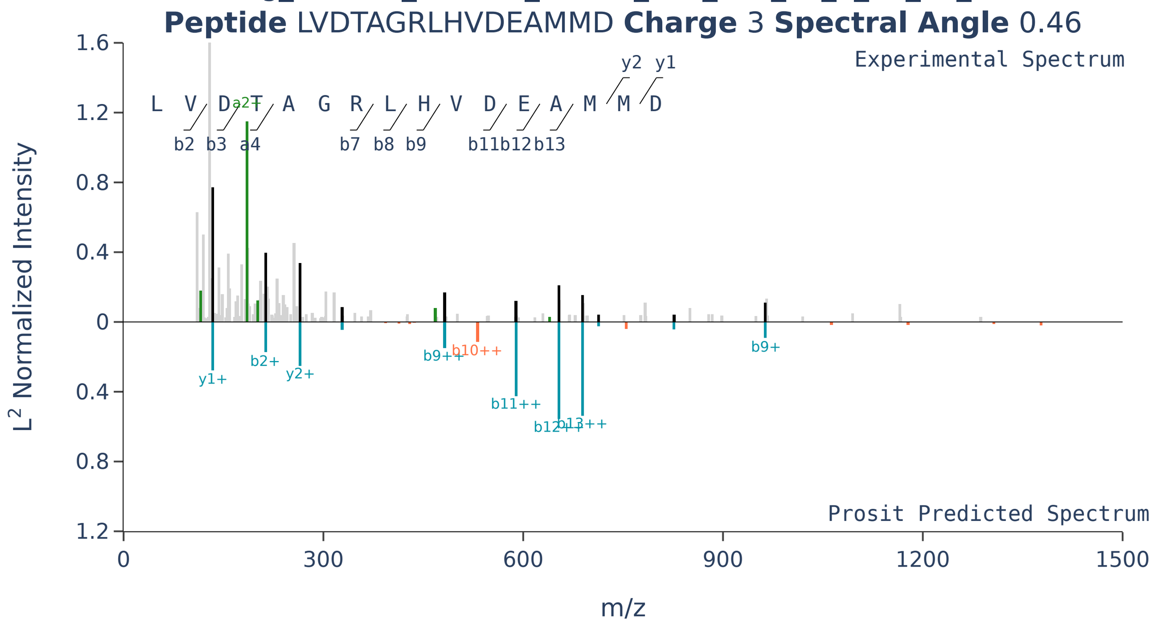


**Source** Ncheng_210623_230623_HFGoe_FFH_20S_25_1_A1_24h_R1 **Scan** 6484
**Peptide** KAVGHEVNKSLT **Charge** 2 **Spectral Angle** 0.69

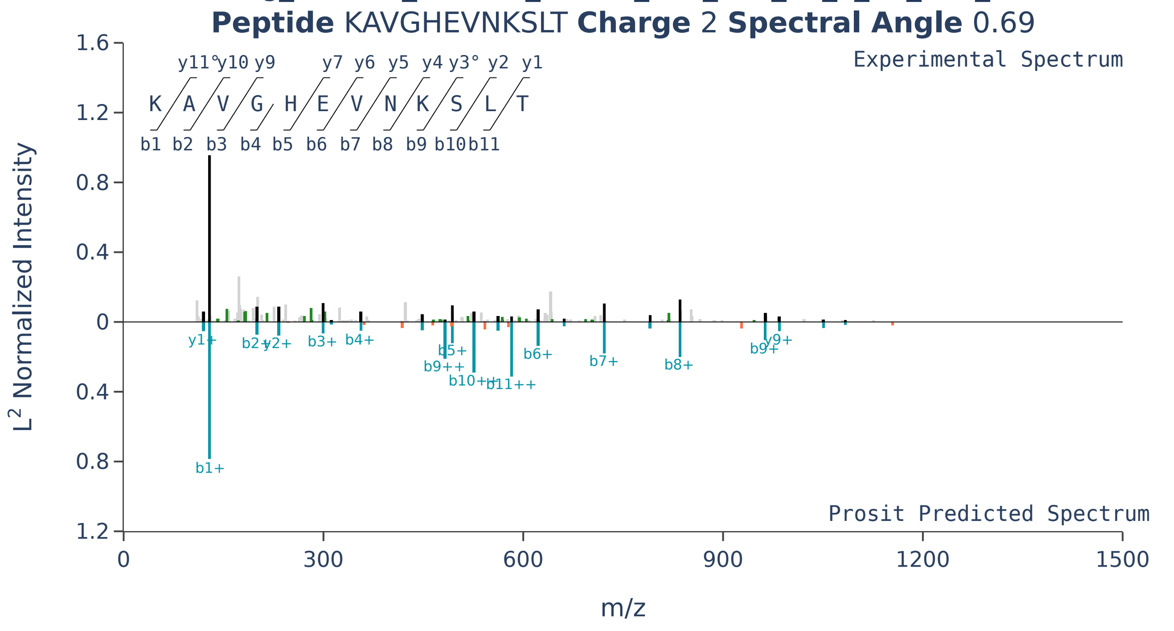


**Source** Ncheng_210623_230623_HFGoe_FFH_20S_25_1_A1_2h_R2 **Scan** 15994
**Peptide** SRTLRNISGRGRLTEDNVKDTLREV **Charge** 5 **Spectral Angle** 0.7

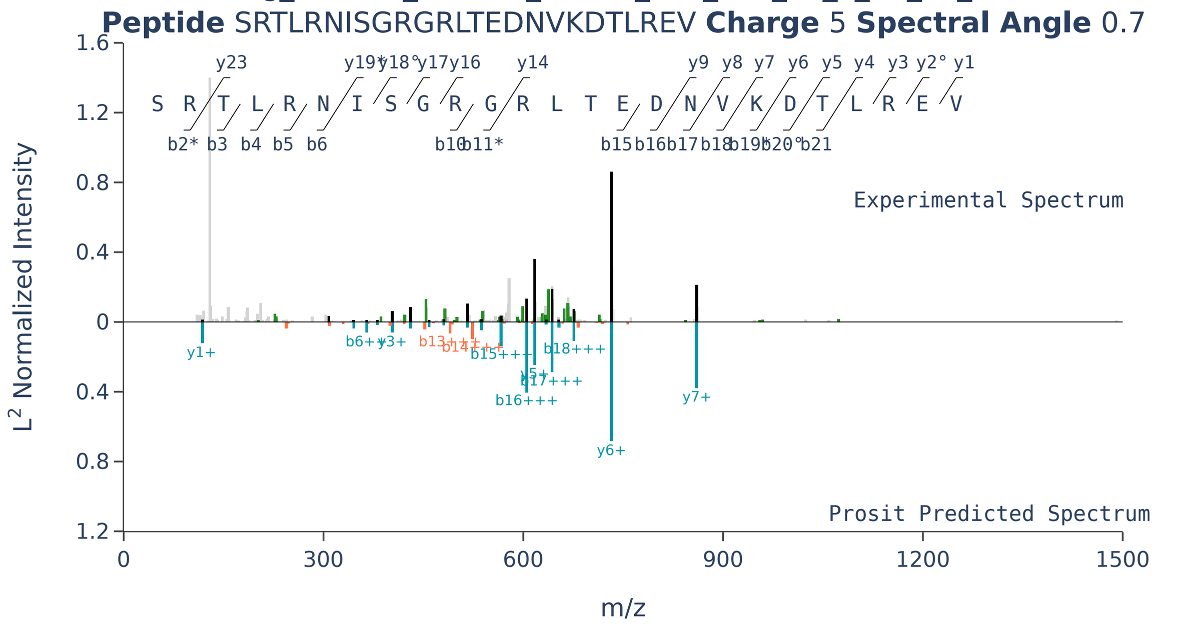

**Source** Ncheng_210623_230623_HFGoe_FFH_20S_25_1_A2_2h_R1 **Scan** 17450
**Peptide** VVLTKVDGDARGGAALSIRHITGK **Charge** 3 **Spectral Angle** 0.62

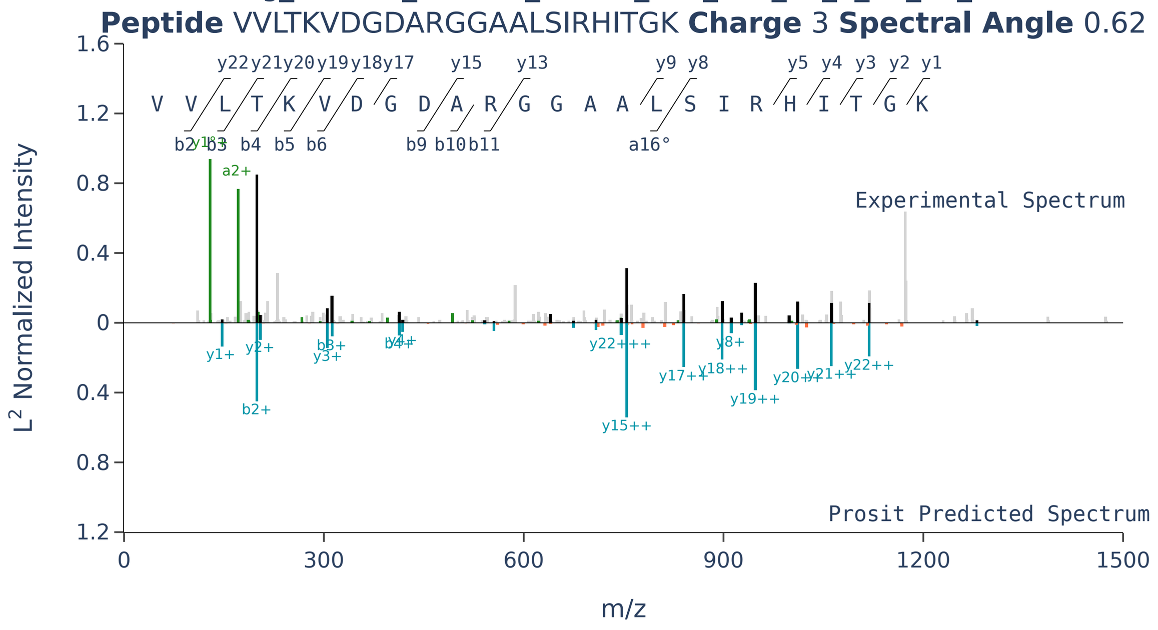


**Source** Ncheng_210623_230623_HFGoe_FFH_20S_25_1_A2_24h_R2 **Scan** 5858
**Peptide** AEKLASKLKK **Charge** 3 **Spectral Angle** 0.71

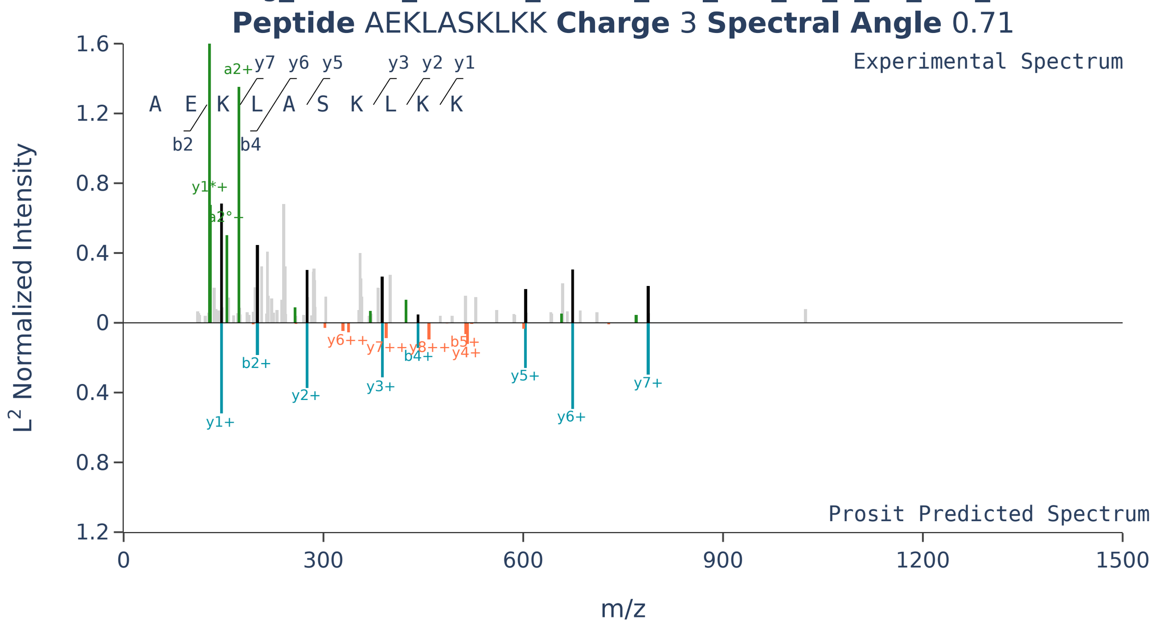


**Source** Ncheng_210623_230623_HFGoe_FFH_20S_25_1_A2_4h_R1 **Scan** 28569
**Peptide** LMGKLPGMGQIPDNVKSQMDDKVLVRM **Charge** 4 **Spectral Angle** 0.67

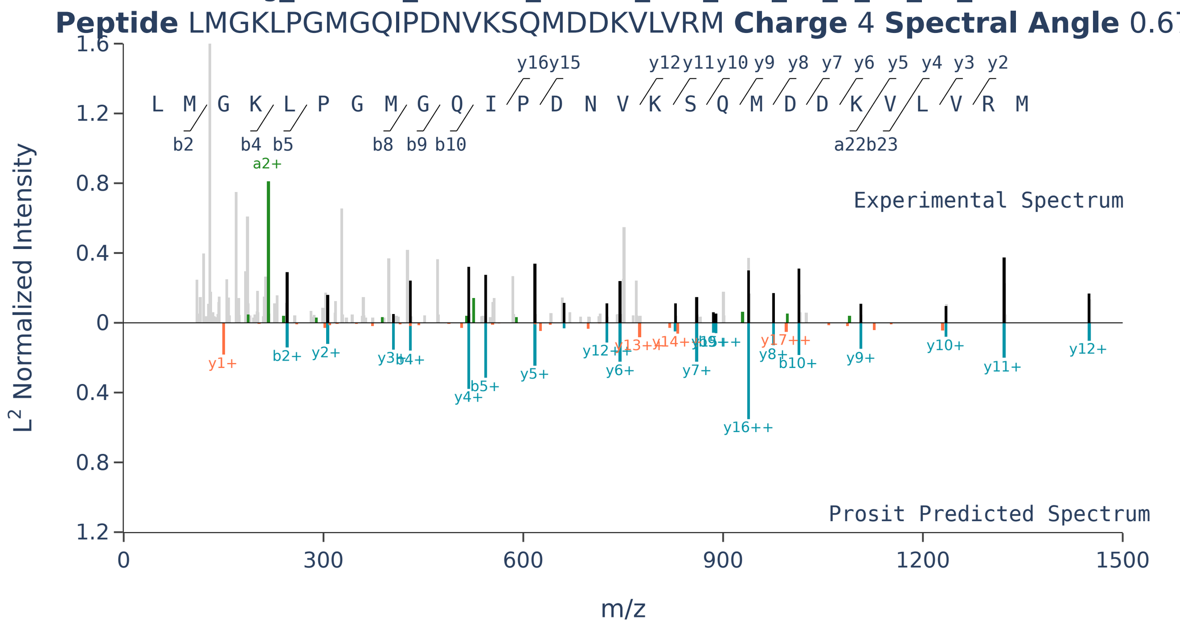


**Source** Ncheng_210623_230623_HFGoe_FFH_20S_25_1_A2_2h_R2 **Scan** 32580
**Peptide** KLPGMGQIPDNVKSQMDDKVLVRMEALL **Charge** 4 **Spectral Angle** 0.86

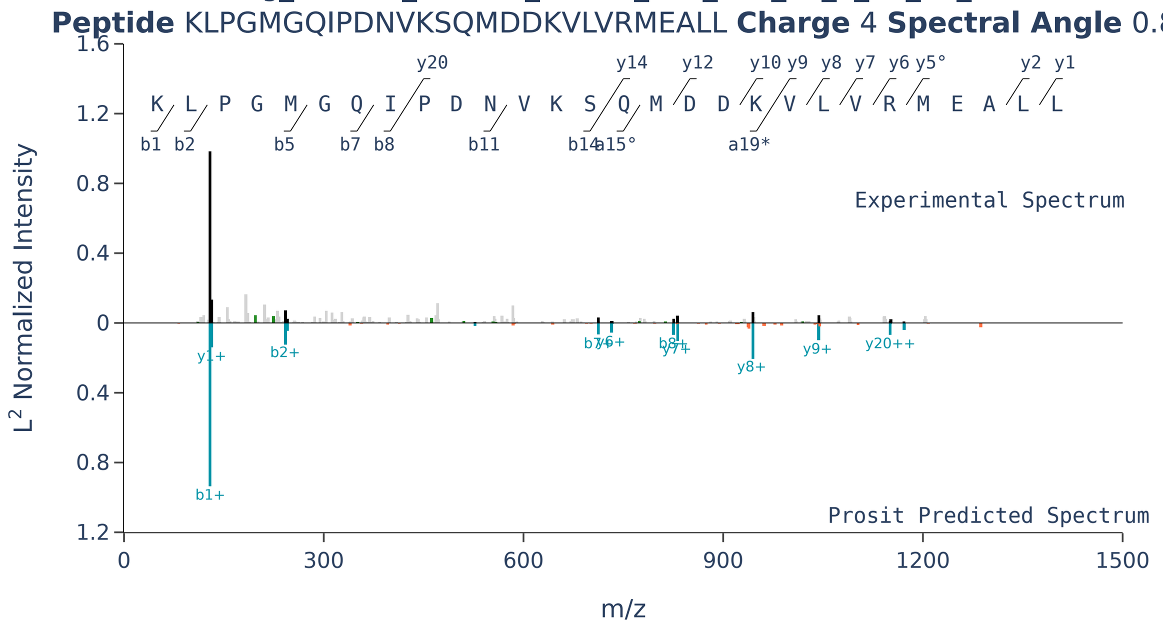


**Source** Ncheng_210623_230623_HFGoe_FFH_20S_25_1_A2_1h_R1 **Scan** 17551
**Peptide** VDTAGRLHVDEAMM **Charge** 3 **Spectral Angle** 0.47

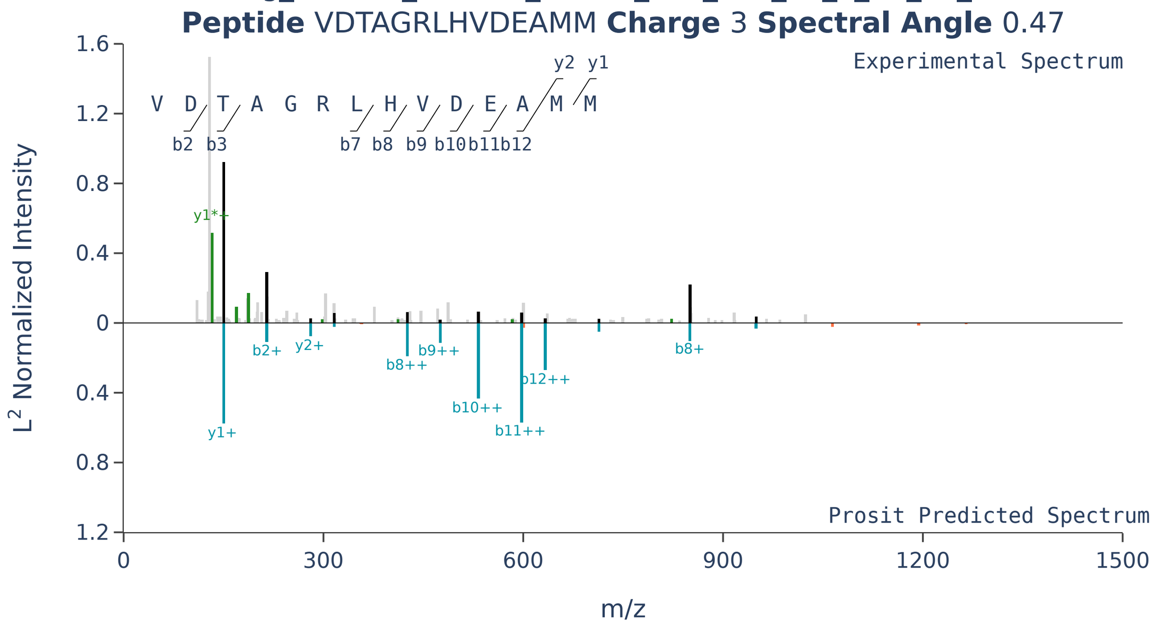


**Source** Ncheng_210623_230623_HFGoe_FFH_20S_25_1_A2_4h_R2 **Scan** 23261
**Peptide** VLTKVDGDARGGAALSIRHITGKPIKFL **Charge** 5 **Spectral Angle** 0.78

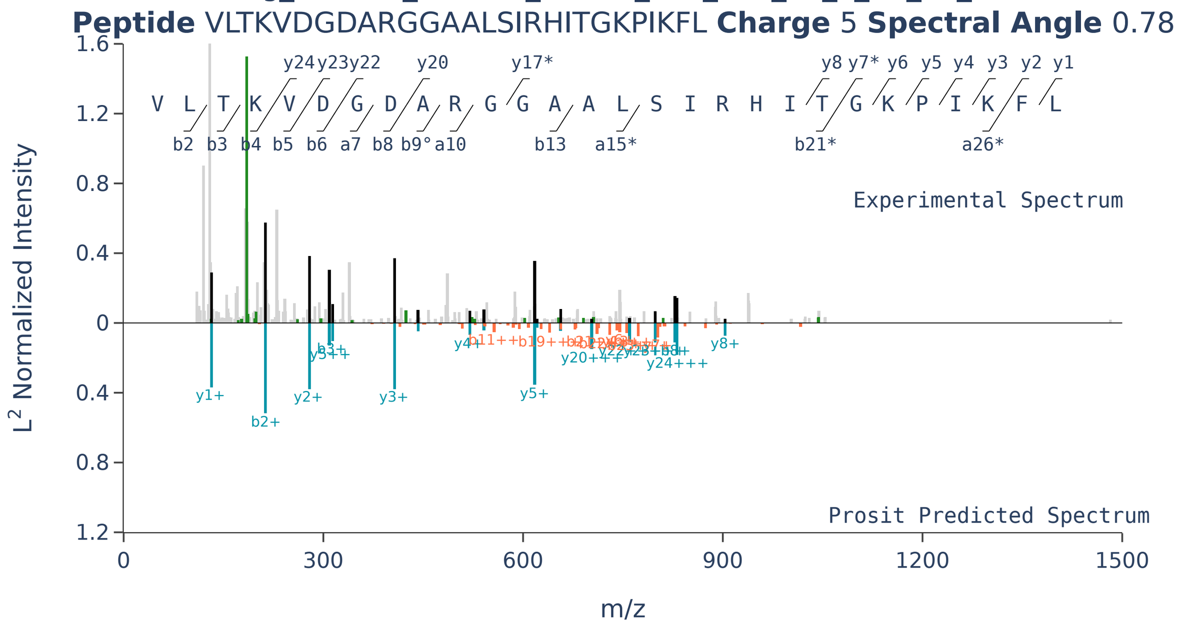


**Source** Ncheng_210623_230623_HFGoe_FFH_20S_25_1_A1_4h_R1 **Scan** 12811
**Peptide** LESKVDRAQAEKLASKLKK **Charge** 6 **Spectral Angle** 0.87

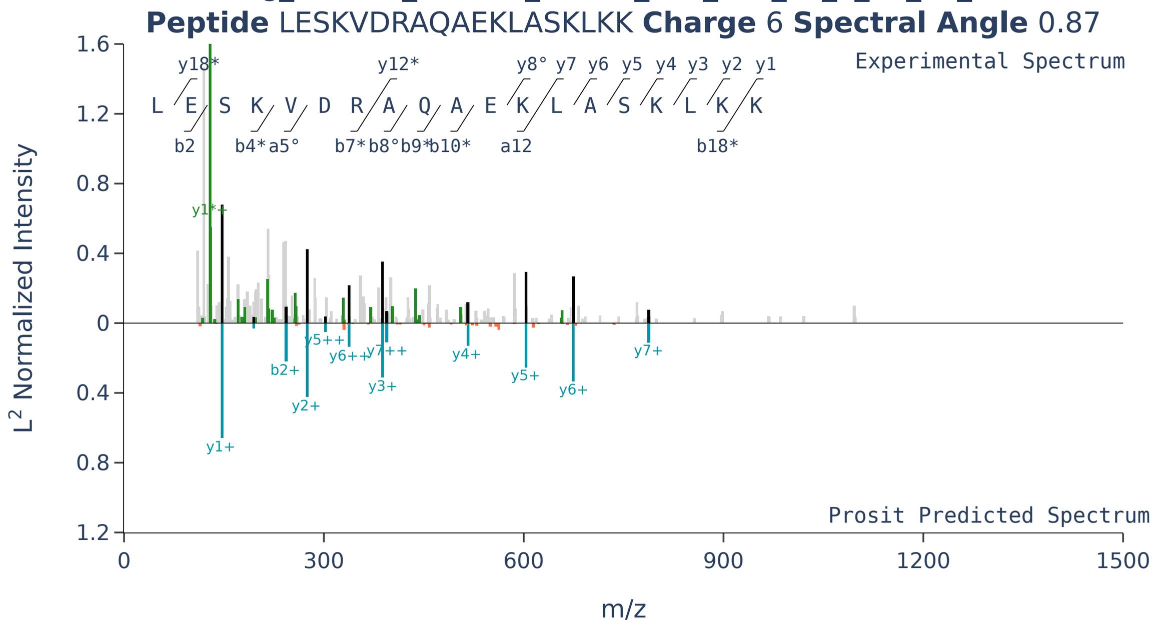


**Source** Ncheng_210623_230623_HFGoe_FFH_20S_25_1_A2_1h_R2 **Scan** 10299
**Peptide** KQLETLAEQ **Charge** 2 **Spectral Angle** 0.67

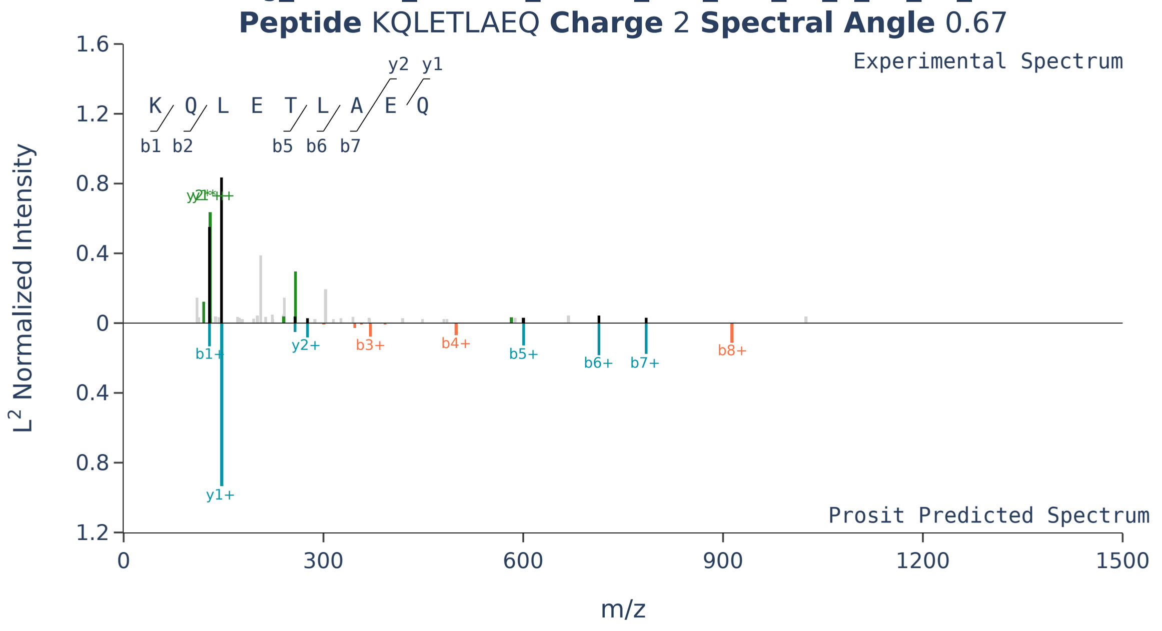


**Source** Ncheng_210623_230623_HFGoe_FFH_20S_25_1_A1_1h_R1 **Scan** 32699
**Peptide** DVYRPAAIKQLETLAEQVG **Charge** 3 **Spectral Angle** 0.74

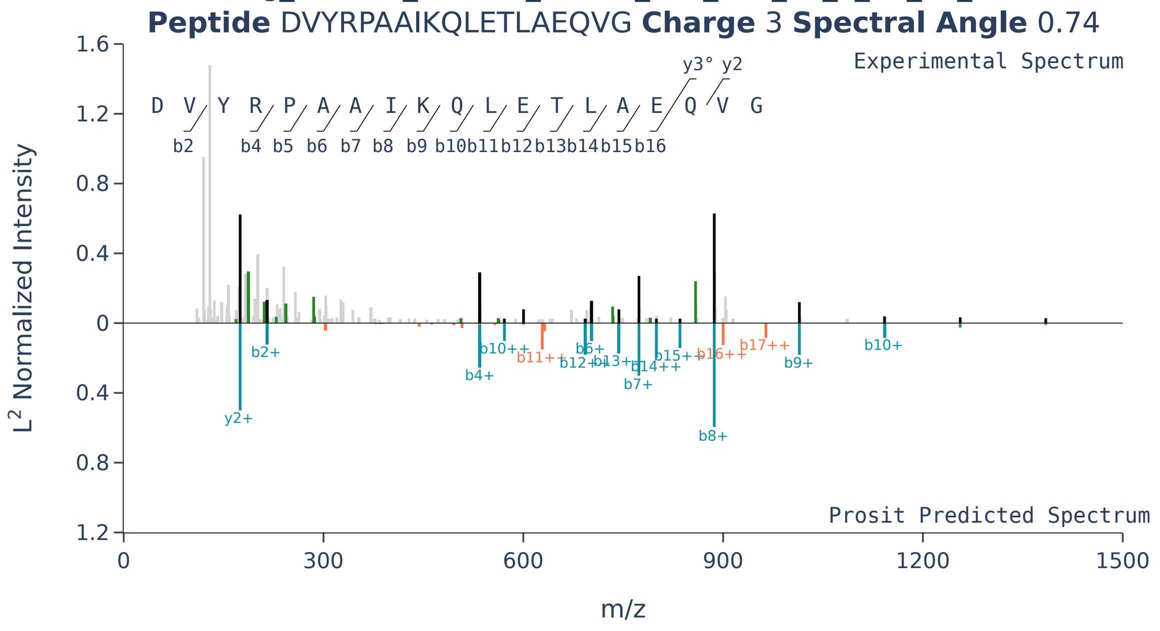


**Source** Ncheng_210623_230623_HFGoe_FFH_20S_25_1_A2_4h_R2 **Scan** 35039
**Peptide** ADVALPVVREFINRVKEKAV **Charge** 3 **Spectral Angle** 0.76

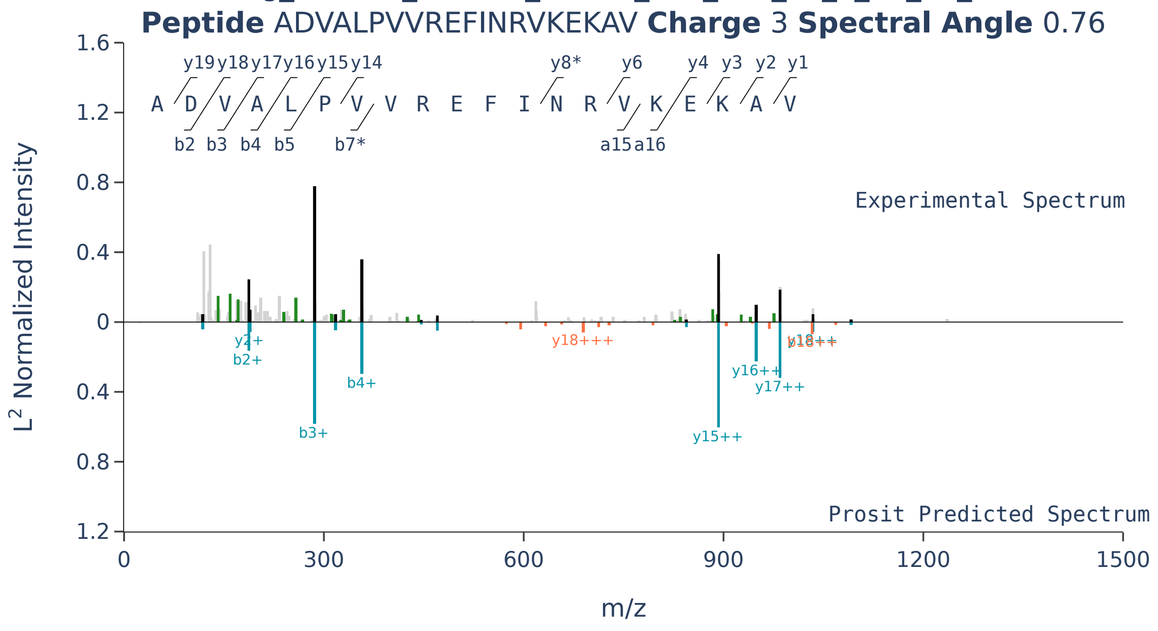


**Source** Ncheng_210623_230623_HFGoe_FFH_20S_25_1_A1_2h_R2 **Scan** 26709
**Peptide** EKAVGHEVNKSLTPGQEFVKIVRNEL **Charge** 4 **Spectral Angle** 0.71

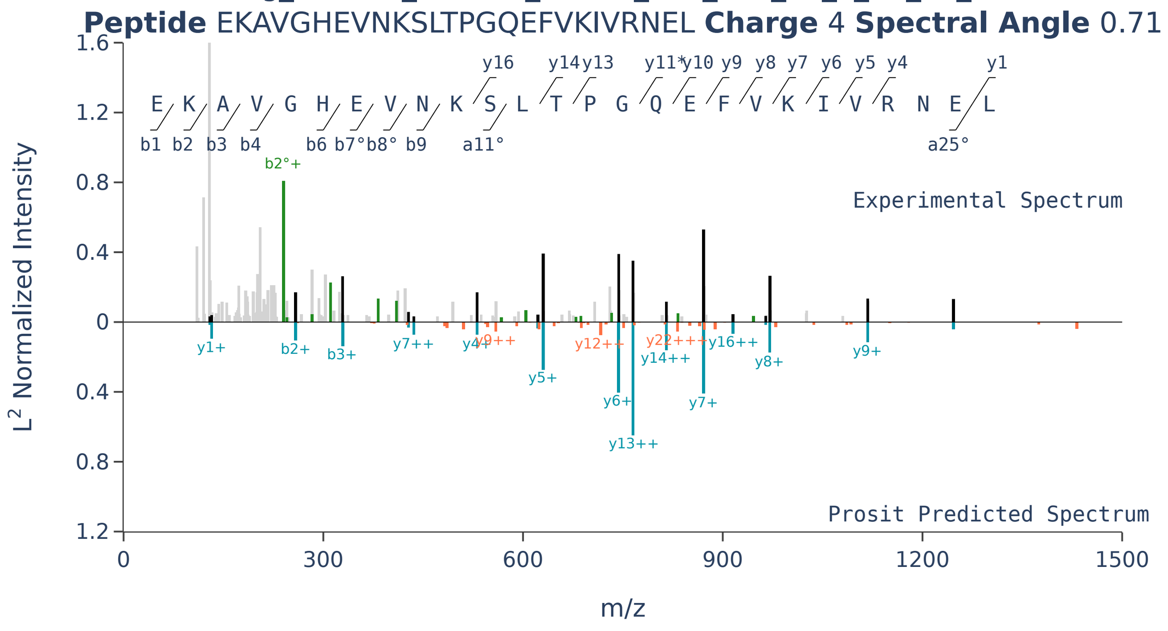


**Source** Ncheng_210623_230623_HFGoe_FFH_20S_25_1_A2_4h_R1 **Scan** 19993
**Peptide** RLTEDNVKDTLREVRMA **Charge** 4 **Spectral Angle** 0.59

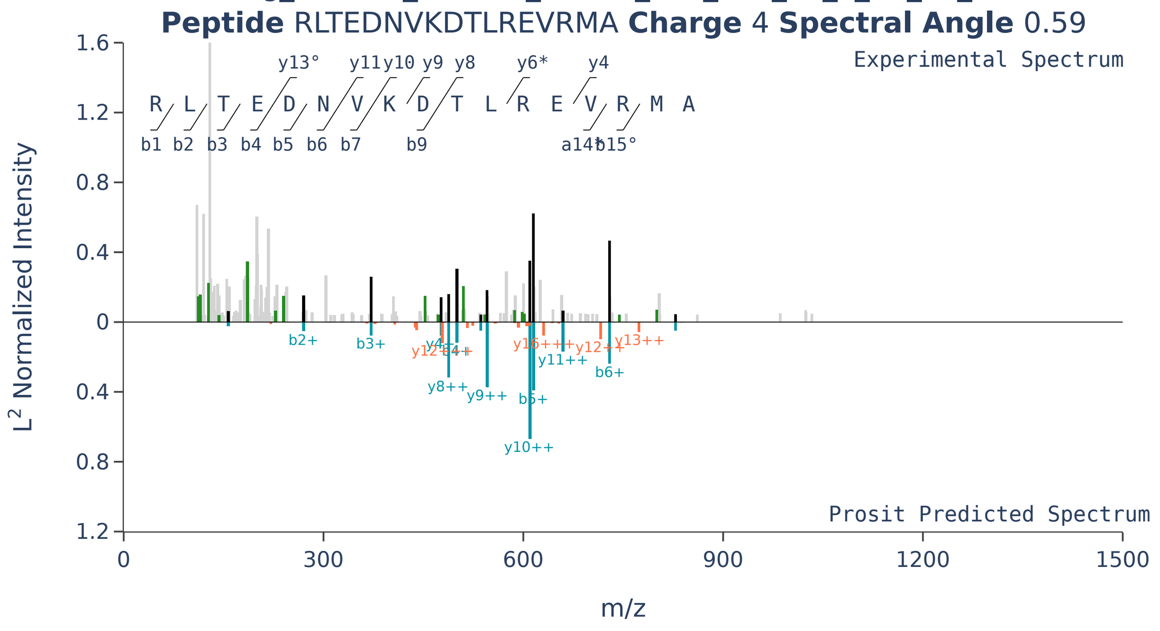


**Source** Ncheng_210623_230623_HFGoe_FFH_20S_25_1_A1_4h_R2 **Scan** 9542
**Peptide** DRAQAEKLASKLKKG **Charge** 4 **Spectral Angle** 0.72

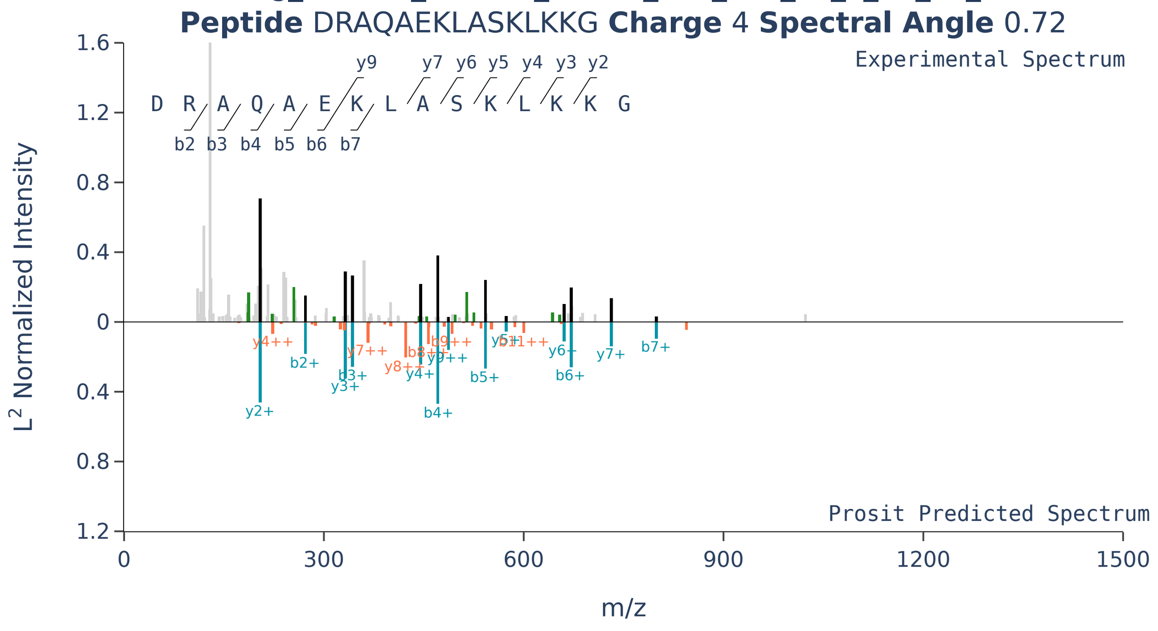


**Source** Ncheng_210623_230623_HFGoe_FFH_20S_25_1_A1_2h_R1 **Scan** 26537
**Peptide** ALEPFHPDRIASRILG **Charge** 3 **Spectral Angle** 0.52

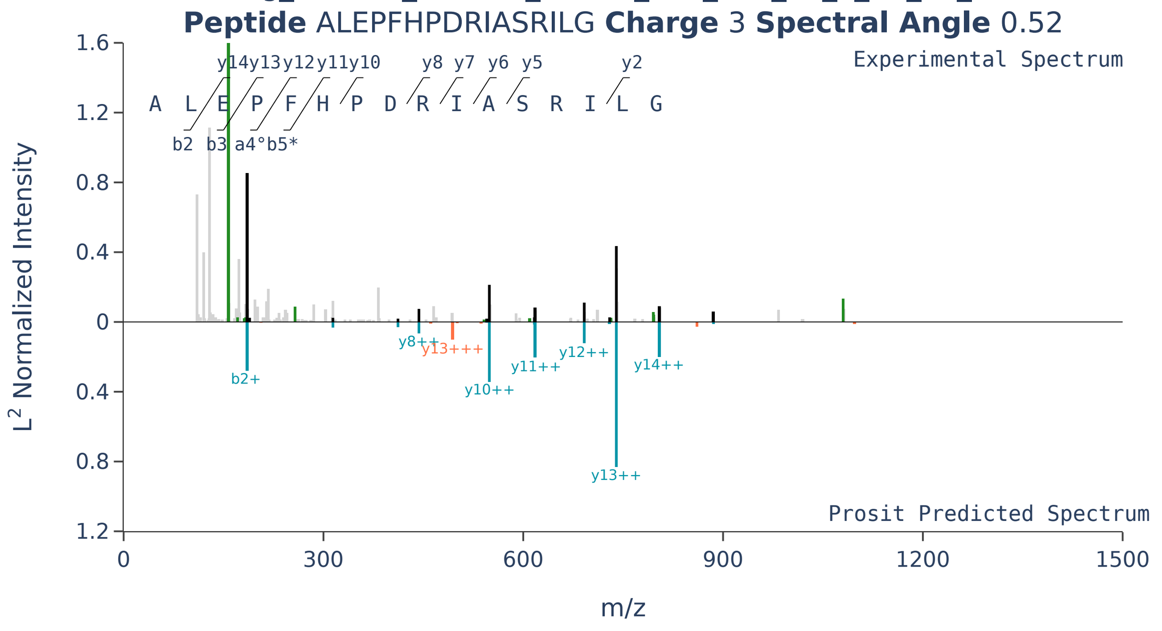


**Source** Ncheng_210623_230623_HFGoe_FFH_20S_25_1_A1_2h_R1 **Scan** 29918
**Peptide** GVGEKTEALEPFHPDRIASRILGM **Charge** 4 **Spectral Angle** 0.71

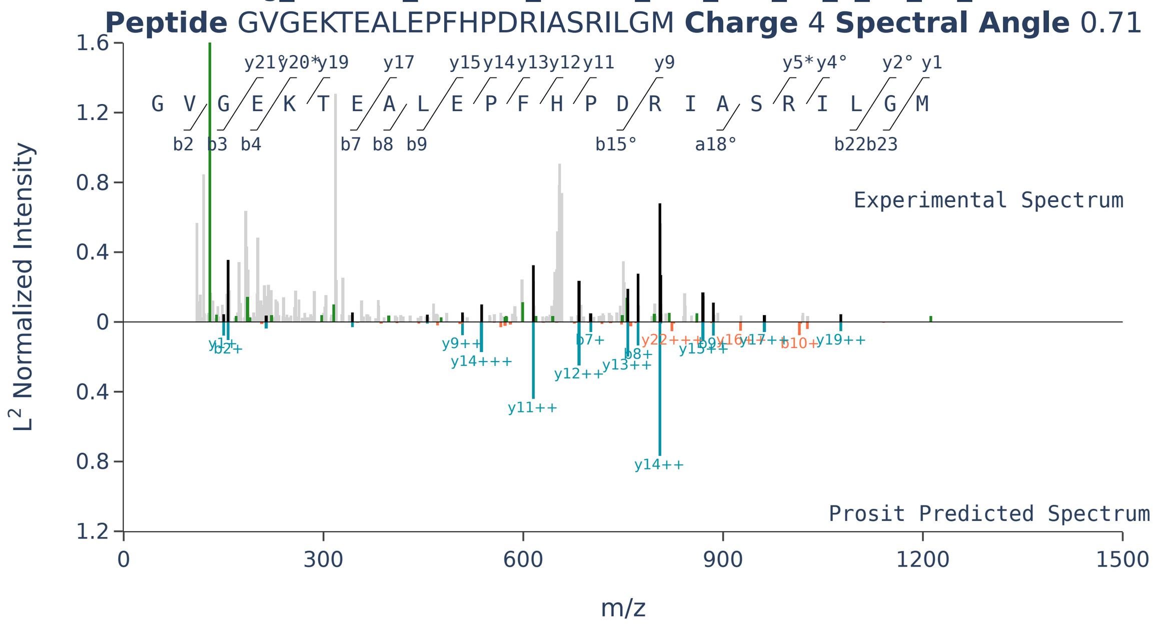

**Source** Ncheng_210623_230623_HFGoe_FFH_20S_25_1_A1_24h_R1 **Scan** 10010
**Peptide** RLSRTLRNI **Charge** 3 **Spectral Angle** 0.54

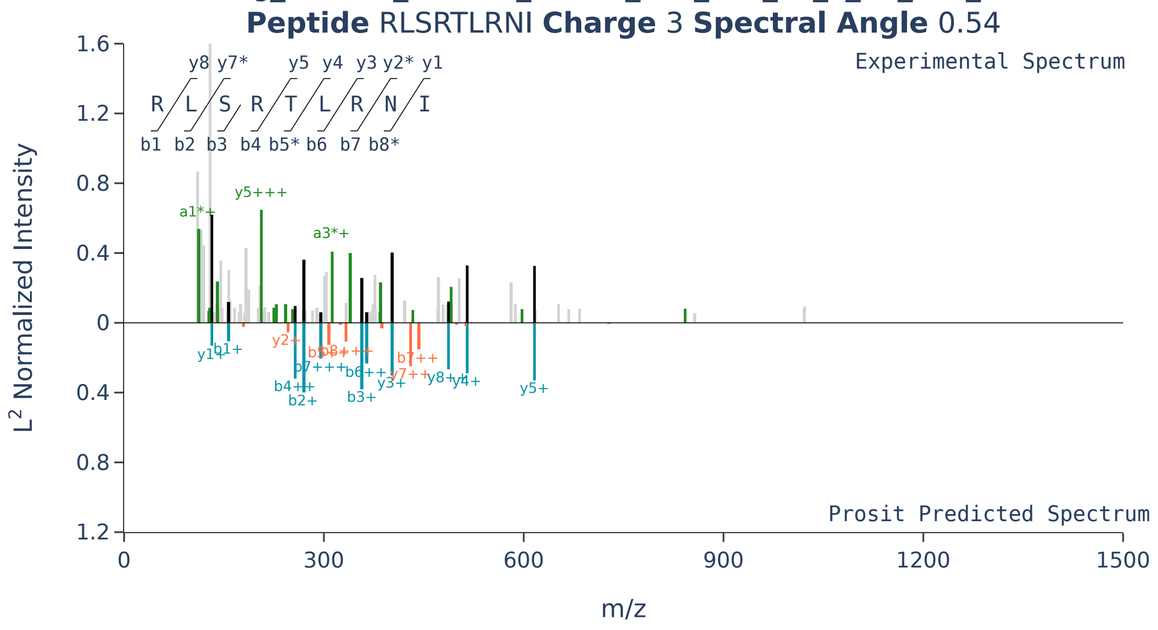


**Source** Ncheng_210623_230623_HFGoe_FFH_20S_25_1_A1_24h_R1 **Scan** 17102
**Peptide** FDDMQRMMKKM **Charge** 3 **Spectral Angle** 0.6

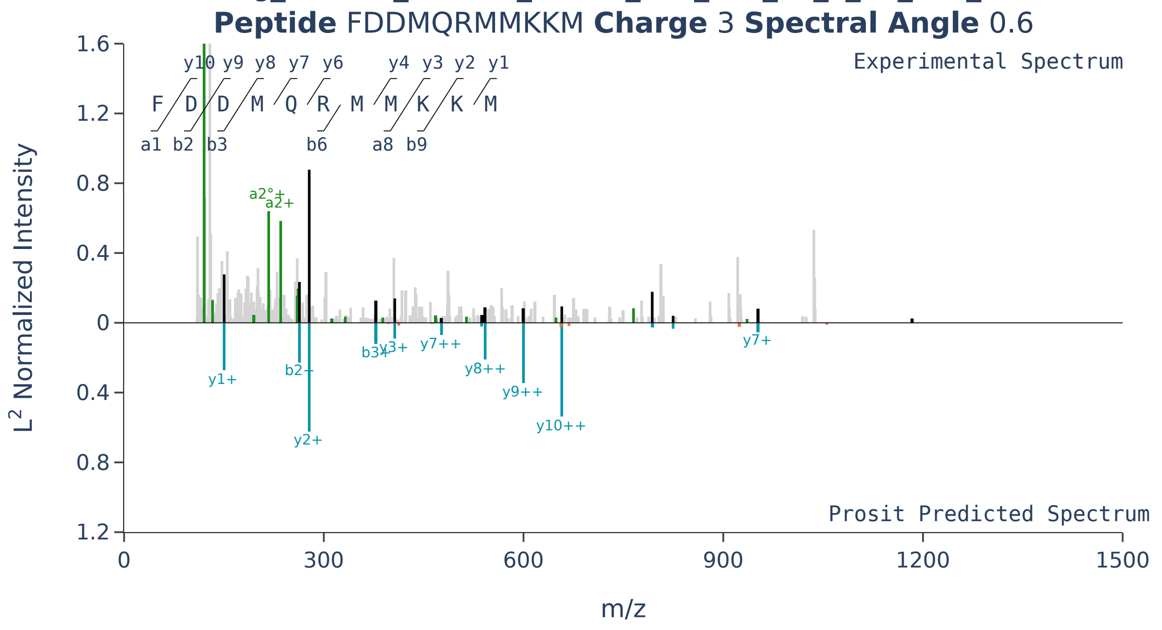


**Source** Ncheng_210623_230623_HFGoe_FFH_20S_25_1_A1_2h_R2 **Scan** 30905
**Peptide** KKGDGFDLNDFL **Charge** 2 **Spectral Angle** 0.39

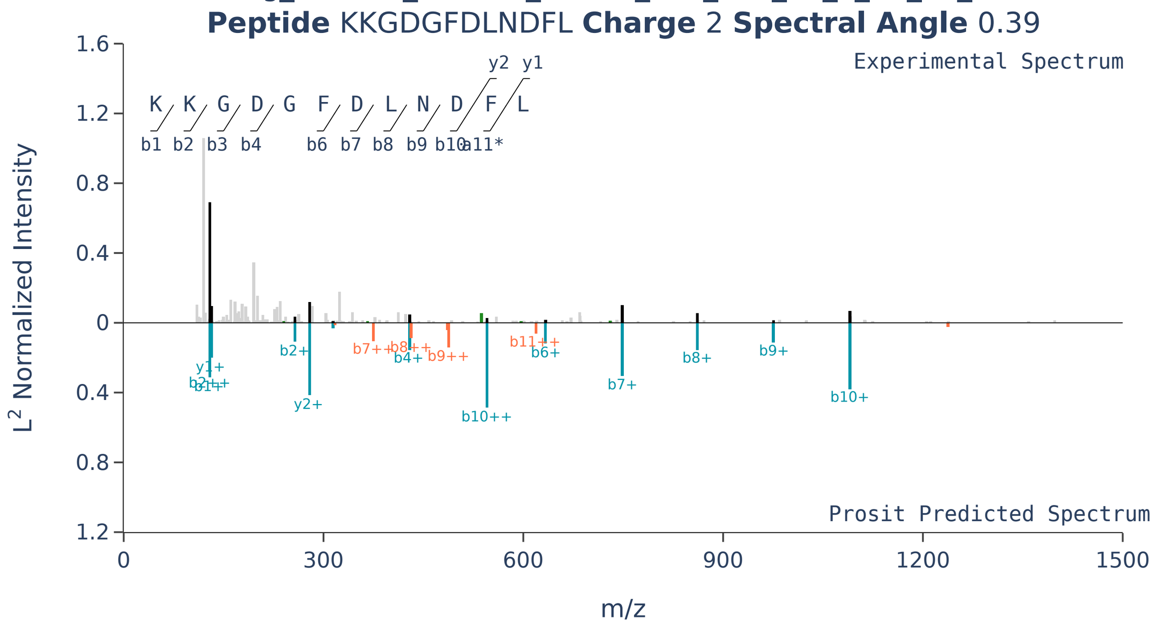


**Source** Ncheng_210623_230623_HFGoe_FFH_20S_25_1_A2_2h_R1 **Scan** 22331
**Peptide** DLESKVDRAQAEKLASKLKKGDGFDL **Charge** 4 **Spectral Angle** 0.8

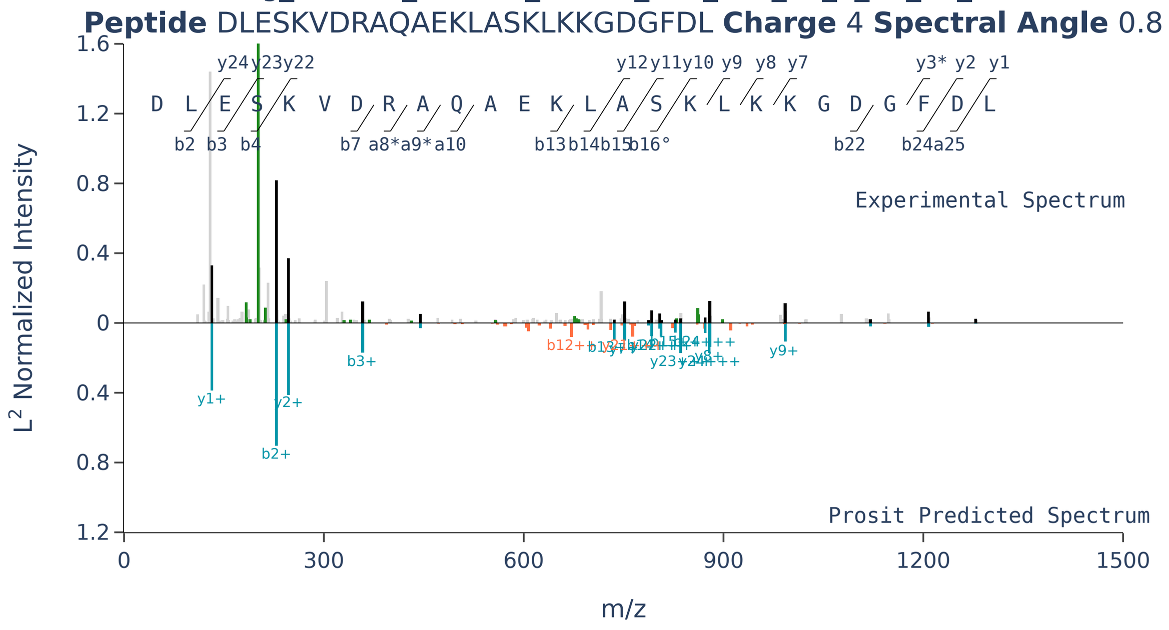


**Source** Ncheng_210623_230623_HFGoe_FFH_20S_25_1_A1_24h_R2 **Scan** 12358
**Peptide** KAVGHEVNKSLTPGQEFVK **Charge** 3 **Spectral Angle** 0.6

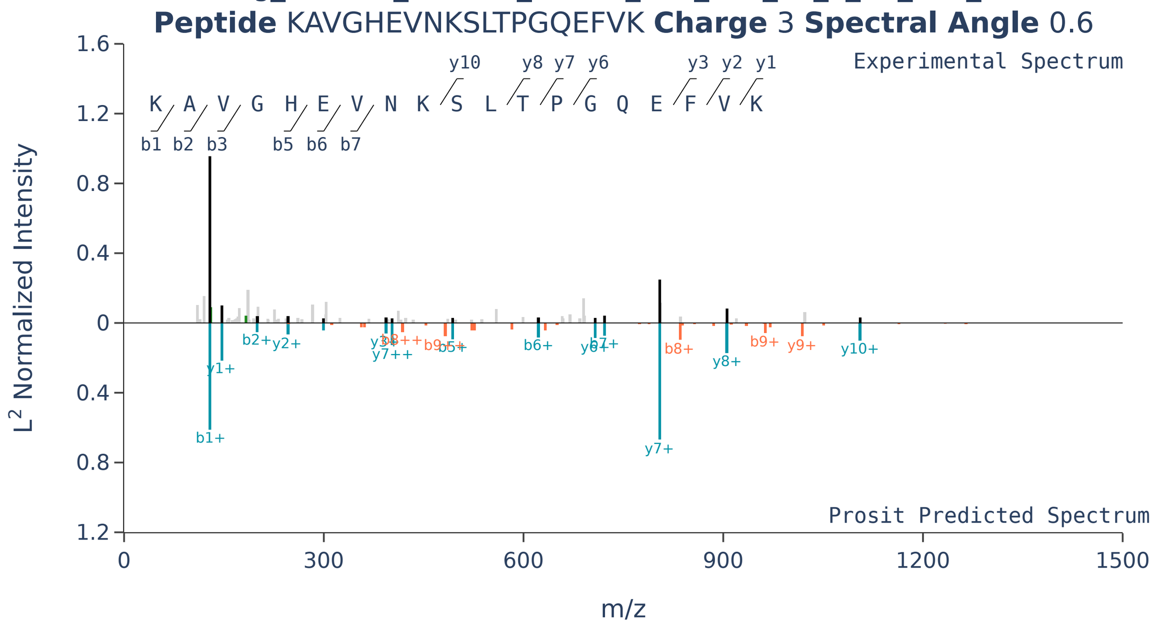


**Source** Ncheng_210623_230623_HFGoe_FFH_20S_25_1_A2_24h_R1 **Scan** 9662
**Peptide** LRNISGRGRLTE **Charge** 3 **Spectral Angle** 0.65

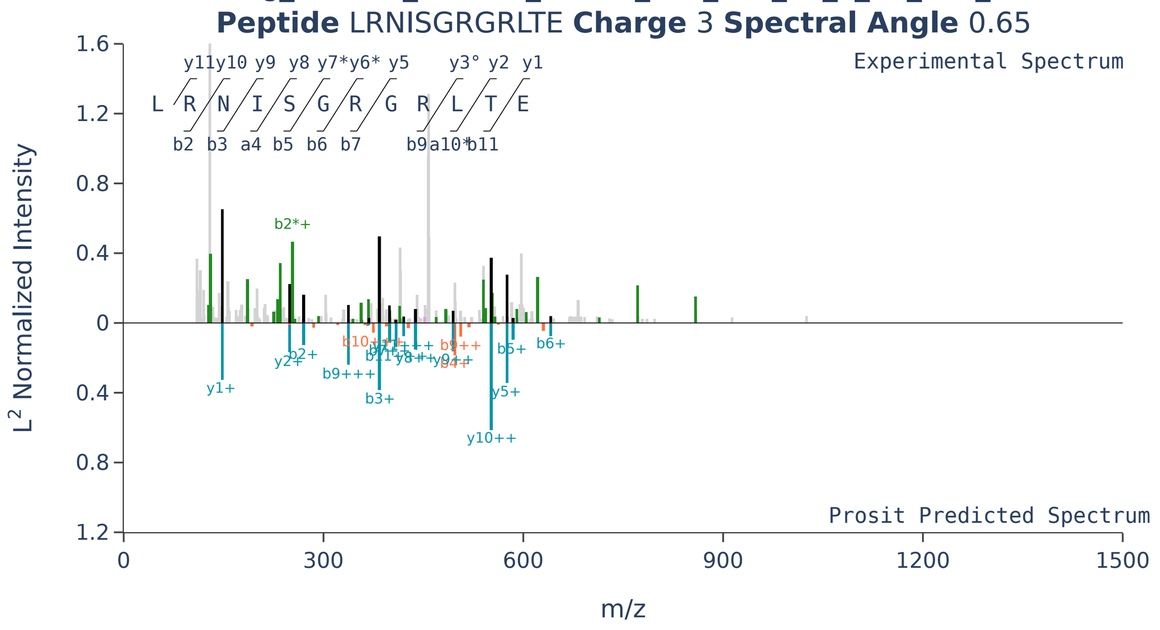


**Source** Ncheng_210623_230623_HFGoe_FFH_20S_25_1_A2_4h_R1 **Scan** 35587
**Peptide** AMTGQDAANTAKAFNEALPLTGVVLT **Charge** 3 **Spectral Angle** 0.7

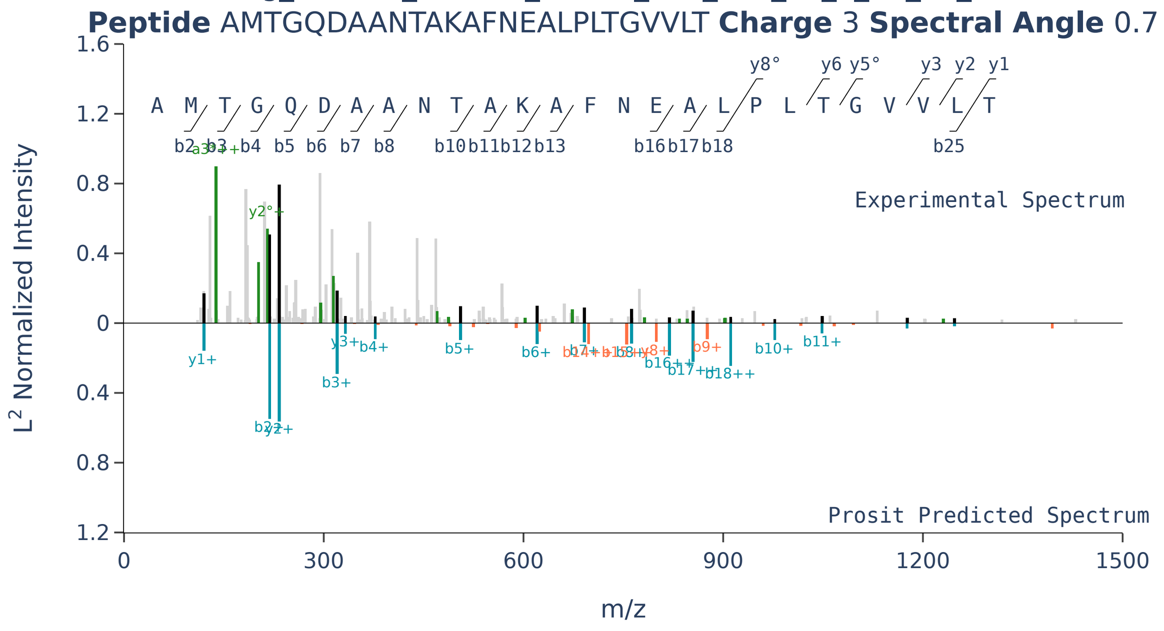


**Source** Ncheng_210623_230623_HFGoe_FFH_20S_25_1_A2_4h_R1 **Scan** 26386
**Peptide** GEKTEALEPFHPDRIASRIL **Charge** 4 **Spectral Angle** 0.8

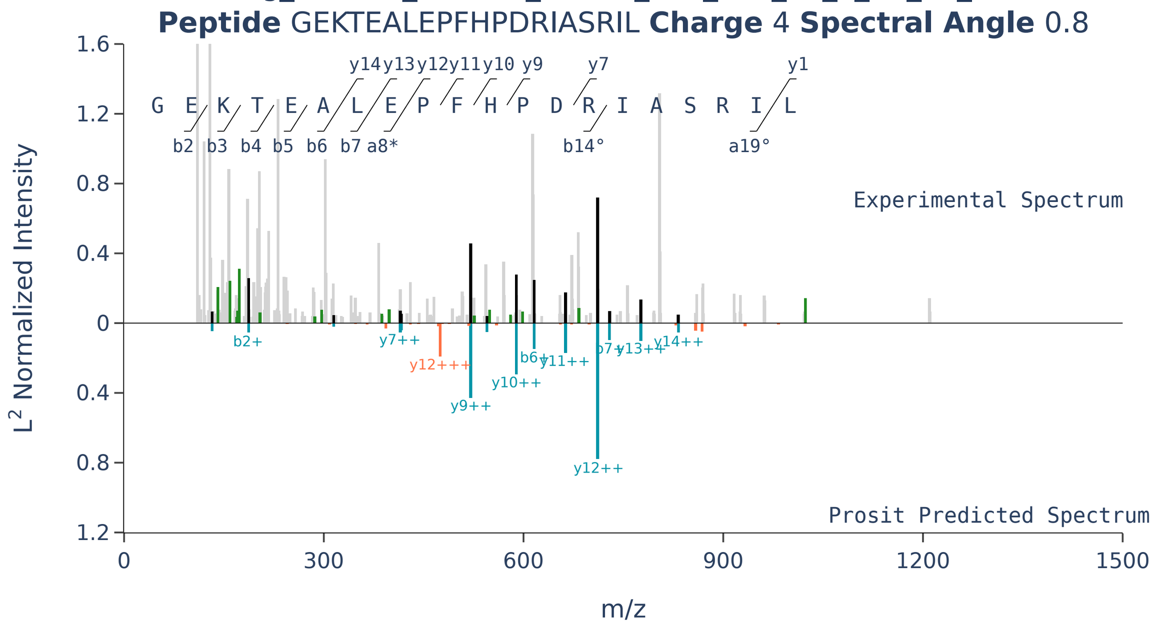


**Source** Ncheng_210623_230623_HFGoe_FFH_20S_25_1_A1_2h_R1 **Scan** 27903
**Peptide** VSADVYRPAAIKQLETLAE **Charge** 3 **Spectral Angle** 0.8

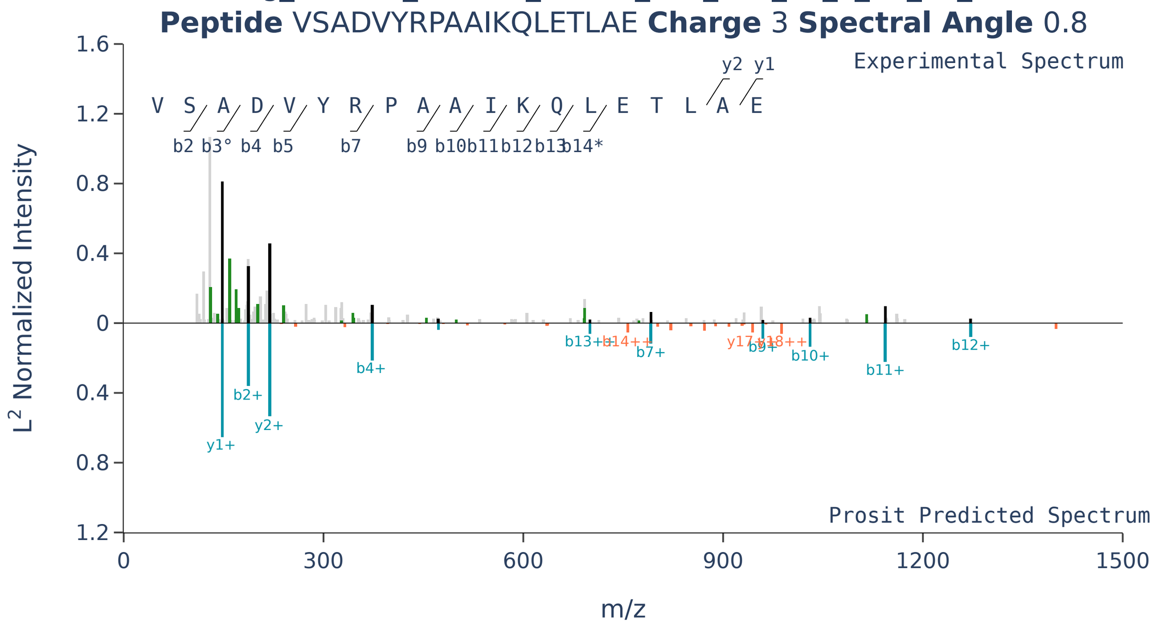


**Source** Ncheng_210623_230623_HFGoe_FFH_20S_25_1_A1_2h_R2 **Scan** 20074
**Peptide** GVVLTKVDGDARGGAALSIRHLTG **Charge** 3 **Spectral Angle** 0.58

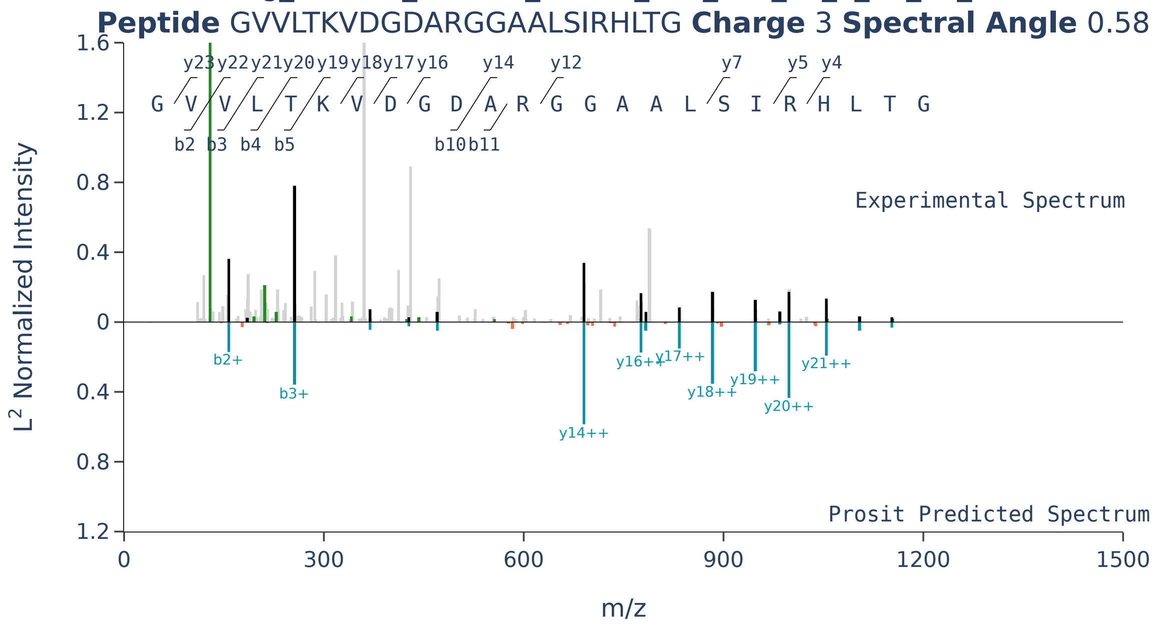


**Source** Ncheng_210623_230623_HFGoe_FFH_20S_25_1_A1_4h_R2 **Scan** 16861
**Peptide** VLTKVDGDARGGAALSIRHL **Charge** 3 **Spectral Angle** 0.66

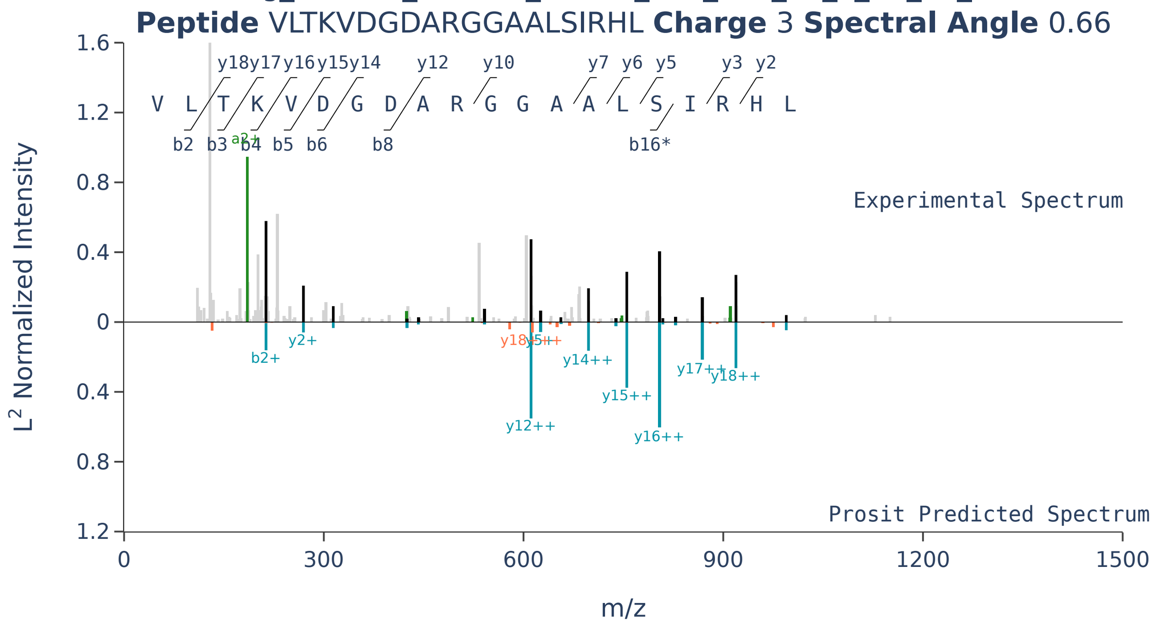


**Source** Ncheng_210623_230623_HFGoe_FFH_20S_25_1_A1_2h_R2 **Scan** 18162
**Peptide** MAGLQGAGKTTSVGKLGKFLREK **Charge** 5 **Spectral Angle** 0.62

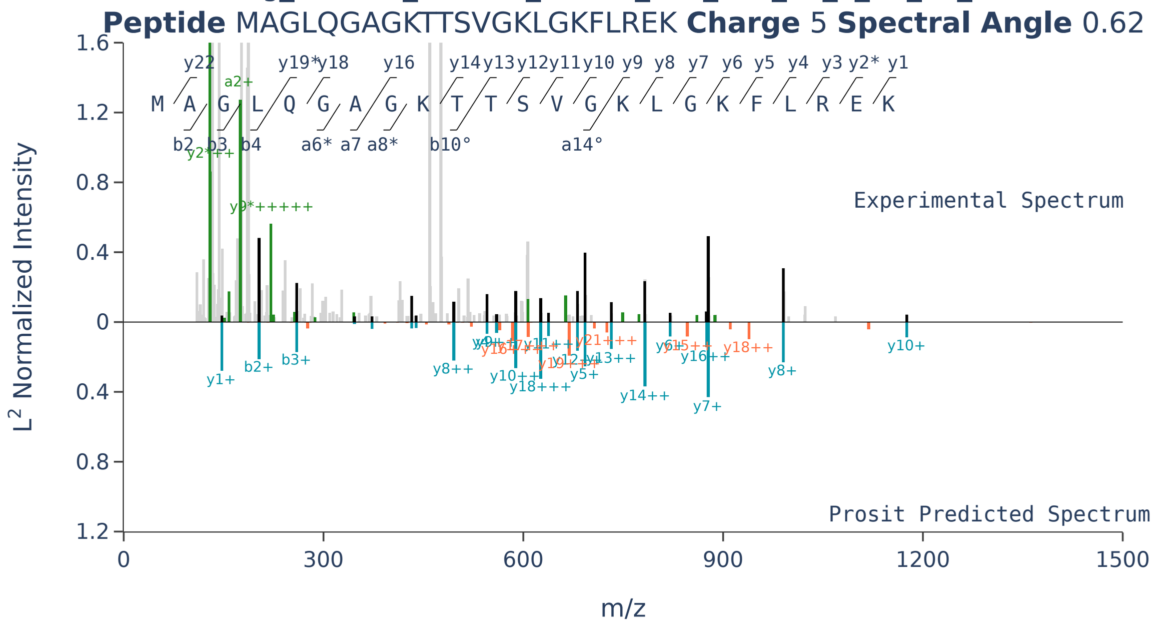


**Source** Ncheng_210623_230623_HFGoe_FFH_20S_25_1_A1_2h_R2 **Scan** 27275
**Peptide** LGVGEKTEALEPFHPDRIASRIL **Charge** 3 **Spectral Angle** 0.68

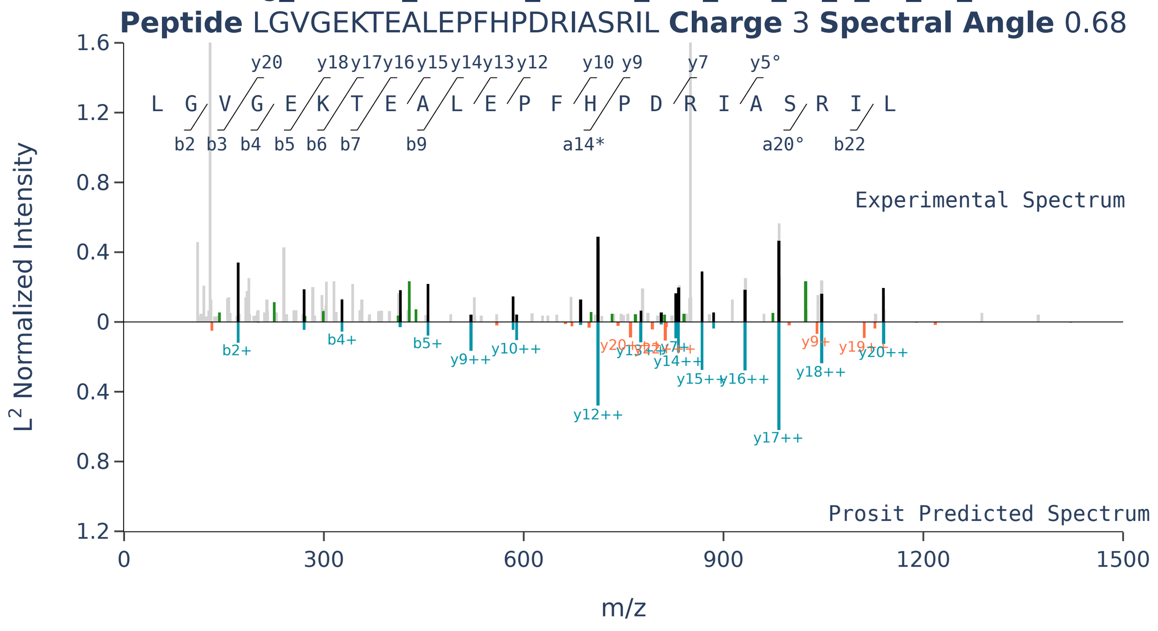


**Source** Ncheng_210623_230623_HFGoe_FFH_20S_25_1_A2_2h_R1 **Scan** 28048
**Peptide** EKAVGHEVNKSLTPGQEFVKIVRNELVA **Charge** 3 **Spectral Angle** 0.71

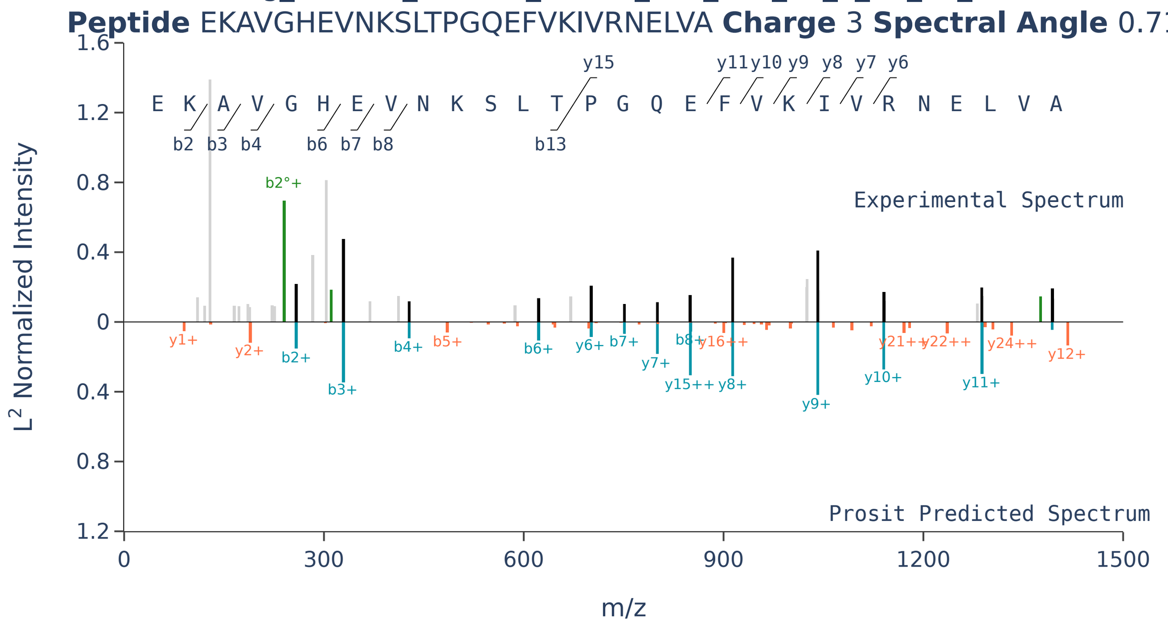


**Source** Ncheng_210623_230623_HFGoe_FFH_20S_25_1_A2_2h_R1 **Scan** 23068
**Peptide** NLTDRLSRTLRNISGRGRLTE **Charge** 6 **Spectral Angle** 0.69

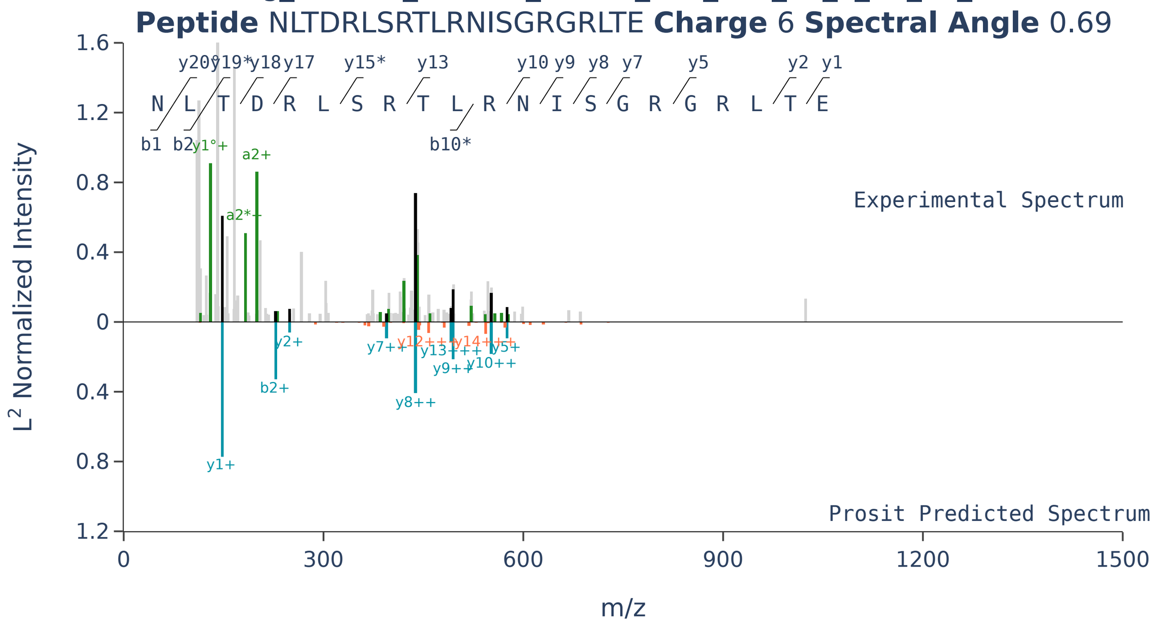

**Source** Ncheng_210623_230623_HFGoe_FFH_20S_25_1_A1_2h_R1 **Scan** 24695
**Peptide** PLTGVVLTKVDGDARGGAALSIRHL **Charge** 5 **Spectral Angle** 0.66

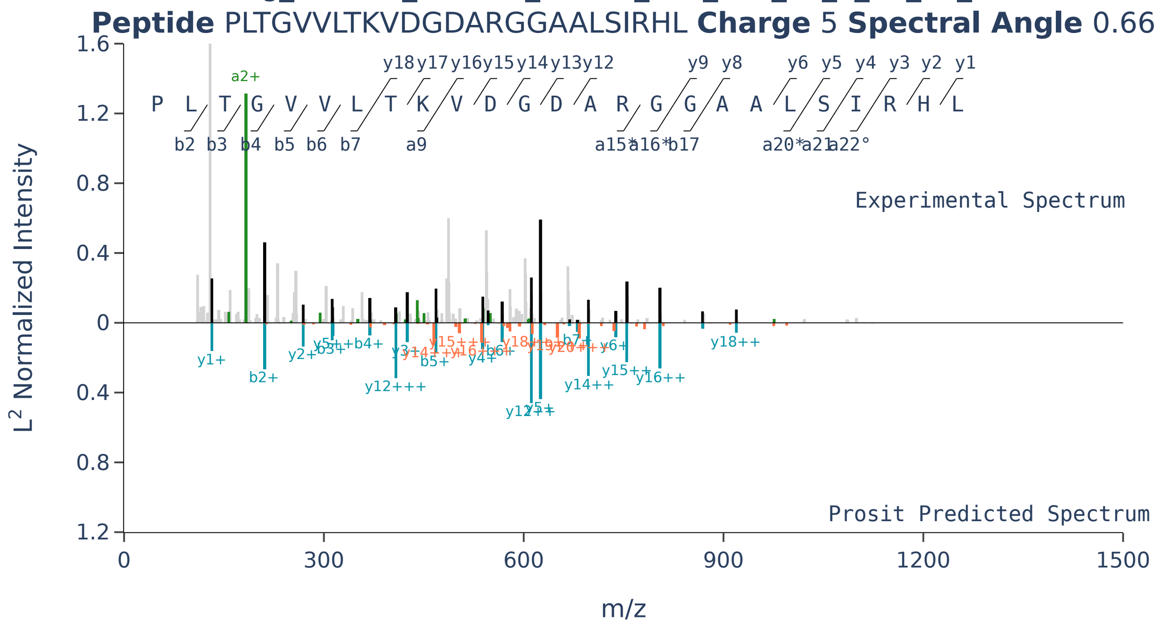


**Source** Ncheng_210623_230623_HFGoe_FFH_20S_25_1_A1_4h_R1 **Scan** 33492
**Peptide** KLPGMGQIPDNVKSQMDDKVLVRMEAII **Charge** 4 **Spectral Angle** 0.87

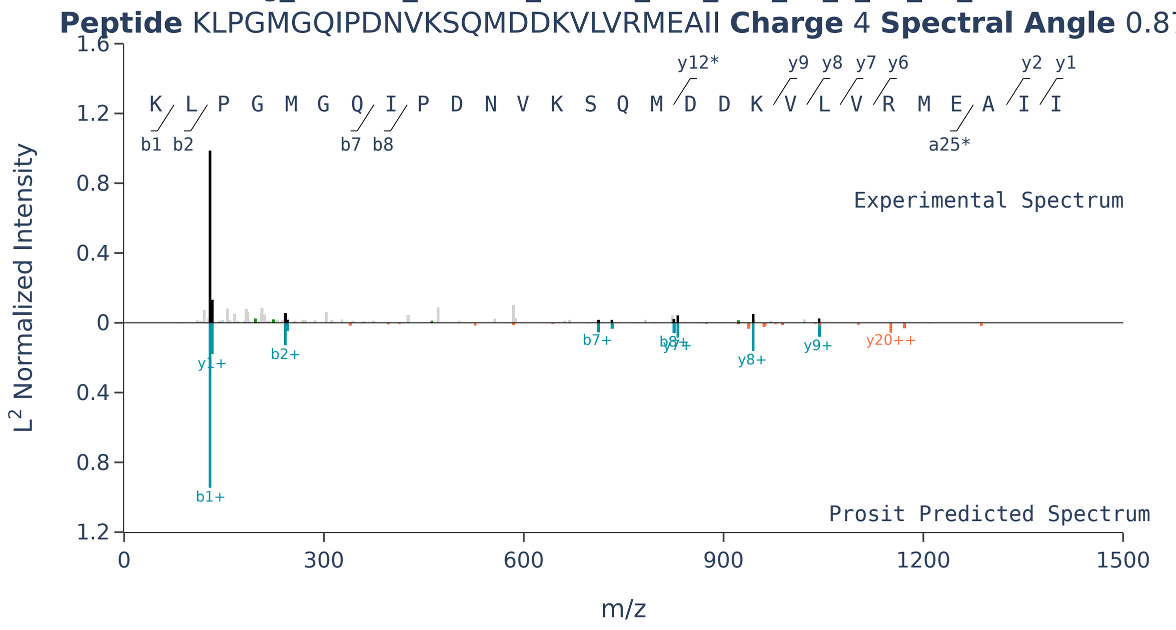


**Source** Ncheng_210623_230623_HFGoe_FFH_20S_25_1_A1_2h_R2 **Scan** 31497
**Peptide** LGVGEKTEALEPFHPDRIASRILGMGD **Charge** 3 **Spectral Angle** 0.78

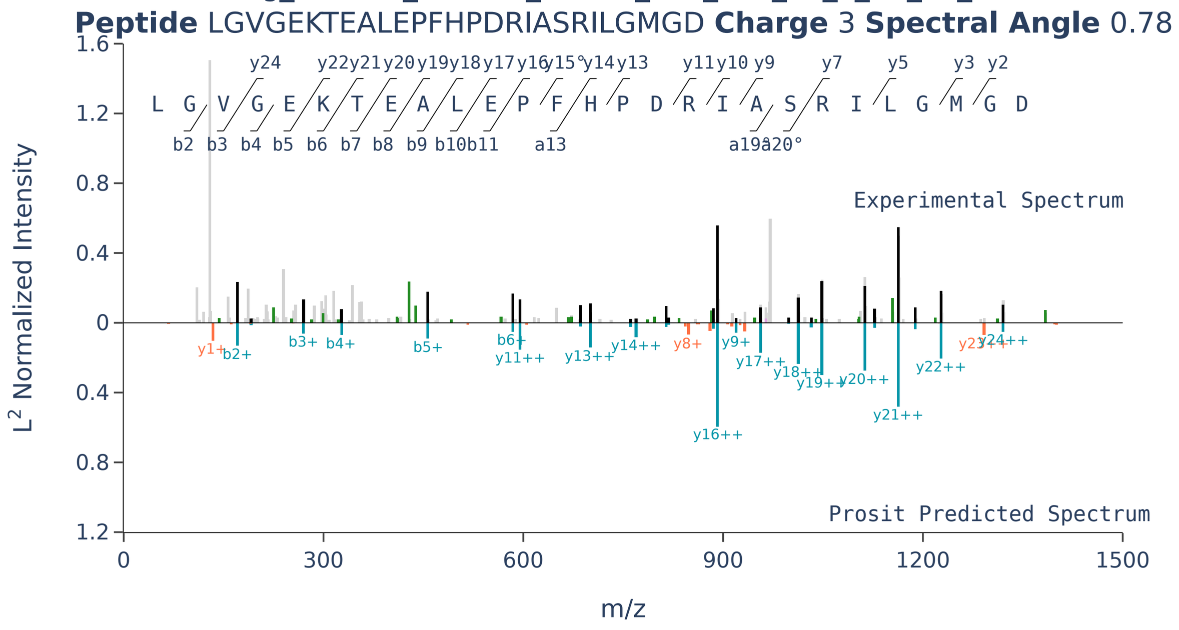


**Source** Ncheng_210623_230623_HFGoe_FFH_20S_25_1_A2_4h_R1 **Scan** 21658
**Peptide** TKVDGDARGGAALSIRHITGKPIKFL **Charge** 3 **Spectral Angle** 0.63

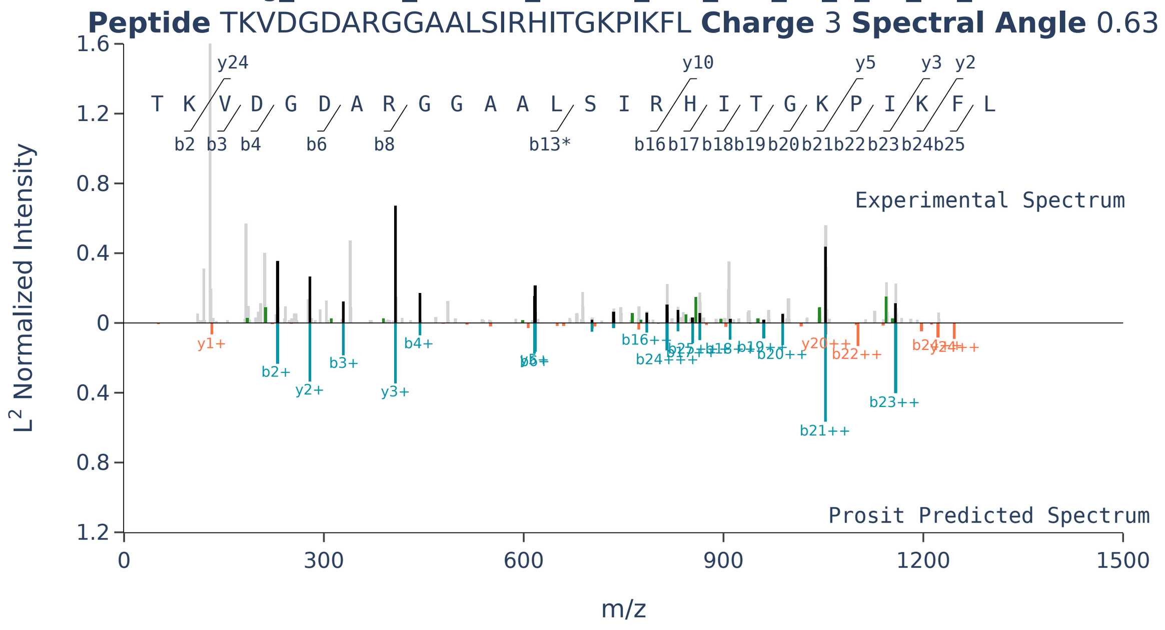


**Source** Ncheng_210623_230623_HFGoe_FFH_20S_25_1_A2_4h_R2 **Scan** 13875
**Peptide** KVDGDARGGAALSIRHLTG **Charge** 3 **Spectral Angle** 0.6

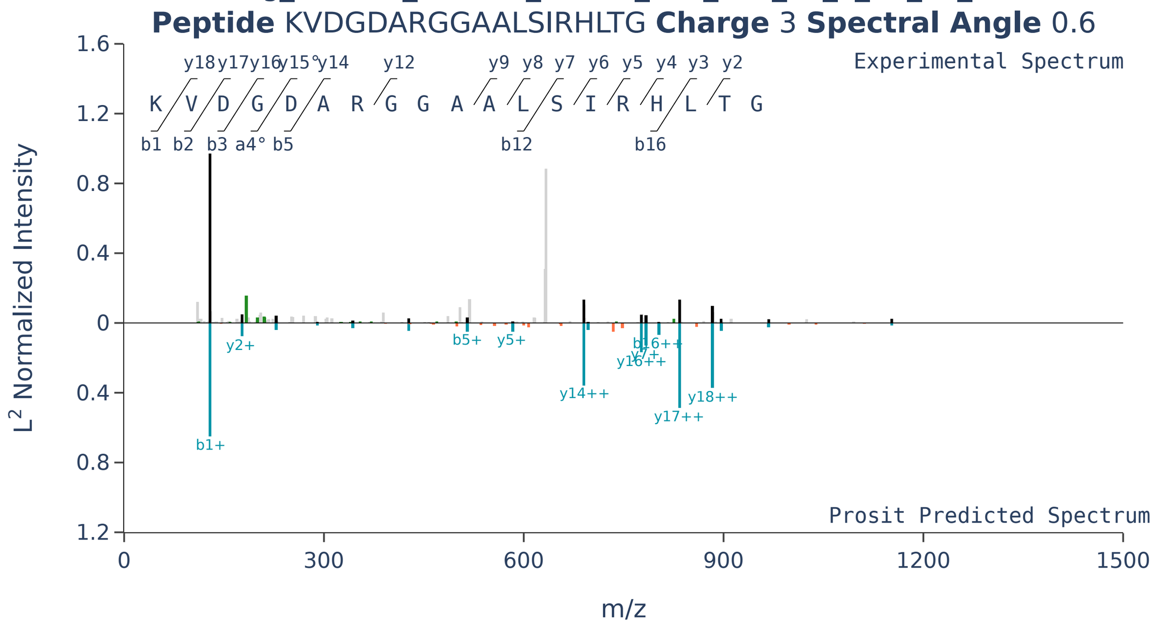


**Source** Ncheng_210623_230623_HFGoe_FFH_20S_25_1_A1_1h_R1 **Scan** 22944
**Peptide** TAGRLHVDEAMMDEIKQV **Charge** 3 **Spectral Angle** 0.55

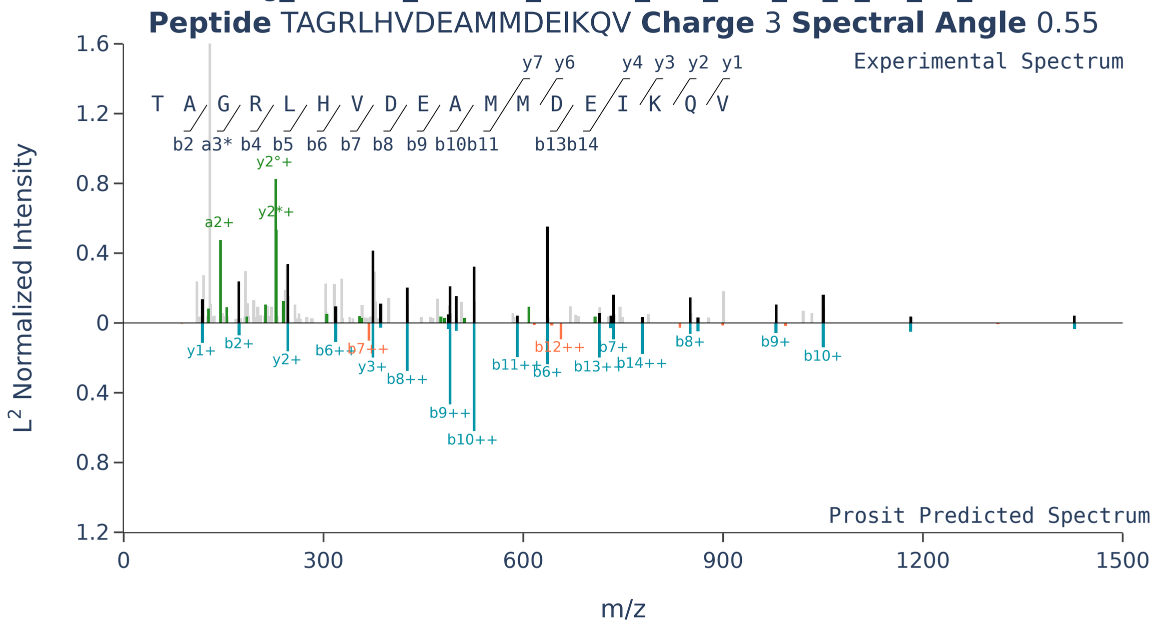


**Source** Ncheng_210623_230623_HFGoe_FFH_20S_25_1_A2_4h_R2 **Scan** 20848
**Peptide** TGVVLTKVDGDARGGAALSIRHI **Charge** 3 **Spectral Angle** 0.58

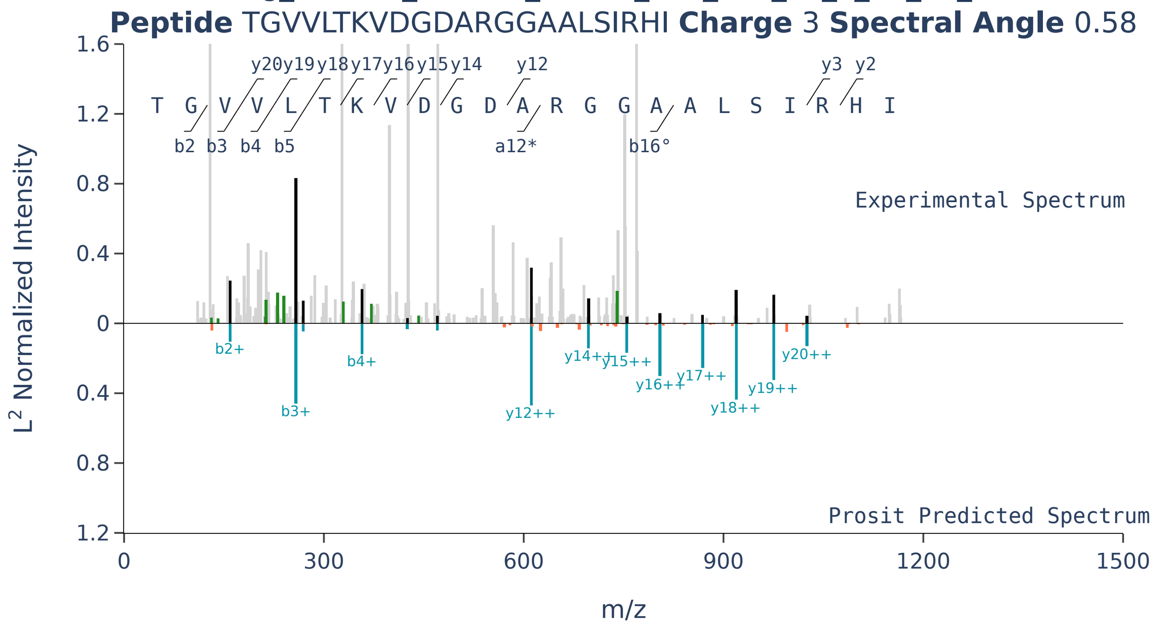


**Source** Ncheng_210623_230623_HFGoe_FFH_20S_25_1_A2_2h_R2 **Scan** 6623
**Peptide** FINRVKEKA **Charge** 3 **Spectral Angle** 0.62

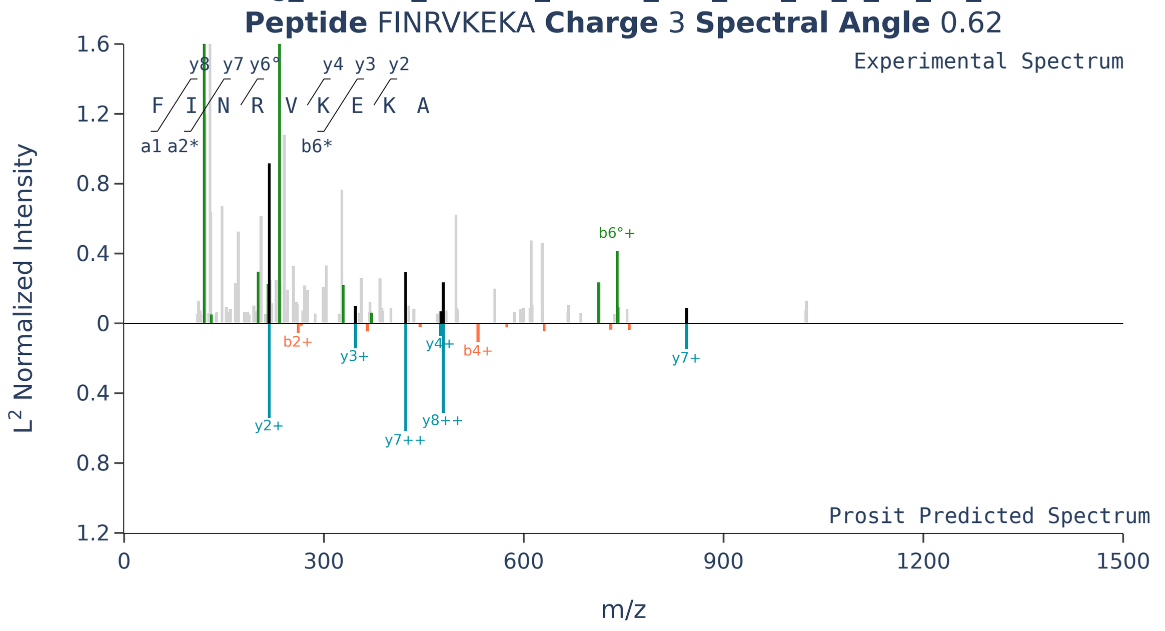


**Source** Ncheng_210623_230623_HFGoe_FFH_20S_25_1_A1_4h_R2 **Scan** 17832
**Peptide** RNISGRGRLTEDNVKDTLREVRMA **Charge** 6 **Spectral Angle** 0.76

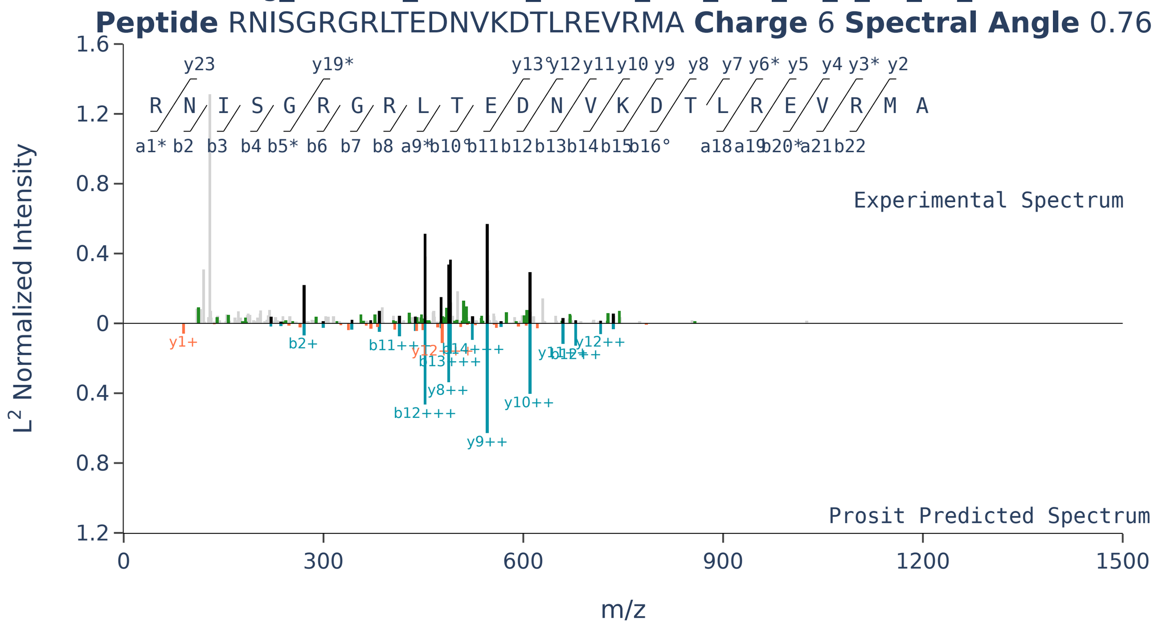


**Source** Ncheng_210623_230623_HFGoe_FFH_20S_25_1_A1_24h_R1 **Scan** 11440
**Peptide** EFINRVKEKAVGHEVN **Charge** 2 **Spectral Angle** 0.46

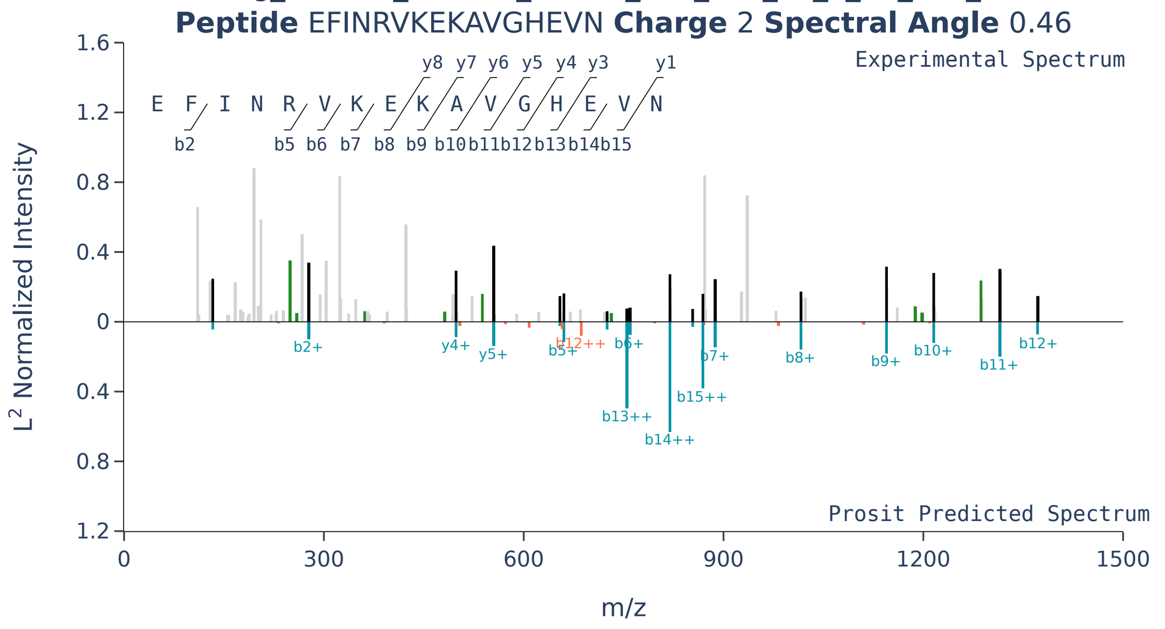


**Source** Ncheng_210623_230623_HFGoe_FFH_20S_25_1_A2_2h_R1 **Scan** 23429
**Peptide** AMMDEIKQVHASINPVET **Charge** 3 **Spectral Angle** 0.45

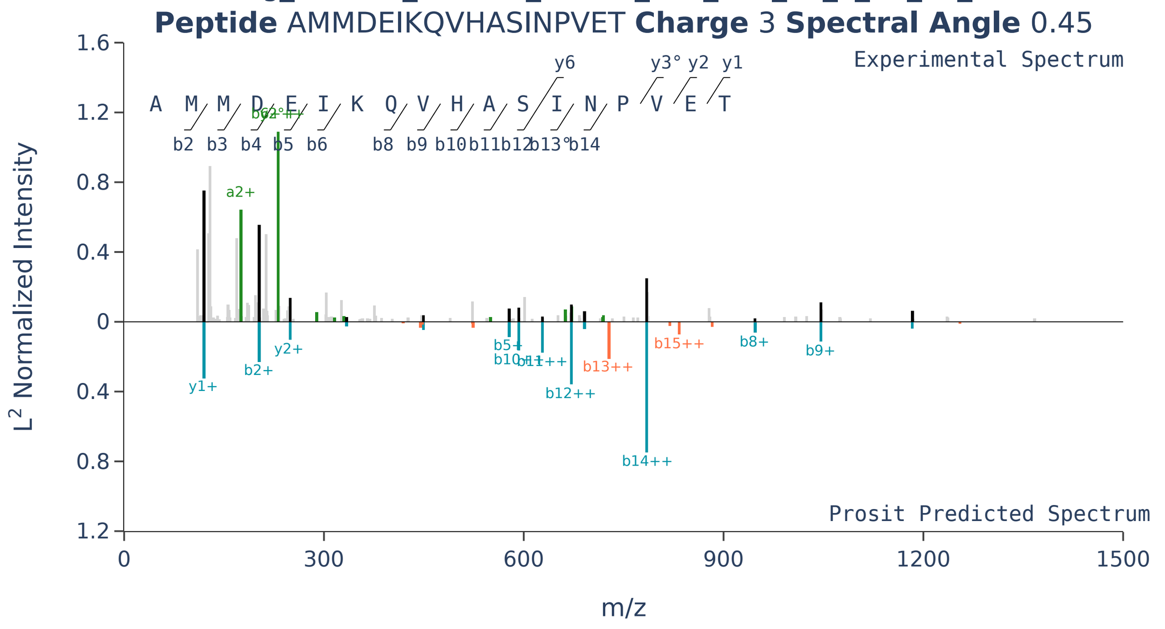


**Source** Ncheng_210623_230623_HFGoe_FFH_20S_25_1_A1_2h_R2 **Scan** 27150
**Peptide** RPAAIKQLETLAEQVG **Charge** 3 **Spectral Angle** 0.58

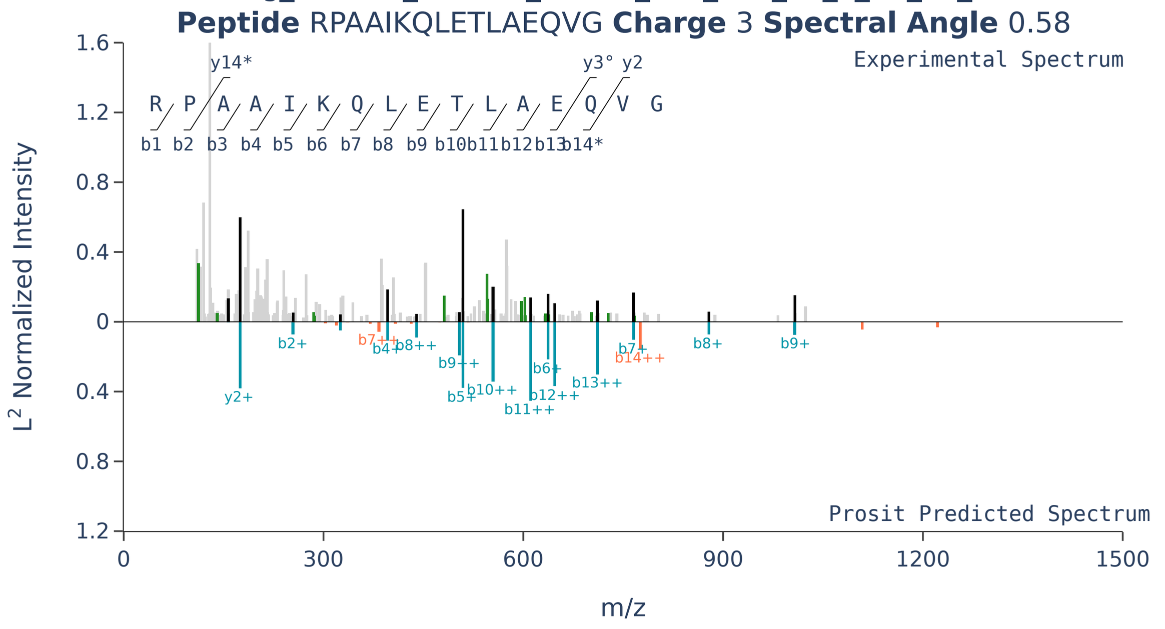


**Source** Ncheng_210623_230623_HFGoe_FFH_20S_25_1_A2_4h_R2 **Scan** 33331
**Peptide** KAFNEALPLTGVVLTKV **Charge** 3 **Spectral Angle** 0.43

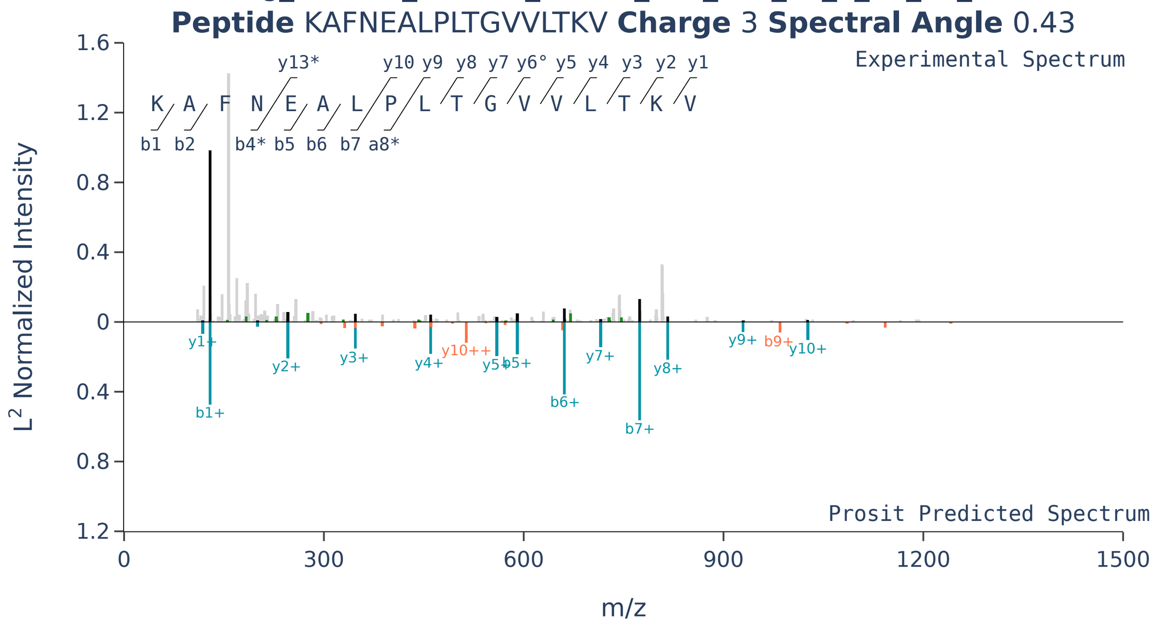


**Source** Ncheng_210623_230623_HFGoe_FFH_20S_25_1_A2_24h_R2 **Scan** 5836
**Peptide** HEVNKSLT **Charge** 2 **Spectral Angle** 0.73

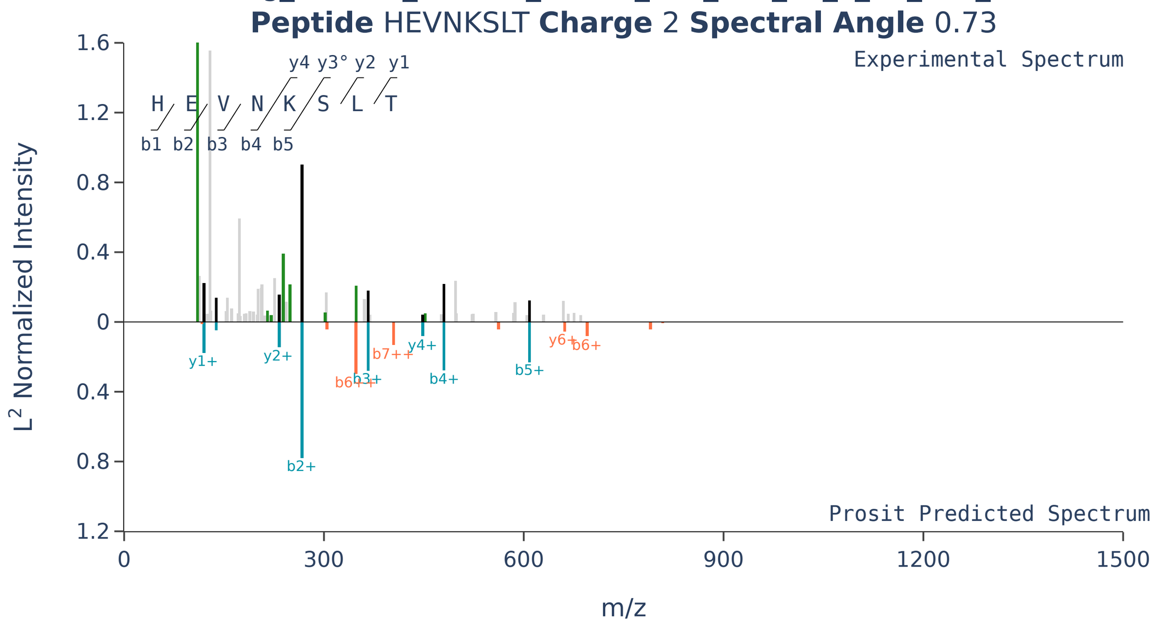


**Source** Ncheng_210623_230623_HFGoe_FFH_20S_25_1_A1_24h_R2 **Scan** 6280
**Peptide** SKLKKGDGF **Charge** 2 **Spectral Angle** 0.77

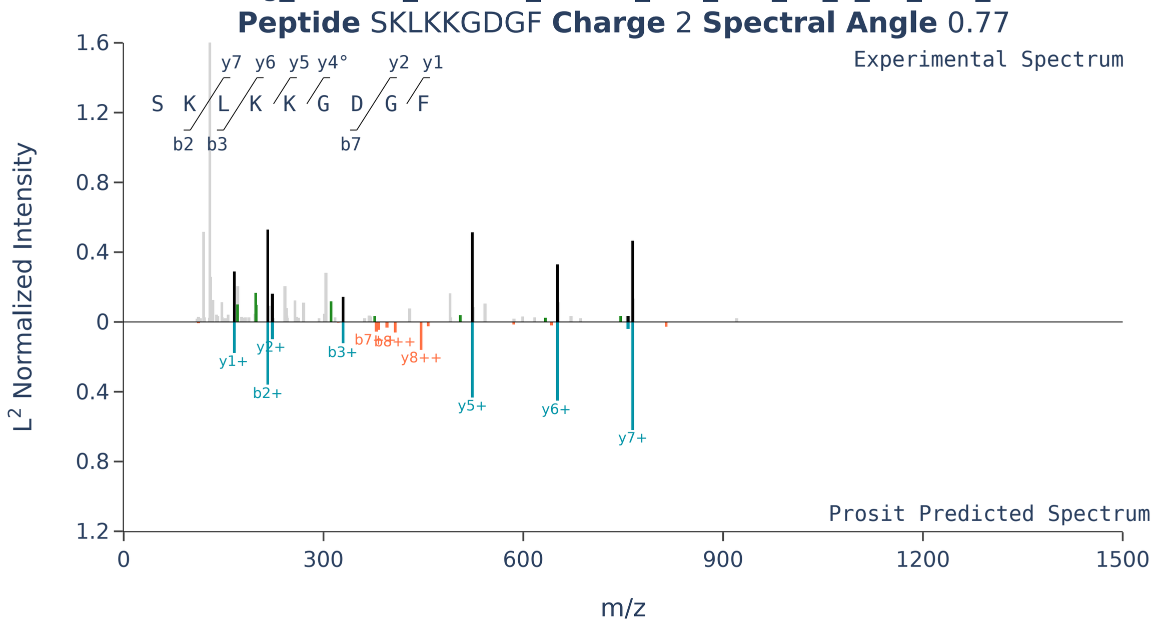

**Source** Ncheng_210623_230623_HFGoe_FFH_20S_25_1_A2_24h_R2 **Scan** 8857
**Peptide** KVDGDARGGAALSIRH **Charge** 3 **Spectral Angle** 0.38

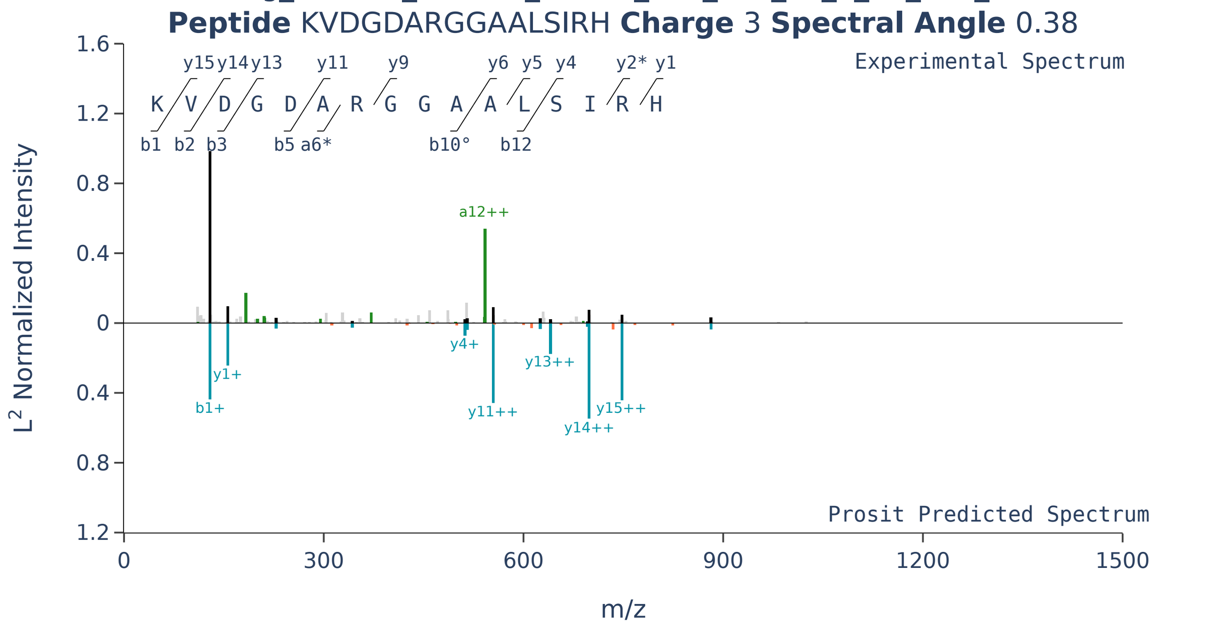


**Source** Ncheng_210623_230623_HFGoe_FFH_20S_25_1_A2_24h_R1 **Scan** 6626
**Peptide** KERAKPEIIK **Charge** 3 **Spectral Angle** 0.77

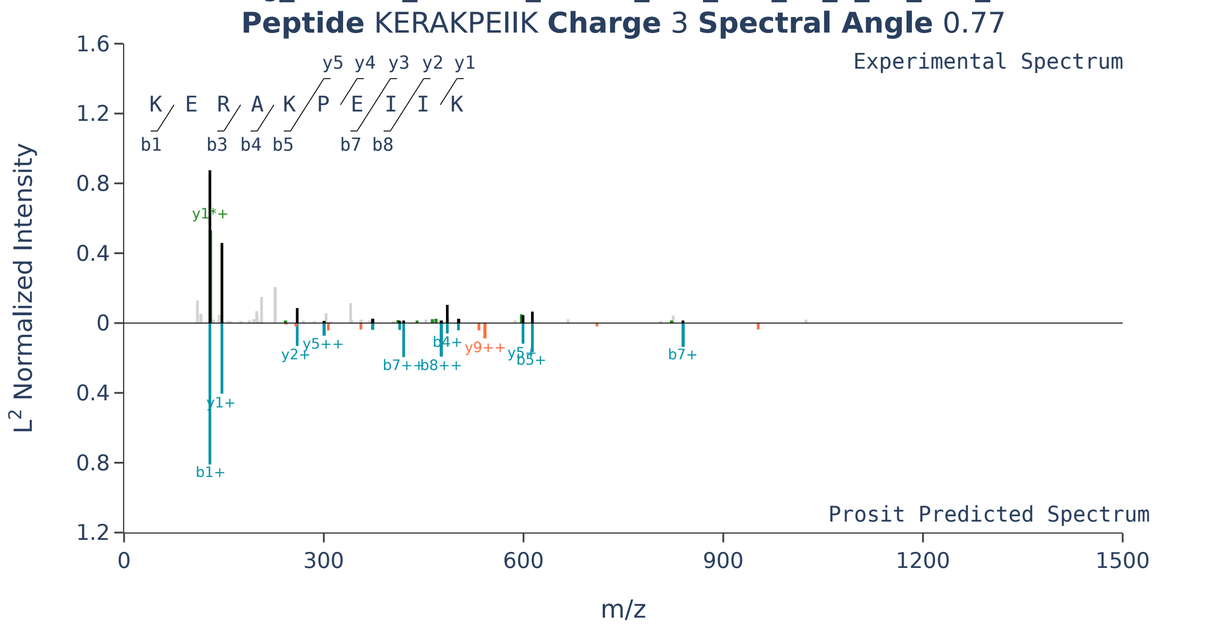


**Source** Ncheng_210623_230623_HFGoe_FFH_20S_25_1_A2_2h_R1 **Scan** 29205
**Peptide** VALPVVREFINRVKE **Charge** 3 **Spectral Angle** 0.43

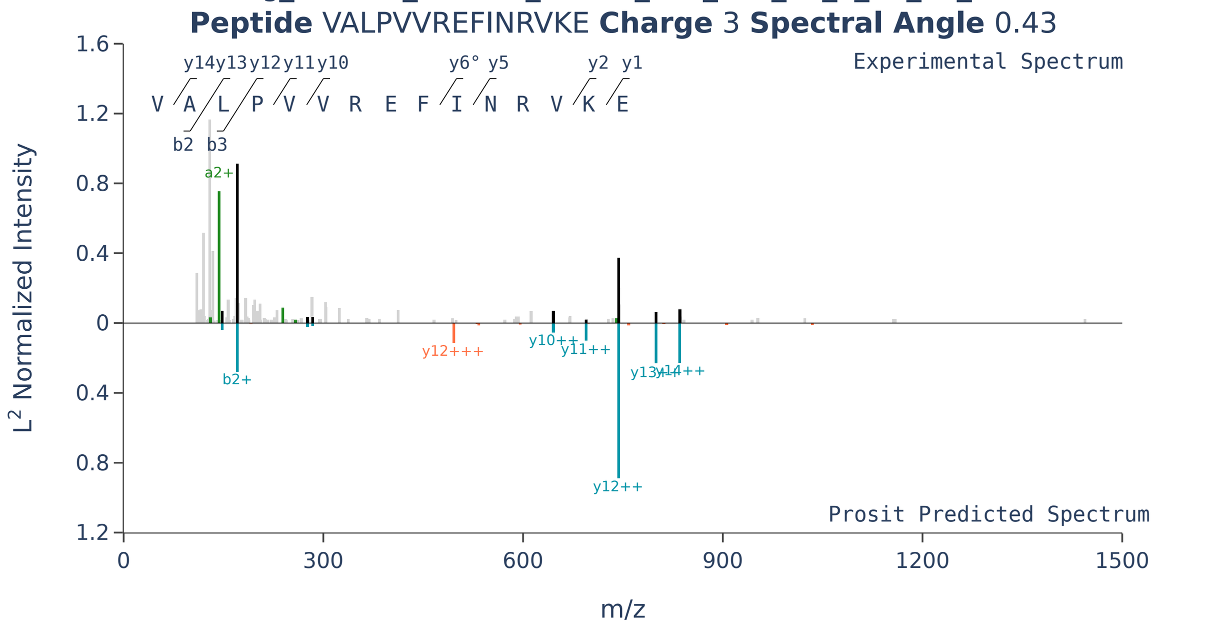


**Source** Ncheng_210623_230623_HFGoe_FFH_20S_25_1_A1_2h_R1 **Scan** 24295
**Peptide** QDVNRLLKQFDDMQR **Charge** 4 **Spectral Angle** 0.81

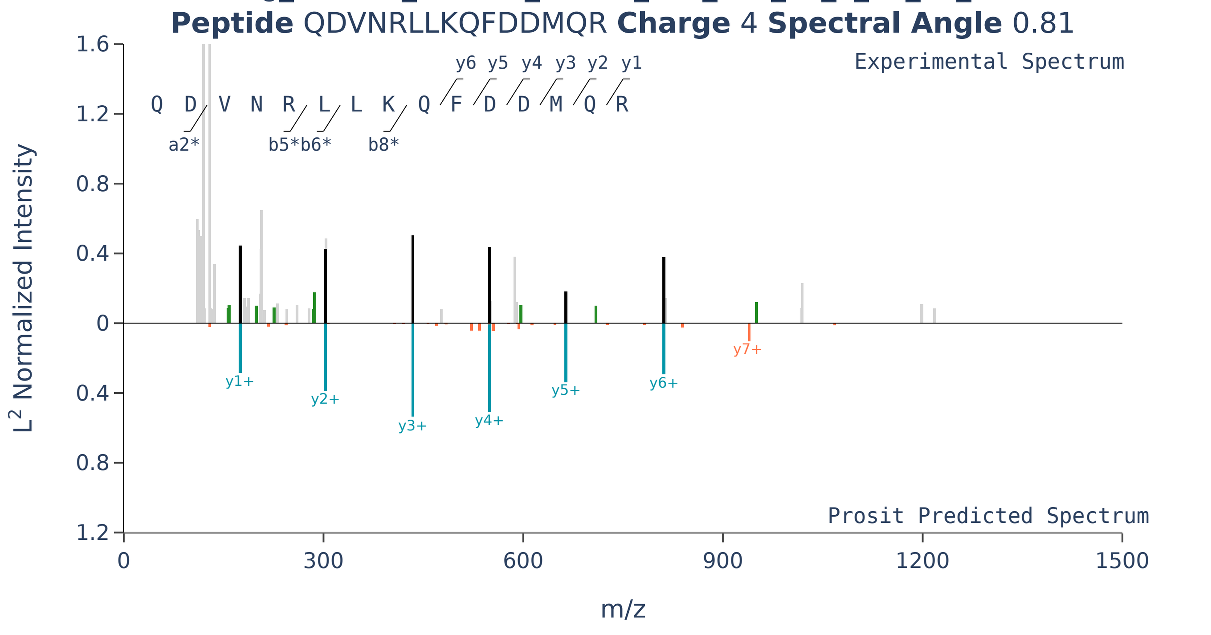


**Source** Ncheng_210623_230623_HFGoe_FFH_20S_25_1_A2_4h_R1 **Scan** 32548
**Peptide** KLASKLKKGDGFDLNDFLEQLRQMKN **Charge** 5 **Spectral Angle** 0.7

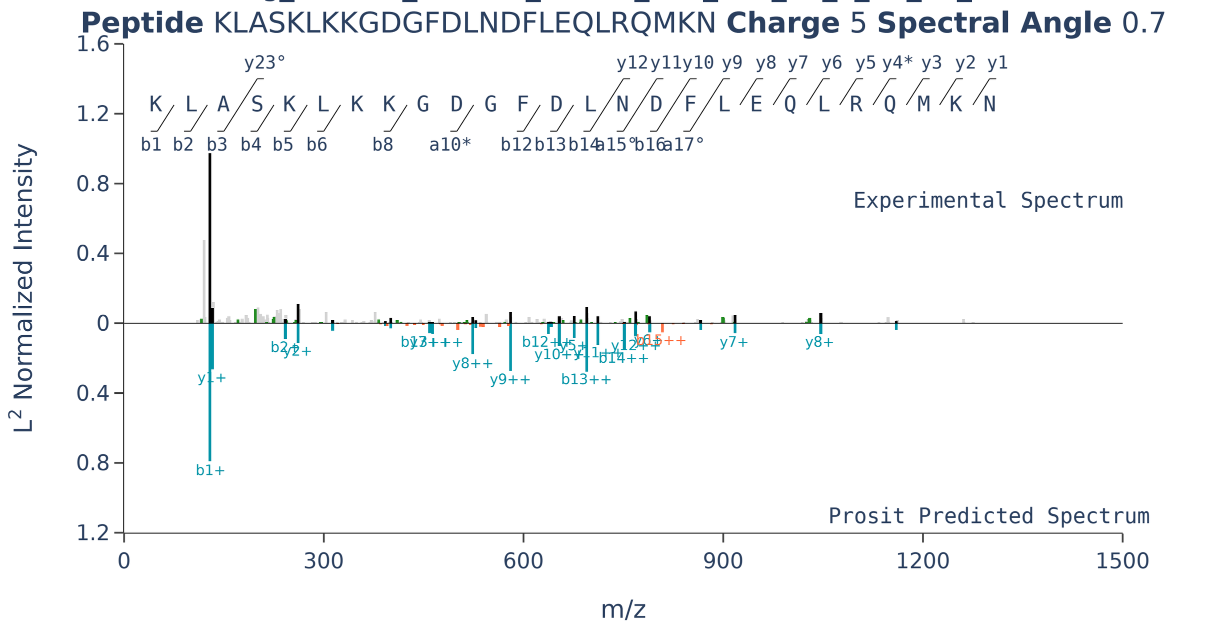


**Source** Ncheng_210623_230623_HFGoe_FFH_20S_25_1_A1_24h_R2 **Scan** 9914
**Peptide** KDTLREVRMA **Charge** 2 **Spectral Angle** 0.28

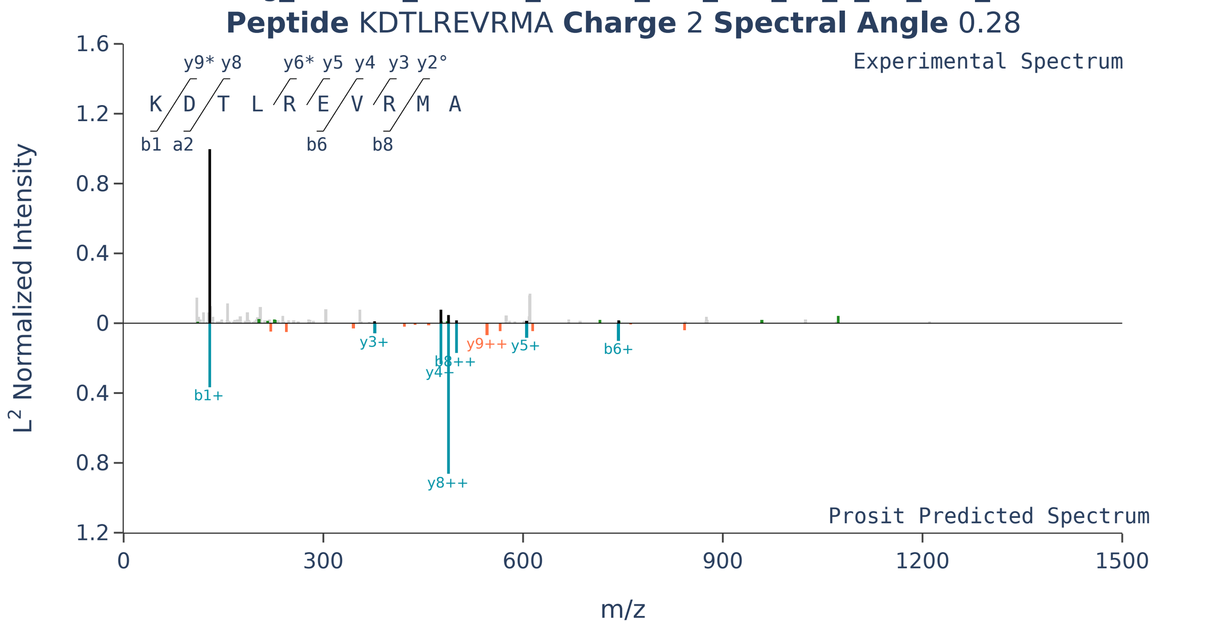


**Source** Ncheng_210623_230623_HFGoe_FFH_20S_25_1_A2_4h_R2 **Scan** 23348
**Peptide** NLTDRLSRTLRNISG **Charge** 2 **Spectral Angle** 0.74

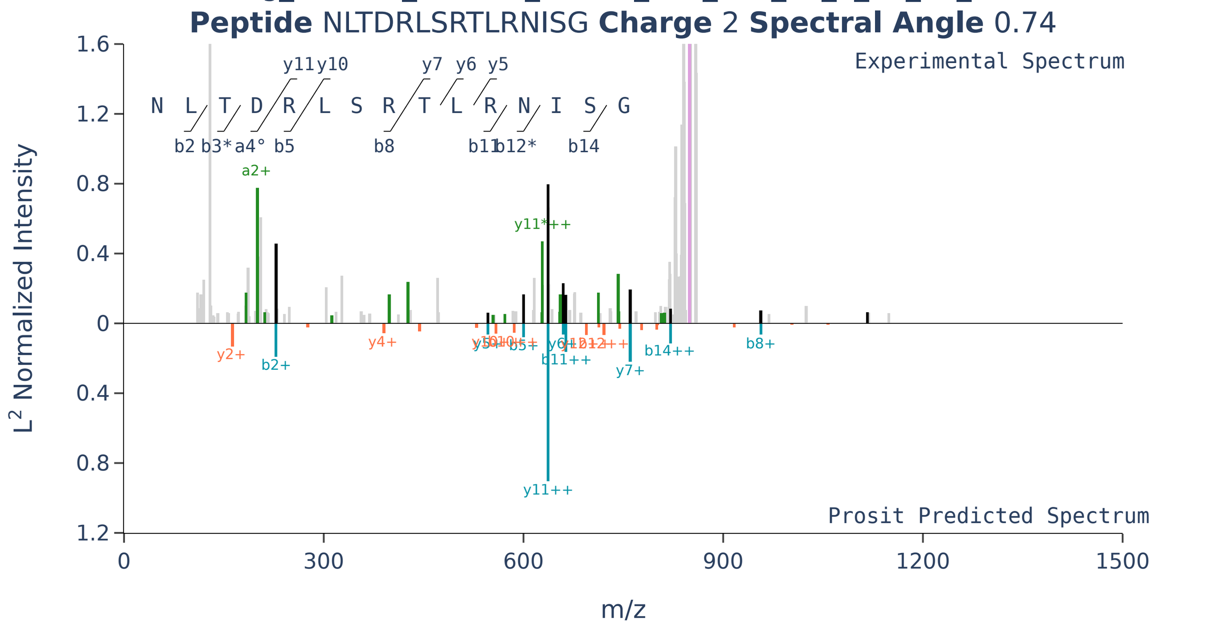


**Source** Ncheng_210623_230623_HFGoe_FFH_20S_25_1_A2_4h_R1 **Scan** 21232
**Peptide** AGLQGAGKTTSVGKLGKFL **Charge** 2 **Spectral Angle** 0.64

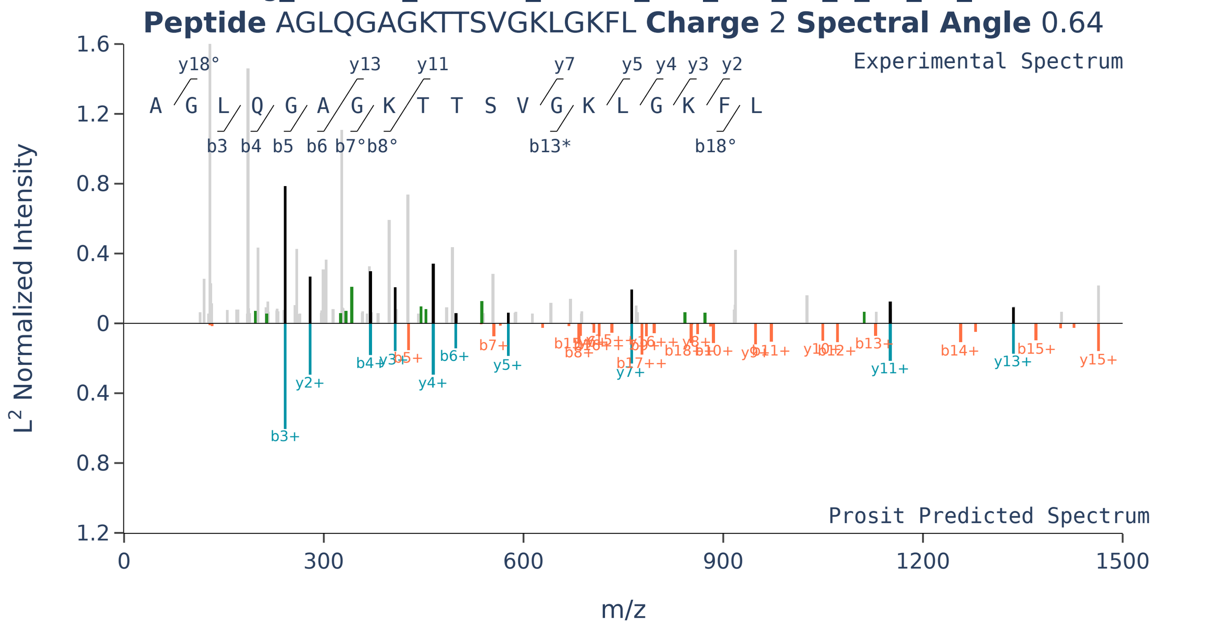


**Source** Ncheng_210623_230623_HFGoe_FFH_20S_25_1_A1_24h_R1 **Scan** 10301
**Peptide** REFINRVKEKAVGHEV **Charge** 3 **Spectral Angle** 0.55

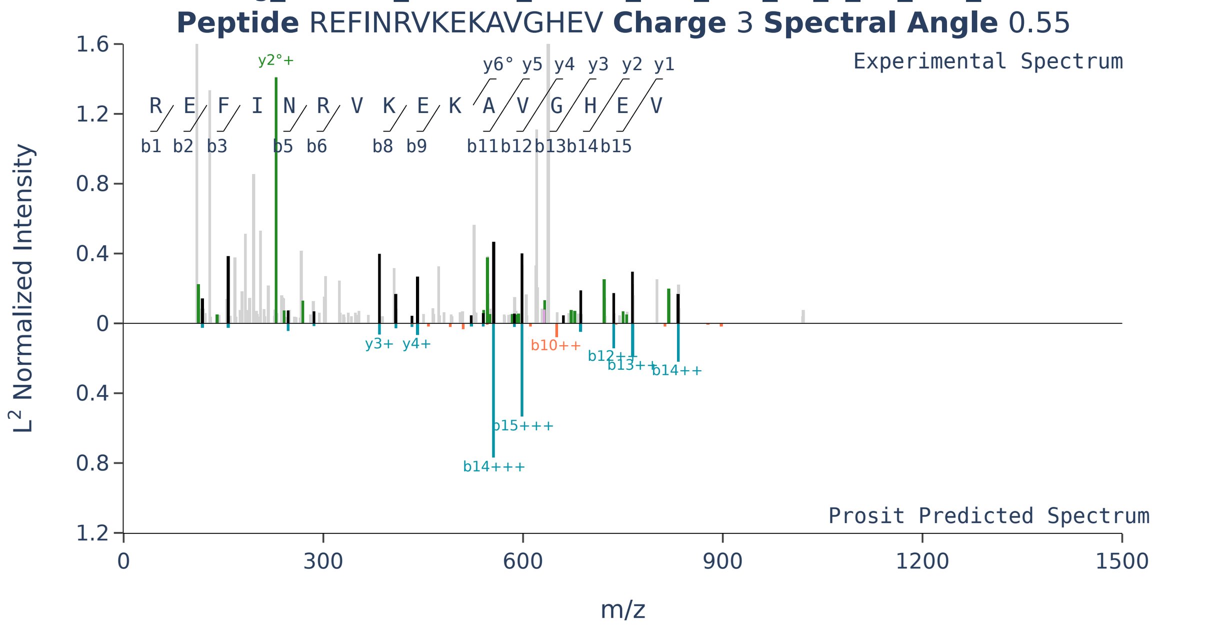


**Source** Ncheng_210623_230623_HFGoe_FFH_20S_25_1_A2_4h_R2 **Scan** 23925
**Peptide** LVNAALKEAKLKFYDV **Charge** 4 **Spectral Angle** 0.65

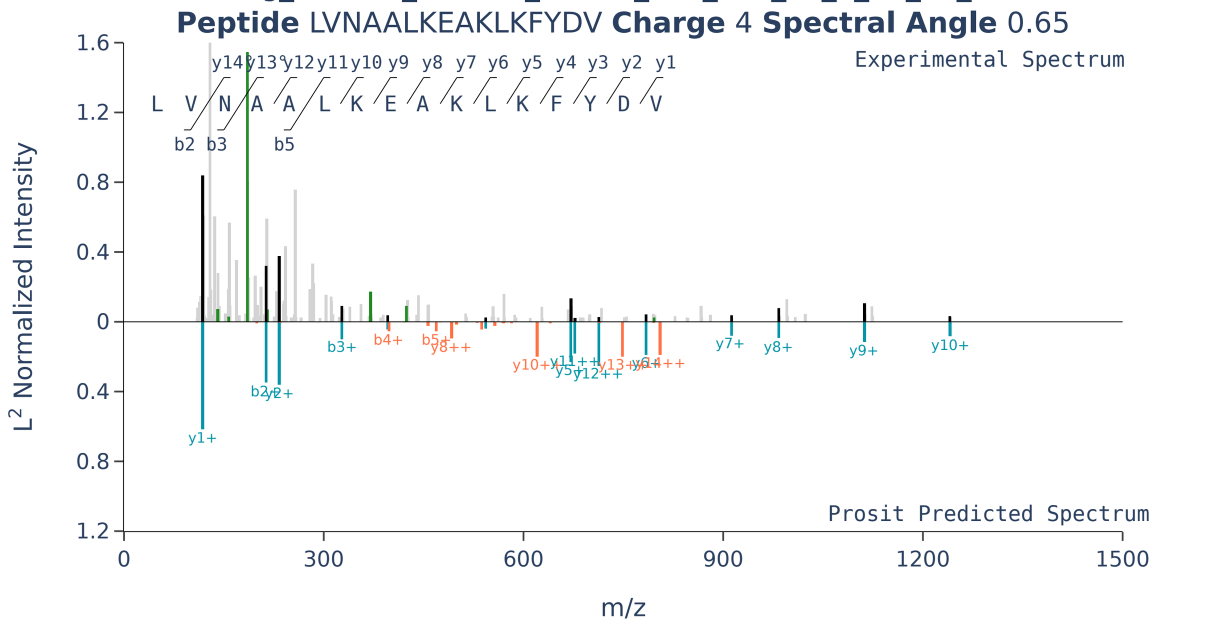


**Source** Ncheng_210623_230623_HFGoe_FFH_20S_25_1_A1_24h_R1 **Scan** 14430
**Peptide** KVDGDARGGAALSIRHITG **Charge** 5 **Spectral Angle** 0.64

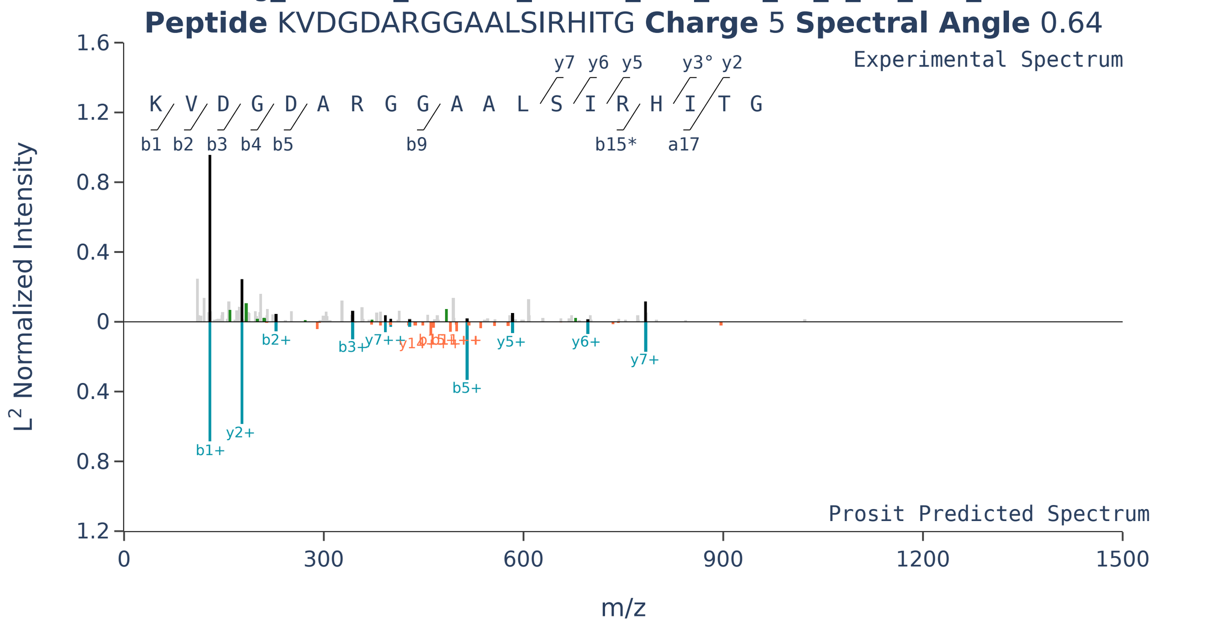


**Source** Ncheng_210623_230623_HFGoe_FFH_20S_25_1_A1_24h_R1 **Scan** 6942
**Peptide** LRQMKNMG **Charge** 2 **Spectral Angle** 0.71

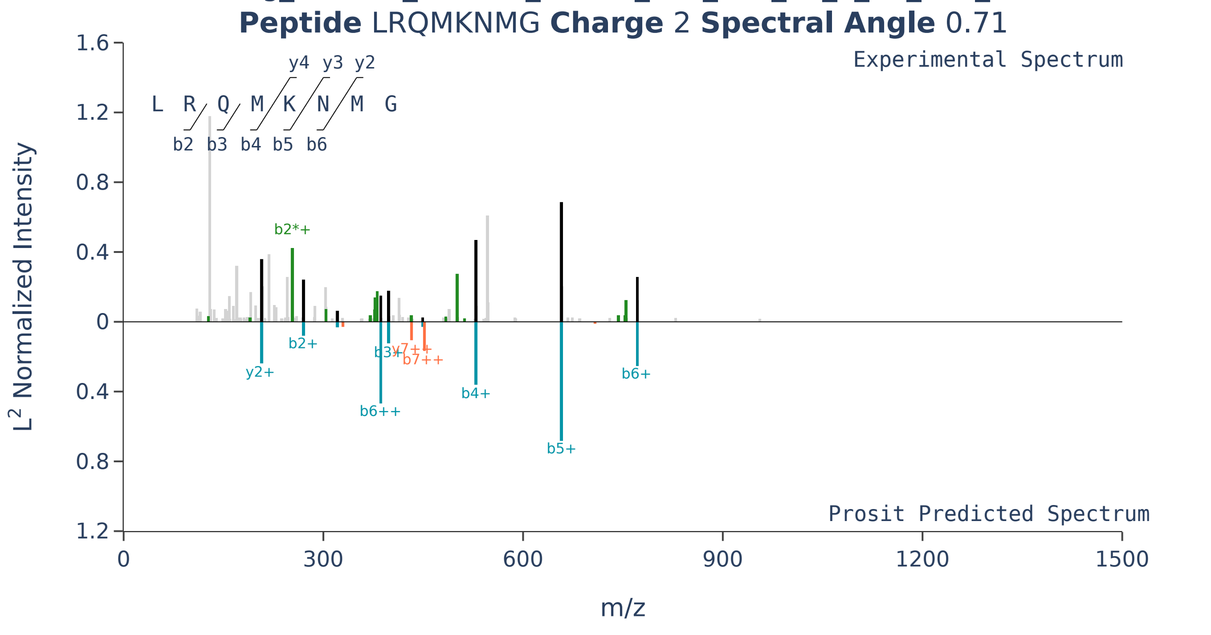


**Source** Ncheng_210623_230623_HFGoe_FFH_20S_25_1_A1_24h_R2 **Scan** 17609
**Peptide** NVKDTLREVRMA **Charge** 3 **Spectral Angle** 0.69

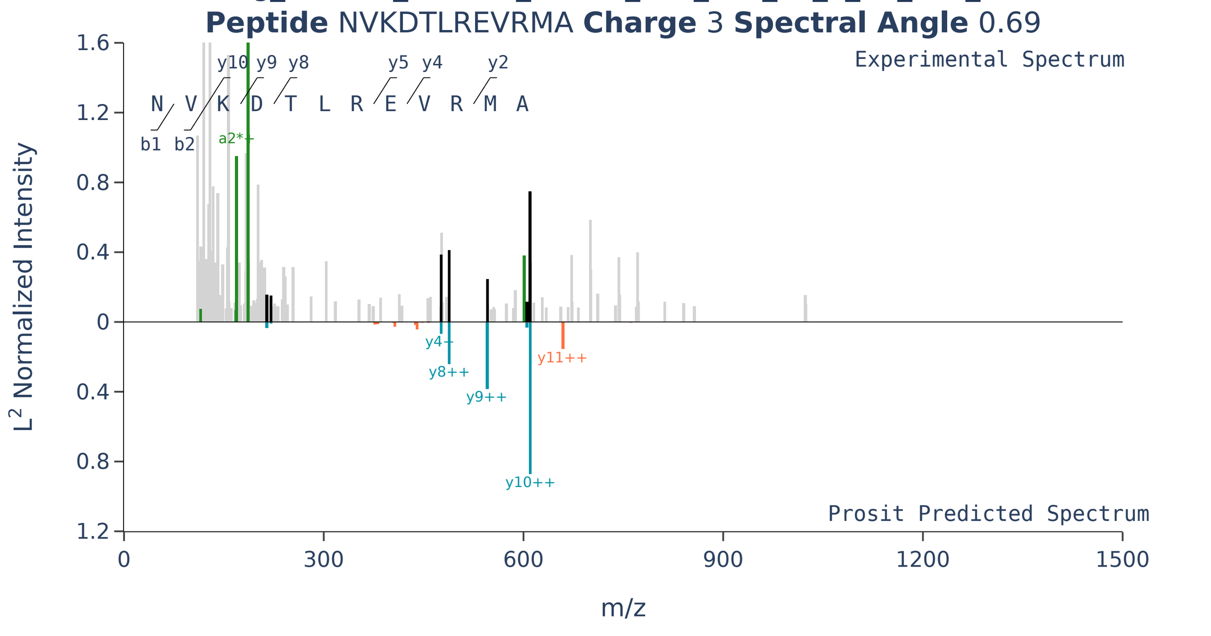


**Source** Ncheng_210623_230623_HFGoe_FFH_20S_25_1_A2_4h_R1 **Scan** 28021
**Peptide** ASLMGKLPGMGQIPDNVKSQM **Charge** 3 **Spectral Angle** 0.65

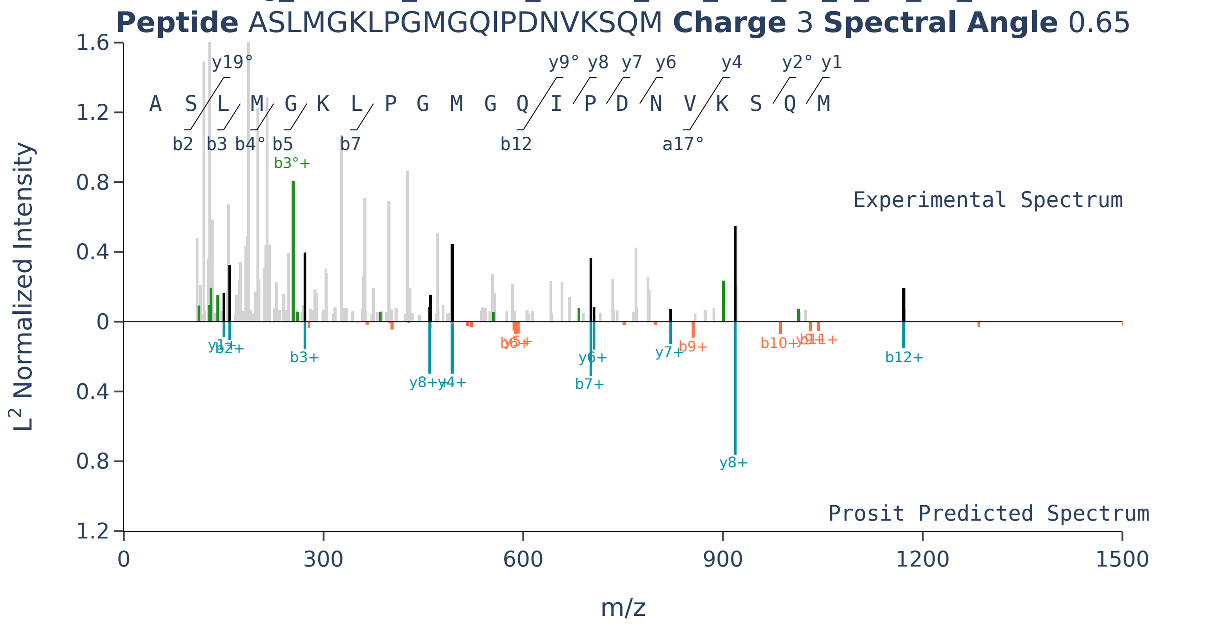


**Source** Ncheng_210623_230623_HFGoe_FFH_20S_25_1_A1_2h_R2 **Scan** 41135
**Peptide** GEENQTLNLAAQPPAVVLMAGLQG **Charge** 3 **Spectral Angle** 0.49

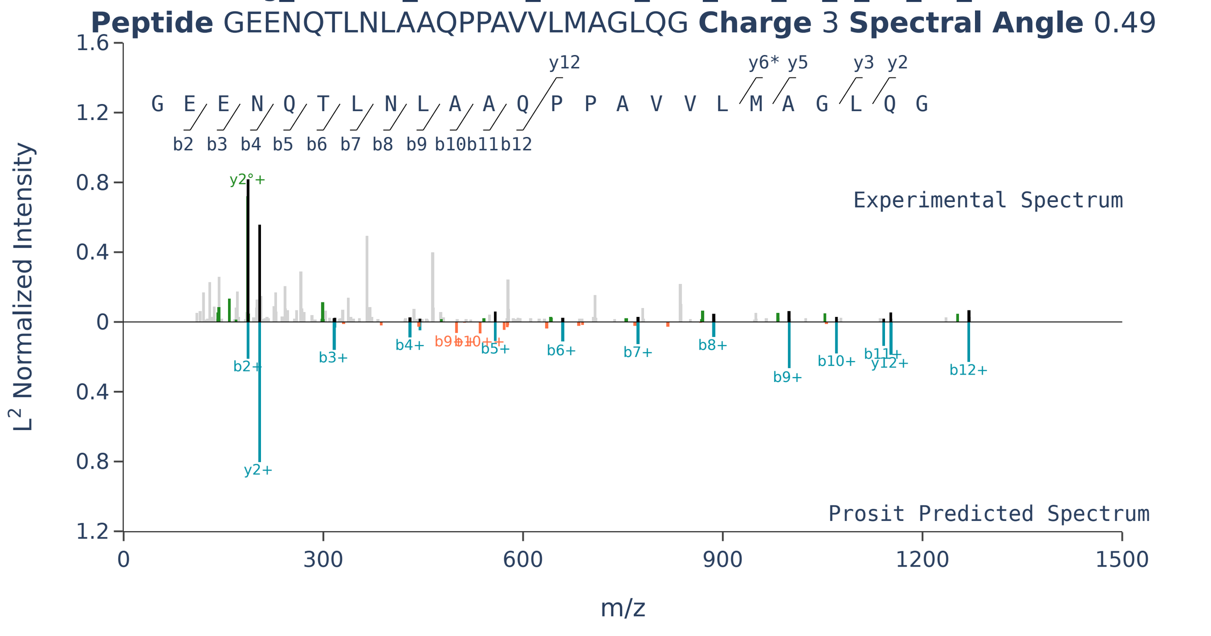

**Source** Ncheng_210623_230623_HFGoe_FFH_20S_25_1_A1_4h_R2 **Scan** 16601
**Peptide** LNRVKEKAVGHEVNKSLTPGQEFVKI **Charge** 6 **Spectral Angle** 0.76

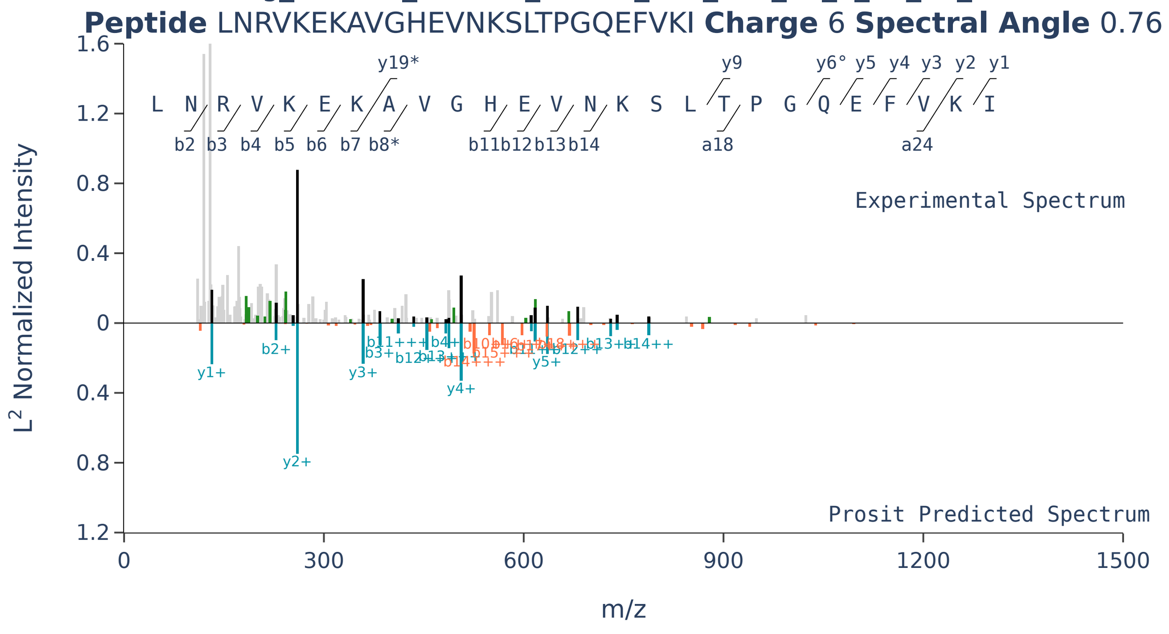


**Source** Ncheng_210623_230623_HFGoe_FFH_20S_25_1_A2_2h_R1 **Scan** 25324
**Peptide** GKLPGMGQIPDNVKSQMDDKVLV **Charge** 3 **Spectral Angle** 0.88

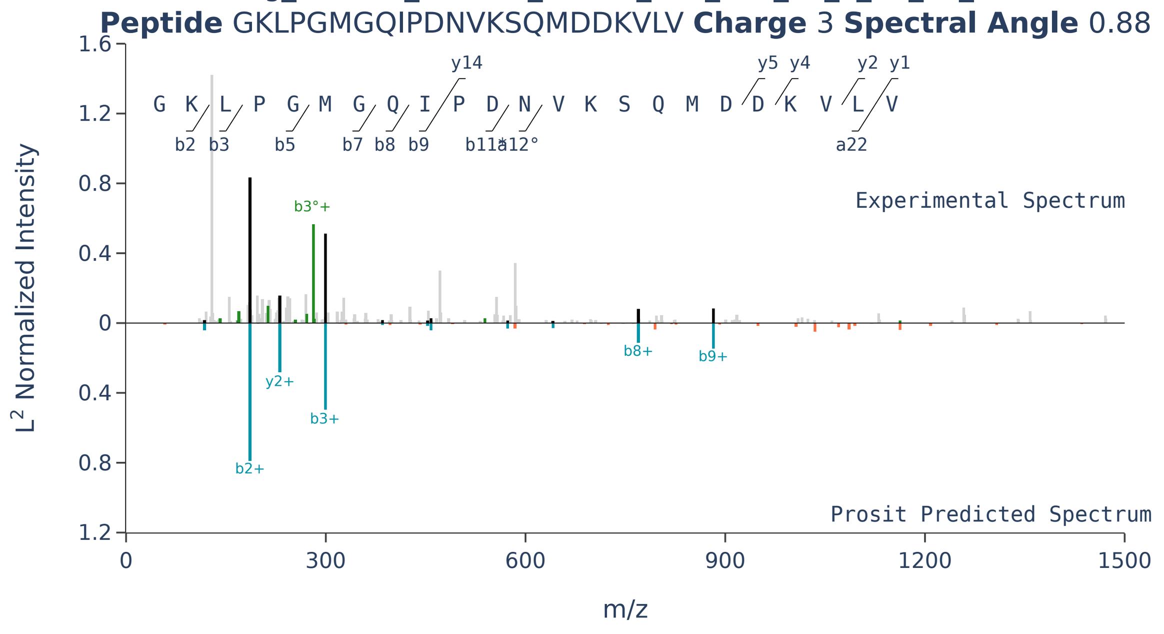


**Source** Ncheng_210623_230623_HFGoe_FFH_20S_25_1_A1_4h_R2 **Scan** 16113
**Peptide** DLESKVDRAQAEKLASKLKKGD **Charge** 6 **Spectral Angle** 0.71

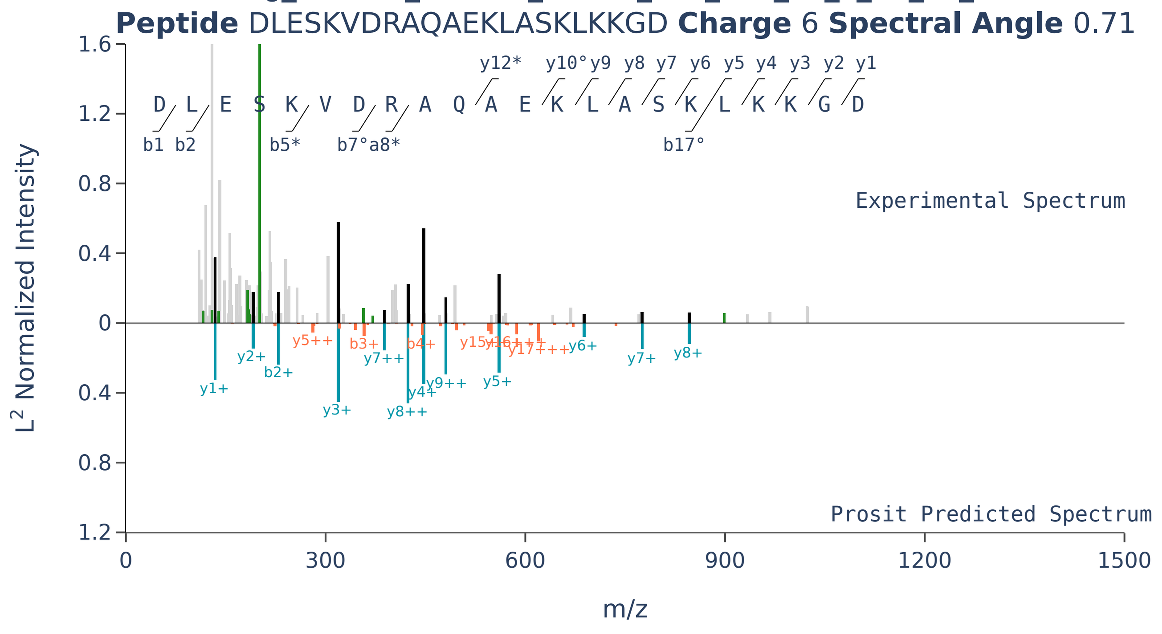


**Source** Ncheng_210623_230623_HFGoe_FFH_20S_25_1_A2_24h_R2 **Scan** 6082
**Peptide** KKMKKGGMAKMM **Charge** 3 **Spectral Angle** 0.91

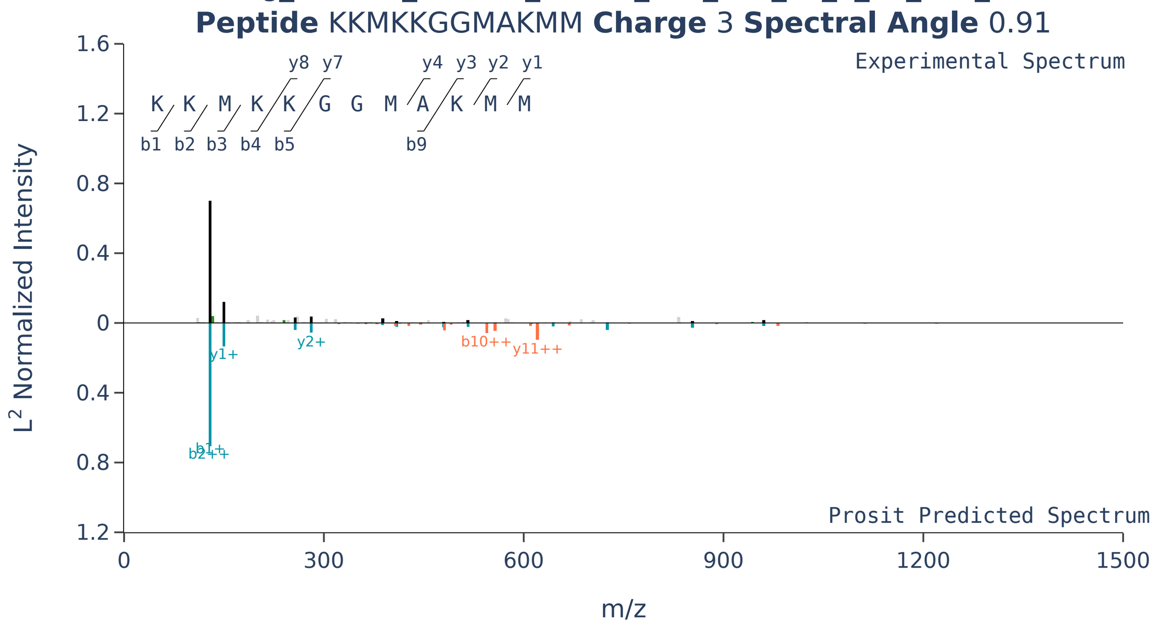


**Source** Ncheng_210623_230623_HFGoe_FFH_20S_25_1_A1_2h_R2 **Scan** 21360
**Peptide** LVNAALKEAKLKFY **Charge** 4 **Spectral Angle** 0.71

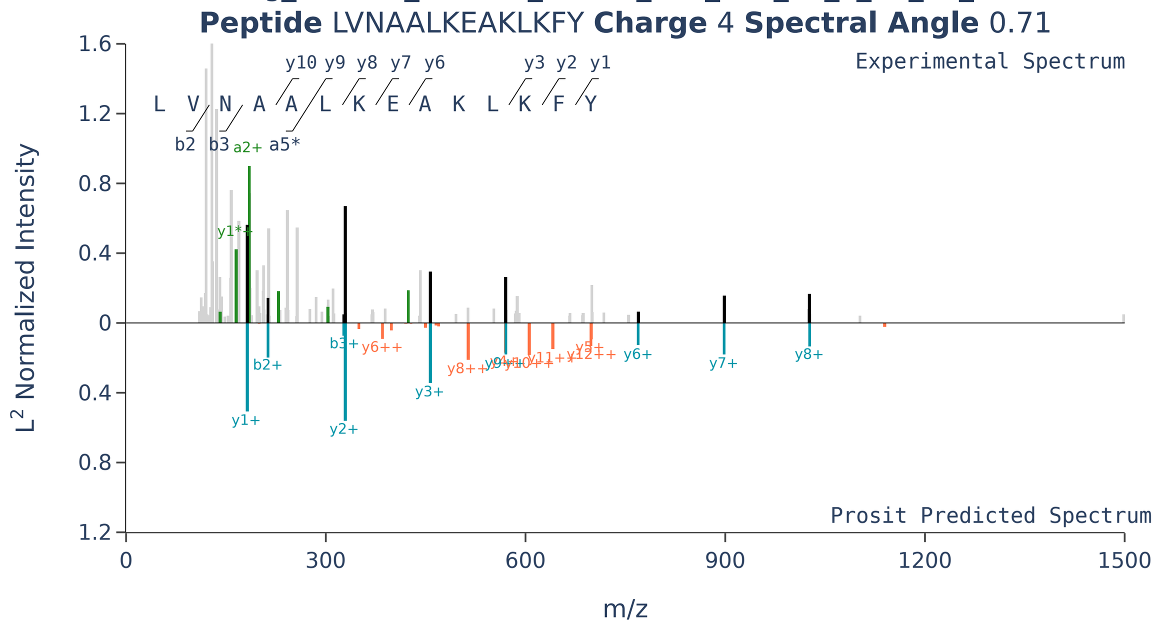


**Source** Ncheng_210623_230623_HFGoe_FFH_20S_25_1_A2_2h_R2 **Scan** 35611
**Peptide** DKVLVRMEAIINSMTMKE **Charge** 3 **Spectral Angle** 0.69

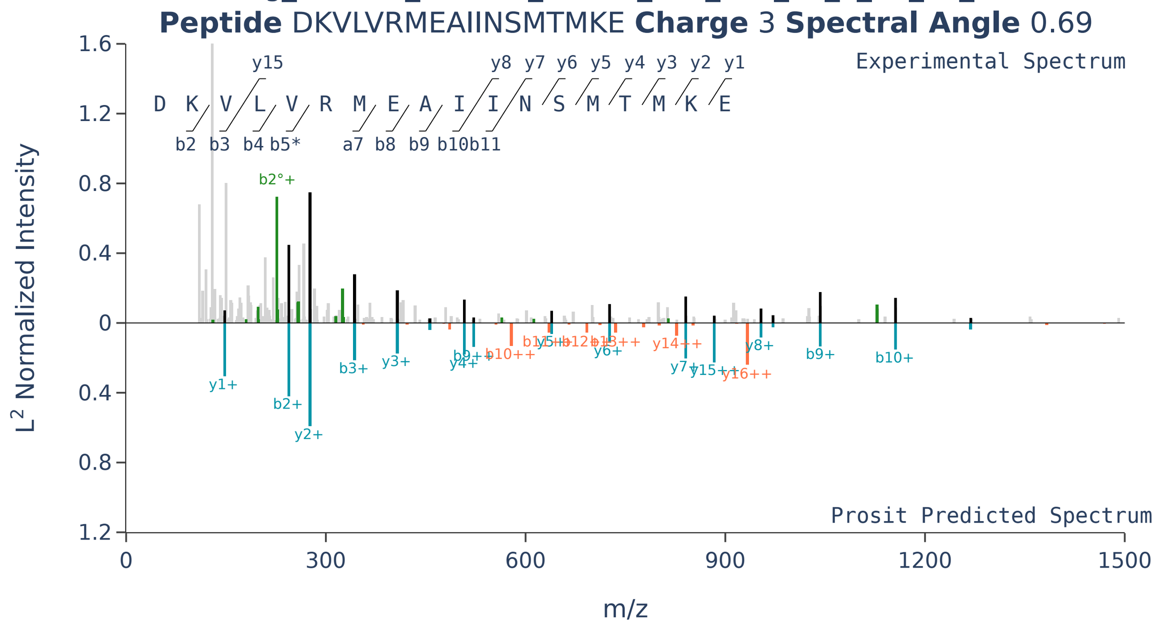


**Source** Ncheng_210623_230623_HFGoe_FFH_20S_25_1_A2_24h_R1 **Scan** 5351
**Peptide** RHITGKPIK **Charge** 3 **Spectral Angle** 0.78

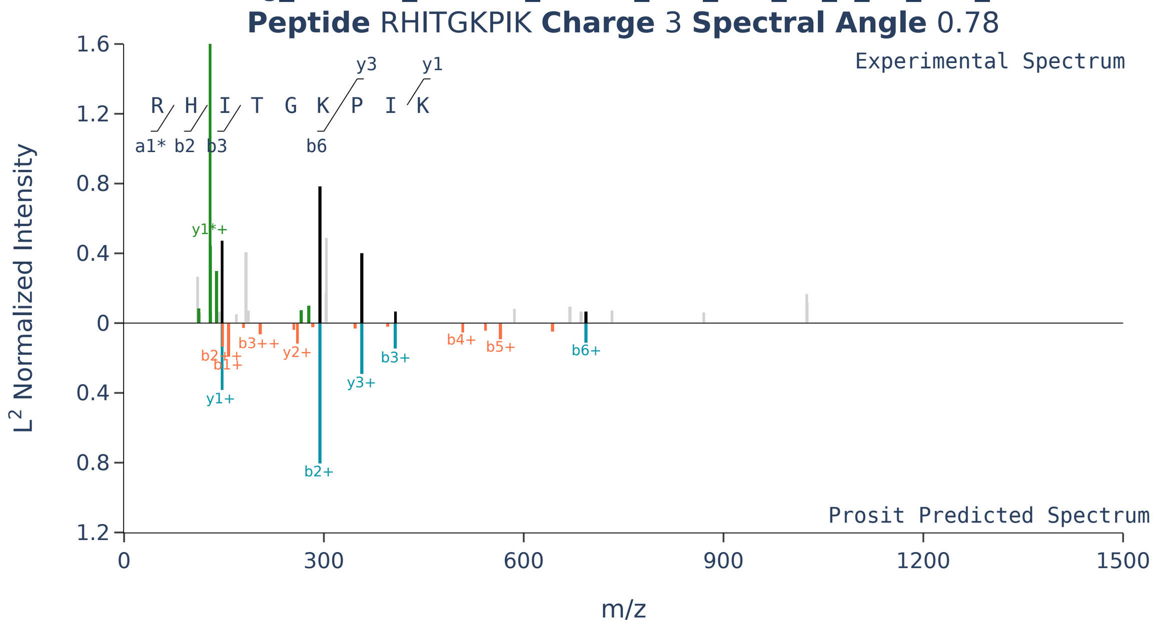


**Source** Ncheng_210623_230623_HFGoe_FFH_20S_25_1_A2_24h_R1 **Scan** 10050
**Peptide** DDMQRMMKKMKKG **Charge** 2 **Spectral Angle** 0.61

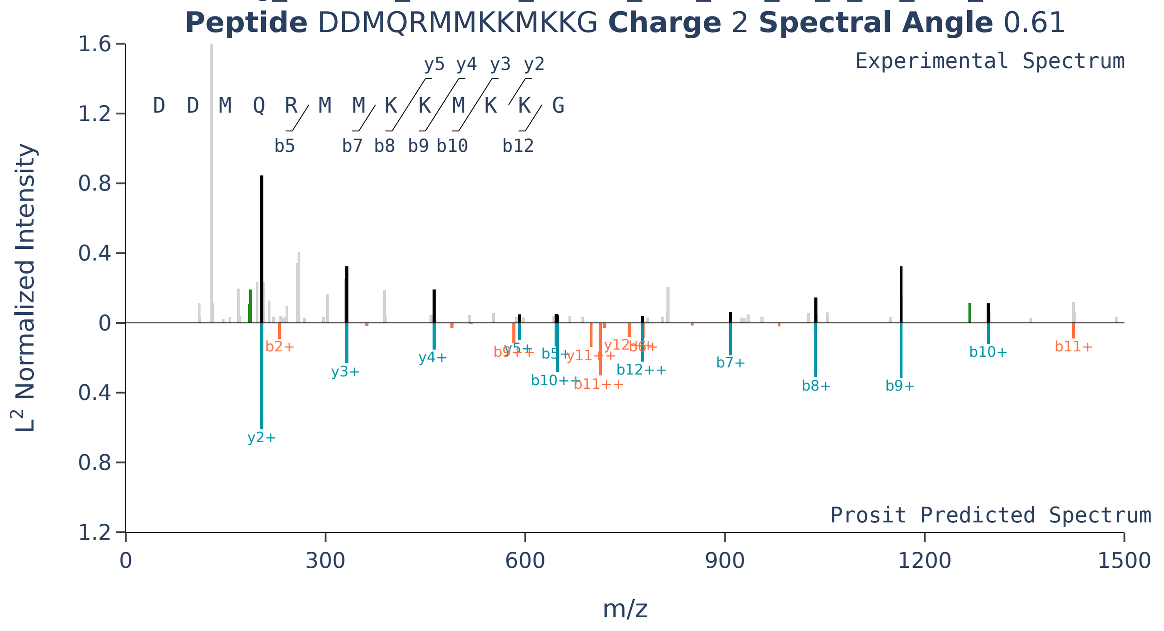


**Source** Ncheng_210623_230623_HFGoe_FFH_20S_25_1_A2_4h_R1 **Scan** 28626
**Peptide** RGRLTEDNVKDTLREVRMALL **Charge** 5 **Spectral Angle** 0.84

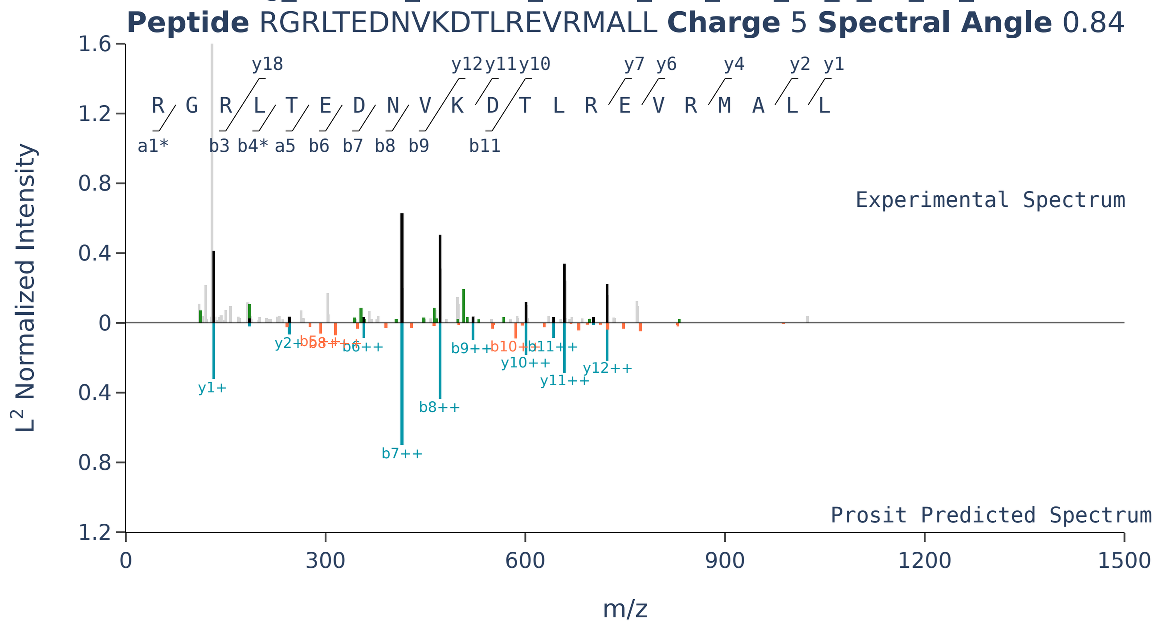


**Source** Ncheng_210623_230623_HFGoe_FFH_20S_25_1_A1_4h_R1 **Scan** 15786
**Peptide** RTLRNISGRGRLTEDNVKDTLREV **Charge** 5 **Spectral Angle** 0.74

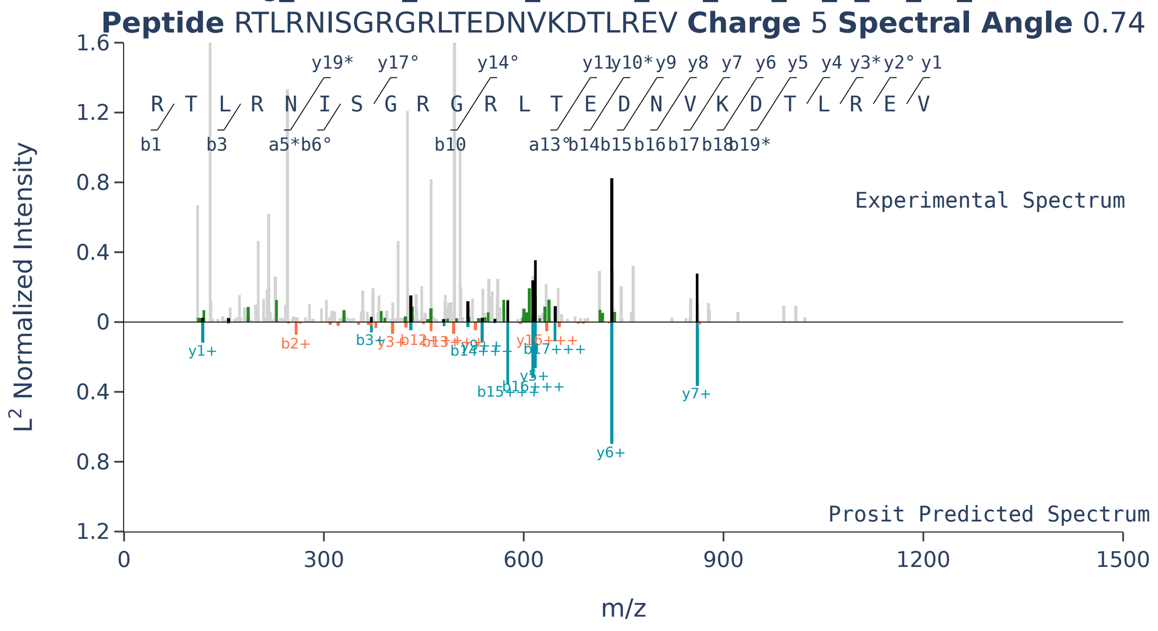


**Source** Ncheng_210623_230623_HFGoe_FFH_20S_25_1_A1_2h_R1 **Scan** 25626
**Peptide** LMGKLPGMGQIPDNVKSQMDDKVLVR **Charge** 4 **Spectral Angle** 0.67

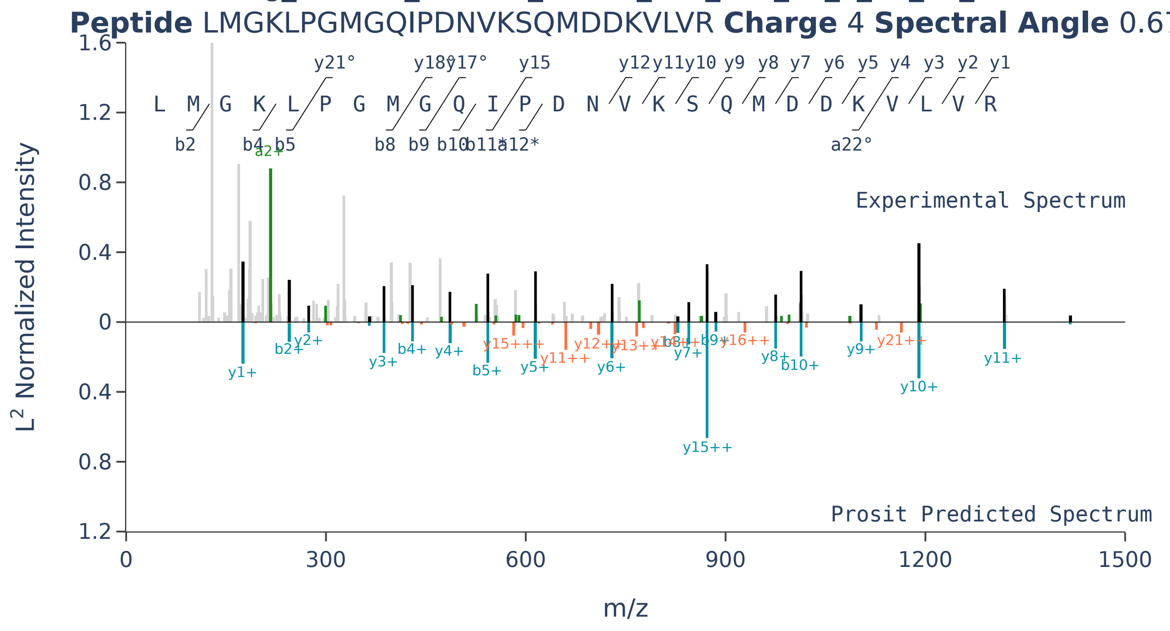


**Source** Ncheng_210623_230623_HFGoe_FFH_20S_25_1_A2_4h_R1 **Scan** 33375
**Peptide** LTEDNVKDTLREVRMALL **Charge** 3 **Spectral Angle** 0.71

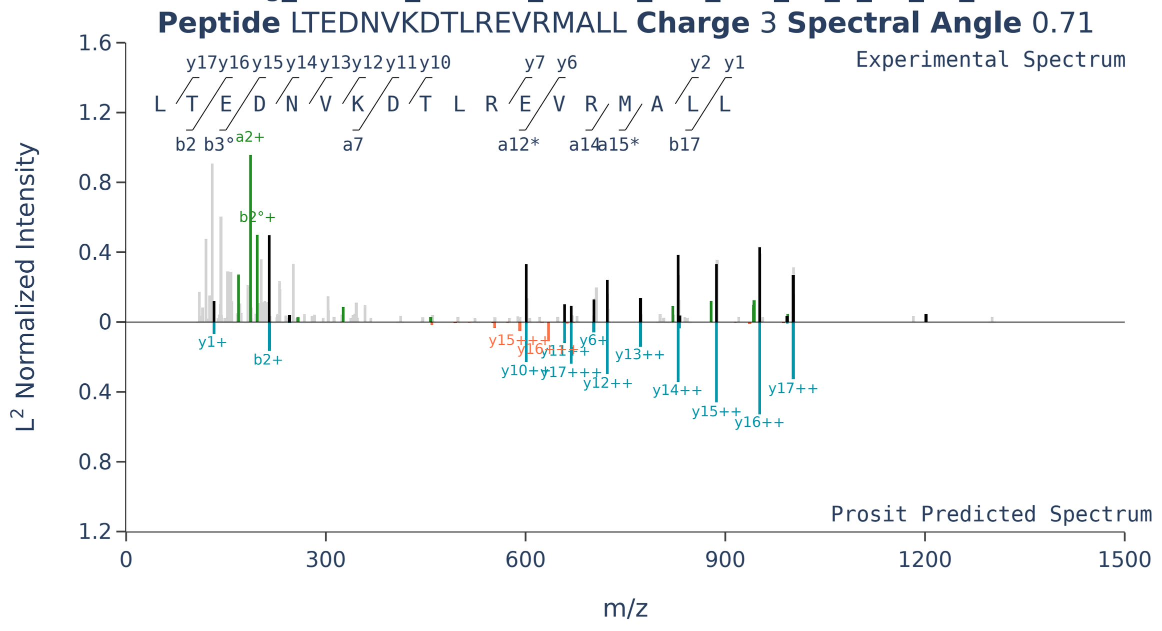


**Source** Ncheng_210623_230623_HFGoe_FFH_20S_25_1_A1_1h_R2 **Scan** 14220
**Peptide** VKIVRNELVA **Charge** 2 **Spectral Angle** 0.69

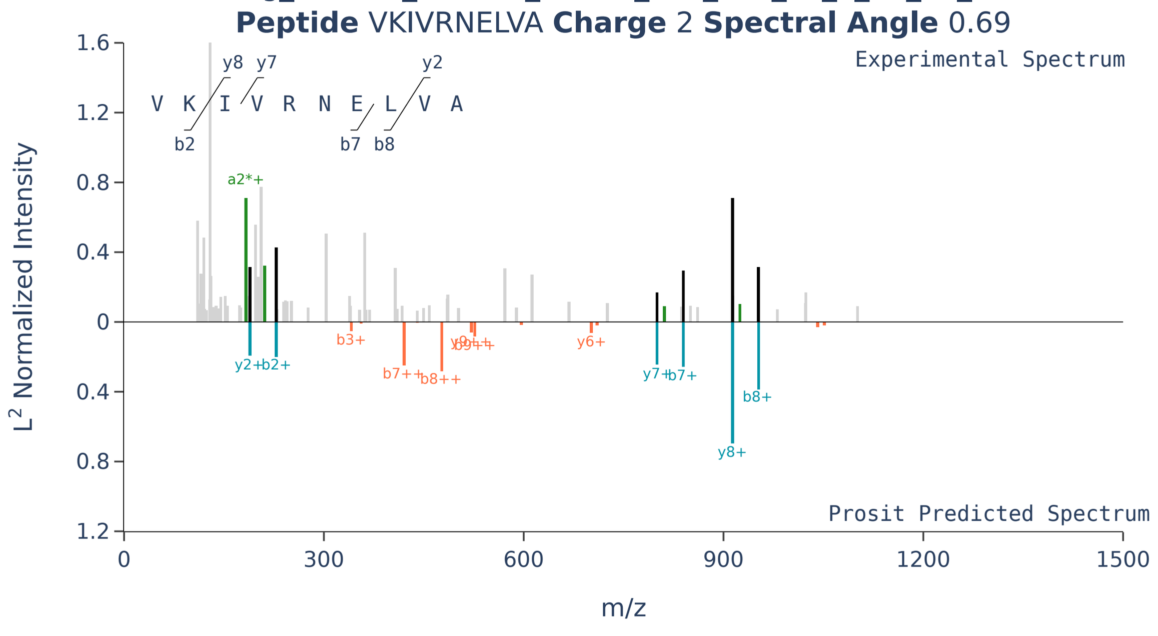


**Source** Ncheng_210623_230623_HFGoe_FFH_20S_25_1_A2_24h_R2 **Scan** 16079
**Peptide** EDIESKVDRAQAEKLASKLKK **Charge** 4 **Spectral Angle** 0.79

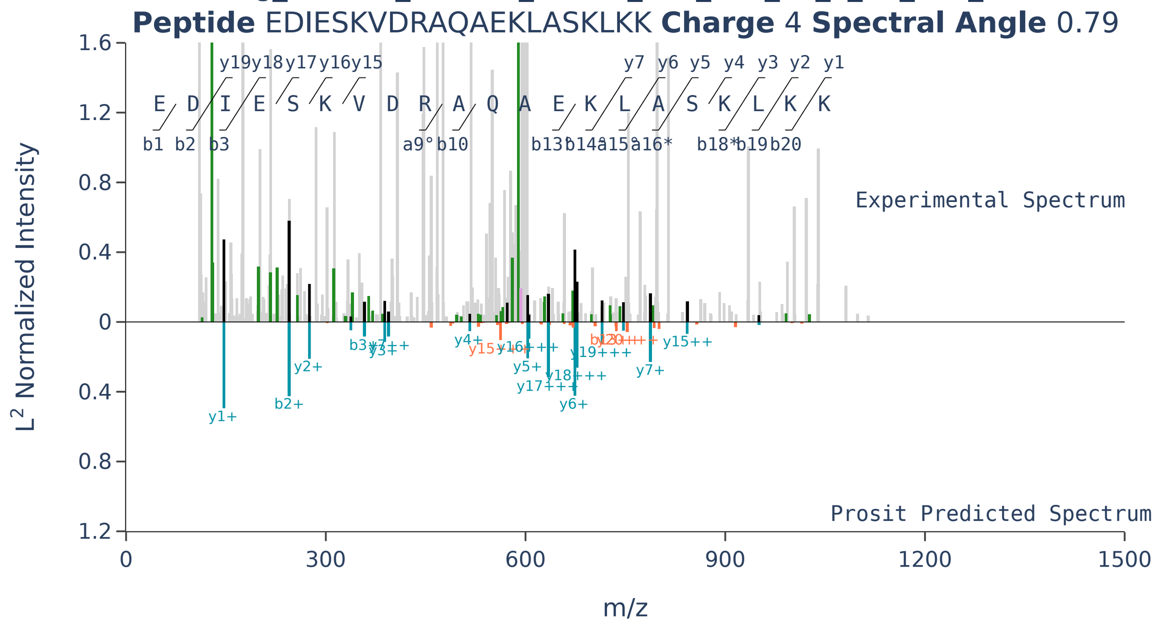


**Source** Ncheng_210623_230623_HFGoe_FFH_20S_25_1_A1_2h_R2 **Scan** 30269
**Peptide** EALPLTGVVLTKVDGDARGGAALSIRHLTG **Charge** 3 **Spectral Angle** 0.58

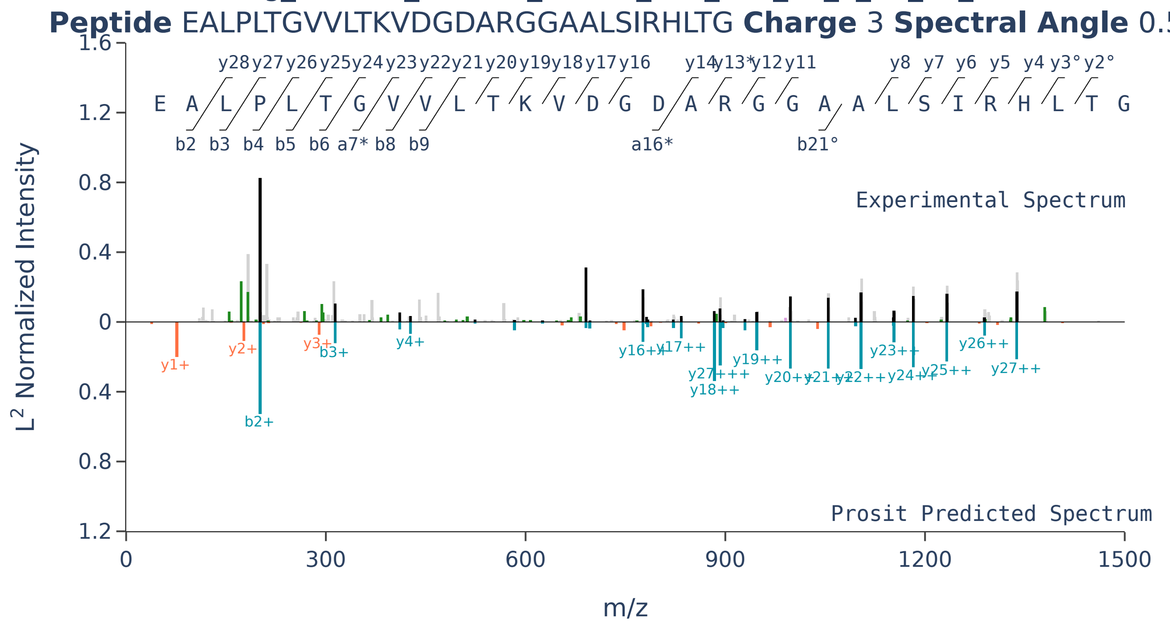

**Source** Ncheng_210623_230623_HFGoe_FFH_20S_25_1_A2_4h_R2 **Scan** 29978
**Peptide** KEAKLKFYDVLLV **Charge** 4 **Spectral Angle** 0.71

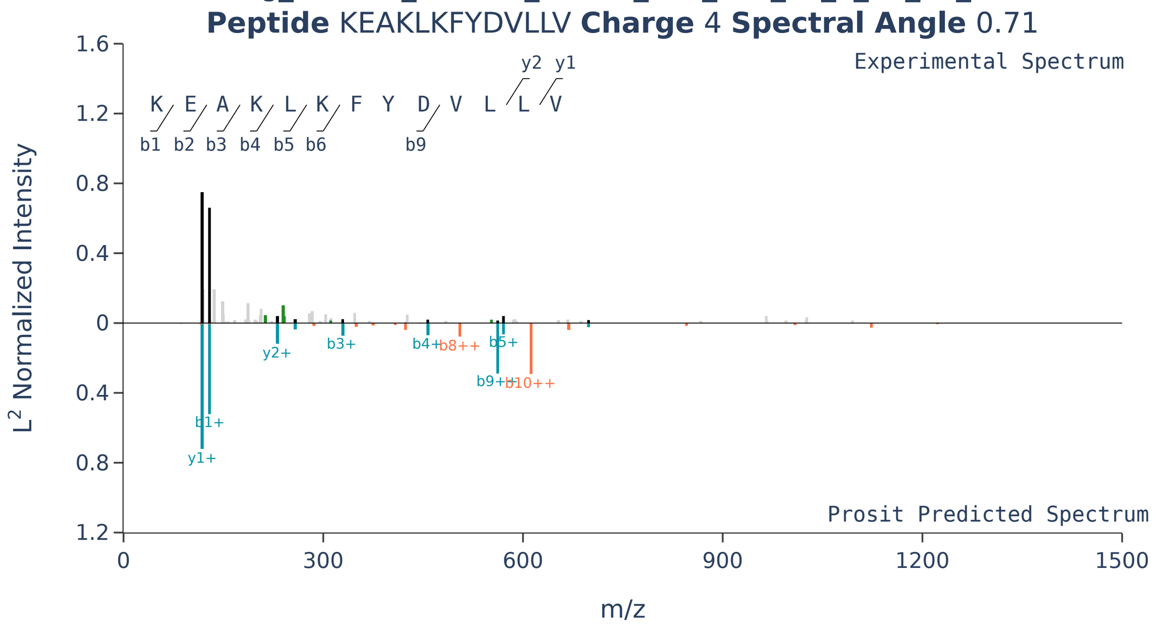


**Source** Ncheng_210623_230623_HFGoe_FFH_20S_25_1_A1_2h_R1 **Scan** 18590
**Peptide** GDARGGAALSIRHITGKPLKF **Charge** 5 **Spectral Angle** 0.66

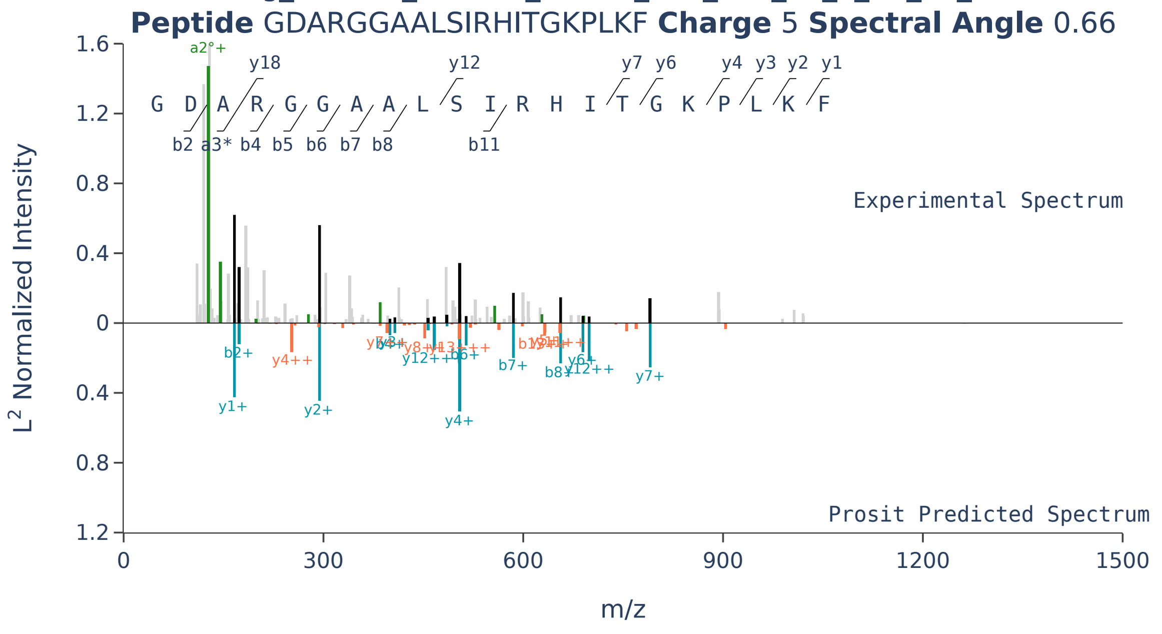


**Source** Ncheng_210623_230623_HFGoe_FFH_20S_25_1_A1_4h_R1 **Scan** 17333
**Peptide** RNISGRGRLTEDNVKDTLREVRM **Charge** 6 **Spectral Angle** 0.67

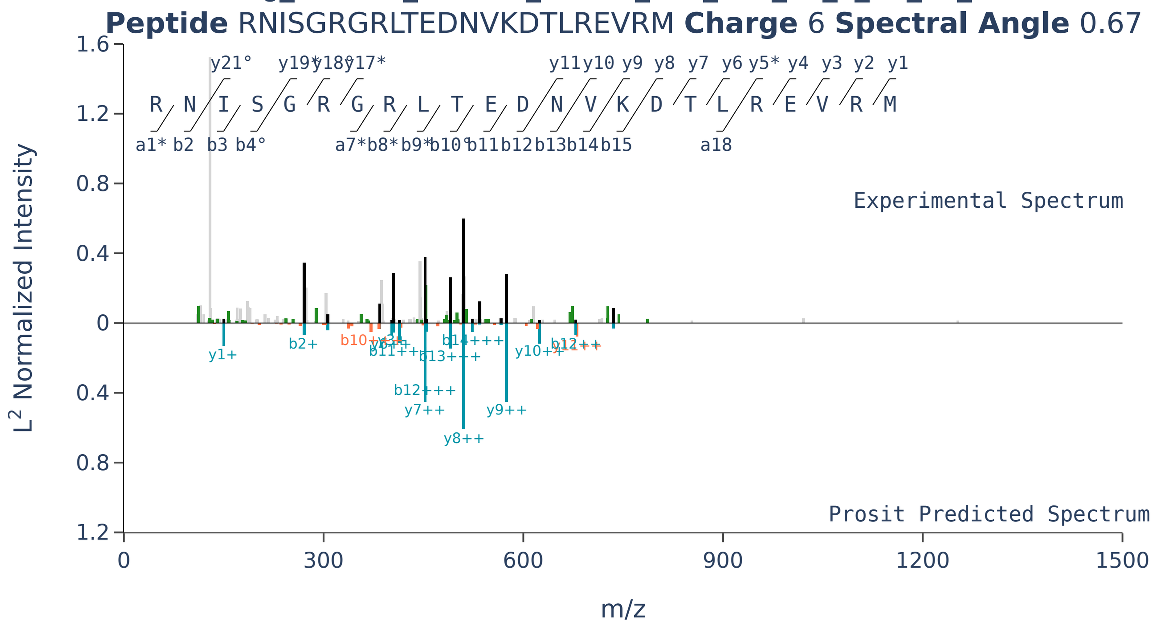


**Source** Ncheng_210623_230623_HFGoe_FFH_20S_25_1_A1_2h_R2 **Scan** 35215
**Peptide** ASKLKKGDGFDLNDFLEQL **Charge** 3 **Spectral Angle** 0.47

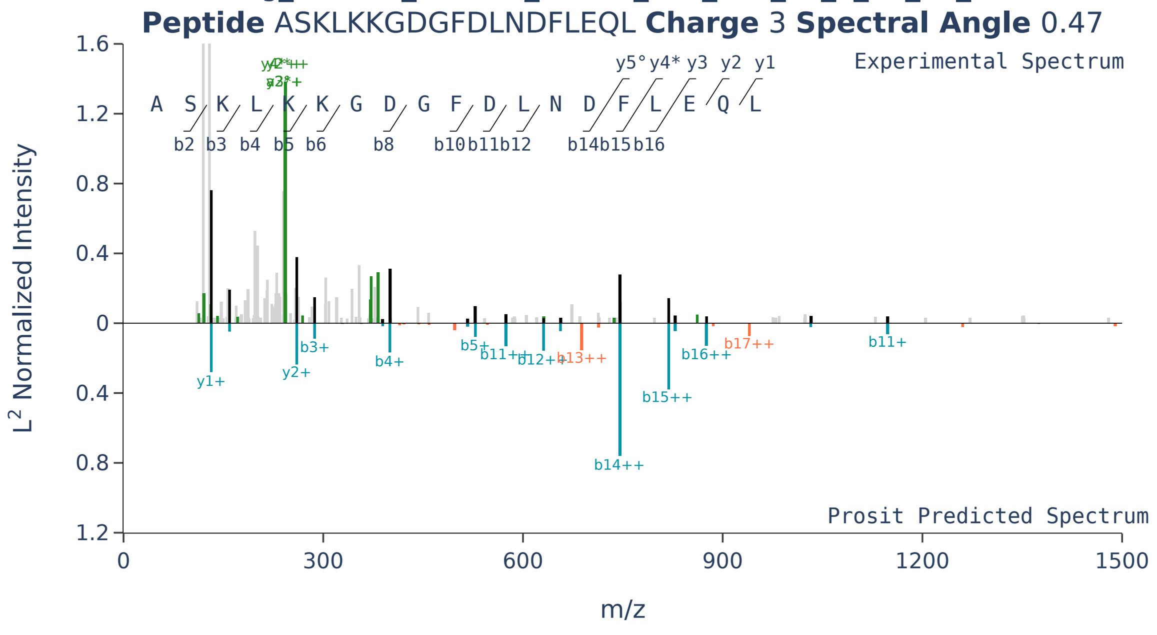


**Source** Ncheng_210623_230623_HFGoe_FFH_20S_25_1_A2_4h_R1 **Scan** 25282
**Peptide** VKEKAVGHEVNKSLTPGQEFVKIVRNEL **Charge** 4 **Spectral Angle** 0.74

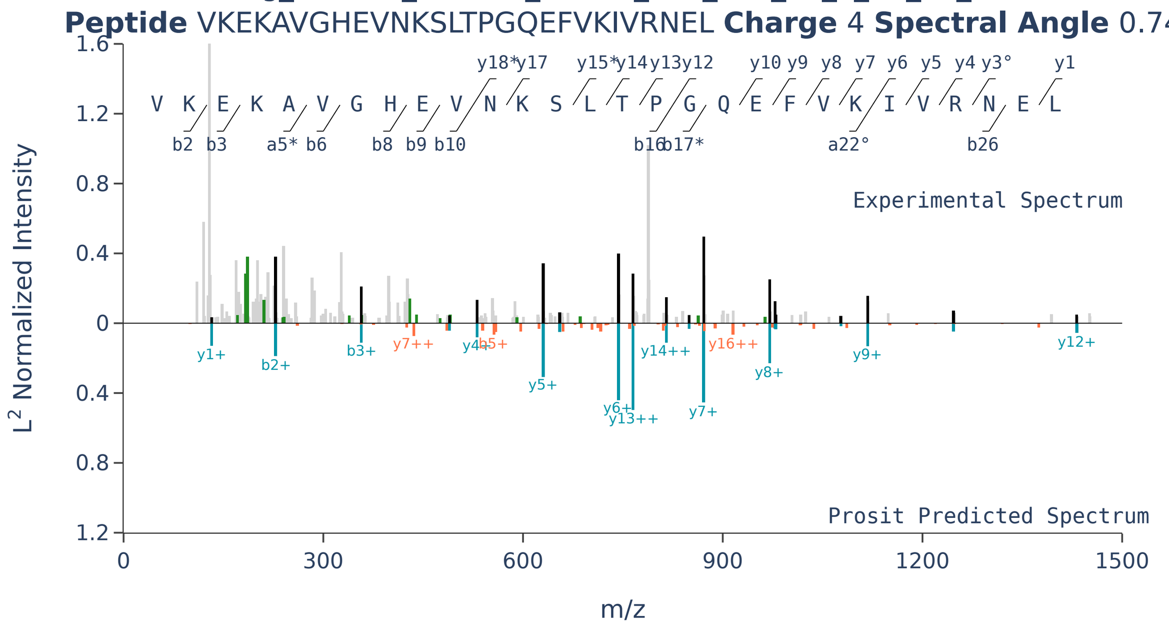


**Source** Ncheng_210623_230623_HFGoe_FFH_20S_25_1_A1_1h_R2 **Scan** 28917
**Peptide** VDFFPSDVGQKPVDIVN **Charge** 2 **Spectral Angle** 0.8

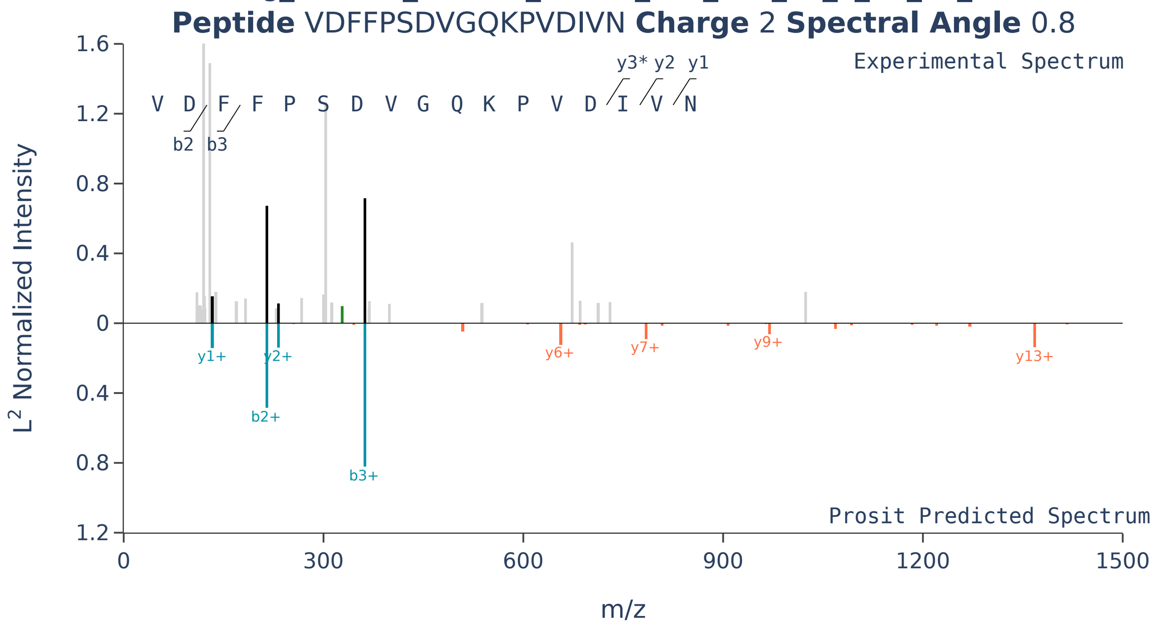


**Source** Ncheng_210623_230623_HFGoe_FFH_20S_25_1_A1_2h_R2 **Scan** 33603
**Peptide** RNELVAAMGEENQTLNLAAQPPAVVLM **Charge** 3 **Spectral Angle** 0.76

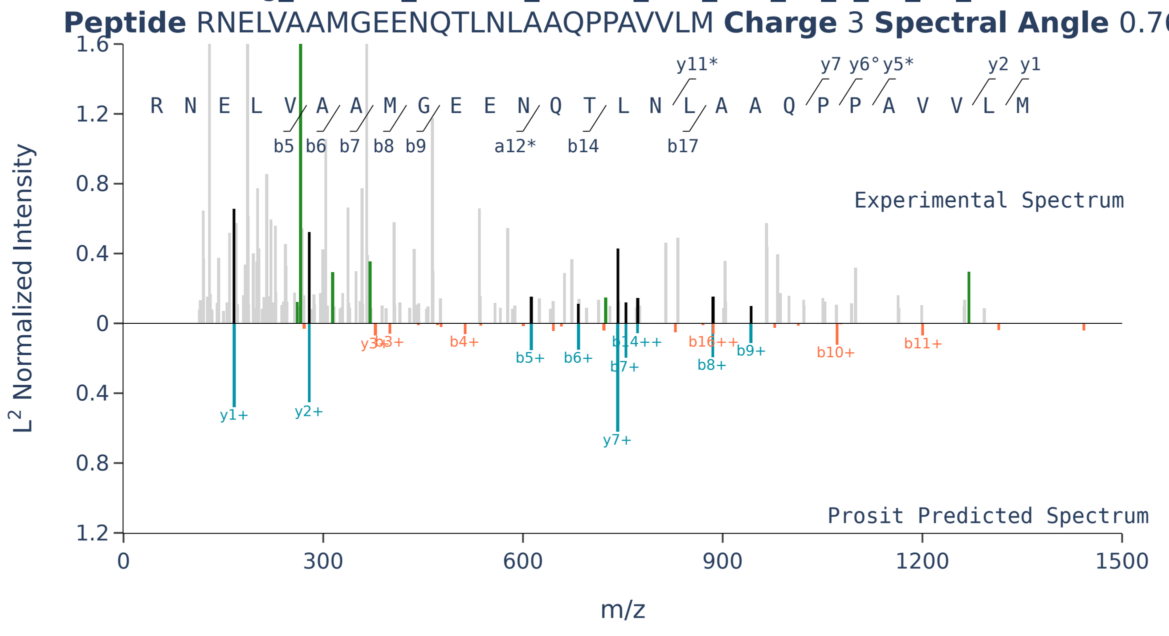


**Source** Ncheng_210623_230623_HFGoe_FFH_20S_25_1_A2_24h_R2 **Scan** 16874
**Peptide** AIINSMTMKERAKPEIIKG **Charge** 2 **Spectral Angle** 0.72

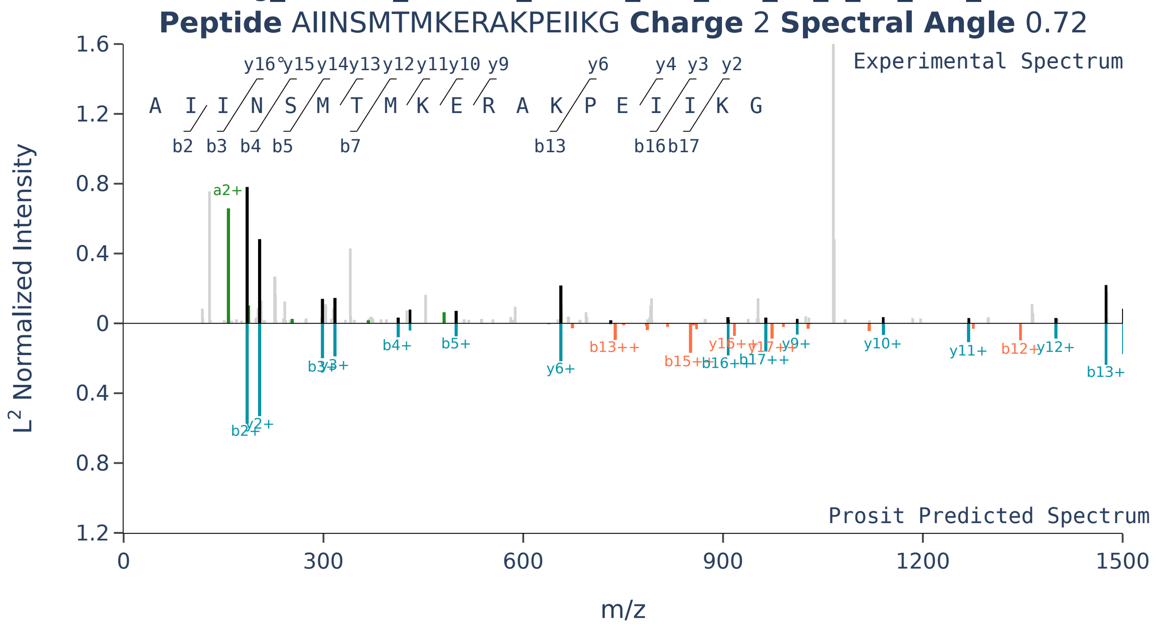


**Source** Ncheng_210623_230623_HFGoe_FFH_20S_25_1_A1_24h_R1 **Scan** 8403
**Peptide** RLSRTLRNISGR **Charge** 4 **Spectral Angle** 0.74

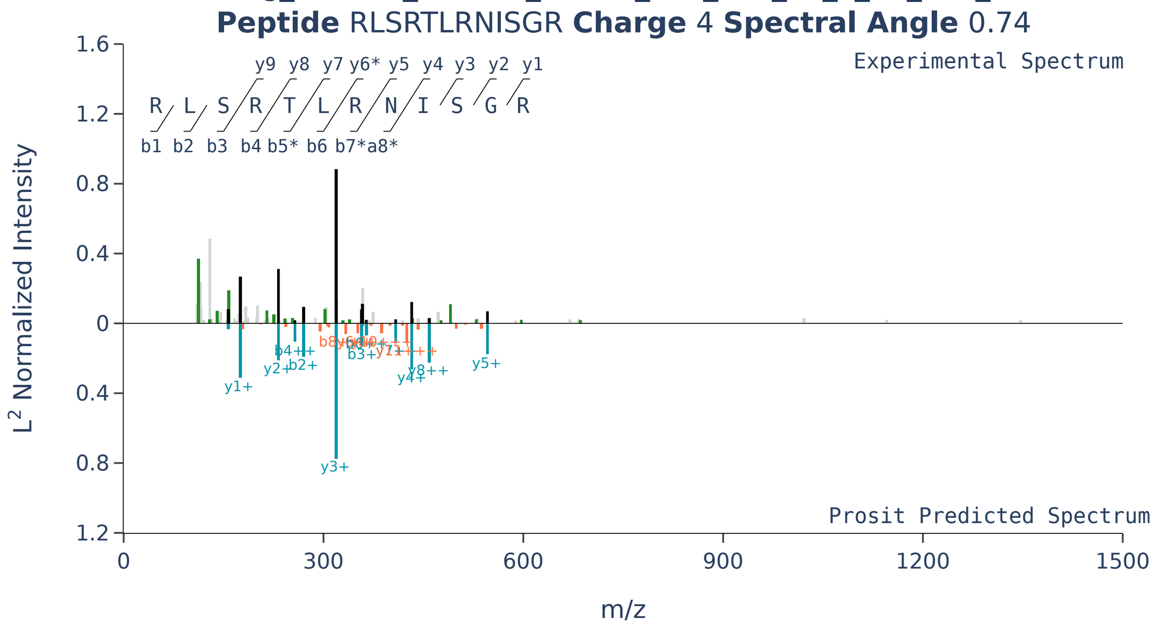


**Source** Ncheng_210623_230623_HFGoe_FFH_20S_25_1_A1_2h_R2 **Scan** 32792
**Peptide** VLVVSADVYRPAAIKQLETLAE **Charge** 3 **Spectral Angle** 0.92

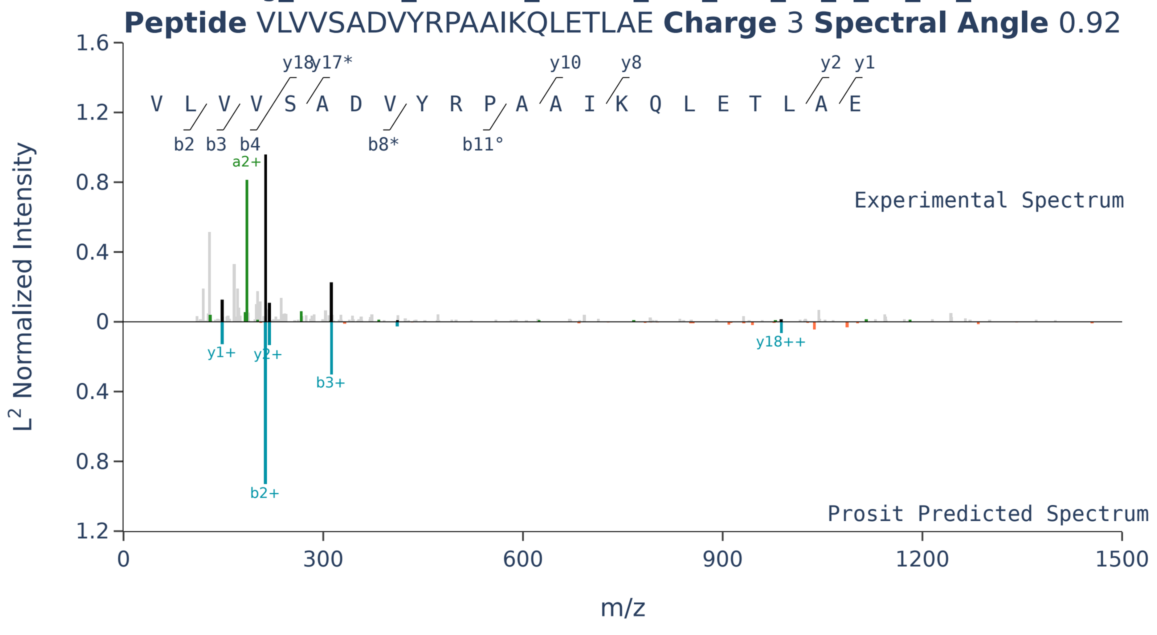


**Source** Ncheng_210623_230623_HFGoe_FFH_20S_25_1_A2_2h_R2 **Scan** 20300
**Peptide** ALSIRHITGKPIKFLGVGEKTE **Charge** 5 **Spectral Angle** 0.68

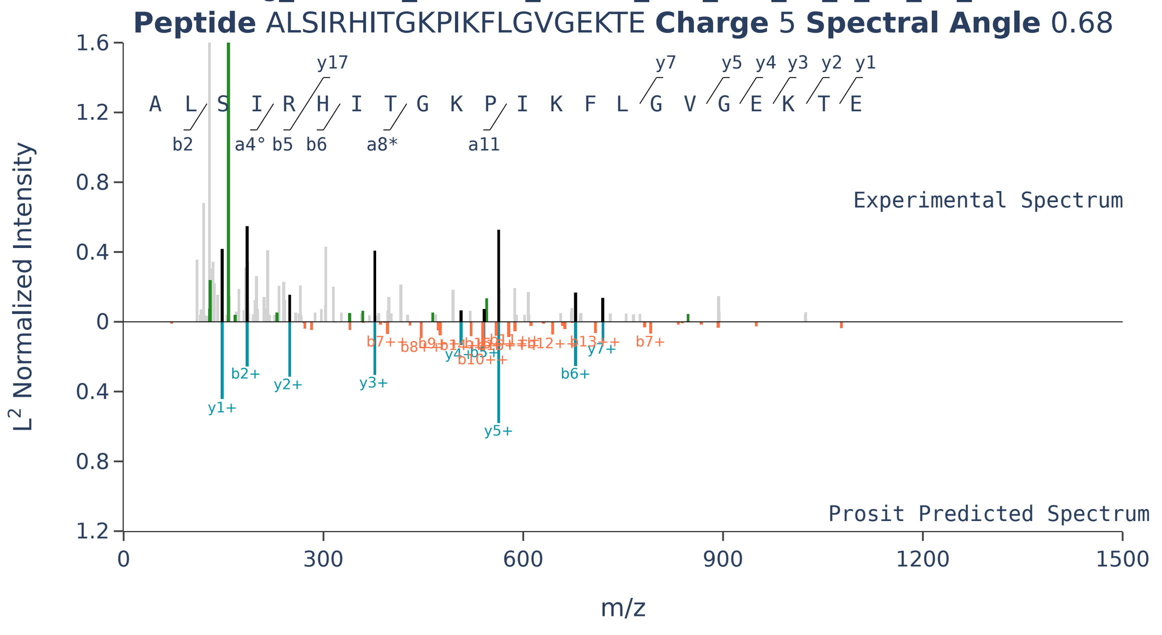


**Source** Ncheng_210623_230623_HFGoe_FFH_20S_25_1_A1_2h_R2 **Scan** 13570
**Peptide** KVDGDARGGAALSIRHL **Charge** 3 **Spectral Angle** 0.49

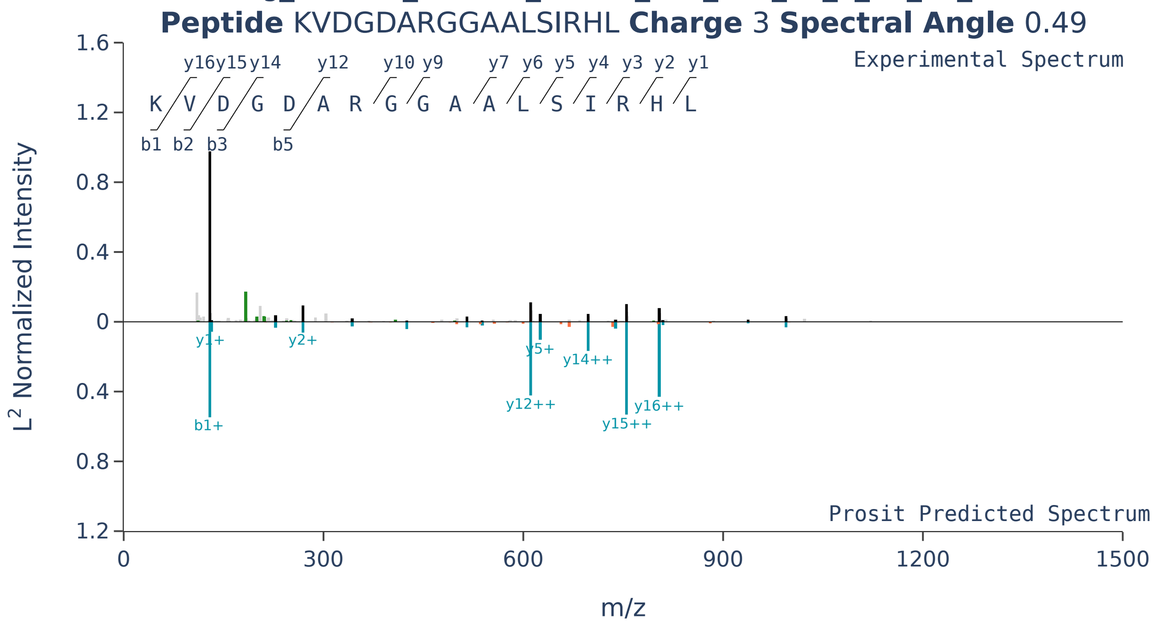


**Source** Ncheng_210623_230623_HFGoe_FFH_20S_25_1_A1_24h_R2 **Scan** 9282
**Peptide** MAGLQGAGKTTS **Charge** 2 **Spectral Angle** 0.69

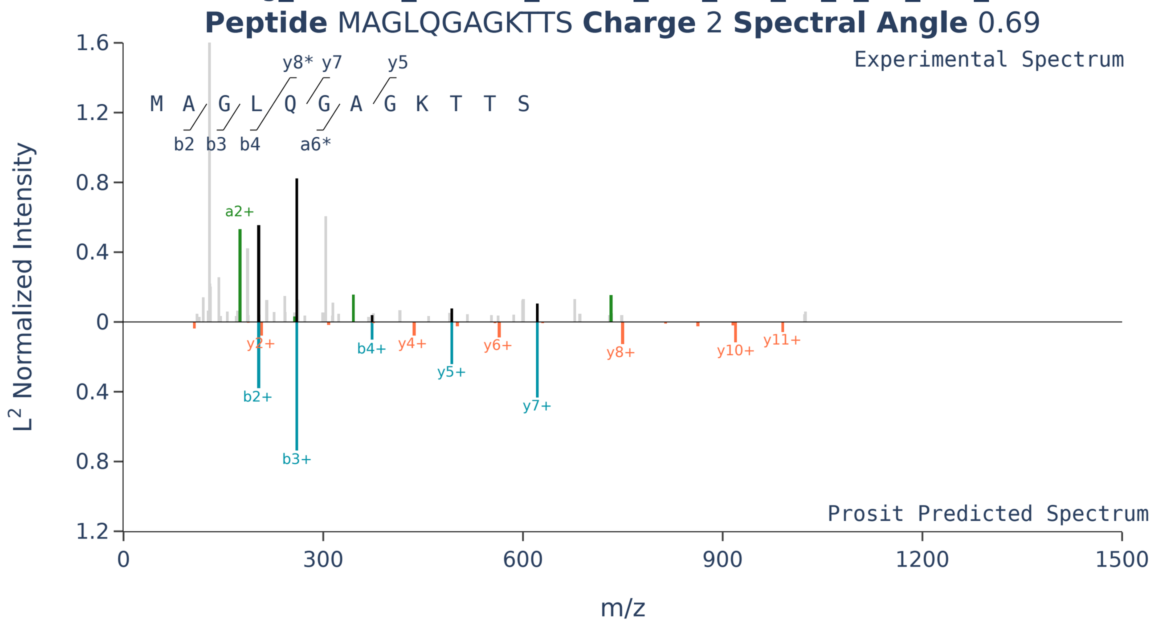


**Source** Ncheng_210623_230623_HFGoe_FFH_20S_25_1_A2_24h_R1 **Scan** 6054
**Peptide** AEKLASKLKKG **Charge** 3 **Spectral Angle** 0.75

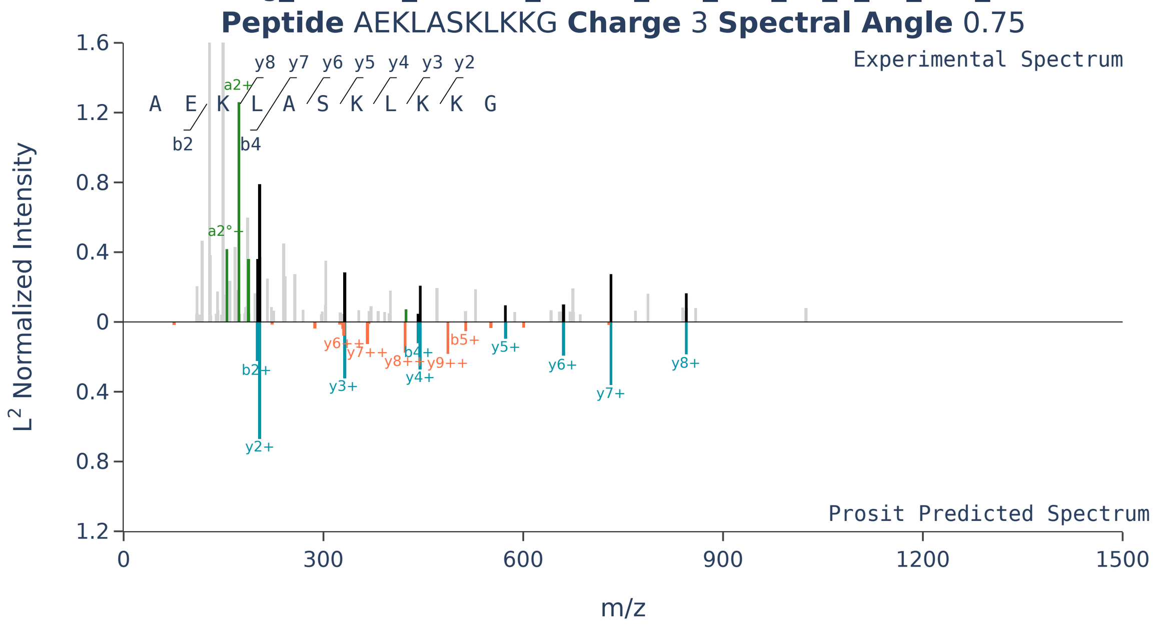


**Source** Ncheng_210623_230623_HFGoe_FFH_20S_25_1_A2_1h_R2 **Scan** 7762
**Peptide** NTAKAFNE **Charge** 1 **Spectral Angle** 0.74

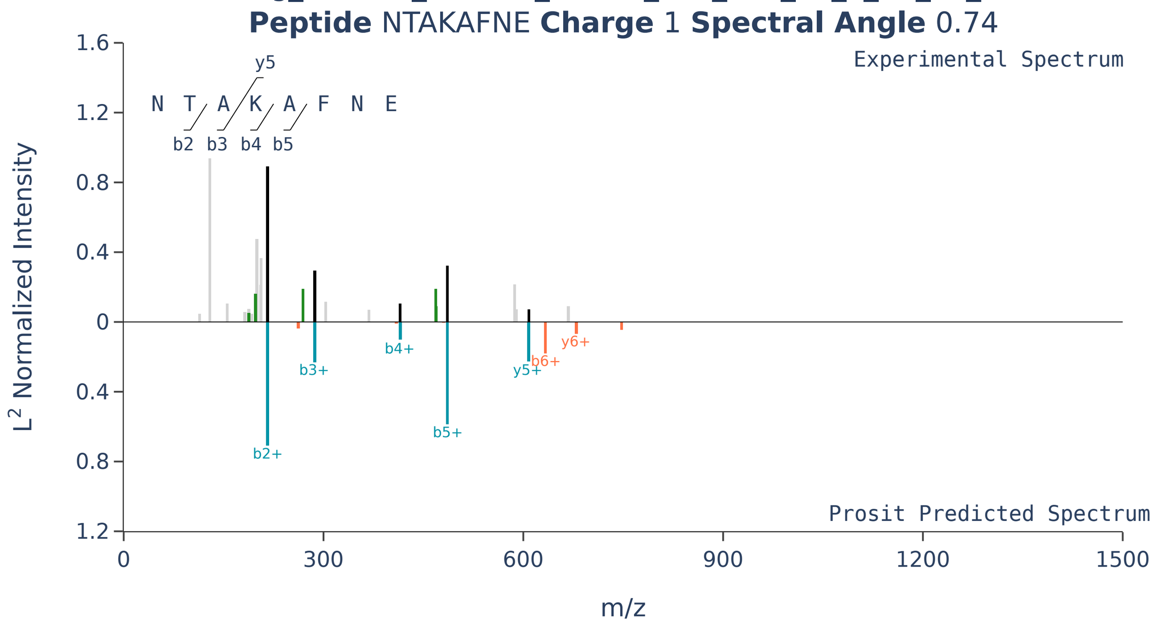

**Source** Ncheng_210623_230623_HFGoe_FFH_20S_25_1_A2_24h_R2 **Scan** 5474
**Peptide** KMKKGGMAKM **Charge** 2 **Spectral Angle** 0.81

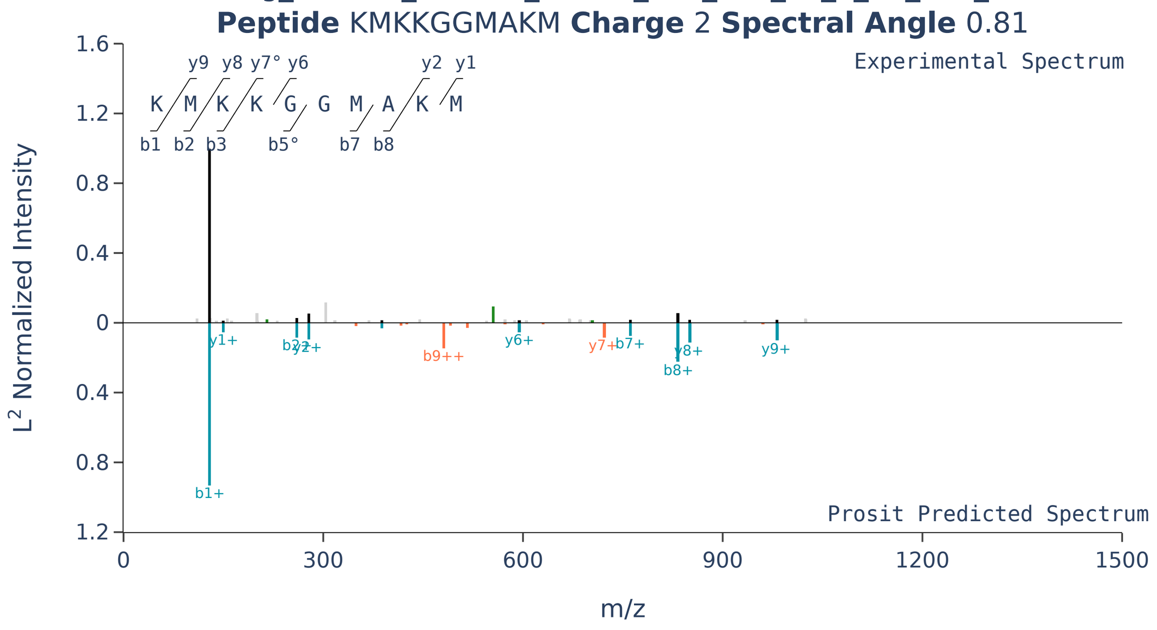


**Source** Ncheng_210623_230623_HFGoe_FFH_20S_25_1_A1_2h_R2 **Scan** 11663
**Peptide** ISGRGRLTEDNVKDTLRE **Charge** 4 **Spectral Angle** 0.58

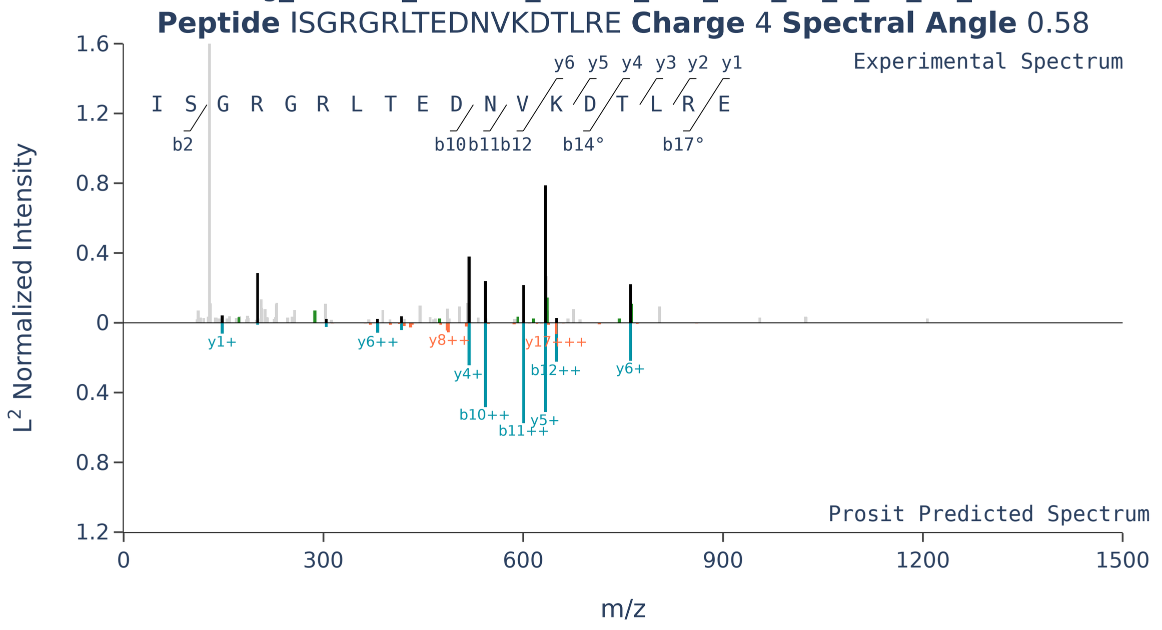


**Source** Ncheng_210623_230623_HFGoe_FFH_20S_25_1_A2_4h_R2 **Scan** 17969
**Peptide** DRAQAEKLASKLKKGDGFDLND **Charge** 5 **Spectral Angle** 0.66

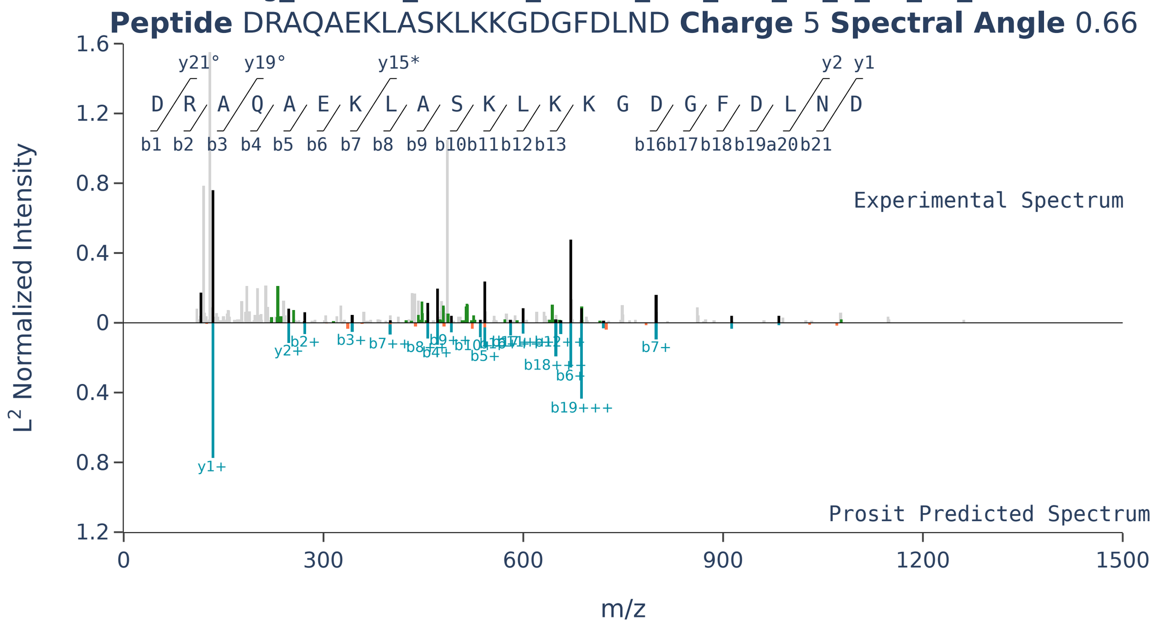


**Source** Ncheng_210623_230623_HFGoe_FFH_20S_25_1_A1_2h_R1 **Scan** 13673
**Peptide** IESKVDRAQAEKLASKLK **Charge** 5 **Spectral Angle** 0.86

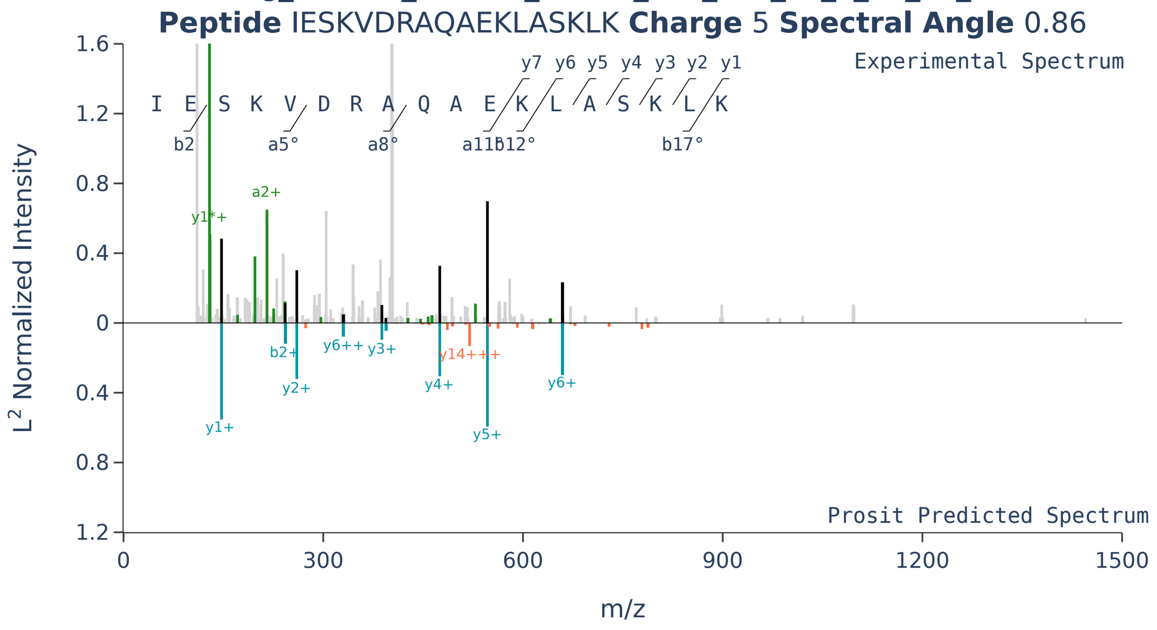


**Source** Ncheng_210623_230623_HFGoe_FFH_20S_25_1_A2_24h_R2 **Scan** 15856
**Peptide** LEDIESKVDRAQAEKLAS **Charge** 3 **Spectral Angle** 0.76

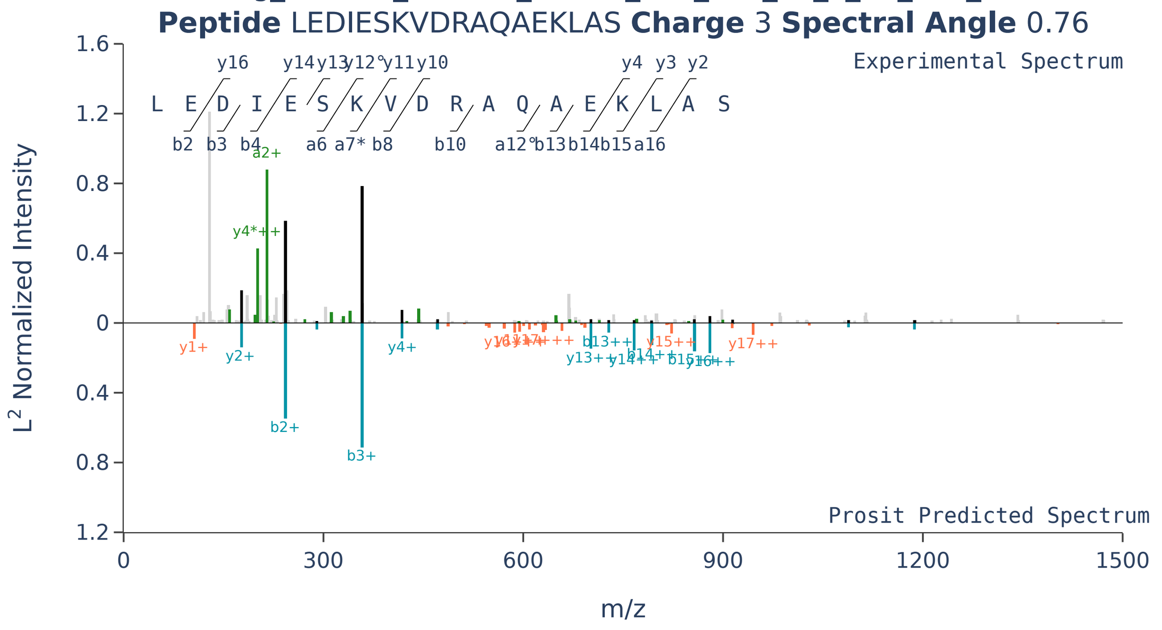


**Source** Ncheng_210623_230623_HFGoe_FFH_20S_25_1_A2_2h_R2 **Scan** 19819
**Peptide** RNELVAAMGEENQTLN **Charge** 2 **Spectral Angle** 0.58

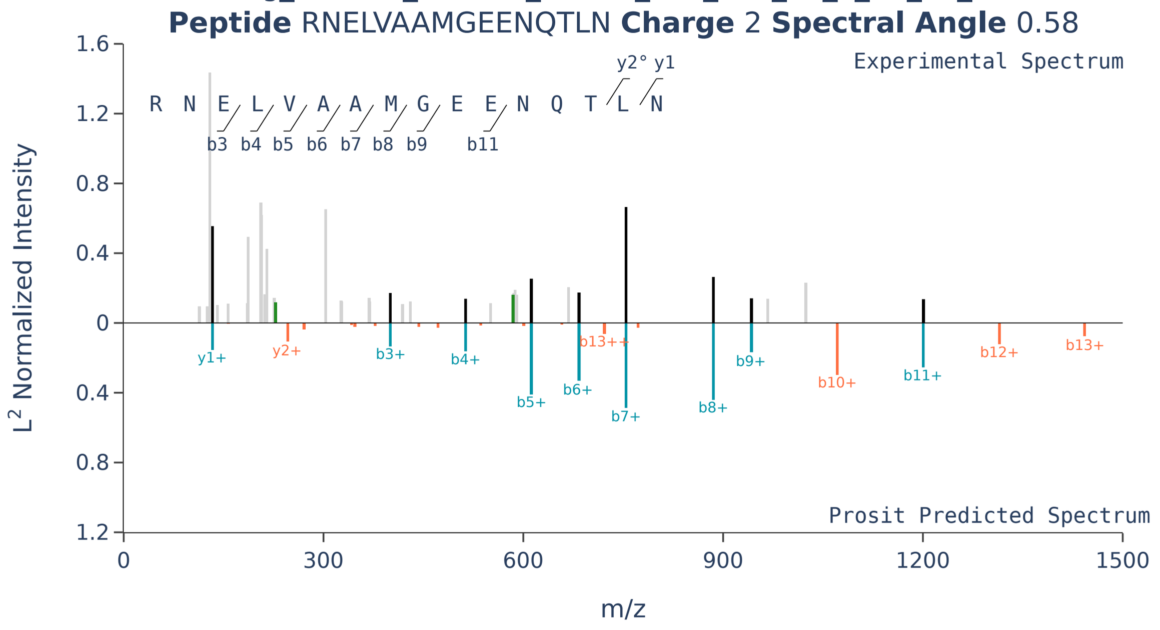


**Source** Ncheng_210623_230623_HFGoe_FFH_20S_25_1_A2_4h_R2 **Scan** 23242
**Peptide** AGRLHVDEAMMDEIKQVHA **Charge** 4 **Spectral Angle** 0.7

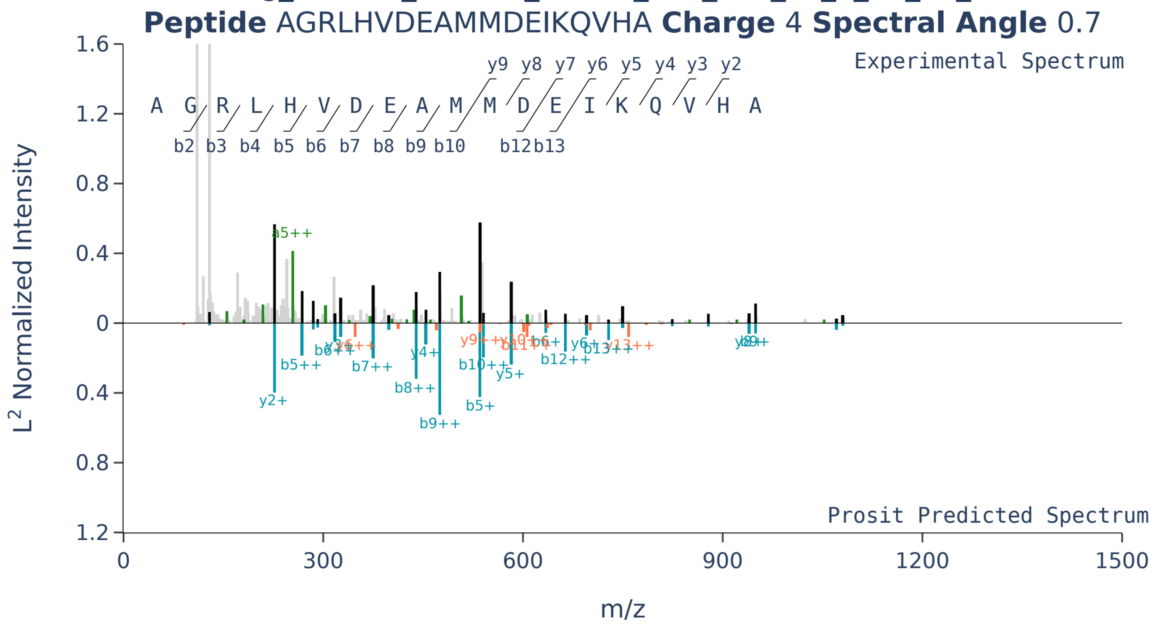


**Source** Ncheng_210623_230623_HFGoe_FFH_20S_25_1_A1_24h_R1 **Scan** 19125
**Peptide** VGEKTEALEPFHPDRL **Charge** 3 **Spectral Angle** 0.77

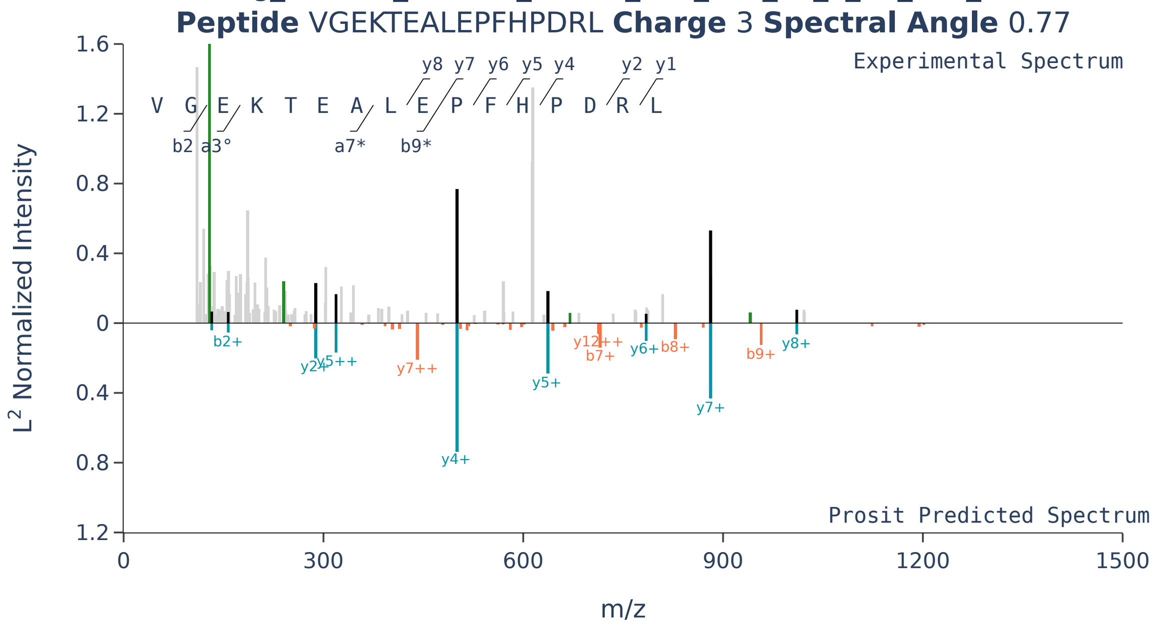


**Source** Ncheng_210623_230623_HFGoe_FFH_20S_25_1_A2_2h_R2 **Scan** 28562
**Peptide** VDTAGRLHVDEAMMDEIKQVHASINPVE **Charge** 4 **Spectral Angle** 0.69

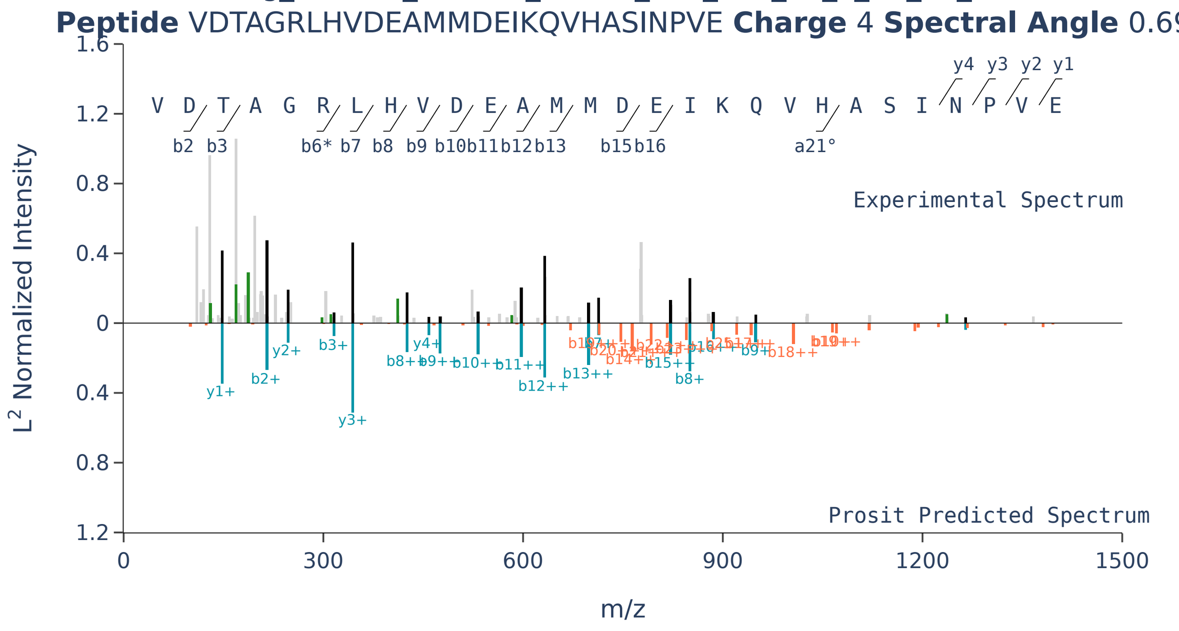


**Source** Ncheng_210623_230623_HFGoe_FFH_20S_25_1_A1_2h_R2 **Scan** 24424
**Peptide** PIKFLGVGEKTEALEPFHPDRIASR **Charge** 5 **Spectral Angle** 0.81

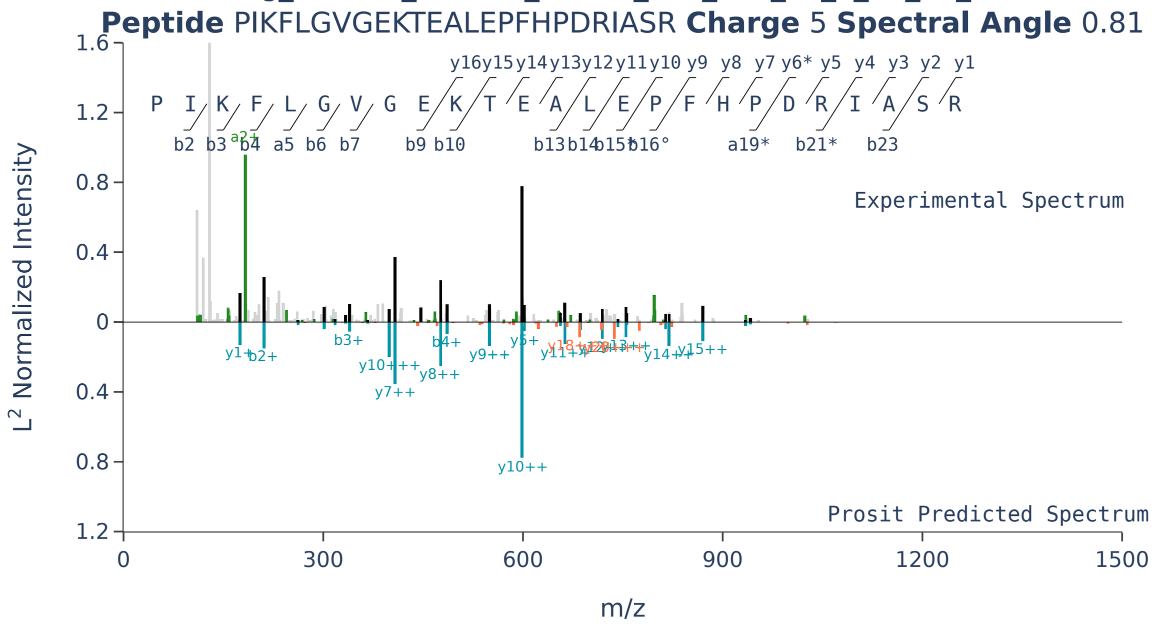


**Source** Ncheng_210623_230623_HFGoe_FFH_20S_25_1_A1_24h_R2 **Scan** 9750
**Peptide** DTAGRLHVDEA **Charge** 1 **Spectral Angle** 0.51

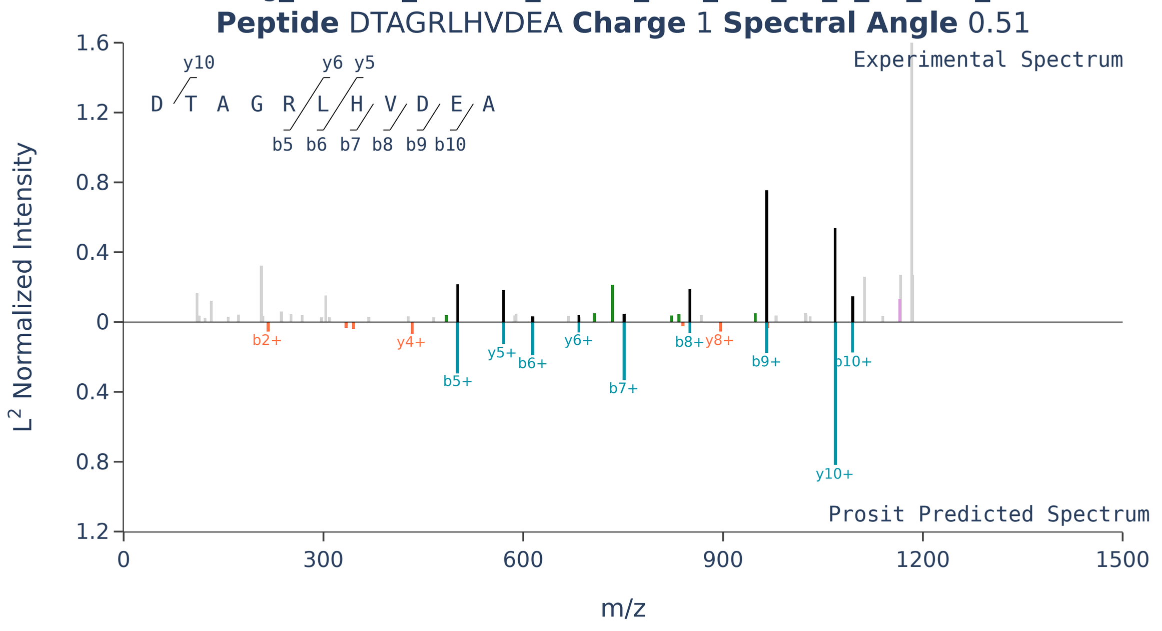


**Source** Ncheng_210623_230623_HFGoe_FFH_20S_25_1_A1_4h_R1 **Scan** 24902
**Peptide** AALKEAKLKFYDVL **Charge** 4 **Spectral Angle** 0.81

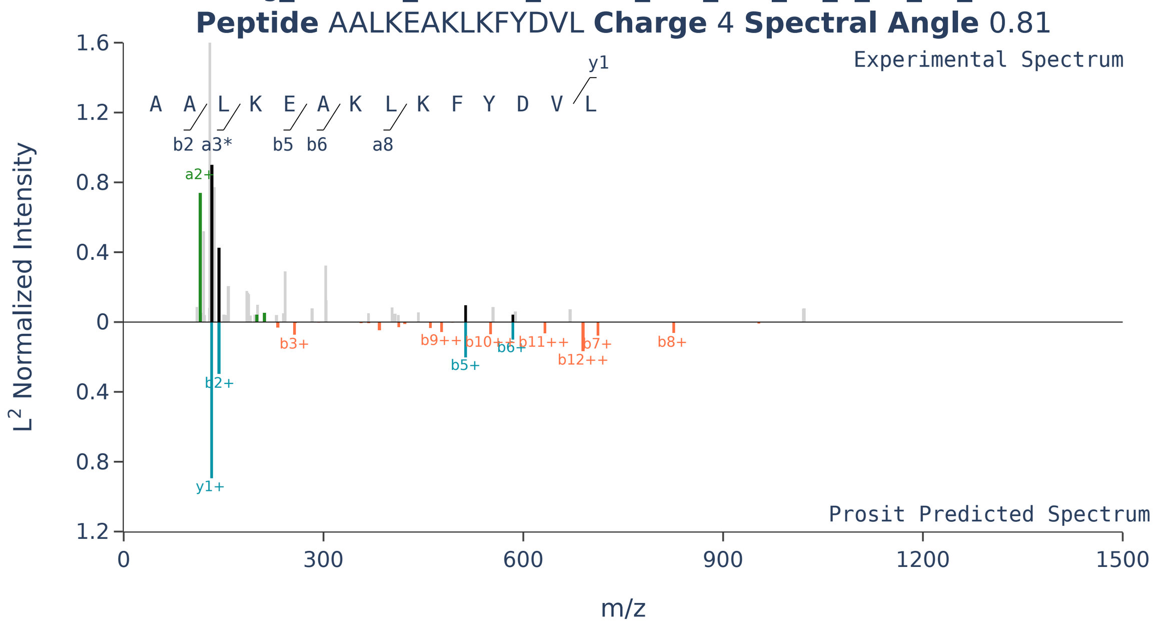


**Source** Ncheng_210623_230623_HFGoe_FFH_20S_25_1_A2_4h_R1 **Scan** 6383
**Peptide** SIRHITG **Charge** 2 **Spectral Angle** 0.84

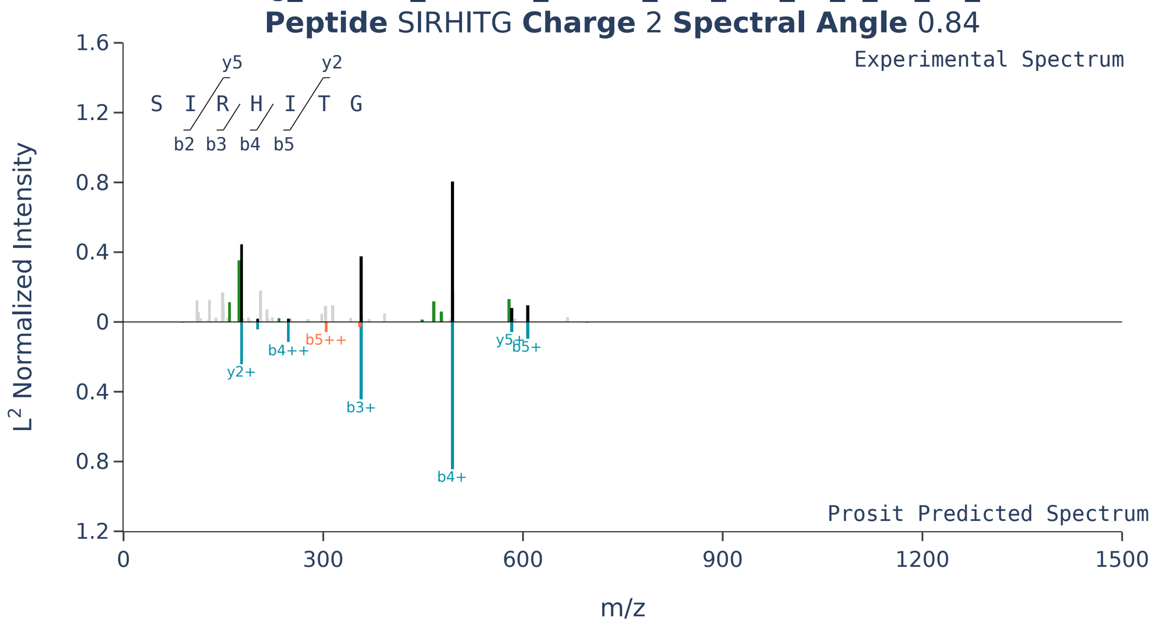


**Source** Ncheng_210623_230623_HFGoe_FFH_20S_25_1_A1_2h_R2 **Scan** 32645
**Peptide** GMASLMGKLPGMGQIPDNVKSQMDDKVLV **Charge** 4 **Spectral Angle** 0.74

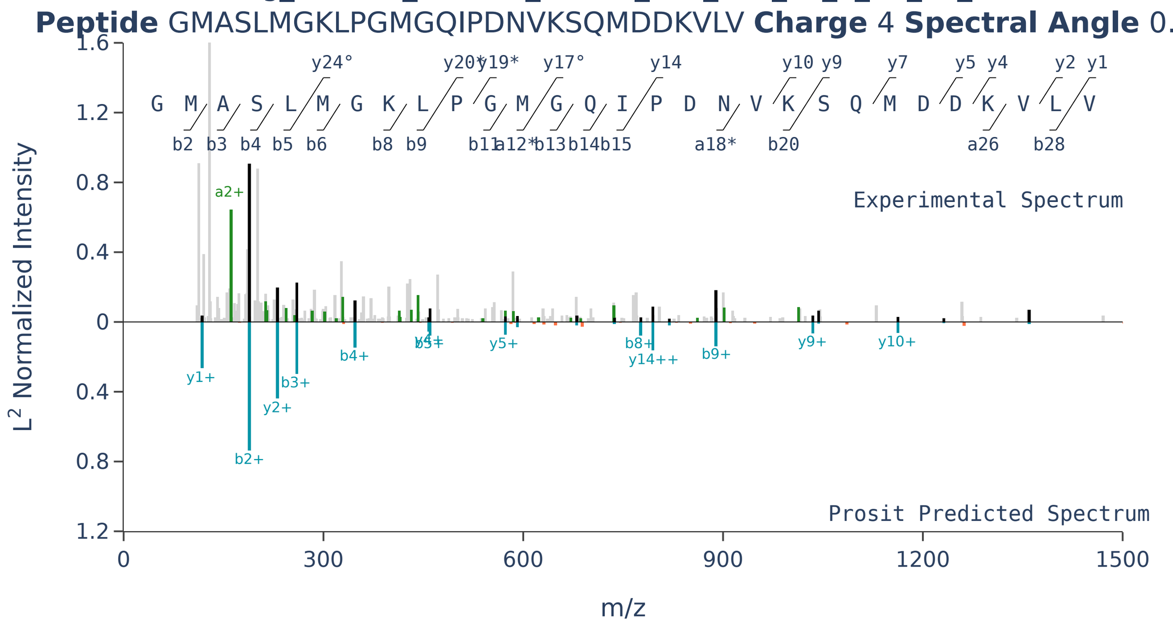


**Source** Ncheng_210623_230623_HFGoe_FFH_20S_25_1_A2_1h_R2 **Scan** 34614
**Peptide** AANTAKAFNEALPLTGVVL **Charge** 2 **Spectral Angle** 0.68

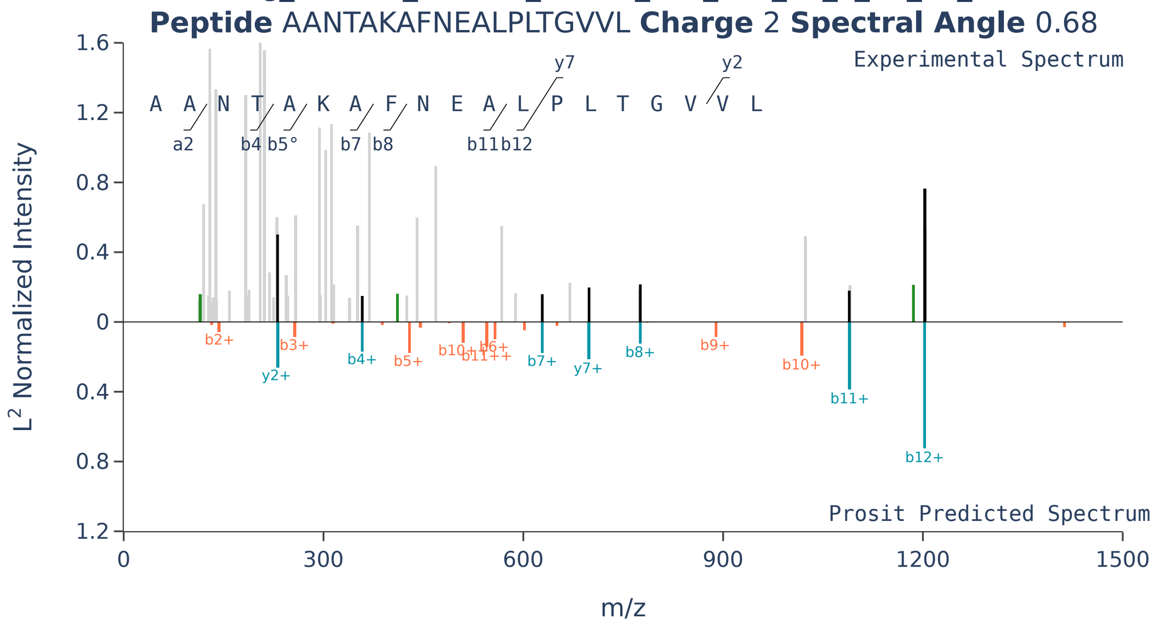

**Source** Ncheng_210623_230623_HFGoe_FFH_20S_25_1_A2_24h_R1 **Scan** 18146
**Peptide** AGLQGAGKTTSVGKLGKFLR **Charge** 4 **Spectral Angle** 0.63

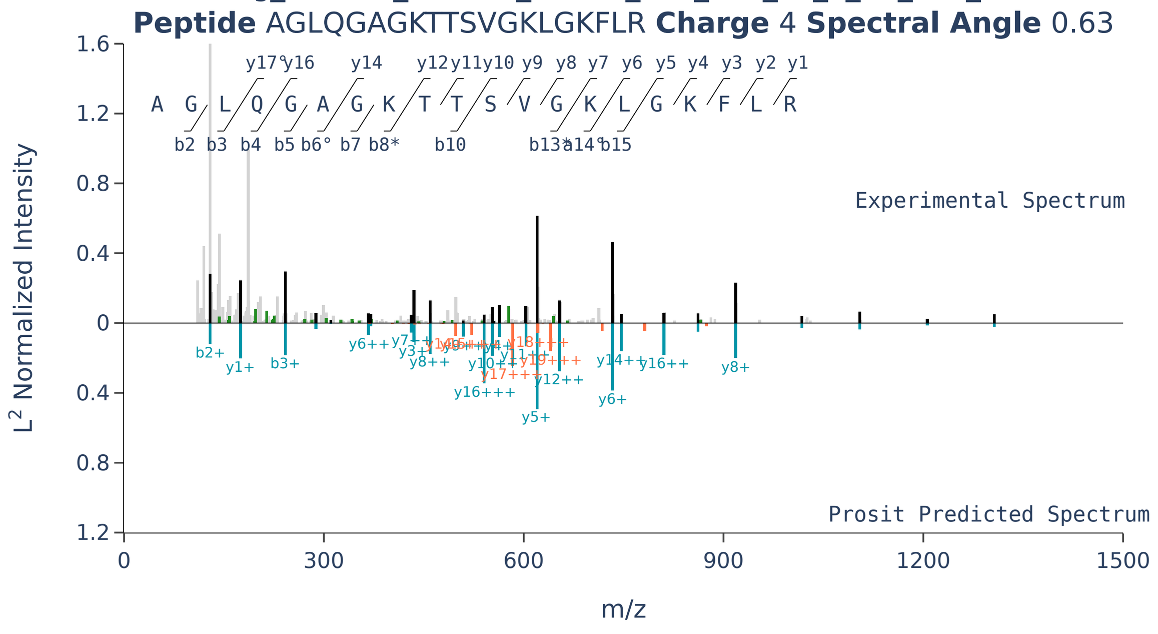


**Source** Ncheng_210623_230623_HFGoe_FFH_20S_25_1_A1_4h_R2 **Scan** 25327
**Peptide** FLEQLRQMKNMGGMA **Charge** 2 **Spectral Angle** 0.67

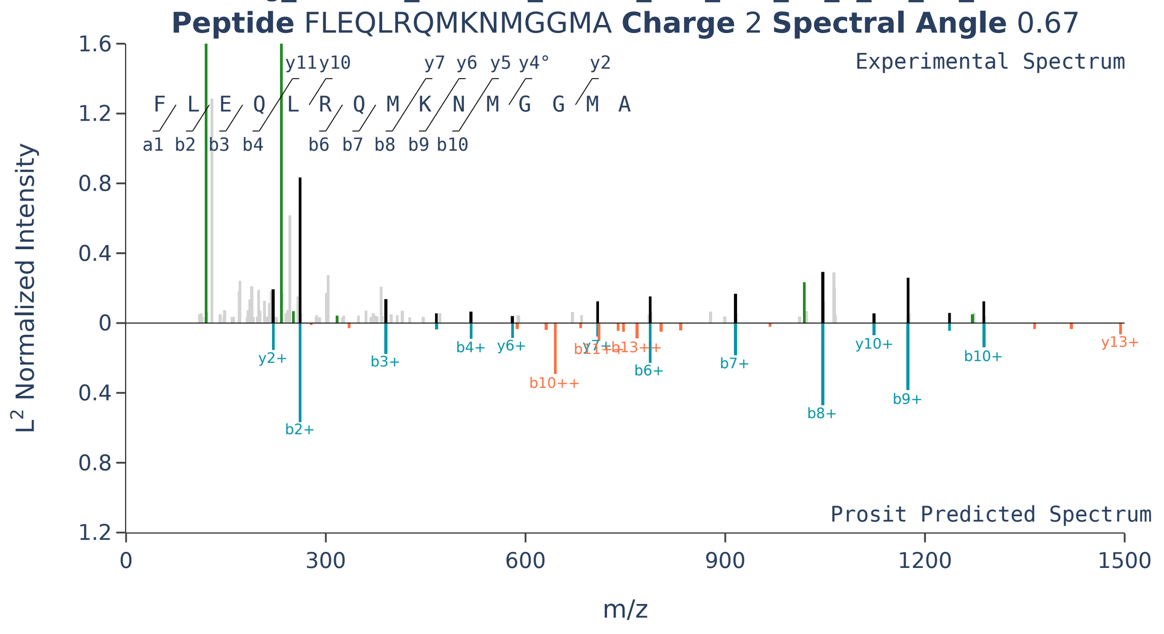


**Source** Ncheng_210623_230623_HFGoe_FFH_20S_25_1_A2_24h_R2 **Scan** 5541
**Peptide** NRVKEKAVGHEV **Charge** 2 **Spectral Angle** 0.43

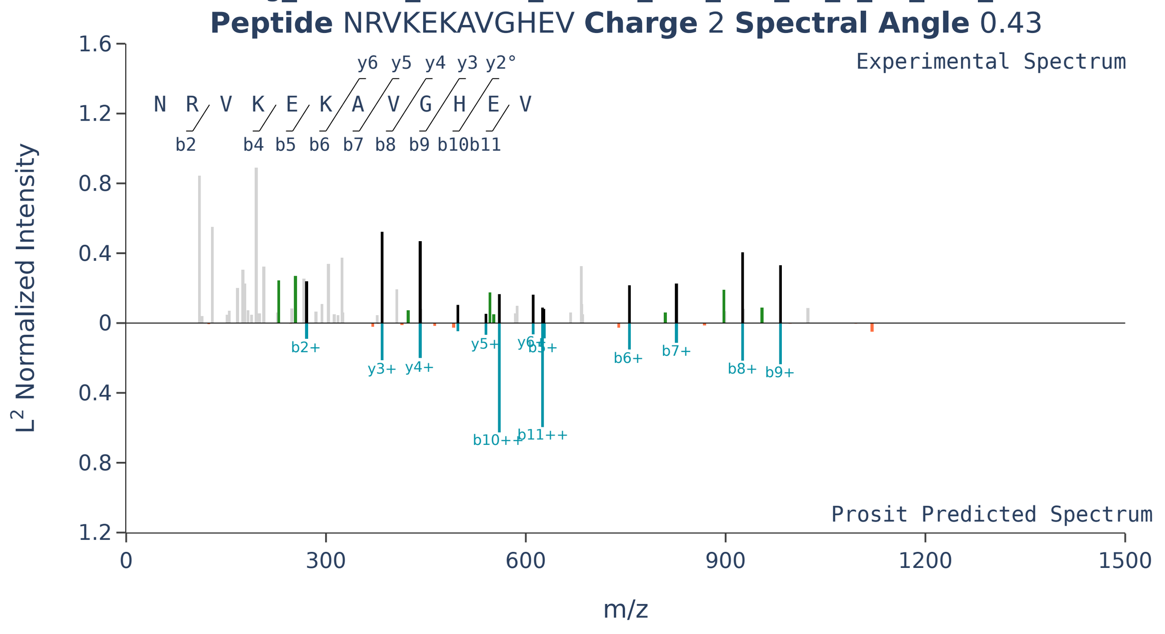


**Source** Ncheng_210623_230623_HFGoe_FFH_20S_25_1_A2_4h_R1 **Scan** 17342
**Peptide** RHITGKPIKFLGVGEKTEAL **Charge** 5 **Spectral Angle** 0.75

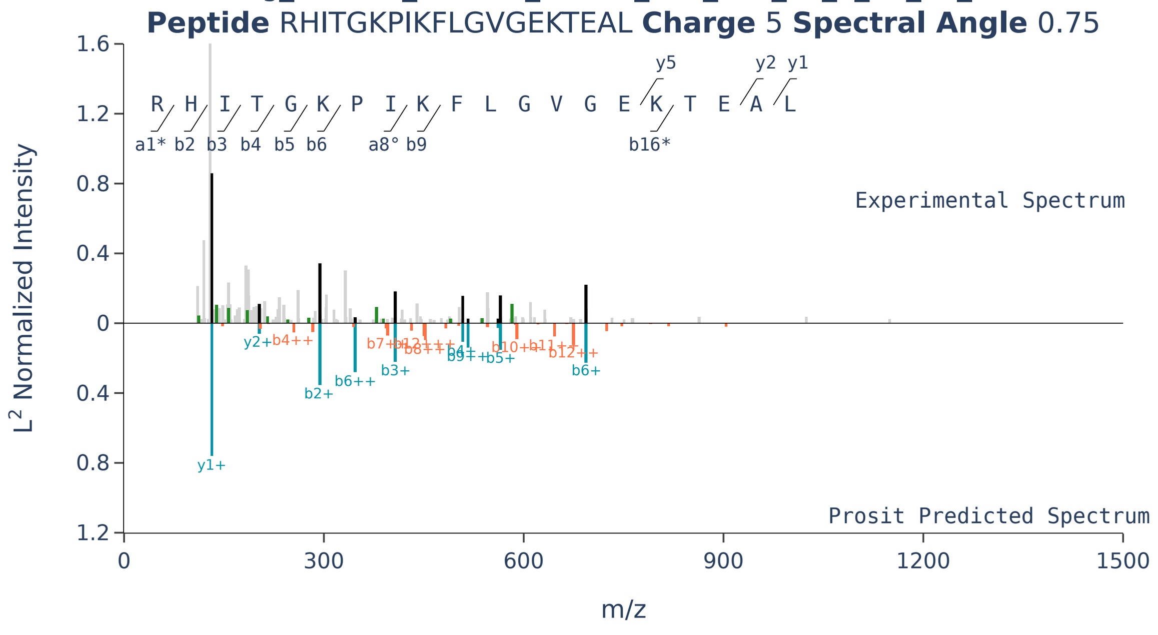


**Source** Ncheng_210623_230623_HFGoe_FFH_20S_25_1_A2_2h_R1 **Scan** 32681
**Peptide** ADVYRPAAIKQLETLAEQV **Charge** 3 **Spectral Angle** 0.73

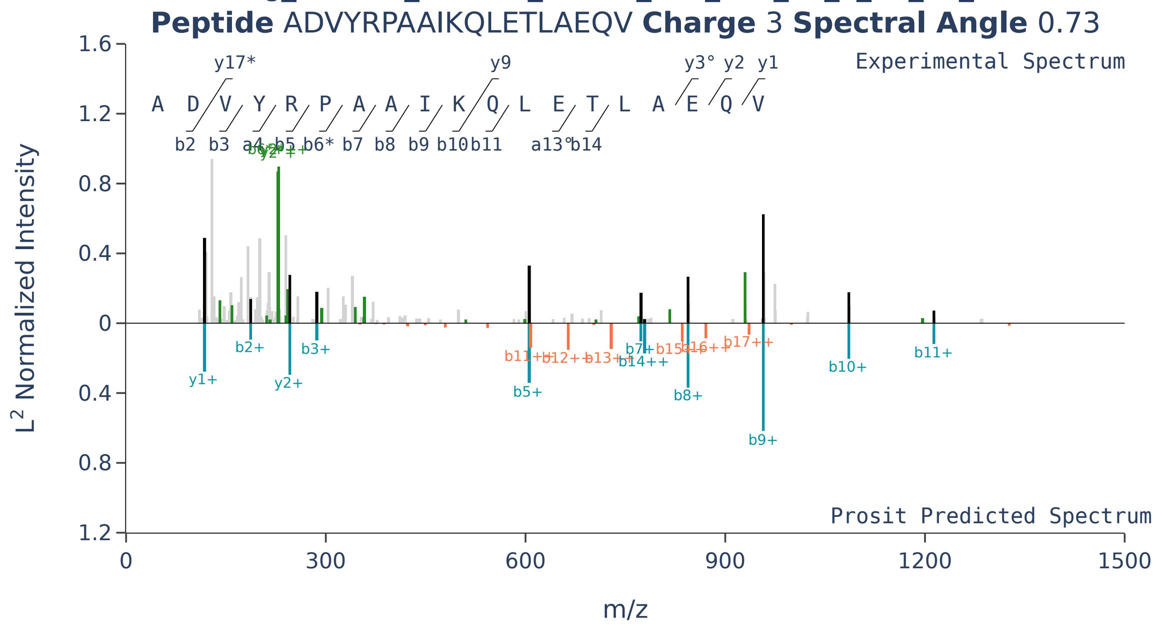


**Source** Ncheng_210623_230623_HFGoe_FFH_20S_25_1_A1_24h_R1 **Scan** 14883
**Peptide** DGDARGGAALSIRHIT **Charge** 4 **Spectral Angle** 0.7

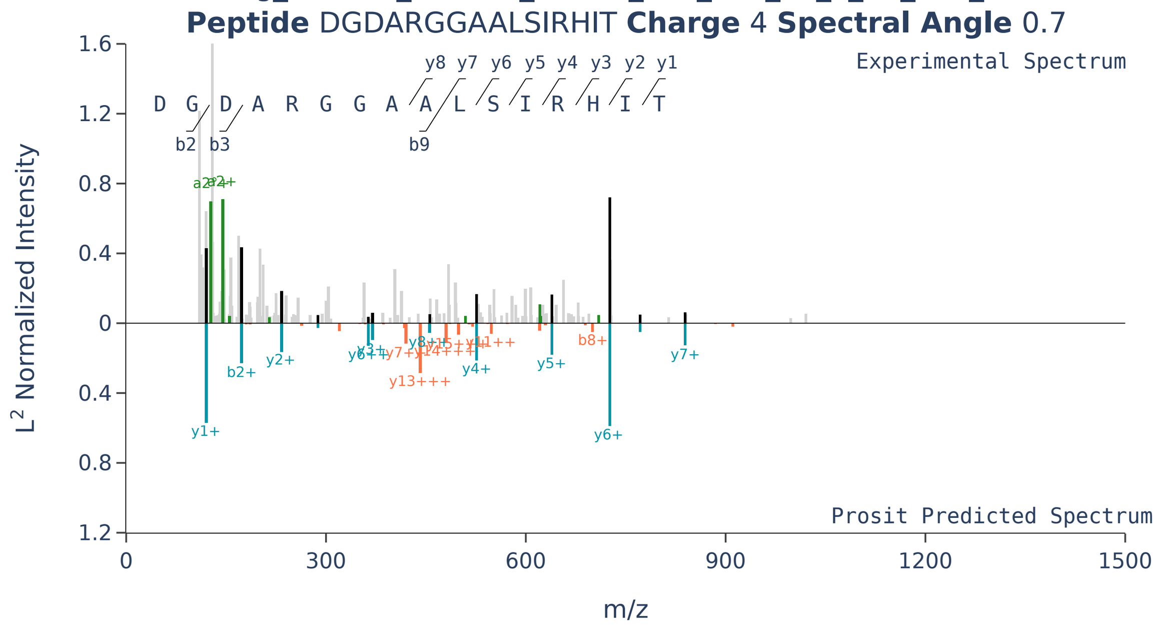


**Source** Ncheng_210623_230623_HFGoe_FFH_20S_25_1_A1_2h_R2 **Scan** 33553
**Peptide** MGKLPGMGQIPDNVKSQMDDKVLVRMEAII **Charge** 4 **Spectral Angle** 0.65

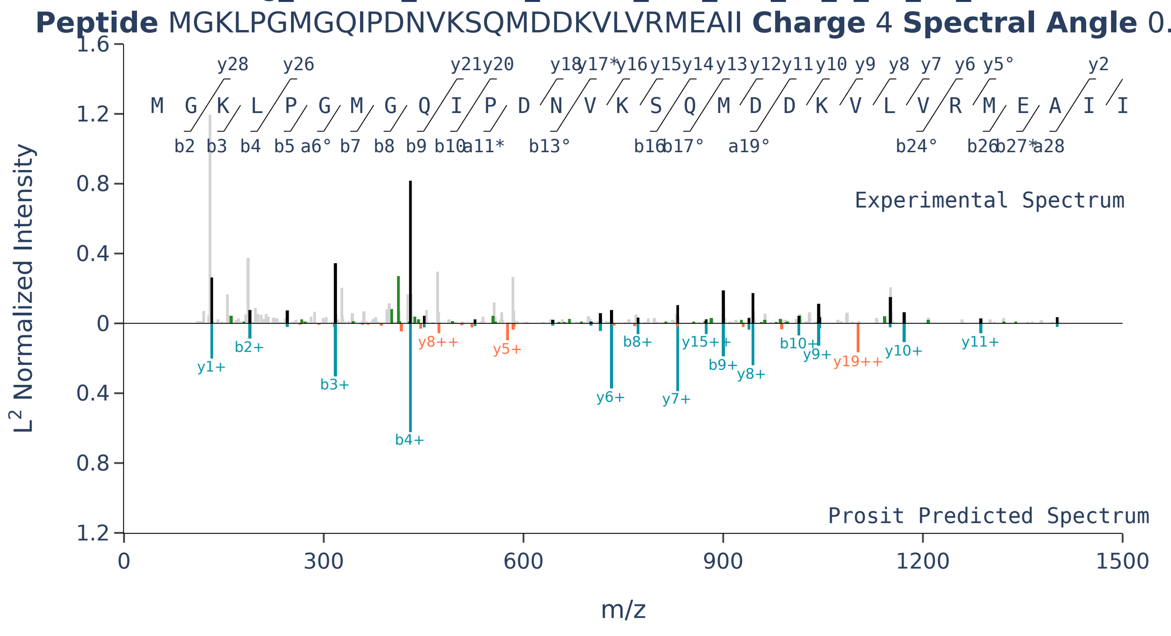


**Source** Ncheng_210623_230623_HFGoe_FFH_20S_25_1_A1_2h_R2 **Scan** 25905
**Peptide** DTAGRLHVDEAMMDEIKQV **Charge** 3 **Spectral Angle** 0.57

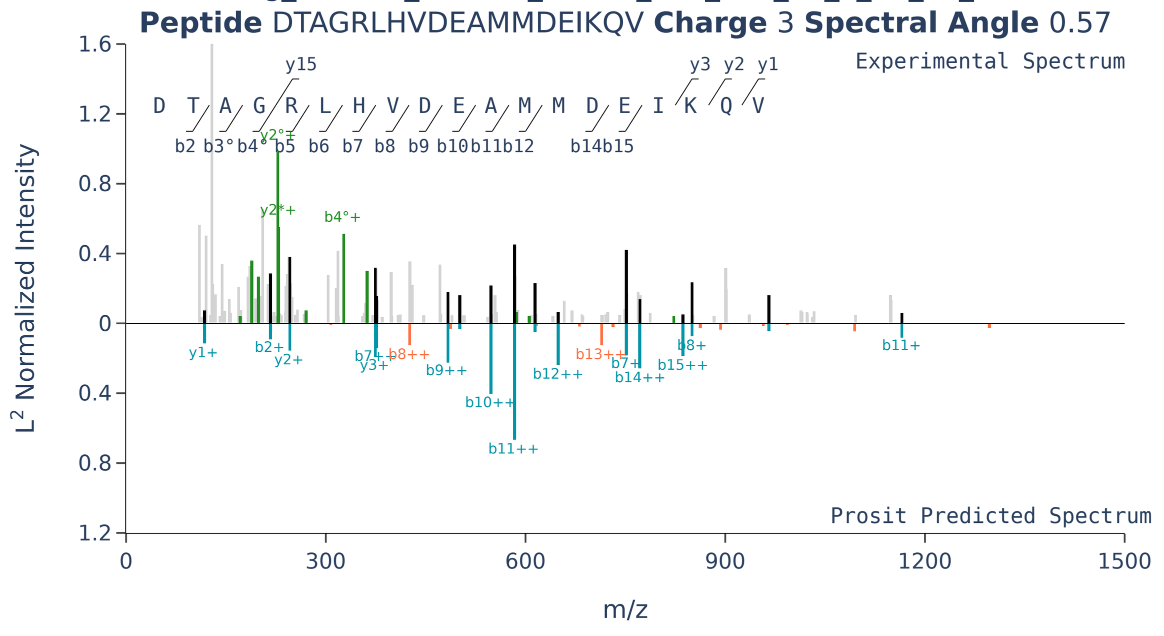


**Source** Ncheng_210623_230623_HFGoe_FFH_20S_25_1_A1_2h_R2 **Scan** 38759
**Peptide** DDKVLVRMEAIINSM **Charge** 2 **Spectral Angle** 0.62

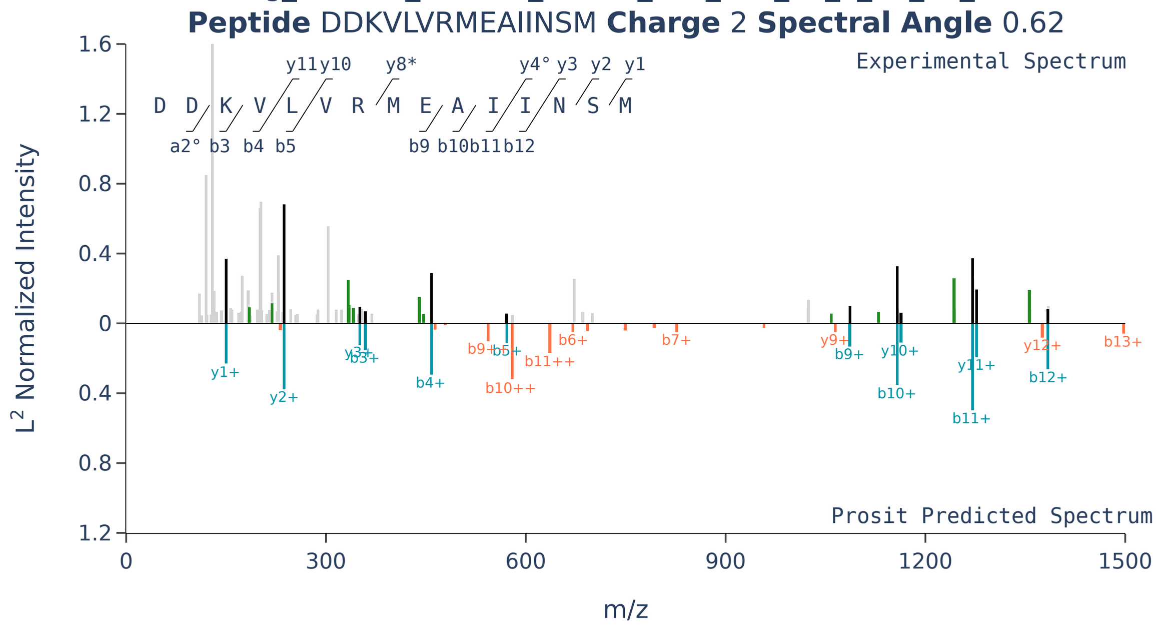


**Source** Ncheng_210623_230623_HFGoe_FFH_20S_25_1_A1_1h_R1 **Scan** 17016
**Peptide** KTEALEPFHPDRIA **Charge** 3 **Spectral Angle** 0.64

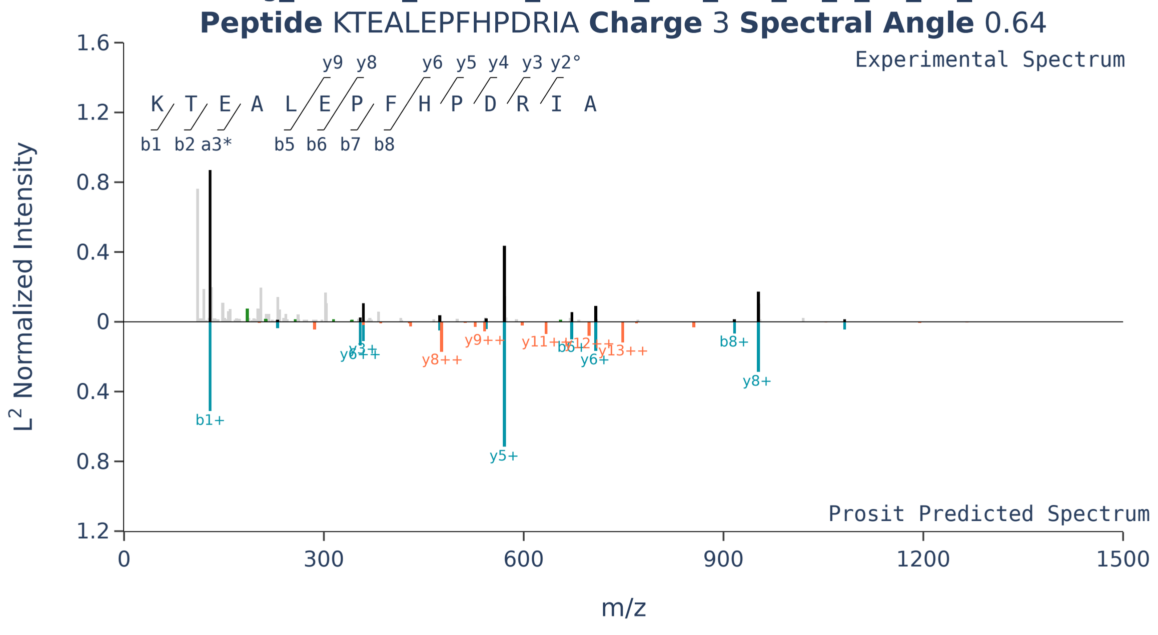


**Source** Ncheng_210623_230623_HFGoe_FFH_20S_25_1_A2_4h_R1 **Scan** 13801
**Peptide** TDRLSRTLRNI **Charge** 2 **Spectral Angle** 0.57

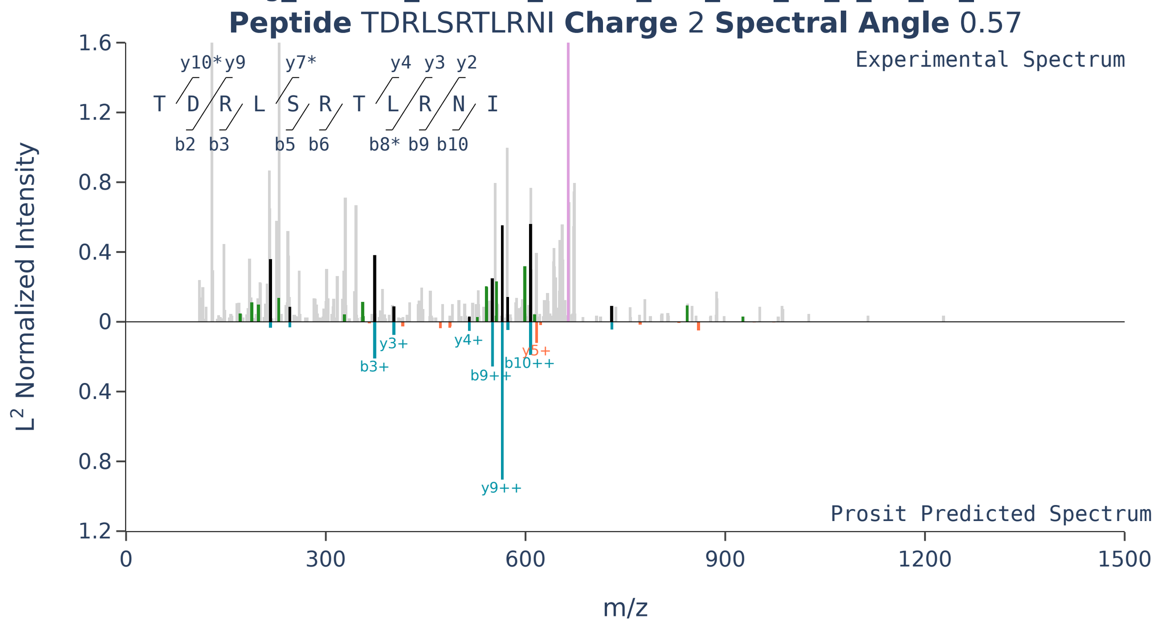


**Source** Ncheng_210623_230623_HFGoe_FFH_20S_25_1_A1_4h_R2 **Scan** 6805
**Peptide** HKKKVLVVS **Charge** 2 **Spectral Angle** 0.55

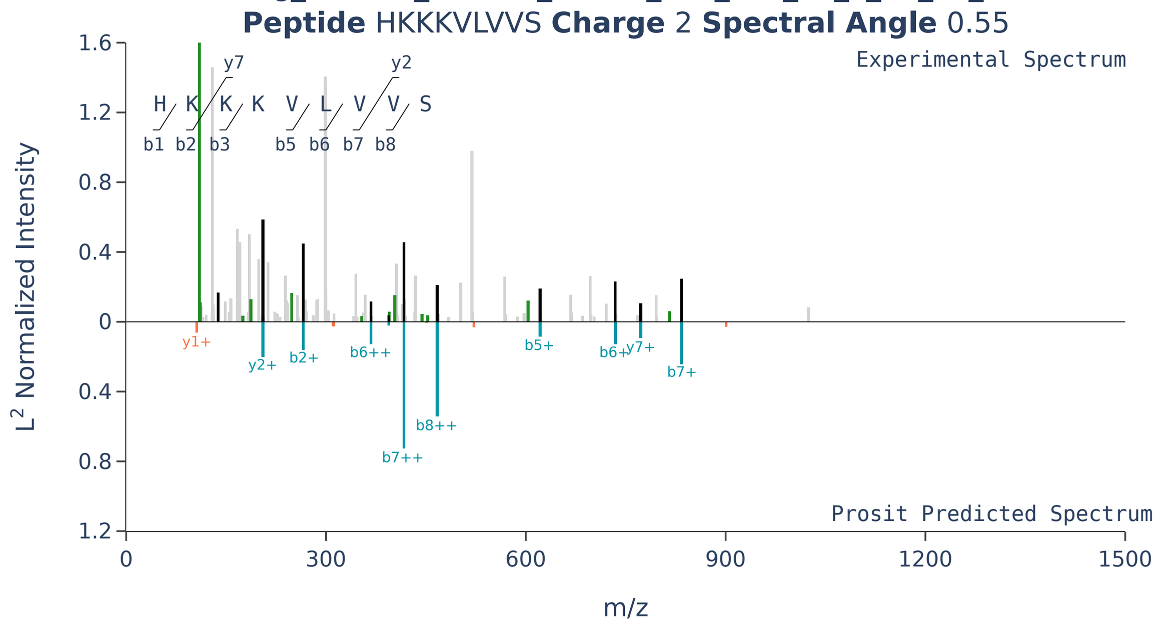


**Source** Ncheng_210623_230623_HFGoe_FFH_20S_25_1_A1_4h_R2 **Scan** 18827
**Peptide** LRNISGRGRLTEDNVKDTLREVRM **Charge** 5 **Spectral Angle** 0.52

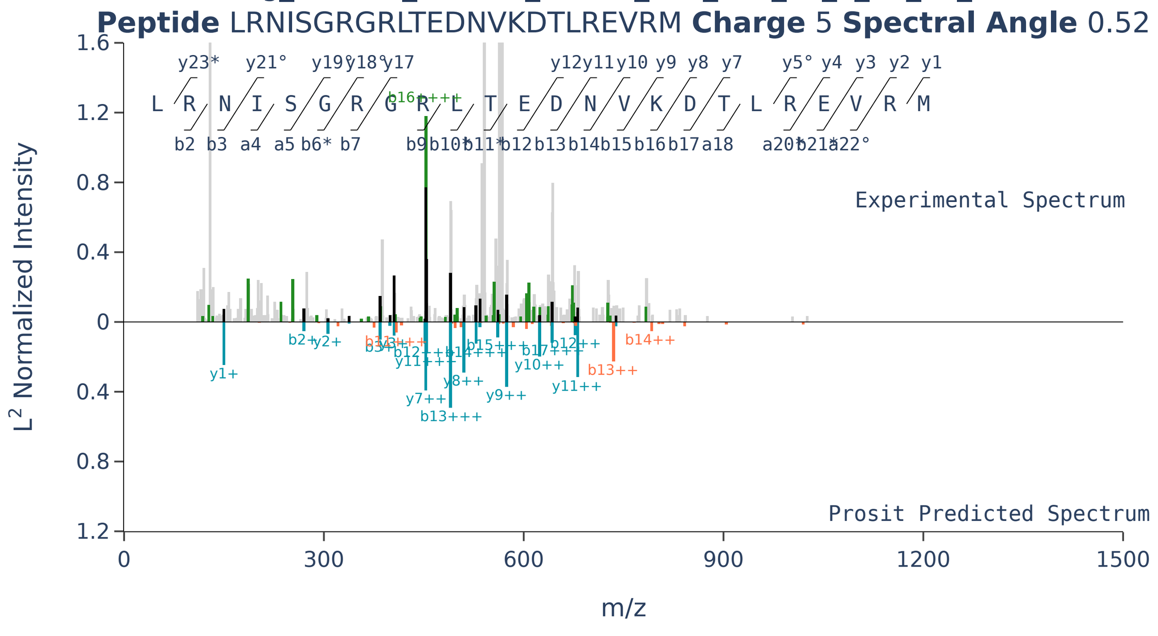


**Source** Ncheng_210623_230623_HFGoe_FFH_20S_25_1_A1_4h_R1 **Scan** 33174
**Peptide** EADVALPVVREFINRVKEK **Charge** 3 **Spectral Angle** 0.65

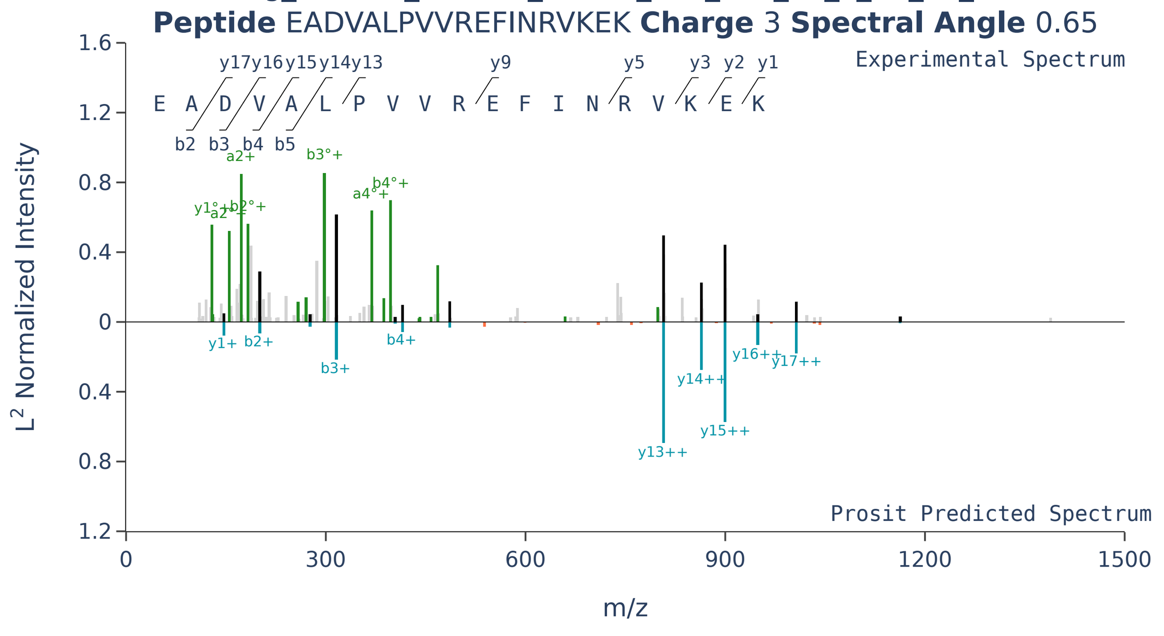


**Source** Ncheng_210623_230623_HFGoe_FFH_20S_25_1_A1_4h_R2 **Scan** 7149
**Peptide** VLTKVDGDAR **Charge** 2 **Spectral Angle** 0.63

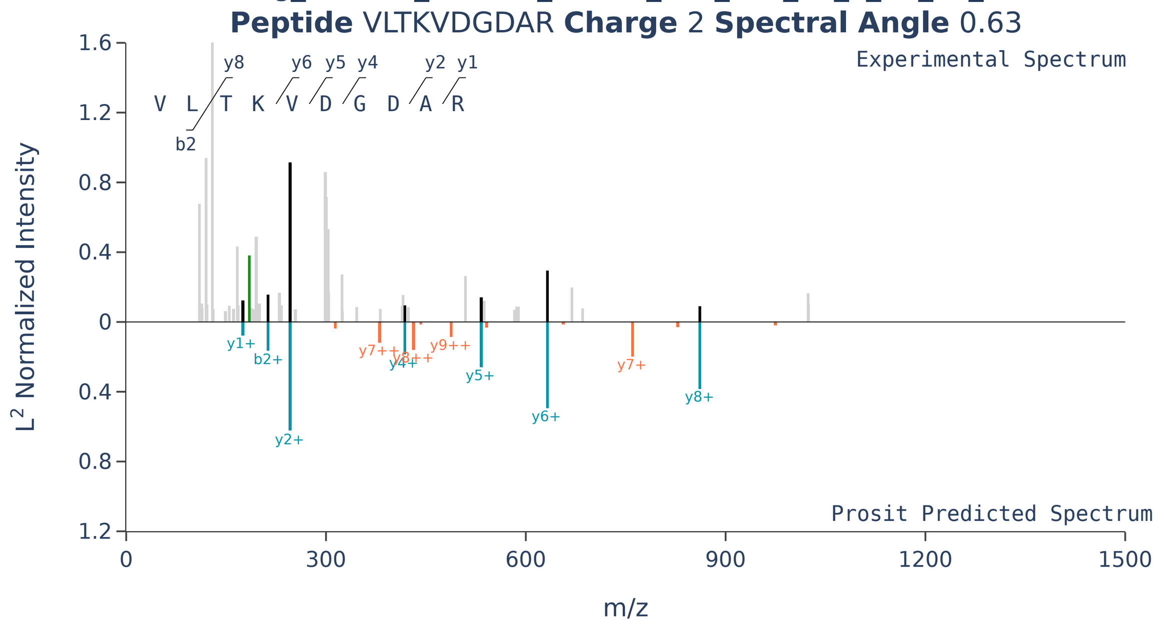

**Source** Ncheng_210623_230623_HFGoe_FFH_20S_25_1_A2_4h_R2 **Scan** 36042
**Peptide** DVALPVVREFINRVKEKAVGH **Charge** 3 **Spectral Angle** 0.78

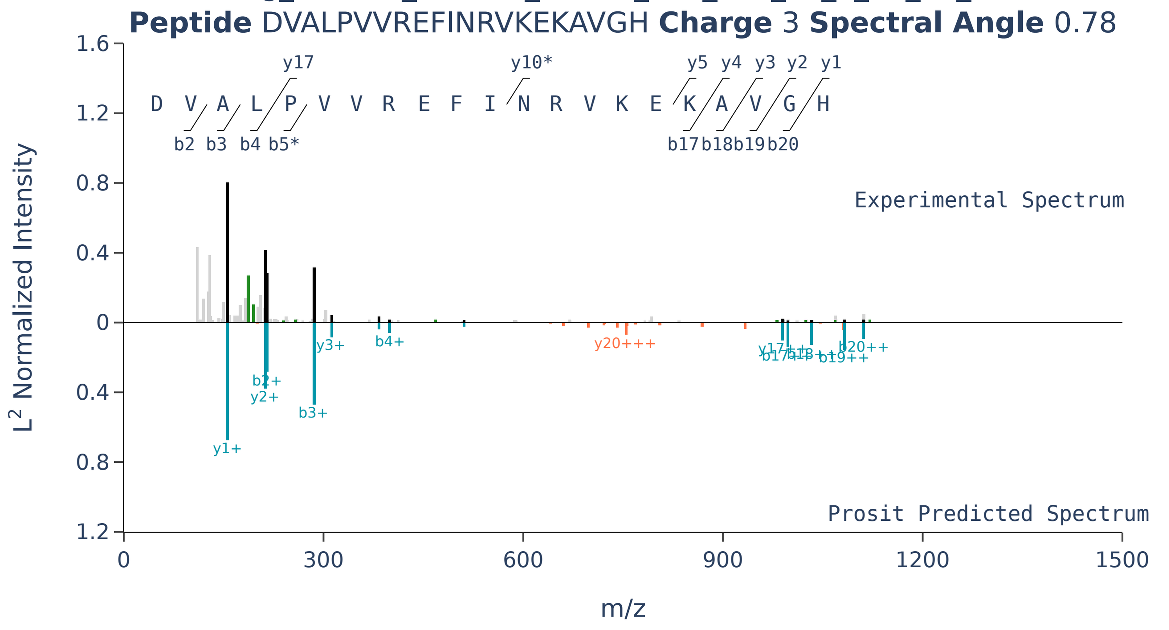


**Source** Ncheng_210623_230623_HFGoe_FFH_20S_25_1_A2_4h_R1 **Scan** 5603
**Peptide** KLASKLK **Charge** 2 **Spectral Angle** 0.89

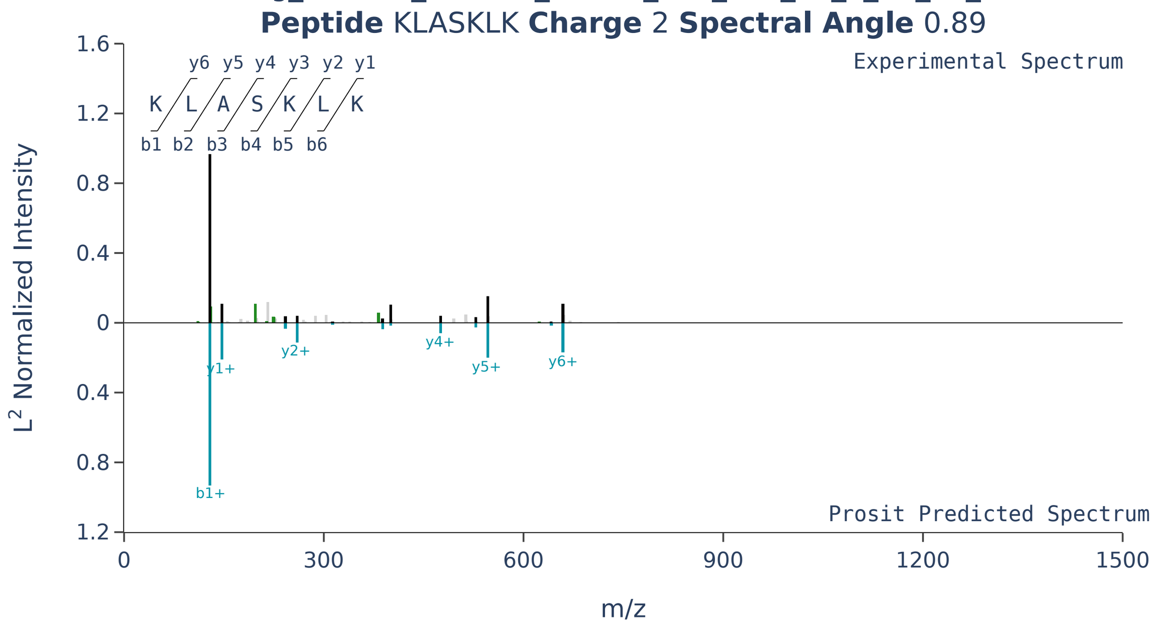


**Source** Ncheng_210623_230623_HFGoe_FFH_20S_25_1_A1_4h_R2 **Scan** 9765
**Peptide** EFINRVKEKAVGHEVNK **Charge** 5 **Spectral Angle** 0.8

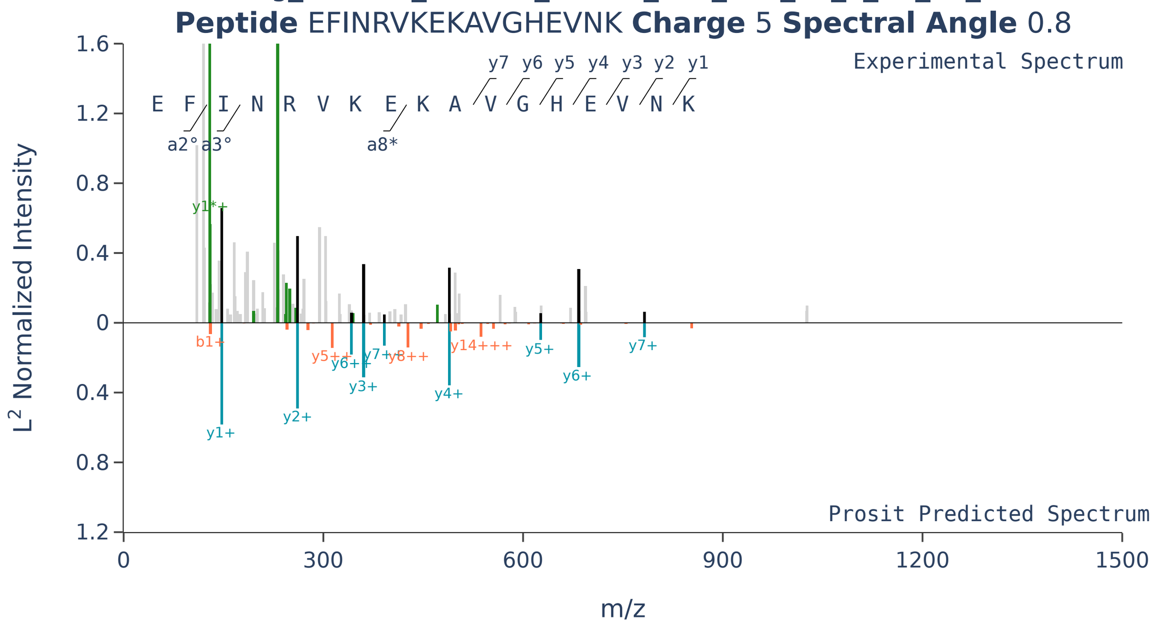


**Source** Ncheng_210623_230623_HFGoe_FFH_20S_25_1_A2_1h_R1 **Scan** 10361
**Peptide** YRPAALKQ **Charge** 2 **Spectral Angle** 0.64

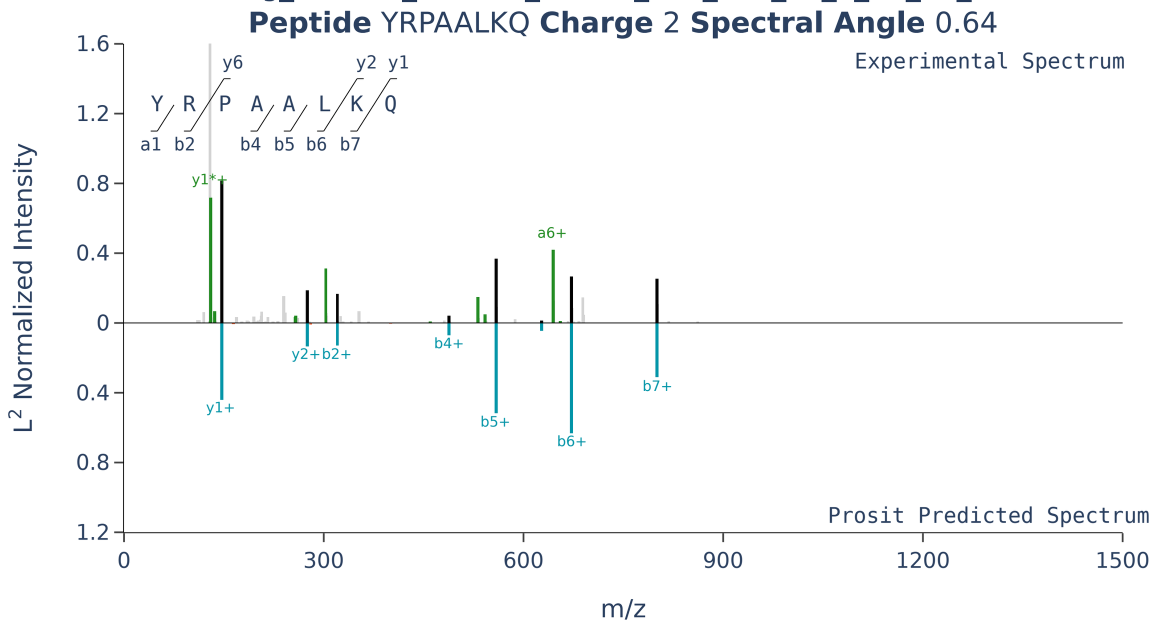


**Source** Ncheng_210623_230623_HFGoe_FFH_20S_25_1_A2_4h_R1 **Scan** 16802
**Peptide** VLTKVDGDARGGAALSIRHI **Charge** 3 **Spectral Angle** 0.66

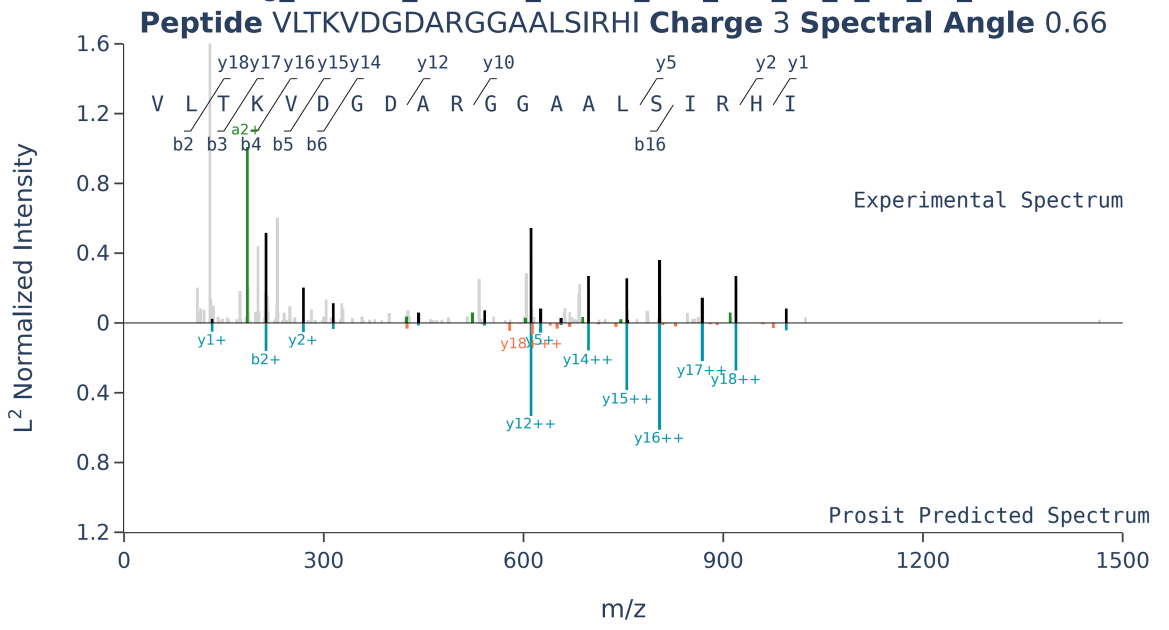


**Source** Ncheng_210623_230623_HFGoe_FFH_20S_25_1_A2_24h_R1 **Scan** 10928
**Peptide** DDMQRMMKKMKKGGMAK **Charge** 3 **Spectral Angle** 0.75

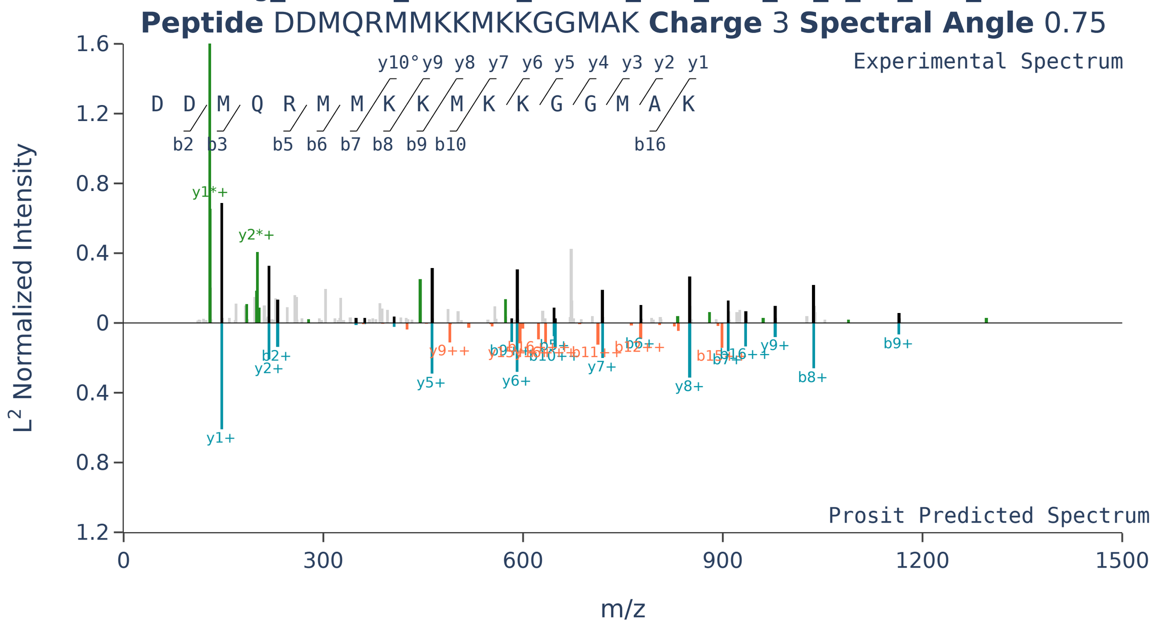


**Source** Ncheng_210623_230623_HFGoe_FFH_20S_25_1_A1_2h_R2 **Scan** 33146
**Peptide** ASKLKKGDGFDLNDFLEQLRQMKN **Charge** 4 **Spectral Angle** 0.68

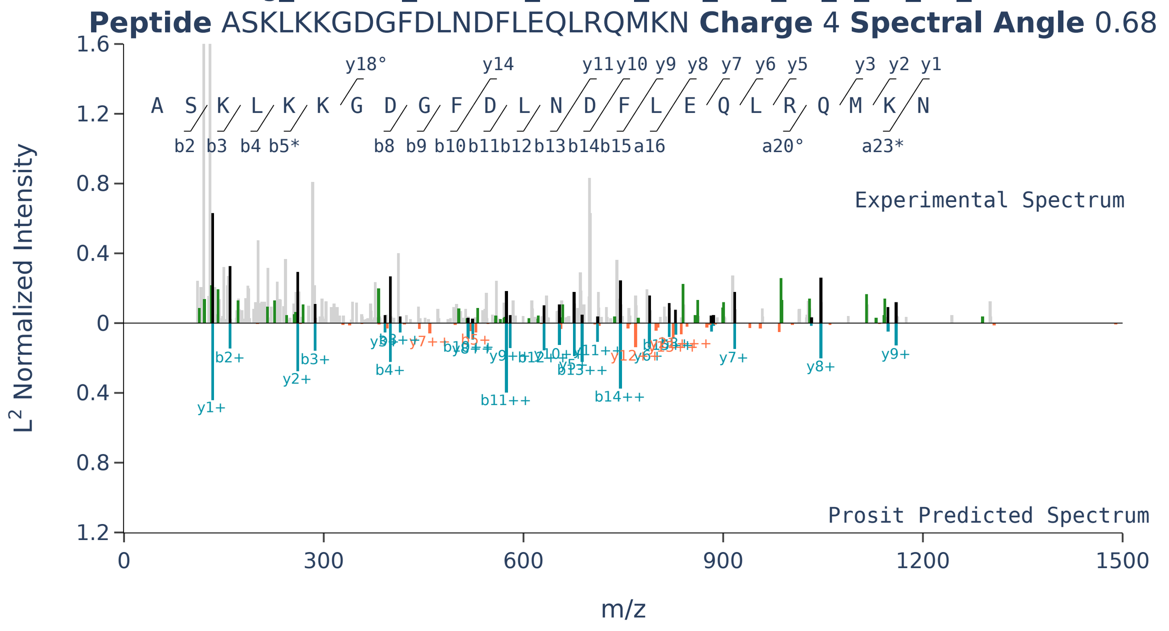


**Source** Ncheng_210623_230623_HFGoe_FFH_20S_25_1_A1_24h_R1 **Scan** 12273
**Peptide** AQAEKLASKLKKGDGF **Charge** 3 **Spectral Angle** 0.55

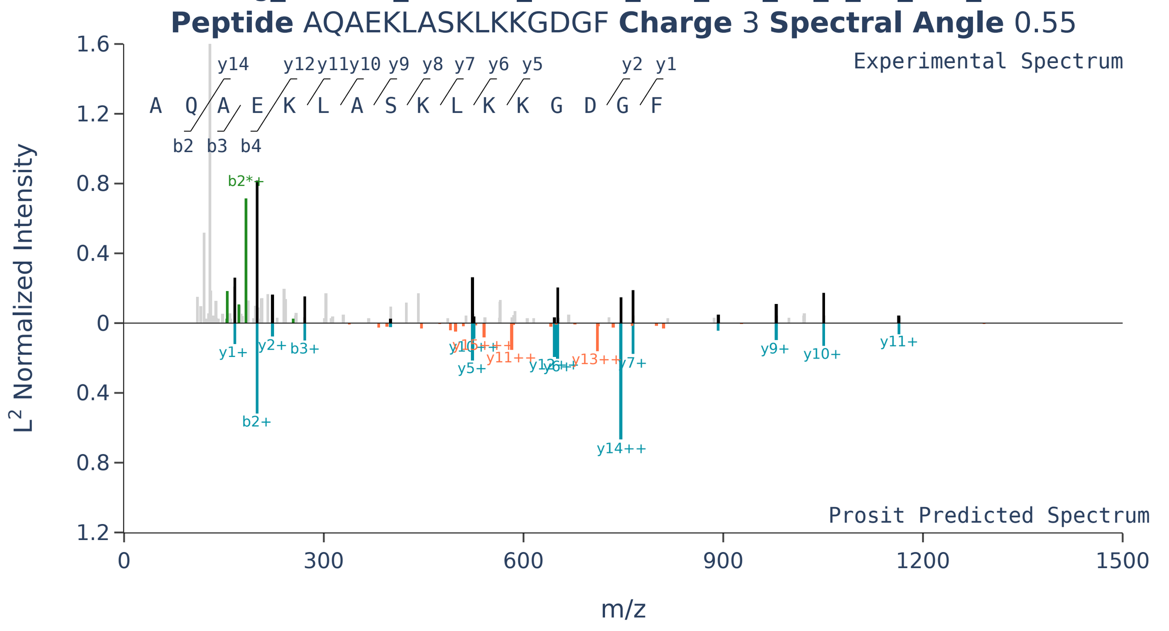


**Source** Ncheng_210623_230623_HFGoe_FFH_20S_25_1_A1_4h_R1 **Scan** 19400
**Peptide** AALKEAKLKFYDV **Charge** 4 **Spectral Angle** 0.77

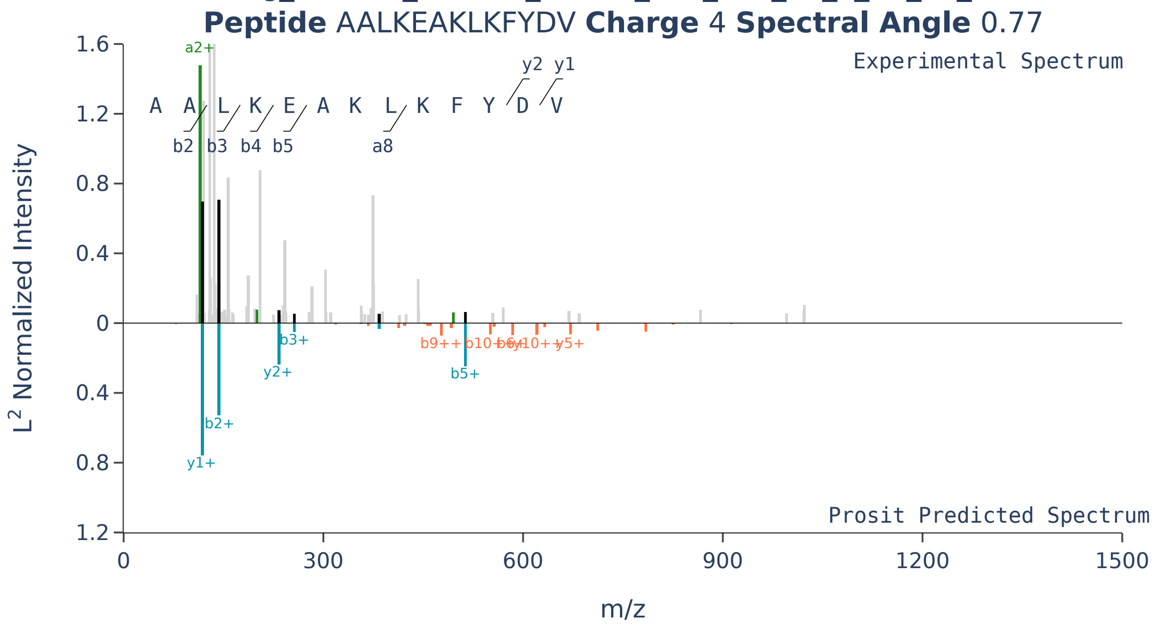


**Source** Ncheng_210623_230623_HFGoe_FFH_20S_25_1_A2_4h_R1 **Scan** 14534
**Peptide** DGDARGGAALSIRHLT **Charge** 3 **Spectral Angle** 0.76

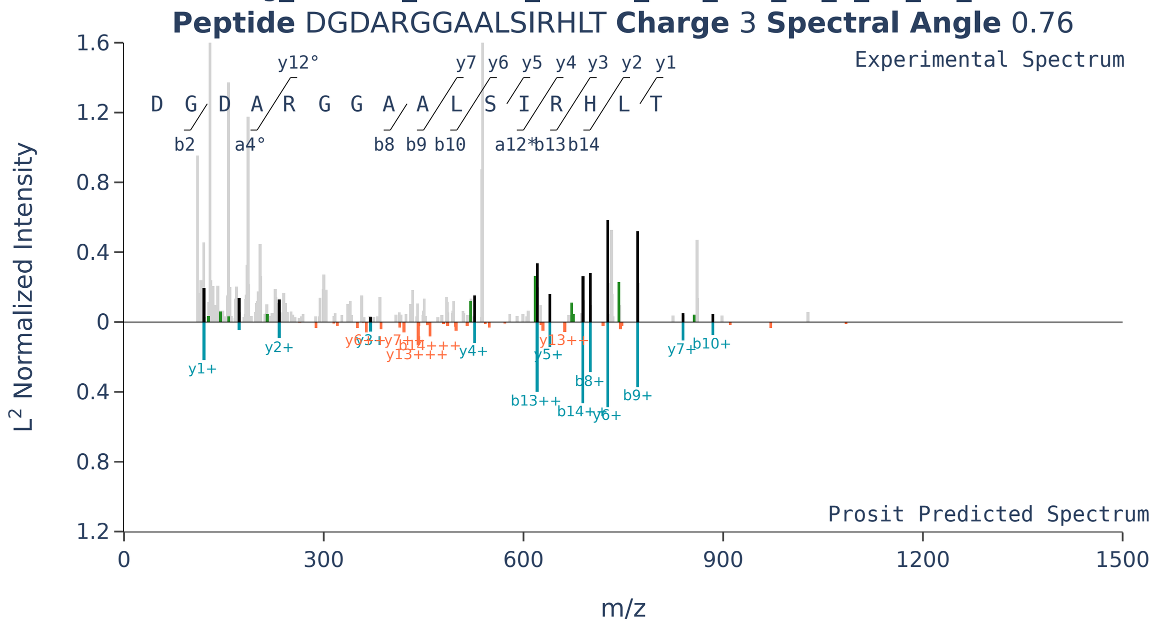


**Source** Ncheng_210623_230623_HFGoe_FFH_20S_25_1_A1_24h_R2 **Scan** 13621
**Peptide** QKPVDIVNA **Charge** 2 **Spectral Angle** 0.83

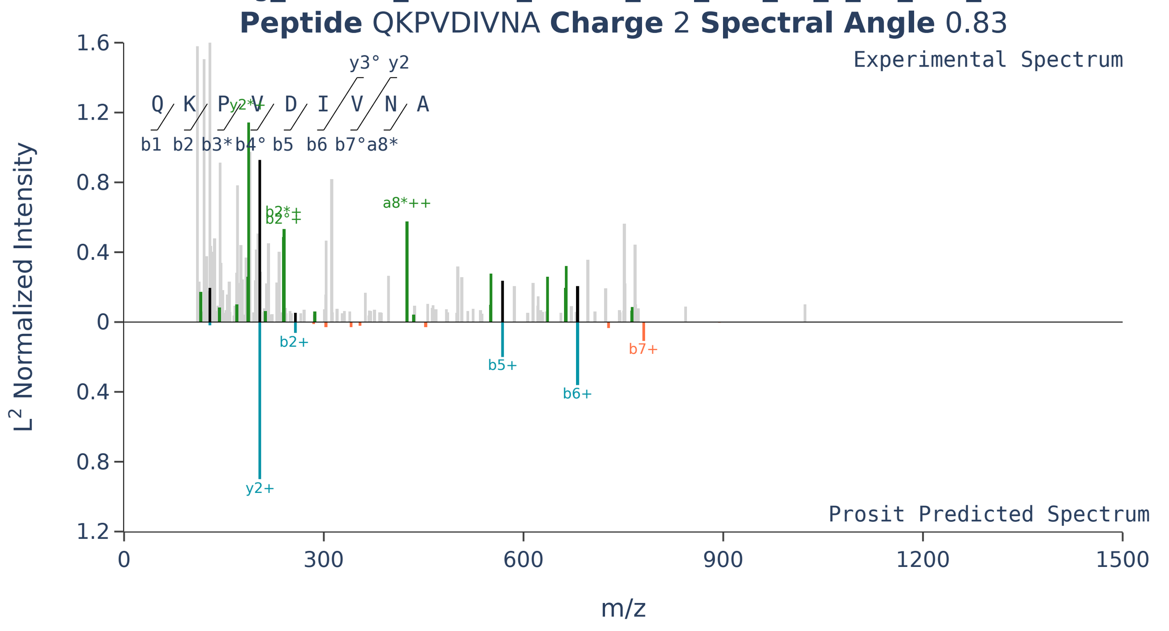


**Source** Ncheng_210623_230623_HFGoe_FFH_20S_25_1_A2_4h_R2 **Scan** 34178
**Peptide** PGMGQIPDNVKSQMDDKVLVRMEAII **Charge** 4 **Spectral Angle** 0.79

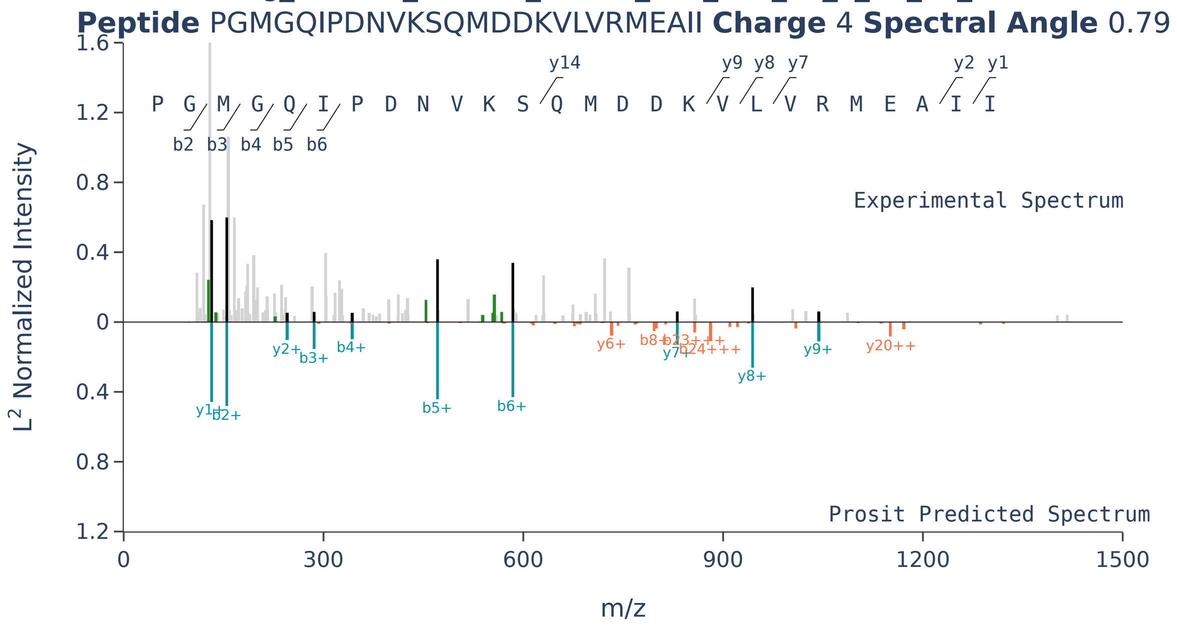


**Source** Ncheng_210623_230623_HFGoe_FFH_20S_25_1_A1_24h_R1 **Scan** 17099
**Peptide** DLESKVDRAQAEKLASKLK **Charge** 5 **Spectral Angle** 0.81

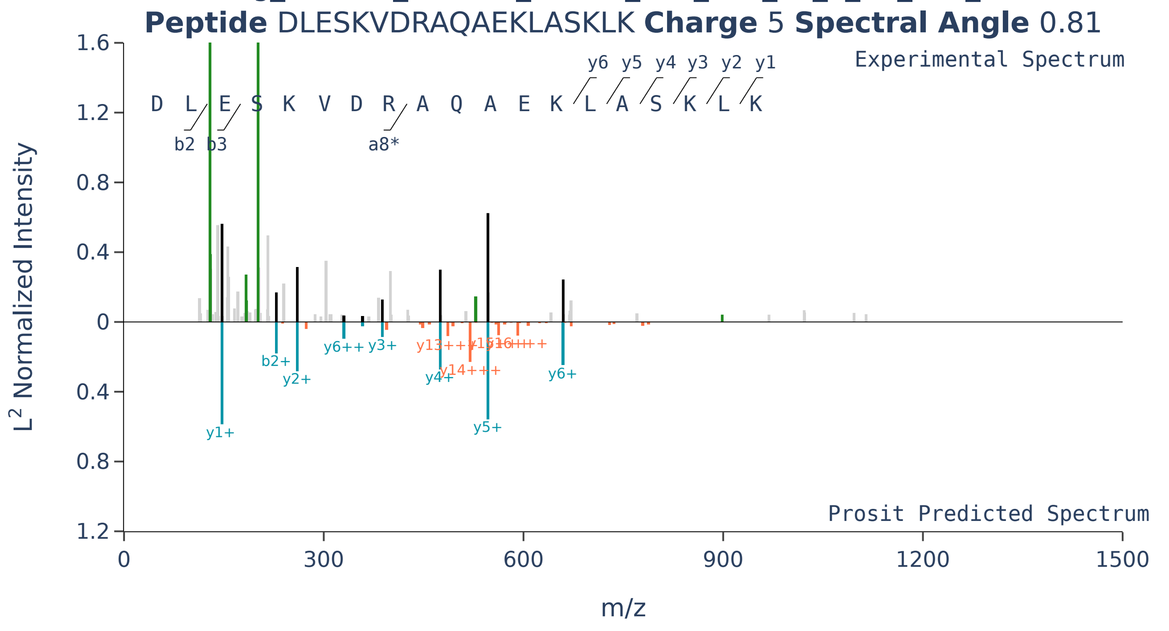


**Source** Ncheng_210623_230623_HFGoe_FFH_20S_25_1_A1_4h_R1 **Scan** 12613
**Peptide** DLESKVDRAQAEKL **Charge** 2 **Spectral Angle** 0.76

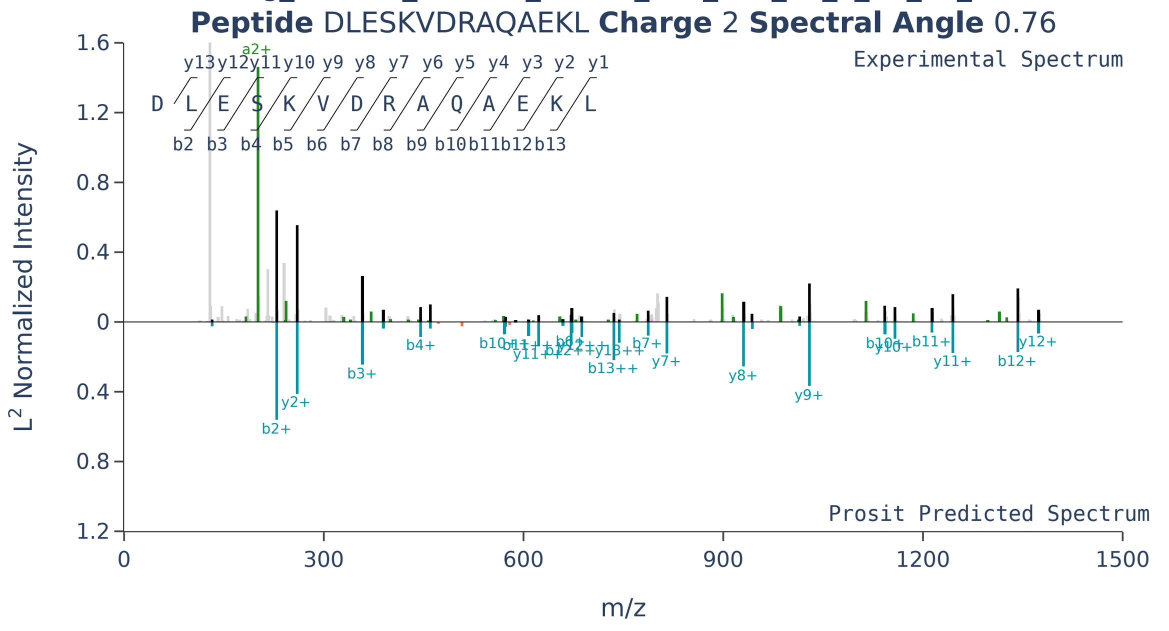


**Source** Ncheng_210623_230623_HFGoe_FFH_20S_25_1_A1_1h_R2 **Scan** 26851
**Peptide** LGVGEKTEALEPFHPDRIASRILG **Charge** 4 **Spectral Angle** 0.75

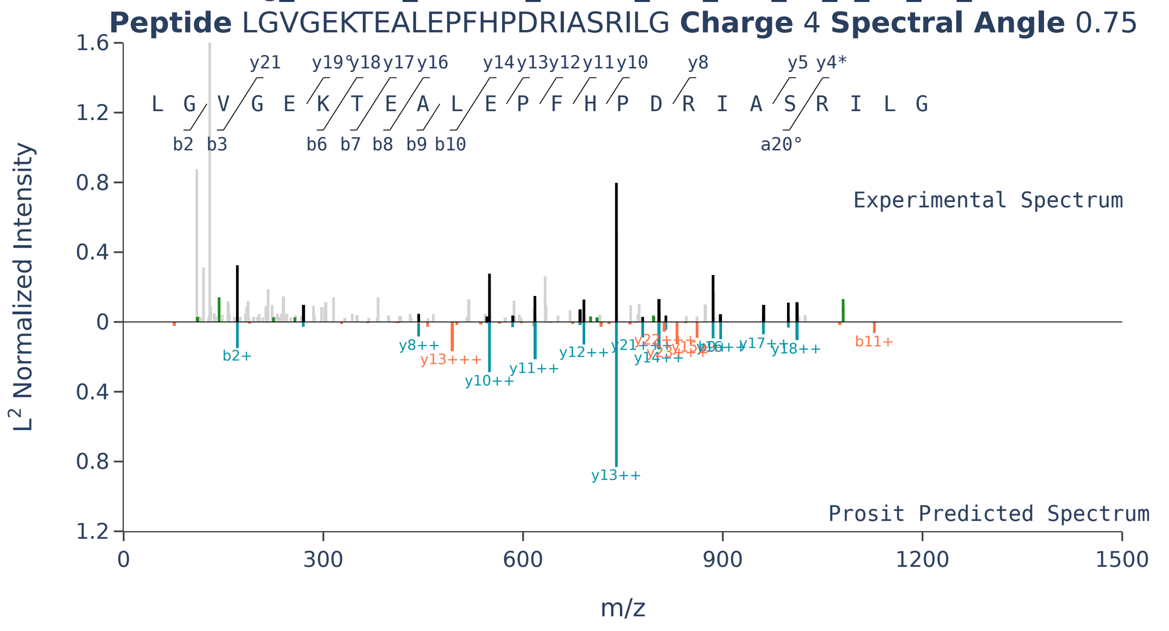

**Source** Ncheng_210623_230623_HFGoe_FFH_20S_25_1_A2_2h_R1 **Scan** 28785
**Peptide** KTEALEPFHPDRIASRILGMGD **Charge** 3 **Spectral Angle** 0.63

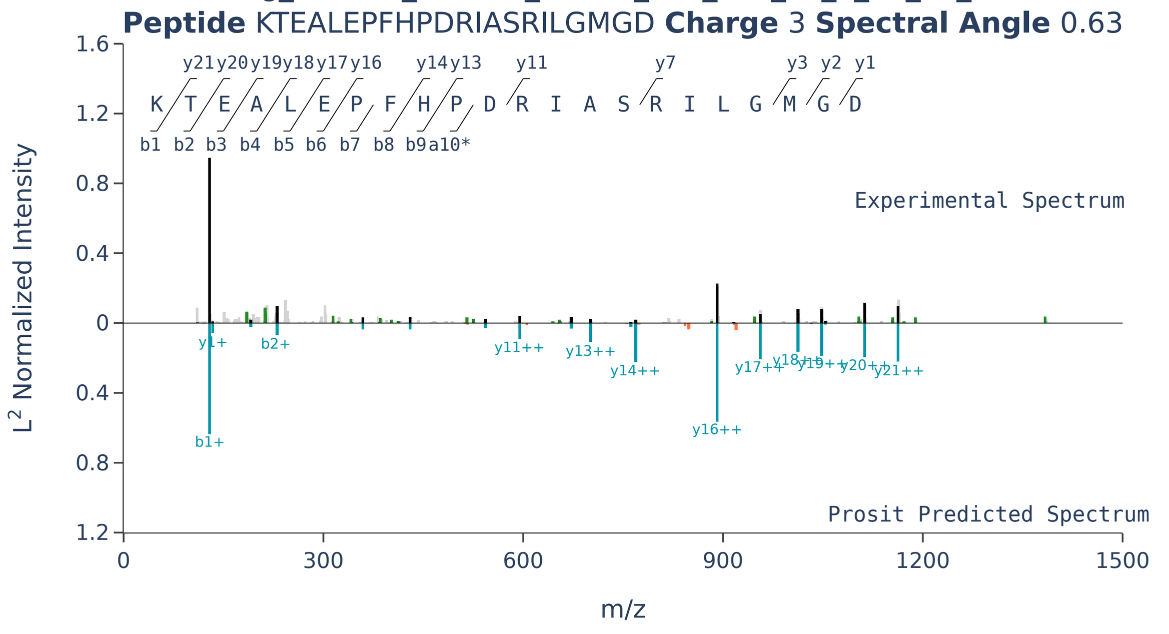


**Source** Ncheng_210623_230623_HFGoe_FFH_20S_25_1_A1_4h_R1 **Scan** 22150
**Peptide** PLTGVVLTKVDGDARGGAALSIRH **Charge** 5 **Spectral Angle** 0.64

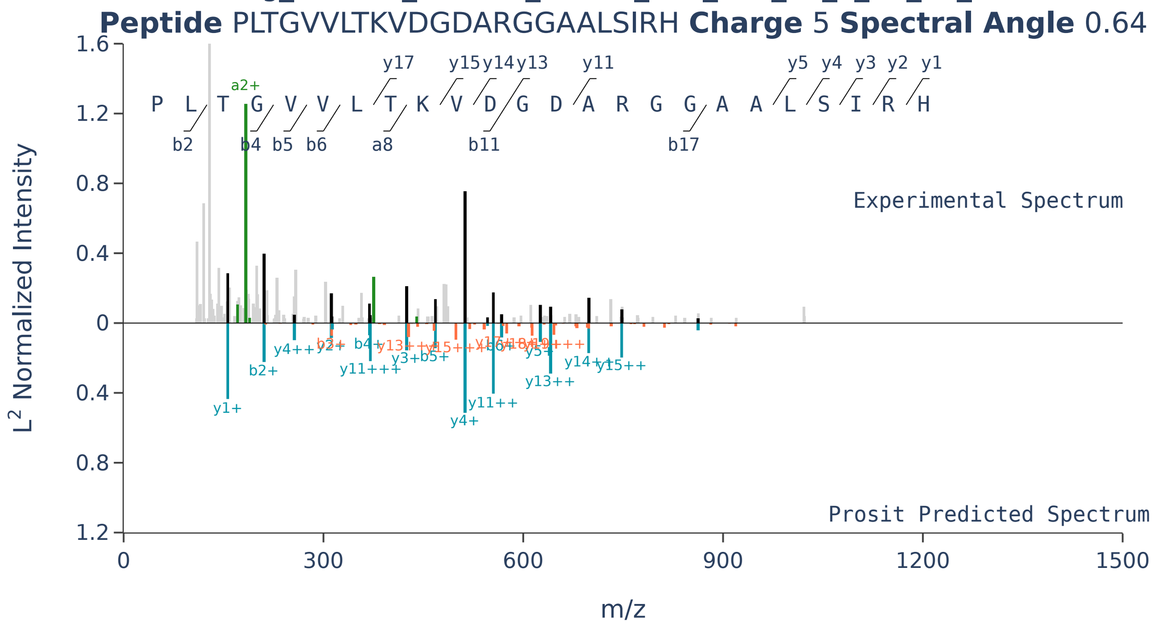


**Source** Ncheng_210623_230623_HFGoe_FFH_20S_25_1_A1_1h_R2 **Scan** 28752
**Peptide** KEAKLKFYDVLLVDTAGRLHVDE **Charge** 3 **Spectral Angle** 0.68

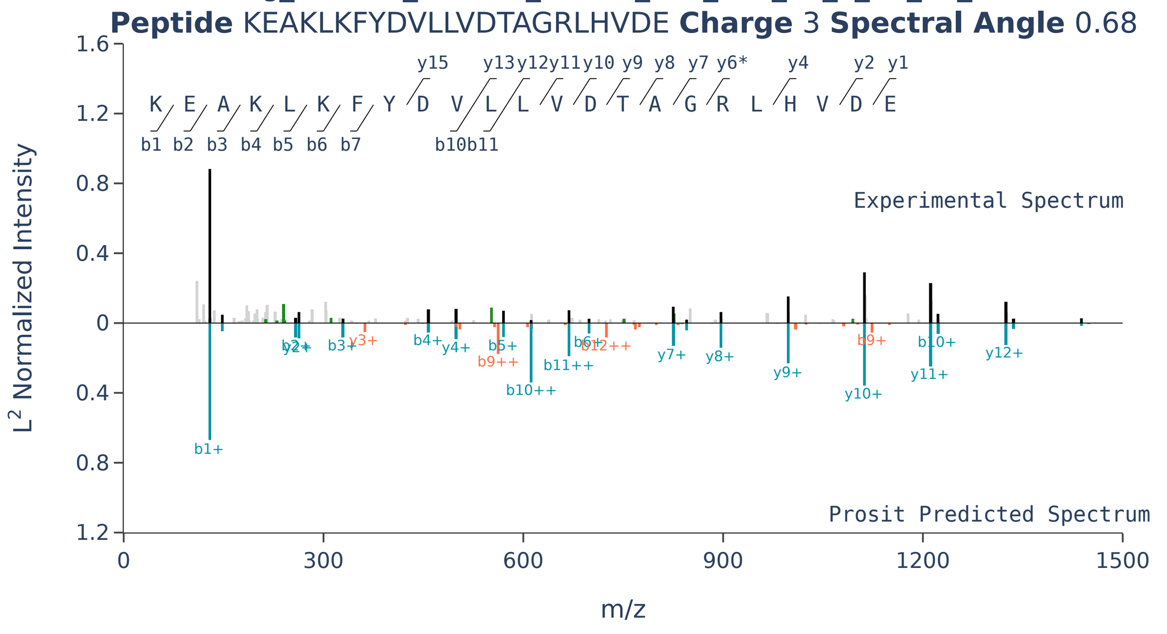


**Source** Ncheng_210623_230623_HFGoe_FFH_20S_25_1_A1_4h_R2 **Scan** 20197
**Peptide** KTEALEPFHPDRIASRI **Charge** 5 **Spectral Angle** 0.51

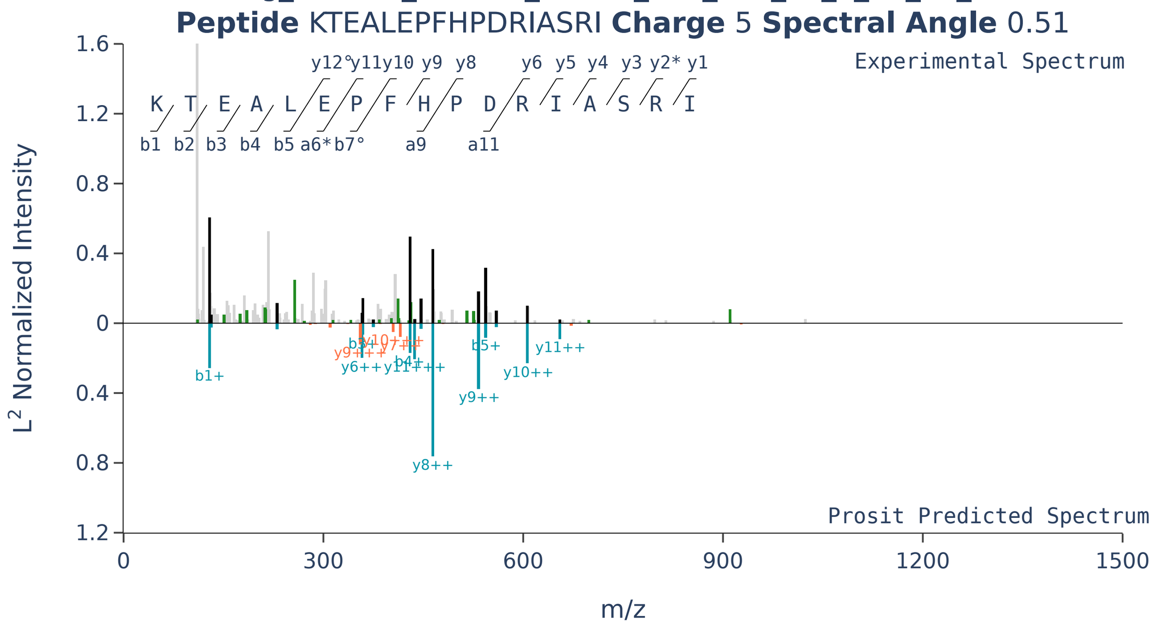


**Source** Ncheng_210623_230623_HFGoe_FFH_20S_25_1_A2_2h_R1 **Scan** 35234
**Peptide** KSQMDDKVLVRMEAIINSM **Charge** 3 **Spectral Angle** 0.51

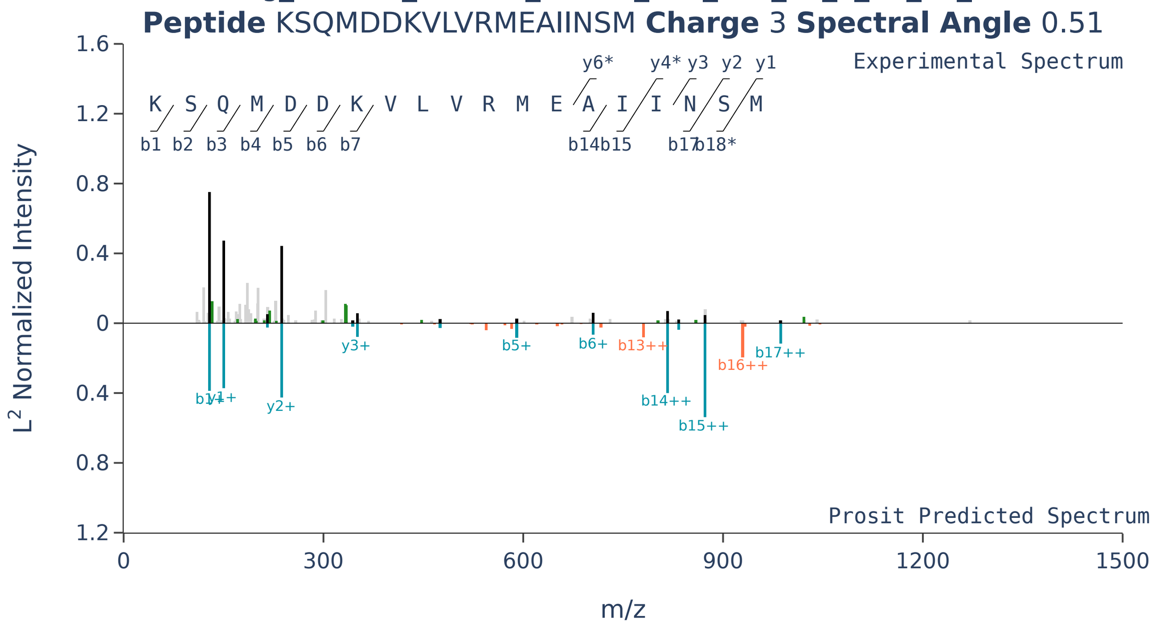


**Source** Ncheng_210623_230623_HFGoe_FFH_20S_25_1_A2_2h_R1 **Scan** 8696
**Peptide** KKKVLVVSA **Charge** 2 **Spectral Angle** 0.35

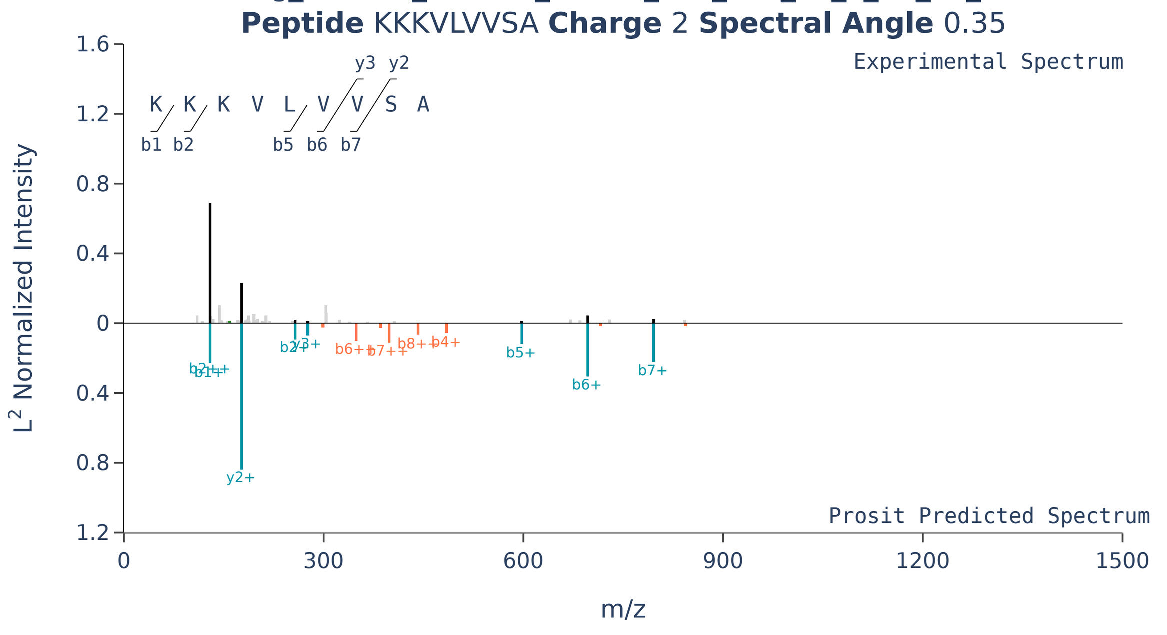


**Source** Ncheng_210623_230623_HFGoe_FFH_20S_25_1_A2_4h_R1 **Scan** 30319
**Peptide** VNAALKEAKLKFYDVLL **Charge** 3 **Spectral Angle** 0.74

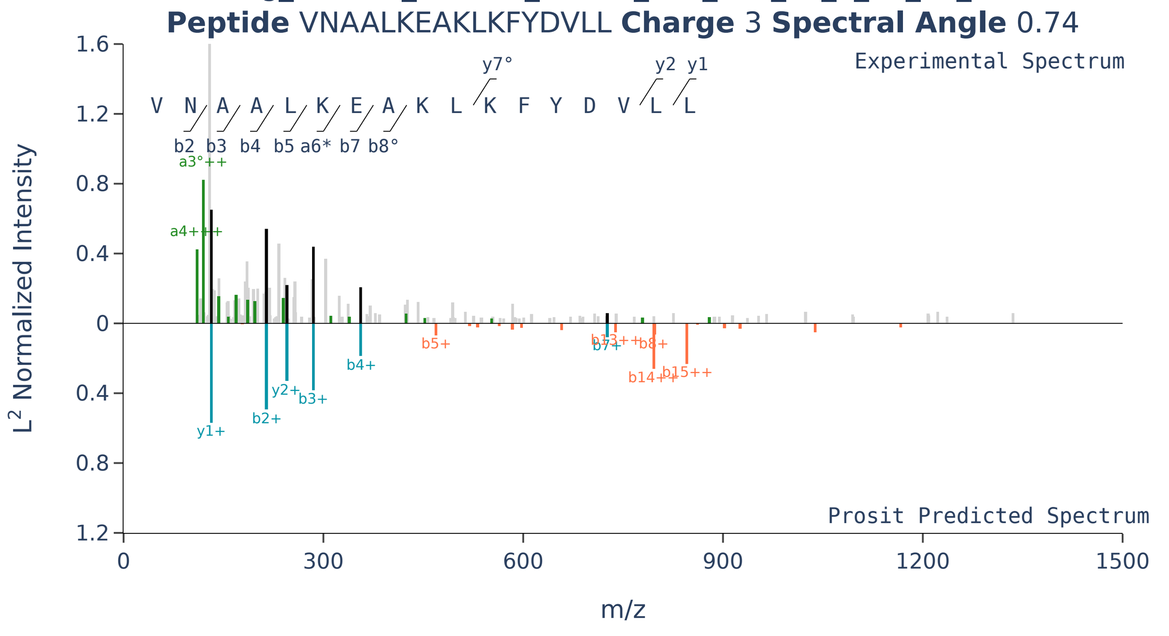


**Source** Ncheng_210623_230623_HFGoe_FFH_20S_25_1_A1_4h_R1 **Scan** 21582
**Peptide** VVLTKVDGDARGGAALSIRHITGKPLKF **Charge** 3 **Spectral Angle** 0.67

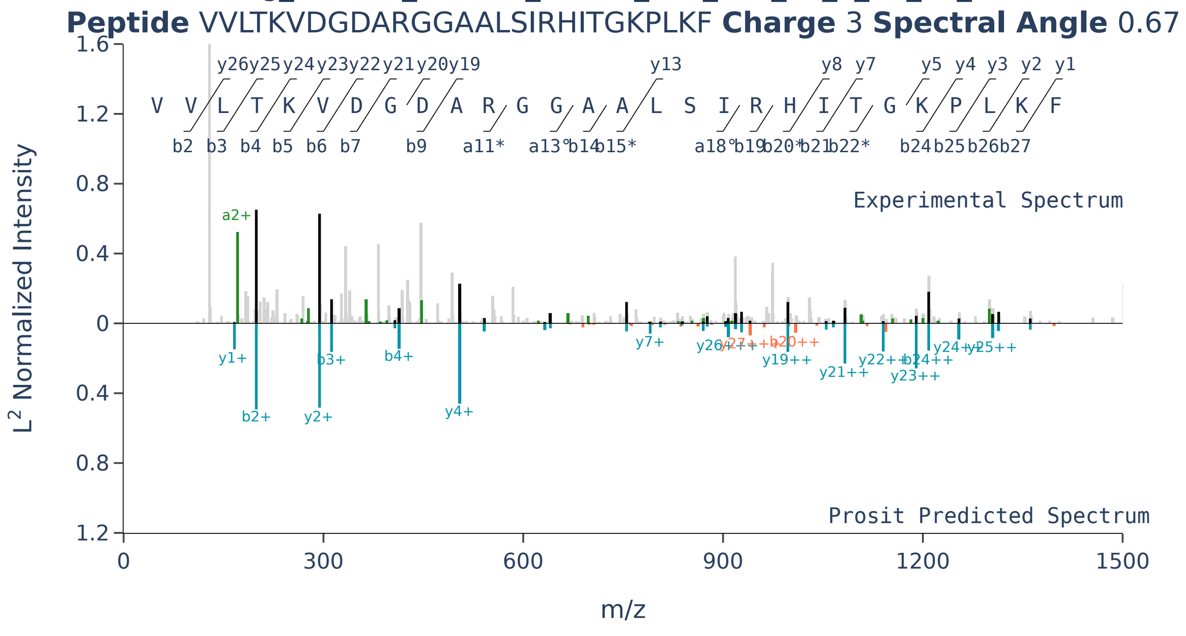


**Source** Ncheng_210623_230623_HFGoe_FFH_20S_25_1_A2_24h_R1 **Scan** 6691
**Peptide** ISGRGRLTED **Charge** 3 **Spectral Angle** 0.63

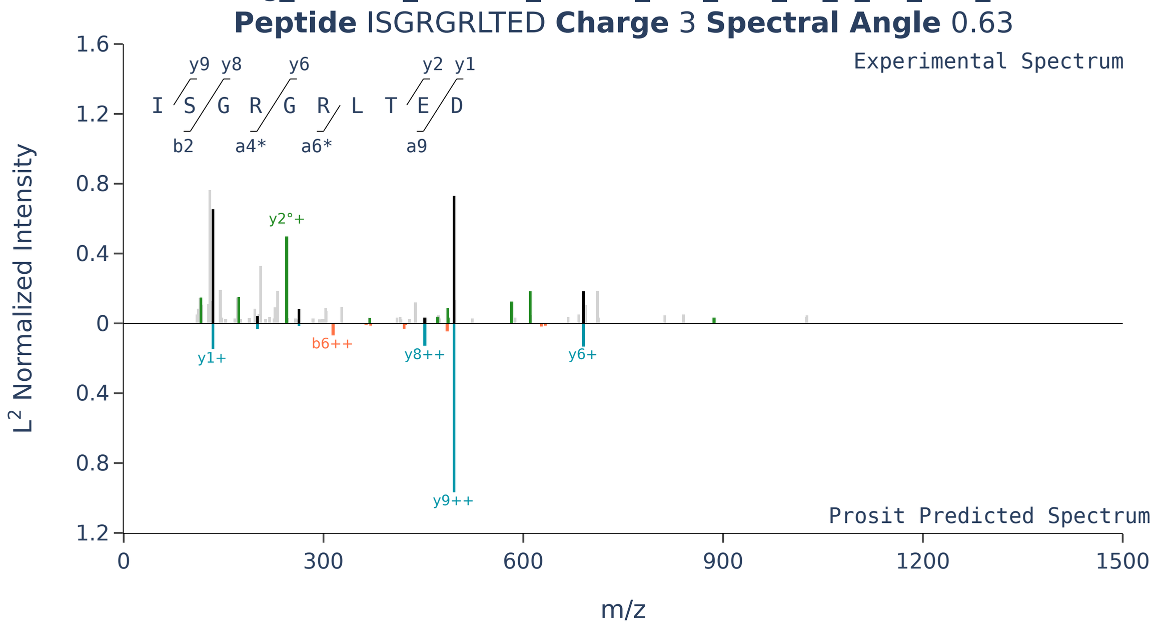


**Source** Ncheng_210623_230623_HFGoe_FFH_20S_25_1_A2_24h_R2 **Scan** 22069
**Peptide** GCGMQVQDVNRLLKQ **Charge** 3 **Spectral Angle** 0.64

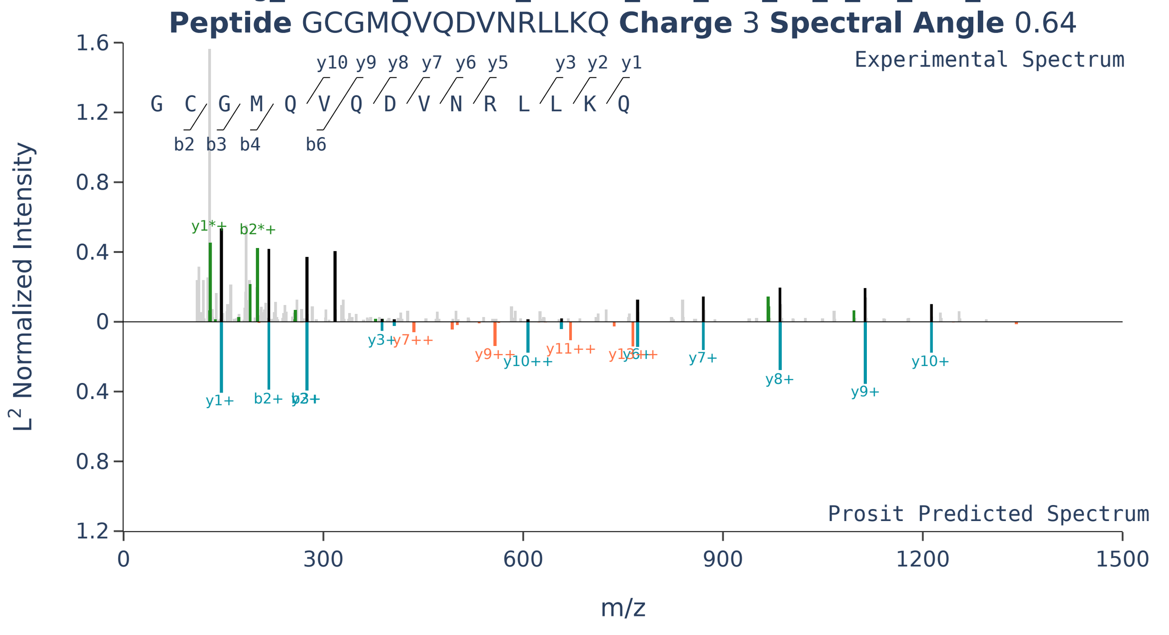


**Source** Ncheng_210623_230623_HFGoe_FFH_20S_25_1_A1_24h_R1 **Scan** 7601
**Peptide** NSMTMKERAKPEIIKGSRK **Charge** 5 **Spectral Angle** 0.71

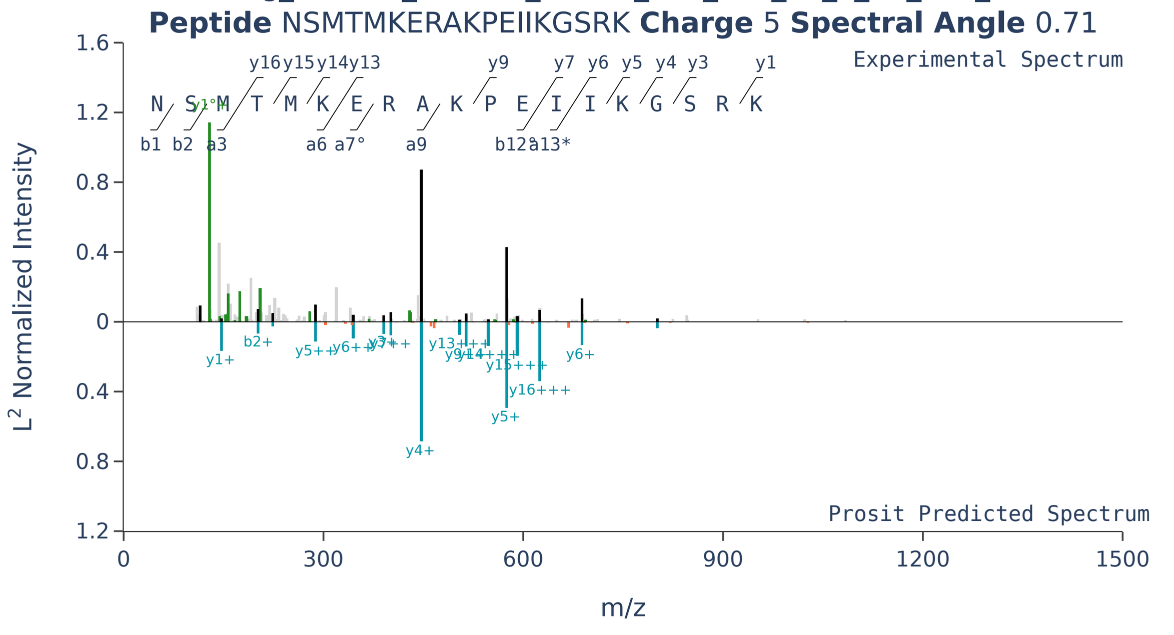


**Source** Ncheng_210623_230623_HFGoe_FFH_20S_25_1_A1_4h_R2 **Scan** 14379
**Peptide** DRAQAEKLASKLKKGDGF **Charge** 4 **Spectral Angle** 0.59

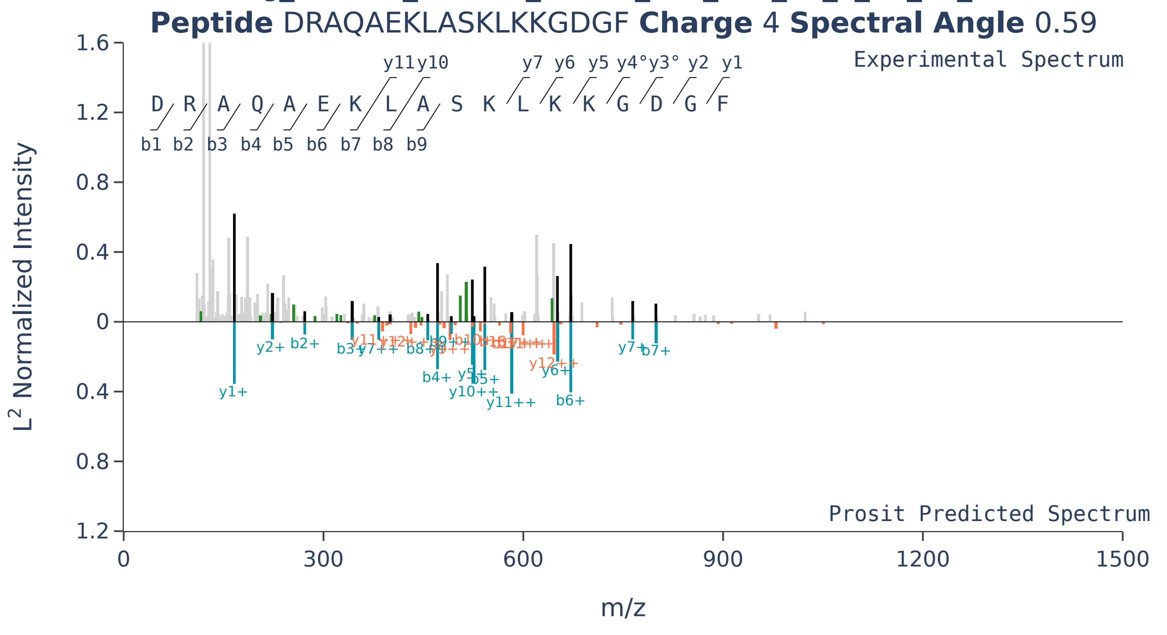


**Source** Ncheng_210623_230623_HFGoe_FFH_20S_25_1_A1_24h_R2 **Scan** 7445
**Peptide** GAGKTTSVGKLG **Charge** 2 **Spectral Angle** 0.56

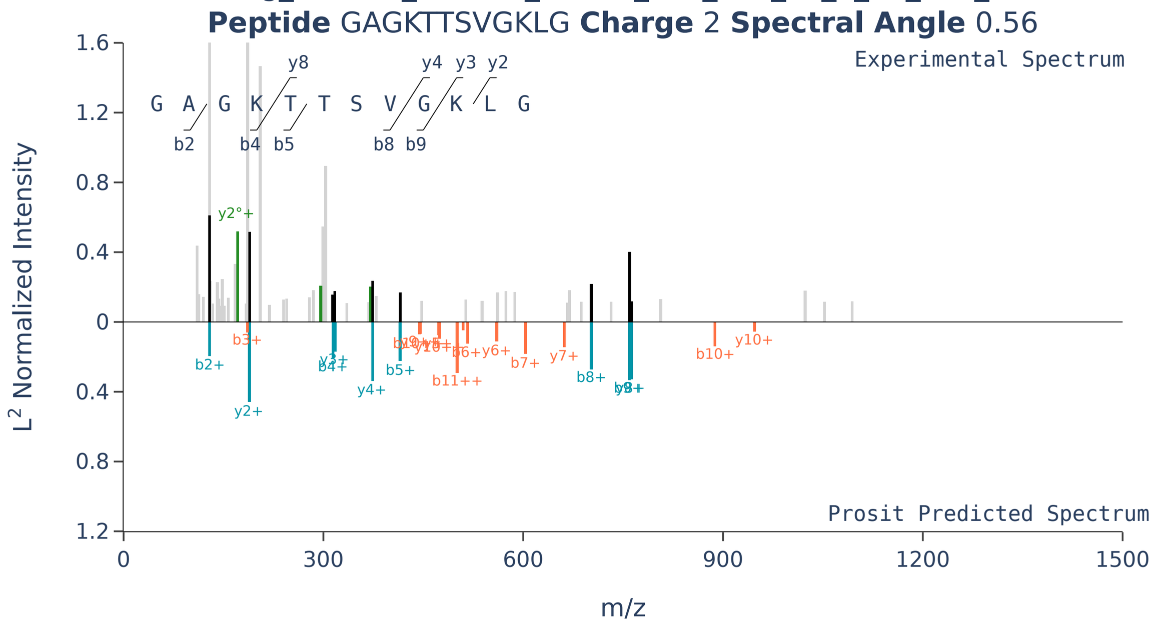


**Source** Ncheng_210623_230623_HFGoe_FFH_20S_25_1_A2_2h_R1 **Scan** 28473
**Peptide** NISGRGRLTEDNVKDTLREVRMALLE **Charge** 5 **Spectral Angle** 0.68

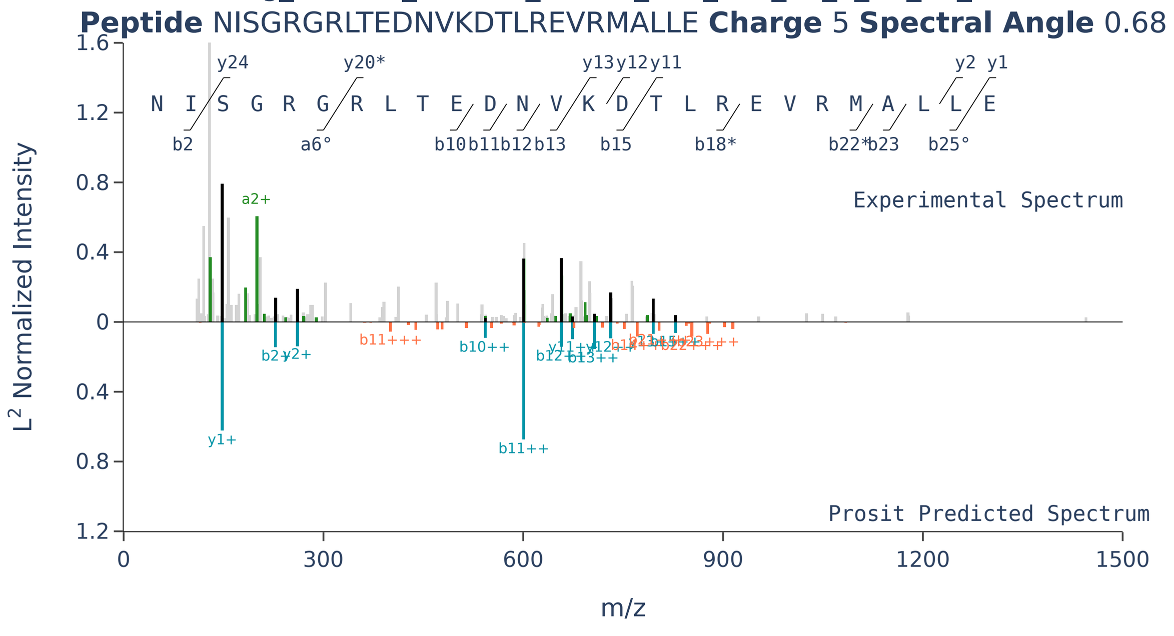


**Source** Ncheng_210623_230623_HFGoe_FFH_20S_25_1_A2_2h_R2 **Scan** 28681
**Peptide** VVSADVYRPAAIKQLETLAE **Charge** 3 **Spectral Angle** 0.82

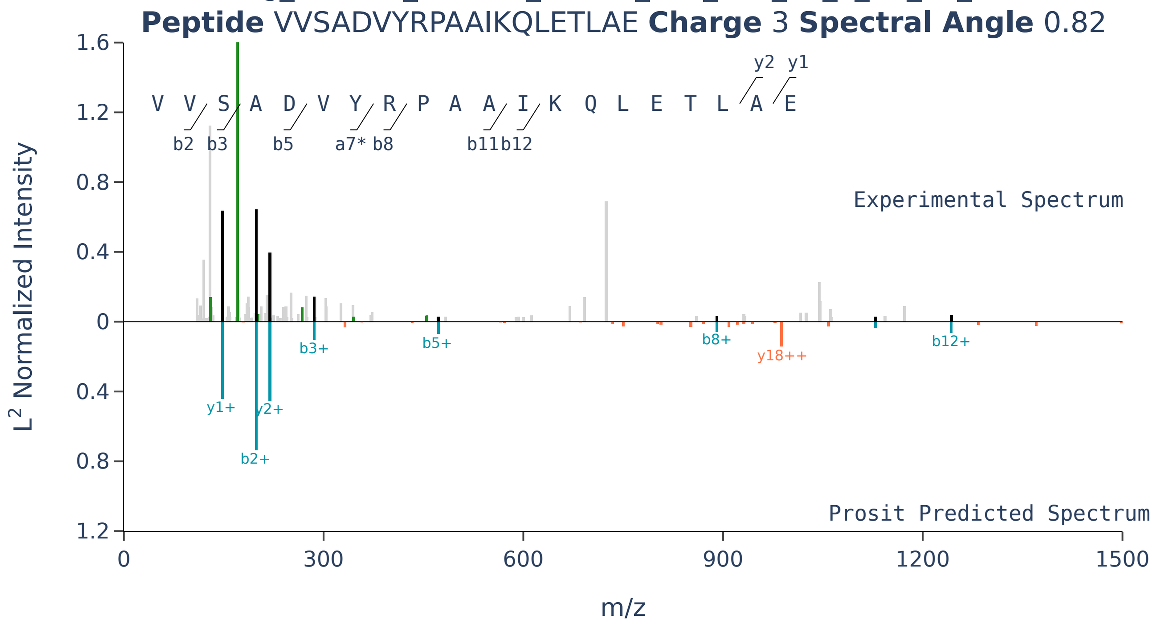

**Source** Ncheng_210623_230623_HFGoe_FFH_20S_25_1_A1_1h_R1 **Scan** 27386
**Peptide** SGRGRLTEDNVKDTLREVRMALL **Charge** 5 **Spectral Angle** 0.83

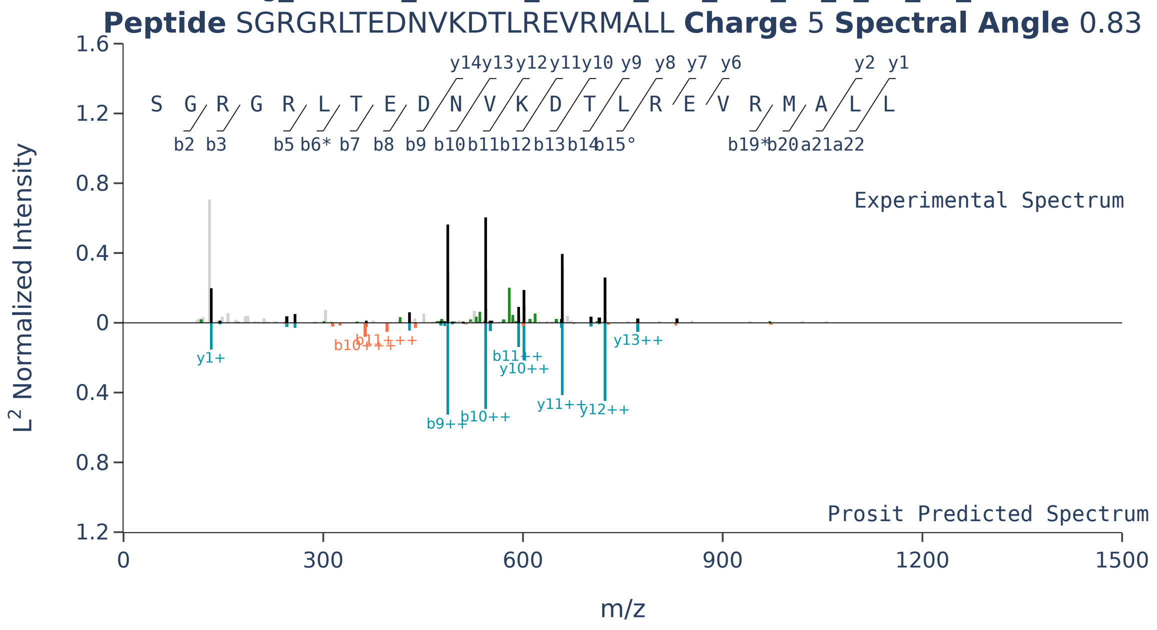


**Source** Ncheng_210623_230623_HFGoe_FFH_20S_25_1_A1_24h_R2 **Scan** 8467
**Peptide** LRNISGRGRLTED **Charge** 2 **Spectral Angle** 0.61

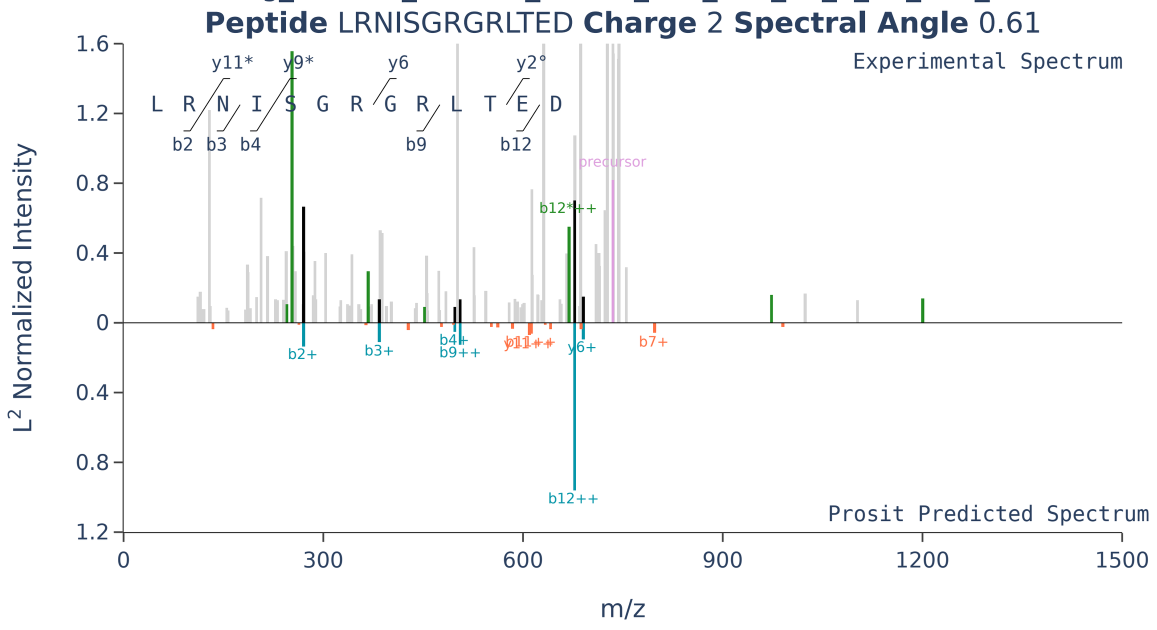


**Source** Ncheng_210623_230623_HFGoe_FFH_20S_25_1_A2_24h_R2 **Scan** 8205
**Peptide** LNRVKEKAVGHEVNKSLTPGQ **Charge** 3 **Spectral Angle** 0.71

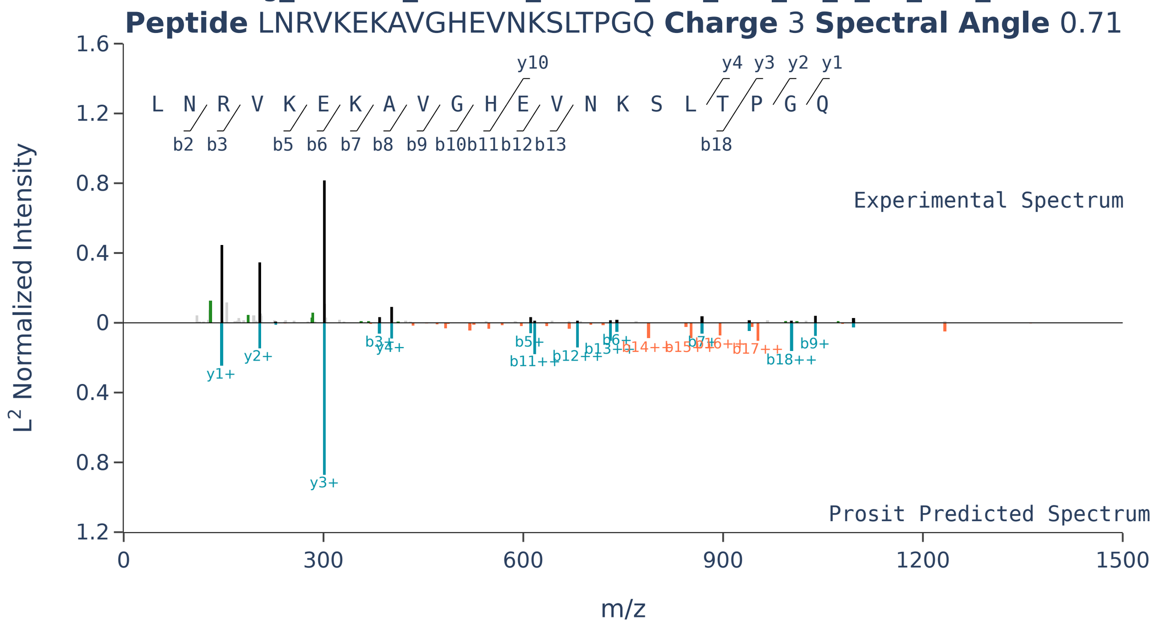


**Source** Ncheng_210623_230623_HFGoe_FFH_20S_25_1_A1_2h_R2 **Scan** 28556
**Peptide** RGRLTEDNVKDTLREVRMALLE **Charge** 5 **Spectral Angle** 0.72

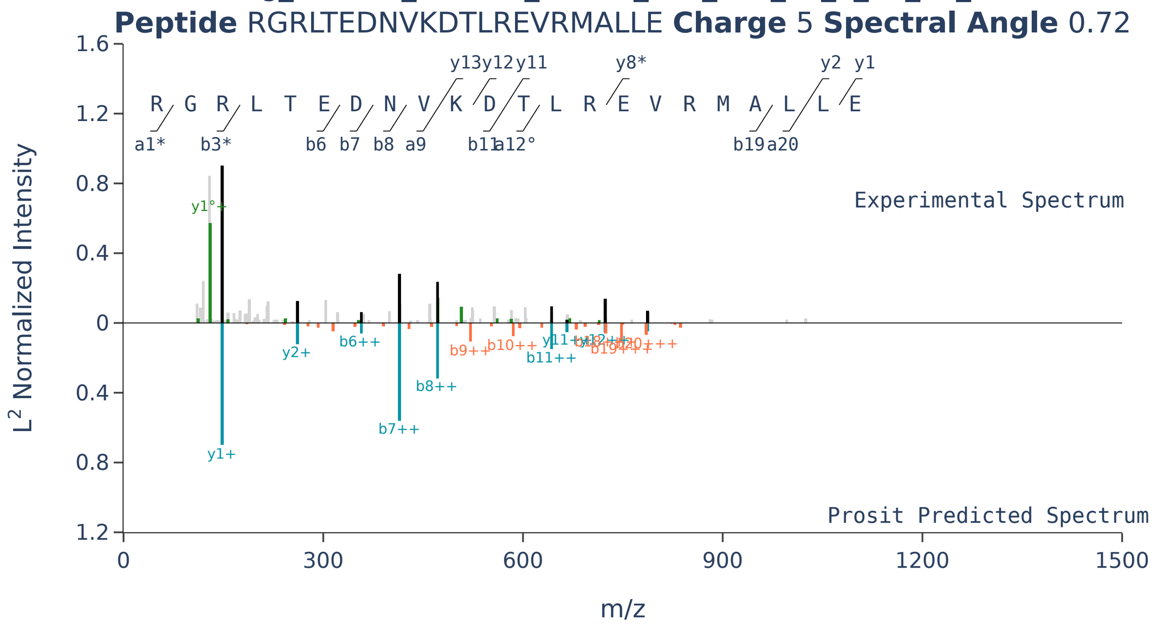


**Source** Ncheng_210623_230623_HFGoe_FFH_20S_25_1_A2_4h_R1 **Scan** 29789
**Peptide** KKGDGFDLNDFLEQ **Charge** 2 **Spectral Angle** 0.52

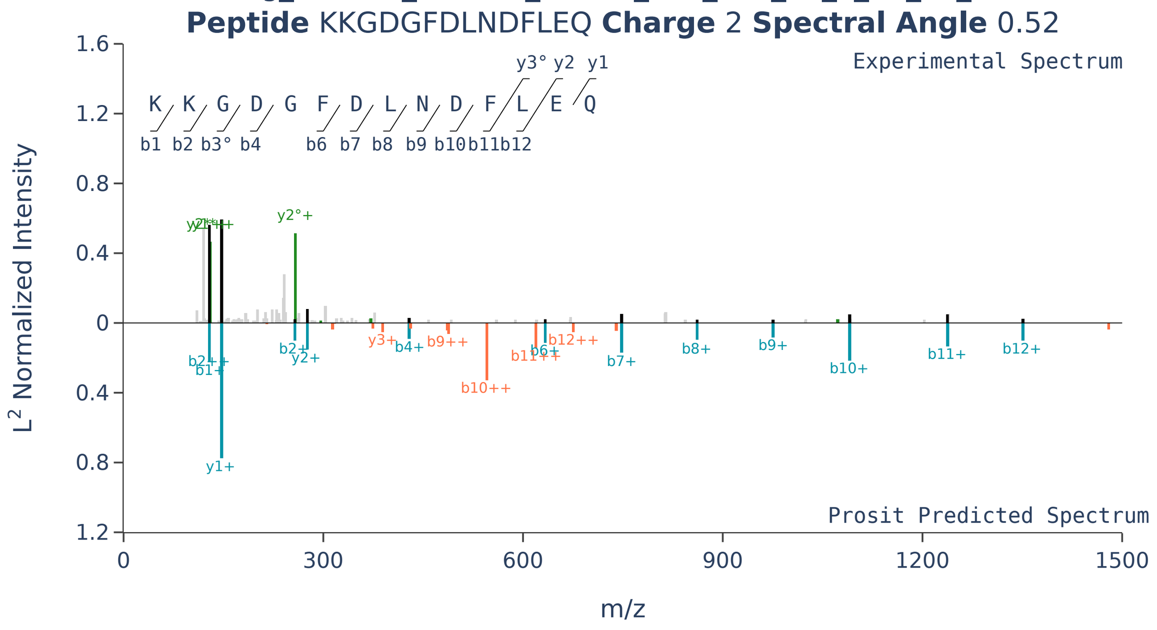


**Source** Ncheng_210623_230623_HFGoe_FFH_20S_25_1_A1_2h_R1 **Scan** 26392
**Peptide** DKVLVRMEAIINS **Charge** 2 **Spectral Angle** 0.65

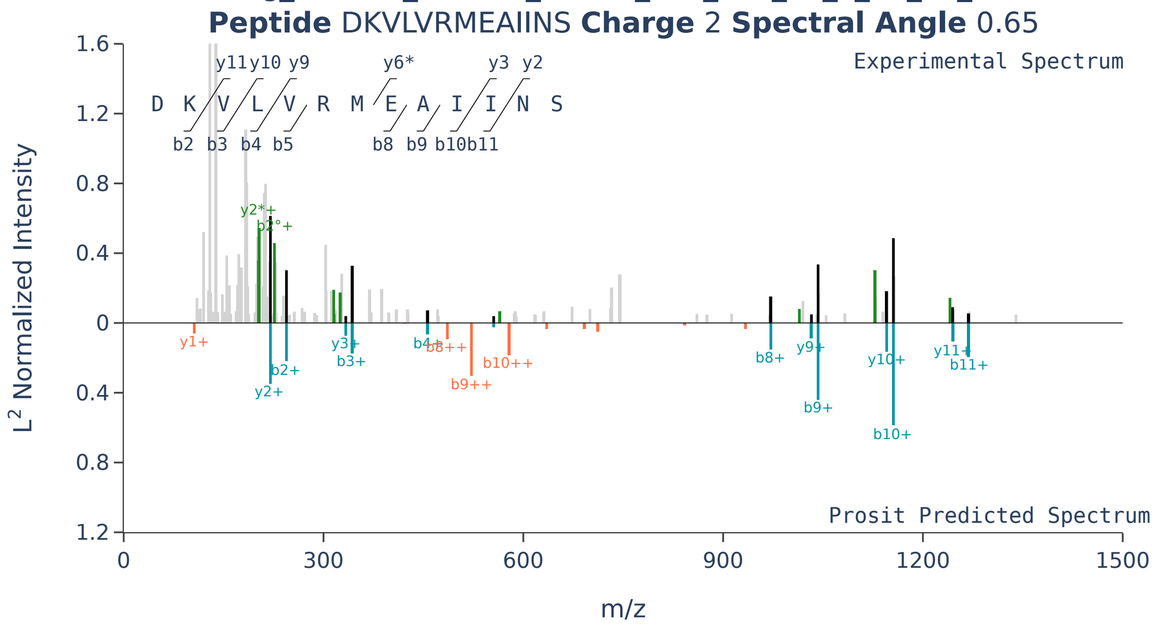


**Source** Ncheng_210623_230623_HFGoe_FFH_20S_25_1_A1_4h_R1 **Scan** 19630
**Peptide** VDRAQAEKLASKLKKGDGFDLND **Charge** 5 **Spectral Angle** 0.74

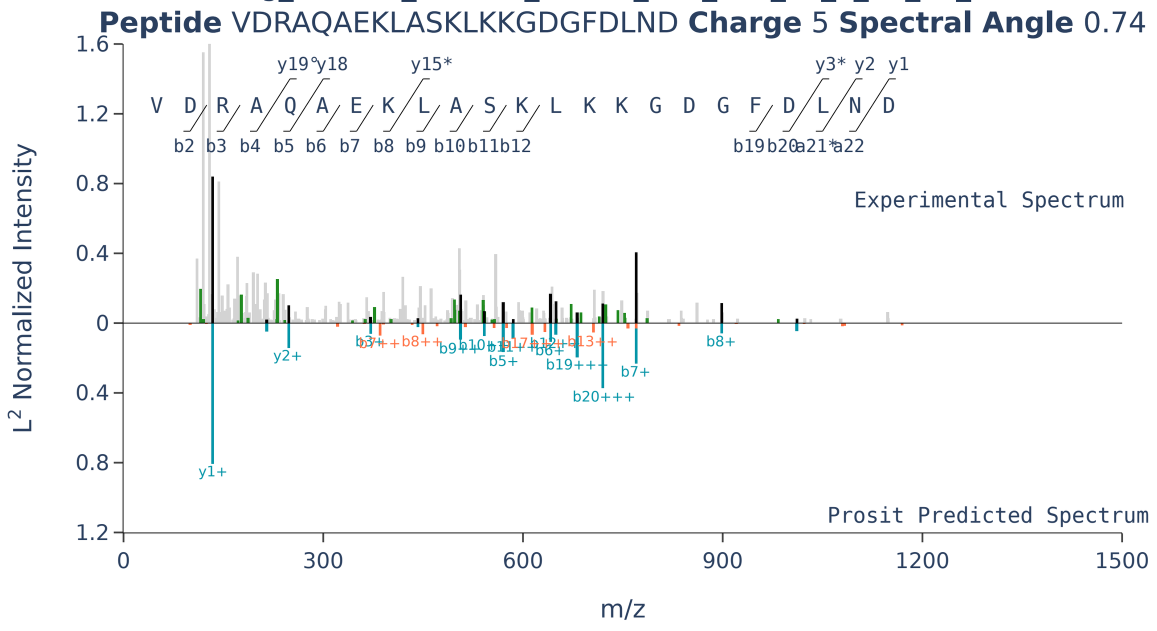


**Source** Ncheng_210623_230623_HFGoe_FFH_20S_25_1_A1_24h_R1 **Scan** 14230
**Peptide** EKAVGHEVNKSLTPGQEF **Charge** 3 **Spectral Angle** 0.81

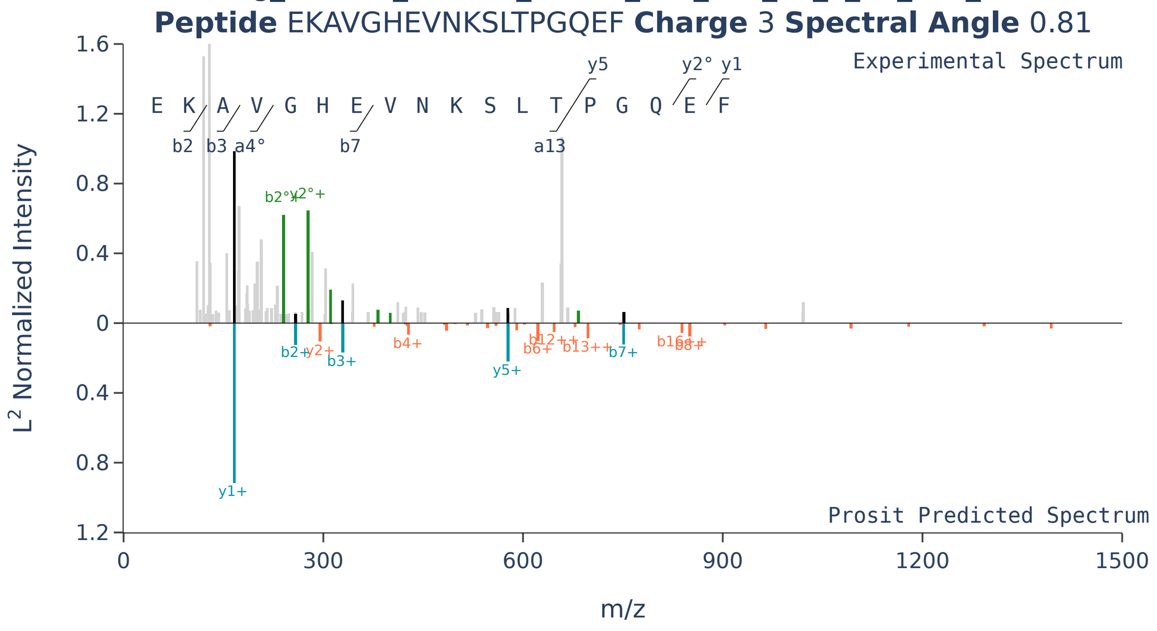


**Source** Ncheng_210623_230623_HFGoe_FFH_20S_25_1_A1_2h_R1 **Scan** 31504
**Peptide** EALEPFHPDRIASRILGMGD **Charge** 3 **Spectral Angle** 0.62

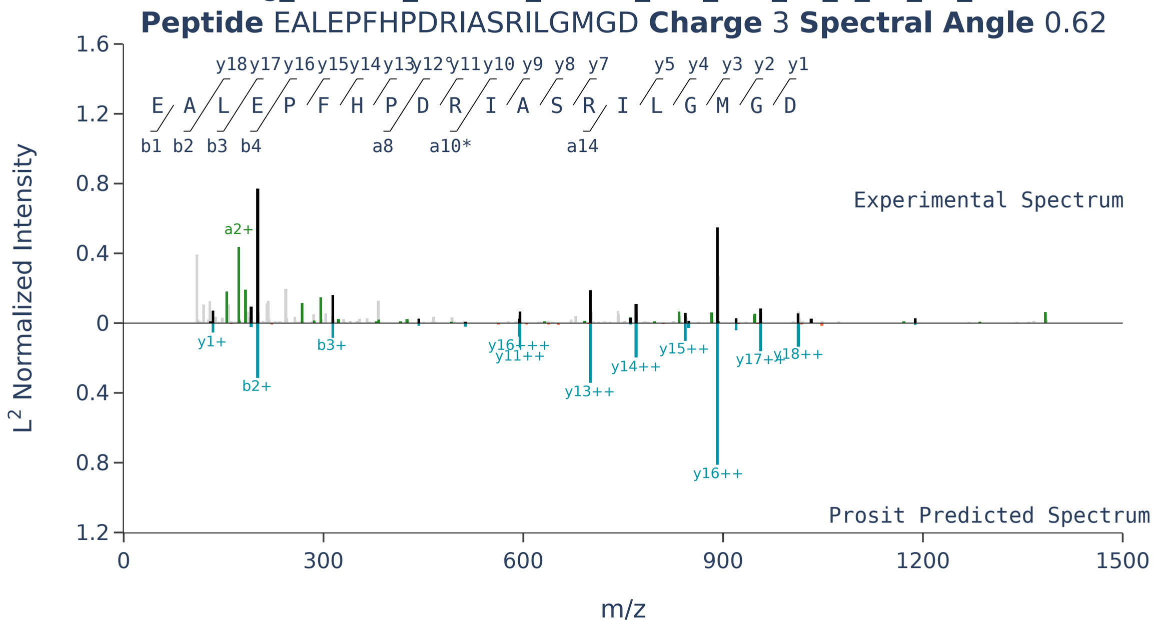


**Source** Ncheng_210623_230623_HFGoe_FFH_20S_25_1_A1_1h_R2 **Scan** 16695
**Peptide** KIVRNELVAAM **Charge** 3 **Spectral Angle** 0.73

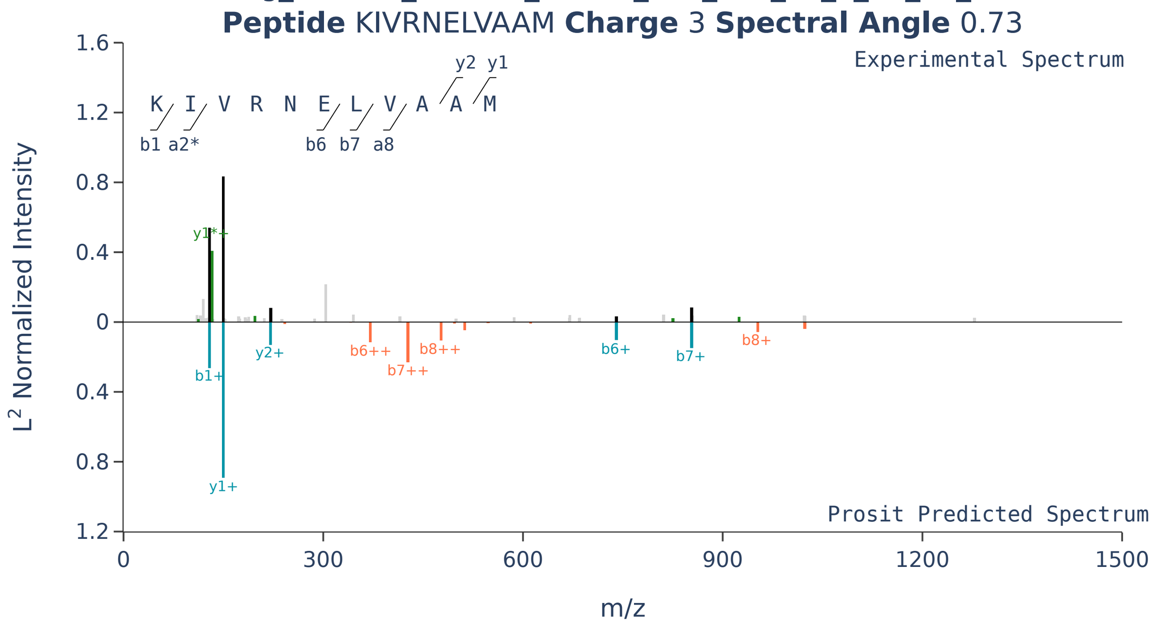


**Source** Ncheng_210623_230623_HFGoe_FFH_20S_25_1_A2_4h_R2 **Scan** 14813
**Peptide** TDRLSRTLRNISGRGRLTE **Charge** 6 **Spectral Angle** 0.73

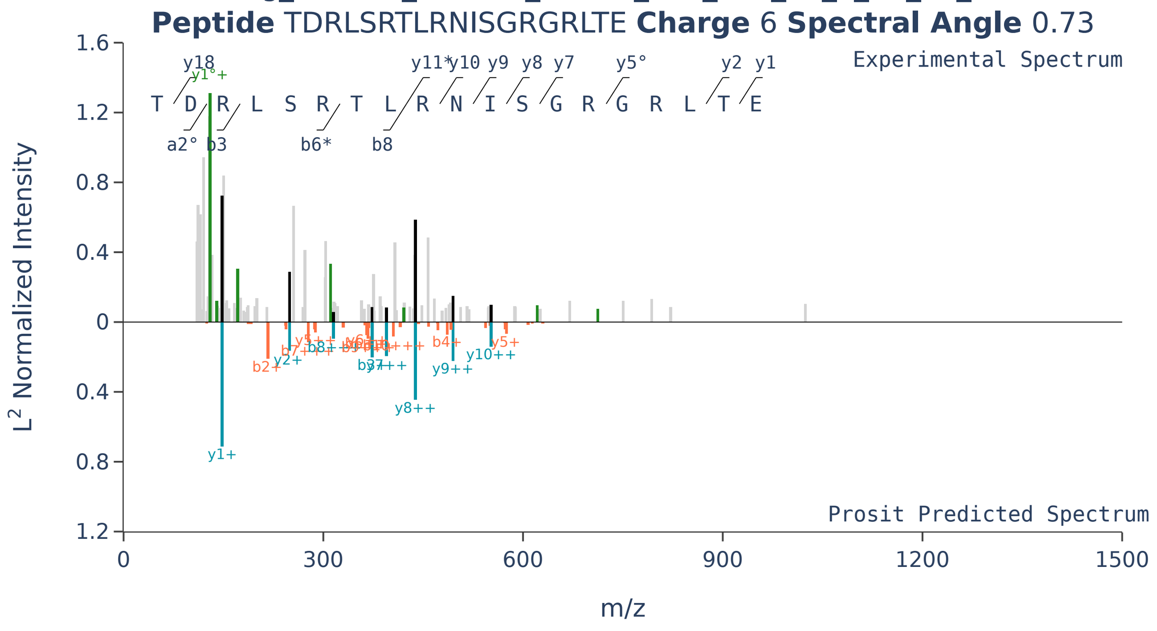


**Source** Ncheng_210623_230623_HFGoe_FFH_20S_25_1_A2_24h_R2 **Scan** 8508
**Peptide** LESKVDRAQAEKLASK **Charge** 3 **Spectral Angle** 0.54

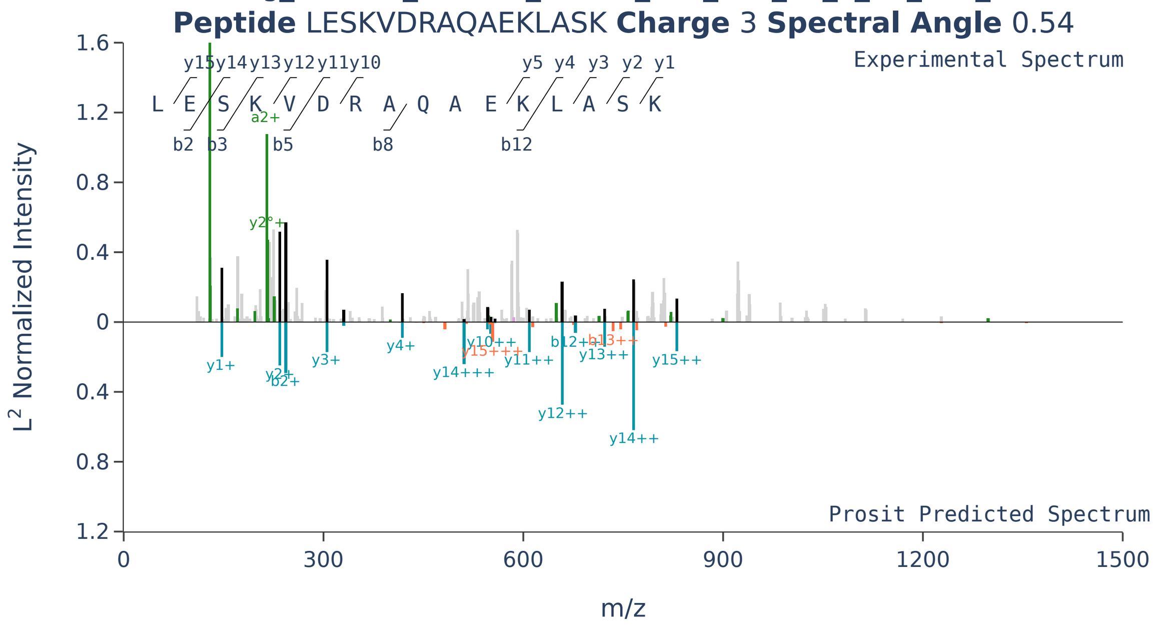


**Source** Ncheng_210623_230623_HFGoe_FFH_20S_25_1_A1_24h_R1 **Scan** 26346
**Peptide** CGMQVQDVNRLL **Charge** 2 **Spectral Angle** 0.65

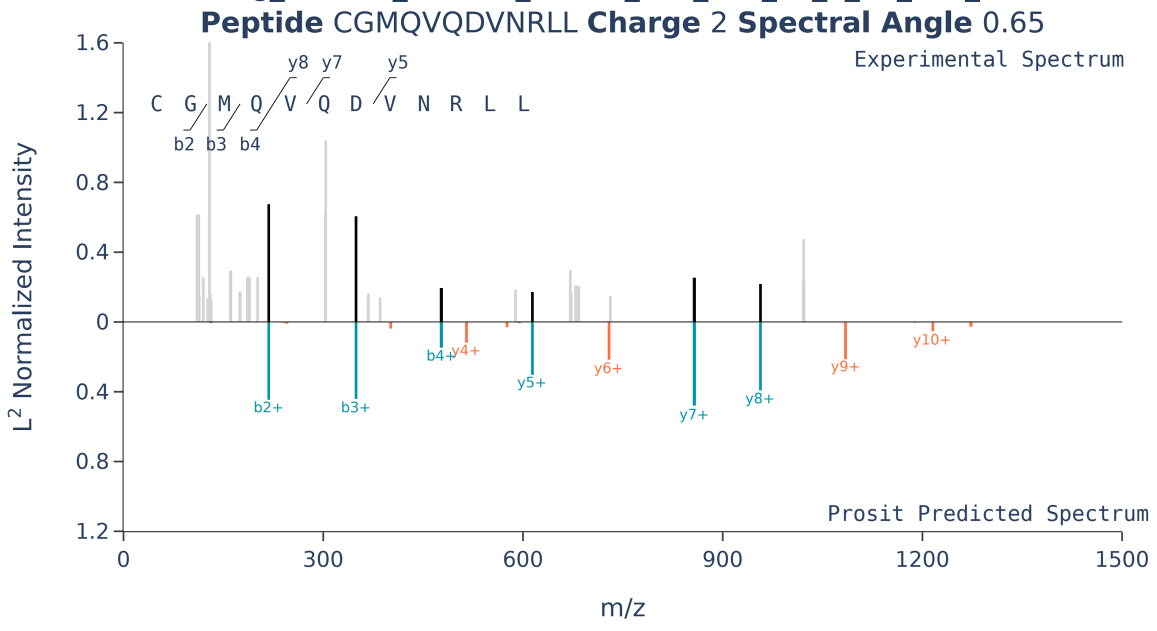


**Source** Ncheng_210623_230623_HFGoe_FFH_20S_25_1_A2_4h_R1 **Scan** 16839
**Peptide** DIESKVDRAQAEKLASKLK **Charge** 5 **Spectral Angle** 0.77

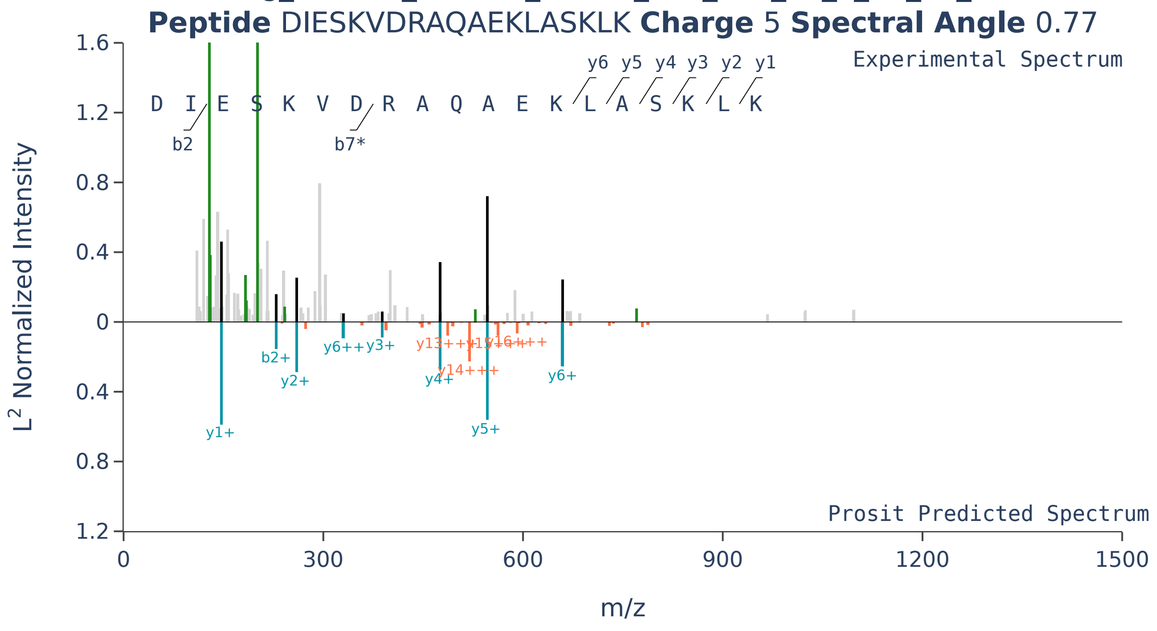


**Source** Ncheng_210623_230623_HFGoe_FFH_20S_25_1_A2_24h_R2 **Scan** 17767
**Peptide** AQAEKLASKLKKGDGFDL **Charge** 3 **Spectral Angle** 0.71

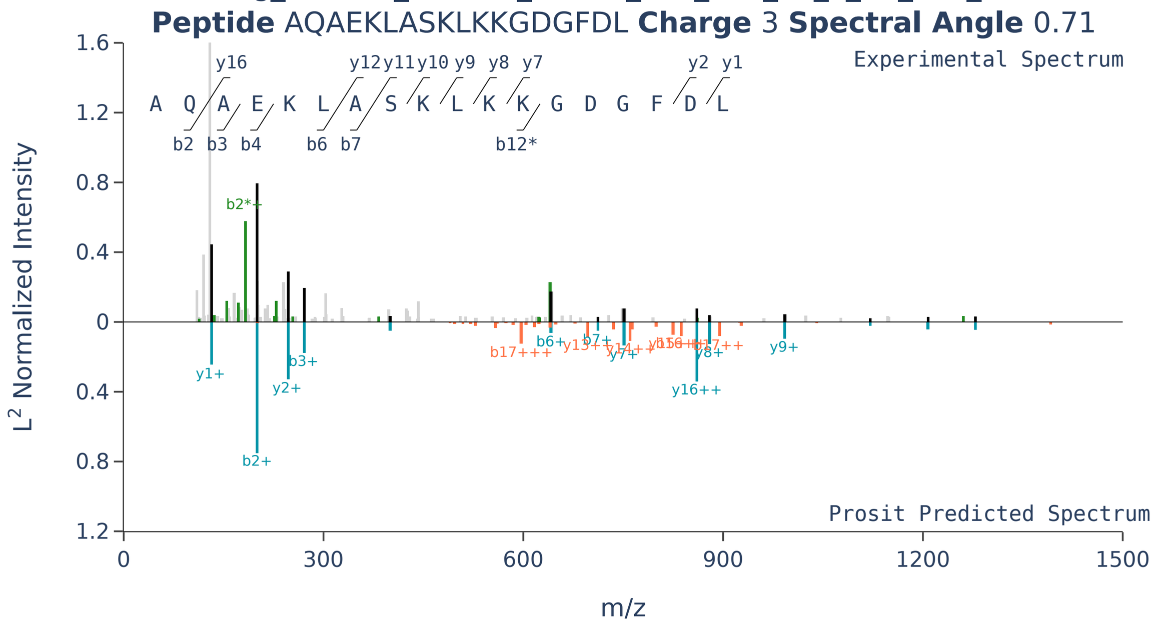

**Source** Ncheng_210623_230623_HFGoe_FFH_20S_25_1_A1_1h_R2 **Scan** 32692
**Peptide** KLASKLKKGDGFDLNDFLEQL **Charge** 3 **Spectral Angle** 0.49

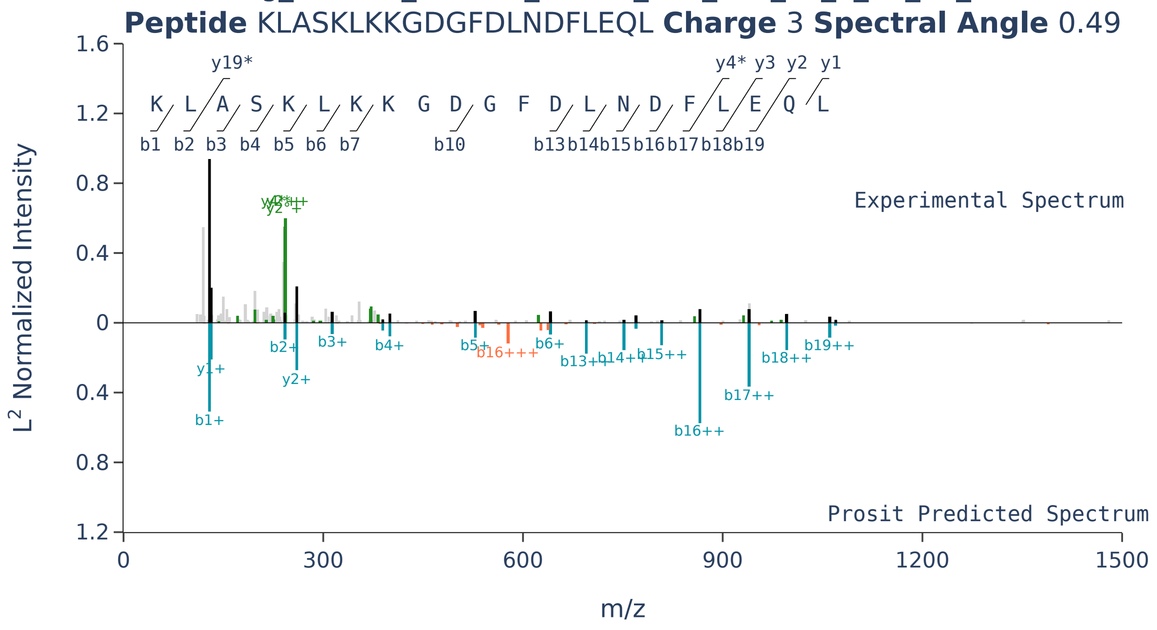


**Source** Ncheng_210623_230623_HFGoe_FFH_20S_25_1_A2_4h_R2 **Scan** 36597
**Peptide** KLASKLKKGDGFDLNDFLEQLRQM **Charge** 3 **Spectral Angle** 0.74

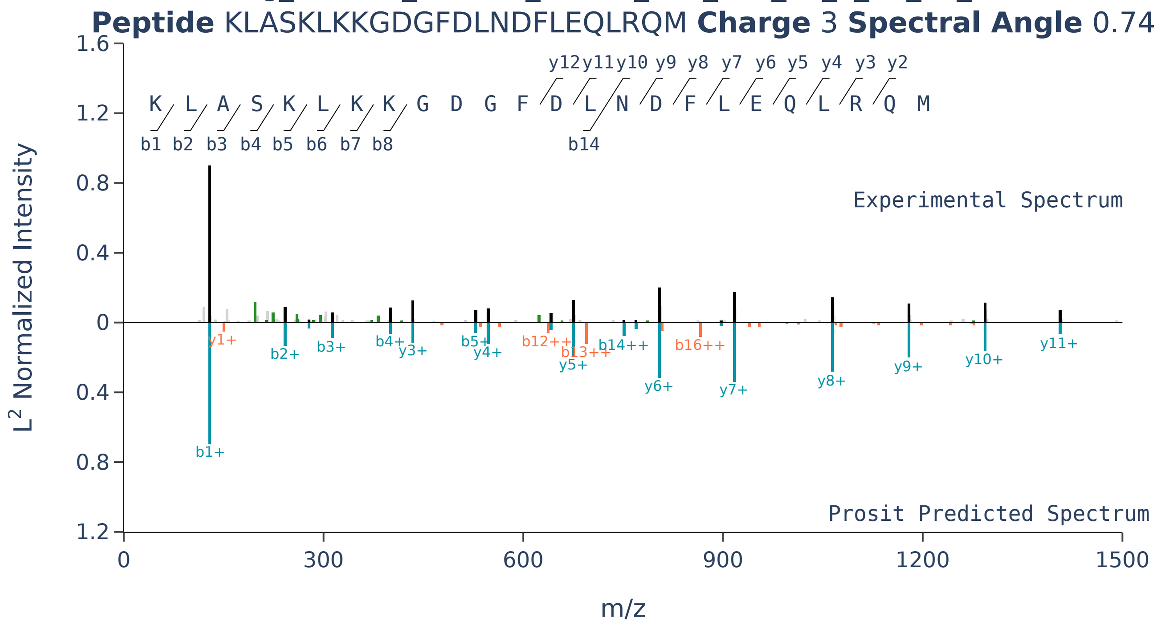


**Source** Ncheng_210623_230623_HFGoe_FFH_20S_25_1_A1_24h_R1 **Scan** 19011
**Peptide** EKAVGHEVNKSLTPGQEFVKLVR **Charge** 4 **Spectral Angle** 0.68

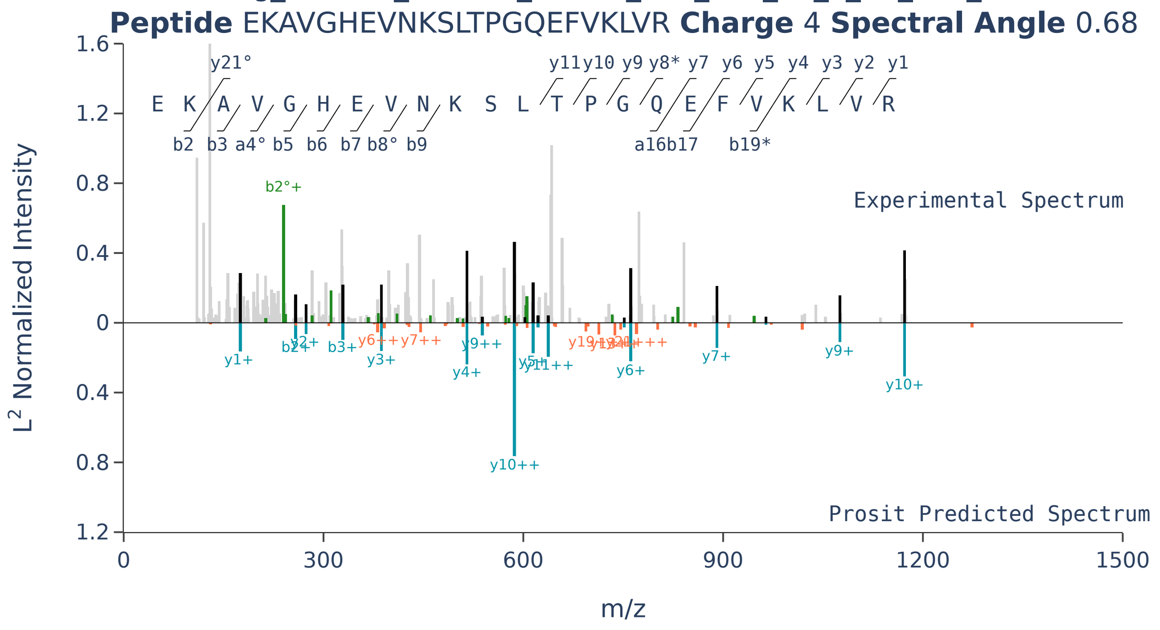


**Source** Ncheng_210623_230623_HFGoe_FFH_20S_25_1_A2_4h_R2 **Scan** 27206
**Peptide** KEKAVGHEVNKSLTPGQEFVKIVRNELVA **Charge** 3 **Spectral Angle** 0.71

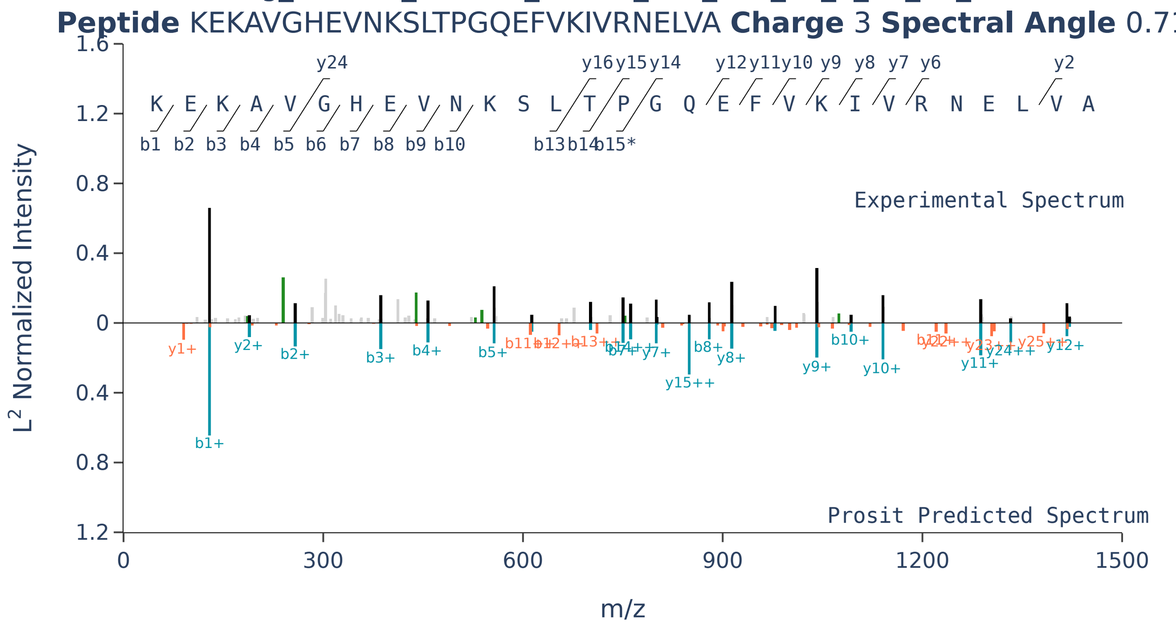


**Source** Ncheng_210623_230623_HFGoe_FFH_20S_25_1_A1_4h_R1 **Scan** 14610
**Peptide** RMEAIINSMTMKERAKPEIIKGSRKR **Charge** 6 **Spectral Angle** 0.79

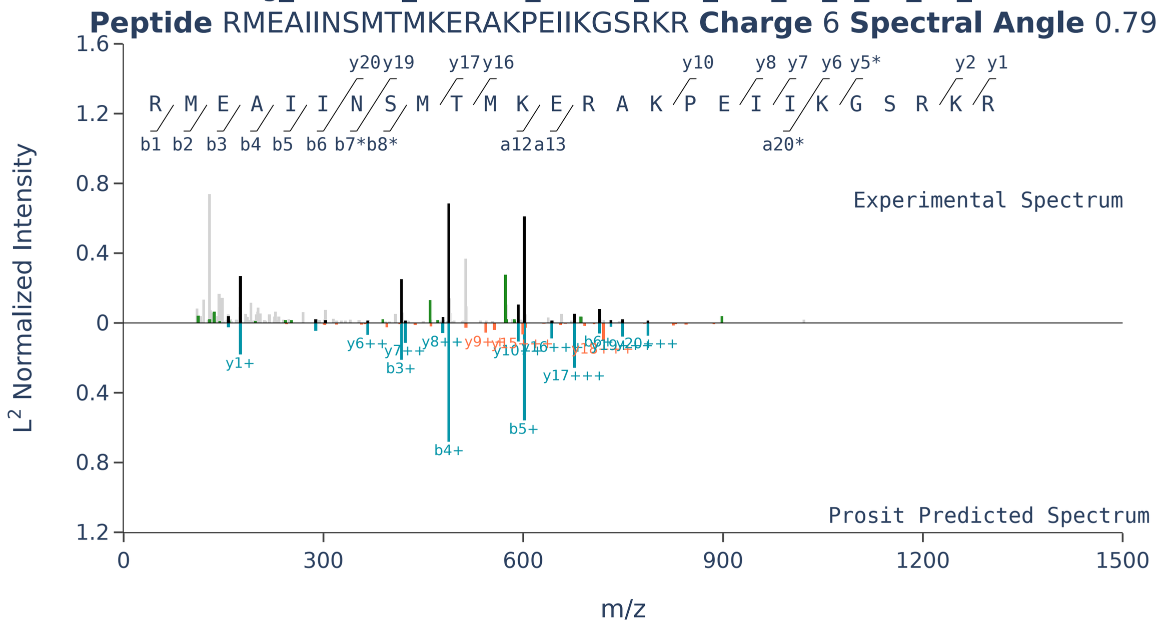


**Source** Ncheng_210623_230623_HFGoe_FFH_20S_25_1_A2_2h_R1 **Scan** 32093
**Peptide** DVALPVVREFINRVKEK **Charge** 4 **Spectral Angle** 0.57

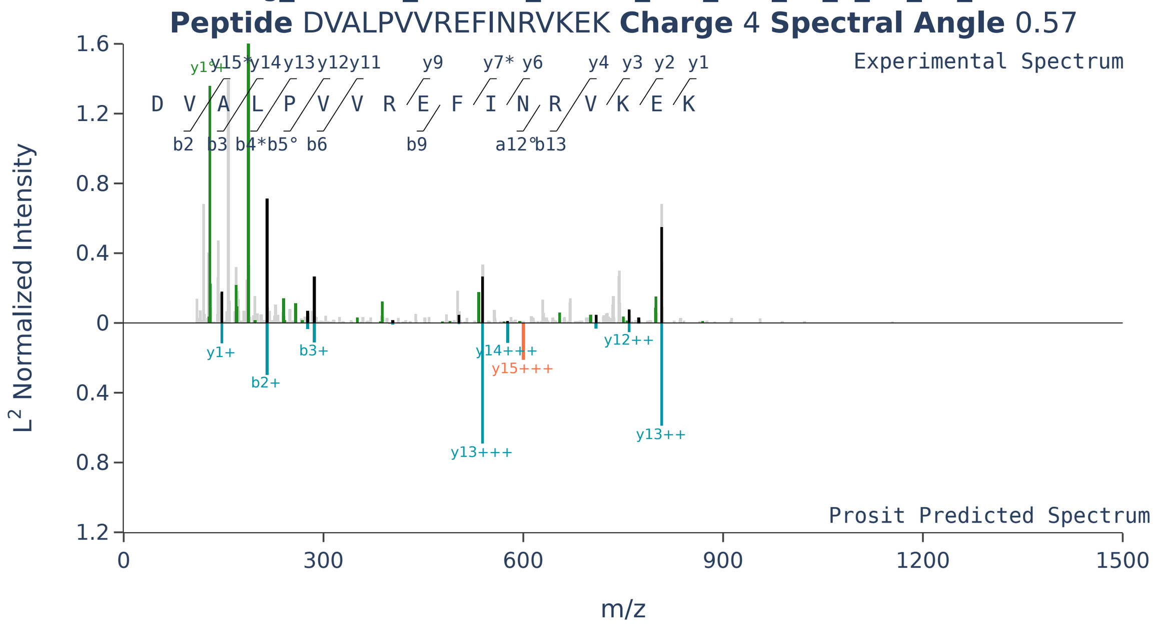


**Source** Ncheng_210623_230623_HFGoe_FFH_20S_25_1_A1_2h_R1 **Scan** 19154
**Peptide** TLRNISGRGRLTEDNVKDTLREVRM **Charge** 6 **Spectral Angle** 0.68

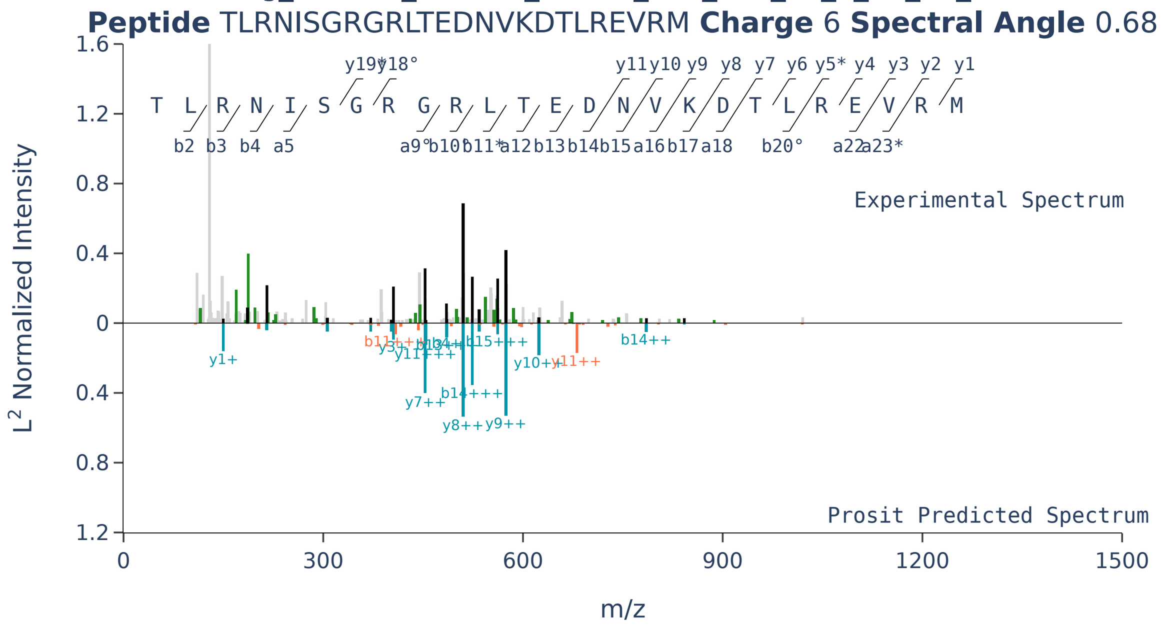


**Source** Ncheng_210623_230623_HFGoe_FFH_20S_25_1_A1_2h_R2 **Scan** 23959
**Peptide** LTKVDGDARGGAALSIRHITGKPIKFL **Charge** 5 **Spectral Angle** 0.77

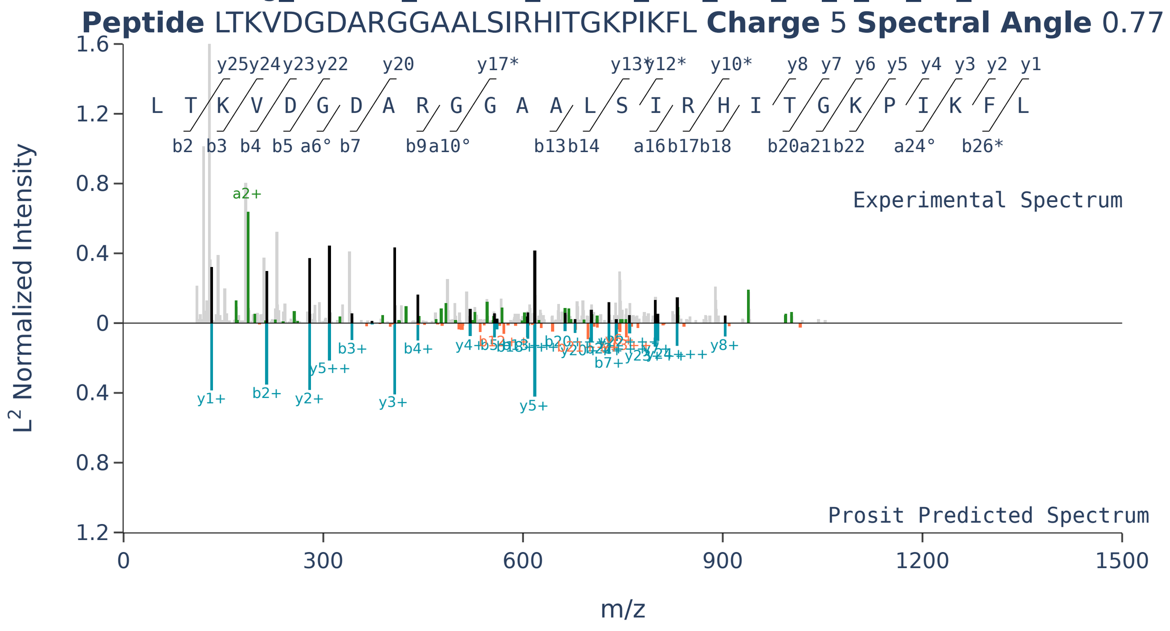


**Source** Ncheng_210623_230623_HFGoe_FFH_20S_25_1_A2_4h_R1 **Scan** 35095
**Peptide** NEALPLTGVVLTKVDGDARGGAALSI **Charge** 3 **Spectral Angle** 0.82

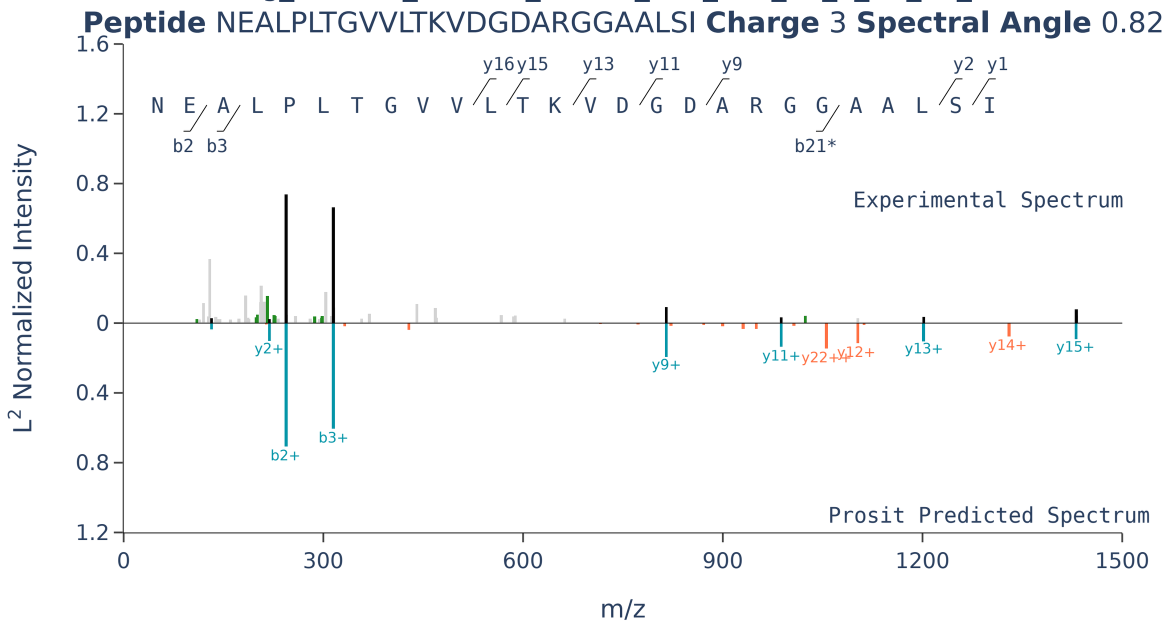


**Source** Ncheng_210623_230623_HFGoe_FFH_20S_25_1_A1_4h_R2 **Scan** 16327
**Peptide** MEAIINSMTMKERAKPEIIKGSRK **Charge** 4 **Spectral Angle** 0.61

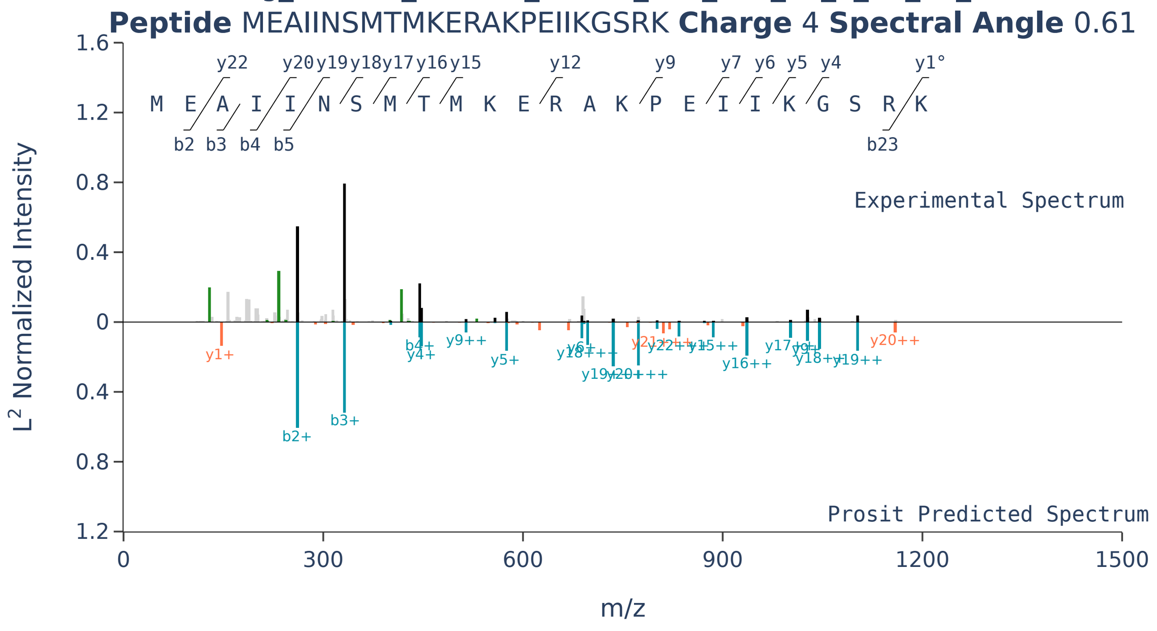


**Source** Ncheng_210623_230623_HFGoe_FFH_20S_25_1_A1_24h_R2 **Scan** 5142
**Peptide** KLASKLKKG **Charge** 2 **Spectral Angle** 0.82

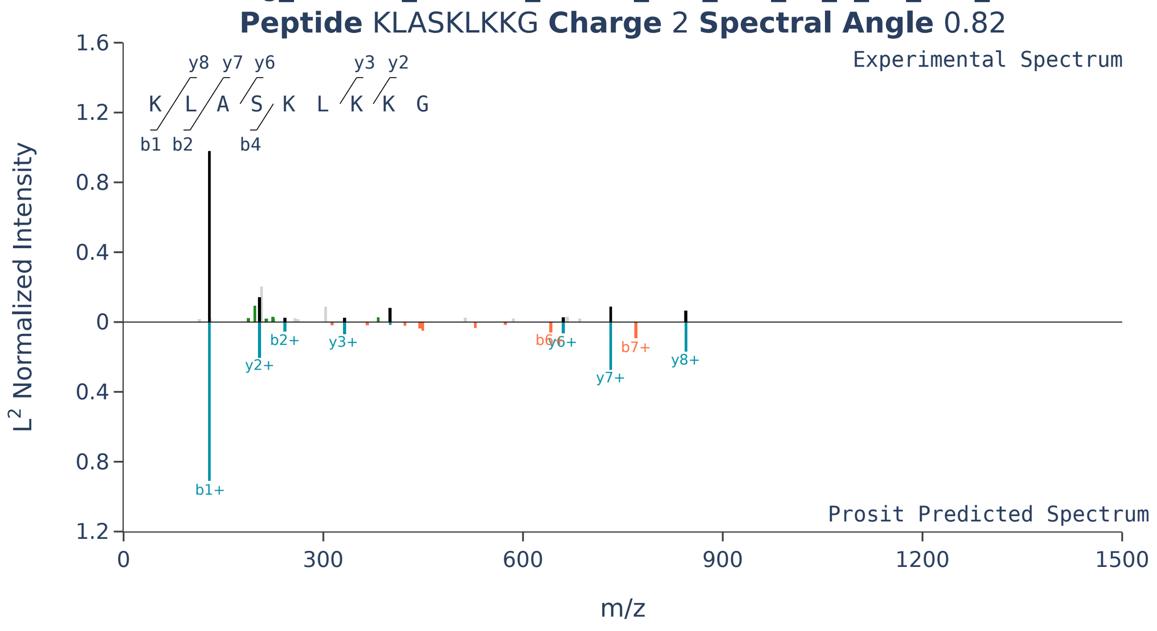


**Source** Ncheng_210623_230623_HFGoe_FFH_20S_25_1_A1_24h_R2 **Scan** 9525
**Peptide** SVGKLGKFLREKHK **Charge** 2 **Spectral Angle** 0.68

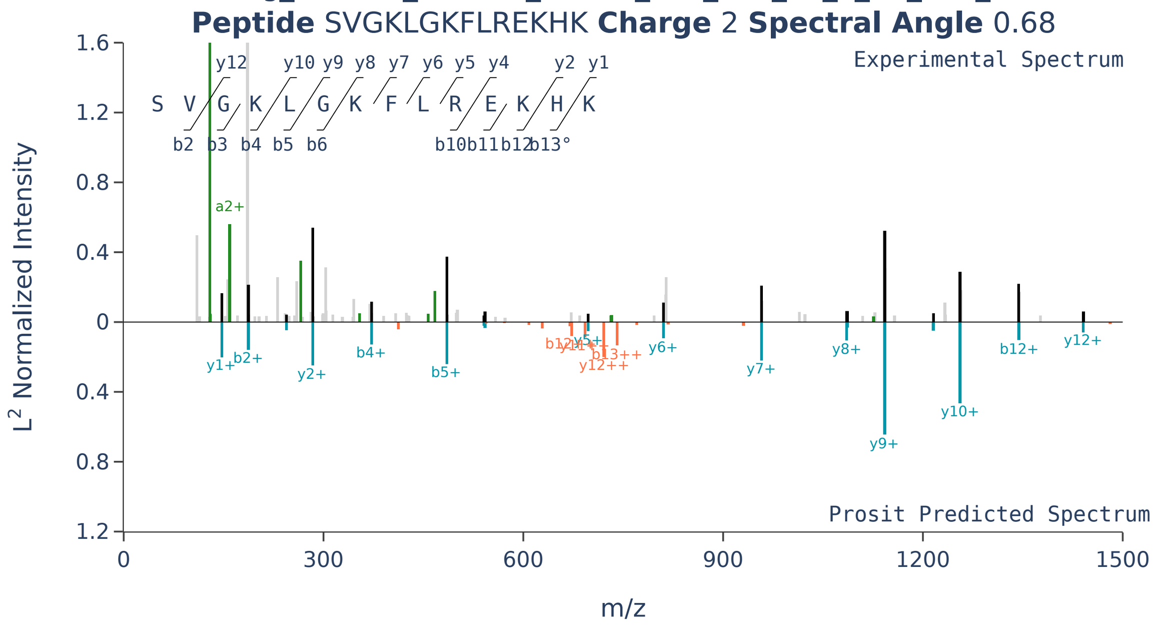


**Source** Ncheng_210623_230623_HFGoe_FFH_20S_25_1_A2_24h_R1 **Scan** 7017
**Peptide** NSMTMKERAKPEIIKGSRKR **Charge** 5 **Spectral Angle** 0.62

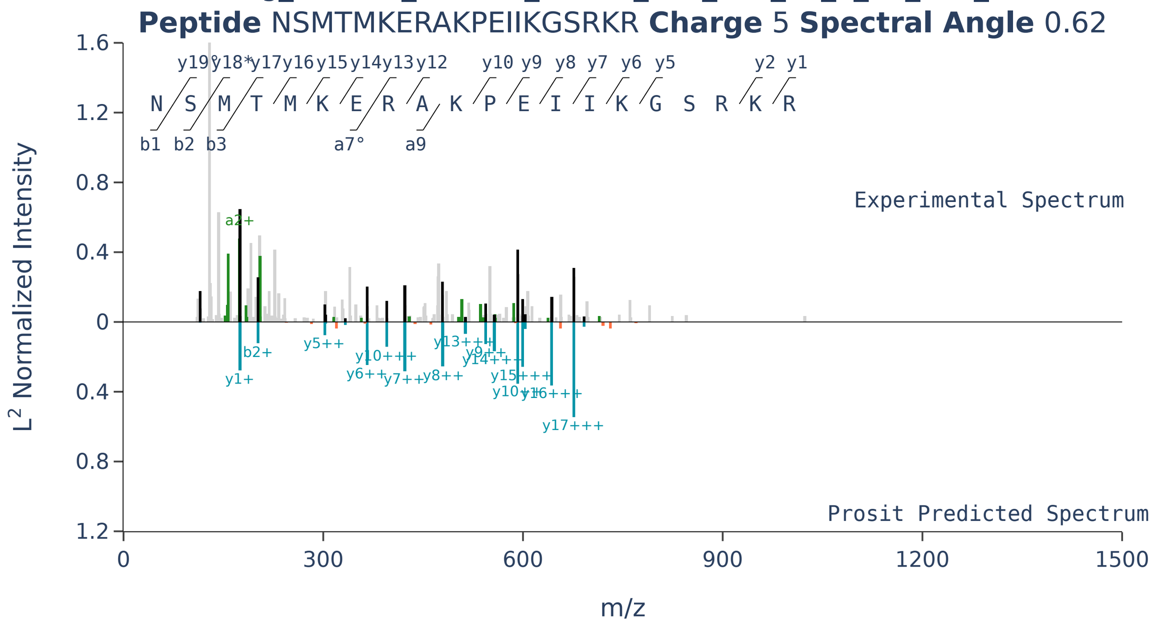


**Source** Ncheng_210623_230623_HFGoe_FFH_20S_25_1_A1_24h_R1 **Scan** 15700
**Peptide** LKQFDDMQRMMKK **Charge** 4 **Spectral Angle** 0.74

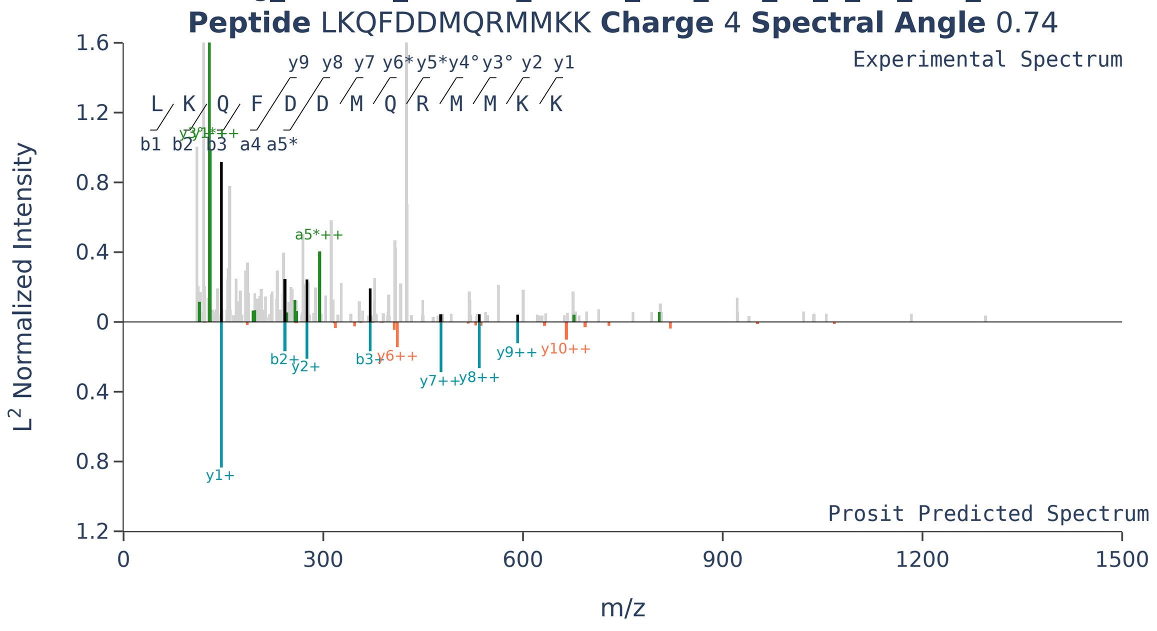


**Source** Ncheng_210623_230623_HFGoe_FFH_20S_25_1_A2_24h_R2 **Scan** 7534
**Peptide** TMKERAKPEI **Charge** 2 **Spectral Angle** 0.71

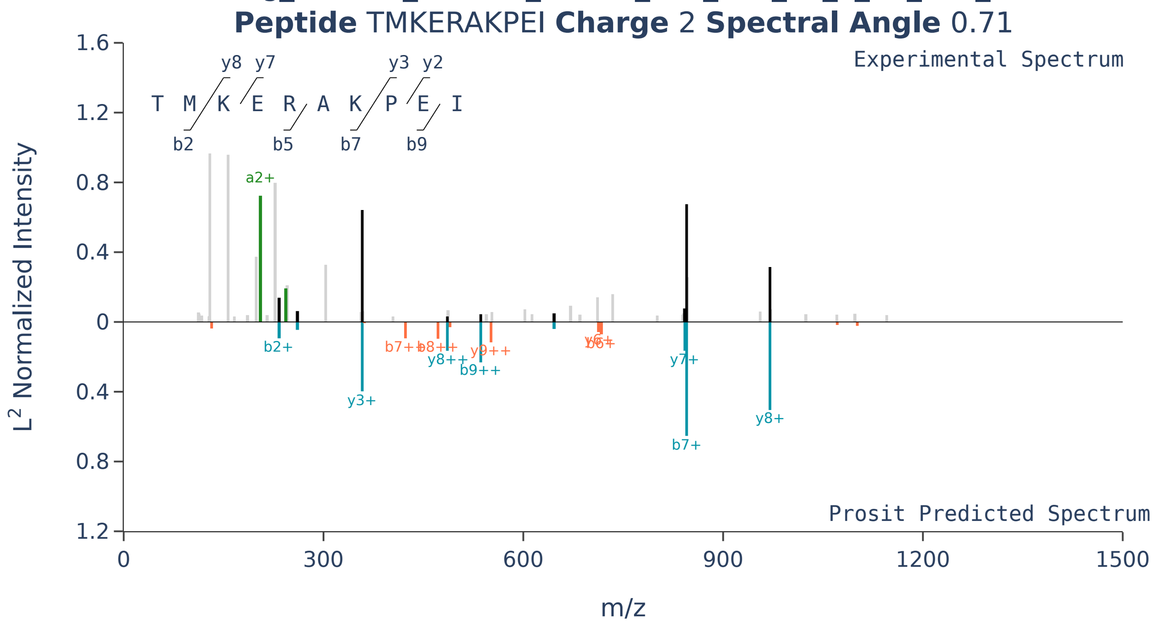

**Source** Ncheng_210623_230623_HFGoe_FFH_20S_25_1_A2_4h_R2 **Scan** 30384
**Peptide** ALKEAKLKFYDVLLVDT **Charge** 3 **Spectral Angle** 0.61

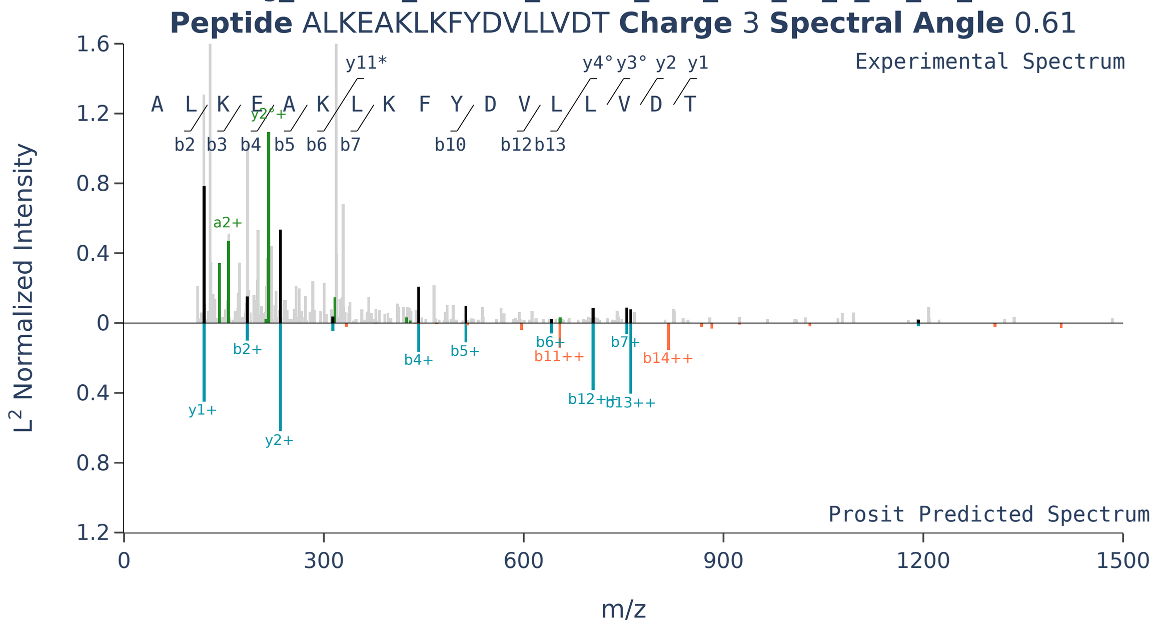


**Source** Ncheng_210623_230623_HFGoe_FFH_20S_25_1_A2_4h_R2 **Scan** 32510
**Peptide** IAAGCGMQVQDVNRLLKQF **Charge** 3 **Spectral Angle** 0.76

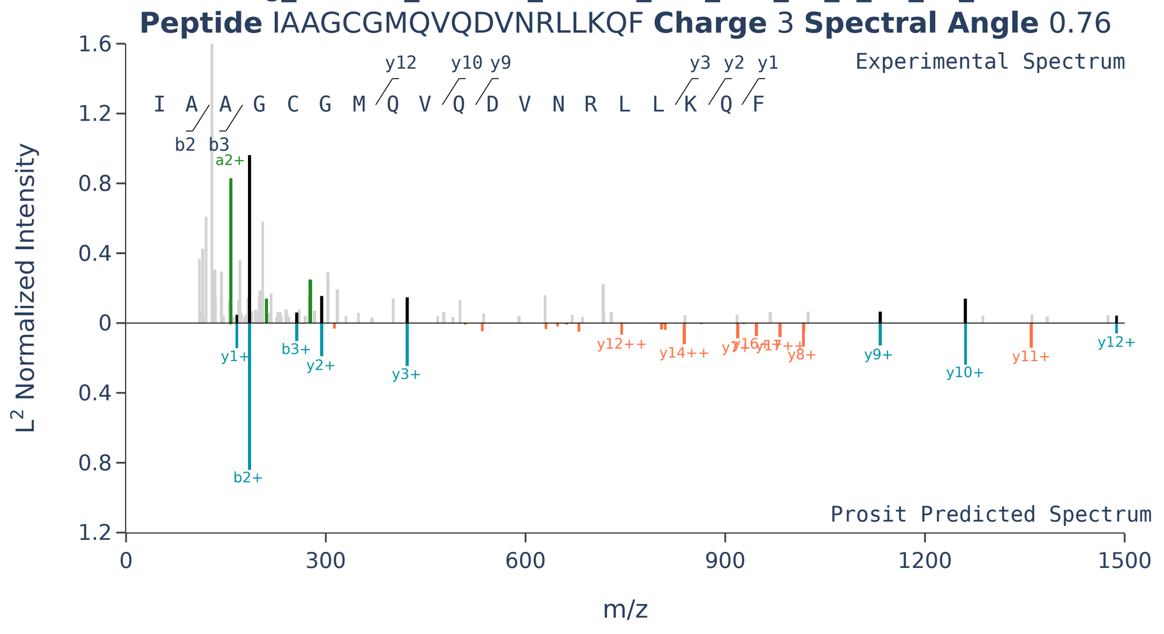


**Source** Ncheng_210623_230623_HFGoe_FFH_20S_25_1_A1_2h_R2 **Scan** 25662
**Peptide** TEDNVKDTLREVRMAL **Charge** 3 **Spectral Angle** 0.54

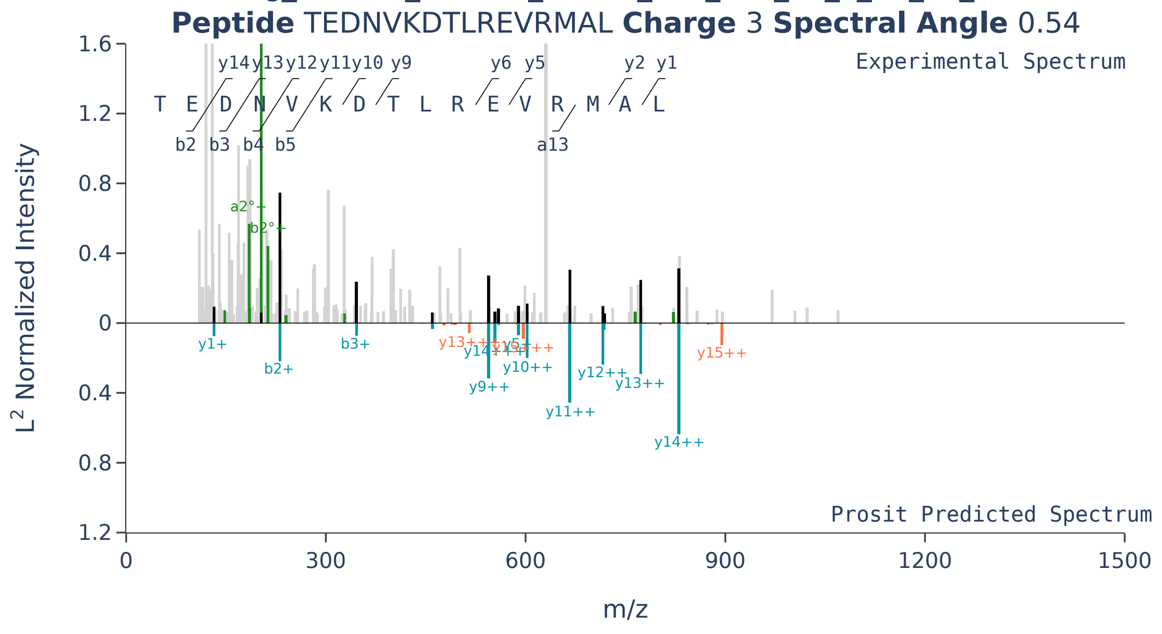


**Source** Ncheng_210623_230623_HFGoe_FFH_20S_25_1_A1_4h_R2 **Scan** 15010
**Peptide** PFHPDRIASRI **Charge** 3 **Spectral Angle** 0.37

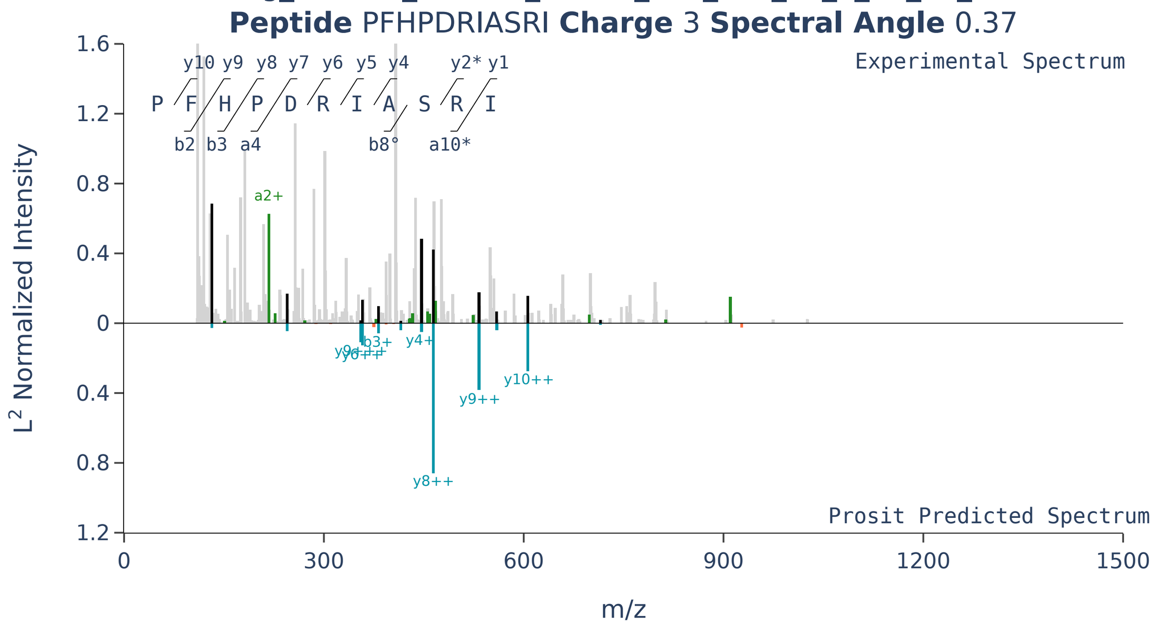


**Source** Ncheng_210623_230623_HFGoe_FFH_20S_25_1_A1_2h_R2 **Scan** 30526
**Peptide** KLASKLKKGDGFDLNDFLEQLRQMKNMG **Charge** 5 **Spectral Angle** 0.67

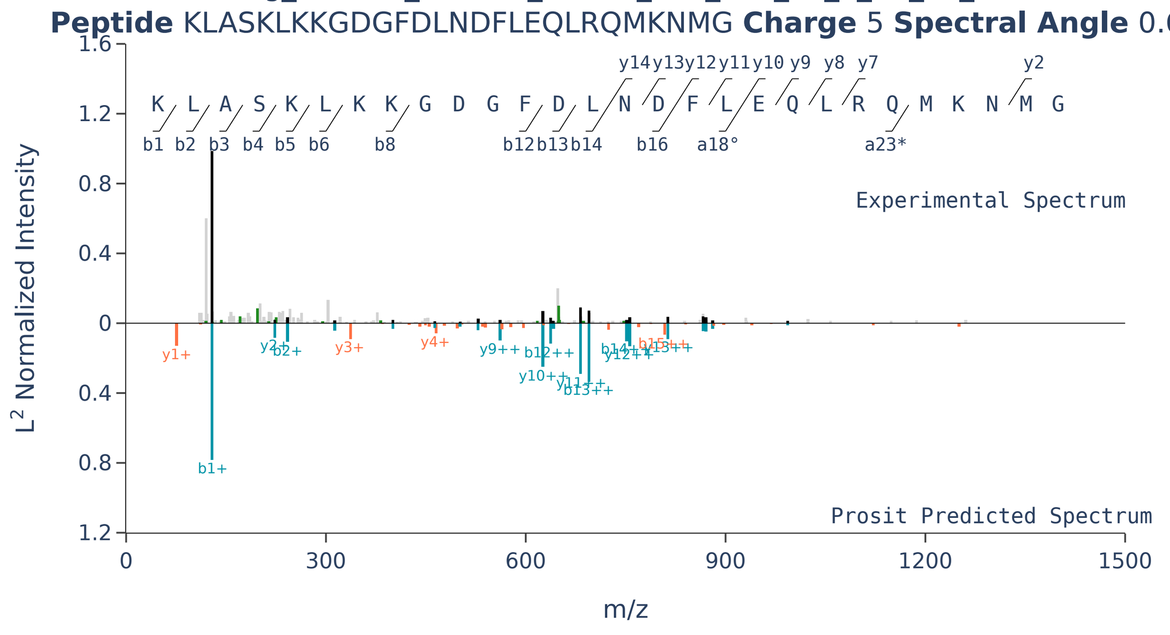


**Source** Ncheng_210623_230623_HFGoe_FFH_20S_25_1_A1_4h_R2 **Scan** 24515
**Peptide** LLEDIESKVDRAQAEKLASKL **Charge** 3 **Spectral Angle** 0.69

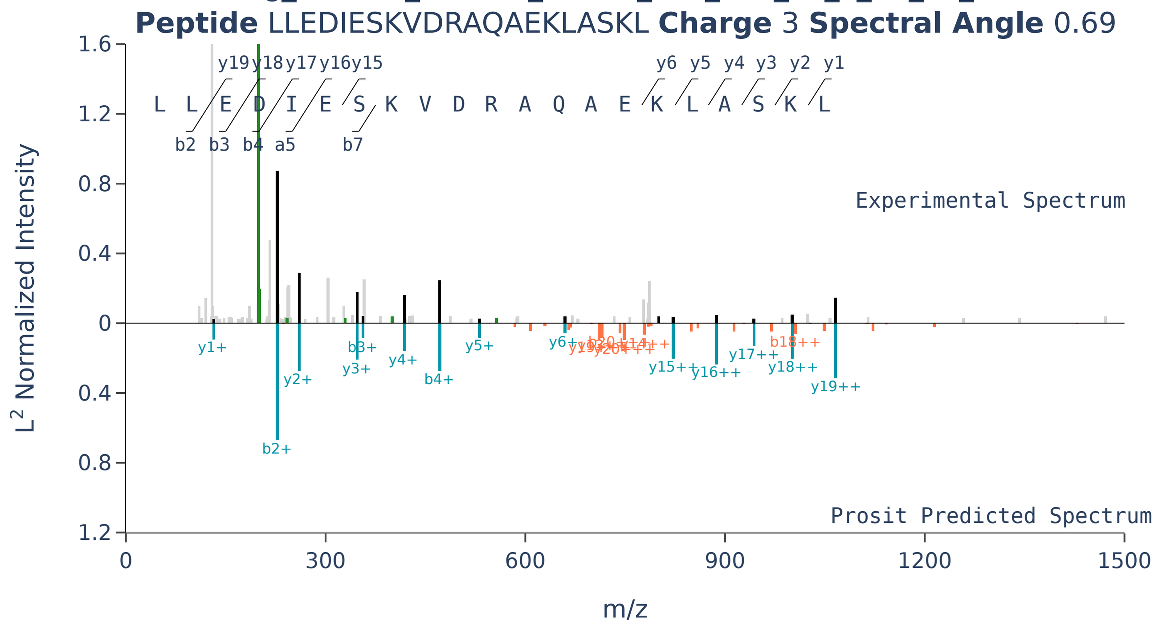


**Source** Ncheng_210623_230623_HFGoe_FFH_20S_25_1_A1_24h_R2 **Scan** 10070
**Peptide** RLSRTLRNISGRGRLTE **Charge** 5 **Spectral Angle** 0.61

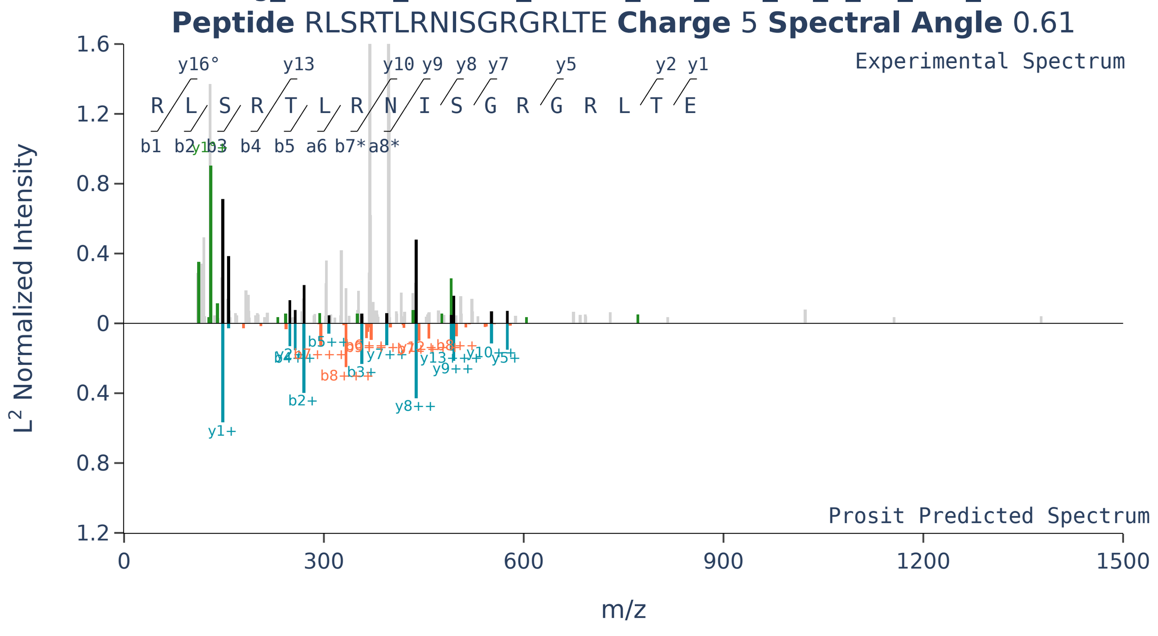


**Source** Ncheng_210623_230623_HFGoe_FFH_20S_25_1_A2_24h_R2 **Scan** 6993
**Peptide** LSGRGRLTEDNVKD **Charge** 3 **Spectral Angle** 0.64

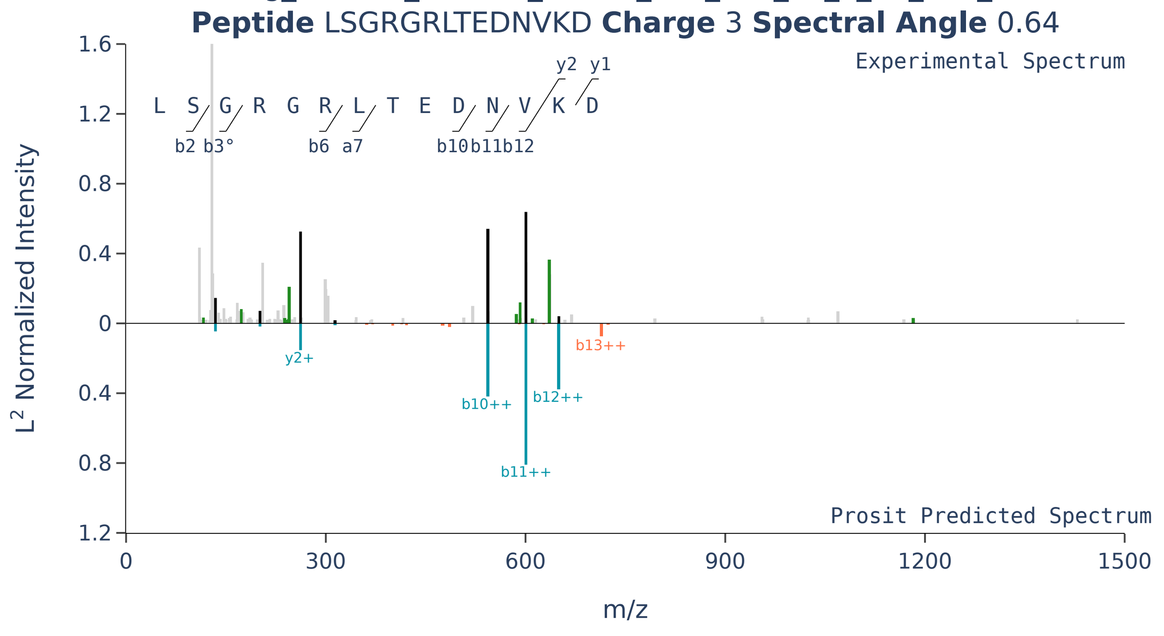


**Source** Ncheng_210623_230623_HFGoe_FFH_20S_25_1_A1_4h_R2 **Scan** 25165
**Peptide** SGRGRLTEDNVKDTLREVRMAL **Charge** 5 **Spectral Angle** 0.77

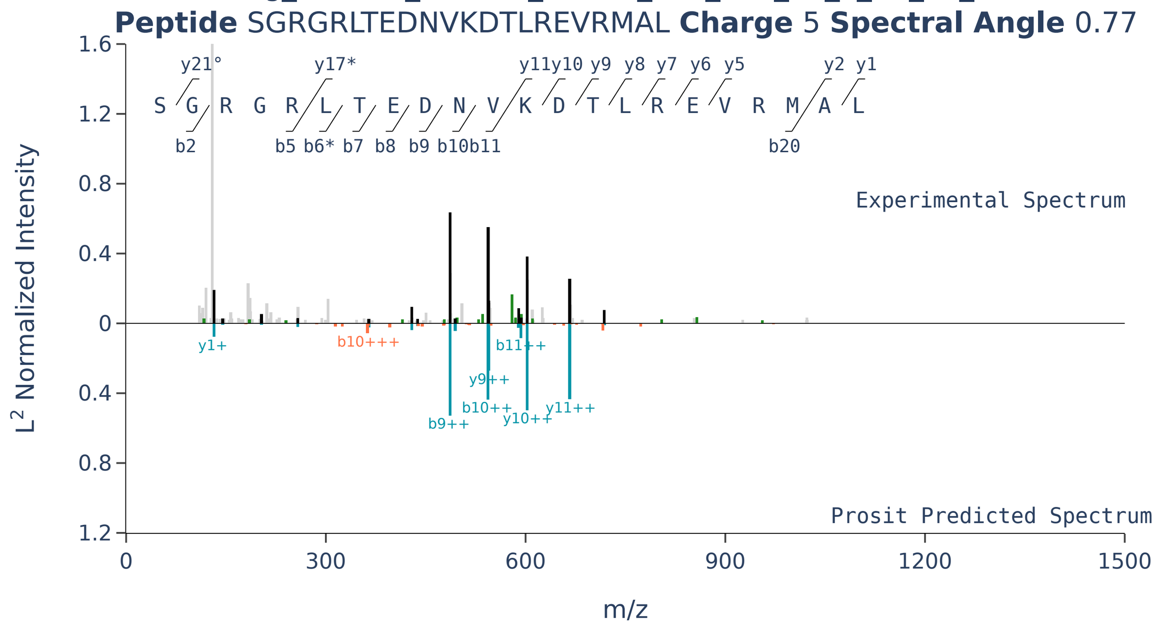


**Source** Ncheng_210623_230623_HFGoe_FFH_20S_25_1_A1_24h_R1 **Scan** 8567
**Peptide** LRNISGRGRL **Charge** 3 **Spectral Angle** 0.65

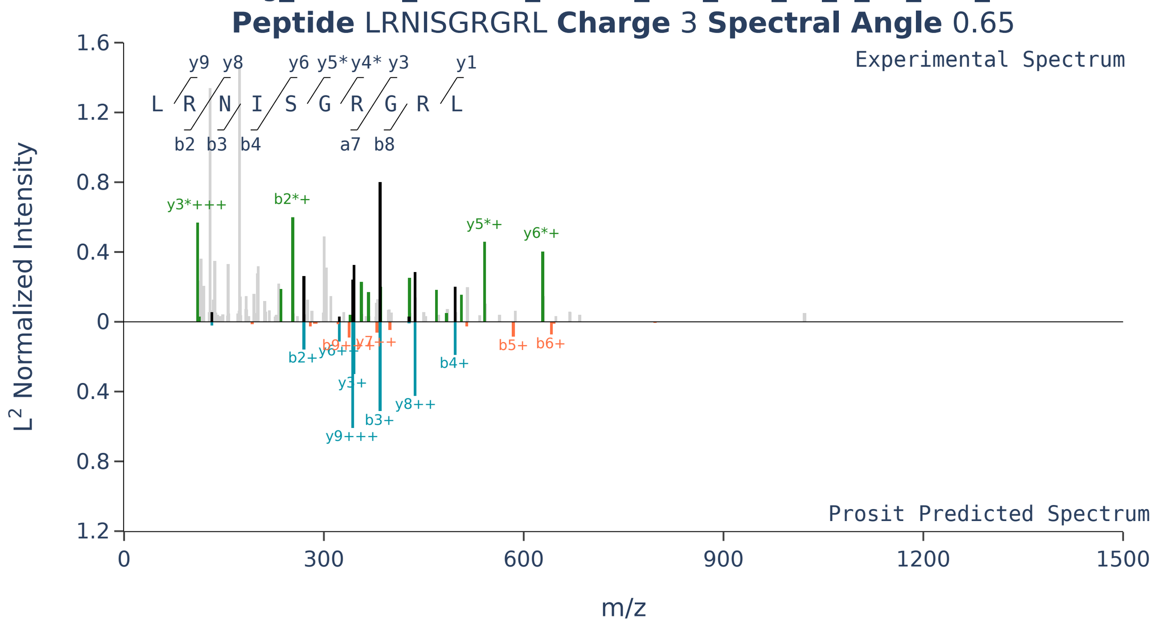


**Source** Ncheng_210623_230623_HFGoe_FFH_20S_25_1_A2_24h_R2 **Scan** 19882
**Peptide** RMEAIINSMTMKERAKPEIIKG **Charge** 4 **Spectral Angle** 0.8

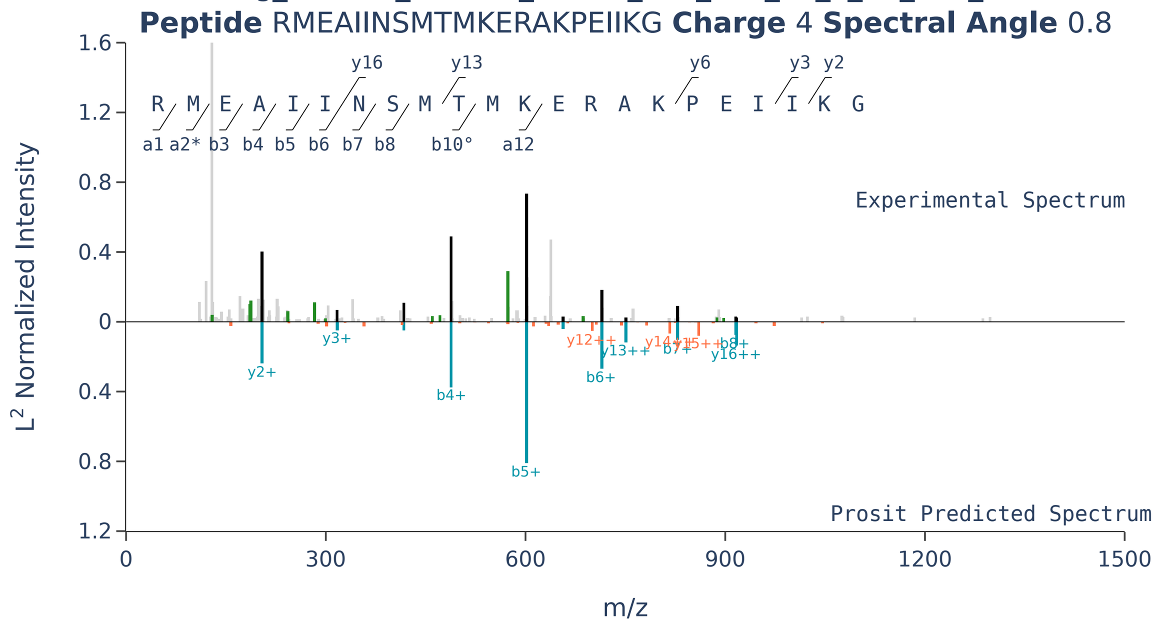


**Source** Ncheng_210623_230623_HFGoe_FFH_20S_25_1_A2_24h_R1 **Scan** 7803
**Peptide** DLESKVDRAQAEK **Charge** 2 **Spectral Angle** 0.56

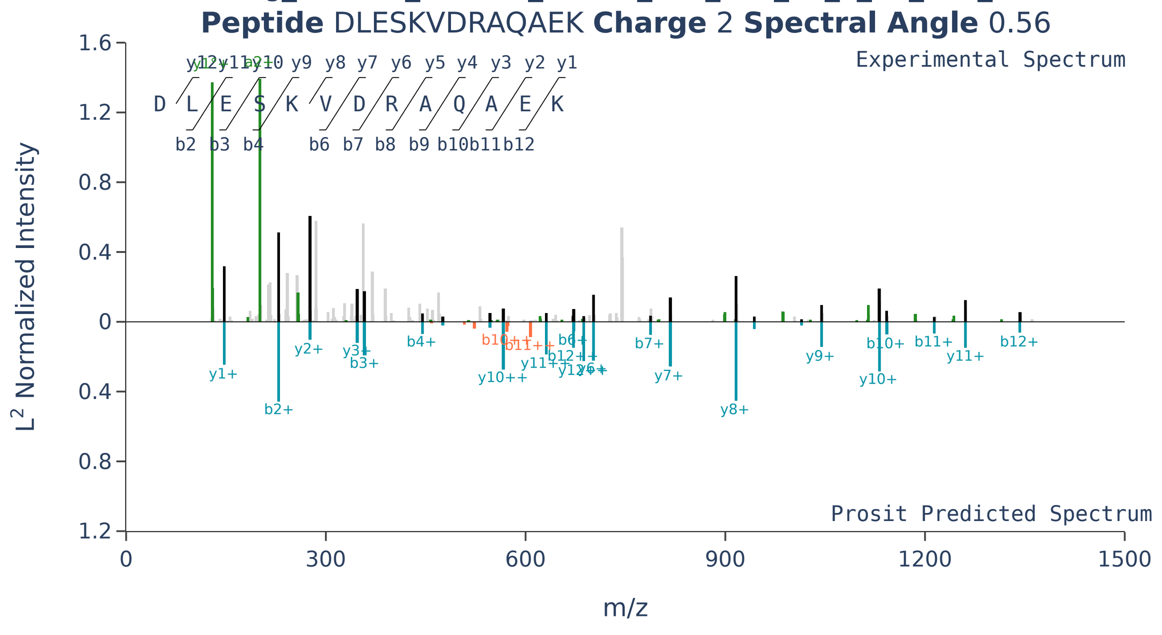


**Source** Ncheng_210623_230623_HFGoe_FFH_20S_25_1_A1_2h_R2 **Scan** 30525
**Peptide** EDNVKDTLREVRMALLE **Charge** 2 **Spectral Angle** 0.61

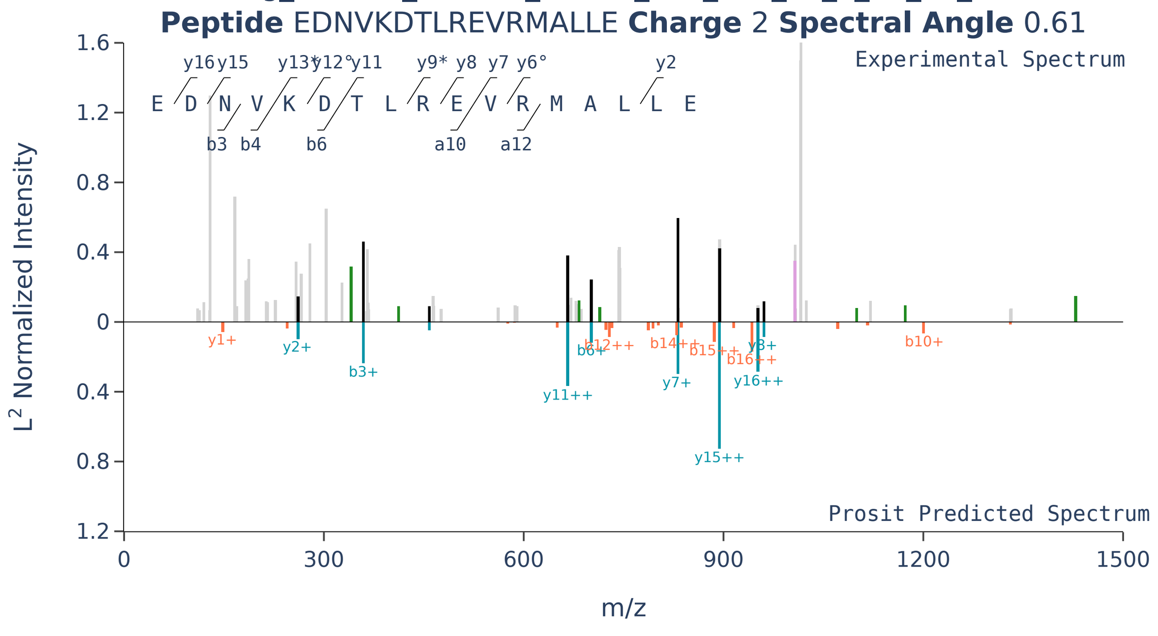


**Source** Ncheng_210623_230623_HFGoe_FFH_20S_25_1_A1_4h_R2 **Scan** 29263
**Peptide** PLTGVVLTKVDGDARGGAALSI **Charge** 3 **Spectral Angle** 0.71

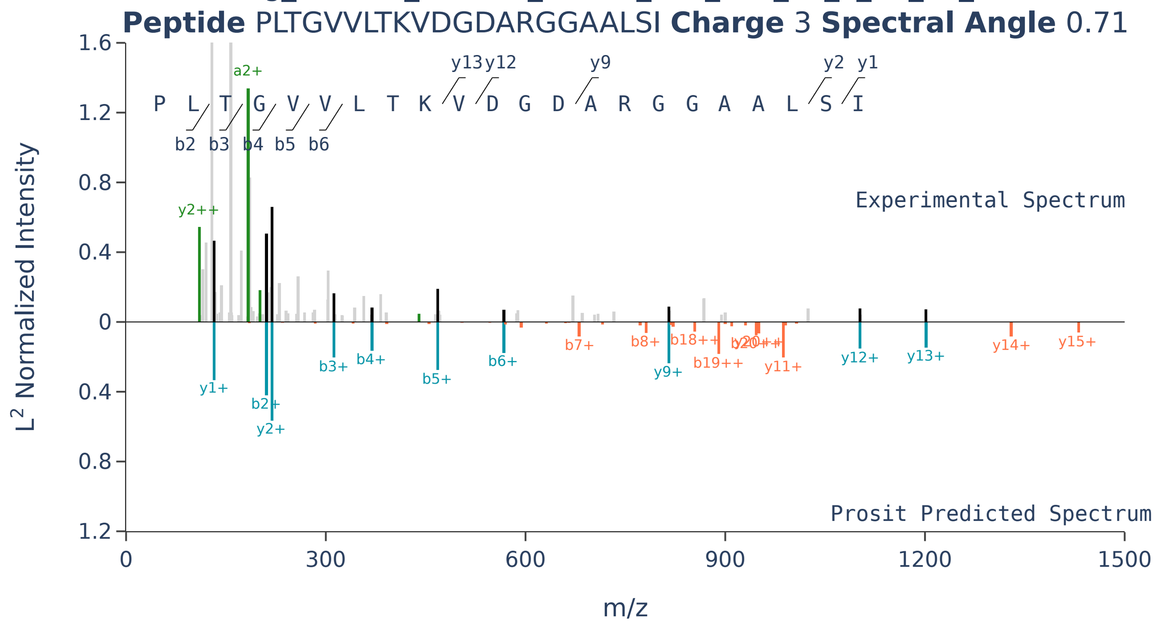


**Source** Ncheng_210623_230623_HFGoe_FFH_20S_25_1_A1_2h_R2 **Scan** 27054
**Peptide** PLKFLGVGEKTEALEPFHPDRIASRI **Charge** 5 **Spectral Angle** 0.76

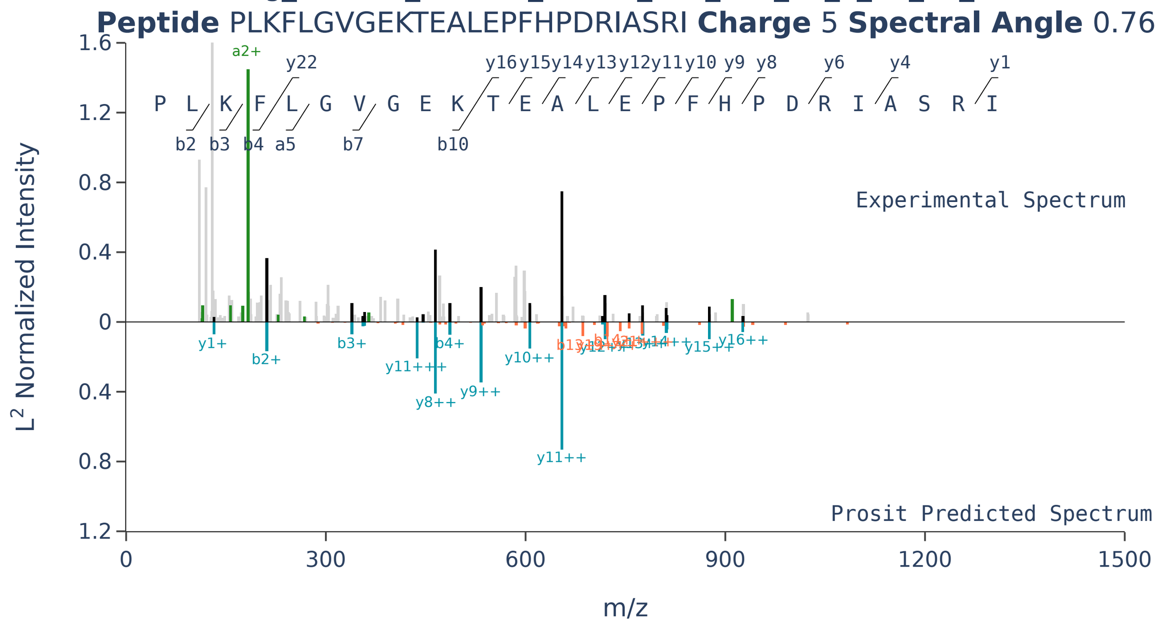

**Source** Ncheng_210623_230623_HFGoe_FFH_20S_25_1_A2_24h_R1 **Scan** 14296
**Peptide** LQGAGKTTSVGKLGKF **Charge** 3 **Spectral Angle** 0.59

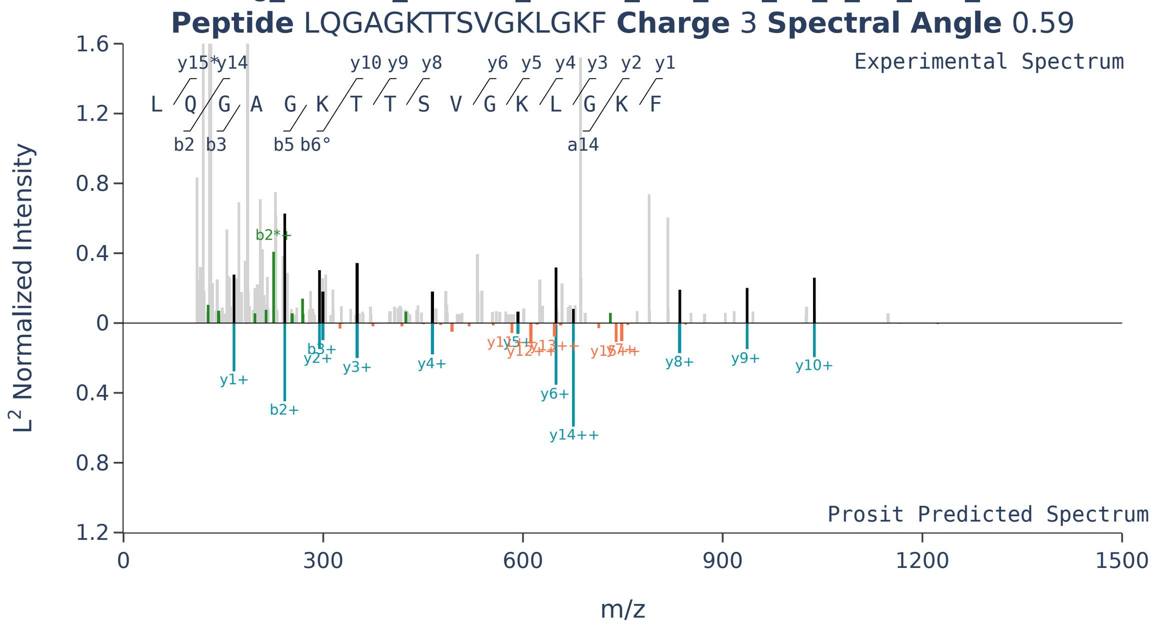


**Source** Ncheng_210623_230623_HFGoe_FFH_20S_25_1_A1_2h_R1 **Scan** 15574
**Peptide** VNKSLTPGQEF **Charge** 2 **Spectral Angle** 0.76

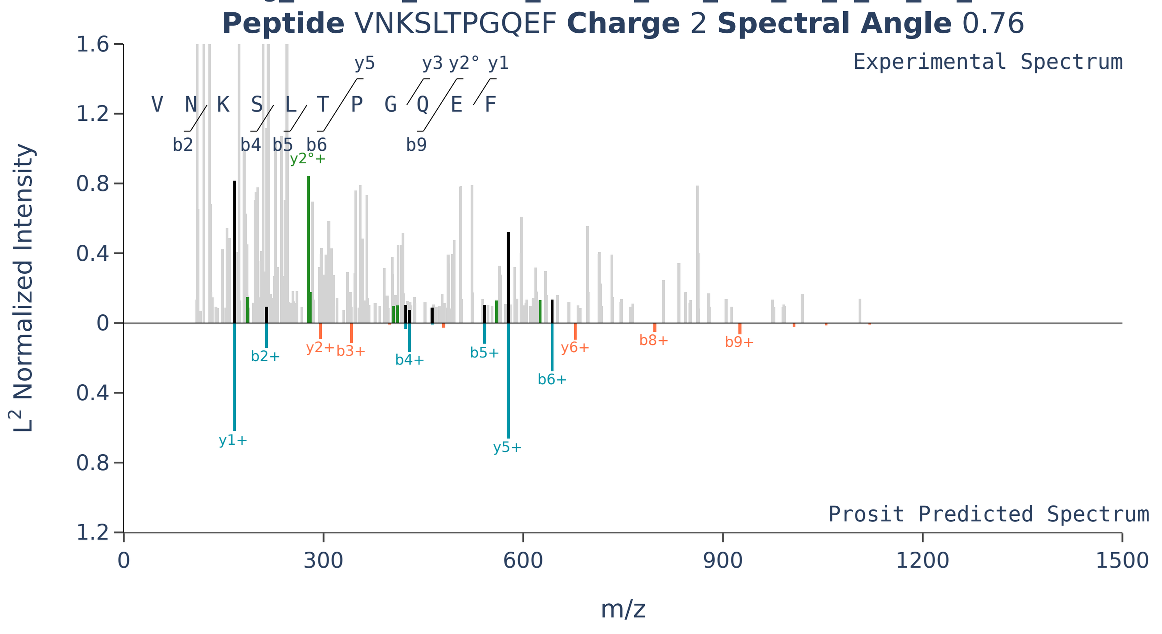


**Source** Ncheng_210623_230623_HFGoe_FFH_20S_25_1_A2_4h_R2 **Scan** 37607
**Peptide** QDAANTAKAFNEALPLTGVVL **Charge** 2 **Spectral Angle** 0.64

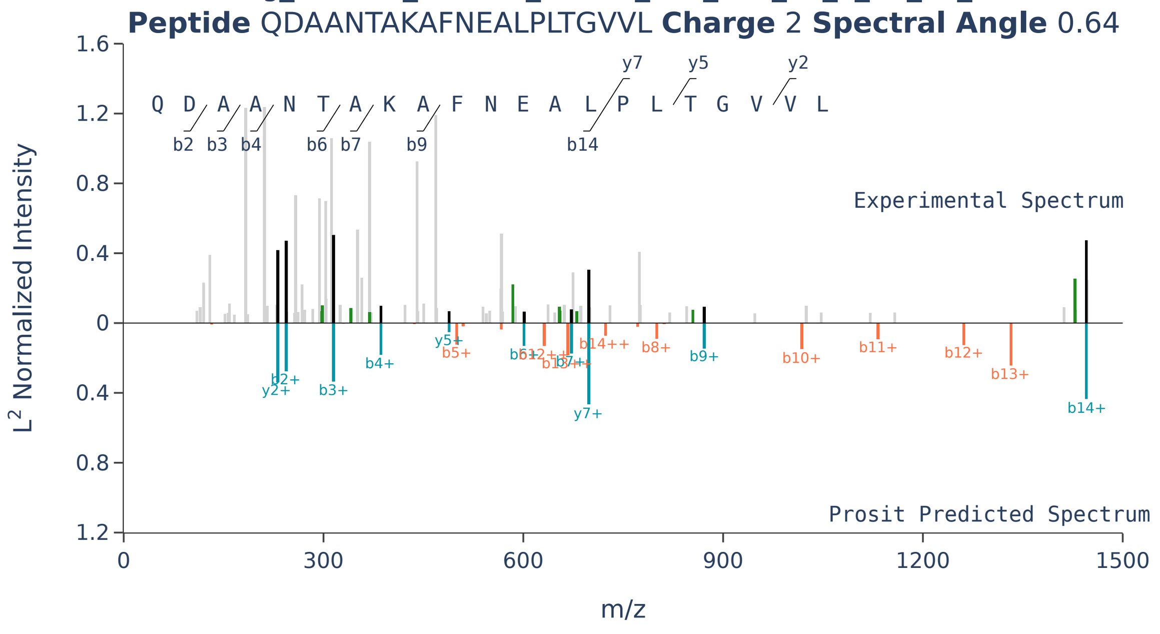


**Source** Ncheng_210623_230623_HFGoe_FFH_20S_25_1_A2_4h_R2 **Scan** 19410
**Peptide** DNLTDRLSRTLRN **Charge** 3 **Spectral Angle** 0.43

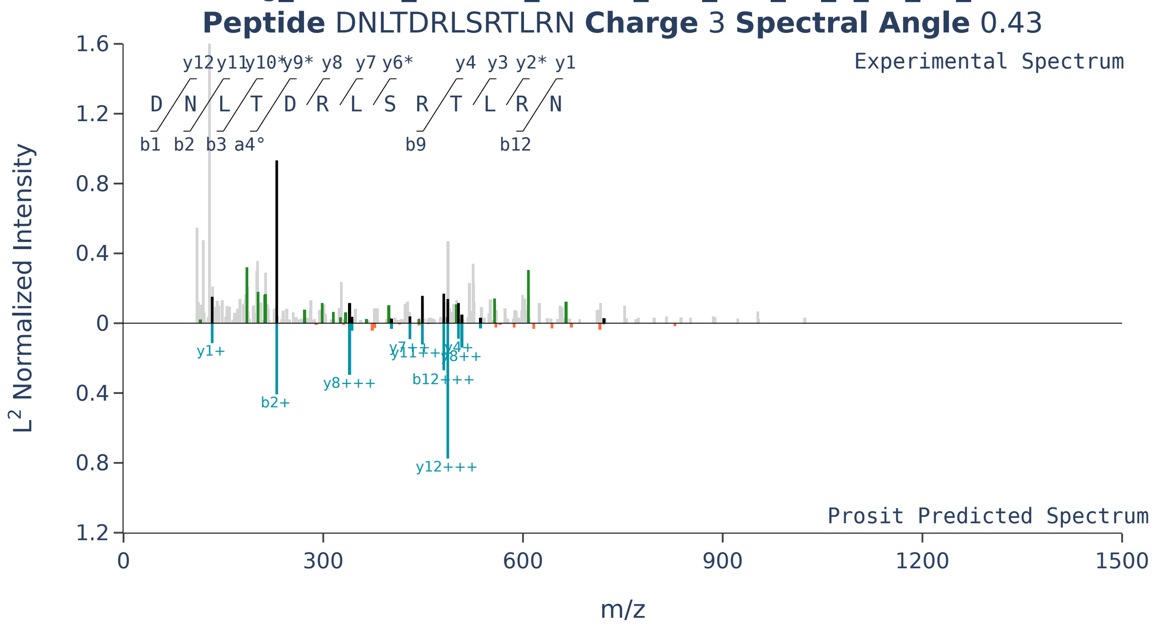


**Source** Ncheng_210623_230623_HFGoe_FFH_20S_25_1_A2_4h_R1 **Scan** 16068
**Peptide** DIESKVDRAQAEKLASKLKKGD **Charge** 6 **Spectral Angle** 0.71

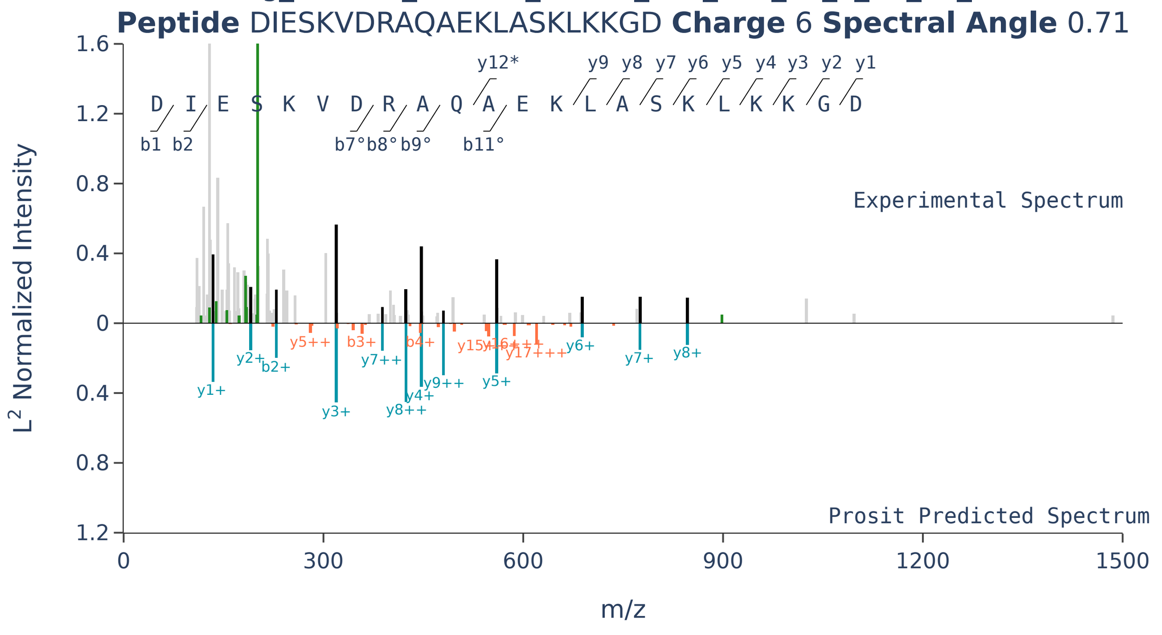


**Source** Ncheng_210623_230623_HFGoe_FFH_20S_25_1_A2_4h_R1 **Scan** 28020
**Peptide** ALPLTGVVLTKVDGDARGGAALSIRH **Charge** 4 **Spectral Angle** 0.64

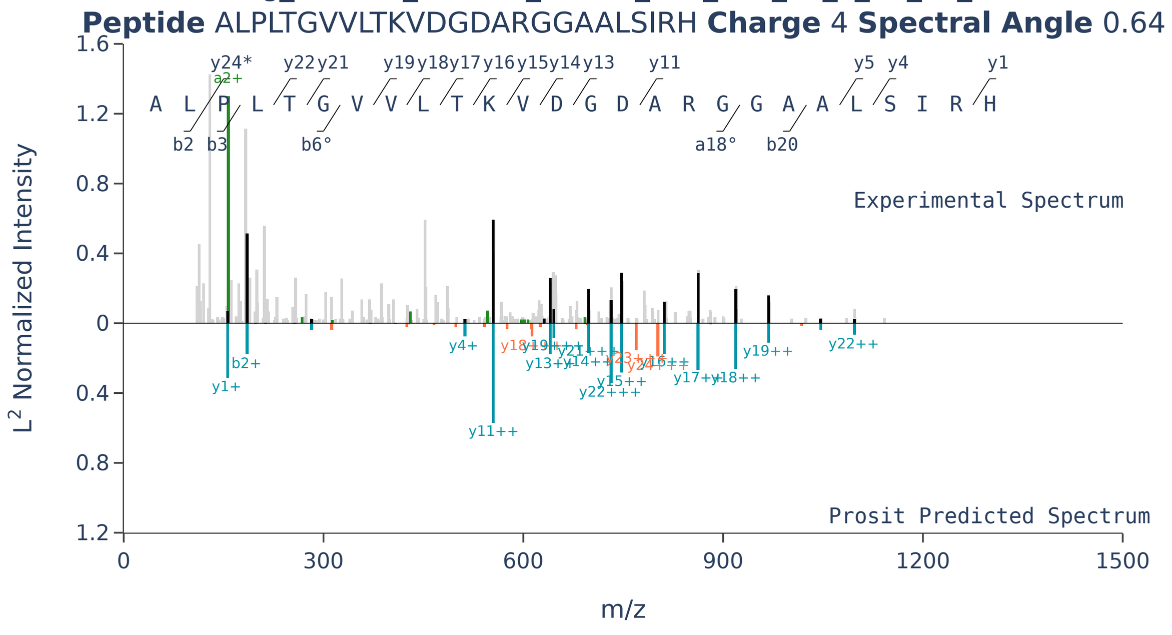


**Source** Ncheng_210623_230623_HFGoe_FFH_20S_25_1_A1_24h_R1 **Scan** 10133
**Peptide** NSMTMKERAKPEIIKGS **Charge** 2 **Spectral Angle** 0.7

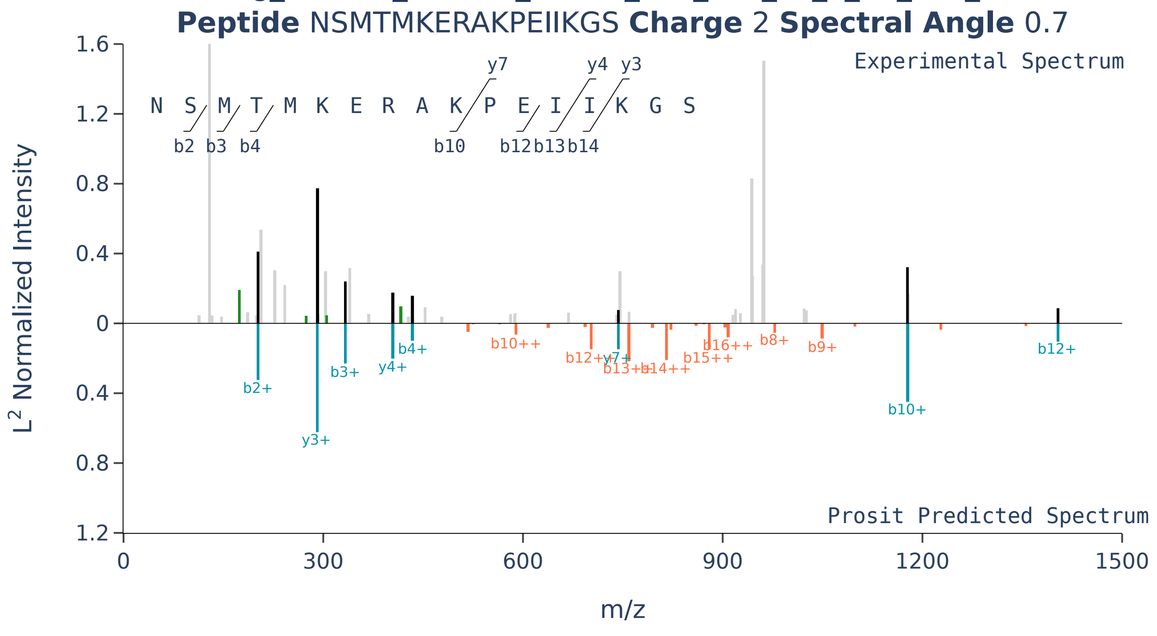


**Source** Ncheng_210623_230623_HFGoe_FFH_20S_25_1_A2_4h_R2 **Scan** 18741
**Peptide** EFINRVKEKAVGHEVNKSLTPGQEFV **Charge** 4 **Spectral Angle** 0.65

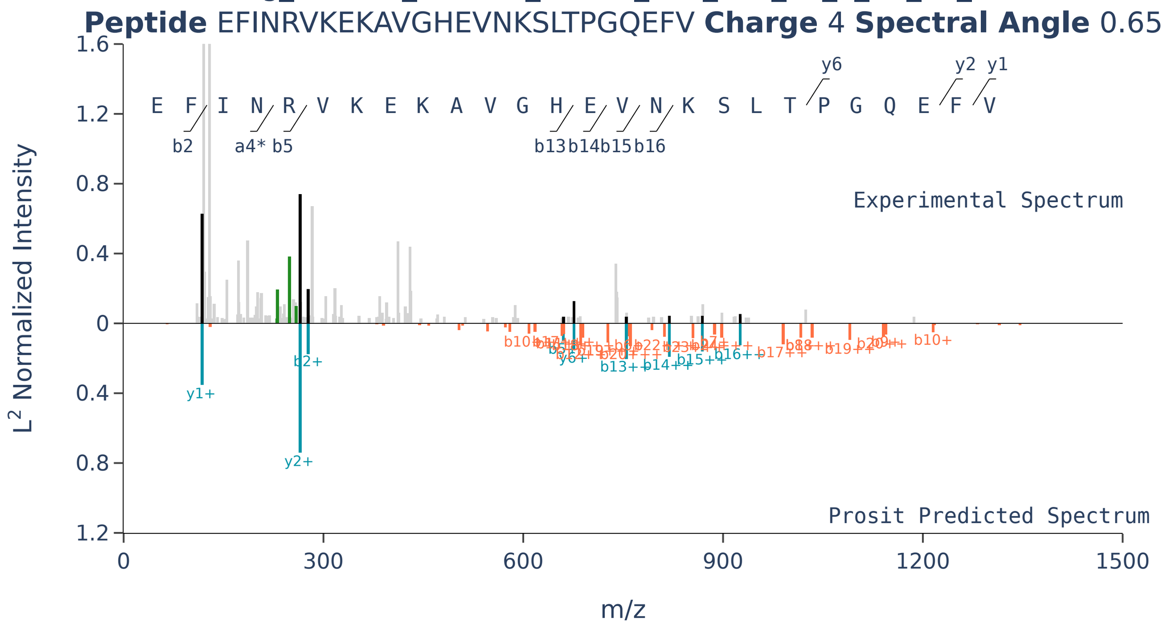


**Source** Ncheng_210623_230623_HFGoe_FFH_20S_25_1_A2_4h_R2 **Scan** 12412
**Peptide** SRTLRNISGRGRLTEDNVKDTLRE **Charge** 5 **Spectral Angle** 0.7

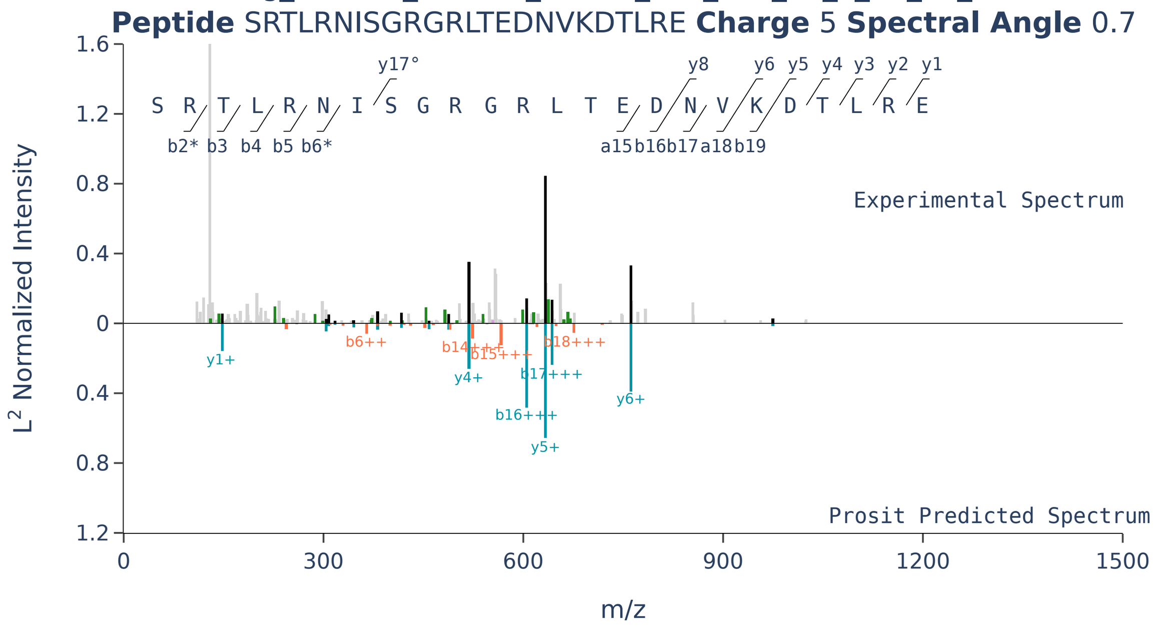


**Source** Ncheng_210623_230623_HFGoe_FFH_20S_25_1_A1_4h_R1 **Scan** 17836
**Peptide** KVDRAQAEKLASKLKKGDGFDLND **Charge** 3 **Spectral Angle** 0.69

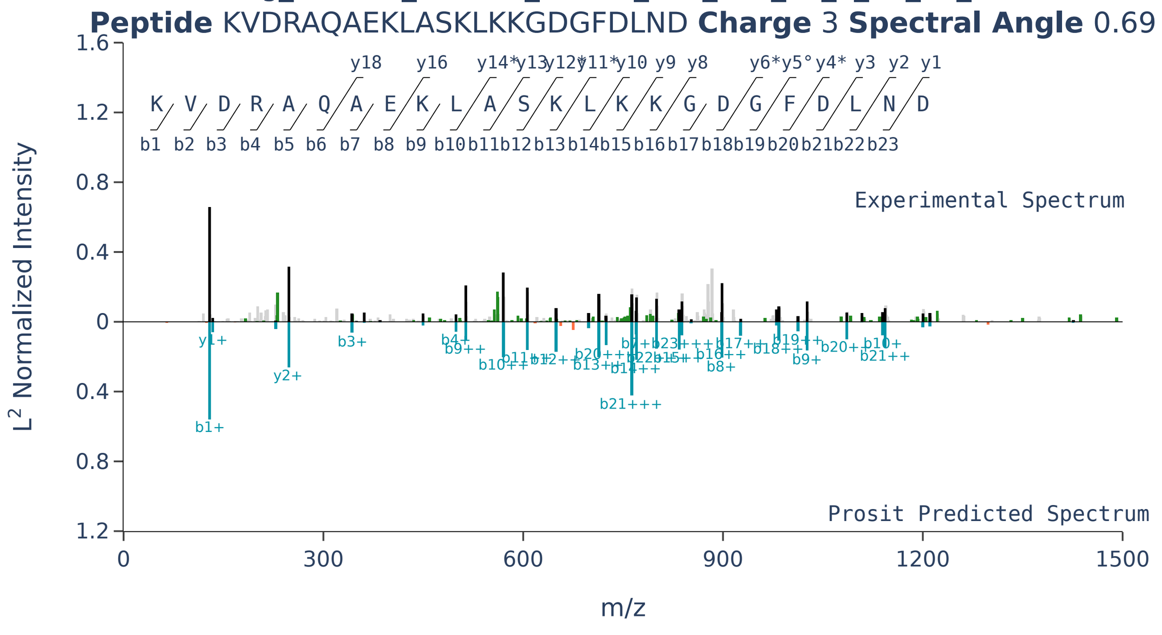


**Source** Ncheng_210623_230623_HFGoe_FFH_20S_25_1_A1_24h_R2 **Scan** 6543
**Peptide** ISGRGRLTE **Charge** 2 **Spectral Angle** 0.52

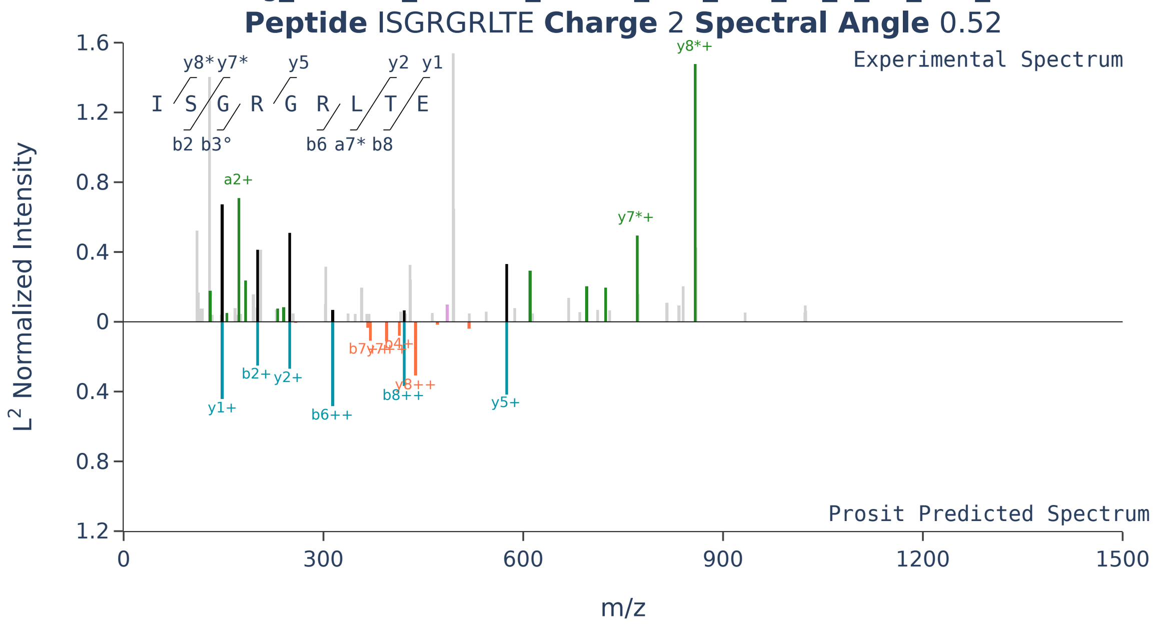


**Source** Ncheng_210623_230623_HFGoe_FFH_20S_25_1_A1_4h_R2 **Scan** 16574
**Peptide** LSGRGRLTEDNVKDTLREV **Charge** 4 **Spectral Angle** 0.67

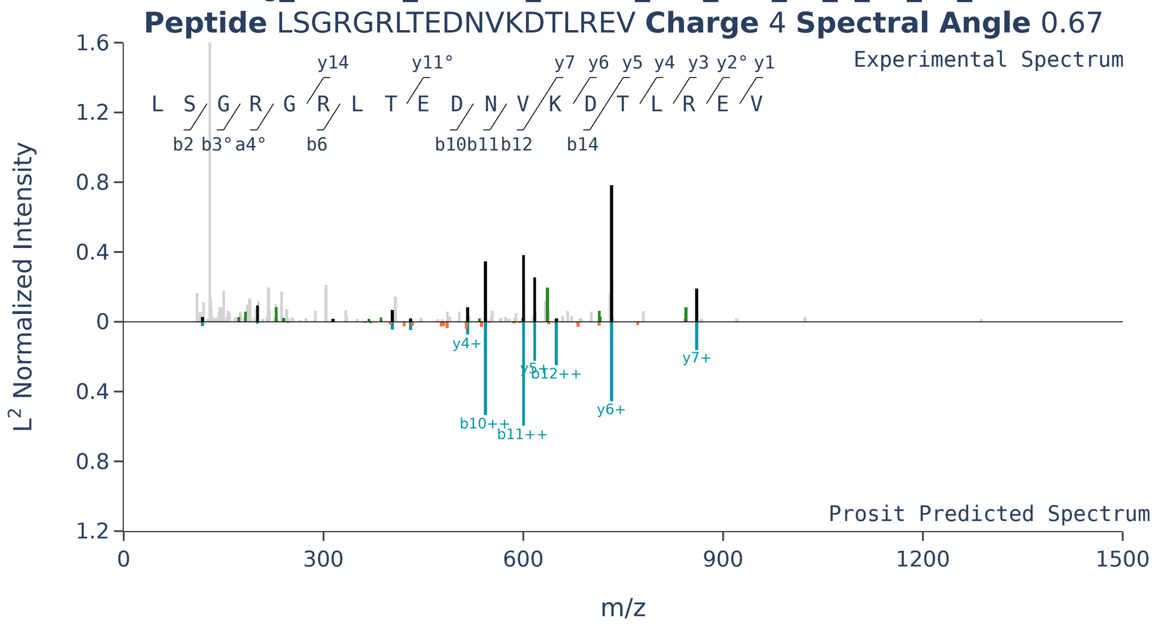


**Source** Ncheng_210623_230623_HFGoe_FFH_20S_25_1_A2_4h_R1 **Scan** 25565
**Peptide** LTPGQEFVKIVRNE **Charge** 3 **Spectral Angle** 0.5

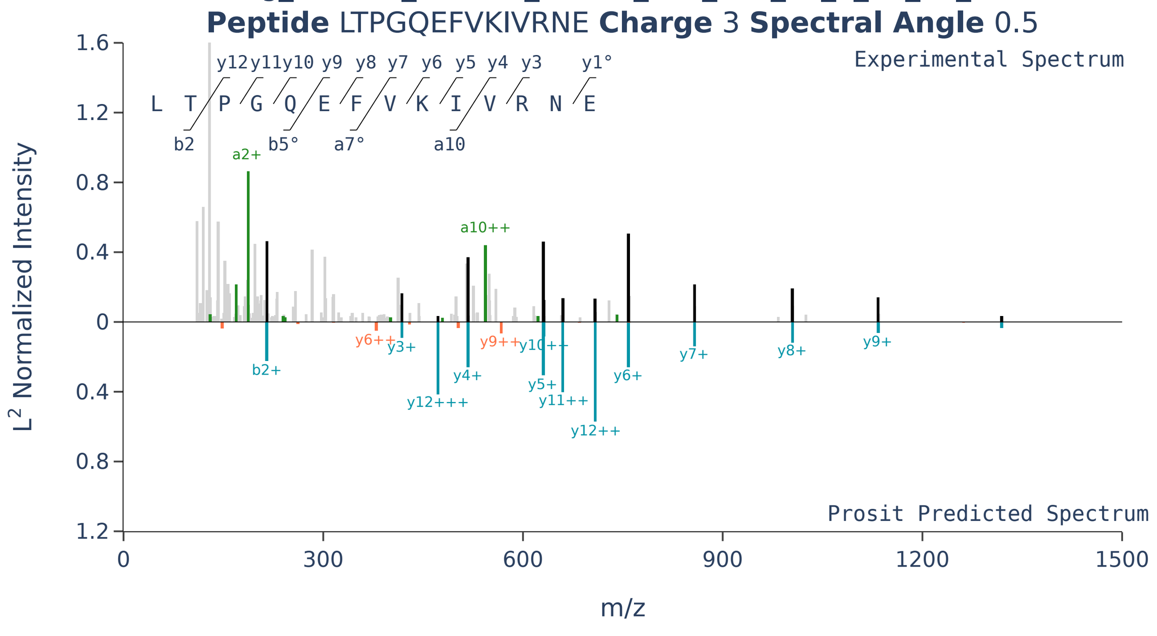


**Source** Ncheng_210623_230623_HFGoe_FFH_20S_25_1_A2_24h_R2 **Scan** 5623
**Peptide** RAKPEIIKGSRK **Charge** 3 **Spectral Angle** 0.74

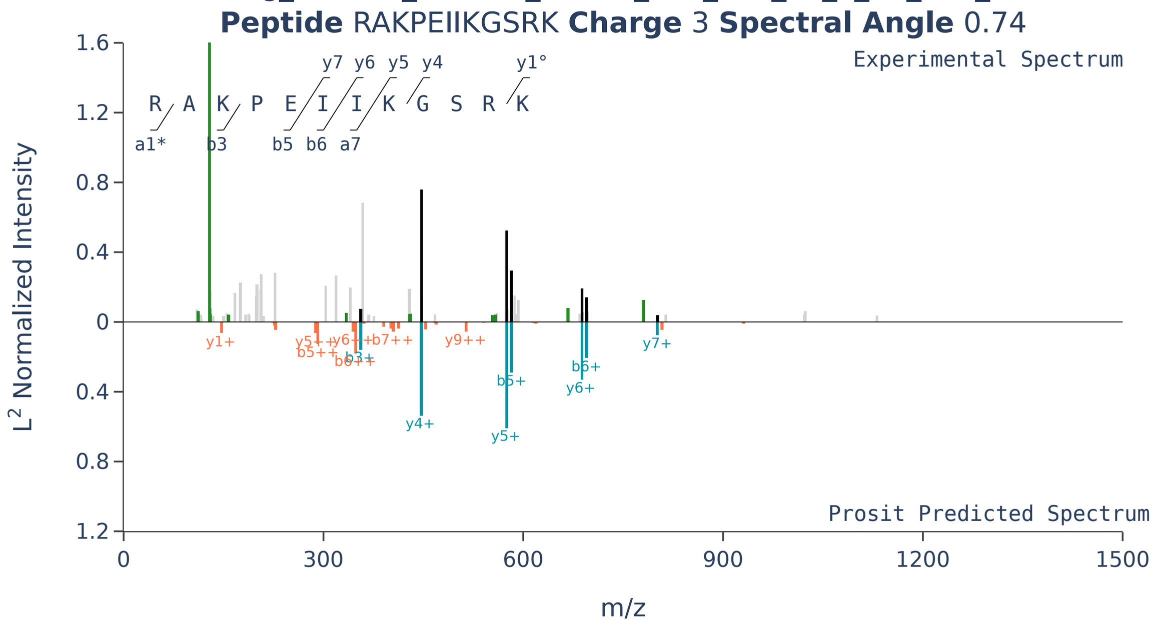


**Source** Ncheng_210623_230623_HFGoe_FFH_20S_25_1_A1_2h_R2 **Scan** 14723
**Peptide** TDRLSRTLRNISGRGRLTED **Charge** 5 **Spectral Angle** 0.76

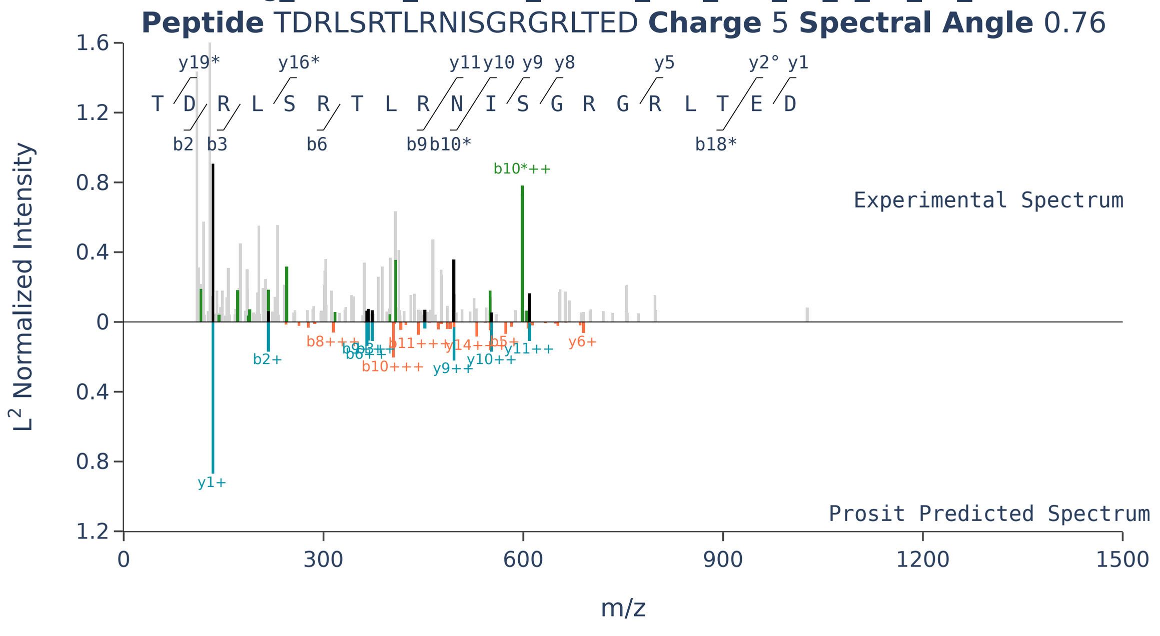

**Source** Ncheng_210623_230623_HFGoe_FFH_20S_25_1_A2_2h_R2 **Scan** 33892
**Peptide** DVYRPAAIKQLETLAEQV **Charge** 2 **Spectral Angle** 0.75

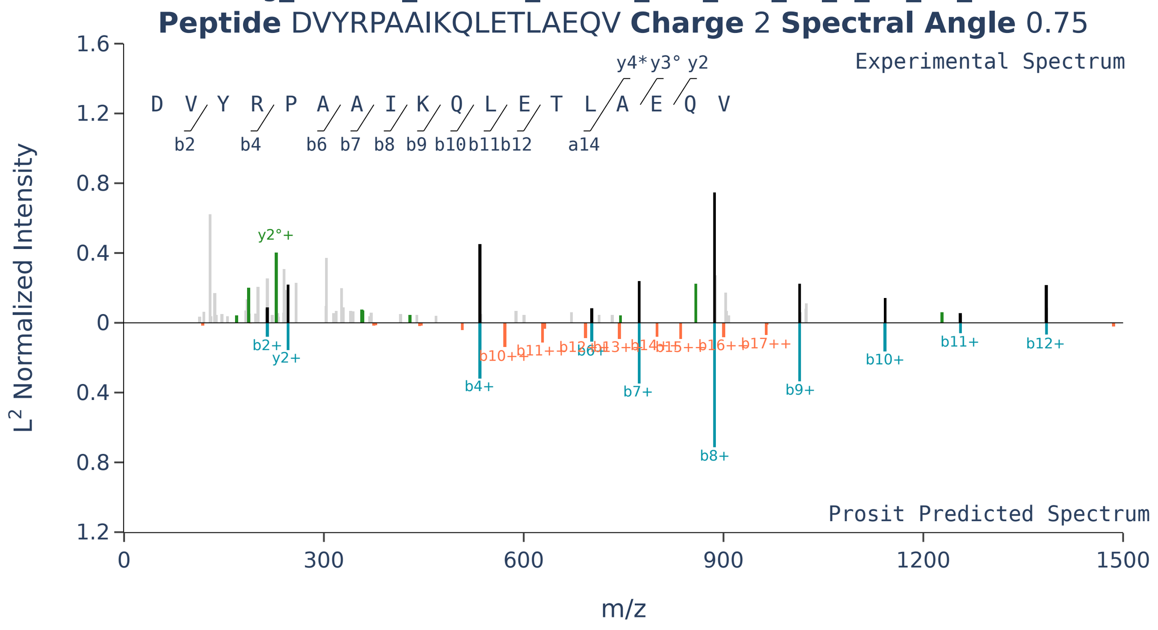


**Source** Ncheng_210623_230623_HFGoe_FFH_20S_25_1_A2_24h_R1 **Scan** 26234
**Peptide** ASLMGKLPGMGQIPDNVKSQ **Charge** 3 **Spectral Angle** 0.66

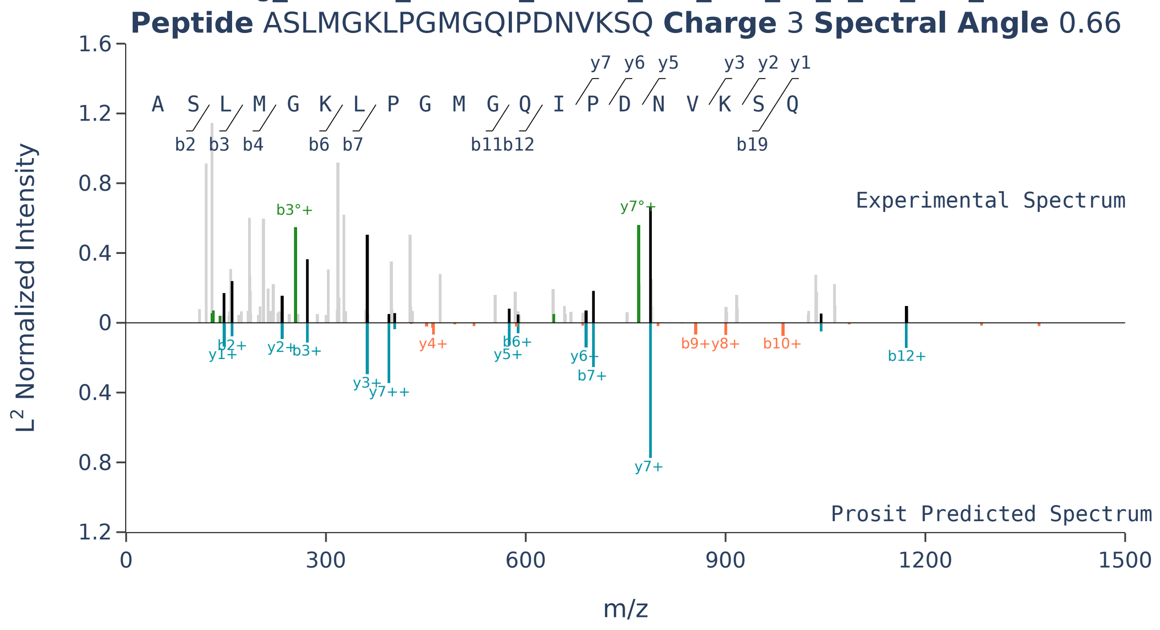


**Source** Ncheng_210623_230623_HFGoe_FFH_20S_25_1_A1_1h_R2 **Scan** 32610
**Peptide** LVNAALKEAKLKFYDVLLVDTAGRLHVD **Charge** 5 **Spectral Angle** 0.66

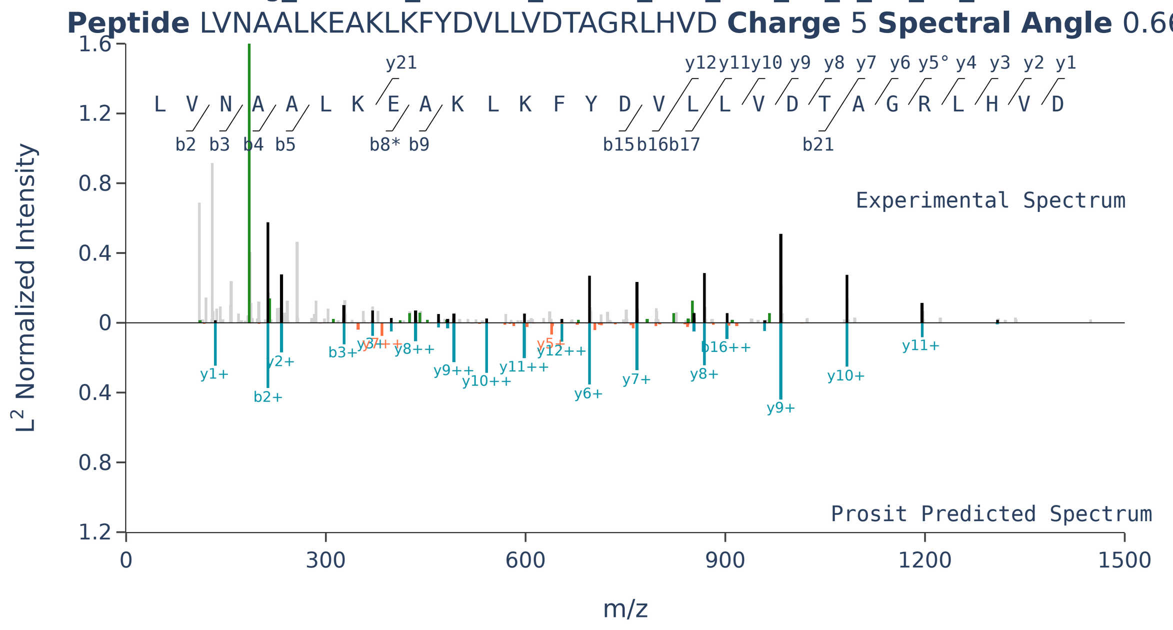


**Source** Ncheng_210623_230623_HFGoe_FFH_20S_25_1_A2_2h_R1 **Scan** 34764
**Peptide** KKGDGFDLNDFLEQLRQMKNMGGMA **Charge** 4 **Spectral Angle** 0.69

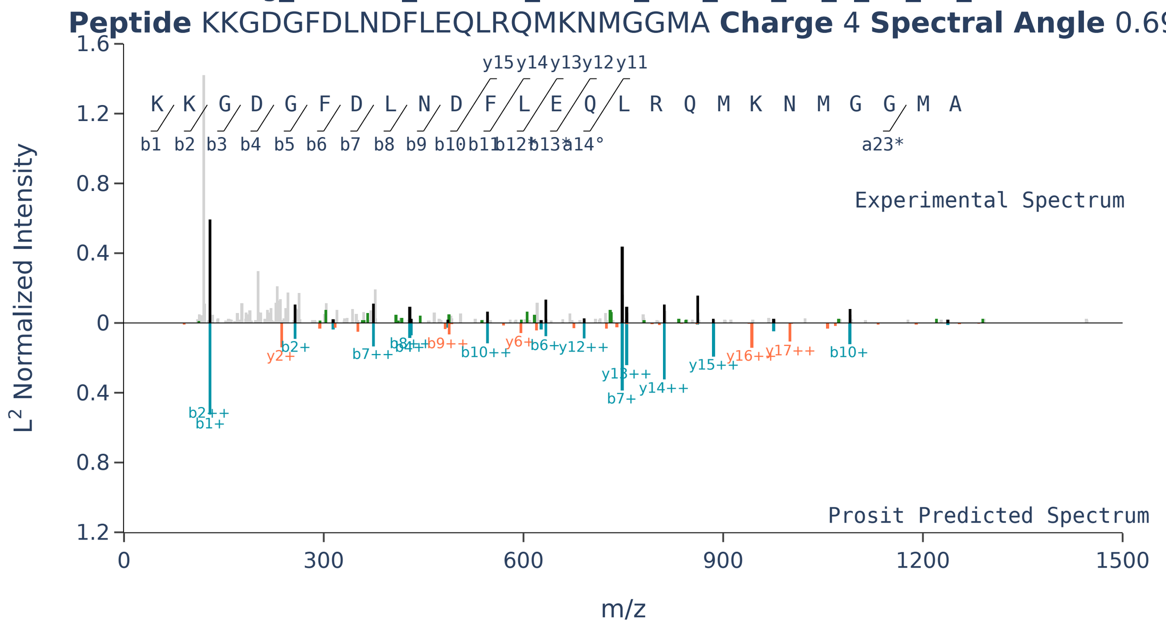


**Source** Ncheng_210623_230623_HFGoe_FFH_20S_25_1_A2_4h_R2 **Scan** 27029
**Peptide** AAMGEENQTLNLAAQPPAV **Charge** 2 **Spectral Angle** 0.96

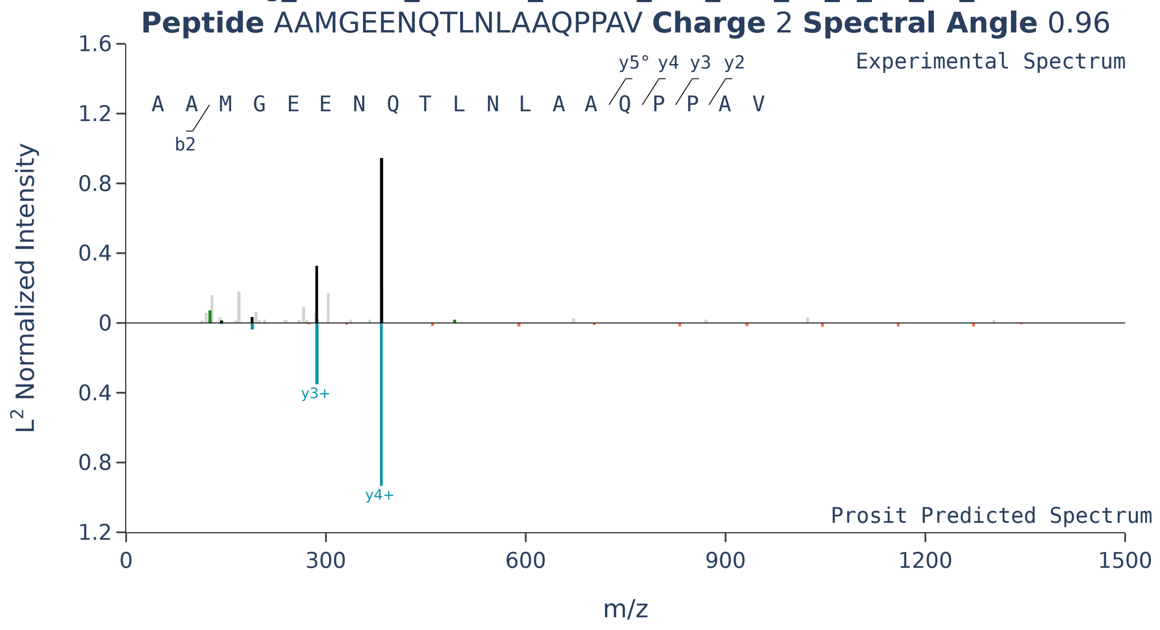


**Source** Ncheng_210623_230623_HFGoe_FFH_20S_25_1_A1_24h_R1 **Scan** 5741
**Peptide** EKLASKLKKG **Charge** 2 **Spectral Angle** 0.66

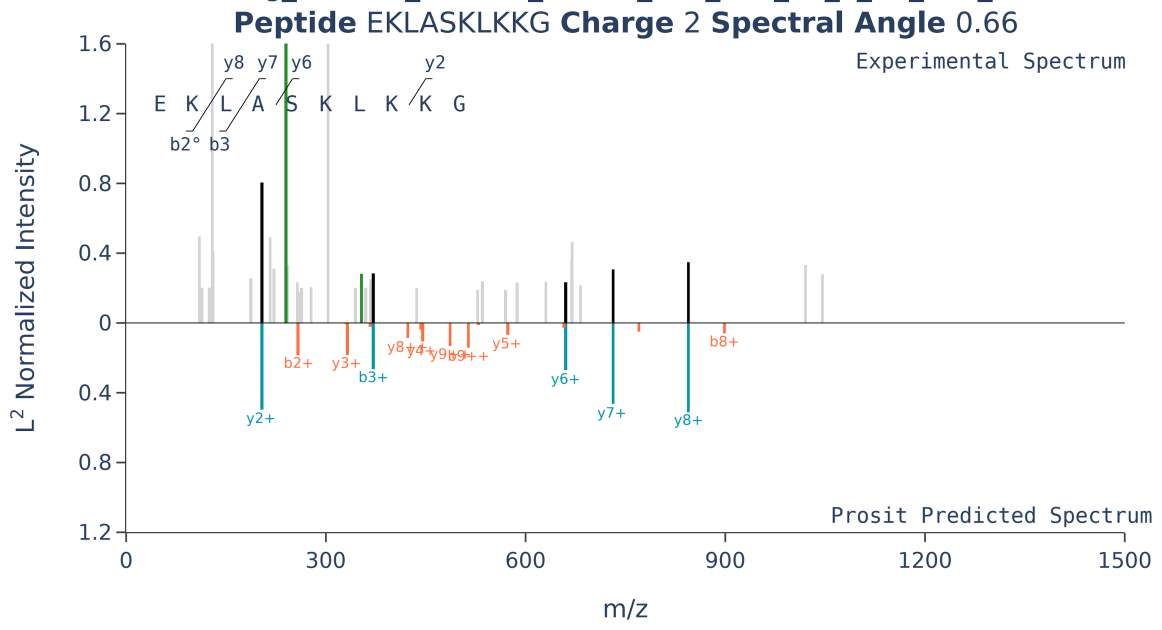


**Source** Ncheng_210623_230623_HFGoe_FFH_20S_25_1_A1_24h_R1 **Scan** 5282
**Peptide** RHITGKPLK **Charge** 2 **Spectral Angle** 0.76

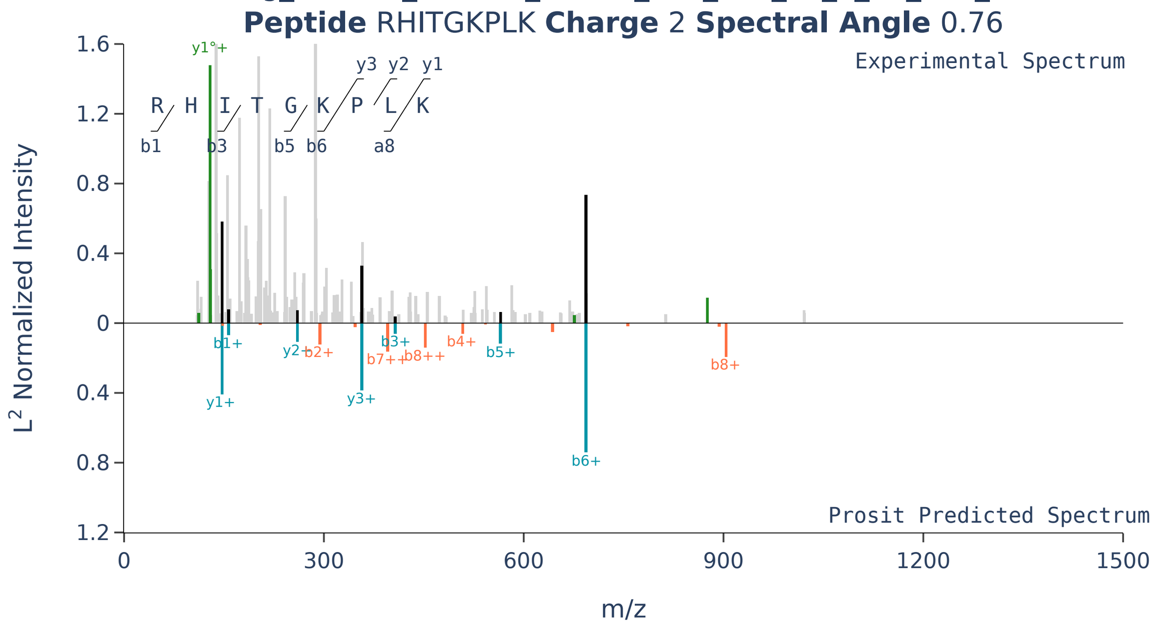


**Source** Ncheng_210623_230623_HFGoe_FFH_20S_25_1_A2_24h_R1 **Scan** 10216
**Peptide** SMTMKERAKPEIIK **Charge** 3 **Spectral Angle** 0.53

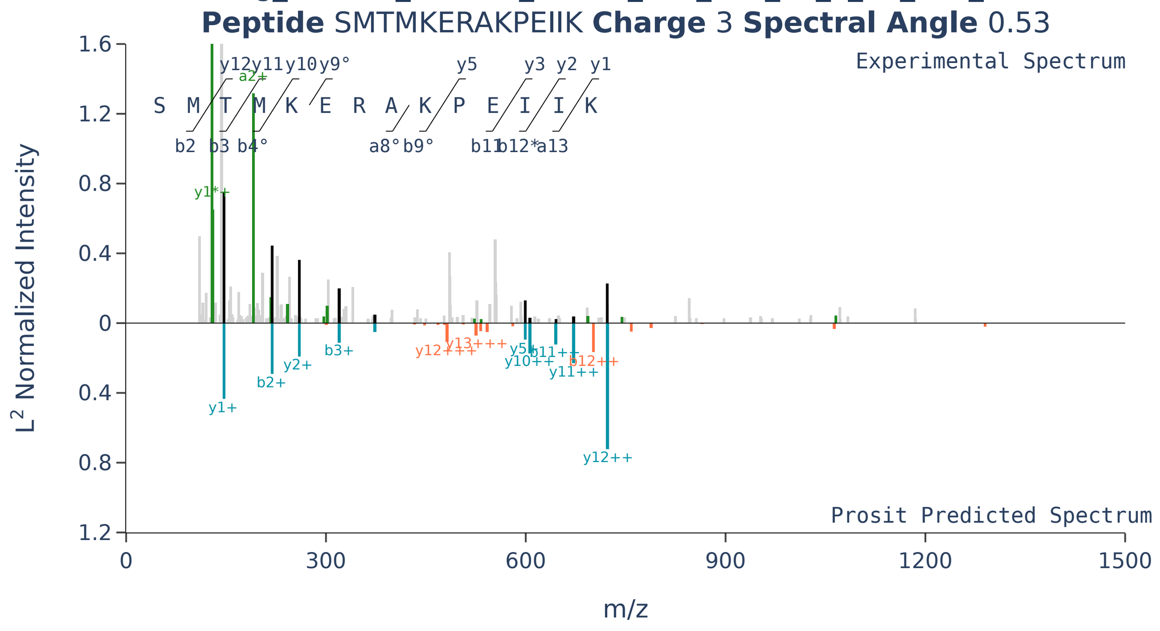


**Source** Ncheng_210623_230623_HFGoe_FFH_20S_25_1_A2_4h_R1 **Scan** 28678
**Peptide** VALPVVREFINRVKEKAVGHEVNK **Charge** 3 **Spectral Angle** 0.67

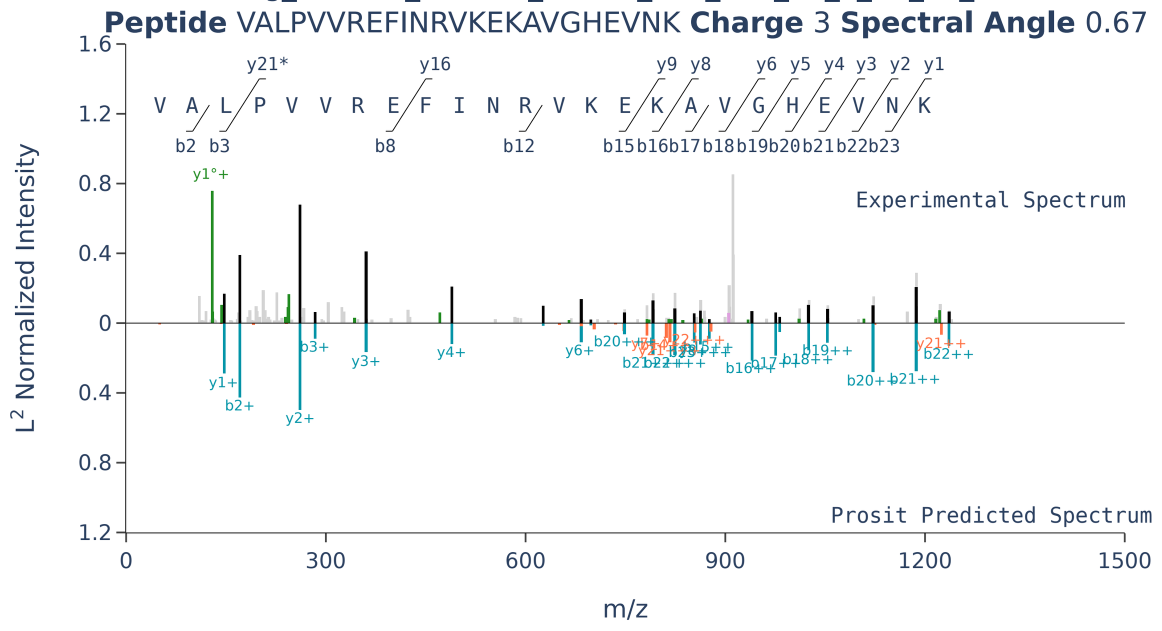


**Source** Ncheng_210623_230623_HFGoe_FFH_20S_25_1_A1_24h_R2 **Scan** 16065
**Peptide** MAGLQGAGKTTSVGKL **Charge** 3 **Spectral Angle** 0.67

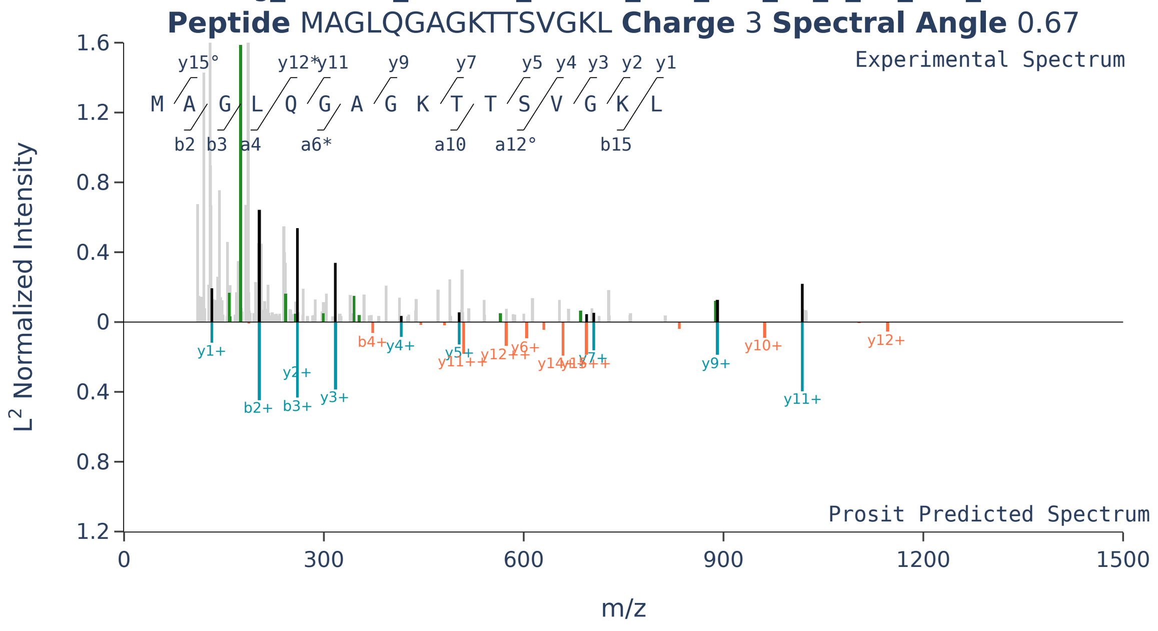


**Source** Ncheng_210623_230623_HFGoe_FFH_20S_25_1_A1_24h_R2 **Scan** 20244
**Peptide** SKLKKGDGFDLNDF **Charge** 3 **Spectral Angle** 0.65

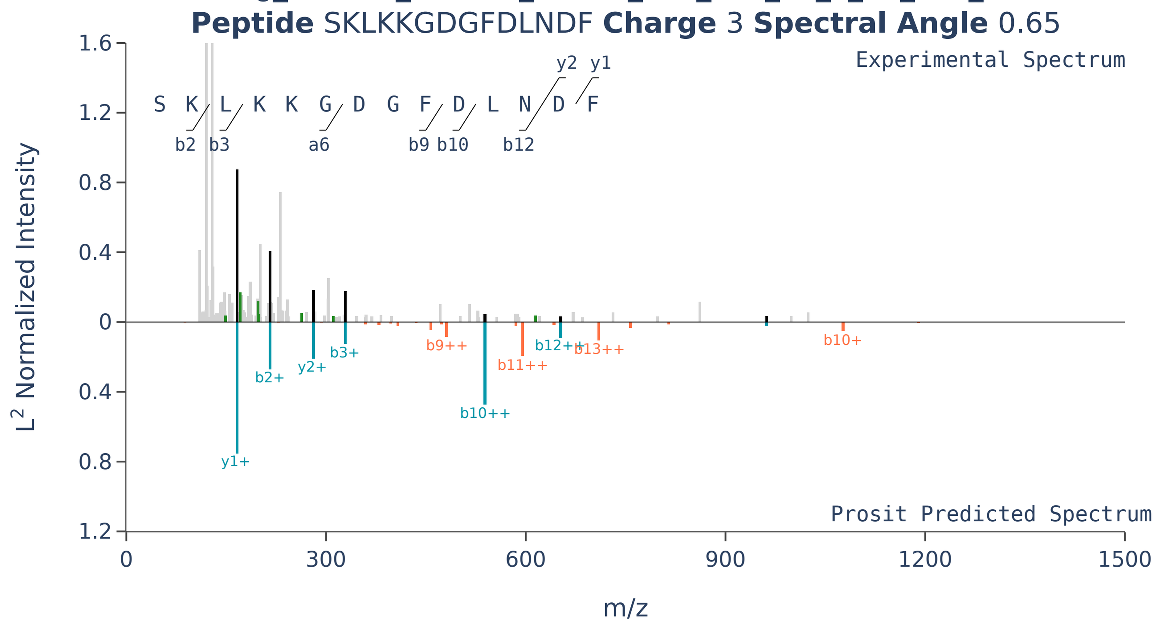


**Source** Ncheng_210623_230623_HFGoe_FFH_20S_25_1_A2_24h_R1 **Scan** 11075
**Peptide** IINSMTMKERAKPEIIKGSRK **Charge** 6 **Spectral Angle** 0.79

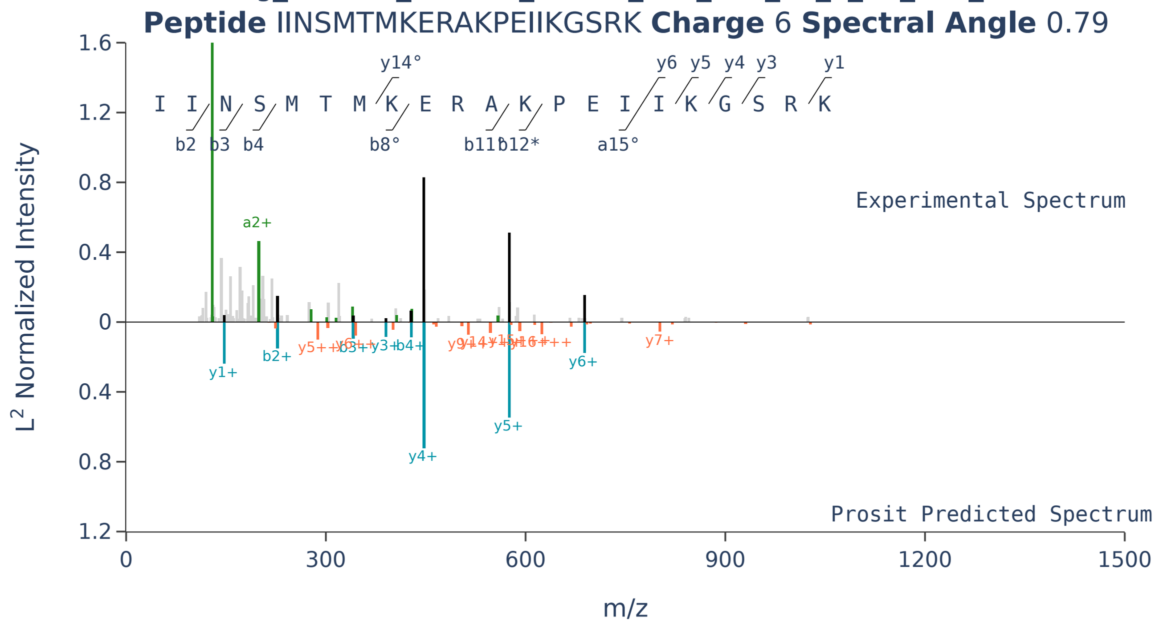


**Source** Ncheng_210623_230623_HFGoe_FFH_20S_25_1_A1_2h_R1 **Scan** 23624
**Peptide** NLTDRLSRTLRNISGRGRLTED **Charge** 5 **Spectral Angle** 0.85

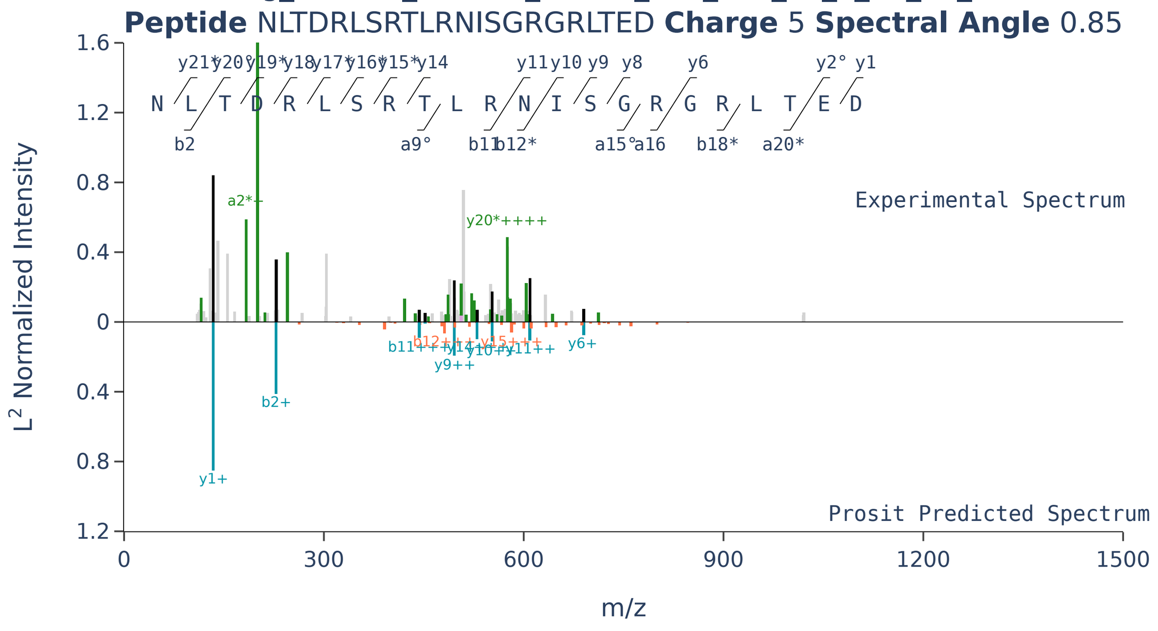


**Source** Ncheng_210623_230623_HFGoe_FFH_20S_25_1_A2_2h_R1 **Scan** 10667
**Peptide** YRPAAIKQ **Charge** 2 **Spectral Angle** 0.65

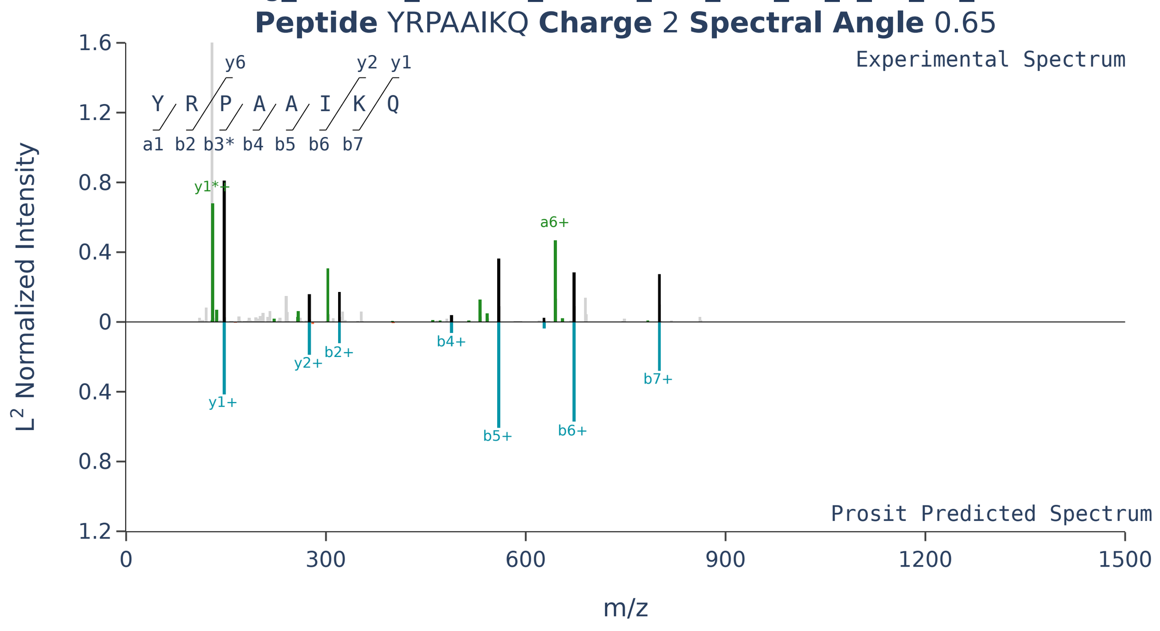


**Source** Ncheng_210623_230623_HFGoe_FFH_20S_25_1_A2_4h_R1 **Scan** 32477
**Peptide** VNAALKEAKLKFYDVLLV **Charge** 3 **Spectral Angle** 0.65

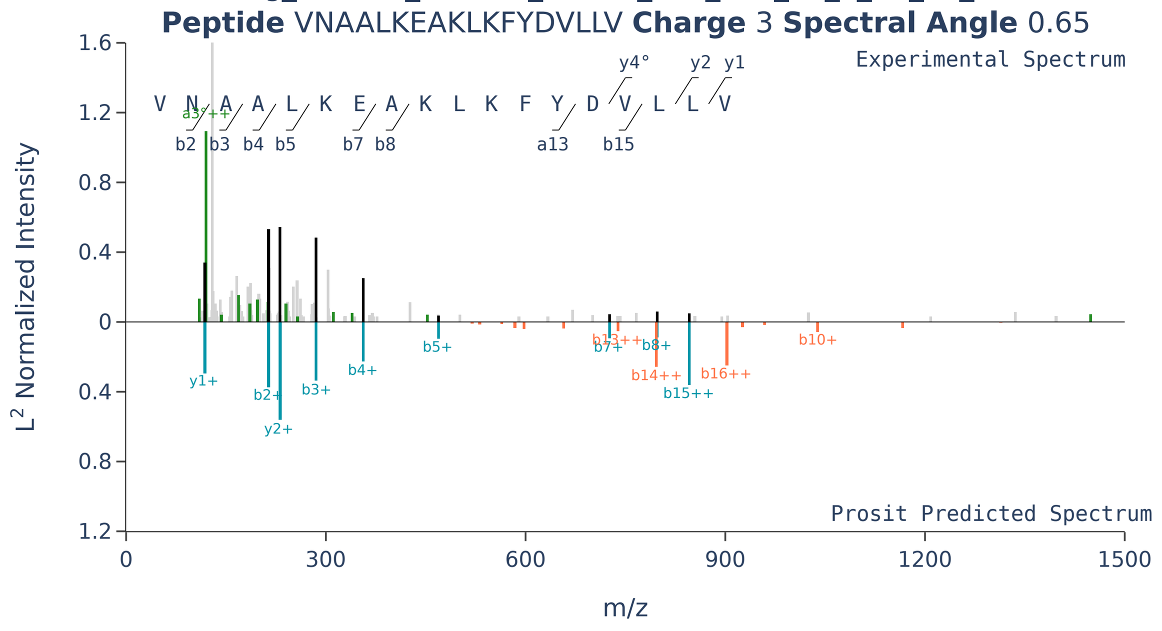

**Source** Ncheng_210623_230623_HFGoe_FFH_20S_25_1_A1_2h_R1 **Scan** 34118
**Peptide** EADVALPVVREFINRVKEKAVGHEVN **Charge** 4 **Spectral Angle** 0.64

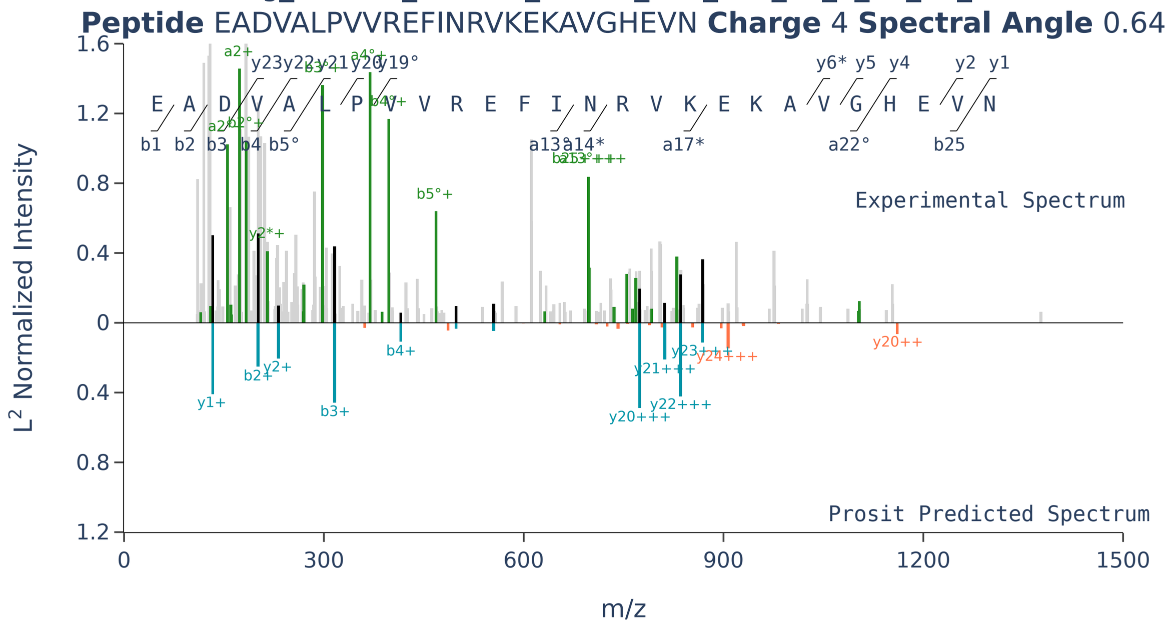


**Source** Ncheng_210623_230623_HFGoe_FFH_20S_25_1_A2_2h_R1 **Scan** 31261
**Peptide** DVALPVVREFINRVKEKAVGHEVNK **Charge** 5 **Spectral Angle** 0.8

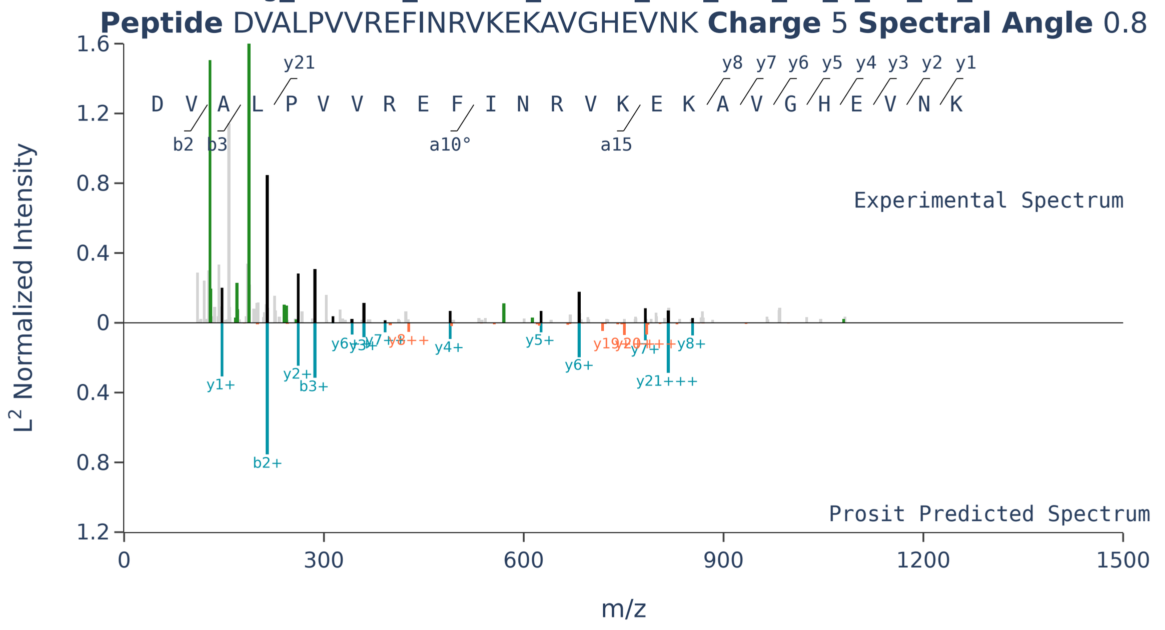


**Source** Ncheng_210623_230623_HFGoe_FFH_20S_25_1_A1_2h_R1 **Scan** 28971
**Peptide** AKLKFYDVLLVDTAGRLH **Charge** 4 **Spectral Angle** 0.69

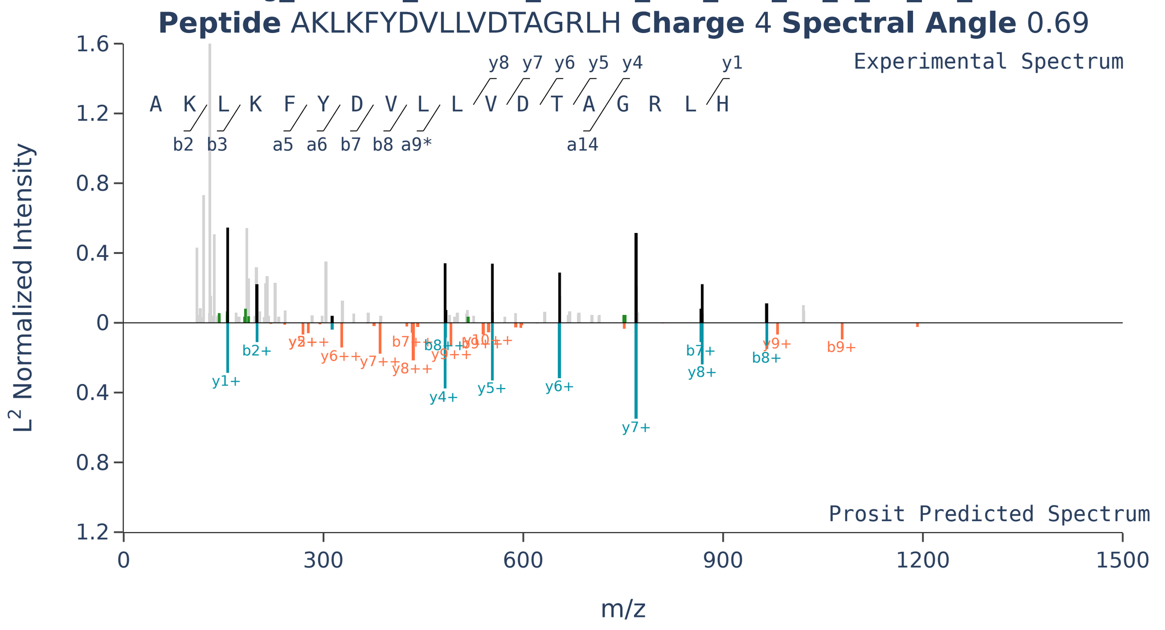


**Source** Ncheng_210623_230623_HFGoe_FFH_20S_25_1_A2_24h_R2 **Scan** 14388
**Peptide** FDDMQRMMKKMK **Charge** 2 **Spectral Angle** 0.65

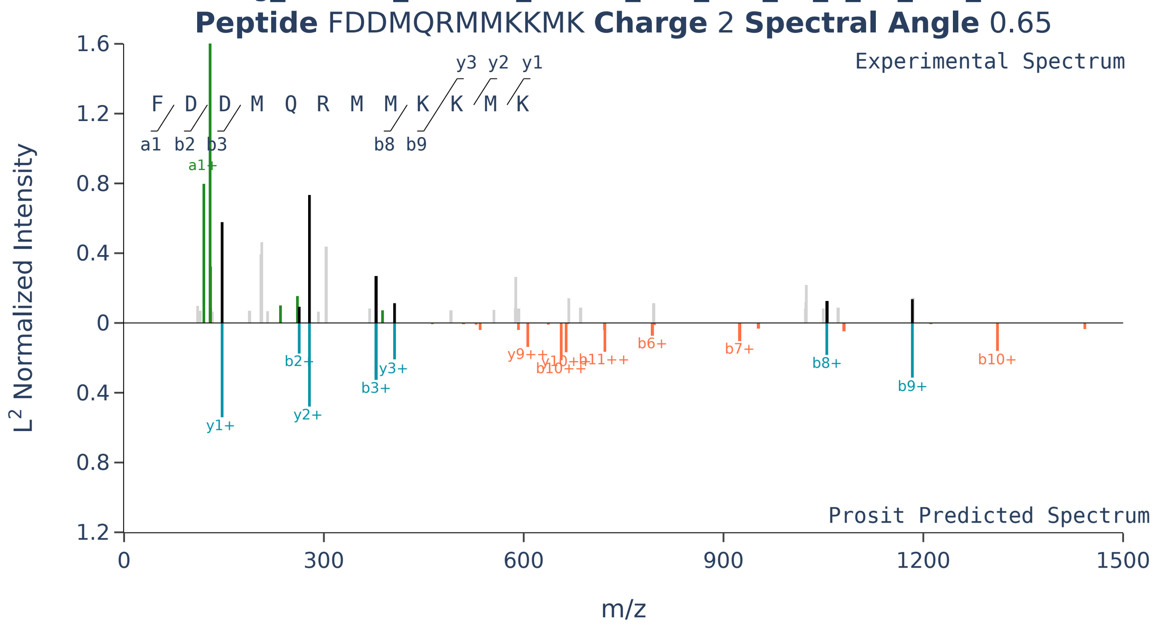


**Source** Ncheng_210623_230623_HFGoe_FFH_20S_25_1_A1_24h_R1 **Scan** 12141
**Peptide** GAGKTTSVGKLGKF **Charge** 3 **Spectral Angle** 0.68

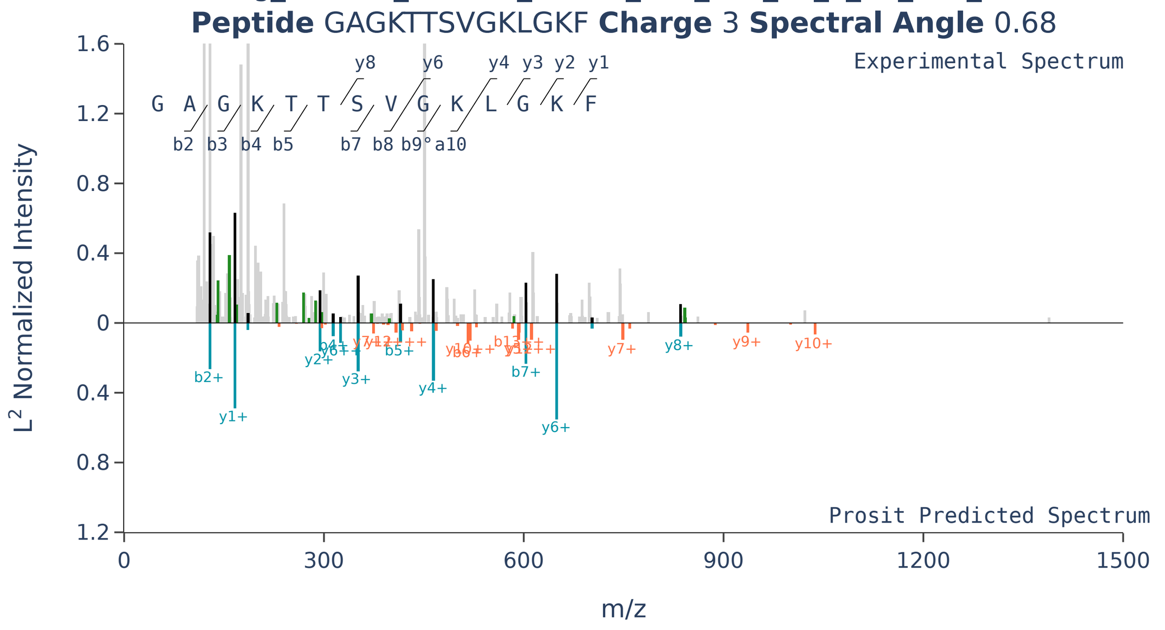


**Source** Ncheng_210623_230623_HFGoe_FFH_20S_25_1_A1_4h_R1 **Scan** 17297
**Peptide** LEDIESKVDRAQAEKLASKLKK **Charge** 5 **Spectral Angle** 0.68

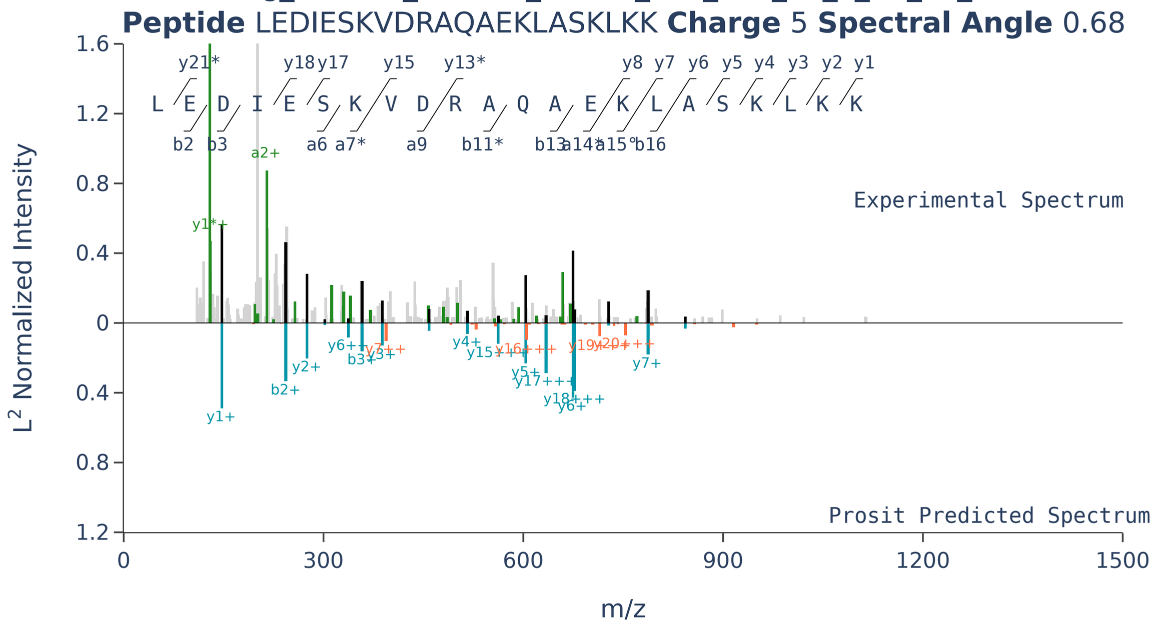


**Source** Ncheng_210623_230623_HFGoe_FFH_20S_25_1_A1_2h_R2 **Scan** 26929
**Peptide** KVLVRMEAIINSMTMKERA **Charge** 4 **Spectral Angle** 0.8

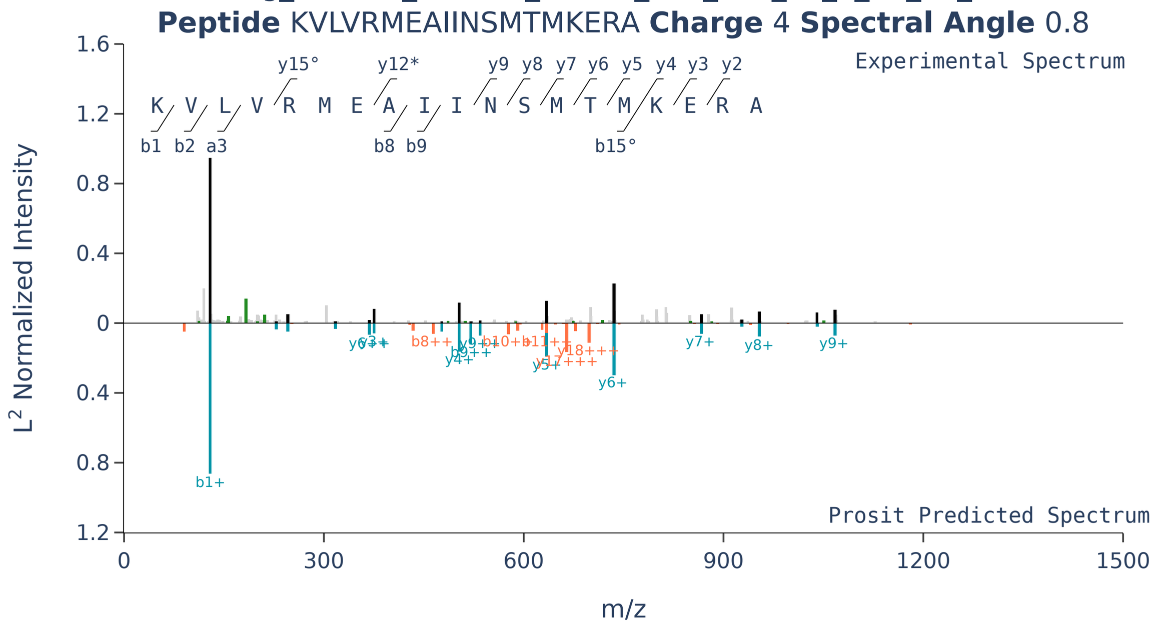


**Source** Ncheng_210623_230623_HFGoe_FFH_20S_25_1_A1_2h_R2 **Scan** 32257
**Peptide** PSDVGQKPVDIVNAALKEAKL **Charge** 3 **Spectral Angle** 0.68

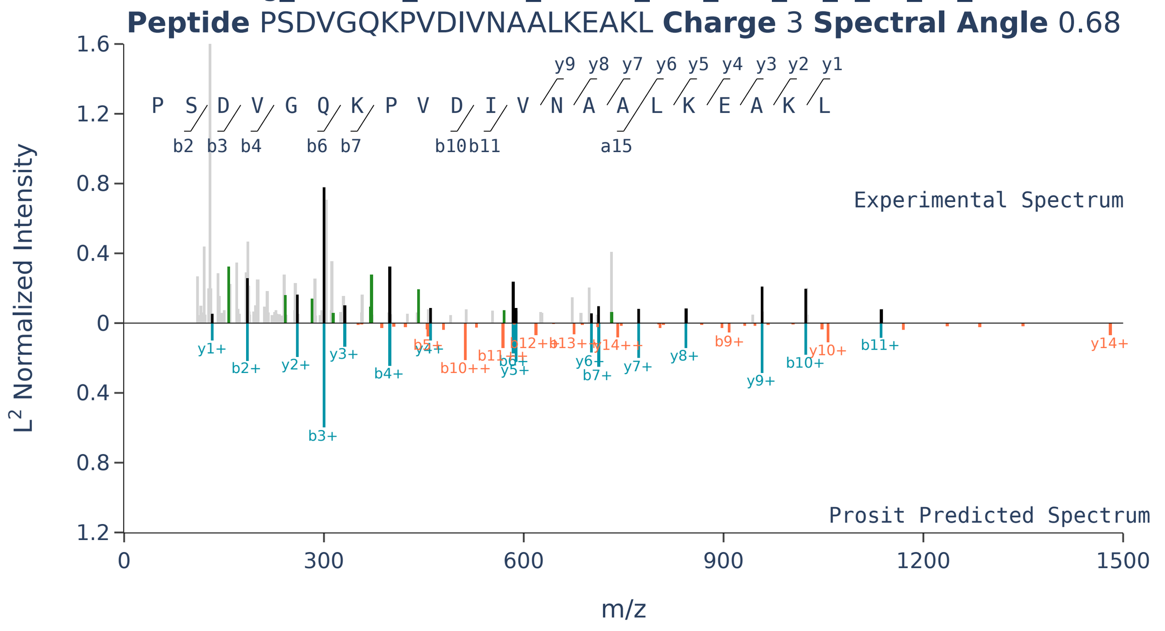


**Source** Ncheng_210623_230623_HFGoe_FFH_20S_25_1_A2_2h_R2 **Scan** 34487
**Peptide** VVSADVYRPAAIKQLETLAEQV **Charge** 3 **Spectral Angle** 0.79

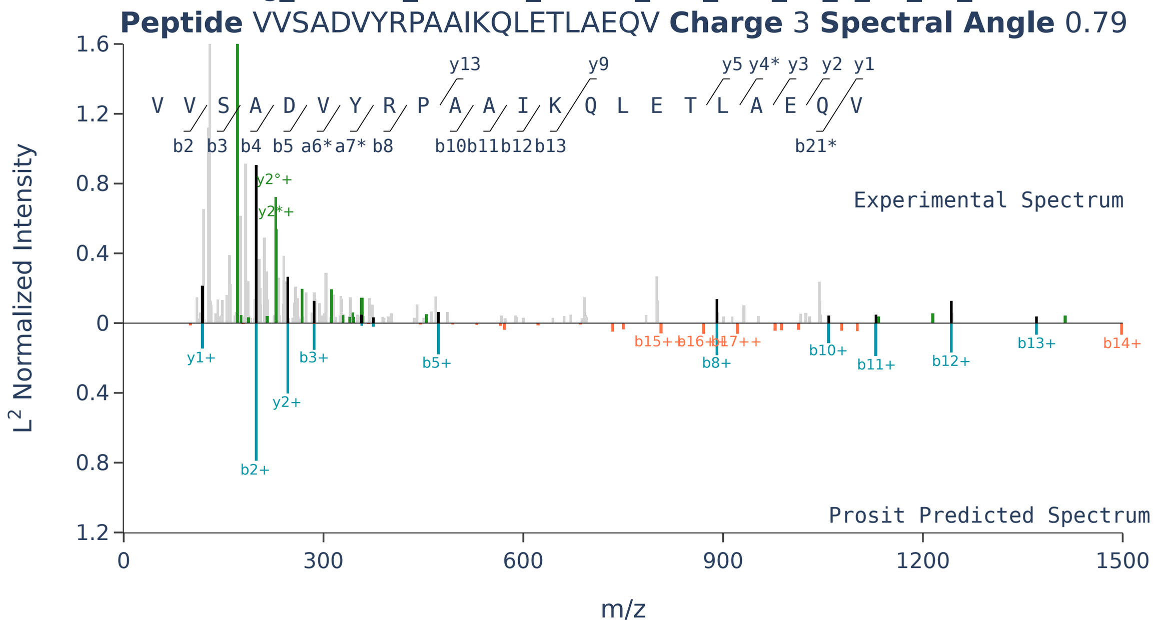


**Source** Ncheng_210623_230623_HFGoe_FFH_20S_25_1_A2_24h_R1 **Scan** 13119
**Peptide** QLPDNVKSQM **Charge** 2 **Spectral Angle** 0.42

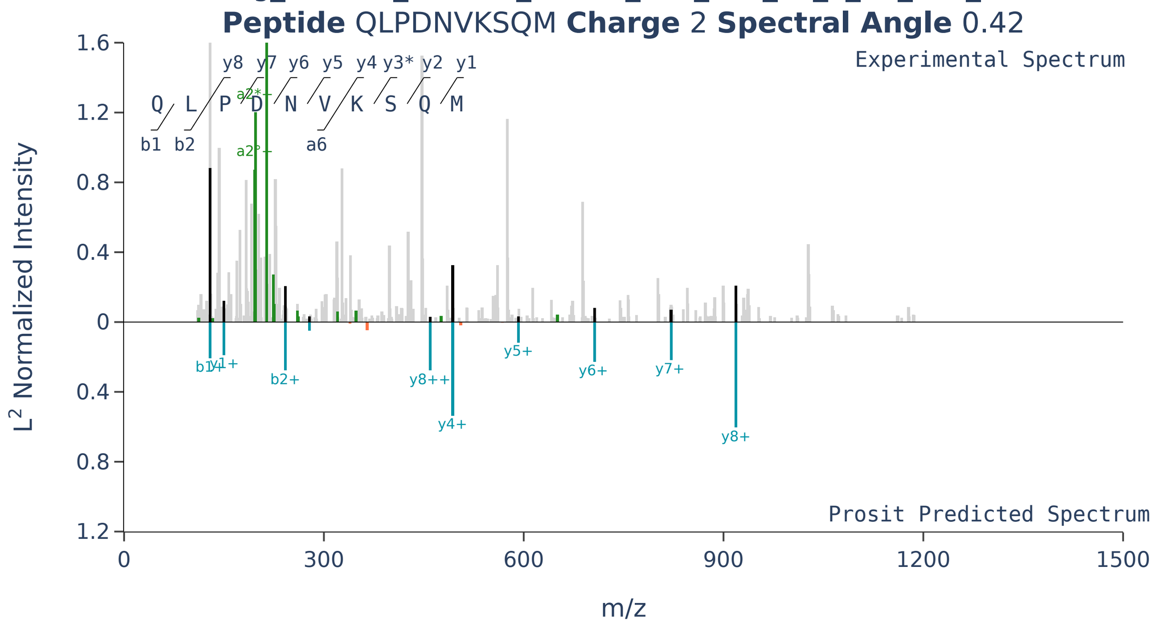


**Source** Ncheng_210623_230623_HFGoe_FFH_20S_25_1_A1_2h_R2 **Scan** 34504
**Peptide** VLSLIEDIESKVDRAQAEKLASKL **Charge** 3 **Spectral Angle** 0.69

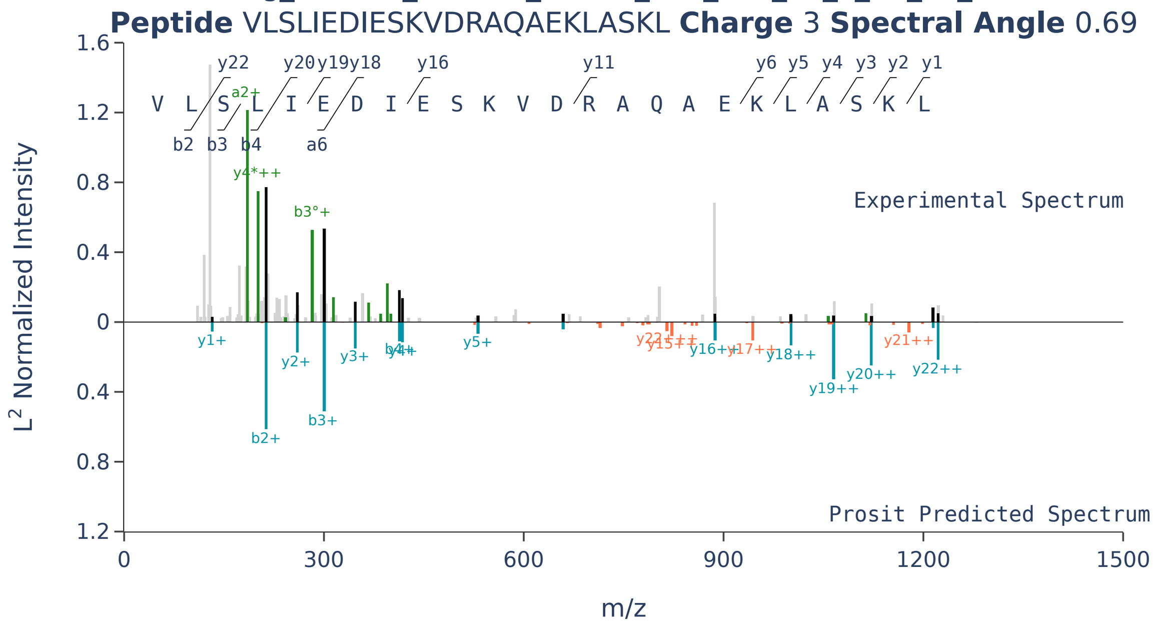


**Source** Ncheng_210623_230623_HFGoe_FFH_20S_25_1_A2_4h_R1 **Scan** 38324
**Peptide** KGDGFDLNDFLEQLRQMKN **Charge** 3 **Spectral Angle** 0.86

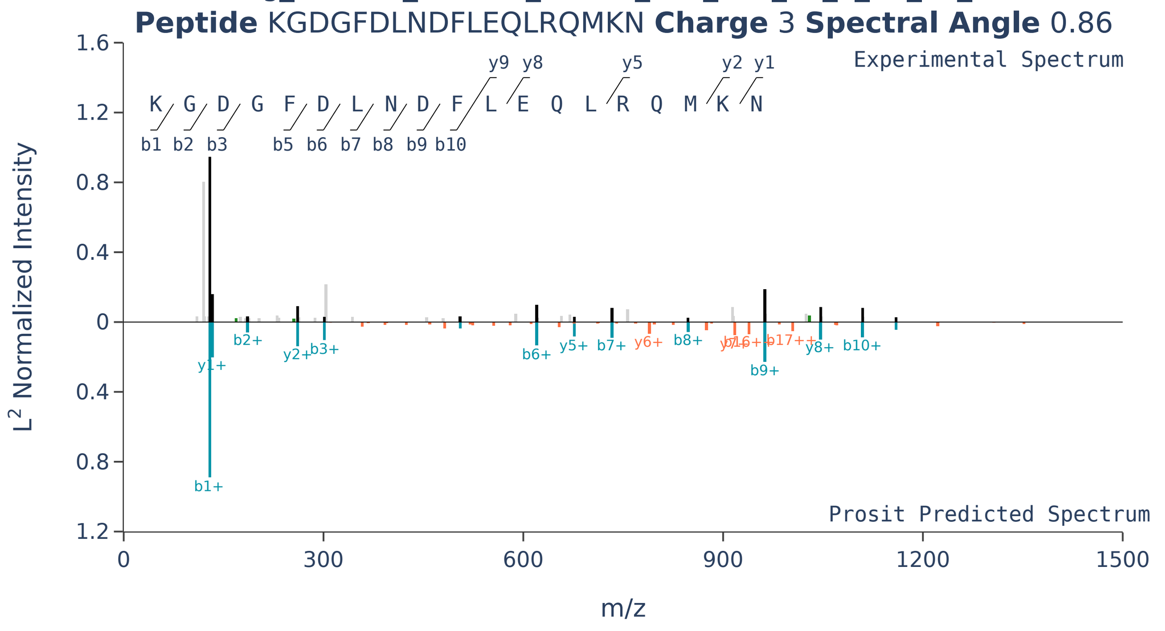


**Source** Ncheng_210623_230623_HFGoe_FFH_20S_25_1_A2_24h_R2 **Scan** 14972
**Peptide** GLQGAGKTTSVGKLGKFLREKH **Charge** 4 **Spectral Angle** 0.79

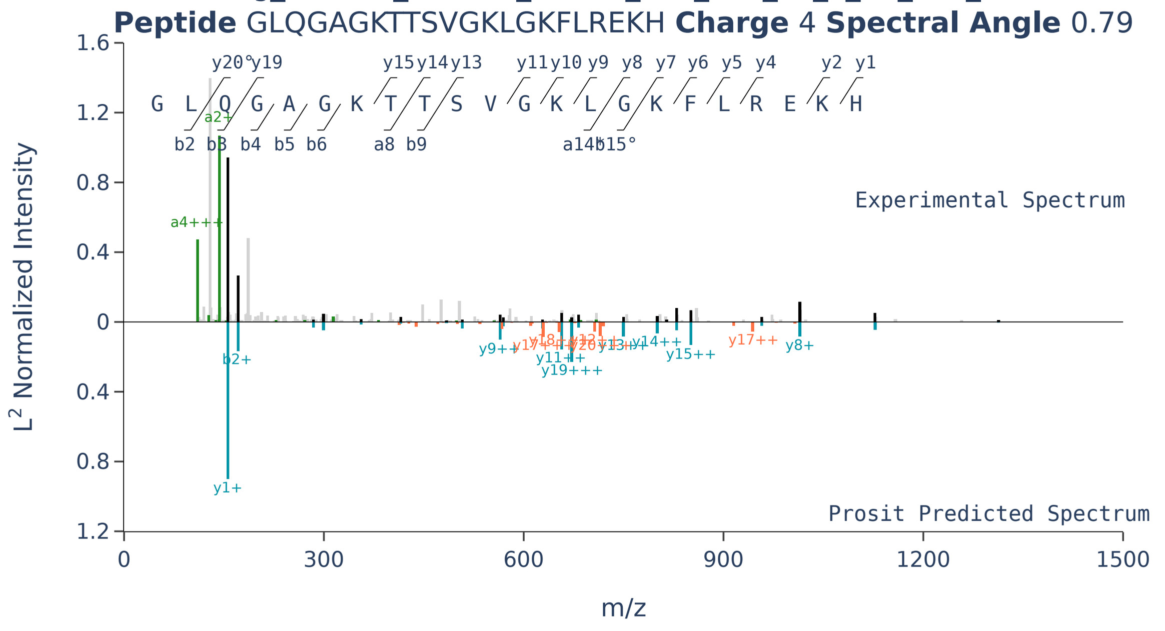


**Source** Ncheng_210623_230623_HFGoe_FFH_20S_25_1_A2_4h_R1 **Scan** 17349
**Peptide** EDNVKDTLREVRMA **Charge** 3 **Spectral Angle** 0.69

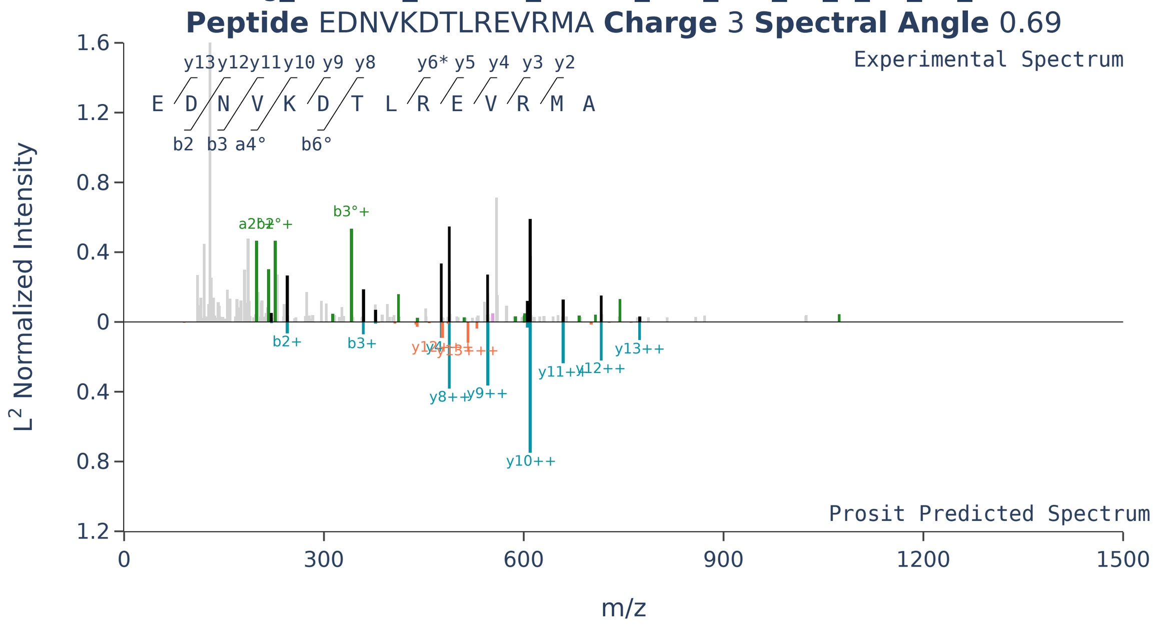


**Source** Ncheng_210623_230623_HFGoe_FFH_20S_25_1_A1_24h_R1 **Scan** 5499
**Peptide** KMMRSMK **Charge** 2 **Spectral Angle** 0.78

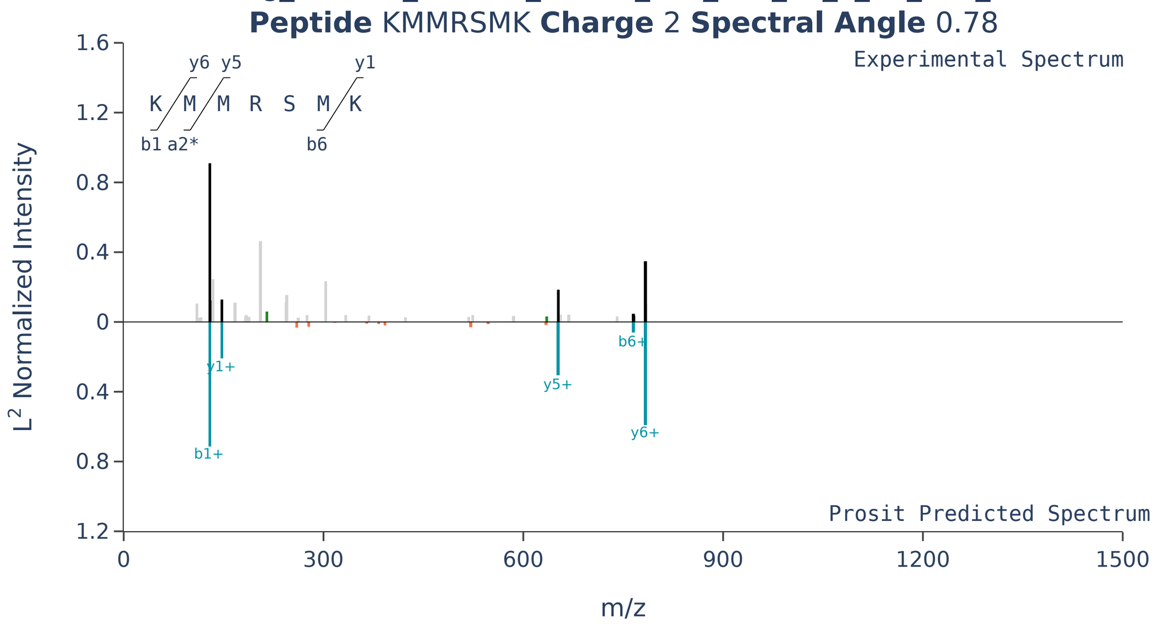

**Source** Ncheng_210623_230623_HFGoe_FFH_20S_25_1_A1_1h_R2 **Scan** 29364
**Peptide** GVDFFPSDVGQKPVDIVN **Charge** 2 **Spectral Angle** 0.75

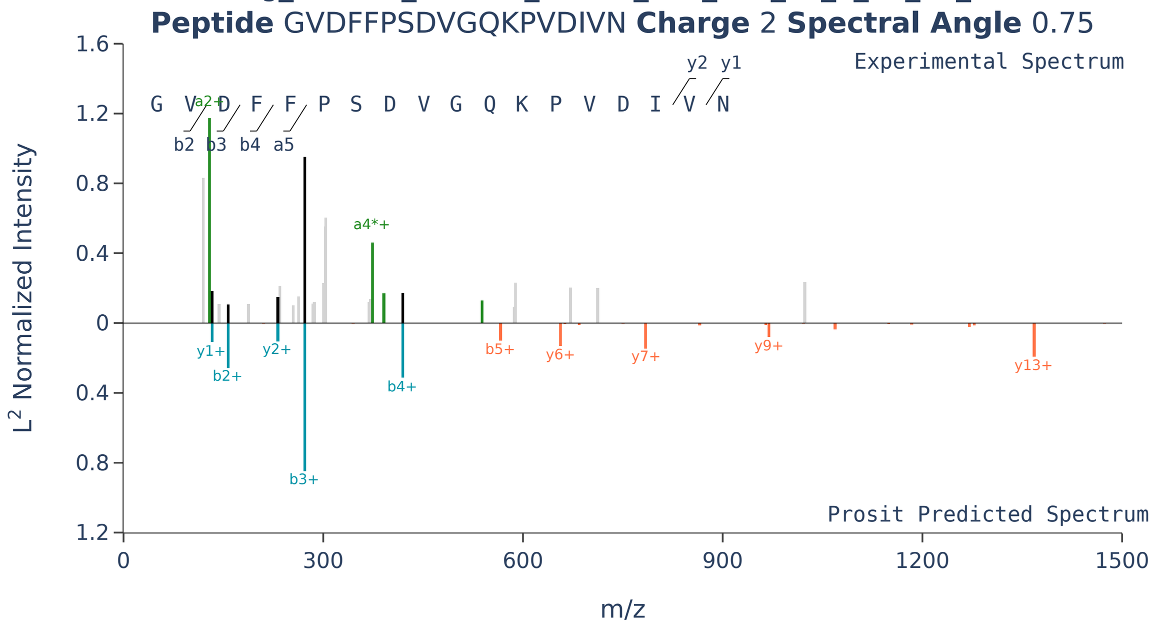


**Source** Ncheng_210623_230623_HFGoe_FFH_20S_25_1_A1_24h_R1 **Scan** 11933
**Peptide** DNLTDRLSR **Charge** 2 **Spectral Angle** 0.33

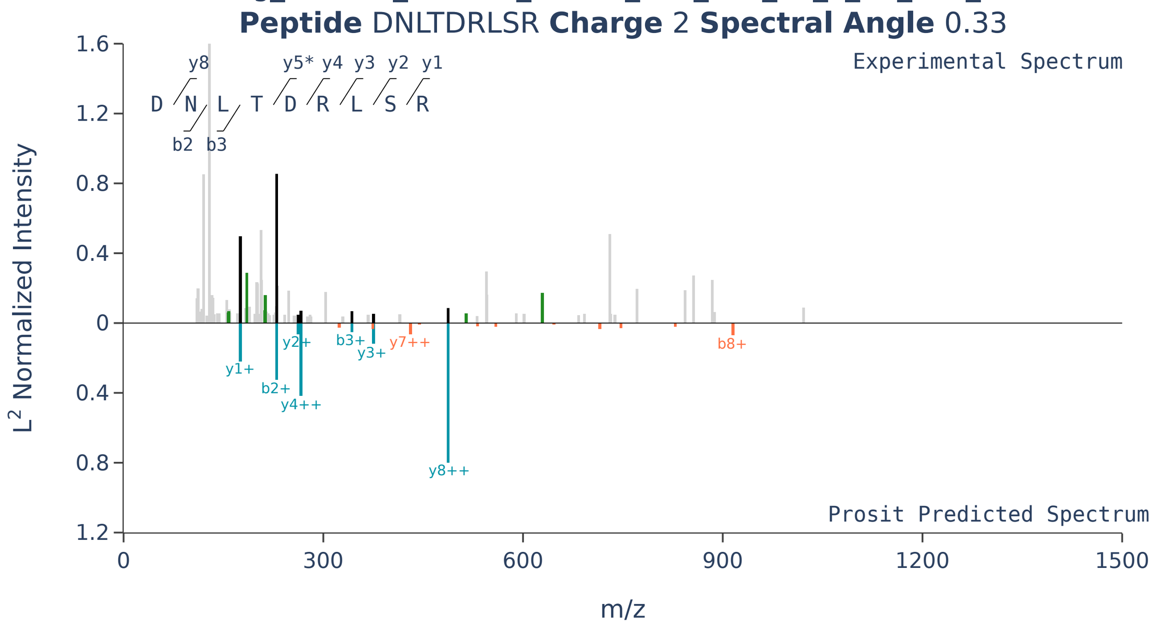


**Source** Ncheng_210623_230623_HFGoe_FFH_20S_25_1_A1_2h_R2 **Scan** 14820
**Peptide** PFHPDRIASRL **Charge** 3 **Spectral Angle** 0.4

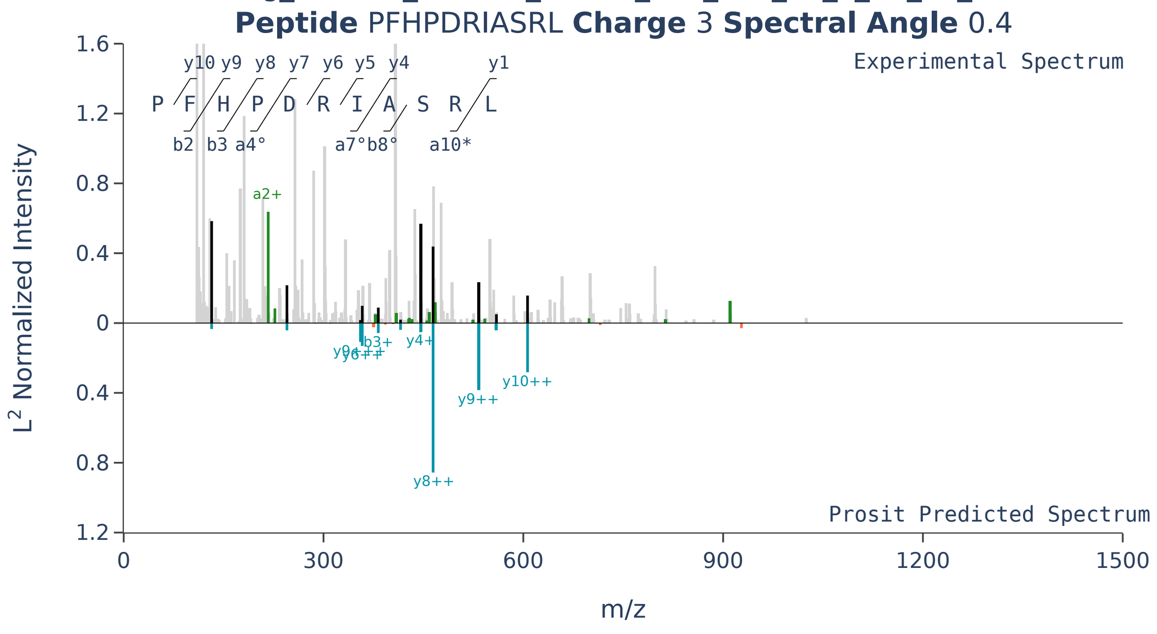


**Source** Ncheng_210623_230623_HFGoe_FFH_20S_25_1_A2_2h_R2 **Scan** 28555
**Peptide** RLAAGCGMQVQDVNRLLKQFD **Charge** 2 **Spectral Angle** 0.4

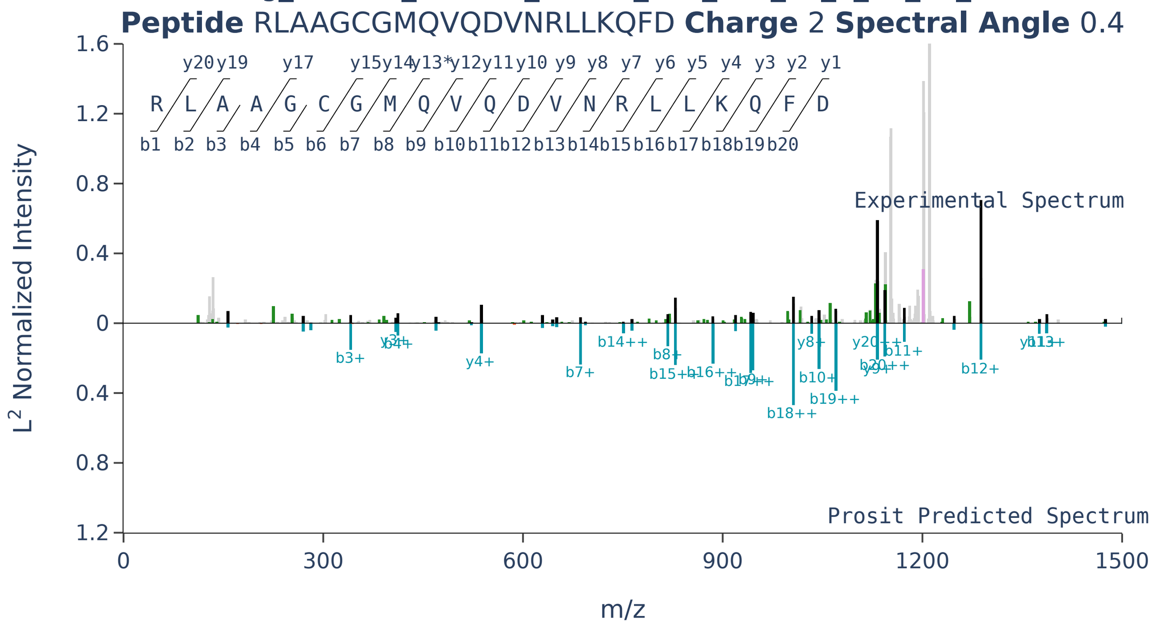


**Source** Ncheng_210623_230623_HFGoe_FFH_20S_25_1_A1_1h_R2 **Scan** 32032
**Peptide** FYDVLLVDTAGRLHVD **Charge** 3 **Spectral Angle** 0.72

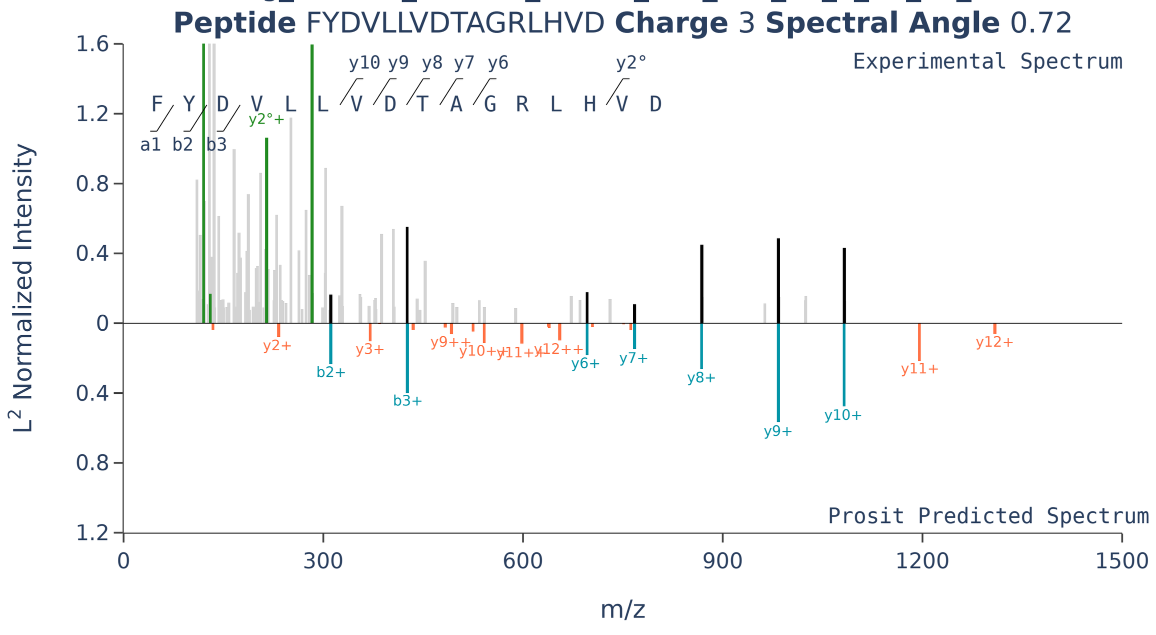


**Source** Ncheng_210623_230623_HFGoe_FFH_20S_25_1_A1_2h_R1 **Scan** 38478
**Peptide** GEENQTLNLAAQPPAVVLMAGLQGAGK **Charge** 3 **Spectral Angle** 0.63

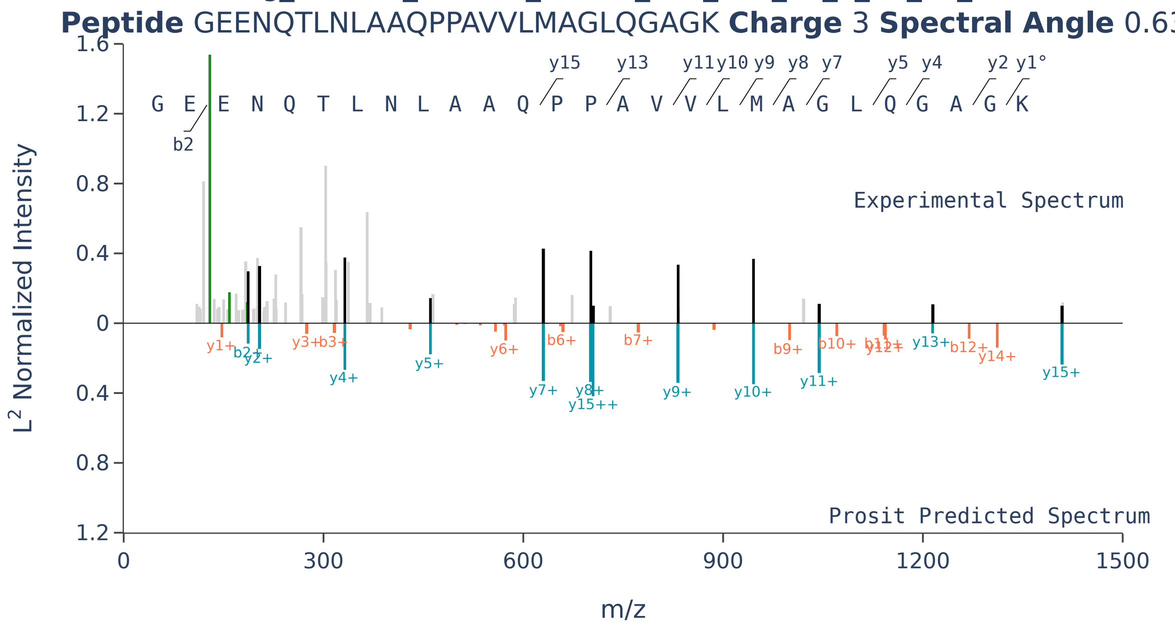


**Source** Ncheng_210623_230623_HFGoe_FFH_20S_25_1_A2_24h_R1 **Scan** 5782
**Peptide** LTKVDGDARG **Charge** 2 **Spectral Angle** 0.68

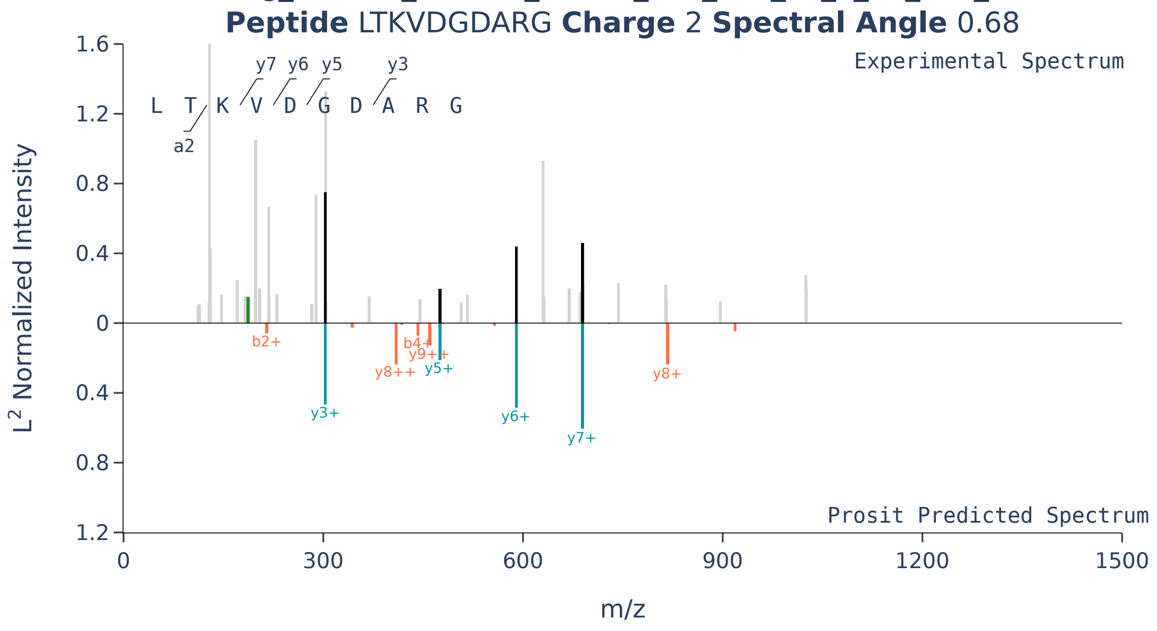


**Source** Ncheng_210623_230623_HFGoe_FFH_20S_25_1_A1_2h_R1 **Scan** 28381
**Peptide** LMGKLPGMGQIPDNVKSQMDDKVLV **Charge** 3 **Spectral Angle** 0.77

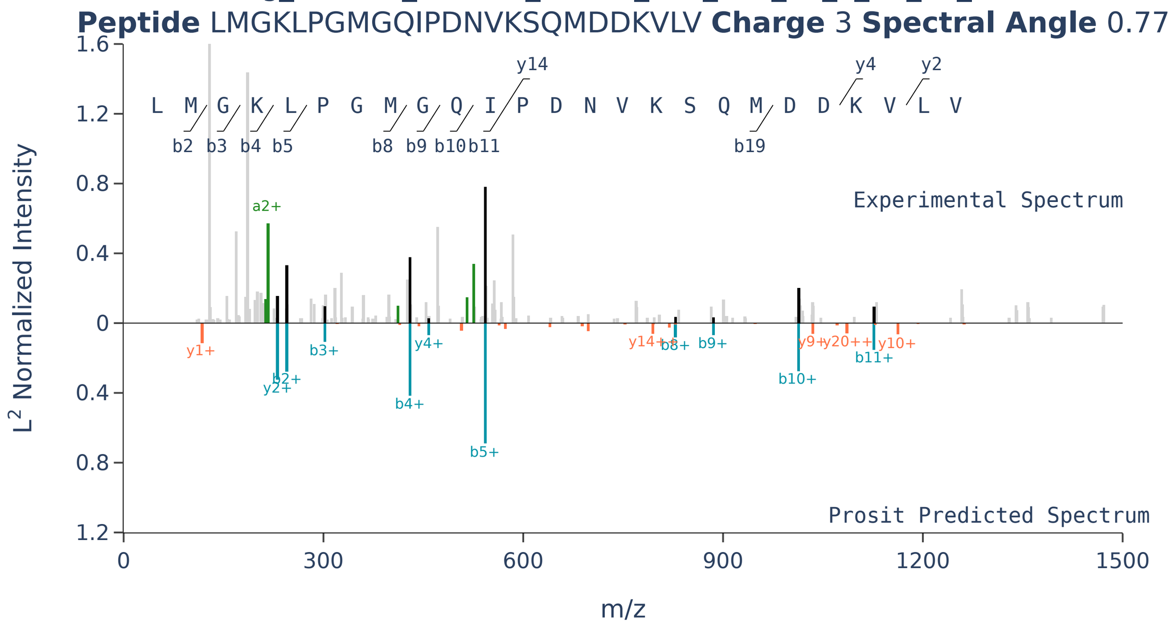


**Source** Ncheng_210623_230623_HFGoe_FFH_20S_25_1_A2_4h_R1 **Scan** 25107
**Peptide** VLLVDTAGRL **Charge** 2 **Spectral Angle** 0.59

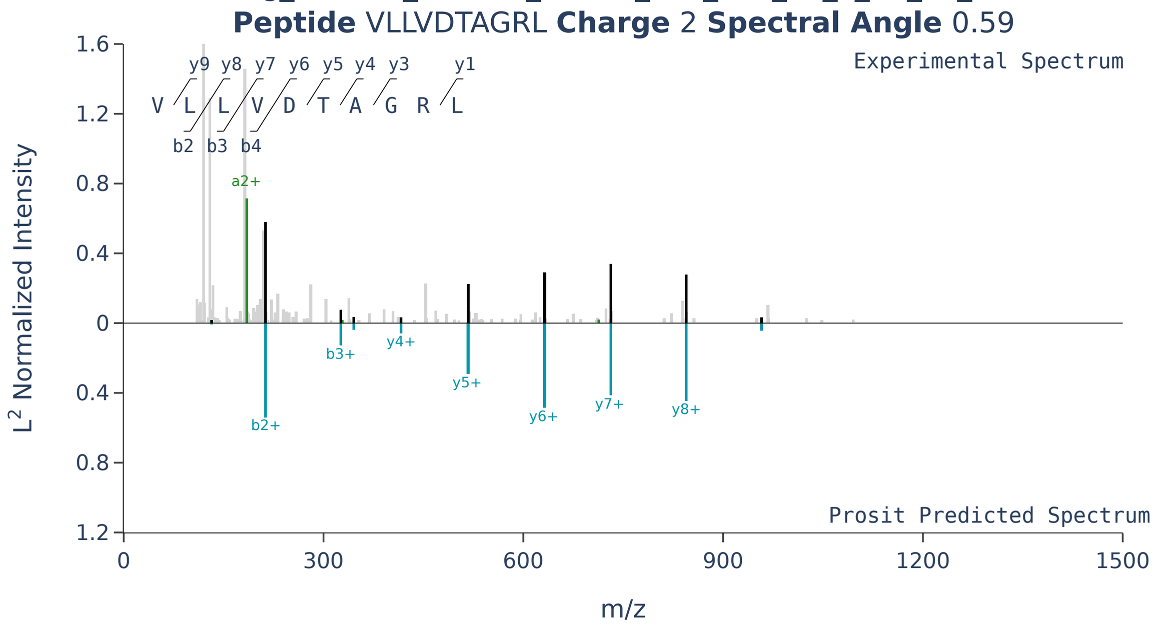


**Source** Ncheng_210623_230623_HFGoe_FFH_20S_25_1_A2_24h_R2 **Scan** 9432
**Peptide** LESKVDRAQAEKLA **Charge** 2 **Spectral Angle** 0.53

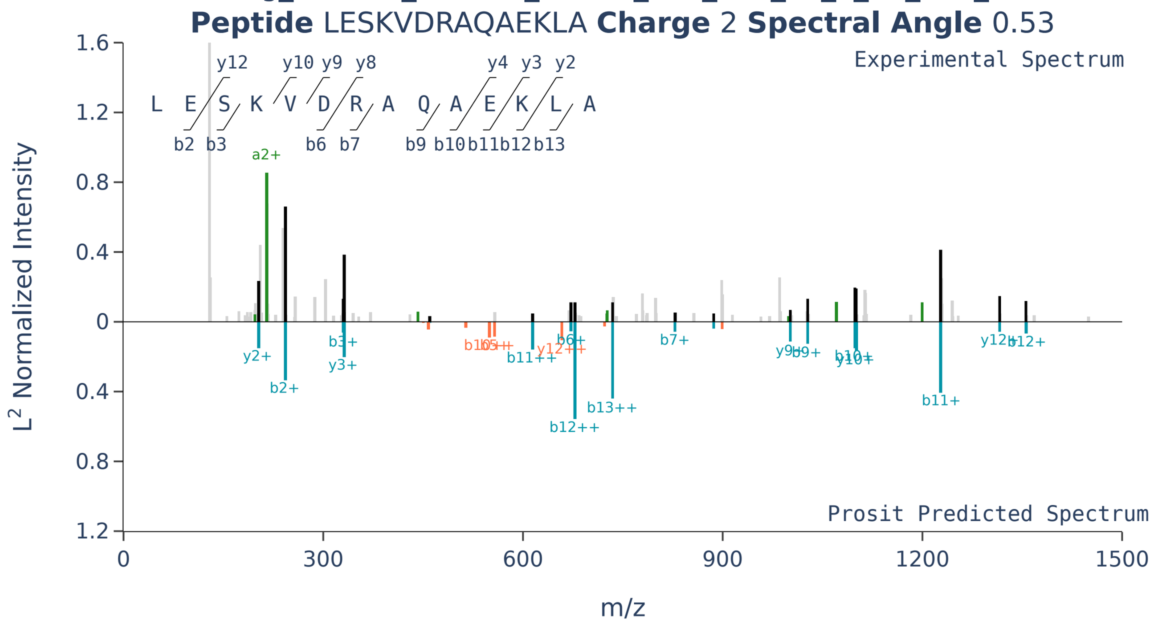


**Source** Ncheng_210623_230623_HFGoe_FFH_20S_25_1_A2_4h_R1 **Scan** 23365
**Peptide** KVDGDARGGAALSIRHITGKPIKFLGV **Charge** 6 **Spectral Angle** 0.81

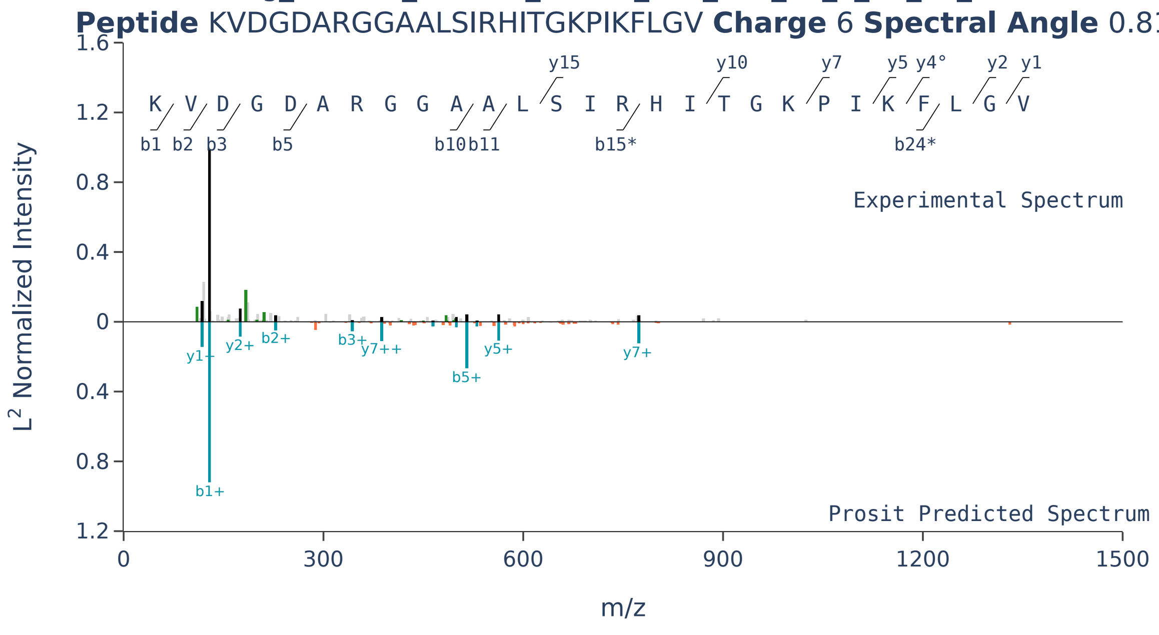


**Source** Ncheng_210623_230623_HFGoe_FFH_20S_25_1_A2_4h_R2 **Scan** 8380
**Peptide** KGGMAKMMRSMKG **Charge** 3 **Spectral Angle** 0.8

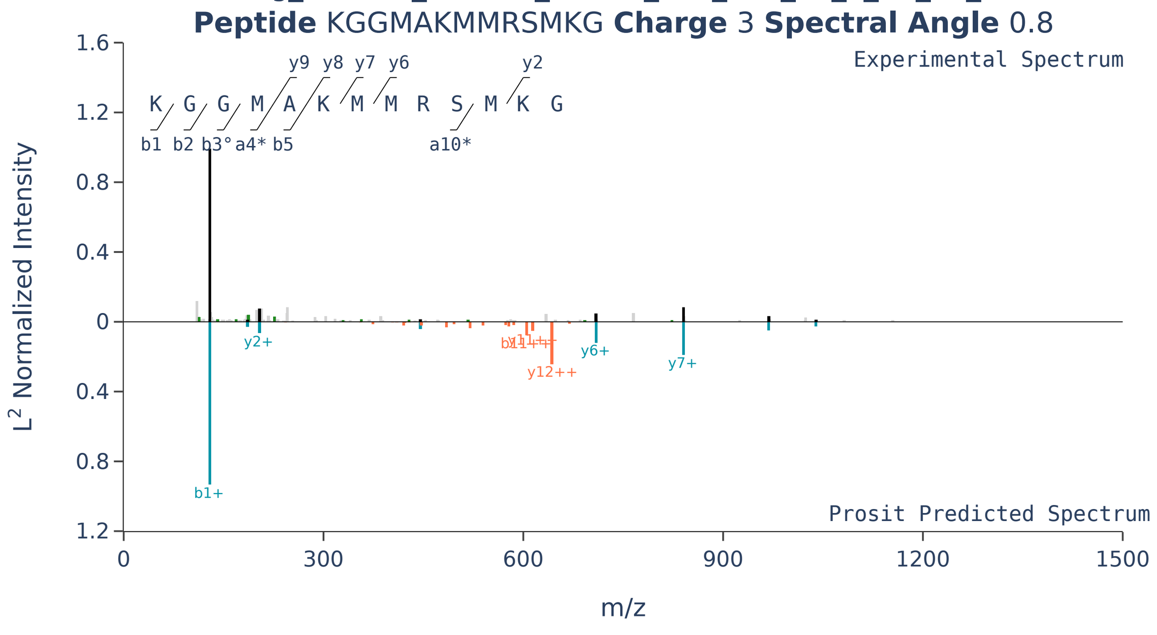


**Source** Ncheng_210623_230623_HFGoe_FFH_20S_25_1_A2_24h_R2 **Scan** 22422
**Peptide** NKSLTPGQEFVKLVR **Charge** 3 **Spectral Angle** 0.53

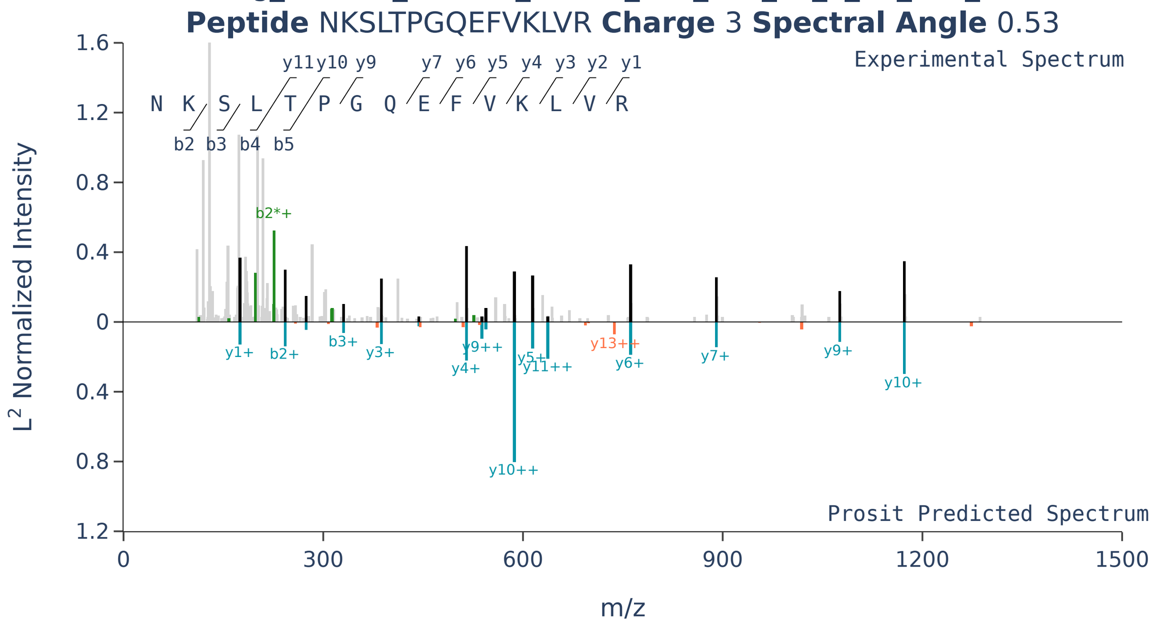


**Source** Ncheng_210623_230623_HFGoe_FFH_20S_25_1_A2_4h_R1 **Scan** 36806
**Peptide** MTGQDAANTAKAFNEALPLTGVVLTKV **Charge** 3 **Spectral Angle** 0.77

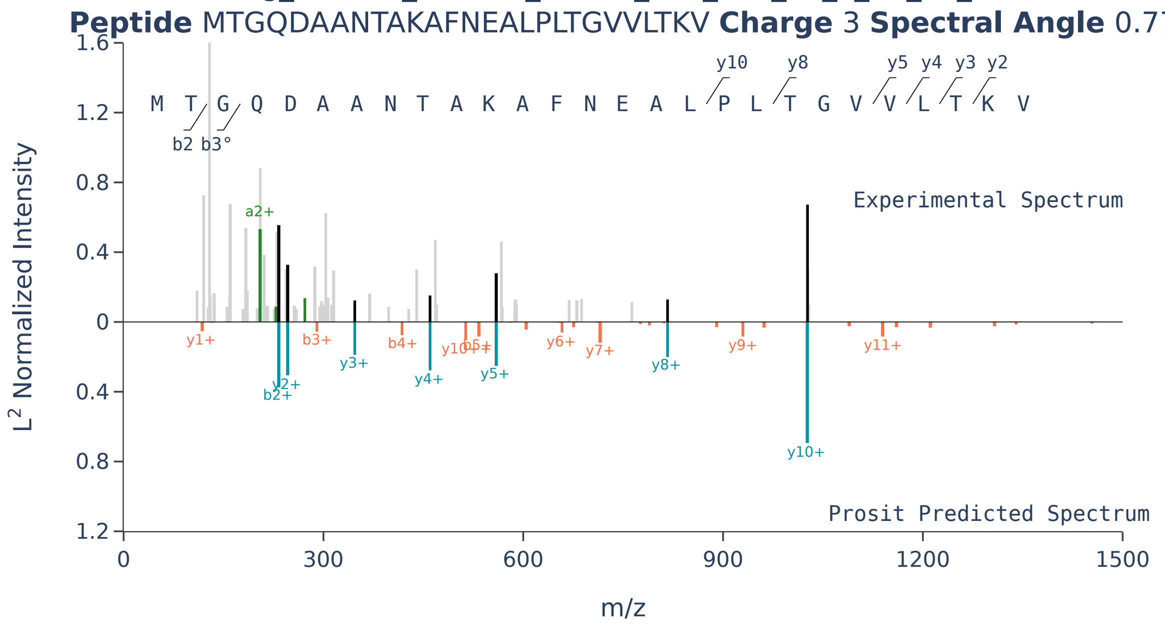


**Source** Ncheng_210623_230623_HFGoe_FFH_20S_25_1_A2_2h_R1 **Scan** 29107
**Peptide** SLMGKLPGMGQIPDNVKSQMDDKVLVRM **Charge** 4 **Spectral Angle** 0.58

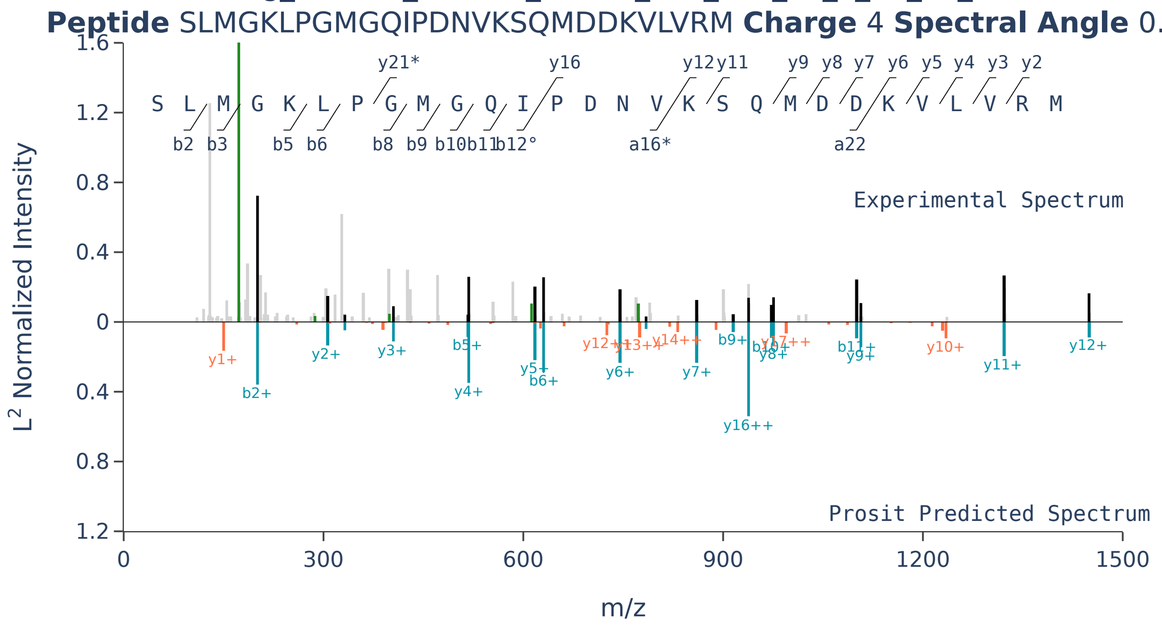

**Source** Ncheng_210623_230623_HFGoe_FFH_20S_25_1_A2_2h_R1 **Scan** 34510
**Peptide** KAVGHEVNKSLTPGQEFVKIVRNELVAAMG **Charge** 4 **Spectral Angle** 0.73

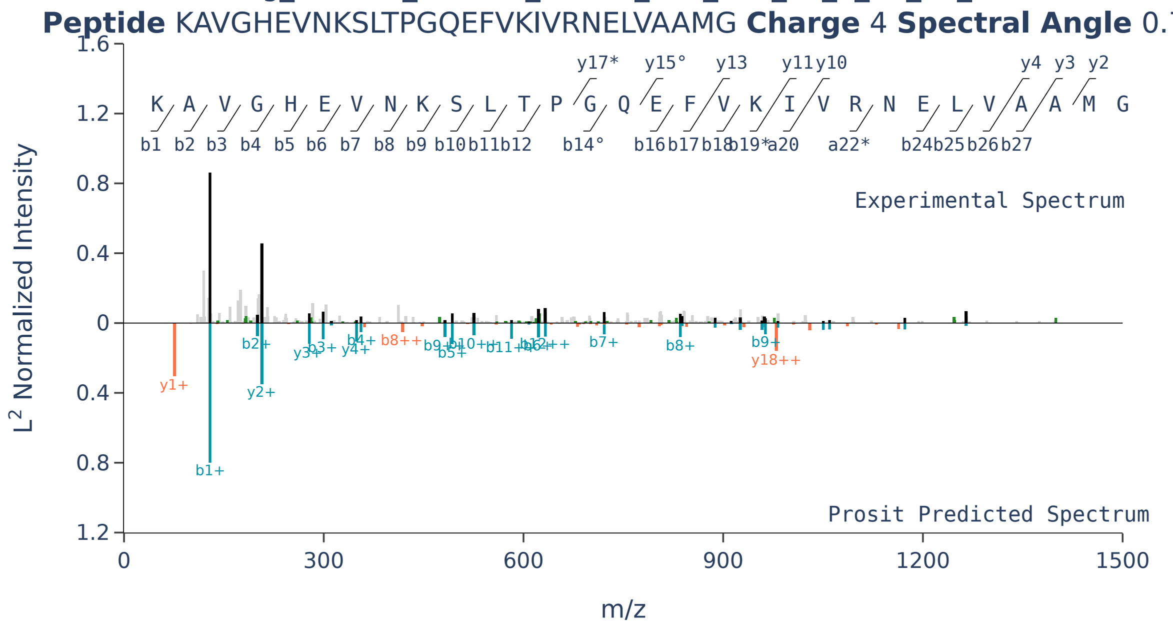


**Source** Ncheng_210623_230623_HFGoe_FFH_20S_25_1_A1_2h_R2 **Scan** 27203
**Peptide** TAGRLHVDEAMMDEIKQVHASINPVE **Charge** 4 **Spectral Angle** 0.65

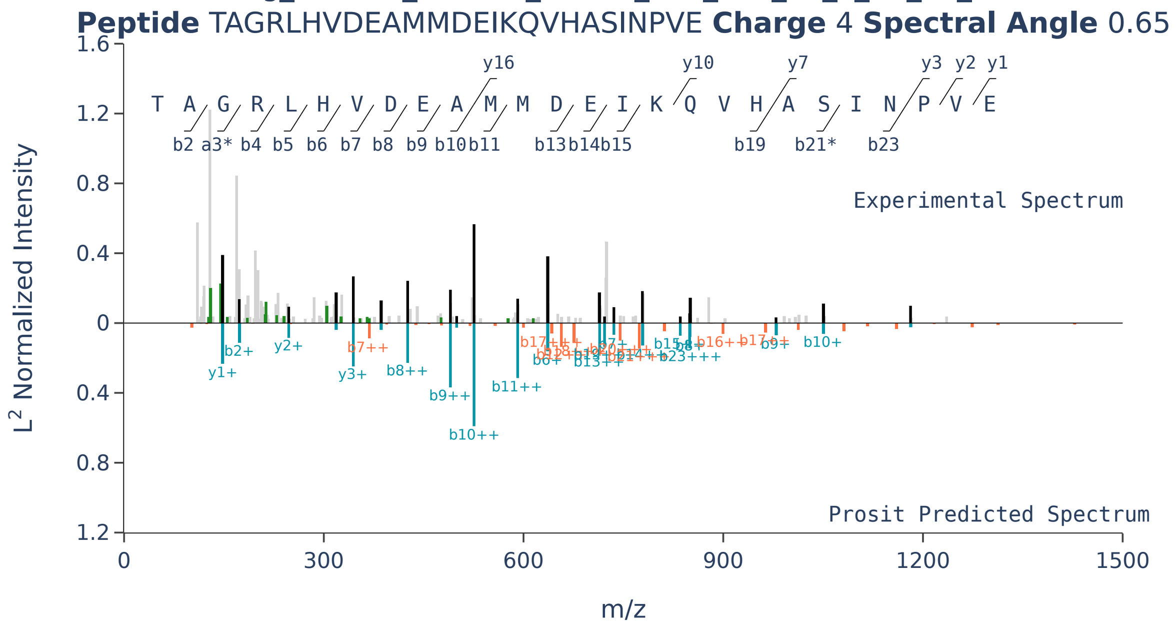


**Source** Ncheng_210623_230623_HFGoe_FFH_20S_25_1_A2_24h_R1 **Scan** 21837
**Peptide** TTSVGKLGKFL **Charge** 3 **Spectral Angle** 0.7

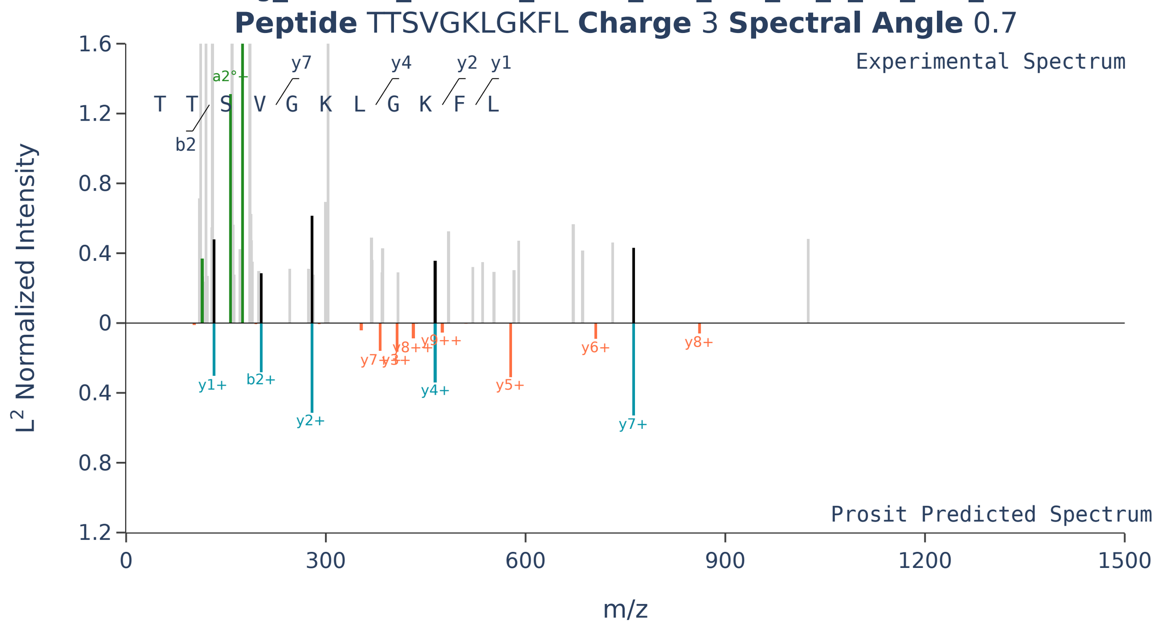


**Source** Ncheng_210623_230623_HFGoe_FFH_20S_25_1_A1_2h_R2 **Scan** 34073
**Peptide** DVALPVVREFINRVKE **Charge** 3 **Spectral Angle** 0.42

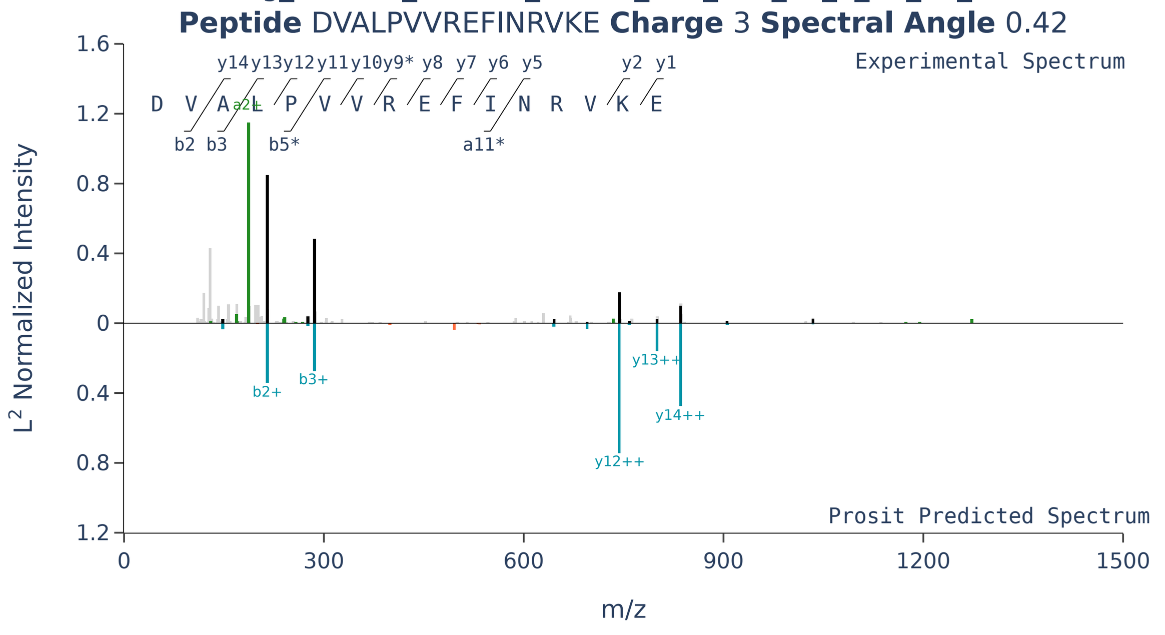


**Source** Ncheng_210623_230623_HFGoe_FFH_20S_25_1_A1_24h_R1 **Scan** 11529
**Peptide** KLVRNELVA **Charge** 2 **Spectral Angle** 0.5

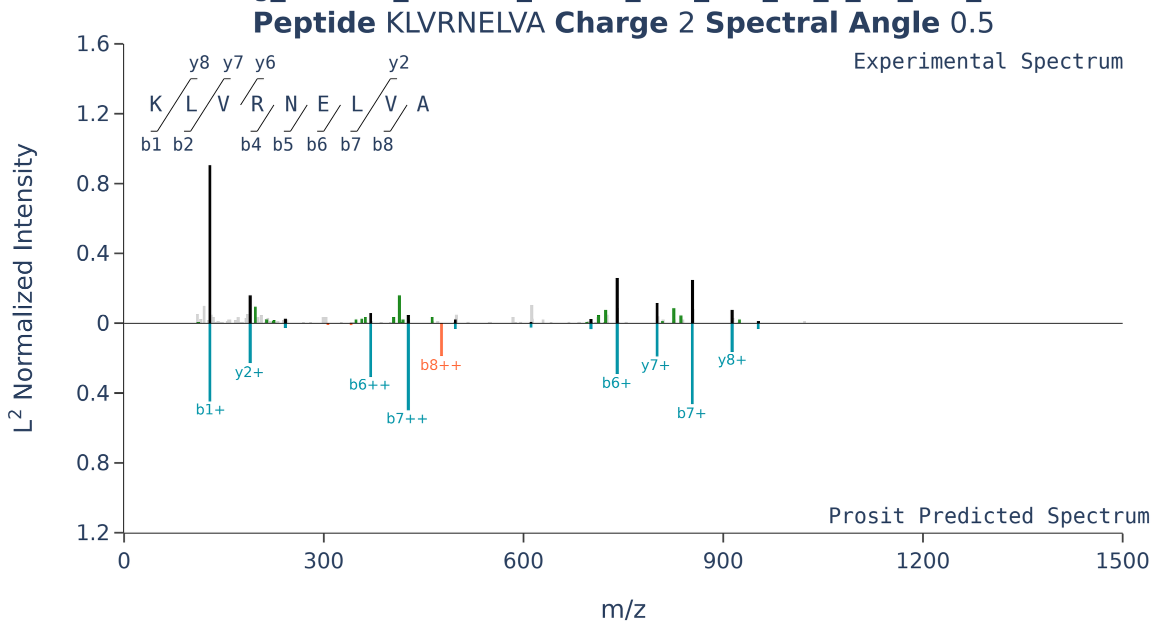


**Source** Ncheng_210623_230623_HFGoe_FFH_20S_25_1_A1_2h_R2 **Scan** 38580
**Peptide** ALLEADVALPVVREFINRVKEK **Charge** 3 **Spectral Angle** 0.55

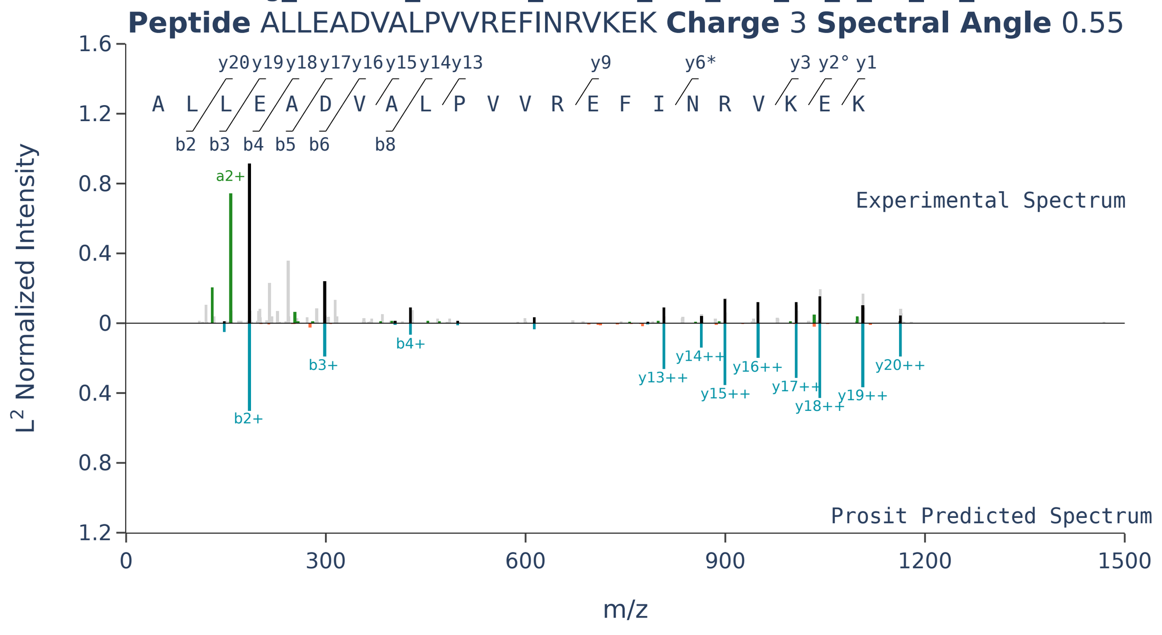


**Source** Ncheng_210623_230623_HFGoe_FFH_20S_25_1_A1_24h_R2 **Scan** 5915
**Peptide** TMKERAKPEIIKGSRK **Charge** 5 **Spectral Angle** 0.82

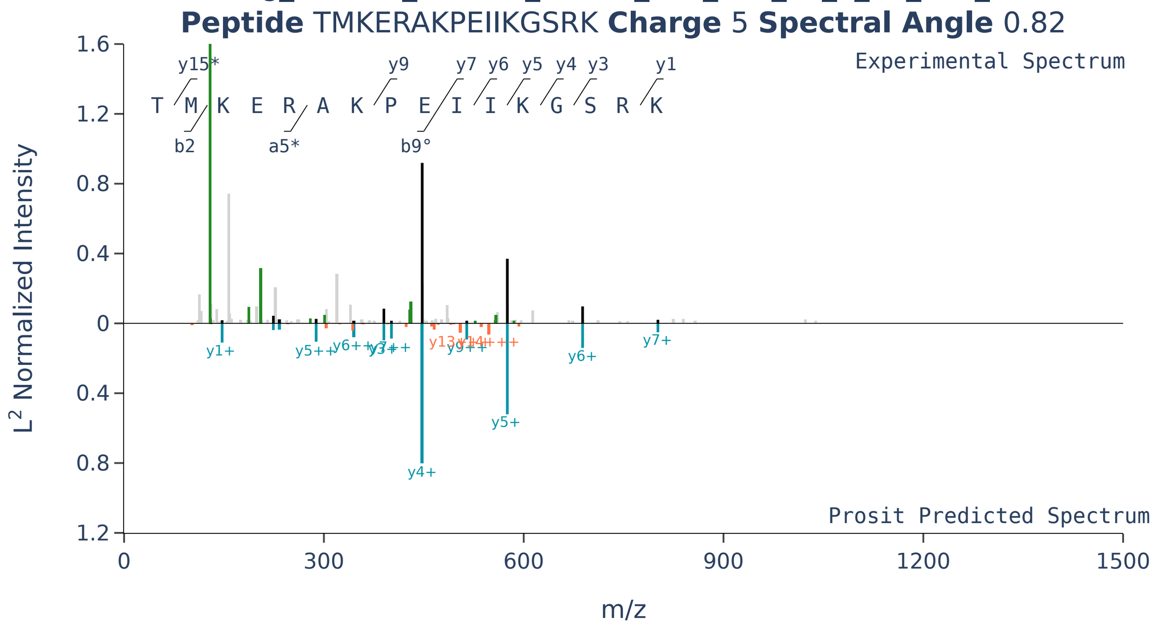


**Source** Ncheng_210623_230623_HFGoe_FFH_20S_25_1_A1_4h_R1 **Scan** 19573
**Peptide** QEFVKIVRN **Charge** 2 **Spectral Angle** 0.85

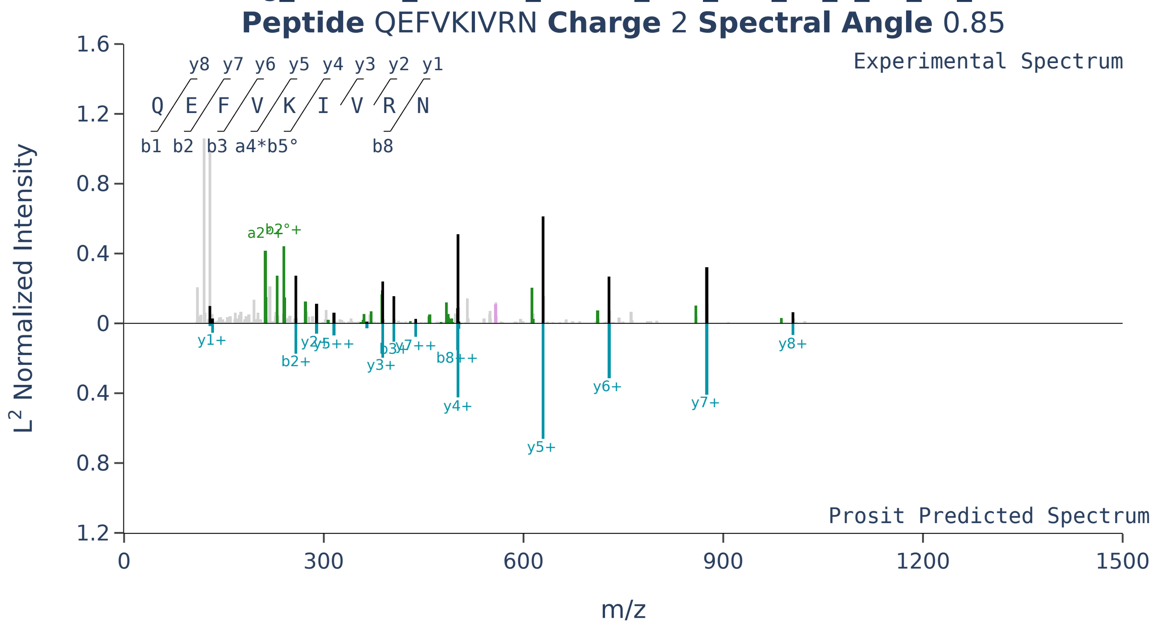


**Source** Ncheng_210623_230623_HFGoe_FFH_20S_25_1_A2_24h_R2 **Scan** 9796
**Peptide** KERAKPELL **Charge** 2 **Spectral Angle** 0.25

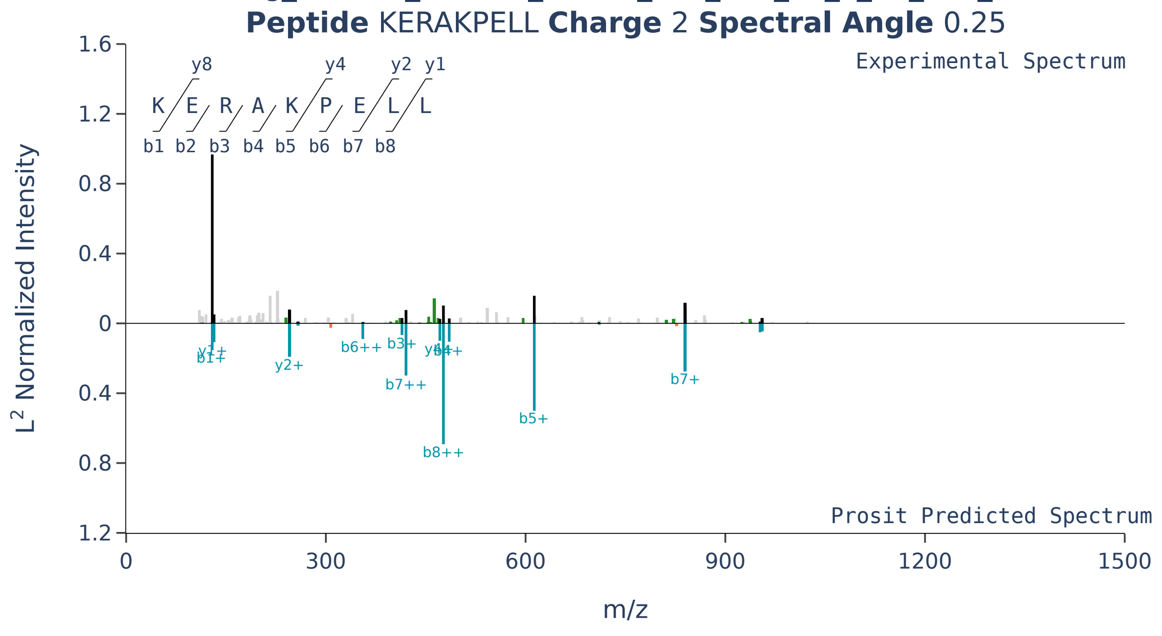


**Source** Ncheng_210623_230623_HFGoe_FFH_20S_25_1_A2_4h_R1 **Scan** 24360
**Peptide** LIEDIESKVDRAQAEKLASKL **Charge** 3 **Spectral Angle** 0.68

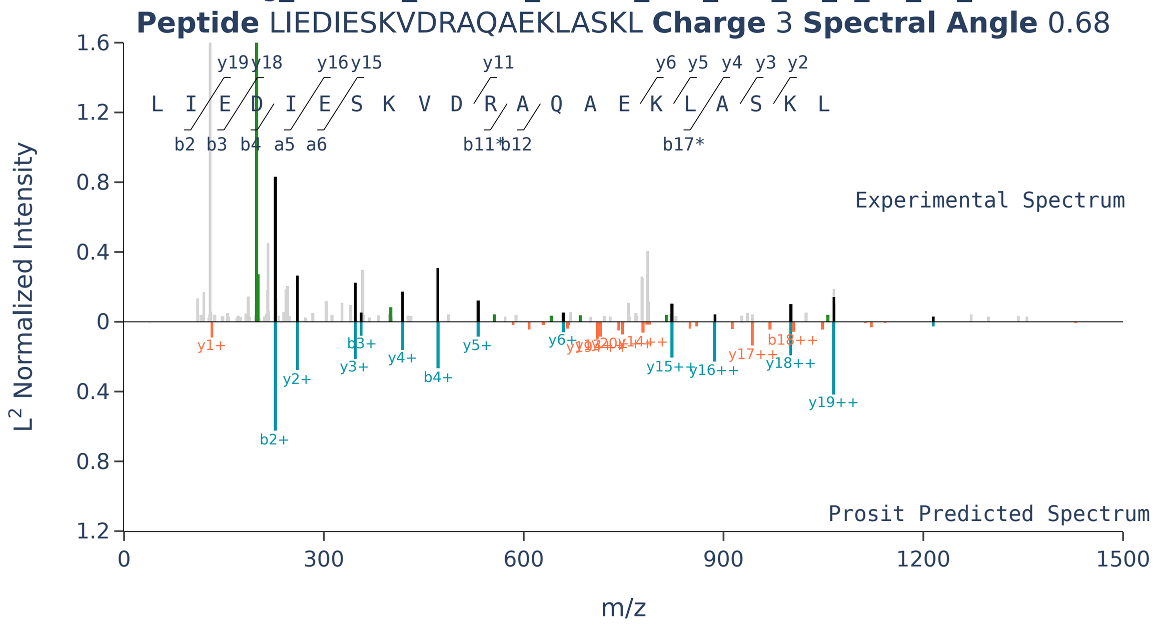


**Source** Ncheng_210623_230623_HFGoe_FFH_20S_25_1_A2_4h_R2 **Scan** 26273
**Peptide** MGKLPGMGQIPDNVKSQMDDKVL **Charge** 4 **Spectral Angle** 0.58

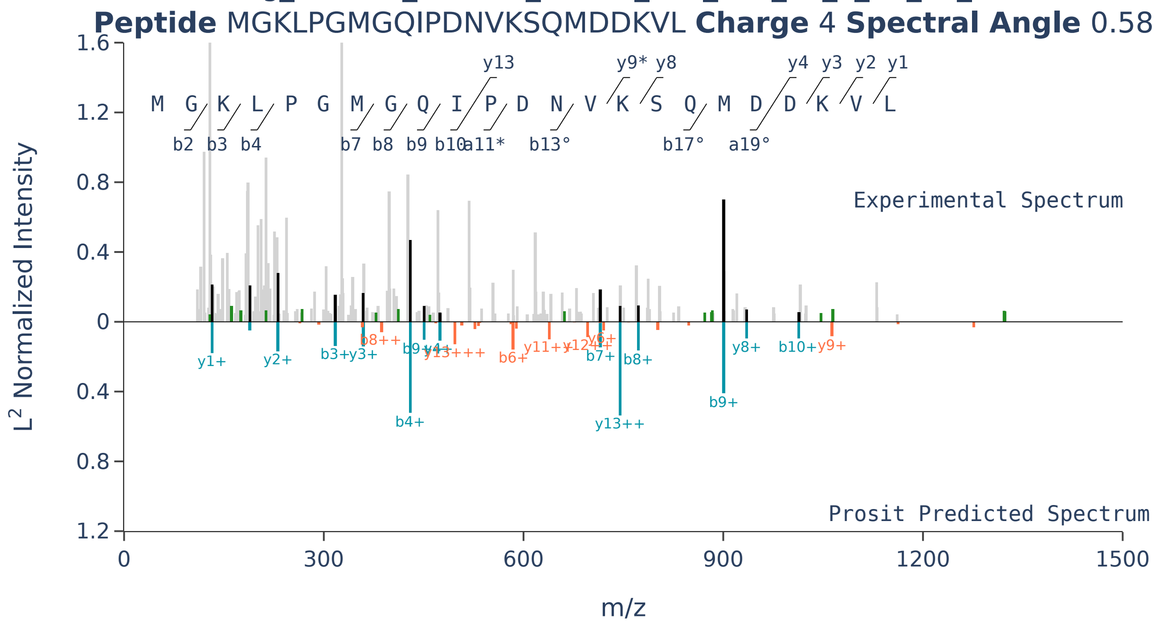


**Source** Ncheng_210623_230623_HFGoe_FFH_20S_25_1_A2_1h_R2 **Scan** 10286
**Peptide** KKGDGFDLN **Charge** 2 **Spectral Angle** 0.42

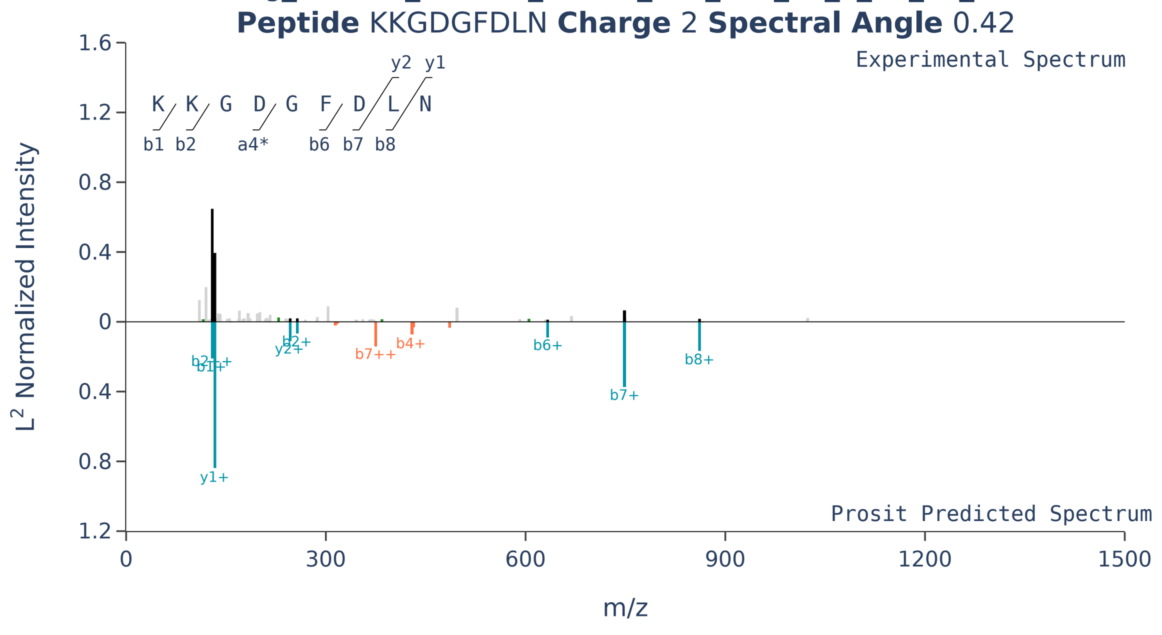


**Source** Ncheng_210623_230623_HFGoe_FFH_20S_25_1_A2_4h_R2 **Scan** 30214
**Peptide** QDAANTAKAFNEALPLTGV **Charge** 2 **Spectral Angle** 0.81

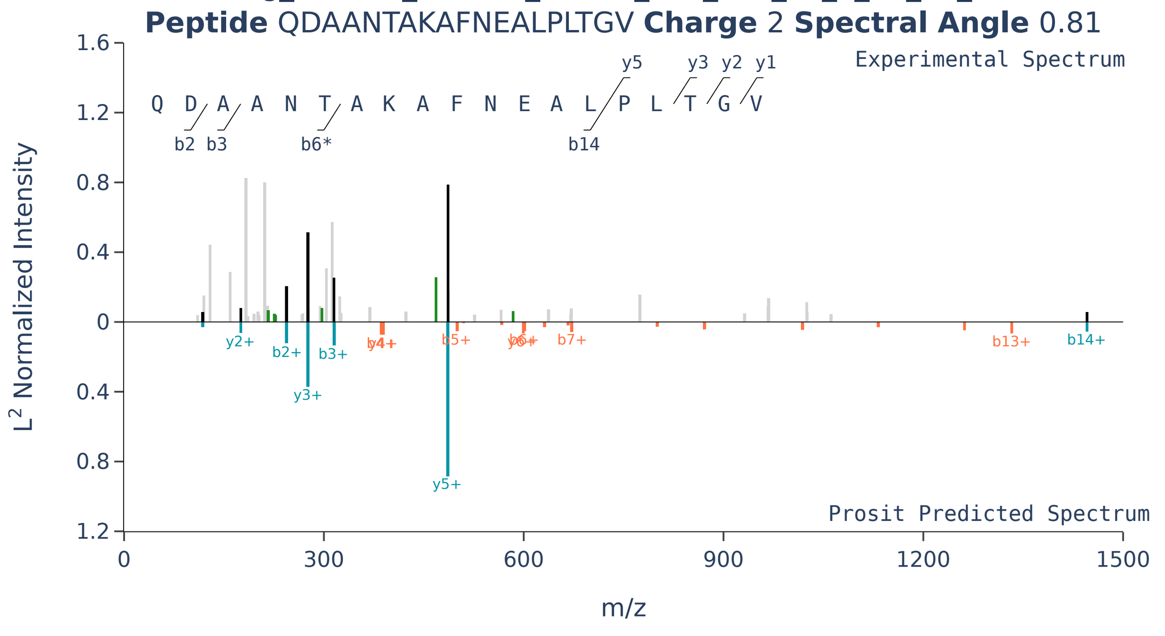


**Source** Ncheng_210623_230623_HFGoe_FFH_20S_25_1_A2_4h_R1 **Scan** 19232
**Peptide** VVLTKVDGDARGGAALSIRHLTG **Charge** 4 **Spectral Angle** 0.54

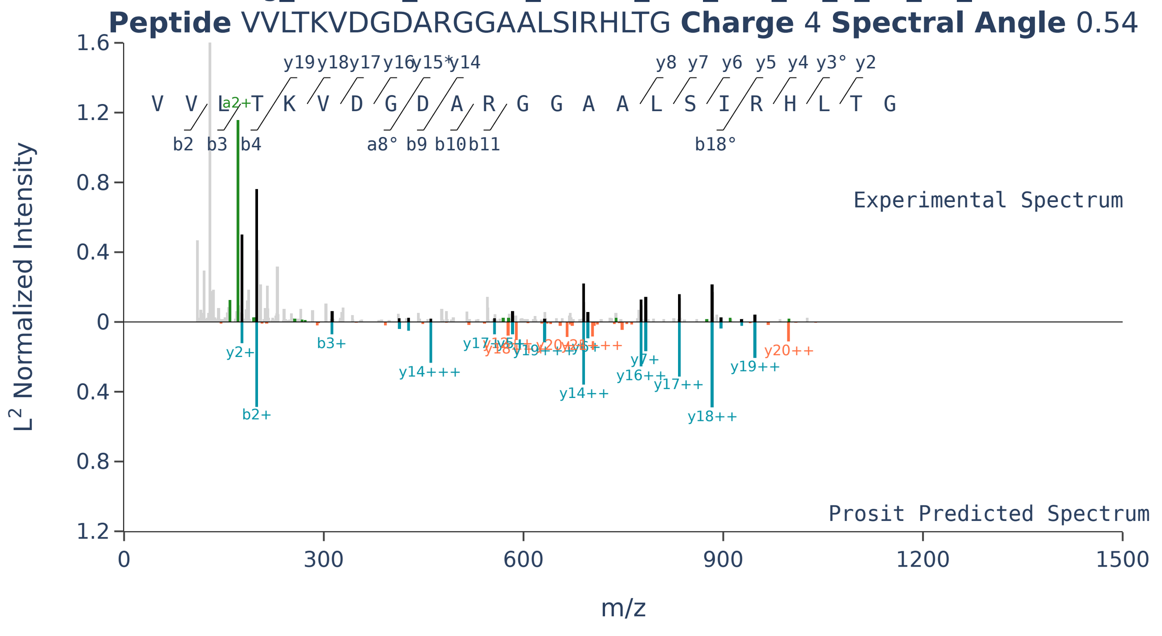


**Source** Ncheng_210623_230623_HFGoe_FFH_20S_25_1_A2_4h_R1 **Scan** 15401
**Peptide** RAQAEKLASKLKKGDGFDL **Charge** 4 **Spectral Angle** 0.54

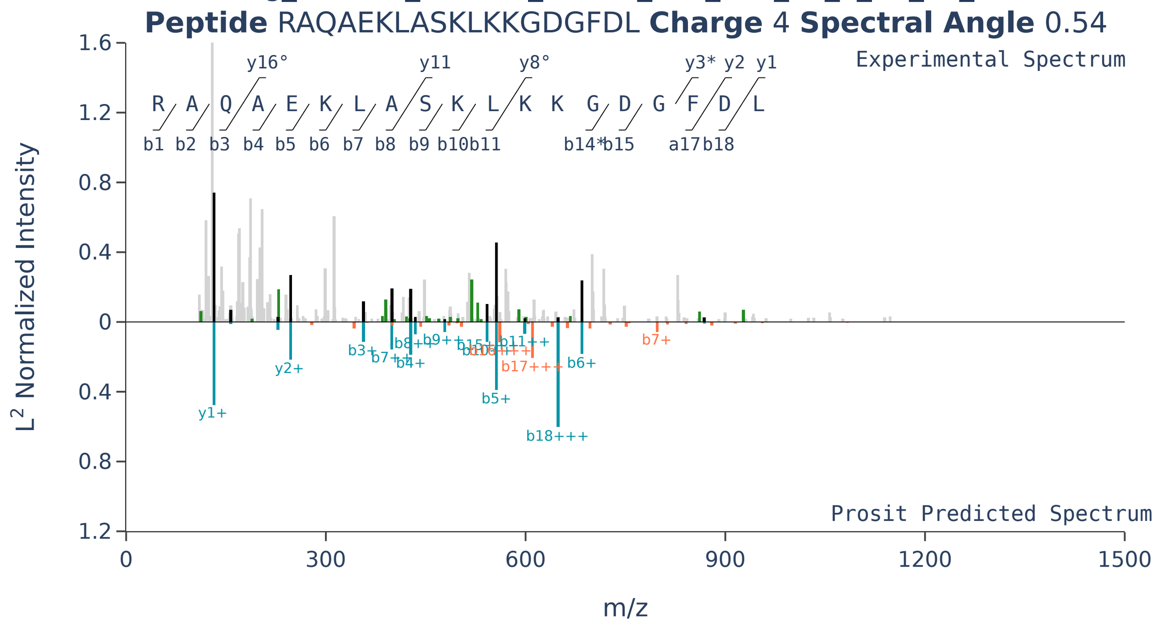

**Source** Ncheng_210623_230623_HFGoe_FFH_20S_25_1_A1_24h_R2 **Scan** 12165
**Peptide** QGAGKTTSVGKLGKFLREKH **Charge** 5 **Spectral Angle** 0.81

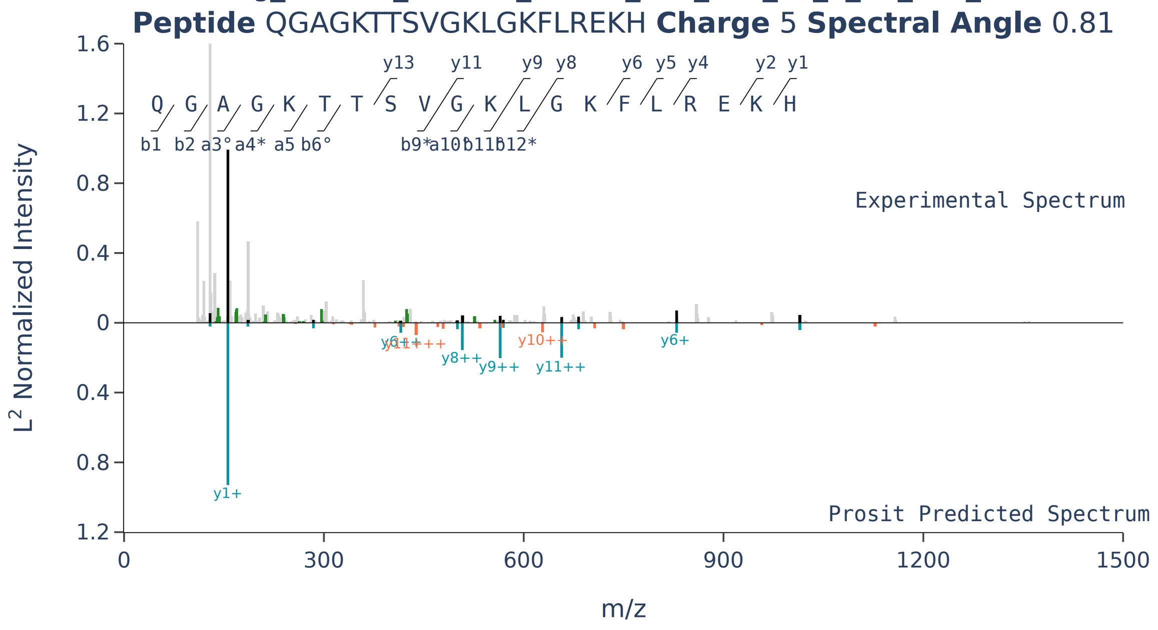


**Source** Ncheng_210623_230623_HFGoe_FFH_20S_25_1_A2_1h_R1 **Scan** 18693
**Peptide** NISGRGRLTEDNVKDTLREVRMA **Charge** 5 **Spectral Angle** 0.7

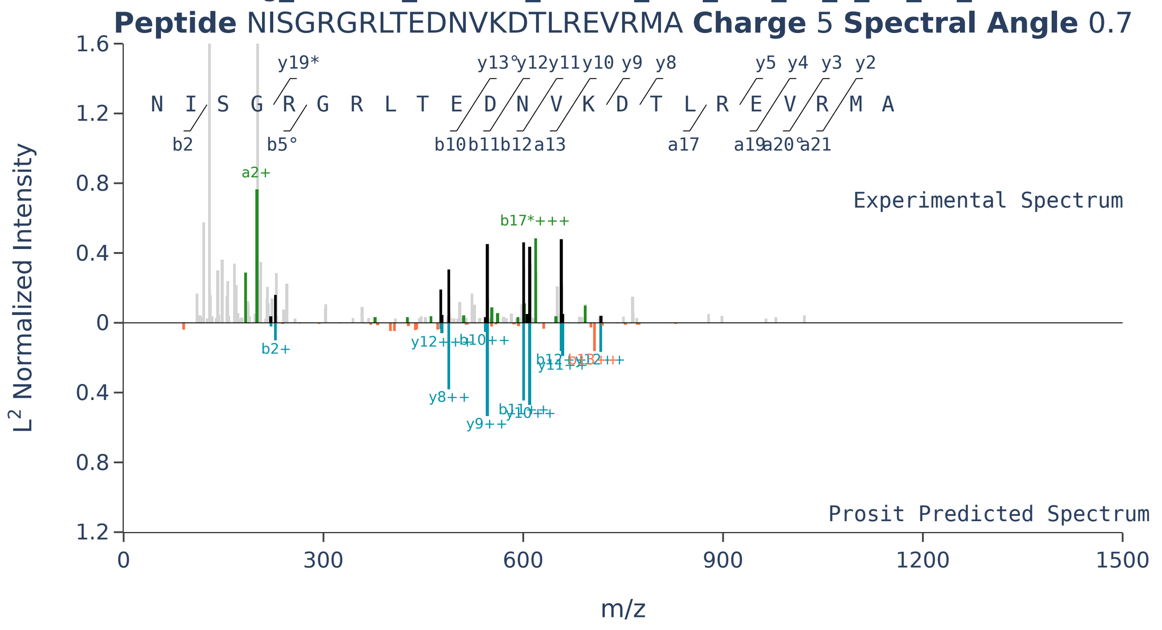


**Source** Ncheng_210623_230623_HFGoe_FFH_20S_25_1_A2_1h_R2 **Scan** 29777
**Peptide** GVDFFPSDVGQKPVDI **Charge** 2 **Spectral Angle** 0.67

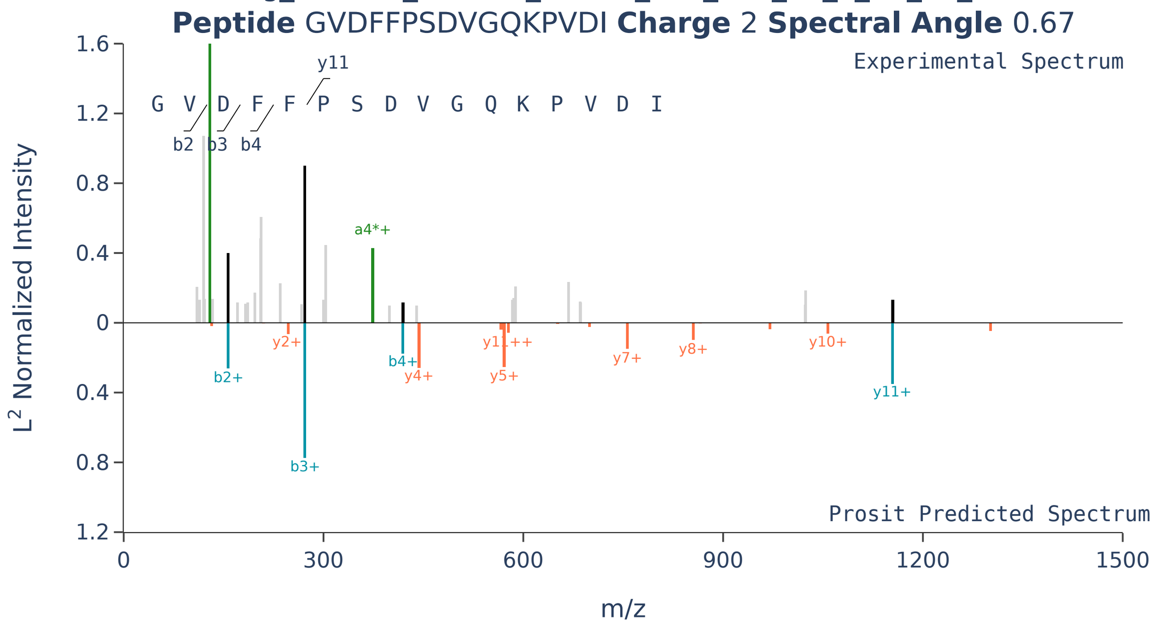


**Source** Ncheng_210623_230623_HFGoe_FFH_20S_25_1_A2_4h_R2 **Scan** 6397
**Peptide** VKIVRNE **Charge** 2 **Spectral Angle** 0.93

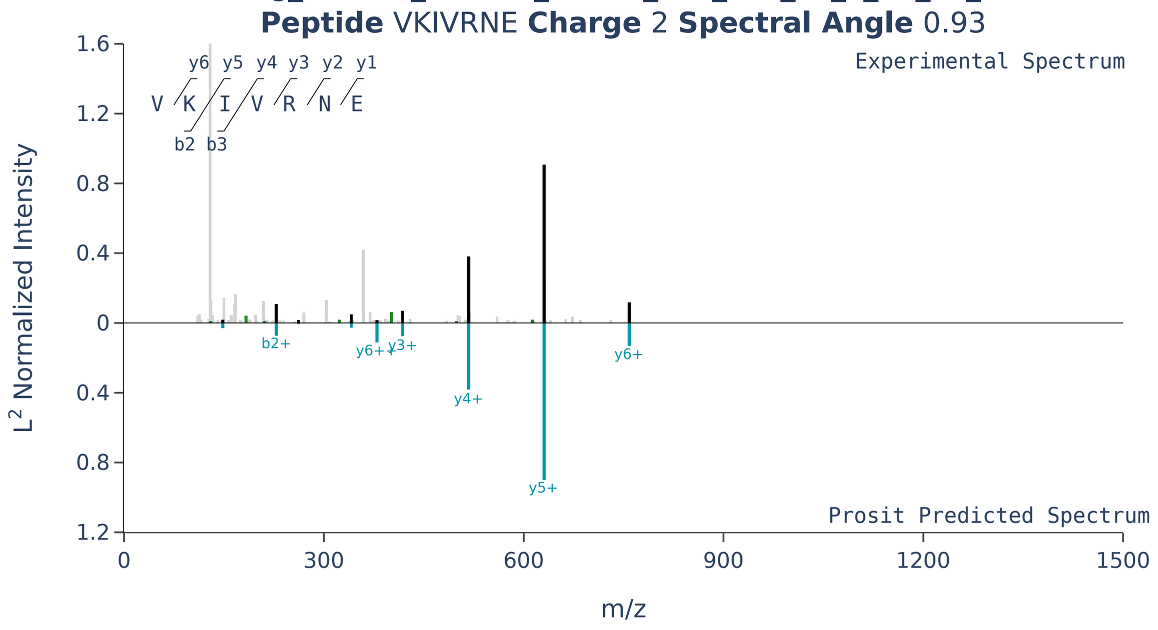


**Source** Ncheng_210623_230623_HFGoe_FFH_20S_25_1_A2_24h_R1 **Scan** 26384
**Peptide** PVDIVNAALKEA **Charge** 2 **Spectral Angle** 0.77

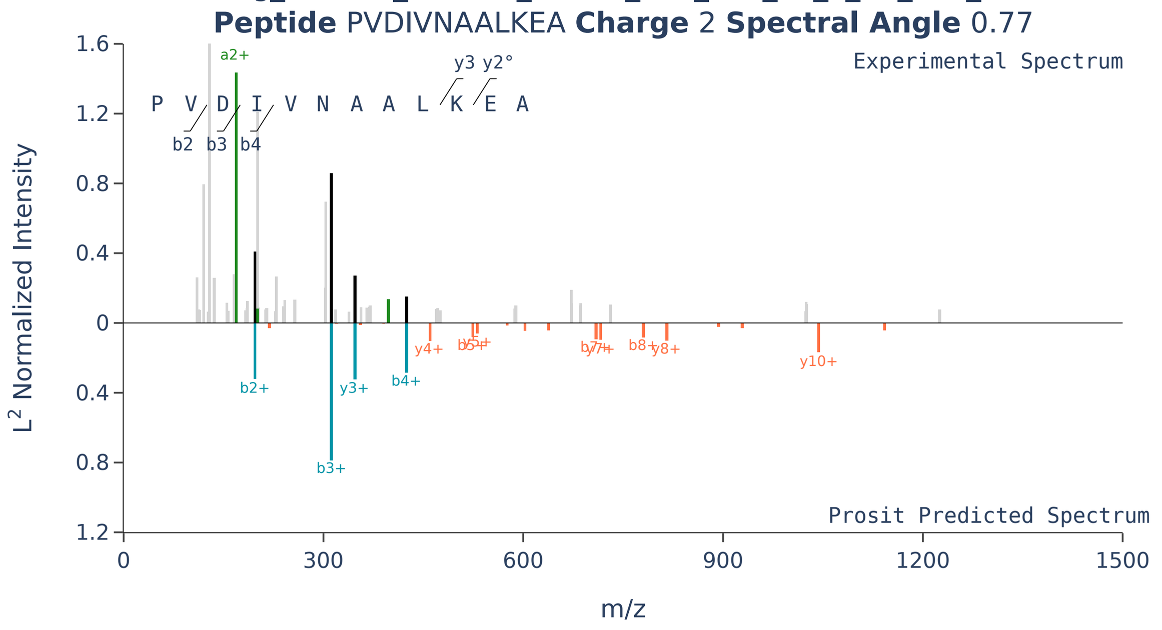


**Source** Ncheng_210623_230623_HFGoe_FFH_20S_25_1_A2_2h_R2 **Scan** 17271
**Peptide** VDAMTGQDAANTAKAF **Charge** 2 **Spectral Angle** 0.85

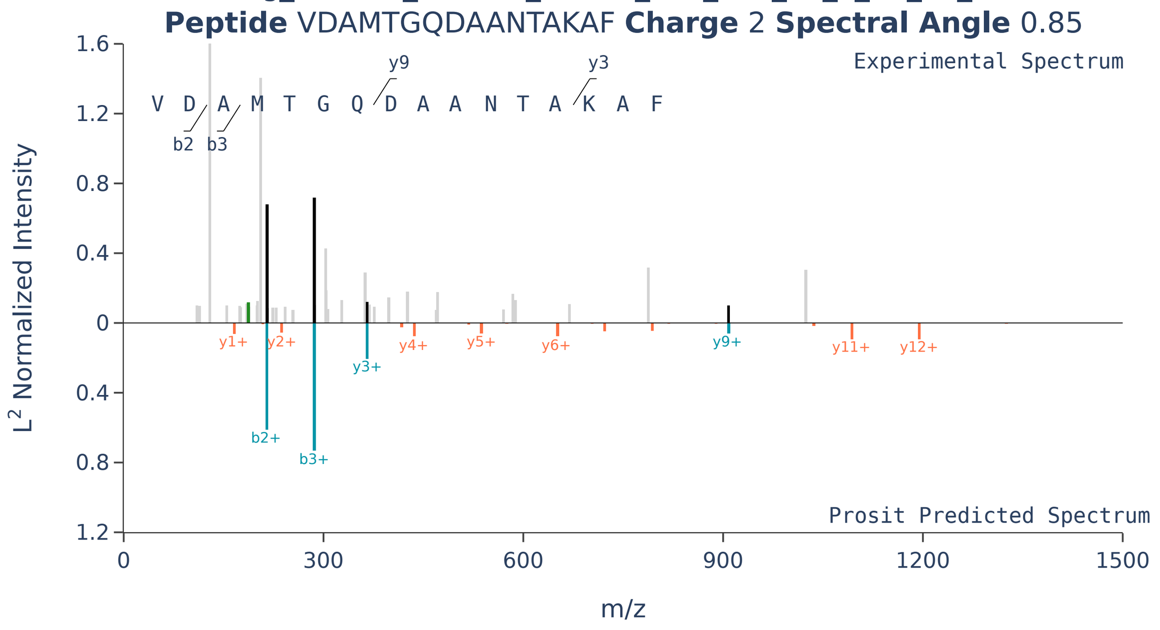


**Source** Ncheng_210623_230623_HFGoe_FFH_20S_25_1_A2_24h_R1 **Scan** 19706
**Peptide** VVLTKVDGDARGGAALSIRHI **Charge** 5 **Spectral Angle** 0.57

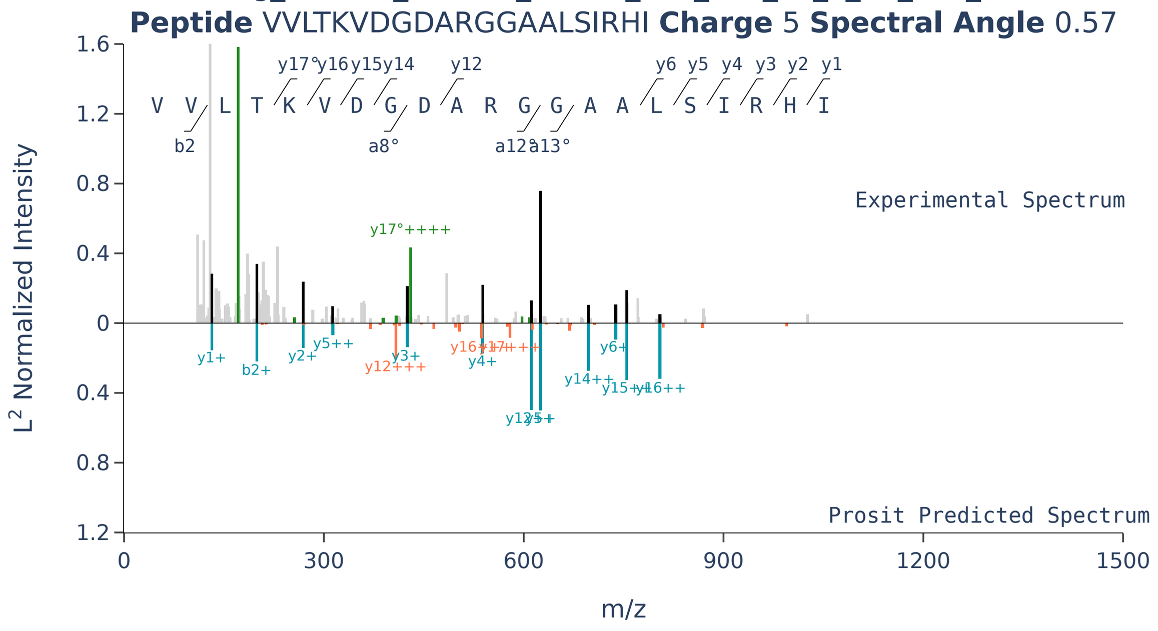


**Source** Ncheng_210623_230623_HFGoe_FFH_20S_25_1_A2_24h_R1 **Scan** 6833
**Peptide** INSMTMKERAK **Charge** 2 **Spectral Angle** 0.62

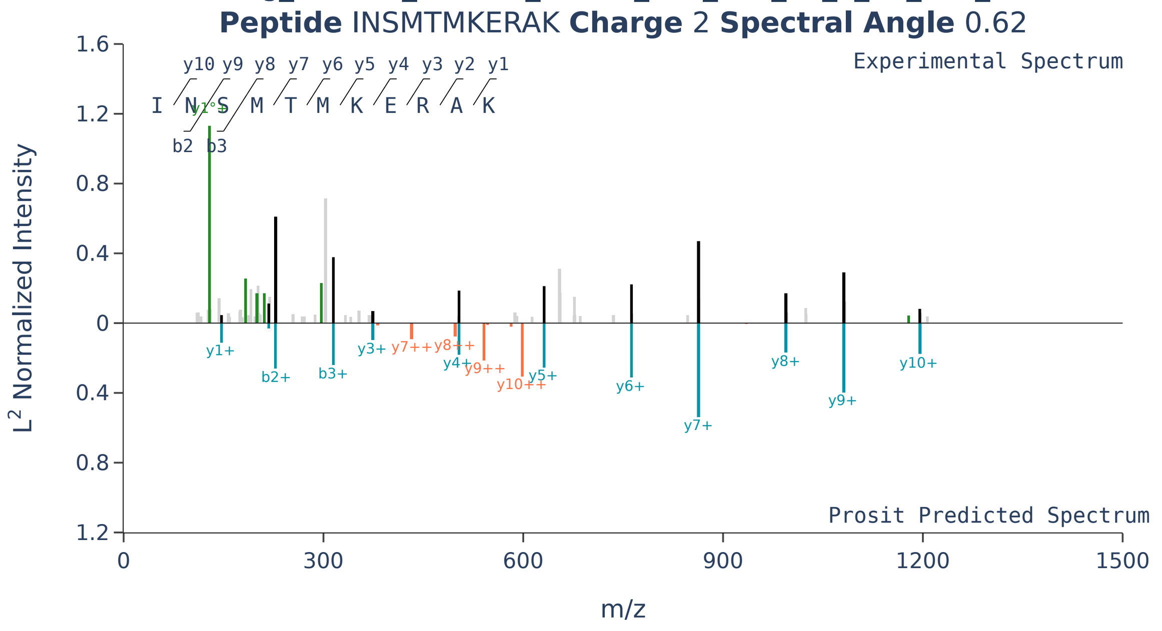


**Source** Ncheng_210623_230623_HFGoe_FFH_20S_25_1_A1_4h_R1 **Scan** 18368
**Peptide** TKVDGDARGGAALSIRHITGKPIKF **Charge** 6 **Spectral Angle** 0.75

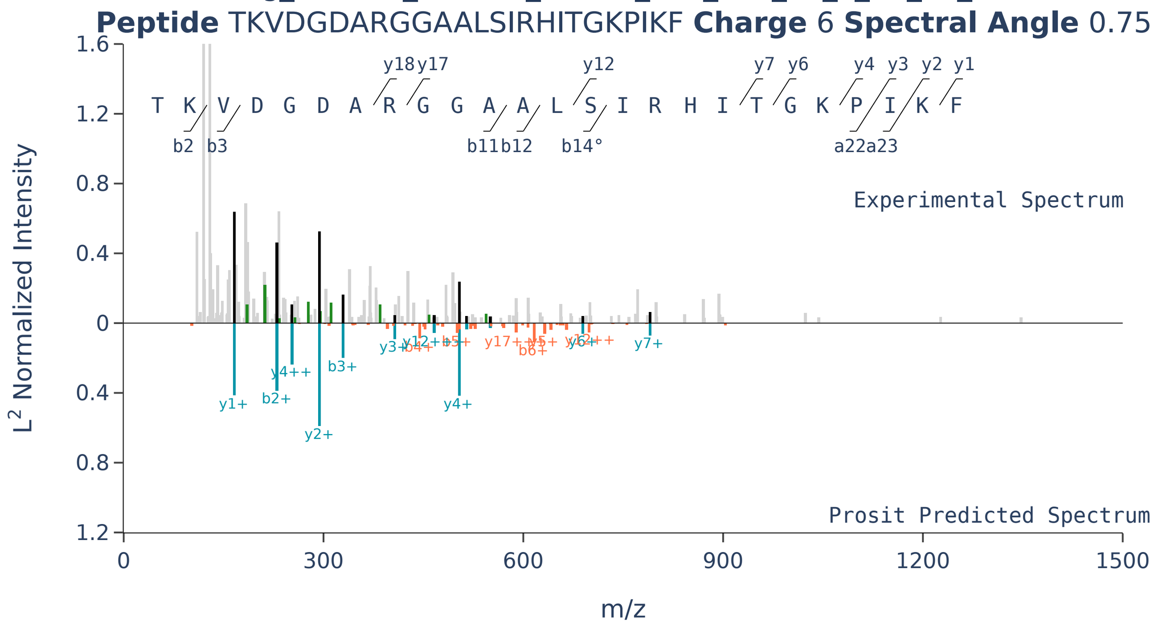


**Source** Ncheng_210623_230623_HFGoe_FFH_20S_25_1_A1_2h_R2 **Scan** 27037
**Peptide** EALEPFHPDRIASRIL **Charge** 3 **Spectral Angle** 0.57

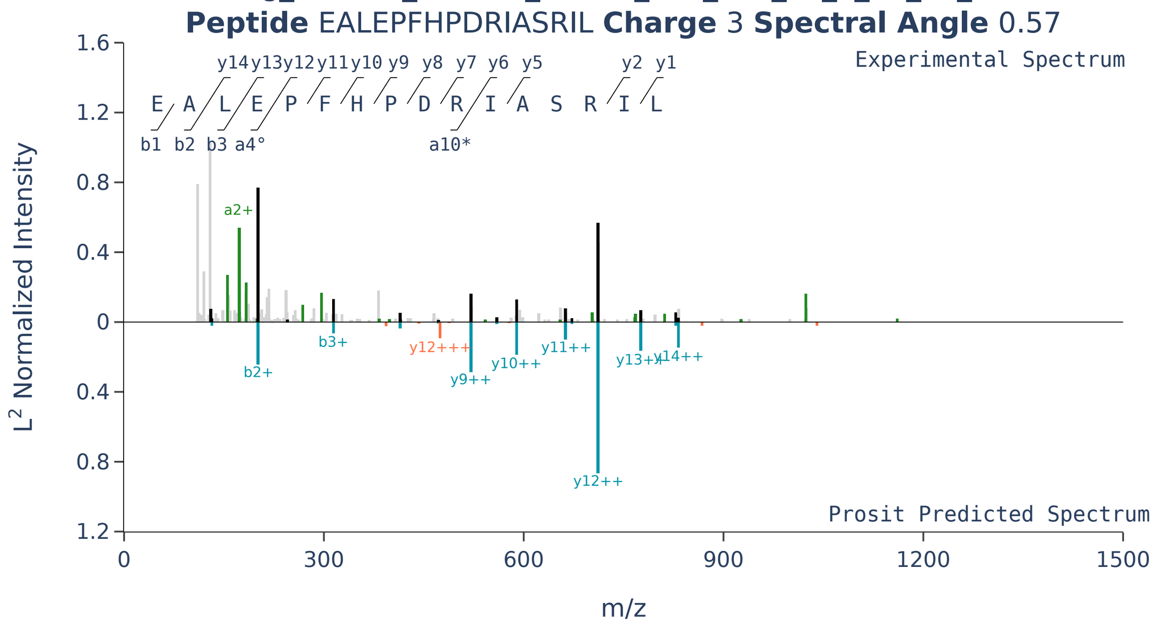


**Source** Ncheng_210623_230623_HFGoe_FFH_20S_25_1_A1_24h_R2 **Scan** 7647
**Peptide** DIESKVDRAQAEK **Charge** 2 **Spectral Angle** 0.55

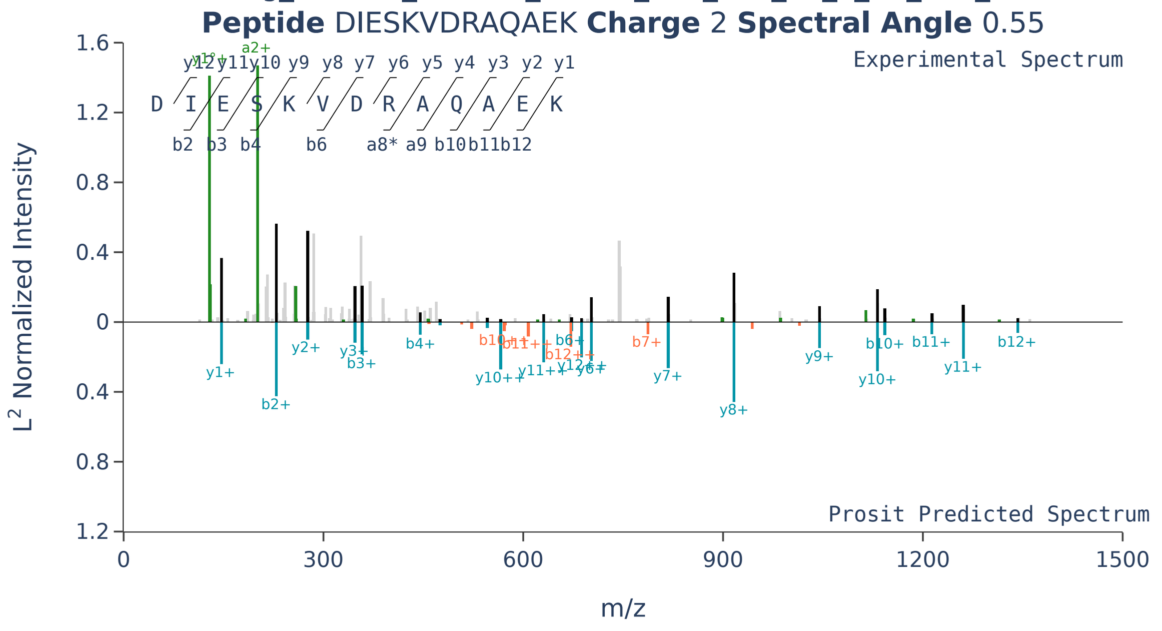


**Source** Ncheng_210623_230623_HFGoe_FFH_20S_25_1_A2_24h_R1 **Scan** 5585
**Peptide** EIKQVHAS **Charge** 2 **Spectral Angle** 0.66

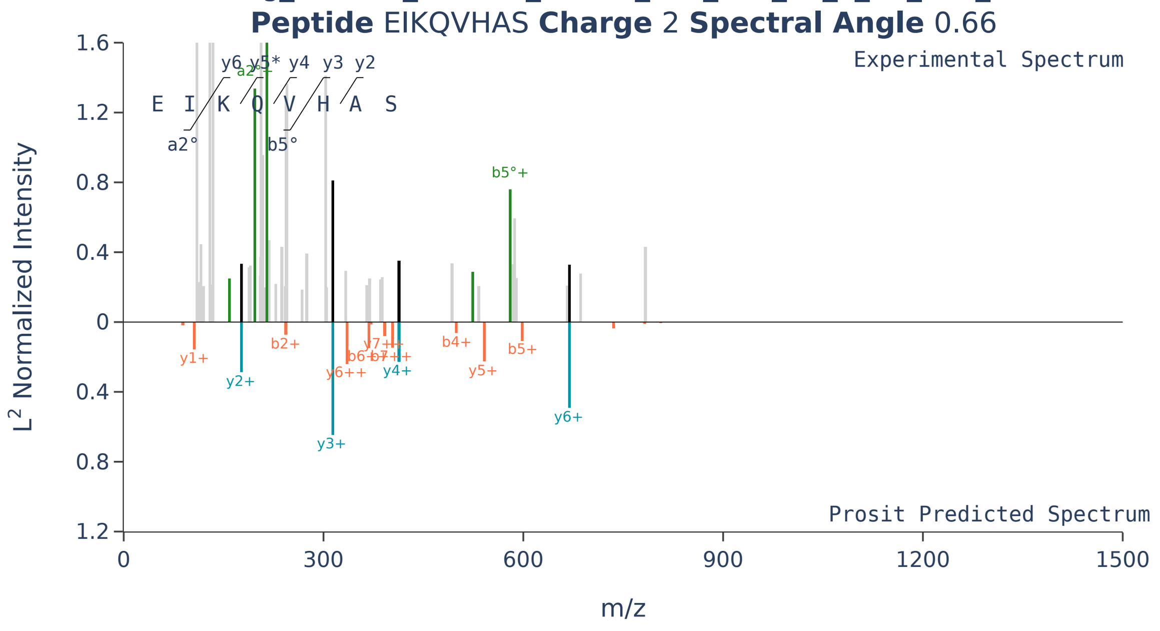


**Source** Ncheng_210623_230623_HFGoe_FFH_20S_25_1_A2_4h_R1 **Scan** 10590
**Peptide** FHPDRIASR **Charge** 2 **Spectral Angle** 0.63

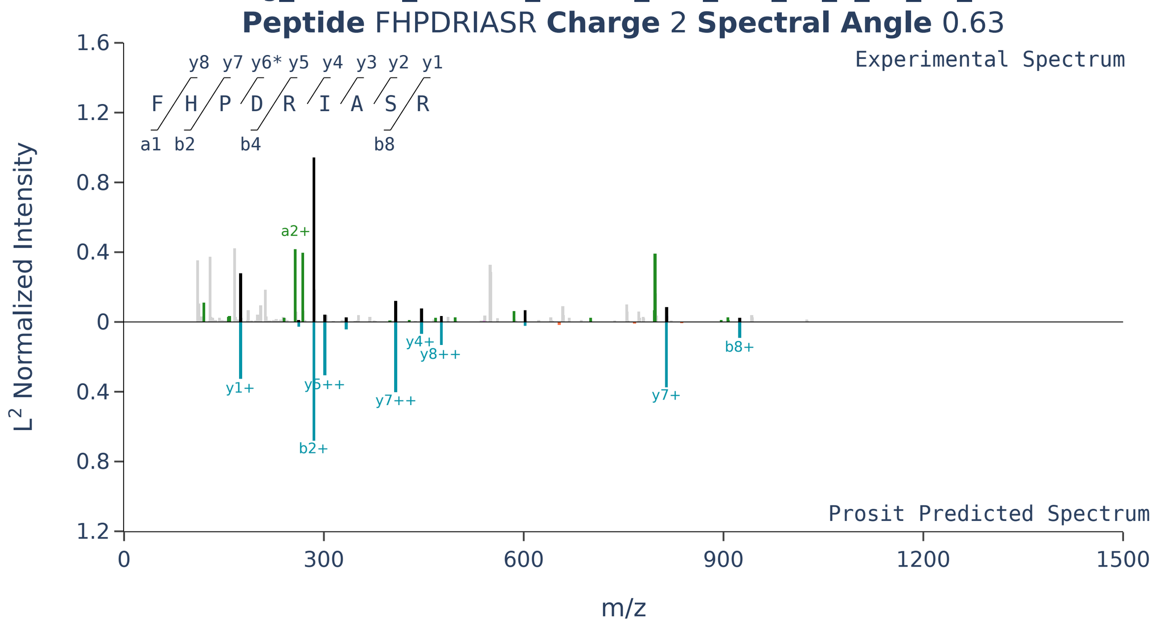


**Source** Ncheng_210623_230623_HFGoe_FFH_20S_25_1_A1_2h_R1 **Scan** 26108
**Peptide** ALEPFHPDRIASRLL **Charge** 3 **Spectral Angle** 0.47

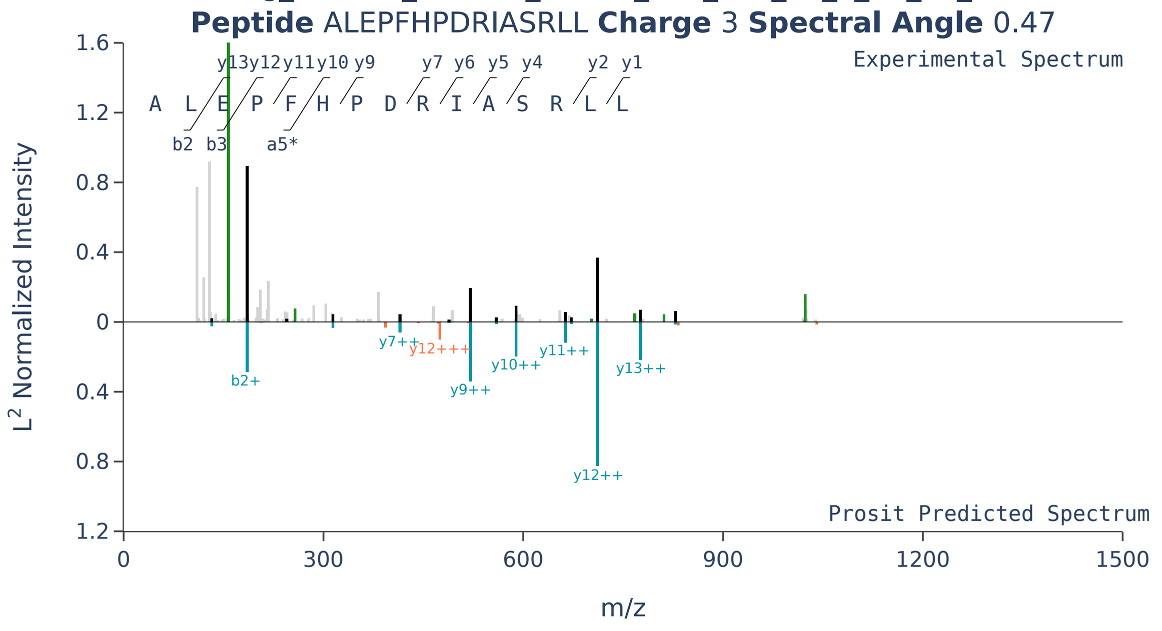


**Source** Ncheng_210623_230623_HFGoe_FFH_20S_25_1_A2_4h_R1 **Scan** 26351
**Peptide** LEPFHPDRIASRILG **Charge** 3 **Spectral Angle** 0.62

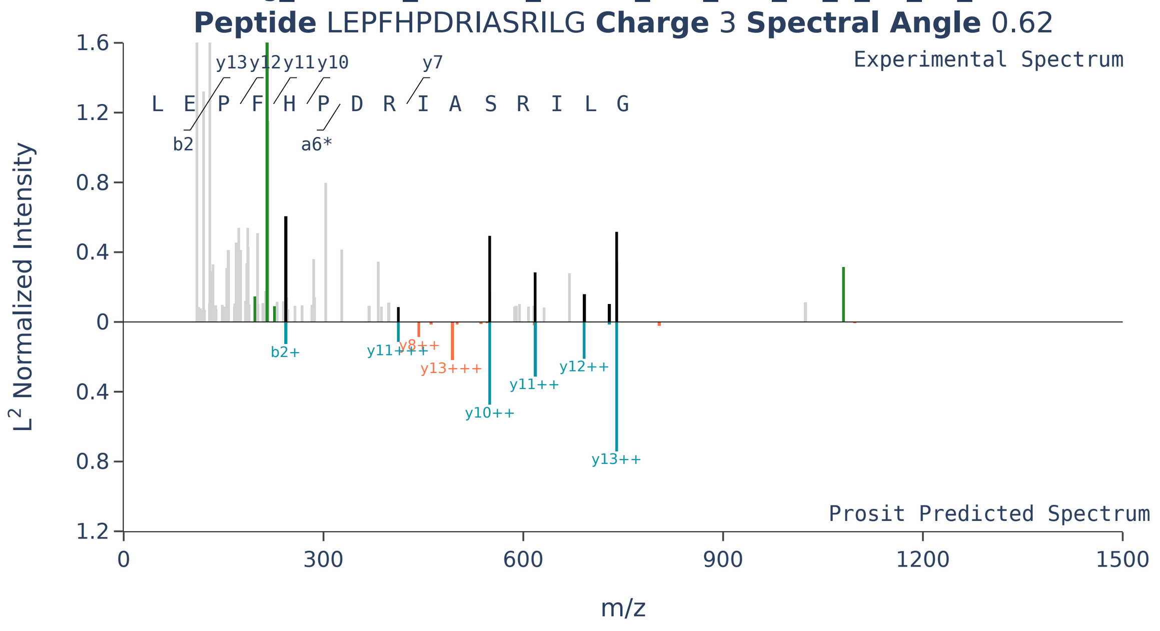

**Source** Ncheng_210623_230623_HFGoe_FFH_20S_25_1_A2_4h_R2 **Scan** 16256
**Peptide** KQFDDMQRMMKKMKKGGMAKM **Charge** 5 **Spectral Angle** 0.93

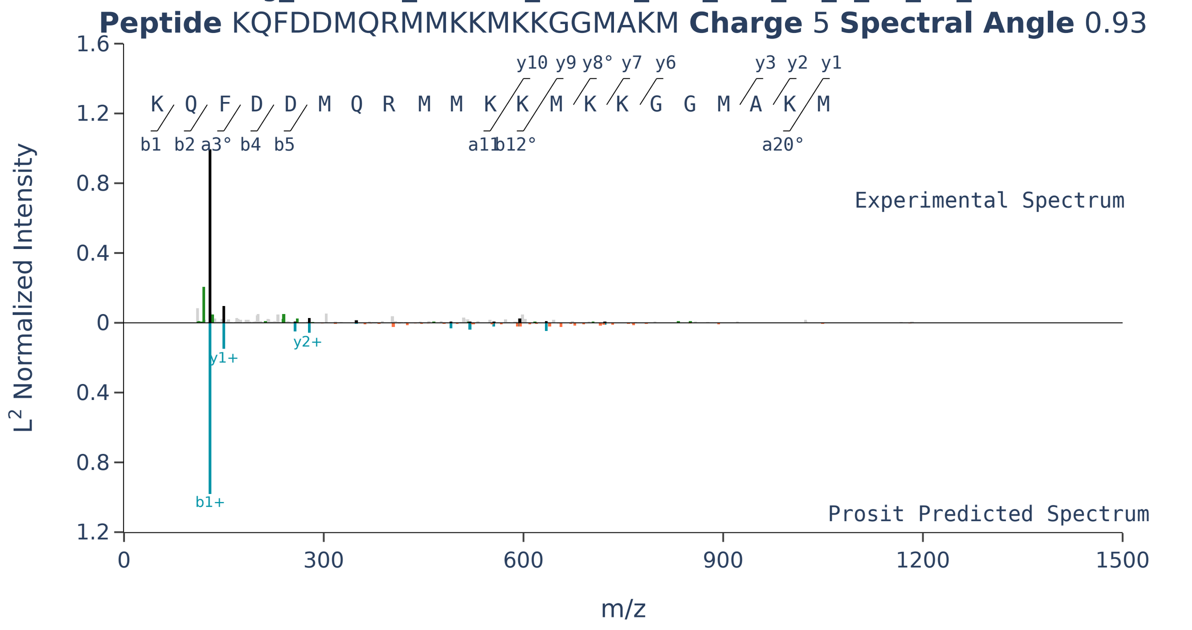


**Source** Ncheng_210623_230623_HFGoe_FFH_20S_25_1_A1_1h_R2 **Scan** 20823
**Peptide** RHITGKPIKFLGVGEKTEALEPFHPDRI **Charge** 5 **Spectral Angle** 0.76

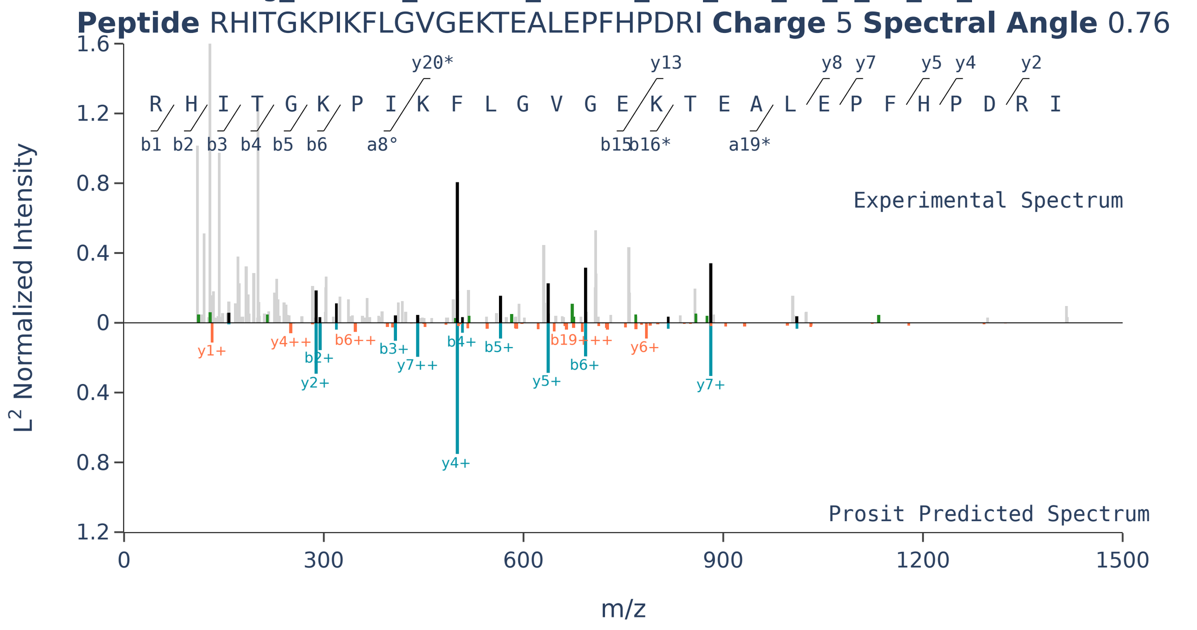


**Source** Ncheng_210623_230623_HFGoe_FFH_20S_25_1_A2_4h_R1 **Scan** 32087
**Peptide** MGGMASLMGKLPGMGQIPDNVKS **Charge** 3 **Spectral Angle** 0.71

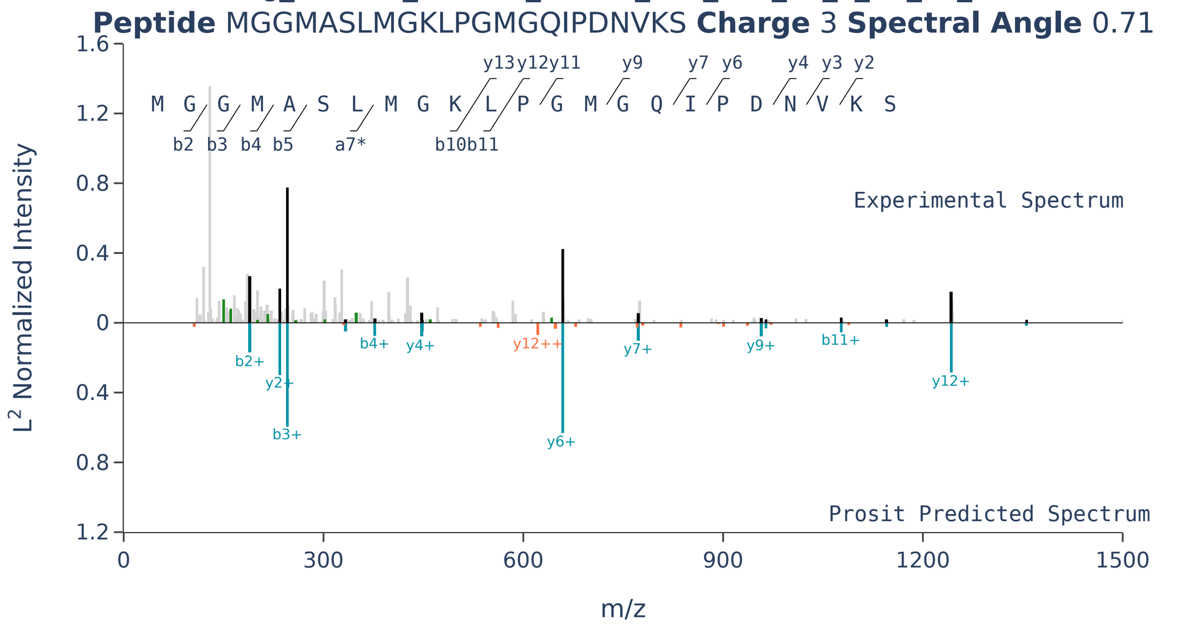


**Source** Ncheng_210623_230623_HFGoe_FFH_20S_25_1_A1_2h_R1 **Scan** 16831
**Peptide** KDTLREVRM **Charge** 2 **Spectral Angle** 0.93

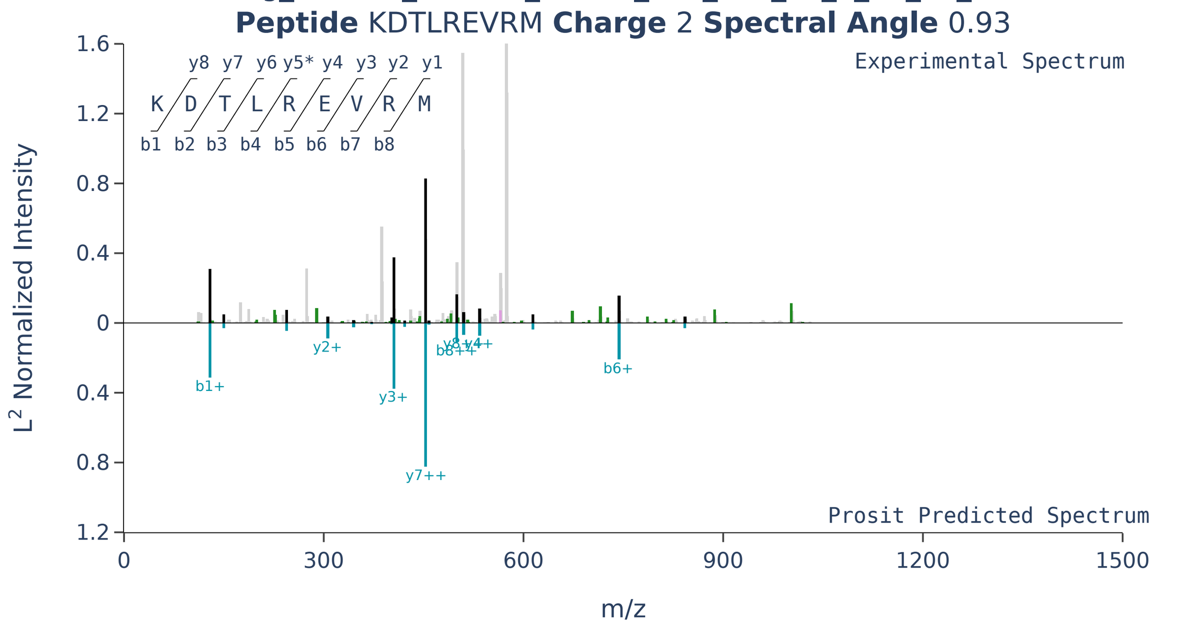


**Source** Ncheng_210623_230623_HFGoe_FFH_20S_25_1_A2_24h_R2 **Scan** 4989
**Peptide** KFLREKHK **Charge** 3 **Spectral Angle** 0.84

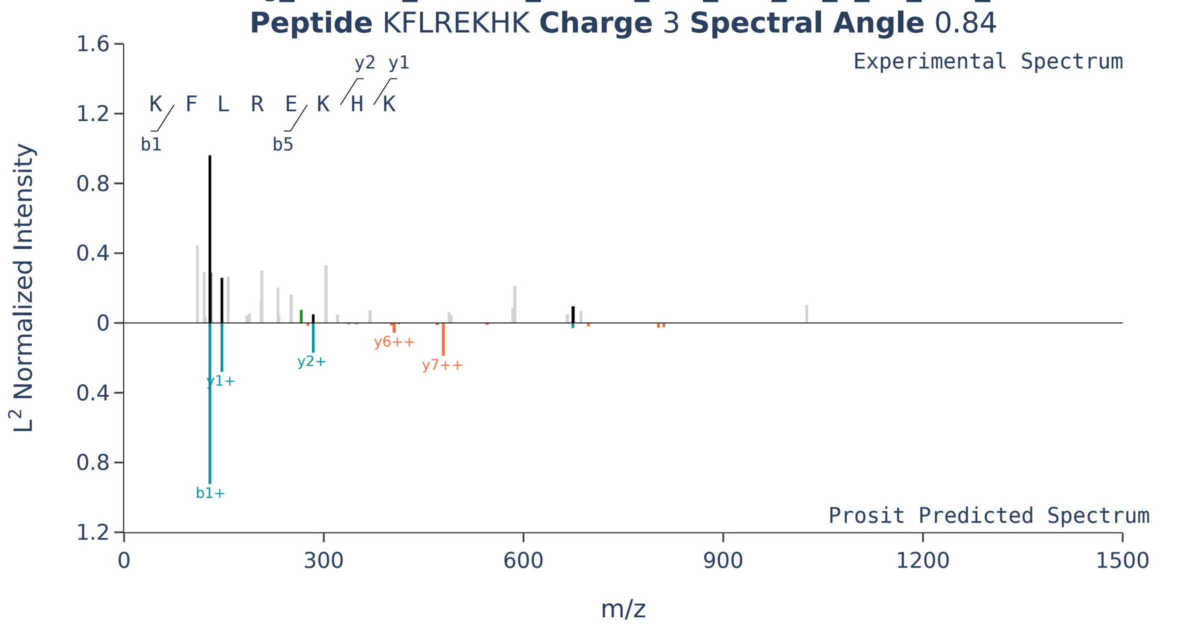


**Source** Ncheng_210623_230623_HFGoe_FFH_20S_25_1_A2_24h_R2 **Scan** 7563
**Peptide** TMKERAKPELLK **Charge** 3 **Spectral Angle** 0.48

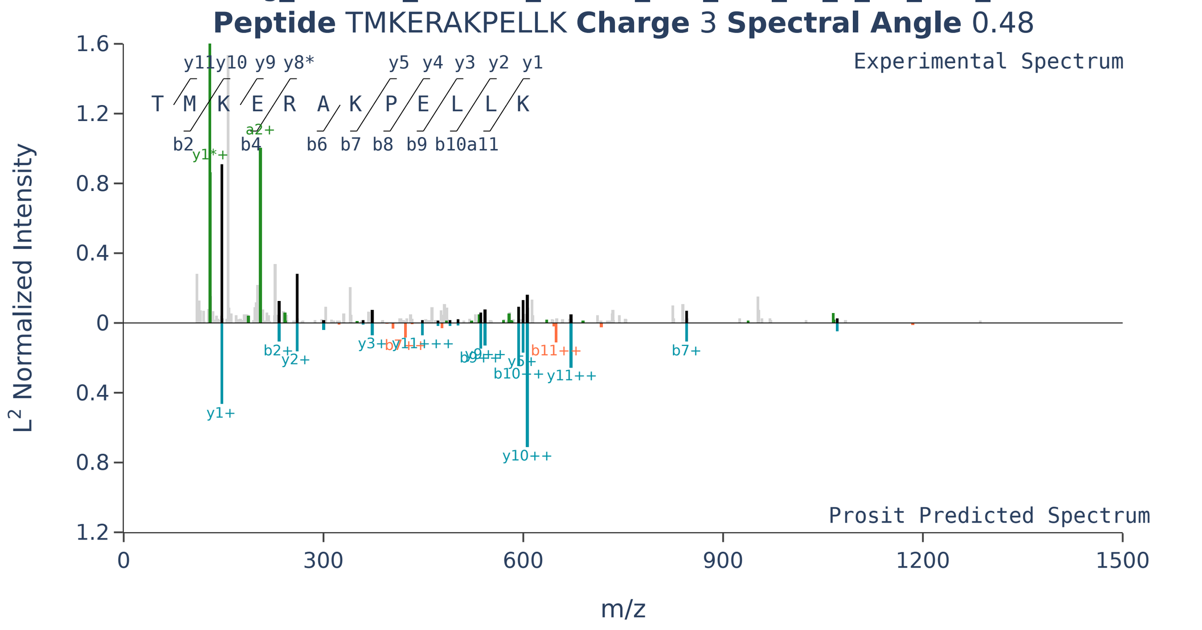


**Source** Ncheng_210623_230623_HFGoe_FFH_20S_25_1_A1_2h_R1 **Scan** 31923
**Peptide** AALKEAKLKFYDVLLVDTAGRLHV **Charge** 5 **Spectral Angle** 0.59

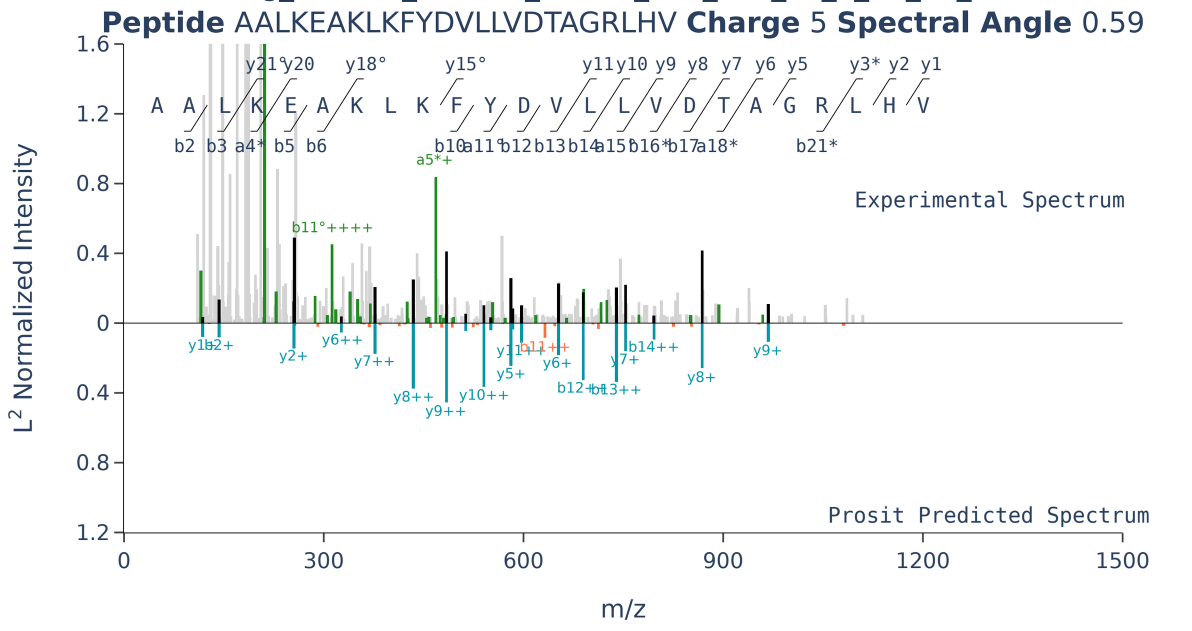


**Source** Ncheng_210623_230623_HFGoe_FFH_20S_25_1_A2_2h_R1 **Scan** 20305
**Peptide** LEDIESKVDRAQAEKLASKL **Charge** 3 **Spectral Angle** 0.76

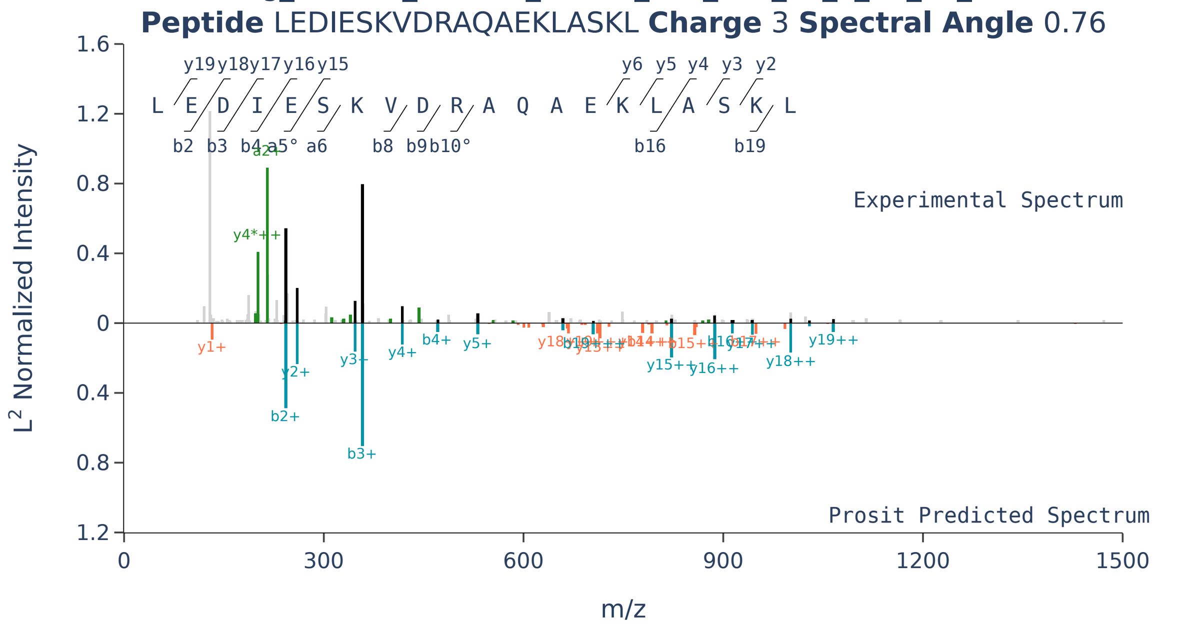


**Source** Ncheng_210623_230623_HFGoe_FFH_20S_25_1_A2_2h_R1 **Scan** 34586
**Peptide** EADVALPVVREFINRVKEKAVGH **Charge** 4 **Spectral Angle** 0.47

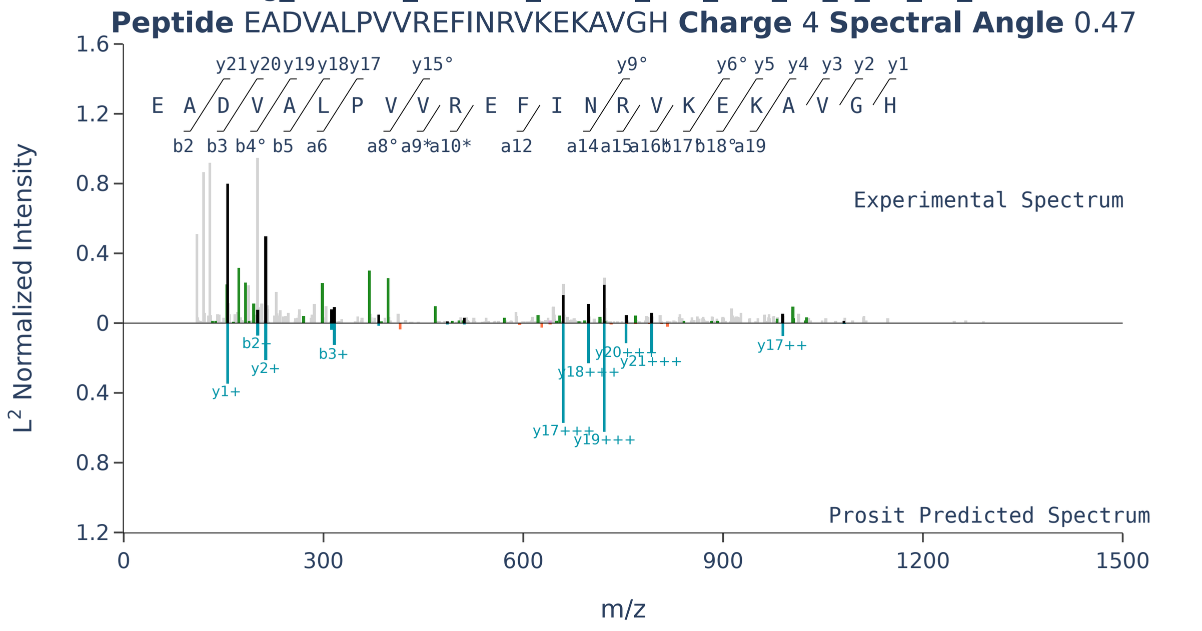


**Source** Ncheng_210623_230623_HFGoe_FFH_20S_25_1_A1_1h_R2 **Scan** 28287
**Peptide** KTEALEPFHPDRIASRILGMG **Charge** 4 **Spectral Angle** 0.51

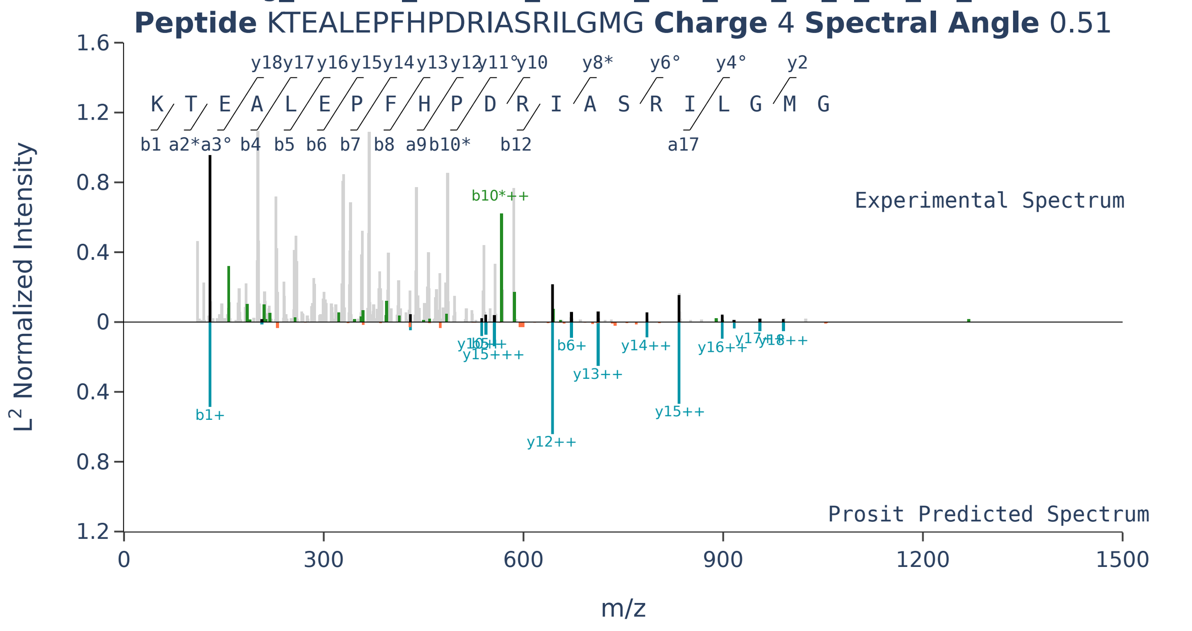


**Source** Ncheng_210623_230623_HFGoe_FFH_20S_25_1_A1_2h_R1 **Scan** 27959
**Peptide** KEAKLKFYDVLLVD **Charge** 3 **Spectral Angle** 0.34

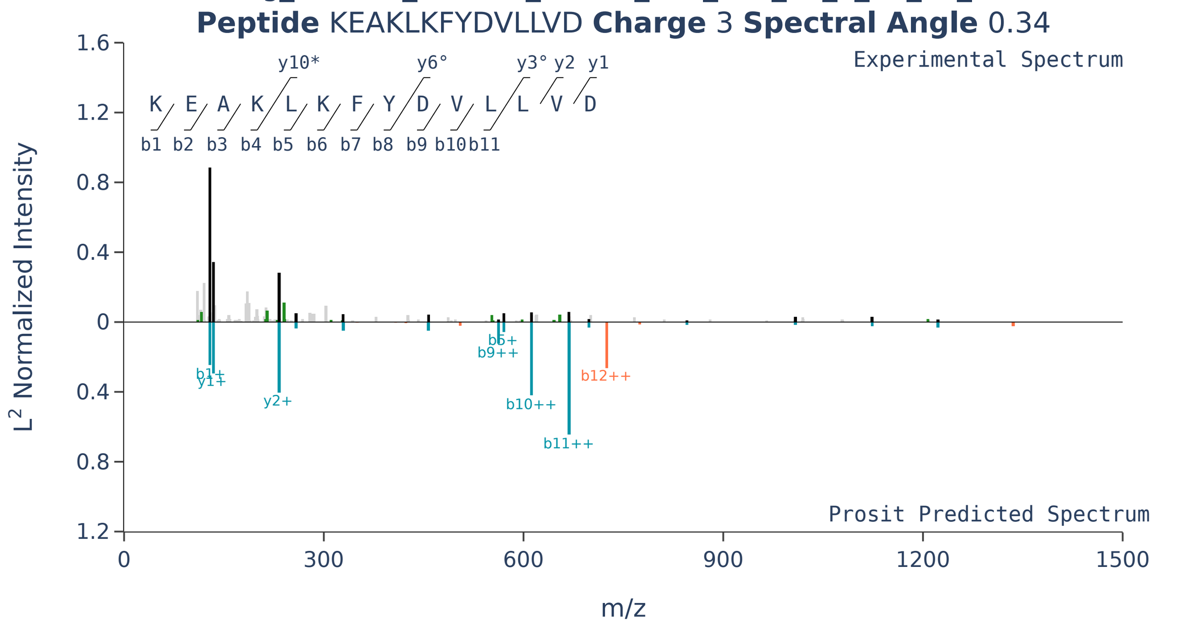


**Source** Ncheng_210623_230623_HFGoe_FFH_20S_25_1_A2_4h_R1 **Scan** 26242
**Peptide** DNVKDTLREVRMAL **Charge** 2 **Spectral Angle** 0.52

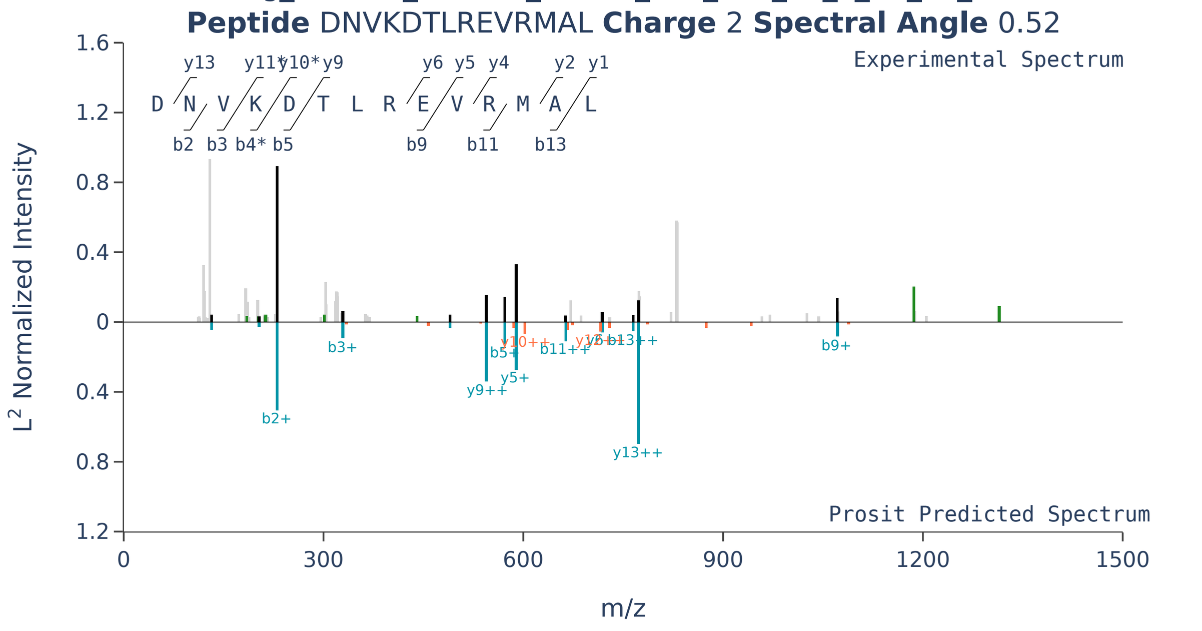


**Source** Ncheng_210623_230623_HFGoe_FFH_20S_25_1_A2_4h_R1 **Scan** 19781
**Peptide** VDGDARGGAALSIRHITGKPLKF **Charge** 5 **Spectral Angle** 0.69

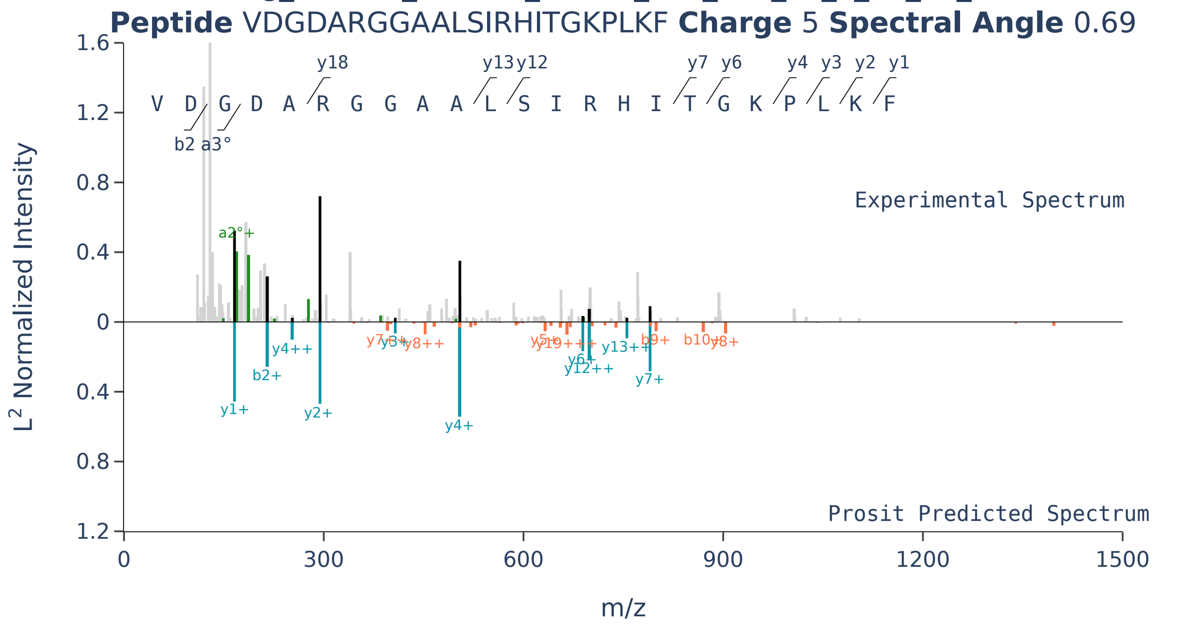


**Source** Ncheng_210623_230623_HFGoe_FFH_20S_25_1_A1_4h_R2 **Scan** 28880
**Peptide** VGVDFFPSDVGQKPVD **Charge** 2 **Spectral Angle** 0.64

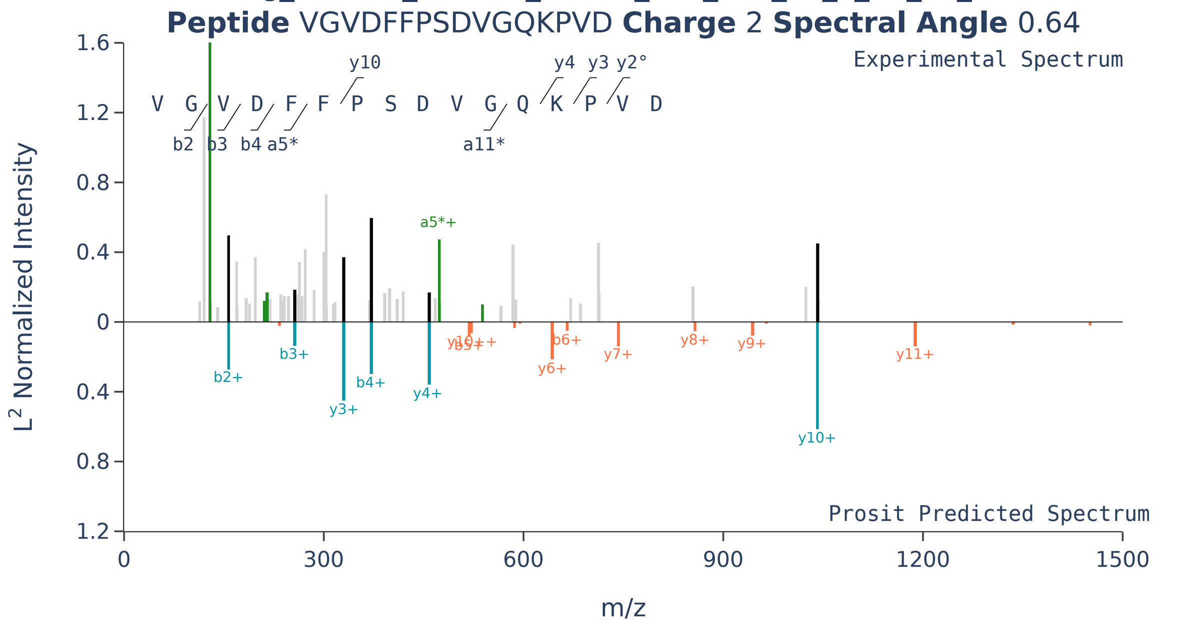


**Source** Ncheng_210623_230623_HFGoe_FFH_20S_25_1_A1_2h_R2 **Scan** 29256
**Peptide** KVLVRMEAIINSMTMKERAKPELL **Charge** 4 **Spectral Angle** 0.82

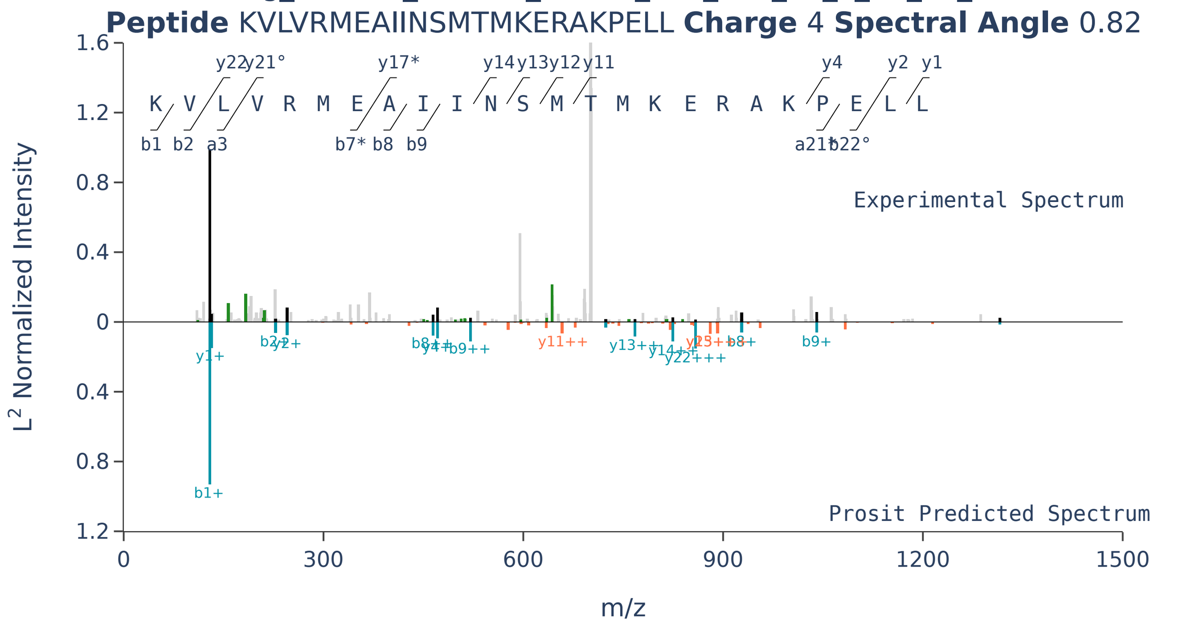

**Source** Ncheng_210623_230623_HFGoe_FFH_20S_25_1_A2_24h_R1 **Scan** 7387
**Peptide** ESKVDRAQAEKL **Charge** 2 **Spectral Angle** 0.65

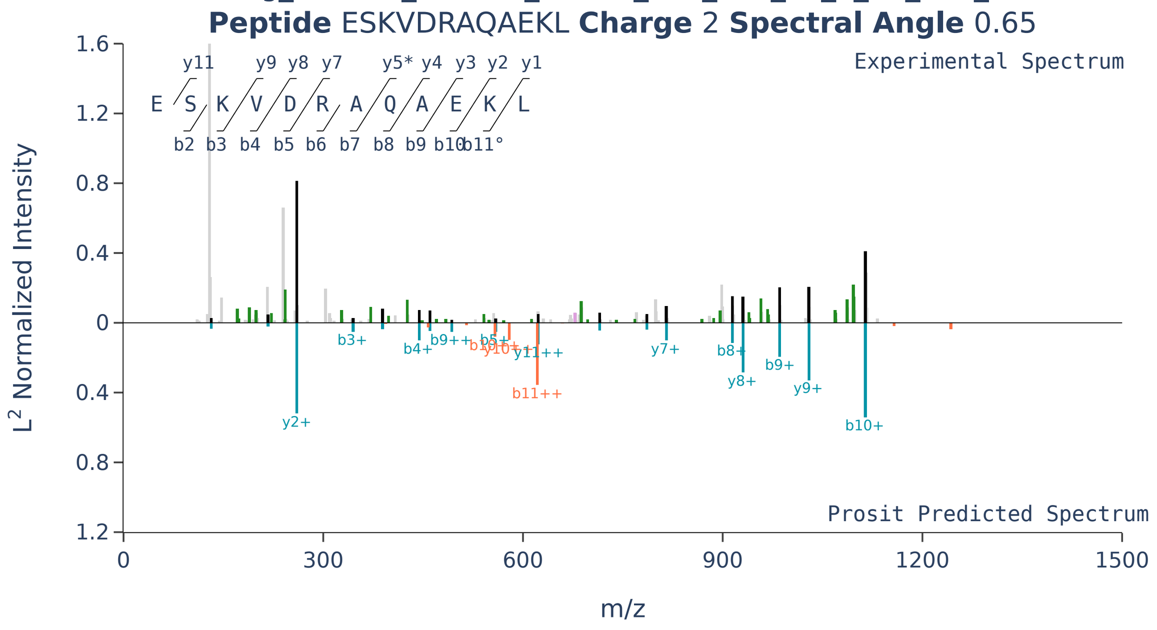


**Source** Ncheng_210623_230623_HFGoe_FFH_20S_25_1_A2_24h_R1 **Scan** 16888
**Peptide** ALLNSMTMKERAKPEIIKG **Charge** 5 **Spectral Angle** 0.83

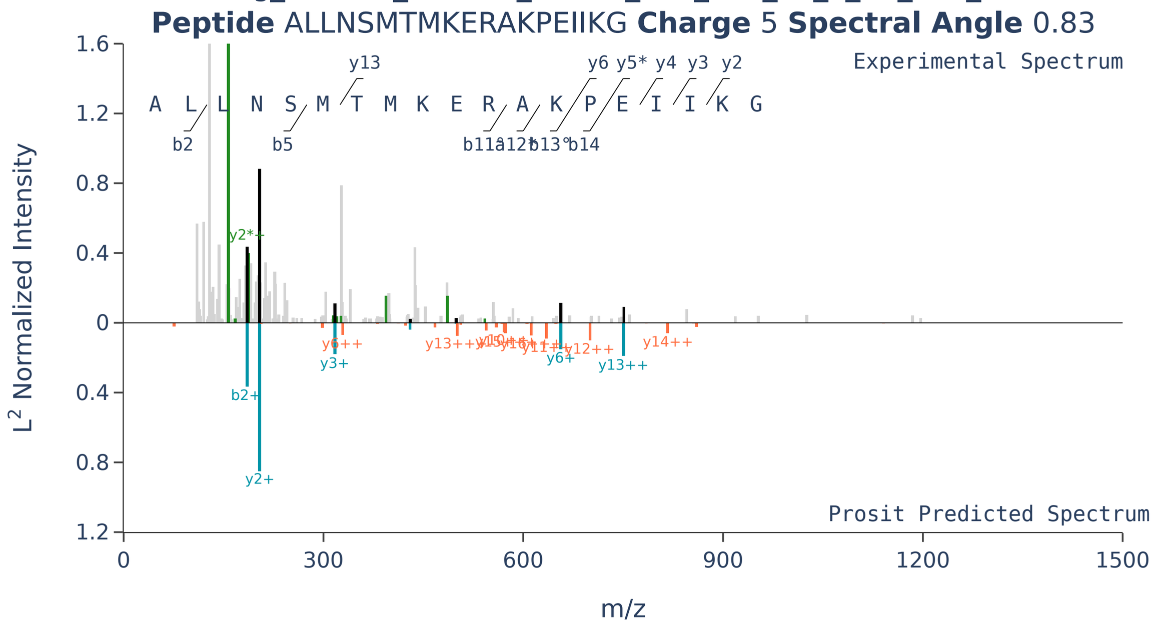


**Source** Ncheng_210623_230623_HFGoe_FFH_20S_25_1_A2_4h_R1 **Scan** 27653
**Peptide** KPIKFLGVGEKTEALEPFHPDRIASRIL **Charge** 4 **Spectral Angle** 0.73

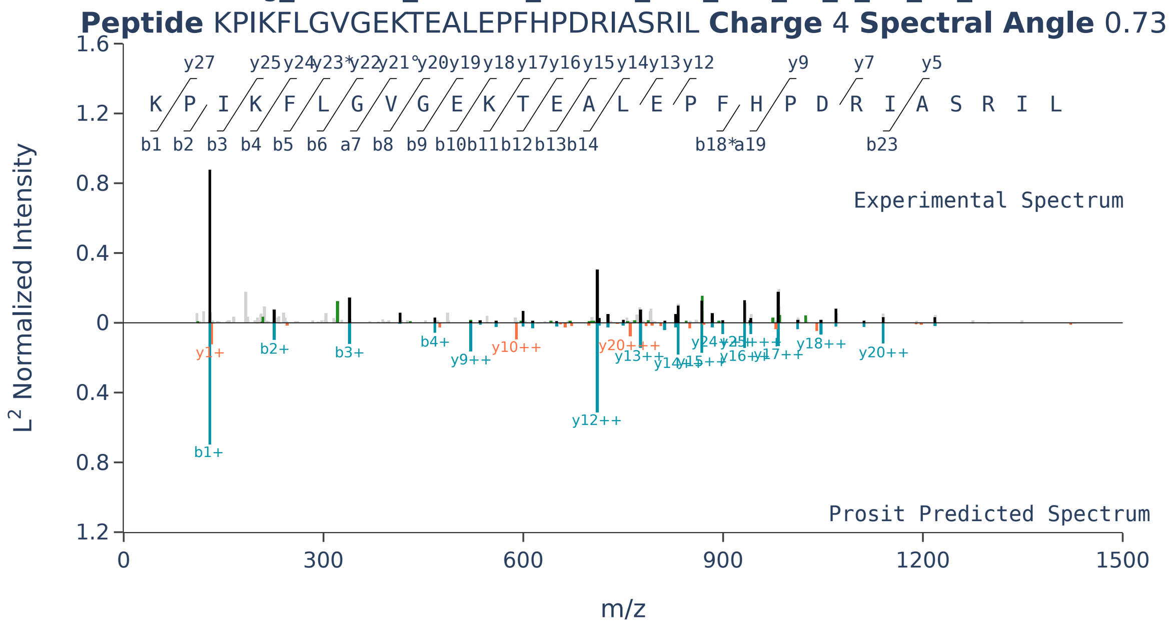


**Source** Ncheng_210623_230623_HFGoe_FFH_20S_25_1_A2_4h_R1 **Scan** 15294
**Peptide** LTKVDGDARGGAALSIRHI **Charge** 5 **Spectral Angle** 0.61

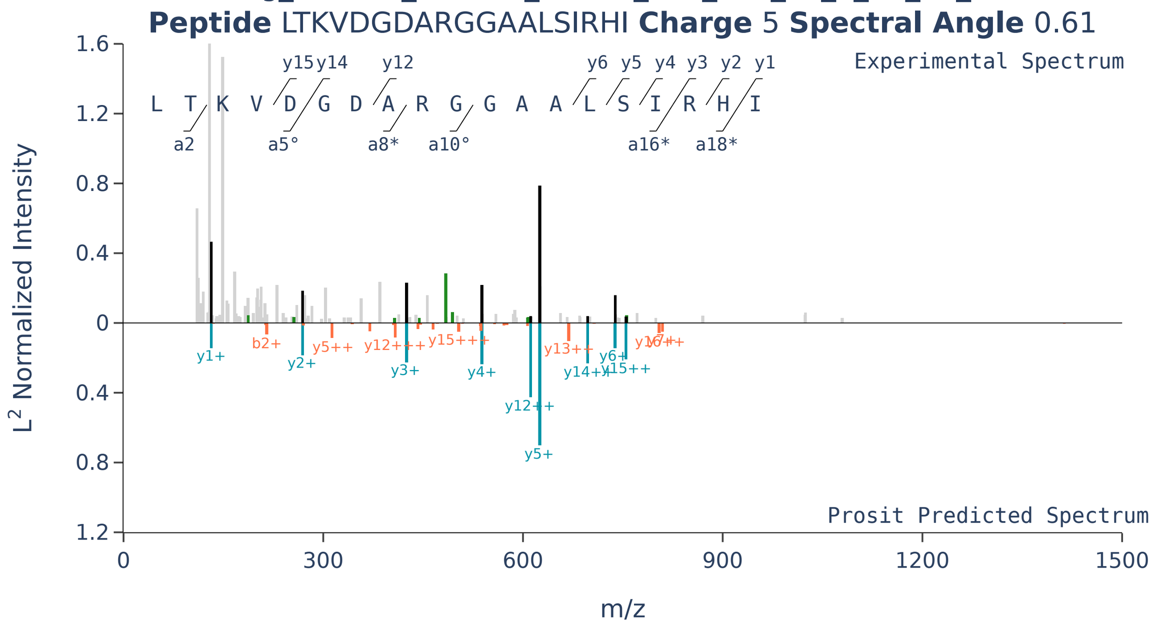


**Source** Ncheng_210623_230623_HFGoe_FFH_20S_25_1_A2_24h_R2 **Scan** 17404
**Peptide** EDIESKVDRAQAEKLASKLK **Charge** 4 **Spectral Angle** 0.75

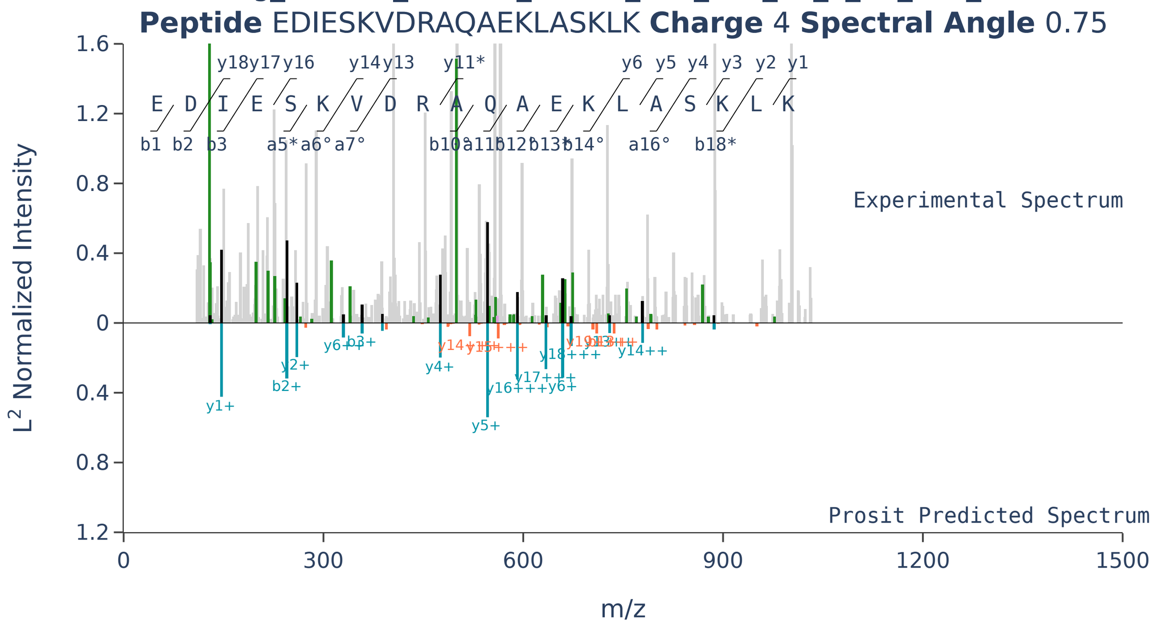


**Source** Ncheng_210623_230623_HFGoe_FFH_20S_25_1_A1_24h_R1 **Scan** 31179
**Peptide** ALPVVREFI **Charge** 2 **Spectral Angle** 0.71

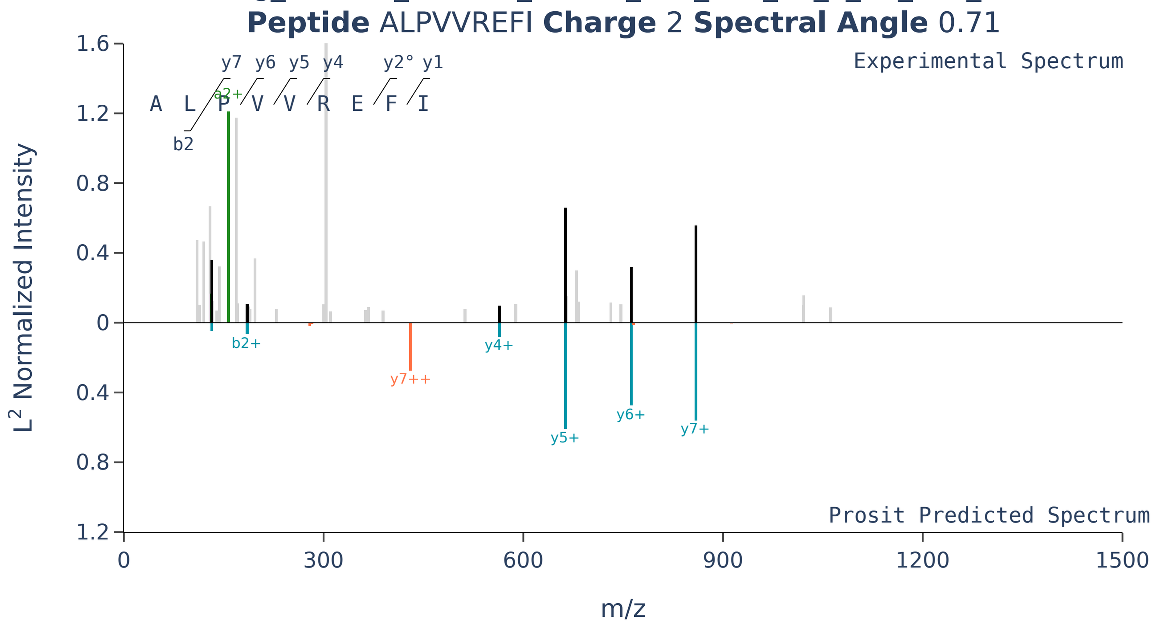


**Source** Ncheng_210623_230623_HFGoe_FFH_20S_25_1_A2_2h_R2 **Scan** 37617
**Peptide** KLKKGDGFDLNDFLEQLRQM **Charge** 3 **Spectral Angle** 0.62

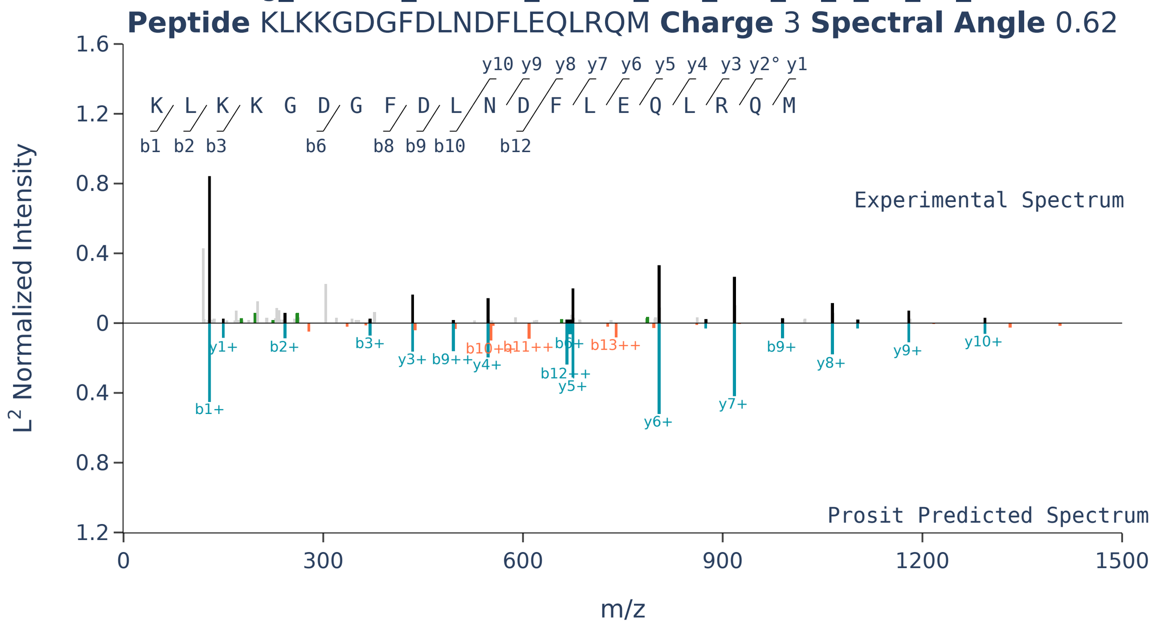


**Source** Ncheng_210623_230623_HFGoe_FFH_20S_25_1_A2_2h_R1 **Scan** 18882
**Peptide** SIRHITGKPIKFLGVGEKTEAL **Charge** 3 **Spectral Angle** 0.57

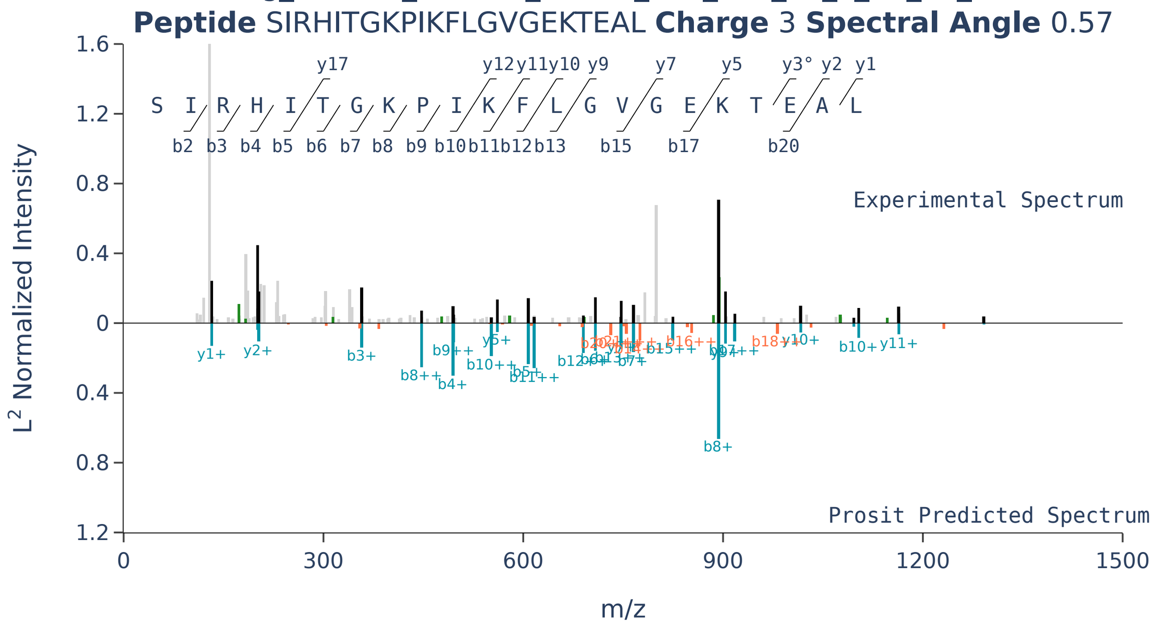


**Source** Ncheng_210623_230623_HFGoe_FFH_20S_25_1_A1_4h_R1 **Scan** 8571
**Peptide** SRTLRNISGRGRL **Charge** 4 **Spectral Angle** 0.64

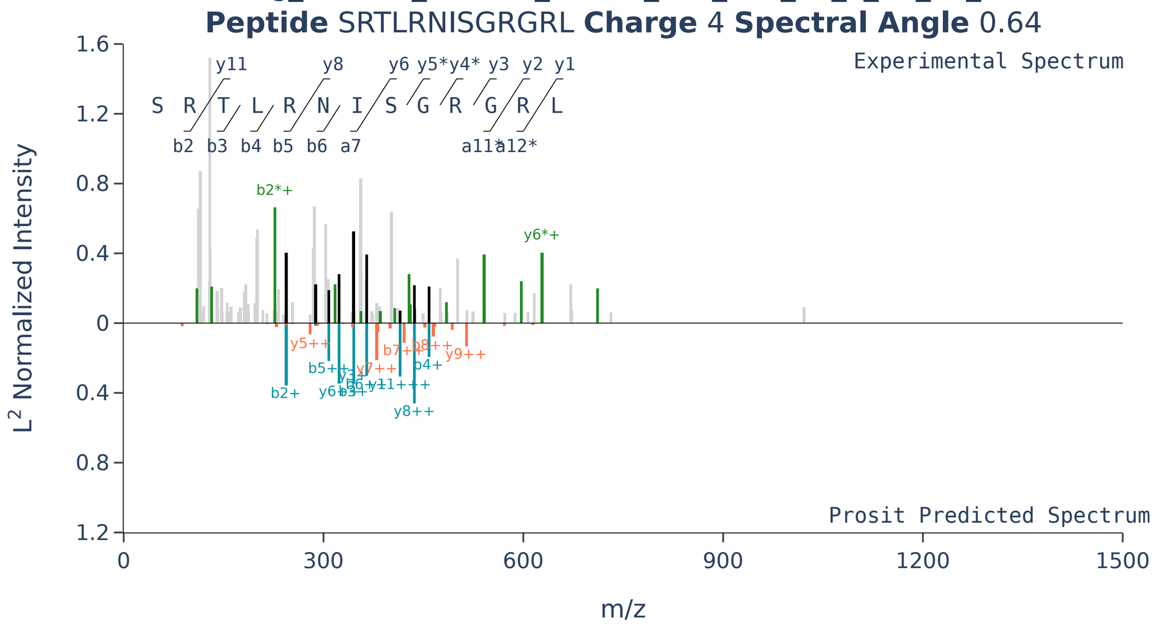


**Source** Ncheng_210623_230623_HFGoe_FFH_20S_25_1_A1_1h_R1 **Scan** 31791
**Peptide** KQLETLAEQVGVDFFPSDVGQKPVD **Charge** 3 **Spectral Angle** 0.62

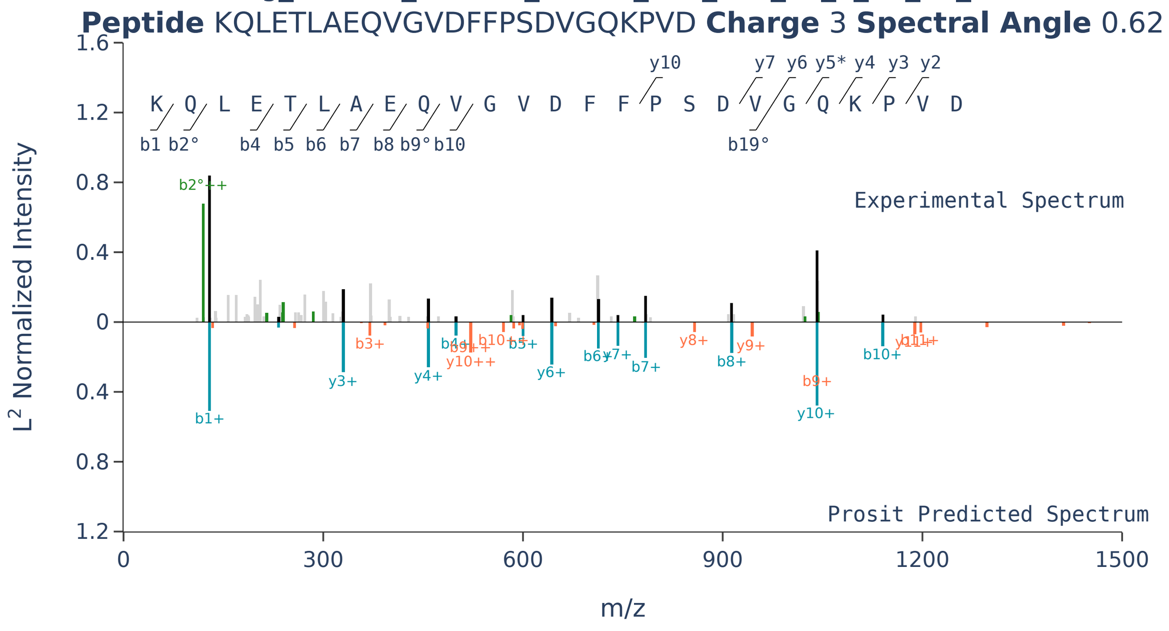


**Source** Ncheng_210623_230623_HFGoe_FFH_20S_25_1_A2_24h_R1 **Scan** 8650
**Peptide** QGAGKTTSVGKL **Charge** 3 **Spectral Angle** 0.72

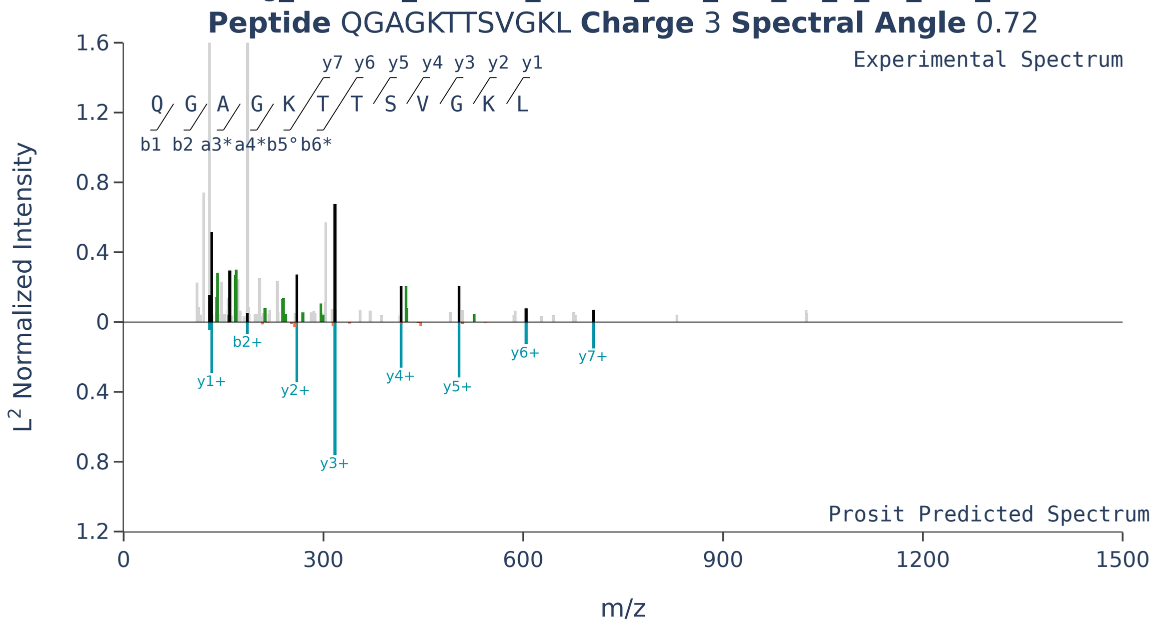


**Source** Ncheng_210623_230623_HFGoe_FFH_20S_25_1_A1_4h_R2 **Scan** 20724
**Peptide** IEDIESKVDRAQAEKLASKLKKGDGFD **Charge** 4 **Spectral Angle** 0.7

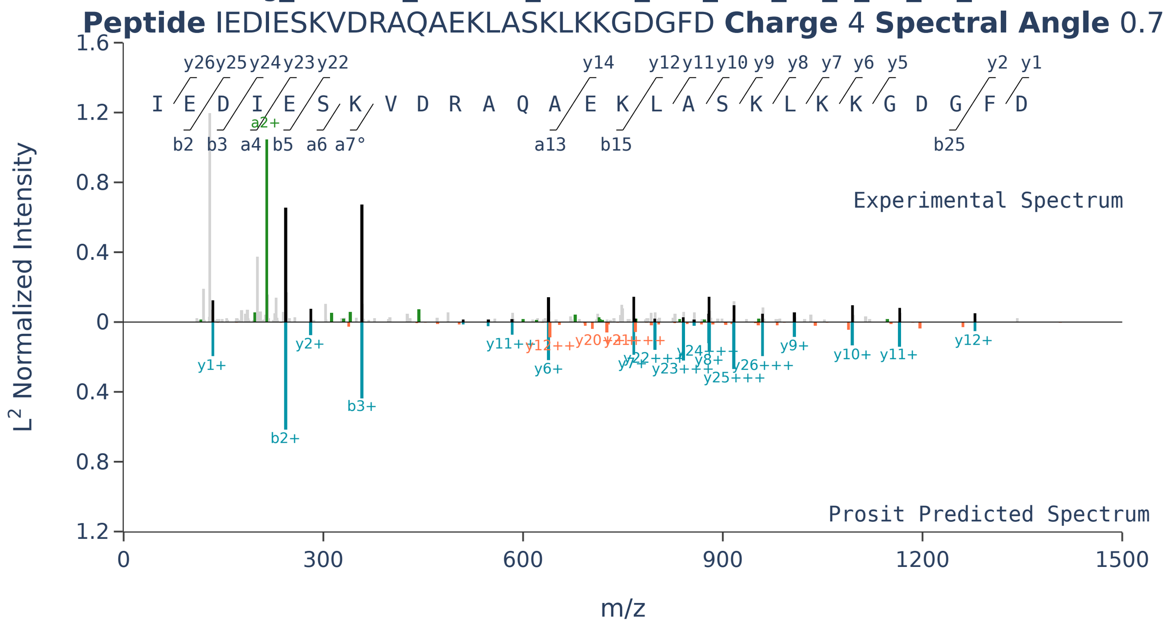


**Source** Ncheng_210623_230623_HFGoe_FFH_20S_25_1_A2_2h_R1 **Scan** 28971
**Peptide** MQVQDVNRLLKQFD **Charge** 3 **Spectral Angle** 0.64

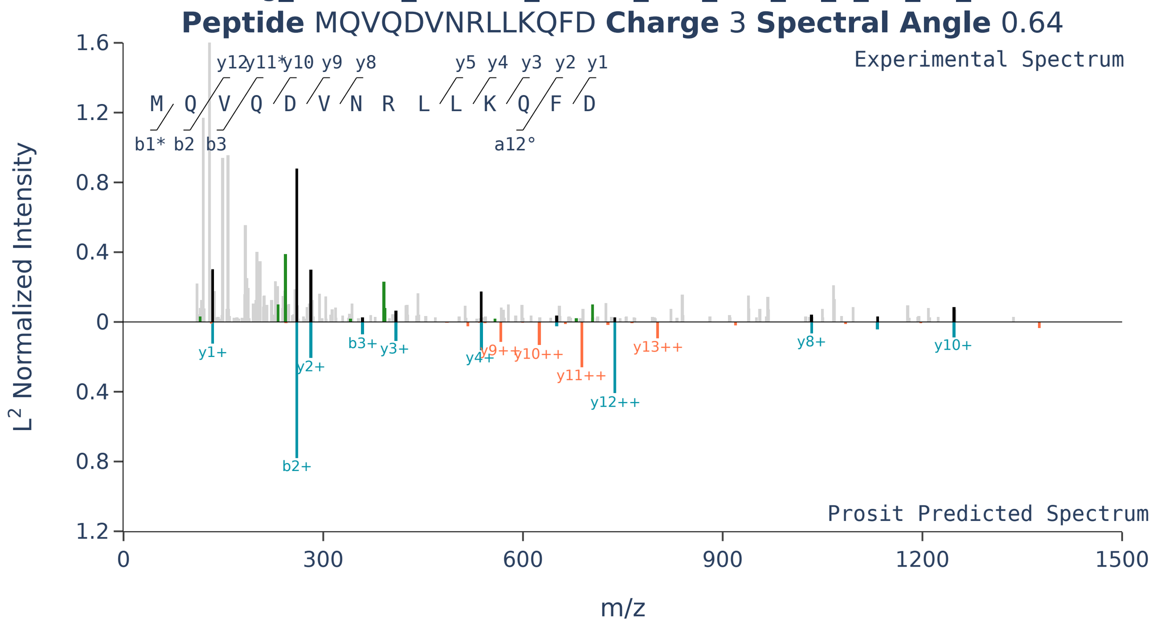


**Source** Ncheng_210623_230623_HFGoe_FFH_20S_25_1_A2_1h_R1 **Scan** 32073
**Peptide** IVNAALKEAKLKFYDVLLVDTAGRLHVDE **Charge** 4 **Spectral Angle** 0.76

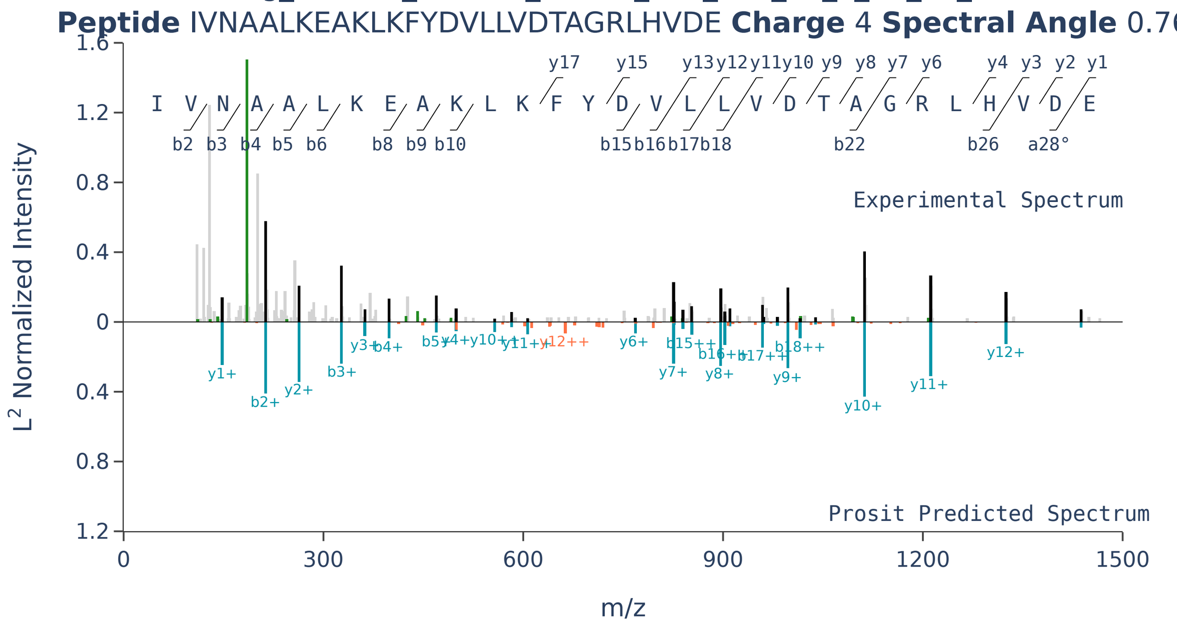


**Source** Ncheng_210623_230623_HFGoe_FFH_20S_25_1_A1_2h_R1 **Scan** 35778
**Peptide** LNDFLEQLRQMKNMGGMASL **Charge** 3 **Spectral Angle** 0.82

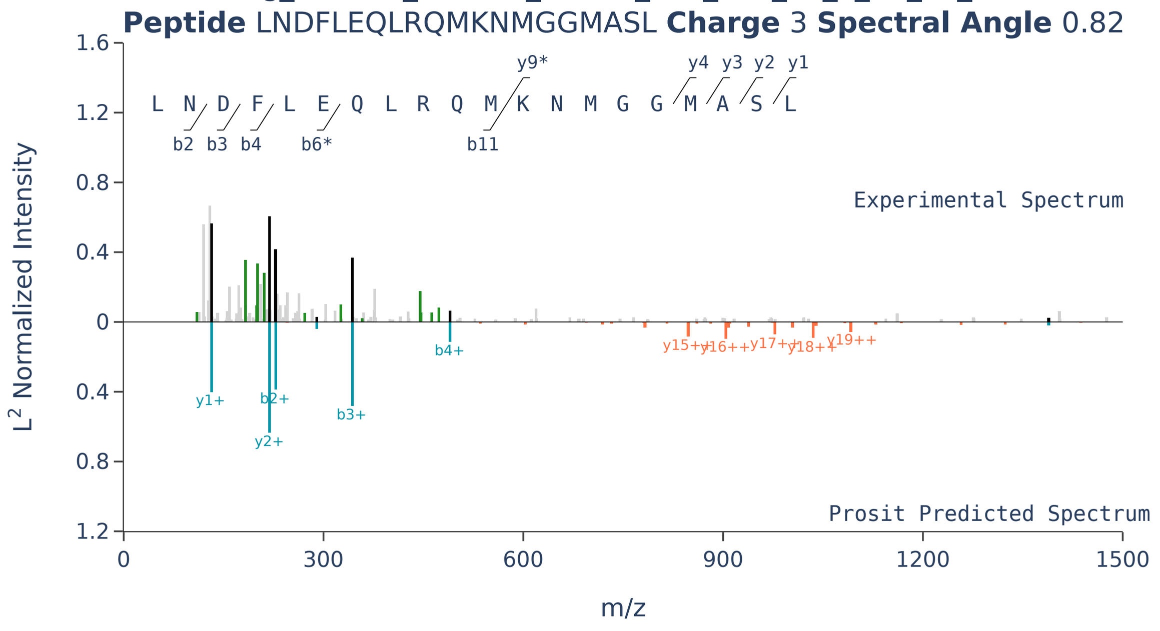

**Source** Ncheng_210623_230623_HFGoe_FFH_20S_25_1_A2_1h_R1 **Scan** 28554
**Peptide** ALPLTGVVLTKVDGDARGGAALSIRHLTG **Charge** 4 **Spectral Angle** 0.65

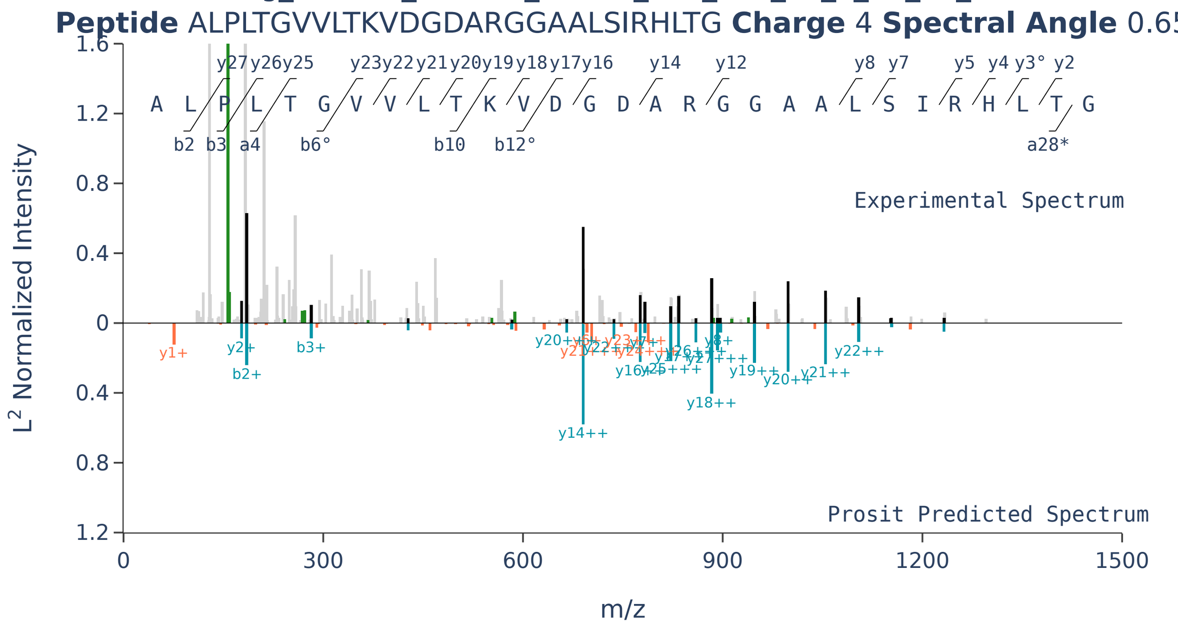


**Source** Ncheng_210623_230623_HFGoe_FFH_20S_25_1_A1_2h_R1 **Scan** 37531
**Peptide** RMALLEADVALPVVREFINRVKEK **Charge** 4 **Spectral Angle** 0.64

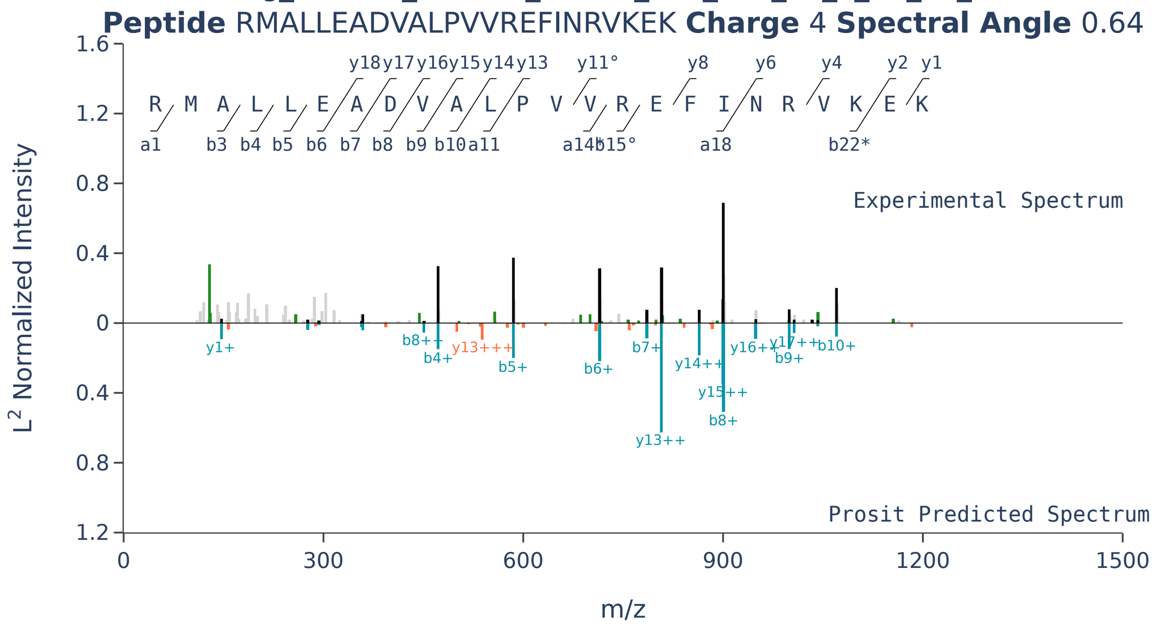


**Source** Ncheng_210623_230623_HFGoe_FFH_20S_25_1_A1_2h_R1 **Scan** 27684
**Peptide** VALPVVREFINRVKEK **Charge** 3 **Spectral Angle** 0.5

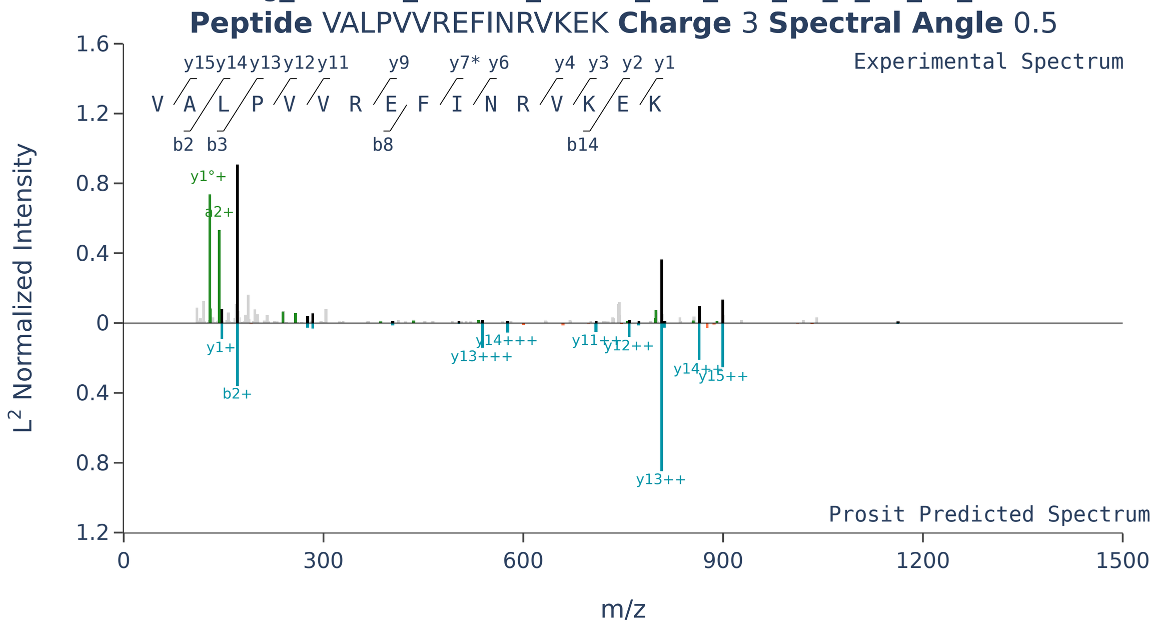


**Source** Ncheng_210623_230623_HFGoe_FFH_20S_25_1_A1_4h_R1 **Scan** 28580
**Peptide** LPLTGVVLTKVDGDARGGAALSIRHI **Charge** 4 **Spectral Angle** 0.67

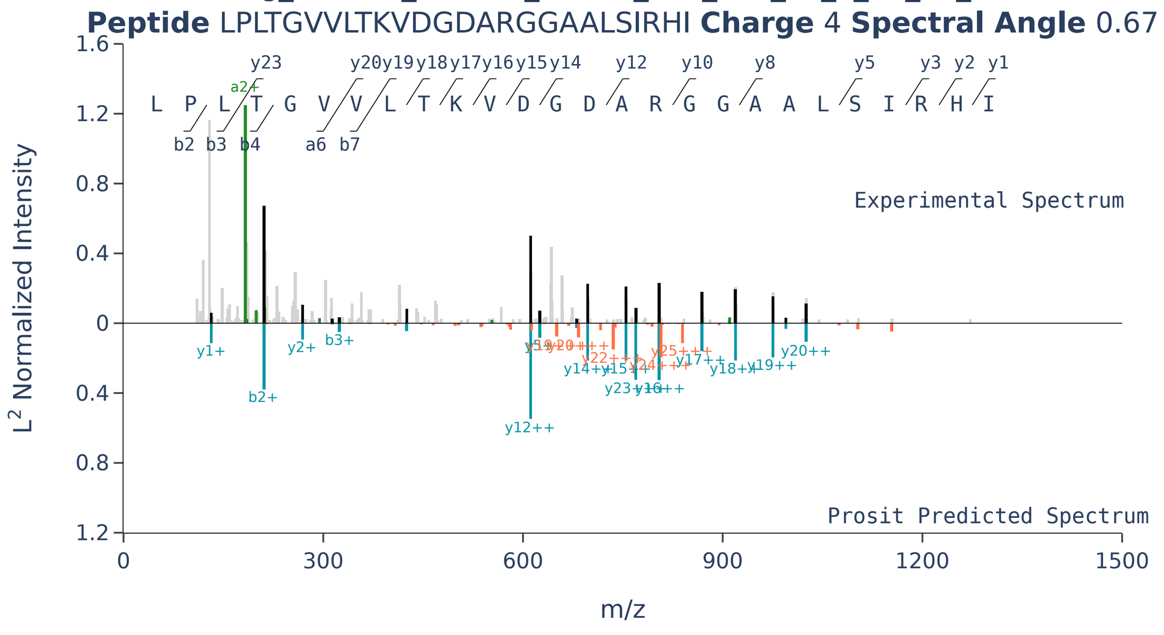


**Source** Ncheng_210623_230623_HFGoe_FFH_20S_25_1_A1_24h_R2 **Scan** 10573
**Peptide** KVDRAQAEKLASKLKK **Charge** 4 **Spectral Angle** 0.87

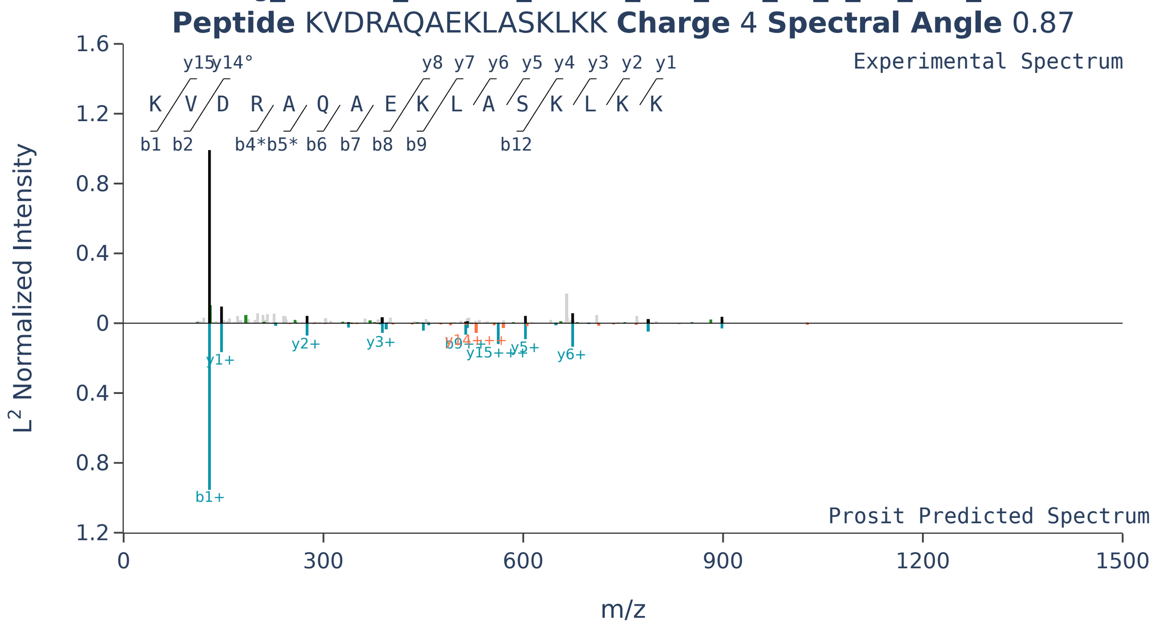


**Source** Ncheng_210623_230623_HFGoe_FFH_20S_25_1_A1_1h_R2 **Scan** 29277
**Peptide** KFYDVLLVDTA **Charge** 2 **Spectral Angle** 0.39

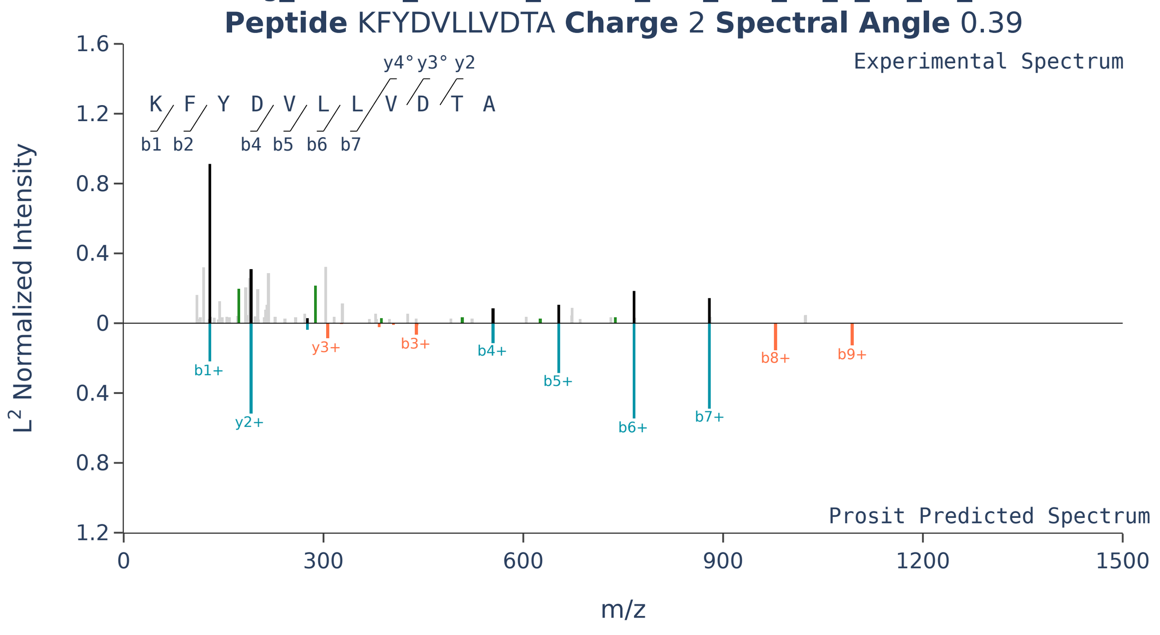


**Source** Ncheng_210623_230623_HFGoe_FFH_20S_25_1_A1_2h_R1 **Scan** 33604
**Peptide** KLKFYDVLLVDTAGRLHVDEAMM **Charge** 4 **Spectral Angle** 0.69

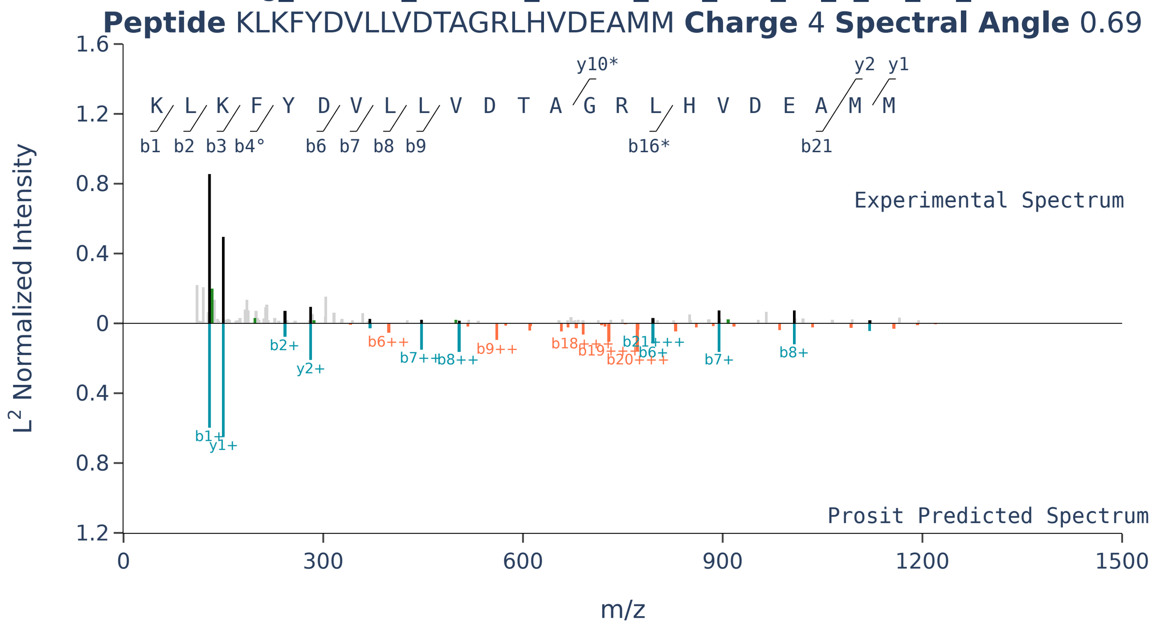


**Source** Ncheng_210623_230623_HFGoe_FFH_20S_25_1_A1_2h_R2 **Scan** 27388
**Peptide** ASLMGKLPGMGQIPDNVKSQMDDKVLVR **Charge** 4 **Spectral Angle** 0.68

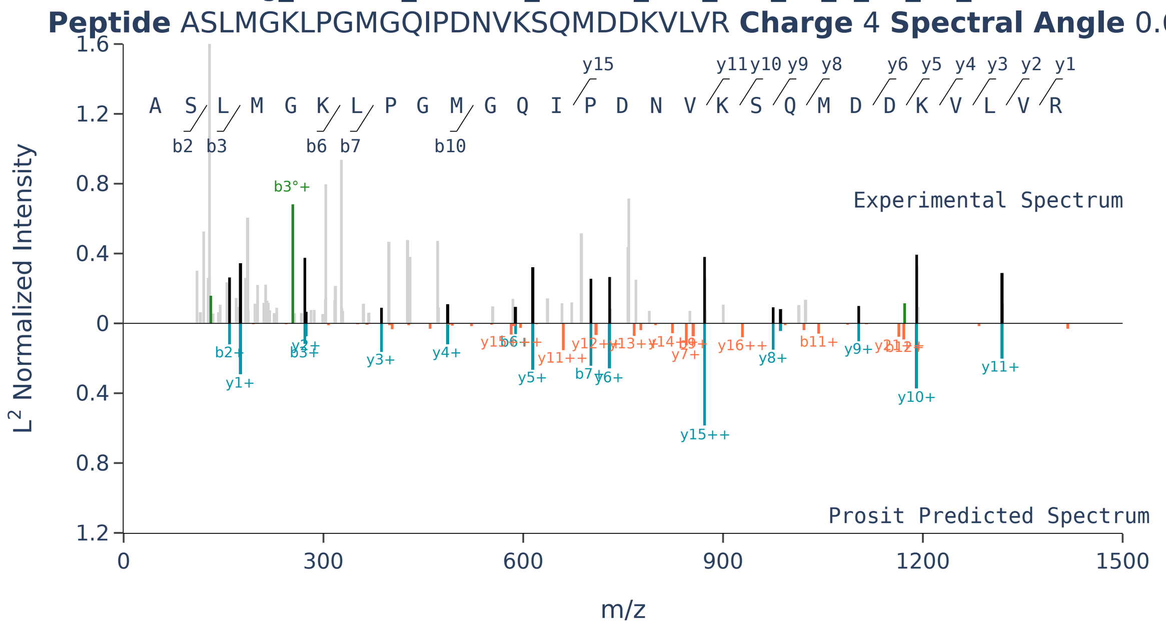


**Source** Ncheng_210623_230623_HFGoe_FFH_20S_25_1_A2_4h_R1 **Scan** 23459
**Peptide** DGDARGGAALSIRHITGKPIKFL **Charge** 3 **Spectral Angle** 0.55

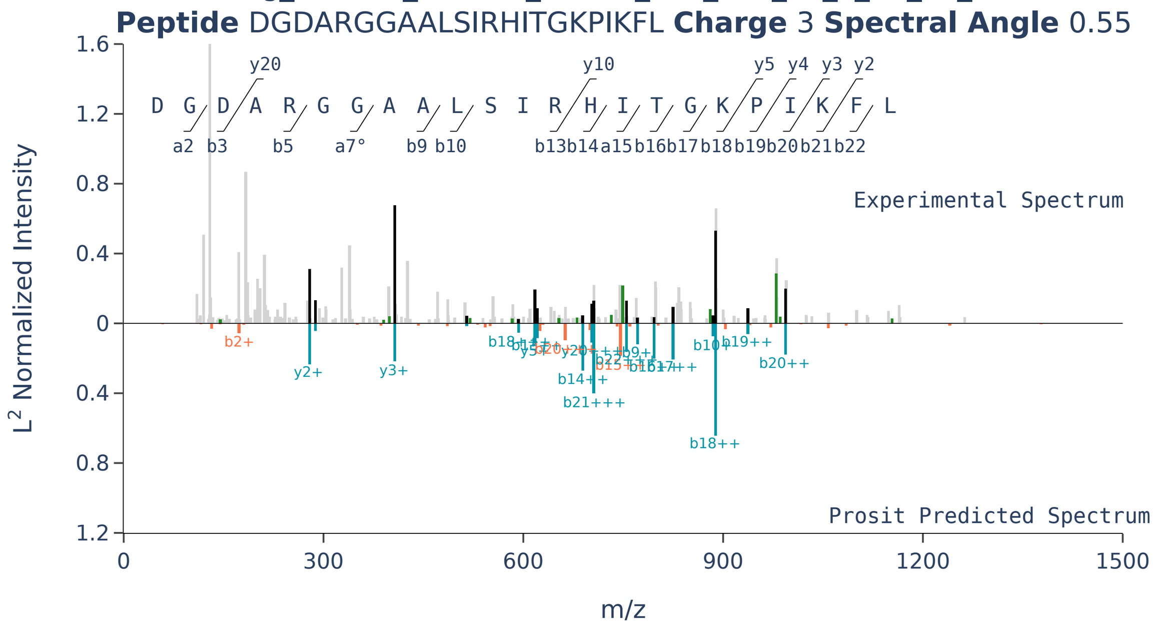


**Source** Ncheng_210623_230623_HFGoe_FFH_20S_25_1_A2_2h_R2 **Scan** 21386
**Peptide** DLESKVDRAQAEKLASKLKKGDGFDLND **Charge** 3 **Spectral Angle** 0.64

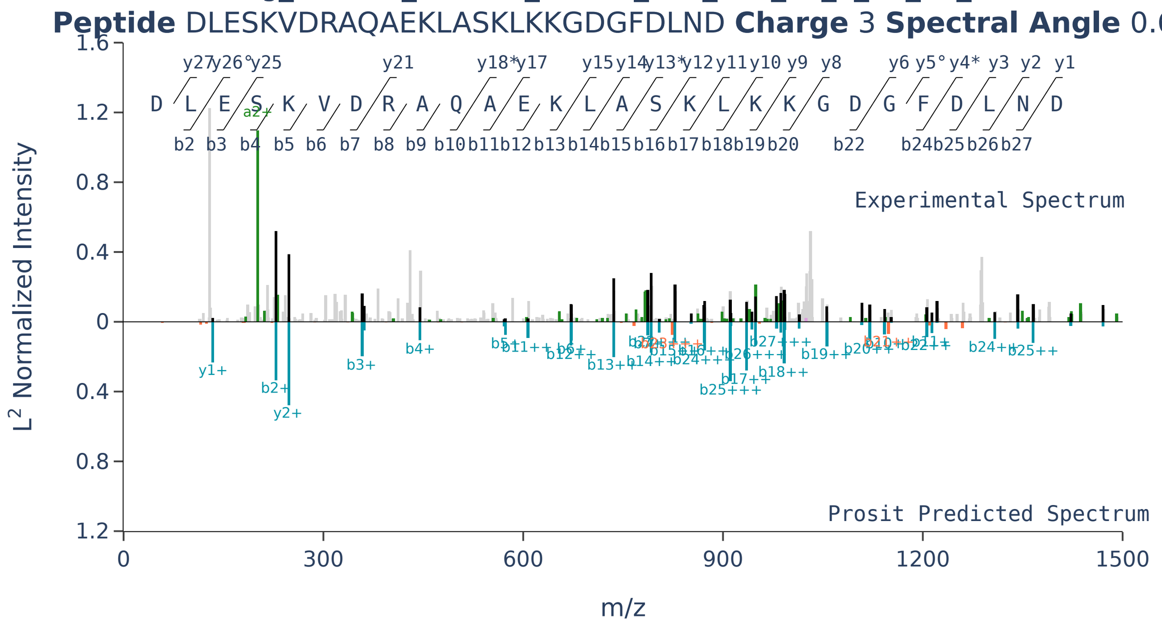


**Source** Ncheng_210623_230623_HFGoe_FFH_20S_25_1_A2_4h_R2 **Scan** 10755
**Peptide** FINRVKEKAVGHEVNKSLTPGQ **Charge** 3 **Spectral Angle** 0.75

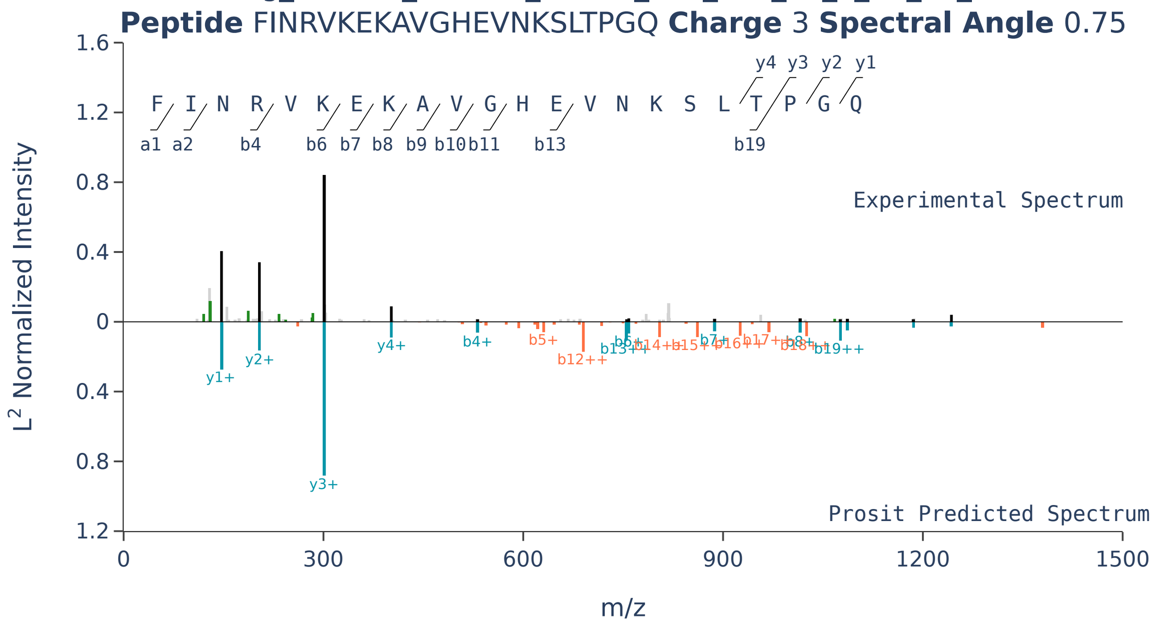


**Source** Ncheng_210623_230623_HFGoe_FFH_20S_25_1_A2_24h_R2 **Scan** 13496
**Peptide** EDIESKVDRAQAEKLAS **Charge** 2 **Spectral Angle** 0.62

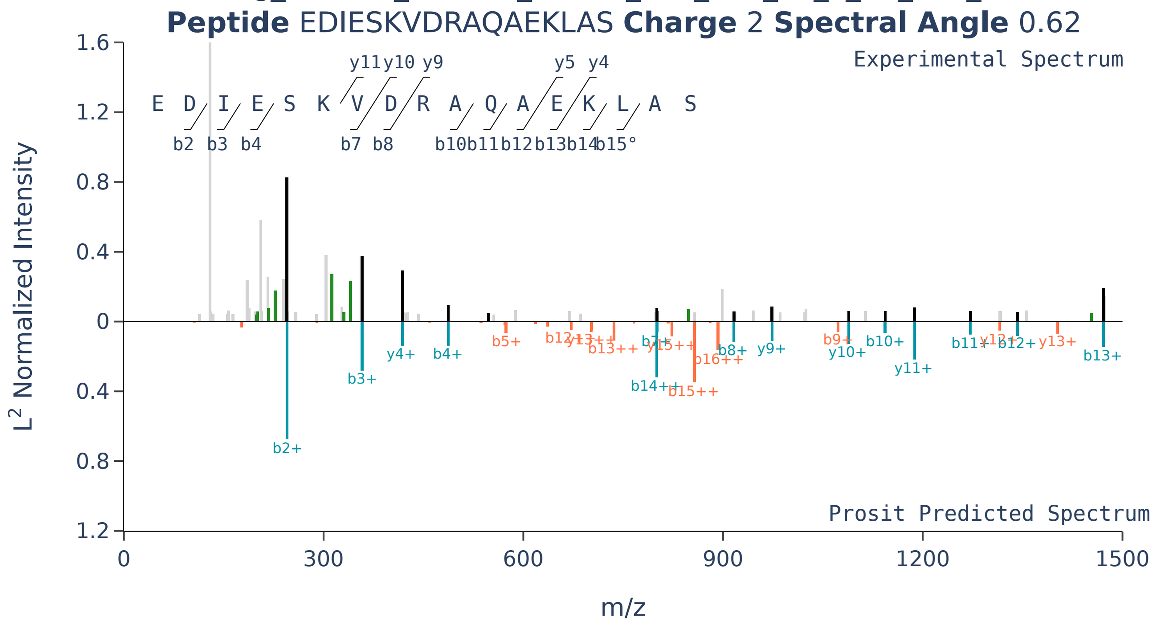


**Source** Ncheng_210623_230623_HFGoe_FFH_20S_25_1_A1_2h_R1 **Scan** 35240
**Peptide** NLAAQPPAVVLMAGLQGAGKTT **Charge** 2 **Spectral Angle** 0.83

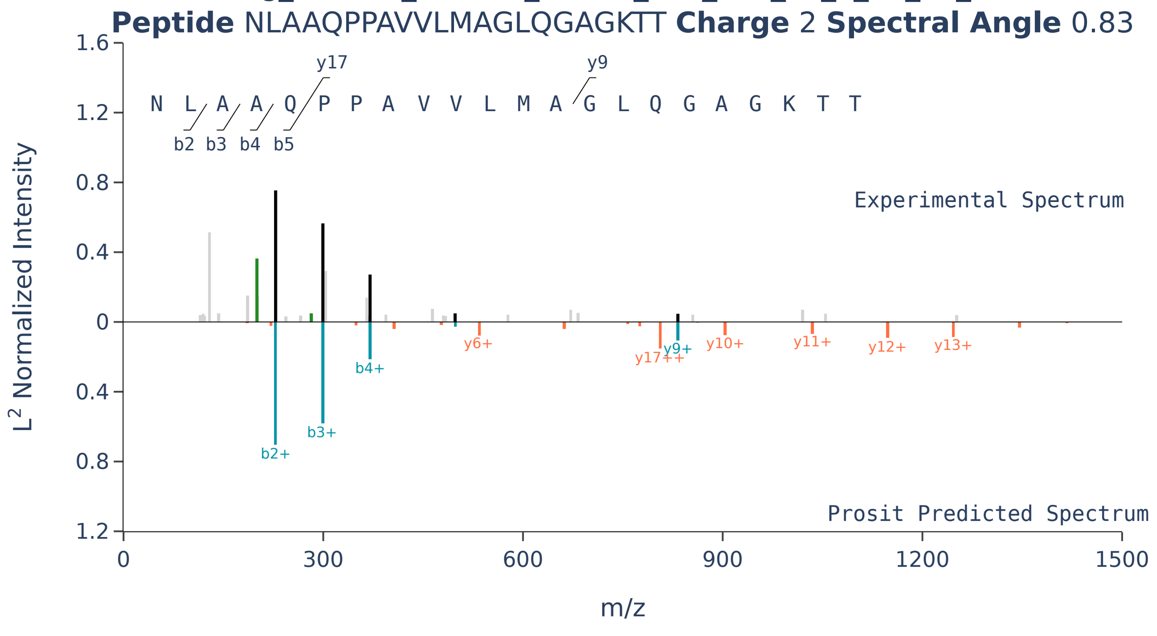


**Source** Ncheng_210623_230623_HFGoe_FFH_20S_25_1_A2_24h_R2 **Scan** 14205
**Peptide** EALINSMTMKERAKPEIIKGSRK **Charge** 5 **Spectral Angle** 0.51

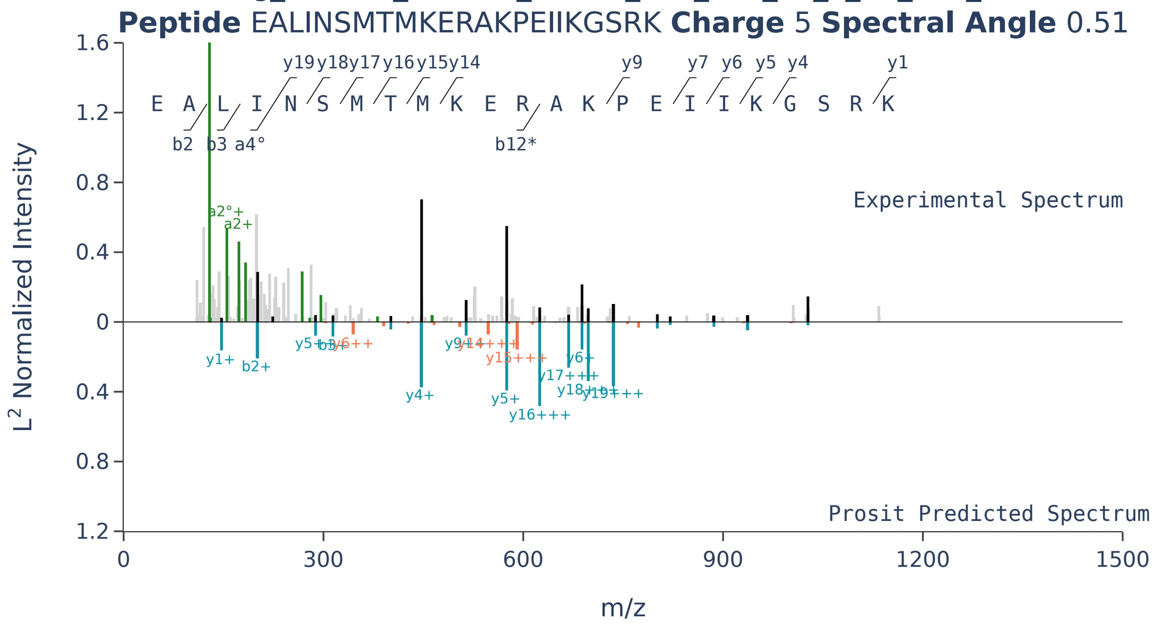


**Source** Ncheng_210623_230623_HFGoe_FFH_20S_25_1_A1_2h_R1 **Scan** 24913
**Peptide** MFDNLTDRLSRTLRN **Charge** 4 **Spectral Angle** 0.47

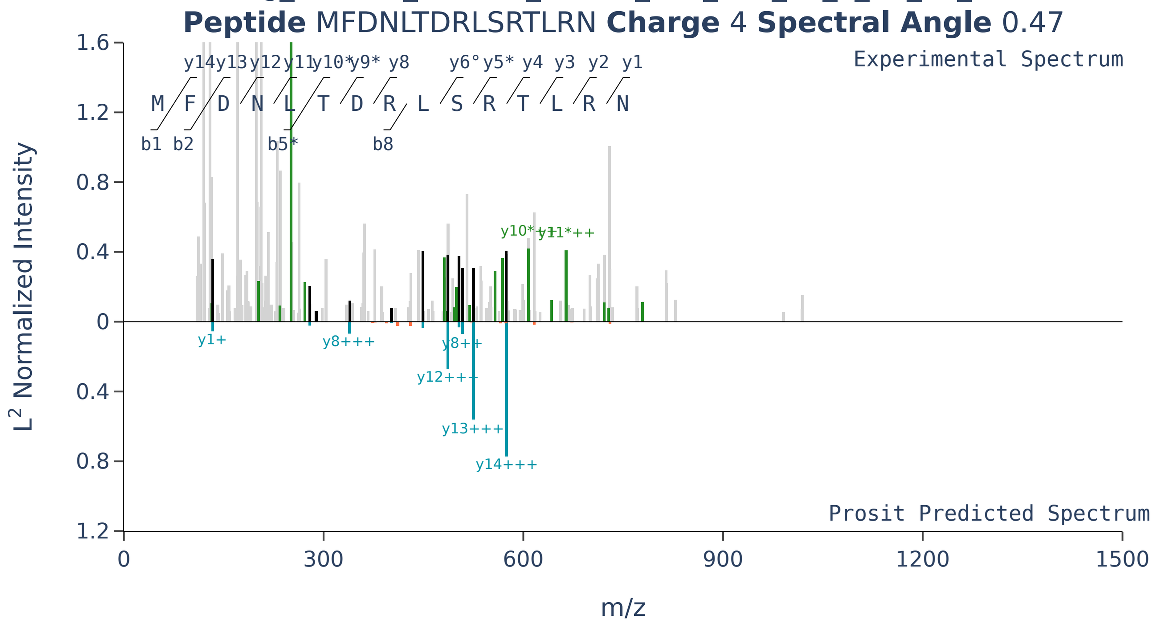

**Source** Ncheng_210623_230623_HFGoe_FFH_20S_25_1_A2_2h_R2 **Scan** 30244
**Peptide** NEALPLTGVVLTKVDGDARGGAALSIRHL **Charge** 4 **Spectral Angle** 0.69

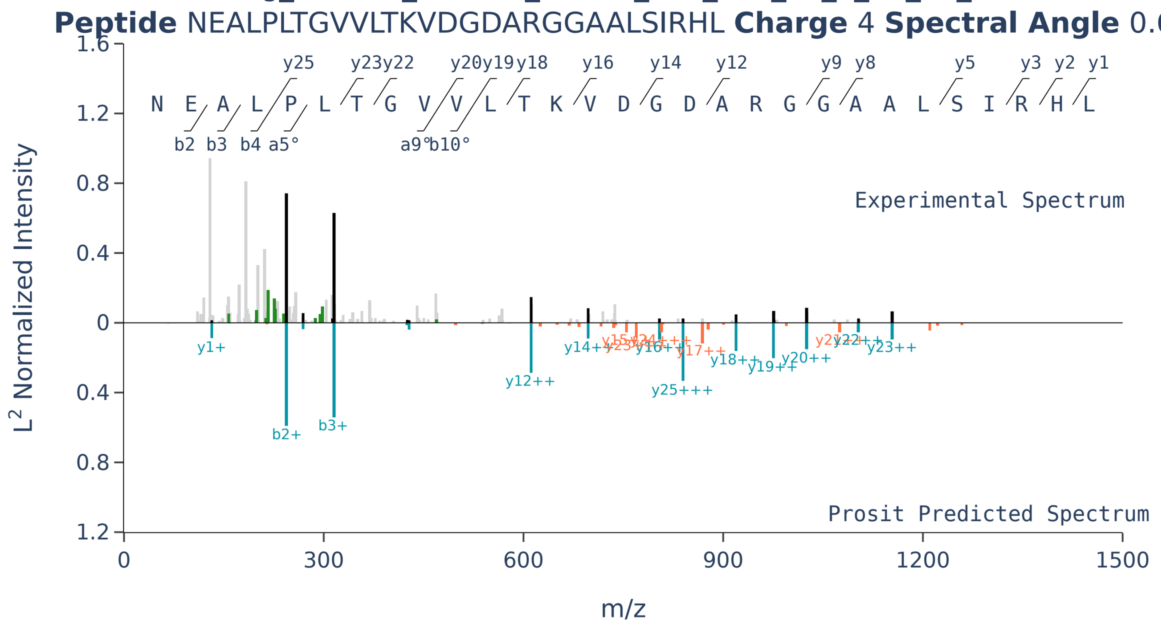

**Source** Ncheng_210623_230623_HFGoe_FFH_20S_25_1_A2_2h_R1 **Scan** 19552
**Peptide** HASINPVETL **Charge** 2 **Spectral Angle** 0.54

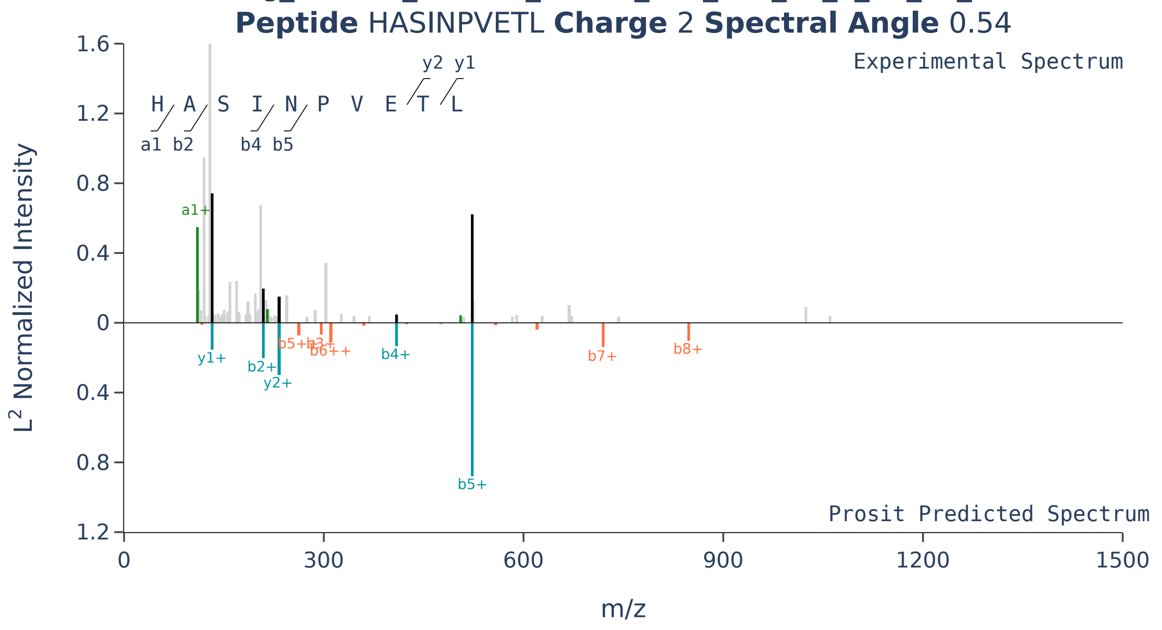

**Source** Ncheng_210623_230623_HFGoe_FFH_20S_25_1_A1_2h_R2 **Scan** 13276
**Peptide** RLSRTLRNISGRGRLTEDNVKDTLRE **Charge** 6 **Spectral Angle** 0.74

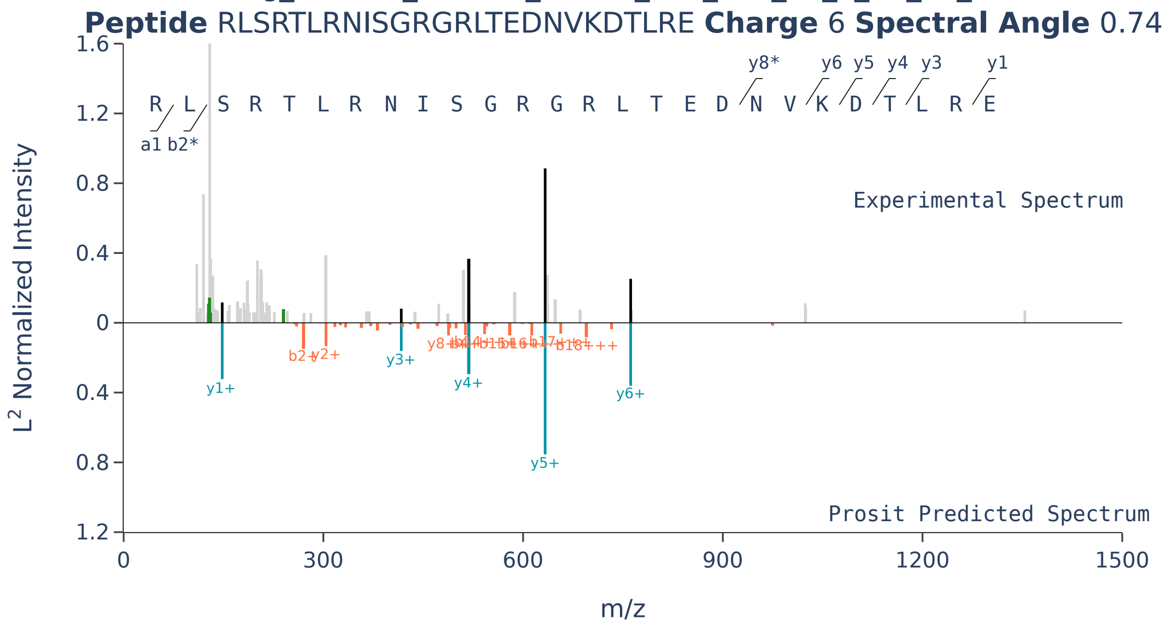

**Source** Ncheng_210623_230623_HFGoe_FFH_20S_25_1_A2_4h_R1 **Scan** 20643
**Peptide** TGVVLTKVDGDARGGAALSIRHL **Charge** 4 **Spectral Angle** 0.55

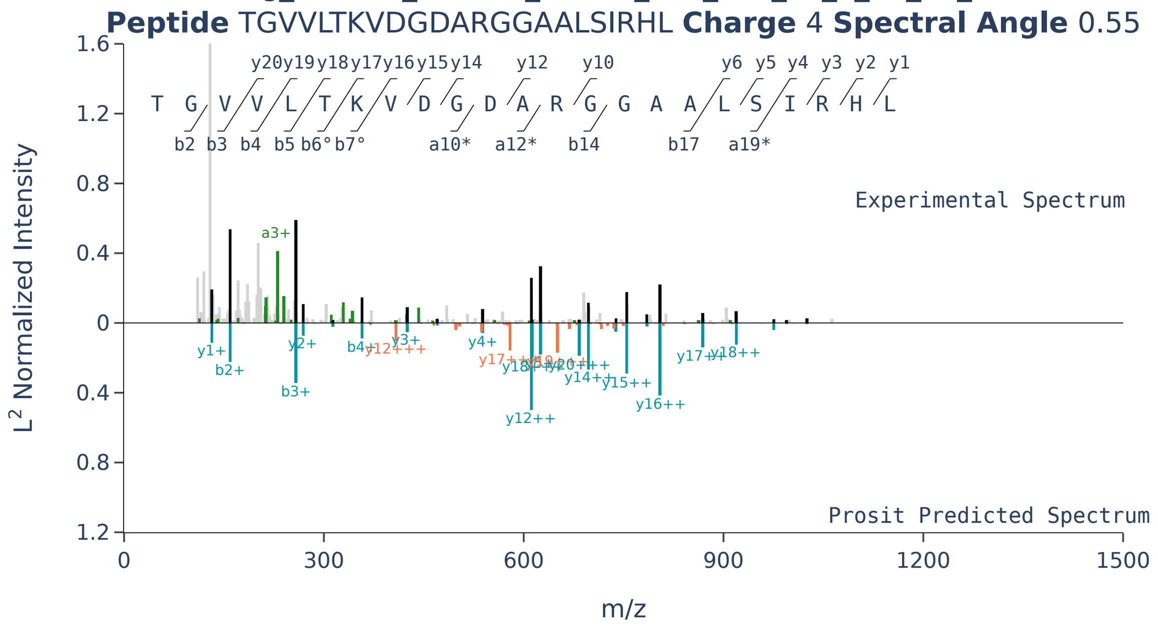

**Source** Ncheng_210623_230623_HFGoe_FFH_20S_25_1_A2_4h_R2 **Scan** 20554
**Peptide** LEPFHPDRIASRL **Charge** 3 **Spectral Angle** 0.72

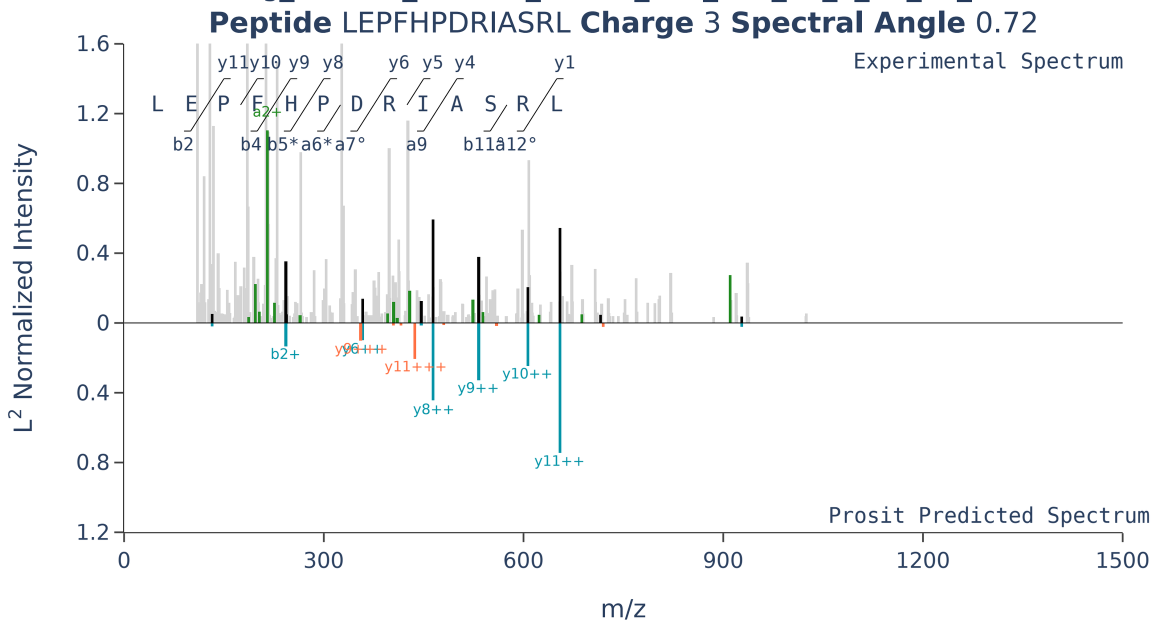

**Source** Ncheng_210623_230623_HFGoe_FFH_20S_25_1_A2_4h_R1 **Scan** 23530
**Peptide** VLLVDTAGRLHVD **Charge** 2 **Spectral Angle** 0.48

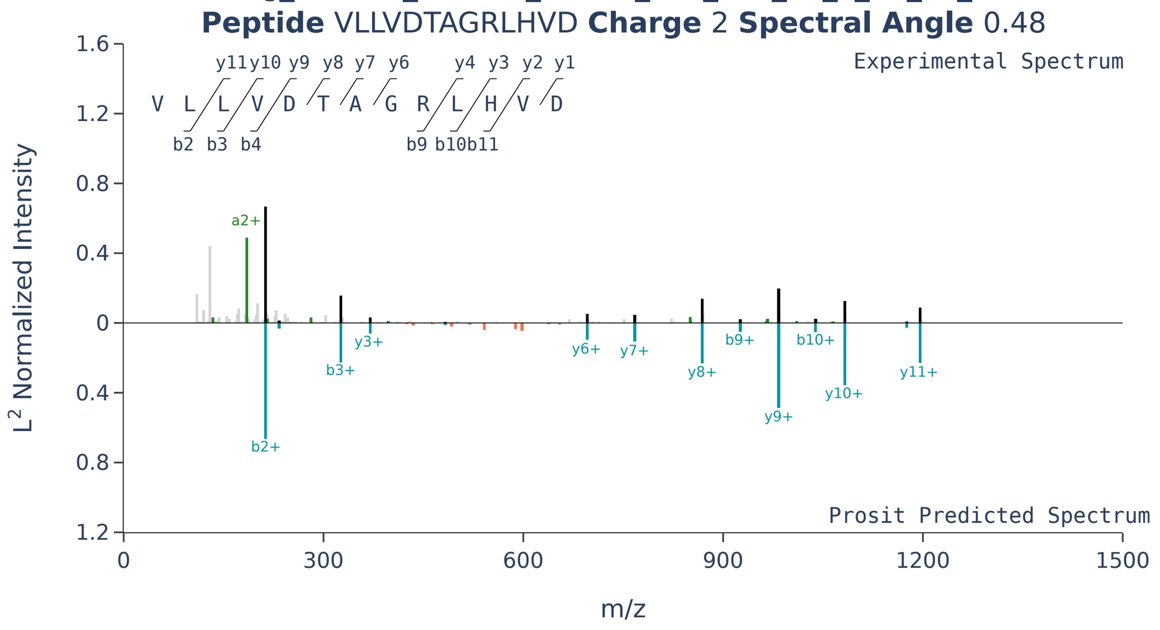

**Source** Ncheng_210623_230623_HFGoe_FFH_20S_25_1_A1_2h_R2 **Scan** 27993
**Peptide** KFYDVLLVDTAGRLH **Charge** 3 **Spectral Angle** 0.35

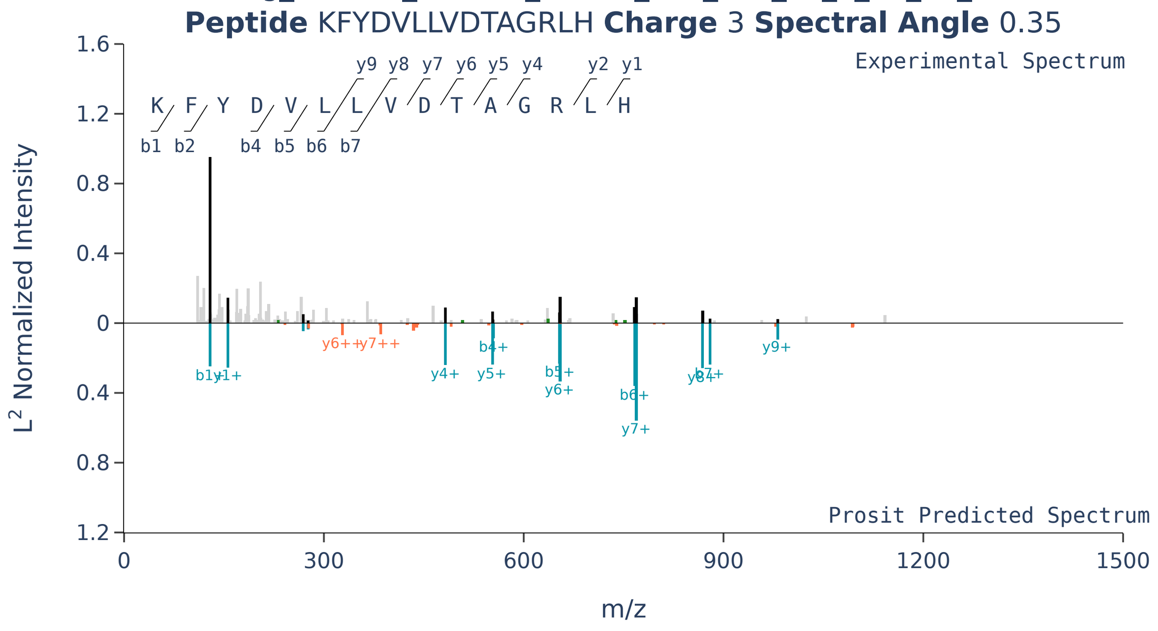

**Source** Ncheng_210623_230623_HFGoe_FFH_20S_25_1_A1_2h_R1 **Scan** 15218
**Peptide** DIESKVDRAQAEKLASKLKK **Charge** 6 **Spectral Angle** 0.8

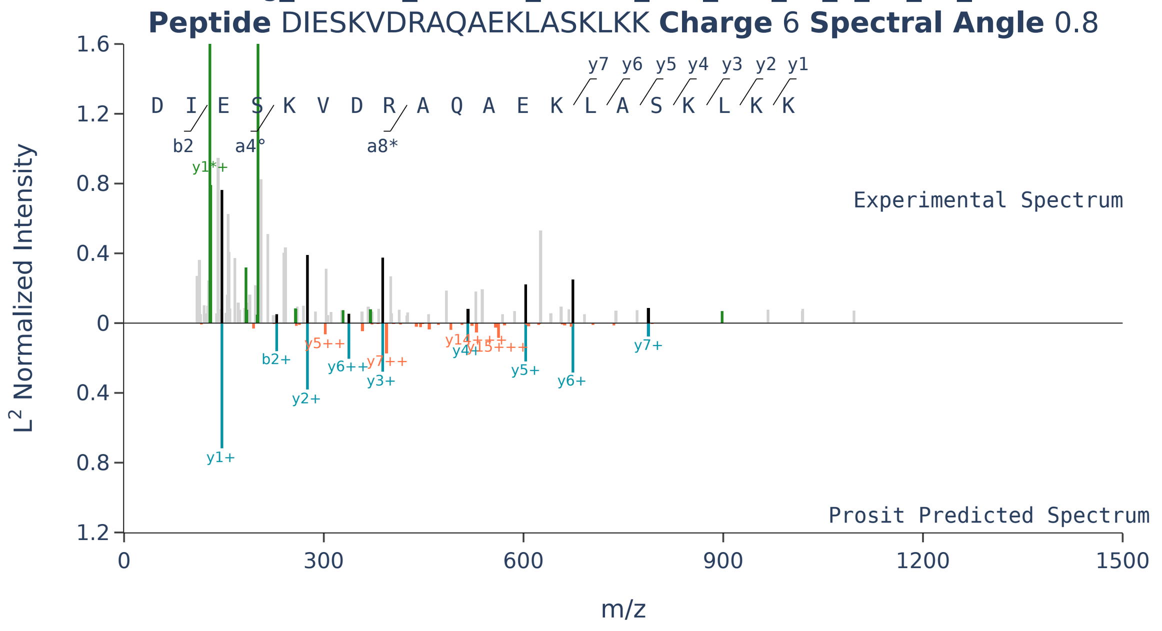

**Source** Ncheng_210623_230623_HFGoe_FFH_20S_25_1_A2_2h_R2 **Scan** 26115
**Peptide** LLVDTAGRLHVDEAMM **Charge** 3 **Spectral Angle** 0.63

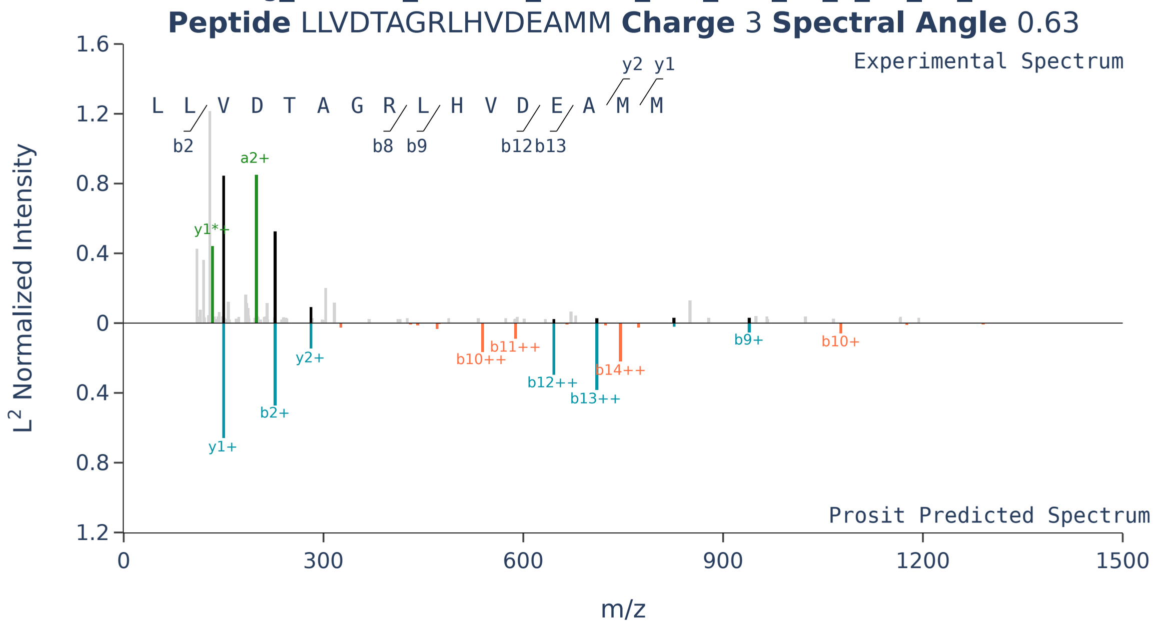

**Source** Ncheng_210623_230623_HFGoe_FFH_20S_25_1_A1_1h_R2 **Scan** 29015
**Peptide** SLMGKLPGMGQIPDNVKSQMDDKVLV **Charge** 4 **Spectral Angle** 0.69

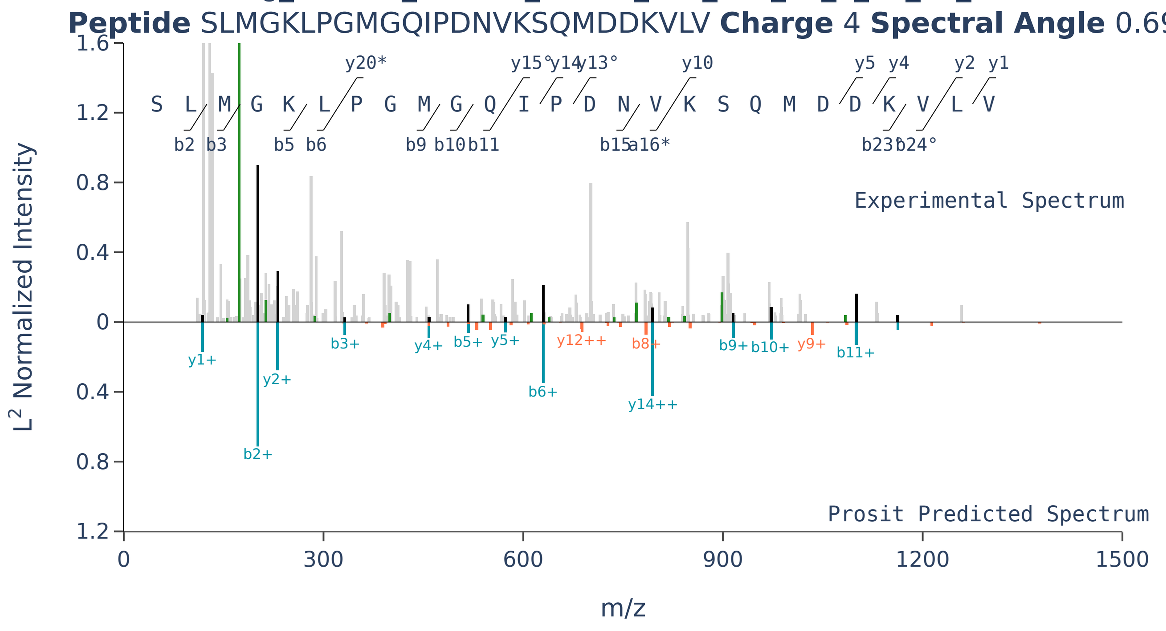

**Source** Ncheng_210623_230623_HFGoe_FFH_20S_25_1_A2_24h_R2 **Scan** 13646
**Peptide** LASKLKKGDGFDLND **Charge** 3 **Spectral Angle** 0.45

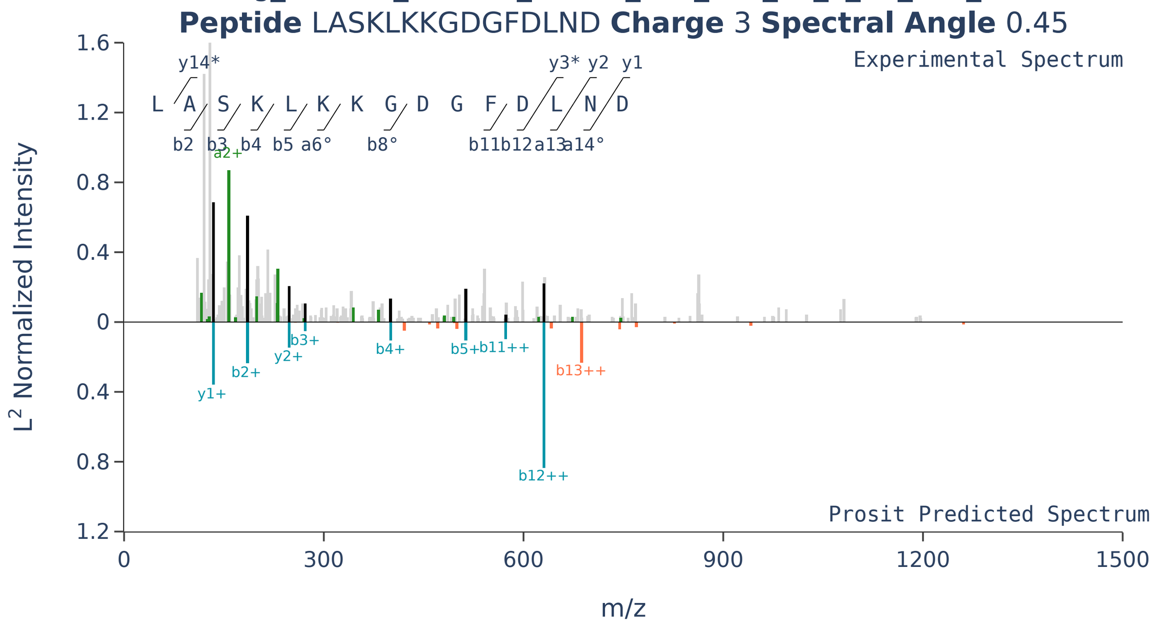

**Source** Ncheng_210623_230623_HFGoe_FFH_20S_25_1_A1_2h_R1 **Scan** 21927
**Peptide** KSQMDDKVLVRMEAI **Charge** 3 **Spectral Angle** 0.58

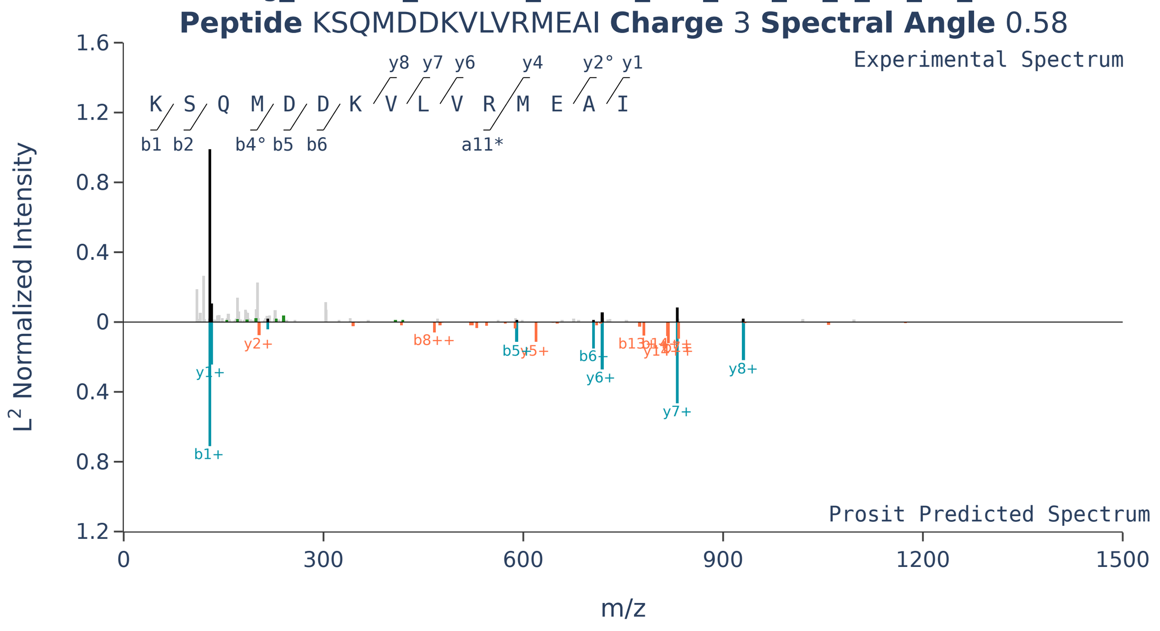

**Source** Ncheng_210623_230623_HFGoe_FFH_20S_25_1_A1_2h_R2 **Scan** 14943
**Peptide** VLTKVDGDARGGAALSIR **Charge** 3 **Spectral Angle** 0.51

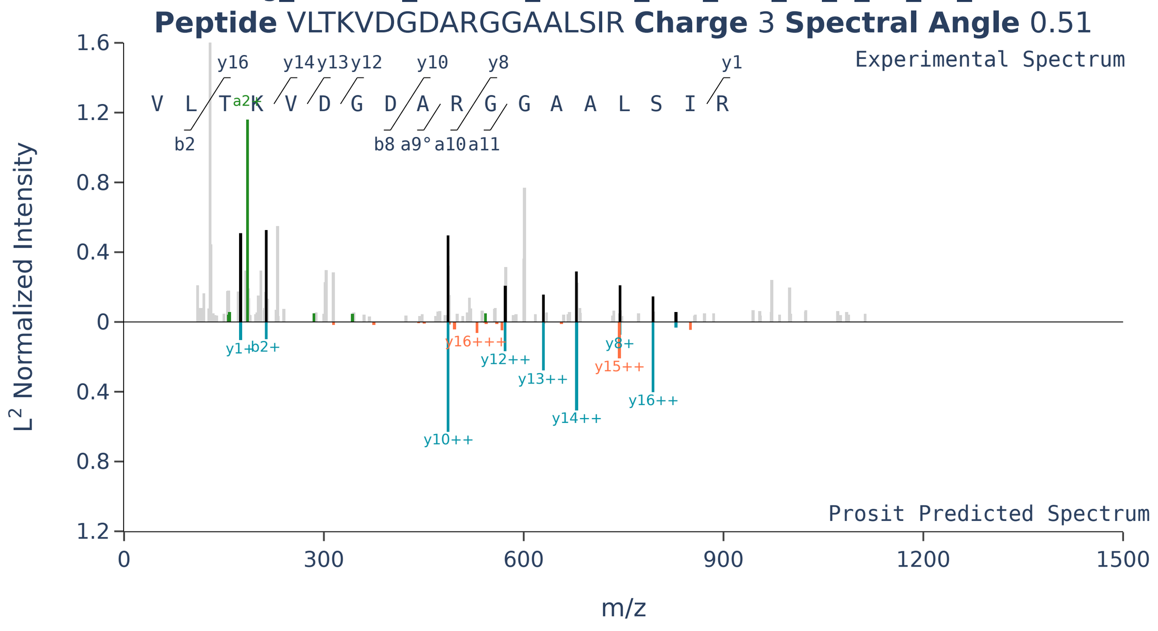

**Source** Ncheng_210623_230623_HFGoe_FFH_20S_25_1_A2_2h_R1 **Scan** 30601
**Peptide** KLKFYDVLLVDTA **Charge** 3 **Spectral Angle** 0.49

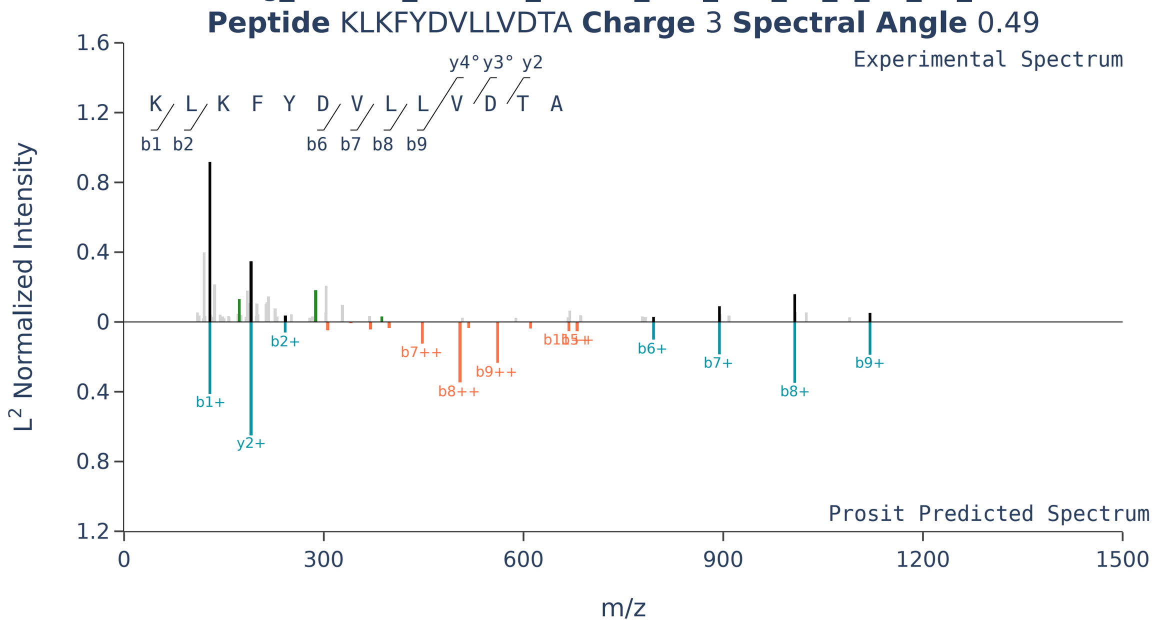

**Source** Ncheng_210623_230623_HFGoe_FFH_20S_25_1_A2_24h_R2 **Scan** 11459
**Peptide** KVDRAQAEKLASKLK **Charge** 3 **Spectral Angle** 0.71

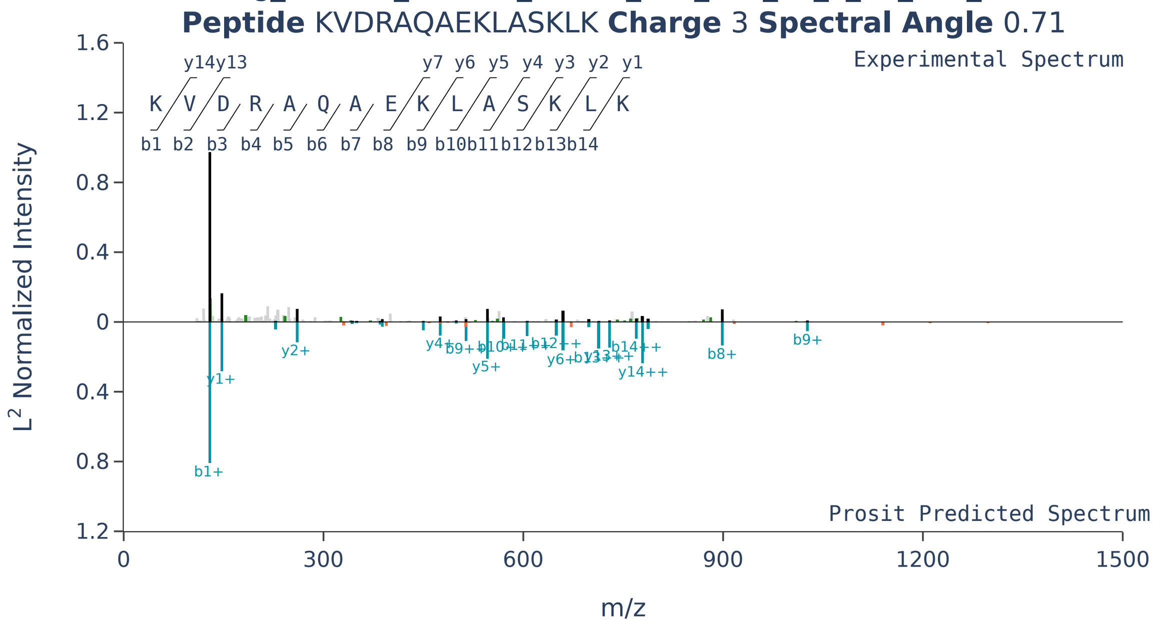

**Source** Ncheng_210623_230623_HFGoe_FFH_20S_25_1_A1_24h_R1 **Scan** 7358
**Peptide** EKHKKKVLVV **Charge** 2 **Spectral Angle** 0.56

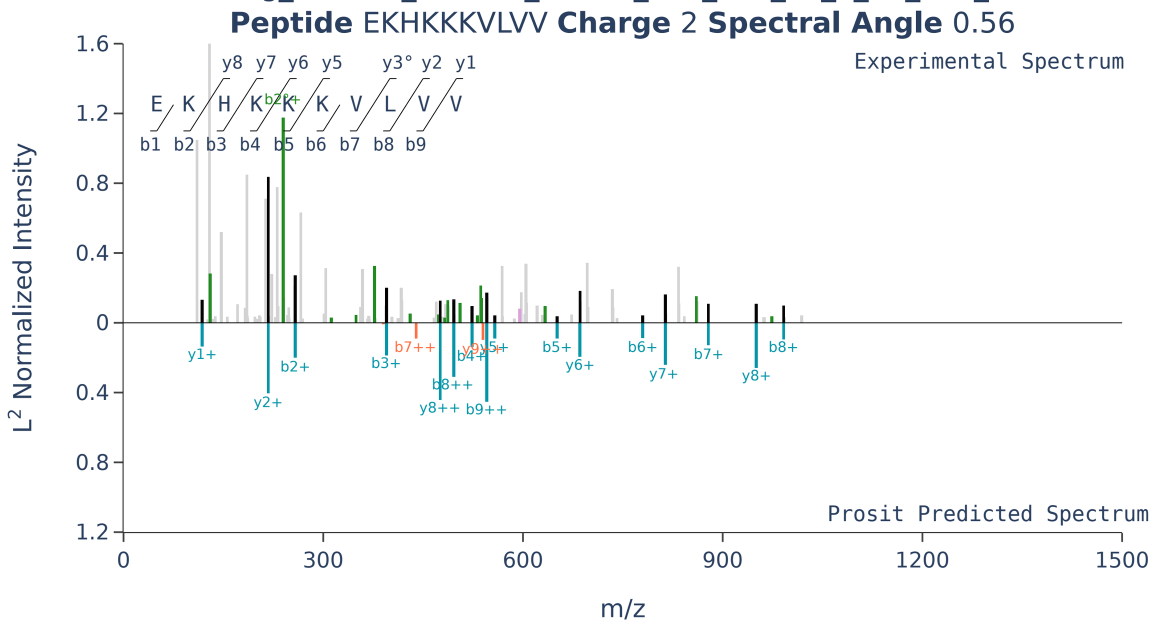


**Source** Ncheng_210623_230623_HFGoe_FFH_20S_25_1_A1_2h_R1 **Scan** 21423
**Peptide** QLPDNVKSQMDDKVLVRM **Charge** 4 **Spectral Angle** 0.6

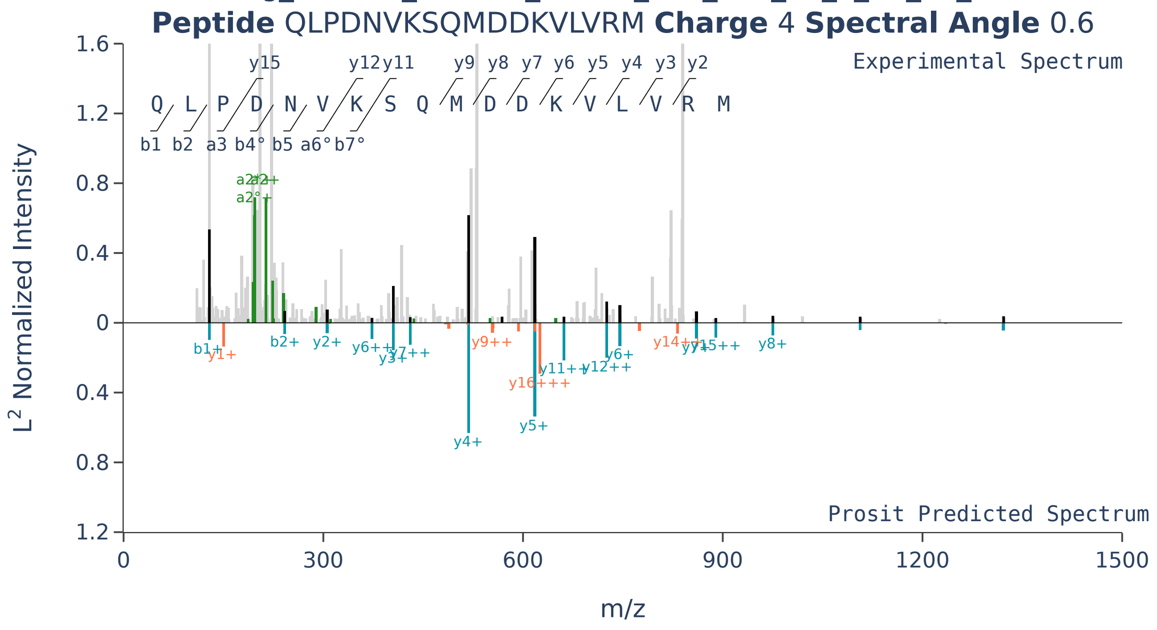


**Source** Ncheng_210623_230623_HFGoe_FFH_20S_25_1_A1_2h_R1 **Scan** 28531
**Peptide** SGRGRLTEDNVKDTLREVRMALLE **Charge** 2 **Spectral Angle** 0.64

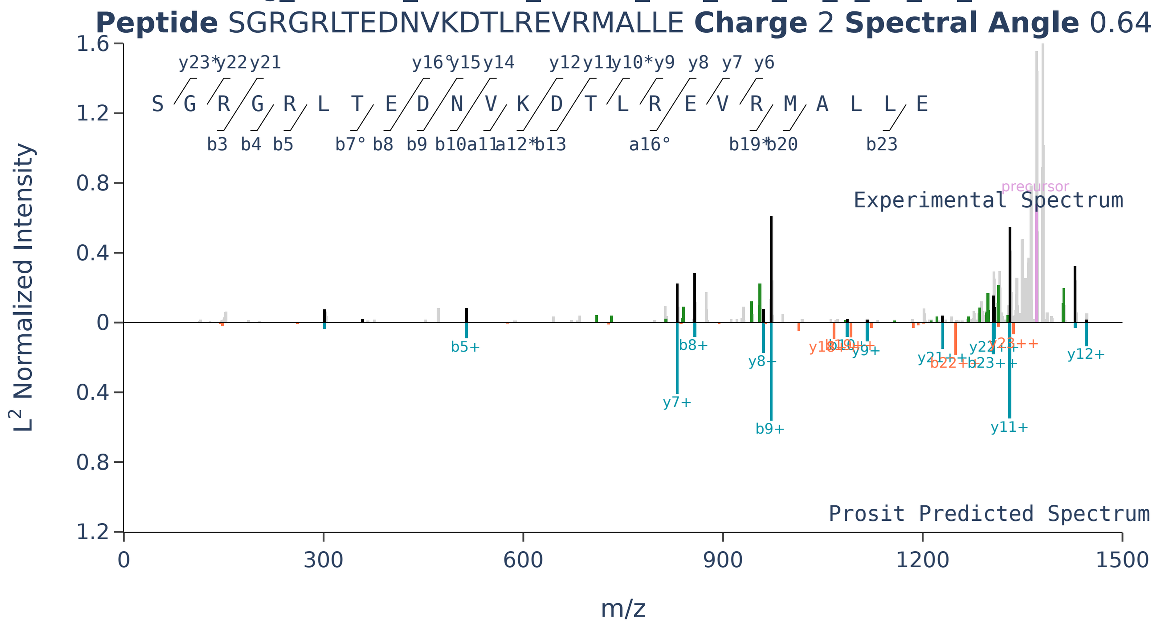


**Source** Ncheng_210623_230623_HFGoe_FFH_20S_25_1_A1_2h_R2 **Scan** 33156
**Peptide** EIKQVHASINPVETLFVVD **Charge** 2 **Spectral Angle** 0.55

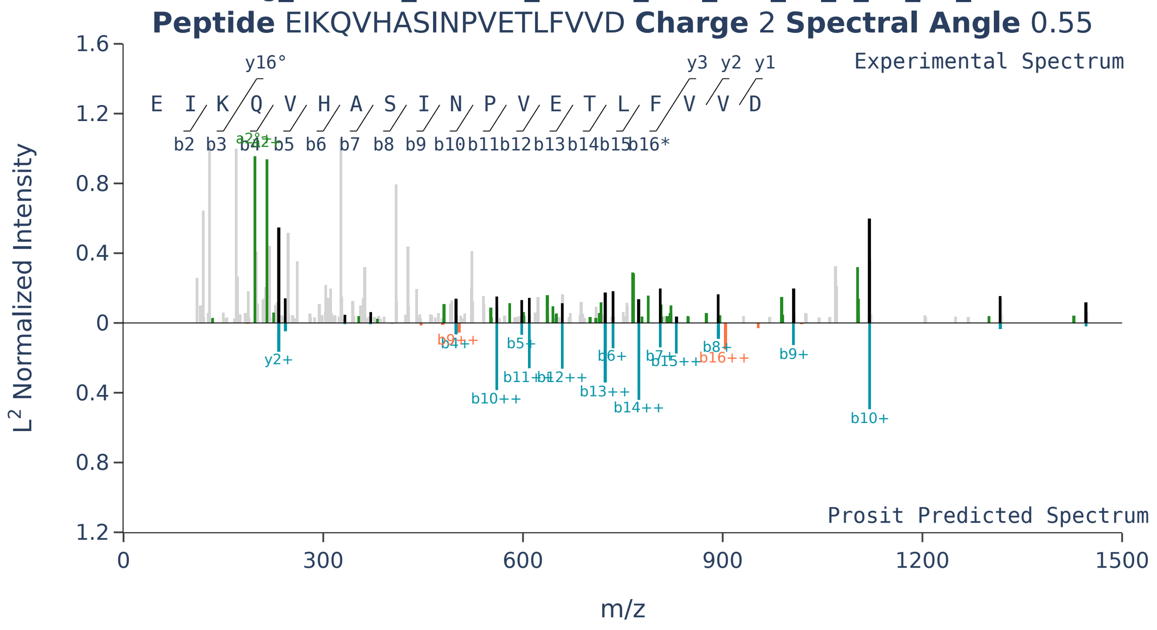


**Source** Ncheng_210623_230623_HFGoe_FFH_20S_25_1_A1_2h_R2 **Scan** 26152
**Peptide** DVLLVDTAGRLHVDE **Charge** 2 **Spectral Angle** 0.62

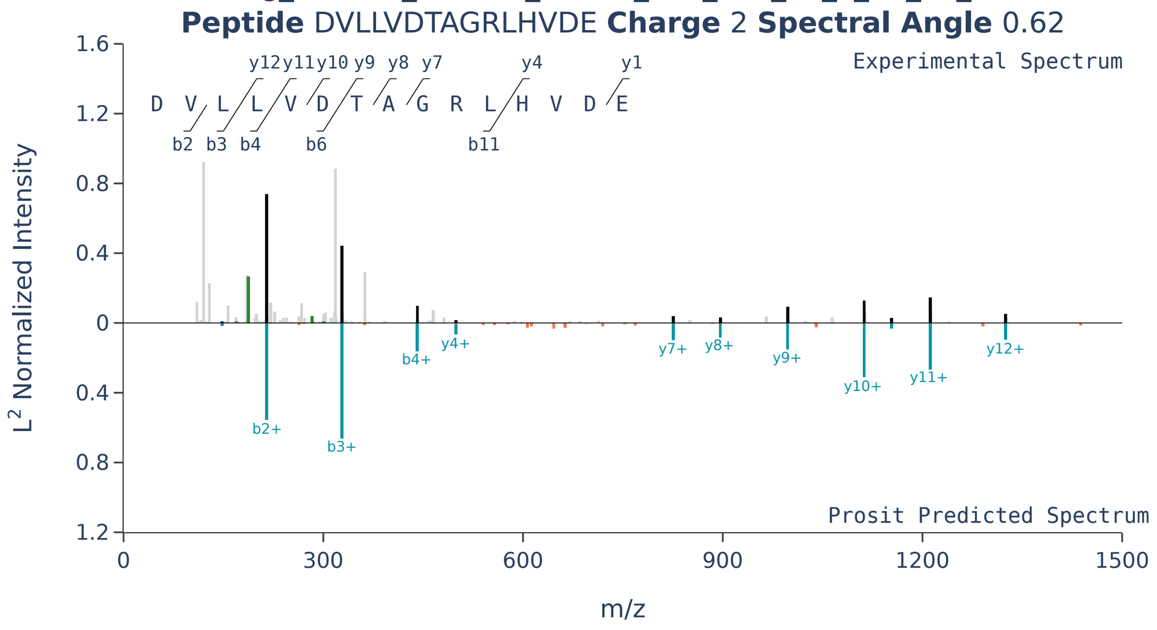


**Source** Ncheng_210623_230623_HFGoe_FFH_20S_25_1_A1_2h_R2 **Scan** 30781
**Peptide** ALEPFHPDRIASRILGM **Charge** 4 **Spectral Angle** 0.64

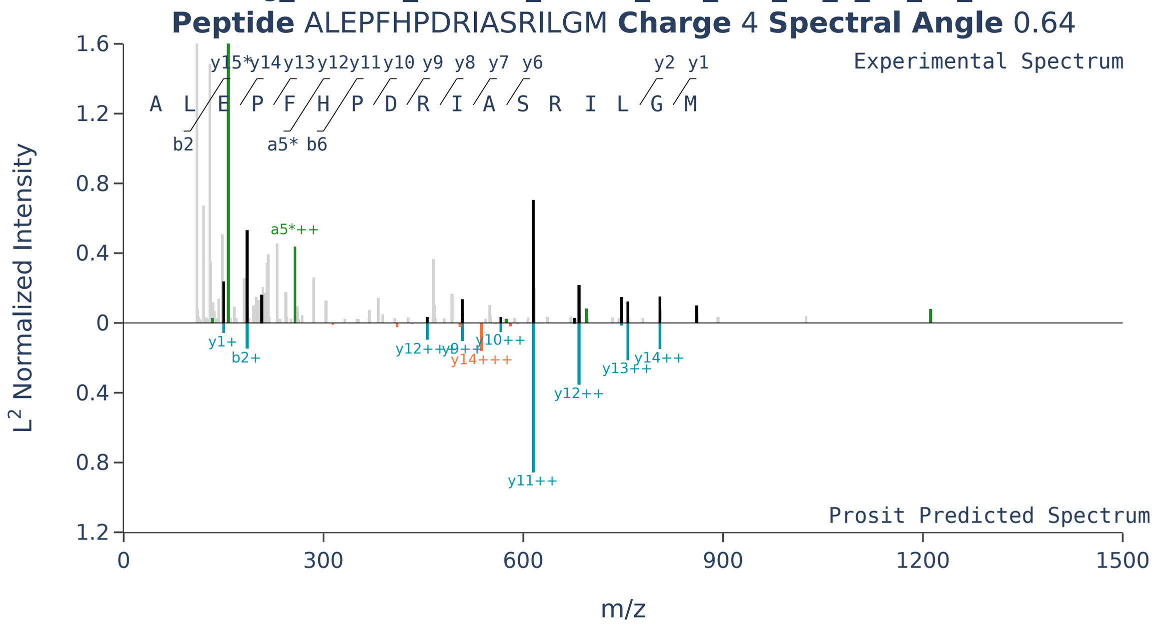


**Source** Ncheng_210623_230623_HFGoe_FFH_20S_25_1_A2_4h_R1 **Scan** 20187
**Peptide** EFINRVKEKAVGHEVNKSLTPGQEFVKI **Charge** 4 **Spectral Angle** 0.72

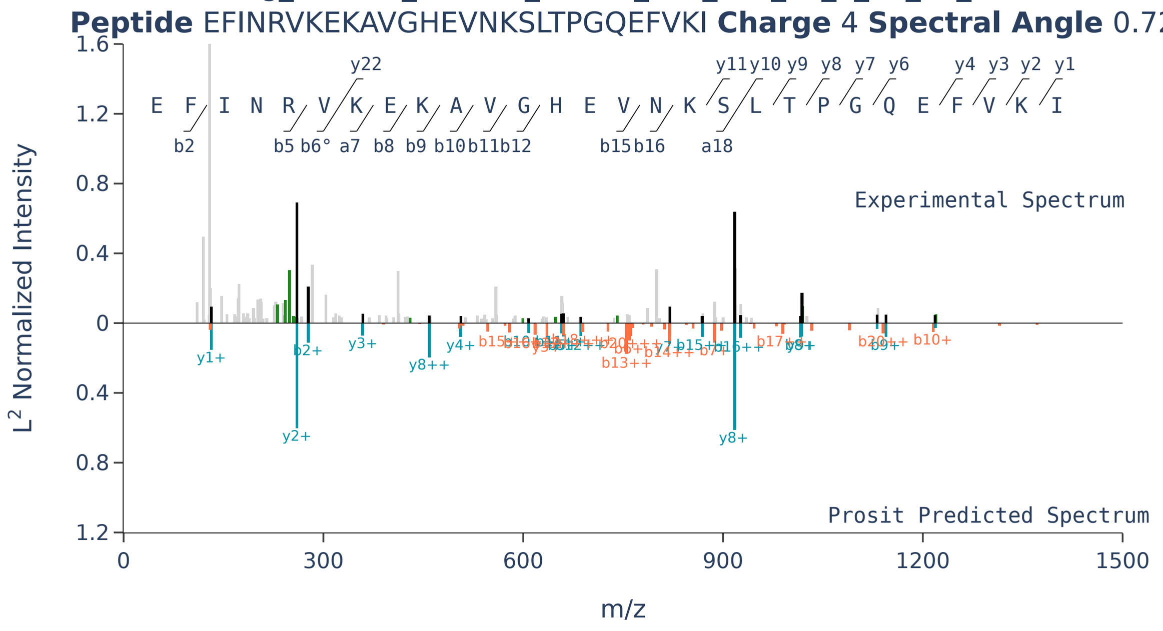


**Source** Ncheng_210623_230623_HFGoe_FFH_20S_25_1_A1_24h_R2 **Scan** 14113
**Peptide** EAIINSMTMKERAKPEIIKGSRK **Charge** 5 **Spectral Angle** 0.53

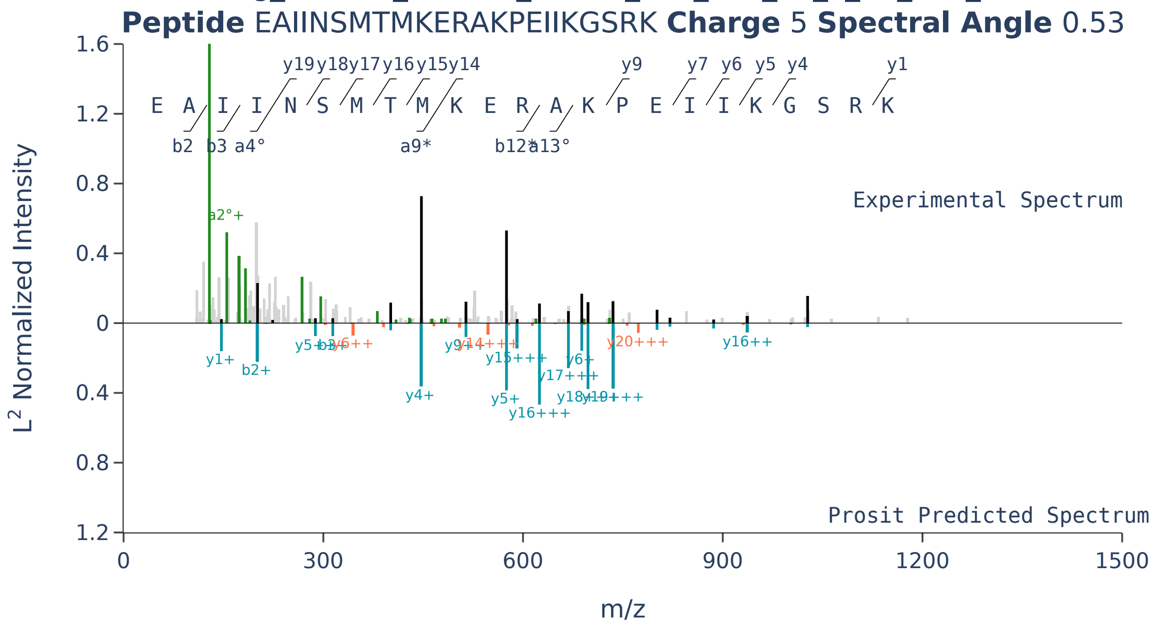


**Source** Ncheng_210623_230623_HFGoe_FFH_20S_25_1_A1_2h_R1 **Scan** 25250
**Peptide** LLVDTAGRLHVDEAMMD **Charge** 3 **Spectral Angle** 0.46

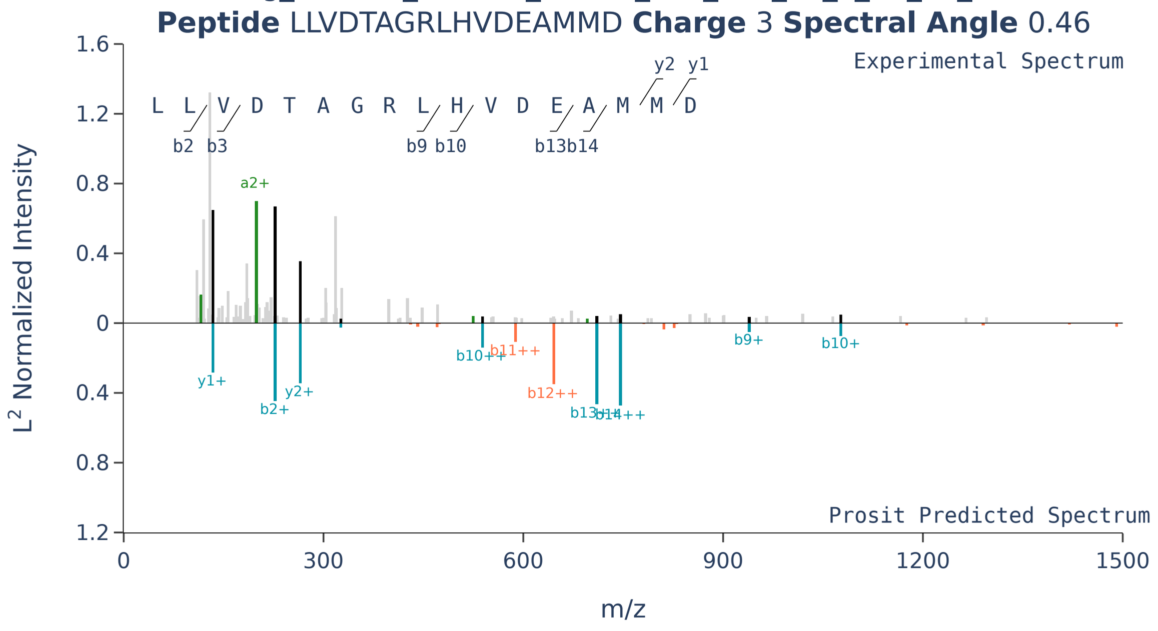


**Source** Ncheng_210623_230623_HFGoe_FFH_20S_25_1_A1_24h_R1 **Scan** 10556
**Peptide** LTKVDGDARGG **Charge** 2 **Spectral Angle** 0.76

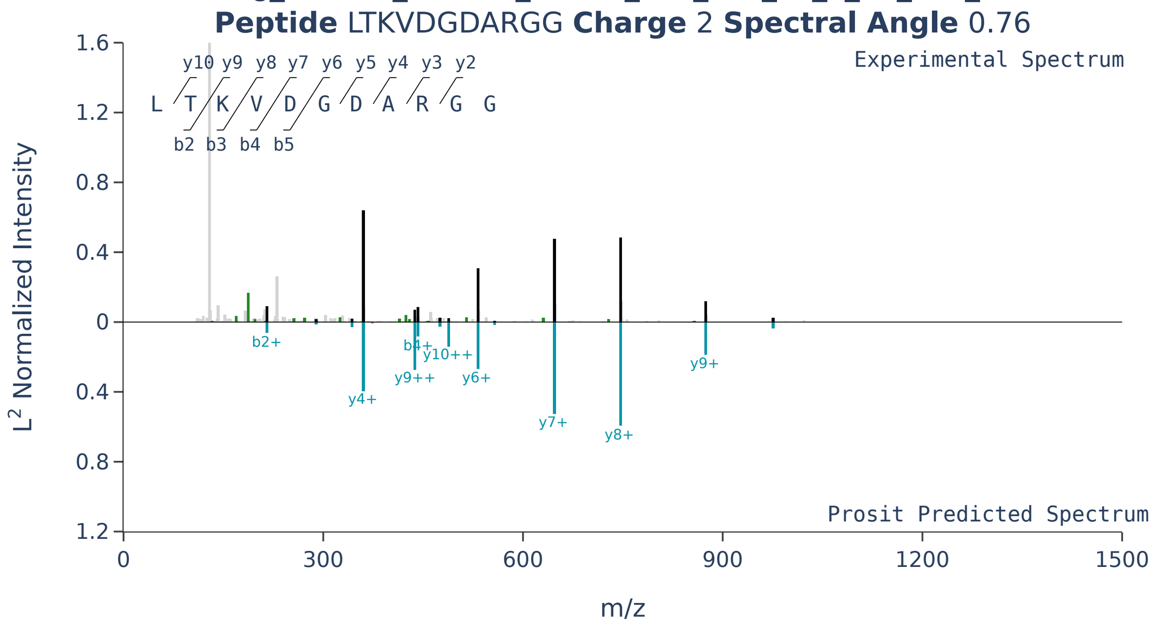


**Source** Ncheng_210623_230623_HFGoe_FFH_20S_25_1_A2_4h_R1 **Scan** 38255
**Peptide** AMTGQDAANTAKAFNEALPLTGVVL **Charge** 3 **Spectral Angle** 0.7

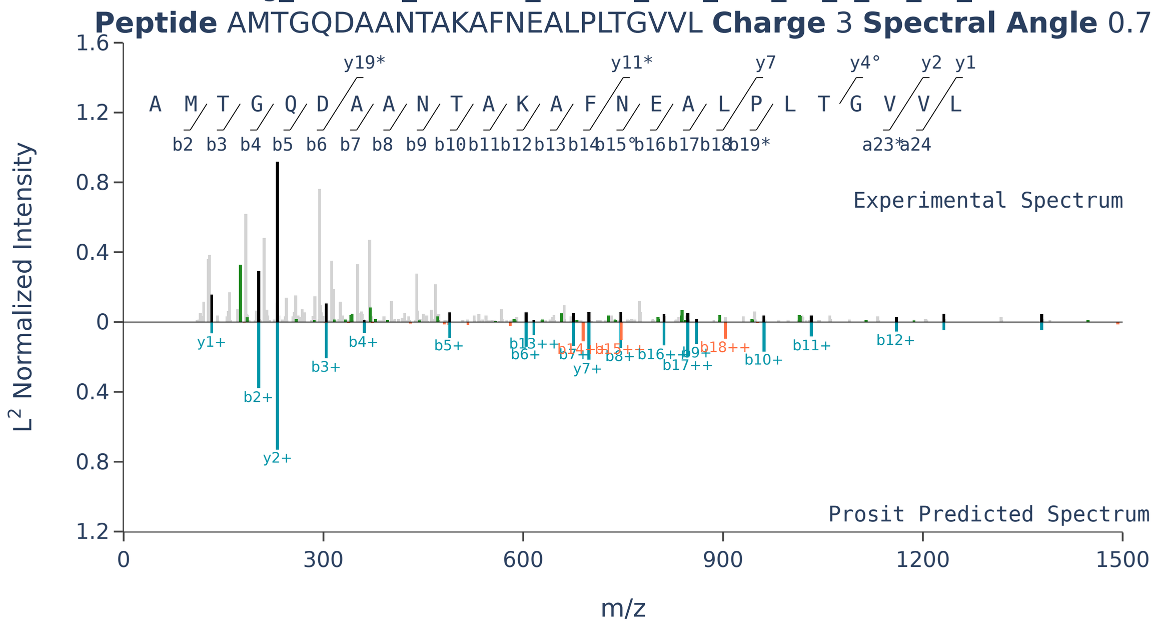


**Source** Ncheng_210623_230623_HFGoe_FFH_20S_25_1_A1_2h_R2 **Scan** 28280
**Peptide** DVLLVDTAGRLHV **Charge** 3 **Spectral Angle** 0.71

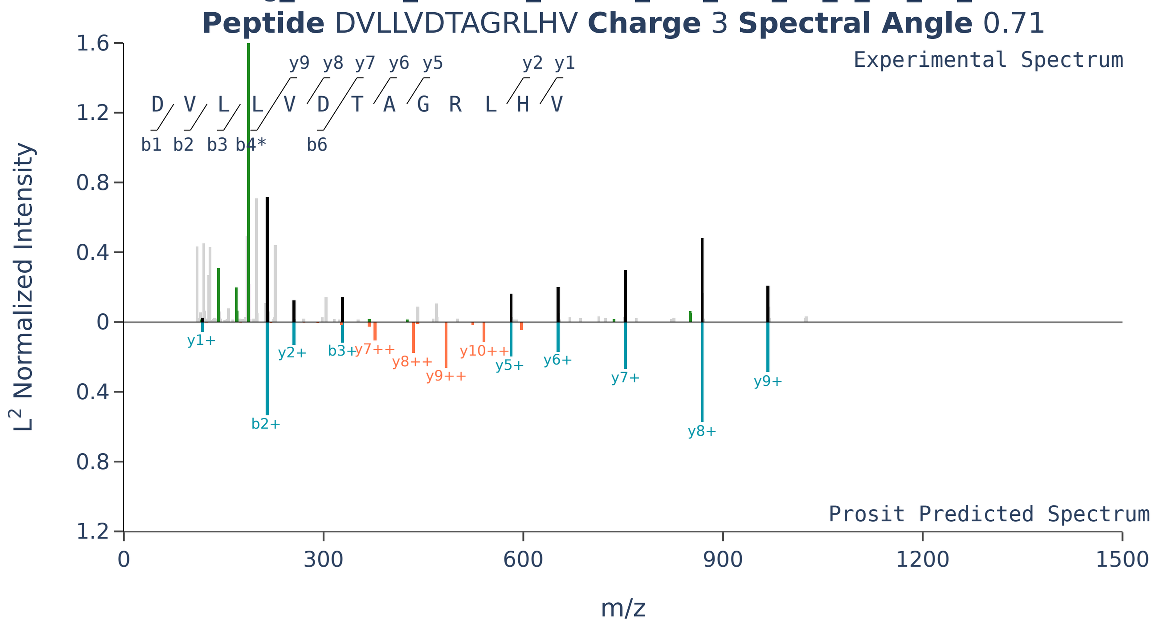


**Source** Ncheng_210623_230623_HFGoe_FFH_20S_25_1_A2_1h_R2 **Scan** 16748
**Peptide** LIEDIESKVDRA **Charge** 3 **Spectral Angle** 0.67

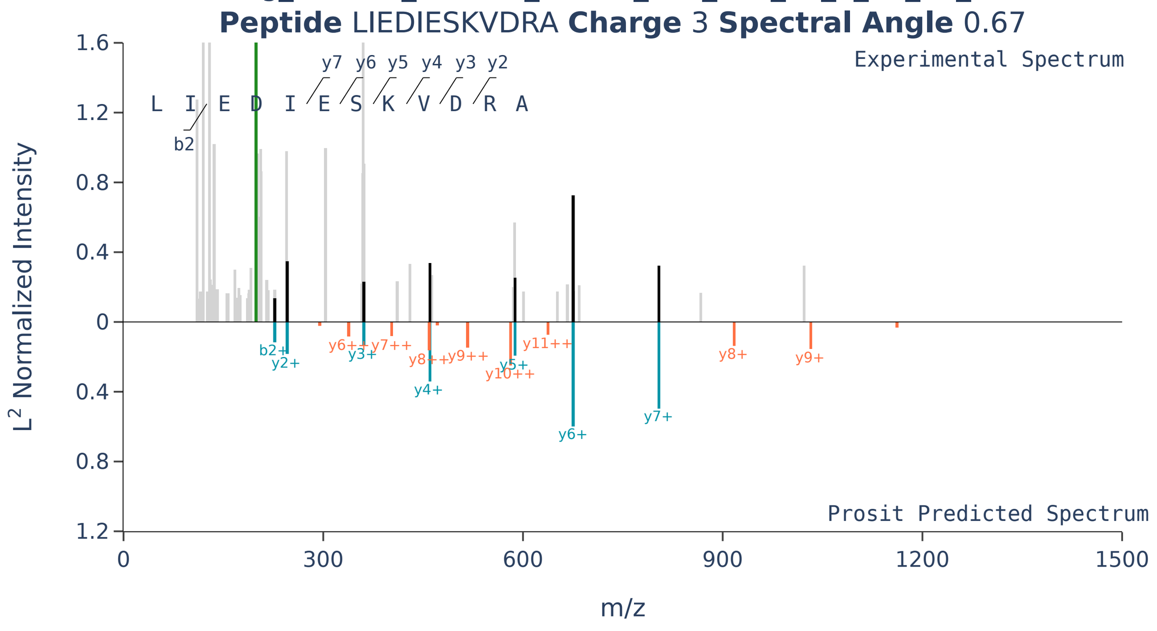


**Source** Ncheng_210623_230623_HFGoe_FFH_20S_25_1_A2_4h_R2 **Scan** 27638
**Peptide** PGQEFVKIVRNELV **Charge** 3 **Spectral Angle** 0.56

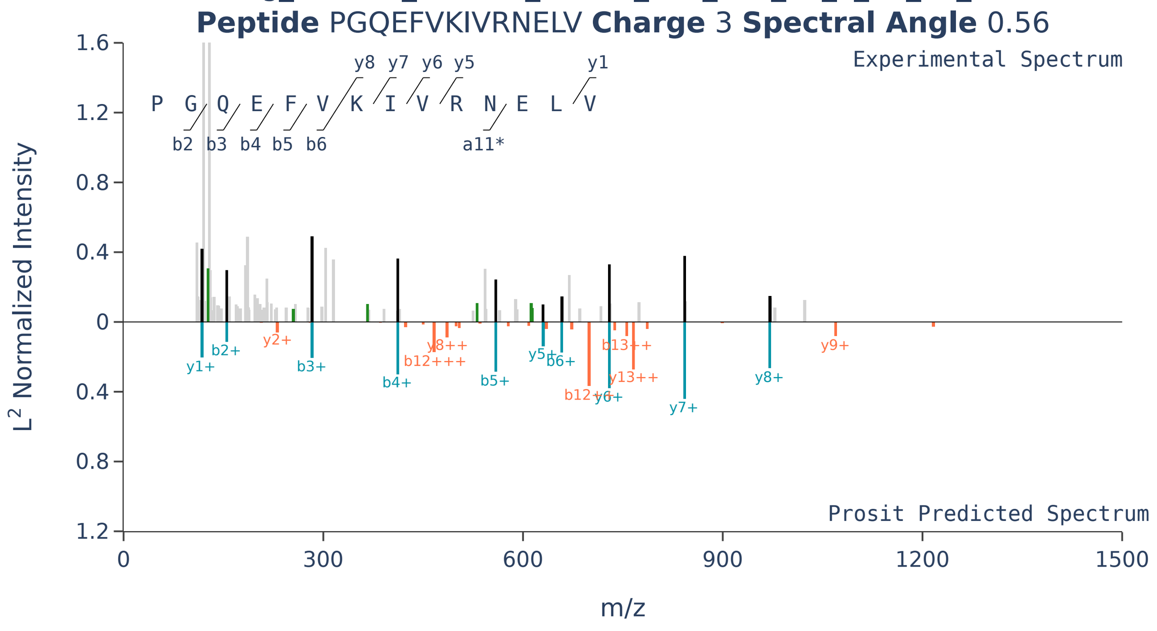


**Source** Ncheng_210623_230623_HFGoe_FFH_20S_25_1_A1_24h_R1 **Scan** 29151
**Peptide** LPVVREFI **Charge** 2 **Spectral Angle** 0.7

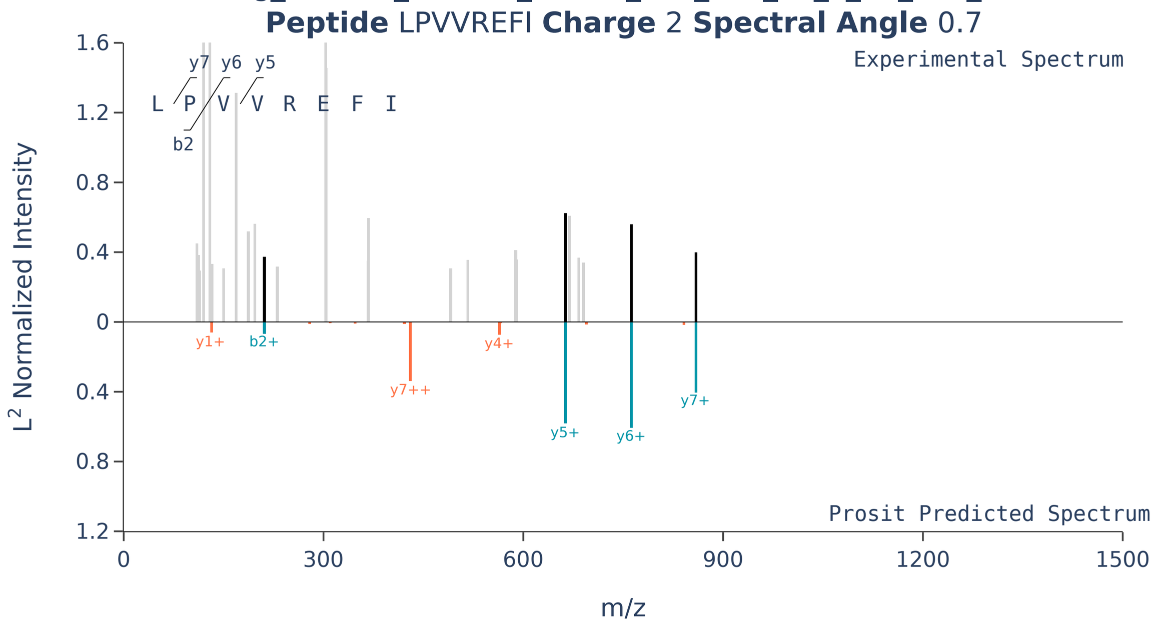

**Source** Ncheng_210623_230623_HFGoe_FFH_20S_25_1_A1_2h_R2 **Scan** 22552
**Peptide** DIESKVDRAQAEKLASKLKKGDGFDL **Charge** 5 **Spectral Angle** 0.9

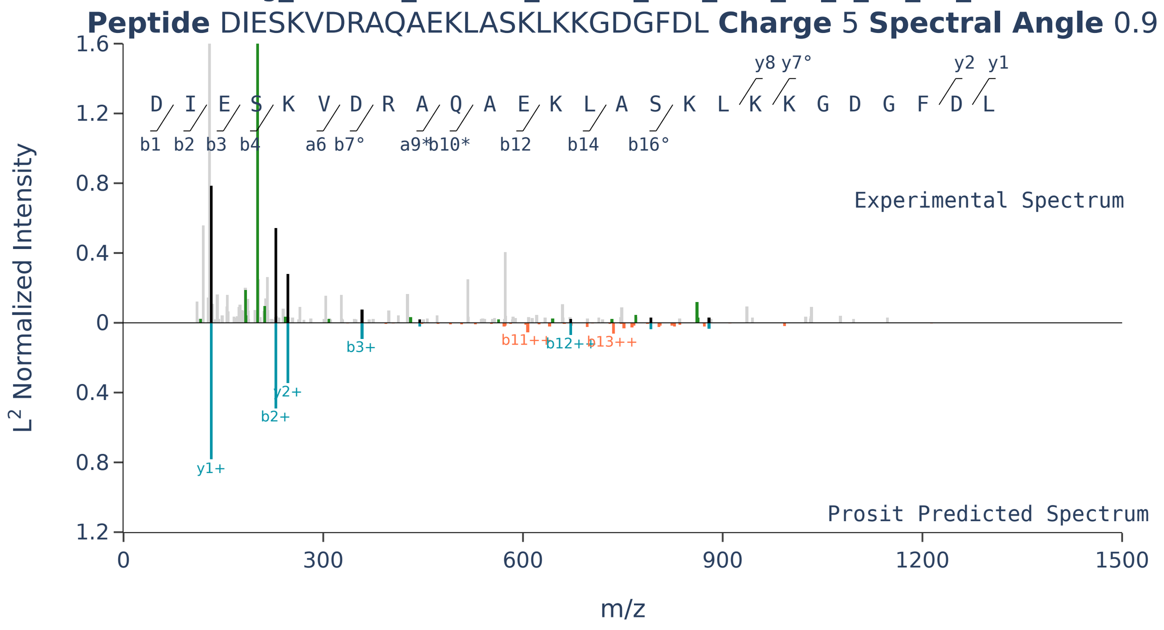


**Source** Ncheng_210623_230623_HFGoe_FFH_20S_25_1_A2_4h_R2 **Scan** 6069
**Peptide** RNISGRGRLTE **Charge** 3 **Spectral Angle** 0.55

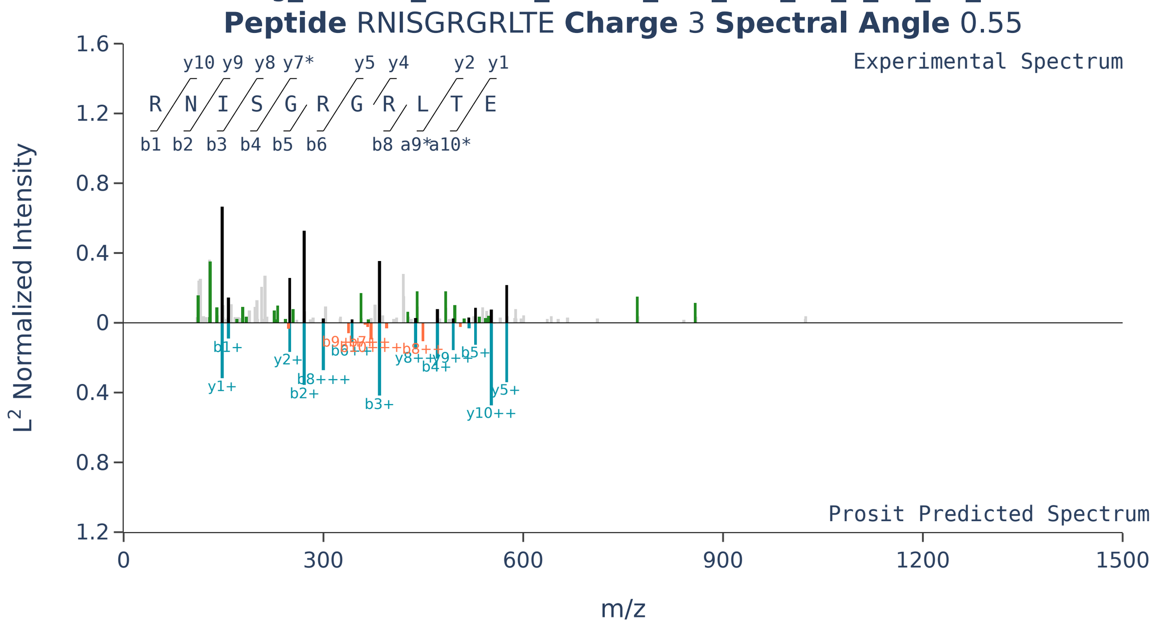


**Source** Ncheng_210623_230623_HFGoe_FFH_20S_25_1_A2_24h_R2 **Scan** 18420
**Peptide** SKVDRAQAEKLASKLKKGDGFDLND **Charge** 5 **Spectral Angle** 0.8

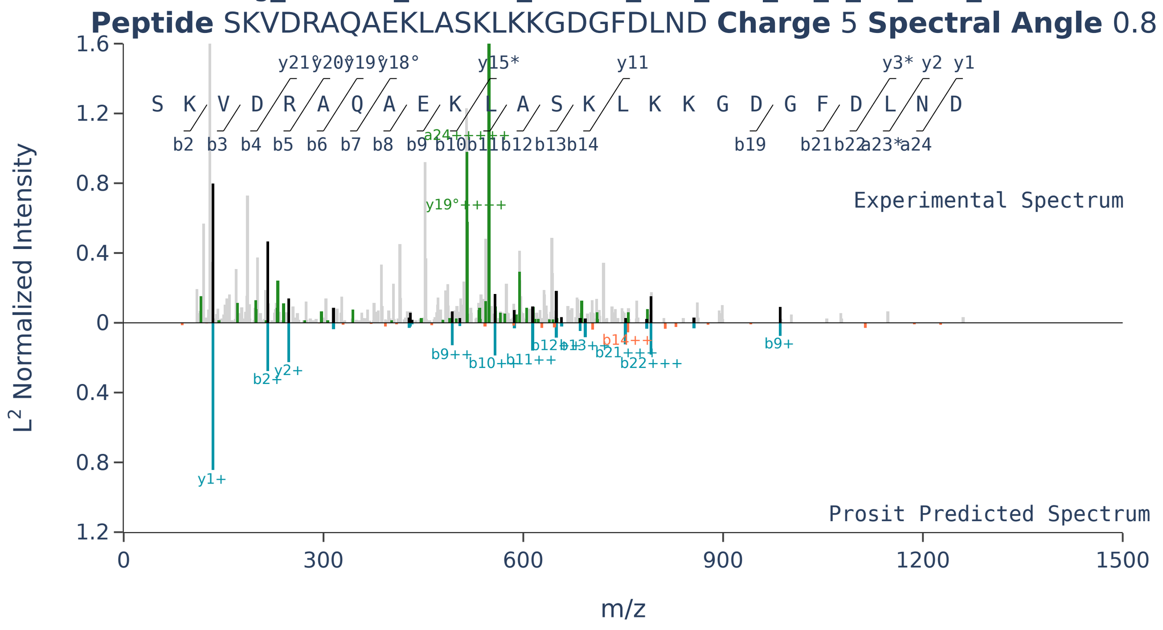


**Source** Ncheng_210623_230623_HFGoe_FFH_20S_25_1_A2_2h_R1 **Scan** 34666
**Peptide** MTGQDAANTAKAFNEALPLTGVVLTKVD **Charge** 3 **Spectral Angle** 0.71

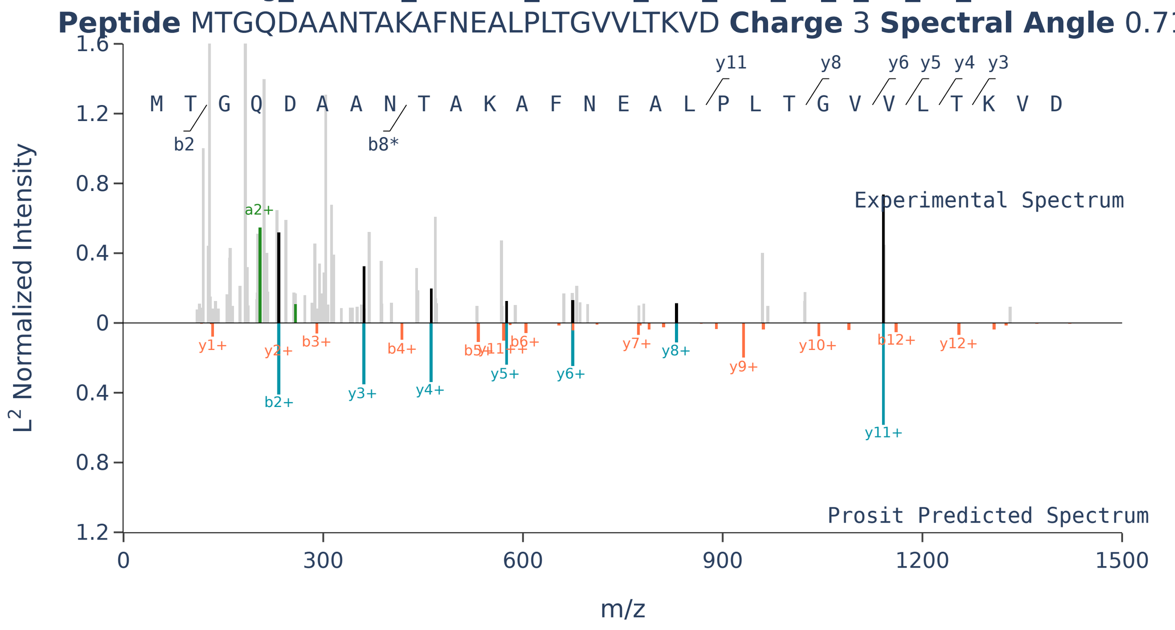


**Source** Ncheng_210623_230623_HFGoe_FFH_20S_25_1_A1_24h_R2 **Scan** 7122
**Peptide** SGRGRLTEDNVKD **Charge** 3 **Spectral Angle** 0.71

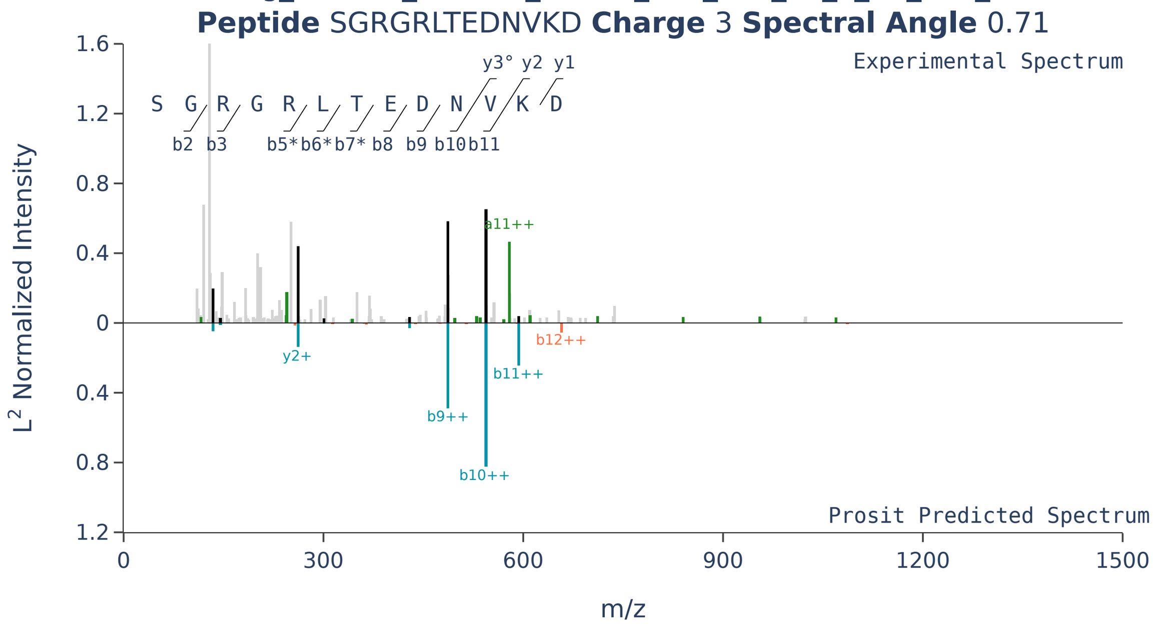


**Source** Ncheng_210623_230623_HFGoe_FFH_20S_25_1_A2_24h_R1 **Scan** 20418
**Peptide** ASKLKKGDGFDLNDF **Charge** 3 **Spectral Angle** 0.67

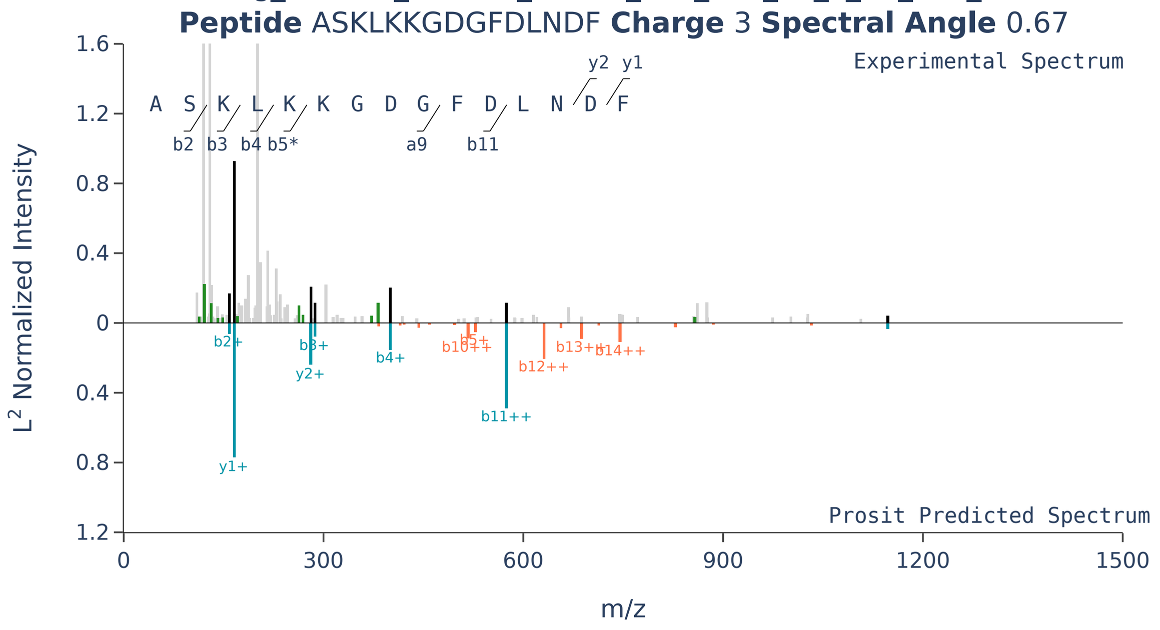


**Source** Ncheng_210623_230623_HFGoe_FFH_20S_25_1_A2_24h_R1 **Scan** 10539
**Peptide** SVGKLGKFLREKHKKKVL **Charge** 5 **Spectral Angle** 0.61

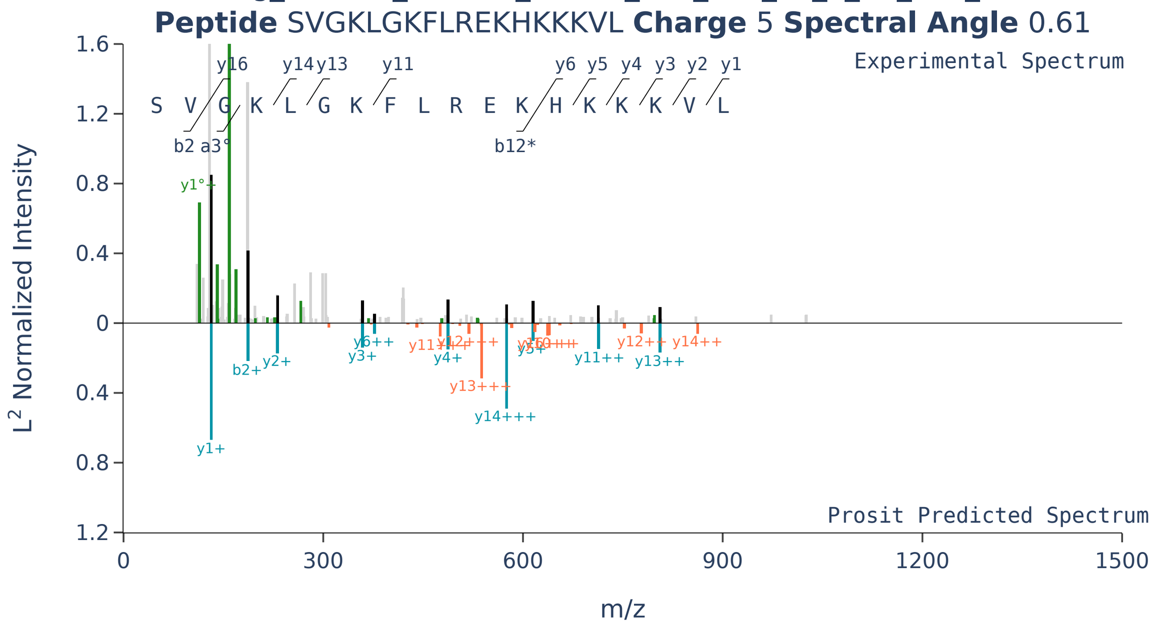


**Source** Ncheng_210623_230623_HFGoe_FFH_20S_25_1_A2_2h_R2 **Scan** 23627
**Peptide** TLREVRMALL **Charge** 3 **Spectral Angle** 0.38

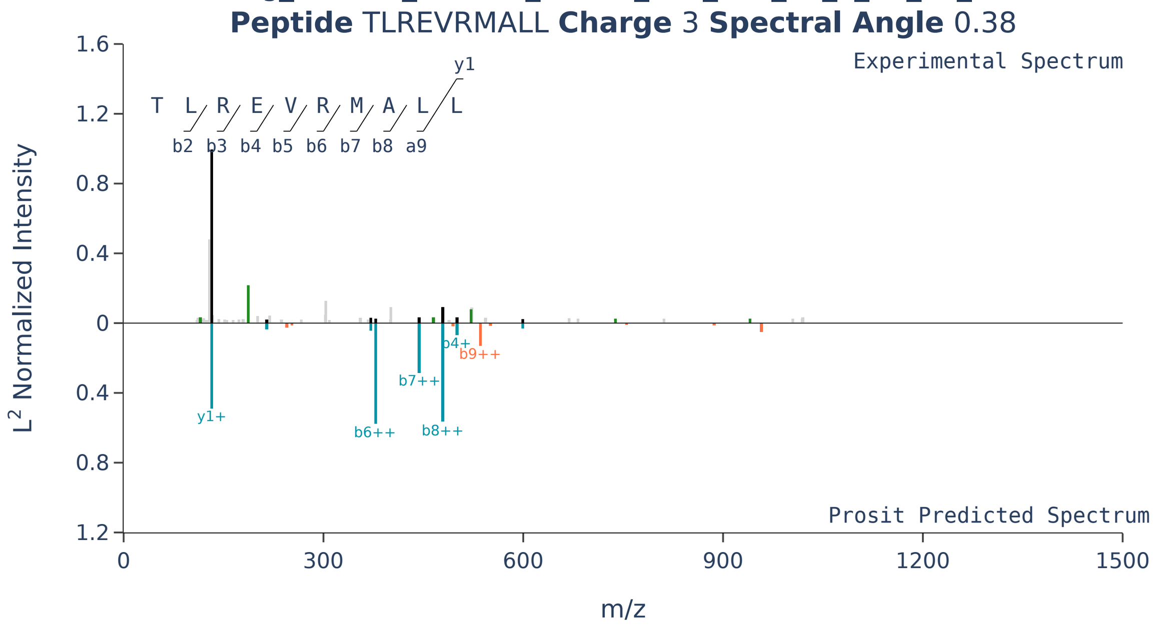


**Source** Ncheng_210623_230623_HFGoe_FFH_20S_25_1_A1_24h_R1 **Scan** 5328
**Peptide** IESKVDRAQ **Charge** 2 **Spectral Angle** 0.74

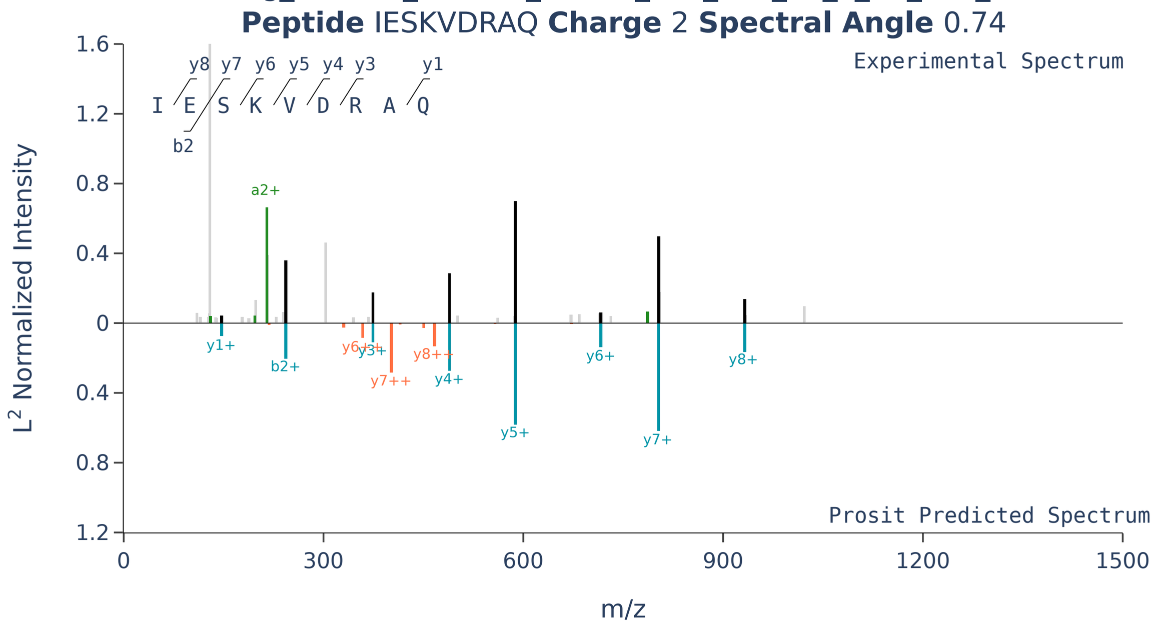


**Source** Ncheng_210623_230623_HFGoe_FFH_20S_25_1_A2_2h_R1 **Scan** 19847
**Peptide** GVVLTKVDGDARGGAALSIRHITG **Charge** 3 **Spectral Angle** 0.53

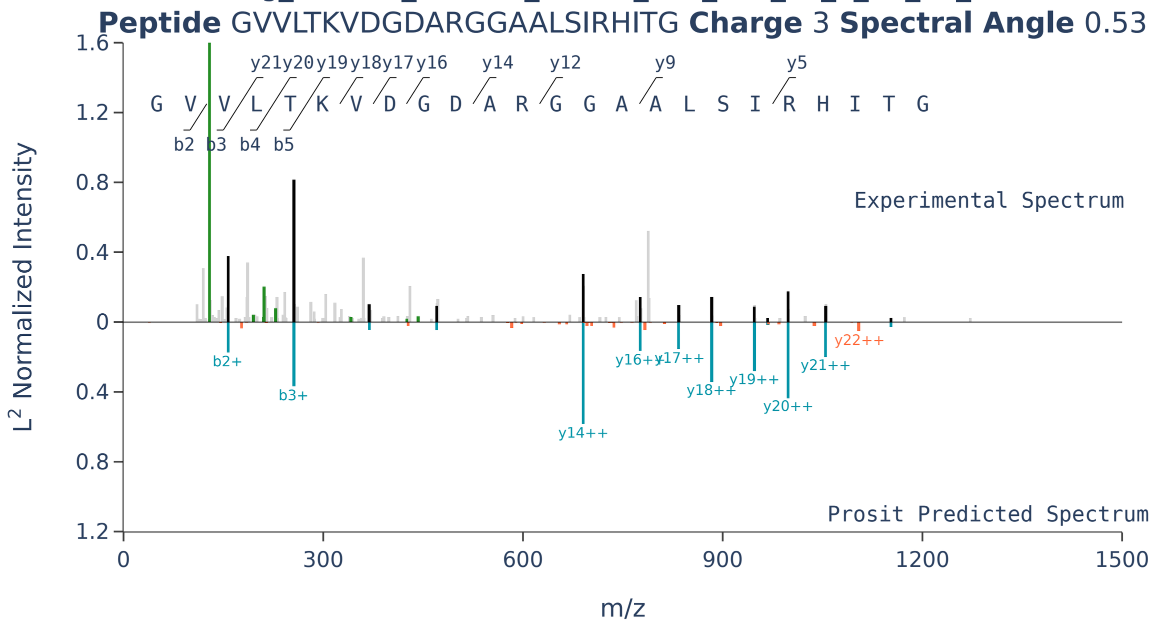


**Source** Ncheng_210623_230623_HFGoe_FFH_20S_25_1_A1_2h_R1 **Scan** 27843
**Peptide** ALPLTGVVLTKVDGDARGG **Charge** 3 **Spectral Angle** 0.56

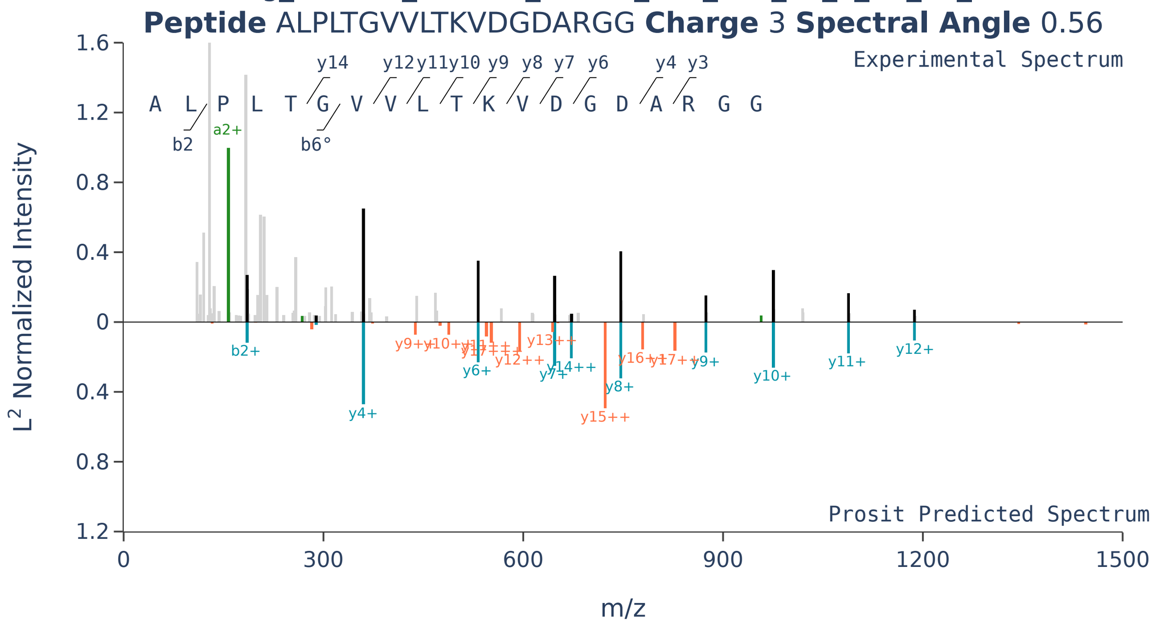


**Source** Ncheng_210623_230623_HFGoe_FFH_20S_25_1_A1_1h_R1 **Scan** 31363
**Peptide** AEQVGVDFFPSDVGQKPVDI **Charge** 2 **Spectral Angle** 0.65

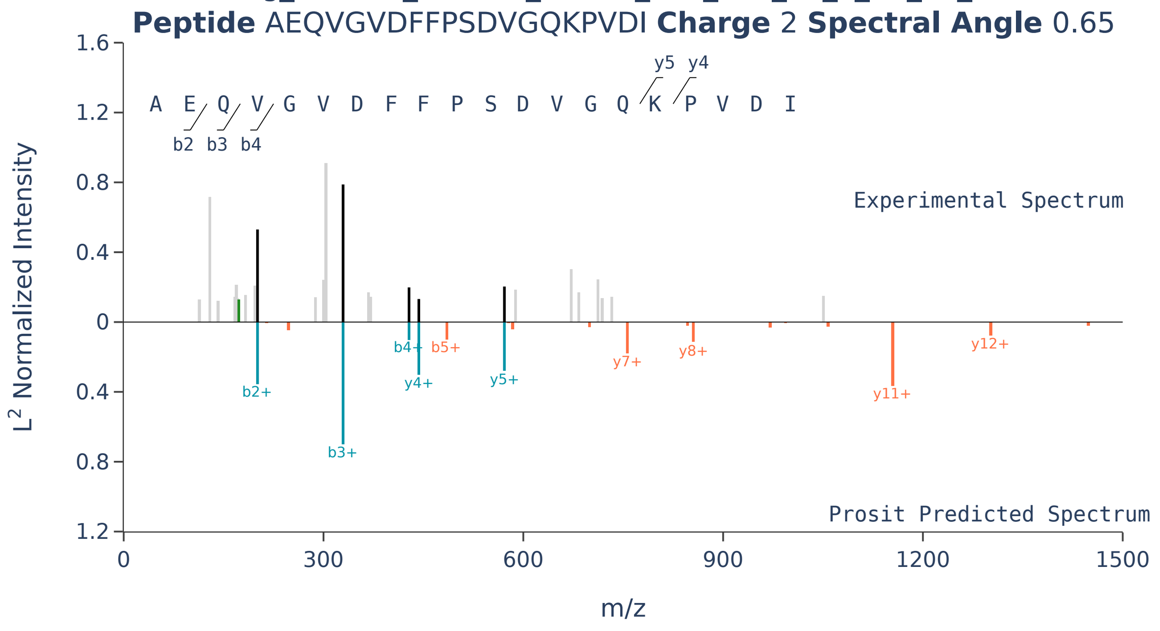


**Source** Ncheng_210623_230623_HFGoe_FFH_20S_25_1_A1_4h_R1 **Scan** 15898
**Peptide** SKVDRAQAEKLASKL **Charge** 3 **Spectral Angle** 0.57

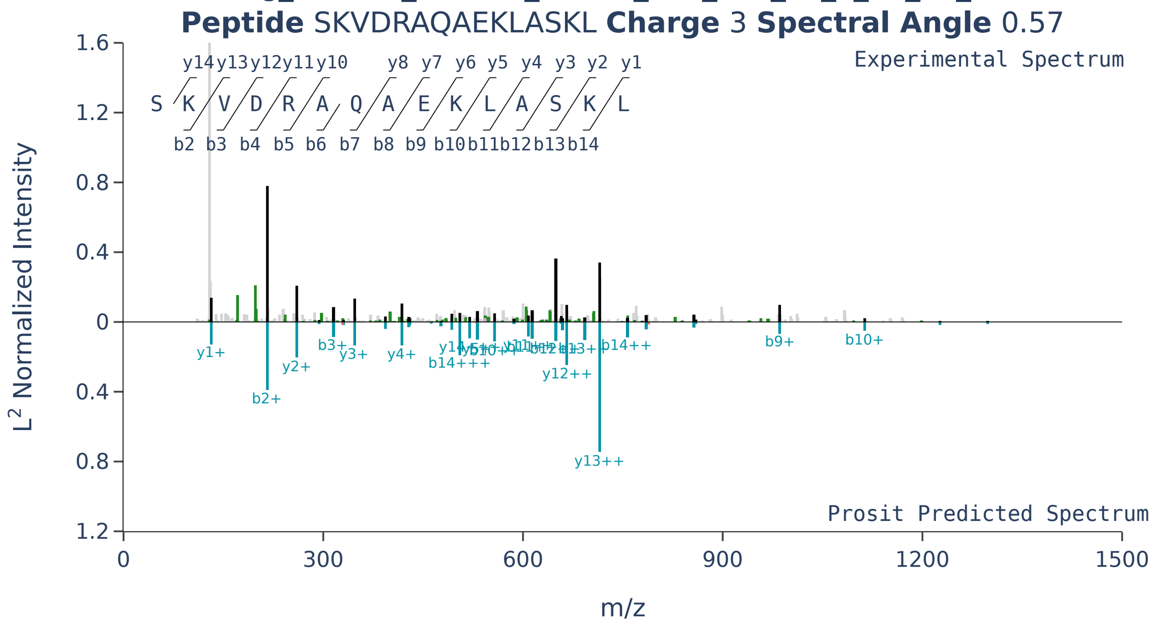


**Source** Ncheng_210623_230623_HFGoe_FFH_20S_25_1_A1_2h_R1 **Scan** 21386
**Peptide** GGAALSIRHITGKPIKFLGVGEKTE **Charge** 3 **Spectral Angle** 0.62

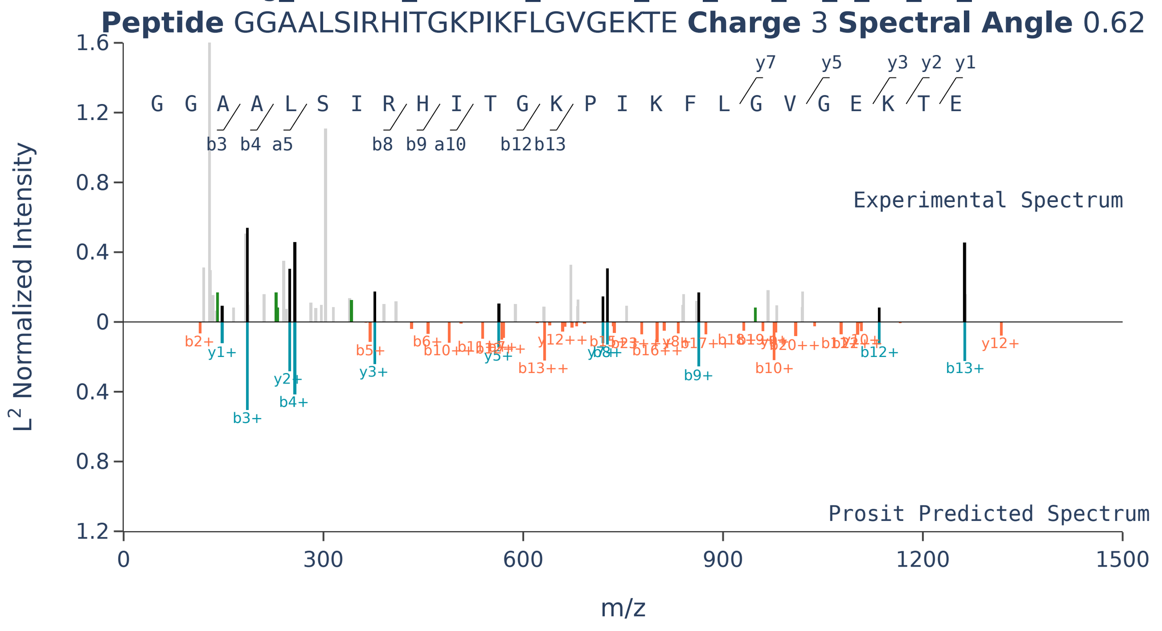


**Source** Ncheng_210623_230623_HFGoe_FFH_20S_25_1_A2_2h_R1 **Scan** 30997
**Peptide** AALKEAKLKFYDVLLVDTAGRLHVD **Charge** 4 **Spectral Angle** 0.62

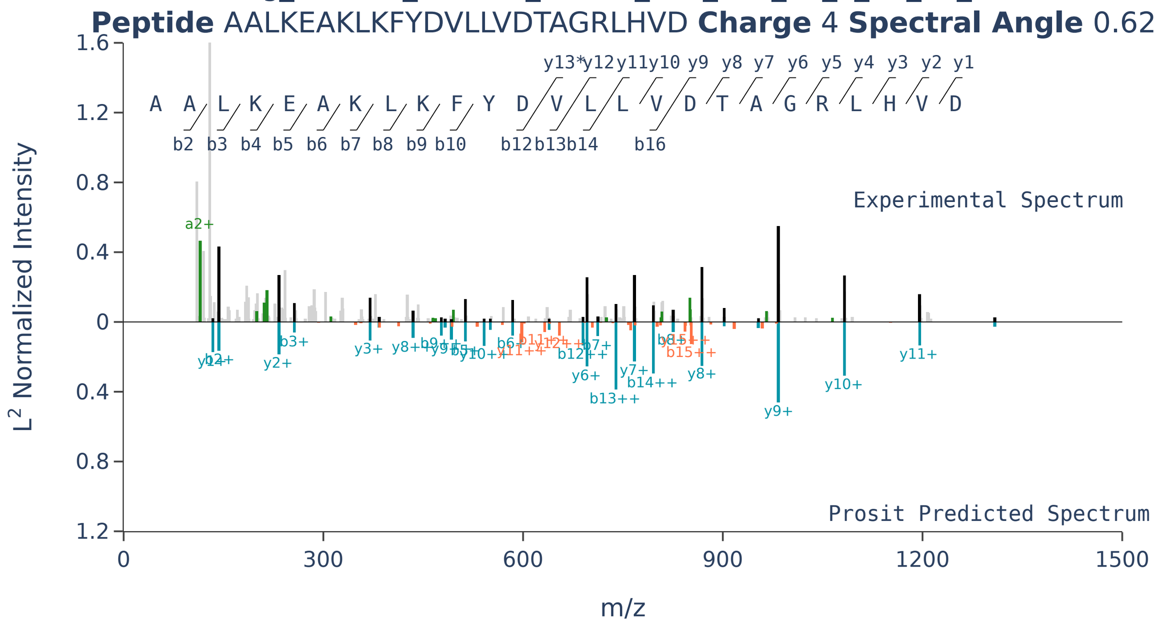

**Source** Ncheng_210623_230623_HFGoe_FFH_20S_25_1_A2_2h_R1 **Scan** 11036
**Peptide** VVDAMTGQDAANTAKA **Charge** 2 **Spectral Angle** 0.62

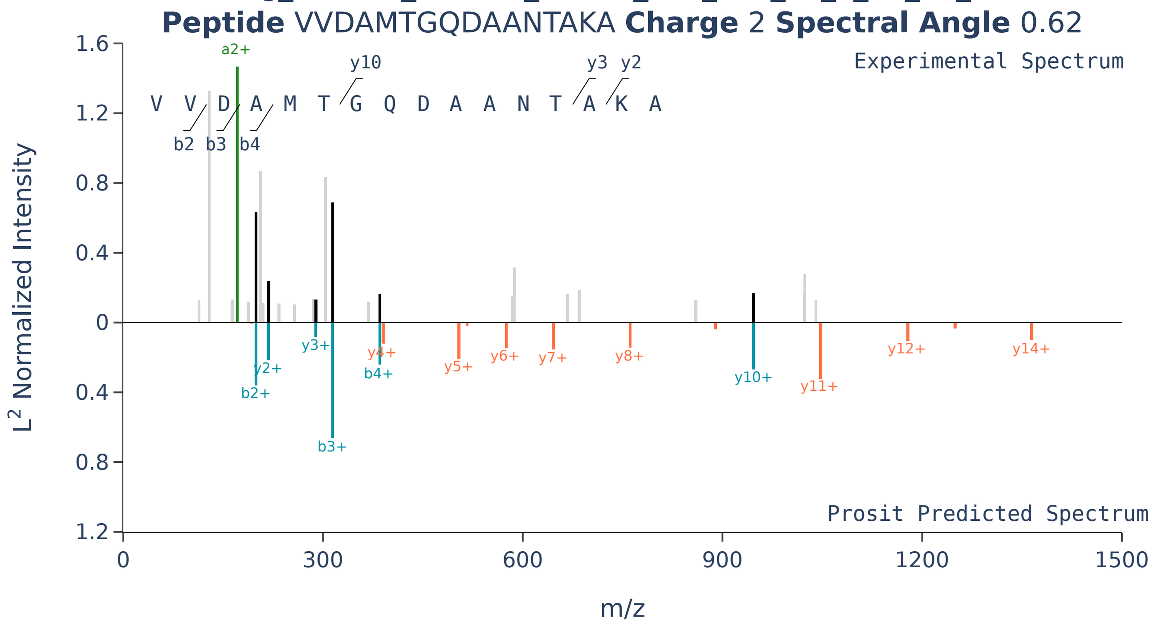


**Source** Ncheng_210623_230623_HFGoe_FFH_20S_25_1_A2_2h_R2 **Scan** 40625
**Peptide** KKGDGFDLNDFLEQLRQMKNMGGMASL **Charge** 4 **Spectral Angle** 0.8

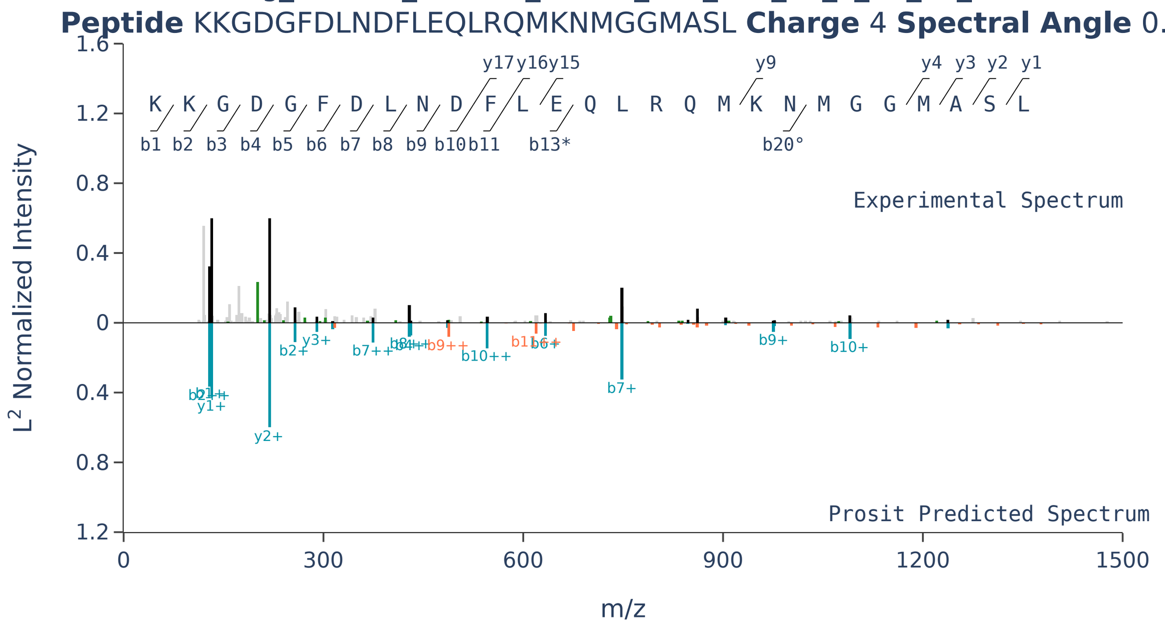


**Source** Ncheng_210623_230623_HFGoe_FFH_20S_25_1_A2_24h_R2 **Scan** 11590
**Peptide** DIESKVDRAQAEKLASK **Charge** 2 **Spectral Angle** 0.66

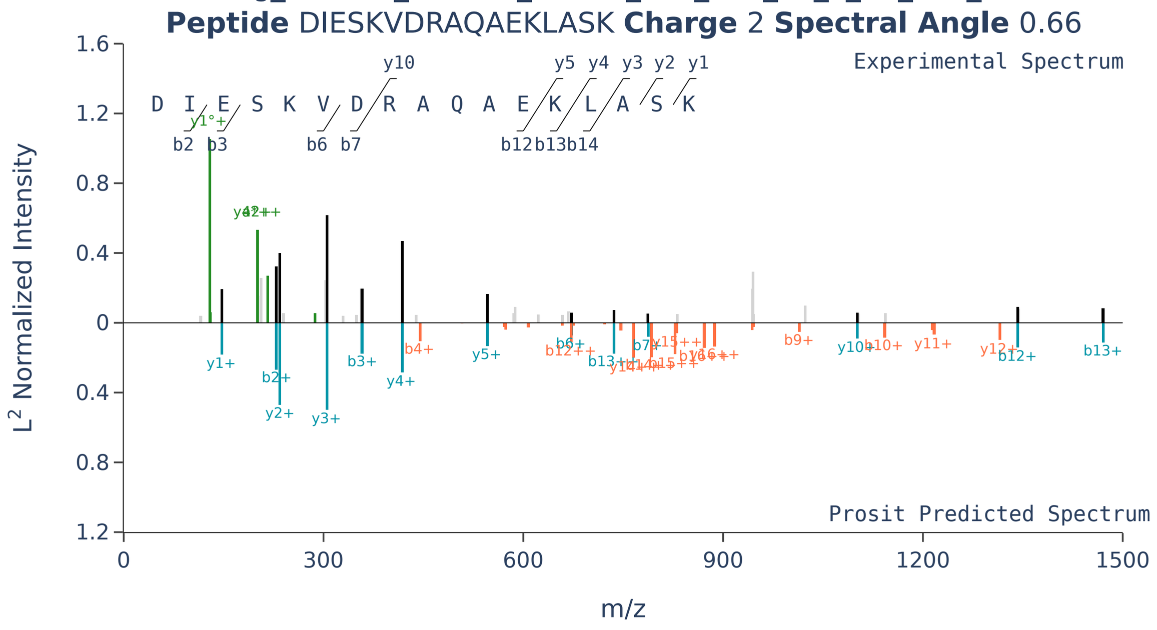


**Source** Ncheng_210623_230623_HFGoe_FFH_20S_25_1_A2_4h_R1 **Scan** 17511
**Peptide** NLSGRGRLTEDNVKDTLREVRMA **Charge** 5 **Spectral Angle** 0.73

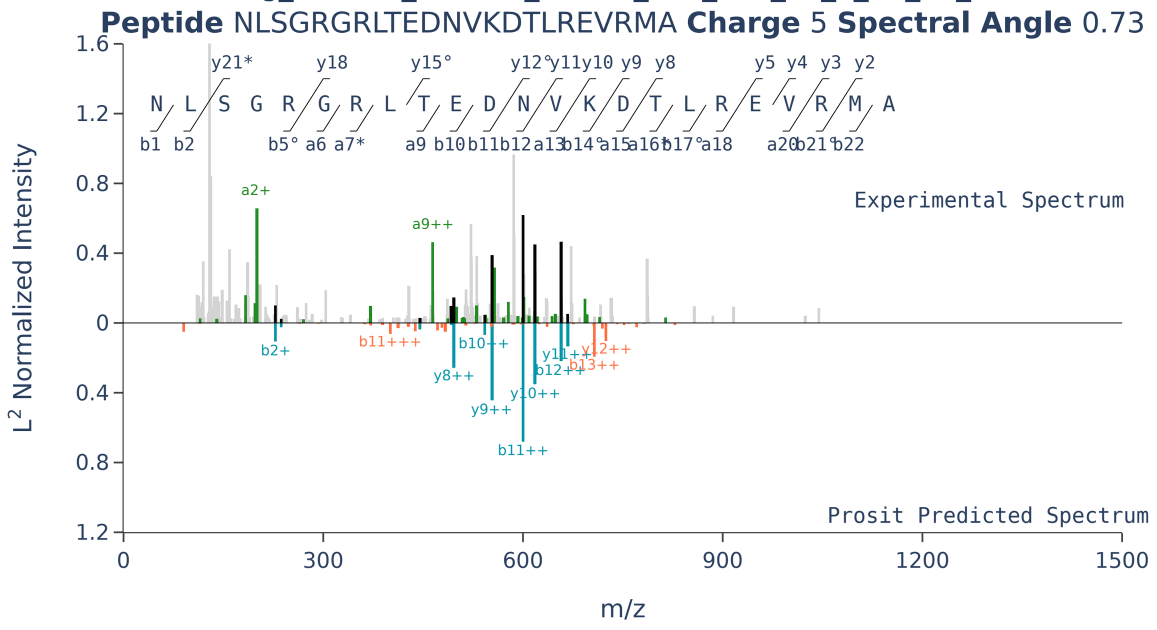


**Source** Ncheng_210623_230623_HFGoe_FFH_20S_25_1_A1_4h_R1 **Scan** 19432
**Peptide** EDIESKVDRAQAEKLASKLKKGDGFD **Charge** 3 **Spectral Angle** 0.78

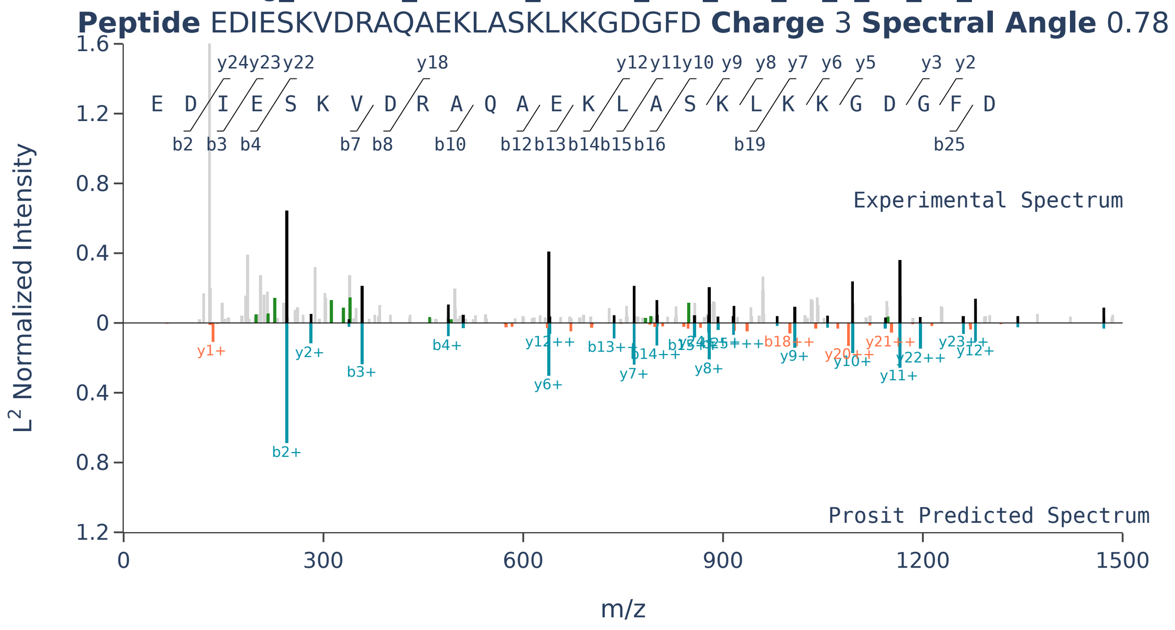


**Source** Ncheng_210623_230623_HFGoe_FFH_20S_25_1_A2_4h_R1 **Scan** 37565
**Peptide** DVALPVVREFINRV **Charge** 3 **Spectral Angle** 0.34

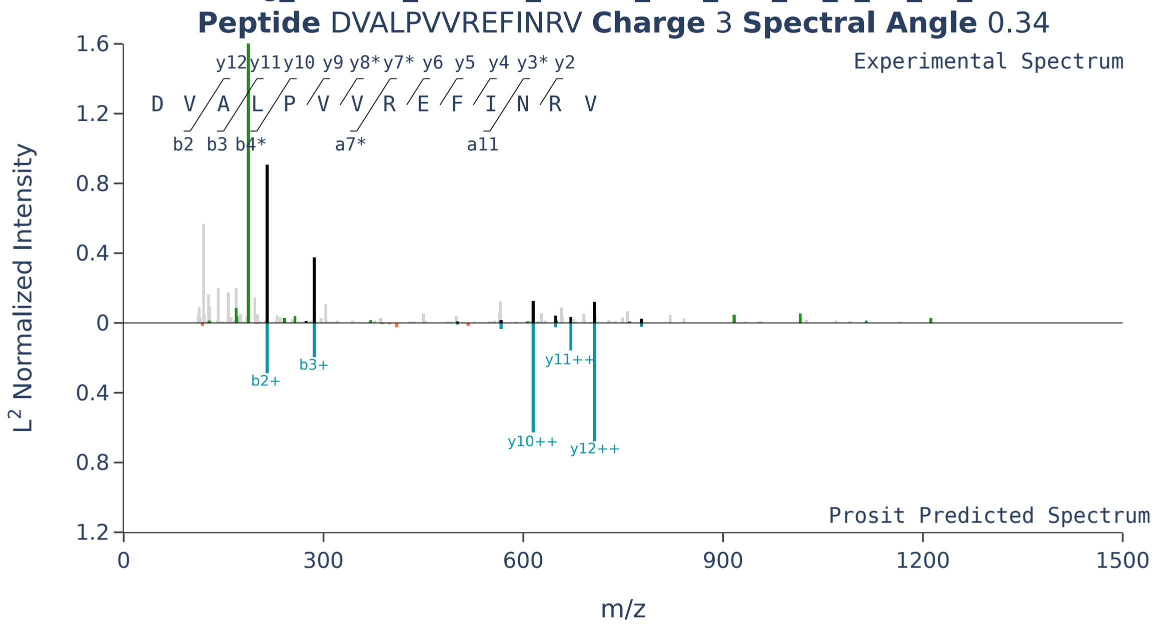


**Source** Ncheng_210623_230623_HFGoe_FFH_20S_25_1_A1_2h_R2 **Scan** 34230
**Peptide** DEIKQVHASINPVETLFVVD **Charge** 3 **Spectral Angle** 0.58

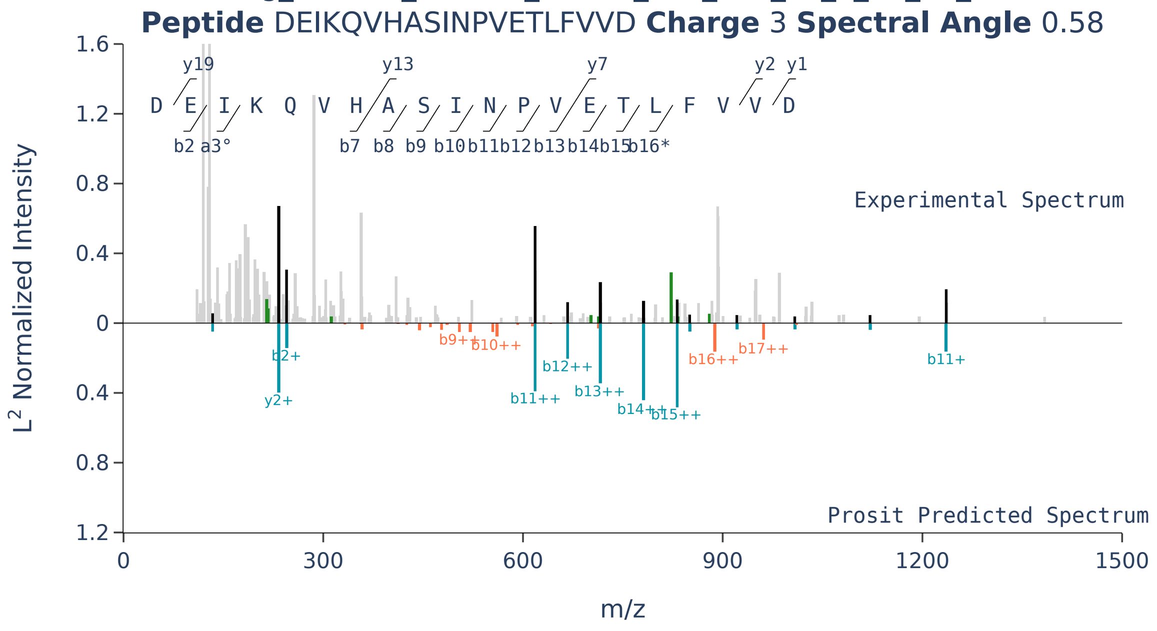


**Source** Ncheng_210623_230623_HFGoe_FFH_20S_25_1_A2_24h_R1 **Scan** 6759
**Peptide** TMKERAKPEIIKGSR **Charge** 3 **Spectral Angle** 0.61

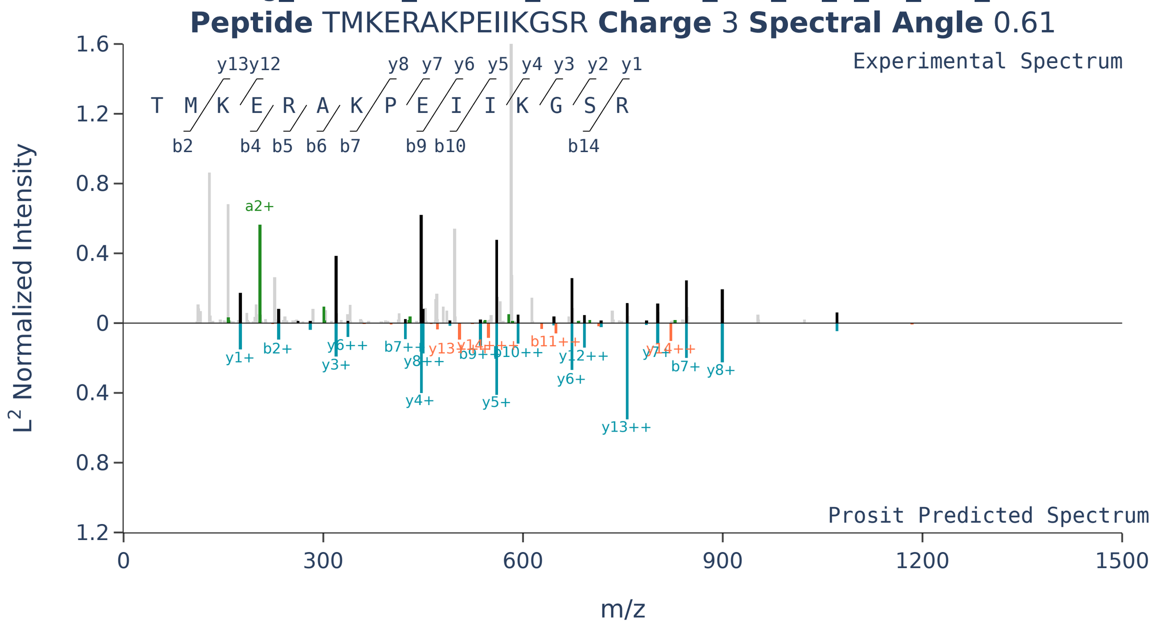


**Source** Ncheng_210623_230623_HFGoe_FFH_20S_25_1_A1_2h_R2 **Scan** 24416
**Peptide** DGDARGGAALSIRHITGKPIKFLGVGE **Charge** 4 **Spectral Angle** 0.57

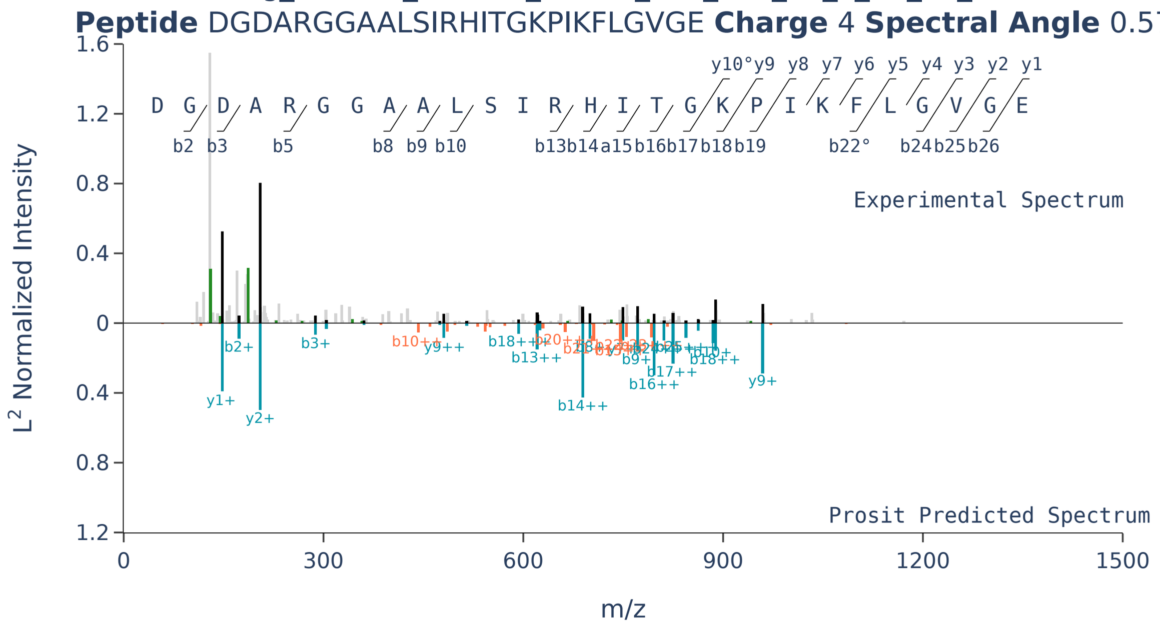


**Source** Ncheng_210623_230623_HFGoe_FFH_20S_25_1_A1_4h_R1 **Scan** 12865
**Peptide** DEIKQVHASL **Charge** 3 **Spectral Angle** 0.29

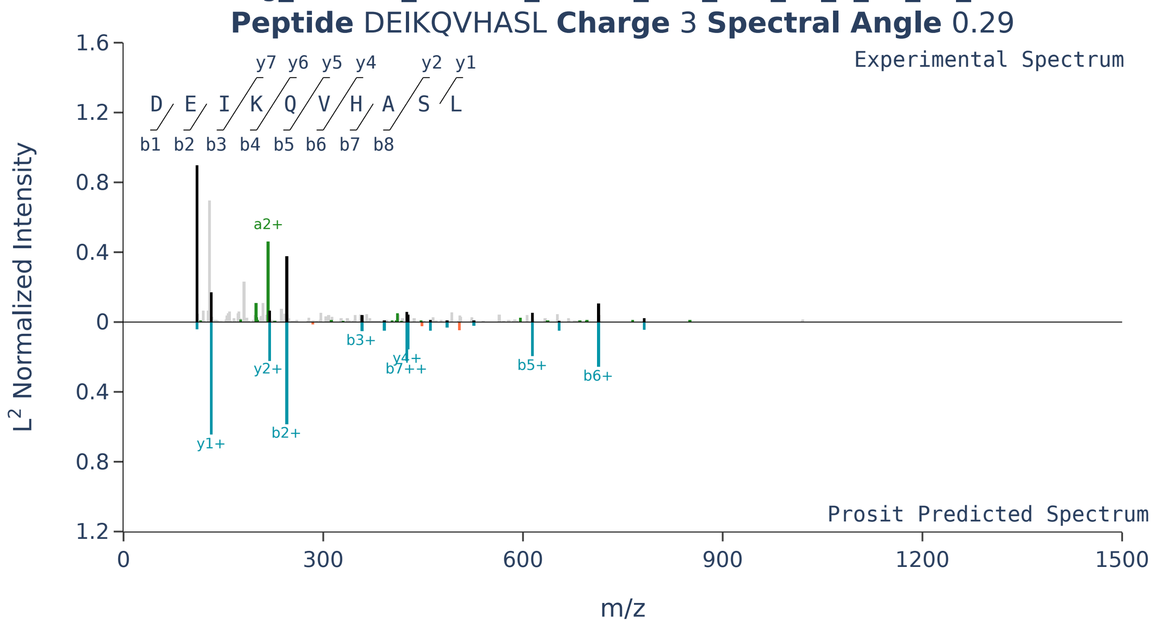


**Source** Ncheng_210623_230623_HFGoe_FFH_20S_25_1_A1_2h_R2 **Scan** 30098
**Peptide** VALPVVREFINRVKEKAVGHEVN **Charge** 5 **Spectral Angle** 0.79

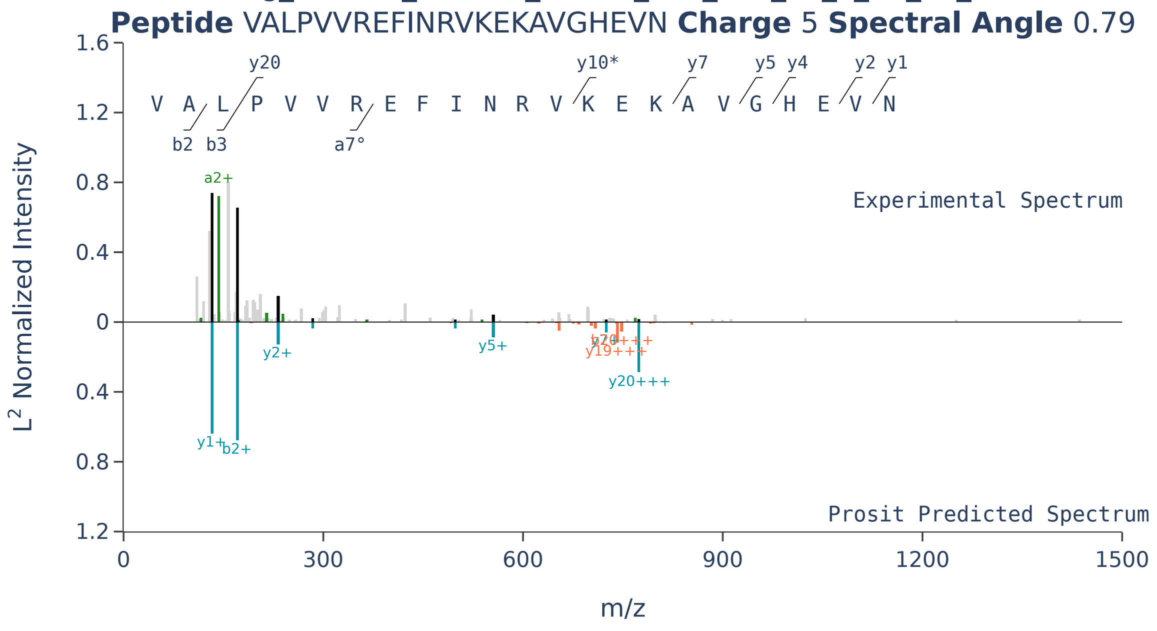


**Source** Ncheng_210623_230623_HFGoe_FFH_20S_25_1_A1_24h_R2 **Scan** 9658
**Peptide** VGKLGKFLREKH **Charge** 3 **Spectral Angle** 0.71

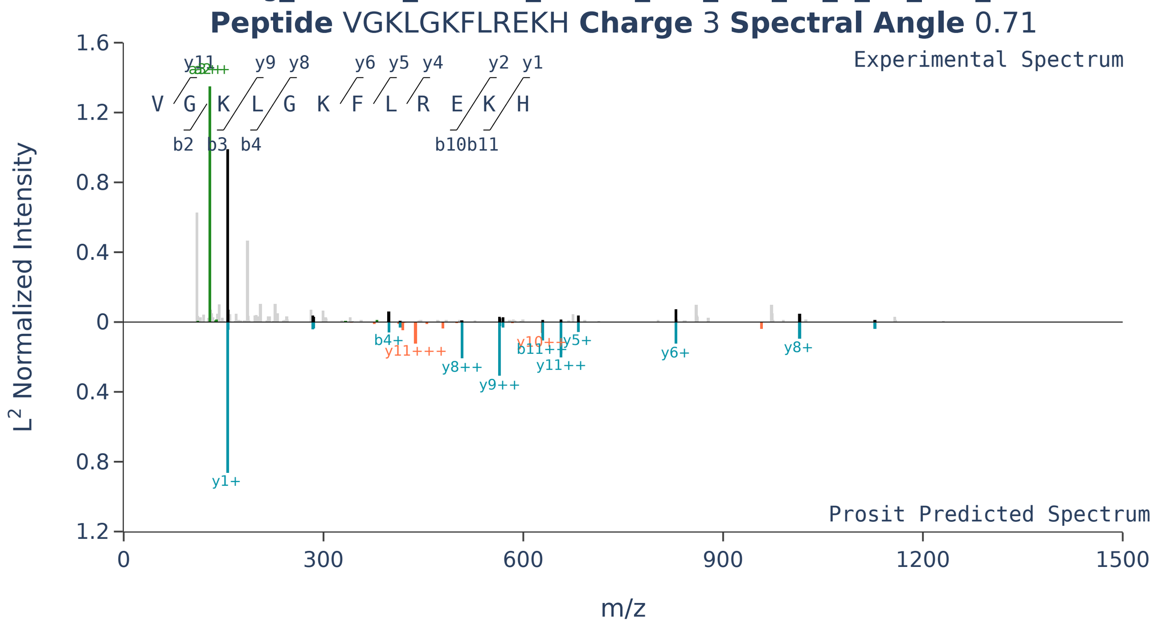


**Source** Ncheng_210623_230623_HFGoe_FFH_20S_25_1_A1_2h_R2 **Scan** 28577
**Peptide** PDNVKSQMDDKVLVRMEAIIN **Charge** 3 **Spectral Angle** 0.72

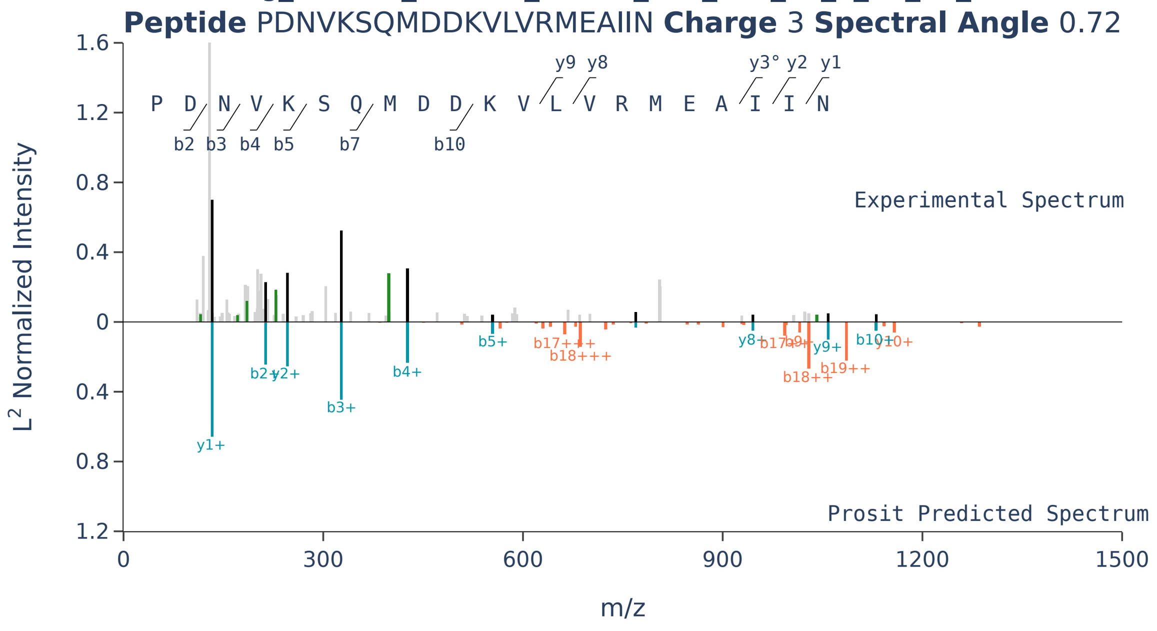


**Source** Ncheng_210623_230623_HFGoe_FFH_20S_25_1_A2_4h_R2 **Scan** 25120
**Peptide** RGRLTEDNVKDTLREVRMAL **Charge** 5 **Spectral Angle** 0.67

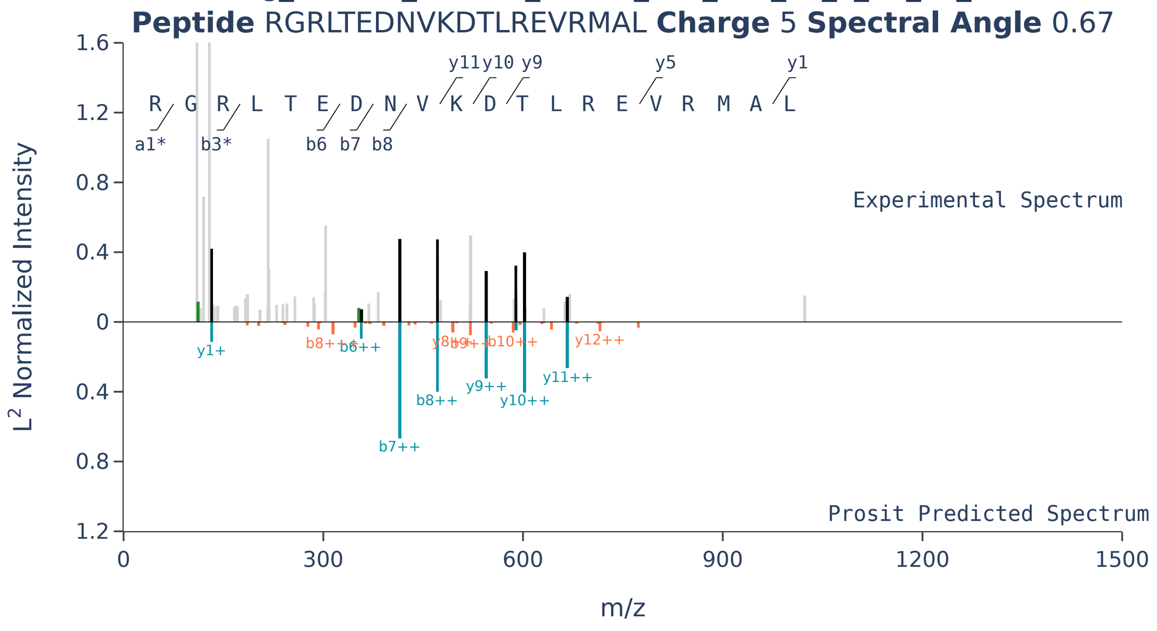


**Source** Ncheng_210623_230623_HFGoe_FFH_20S_25_1_A2_4h_R1 **Scan** 30330
**Peptide** NAALKEAKLKFYDVLLVD **Charge** 3 **Spectral Angle** 0.53

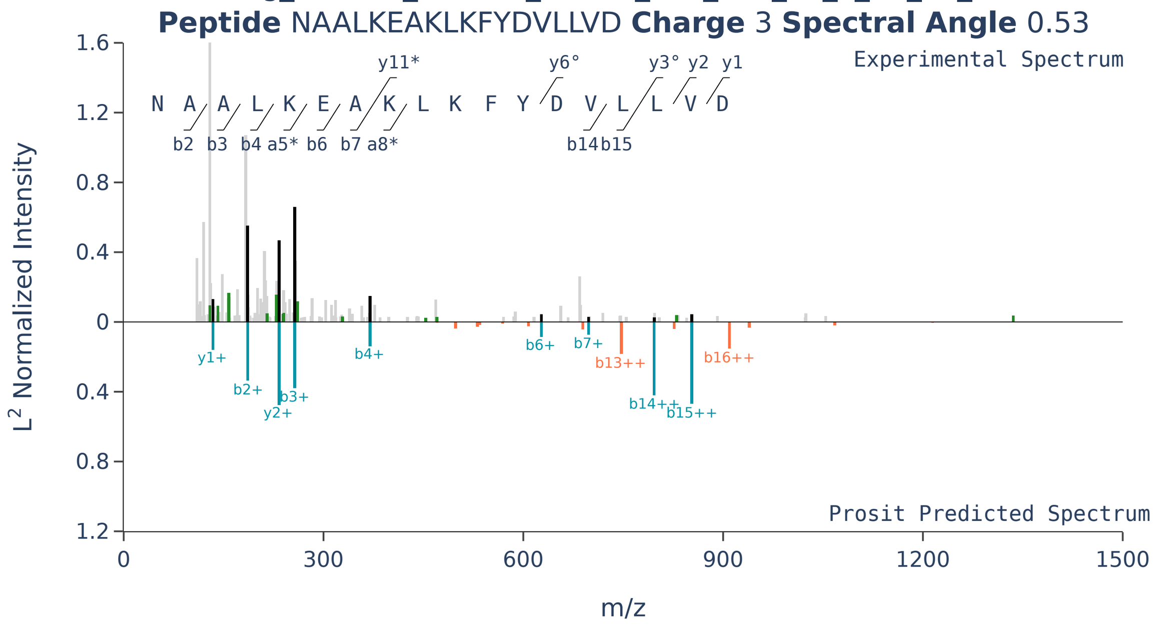

**Source** Ncheng_210623_230623_HFGoe_FFH_20S_25_1_A2_24h_R1 **Scan** 7202
**Peptide** RQMKNMGGMAS **Charge** 2 **Spectral Angle** 0.62

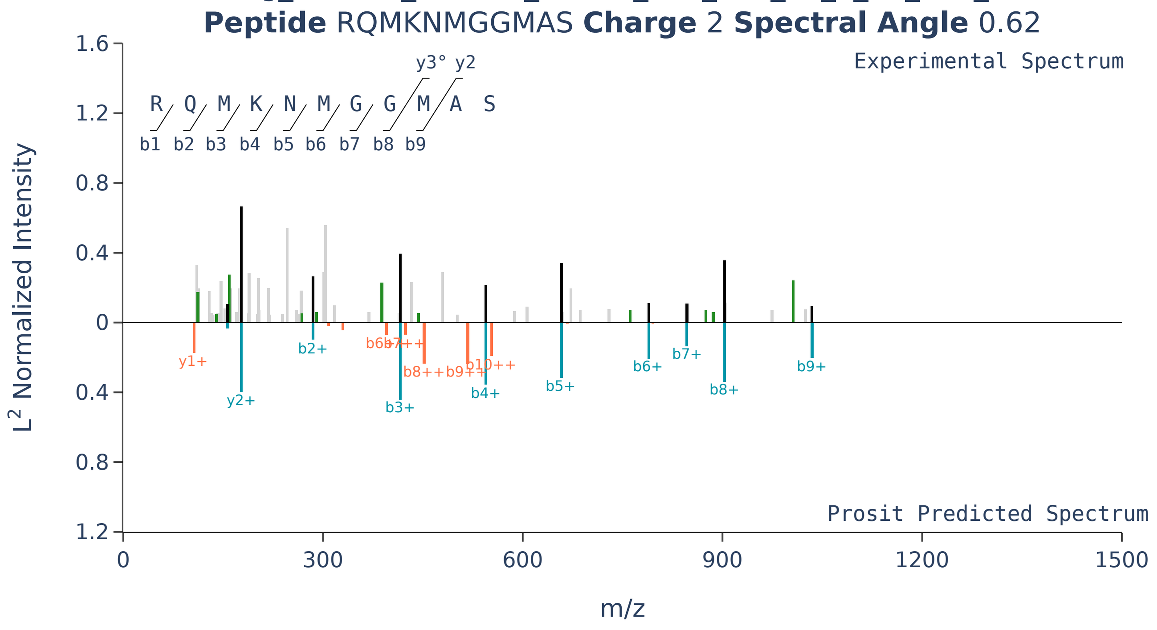


**Source** Ncheng_210623_230623_HFGoe_FFH_20S_25_1_A2_4h_R2 **Scan** 16769
**Peptide** KKVLVVSADVYRPAAIKQ **Charge** 4 **Spectral Angle** 0.8

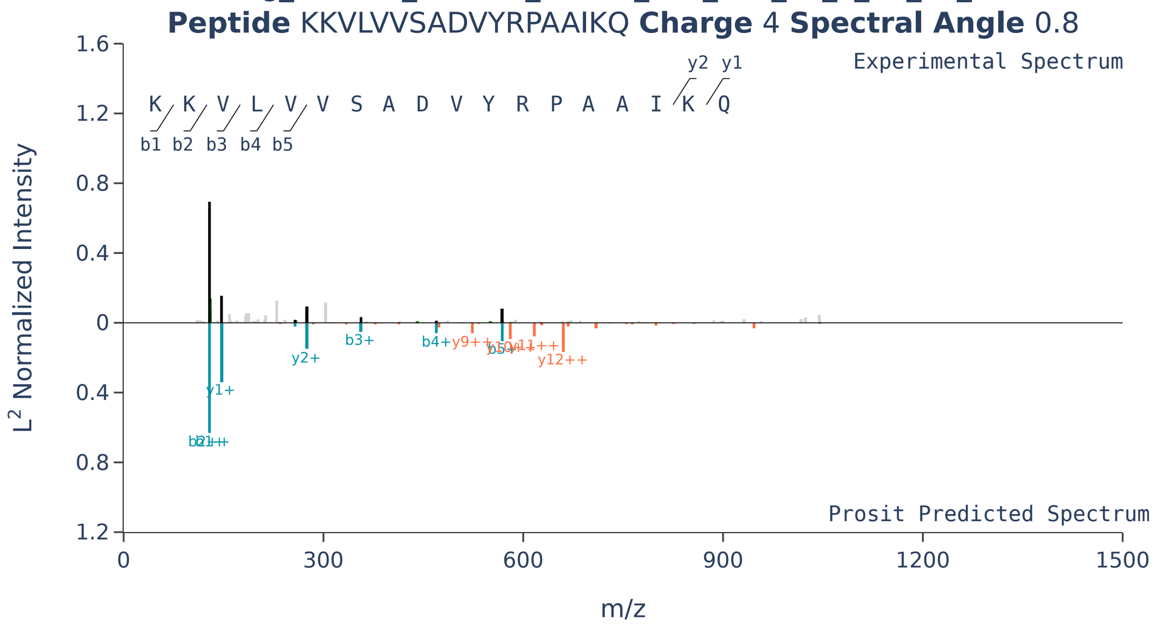


**Source** Ncheng_210623_230623_HFGoe_FFH_20S_25_1_A2_2h_R1 **Scan** 22346
**Peptide** KVDGDARGGAALSIRHITGKPIKFLGVGE **Charge** 5 **Spectral Angle** 0.88

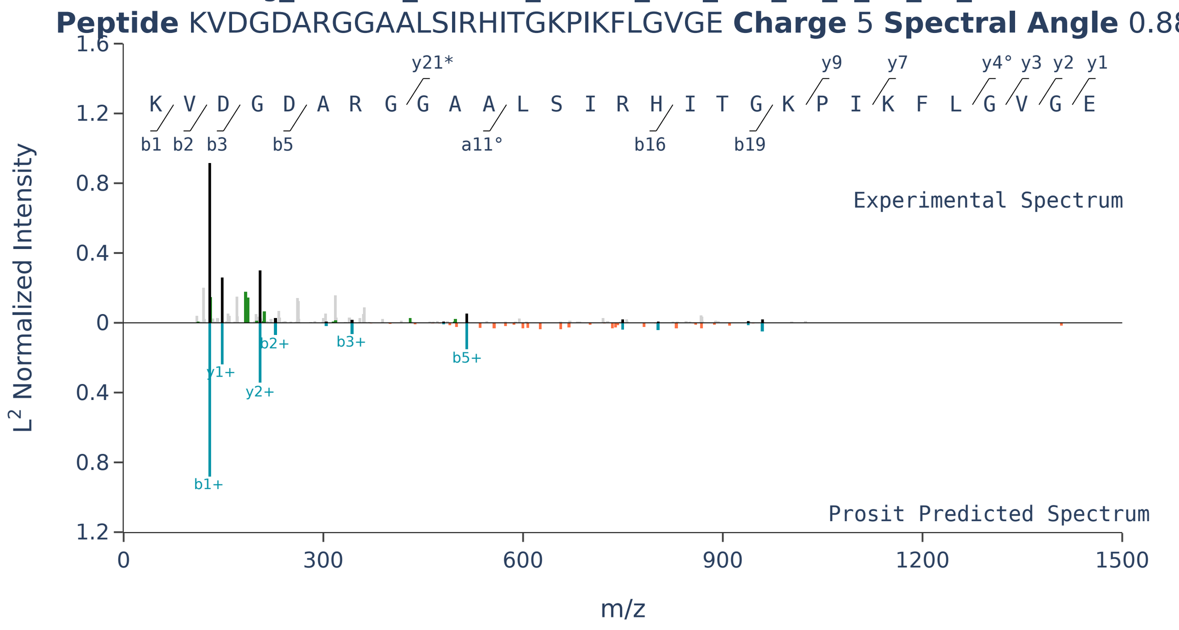


**Source** Ncheng_210623_230623_HFGoe_FFH_20S_25_1_A2_4h_R1 **Scan** 5096
**Peptide** IESKVDR **Charge** 2 **Spectral Angle** 0.79

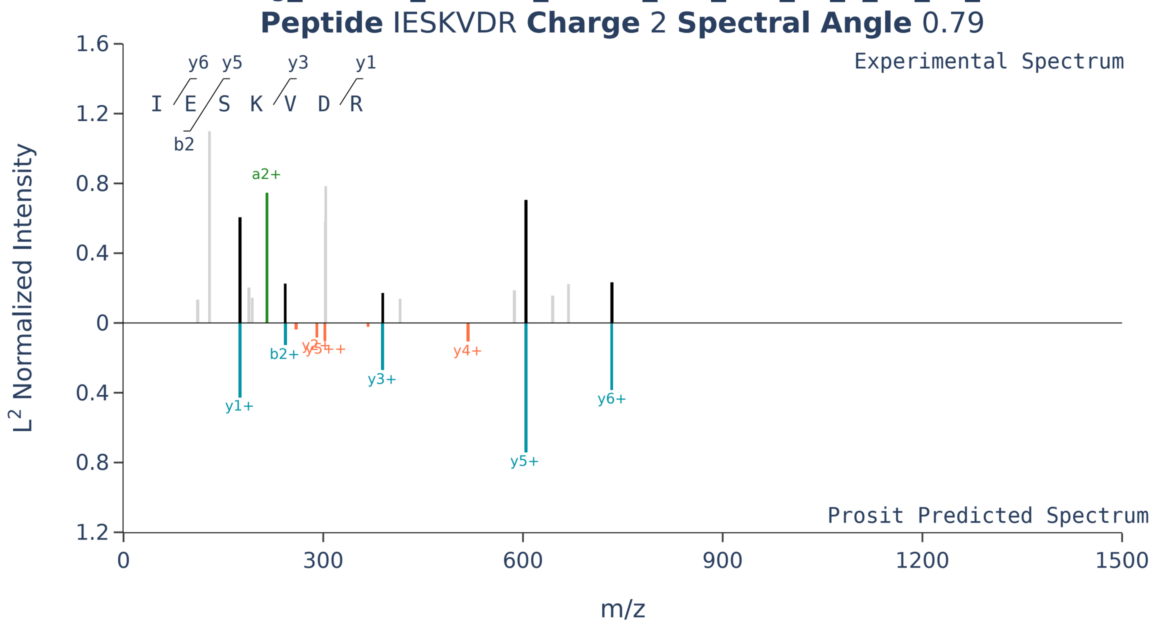


**Source** Ncheng_210623_230623_HFGoe_FFH_20S_25_1_A2_4h_R2 **Scan** 33586
**Peptide** ADVALPVVREFINRVKEKA **Charge** 3 **Spectral Angle** 0.57

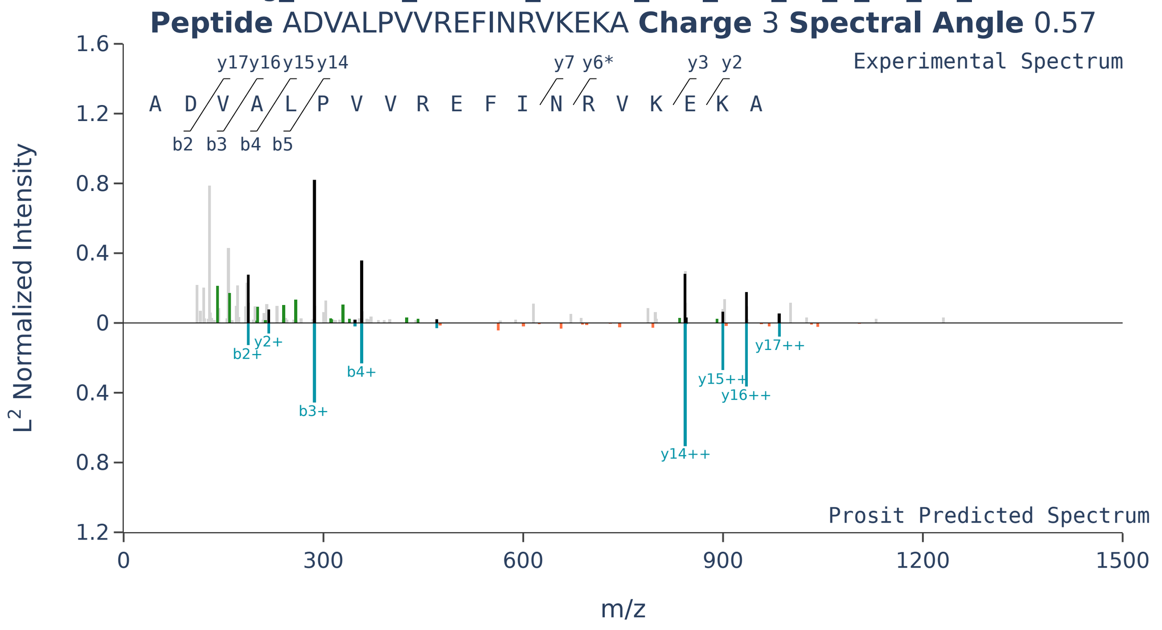


**Source** Ncheng_210623_230623_HFGoe_FFH_20S_25_1_A1_24h_R1 **Scan** 7815
**Peptide** QGAGKTTSVGKLG **Charge** 3 **Spectral Angle** 0.82

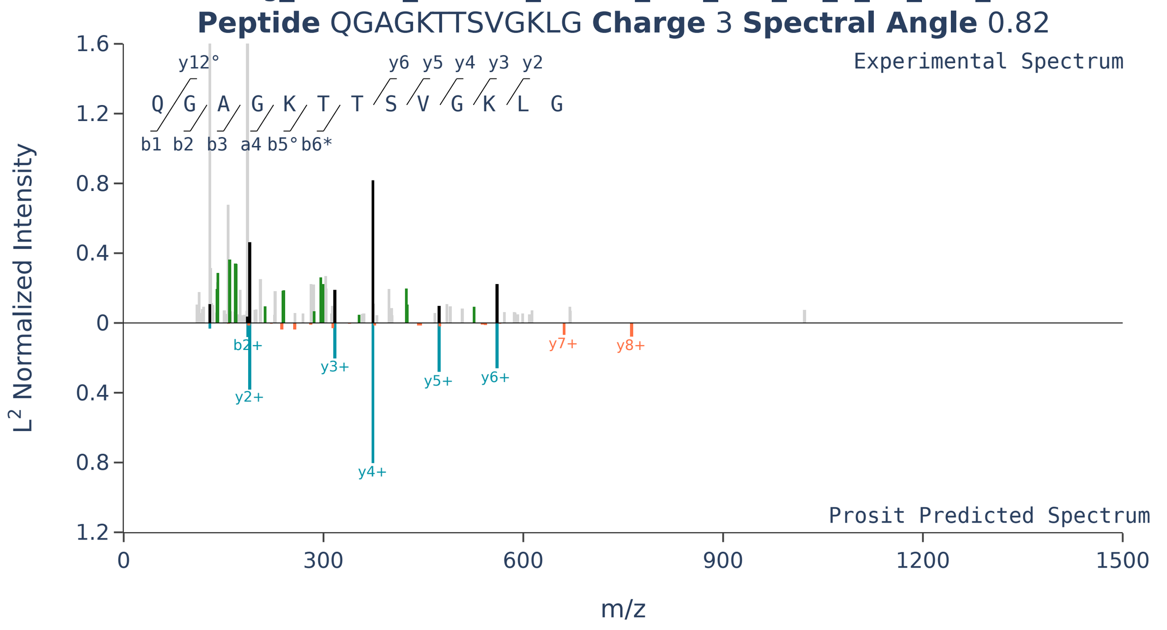


**Source** Ncheng_210623_230623_HFGoe_FFH_20S_25_1_A1_1h_R1 **Scan** 26332
**Peptide** VDEAMMDEIKQVHASINPVE **Charge** 3 **Spectral Angle** 0.63

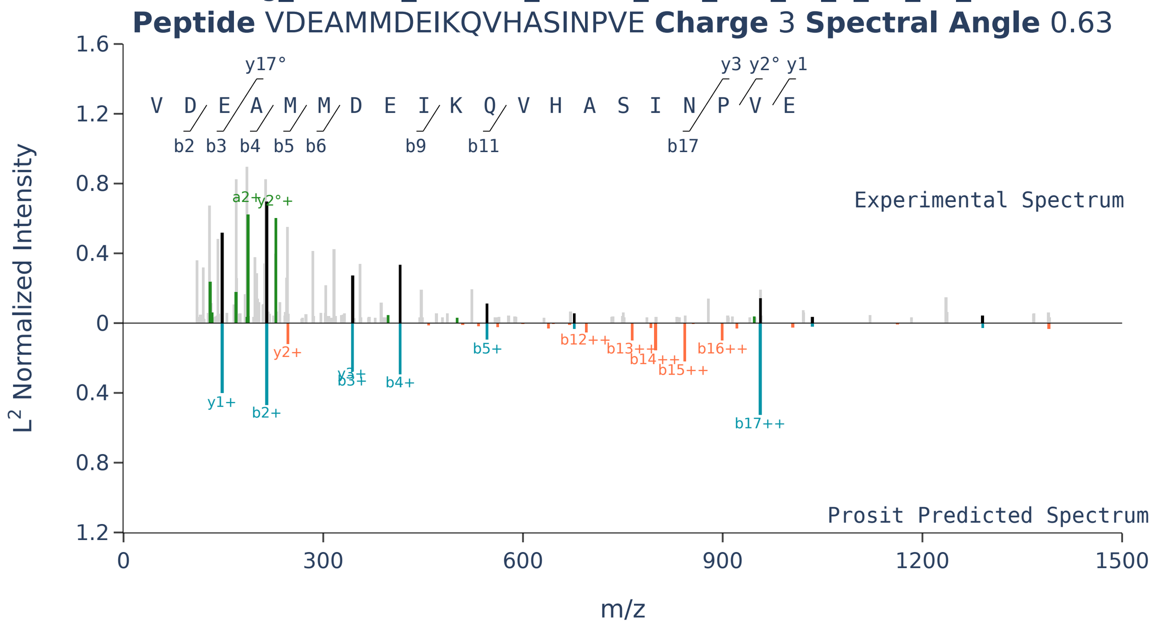


**Source** Ncheng_210623_230623_HFGoe_FFH_20S_25_1_A2_4h_R1 **Scan** 21177
**Peptide** KVLVRMEAILN **Charge** 3 **Spectral Angle** 0.78

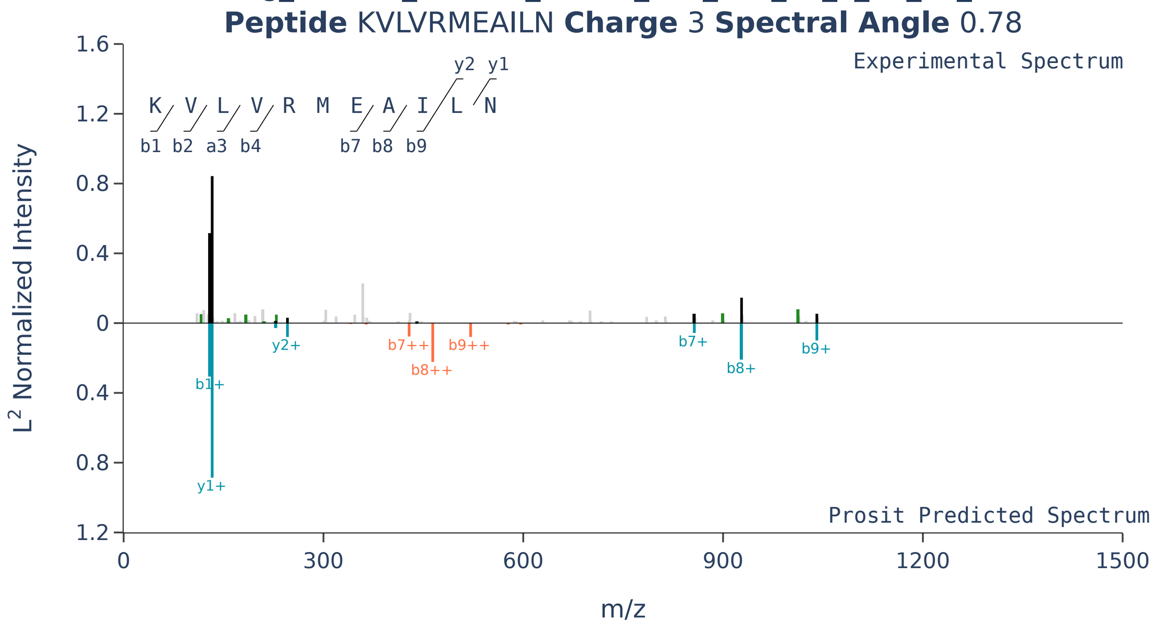


**Source** Ncheng_210623_230623_HFGoe_FFH_20S_25_1_A2_24h_R2 **Scan** 7983
**Peptide** IESKVDRA **Charge** 2 **Spectral Angle** 0.63

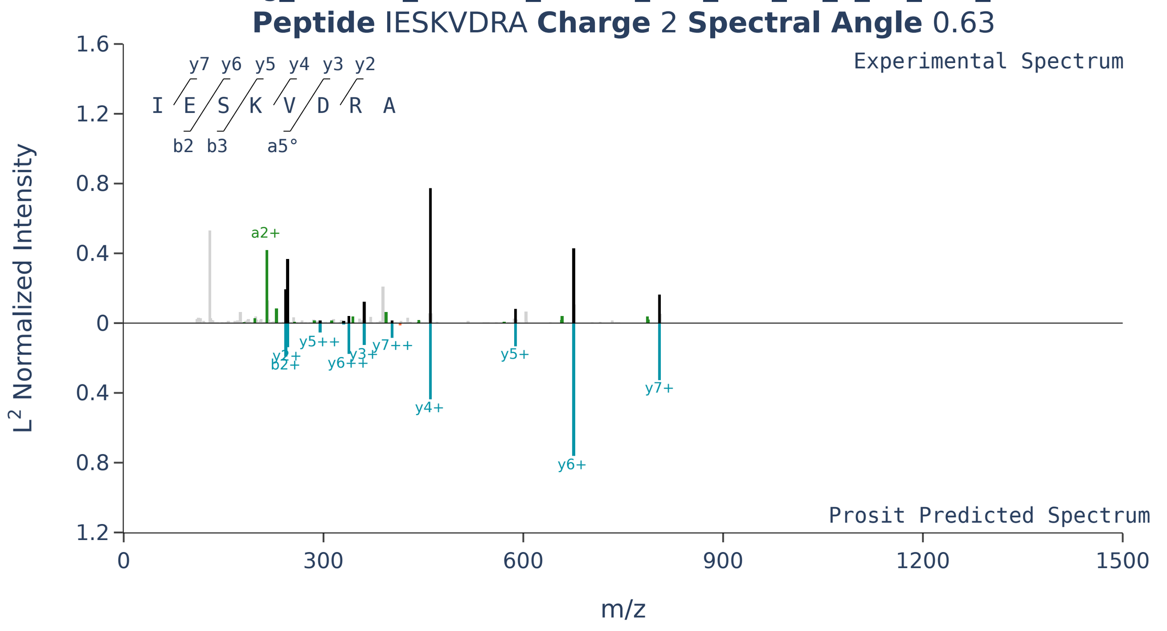


**Source** Ncheng_210623_230623_HFGoe_FFH_20S_25_1_A1_24h_R1 **Scan** 22196
**Peptide** EKAVGHEVNKSLTPGQEFVKIV **Charge** 3 **Spectral Angle** 0.74

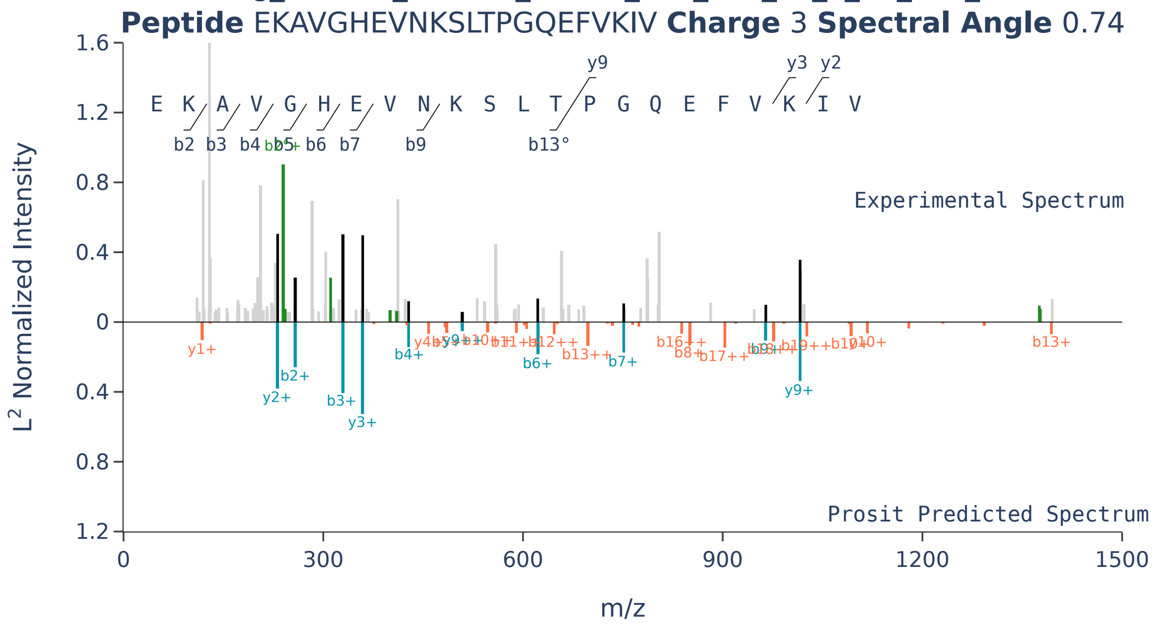


**Source** Ncheng_210623_230623_HFGoe_FFH_20S_25_1_A2_2h_R1 **Scan** 28930
**Peptide** KVLVRMEAIINSMTMKERAKPEII **Charge** 4 **Spectral Angle** 0.81

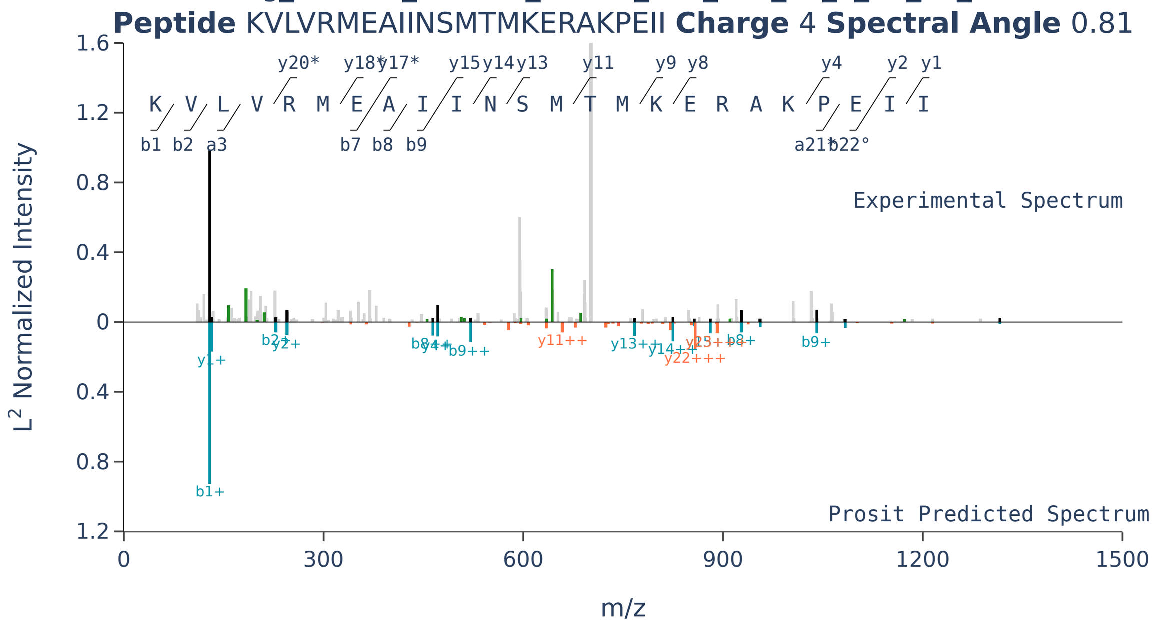


**Source** Ncheng_210623_230623_HFGoe_FFH_20S_25_1_A2_24h_R1 **Scan** 13856
**Peptide** LEPFHPDR **Charge** 1 **Spectral Angle** 0.6

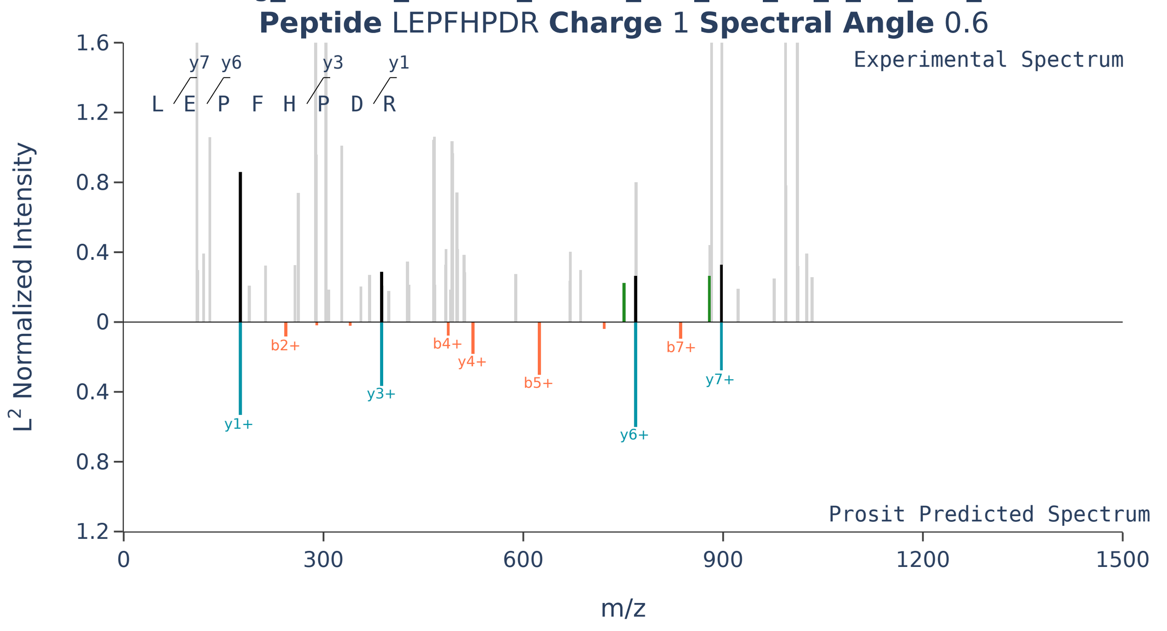


**Source** Ncheng_210623_230623_HFGoe_FFH_20S_25_1_A1_4h_R1 **Scan** 21354
**Peptide** EIKQVHASINPVETL **Charge** 2 **Spectral Angle** 0.47

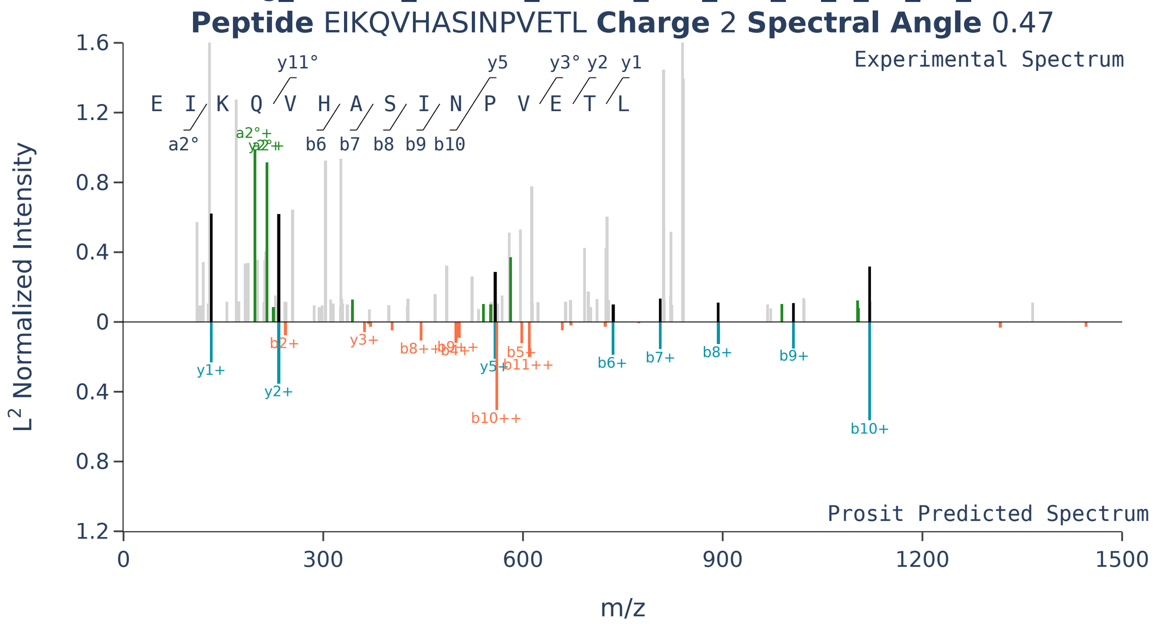


**Source** Ncheng_210623_230623_HFGoe_FFH_20S_25_1_A2_2h_R1 **Scan** 14887
**Peptide** KPIKFLGVGEKTEA **Charge** 3 **Spectral Angle** 0.73

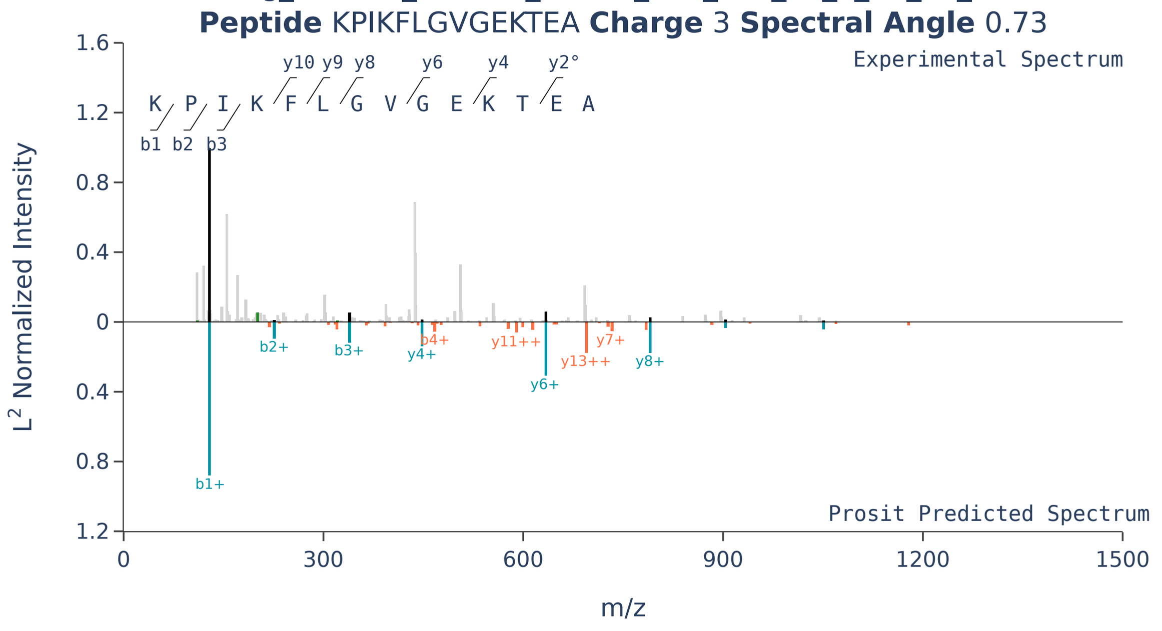


**Source** Ncheng_210623_230623_HFGoe_FFH_20S_25_1_A2_4h_R2 **Scan** 23896
**Peptide** GDARGGAALSIRHITGKPIKFLGVGE **Charge** 5 **Spectral Angle** 0.71

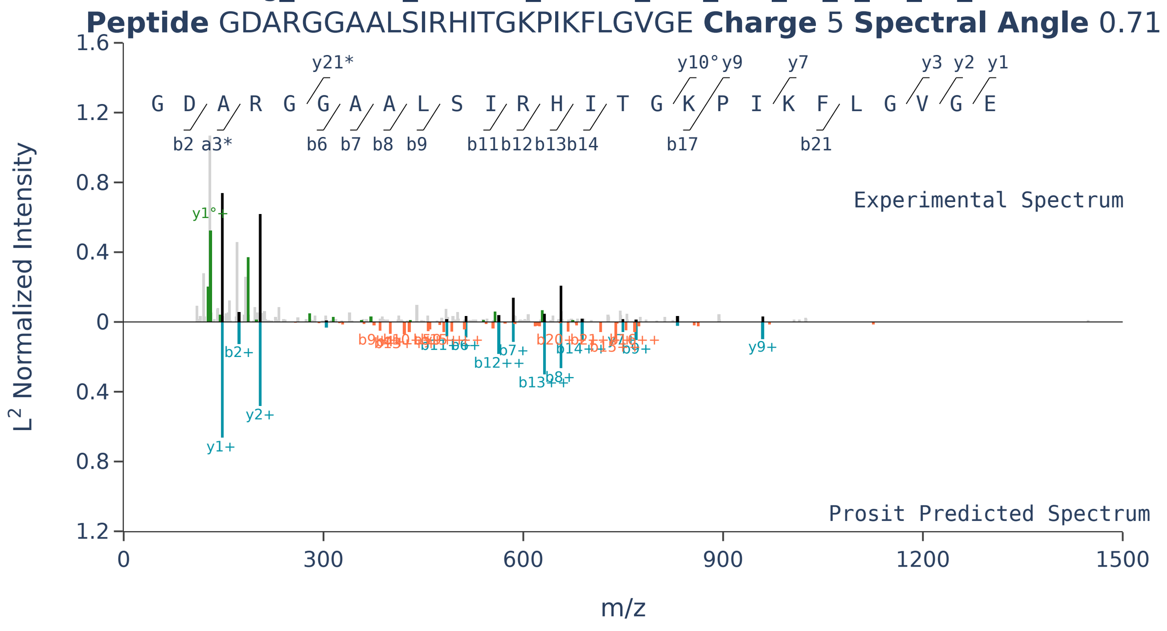

**Source** Ncheng_210623_230623_HFGoe_FFH_20S_25_1_A1_24h_R2 **Scan** 29461
**Peptide** KAFNEALPLTGVVLTK **Charge** 3 **Spectral Angle** 0.61

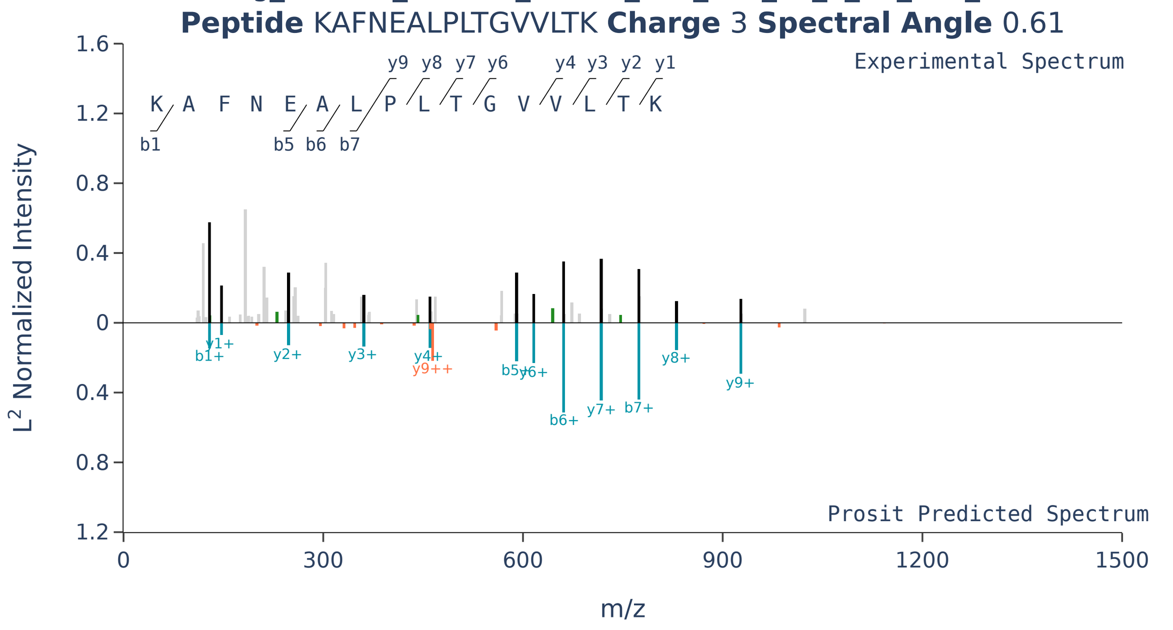


**Source** Ncheng_210623_230623_HFGoe_FFH_20S_25_1_A2_4h_R2 **Scan** 23691
**Peptide** LIEDIESKVDRAQAEKLASKLKKGDGFD **Charge** 4 **Spectral Angle** 0.67

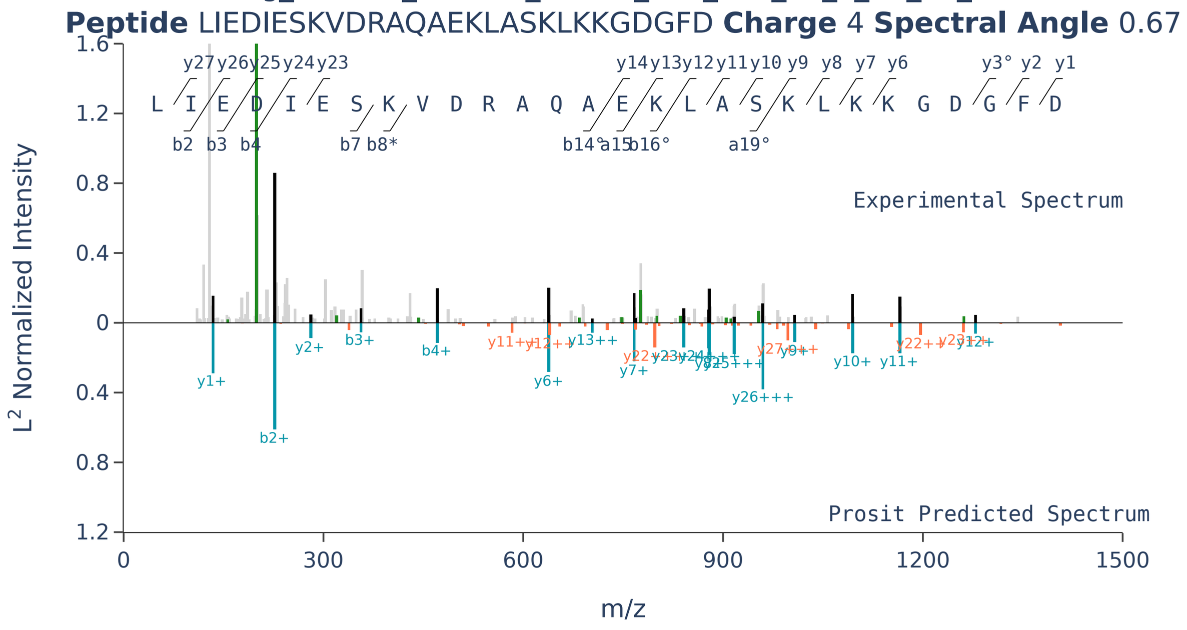


**Source** Ncheng_210623_230623_HFGoe_FFH_20S_25_1_A1_2h_R2 **Scan** 30808
**Peptide** AALKEAKLKFYDVLLV **Charge** 4 **Spectral Angle** 0.61

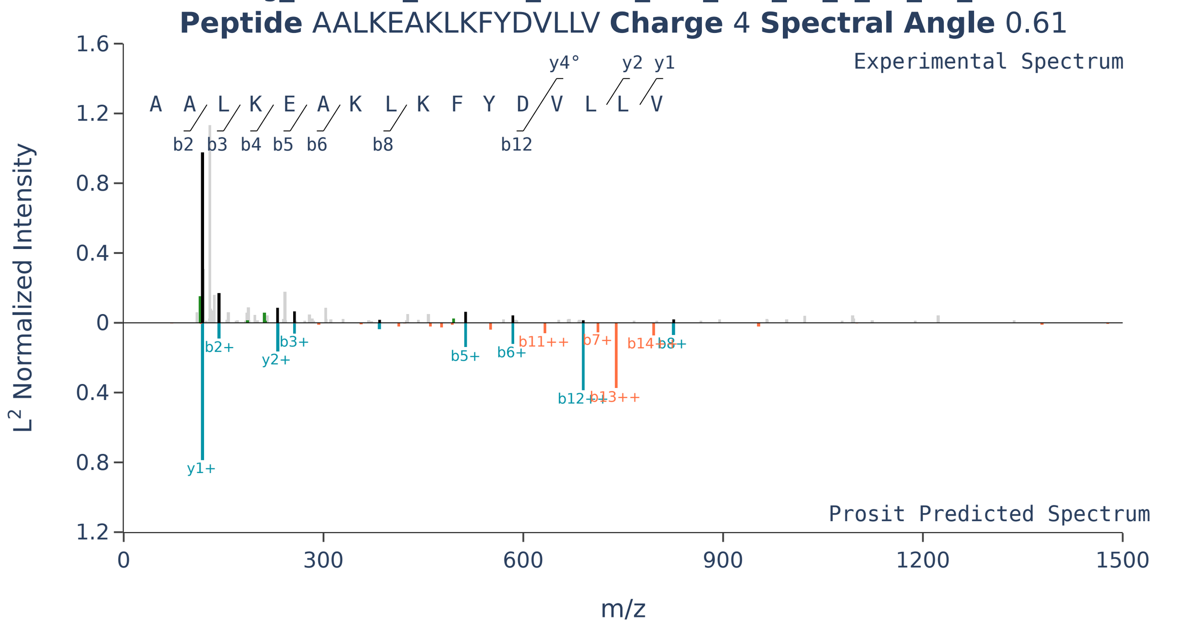


**Source** Ncheng_210623_230623_HFGoe_FFH_20S_25_1_A2_24h_R1 **Scan** 5149
**Peptide** INRVKEKAVGH **Charge** 3 **Spectral Angle** 0.77

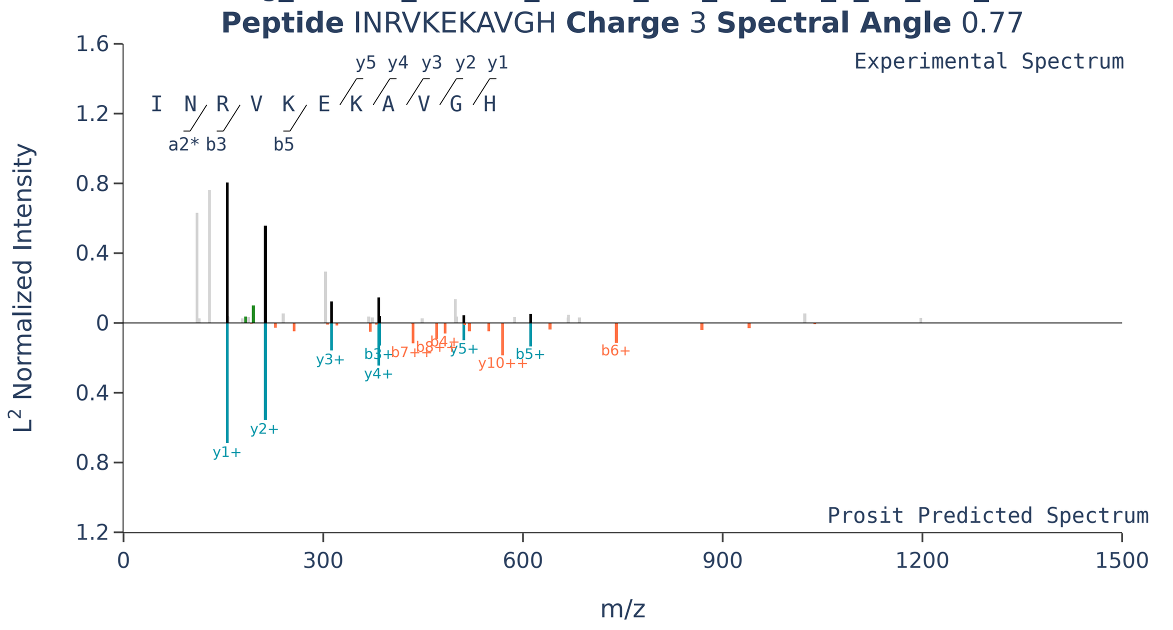


**Source** Ncheng_210623_230623_HFGoe_FFH_20S_25_1_A1_2h_R1 **Scan** 4887
**Peptide** AVGHEVNK **Charge** 2 **Spectral Angle** 0.59

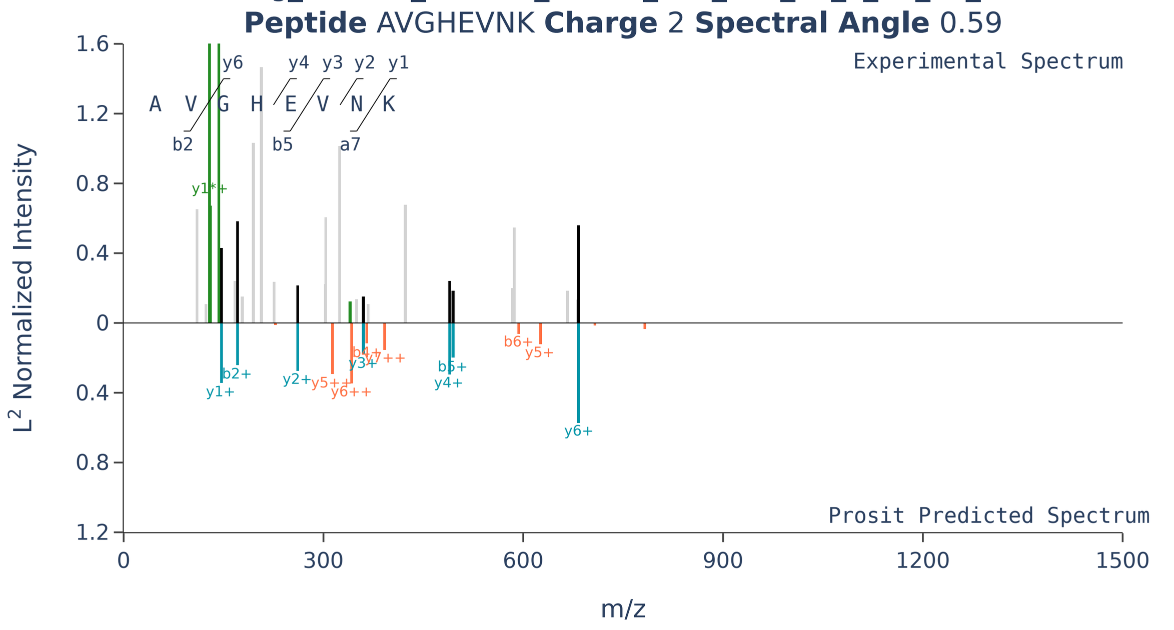


**Source** Ncheng_210623_230623_HFGoe_FFH_20S_25_1_A2_24h_R1 **Scan** 15514
**Peptide** AGRLHVDEAMM **Charge** 3 **Spectral Angle** 0.46

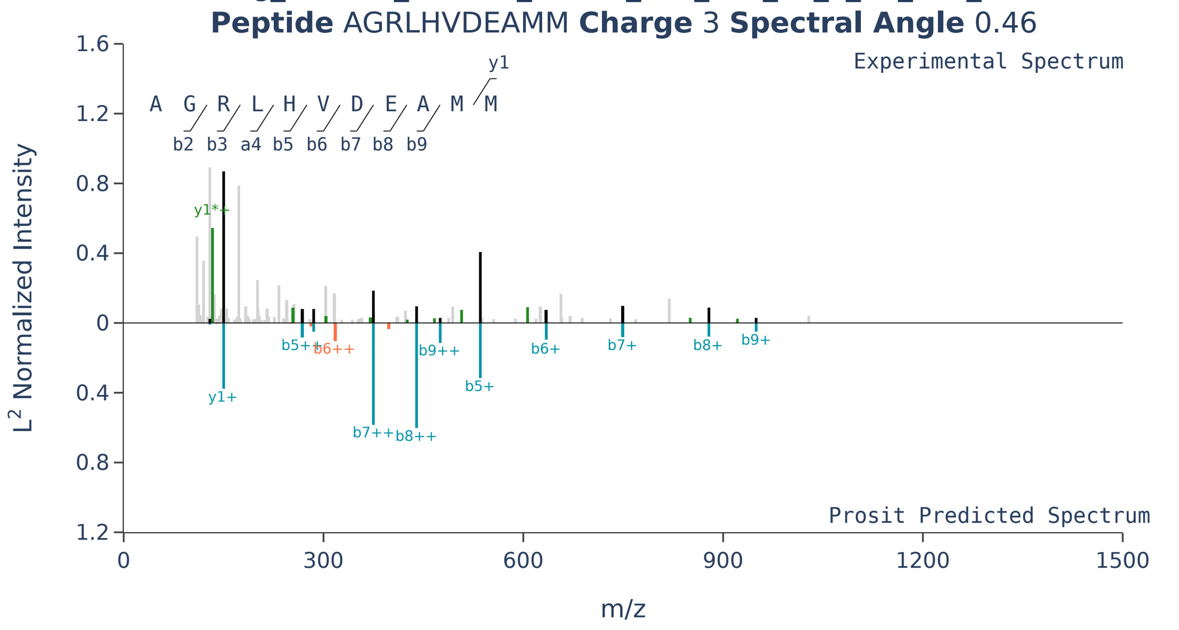


**Source** Ncheng_210623_230623_HFGoe_FFH_20S_25_1_A1_1h_R2 **Scan** 37065
**Peptide** HEVNKSLTPGQEFVKIVRNELVAAMGE **Charge** 4 **Spectral Angle** 0.79

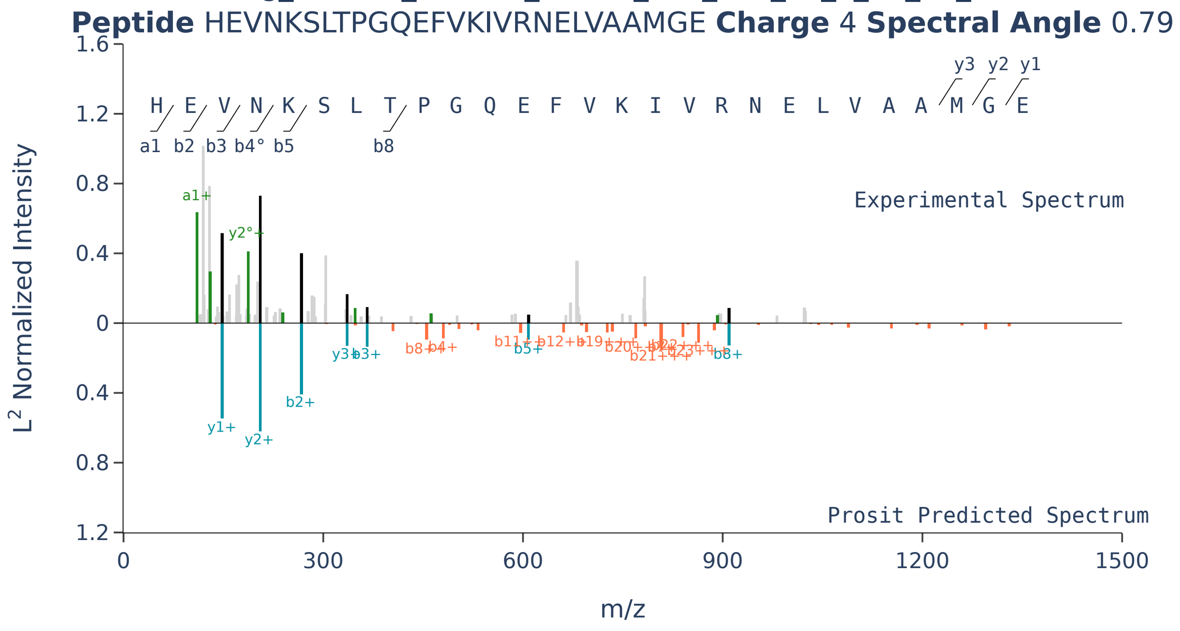


**Source** Ncheng_210623_230623_HFGoe_FFH_20S_25_1_A1_24h_R2 **Scan** 6626
**Peptide** MTMKERAKPEIIKGSRKR **Charge** 5 **Spectral Angle** 0.59

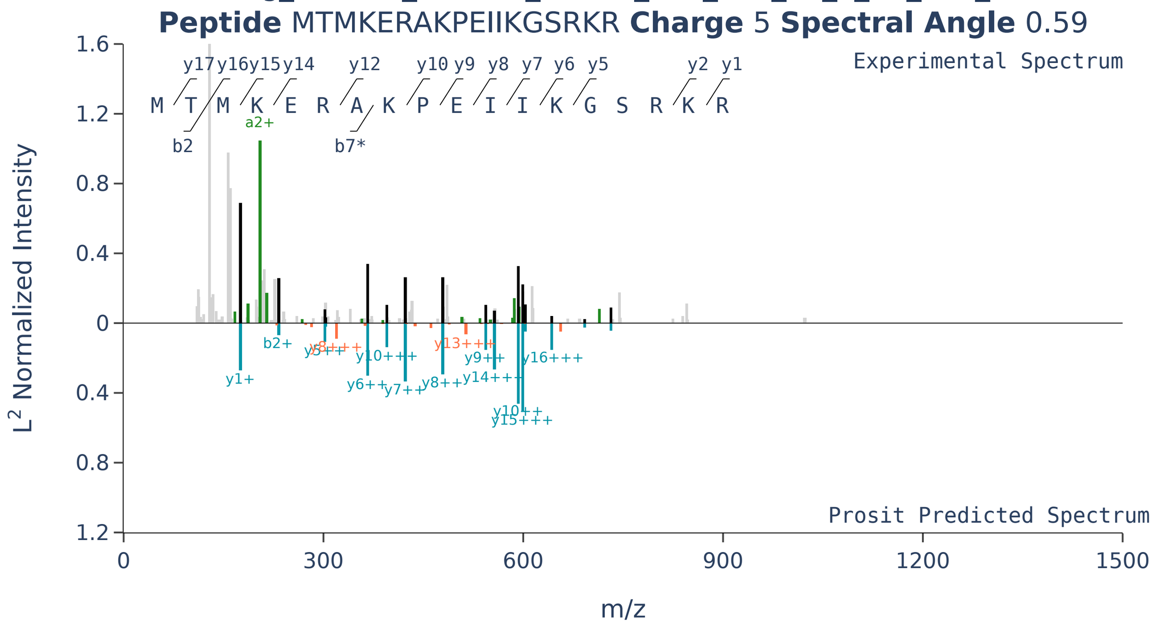


**Source** Ncheng_210623_230623_HFGoe_FFH_20S_25_1_A2_24h_R1 **Scan** 6351
**Peptide** KEKAVGHEVNKSL **Charge** 2 **Spectral Angle** 0.76

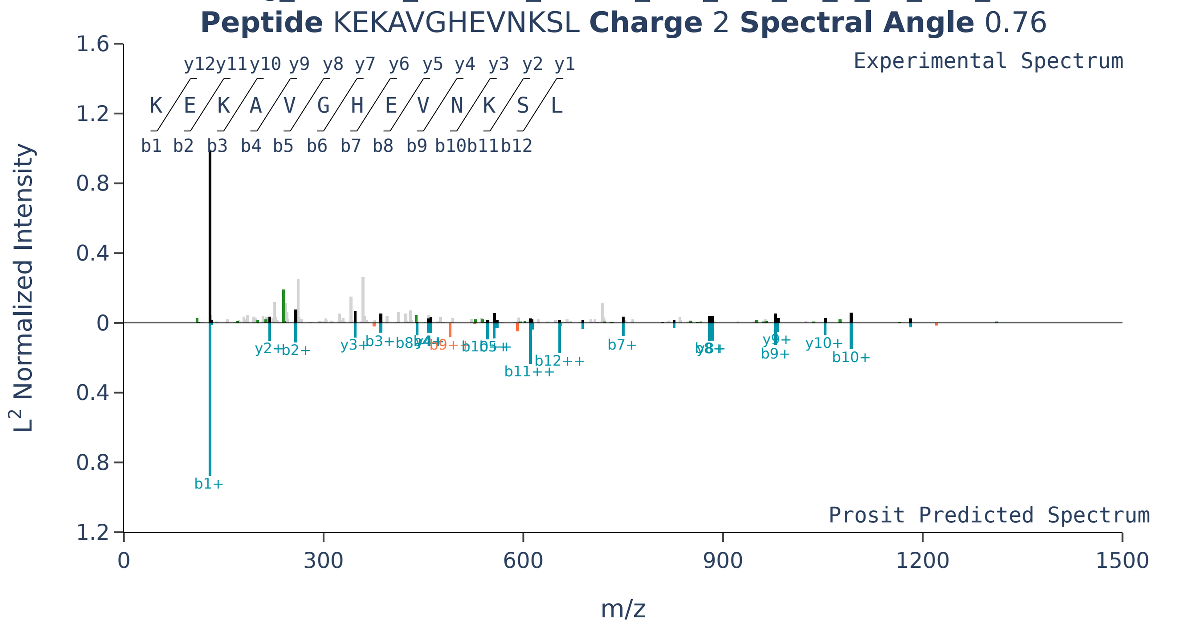


**Source** Ncheng_210623_230623_HFGoe_FFH_20S_25_1_A2_4h_R2 **Scan** 14745
**Peptide** DMQRMMKKMKKGGMAKMMRSM **Charge** 3 **Spectral Angle** 0.65

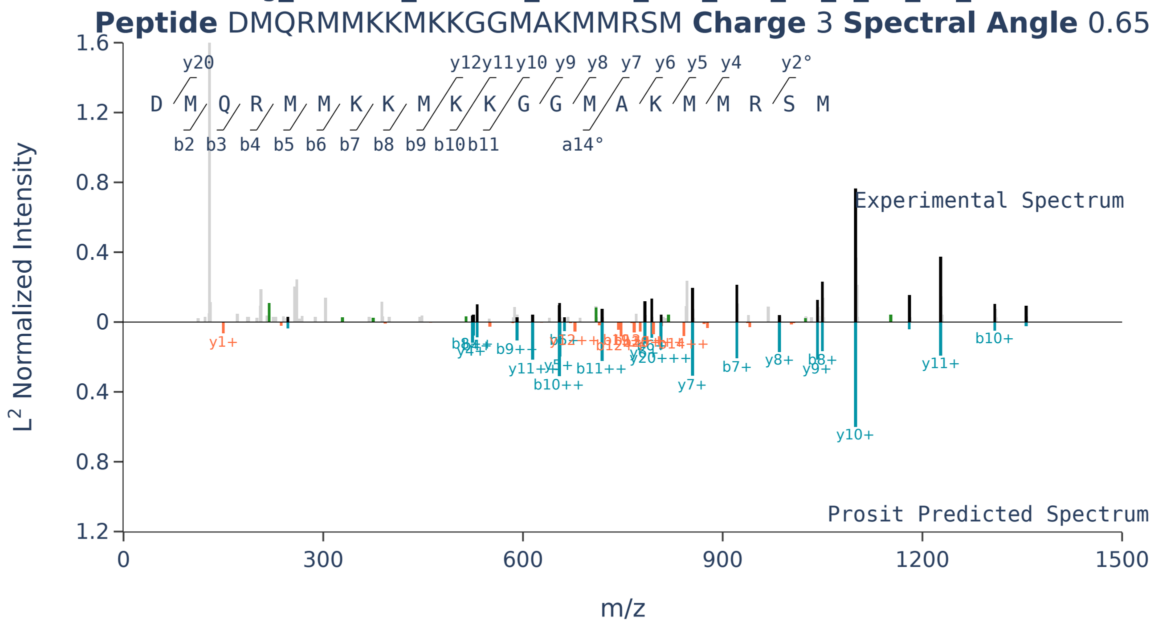


**Source** Ncheng_210623_230623_HFGoe_FFH_20S_25_1_A1_24h_R1 **Scan** 8265
**Peptide** RTLRNISGRGRLTE **Charge** 4 **Spectral Angle** 0.6

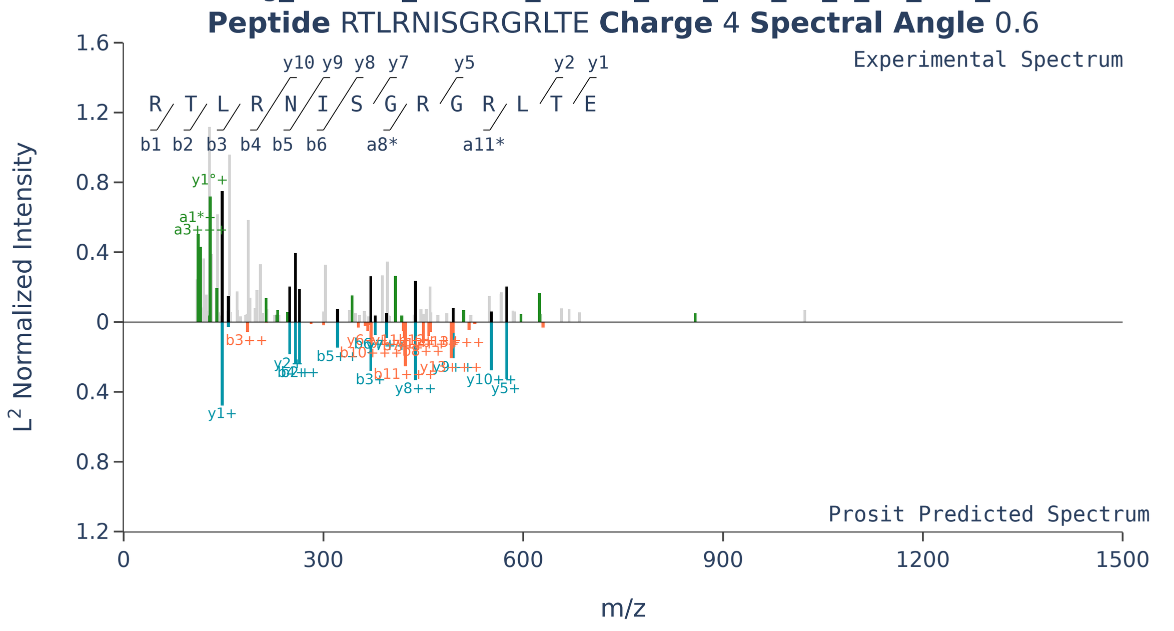


**Source** Ncheng_210623_230623_HFGoe_FFH_20S_25_1_A2_24h_R1 **Scan** 5769
**Peptide** INRVKEKAV **Charge** 3 **Spectral Angle** 0.54

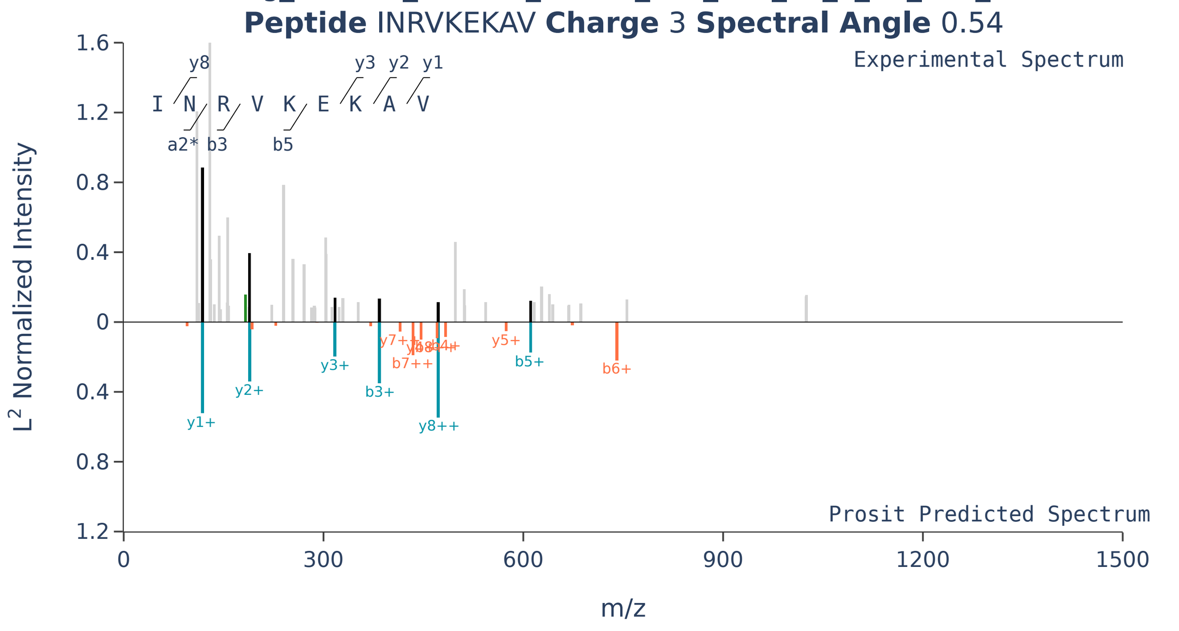


**Source** Ncheng_210623_230623_HFGoe_FFH_20S_25_1_A2_24h_R2 **Scan** 6443
**Peptide** KERAKPEIIKGS **Charge** 2 **Spectral Angle** 0.62

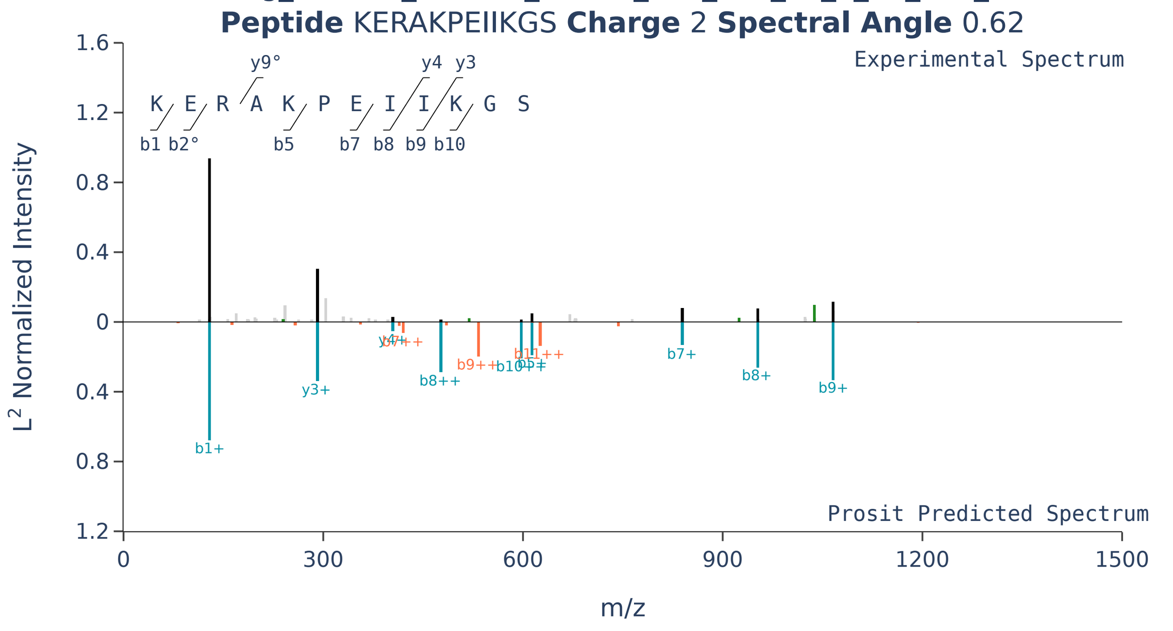


**Source** Ncheng_210623_230623_HFGoe_FFH_20S_25_1_A1_2h_R2 **Scan** 24952
**Peptide** PLTGVVLTKVDGDARGGAALSIRHITG **Charge** 4 **Spectral Angle** 0.69

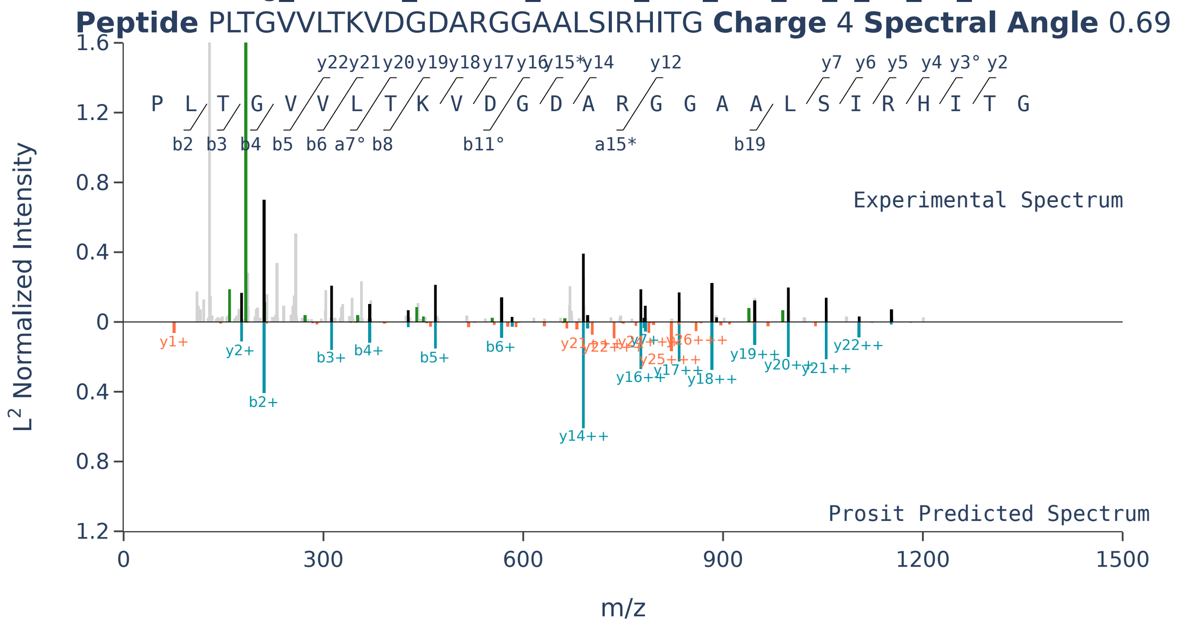


**Source** Ncheng_210623_230623_HFGoe_FFH_20S_25_1_A2_2h_R1 **Scan** 20861
**Peptide** VRMEAIINSMTMKERAK **Charge** 4 **Spectral Angle** 0.63

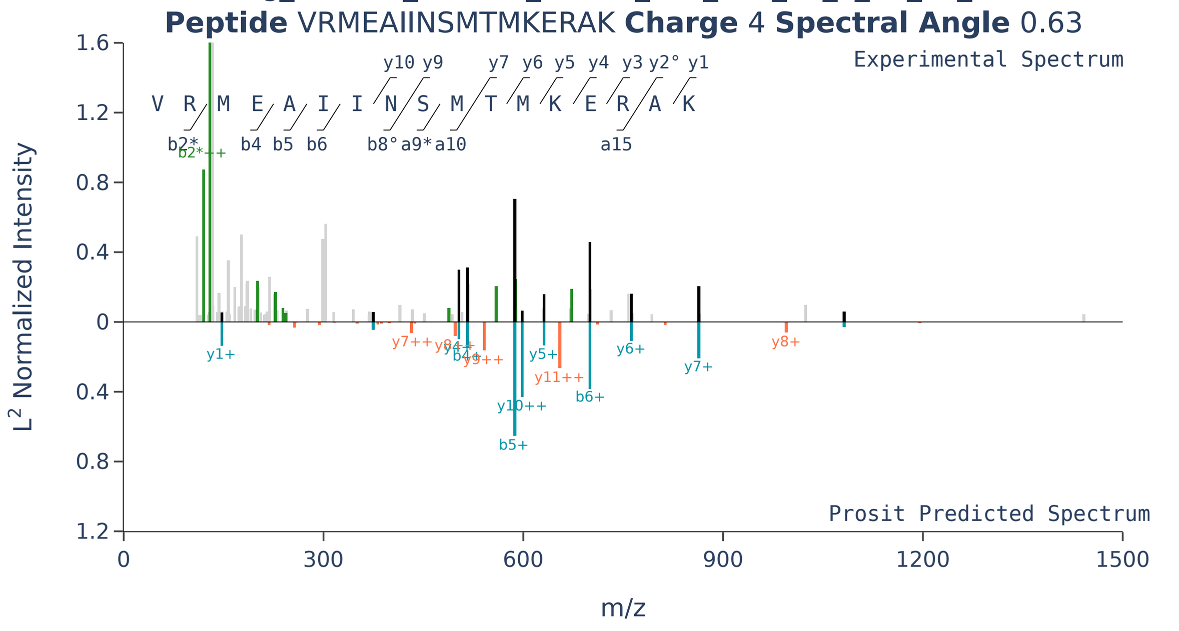

**Source** Ncheng_210623_230623_HFGoe_FFH_20S_25_1_A1_1h_R1 **Scan** 28424
**Peptide** ISGRGRLTEDNVKDTLREVRMALLE **Charge** 5 **Spectral Angle** 0.58

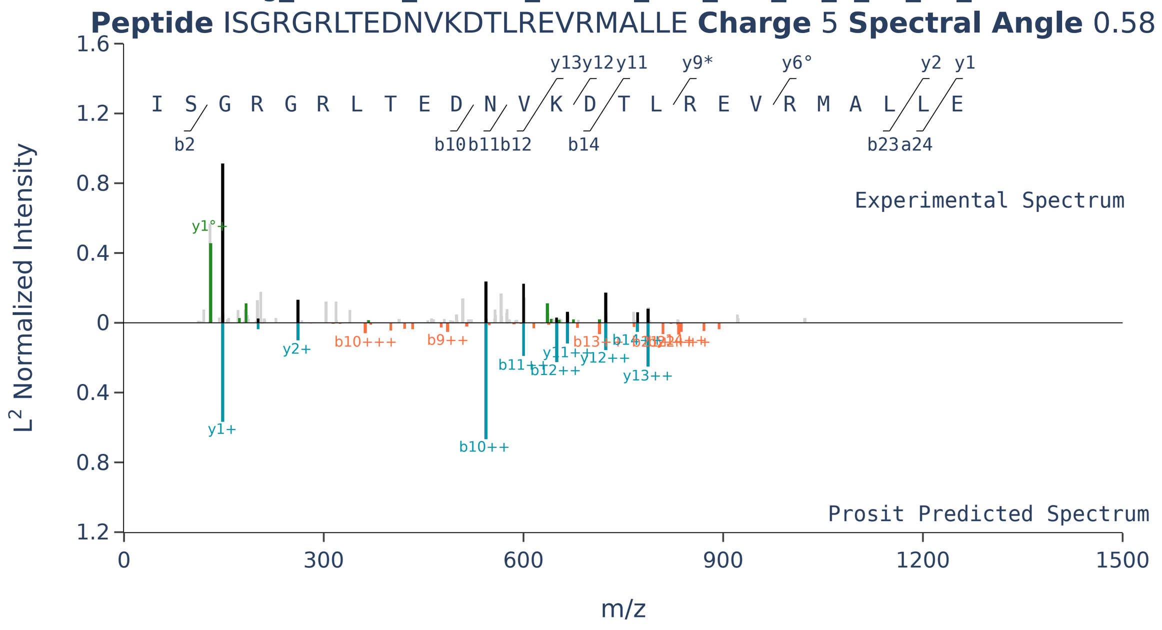


**Source** Ncheng_210623_230623_HFGoe_FFH_20S_25_1_A1_2h_R1 **Scan** 31943
**Peptide** VNAALKEAKLKFYDVLLVDTAGRLHVDE **Charge** 4 **Spectral Angle** 0.74

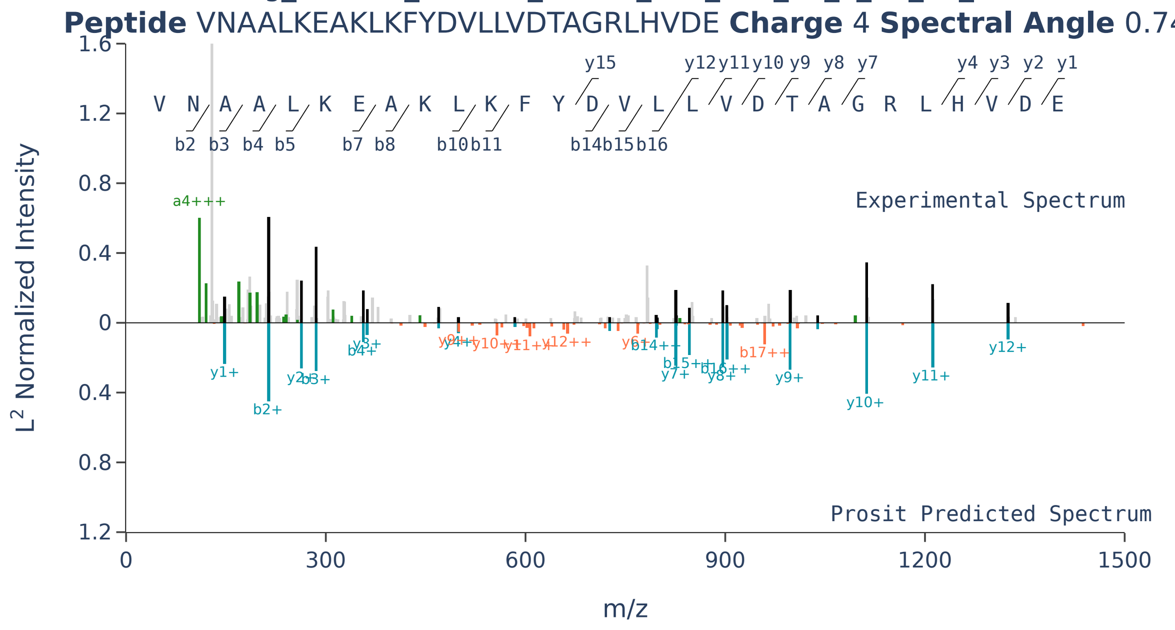


**Source** Ncheng_210623_230623_HFGoe_FFH_20S_25_1_A1_4h_R1 **Scan** 34632
**Peptide** KAFNEALPLTGVVLTKVDGDARGGAALSI **Charge** 4 **Spectral Angle** 0.65

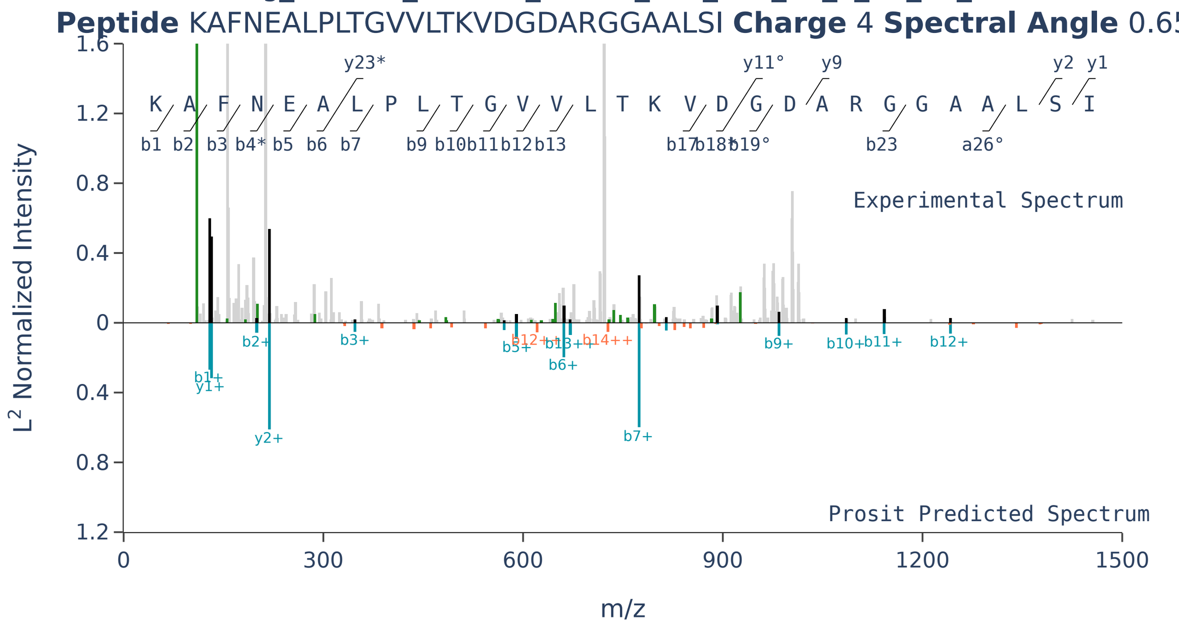


**Source** Ncheng_210623_230623_HFGoe_FFH_20S_25_1_A2_24h_R1 **Scan** 18222
**Peptide** LEDIESKVDRAQAEKLASKLKKG **Charge** 6 **Spectral Angle** 0.79

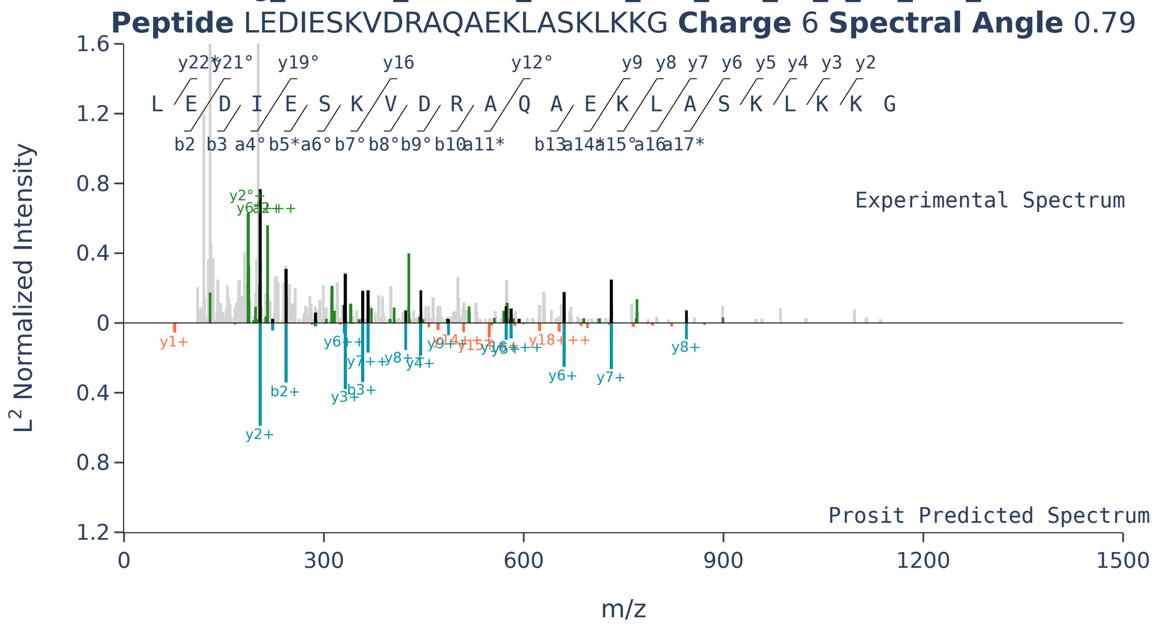


**Source** Ncheng_210623_230623_HFGoe_FFH_20S_25_1_A2_4h_R2 **Scan** 25105
**Peptide** KTEALEPFHPDRIASRIL **Charge** 5 **Spectral Angle** 0.59

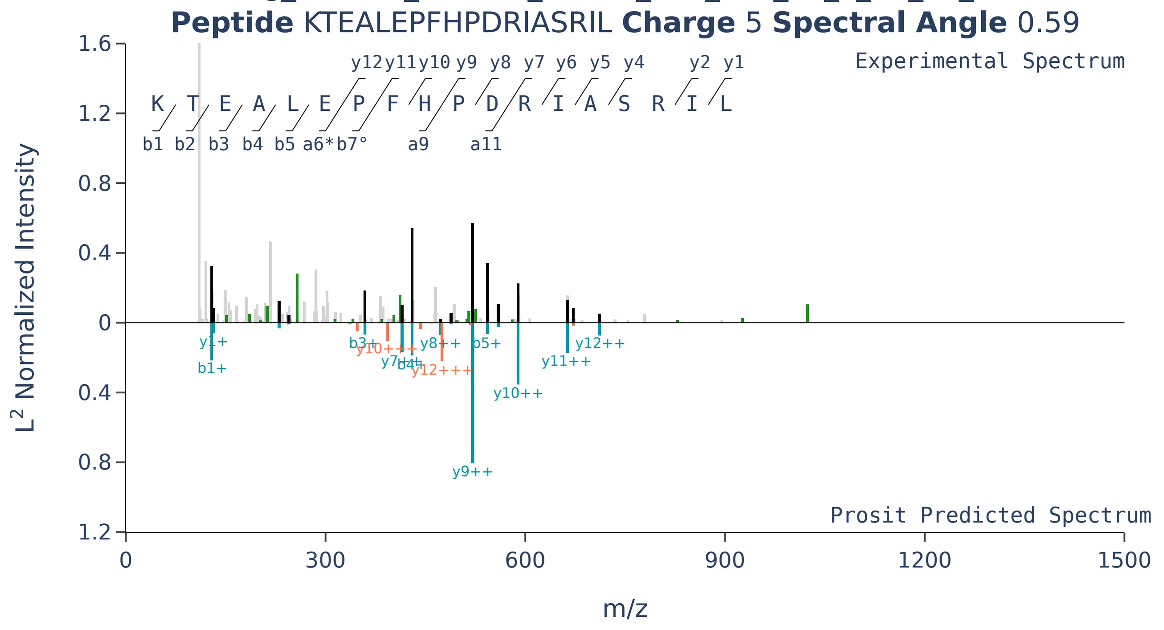


**Source** Ncheng_210623_230623_HFGoe_FFH_20S_25_1_A2_2h_R1 **Scan** 31151
**Peptide** ADVALPVVREFINRVKEKAVGHEVNKS **Charge** 4 **Spectral Angle** 0.79

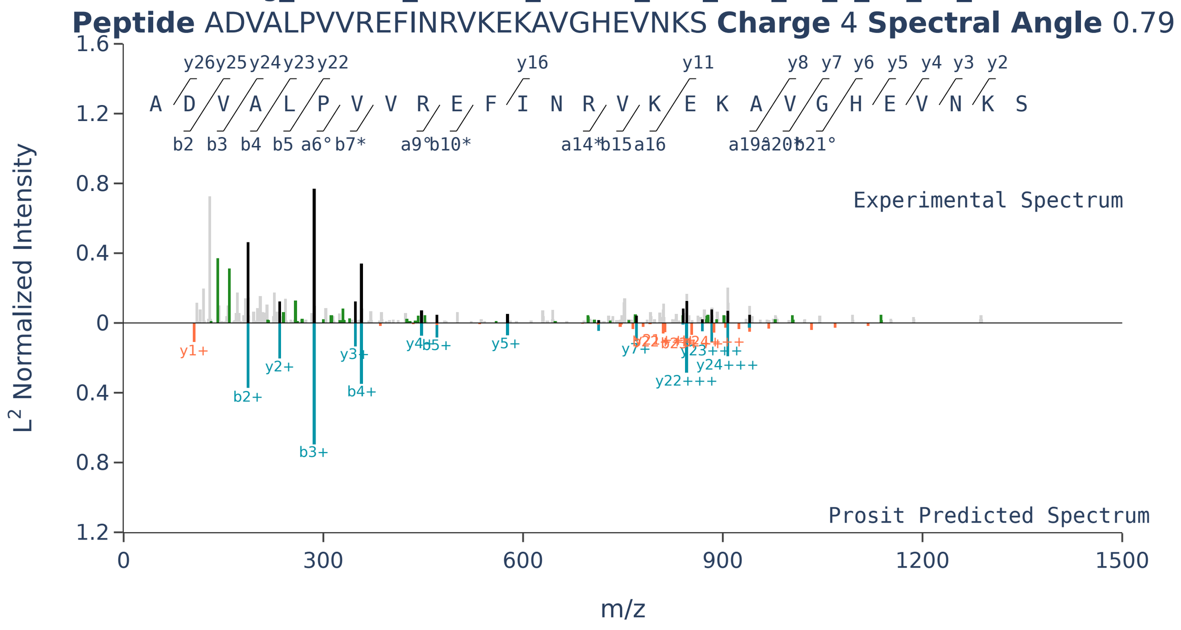


**Source** Ncheng_210623_230623_HFGoe_FFH_20S_25_1_A2_24h_R2 **Scan** 22950
**Peptide** KAFNEALPLT **Charge** 2 **Spectral Angle** 0.24

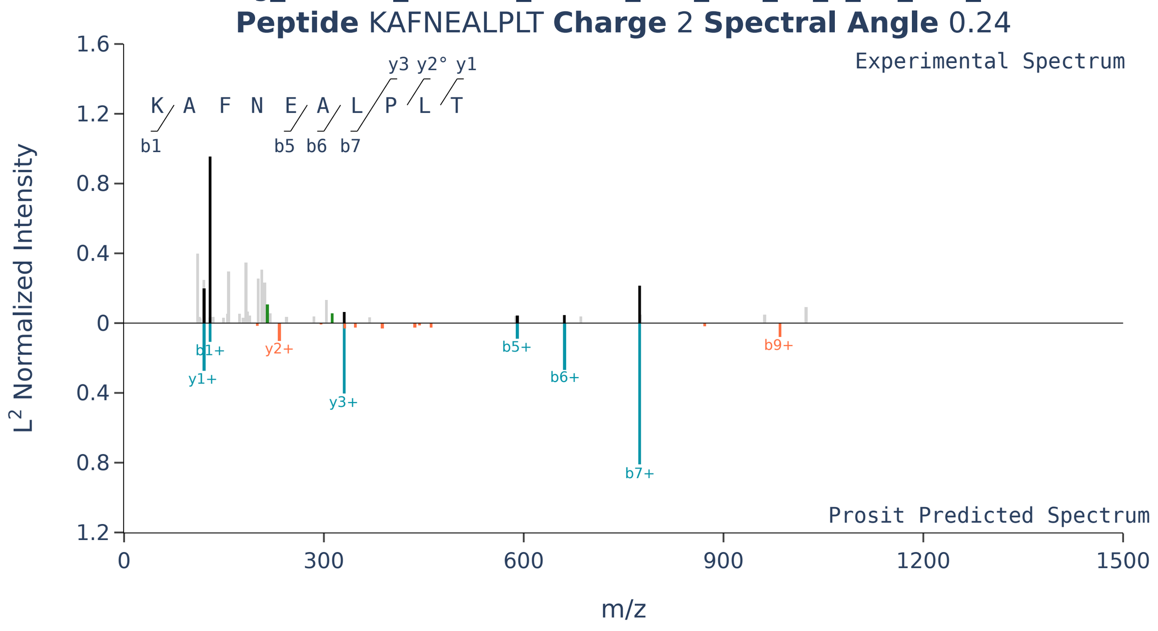


**Source** Ncheng_210623_230623_HFGoe_FFH_20S_25_1_A1_2h_R1 **Scan** 6978
**Peptide** TDRLSRTLRN **Charge** 2 **Spectral Angle** 0.54

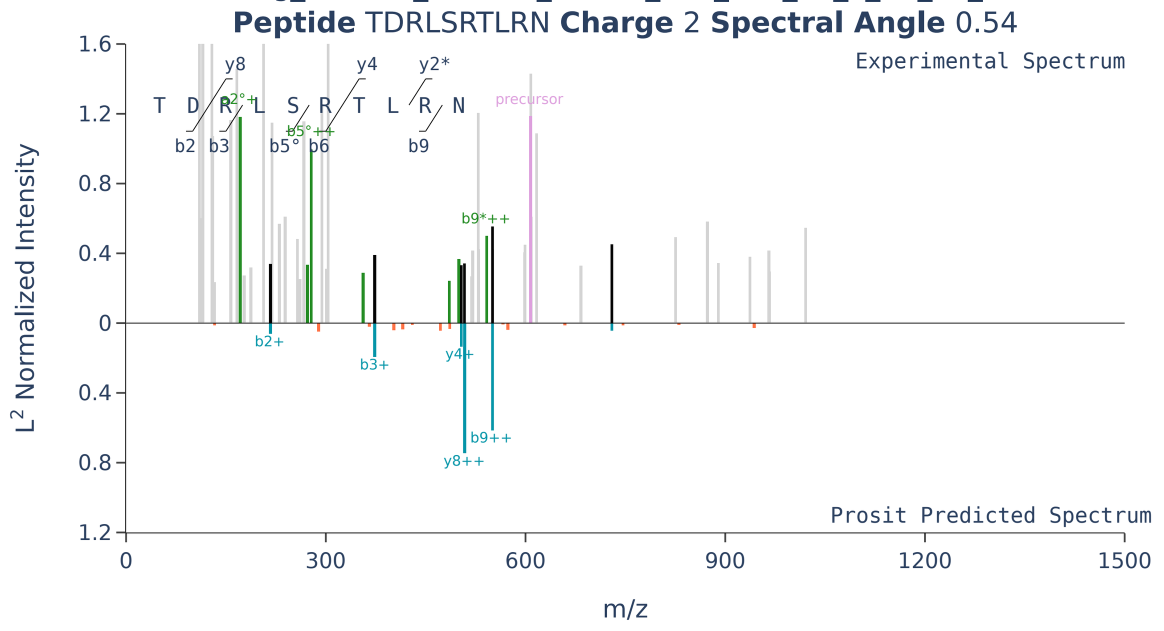


**Source** Ncheng_210623_230623_HFGoe_FFH_20S_25_1_A1_2h_R2 **Scan** 10295
**Peptide** MKERAKPELL **Charge** 3 **Spectral Angle** 0.7

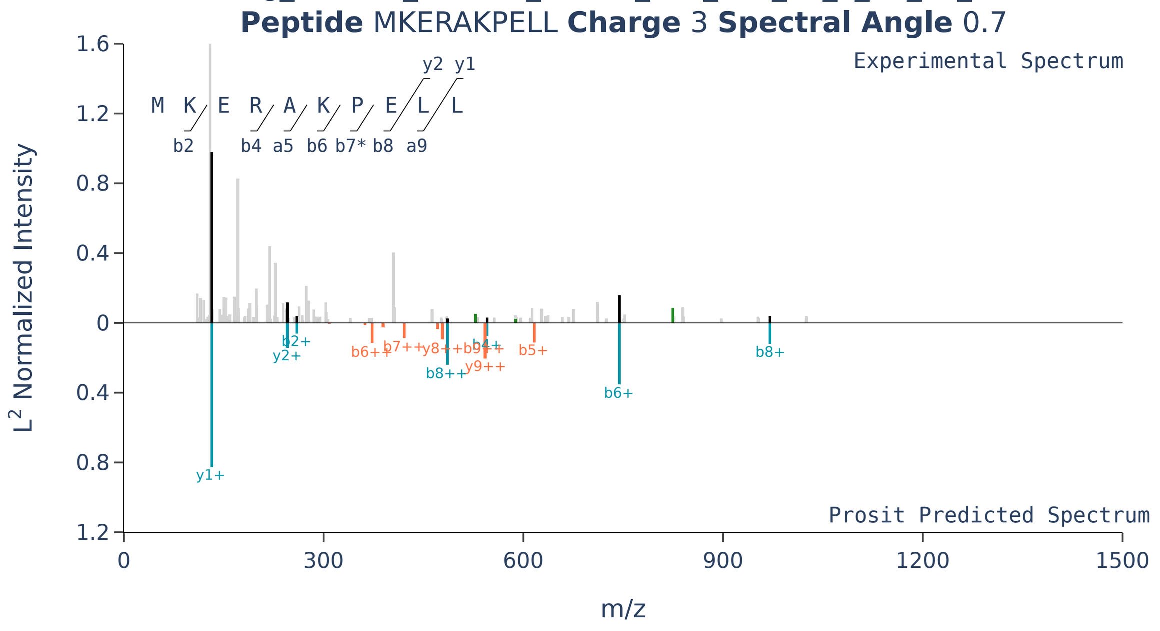


**Source** Ncheng_210623_230623_HFGoe_FFH_20S_25_1_A2_2h_R1 **Scan** 24537
**Peptide** VLLVDTAGRLHV **Charge** 3 **Spectral Angle** 0.55

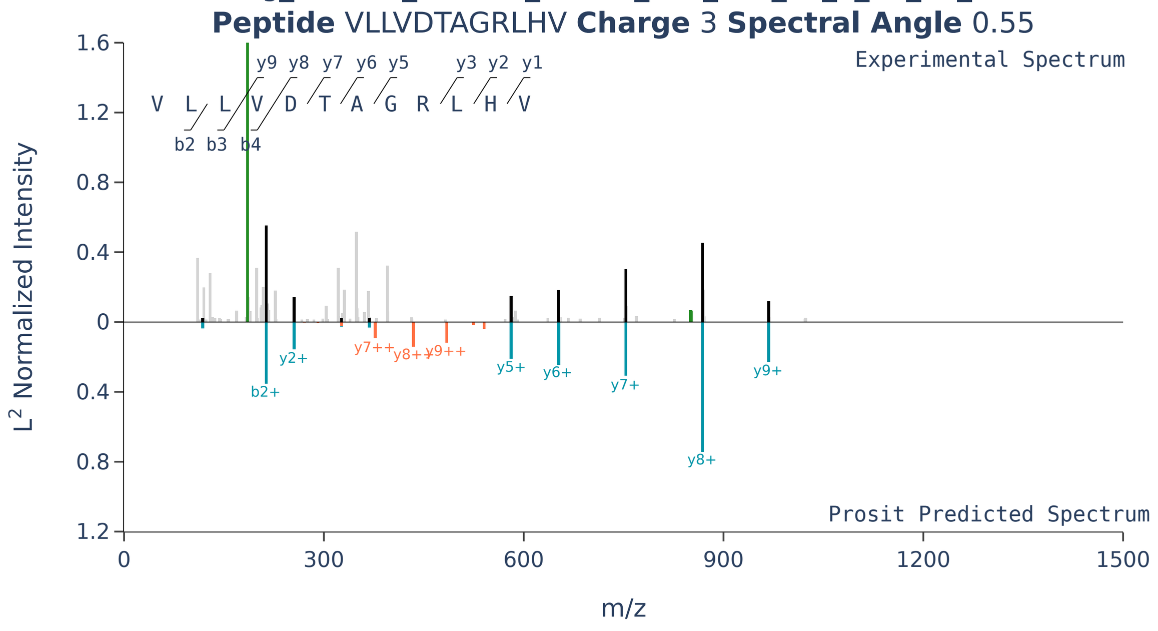


**Source** Ncheng_210623_230623_HFGoe_FFH_20S_25_1_A2_2h_R1 **Scan** 28329
**Peptide** ALEPFHPDRIASRILGMGD **Charge** 3 **Spectral Angle** 0.62

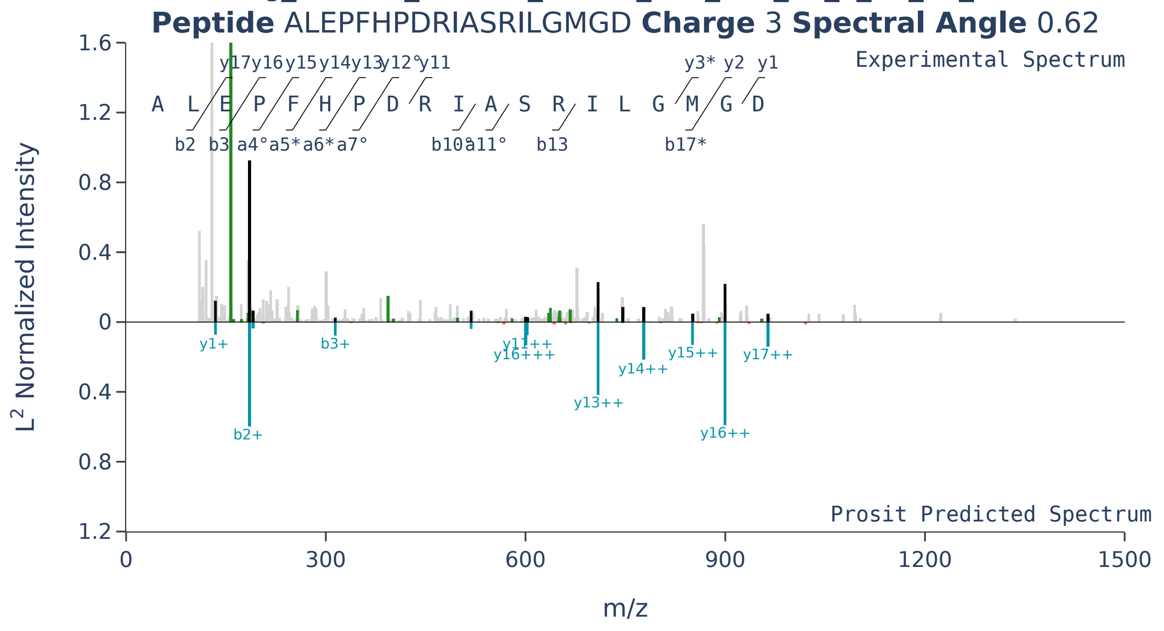


**Source** Ncheng_210623_230623_HFGoe_FFH_20S_25_1_A1_4h_R1 **Scan** 26653
**Peptide** VNAALKEAKLKFYDVL **Charge** 4 **Spectral Angle** 0.85

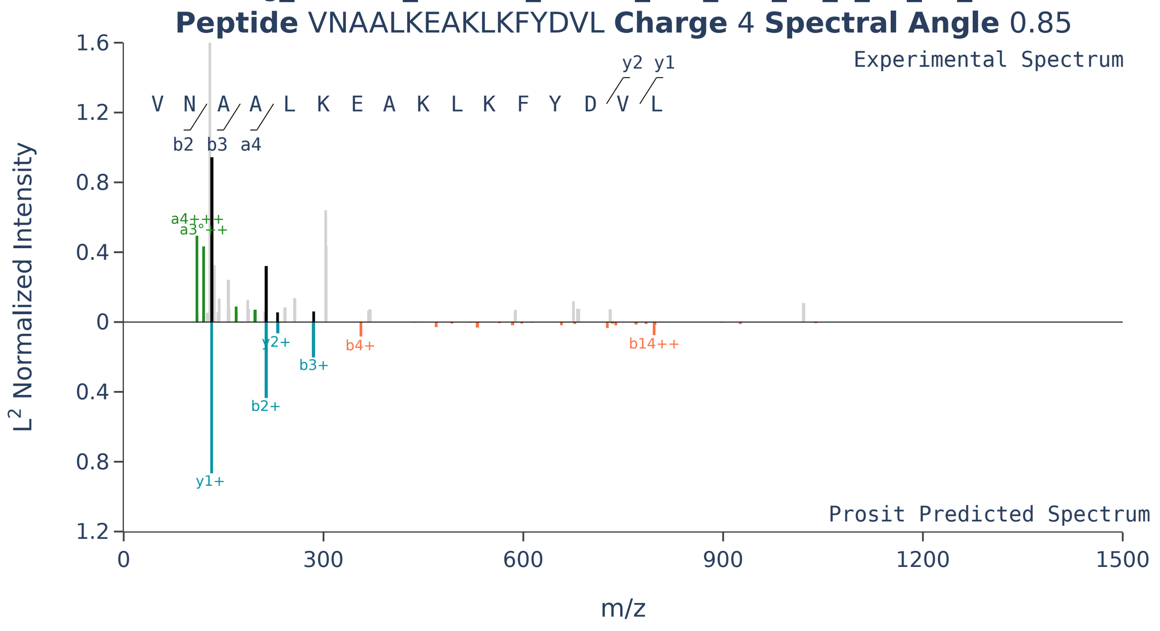


**Source** Ncheng_210623_230623_HFGoe_FFH_20S_25_1_A2_4h_R2 **Scan** 10418
**Peptide** IPDNVKSQMDD **Charge** 2 **Spectral Angle** 0.46

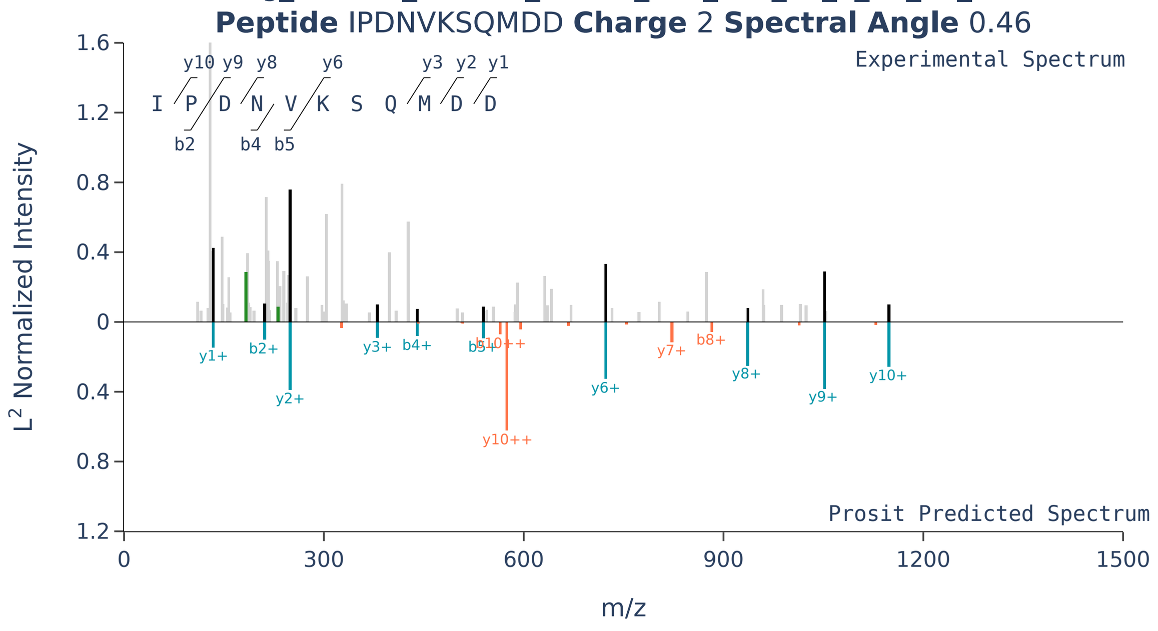


**Source** Ncheng_210623_230623_HFGoe_FFH_20S_25_1_A2_24h_R2 **Scan** 15626
**Peptide** AGLQGAGKTTSVGKLGKFLREKH **Charge** 4 **Spectral Angle** 0.8

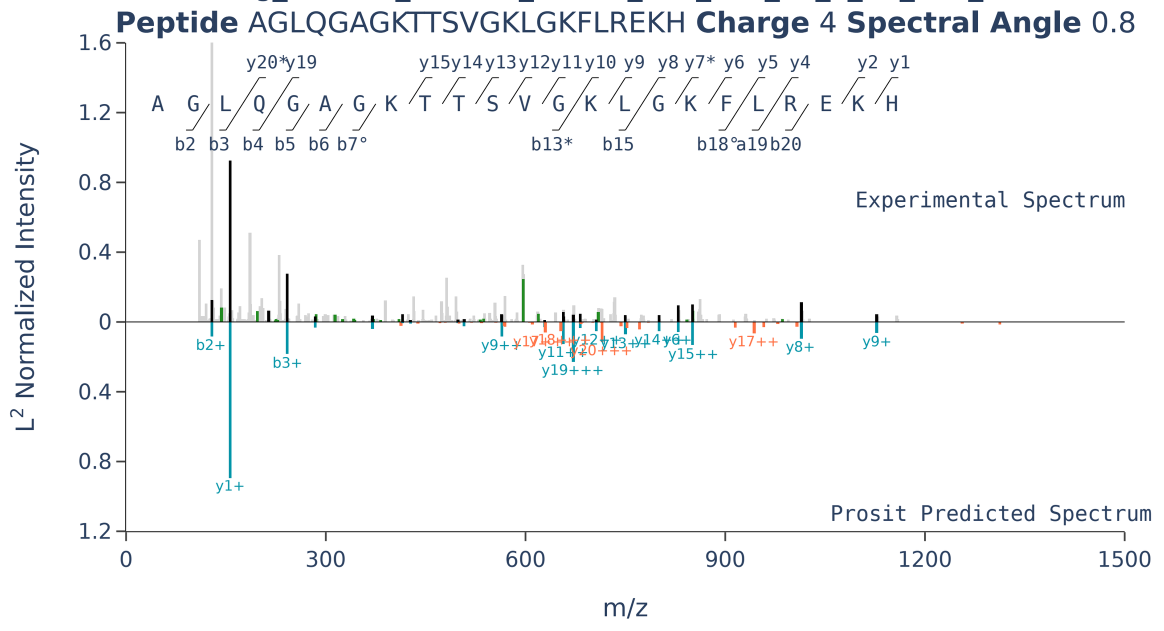


**Source** Ncheng_210623_230623_HFGoe_FFH_20S_25_1_A2_4h_R1 **Scan** 28168
**Peptide** RPAAIKQLETLAEQV **Charge** 3 **Spectral Angle** 0.57

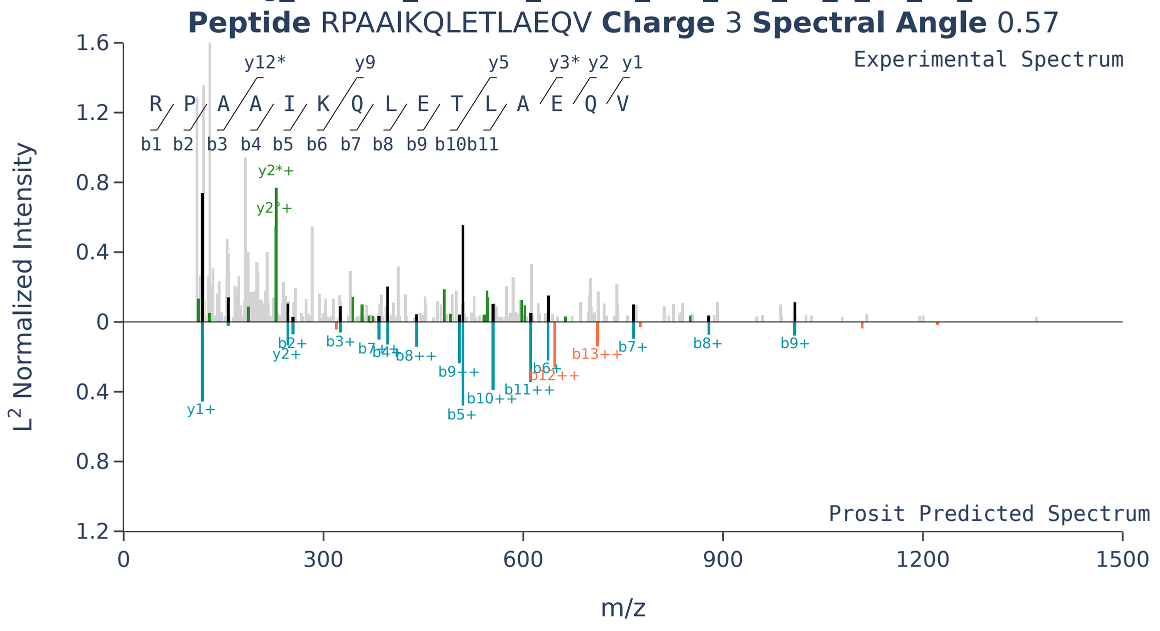

**Source** Ncheng_210623_230623_HFGoe_FFH_20S_25_1_A1_2h_R2 **Scan** 26593
**Peptide** VKEKAVGHEVNKSLTPGQEFVKIVRNELVA **Charge** 4 **Spectral Angle** 0.54

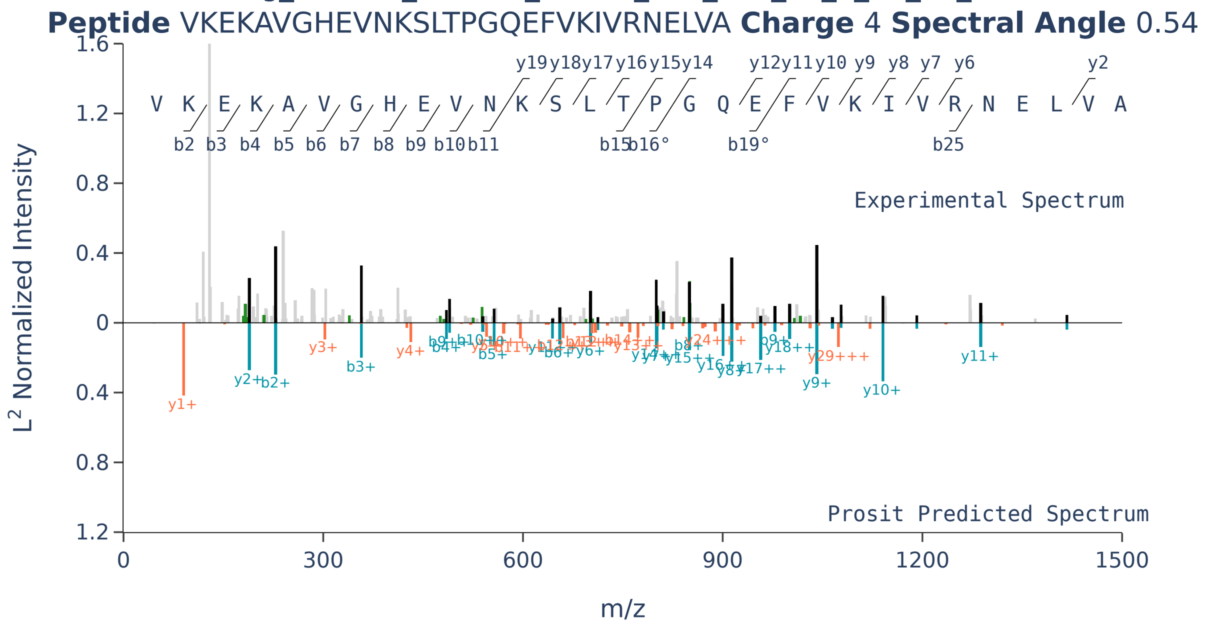


**Source** Ncheng_210623_230623_HFGoe_FFH_20S_25_1_A2_4h_R2 **Scan** 24704
**Peptide** RQMKNMGGMASLMGKLPGMGQIPDNVKS **Charge** 5 **Spectral Angle** 0.79

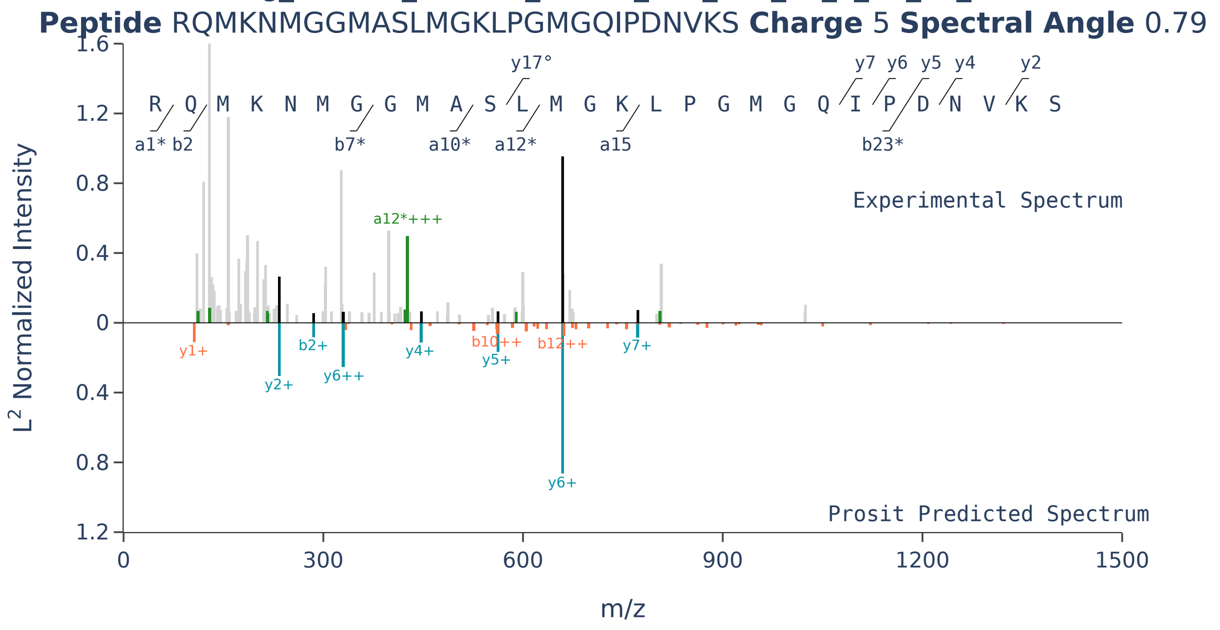


**Source** Ncheng_210623_230623_HFGoe_FFH_20S_25_1_A1_24h_R2 **Scan** 8676
**Peptide** SMTMKERAKPEIIKGSR **Charge** 5 **Spectral Angle** 0.75

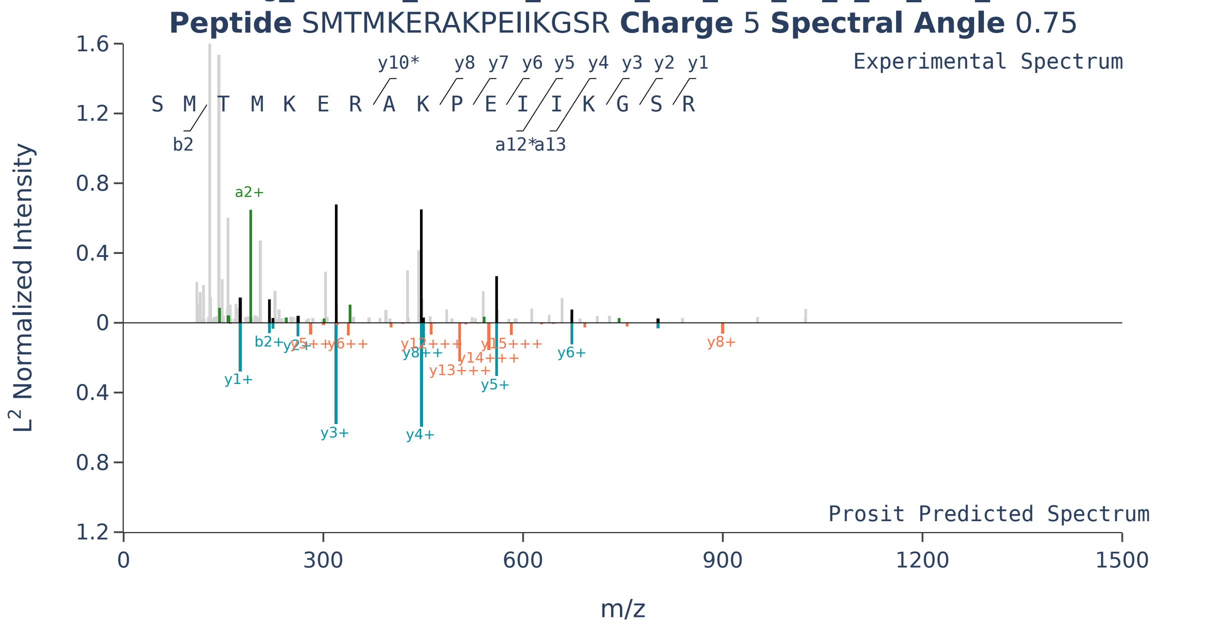


**Source** Ncheng_210623_230623_HFGoe_FFH_20S_25_1_A1_24h_R2 **Scan** 6401
**Peptide** SLRHITG **Charge** 2 **Spectral Angle** 0.88

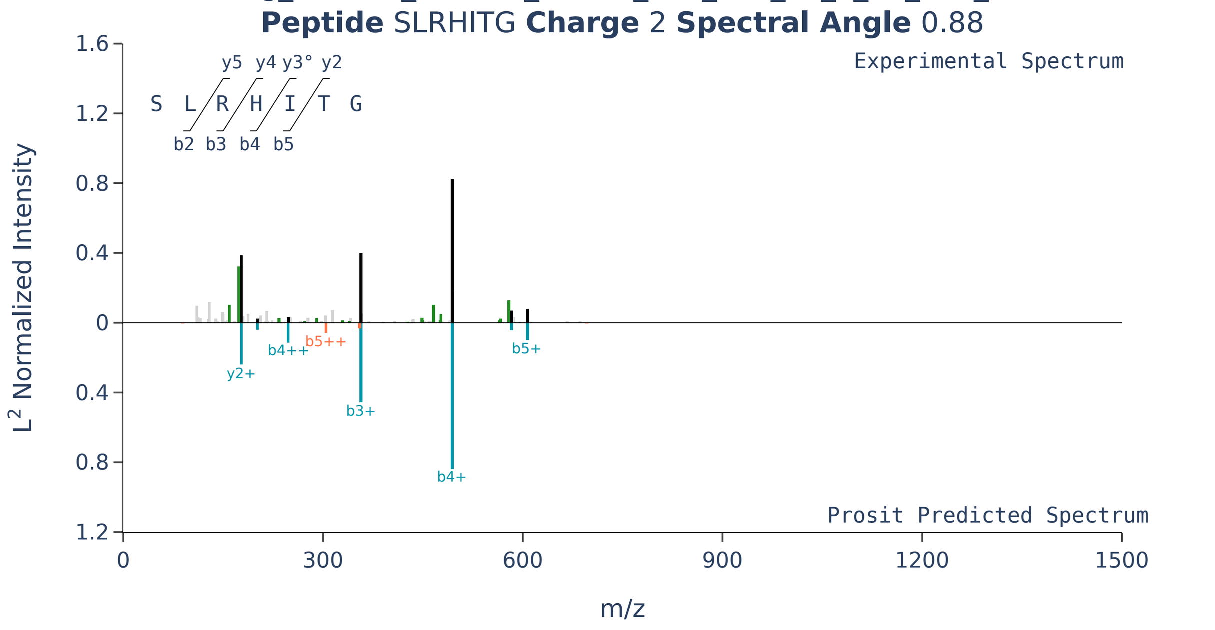


**Source** Ncheng_210623_230623_HFGoe_FFH_20S_25_1_A1_2h_R1 **Scan** 28355
**Peptide** QDVNRLLKQFDDMQRMMKKMK **Charge** 5 **Spectral Angle** 0.75

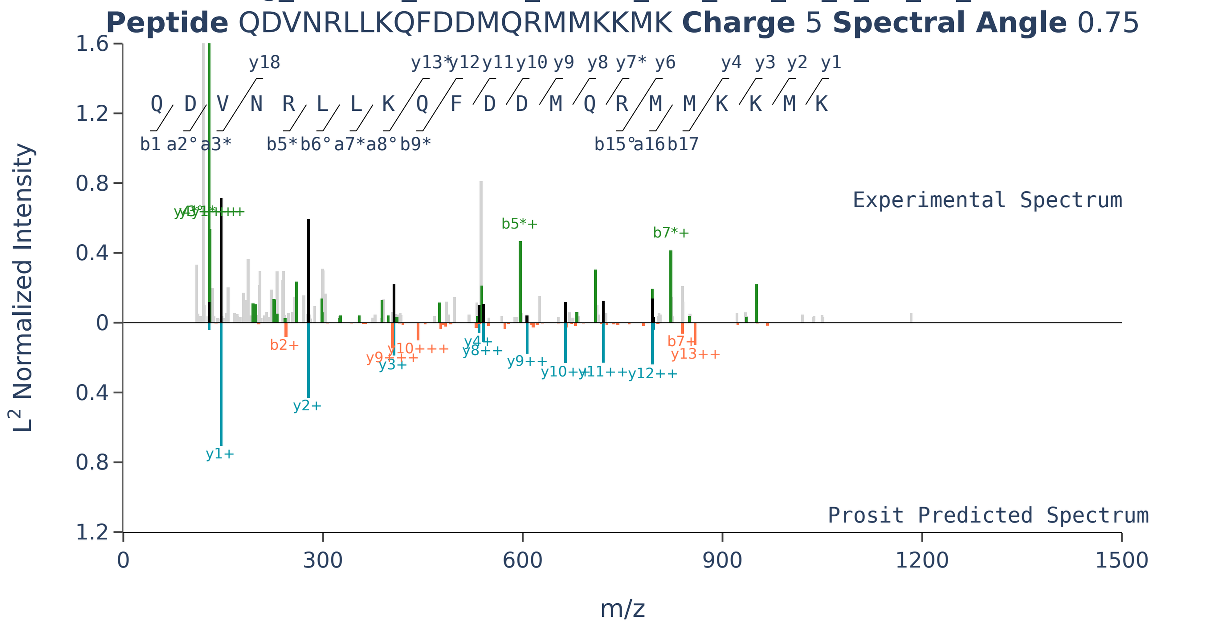


**Source** Ncheng_210623_230623_HFGoe_FFH_20S_25_1_A2_4h_R2 **Scan** 18654
**Peptide** FDDMQRMMKKMKKGGMAKMM **Charge** 5 **Spectral Angle** 0.83

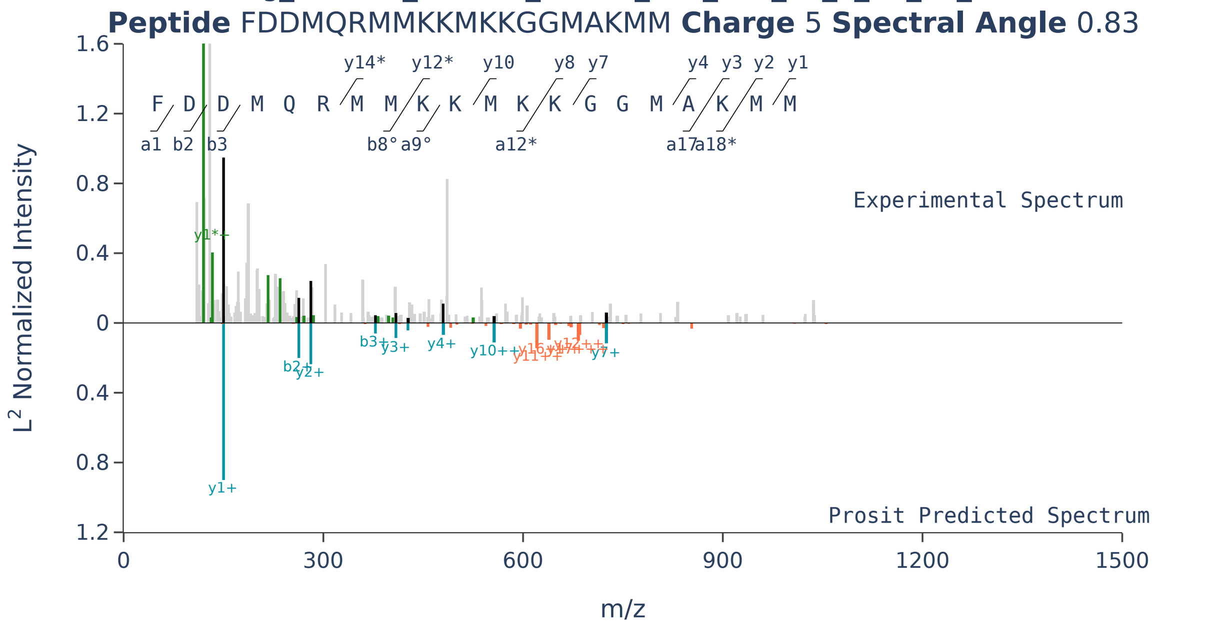


**Source** Ncheng_210623_230623_HFGoe_FFH_20S_25_1_A1_4h_R1 **Scan** 28512
**Peptide** LPGMGQIPDNVKSQMDDKVLV **Charge** 3 **Spectral Angle** 0.8

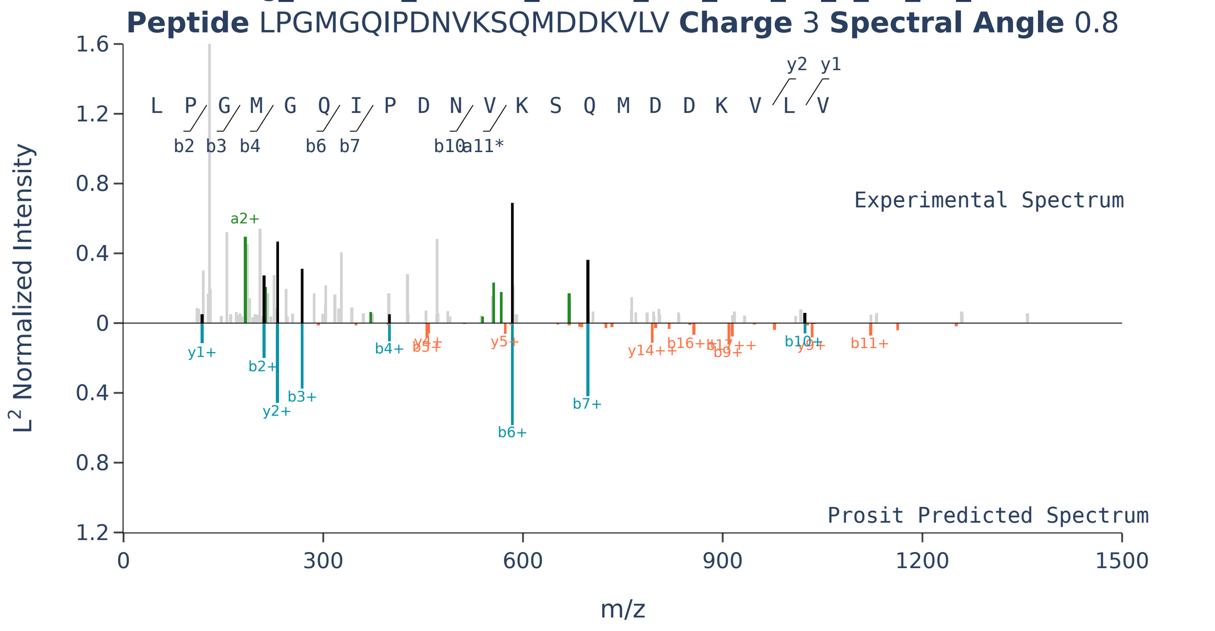


**Source** Ncheng_210623_230623_HFGoe_FFH_20S_25_1_A2_24h_R2 **Scan** 5720
**Peptide** VKEKAVGHEV **Charge** 3 **Spectral Angle** 0.58

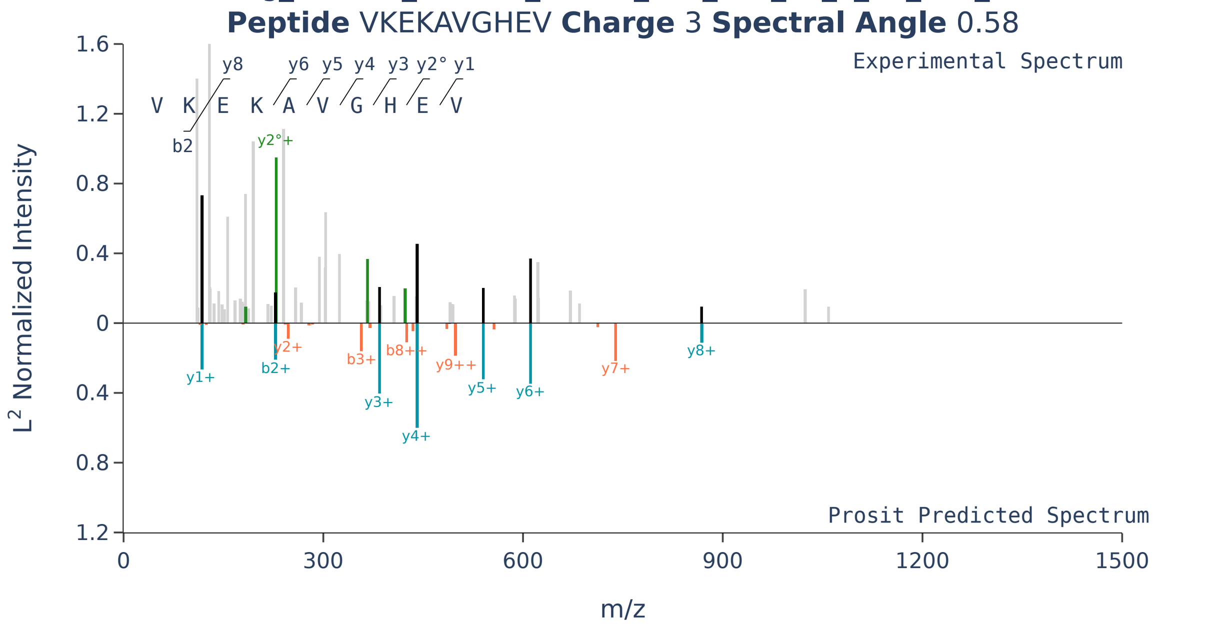


**Source** Ncheng_210623_230623_HFGoe_FFH_20S_25_1_A2_24h_R2 **Scan** 11800
**Peptide** ESKVDRAQAEKLASKLKKG **Charge** 5 **Spectral Angle** 0.81

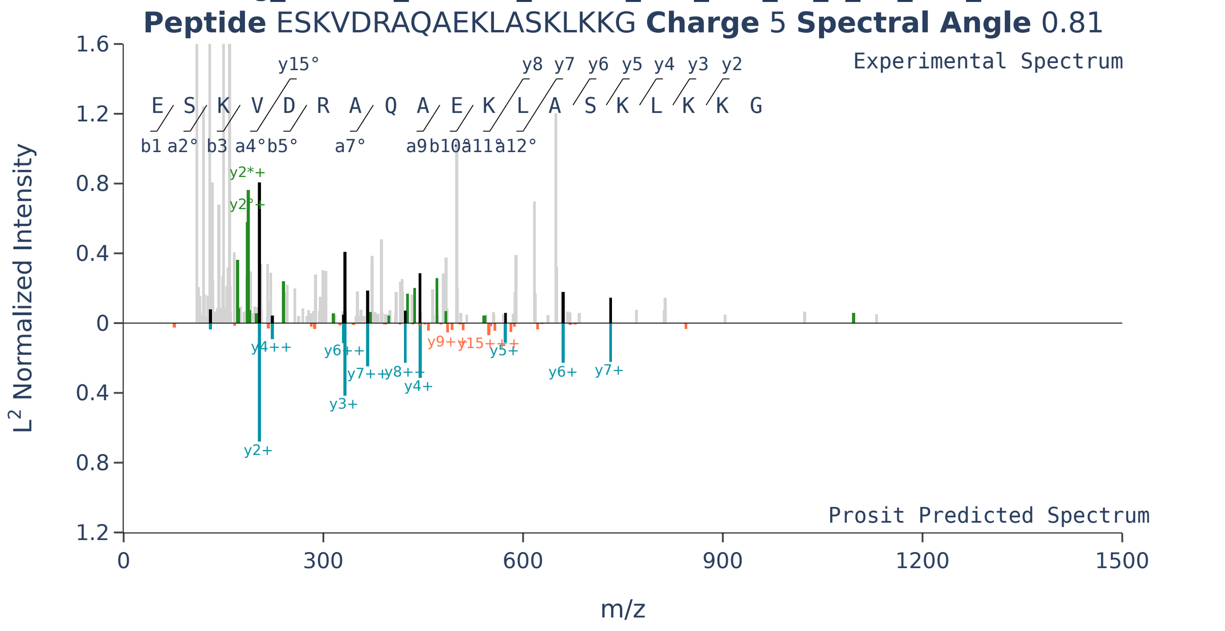


**Source** Ncheng_210623_230623_HFGoe_FFH_20S_25_1_A2_4h_R1 **Scan** 36574
**Peptide** AEKLASKLKKGDGFDLNDFLEQLRQM **Charge** 4 **Spectral Angle** 0.76

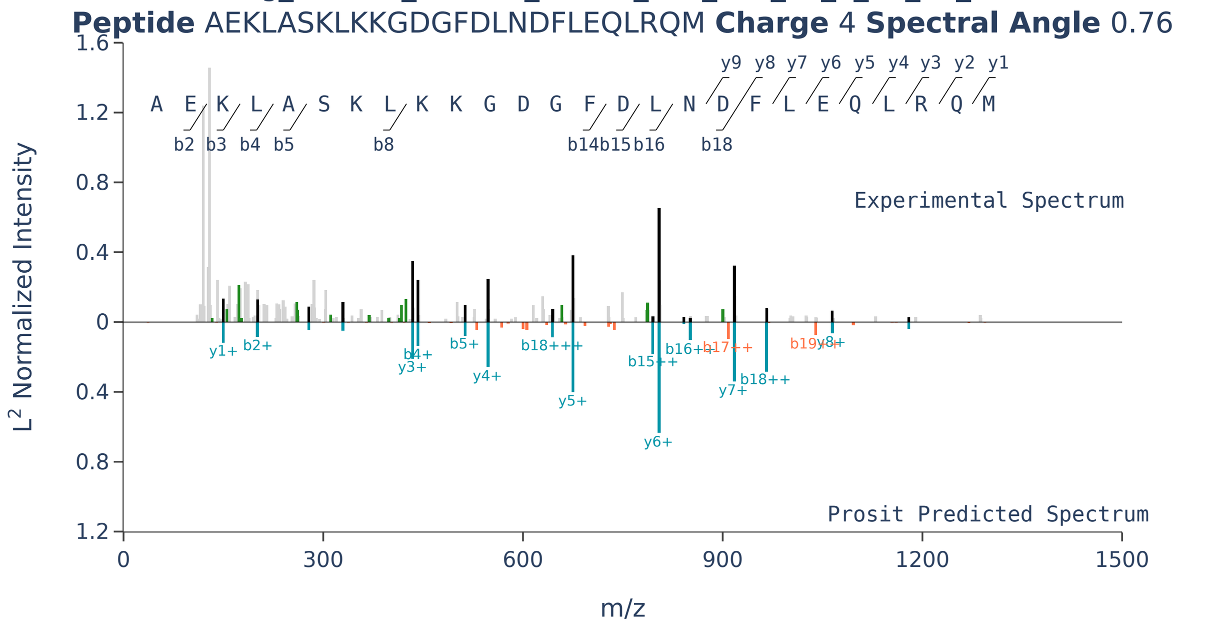


**Source** Ncheng_210623_230623_HFGoe_FFH_20S_25_1_A2_2h_R2 **Scan** 12646
**Peptide** IESKVDRAQAEKLASKLKK **Charge** 6 **Spectral Angle** 0.85

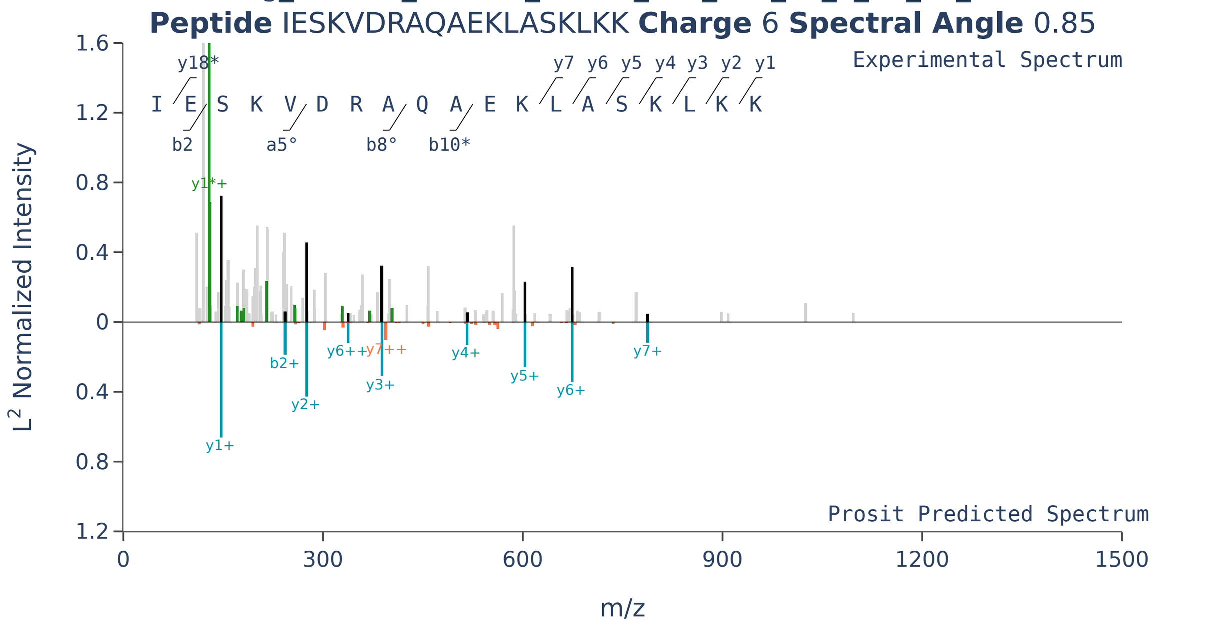


**Source** Ncheng_210623_230623_HFGoe_FFH_20S_25_1_A1_24h_R2 **Scan** 18289
**Peptide** GAGKTTSVGKLGKFL **Charge** 4 **Spectral Angle** 0.57

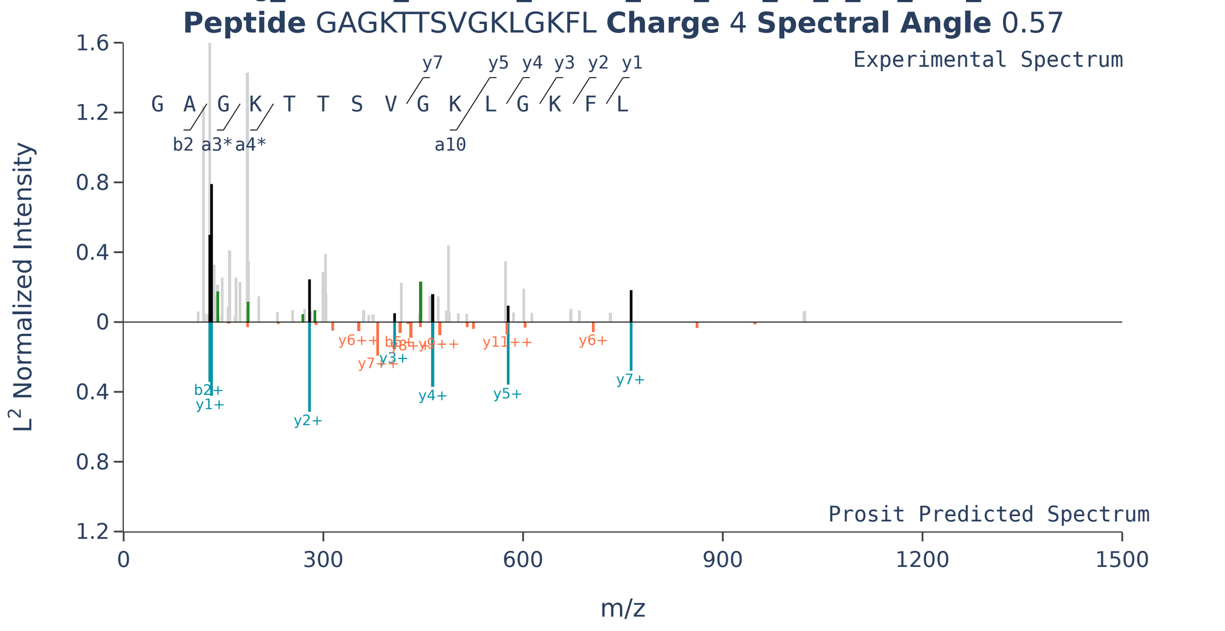


**Source** Ncheng_210623_230623_HFGoe_FFH_20S_25_1_A2_24h_R2 **Scan** 19117
**Peptide** LNRVKEKAVGHEVNKSLTPGQEFVKIV **Charge** 6 **Spectral Angle** 0.61

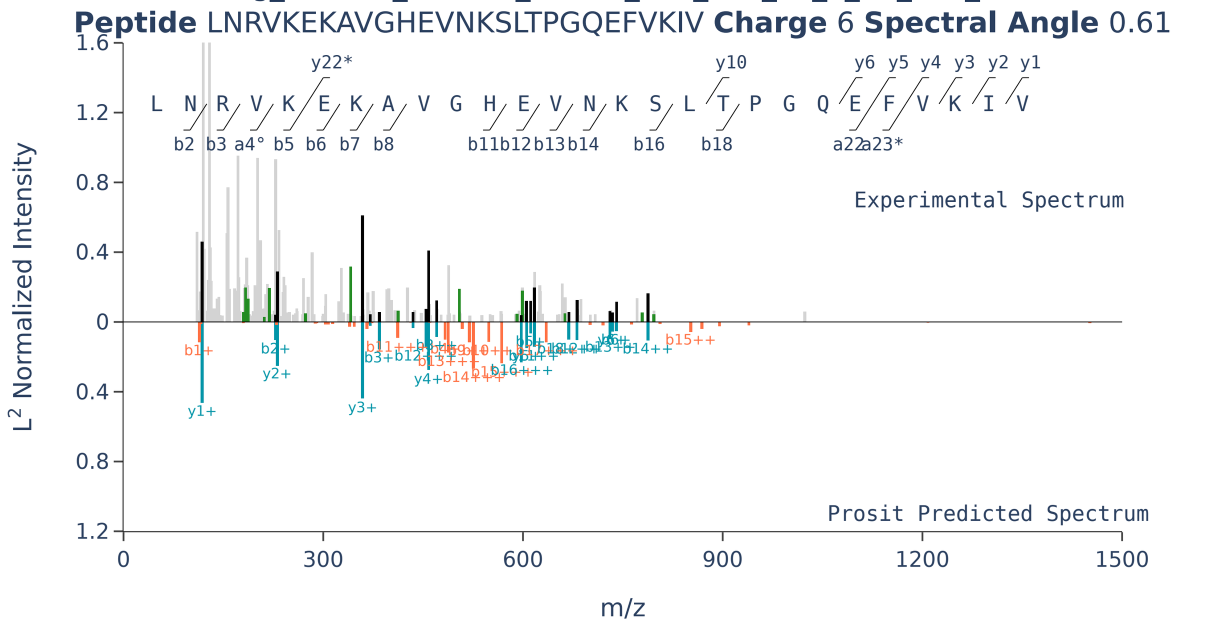


**Source** Ncheng_210623_230623_HFGoe_FFH_20S_25_1_A1_4h_R2 **Scan** 11086
**Peptide** EFINRVKEKAVG **Charge** 2 **Spectral Angle** 0.41

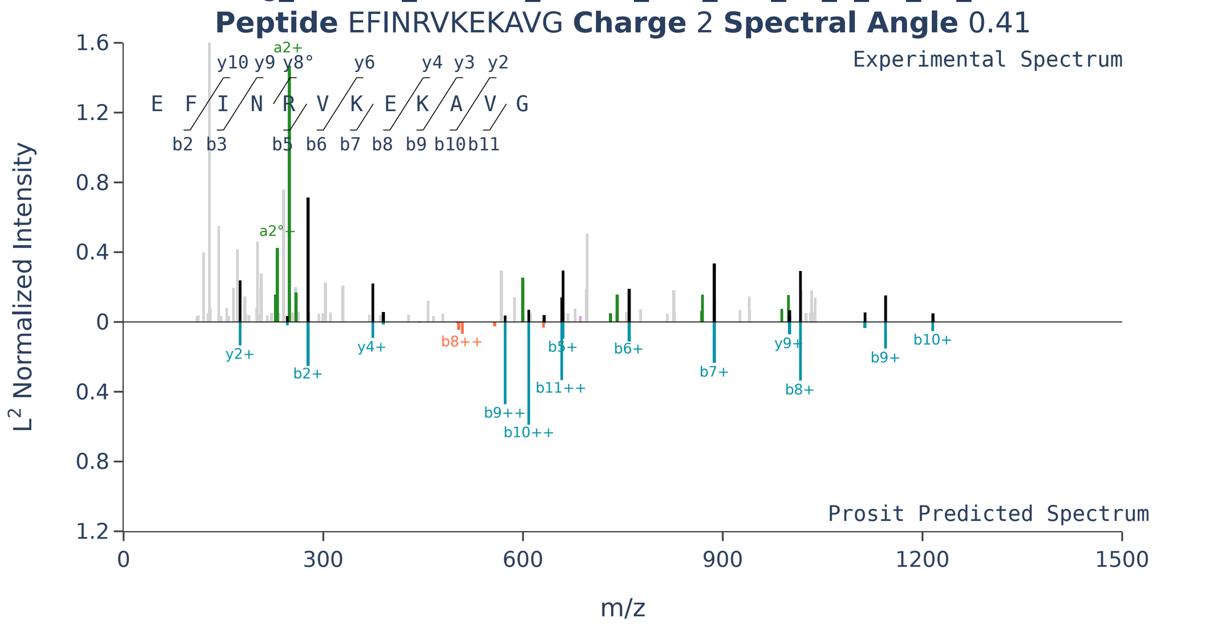


**Source** Ncheng_210623_230623_HFGoe_FFH_20S_25_1_A2_24h_R1 **Scan** 17116
**Peptide** IESKVDRAQAEKLASKLKKGDGFD **Charge** 3 **Spectral Angle** 0.71

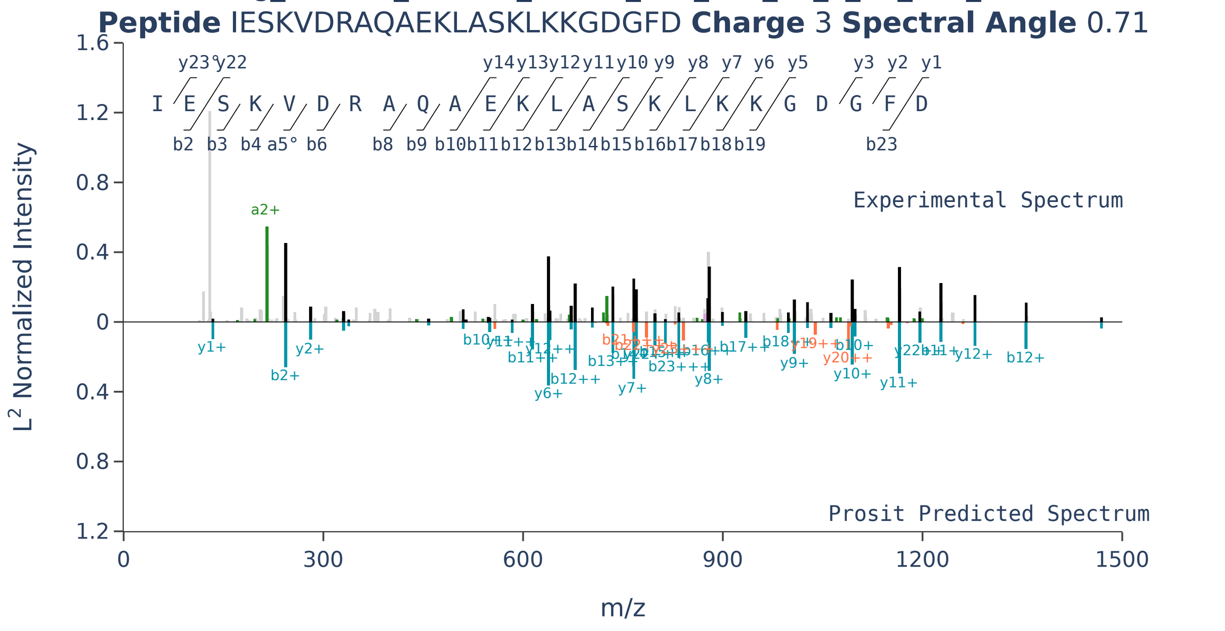

**Source** Ncheng_210623_230623_HFGoe_FFH_20S_25_1_A1_1h_R2 **Scan** 20730
**Peptide** DARGGAALSIRHITGKPIKFLGVGEKTE **Charge** 5 **Spectral Angle** 0.59

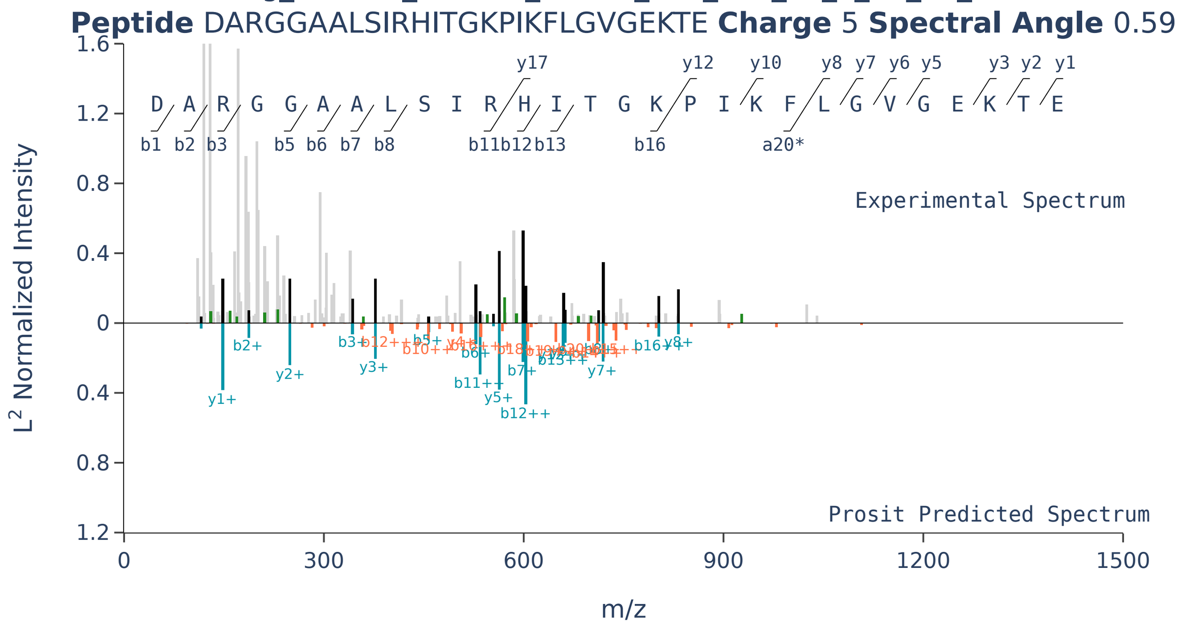


**Source** Ncheng_210623_230623_HFGoe_FFH_20S_25_1_A2_4h_R2 **Scan** 22195
**Peptide** NRLLKQFDDMQRMMKKMK **Charge** 5 **Spectral Angle** 0.75

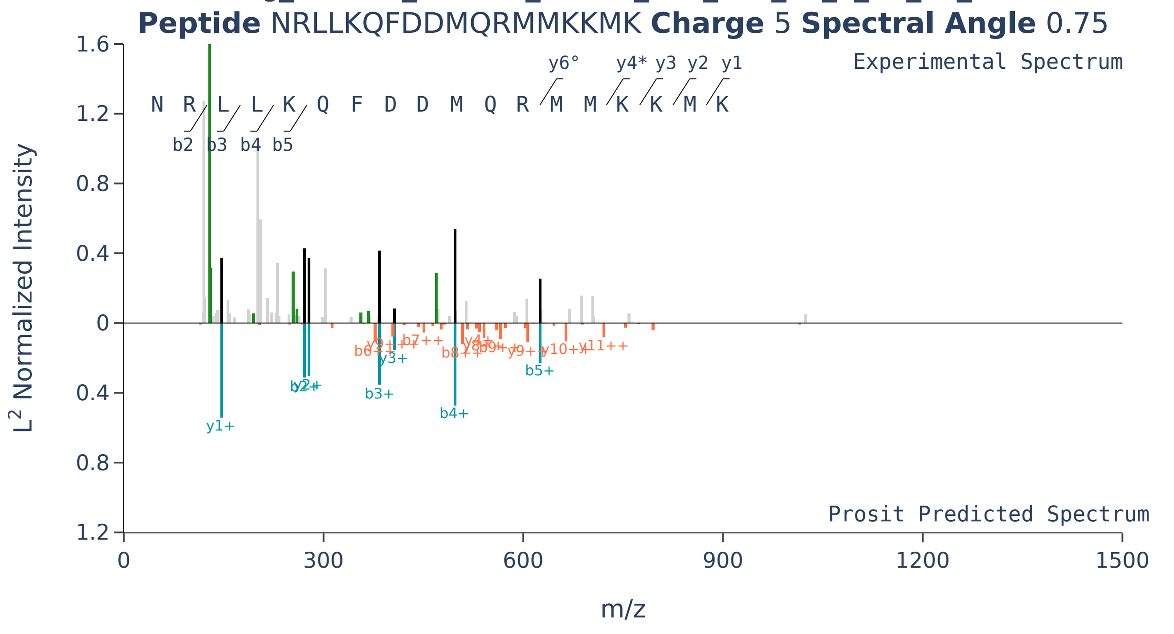


**Source** Ncheng_210623_230623_HFGoe_FFH_20S_25_1_A1_2h_R1 **Scan** 34806
**Peptide** LVAAMGEENQTLNLAAQPPAVVLM **Charge** 3 **Spectral Angle** 0.69

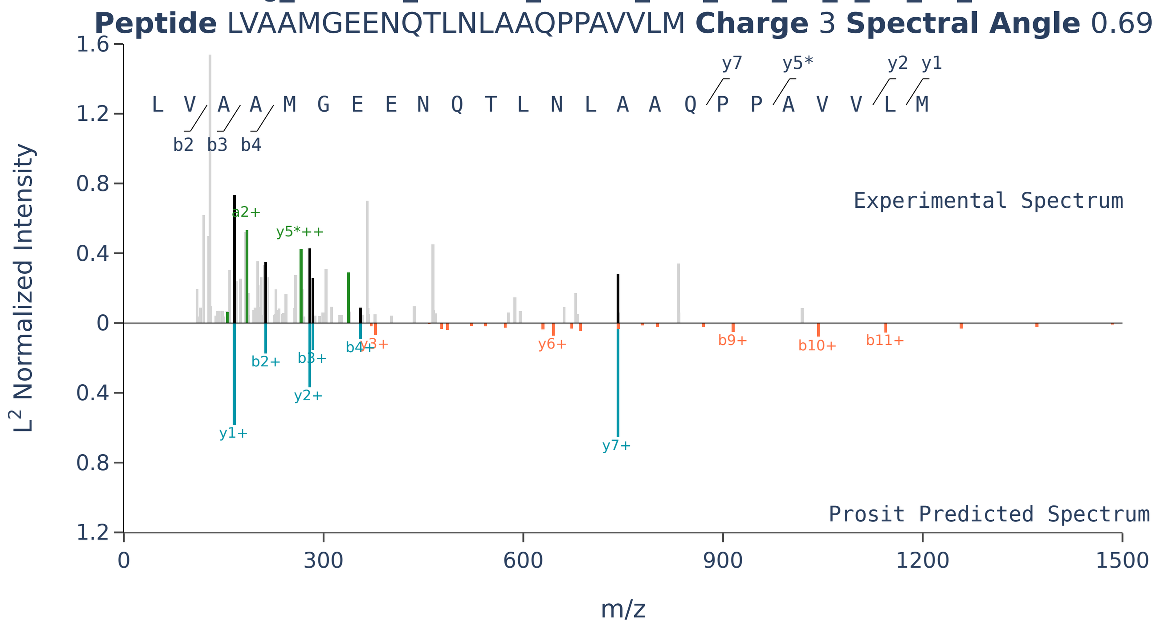


**Source** Ncheng_210623_230623_HFGoe_FFH_20S_25_1_A1_24h_R1 **Scan** 5952
**Peptide** EQLRQMKN **Charge** 2 **Spectral Angle** 0.52

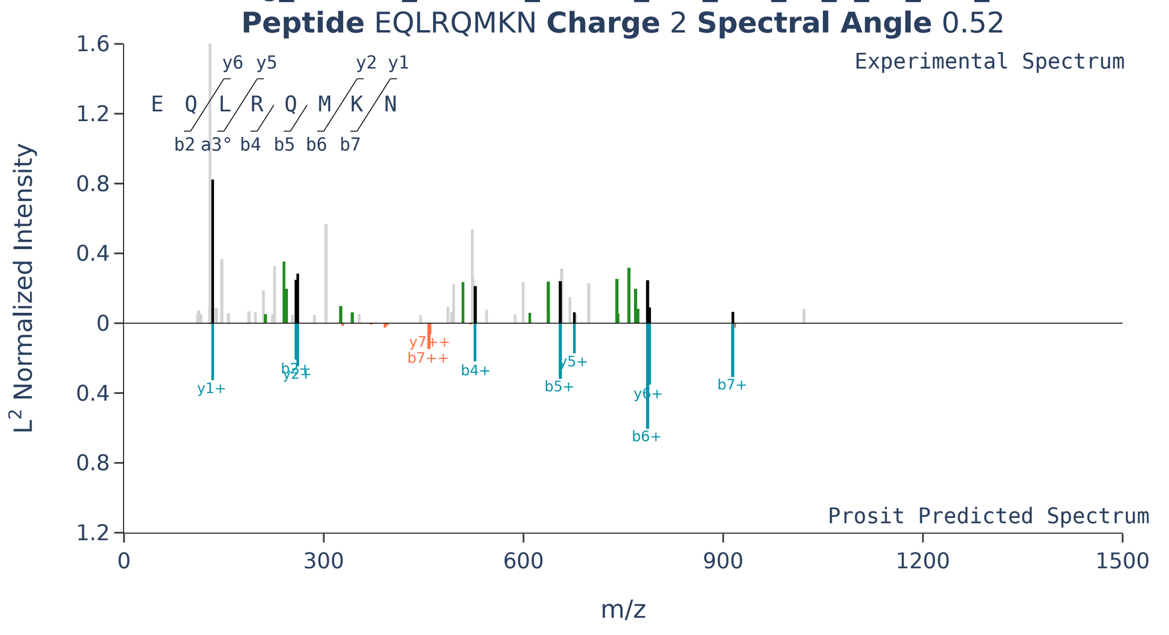


**Source** Ncheng_210623_230623_HFGoe_FFH_20S_25_1_A2_2h_R1 **Scan** 29911
**Peptide** EALPLTGVVLTKVDGDARGGAALSIRHITG **Charge** 3 **Spectral Angle** 0.57

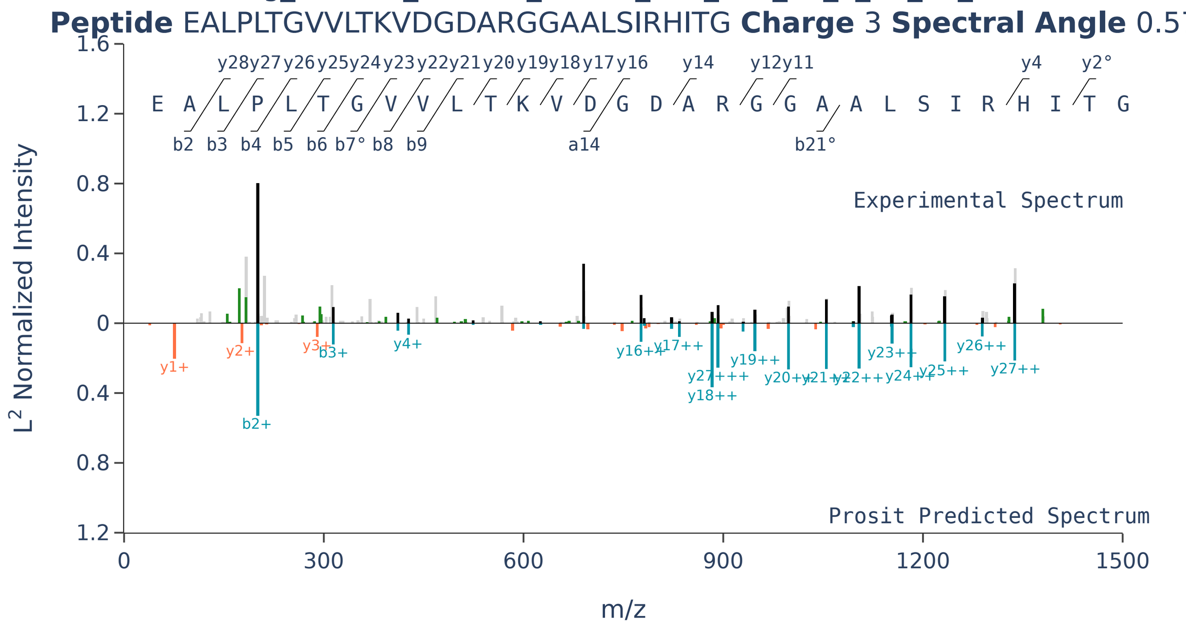


**Source** Ncheng_210623_230623_HFGoe_FFH_20S_25_1_A1_24h_R2 **Scan** 6829
**Peptide** SMTMKERAKPEIIKGSRKR **Charge** 5 **Spectral Angle** 0.53

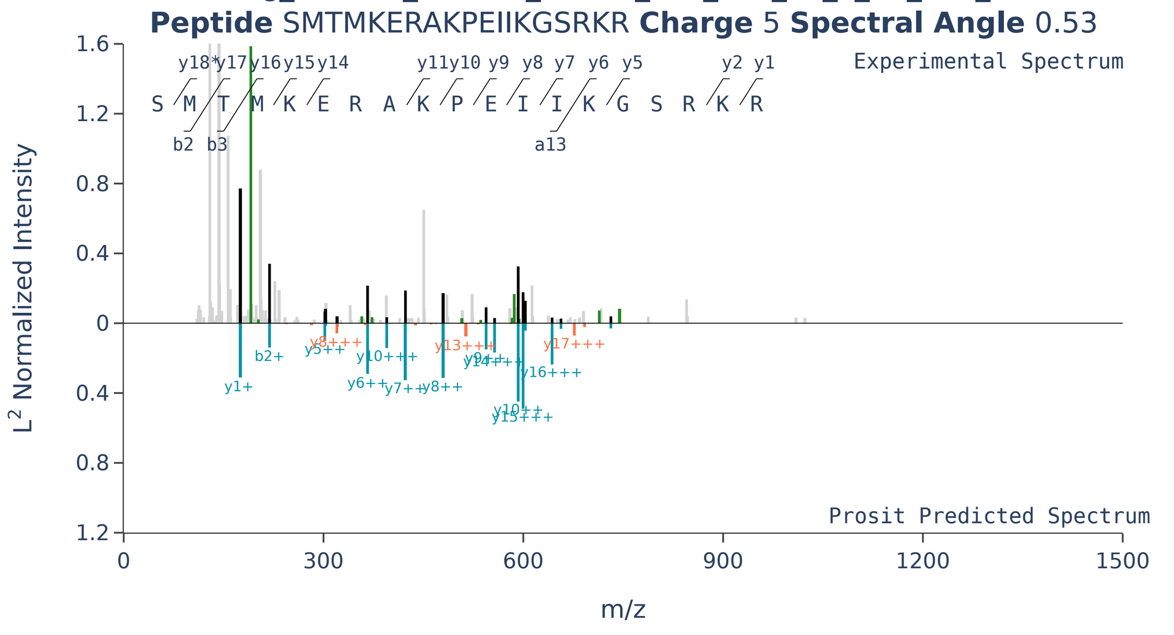


**Source** Ncheng_210623_230623_HFGoe_FFH_20S_25_1_A2_4h_R2 **Scan** 19053
**Peptide** GVVLTKVDGDARGGAALSLR **Charge** 3 **Spectral Angle** 0.49

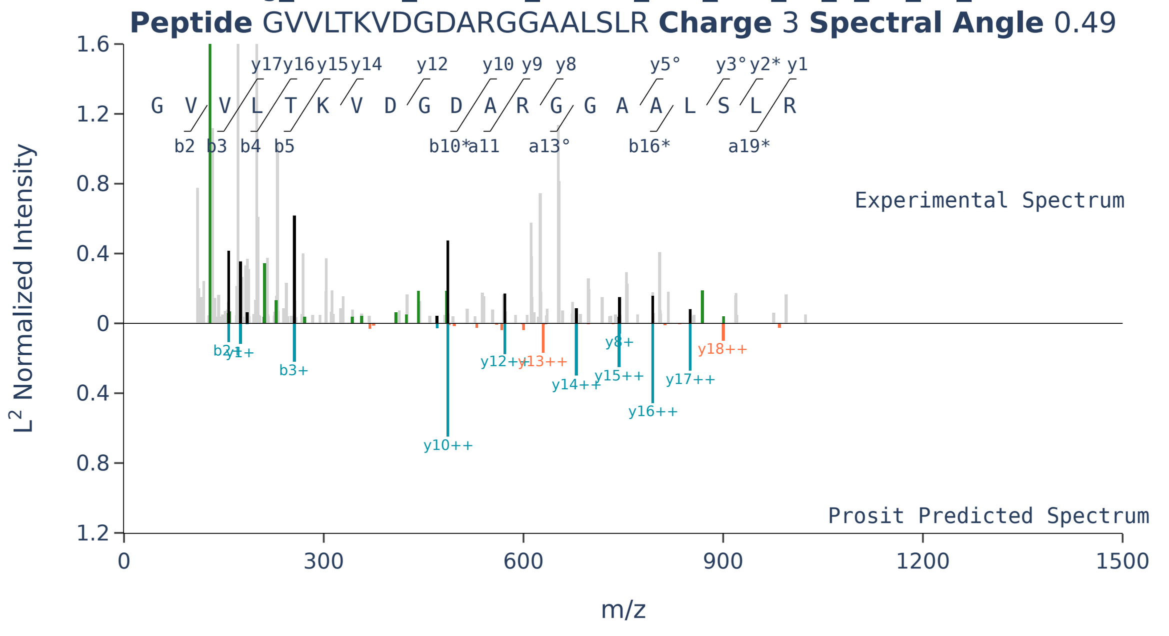


**Source** Ncheng_210623_230623_HFGoe_FFH_20S_25_1_A1_1h_R2 **Scan** 7879
**Peptide** REFINRVKEKAVGH **Charge** 4 **Spectral Angle** 0.7

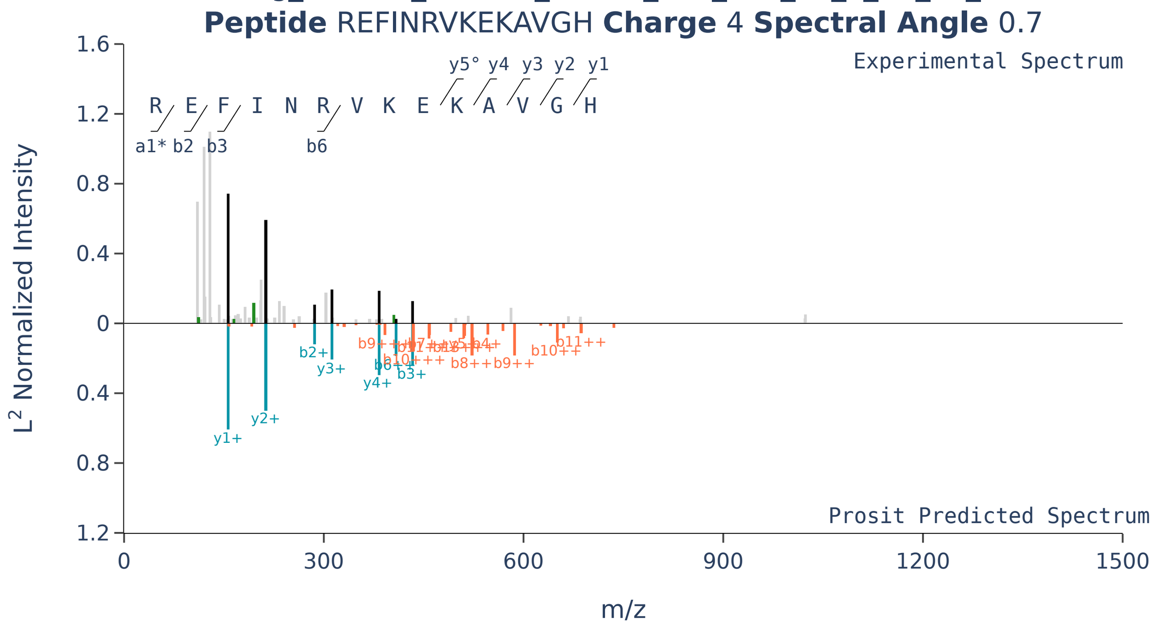


**Source** Ncheng_210623_230623_HFGoe_FFH_20S_25_1_A1_2h_R2 **Scan** 12738
**Peptide** DEIKQVHASI **Charge** 3 **Spectral Angle** 0.3

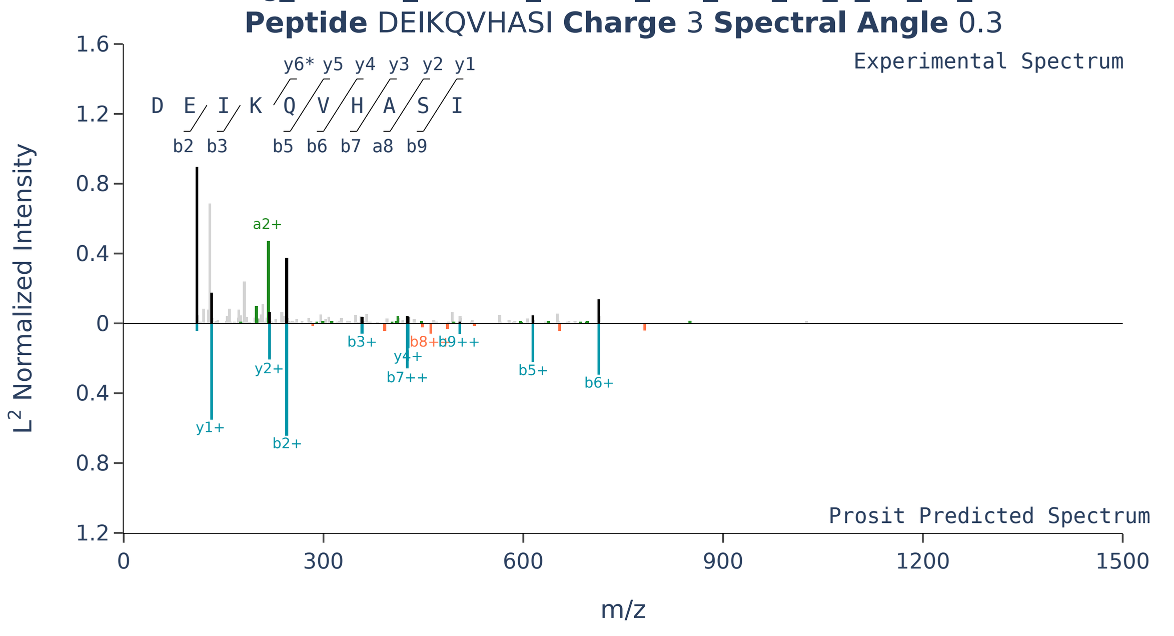


**Source** Ncheng_210623_230623_HFGoe_FFH_20S_25_1_A2_24h_R2 **Scan** 6080
**Peptide** KEKAVGHEVNKSLT **Charge** 4 **Spectral Angle** 0.81

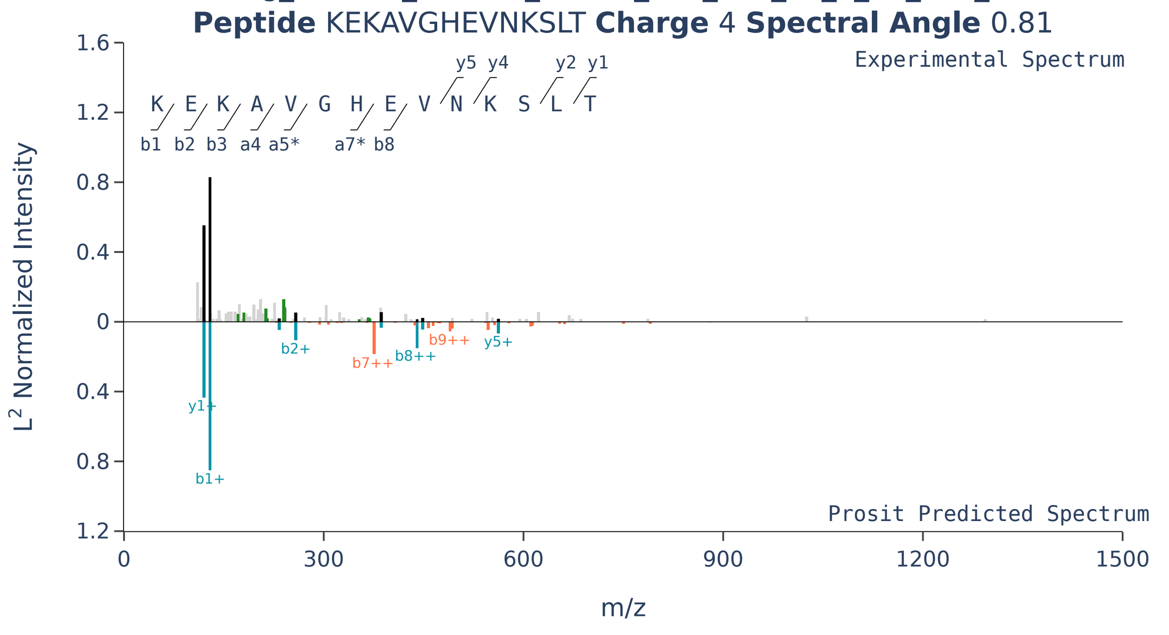


**Source** Ncheng_210623_230623_HFGoe_FFH_20S_25_1_A1_1h_R1 **Scan** 23748
**Peptide** TGKPIKFLGVGEKTEALEPFHPDRIASRL **Charge** 5 **Spectral Angle** 0.73

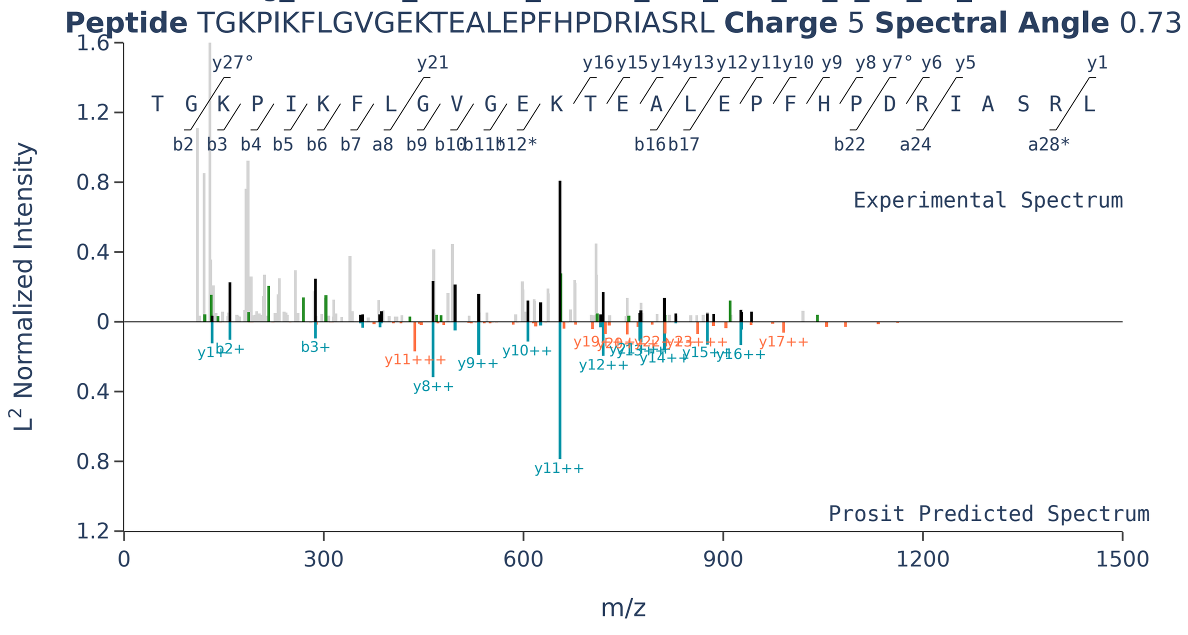


**Source** Ncheng_210623_230623_HFGoe_FFH_20S_25_1_A1_1h_R2 **Scan** 27744
**Peptide** KFYDVLL **Charge** 2 **Spectral Angle** 0.53

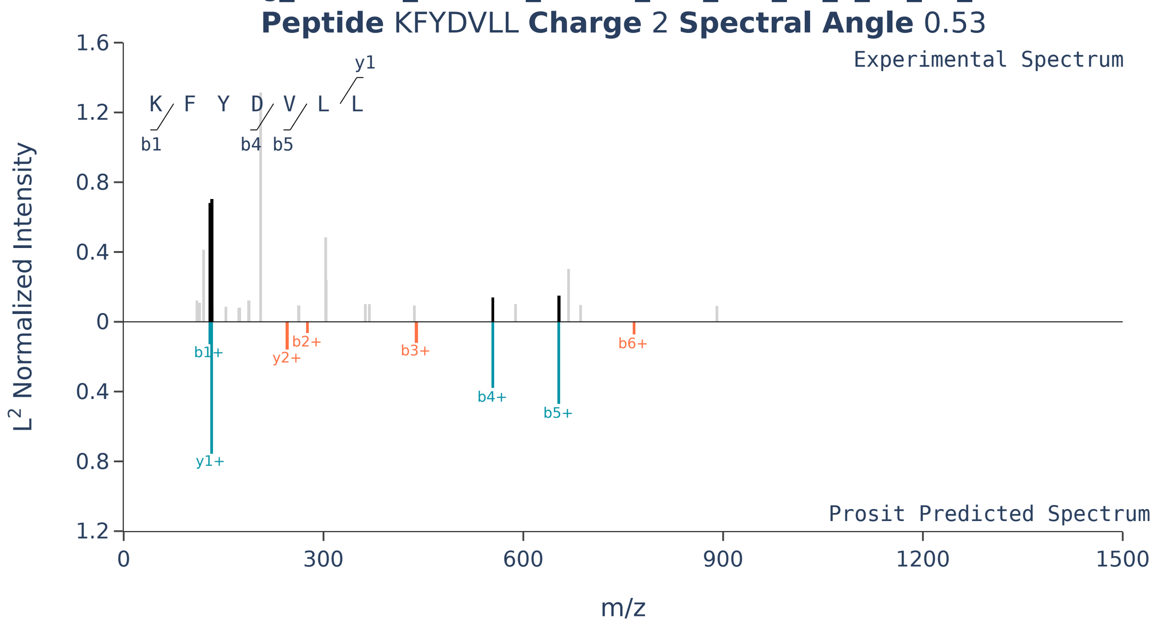


**Source** Ncheng_210623_230623_HFGoe_FFH_20S_25_1_A2_2h_R1 **Scan** 34680
**Peptide** ASKLKKGDGFDLNDFLEQLRQMKNMG **Charge** 4 **Spectral Angle** 0.6

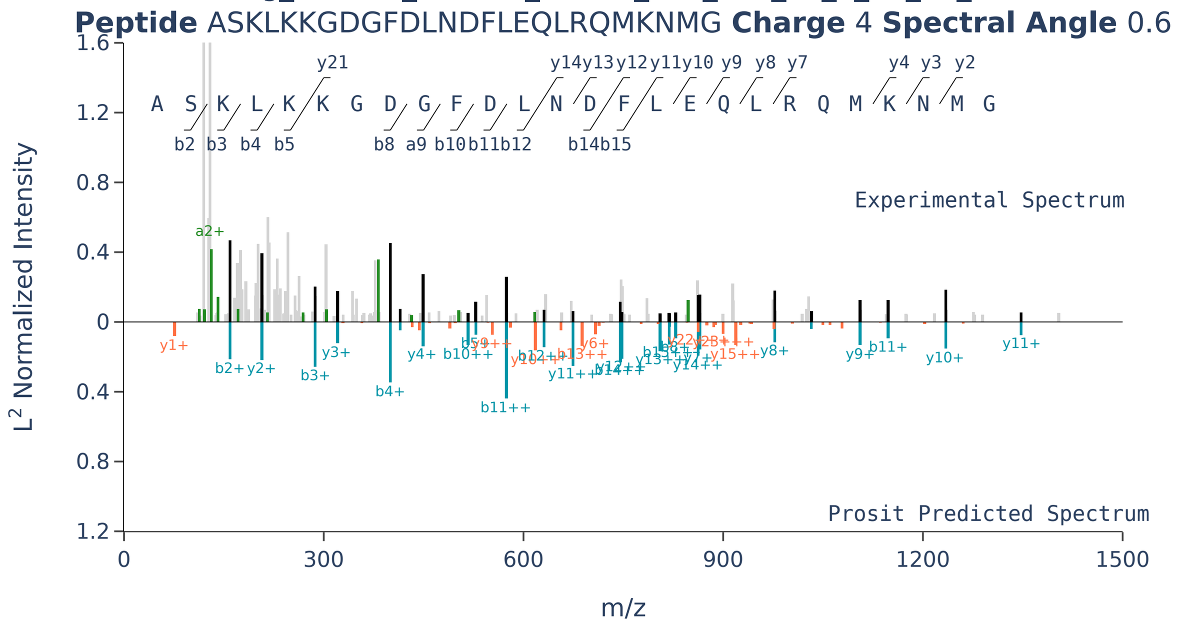


**Source** Ncheng_210623_230623_HFGoe_FFH_20S_25_1_A1_2h_R1 **Scan** 26732
**Peptide** GRLTEDNVKDTLREVRMAL **Charge** 4 **Spectral Angle** 0.48

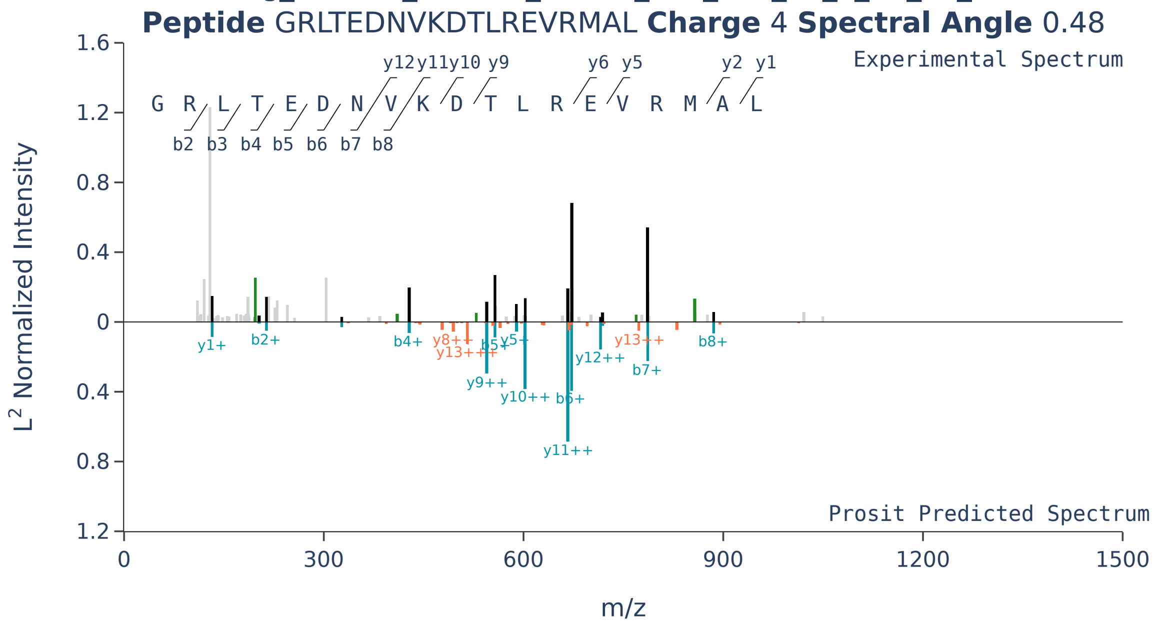


**Source** Ncheng_210623_230623_HFGoe_FFH_20S_25_1_A2_2h_R2 **Scan** 18531
**Peptide** GDARGGAALSIRHITGKPIKF **Charge** 5 **Spectral Angle** 0.67

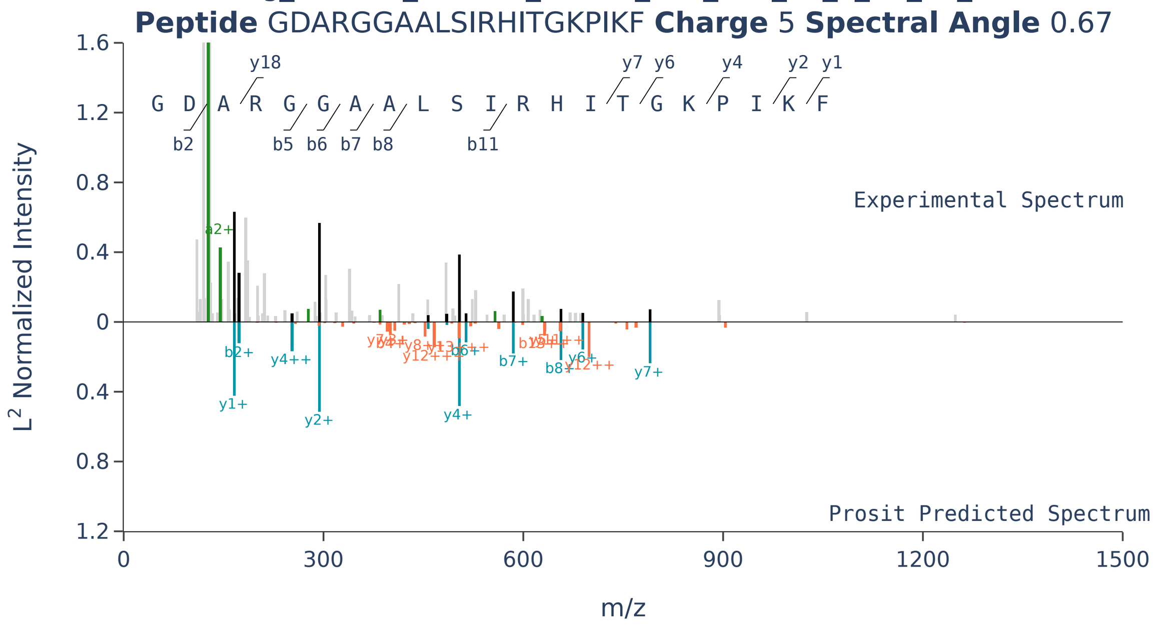

**Source** Ncheng_210623_230623_HFGoe_FFH_20S_25_1_A2_2h_R2 **Scan** 32293
**Peptide** GRLTEDNVKDTLREVRMALLEAD **Charge** 4 **Spectral Angle** 0.54

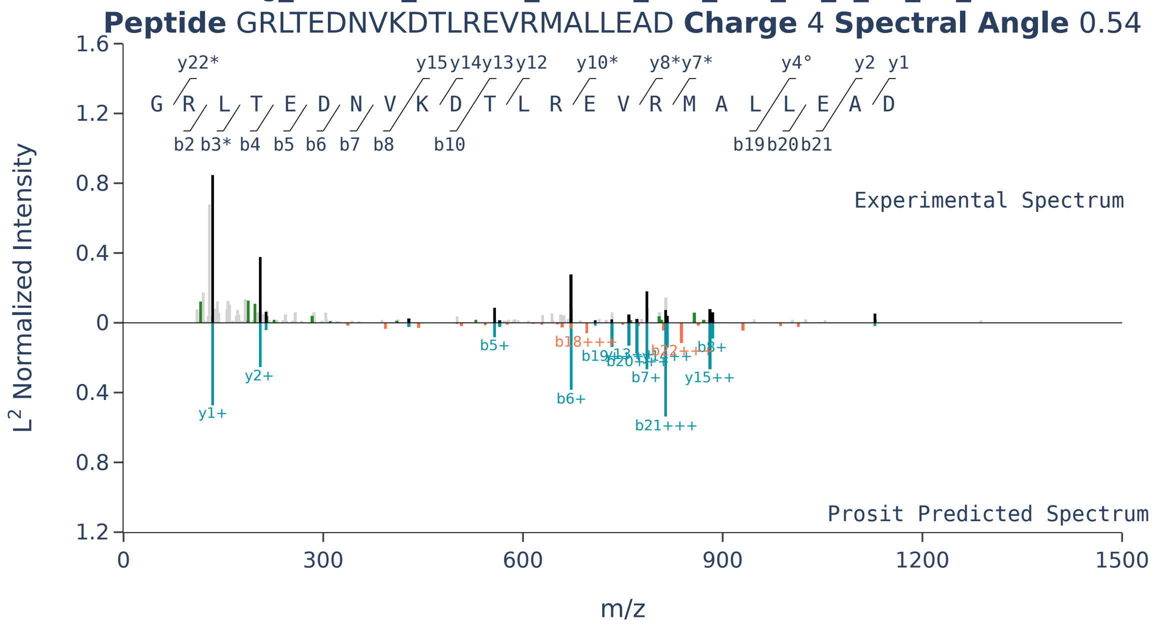


**Source** Ncheng_210623_230623_HFGoe_FFH_20S_25_1_A2_2h_R2 **Scan** 18783
**Peptide** LRNISGRGRLTEDNVKDTLREVRMA **Charge** 6 **Spectral Angle** 0.77

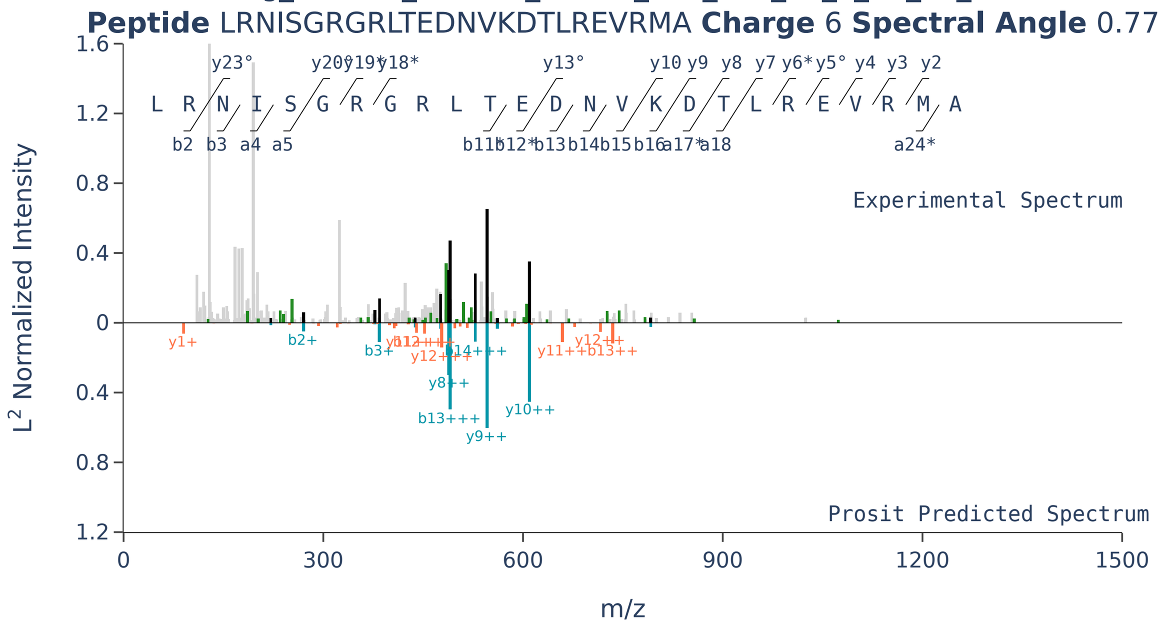


**Source** Ncheng_210623_230623_HFGoe_FFH_20S_25_1_A1_2h_R2 **Scan** 31022
**Peptide** AALKEAKLKFYDVLLVDTAGRLHVDE **Charge** 5 **Spectral Angle** 0.55

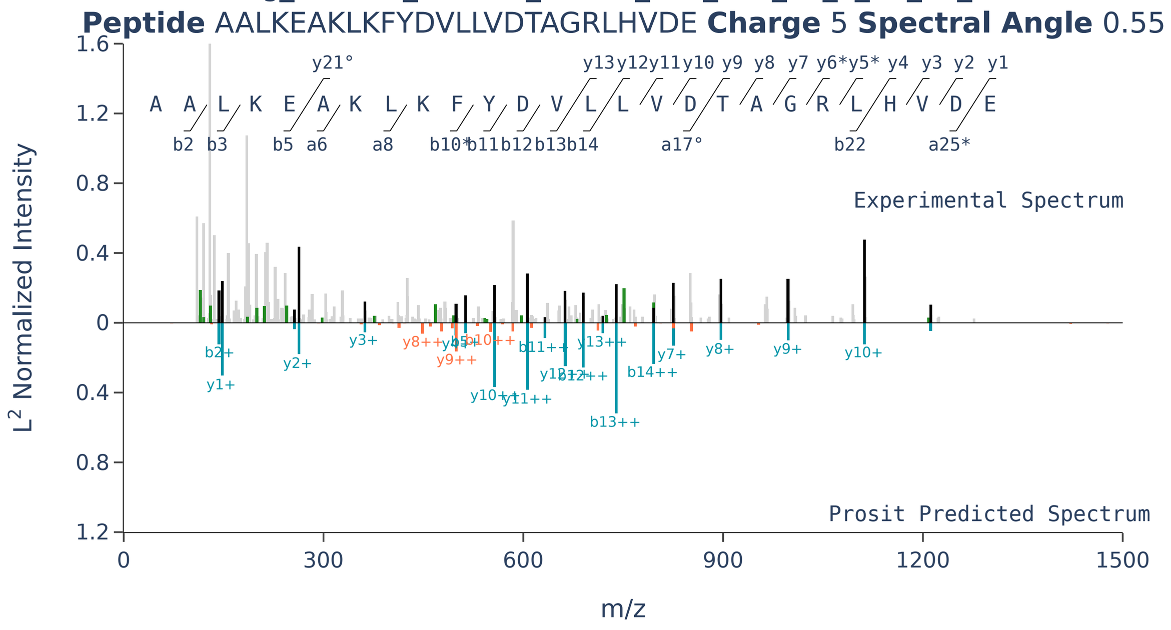


**Source** Ncheng_210623_230623_HFGoe_FFH_20S_25_1_A2_24h_R2 **Scan** 12780
**Peptide** GKTTSVGKLGKFLREK **Charge** 4 **Spectral Angle** 0.47

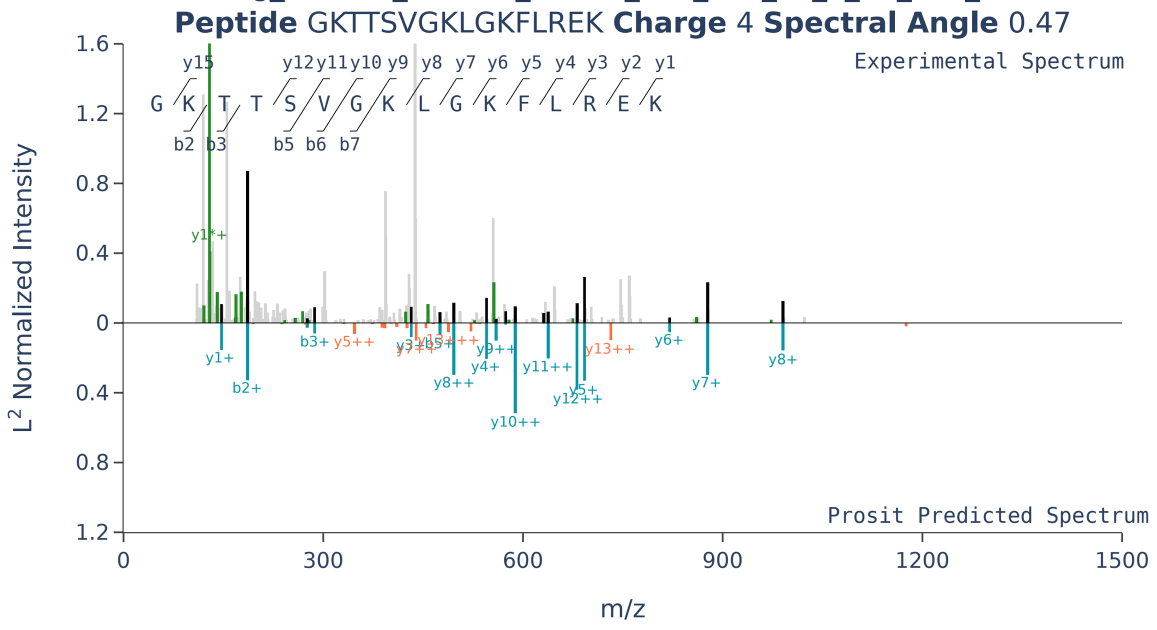


**Source** Ncheng_210623_230623_HFGoe_FFH_20S_25_1_A1_2h_R1 **Scan** 31707
**Peptide** KNMGGMASLMGKLPGMGQI **Charge** 3 **Spectral Angle** 0.72

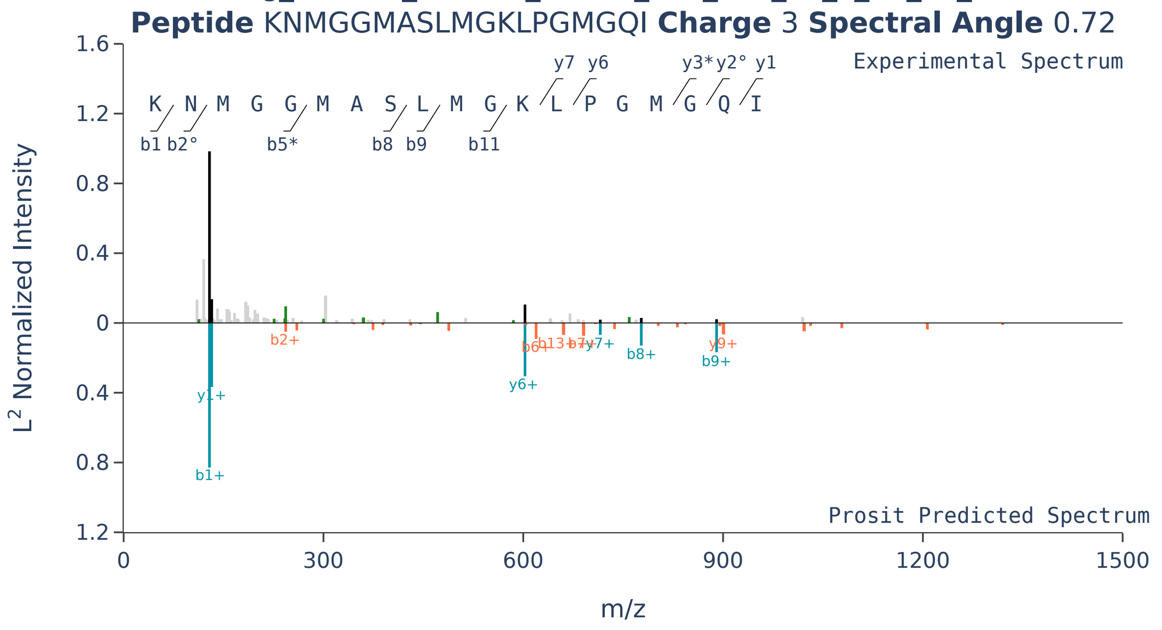


**Source** Ncheng_210623_230623_HFGoe_FFH_20S_25_1_A2_2h_R1 **Scan** 32027
**Peptide** AGCGMQVQDVNRLLKQFDDMQRMMKKMKK **Charge** 5 **Spectral Angle** 0.66

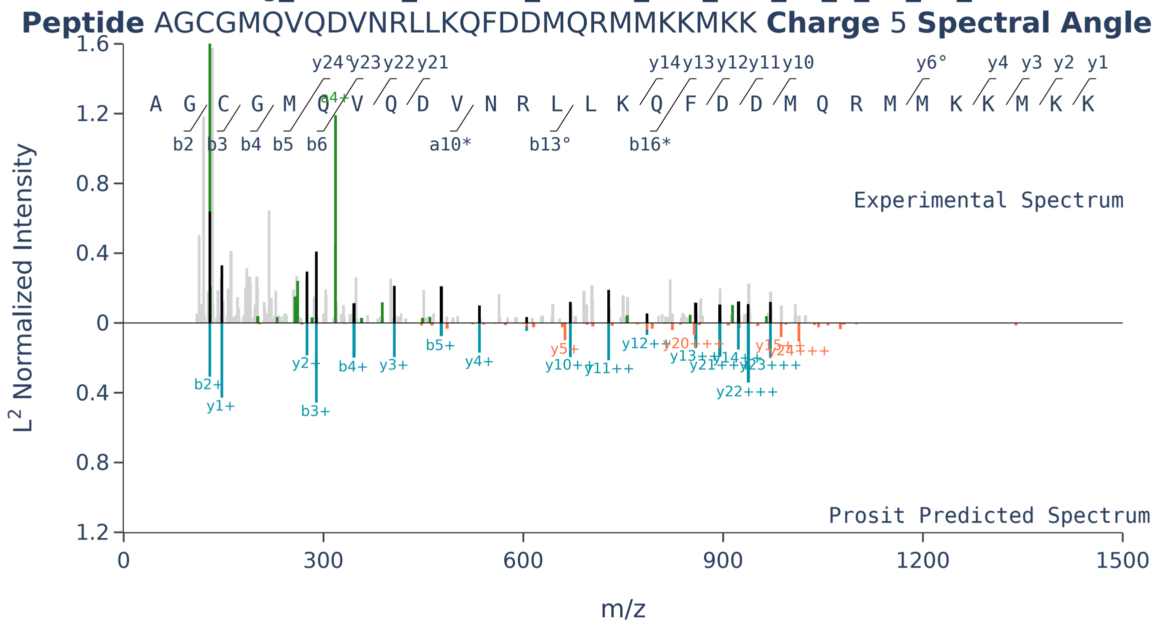


**Source** Ncheng_210623_230623_HFGoe_FFH_20S_25_1_A2_4h_R1 **Scan** 4752
**Peptide** MMKKMKK **Charge** 2 **Spectral Angle** 0.63

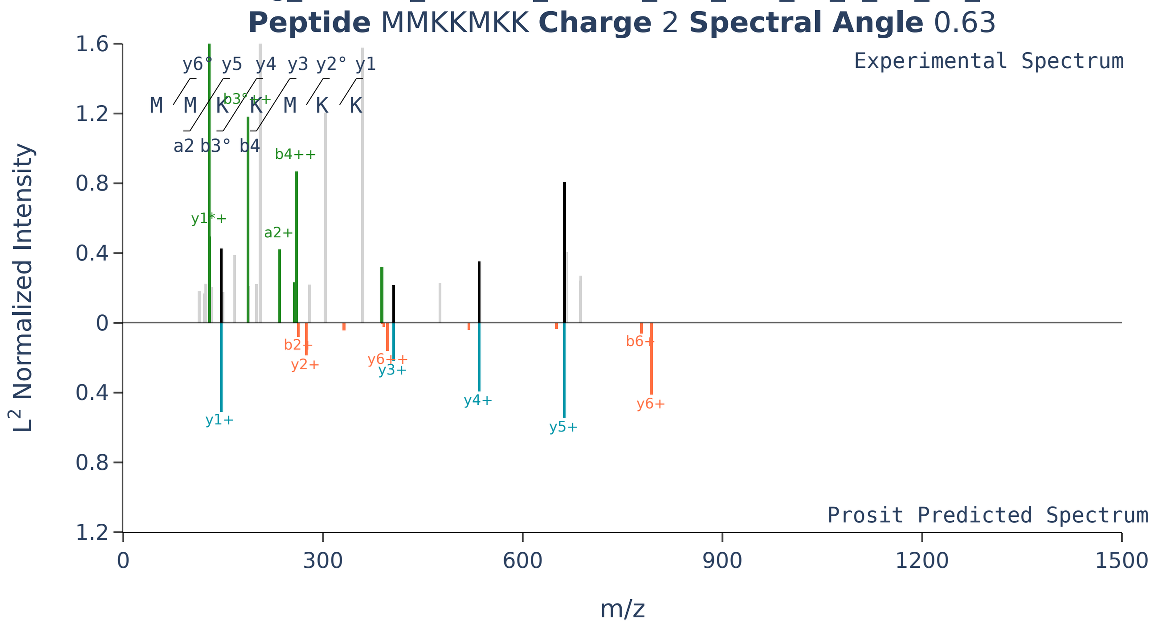


**Source** Ncheng_210623_230623_HFGoe_FFH_20S_25_1_A2_4h_R2 **Scan** 15874
**Peptide** VLTKVDGDARGGAALSIRHITGK **Charge** 4 **Spectral Angle** 0.45

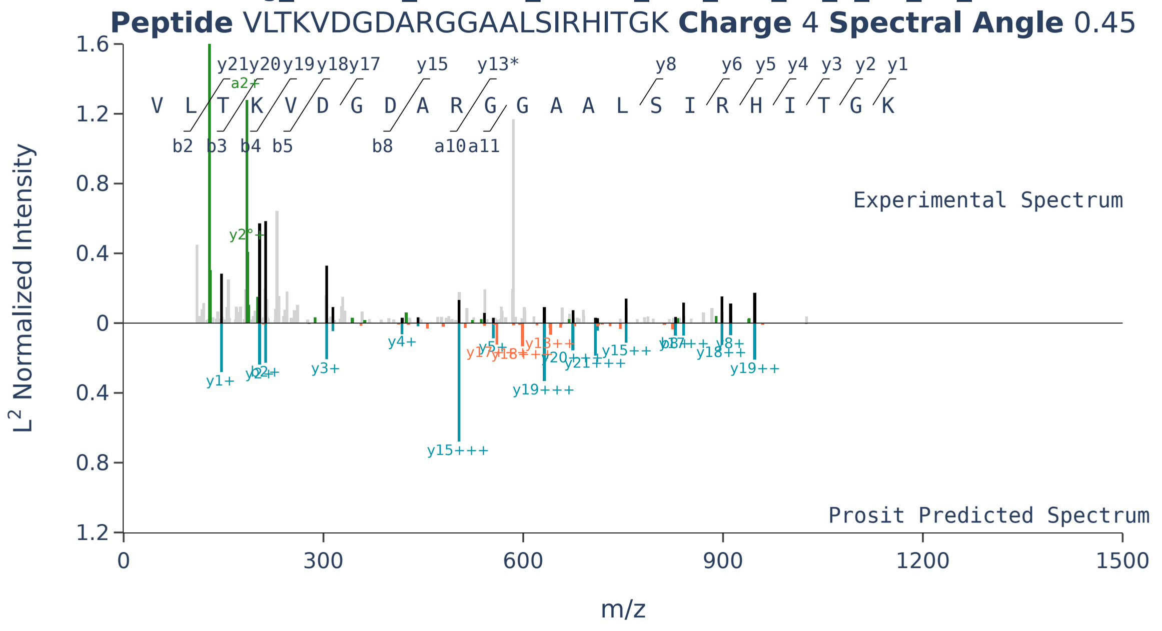


**Source** Ncheng_210623_230623_HFGoe_FFH_20S_25_1_A2_2h_R2 **Scan** 24294
**Peptide** PLKFLGVGEKTEALEPFHPDRIASR **Charge** 5 **Spectral Angle** 0.73

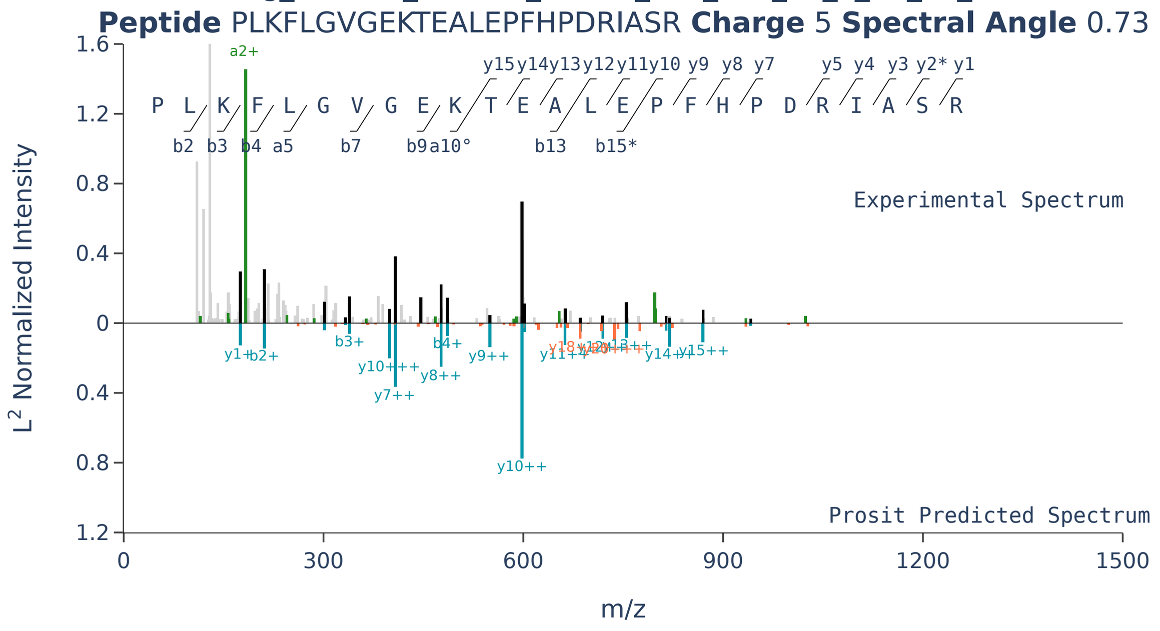


**Source** Ncheng_210623_230623_HFGoe_FFH_20S_25_1_A2_2h_R2 **Scan** 23118
**Peptide** EFVKIVRNELVAA **Charge** 3 **Spectral Angle** 0.65

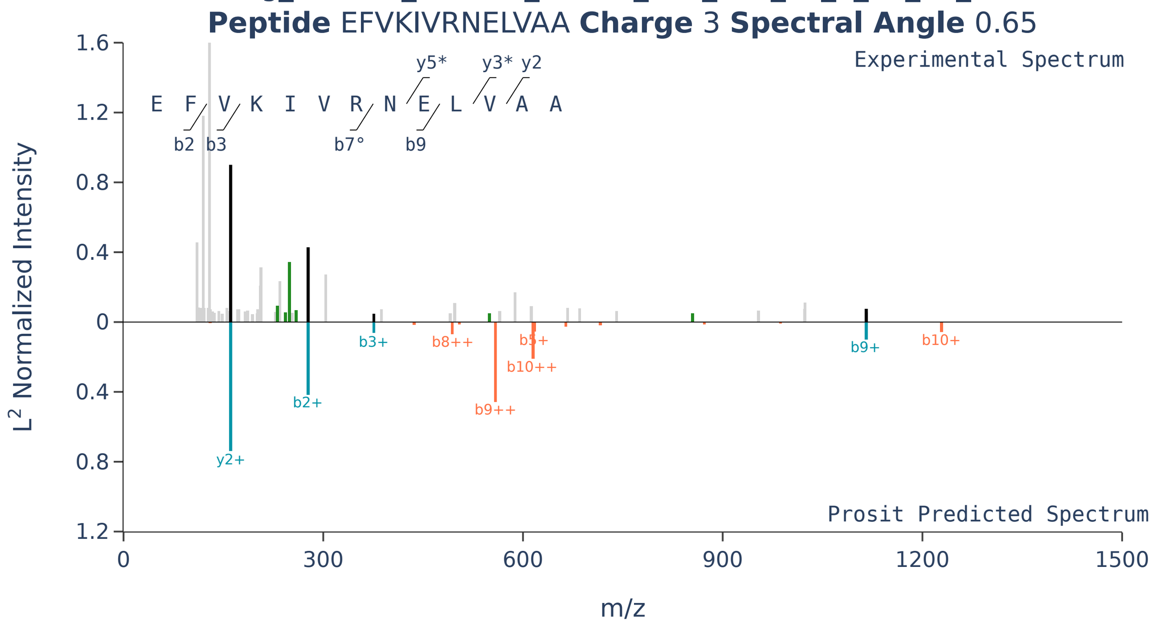


**Source** Ncheng_210623_230623_HFGoe_FFH_20S_25_1_A1_4h_R1 **Scan** 20982
**Peptide** GDARGGAALSIRHITGKPIKFLGVGEKTE **Charge** 5 **Spectral Angle** 0.62

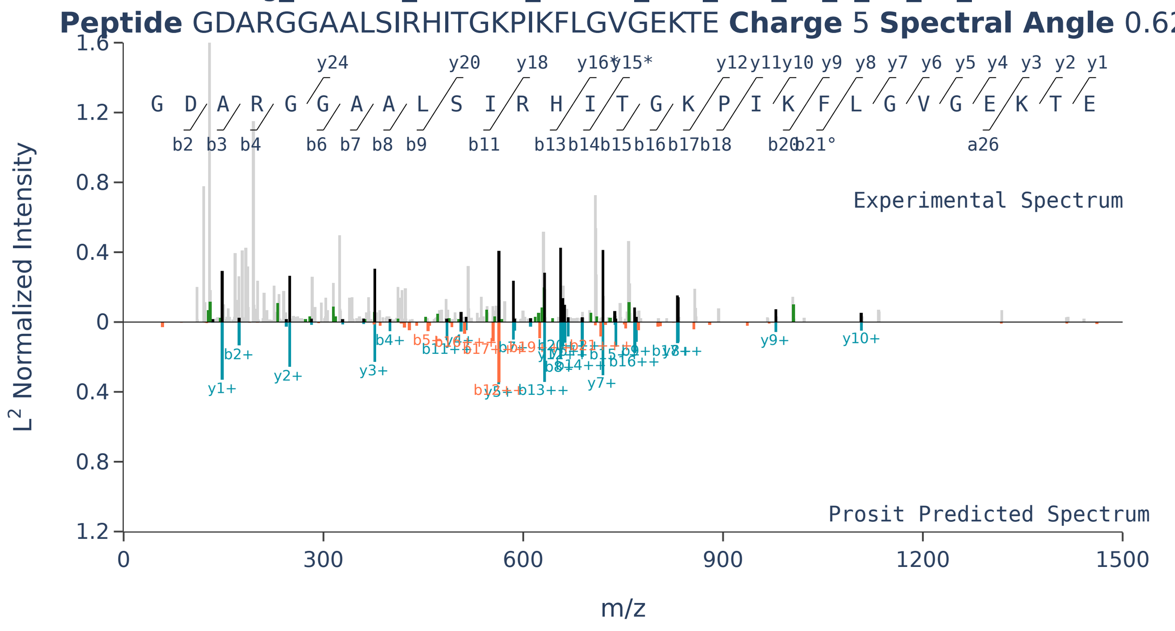


**Source** Ncheng_210623_230623_HFGoe_FFH_20S_25_1_A2_4h_R1 **Scan** 35252
**Peptide** KKGDGFDLNDFLEQLR **Charge** 4 **Spectral Angle** 0.68

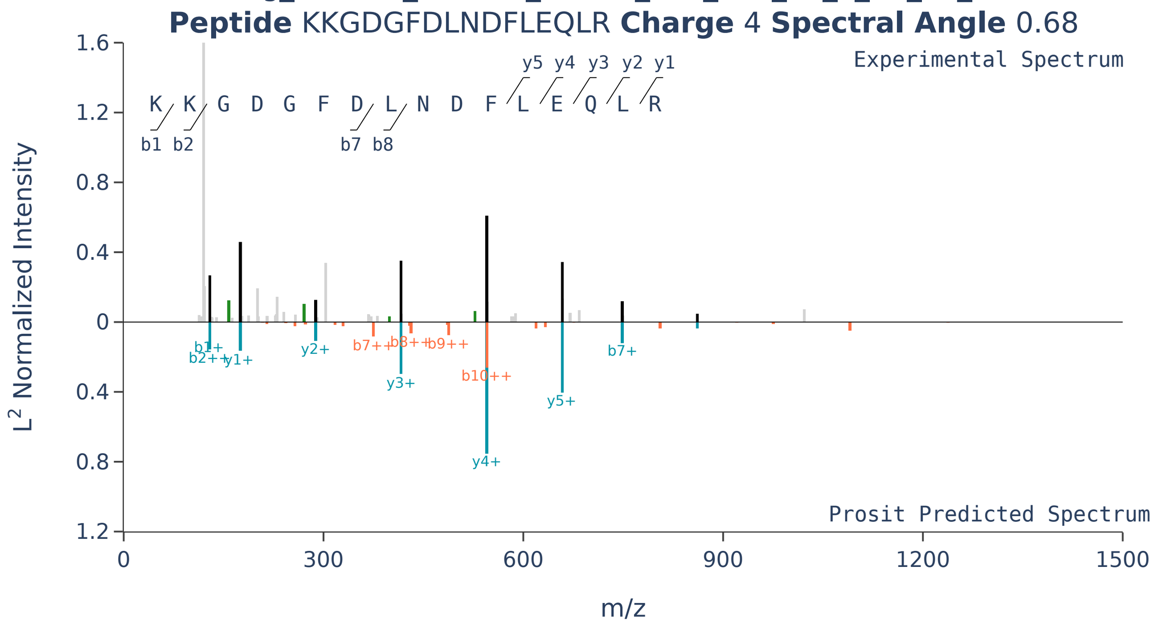


**Source** Ncheng_210623_230623_HFGoe_FFH_20S_25_1_A2_4h_R2 **Scan** 9648
**Peptide** MKKGGMAKMMRSM **Charge** 4 **Spectral Angle** 0.77

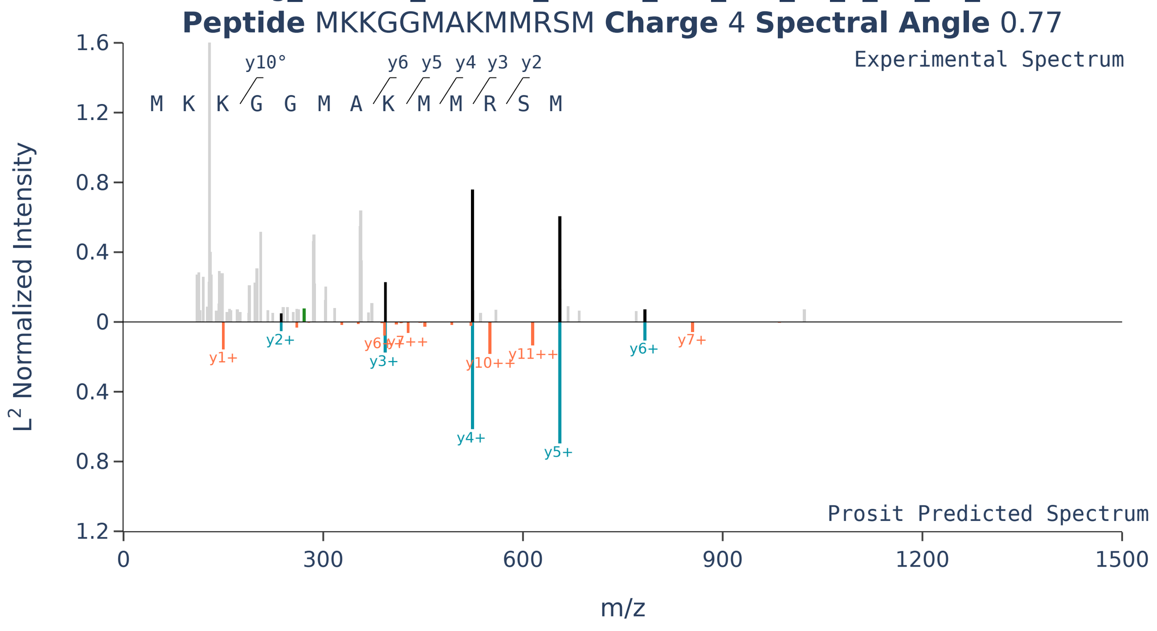


**Source** Ncheng_210623_230623_HFGoe_FFH_20S_25_1_A1_4h_R2 **Scan** 19425
**Peptide** LTKVDGDARGGAALSIRHLTG **Charge** 4 **Spectral Angle** 0.55

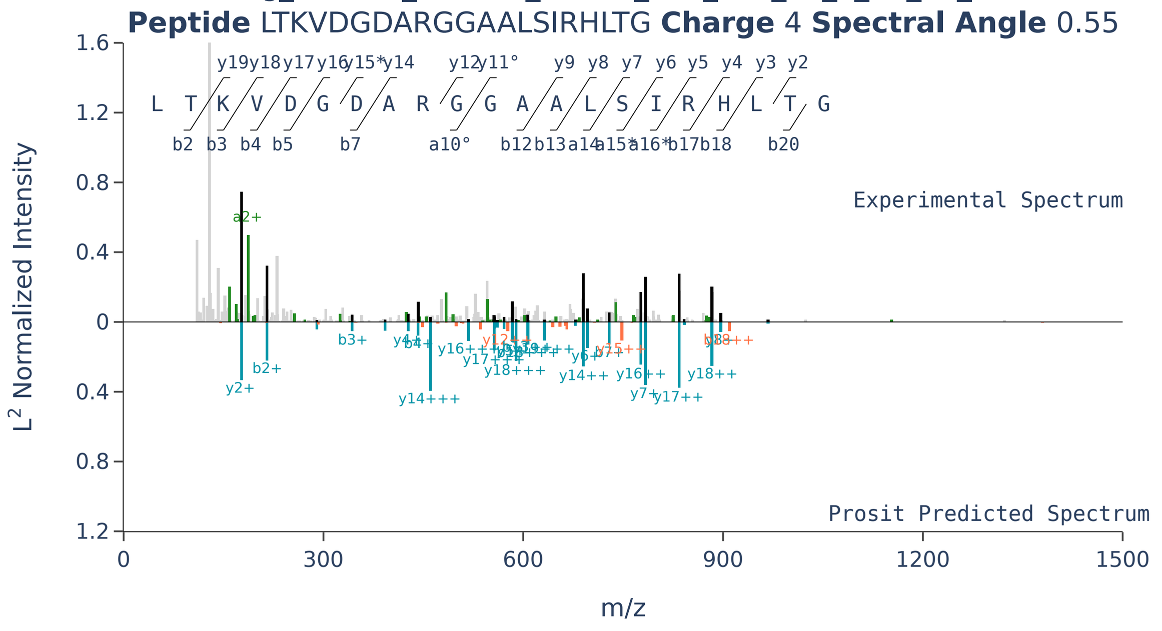


**Source** Ncheng_210623_230623_HFGoe_FFH_20S_25_1_A2_4h_R2 **Scan** 21369
**Peptide** VVSADVYRPAAIKQL **Charge** 3 **Spectral Angle** 0.67

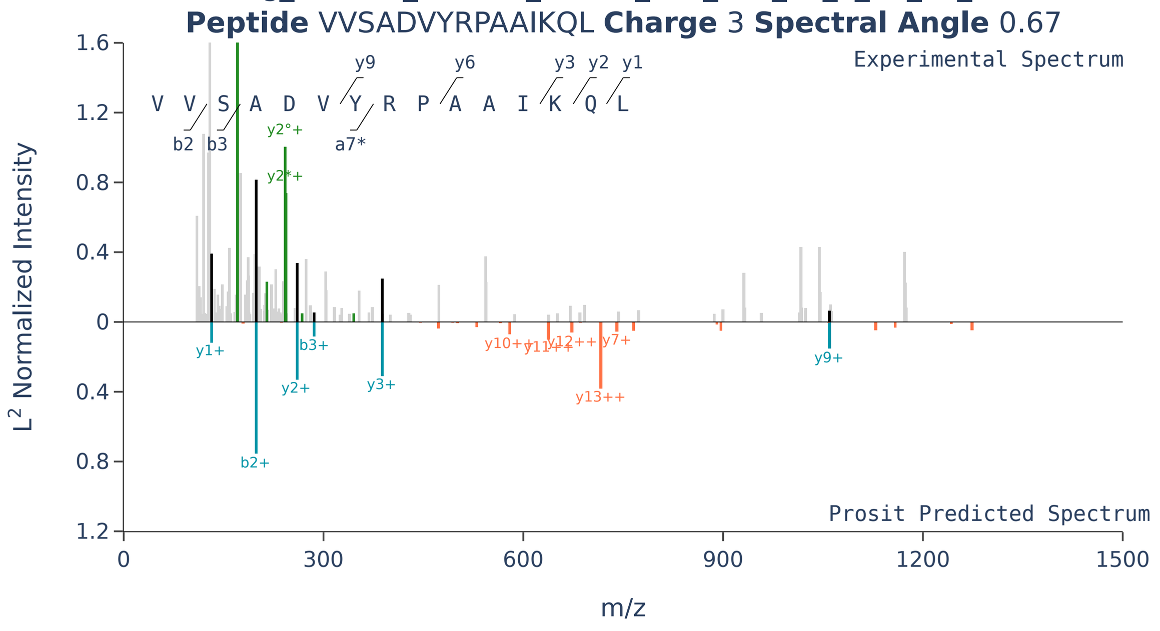

**Source** Ncheng_210623_230623_HFGoe_FFH_20S_25_1_A2_2h_R1 **Scan** 34297
**Peptide** DVALPVVREFINRVKEKAVGHEVN **Charge** 4 **Spectral Angle** 0.67

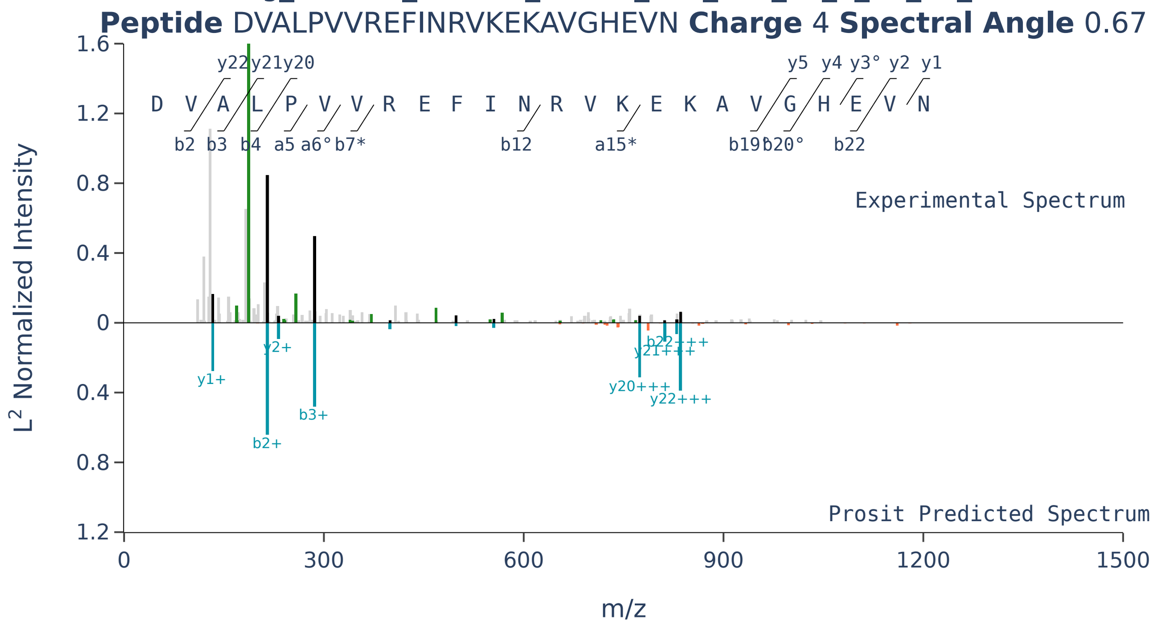


**Source** Ncheng_210623_230623_HFGoe_FFH_20S_25_1_A2_1h_R1 **Scan** 24488
**Peptide** HVDEAMMDEIKQVHASINPVE **Charge** 3 **Spectral Angle** 0.7

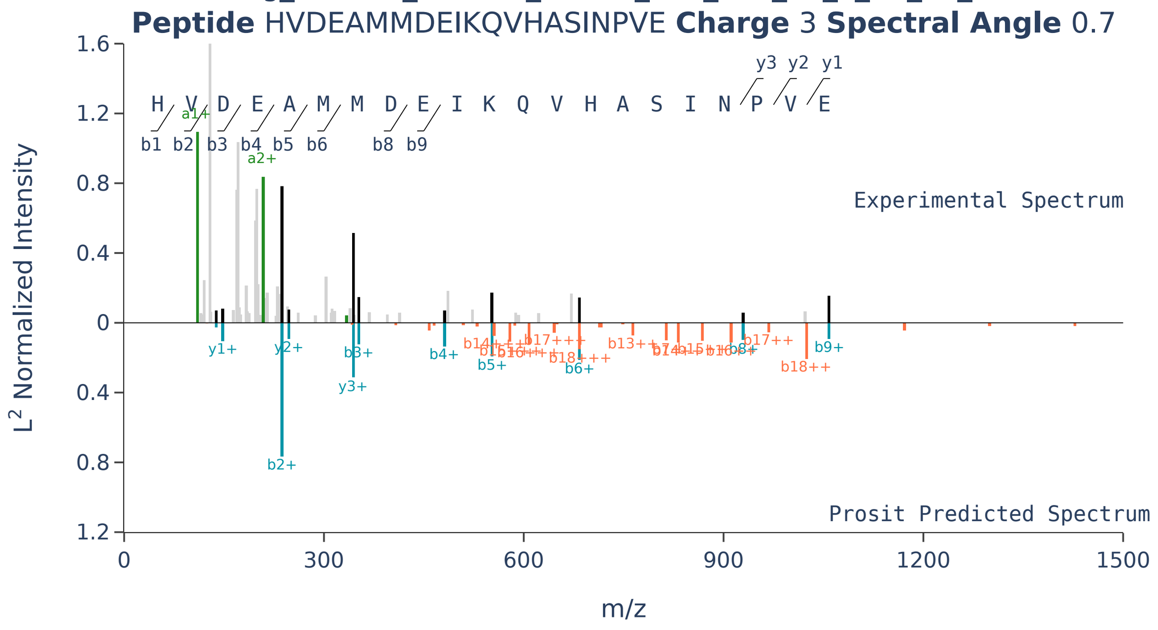


**Source** Ncheng_210623_230623_HFGoe_FFH_20S_25_1_A2_4h_R1 **Scan** 22117
**Peptide** NLTDRLSRTLRNISGRGRLTEDNVKD **Charge** 4 **Spectral Angle** 0.41

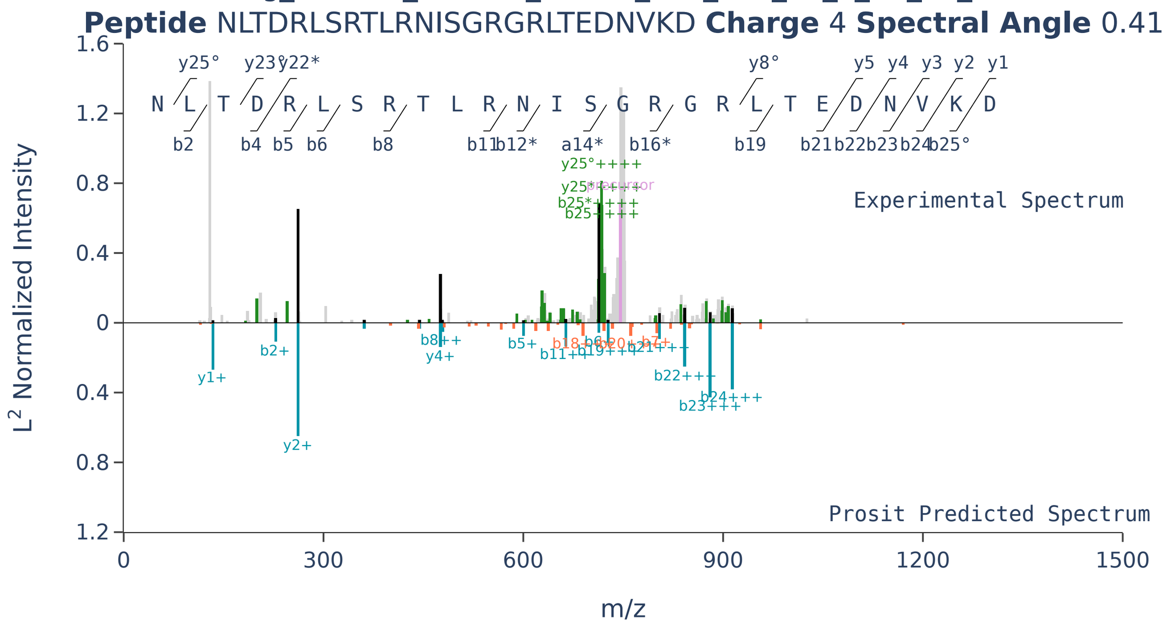


**Source** Ncheng_210623_230623_HFGoe_FFH_20S_25_1_A2_2h_R1 **Scan** 27208
**Peptide** QDVNRLLKQFDDMQRMMKKMKKG **Charge** 6 **Spectral Angle** 0.66

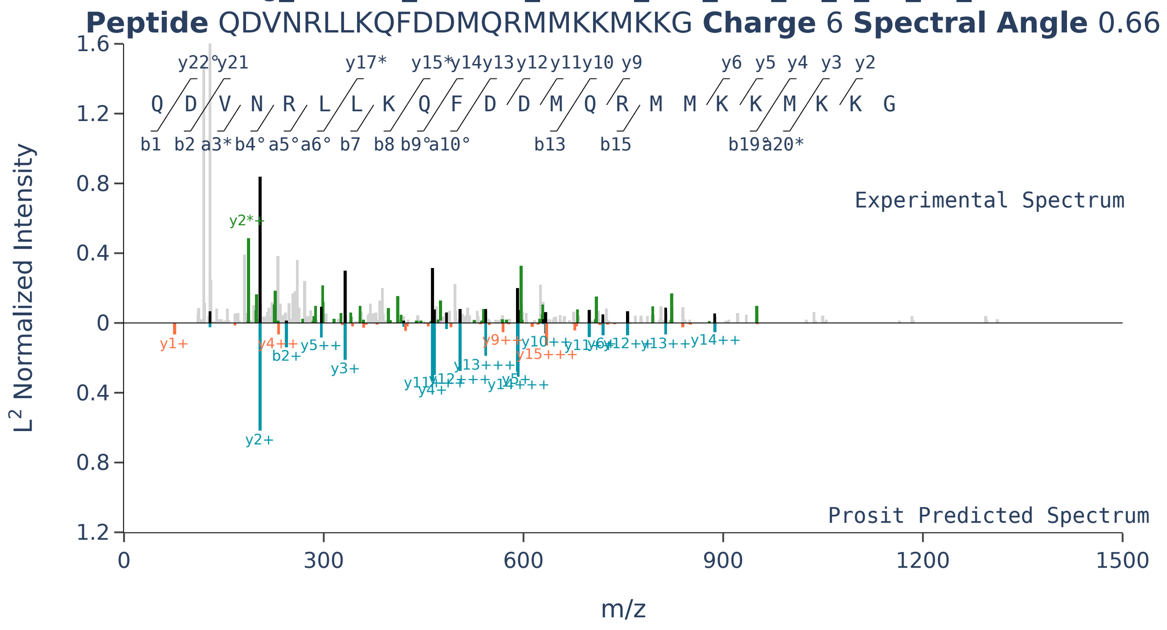


**Source** Ncheng_210623_230623_HFGoe_FFH_20S_25_1_A2_4h_R1 **Scan** 11123
**Peptide** ASKLKKGDGFDLND **Charge** 4 **Spectral Angle** 0.57

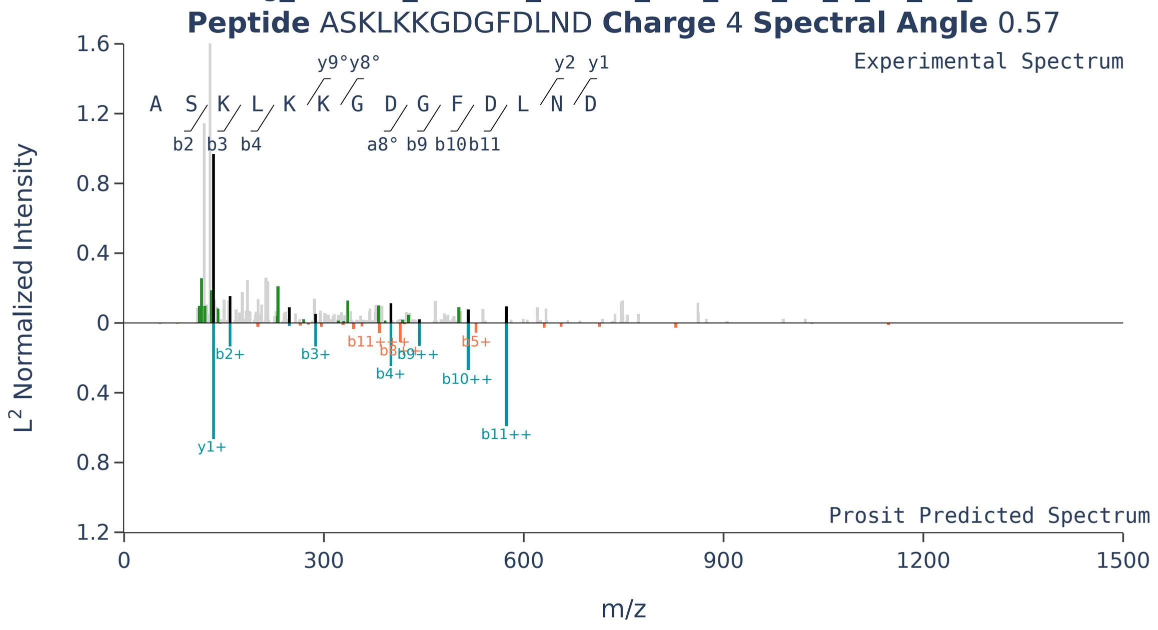


**Source** Ncheng_210623_230623_HFGoe_FFH_20S_25_1_A1_2h_R2 **Scan** 33769
**Peptide** EKAVGHEVNKSLTPGQEFVKIVRNELVAAM **Charge** 4 **Spectral Angle** 0.57

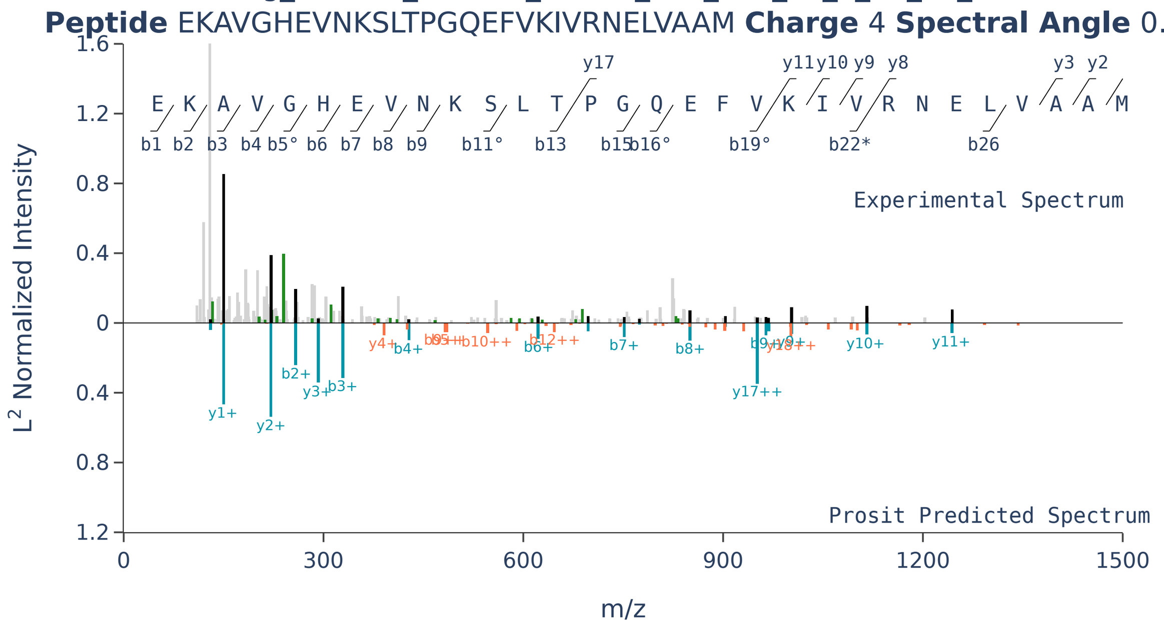


**Source** Ncheng_210623_230623_HFGoe_FFH_20S_25_1_A2_4h_R1 **Scan** 5285
**Peptide** KKGGMAKM **Charge** 2 **Spectral Angle** 0.91

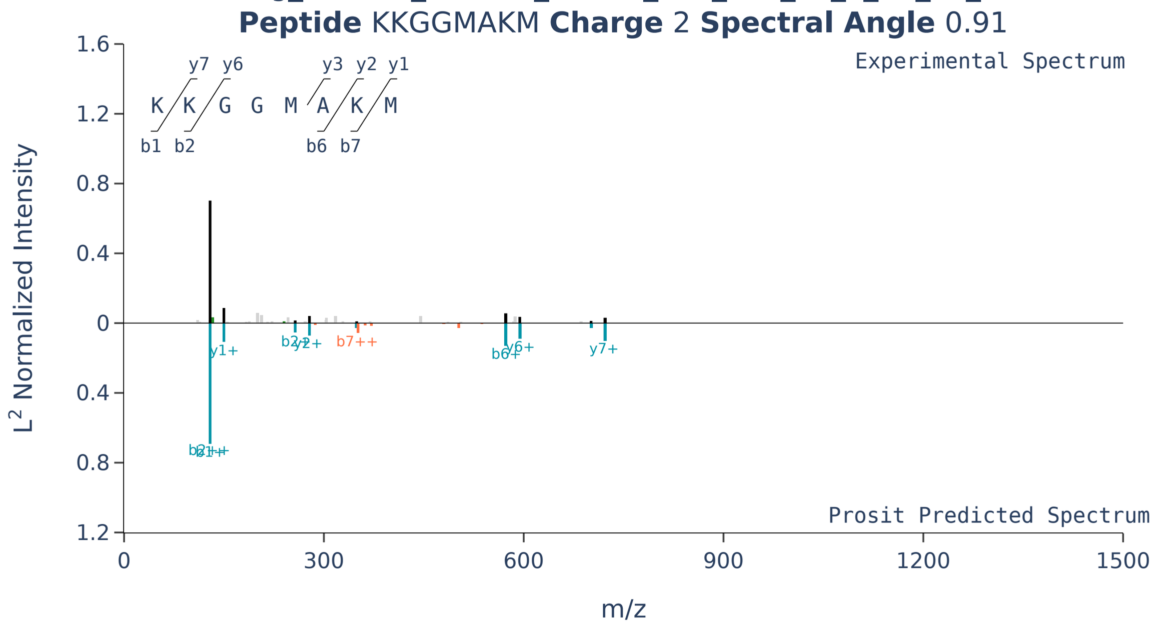


**Source** Ncheng_210623_230623_HFGoe_FFH_20S_25_1_A2_24h_R2 **Scan** 24664
**Peptide** ANTAKAFNEALPLTG **Charge** 2 **Spectral Angle** 0.71

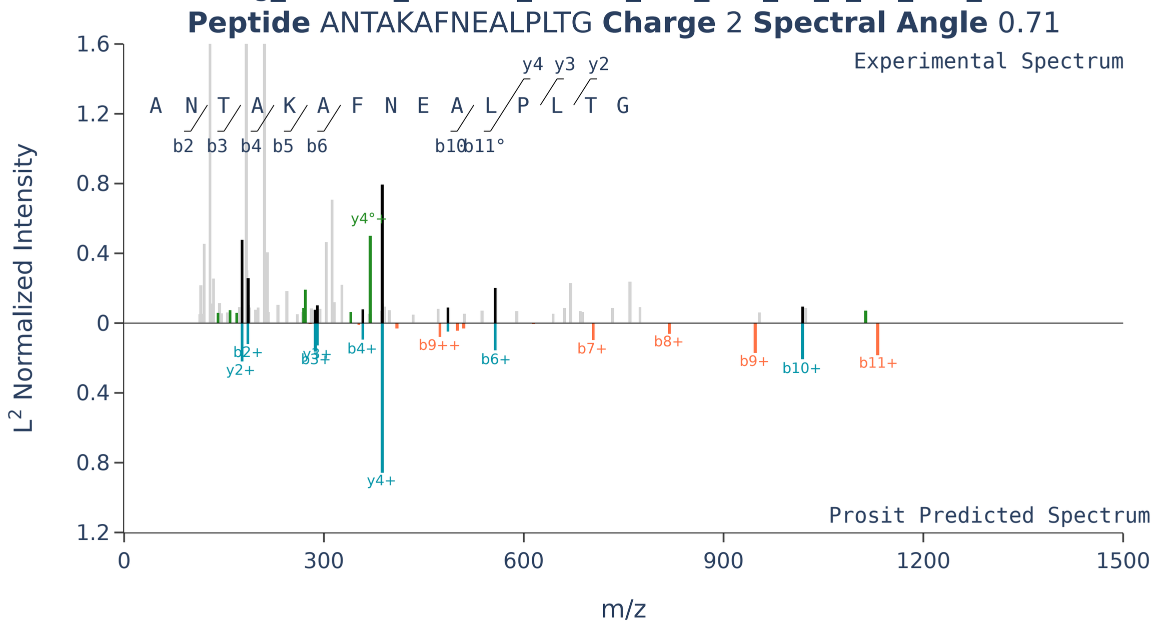


**Source** Ncheng_210623_230623_HFGoe_FFH_20S_25_1_A1_2h_R1 **Scan** 32021
**Peptide** ADVALPVVREFINRVKEKAVGHEVNKSLT **Charge** 5 **Spectral Angle** 0.84

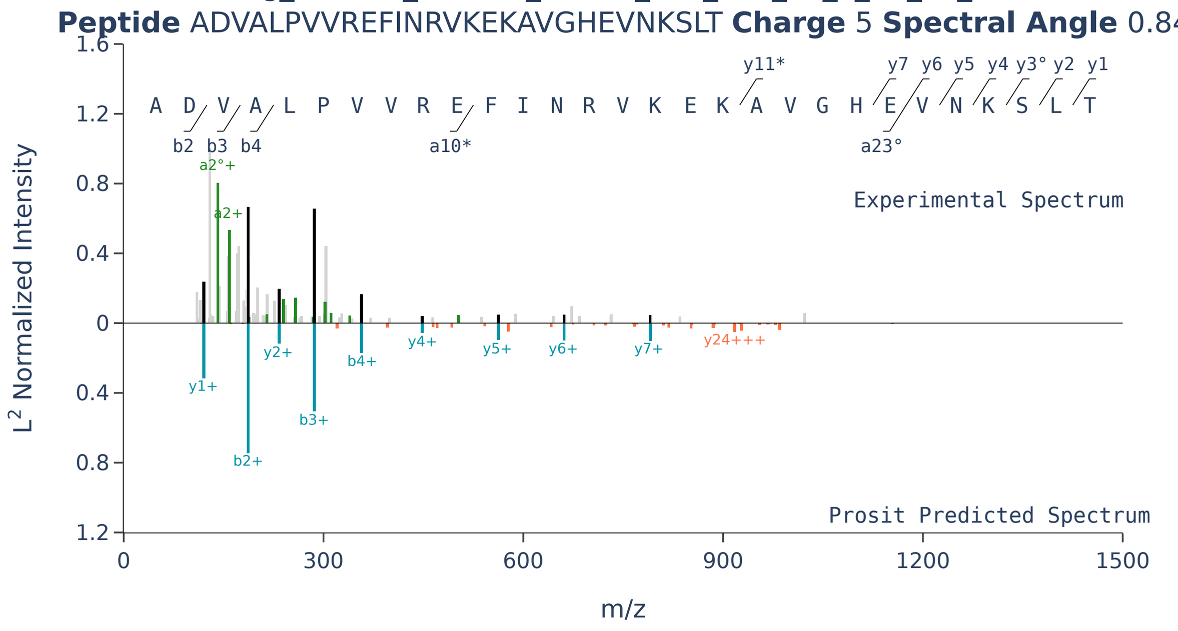


**Source** Ncheng_210623_230623_HFGoe_FFH_20S_25_1_A1_2h_R2 **Scan** 8301
**Peptide** FINRVKEKAV **Charge** 3 **Spectral Angle** 0.46

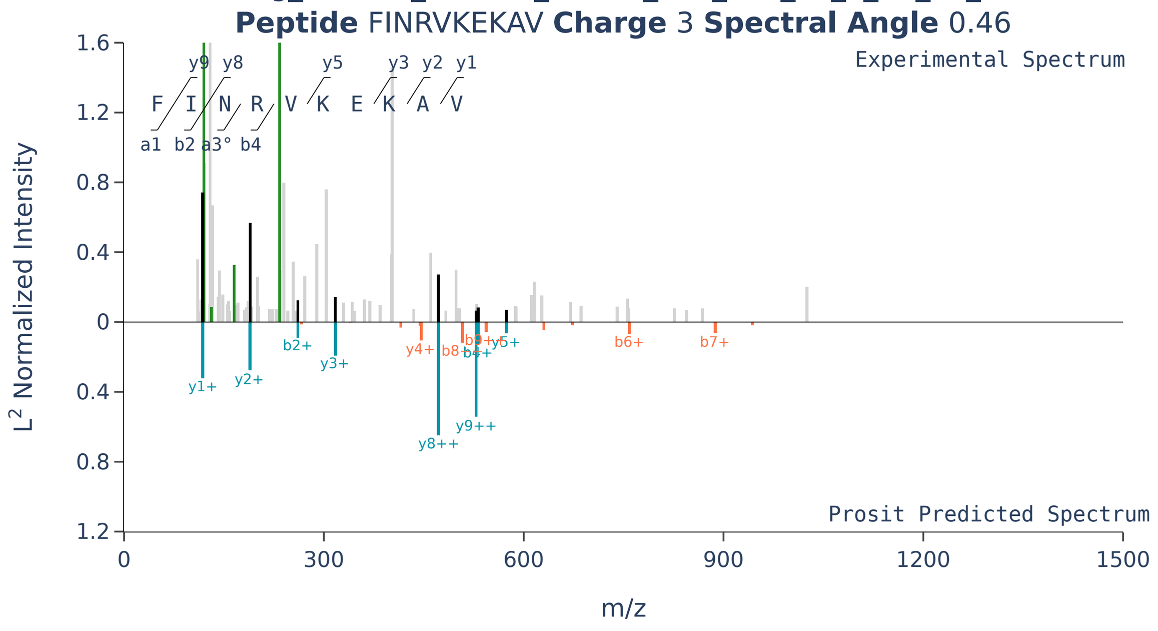


**Source** Ncheng_210623_230623_HFGoe_FFH_20S_25_1_A1_1h_R1 **Scan** 31406
**Peptide** KLKFYDVLLVDTAGRL **Charge** 4 **Spectral Angle** 0.58

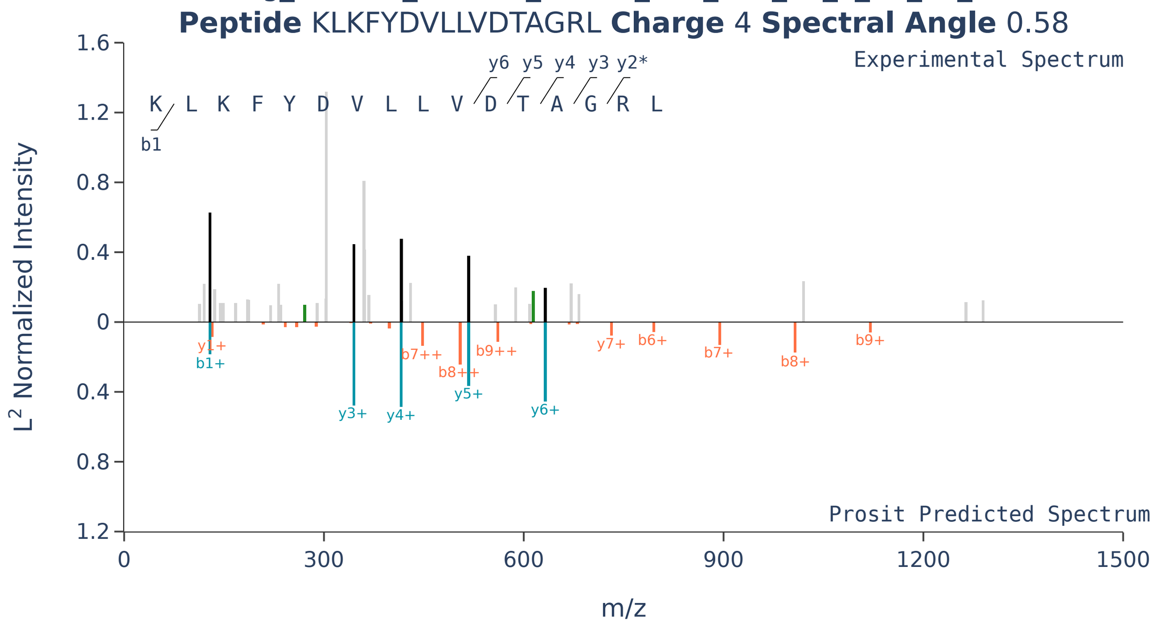


**Source** Ncheng_210623_230623_HFGoe_FFH_20S_25_1_A2_1h_R1 **Scan** 20919
**Peptide** TGKPIKFLGVGEKTEALEPFHPDRIASR **Charge** 3 **Spectral Angle** 0.6

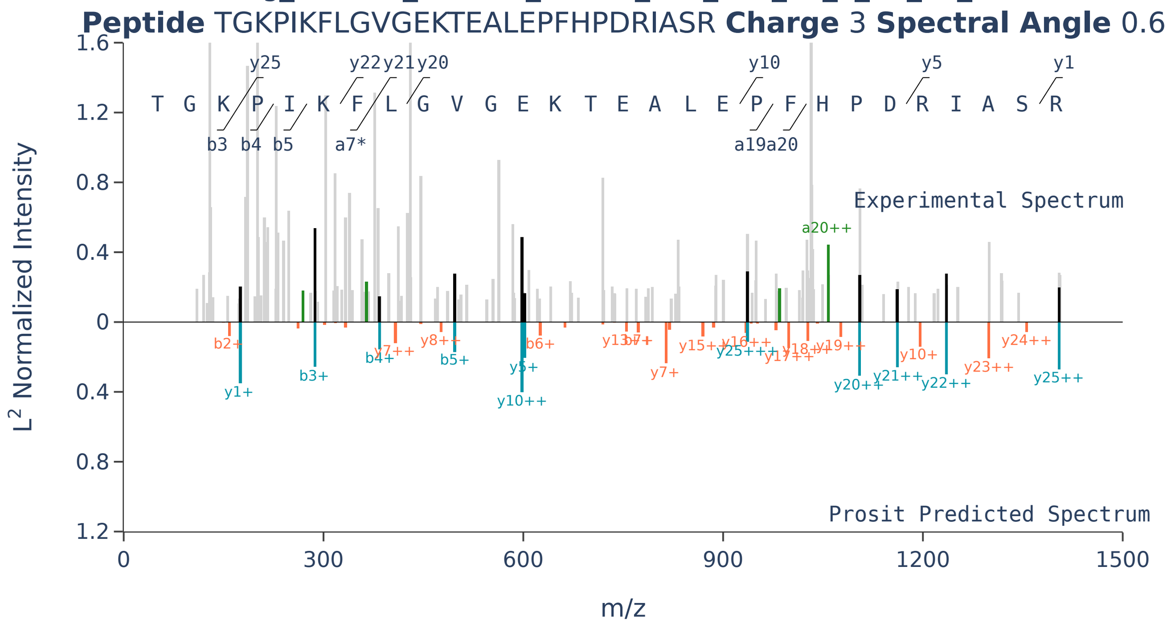


**Source** Ncheng_210623_230623_HFGoe_FFH_20S_25_1_A1_2h_R1 **Scan** 34011
**Peptide** PSDVGQKPVDIVNAALKEAKLKFYD **Charge** 4 **Spectral Angle** 0.71

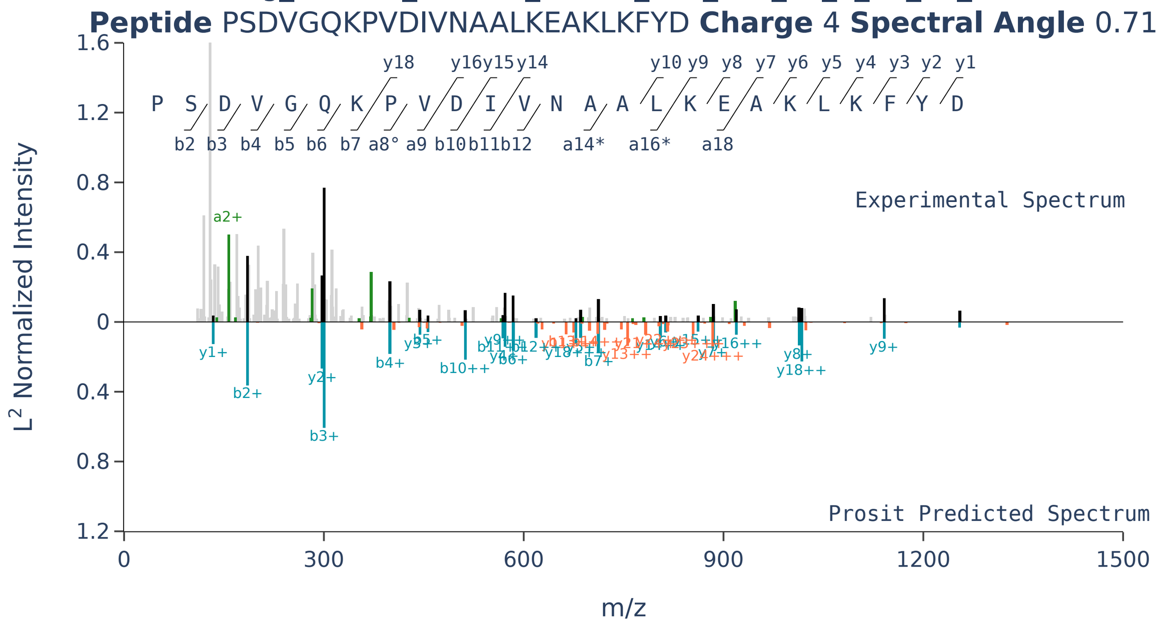


**Source** Ncheng_210623_230623_HFGoe_FFH_20S_25_1_A2_24h_R2 **Scan** 8157
**Peptide** FLNRVKEKAVG **Charge** 3 **Spectral Angle** 0.48

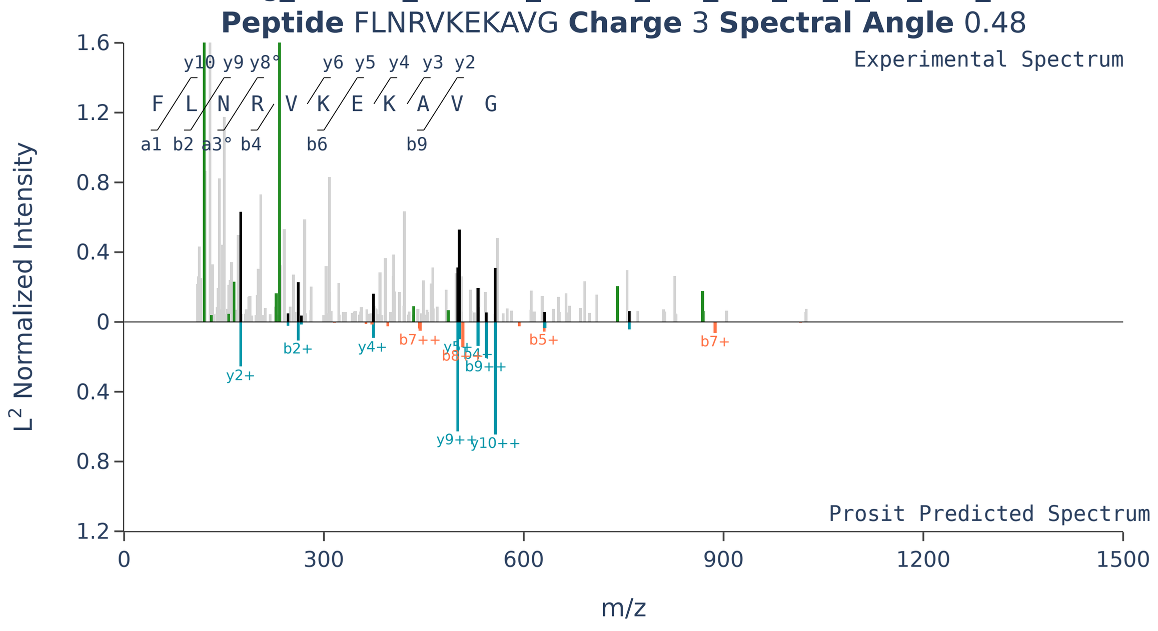


**Source** Ncheng_210623_230623_HFGoe_FFH_20S_25_1_A1_2h_R2 **Scan** 33628
**Peptide** PGQEFVKIVRNELVAAM **Charge** 2 **Spectral Angle** 0.62

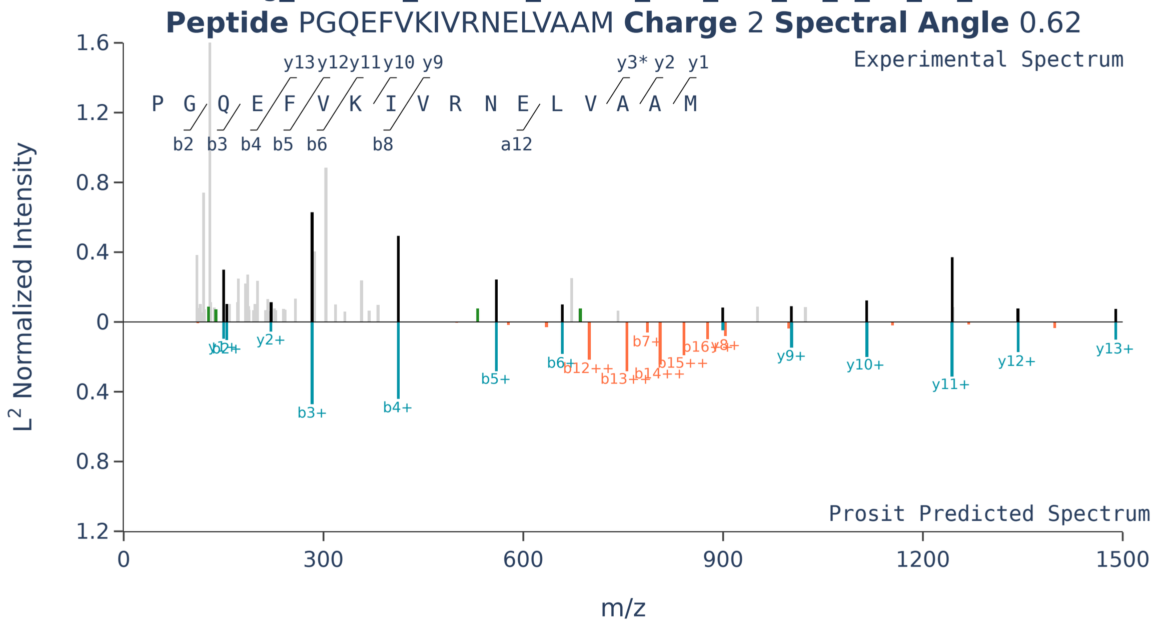

**Source** Ncheng_210623_230623_HFGoe_FFH_20S_25_1_A2_1h_R1 **Scan** 26328
**Peptide** KPIKFLGVGEKTEALEPFHPDRIASRLL **Charge** 5 **Spectral Angle** 0.59

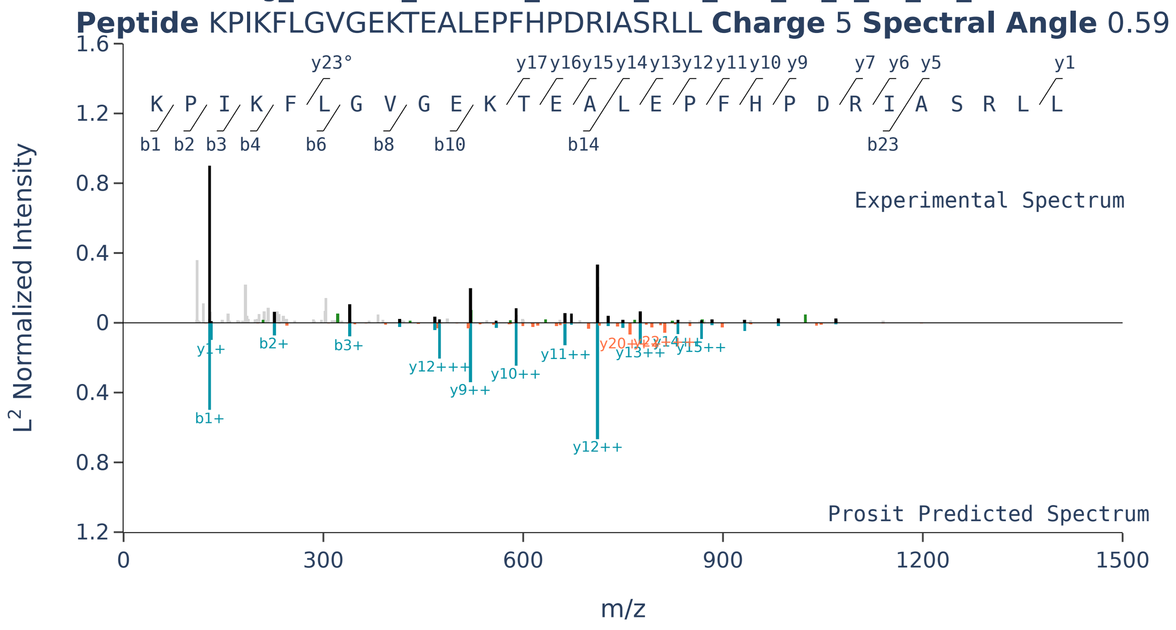


**Source** Ncheng_210623_230623_HFGoe_FFH_20S_25_1_A1_2h_R2 **Scan** 39583
**Peptide** QVGVDFFPSDVGQKPVDIVNAALKEAKL **Charge** 4 **Spectral Angle** 0.65

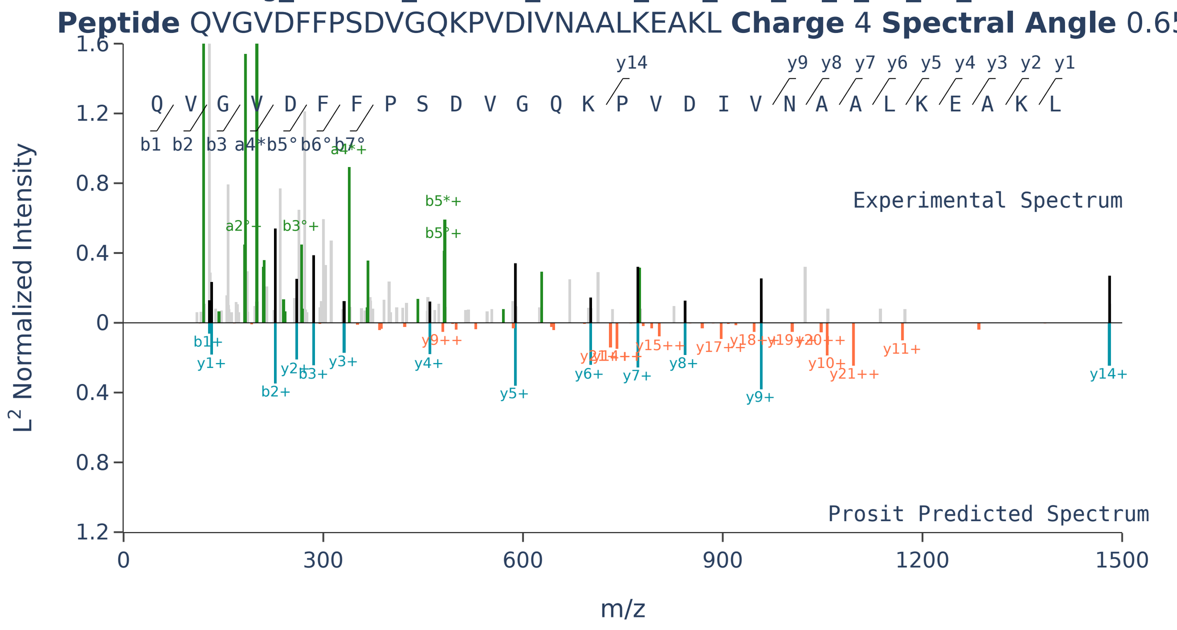


**Source** Ncheng_210623_230623_HFGoe_FFH_20S_25_1_A1_2h_R2 **Scan** 22575
**Peptide** SLRHITGKPIKFLGVGEKTEALEPFHPDRI **Charge** 5 **Spectral Angle** 0.65

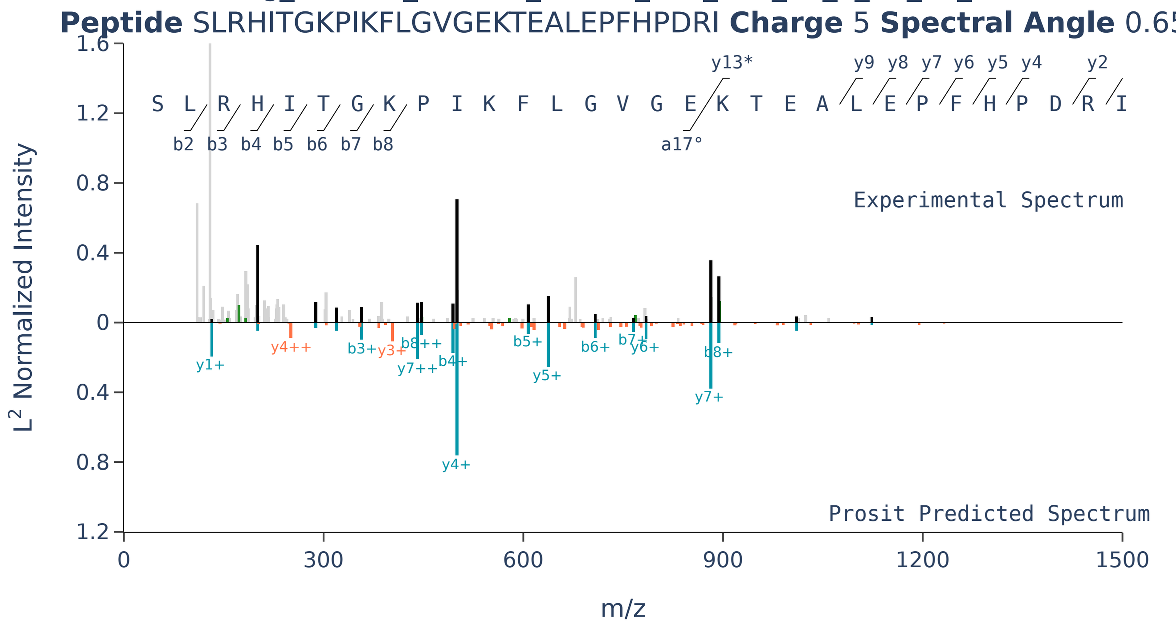


**Source** Ncheng_210623_230623_HFGoe_FFH_20S_25_1_A2_2h_R2 **Scan** 37509
**Peptide** ALLEADVALPVVREFINRVKEKAVGHEVNK **Charge** 4 **Spectral Angle** 0.69

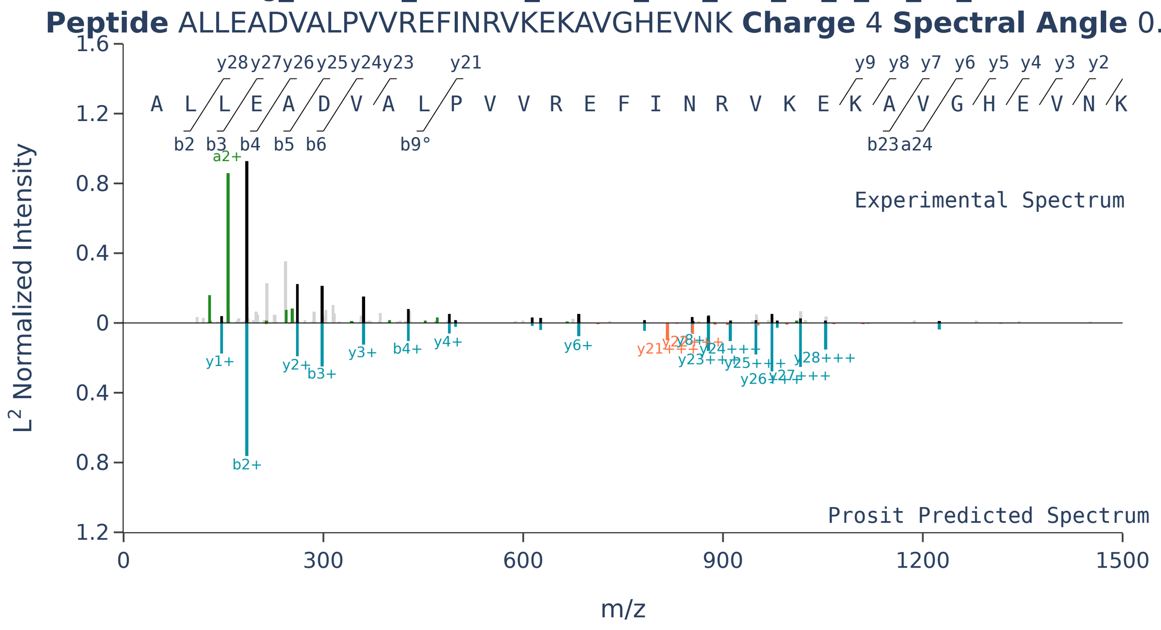


**Source** Ncheng_210623_230623_HFGoe_FFH_20S_25_1_A2_4h_R1 **Scan** 35445
**Peptide** KKGDGFDLNDFLEQLRQ **Charge** 3 **Spectral Angle** 0.51

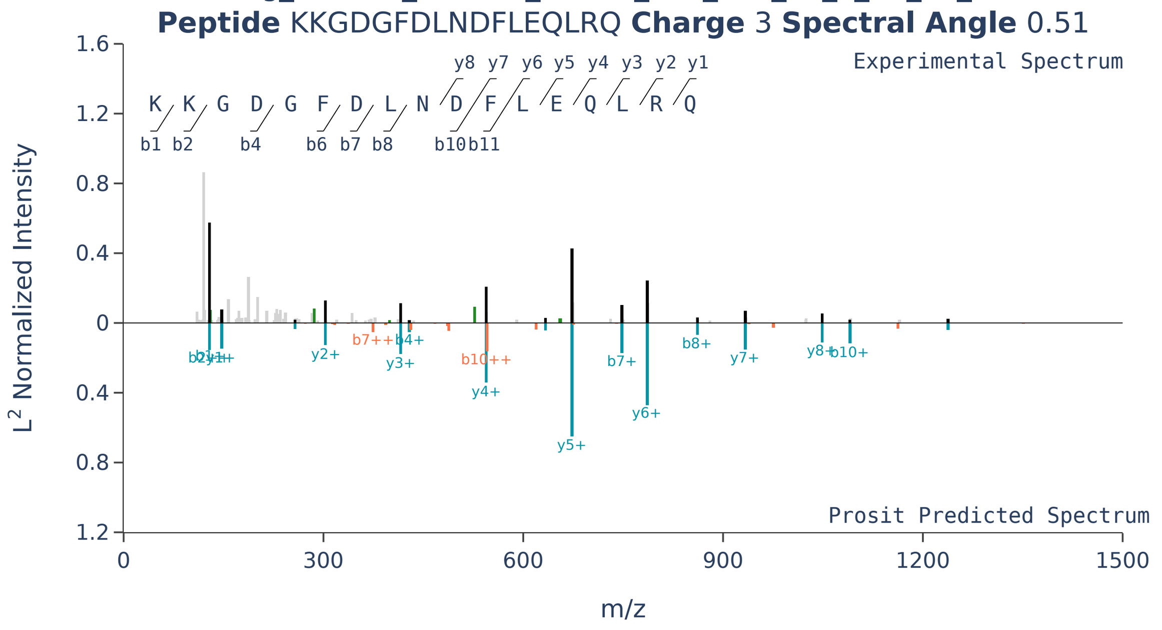


**Source** Ncheng_210623_230623_HFGoe_FFH_20S_25_1_A1_24h_R2 **Scan** 13928
**Peptide** DGDARGGAALSIRHITGKPIK **Charge** 4 **Spectral Angle** 0.57

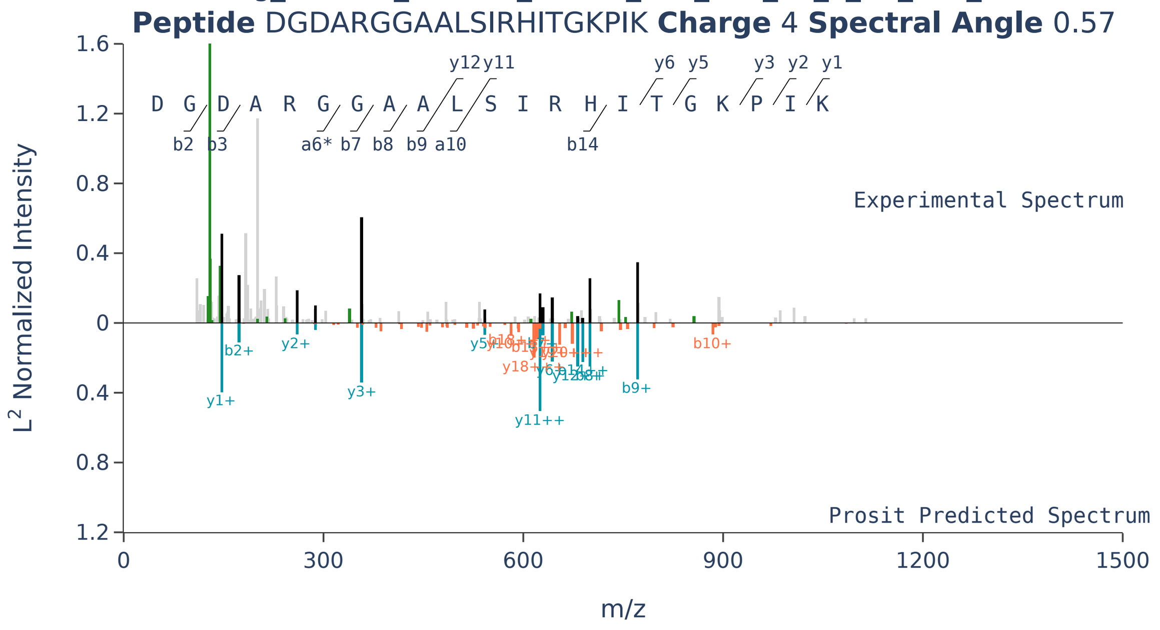


**Source** Ncheng_210623_230623_HFGoe_FFH_20S_25_1_A2_1h_R1 **Scan** 12042
**Peptide** GAALSIRHITGK **Charge** 3 **Spectral Angle** 0.55

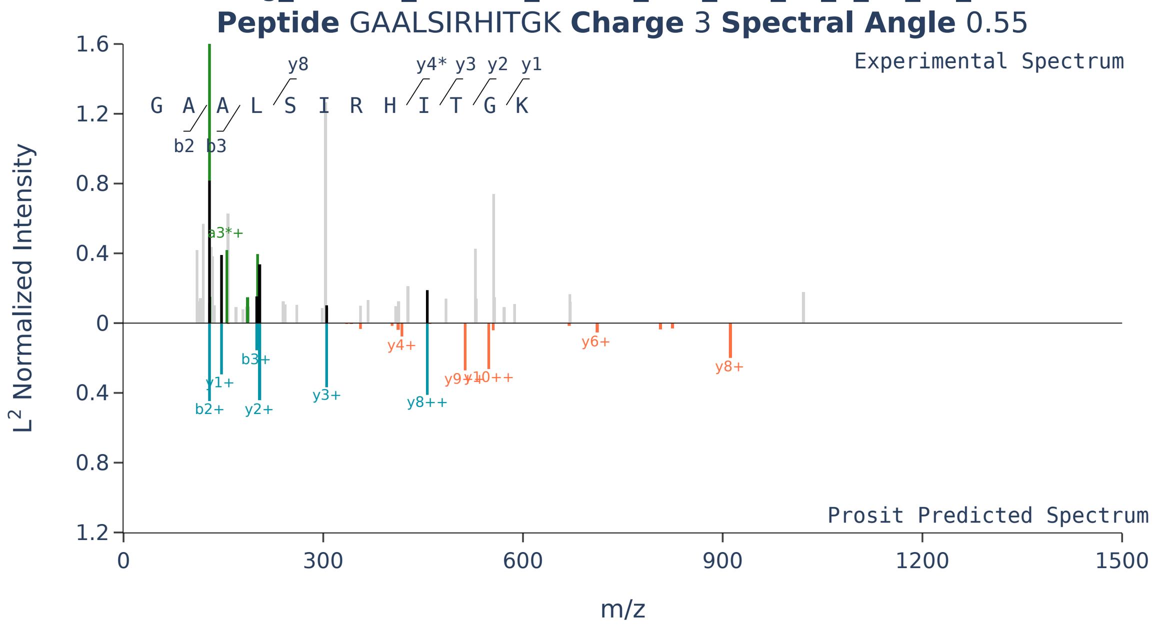


**Source** Ncheng_210623_230623_HFGoe_FFH_20S_25_1_A2_24h_R1 **Scan** 11616
**Peptide** TDRLSRTLRNISGRGR **Charge** 5 **Spectral Angle** 0.63

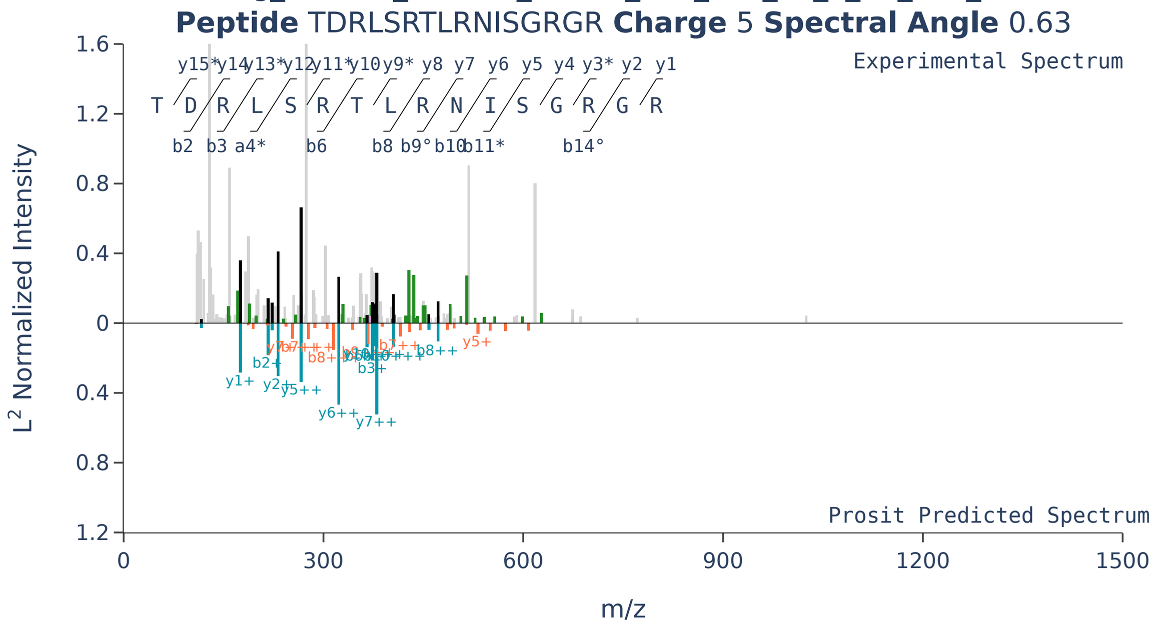


**Source** Ncheng_210623_230623_HFGoe_FFH_20S_25_1_A2_2h_R2 **Scan** 24089
**Peptide** GAALSIRHITGKPIKFLGVGE **Charge** 3 **Spectral Angle** 0.74

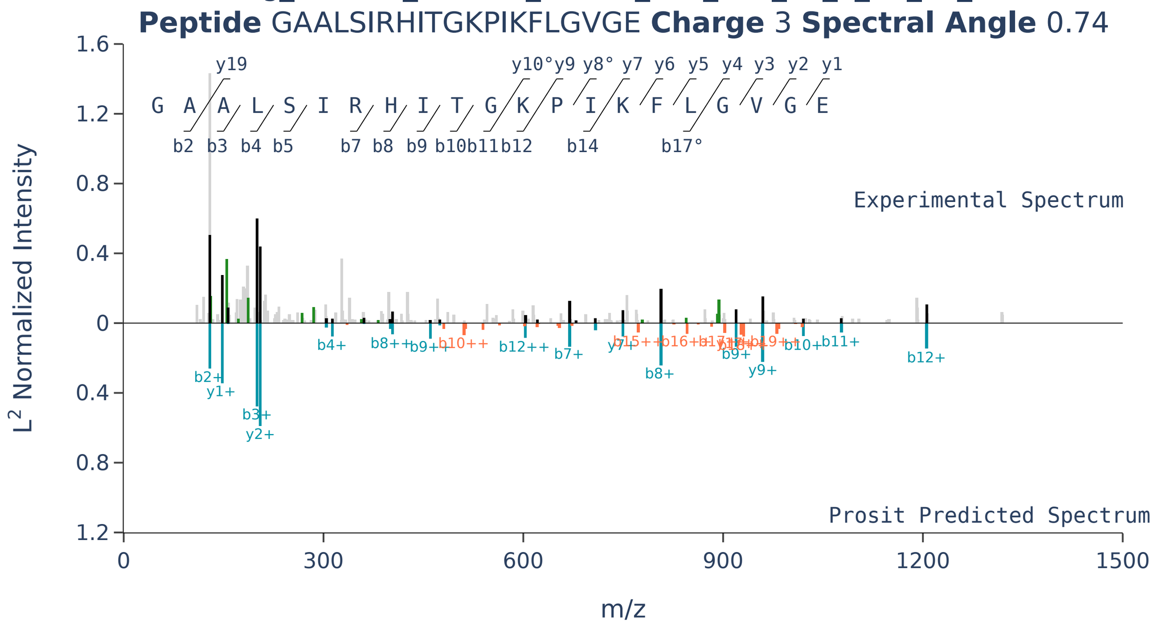


**Source** Ncheng_210623_230623_HFGoe_FFH_20S_25_1_A1_1h_R1 **Scan** 26630
**Peptide** EALEPFHPDRIASRILG **Charge** 3 **Spectral Angle** 0.53

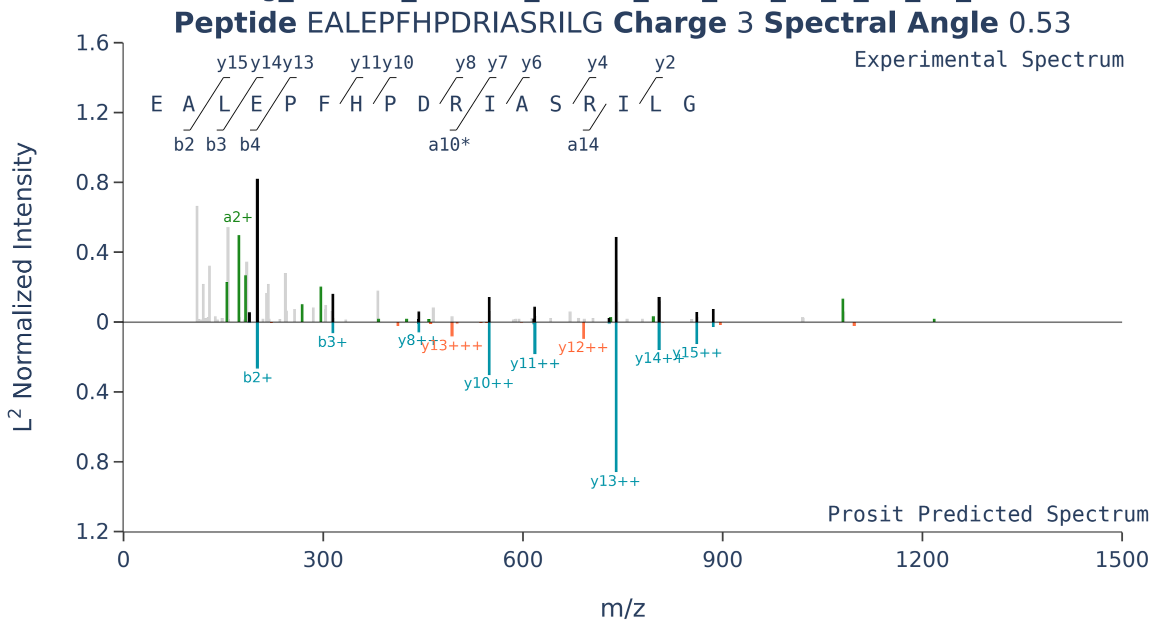


**Source** Ncheng_210623_230623_HFGoe_FFH_20S_25_1_A2_2h_R1 **Scan** 7747
**Peptide** QRMMKKMKKG **Charge** 3 **Spectral Angle** 0.77

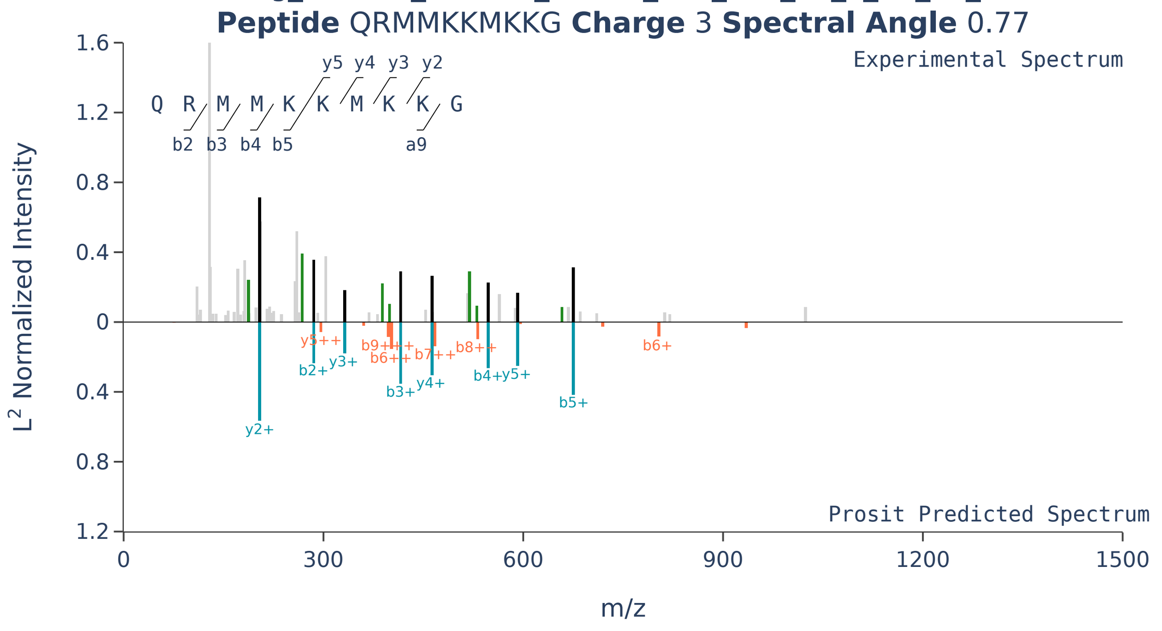


**Source** Ncheng_210623_230623_HFGoe_FFH_20S_25_1_A2_2h_R1 **Scan** 20197
**Peptide** LESKVDRAQAEKLASKLKKGDGFDL **Charge** 5 **Spectral Angle** 0.88

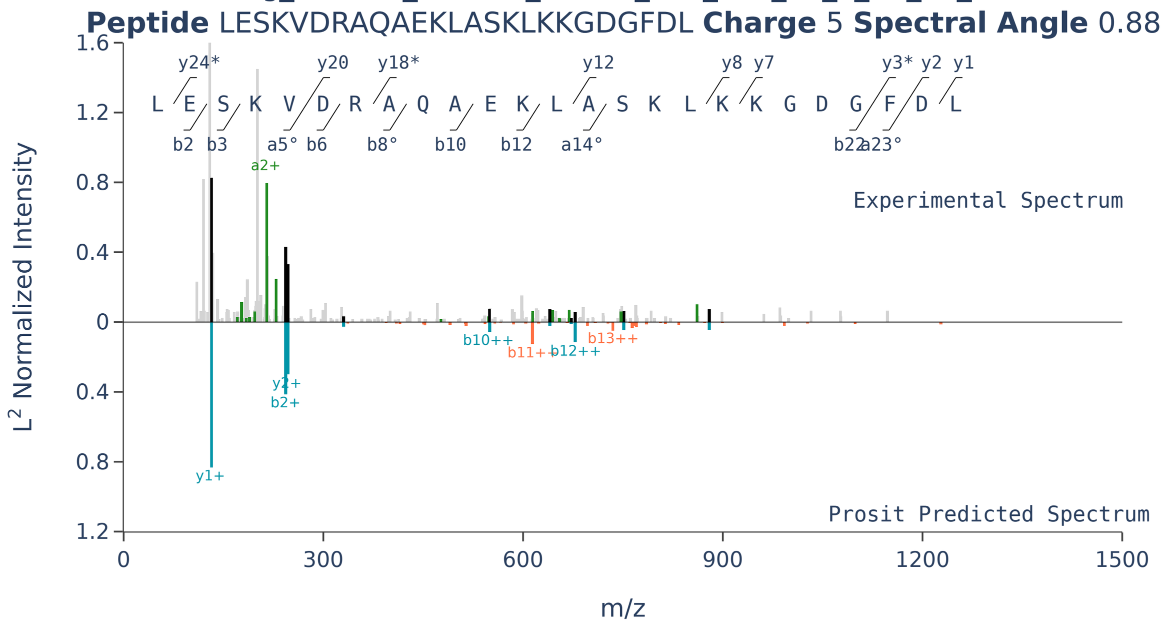


**Source** Ncheng_210623_230623_HFGoe_FFH_20S_25_1_A1_2h_R2 **Scan** 20000
**Peptide** VLTKVDGDARGGAALSIRHITGKPIKF **Charge** 6 **Spectral Angle** 0.75

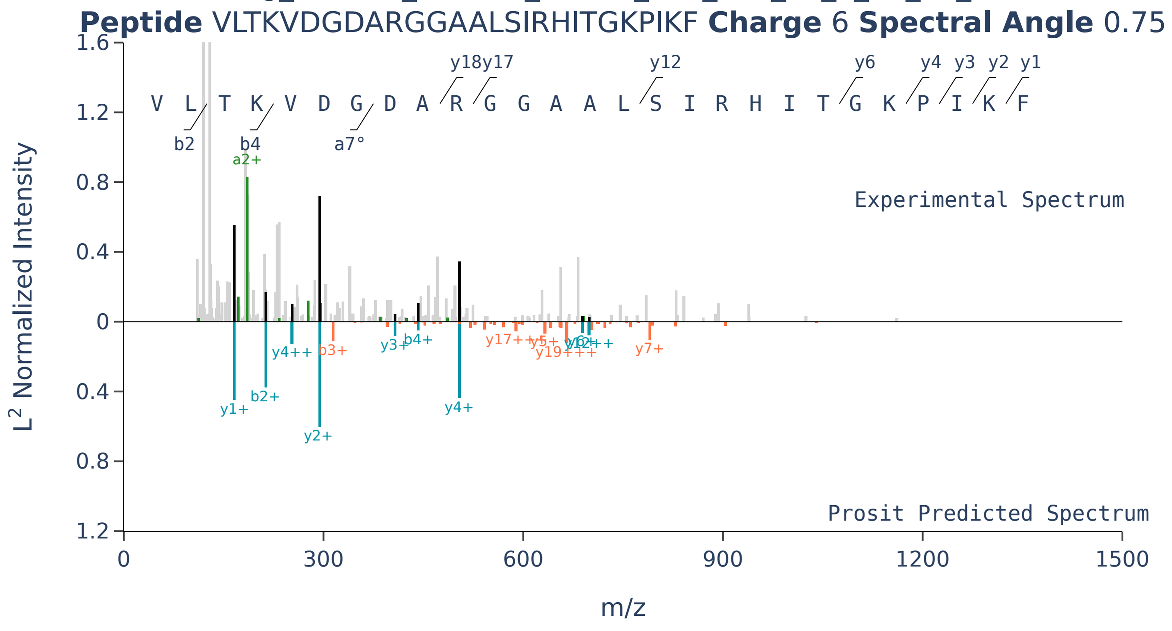


**Source** Ncheng_210623_230623_HFGoe_FFH_20S_25_1_A1_2h_R2 **Scan** 28645
**Peptide** KEAKLKFYDVLLVDT **Charge** 3 **Spectral Angle** 0.42

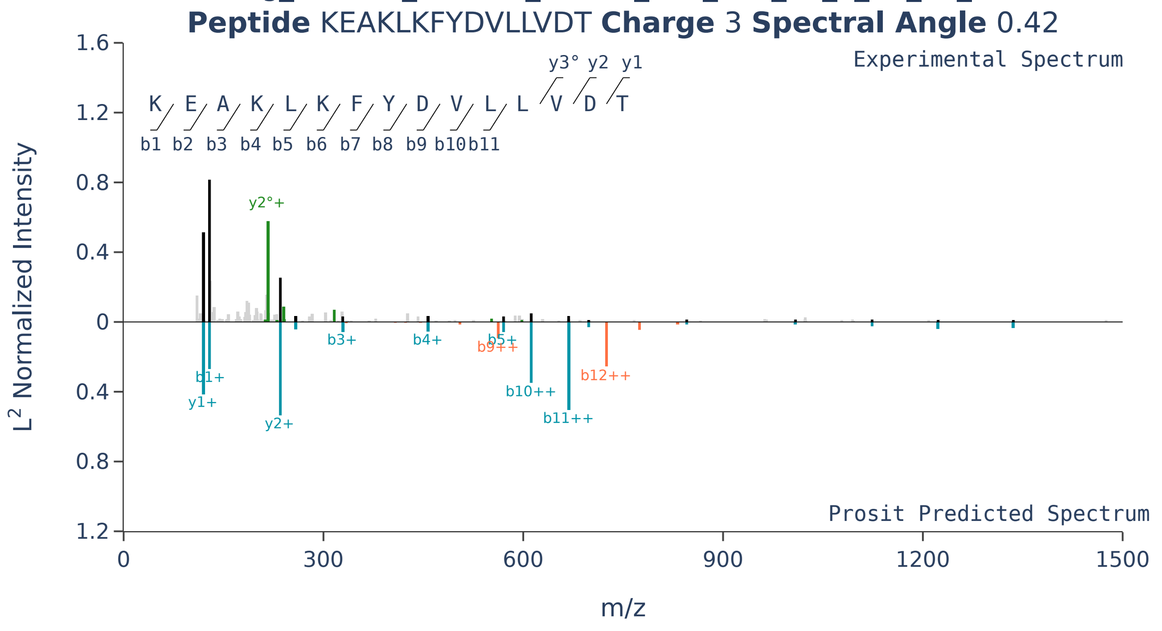


**Source** Ncheng_210623_230623_HFGoe_FFH_20S_25_1_A1_24h_R2 **Scan** 16624
**Peptide** LNRVKEKAVGHEVNKSLTPQEFVKIVRN **Charge** 6 **Spectral Angle** 0.66

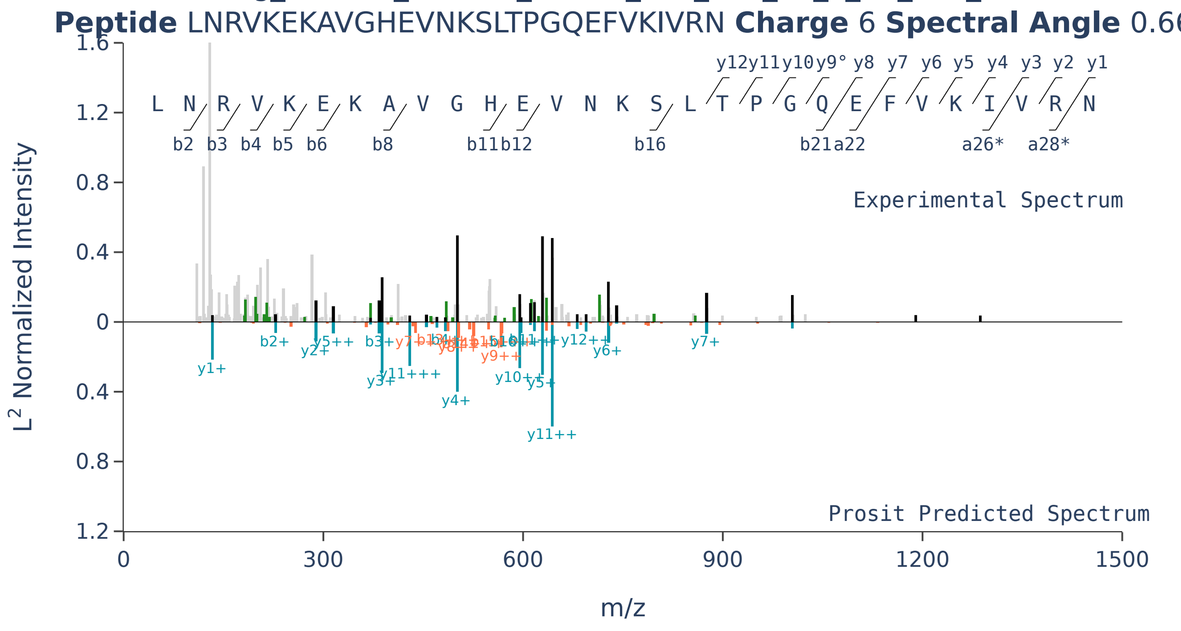

**Source** Ncheng_210623_230623_HFGoe_FFH_20S_25_1_A2_2h_R2 **Scan** 33831
**Peptide** VSADVYRPAAIKQLETLAEQV **Charge** 3 **Spectral Angle** 0.65

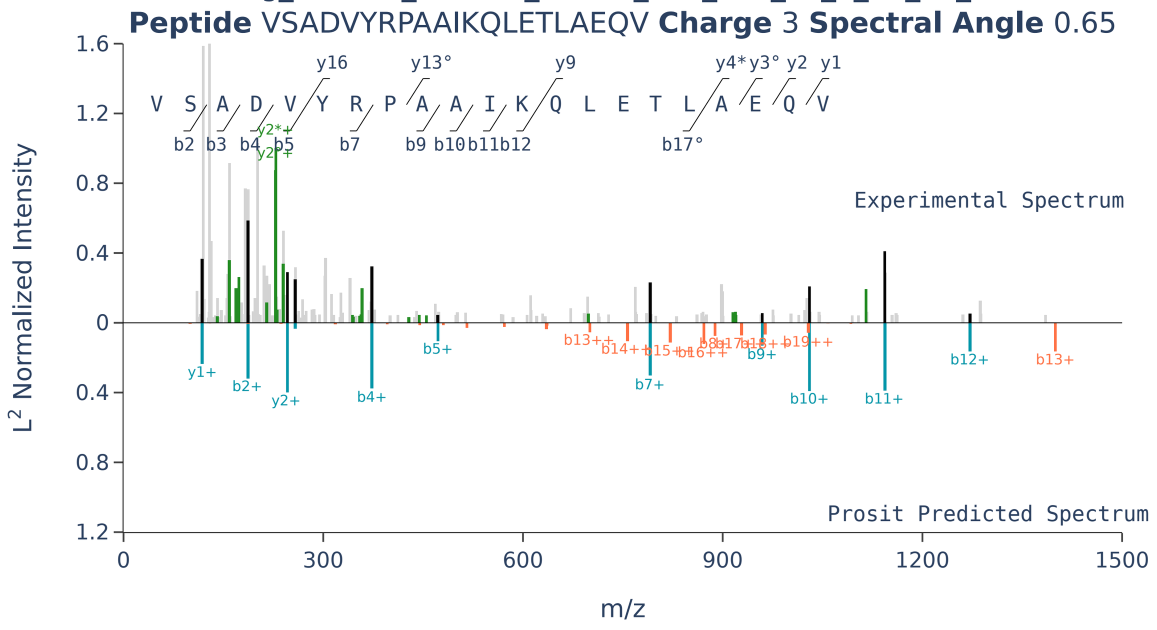


**Source** Ncheng_210623_230623_HFGoe_FFH_20S_25_1_A2_2h_R2 **Scan** 40524
**Peptide** GQIPDNVKSQMDDKVLVRMEAIINSMTM **Charge** 4 **Spectral Angle** 0.83

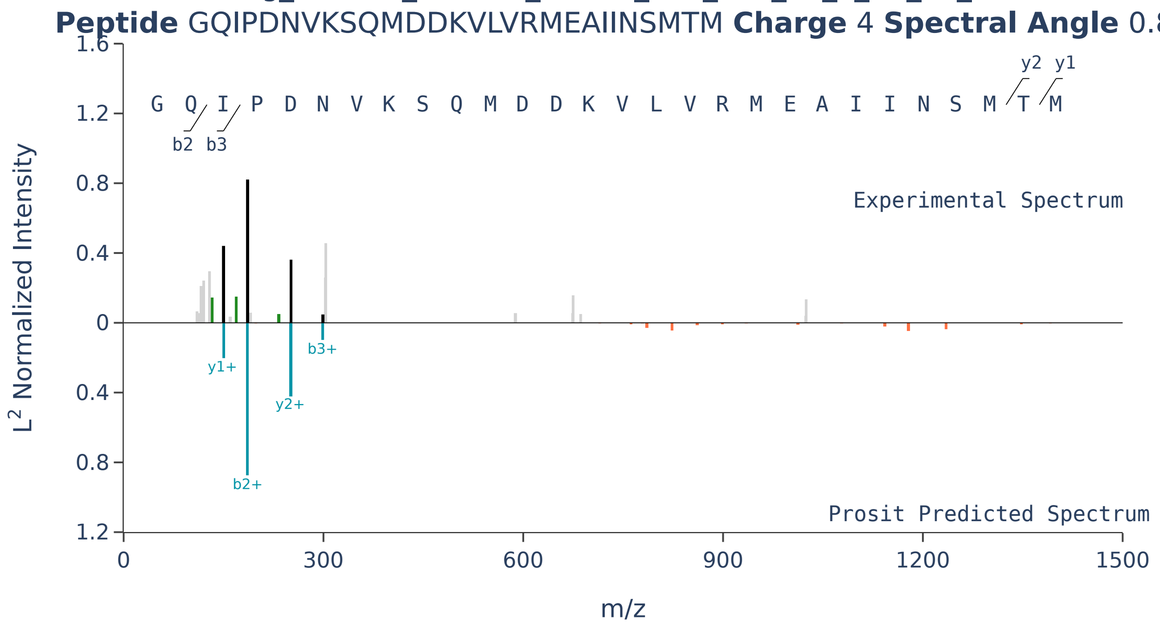


**Source** Ncheng_210623_230623_HFGoe_FFH_20S_25_1_A2_1h_R2 **Scan** 20689
**Peptide** EFINRVKEKAVGHEVNKSLTPGQEFVKIV **Charge** 6 **Spectral Angle** 0.77

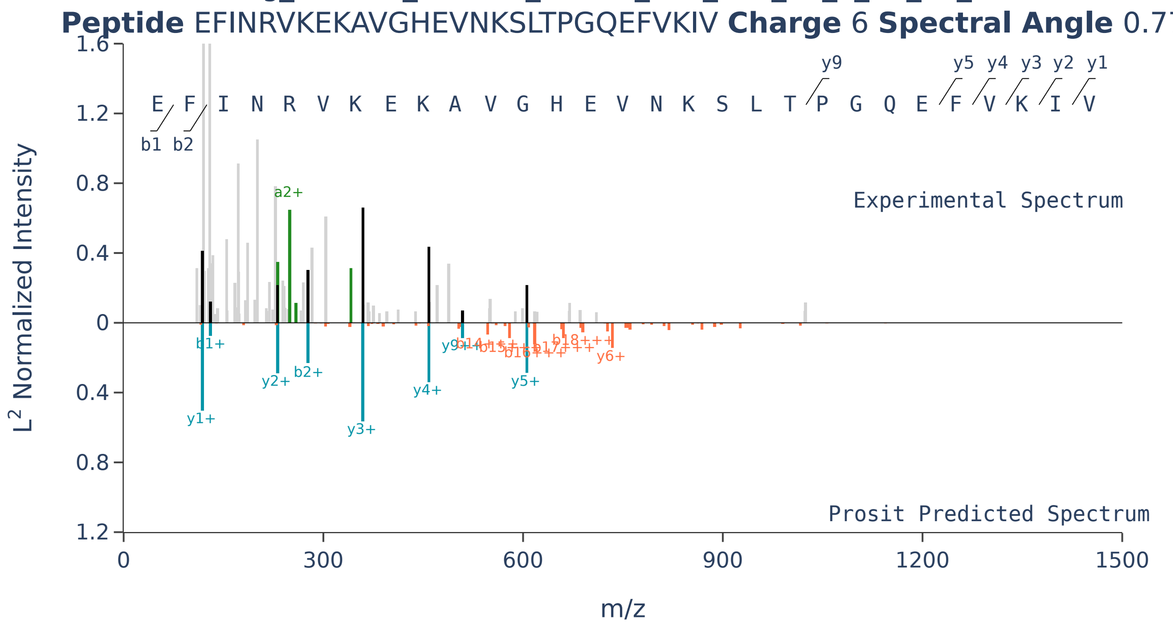


**Source** Ncheng_210623_230623_HFGoe_FFH_20S_25_1_A1_4h_R1 **Scan** 9469
**Peptide** DDMQRMMKKMKK **Charge** 4 **Spectral Angle** 0.7

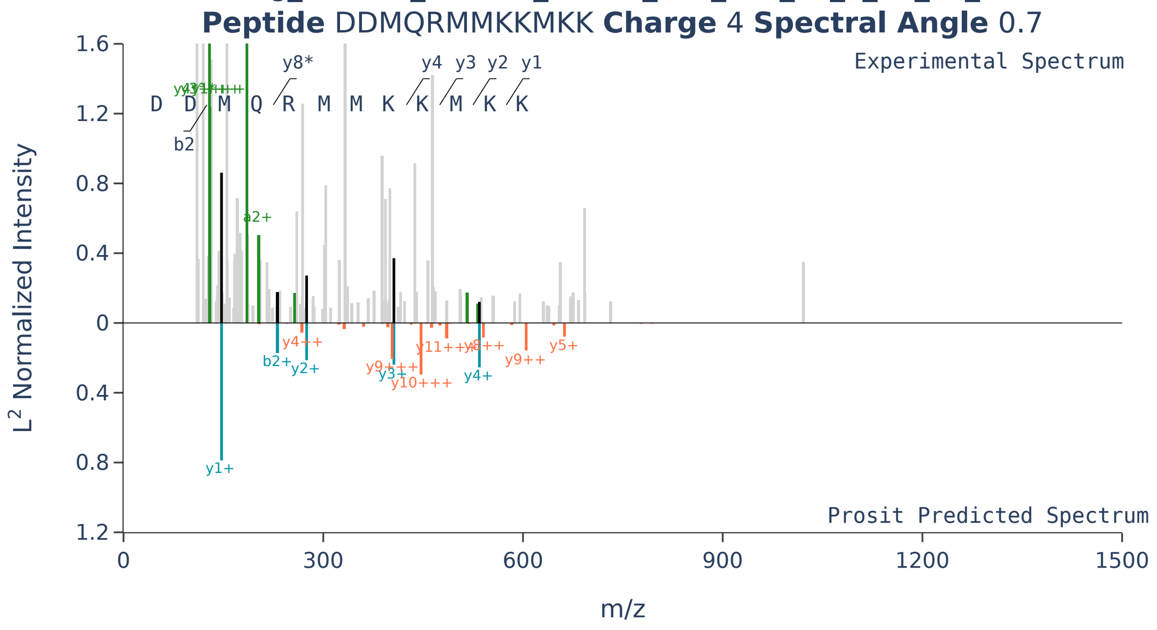


**Source** Ncheng_210623_230623_HFGoe_FFH_20S_25_1_A2_24h_R1 **Scan** 5199
**Peptide** GKFLREKHK **Charge** 2 **Spectral Angle** 0.63

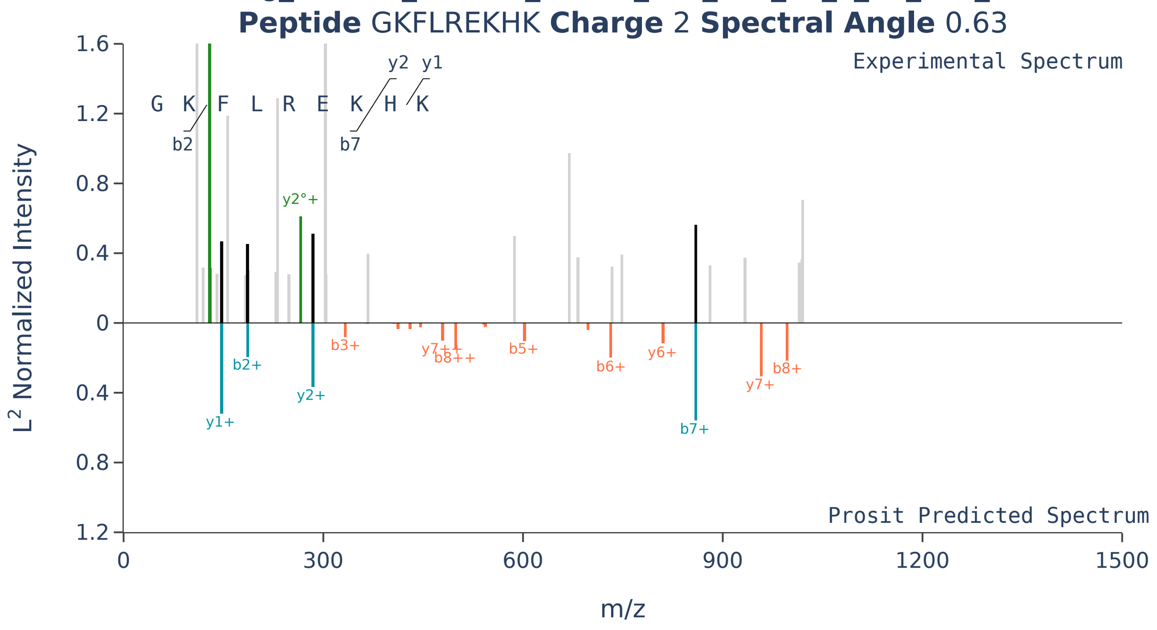


**Source** Ncheng_210623_230623_HFGoe_FFH_20S_25_1_A1_24h_R2 **Scan** 5665
**Peptide** ERAKPEIIKGSRK **Charge** 3 **Spectral Angle** 0.72

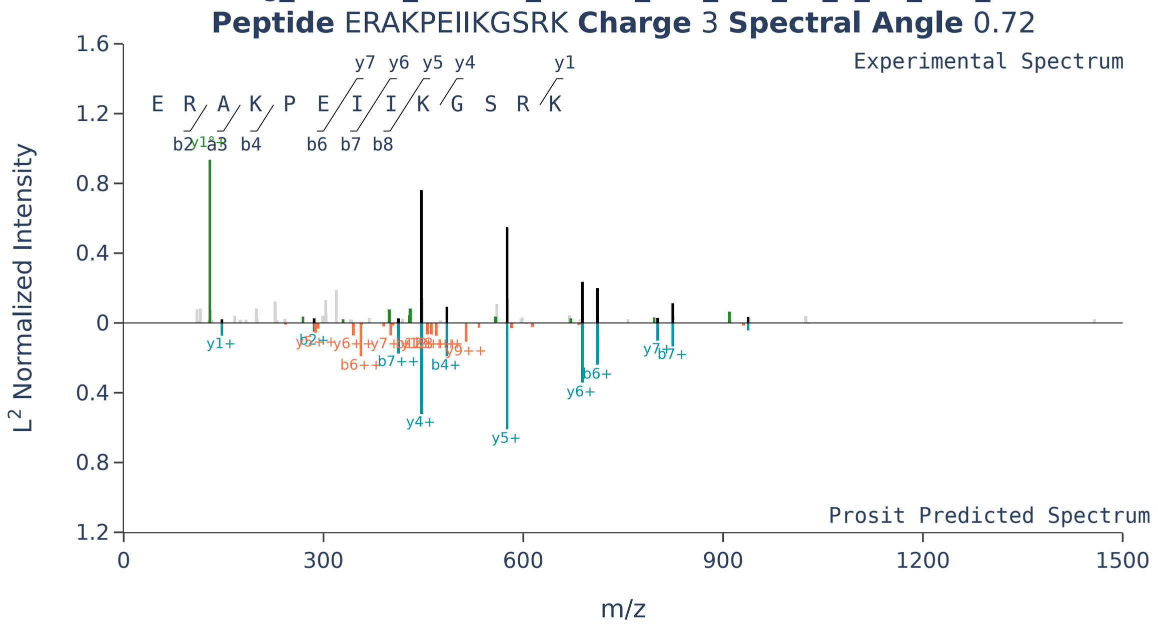


**Source** Ncheng_210623_230623_HFGoe_FFH_20S_25_1_A1_4h_R1 **Scan** 16522
**Peptide** INRVKEKAVGHEVNKSLTPGQEFVKI **Charge** 6 **Spectral Angle** 0.72

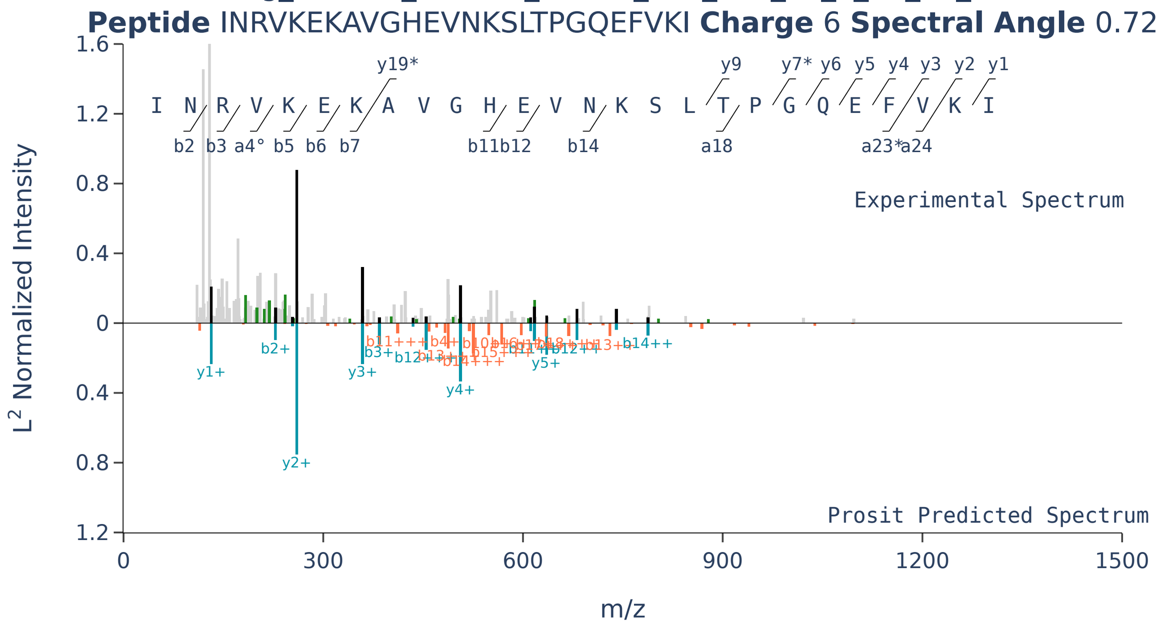


**Source** Ncheng_210623_230623_HFGoe_FFH_20S_25_1_A2_4h_R2 **Scan** 12577
**Peptide** FDDMQRMMKKMKK **Charge** 3 **Spectral Angle** 0.45

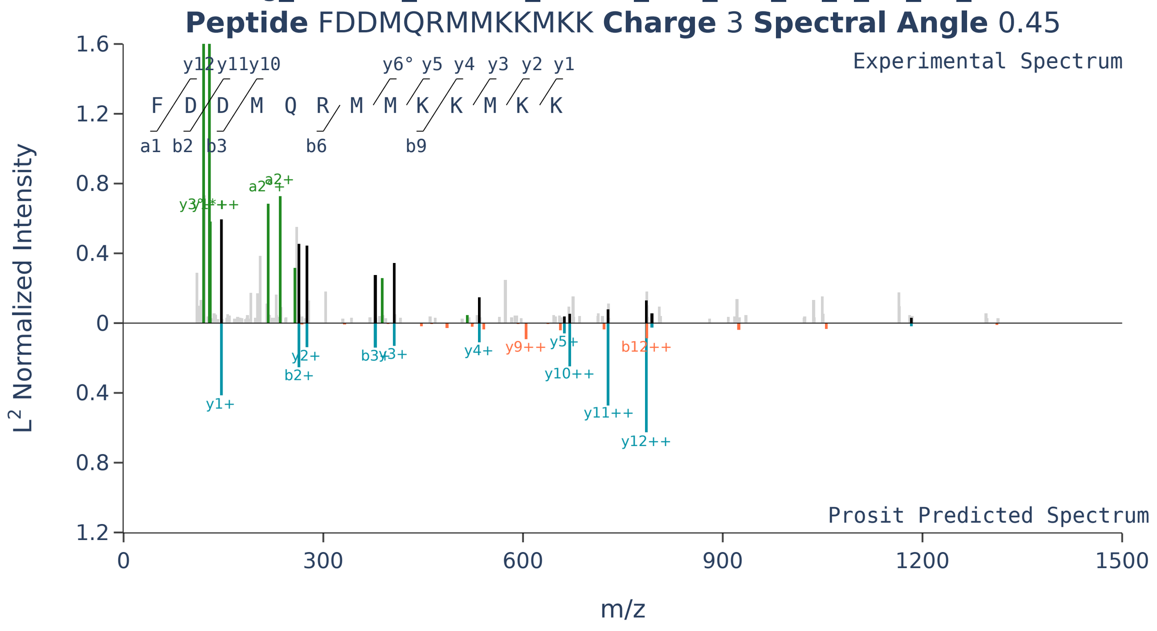


**Source** Ncheng_210623_230623_HFGoe_FFH_20S_25_1_A1_2h_R1 **Scan** 31577
**Peptide** EAKLKFYDVLLVD **Charge** 3 **Spectral Angle** 0.72

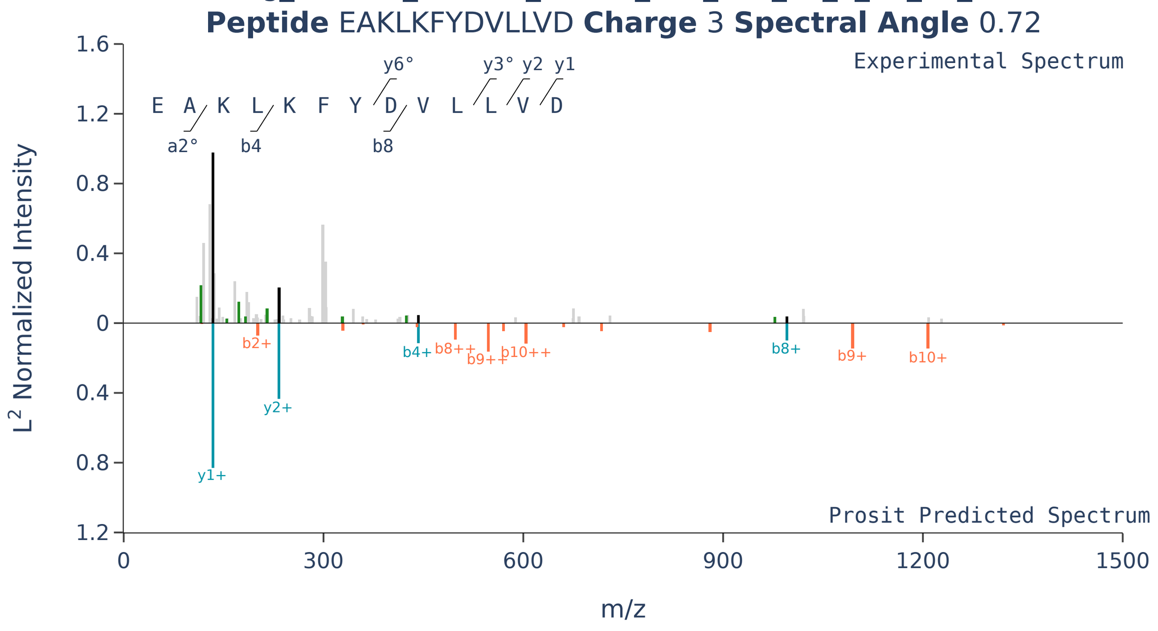


**Source** Ncheng_210623_230623_HFGoe_FFH_20S_25_1_A1_2h_R1 **Scan** 32833
**Peptide** VGQKPVDIVNAALKEAKLKFYD **Charge** 5 **Spectral Angle** 0.62

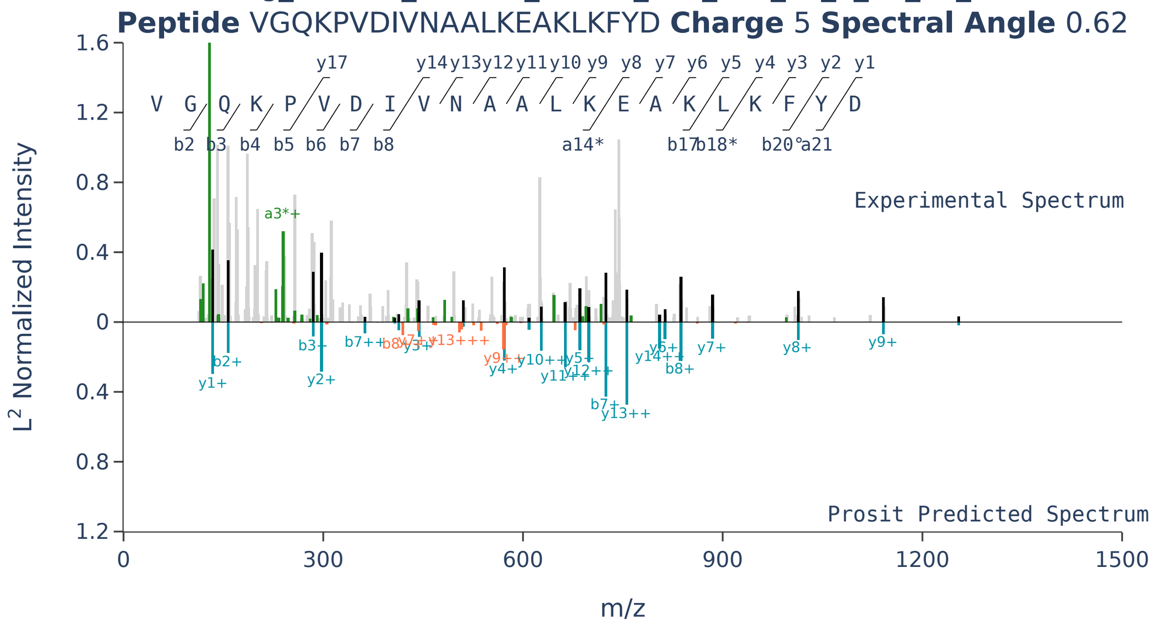


**Source** Ncheng_210623_230623_HFGoe_FFH_20S_25_1_A2_4h_R1 **Scan** 20773
**Peptide** TDRLSRTLRNISGRGRLTEDNVKDTLREV **Charge** 6 **Spectral Angle** 0.69

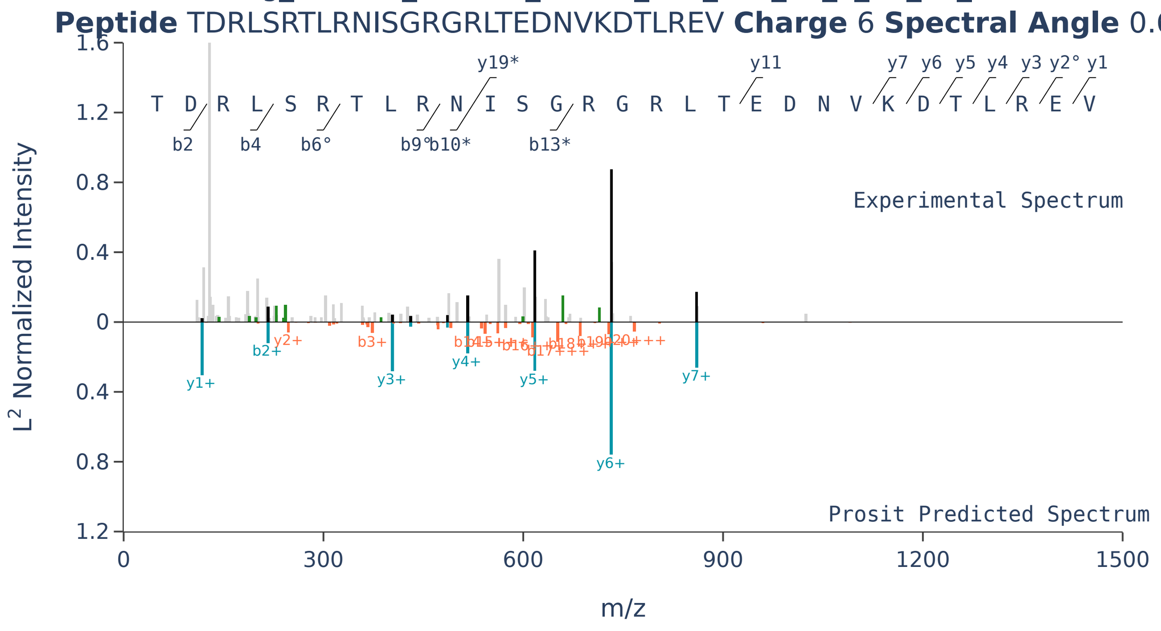


**Source** Ncheng_210623_230623_HFGoe_FFH_20S_25_1_A1_2h_R2 **Scan** 14539
**Peptide** RLHVDEAMM **Charge** 3 **Spectral Angle** 0.52

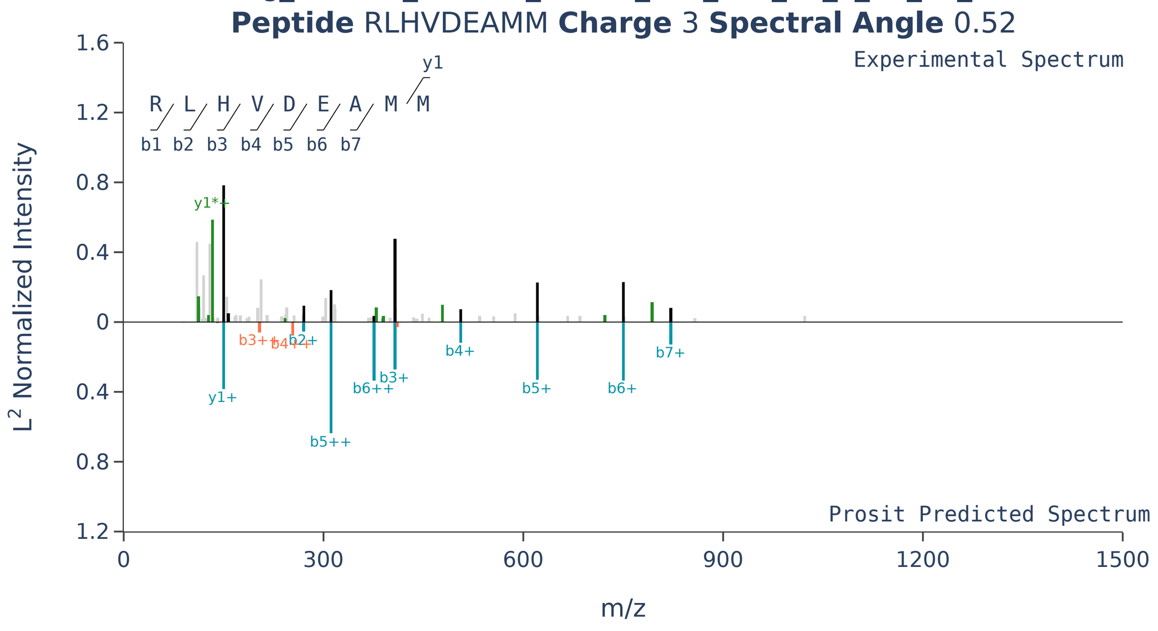


**Source** Ncheng_210623_230623_HFGoe_FFH_20S_25_1_A1_24h_R2 **Scan** 11032
**Peptide** KVDRAQAEKLASKLKKG **Charge** 4 **Spectral Angle** 0.85

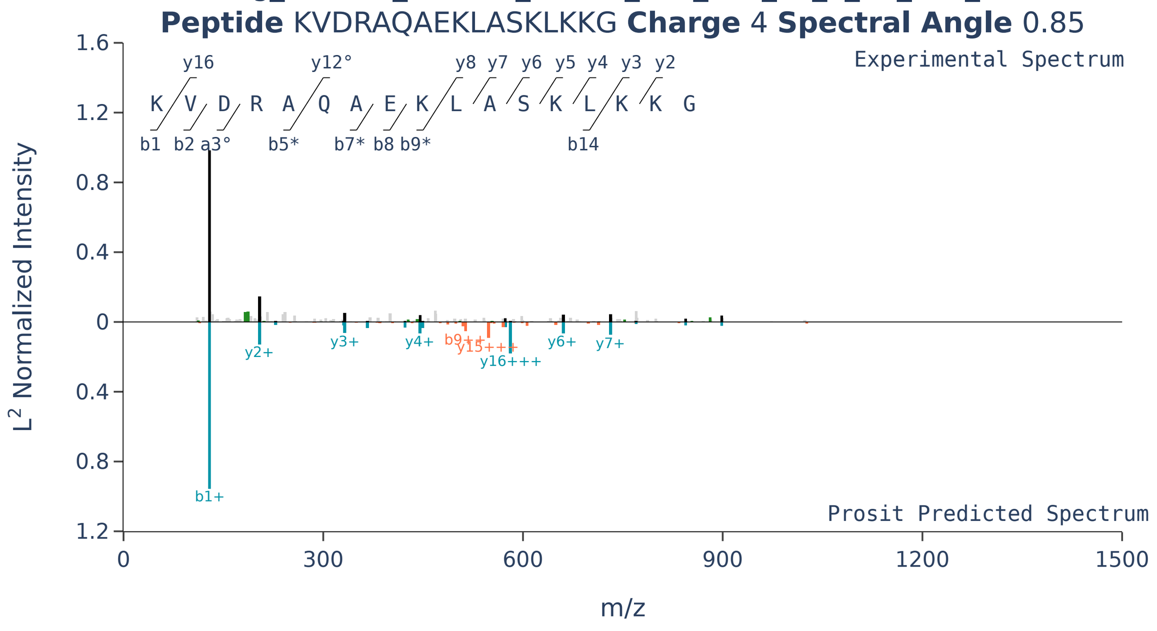


**Source** Ncheng_210623_230623_HFGoe_FFH_20S_25_1_A1_4h_R2 **Scan** 10623
**Peptide** QRMMKKM **Charge** 2 **Spectral Angle** 0.85

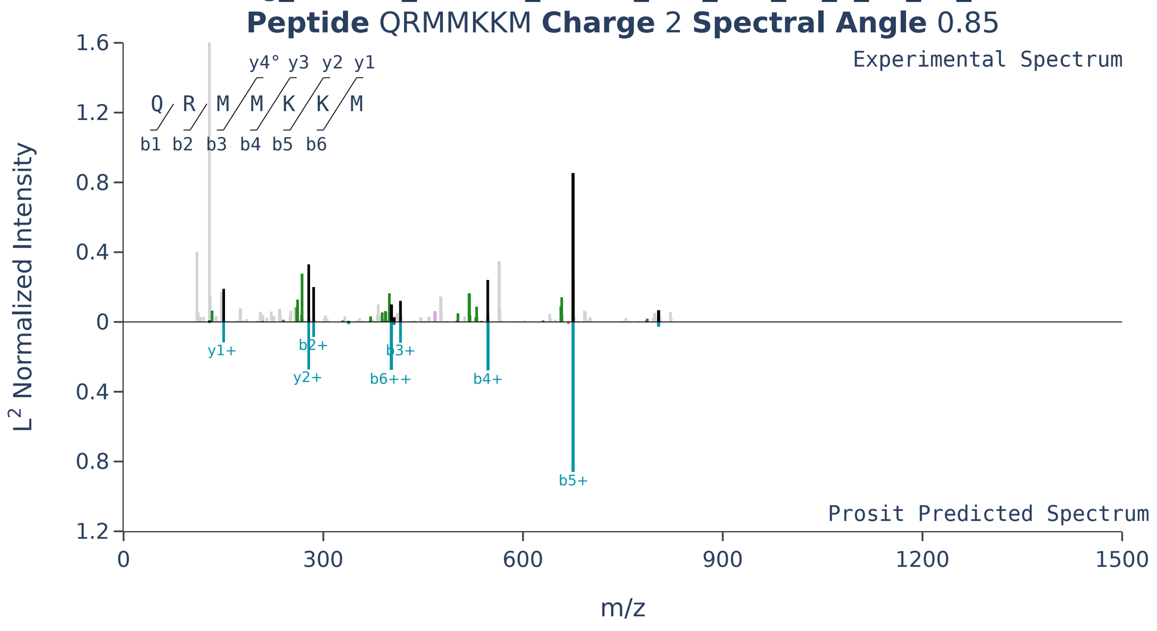


**Source** Ncheng_210623_230623_HFGoe_FFH_20S_25_1_A2_24h_R2 **Scan** 7341
**Peptide** ESKVDRAQAEKLAS **Charge** 2 **Spectral Angle** 0.44

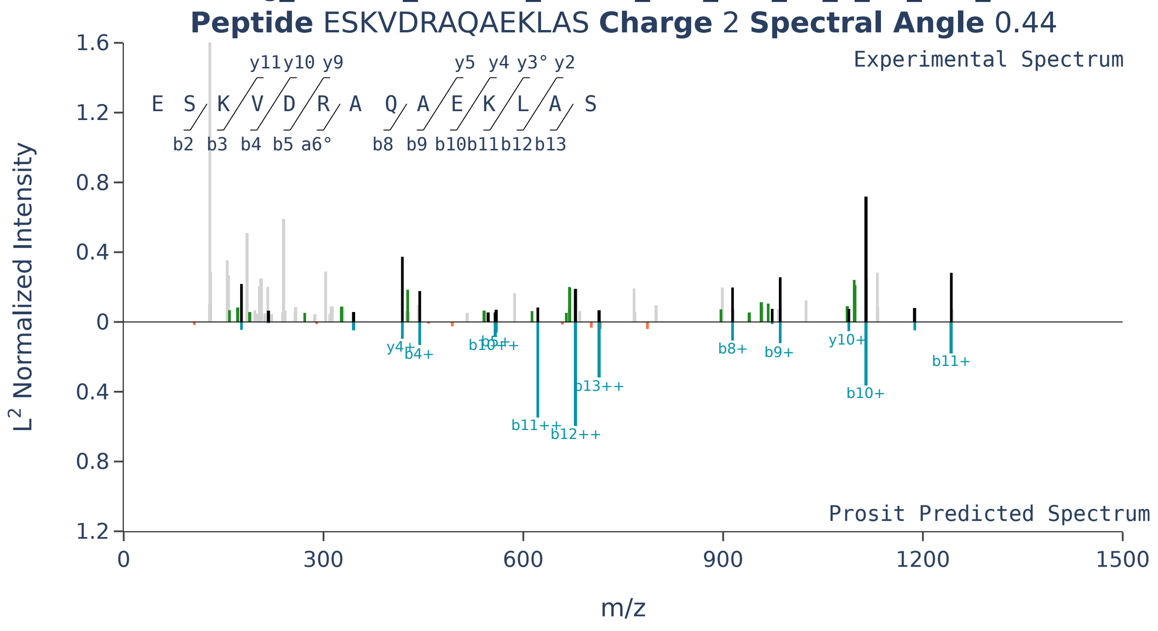

**Source** Ncheng_210623_230623_HFGoe_FFH_20S_25_1_A1_24h_R1 **Scan** 28373
**Peptide** AKAFNEALPLTGV **Charge** 2 **Spectral Angle** 0.72

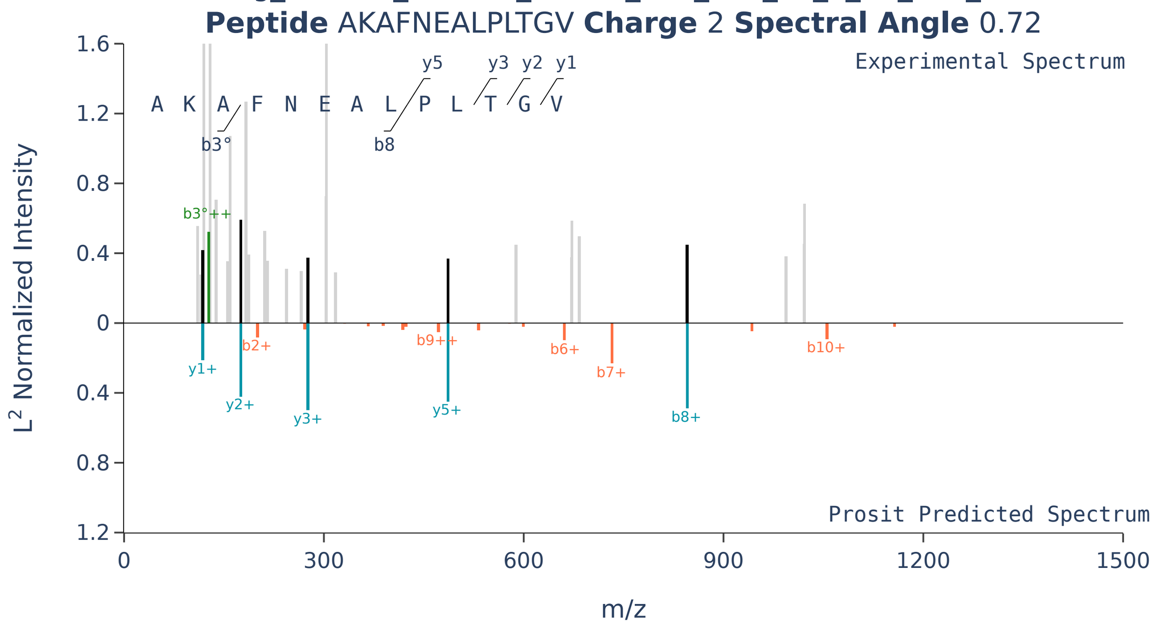


**Source** Ncheng_210623_230623_HFGoe_FFH_20S_25_1_A2_2h_R1 **Scan** 23042
**Peptide** RMEAIINSMTMKERAKPELL **Charge** 4 **Spectral Angle** 0.75

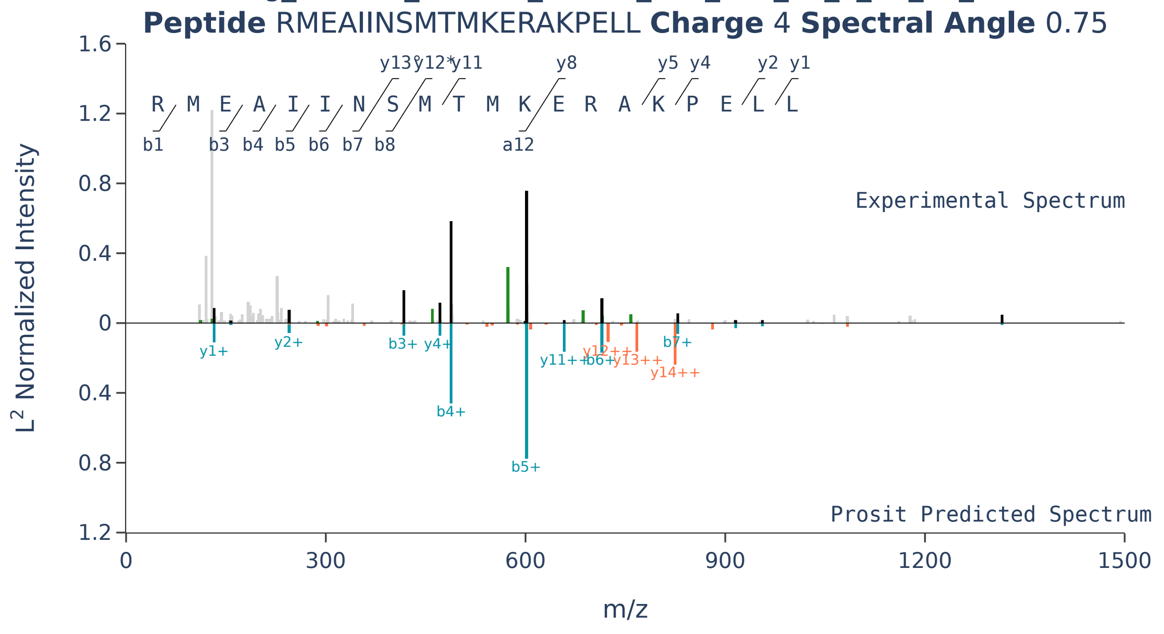


**Source** Ncheng_210623_230623_HFGoe_FFH_20S_25_1_A2_24h_R2 **Scan** 16803
**Peptide** AGLQGAGKTTSVGKLGKFLREK **Charge** 6 **Spectral Angle** 0.57

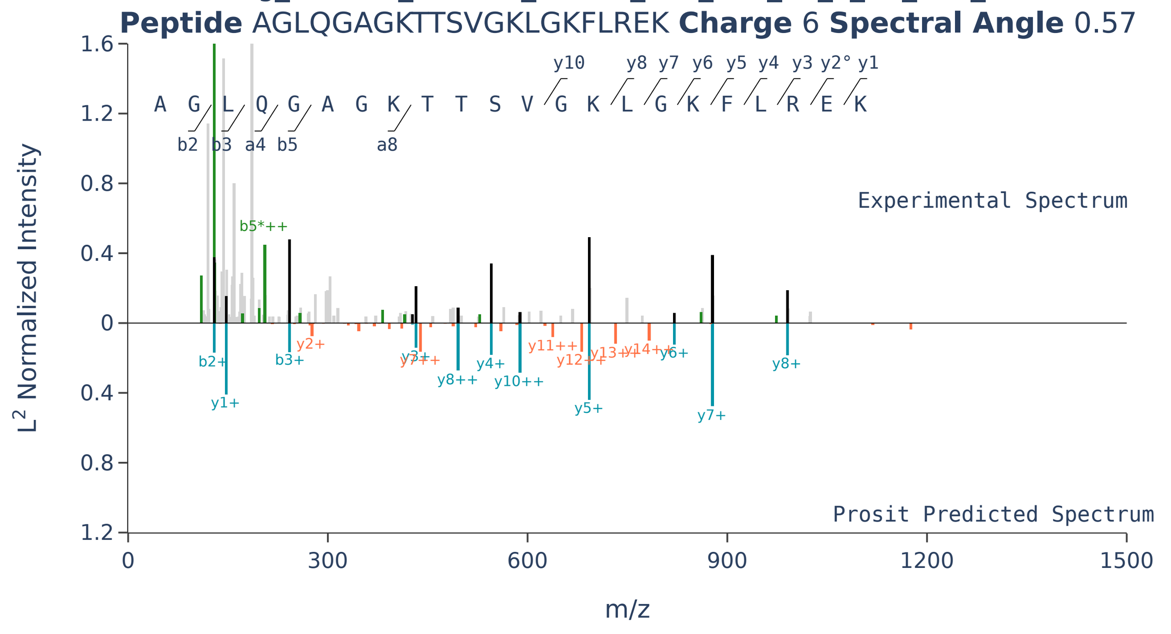


**Source** Ncheng_210623_230623_HFGoe_FFH_20S_25_1_A1_24h_R2 **Scan** 17460
**Peptide** GLQGAGKTTSVGKLGKFLR **Charge** 4 **Spectral Angle** 0.64

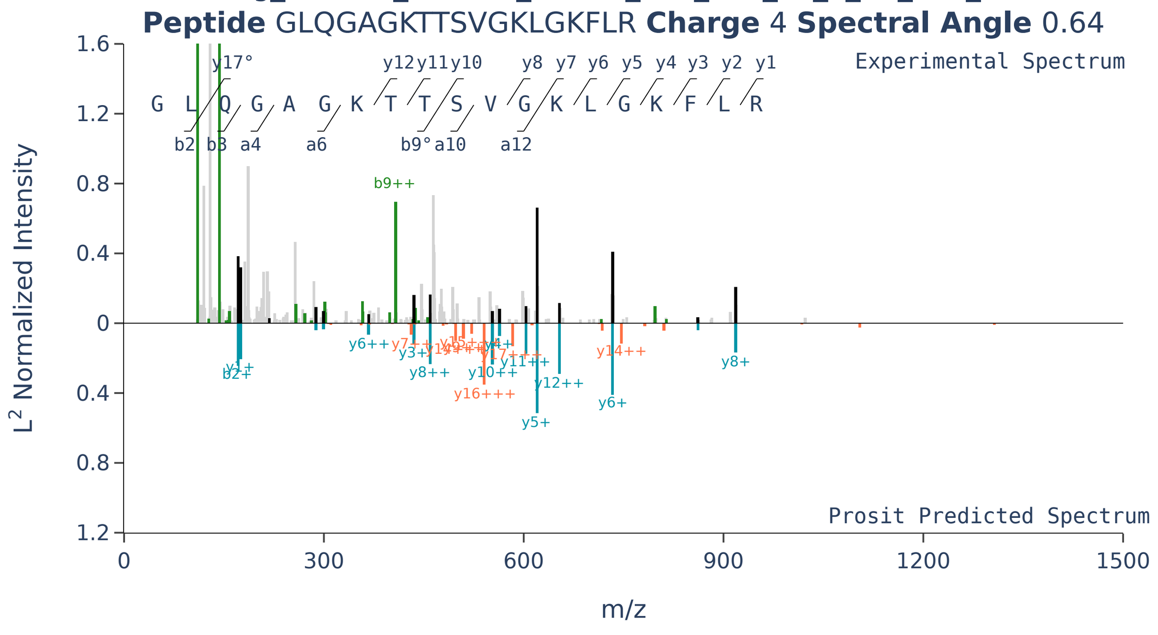


**Source** Ncheng_210623_230623_HFGoe_FFH_20S_25_1_A1_2h_R1 **Scan** 33333
**Peptide** MGKLPGMGQIPDNVKSQMDDKVLVRMEALL **Charge** 4 **Spectral Angle** 0.64

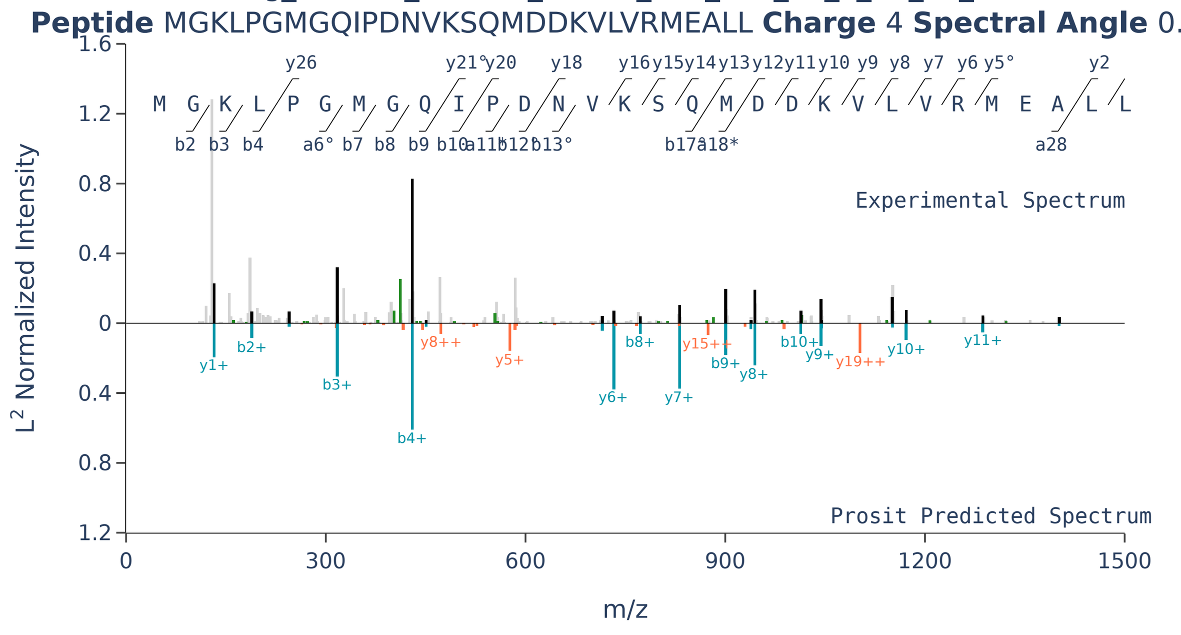


**Source** Ncheng_210623_230623_HFGoe_FFH_20S_25_1_A2_4h_R2 **Scan** 12795
**Peptide** MTMKERAKPEII **Charge** 3 **Spectral Angle** 0.44

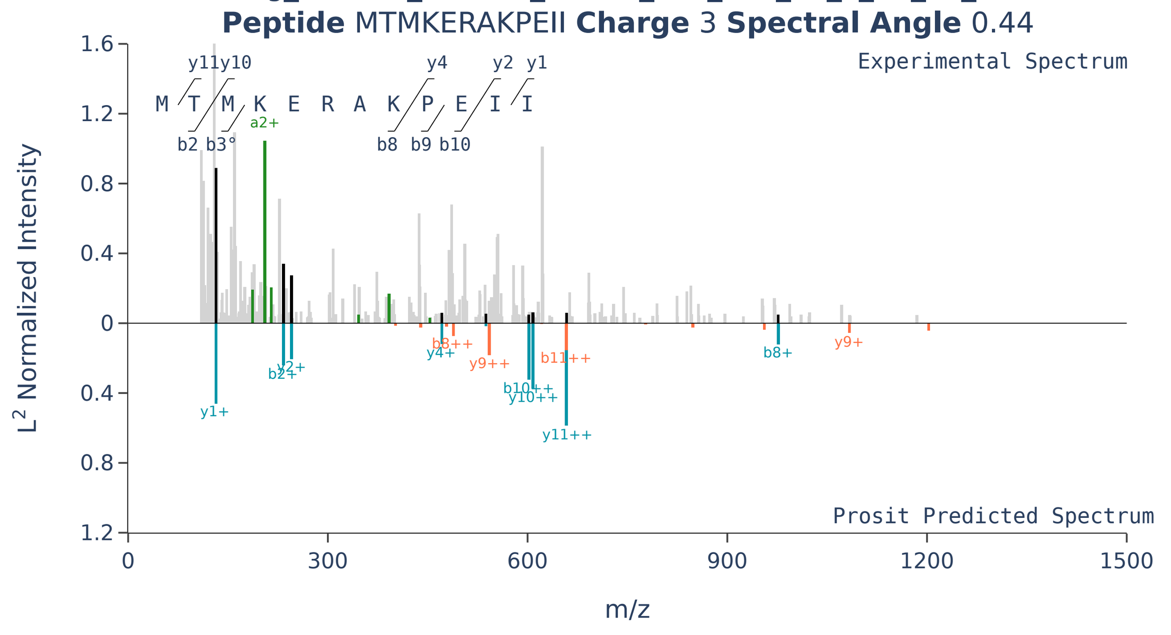


**Source** Ncheng_210623_230623_HFGoe_FFH_20S_25_1_A2_1h_R2 **Scan** 20819
**Peptide** DGDARGGAALSIRHITGKPIKFLGVGEKTE **Charge** 5 **Spectral Angle** 0.6

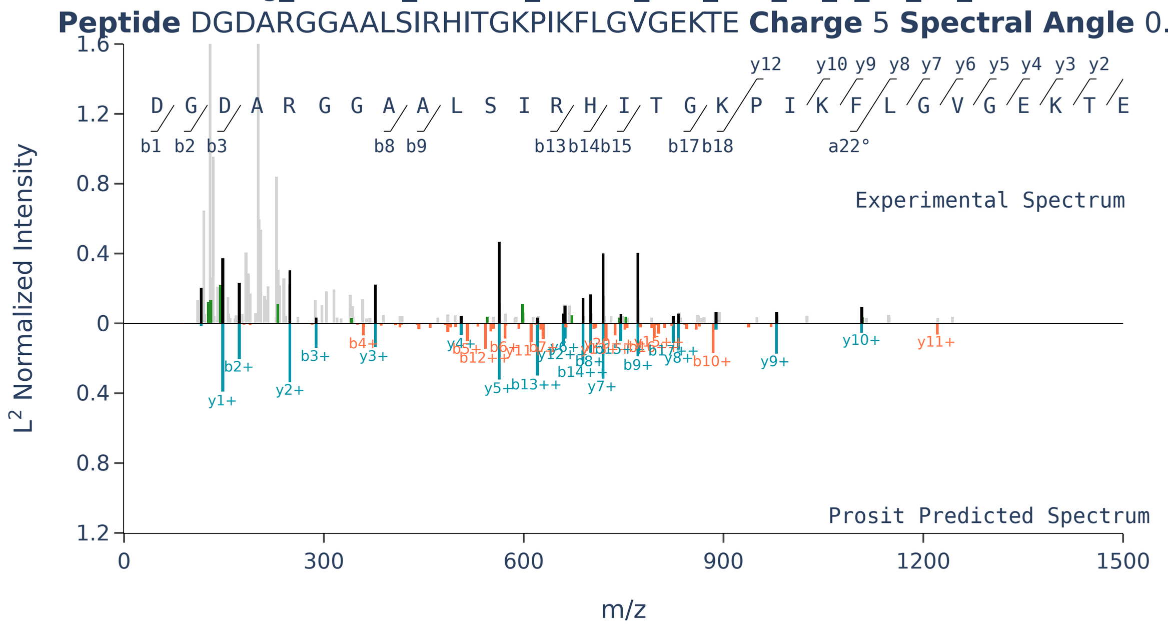


**Source** Ncheng_210623_230623_HFGoe_FFH_20S_25_1_A2_4h_R2 **Scan** 37457
**Peptide** GQDAANTAKAFNEALPLTGVVL **Charge** 2 **Spectral Angle** 0.58

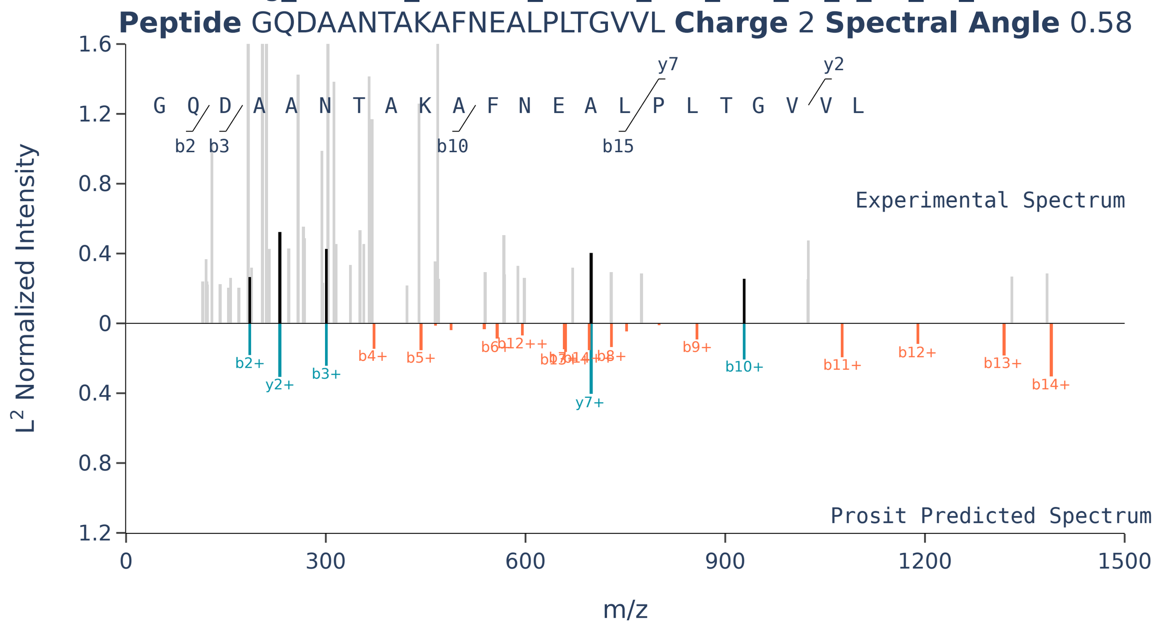


**Source** Ncheng_210623_230623_HFGoe_FFH_20S_25_1_A1_24h_R1 **Scan** 6262
**Peptide** RNISGRGRLTED **Charge** 3 **Spectral Angle** 0.46

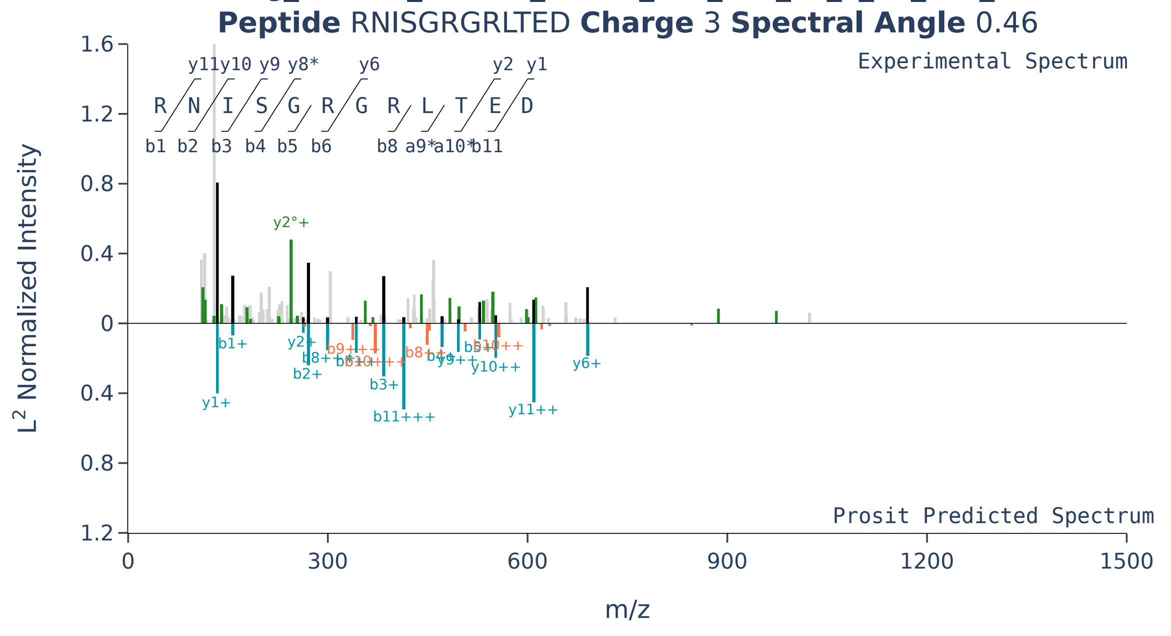


**Source** Ncheng_210623_230623_HFGoe_FFH_20S_25_1_A2_2h_R1 **Scan** 23598
**Peptide** AALSIRHITGKPIKFLGVGE **Charge** 4 **Spectral Angle** 0.84

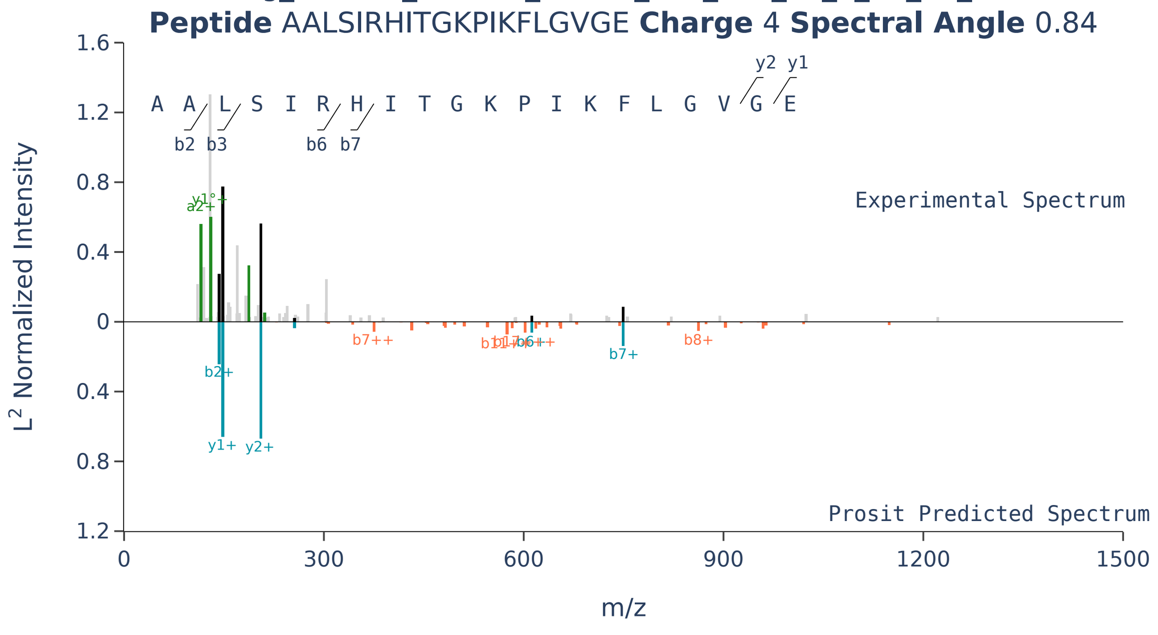


**Source** Ncheng_210623_230623_HFGoe_FFH_20S_25_1_A1_24h_R1 **Scan** 8532
**Peptide** REFINRVKEKAVGHEVNK **Charge** 5 **Spectral Angle** 0.61

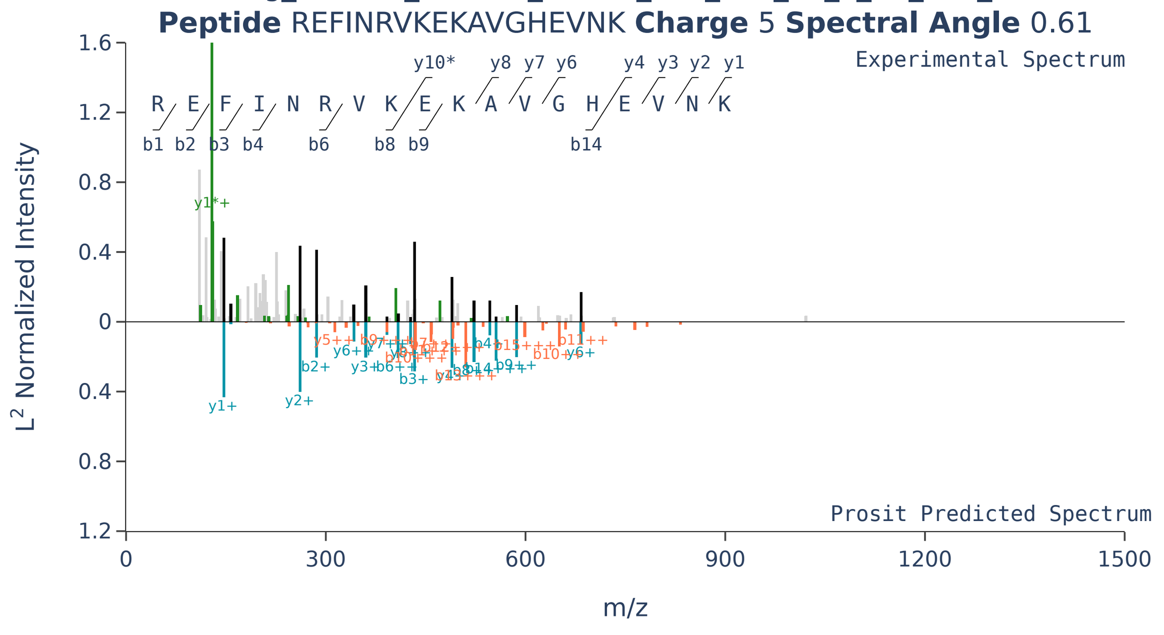


**Source** Ncheng_210623_230623_HFGoe_FFH_20S_25_1_A1_2h_R1 **Scan** 28307
**Peptide** MGGMASLMGKLPGMGQIPDNVKSQMD **Charge** 3 **Spectral Angle** 0.81

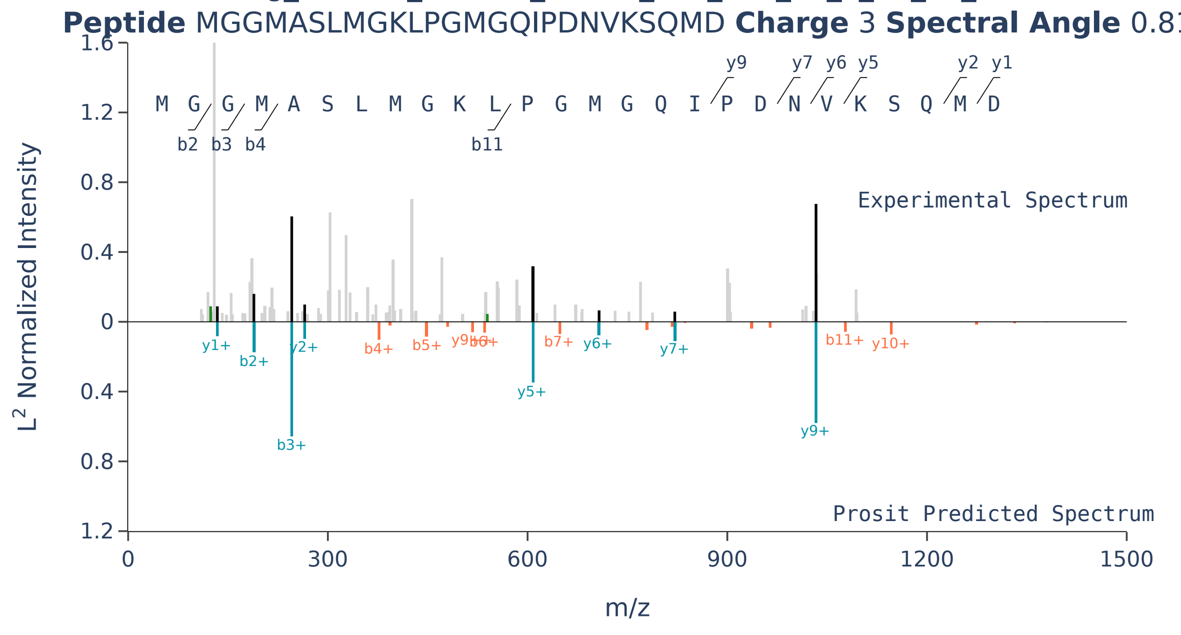


**Source** Ncheng_210623_230623_HFGoe_FFH_20S_25_1_A2_4h_R1 **Scan** 34259
**Peptide** DAANTAKAFNEALPLTGVVLTKVDGDARG **Charge** 4 **Spectral Angle** 0.5

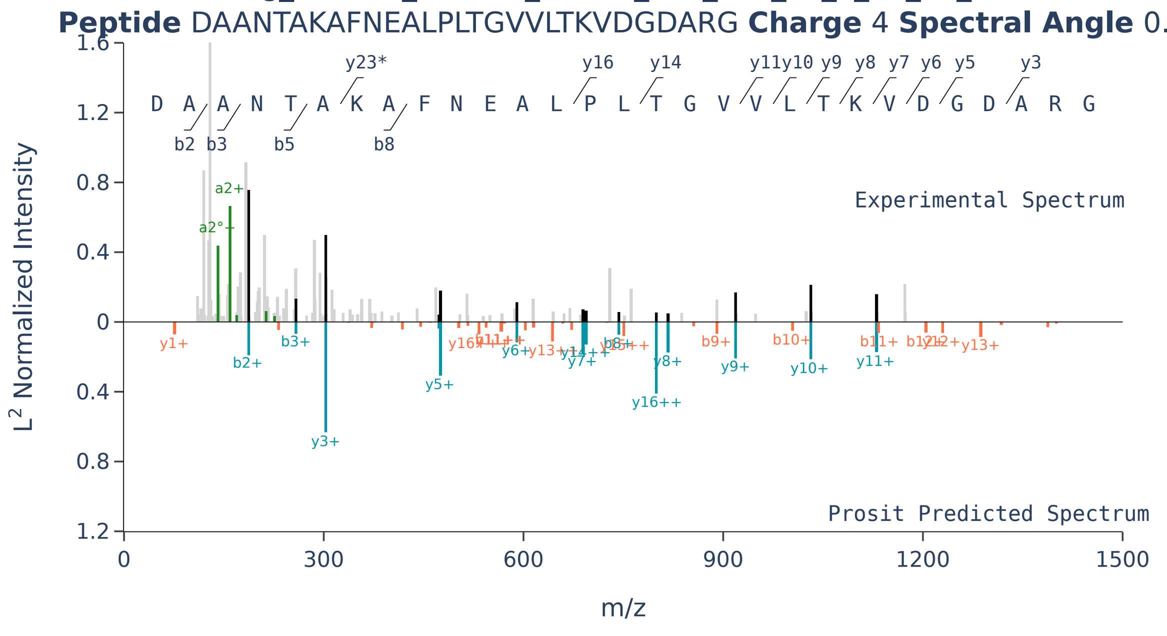


**Source** Ncheng_210623_230623_HFGoe_FFH_20S_25_1_A1_4h_R1 **Scan** 29284
**Peptide** AAMGEENQTLNLAAQPPAVV **Charge** 2 **Spectral Angle** 0.93

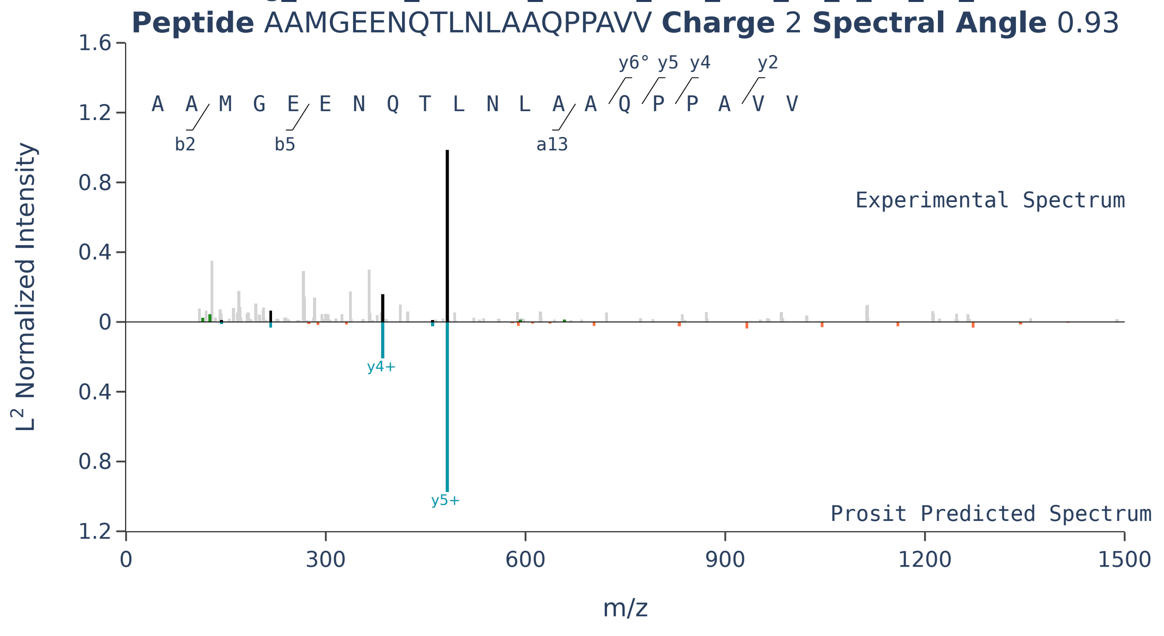


**Source** Ncheng_210623_230623_HFGoe_FFH_20S_25_1_A1_24h_R2 **Scan** 6778
**Peptide** MKKMKKGGMAKMM **Charge** 4 **Spectral Angle** 0.76

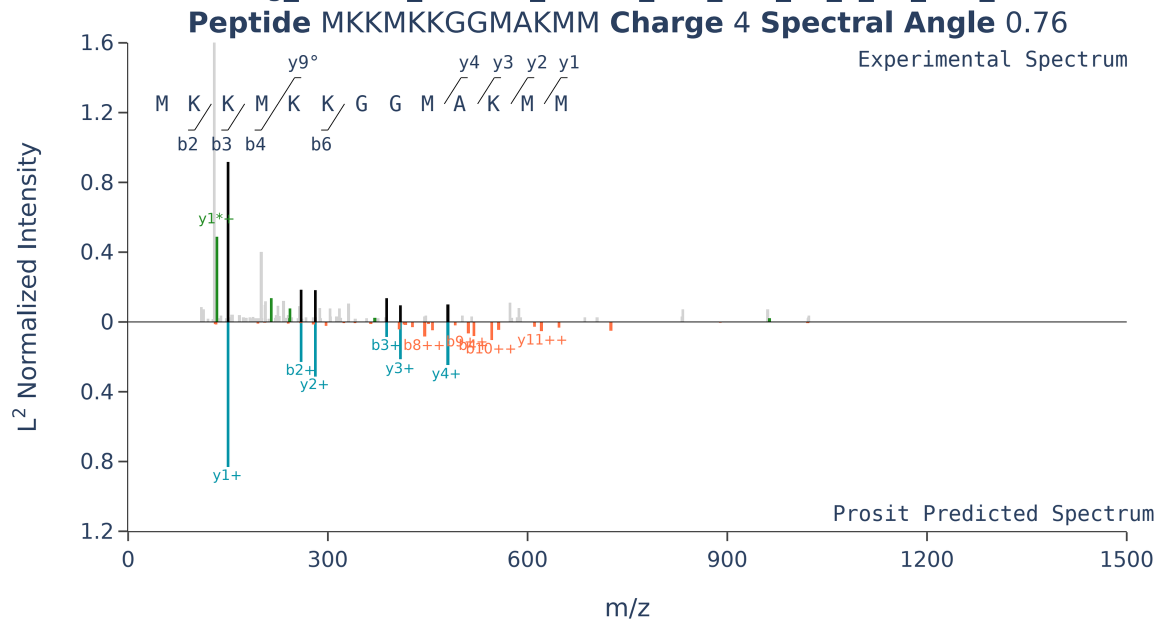

**Source** Ncheng_210623_230623_HFGoe_FFH_20S_25_1_A2_2h_R2 **Scan** 11004
**Peptide** LMGKLPGM **Charge** 1 **Spectral Angle** 0.69

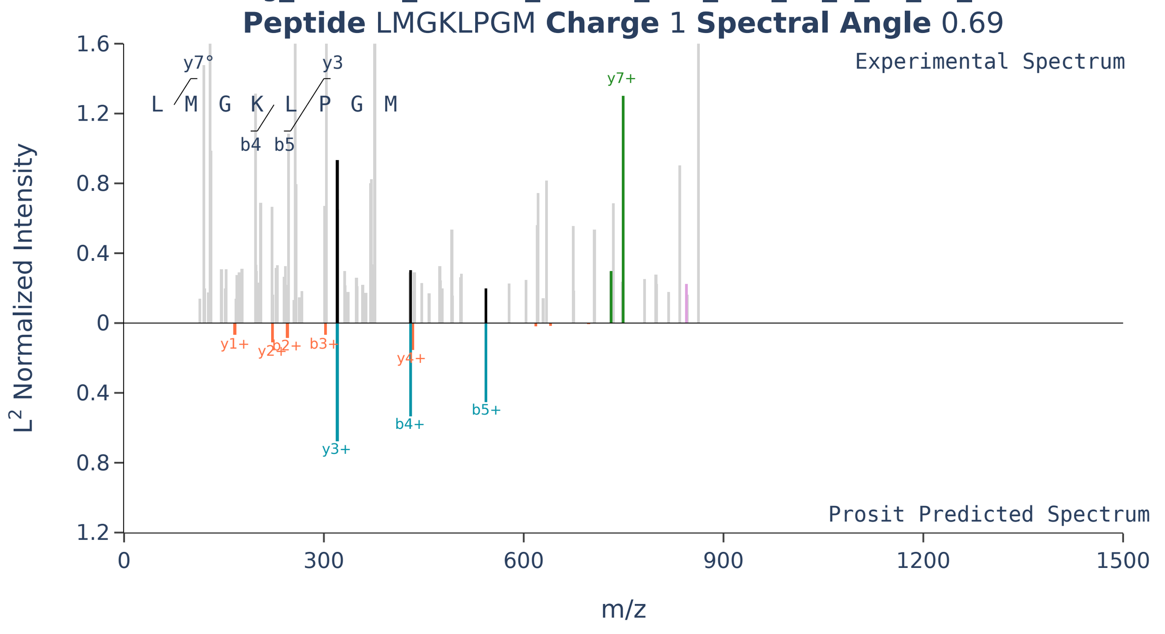


**Source** Ncheng_210623_230623_HFGoe_FFH_20S_25_1_A1_24h_R2 **Scan** 8210
**Peptide** DARGGAAL **Charge** 1 **Spectral Angle** 0.87

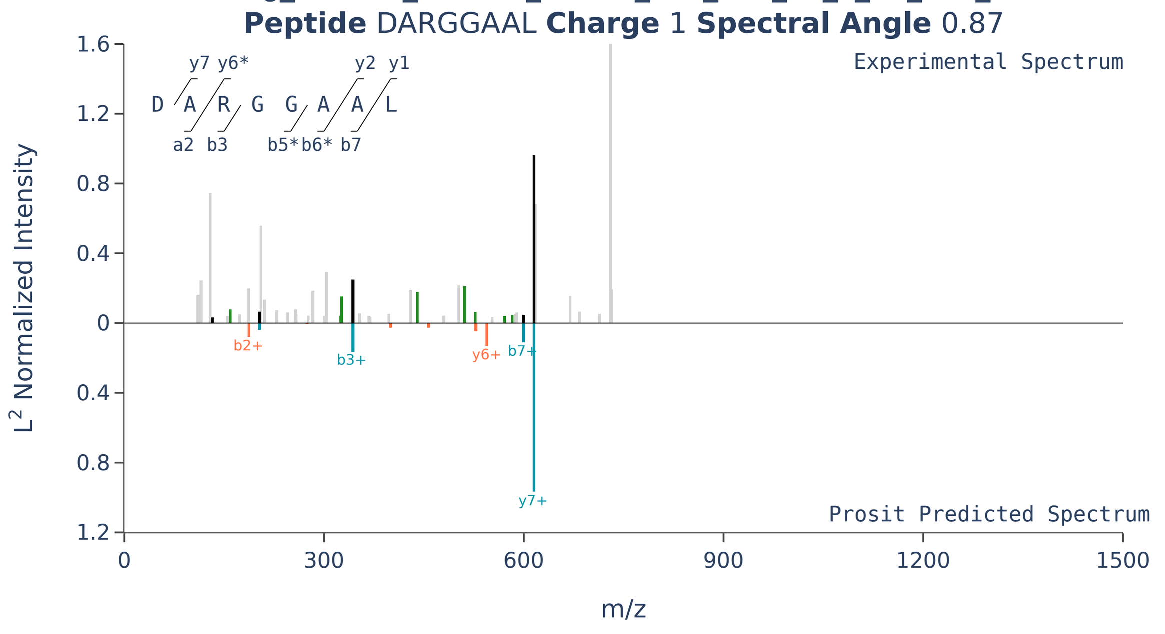


**Source** Ncheng_210623_230623_HFGoe_FFH_20S_25_1_A1_2h_R2 **Scan** 30948
**Peptide** ALEPFHPDRIASRILGMG **Charge** 3 **Spectral Angle** 0.46

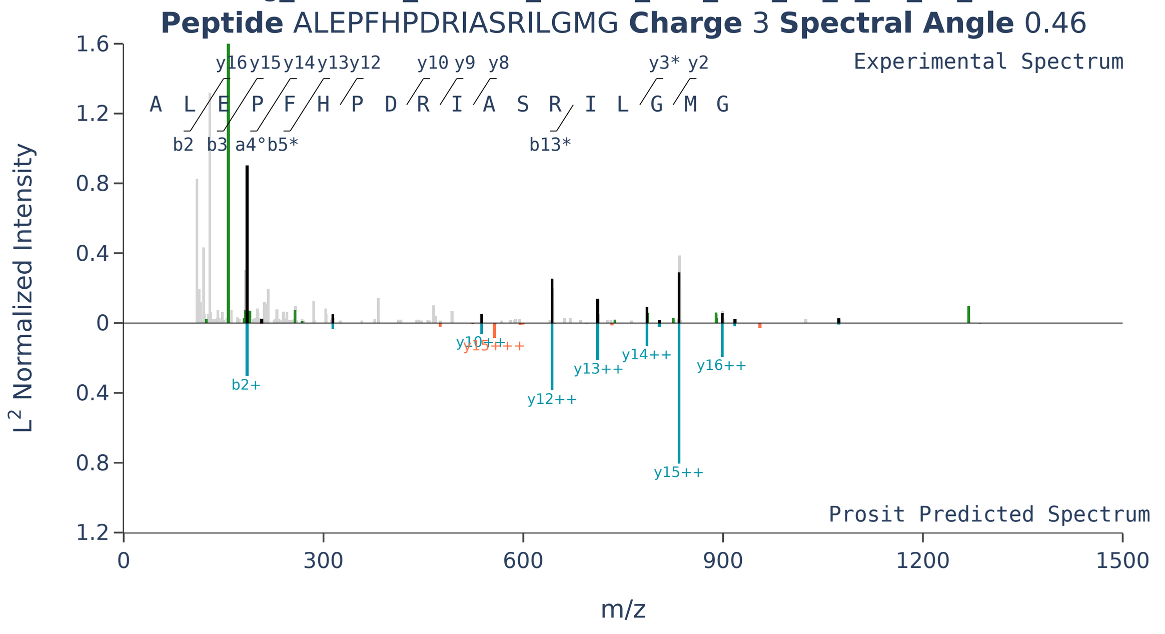


**Source** Ncheng_210623_230623_HFGoe_FFH_20S_25_1_A1_2h_R1 **Scan** 15865
**Peptide** DDMQRMMKKMKKGGMAKMM **Charge** 5 **Spectral Angle** 0.87

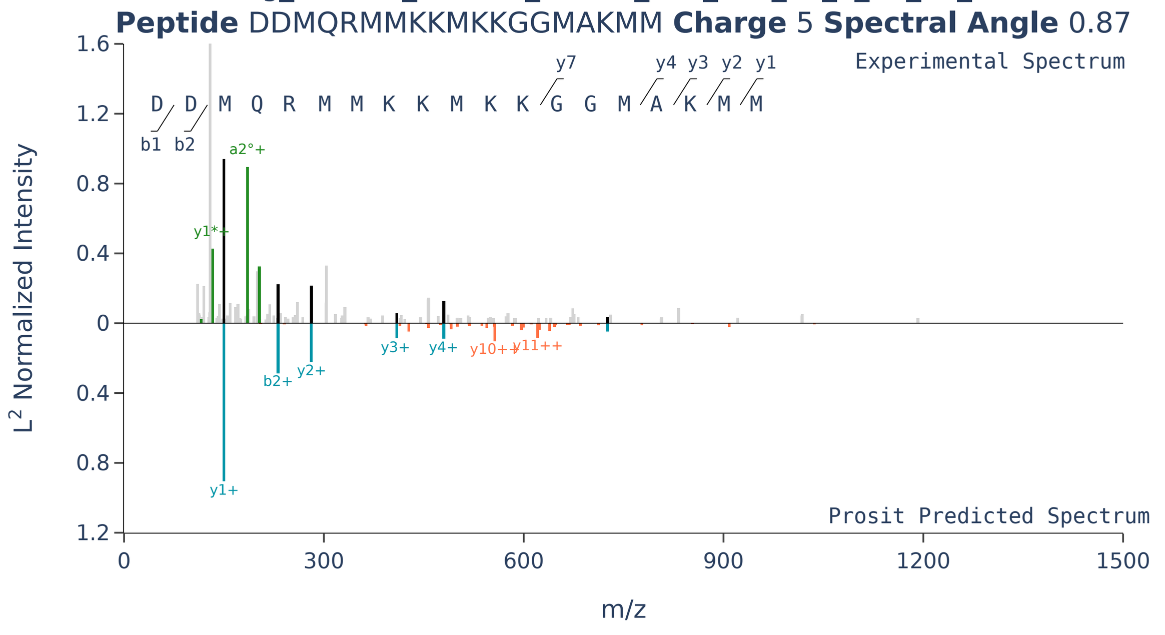


**Source** Ncheng_210623_230623_HFGoe_FFH_20S_25_1_A2_1h_R1 **Scan** 13213
**Peptide** DRAQAEKLASKLKKGDGFD **Charge** 4 **Spectral Angle** 0.55

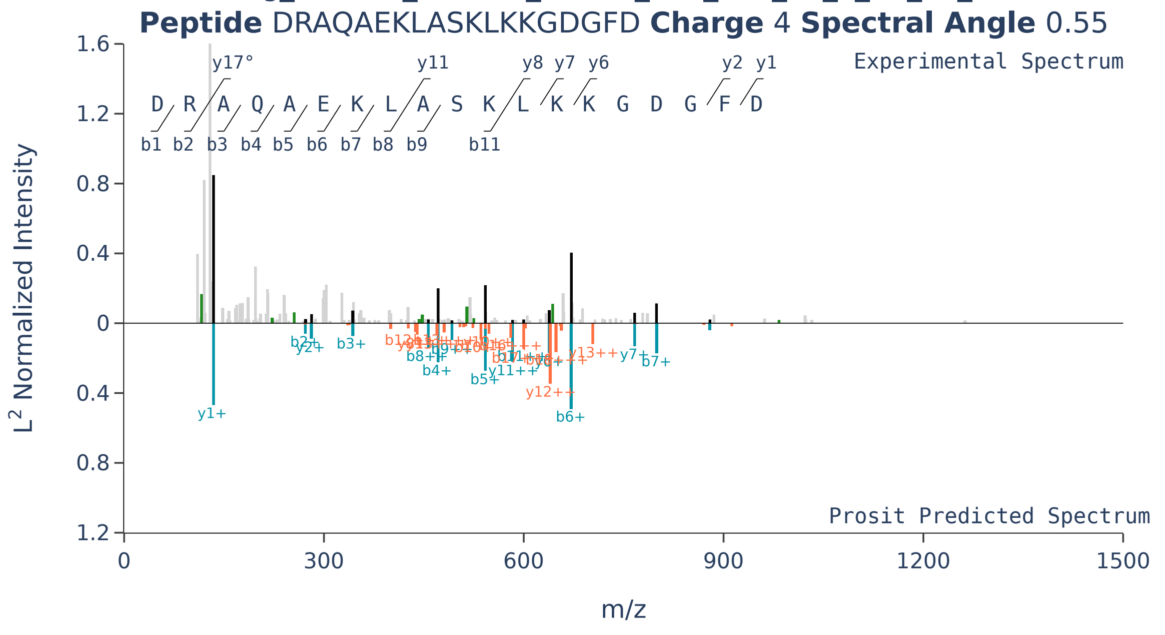


**Source** Ncheng_210623_230623_HFGoe_FFH_20S_25_1_A1_2h_R2 **Scan** 27399
**Peptide** FDNLTDRLSRTLRNISGRGRLTE **Charge** 5 **Spectral Angle** 0.73

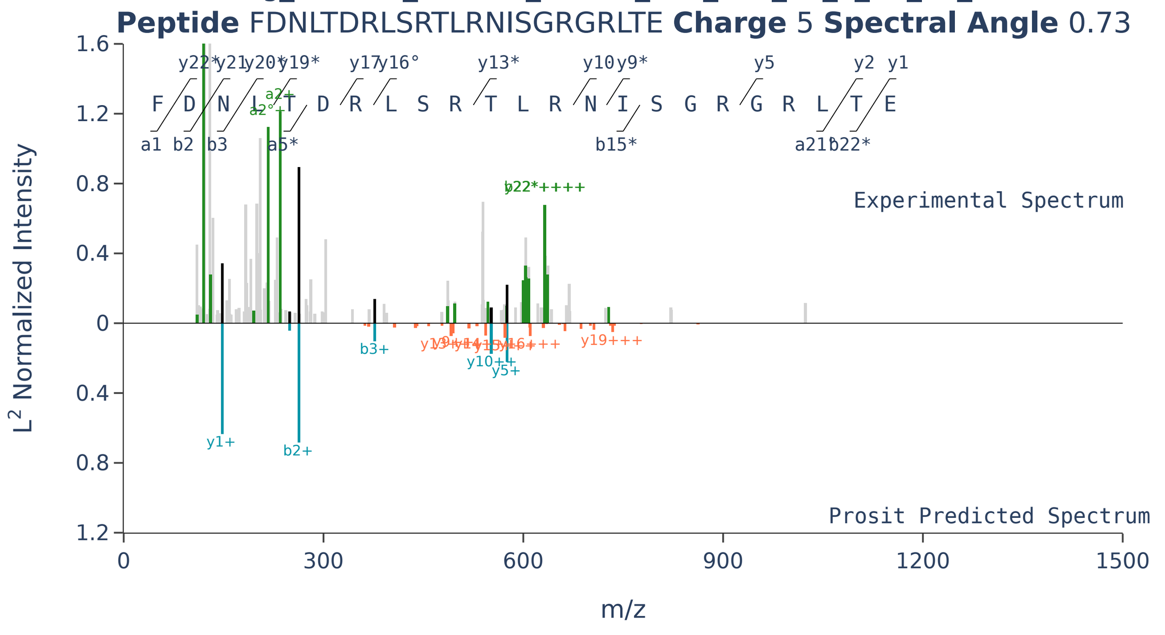


**Source** Ncheng_210623_230623_HFGoe_FFH_20S_25_1_A2_4h_R1 **Scan** 30358
**Peptide** QMDDKVLVRMEAIIN **Charge** 3 **Spectral Angle** 0.81

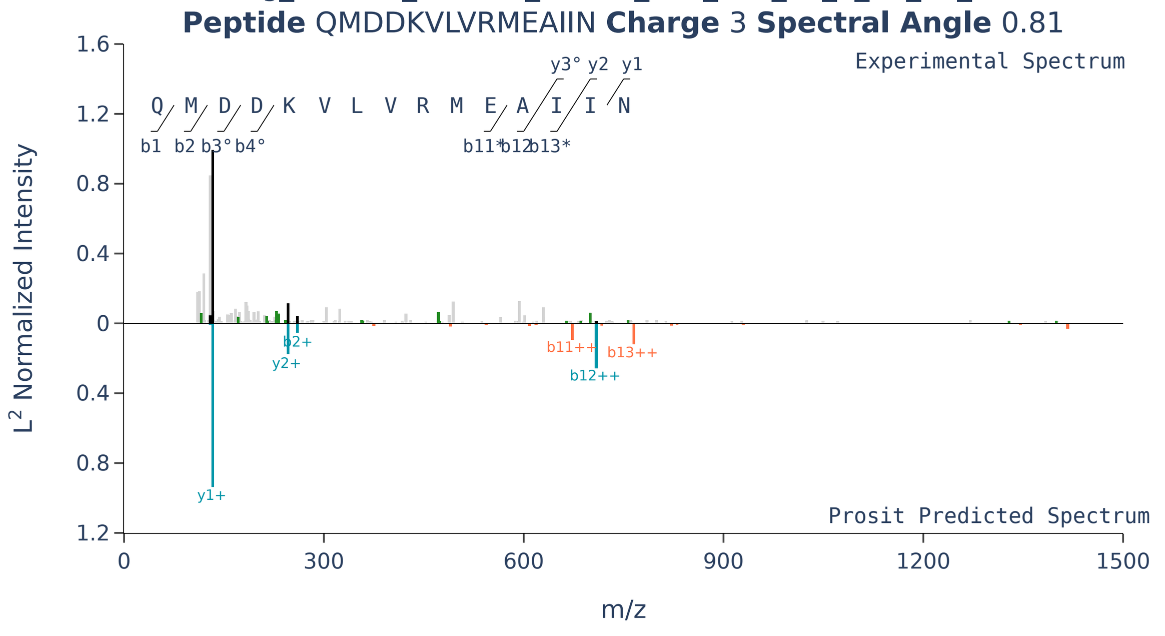


**Source** Ncheng_210623_230623_HFGoe_FFH_20S_25_1_A1_2h_R2 **Scan** 31719
**Peptide** KQVHASINPVETLFVVD **Charge** 3 **Spectral Angle** 0.31

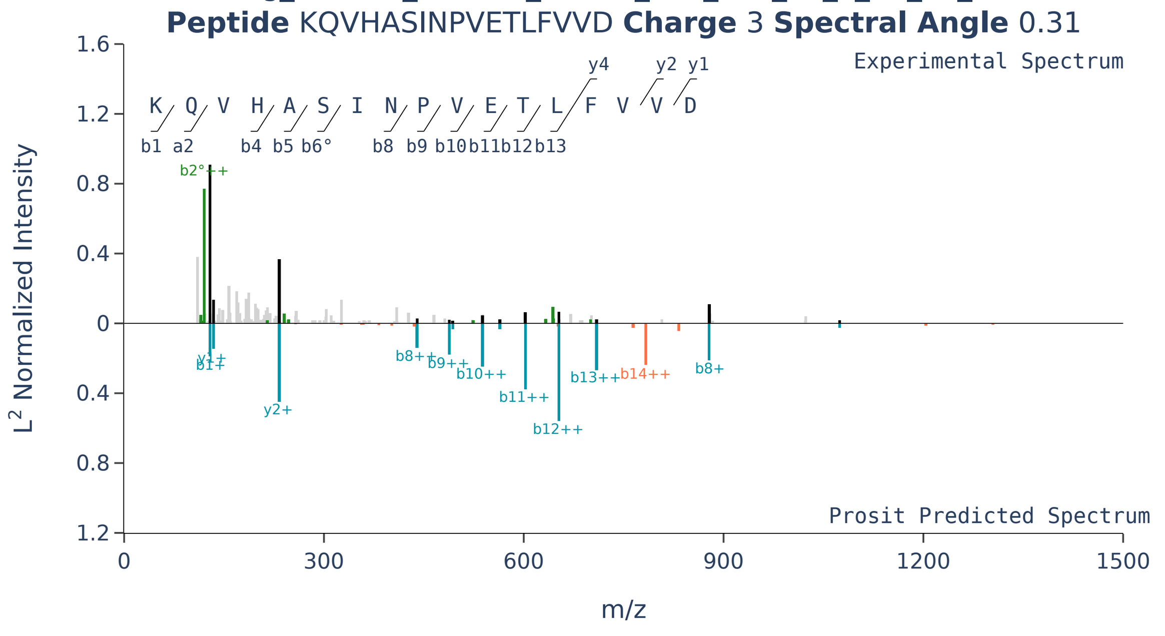


**Source** Ncheng_210623_230623_HFGoe_FFH_20S_25_1_A2_4h_R2 **Scan** 24536
**Peptide** GMGQIPDNVKSQMDDKVLVRME **Charge** 3 **Spectral Angle** 0.48

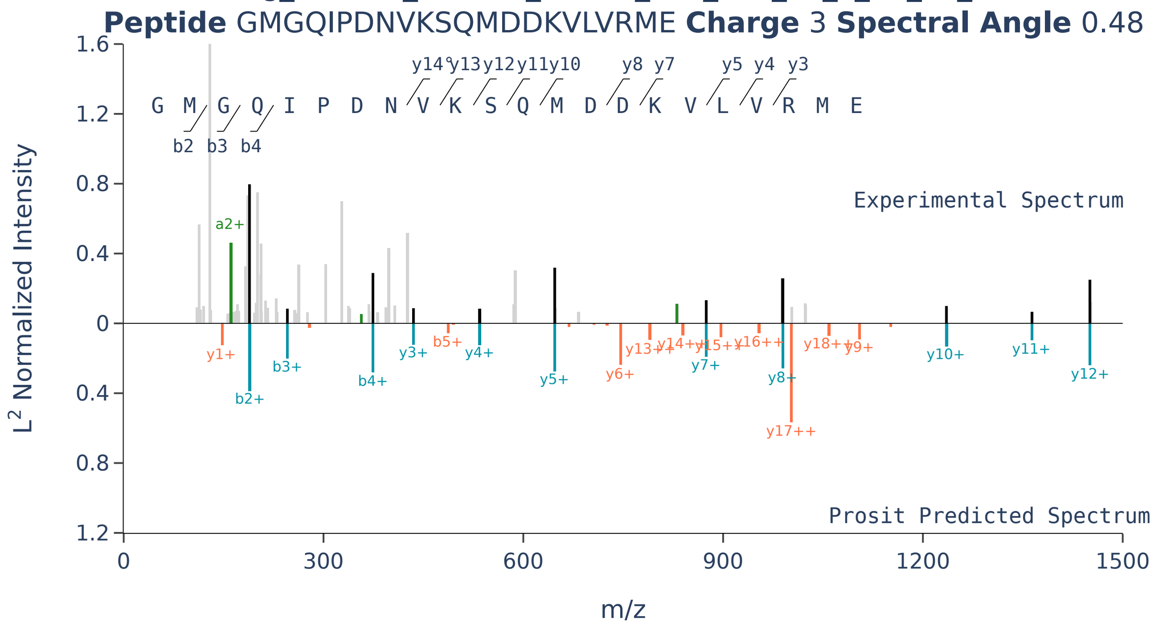


**Source** Ncheng_210623_230623_HFGoe_FFH_20S_25_1_A2_2h_R1 **Scan** 26576
**Peptide** VRMEAIINSMTMKERAKPEII **Charge** 4 **Spectral Angle** 0.73

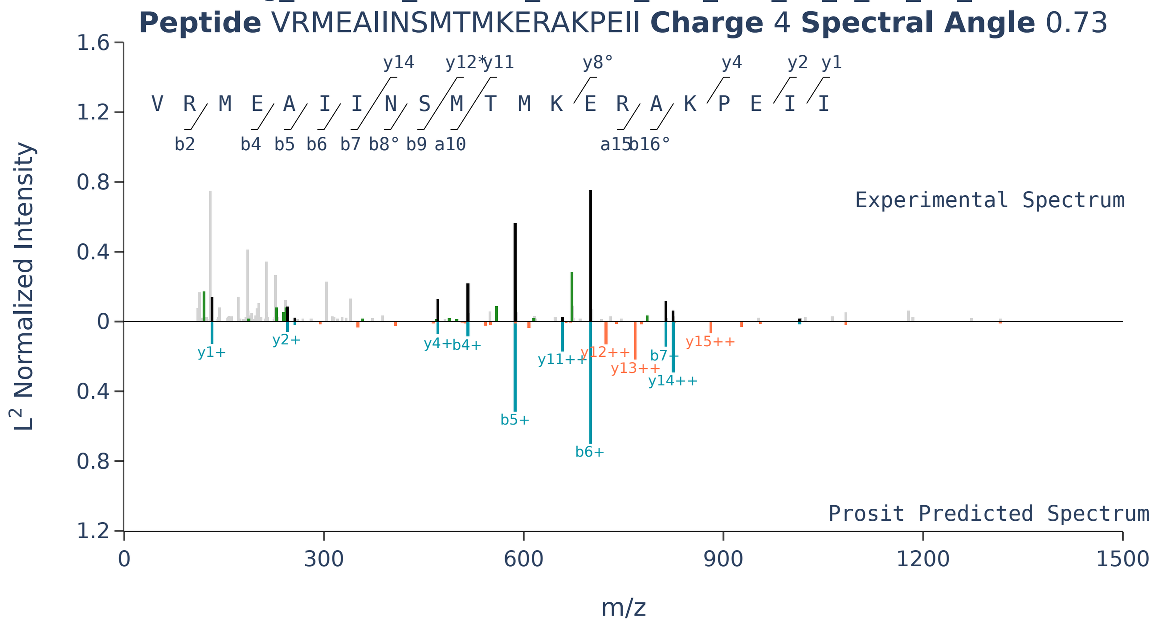


**Source** Ncheng_210623_230623_HFGoe_FFH_20S_25_1_A1_2h_R2 **Scan** 30829
**Peptide** QDVNRLLKQFDDMQRMMKKM **Charge** 4 **Spectral Angle** 0.71

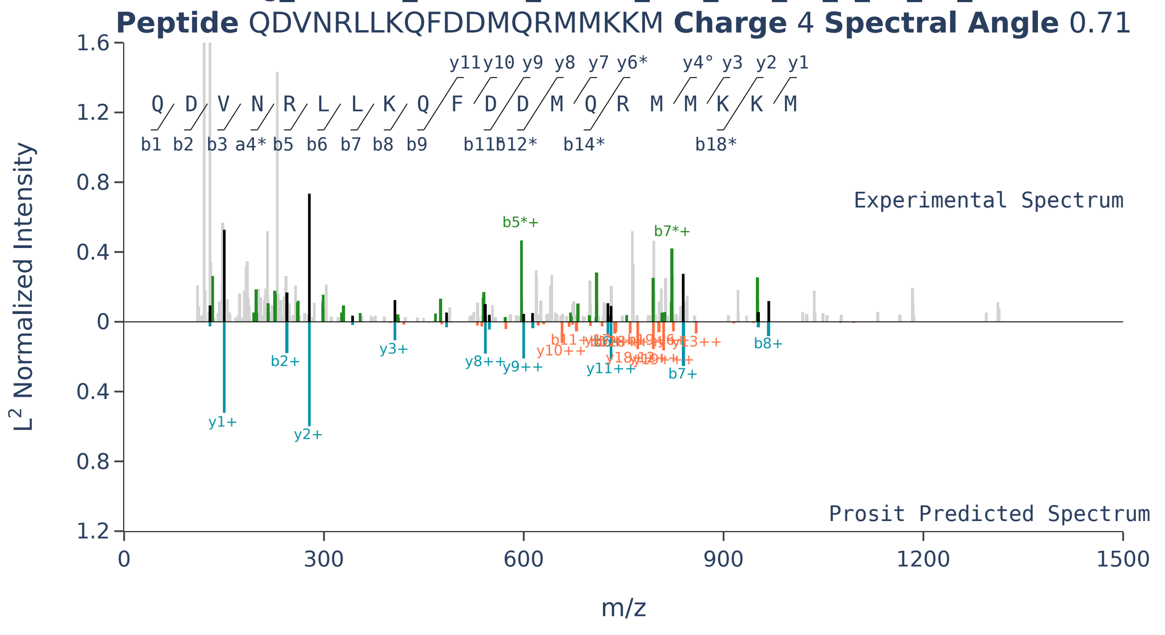


**Source** Ncheng_210623_230623_HFGoe_FFH_20S_25_1_A2_2h_R2 **Scan** 10094
**Peptide** LPDNVKSQMD **Charge** 2 **Spectral Angle** 0.47

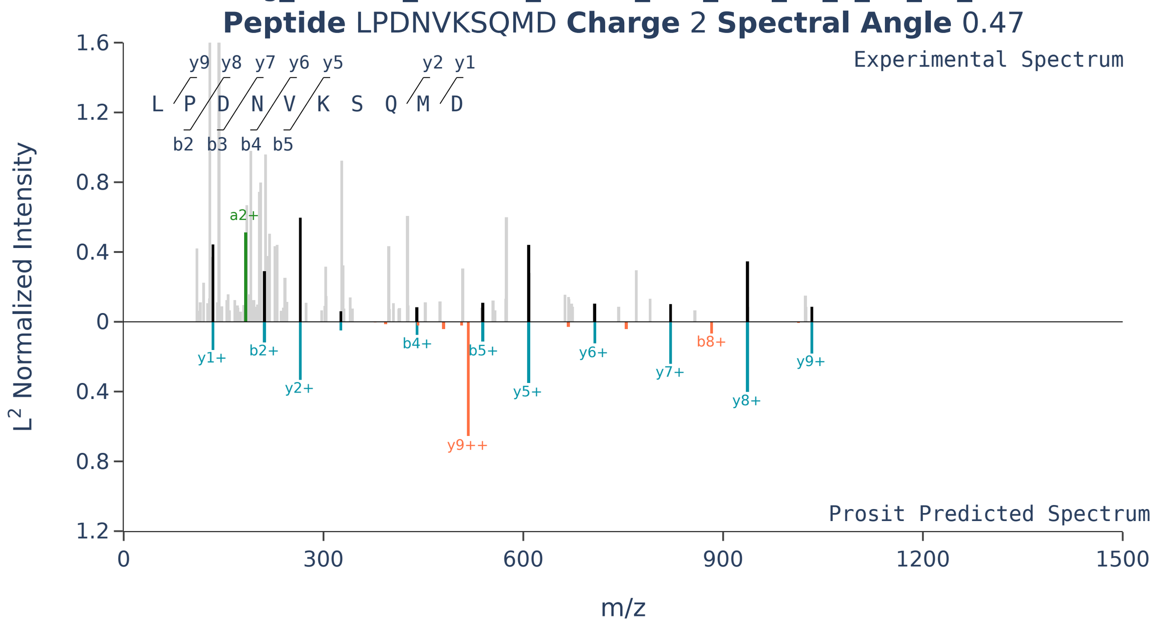


**Source** Ncheng_210623_230623_HFGoe_FFH_20S_25_1_A2_4h_R2 **Scan** 13198
**Peptide** LESKVDRAQAEKLASKLKKG **Charge** 6 **Spectral Angle** 0.81

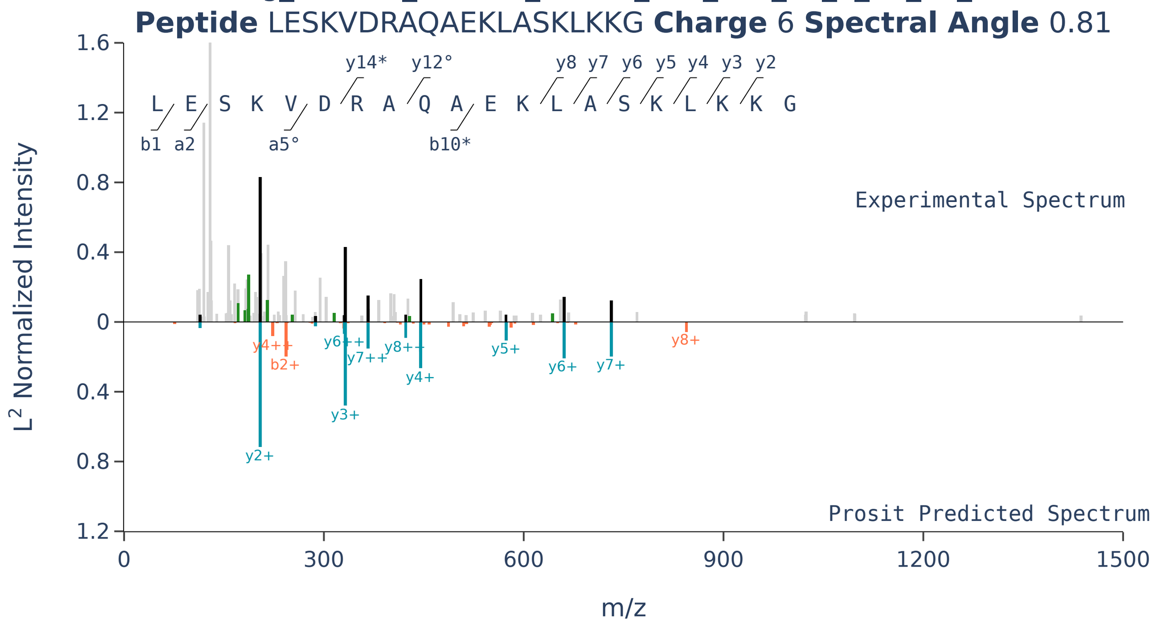


**Source** Ncheng_210623_230623_HFGoe_FFH_20S_25_1_A1_2h_R1 **Scan** 27767
**Peptide** EPFHPDRIASRILGMGD **Charge** 4 **Spectral Angle** 0.84

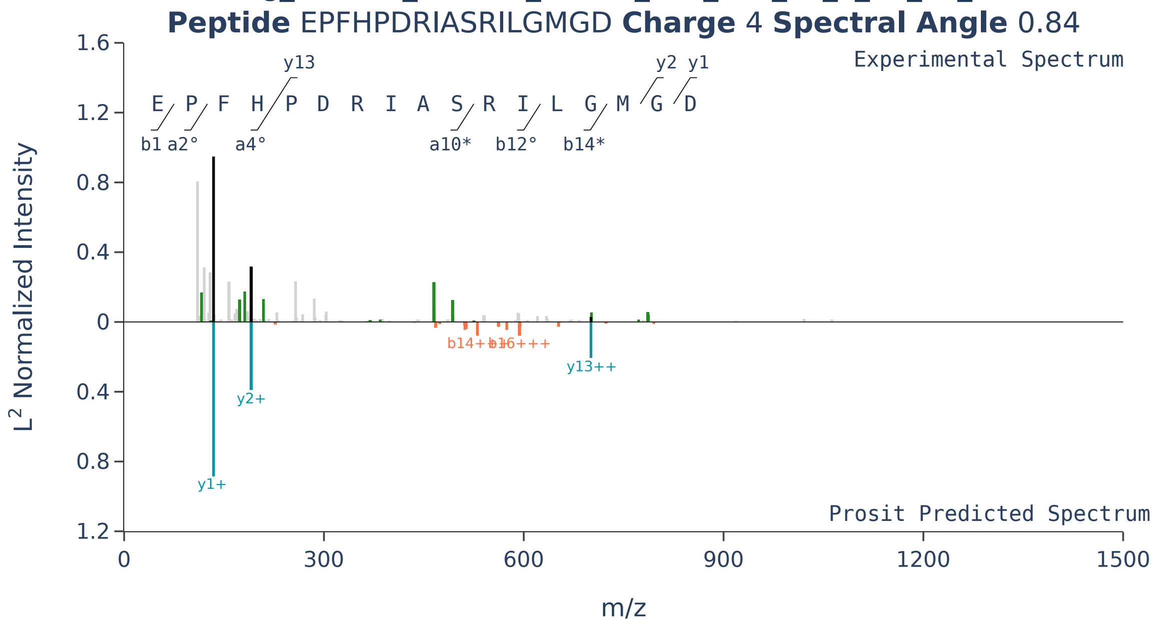


**Source** Ncheng_210623_230623_HFGoe_FFH_20S_25_1_A1_2h_R1 **Scan** 30166
**Peptide** LEPFHPDRIASRILGMGD **Charge** 3 **Spectral Angle** 0.63

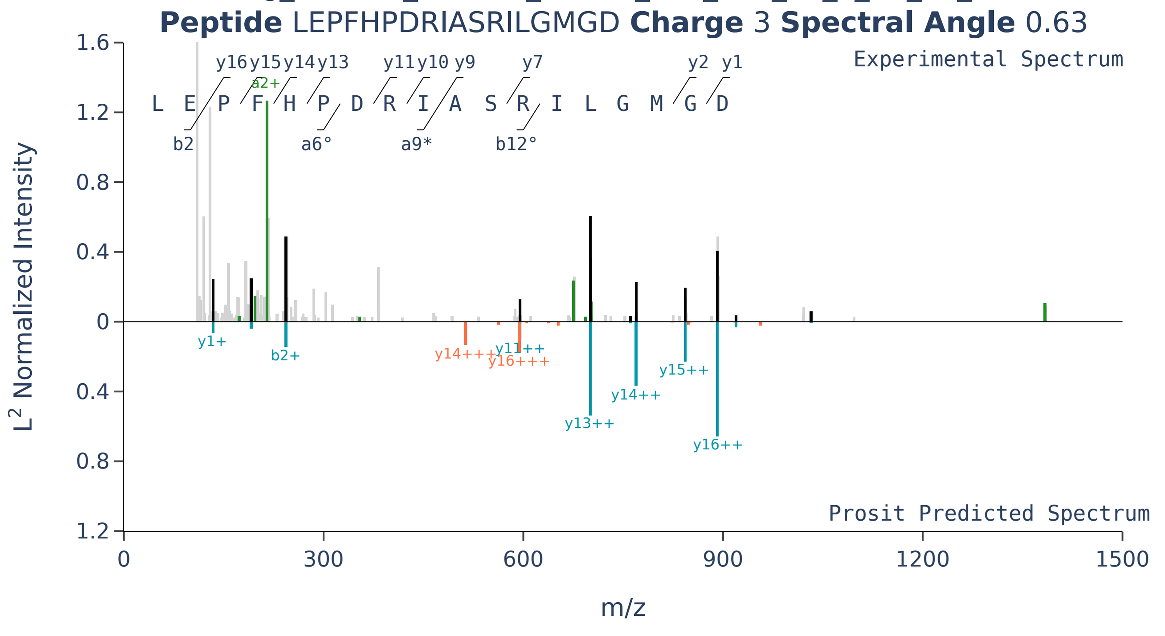

**Source** Ncheng_210623_230623_HFGoe_FFH_20S_25_1_A2_4h_R2 **Scan** 22960
**Peptide** KDTLREVRMALLE **Charge** 3 **Spectral Angle** 0.29

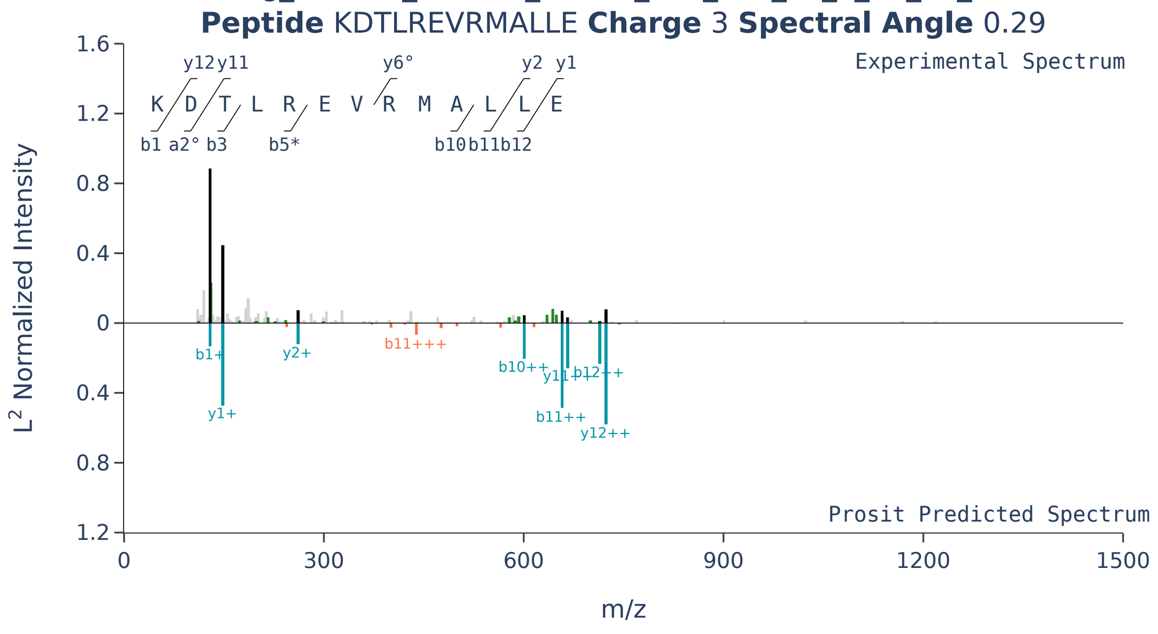


**Source** Ncheng_210623_230623_HFGoe_FFH_20S_25_1_A1_1h_R2 **Scan** 19011
**Peptide** DGDARGGAALSIRHITGKPLKF **Charge** 4 **Spectral Angle** 0.58

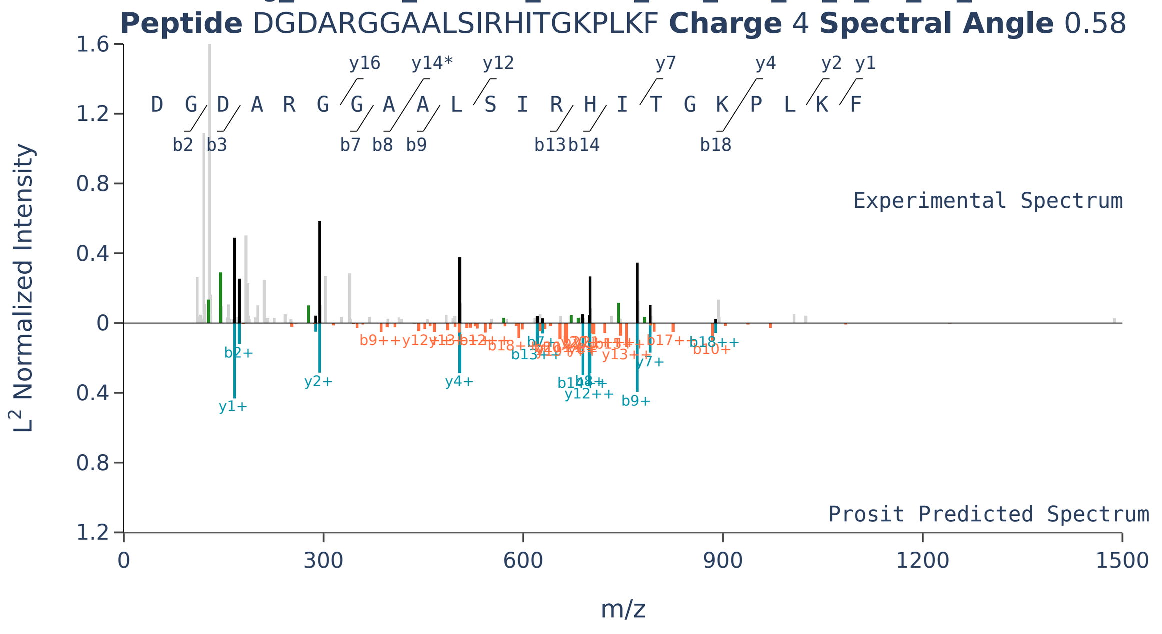


**Source** Ncheng_210623_230623_HFGoe_FFH_20S_25_1_A2_2h_R1 **Scan** 37541
**Peptide** NELVAAMGEENQTLNLAAQPPAVVL **Charge** 3 **Spectral Angle** 0.67

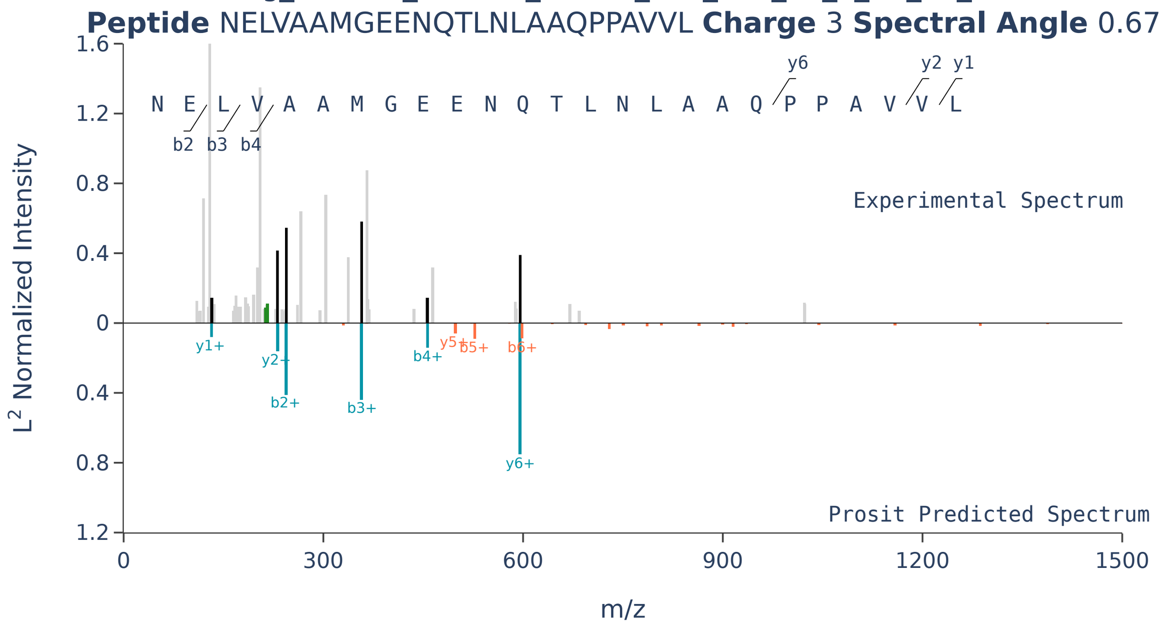


**Source** Ncheng_210623_230623_HFGoe_FFH_20S_25_1_A2_24h_R1 **Scan** 11342
**Peptide** QDAANTAKAFNEA **Charge** 2 **Spectral Angle** 0.76

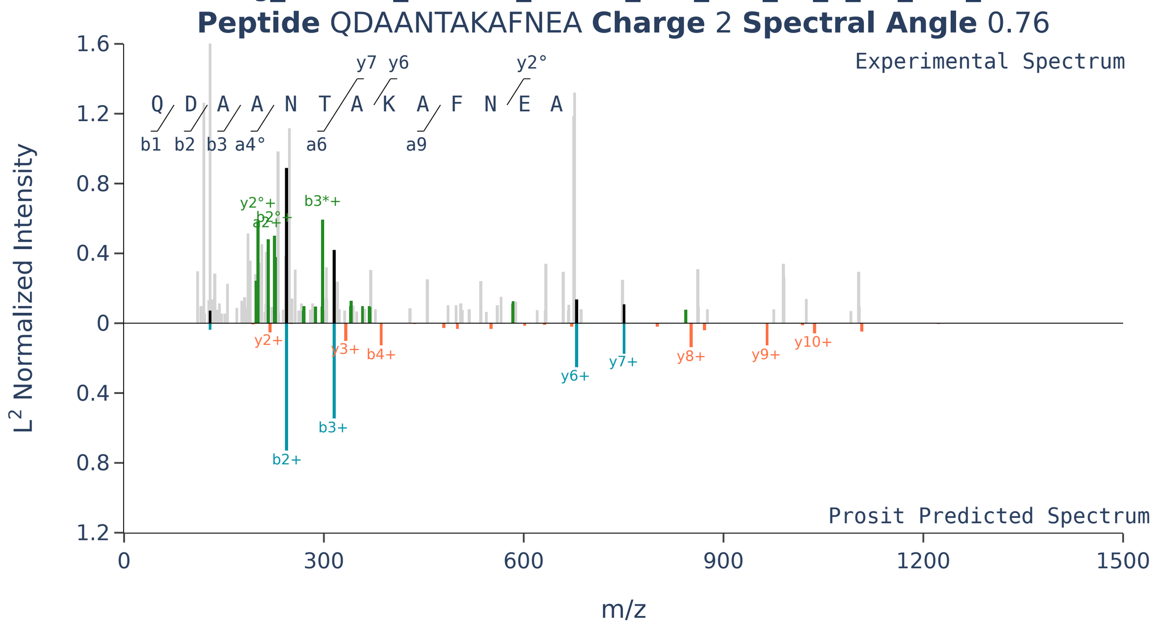


**Source** Ncheng_210623_230623_HFGoe_FFH_20S_25_1_A2_24h_R2 **Scan** 8152
**Peptide** FINRVKEKAVG **Charge** 2 **Spectral Angle** 0.4

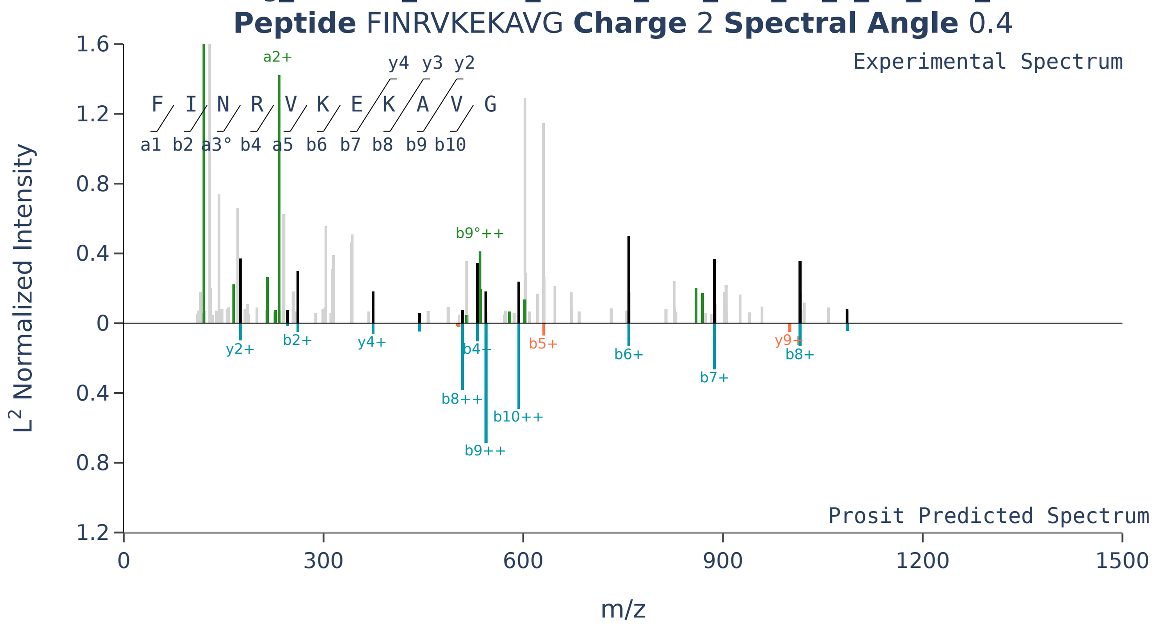


**Source** Ncheng_210623_230623_HFGoe_FFH_20S_25_1_A1_4h_R2 **Scan** 12958
**Peptide** SVGKLGKFLREKHKKKVLVV **Charge** 5 **Spectral Angle** 0.89

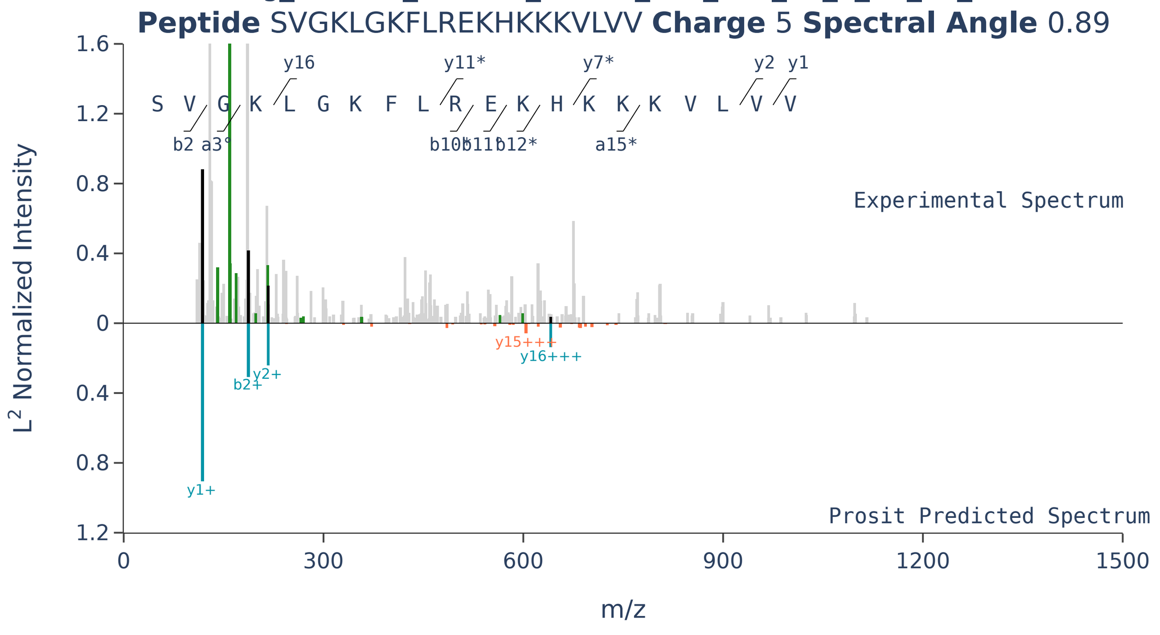


**Source** Ncheng_210623_230623_HFGoe_FFH_20S_25_1_A2_24h_R1 **Scan** 7824
**Peptide** MQRMMKKMKK **Charge** 3 **Spectral Angle** 0.74

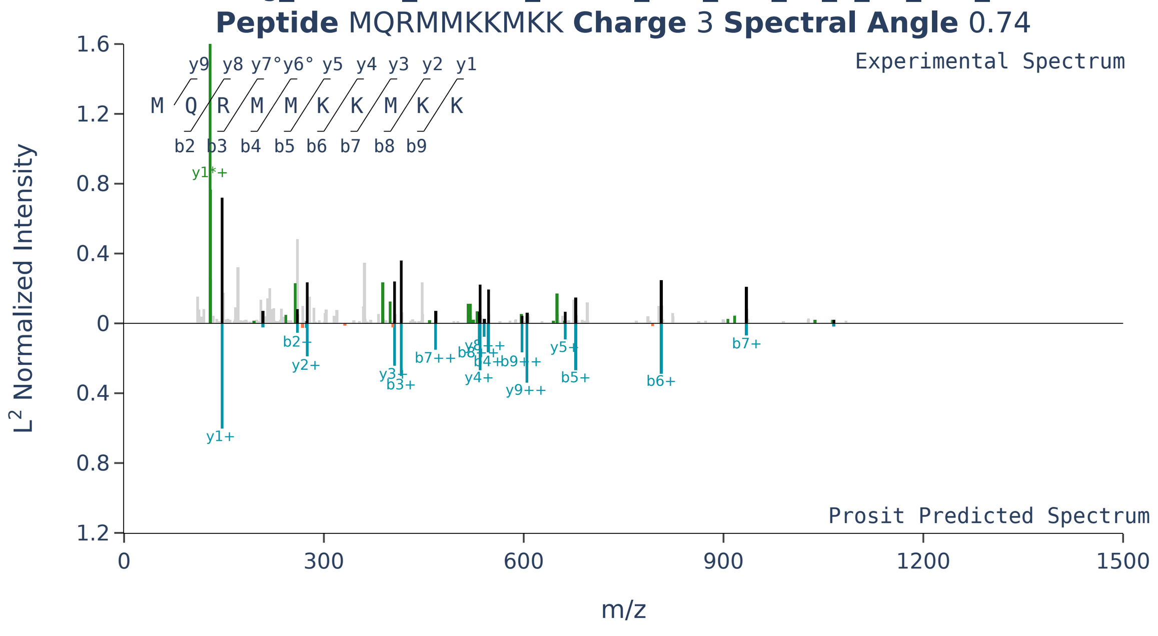


**Source** Ncheng_210623_230623_HFGoe_FFH_20S_25_1_A2_4h_R1 **Scan** 32555
**Peptide** KFYDVLLVDTAGRL **Charge** 3 **Spectral Angle** 0.25

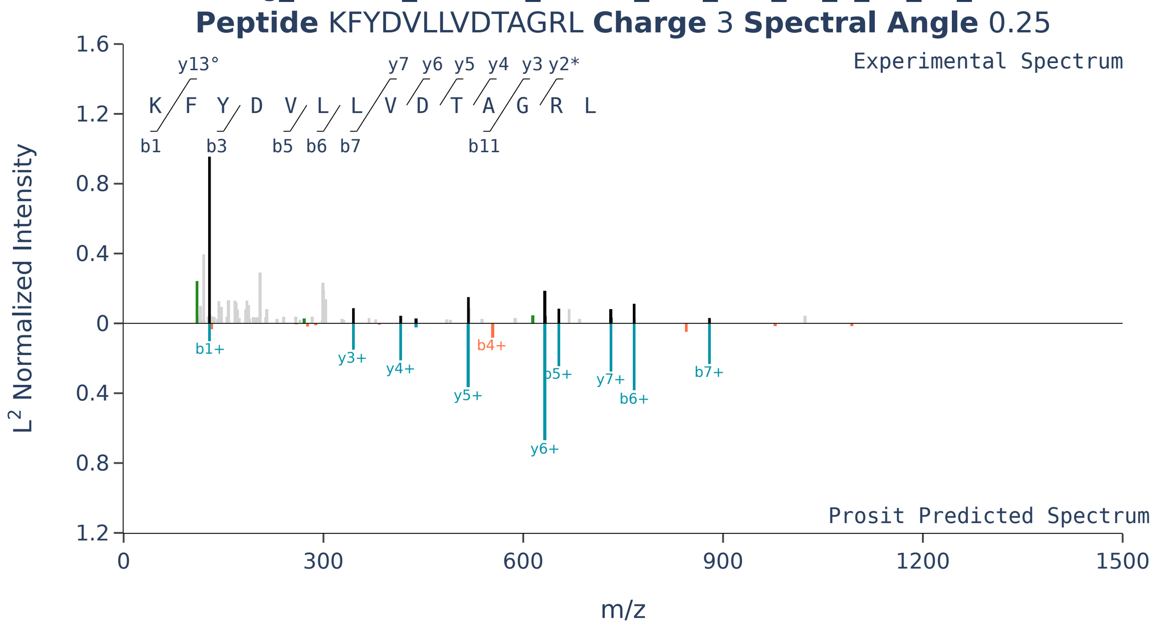


**Source** Ncheng_210623_230623_HFGoe_FFH_20S_25_1_A2_4h_R2 **Scan** 13510
**Peptide** LESKVDRAQAEKLASKLKKGD **Charge** 5 **Spectral Angle** 0.61

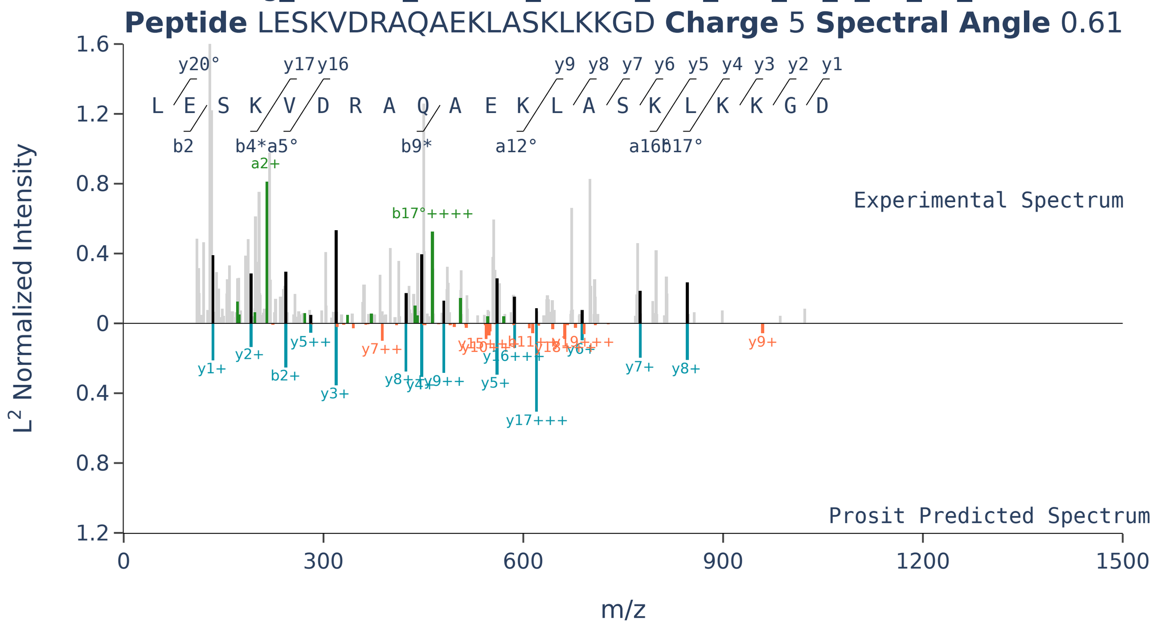


**Source** Ncheng_210623_230623_HFGoe_FFH_20S_25_1_A1_2h_R2 **Scan** 29950
**Peptide** PLKFLGVGEKTEALEPFHPDRIASRIL **Charge** 5 **Spectral Angle** 0.71

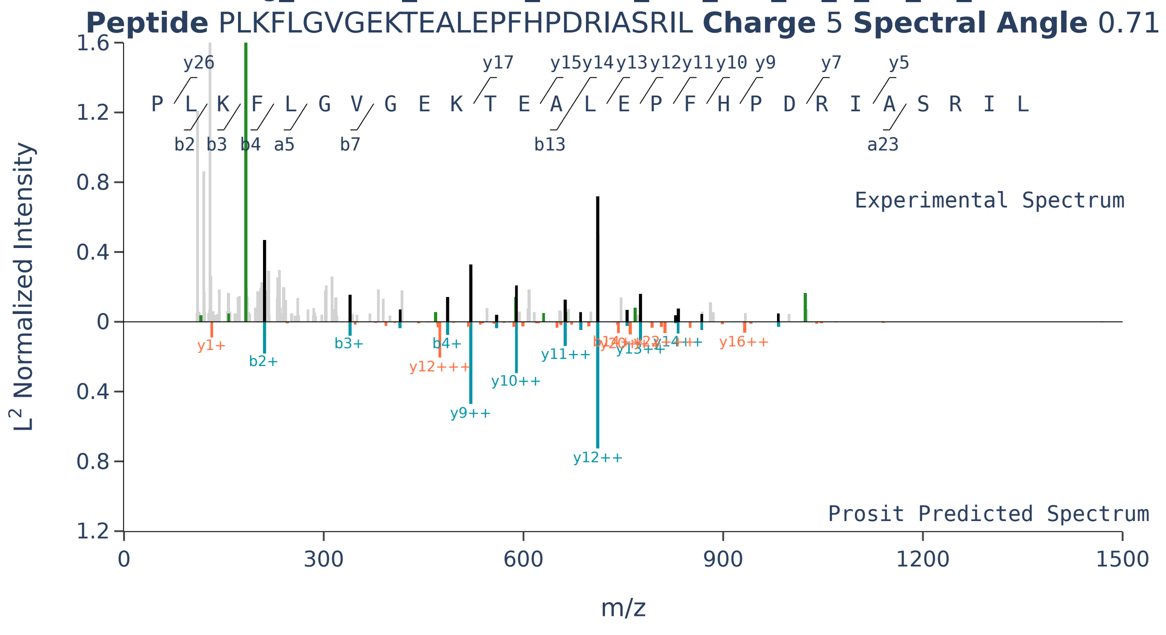


**Source** Ncheng_210623_230623_HFGoe_FFH_20S_25_1_A1_2h_R1 **Scan** 29381
**Peptide** VALPVVREFINRVKEKAVGHEVNKSL **Charge** 5 **Spectral Angle** 0.82

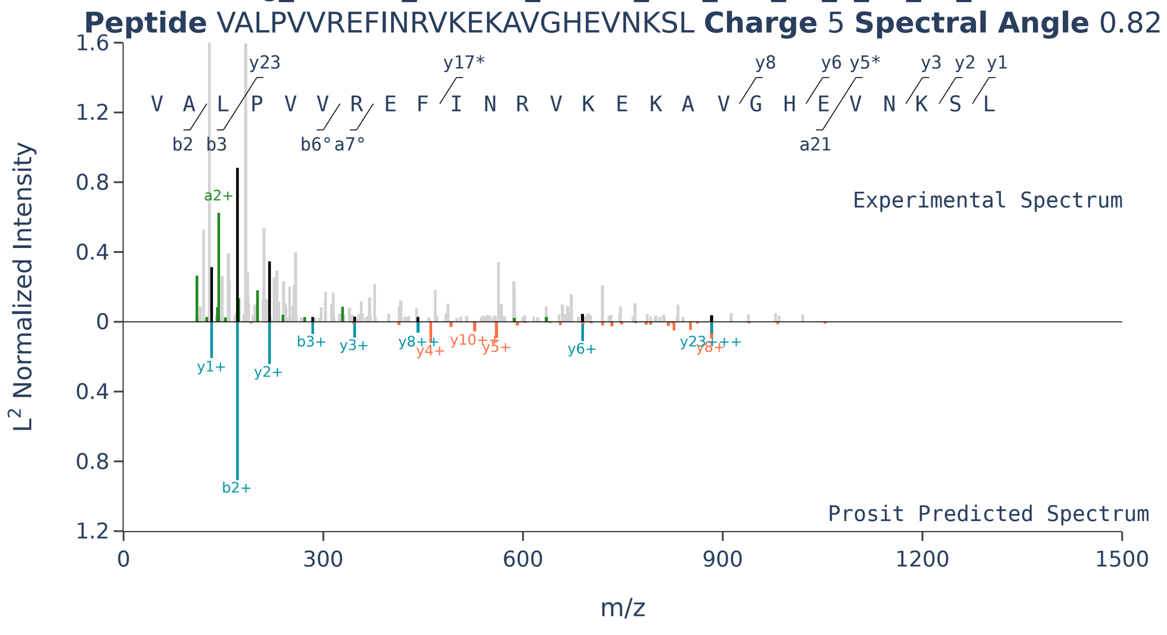


**Source** Ncheng_210623_230623_HFGoe_FFH_20S_25_1_A2_4h_R1 **Scan** 21548
**Peptide** VNAALKEAKLKFYDV **Charge** 3 **Spectral Angle** 0.61

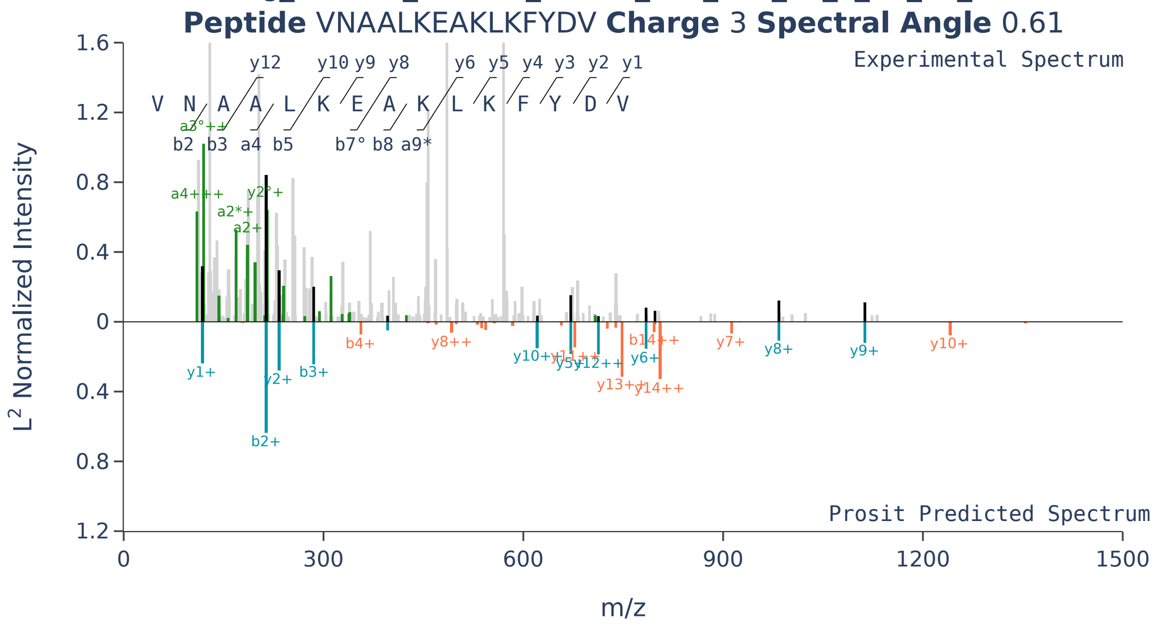


**Source** Ncheng_210623_230623_HFGoe_FFH_20S_25_1_A1_2h_R2 **Scan** 34993
**Peptide** TGQDAANTAKAFNEALPLTGVVLT **Charge** 2 **Spectral Angle** 0.6

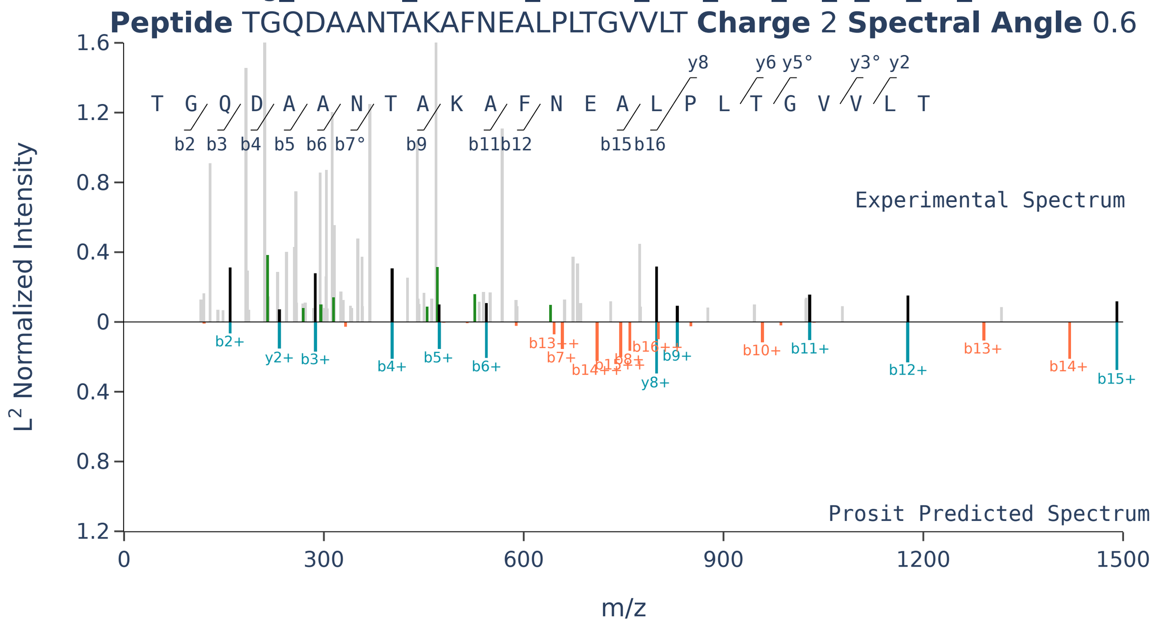


**Source** Ncheng_210623_230623_HFGoe_FFH_20S_25_1_A1_4h_R1 **Scan** 27749
**Peptide** RQMKNMGGMASLMGKLPGMGQIPDNVKSQ **Charge** 4 **Spectral Angle** 0.81

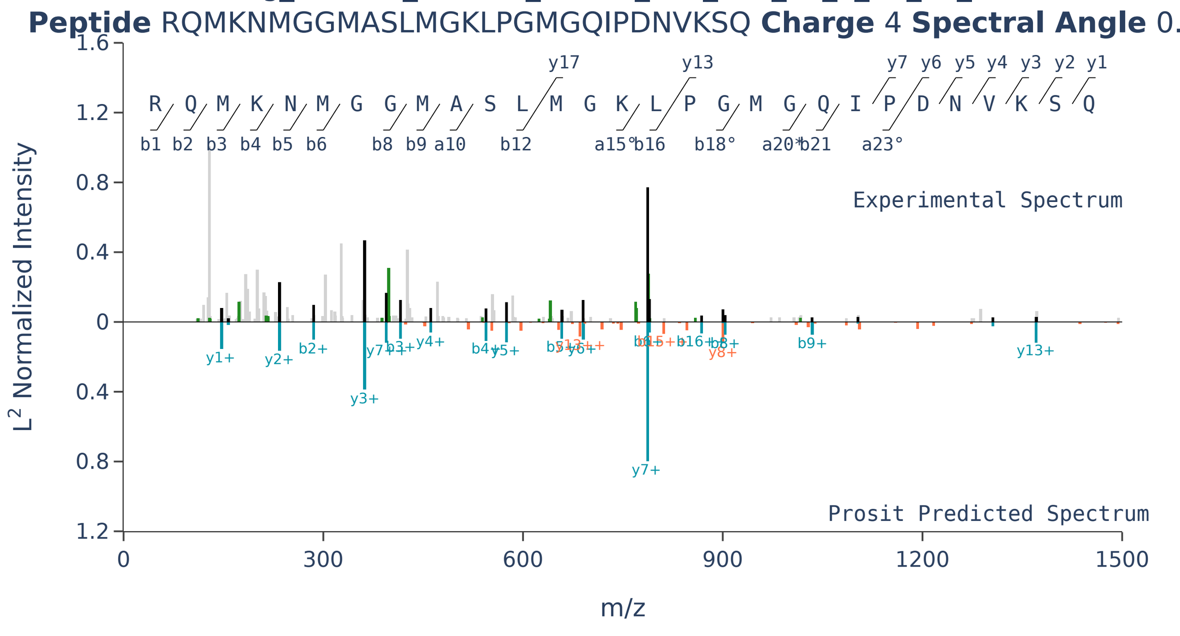


**Source** Ncheng_210623_230623_HFGoe_FFH_20S_25_1_A2_4h_R1 **Scan** 18597
**Peptide** RQMKNMGGMASLMG **Charge** 2 **Spectral Angle** 0.56

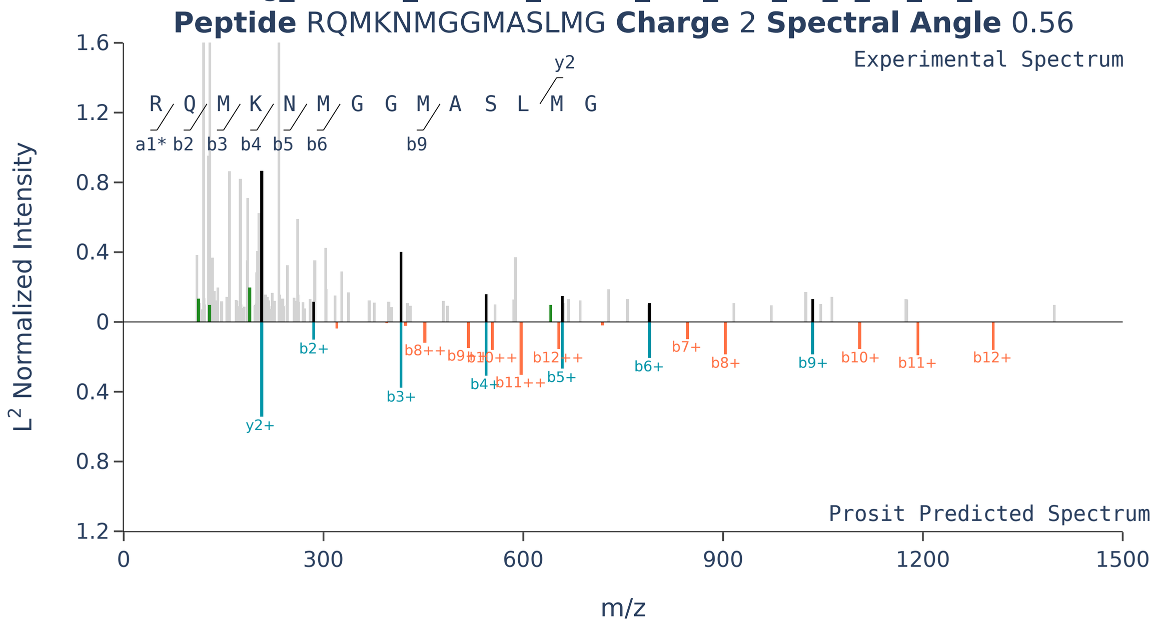

**Source** Ncheng_210623_230623_HFGoe_FFH_20S_25_1_A2_24h_R2 **Scan** 15824
**Peptide** IEDIESKVDRAQAEKLAS **Charge** 2 **Spectral Angle** 0.75

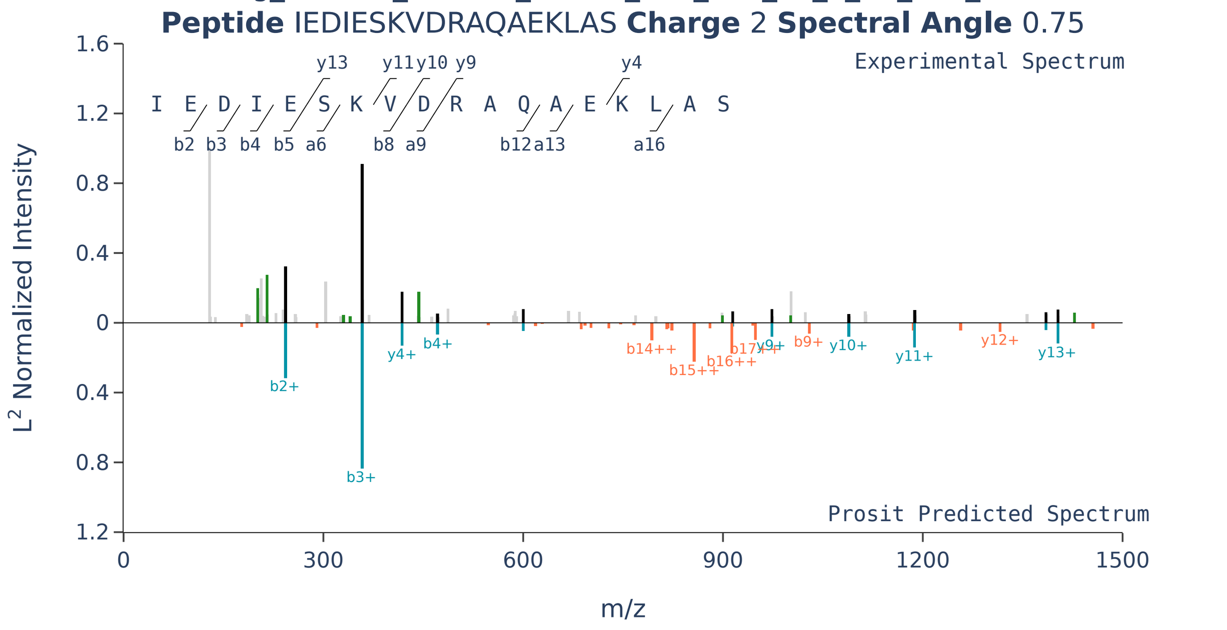


**Source** Ncheng_210623_230623_HFGoe_FFH_20S_25_1_A2_2h_R2 **Scan** 35807
**Peptide** AQAEKLASKLKKGDGFDLNDFLEQLRQM **Charge** 4 **Spectral Angle** 0.77

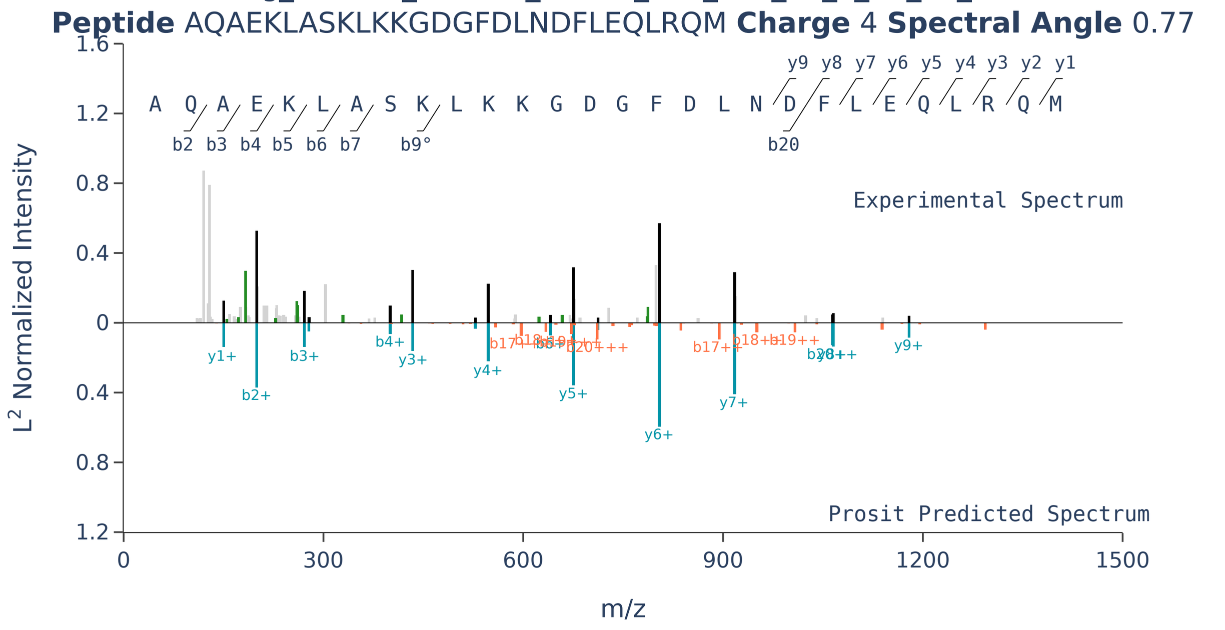


**Source** Ncheng_210623_230623_HFGoe_FFH_20S_25_1_A2_24h_R1 **Scan** 14572
**Peptide** TSVGKLGKFLREK **Charge** 4 **Spectral Angle** 0.53

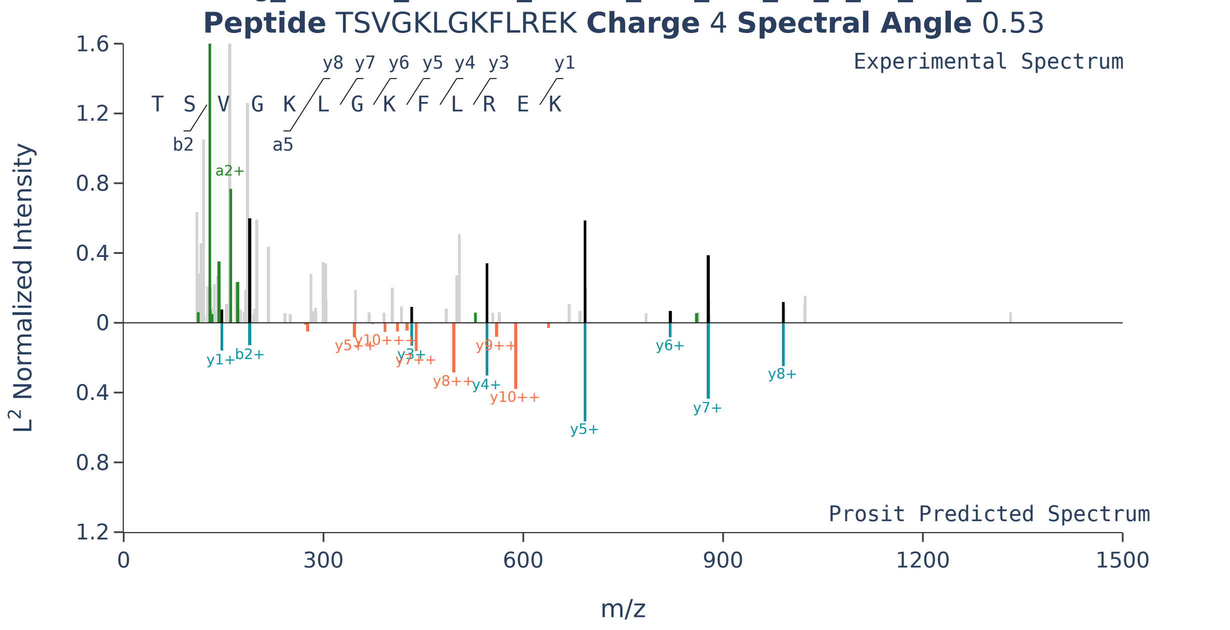


**Source** Ncheng_210623_230623_HFGoe_FFH_20S_25_1_A2_4h_R2 **Scan** 24848
**Peptide** DARGGAALSIRHITGKPIKFLGVGE **Charge** 5 **Spectral Angle** 0.69

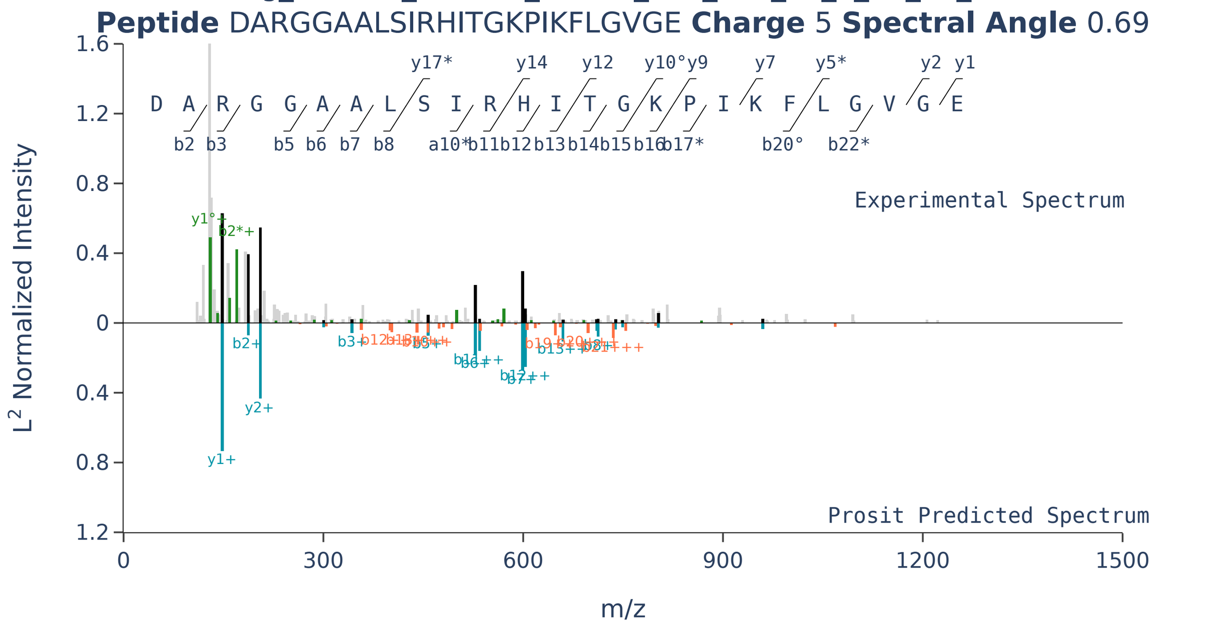


**Source** Ncheng_210623_230623_HFGoe_FFH_20S_25_1_A1_1h_R2 **Scan** 18284
**Peptide** GVGEKTEALEPFHPDRLAS **Charge** 4 **Spectral Angle** 0.65

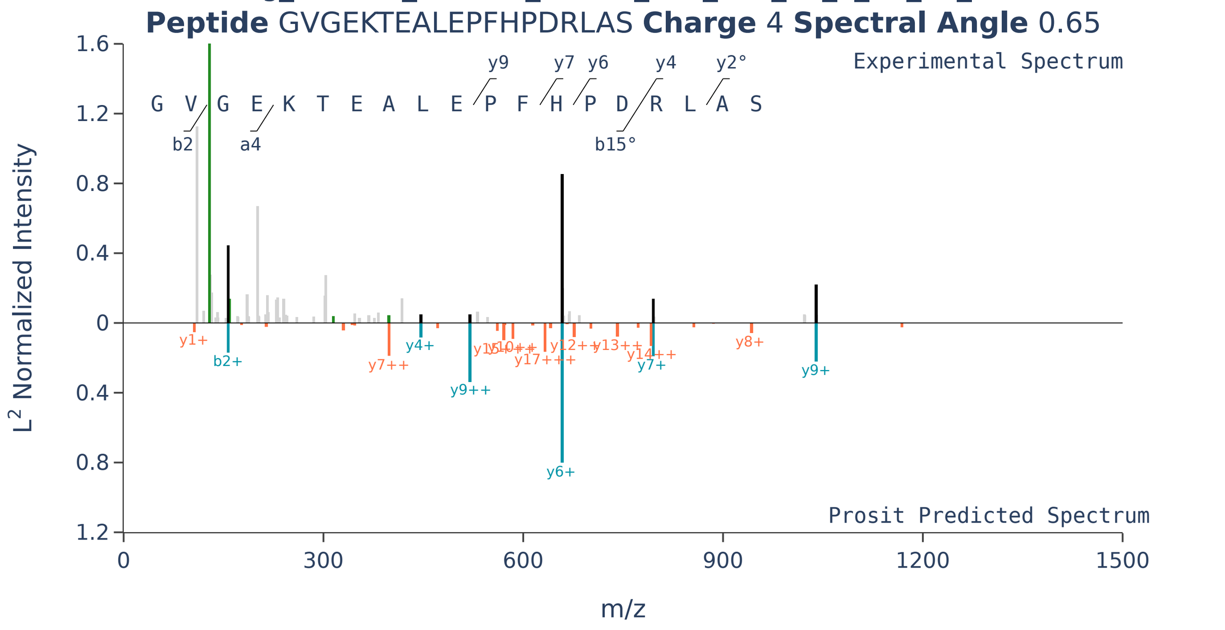


**Source** Ncheng_210623_230623_HFGoe_FFH_20S_25_1_A2_24h_R2 **Scan** 19984
**Peptide** VKEKAVGHEVNKSLTPGQEFVKLV **Charge** 5 **Spectral Angle** 0.54

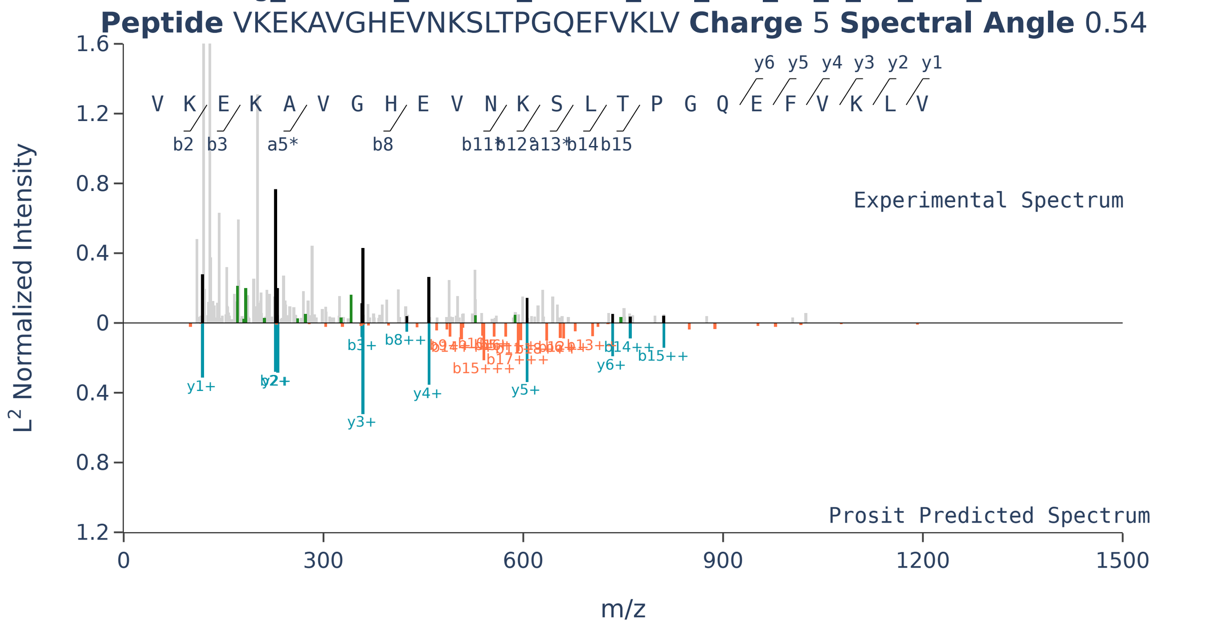


**Source** Ncheng_210623_230623_HFGoe_FFH_20S_25_1_A1_2h_R2 **Scan** 26165
**Peptide** ALPVVREFINRVKE **Charge** 4 **Spectral Angle** 0.43

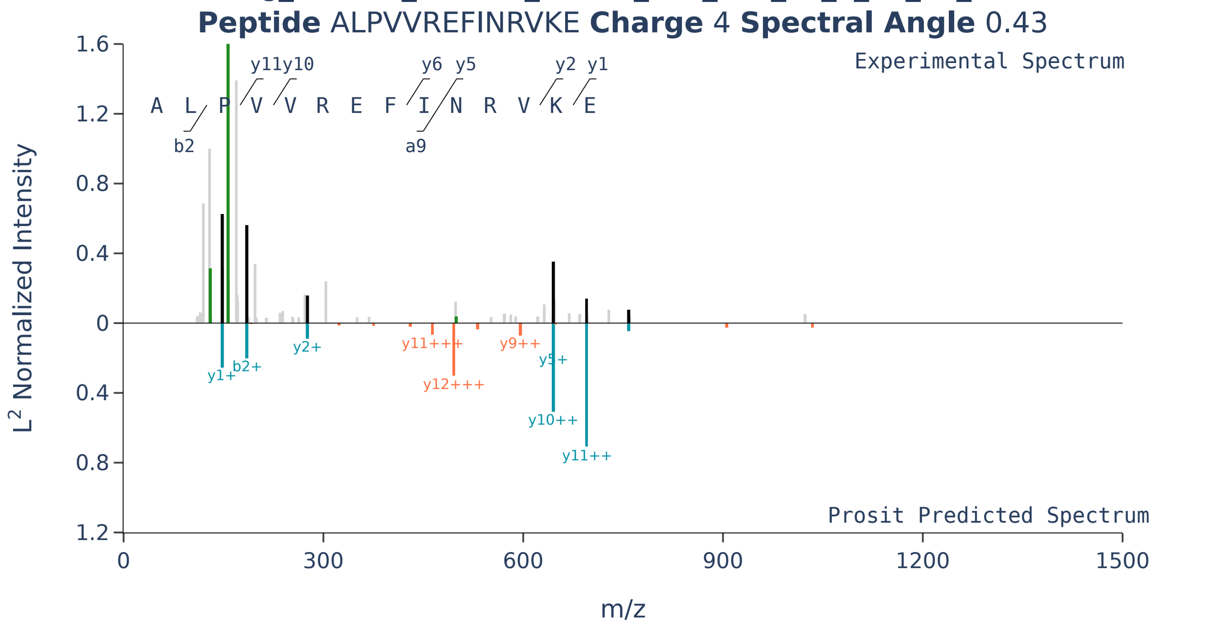


**Source** Ncheng_210623_230623_HFGoe_FFH_20S_25_1_A2_2h_R1 **Scan** 22891
**Peptide** TKVDGDARGGAALSIRHITGKPIKFLGV **Charge** 5 **Spectral Angle** 0.77

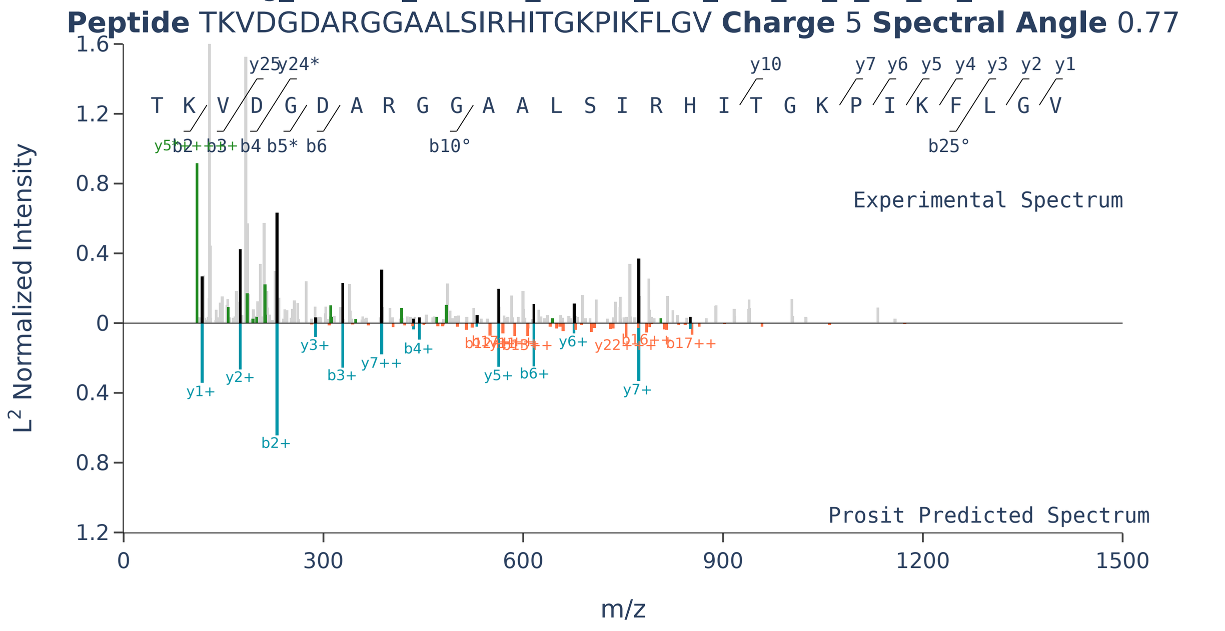


**Source** Ncheng_210623_230623_HFGoe_FFH_20S_25_1_A2_24h_R2 **Scan** 10210
**Peptide** QAEKLASK **Charge** 2 **Spectral Angle** 0.79

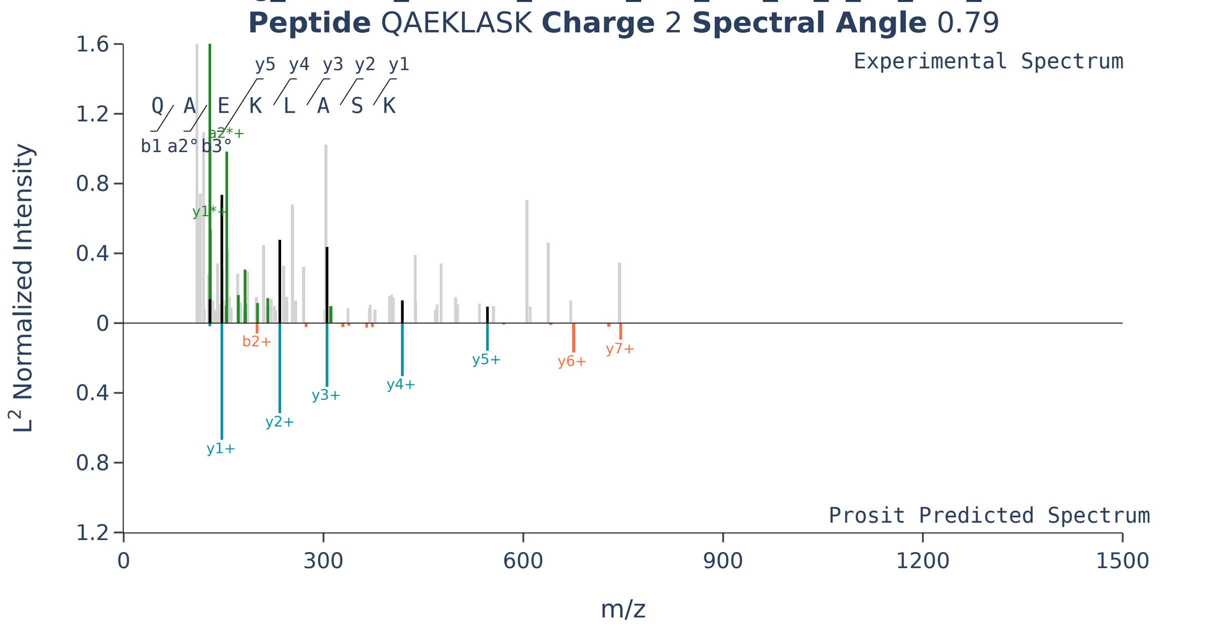


**Source** Ncheng_210623_230623_HFGoe_FFH_20S_25_1_A1_4h_R1 **Scan** 25864
**Peptide** ALPVVREFINRVKEKAVGHEVNK **Charge** 5 **Spectral Angle** 0.75

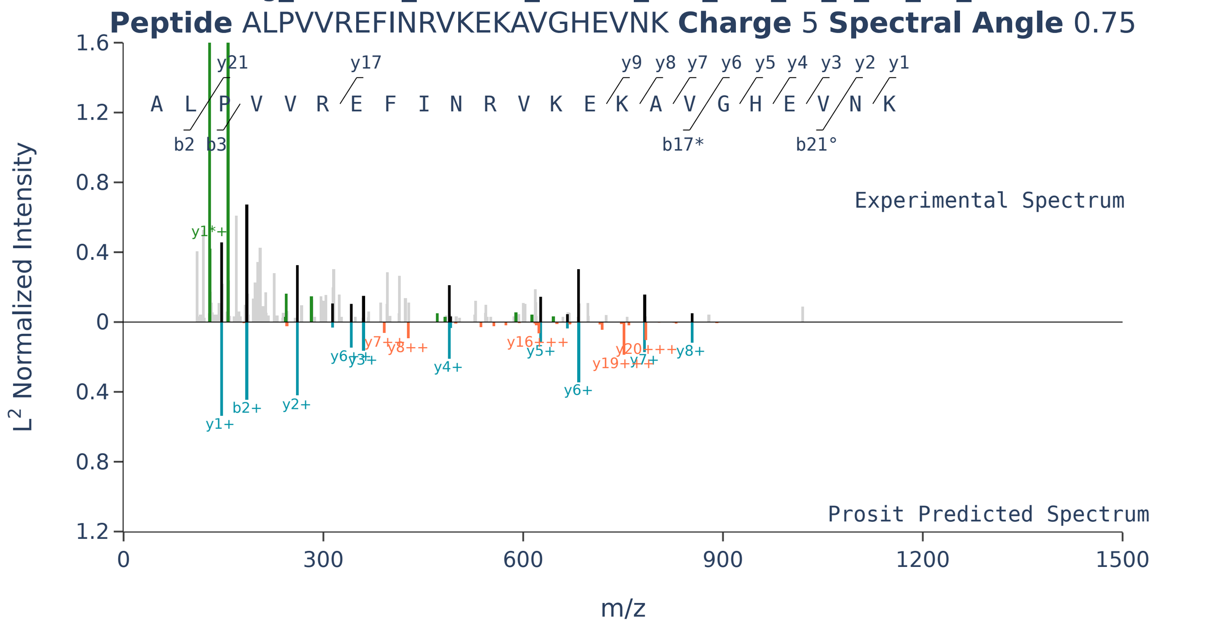


**Source** Ncheng_210623_230623_HFGoe_FFH_20S_25_1_A2_4h_R2 **Scan** 20793
**Peptide** LEDIESKVDRAQAEKLASKLKKGDGFD **Charge** 4 **Spectral Angle** 0.67

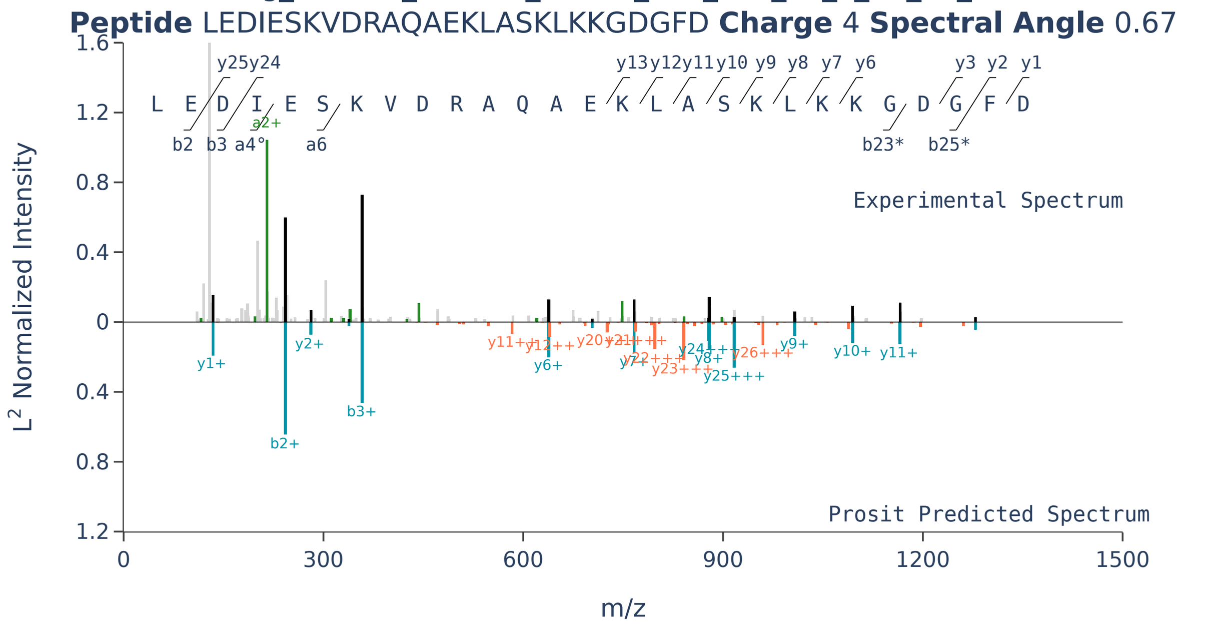


**Source** Ncheng_210623_230623_HFGoe_FFH_20S_25_1_A2_2h_R2 **Scan** 34108
**Peptide** VVSADVYRPAAIKQLETLAEQVG **Charge** 3 **Spectral Angle** 0.75

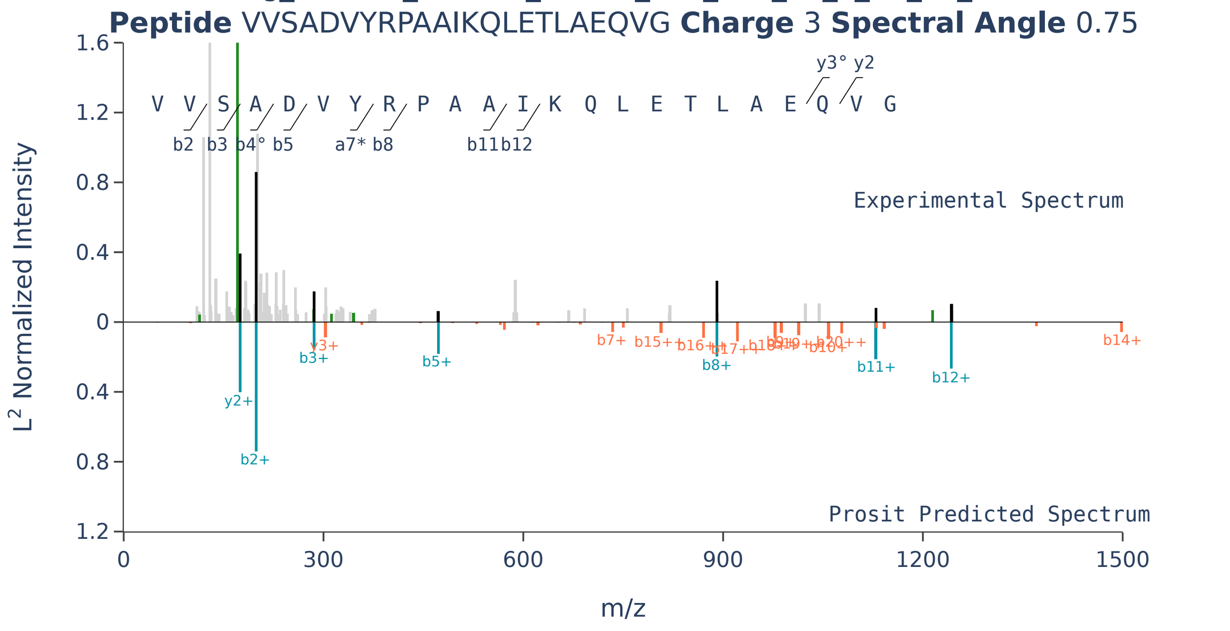


**Source** Ncheng_210623_230623_HFGoe_FFH_20S_25_1_A1_24h_R2 **Scan** 19653
**Peptide** VNKSLTPGQEFV **Charge** 2 **Spectral Angle** 0.37

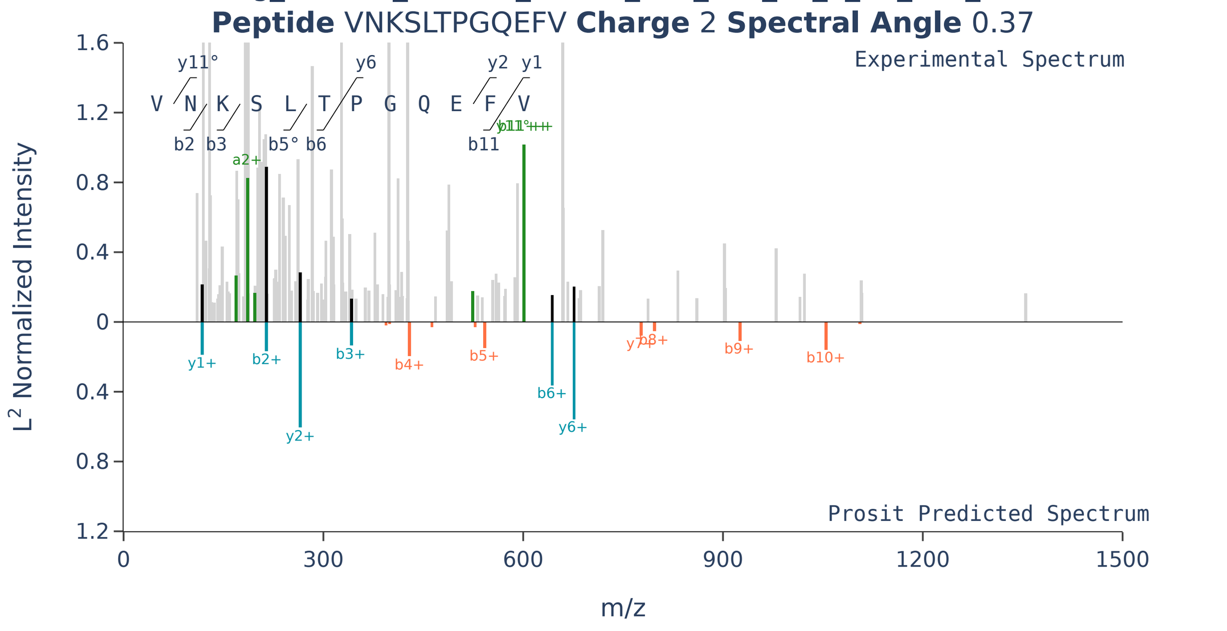


**Source** Ncheng_210623_230623_HFGoe_FFH_20S_25_1_A2_4h_R1 **Scan** 20175
**Peptide** VLTKVDGDARGGAALSIRHITGKPLKF **Charge** 5 **Spectral Angle** 0.68

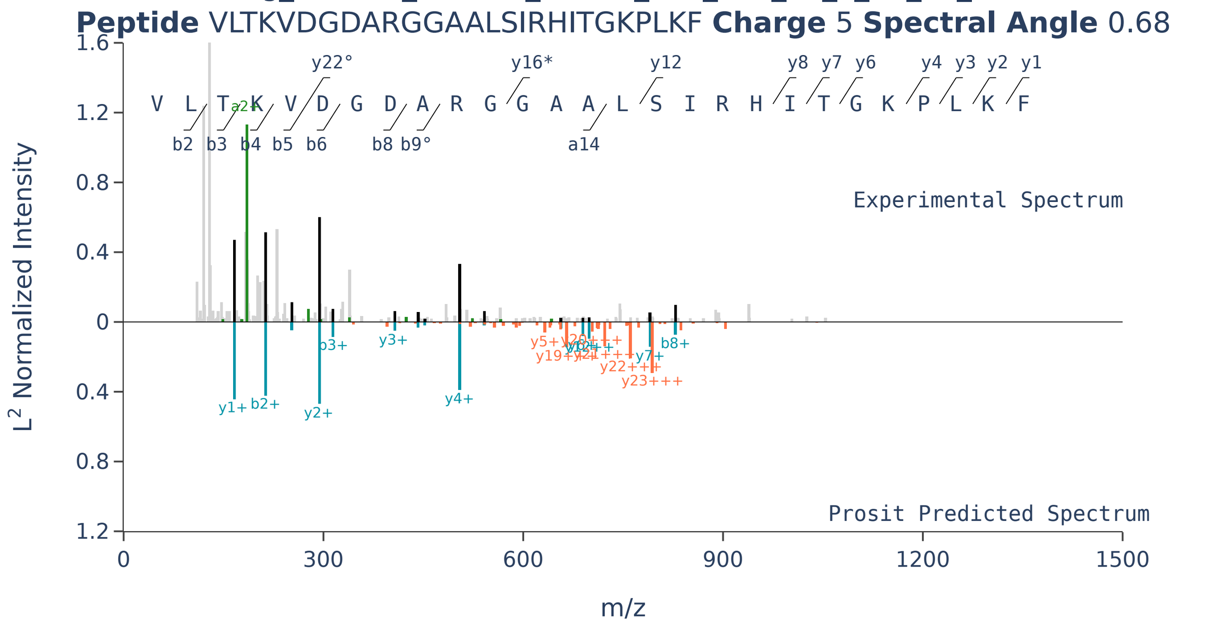


**Source** Ncheng_210623_230623_HFGoe_FFH_20S_25_1_A1_24h_R2 **Scan** 12171
**Peptide** INRVKEKAVGHEVNKSLTPGQEF **Charge** 5 **Spectral Angle** 0.83

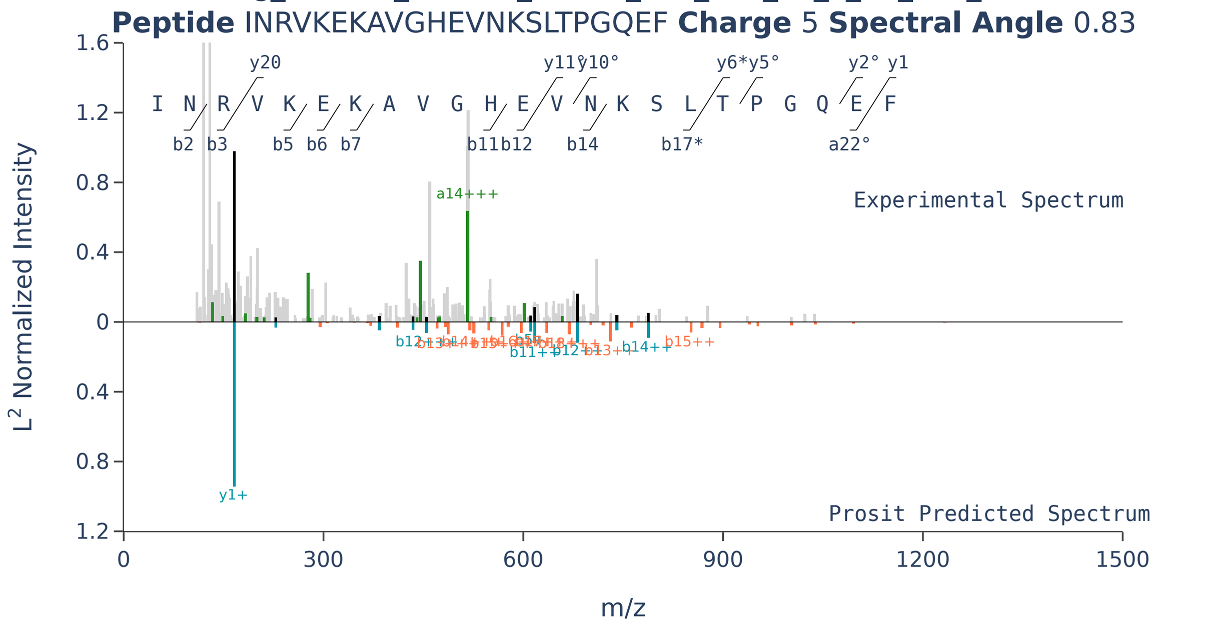

**Source** Ncheng_210623_230623_HFGoe_FFH_20S_25_1_A1_2h_R2 **Scan** 11499
**Peptide** REFINRVKEKAVGHEVNKSLTPGQ **Charge** 4 **Spectral Angle** 0.68

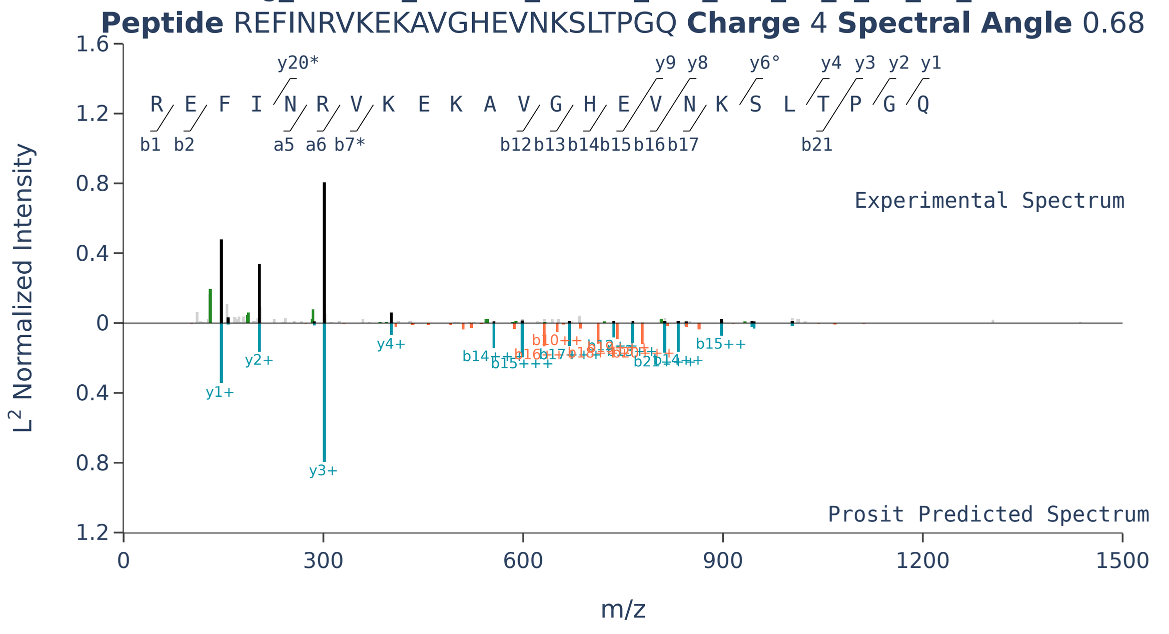


**Source** Ncheng_210623_230623_HFGoe_FFH_20S_25_1_A1_24h_R2 **Scan** 19478
**Peptide** ISGRGRLTEDNVKDTLREVRMA **Charge** 5 **Spectral Angle** 0.65

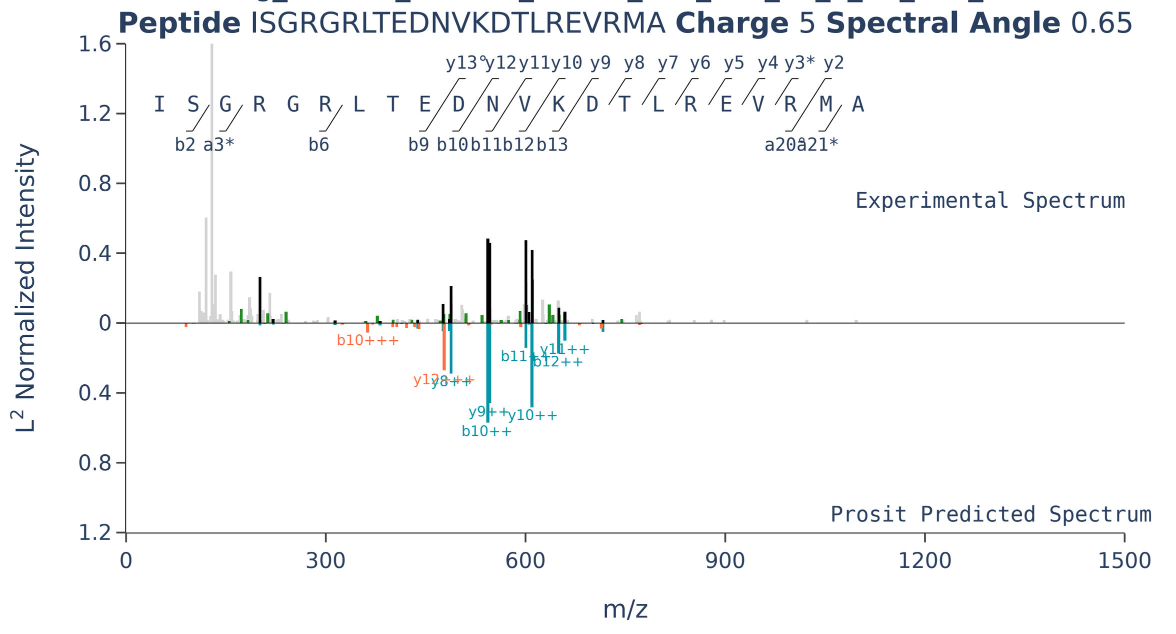


**Source** Ncheng_210623_230623_HFGoe_FFH_20S_25_1_A1_2h_R1 **Scan** 27606
**Peptide** VLLVDTAGRLHVDEAMMD **Charge** 3 **Spectral Angle** 0.43

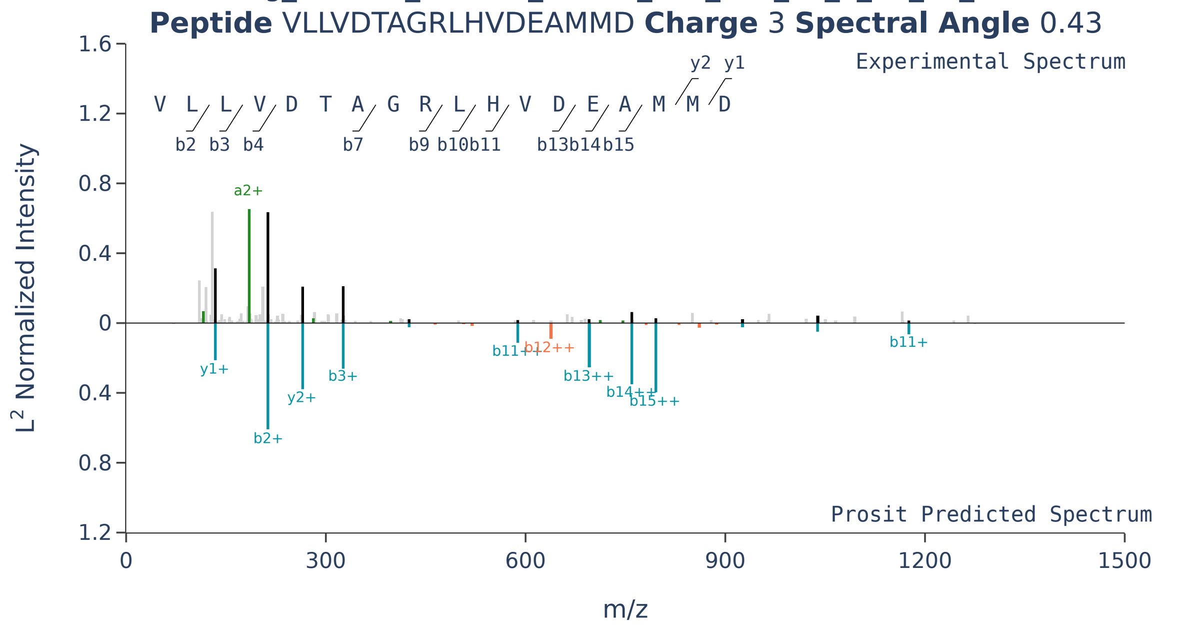


**Source** Ncheng_210623_230623_HFGoe_FFH_20S_25_1_A2_4h_R2 **Scan** 35608
**Peptide** SLTPGQEFVKIVRNELV **Charge** 3 **Spectral Angle** 0.55

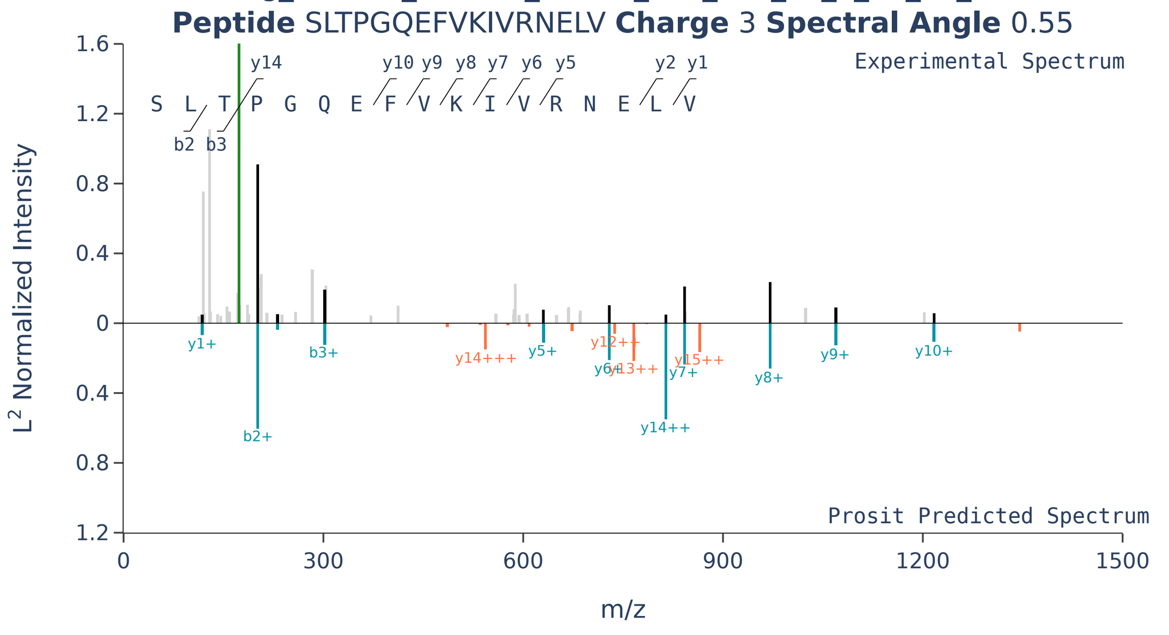


**Source** Ncheng_210623_230623_HFGoe_FFH_20S_25_1_A2_4h_R1 **Scan** 24187
**Peptide** IEDIESKVDRAQAEKLASKLKKGDGFDL **Charge** 5 **Spectral Angle** 0.84

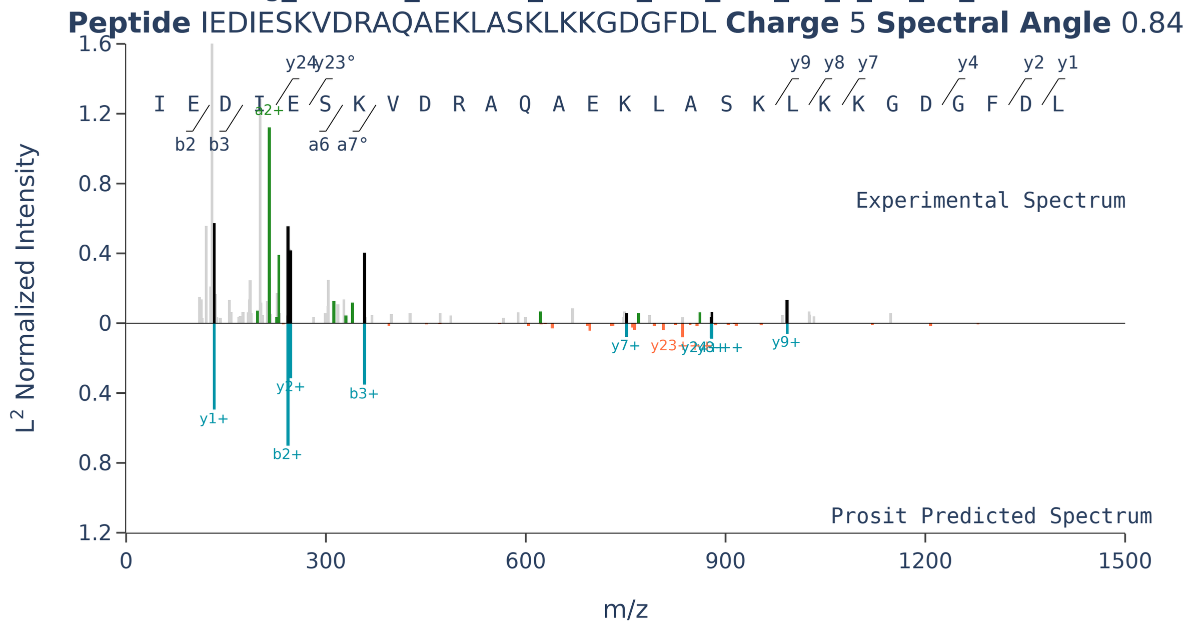


**Source** Ncheng_210623_230623_HFGoe_FFH_20S_25_1_A2_24h_R1 **Scan** 19050
**Peptide** LEDIESKVDRAQAEKLASKLK **Charge** 5 **Spectral Angle** 0.73

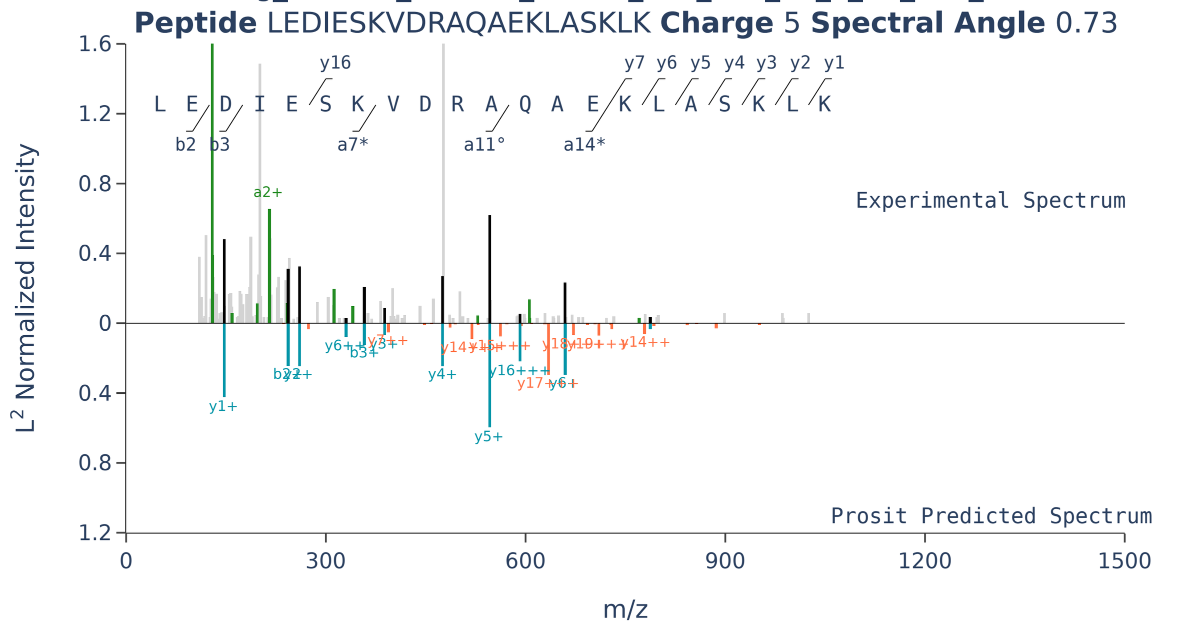


**Source** Ncheng_210623_230623_HFGoe_FFH_20S_25_1_A1_2h_R1 **Scan** 28589
**Peptide** ISGRGRLTEDNVKDTLREVRMALL **Charge** 5 **Spectral Angle** 0.71

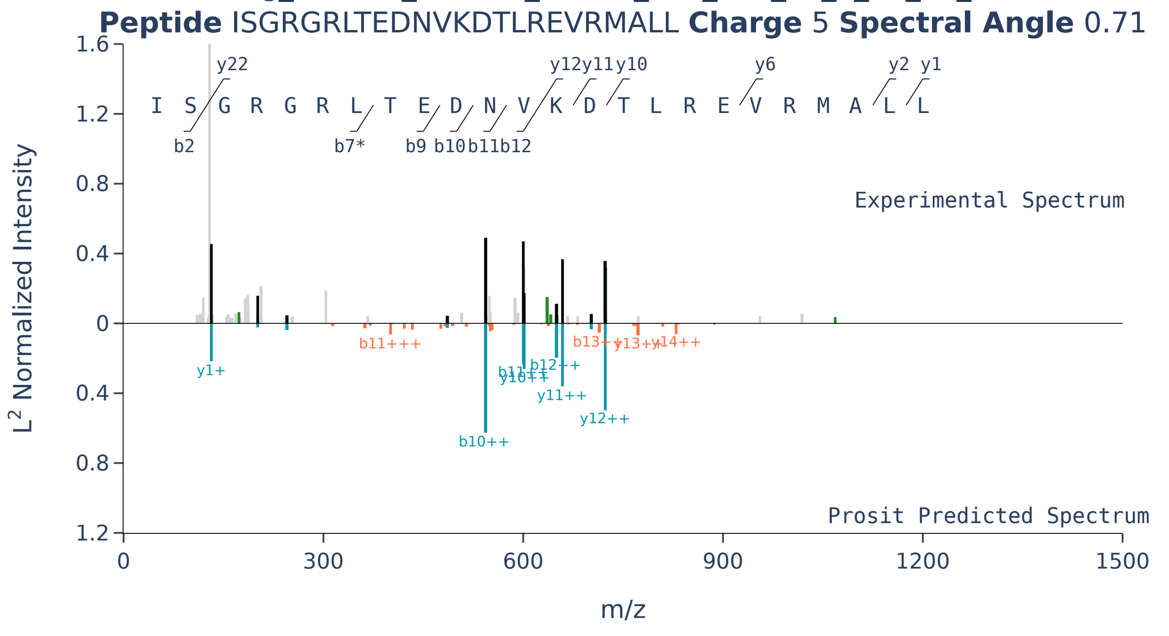


**Source** Ncheng_210623_230623_HFGoe_FFH_20S_25_1_A1_24h_R1 **Scan** 18365
**Peptide** EAIINSMTMKERAKPEIIKG **Charge** 3 **Spectral Angle** 0.74

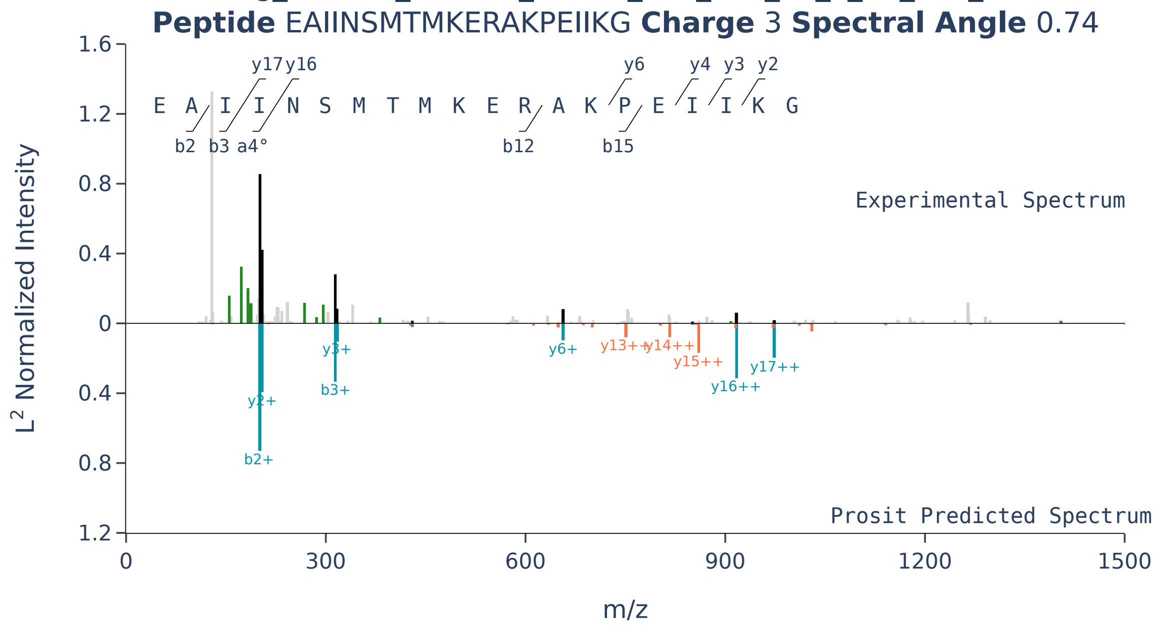


**Source** Ncheng_210623_230623_HFGoe_FFH_20S_25_1_A1_2h_R2 **Scan** 23350
**Peptide** NLTDRLSRTLRNISGRGRLT **Charge** 5 **Spectral Angle** 0.68

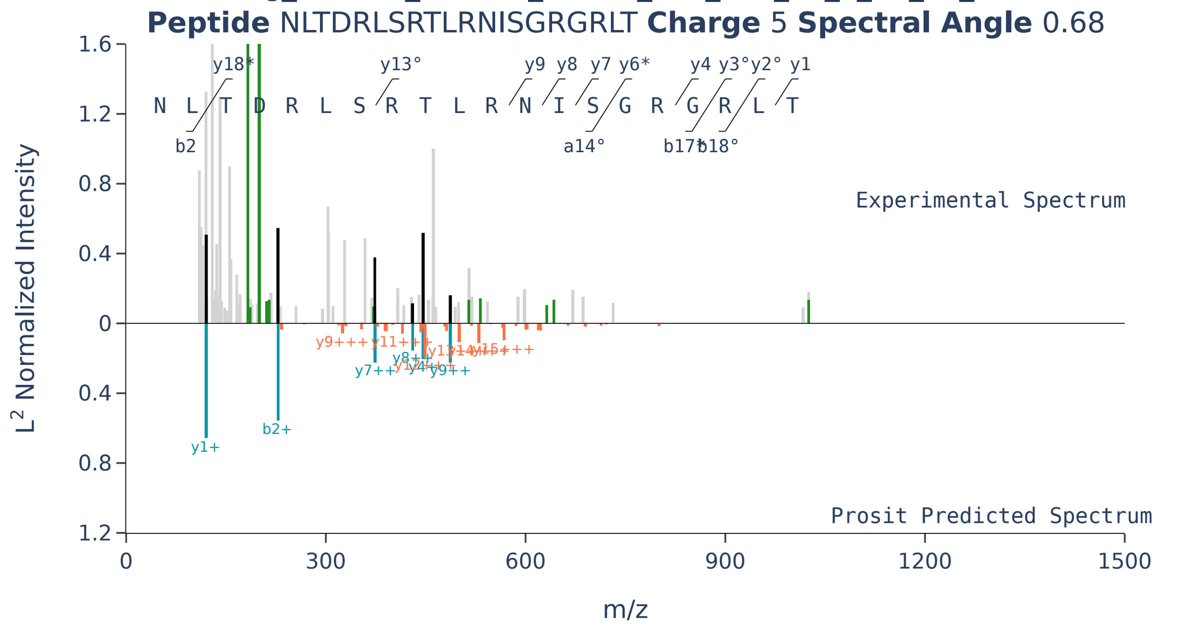


**Source** Ncheng_210623_230623_HFGoe_FFH_20S_25_1_A1_2h_R1 **Scan** 39384
**Peptide** KGDGFDLNDFLEQLRQMKNMG **Charge** 3 **Spectral Angle** 0.77

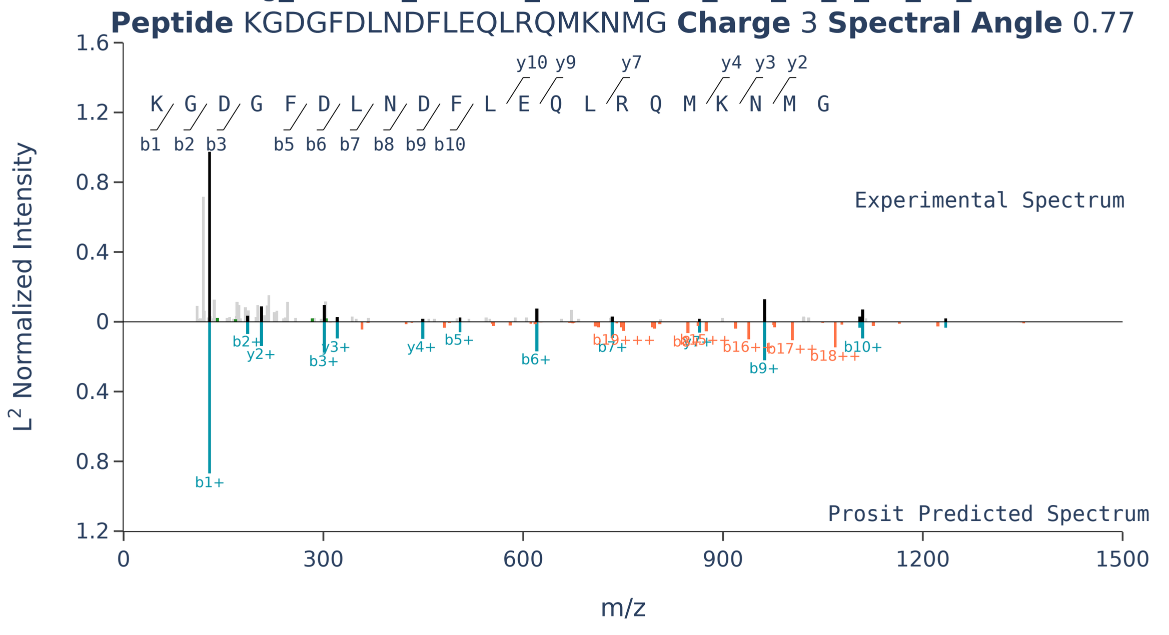


**Source** Ncheng_210623_230623_HFGoe_FFH_20S_25_1_A2_4h_R2 **Scan** 24849
**Peptide** DAMTGQDAANTAKAFNEAL **Charge** 2 **Spectral Angle** 0.64

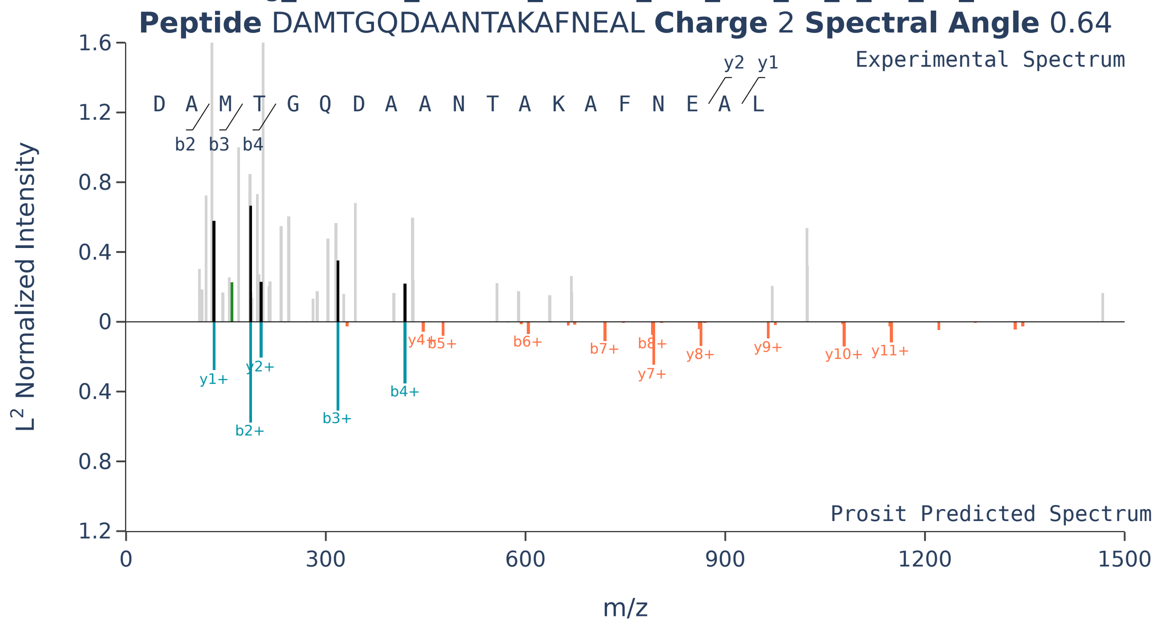


**Source** Ncheng_210623_230623_HFGoe_FFH_20S_25_1_A1_2h_R1 **Scan** 24876
**Peptide** PLTGVVLTKVDGDARGGAALSIRHLTG **Charge** 4 **Spectral Angle** 0.57

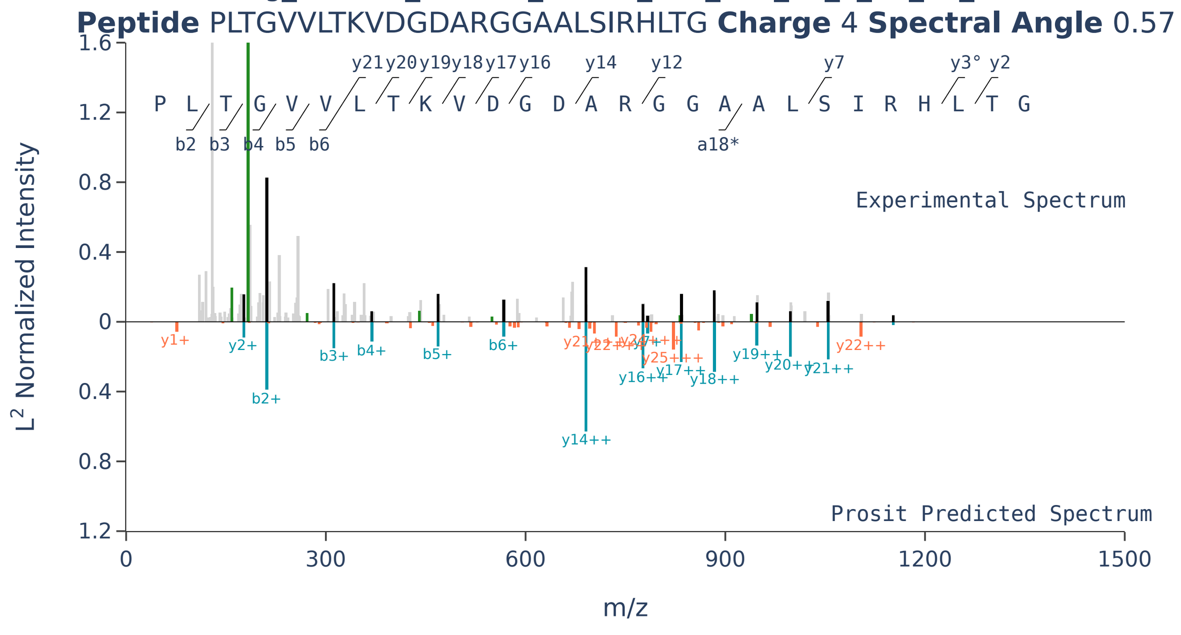


**Source** Ncheng_210623_230623_HFGoe_FFH_20S_25_1_A1_2h_R2 **Scan** 12618
**Peptide** ASKLKKGDGFDL **Charge** 3 **Spectral Angle** 0.71

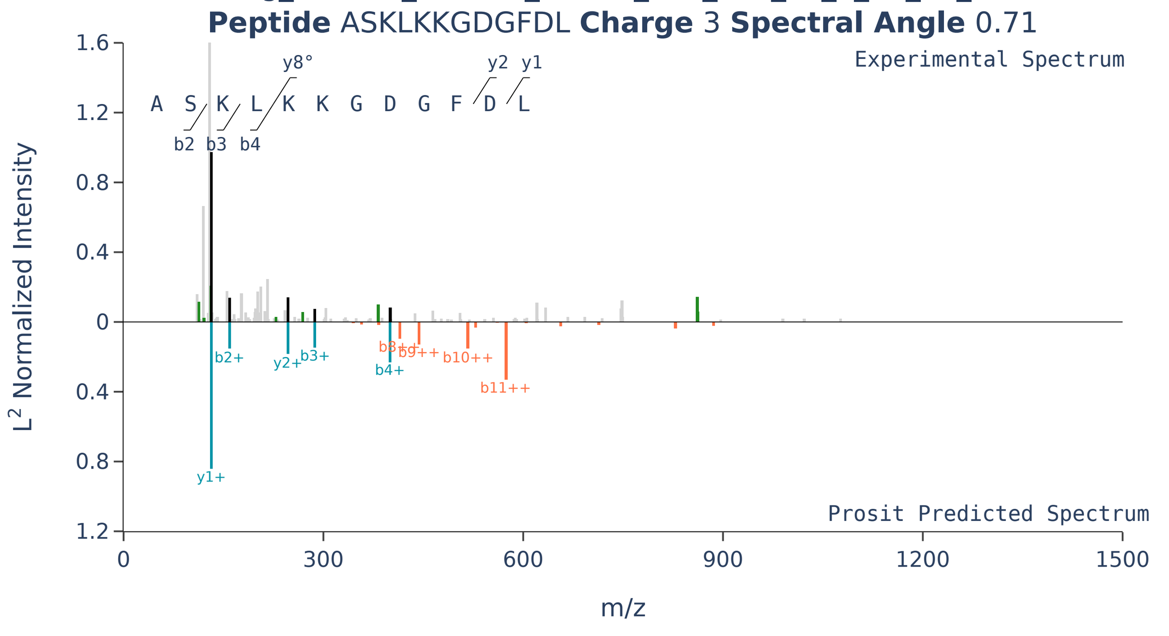


**Source** Ncheng_210623_230623_HFGoe_FFH_20S_25_1_A1_1h_R2 **Scan** 10931
**Peptide** RLTEDNVKDTLRE **Charge** 3 **Spectral Angle** 0.76

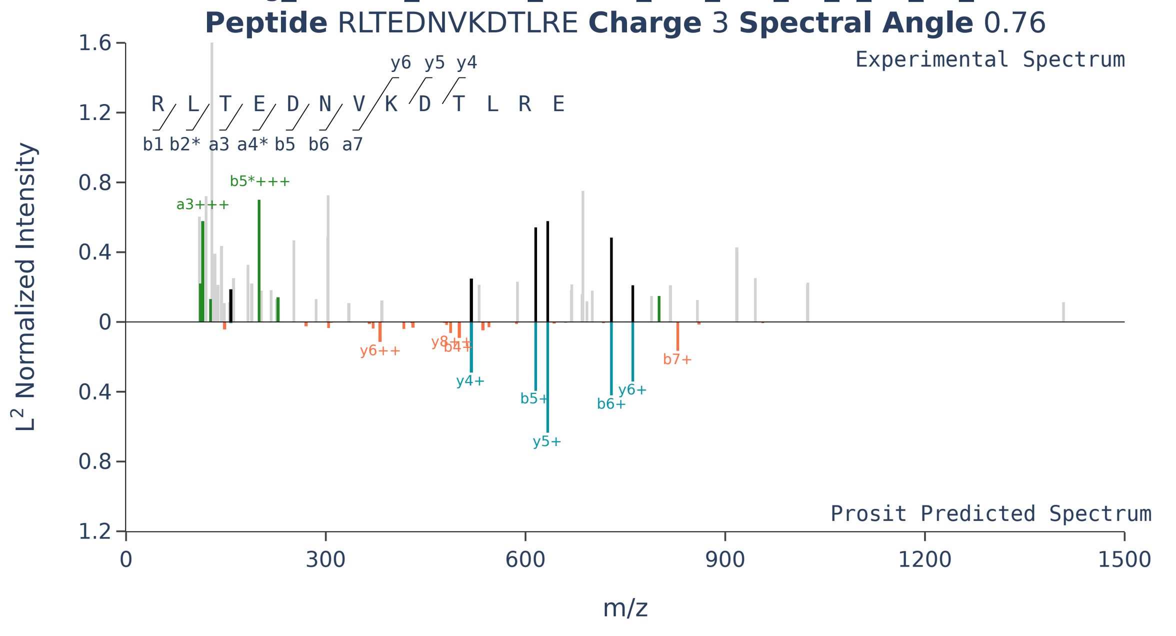


**Source** Ncheng_210623_230623_HFGoe_FFH_20S_25_1_A1_4h_R2 **Scan** 25641
**Peptide** QVQDVNRLLKQF **Charge** 3 **Spectral Angle** 0.66

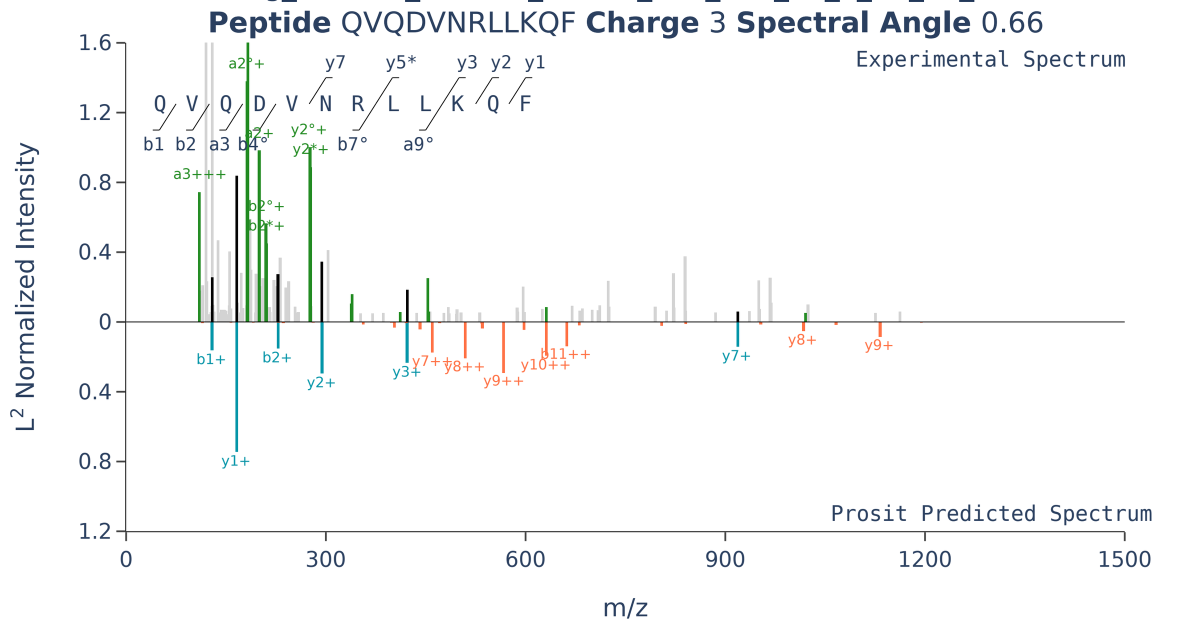

**Source** Ncheng_210623_230623_HFGoe_FFH_20S_25_1_A2_2h_R2 **Scan** 26123
**Peptide** RRIAAGCGMQVQDVNRLLKQFD **Charge** 4 **Spectral Angle** 0.5

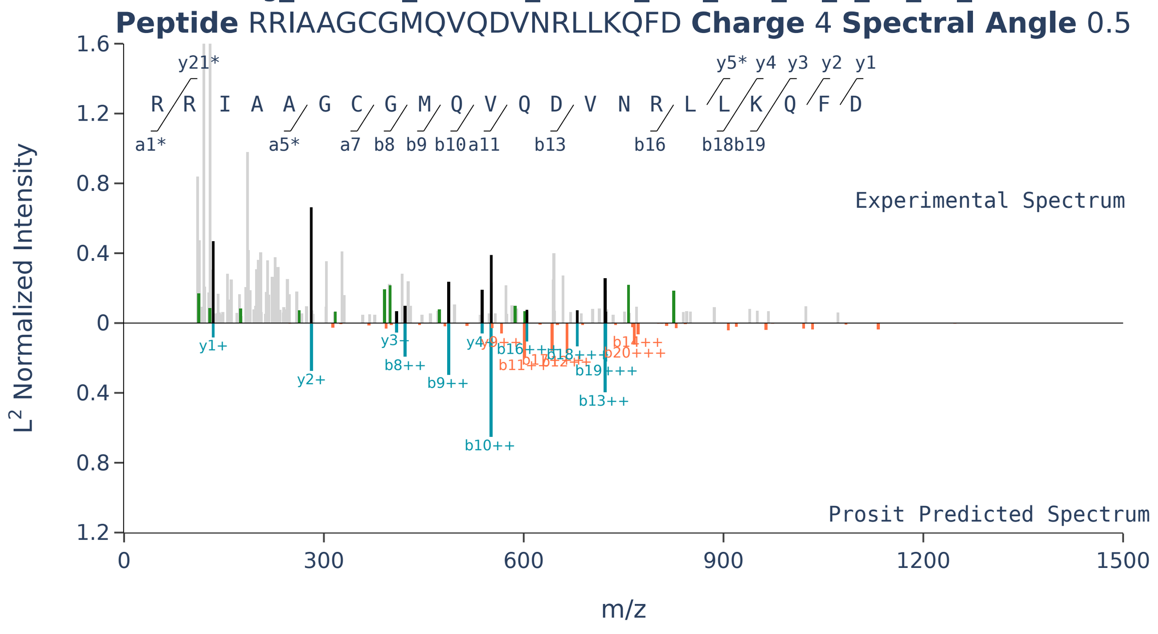


**Source** Ncheng_210623_230623_HFGoe_FFH_20S_25_1_A1_4h_R2 **Scan** 18306
**Peptide** FINRVKEKAVGHEVNKSLTPGQEFVKI **Charge** 5 **Spectral Angle** 0.61

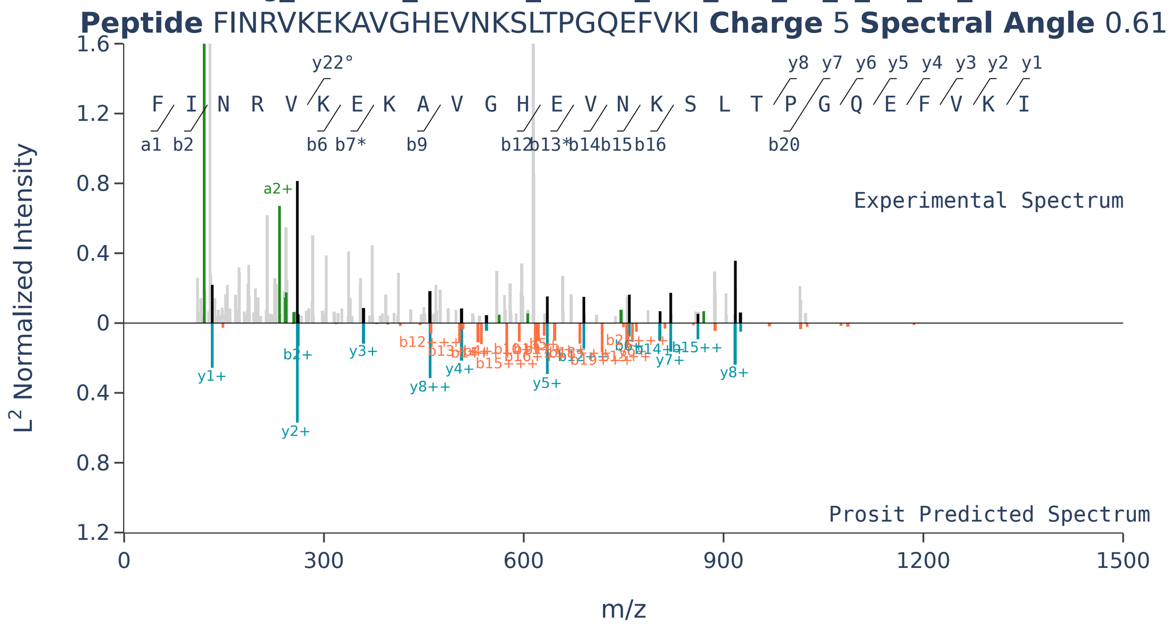


**Source** Ncheng_210623_230623_HFGoe_FFH_20S_25_1_A2_2h_R1 **Scan** 37696
**Peptide** QMDDKVLVRMEAIINSMTMKER **Charge** 4 **Spectral Angle** 0.73

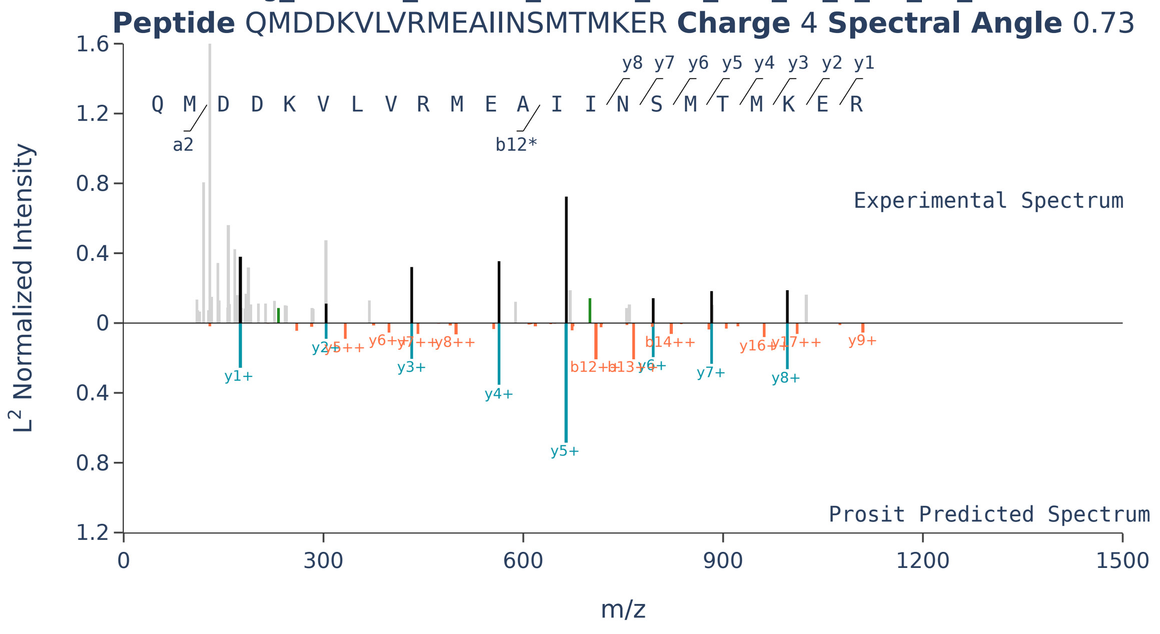


**Source** Ncheng_210623_230623_HFGoe_FFH_20S_25_1_A2_4h_R2 **Scan** 14233
**Peptide** TKVDGDARGGAALSIRHLTG **Charge** 3 **Spectral Angle** 0.52

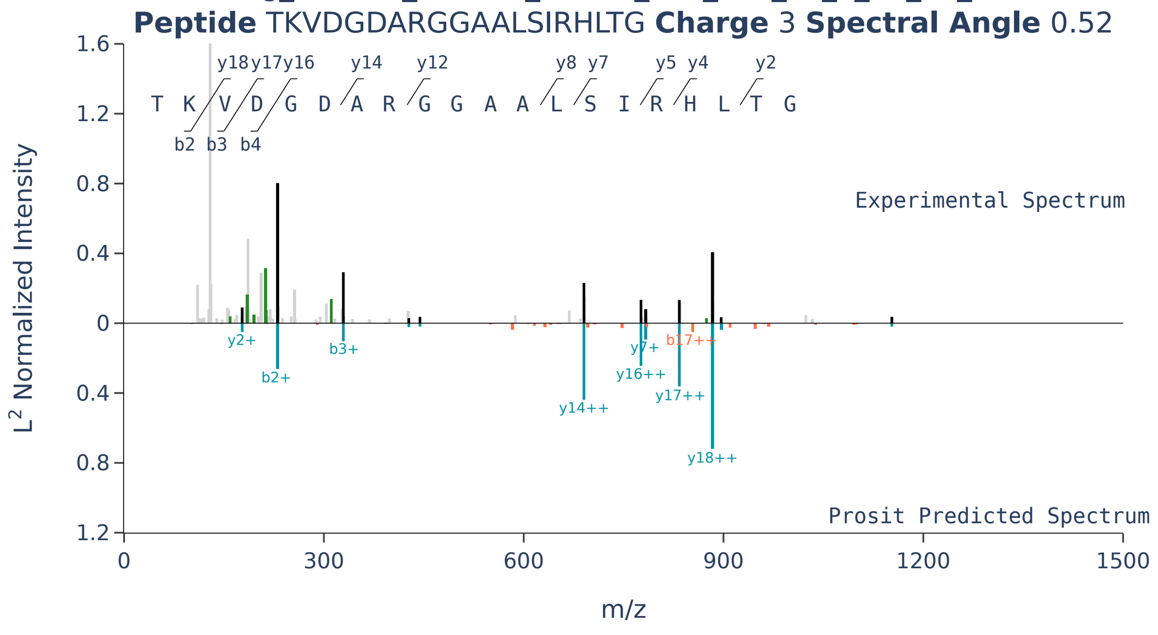


**Source** Ncheng_210623_230623_HFGoe_FFH_20S_25_1_A2_4h_R2 **Scan** 28524
**Peptide** EPFHPDRIASRILGM **Charge** 3 **Spectral Angle** 0.82

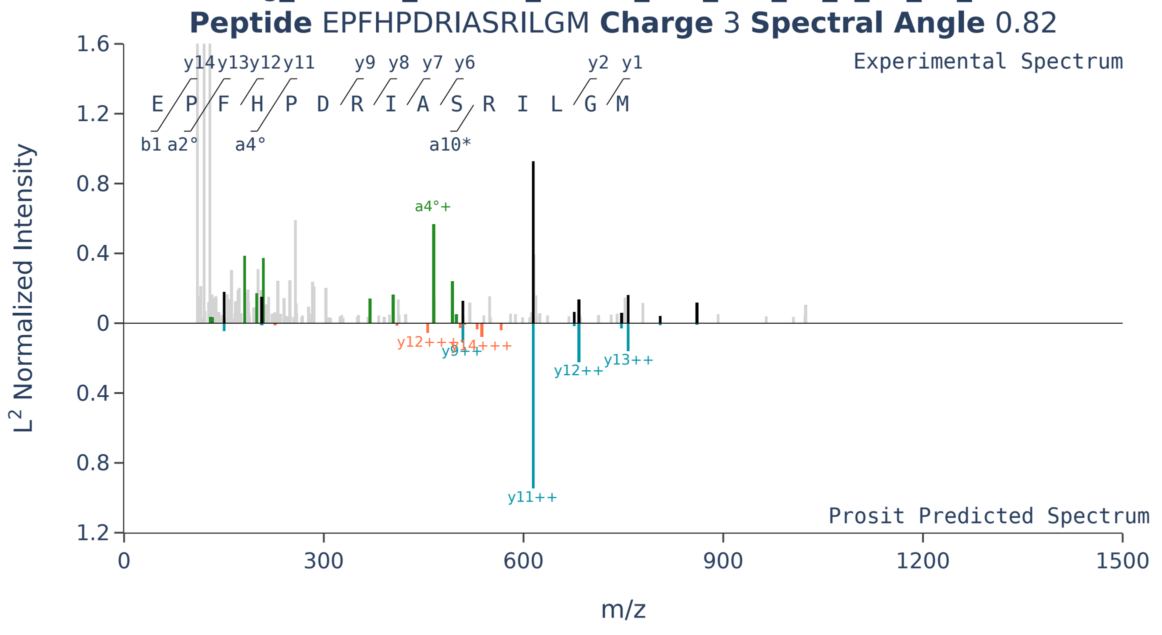


**Source** Ncheng_210623_230623_HFGoe_FFH_20S_25_1_A1_24h_R1 **Scan** 6806
**Peptide** SRTLRNISGRG **Charge** 3 **Spectral Angle** 0.6

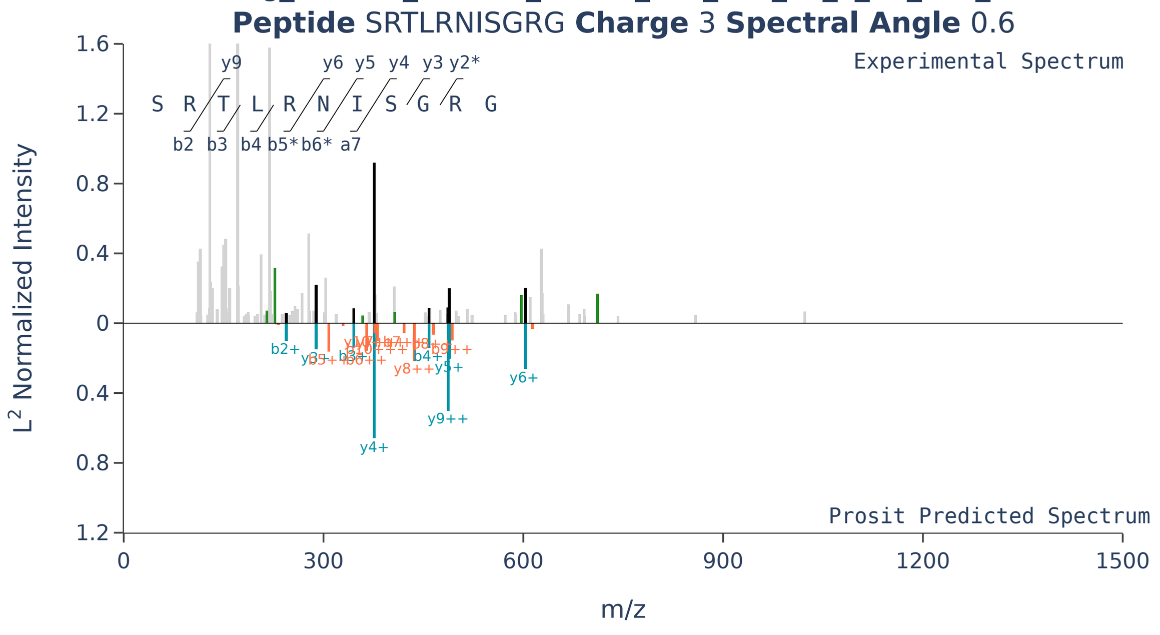


**Source** Ncheng_210623_230623_HFGoe_FFH_20S_25_1_A1_2h_R1 **Scan** 30467
**Peptide** KEAKLKFYDVLLVDTAGRL **Charge** 4 **Spectral Angle** 0.4

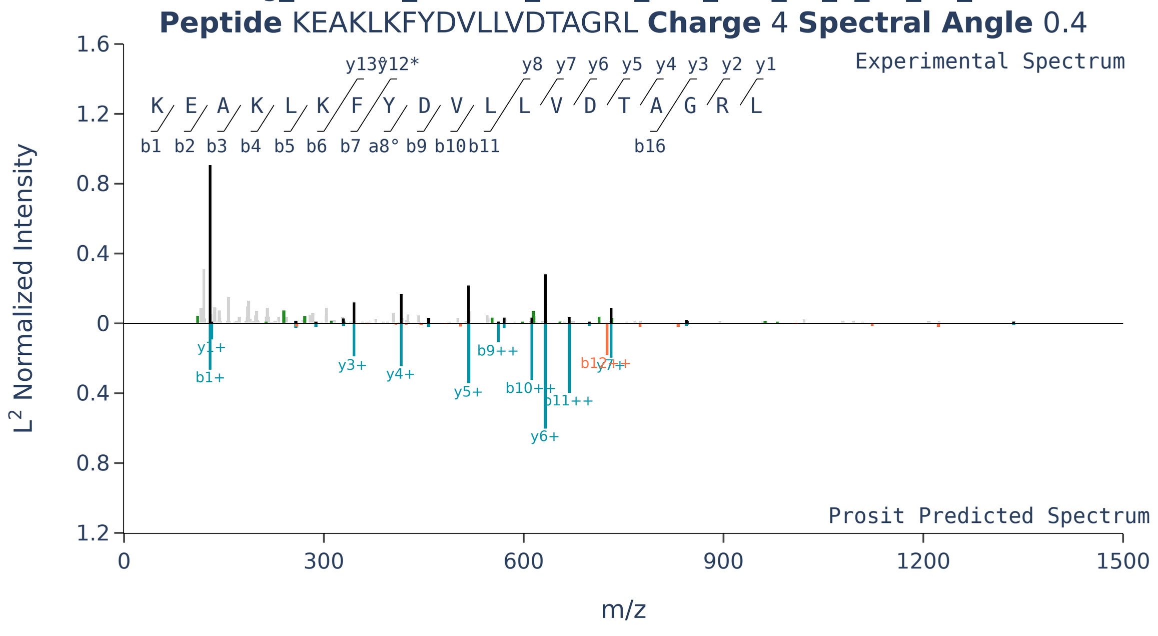


**Source** Ncheng_210623_230623_HFGoe_FFH_20S_25_1_A2_24h_R1 **Scan** 9166
**Peptide** LEQLRQMKN **Charge** 2 **Spectral Angle** 0.52

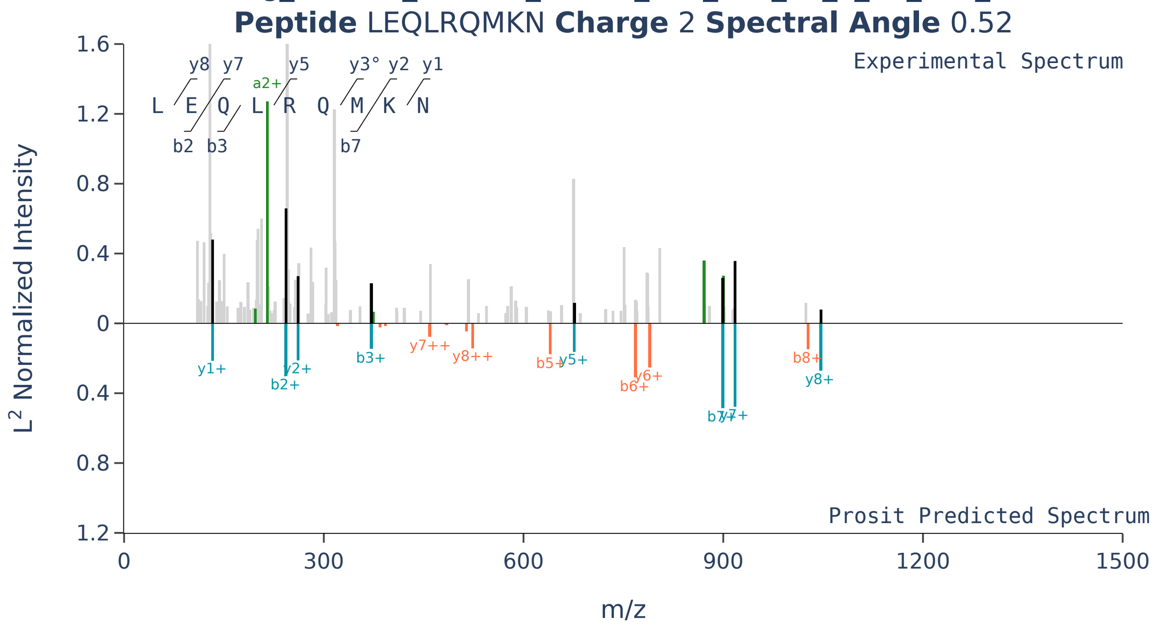


**Source** Ncheng_210623_230623_HFGoe_FFH_20S_25_1_A1_1h_R2 **Scan** 21834
**Peptide** SIRHITGKPIKFLGVGEKTEALEPFHPDRI **Charge** 5 **Spectral Angle** 0.68

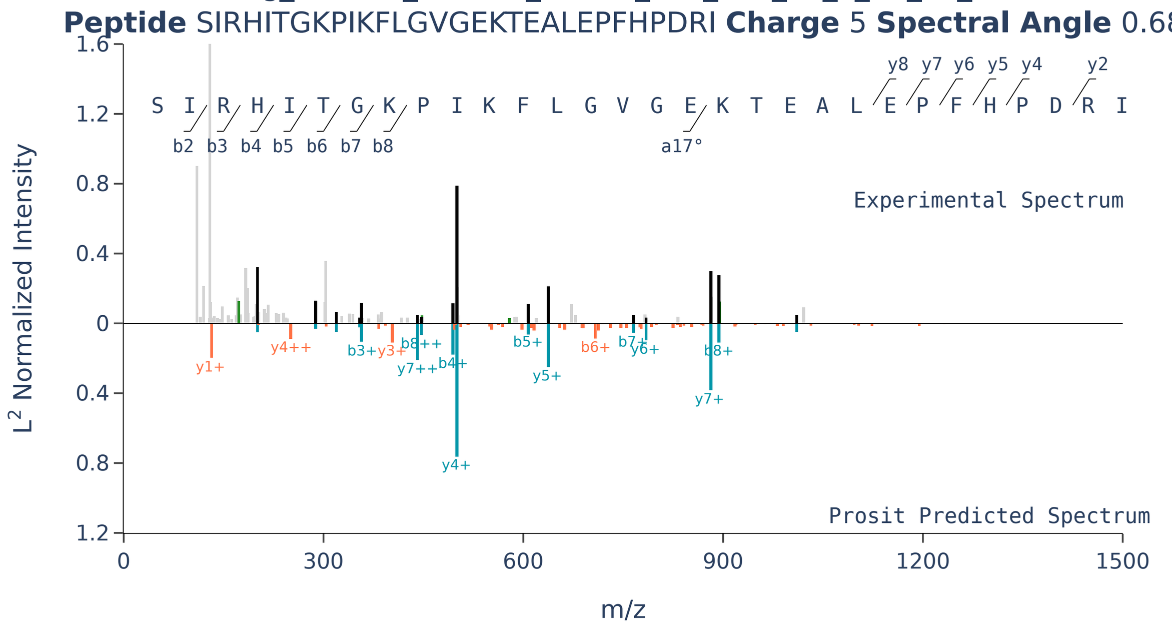


**Source** Ncheng_210623_230623_HFGoe_FFH_20S_25_1_A2_24h_R2 **Scan** 17817
**Peptide** VKEKAVGHEVNKSLTPGQEFVKI **Charge** 5 **Spectral Angle** 0.6

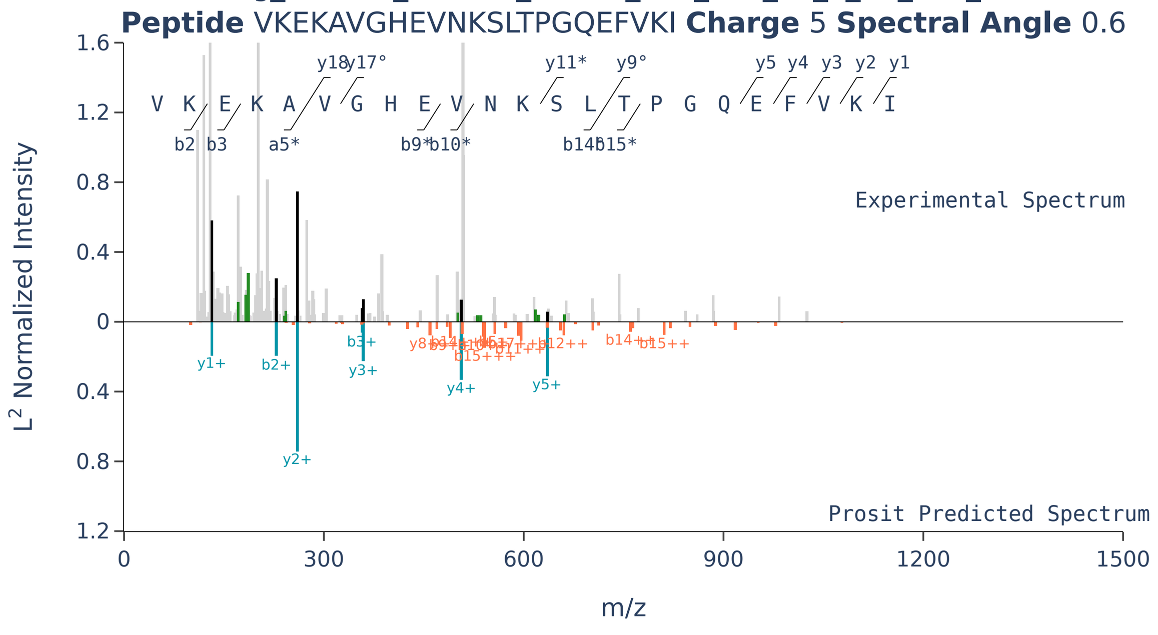


**Source** Ncheng_210623_230623_HFGoe_FFH_20S_25_1_A2_2h_R1 **Scan** 37837
**Peptide** TLAEQVGVDFFPSDVGQKPVDIVNAALKEA **Charge** 3 **Spectral Angle** 0.69

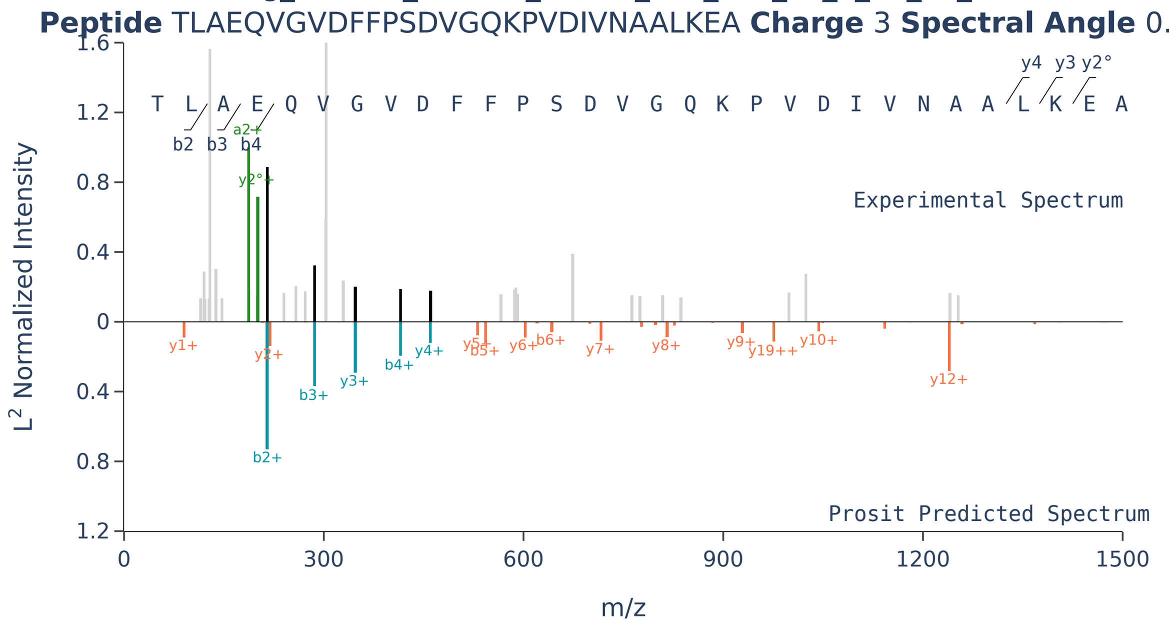


**Source** Ncheng_210623_230623_HFGoe_FFH_20S_25_1_A2_24h_R2 **Scan** 5128
**Peptide** LREKHKKKVL **Charge** 3 **Spectral Angle** 0.72

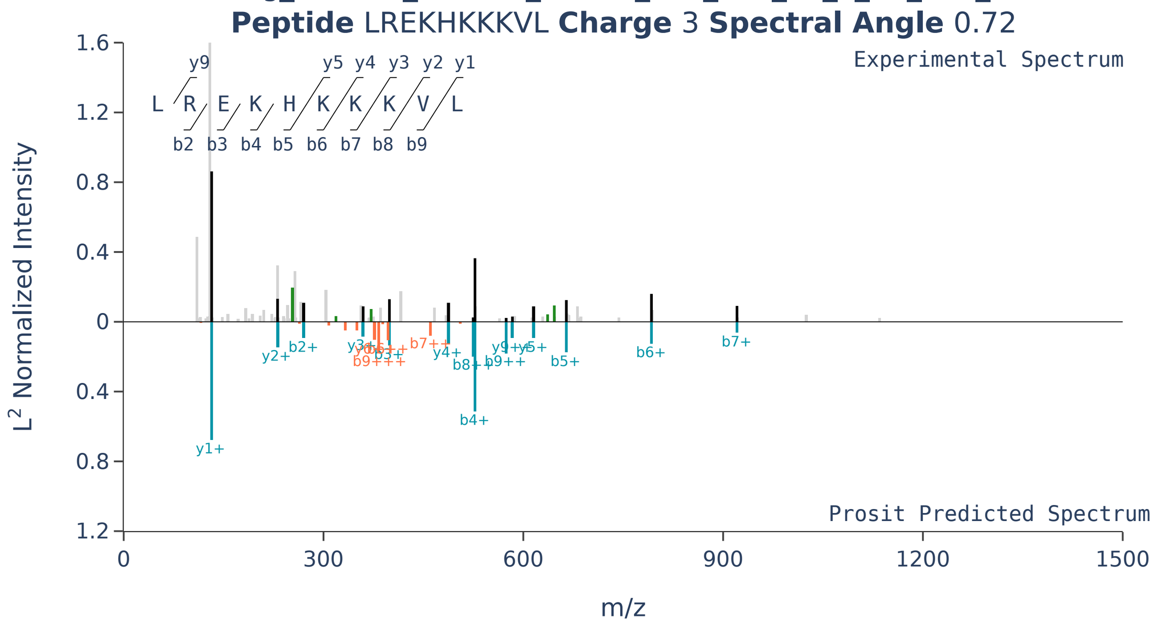


**Source** Ncheng_210623_230623_HFGoe_FFH_20S_25_1_A1_4h_R2 **Scan** 8641
**Peptide** SRTLRNISGRGRLTED **Charge** 4 **Spectral Angle** 0.59

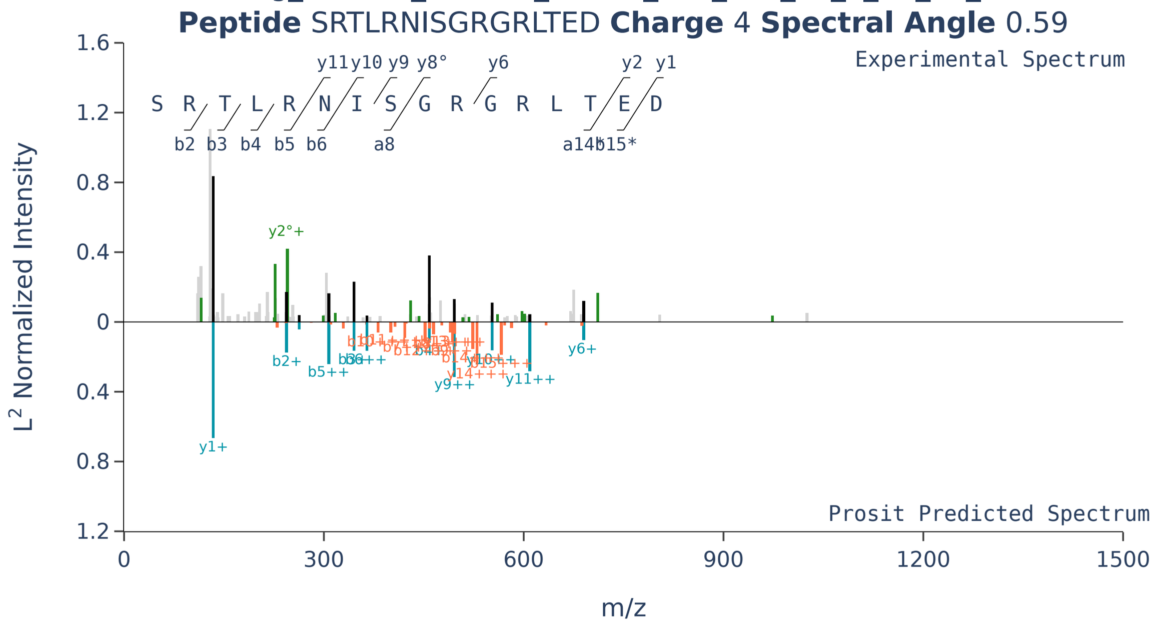


**Source** Ncheng_210623_230623_HFGoe_FFH_20S_25_1_A2_2h_R1 **Scan** 15869
**Peptide** EDIESKVDRAQAEKLASKLKKG **Charge** 5 **Spectral Angle** 0.65

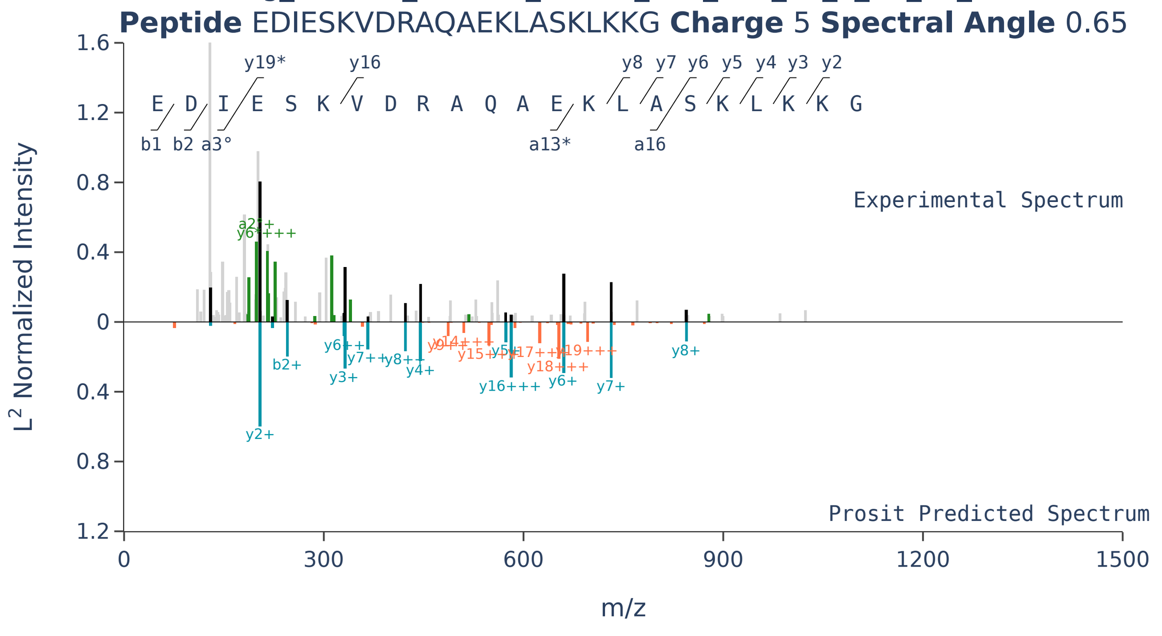


**Source** Ncheng_210623_230623_HFGoe_FFH_20S_25_1_A1_2h_R1 **Scan** 23046
**Peptide** KPIKFLGVGEKTEALEPFHPDRIAS **Charge** 3 **Spectral Angle** 0.66

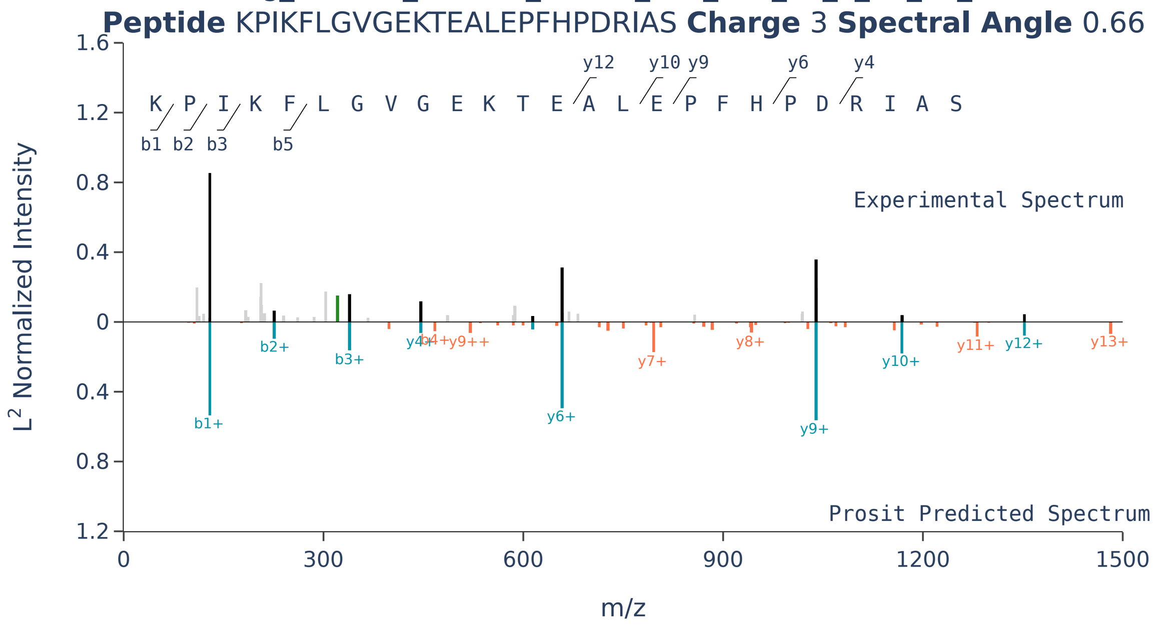

**Source** Ncheng_210623_230623_HFGoe_FFH_20S_25_1_A1_4h_R1 **Scan** 13525
**Peptide** TLRNISGRGRLTEDNVKDTLRE **Charge** 5 **Spectral Angle** 0.61

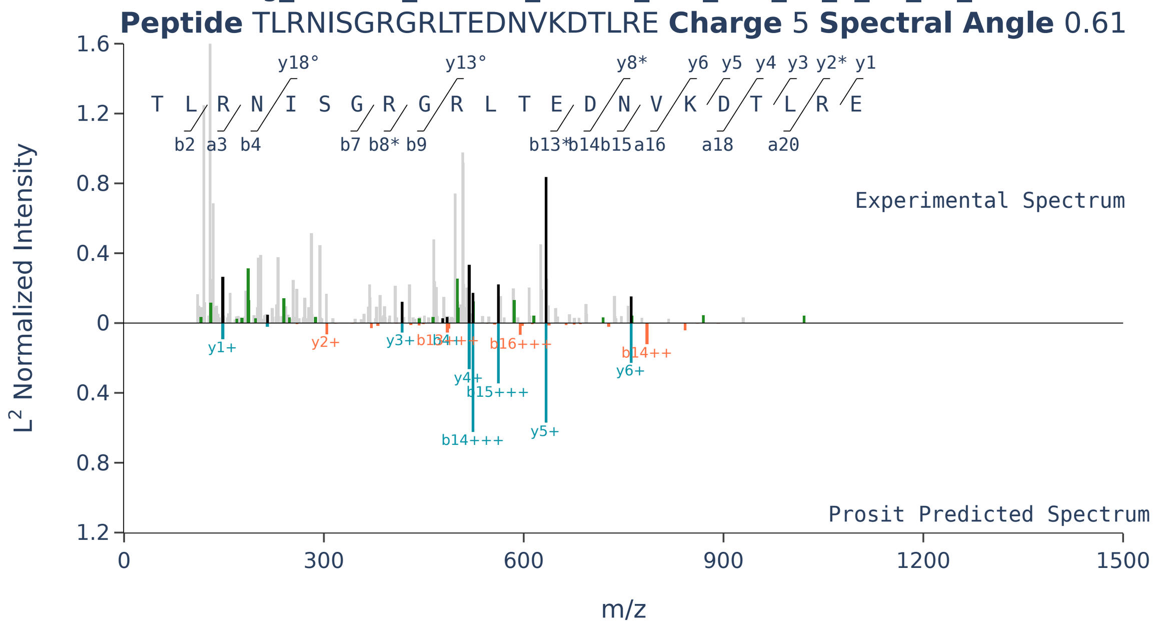


**Source** Ncheng_210623_230623_HFGoe_FFH_20S_25_1_A1_24h_R1 **Scan** 13333
**Peptide** FDDMQRMMKKMKKG **Charge** 3 **Spectral Angle** 0.48

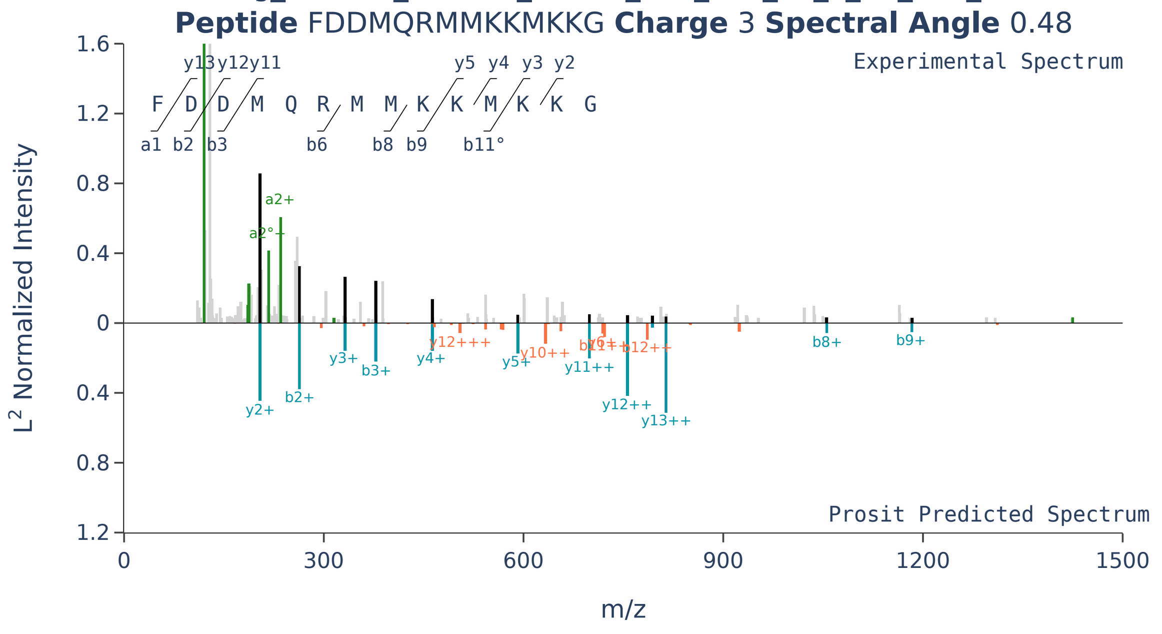


**Source** Ncheng_210623_230623_HFGoe_FFH_20S_25_1_A2_4h_R1 **Scan** 9678
**Peptide** DMQRMMKKMKKGGMAKMM **Charge** 5 **Spectral Angle** 0.84

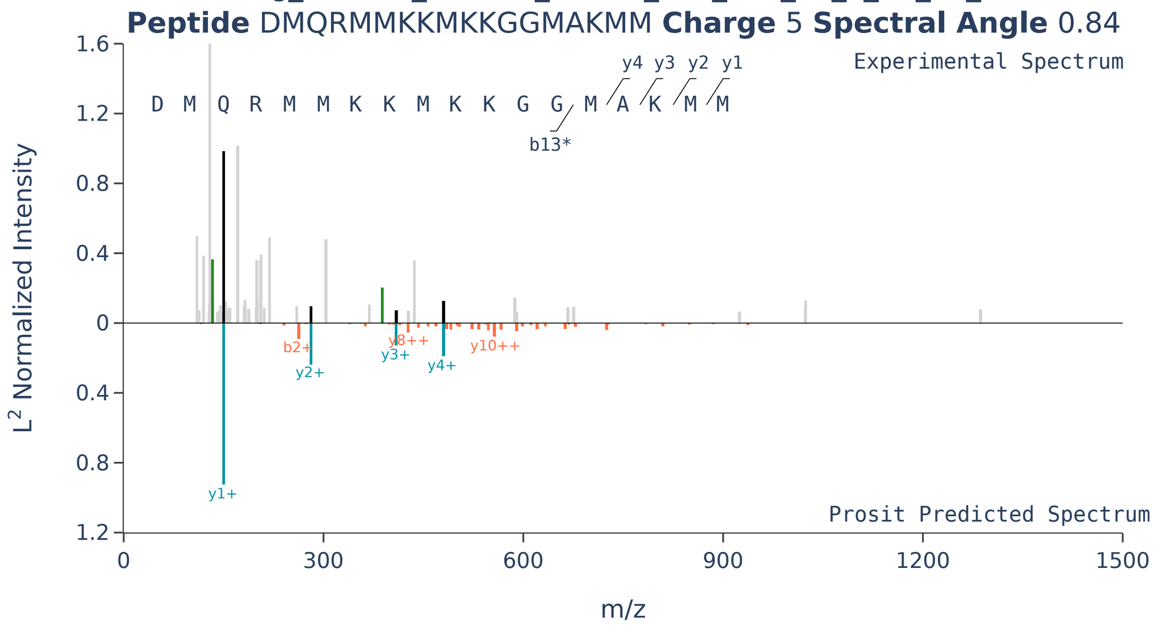


**Source** Ncheng_210623_230623_HFGoe_FFH_20S_25_1_A2_24h_R1 **Scan** 15365
**Peptide** VVLTKVDGDARGGAALS **Charge** 3 **Spectral Angle** 0.45

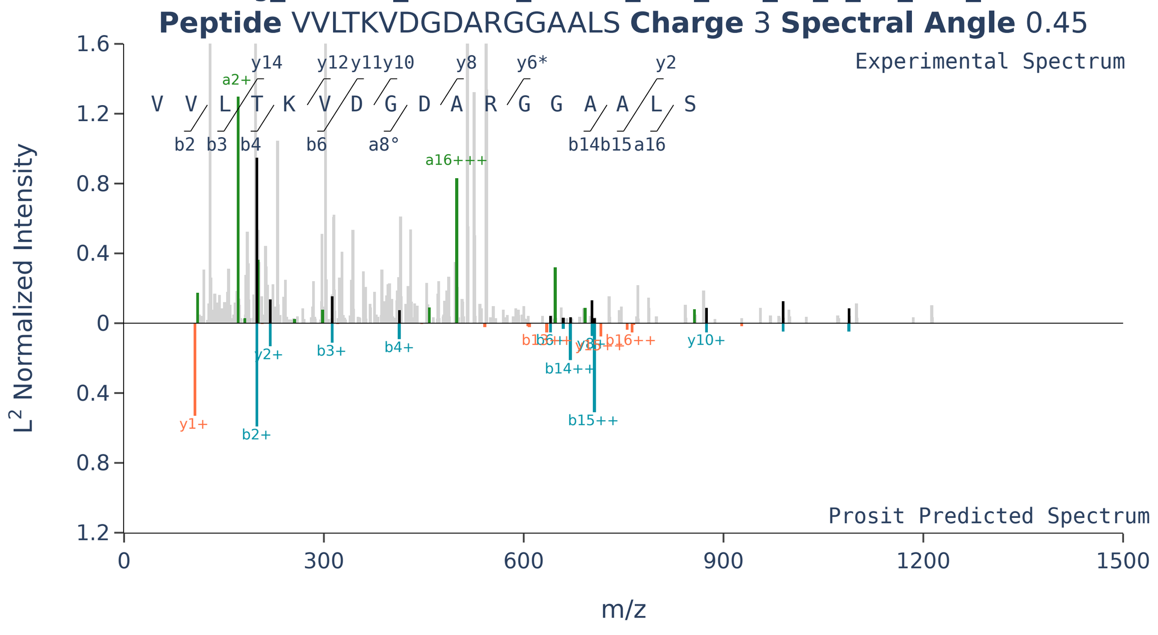


**Source** Ncheng_210623_230623_HFGoe_FFH_20S_25_1_A2_24h_R1 **Scan** 16522
**Peptide** DGDARGGAALSIRHLTG **Charge** 3 **Spectral Angle** 0.58

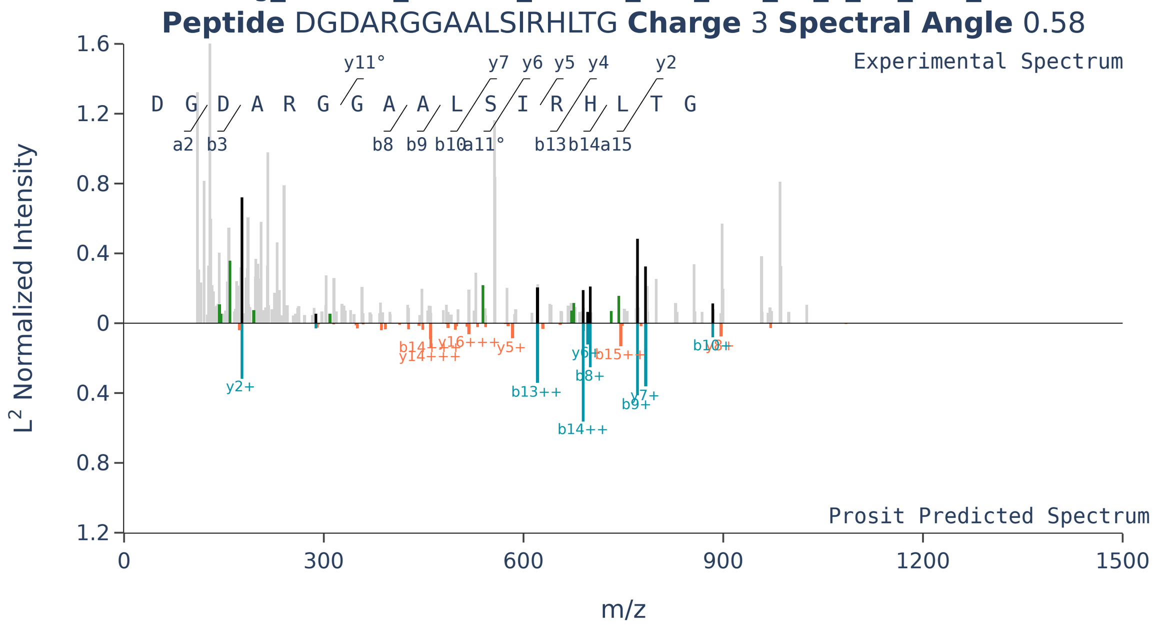


**Source** Ncheng_210623_230623_HFGoe_FFH_20S_25_1_A1_24h_R2 **Scan** 12584
**Peptide** DDMQRMMKKMKKGGMA **Charge** 4 **Spectral Angle** 0.39

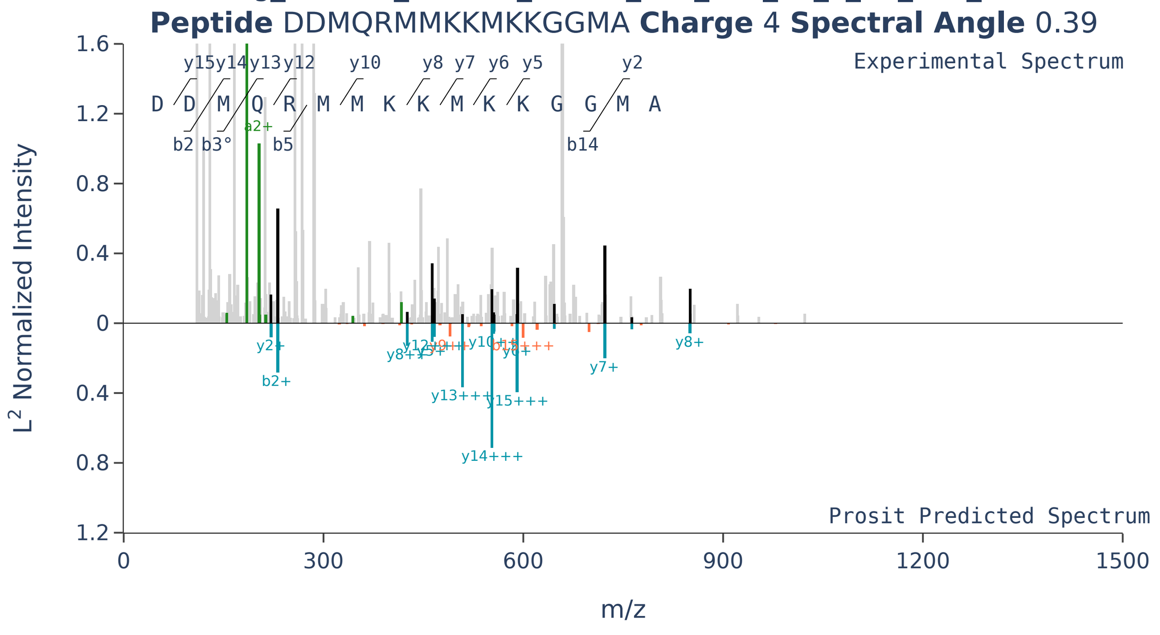


**Source** Ncheng_210623_230623_HFGoe_FFH_20S_25_1_A2_24h_R2 **Scan** 8535
**Peptide** IESKVDRAQAEKLASK **Charge** 2 **Spectral Angle** 0.64

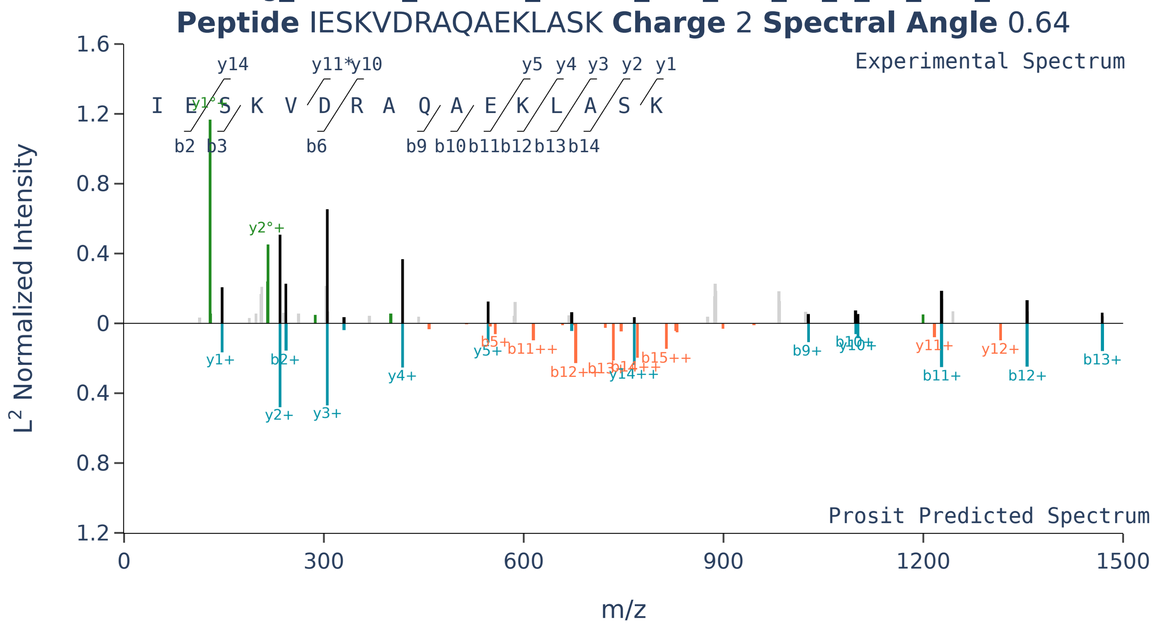


**Source** Ncheng_210623_230623_HFGoe_FFH_20S_25_1_A2_2h_R1 **Scan** 25229
**Peptide** DVLLVDTAGRLHVDEAMM **Charge** 3 **Spectral Angle** 0.79

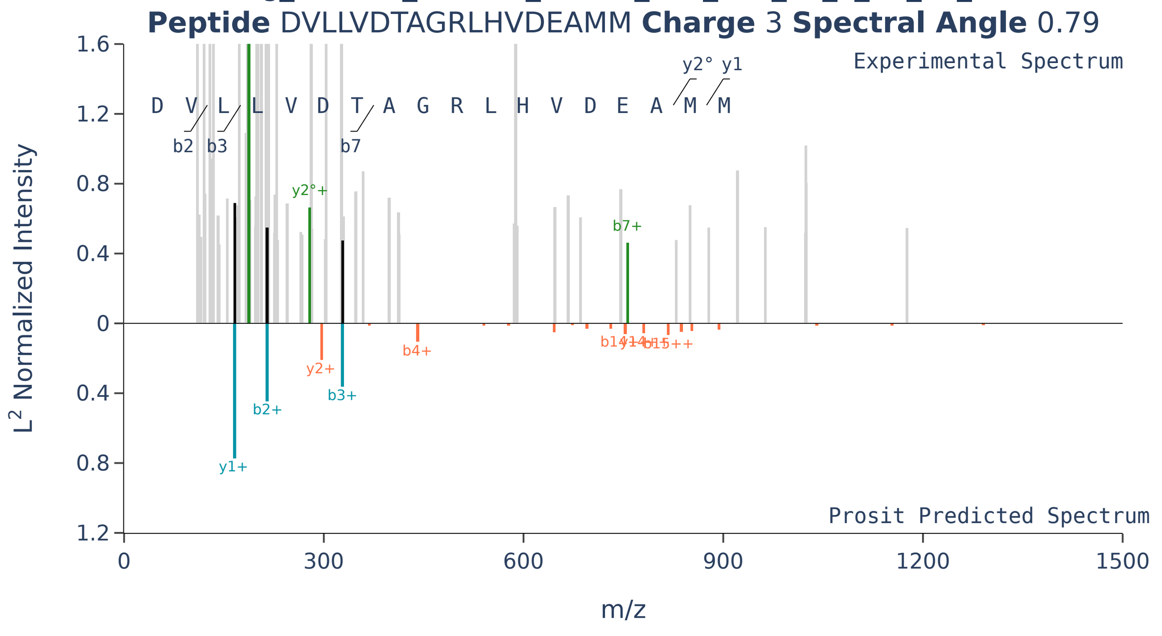


**Source** Ncheng_210623_230623_HFGoe_FFH_20S_25_1_A2_4h_R2 **Scan** 24530
**Peptide** VVLTKVDGDARGGAALSIRHITGKPIKFL **Charge** 3 **Spectral Angle** 0.63

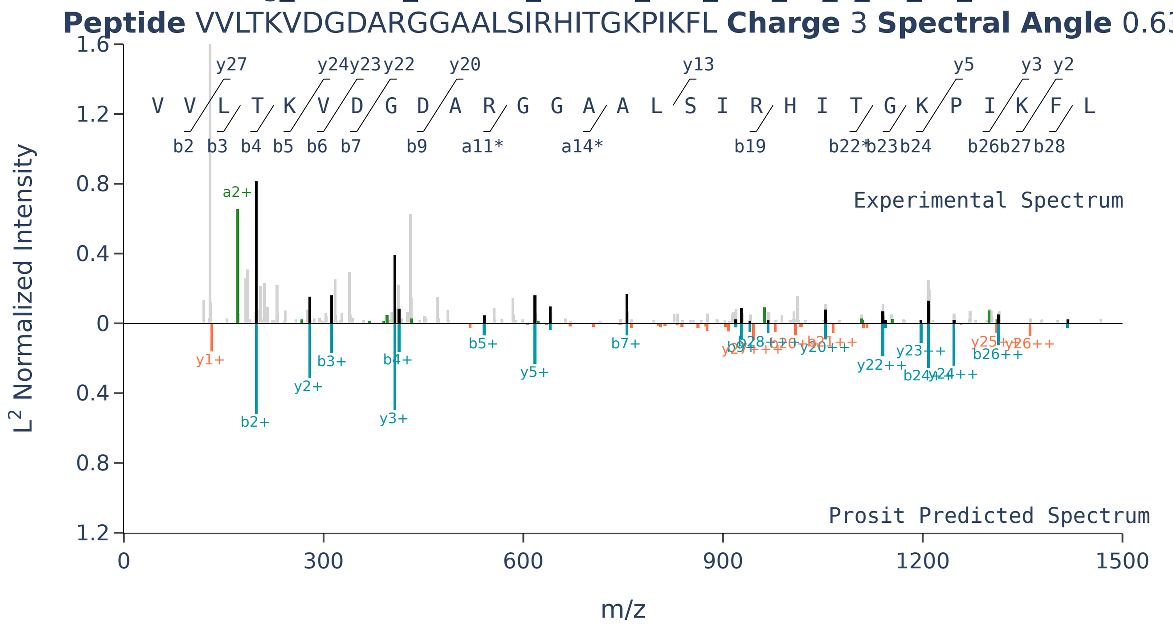


**Source** Ncheng_210623_230623_HFGoe_FFH_20S_25_1_A1_2h_R2 **Scan** 38849
**Peptide** VDFFPSDVGQKPVDIVNAALKEAKL **Charge** 3 **Spectral Angle** 0.76

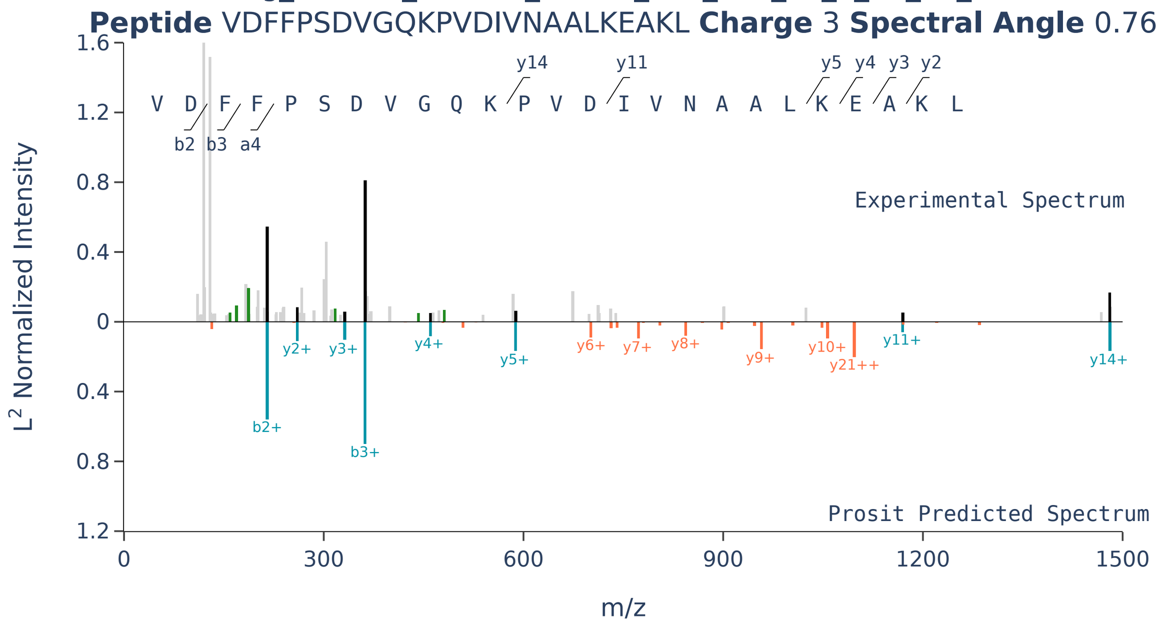


**Source** Ncheng_210623_230623_HFGoe_FFH_20S_25_1_A2_2h_R2 **Scan** 37569
**Peptide** AVGHEVNKSLTPGQEFVKIVRNELVAAMGE **Charge** 3 **Spectral Angle** 0.68

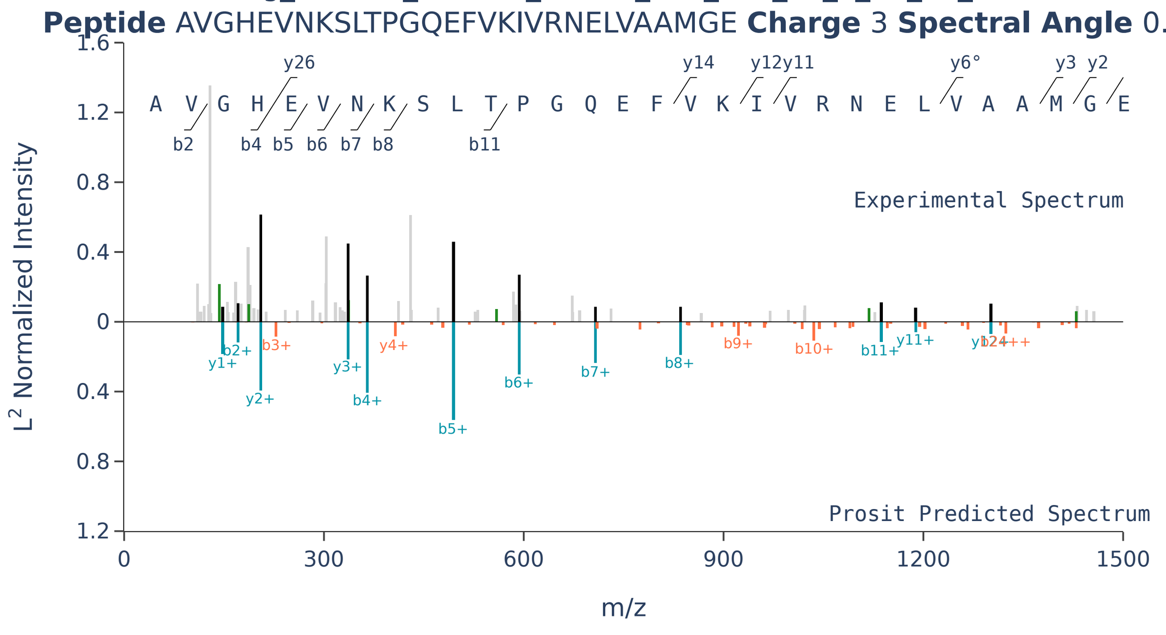


**Source** Ncheng_210623_230623_HFGoe_FFH_20S_25_1_A1_2h_R1 **Scan** 13228
**Peptide** DDMQRMMKKMKKGGMAKM **Charge** 5 **Spectral Angle** 0.86

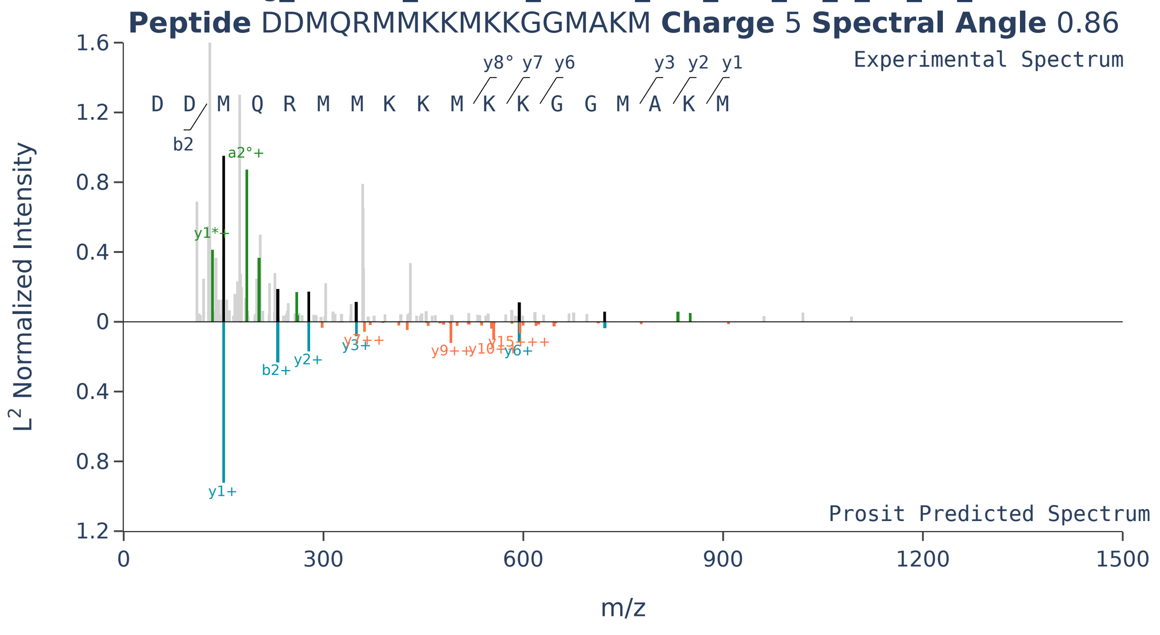


**Source** Ncheng_210623_230623_HFGoe_FFH_20S_25_1_A1_24h_R2 **Scan** 4787
**Peptide** MKKMKKGGMAK **Charge** 2 **Spectral Angle** 0.65

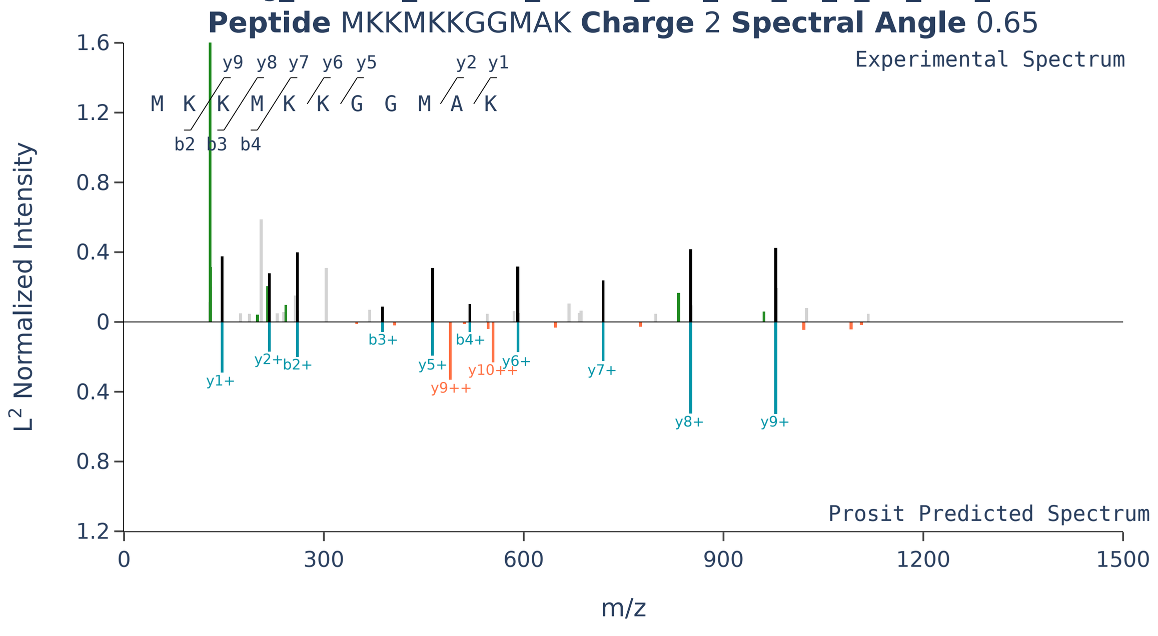


**Source** Ncheng_210623_230623_HFGoe_FFH_20S_25_1_A1_24h_R1 **Scan** 15280
**Peptide** GRLTEDNVKDTLREVR **Charge** 3 **Spectral Angle** 0.57

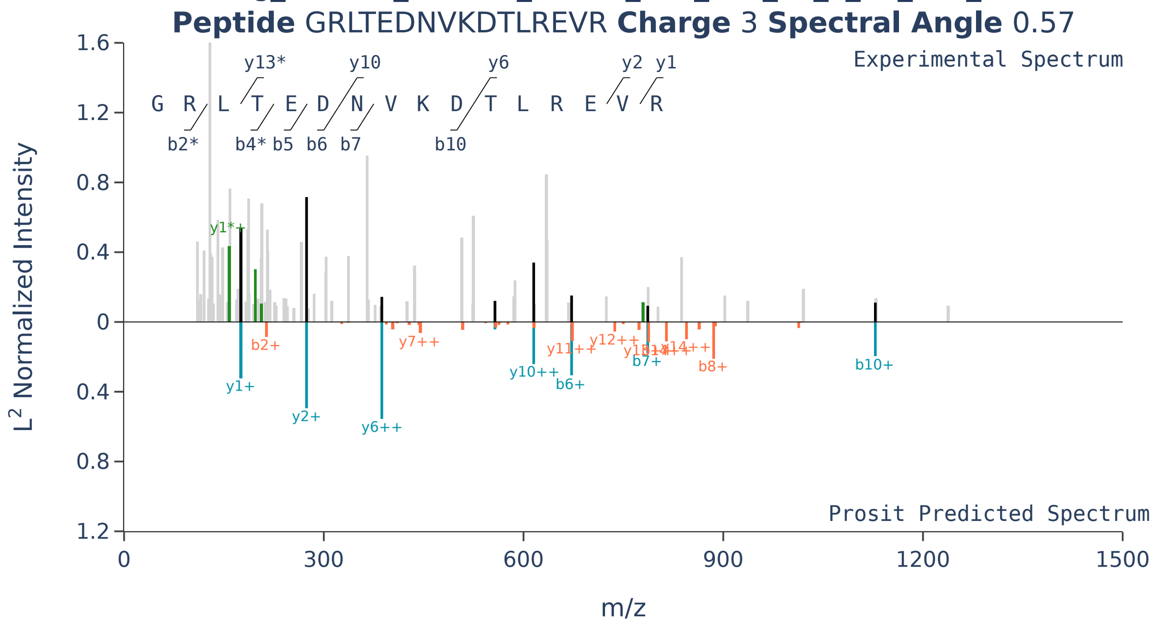


**Source** Ncheng_210623_230623_HFGoe_FFH_20S_25_1_A1_1h_R1 **Scan** 38608
**Peptide** VDFFPSDVGQKPVDIVNAALKEAKLKFYD **Charge** 4 **Spectral Angle** 0.75

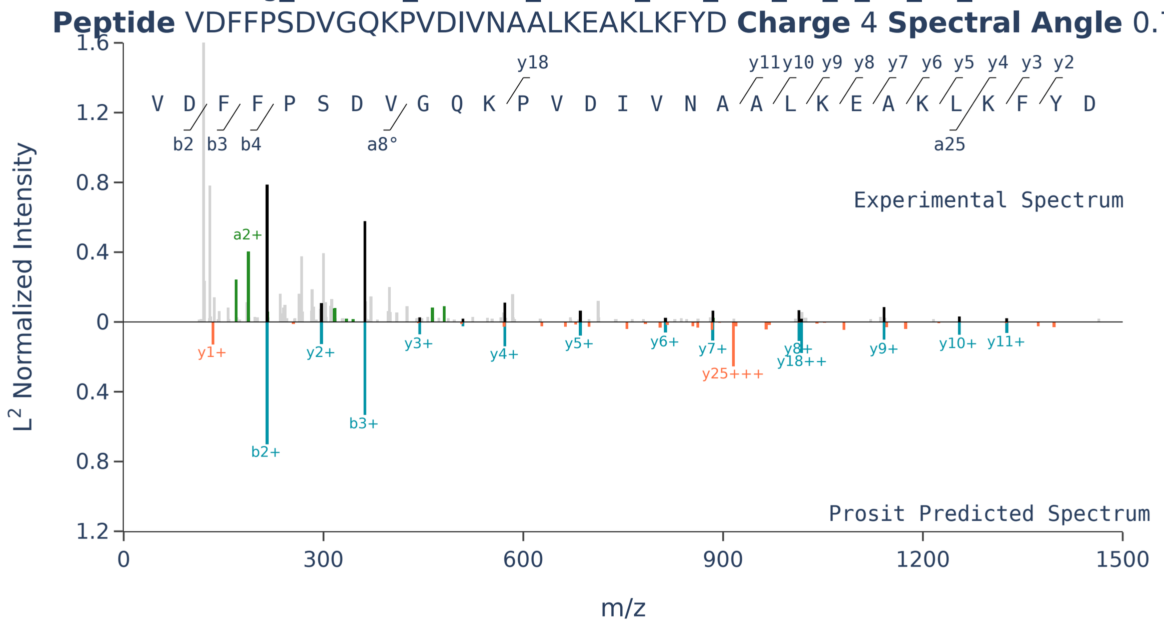

**Source** Ncheng_210623_230623_HFGoe_FFH_20S_25_1_A2_2h_R2 **Scan** 35722
**Peptide** QAEKLASKLKKGDGFDLNDFLEQLRQM **Charge** 4 **Spectral Angle** 0.82

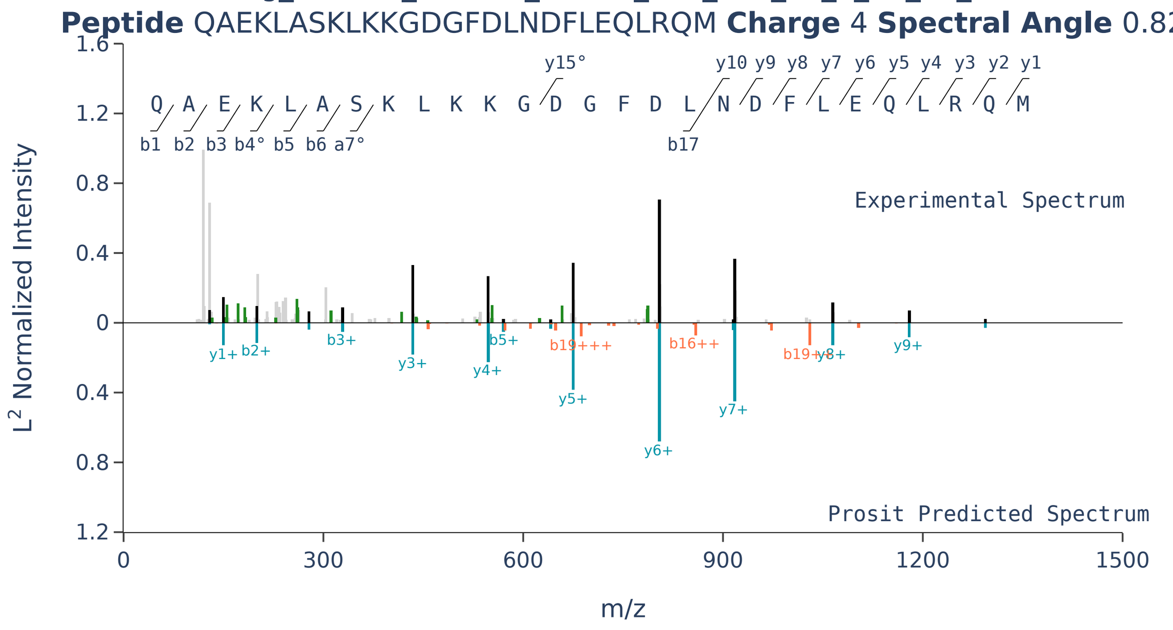


**Source** Ncheng_210623_230623_HFGoe_FFH_20S_25_1_A1_2h_R1 **Scan** 35190
**Peptide** AMTGQDAANTAKAFNEALPLTGVVLTKVDG **Charge** 3 **Spectral Angle** 0.42

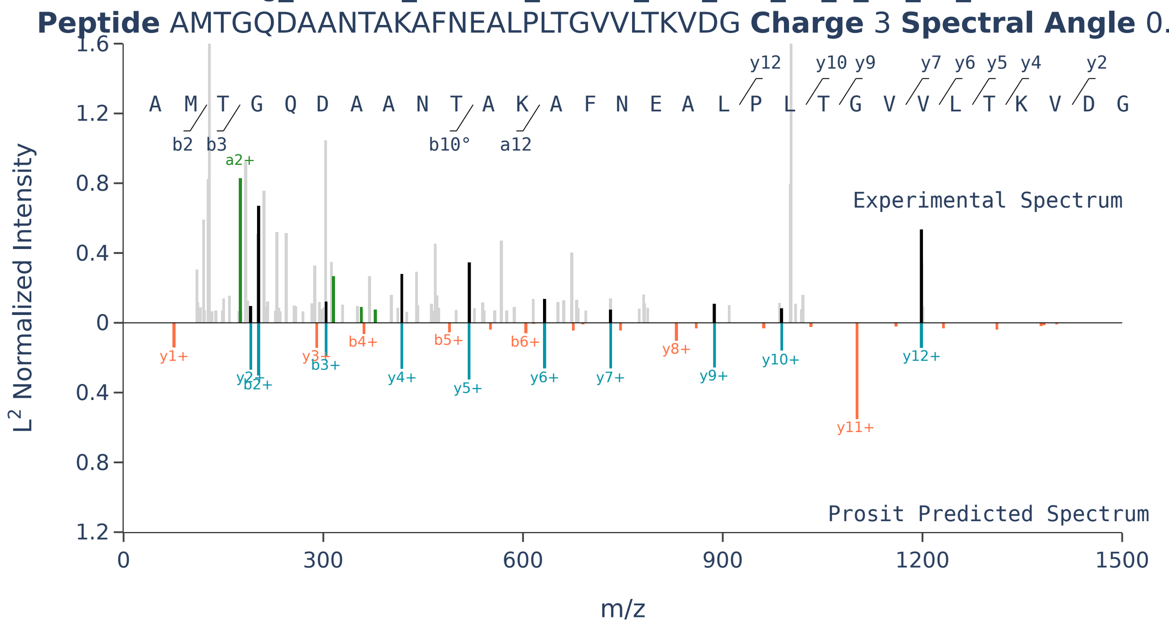


**Source** Ncheng_210623_230623_HFGoe_FFH_20S_25_1_A2_4h_R2 **Scan** 23756
**Peptide** FDNLTDRLSRTL **Charge** 3 **Spectral Angle** 0.5

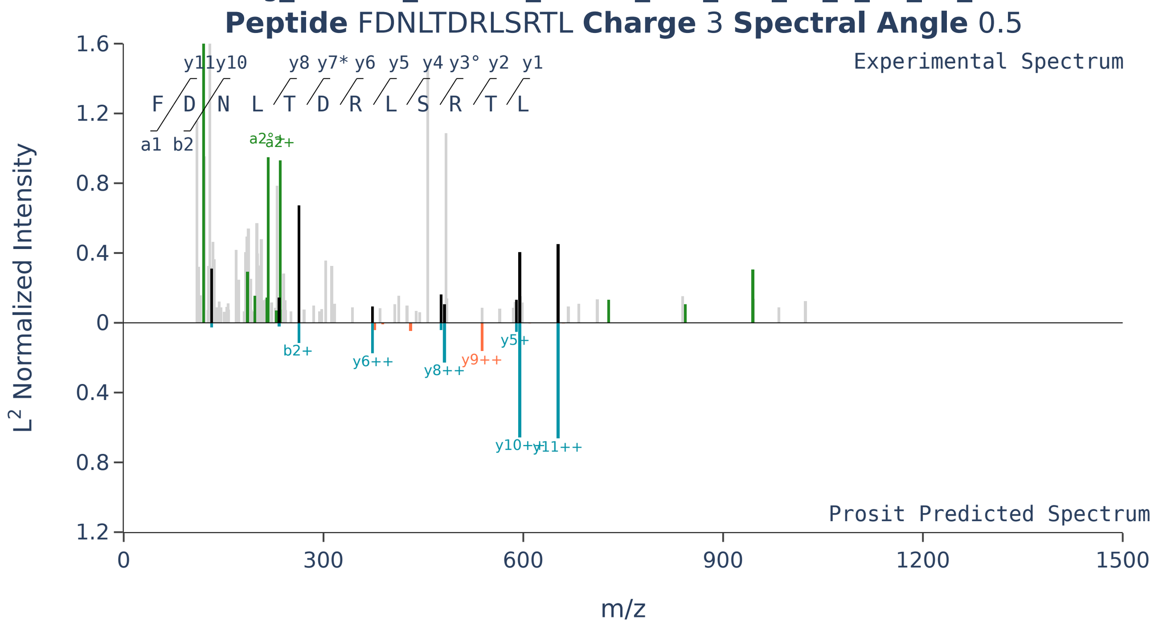


**Source** Ncheng_210623_230623_HFGoe_FFH_20S_25_1_A2_24h_R1 **Scan** 8394
**Peptide** INRVKEKAVGHEVNKSLTPGQ **Charge** 3 **Spectral Angle** 0.71

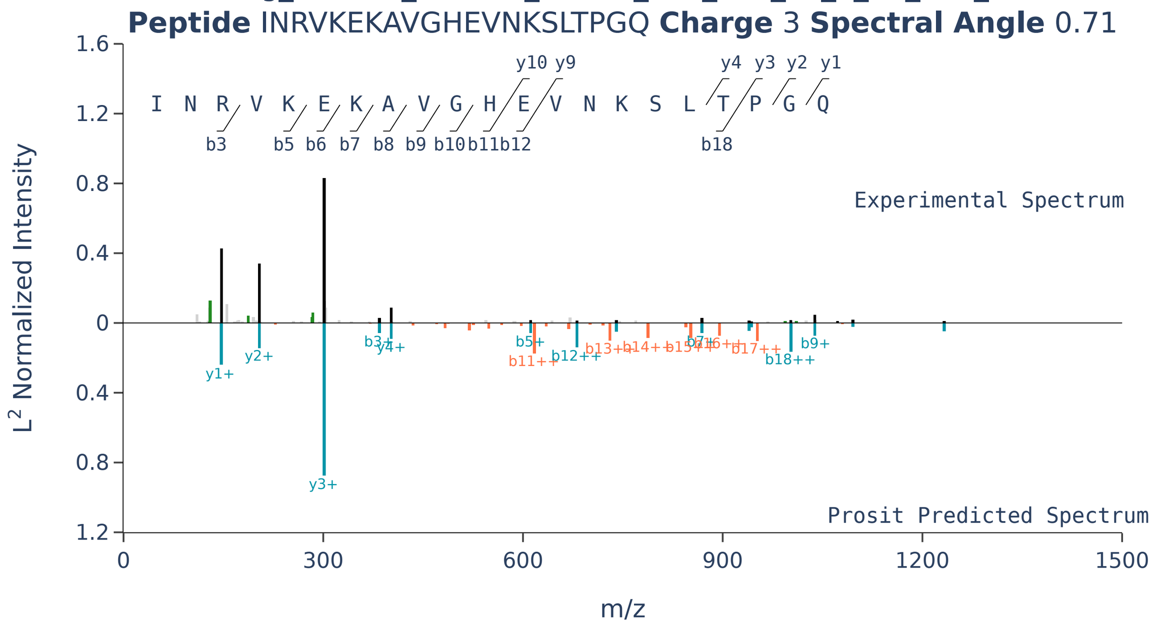


**Source** Ncheng_210623_230623_HFGoe_FFH_20S_25_1_A2_24h_R2 **Scan** 8264
**Peptide** NLSGRGRLTEDNVKDT **Charge** 3 **Spectral Angle** 0.44

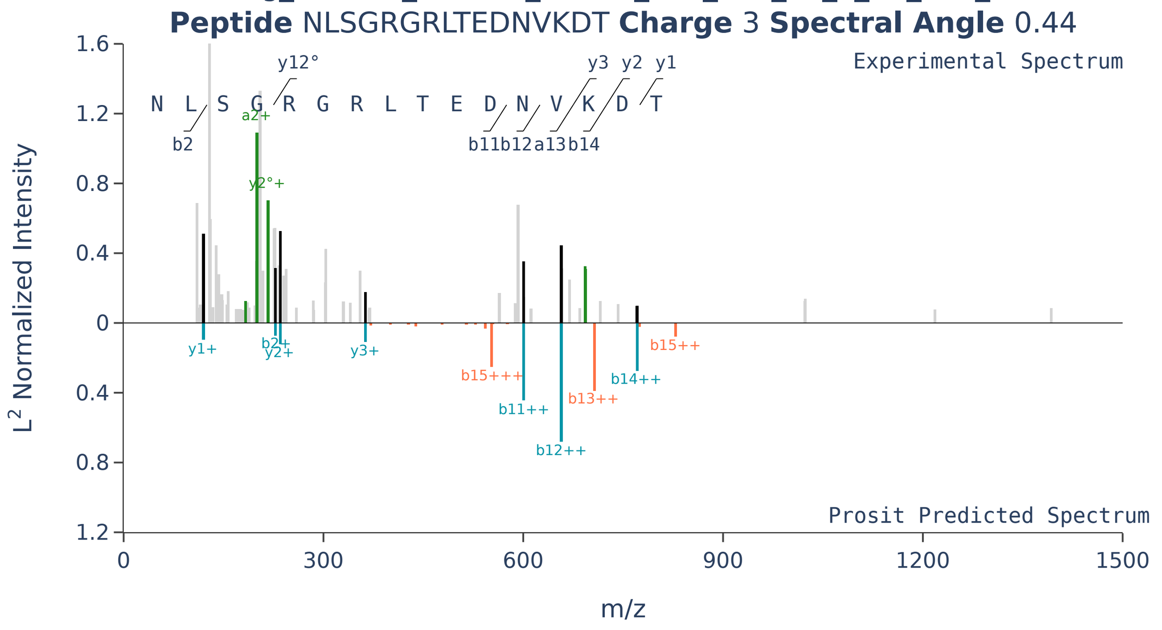


**Source** Ncheng_210623_230623_HFGoe_FFH_20S_25_1_A1_2h_R2 **Scan** 31098
**Peptide** MFDNLTDRLSRTLRNISG **Charge** 4 **Spectral Angle** 0.43

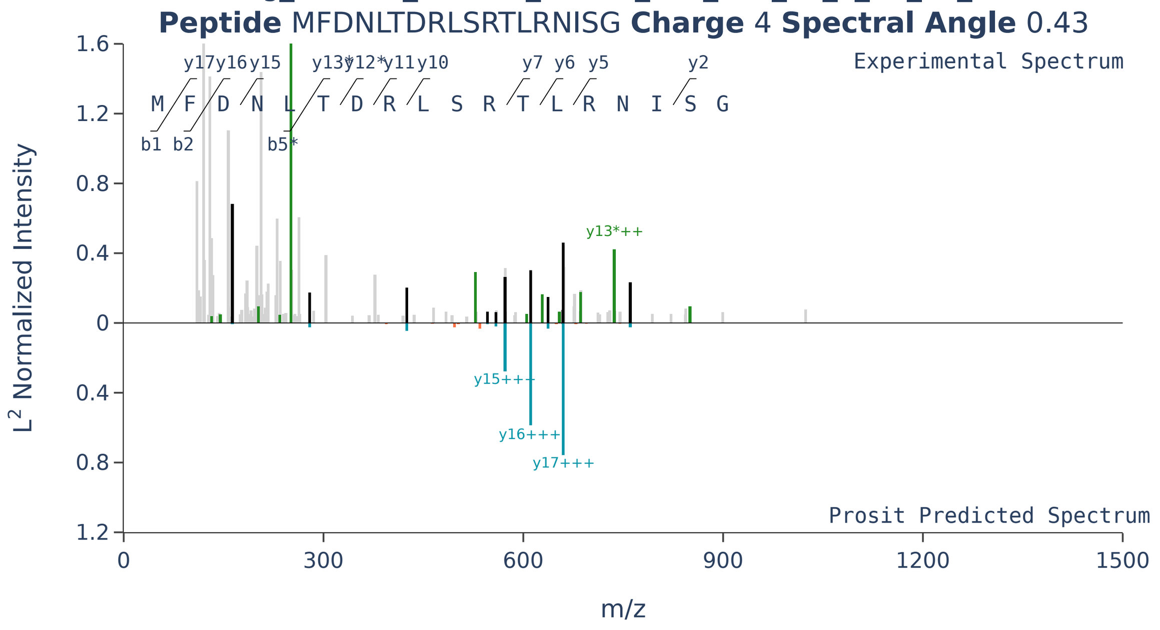


**Source** Ncheng_210623_230623_HFGoe_FFH_20S_25_1_A1_2h_R1 **Scan** 21351
**Peptide** VVLTKVDGDARGGAALSIRHITGKPIKF **Charge** 3 **Spectral Angle** 0.69

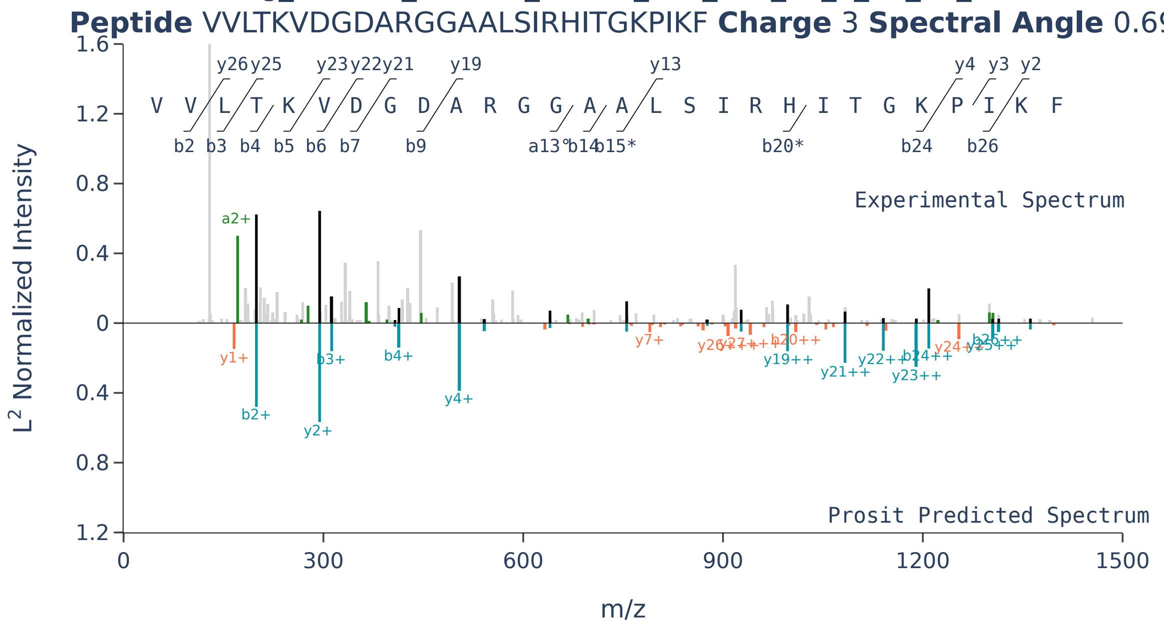


**Source** Ncheng_210623_230623_HFGoe_FFH_20S_25_1_A1_24h_R2 **Scan** 8380
**Peptide** INRVKEKAVGHEVNKSLTPGQE **Charge** 3 **Spectral Angle** 0.63

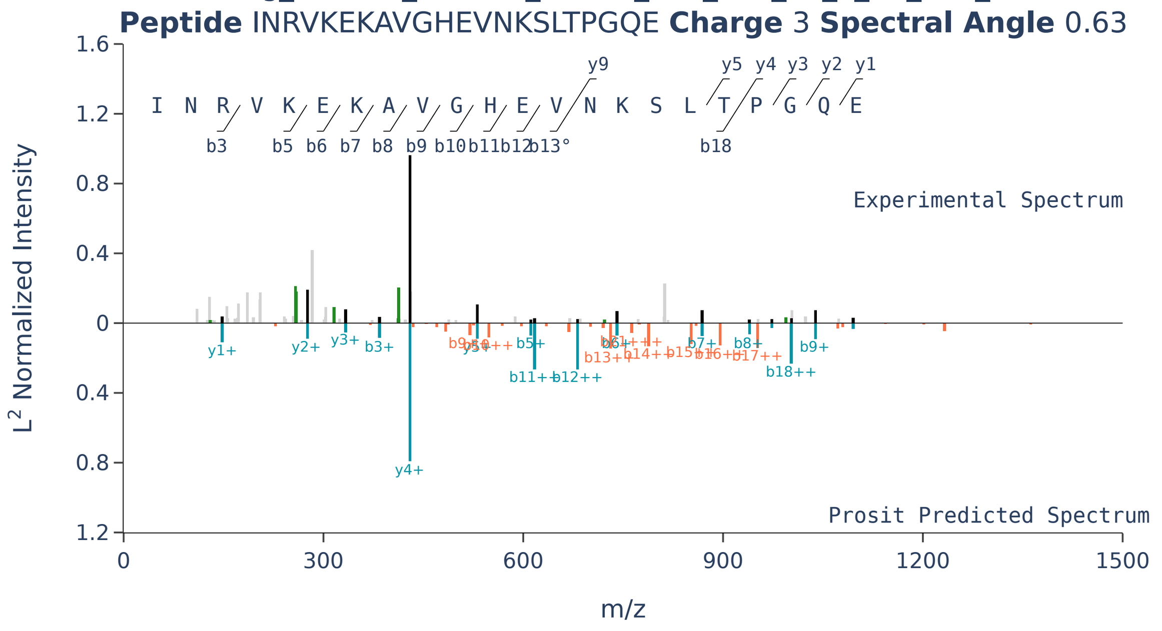


**Source** Ncheng_210623_230623_HFGoe_FFH_20S_25_1_A2_2h_R1 **Scan** 31894
**Peptide** FNEALPLTGVVLTKVDGDARGGAALSIRHI **Charge** 4 **Spectral Angle** 0.58

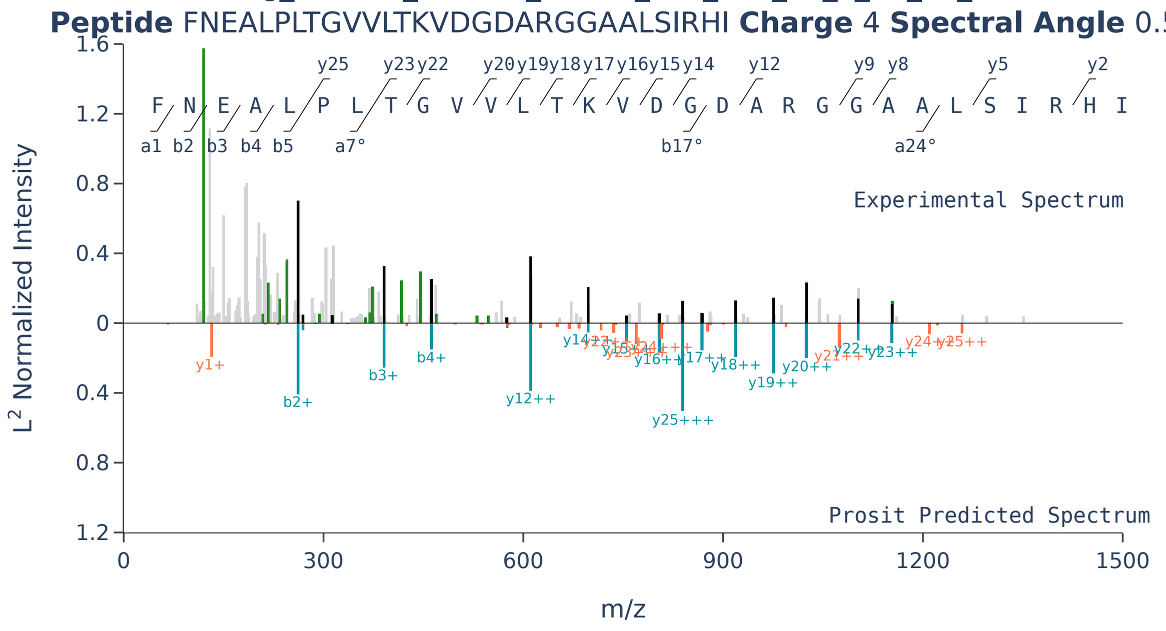


**Source** Ncheng_210623_230623_HFGoe_FFH_20S_25_1_A1_4h_R2 **Scan** 11410
**Peptide** DLESKVDRAQAEKLASK **Charge** 3 **Spectral Angle** 0.53

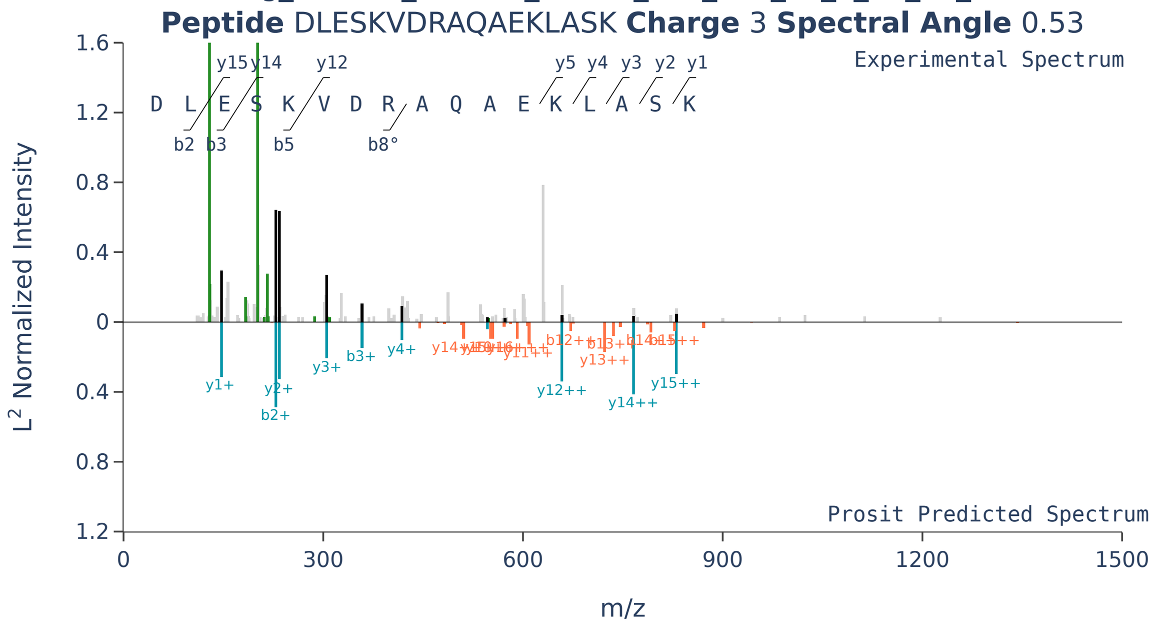


**Source** Ncheng_210623_230623_HFGoe_FFH_20S_25_1_A2_24h_R1 **Scan** 9186
**Peptide** SRTLRNISGRGRLTE **Charge** 4 **Spectral Angle** 0.51

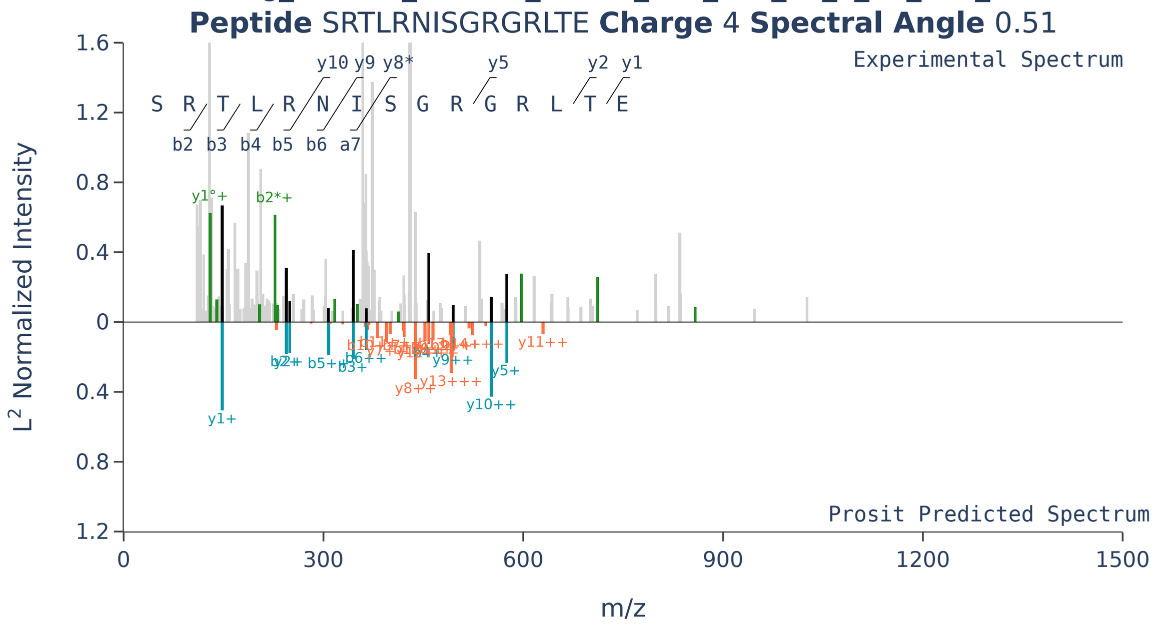


**Source** Ncheng_210623_230623_HFGoe_FFH_20S_25_1_A1_2h_R1 **Scan** 32615
**Peptide** VGQKPVDIVNAALKEAKLKF **Charge** 3 **Spectral Angle** 0.63

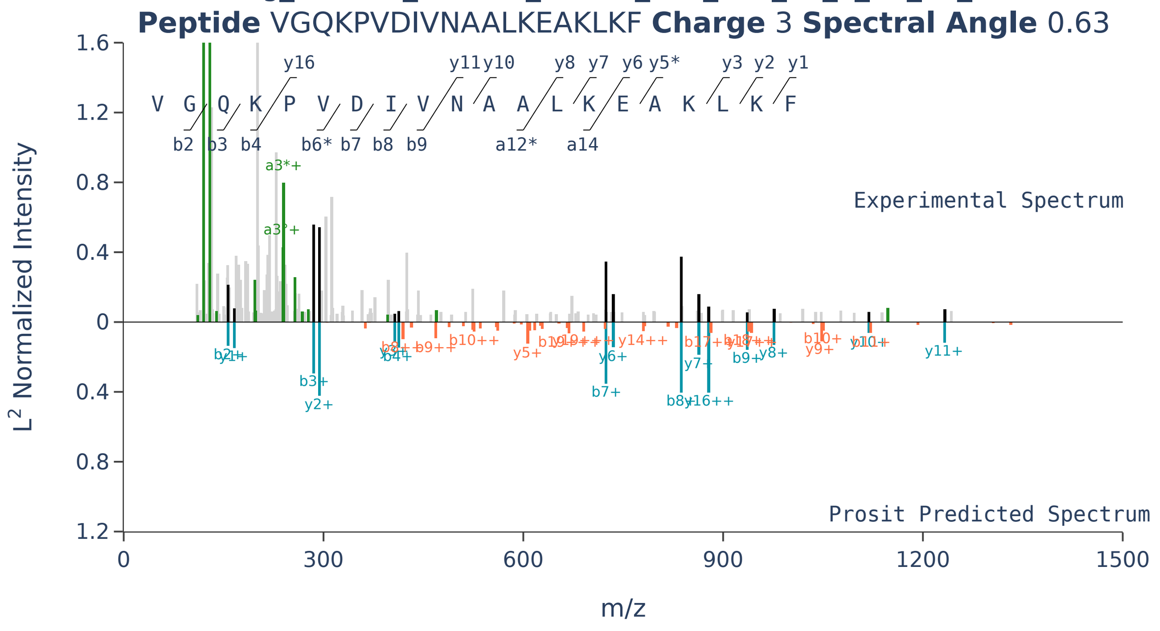


**Source** Ncheng_210623_230623_HFGoe_FFH_20S_25_1_A2_2h_R2 **Scan** 22583
**Peptide** TKVDGDARGGAALSIRHITGKPIKFLGVGE **Charge** 6 **Spectral Angle** 0.77

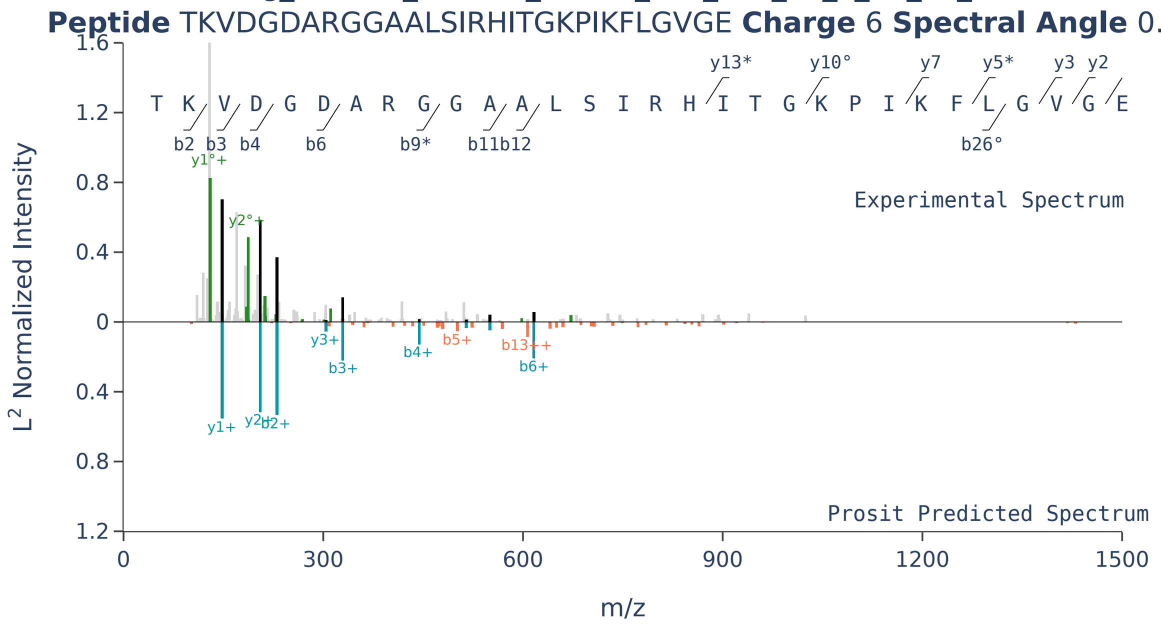


**Source** Ncheng_210623_230623_HFGoe_FFH_20S_25_1_A2_4h_R2 **Scan** 24922
**Peptide** GGAALSIRHITGKPIKFLGVGE **Charge** 3 **Spectral Angle** 0.76

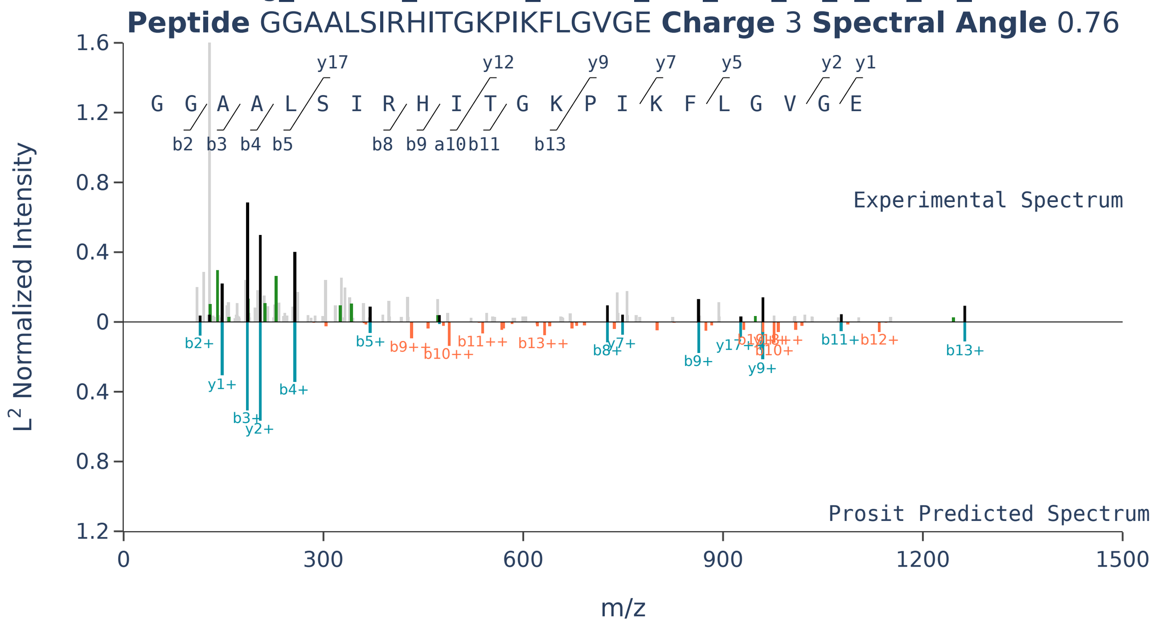


**Source** Ncheng_210623_230623_HFGoe_FFH_20S_25_1_A1_2h_R1 **Scan** 40051
**Peptide** QMDDKVLVRMEAIINSMTMKE **Charge** 4 **Spectral Angle** 0.56

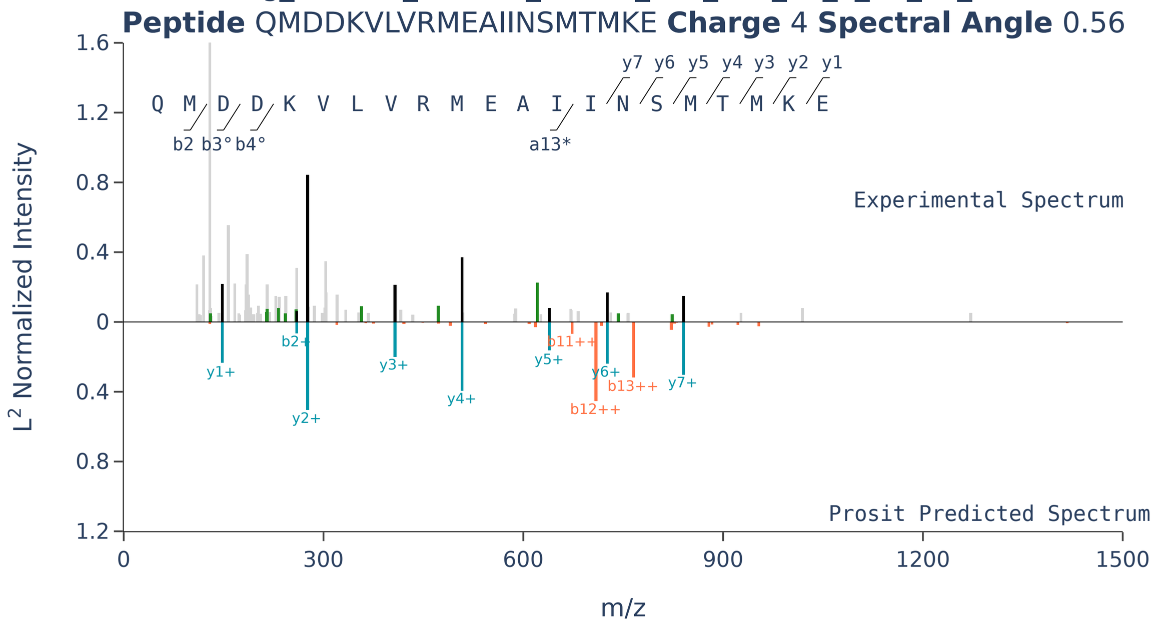

**Source** Ncheng_210623_230623_HFGoe_FFH_20S_25_1_A1_24h_R1 **Scan** 16850
**Peptide** ALLNSMTMKERAKPEIIKGS **Charge** 3 **Spectral Angle** 0.7

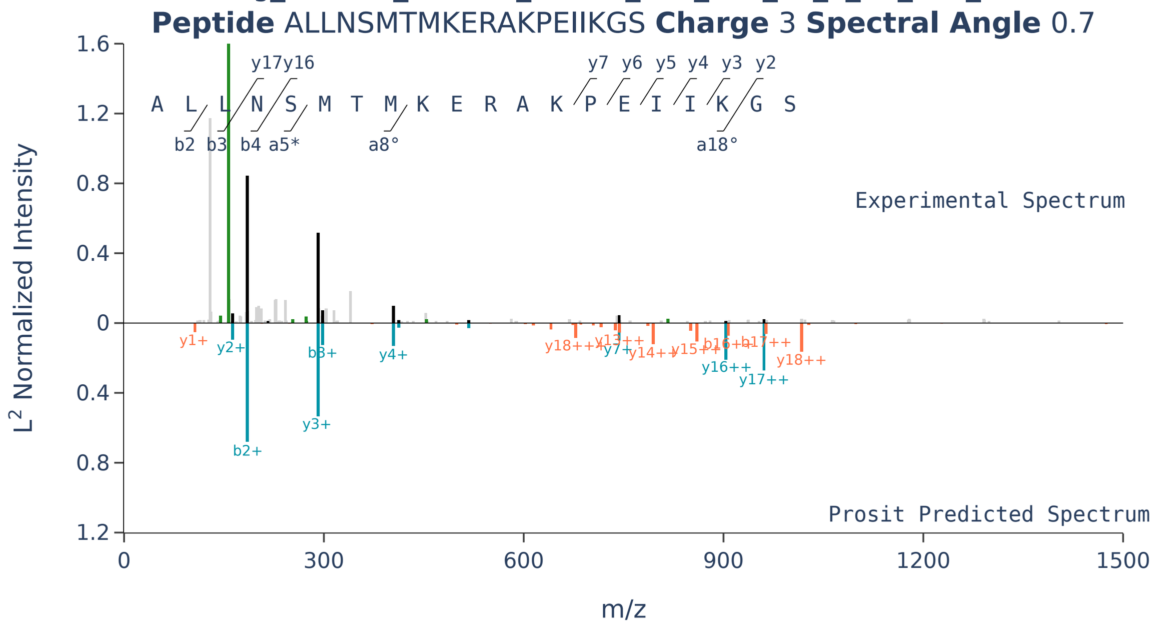


**Source** Ncheng_210623_230623_HFGoe_FFH_20S_25_1_A2_2h_R2 **Scan** 8940
**Peptide** FLNRVKEKAVGHEV **Charge** 4 **Spectral Angle** 0.56

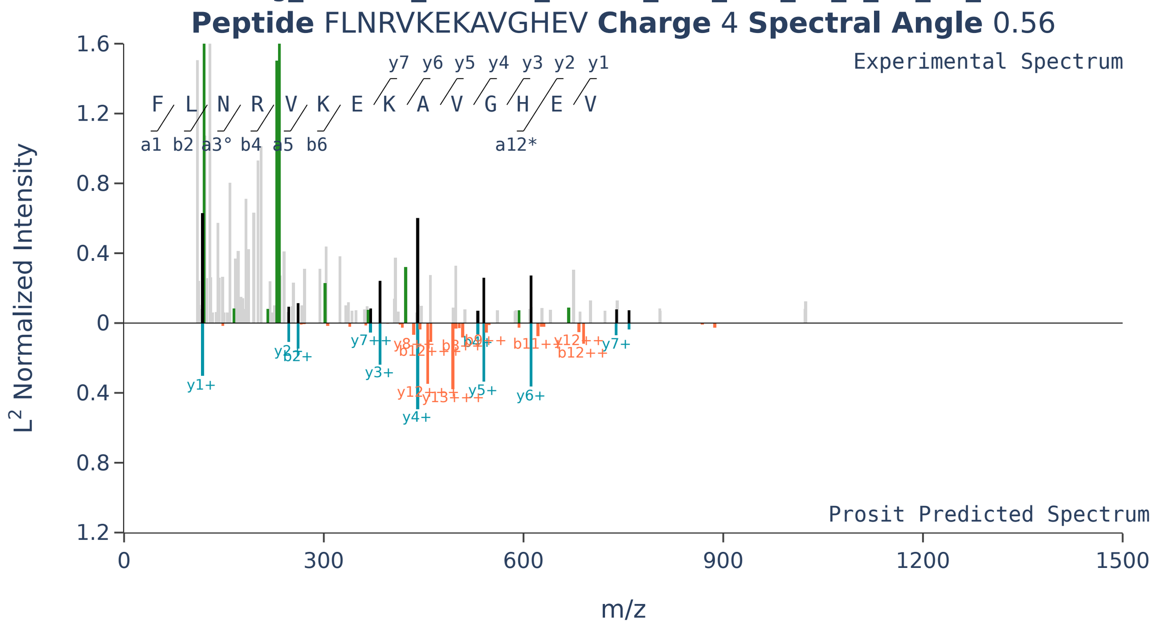


**Source** Ncheng_210623_230623_HFGoe_FFH_20S_25_1_A2_4h_R2 **Scan** 18971
**Peptide** AALSIRHITGKPIKF **Charge** 4 **Spectral Angle** 0.64

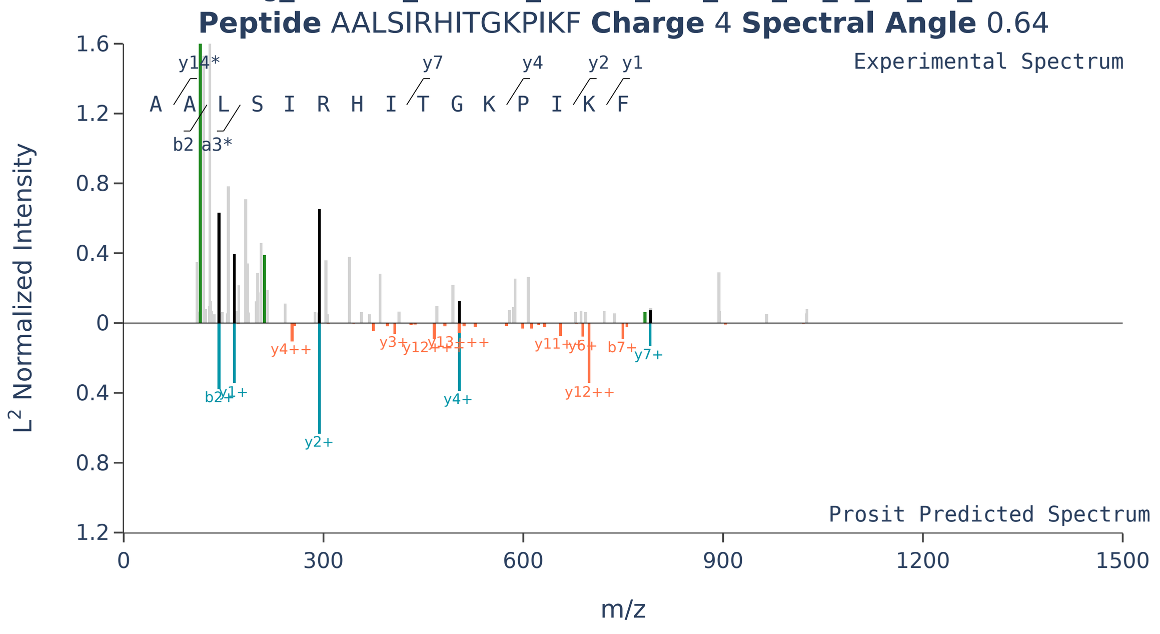


**Source** Ncheng_210623_230623_HFGoe_FFH_20S_25_1_A1_24h_R1 **Scan** 9409
**Peptide** KKVLVVS **Charge** 2 **Spectral Angle** 0.58

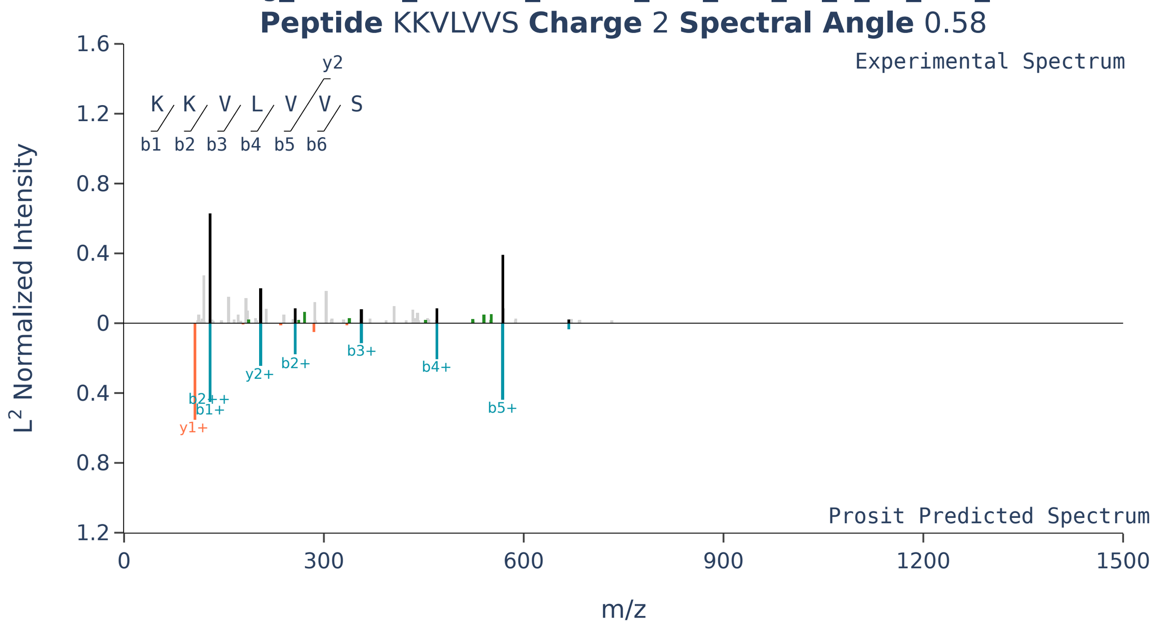


**Source** Ncheng_210623_230623_HFGoe_FFH_20S_25_1_A2_24h_R1 **Scan** 12820
**Peptide** DDMQRMMKKMKKGGM **Charge** 3 **Spectral Angle** 0.59

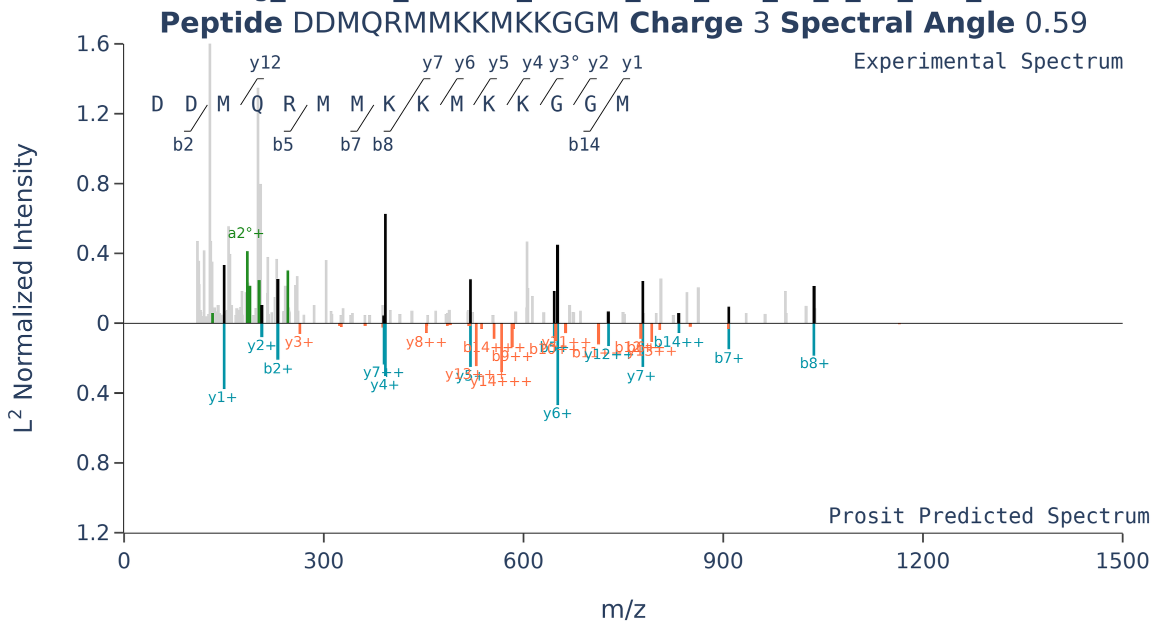


**Source** Ncheng_210623_230623_HFGoe_FFH_20S_25_1_A2_24h_R1 **Scan** 5037
**Peptide** MKKMKKGGM **Charge** 2 **Spectral Angle** 0.65

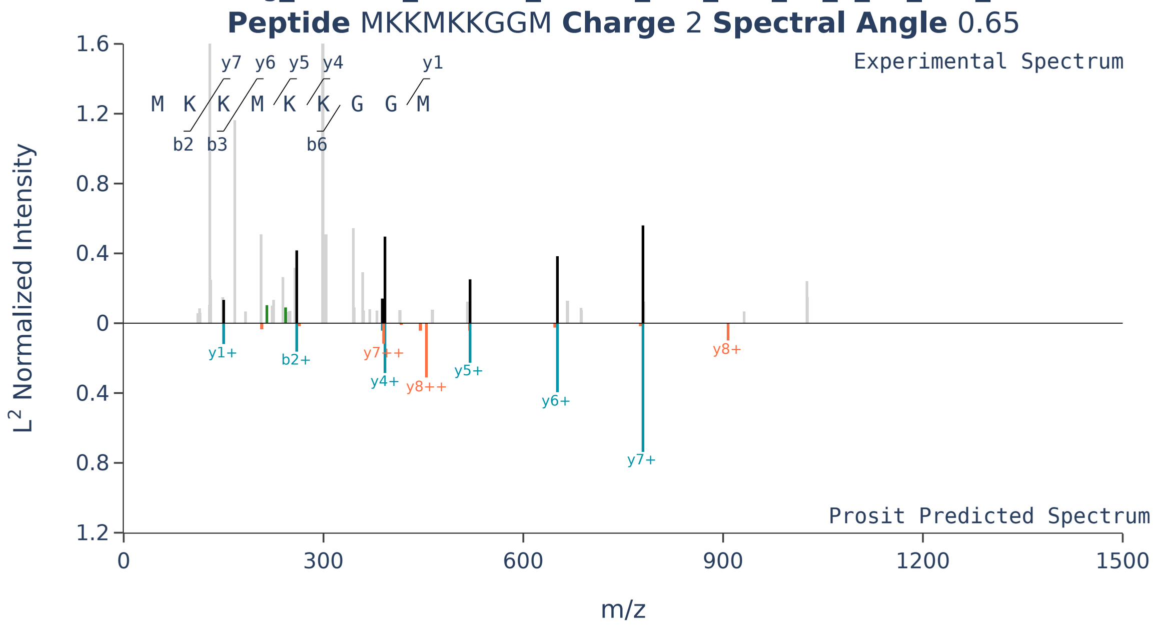


**Source** Ncheng_210623_230623_HFGoe_FFH_20S_25_1_A2_4h_R2 **Scan** 21922
**Peptide** LGVGEKTEALEPFHPDRLAS **Charge** 4 **Spectral Angle** 0.71

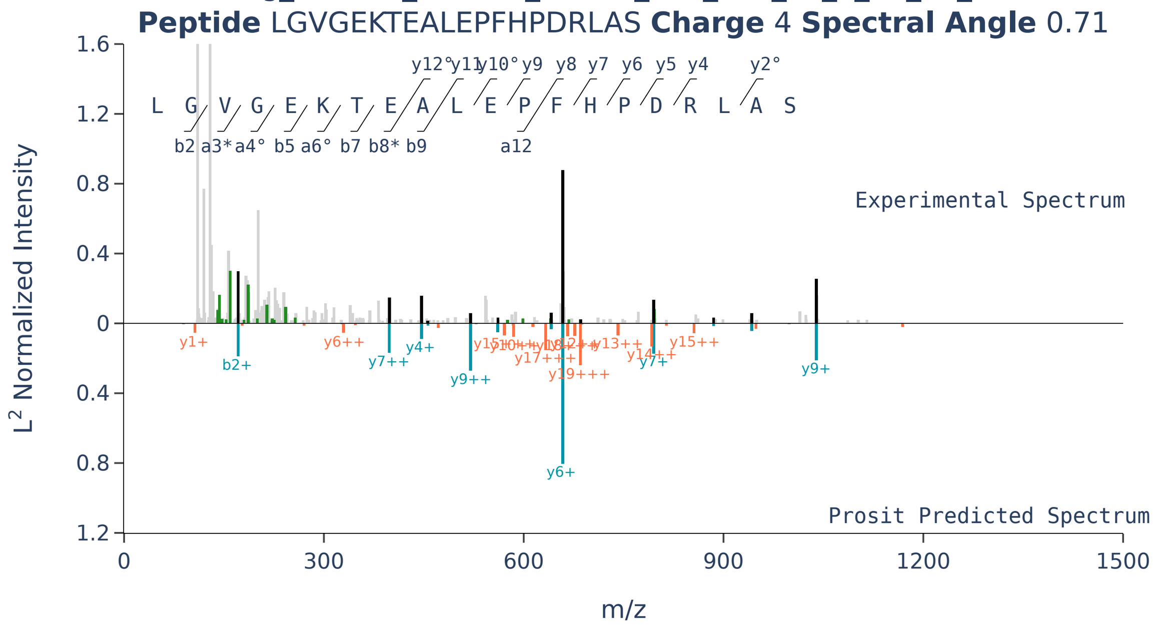


**Source** Ncheng_210623_230623_HFGoe_FFH_20S_25_1_A2_4h_R2 **Scan** 28159
**Peptide** MASLMGKLPGMGQIPDNVKSQ **Charge** 3 **Spectral Angle** 0.63

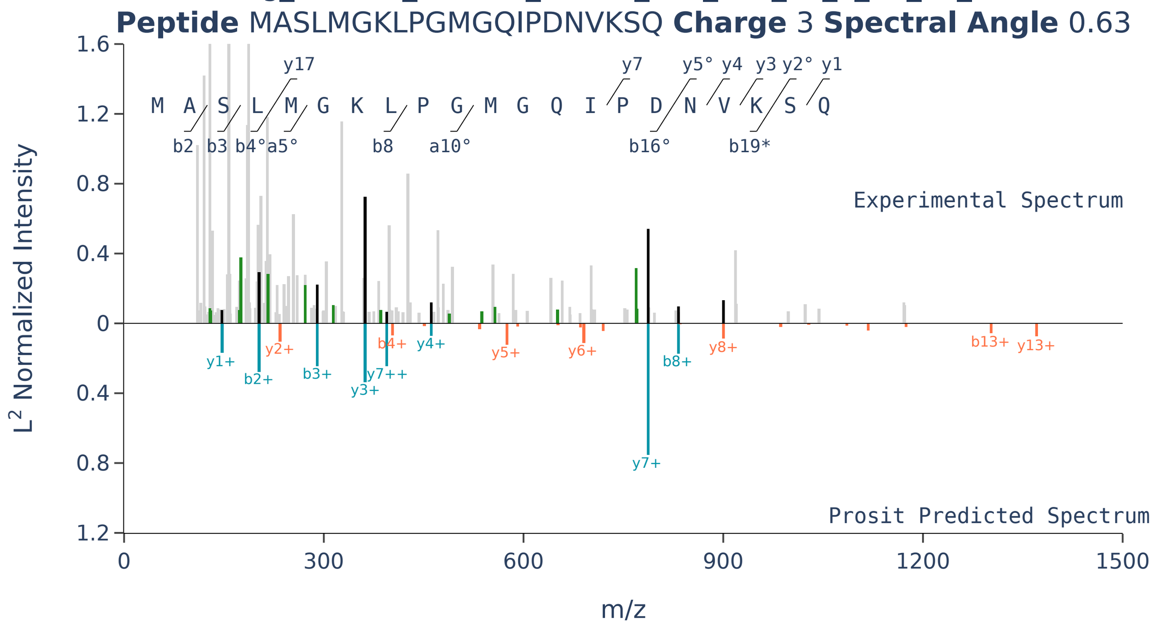


**Source** Ncheng_210623_230623_HFGoe_FFH_20S_25_1_A2_2h_R2 **Scan** 5087
**Peptide** KLASKLKKGD **Charge** 2 **Spectral Angle** 0.68

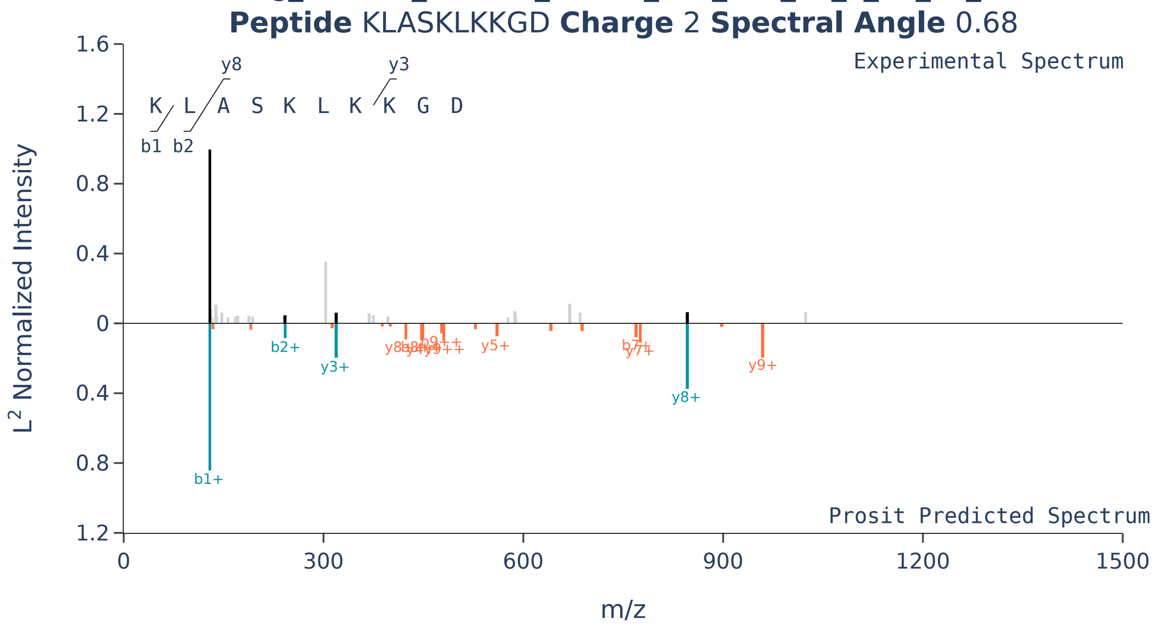


**Source** Ncheng_210623_230623_HFGoe_FFH_20S_25_1_A1_2h_R2 **Scan** 28647
**Peptide** MFDNLTDRLSRTLRNISGRGRLTEDNVKD **Charge** 6 **Spectral Angle** 0.75

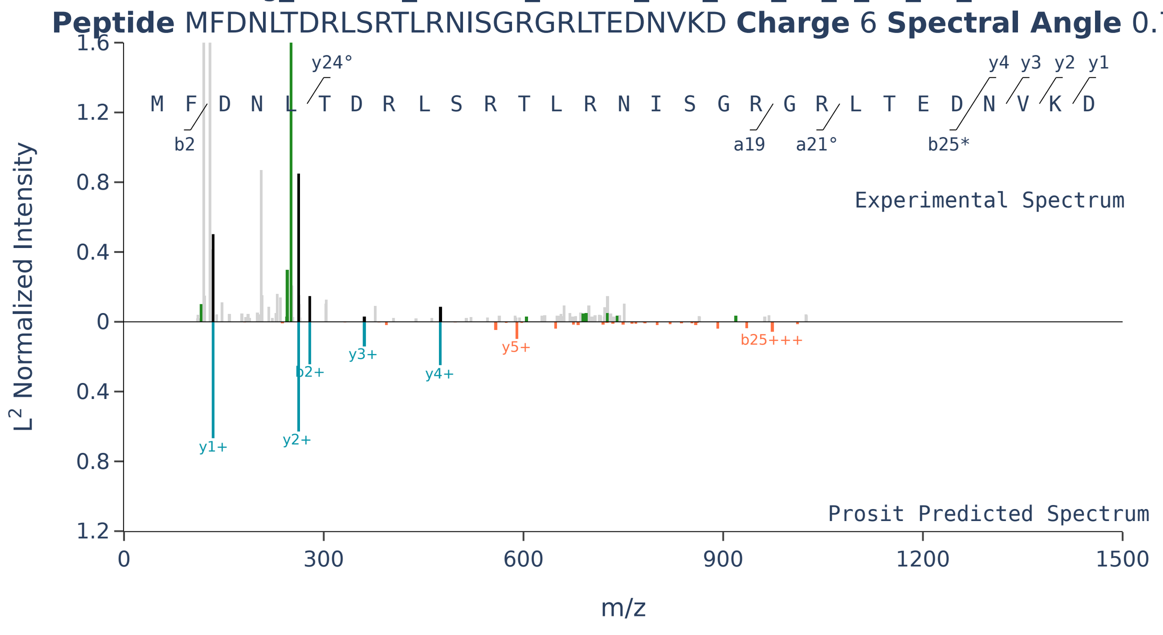


**Source** Ncheng_210623_230623_HFGoe_FFH_20S_25_1_A1_2h_R2 **Scan** 29115
**Peptide** NISGRGRLTEDNVKDTLREVRMALL **Charge** 5 **Spectral Angle** 0.72

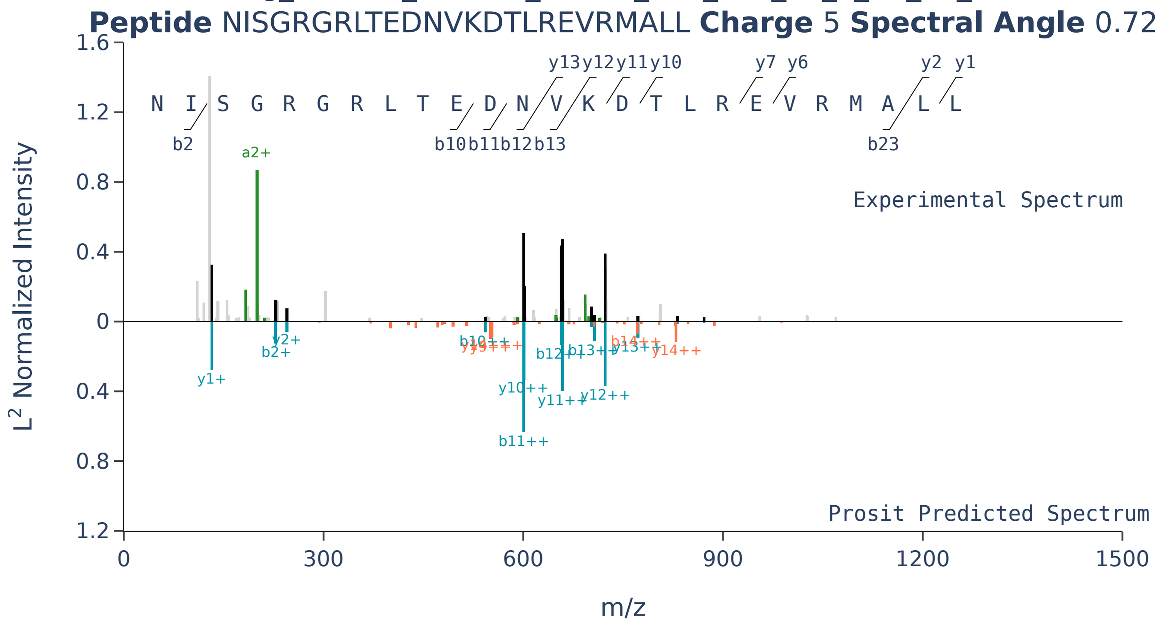


**Source** Ncheng_210623_230623_HFGoe_FFH_20S_25_1_A2_2h_R1 **Scan** 19486
**Peptide** DGDARGGAALSIRHITGKPIKF **Charge** 6 **Spectral Angle** 0.68

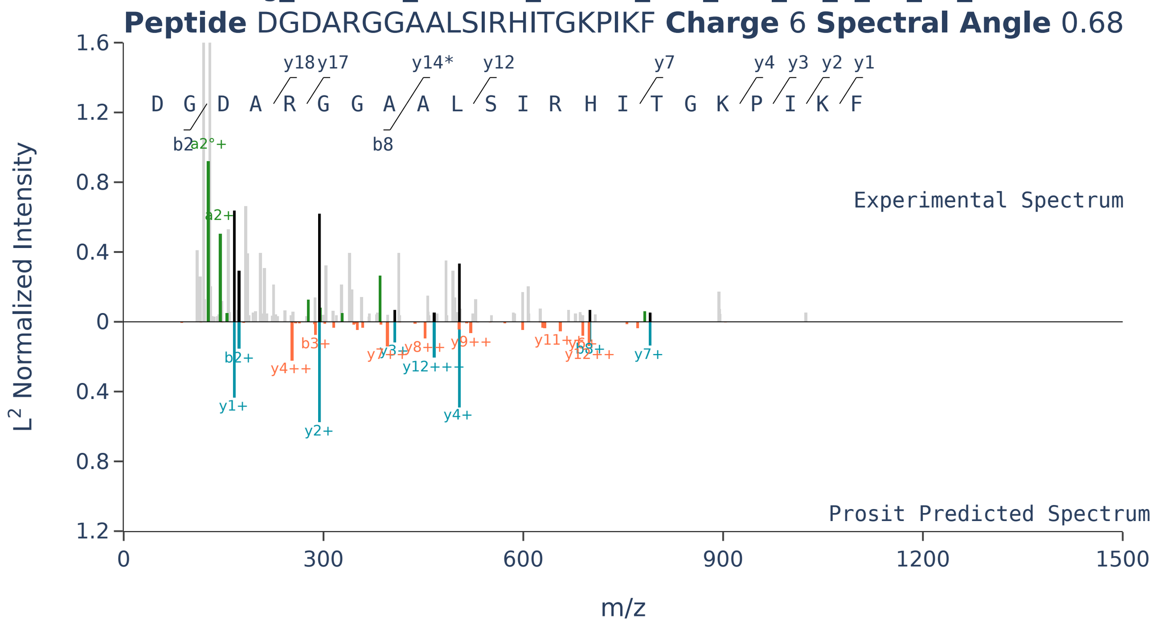


**Source** Ncheng_210623_230623_HFGoe_FFH_20S_25_1_A1_24h_R2 **Scan** 8326
**Peptide** GAGKTTSVGKL **Charge** 2 **Spectral Angle** 0.59

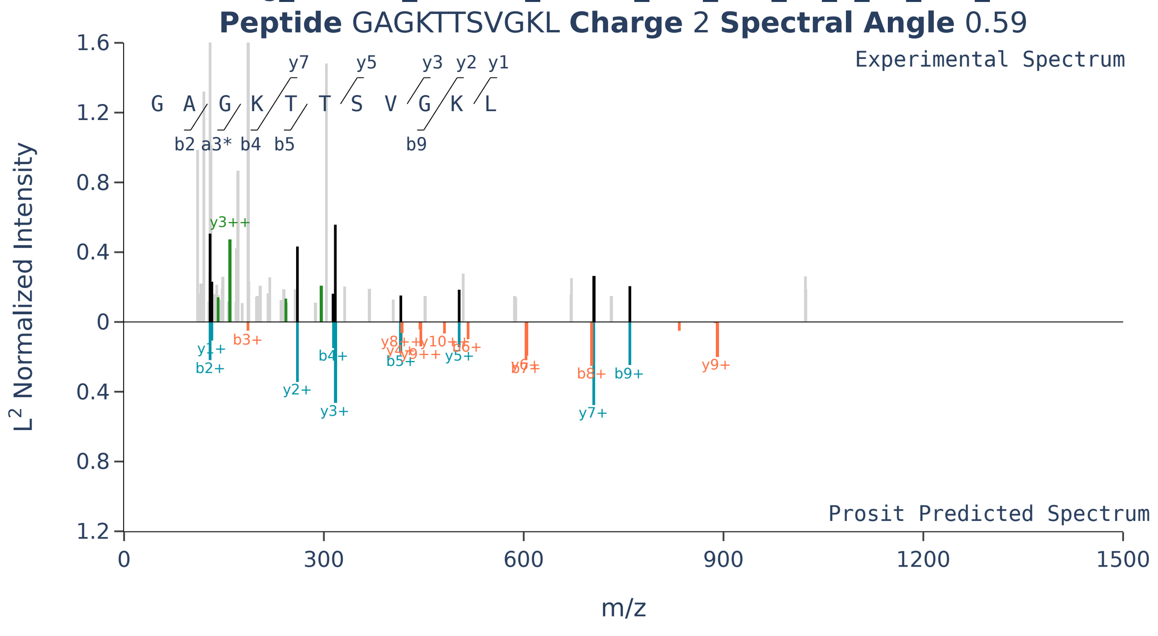


**Source** Ncheng_210623_230623_HFGoe_FFH_20S_25_1_A2_24h_R1 **Scan** 6491
**Peptide** KVDRAQAEKLASK **Charge** 3 **Spectral Angle** 0.61

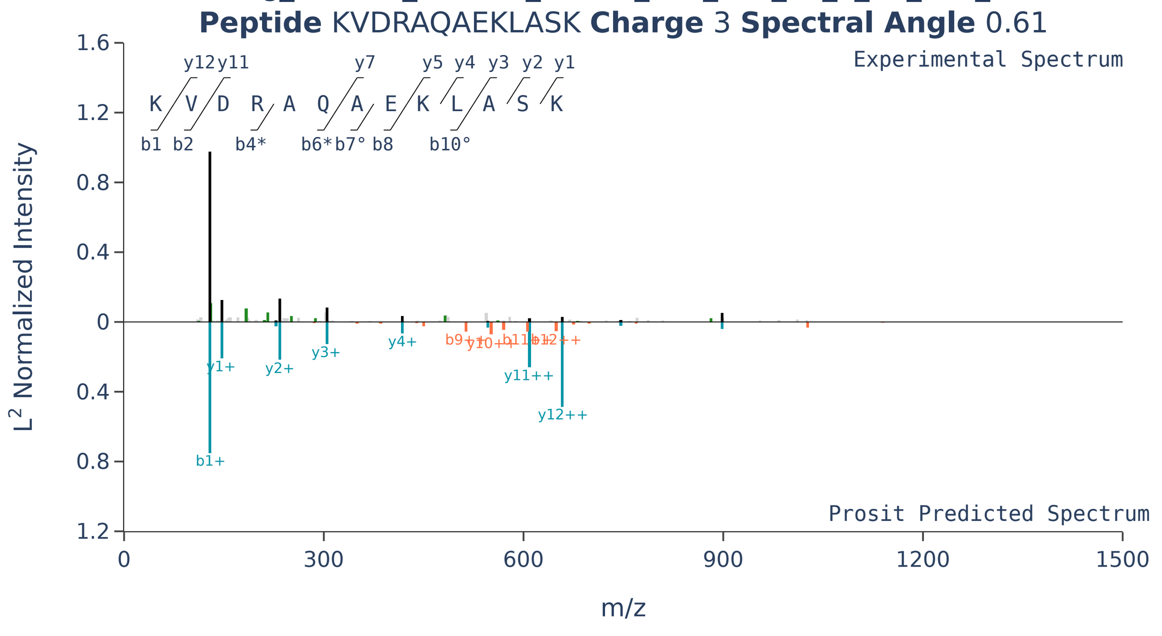


**Source** Ncheng_210623_230623_HFGoe_FFH_20S_25_1_A2_4h_R2 **Scan** 20051
**Peptide** FLNRVKEKAVGHEVNKSLTPGQEFVKIV **Charge** 6 **Spectral Angle** 0.62

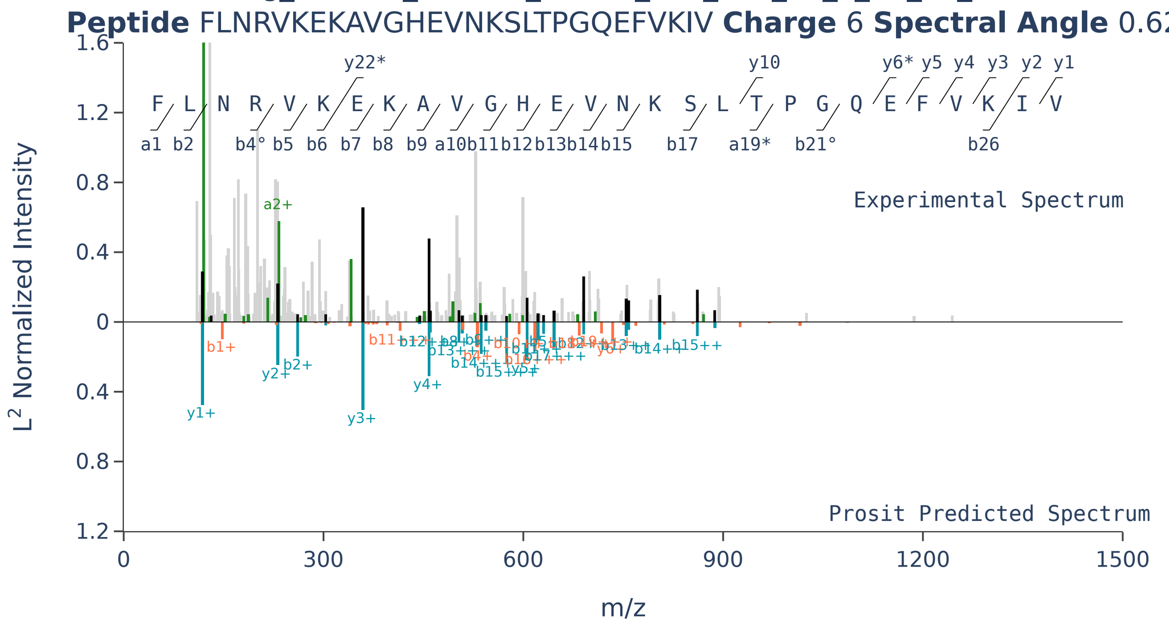

**Source** Ncheng_210623_230623_HFGoe_FFH_20S_25_1_A2_4h_R2 **Scan** 10122
**Peptide** DMQRMMKKMKKGGM **Charge** 3 **Spectral Angle** 0.59

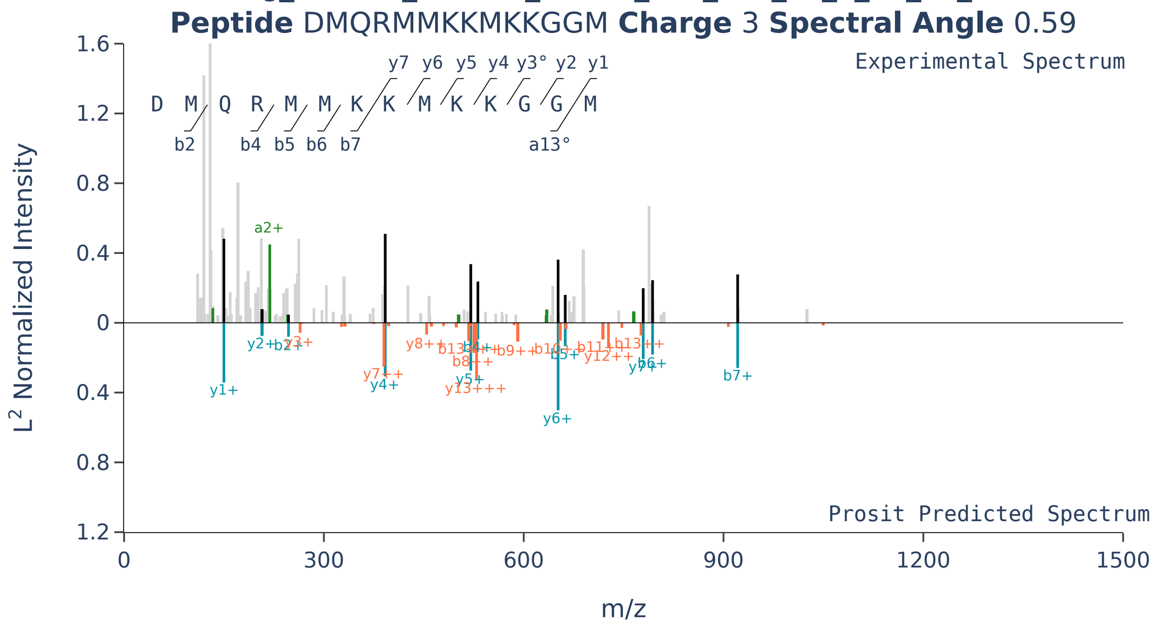


**Source** Ncheng_210623_230623_HFGoe_FFH_20S_25_1_A2_4h_R1 **Scan** 24463
**Peptide** VNRLLKQFDDMQRMMKKMK **Charge** 5 **Spectral Angle** 0.68

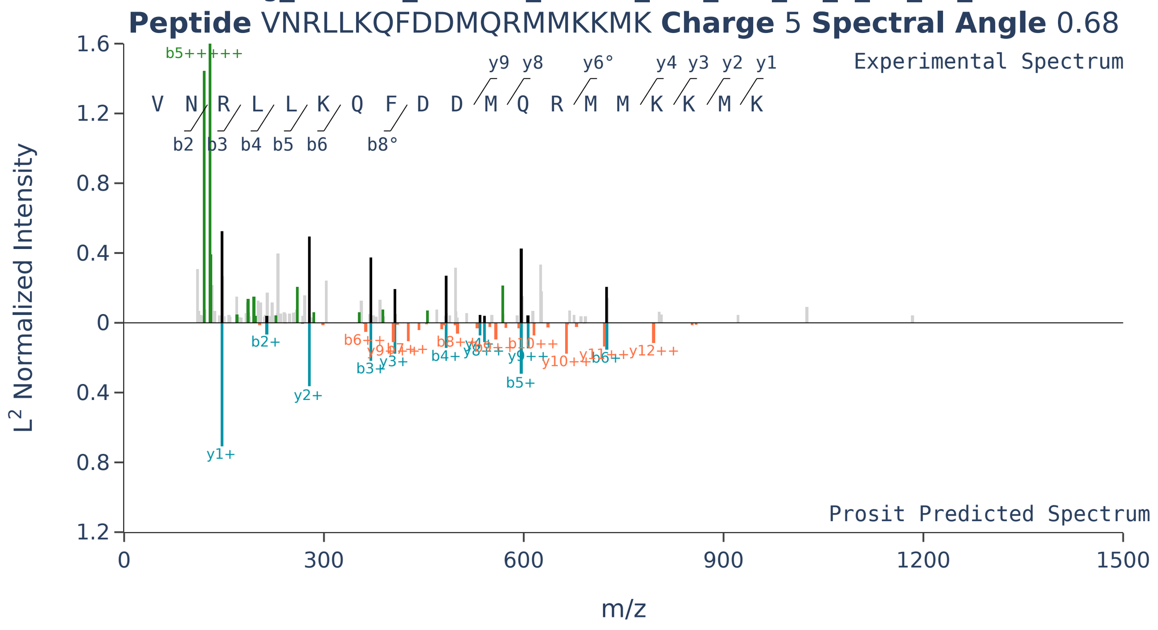


**Source** Ncheng_210623_230623_HFGoe_FFH_20S_25_1_A2_4h_R1 **Scan** 13229
**Peptide** SRTLRNISGRGRLTEDNVKDTL **Charge** 5 **Spectral Angle** 0.43

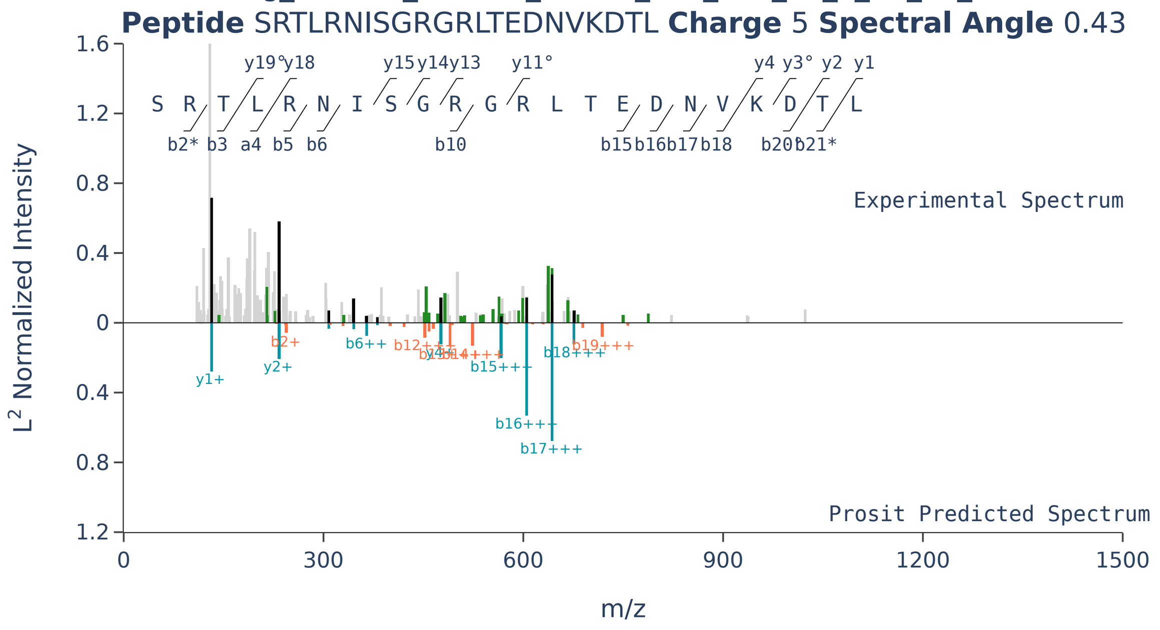


**Source** Ncheng_210623_230623_HFGoe_FFH_20S_25_1_A2_4h_R2 **Scan** 21636
**Peptide** VVSADVYRPAAIKQLET **Charge** 3 **Spectral Angle** 0.63

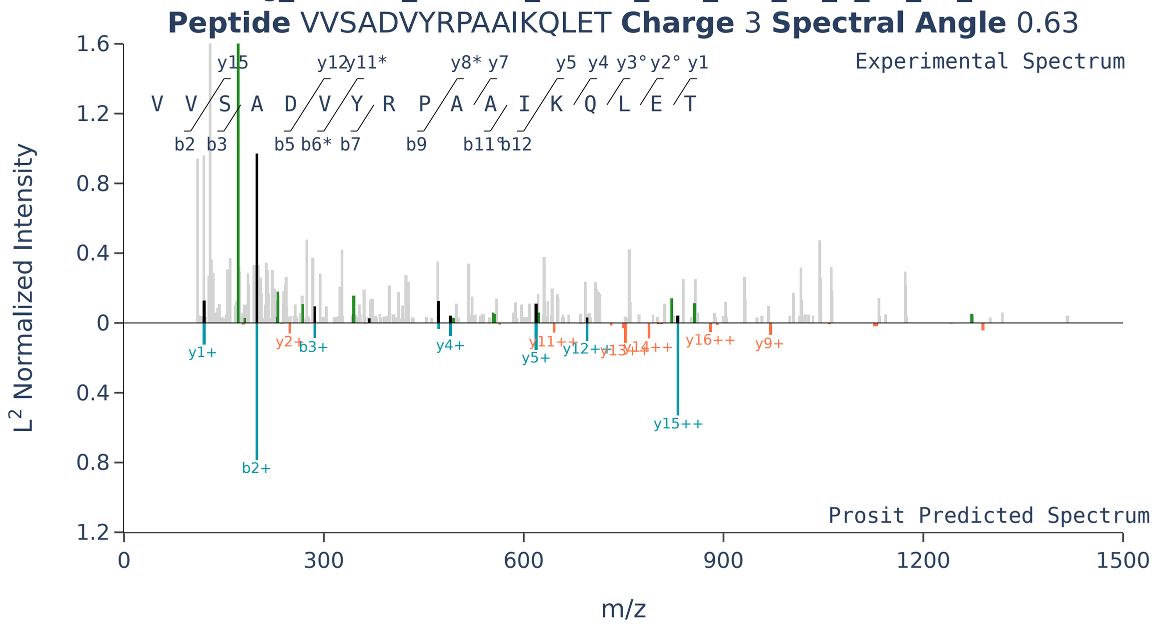


**Source** Ncheng_210623_230623_HFGoe_FFH_20S_25_1_A1_1h_R1 **Scan** 32432
**Peptide** IVNAALKEAKLKFYDVLLVDTAGRLHVD **Charge** 5 **Spectral Angle** 0.64

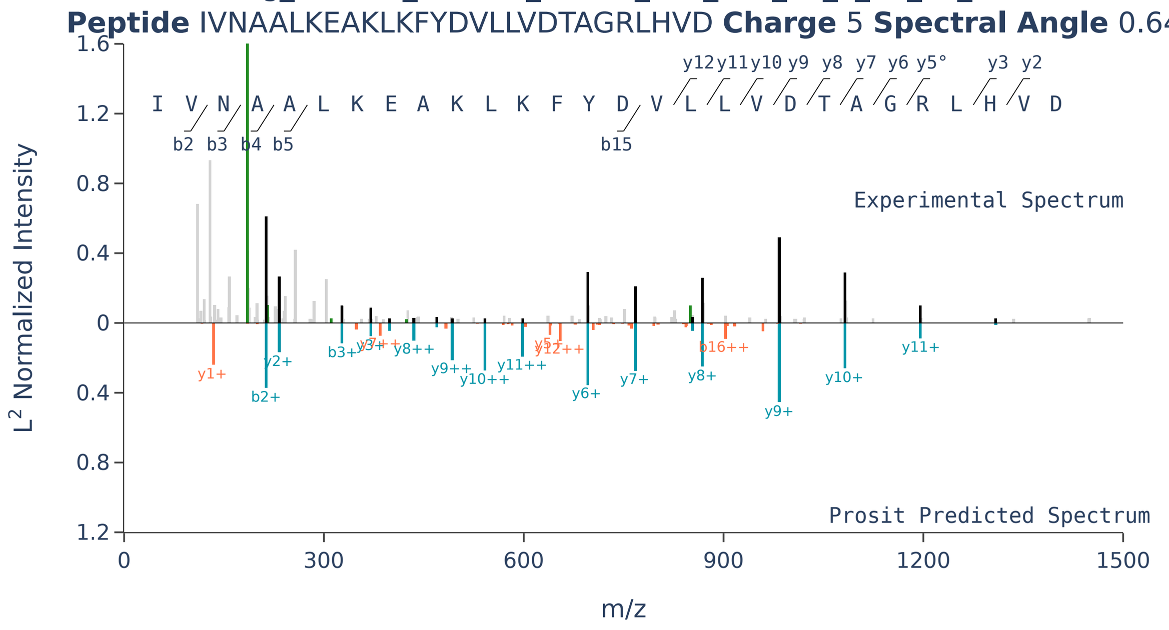


**Source** Ncheng_210623_230623_HFGoe_FFH_20S_25_1_A2_4h_R1 **Scan** 7091
**Peptide** REFINRVKEK **Charge** 3 **Spectral Angle** 0.48

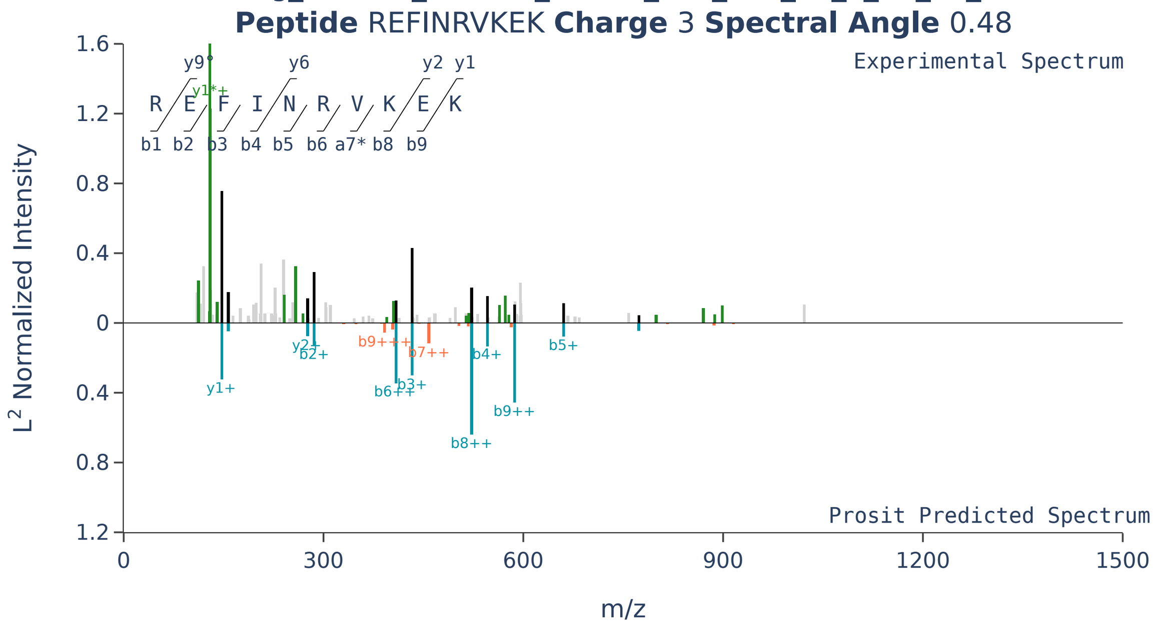


**Source** Ncheng_210623_230623_HFGoe_FFH_20S_25_1_A1_2h_R1 **Scan** 26929
**Peptide** PIKFLGVGEKTEALEPFHPDRIASRI **Charge** 5 **Spectral Angle** 0.68

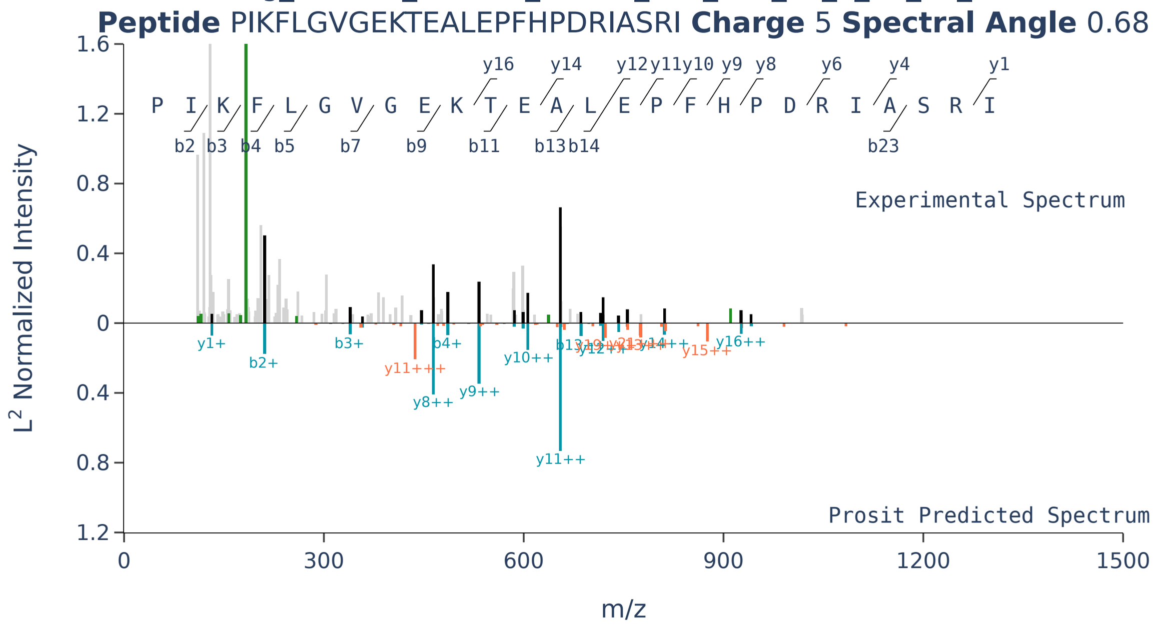


**Source** Ncheng_210623_230623_HFGoe_FFH_20S_25_1_A2_24h_R1 **Scan** 9648
**Peptide** IESKVDRAQAEKLA **Charge** 2 **Spectral Angle** 0.46

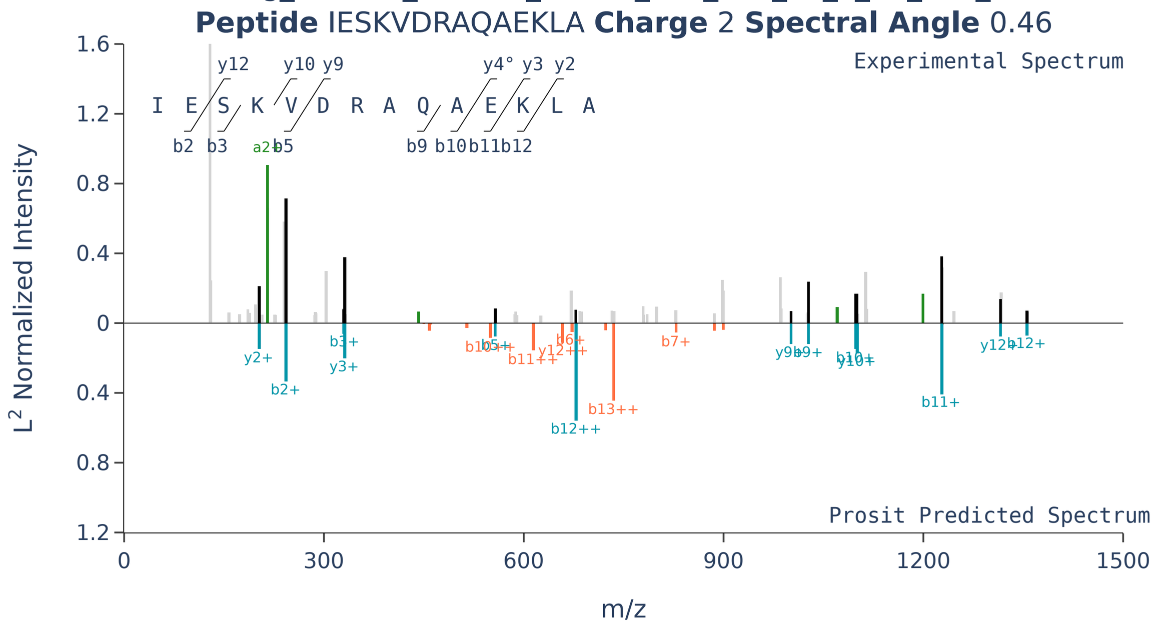


**Source** Ncheng_210623_230623_HFGoe_FFH_20S_25_1_A2_2h_R1 **Scan** 19431
**Peptide** VSADVYRPAAIKQL **Charge** 3 **Spectral Angle** 0.66

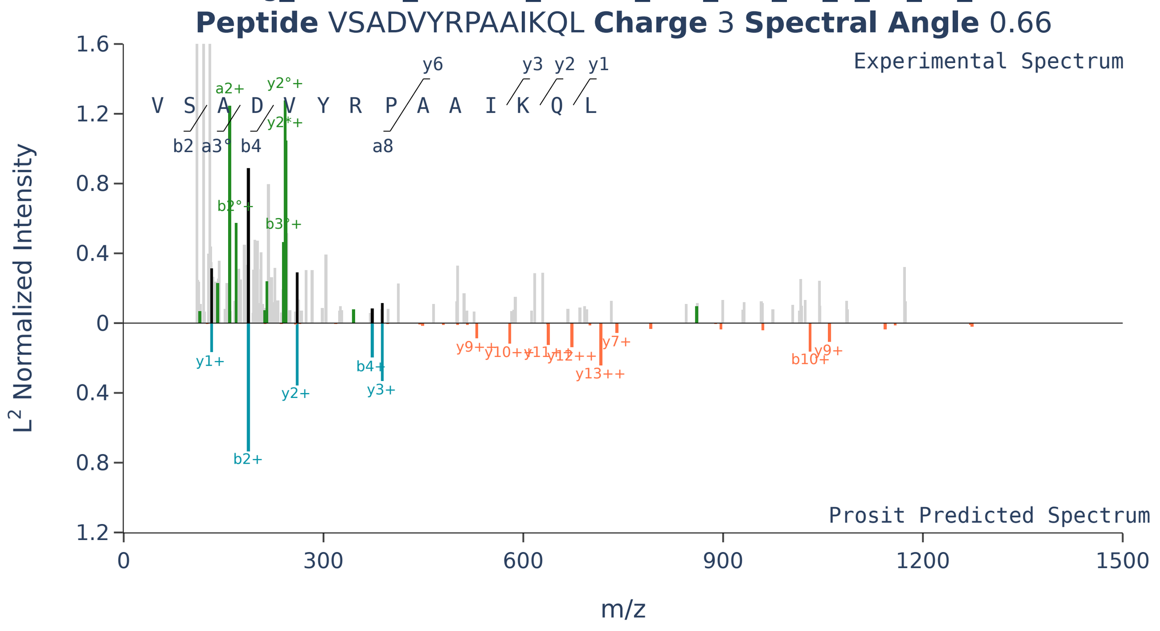


**Source** Ncheng_210623_230623_HFGoe_FFH_20S_25_1_A1_24h_R1 **Scan** 5116
**Peptide** KKMKKGGMAKM **Charge** 2 **Spectral Angle** 0.73

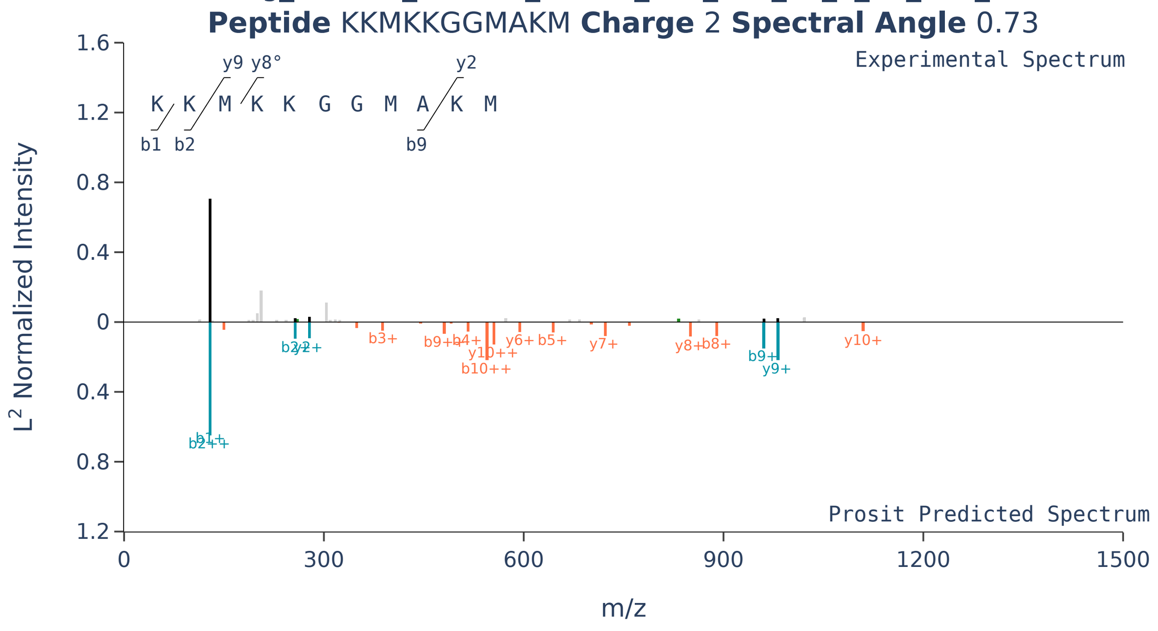


**Source** Ncheng_210623_230623_HFGoe_FFH_20S_25_1_A2_4h_R1 **Scan** 36003
**Peptide** KQLETLAEQVGVDFF **Charge** 2 **Spectral Angle** 0.36

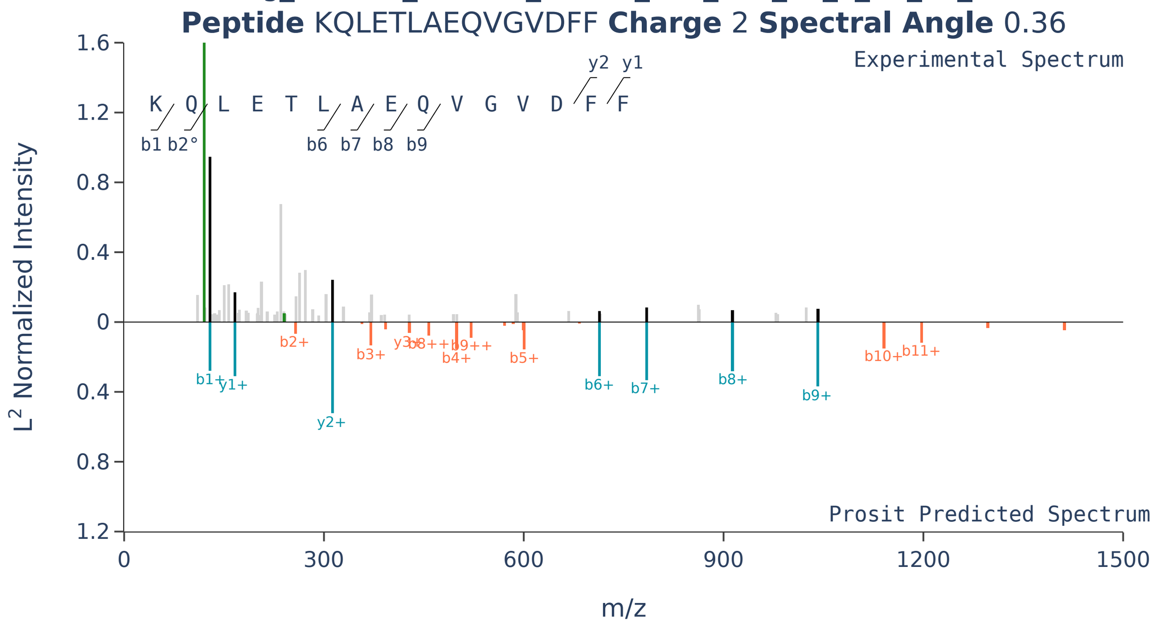


**Source** Ncheng_210623_230623_HFGoe_FFH_20S_25_1_A1_2h_R2 **Scan** 19781
**Peptide** GVVLTKVDGDARGGAALSIRHL **Charge** 5 **Spectral Angle** 0.51

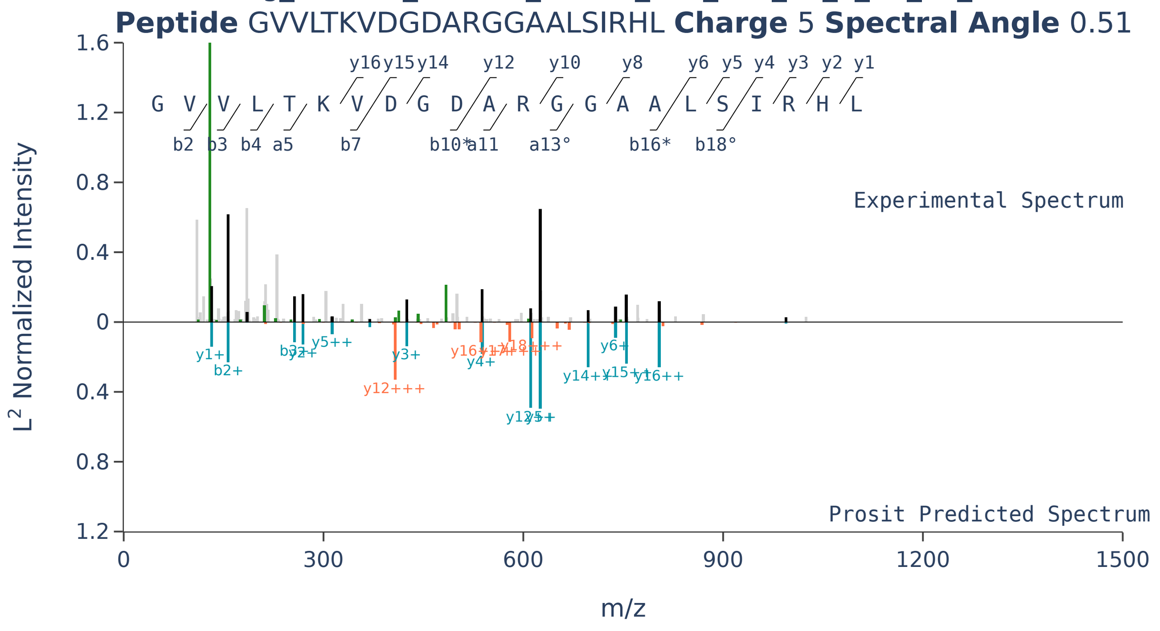


**Source** Ncheng_210623_230623_HFGoe_FFH_20S_25_1_A1_4h_R2 **Scan** 14095
**Peptide** INRVKEKAVGHEVNKSLTPGQEFV **Charge** 5 **Spectral Angle** 0.7

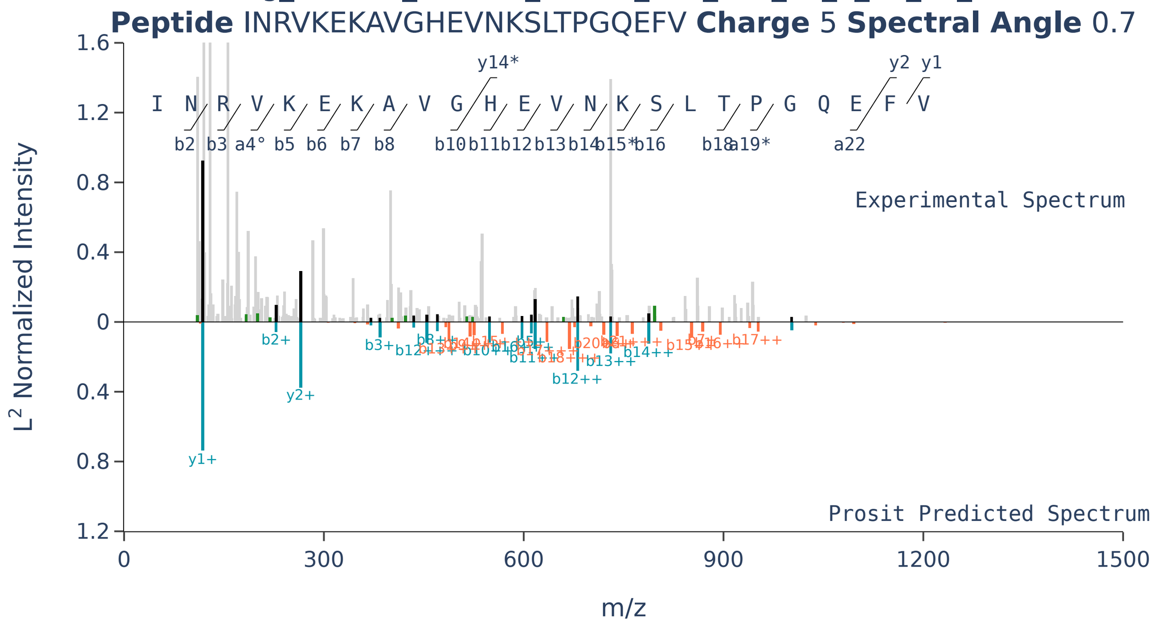


**Source** Ncheng_210623_230623_HFGoe_FFH_20S_25_1_A1_2h_R2 **Scan** 19567
**Peptide** PVVREFINRVKEKAVGHEV **Charge** 5 **Spectral Angle** 0.64

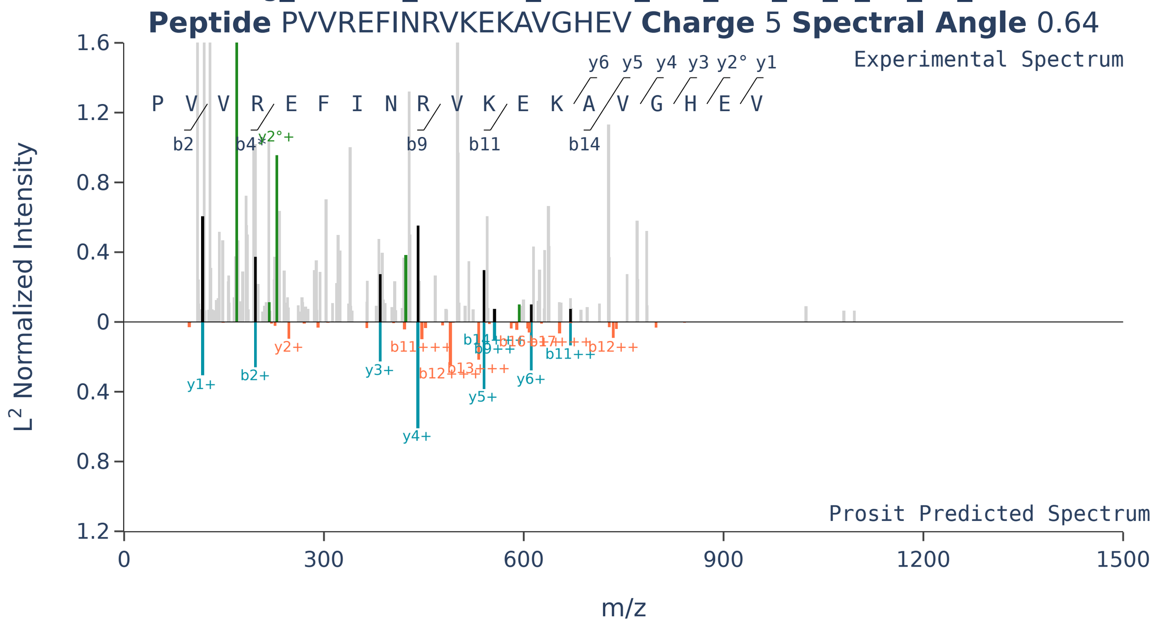


**Source** Ncheng_210623_230623_HFGoe_FFH_20S_25_1_A2_1h_R1 **Scan** 16141
**Peptide** RHITGKPIKFLGVGEKTEALE **Charge** 4 **Spectral Angle** 0.78

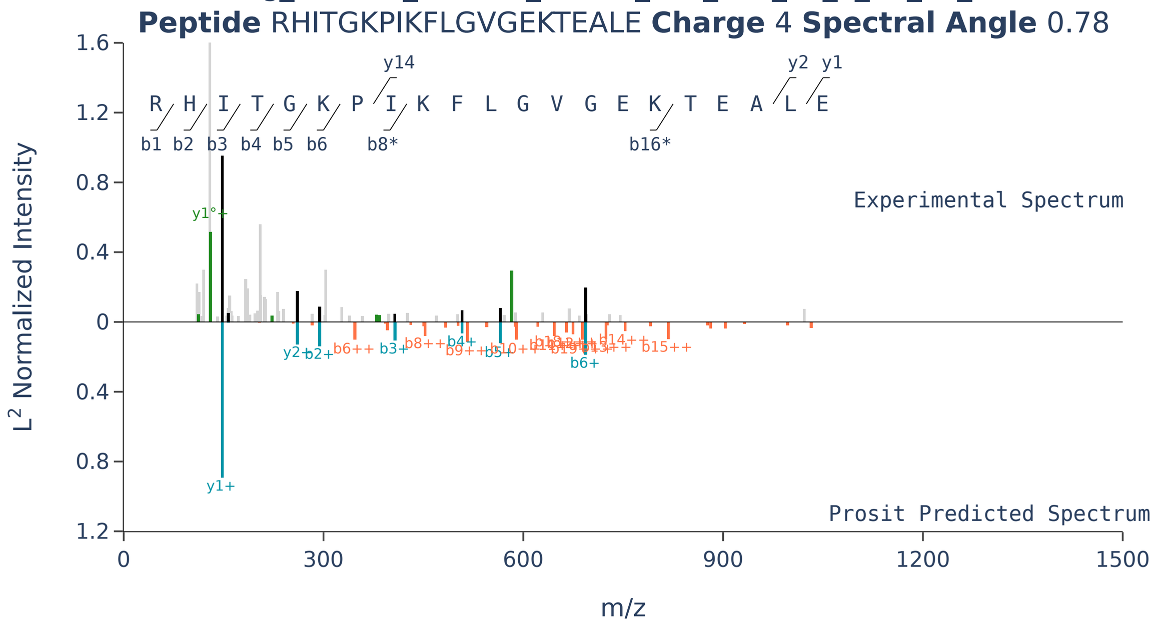

**Source** Ncheng_210623_230623_HFGoe_FFH_20S_25_1_A1_1h_R1 **Scan** 27562
**Peptide** EALPLTGVVLTKVDGDARGGAALSIRH **Charge** 5 **Spectral Angle** 0.54

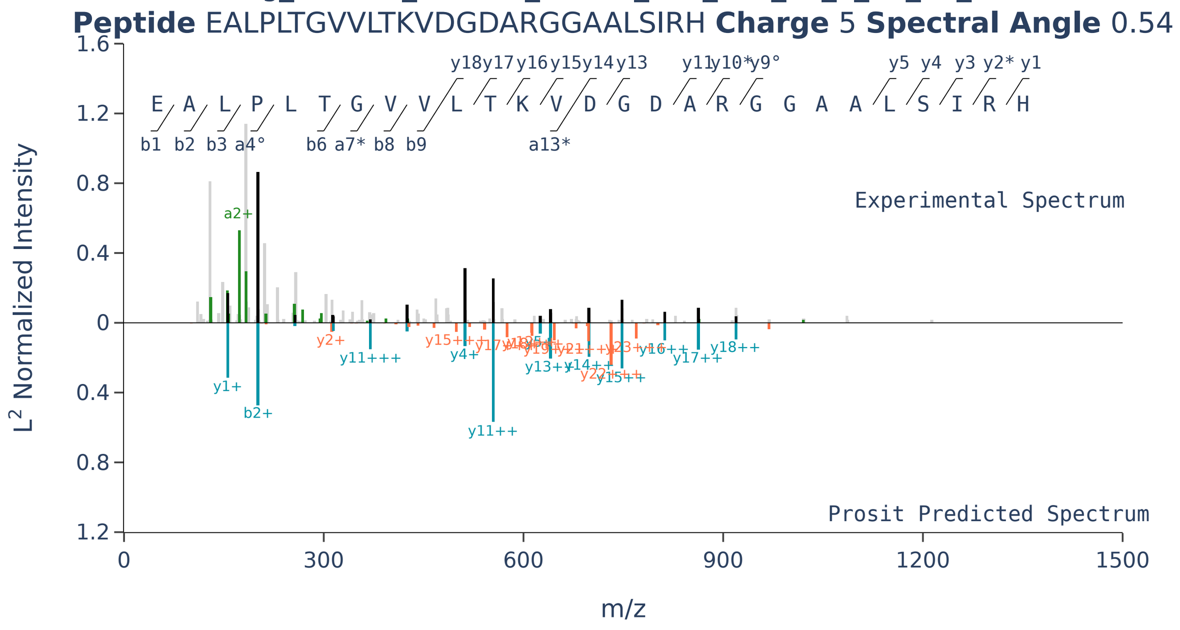


**Source** Ncheng_210623_230623_HFGoe_FFH_20S_25_1_A1_24h_R2 **Scan** 20504
**Peptide** LKQFDDMQRMMKKM **Charge** 5 **Spectral Angle** 0.66

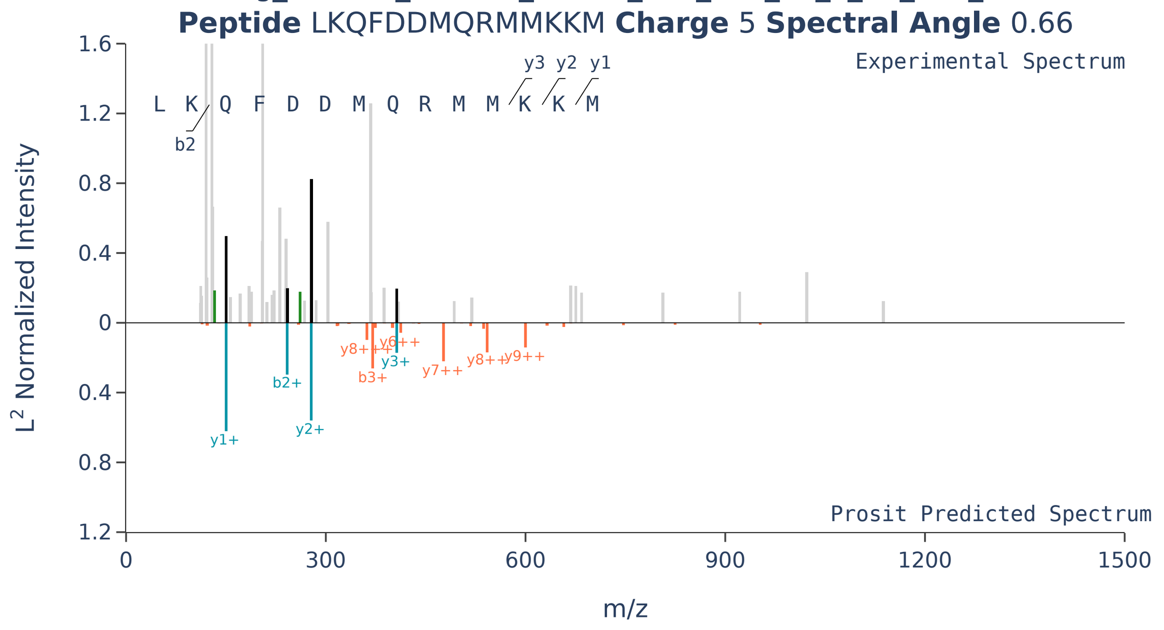


**Source** Ncheng_210623_230623_HFGoe_FFH_20S_25_1_A1_4h_R2 **Scan** 13182
**Peptide** IESKVDRAQAEKLASKLKKG **Charge** 6 **Spectral Angle** 0.81

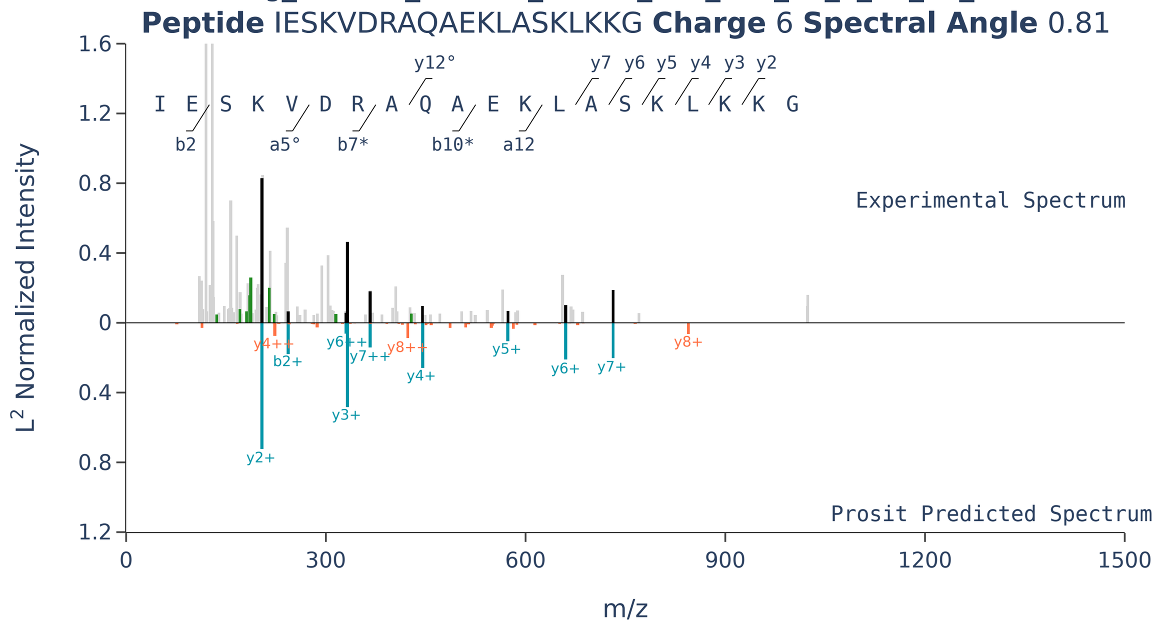


**Source** Ncheng_210623_230623_HFGoe_FFH_20S_25_1_A1_2h_R1 **Scan** 34769
**Peptide** LTEDNVKDTLREVRMALLEAD **Charge** 3 **Spectral Angle** 0.42

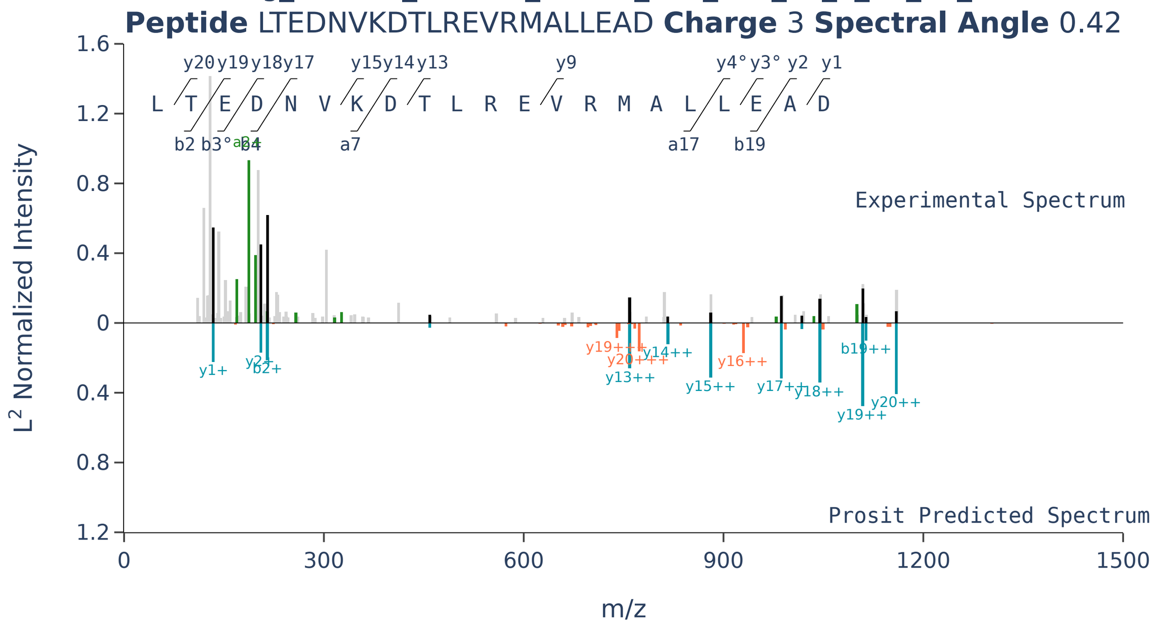


**Source** Ncheng_210623_230623_HFGoe_FFH_20S_25_1_A2_4h_R1 **Scan** 42778
**Peptide** KGDGFDLNDFLEQLRQM **Charge** 3 **Spectral Angle** 0.51

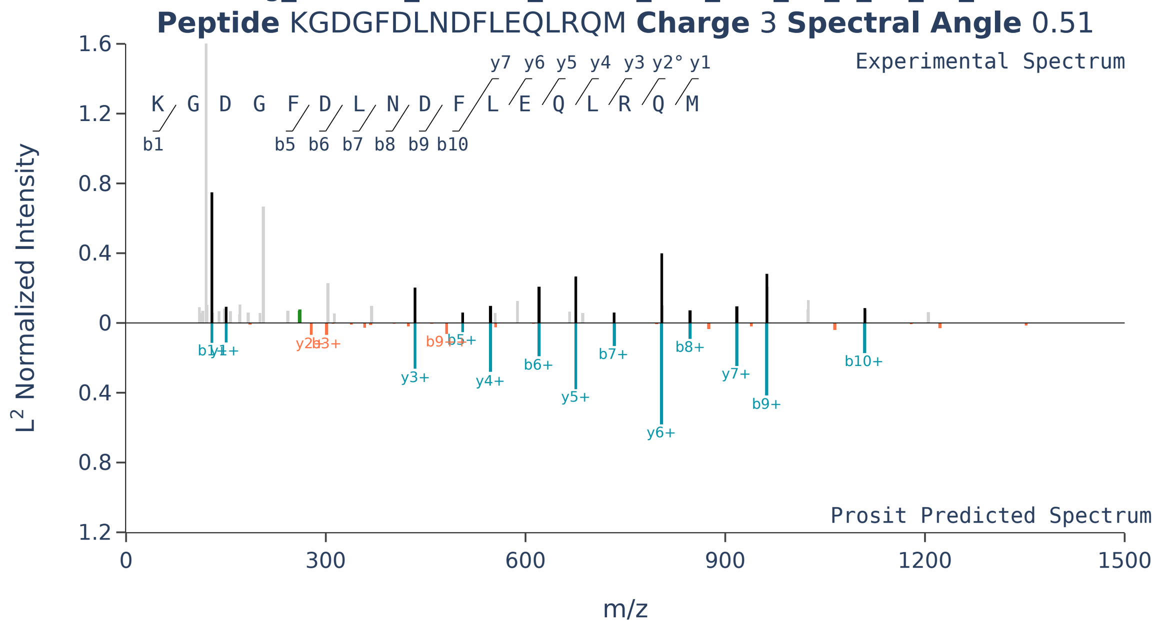


**Source** Ncheng_210623_230623_HFGoe_FFH_20S_25_1_A2_4h_R1 **Scan** 7330
**Peptide** NISGRGRLTED **Charge** 3 **Spectral Angle** 0.31

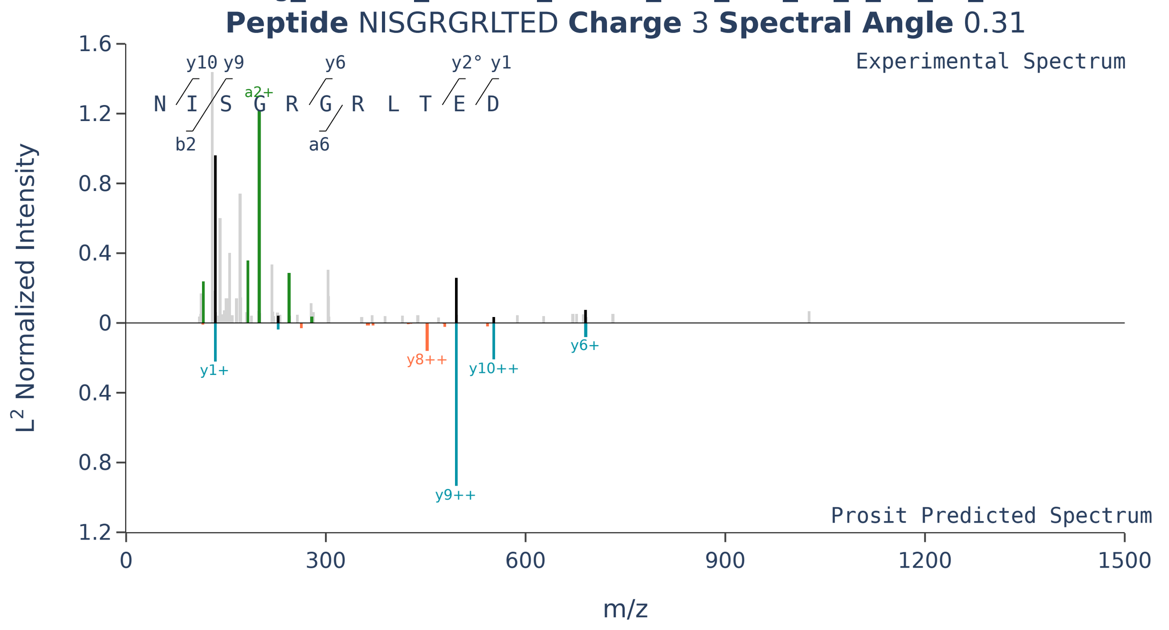


**Source** Ncheng_210623_230623_HFGoe_FFH_20S_25_1_A2_4h_R1 **Scan** 35925
**Peptide** SKLKKGDGFDLNDFLEQL **Charge** 3 **Spectral Angle** 0.39

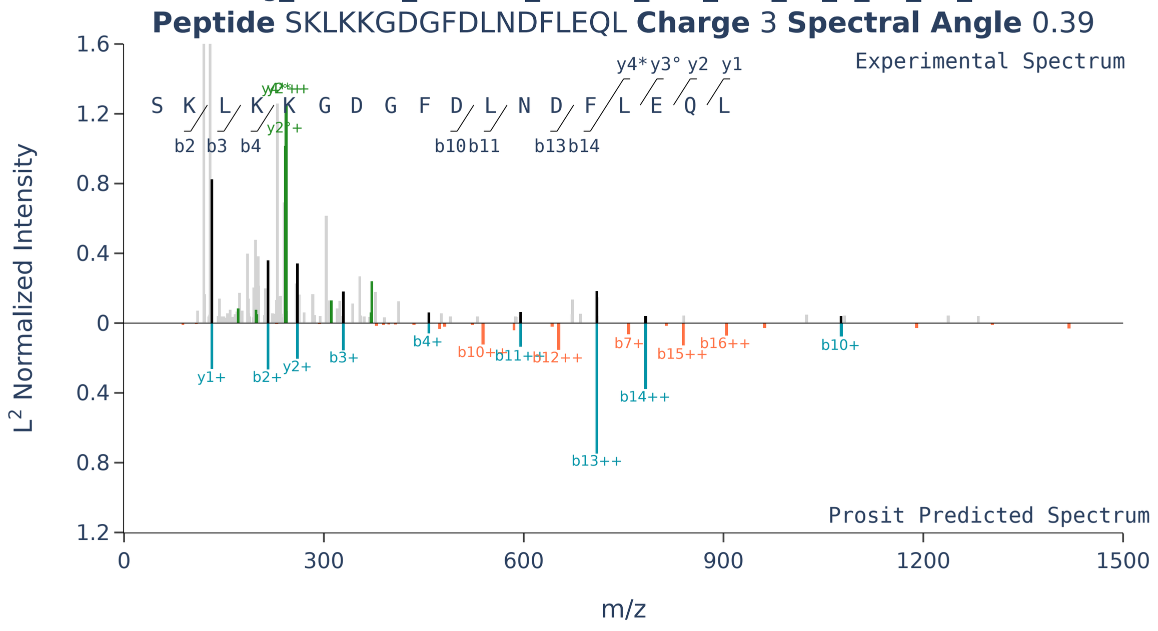


**Source** Ncheng_210623_230623_HFGoe_FFH_20S_25_1_A1_2h_R1 **Scan** 37232
**Peptide** VDAMTGQDAANTAKAFNEALPLTGVVLTKV **Charge** 3 **Spectral Angle** 0.7

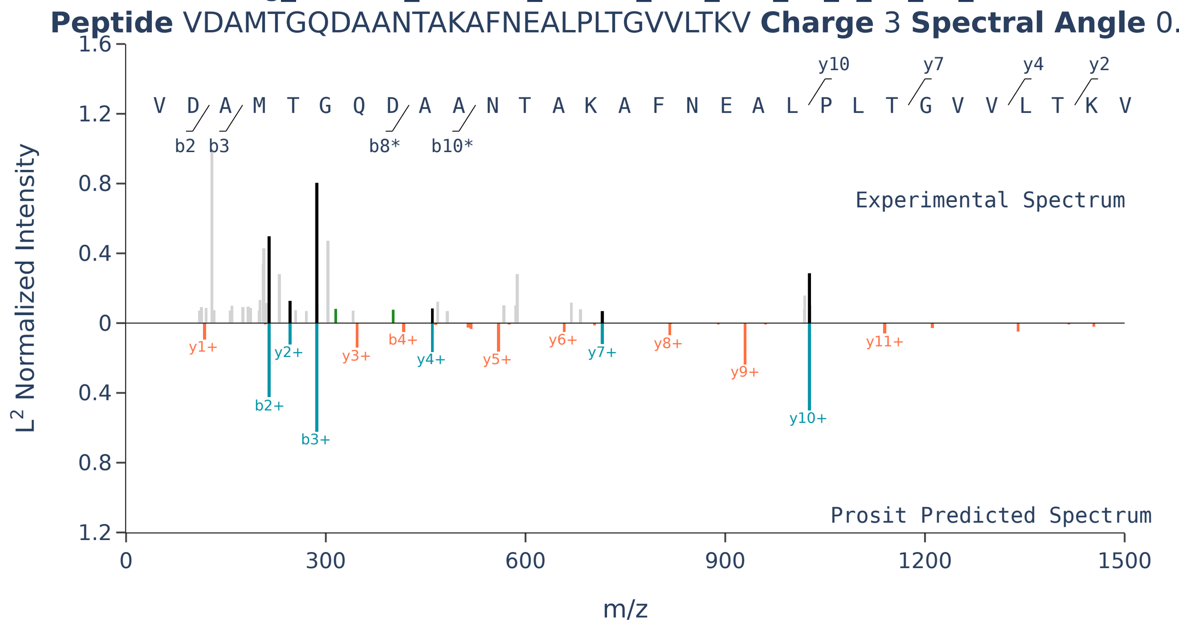


**Source** Ncheng_210623_230623_HFGoe_FFH_20S_25_1_A2_4h_R1 **Scan** 24684
**Peptide** KPIKFLGVGEKTEALEPFHPDRIASRL **Charge** 4 **Spectral Angle** 0.63

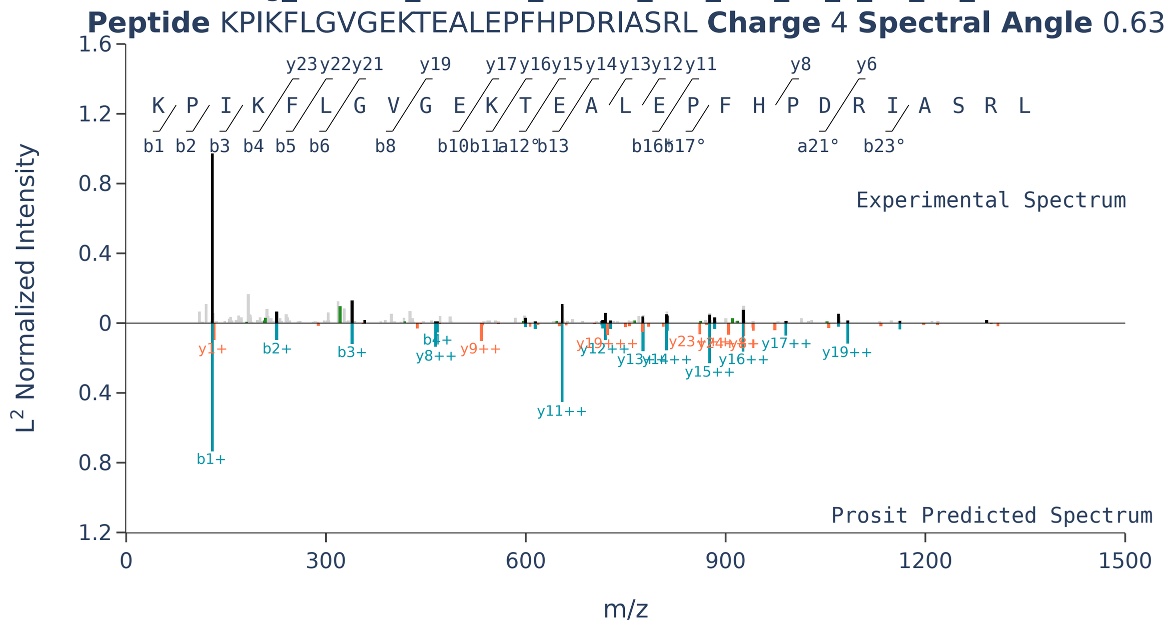


**Source** Ncheng_210623_230623_HFGoe_FFH_20S_25_1_A1_2h_R1 **Scan** 21996
**Peptide** ARGGAALSIRHITGKPIKFLGVGE **Charge** 4 **Spectral Angle** 0.56

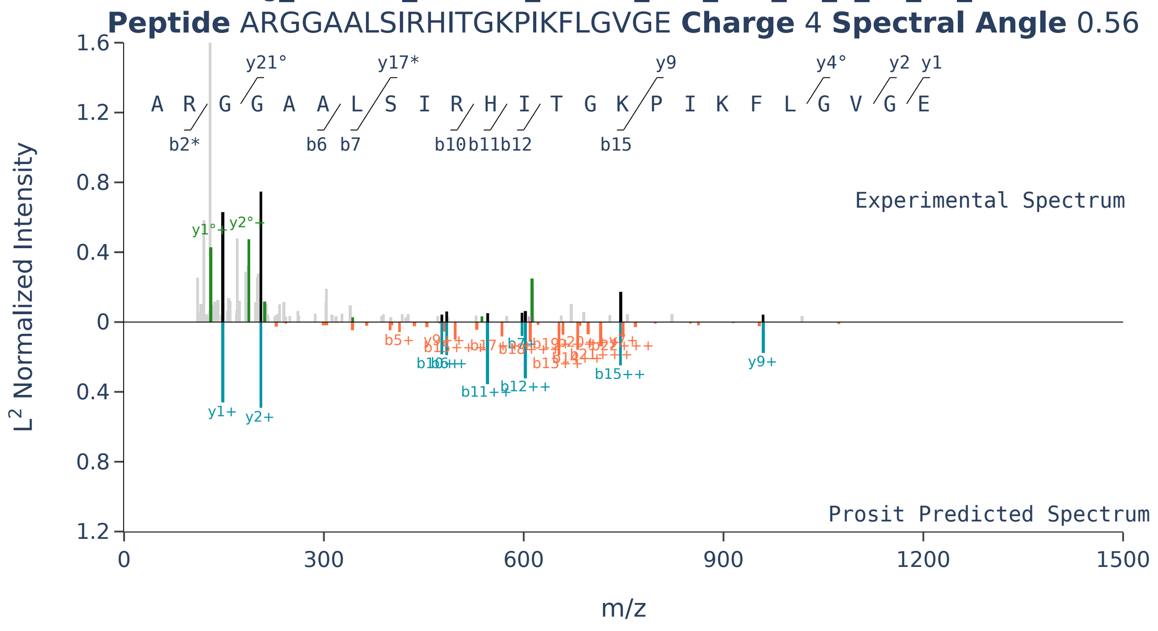


**Source** Ncheng_210623_230623_HFGoe_FFH_20S_25_1_A2_4h_R2 **Scan** 18592
**Peptide** NRVKEKAVGHEVNKSLTPGQEFVKLV **Charge** 6 **Spectral Angle** 0.6

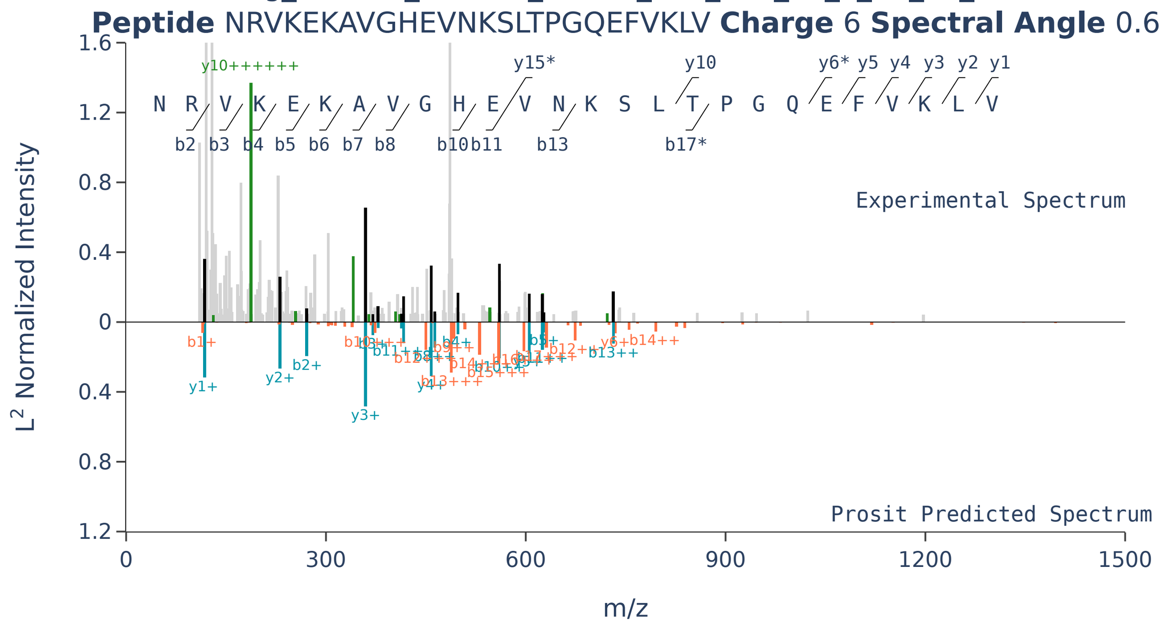


**Source** Ncheng_210623_230623_HFGoe_FFH_20S_25_1_A2_4h_R1 **Scan** 37131
**Peptide** MGEENQTLNLAAQPPAVVLM **Charge** 2 **Spectral Angle** 0.8

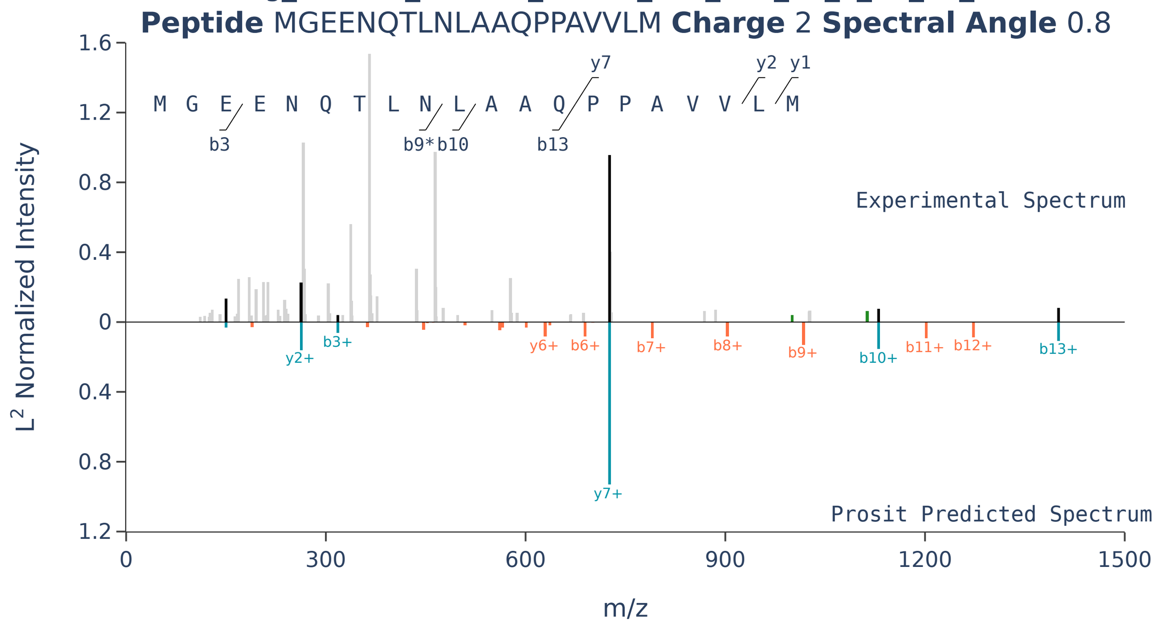


**Source** Ncheng_210623_230623_HFGoe_FFH_20S_25_1_A2_2h_R2 **Scan** 37727
**Peptide** PDNVKSQMDDKVLVRMEAIINSMTMKE **Charge** 4 **Spectral Angle** 0.77

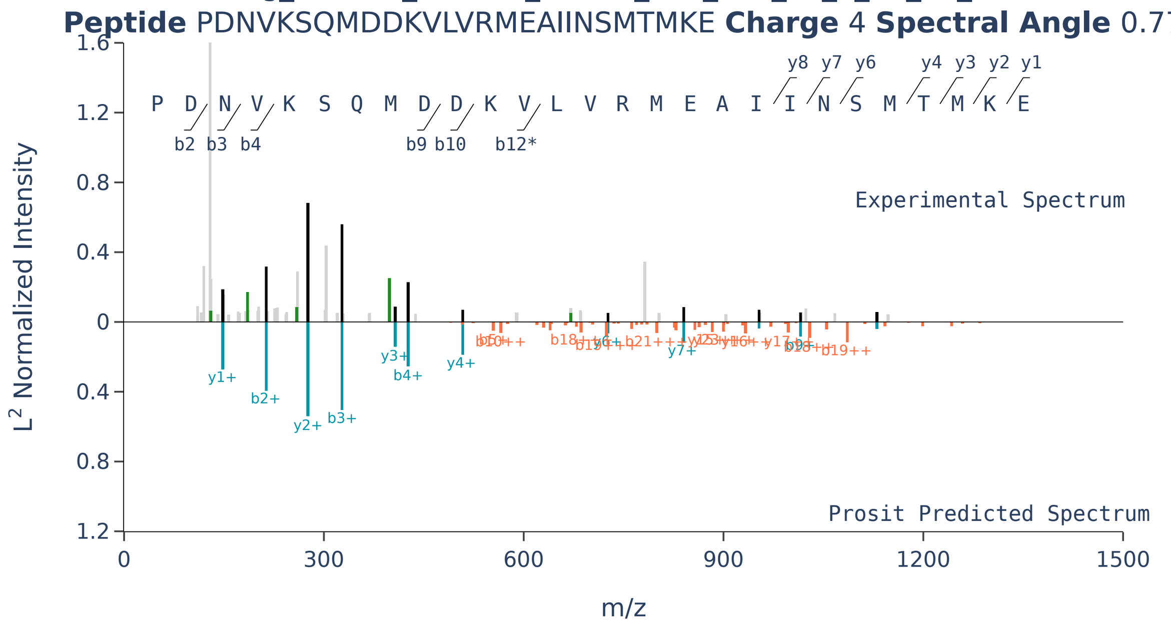


**Source** Ncheng_210623_230623_HFGoe_FFH_20S_25_1_A2_2h_R1 **Scan** 26557
**Peptide** TAGRLHVDEAMMDEIKQVHASI **Charge** 4 **Spectral Angle** 0.59

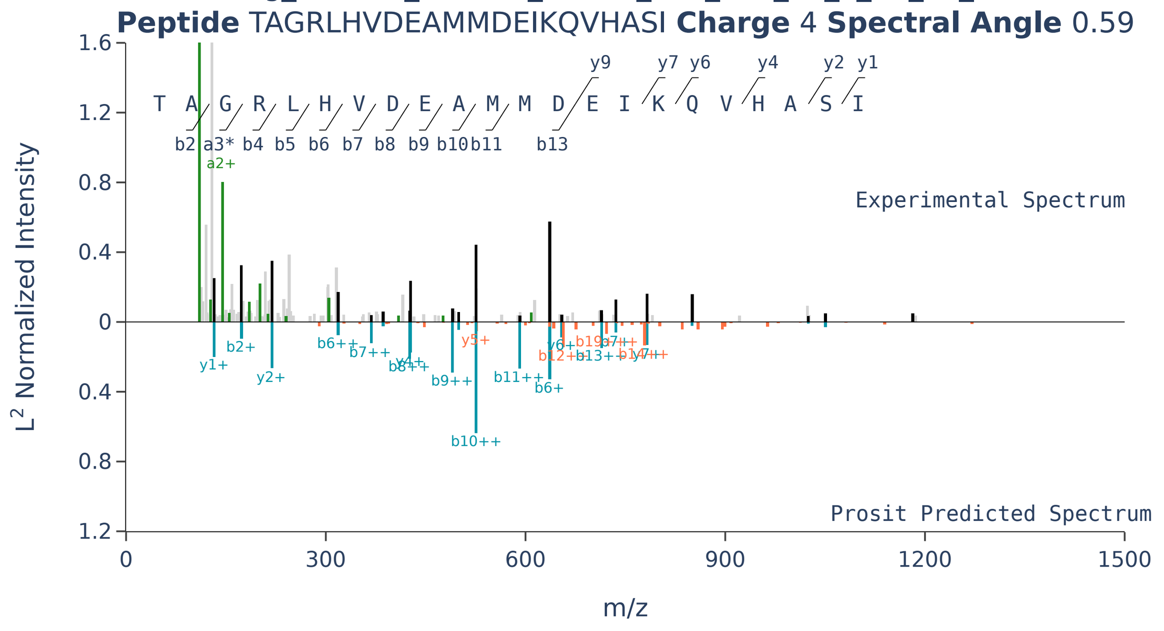


**Source** Ncheng_210623_230623_HFGoe_FFH_20S_25_1_A2_4h_R1 **Scan** 28313
**Peptide** ALPLTGVVLTKVDGDARGGAALSIRHITGK **Charge** 5 **Spectral Angle** 0.51

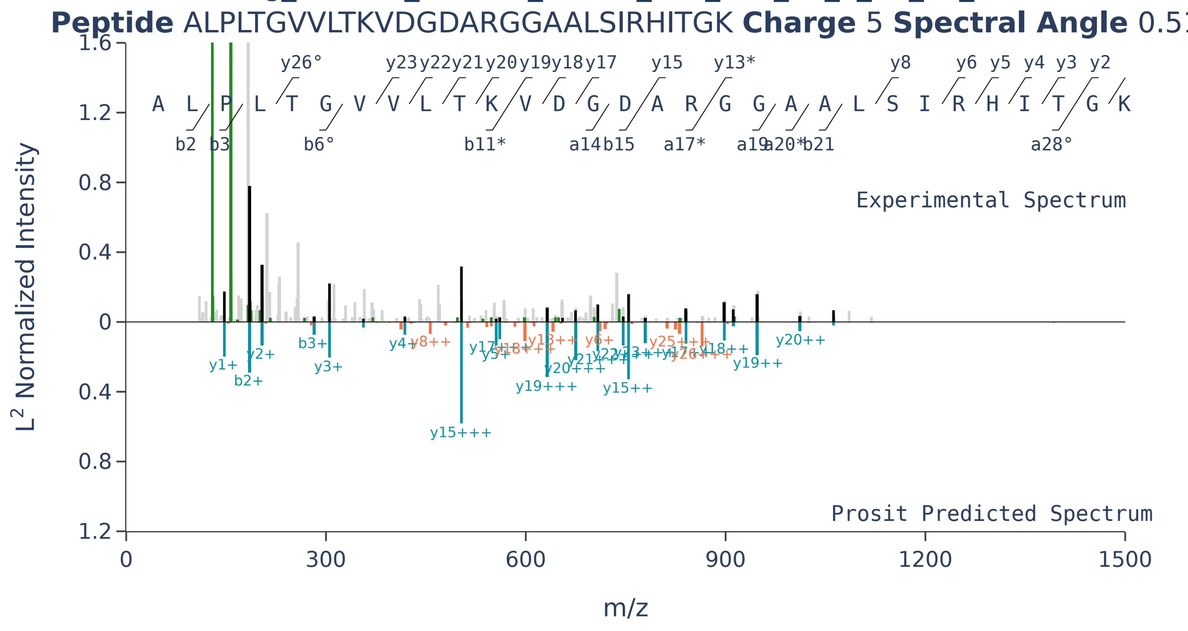

**Source** Ncheng_210623_230623_HFGoe_FFH_20S_25_1_A2_4h_R2 **Scan** 19761
**Peptide** LESKVDRAQAEKLASKLKKGDGFDLND **Charge** 4 **Spectral Angle** 0.63

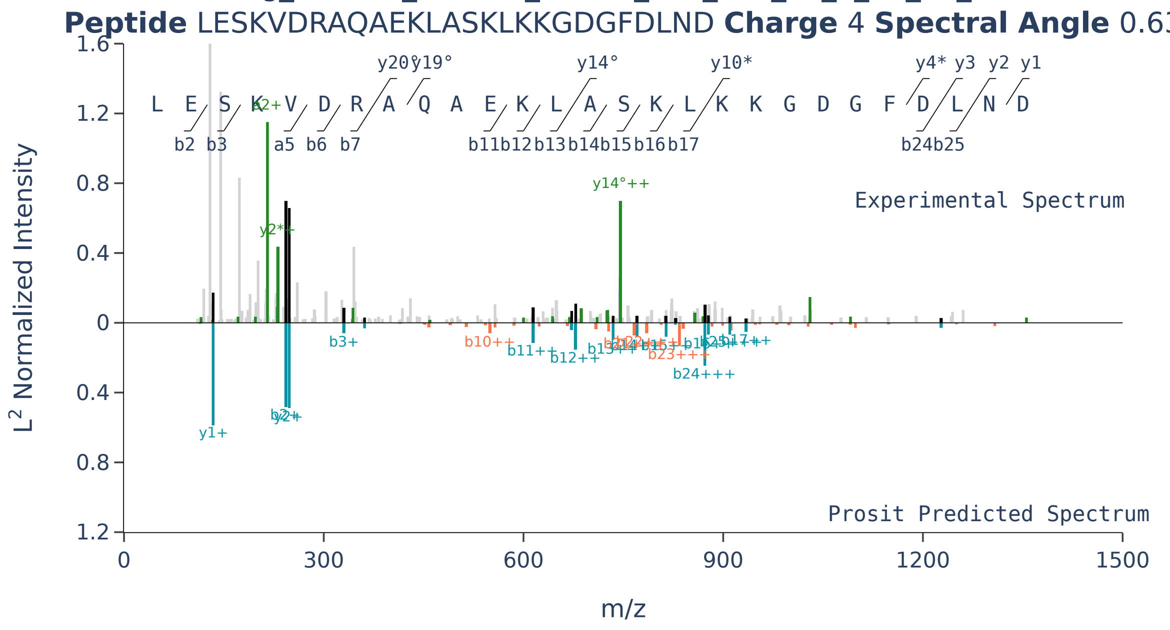


**Source** Ncheng_210623_230623_HFGoe_FFH_20S_25_1_A2_2h_R2 **Scan** 13651
**Peptide** LESKVDRAQAEKLASKLK **Charge** 5 **Spectral Angle** 0.84

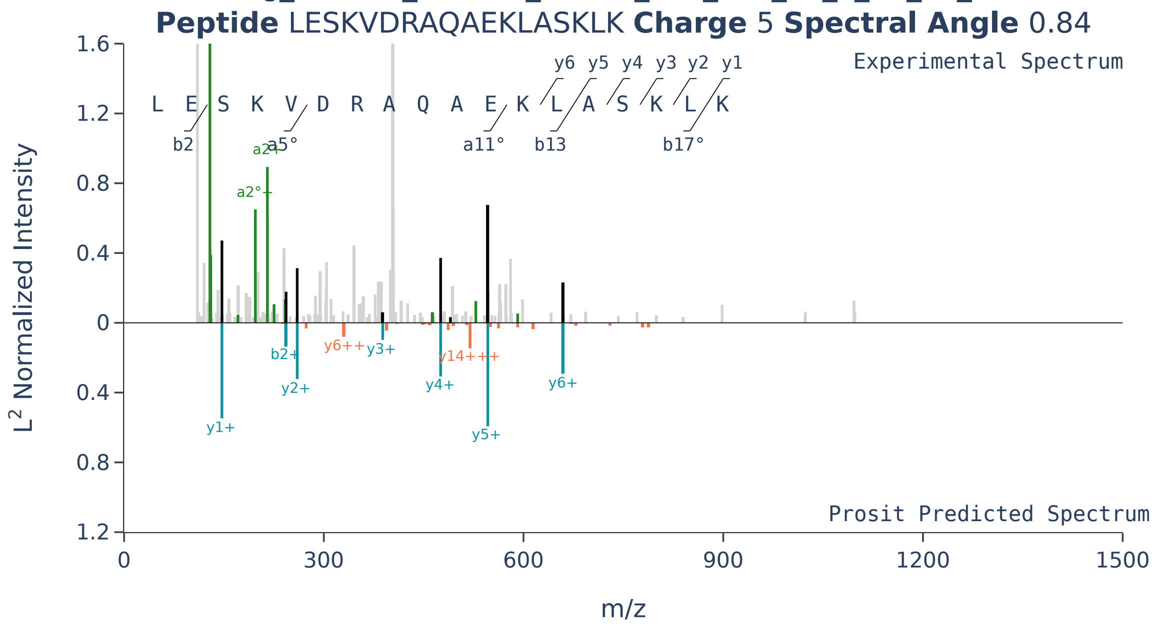


**Source** Ncheng_210623_230623_HFGoe_FFH_20S_25_1_A2_4h_R2 **Scan** 13494
**Peptide** SMTMKERAKPELL **Charge** 3 **Spectral Angle** 0.39

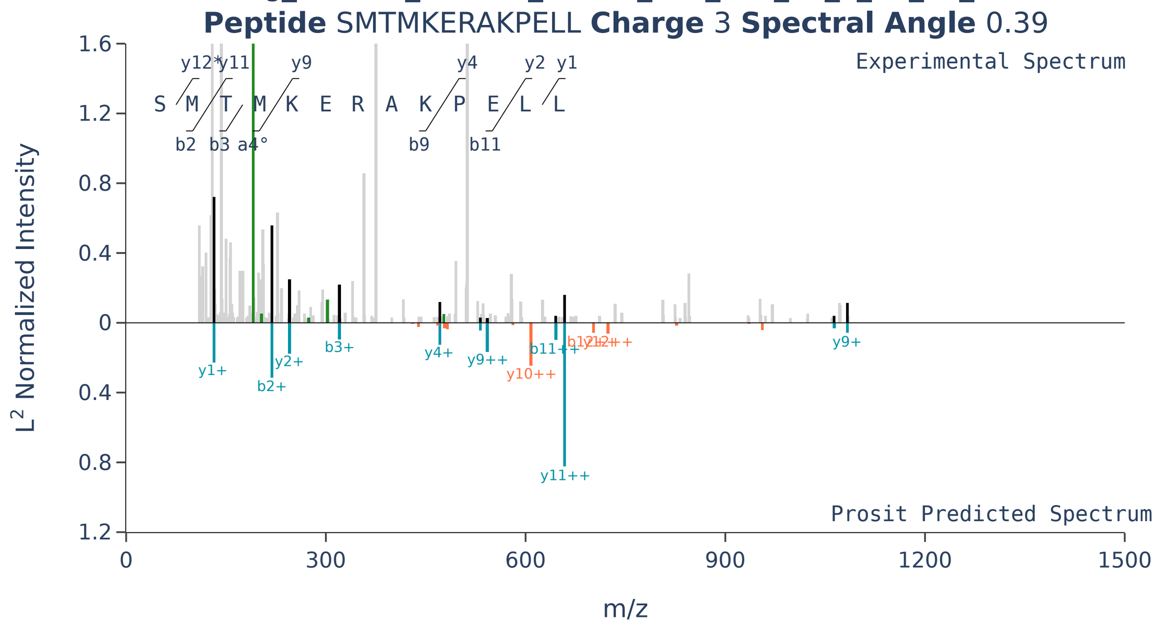


**Source** Ncheng_210623_230623_HFGoe_FFH_20S_25_1_A2_24h_R2 **Scan** 9475
**Peptide** TLRNISGRGRLTEDNVKD **Charge** 2 **Spectral Angle** 0.41

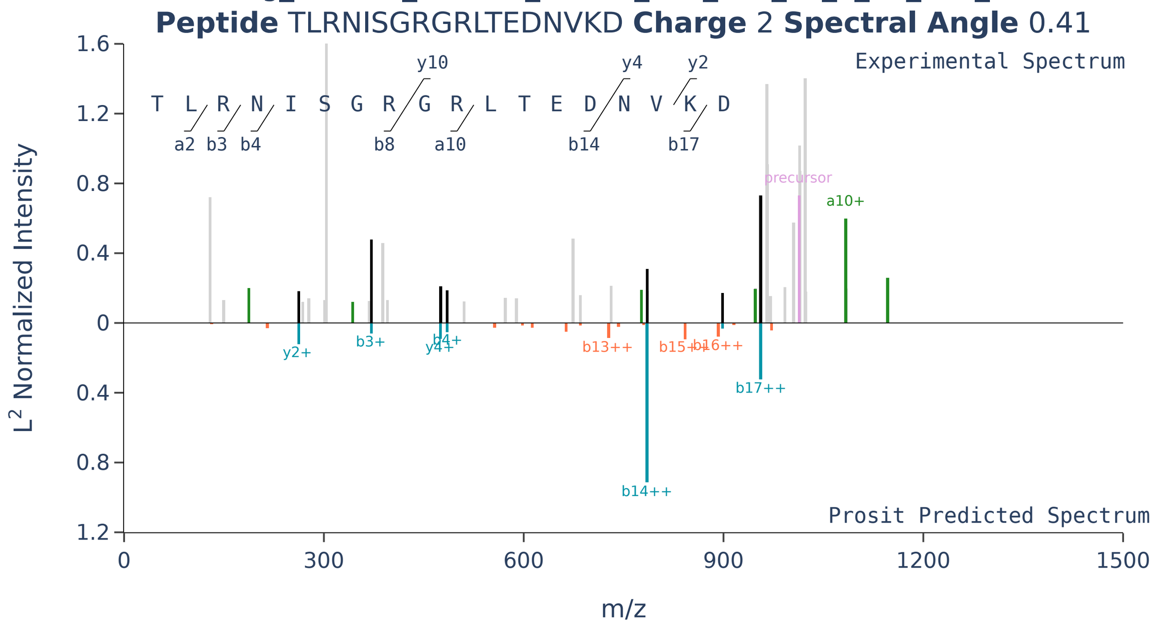


**Source** Ncheng_210623_230623_HFGoe_FFH_20S_25_1_A2_2h_R1 **Scan** 36220
**Peptide** KKGDGFDLNDFLEQLRQMK **Charge** 4 **Spectral Angle** 0.52

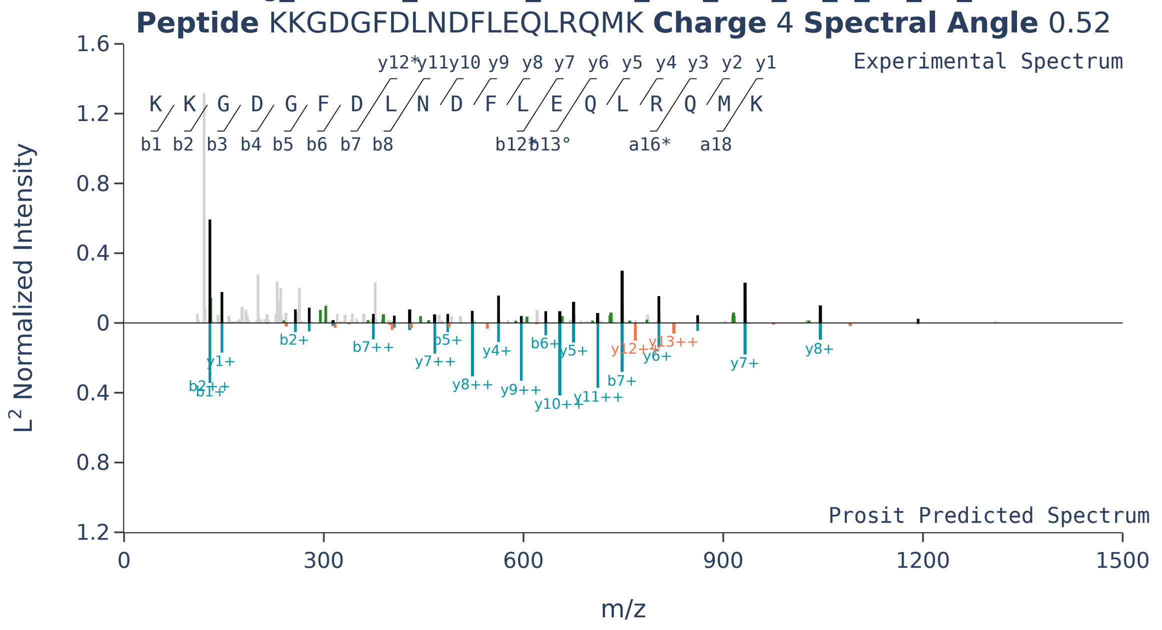


**Source** Ncheng_210623_230623_HFGoe_FFH_20S_25_1_A1_1h_R2 **Scan** 16937
**Peptide** RSMKGMMPPGFPGR **Charge** 4 **Spectral Angle** 0.43

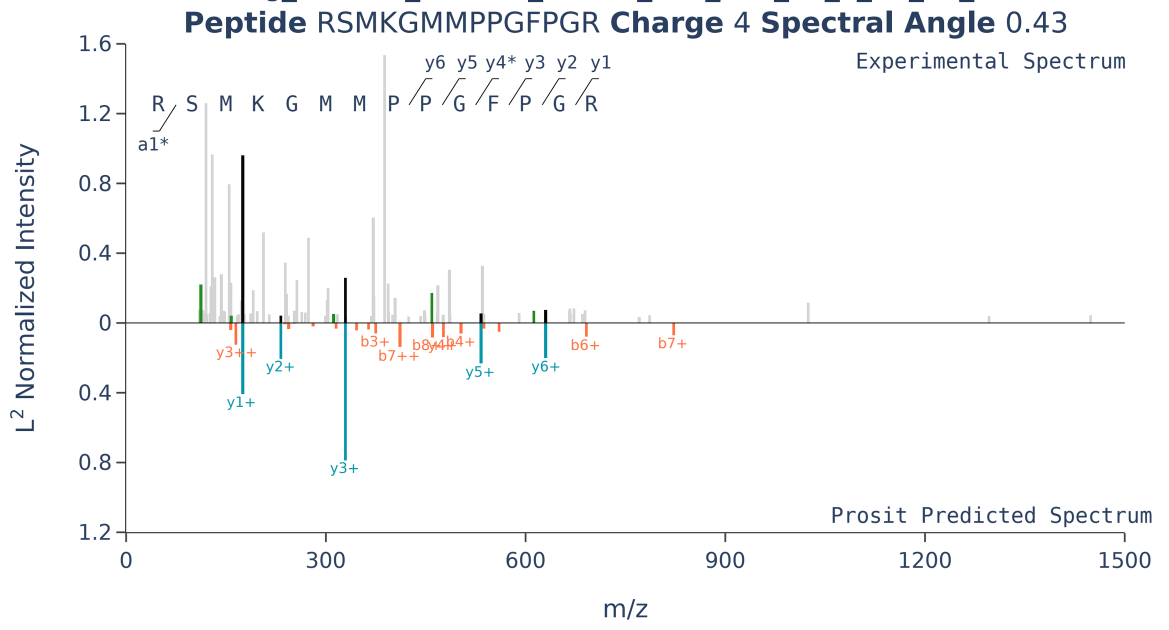


**Source** Ncheng_210623_230623_HFGoe_FFH_20S_25_1_A2_24h_R1 **Scan** 5539
**Peptide** MMKKMKKGGMA **Charge** 3 **Spectral Angle** 0.66

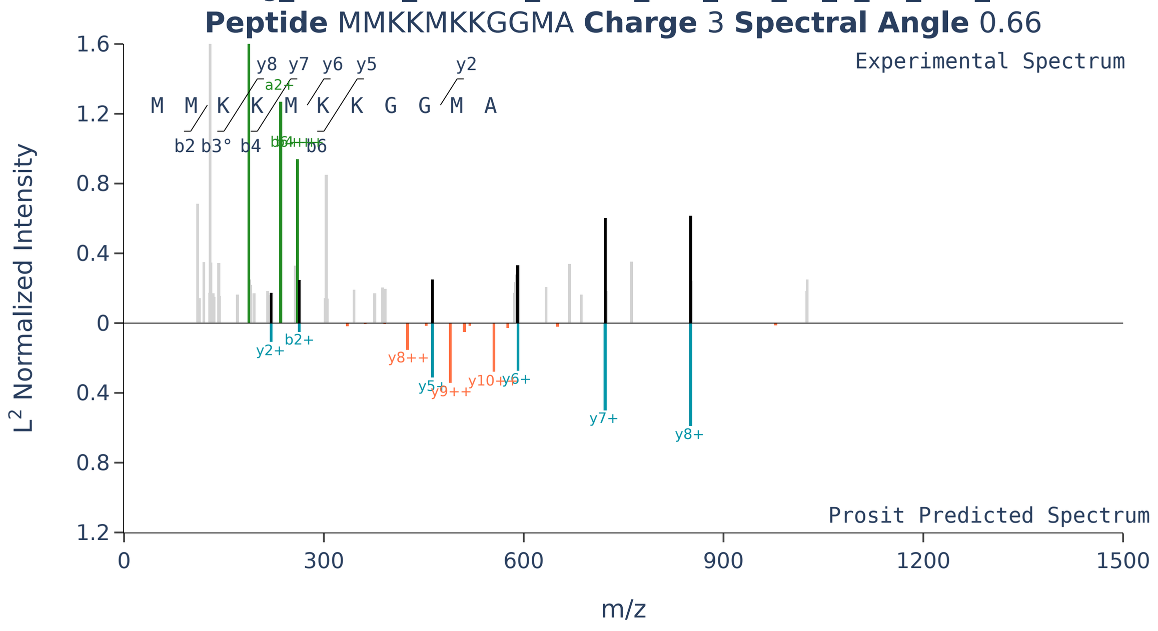


**Source** Ncheng_210623_230623_HFGoe_FFH_20S_25_1_A1_2h_R1 **Scan** 26614
**Peptide** VLVVSADVYRPAAIKQLET **Charge** 2 **Spectral Angle** 0.84

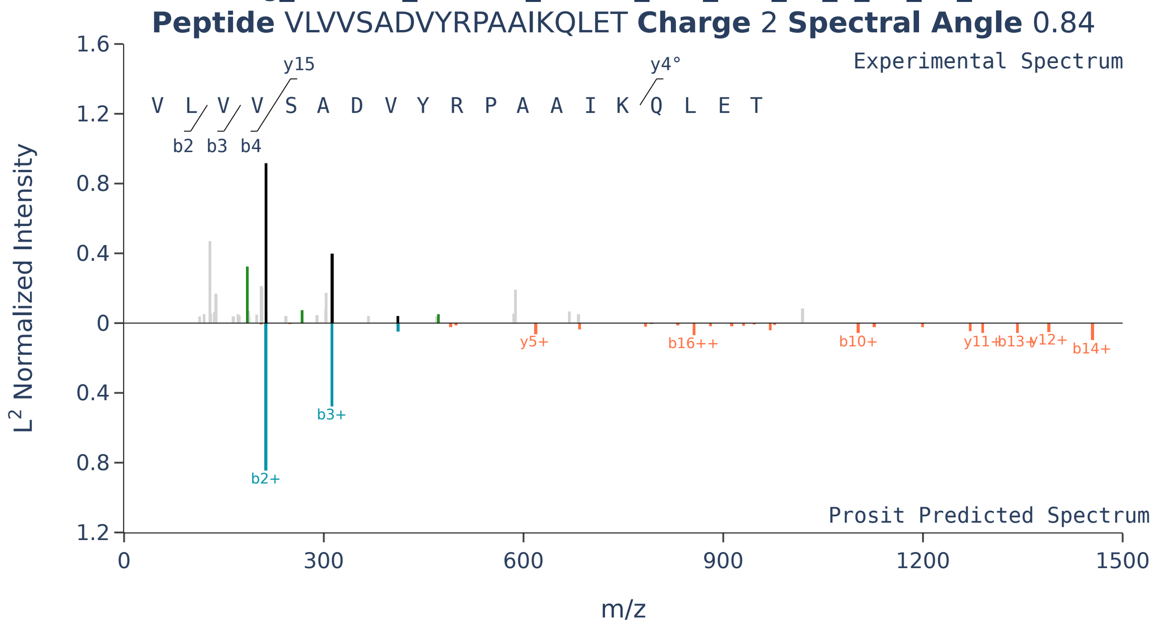


**Source** Ncheng_210623_230623_HFGoe_FFH_20S_25_1_A2_24h_R1 **Scan** 5881
**Peptide** INRVKEKAVGHEVN **Charge** 4 **Spectral Angle** 0.57

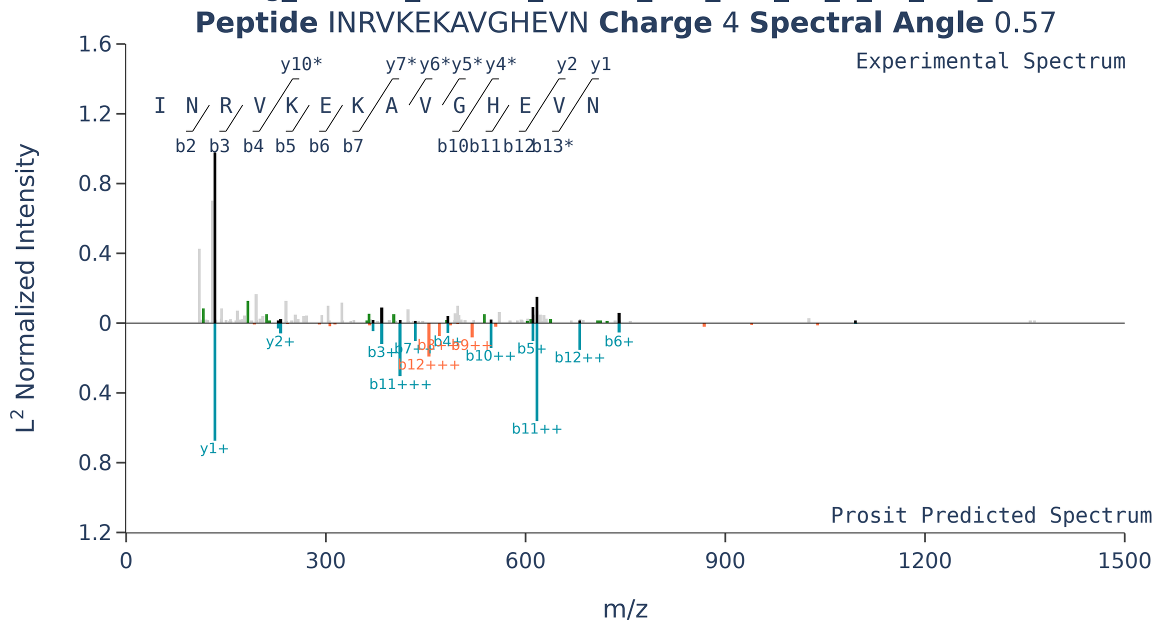


**Source** Ncheng_210623_230623_HFGoe_FFH_20S_25_1_A2_2h_R2 **Scan** 22963
**Peptide** KPIKFLGVGEKTEALEPFHPDRLAS **Charge** 5 **Spectral Angle** 0.64

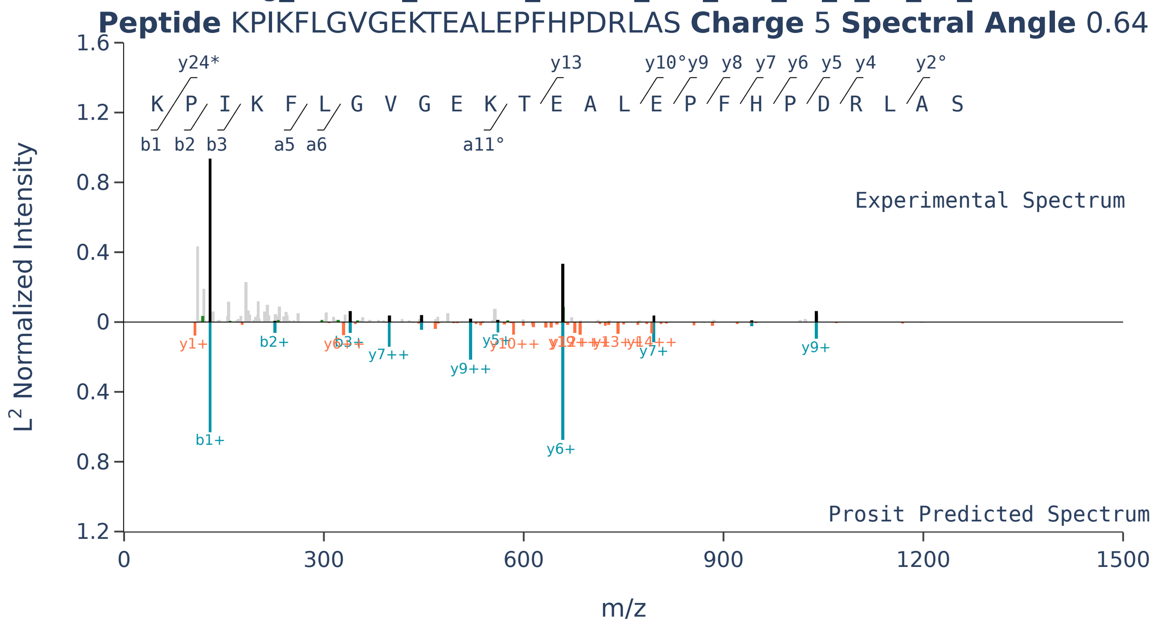


**Source** Ncheng_210623_230623_HFGoe_FFH_20S_25_1_A1_24h_R1 **Scan** 13180
**Peptide** EFINRVKEKAVGHEVNKSL **Charge** 3 **Spectral Angle** 0.48

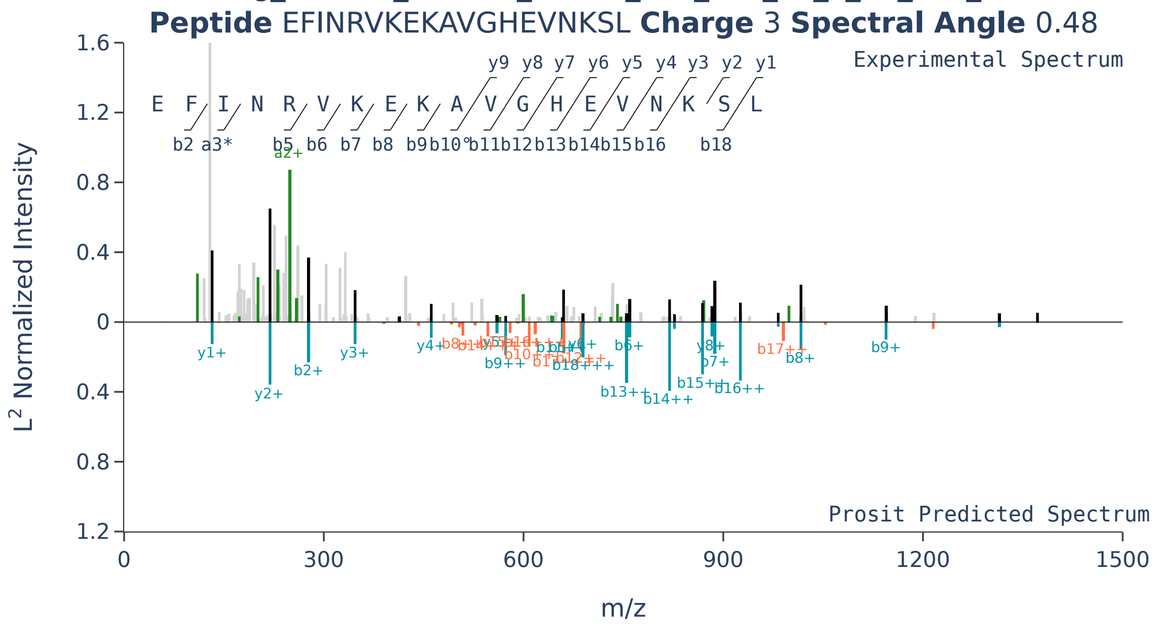


**Source** Ncheng_210623_230623_HFGoe_FFH_20S_25_1_A2_4h_R1 **Scan** 10205
**Peptide** DMQRMMKKMKKGGMA **Charge** 3 **Spectral Angle** 0.55

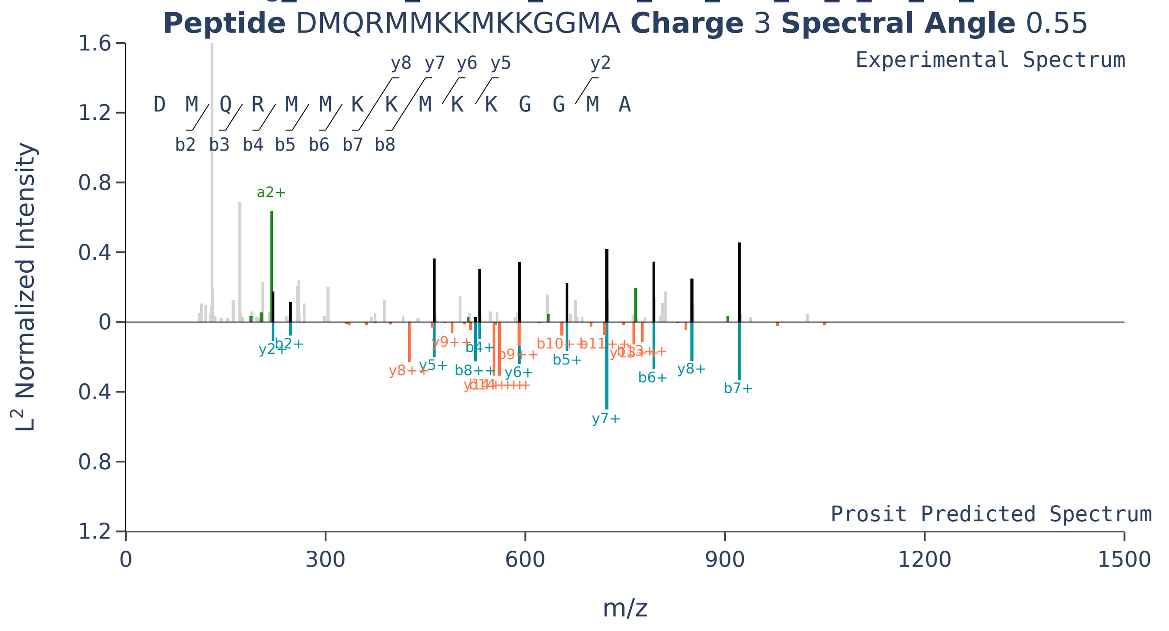


**Source** Ncheng_210623_230623_HFGoe_FFH_20S_25_1_A1_2h_R2 **Scan** 29551
**Peptide** ALPLTGVVLTKVDGDARGGAALSIRHITG **Charge** 3 **Spectral Angle** 0.52

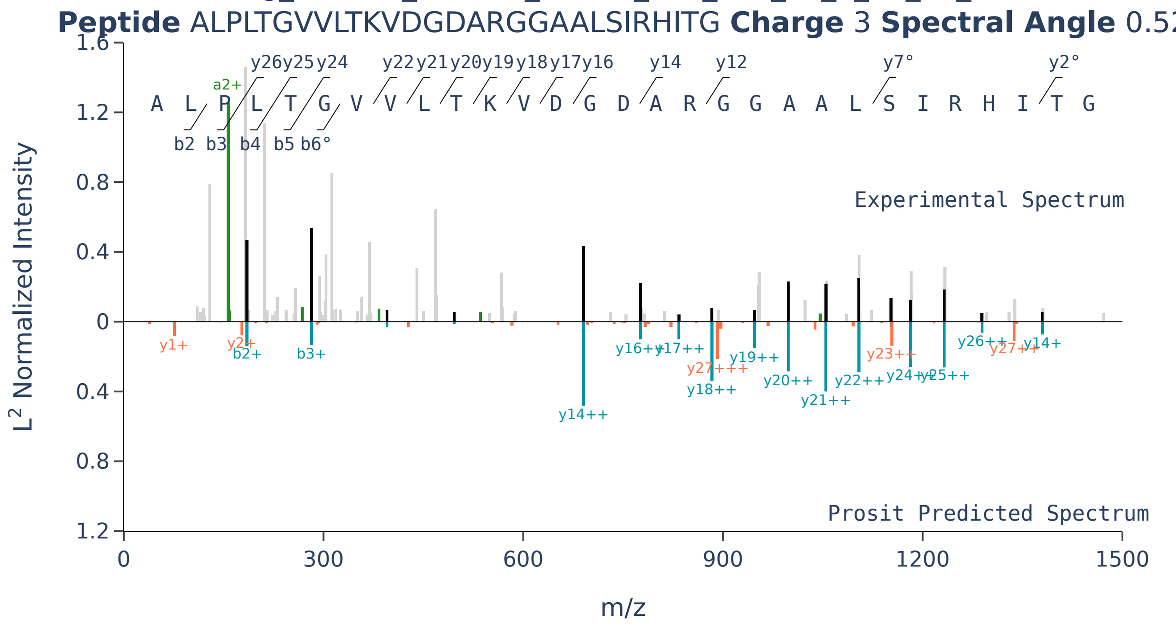


**Source** Ncheng_210623_230623_HFGoe_FFH_20S_25_1_A1_4h_R1 **Scan** 16862
**Peptide** RLSRTLRNISGRGRLTEDNVKDTLREV **Charge** 6 **Spectral Angle** 0.59

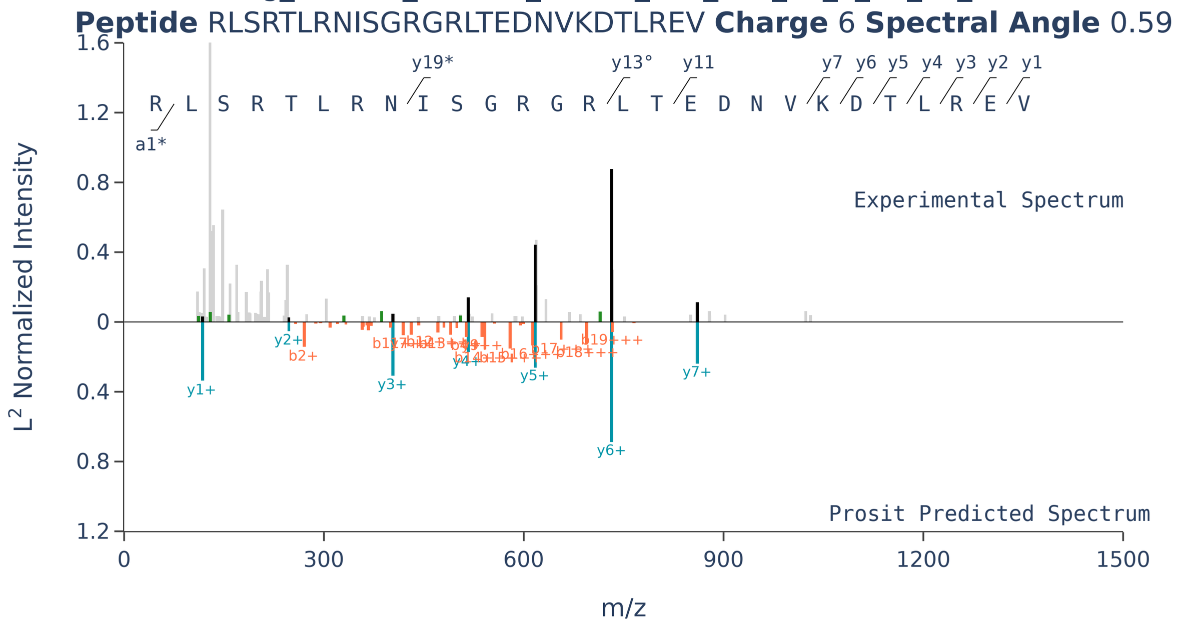


**Source** Ncheng_210623_230623_HFGoe_FFH_20S_25_1_A2_4h_R2 **Scan** 28002
**Peptide** TAGRLHVDEAMMDEIKQVHASINPVET **Charge** 4 **Spectral Angle** 0.52

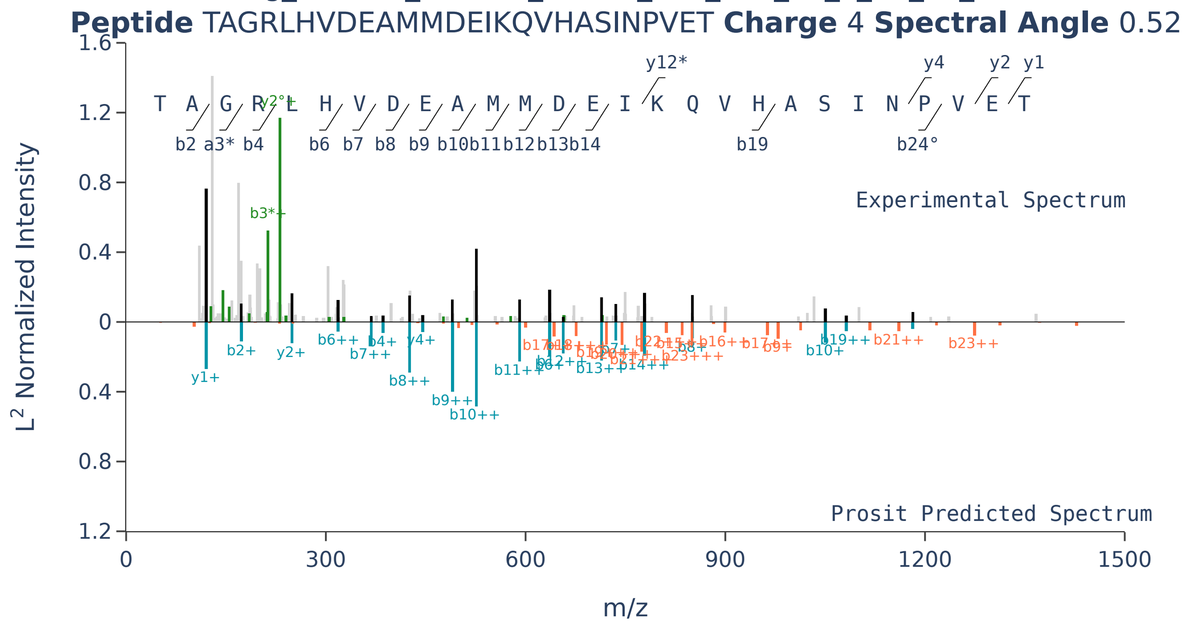

**Source** Ncheng_210623_230623_HFGoe_FFH_20S_25_1_A2_24h_R2 **Scan** 18956
**Peptide** GKLPGMGQIPDN **Charge** 2 **Spectral Angle** 0.5

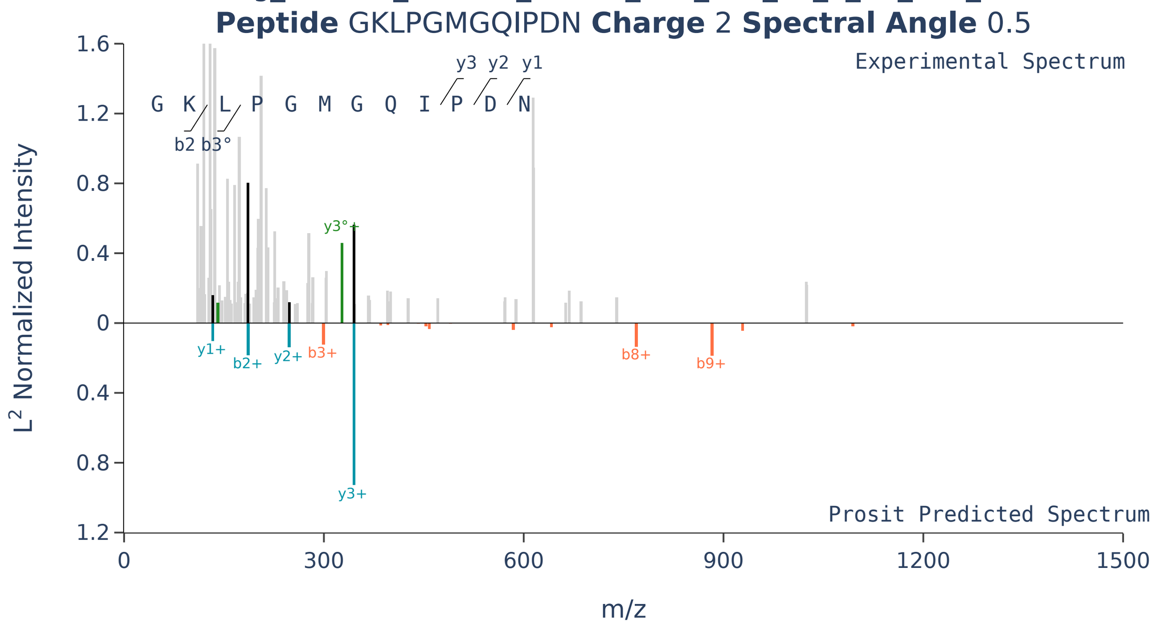


**Source** Ncheng_210623_230623_HFGoe_FFH_20S_25_1_A1_4h_R2 **Scan** 15014
**Peptide** DRLSRTLRNISGRGRLTE **Charge** 5 **Spectral Angle** 0.64

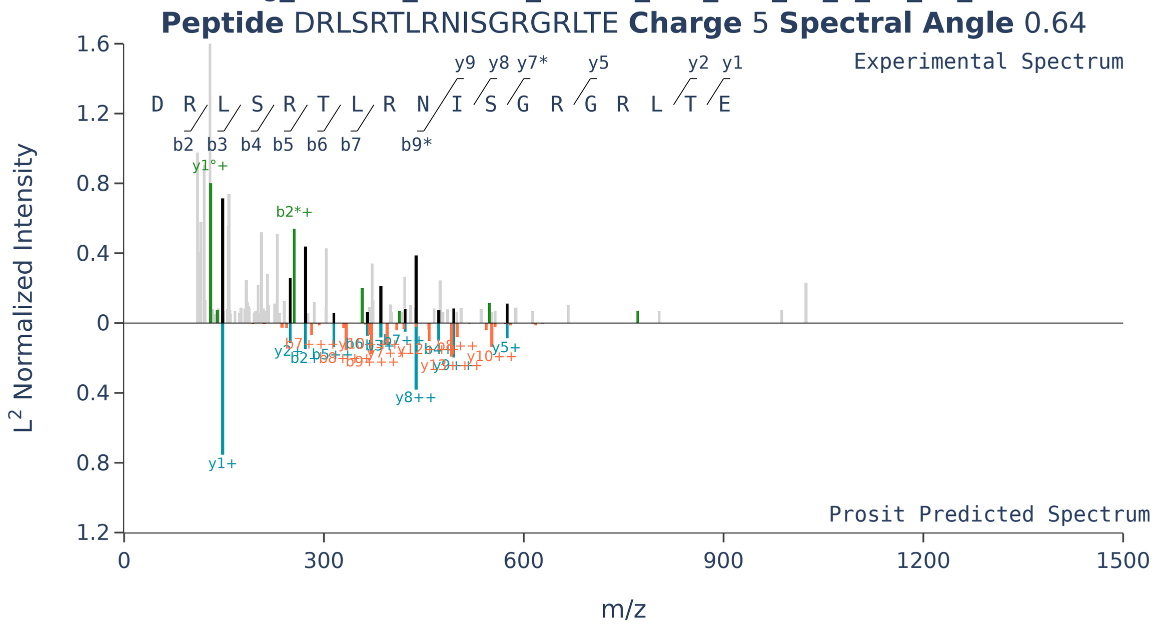


**Source** Ncheng_210623_230623_HFGoe_FFH_20S_25_1_A2_1h_R1 **Scan** 6695
**Peptide** RTLRNISG **Charge** 2 **Spectral Angle** 0.56

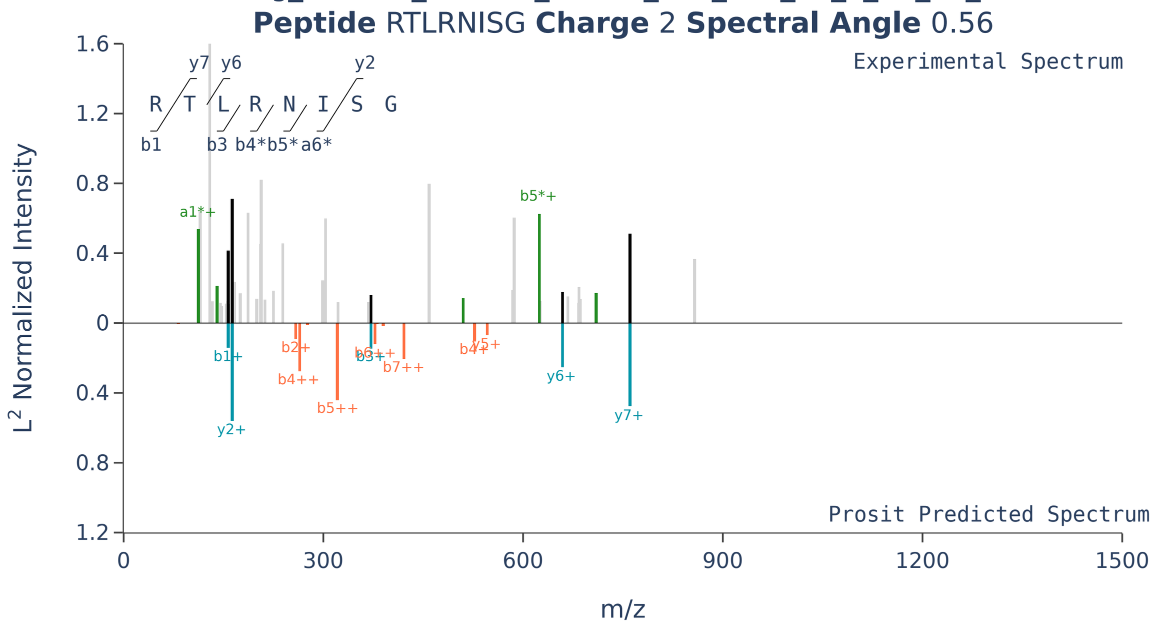


**Source** Ncheng_210623_230623_HFGoe_FFH_20S_25_1_A2_1h_R1 **Scan** 4681
**Peptide** KAVGHEVNK **Charge** 2 **Spectral Angle** 0.76

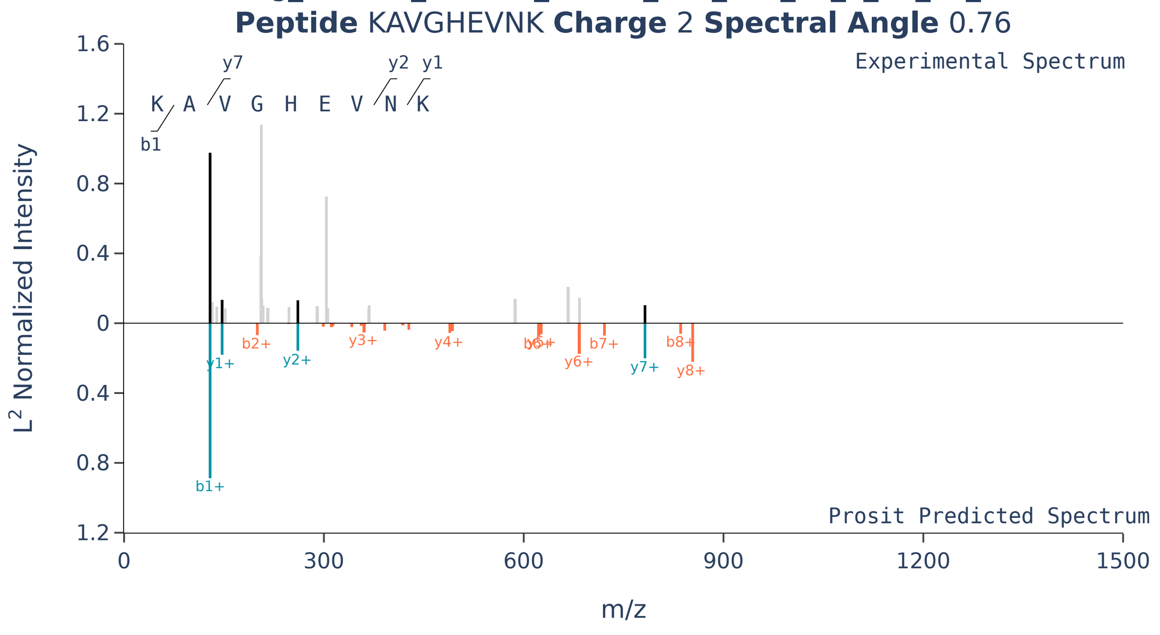


**Source** Ncheng_210623_230623_HFGoe_FFH_20S_25_1_A1_24h_R2 **Scan** 6470
**Peptide** PEIIKGSRKRRIA **Charge** 3 **Spectral Angle** 0.42

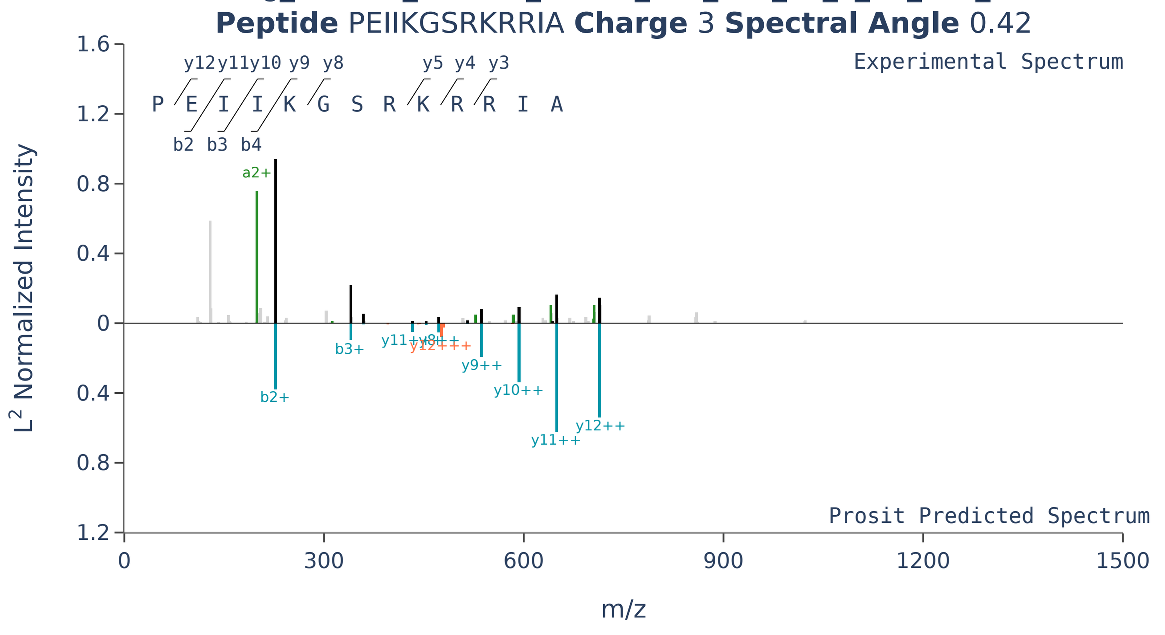


**Source** Ncheng_210623_230623_HFGoe_FFH_20S_25_1_A2_2h_R2 **Scan** 8906
**Peptide** REFINRVKEKAVG **Charge** 4 **Spectral Angle** 0.75

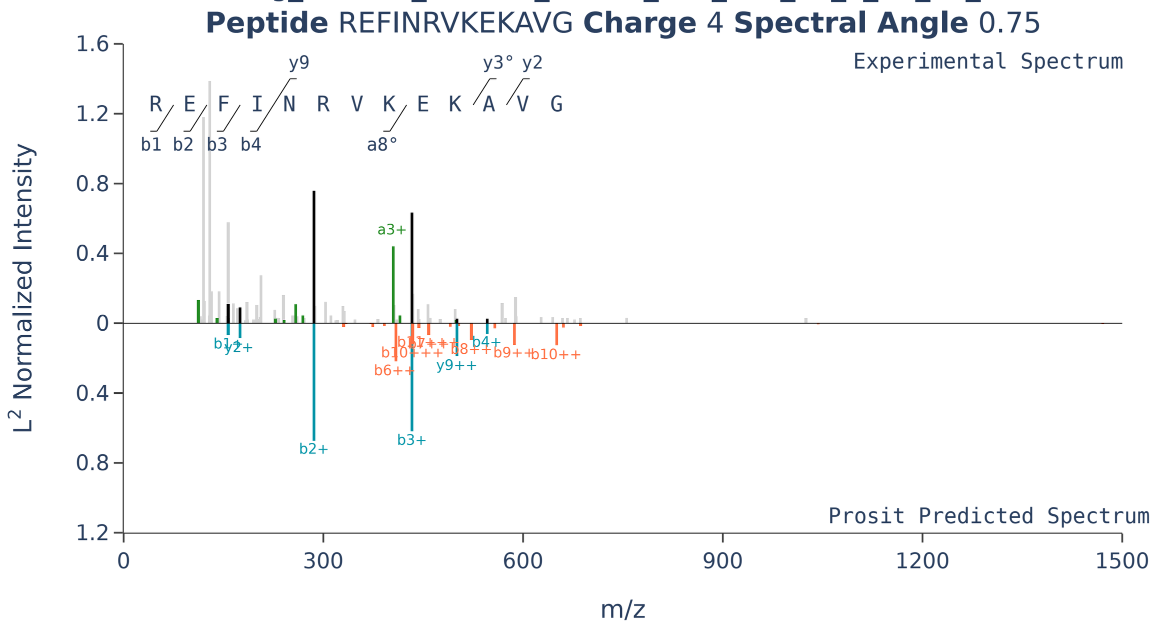


**Source** Ncheng_210623_230623_HFGoe_FFH_20S_25_1_A2_24h_R1 **Scan** 12103
**Peptide** REFINRVKEKAVGHEVNKSLTPGQE **Charge** 5 **Spectral Angle** 0.73

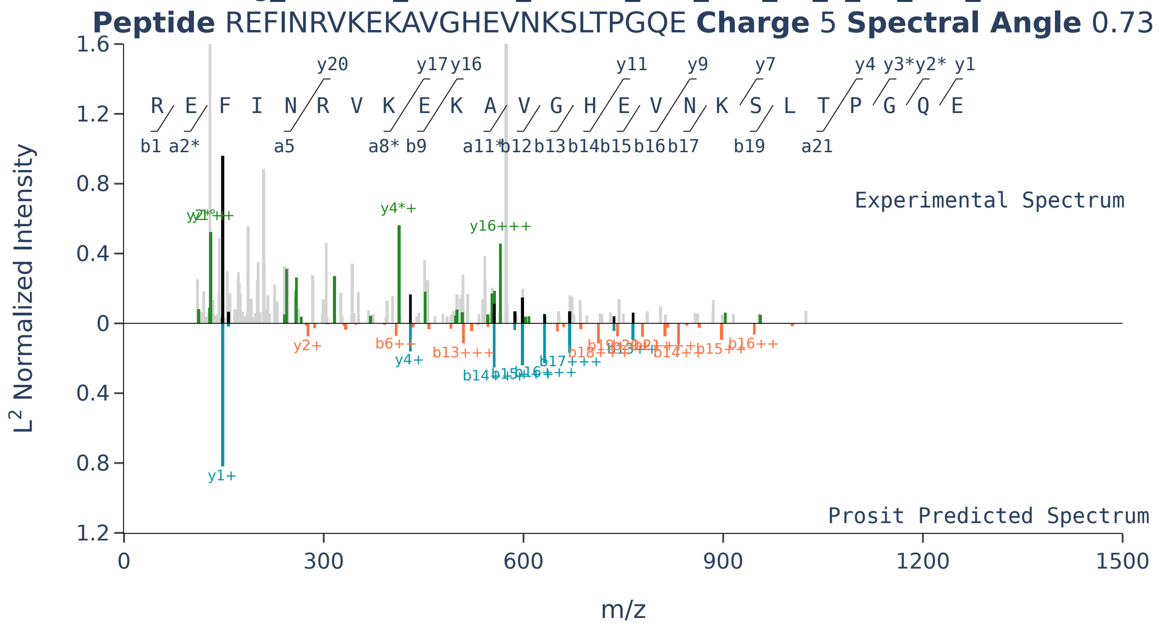


**Source** Ncheng_210623_230623_HFGoe_FFH_20S_25_1_A1_1h_R2 **Scan** 33091
**Peptide** DVALPVVREFINRVKEKA **Charge** 3 **Spectral Angle** 0.54

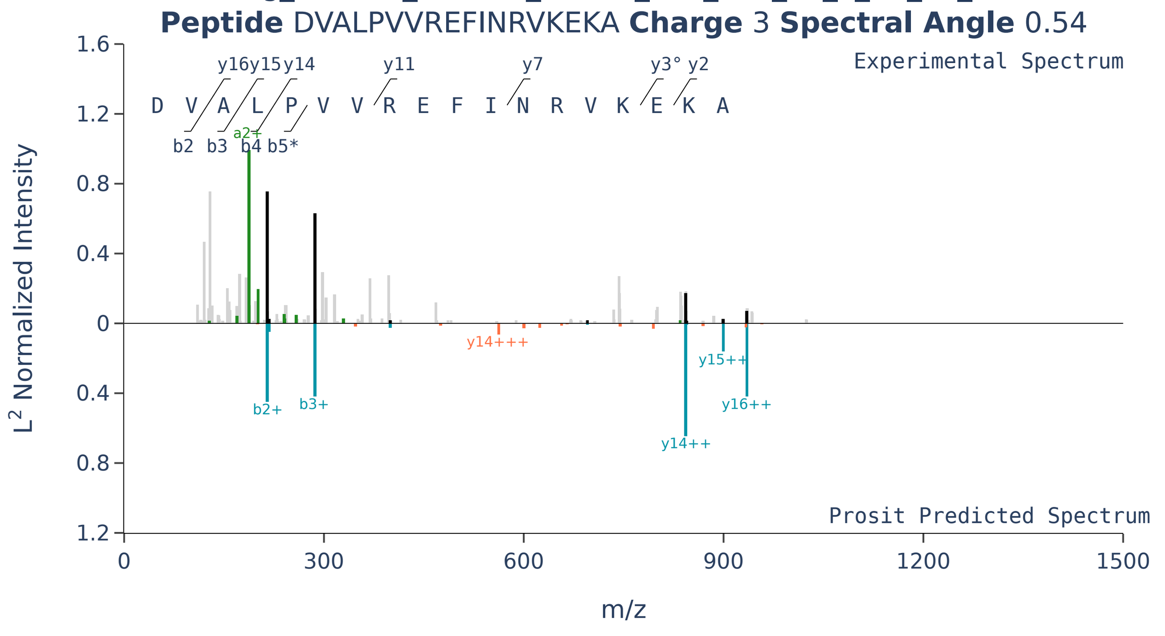


**Source** Ncheng_210623_230623_HFGoe_FFH_20S_25_1_A2_4h_R1 **Scan** 20869
**Peptide** NLTDRLSRTLRNISGRGR **Charge** 5 **Spectral Angle** 0.62

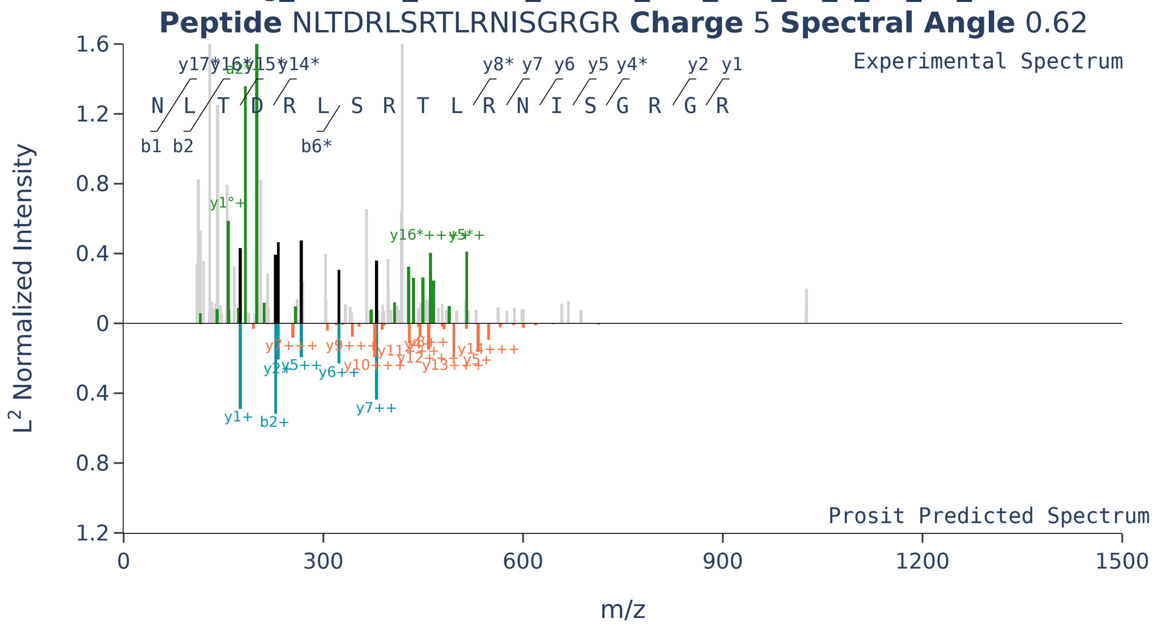


**Source** Ncheng_210623_230623_HFGoe_FFH_20S_25_1_A1_24h_R2 **Scan** 11729
**Peptide** AGKTTSVGKLGKFLREKH **Charge** 4 **Spectral Angle** 0.74

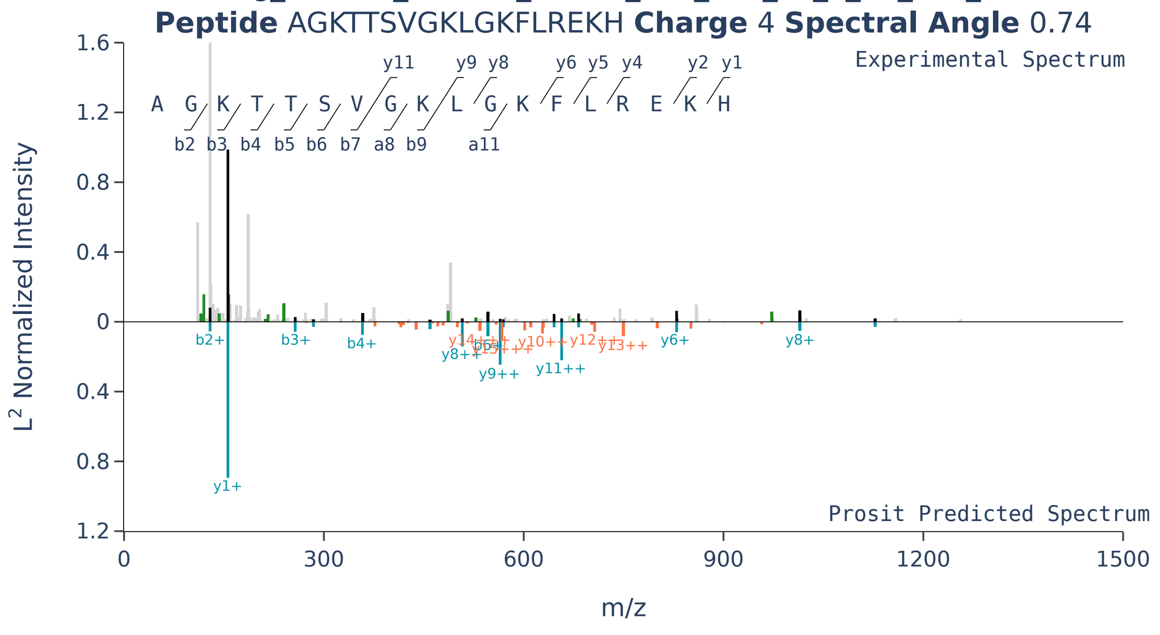


**Source** Ncheng_210623_230623_HFGoe_FFH_20S_25_1_A2_4h_R1 **Scan** 14940
**Peptide** REFINRVKEKAVGHEVNKSLTPGQEF **Charge** 5 **Spectral Angle** 0.67

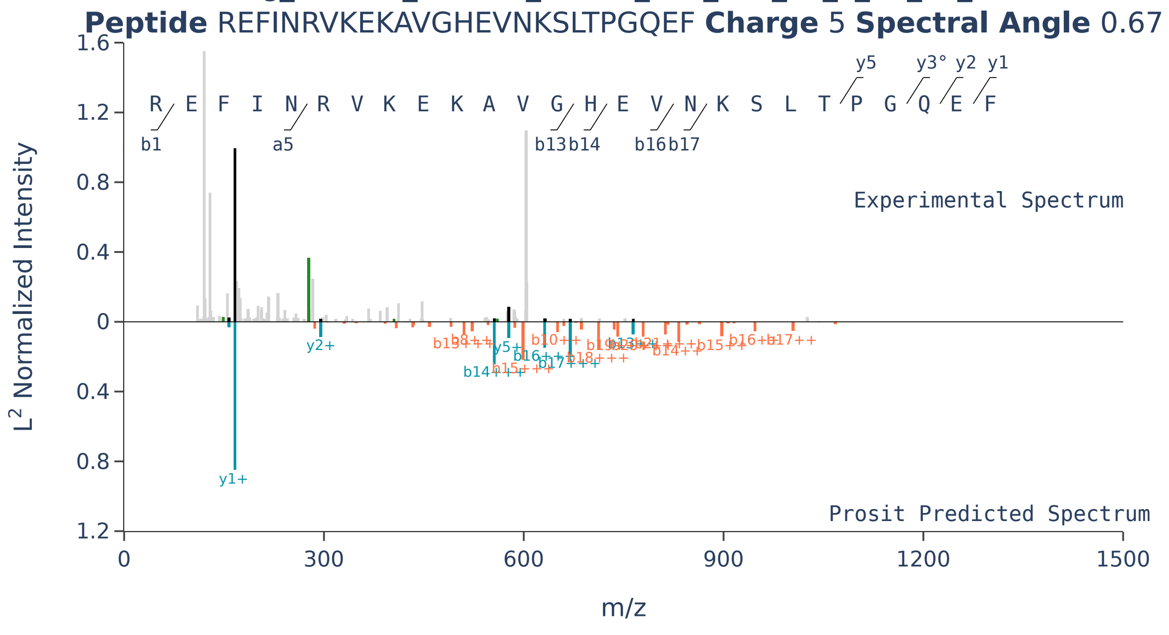


**Source** Ncheng_210623_230623_HFGoe_FFH_20S_25_1_A2_4h_R1 **Scan** 25447
**Peptide** DGDARGGAALSIRHITGKPIKFLGV **Charge** 4 **Spectral Angle** 0.59

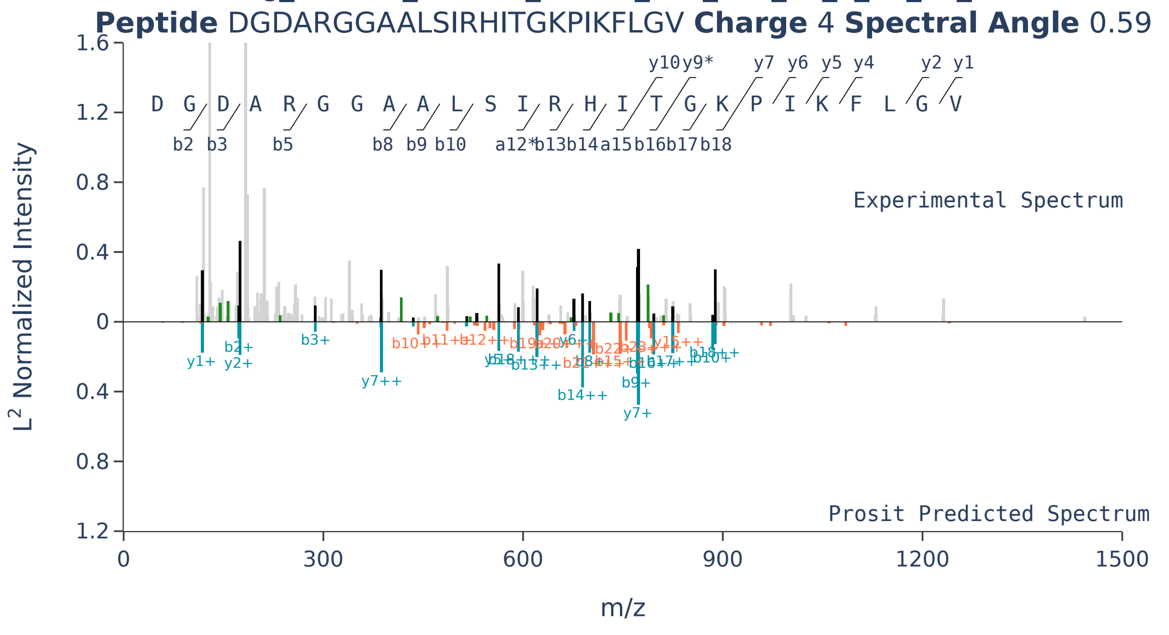


**Source** Ncheng_210623_230623_HFGoe_FFH_20S_25_1_A1_24h_R1 **Scan** 11811
**Peptide** GKTTSVGKLGKFLREKH **Charge** 4 **Spectral Angle** 0.71

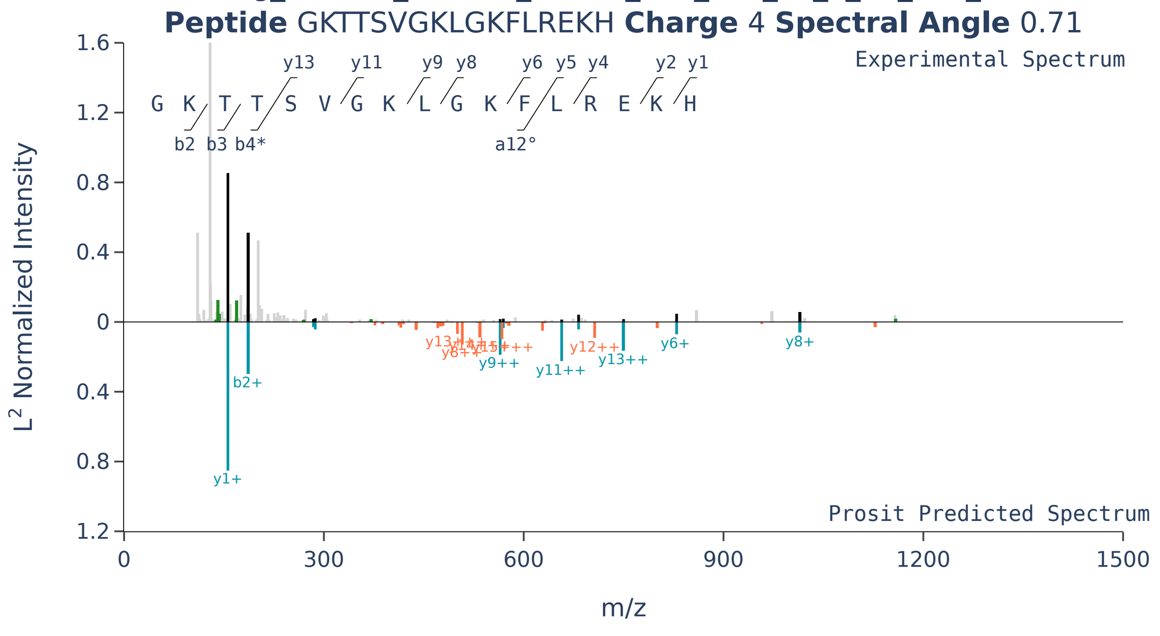


**Source** Ncheng_210623_230623_HFGoe_FFH_20S_25_1_A1_2h_R2 **Scan** 33467
**Peptide** VGQKPVDIVNAALKEAKLKFY **Charge** 5 **Spectral Angle** 0.68

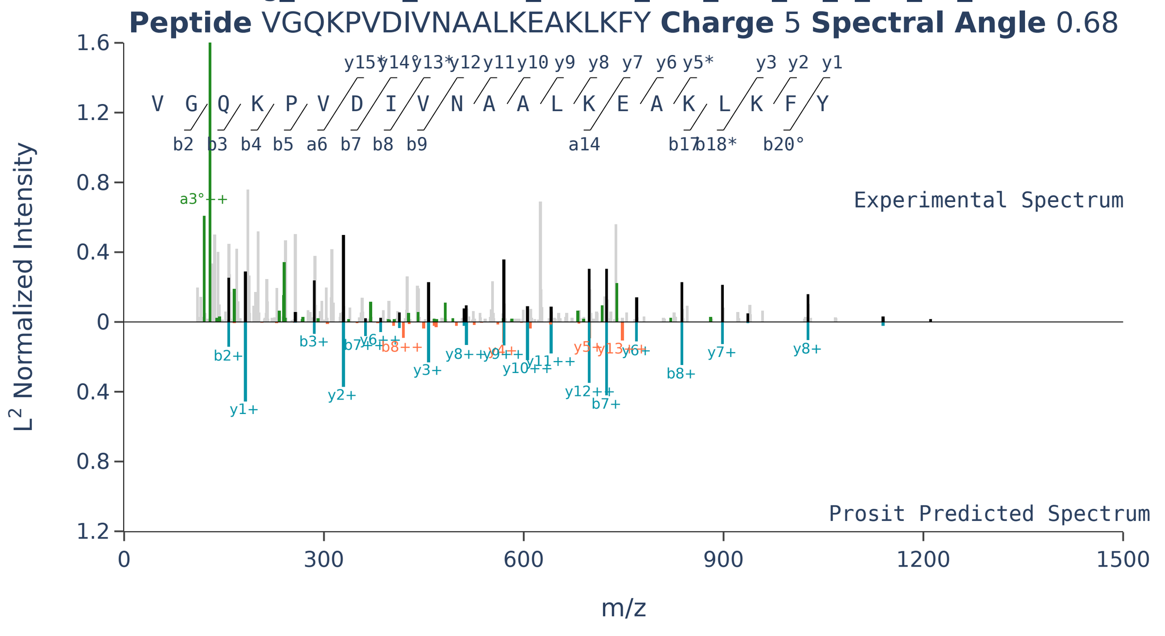


**Source** Ncheng_210623_230623_HFGoe_FFH_20S_25_1_A2_4h_R1 **Scan** 39947
**Peptide** MGEENQTLNLAAQPPAVVLMAGLQGAGKTT **Charge** 3 **Spectral Angle** 0.52

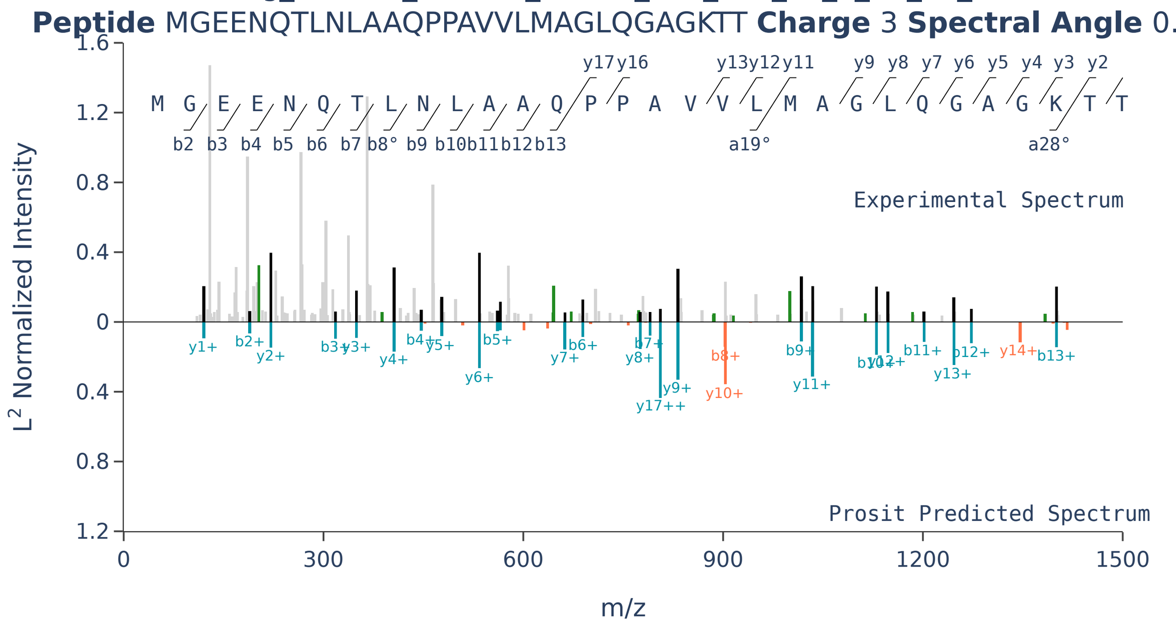

**Source** Ncheng_210623_230623_HFGoe_FFH_20S_25_1_A2_2h_R1 **Scan** 14352
**Peptide** PSDVGQKPVDIVN **Charge** 2 **Spectral Angle** 0.58

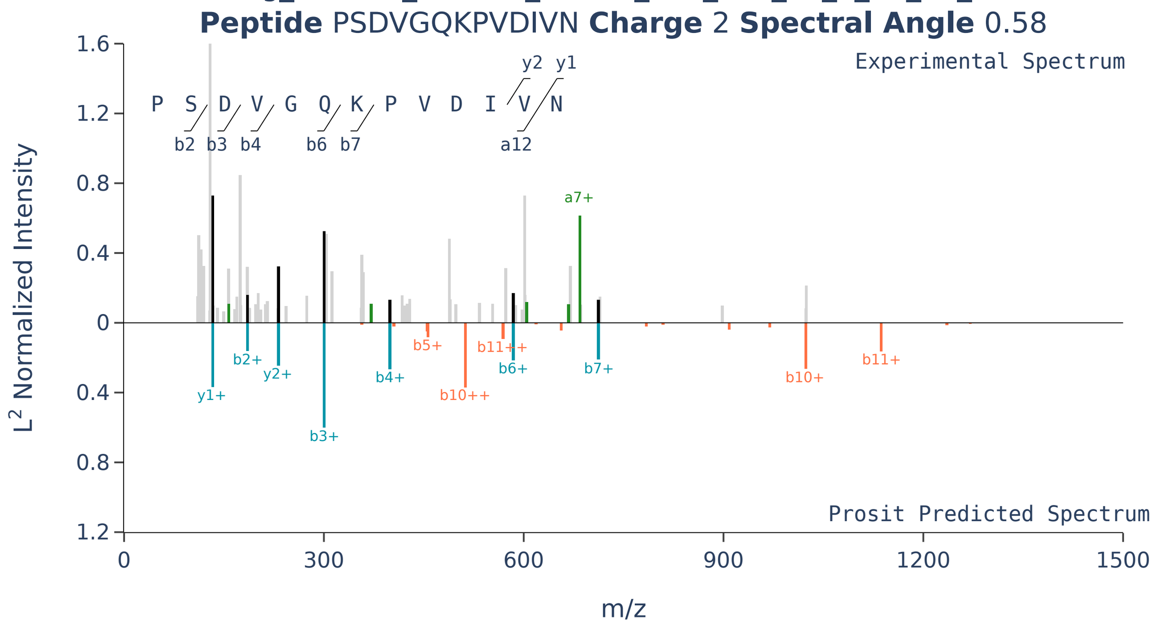


**Source** Ncheng_210623_230623_HFGoe_FFH_20S_25_1_A1_24h_R1 **Scan** 7644
**Peptide** KKKVLVVS **Charge** 2 **Spectral Angle** 0.6

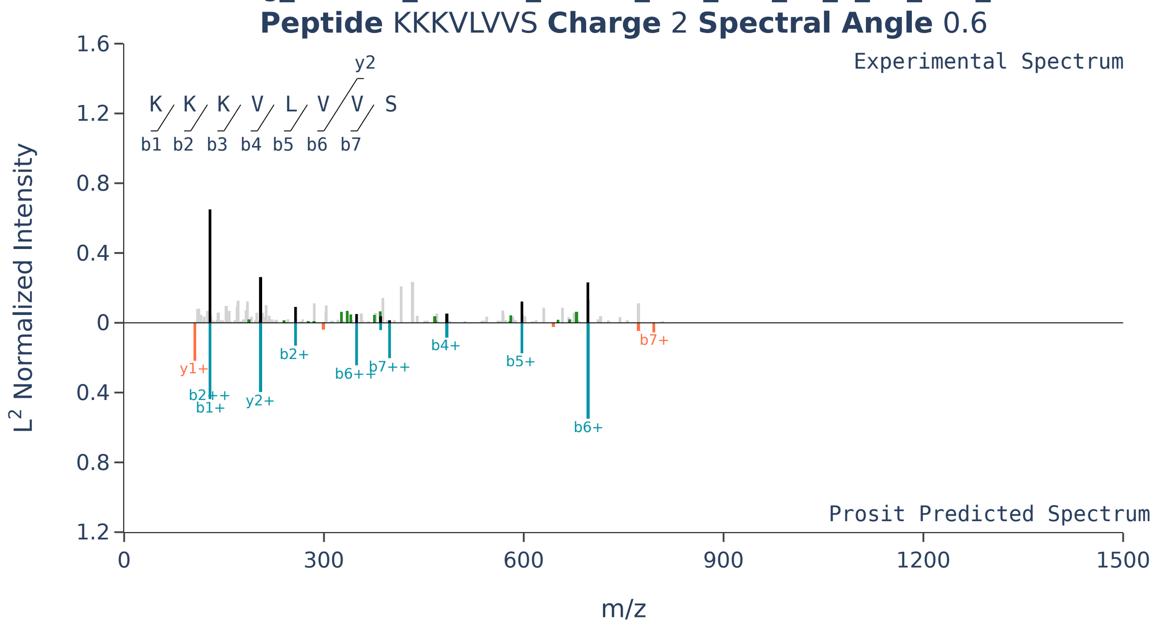


**Source** Ncheng_210623_230623_HFGoe_FFH_20S_25_1_A2_24h_R2 **Scan** 15237
**Peptide** KVDRAQAEKLASKLKKGDGF **Charge** 4 **Spectral Angle** 0.67

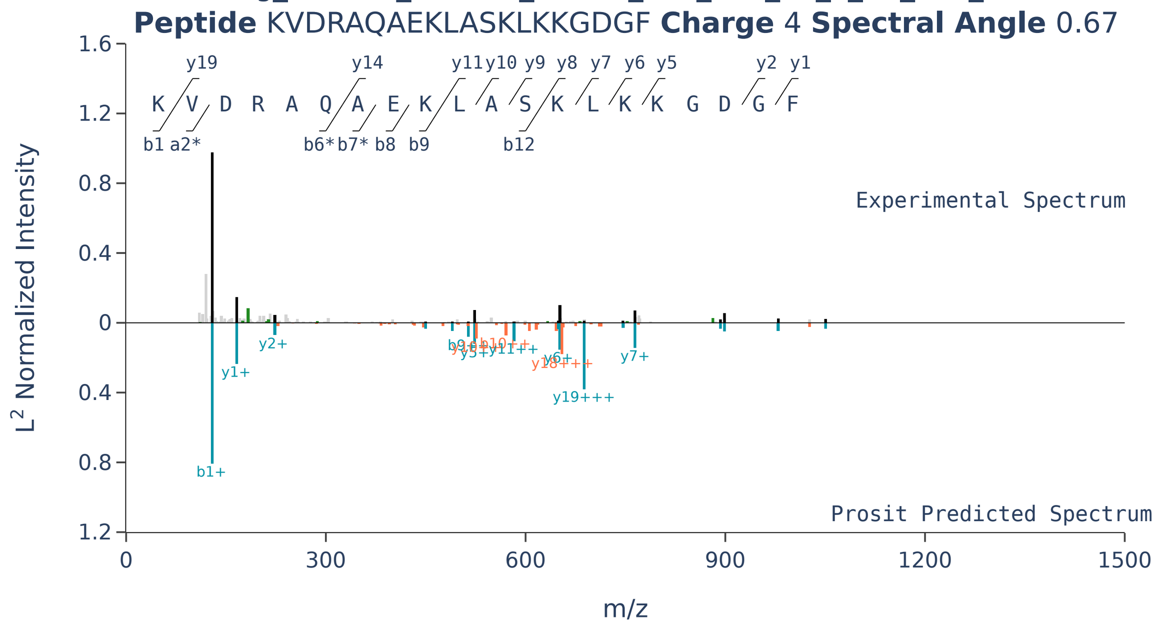


**Source** Ncheng_210623_230623_HFGoe_FFH_20S_25_1_A2_2h_R1 **Scan** 24649
**Peptide** VDGDARGGAALSIRHITGKPIKFLGV **Charge** 5 **Spectral Angle** 0.72

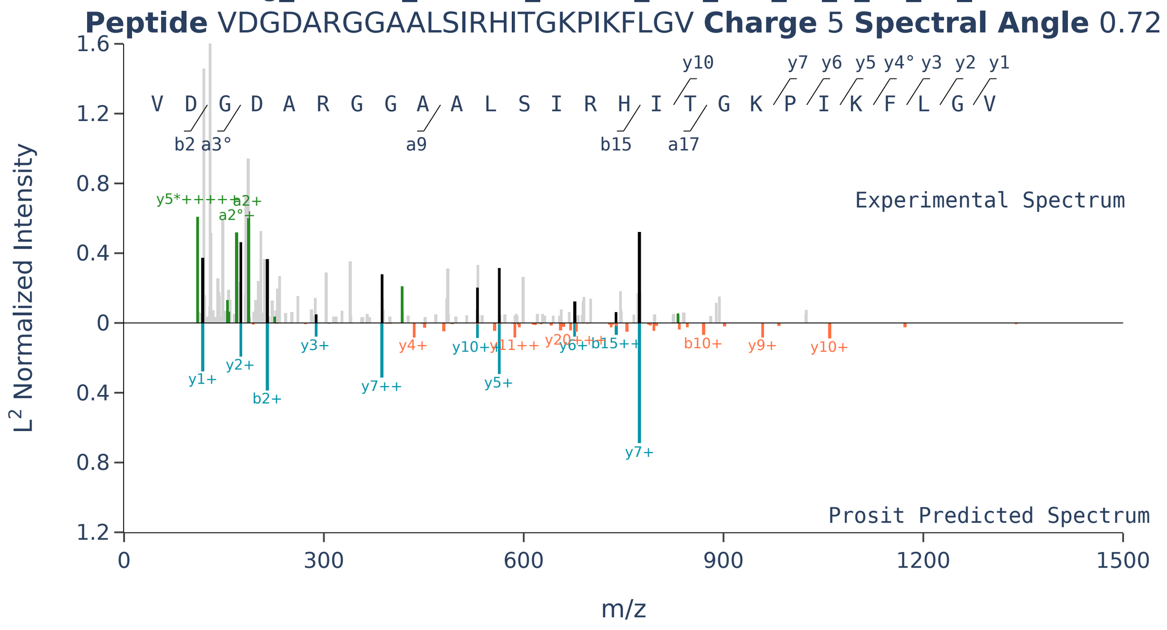


**Source** Ncheng_210623_230623_HFGoe_FFH_20S_25_1_A2_24h_R2 **Scan** 17474
**Peptide** LESKVDRAQAEKLASKLKKGDGF **Charge** 6 **Spectral Angle** 0.71

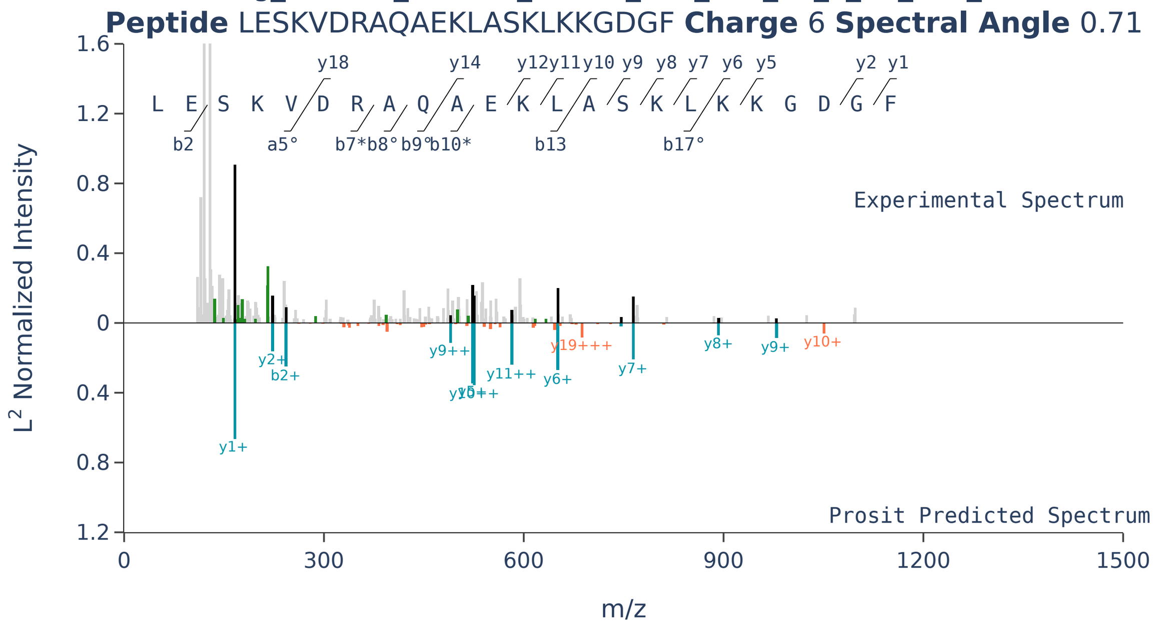


**Source** Ncheng_210623_230623_HFGoe_FFH_20S_25_1_A1_2h_R2 **Scan** 24497
**Peptide** VKIVRNELVAAMGEENQTL **Charge** 3 **Spectral Angle** 0.51

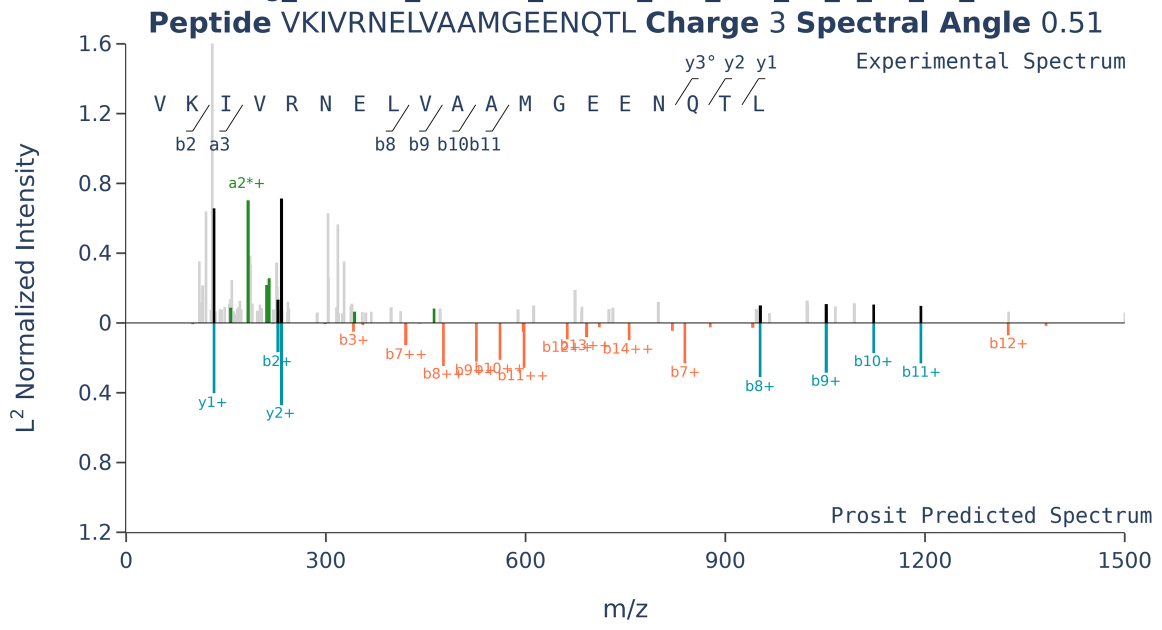


**Source** Ncheng_210623_230623_HFGoe_FFH_20S_25_1_A1_1h_R1 **Scan** 39575
**Peptide** TLFVVDAMTGQDAANTAKAFNEALPLTGV **Charge** 3 **Spectral Angle** 0.7

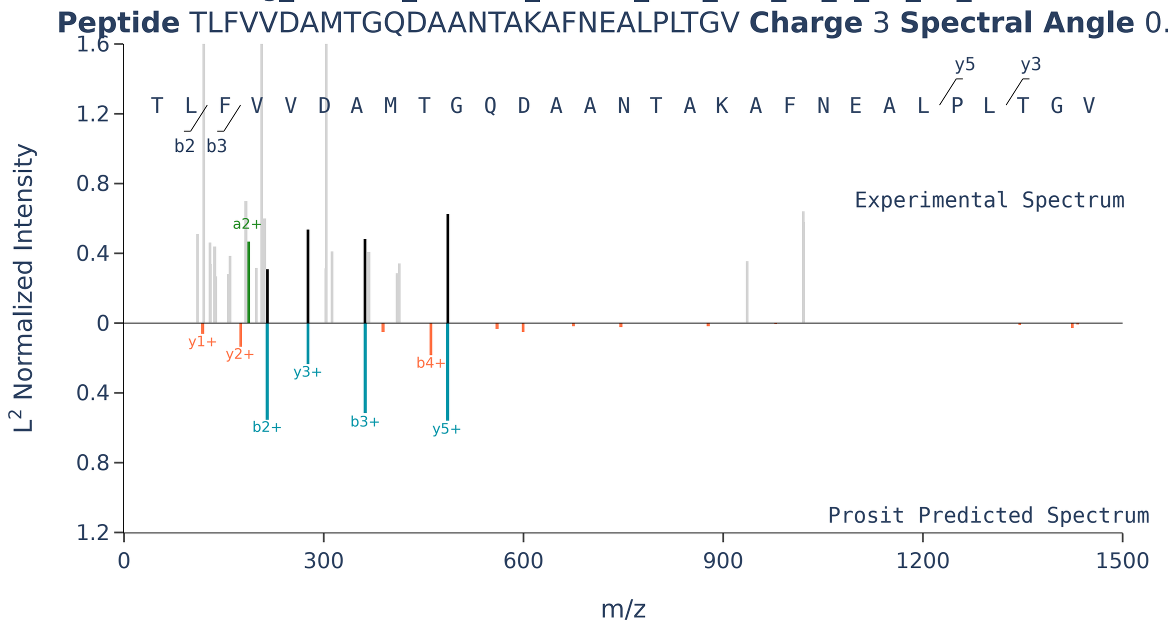


**Source** Ncheng_210623_230623_HFGoe_FFH_20S_25_1_A2_24h_R2 **Scan** 7457
**Peptide** INRVKEKAVGHEVNKSL **Charge** 2 **Spectral Angle** 0.53

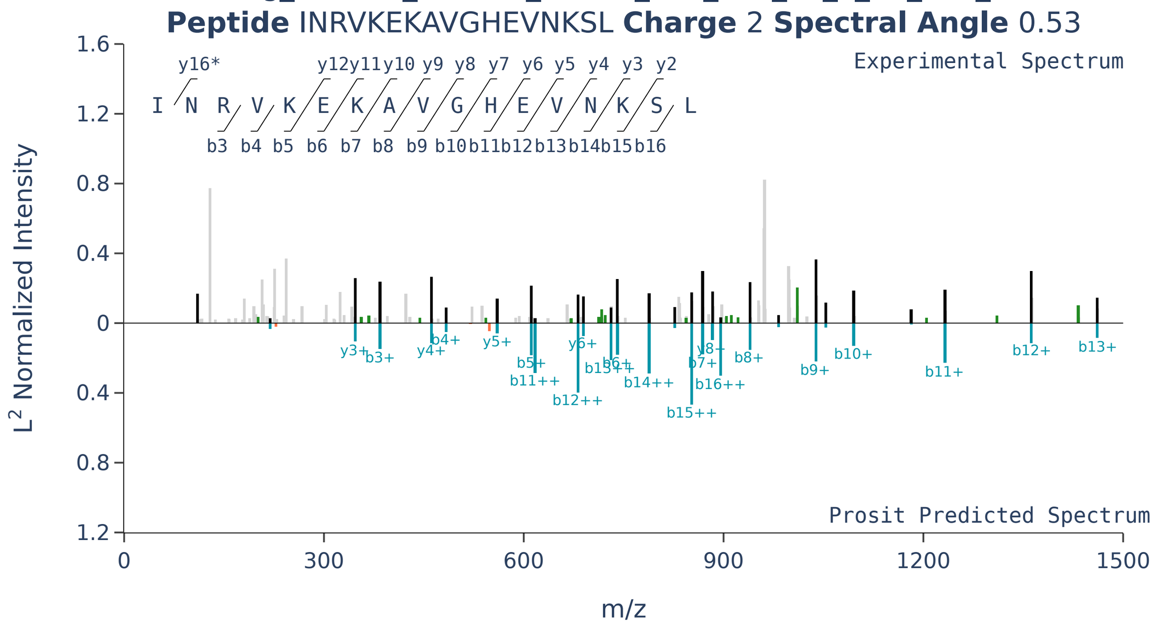


**Source** Ncheng_210623_230623_HFGoe_FFH_20S_25_1_A1_4h_R2 **Scan** 33718
**Peptide** KAFNEALPLTGVVLT **Charge** 2 **Spectral Angle** 0.49

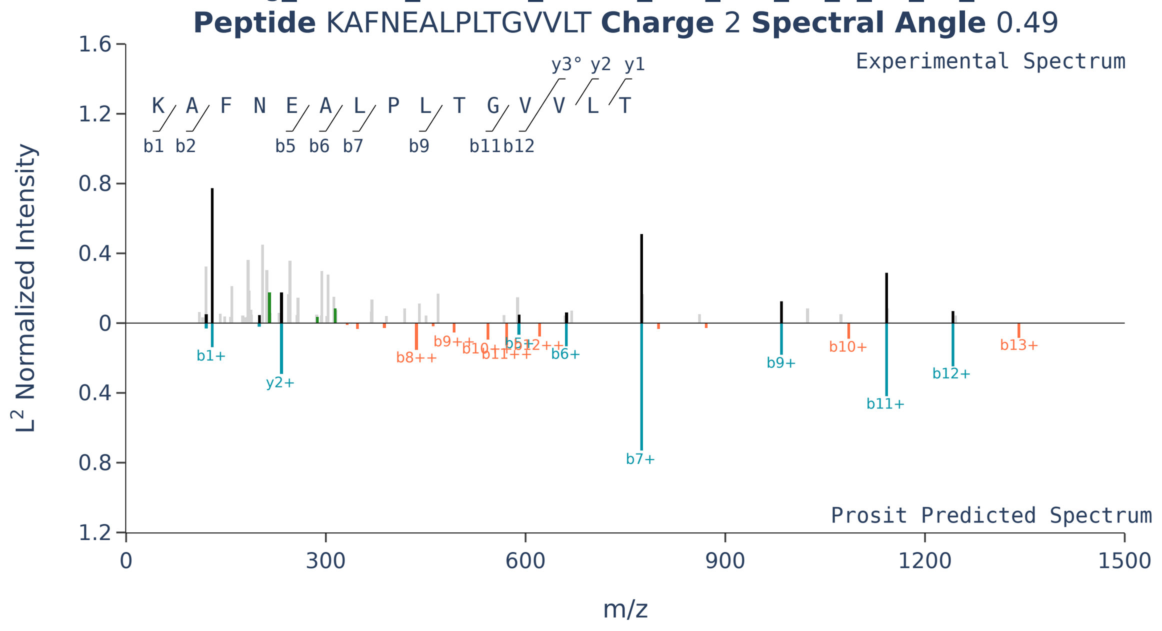


**Source** Ncheng_210623_230623_HFGoe_FFH_20S_25_1_A2_24h_R2 **Scan** 22509
**Peptide** GVGEKTEALEPFHPDRIASRI **Charge** 5 **Spectral Angle** 0.54

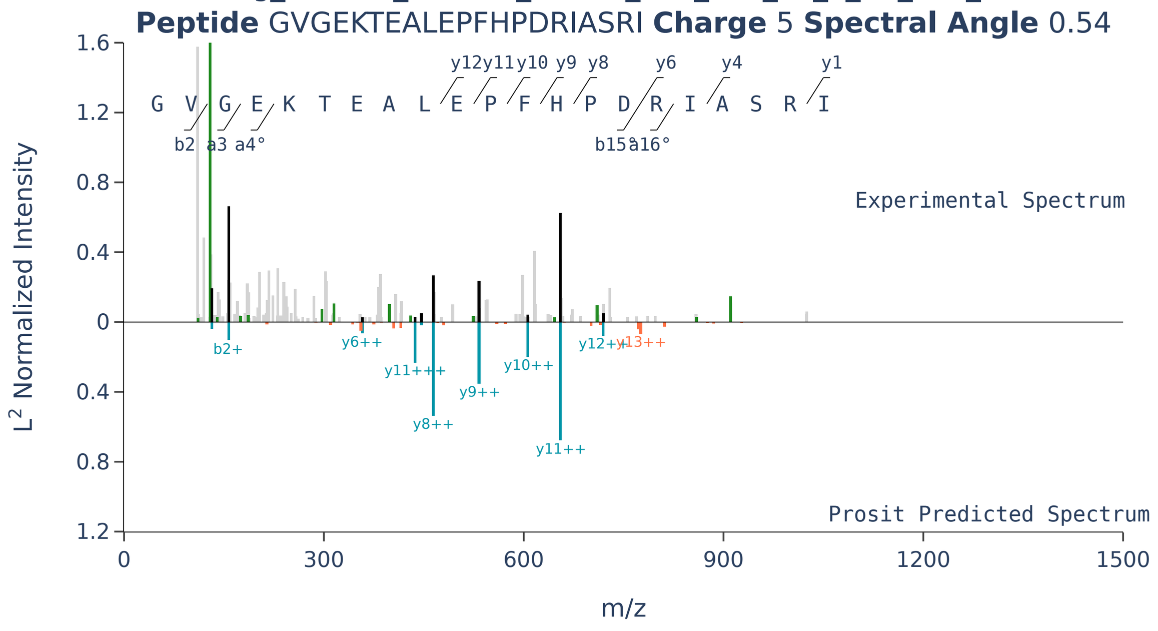


**Source** Ncheng_210623_230623_HFGoe_FFH_20S_25_1_A1_2h_R2 **Scan** 21538
**Peptide** DIESKVDRAQAEKLASKLKKGDGFDLND **Charge** 5 **Spectral Angle** 0.78

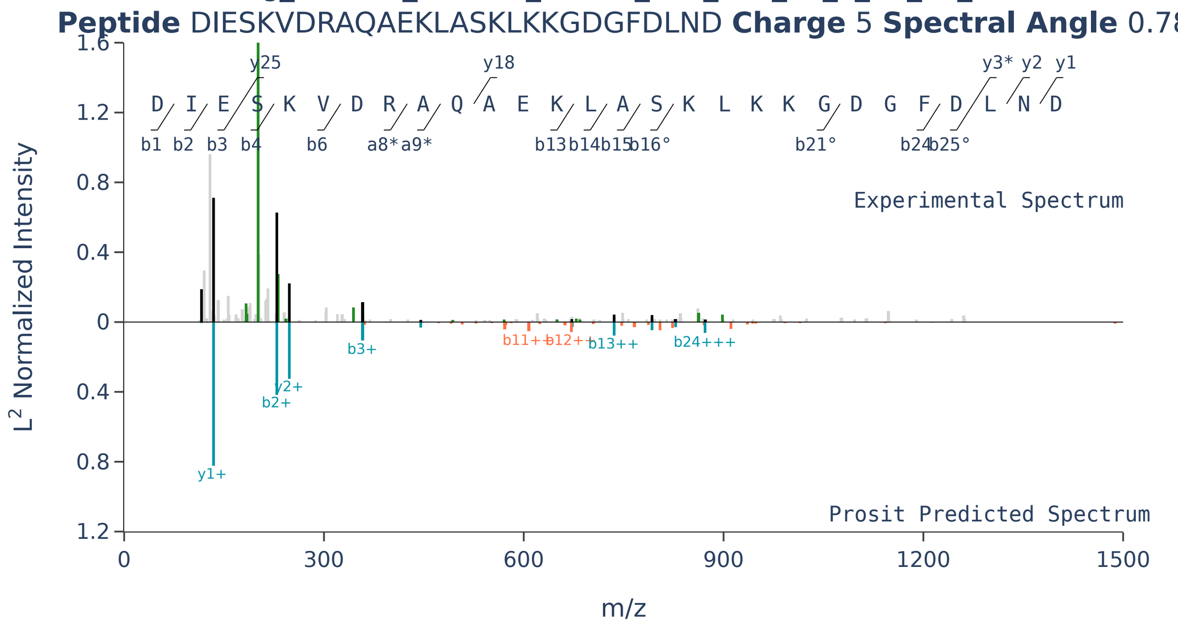


**Source** Ncheng_210623_230623_HFGoe_FFH_20S_25_1_A1_24h_R2 **Scan** 4999
**Peptide** MKKMKKGGMA **Charge** 2 **Spectral Angle** 0.57

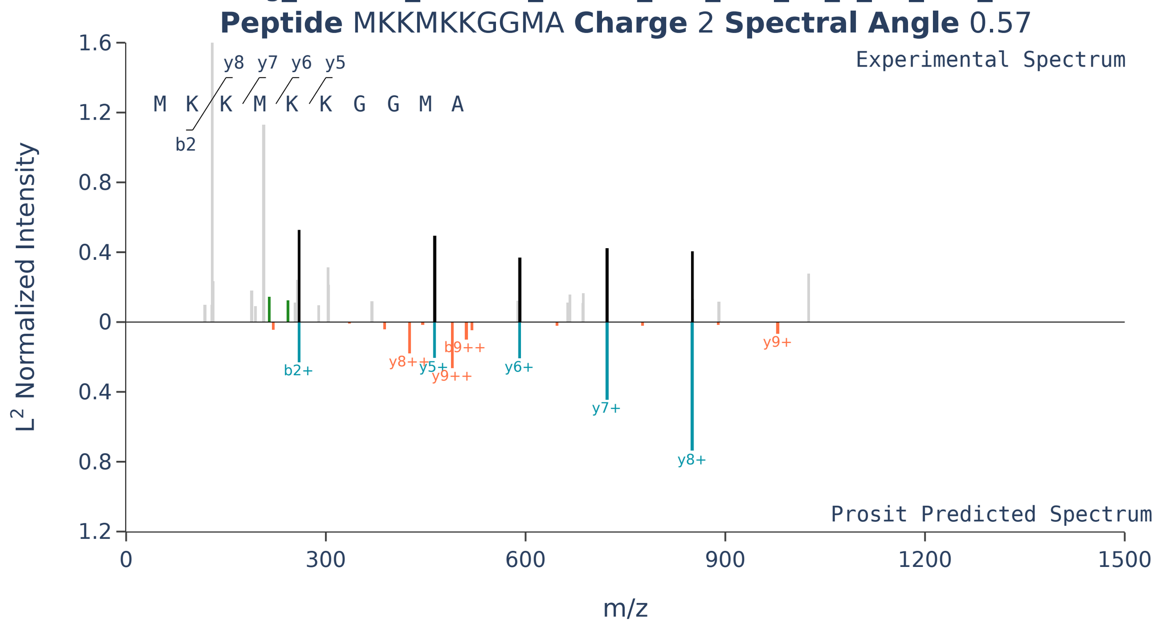


**Source** Ncheng_210623_230623_HFGoe_FFH_20S_25_1_A2_24h_R2 **Scan** 7733
**Peptide** SVGKLGKFLREKHKKK **Charge** 2 **Spectral Angle** 0.64

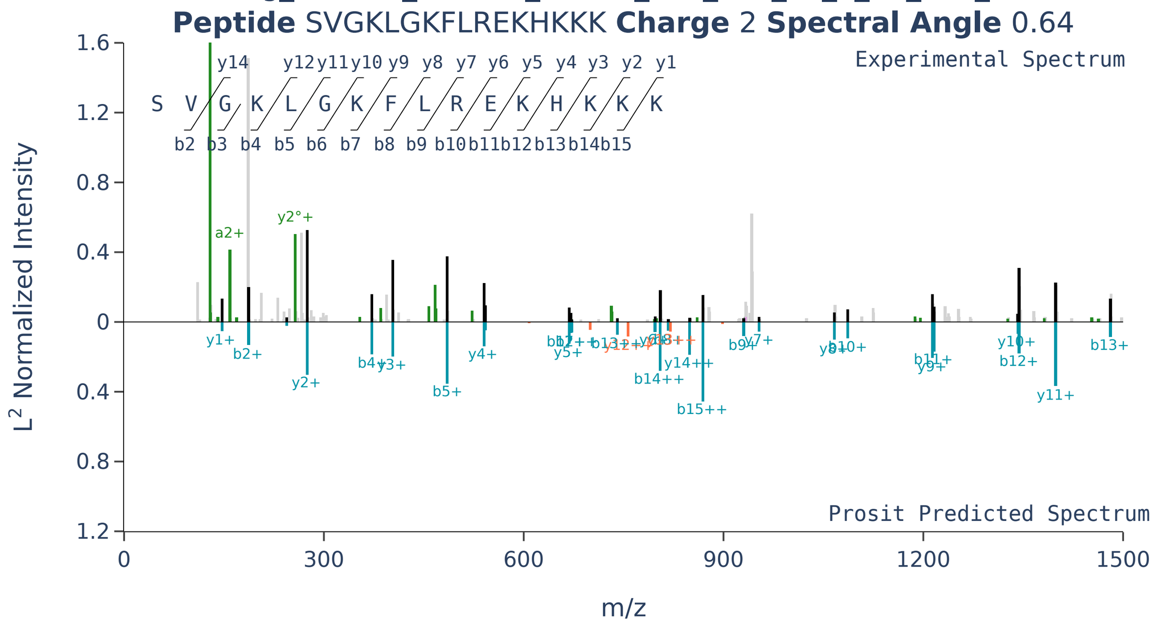


**Source** Ncheng_210623_230623_HFGoe_FFH_20S_25_1_A2_4h_R1 **Scan** 24262
**Peptide** GDARGGAALSIRHITGKPIKFLGV **Charge** 4 **Spectral Angle** 0.59

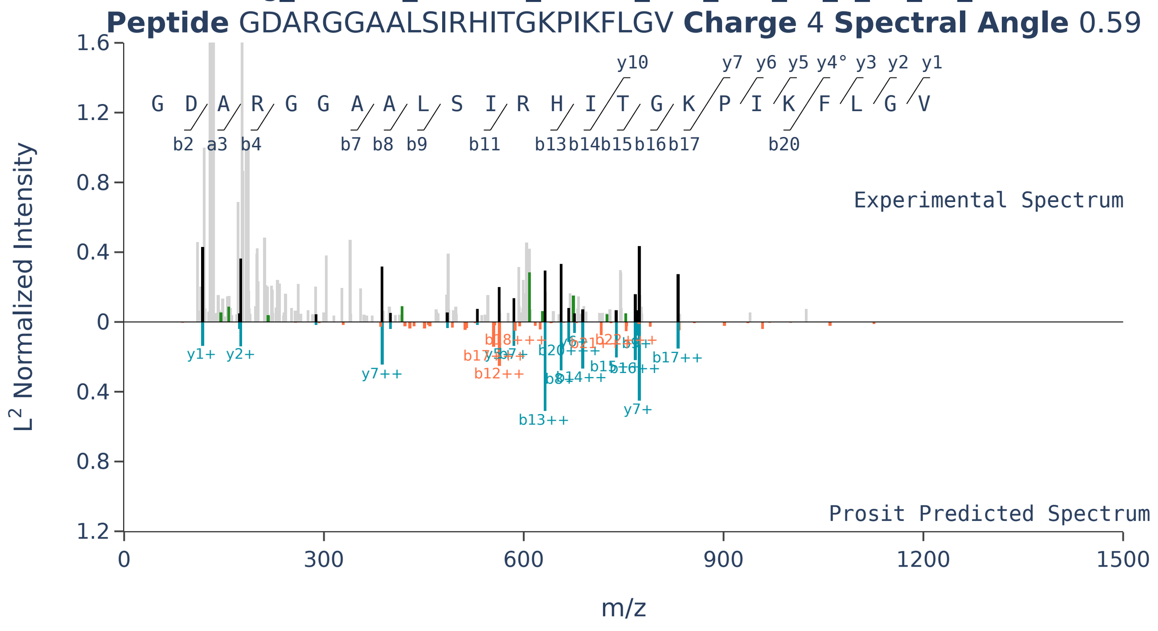


**Source** Ncheng_210623_230623_HFGoe_FFH_20S_25_1_A2_24h_R1 **Scan** 7327
**Peptide** FINRVKEKAVGHEVNK **Charge** 5 **Spectral Angle** 0.64

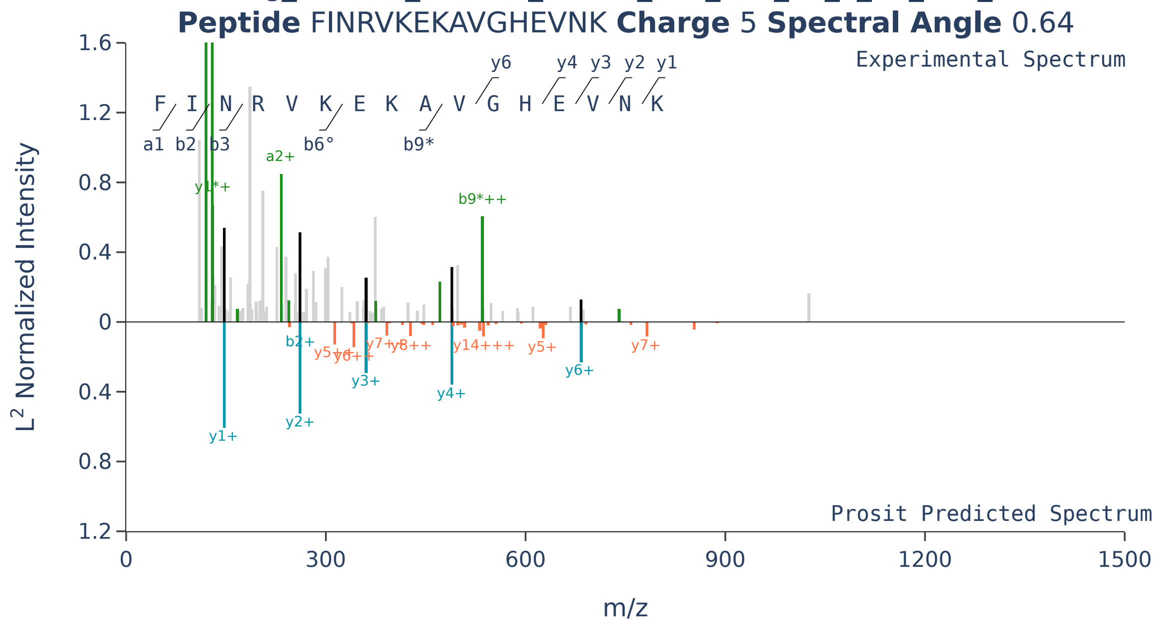

**Source** Ncheng_210623_230623_HFGoe_FFH_20S_25_1_A1_2h_R1 **Scan** 20491
**Peptide** IESKVDRAQAEKLASKLKKGDGFDL **Charge** 5 **Spectral Angle** 0.88

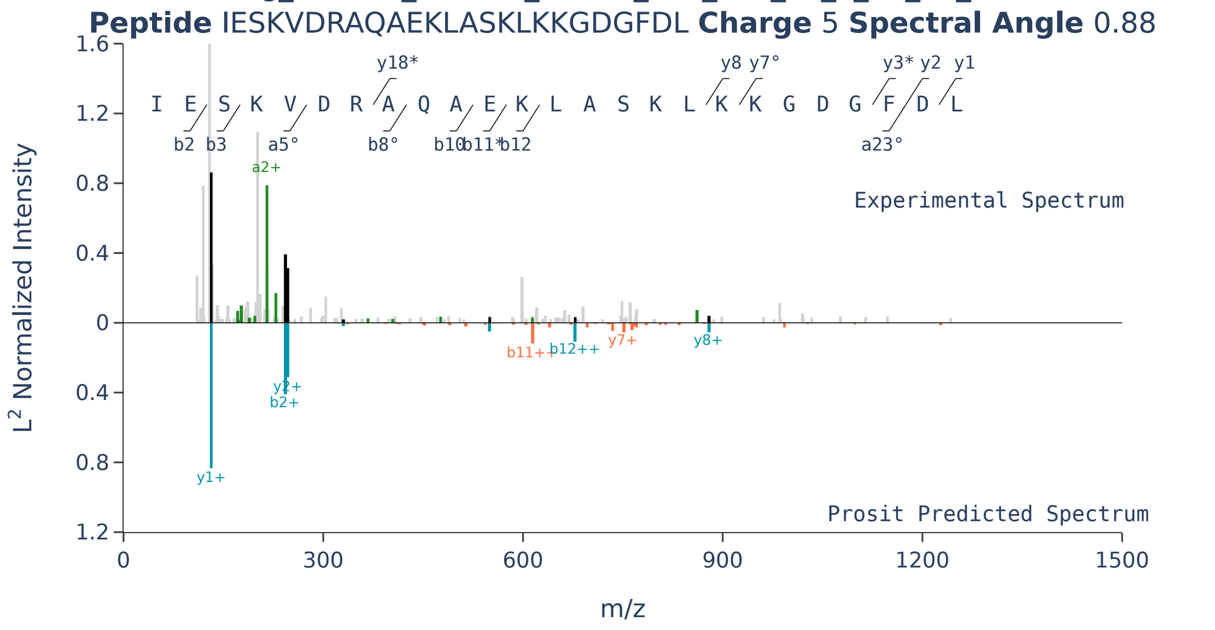


**Source** Ncheng_210623_230623_HFGoe_FFH_20S_25_1_A2_24h_R2 **Scan** 6565
**Peptide** RNISGRGRLTEDNVKD **Charge** 4 **Spectral Angle** 0.49

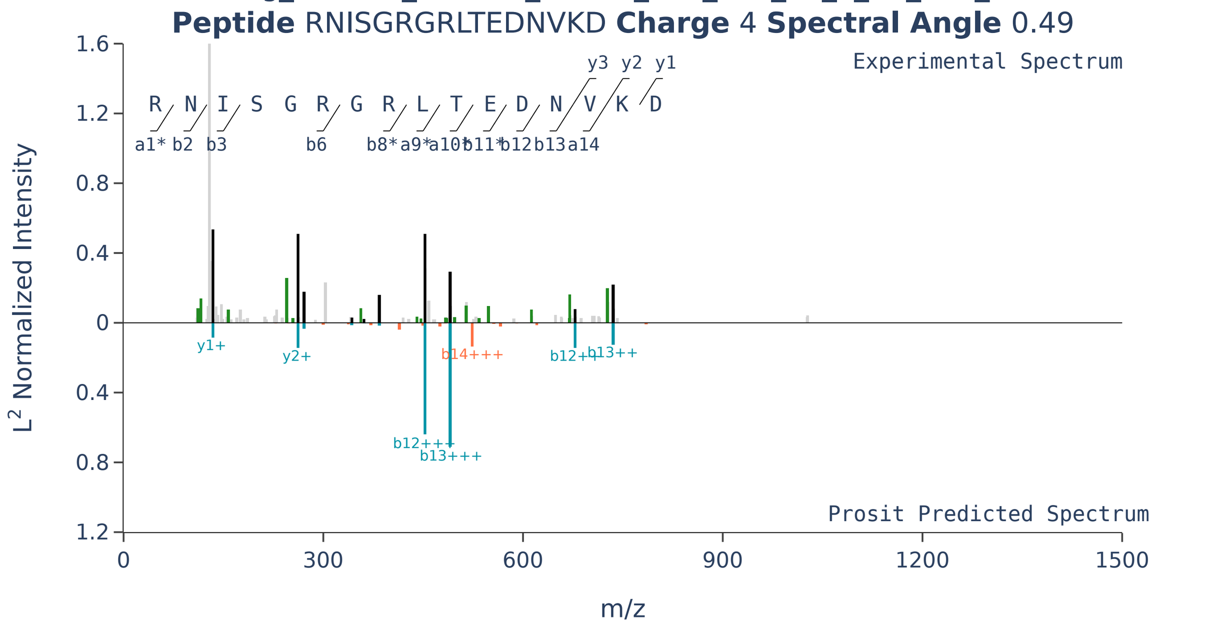


**Source** Ncheng_210623_230623_HFGoe_FFH_20S_25_1_A1_2h_R1 **Scan** 35511
**Peptide** KLKKGDGFDLNDFLEQL **Charge** 3 **Spectral Angle** 0.35

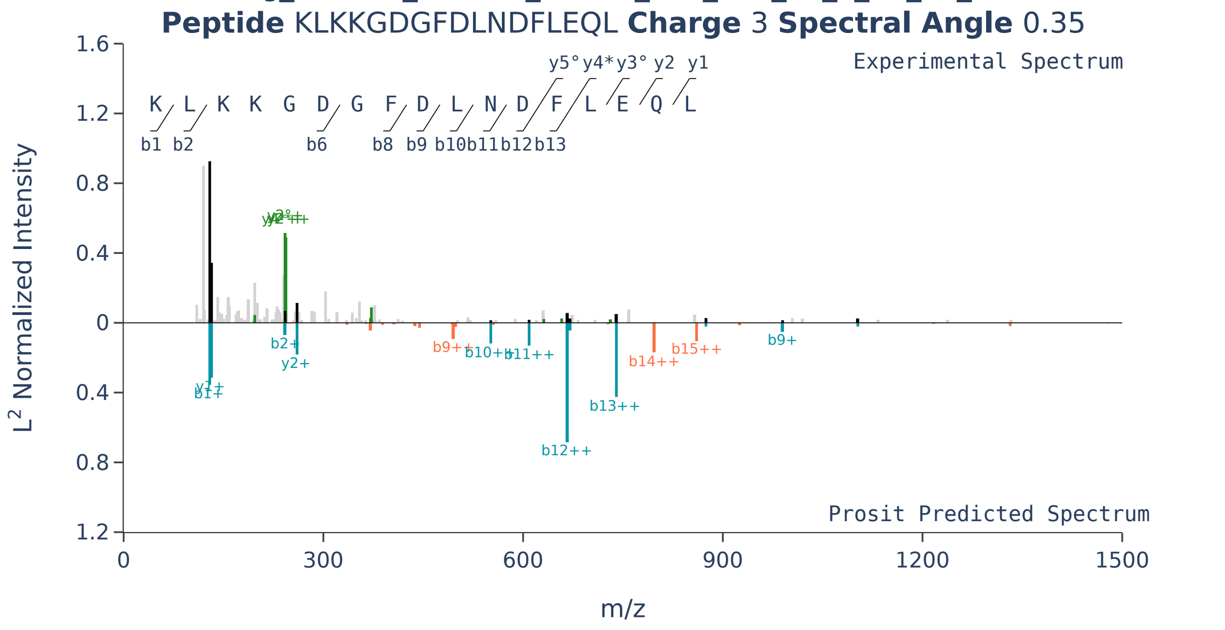


**Source** Ncheng_210623_230623_HFGoe_FFH_20S_25_1_A2_4h_R1 **Scan** 23715
**Peptide** PLTGVVLTKVDGDARGGAALSIRHITGK **Charge** 5 **Spectral Angle** 0.54

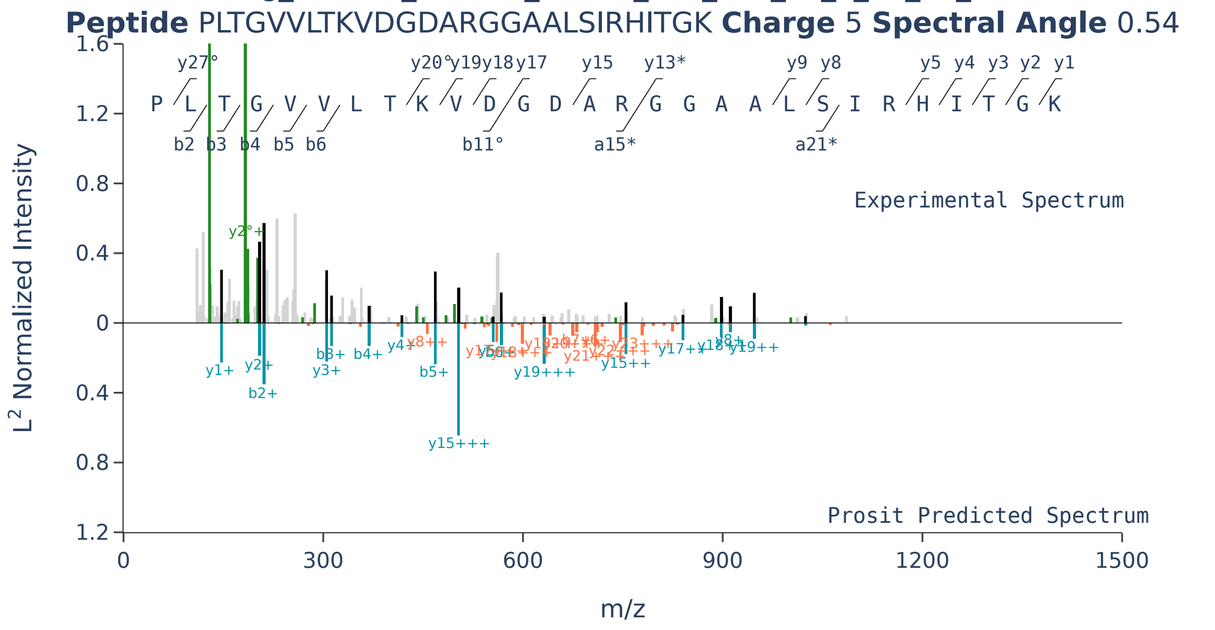


**Source** Ncheng_210623_230623_HFGoe_FFH_20S_25_1_A1_1h_R2 **Scan** 18463
**Peptide** REVRMALLEAD **Charge** 3 **Spectral Angle** 0.4

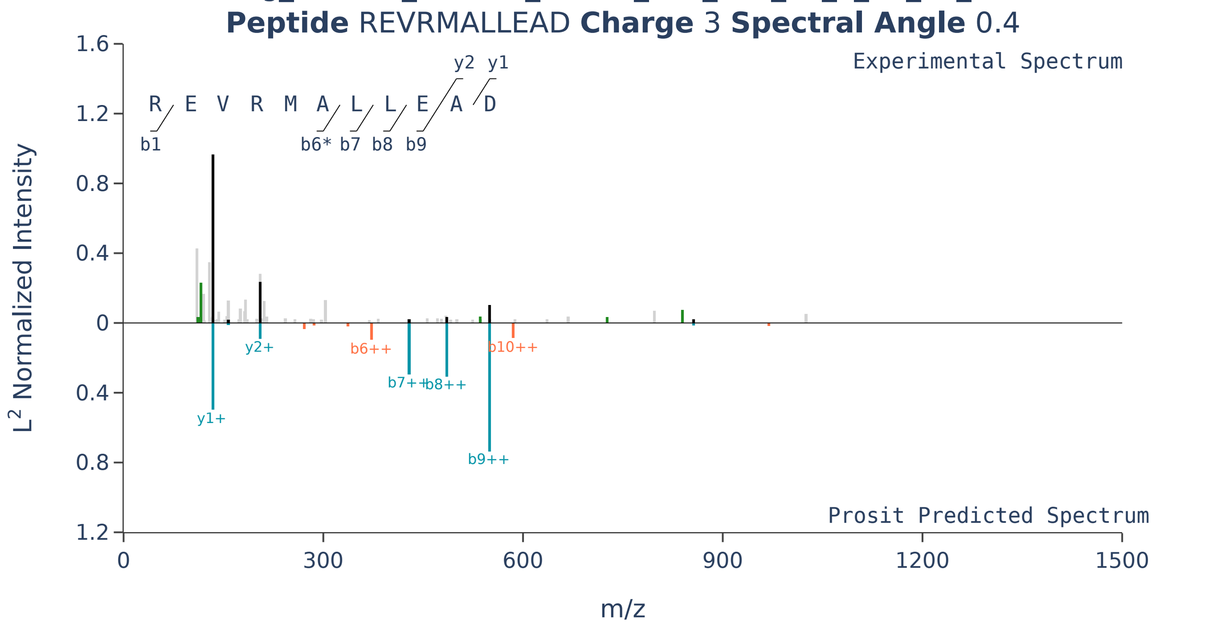


**Source** Ncheng_210623_230623_HFGoe_FFH_20S_25_1_A2_4h_R2 **Scan** 33378
**Peptide** AQAEKLASKLKKGDGFDLNDFLEQLRQMKN **Charge** 5 **Spectral Angle** 0.71

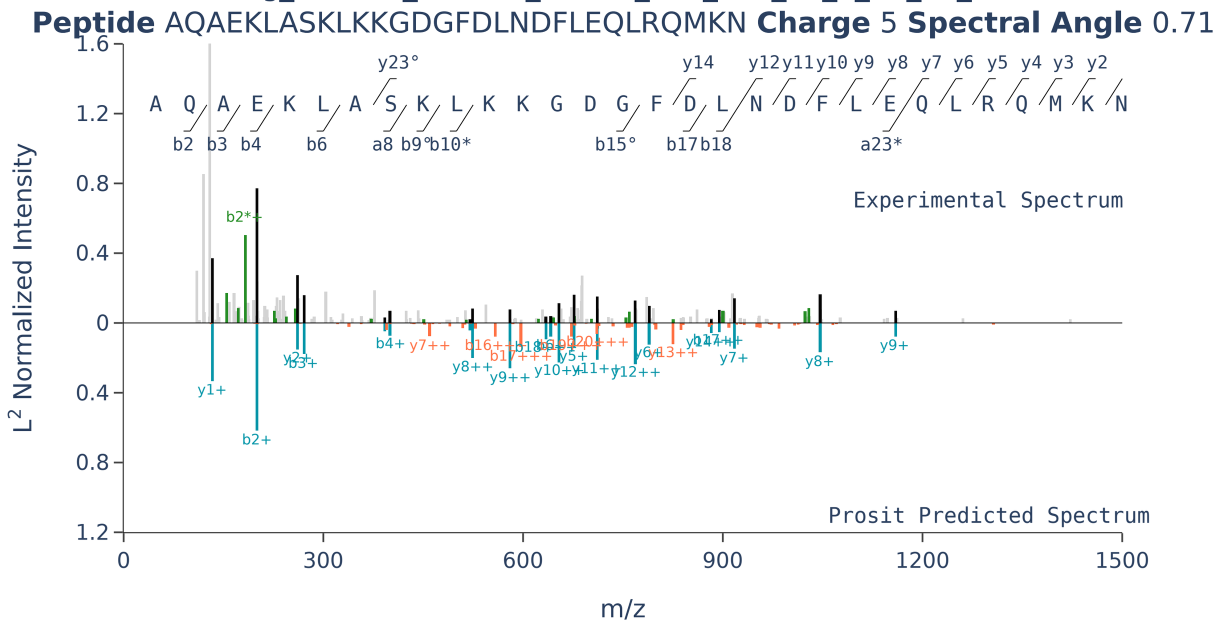


**Source** Ncheng_210623_230623_HFGoe_FFH_20S_25_1_A1_2h_R1 **Scan** 23165
**Peptide** AALSIRHITGKPIKFLGVGEKTEAL **Charge** 5 **Spectral Angle** 0.75

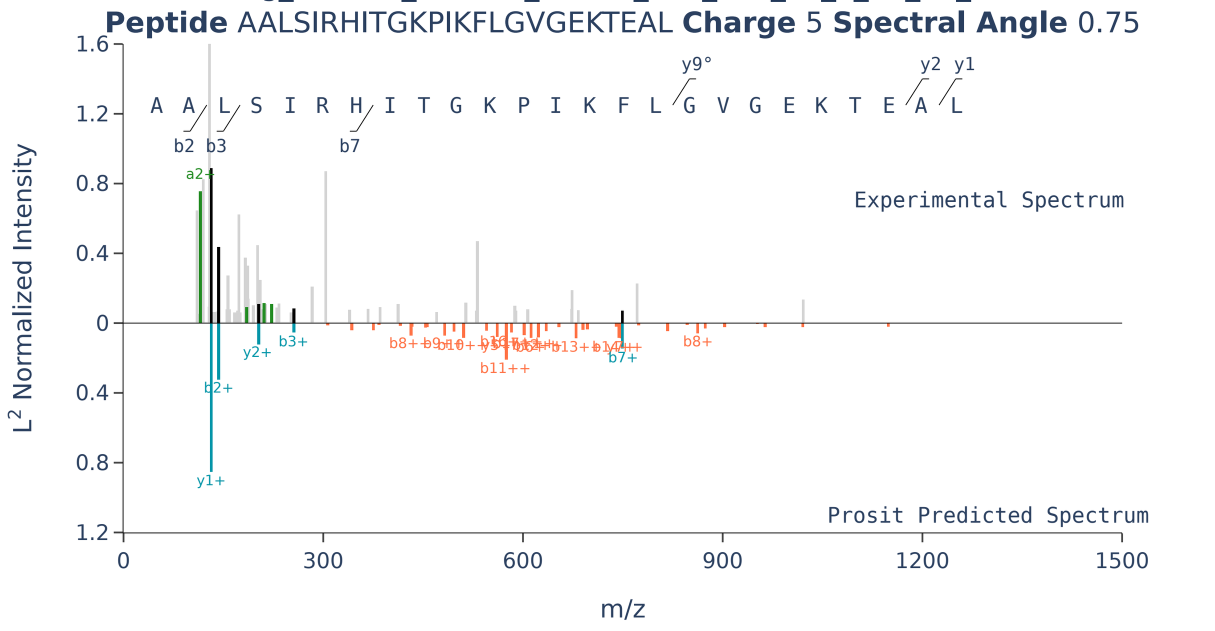


**Source** Ncheng_210623_230623_HFGoe_FFH_20S_25_1_A1_4h_R2 **Scan** 8725
**Peptide** SRTLRNISGRGRLTEDNVKD **Charge** 4 **Spectral Angle** 0.3

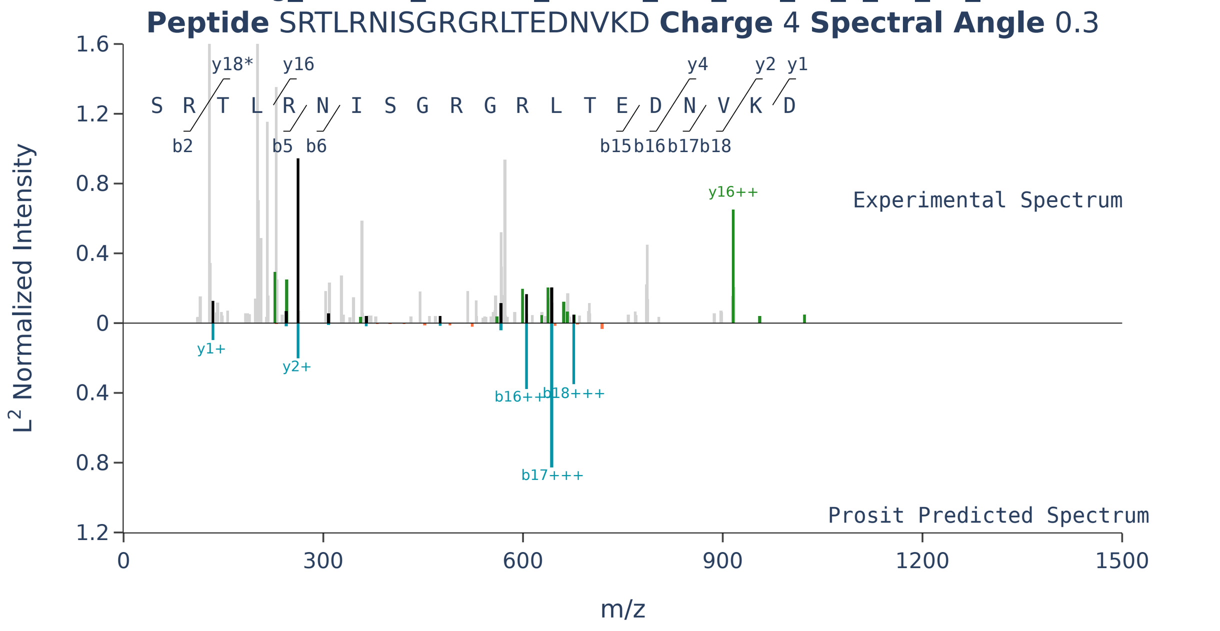


**Source** Ncheng_210623_230623_HFGoe_FFH_20S_25_1_A2_2h_R2 **Scan** 27099
**Peptide** QDVNRLLKQFDDMQRMMKKMKK **Charge** 5 **Spectral Angle** 0.69

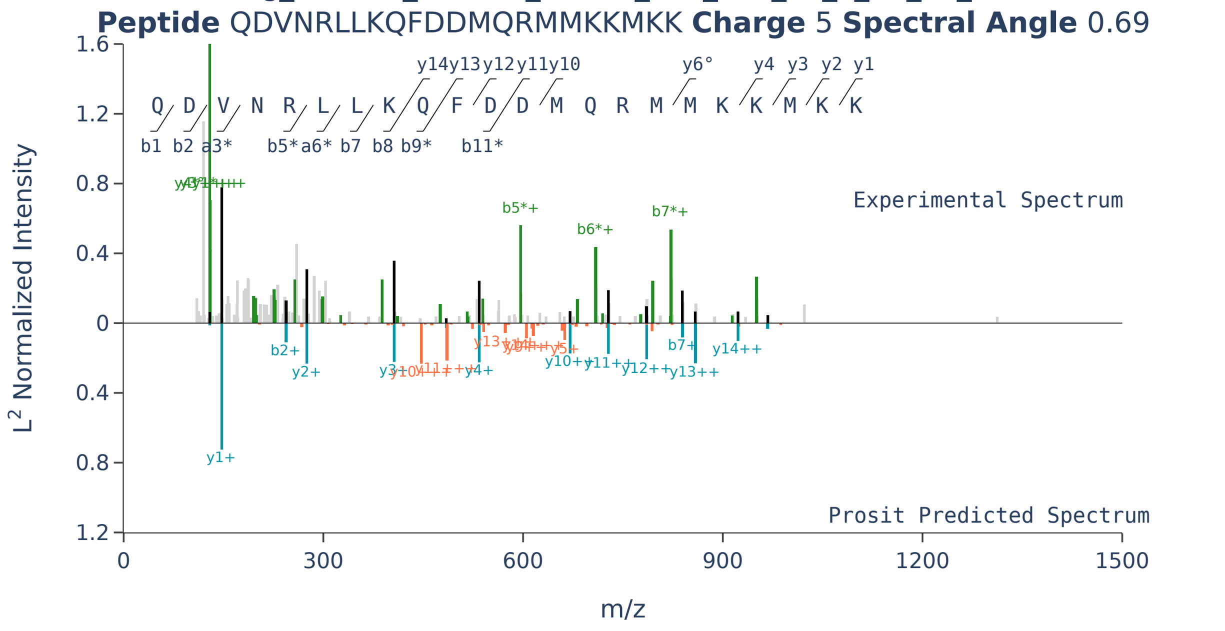


**Source** Ncheng_210623_230623_HFGoe_FFH_20S_25_1_A2_4h_R2 **Scan** 24767
**Peptide** VDGDARGGAALSIRHITGKPIKFLGVGE **Charge** 5 **Spectral Angle** 0.72

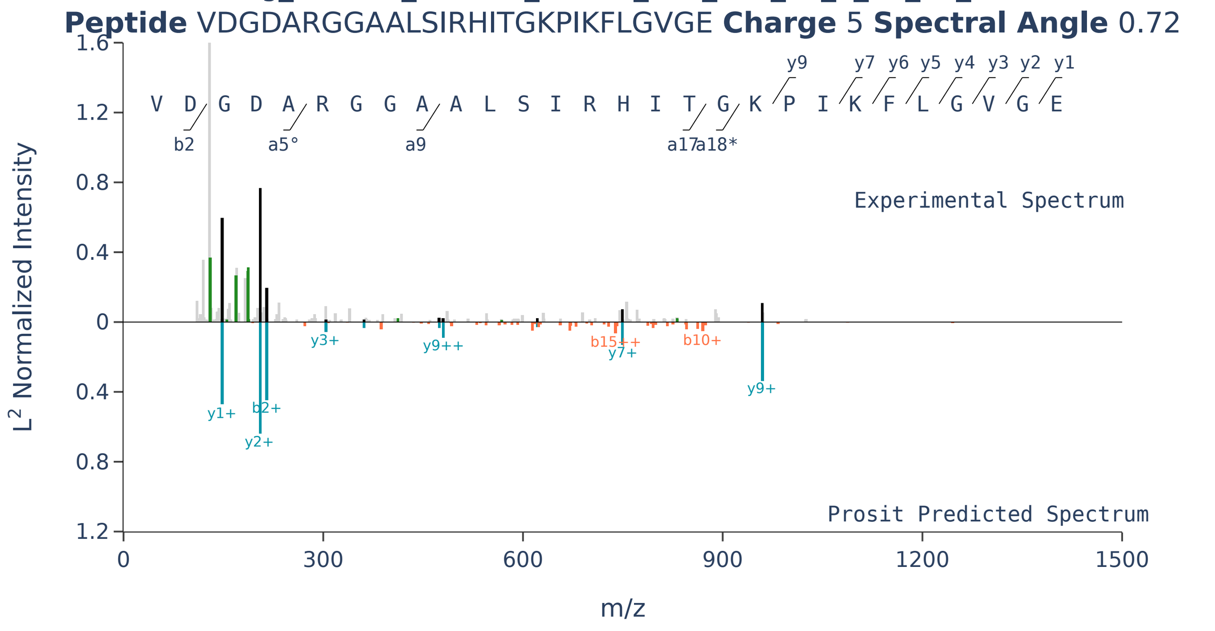


**Source** Ncheng_210623_230623_HFGoe_FFH_20S_25_1_A2_4h_R1 **Scan** 30381
**Peptide** MDDKVLVRMEAILN **Charge** 3 **Spectral Angle** 0.67

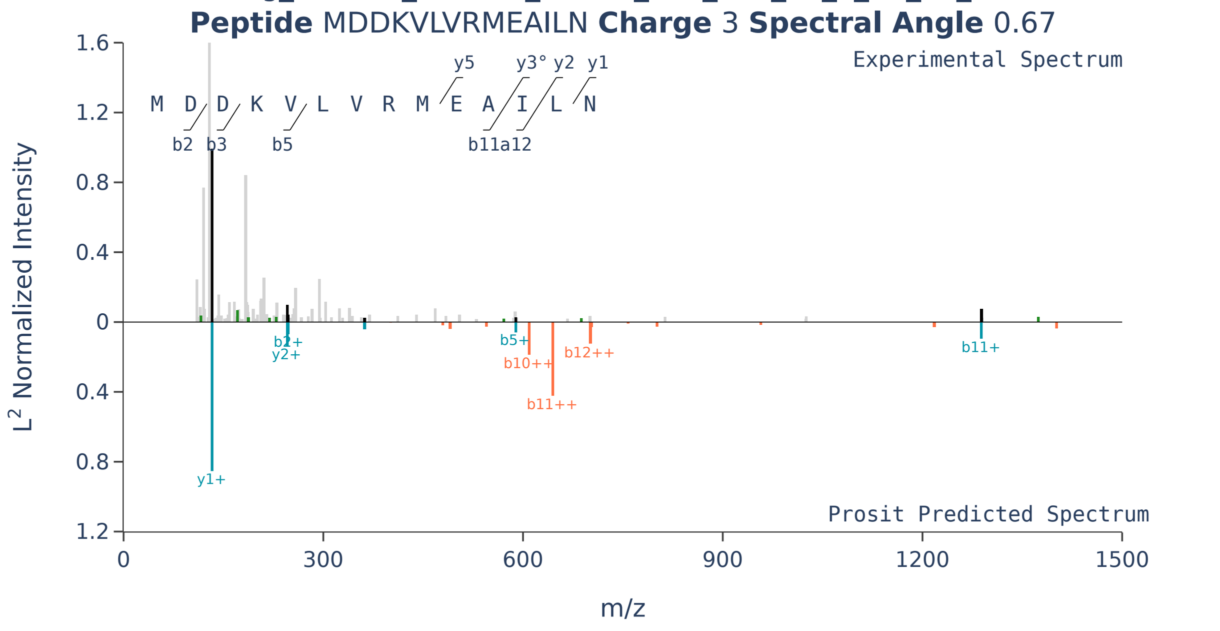


**Source** Ncheng_210623_230623_HFGoe_FFH_20S_25_1_A2_4h_R1 **Scan** 29348
**Peptide** LTEDNVKDTLREVRMAL **Charge** 4 **Spectral Angle** 0.48

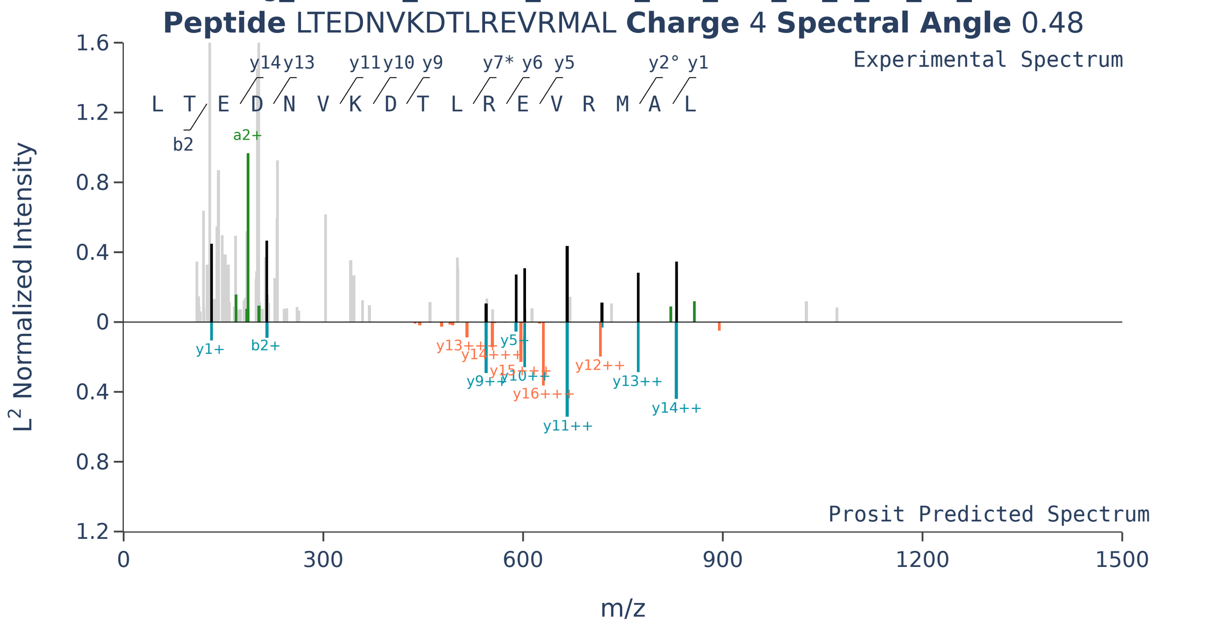


**Source** Ncheng_210623_230623_HFGoe_FFH_20S_25_1_A2_24h_R1 **Scan** 21350
**Peptide** VNKSLTPGQEFVKIVR **Charge** 4 **Spectral Angle** 0.46

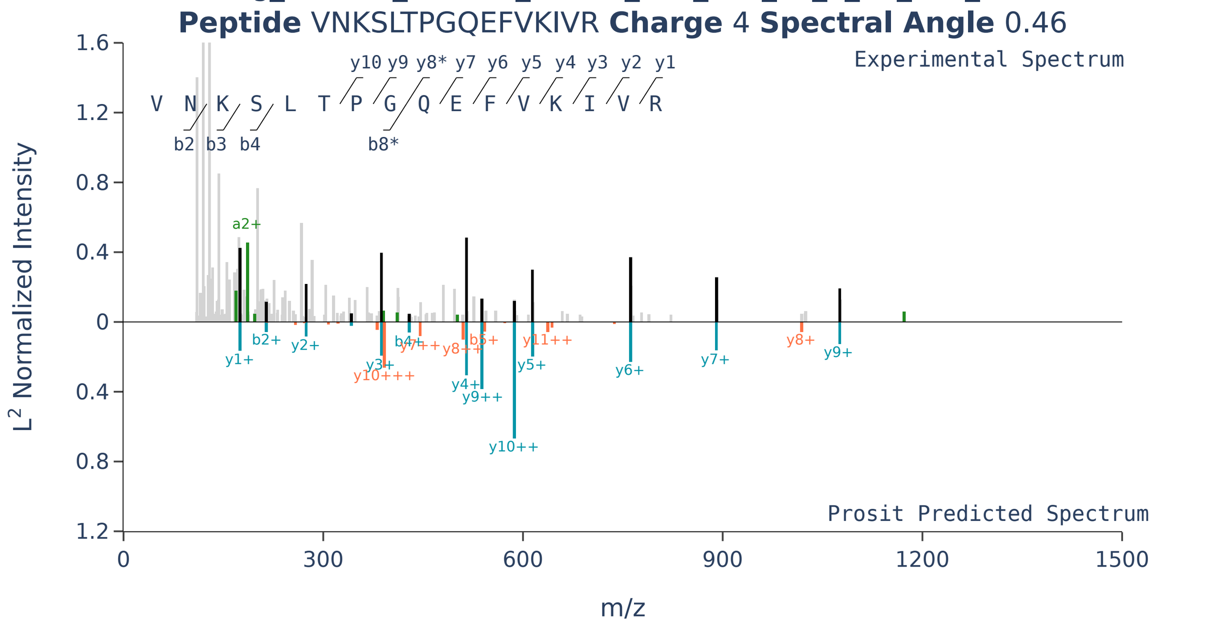


**Source** Ncheng_210623_230623_HFGoe_FFH_20S_25_1_A2_4h_R2 **Scan** 36489
**Peptide** KLKKGDGFDLNDFLEQLRQMKNMG **Charge** 4 **Spectral Angle** 0.8

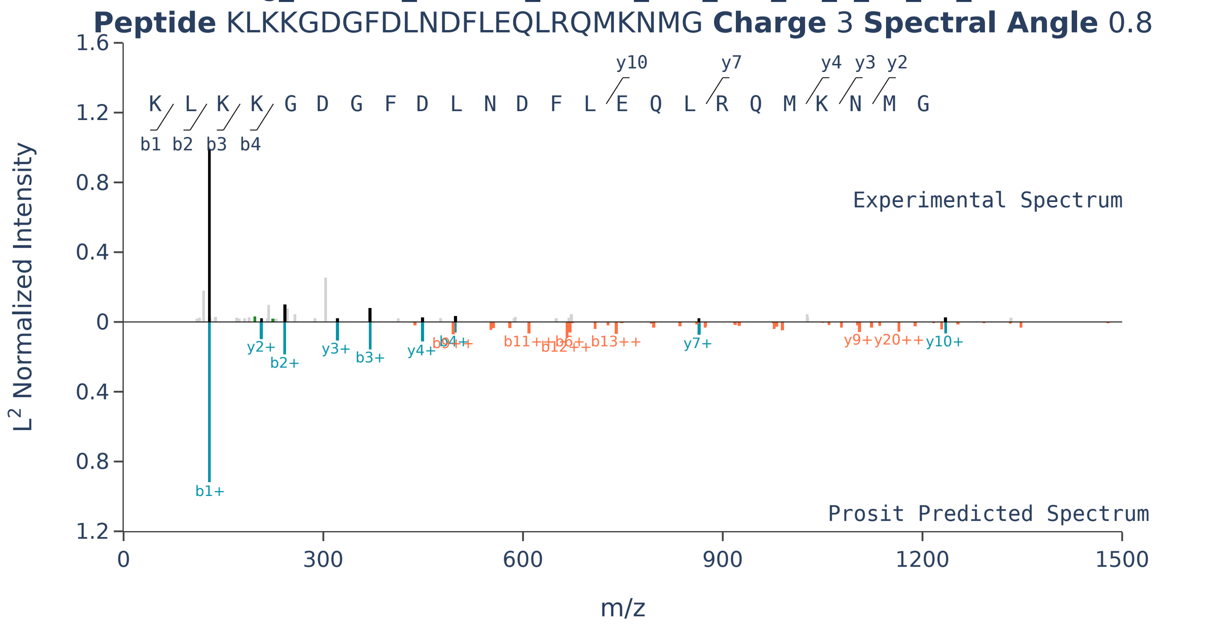


**Source** Ncheng_210623_230623_HFGoe_FFH_20S_25_1_A2_2h_R2 **Scan** 33239
**Peptide** KTEALEPFHPDRIASRILGMGDVL **Charge** 4 **Spectral Angle** 0.62

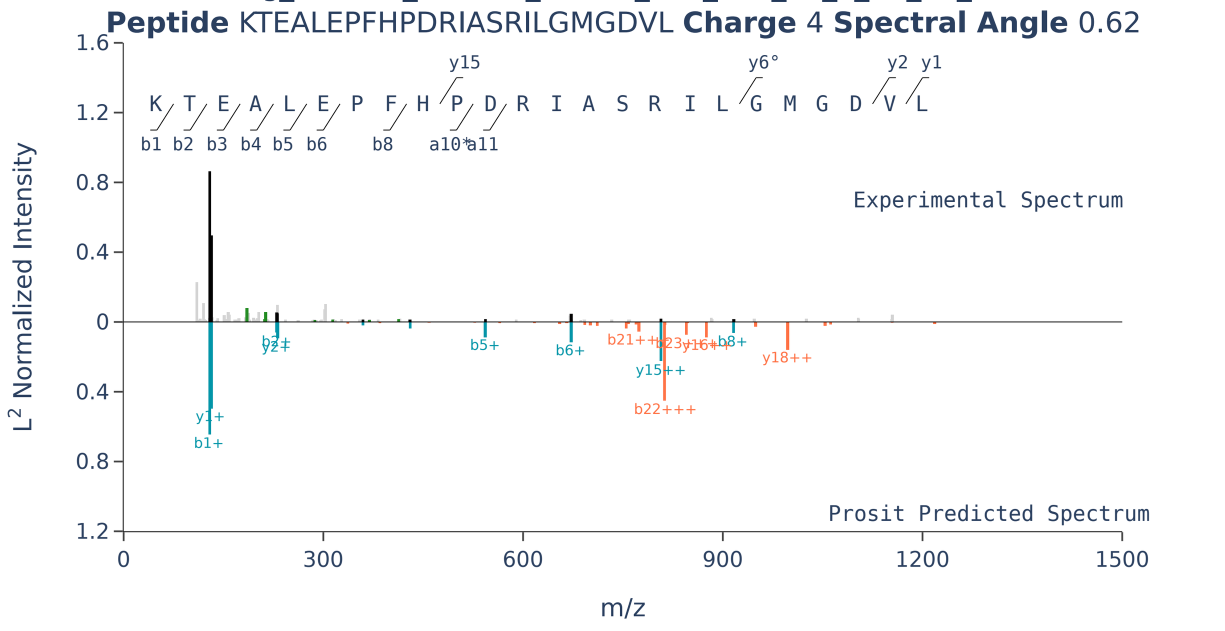

**Source** Ncheng_210623_230623_HFGoe_FFH_20S_25_1_A2_4h_R1 **Scan** 35856
**Peptide** SKLKKGDGFDLNDFLEQLRQMKNMG **Charge** 3 **Spectral Angle** 0.73

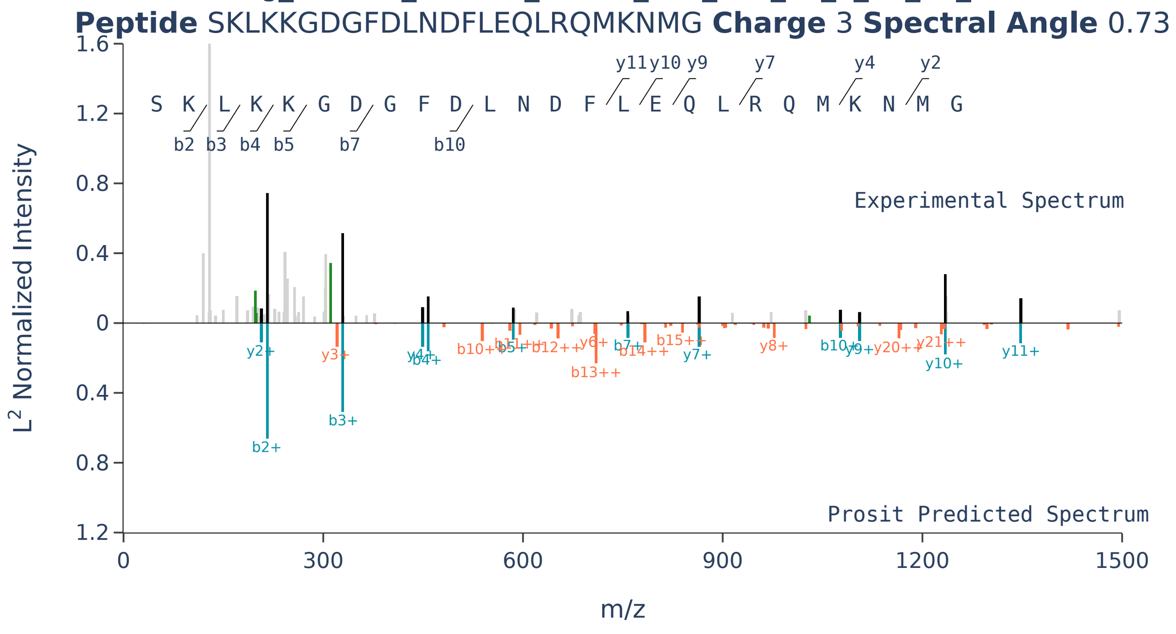


**Source** Ncheng_210623_230623_HFGoe_FFH_20S_25_1_A1_2h_R2 **Scan** 37365
**Peptide** TPGQEFVKIVRNELVAAM **Charge** 3 **Spectral Angle** 0.68

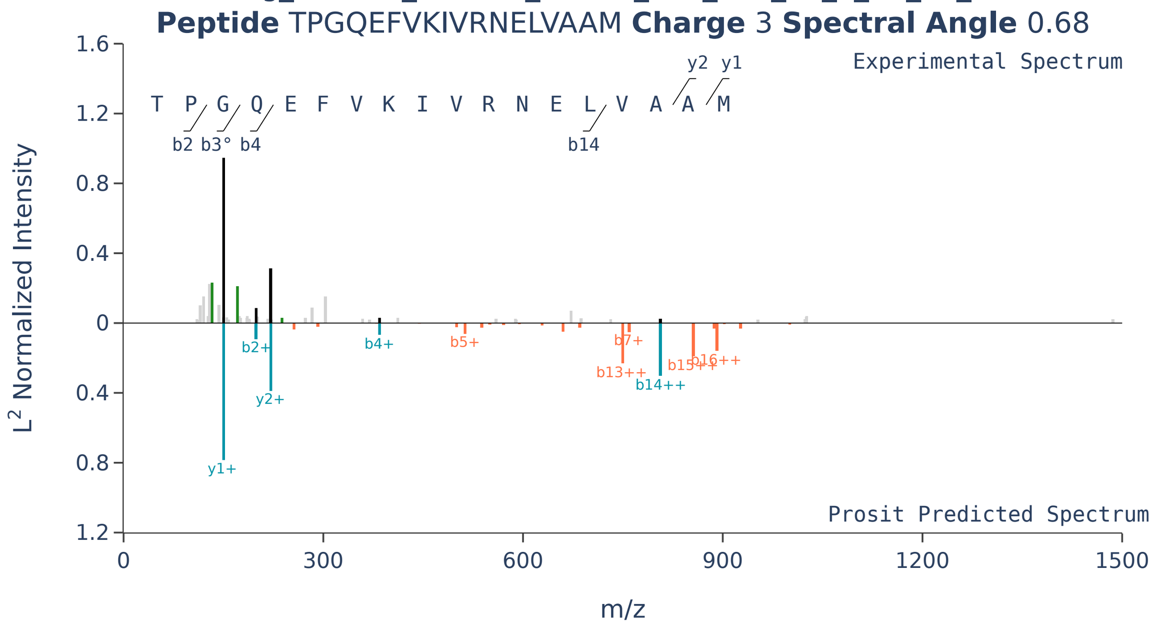


**Source** Ncheng_210623_230623_HFGoe_FFH_20S_25_1_A1_2h_R2 **Scan** 36365
**Peptide** KTEALEPFHPDRIASRILGMGDVLSLIE **Charge** 4 **Spectral Angle** 0.64

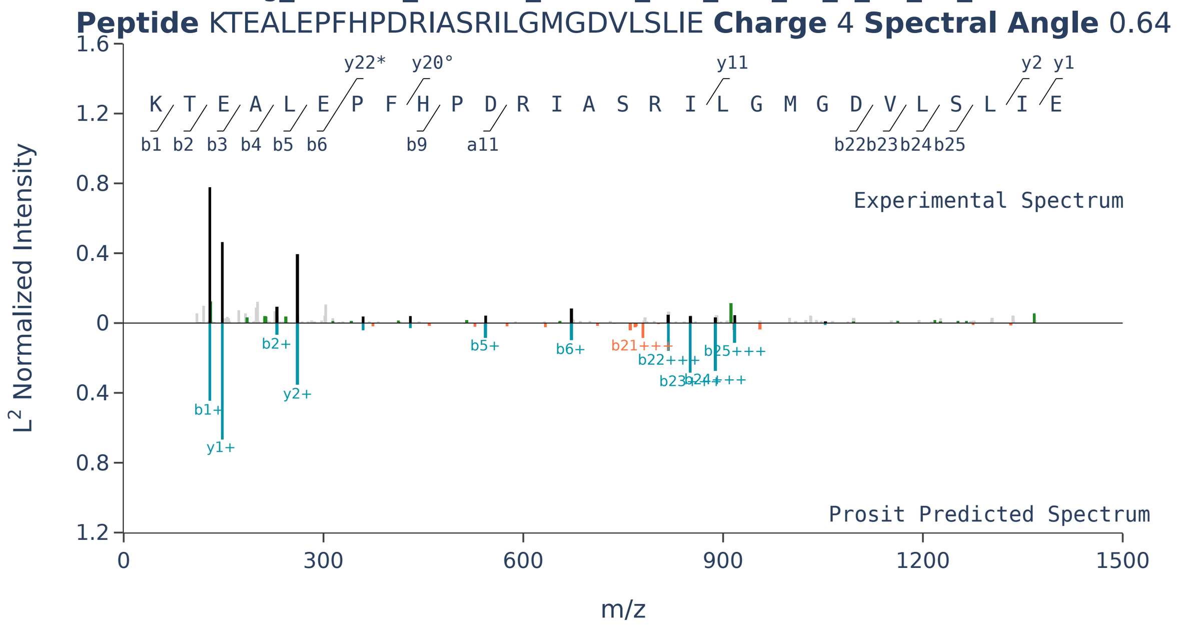


**Source** Ncheng_210623_230623_HFGoe_FFH_20S_25_1_A1_2h_R1 **Scan** 38771
**Peptide** KKGDGFDLNDFLEQLRQMKNMGGM **Charge** 3 **Spectral Angle** 0.75

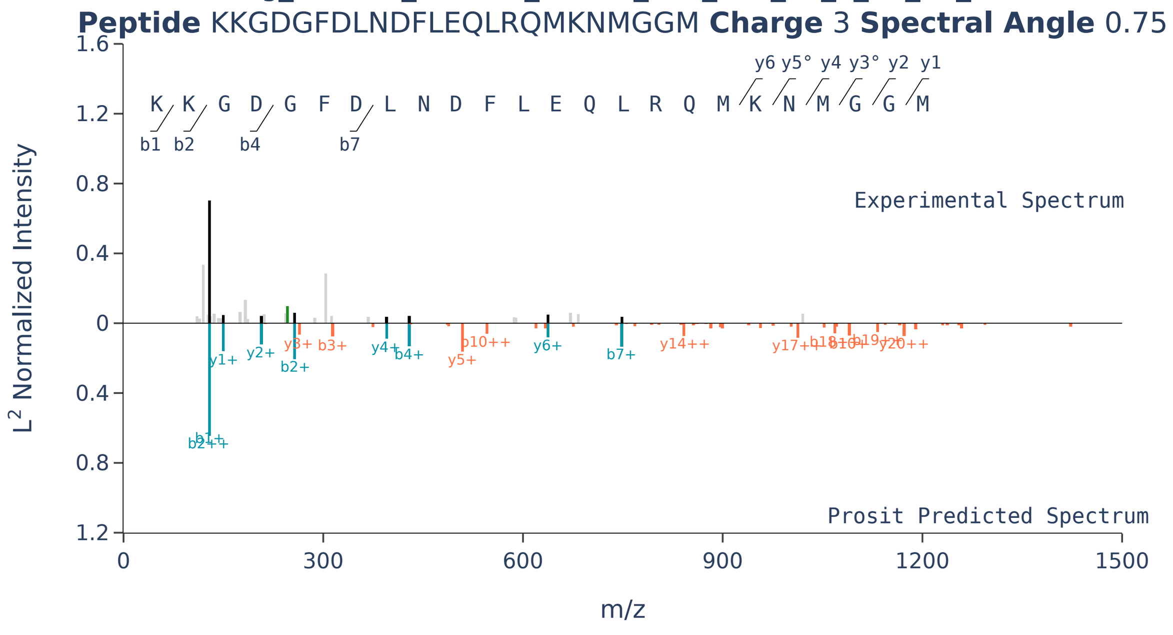


**Source** Ncheng_210623_230623_HFGoe_FFH_20S_25_1_A1_24h_R2 **Scan** 18826
**Peptide** IEDIESKVDRAQAEKLASKLK **Charge** 5 **Spectral Angle** 0.71

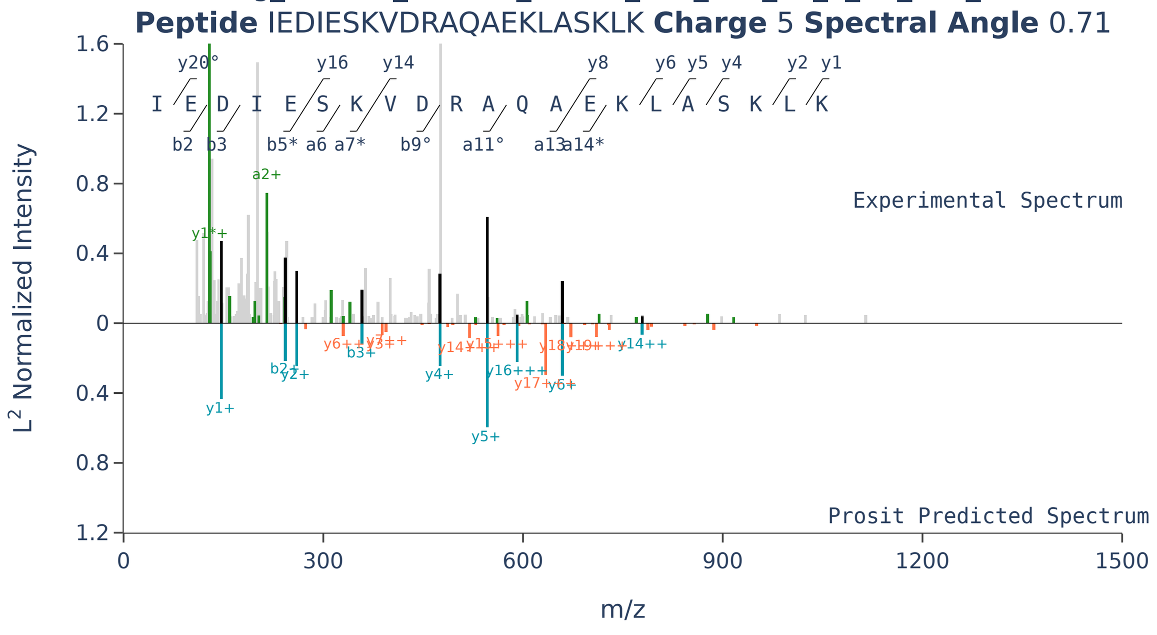


**Source** Ncheng_210623_230623_HFGoe_FFH_20S_25_1_A2_2h_R2 **Scan** 23675
**Peptide** TLREVRMALLEAD **Charge** 3 **Spectral Angle** 0.36

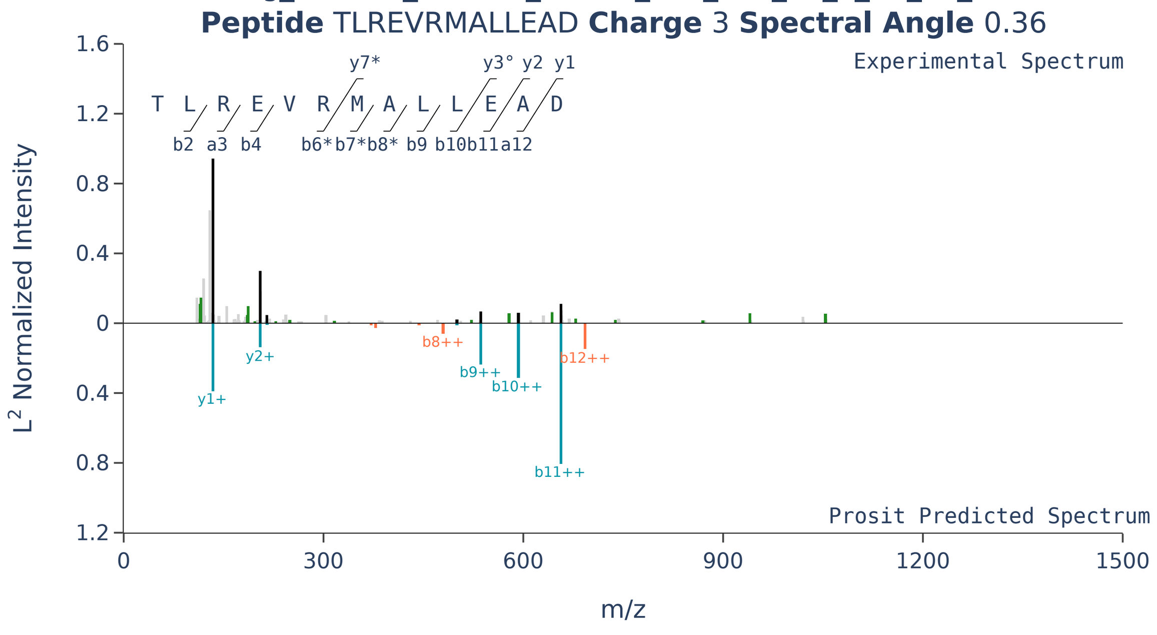


**Source** Ncheng_210623_230623_HFGoe_FFH_20S_25_1_A2_4h_R1 **Scan** 17742
**Peptide** FLNRVKEKAVGHEVNKSLTPGQEFVKIVRN **Charge** 5 **Spectral Angle** 0.68

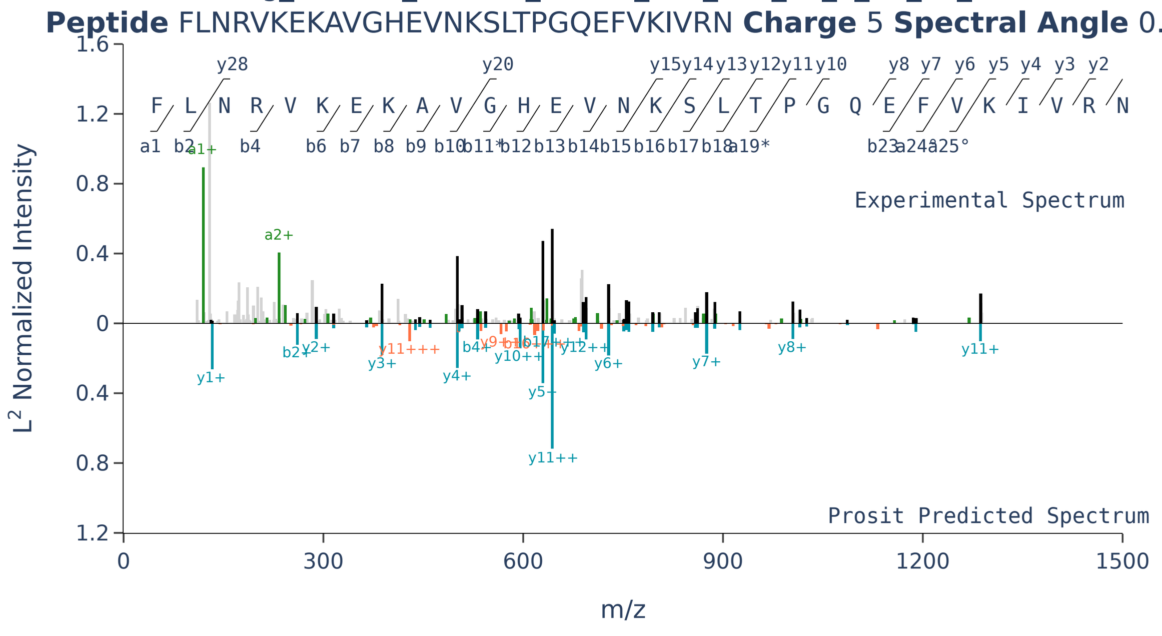


**Source** Ncheng_210623_230623_HFGoe_FFH_20S_25_1_A1_24h_R2 **Scan** 10047
**Peptide** SMTMKERAKPEIIKGS **Charge** 3 **Spectral Angle** 0.58

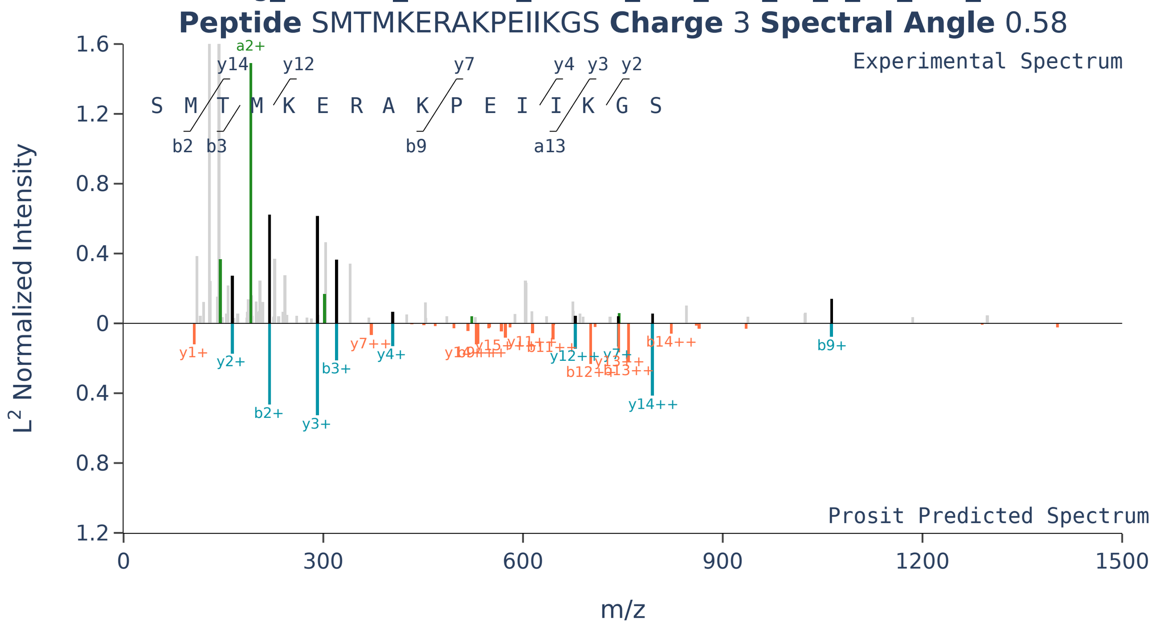


**Source** Ncheng_210623_230623_HFGoe_FFH_20S_25_1_A1_2h_R2 **Scan** 32343
**Peptide** GMQVQDVNRLLKQFDDMQRMMKKMKKG **Charge** 5 **Spectral Angle** 0.7

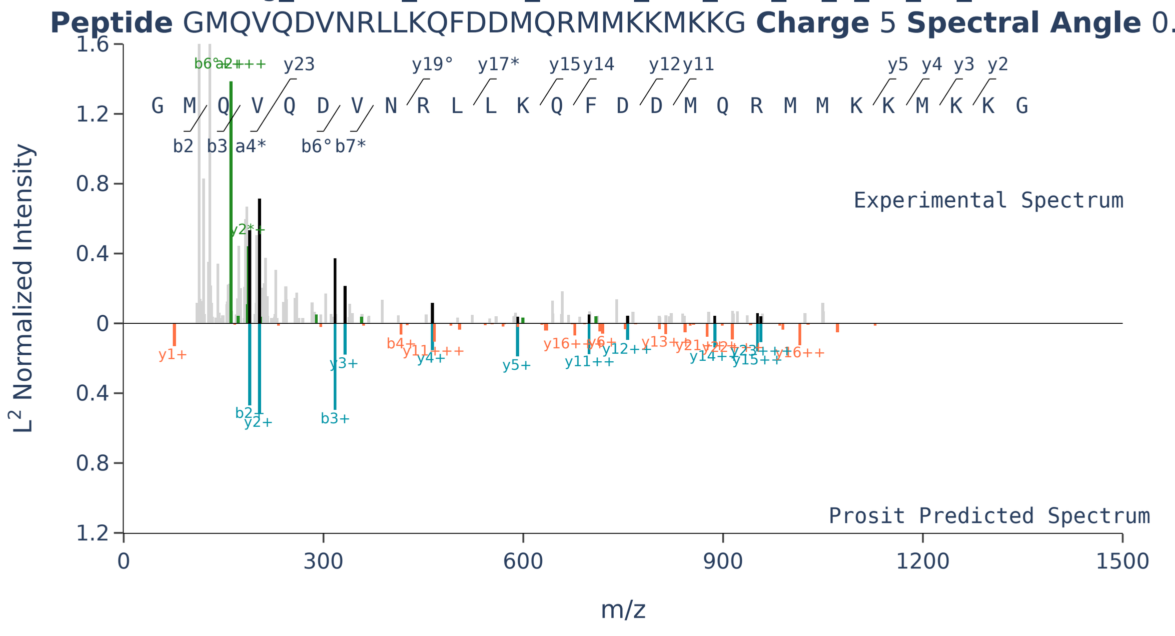


**Source** Ncheng_210623_230623_HFGoe_FFH_20S_25_1_A2_2h_R1 **Scan** 35166
**Peptide** VDFFPSDVGQKPVDIVNAALKEAKLK **Charge** 5 **Spectral Angle** 0.63

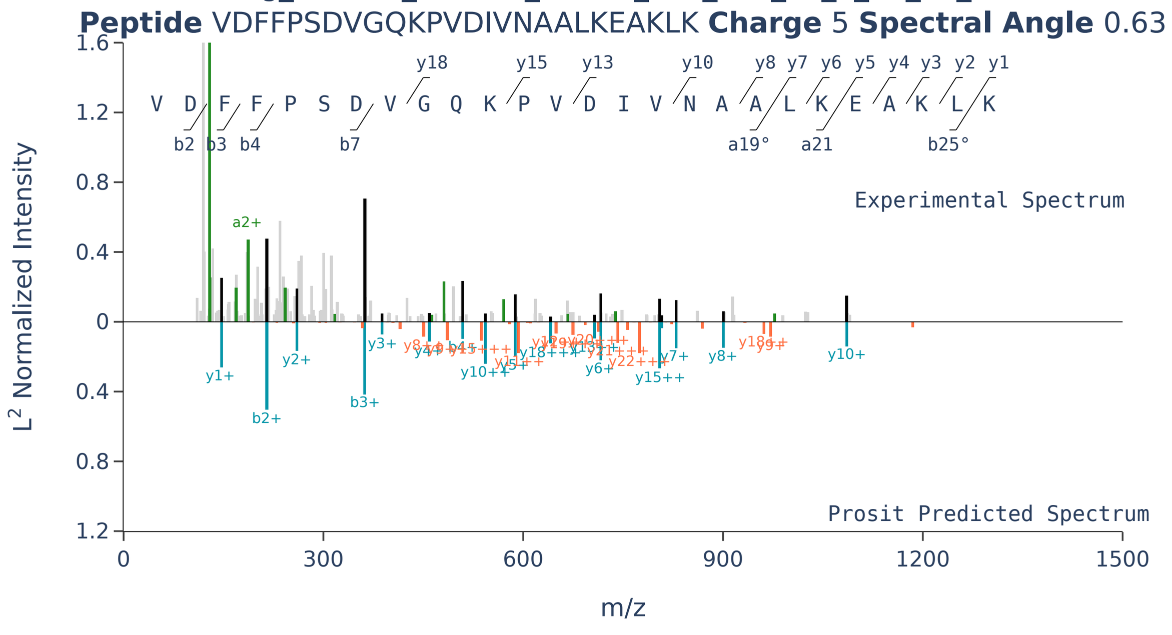


**Source** Ncheng_210623_230623_HFGoe_FFH_20S_25_1_A2_4h_R2 **Scan** 9684
**Peptide** MTMKERAKPEI **Charge** 2 **Spectral Angle** 0.61

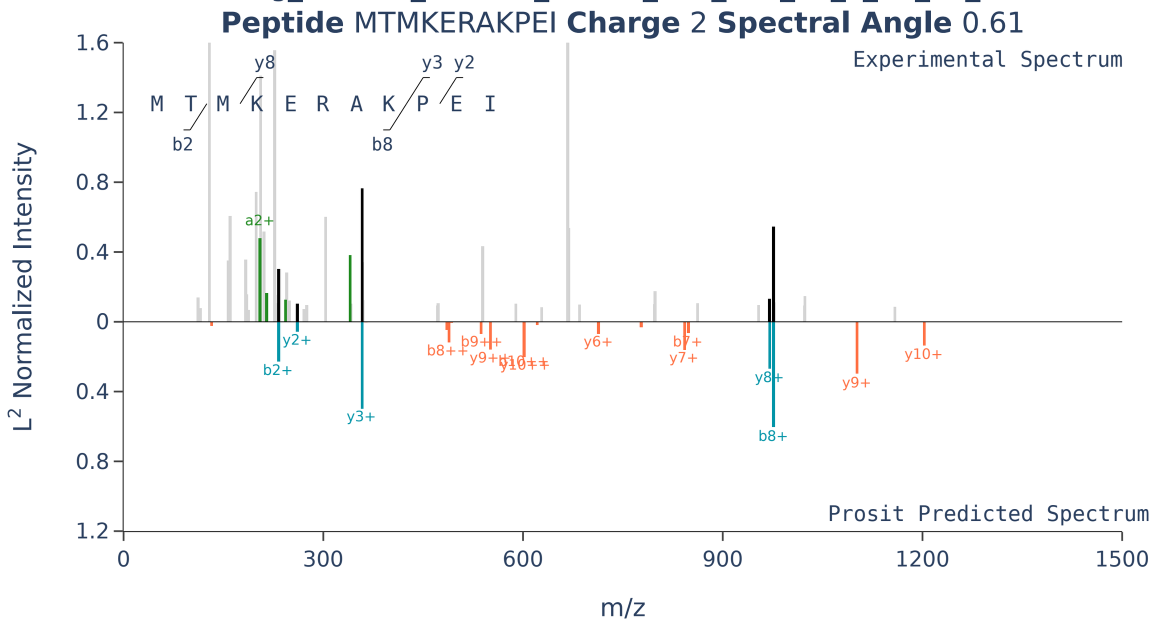


**Source** Ncheng_210623_230623_HFGoe_FFH_20S_25_1_A2_2h_R2 **Scan** 19523
**Peptide** LSLRHITGKPIKFLGVGEKTE **Charge** 4 **Spectral Angle** 0.55

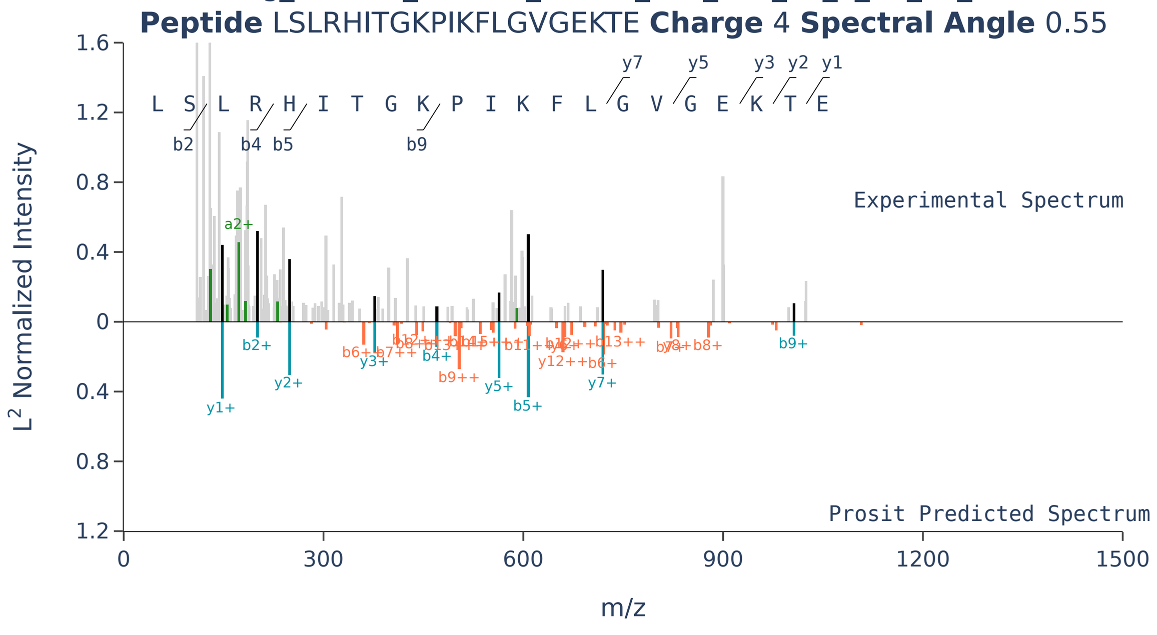


**Source** Ncheng_210623_230623_HFGoe_FFH_20S_25_1_A2_2h_R1 **Scan** 25480
**Peptide** KLASKLKKGDGFDLNDFLE **Charge** 3 **Spectral Angle** 0.4

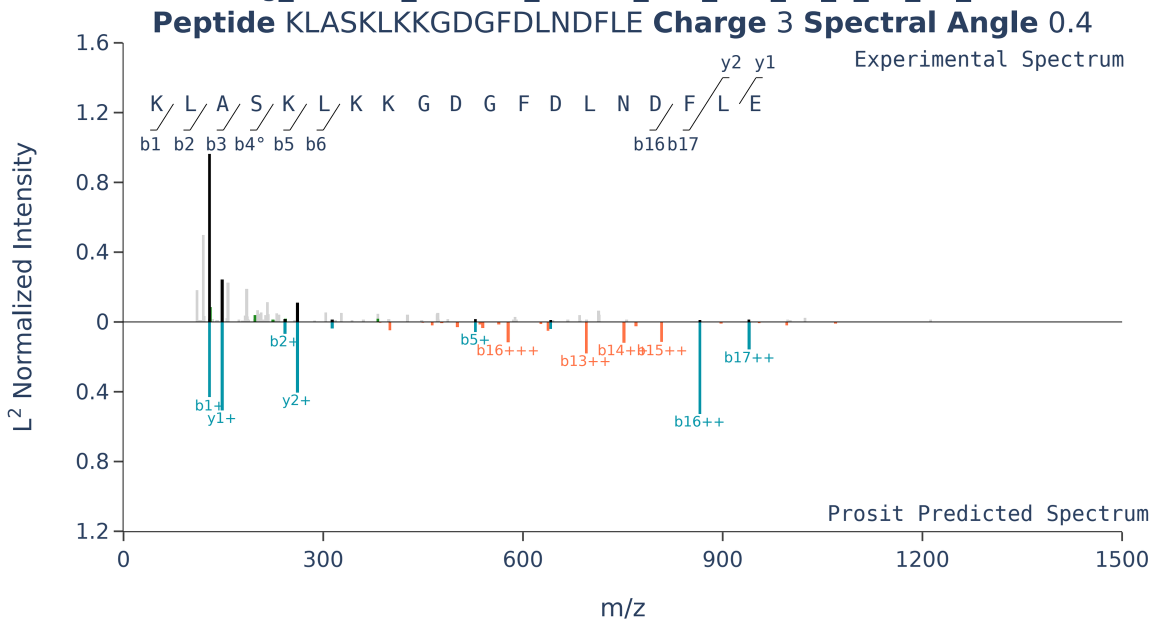


**Source** Ncheng_210623_230623_HFGoe_FFH_20S_25_1_A1_2h_R1 **Scan** 18963
**Peptide** LKQFDDMQRMMKKMKKGGMA **Charge** 5 **Spectral Angle** 0.56

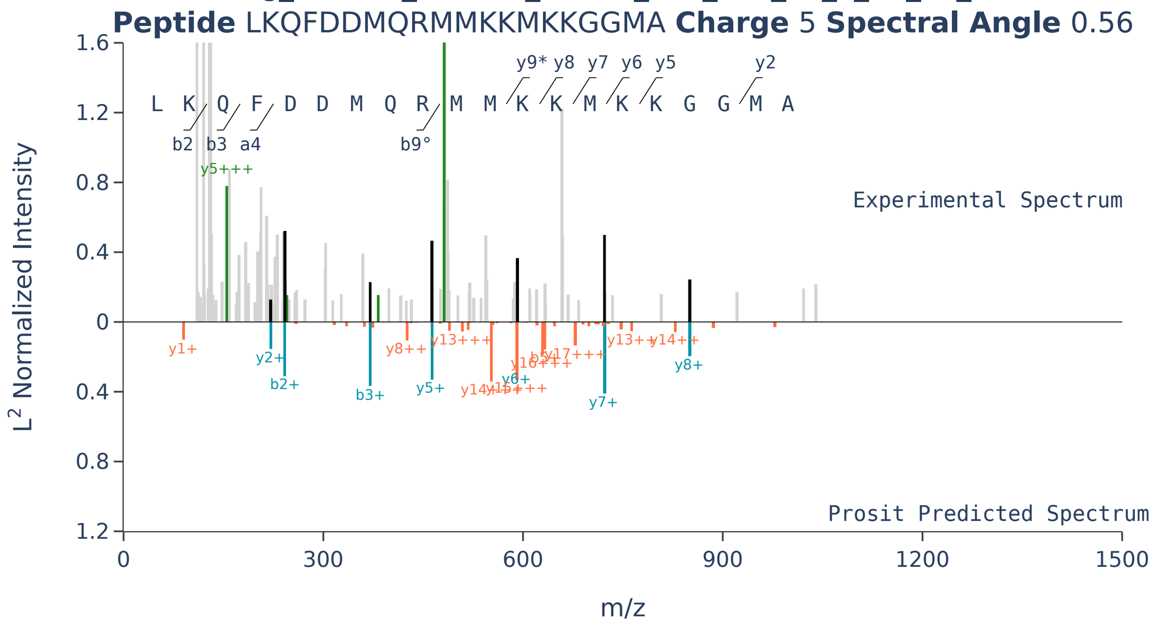


**Source** Ncheng_210623_230623_HFGoe_FFH_20S_25_1_A1_2h_R2 **Scan** 15930
**Peptide** SIRHITGKPIKFLGVGEKTEA **Charge** 4 **Spectral Angle** 0.46

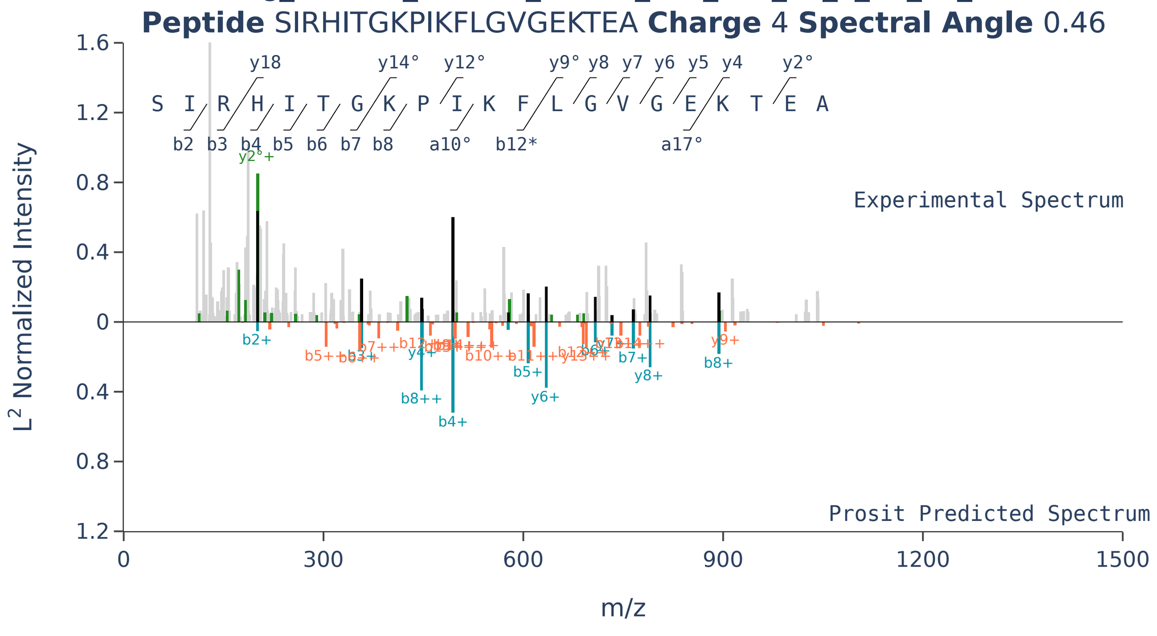

**Source** Ncheng_210623_230623_HFGoe_FFH_20S_25_1_A2_24h_R1 **Scan** 9409
**Peptide** IESKVDRAQAEKLAS **Charge** 2 **Spectral Angle** 0.43

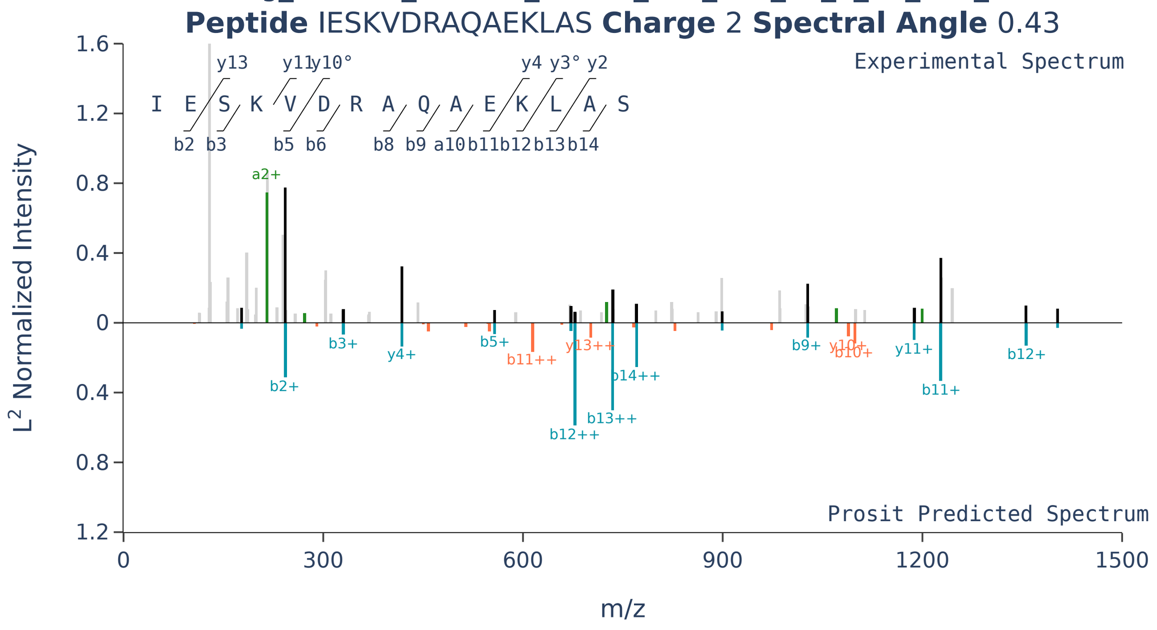


**Source** Ncheng_210623_230623_HFGoe_FFH_20S_25_1_A2_2h_R1 **Scan** 34480
**Peptide** DVALPVVREFINRVKEKAVGHEV **Charge** 4 **Spectral Angle** 0.46

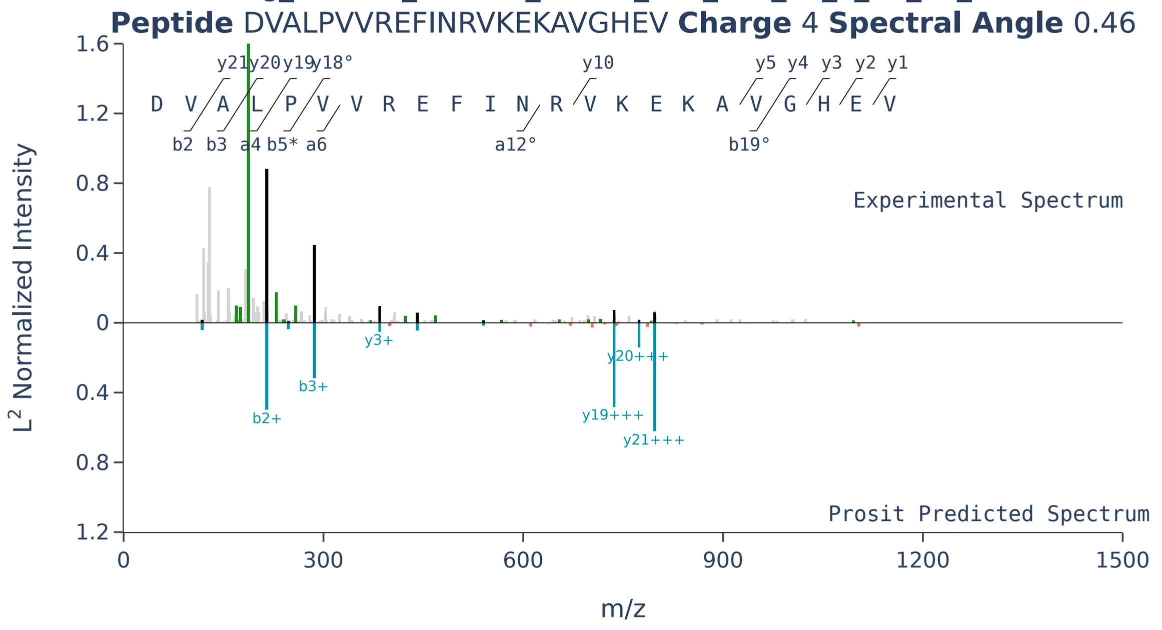


**Source** Ncheng_210623_230623_HFGoe_FFH_20S_25_1_A1_4h_R2 **Scan** 24845
**Peptide** KPIKFLGVGEKTEALEPFHPDRIASRI **Charge** 4 **Spectral Angle** 0.66

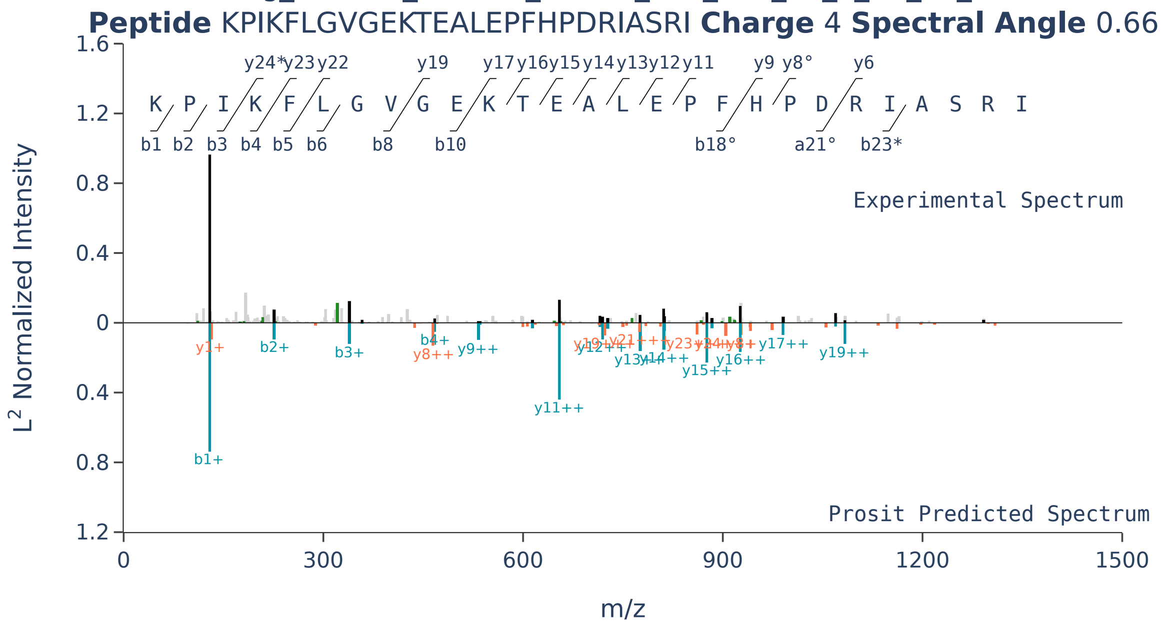


**Source** Ncheng_210623_230623_HFGoe_FFH_20S_25_1_A1_24h_R1 **Scan** 7555
**Peptide** SGRGRLTEDNV **Charge** 2 **Spectral Angle** 0.67

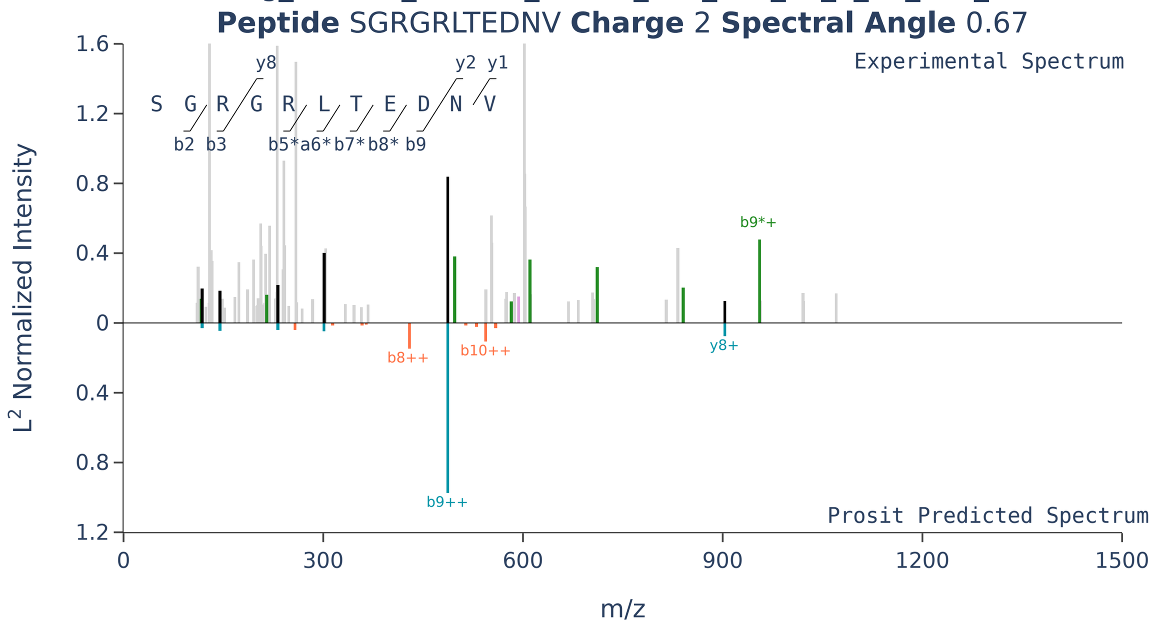


**Source** Ncheng_210623_230623_HFGoe_FFH_20S_25_1_A2_2h_R2 **Scan** 27376
**Peptide** RNISGRGRLTEDNVKDTLREVRMALLE **Charge** 5 **Spectral Angle** 0.52

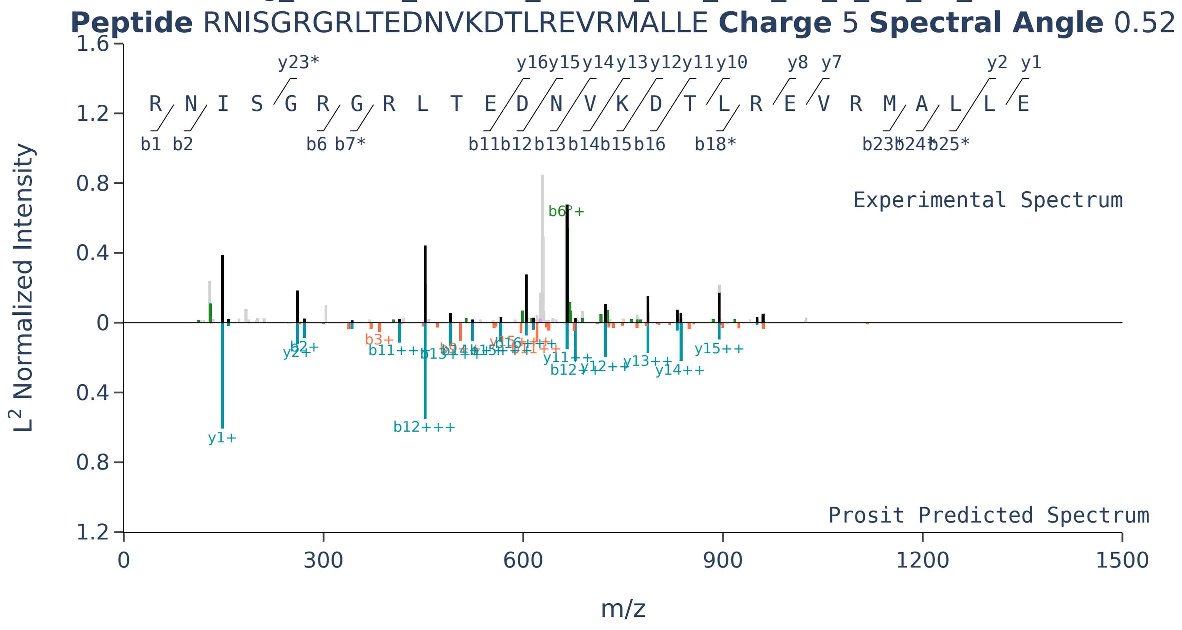


**Source** Ncheng_210623_230623_HFGoe_FFH_20S_25_1_A1_4h_R1 **Scan** 11616
**Peptide** RNISGRGRLTEDNVKDTL **Charge** 4 **Spectral Angle** 0.37

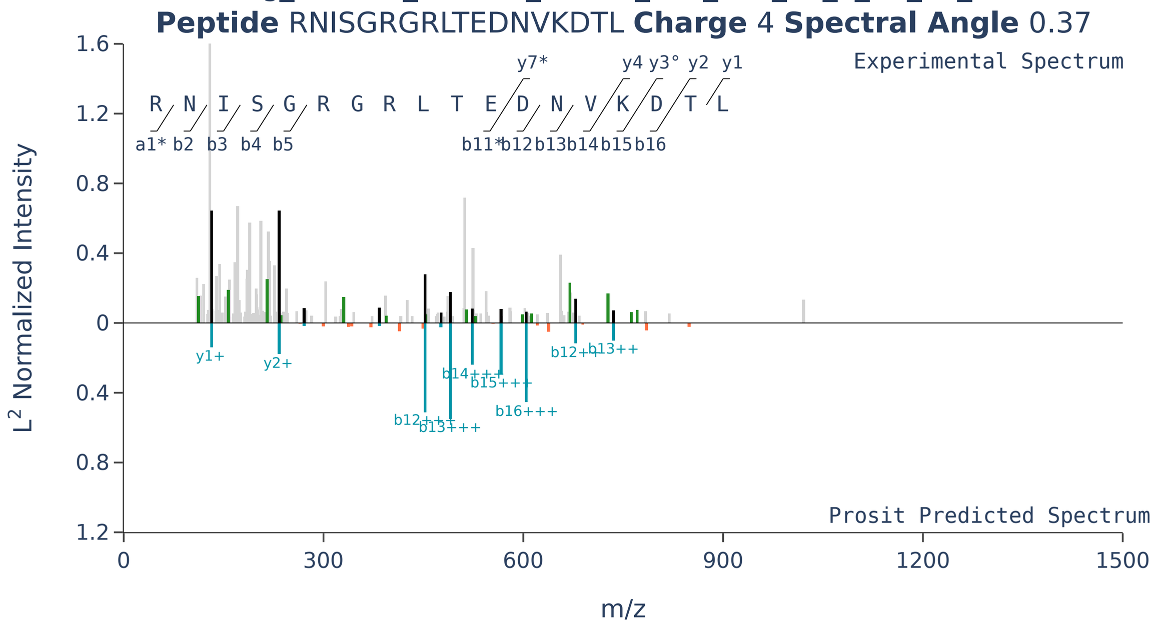


**Source** Ncheng_210623_230623_HFGoe_FFH_20S_25_1_A1_2h_R2 **Scan** 30097
**Peptide** MFDNLTDRLSRTLRNISGRGRLTE **Charge** 5 **Spectral Angle** 0.68

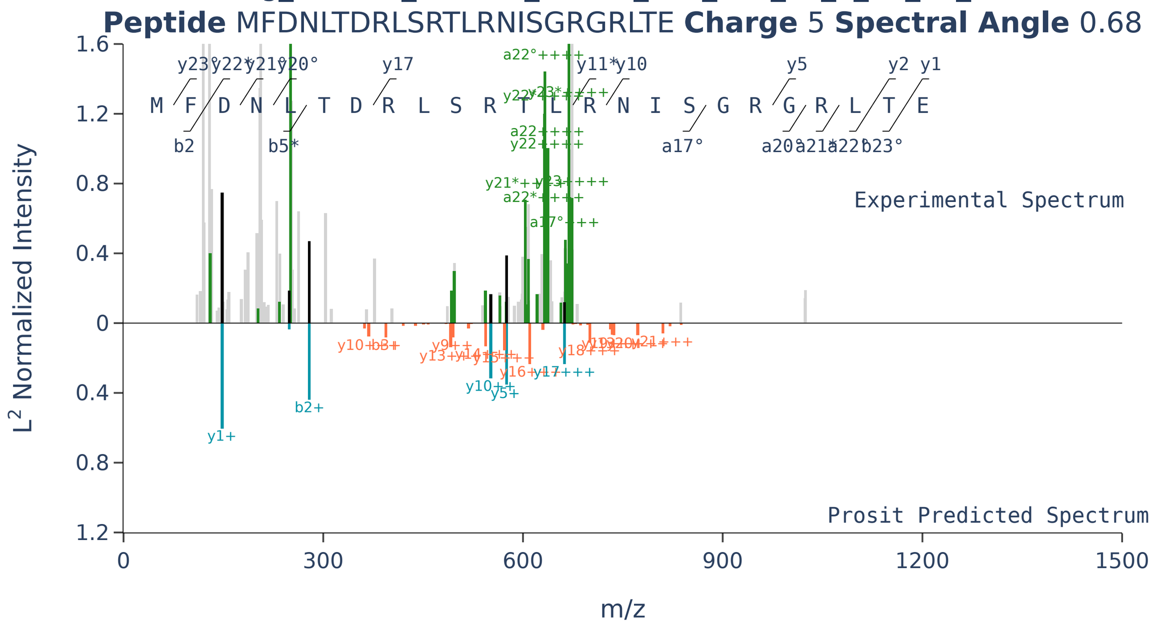


**Source** Ncheng_210623_230623_HFGoe_FFH_20S_25_1_A1_2h_R2 **Scan** 31081
**Peptide** NDFLEQLRQMKNMGGMA **Charge** 3 **Spectral Angle** 0.62

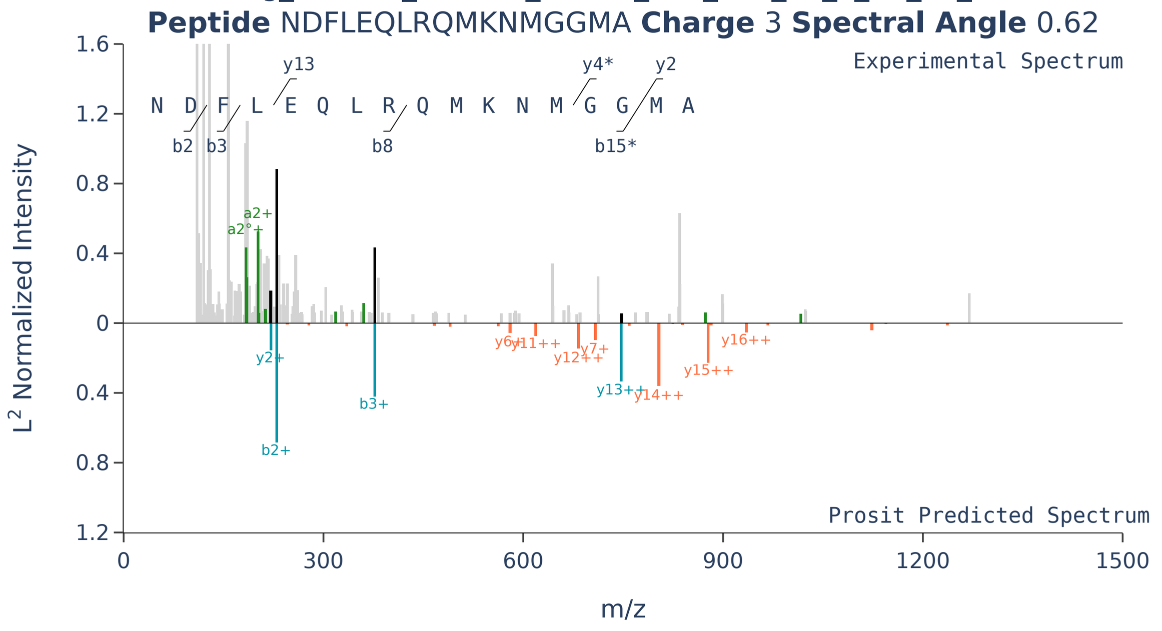


**Source** Ncheng_210623_230623_HFGoe_FFH_20S_25_1_A2_24h_R2 **Scan** 9740
**Peptide** AEKLASKLKKGDGF **Charge** 3 **Spectral Angle** 0.37

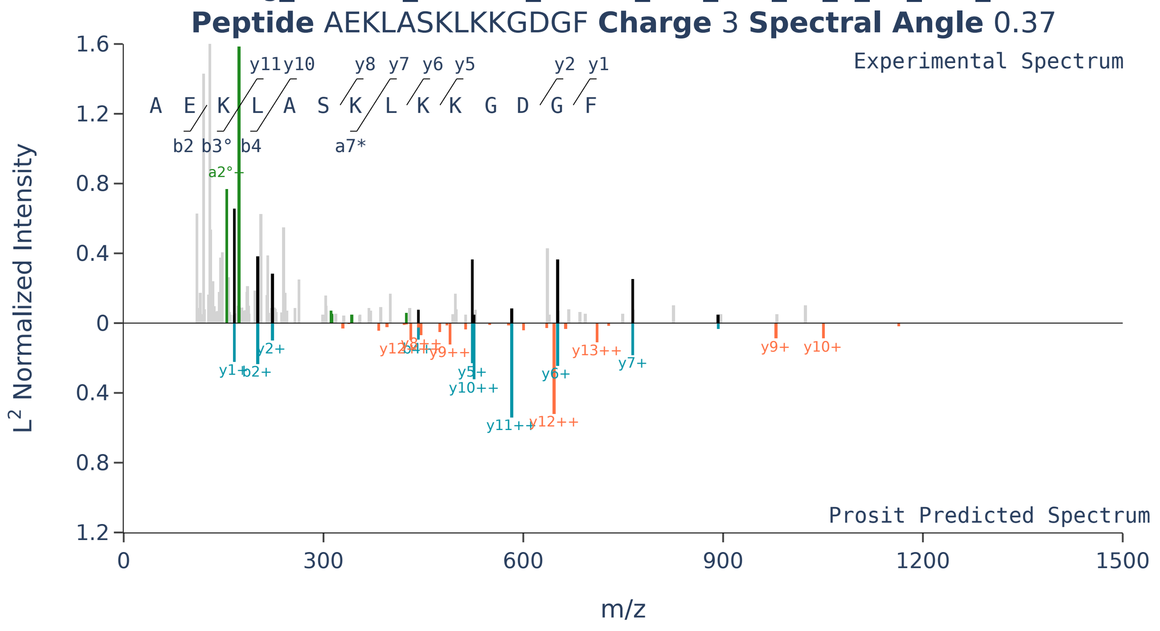


**Source** Ncheng_210623_230623_HFGoe_FFH_20S_25_1_A2_2h_R1 **Scan** 18348
**Peptide** VRNELVAAMGEENQT **Charge** 2 **Spectral Angle** 0.56

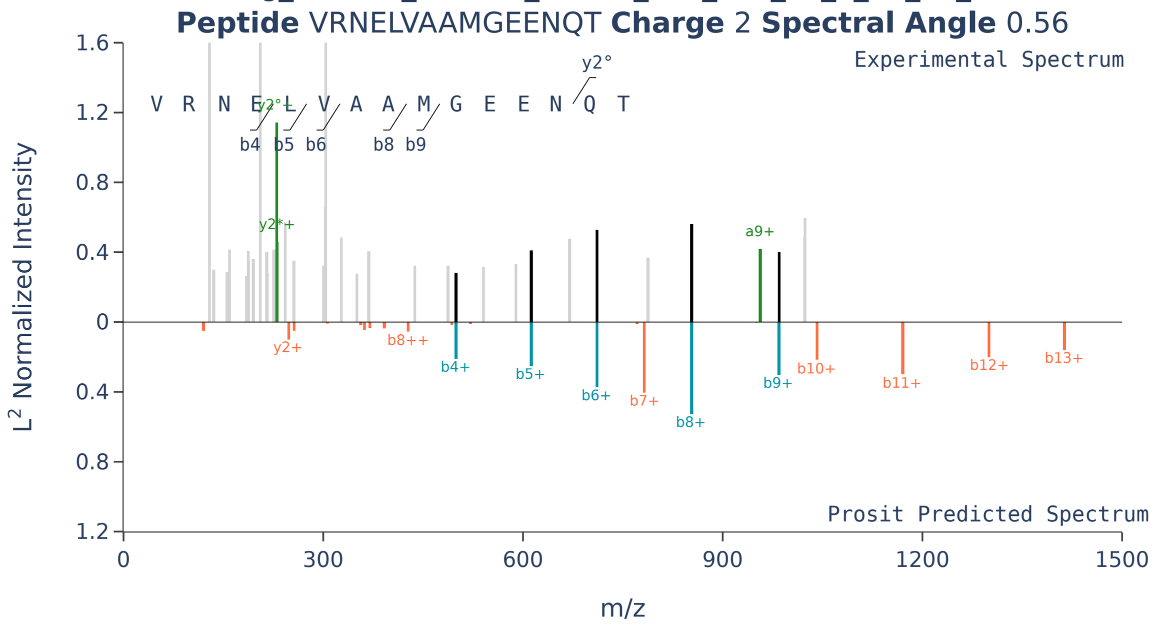


**Source** Ncheng_210623_230623_HFGoe_FFH_20S_25_1_A1_2h_R2 **Scan** 31820
**Peptide** MFDNLTDRLSRTLRNI **Charge** 2 **Spectral Angle** 0.6

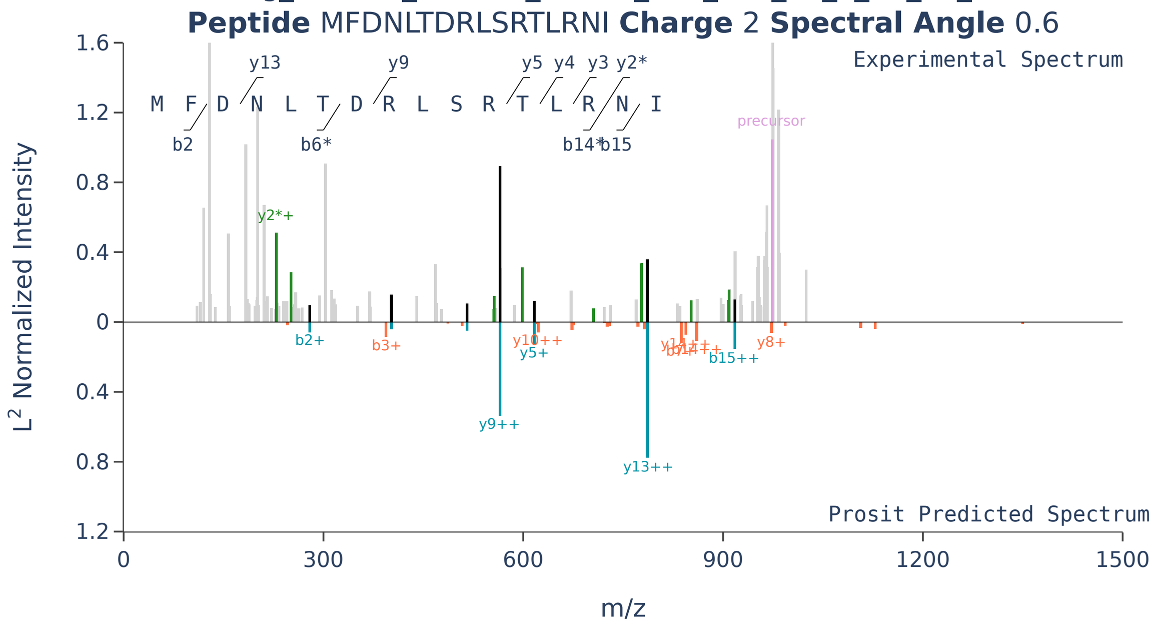


**Source** Ncheng_210623_230623_HFGoe_FFH_20S_25_1_A1_2h_R1 **Scan** 38664
**Peptide** GVDFFPSDVGQKPVDIVNAALKEAKL **Charge** 4 **Spectral Angle** 0.65

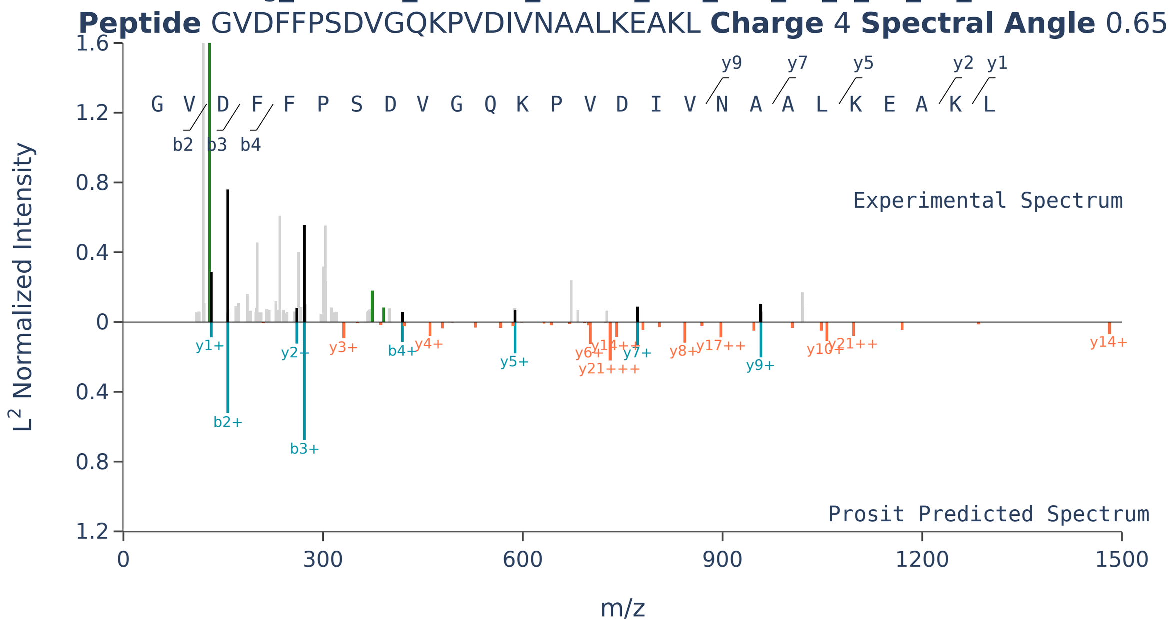


**Source** Ncheng_210623_230623_HFGoe_FFH_20S_25_1_A1_4h_R1 **Scan** 26215
**Peptide** GVGEKTEALEPFHPDRIASRIL **Charge** 3 **Spectral Angle** 0.39

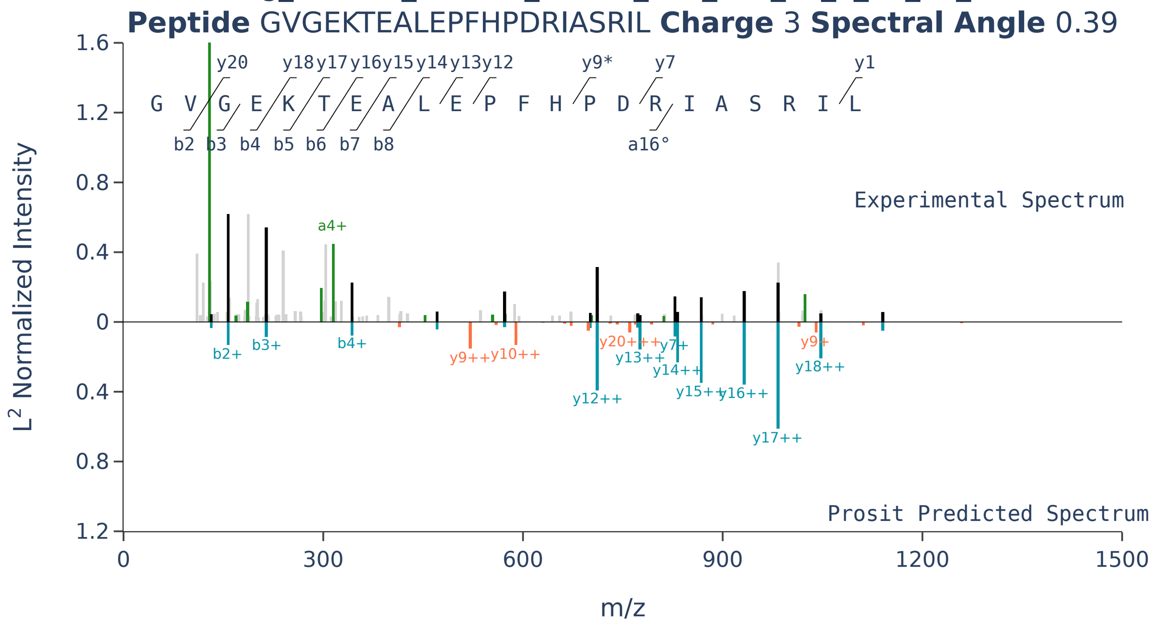


**Source** Ncheng_210623_230623_HFGoe_FFH_20S_25_1_A2_4h_R1 **Scan** 33502
**Peptide** NMGGMASLMGKLPGMGQIPDNVKSQMDD **Charge** 3 **Spectral Angle** 0.6

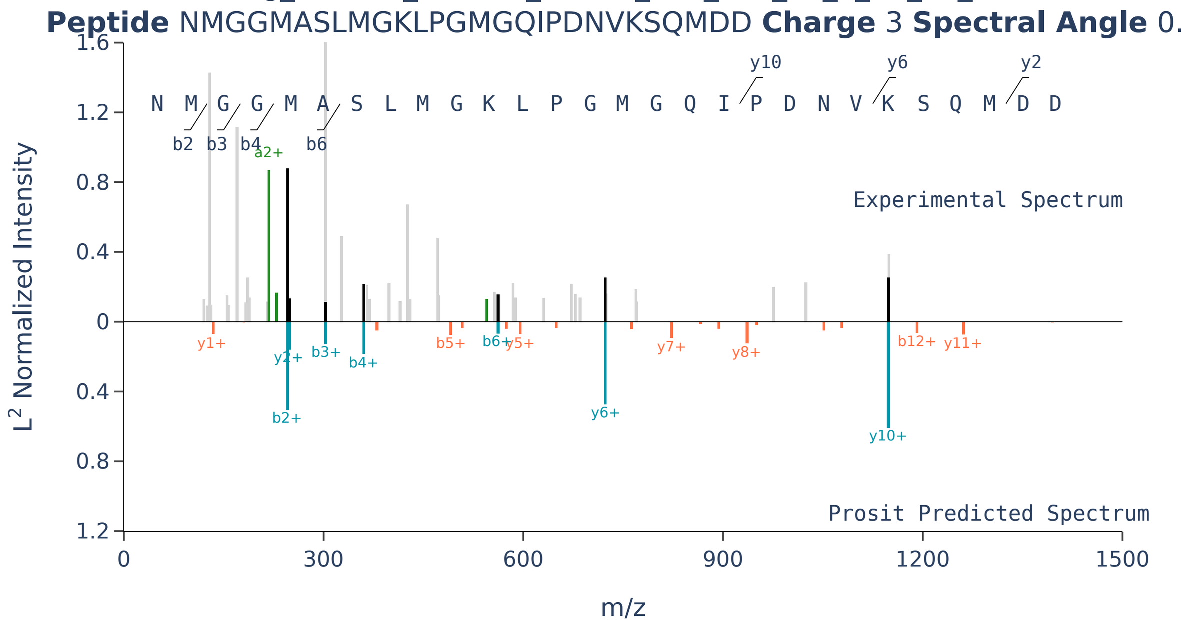


**Source** Ncheng_210623_230623_HFGoe_FFH_20S_25_1_A1_2h_R2 **Scan** 39857
**Peptide** DDKVLVRMEAIINSMTMKE **Charge** 3 **Spectral Angle** 0.63

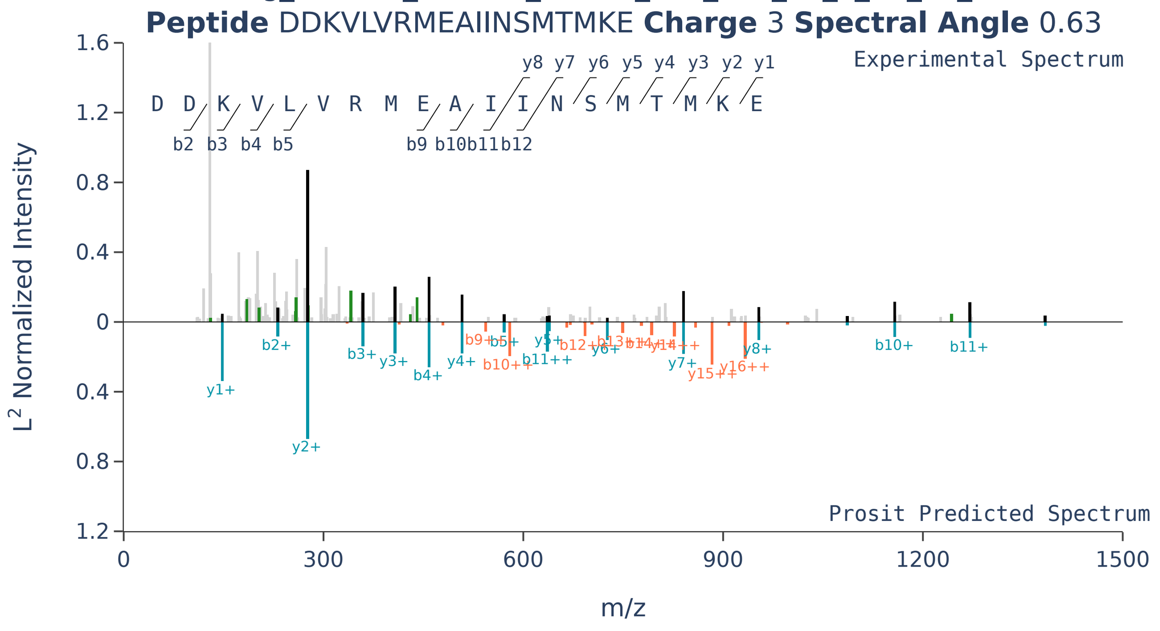

**Source** Ncheng_210623_230623_HFGoe_FFH_20S_25_1_A2_24h_R2 **Scan** 17478
**Peptide** IESKVDRAQAEKLASKLKKGDGF **Charge** 3 **Spectral Angle** 0.59

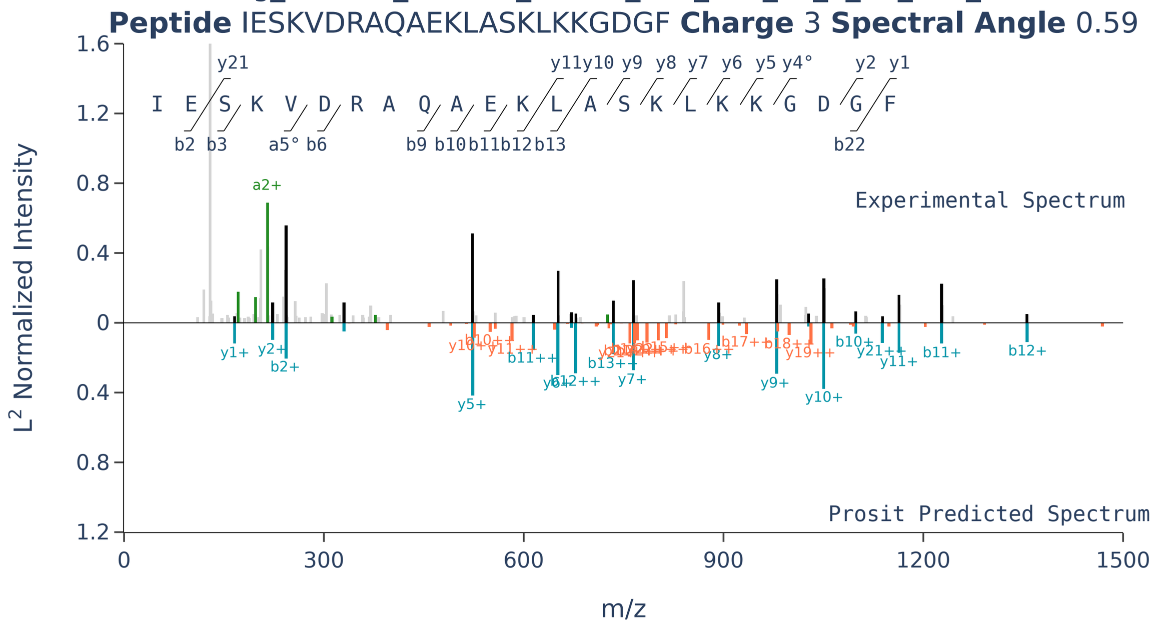


**Source** Ncheng_210623_230623_HFGoe_FFH_20S_25_1_A1_1h_R2 **Scan** 29334
**Peptide** SGRGRLTEDNVKDTLREVRMALLEAD **Charge** 5 **Spectral Angle** 0.64

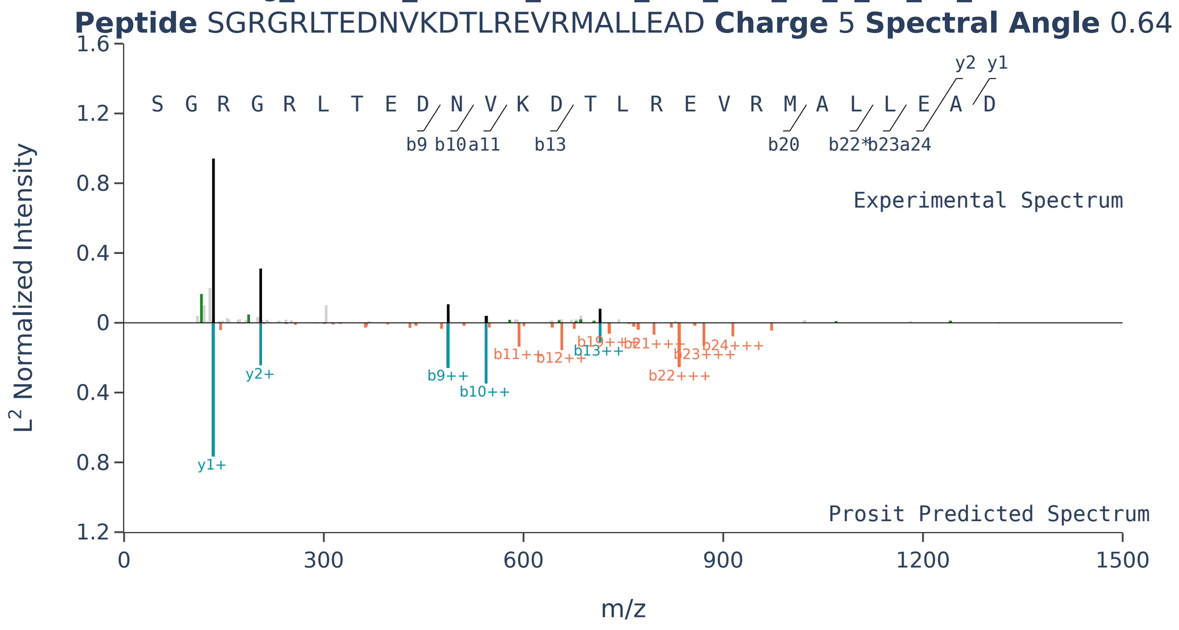


**Source** Ncheng_210623_230623_HFGoe_FFH_20S_25_1_A1_2h_R1 **Scan** 28358
**Peptide** PSDVGQKPVDIVNAALKEAKLK **Charge** 5 **Spectral Angle** 0.51

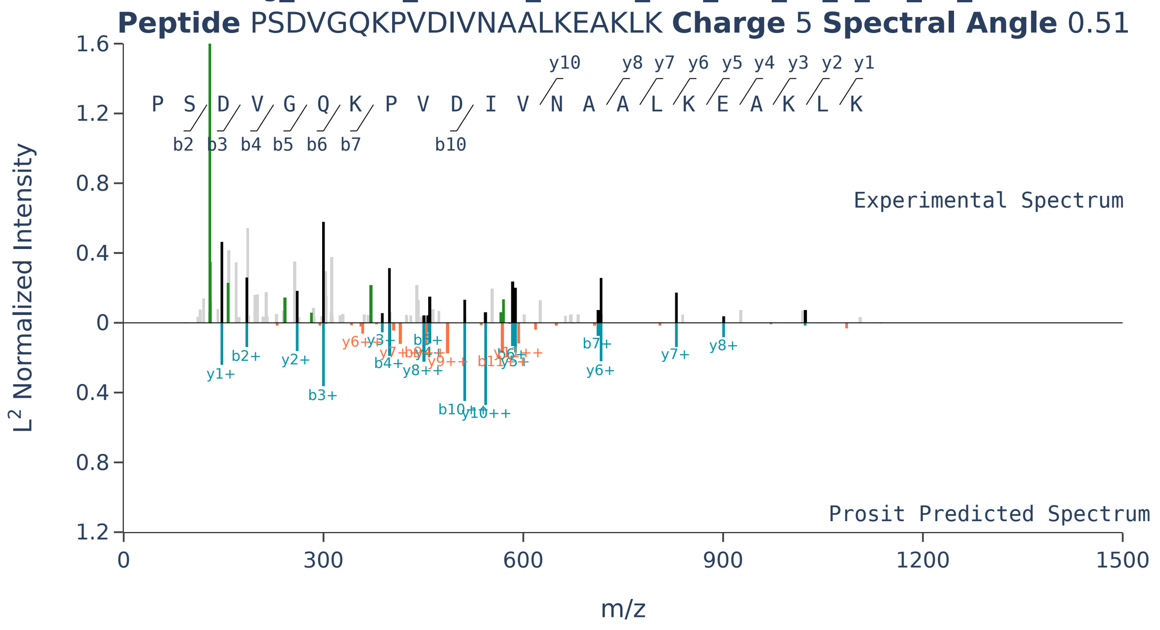


**Source** Ncheng_210623_230623_HFGoe_FFH_20S_25_1_A1_4h_R2 **Scan** 19741
**Peptide** IESKVDRAQAEKLASKLKKGDGFDLND **Charge** 3 **Spectral Angle** 0.57

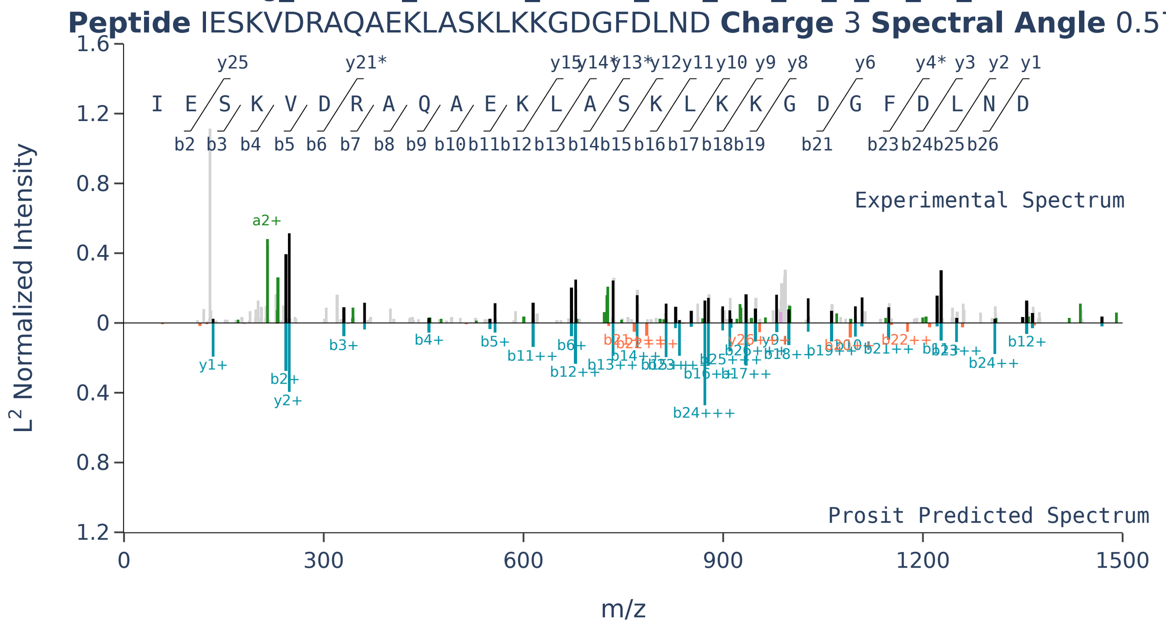


**Source** Ncheng_210623_230623_HFGoe_FFH_20S_25_1_A1_2h_R2 **Scan** 38149
**Peptide** KKGDGFDLNDFLEQLRQMKNMGGMAS **Charge** 4 **Spectral Angle** 0.65

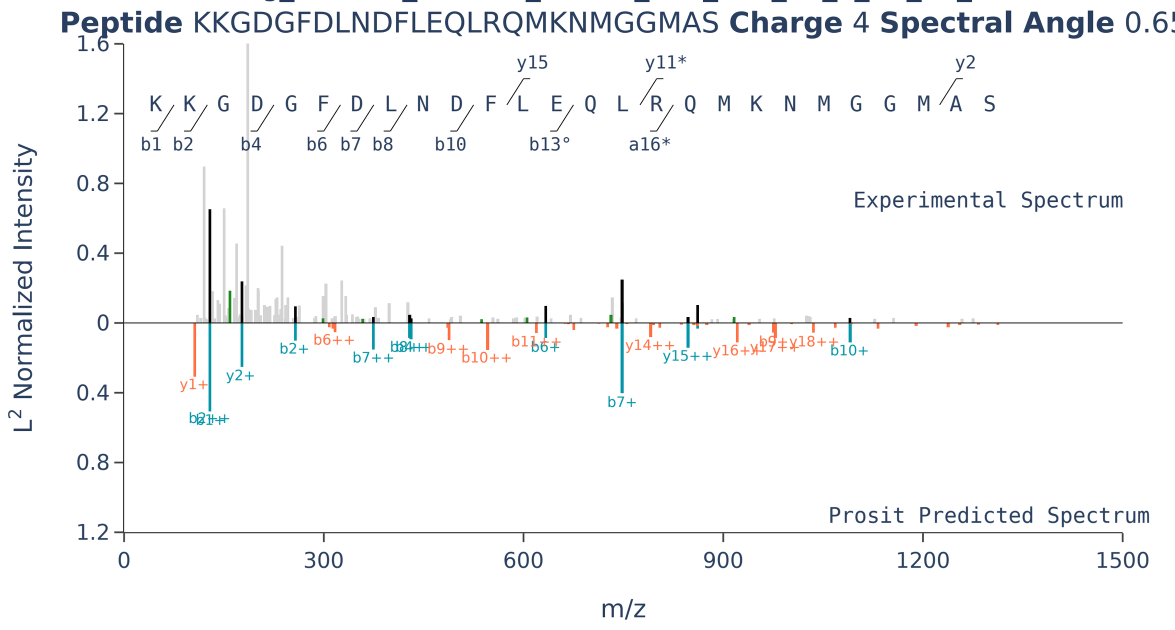


**Source** Ncheng_210623_230623_HFGoe_FFH_20S_25_1_A2_24h_R2 **Scan** 7118
**Peptide** MKERAKPEL **Charge** 2 **Spectral Angle** 0.7

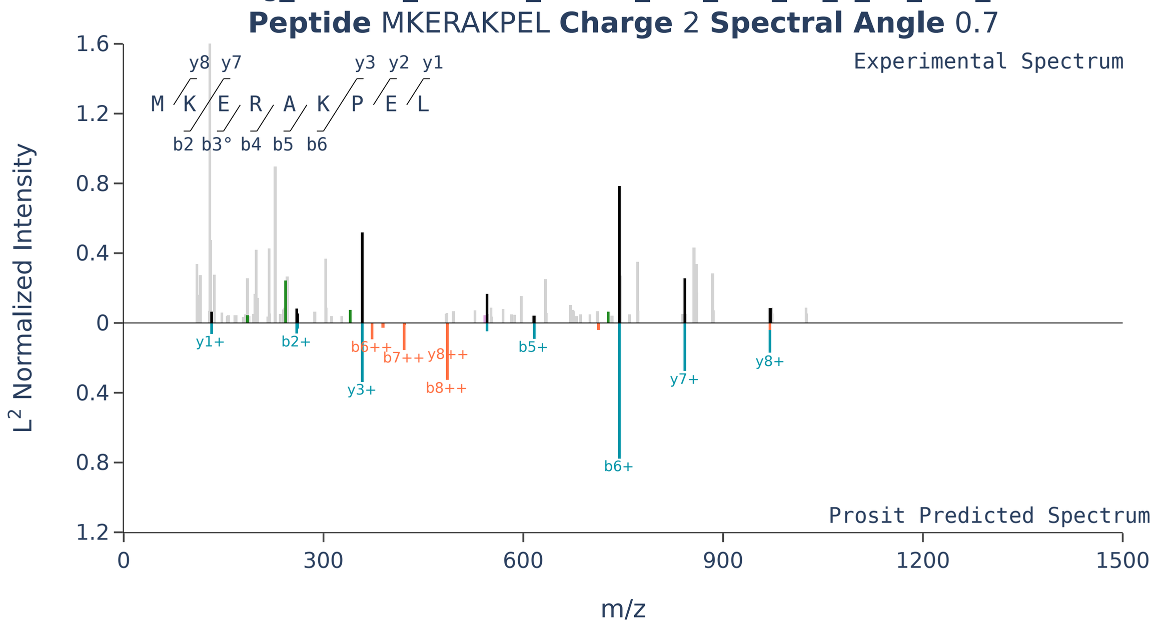


**Source** Ncheng_210623_230623_HFGoe_FFH_20S_25_1_A2_4h_R1 **Scan** 30880
**Peptide** VLSLIEDIESKVDRAQAEKLASKLKKG **Charge** 5 **Spectral Angle** 0.62

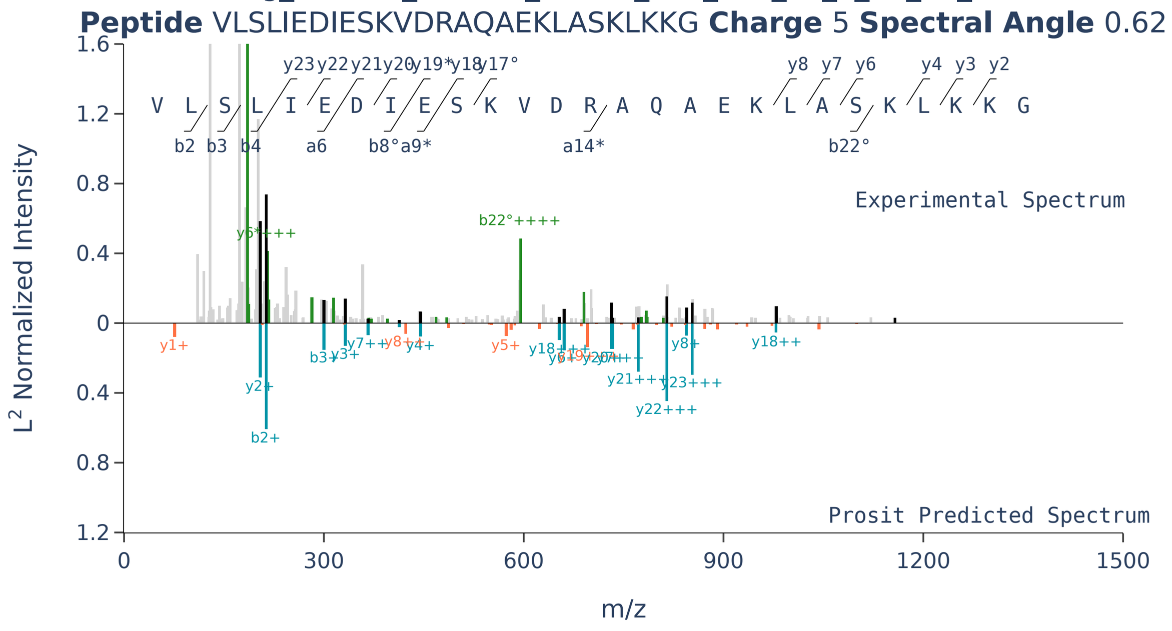


**Source** Ncheng_210623_230623_HFGoe_FFH_20S_25_1_A2_4h_R2 **Scan** 9048
**Peptide** RLSRTLRNISG **Charge** 3 **Spectral Angle** 0.53

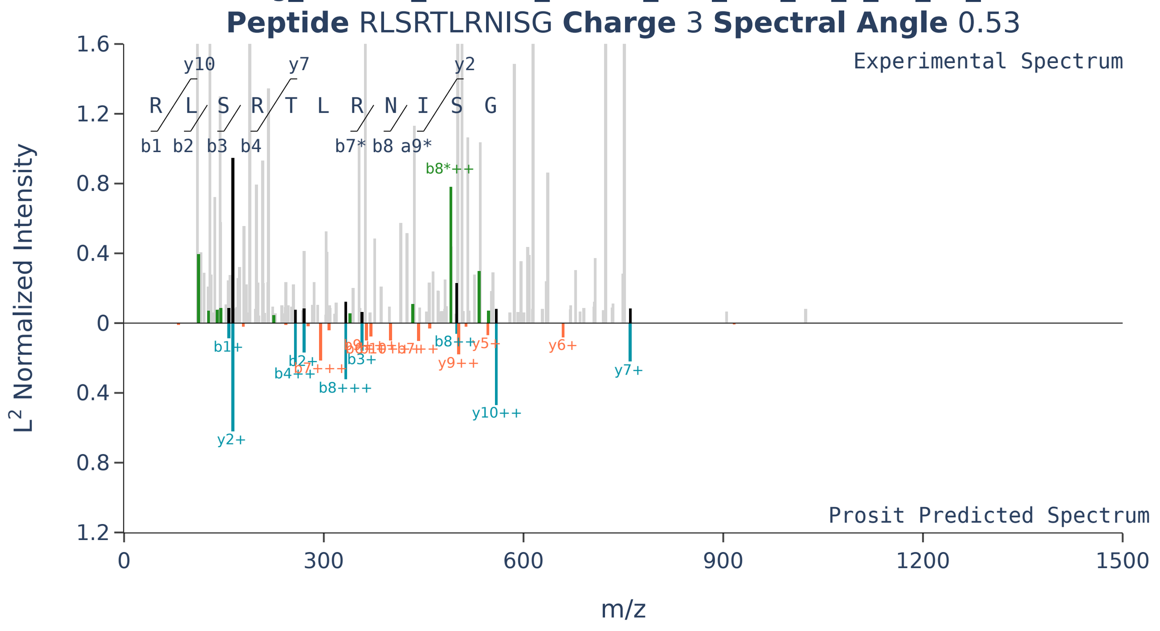


**Source** Ncheng_210623_230623_HFGoe_FFH_20S_25_1_A2_4h_R2 **Scan** 15937
**Peptide** IEDIESKVDRAQAEKLA **Charge** 3 **Spectral Angle** 0.66

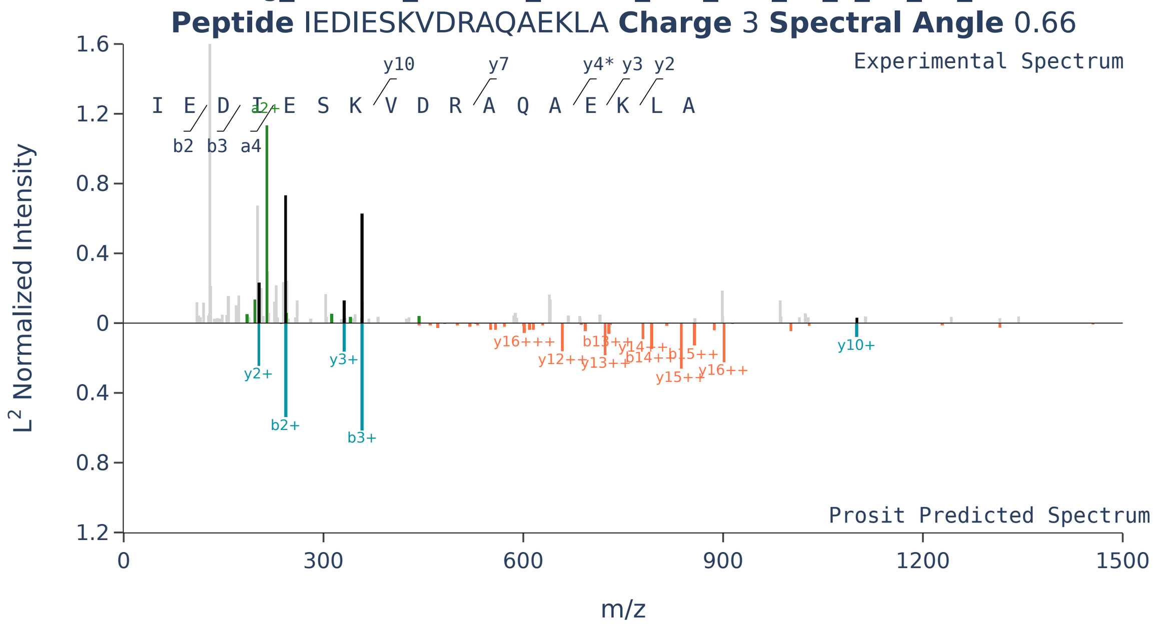


**Source** Ncheng_210623_230623_HFGoe_FFH_20S_25_1_A1_2h_R2 **Scan** 40121
**Peptide** NELVAAMGEENQTLNLAAQPPAVVLM **Charge** 3 **Spectral Angle** 0.62

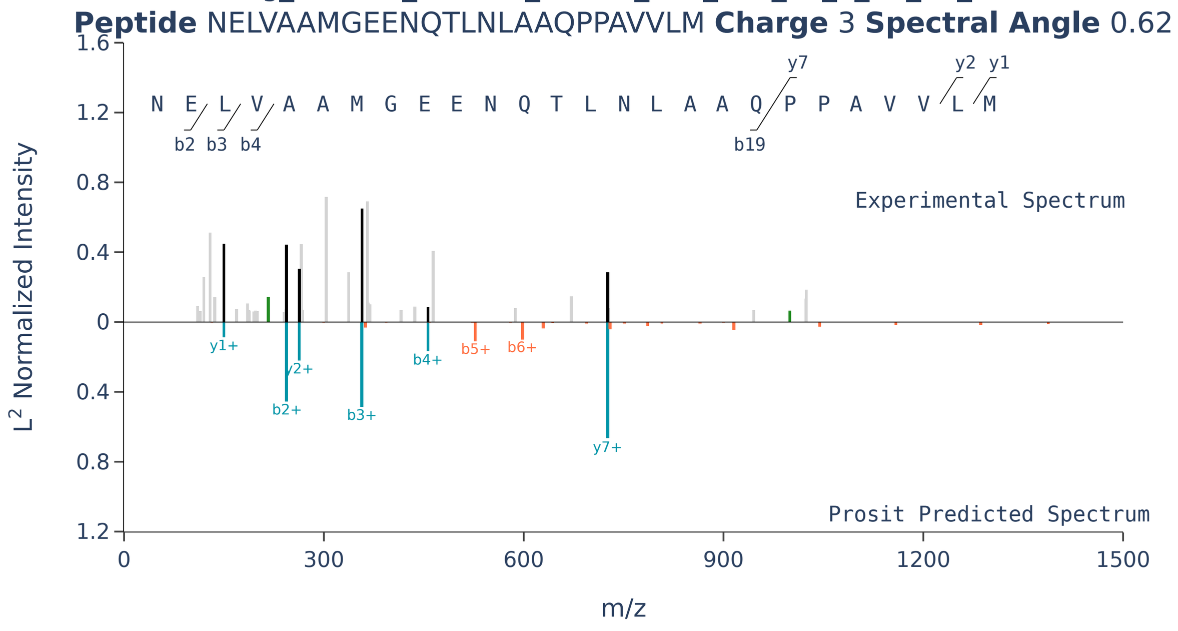


**Source** Ncheng_210623_230623_HFGoe_FFH_20S_25_1_A2_4h_R2 **Scan** 10601
**Peptide** HPDRIASR **Charge** 2 **Spectral Angle** 0.59

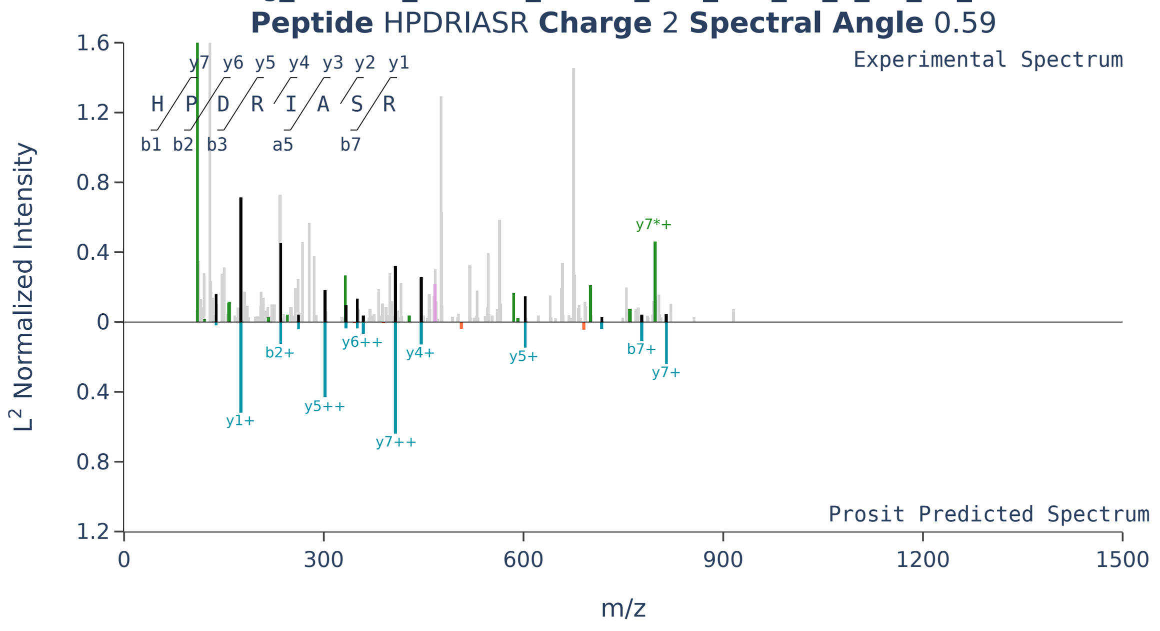


**Source** Ncheng_210623_230623_HFGoe_FFH_20S_25_1_A1_1h_R2 **Scan** 27481
**Peptide** LRNISGRGRLTEDNVKDTLREVRMALLE **Charge** 6 **Spectral Angle** 0.75

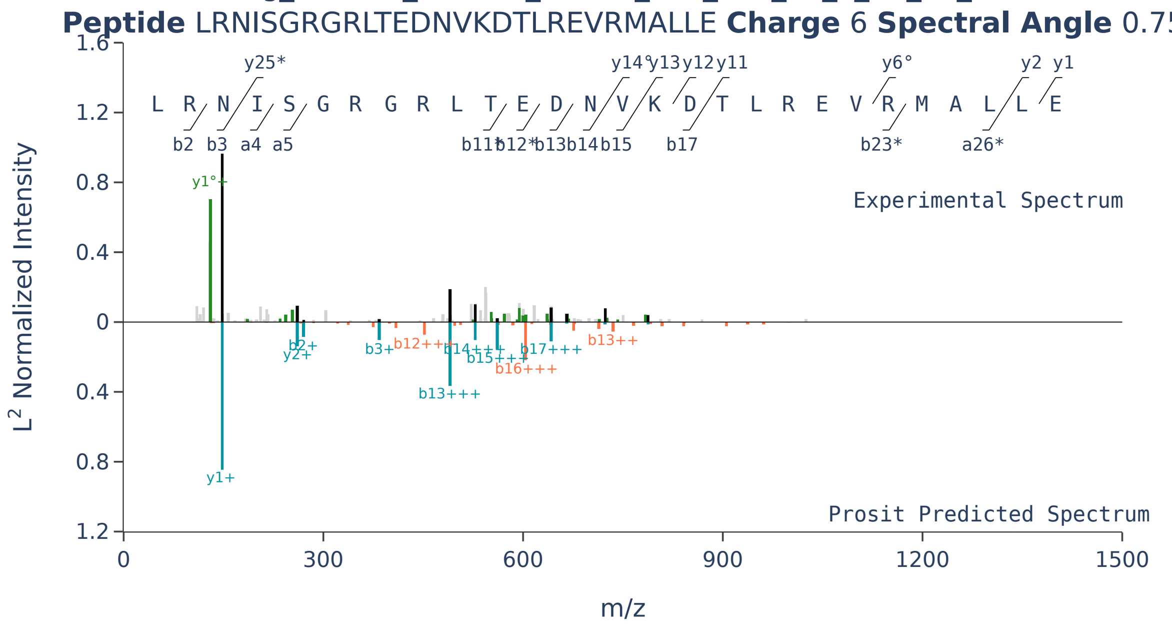


**Source** Ncheng_210623_230623_HFGoe_FFH_20S_25_1_A2_24h_R2 **Scan** 24640
**Peptide** KPVDIVNAALKEA **Charge** 3 **Spectral Angle** 0.55

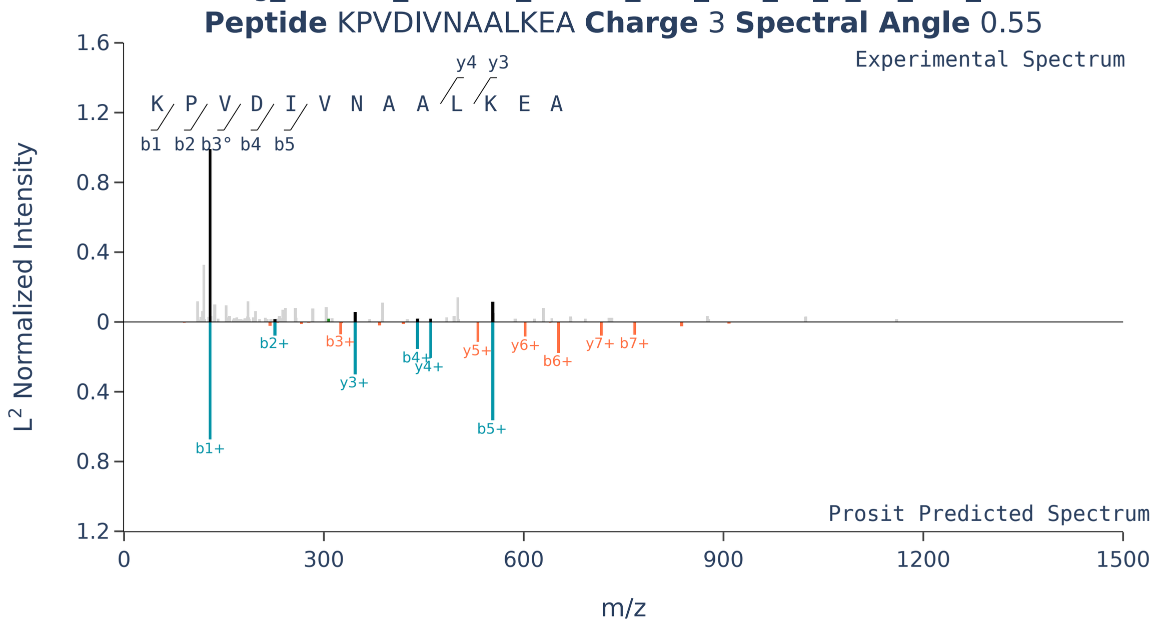


**Source** Ncheng_210623_230623_HFGoe_FFH_20S_25_1_A1_24h_R1 **Scan** 5613
**Peptide** REKHKKKVLV **Charge** 2 **Spectral Angle** 0.49

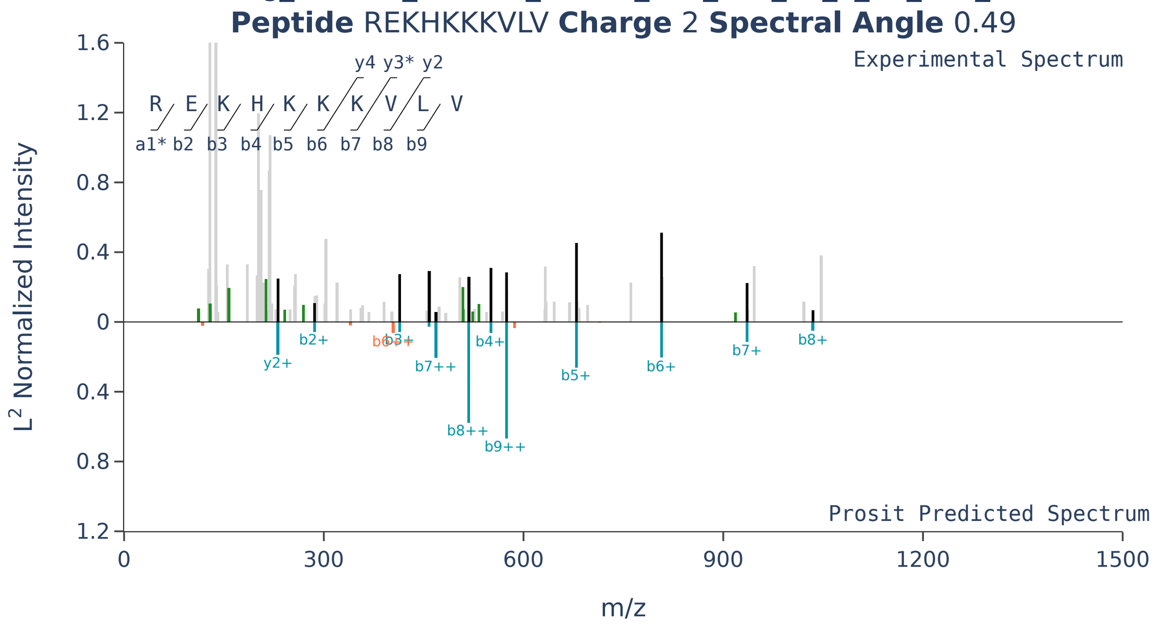


**Source** Ncheng_210623_230623_HFGoe_FFH_20S_25_1_A1_2h_R1 **Scan** 38247
**Peptide** DKVLVRMEAIINSMTM **Charge** 3 **Spectral Angle** 0.65

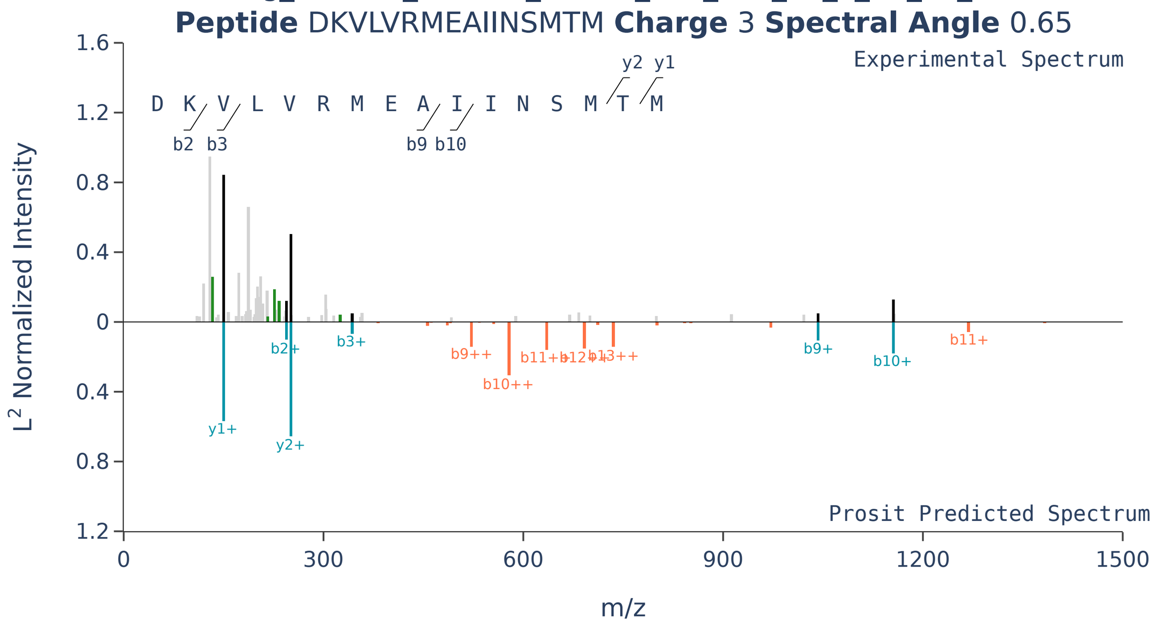

**Source** Ncheng_210623_230623_HFGoe_FFH_20S_25_1_A1_24h_R2 **Scan** 15440
**Peptide** LSGRGRLTEDNVKDTLREVRM **Charge** 5 **Spectral Angle** 0.62

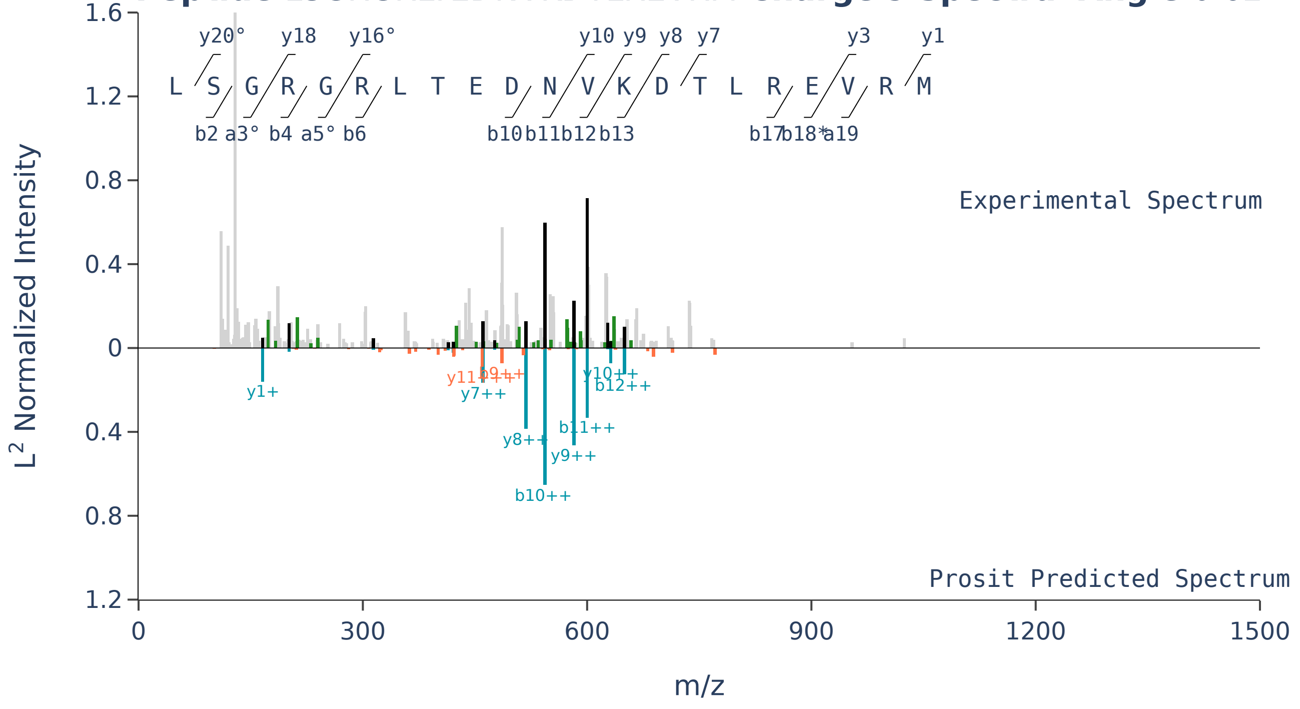


**Source** Ncheng_210623_230623_HFGoe_FFH_20S_25_1_A1_2h_R2 **Scan** 28936
**Peptide** NVKDTLREVRMALLE **Charge** 2 **Spectral Angle** 0.62

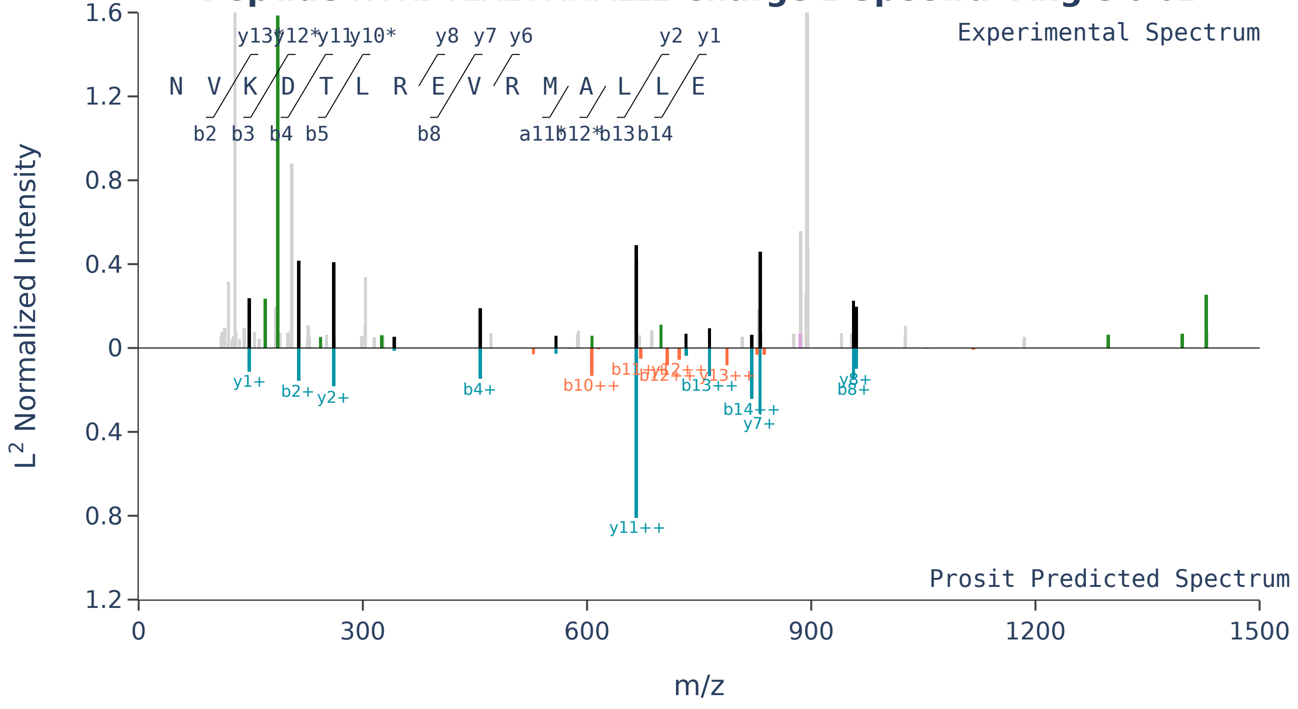


**Source** Ncheng_210623_230623_HFGoe_FFH_20S_25_1_A1_4h_R1 **Scan** 17066
**Peptide** EFINRVKEKAVGHEVNKSLTPGQEF **Charge** 4 **Spectral Angle** 0.59

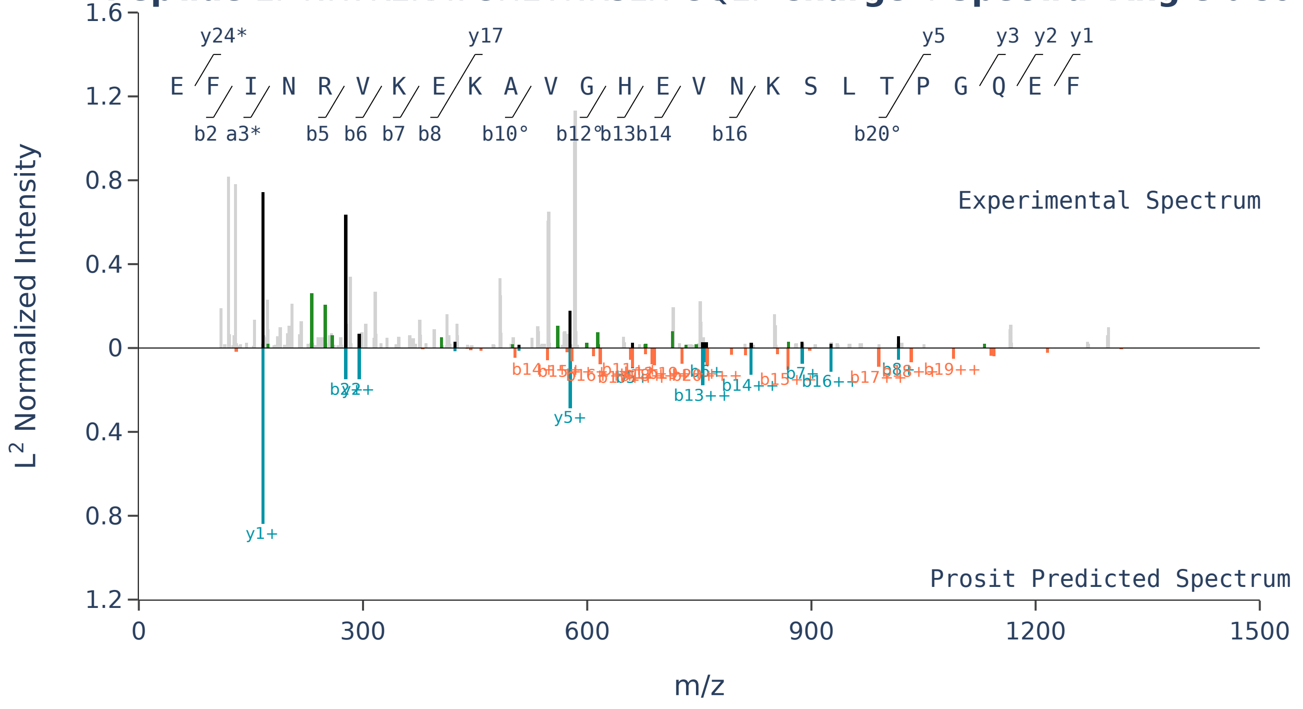


**Source** Ncheng_210623_230623_HFGoe_FFH_20S_25_1_A2_4h_R2 **Scan** 23353
**Peptide** IEDIESKVDRAQAEKLASKLKKGDGFDLND **Charge** 4 **Spectral Angle** 0.8

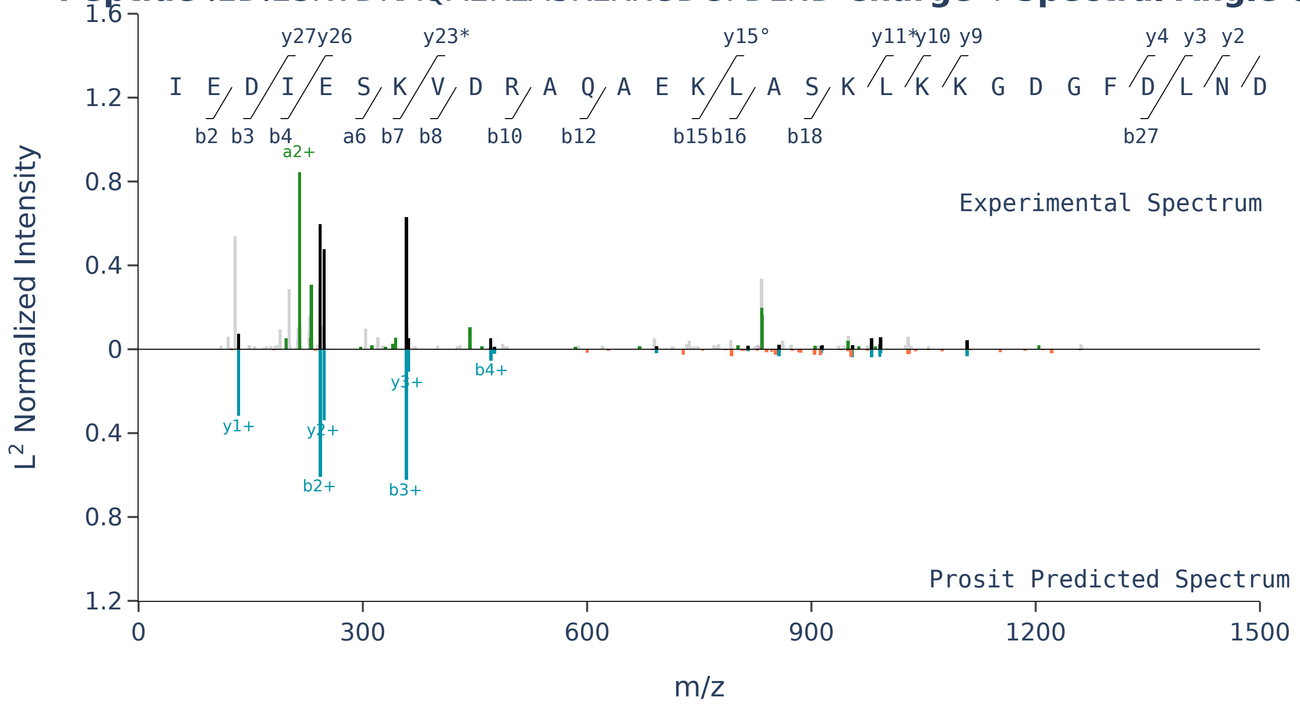


**Source** Ncheng_210623_230623_HFGoe_FFH_20S_25_1_A2_4h_R2 **Scan** 33243
**Peptide** AAGCGMQVQDVNRLLKQFDDMQRMMKKMKK **Charge** 5 **Spectral Angle** 0.62

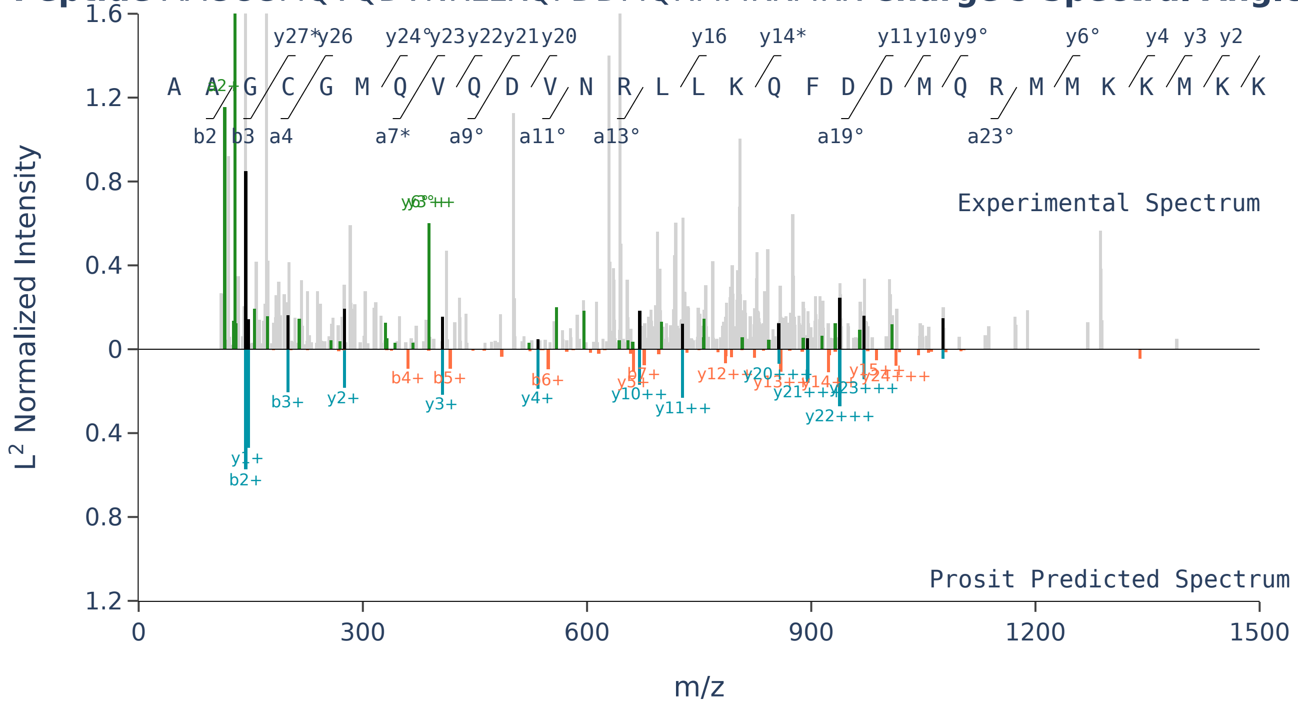


**Source** Ncheng_210623_230623_HFGoe_FFH_20S_25_1_A2_4h_R1 **Scan** 16822
**Peptide** LKQFDDMQRMMKKMKK **Charge** 4 **Spectral Angle** 0.62

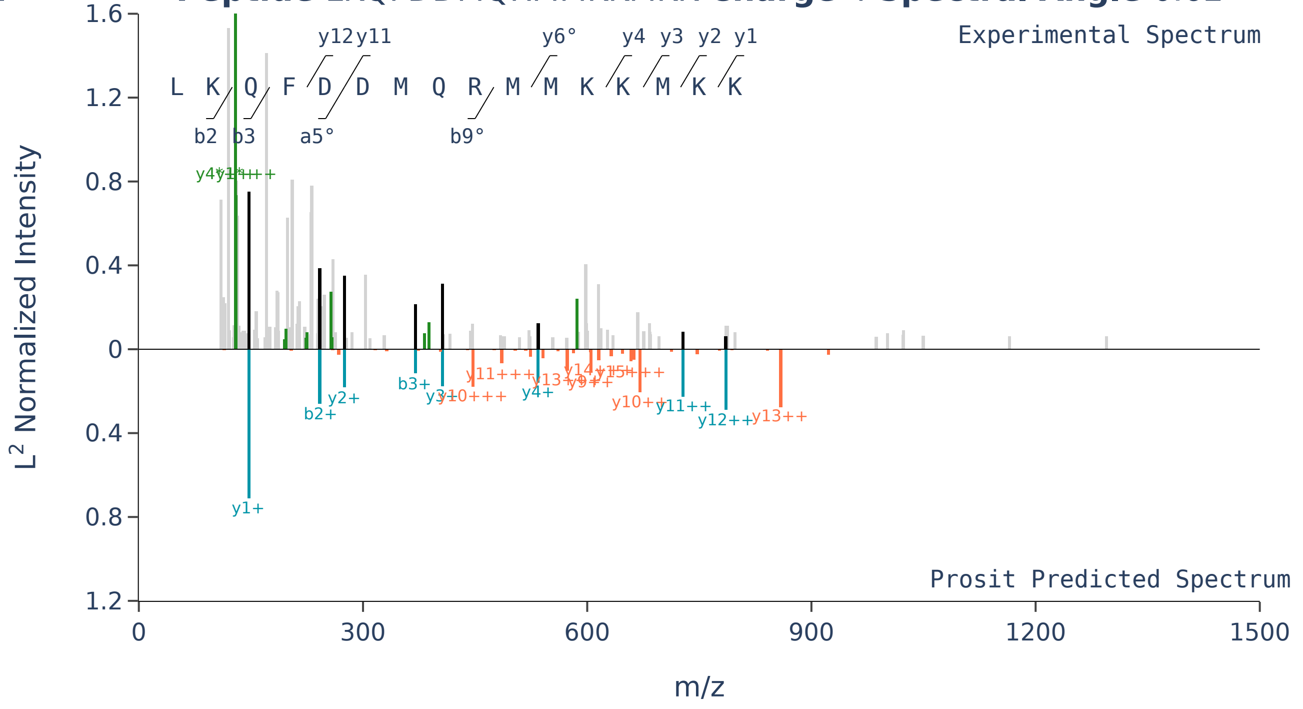


**Source** Ncheng_210623_230623_HFGoe_FFH_20S_25_1_A1_1h_R1 **Scan** 36094
**Peptide** GHEVNKSLTPGQEFVKIVRNELVAAMG **Charge** 4 **Spectral Angle** 0.79

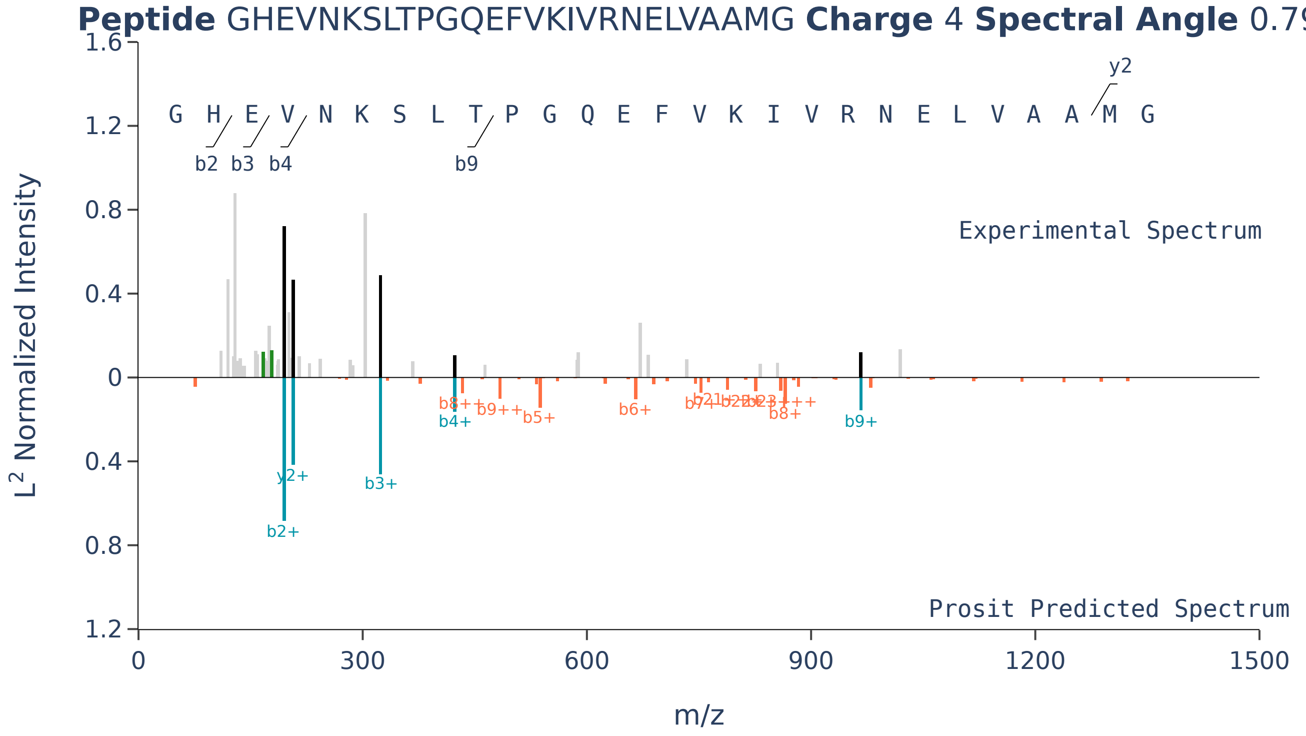


**Source** Ncheng_210623_230623_HFGoe_FFH_20S_25_1_A1_4h_R1 **Scan** 23194
**Peptide** VDGDARGGAALSIRHITGKPIKFL **Charge** 5 **Spectral Angle** 0.72

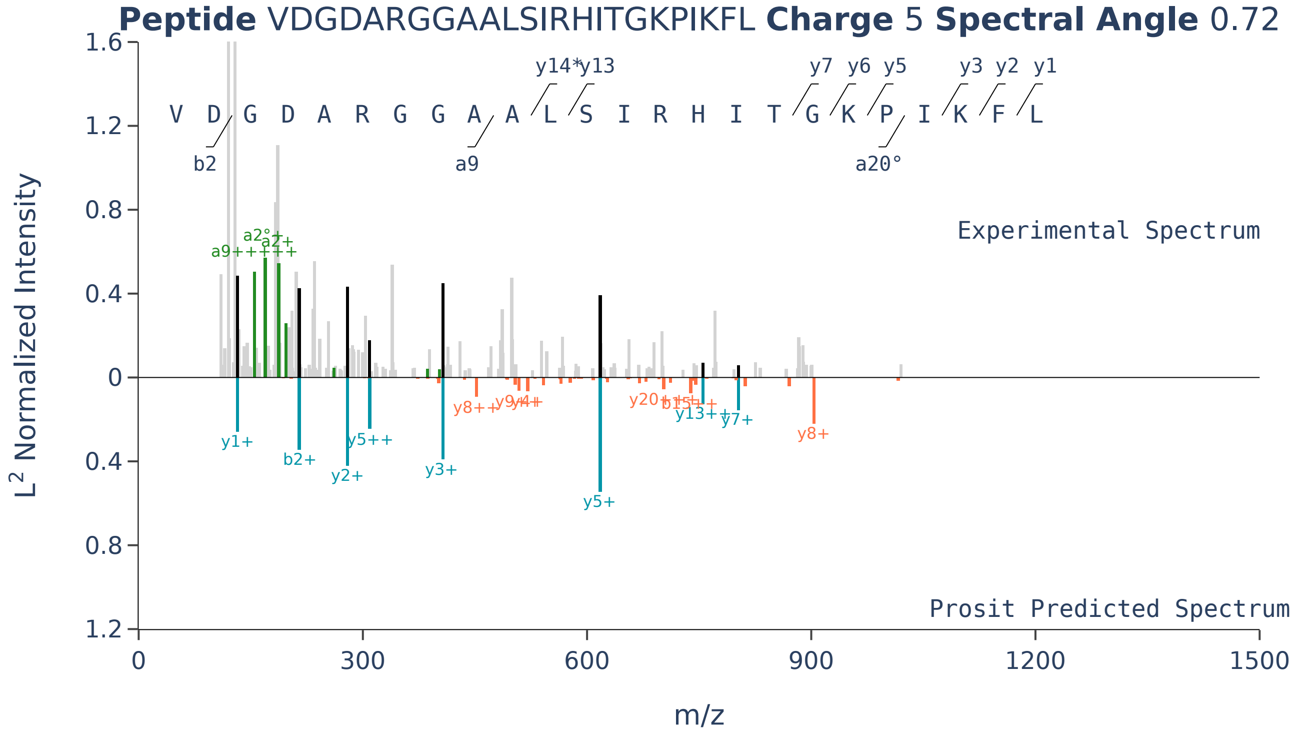

**Source** HRoetschke_291222_060123_Fu_H2A_b1R1_1h **Scan** 12191
**Peptide** AQGGVLPNLQ **Charge** 2 **Spectral Angle** 0.94

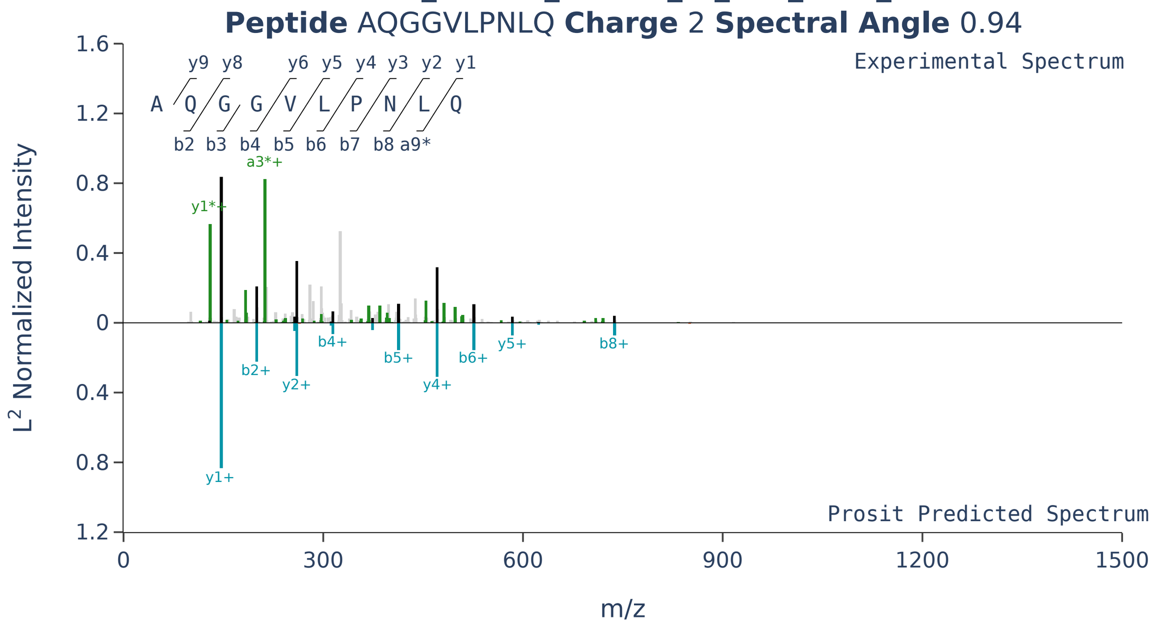


**Source** HRoetschke_291222_060123_Fu_H2A_b1R2_4h **Scan** 13004
**Peptide** GLQFPVGRVH **Charge** 2 **Spectral Angle** 0.94

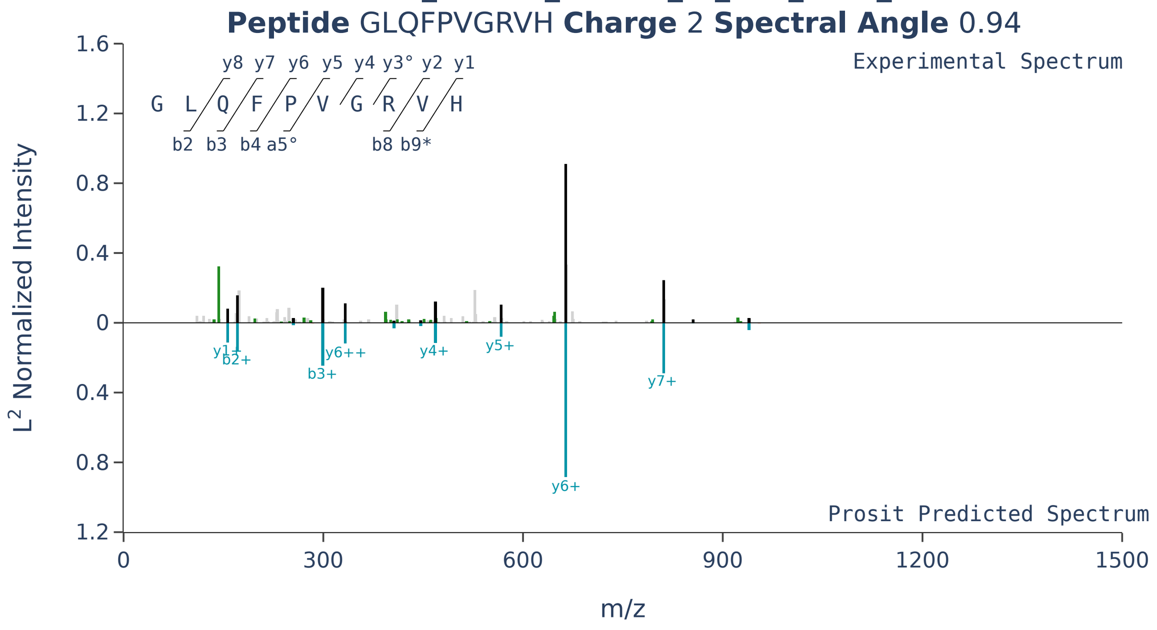


**Source** HRoetschke_291222_060123_Fu_H2A_b2R2_24h **Scan** 13035
**Peptide** AGLQFPVGR **Charge** 2 **Spectral Angle** 0.91

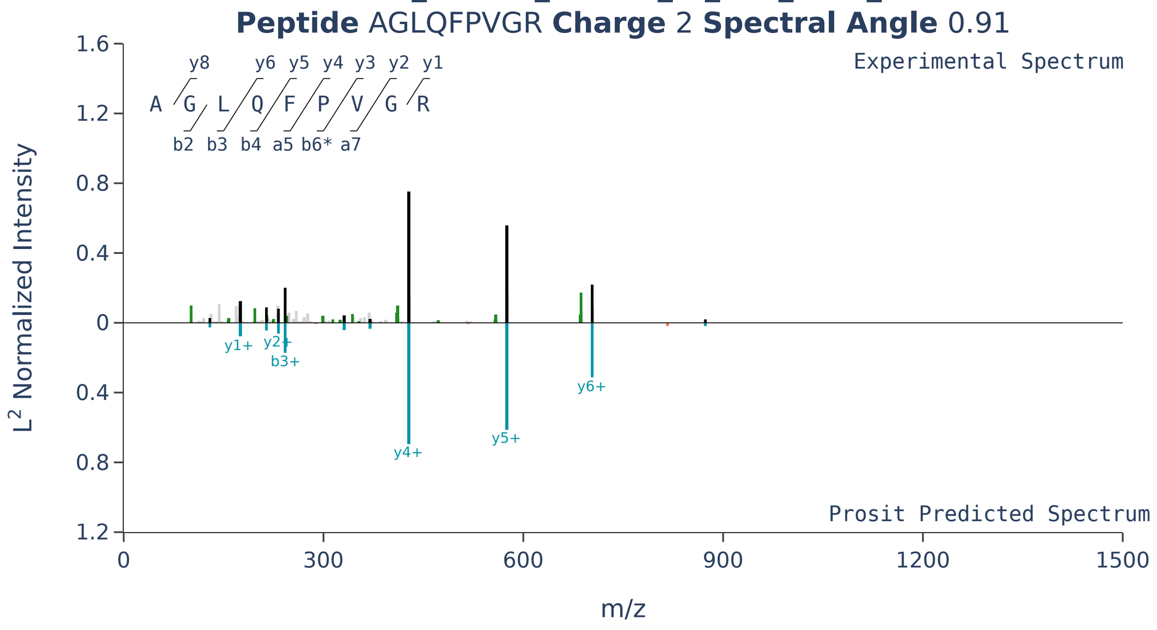


**Source** HRoetschke_291222_060123_Fu_H2A_b1R1_1h **Scan** 16179
**Peptide** TAEILEL **Charge** 2 **Spectral Angle** 0.97

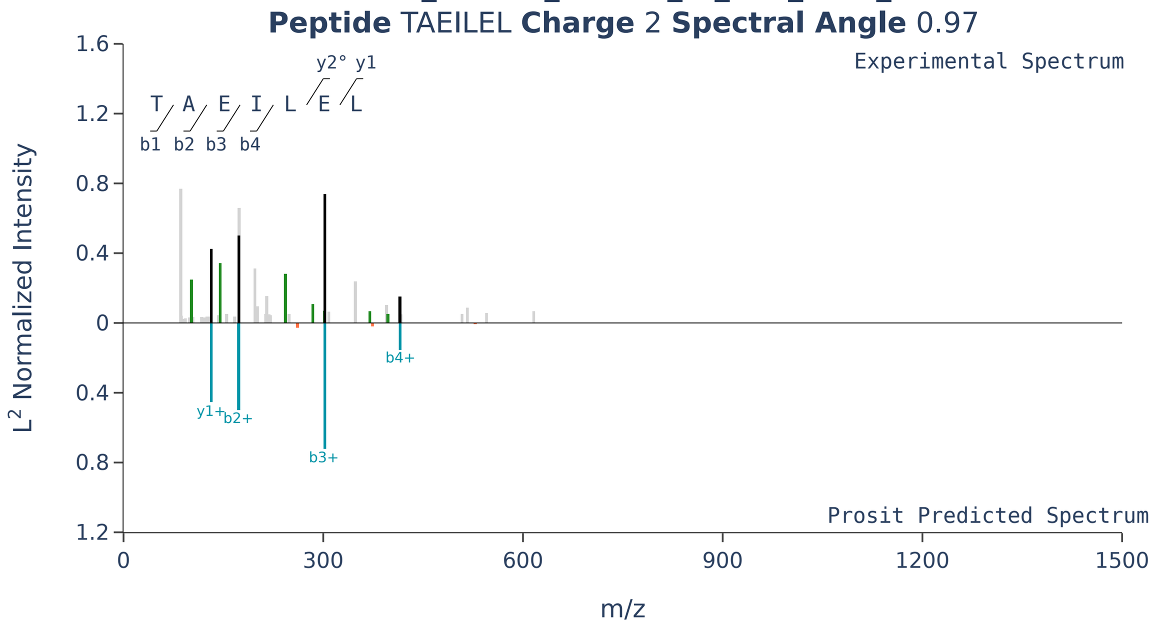


**Source** HRoetschke_291222_060123_Fu_H2A_b2R1_2h **Scan** 12159
**Peptide** AQGGVLPNIQ **Charge** 2 **Spectral Angle** 0.92

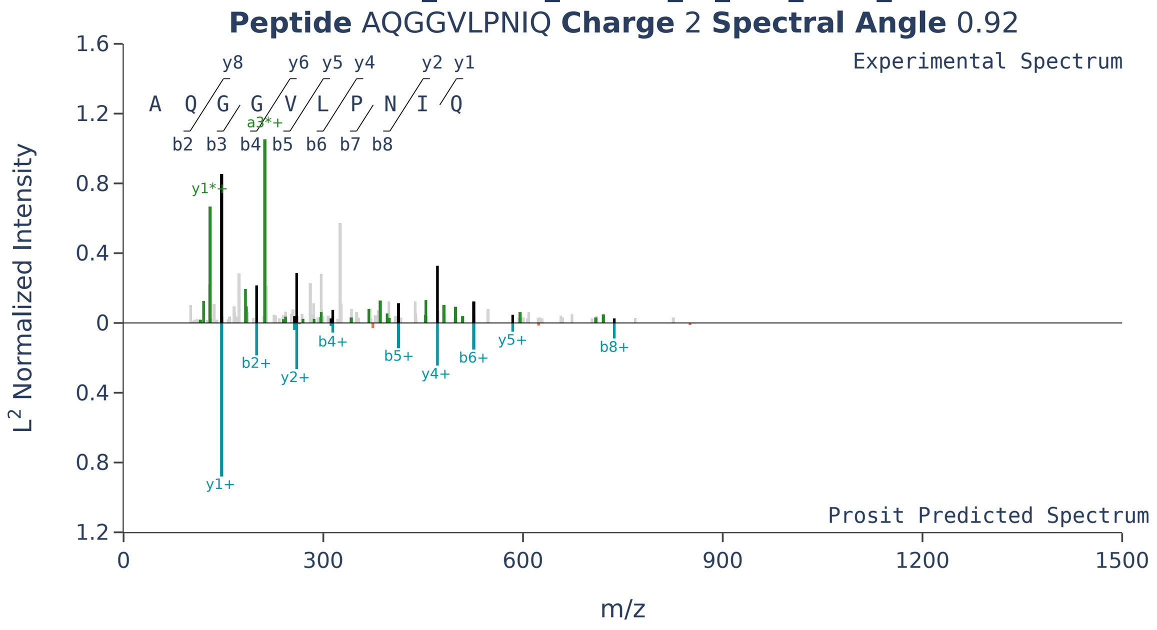


**Source** HRoetschke_291222_060123_Fu_H2A_b2R2_1h **Scan** 9192
**Peptide** RAGLQFPVGR **Charge** 3 **Spectral Angle** 0.89

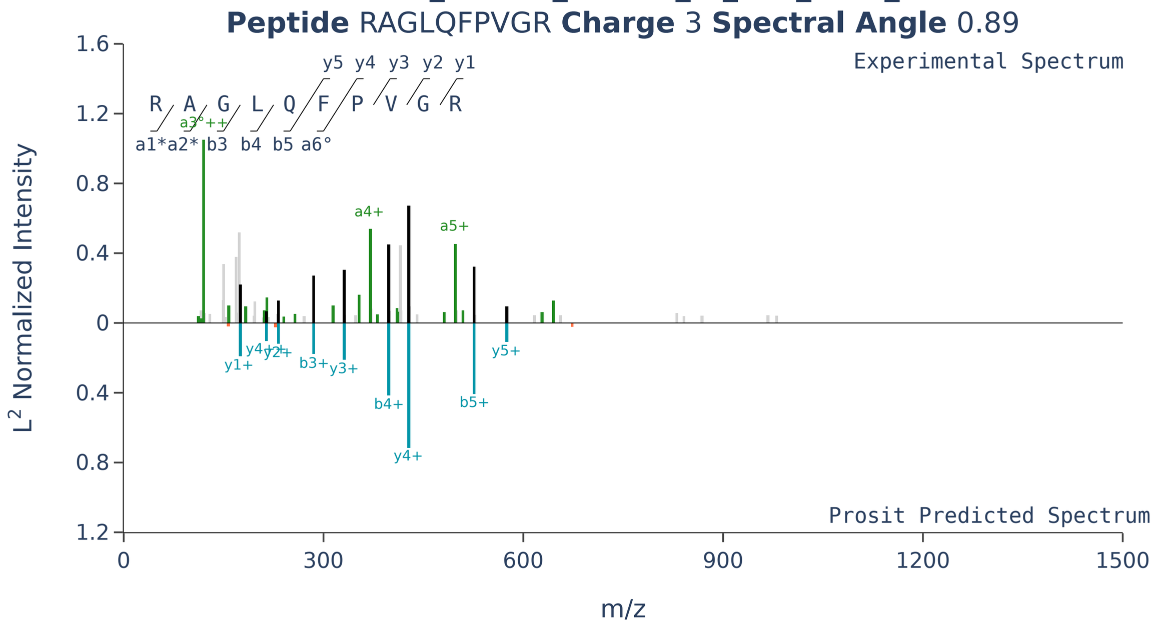


**Source** HRoetschke_291222_060123_Fu_H2A_b1R1_2h **Scan** 12868
**Peptide** NDEELNKLLGR **Charge** 2 **Spectral Angle** 0.88

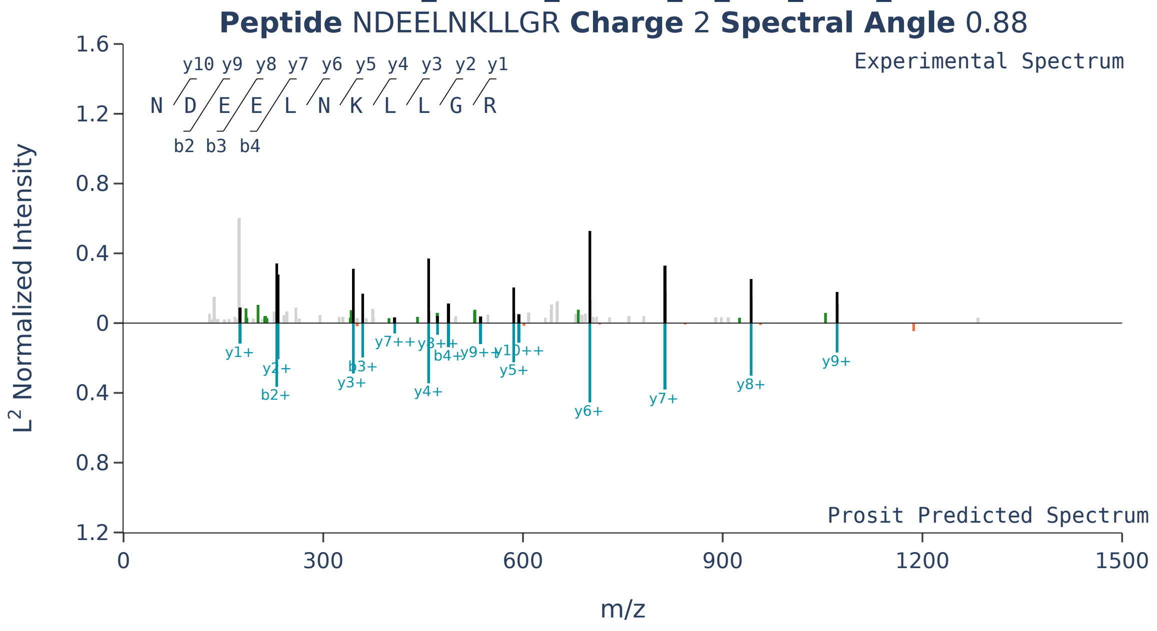


**Source** HRoetschke_291222_060123_Fu_H2A_b1R2_4h **Scan** 11172
**Peptide** RNDEELNKLLGR **Charge** 4 **Spectral Angle** 0.91

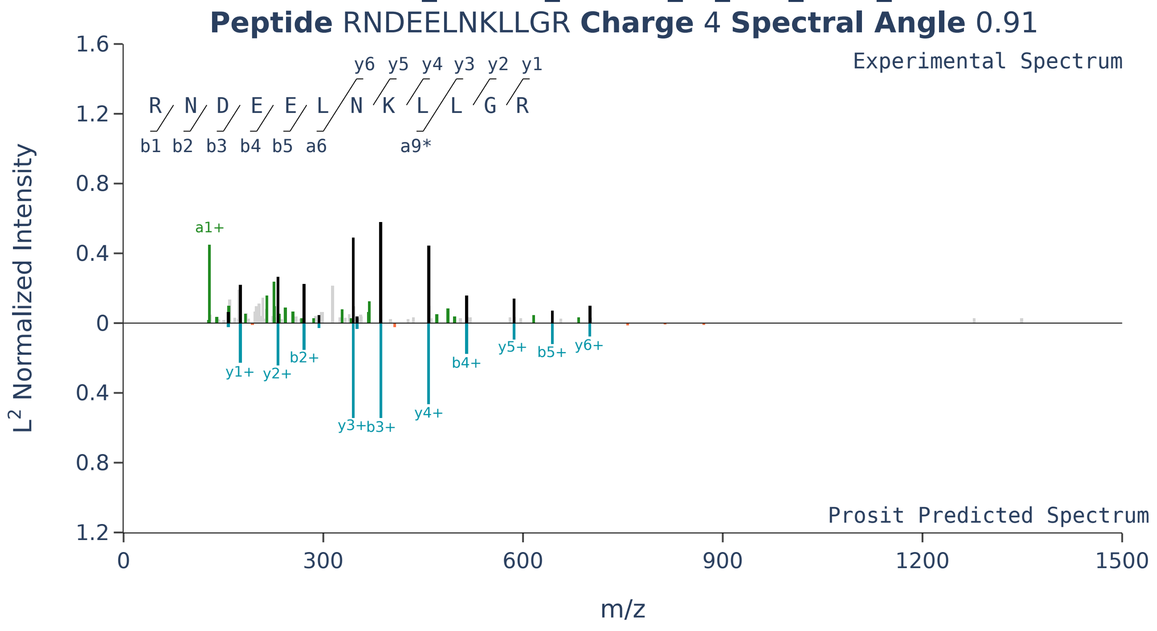


**Source** HRoetschke_291222_060123_Fu_H2A_b2R2_24h **Scan** 10855
**Peptide** SRAGLQFPVGR **Charge** 3 **Spectral Angle** 0.92

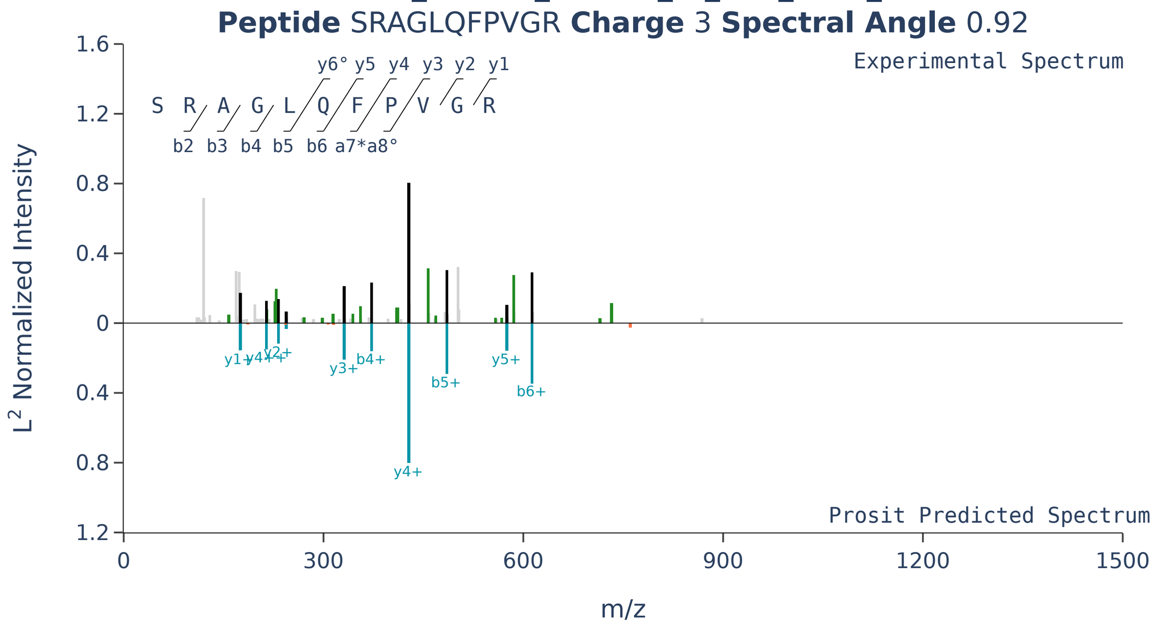


**Source** HRoetschke_291222_060123_Fu_H2A_b1R1_1h **Scan** 9521
**Peptide** EELNKLLGR **Charge** 3 **Spectral Angle** 0.94

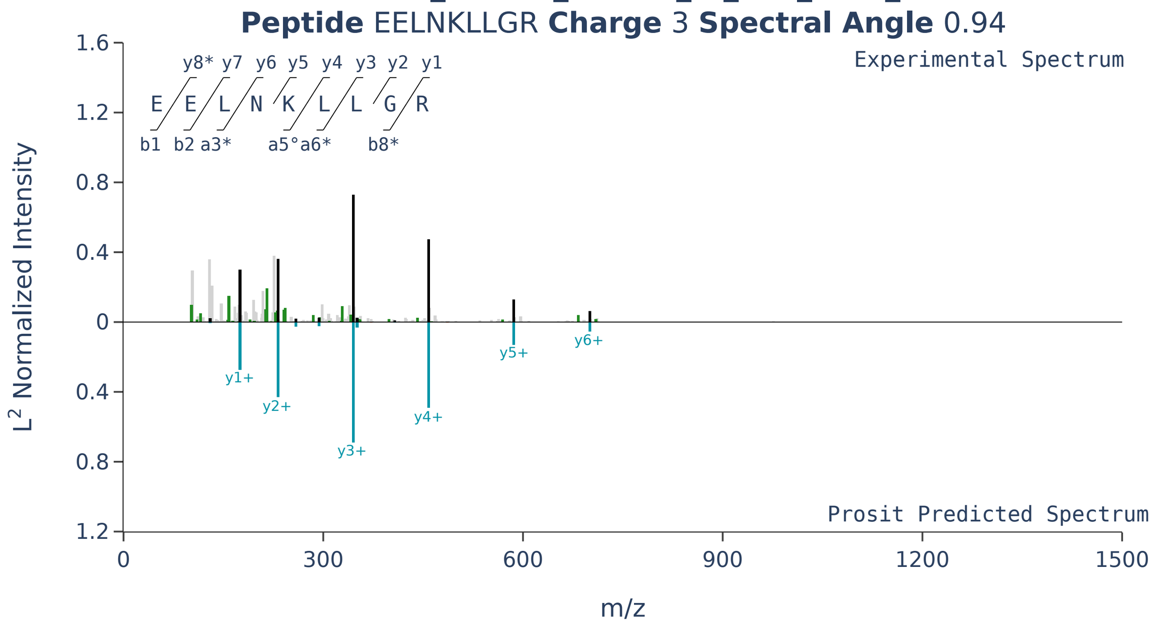


**Source** HRoetschke_291222_060123_Fu_H2A_b1R1_24h **Scan** 14156
**Peptide** LAQGGVLPNIQ **Charge** 2 **Spectral Angle** 0.94

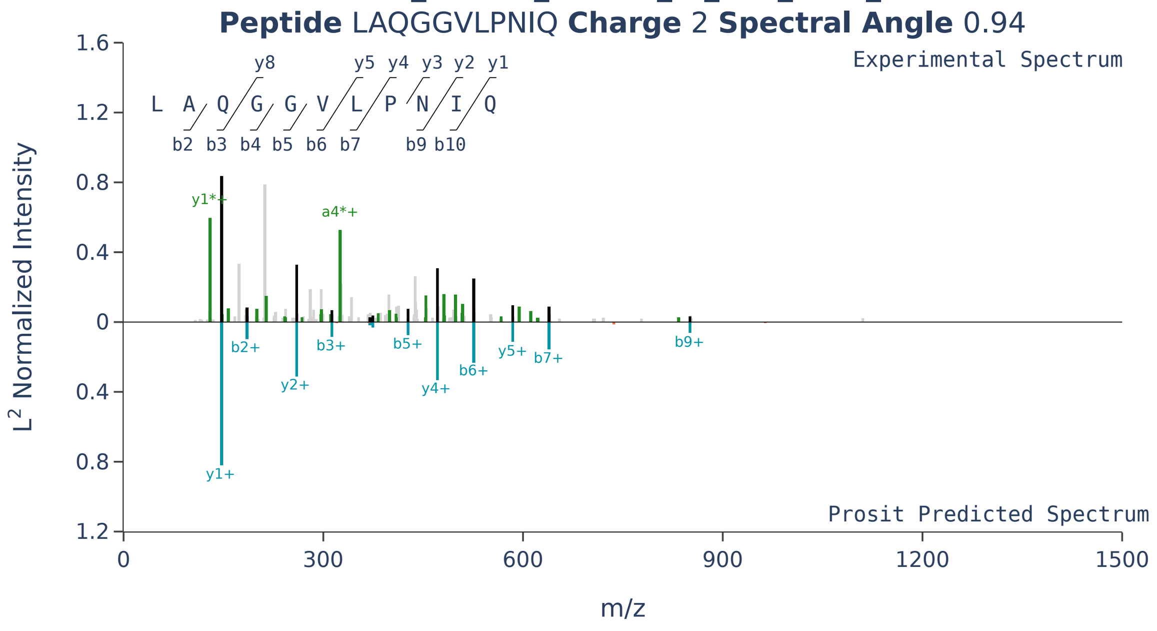


**Source** HRoetschke_291222_060123_Fu_H2A_b1R1_1h **Scan** 7756
**Peptide** LQLAVRND **Charge** 2 **Spectral Angle** 0.95

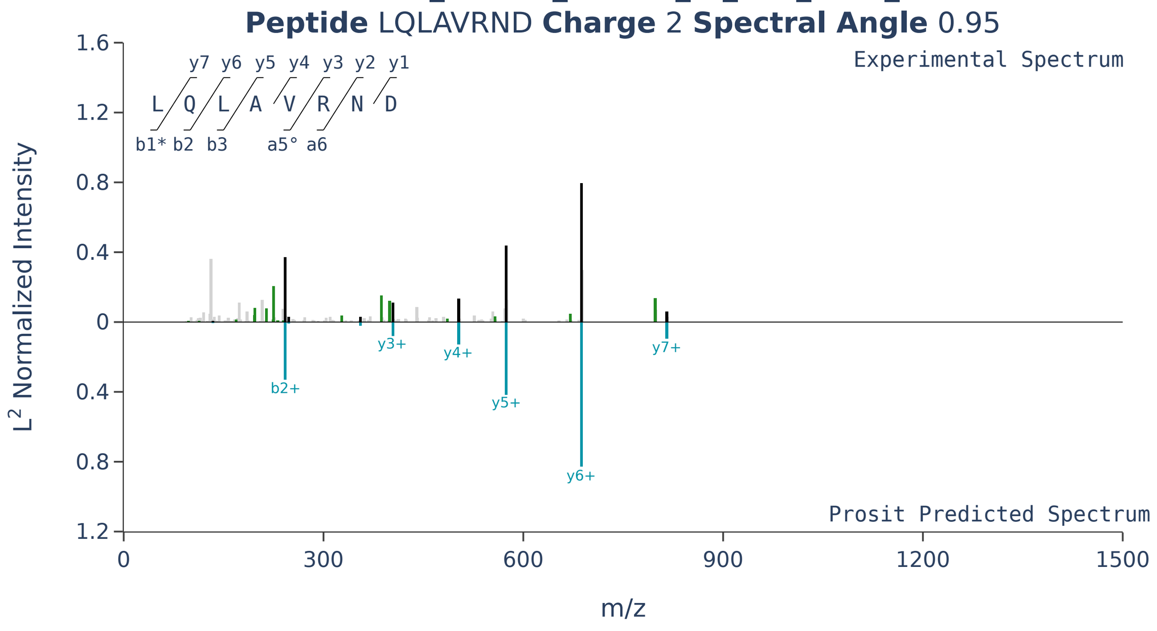


**Source** HRoetschke_291222_060123_Fu_H2A_b1R1_4h **Scan** 17151
**Peptide** AQGGVLPNIQSV **Charge** 2 **Spectral Angle** 0.9

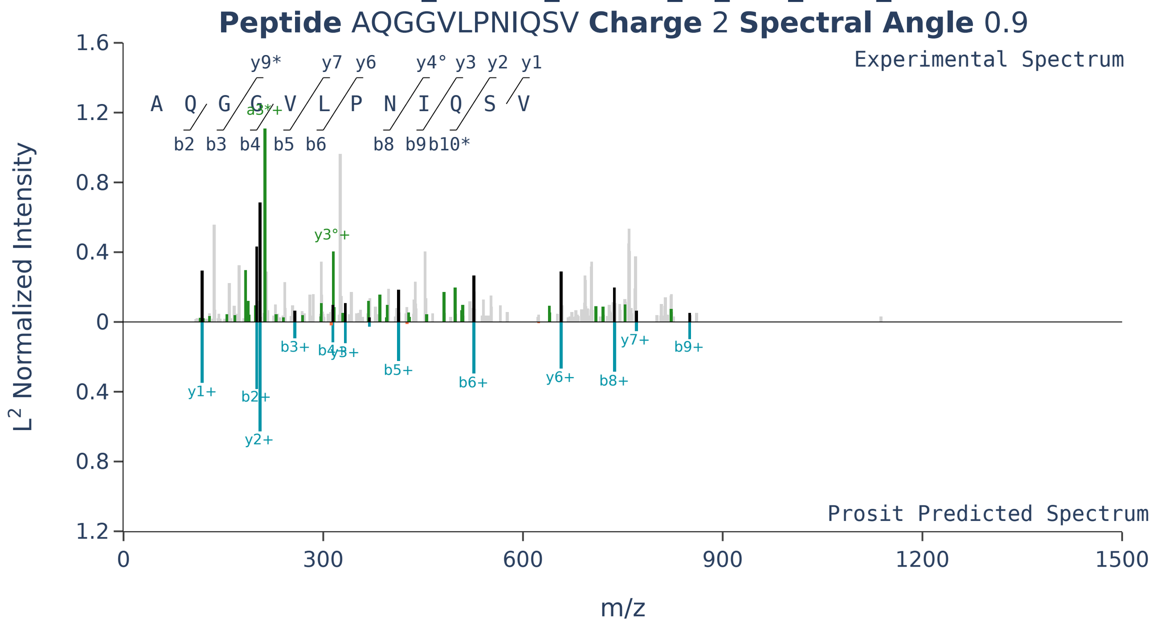


**Source** HRoetschke_291222_060123_Fu_H2A_b2R2_2h **Scan** 12985
**Peptide** GVLPNLQ **Charge** 2 **Spectral Angle** 0.89

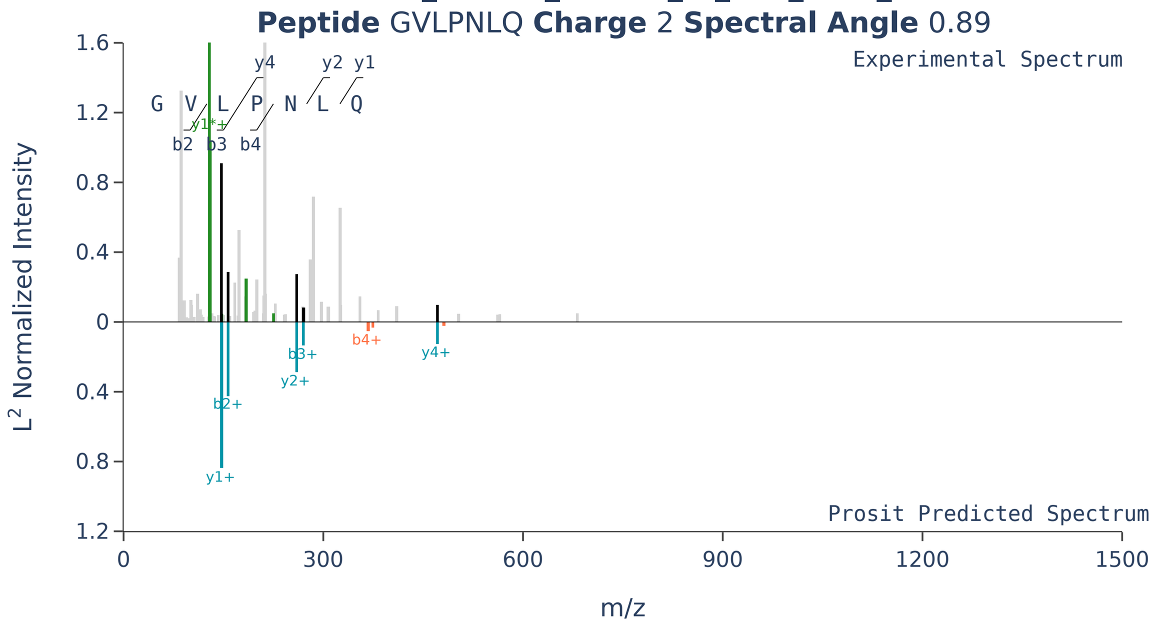


**Source** HRoetschke_291222_060123_Fu_H2A_b2R2_24h **Scan** 7235
**Peptide** VRNDEELNKL **Charge** 3 **Spectral Angle** 0.92

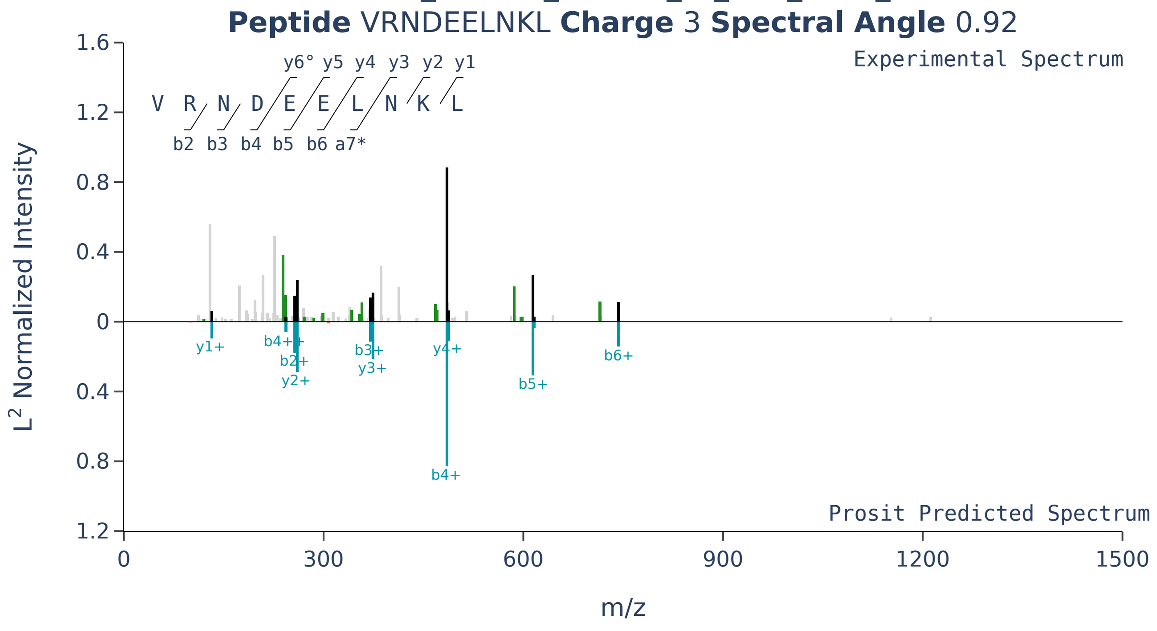

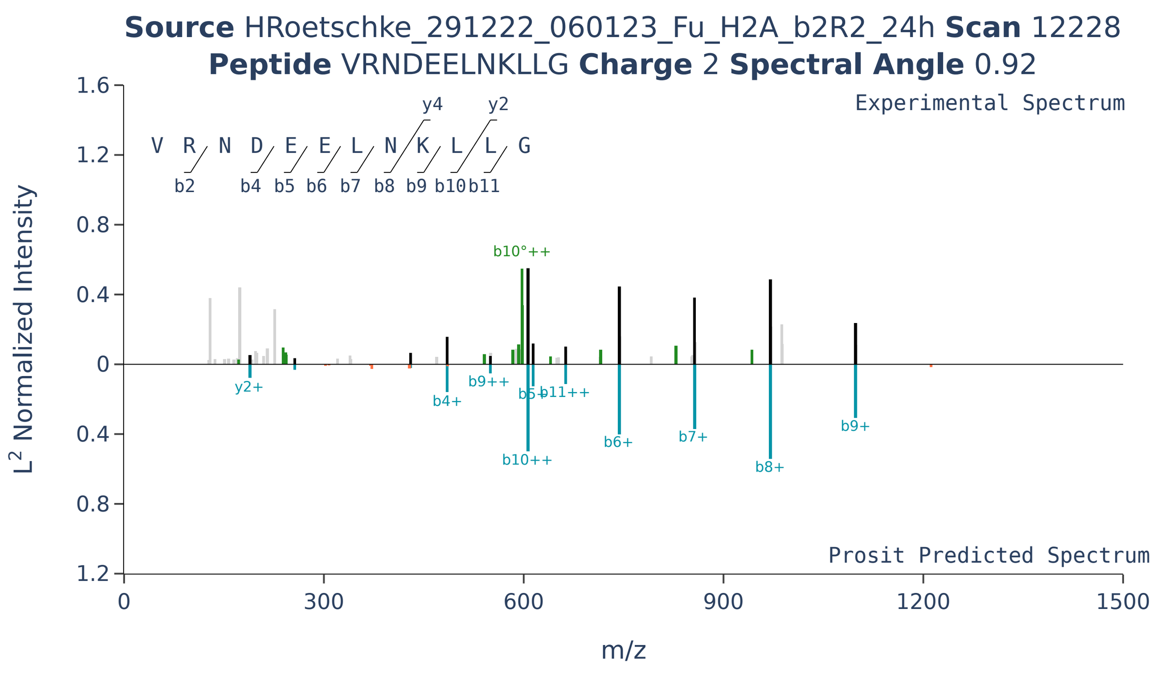
**Source** HRoetschke_291222_060123_Fu_H2A_b2R2_24h **Scan** 12228
**Peptide** VRNDEELNKLLG **Charge** 2 **Spectral Angle** 0.92
Experimental Spectrum
Prosit Predicted Spectrum
L² Normalized Intensity
m/z

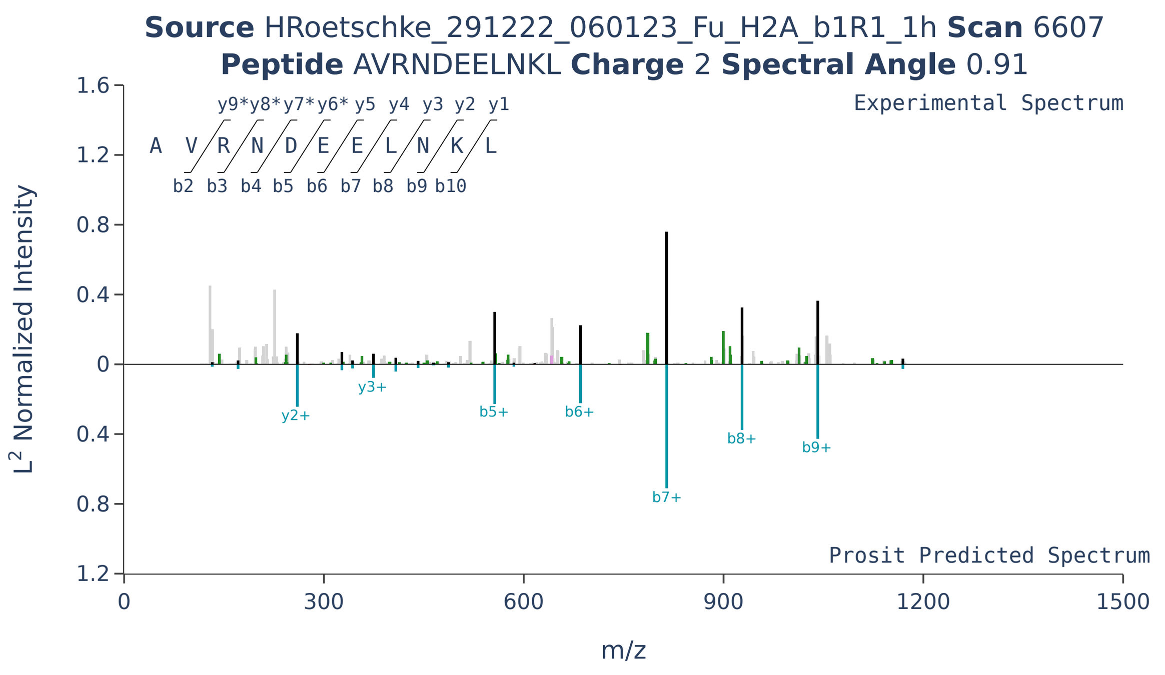
**Source** HRoetschke_291222_060123_Fu_H2A_b1R1_1h **Scan** 6607
**Peptide** AVRNDEELNKL **Charge** 2 **Spectral Angle** 0.91
Experimental Spectrum
Prosit Predicted Spectrum
L² Normalized Intensity
m/z

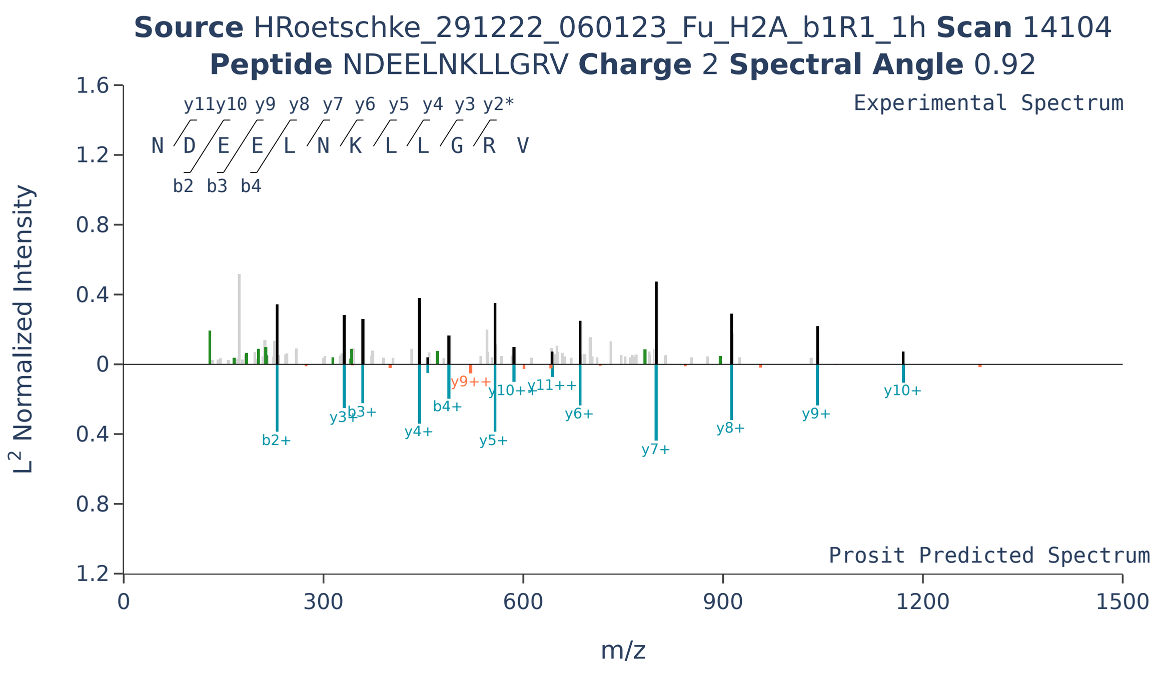
**Source** HRoetschke_291222_060123_Fu_H2A_b1R1_1h **Scan** 14104
**Peptide** NDEELNKLLGRV **Charge** 2 **Spectral Angle** 0.92
Experimental Spectrum
Prosit Predicted Spectrum
L² Normalized Intensity
m/z

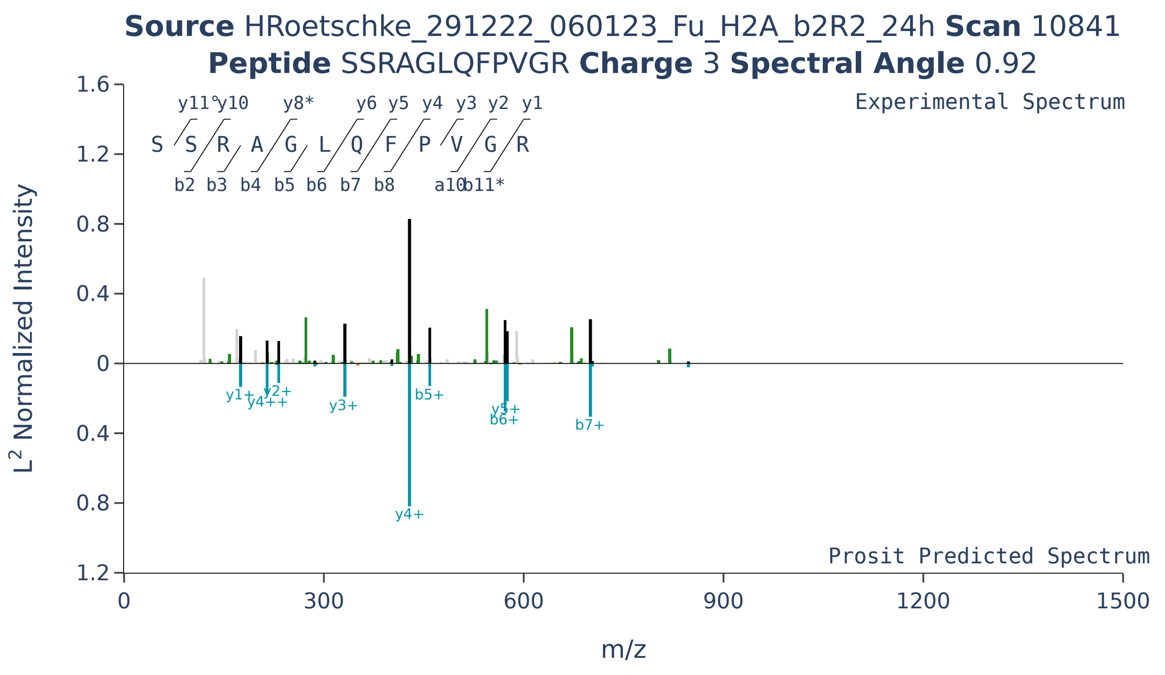
**Source** HRoetschke_291222_060123_Fu_H2A_b2R2_24h **Scan** 10841
**Peptide** SSRAGLQFPVGR **Charge** 3 **Spectral Angle** 0.92
Experimental Spectrum
Prosit Predicted Spectrum
L² Normalized Intensity
m/z

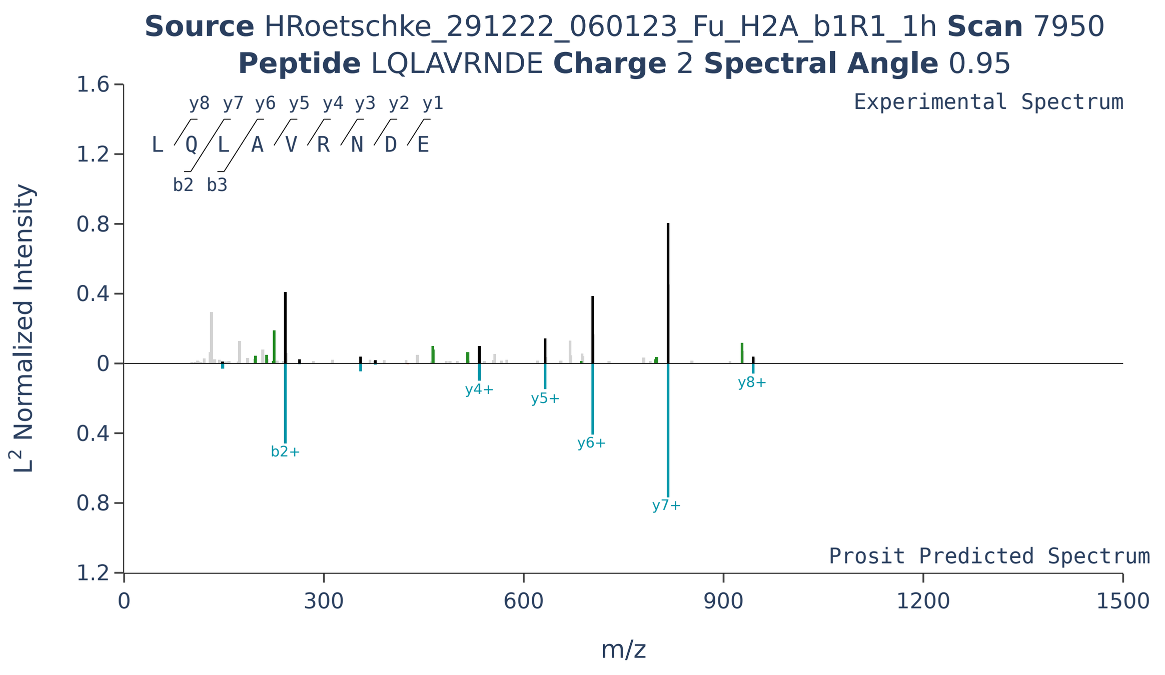
**Source** HRoetschke_291222_060123_Fu_H2A_b1R1_1h **Scan** 7950
**Peptide** LQLAVRNDE **Charge** 2 **Spectral Angle** 0.95
Experimental Spectrum
Prosit Predicted Spectrum
L² Normalized Intensity
m/z

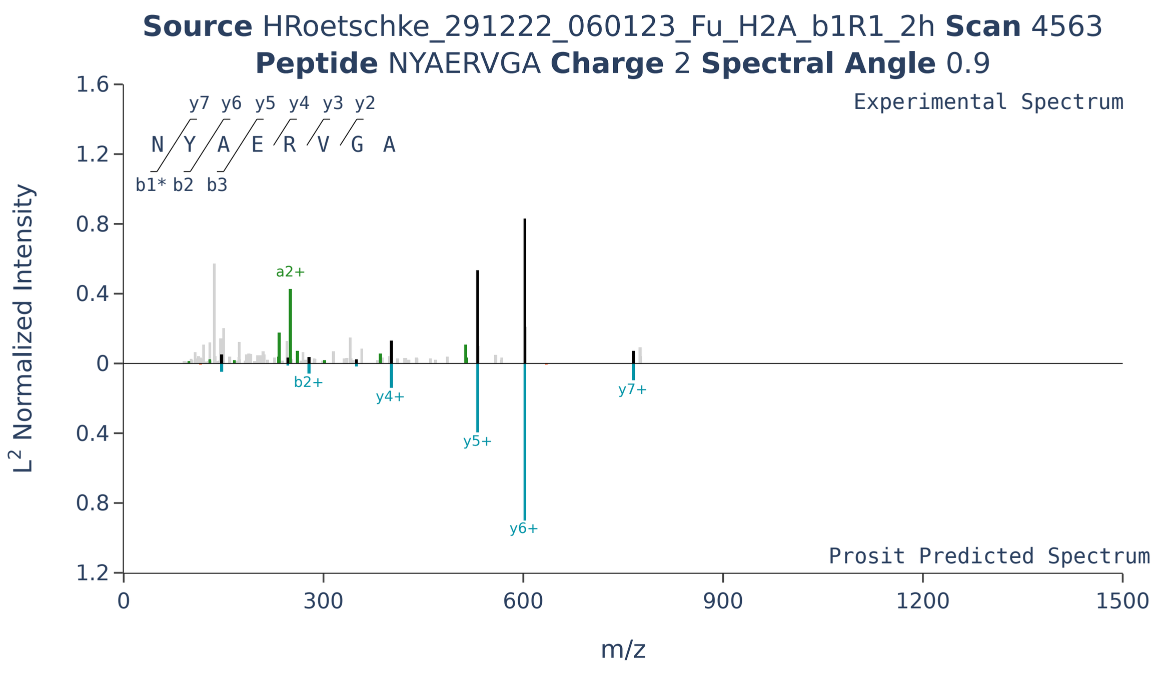
**Source** HRoetschke_291222_060123_Fu_H2A_b1R1_2h **Scan** 4563
**Peptide** NYAERVGA **Charge** 2 **Spectral Angle** 0.9
Experimental Spectrum
Prosit Predicted Spectrum
L² Normalized Intensity
m/z

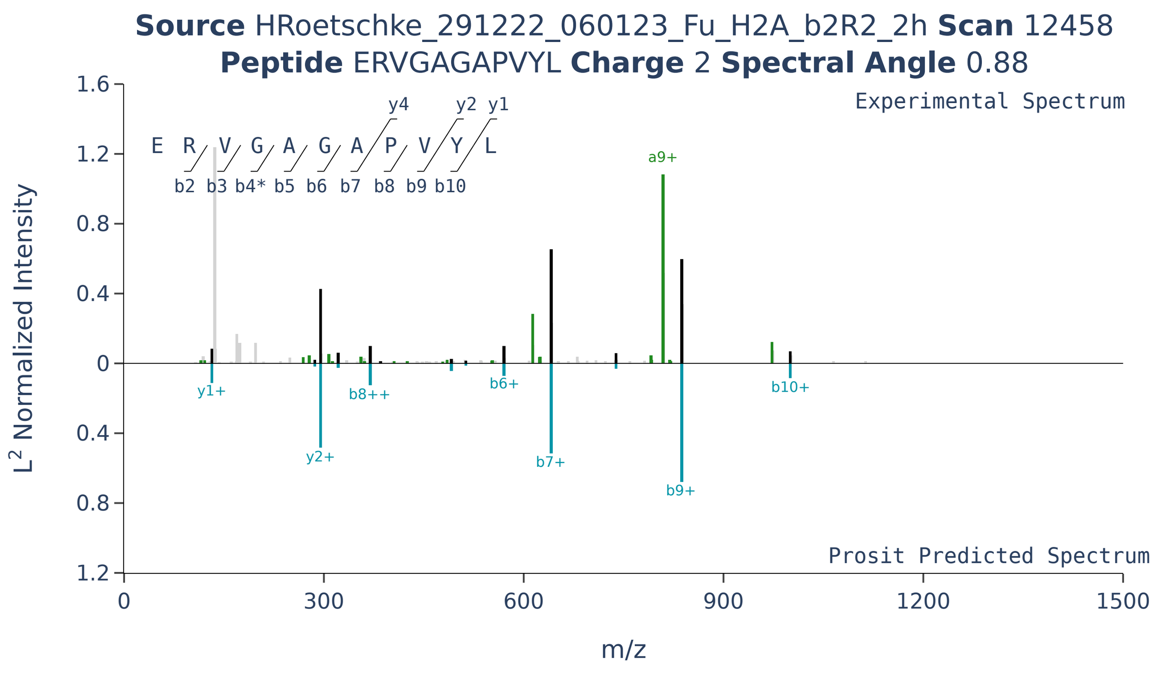
**Source** HRoetschke_291222_060123_Fu_H2A_b2R2_2h **Scan** 12458
**Peptide** ERVGAGAPVYL **Charge** 2 **Spectral Angle** 0.88
Experimental Spectrum
Prosit Predicted Spectrum
L² Normalized Intensity
m/z

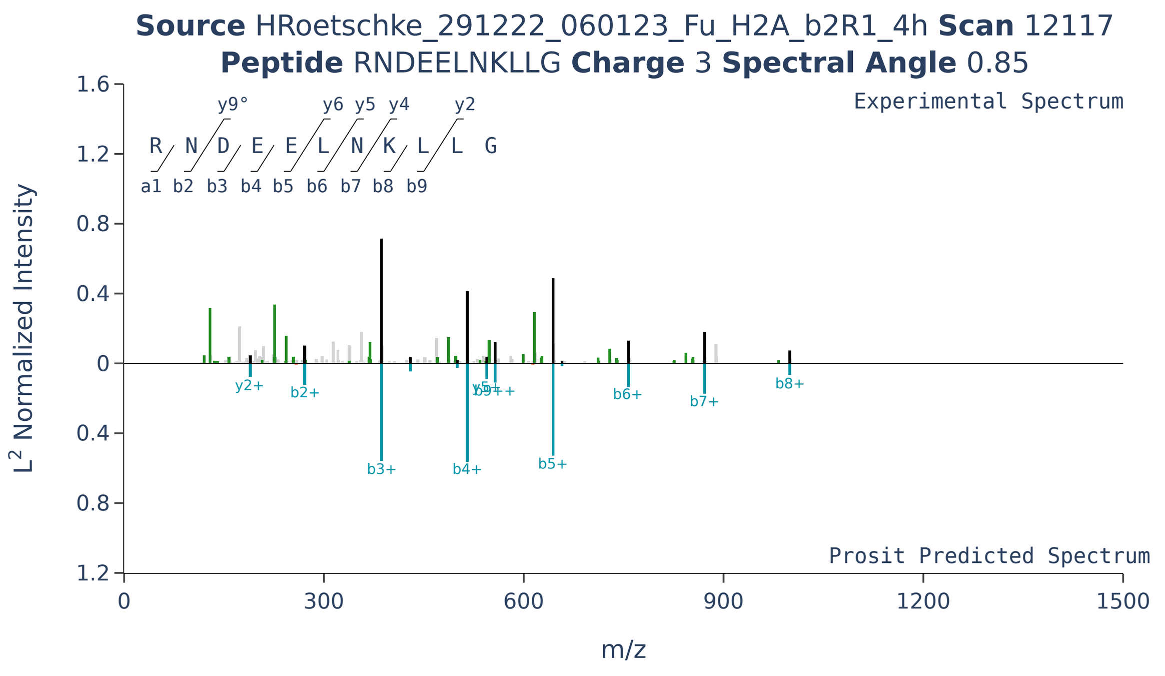
**Source** HRoetschke_291222_060123_Fu_H2A_b2R1_4h **Scan** 12117
**Peptide** RNDEELNKLLG **Charge** 3 **Spectral Angle** 0.85
Experimental Spectrum
Prosit Predicted Spectrum
L² Normalized Intensity
m/z

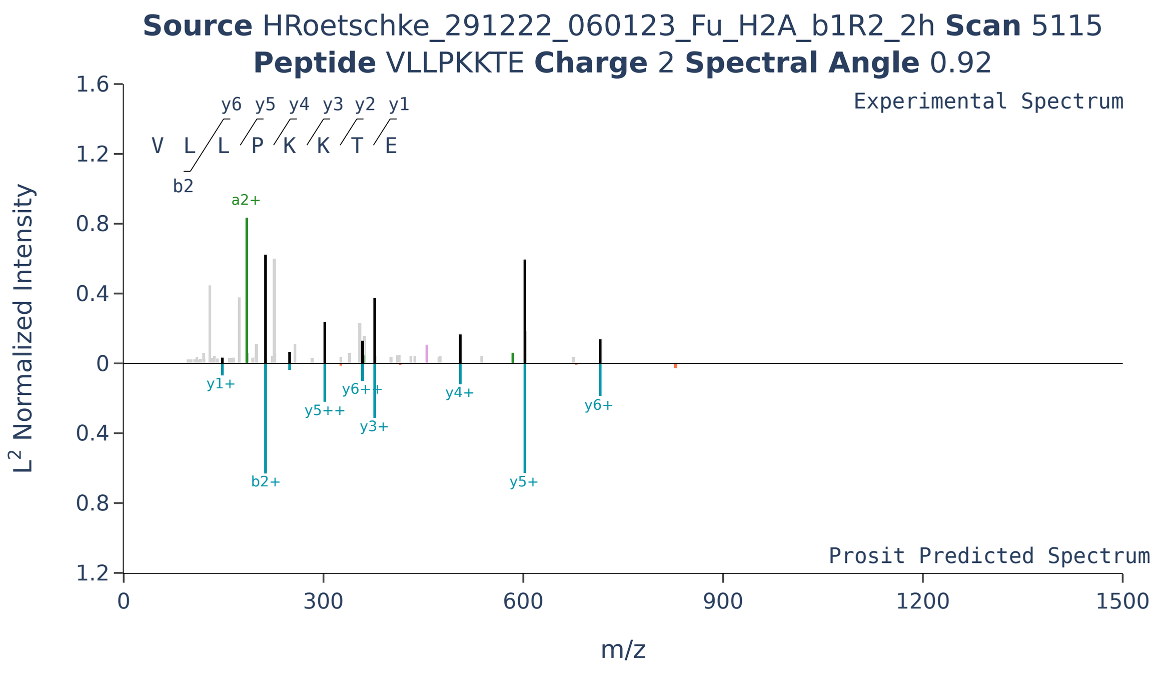
**Source** HRoetschke_291222_060123_Fu_H2A_b1R2_2h **Scan** 5115
**Peptide** VLLPKKTE **Charge** 2 **Spectral Angle** 0.92
Experimental Spectrum
Prosit Predicted Spectrum
L² Normalized Intensity
m/z

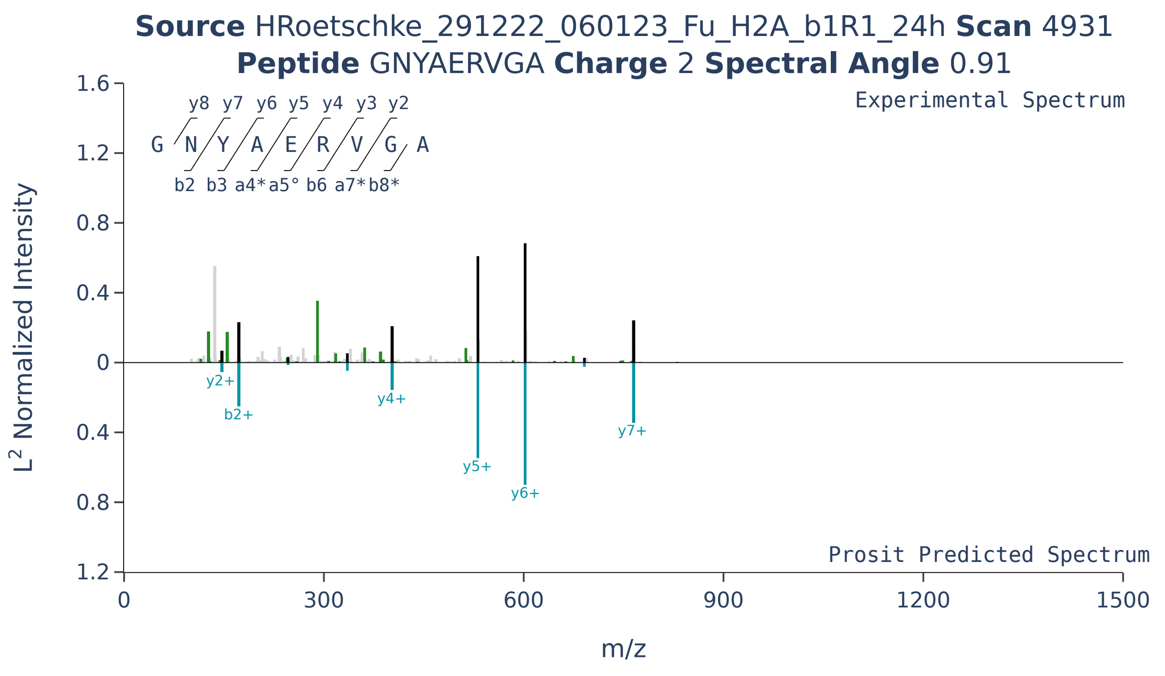
**Source** HRoetschke_291222_060123_Fu_H2A_b1R1_24h **Scan** 4931
**Peptide** GNYAERVGA **Charge** 2 **Spectral Angle** 0.91
Experimental Spectrum
Prosit Predicted Spectrum
L² Normalized Intensity
m/z

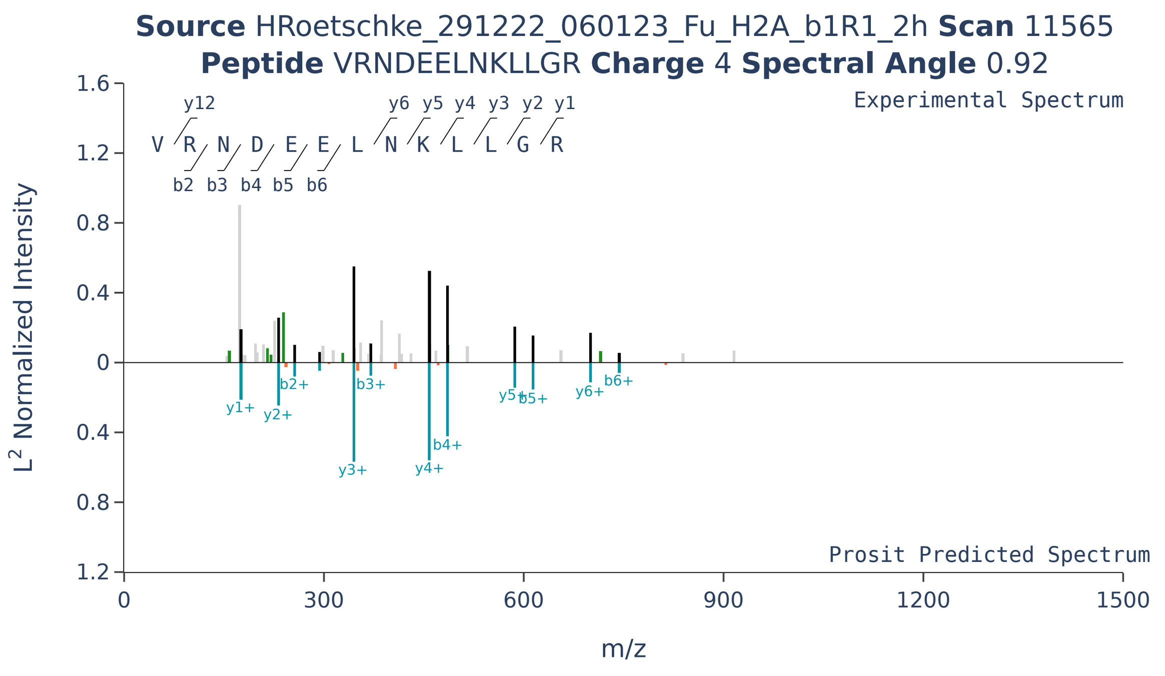
**Source** HRoetschke_291222_060123_Fu_H2A_b1R1_2h **Scan** 11565
**Peptide** VRNDEELNKLLGR **Charge** 4 **Spectral Angle** 0.92
Experimental Spectrum
Prosit Predicted Spectrum
L² Normalized Intensity
m/z

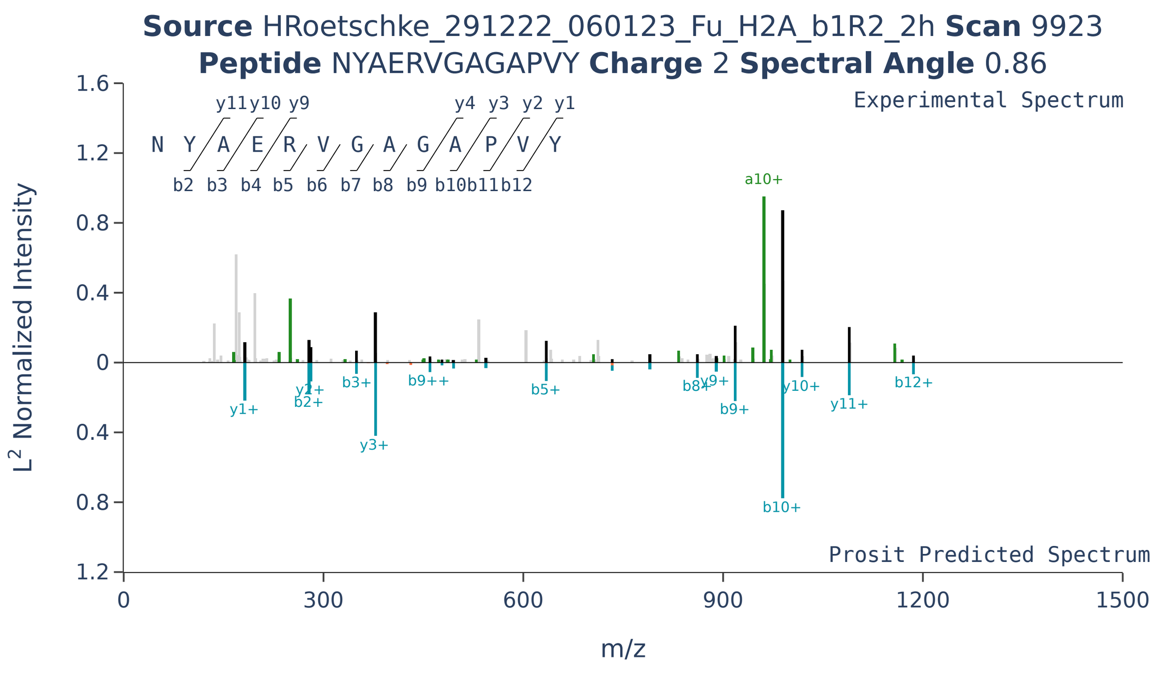
**Source** HRoetschke_291222_060123_Fu_H2A_b1R2_2h **Scan** 9923
**Peptide** NYAERVGAGAPVY **Charge** 2 **Spectral Angle** 0.86
Experimental Spectrum
Prosit Predicted Spectrum
L² Normalized Intensity
m/z

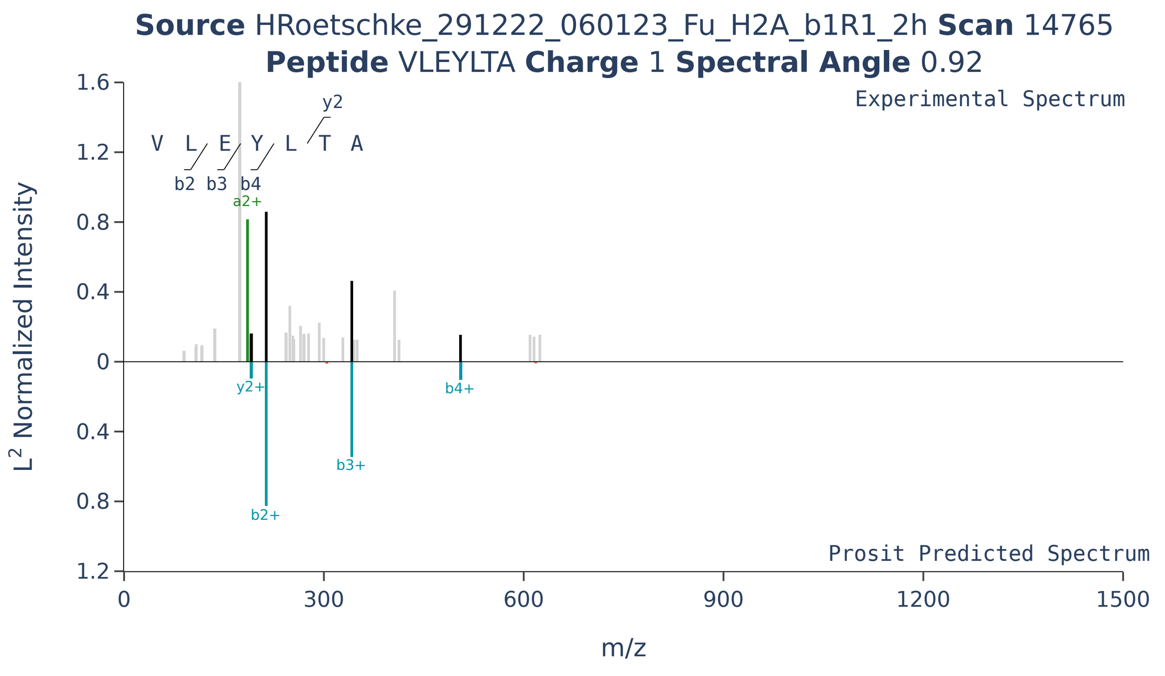
**Source** HRoetschke_291222_060123_Fu_H2A_b1R1_2h **Scan** 14765
**Peptide** VLEYLTA **Charge** 1 **Spectral Angle** 0.92
Experimental Spectrum
Prosit Predicted Spectrum
L² Normalized Intensity
m/z

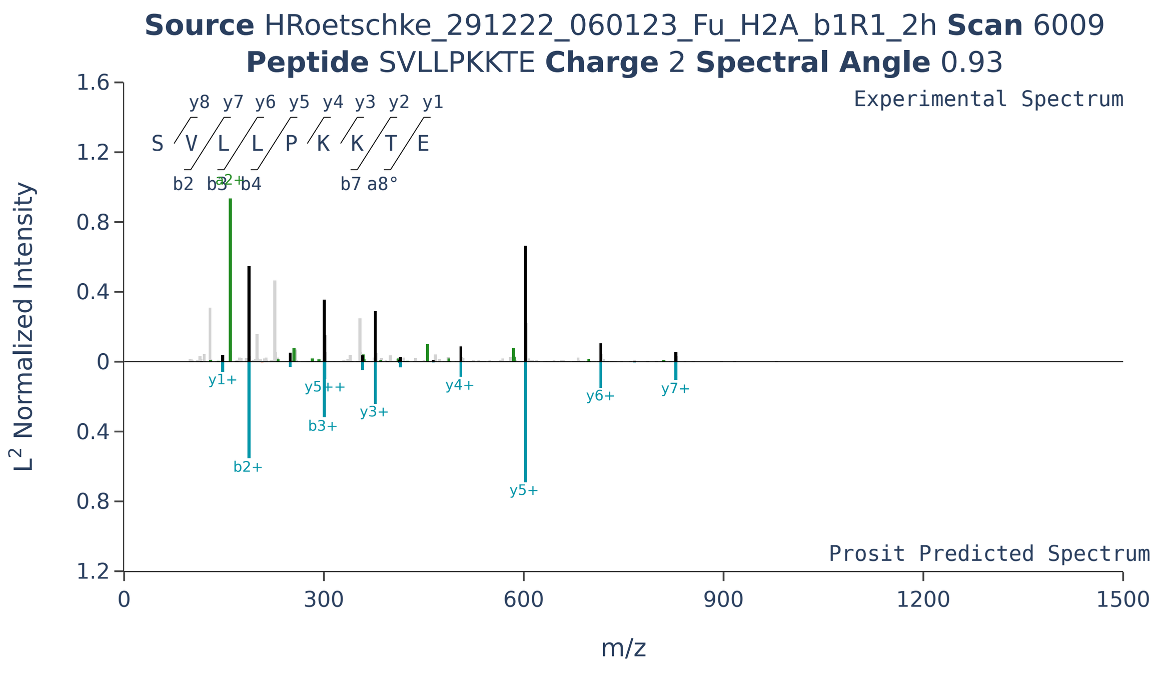
**Source** HRoetschke_291222_060123_Fu_H2A_b1R1_2h **Scan** 6009
**Peptide** SVLLPKKTE **Charge** 2 **Spectral Angle** 0.93
Experimental Spectrum
Prosit Predicted Spectrum
L² Normalized Intensity
m/z

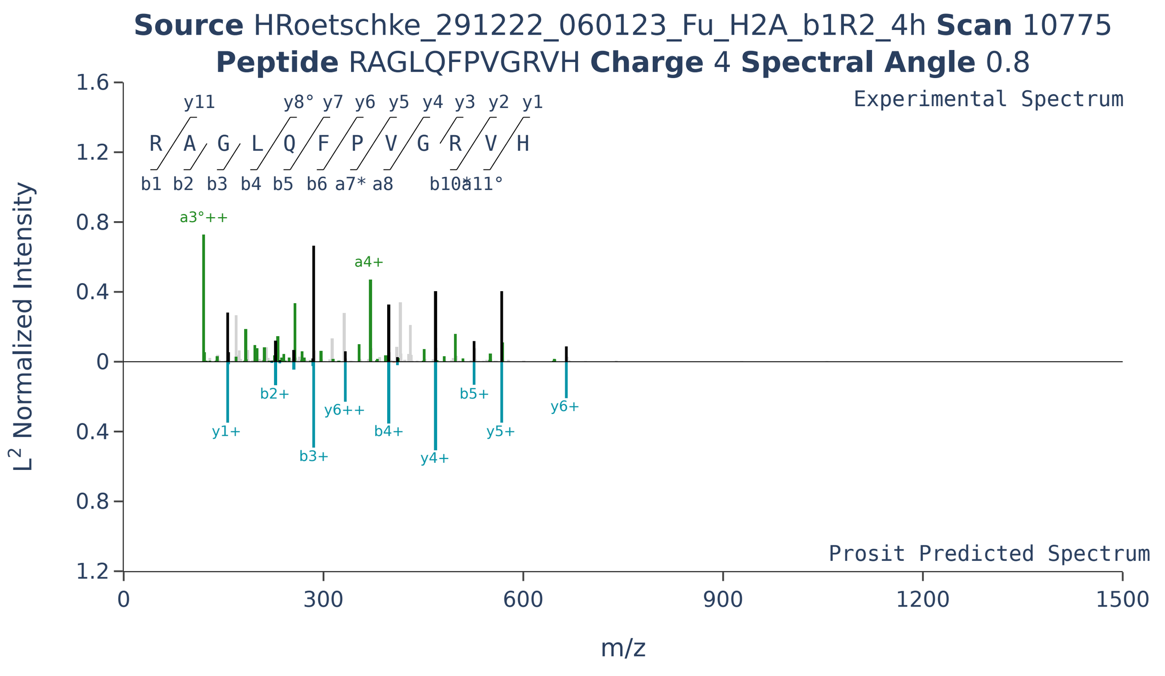
**Source** HRoetschke_291222_060123_Fu_H2A_b1R2_4h **Scan** 10775
**Peptide** RAGLQFPVGRVH **Charge** 4 **Spectral Angle** 0.8
Experimental Spectrum
Prosit Predicted Spectrum
L² Normalized Intensity
m/z

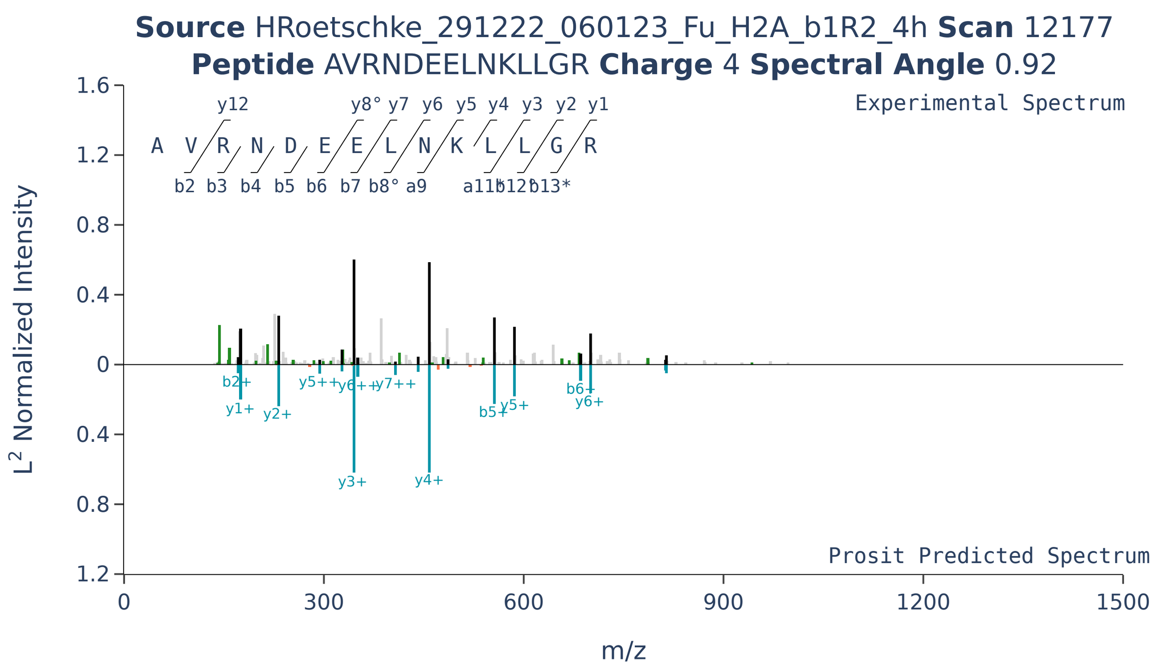
**Source** HRoetschke_291222_060123_Fu_H2A_b1R2_4h **Scan** 12177
**Peptide** AVRNDEELNKLLGR **Charge** 4 **Spectral Angle** 0.92
Experimental Spectrum
Prosit Predicted Spectrum
L² Normalized Intensity
m/z

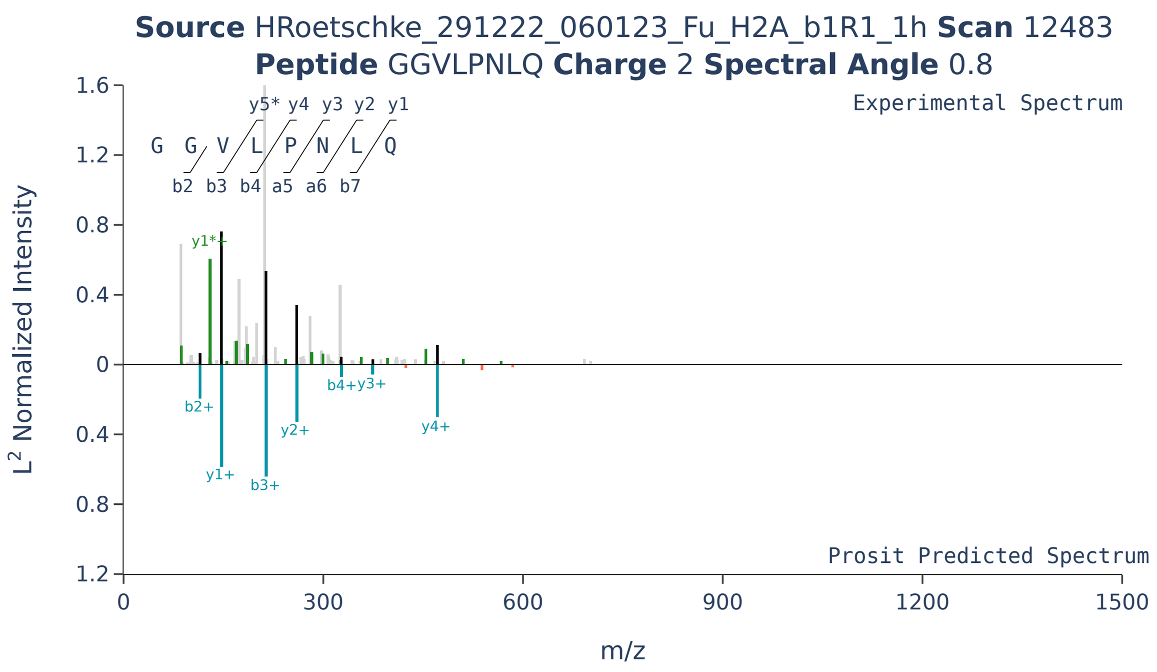
**Source** HRoetschke_291222_060123_Fu_H2A_b1R1_1h **Scan** 12483
**Peptide** GGVLPNLQ **Charge** 2 **Spectral Angle** 0.8
Experimental Spectrum
Prosit Predicted Spectrum
L² Normalized Intensity
m/z

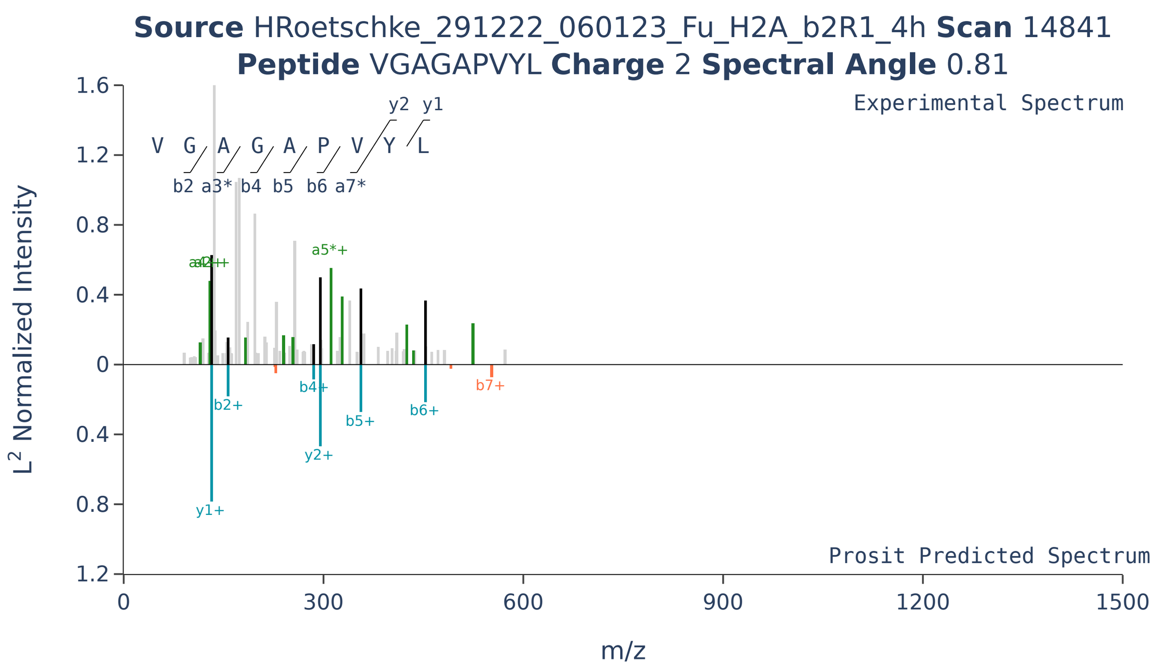
**Source** HRoetschke_291222_060123_Fu_H2A_b2R1_4h **Scan** 14841
**Peptide** VGAGAPVYL **Charge** 2 **Spectral Angle** 0.81
Experimental Spectrum
Prosit Predicted Spectrum
L² Normalized Intensity
m/z

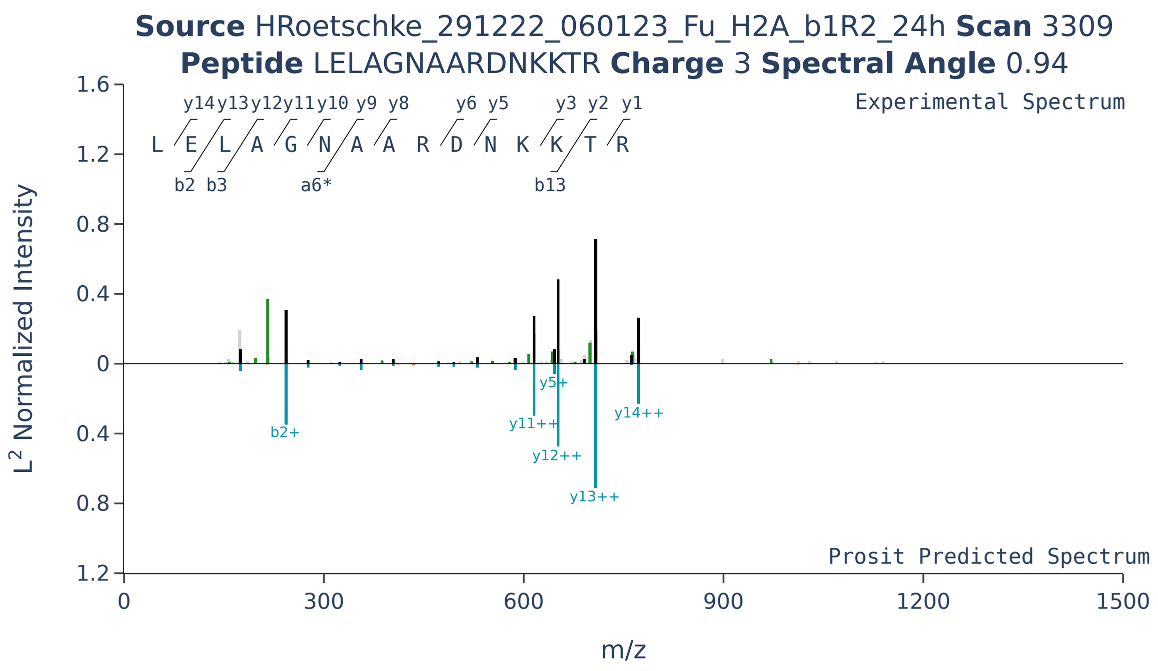
**Source** HRoetschke_291222_060123_Fu_H2A_b1R2_24h **Scan** 3309
**Peptide** LELAGNAARDNKKTR **Charge** 3 **Spectral Angle** 0.94
Experimental Spectrum
Prosit Predicted Spectrum
L² Normalized Intensity
m/z

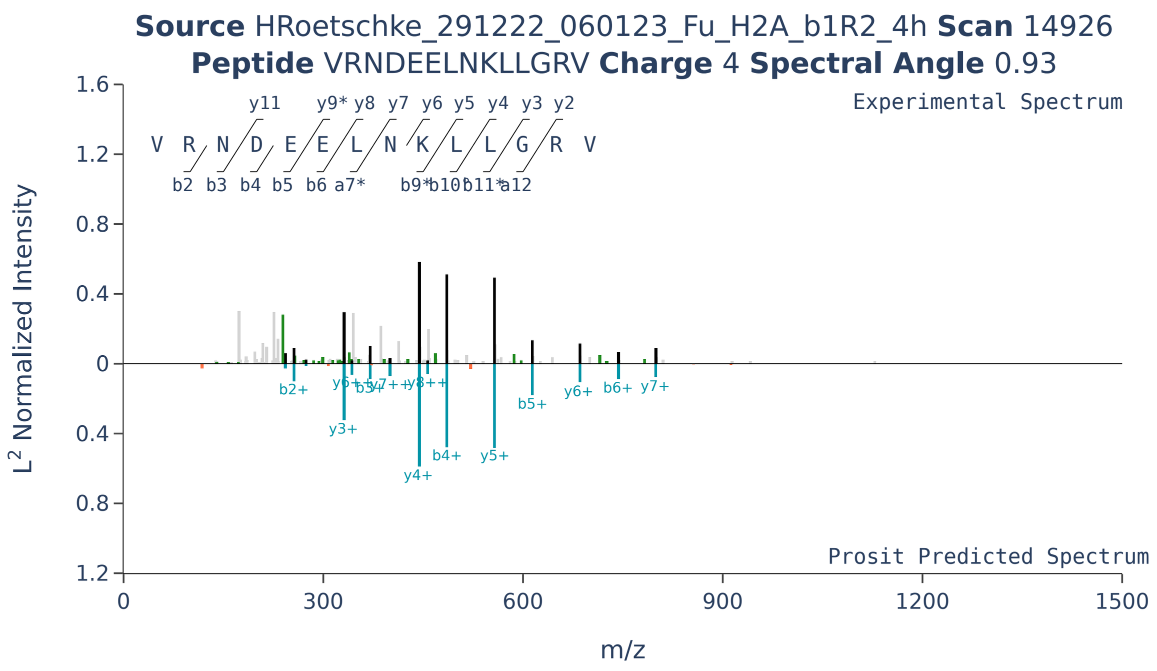
**Source** HRoetschke_291222_060123_Fu_H2A_b1R2_4h **Scan** 14926
**Peptide** VRNDEELNKLLGRV **Charge** 4 **Spectral Angle** 0.93
Experimental Spectrum
Prosit Predicted Spectrum
L² Normalized Intensity
m/z

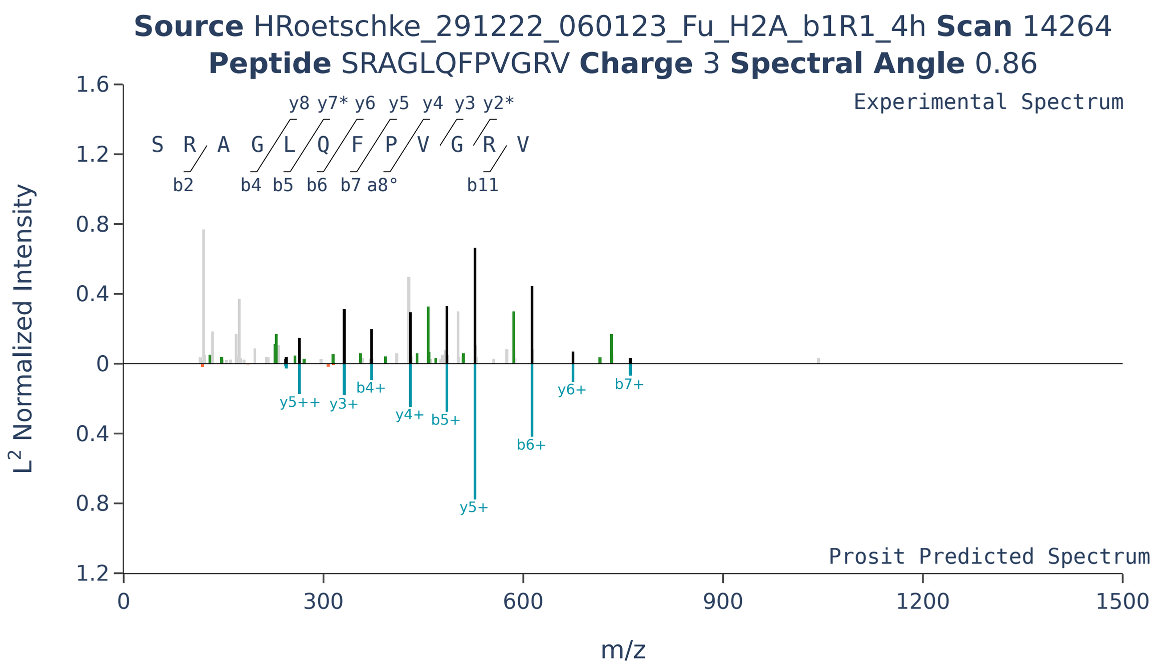
**Source** HRoetschke_291222_060123_Fu_H2A_b1R1_4h **Scan** 14264
**Peptide** SRAGLQFPVGRV **Charge** 3 **Spectral Angle** 0.86
Experimental Spectrum
Prosit Predicted Spectrum
L² Normalized Intensity
m/z

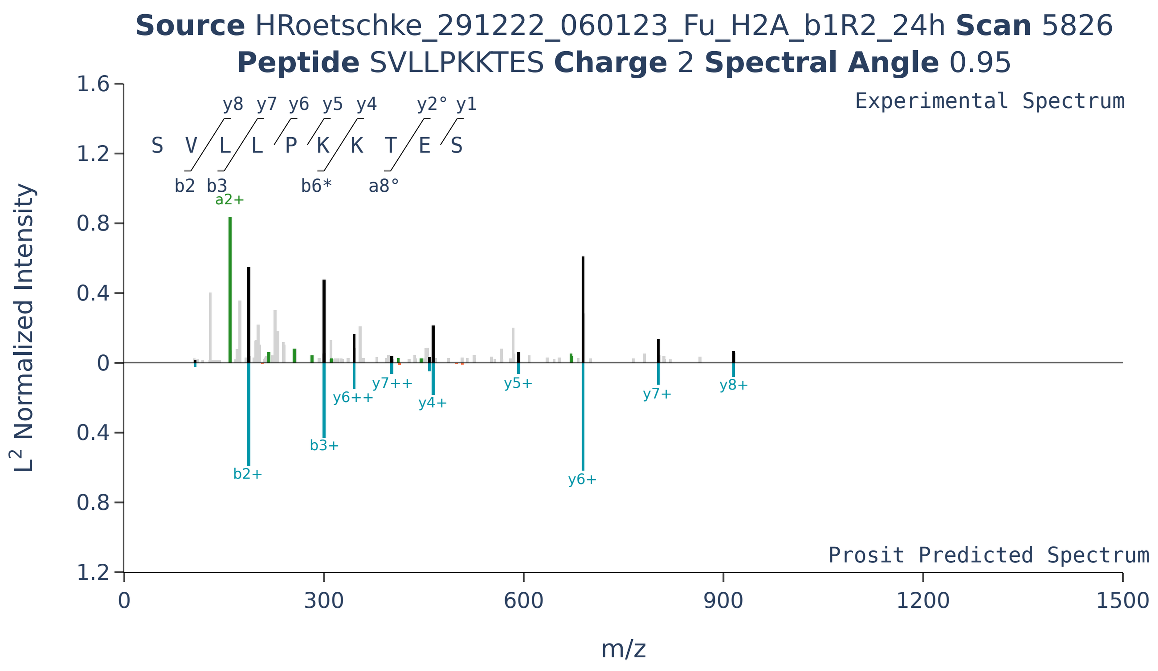
**Source** HRoetschke_291222_060123_Fu_H2A_b1R2_24h **Scan** 5826
**Peptide** SVLLPKKTES **Charge** 2 **Spectral Angle** 0.95
Experimental Spectrum
Prosit Predicted Spectrum
L² Normalized Intensity
m/z

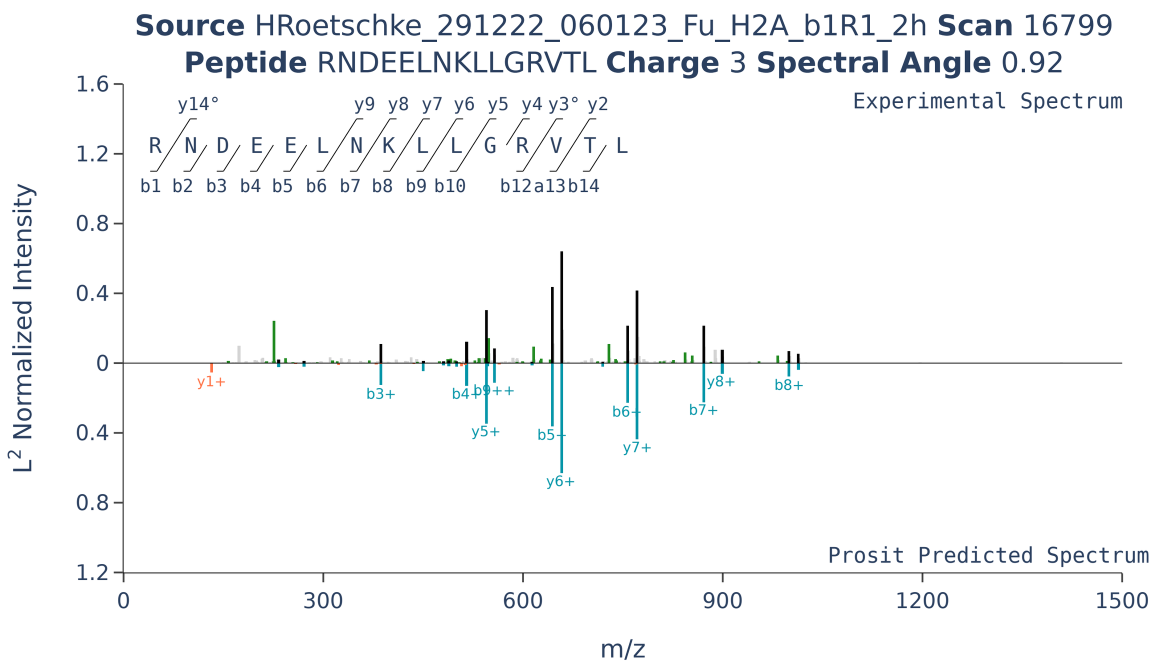
**Source** HRoetschke_291222_060123_Fu_H2A_b1R1_2h **Scan** 16799
**Peptide** RNDEELNKLLGRVTL **Charge** 3 **Spectral Angle** 0.92
Experimental Spectrum
Prosit Predicted Spectrum
L² Normalized Intensity
m/z

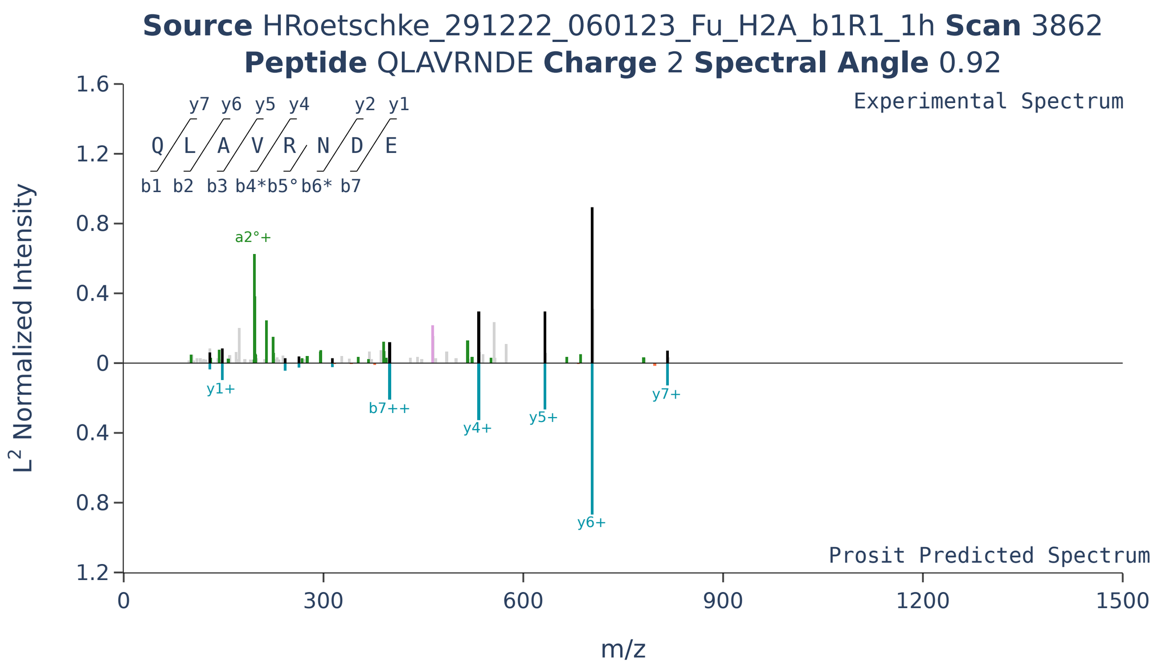
**Source** HRoetschke_291222_060123_Fu_H2A_b1R1_1h **Scan** 3862
**Peptide** QLAVRNDE **Charge** 2 **Spectral Angle** 0.92
Experimental Spectrum
Prosit Predicted Spectrum
L² Normalized Intensity
m/z

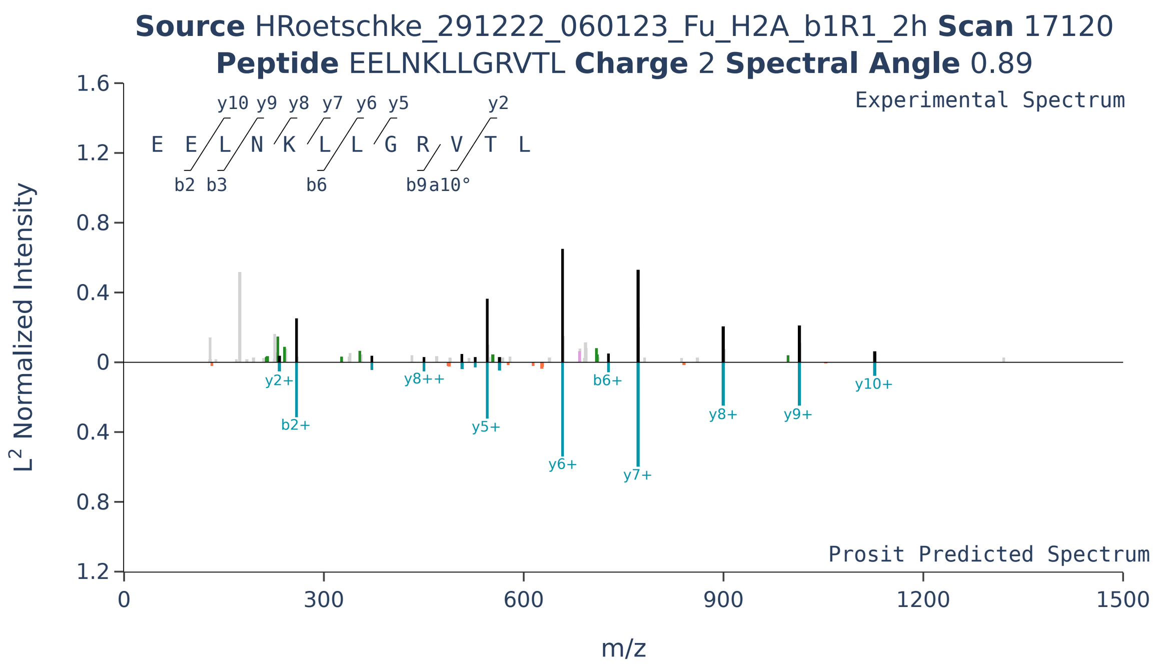
**Source** HRoetschke_291222_060123_Fu_H2A_b1R1_2h **Scan** 17120
**Peptide** EELNKLLGRVTL **Charge** 2 **Spectral Angle** 0.89
Experimental Spectrum
Prosit Predicted Spectrum
L² Normalized Intensity
m/z

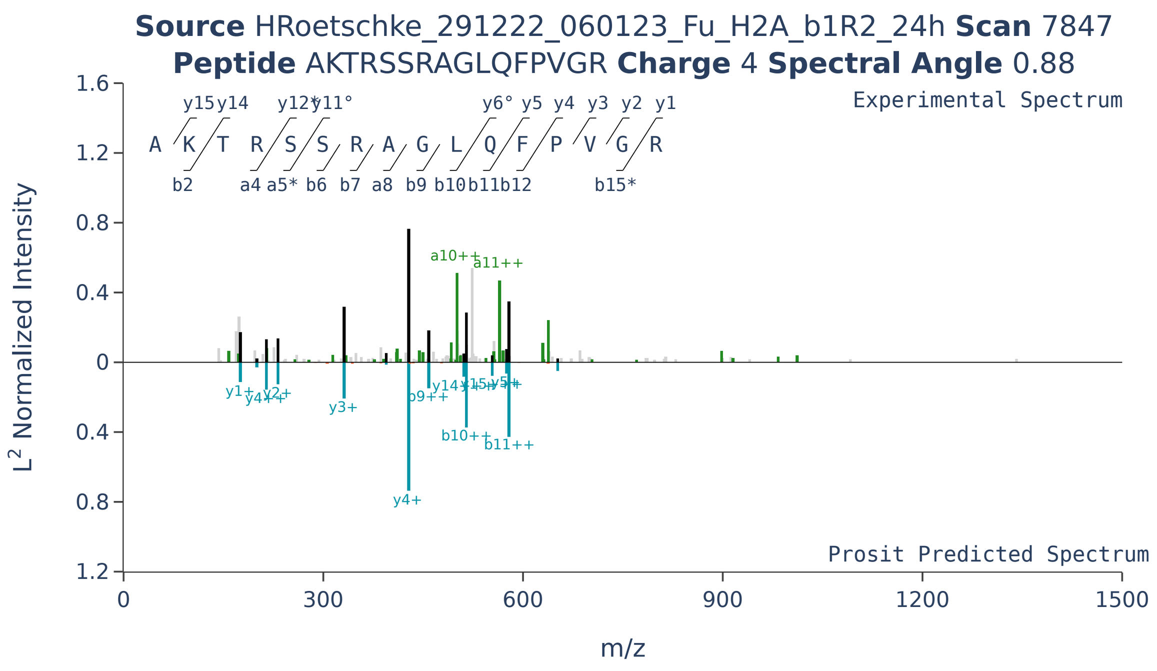
**Source** HRoetschke_291222_060123_Fu_H2A_b1R2_24h **Scan** 7847
**Peptide** AKTRSSRAGLQFPVGR **Charge** 4 **Spectral Angle** 0.88
Experimental Spectrum
Prosit Predicted Spectrum
L² Normalized Intensity
m/z

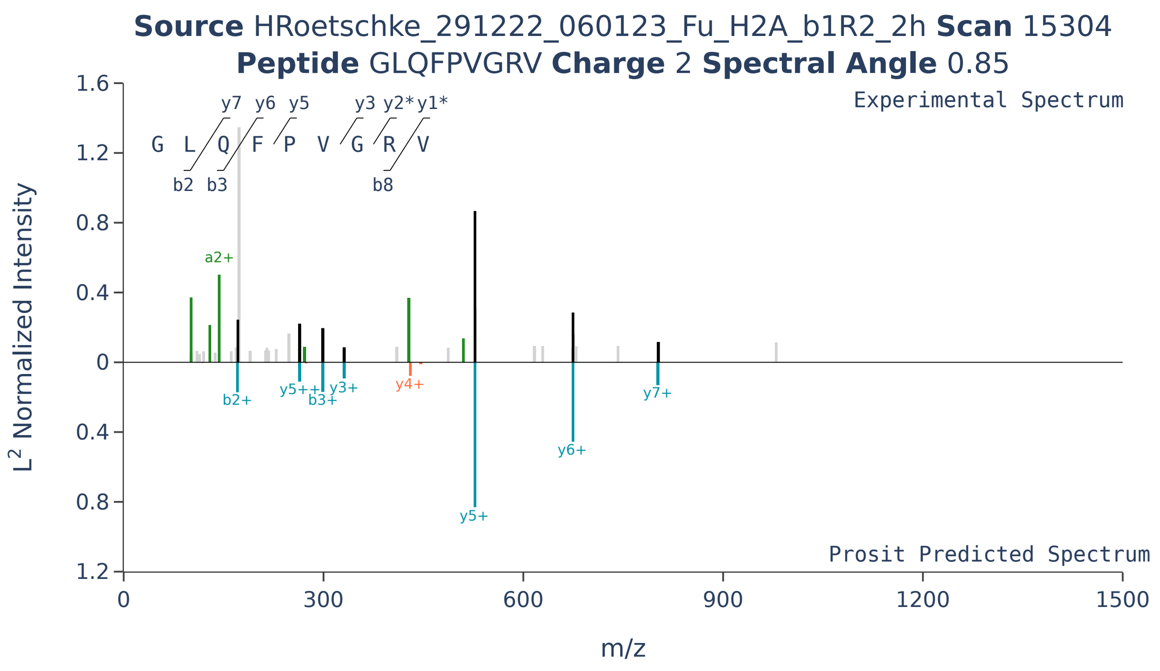
**Source** HRoetschke_291222_060123_Fu_H2A_b1R2_2h **Scan** 15304
**Peptide** GLQFPVGRV **Charge** 2 **Spectral Angle** 0.85
Experimental Spectrum
Prosit Predicted Spectrum
L² Normalized Intensity
m/z

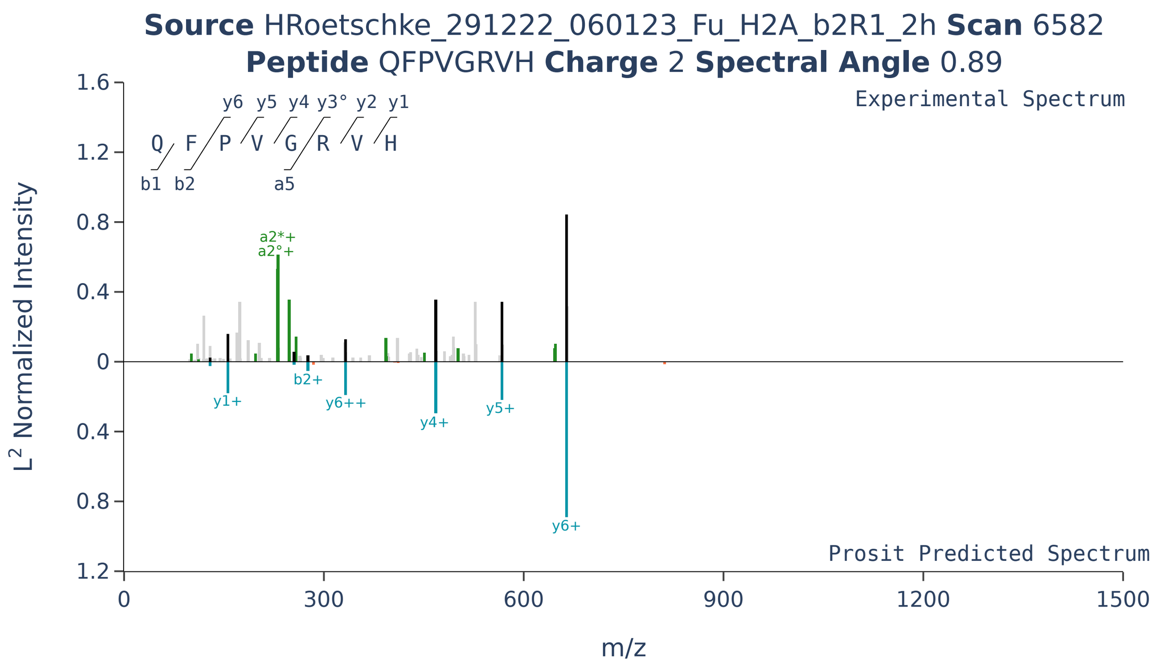
**Source** HRoetschke_291222_060123_Fu_H2A_b2R1_2h **Scan** 6582
**Peptide** QFPVGRVH **Charge** 2 **Spectral Angle** 0.89
Experimental Spectrum
Prosit Predicted Spectrum
L² Normalized Intensity
m/z

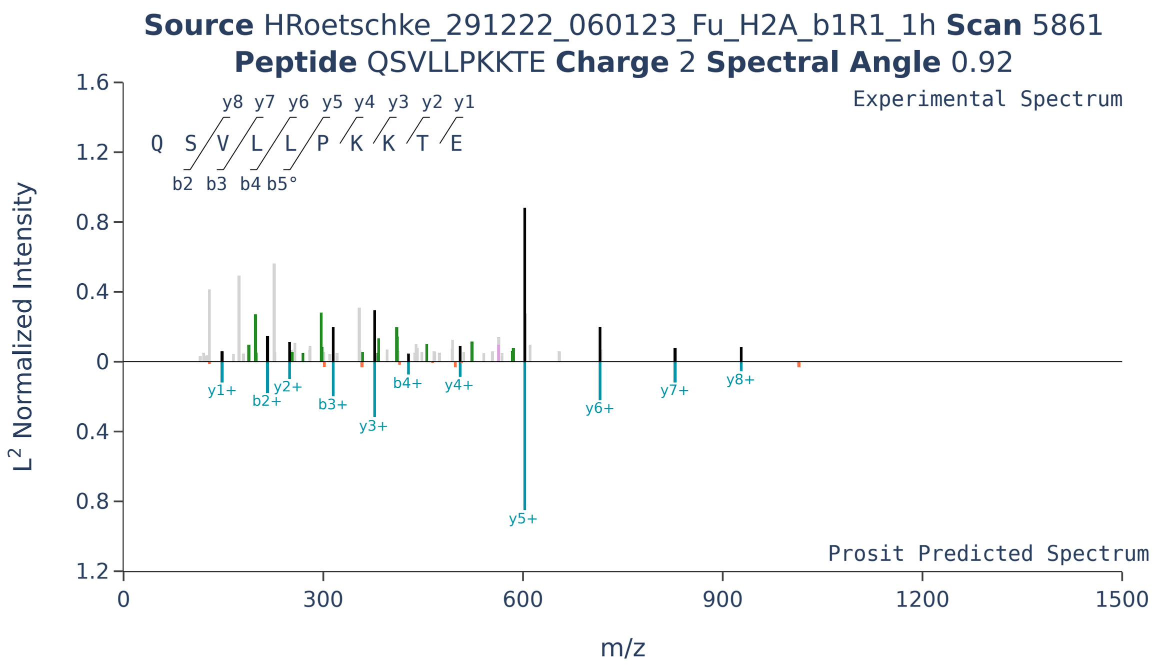
**Source** HRoetschke_291222_060123_Fu_H2A_b1R1_1h **Scan** 5861
**Peptide** QSVLLPKKTE **Charge** 2 **Spectral Angle** 0.92
Experimental Spectrum
Prosit Predicted Spectrum
L² Normalized Intensity
m/z

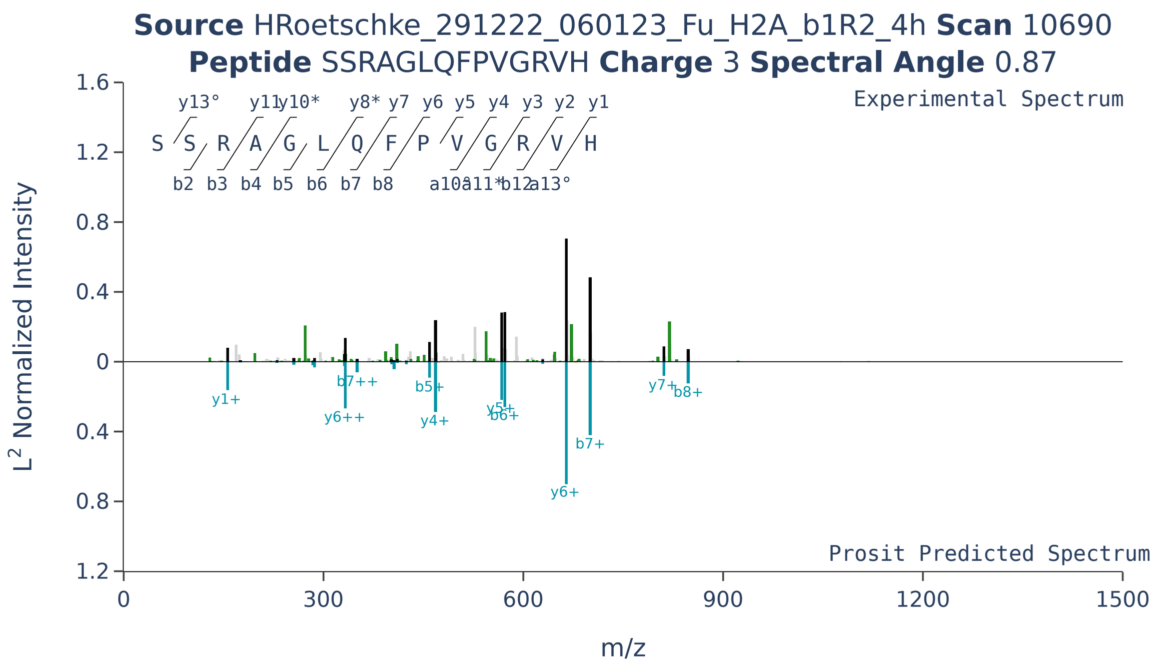
**Source** HRoetschke_291222_060123_Fu_H2A_b1R2_4h **Scan** 10690
**Peptide** SSRAGLQFPVGRVH **Charge** 3 **Spectral Angle** 0.87
Experimental Spectrum
Prosit Predicted Spectrum
L² Normalized Intensity
m/z

**Source** HRoetschke_291222_060123_Fu_H2A_b2R2_24h **Scan** 13064
**Peptide** QGGVLPNLQ **Charge** 2 **Spectral Angle** 0.91

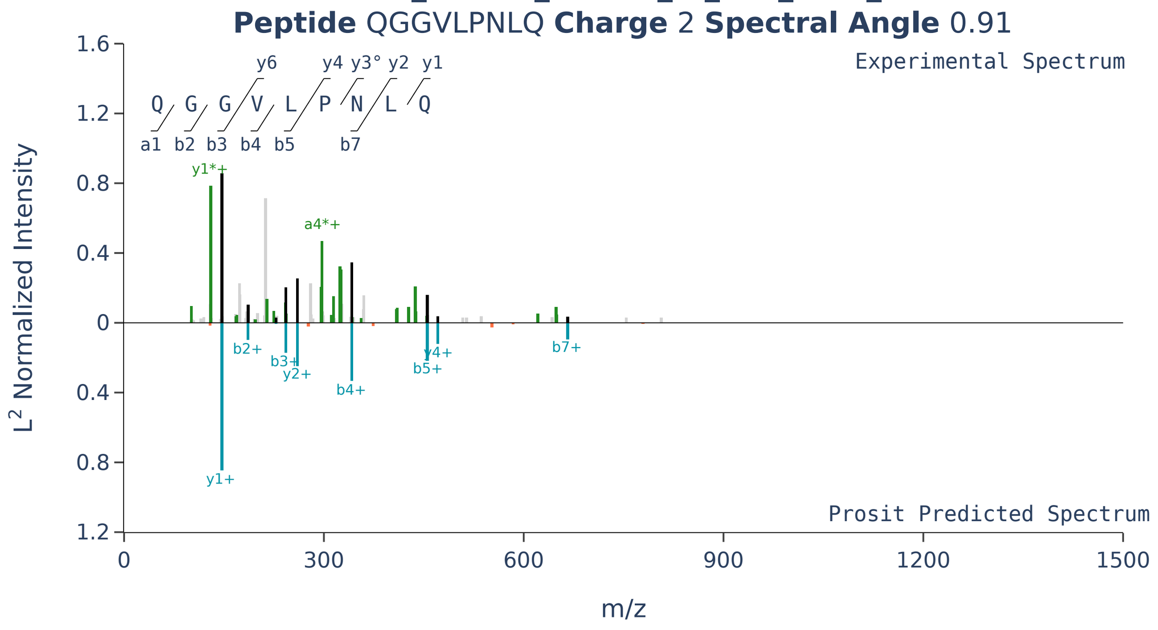


**Source** HRoetschke_291222_060123_Fu_H2A_b2R1_24h **Scan** 8869
**Peptide** ELNKLLGR **Charge** 3 **Spectral Angle** 0.91

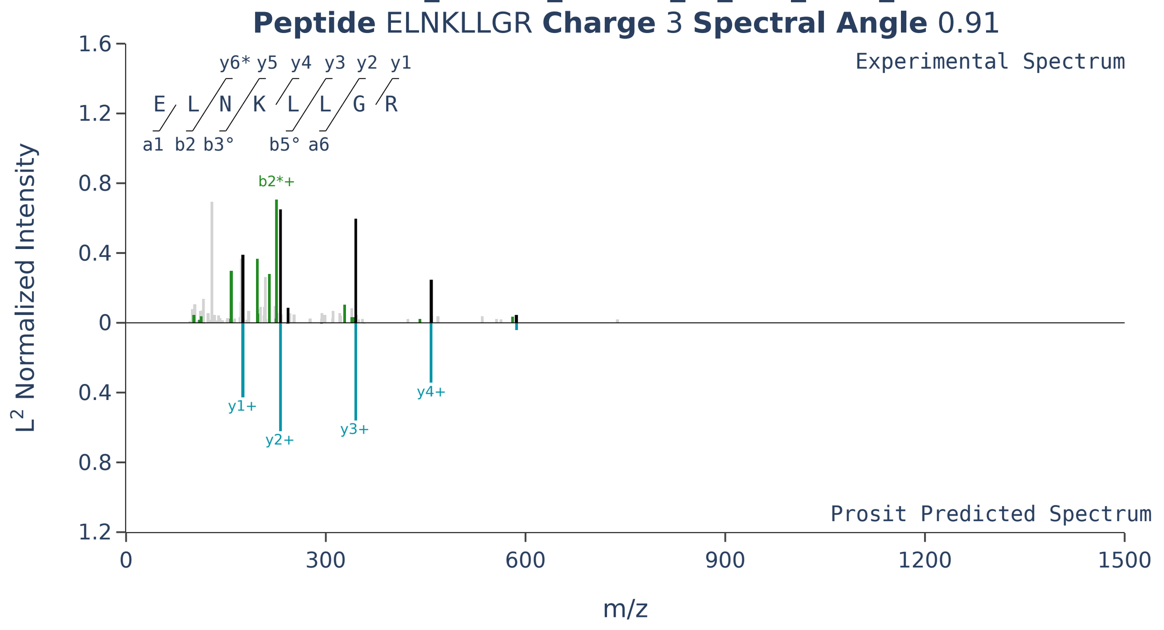


**Source** HRoetschke_291222_060123_Fu_H2A_b1R2_2h **Scan** 12590
**Peptide** AQGGVLPNIQS **Charge** 2 **Spectral Angle** 0.89

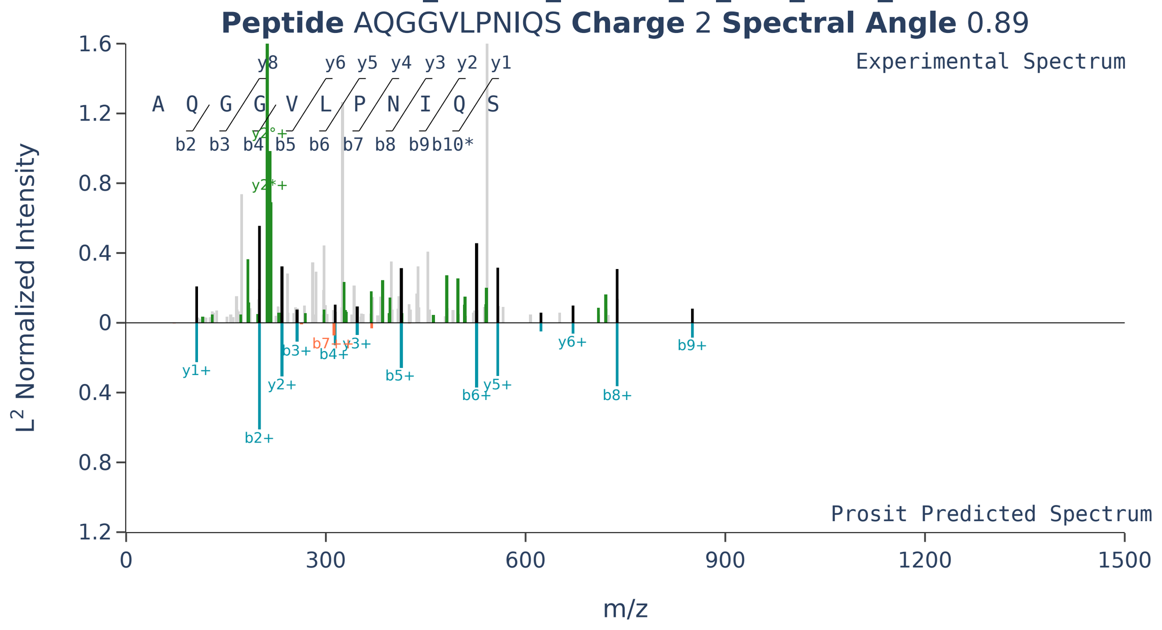


**Source** HRoetschke_291222_060123_Fu_H2A_b2R2_4h **Scan** 2267
**Peptide** RLLRKGNYAER **Charge** 3 **Spectral Angle** 0.83

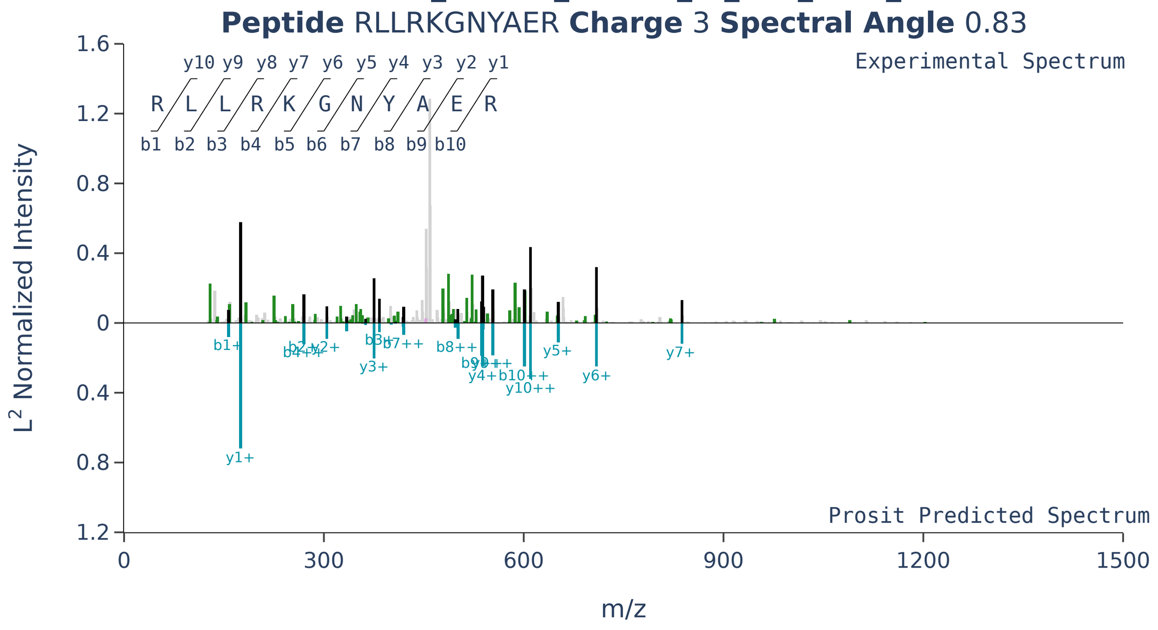


**Source** HRoetschke_291222_060123_Fu_H2A_b1R1_4h **Scan** 14582
**Peptide** RNDEELNKLLGRV **Charge** 3 **Spectral Angle** 0.86

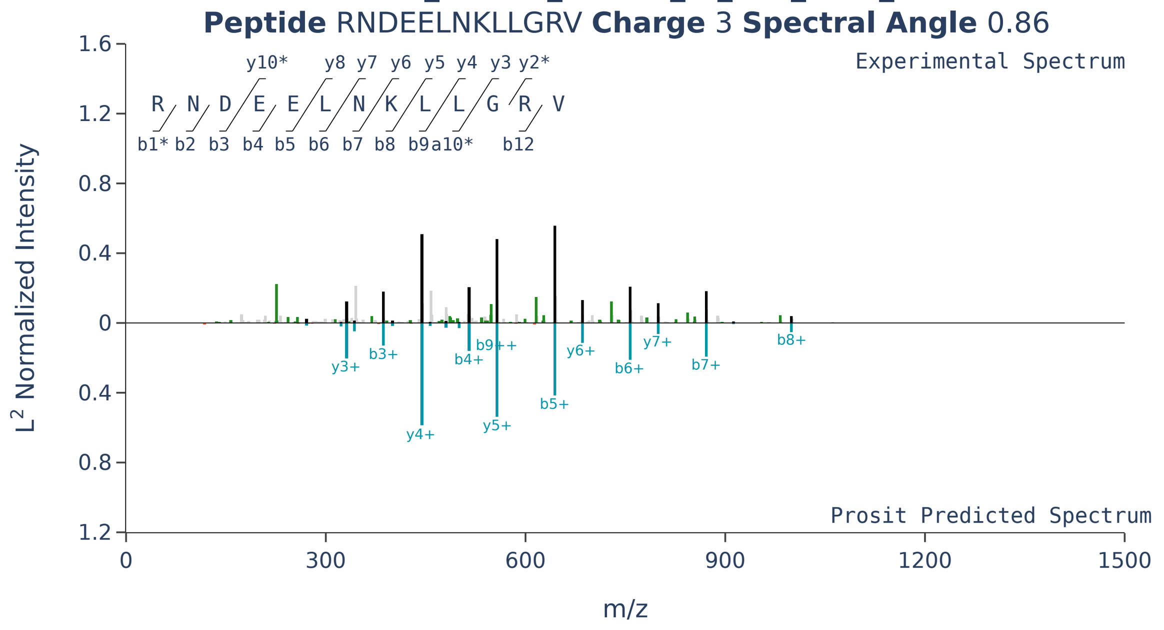


**Source** HRoetschke_291222_060123_Fu_H2A_b2R2_24h **Scan** 13427
**Peptide** AGAPVYL **Charge** 1 **Spectral Angle** 0.83

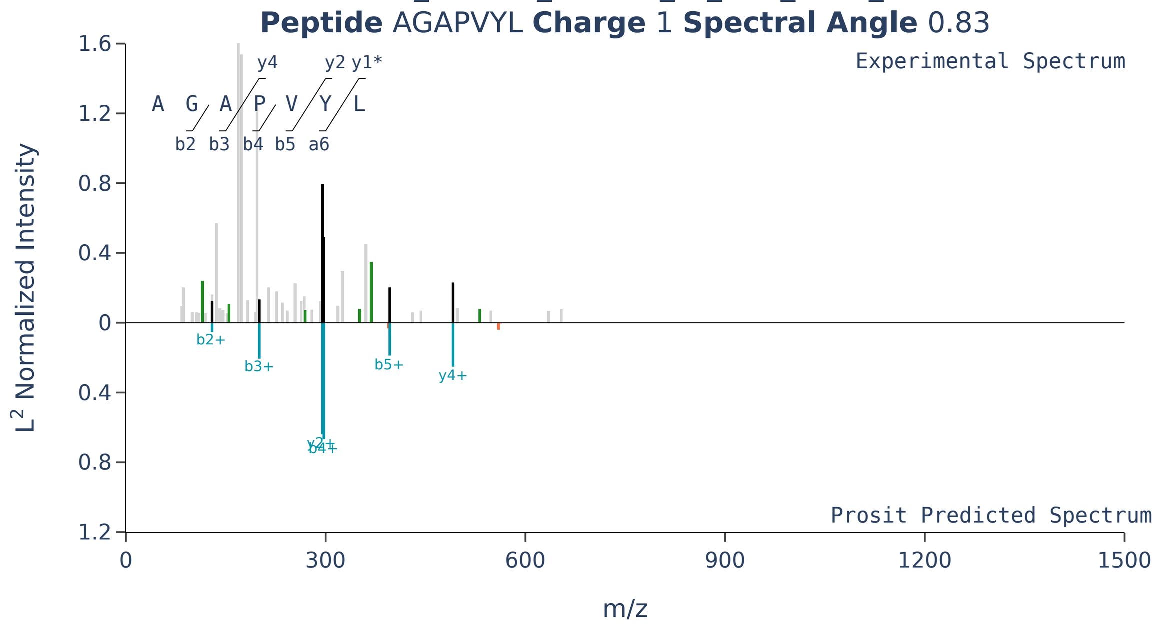


**Source** HRoetschke_291222_060123_Fu_H2A_b2R2_4h **Scan** 13026
**Peptide** SSRAGLQFPVG **Charge** 2 **Spectral Angle** 0.88

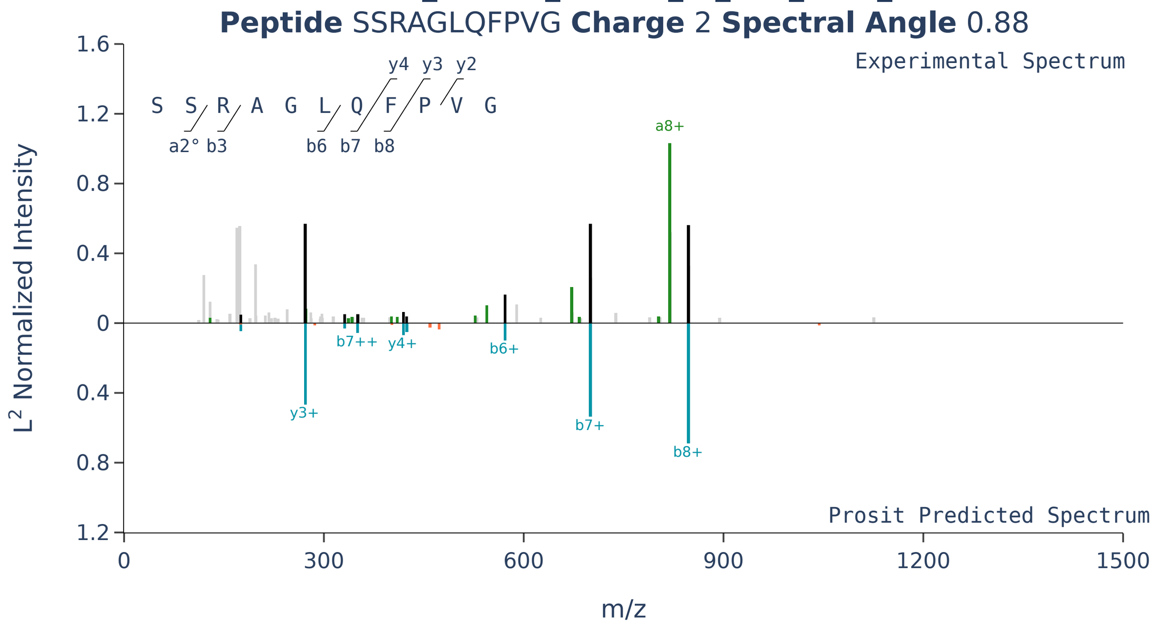


**Source** HRoetschke_291222_060123_Fu_H2A_b1R1_24h **Scan** 6737
**Peptide** NKLLGRV **Charge** 2 **Spectral Angle** 0.94

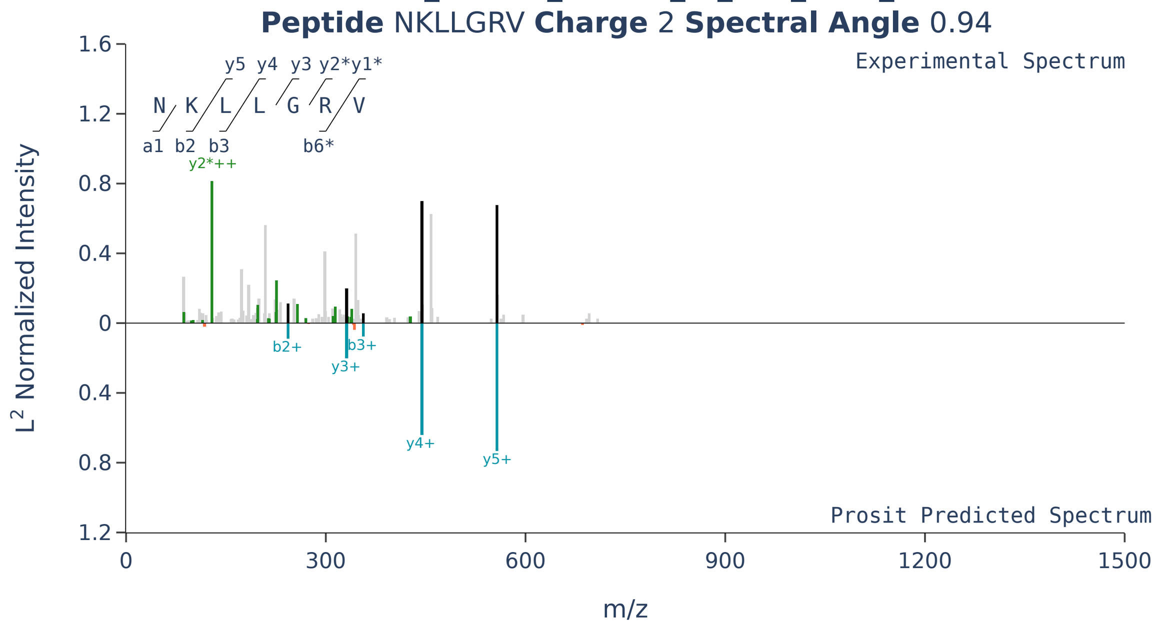


**Source** HRoetschke_291222_060123_Fu_H2A_b2R2_24h **Scan** 7058
**Peptide** LELAGNAARD **Charge** 2 **Spectral Angle** 0.96

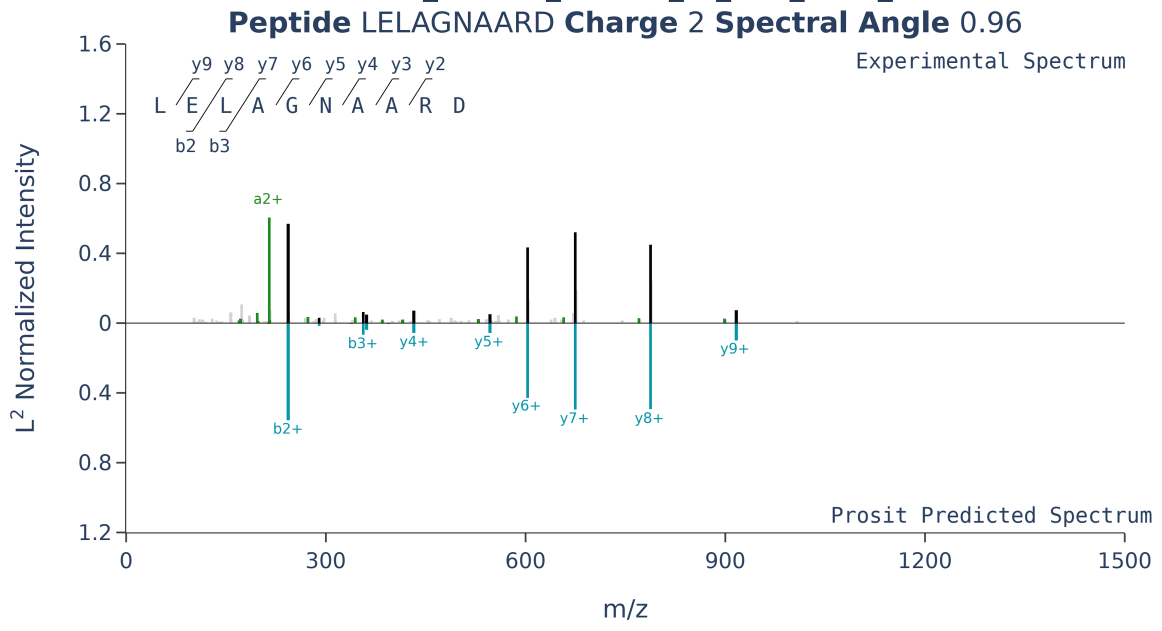


**Source** HRoetschke_291222_060123_Fu_H2A_b1R1_1h **Scan** 12708
**Peptide** RAGLQFPVG **Charge** 2 **Spectral Angle** 0.9

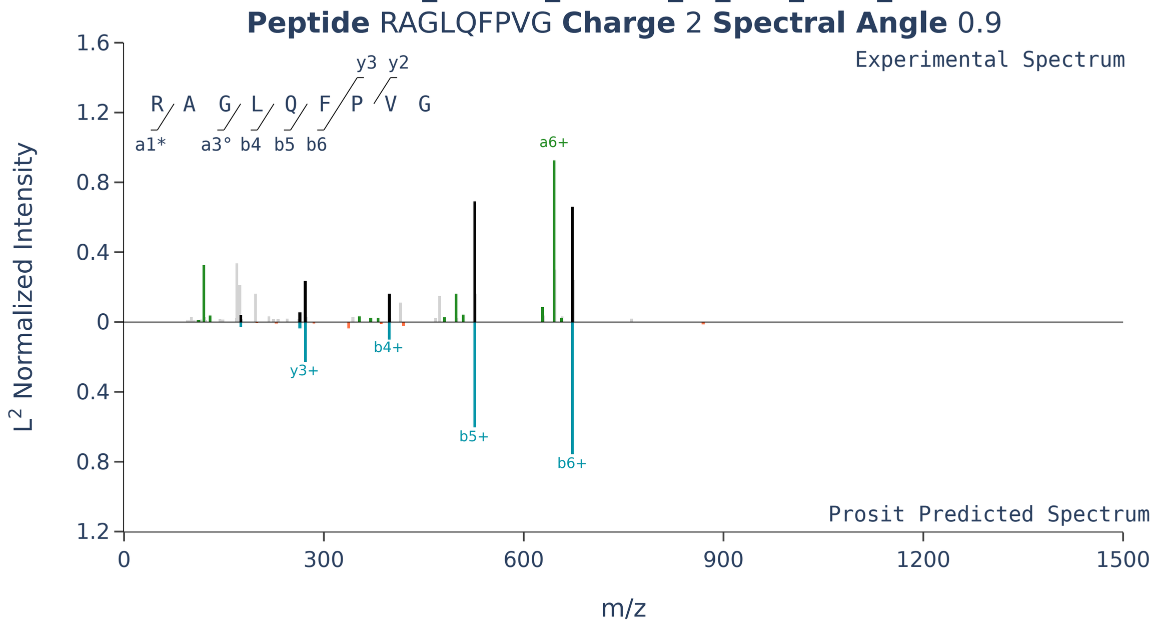


**Source** HRoetschke_291222_060123_Fu_H2A_b1R1_1h **Scan** 4854
**Peptide** VLLPKKT **Charge** 2 **Spectral Angle** 0.91

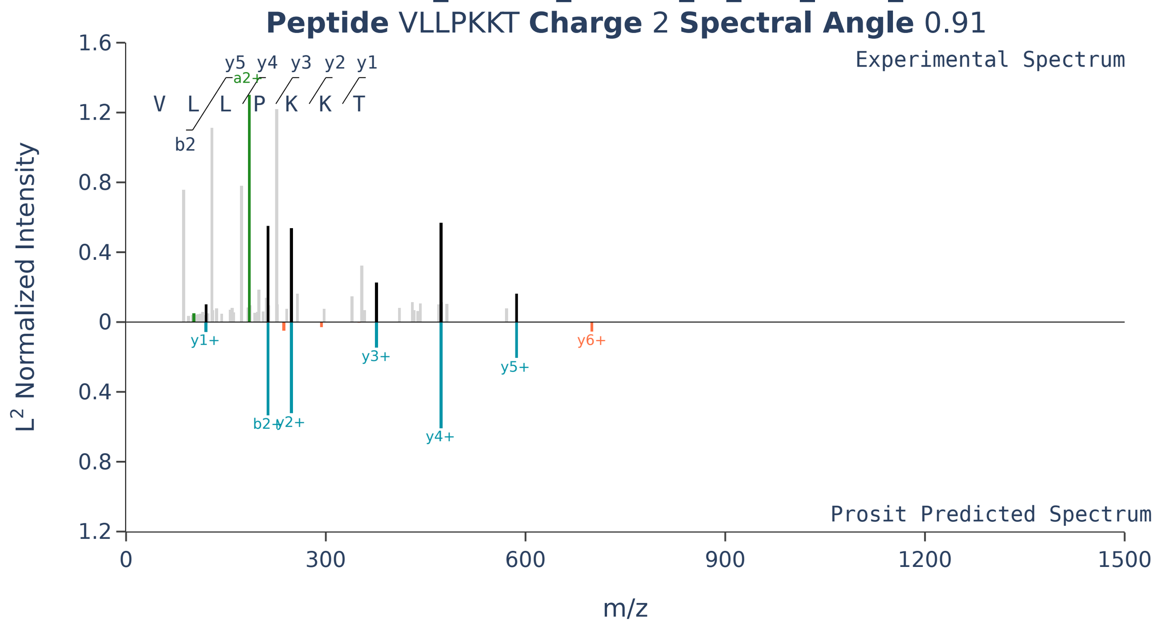


**Source** HRoetschke_291222_060123_Fu_H2A_b1R1_1h **Scan** 4694
**Peptide** VLLPKKTES **Charge** 2 **Spectral Angle** 0.94

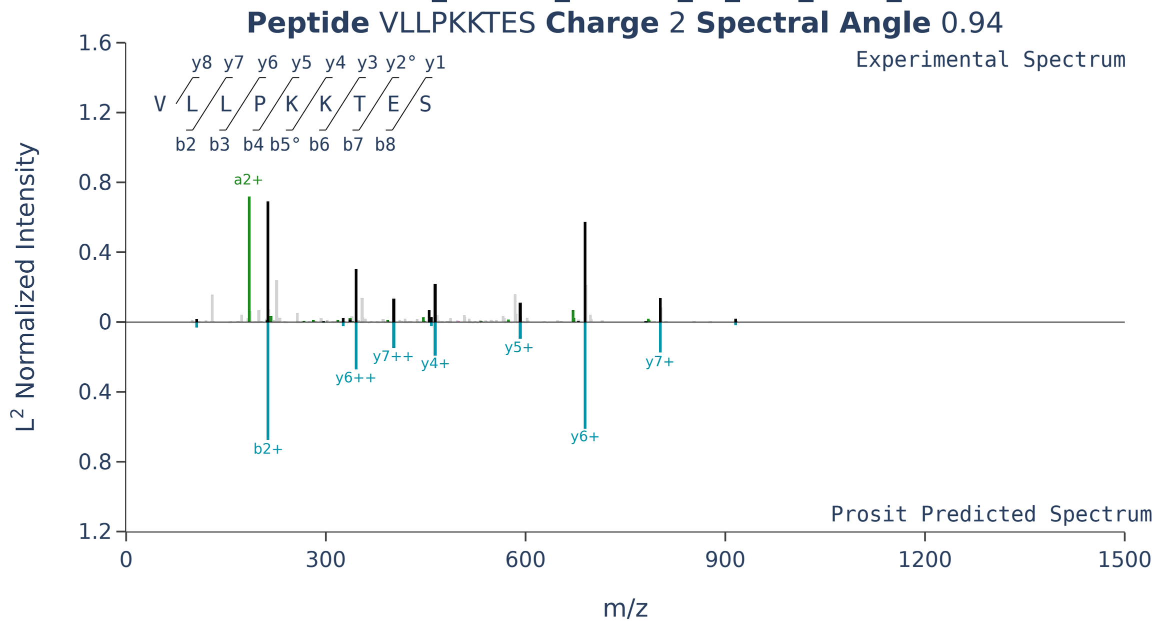


**Source** HRoetschke_291222_060123_Fu_H2A_b1R2_1h **Scan** 12537
**Peptide** DEELNKLLG **Charge** 2 **Spectral Angle** 0.91

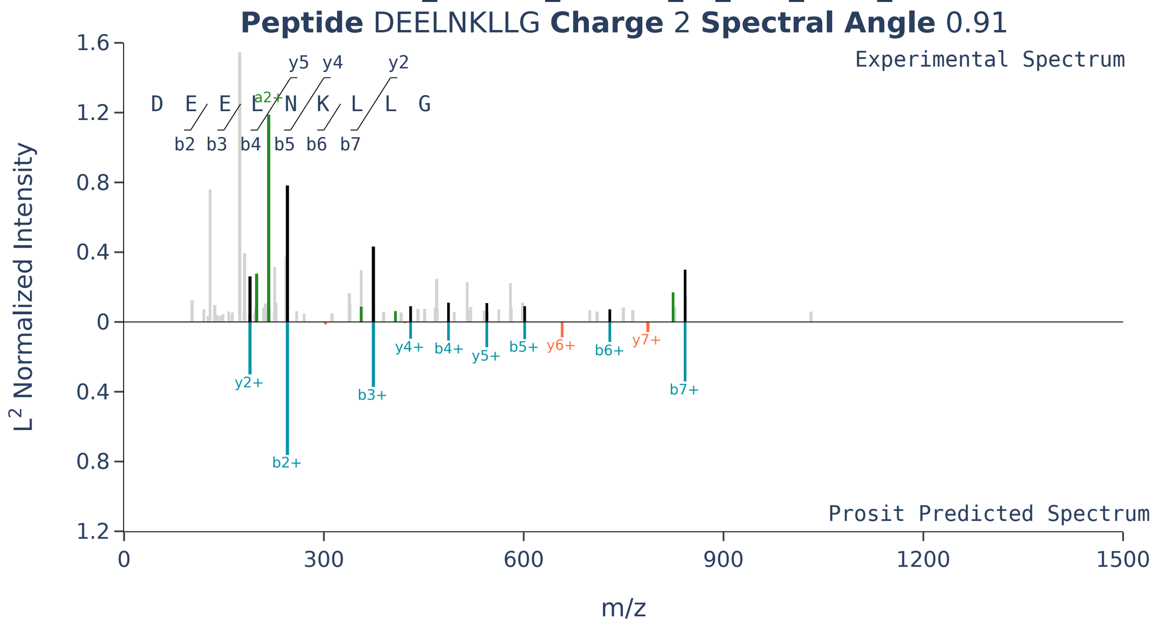


**Source** HRoetschke_291222_060123_Fu_H2A_b2R1_24h **Scan** 9448
**Peptide** LELAGNAA **Charge** 2 **Spectral Angle** 0.95

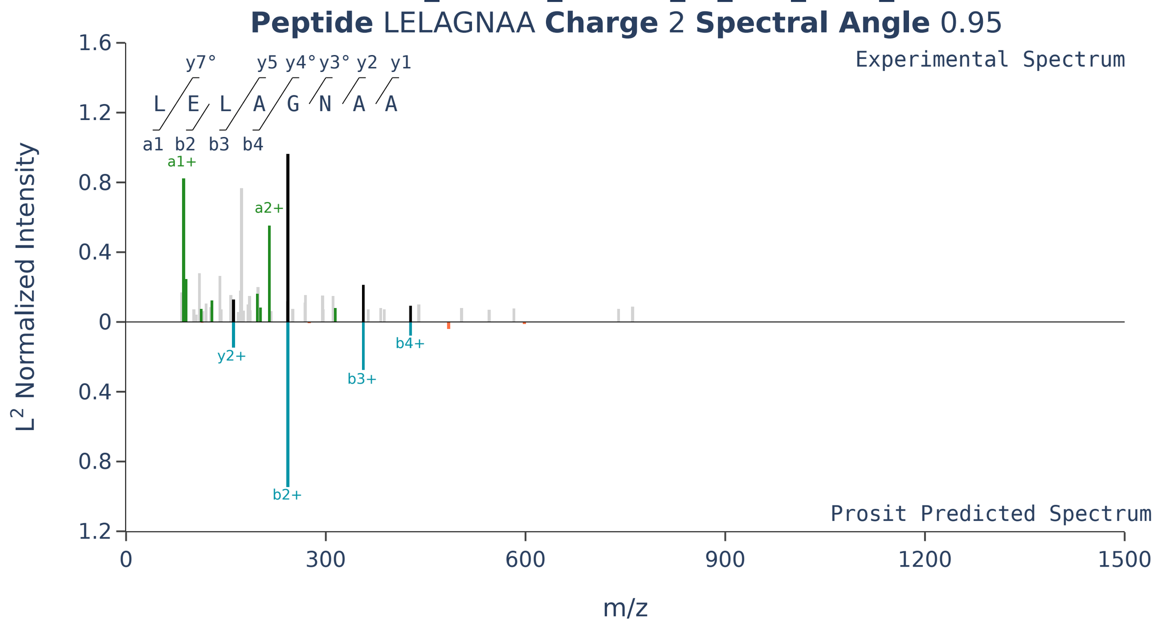


**Source** HRoetschke_291222_060123_Fu_H2A_b1R2_4h **Scan** 17033
**Peptide** AVLEYLTAE **Charge** 2 **Spectral Angle** 0.88

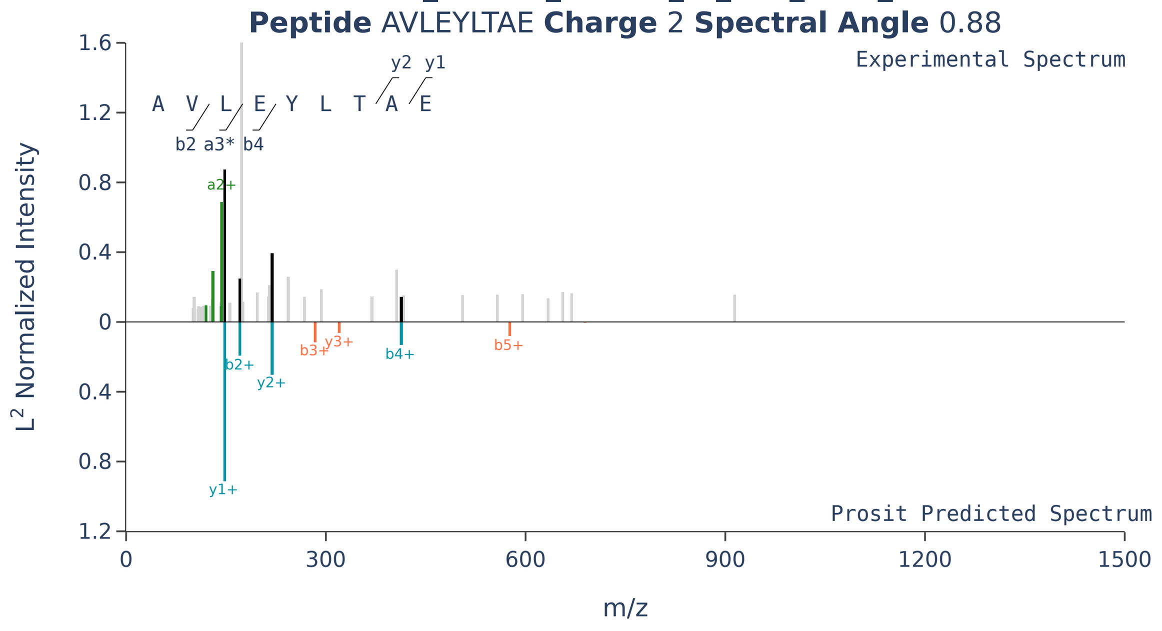

**Source** HRoetschke_291222_060123_Fu_H2A_b1R1_4h **Scan** 14265
**Peptide** RAGLQFPVGRV **Charge** 3 **Spectral Angle** 0.84

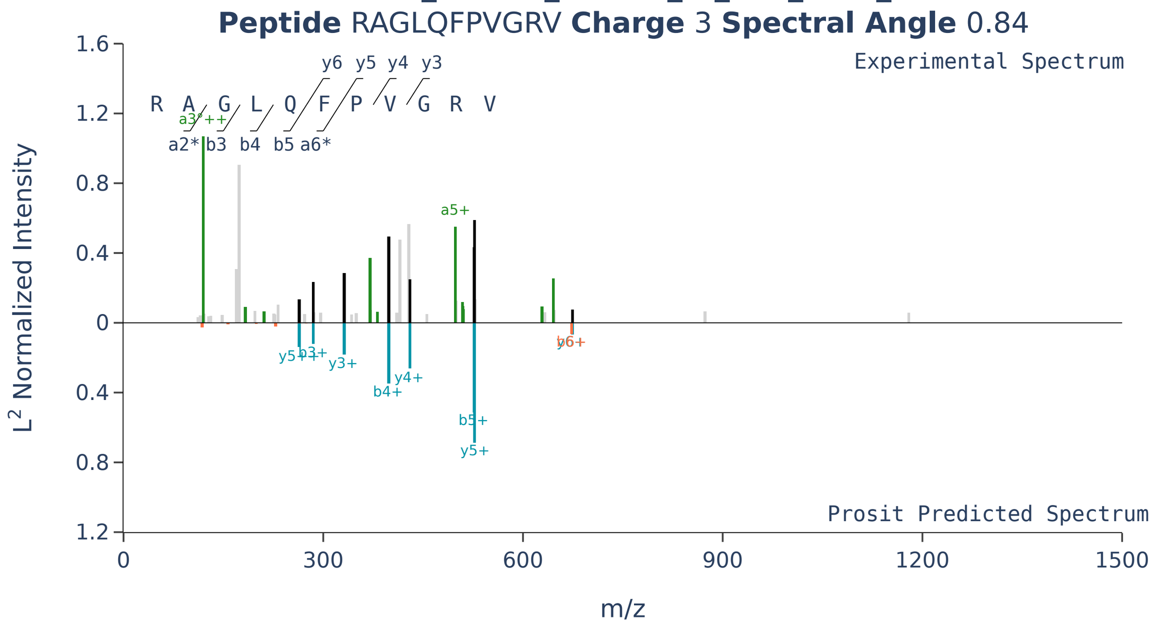


**Source** HRoetschke_291222_060123_Fu_H2A_b2R1_2h **Scan** 13157
**Peptide** AVRNDEELNKLLGRV **Charge** 4 **Spectral Angle** 0.9

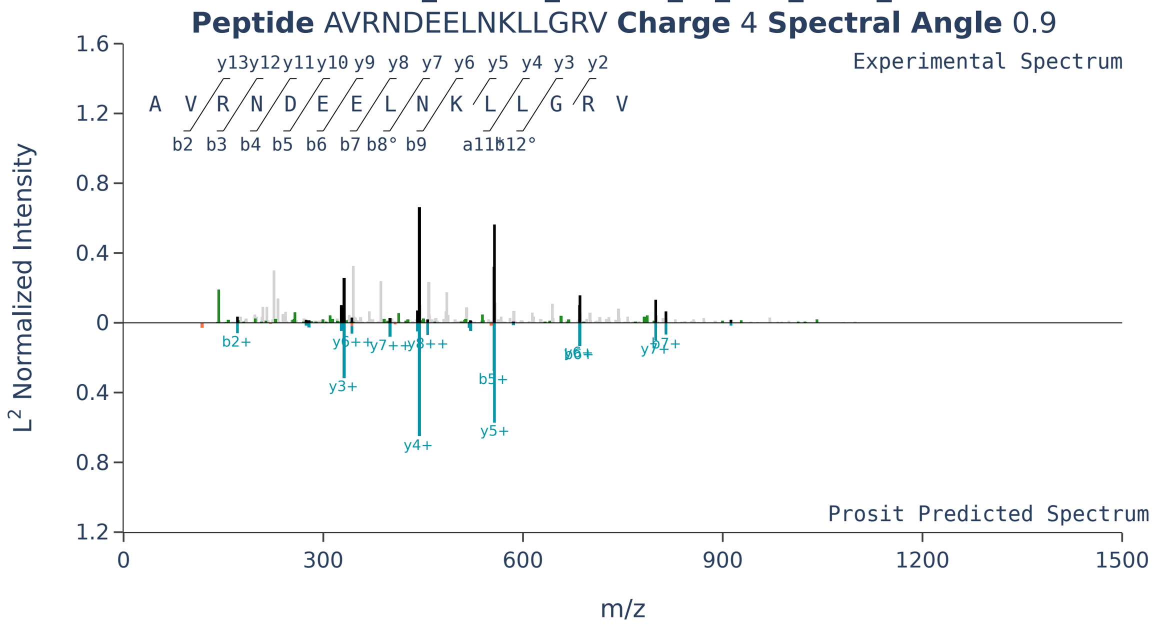


**Source** HRoetschke_291222_060123_Fu_H2A_b1R1_2h **Scan** 16158
**Peptide** DEELNKLLGRV **Charge** 2 **Spectral Angle** 0.9

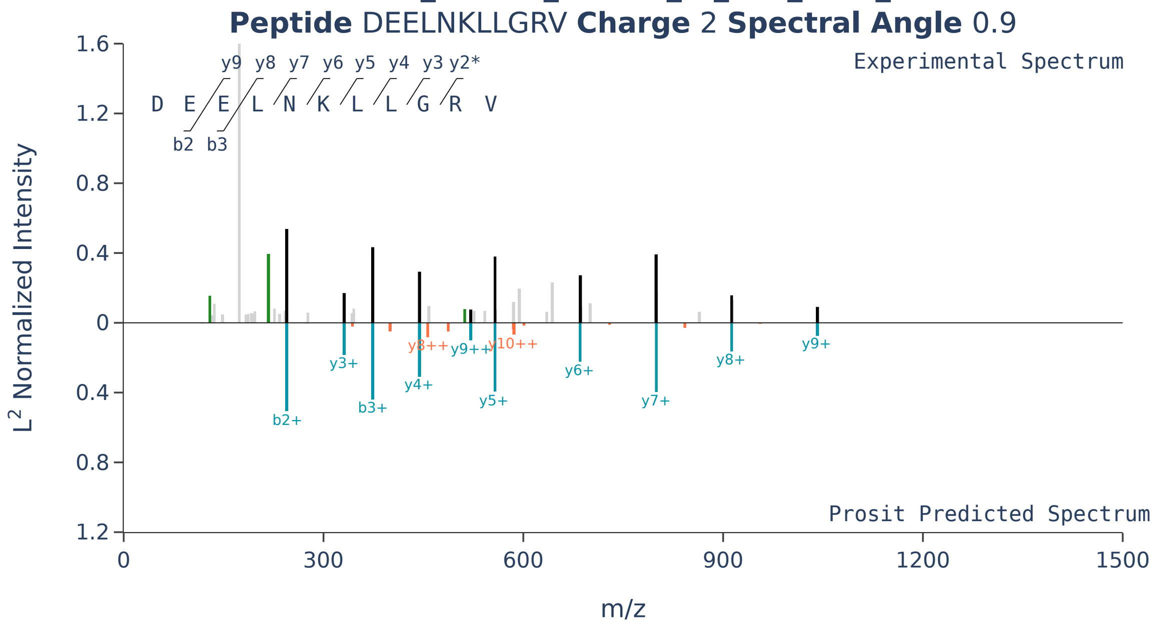


**Source** HRoetschke_291222_060123_Fu_H2A_b1R1_2h **Scan** 15646
**Peptide** QGGVLPNL **Charge** 1 **Spectral Angle** 0.82

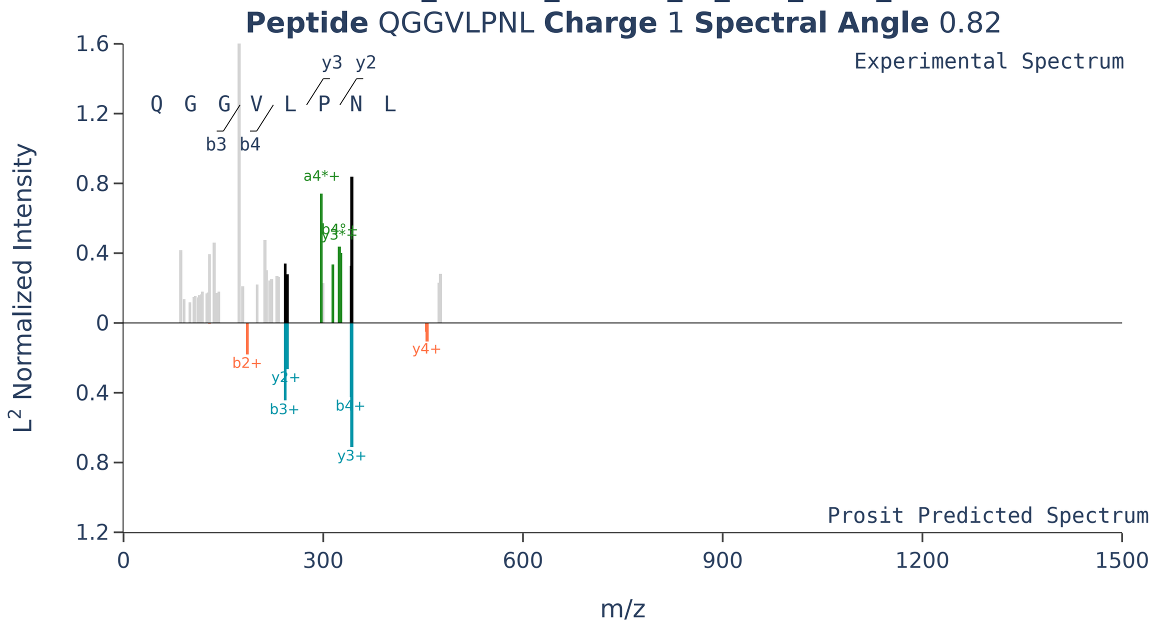


**Source** HRoetschke_291222_060123_Fu_H2A_b1R1_1h **Scan** 5394
**Peptide** PRHLQLAVRND **Charge** 4 **Spectral Angle** 0.81

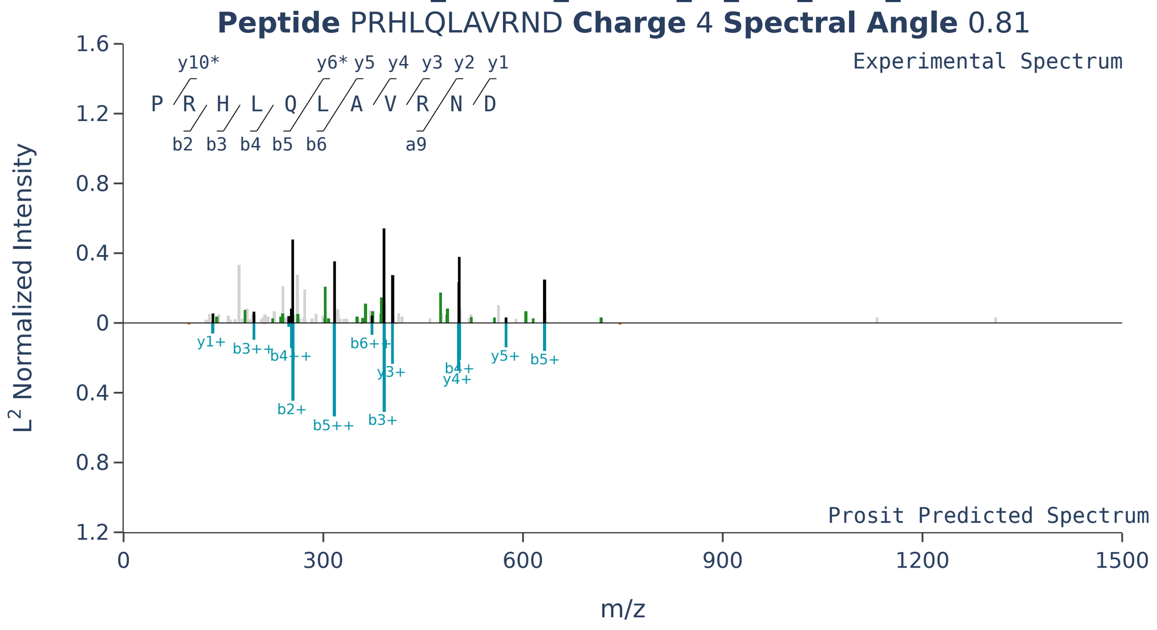


**Source** HRoetschke_291222_060123_Fu_H2A_b2R2_24h **Scan** 11698
**Peptide** LQFPVGRVH **Charge** 3 **Spectral Angle** 0.83

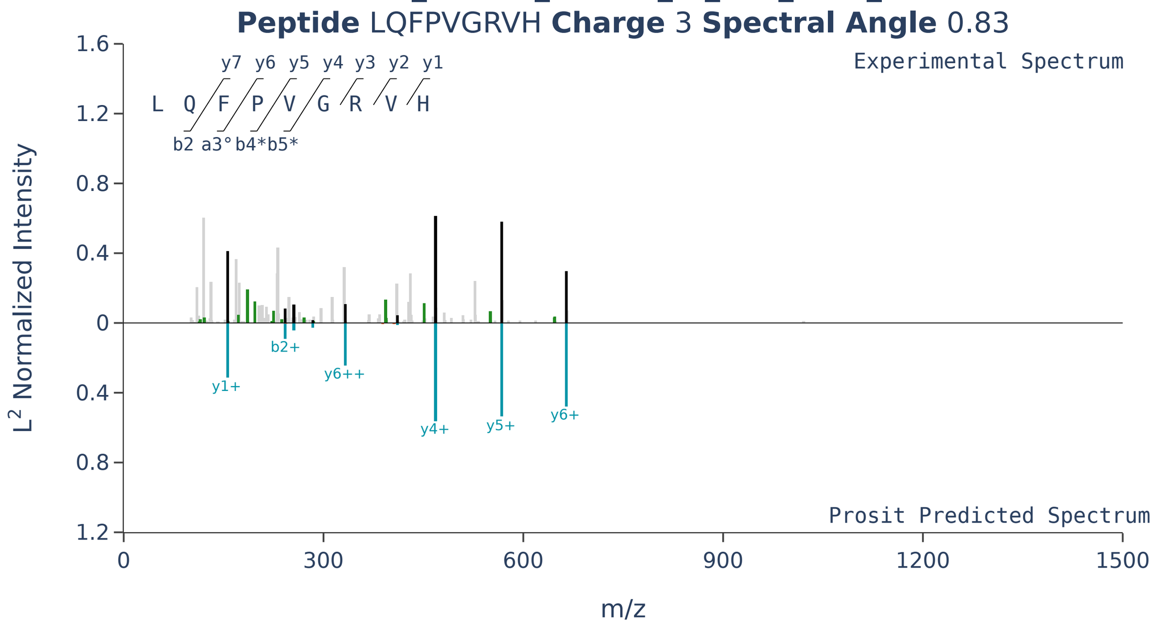


**Source** HRoetschke_291222_060123_Fu_H2A_b1R1_1h **Scan** 6971
**Peptide** ERVGAGAPVY **Charge** 2 **Spectral Angle** 0.9

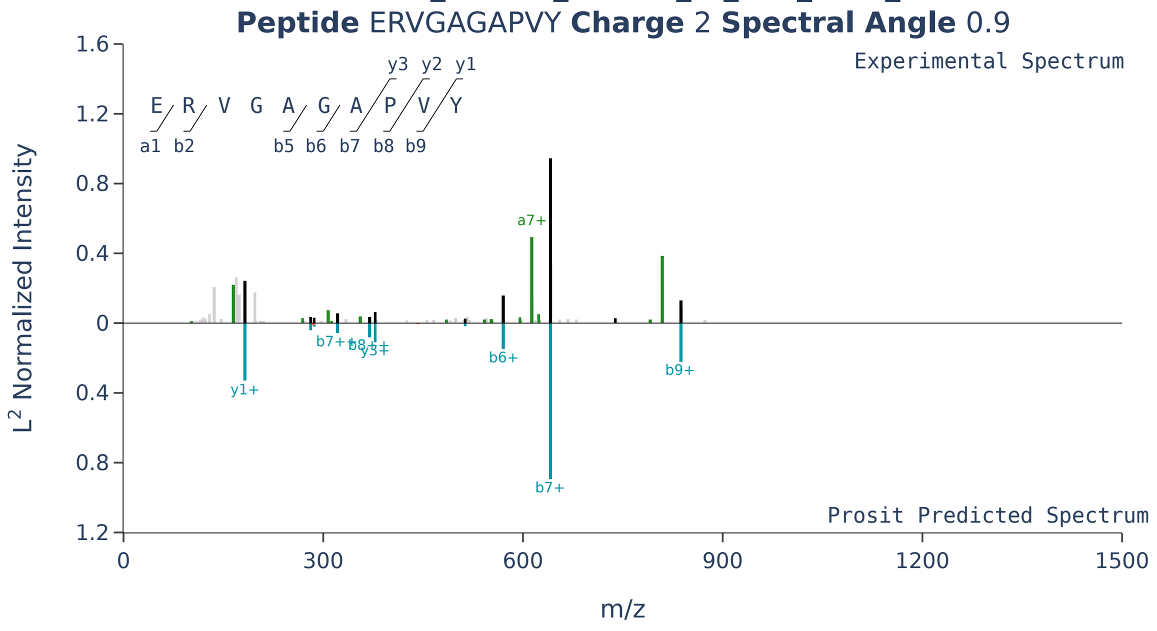


**Source** HRoetschke_291222_060123_Fu_H2A_b1R1_24h **Scan** 8359
**Peptide** NDEELNKL **Charge** 2 **Spectral Angle** 0.89

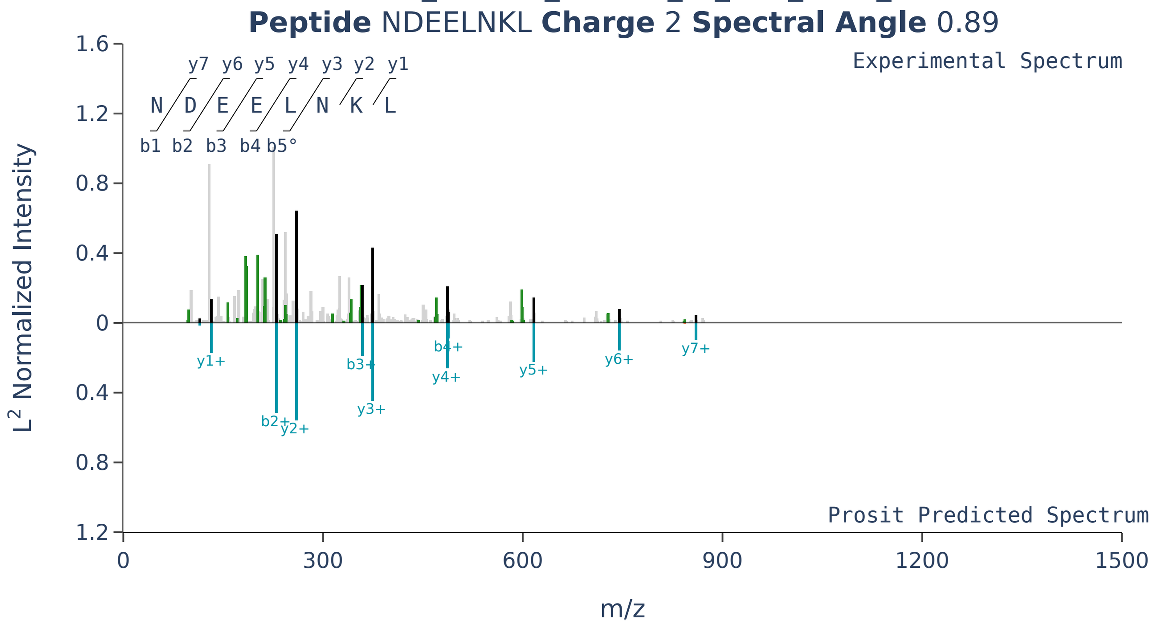


**Source** HRoetschke_291222_060123_Fu_H2A_b1R2_4h **Scan** 16938
**Peptide** RNDEELNKLLGRVTLA **Charge** 3 **Spectral Angle** 0.89

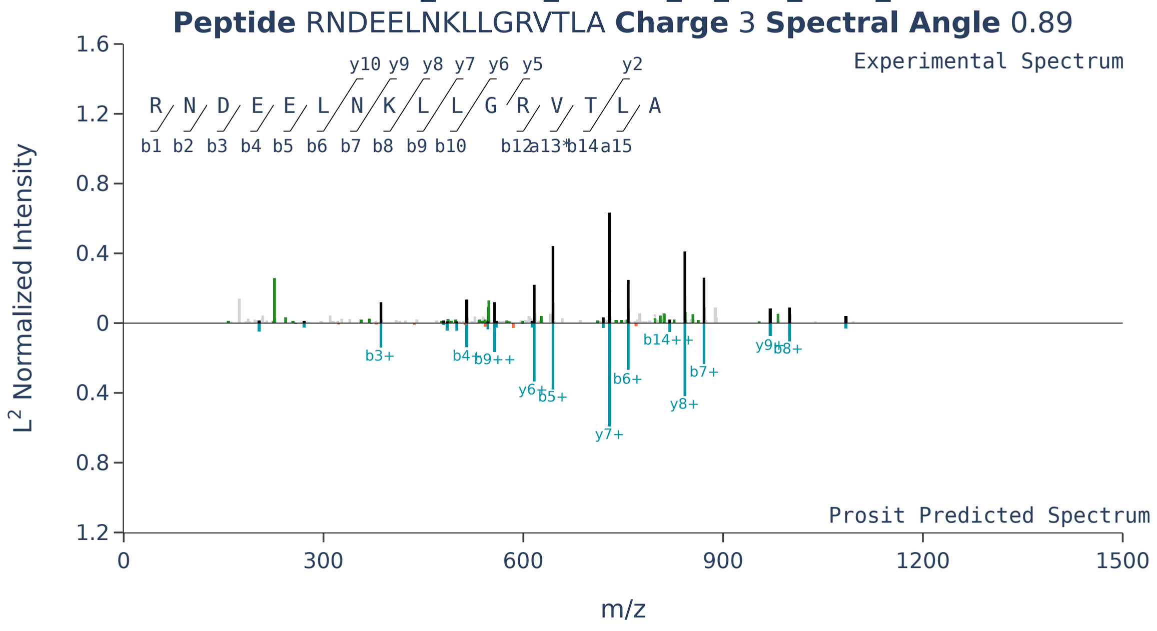


**Source** HRoetschke_291222_060123_Fu_H2A_b1R2_1h **Scan** 10791
**Peptide** ERVGAGAPVYLA **Charge** 2 **Spectral Angle** 0.91

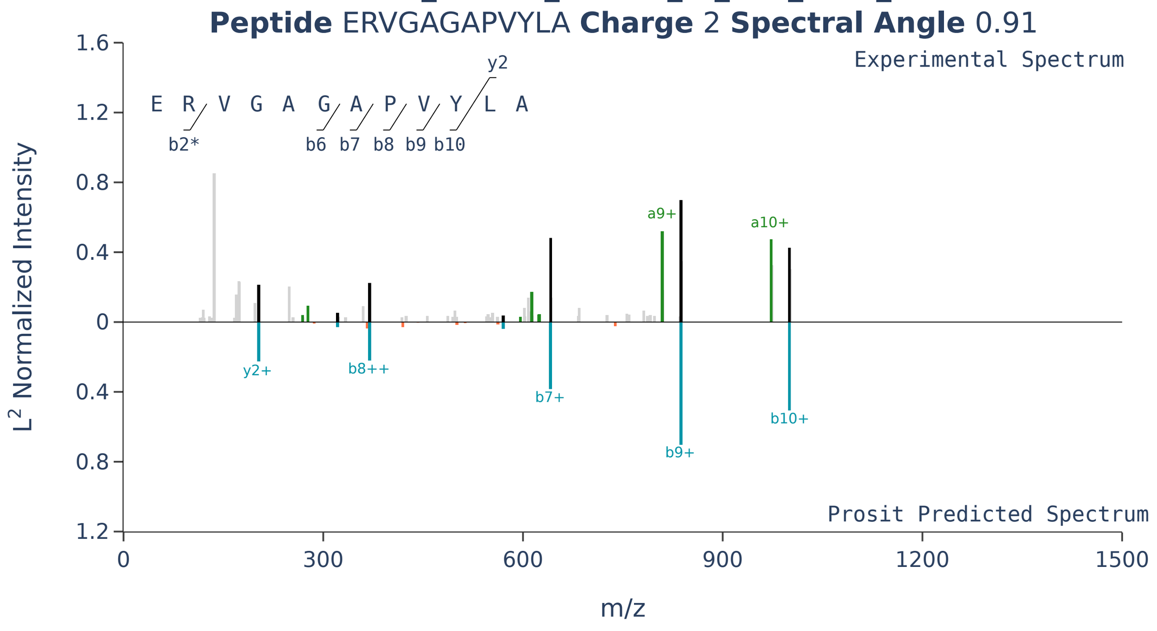


**Source** HRoetschke_291222_060123_Fu_H2A_b2R1_2h **Scan** 12129
**Peptide** DEELNKLLGR **Charge** 2 **Spectral Angle** 0.85

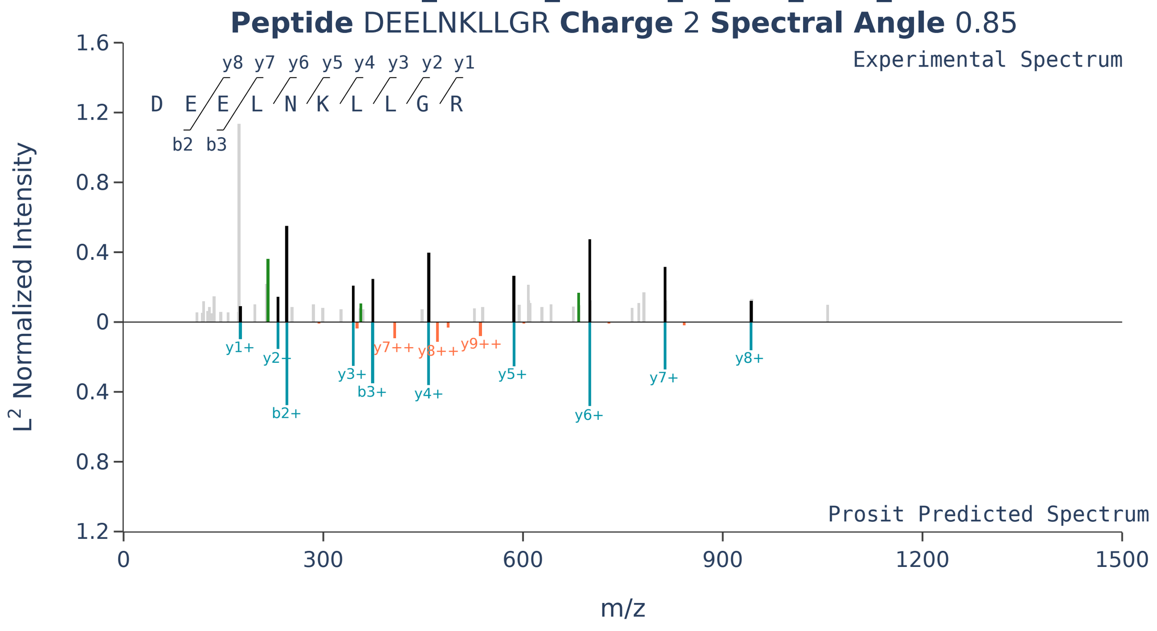


**Source** HRoetschke_291222_060123_Fu_H2A_b2R2_4h **Scan** 2421
**Peptide** VHRLLRKGNYAER **Charge** 3 **Spectral Angle** 0.82

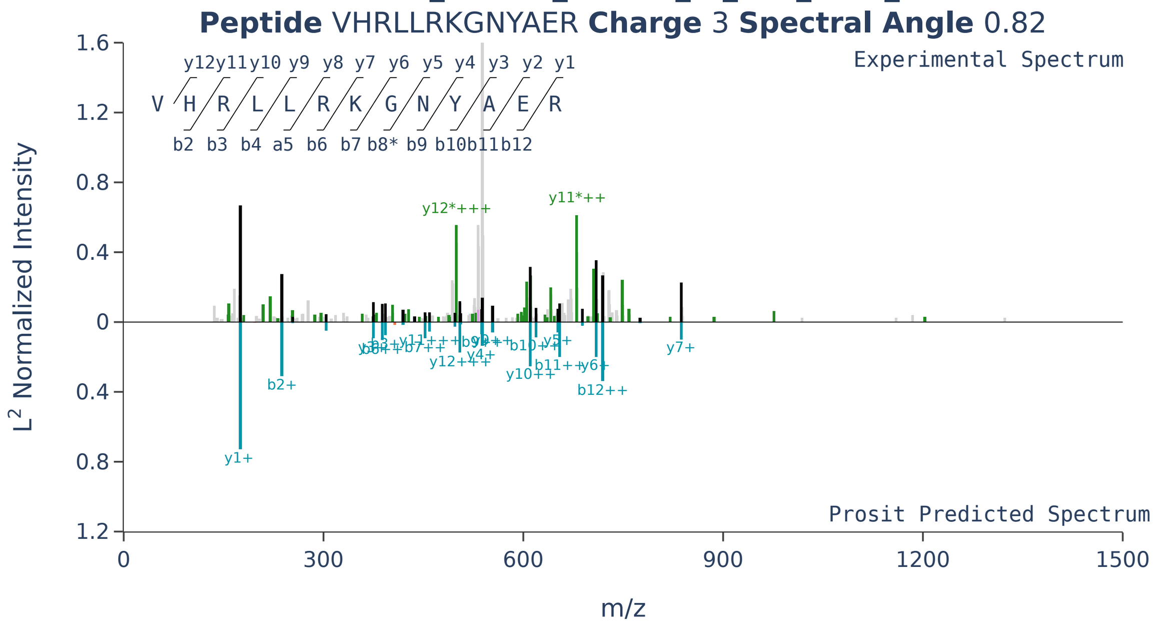


**Source** HRoetschke_291222_060123_Fu_H2A_b2R2_1h **Scan** 14345
**Peptide** AAVLEYLTA **Charge** 2 **Spectral Angle** 0.89

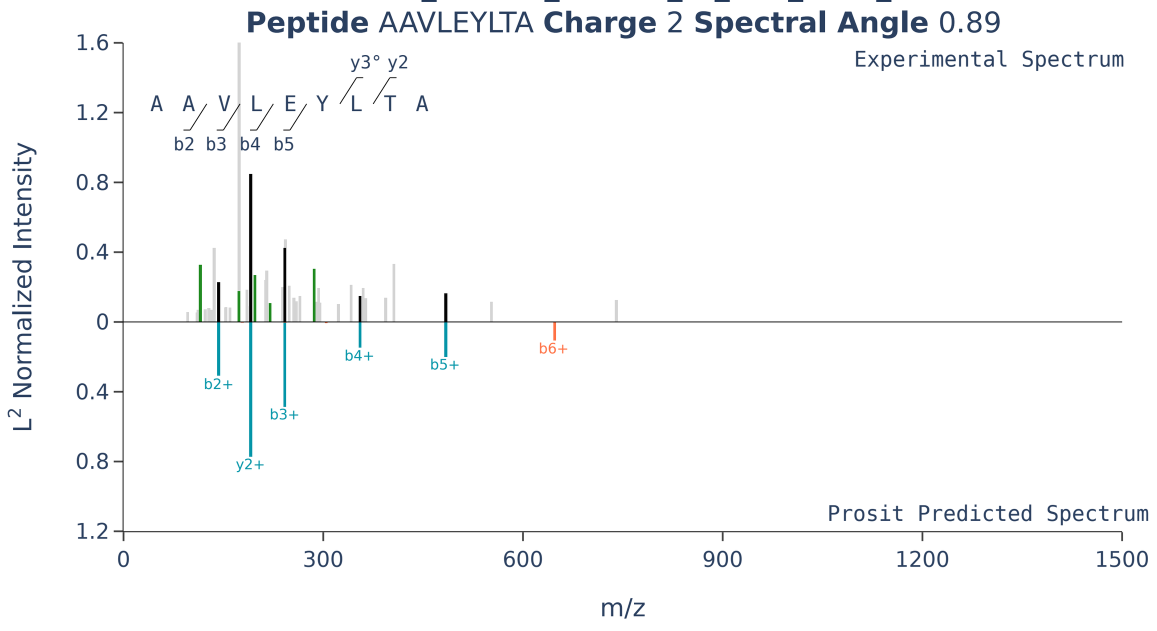


**Source** HRoetschke_291222_060123_Fu_H2A_b1R1_4h **Scan** 14333
**Peptide** EELNKLLGRV **Charge** 2 **Spectral Angle** 0.91

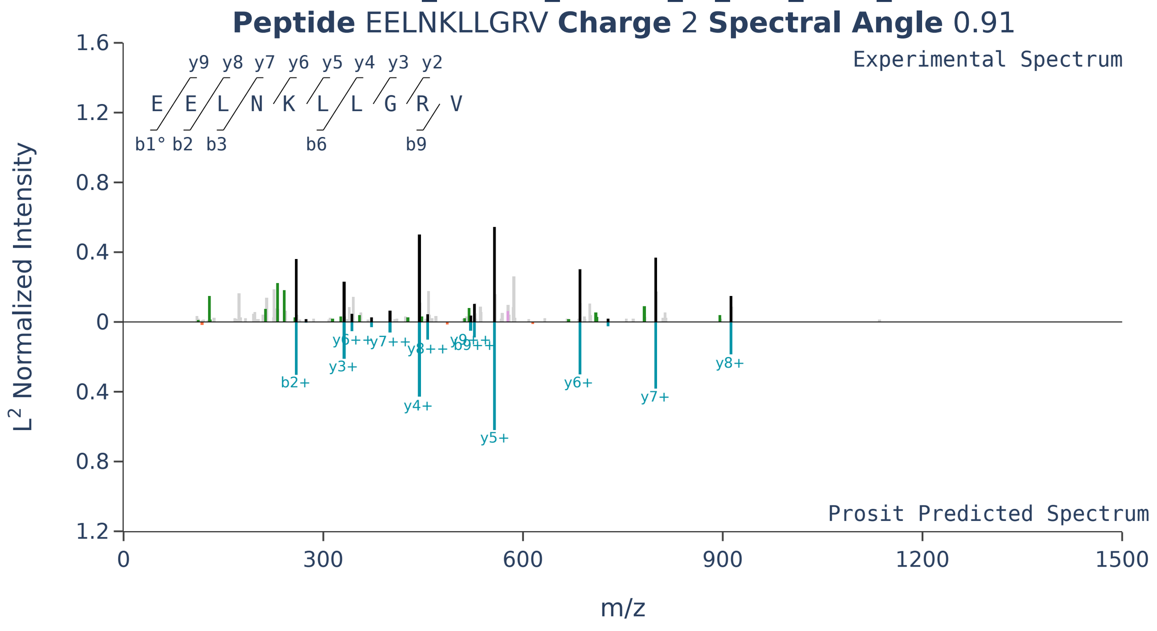


**Source** HRoetschke_291222_060123_Fu_H2A_b2R1_1h **Scan** 12149
**Peptide** AQGGVLPNL **Charge** 2 **Spectral Angle** 0.79

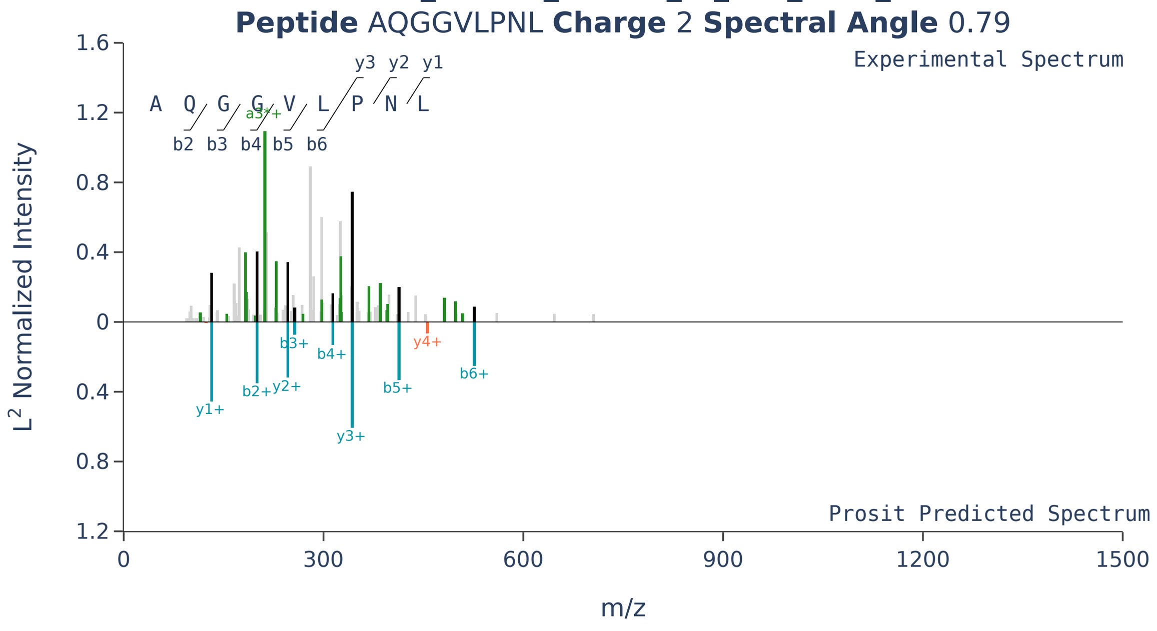

**Source** HRoetschke_291222_060123_Fu_H2A_b2R1_24h **Scan** 12047
**Peptide** RNDEELNKLL **Charge** 2 **Spectral Angle** 0.89

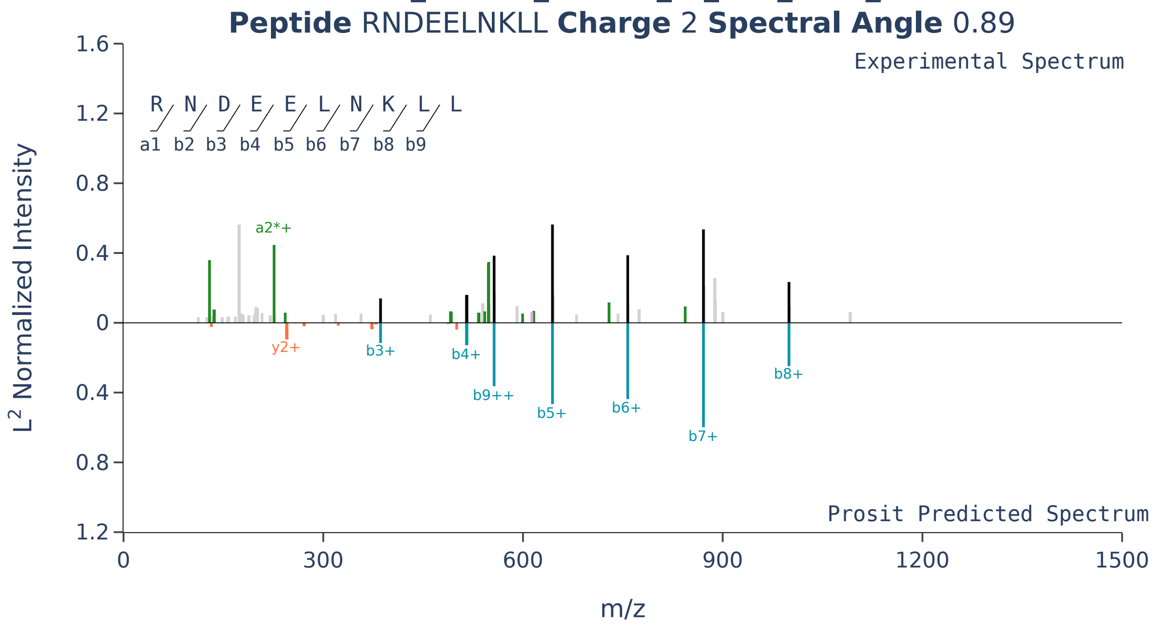


**Source** HRoetschke_291222_060123_Fu_H2A_b1R1_1h **Scan** 2444
**Peptide** SVLLPKKTESSKSAKSK **Charge** 3 **Spectral Angle** 0.87

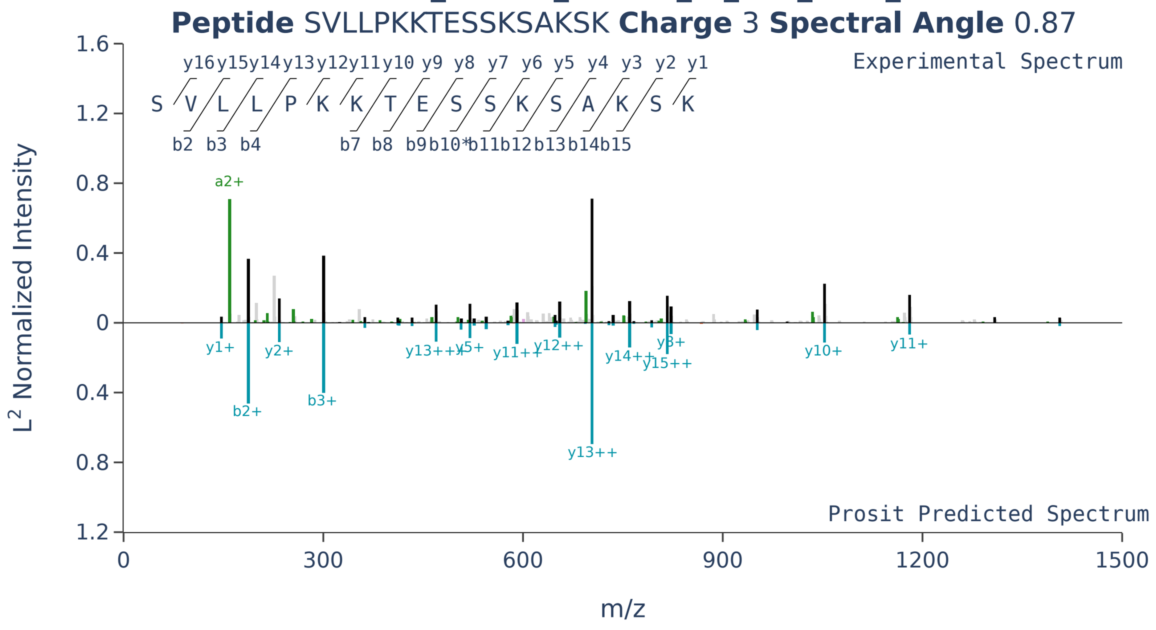


**Source** HRoetschke_291222_060123_Fu_H2A_b1R2_1h **Scan** 9131
**Peptide** QLAVRNDEELNKL **Charge** 2 **Spectral Angle** 0.9

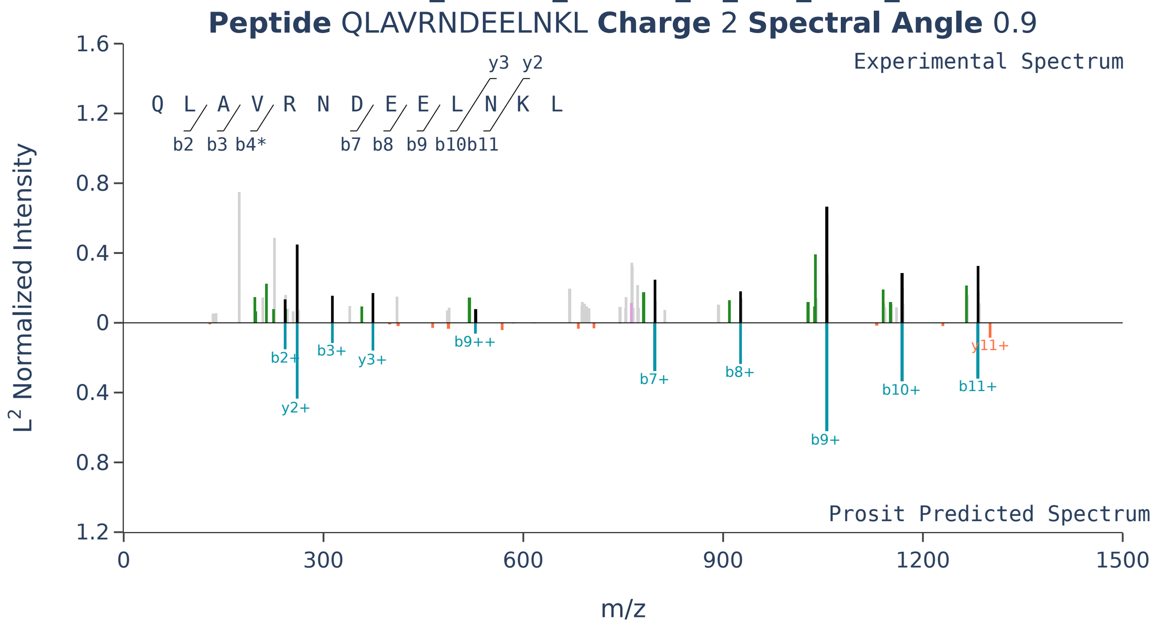


**Source** HRoetschke_291222_060123_Fu_H2A_b1R1_1h **Scan** 8894
**Peptide** SRAGLQF **Charge** 2 **Spectral Angle** 0.93

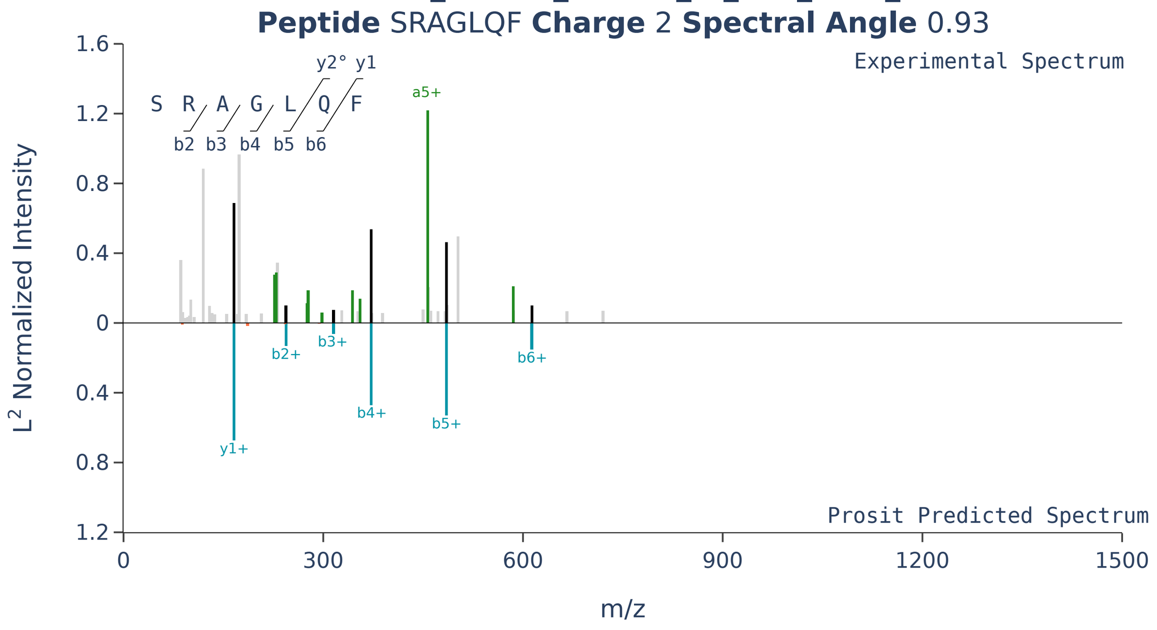


**Source** HRoetschke_291222_060123_Fu_H2A_b2R1_4h **Scan** 13225
**Peptide** AERVGAGAPVYL **Charge** 2 **Spectral Angle** 0.86

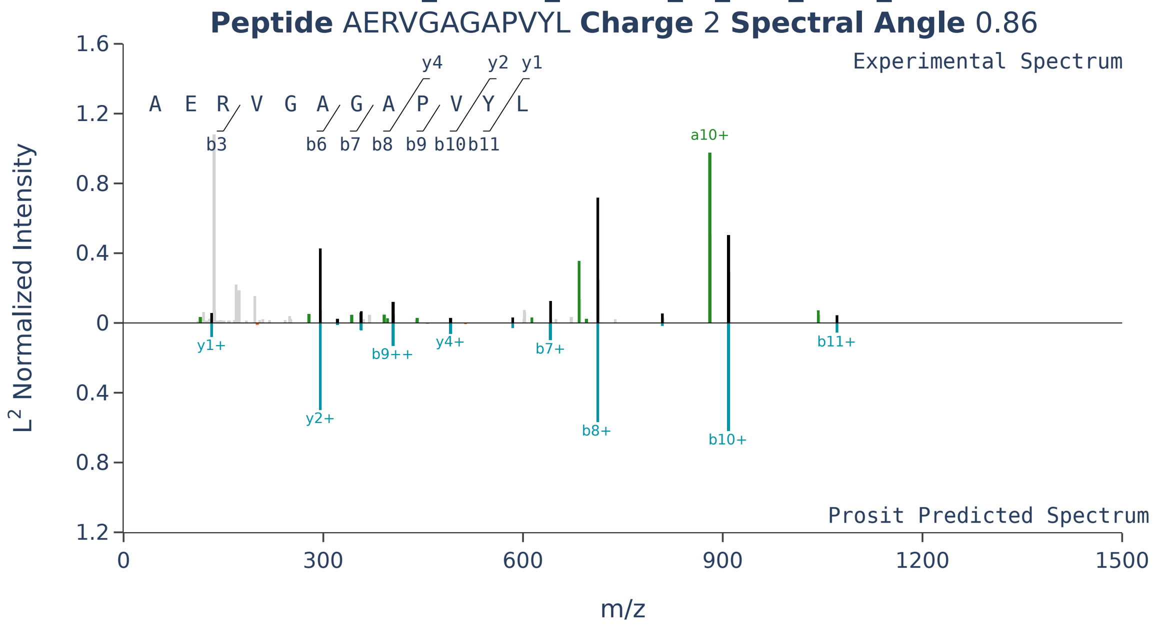


**Source** HRoetschke_291222_060123_Fu_H2A_b1R1_4h **Scan** 14125
**Peptide** SSRAGLQFPVGRV **Charge** 3 **Spectral Angle** 0.89

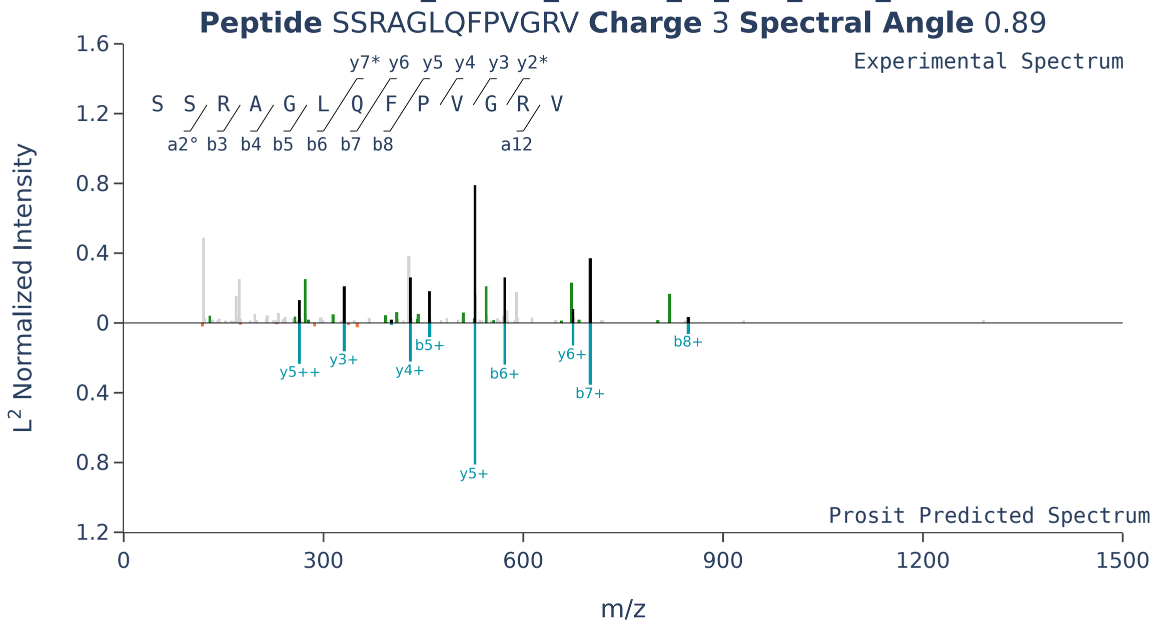


**Source** HRoetschke_291222_060123_Fu_H2A_b1R2_4h **Scan** 10767
**Peptide** SRAGLQFPVGRVH **Charge** 4 **Spectral Angle** 0.77

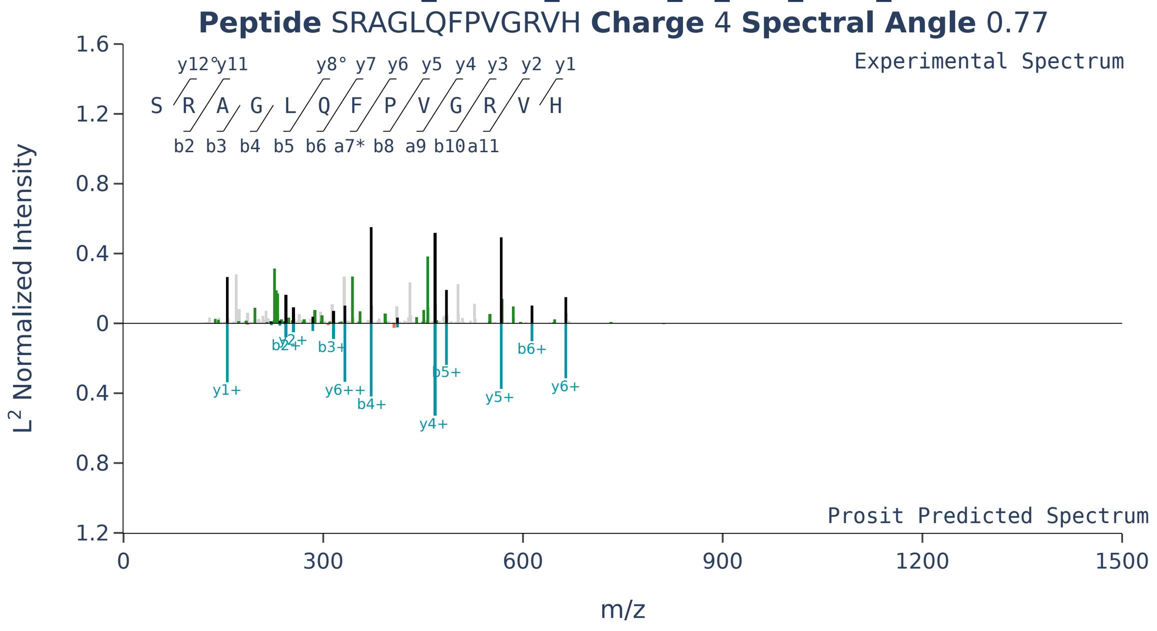


**Source** HRoetschke_291222_060123_Fu_H2A_b2R2_24h **Scan** 3739
**Peptide** VLLPKKTESSKSA **Charge** 2 **Spectral Angle** 0.92

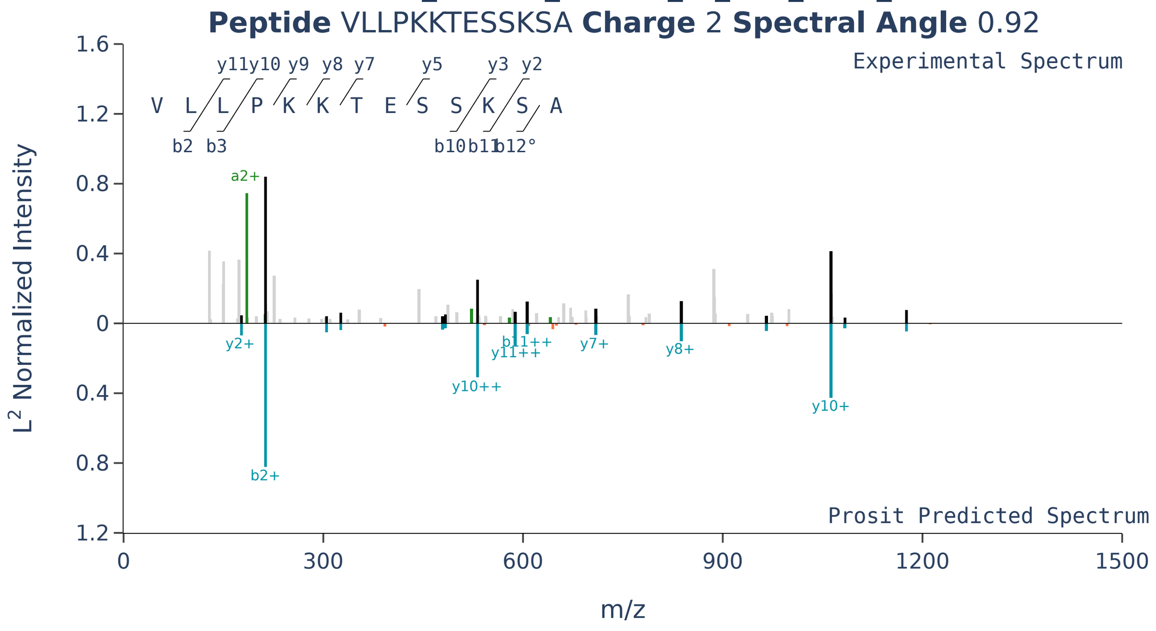


**Source** HRoetschke_291222_060123_Fu_H2A_b1R2_4h **Scan** 17835
**Peptide** AVRNDEELNKLLGRVTL **Charge** 3 **Spectral Angle** 0.89

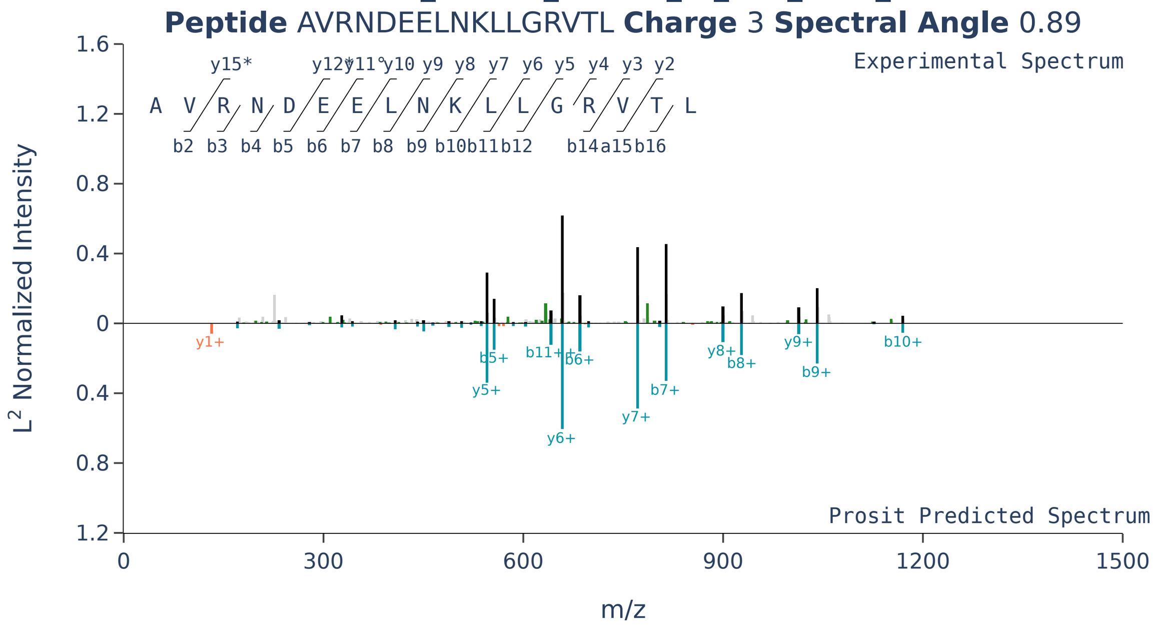


**Source** HRoetschke_291222_060123_Fu_H2A_b1R1_24h **Scan** 10592
**Peptide** LLELAGNAARD **Charge** 2 **Spectral Angle** 0.91

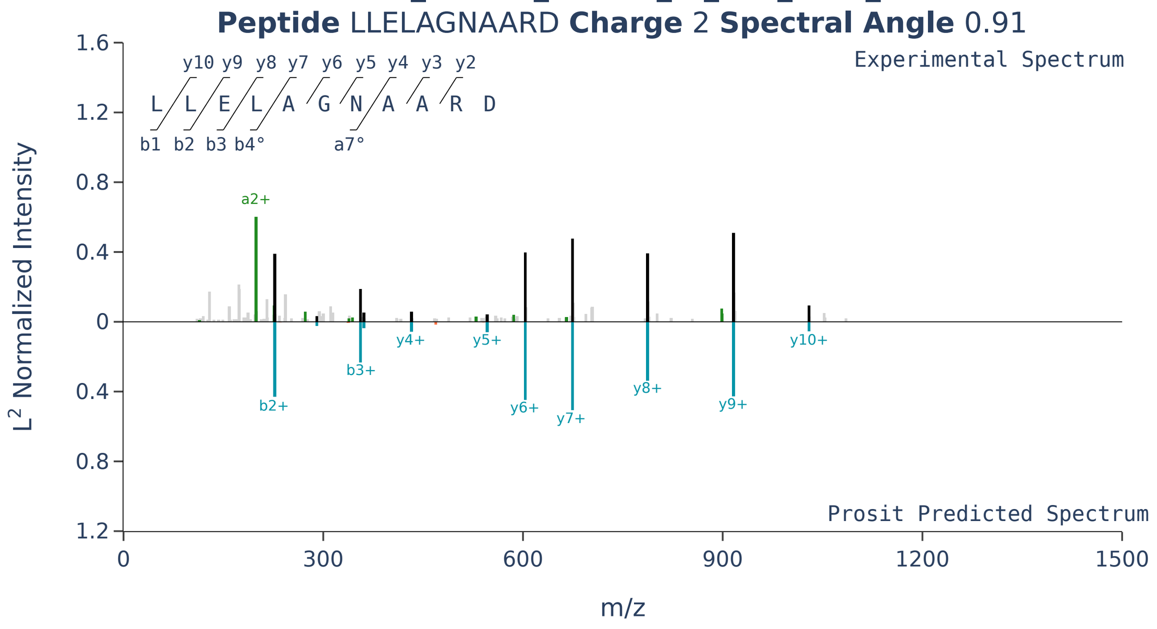


**Source** HRoetschke_291222_060123_Fu_H2A_b1R1_24h **Scan** 10196
**Peptide** SSRAGLQF **Charge** 2 **Spectral Angle** 0.86

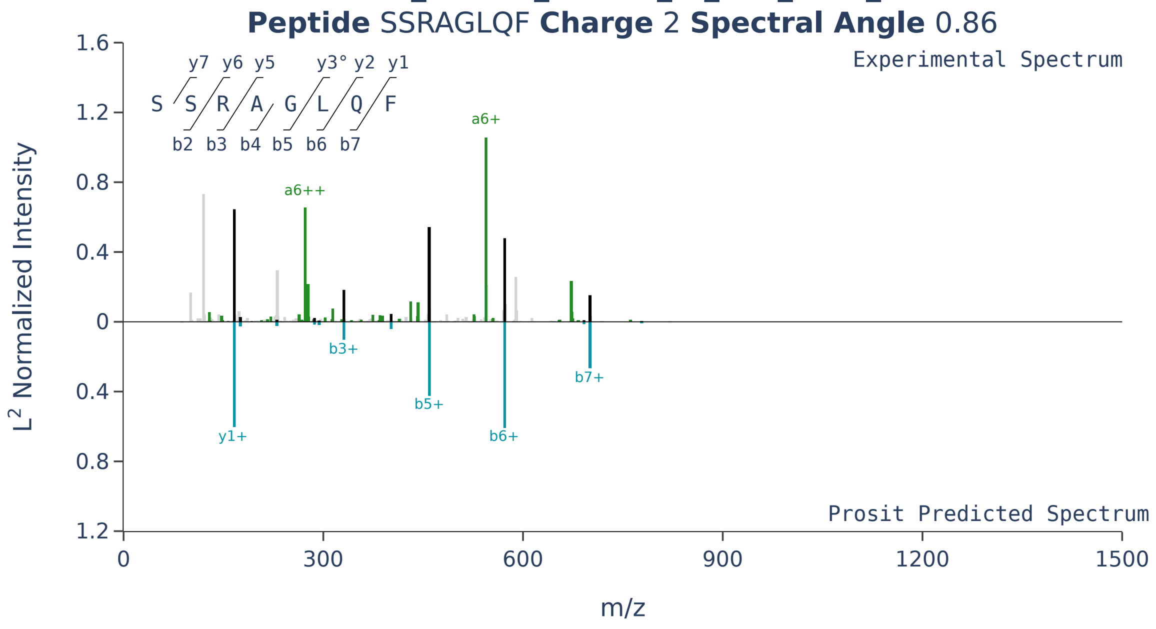


**Source** HRoetschke_291222_060123_Fu_H2A_b2R2_24h **Scan** 3422
**Peptide** LAGNAARDNKKTRIIPR **Charge** 4 **Spectral Angle** 0.92

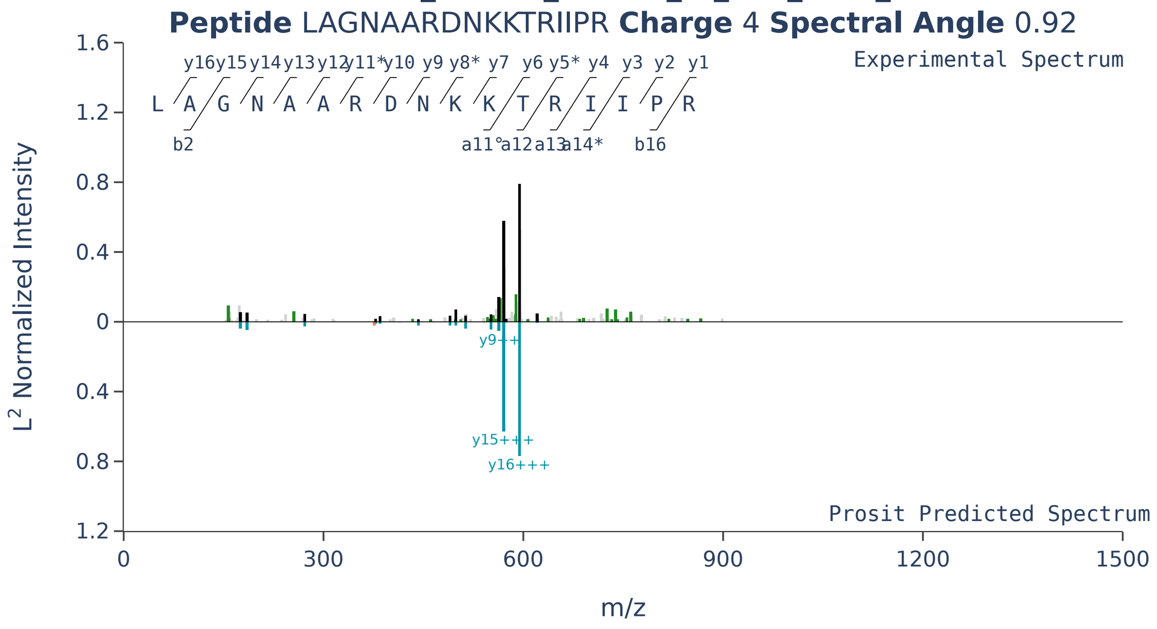


**Source** HRoetschke_291222_060123_Fu_H2A_b1R2_1h **Scan** 3799
**Peptide** LAGNAARDNKKTRLL **Charge** 3 **Spectral Angle** 0.95

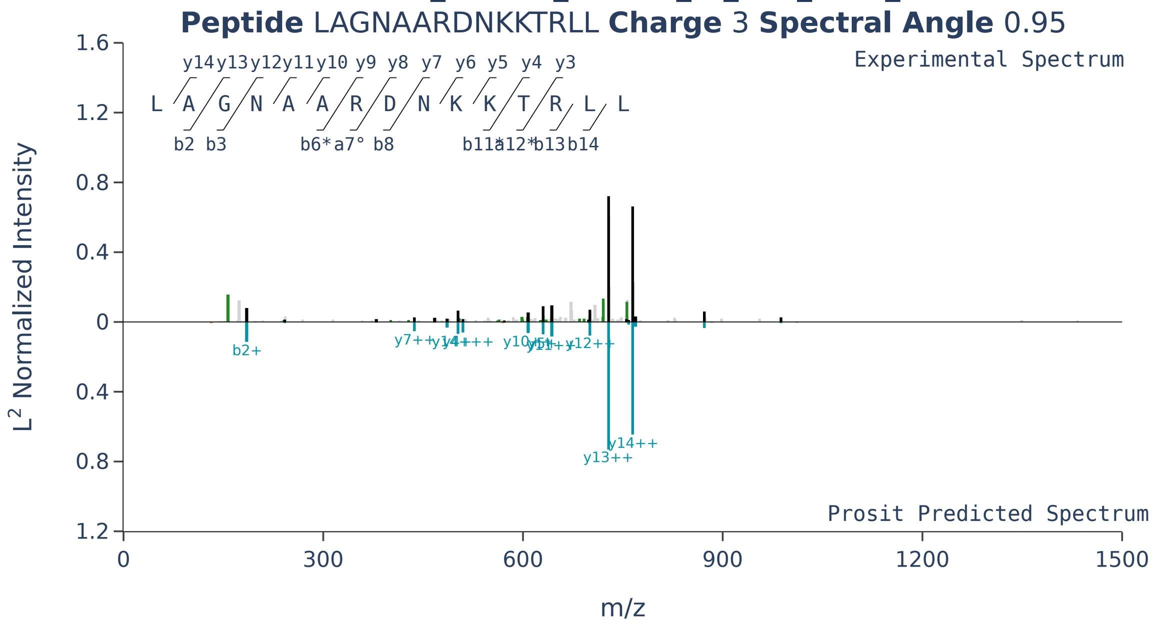


**Source** HRoetschke_291222_060123_Fu_H2A_b2R2_24h **Scan** 6580
**Peptide** RNDEELNKL **Charge** 3 **Spectral Angle** 0.88

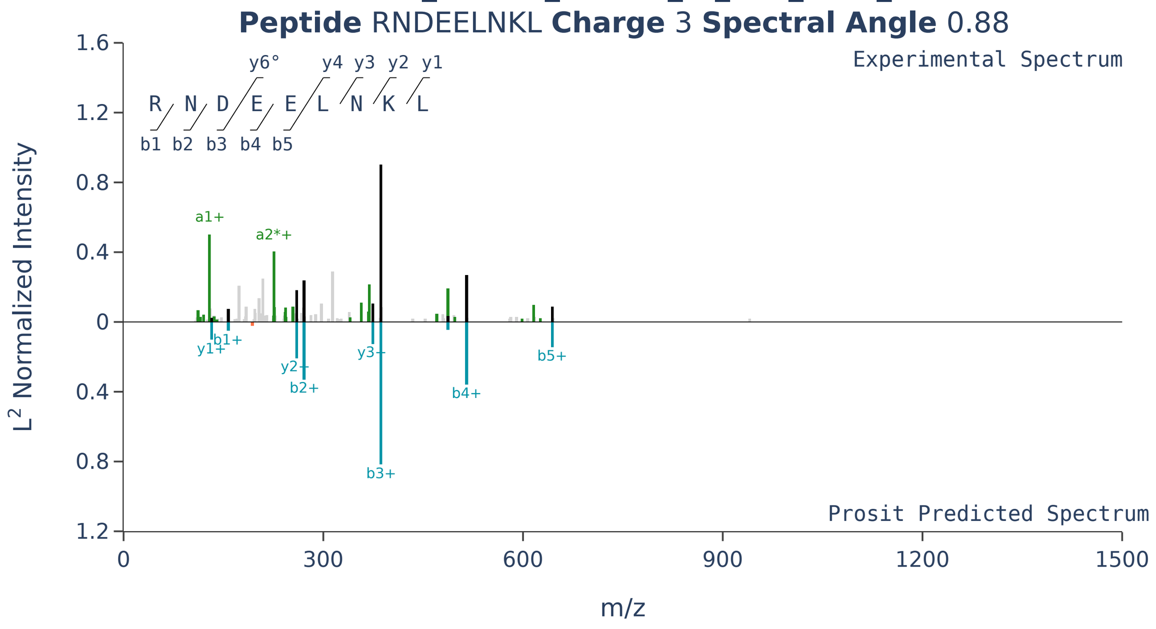


**Source** HRoetschke_291222_060123_Fu_H2A_b1R1_4h **Scan** 12107
**Peptide** RLLRKGNYAERVGAGAPVYLAA **Charge** 4 **Spectral Angle** 0.83

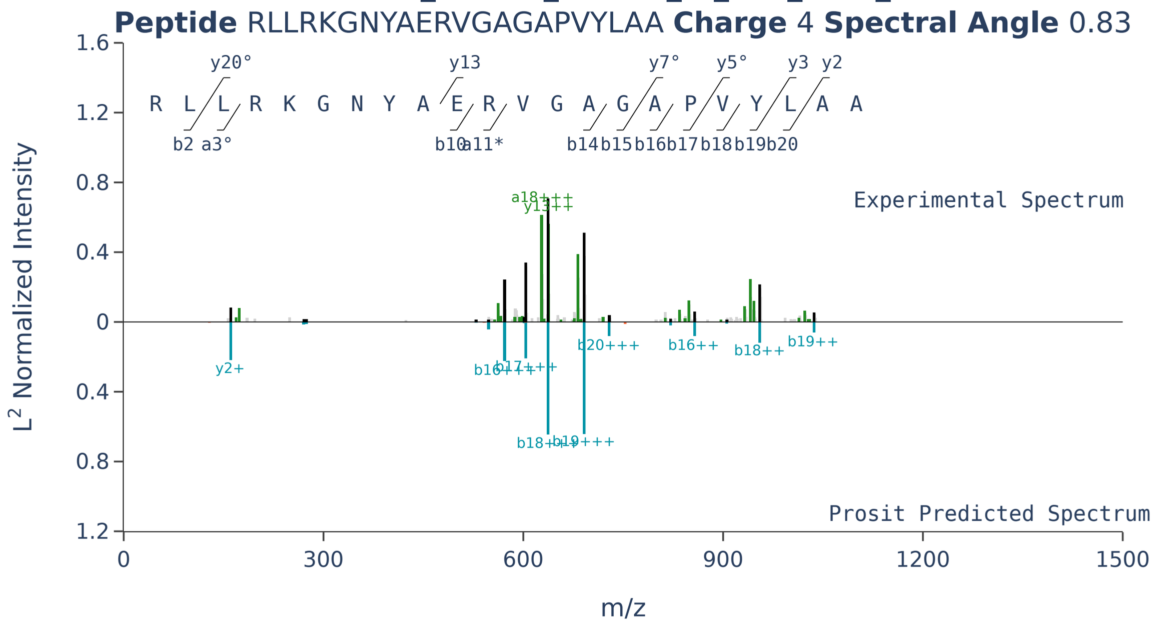

**Source** HRoetschke_291222_060123_Fu_H2A_b2R1_24h **Scan** 15085
**Peptide** AQGGVLPNI **Charge** 2 **Spectral Angle** 0.87

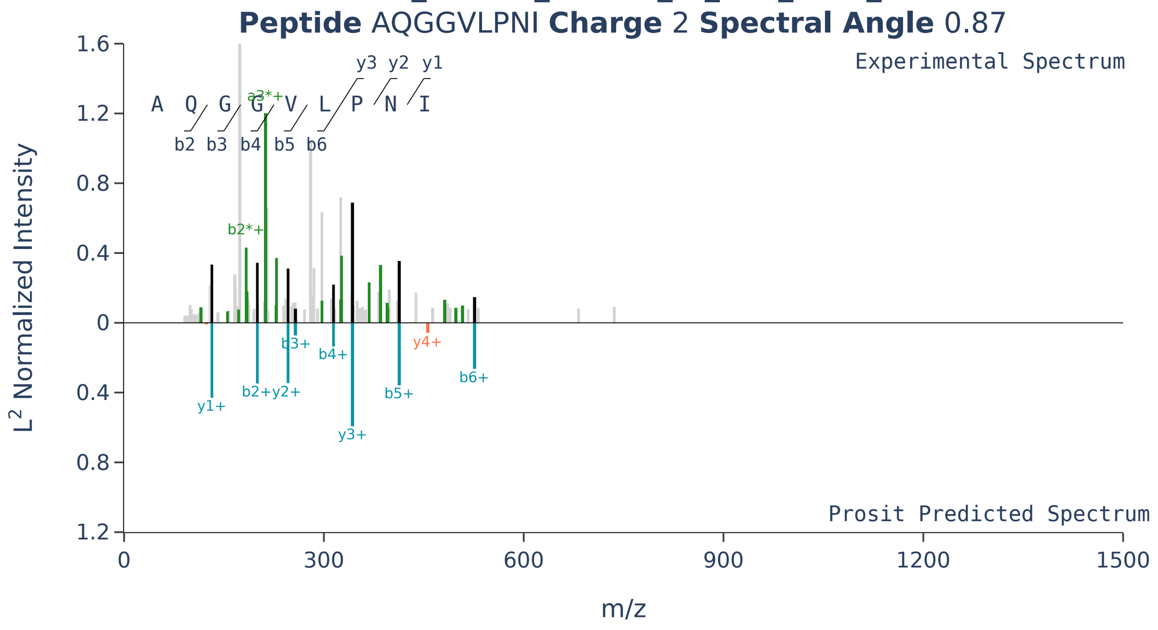


**Source** HRoetschke_291222_060123_Fu_H2A_b1R1_1h **Scan** 3191
**Peptide** RVHRLLRKGNYAERVG **Charge** 5 **Spectral Angle** 0.86

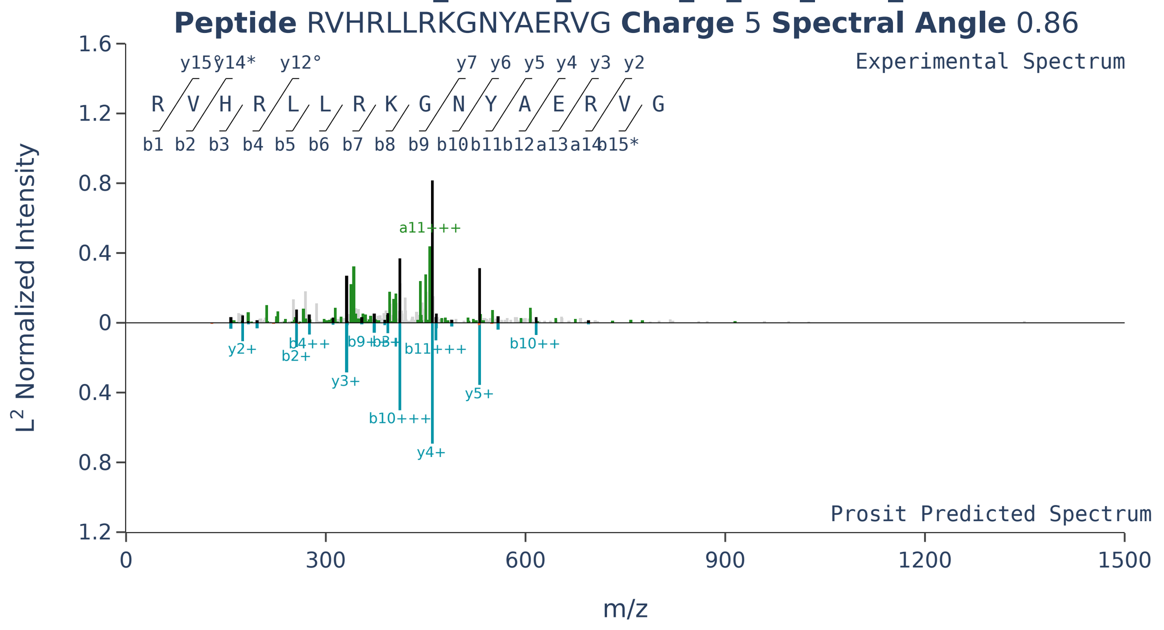


**Source** HRoetschke_291222_060123_Fu_H2A_b1R2_2h **Scan** 3857
**Peptide** GNYAERVG **Charge** 2 **Spectral Angle** 0.88

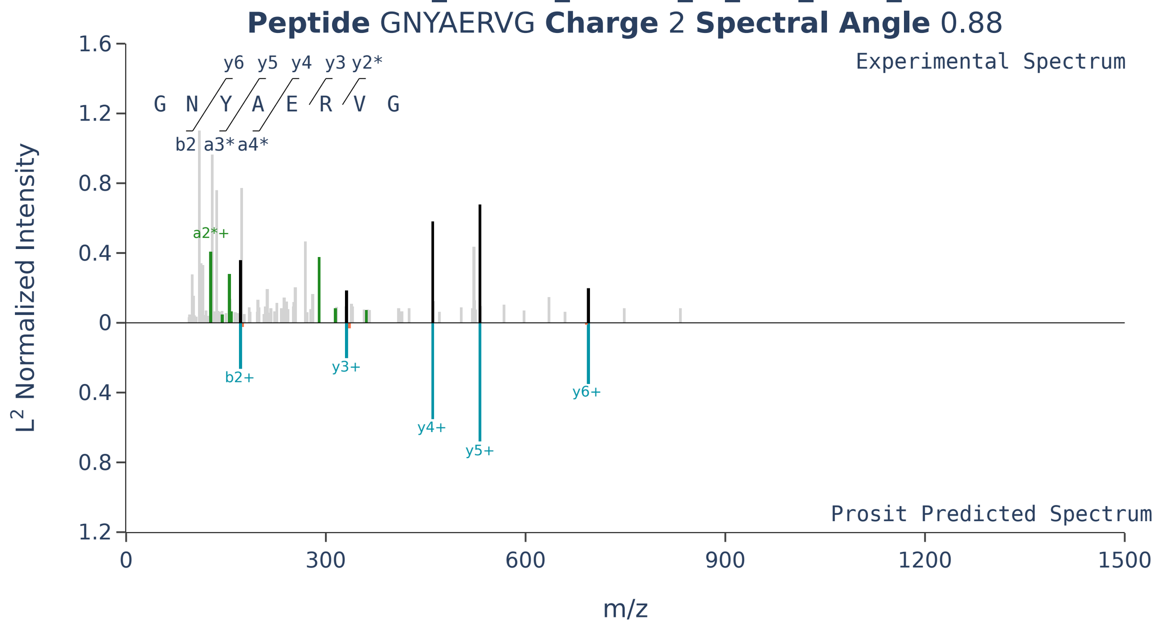


**Source** HRoetschke_291222_060123_Fu_H2A_b1R2_4h **Scan** 15746
**Peptide** GRVTIAQGGVLPNLQ **Charge** 2 **Spectral Angle** 0.89

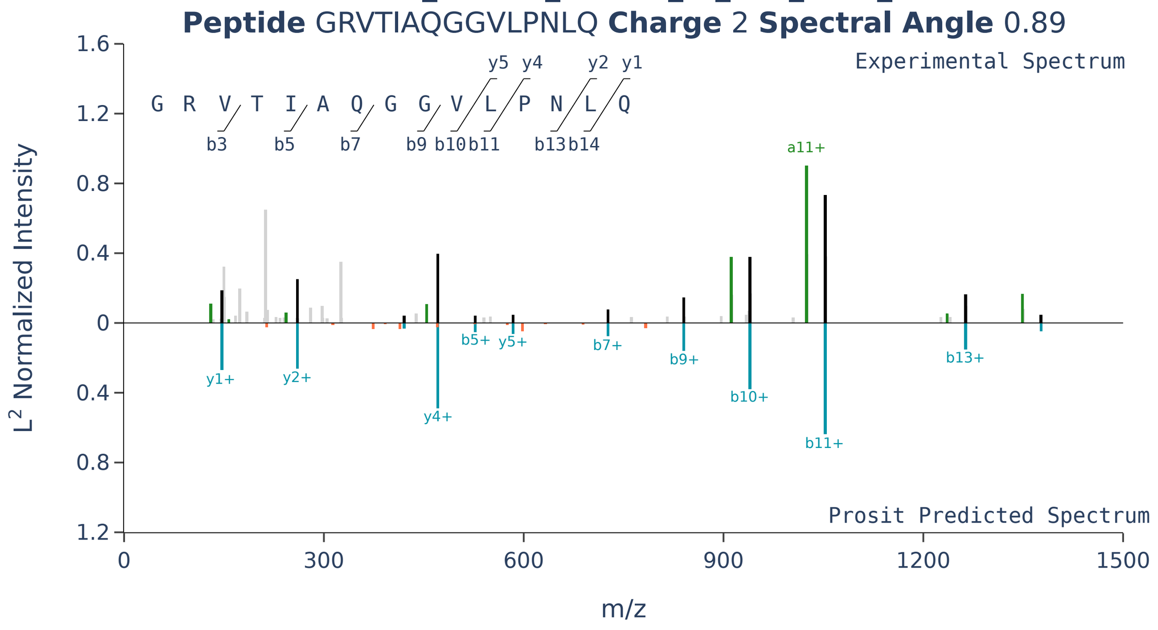


**Source** HRoetschke_291222_060123_Fu_H2A_b1R1_2h **Scan** 13013
**Peptide** QGGVLPNIQS **Charge** 2 **Spectral Angle** 0.86

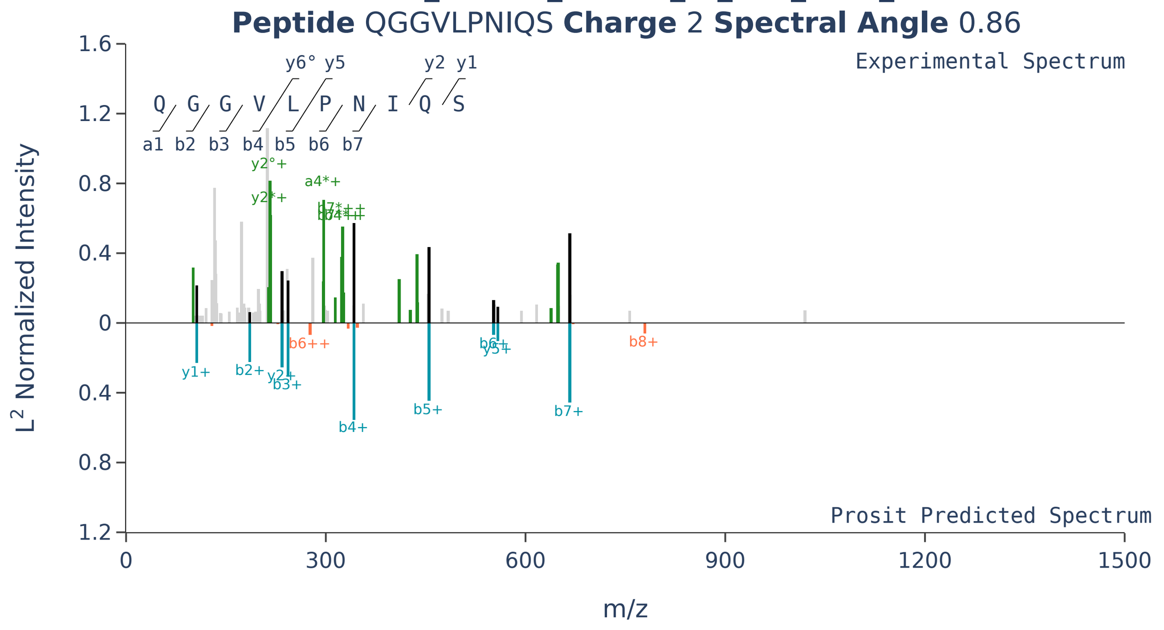


**Source** HRoetschke_291222_060123_Fu_H2A_b1R2_2h **Scan** 4367
**Peptide** GNYAERV **Charge** 2 **Spectral Angle** 0.91

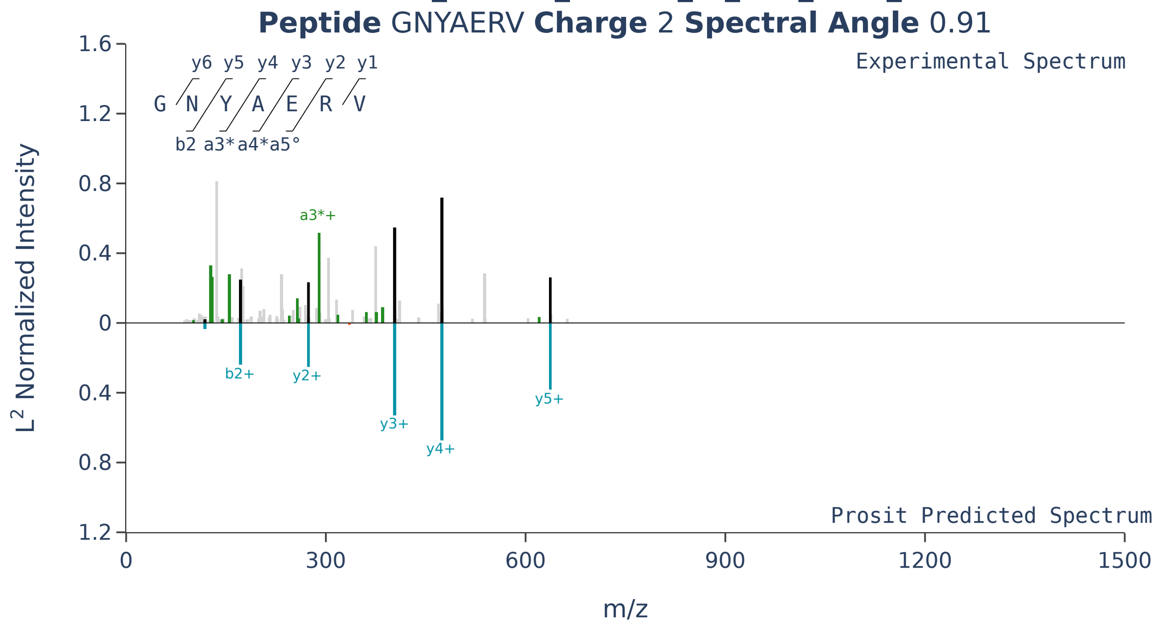


**Source** HRoetschke_291222_060123_Fu_H2A_b1R2_4h **Scan** 13625
**Peptide** AGLQFPVGRVH **Charge** 3 **Spectral Angle** 0.86

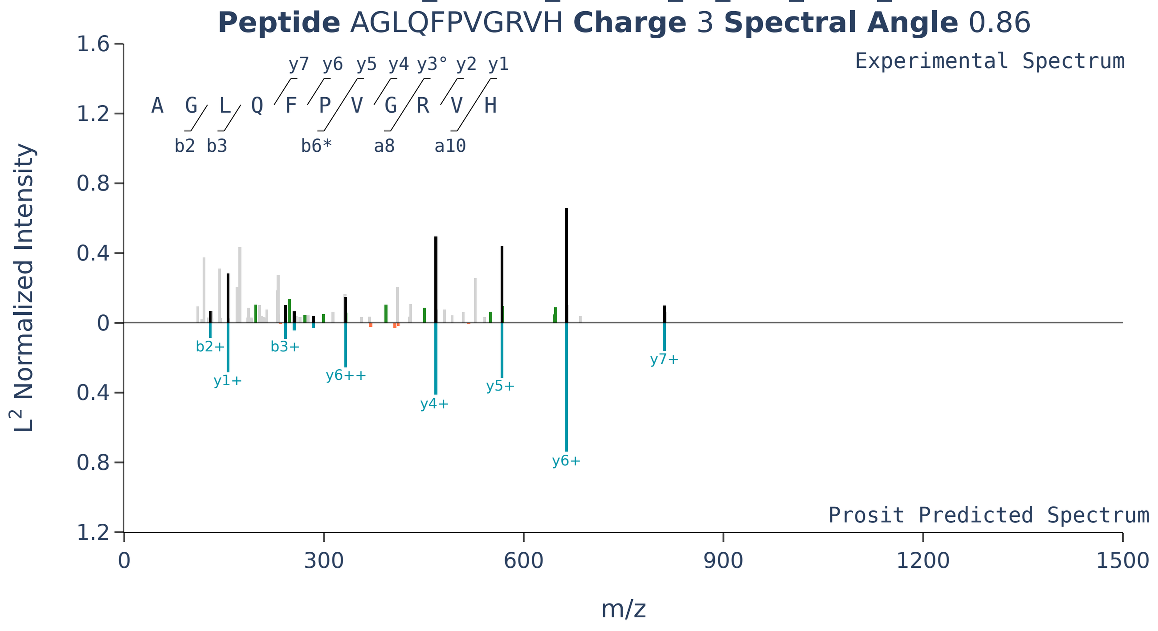


**Source** HRoetschke_291222_060123_Fu_H2A_b1R1_1h **Scan** 12675
**Peptide** NDEELNKLLG **Charge** 2 **Spectral Angle** 0.81

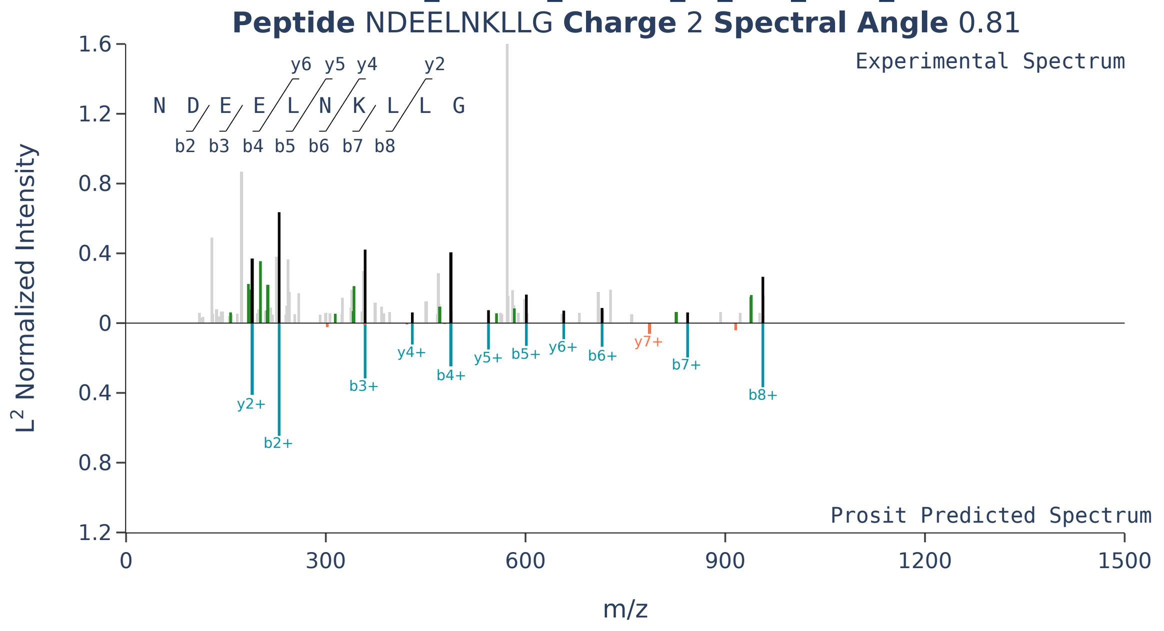


**Source** HRoetschke_291222_060123_Fu_H2A_b1R1_4h **Scan** 17622
**Peptide** AVRNDEELNKLLGRVTLA **Charge** 3 **Spectral Angle** 0.85

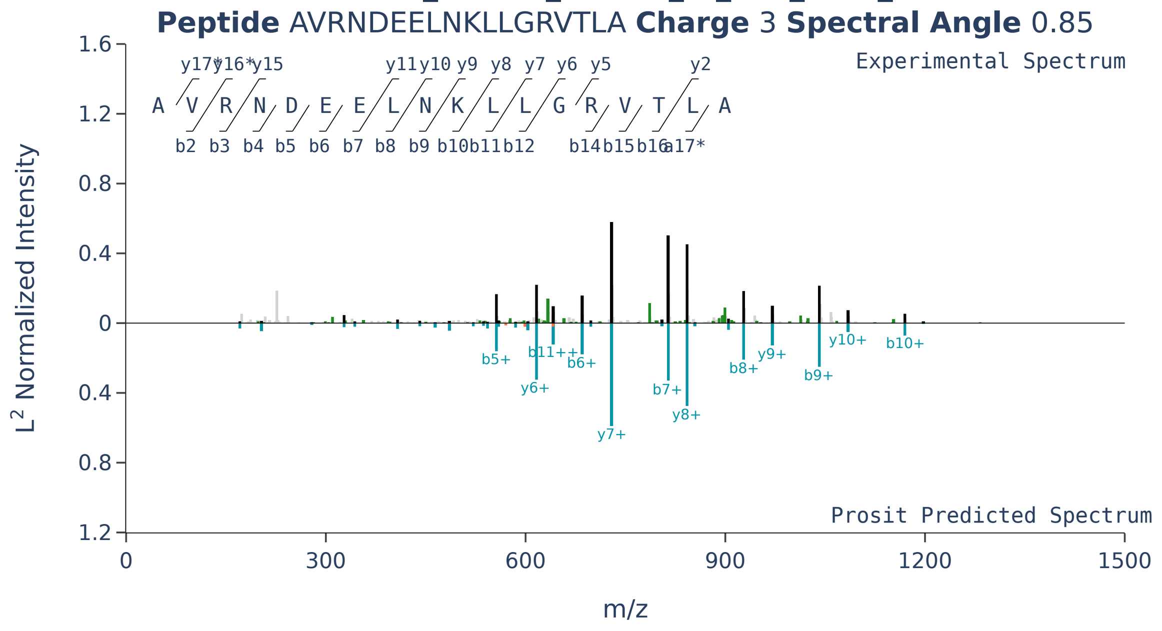


**Source** HRoetschke_291222_060123_Fu_H2A_b2R2_24h **Scan** 8569
**Peptide** LELAGNAARDNKKTRLL **Charge** 3 **Spectral Angle** 0.89

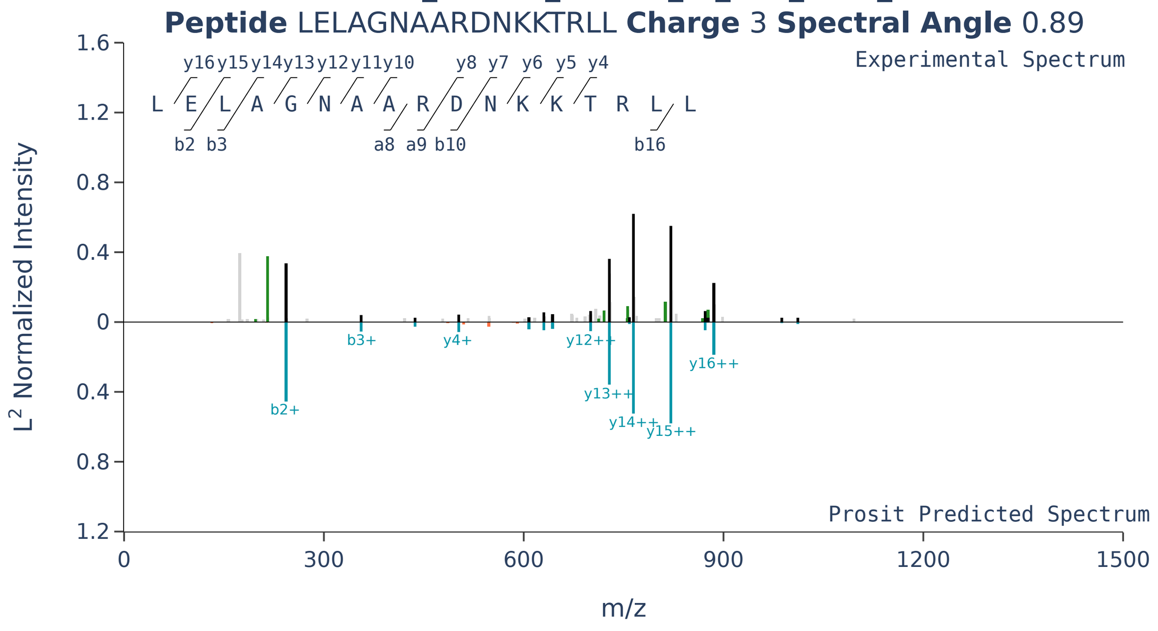


**Source** HRoetschke_291222_060123_Fu_H2A_b2R1_24h **Scan** 9311
**Peptide** TRSSRAGLQFPVGR **Charge** 4 **Spectral Angle** 0.79

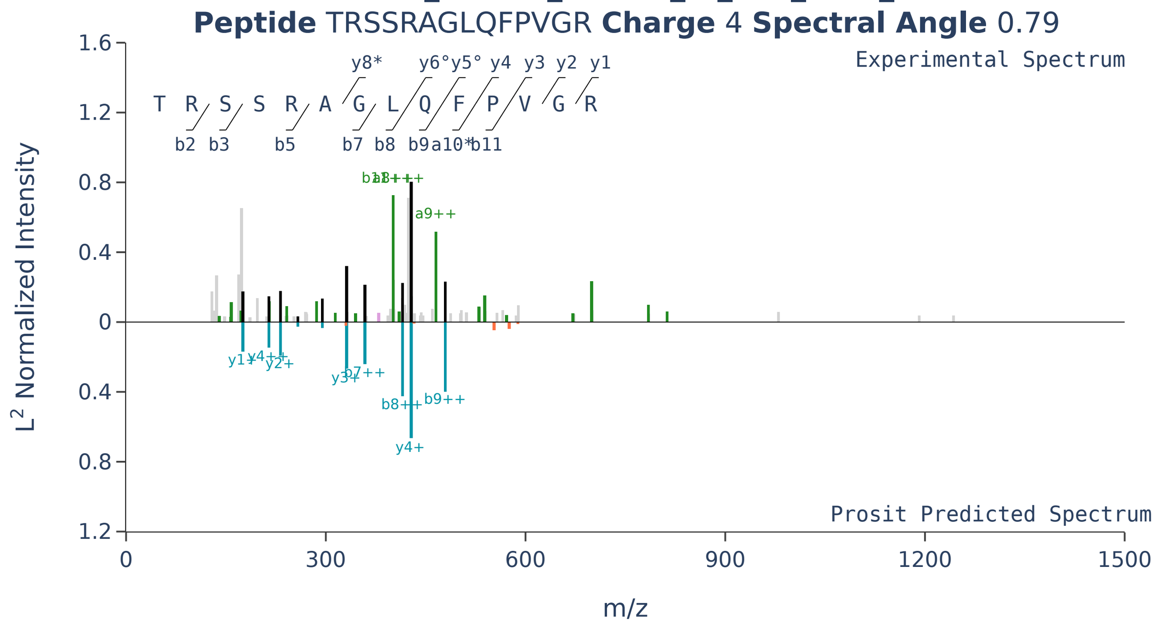


**Source** HRoetschke_291222_060123_Fu_H2A_b1R1_4h **Scan** 16601
**Peptide** EELNKLLGRVTIAQG **Charge** 2 **Spectral Angle** 0.88

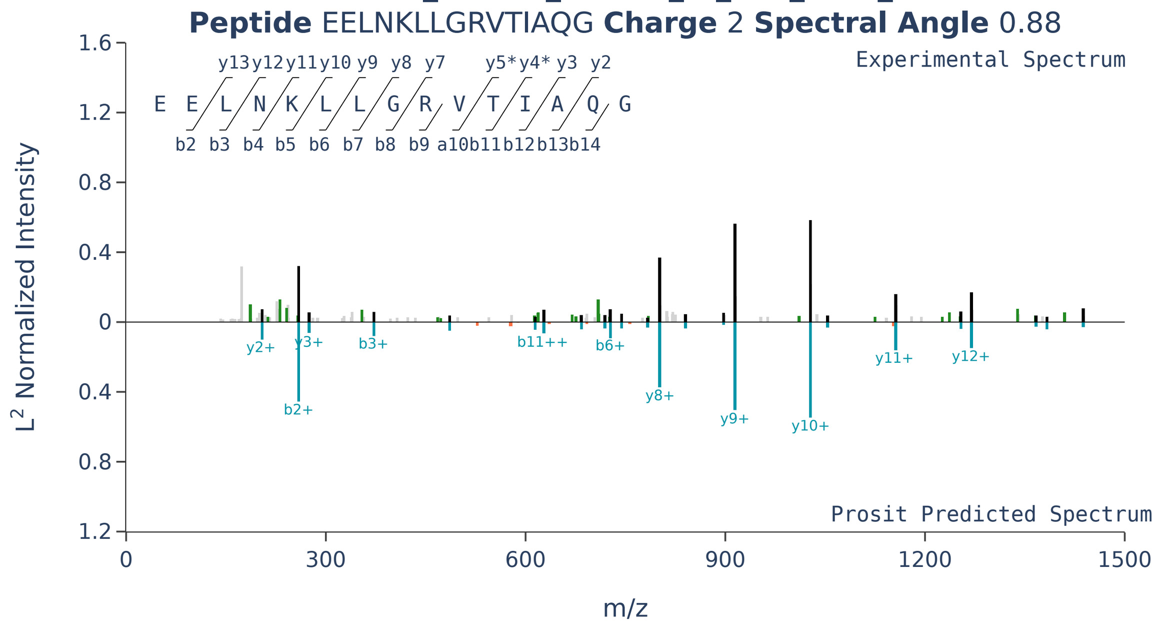


**Source** HRoetschke_291222_060123_Fu_H2A_b2R1_24h **Scan** 16302
**Peptide** VTIAQGGVLPNLQ **Charge** 2 **Spectral Angle** 0.89

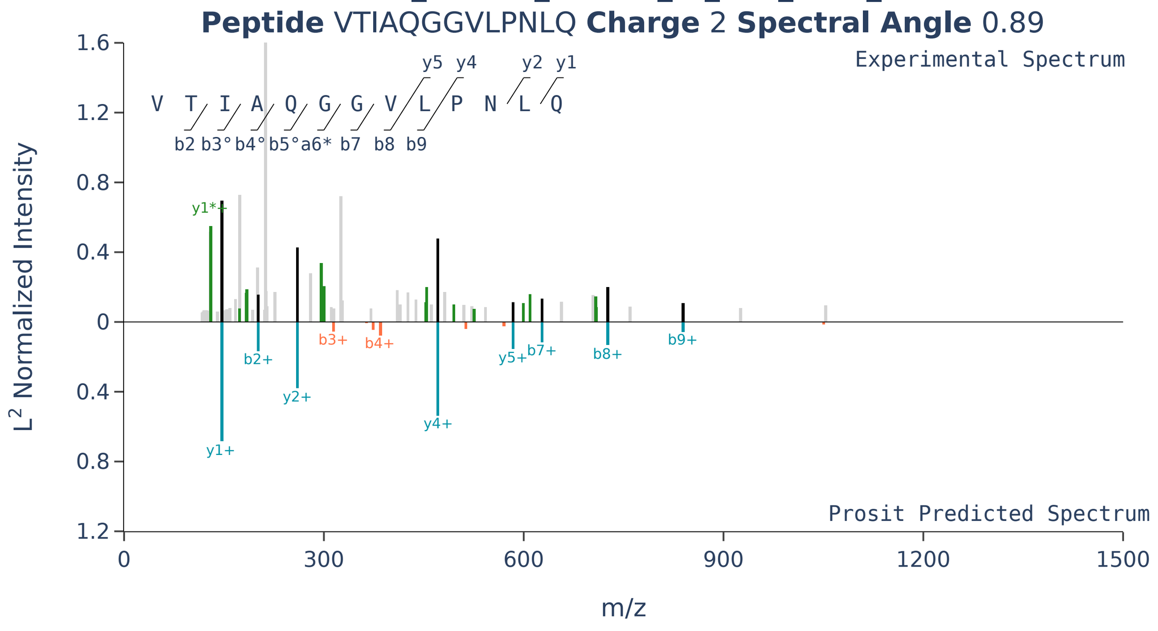


**Source** HRoetschke_291222_060123_Fu_H2A_b1R1_1h **Scan** 2807
**Peptide** RLLRKGNYAE **Charge** 3 **Spectral Angle** 0.79

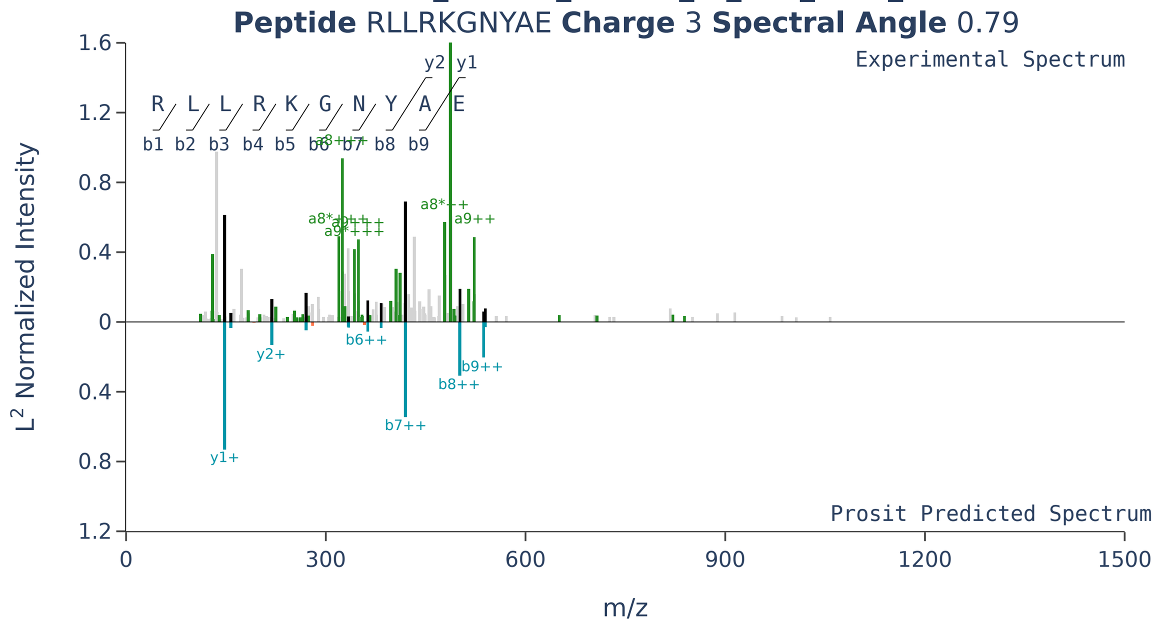


**Source** HRoetschke_291222_060123_Fu_H2A_b1R1_1h **Scan** 5408
**Peptide** LLELAGNAARDNKKTR **Charge** 3 **Spectral Angle** 0.89

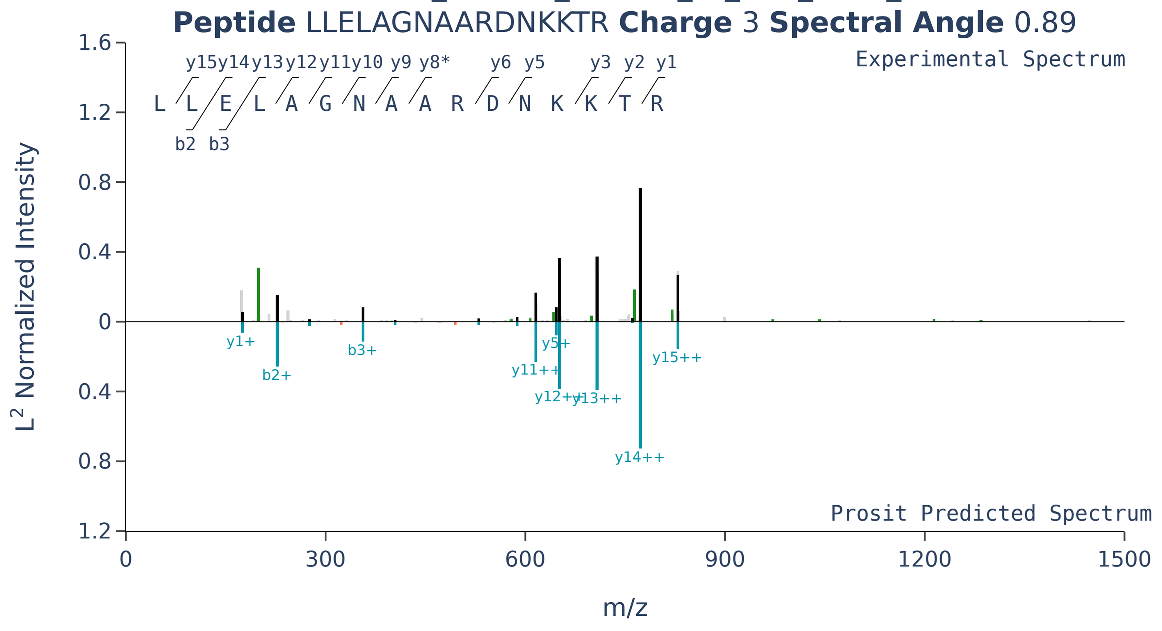

**Source** HRoetschke_291222_060123_Fu_H2A_b2R1_2h **Scan** 4008
**Peptide** RLLRKGNYAERVG **Charge** 3 **Spectral Angle** 0.74

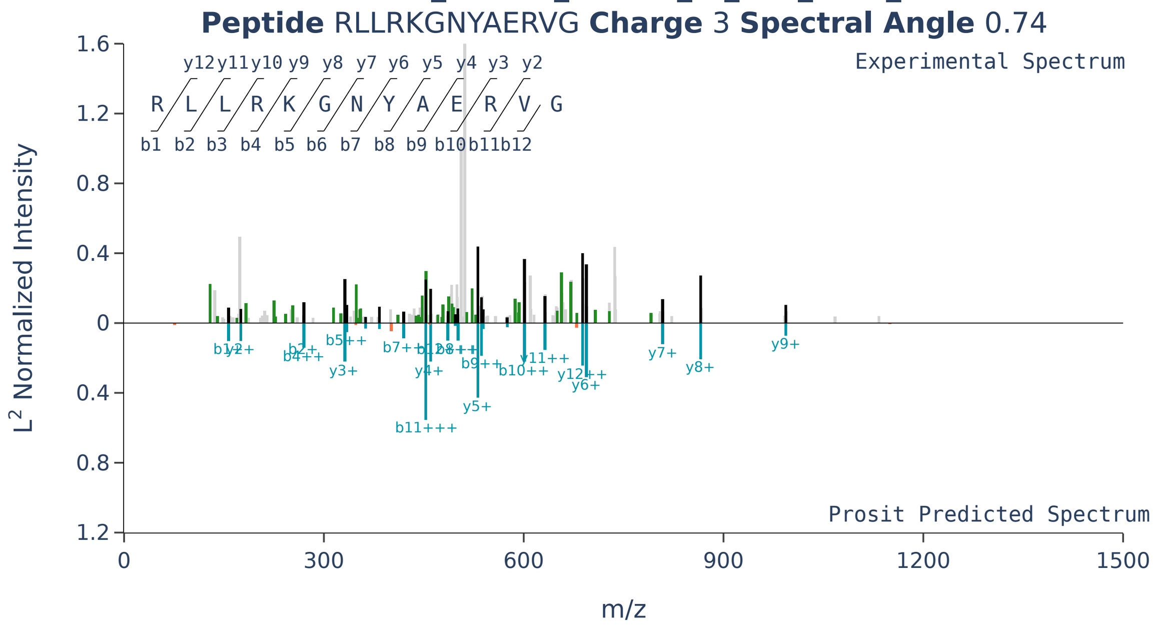


**Source** HRoetschke_291222_060123_Fu_H2A_b1R1_1h **Scan** 3127
**Peptide** SVLLPKKTESSKSAKS **Charge** 3 **Spectral Angle** 0.85

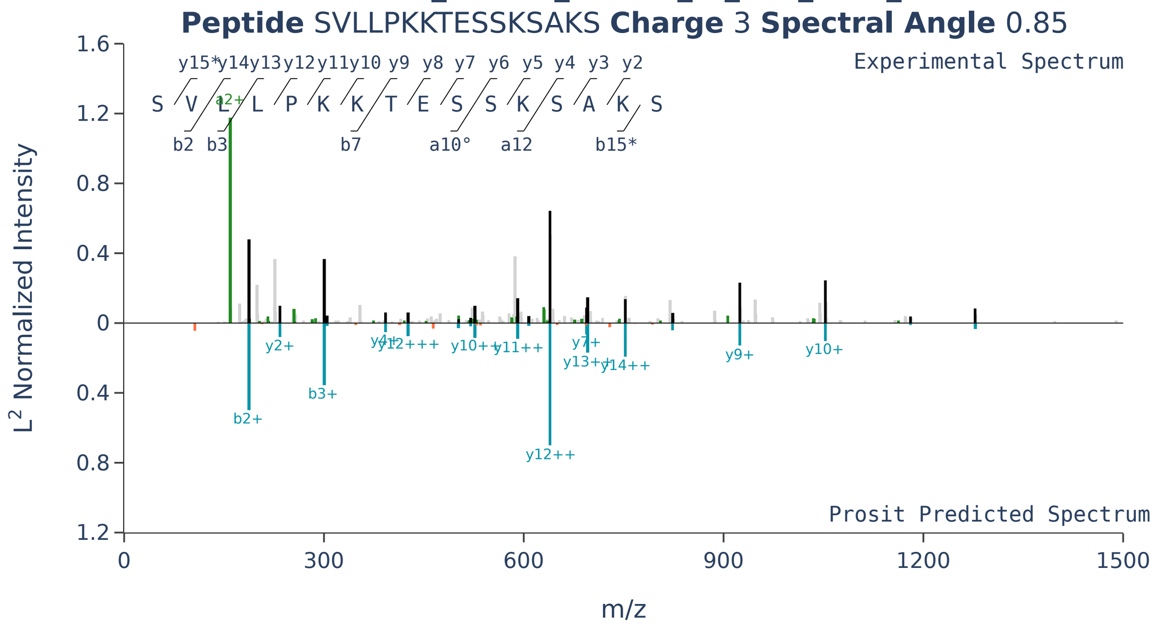


**Source** HRoetschke_291222_060123_Fu_H2A_b1R1_4h **Scan** 11810
**Peptide** KLLGRVTL **Charge** 2 **Spectral Angle** 0.91

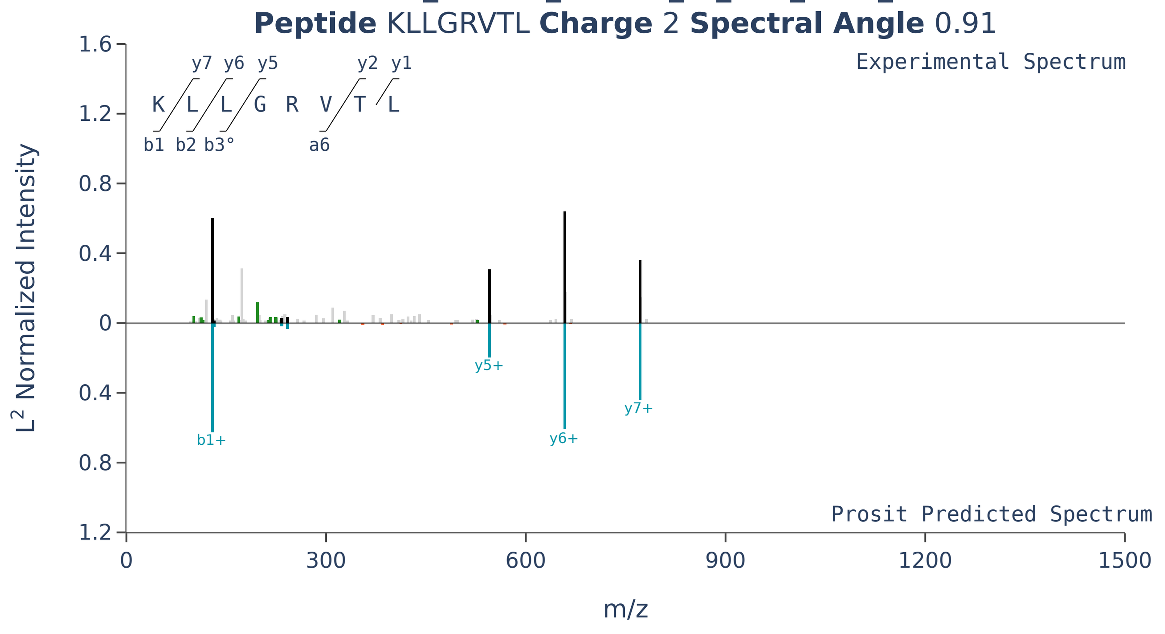


**Source** HRoetschke_291222_060123_Fu_H2A_b1R1_2h **Scan** 17742
**Peptide** EYLTAEILE **Charge** 2 **Spectral Angle** 0.86

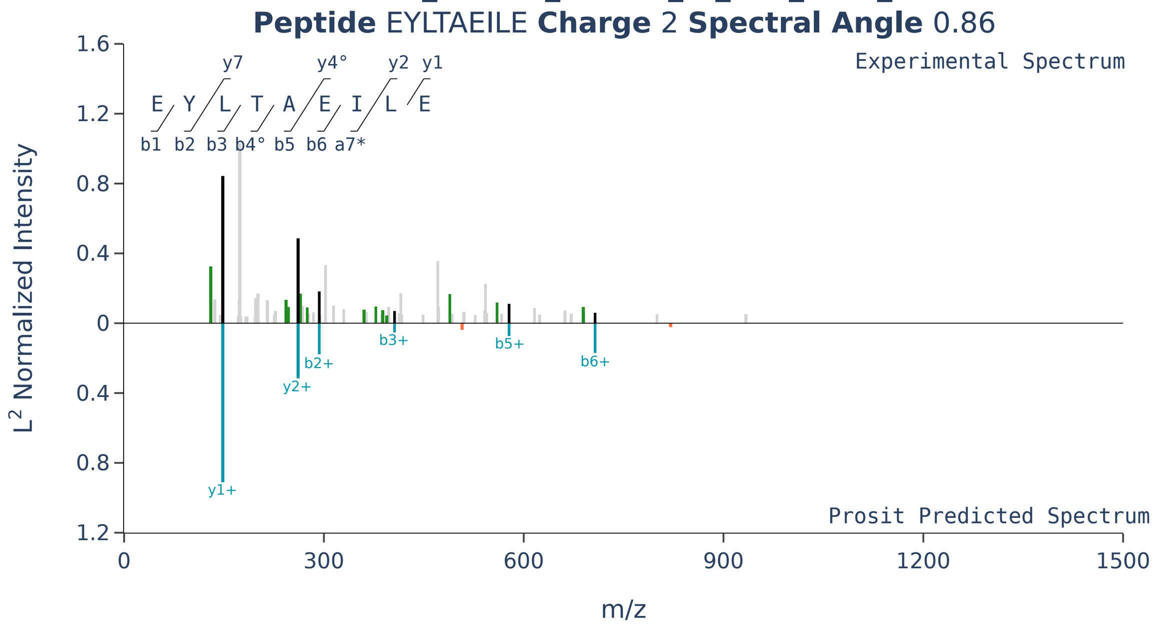


**Source** HRoetschke_291222_060123_Fu_H2A_b1R1_2h **Scan** 12891
**Peptide** AVRNDEELNKLL **Charge** 3 **Spectral Angle** 0.83

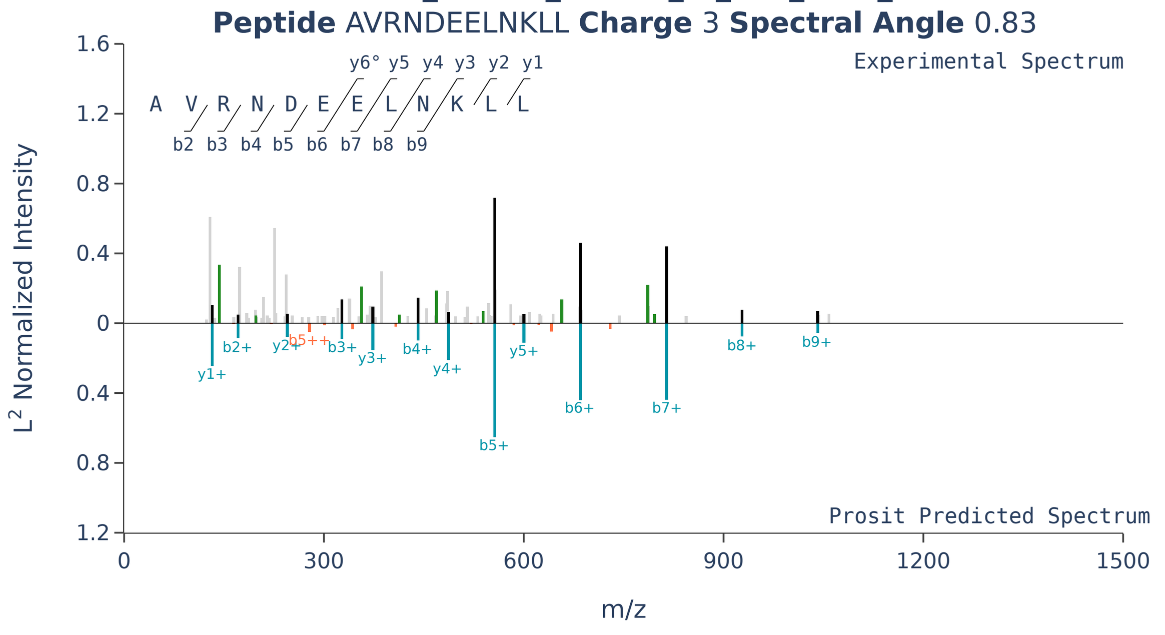


**Source** HRoetschke_291222_060123_Fu_H2A_b1R1_1h **Scan** 4095
**Peptide** SVLLPKKTESSKSA **Charge** 3 **Spectral Angle** 0.83

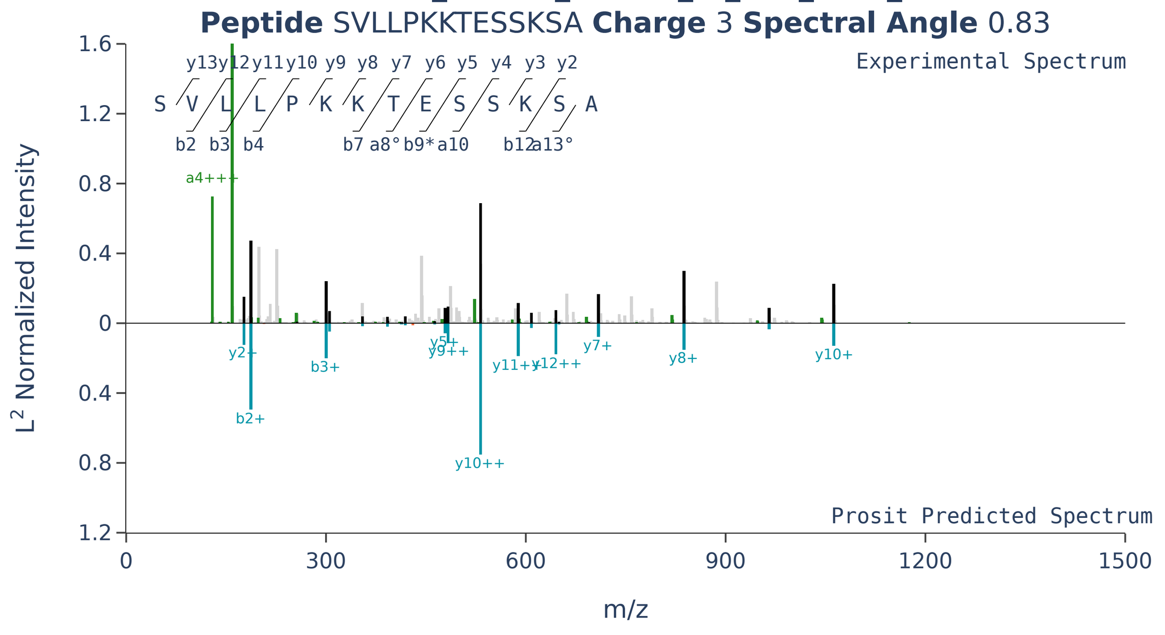


**Source** HRoetschke_291222_060123_Fu_H2A_b1R1_2h **Scan** 16697
**Peptide** YLTAEILE **Charge** 2 **Spectral Angle** 0.88

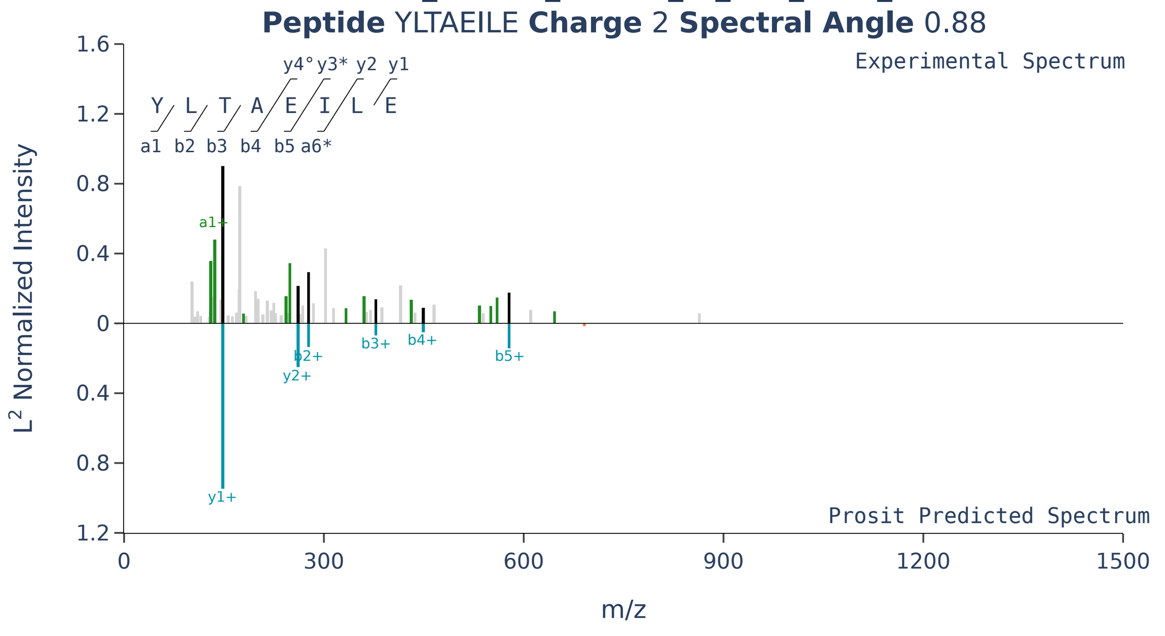


**Source** HRoetschke_291222_060123_Fu_H2A_b1R2_2h **Scan** 9248
**Peptide** KLLGRVTIAQG **Charge** 2 **Spectral Angle** 0.87

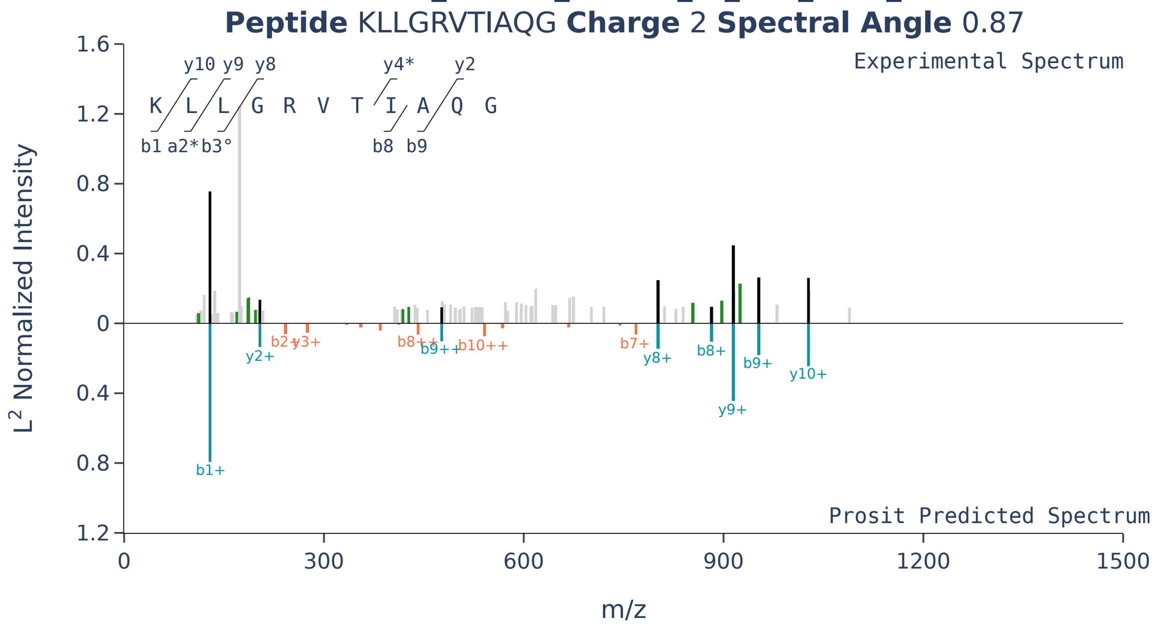


**Source** HRoetschke_291222_060123_Fu_H2A_b1R2_4h **Scan** 17654
**Peptide** EELNKLLGRVTI **Charge** 2 **Spectral Angle** 0.89

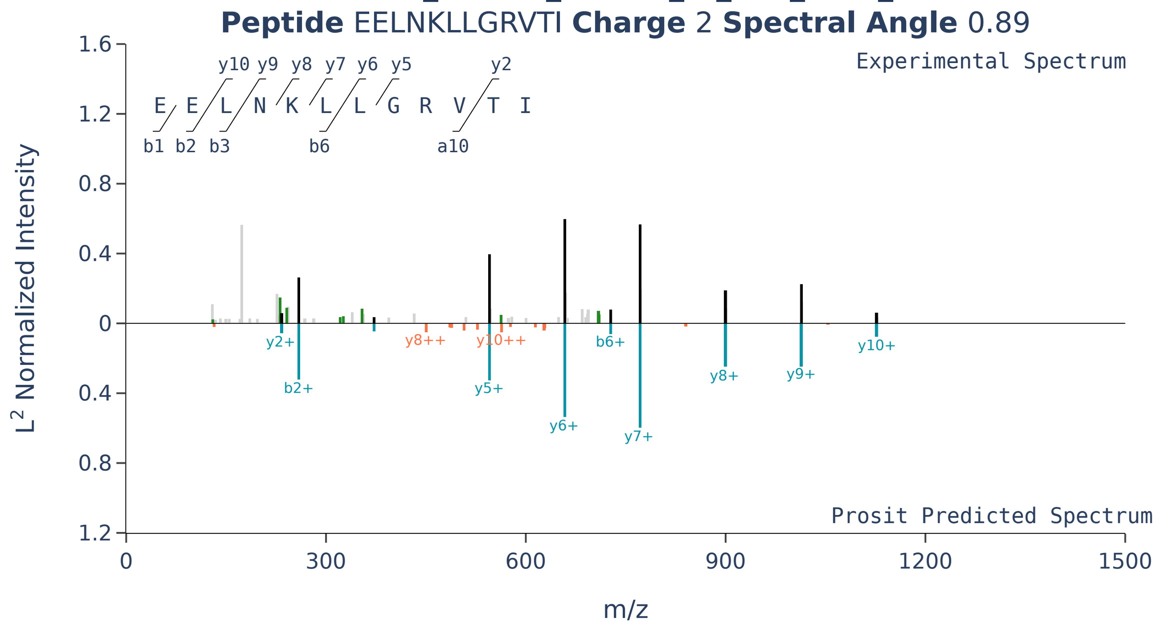


**Source** HRoetschke_291222_060123_Fu_H2A_b1R1_4h **Scan** 8173
**Peptide** AERVGAGAPVY **Charge** 2 **Spectral Angle** 0.91

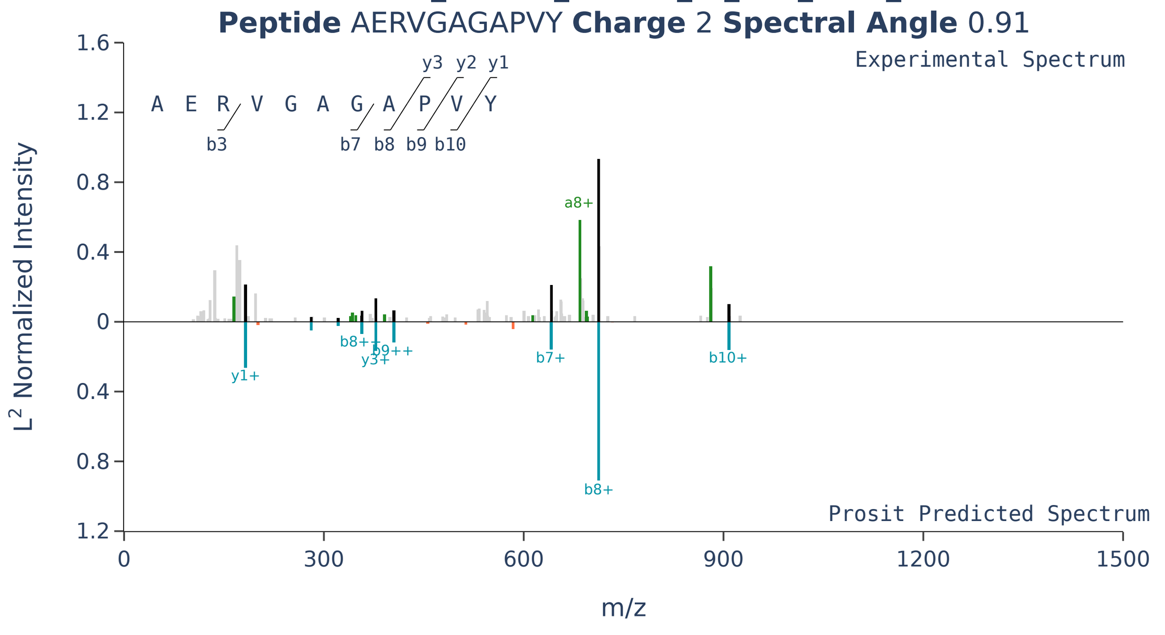


**Source** HRoetschke_291222_060123_Fu_H2A_b1R1_24h **Scan** 15652
**Peptide** AGLQFPVG **Charge** 1 **Spectral Angle** 0.86

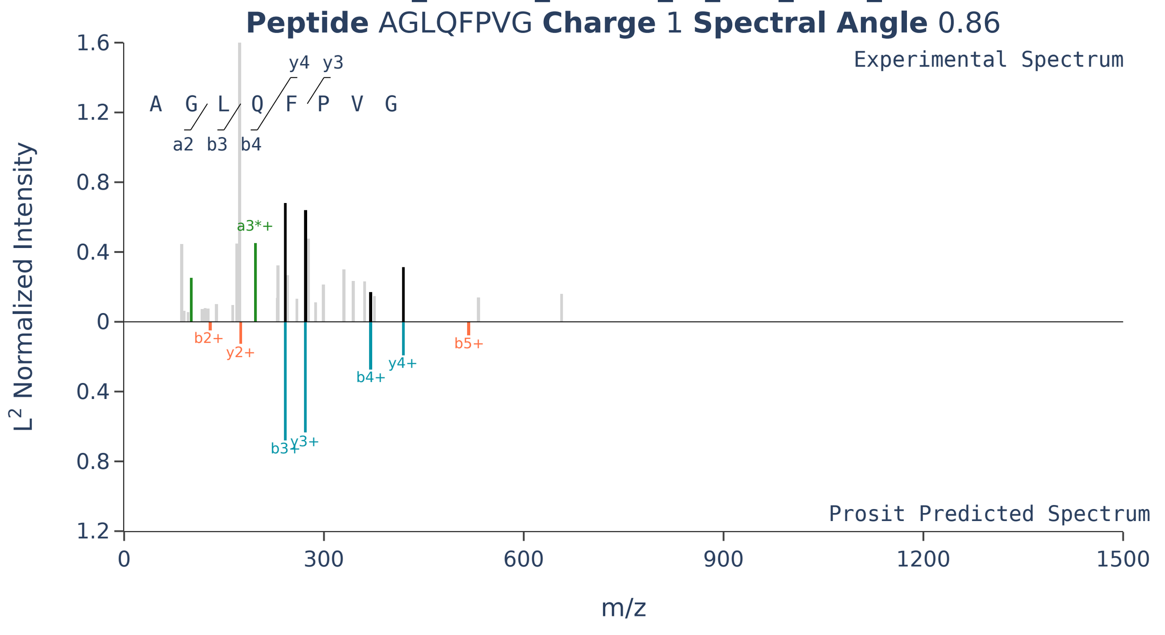


**Source** HRoetschke_291222_060123_Fu_H2A_b1R2_4h **Scan** 10288
**Peptide** AKTRSSRAGLQFPVGRV **Charge** 4 **Spectral Angle** 0.86

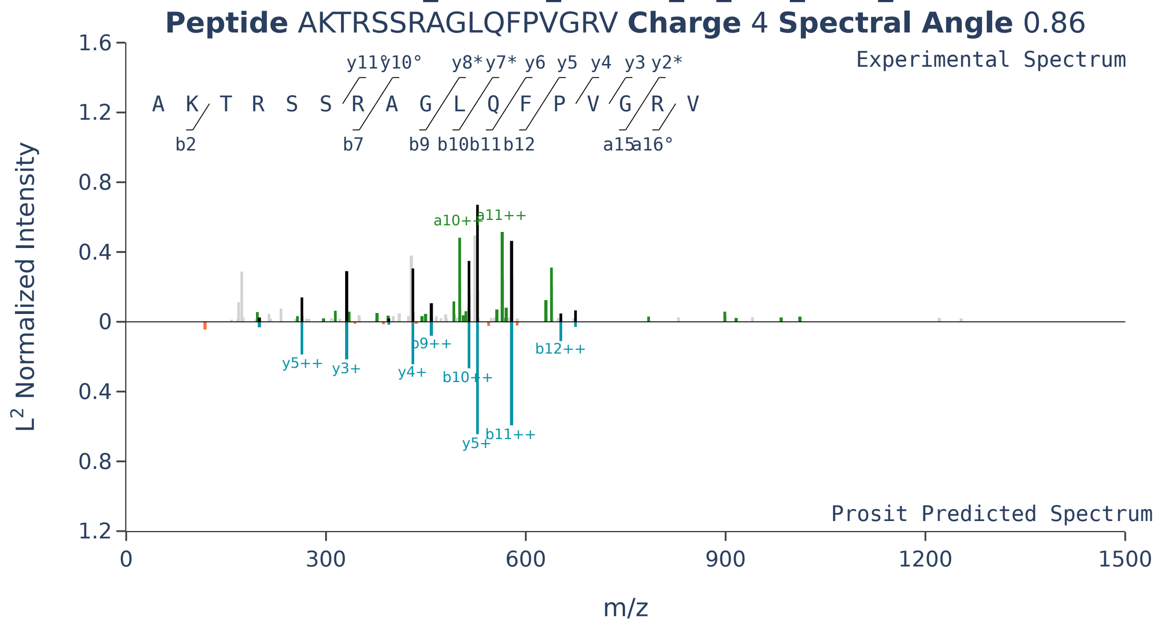


**Source** HRoetschke_291222_060123_Fu_H2A_b2R2_2h **Scan** 12656
**Peptide** AGAPVYLAA **Charge** 2 **Spectral Angle** 0.8

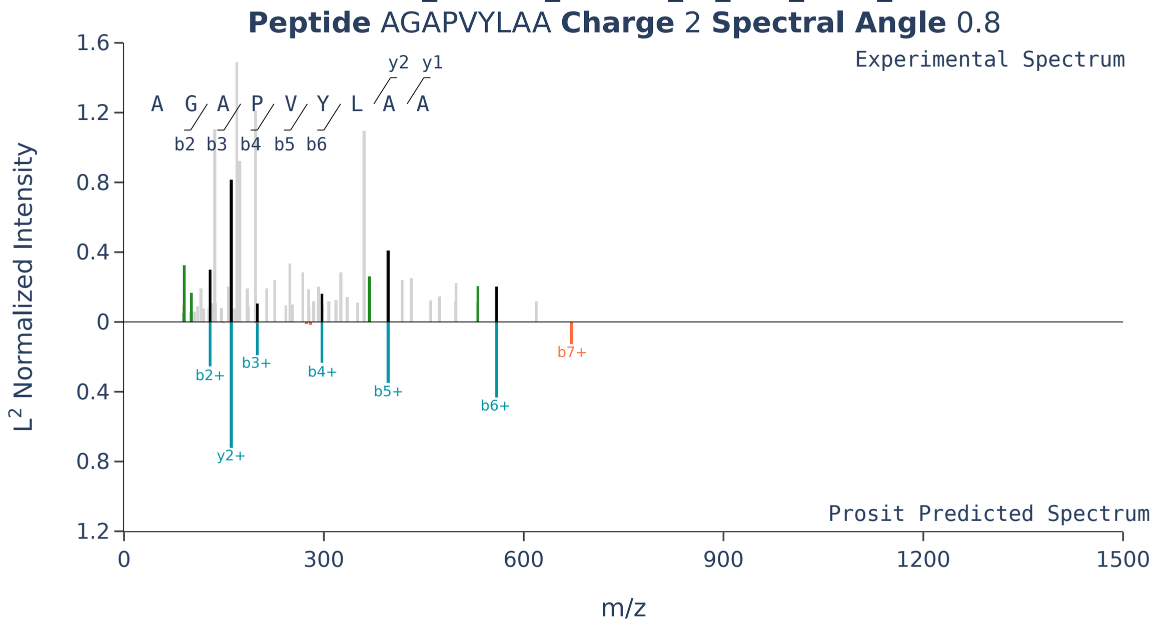


**Source** HRoetschke_291222_060123_Fu_H2A_b1R2_24h **Scan** 3610
**Peptide** LLRKGNYAE **Charge** 2 **Spectral Angle** 0.84

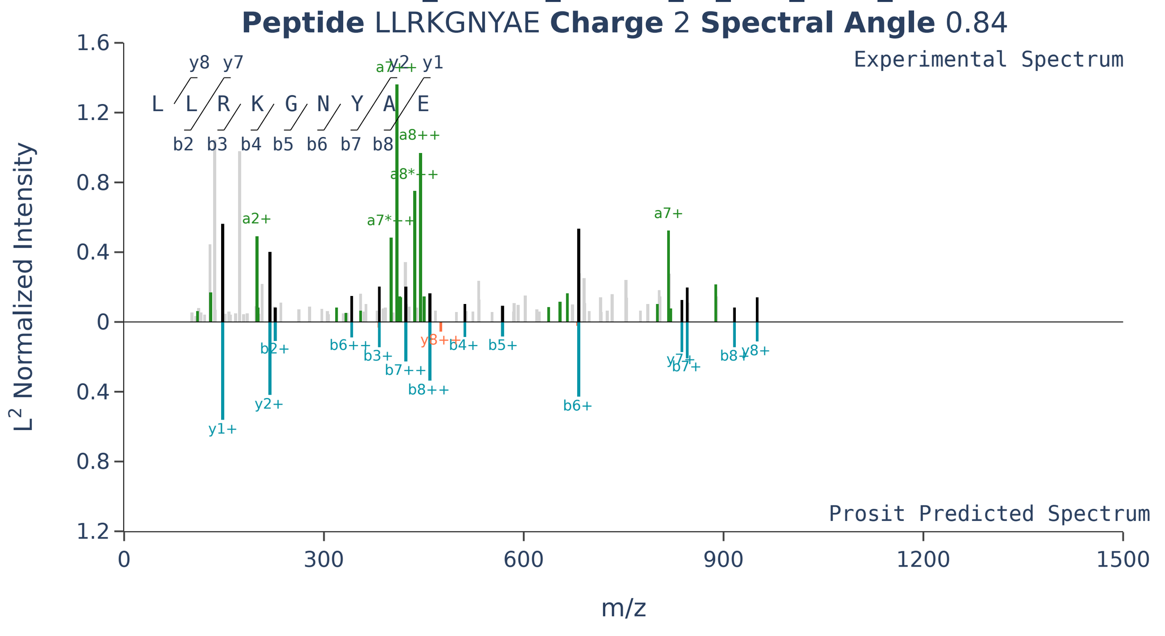


**Source** HRoetschke_291222_060123_Fu_H2A_b1R1_2h **Scan** 16475
**Peptide** VRNDEELNKLLGRVTLA **Charge** 3 **Spectral Angle** 0.89

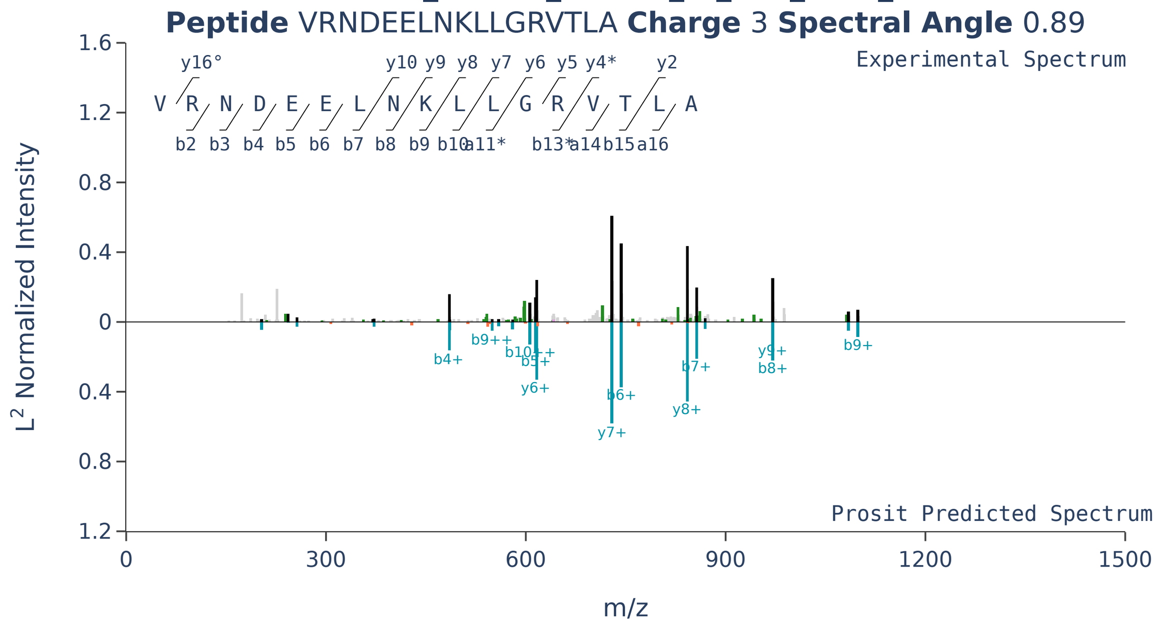

**Source** HRoetschke_291222_060123_Fu_H2A_b1R2_24h **Scan** 3023
**Peptide** AGNAARDNKKTRII **Charge** 3 **Spectral Angle** 0.86

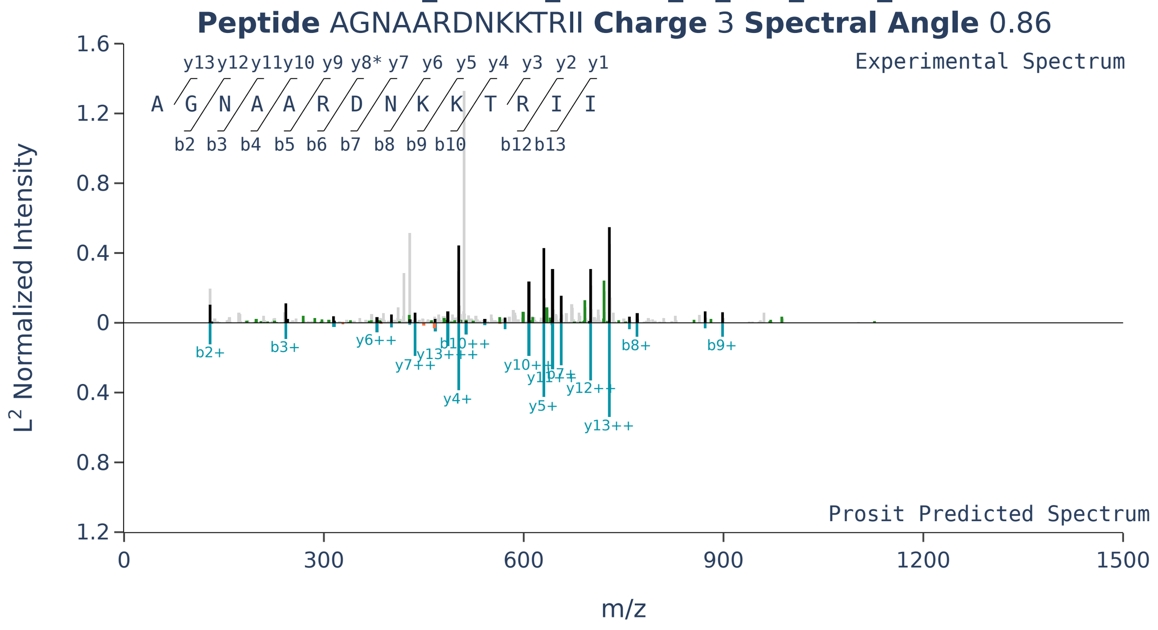


**Source** HRoetschke_291222_060123_Fu_H2A_b1R1_4h **Scan** 13299
**Peptide** GLQFPVGRVHRL **Charge** 3 **Spectral Angle** 0.86

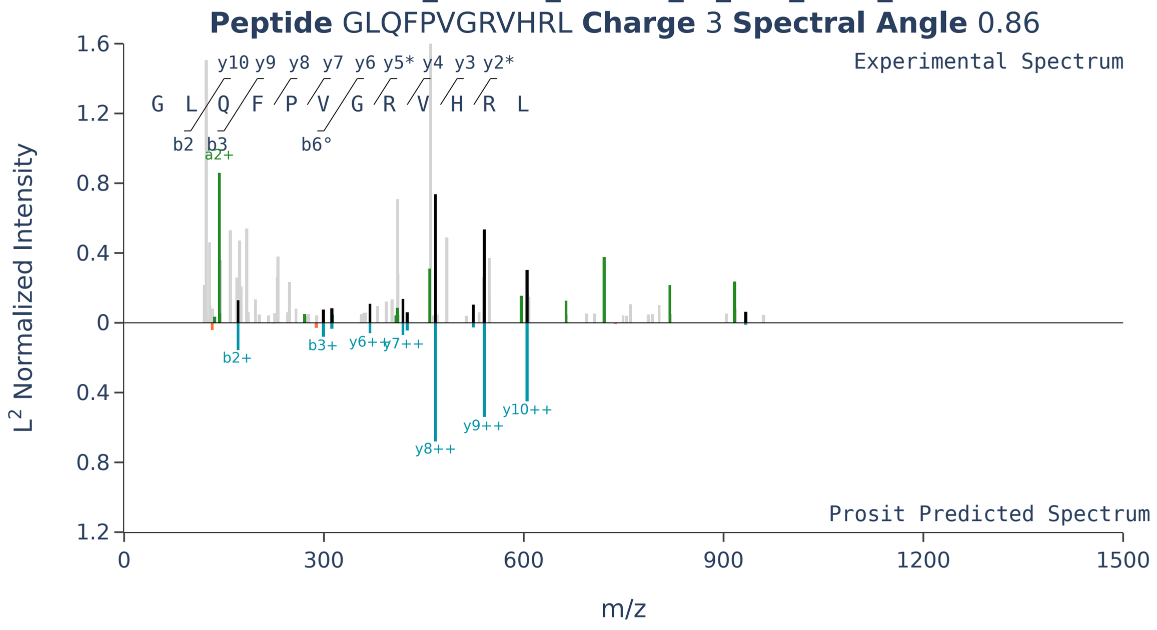


**Source** HRoetschke_291222_060123_Fu_H2A_b1R1_24h **Scan** 2618
**Peptide** LAGNAARDNKKTRIIPRH **Charge** 4 **Spectral Angle** 0.95

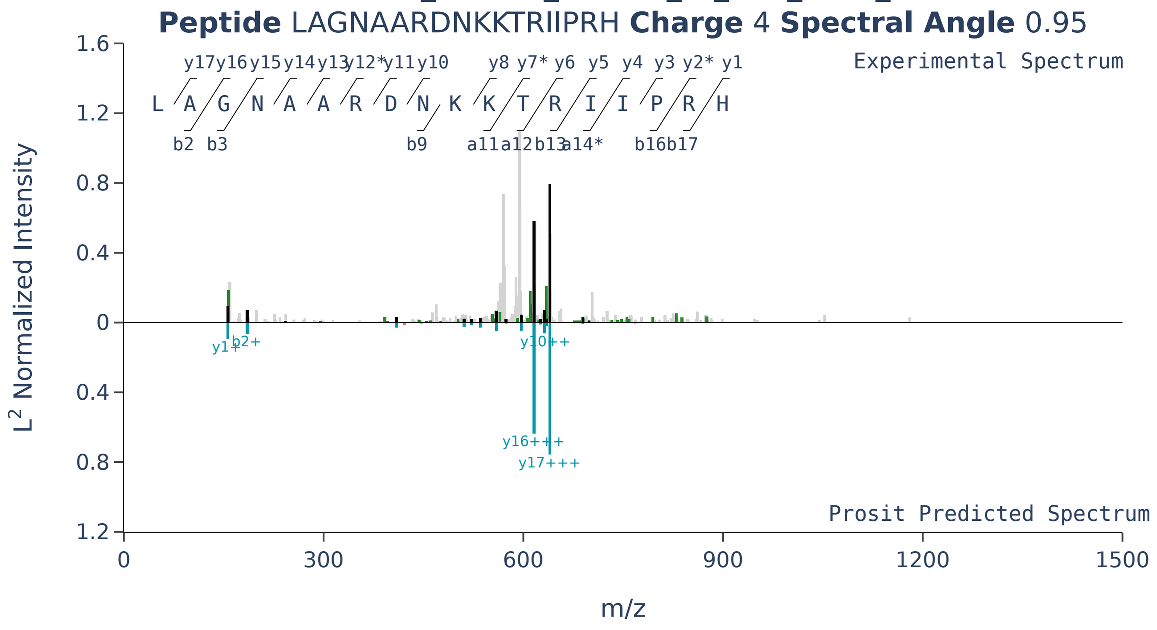


**Source** HRoetschke_291222_060123_Fu_H2A_b1R2_1h **Scan** 7402
**Peptide** DEELNKL **Charge** 2 **Spectral Angle** 0.91

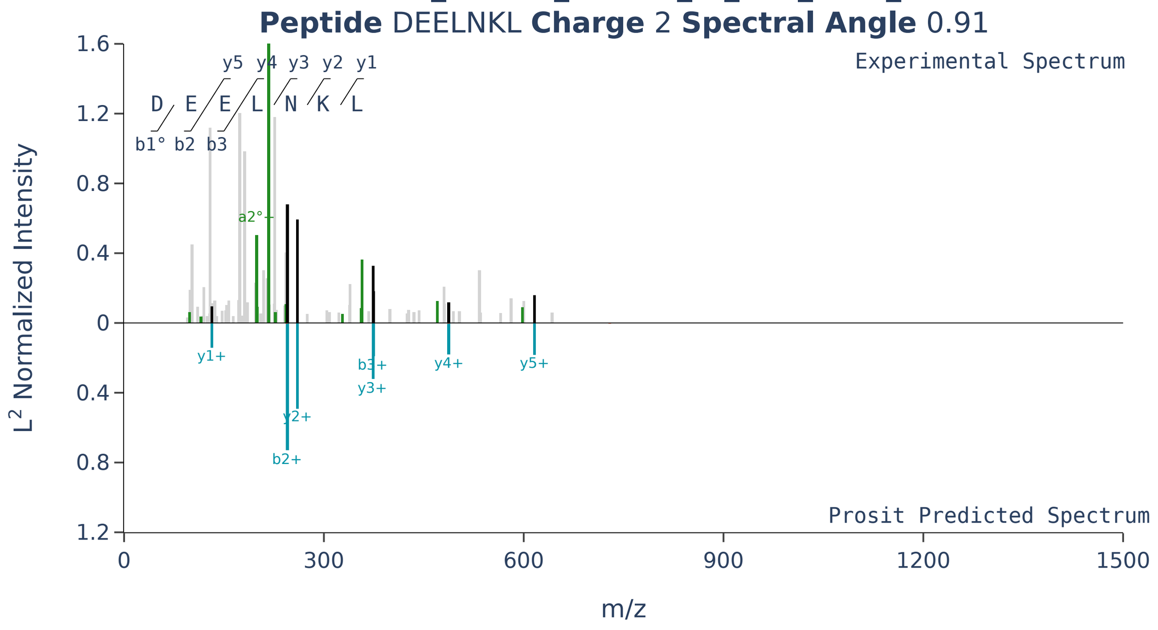


**Source** HRoetschke_291222_060123_Fu_H2A_b2R2_24h **Scan** 3428
**Peptide** KGNYAERVGA **Charge** 2 **Spectral Angle** 0.89

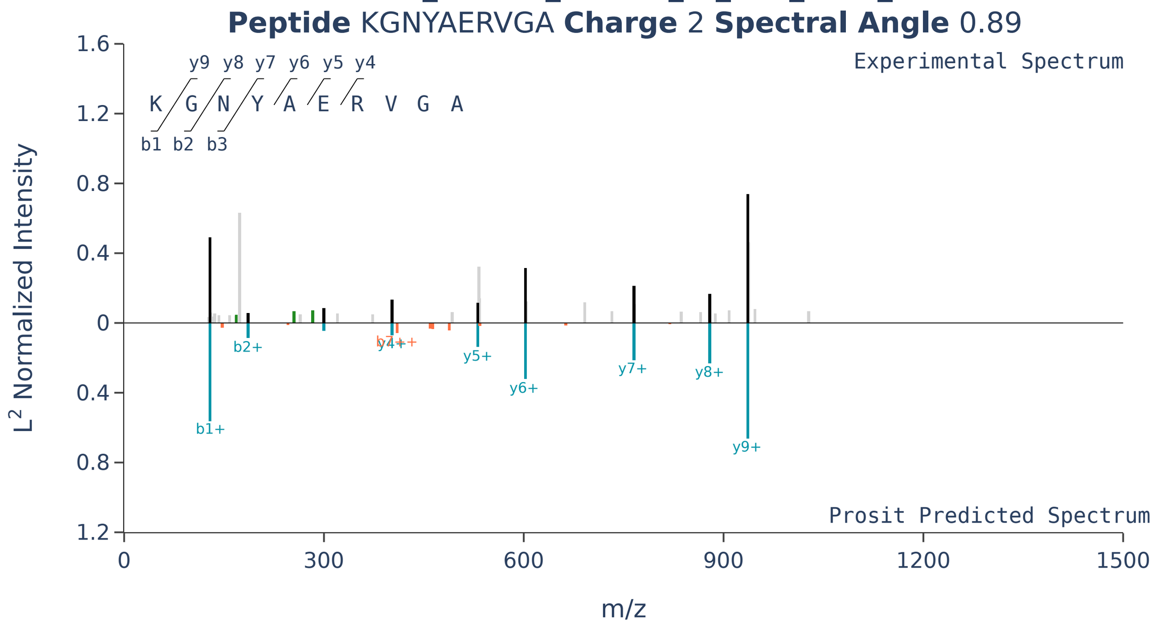


**Source** HRoetschke_291222_060123_Fu_H2A_b2R2_2h **Scan** 5667
**Peptide** ELAGNAARDNKKTRIIPRHL **Charge** 4 **Spectral Angle** 0.94

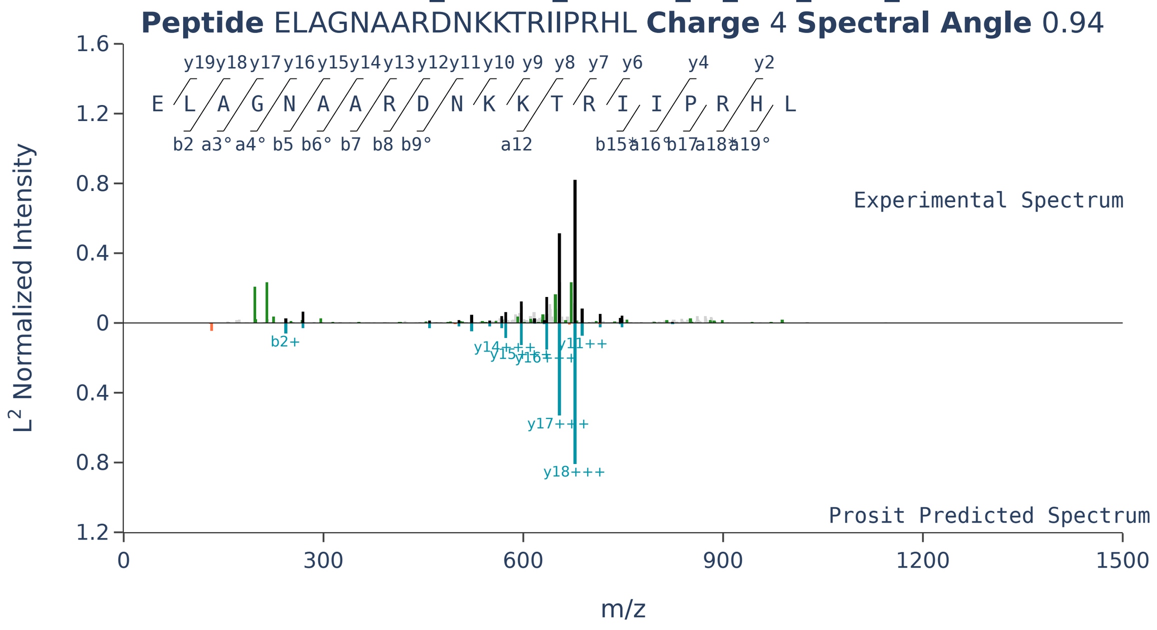


**Source** HRoetschke_291222_060123_Fu_H2A_b2R1_1h **Scan** 7641
**Peptide** RSSRAGLQFPVGRVH **Charge** 4 **Spectral Angle** 0.82

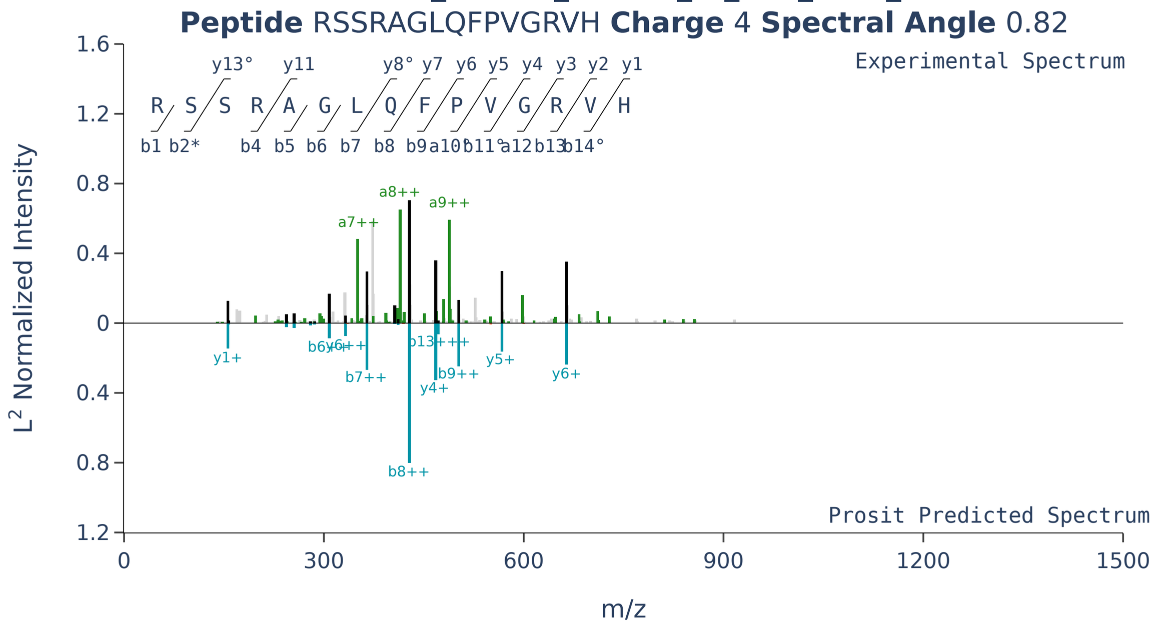


**Source** HRoetschke_291222_060123_Fu_H2A_b2R1_24h **Scan** 7898
**Peptide** KTRSSRAGLQFPVGR **Charge** 4 **Spectral Angle** 0.85

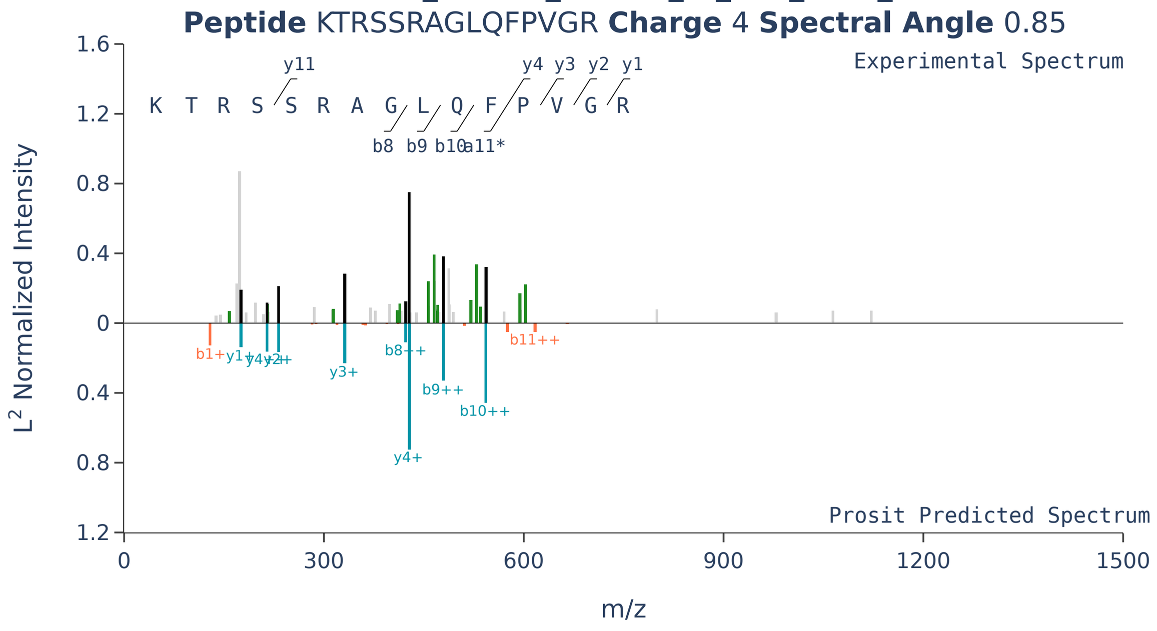


**Source** HRoetschke_291222_060123_Fu_H2A_b2R1_24h **Scan** 5314
**Peptide** GNYAERVGAGA **Charge** 2 **Spectral Angle** 0.94

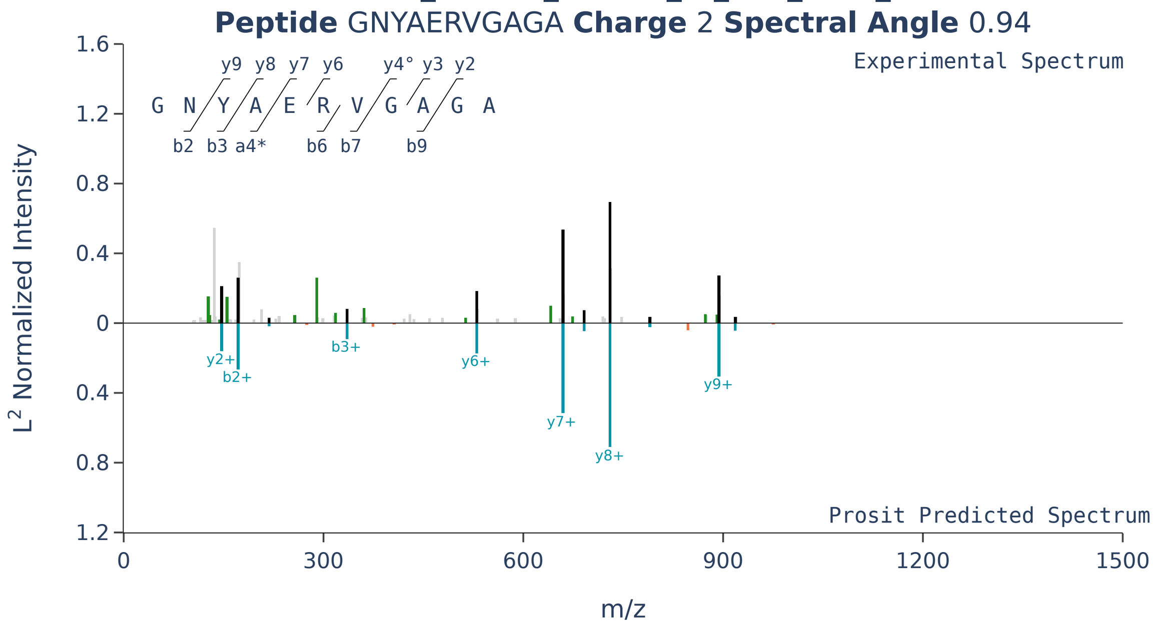


**Source** HRoetschke_291222_060123_Fu_H2A_b2R1_24h **Scan** 12684
**Peptide** GLQFPVGR **Charge** 2 **Spectral Angle** 0.91

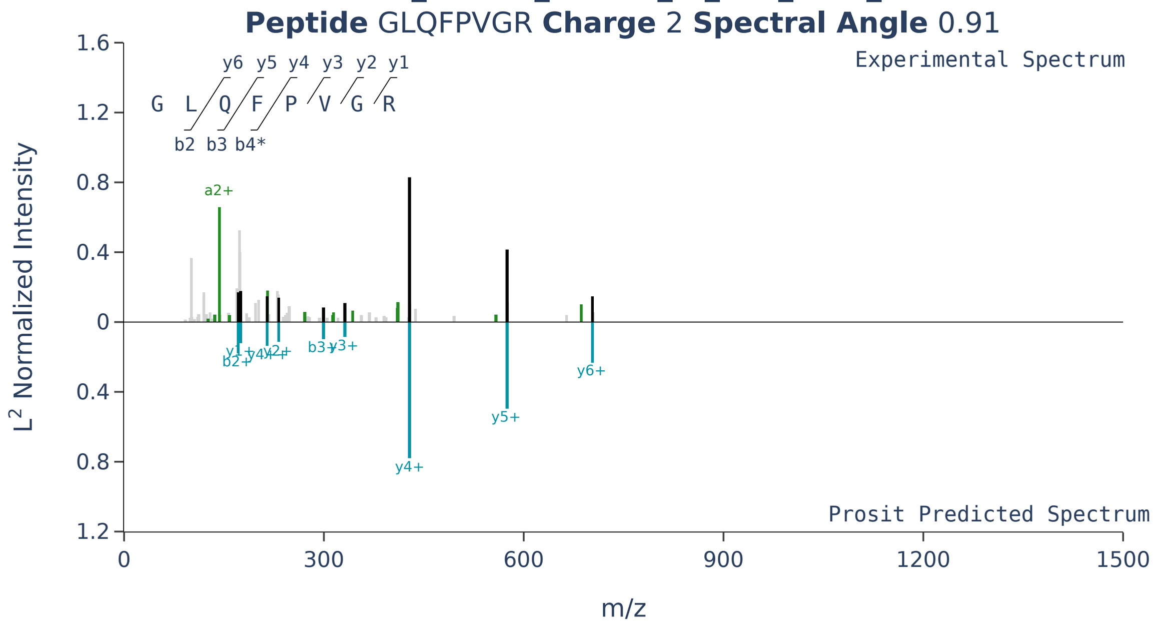


**Source** HRoetschke_291222_060123_Fu_H2A_b1R1_24h **Scan** 6305
**Peptide** PRHLQLAVRNDE **Charge** 3 **Spectral Angle** 0.83

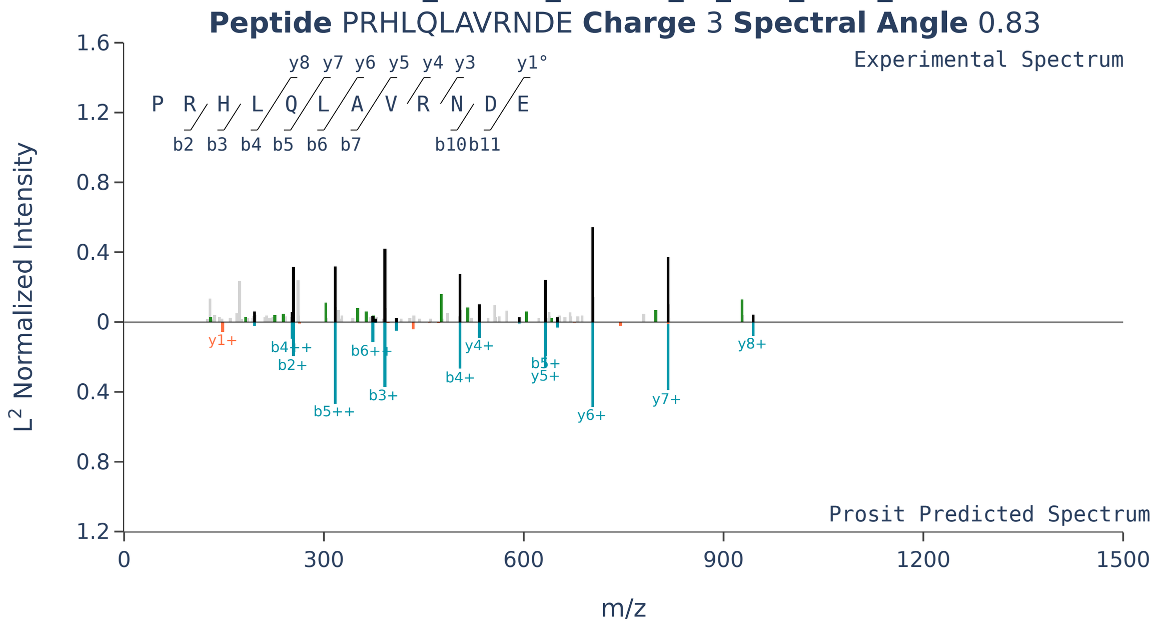


**Source** HRoetschke_291222_060123_Fu_H2A_b1R1_1h **Scan** 1997
**Peptide** RKGNYAERVG **Charge** 3 **Spectral Angle** 0.87

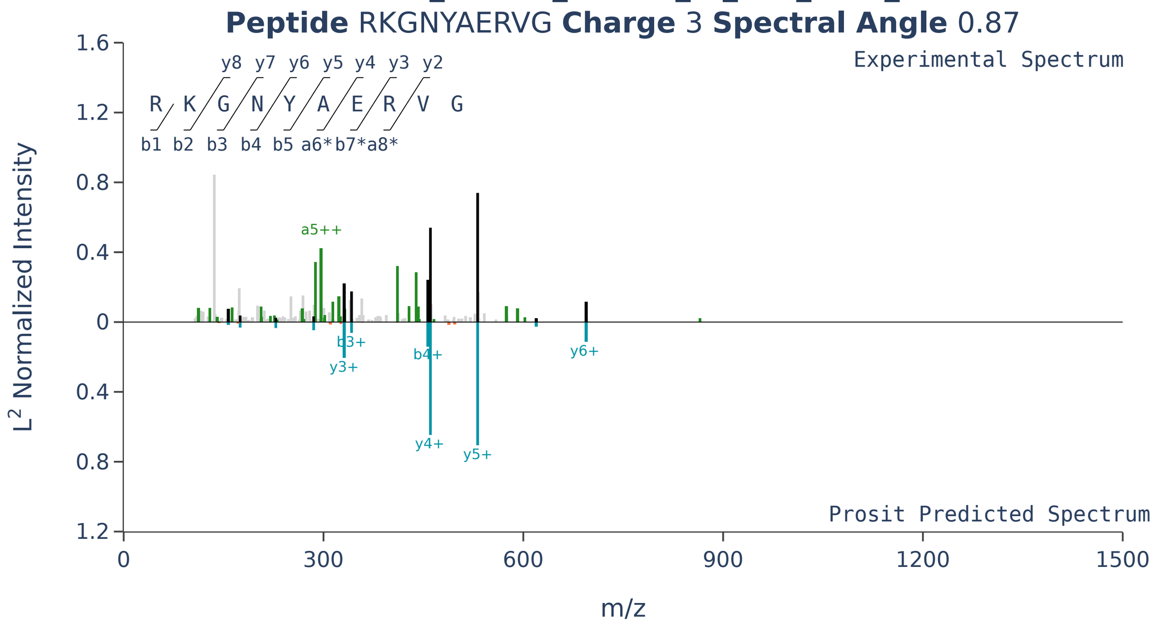


**Source** HRoetschke_291222_060123_Fu_H2A_b2R1_4h **Scan** 2153
**Peptide** RVHRLLRKGNYAER **Charge** 5 **Spectral Angle** 0.88

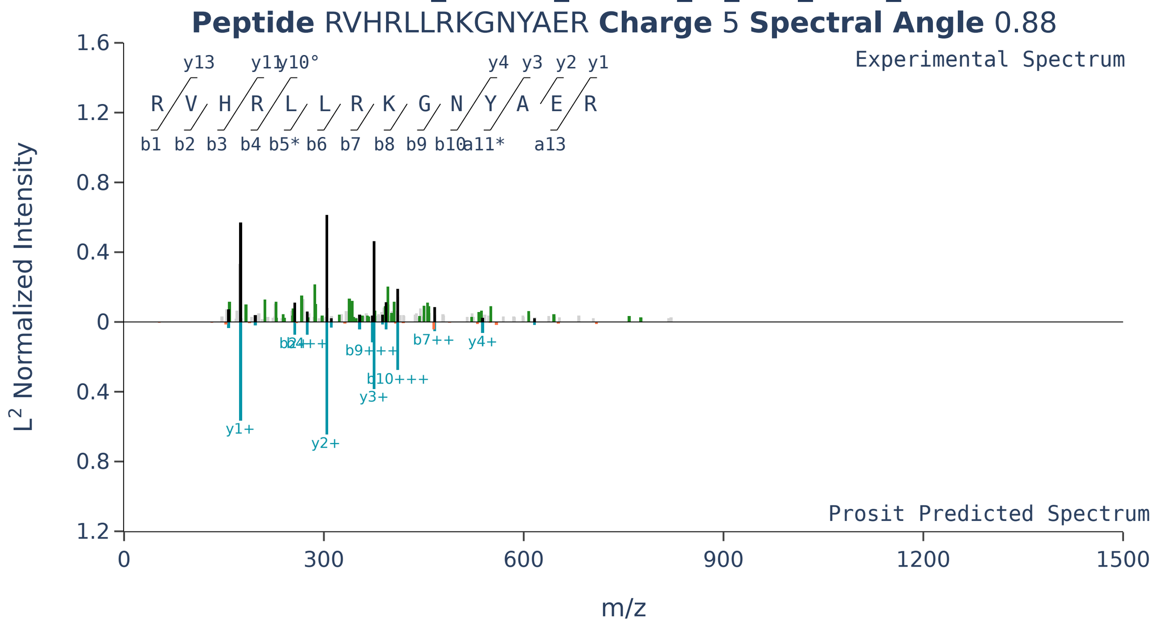


**Source** HRoetschke_291222_060123_Fu_H2A_b1R1_2h **Scan** 16991
**Peptide** VRNDEELNKLLGRVTL **Charge** 3 **Spectral Angle** 0.91

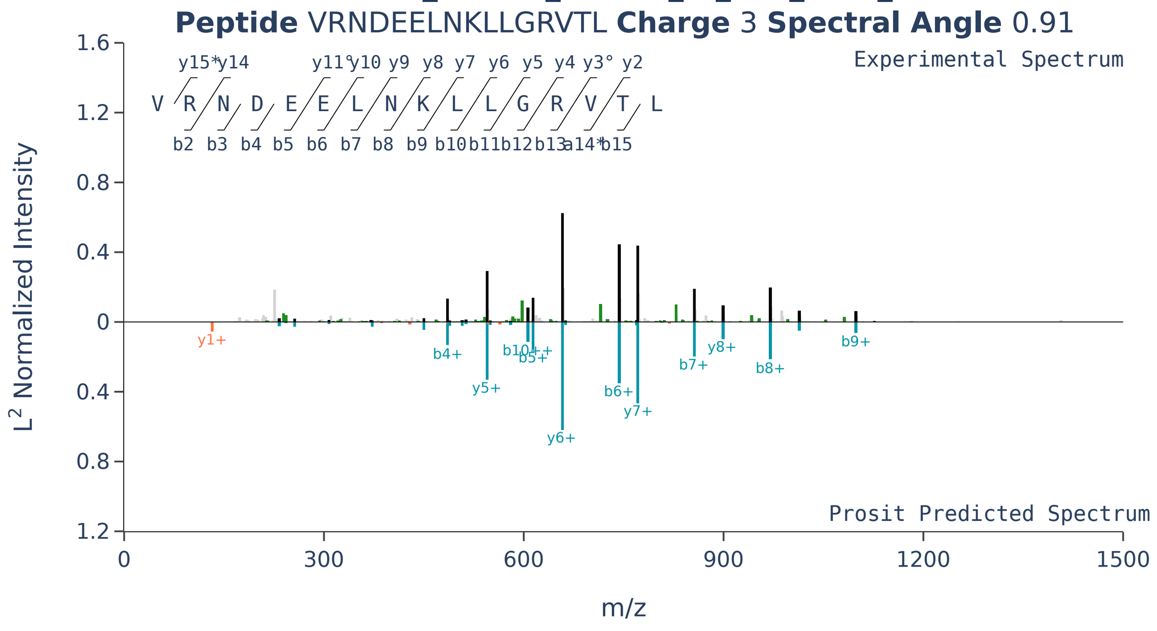


**Source** HRoetschke_291222_060123_Fu_H2A_b1R1_2h **Scan** 12927
**Peptide** AVRNDEELNKLLG **Charge** 3 **Spectral Angle** 0.82

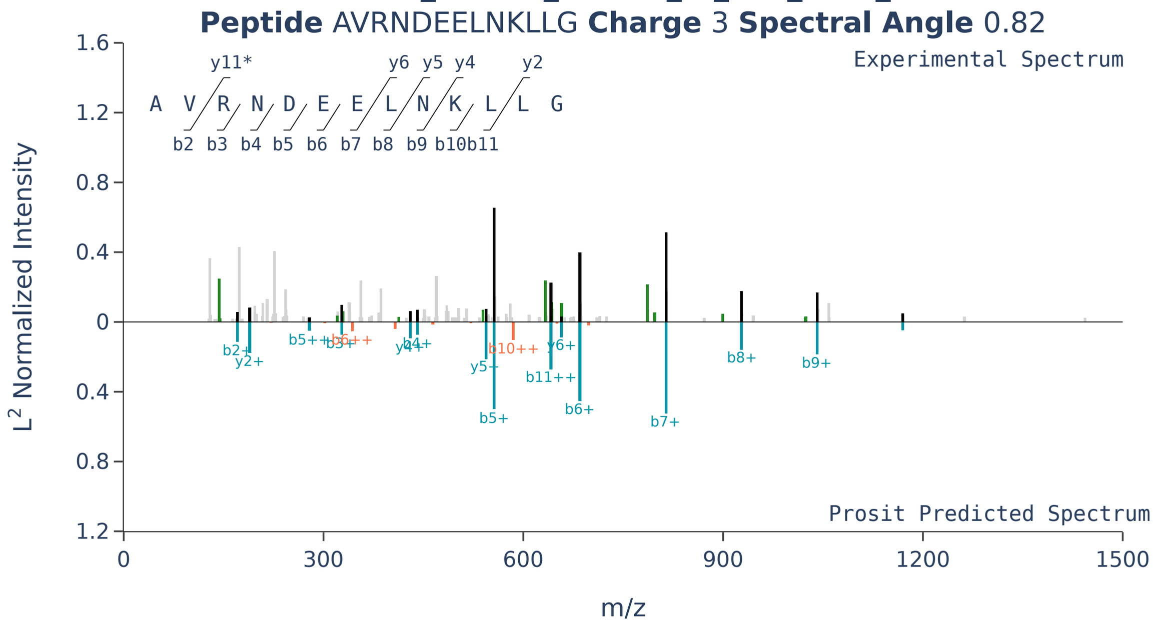

**Source** HRoetschke_291222_060123_Fu_H2A_b1R2_1h **Scan** 10803
**Peptide** VRNDEELNKLL **Charge** 2 **Spectral Angle** 0.86

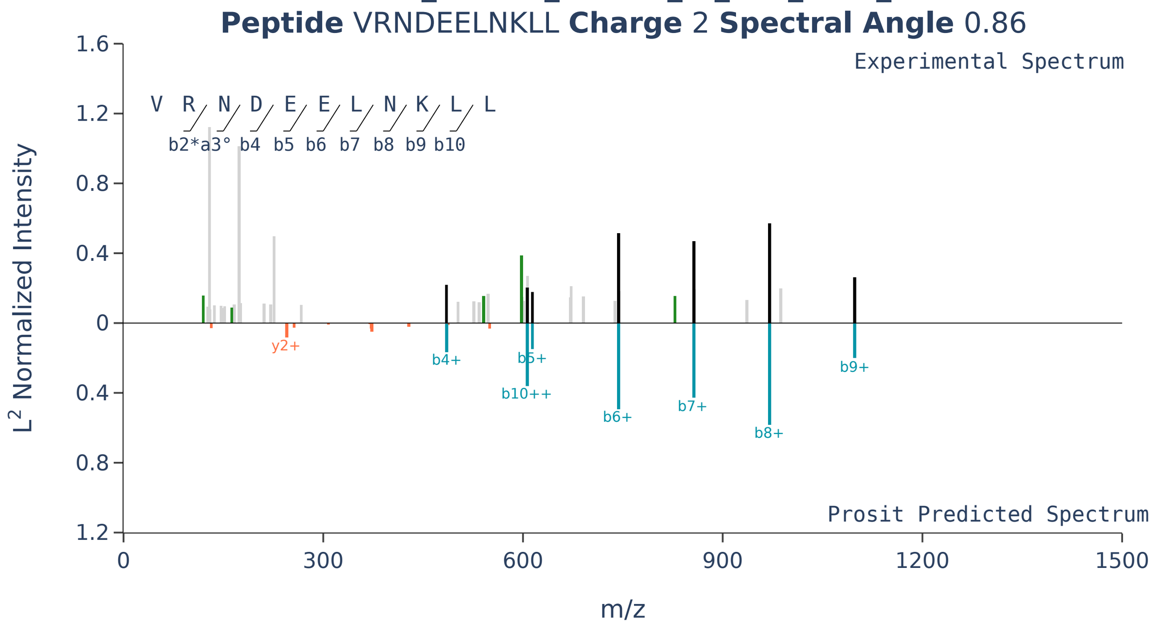


**Source** HRoetschke_291222_060123_Fu_H2A_b1R1_2h **Scan** 13565
**Peptide** RNDEELNKLLGRVT **Charge** 3 **Spectral Angle** 0.89

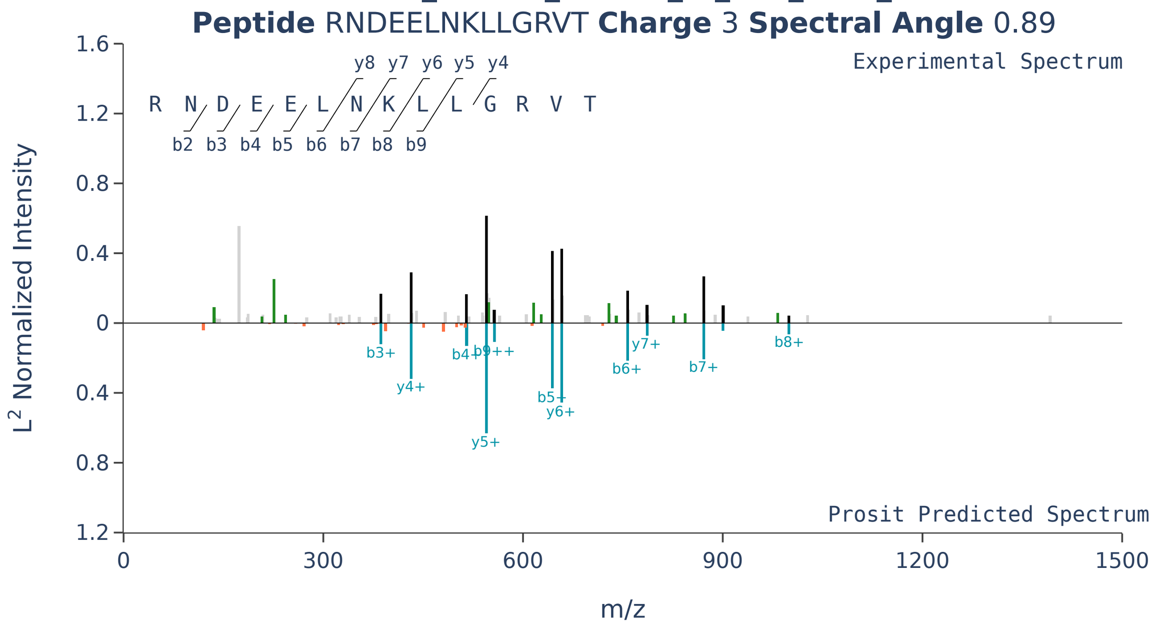


**Source** HRoetschke_291222_060123_Fu_H2A_b1R1_24h **Scan** 2677
**Peptide** KGNYAERVG **Charge** 2 **Spectral Angle** 0.94

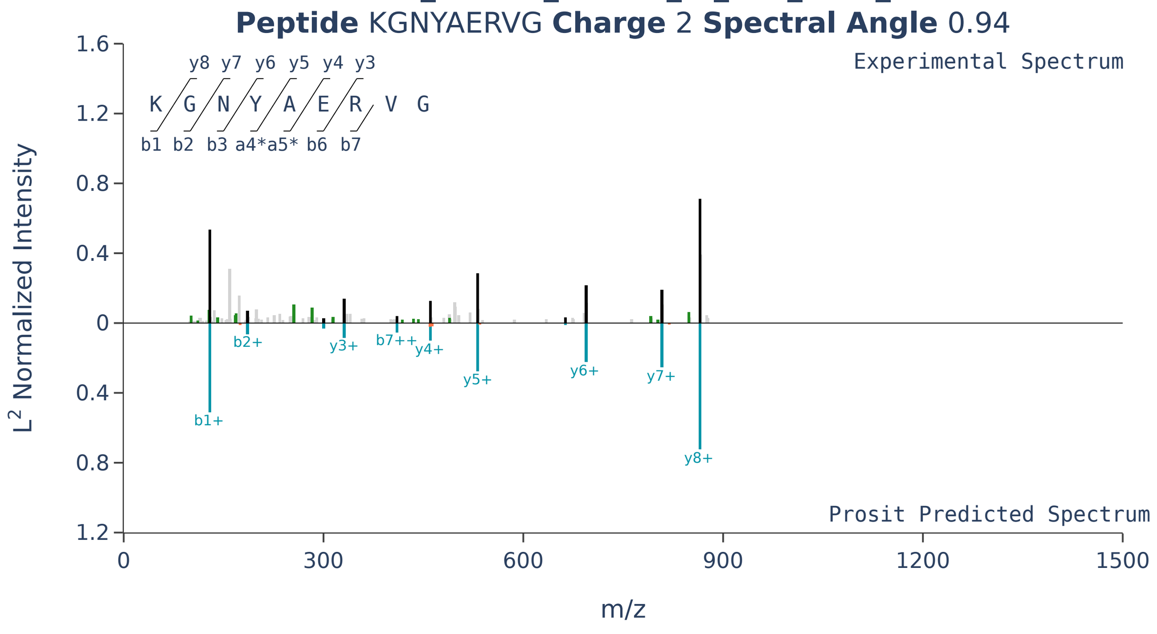


**Source** HRoetschke_291222_060123_Fu_H2A_b1R2_1h **Scan** 2688
**Peptide** LRKGNYAERVG **Charge** 3 **Spectral Angle** 0.88

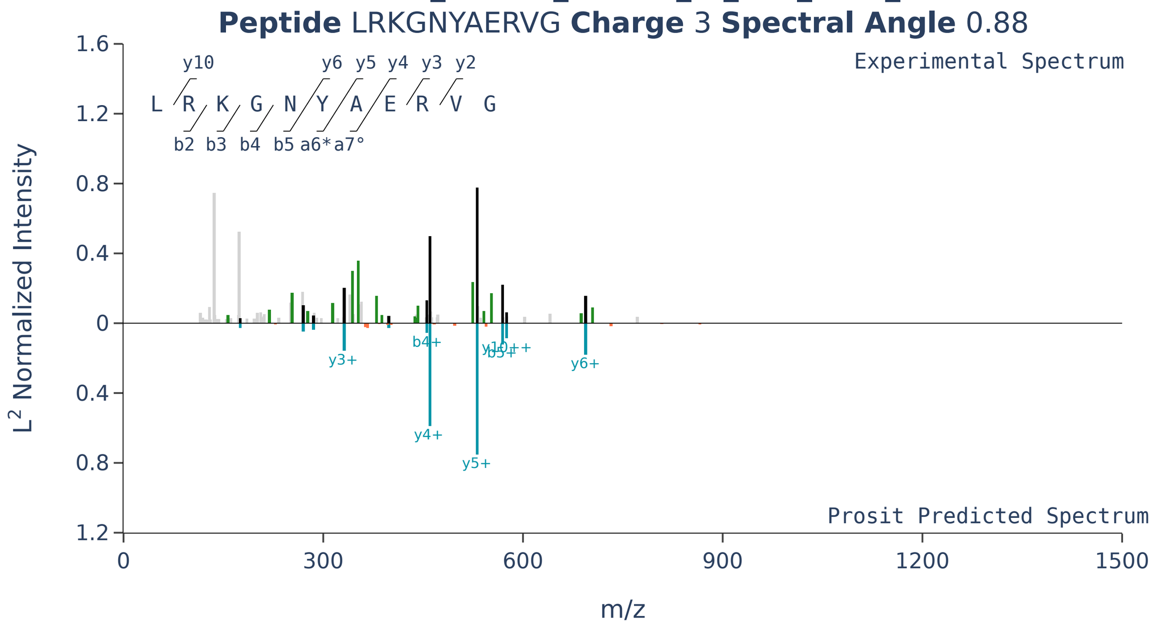


**Source** HRoetschke_291222_060123_Fu_H2A_b1R1_2h **Scan** 15610
**Peptide** TAEILELAGNAARD **Charge** 2 **Spectral Angle** 0.85

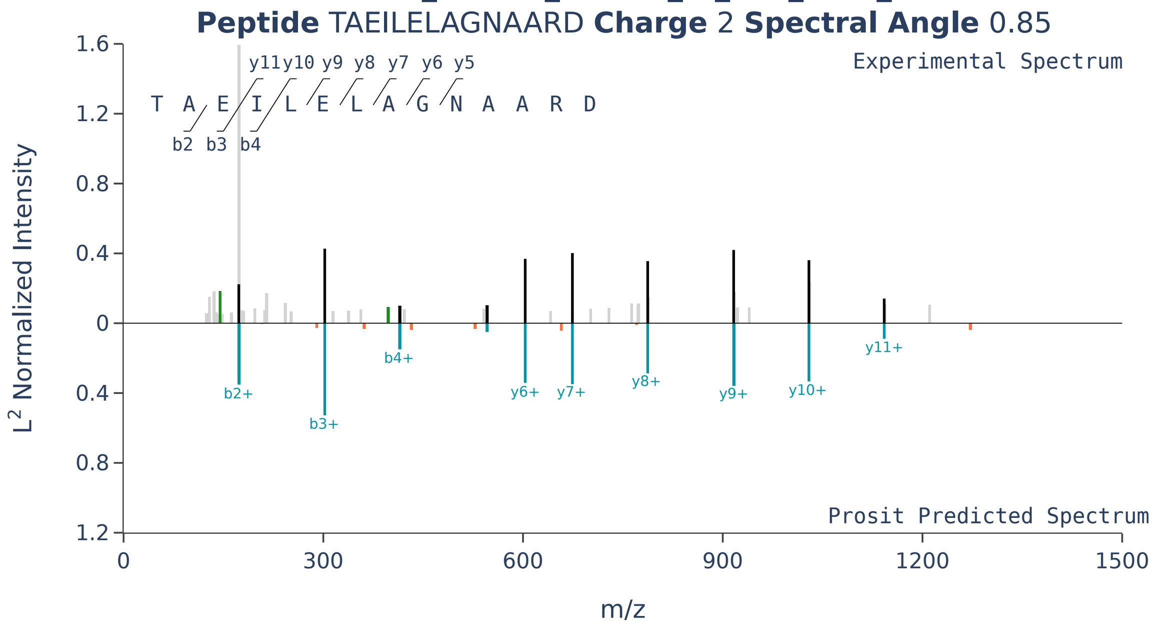


**Source** HRoetschke_291222_060123_Fu_H2A_b2R2_4h **Scan** 2695
**Peptide** QSVLLPKKTESSKSAKSK **Charge** 2 **Spectral Angle** 0.81

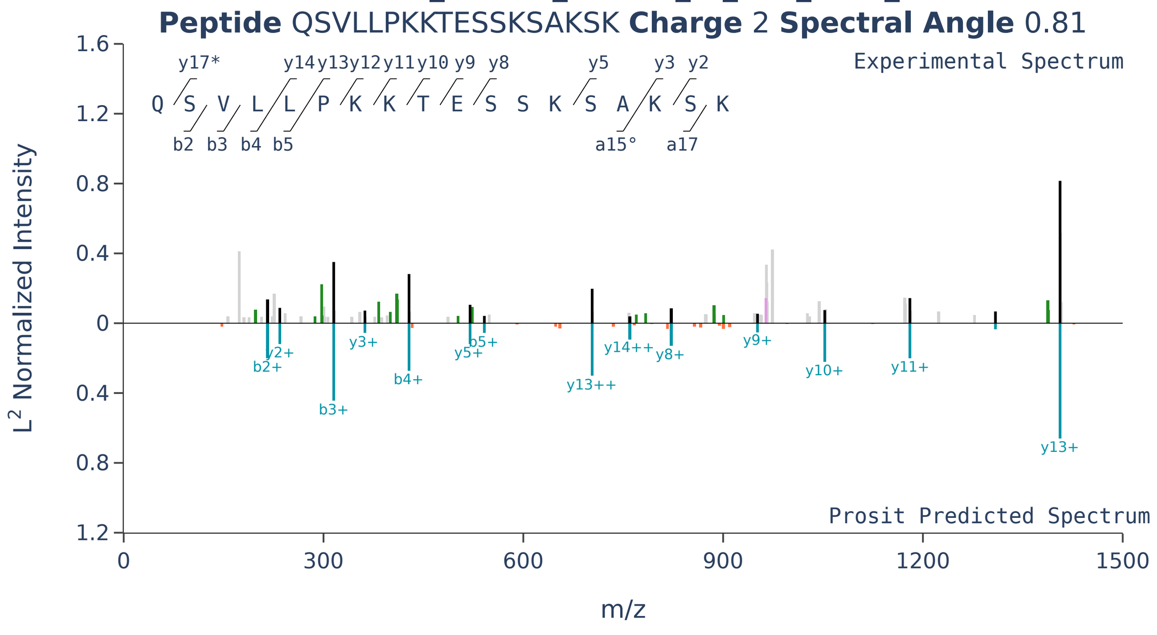


**Source** HRoetschke_291222_060123_Fu_H2A_b1R2_2h **Scan** 2080
**Peptide** LAGNAARDNKKTRL **Charge** 3 **Spectral Angle** 0.95

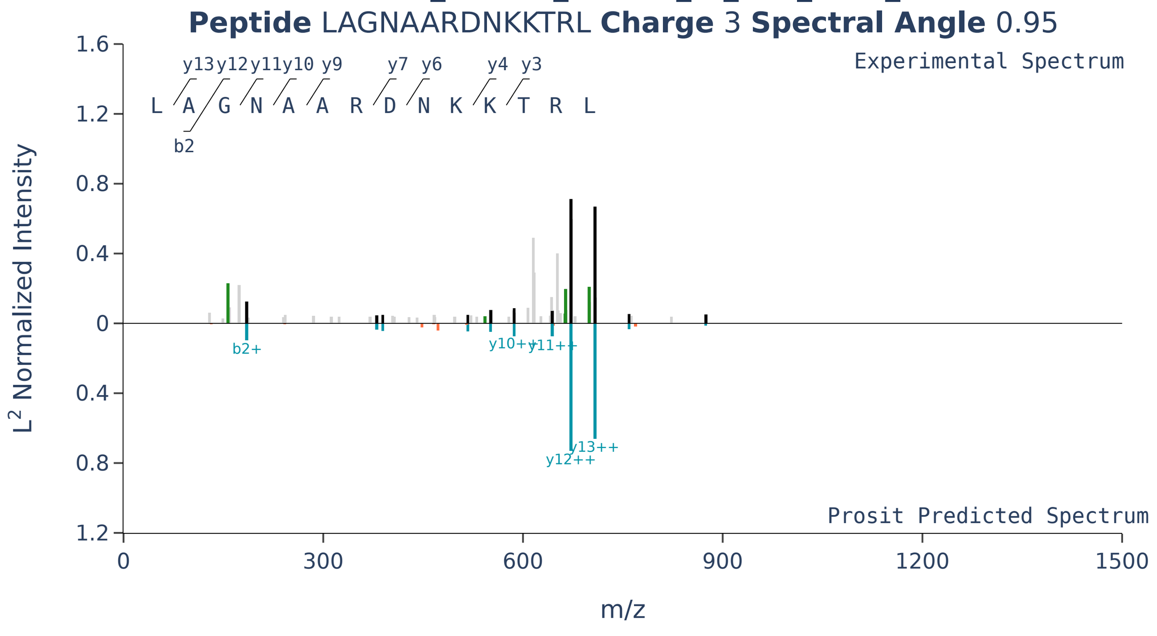


**Source** HRoetschke_291222_060123_Fu_H2A_b1R1_2h **Scan** 17834
**Peptide** AAVLEYLTAE **Charge** 2 **Spectral Angle** 0.86

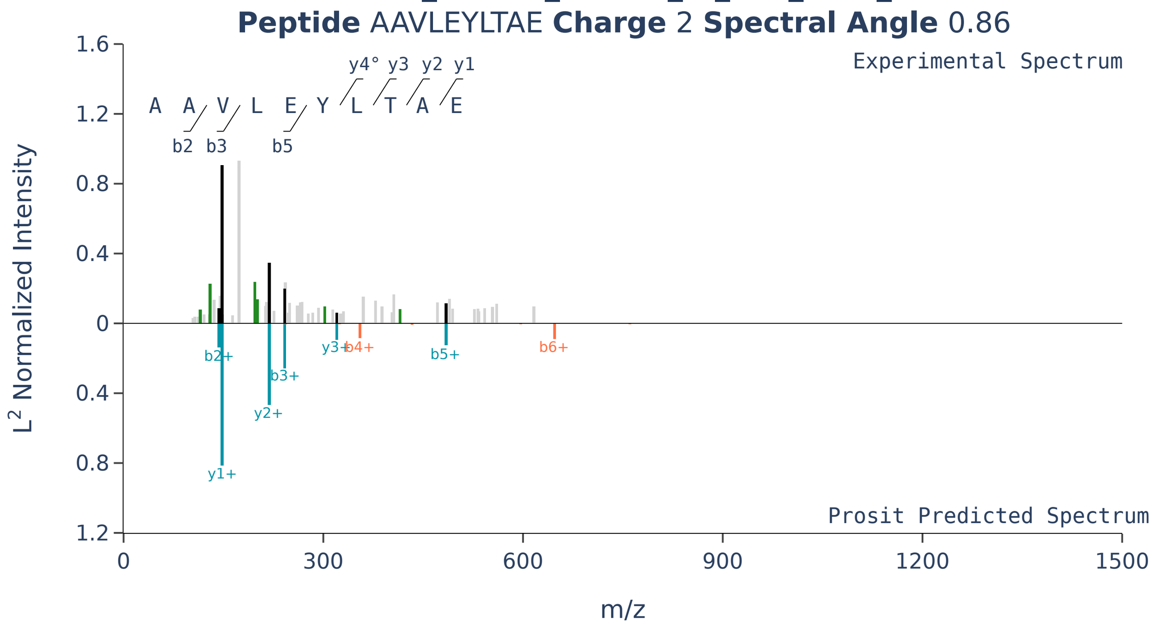


**Source** HRoetschke_291222_060123_Fu_H2A_b1R1_1h **Scan** 3969
**Peptide** RLLRKGNYAERV **Charge** 4 **Spectral Angle** 0.76

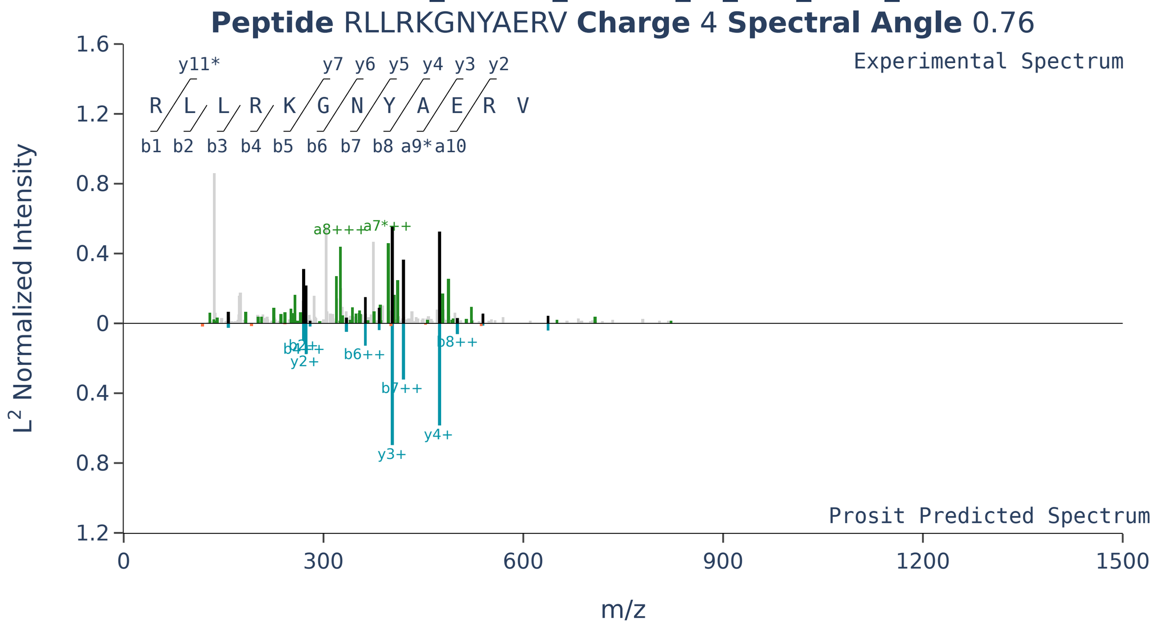


**Source** HRoetschke_291222_060123_Fu_H2A_b1R1_4h **Scan** 12268
**Peptide** TRSSRAGLQFPVGRV **Charge** 4 **Spectral Angle** 0.82

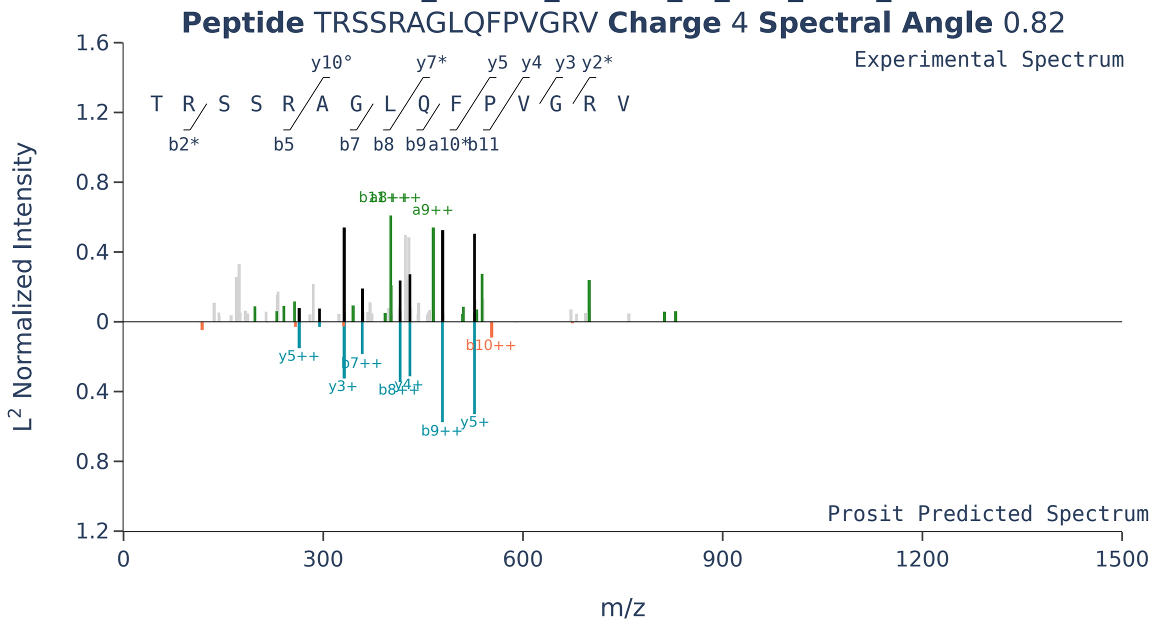


**Source** HRoetschke_291222_060123_Fu_H2A_b2R1_2h **Scan** 5485
**Peptide** PRHLQLAVRN **Charge** 3 **Spectral Angle** 0.73

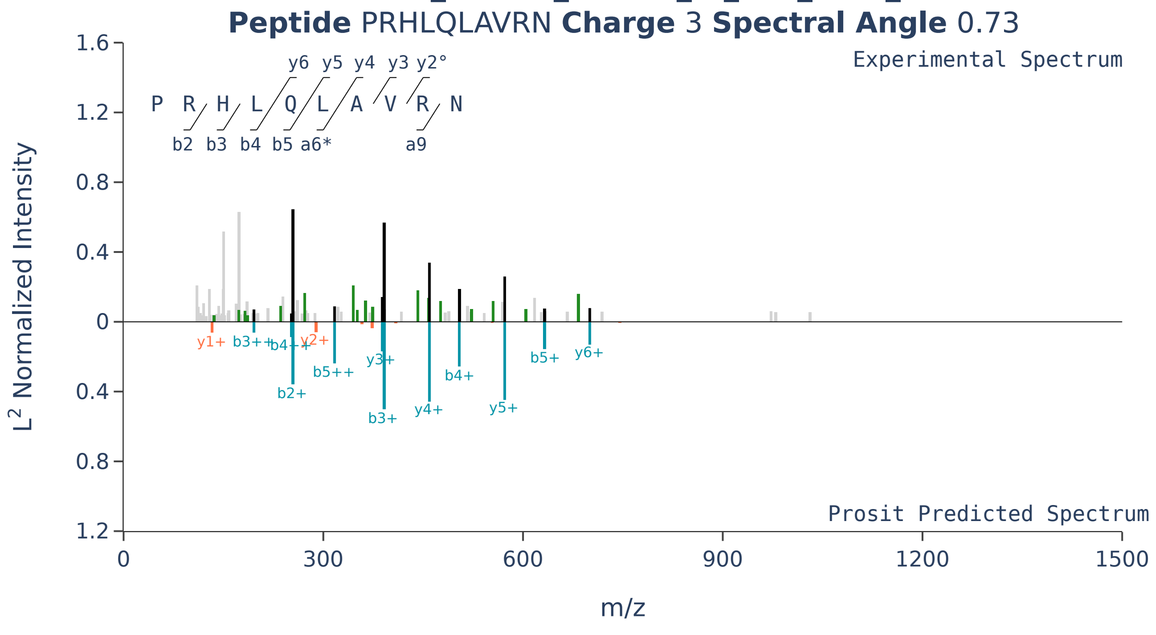


**Source** HRoetschke_291222_060123_Fu_H2A_b2R2_4h **Scan** 8134
**Peptide** QFPVGRVHRL **Charge** 3 **Spectral Angle** 0.83

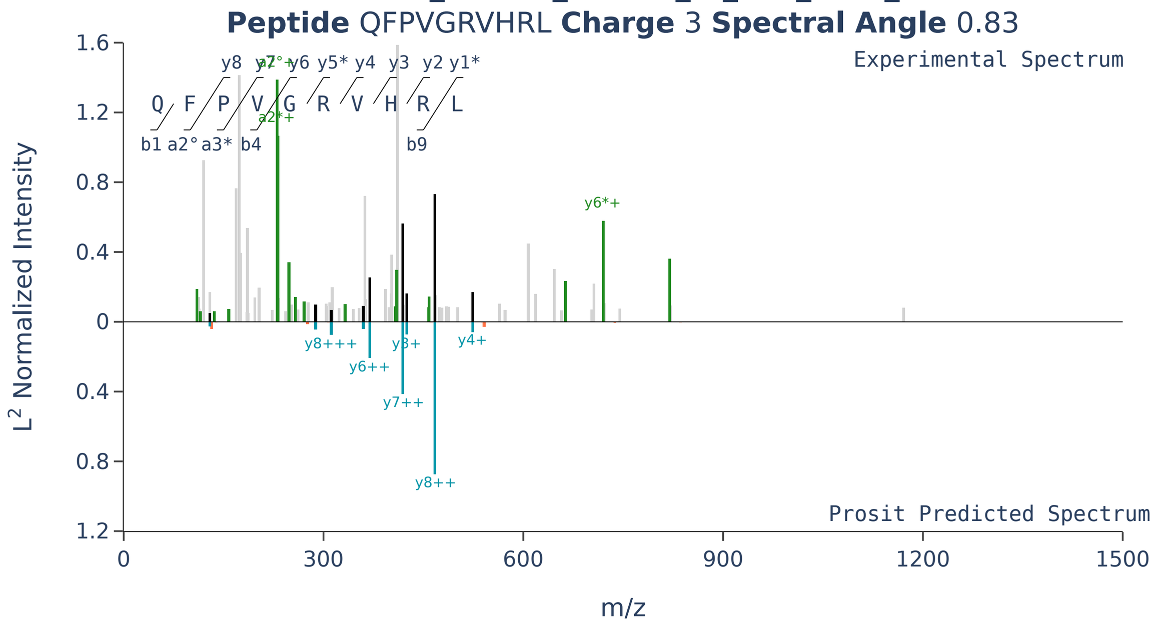


**Source** HRoetschke_291222_060123_Fu_H2A_b1R1_1h **Scan** 10833
**Peptide** NKLLGRVTL **Charge** 2 **Spectral Angle** 0.94

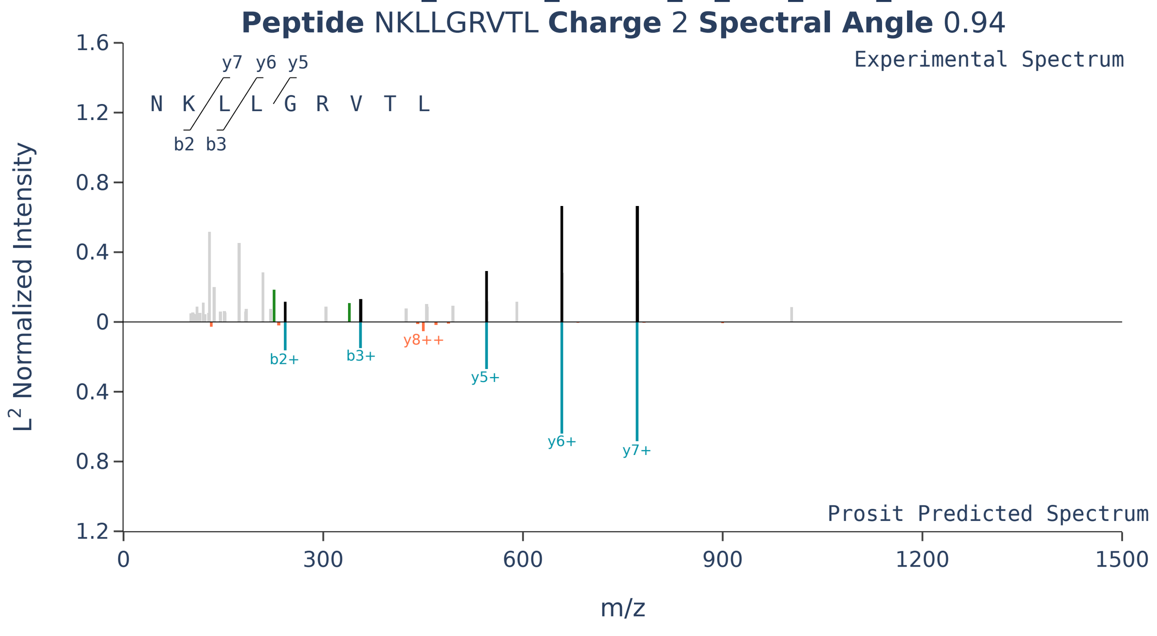


**Source** HRoetschke_291222_060123_Fu_H2A_b1R1_4h **Scan** 19167
**Peptide** QLAVRNDEELNKLLGRVTL **Charge** 3 **Spectral Angle** 0.89

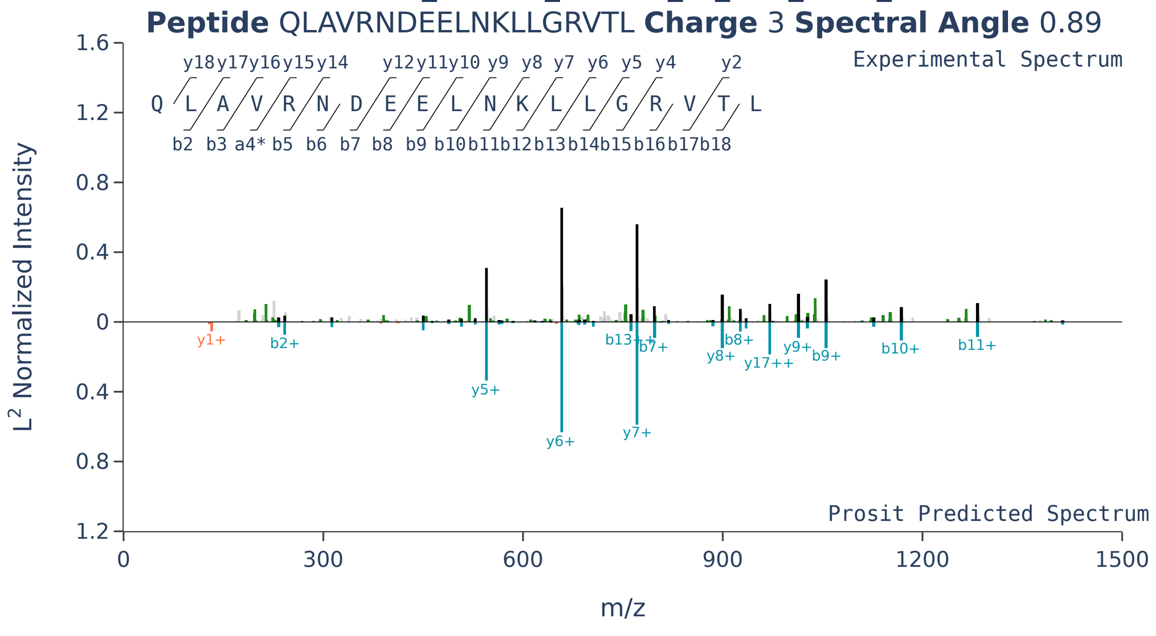


**Source** HRoetschke_291222_060123_Fu_H2A_b1R1_4h **Scan** 16203
**Peptide** NDEELNKLLGRVT **Charge** 2 **Spectral Angle** 0.89

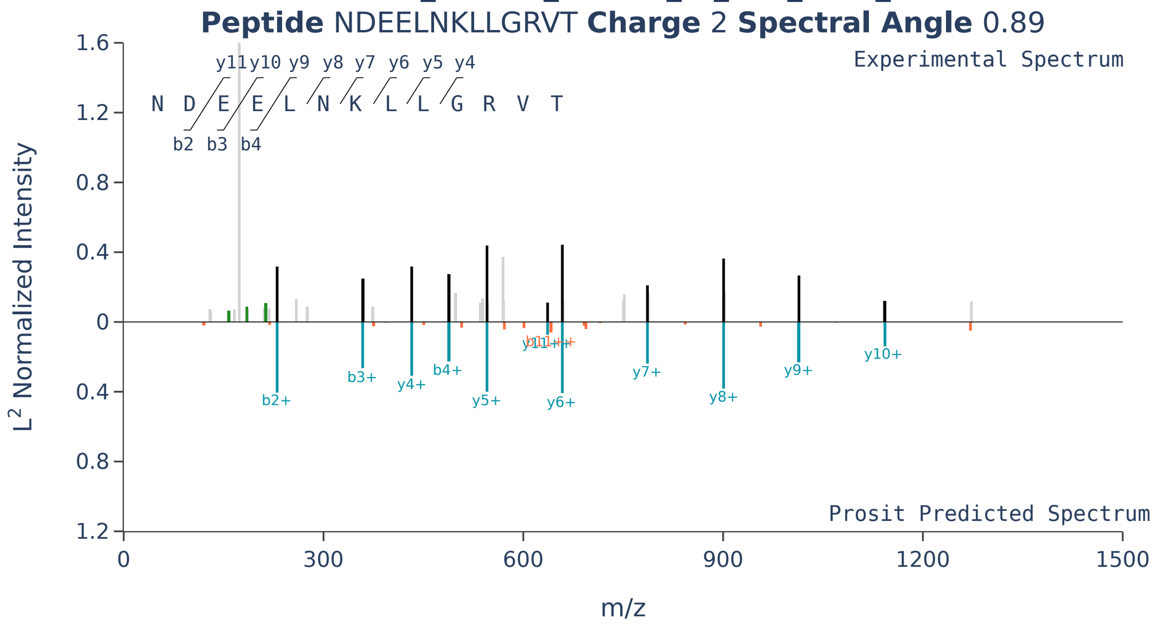

**Source** HRoetschke_291222_060123_Fu_H2A_b1R2_4h **Scan** 10693
**Peptide** SSRAGLQFPVGRVHRLLRKG **Charge** 4 **Spectral Angle** 0.8

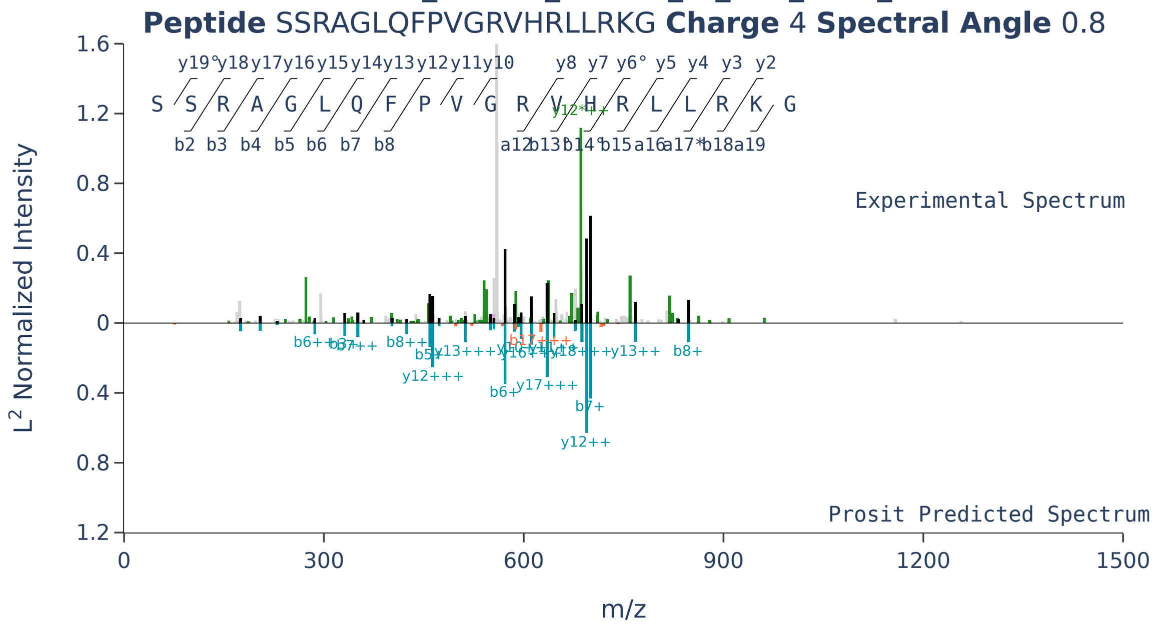


**Source** HRoetschke_291222_060123_Fu_H2A_b1R1_2h **Scan** 9151
**Peptide** QLAVRNDEEL **Charge** 2 **Spectral Angle** 0.9

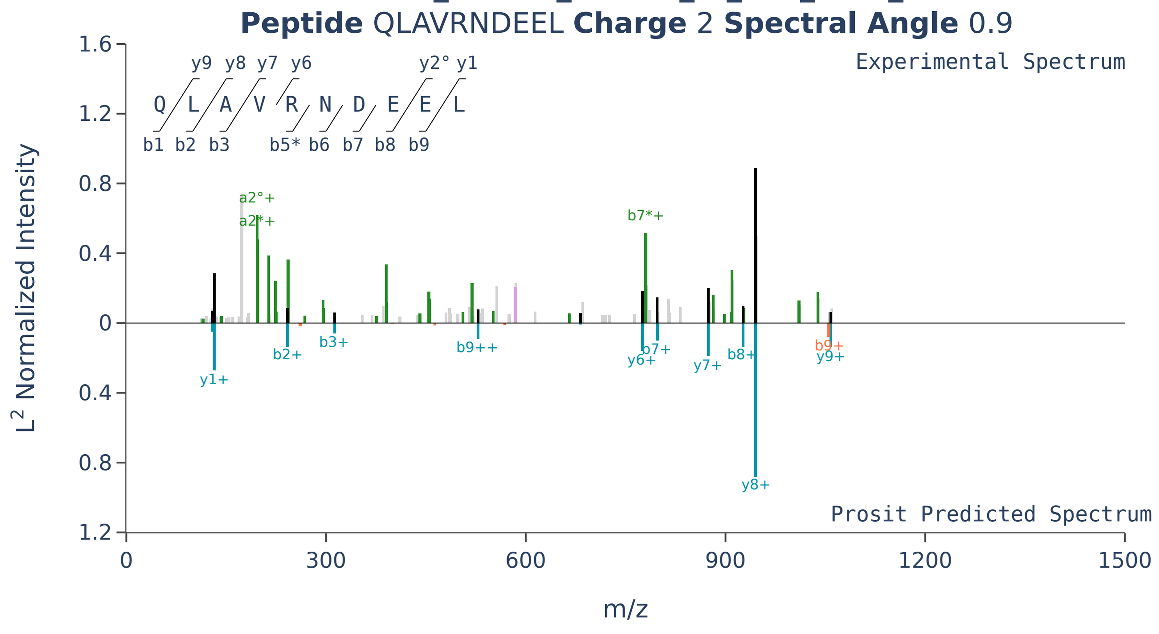


**Source** HRoetschke_291222_060123_Fu_H2A_b1R1_24h **Scan** 2262
**Peptide** VLLPKKTESSKSAKSK **Charge** 3 **Spectral Angle** 0.81

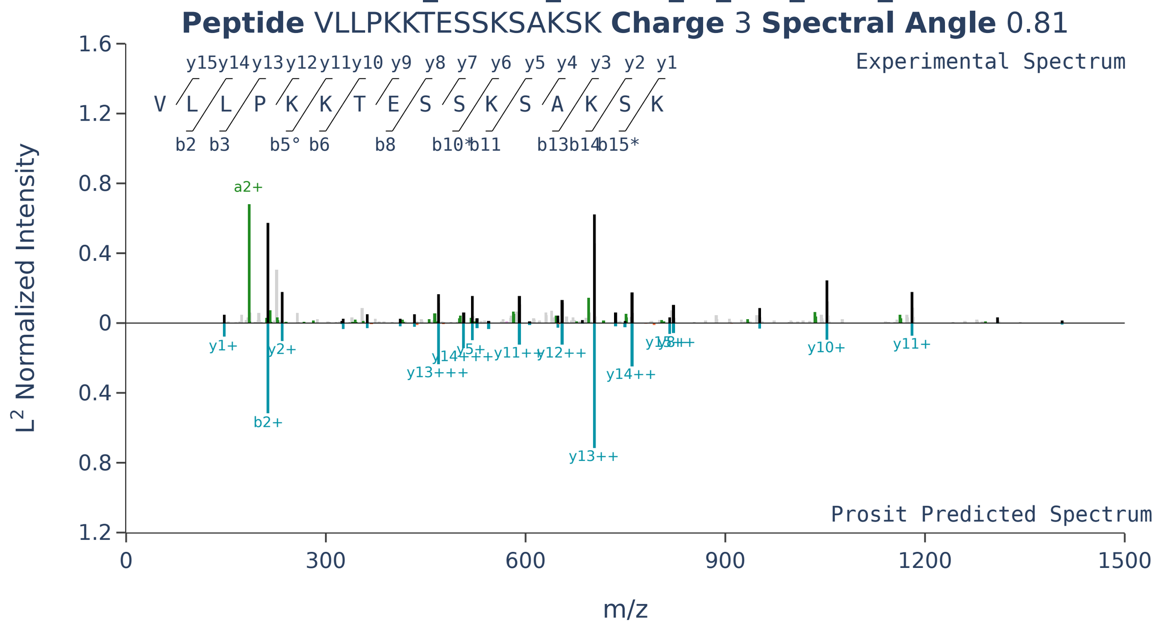


**Source** HRoetschke_291222_060123_Fu_H2A_b1R1_2h **Scan** 4162
**Peptide** SVLLPKKTESSK **Charge** 3 **Spectral Angle** 0.79

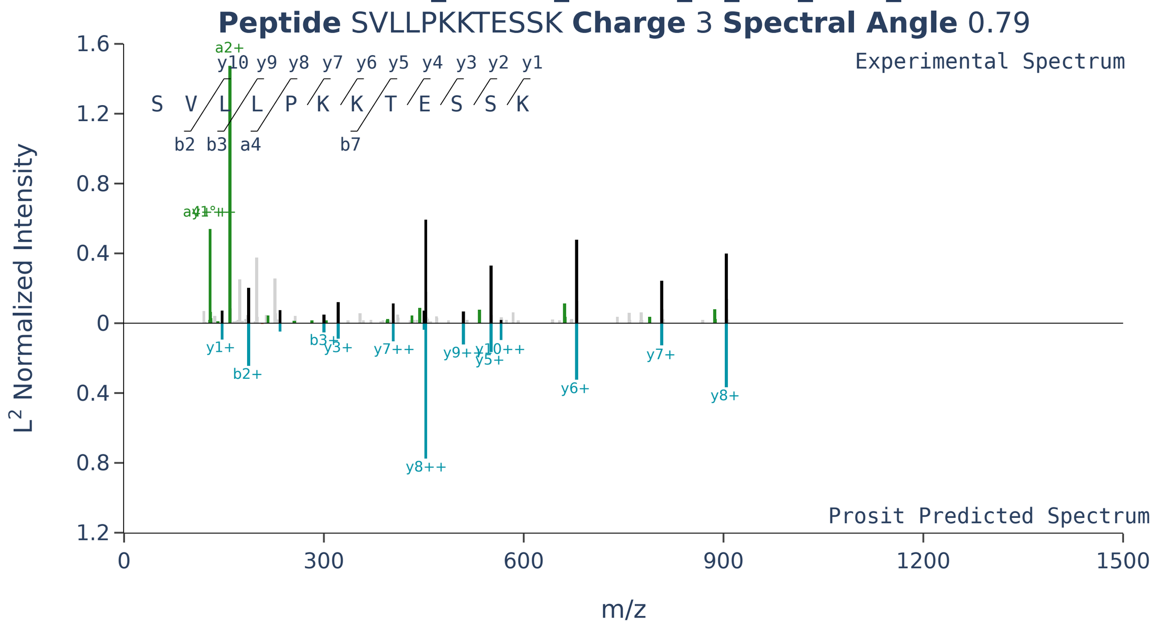


**Source** HRoetschke_291222_060123_Fu_H2A_b1R2_24h **Scan** 4527
**Peptide** GNYAERVGAG **Charge** 2 **Spectral Angle** 0.93

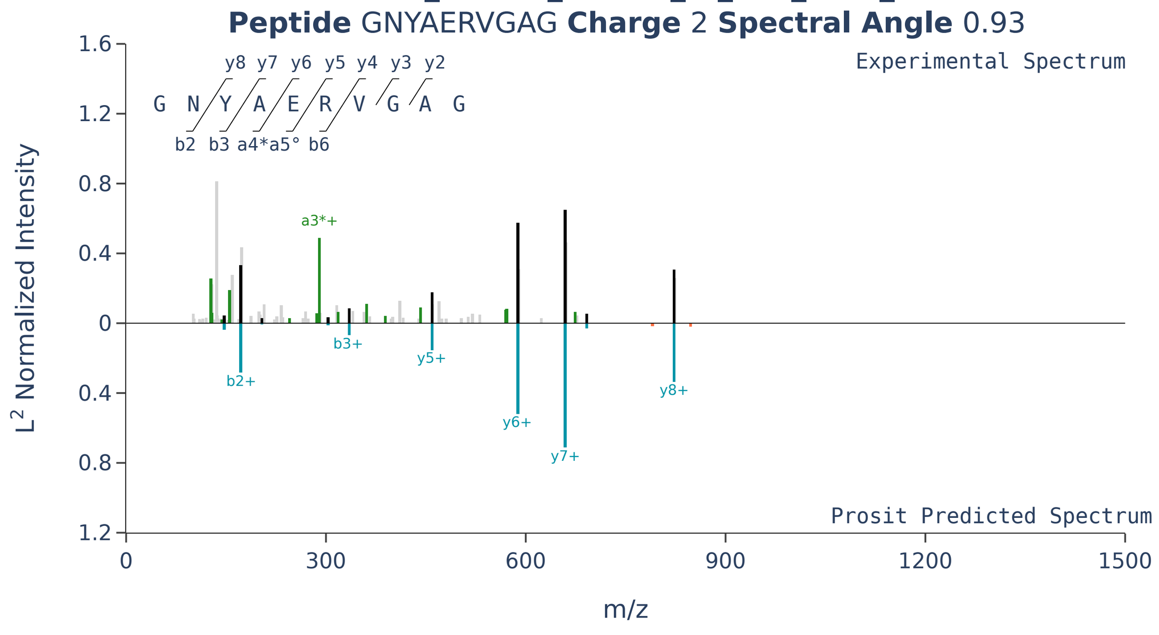


**Source** HRoetschke_291222_060123_Fu_H2A_b2R1_2h **Scan** 5521
**Peptide** LELAGNAARDNKKTRL **Charge** 4 **Spectral Angle** 0.89

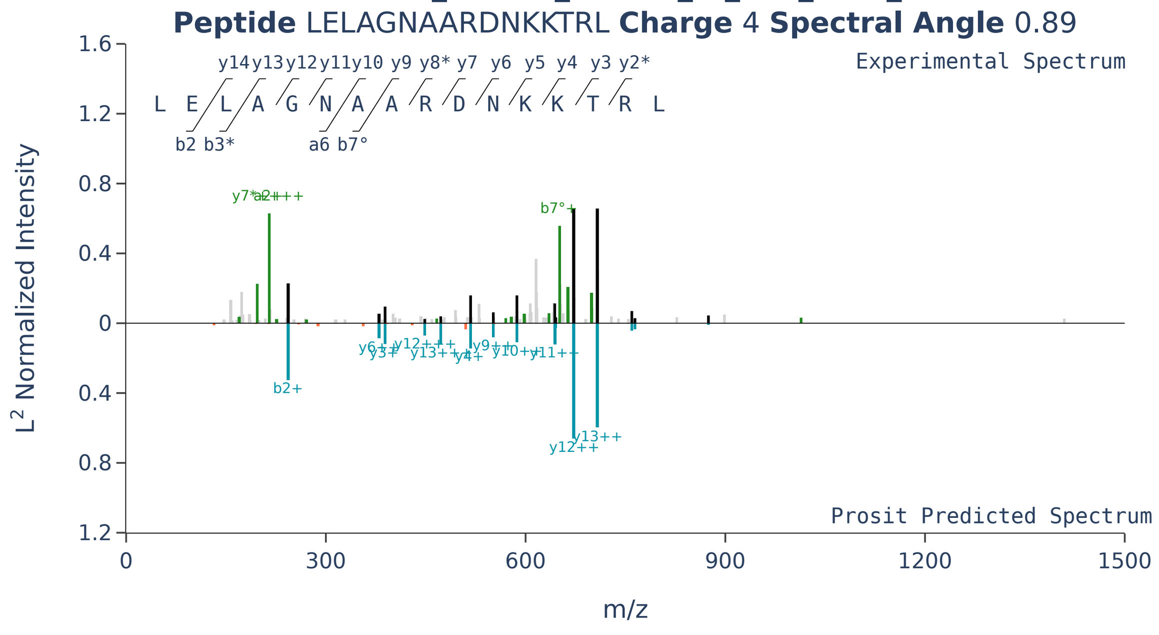


**Source** HRoetschke_291222_060123_Fu_H2A_b2R2_2h **Scan** 10458
**Peptide** RKGNYAERVGAGAPVYLAA **Charge** 3 **Spectral Angle** 0.91

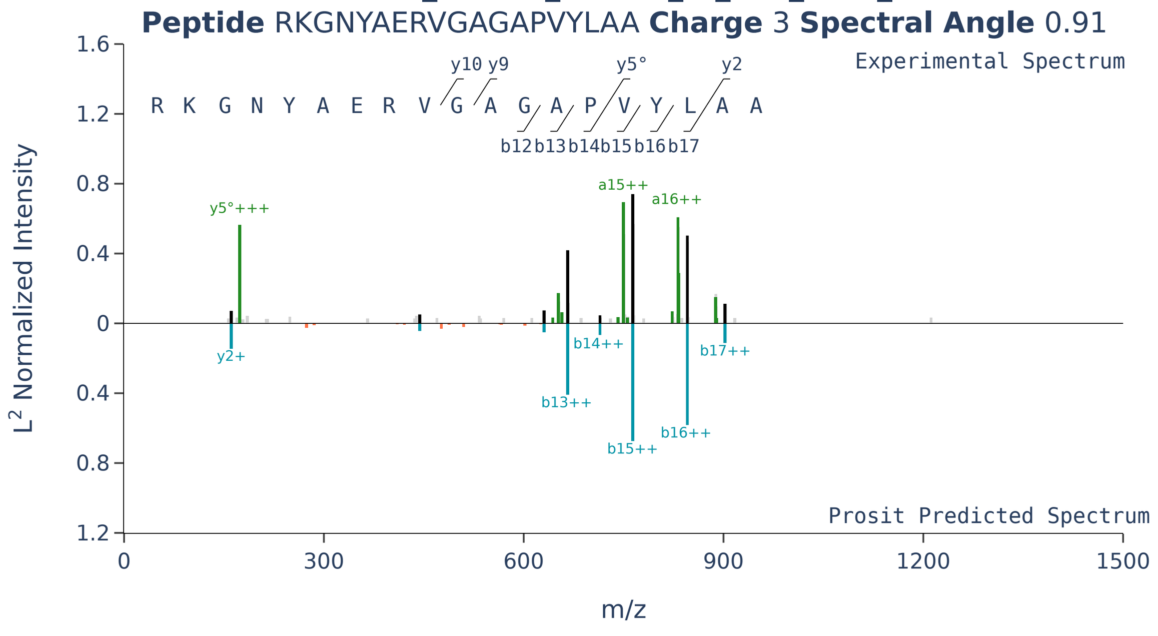


**Source** HRoetschke_291222_060123_Fu_H2A_b1R1_1h **Scan** 5979
**Peptide** LAVRNDEELN **Charge** 2 **Spectral Angle** 0.91

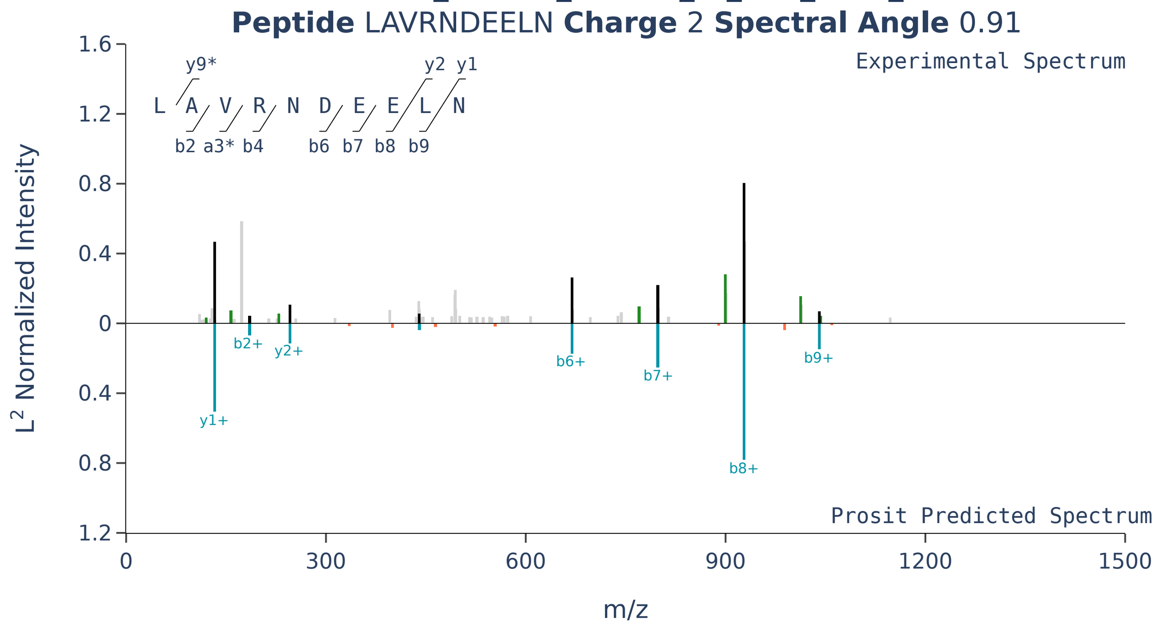


**Source** HRoetschke_291222_060123_Fu_H2A_b1R2_24h **Scan** 12422
**Peptide** RVGAGAPVYLAA **Charge** 2 **Spectral Angle** 0.89

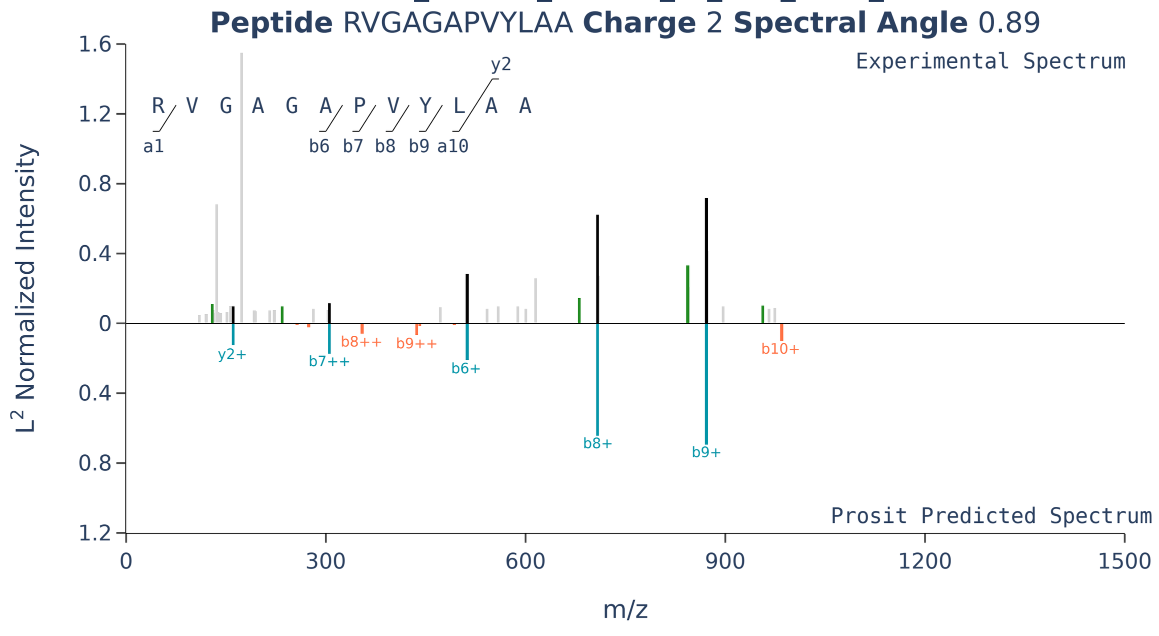


**Source** HRoetschke_291222_060123_Fu_H2A_b2R2_2h **Scan** 3034
**Peptide** HRLLRKGNYAERVG **Charge** 5 **Spectral Angle** 0.87

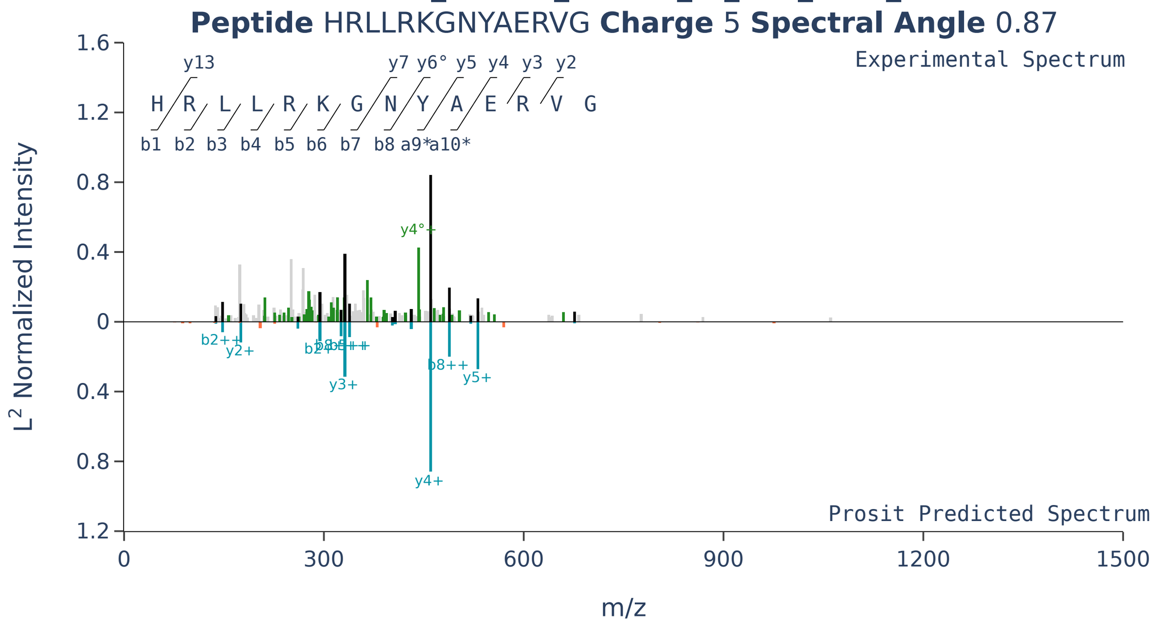


**Source** HRoetschke_291222_060123_Fu_H2A_b1R1_1h **Scan** 6952
**Peptide** LGRVTIAQG **Charge** 2 **Spectral Angle** 0.94

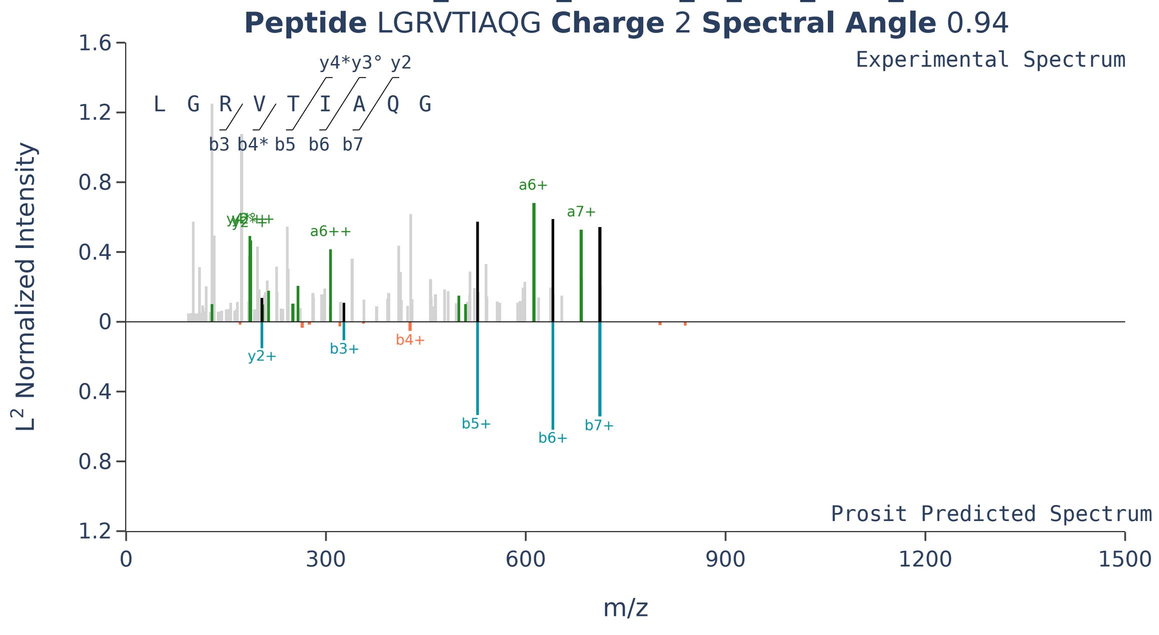


**Source** HRoetschke_291222_060123_Fu_H2A_b2R1_1h **Scan** 12343
**Peptide** ELNKLLGRVTI **Charge** 2 **Spectral Angle** 0.91

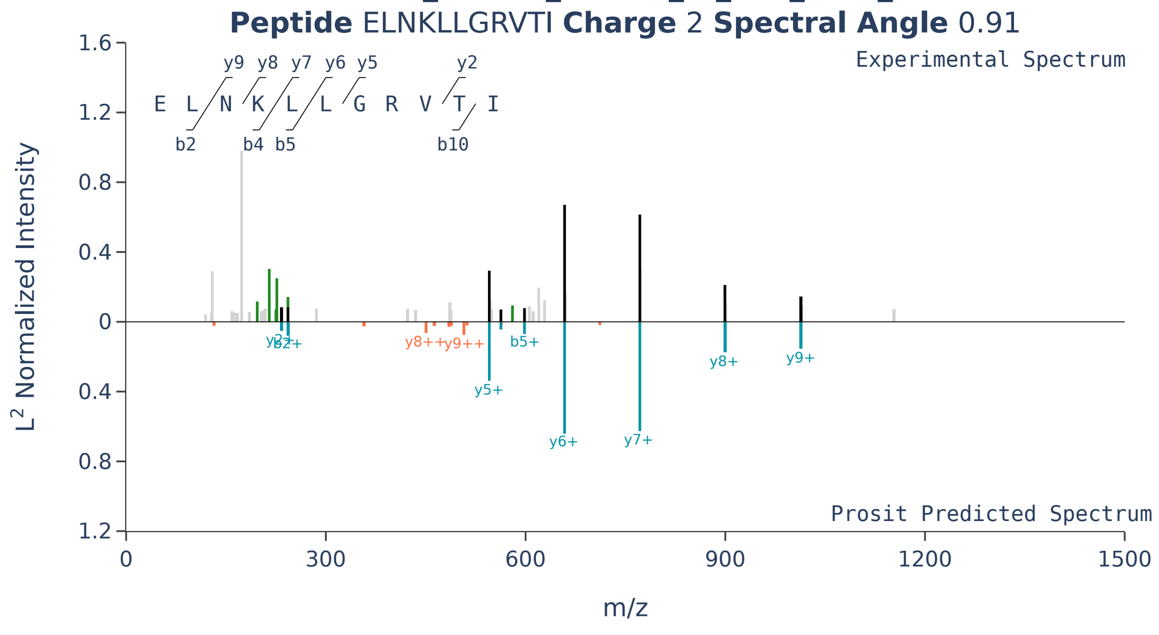


**Source** HRoetschke_291222_060123_Fu_H2A_b1R1_24h **Scan** 6524
**Peptide** TRIIPRHL **Charge** 3 **Spectral Angle** 0.76

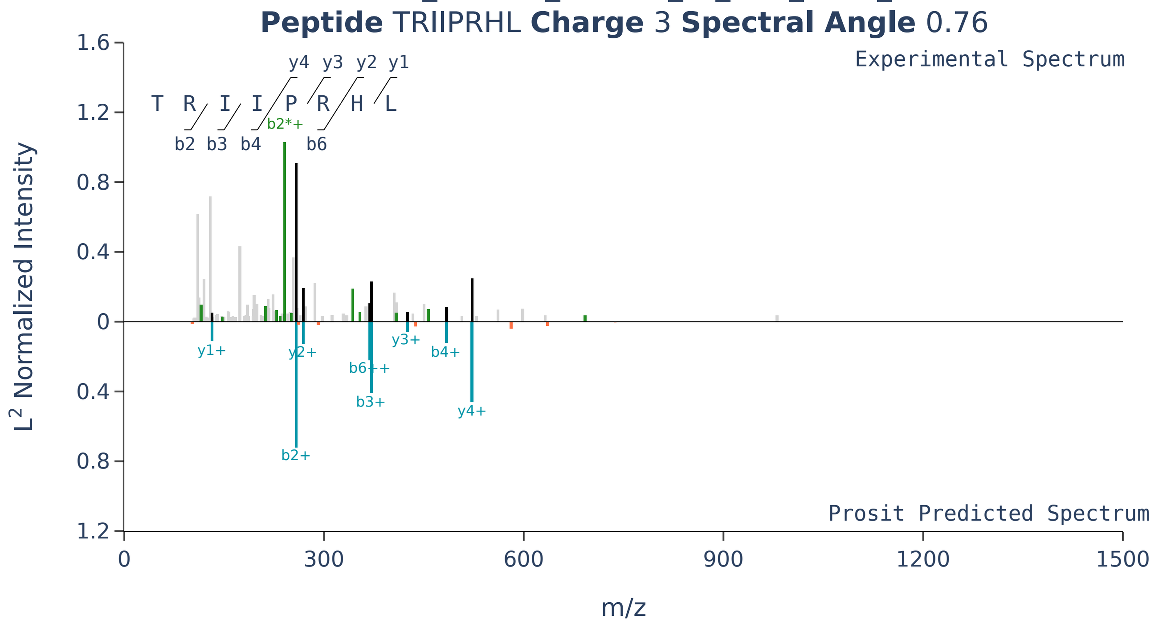


**Source** HRoetschke_291222_060123_Fu_H2A_b1R1_2h **Scan** 17108
**Peptide** NDEELNKLLGRVTIAQG **Charge** 3 **Spectral Angle** 0.75

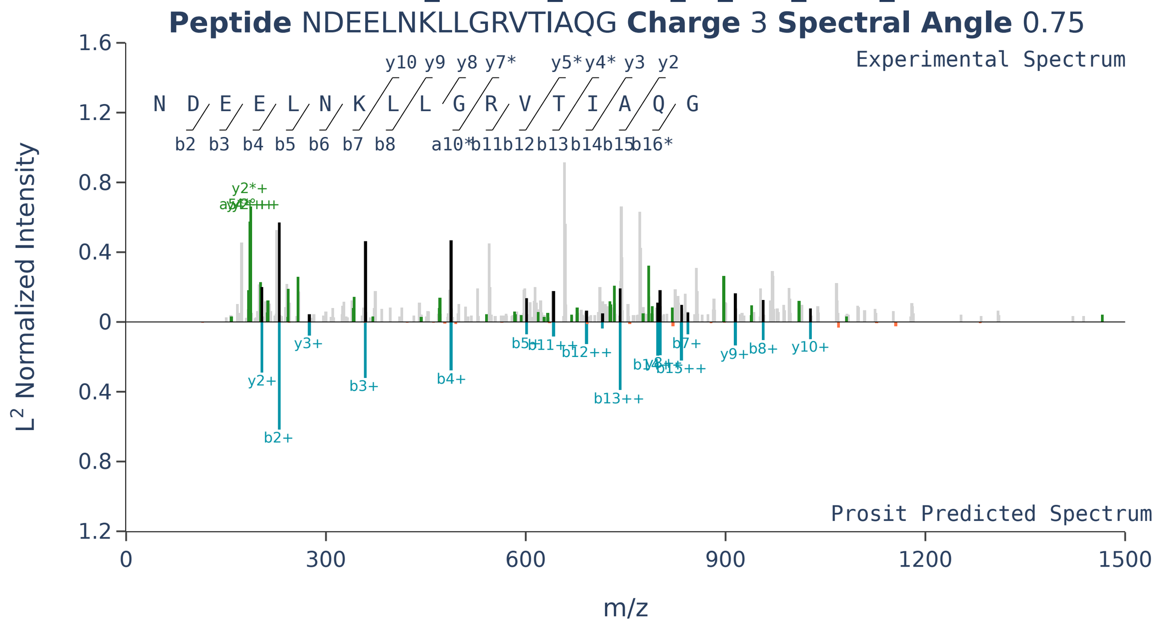


**Source** HRoetschke_291222_060123_Fu_H2A_b1R1_4h **Scan** 7364
**Peptide** QLAVRNDEELN **Charge** 2 **Spectral Angle** 0.89

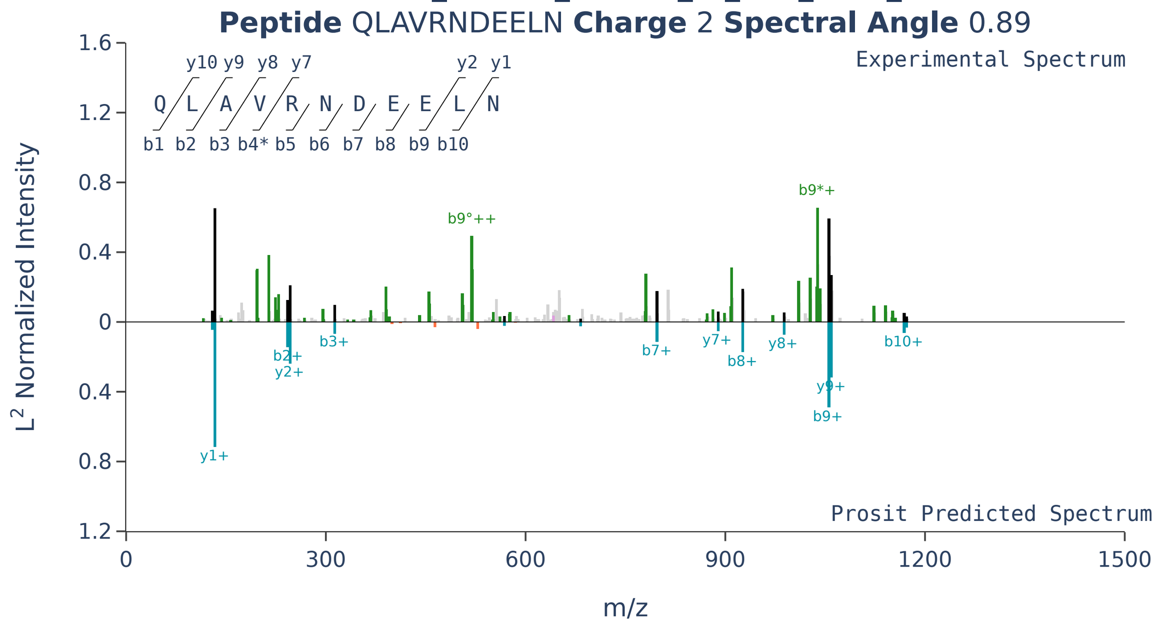

**Source** HRoetschke_291222_060123_Fu_H2A_b1R2_4h **Scan** 8878
**Peptide** RAGLQFPVGRVHR **Charge** 4 **Spectral Angle** 0.75

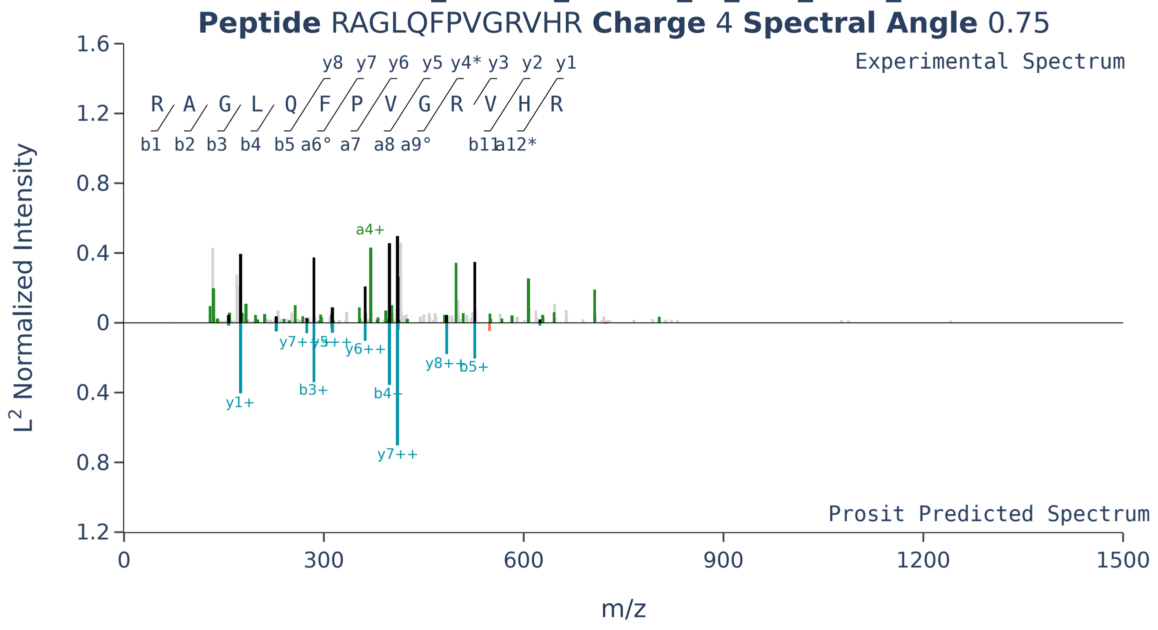


**Source** HRoetschke_291222_060123_Fu_H2A_b1R1_4h **Scan** 13830
**Peptide** ERVGAGAPVYLAA **Charge** 2 **Spectral Angle** 0.89

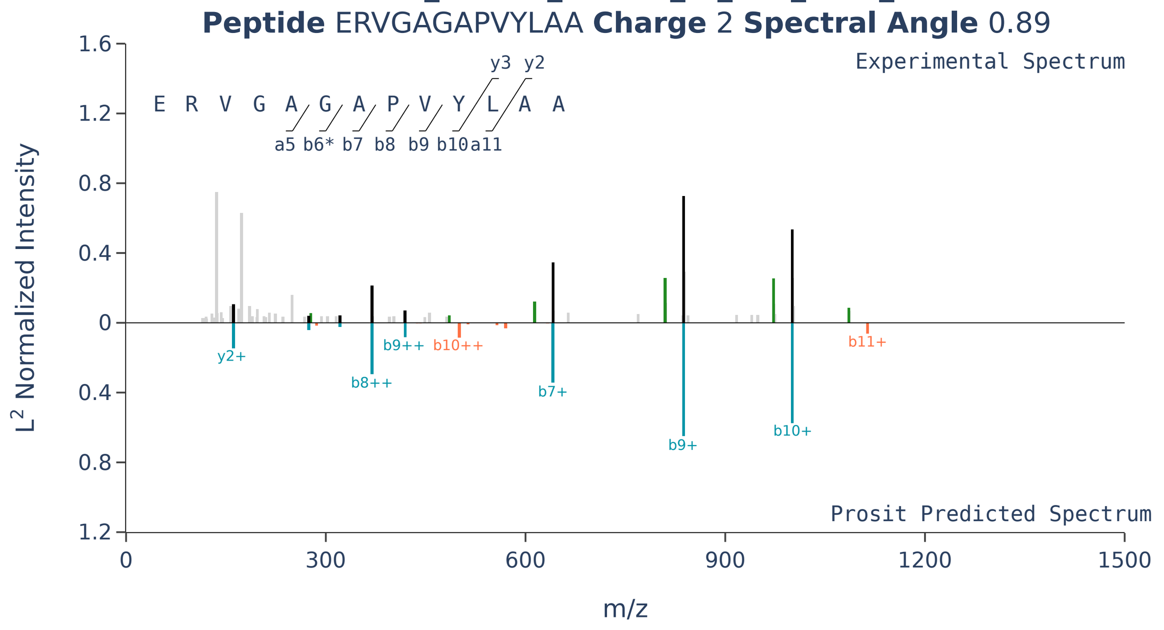


**Source** HRoetschke_291222_060123_Fu_H2A_b2R2_4h **Scan** 2803
**Peptide** ELAGNAARDNKKTRL **Charge** 3 **Spectral Angle** 0.92

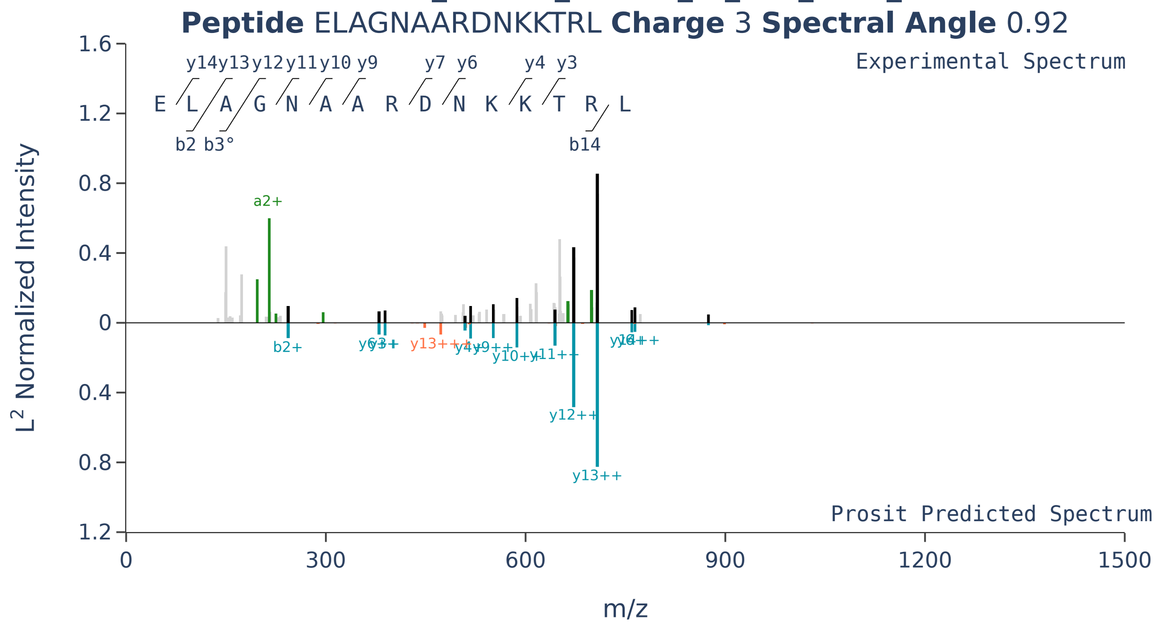


**Source** HRoetschke_291222_060123_Fu_H2A_b2R1_2h **Scan** 13456
**Peptide** GNYAERVGAGAPVYL **Charge** 2 **Spectral Angle** 0.86

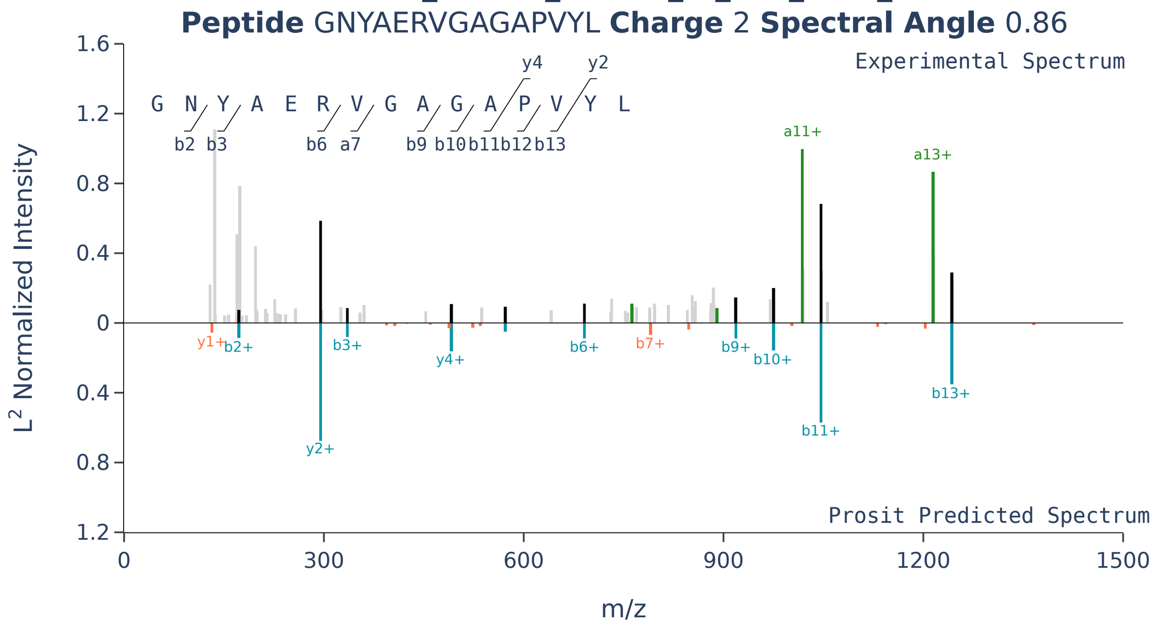


**Source** HRoetschke_291222_060123_Fu_H2A_b1R1_4h **Scan** 16621
**Peptide** RNDEELNKLLGRVTIAQG **Charge** 3 **Spectral Angle** 0.84

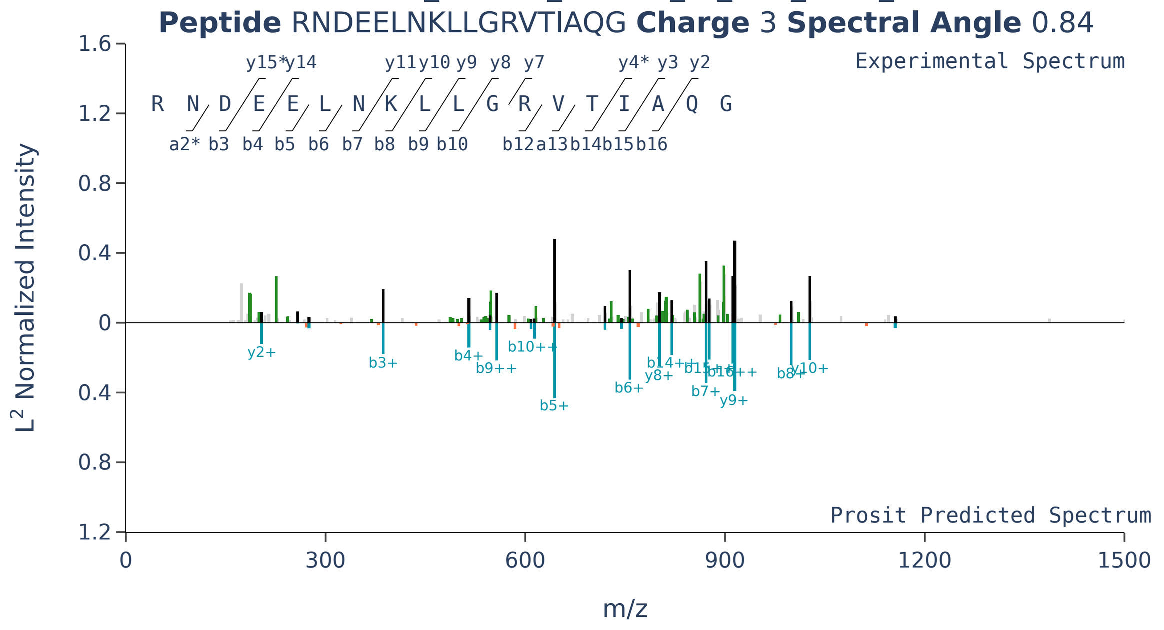


**Source** HRoetschke_291222_060123_Fu_H2A_b1R2_1h **Scan** 11969
**Peptide** VRNDEELNKLLGRVT **Charge** 3 **Spectral Angle** 0.84

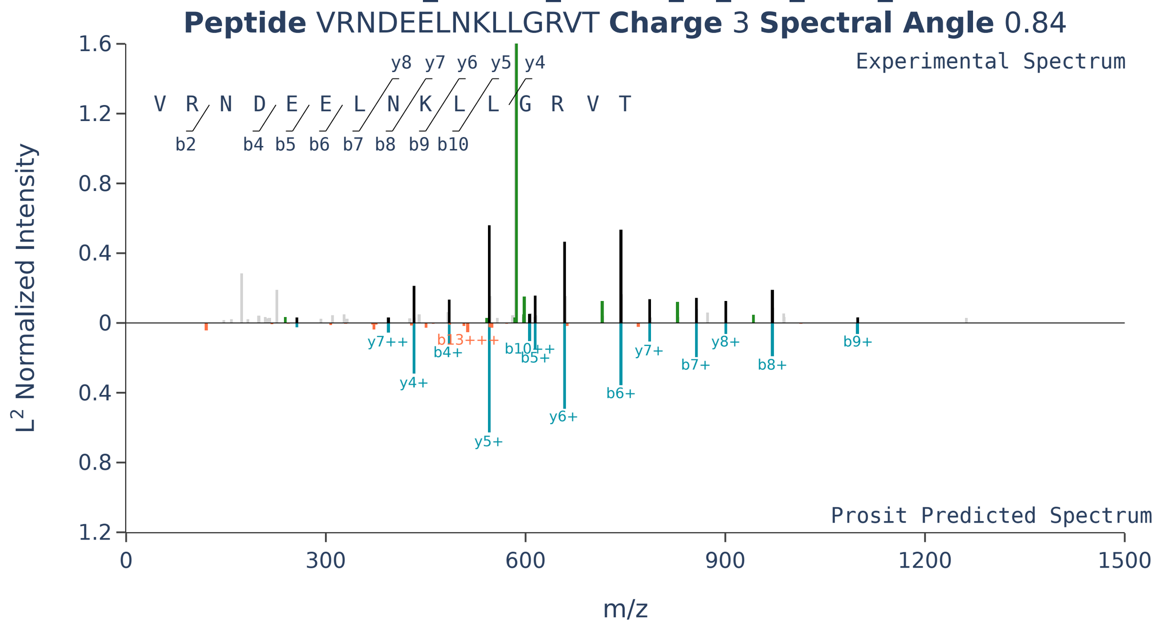


**Source** HRoetschke_291222_060123_Fu_H2A_b2R1_24h **Scan** 4496
**Peptide** LLRKGNYAERVG **Charge** 3 **Spectral Angle** 0.9

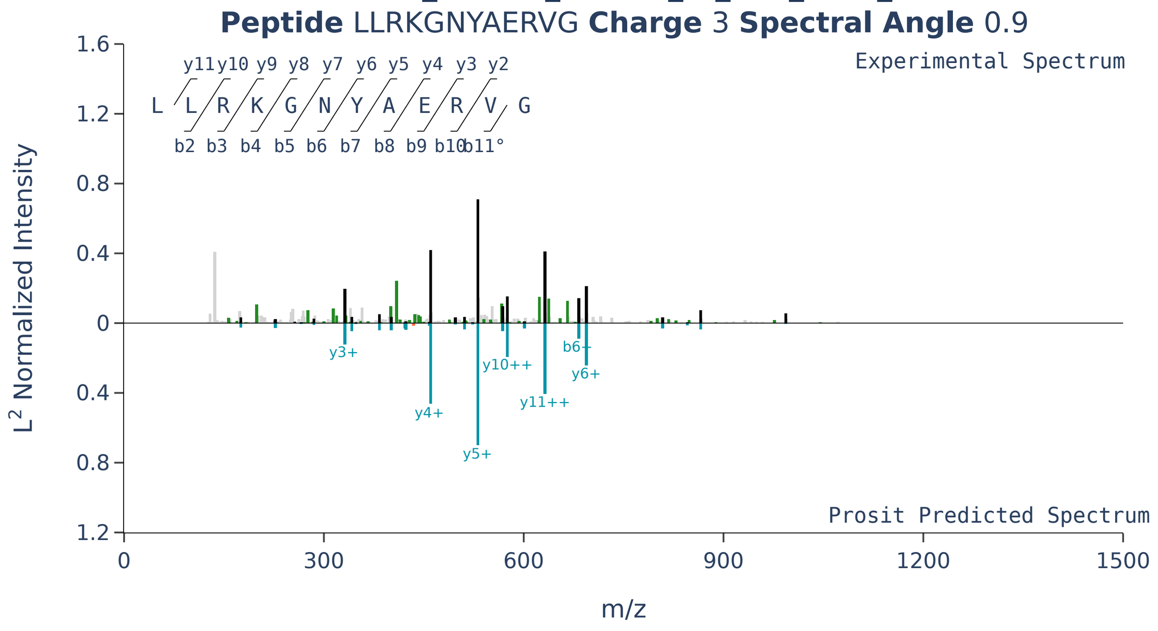


**Source** HRoetschke_291222_060123_Fu_H2A_b2R1_2h **Scan** 10721
**Peptide** TAEILELAGNAARDNKKTR **Charge** 5 **Spectral Angle** 0.86

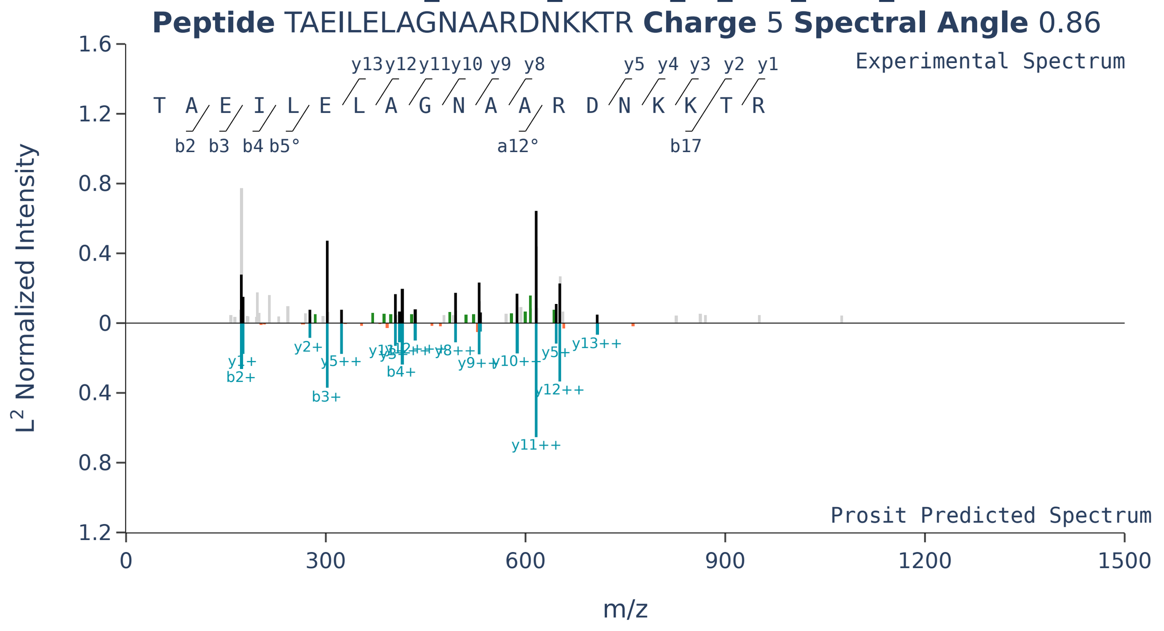


**Source** HRoetschke_291222_060123_Fu_H2A_b1R1_2h **Scan** 2310
**Peptide** HRLLRKGNY **Charge** 3 **Spectral Angle** 0.78

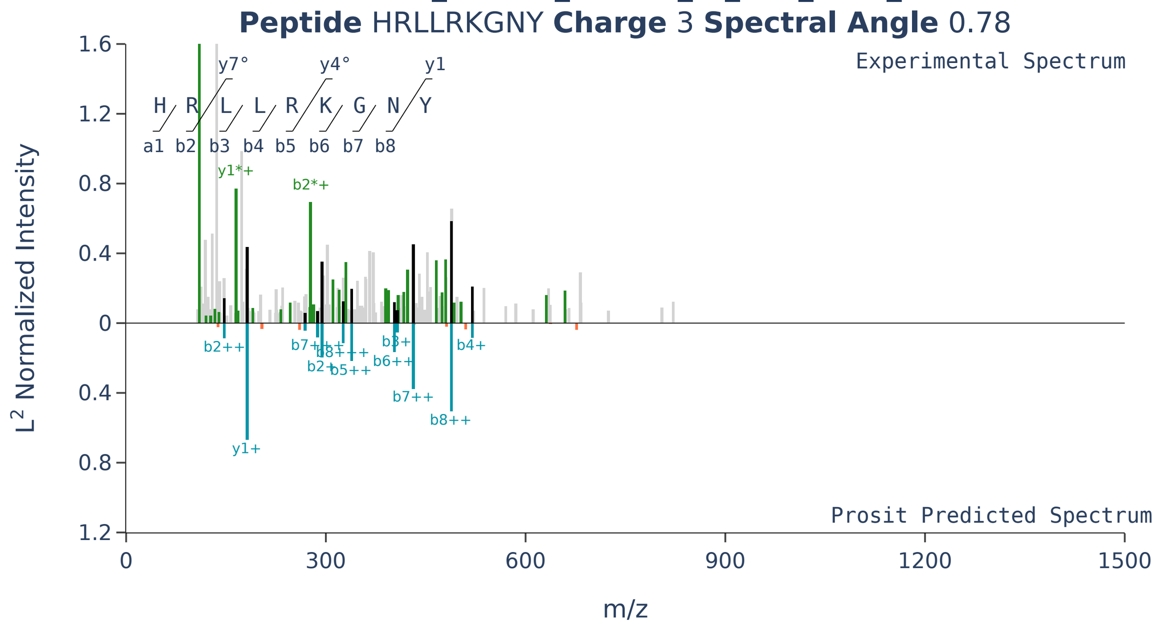


**Source** HRoetschke_291222_060123_Fu_H2A_b1R2_24h **Scan** 7759
**Peptide** AKTRSSRAGLQFPVGRVH **Charge** 4 **Spectral Angle** 0.82

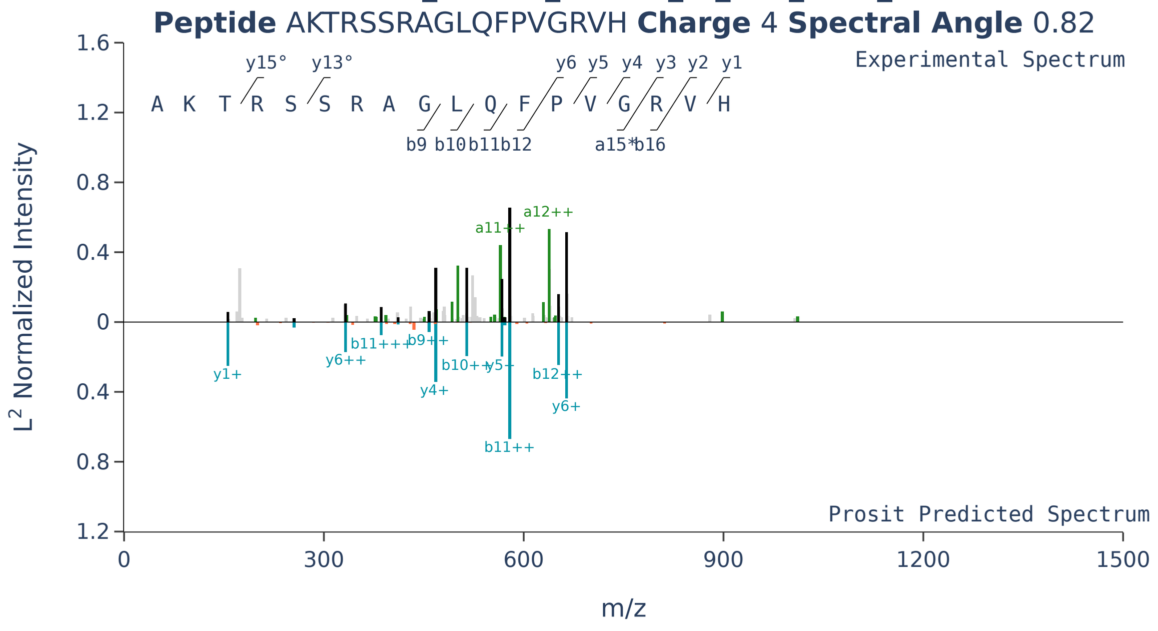


**Source** HRoetschke_291222_060123_Fu_H2A_b1R1_2h **Scan** 14344
**Peptide** AVRNDEELNKLLGRVT **Charge** 3 **Spectral Angle** 0.84

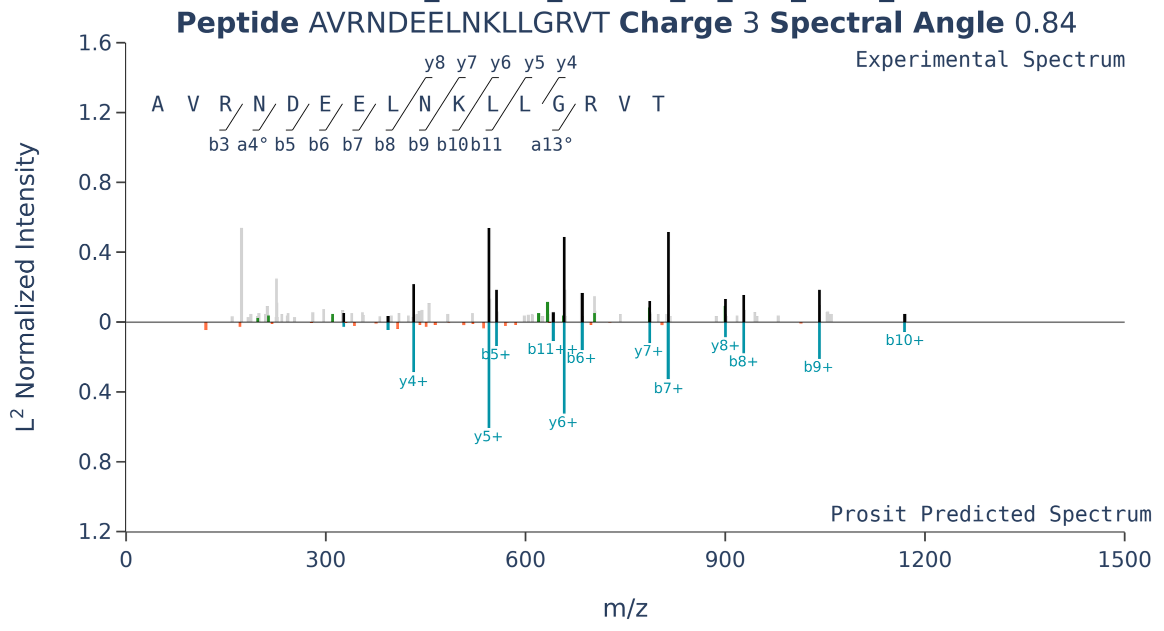


**Source** HRoetschke_291222_060123_Fu_H2A_b1R1_24h **Scan** 5235
**Peptide** LAGNAARDNKKTRIIPRHL **Charge** 4 **Spectral Angle** 0.95

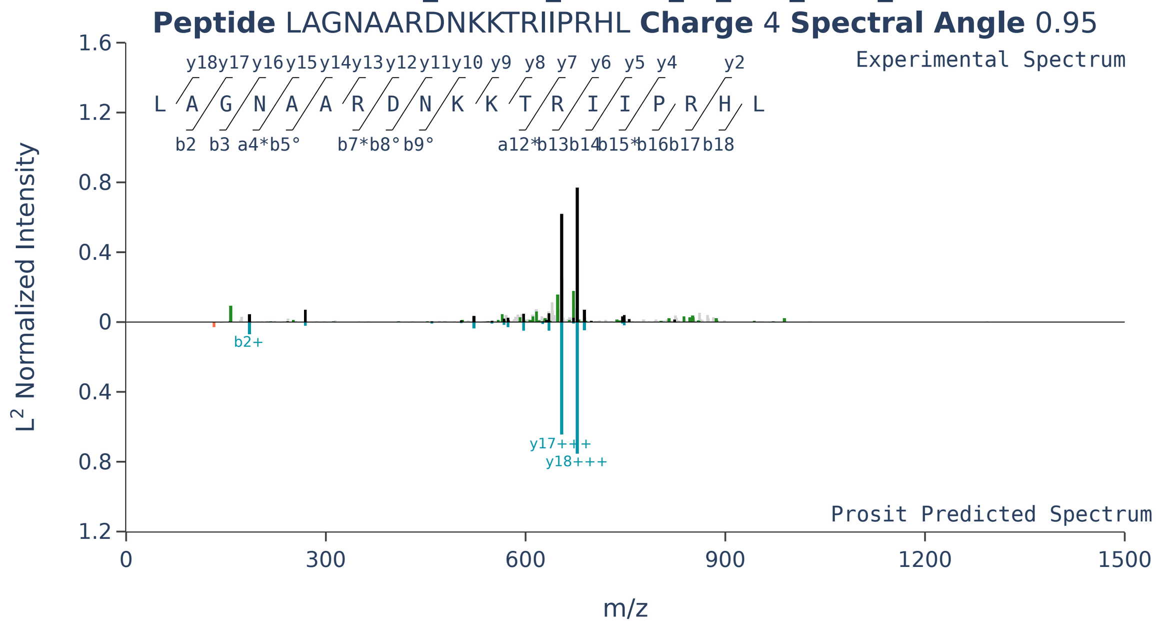


**Source** HRoetschke_291222_060123_Fu_H2A_b1R1_4h **Scan** 18887
**Peptide** QLAVRNDEELNKLLGRVTLA **Charge** 3 **Spectral Angle** 0.85

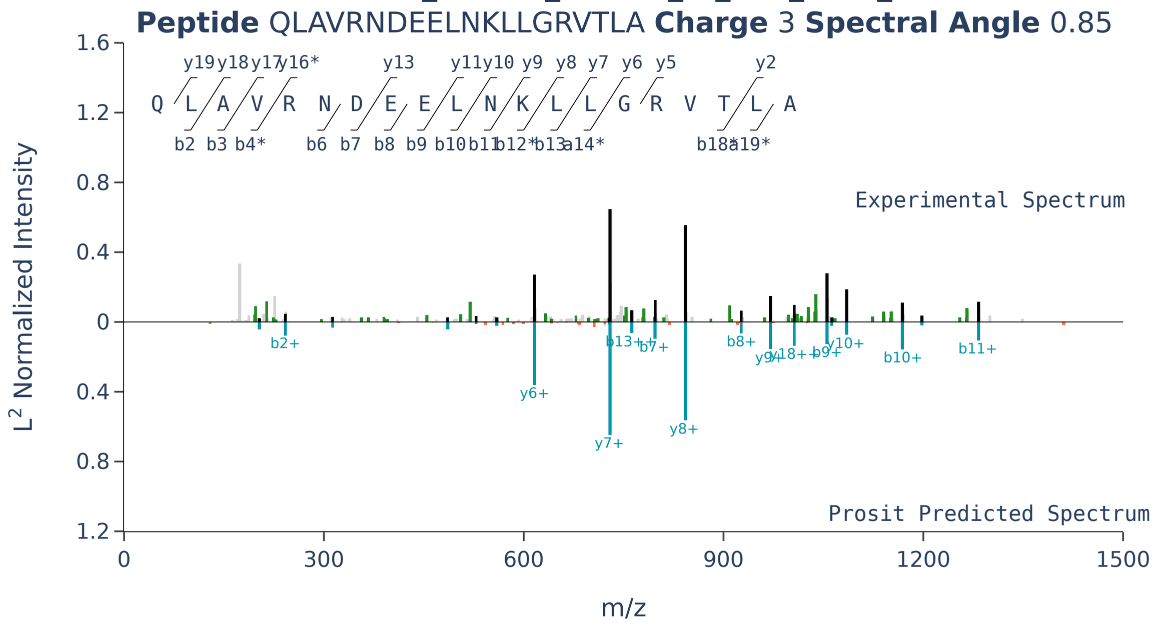


**Source** HRoetschke_291222_060123_Fu_H2A_b1R2_4h **Scan** 19594
**Peptide** LEYLTAEILE **Charge** 2 **Spectral Angle** 0.81

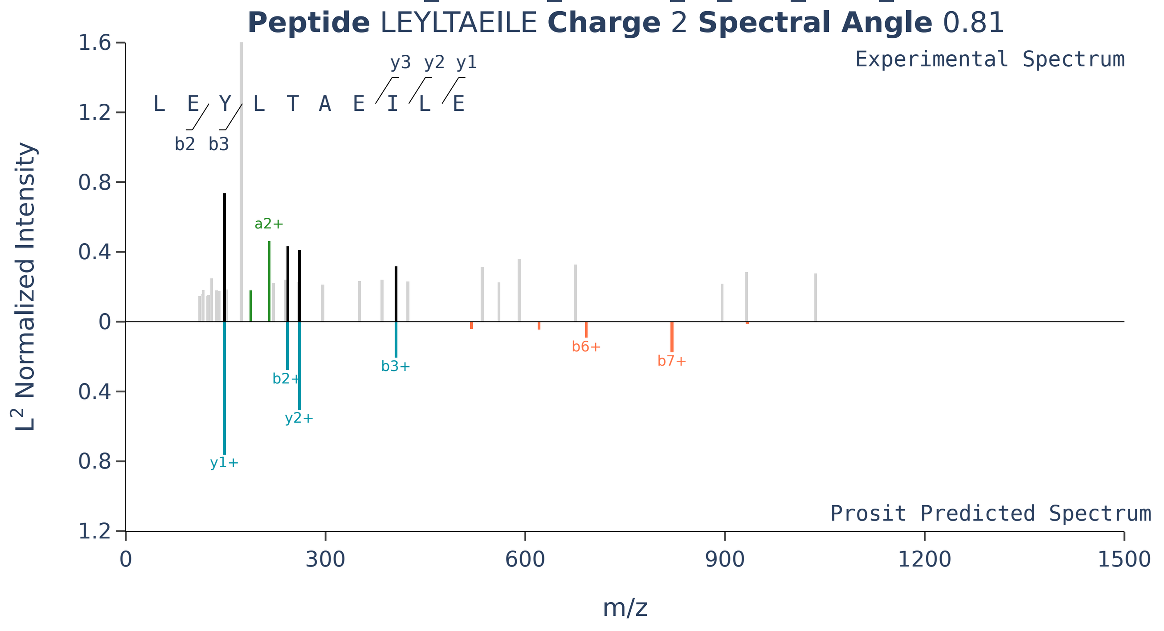


**Source** HRoetschke_291222_060123_Fu_H2A_b2R1_24h **Scan** 14789
**Peptide** RVTIAQGGVLPNLQ **Charge** 2 **Spectral Angle** 0.87

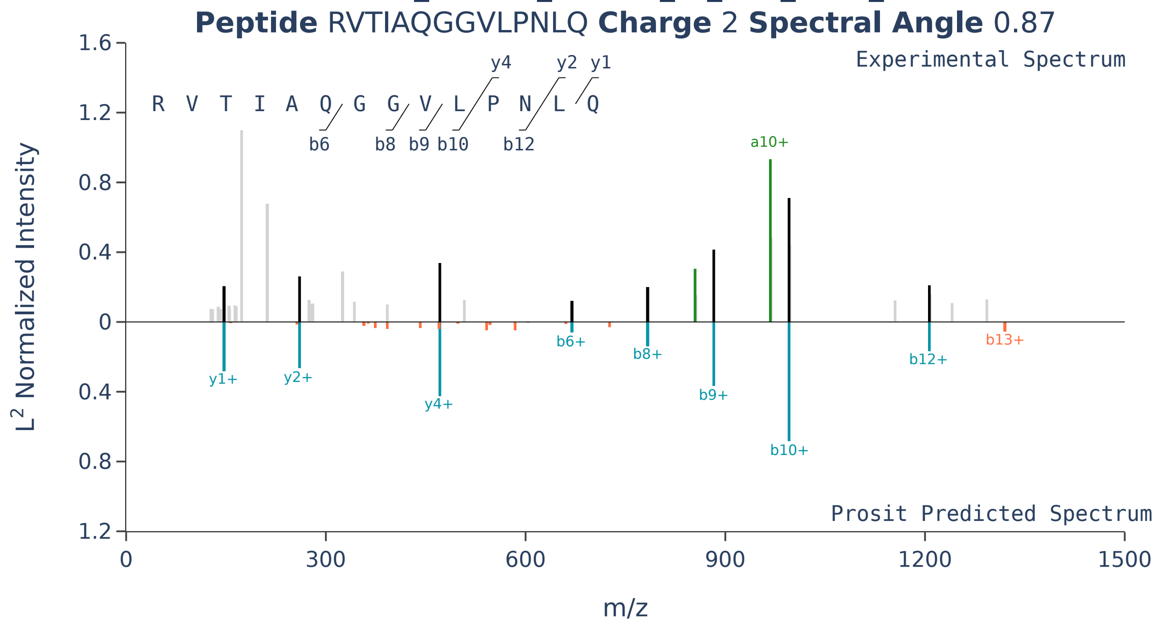

**Source** HRoetschke_291222_060123_Fu_H2A_b2R1_24h **Scan** 14755
**Peptide** TIAQGGVLPNIQS **Charge** 2 **Spectral Angle** 0.84

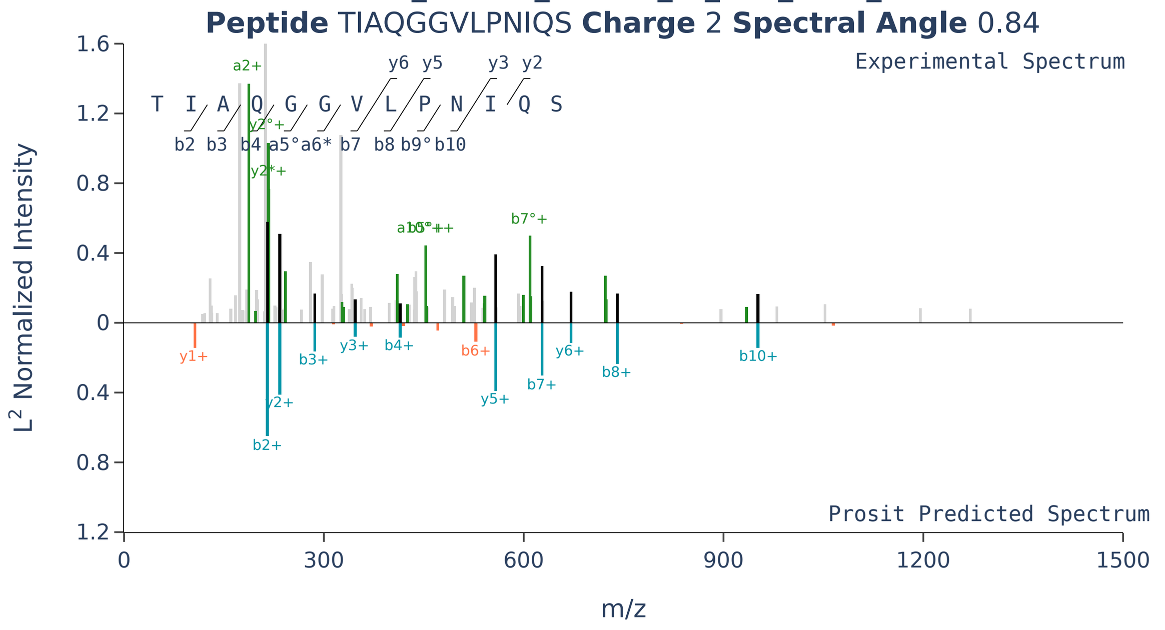


**Source** HRoetschke_291222_060123_Fu_H2A_b2R1_4h **Scan** 6705
**Peptide** AKAKTRSSRAGLQFPVGR **Charge** 4 **Spectral Angle** 0.85

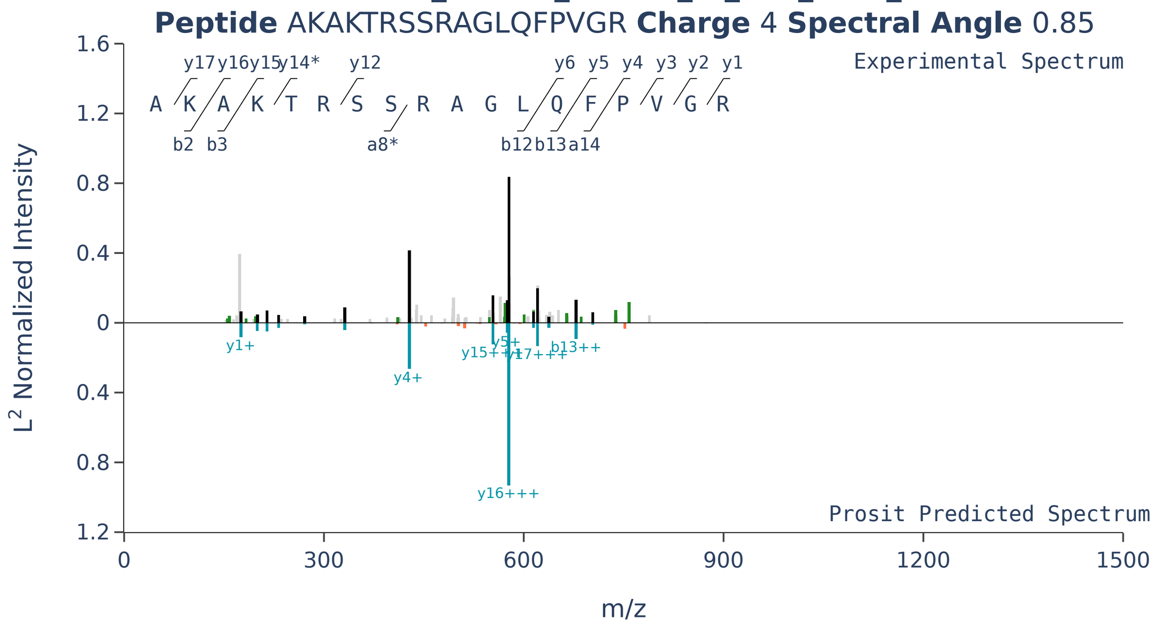


**Source** HRoetschke_291222_060123_Fu_H2A_b1R1_1h **Scan** 4535
**Peptide** AVRNDEEL **Charge** 2 **Spectral Angle** 0.88

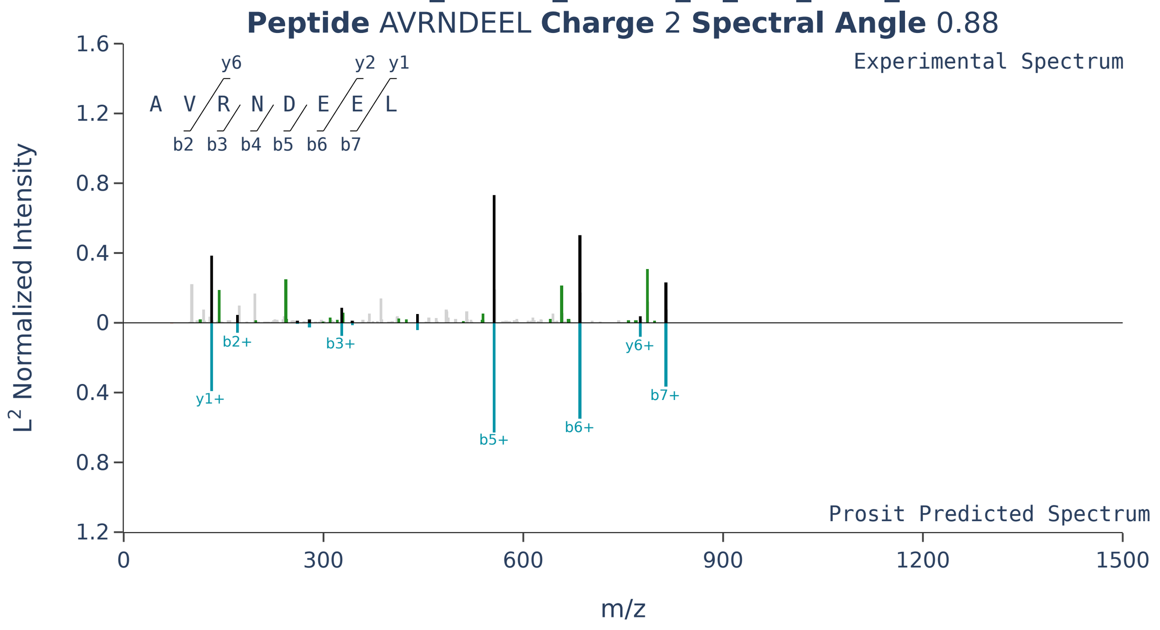


**Source** HRoetschke_291222_060123_Fu_H2A_b1R1_4h **Scan** 12567
**Peptide** SSRAGLQFPVGRVHRLLR **Charge** 5 **Spectral Angle** 0.78

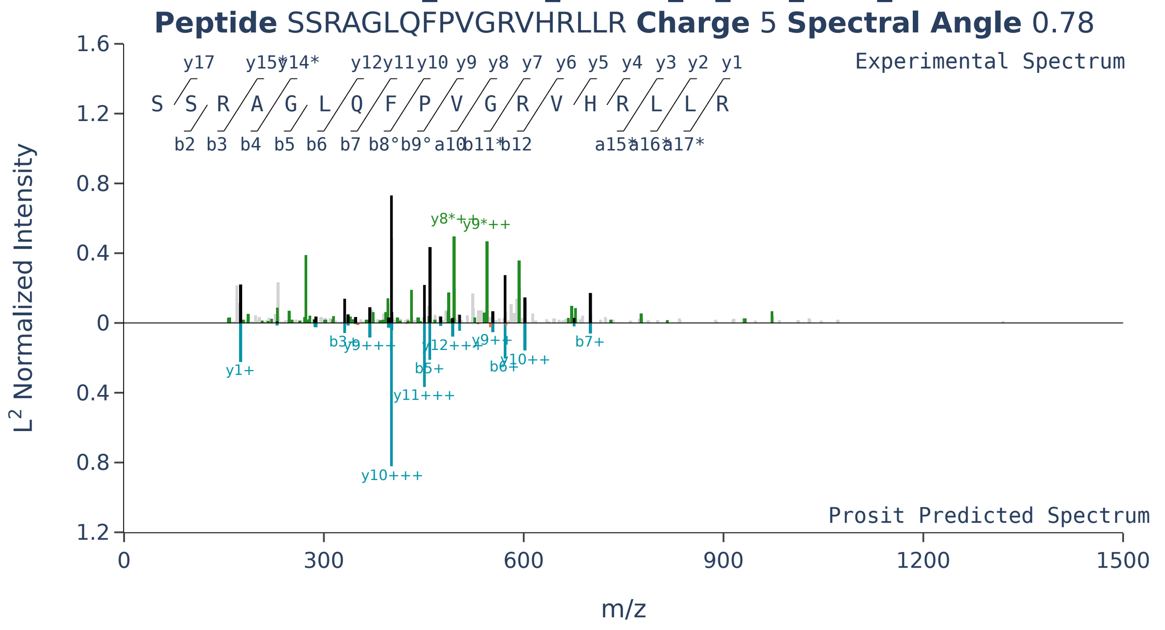


**Source** HRoetschke_291222_060123_Fu_H2A_b1R1_2h **Scan** 4856
**Peptide** NYAERVGAGA **Charge** 2 **Spectral Angle** 0.91

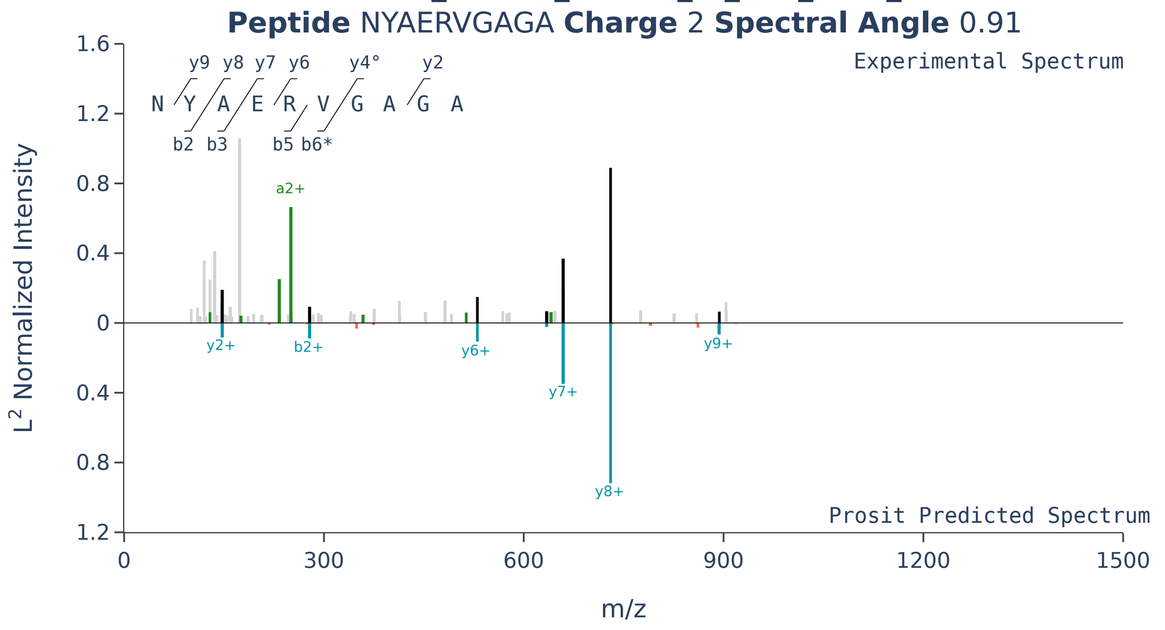


**Source** HRoetschke_291222_060123_Fu_H2A_b1R1_4h **Scan** 12089
**Peptide** LRKGNYAERVGAGAPVYLAA **Charge** 3 **Spectral Angle** 0.89

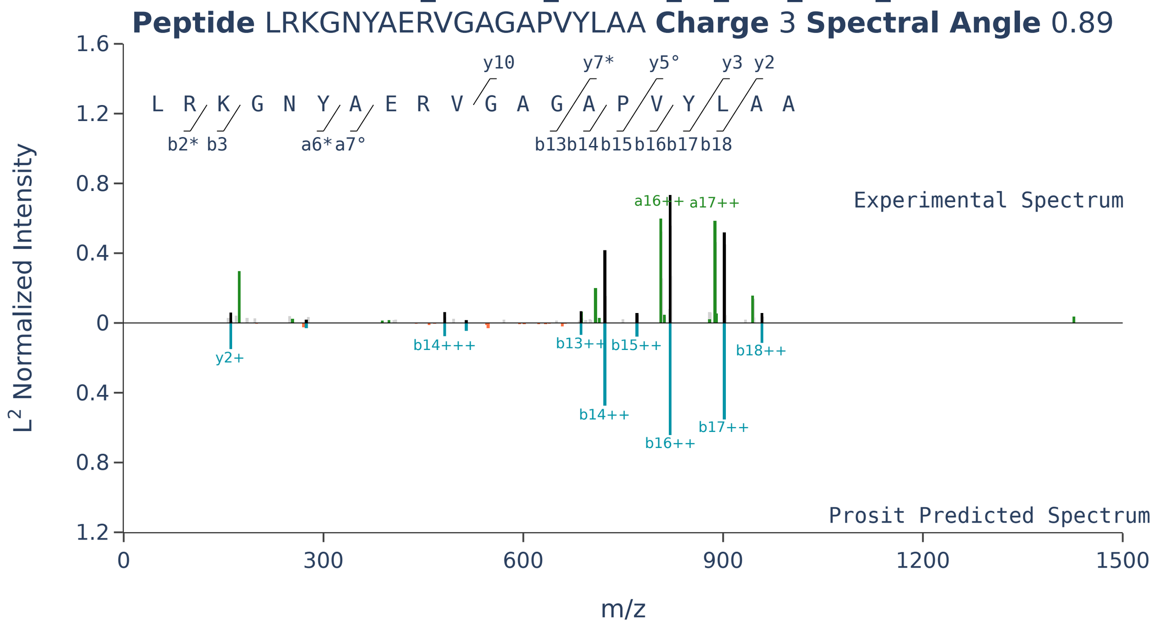


**Source** HRoetschke_291222_060123_Fu_H2A_b1R1_2h **Scan** 2914
**Peptide** AGNAARDNKKTRLL **Charge** 3 **Spectral Angle** 0.86

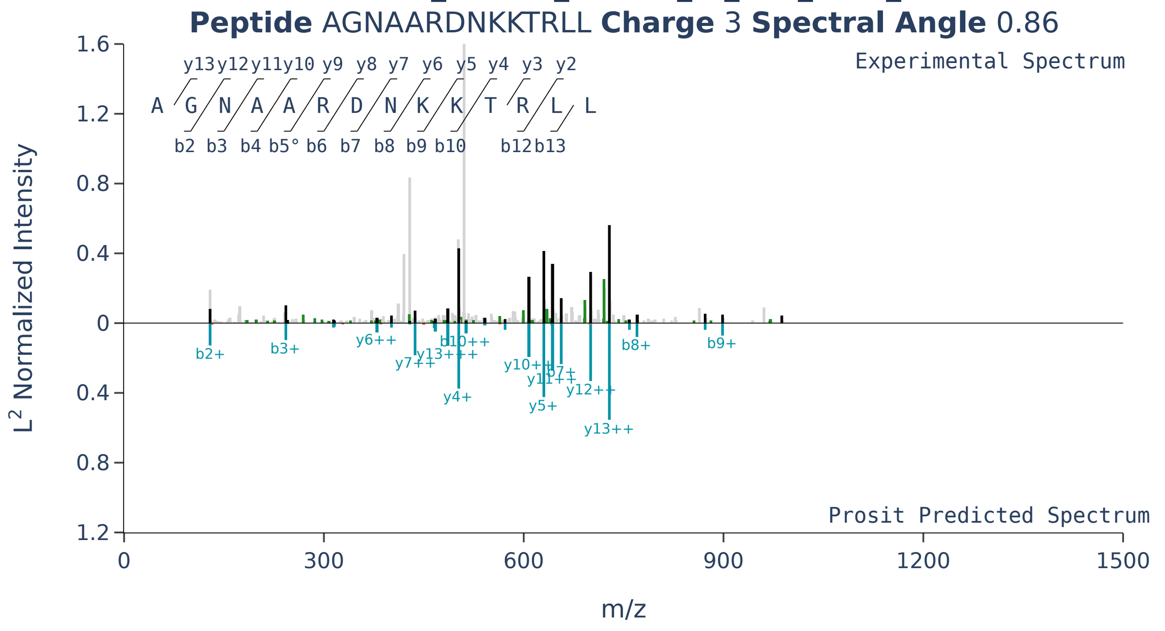


**Source** HRoetschke_291222_060123_Fu_H2A_b1R1_4h **Scan** 18406
**Peptide** QLAVRNDEELNKLLGRVTIAQG **Charge** 3 **Spectral Angle** 0.83

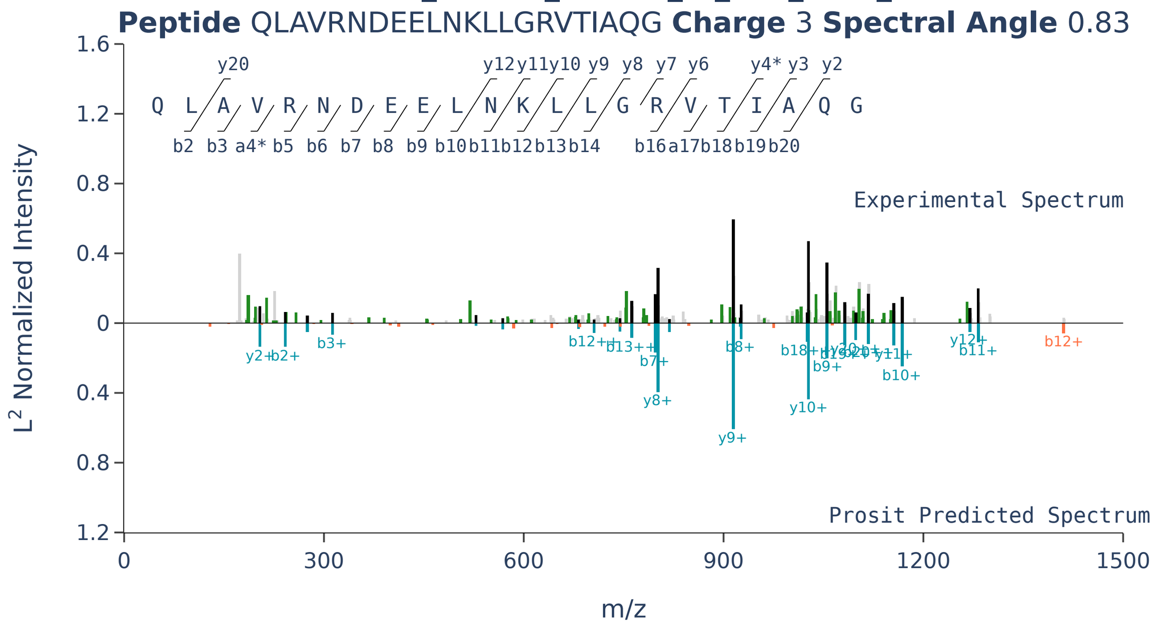


**Source** HRoetschke_291222_060123_Fu_H2A_b1R1_1h **Scan** 12603
**Peptide** SRAGLQFPVG **Charge** 2 **Spectral Angle** 0.86

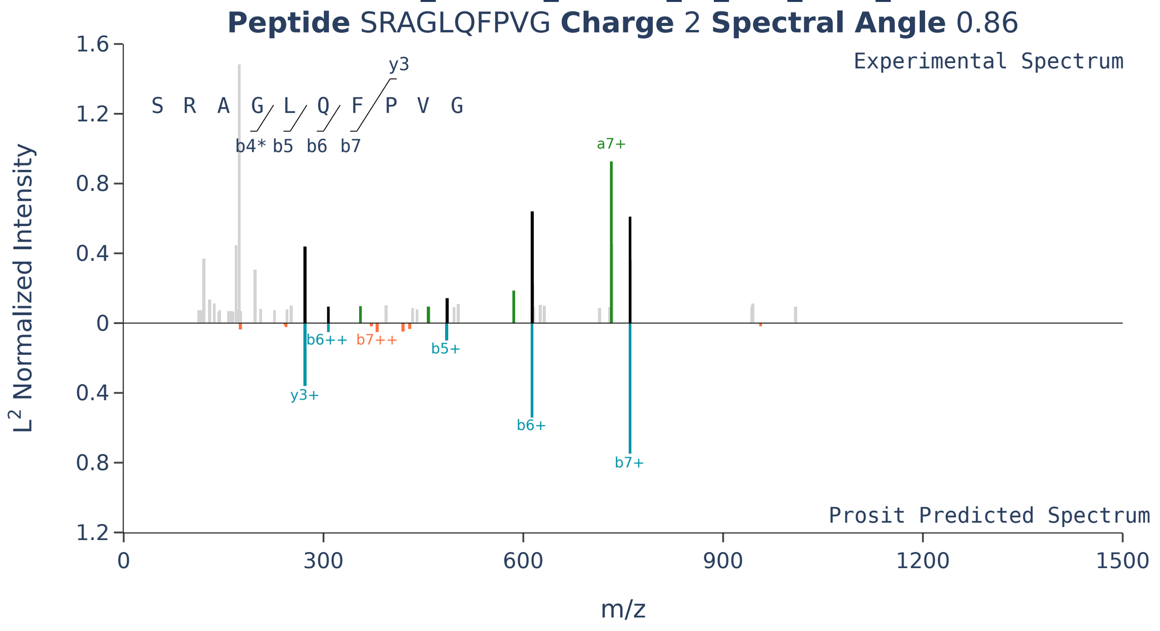


**Source** HRoetschke_291222_060123_Fu_H2A_b1R1_4h **Scan** 17035
**Peptide** AVRNDEELNKLLGRVTIAQG **Charge** 3 **Spectral Angle** 0.82

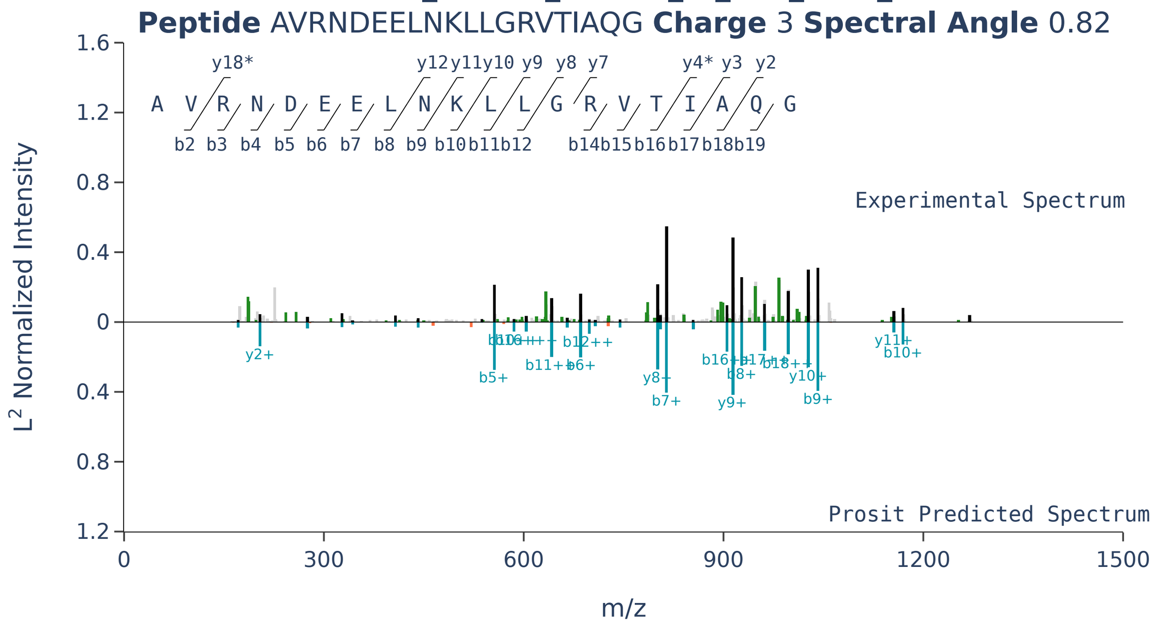


**Source** HRoetschke_291222_060123_Fu_H2A_b1R1_2h **Scan** 4581
**Peptide** KAKTRSSRAGLQF **Charge** 3 **Spectral Angle** 0.9

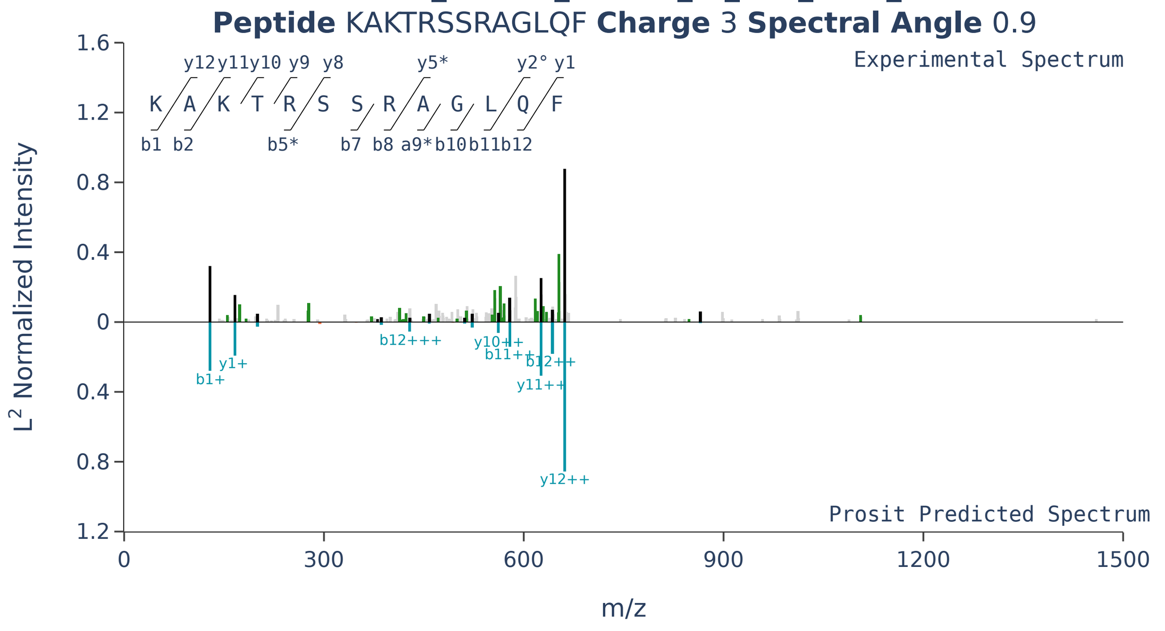


**Source** HRoetschke_291222_060123_Fu_H2A_b1R1_4h **Scan** 17117
**Peptide** QLAVRNDEELNKLLGRV **Charge** 3 **Spectral Angle** 0.81

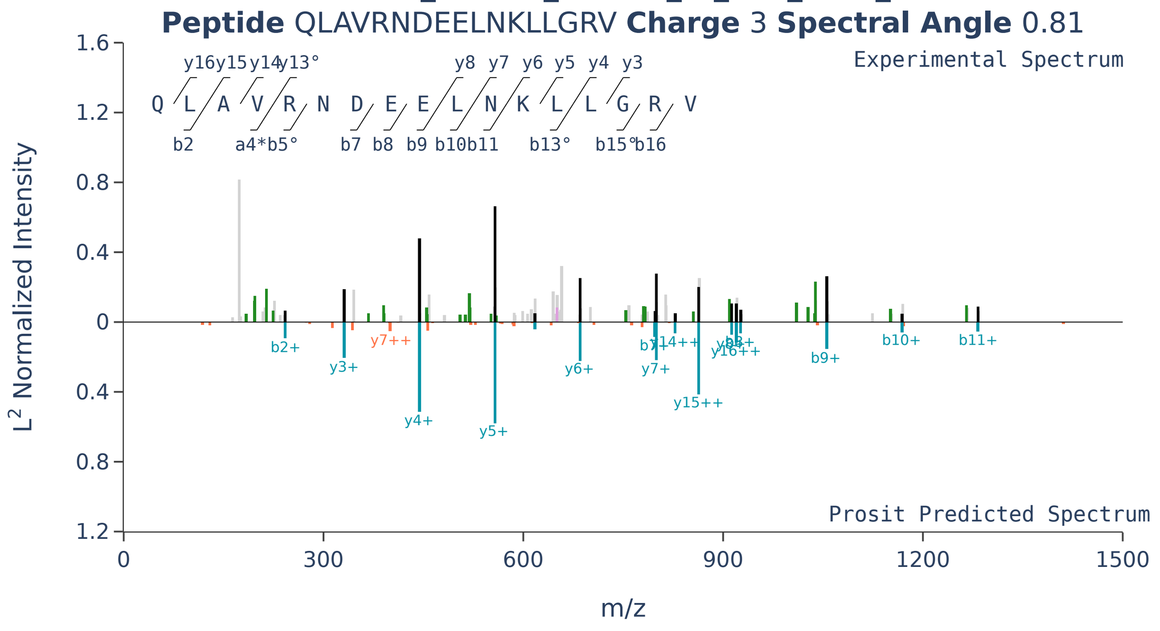


**Source** HRoetschke_291222_060123_Fu_H2A_b1R2_2h **Scan** 10778
**Peptide** SSRAGLQFPVGRVHRL **Charge** 4 **Spectral Angle** 0.79

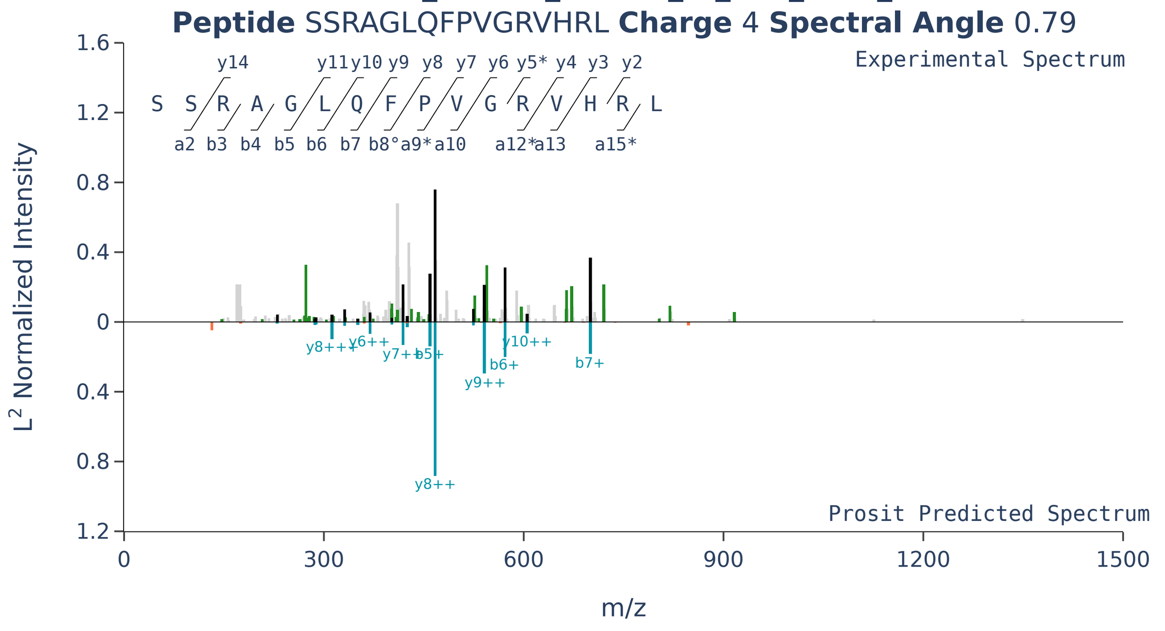


**Source** HRoetschke_291222_060123_Fu_H2A_b1R1_1h **Scan** 3904
**Peptide** SVLLPKKTESSKS **Charge** 3 **Spectral Angle** 0.79

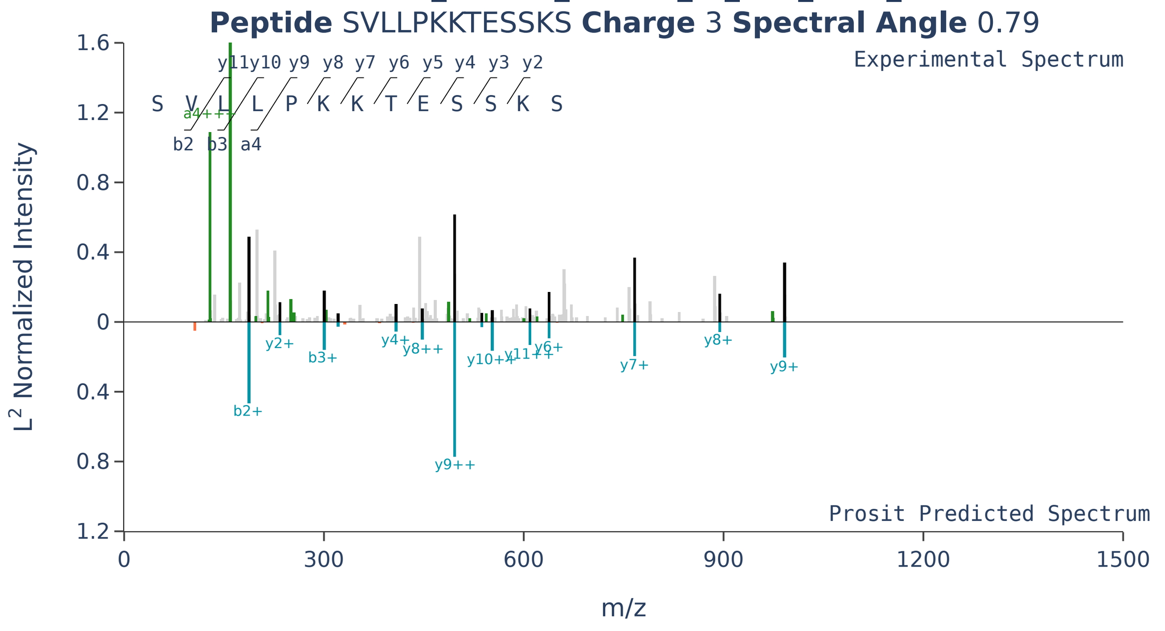


**Source** HRoetschke_291222_060123_Fu_H2A_b1R1_2h **Scan** 16463
**Peptide** LGRVTIAQGGVLPNLQ **Charge** 2 **Spectral Angle** 0.85

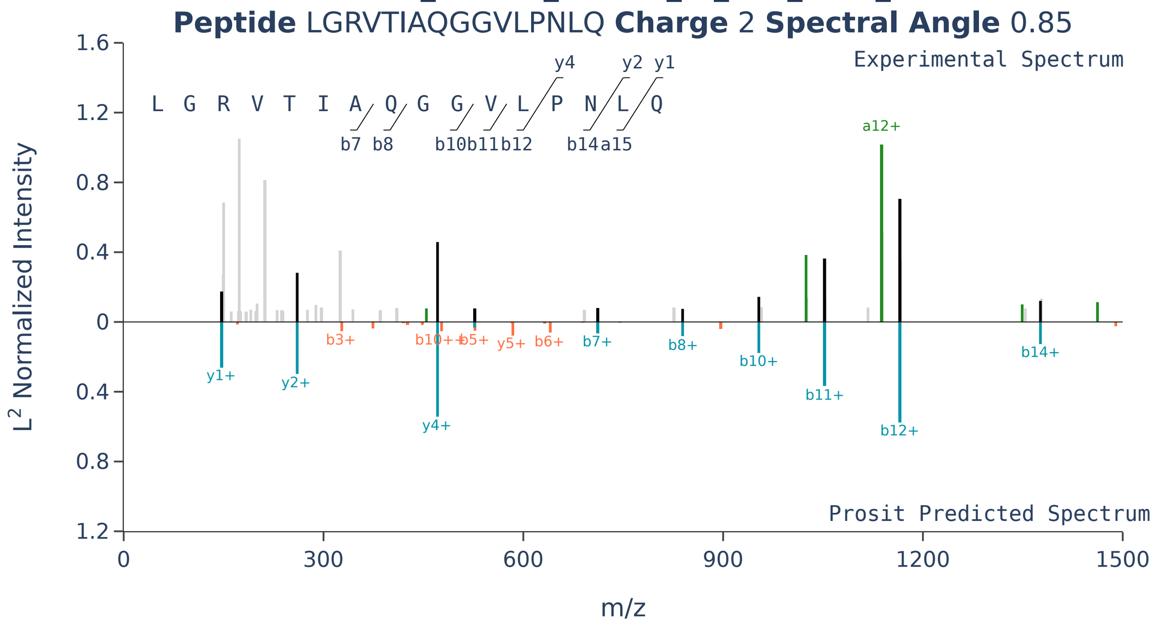

**Source** HRoetschke_291222_060123_Fu_H2A_b1R1_2h **Scan** 6602
**Peptide** AKAKTRSSRAGLQFPVGRVH **Charge** 4 **Spectral Angle** 0.75

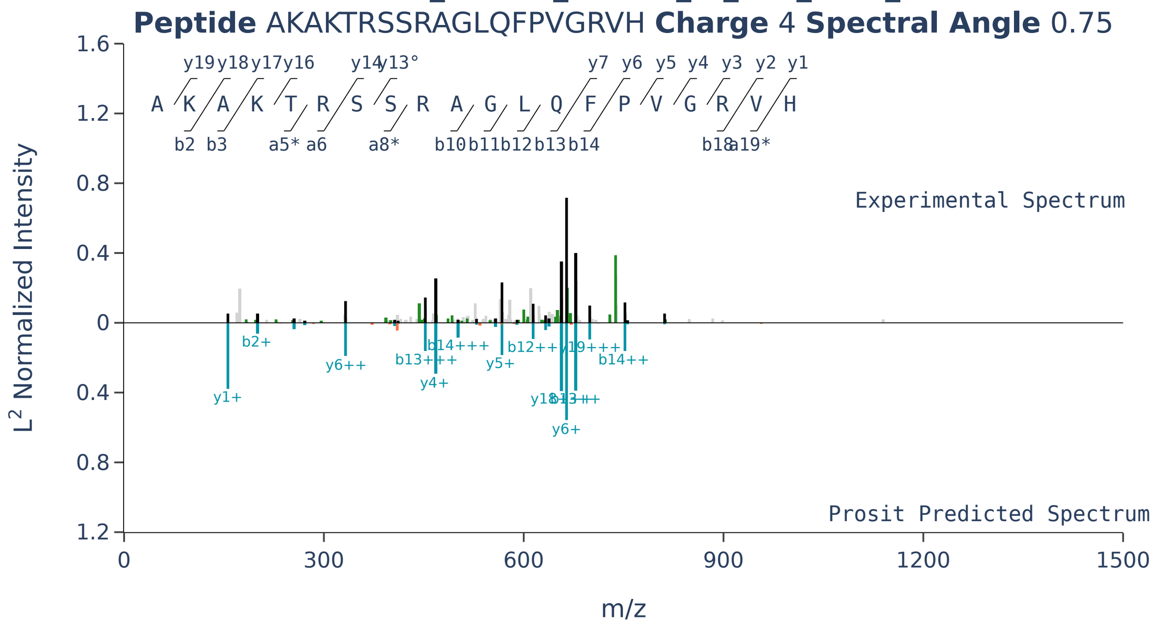


**Source** HRoetschke_291222_060123_Fu_H2A_b1R2_4h **Scan** 11484
**Peptide** GLQFPVGRVHRLLRKGNYAER **Charge** 4 **Spectral Angle** 0.76

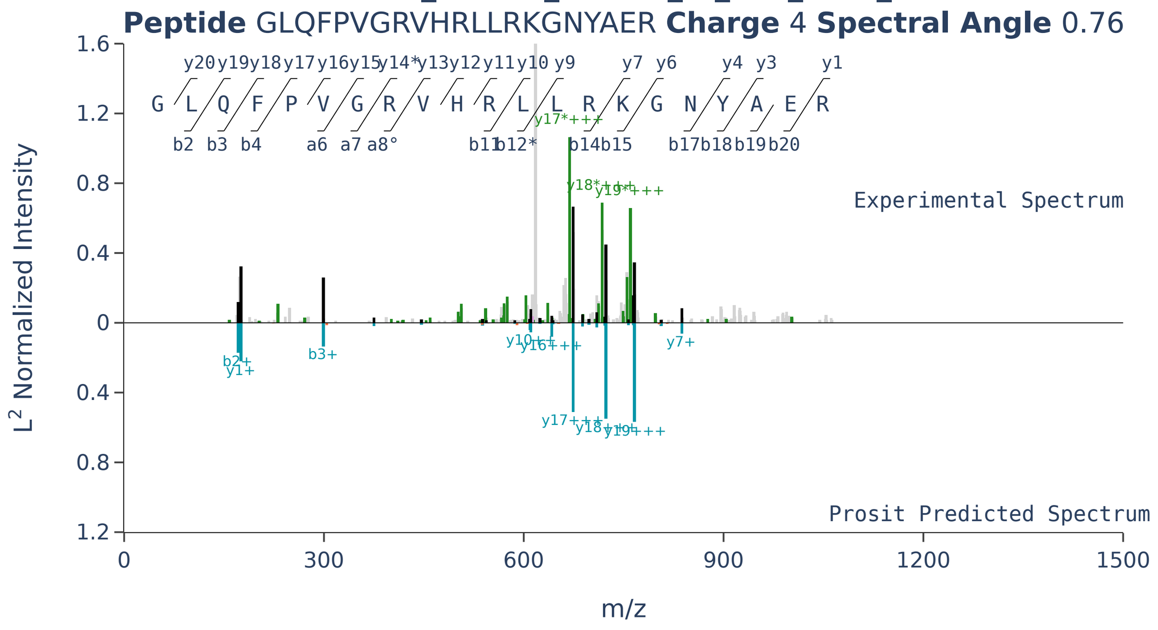


**Source** HRoetschke_291222_060123_Fu_H2A_b1R1_2h **Scan** 10027
**Peptide** LLELAGNAARDN **Charge** 2 **Spectral Angle** 0.91

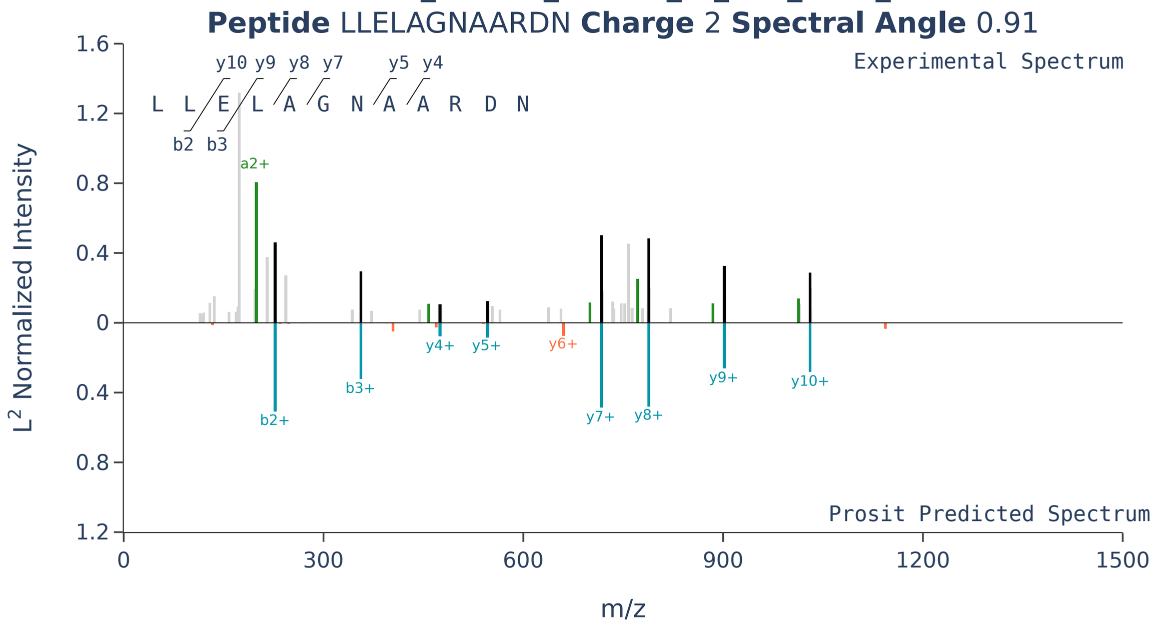


**Source** HRoetschke_291222_060123_Fu_H2A_b2R2_24h **Scan** 12741
**Peptide** EELNKLL **Charge** 2 **Spectral Angle** 0.86

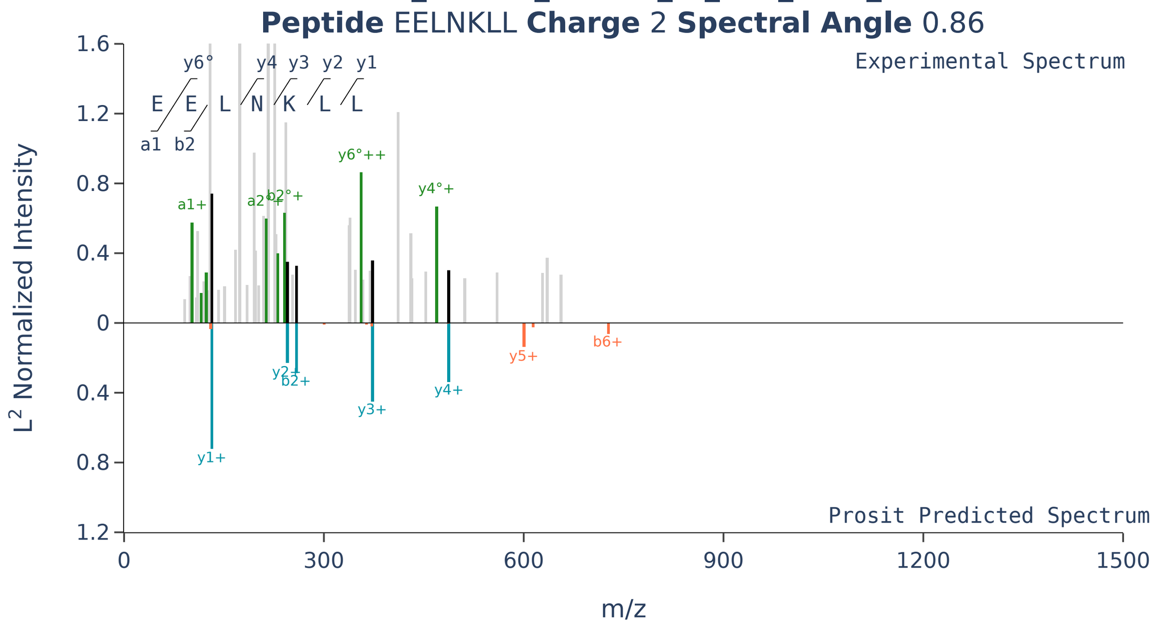


**Source** HRoetschke_291222_060123_Fu_H2A_b1R1_2h **Scan** 3507
**Peptide** VLLPKKTESSK **Charge** 4 **Spectral Angle** 0.74

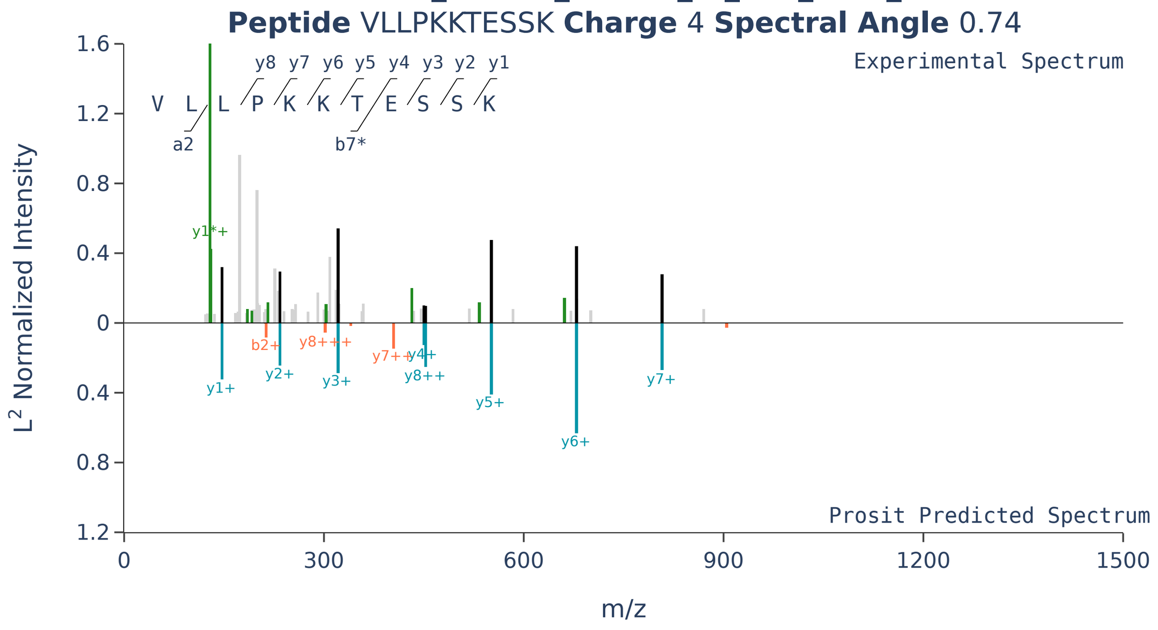


**Source** HRoetschke_291222_060123_Fu_H2A_b1R1_4h **Scan** 12763
**Peptide** TAEILELAGNAARDNKKTRIIPRH **Charge** 5 **Spectral Angle** 0.82

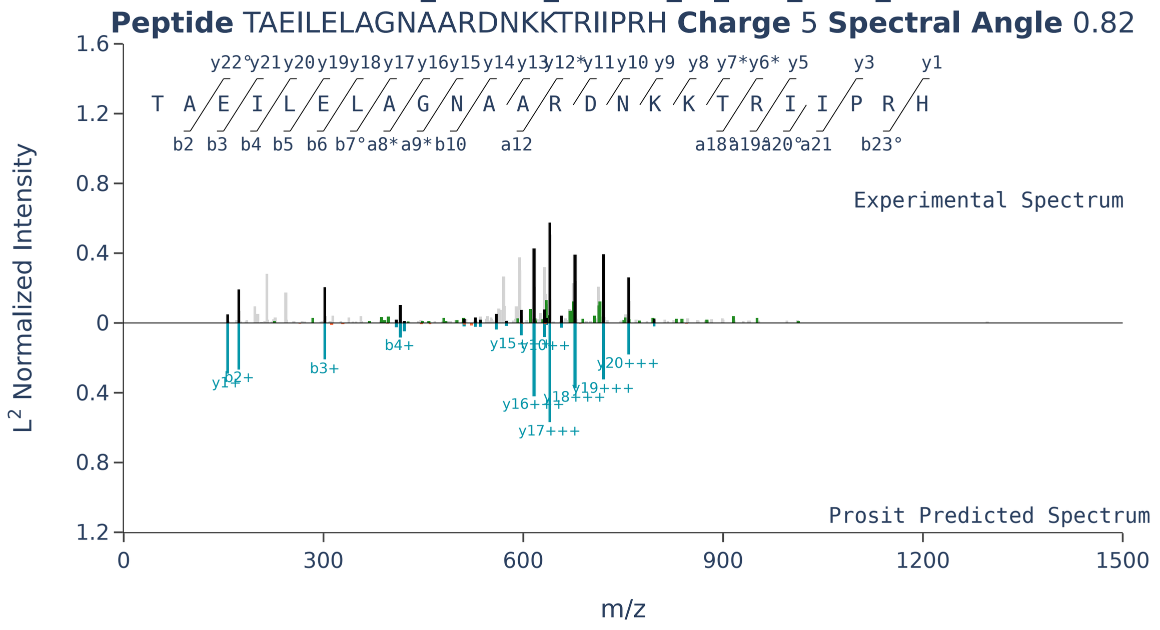


**Source** HRoetschke_291222_060123_Fu_H2A_b2R2_24h **Scan** 10534
**Peptide** RKGNYAERVGAGAPVYLA **Charge** 3 **Spectral Angle** 0.87

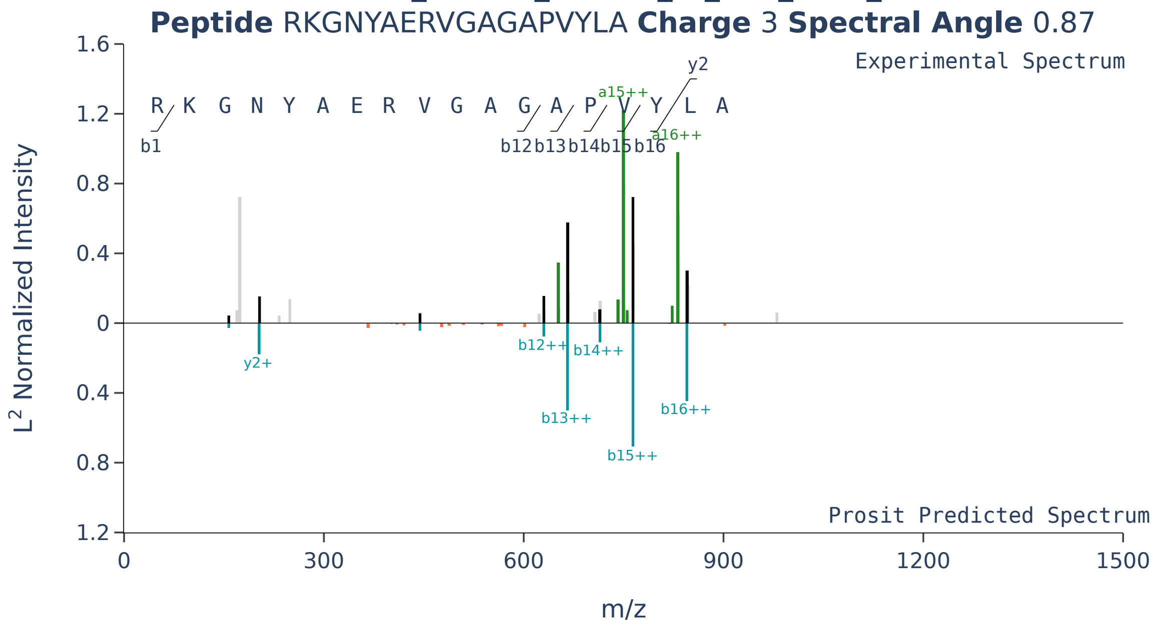


**Source** HRoetschke_291222_060123_Fu_H2A_b1R1_1h **Scan** 4549
**Peptide** VLLPKKTESS **Charge** 3 **Spectral Angle** 0.73

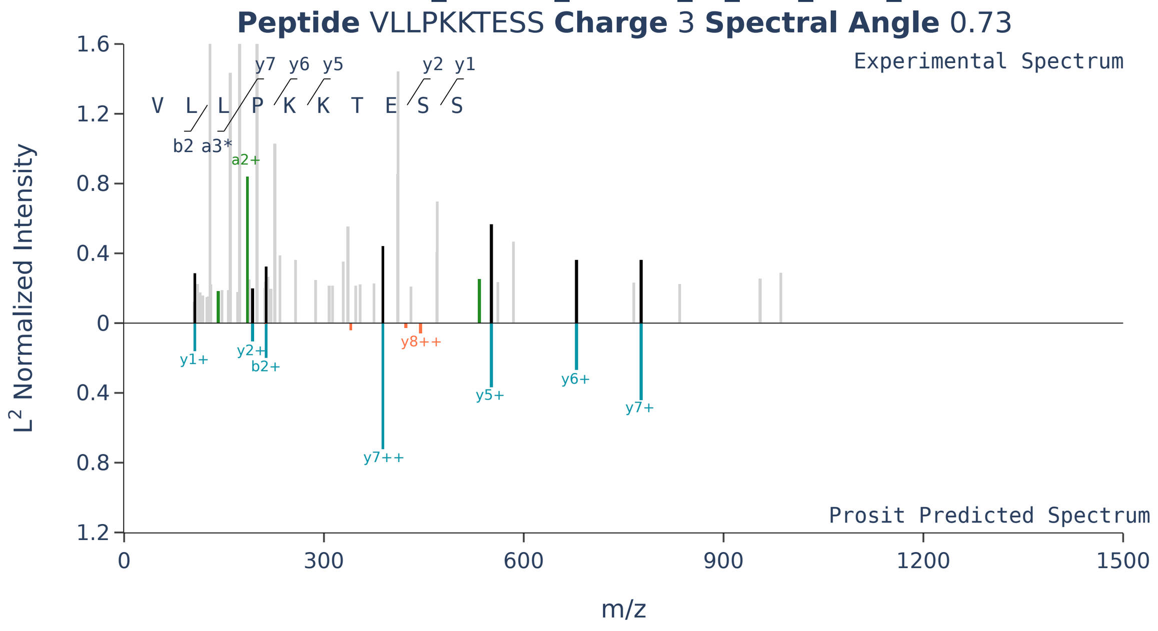


**Source** HRoetschke_291222_060123_Fu_H2A_b1R2_1h **Scan** 9581
**Peptide** LRKGNYAERVGAGAPVYLA **Charge** 3 **Spectral Angle** 0.89

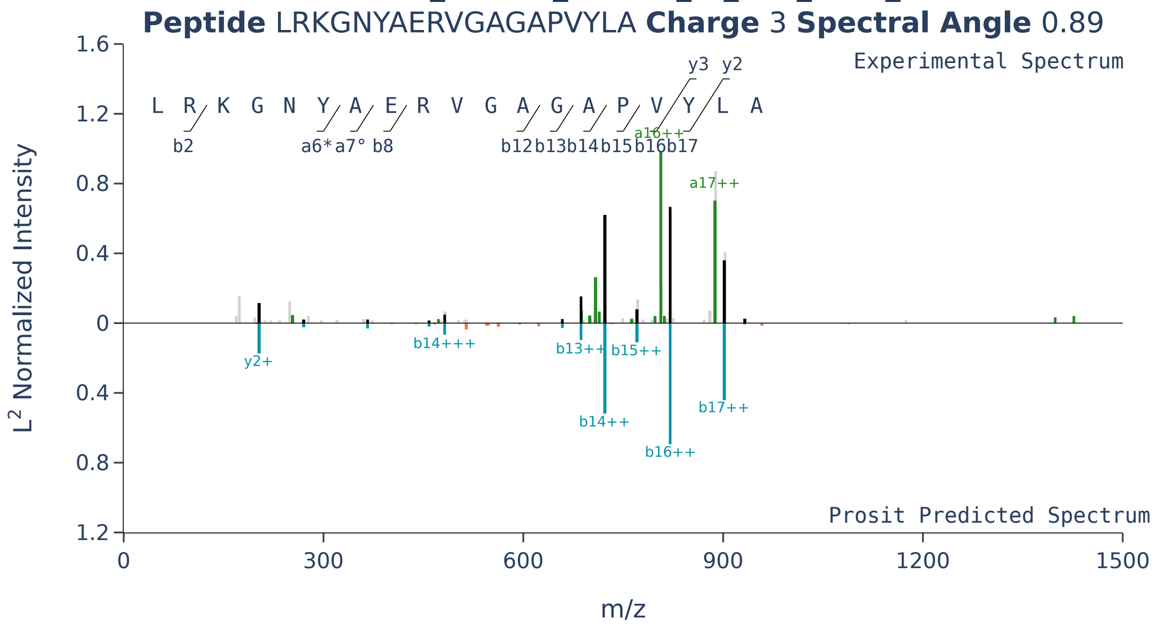


**Source** HRoetschke_291222_060123_Fu_H2A_b1R1_4h **Scan** 15810
**Peptide** NYAERVGAGAPVYL **Charge** 2 **Spectral Angle** 0.81

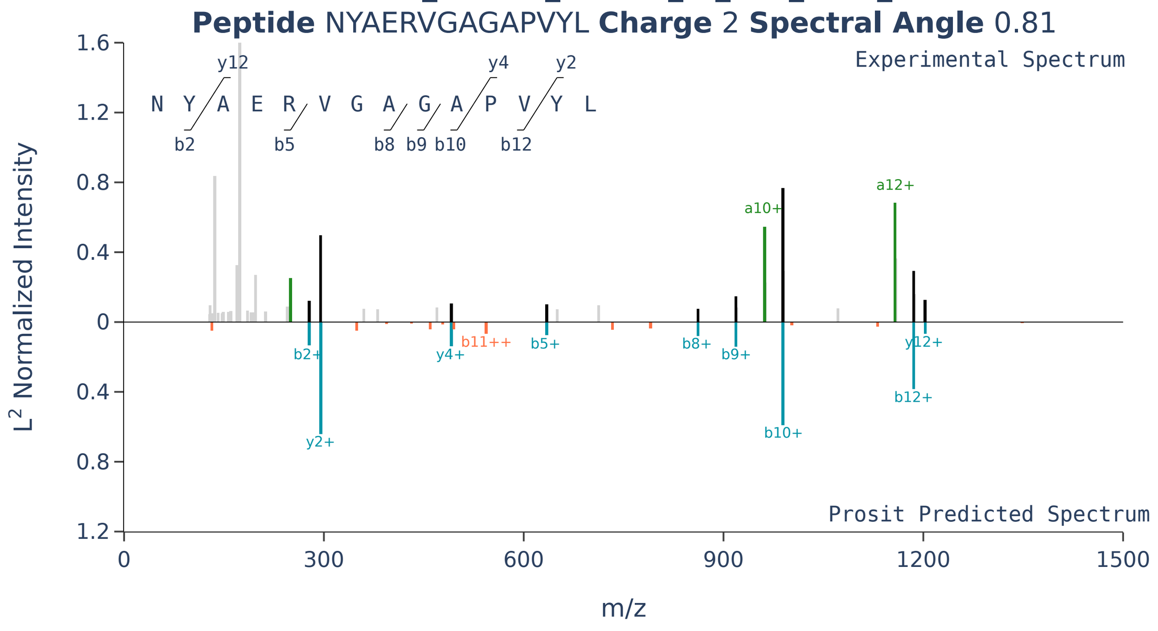


**Source** HRoetschke_291222_060123_Fu_H2A_b1R2_24h **Scan** 12193
**Peptide** EELNKLLG **Charge** 2 **Spectral Angle** 0.83

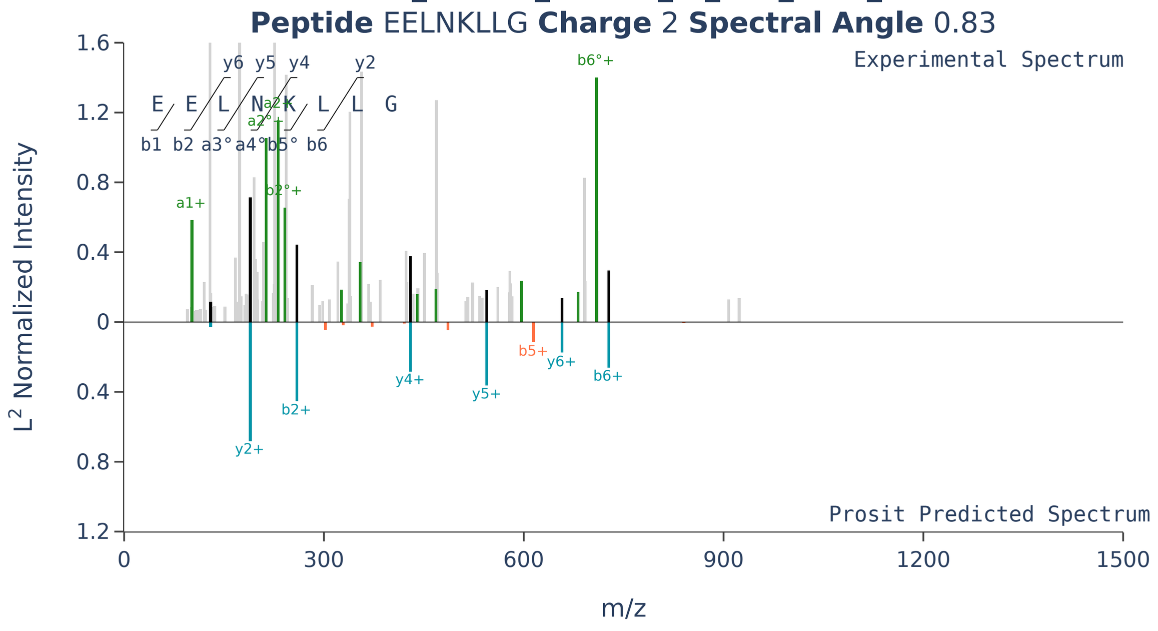


**Source** HRoetschke_291222_060123_Fu_H2A_b1R2_2h **Scan** 15713
**Peptide** QGGVLPNIQSV **Charge** 2 **Spectral Angle** 0.83

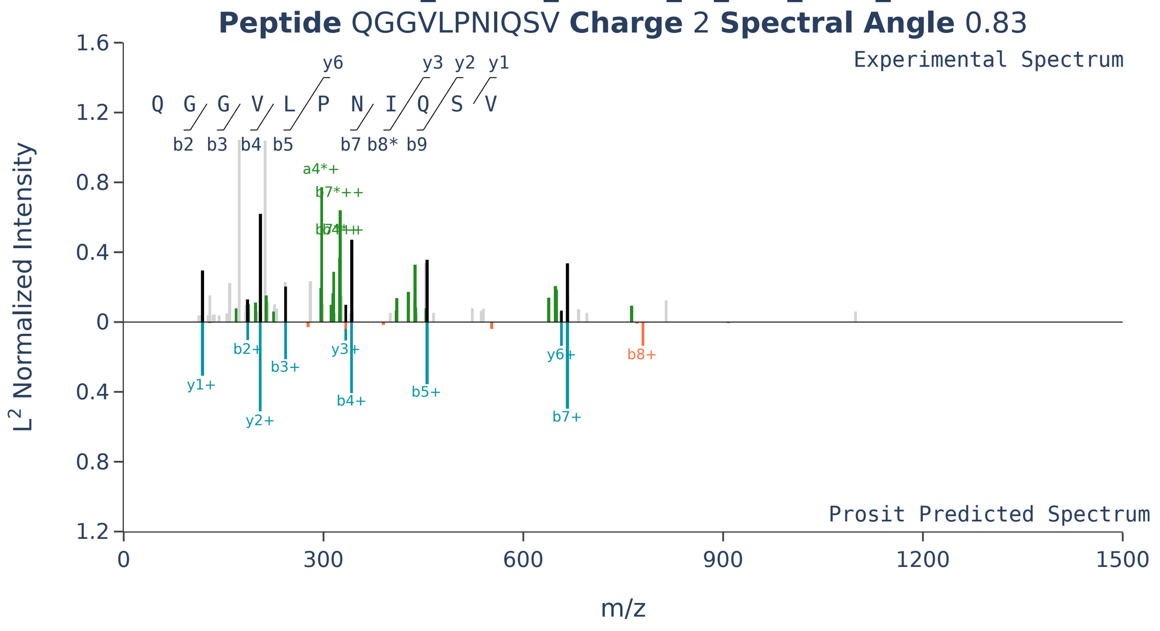


**Source** HRoetschke_291222_060123_Fu_H2A_b2R1_2h **Scan** 3076
**Peptide** SVLLPKKTESSKSAK **Charge** 3 **Spectral Angle** 0.77

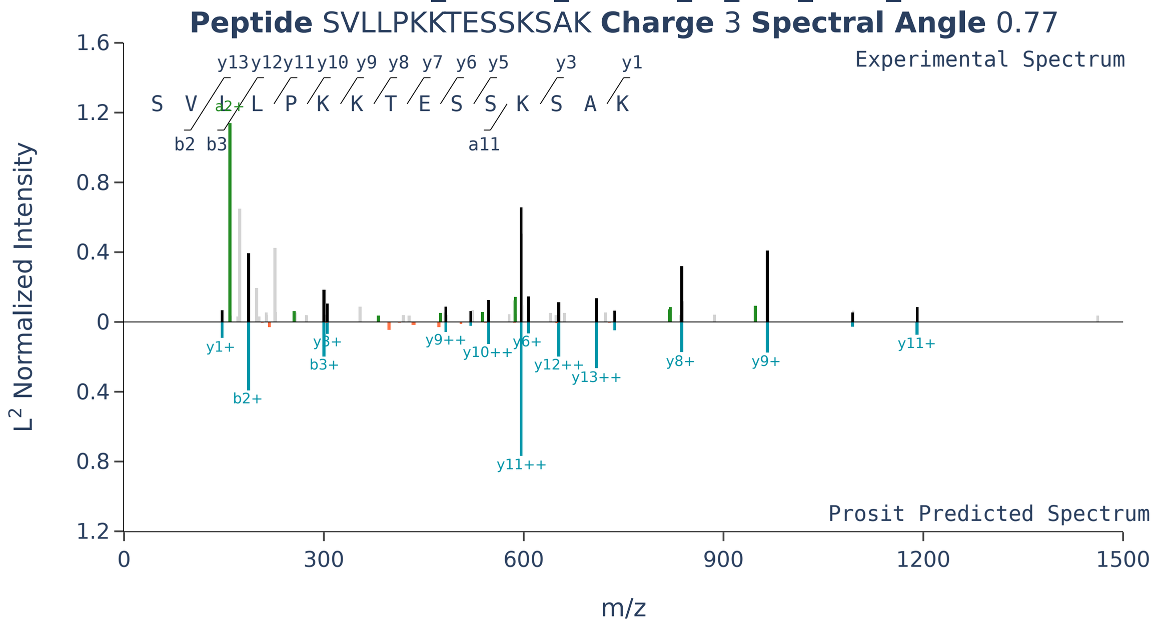


**Source** HRoetschke_291222_060123_Fu_H2A_b1R2_1h **Scan** 13418
**Peptide** ELNKLLGRVTL **Charge** 2 **Spectral Angle** 0.9

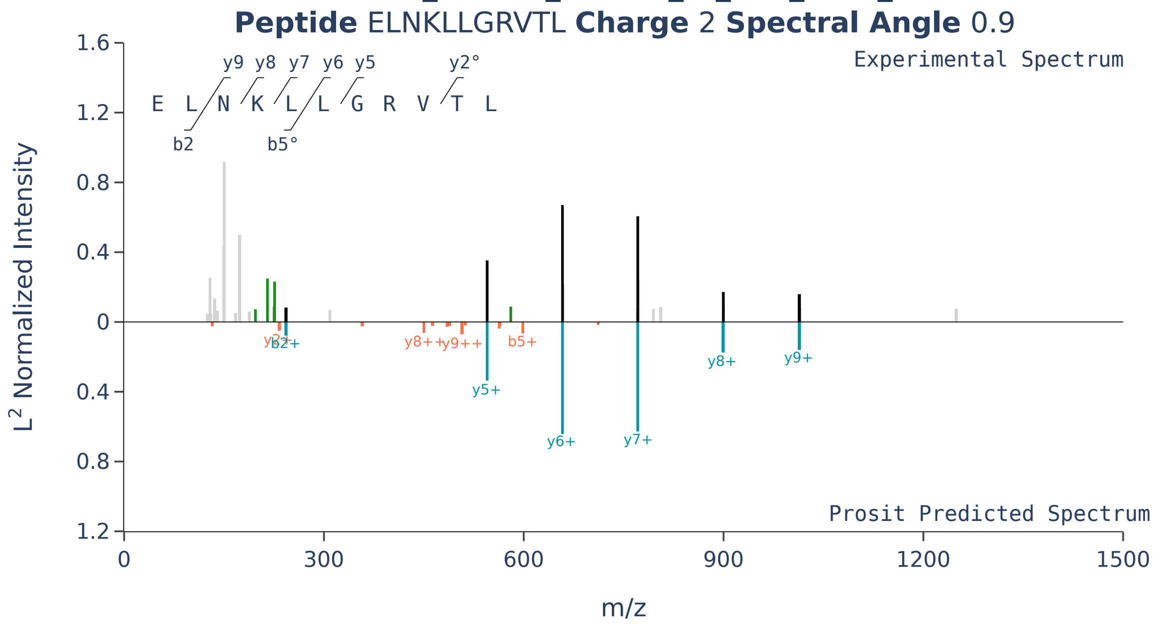


**Source** HRoetschke_291222_060123_Fu_H2A_b1R2_4h **Scan** 11355
**Peptide** SRAGLQFPVGRVHRL **Charge** 4 **Spectral Angle** 0.78

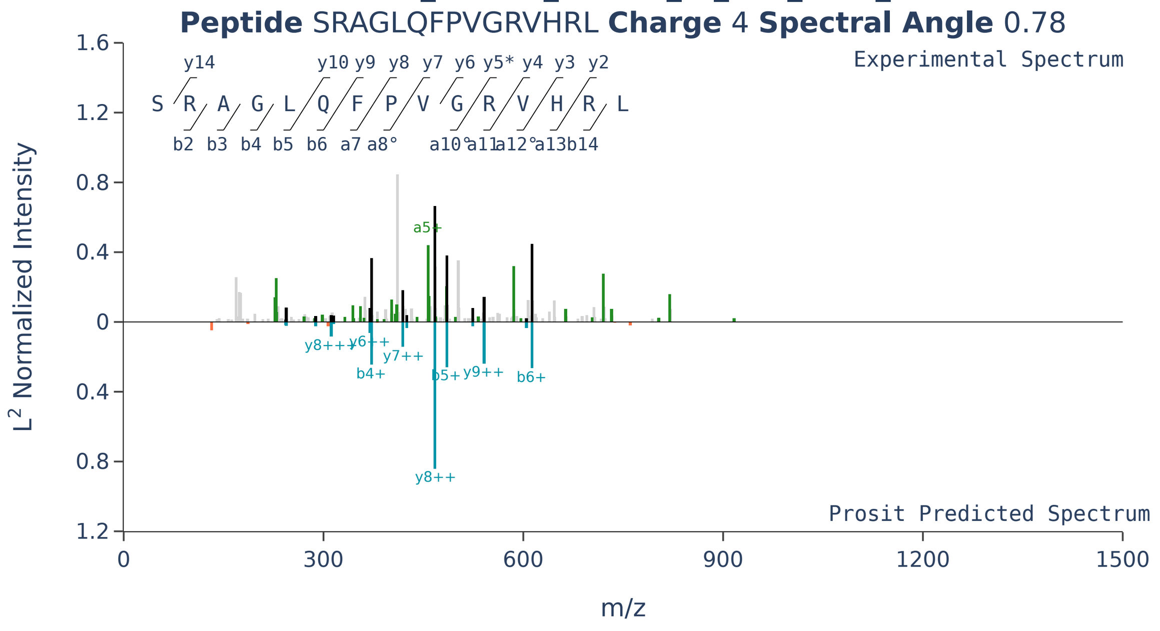

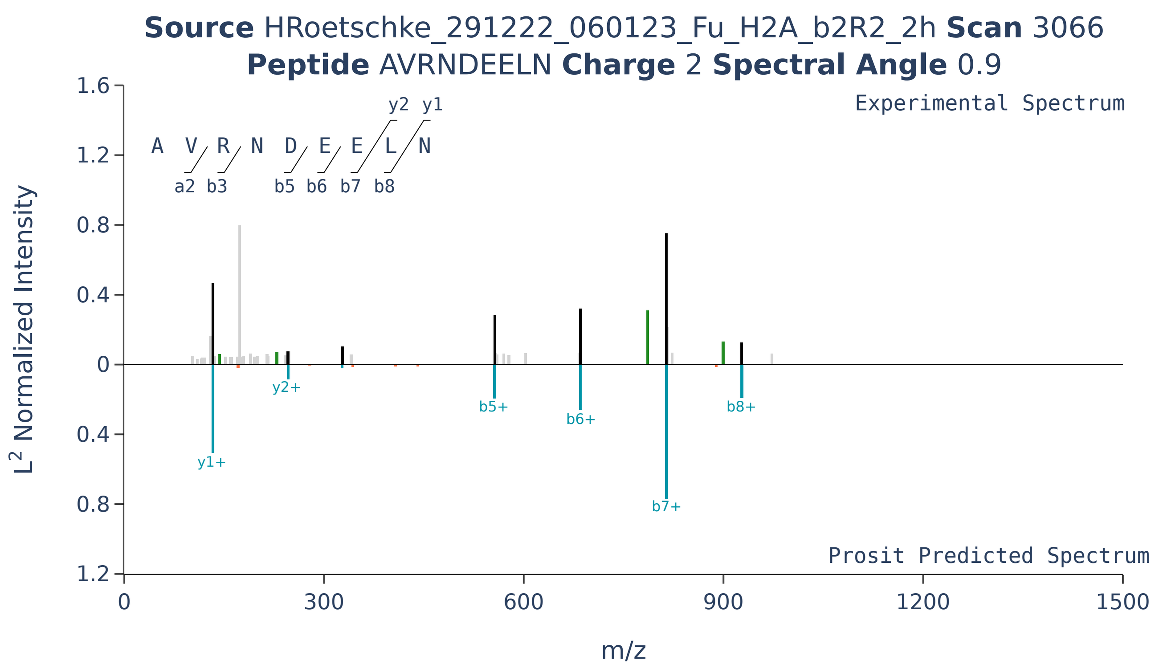
Source HRoetschke_291222_060123_Fu_H2A_b2R2_2h Scan 3066
Peptide AVRNDEELN Charge 2 Spectral Angle 0.9
Experimental Spectrum
Prosit Predicted Spectrum
L2 Normalized Intensity
m/z

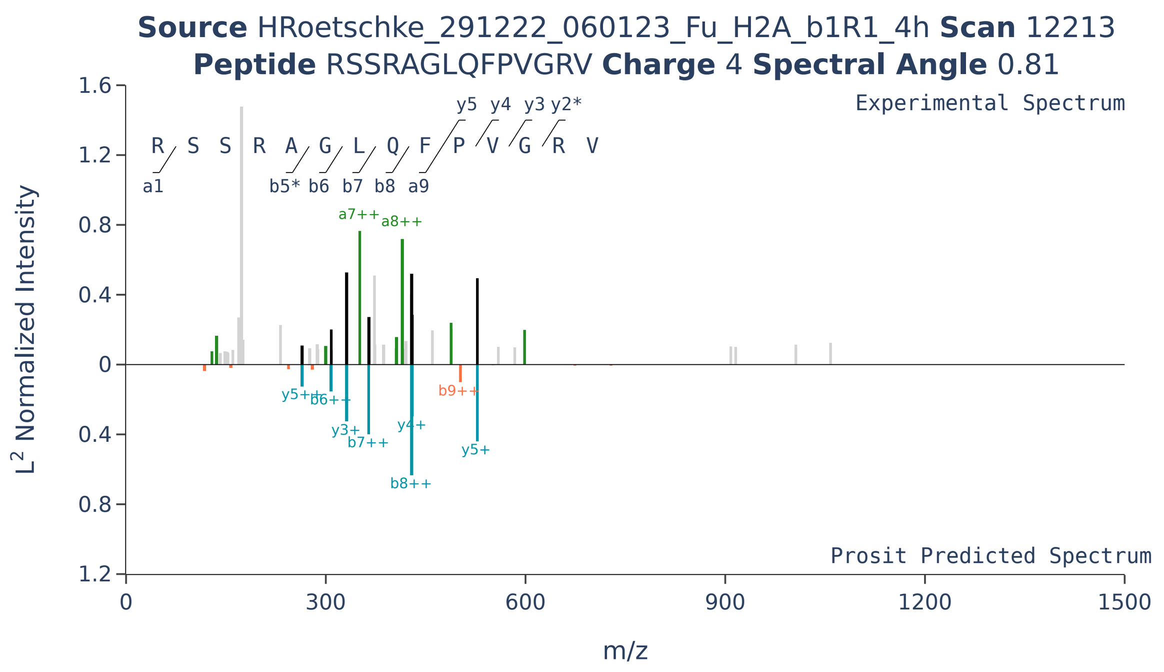
Source HRoetschke_291222_060123_Fu_H2A_b1R1_4h Scan 12213
Peptide RSSRAGLQFPVGRV Charge 4 Spectral Angle 0.81
Experimental Spectrum
Prosit Predicted Spectrum
L2 Normalized Intensity
m/z

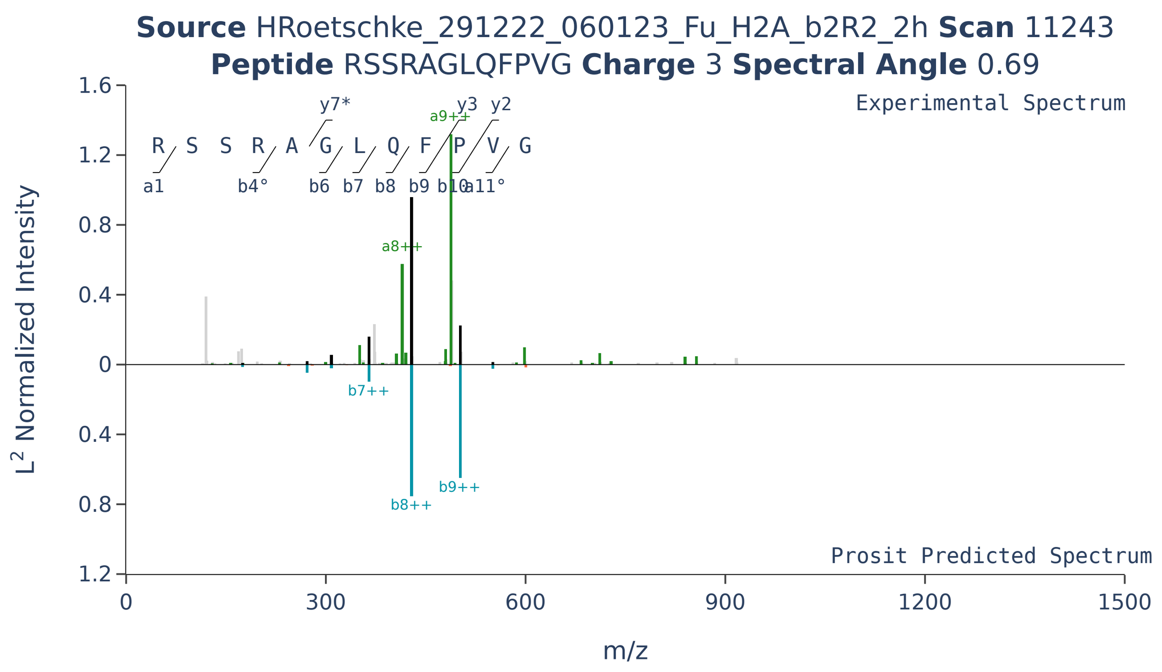
Source HRoetschke_291222_060123_Fu_H2A_b2R2_2h Scan 11243
Peptide RSSRAGLQFPVG Charge 3 Spectral Angle 0.69
Experimental Spectrum
Prosit Predicted Spectrum
L2 Normalized Intensity
m/z

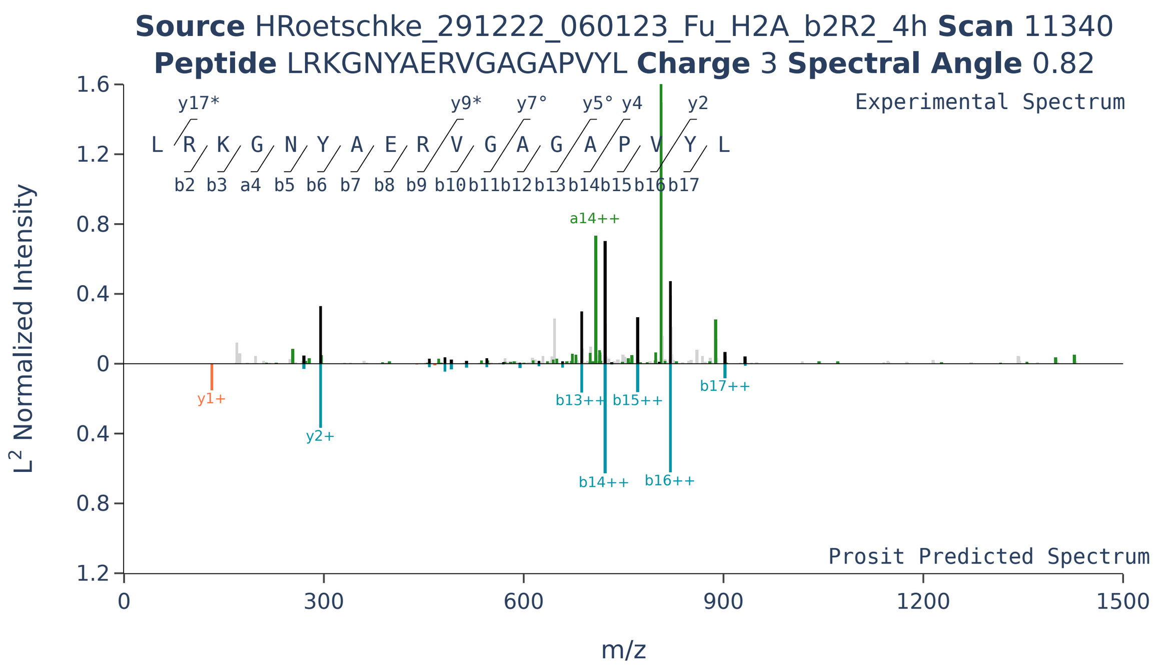
Source HRoetschke_291222_060123_Fu_H2A_b2R2_4h Scan 11340
Peptide LRKGNYAERVGAGAPVYL Charge 3 Spectral Angle 0.82
Experimental Spectrum
Prosit Predicted Spectrum
L2 Normalized Intensity
m/z

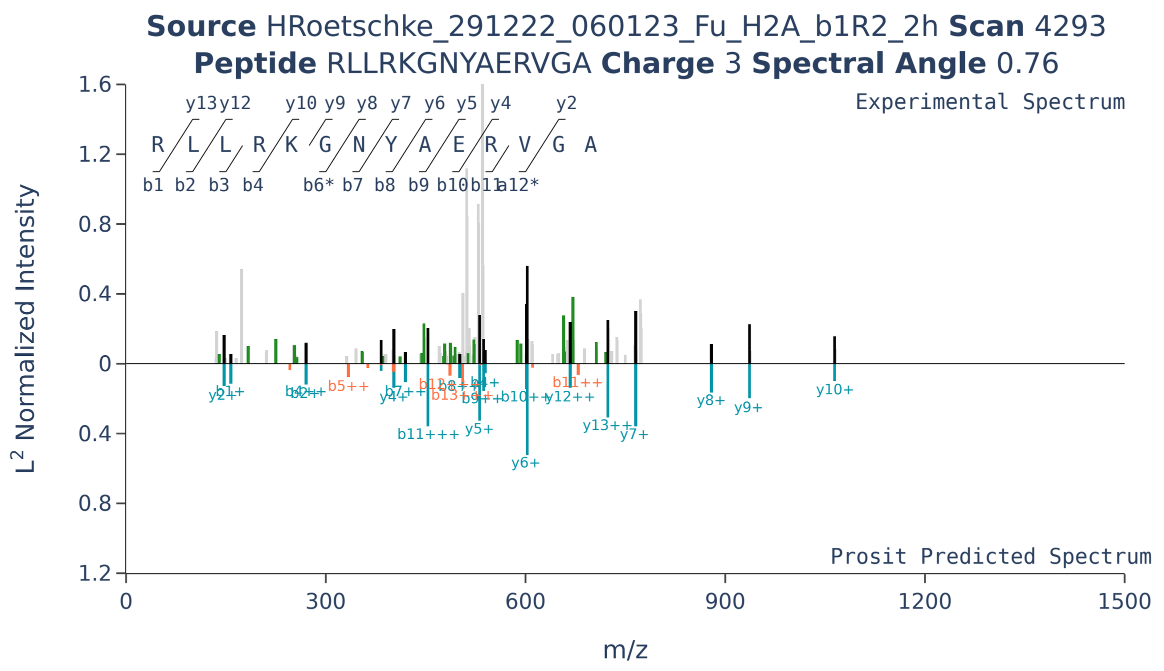
Source HRoetschke_291222_060123_Fu_H2A_b1R2_2h Scan 4293
Peptide RLLRKGNYAERVGA Charge 3 Spectral Angle 0.76
Experimental Spectrum
Prosit Predicted Spectrum
L2 Normalized Intensity
m/z

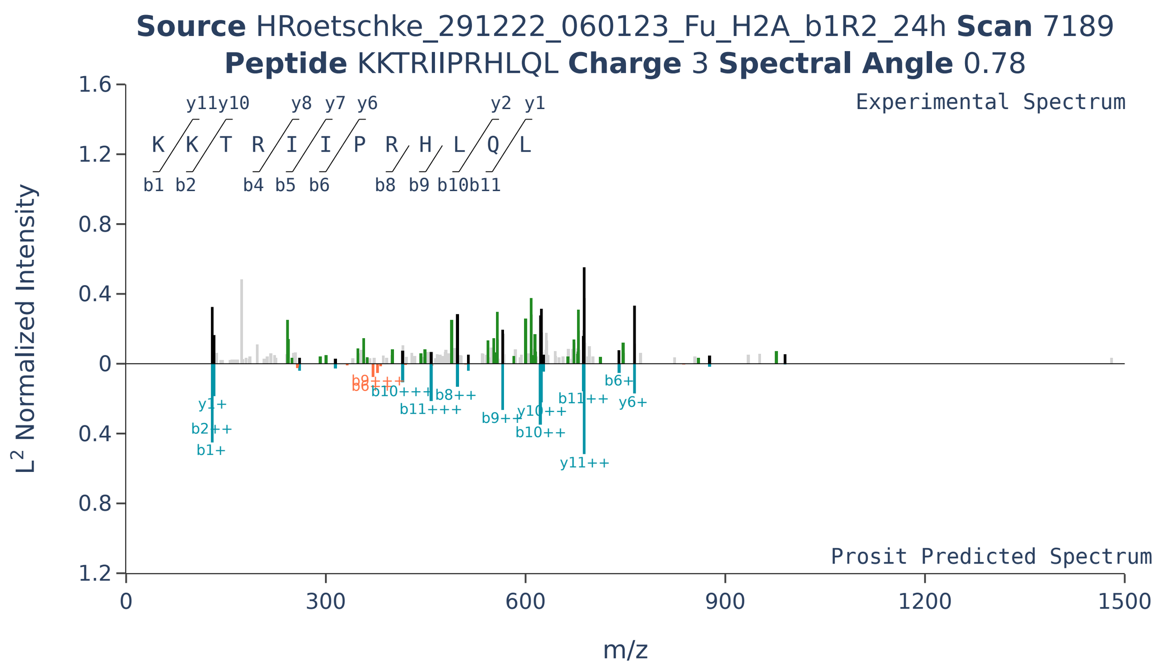
Source HRoetschke_291222_060123_Fu_H2A_b1R2_24h Scan 7189
Peptide KKTRIIPRHLQL Charge 3 Spectral Angle 0.78
Experimental Spectrum
Prosit Predicted Spectrum
L2 Normalized Intensity
m/z

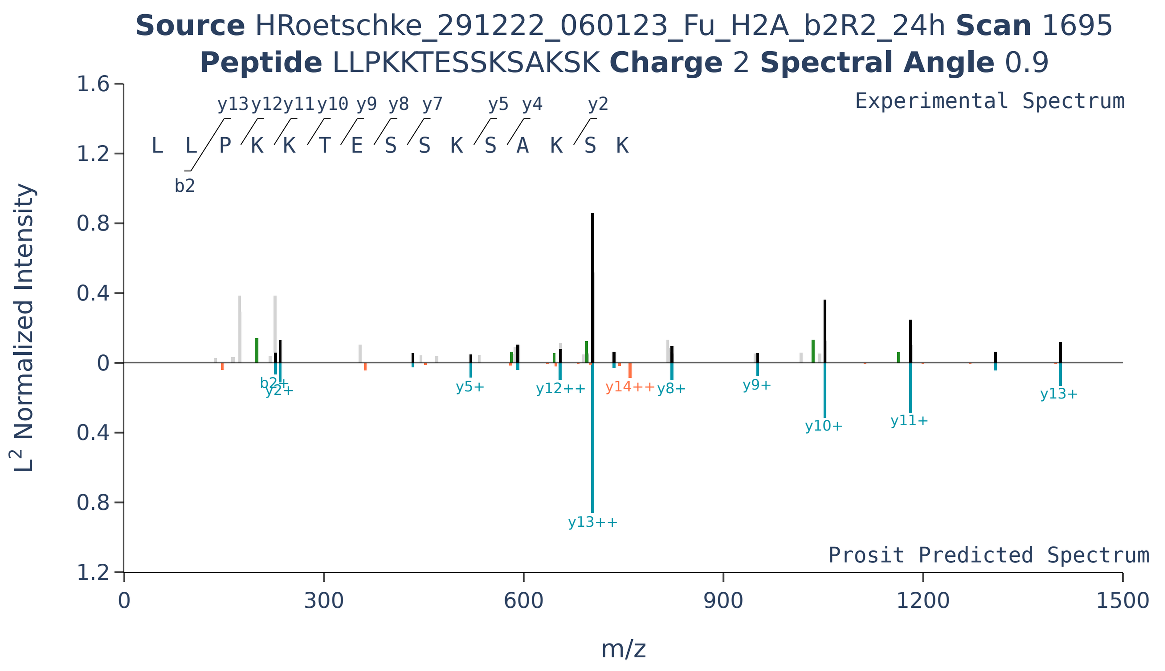
Source HRoetschke_291222_060123_Fu_H2A_b2R2_24h Scan 1695
Peptide LLPKKTESSKSAKSK Charge 2 Spectral Angle 0.9
Experimental Spectrum
Prosit Predicted Spectrum
L2 Normalized Intensity
m/z

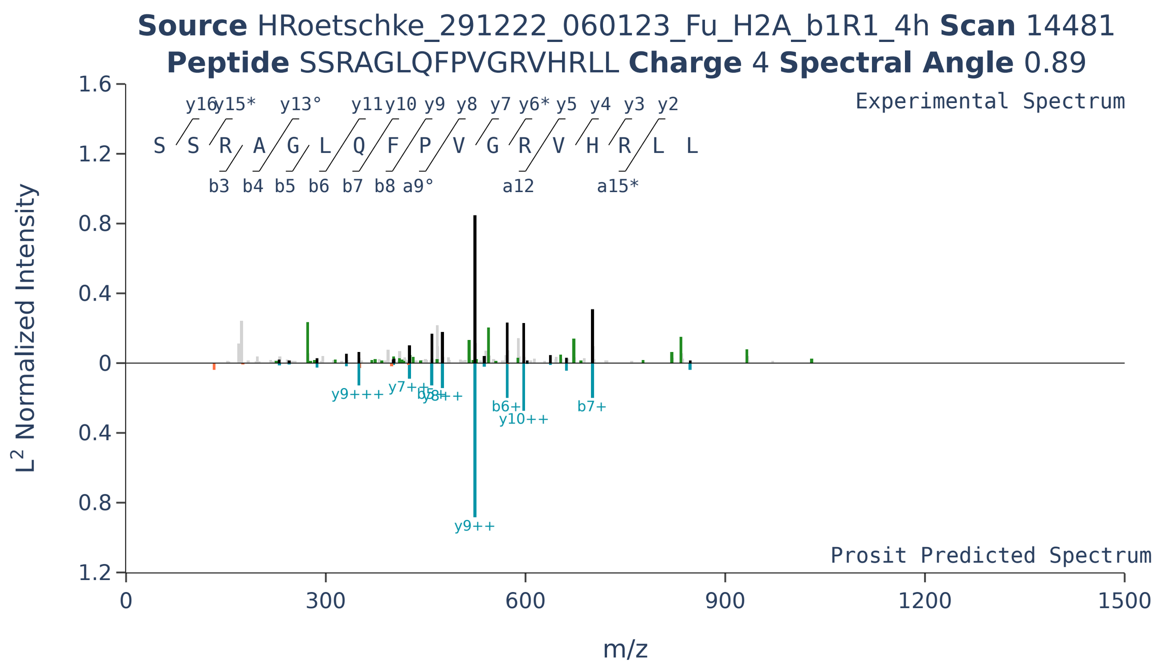
Source HRoetschke_291222_060123_Fu_H2A_b1R1_4h Scan 14481
Peptide SSRAGLQFPVGRVHRLL Charge 4 Spectral Angle 0.89
Experimental Spectrum
Prosit Predicted Spectrum
L2 Normalized Intensity
m/z

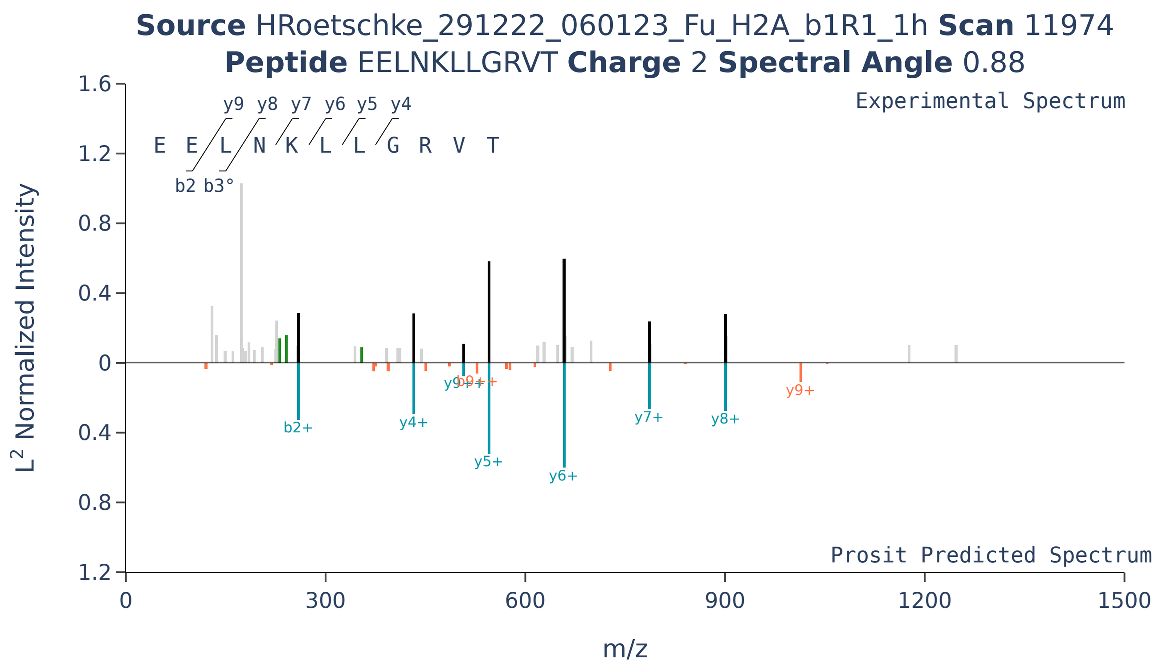
Source HRoetschke_291222_060123_Fu_H2A_b1R1_1h Scan 11974
Peptide EELNKLLGRVT Charge 2 Spectral Angle 0.88
Experimental Spectrum
Prosit Predicted Spectrum
L2 Normalized Intensity
m/z

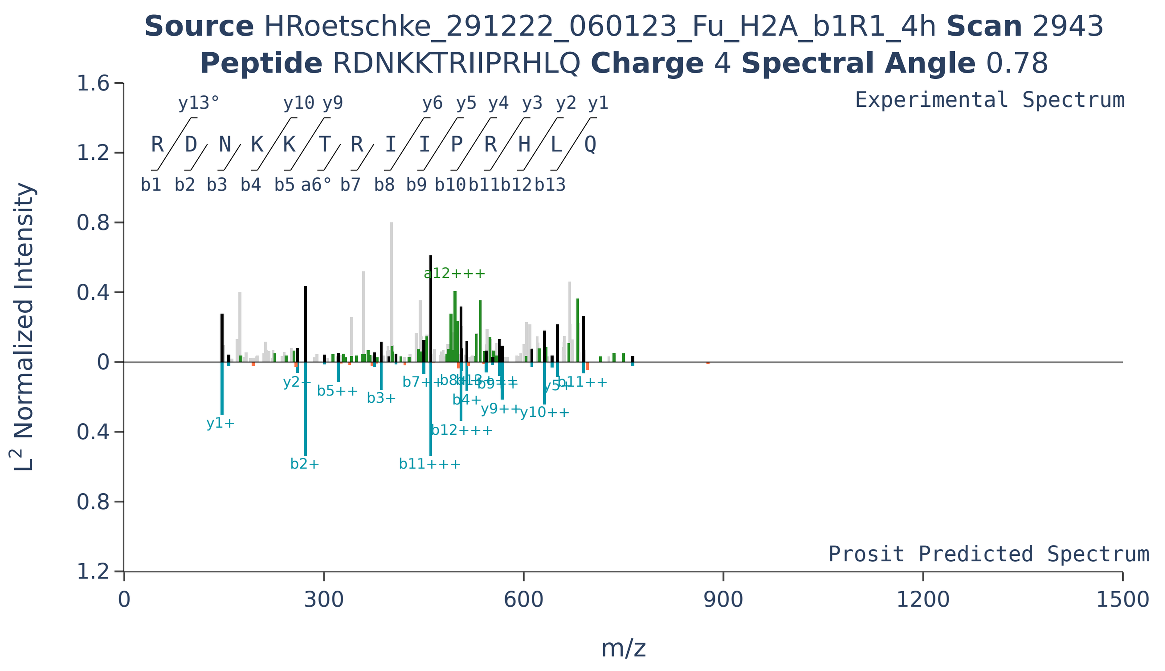
Source HRoetschke_291222_060123_Fu_H2A_b1R1_4h Scan 2943
Peptide RDNKKTRIIPRHLQ Charge 4 Spectral Angle 0.78
Experimental Spectrum
Prosit Predicted Spectrum
L2 Normalized Intensity
m/z

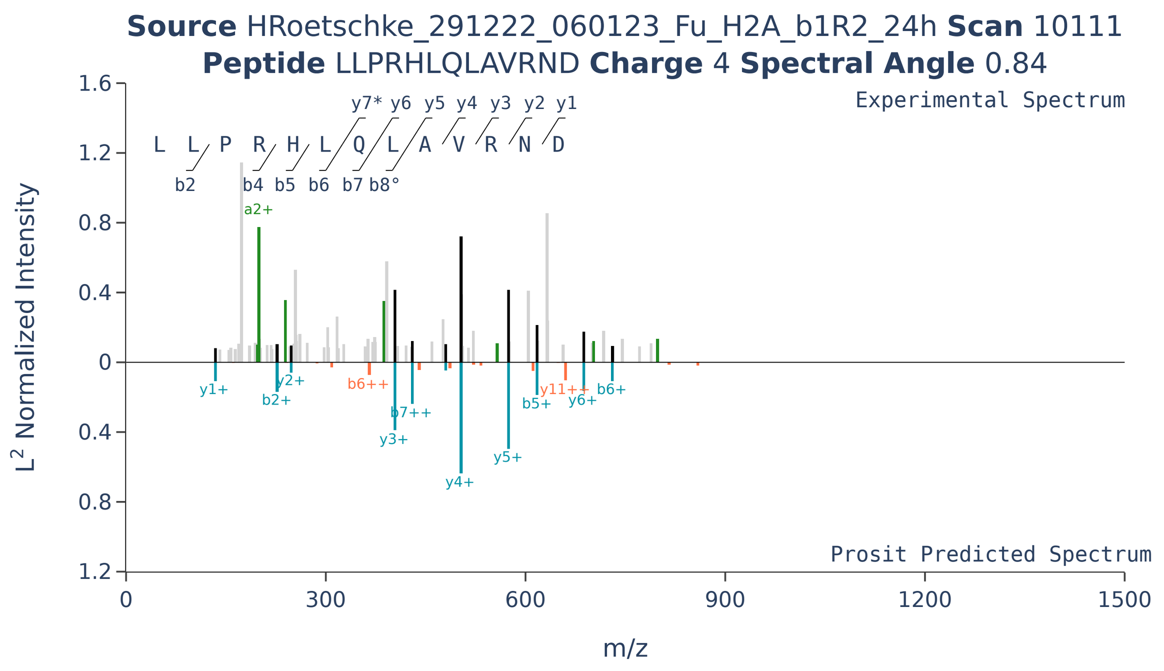
Source HRoetschke_291222_060123_Fu_H2A_b1R2_24h Scan 10111
Peptide LLPRHLQLAVRND Charge 4 Spectral Angle 0.84
Experimental Spectrum
Prosit Predicted Spectrum
L2 Normalized Intensity
m/z

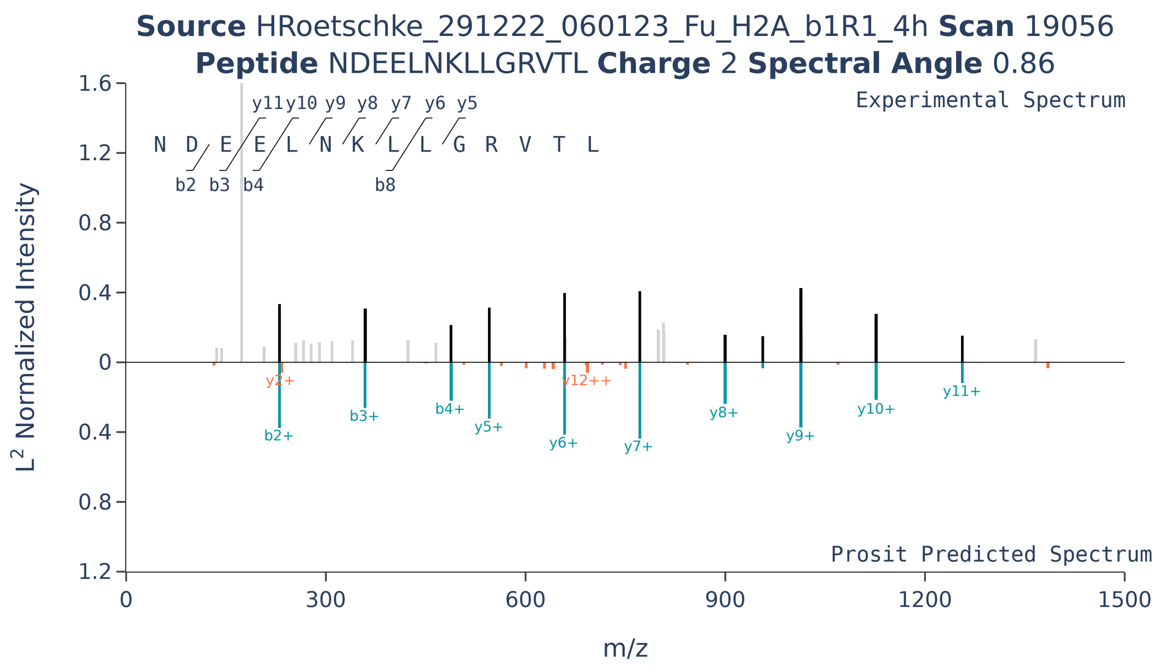
Source HRoetschke_291222_060123_Fu_H2A_b1R1_4h Scan 19056
Peptide NDEELNKLLGRVTL Charge 2 Spectral Angle 0.86
Experimental Spectrum
Prosit Predicted Spectrum
L2 Normalized Intensity
m/z

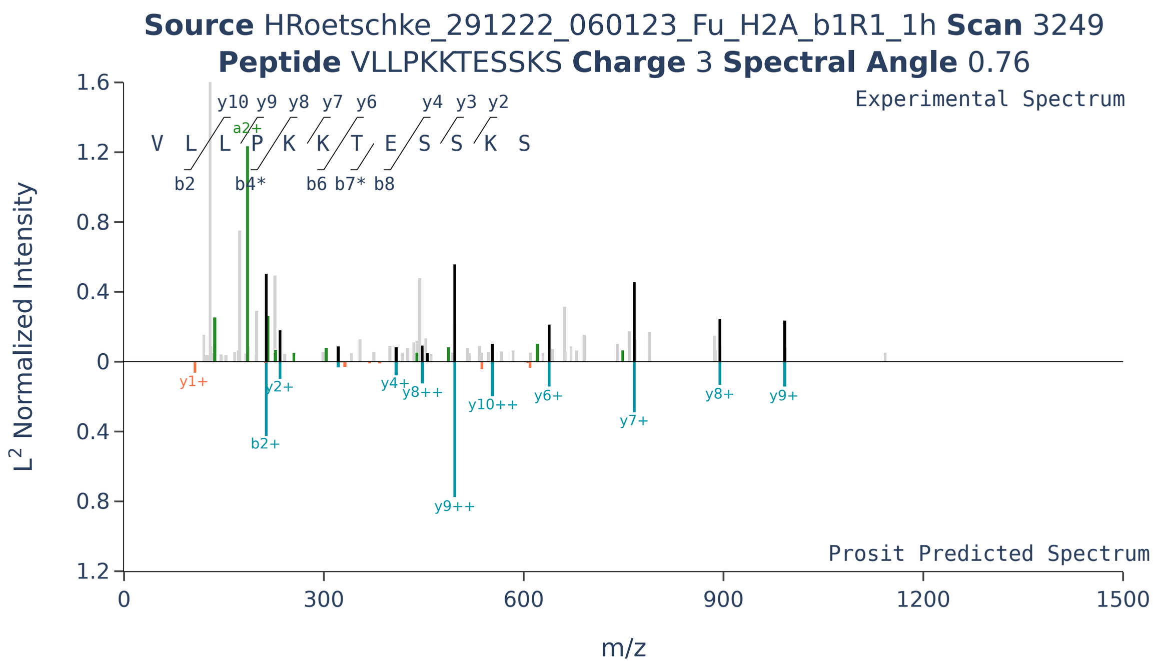
Source HRoetschke_291222_060123_Fu_H2A_b1R1_1h Scan 3249
Peptide VLLPKKTESSKS Charge 3 Spectral Angle 0.76
Experimental Spectrum
Prosit Predicted Spectrum
L2 Normalized Intensity
m/z

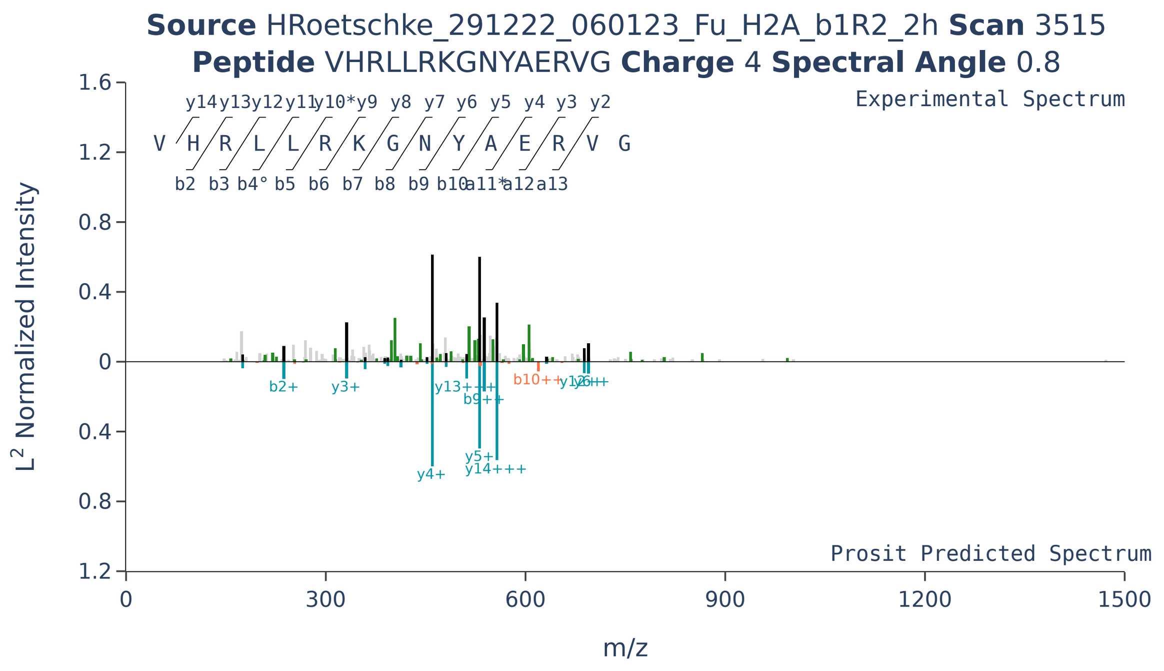
Source HRoetschke_291222_060123_Fu_H2A_b1R2_2h Scan 3515
Peptide VHRLLRKGNYAERVG Charge 4 Spectral Angle 0.8
Experimental Spectrum
Prosit Predicted Spectrum
L2 Normalized Intensity
m/z

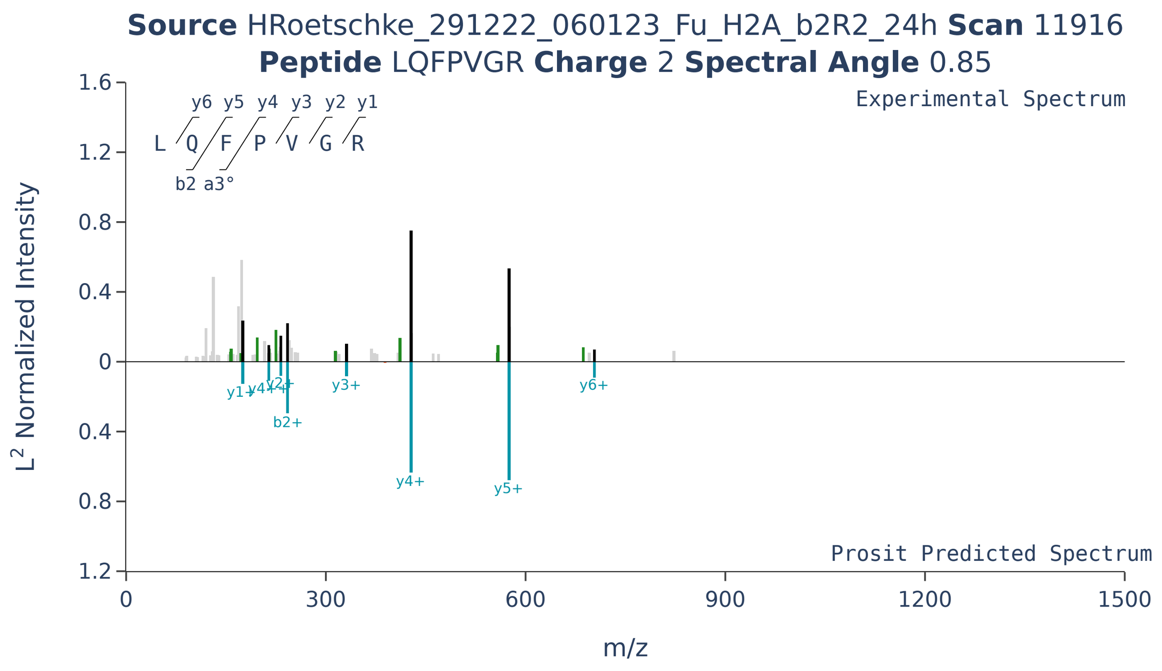
Source HRoetschke_291222_060123_Fu_H2A_b2R2_24h Scan 11916
Peptide LQFPVGR Charge 2 Spectral Angle 0.85
Experimental Spectrum
Prosit Predicted Spectrum
L2 Normalized Intensity
m/z

**Source** HRoetschke_291222_060123_Fu_H2A_b2R2_24h **Scan** 13519
**Peptide** AEILELAGNAARD **Charge** 2 **Spectral Angle** 0.83

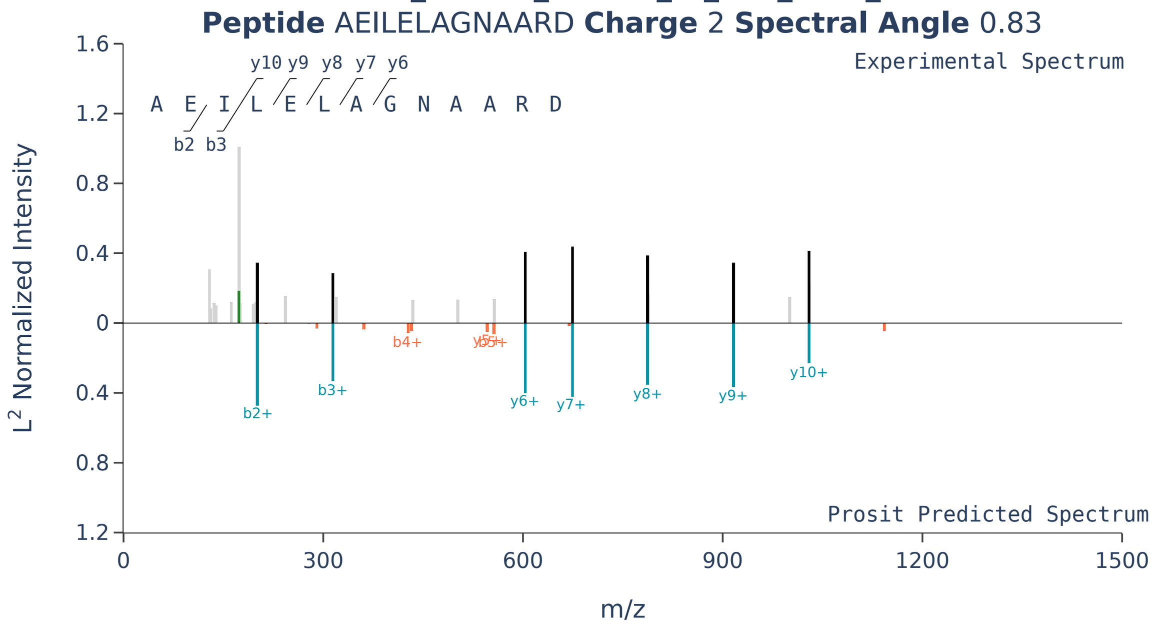


**Source** HRoetschke_291222_060123_Fu_H2A_b1R2_2h **Scan** 2986
**Peptide** LRKGNYAERV **Charge** 4 **Spectral Angle** 0.71

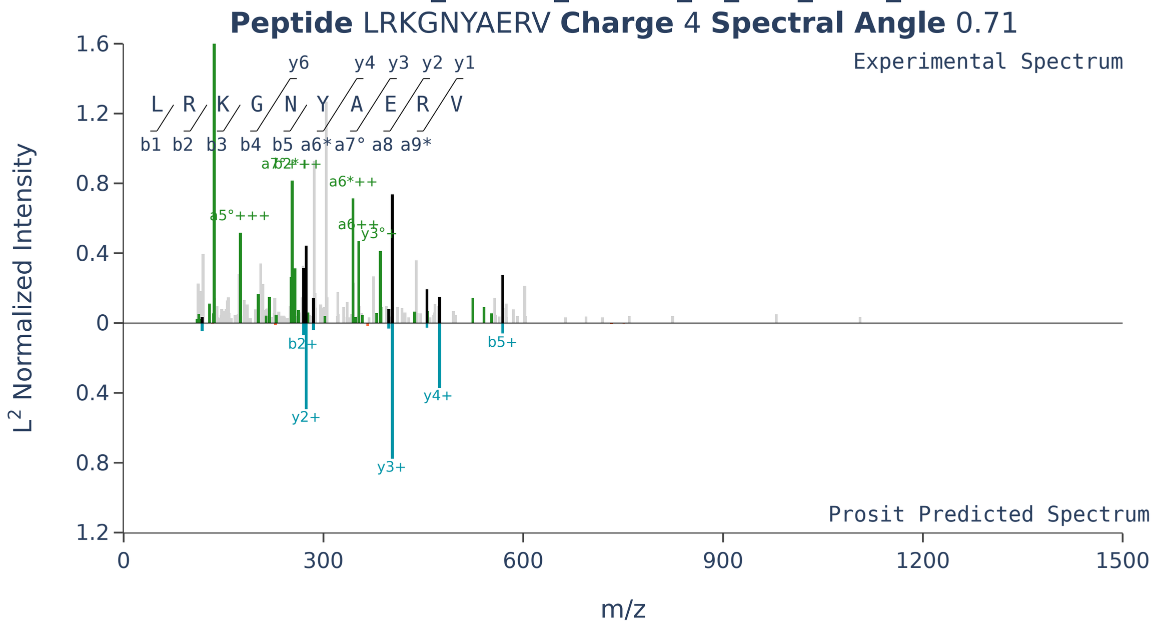


**Source** HRoetschke_291222_060123_Fu_H2A_b1R2_4h **Scan** 11699
**Peptide** SSRAGLQFPVGRVHRLLRKGNYA **Charge** 5 **Spectral Angle** 0.84

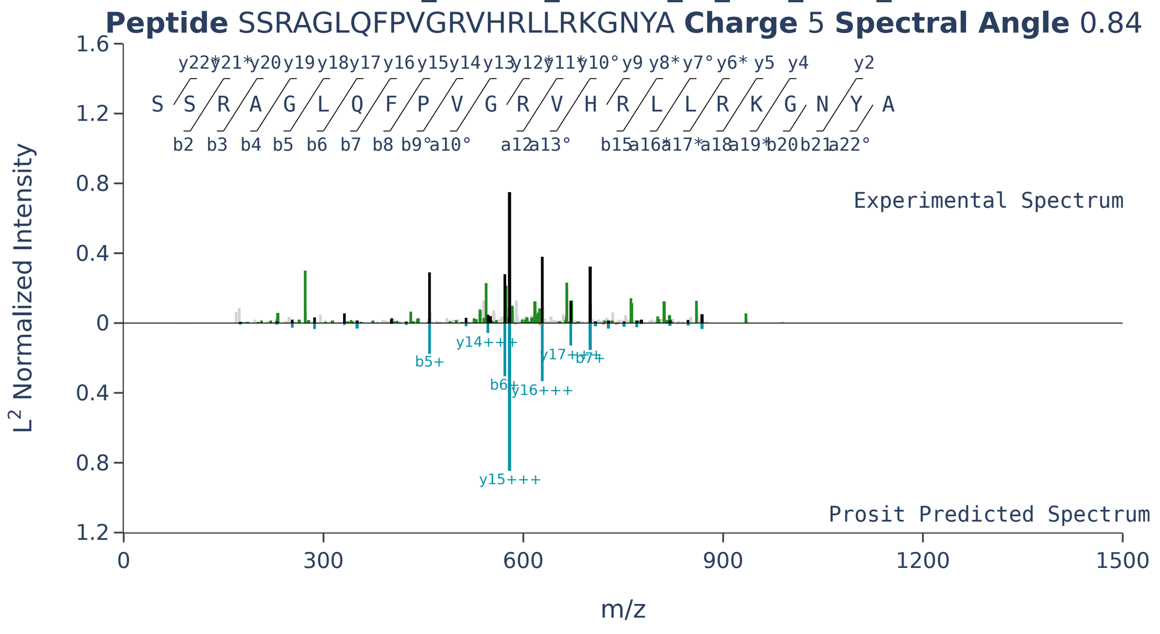


**Source** HRoetschke_291222_060123_Fu_H2A_b1R1_2h **Scan** 3351
**Peptide** HRLLRKGNYAERV **Charge** 5 **Spectral Angle** 0.82

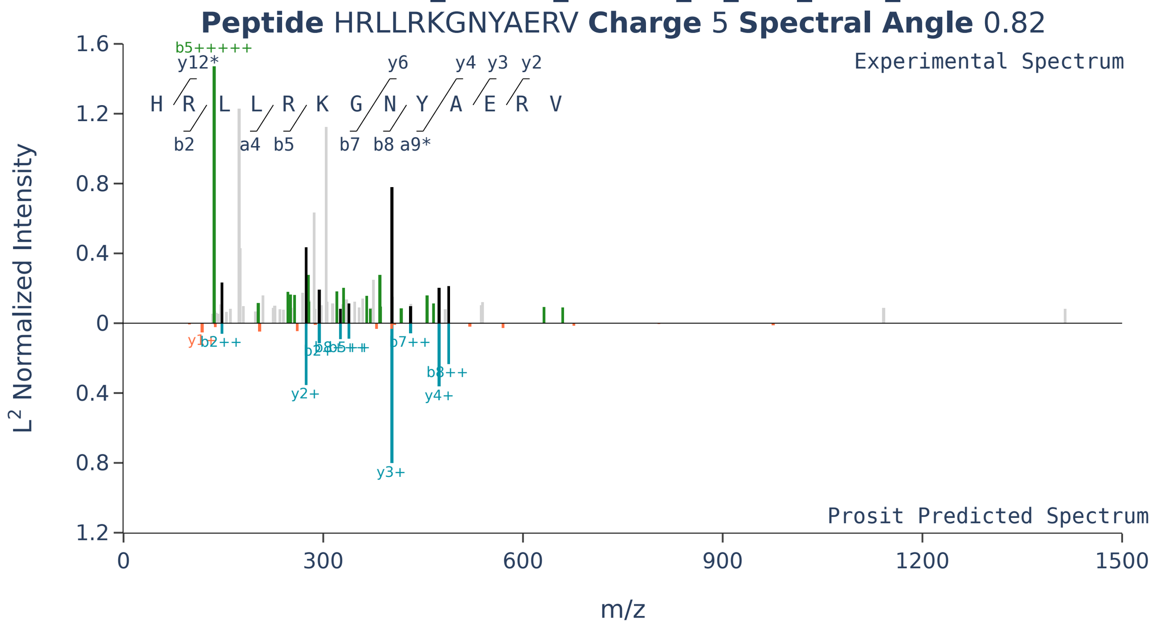


**Source** HRoetschke_291222_060123_Fu_H2A_b1R1_2h **Scan** 13964
**Peptide** QLAVRNDEELNKLLGR **Charge** 3 **Spectral Angle** 0.87

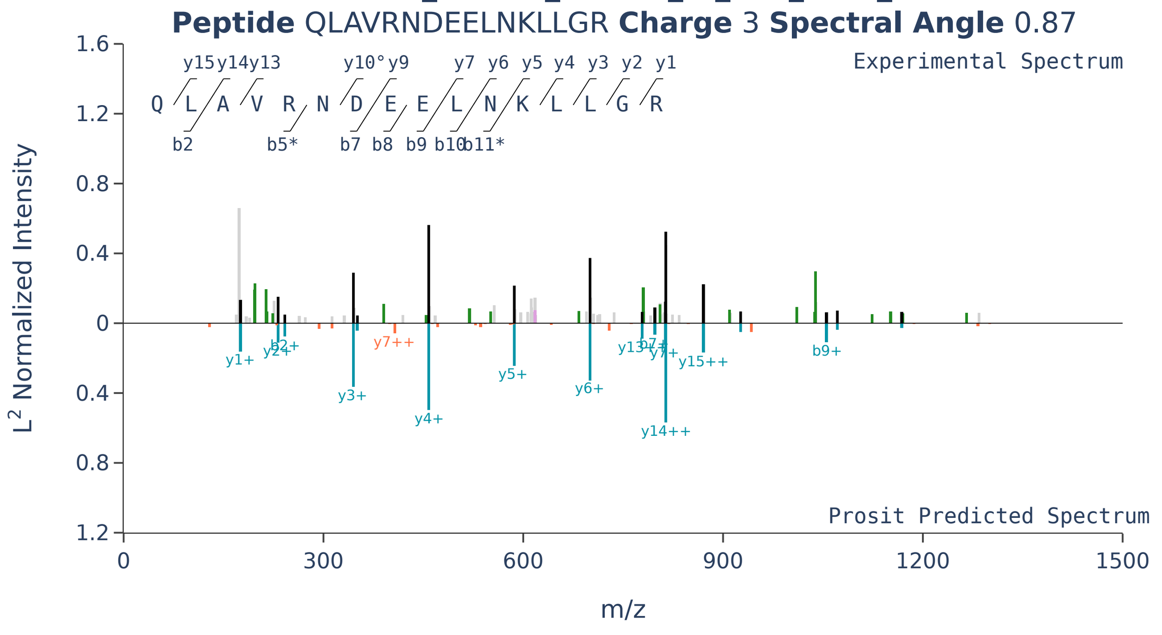


**Source** HRoetschke_291222_060123_Fu_H2A_b2R2_2h **Scan** 3599
**Peptide** RVHRLLRKGNYAERVGA **Charge** 5 **Spectral Angle** 0.83

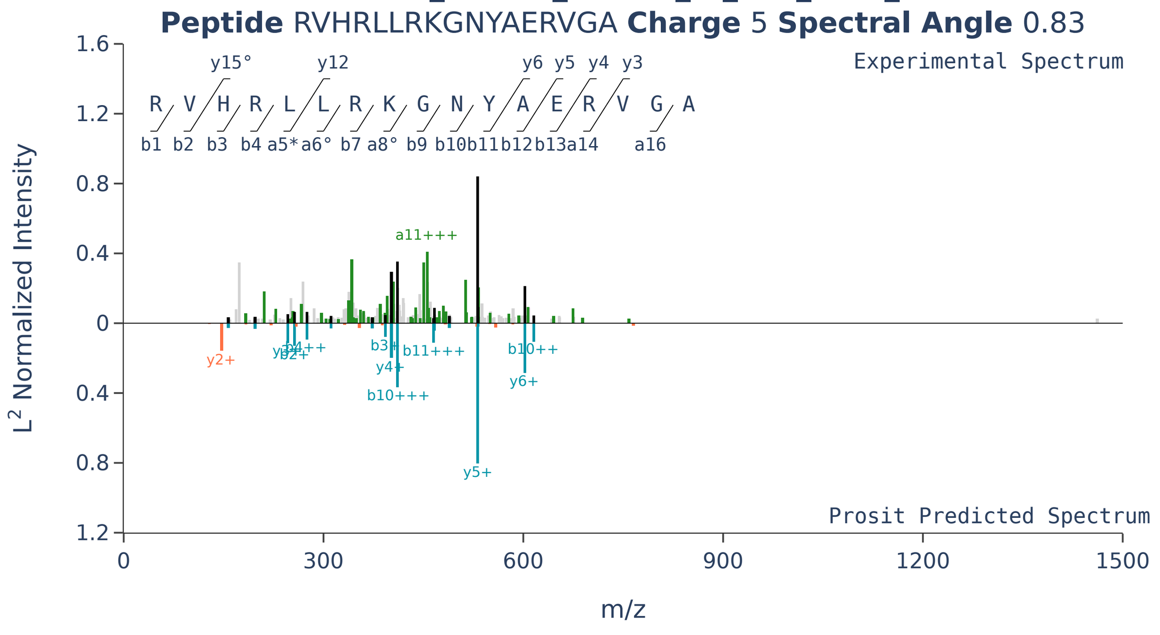


**Source** HRoetschke_291222_060123_Fu_H2A_b1R2_4h **Scan** 16784
**Peptide** AGLQFPVGRV **Charge** 2 **Spectral Angle** 0.85

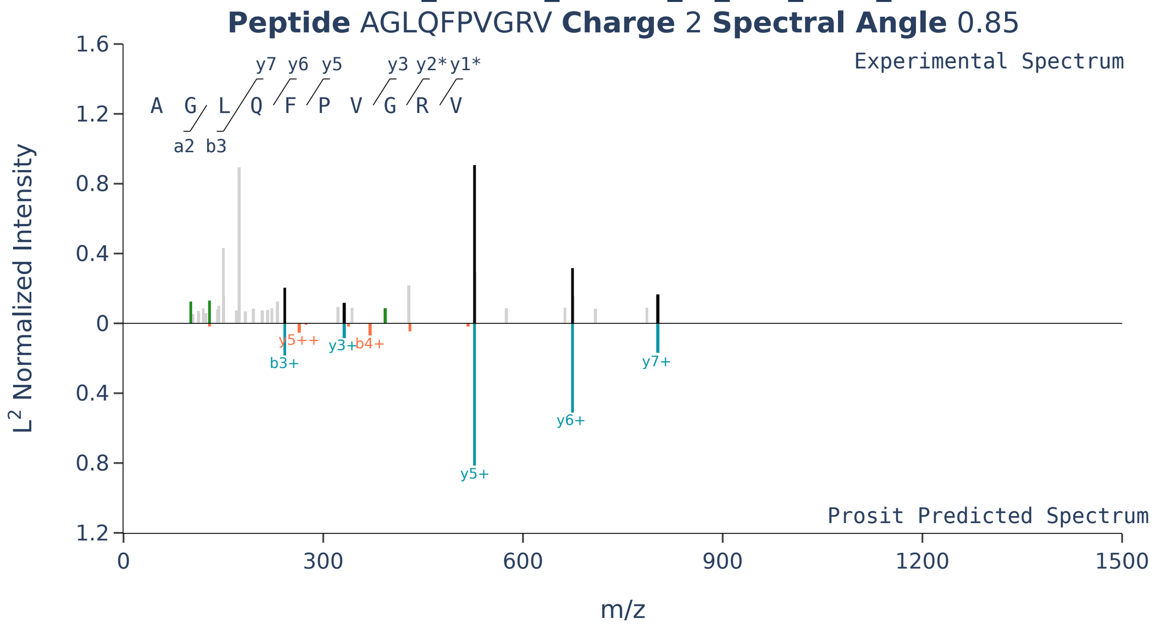


**Source** HRoetschke_291222_060123_Fu_H2A_b1R2_4h **Scan** 11286
**Peptide** RLLRKGNYAERVGAGAPVYLA **Charge** 4 **Spectral Angle** 0.73

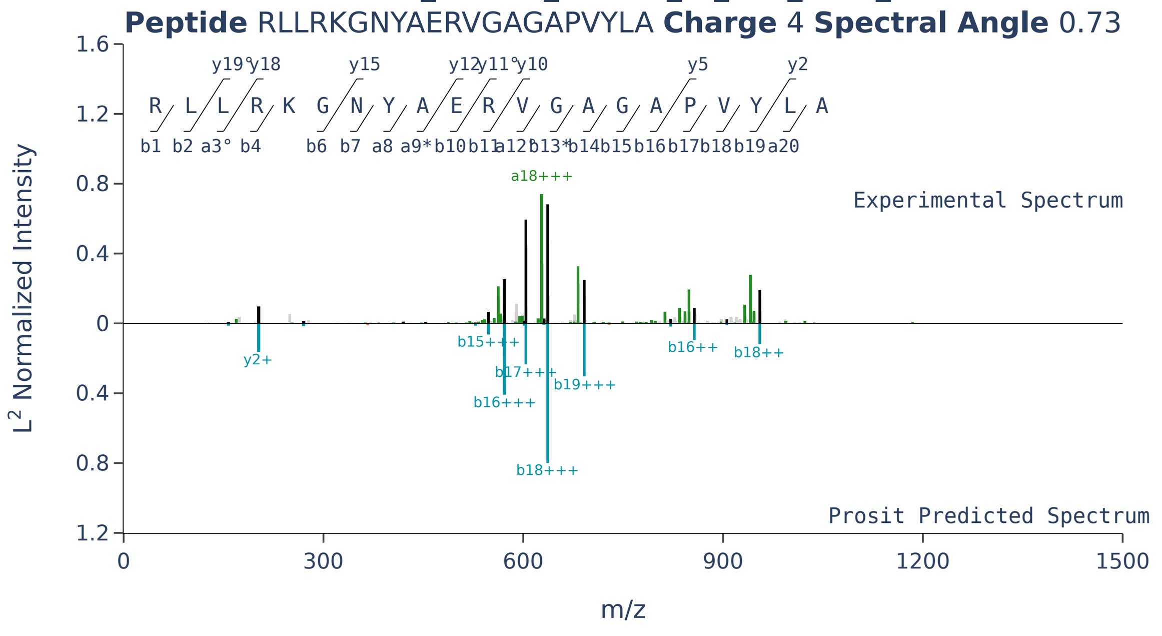


**Source** HRoetschke_291222_060123_Fu_H2A_b2R2_24h **Scan** 5710
**Peptide** SVLLPKKTESS **Charge** 2 **Spectral Angle** 0.92

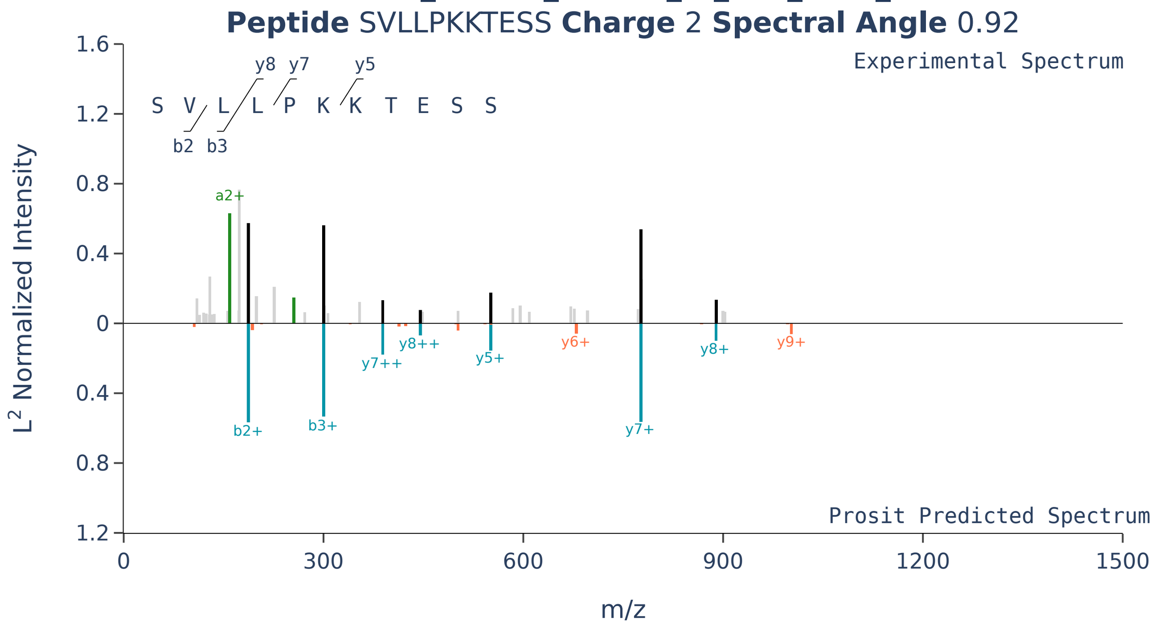


**Source** HRoetschke_291222_060123_Fu_H2A_b1R1_2h **Scan** 3274
**Peptide** ELAGNAARDNKKTRIIPRH **Charge** 5 **Spectral Angle** 0.7

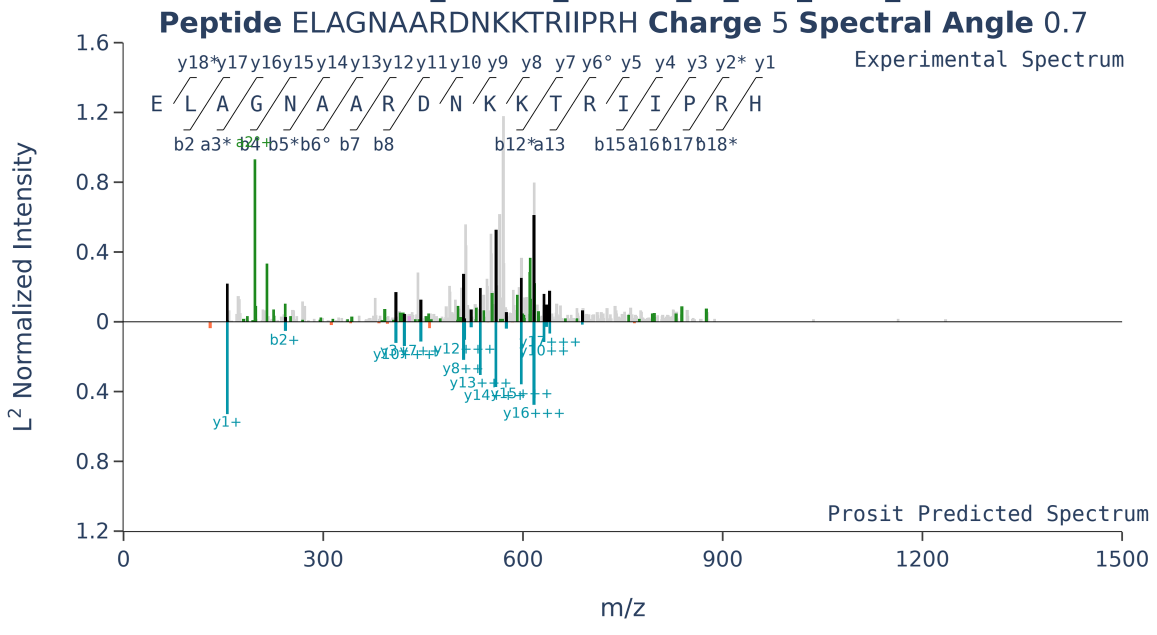


**Source** HRoetschke_291222_060123_Fu_H2A_b1R2_24h **Scan** 7292
**Peptide** LELAGNAARDNKKTRIIPRHLQ **Charge** 5 **Spectral Angle** 0.81

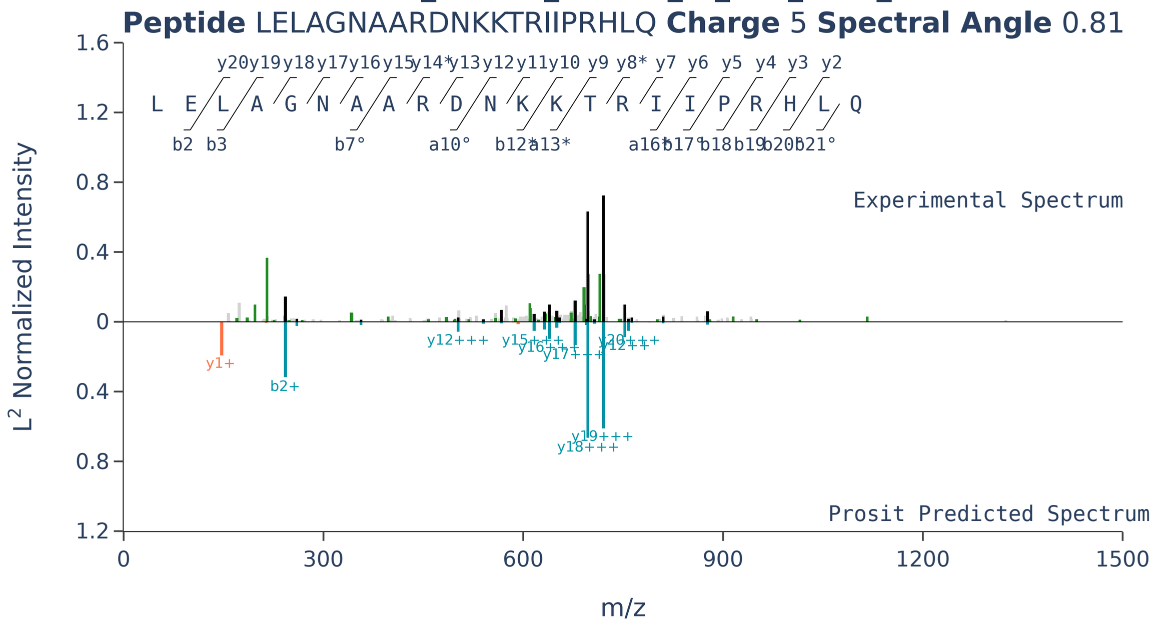


**Source** HRoetschke_291222_060123_Fu_H2A_b2R2_24h **Scan** 15700
**Peptide** RVTIAQGGVLPNL **Charge** 2 **Spectral Angle** 0.89

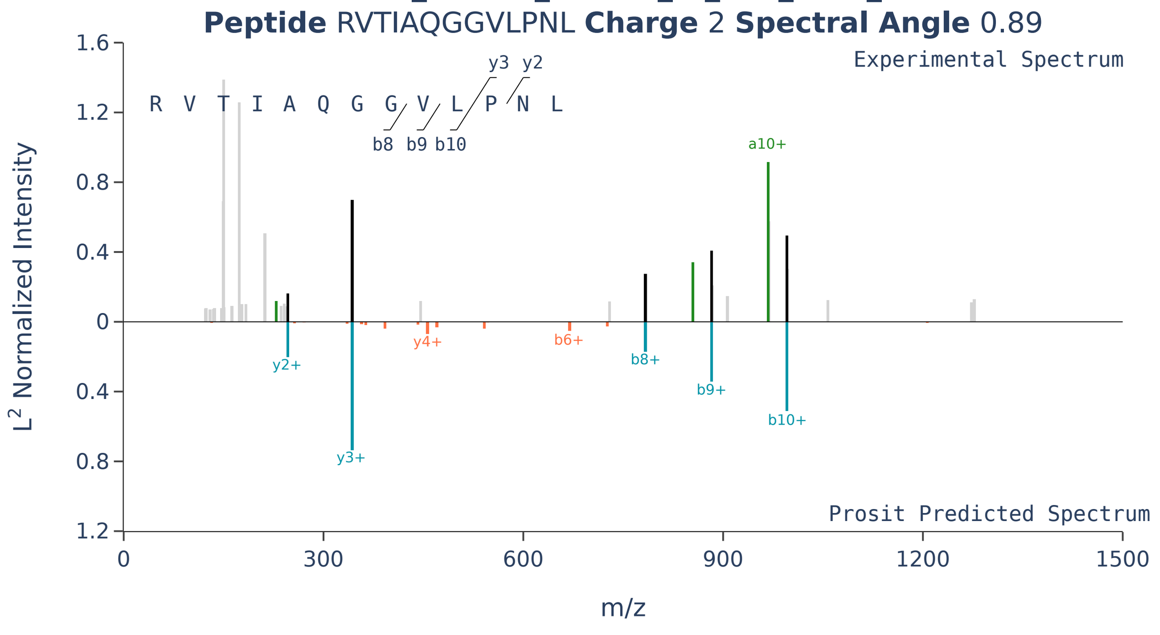


**Source** HRoetschke_291222_060123_Fu_H2A_b1R1_1h **Scan** 10237
**Peptide** LQLAVRNDEELN **Charge** 2 **Spectral Angle** 0.83

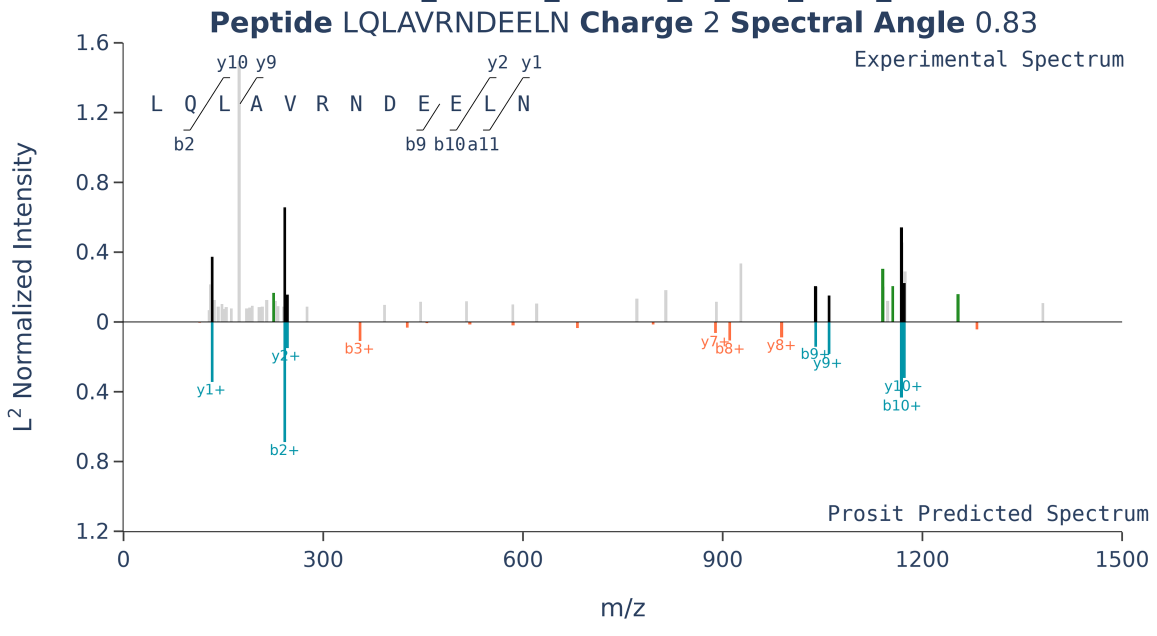


**Source** HRoetschke_291222_060123_Fu_H2A_b1R1_4h **Scan** 8917
**Peptide** SSRAGLQFPVGRVHR **Charge** 4 **Spectral Angle** 0.71

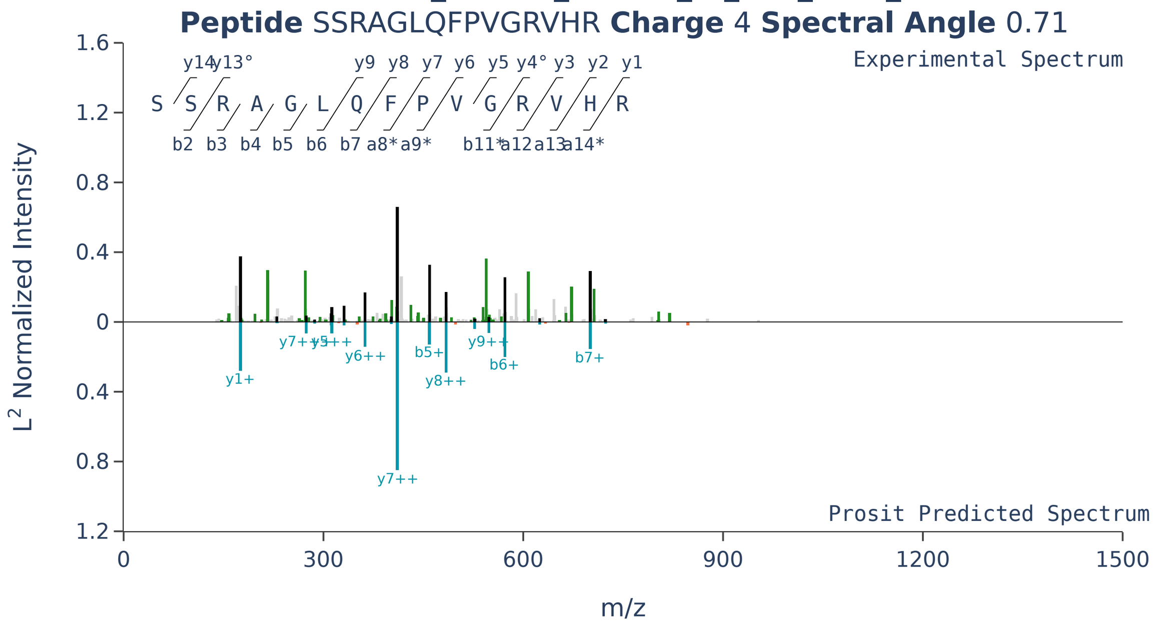


**Source** HRoetschke_291222_060123_Fu_H2A_b1R2_1h **Scan** 14996
**Peptide** DEELNKLLGRVTIAQG **Charge** 3 **Spectral Angle** 0.79

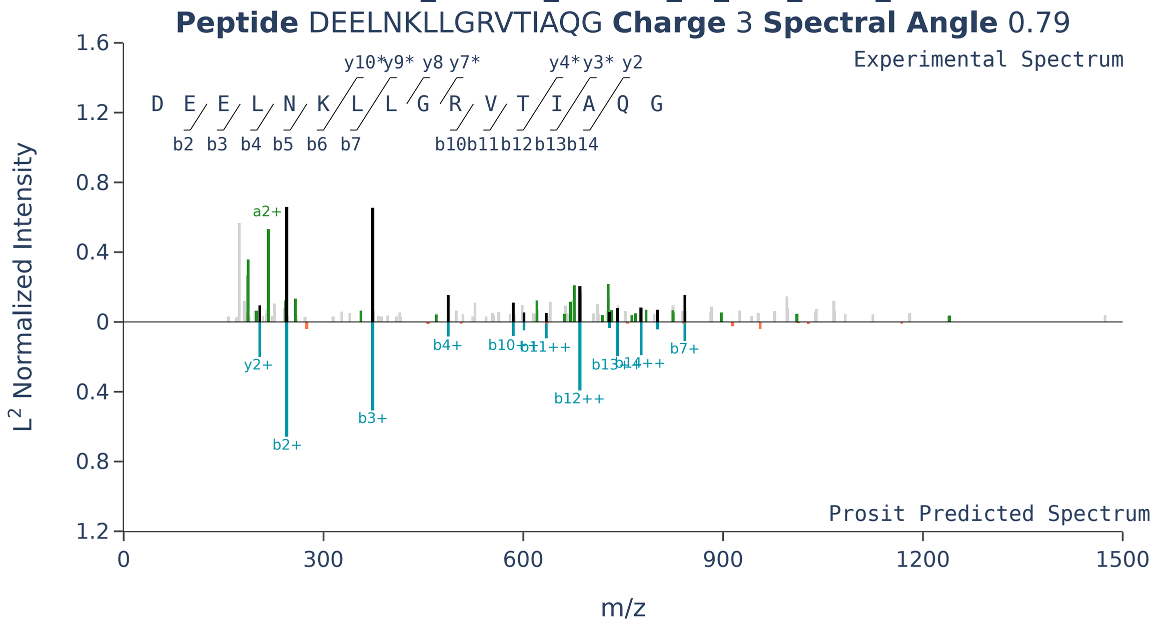

**Source** HRoetschke_291222_060123_Fu_H2A_b2R2_24h **Scan** 6473
**Peptide** NYAERVGAGAP **Charge** 2 **Spectral Angle** 0.89

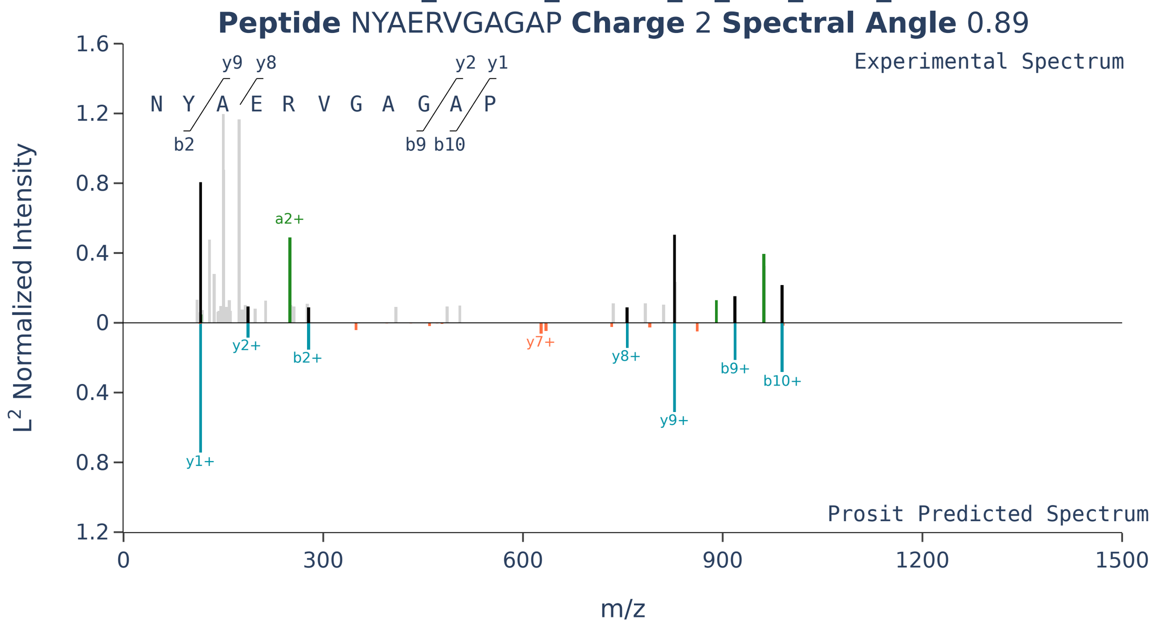


**Source** HRoetschke_291222_060123_Fu_H2A_b1R1_4h **Scan** 11124
**Peptide** LLELAGNAARDNKKTRII **Charge** 4 **Spectral Angle** 0.79

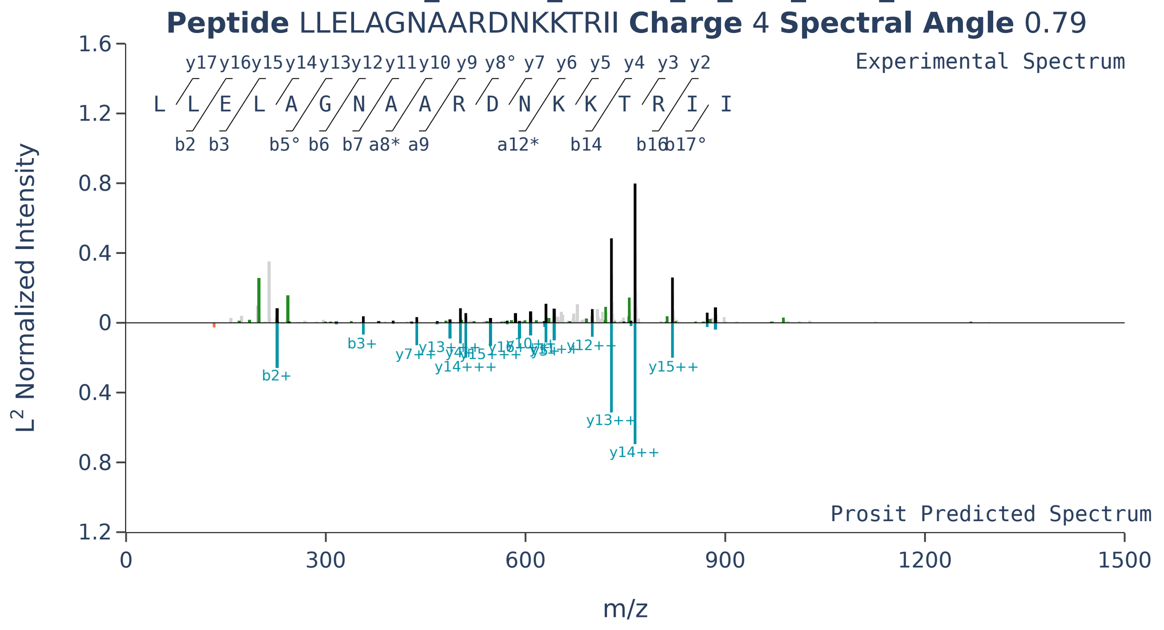


**Source** HRoetschke_291222_060123_Fu_H2A_b1R1_24h **Scan** 10370
**Peptide** GLQFPVGRVHR **Charge** 3 **Spectral Angle** 0.76

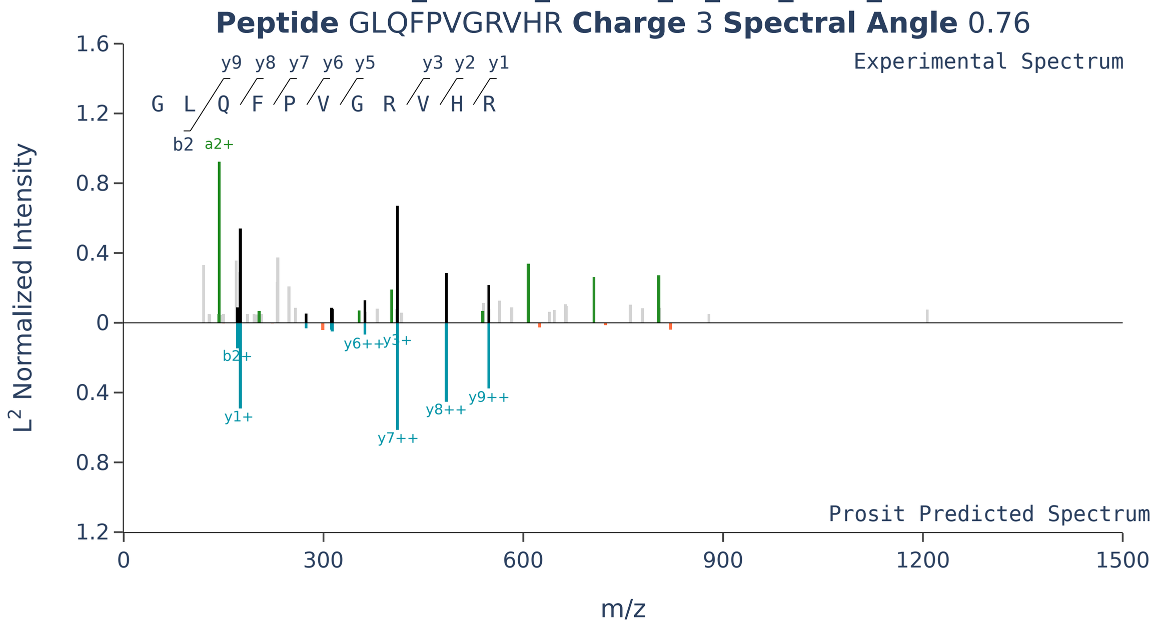


**Source** HRoetschke_291222_060123_Fu_H2A_b2R2_24h **Scan** 2981
**Peptide** RLLRKGNYA **Charge** 2 **Spectral Angle** 0.78

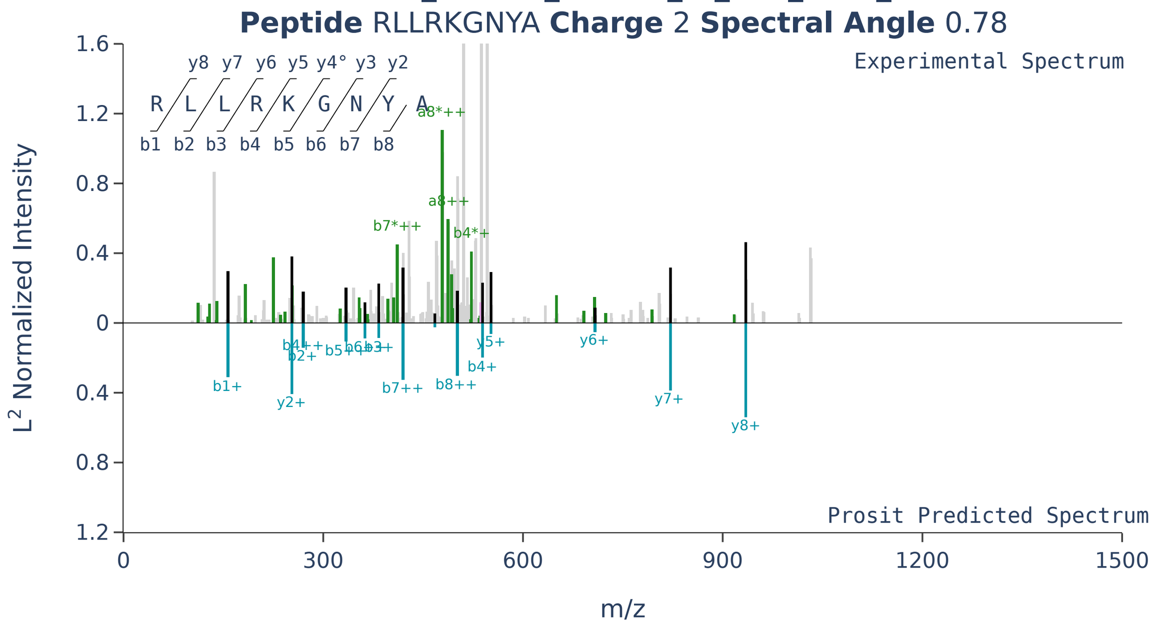


**Source** HRoetschke_291222_060123_Fu_H2A_b1R2_24h **Scan** 9713
**Peptide** AEILELAGNAARDNKKTR **Charge** 5 **Spectral Angle** 0.82

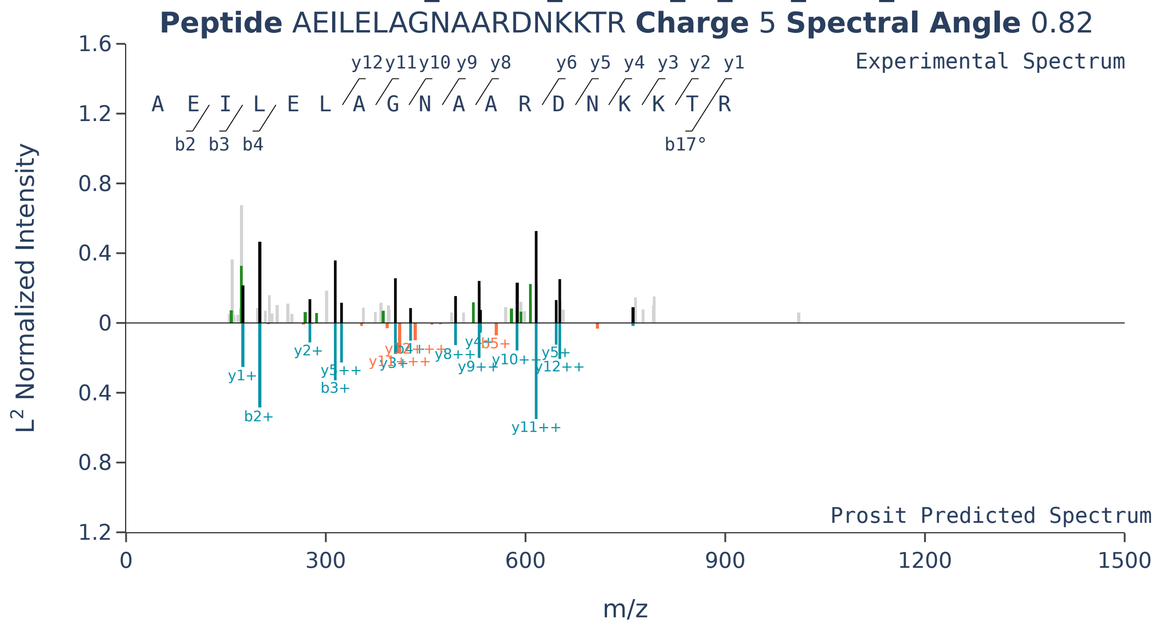


**Source** HRoetschke_291222_060123_Fu_H2A_b2R2_24h **Scan** 11787
**Peptide** ELNKLLGRV **Charge** 2 **Spectral Angle** 0.83

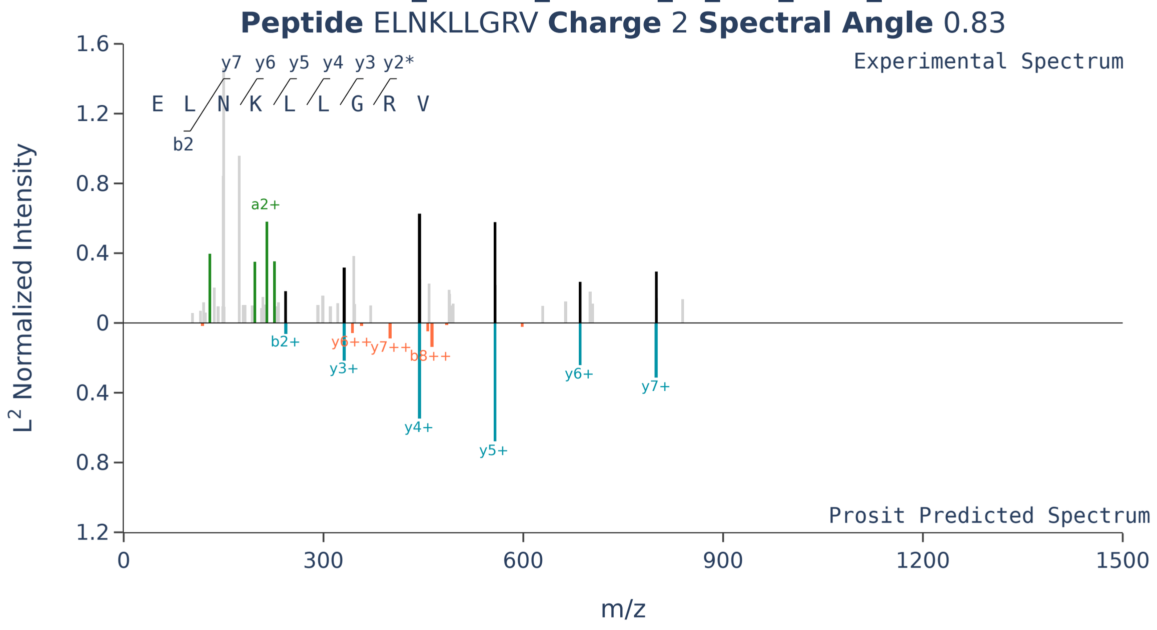


**Source** HRoetschke_291222_060123_Fu_H2A_b1R1_1h **Scan** 2569
**Peptide** RVHRLLRKGNYAE **Charge** 4 **Spectral Angle** 0.79

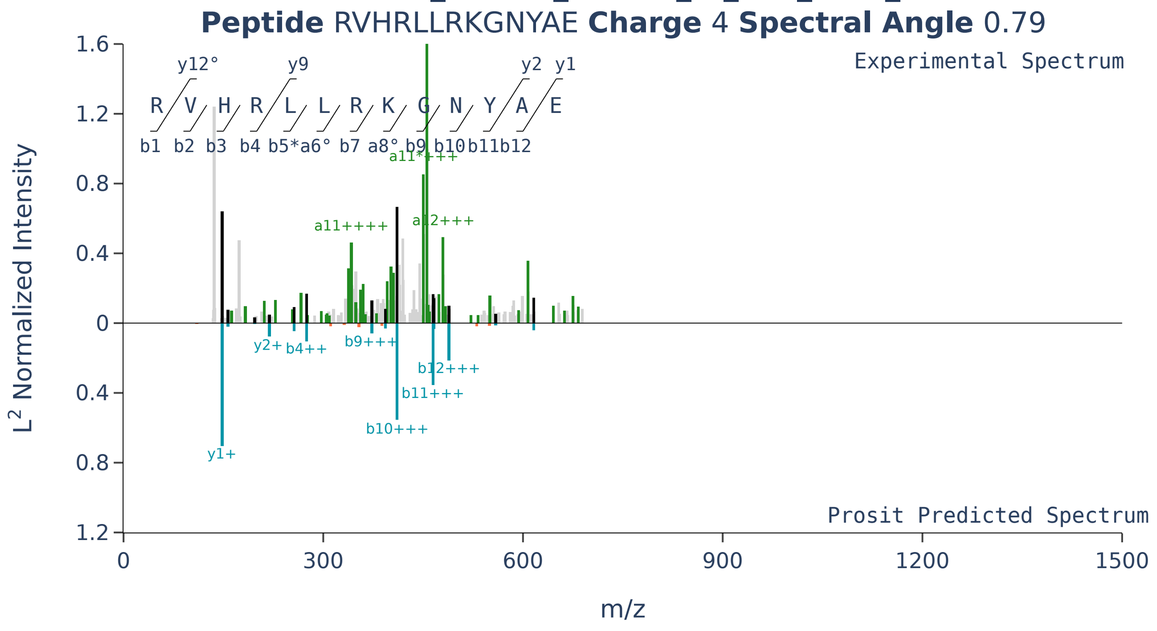


**Source** HRoetschke_291222_060123_Fu_H2A_b2R2_2h **Scan** 7872
**Peptide** LAGNAARDNKKTRIIPRHLQL **Charge** 4 **Spectral Angle** 0.93

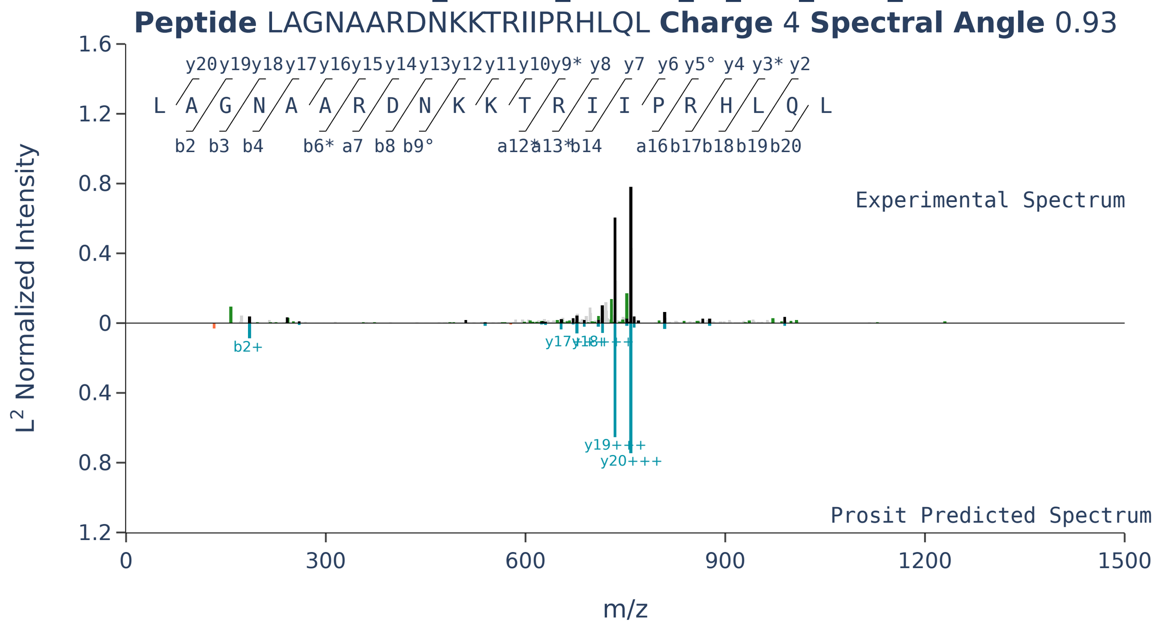


**Source** HRoetschke_291222_060123_Fu_H2A_b1R1_2h **Scan** 20199
**Peptide** LAAVLEYLTAE **Charge** 2 **Spectral Angle** 0.82

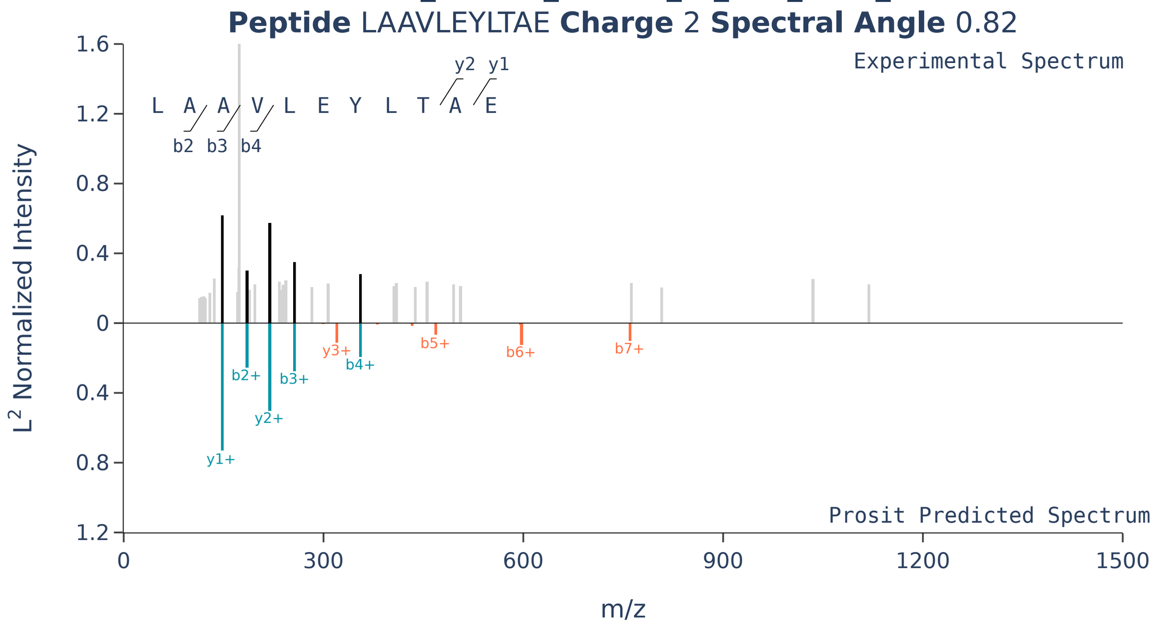


**Source** HRoetschke_291222_060123_Fu_H2A_b2R2_4h **Scan** 6150
**Peptide** QSVLLPKKTES **Charge** 3 **Spectral Angle** 0.86

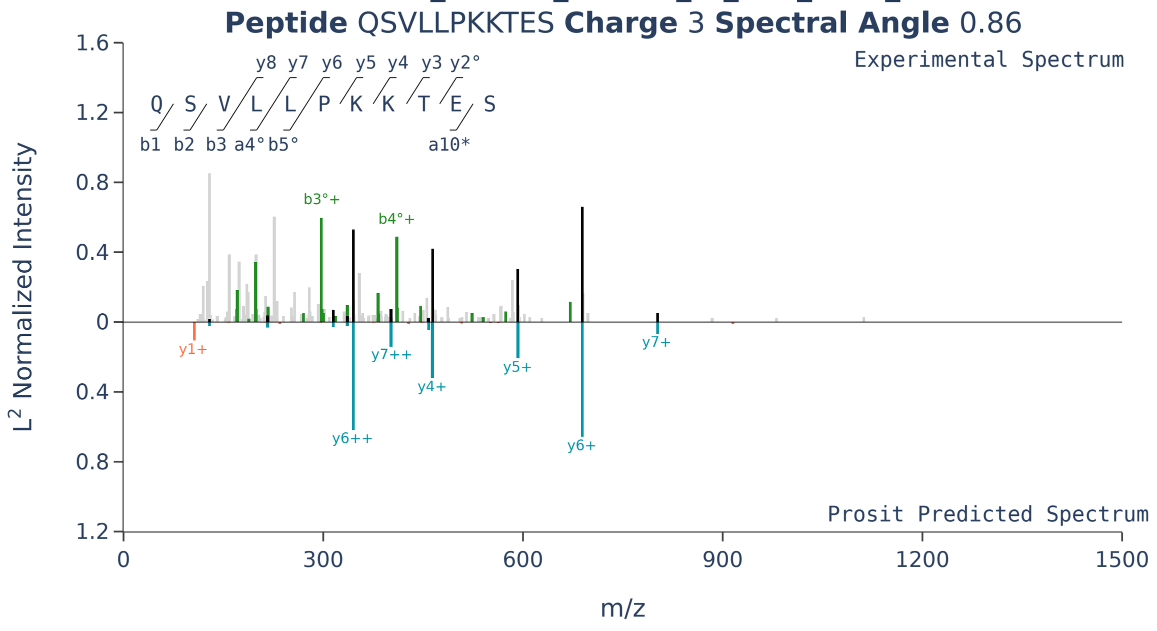


**Source** HRoetschke_291222_060123_Fu_H2A_b1R1_4h **Scan** 15744
**Peptide** VGAGAPVYLAA **Charge** 2 **Spectral Angle** 0.8

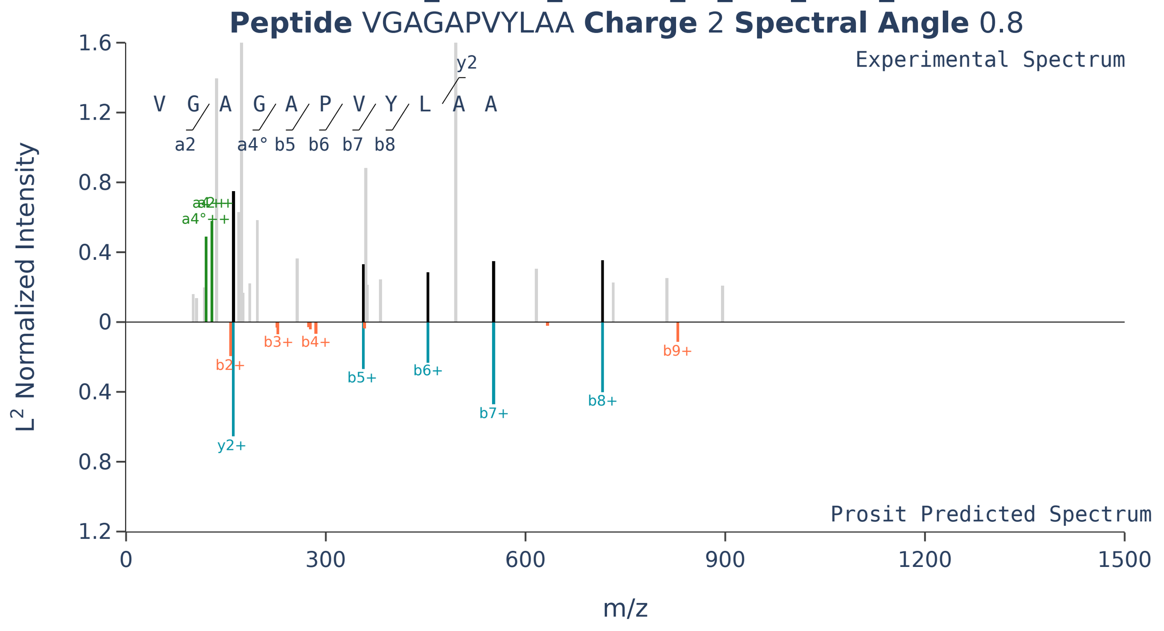


**Source** HRoetschke_291222_060123_Fu_H2A_b1R2_4h **Scan** 9969
**Peptide** TRSSRAGLQFPVGRVHRL **Charge** 5 **Spectral Angle** 0.83

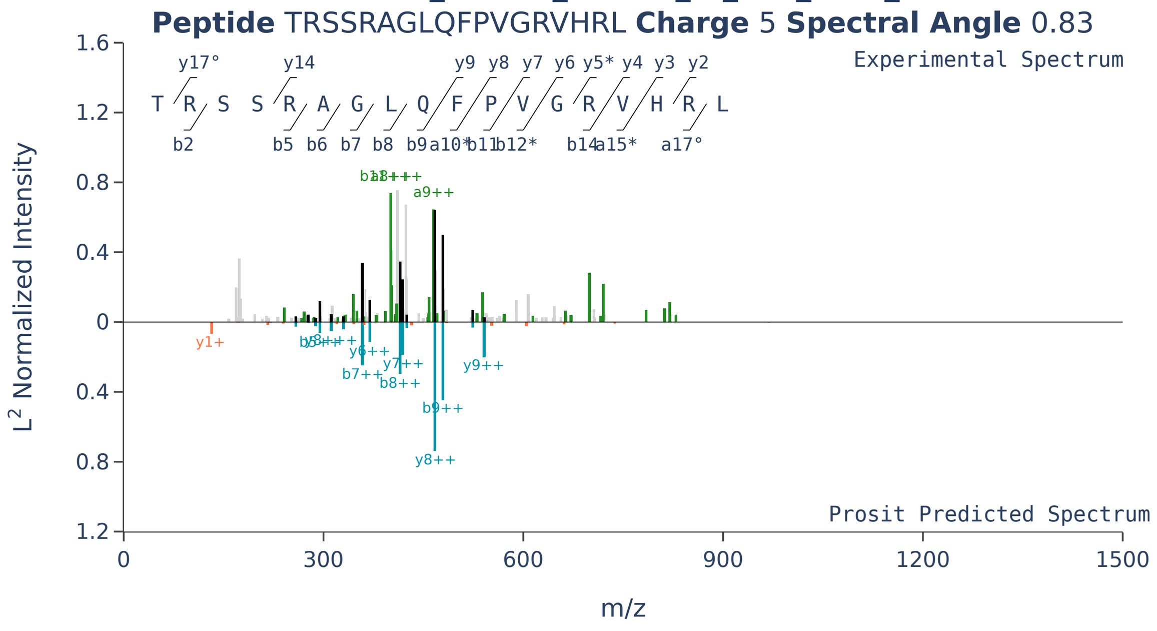


**Source** HRoetschke_291222_060123_Fu_H2A_b1R2_4h **Scan** 4697
**Peptide** LLRKGNYAERV **Charge** 3 **Spectral Angle** 0.81

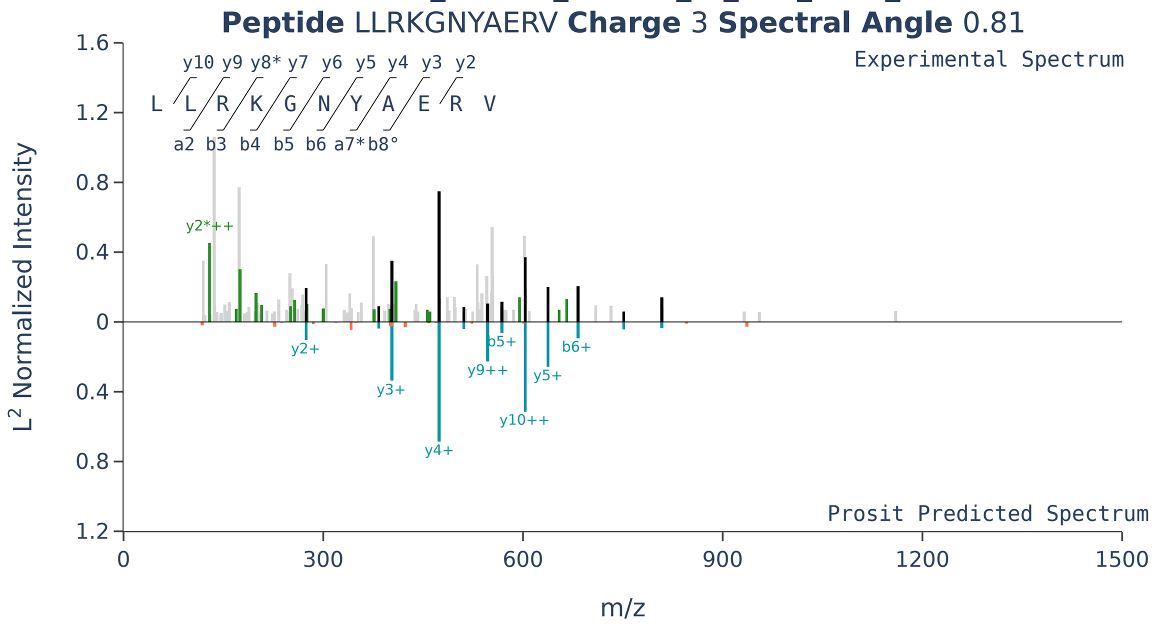


**Source** HRoetschke_291222_060123_Fu_H2A_b2R1_4h **Scan** 10399
**Peptide** GNYAERVGAGAPVY **Charge** 2 **Spectral Angle** 0.81

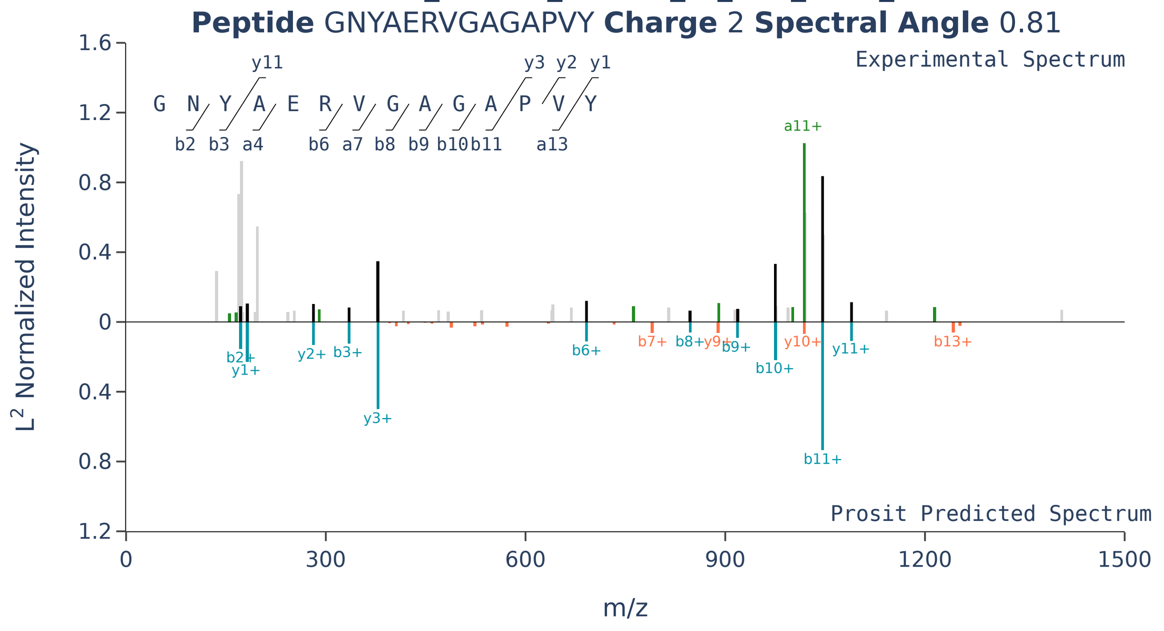


**Source** HRoetschke_291222_060123_Fu_H2A_b1R1_2h **Scan** 13917
**Peptide** TAEILELAGNAARDNKKTRIIPRHL **Charge** 5 **Spectral Angle** 0.88

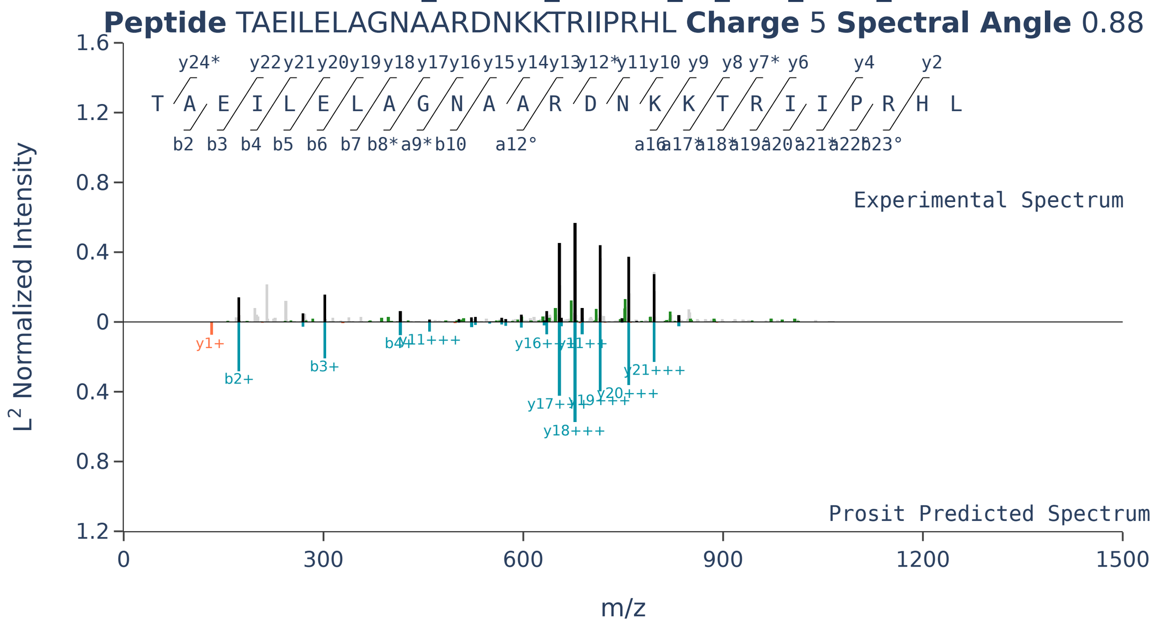

**Source** HRoetschke_291222_060123_Fu_H2A_b2R1_4h **Scan** 10118
**Peptide** LLELAGNAARDNKKTRIIPRHL **Charge** 5 **Spectral Angle** 0.82

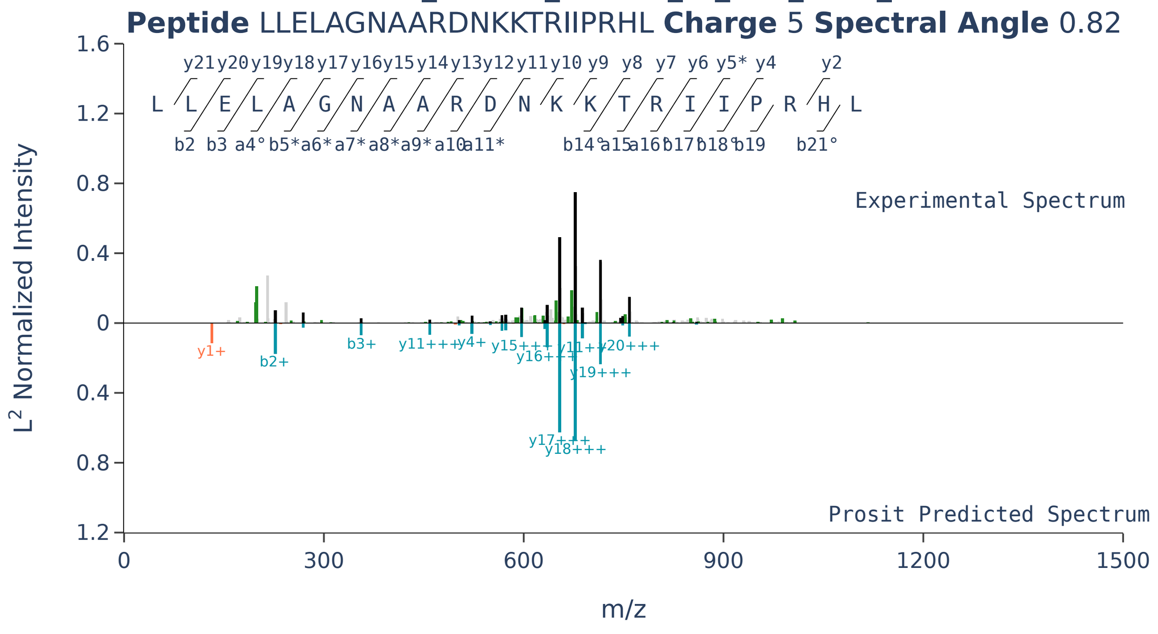


**Source** HRoetschke_291222_060123_Fu_H2A_b2R1_2h **Scan** 6872
**Peptide** RVHRLLRKGNYAERVGAGAPVY **Charge** 4 **Spectral Angle** 0.84

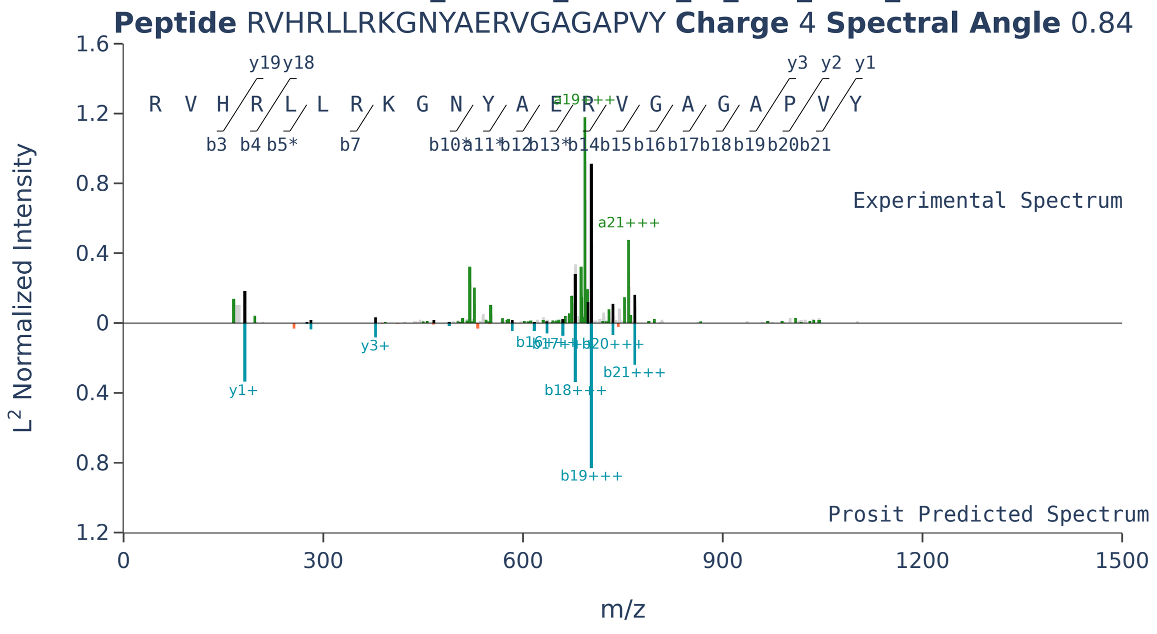


**Source** HRoetschke_291222_060123_Fu_H2A_b2R1_2h **Scan** 2423
**Peptide** RVHRLLRKGNYA **Charge** 3 **Spectral Angle** 0.68

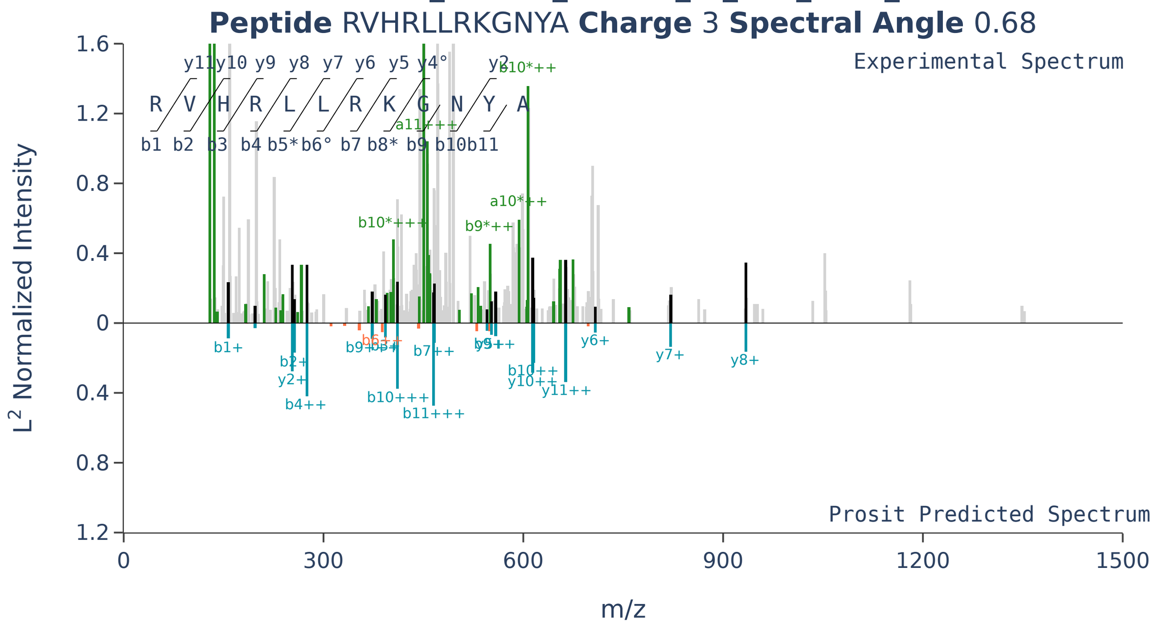


**Source** HRoetschke_291222_060123_Fu_H2A_b1R2_2h **Scan** 3483
**Peptide** PVGRVHRLLRKGNYAER **Charge** 4 **Spectral Angle** 0.69

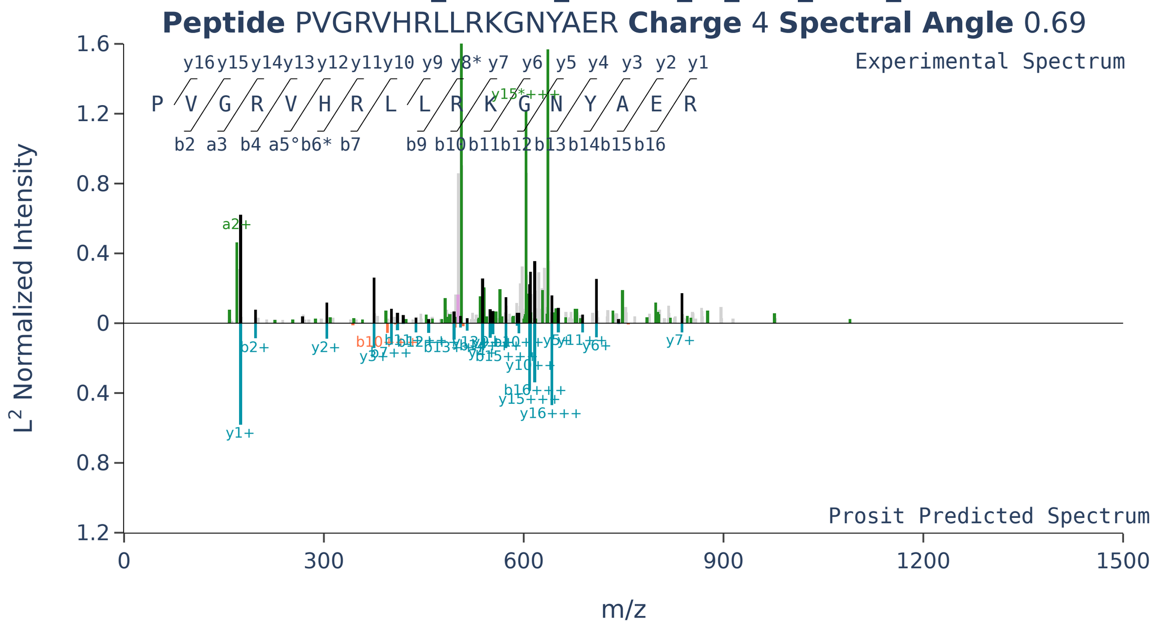


**Source** HRoetschke_291222_060123_Fu_H2A_b1R1_2h **Scan** 17841
**Peptide** LGRVTIAQGGVLPNL **Charge** 2 **Spectral Angle** 0.81

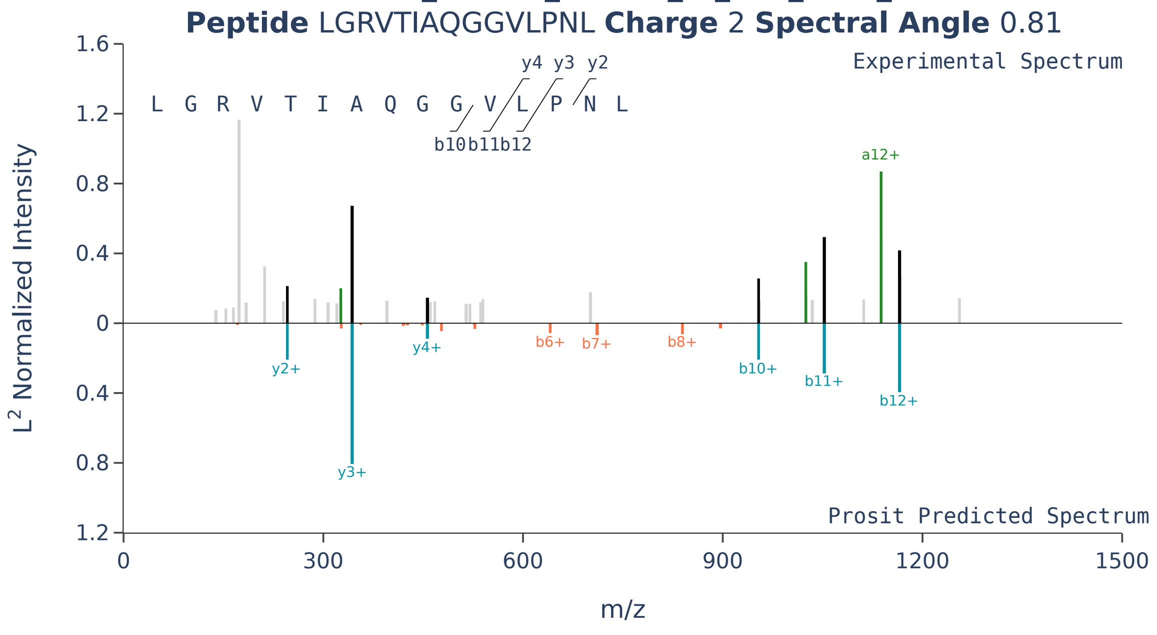


**Source** HRoetschke_291222_060123_Fu_H2A_b1R1_2h **Scan** 11333
**Peptide** SRAGLQFPVGRVHRLLRKGNYA **Charge** 5 **Spectral Angle** 0.87

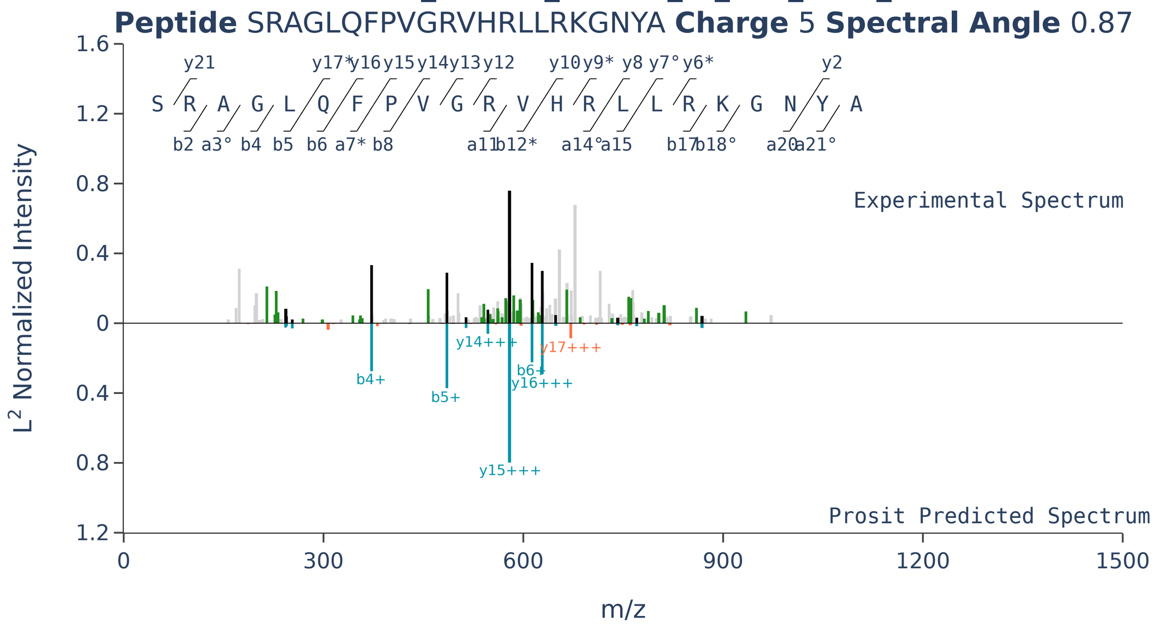


**Source** HRoetschke_291222_060123_Fu_H2A_b2R1_2h **Scan** 9229
**Peptide** AKTRSSRAGLQFPVG **Charge** 3 **Spectral Angle** 0.9

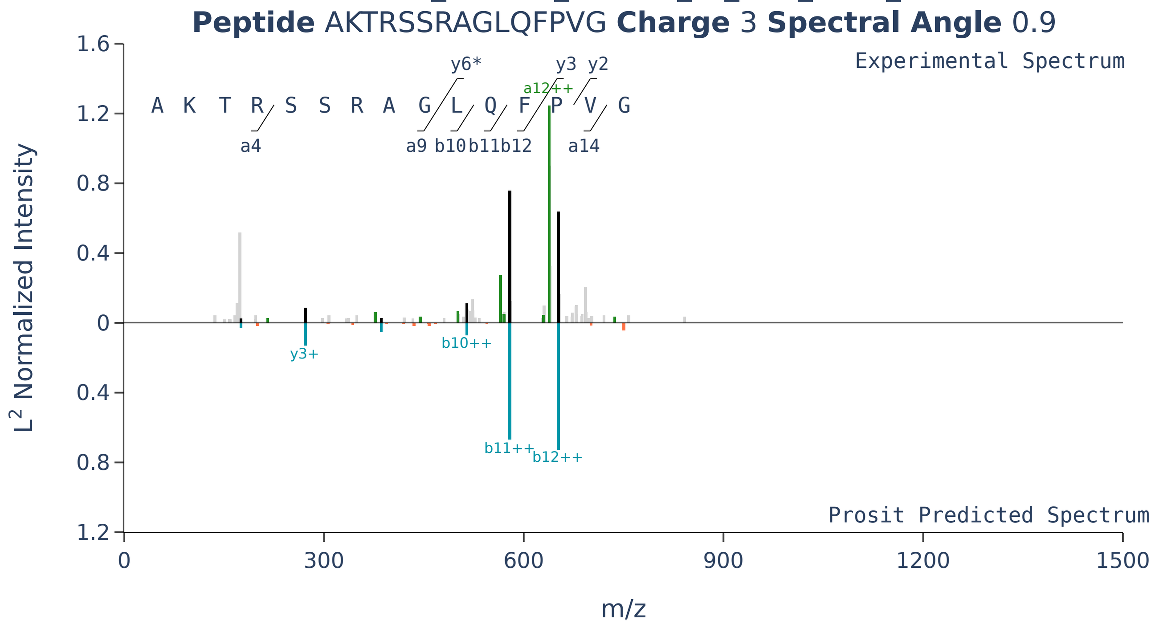


**Source** HRoetschke_291222_060123_Fu_H2A_b2R1_24h **Scan** 10083
**Peptide** KTRSSRAGLQFPVG **Charge** 3 **Spectral Angle** 0.8

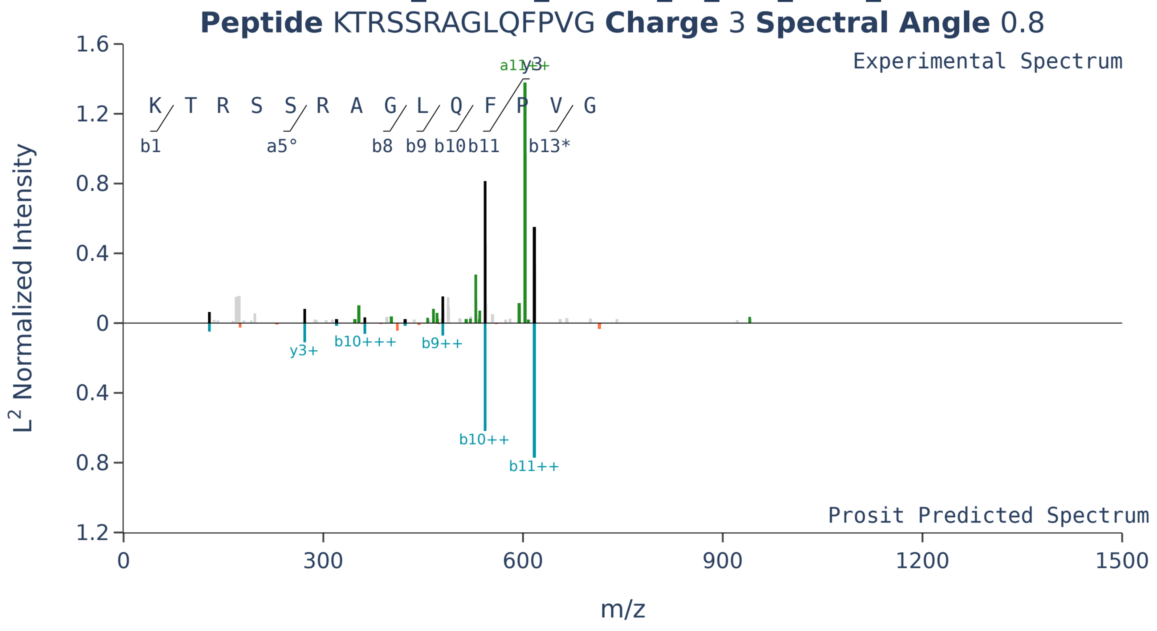


**Source** HRoetschke_291222_060123_Fu_H2A_b2R1_2h **Scan** 10828
**Peptide** ELLELAGNAARDNKKTRIIPRHL **Charge** 5 **Spectral Angle** 0.79

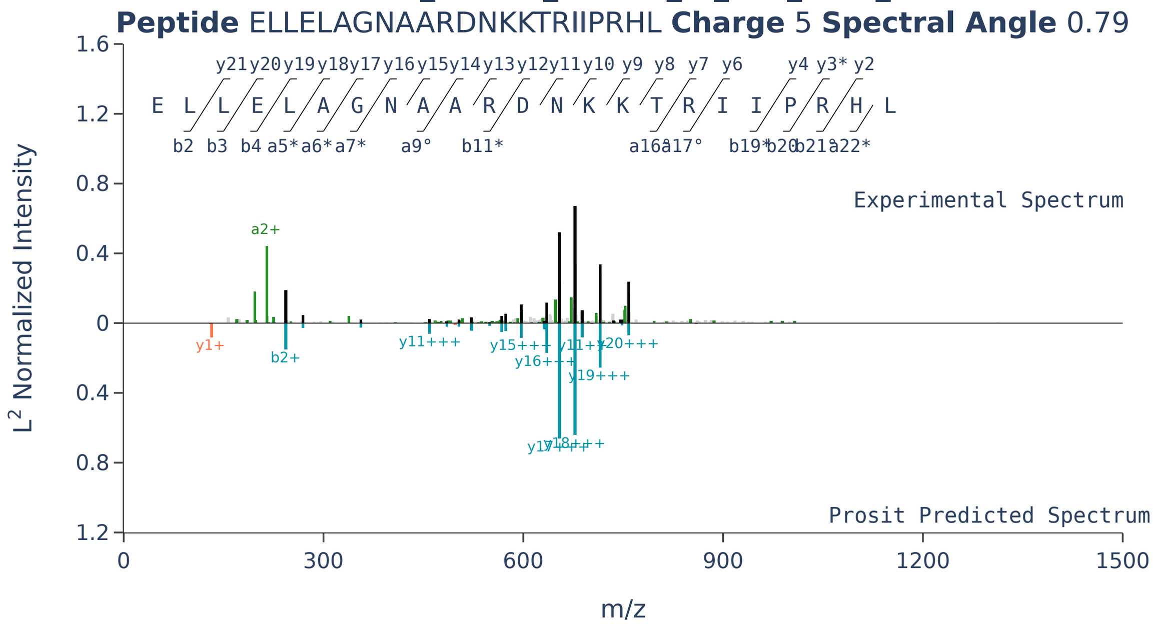


**Source** HRoetschke_291222_060123_Fu_H2A_b1R1_4h **Scan** 11486
**Peptide** RAGLQFPVGRVHRL **Charge** 3 **Spectral Angle** 0.77

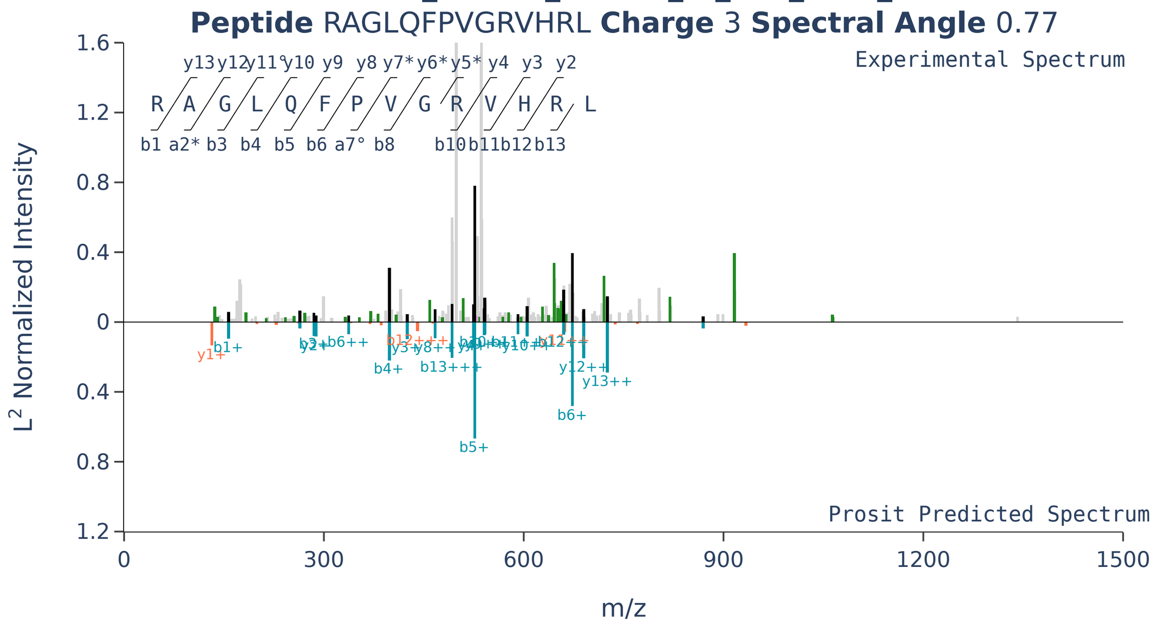


**Source** HRoetschke_291222_060123_Fu_H2A_b2R2_1h **Scan** 4834
**Peptide** KLLGRVT **Charge** 2 **Spectral Angle** 0.91

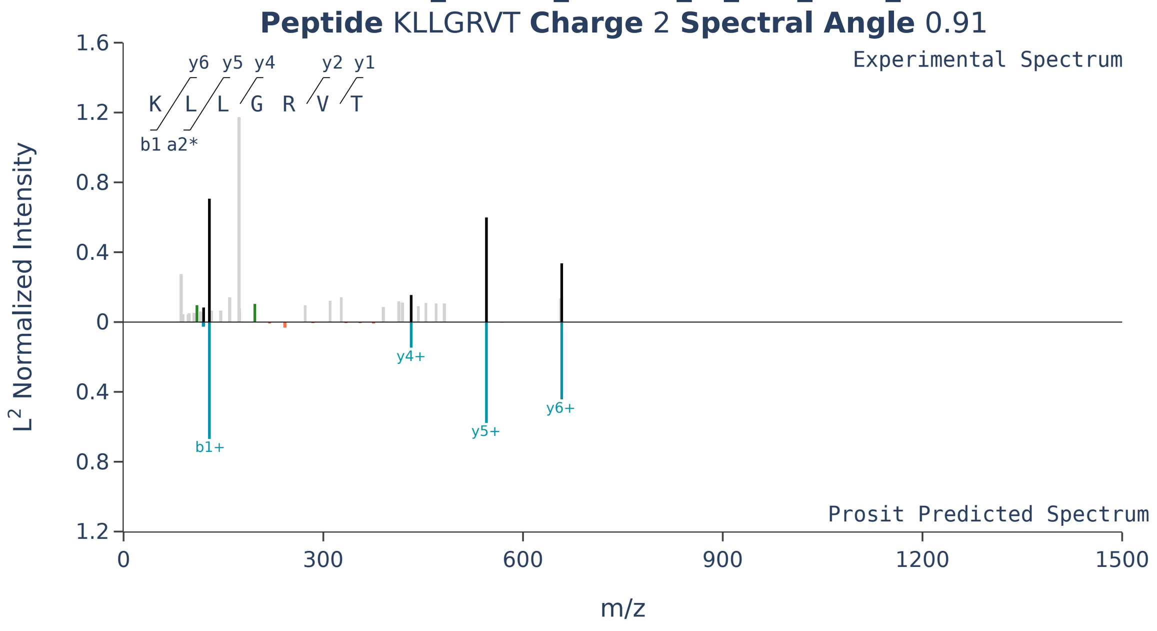


**Source** HRoetschke_291222_060123_Fu_H2A_b2R1_24h **Scan** 7802
**Peptide** KTRSSRAGLQFPVGRVH **Charge** 4 **Spectral Angle** 0.77

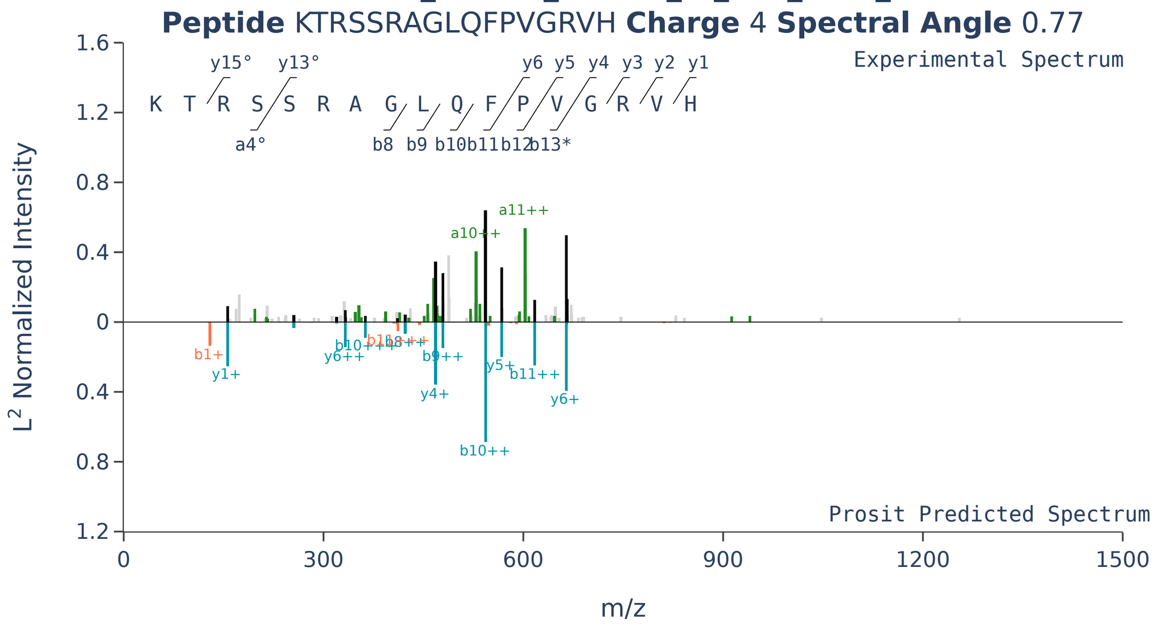


**Source** HRoetschke_291222_060123_Fu_H2A_b1R1_4h **Scan** 17008
**Peptide** QLAVRNDEELNKLLGRVT **Charge** 3 **Spectral Angle** 0.84

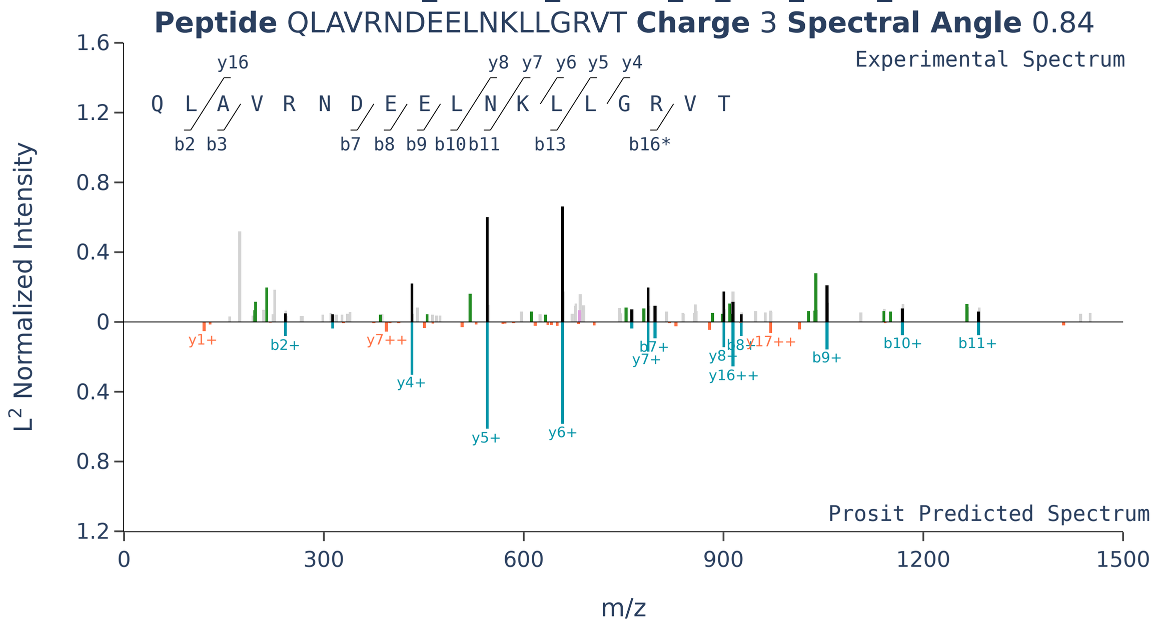


**Source** HRoetschke_291222_060123_Fu_H2A_b1R1_2h **Scan** 7989
**Peptide** QFPVGRVHRLLRKGNYAER **Charge** 5 **Spectral Angle** 0.72

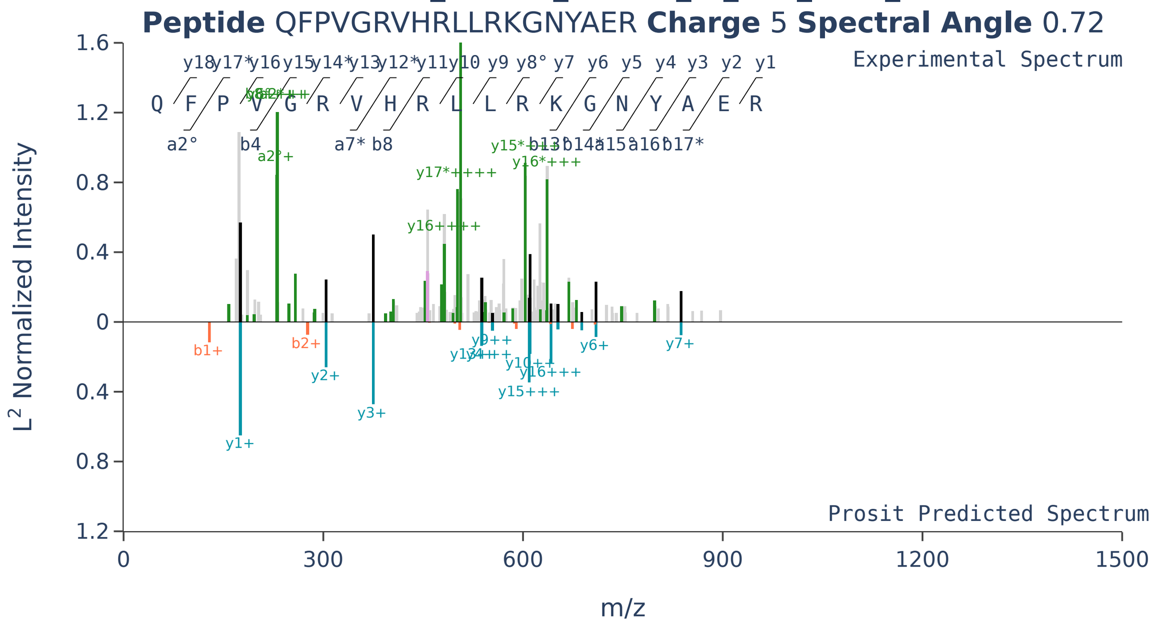


**Source** HRoetschke_291222_060123_Fu_H2A_b1R1_1h **Scan** 14161
**Peptide** EELNKLLGRVTIAQ **Charge** 2 **Spectral Angle** 0.83

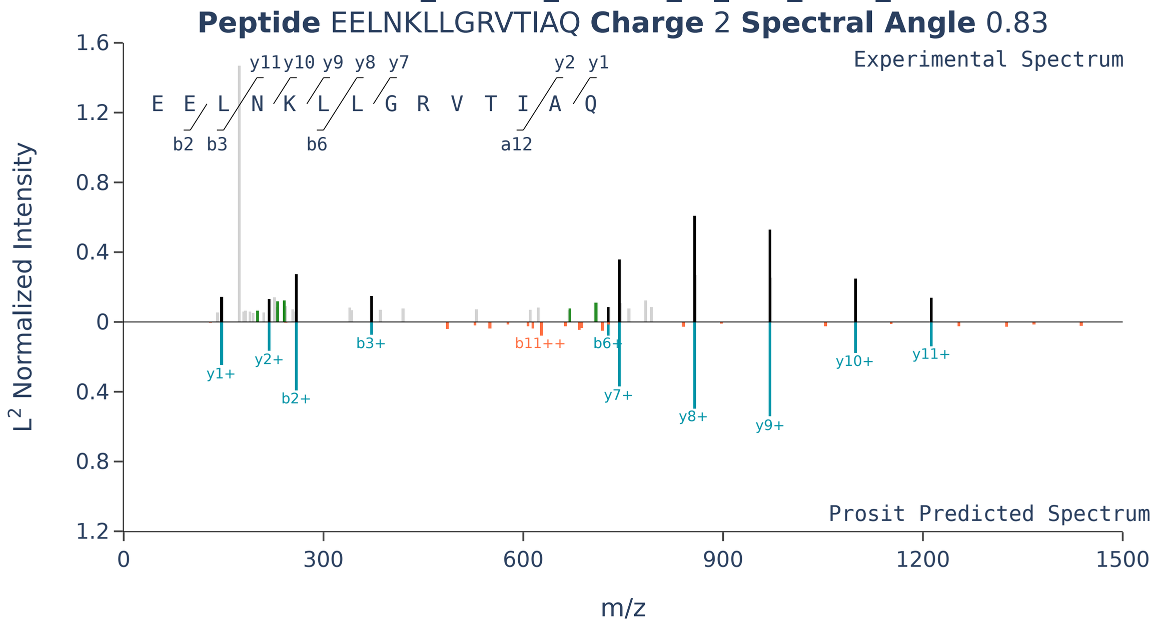

**Source** HRoetschke_291222_060123_Fu_H2A_b1R1_24h **Scan** 11360
**Peptide** ELNKLLGRVT **Charge** 3 **Spectral Angle** 0.89

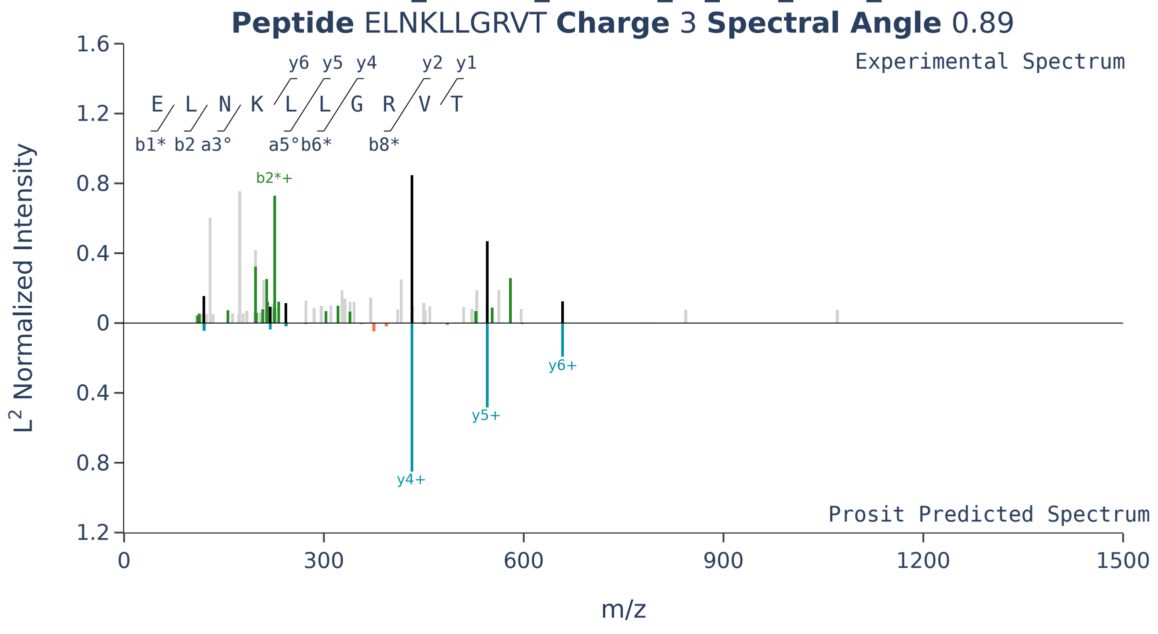


**Source** HRoetschke_291222_060123_Fu_H2A_b2R2_4h **Scan** 6848
**Peptide** RKGNYAERVGAGAPVY **Charge** 3 **Spectral Angle** 0.8

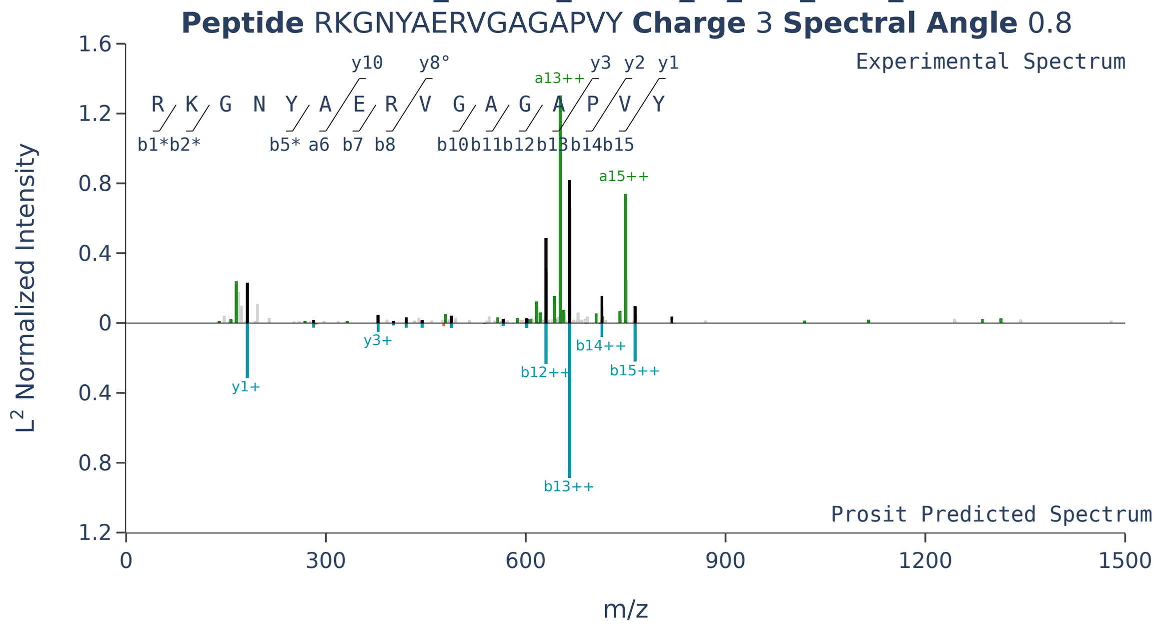


**Source** HRoetschke_291222_060123_Fu_H2A_b1R2_1h **Scan** 15849
**Peptide** DEELNKLLGRVTL **Charge** 2 **Spectral Angle** 0.82

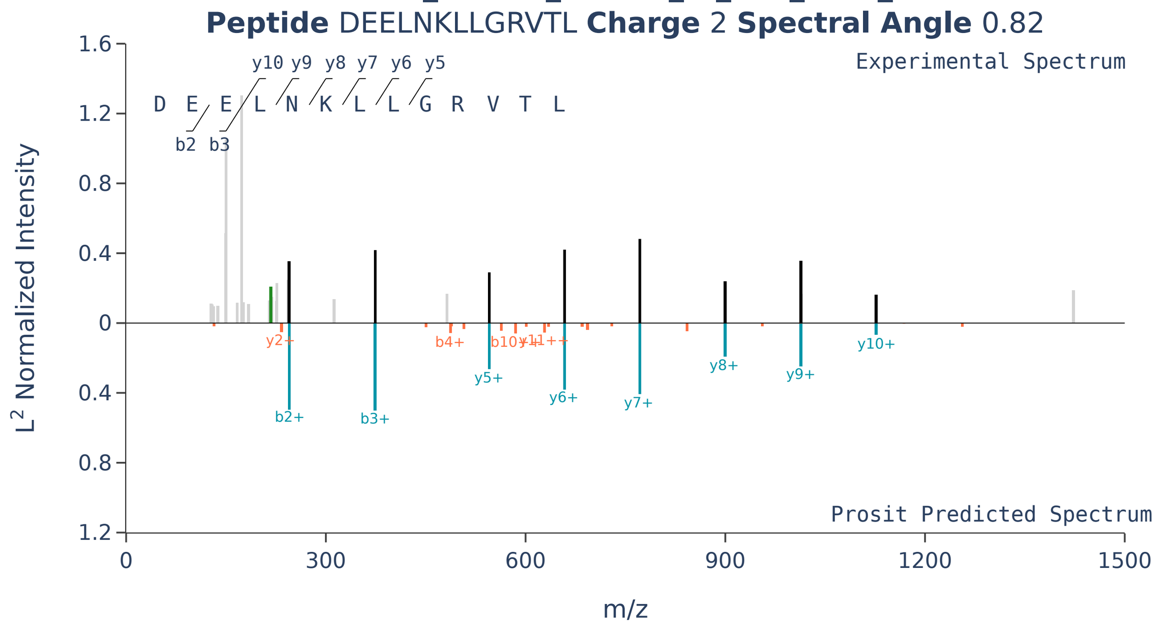


**Source** HRoetschke_291222_060123_Fu_H2A_b1R1_2h **Scan** 11719
**Peptide** AGLQFPVGRVHRLLRKGNYAER **Charge** 5 **Spectral Angle** 0.77

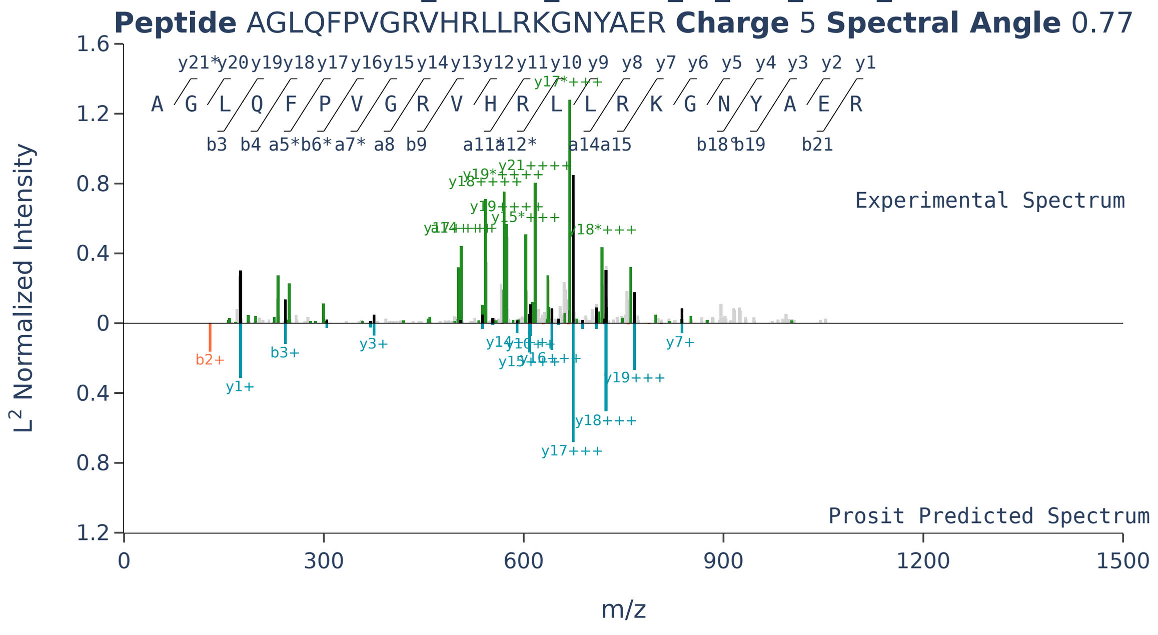


**Source** HRoetschke_291222_060123_Fu_H2A_b2R2_4h **Scan** 15687
**Peptide** TIAQGGVLPNL **Charge** 2 **Spectral Angle** 0.85

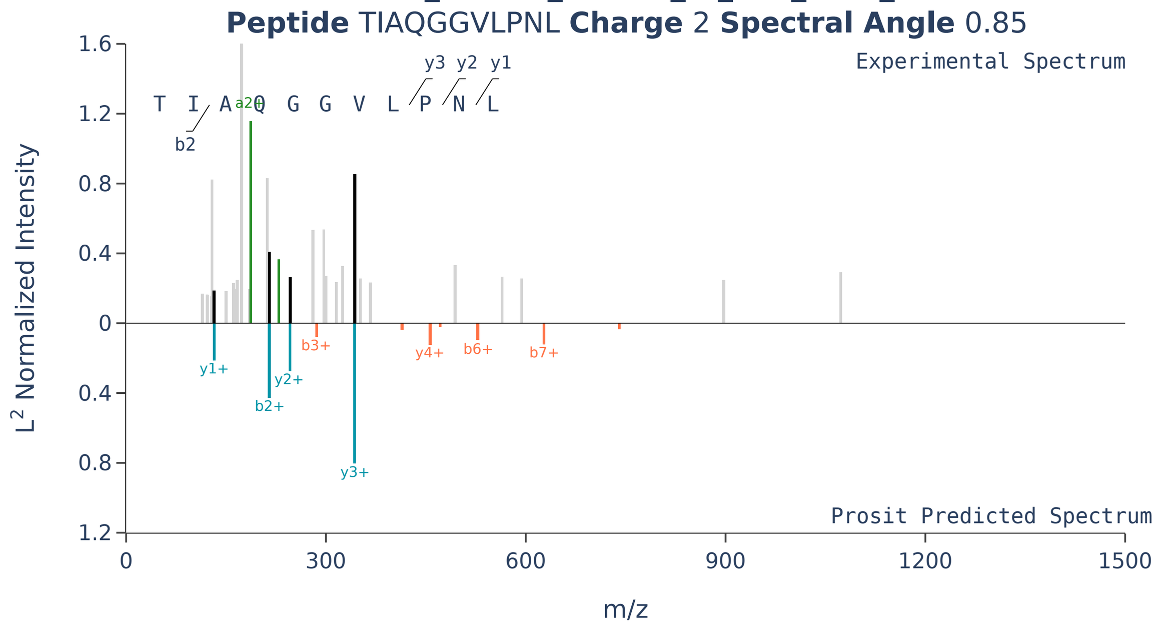


**Source** HRoetschke_291222_060123_Fu_H2A_b1R1_2h **Scan** 10436
**Peptide** HRLLRKGNYAERVGAGAPVYLAA **Charge** 4 **Spectral Angle** 0.86

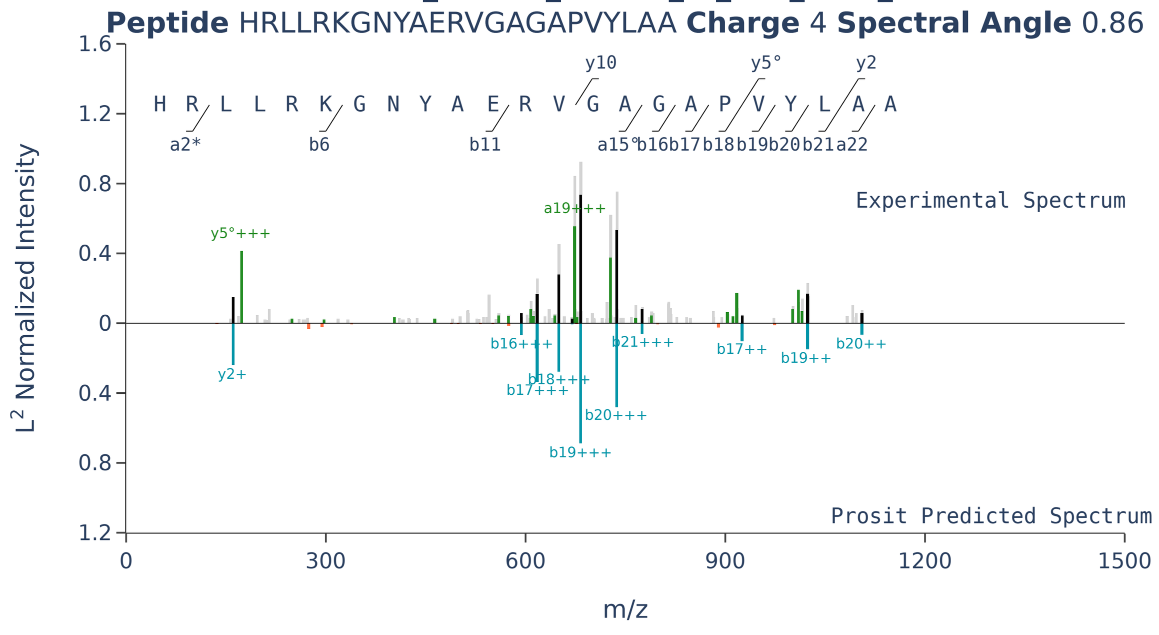


**Source** HRoetschke_291222_060123_Fu_H2A_b2R2_1h **Scan** 3378
**Peptide** RVHRLLRKGNYAERV **Charge** 5 **Spectral Angle** 0.83

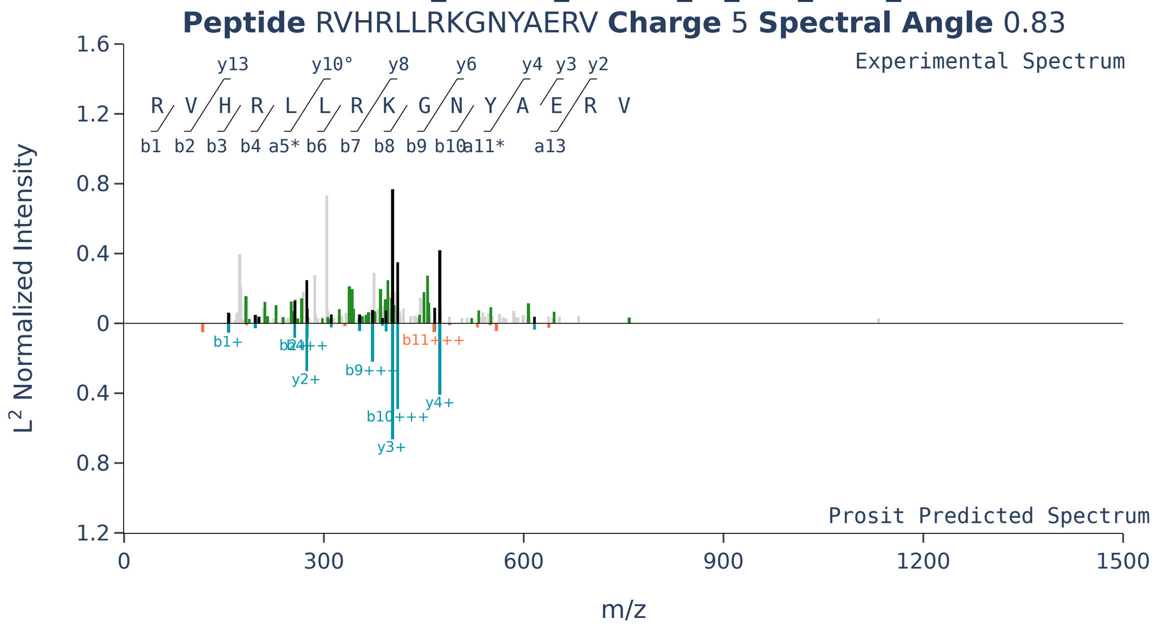


**Source** HRoetschke_291222_060123_Fu_H2A_b1R2_4h **Scan** 11867
**Peptide** EILELAGNAARDNKKTRIIPRHL **Charge** 5 **Spectral Angle** 0.79

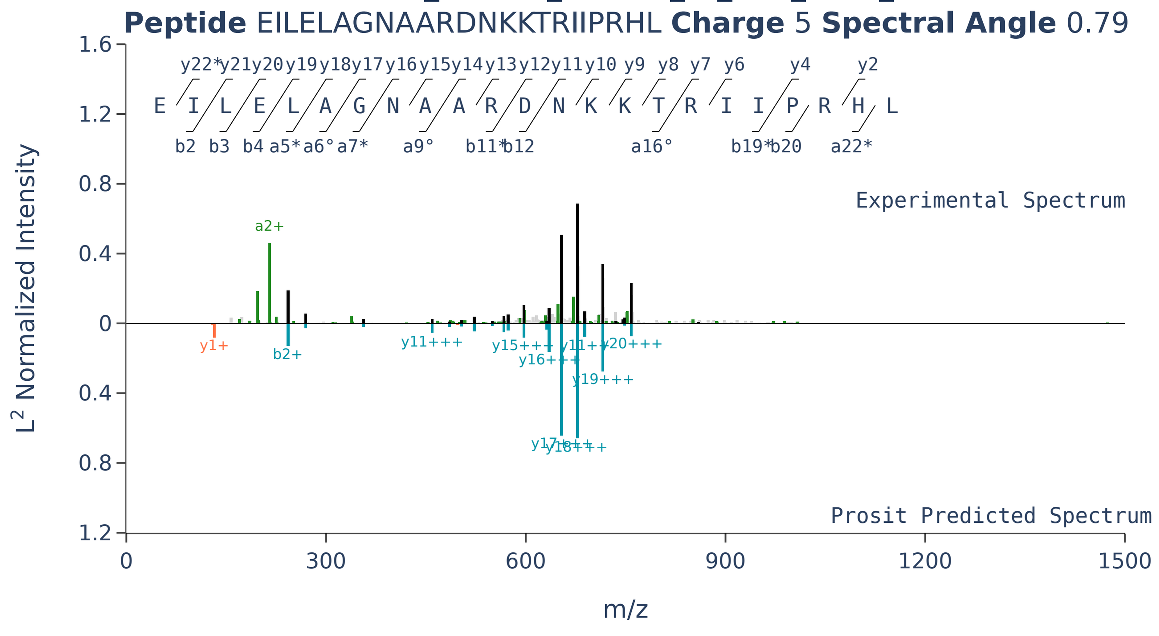


**Source** HRoetschke_291222_060123_Fu_H2A_b1R1_4h **Scan** 14285
**Peptide** RLLRKGNYAERVGAGAPVYLAAV **Charge** 4 **Spectral Angle** 0.85

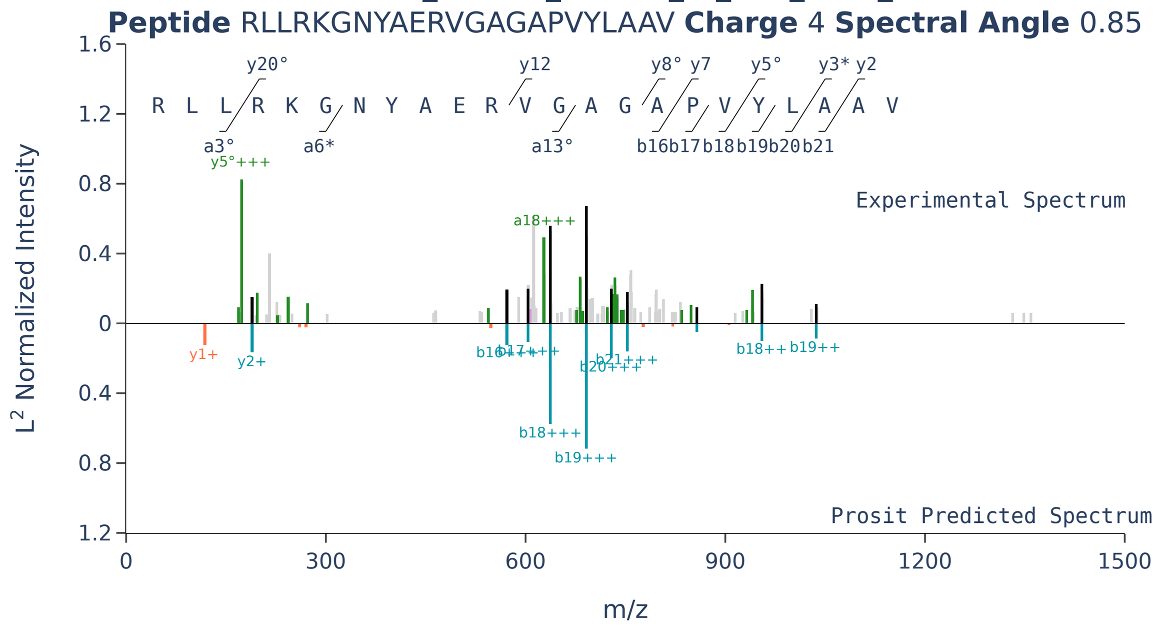


**Source** HRoetschke_291222_060123_Fu_H2A_b1R2_2h **Scan** 8615
**Peptide** ELAGNAARDNKKTRIIPRHLQL **Charge** 4 **Spectral Angle** 0.87

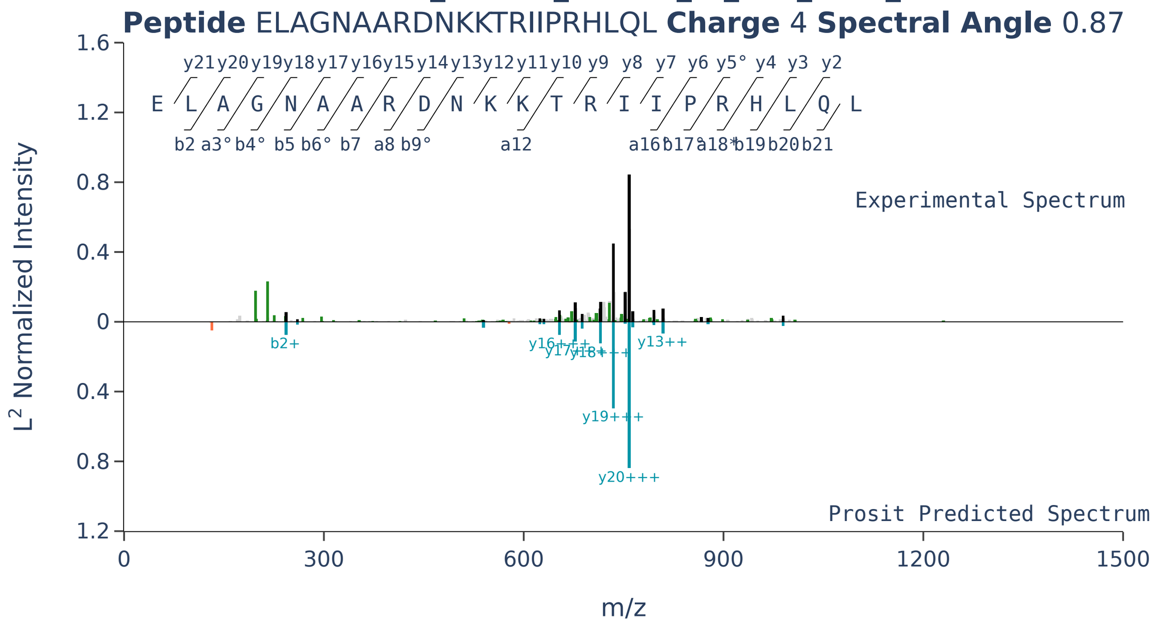


**Source** HRoetschke_291222_060123_Fu_H2A_b1R2_4h **Scan** 7150
**Peptide** NKKTRIIPRHLQL **Charge** 3 **Spectral Angle** 0.8

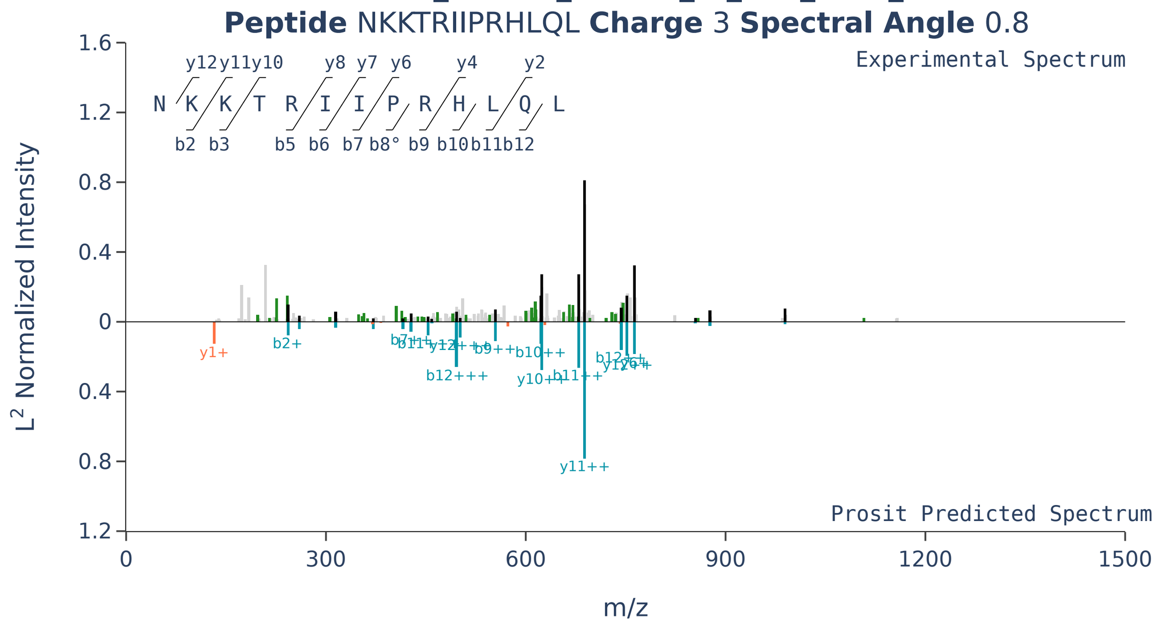


**Source** HRoetschke_291222_060123_Fu_H2A_b1R2_2h **Scan** 12855
**Peptide** AERVGAGAPVYLAA **Charge** 2 **Spectral Angle** 0.85

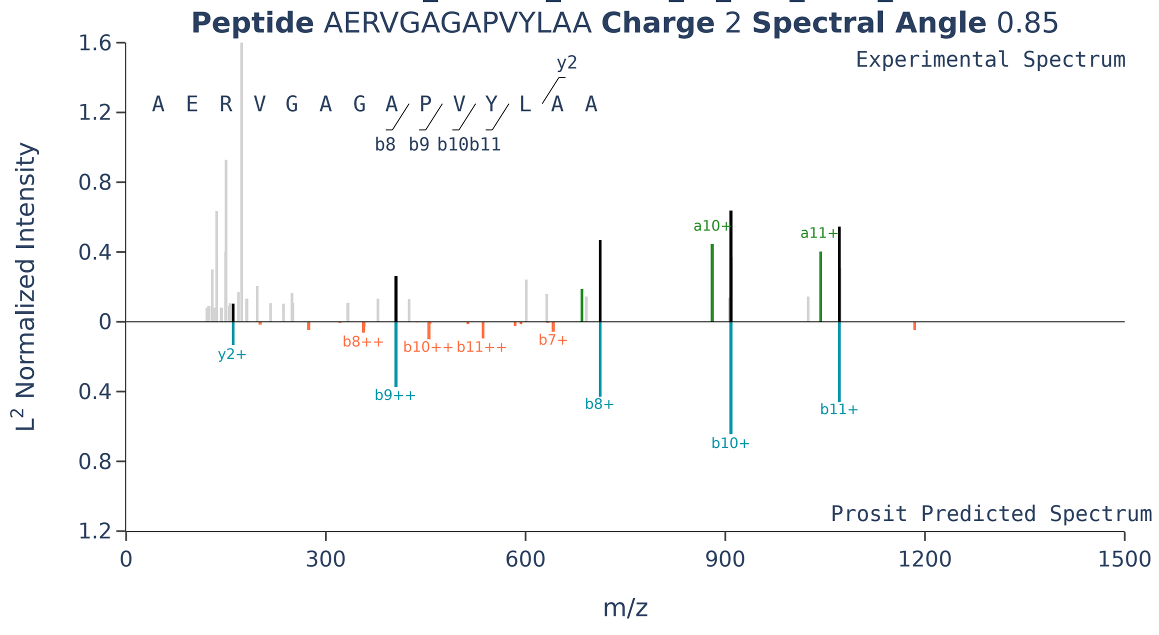


**Source** HRoetschke_291222_060123_Fu_H2A_b1R1_4h **Scan** 10509
**Peptide** RAGLQFPVGRVHRLLRKG **Charge** 4 **Spectral Angle** 0.77

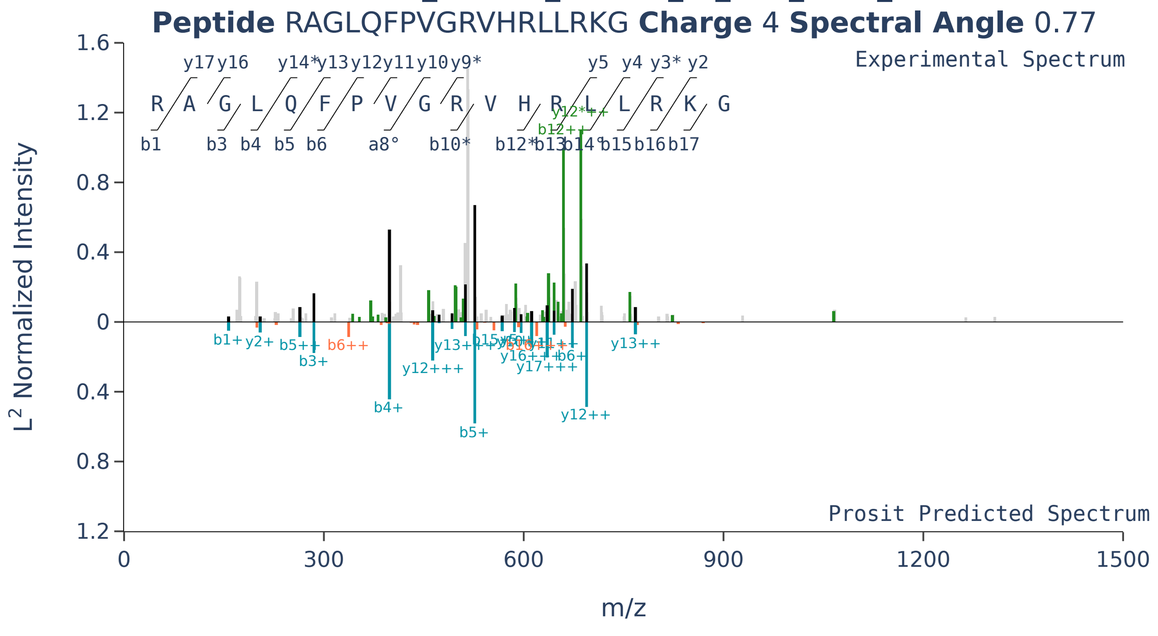


**Source** HRoetschke_291222_060123_Fu_H2A_b1R1_24h **Scan** 10259
**Peptide** RLLPRHLQL **Charge** 3 **Spectral Angle** 0.64

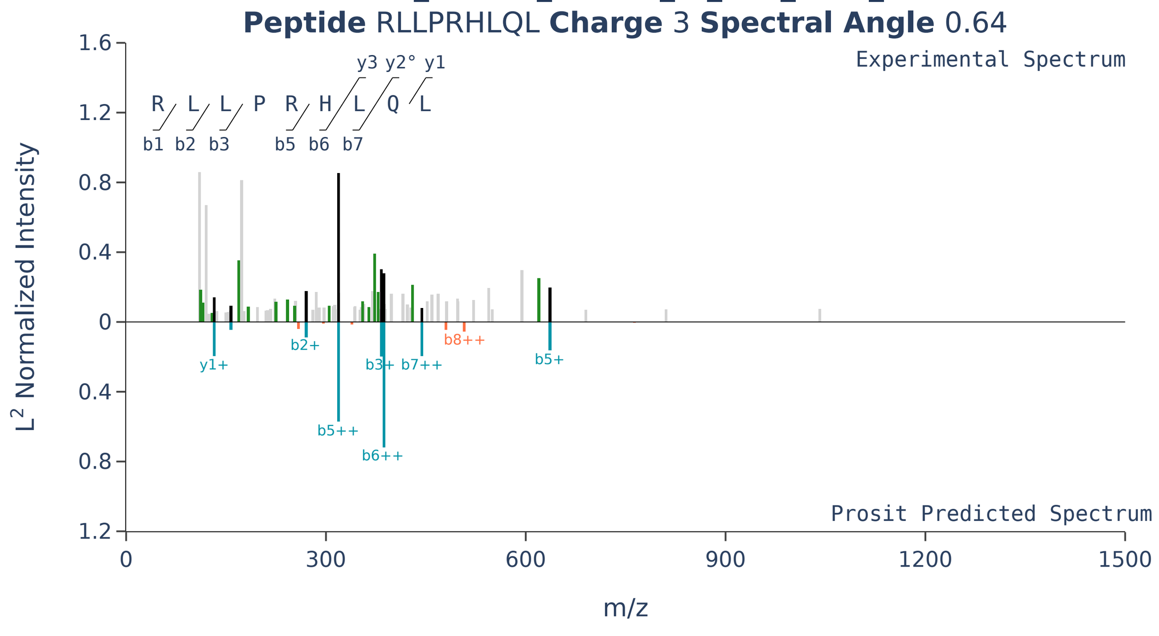


**Source** HRoetschke_291222_060123_Fu_H2A_b2R1_24h **Scan** 3109
**Peptide** VHRLLRKGNYAE **Charge** 3 **Spectral Angle** 0.71

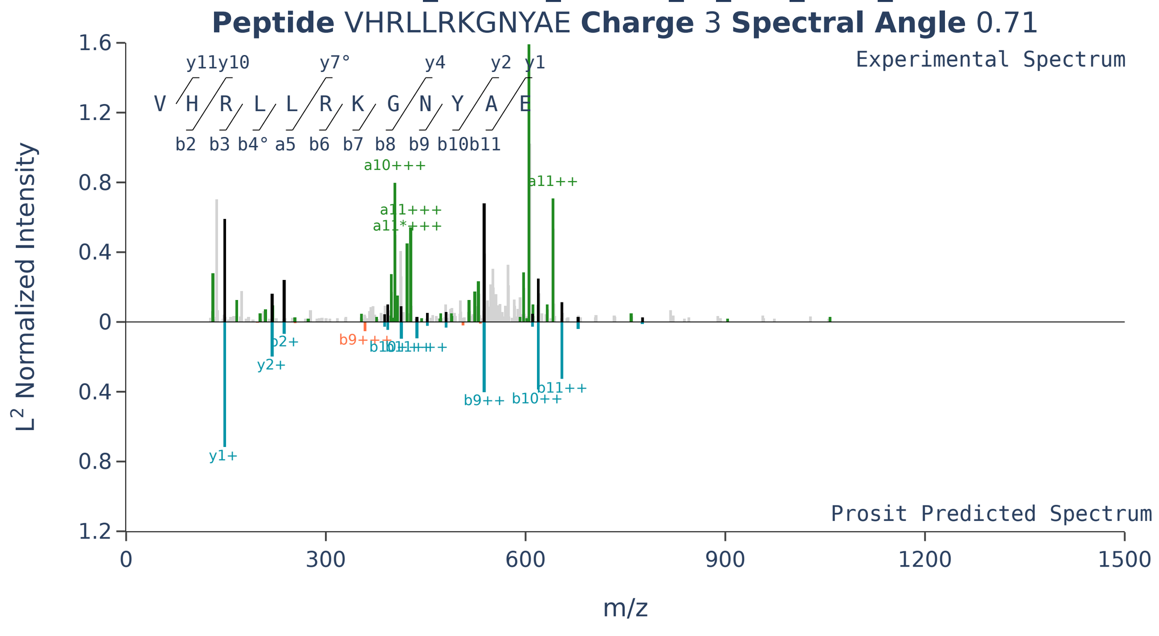

**Source** HRoetschke_291222_060123_Fu_H2A_b1R1_4h **Scan** 10814
**Peptide** SSRAGLQFPVGRVHRLLRKGNYAER **Charge** 5 **Spectral Angle** 0.71

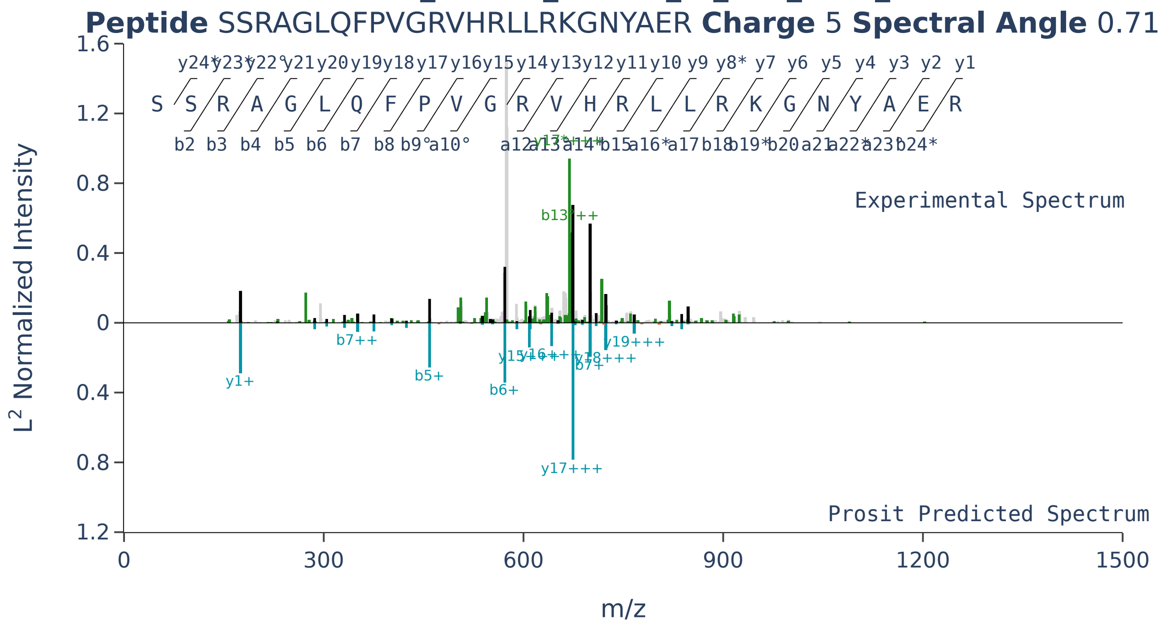


**Source** HRoetschke_291222_060123_Fu_H2A_b2R1_4h **Scan** 9284
**Peptide** RLLPRHLQLAVRND **Charge** 4 **Spectral Angle** 0.82

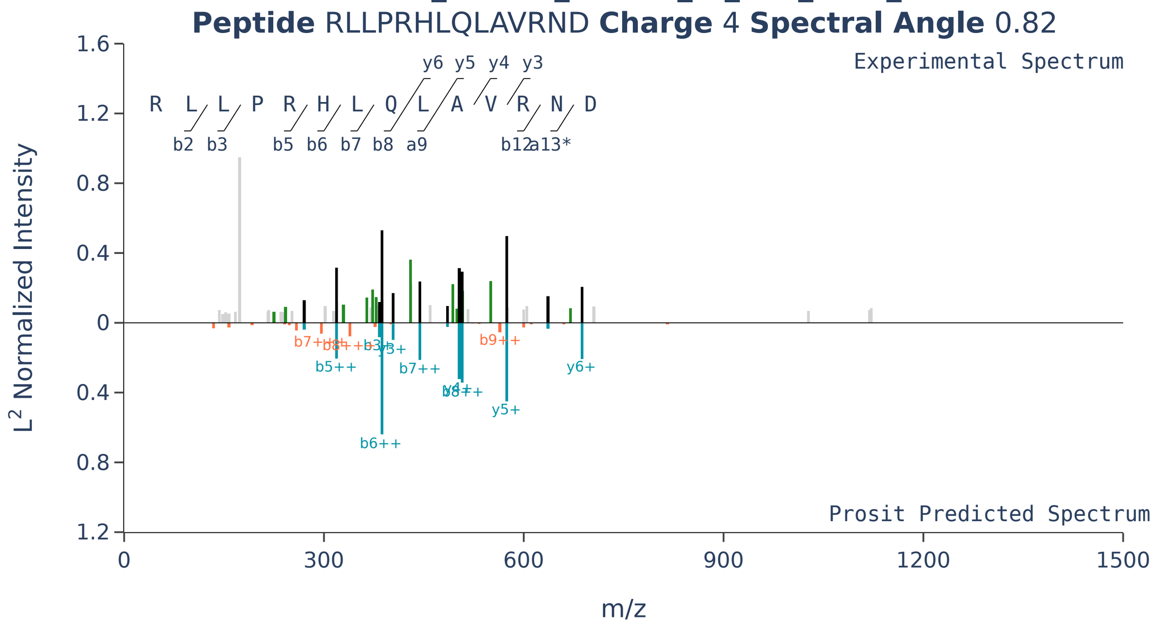


**Source** HRoetschke_291222_060123_Fu_H2A_b2R1_2h **Scan** 5848
**Peptide** AKTRSSRAGLQF **Charge** 3 **Spectral Angle** 0.73

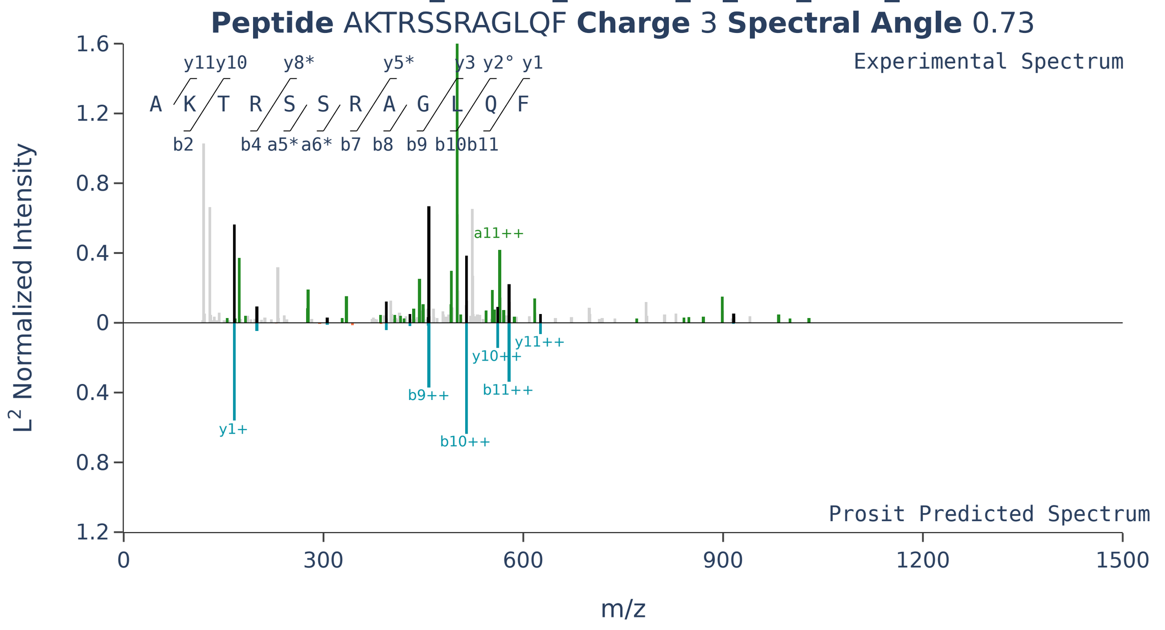


**Source** HRoetschke_291222_060123_Fu_H2A_b1R1_4h **Scan** 10362
**Peptide** IIPRHLQLAVRND **Charge** 4 **Spectral Angle** 0.82

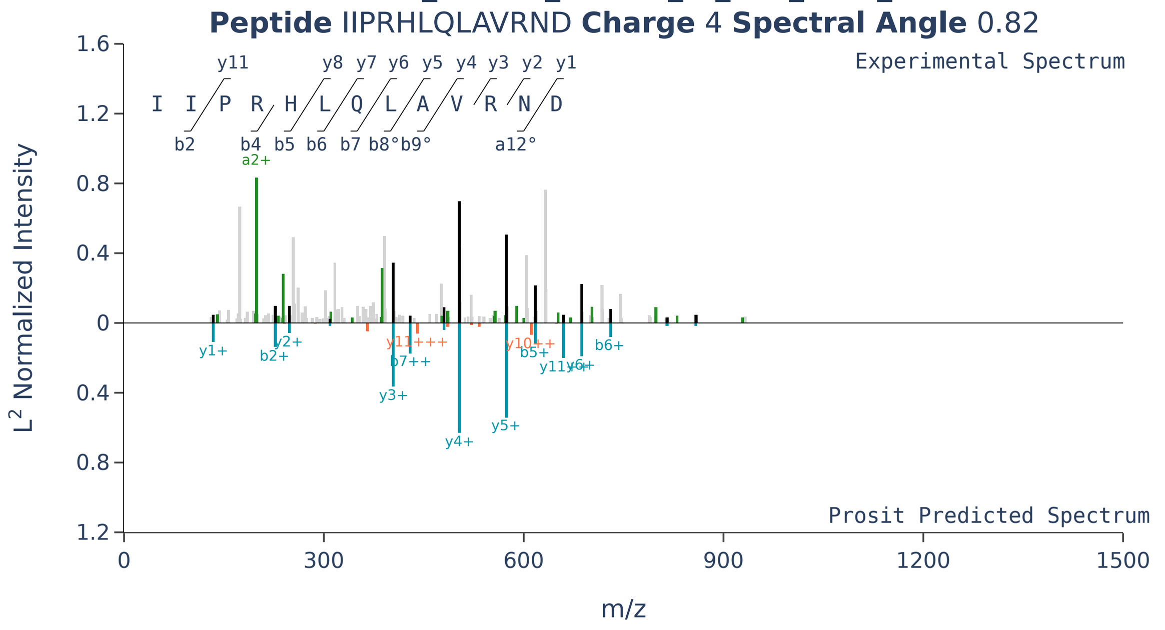


**Source** HRoetschke_291222_060123_Fu_H2A_b1R2_24h **Scan** 3536
**Peptide** QSVLLPKKTESSKSAKS **Charge** 3 **Spectral Angle** 0.74

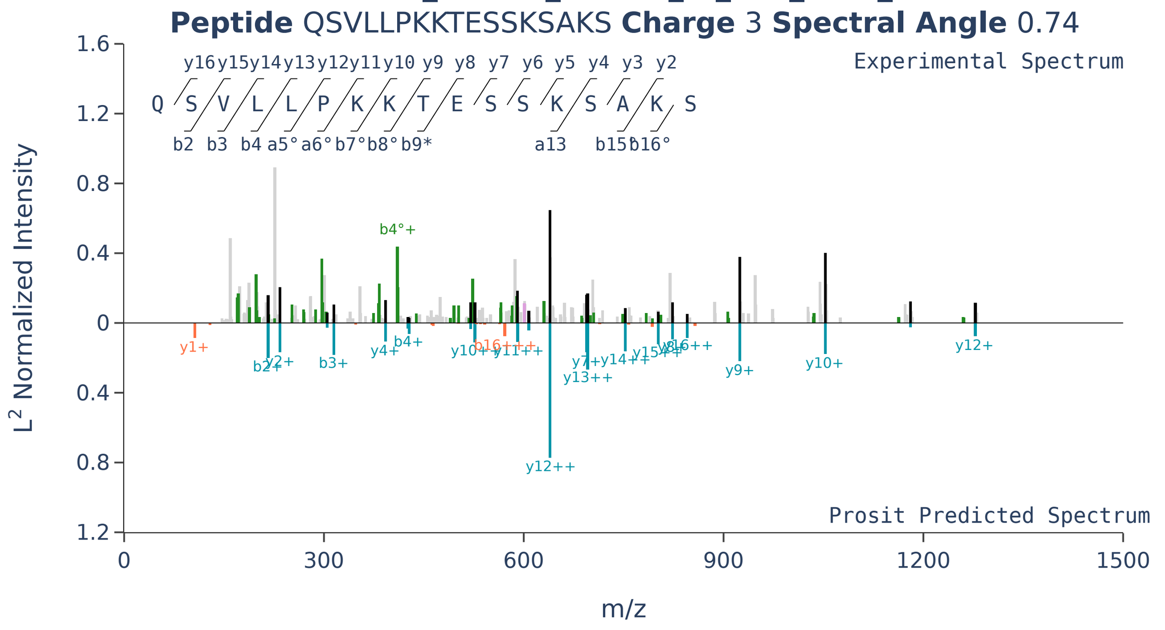


**Source** HRoetschke_291222_060123_Fu_H2A_b1R1_24h **Scan** 4866
**Peptide** QSVLLPKKTESSKSA **Charge** 3 **Spectral Angle** 0.76

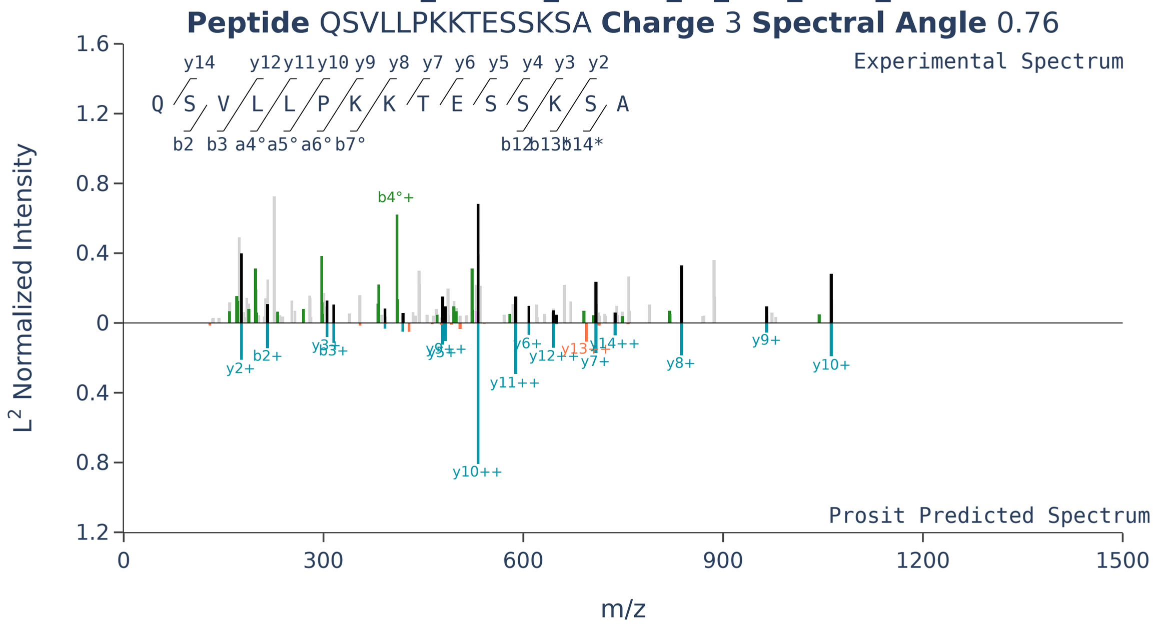


**Source** HRoetschke_291222_060123_Fu_H2A_b2R2_24h **Scan** 7928
**Peptide** LLELAGNAARDNKKTRIIPRH **Charge** 5 **Spectral Angle** 0.78

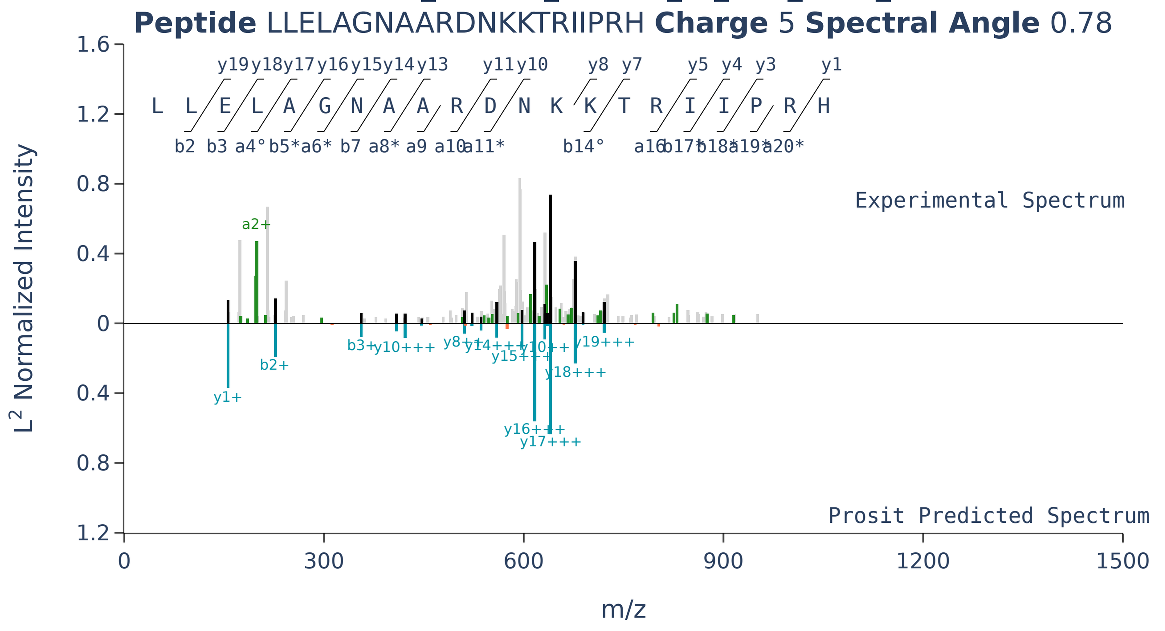


**Source** HRoetschke_291222_060123_Fu_H2A_b1R2_1h **Scan** 4821
**Peptide** ELAGNAARDNKKTRLL **Charge** 3 **Spectral Angle** 0.87

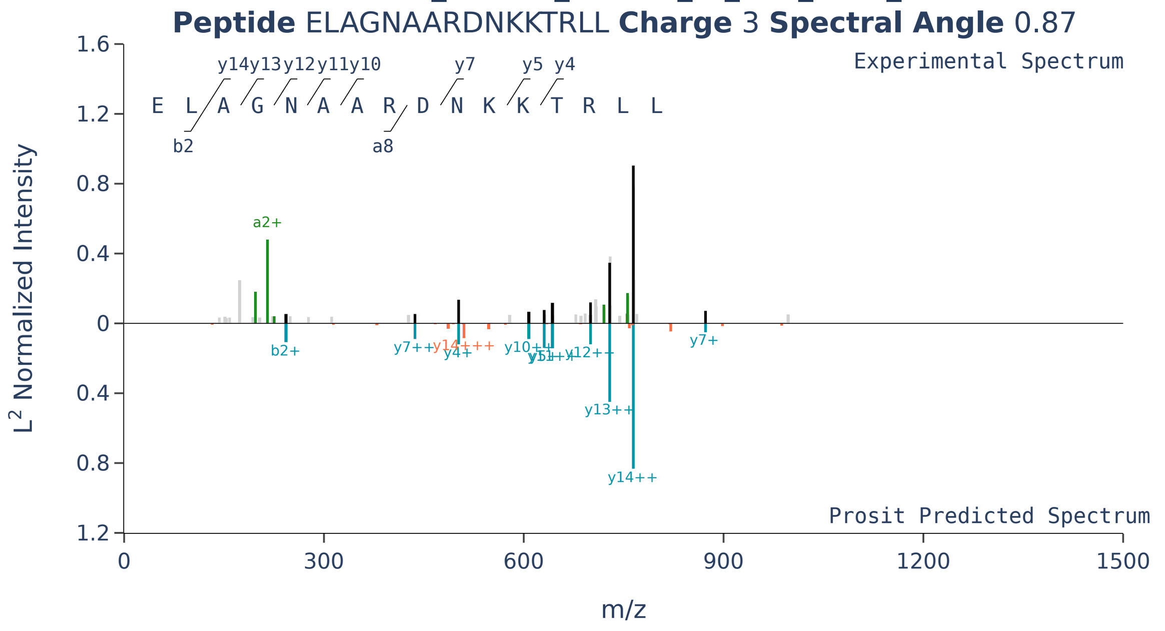


**Source** HRoetschke_291222_060123_Fu_H2A_b1R2_1h **Scan** 7381
**Peptide** LQLAVRN **Charge** 2 **Spectral Angle** 0.85

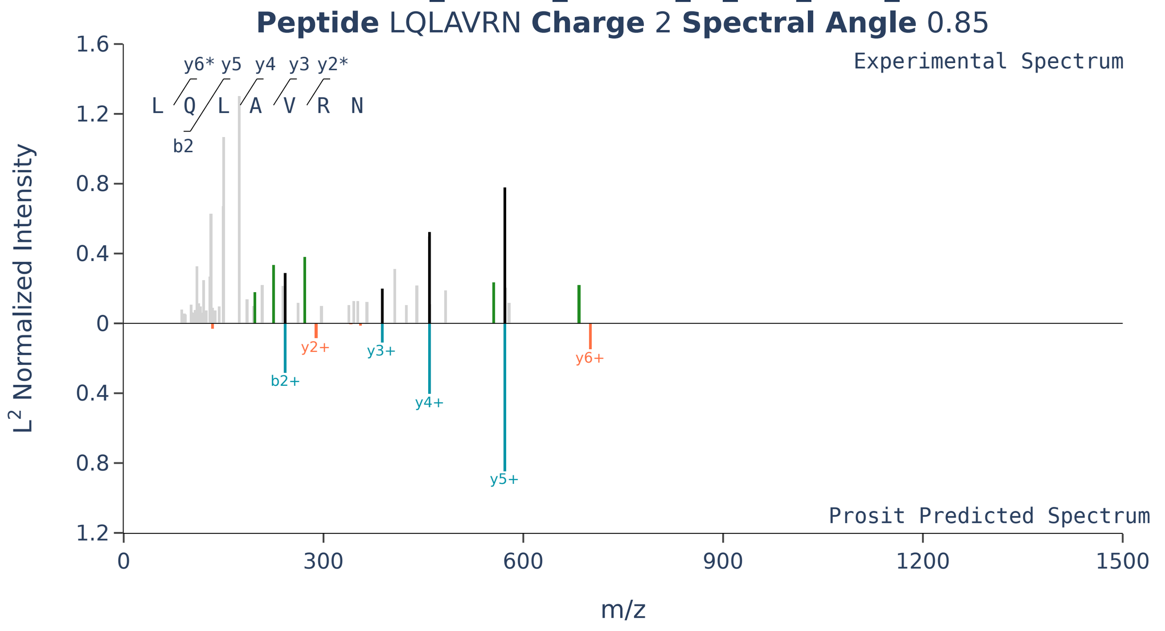


**Source** HRoetschke_291222_060123_Fu_H2A_b1R1_2h **Scan** 20311
**Peptide** AGAPVYLAAVL **Charge** 2 **Spectral Angle** 0.78

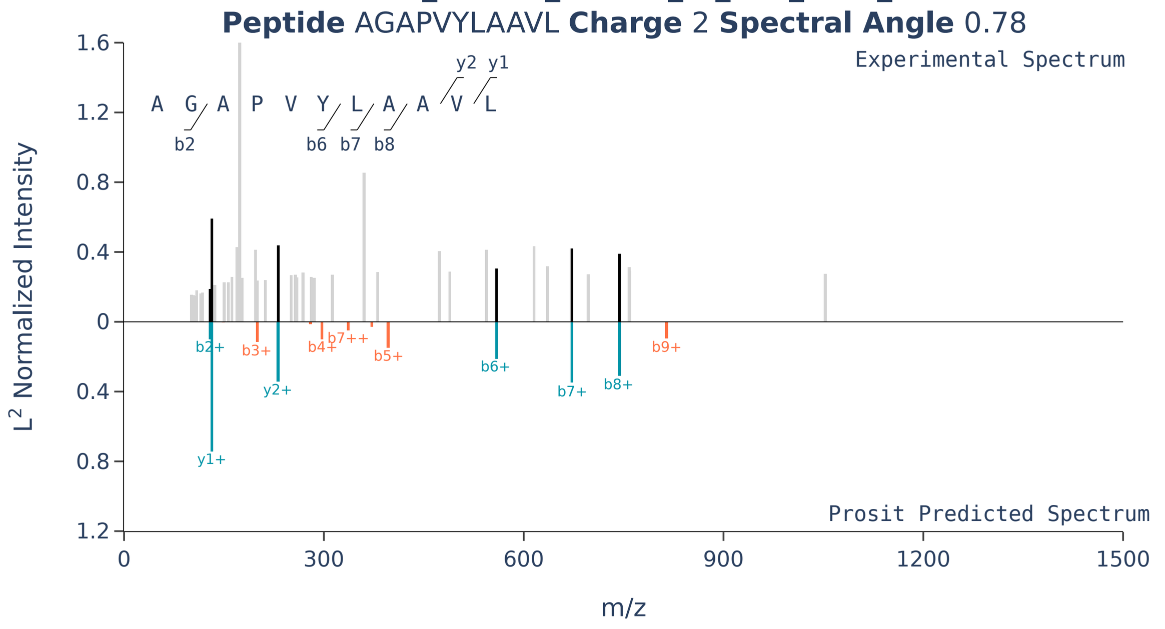


**Source** HRoetschke_291222_060123_Fu_H2A_b1R1_2h **Scan** 6641
**Peptide** RDNKKTRIIPRHLQLA **Charge** 4 **Spectral Angle** 0.8

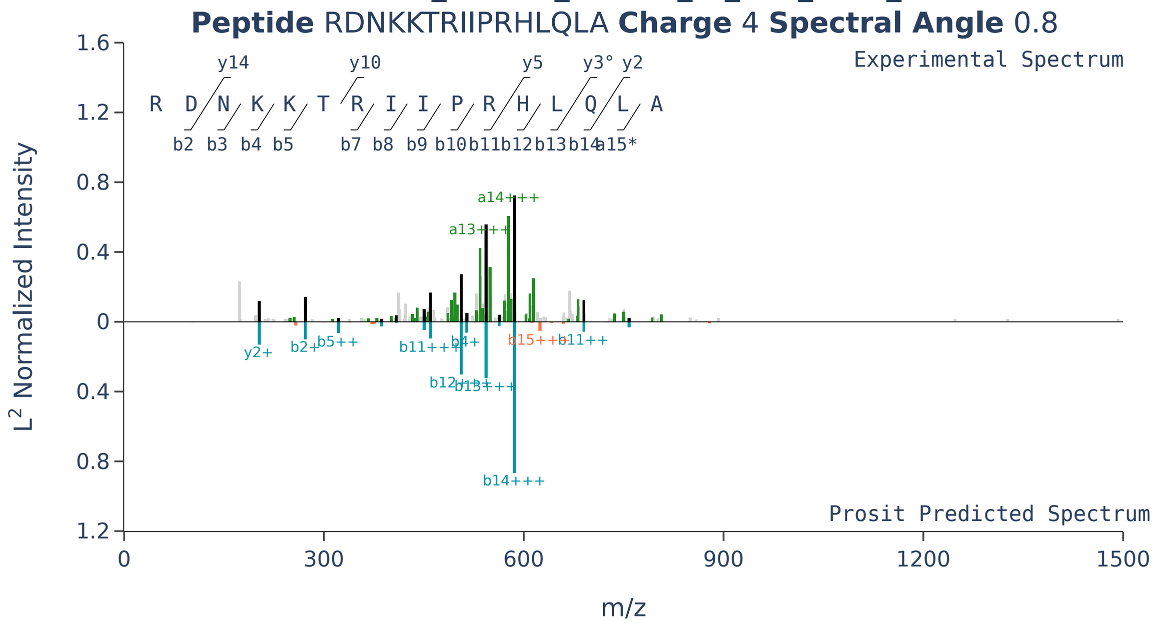


**Source** HRoetschke_291222_060123_Fu_H2A_b2R1_1h **Scan** 5612
**Peptide** KTRSSRAGLQF **Charge** 3 **Spectral Angle** 0.79

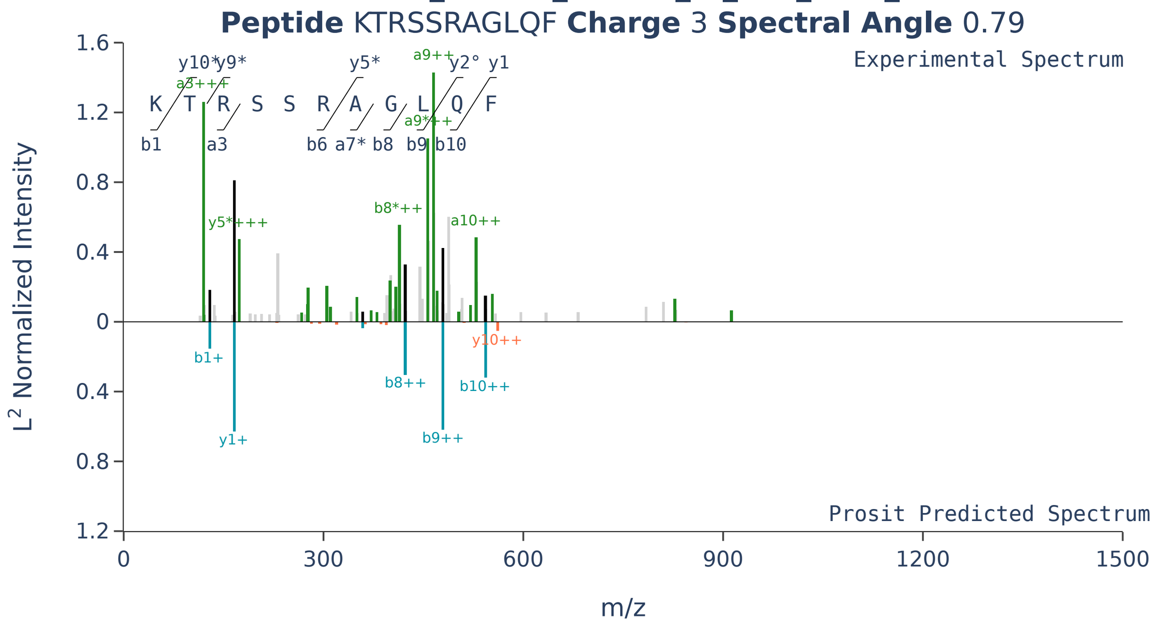


**Source** HRoetschke_291222_060123_Fu_H2A_b1R2_4h **Scan** 15180
**Peptide** NYAERVGAGAPVYLAA **Charge** 2 **Spectral Angle** 0.84

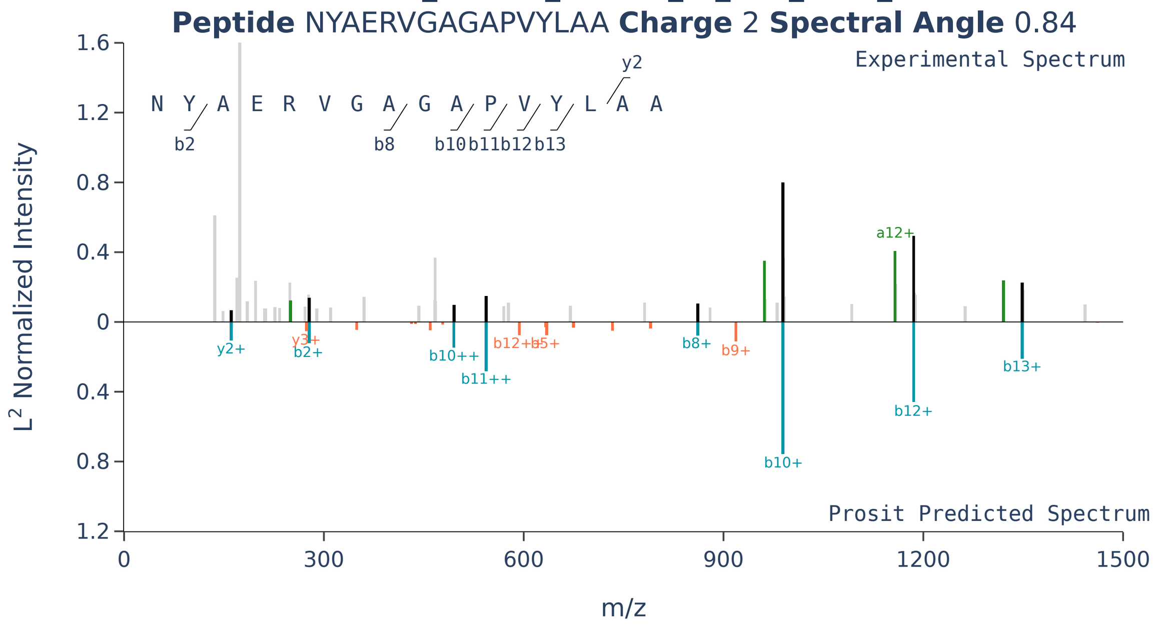


**Source** HRoetschke_291222_060123_Fu_H2A_b1R2_2h **Scan** 15368
**Peptide** DEELNKLLGRVT **Charge** 2 **Spectral Angle** 0.83

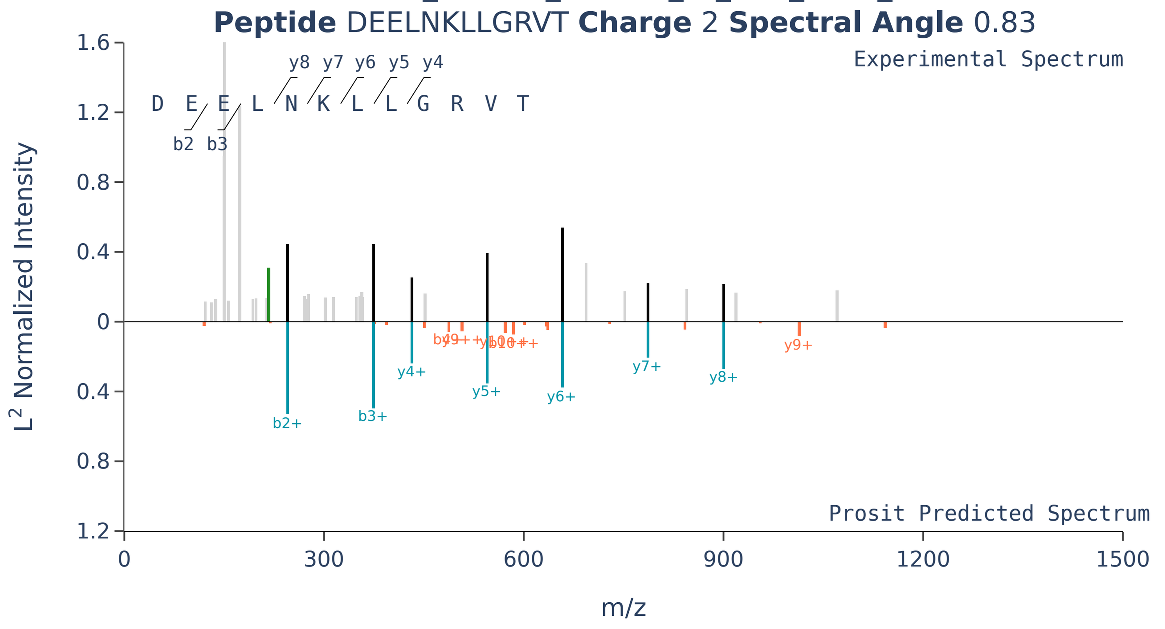


**Source** HRoetschke_291222_060123_Fu_H2A_b1R1_4h **Scan** 6997
**Peptide** NKKTRIIPRHLQLAVRND **Charge** 5 **Spectral Angle** 0.75

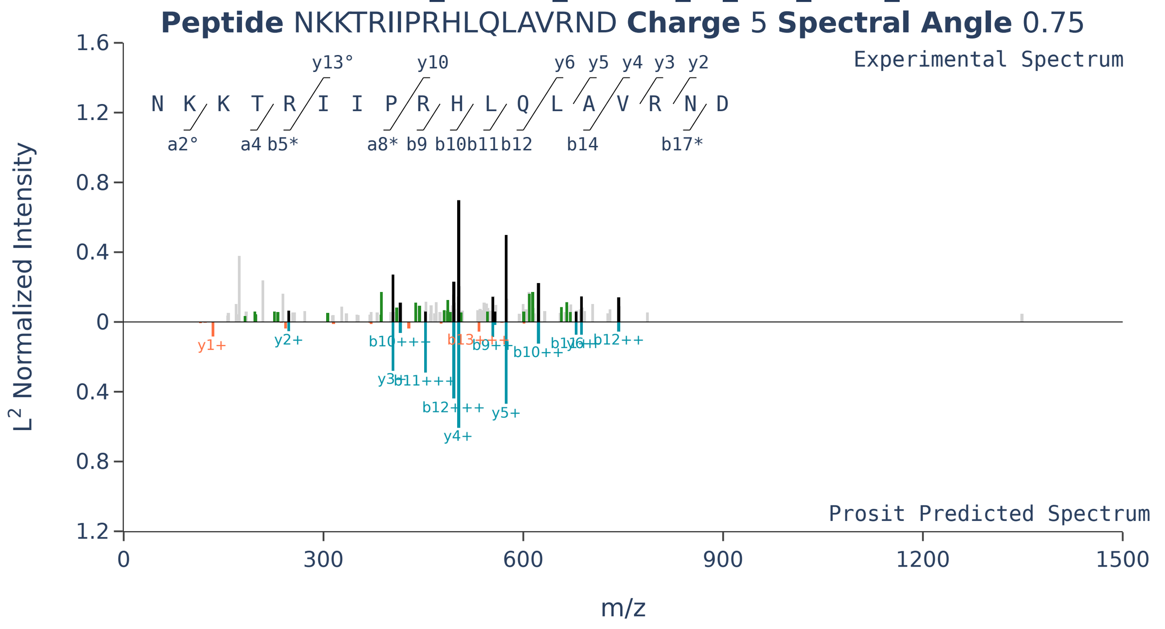

**Source** HRoetschke_291222_060123_Fu_H2A_b2R1_4h **Scan** 4085
**Peptide** AGNAARDNKKTRIIPRHL **Charge** 4 **Spectral Angle** 0.83

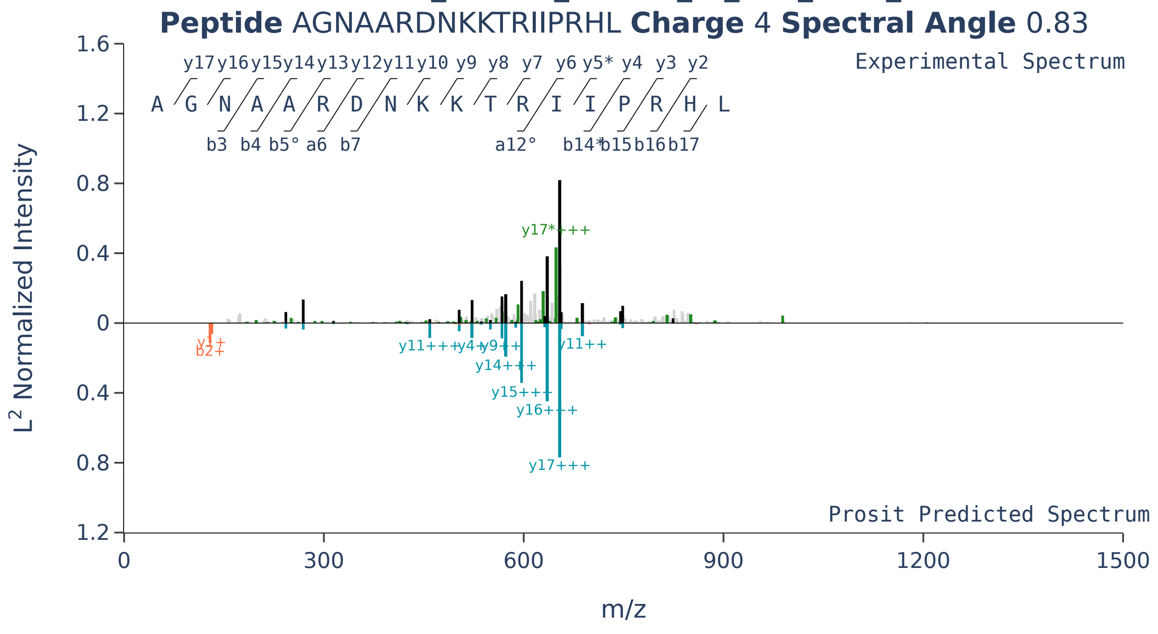


**Source** HRoetschke_291222_060123_Fu_H2A_b1R2_4h **Scan** 3862
**Peptide** RDNKKTRIIPRHL **Charge** 5 **Spectral Angle** 0.87

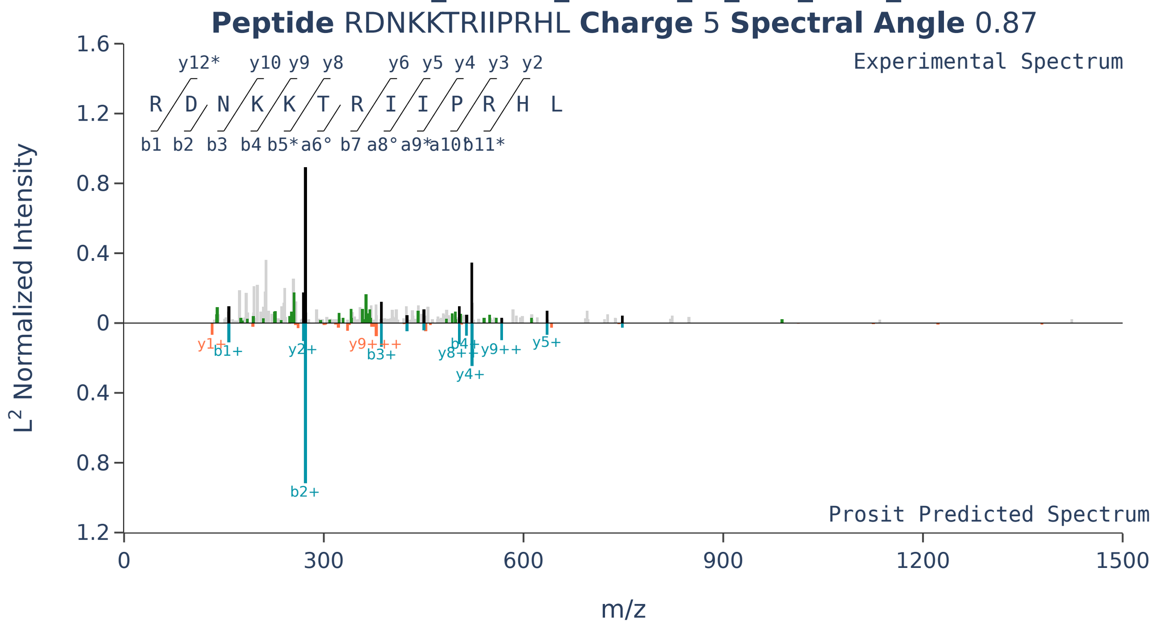


**Source** HRoetschke_291222_060123_Fu_H2A_b1R1_2h **Scan** 4183
**Peptide** LAGNAARDNKKTRIIPRHLQ **Charge** 4 **Spectral Angle** 0.93

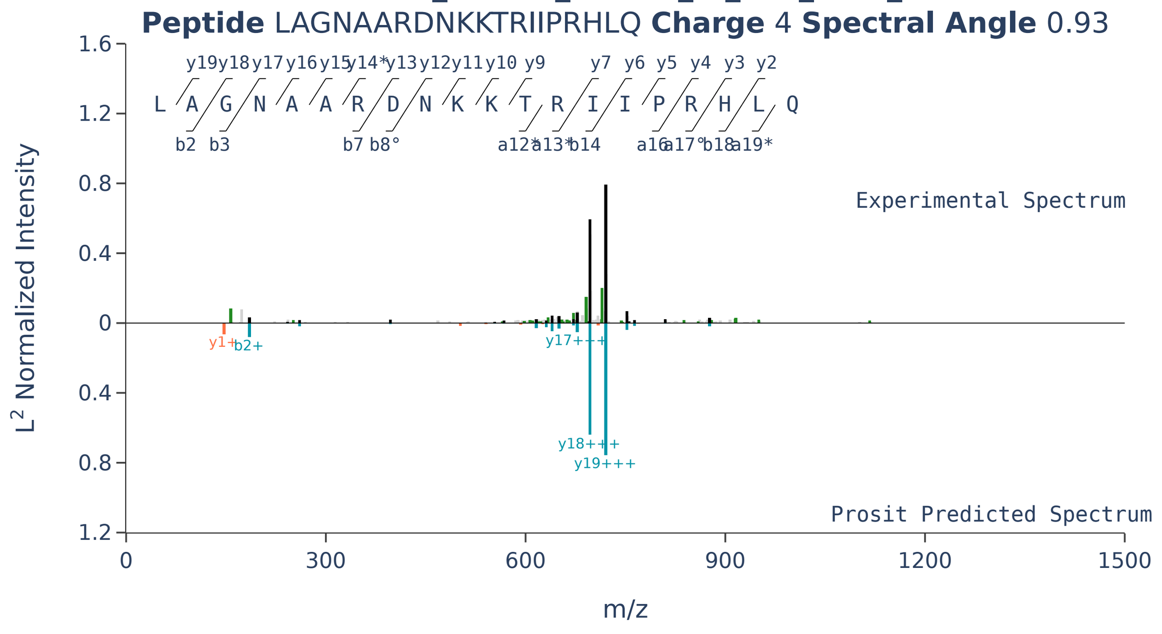


**Source** HRoetschke_291222_060123_Fu_H2A_b1R1_4h **Scan** 18053
**Peptide** NDEELNKLLGRVTIAQ **Charge** 3 **Spectral Angle** 0.81

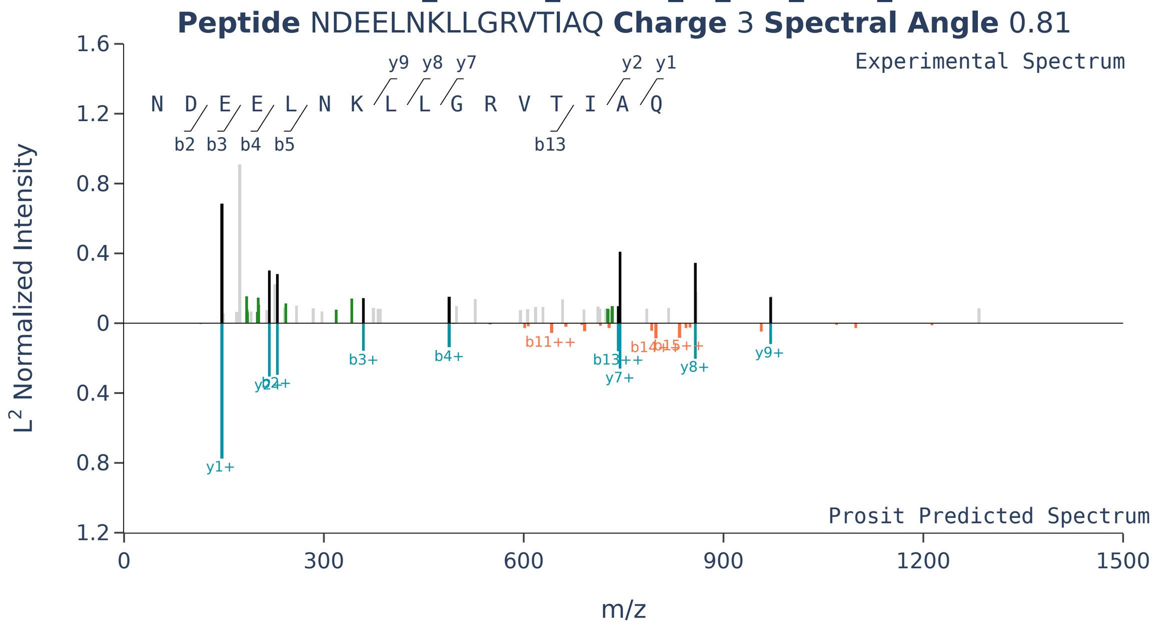


**Source** HRoetschke_291222_060123_Fu_H2A_b1R1_4h **Scan** 15711
**Peptide** RVTIAQGGVLPNIQS **Charge** 2 **Spectral Angle** 0.87

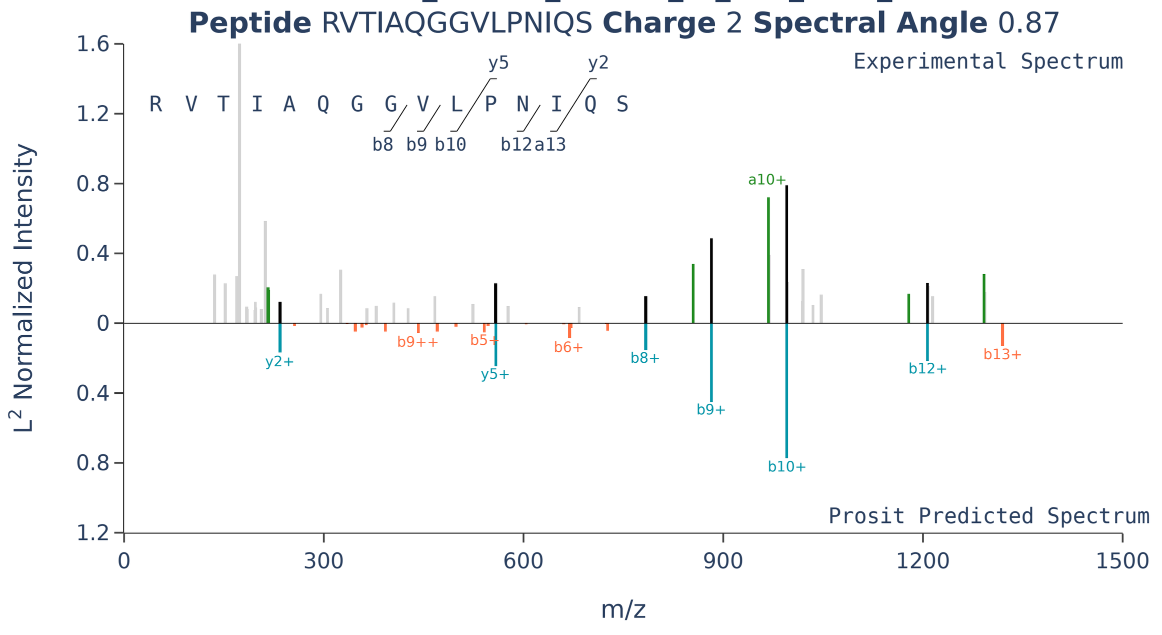


**Source** HRoetschke_291222_060123_Fu_H2A_b1R2_4h **Scan** 13017
**Peptide** AEILELAGNAARDNKKTRIIPRHL **Charge** 6 **Spectral Angle** 0.78

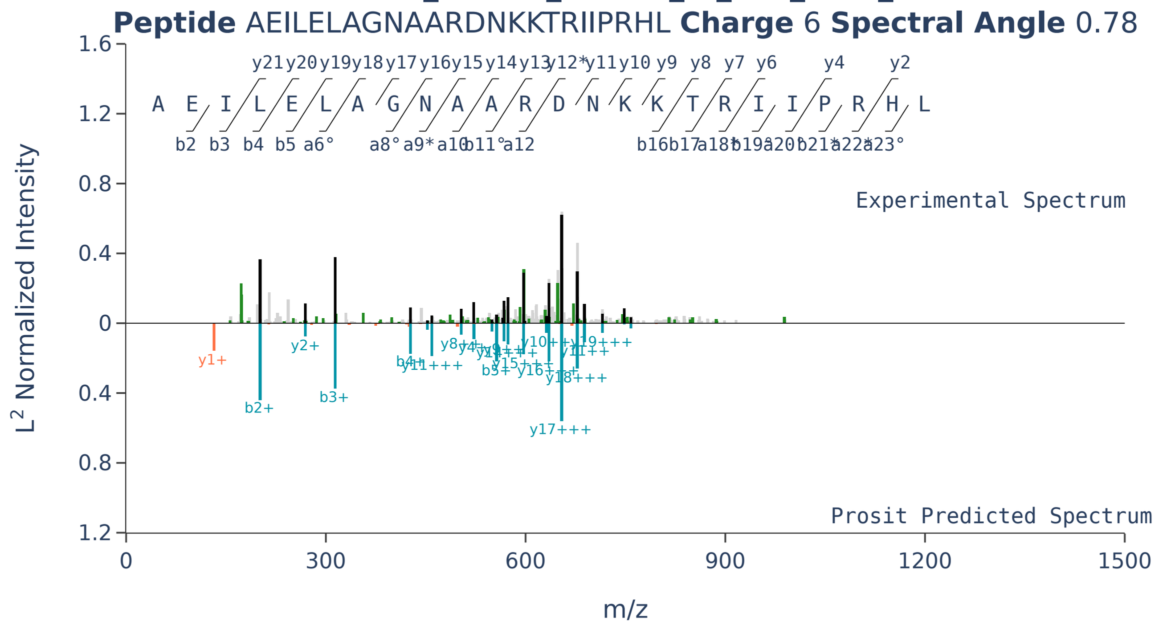


**Source** HRoetschke_291222_060123_Fu_H2A_b1R1_24h **Scan** 12523
**Peptide** AGAPVYLA **Charge** 2 **Spectral Angle** 0.67

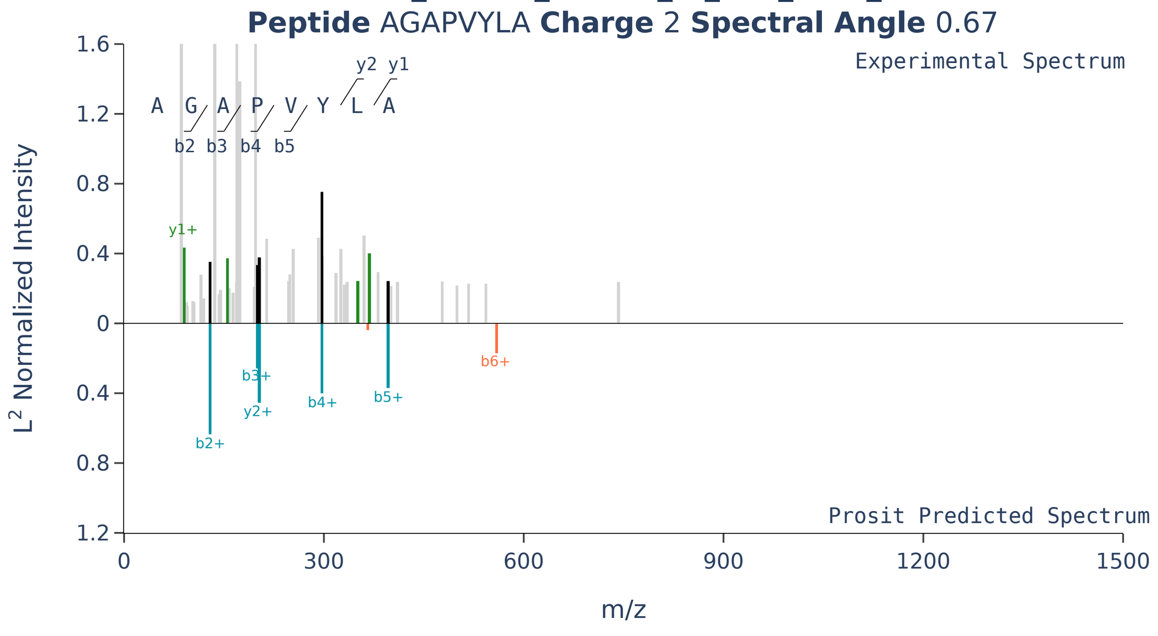


**Source** HRoetschke_291222_060123_Fu_H2A_b2R2_24h **Scan** 9840
**Peptide** TRIIPRHLQLAVRND **Charge** 4 **Spectral Angle** 0.84

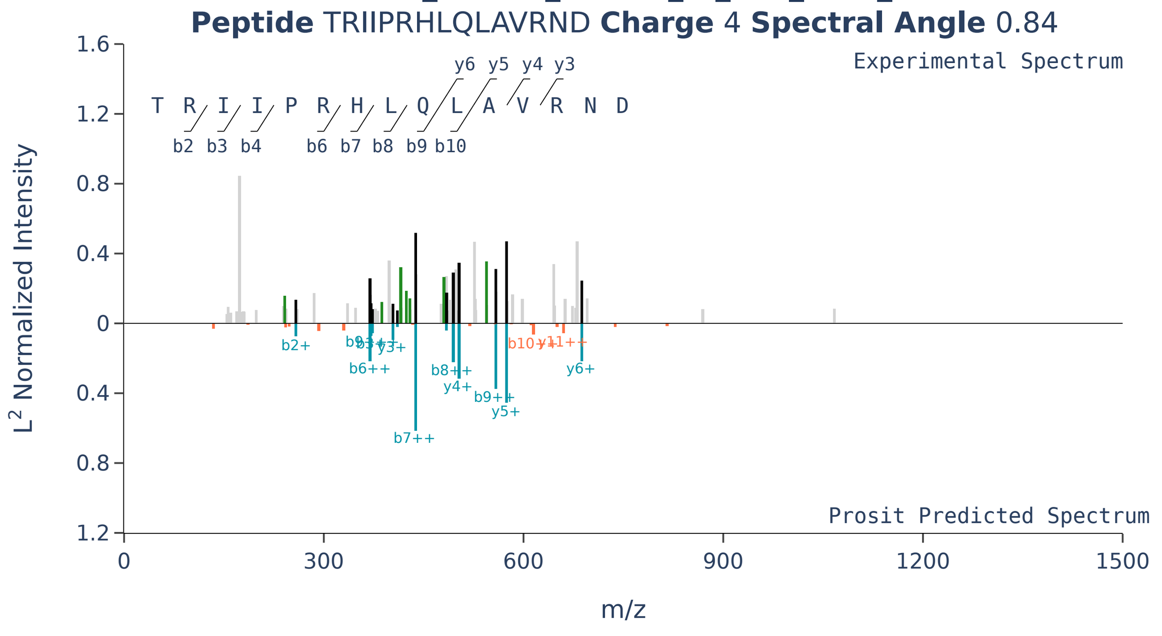


**Source** HRoetschke_291222_060123_Fu_H2A_b1R2_1h **Scan** 3075
**Peptide** LRKGNYAERVGA **Charge** 3 **Spectral Angle** 0.85

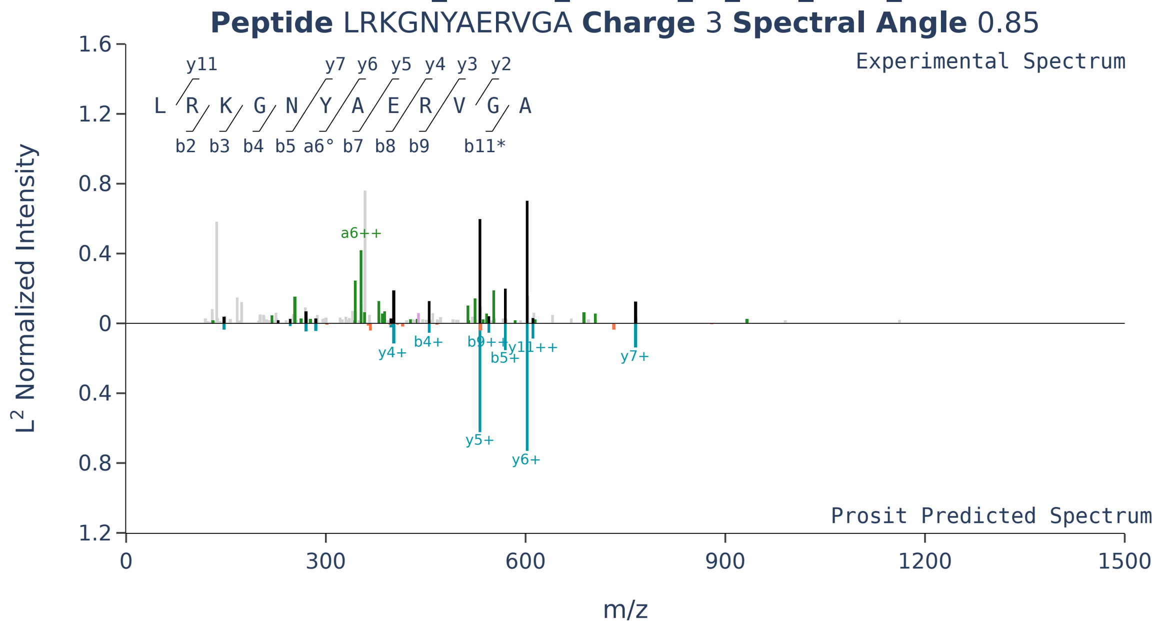


**Source** HRoetschke_291222_060123_Fu_H2A_b2R1_4h **Scan** 10227
**Peptide** KTRSSRAGLQFPVGRV **Charge** 4 **Spectral Angle** 0.83

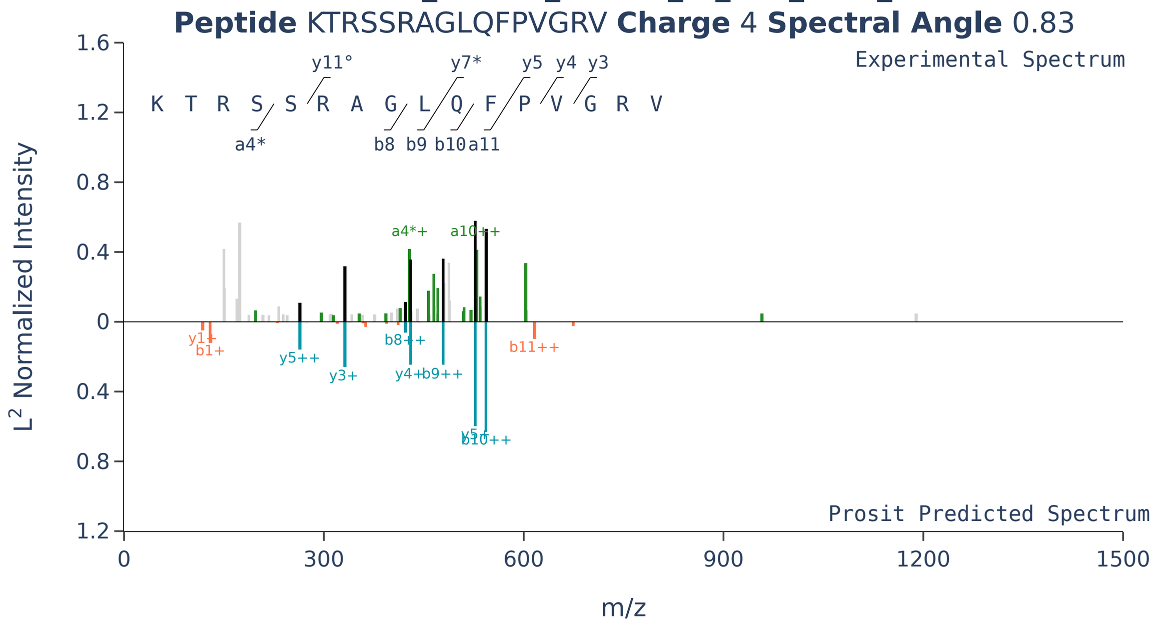


**Source** HRoetschke_291222_060123_Fu_H2A_b1R1_4h **Scan** 2024
**Peptide** HRLLRKGNYAER **Charge** 3 **Spectral Angle** 0.79

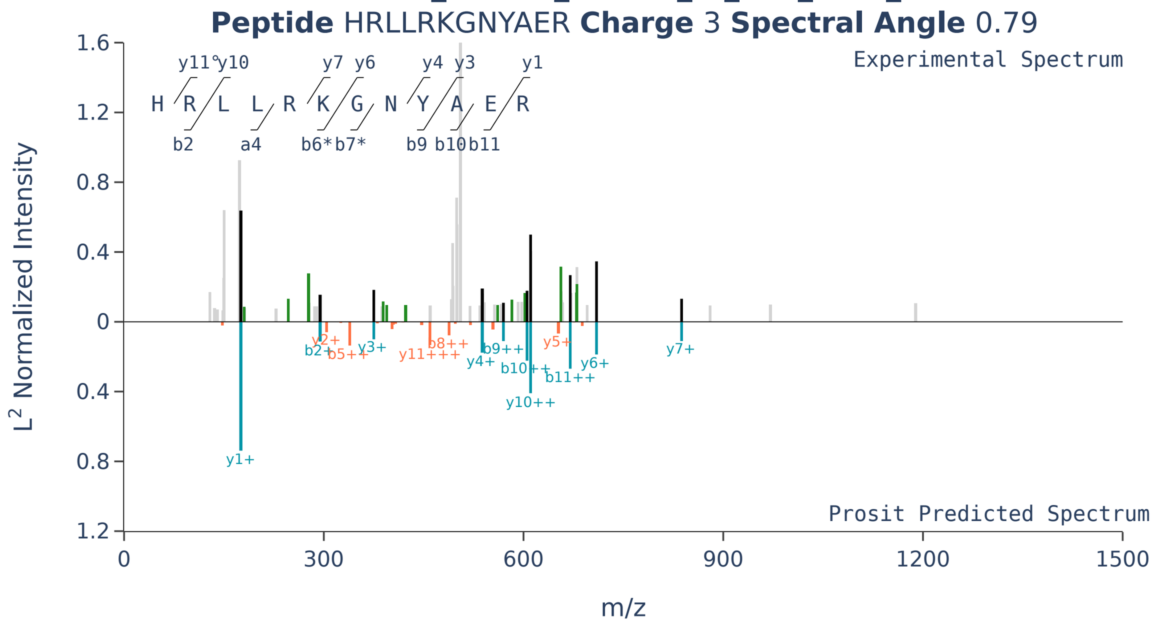


**Source** HRoetschke_291222_060123_Fu_H2A_b2R1_24h **Scan** 11017
**Peptide** RKGNYAERVGAGAPVYL **Charge** 3 **Spectral Angle** 0.85

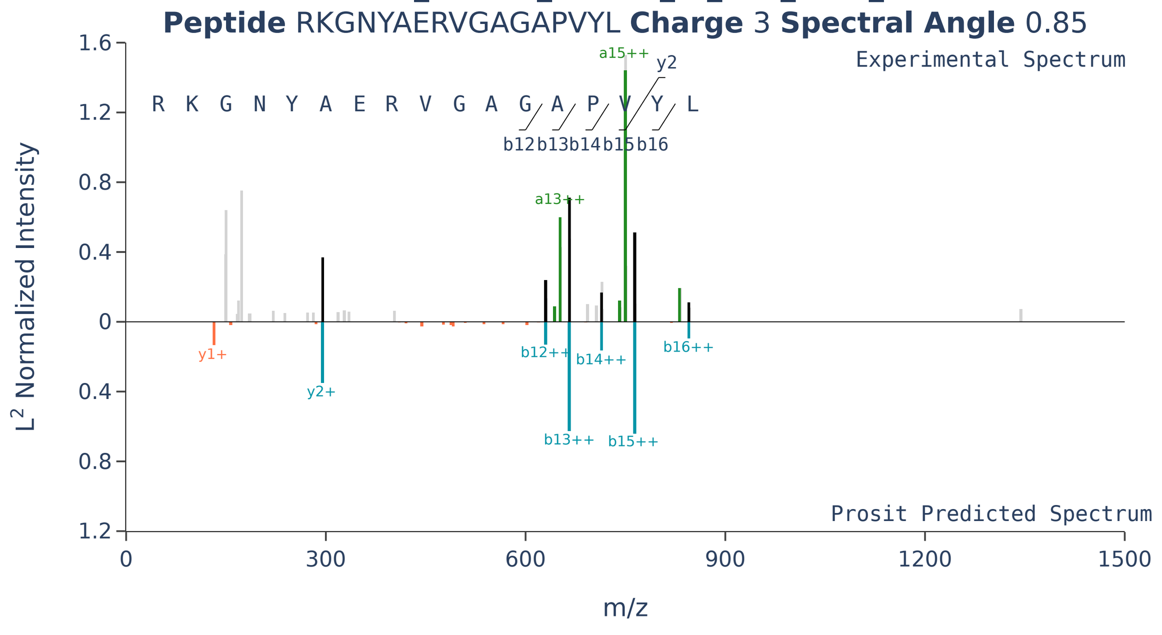


**Source** HRoetschke_291222_060123_Fu_H2A_b2R1_2h **Scan** 3403
**Peptide** ARDNKKTRIIPRHL **Charge** 3 **Spectral Angle** 0.68

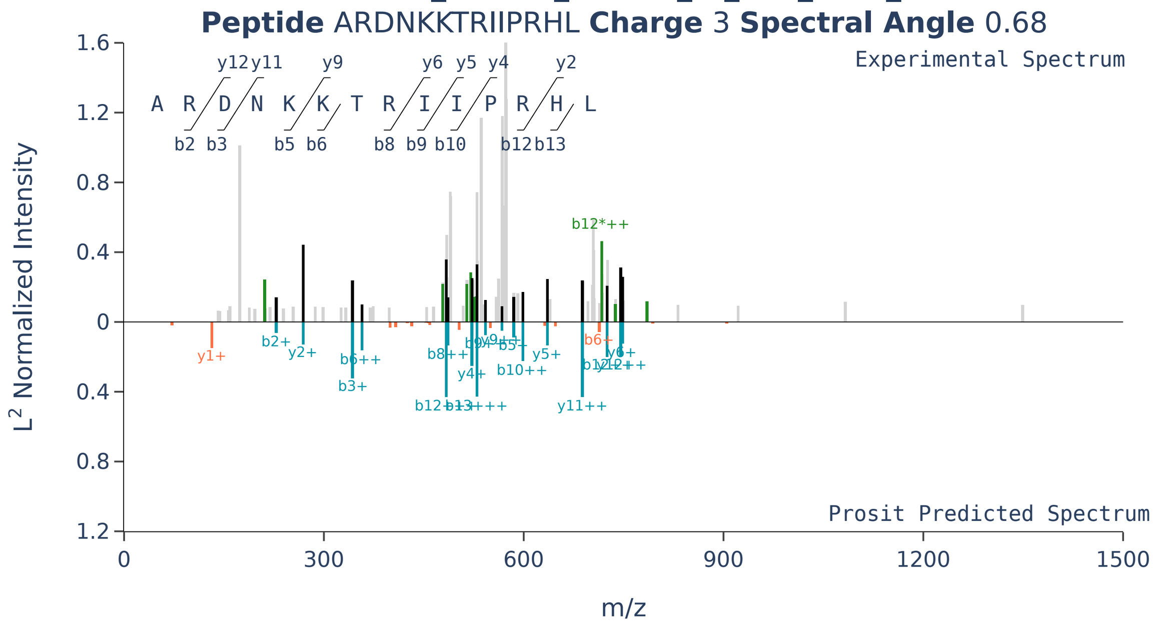


**Source** HRoetschke_291222_060123_Fu_H2A_b2R1_4h **Scan** 7379
**Peptide** NAARDNKKTRIIPRHLQL **Charge** 4 **Spectral Angle** 0.71

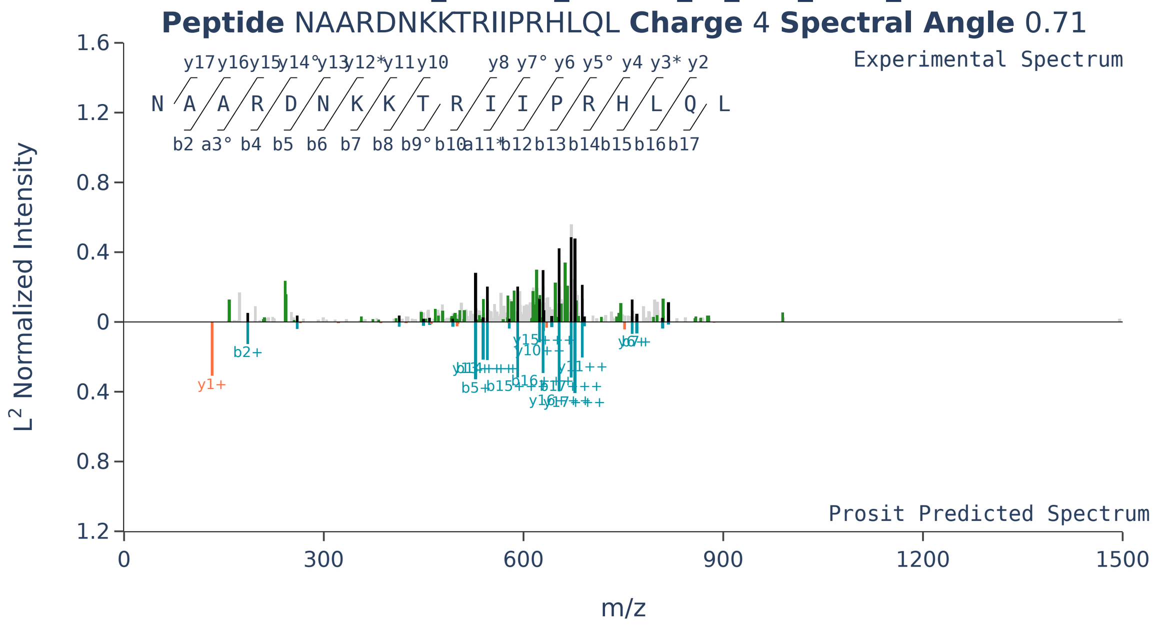


**Source** HRoetschke_291222_060123_Fu_H2A_b2R2_2h **Scan** 5459
**Peptide** LELAGNAARDNKKTRIIPRH **Charge** 4 **Spectral Angle** 0.87

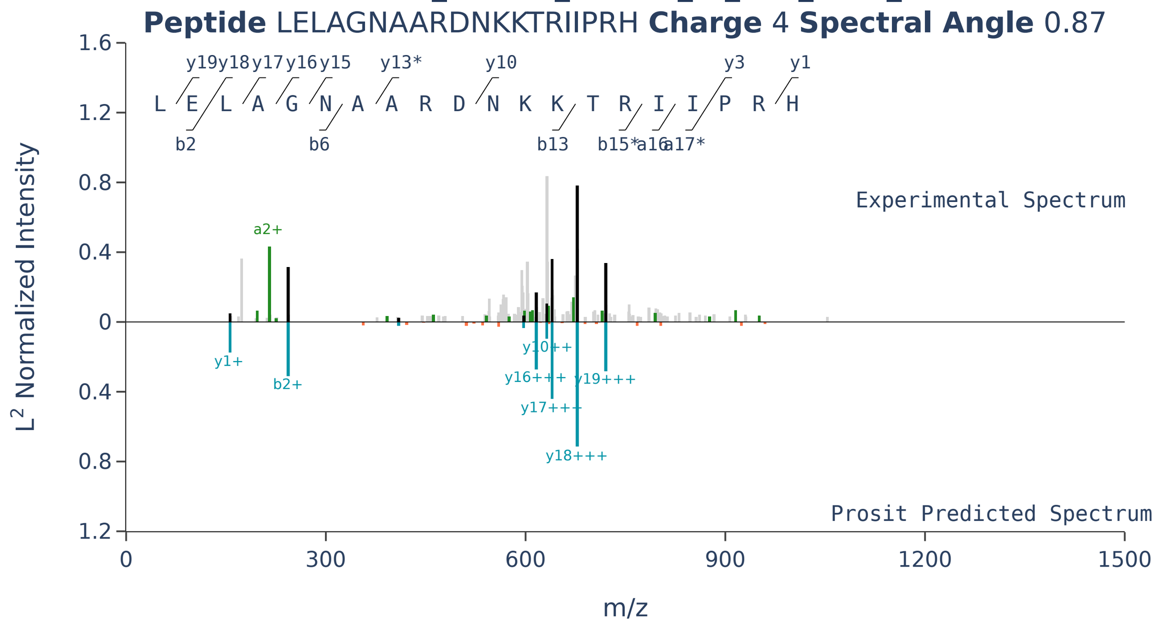

**Source** HRoetschke_291222_060123_Fu_H2A_b1R1_4h **Scan** 11255
**Peptide** LELAGNAARDNKKTRIIPRHLQL **Charge** 5 **Spectral Angle** 0.78

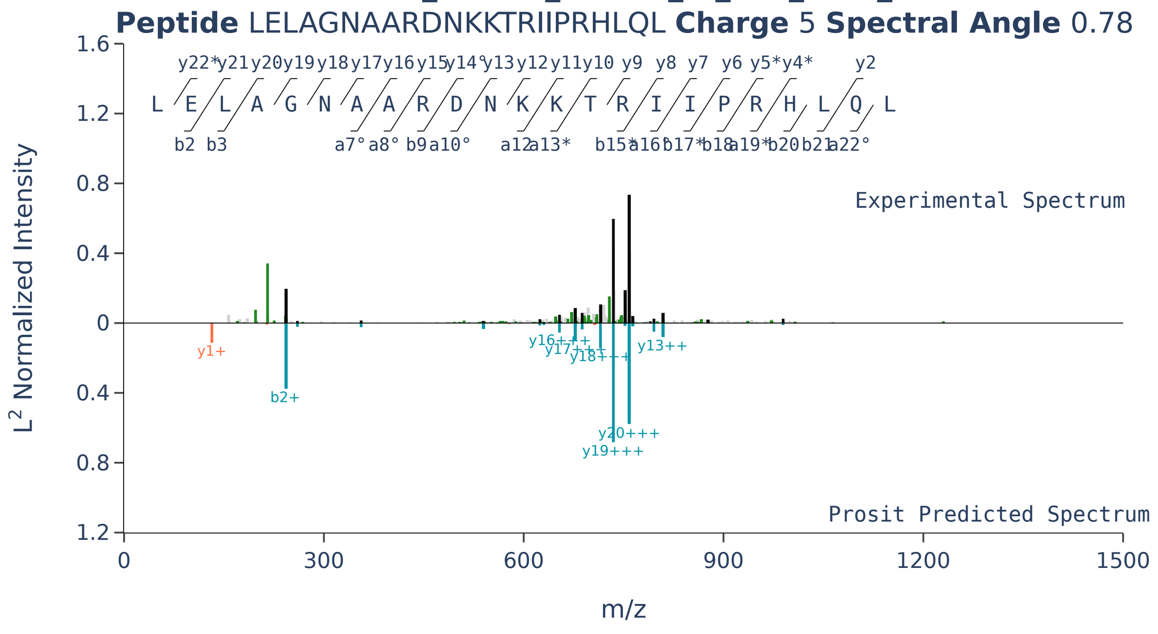


**Source** HRoetschke_291222_060123_Fu_H2A_b1R1_4h **Scan** 15283
**Peptide** QGGVLPNIQSVLLPKKTESSKSAKSK **Charge** 3 **Spectral Angle** 0.8

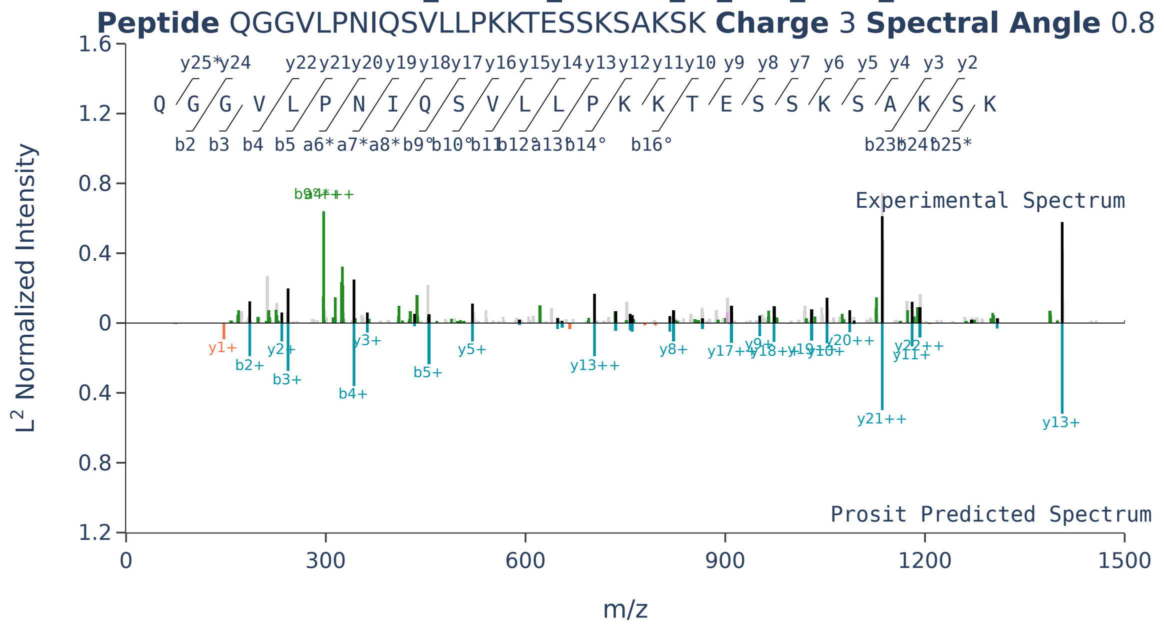


**Source** HRoetschke_291222_060123_Fu_H2A_b1R1_24h **Scan** 8440
**Peptide** LLELAGNAARDNKKTRI **Charge** 4 **Spectral Angle** 0.86

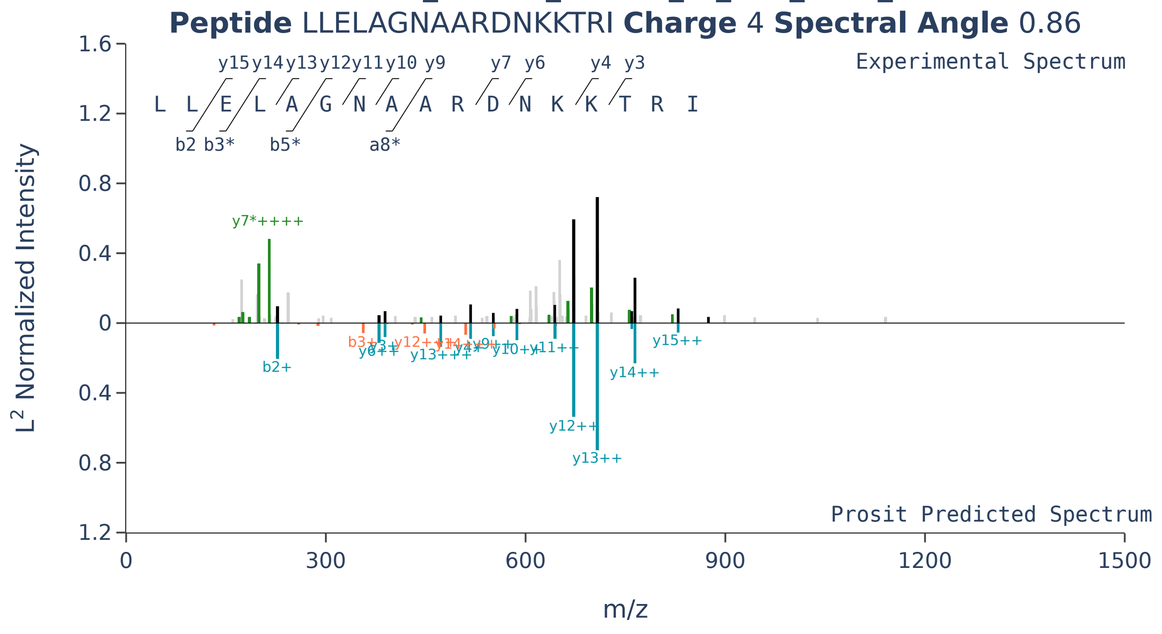


**Source** HRoetschke_291222_060123_Fu_H2A_b2R1_24h **Scan** 9731
**Peptide** LQFPVGRVHR **Charge** 3 **Spectral Angle** 0.8

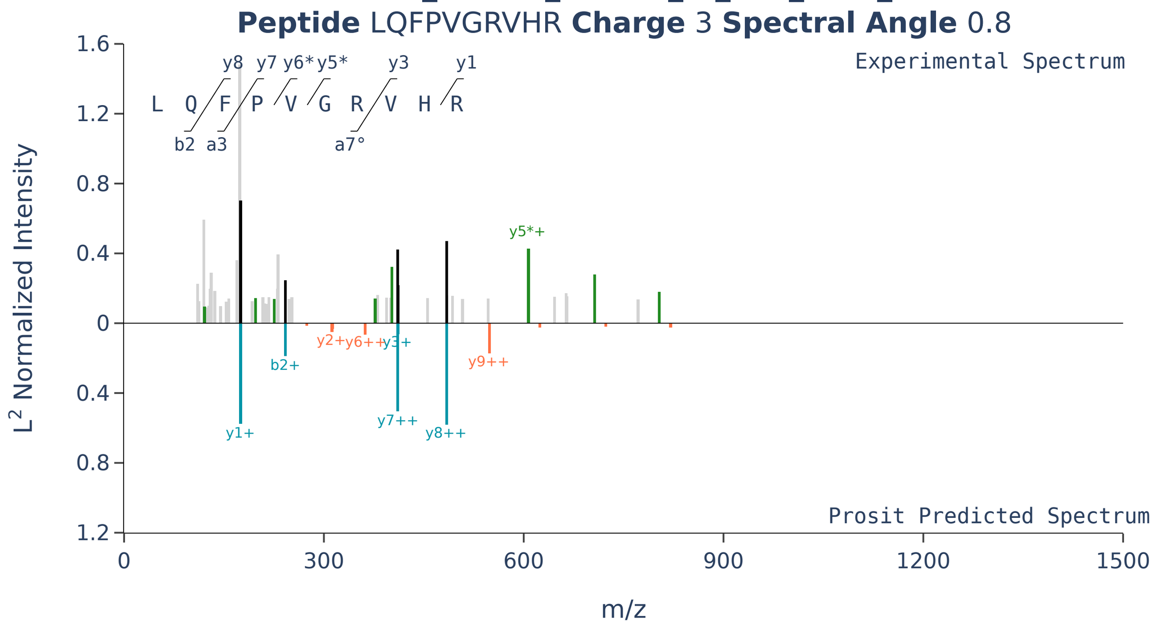


**Source** HRoetschke_291222_060123_Fu_H2A_b1R2_2h **Scan** 9005
**Peptide** QFPVGRVHRLLRKGNYAERVG **Charge** 4 **Spectral Angle** 0.83

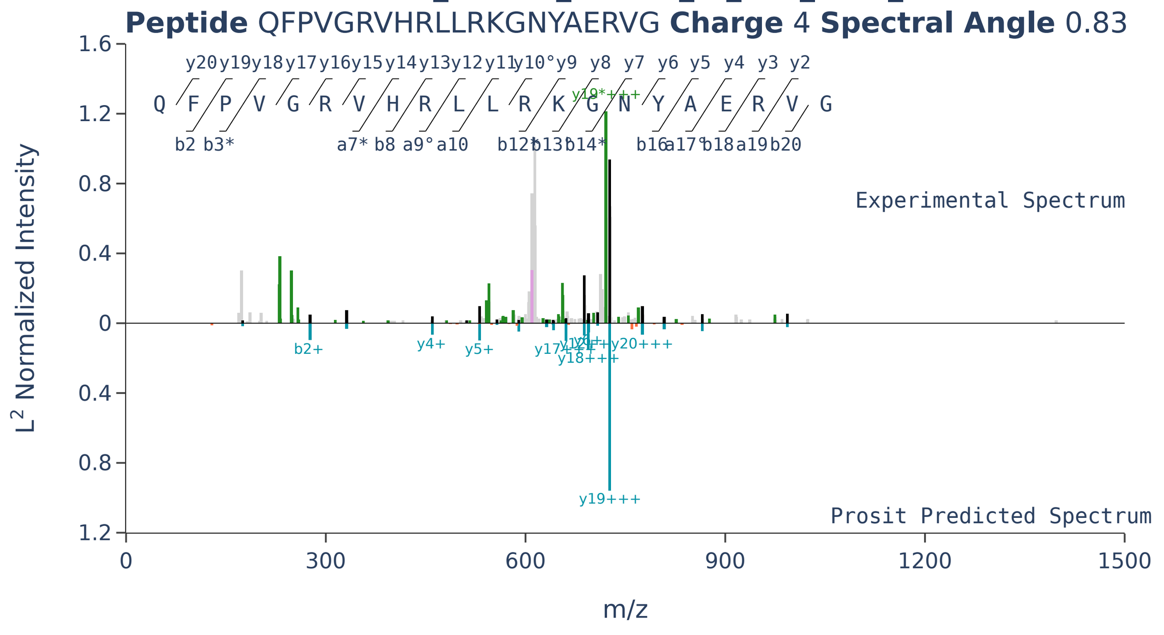


**Source** HRoetschke_291222_060123_Fu_H2A_b1R1_1h **Scan** 7185
**Peptide** ELLELAGNAARDNKKTR **Charge** 4 **Spectral Angle** 0.77

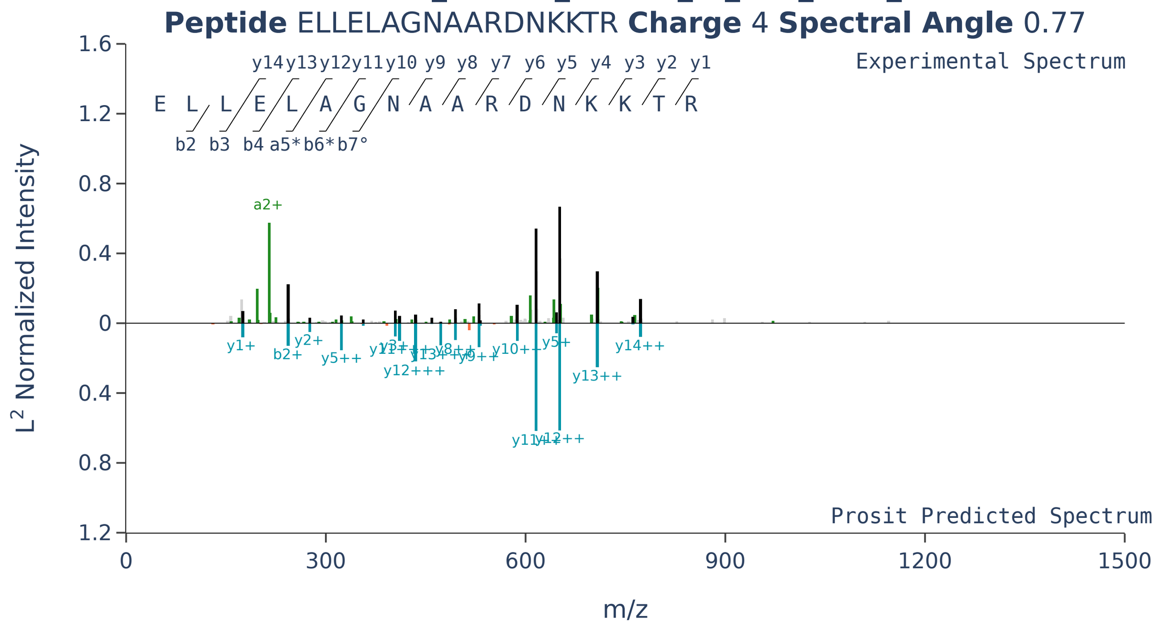


**Source** HRoetschke_291222_060123_Fu_H2A_b2R2_2h **Scan** 2246
**Peptide** HRLLRKGNYA **Charge** 3 **Spectral Angle** 0.85

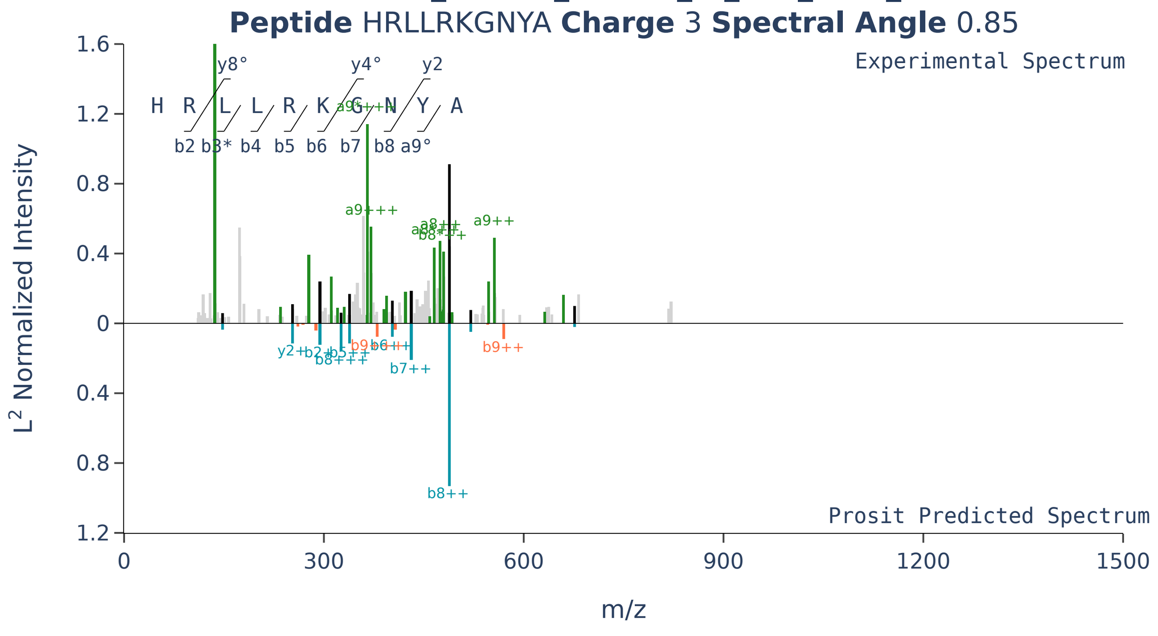


**Source** HRoetschke_291222_060123_Fu_H2A_b1R1_24h **Scan** 7710
**Peptide** LGRVTIAQGG **Charge** 2 **Spectral Angle** 0.83

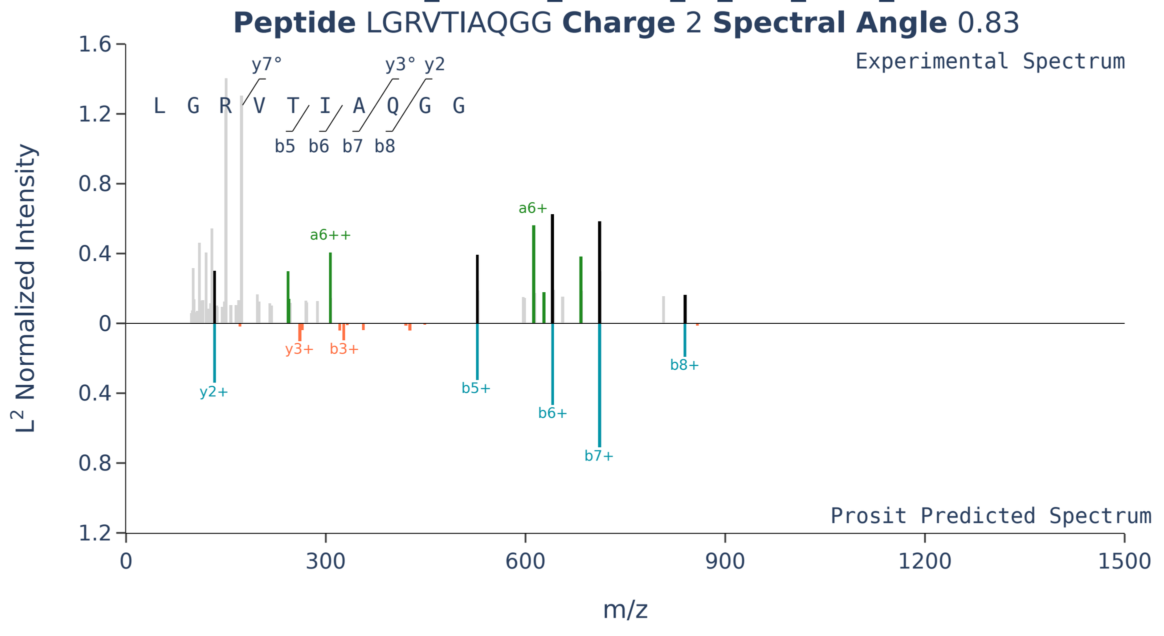


**Source** HRoetschke_291222_060123_Fu_H2A_b2R1_24h **Scan** 2385
**Peptide** RDNKKTRIIPR **Charge** 4 **Spectral Angle** 0.78

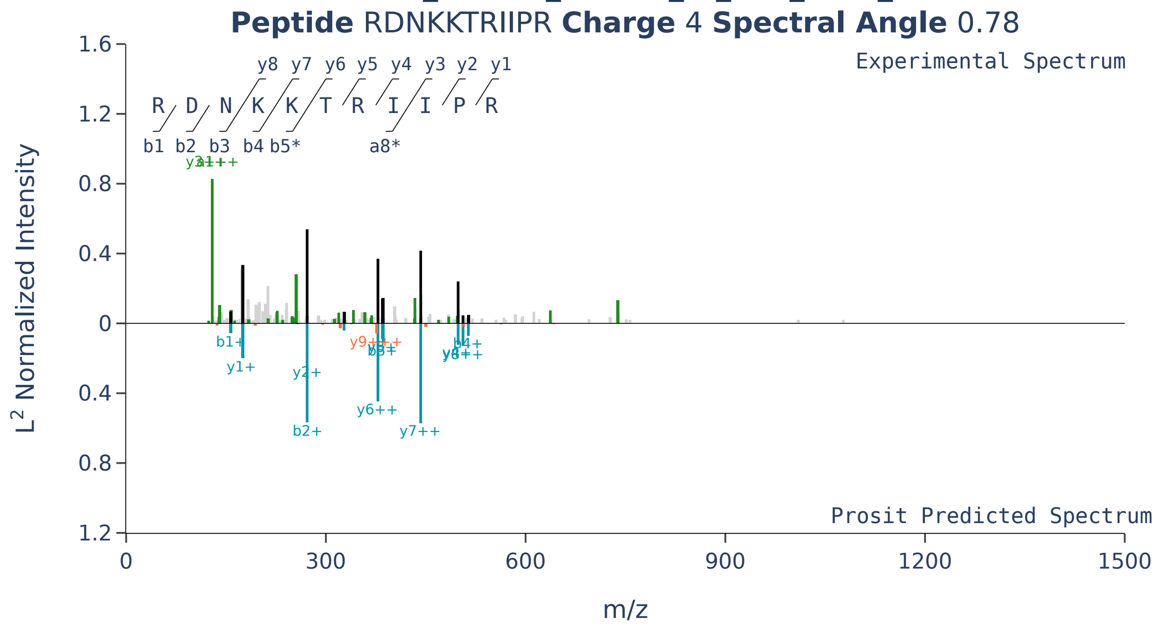


**Source** HRoetschke_291222_060123_Fu_H2A_b1R1_4h **Scan** 8583
**Peptide** ELAGNAARDNKKTRIIPRHLQLA **Charge** 4 **Spectral Angle** 0.86

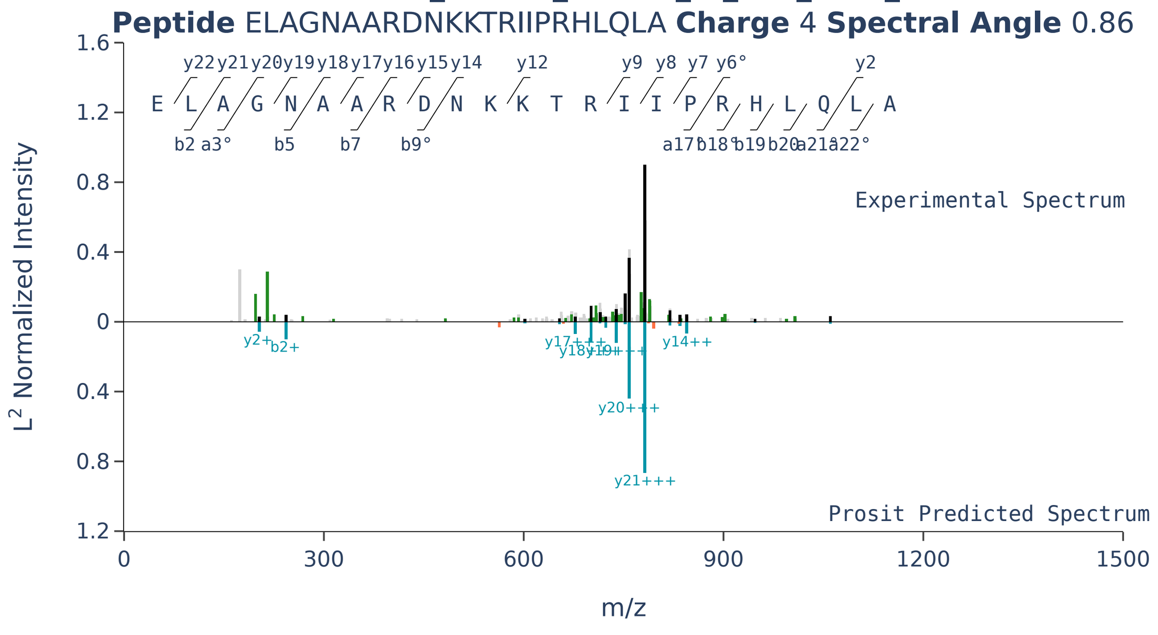


**Source** HRoetschke_291222_060123_Fu_H2A_b1R2_4h **Scan** 11095
**Peptide** LQFPVGRVHRLLRKGNYAER **Charge** 5 **Spectral Angle** 0.74

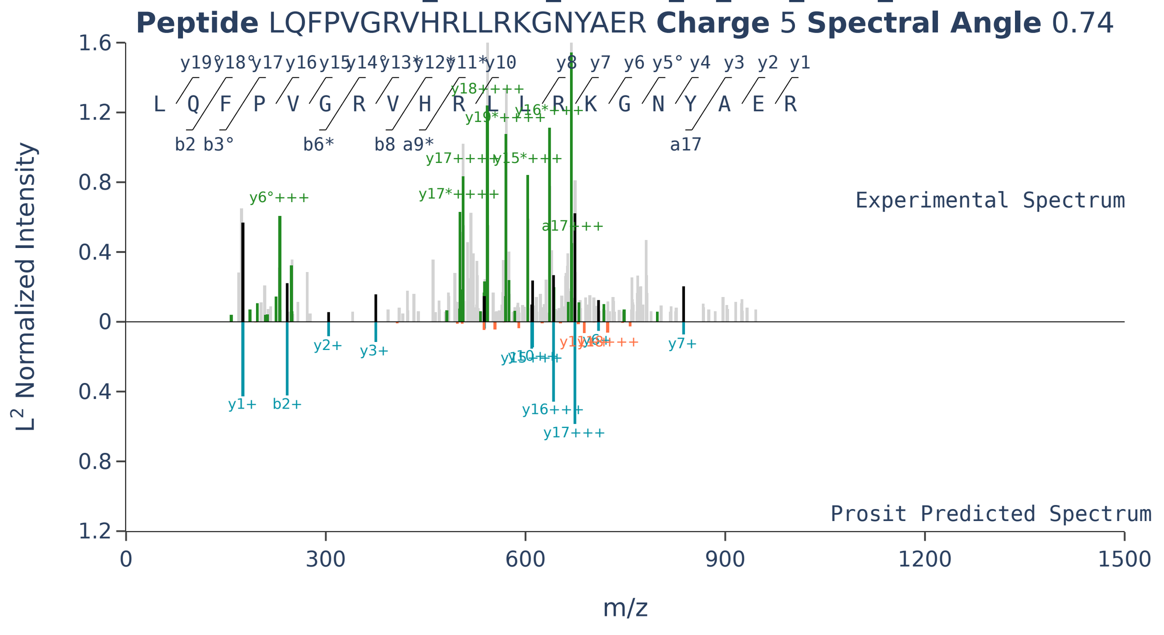


**Source** HRoetschke_291222_060123_Fu_H2A_b2R2_4h **Scan** 9171
**Peptide** LLELAGNAARDNKKTRIIPRHLQ **Charge** 5 **Spectral Angle** 0.8

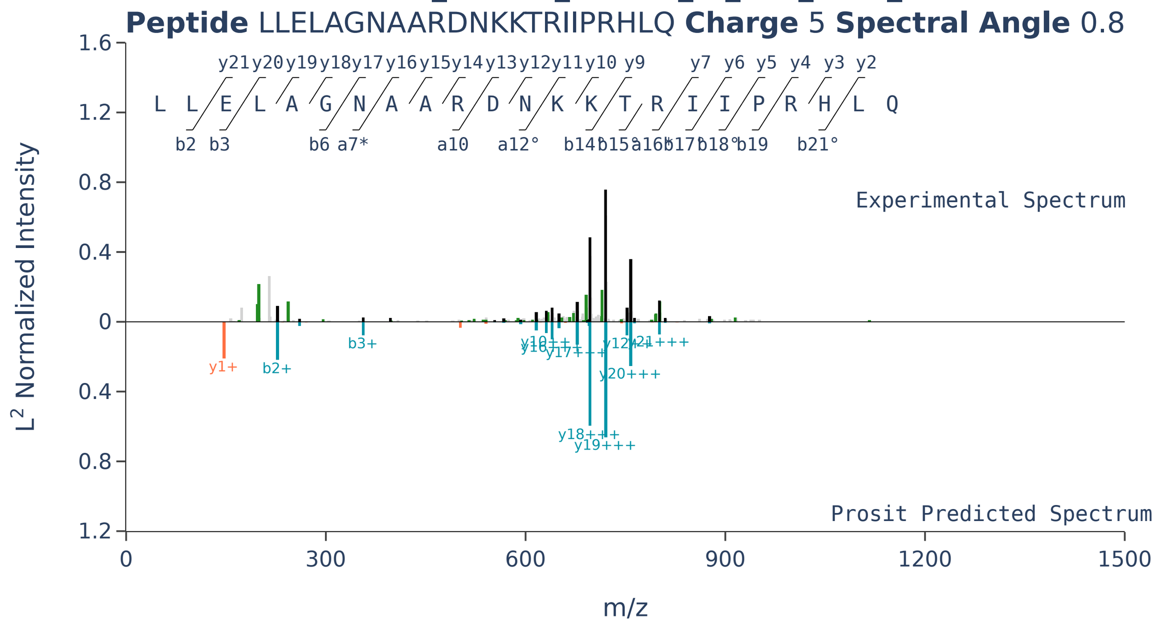


**Source** HRoetschke_291222_060123_Fu_H2A_b1R1_2h **Scan** 18375
**Peptide** AQGGVLPNIQSVLLPKKTE **Charge** 3 **Spectral Angle** 0.82

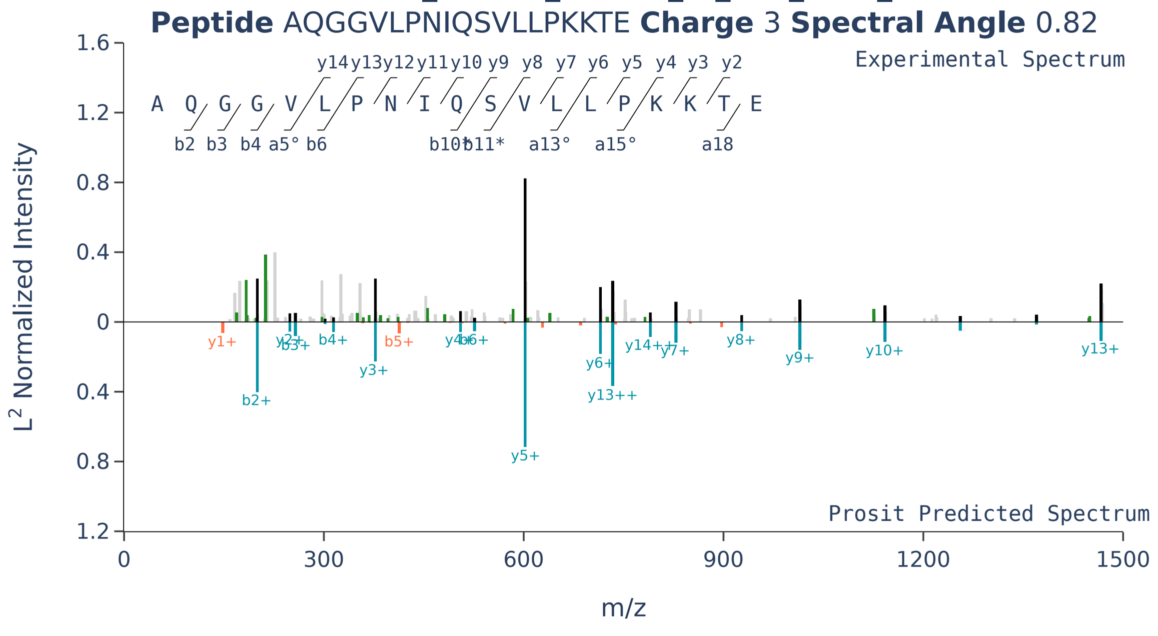


**Source** HRoetschke_291222_060123_Fu_H2A_b2R2_24h **Scan** 6665
**Peptide** GNYAERVGAGAP **Charge** 2 **Spectral Angle** 0.82

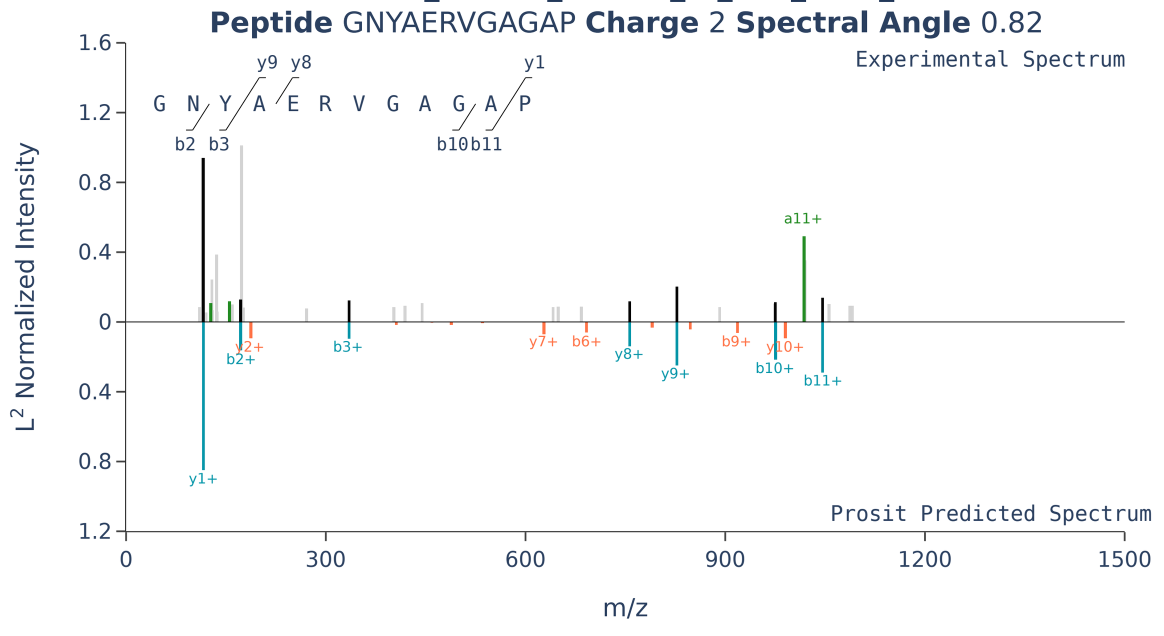


**Source** HRoetschke_291222_060123_Fu_H2A_b1R1_4h **Scan** 17688
**Peptide** QGGVLPNIQSVLLPKKTESSKSA **Charge** 4 **Spectral Angle** 0.76

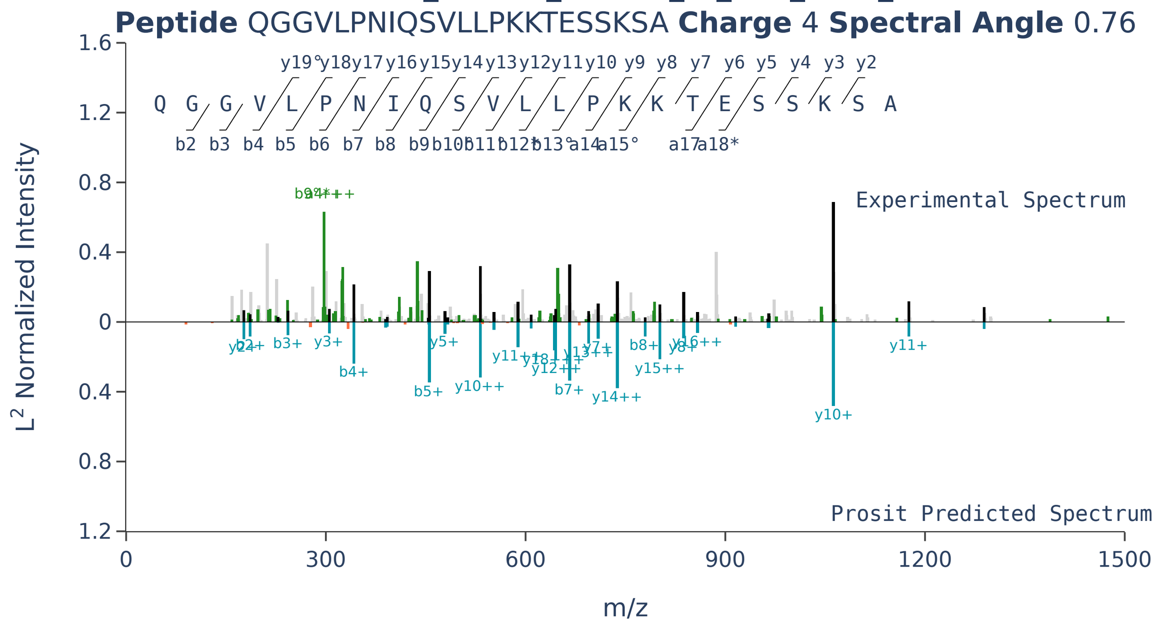

**Source** HRoetschke_291222_060123_Fu_H2A_b2R1_2h **Scan** 7585
**Peptide** LELAGNAARDNKKTRIIPRHL **Charge** 4 **Spectral Angle** 0.83

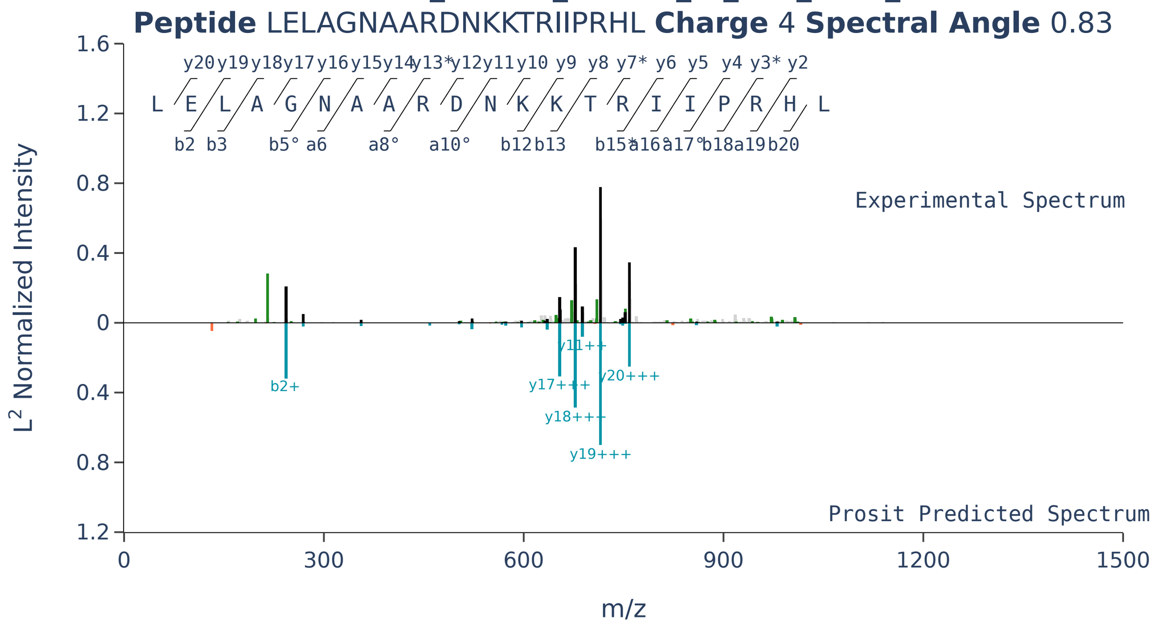


**Source** HRoetschke_291222_060123_Fu_H2A_b1R1_4h **Scan** 5776
**Peptide** QLAVRNDEELNK **Charge** 3 **Spectral Angle** 0.85

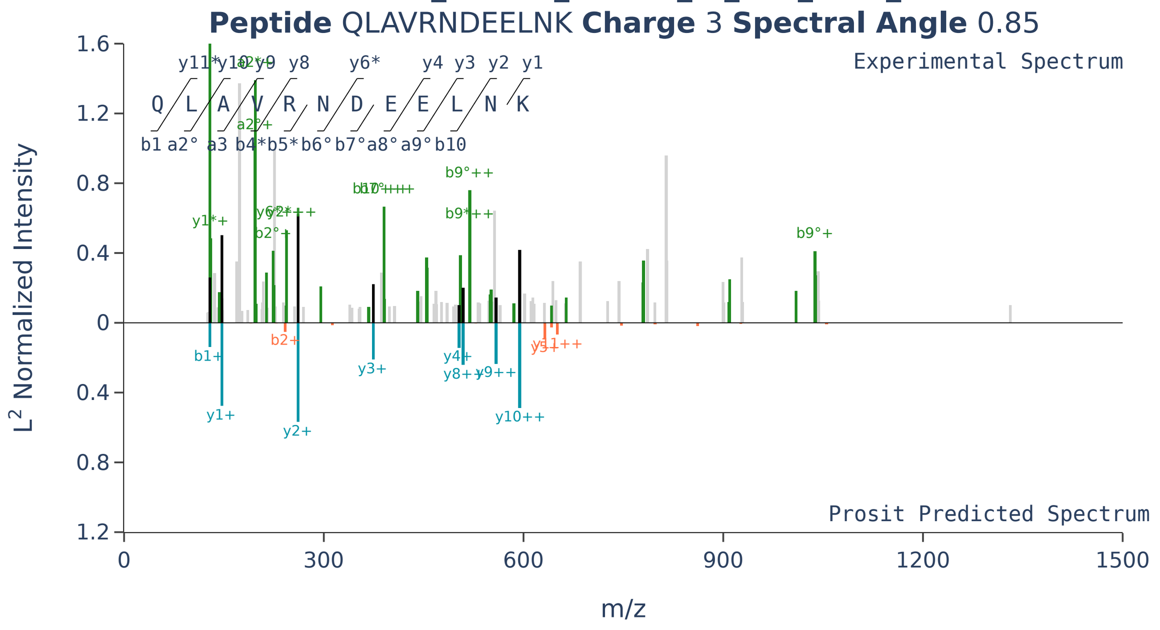


**Source** HRoetschke_291222_060123_Fu_H2A_b1R1_4h **Scan** 14588
**Peptide** RAGLQFPVGRVHRLL **Charge** 4 **Spectral Angle** 0.77

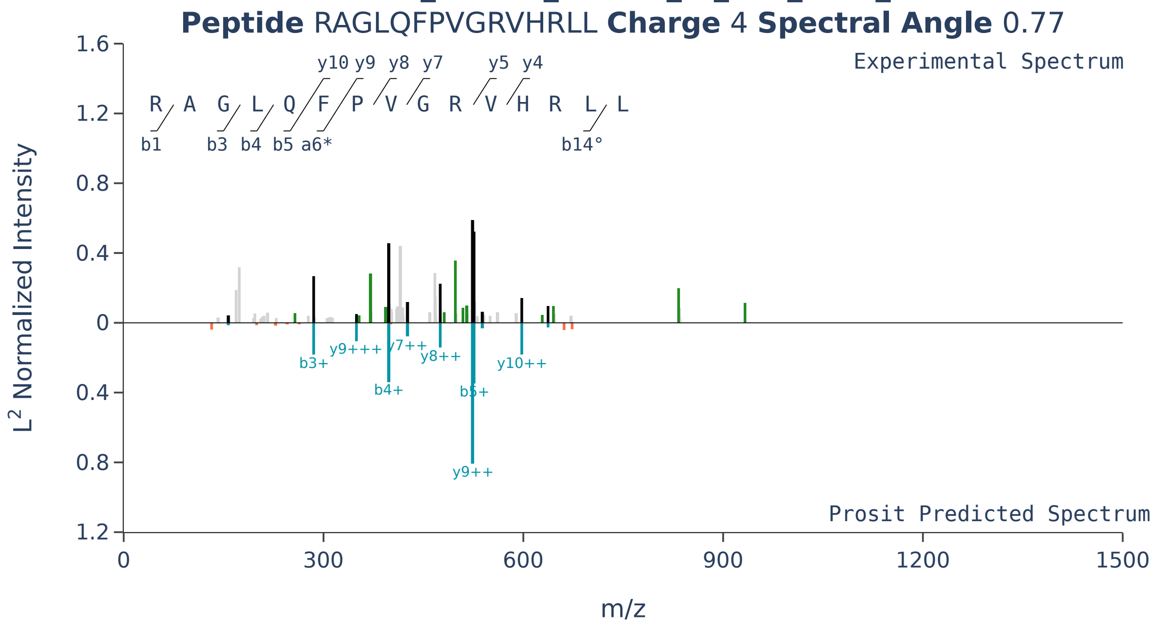


**Source** HRoetschke_291222_060123_Fu_H2A_b1R2_1h **Scan** 3093
**Peptide** KKTRIIPRHL **Charge** 3 **Spectral Angle** 0.85

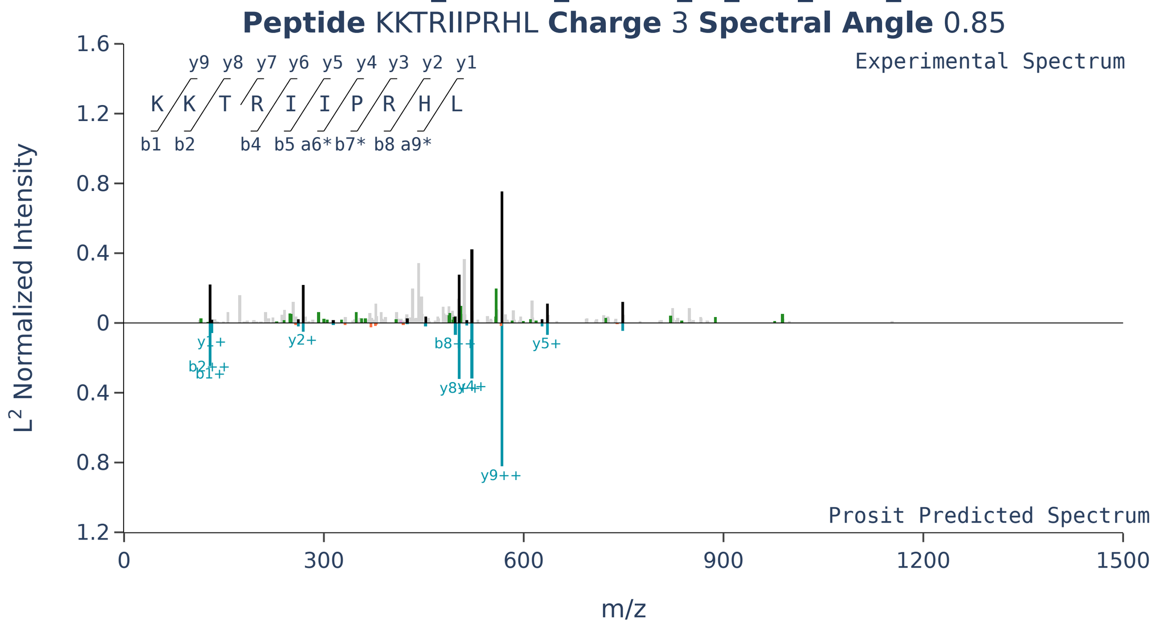


**Source** HRoetschke_291222_060123_Fu_H2A_b1R1_24h **Scan** 2713
**Peptide** VLLPKKTESSKSAKS **Charge** 3 **Spectral Angle** 0.71

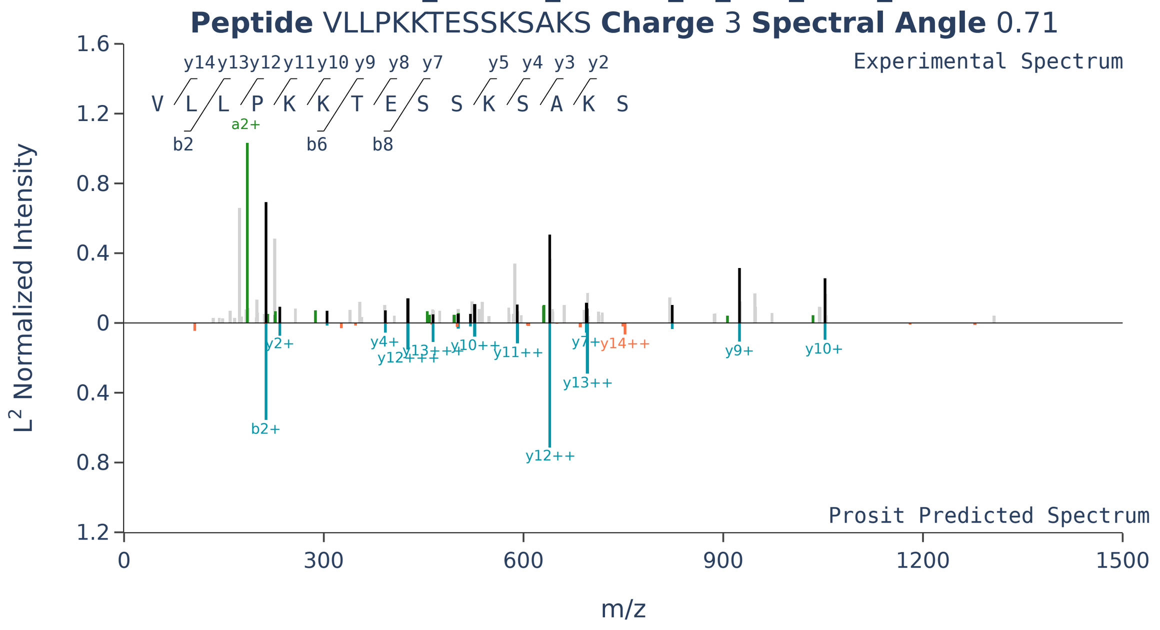


**Source** HRoetschke_291222_060123_Fu_H2A_b1R2_24h **Scan** 15805
**Peptide** TAEILELAGNA **Charge** 2 **Spectral Angle** 0.84

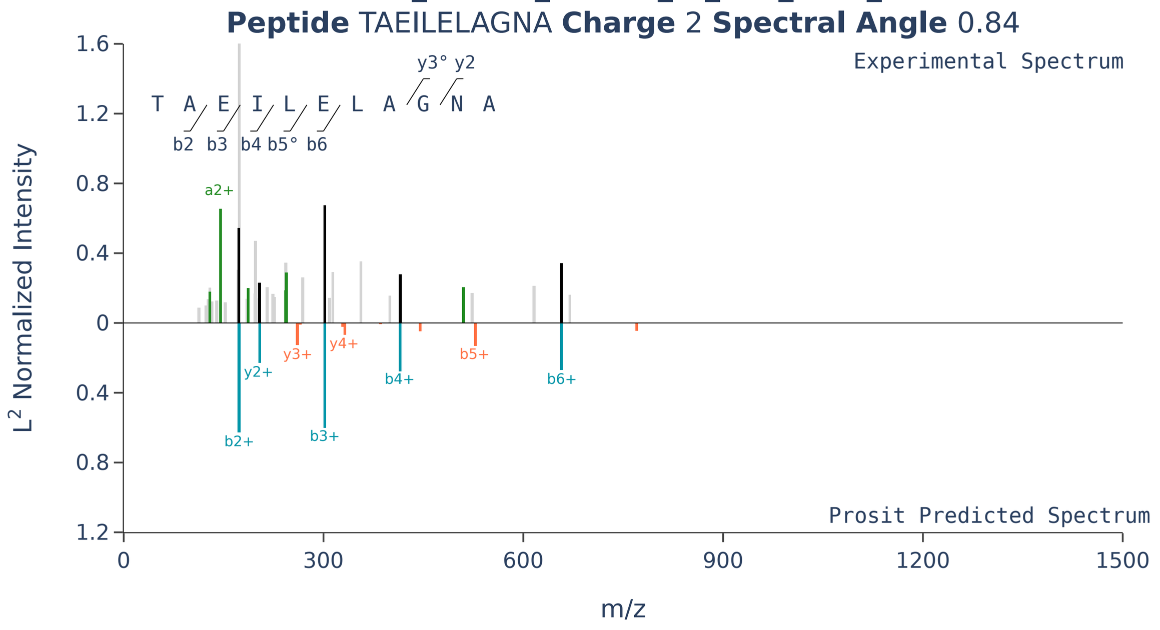


**Source** HRoetschke_291222_060123_Fu_H2A_b1R1_2h **Scan** 17225
**Peptide** LAVRNDEELNKLLGRVTIAQG **Charge** 3 **Spectral Angle** 0.75

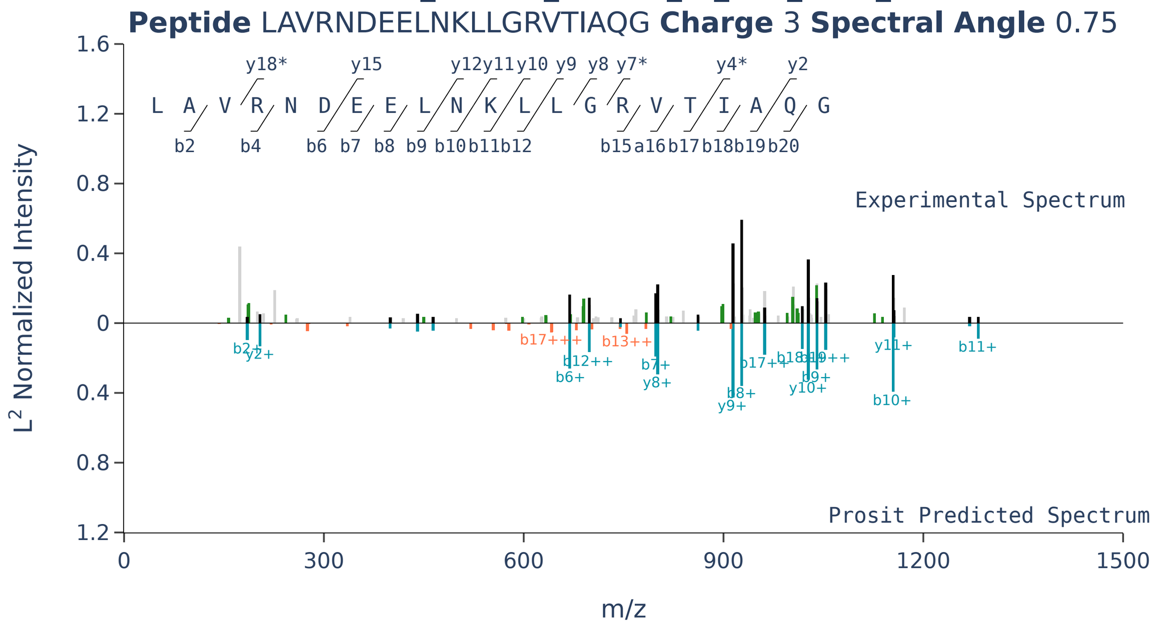


**Source** HRoetschke_291222_060123_Fu_H2A_b1R1_4h **Scan** 19322
**Peptide** AQGGVLPNIQSVLLPKKT **Charge** 3 **Spectral Angle** 0.78

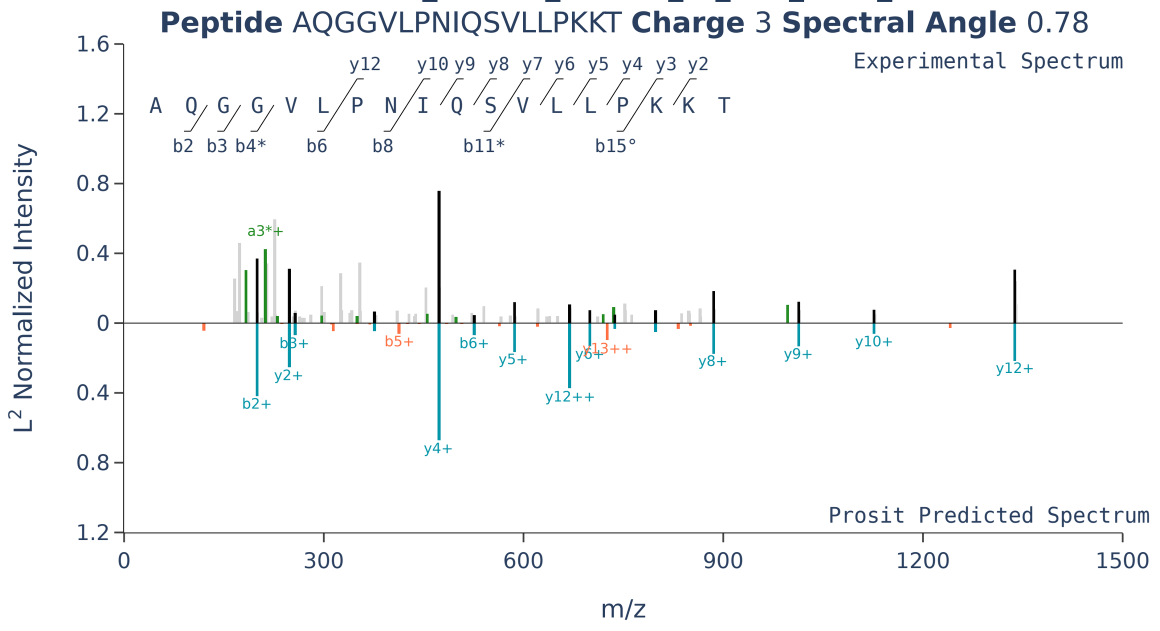


**Source** HRoetschke_291222_060123_Fu_H2A_b2R1_24h **Scan** 15898
**Peptide** EELNKLLGRVTLA **Charge** 2 **Spectral Angle** 0.86

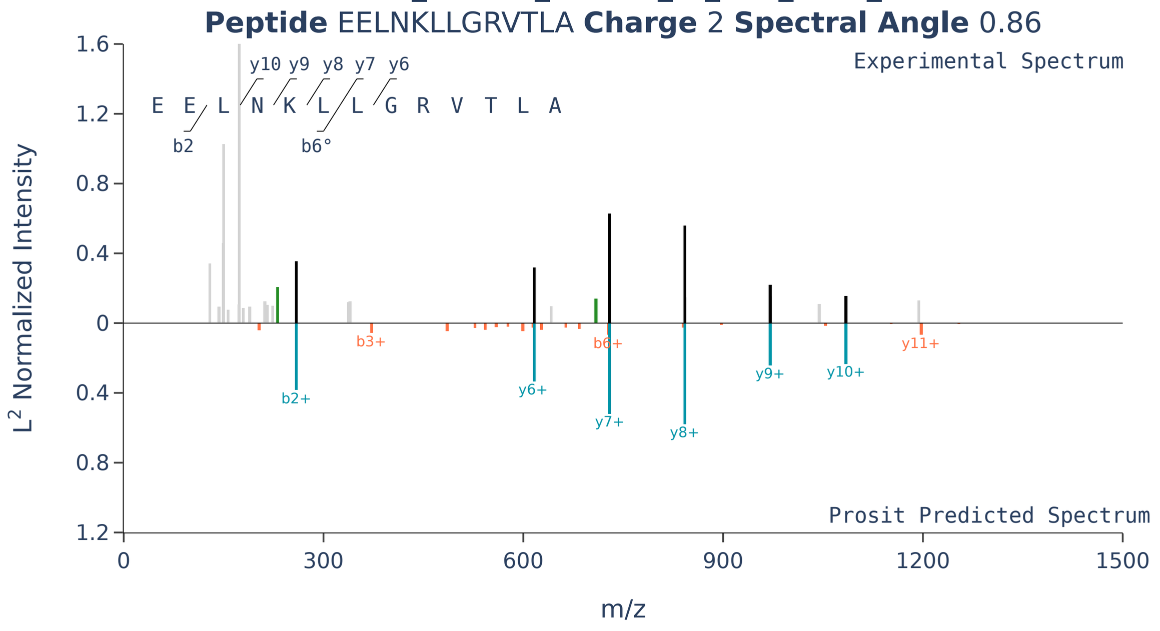


**Source** HRoetschke_291222_060123_Fu_H2A_b1R1_2h **Scan** 7343
**Peptide** TRAKAKTRSSRAGLQFPVG **Charge** 4 **Spectral Angle** 0.79

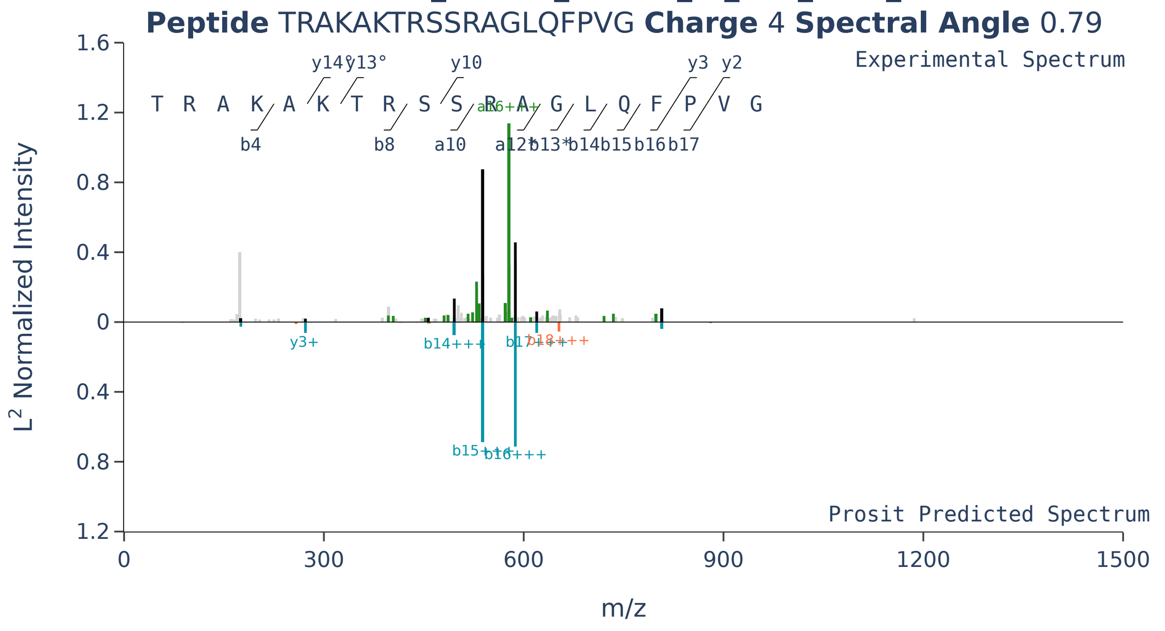


**Source** HRoetschke_291222_060123_Fu_H2A_b1R1_4h **Scan** 10641
**Peptide** SRAGLQFPVGRVHRLLRKGNYAER **Charge** 6 **Spectral Angle** 0.75

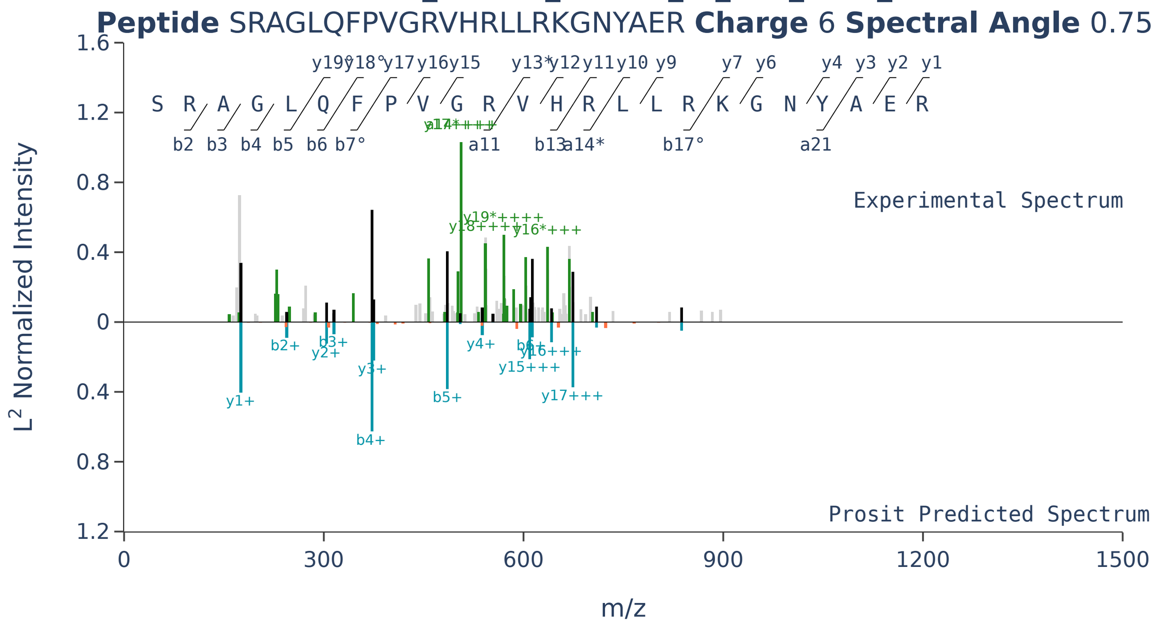


**Source** HRoetschke_291222_060123_Fu_H2A_b1R1_24h **Scan** 7076
**Peptide** LELAGNAARDNKKTRIIPR **Charge** 4 **Spectral Angle** 0.87

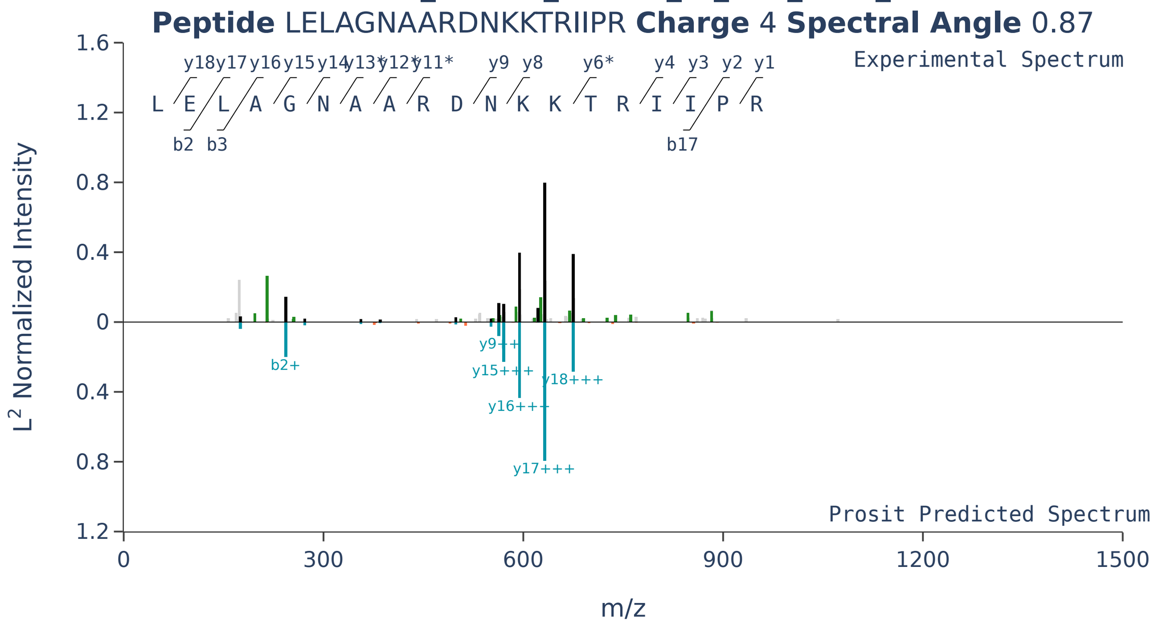


**Source** HRoetschke_291222_060123_Fu_H2A_b1R1_4h **Scan** 10585
**Peptide** RAGLQFPVGRVHRLLRKGNYAER **Charge** 6 **Spectral Angle** 0.78

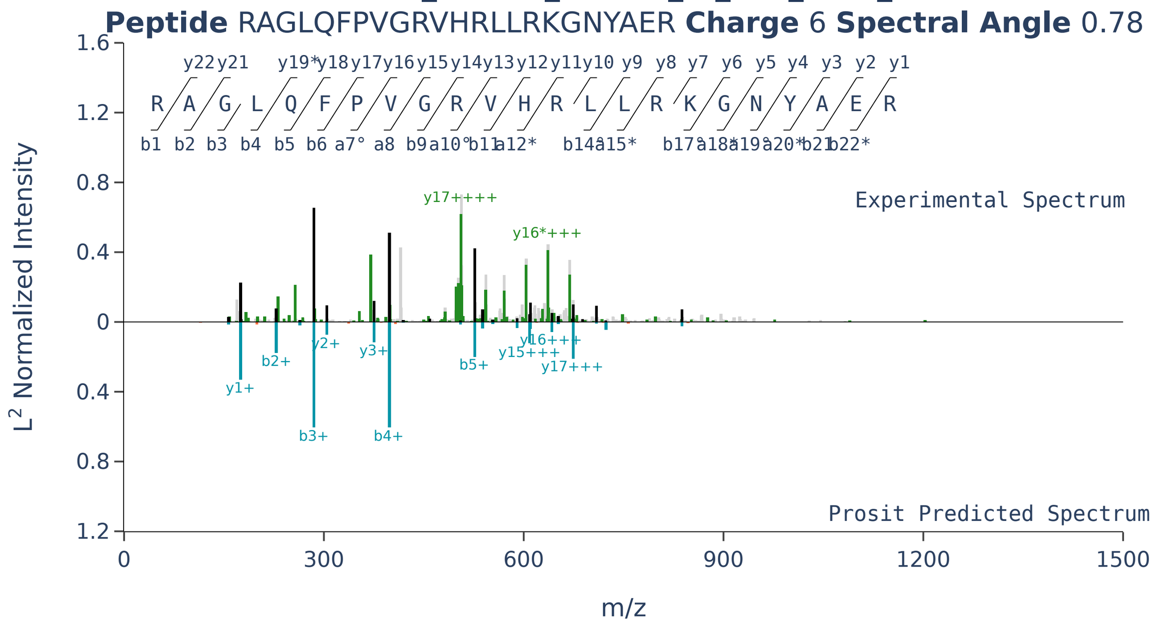


**Source** HRoetschke_291222_060123_Fu_H2A_b2R1_4h **Scan** 6851
**Peptide** ARDNKKTRIIPRHLQLA **Charge** 5 **Spectral Angle** 0.7

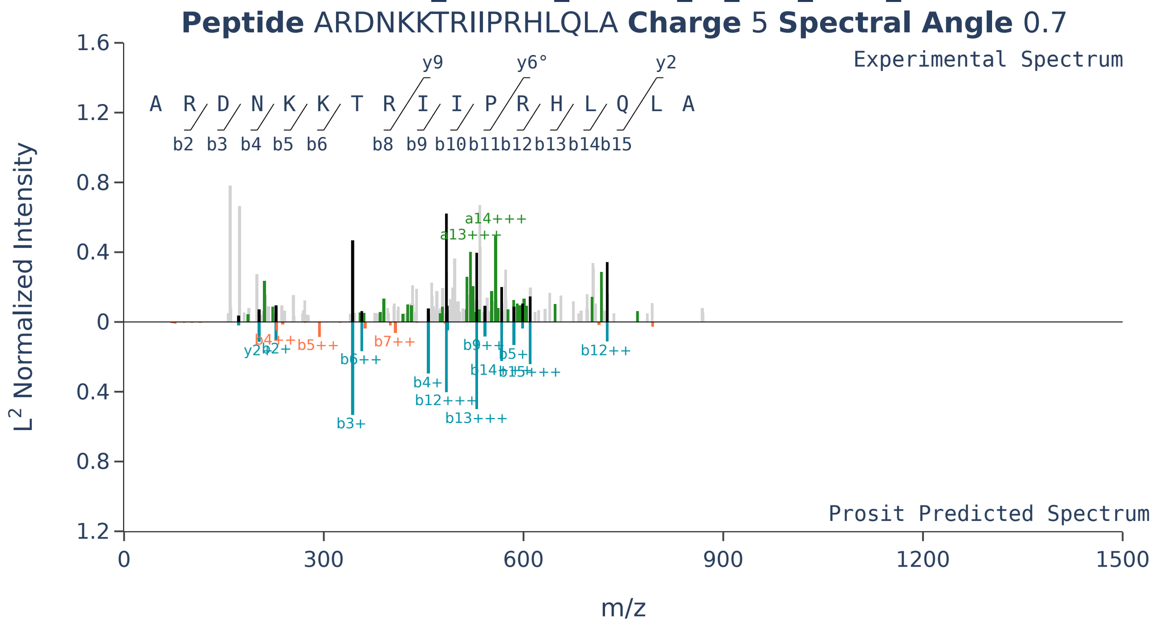


**Source** HRoetschke_291222_060123_Fu_H2A_b1R2_2h **Scan** 11299
**Peptide** SSRAGLQFPVGRVHRLLRKGNYAERVG **Charge** 5 **Spectral Angle** 0.71

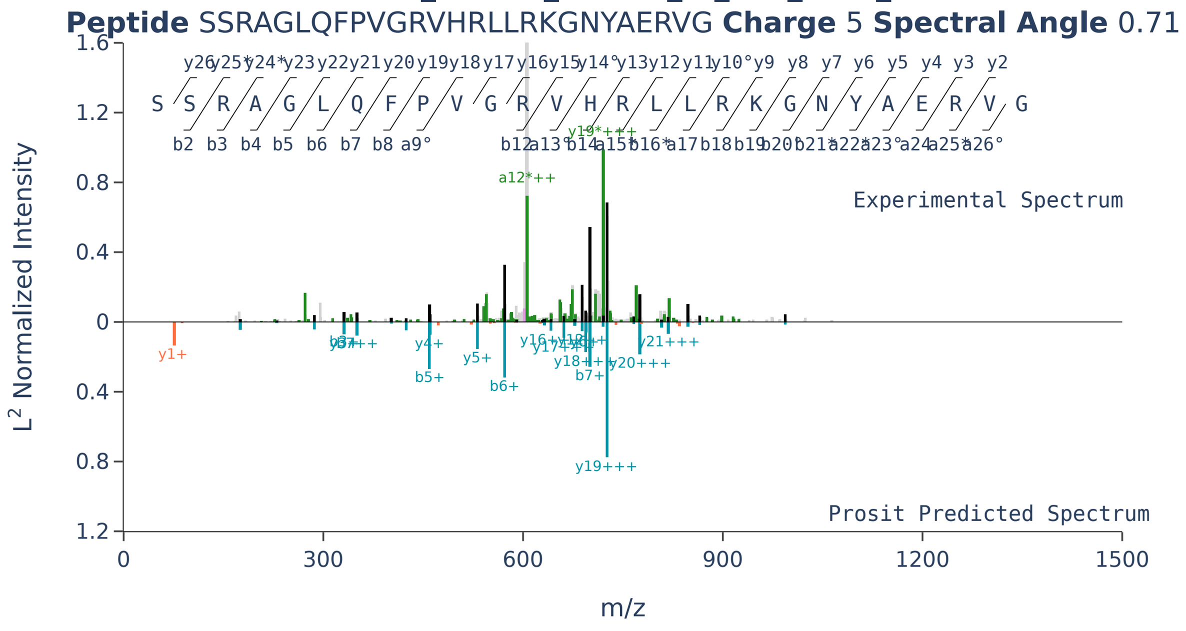

**Source** HRoetschke_291222_060123_Fu_H2A_b1R1_4h **Scan** 17748
**Peptide** AQGGVLPNIQSVLLPKKTESSKSA **Charge** 4 **Spectral Angle** 0.69

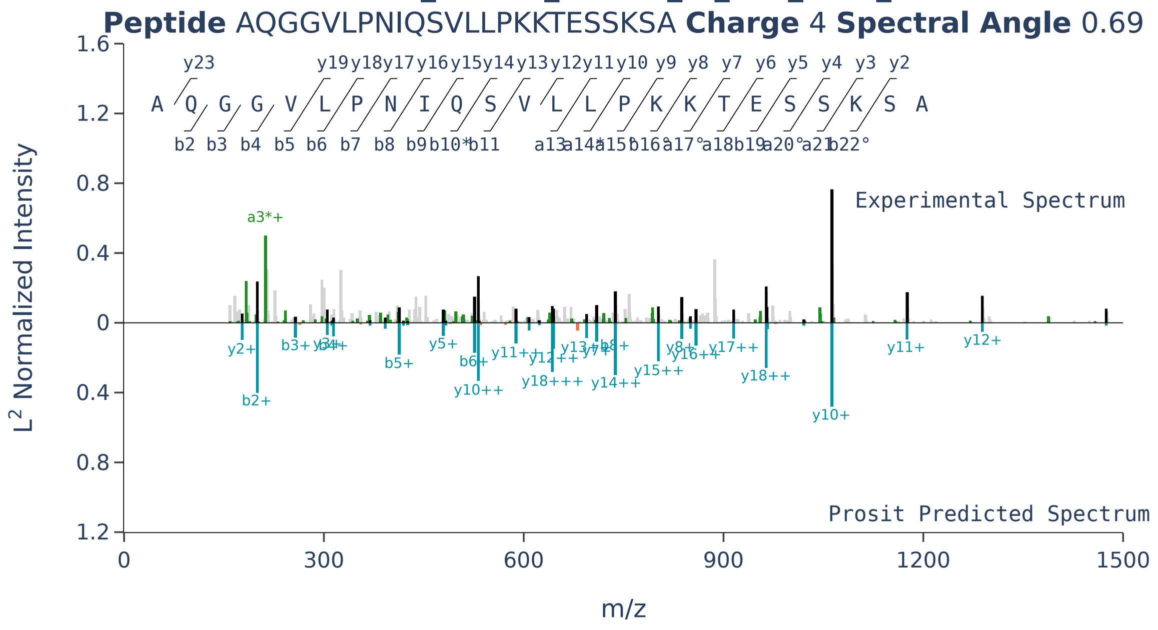


**Source** HRoetschke_291222_060123_Fu_H2A_b2R1_24h **Scan** 13620
**Peptide** LTAEILE **Charge** 1 **Spectral Angle** 0.8

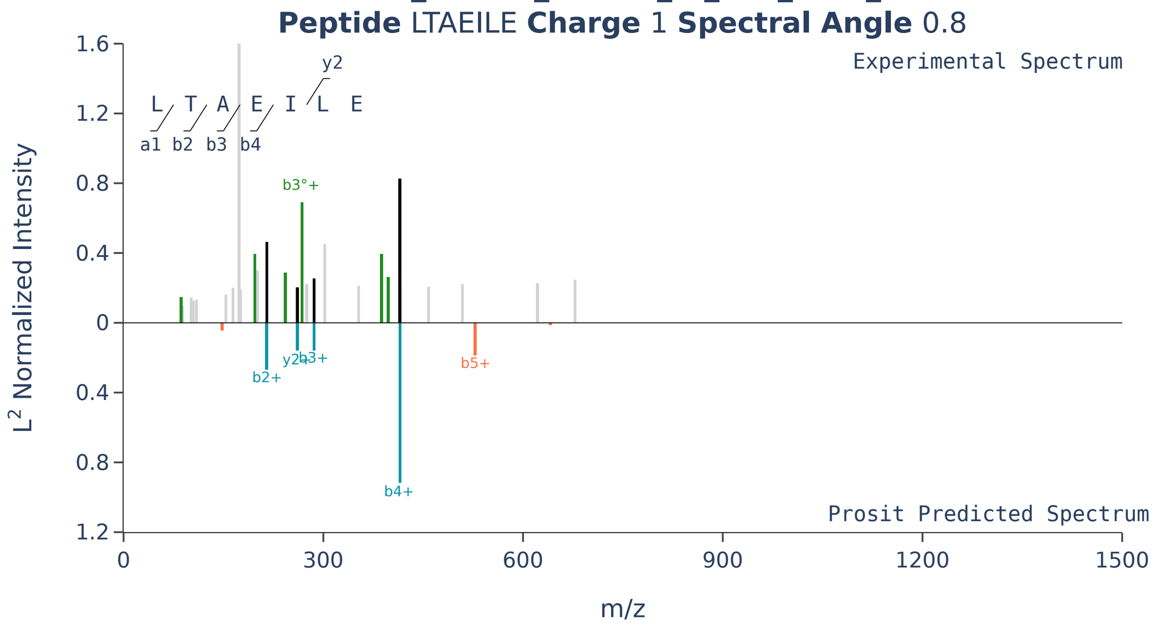


**Source** HRoetschke_291222_060123_Fu_H2A_b1R1_2h **Scan** 6734
**Peptide** AGNAARDNKKTRIIPRHLQLA **Charge** 4 **Spectral Angle** 0.83

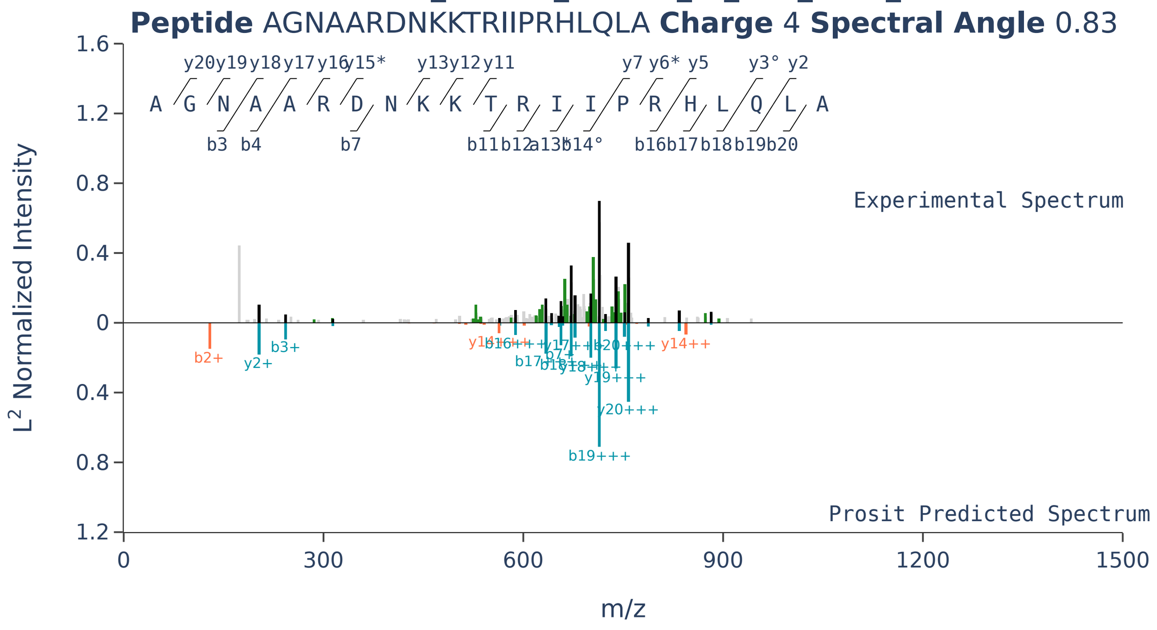


**Source** HRoetschke_291222_060123_Fu_H2A_b1R1_4h **Scan** 10546
**Peptide** SSRAGLQFPVGRVHRLLRK **Charge** 5 **Spectral Angle** 0.64

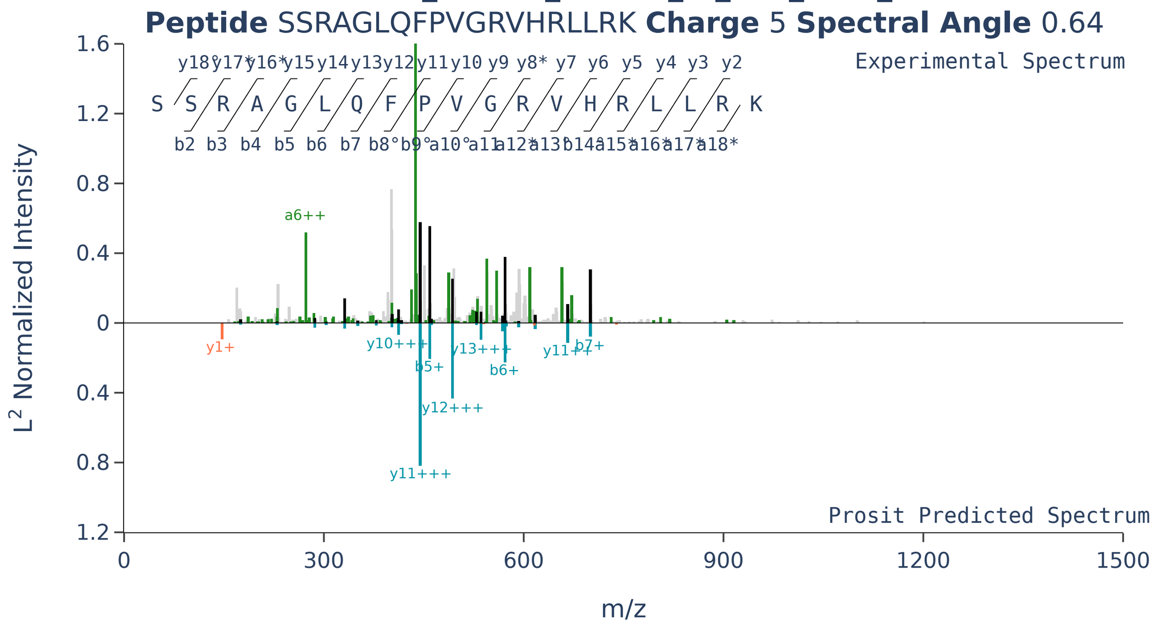


**Source** HRoetschke_291222_060123_Fu_H2A_b1R1_4h **Scan** 2666
**Peptide** VLLPKKTESSKSAK **Charge** 3 **Spectral Angle** 0.77

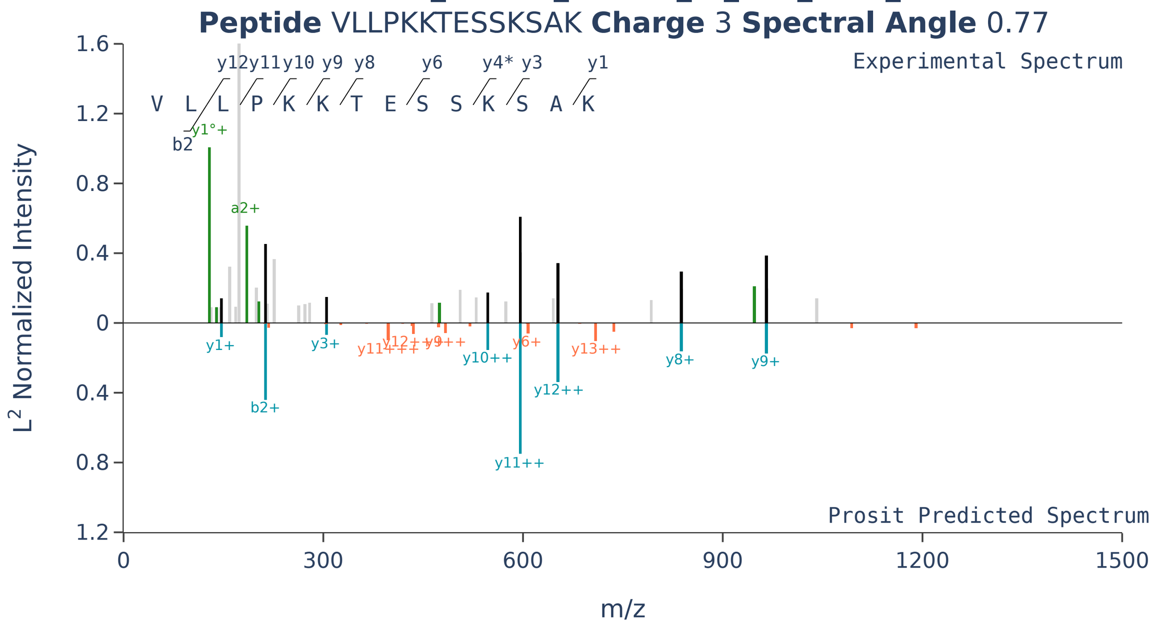


**Source** HRoetschke_291222_060123_Fu_H2A_b2R1_24h **Scan** 1939
**Peptide** RDNKKTRIIPRH **Charge** 4 **Spectral Angle** 0.69

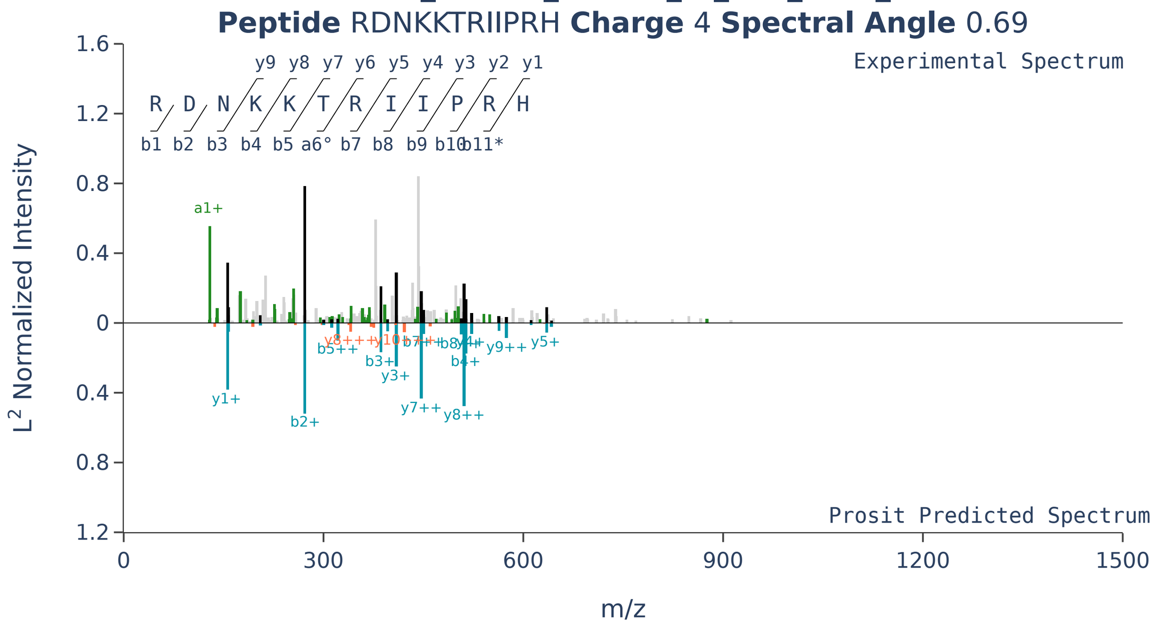


**Source** HRoetschke_291222_060123_Fu_H2A_b1R1_4h **Scan** 11682
**Peptide** RAGLQFPVGRVHRLLRKGNYAERVG **Charge** 6 **Spectral Angle** 0.7

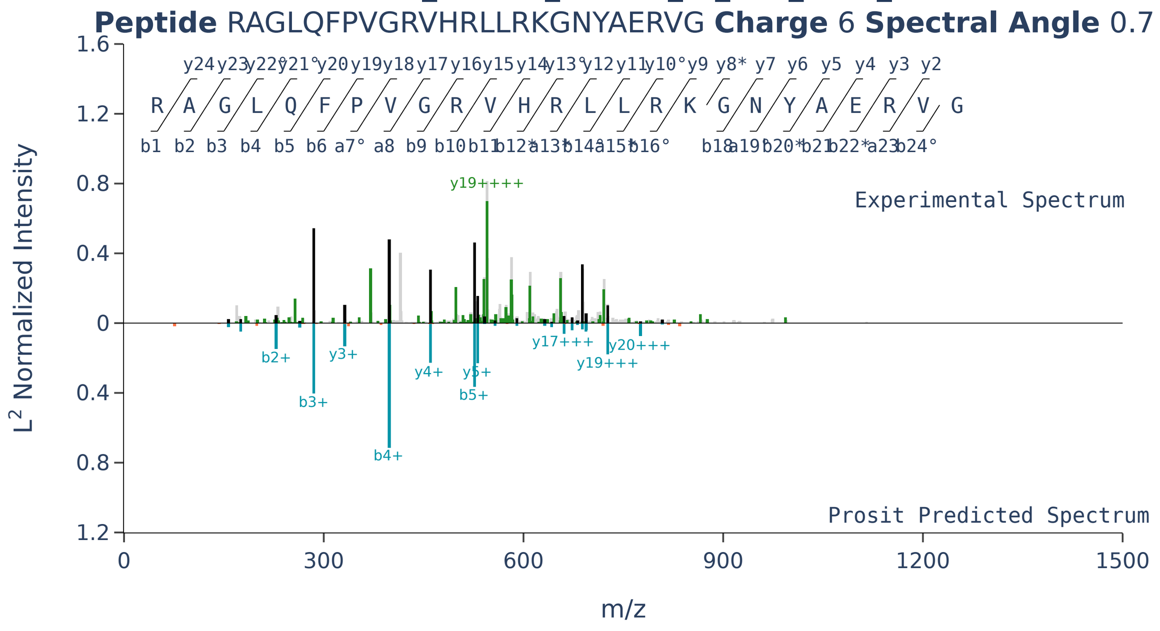


**Source** HRoetschke_291222_060123_Fu_H2A_b1R2_4h **Scan** 8948
**Peptide** AKAKTRSSRAGLQFPVGRV **Charge** 4 **Spectral Angle** 0.78

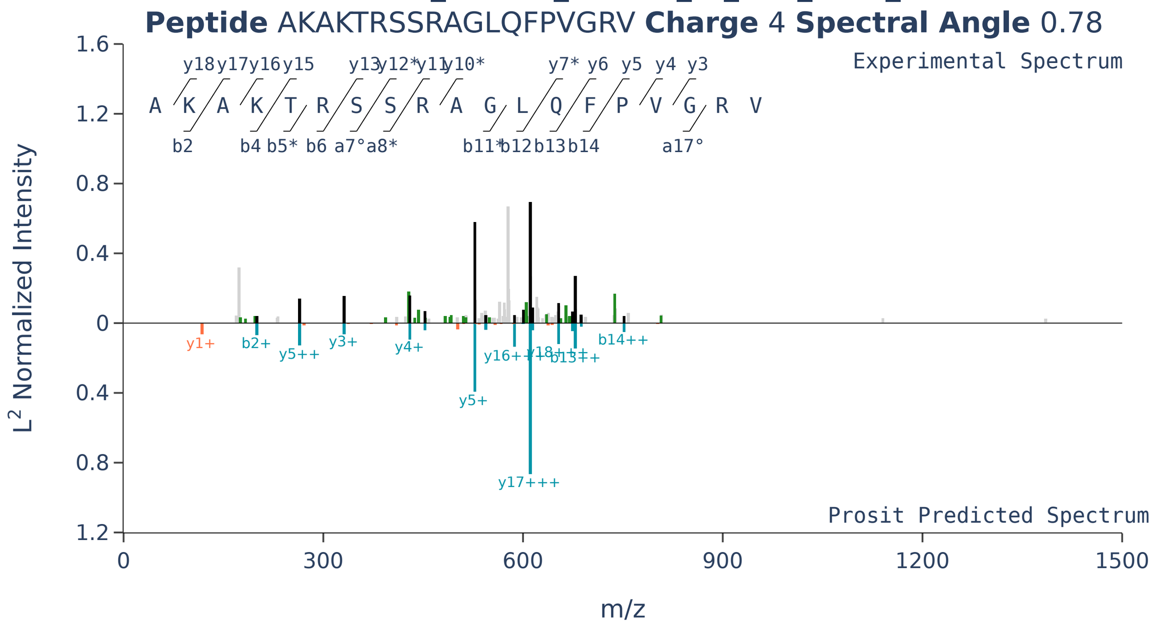


**Source** HRoetschke_291222_060123_Fu_H2A_b1R2_2h **Scan** 7463
**Peptide** RSSRAGLQF **Charge** 2 **Spectral Angle** 0.79

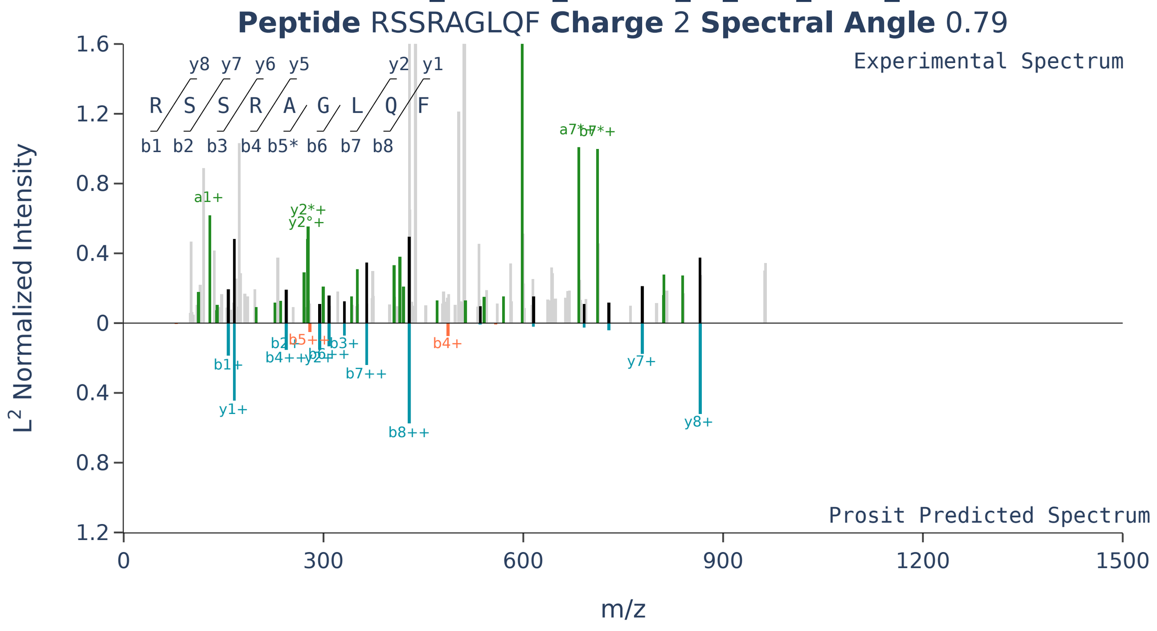


**Source** HRoetschke_291222_060123_Fu_H2A_b1R2_4h **Scan** 7177
**Peptide** GNAARDNKKTRIIPRHLQL **Charge** 4 **Spectral Angle** 0.74

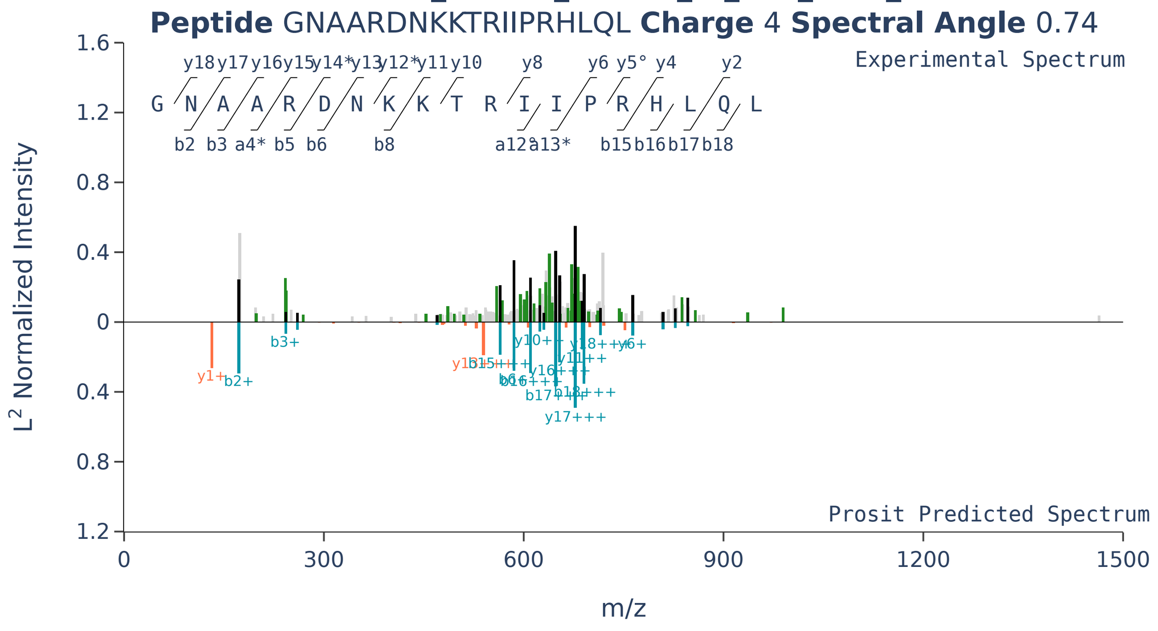


**Source** HRoetschke_291222_060123_Fu_H2A_b2R1_1h **Scan** 4921
**Peptide** ELAGNAARDNKKTRIIPRHLQ **Charge** 4 **Spectral Angle** 0.87

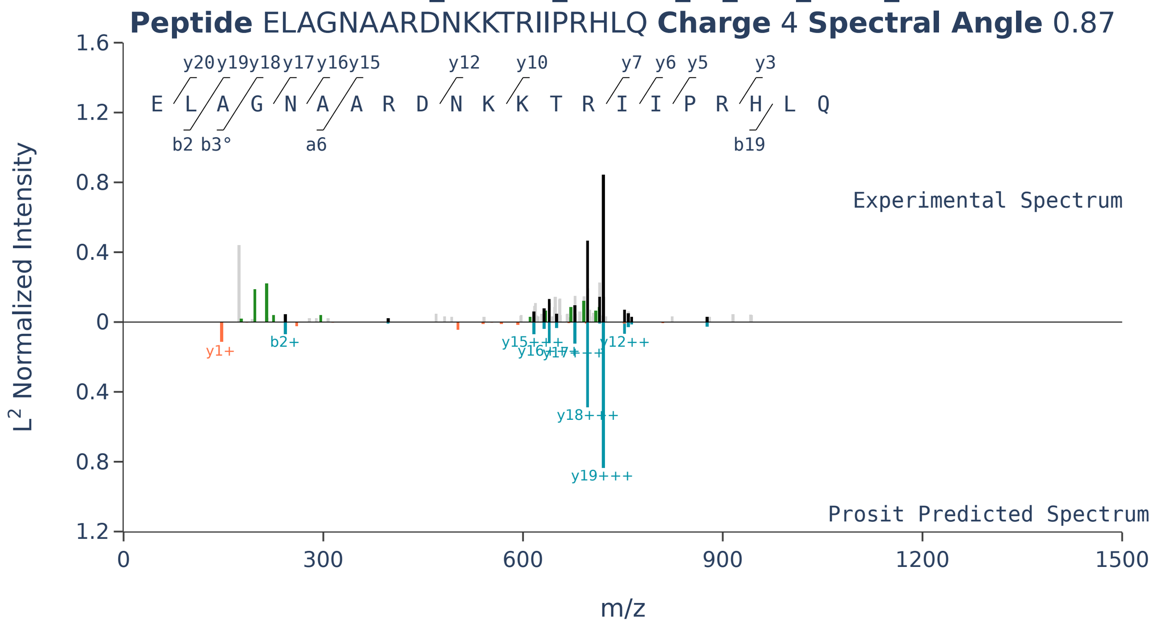


**Source** HRoetschke_291222_060123_Fu_H2A_b2R1_24h **Scan** 12878
**Peptide** AGLQFPVGRVHRL **Charge** 3 **Spectral Angle** 0.88

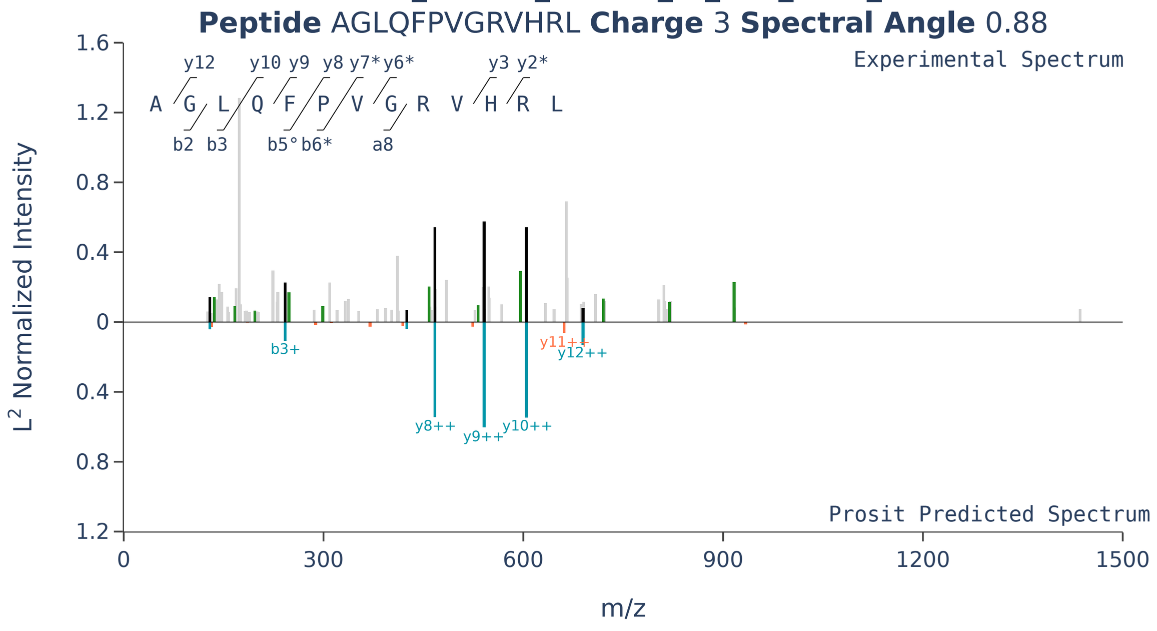


**Source** HRoetschke_291222_060123_Fu_H2A_b1R1_4h **Scan** 16307
**Peptide** RNDEELNKLLGRVTIAQGG **Charge** 3 **Spectral Angle** 0.75

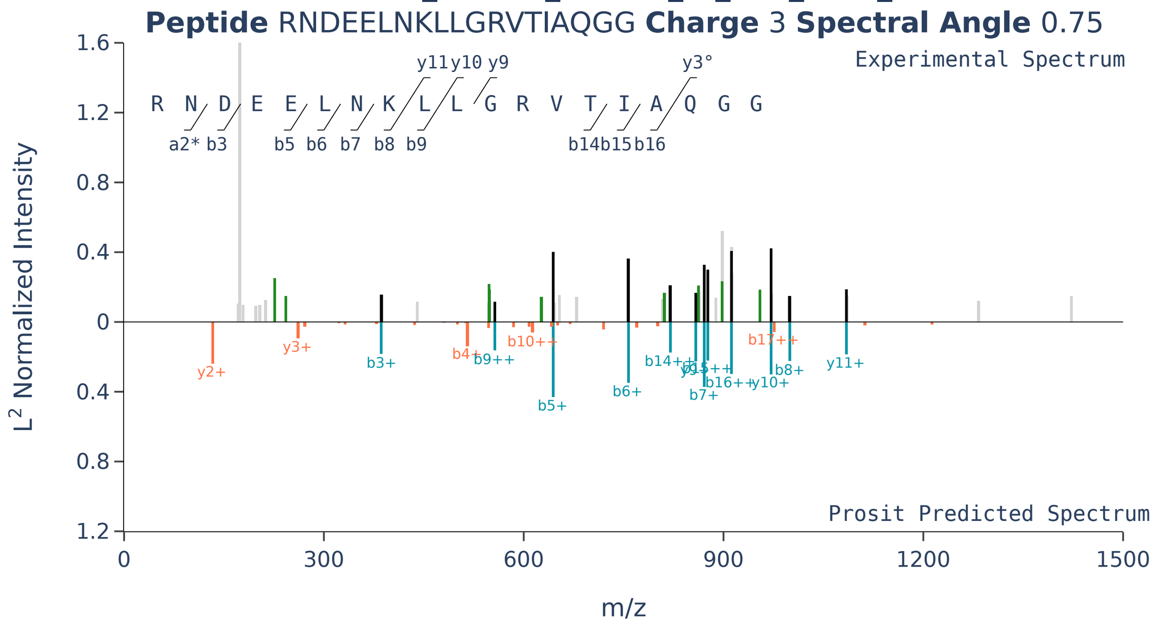


**Source** HRoetschke_291222_060123_Fu_H2A_b1R1_2h **Scan** 7658
**Peptide** LRKGNYAERVGAGAPVY **Charge** 3 **Spectral Angle** 0.83

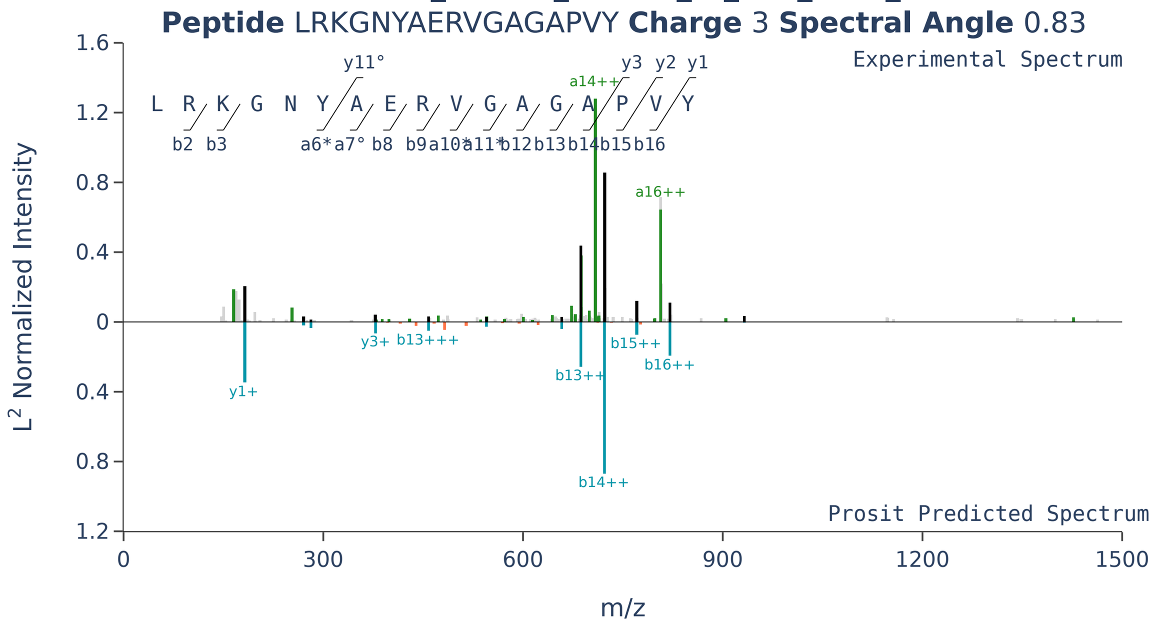


**Source** HRoetschke_291222_060123_Fu_H2A_b2R2_1h **Scan** 7993
**Peptide** KLLGRVTIAQGG **Charge** 3 **Spectral Angle** 0.82

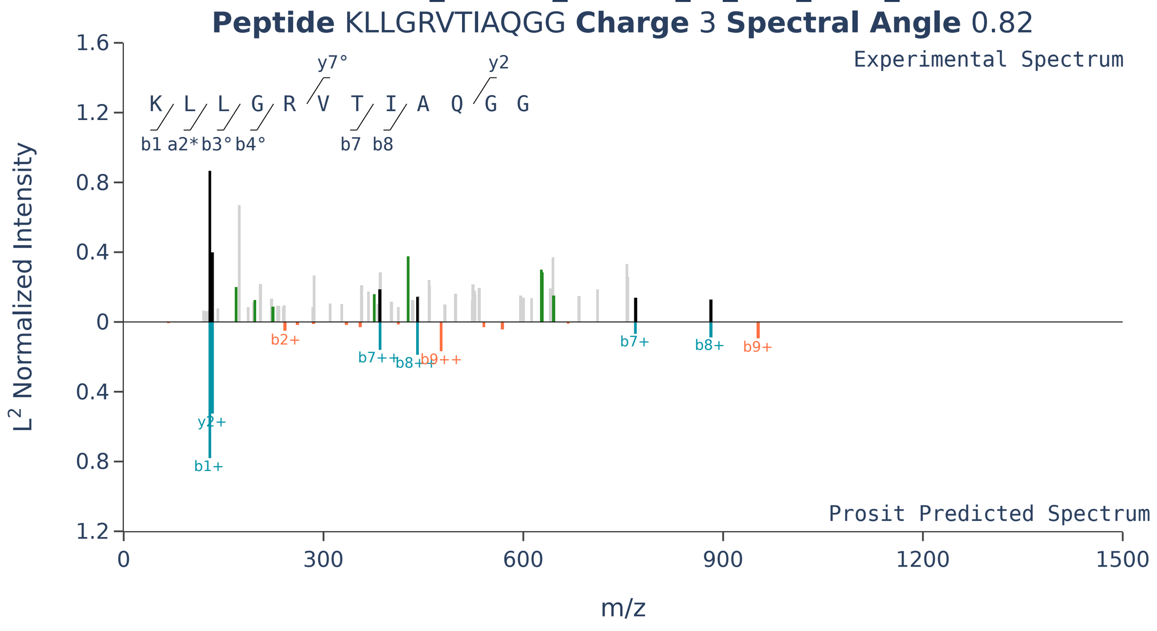

**Source** HRoetschke_291222_060123_Fu_H2A_b2R1_1h **Scan** 5767
**Peptide** LELAGNAARDN **Charge** 2 **Spectral Angle** 0.87

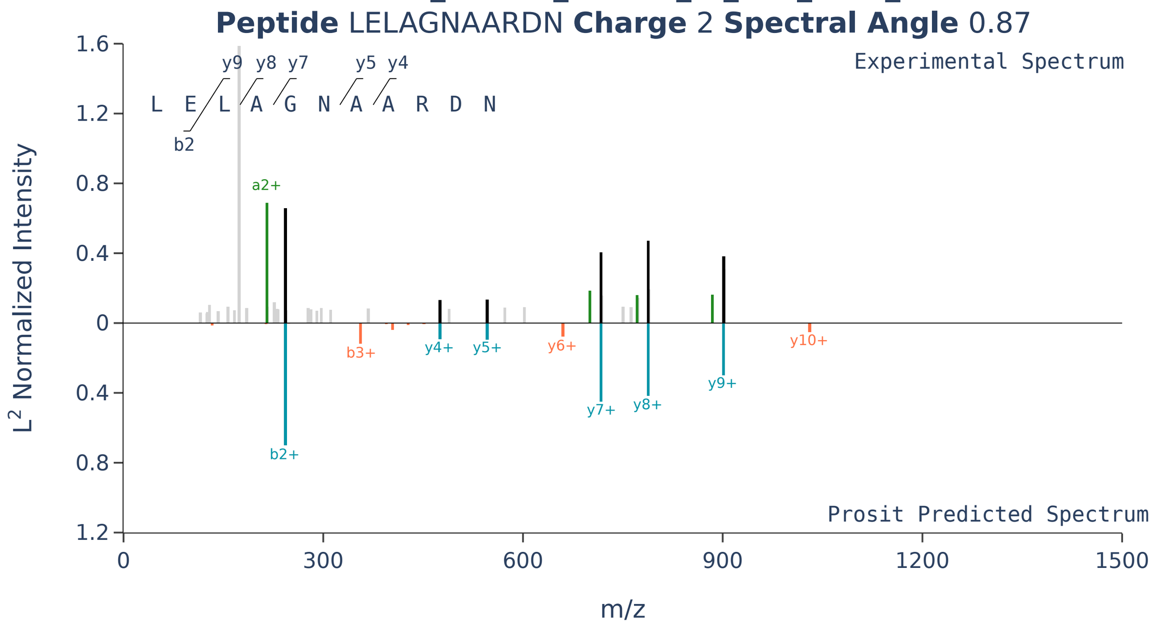


**Source** HRoetschke_291222_060123_Fu_H2A_b1R2_4h **Scan** 7080
**Peptide** RDNKKTRIIPRHLQLAVR **Charge** 5 **Spectral Angle** 0.8

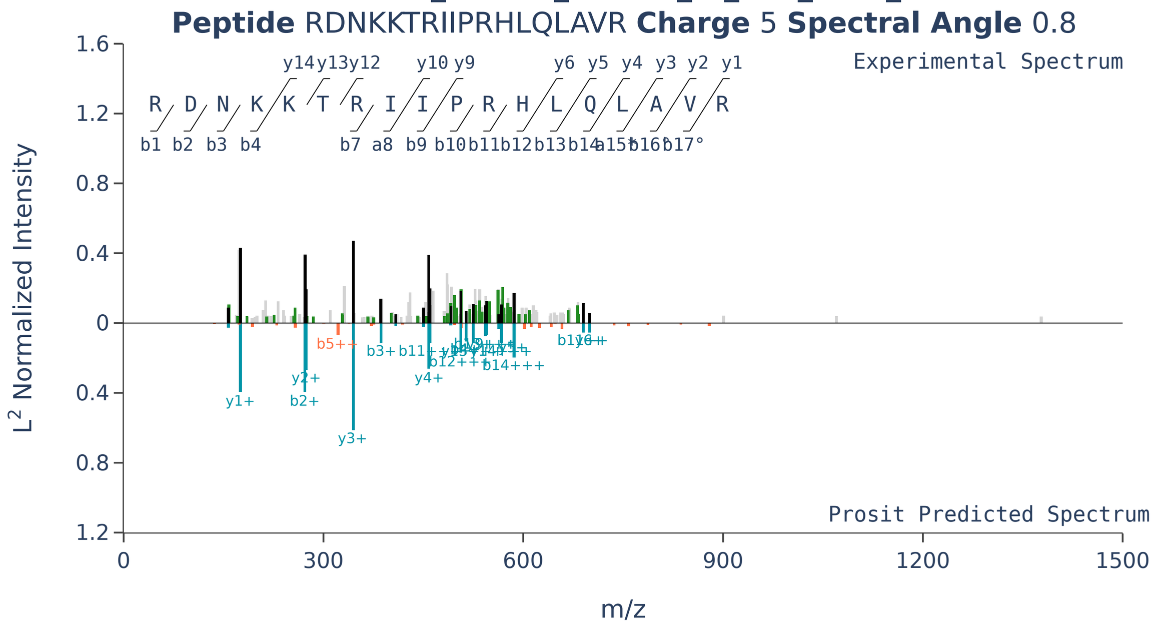


**Source** HRoetschke_291222_060123_Fu_H2A_b2R1_24h **Scan** 3744
**Peptide** AARDNKKTRIIPRHL **Charge** 3 **Spectral Angle** 0.69

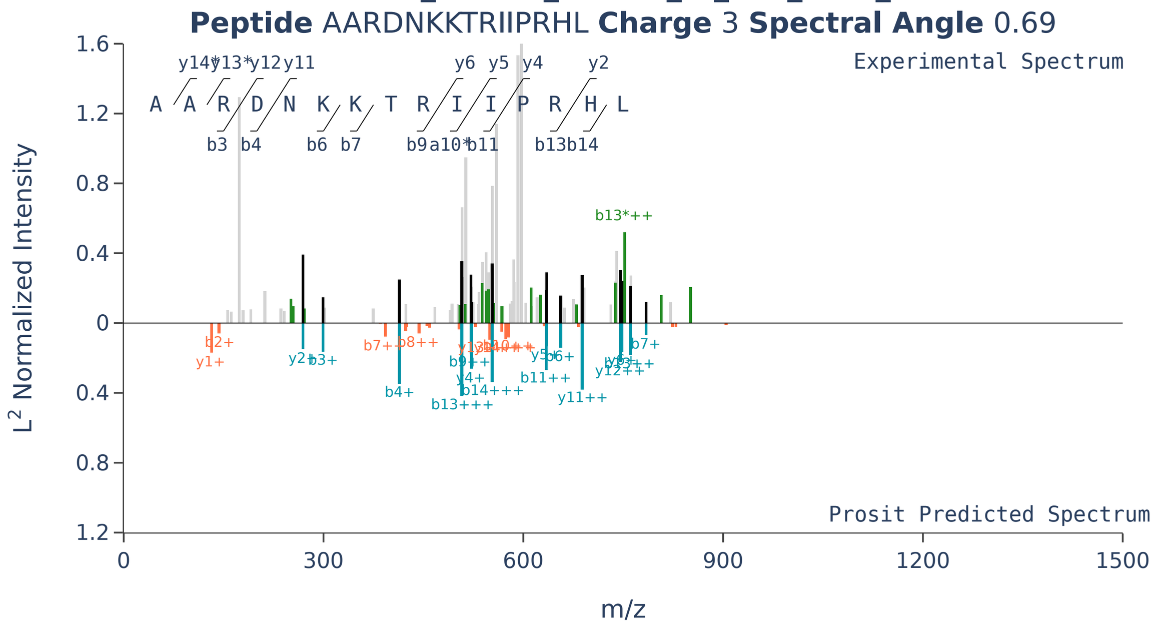


**Source** HRoetschke_291222_060123_Fu_H2A_b1R1_1h **Scan** 7301
**Peptide** RLLRKGNYAERVGAGAPVY **Charge** 4 **Spectral Angle** 0.68

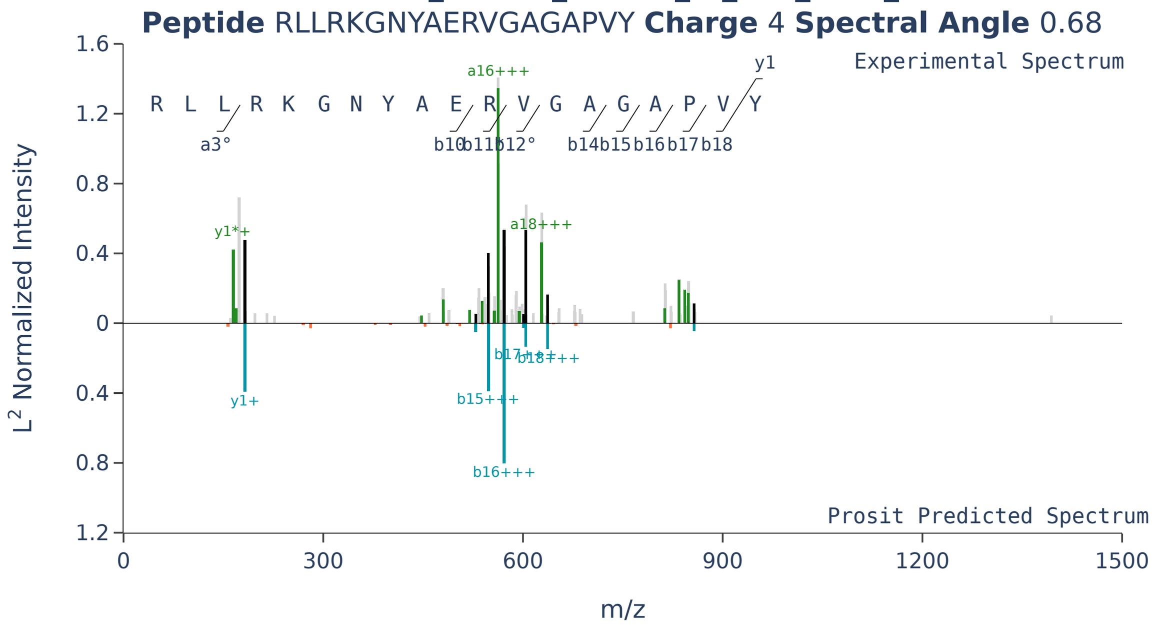


**Source** HRoetschke_291222_060123_Fu_H2A_b2R2_1h **Scan** 6716
**Peptide** TRSSRAGLQF **Charge** 3 **Spectral Angle** 0.77

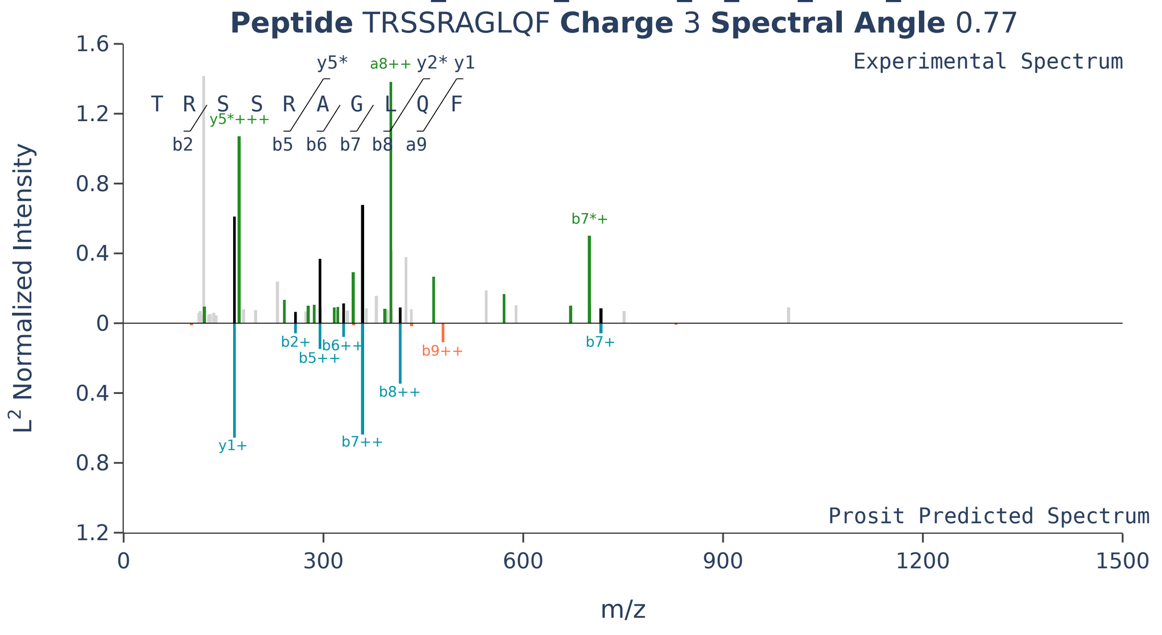


**Source** HRoetschke_291222_060123_Fu_H2A_b1R1_1h **Scan** 6179
**Peptide** LLELAGNAARDNKKT **Charge** 3 **Spectral Angle** 0.75

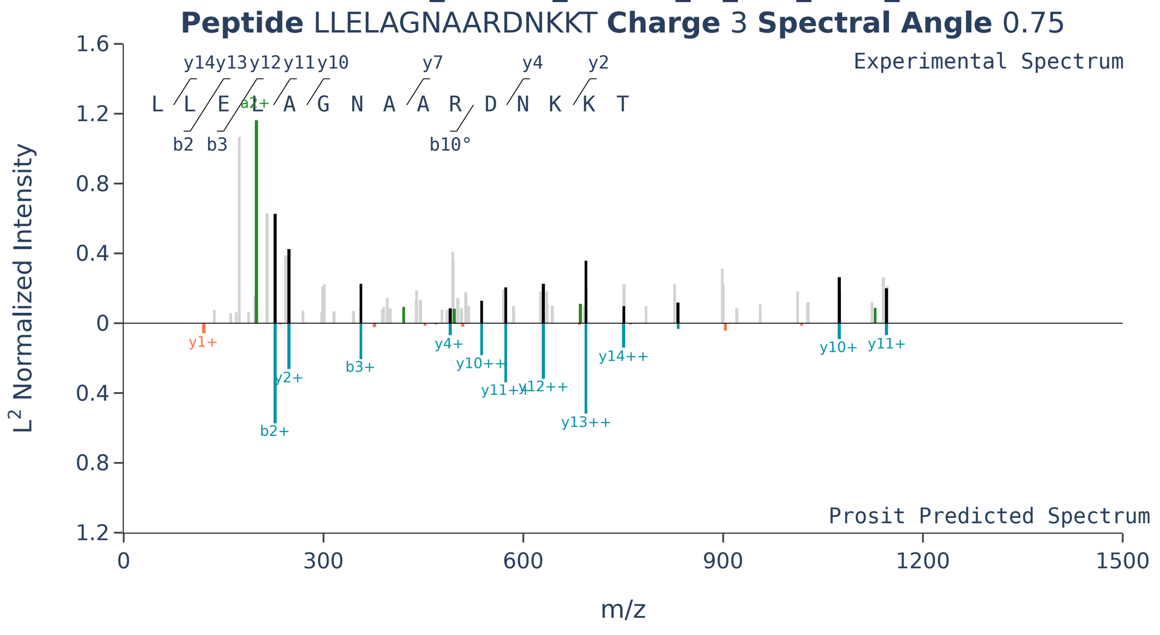


**Source** HRoetschke_291222_060123_Fu_H2A_b1R2_2h **Scan** 14152
**Peptide** GNYAERVGAGAPVYLAA **Charge** 2 **Spectral Angle** 0.83

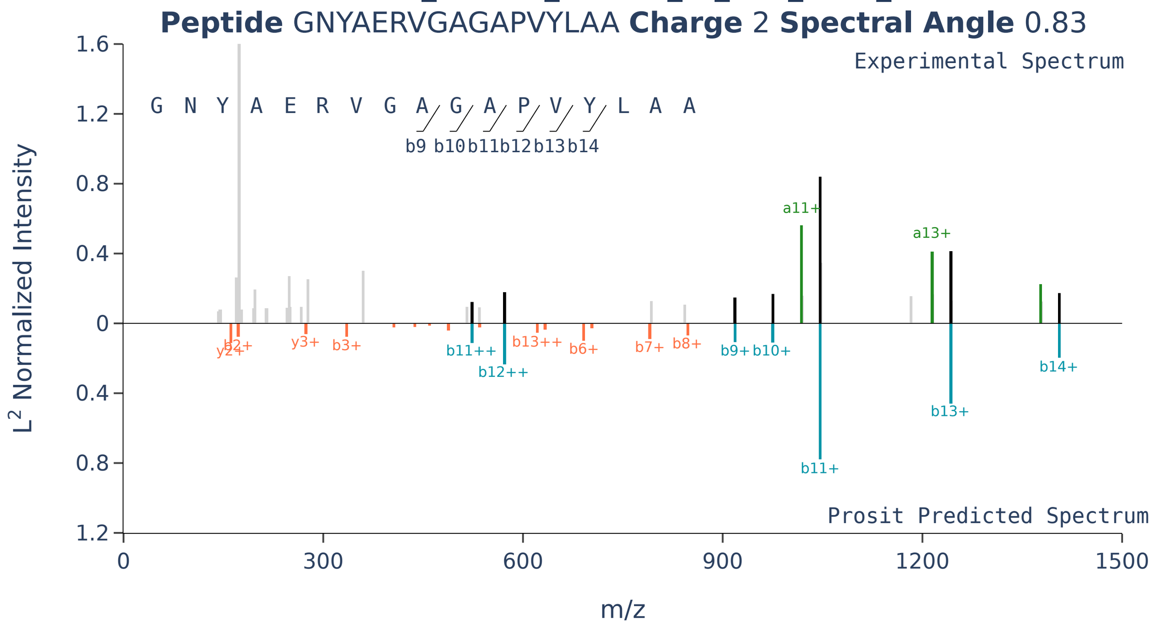


**Source** HRoetschke_291222_060123_Fu_H2A_b1R2_4h **Scan** 8905
**Peptide** QFPVGRVHRLLRKGNYA **Charge** 4 **Spectral Angle** 0.92

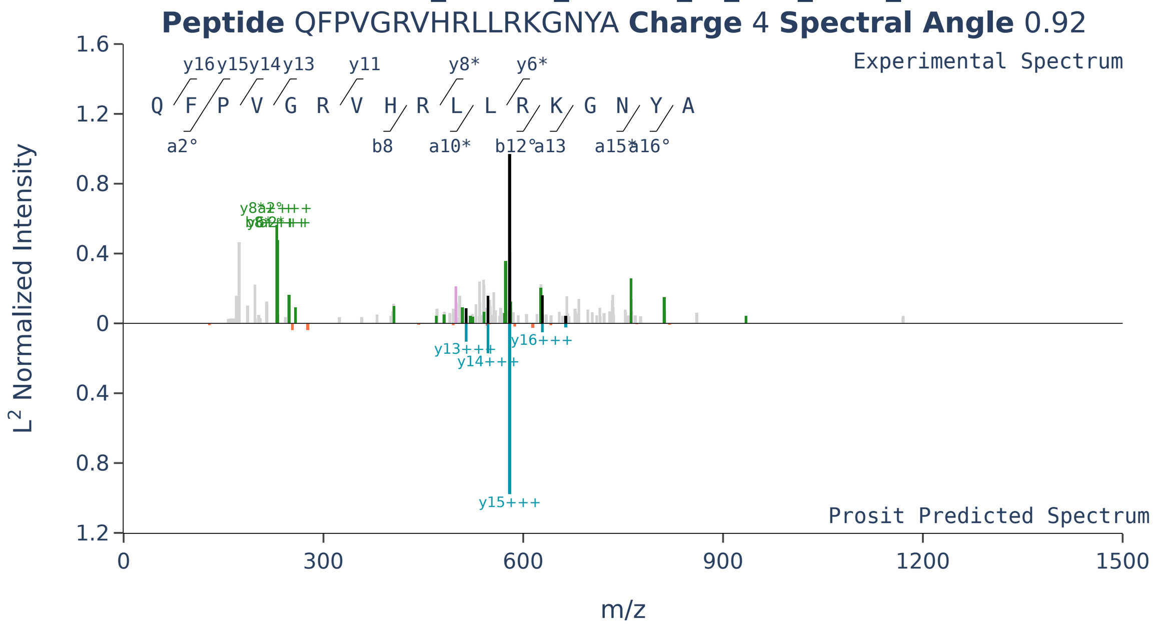


**Source** HRoetschke_291222_060123_Fu_H2A_b1R2_4h **Scan** 13221
**Peptide** AGLQFPVGRVHRLLRKGNYA **Charge** 5 **Spectral Angle** 0.81

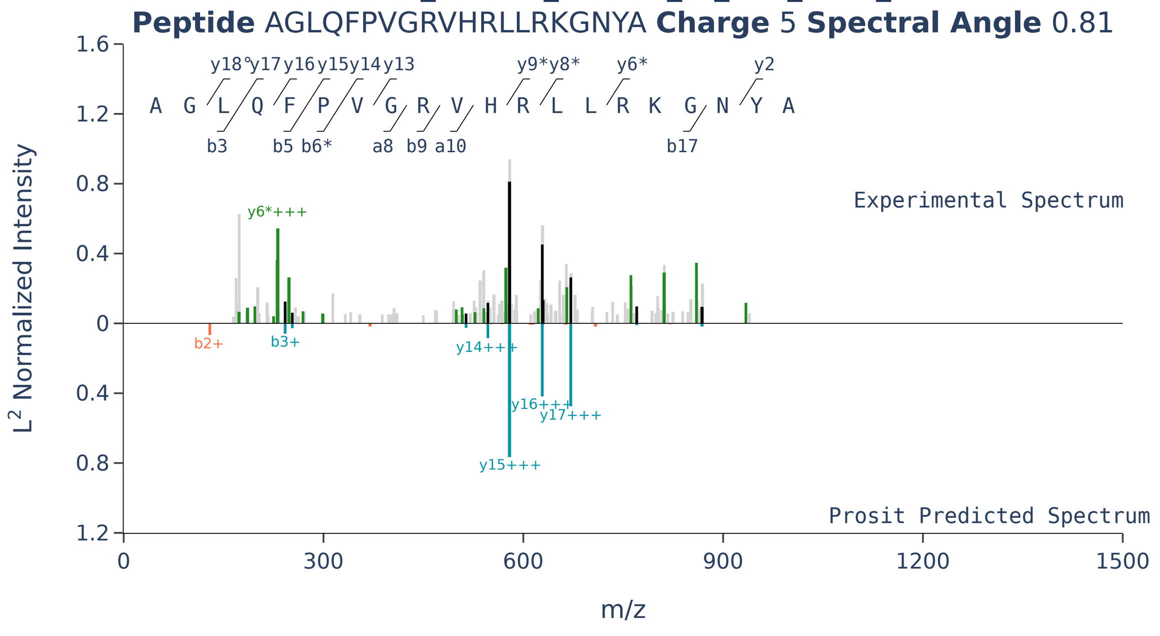


**Source** HRoetschke_291222_060123_Fu_H2A_b1R1_2h **Scan** 9156
**Peptide** RLLPRHLQLAVRNDE **Charge** 4 **Spectral Angle** 0.76

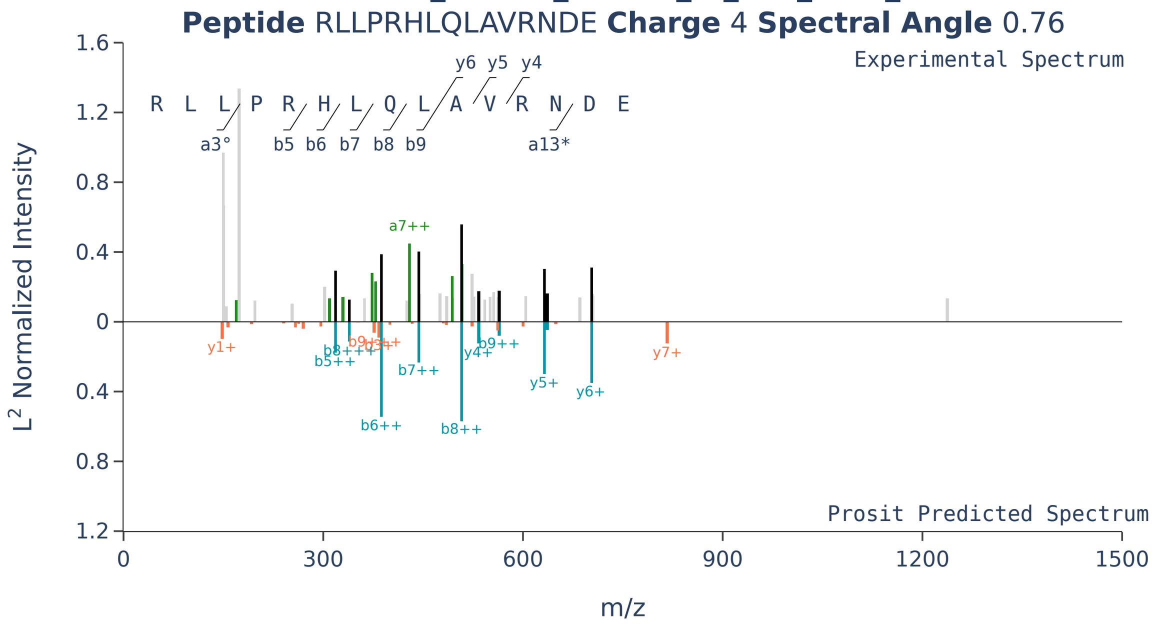


**Source** HRoetschke_291222_060123_Fu_H2A_b1R1_2h **Scan** 6780
**Peptide** RDNKKTRIIPRHLQLAVRND **Charge** 5 **Spectral Angle** 0.81

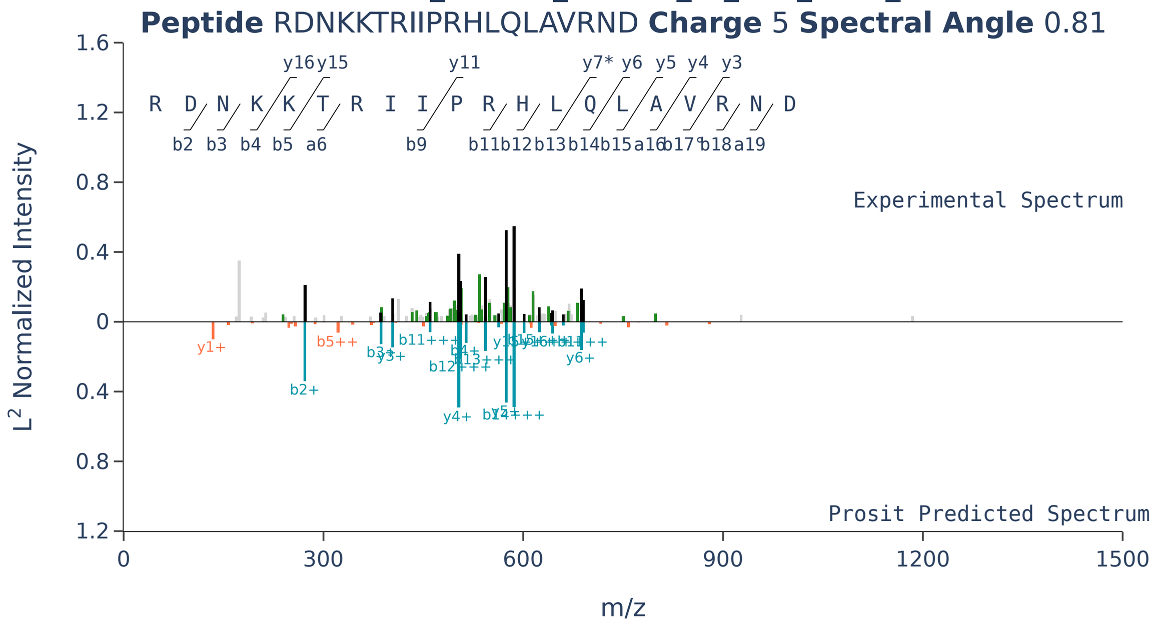


**Source** HRoetschke_291222_060123_Fu_H2A_b1R1_4h **Scan** 9312
**Peptide** TRSSRAGLQFPVGRVH **Charge** 4 **Spectral Angle** 0.72

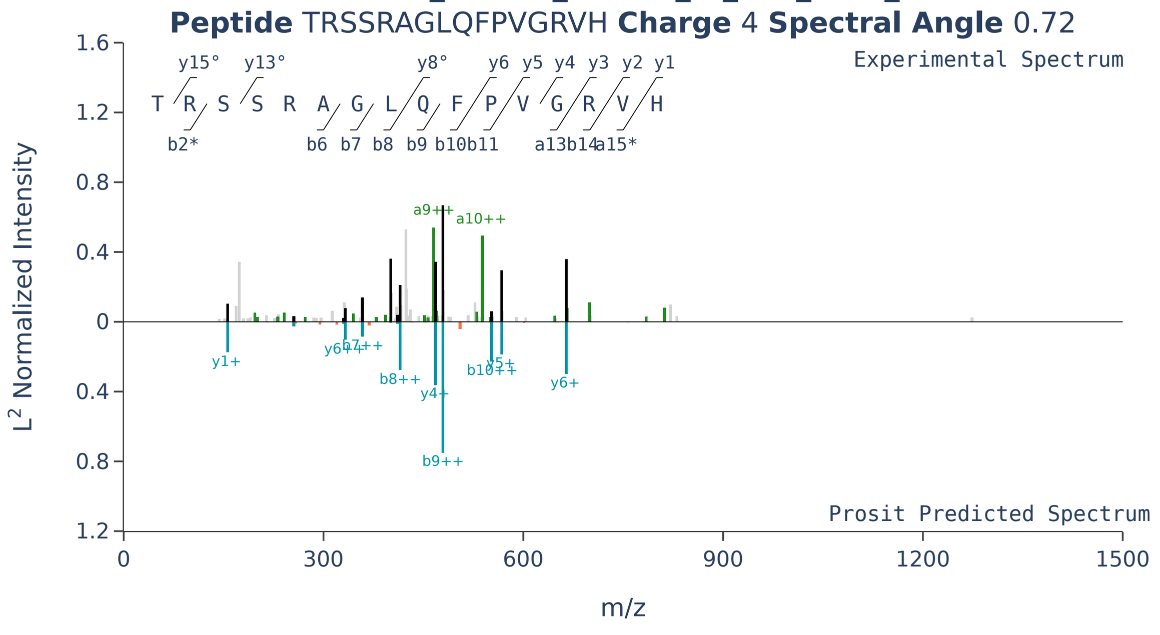


**Source** HRoetschke_291222_060123_Fu_H2A_b1R1_4h **Scan** 12525
**Peptide** AGLQFPVGRVHRLLRKG **Charge** 3 **Spectral Angle** 0.72

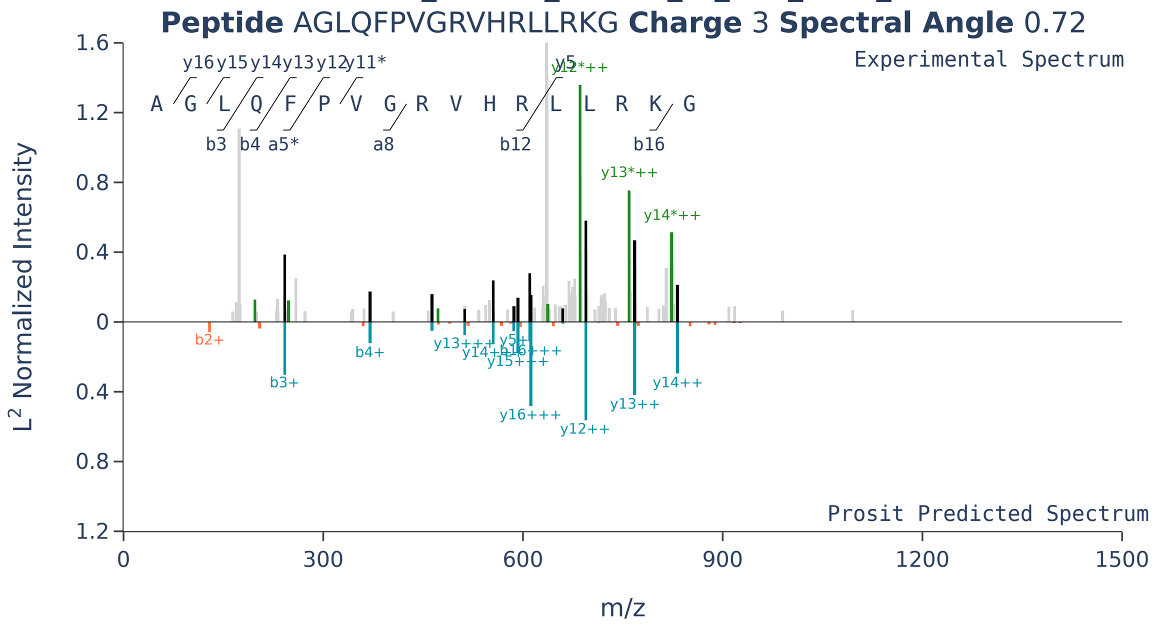


**Source** HRoetschke_291222_060123_Fu_H2A_b1R1_4h **Scan** 19194
**Peptide** AQGGVLPNIQSVLLPKKTES **Charge** 3 **Spectral Angle** 0.82

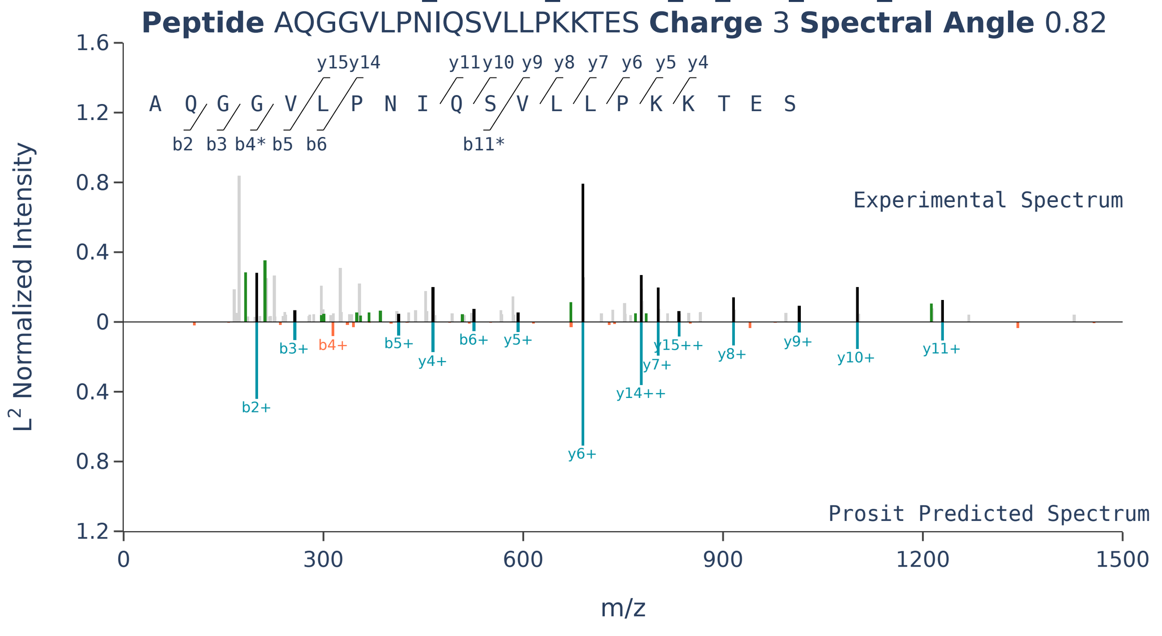


**Source** HRoetschke_291222_060123_Fu_H2A_b2R2_4h **Scan** 2095
**Peptide** LLPKKTESSKSA **Charge** 2 **Spectral Angle** 0.87

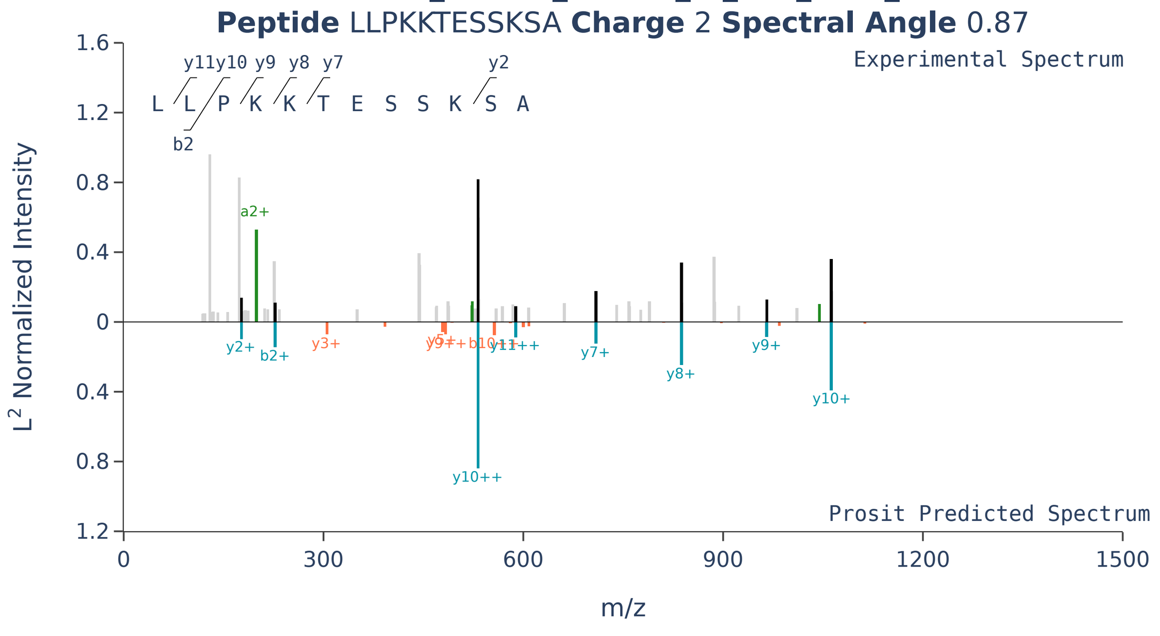

**Source** HRoetschke_291222_060123_Fu_H2A_b2R2_24h **Scan** 9208
**Peptide** RSSRAGLQFPVGR **Charge** 4 **Spectral Angle** 0.71

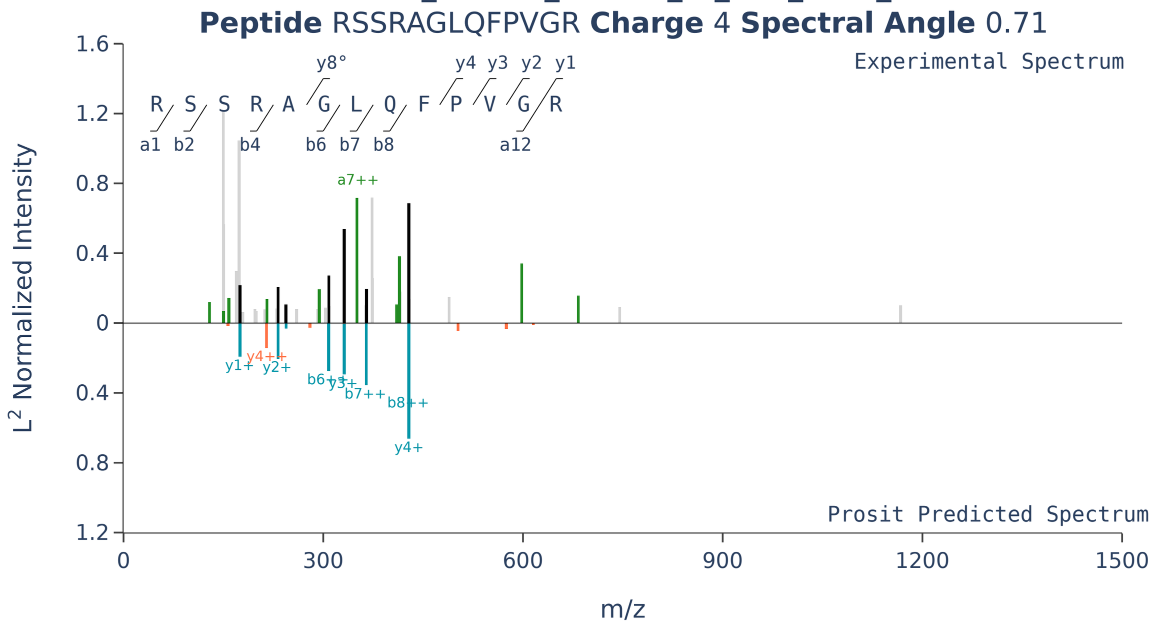


**Source** HRoetschke_291222_060123_Fu_H2A_b1R1_2h **Scan** 12446
**Peptide** GLQFPVGRVHRLLRKGNYA **Charge** 4 **Spectral Angle** 0.84

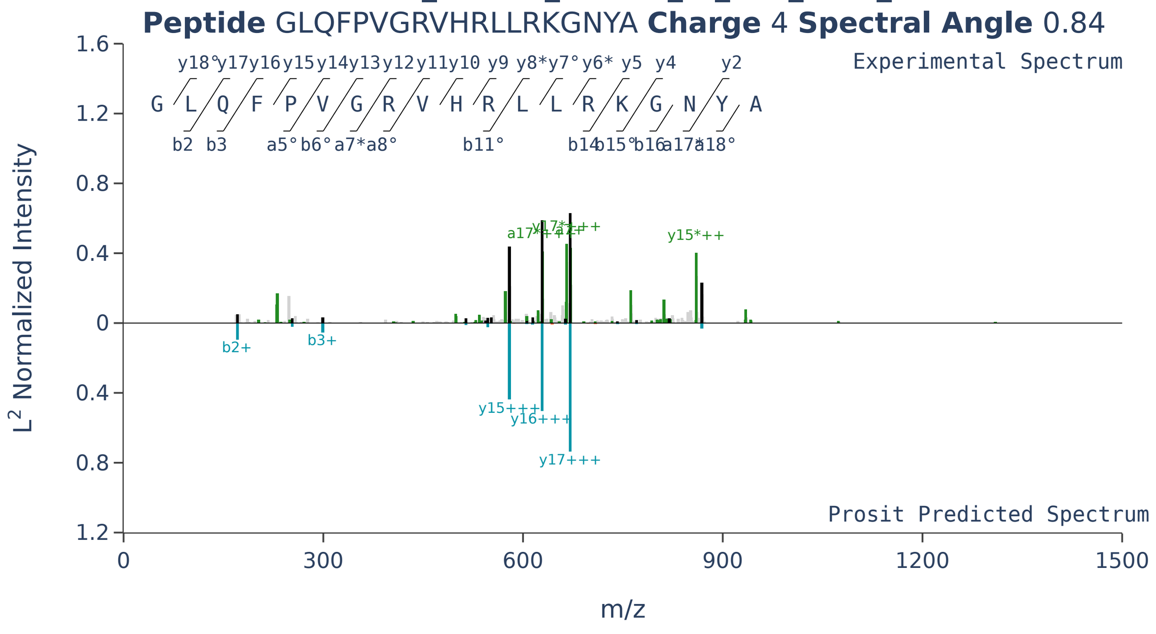


**Source** HRoetschke_291222_060123_Fu_H2A_b1R1_4h **Scan** 12023
**Peptide** RLLRKGNYAERVGAGAPVYL **Charge** 3 **Spectral Angle** 0.68

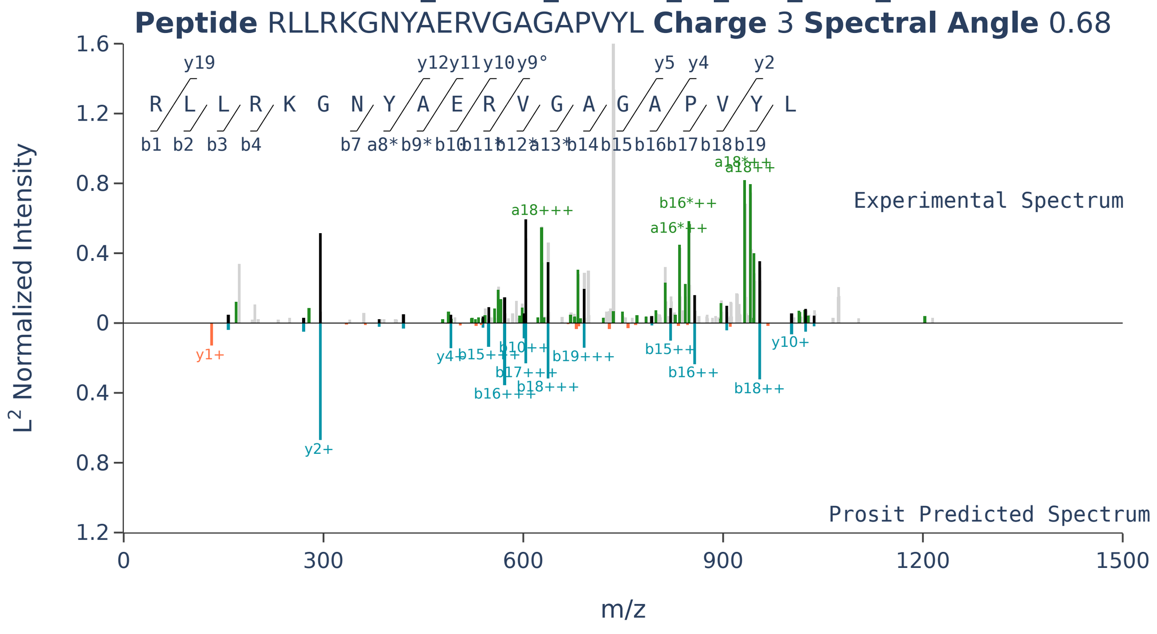


**Source** HRoetschke_291222_060123_Fu_H2A_b1R1_4h **Scan** 13147
**Peptide** LLELAGNAARDNKKTRIIPRHLQL **Charge** 5 **Spectral Angle** 0.81

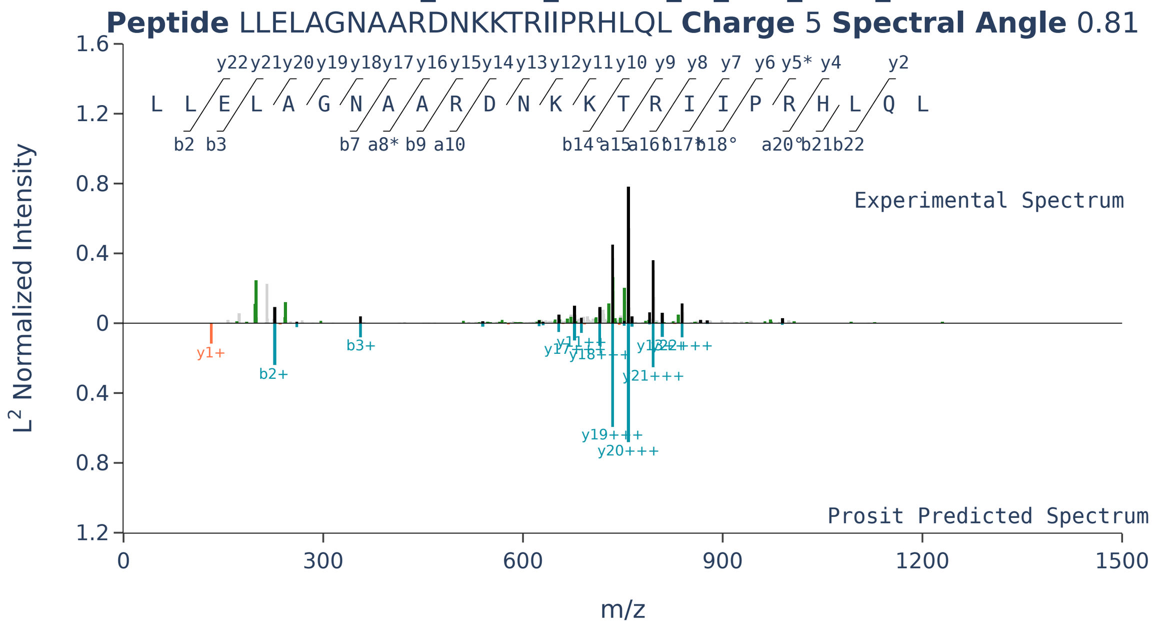


**Source** HRoetschke_291222_060123_Fu_H2A_b1R2_4h **Scan** 12792
**Peptide** GLQFPVGRVHRLLRKGNYAE **Charge** 4 **Spectral Angle** 0.82

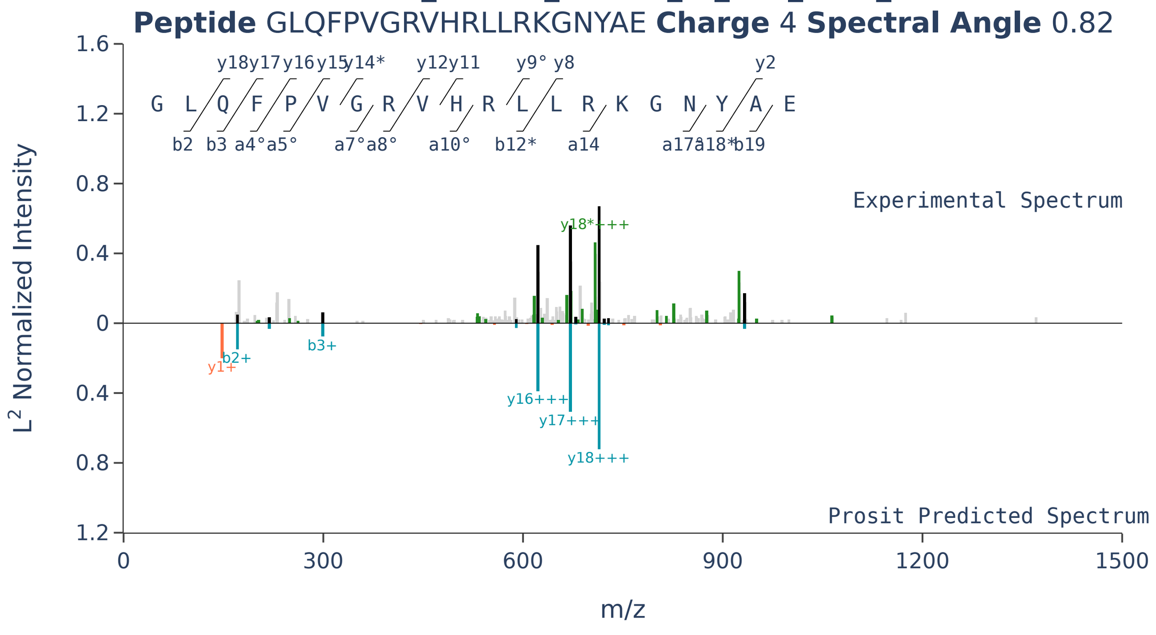


**Source** HRoetschke_291222_060123_Fu_H2A_b2R1_2h **Scan** 9617
**Peptide** HRLLRKGNYAERVGAGAPVYLA **Charge** 4 **Spectral Angle** 0.74

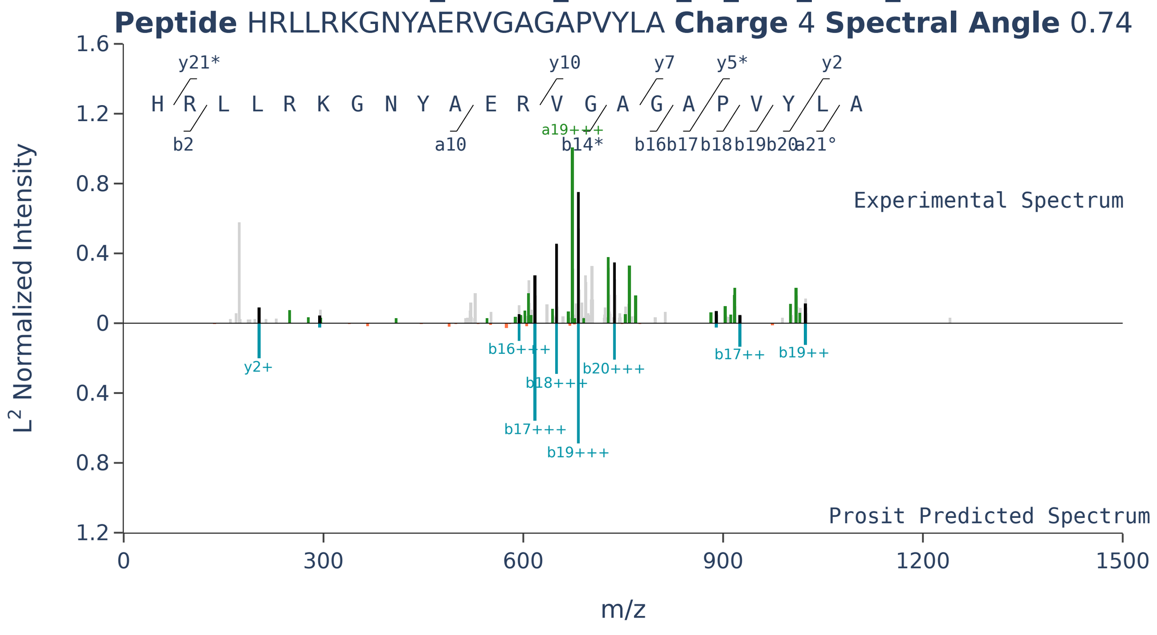


**Source** HRoetschke_291222_060123_Fu_H2A_b1R2_1h **Scan** 6462
**Peptide** HRLLRKGNYAERVGAGAPVY **Charge** 4 **Spectral Angle** 0.77

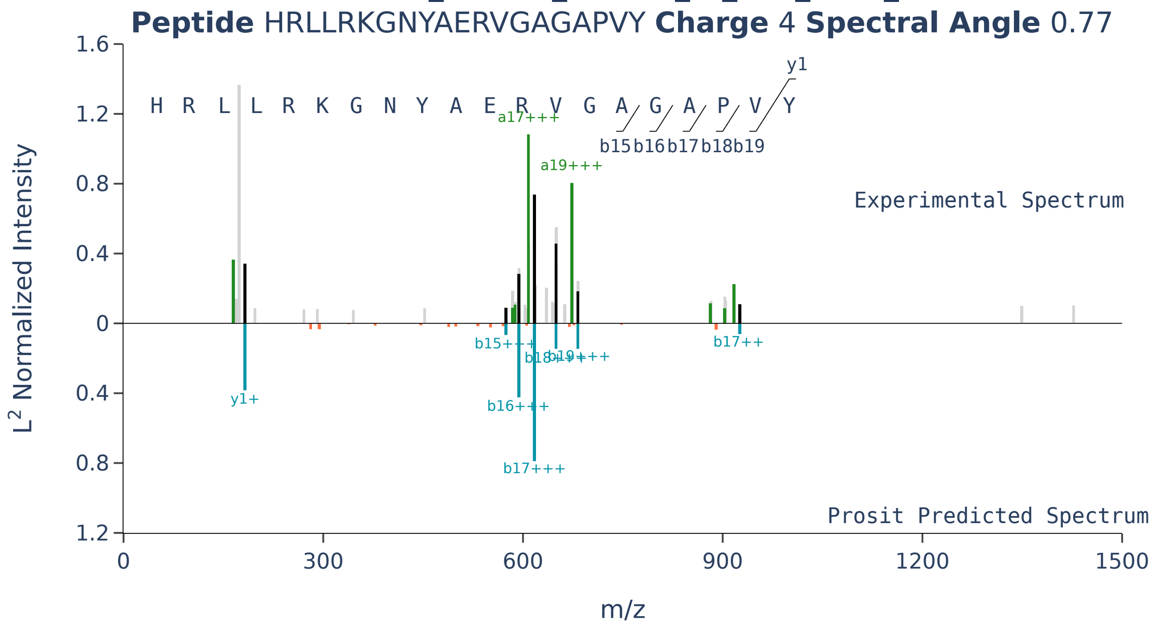


**Source** HRoetschke_291222_060123_Fu_H2A_b1R2_4h **Scan** 16188
**Peptide** RNDEELNKLLGRVTIAQ **Charge** 3 **Spectral Angle** 0.69

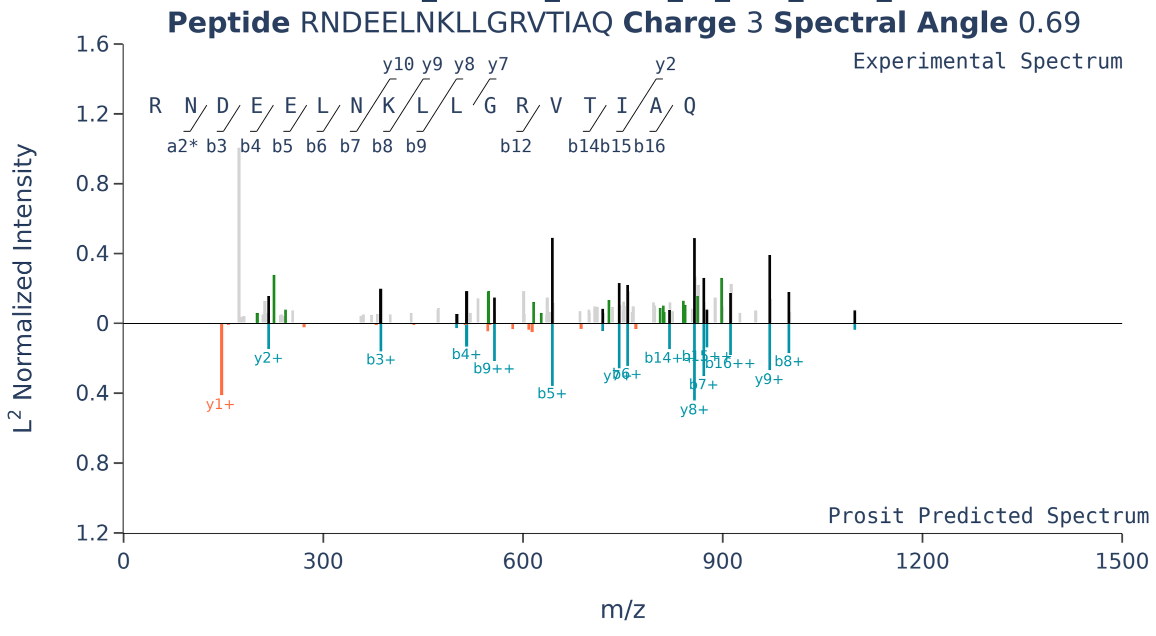


**Source** HRoetschke_291222_060123_Fu_H2A_b1R1_4h **Scan** 18514
**Peptide** AVRNDEELNKLLGRVTI **Charge** 3 **Spectral Angle** 0.86

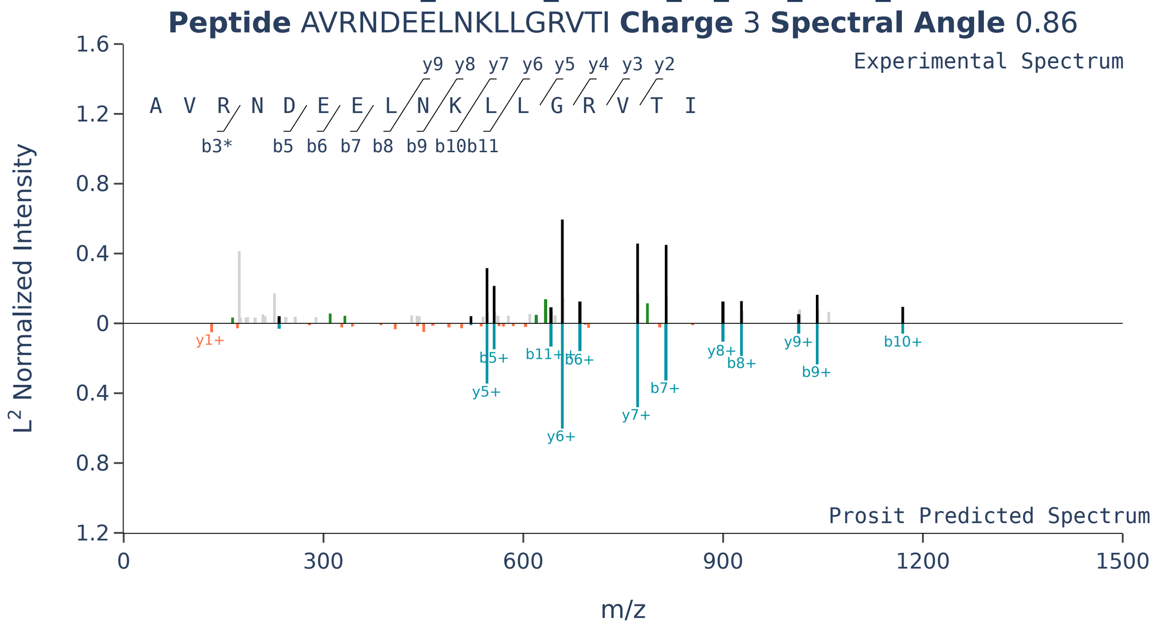


**Source** HRoetschke_291222_060123_Fu_H2A_b1R1_4h **Scan** 7324
**Peptide** ARDNKKTRIIPRHLQL **Charge** 4 **Spectral Angle** 0.72

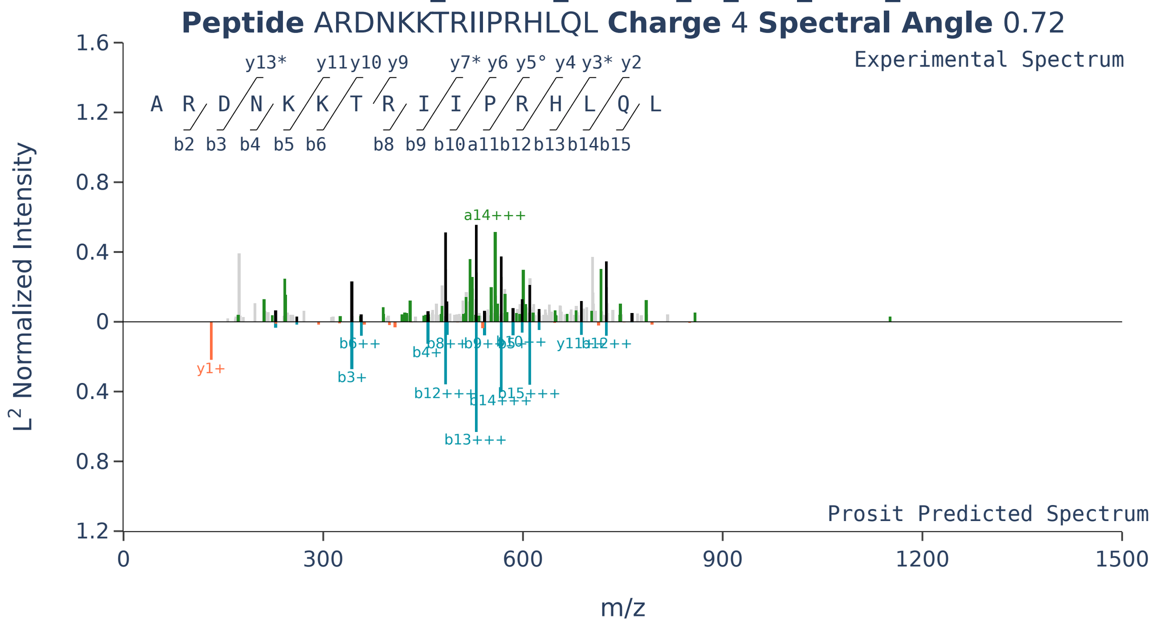


**Source** HRoetschke_291222_060123_Fu_H2A_b1R2_1h **Scan** 7491
**Peptide** KAKTRSSRAGLQFPVG **Charge** 3 **Spectral Angle** 0.82

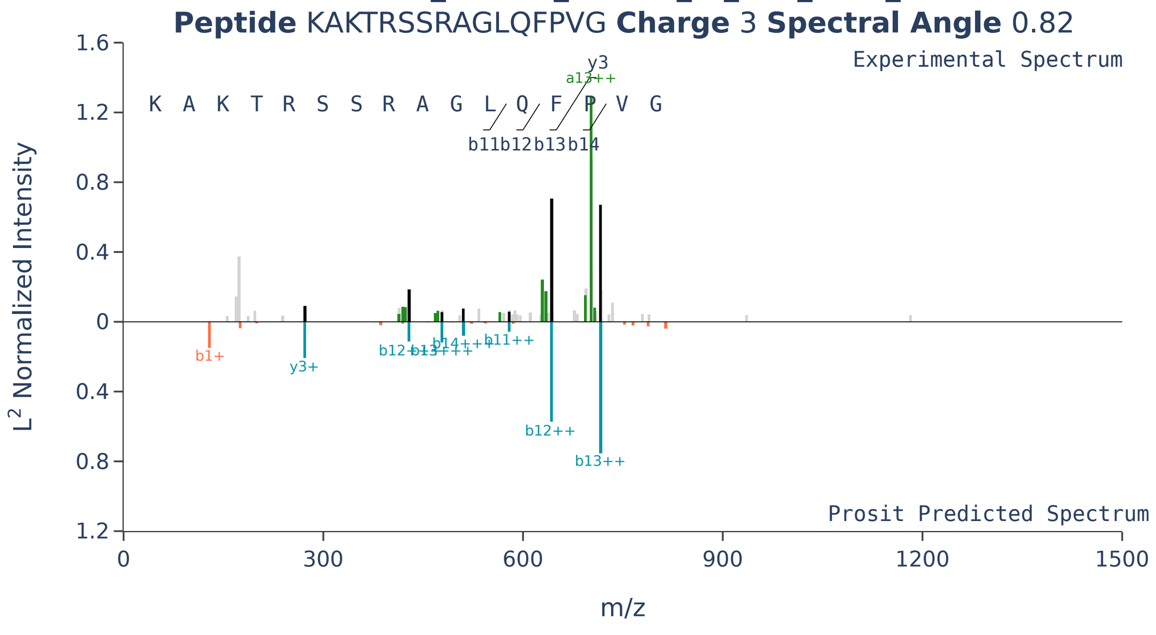


**Source** HRoetschke_291222_060123_Fu_H2A_b1R2_4h **Scan** 14749
**Peptide** LAQGGVLPNIQS **Charge** 2 **Spectral Angle** 0.74

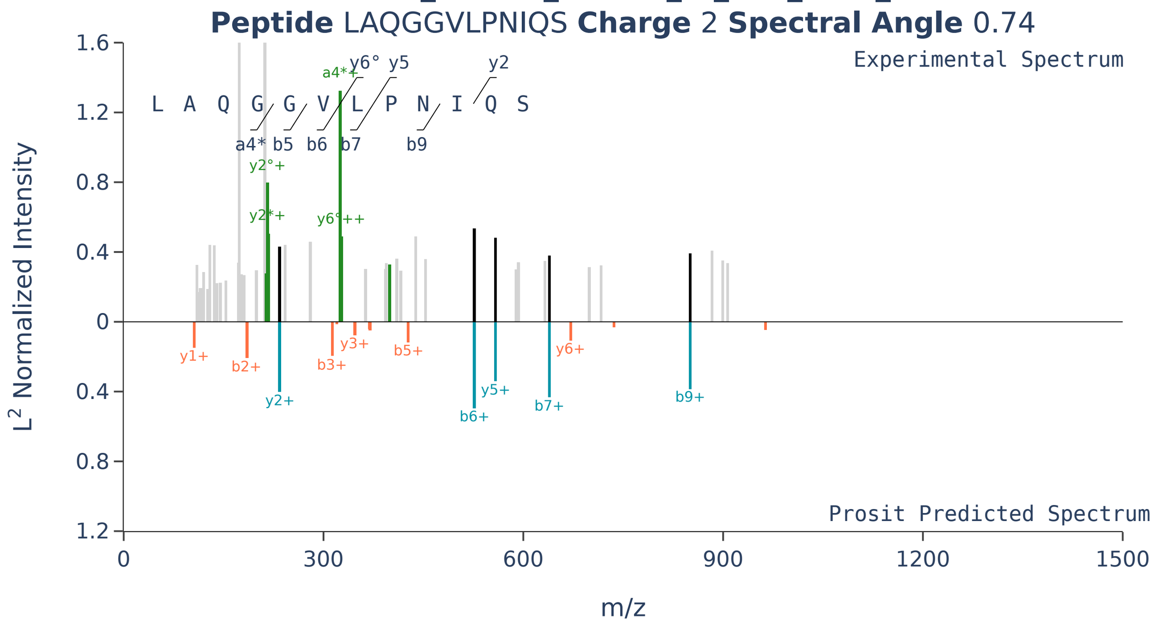


**Source** HRoetschke_291222_060123_Fu_H2A_b1R1_4h **Scan** 12932
**Peptide** ELLELAGNAARDNKKTRII **Charge** 3 **Spectral Angle** 0.78

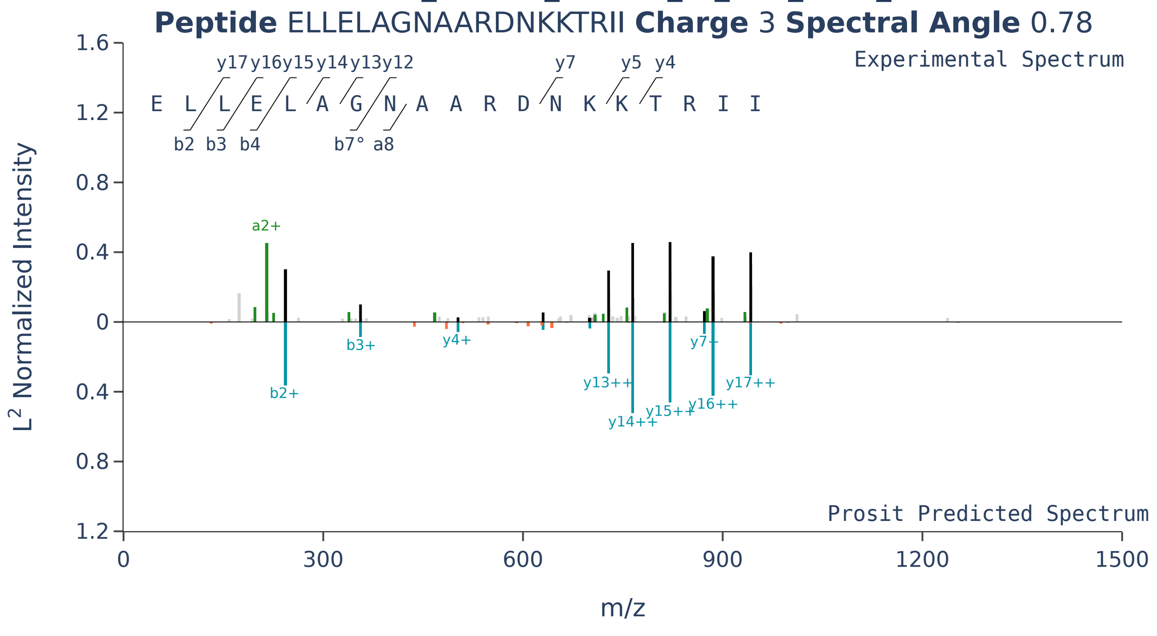


**Source** HRoetschke_291222_060123_Fu_H2A_b2R2_2h **Scan** 6785
**Peptide** AGNAARDNKKTRIIPRHLQL **Charge** 4 **Spectral Angle** 0.84

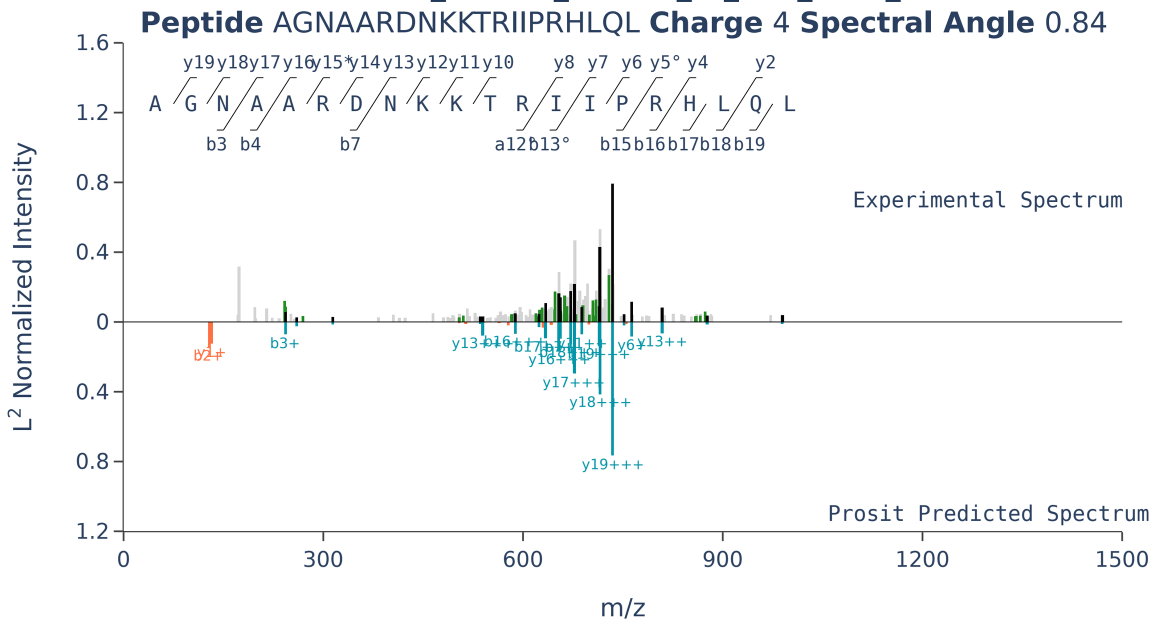


**Source** HRoetschke_291222_060123_Fu_H2A_b2R2_4h **Scan** 2599
**Peptide** LLRKGNYAER **Charge** 4 **Spectral Angle** 0.85

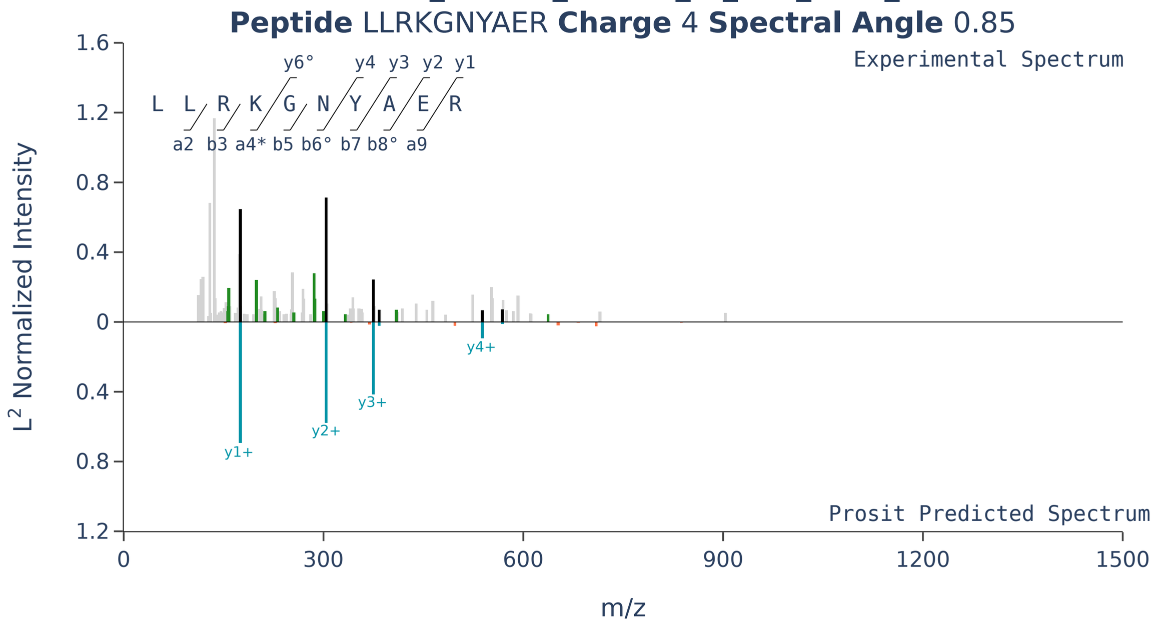

**Source** HRoetschke_291222_060123_Fu_H2A_b2R1_4h **Scan** 10490
**Peptide** HRLLRKGNYAERVGAGAPVYL **Charge** 4 **Spectral Angle** 0.6

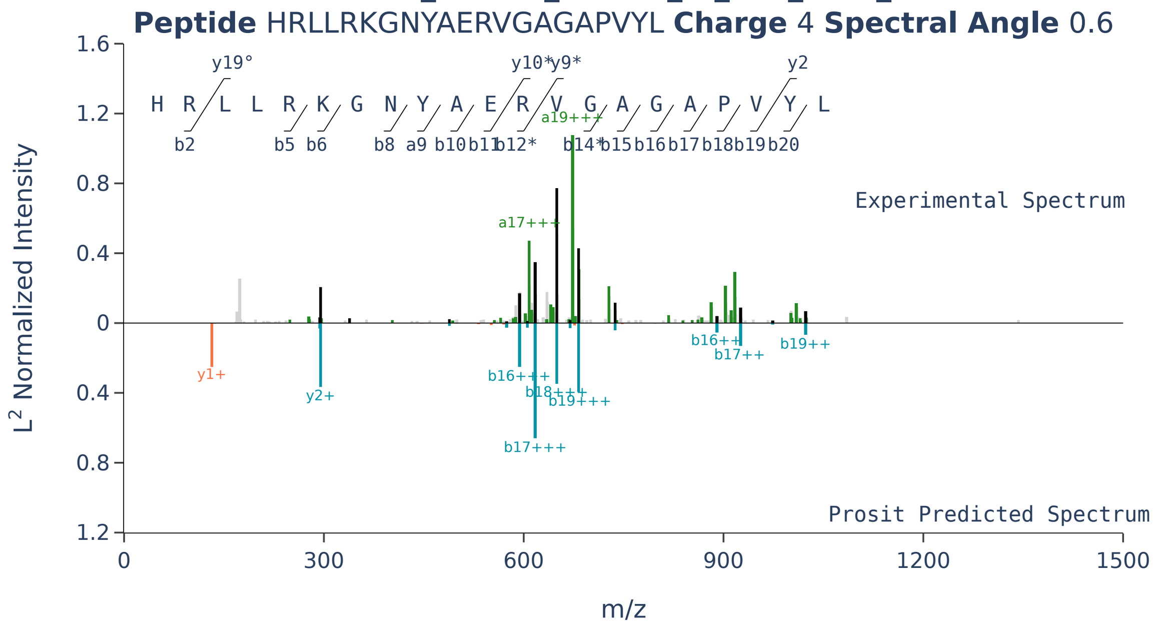


**Source** HRoetschke_291222_060123_Fu_H2A_b1R1_4h **Scan** 12651
**Peptide** RAGLQFPVGRVHRLLR **Charge** 4 **Spectral Angle** 0.83

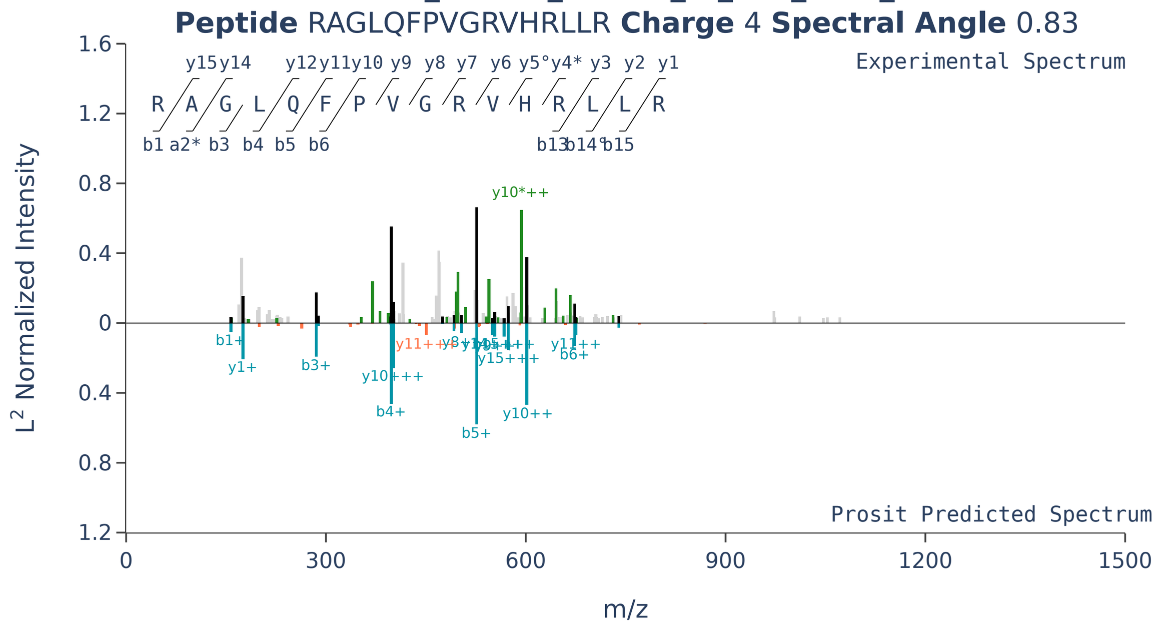


**Source** HRoetschke_291222_060123_Fu_H2A_b1R1_4h **Scan** 10143
**Peptide** LAGNAARDNKKTRIIPRHLQLAV **Charge** 4 **Spectral Angle** 0.78

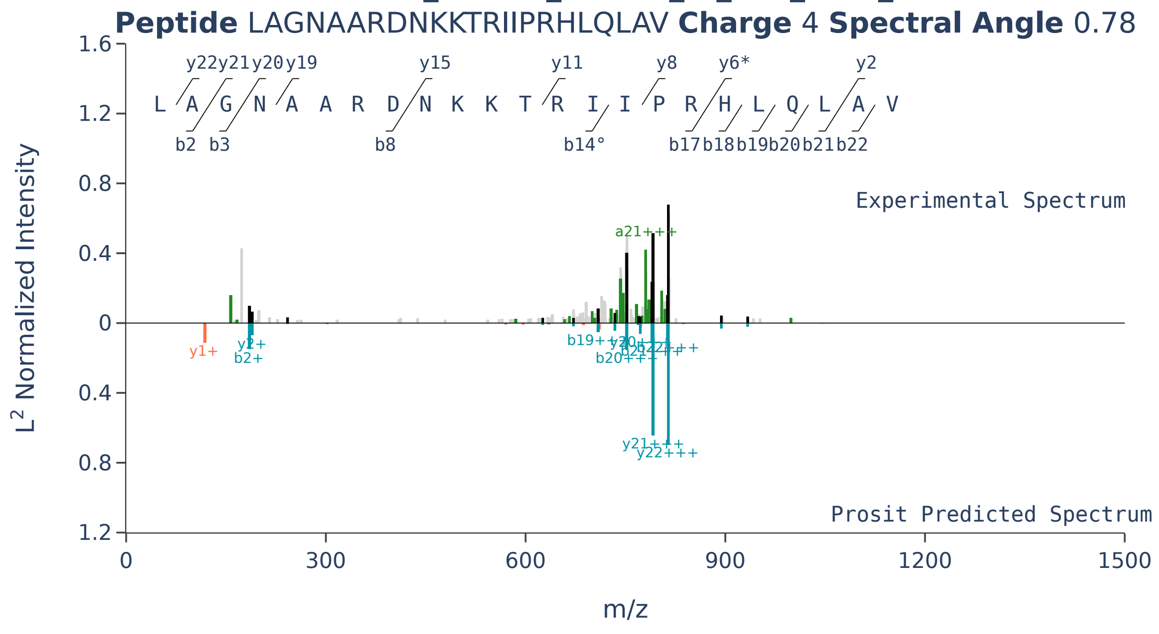


**Source** HRoetschke_291222_060123_Fu_H2A_b1R1_4h **Scan** 16063
**Peptide** TAEILELAGNAARDNKKTRLL **Charge** 4 **Spectral Angle** 0.73

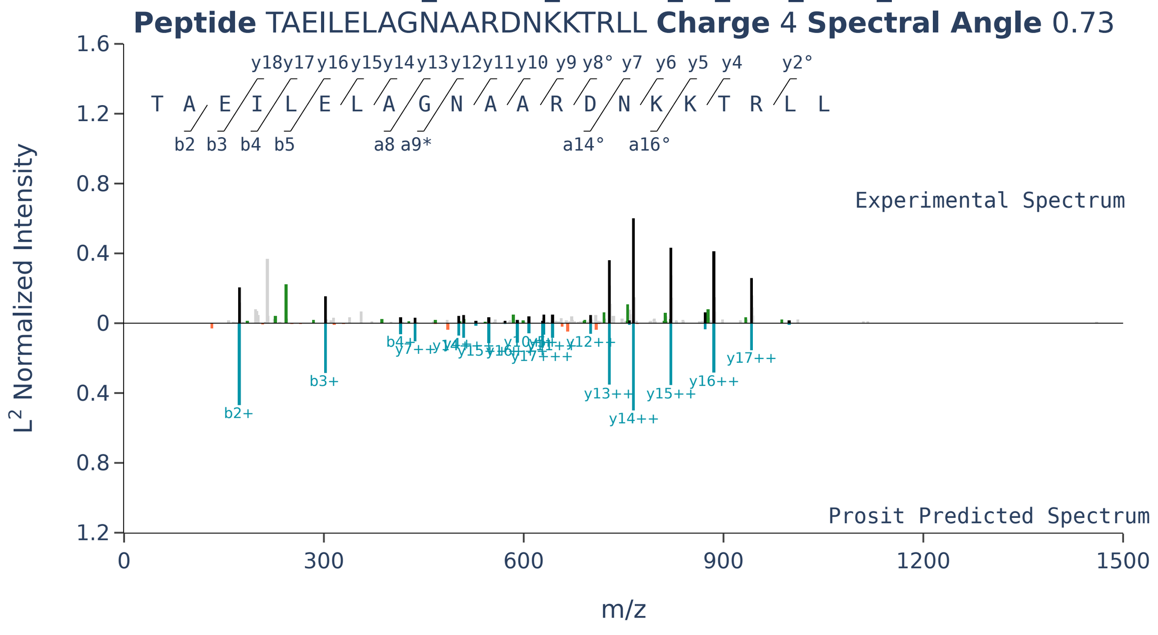


**Source** HRoetschke_291222_060123_Fu_H2A_b2R2_4h **Scan** 8471
**Peptide** AKAKTRSSRAGLQFPVG **Charge** 3 **Spectral Angle** 0.65

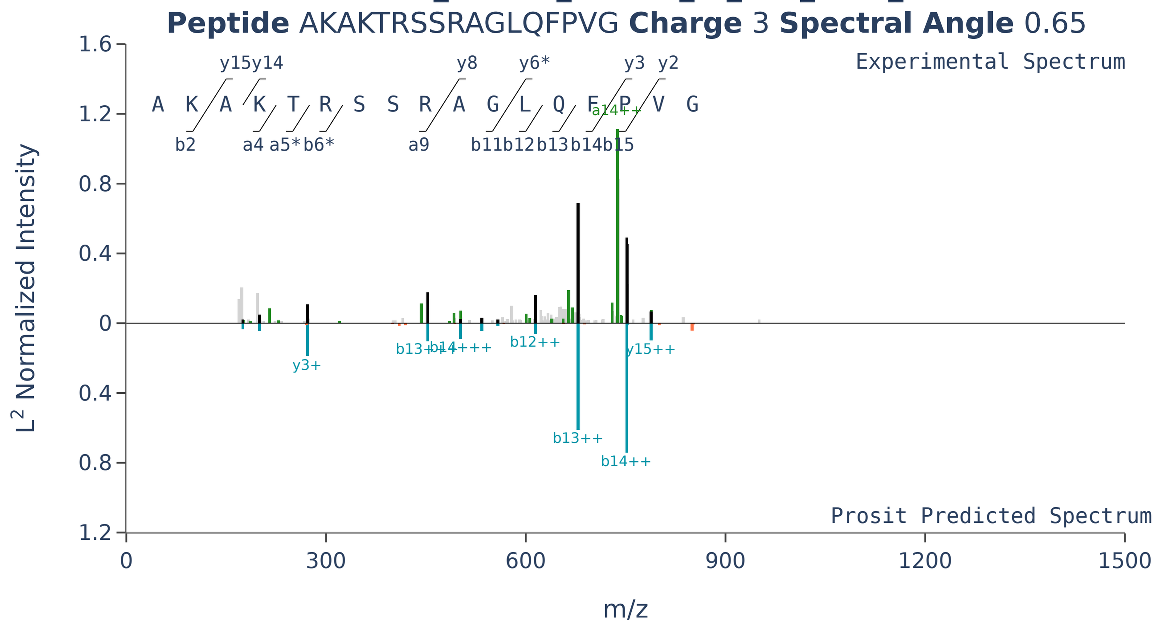


**Source** HRoetschke_291222_060123_Fu_H2A_b1R1_4h **Scan** 6851
**Peptide** NAARDNKKTRIIPRHLQLA **Charge** 4 **Spectral Angle** 0.78

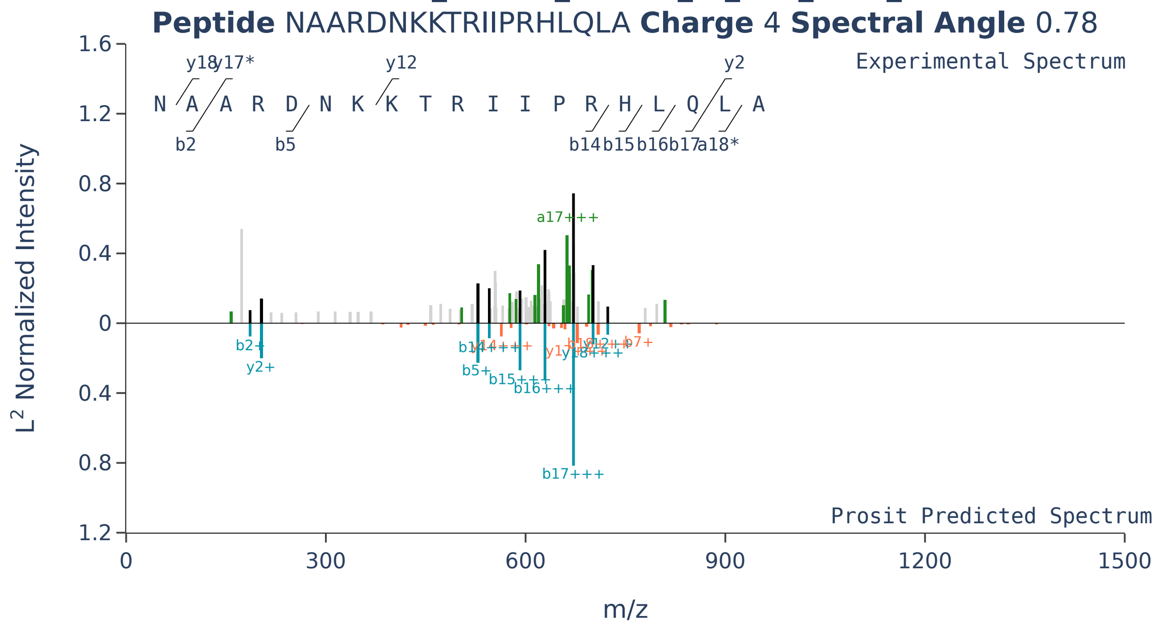


**Source** HRoetschke_291222_060123_Fu_H2A_b1R1_1h **Scan** 2647
**Peptide** LLPKKTE **Charge** 2 **Spectral Angle** 0.83

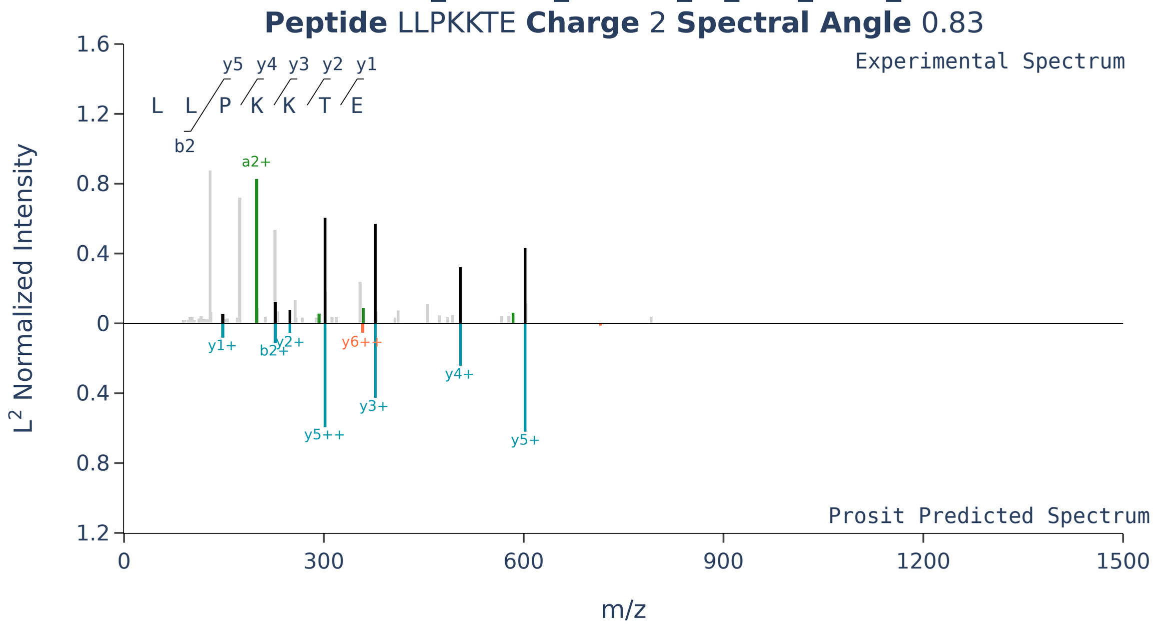


**Source** HRoetschke_291222_060123_Fu_H2A_b1R1_4h **Scan** 12142
**Peptide** LQFPVGRVHRLLRKGNYAERVG **Charge** 4 **Spectral Angle** 0.78

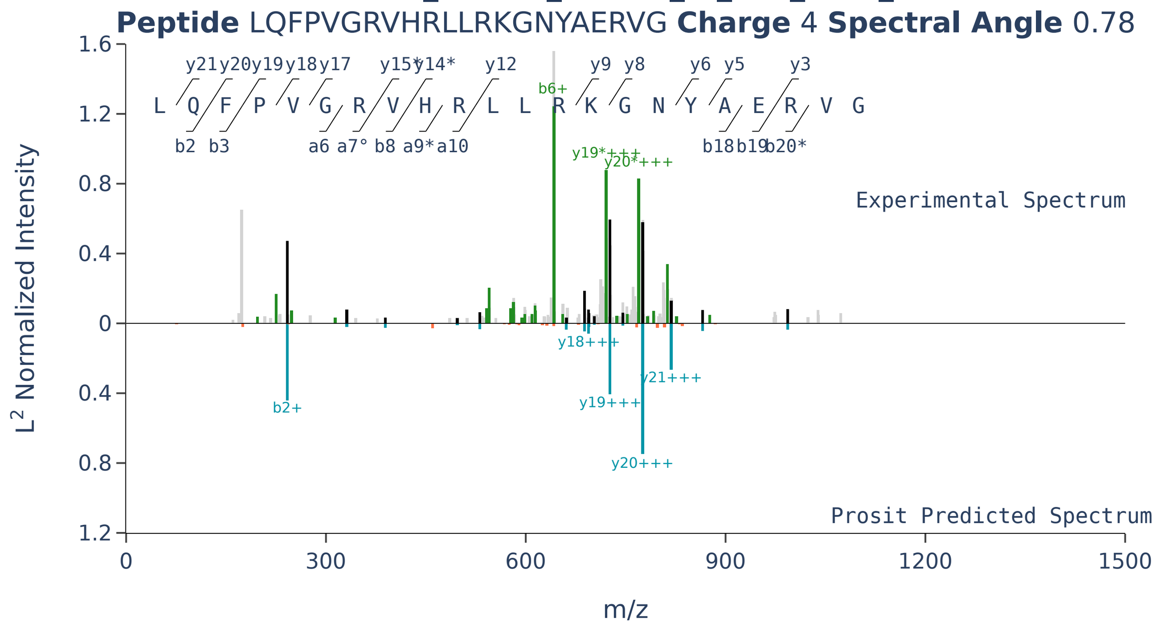


**Source** HRoetschke_291222_060123_Fu_H2A_b2R1_2h **Scan** 3567
**Peptide** LRKGNYAERVGAGA **Charge** 3 **Spectral Angle** 0.77

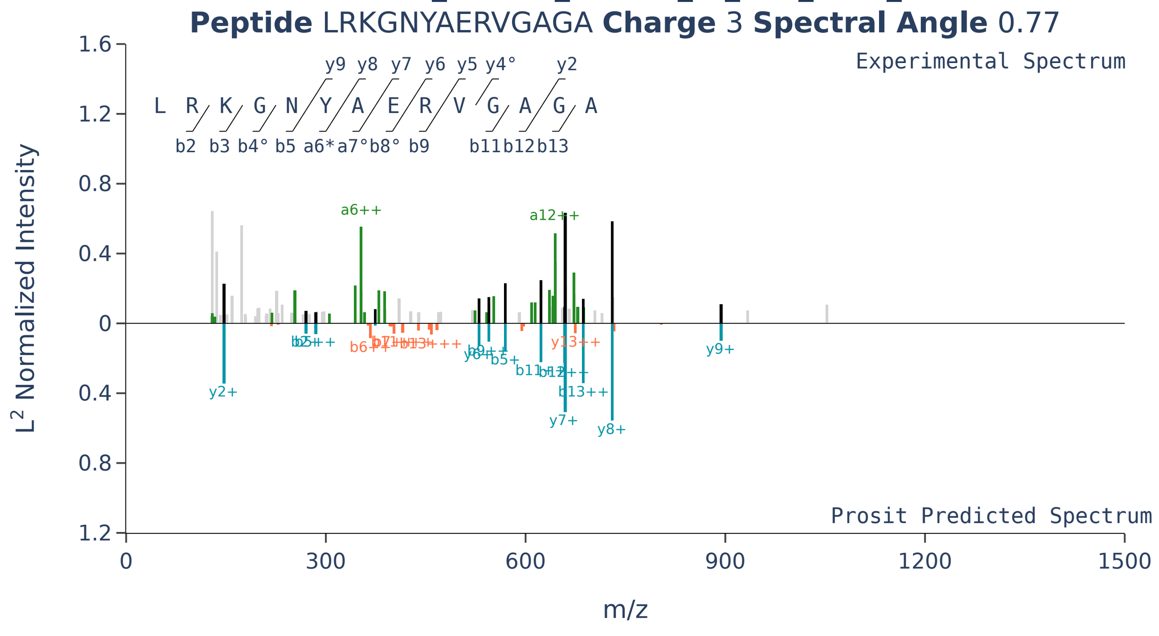


**Source** HRoetschke_291222_060123_Fu_H2A_b1R2_24h **Scan** 4104
**Peptide** LELAGNAARDNKKT **Charge** 2 **Spectral Angle** 0.79

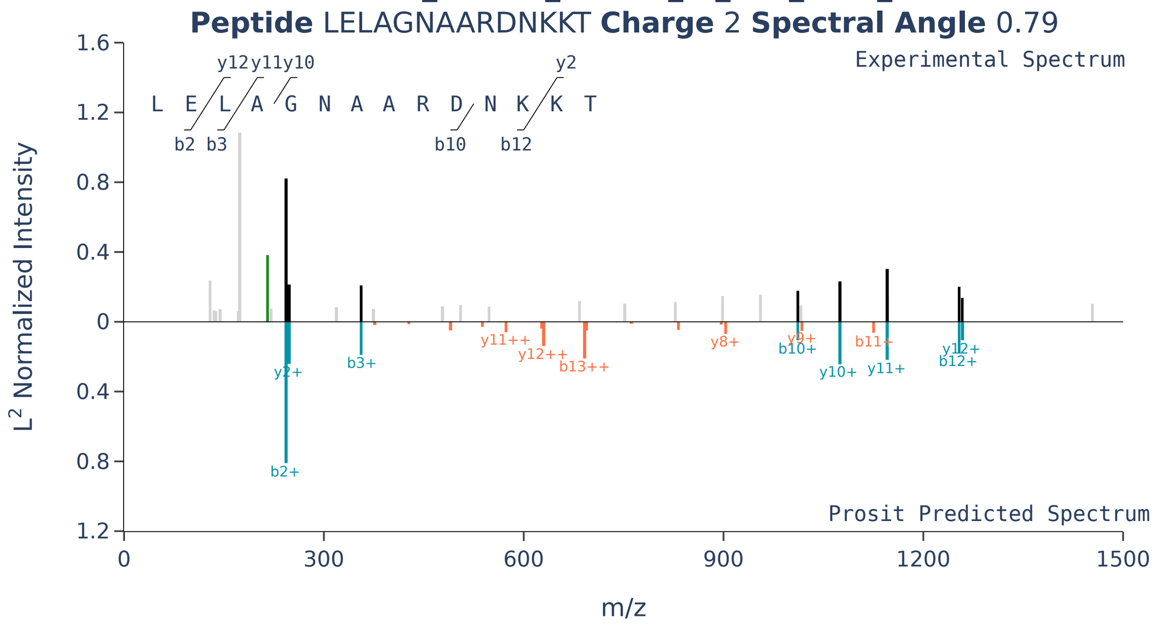


**Source** HRoetschke_291222_060123_Fu_H2A_b2R1_2h **Scan** 3136
**Peptide** LRKGNYAERVGAG **Charge** 3 **Spectral Angle** 0.73

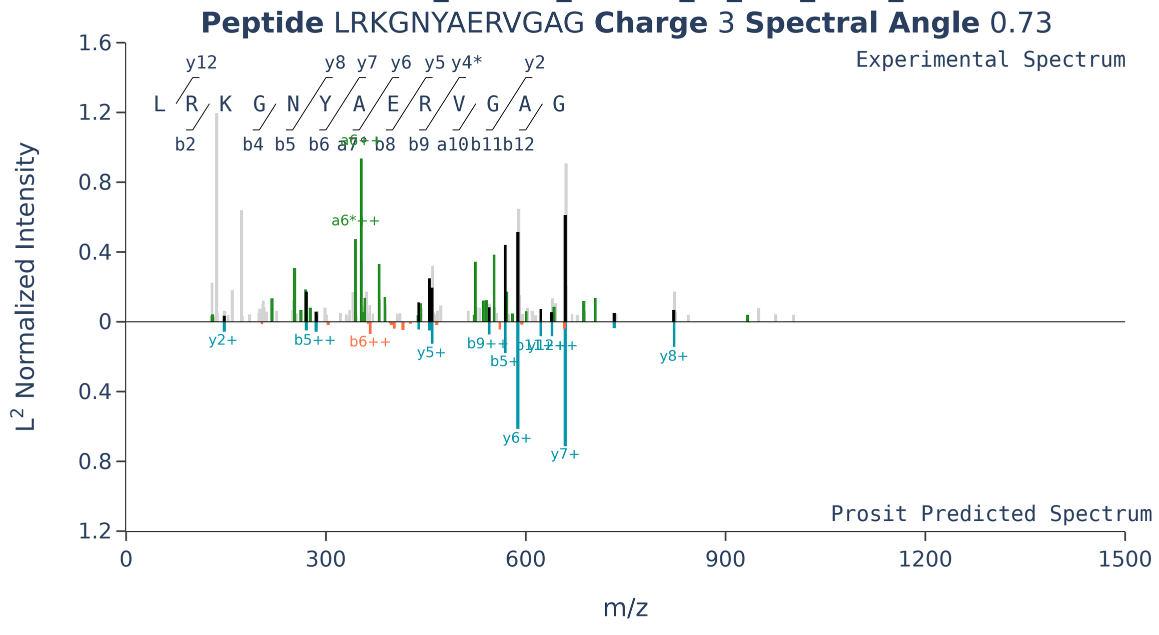


**Source** HRoetschke_291222_060123_Fu_H2A_b2R2_24h **Scan** 7237
**Peptide** RVGAGAPVY **Charge** 2 **Spectral Angle** 0.86

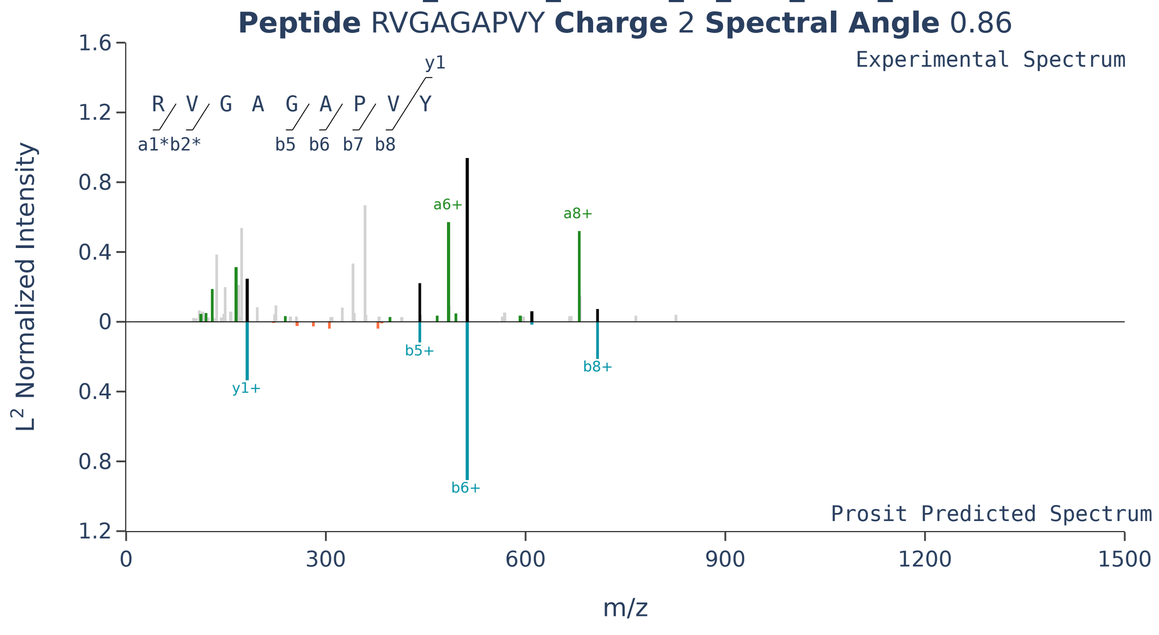


**Source** HRoetschke_291222_060123_Fu_H2A_b1R1_24h **Scan** 8722
**Peptide** KGNYAERVGAGAPVY **Charge** 3 **Spectral Angle** 0.8

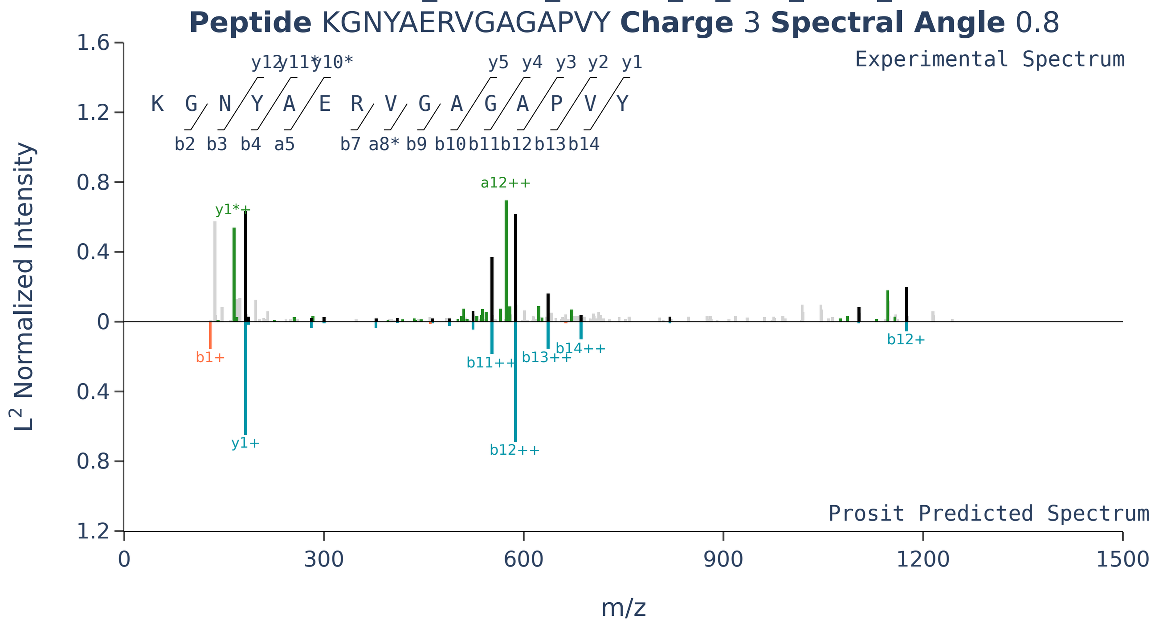


**Source** HRoetschke_291222_060123_Fu_H2A_b2R1_24h **Scan** 14256
**Peptide** LTAEILELAGNAARDNKKTR **Charge** 5 **Spectral Angle** 0.82

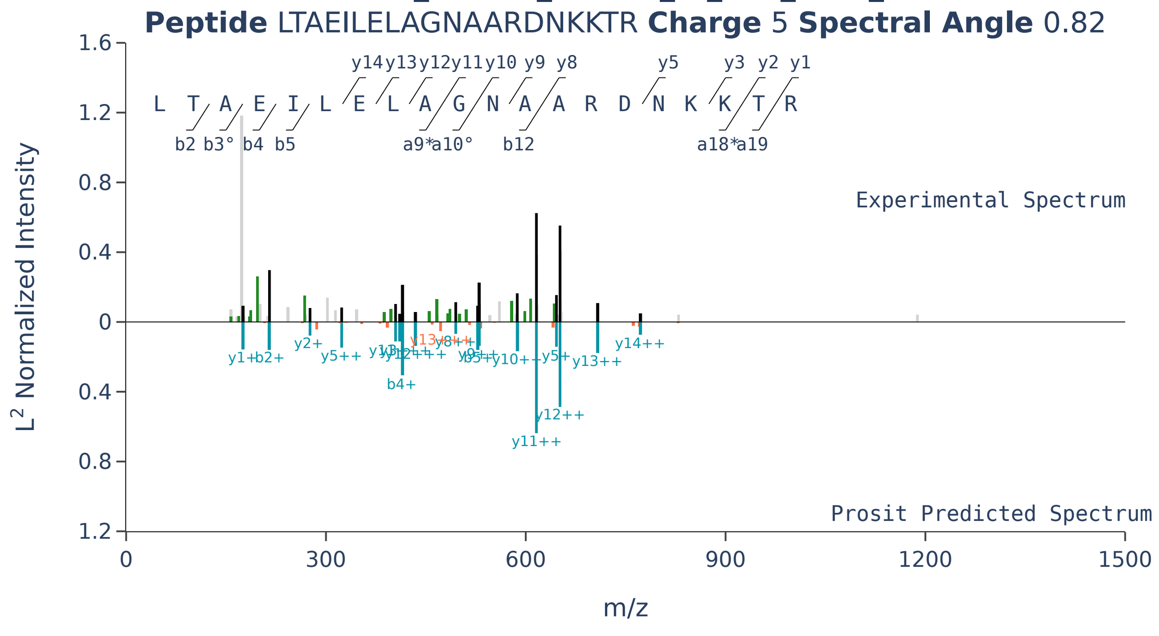


**Source** HRoetschke_291222_060123_Fu_H2A_b1R1_4h **Scan** 11791
**Peptide** RAGLQFPVGRVHRLLRKGNYA **Charge** 5 **Spectral Angle** 0.81

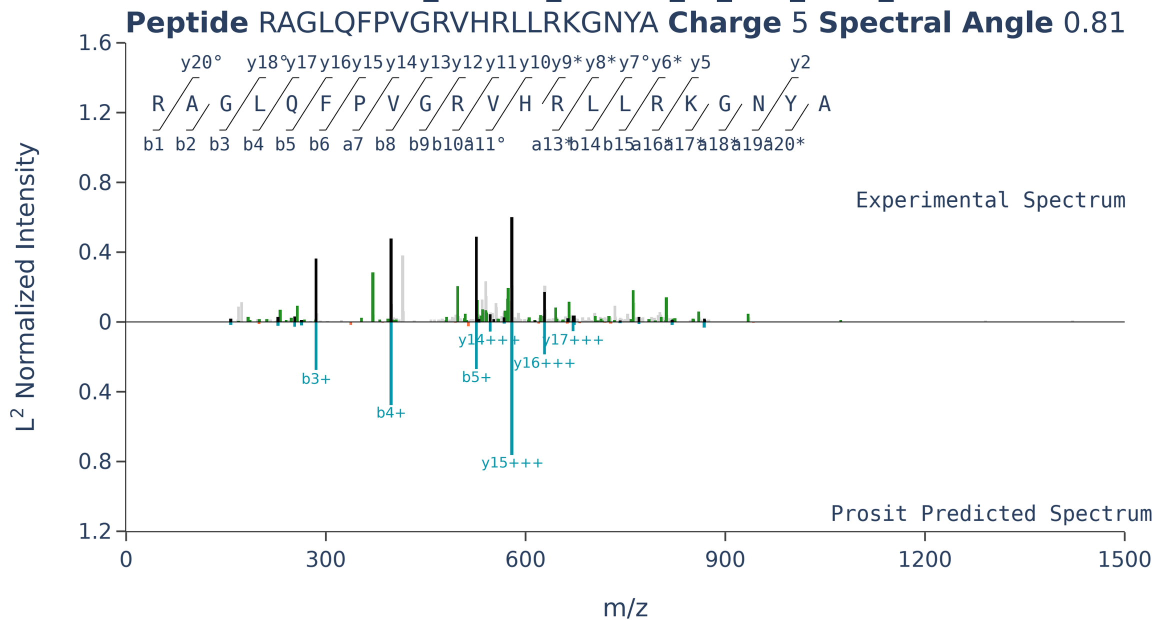

**Source** HRoetschke_291222_060123_Fu_H2A_b1R1_4h **Scan** 16889
**Peptide** AVRNDEELNKLLGRVTIAQ **Charge** 3 **Spectral Angle** 0.67

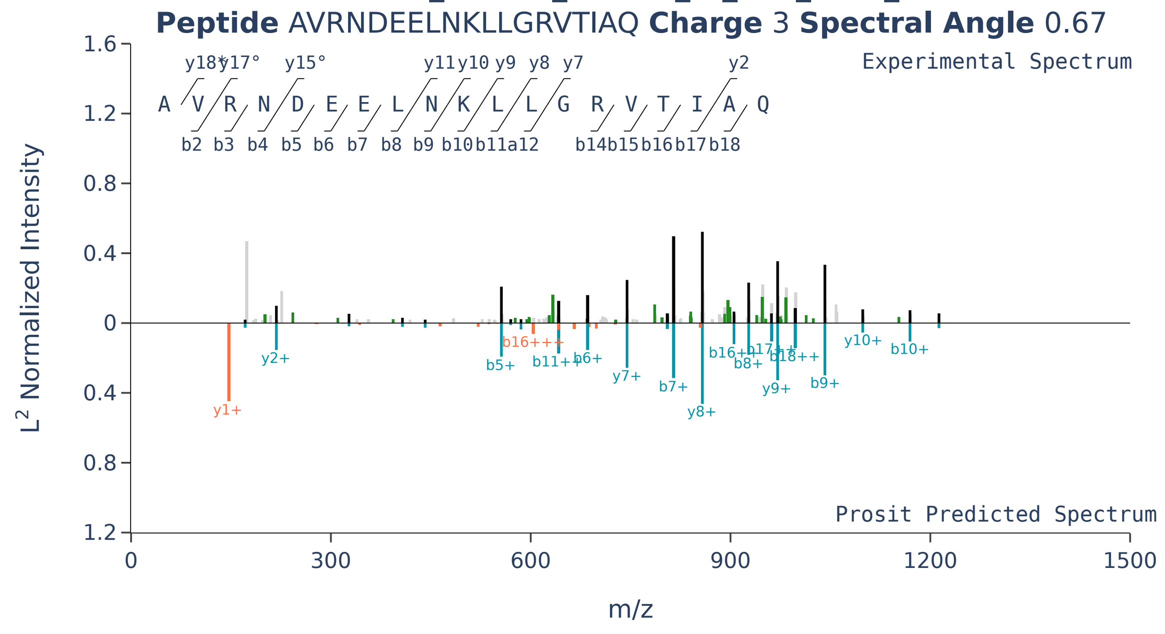


**Source** HRoetschke_291222_060123_Fu_H2A_b1R1_2h **Scan** 9071
**Peptide** LLELAGNAARDNKKTRIIPR **Charge** 4 **Spectral Angle** 0.87

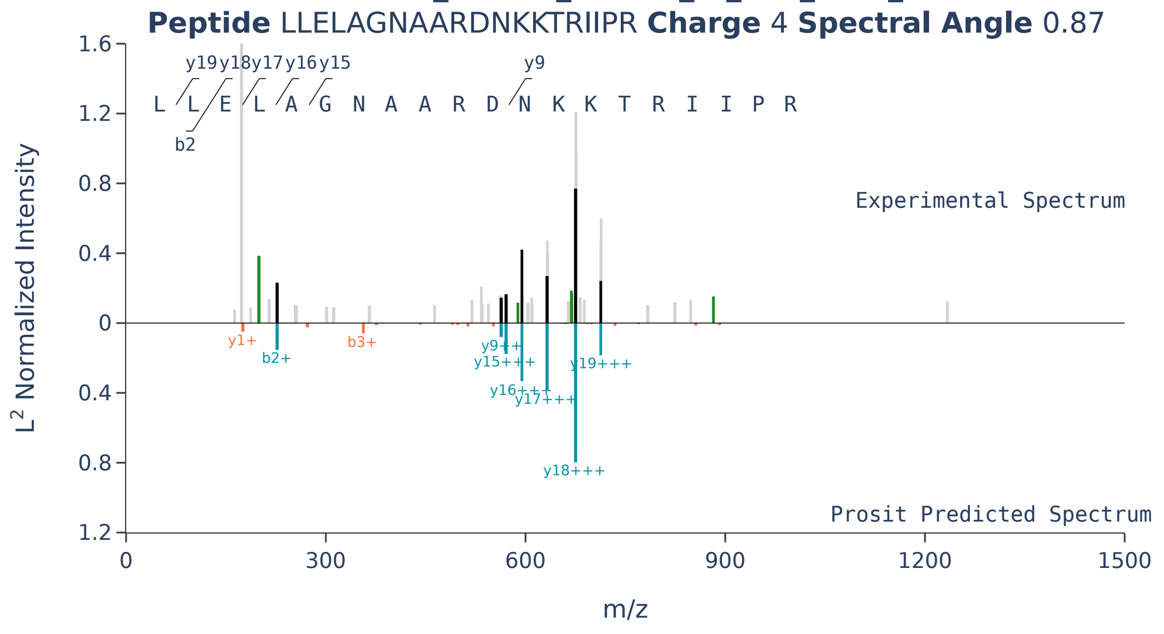


**Source** HRoetschke_291222_060123_Fu_H2A_b1R1_4h **Scan** 13151
**Peptide** LLRKGNYAERVGAGAPVYL **Charge** 4 **Spectral Angle** 0.75

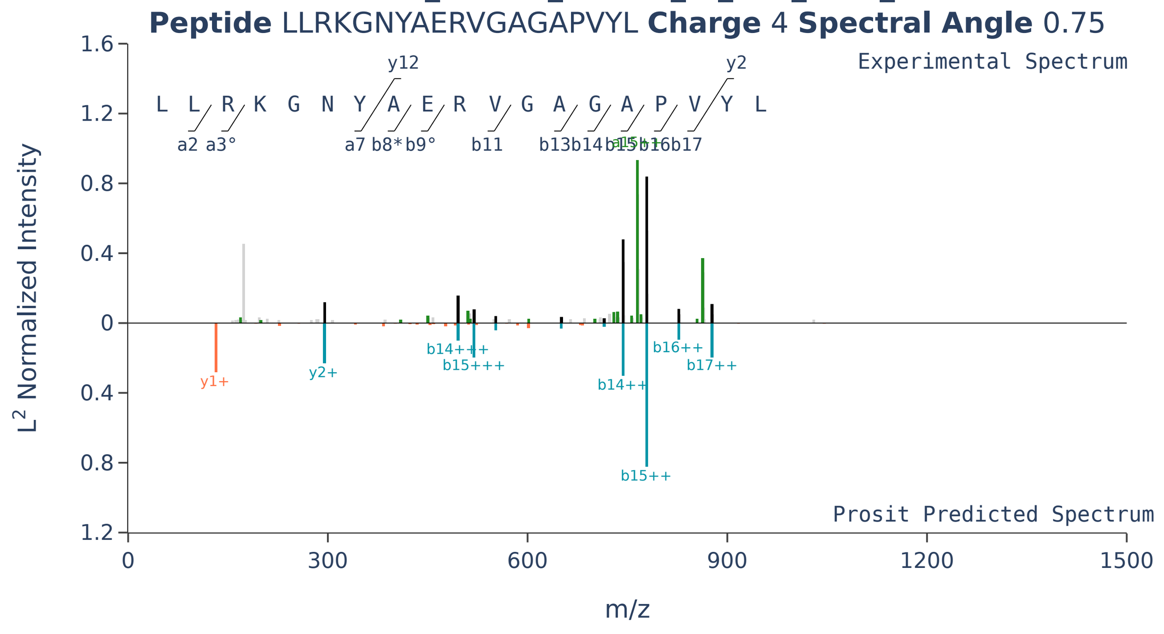


**Source** HRoetschke_291222_060123_Fu_H2A_b1R2_4h **Scan** 10551
**Peptide** ELAGNAARDNKKTRIIPRHLQLAV **Charge** 4 **Spectral Angle** 0.81

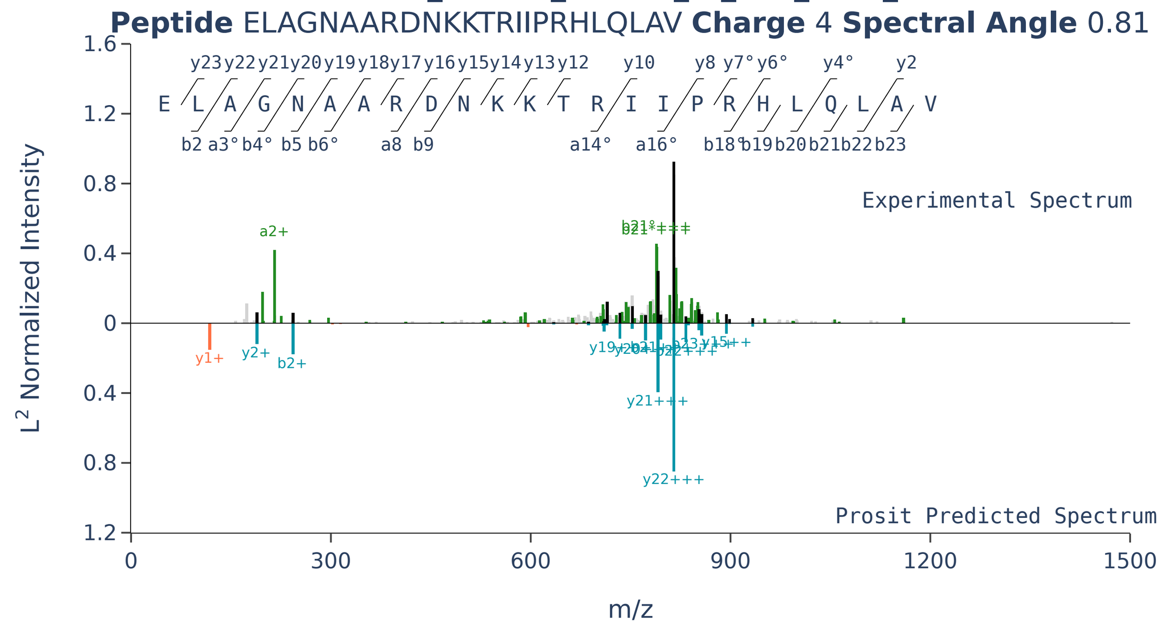


**Source** HRoetschke_291222_060123_Fu_H2A_b1R1_1h **Scan** 15722
**Peptide** TAEILELAGNAA **Charge** 2 **Spectral Angle** 0.77

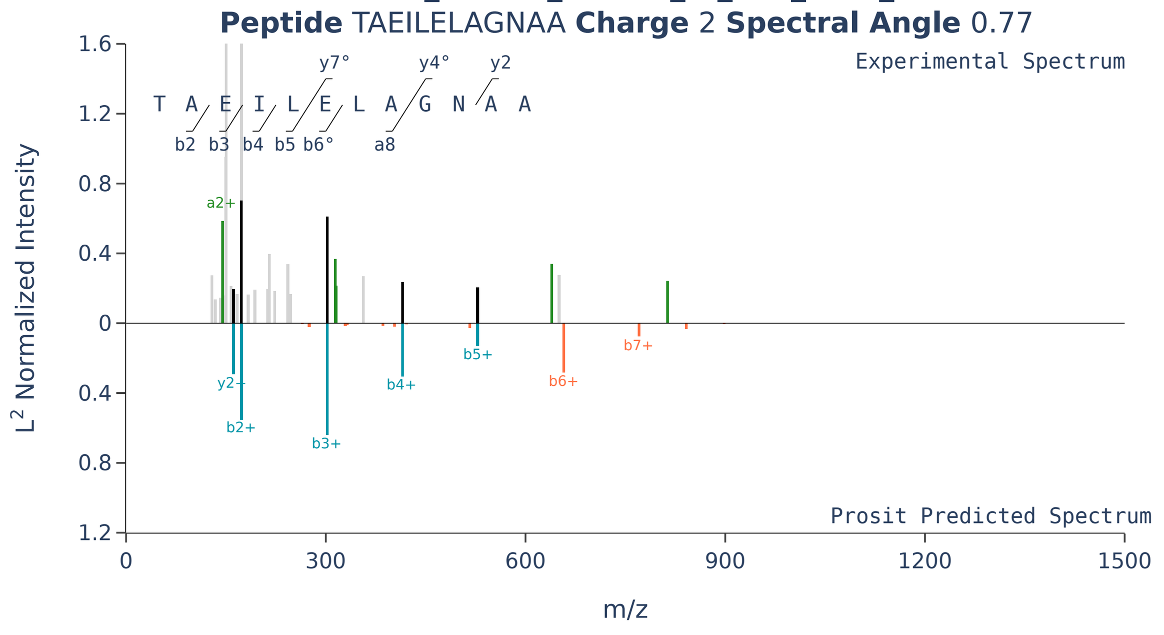


**Source** HRoetschke_291222_060123_Fu_H2A_b1R1_4h **Scan** 15095
**Peptide** AQGGVLPNIQSVLLPKKTESSKSAKSK **Charge** 5 **Spectral Angle** 0.73

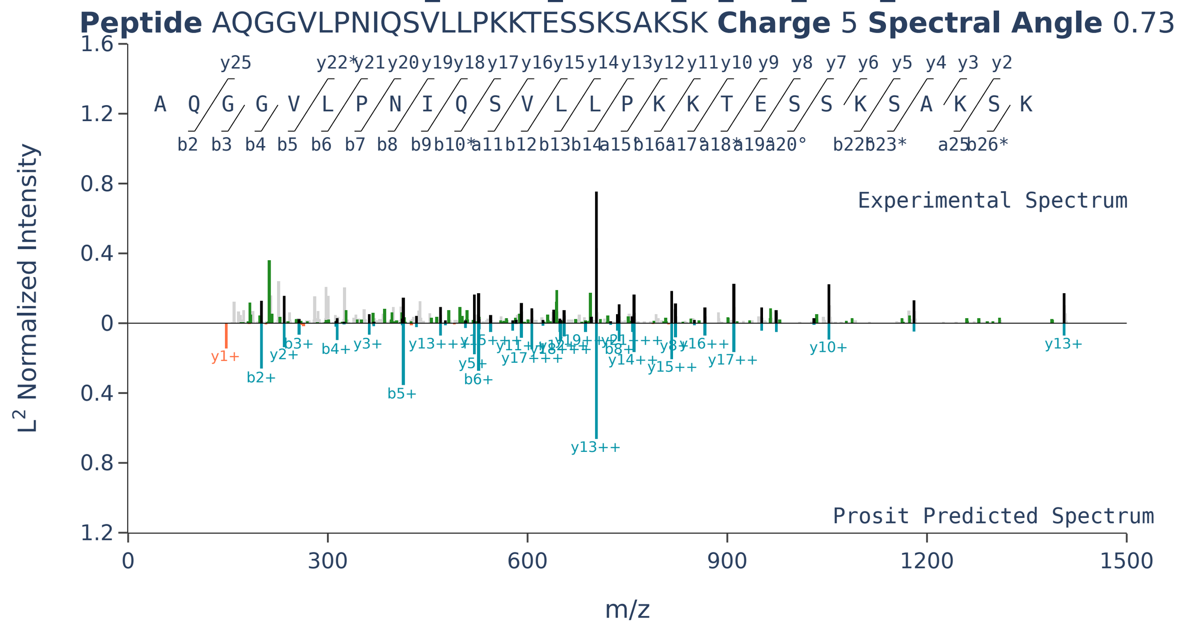


**Source** HRoetschke_291222_060123_Fu_H2A_b1R2_2h **Scan** 5606
**Peptide** PVGRVHRLLRKGNYAERVG **Charge** 5 **Spectral Angle** 0.87

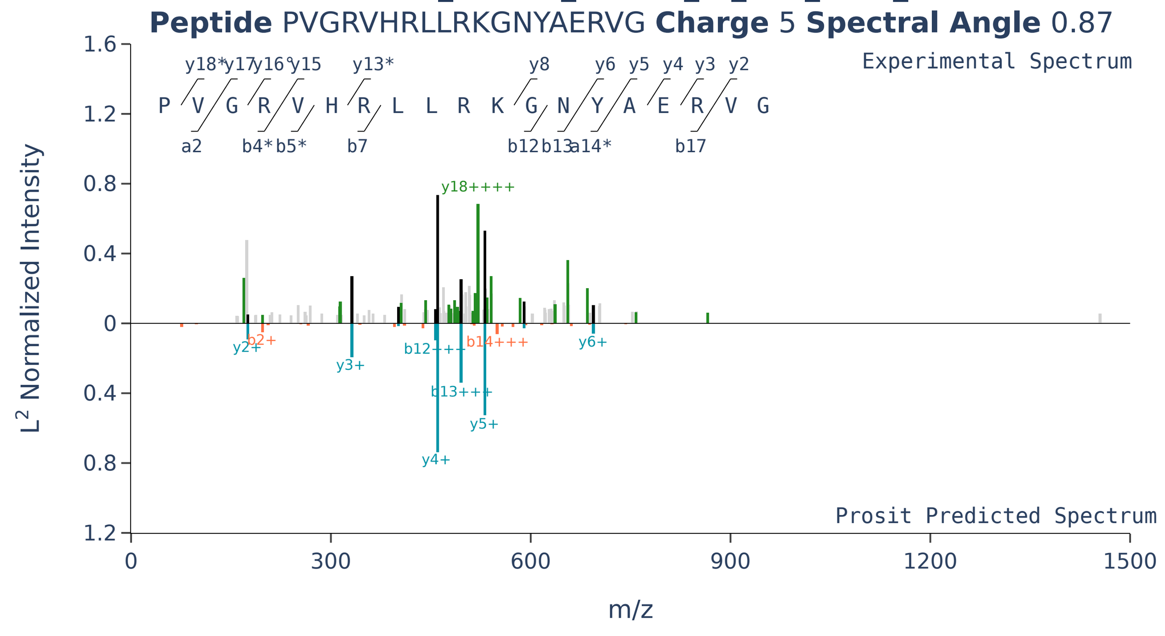


**Source** HRoetschke_291222_060123_Fu_H2A_b1R1_4h **Scan** 12188
**Peptide** LQFPVGRVHRLLRKGNYA **Charge** 4 **Spectral Angle** 0.92

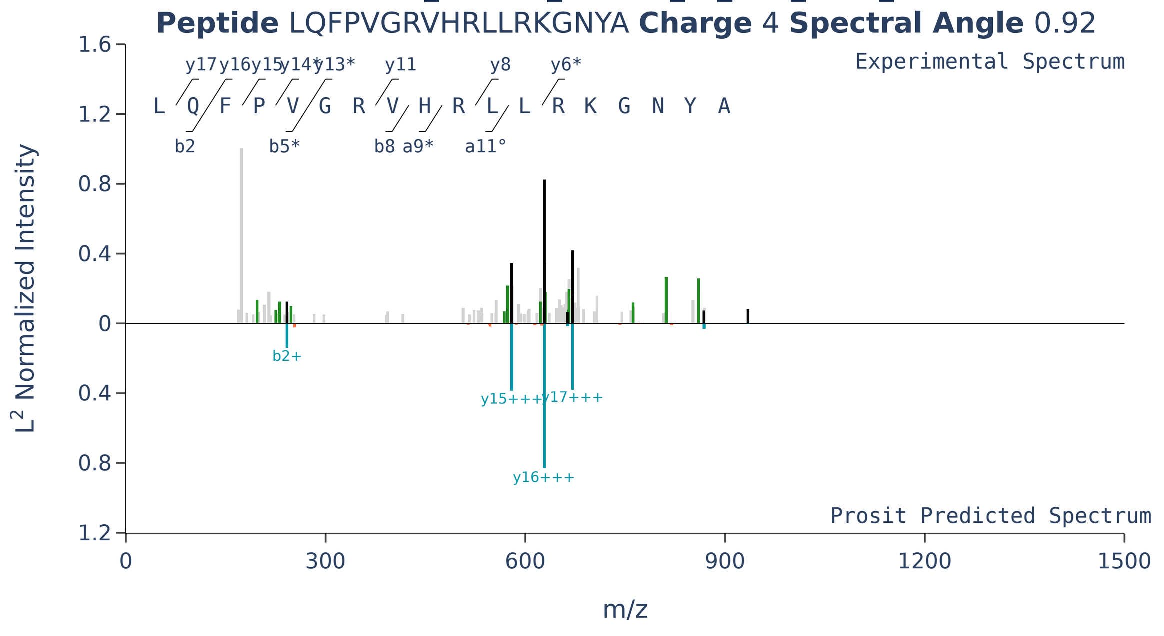


**Source** HRoetschke_291222_060123_Fu_H2A_b1R1_2h **Scan** 14995
**Peptide** QLAVRNDEELNKLLG **Charge** 2 **Spectral Angle** 0.77

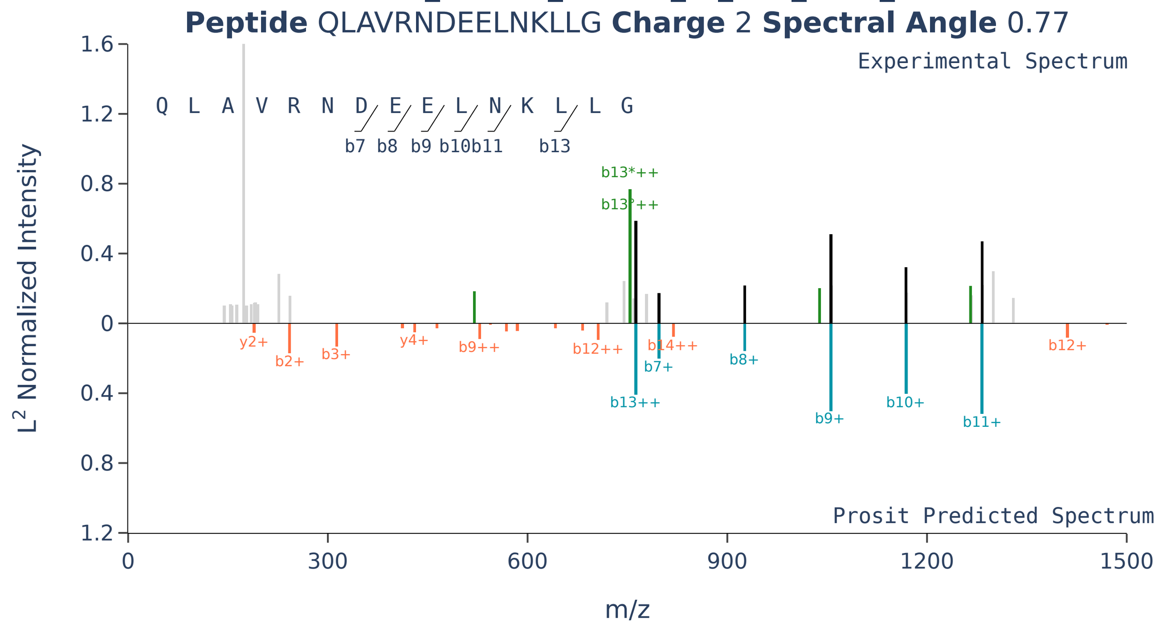


**Source** HRoetschke_291222_060123_Fu_H2A_b1R2_24h **Scan** 15474
**Peptide** LAQGGVLPNI **Charge** 2 **Spectral Angle** 0.76

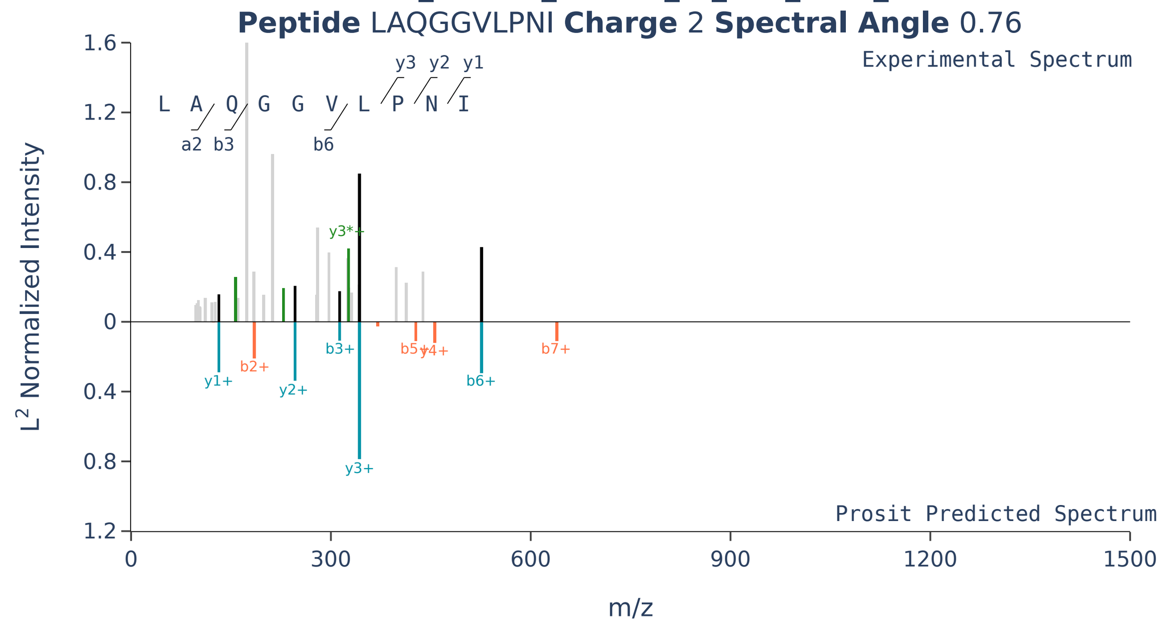


**Source** HRoetschke_291222_060123_Fu_H2A_b1R1_2h **Scan** 6340
**Peptide** GKTRAKAKTRSSRAGLQFPVG **Charge** 4 **Spectral Angle** 0.82

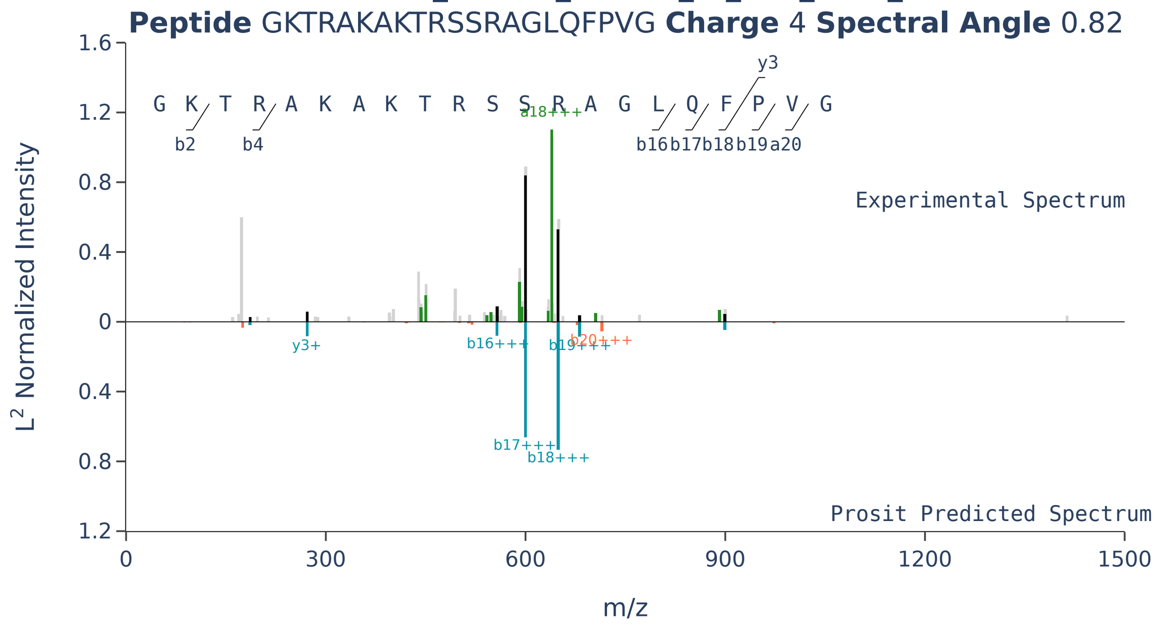


**Source** HRoetschke_291222_060123_Fu_H2A_b2R1_2h **Scan** 9603
**Peptide** RVHRLLRKGNYAERVGAGAPVYLAA **Charge** 4 **Spectral Angle** 0.8

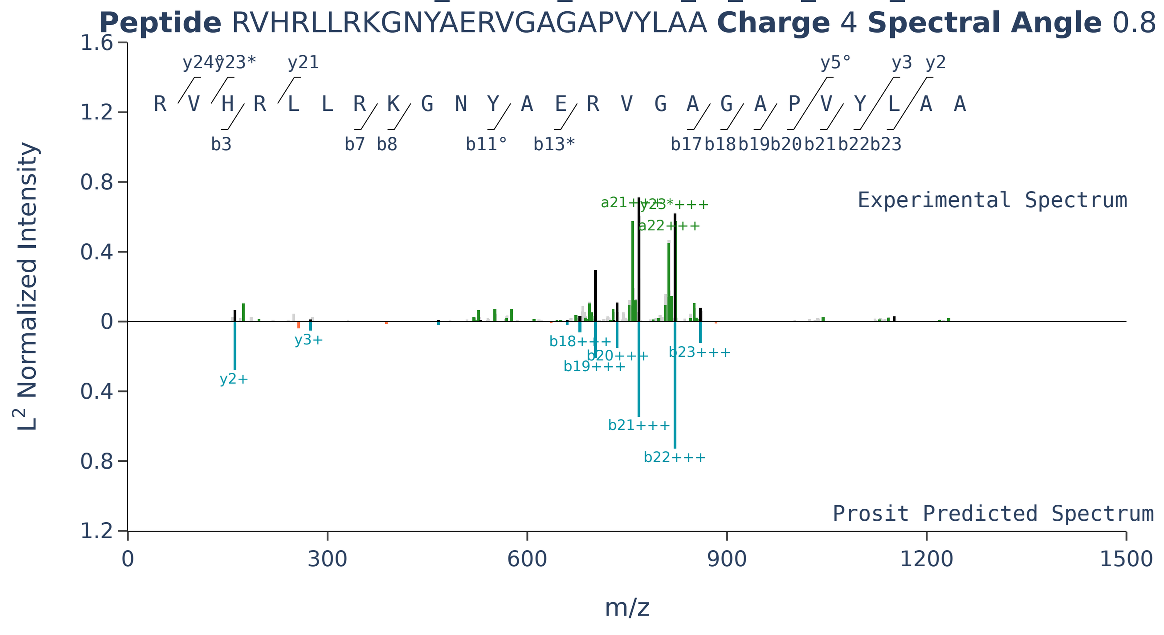


**Source** HRoetschke_291222_060123_Fu_H2A_b2R2_24h **Scan** 10084
**Peptide** LAVRNDEELNKL **Charge** 3 **Spectral Angle** 0.73

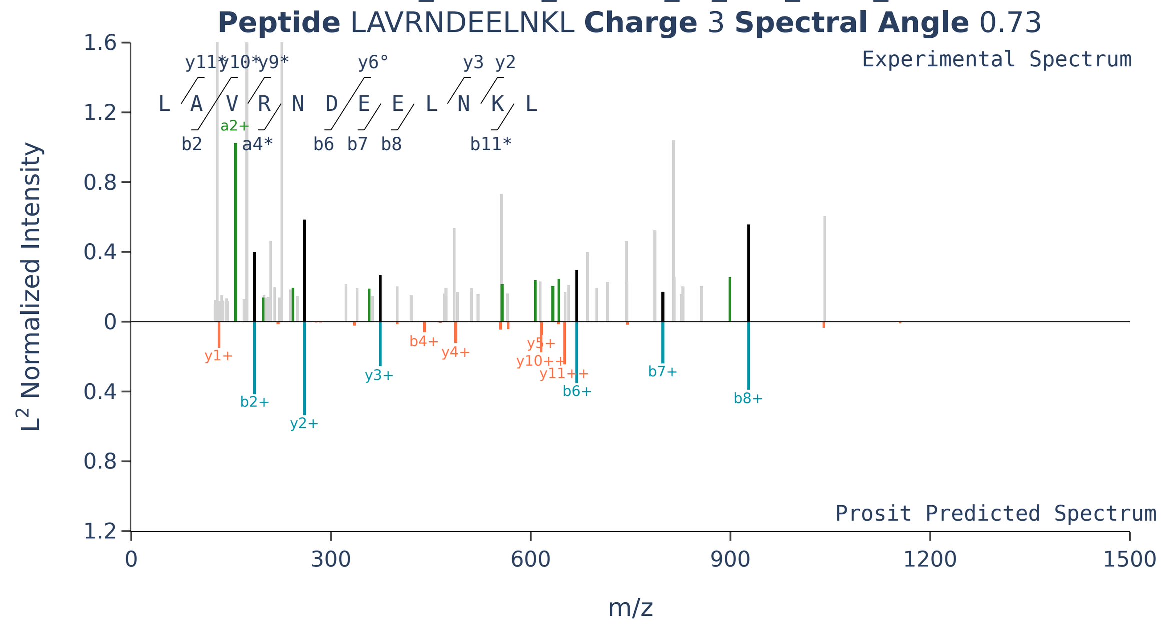


**Source** HRoetschke_291222_060123_Fu_H2A_b1R2_4h **Scan** 13360
**Peptide** AGLQFPVGRVHRLLRKGNY **Charge** 4 **Spectral Angle** 0.81

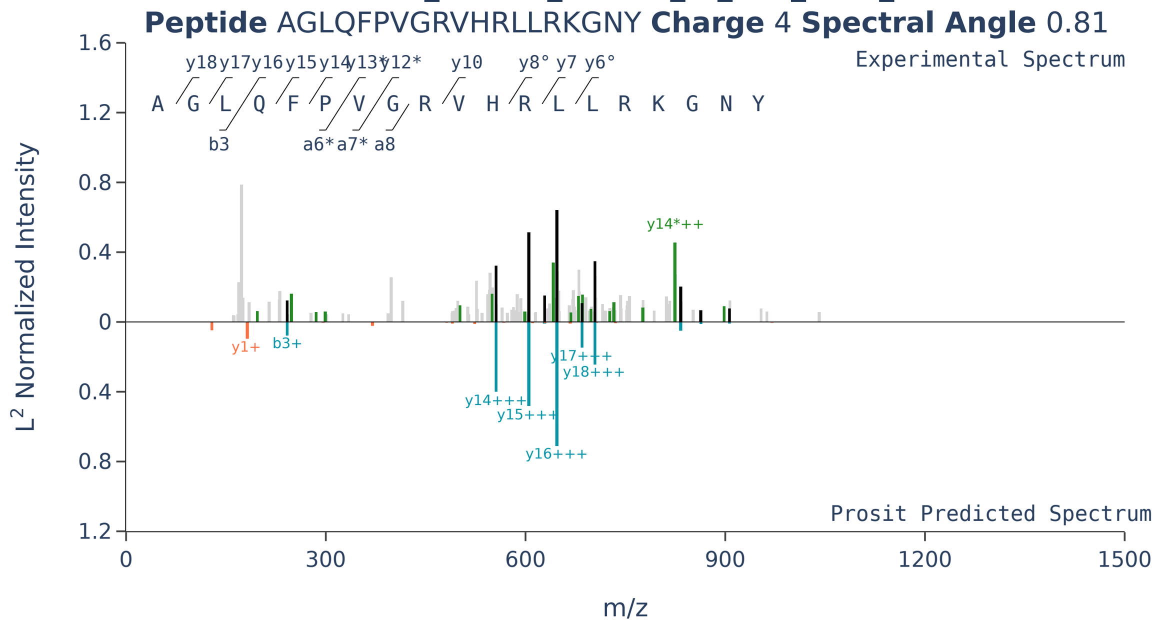


**Source** HRoetschke_291222_060123_Fu_H2A_b1R1_4h **Scan** 14388
**Peptide** ELLELAGNAARDNKKTRIIPRHLQL **Charge** 5 **Spectral Angle** 0.75

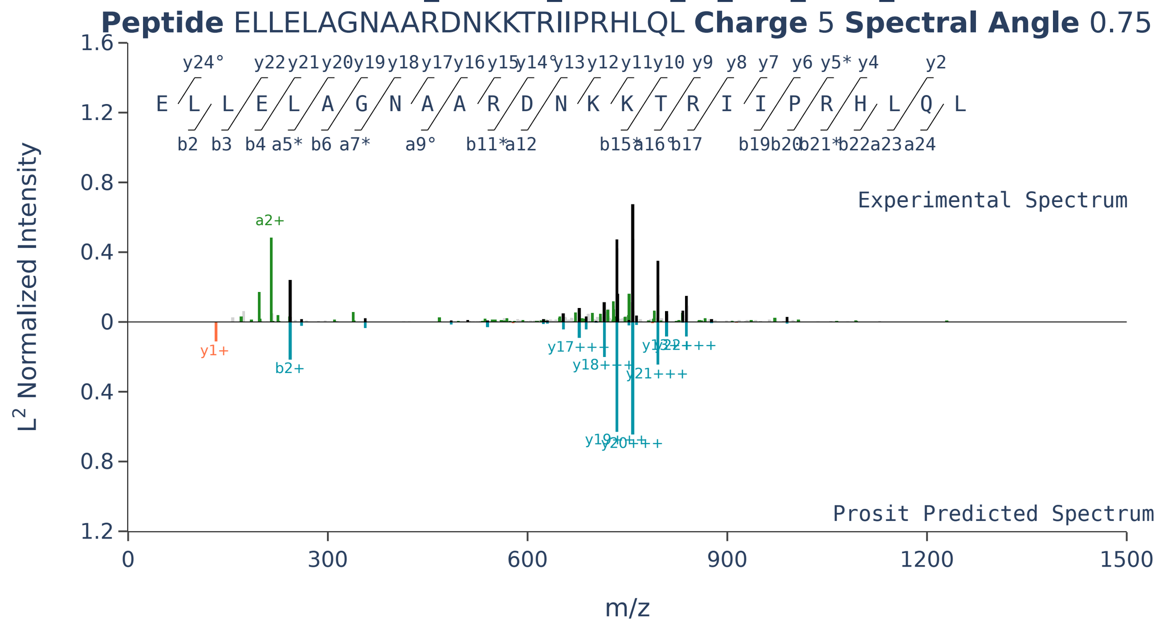

**Source** HRoetschke_291222_060123_Fu_H2A_b1R1_4h **Scan** 12080
**Peptide** AKTRSSRAGLQFPVGRVHRLL **Charge** 5 **Spectral Angle** 0.89

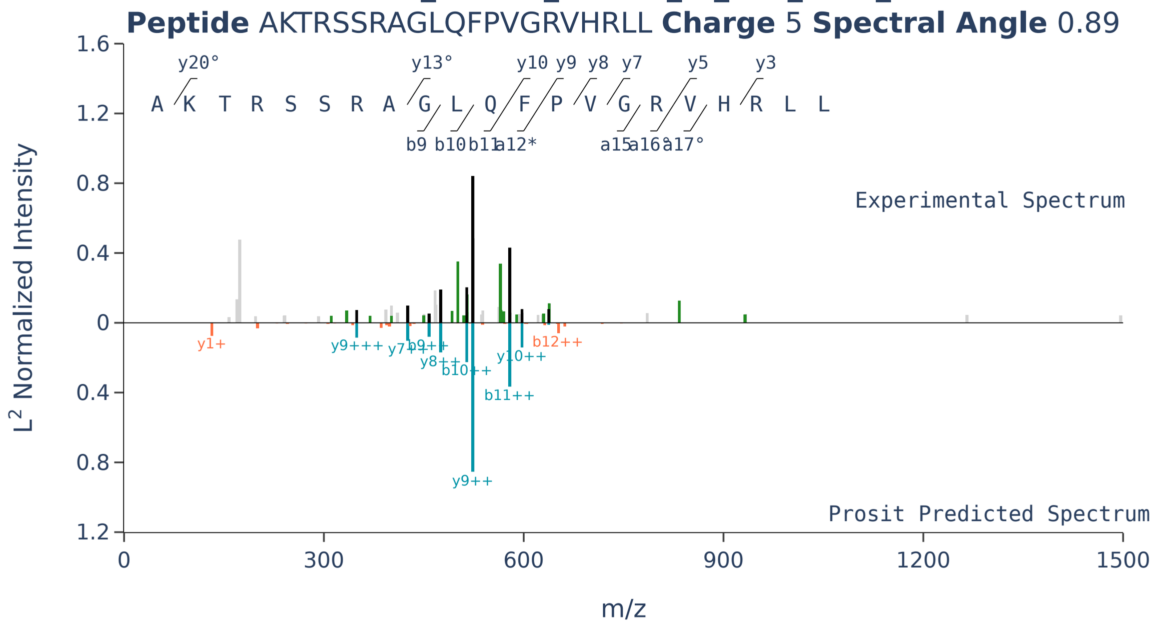


**Source** HRoetschke_291222_060123_Fu_H2A_b2R1_24h **Scan** 1540
**Peptide** SGRGKQGGKTRAKAKTR **Charge** 4 **Spectral Angle** 0.82

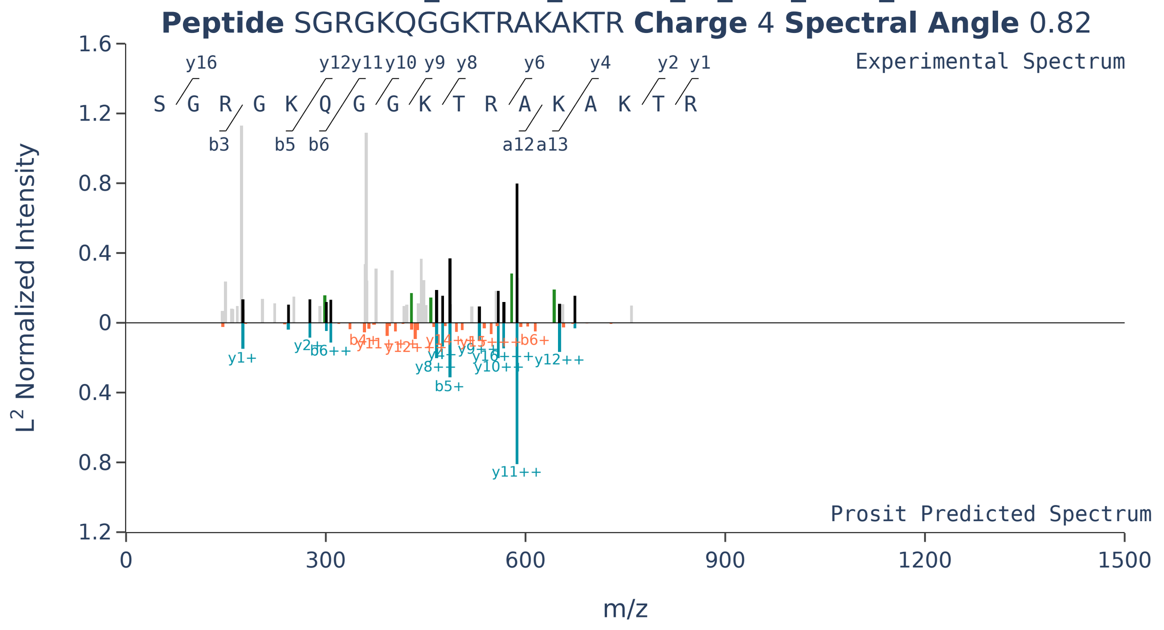


**Source** HRoetschke_291222_060123_Fu_H2A_b1R1_4h **Scan** 18590
**Peptide** YLTAEILELAGNAARDNKKTR **Charge** 5 **Spectral Angle** 0.8

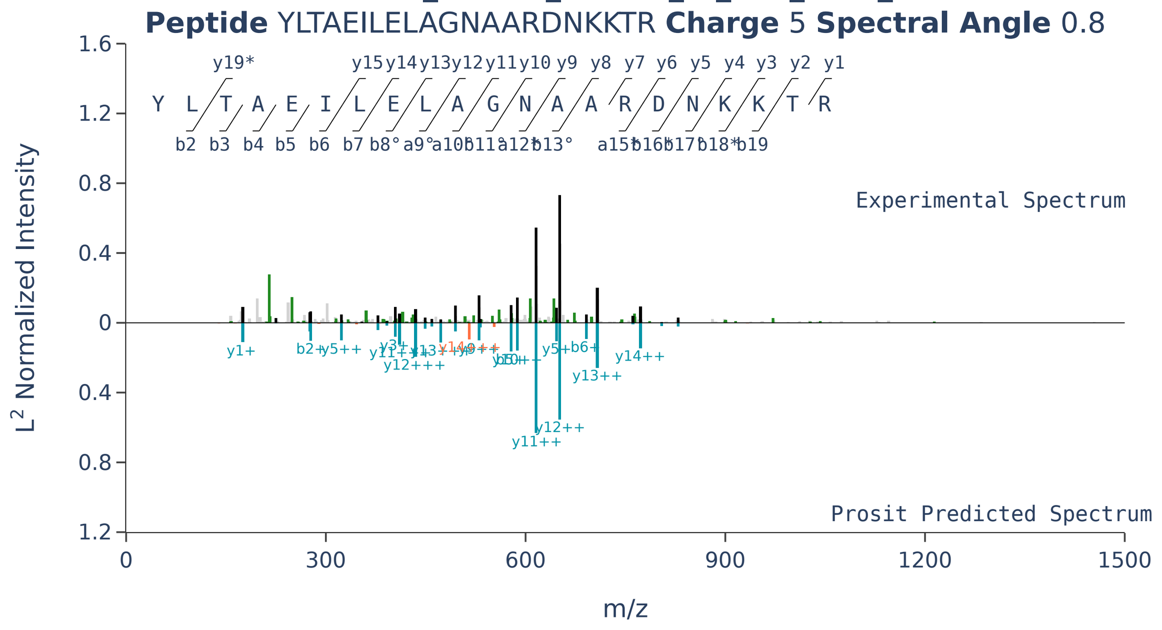


**Source** HRoetschke_291222_060123_Fu_H2A_b1R1_2h **Scan** 7575
**Peptide** QFPVGRVHRLLRKG **Charge** 4 **Spectral Angle** 0.82

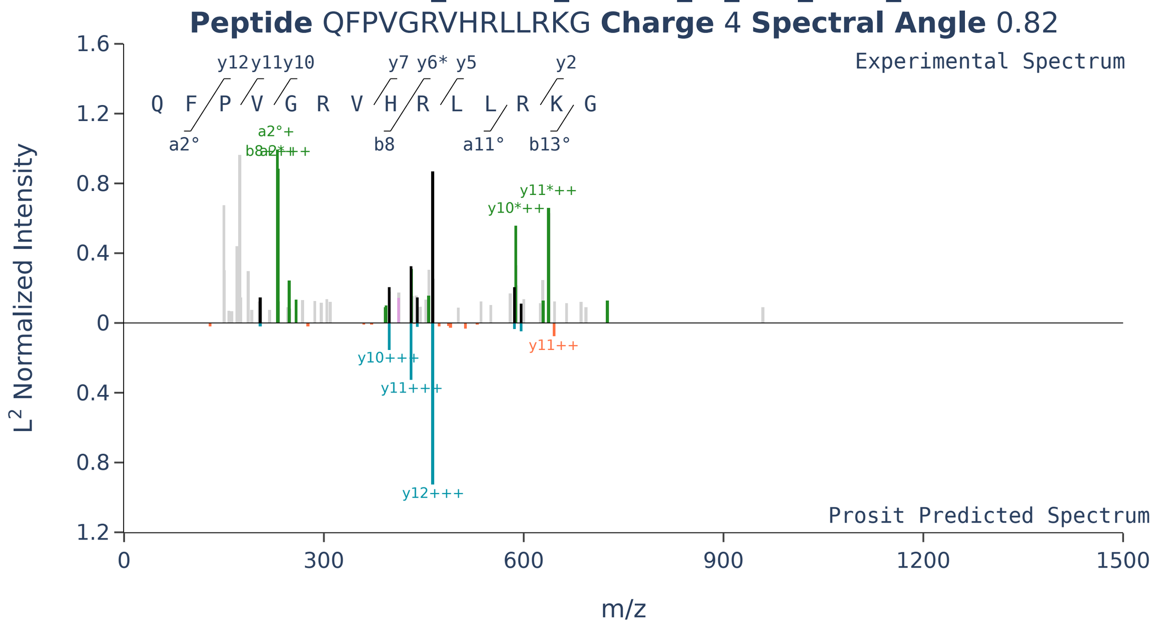


**Source** HRoetschke_291222_060123_Fu_H2A_b1R1_1h **Scan** 3329
**Peptide** QLAVRND **Charge** 2 **Spectral Angle** 0.94

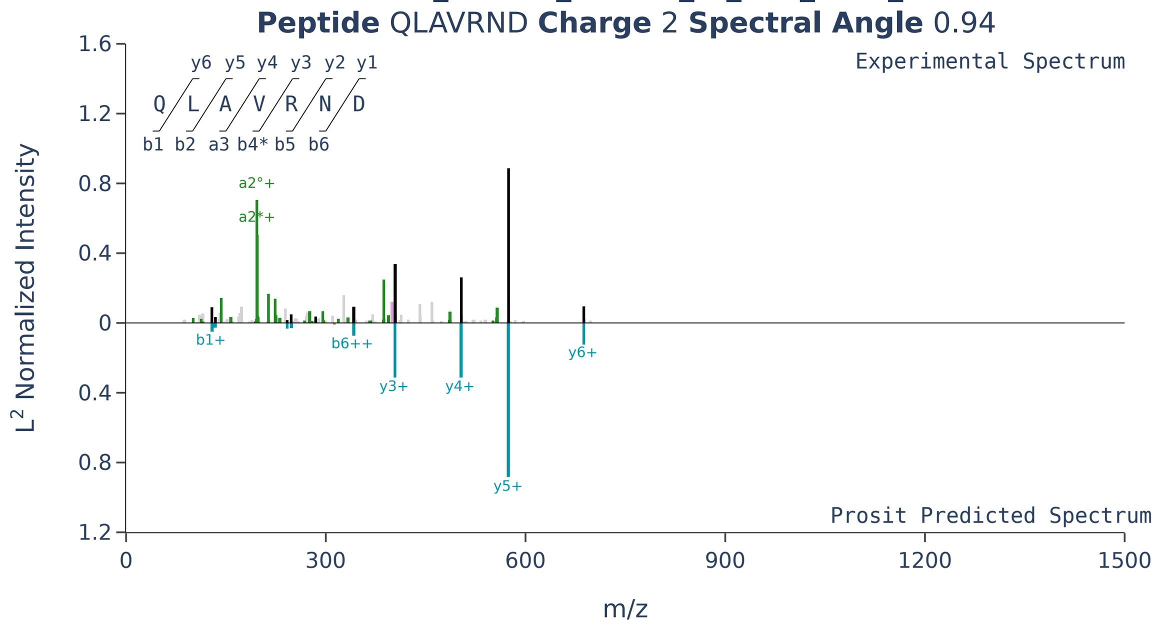


**Source** HRoetschke_291222_060123_Fu_H2A_b2R1_1h **Scan** 5028
**Peptide** RLLPRHL **Charge** 3 **Spectral Angle** 0.75

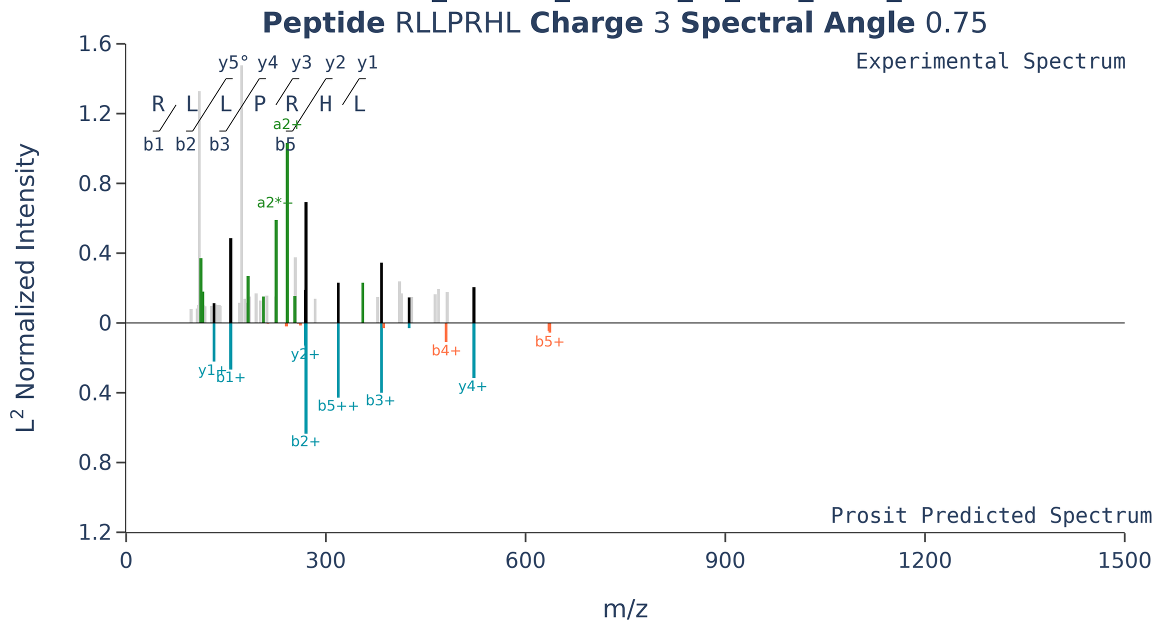


**Source** HRoetschke_291222_060123_Fu_H2A_b1R1_2h **Scan** 10662
**Peptide** VHRLLRKGNYAERVGAGAPVYLAA **Charge** 4 **Spectral Angle** 0.83

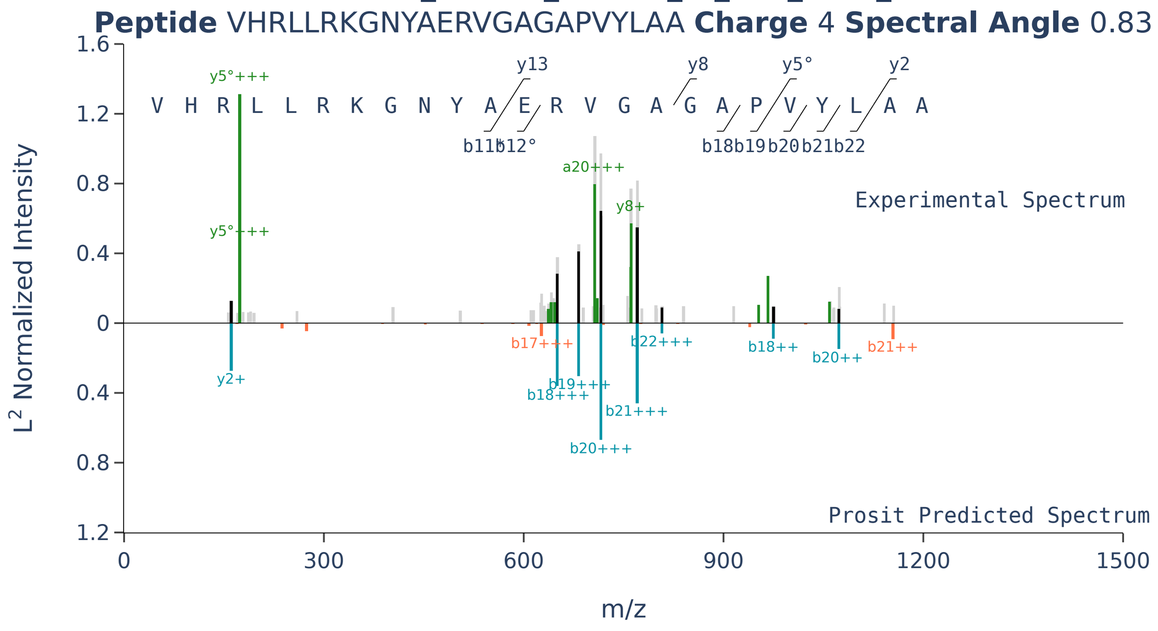


**Source** HRoetschke_291222_060123_Fu_H2A_b2R1_24h **Scan** 8416
**Peptide** DNKKTRIIPRHLQLAVRND **Charge** 5 **Spectral Angle** 0.74

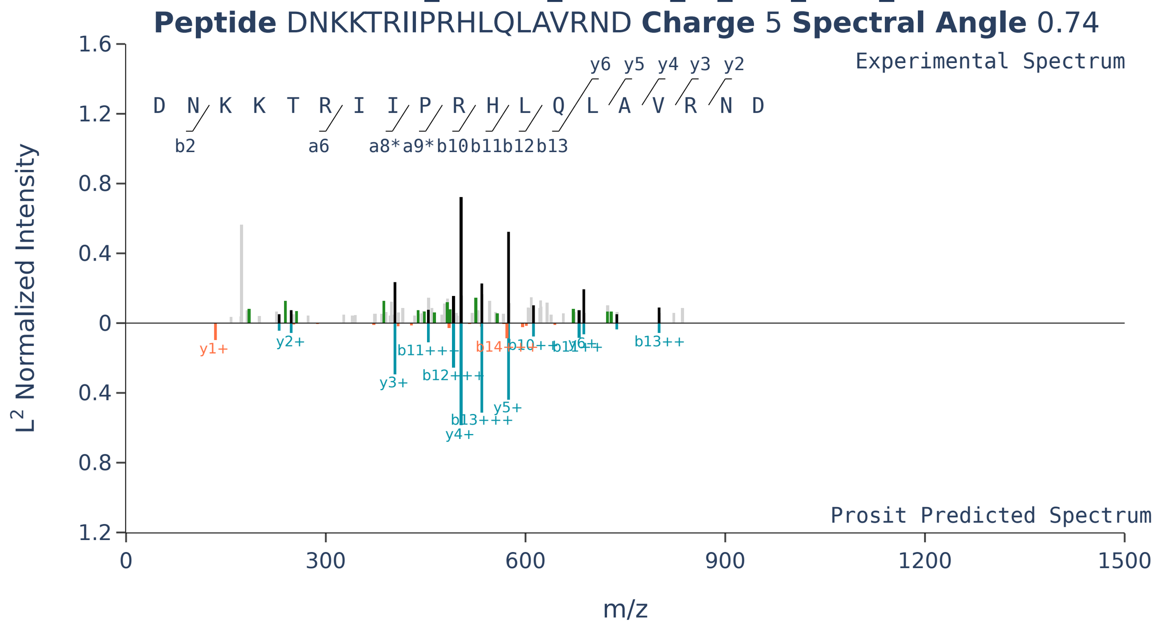


**Source** HRoetschke_291222_060123_Fu_H2A_b1R1_24h **Scan** 7578
**Peptide** AQGGVLPN **Charge** 1 **Spectral Angle** 0.87

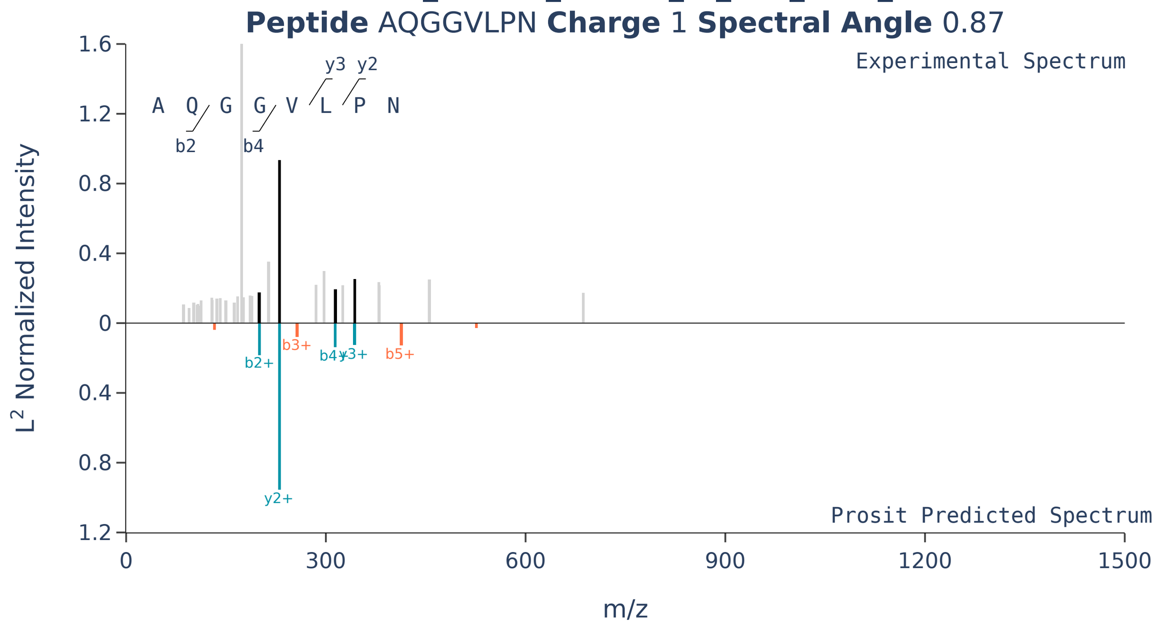


**Source** HRoetschke_291222_060123_Fu_H2A_b2R1_4h **Scan** 2523
**Peptide** RVHRLLRKGNY **Charge** 3 **Spectral Angle** 0.62

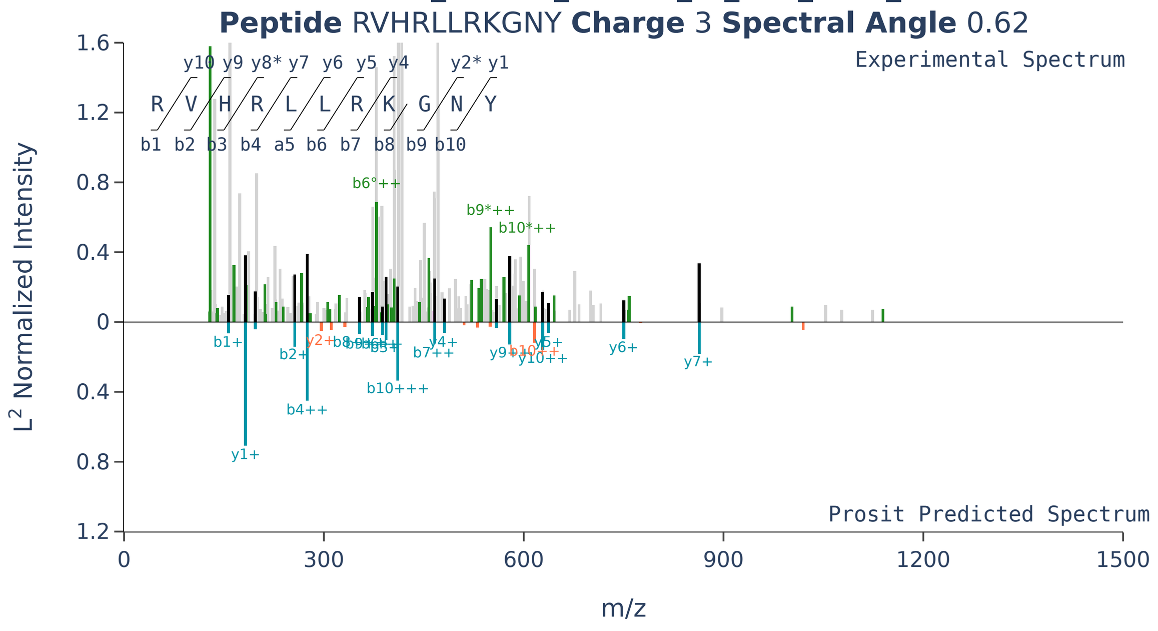


**Source** HRoetschke_291222_060123_Fu_H2A_b2R2_24h **Scan** 3244
**Peptide** AGNAARDNKKTRIIPRHLQ **Charge** 4 **Spectral Angle** 0.83

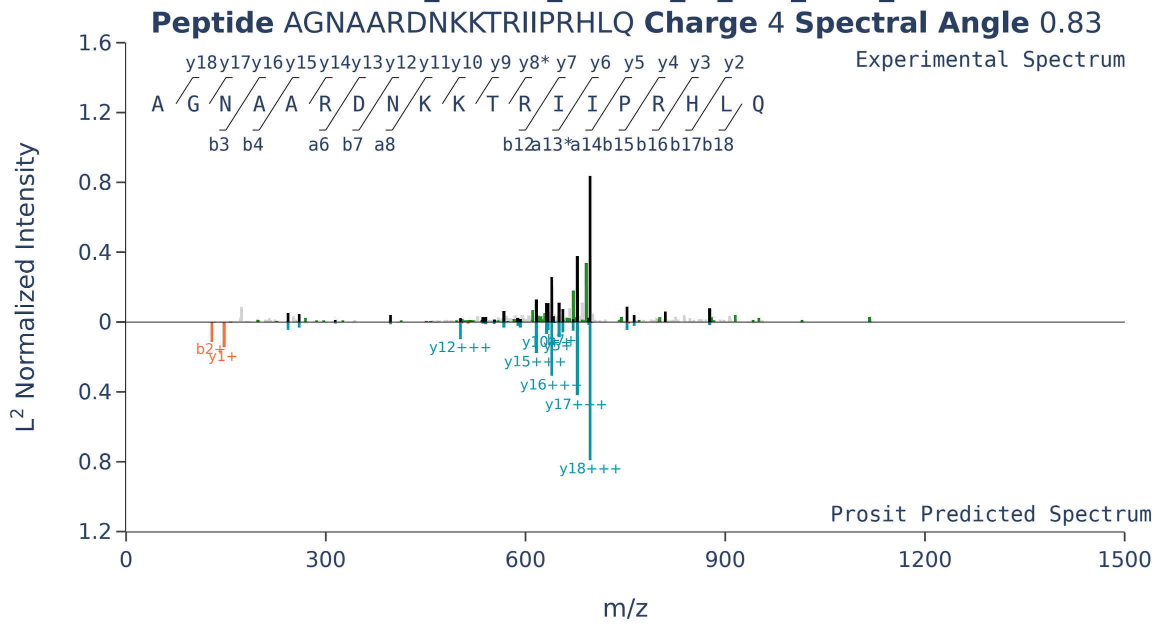


**Source** HRoetschke_291222_060123_Fu_H2A_b1R2_2h **Scan** 9782
**Peptide** LLPRHLQLAVRNDE **Charge** 4 **Spectral Angle** 0.74

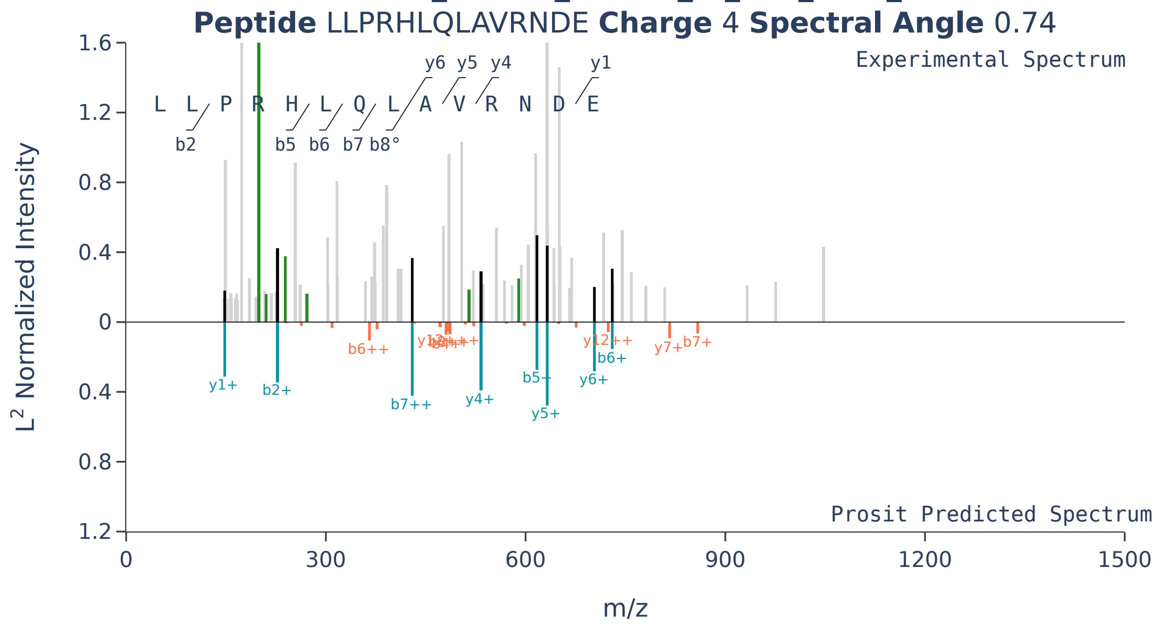


**Source** HRoetschke_291222_060123_Fu_H2A_b2R2_1h **Scan** 3555
**Peptide** GNAARDNKKTRIIPRHL **Charge** 4 **Spectral Angle** 0.67

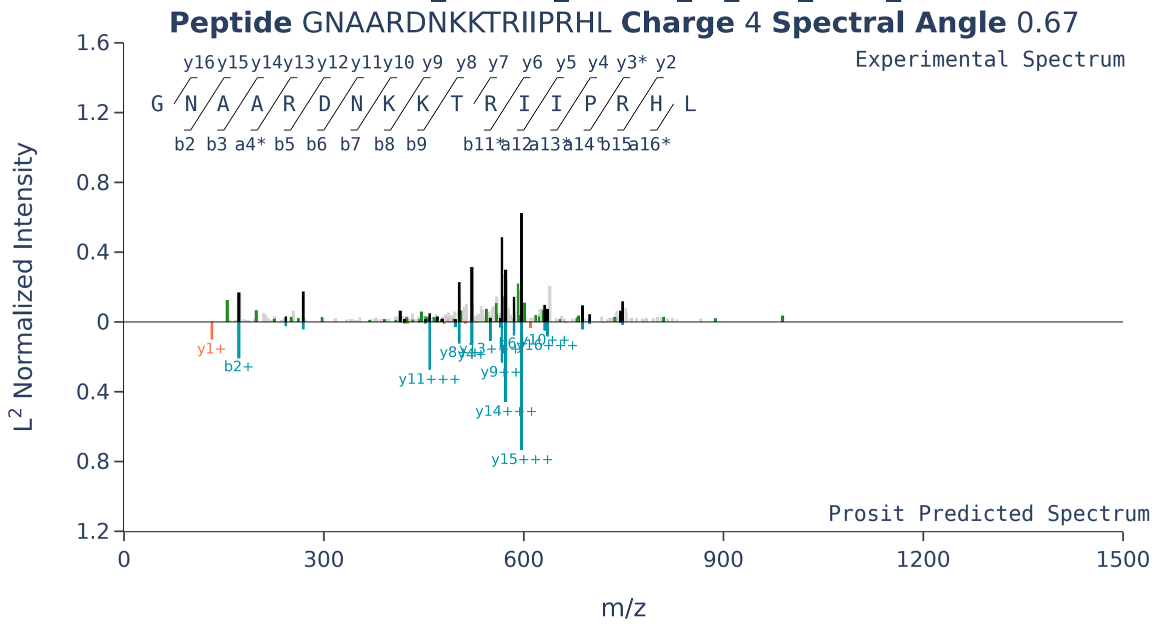


**Source** HRoetschke_291222_060123_Fu_H2A_b1R2_4h **Scan** 8101
**Peptide** PNIQSVLLPKKTESSKSAKSK **Charge** 4 **Spectral Angle** 0.72

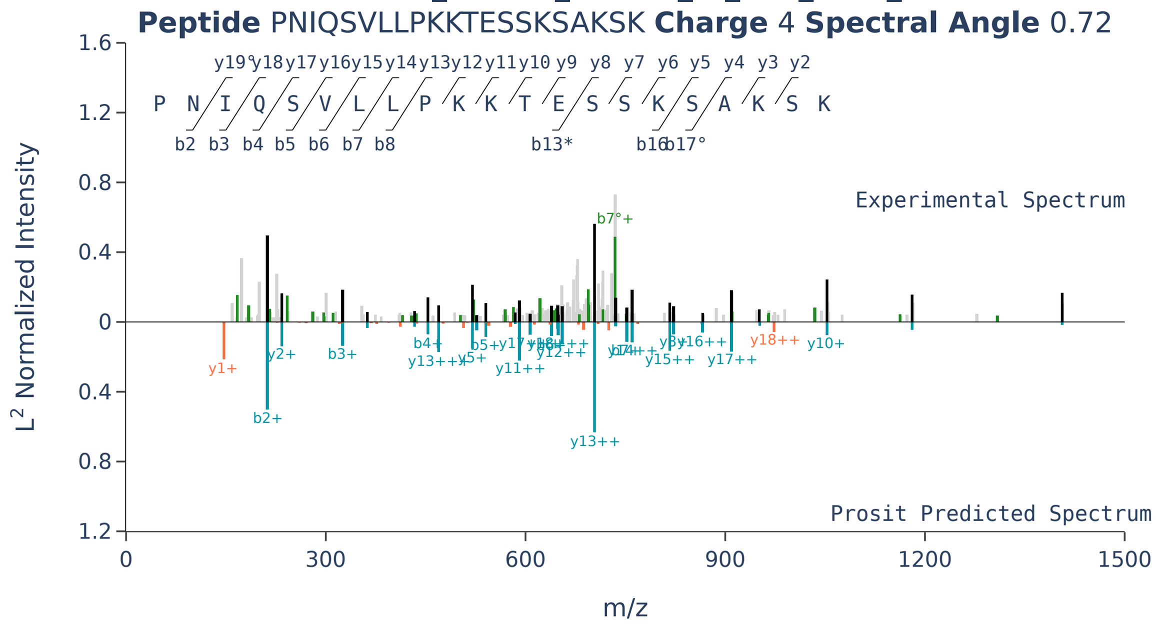


**Source** HRoetschke_291222_060123_Fu_H2A_b1R1_24h **Scan** 4000
**Peptide** LELAGNAARDNKK **Charge** 3 **Spectral Angle** 0.64

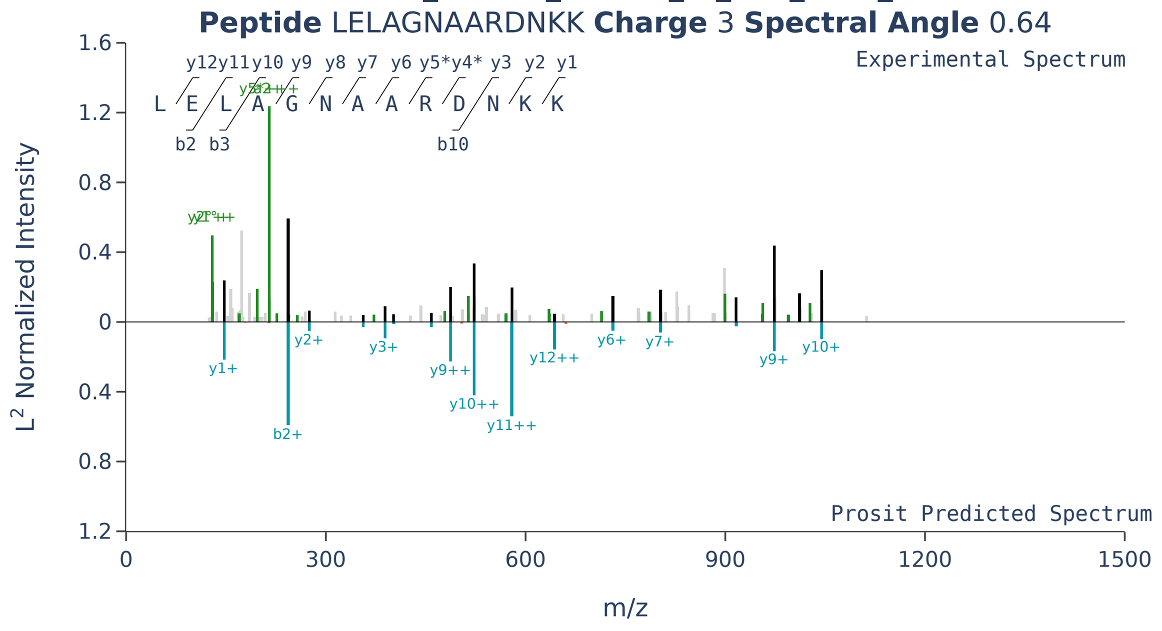

**Source** HRoetschke_291222_060123_Fu_H2A_b1R1_2h **Scan** 11767
**Peptide** LQFPVGRVHRLLRKGNYAE **Charge** 4 **Spectral Angle** 0.87

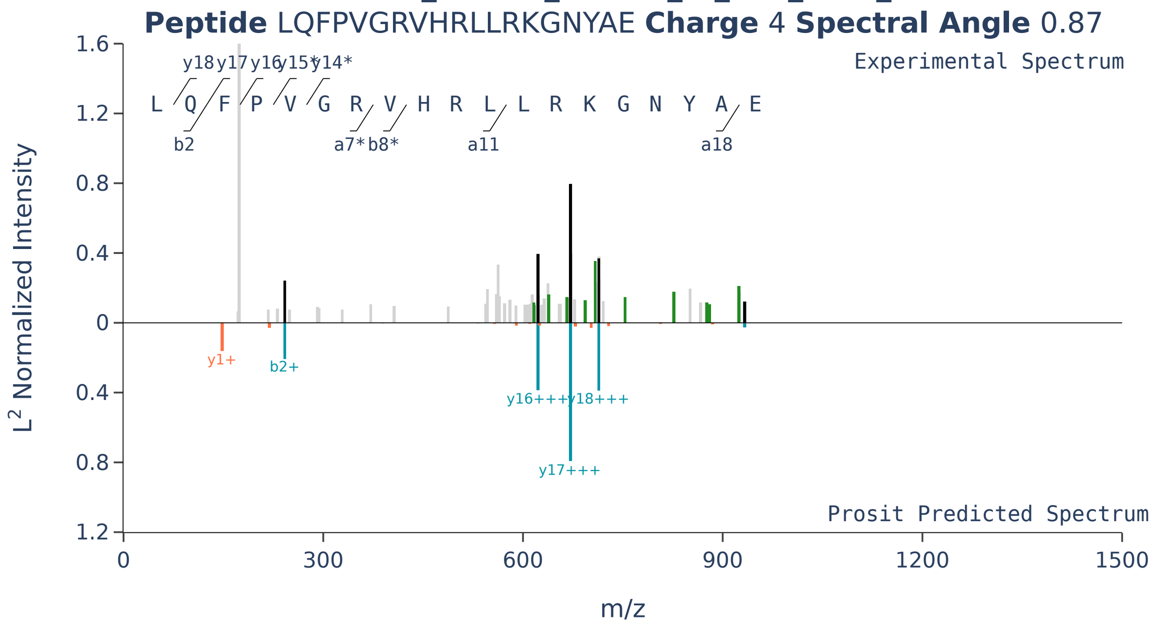


**Source** HRoetschke_291222_060123_Fu_H2A_b1R1_4h **Scan** 13942
**Peptide** ELLELAGNAARDNKKTRIIPRHLQLA **Charge** 5 **Spectral Angle** 0.76

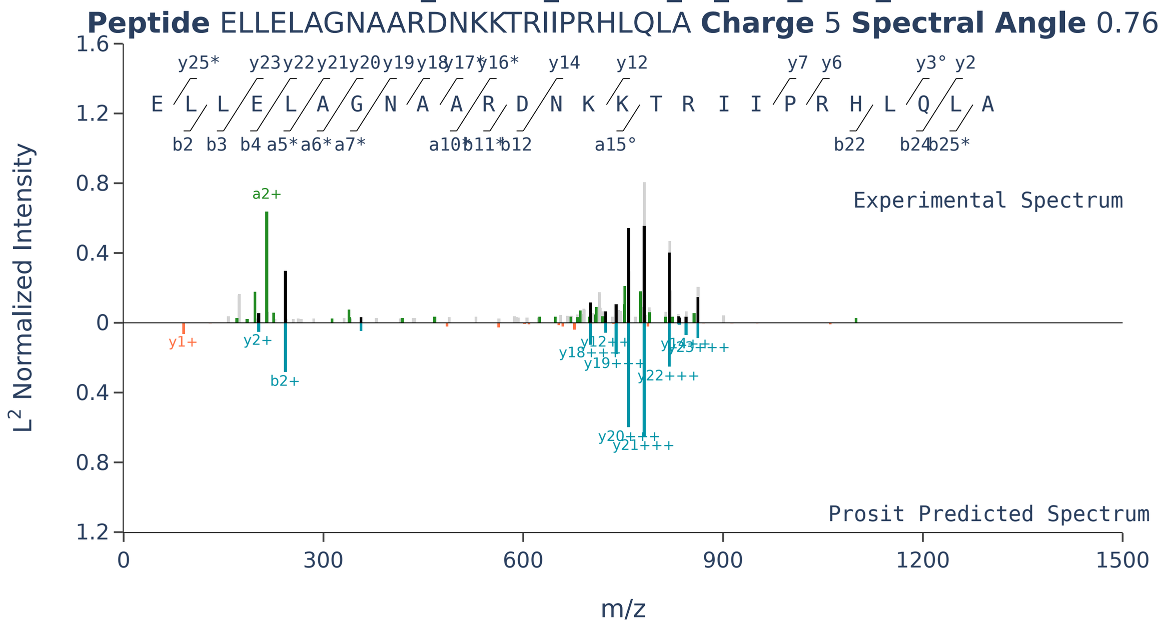


**Source** HRoetschke_291222_060123_Fu_H2A_b1R2_2h **Scan** 15382
**Peptide** TAEILELAGNAARDNKKTRIIPRHLQL **Charge** 5 **Spectral Angle** 0.79

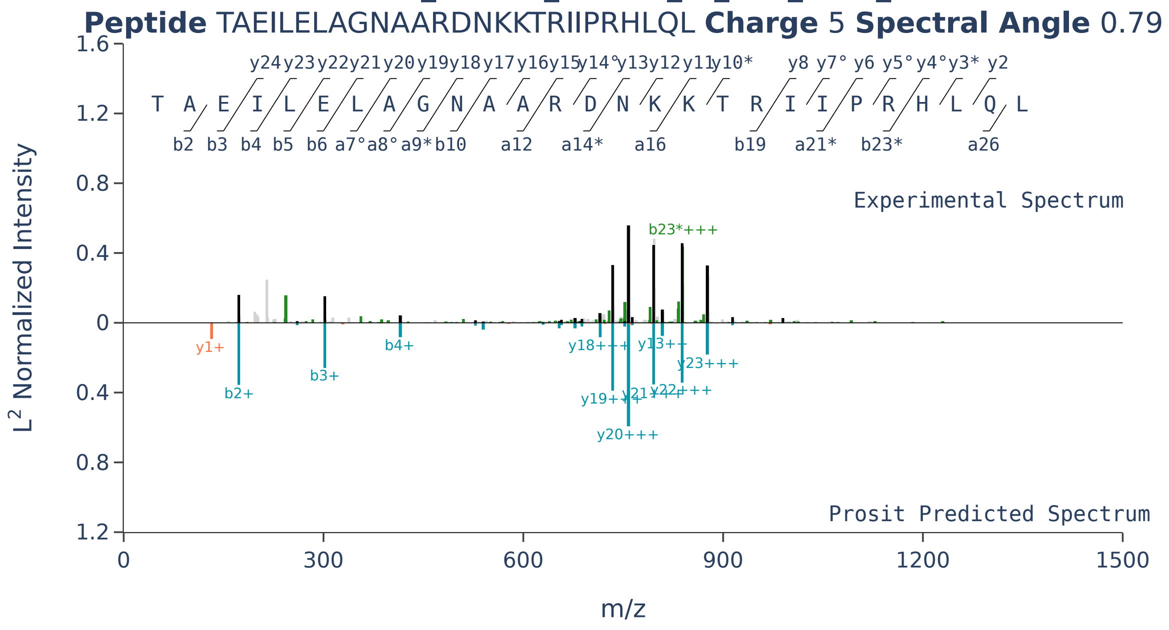


**Source** HRoetschke_291222_060123_Fu_H2A_b1R1_4h **Scan** 8929
**Peptide** KTRSSRAGLQFPVGRVHRL **Charge** 5 **Spectral Angle** 0.81

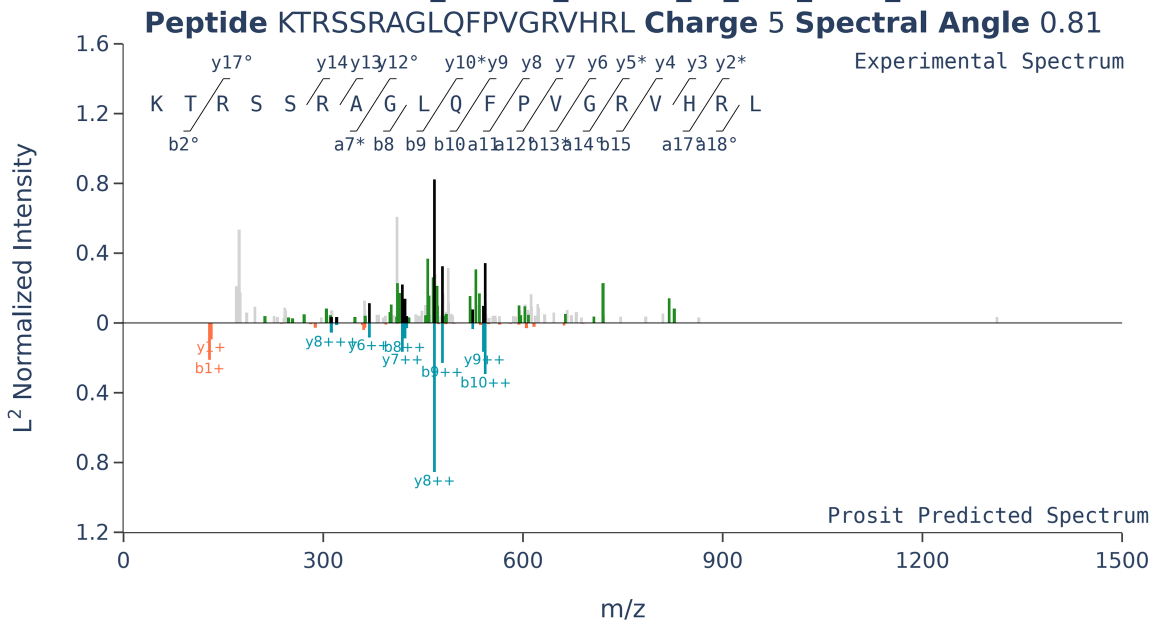


**Source** HRoetschke_291222_060123_Fu_H2A_b1R1_4h **Scan** 12631
**Peptide** LLELAGNAARDNKKTRIIPRHLQLA **Charge** 4 **Spectral Angle** 0.79

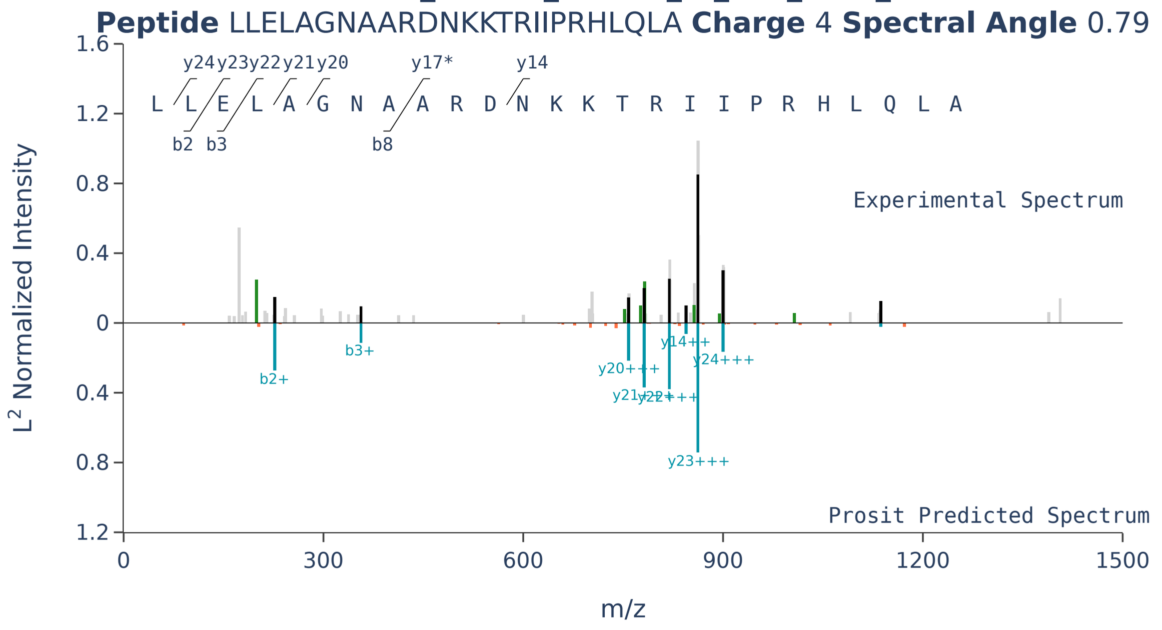


**Source** HRoetschke_291222_060123_Fu_H2A_b1R2_4h **Scan** 6521
**Peptide** AKTRSSRAGLQFPVGRVHR **Charge** 5 **Spectral Angle** 0.75

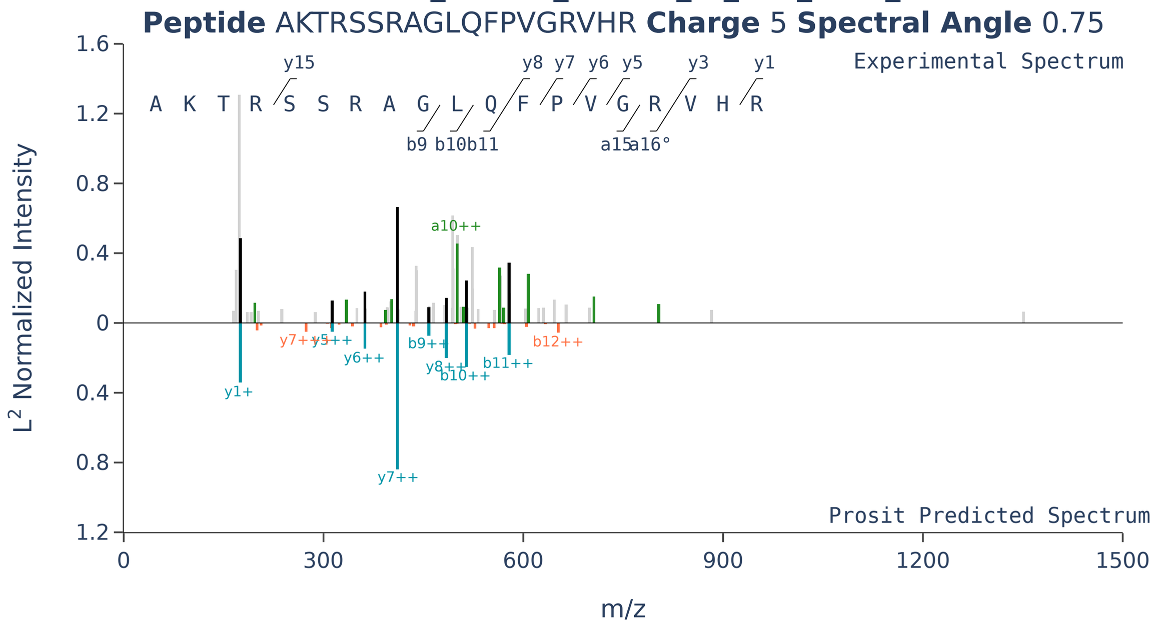


**Source** HRoetschke_291222_060123_Fu_H2A_b2R1_24h **Scan** 3110
**Peptide** ARDNKKTRIIPRHLQ **Charge** 4 **Spectral Angle** 0.69

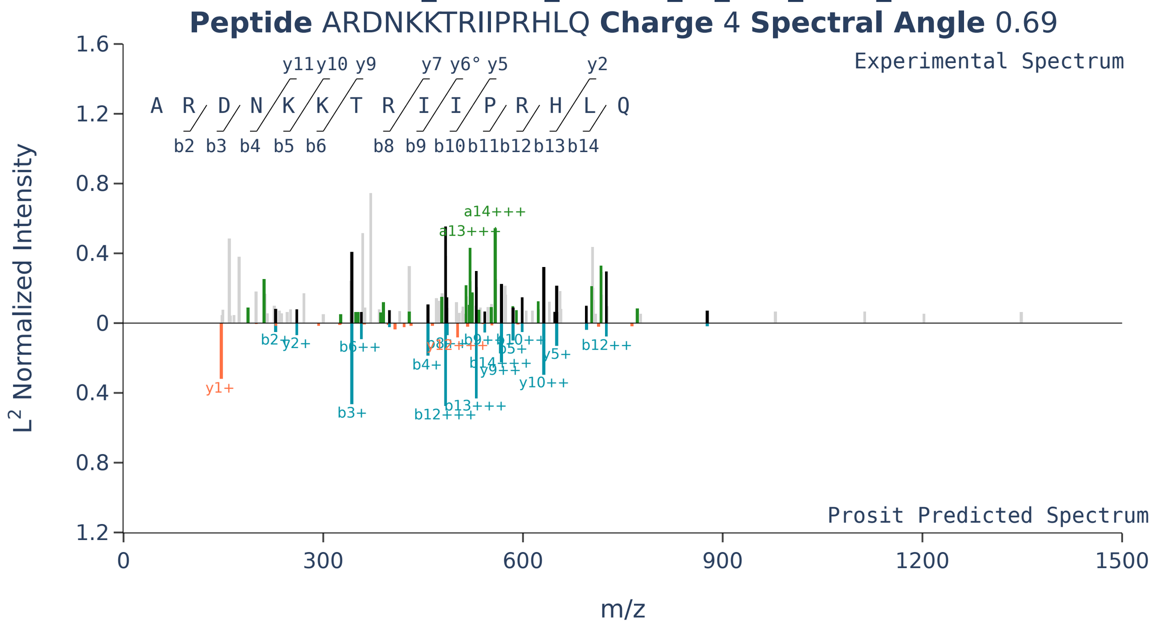


**Source** HRoetschke_291222_060123_Fu_H2A_b2R1_24h **Scan** 2040
**Peptide** AGNAARDNKKTRIIPRH **Charge** 4 **Spectral Angle** 0.75

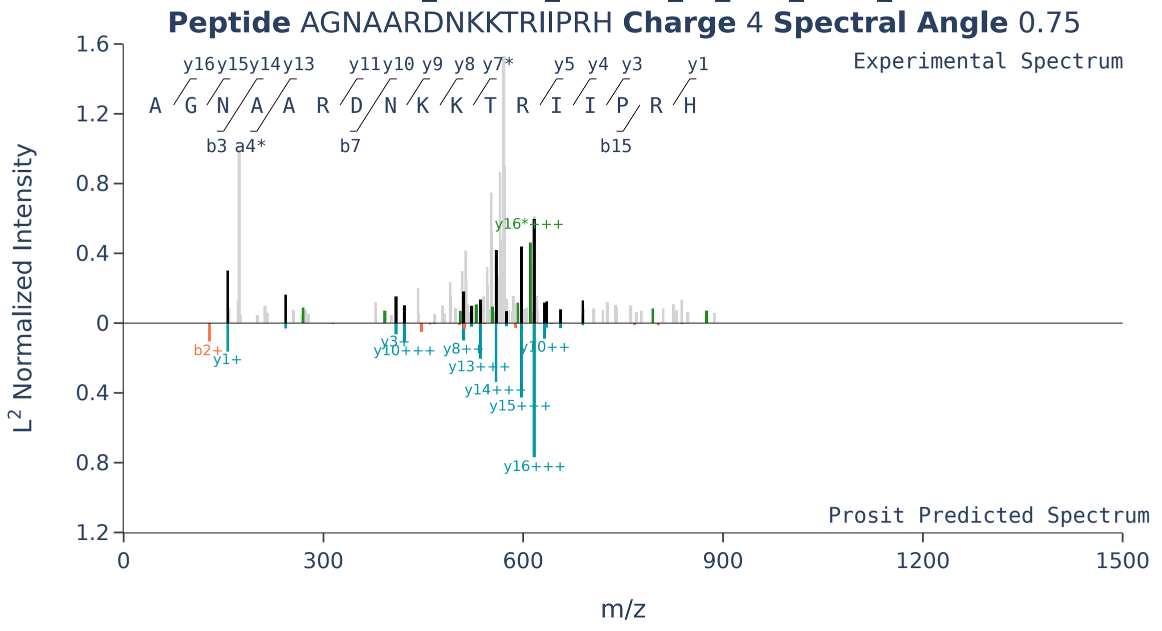


**Source** HRoetschke_291222_060123_Fu_H2A_b2R2_24h **Scan** 8001
**Peptide** LAGNAARDNKKTRIIPRHLQLA **Charge** 4 **Spectral Angle** 0.93

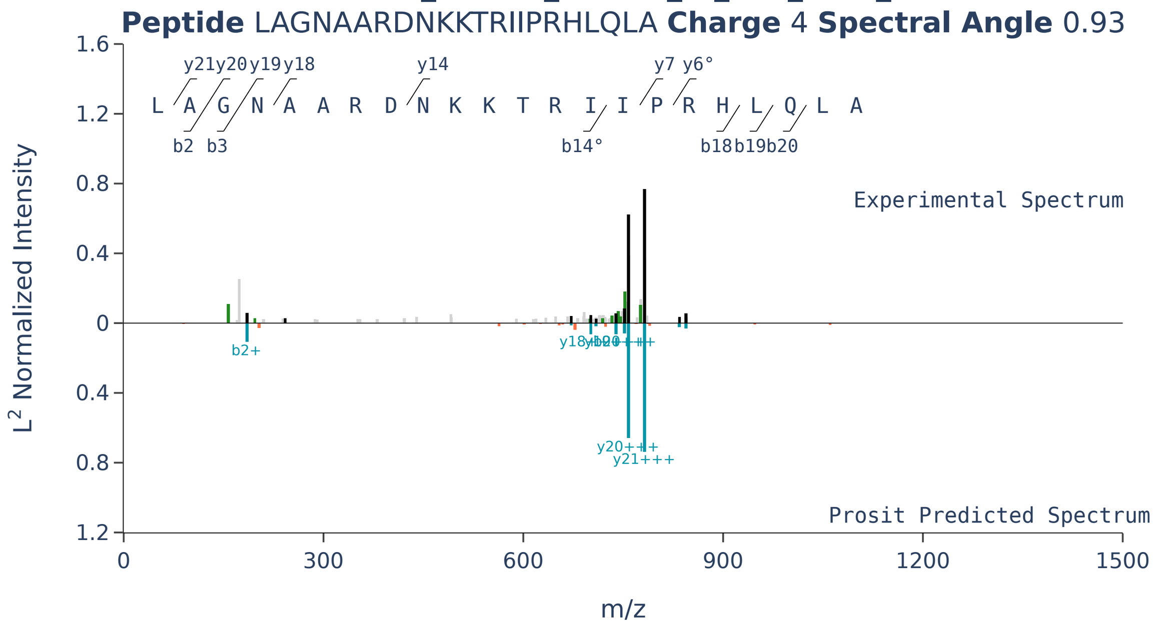


**Source** HRoetschke_291222_060123_Fu_H2A_b1R1_4h **Scan** 15694
**Peptide** GRVTIAQGGVLPNIQS **Charge** 2 **Spectral Angle** 0.81

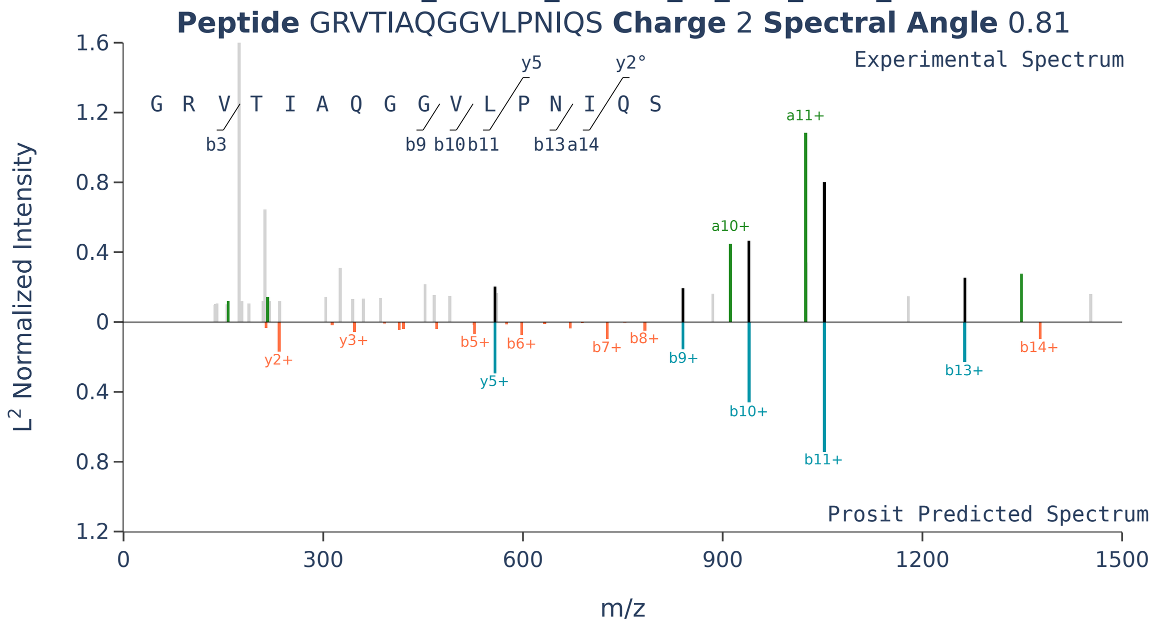


**Source** HRoetschke_291222_060123_Fu_H2A_b1R1_24h **Scan** 6949
**Peptide** LLELAGNAARDNKK **Charge** 3 **Spectral Angle** 0.64

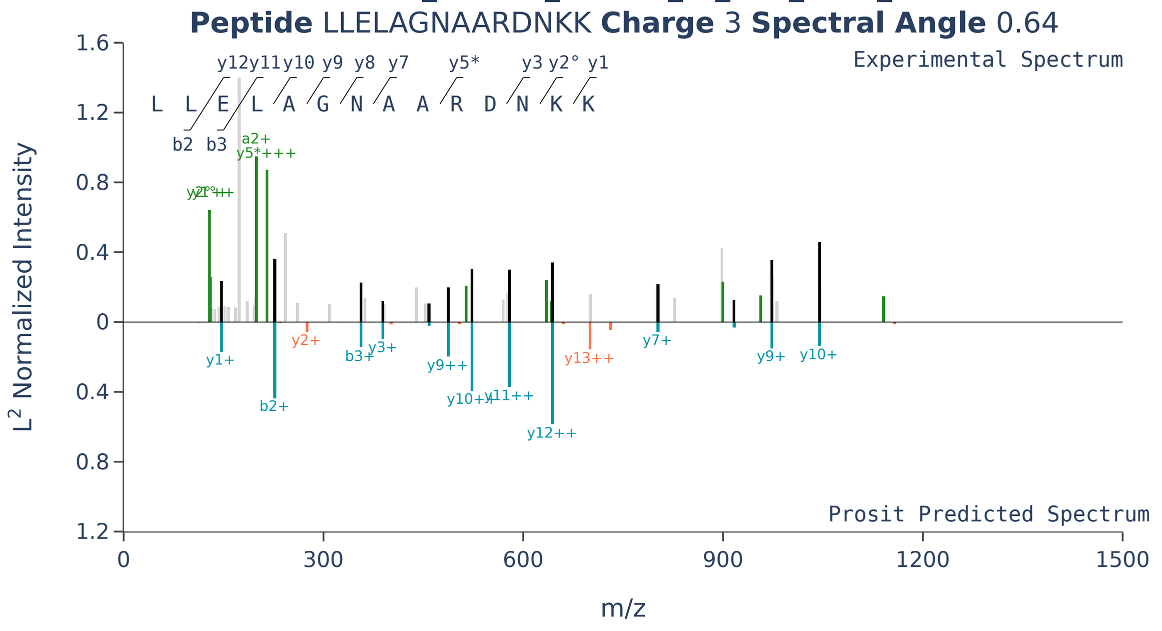


**Source** HRoetschke_291222_060123_Fu_H2A_b1R1_4h **Scan** 10755
**Peptide** RAGLQFPVGRVHRLLRK **Charge** 5 **Spectral Angle** 0.67

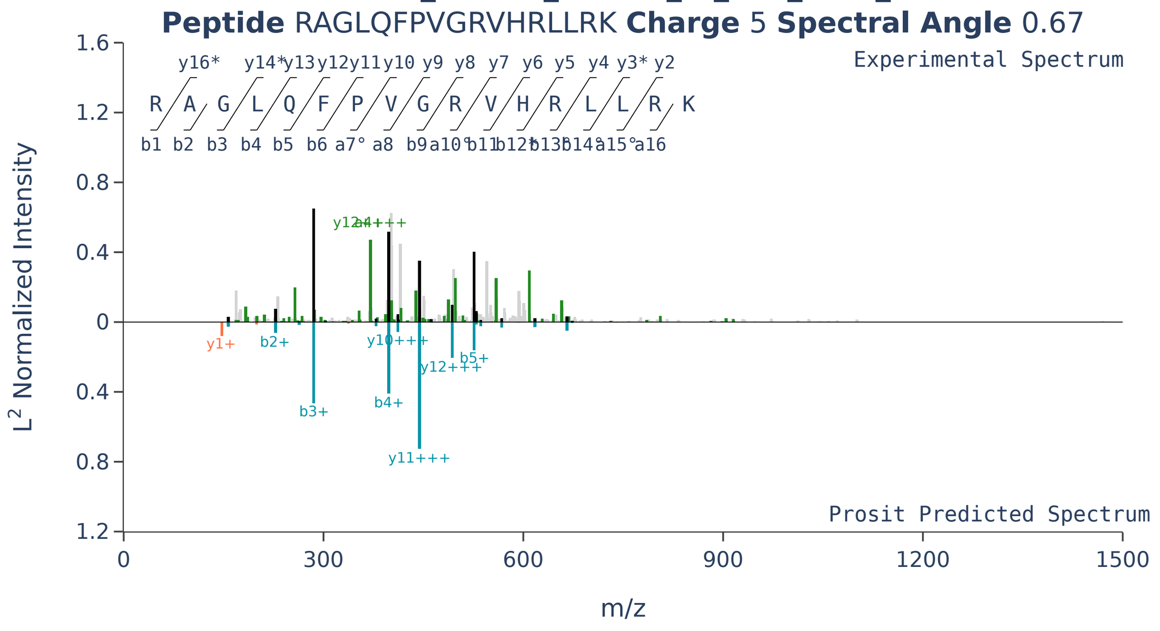


**Source** HRoetschke_291222_060123_Fu_H2A_b2R1_4h **Scan** 7170
**Peptide** NAARDNKKTRIIPRHLQLAVRND **Charge** 6 **Spectral Angle** 0.86

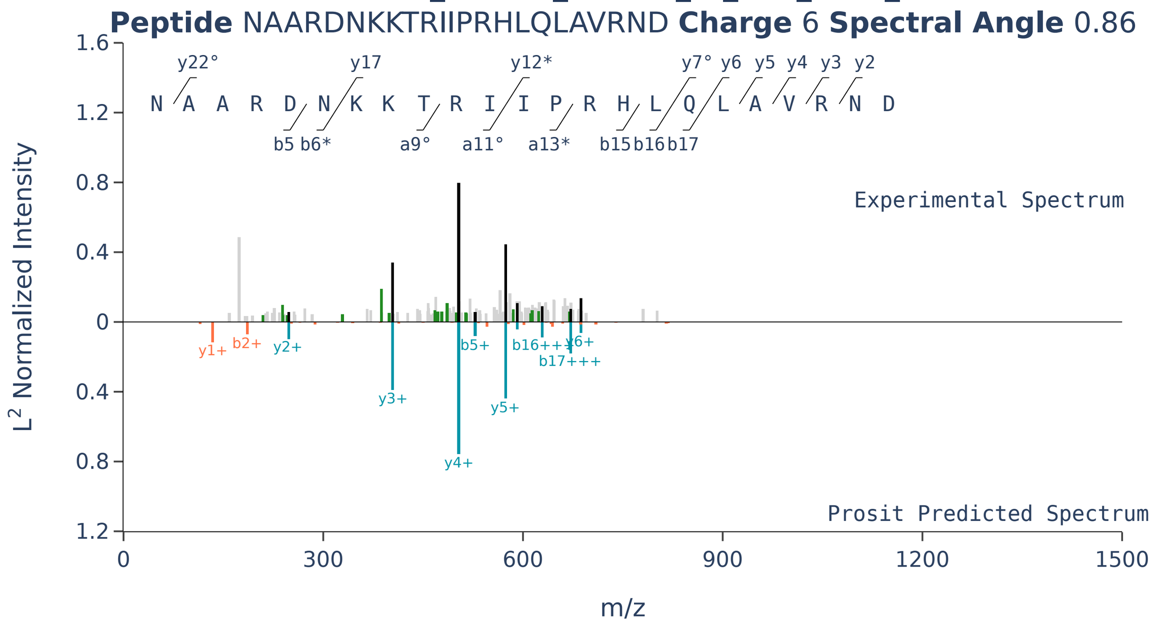


**Source** HRoetschke_291222_060123_Fu_H2A_b1R1_4h **Scan** 11654
**Peptide** RAGLQFPVGRVHRLLRKGNY **Charge** 5 **Spectral Angle** 0.73

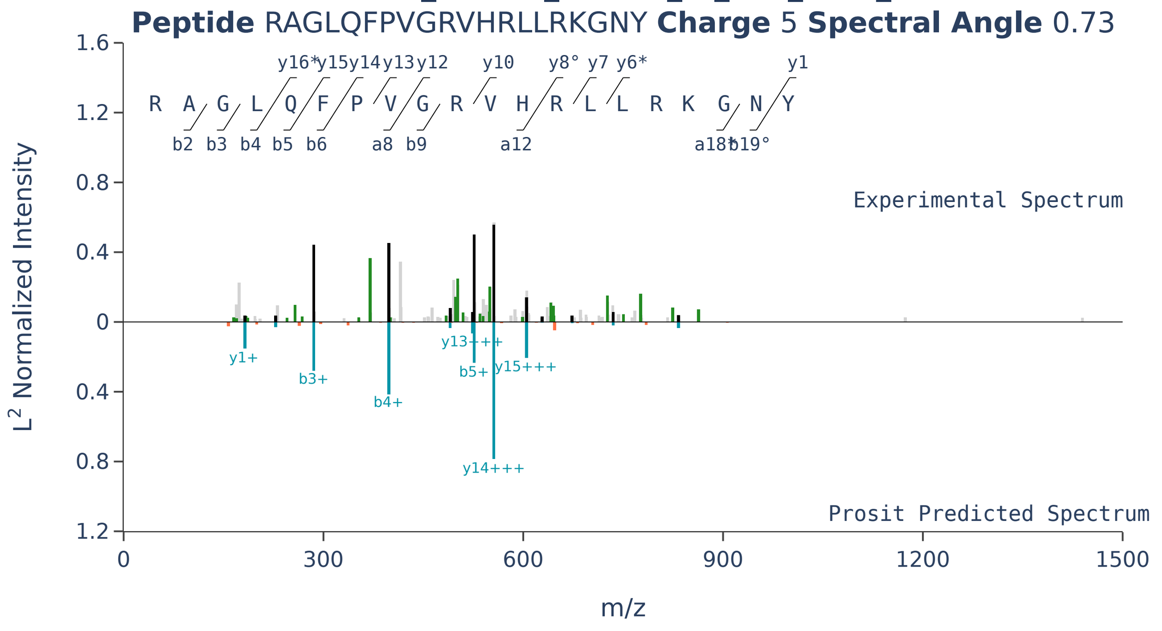


**Source** HRoetschke_291222_060123_Fu_H2A_b2R1_24h **Scan** 5176
**Peptide** GKTRAKAKTRSSRAGLQFPVGR **Charge** 5 **Spectral Angle** 0.81

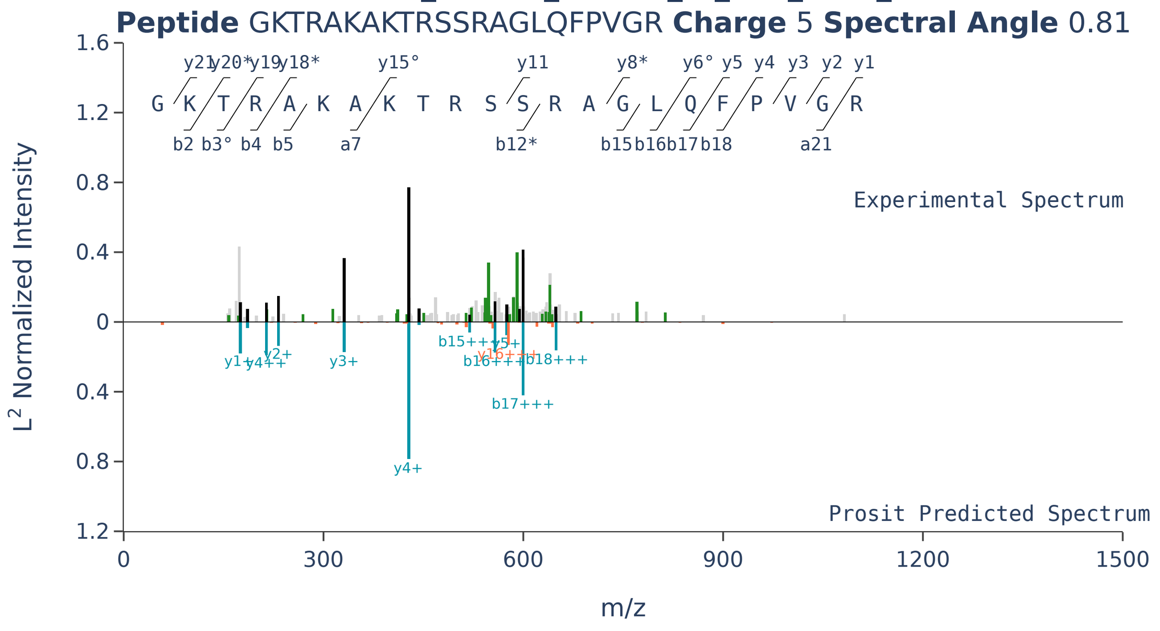

**Source** HRoetschke_291222_060123_Fu_H2A_b1R2_4h **Scan** 2885
**Peptide** RLLRKGNY **Charge** 2 **Spectral Angle** 0.7

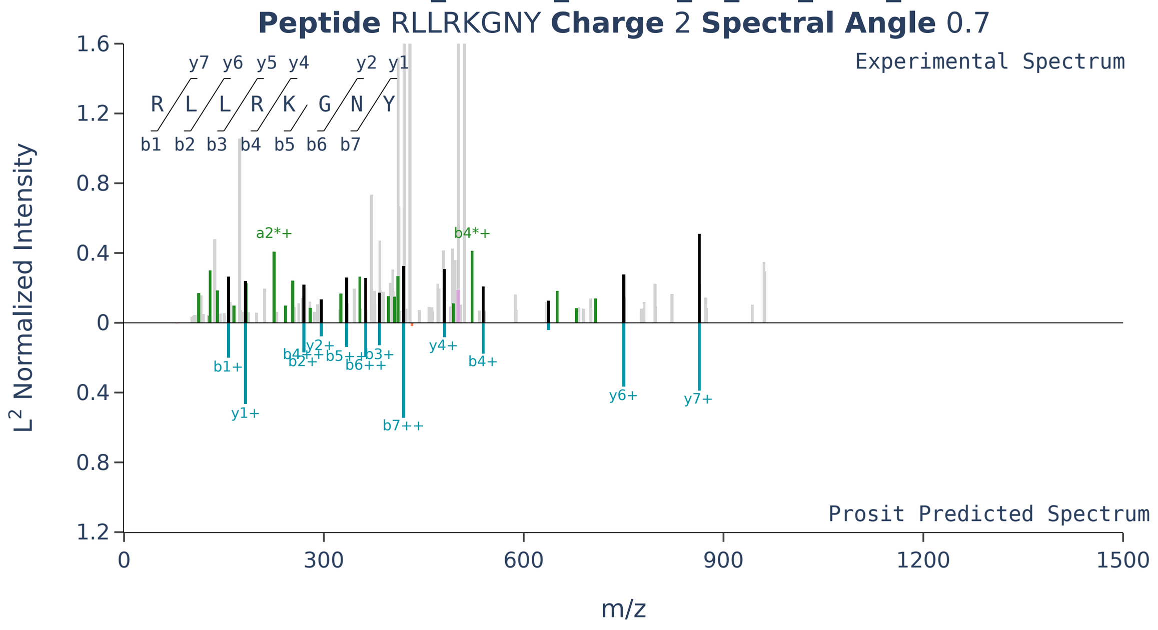


**Source** HRoetschke_291222_060123_Fu_H2A_b1R1_24h **Scan** 9670
**Peptide** YAERVGAGAPVY **Charge** 2 **Spectral Angle** 0.82

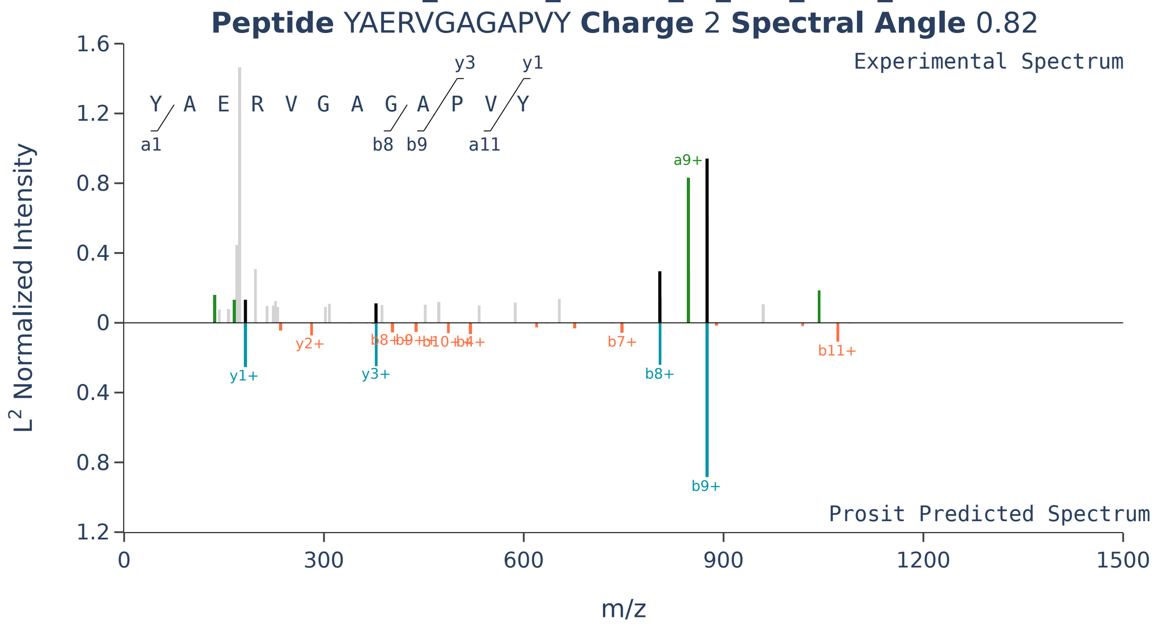


**Source** HRoetschke_291222_060123_Fu_H2A_b1R1_4h **Scan** 17086
**Peptide** LTAEILELAGNAARDNKKTRIIPRHL **Charge** 5 **Spectral Angle** 0.89

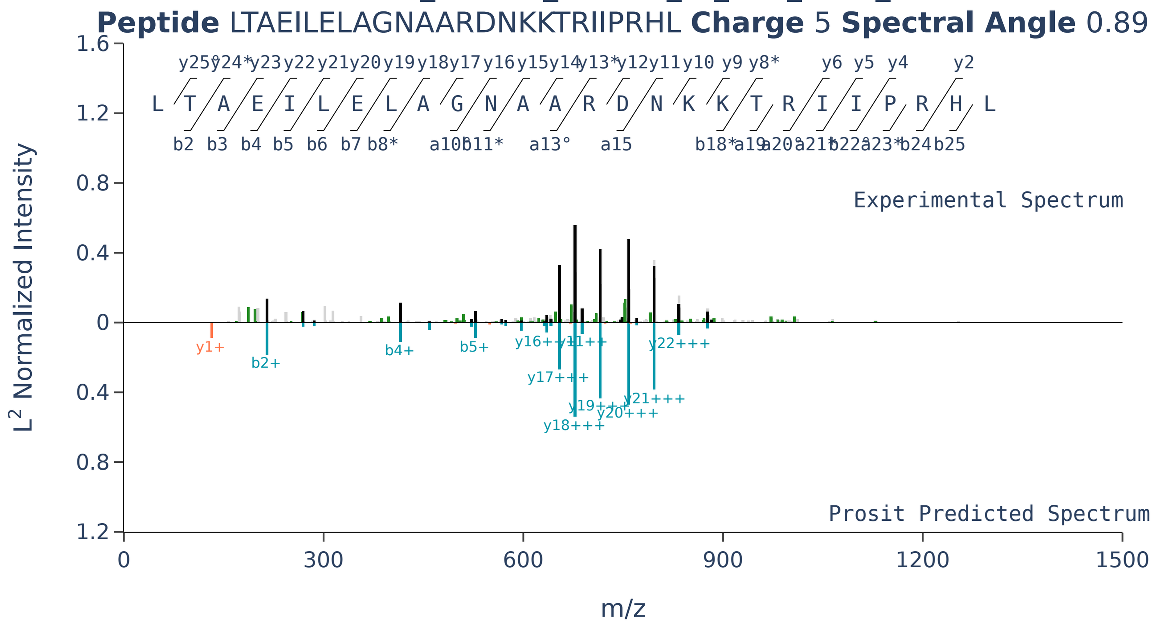


**Source** HRoetschke_291222_060123_Fu_H2A_b1R2_4h **Scan** 18241
**Peptide** RKGNYAERVGAGAPVYLAAVL **Charge** 4 **Spectral Angle** 0.85

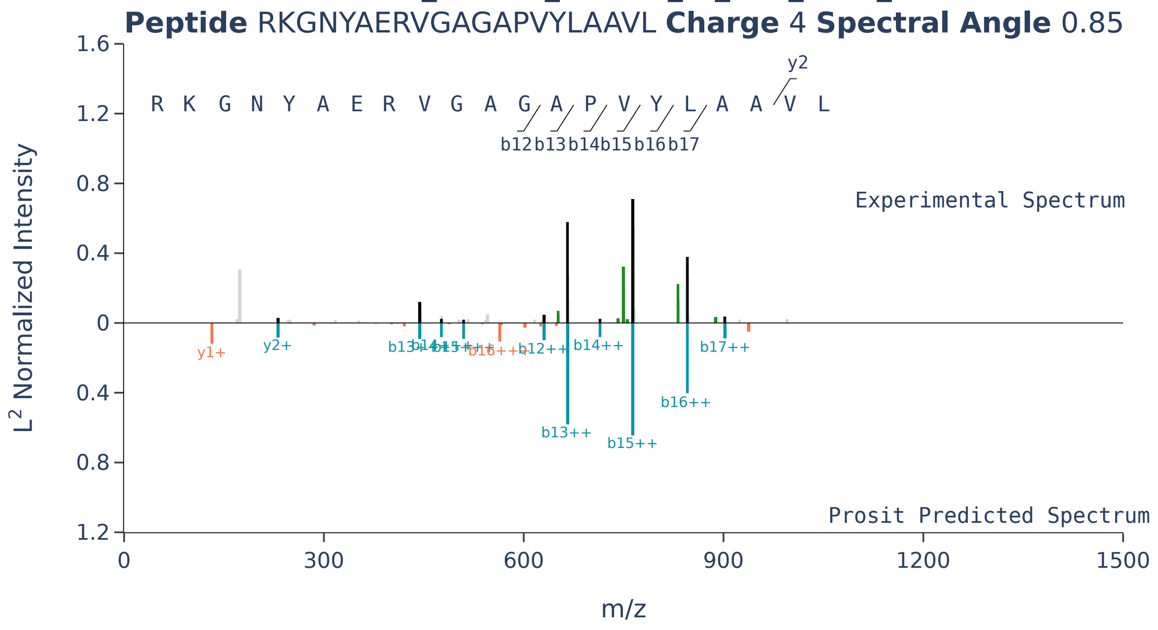


**Source** HRoetschke_291222_060123_Fu_H2A_b2R1_2h **Scan** 4637
**Peptide** AKAKTRSSRAGLQF **Charge** 3 **Spectral Angle** 0.91

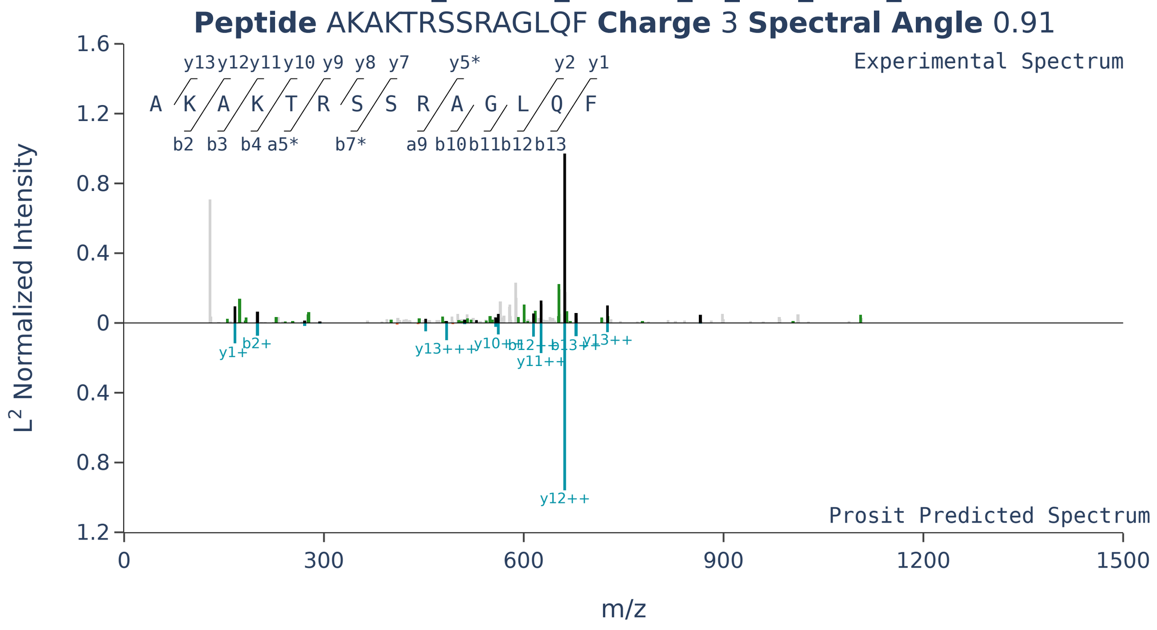


**Source** HRoetschke_291222_060123_Fu_H2A_b2R2_24h **Scan** 4719
**Peptide** RLLRKGNYAERVGAGA **Charge** 3 **Spectral Angle** 0.72

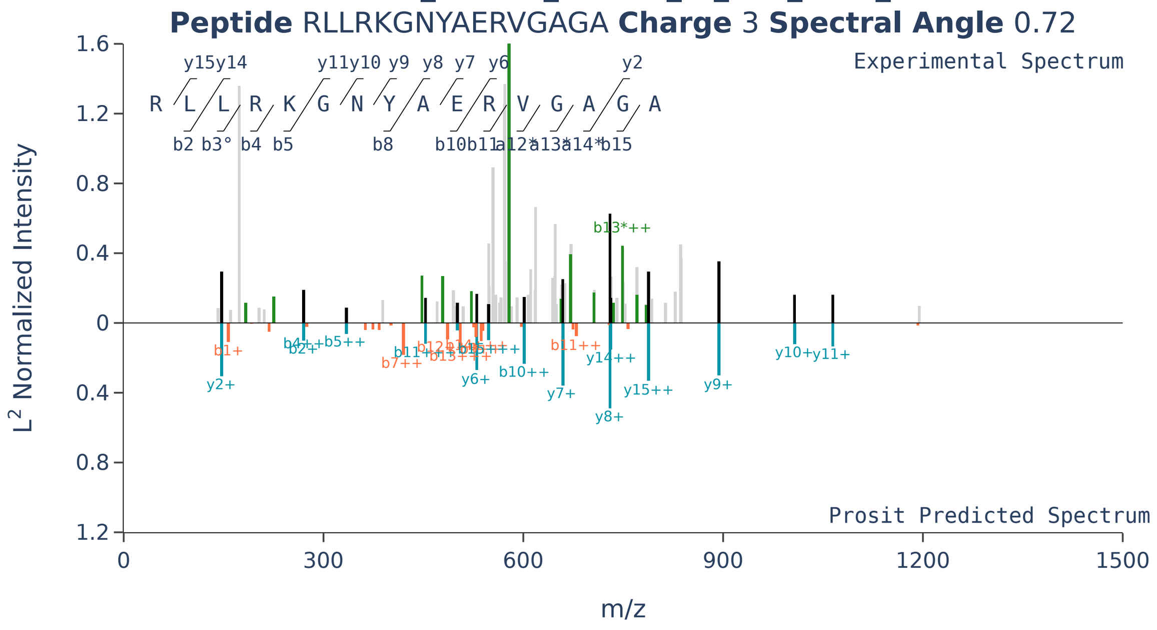


**Source** HRoetschke_291222_060123_Fu_H2A_b1R1_24h **Scan** 6777
**Peptide** KAKTRSSRAGLQFPVGR **Charge** 4 **Spectral Angle** 0.75

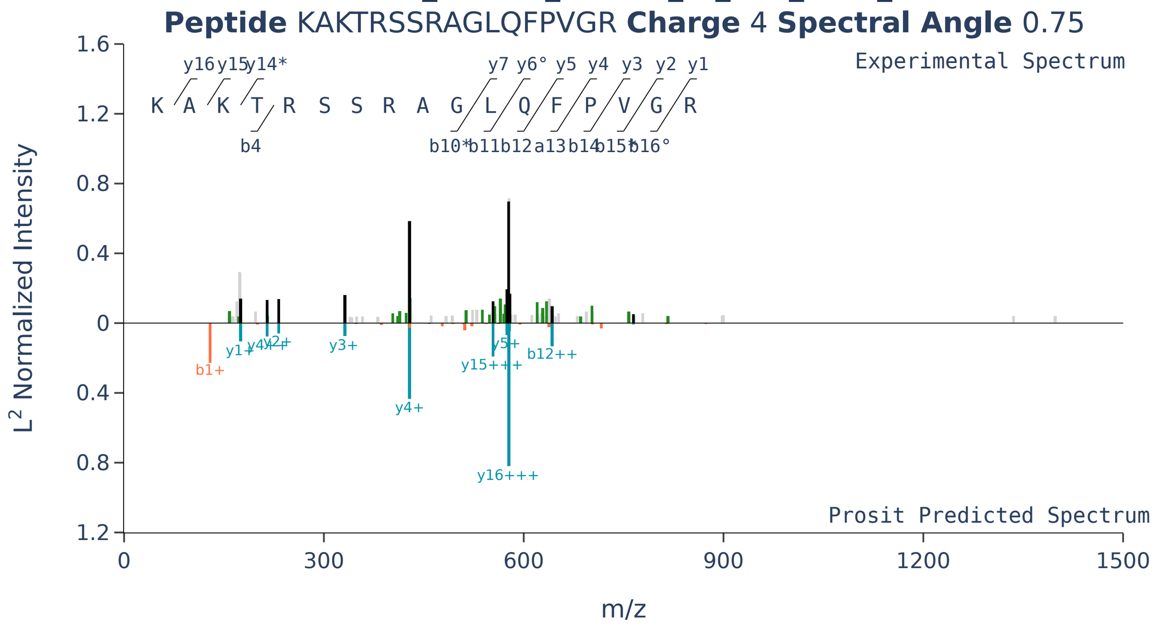


**Source** HRoetschke_291222_060123_Fu_H2A_b2R1_4h **Scan** 13061
**Peptide** TAEILELAGNAARDNKKTRIIPRHLQ **Charge** 4 **Spectral Angle** 0.83

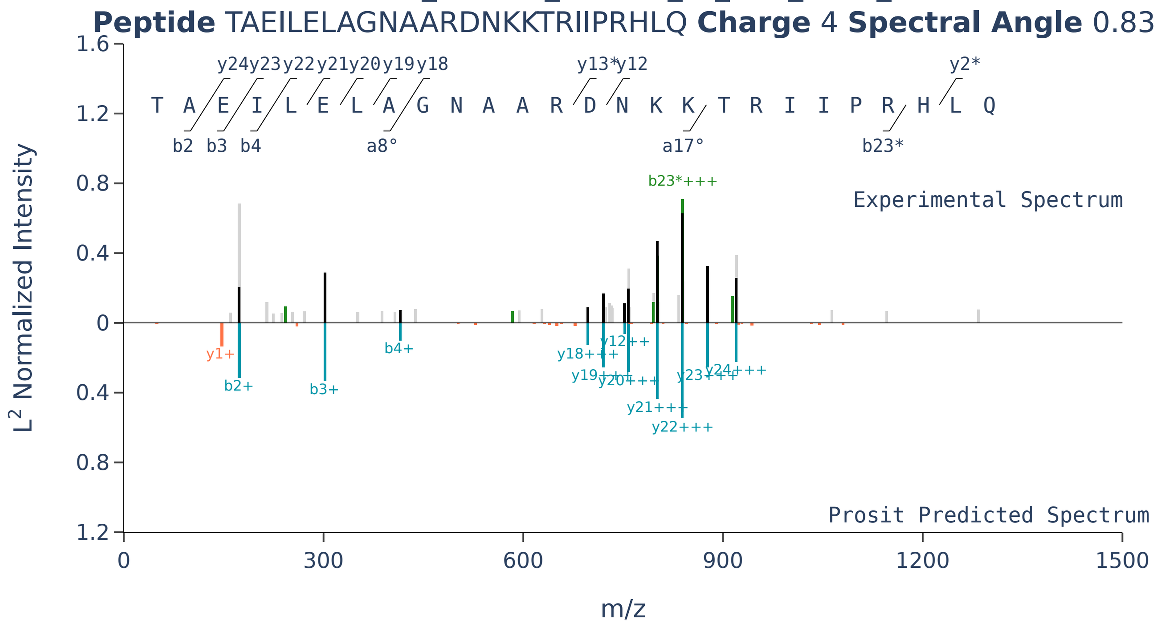


**Source** HRoetschke_291222_060123_Fu_H2A_b1R1_2h **Scan** 12186
**Peptide** GLQFPVGRVHRLLRKGNYAERVG **Charge** 5 **Spectral Angle** 0.57

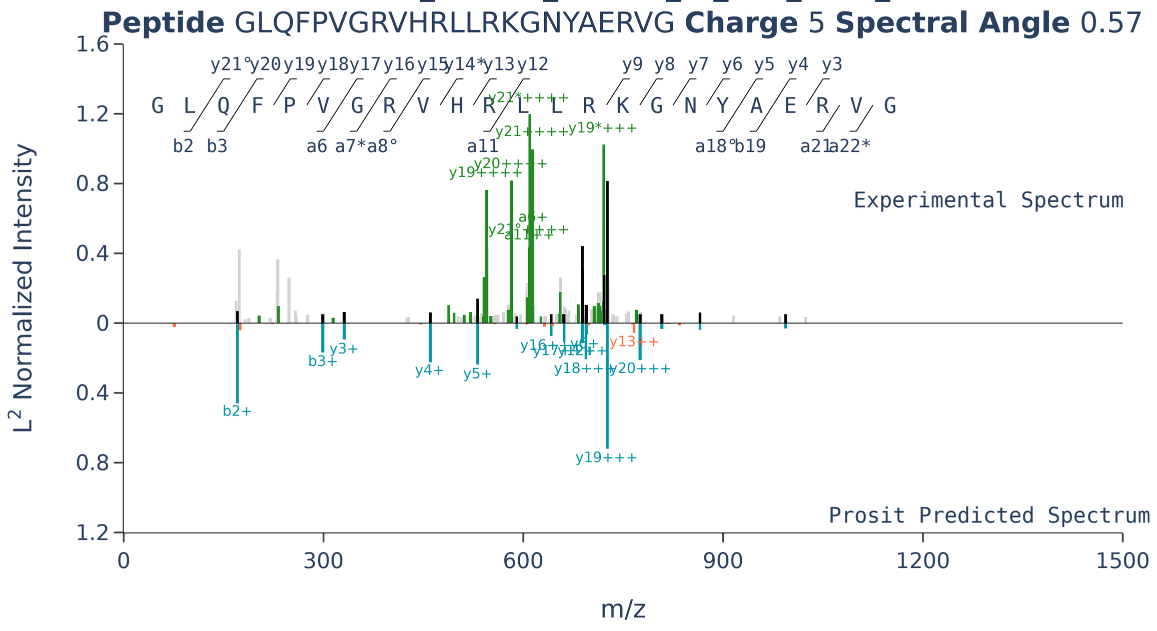


**Source** HRoetschke_291222_060123_Fu_H2A_b1R2_2h **Scan** 12203
**Peptide** GLQFPVGRVHRLLRKGNY **Charge** 4 **Spectral Angle** 0.83

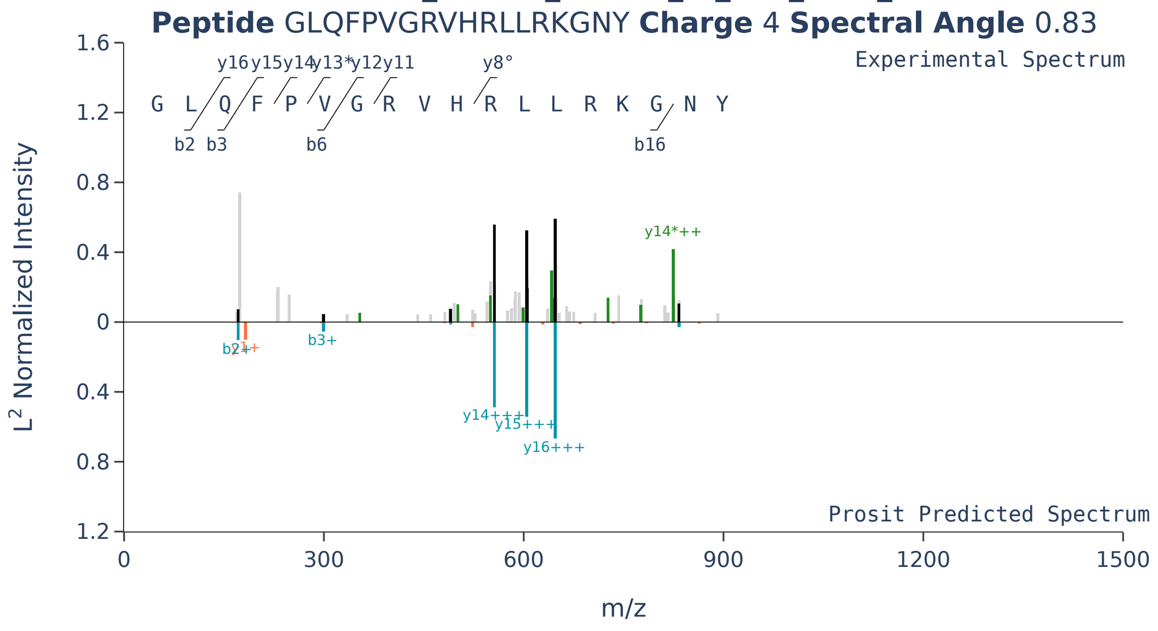


**Source** HRoetschke_291222_060123_Fu_H2A_b1R1_1h **Scan** 1788
**Peptide** LLPKKTESSKSAKS **Charge** 3 **Spectral Angle** 0.73

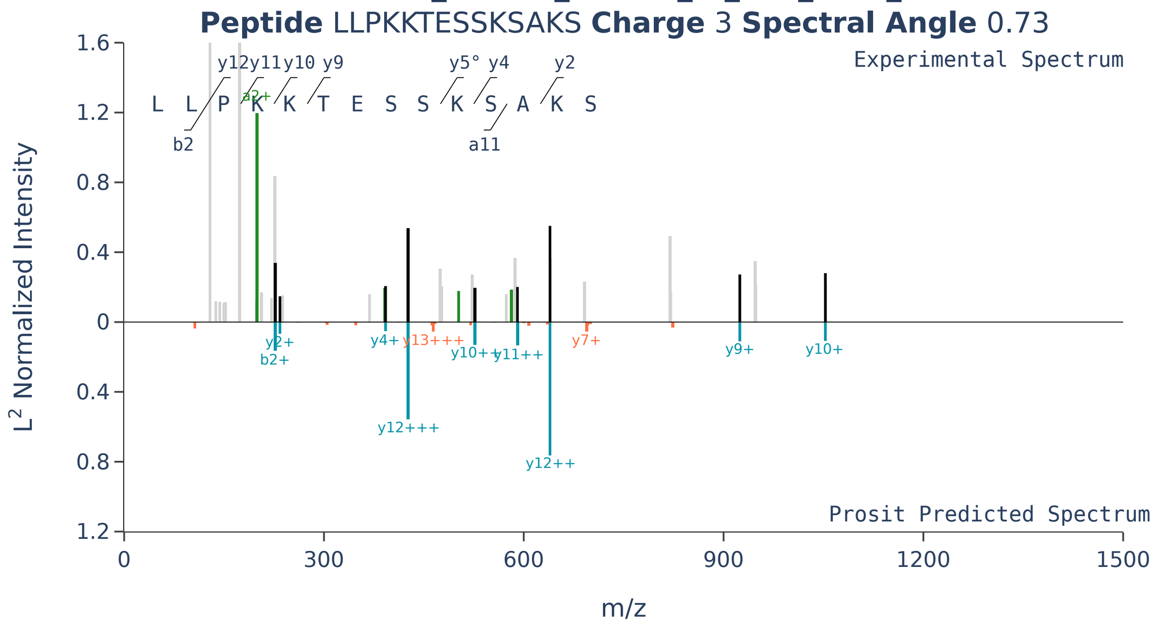


**Source** HRoetschke_291222_060123_Fu_H2A_b2R2_4h **Scan** 7248
**Peptide** AARDNKKTRIIPRHLQL **Charge** 4 **Spectral Angle** 0.66

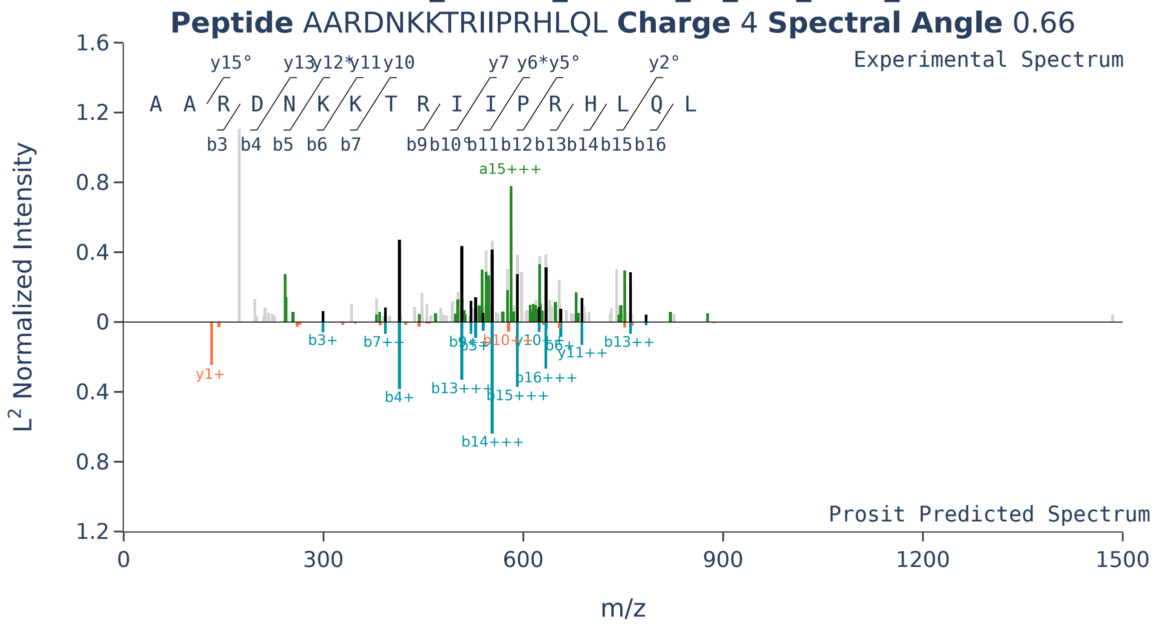


**Source** HRoetschke_291222_060123_Fu_H2A_b1R2_2h **Scan** 8546
**Peptide** AKTRSSRAGLQFPVGRVHRL **Charge** 5 **Spectral Angle** 0.85

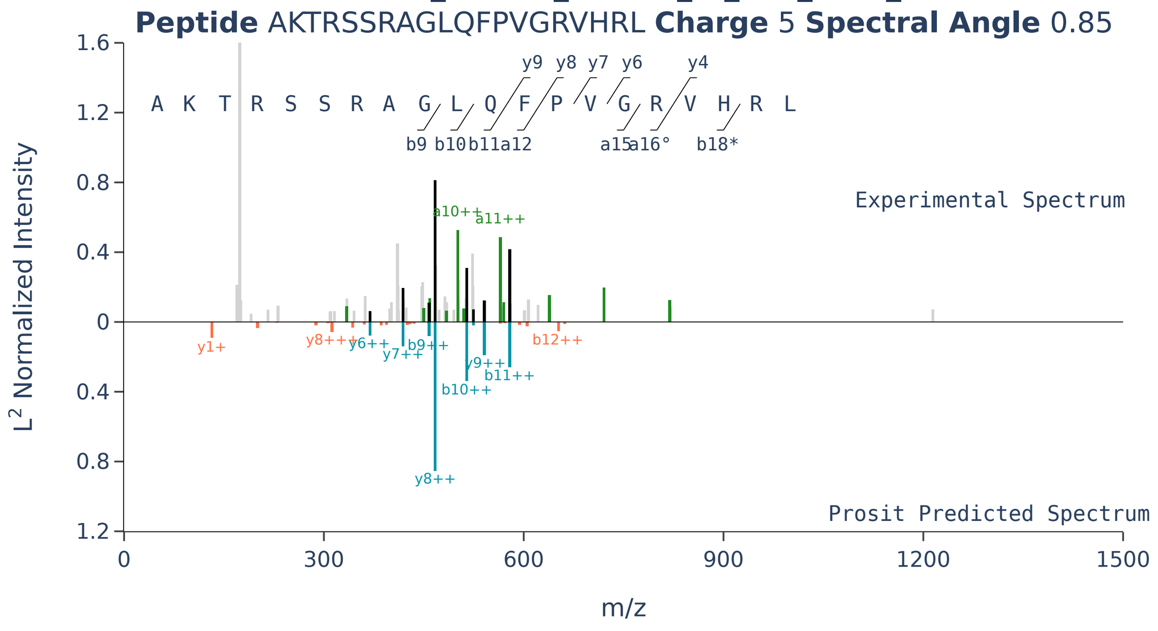


**Source** HRoetschke_291222_060123_Fu_H2A_b1R2_4h **Scan** 8447
**Peptide** PVGRVHRLLRKGNYAERVGAGAPVY **Charge** 4 **Spectral Angle** 0.82

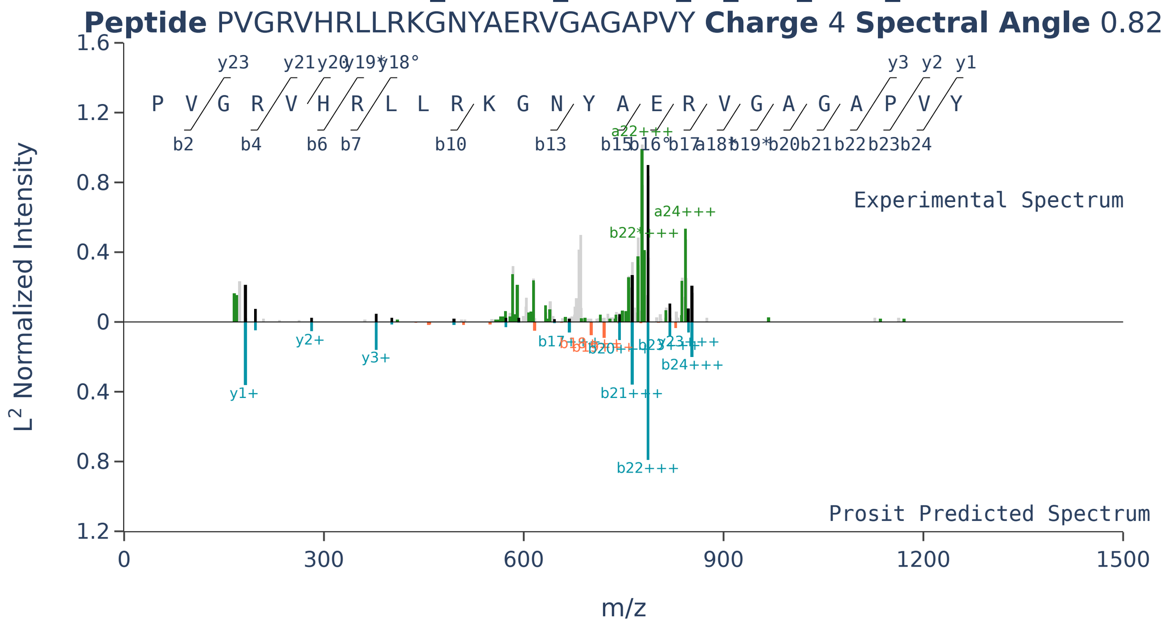


**Source** HRoetschke_291222_060123_Fu_H2A_b1R2_4h **Scan** 16500
**Peptide** VRNDEELNKLLGRVTIAQG **Charge** 3 **Spectral Angle** 0.72

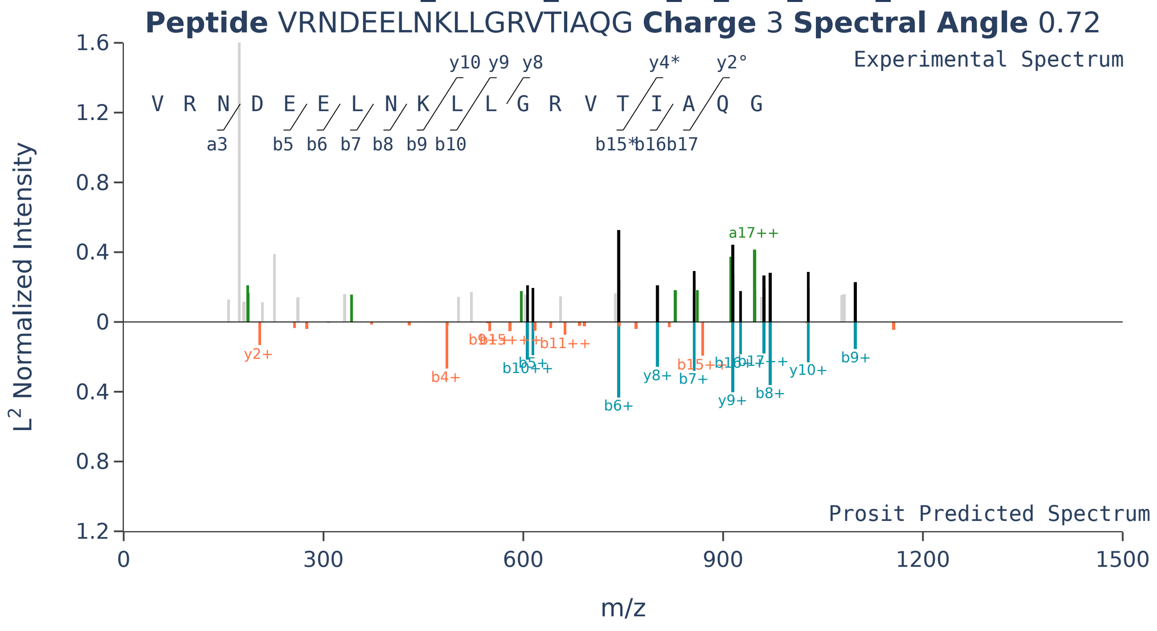

**Source** HRoetschke_291222_060123_Fu_H2A_b1R2_24h **Scan** 7931
**Peptide** QFPVGRVHRLLRK **Charge** 4 **Spectral Angle** 0.76

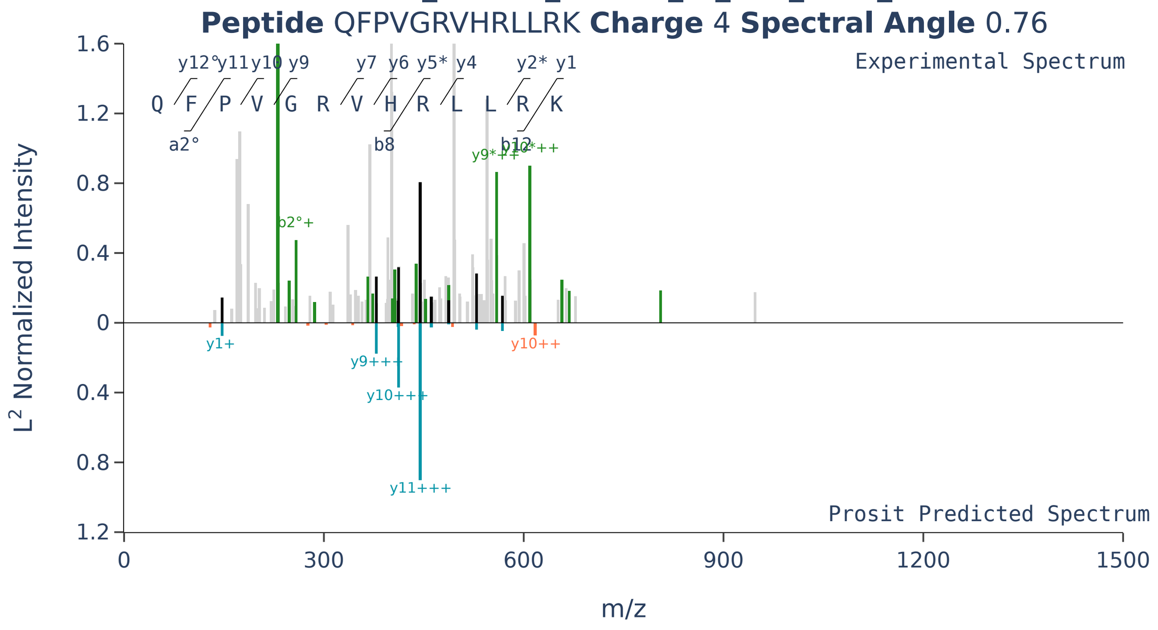


**Source** HRoetschke_291222_060123_Fu_H2A_b1R2_2h **Scan** 6594
**Peptide** GNAARDNKKTRIIPRHLQLA **Charge** 4 **Spectral Angle** 0.85

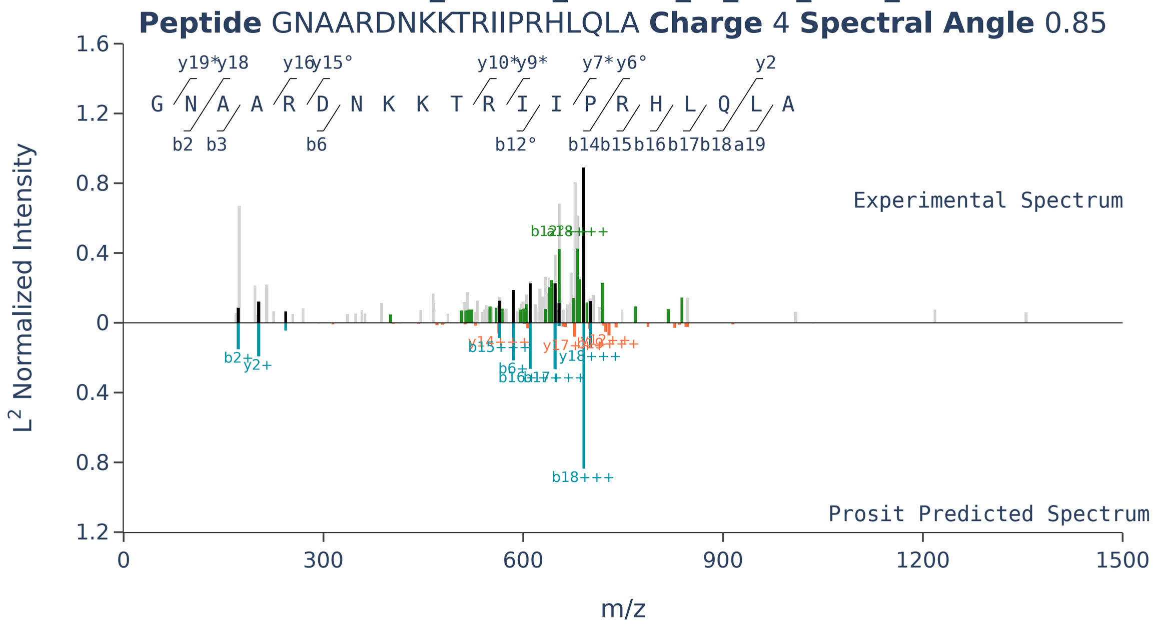


**Source** HRoetschke_291222_060123_Fu_H2A_b1R1_2h **Scan** 17453
**Peptide** RLLRKGNYAERVGAGAPVYLAAVL **Charge** 4 **Spectral Angle** 0.72

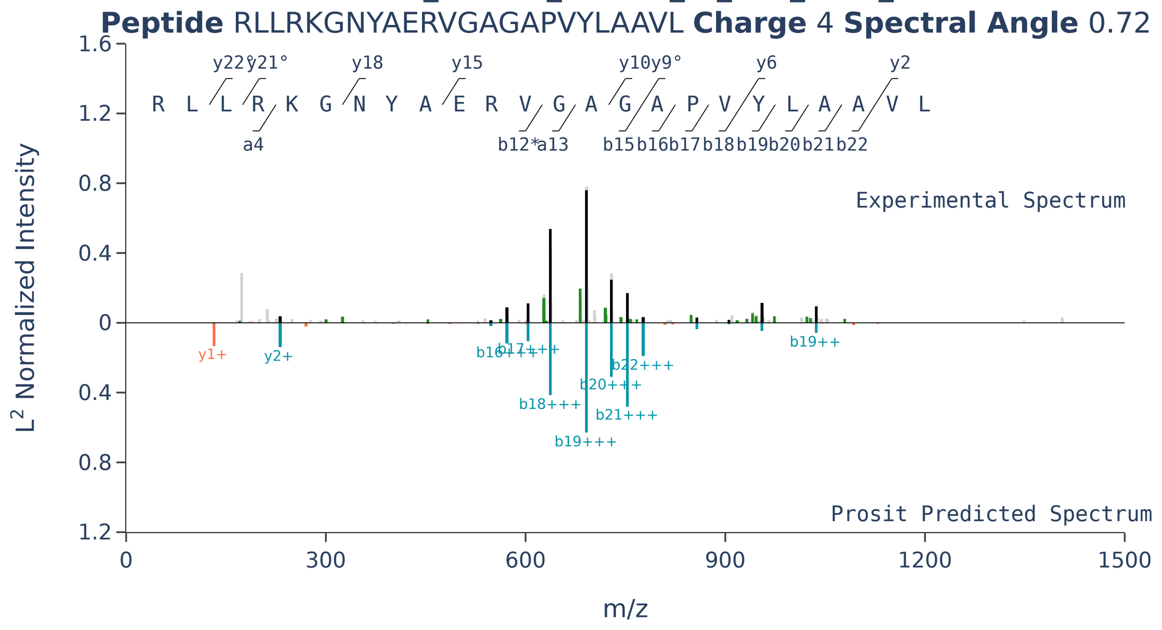


**Source** HRoetschke_291222_060123_Fu_H2A_b1R1_24h **Scan** 12252
**Peptide** ELLELAGNAARD **Charge** 2 **Spectral Angle** 0.67

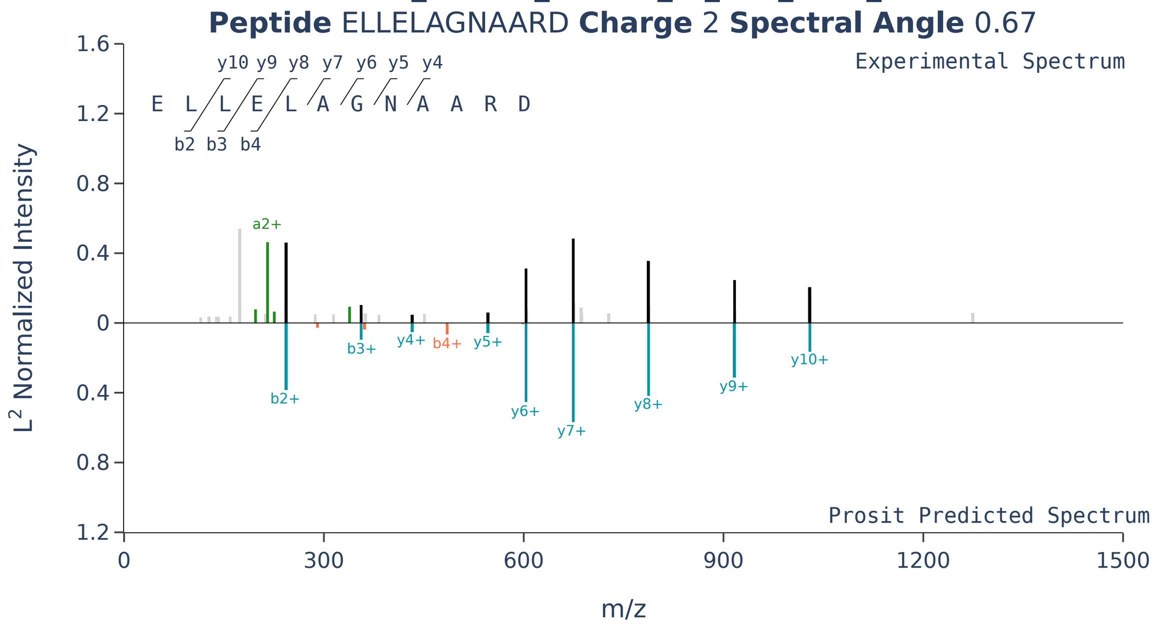


**Source** HRoetschke_291222_060123_Fu_H2A_b1R2_2h **Scan** 9707
**Peptide** RVHRLLRKGNYAERVGAGAPVYL **Charge** 5 **Spectral Angle** 0.68

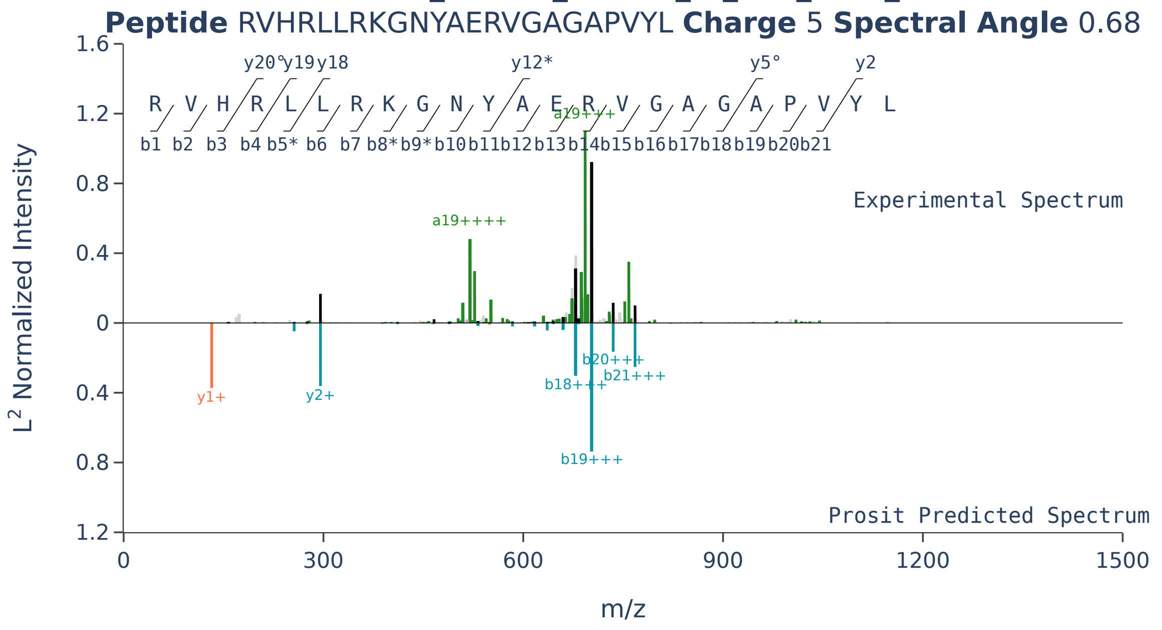


**Source** HRoetschke_291222_060123_Fu_H2A_b1R1_1h **Scan** 9485
**Peptide** ELLELAGNAARDNKKTRIIPRHLQ **Charge** 5 **Spectral Angle** 0.75

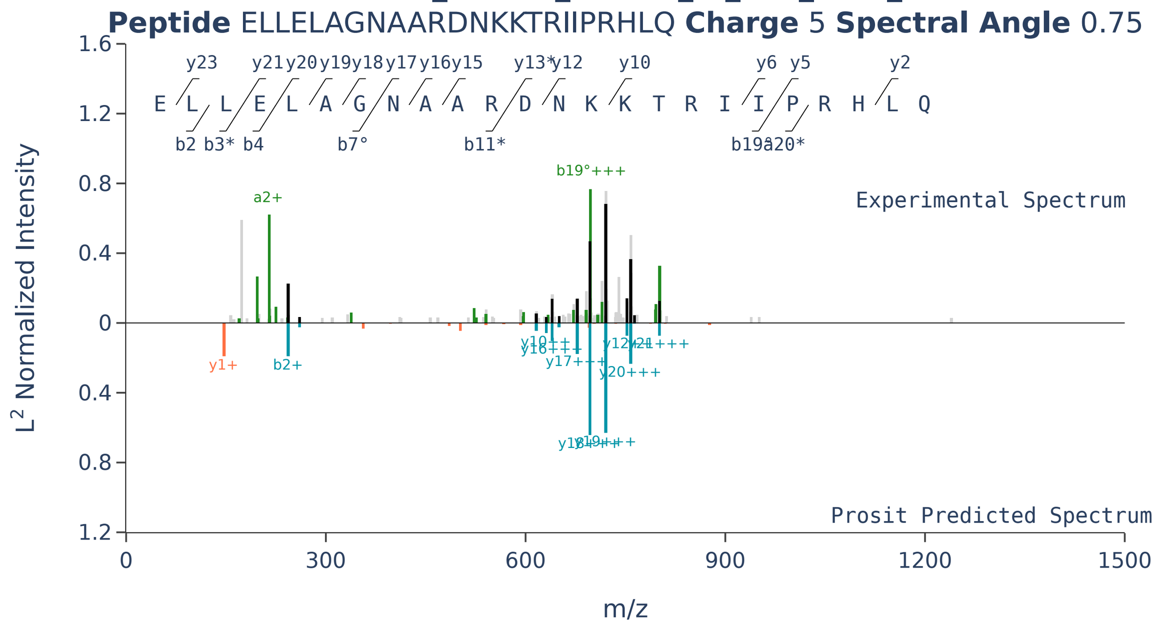


**Source** HRoetschke_291222_060123_Fu_H2A_b1R2_1h **Scan** 8855
**Peptide** LELAGNAARDNKKTRIIPRHLQLA **Charge** 5 **Spectral Angle** 0.79

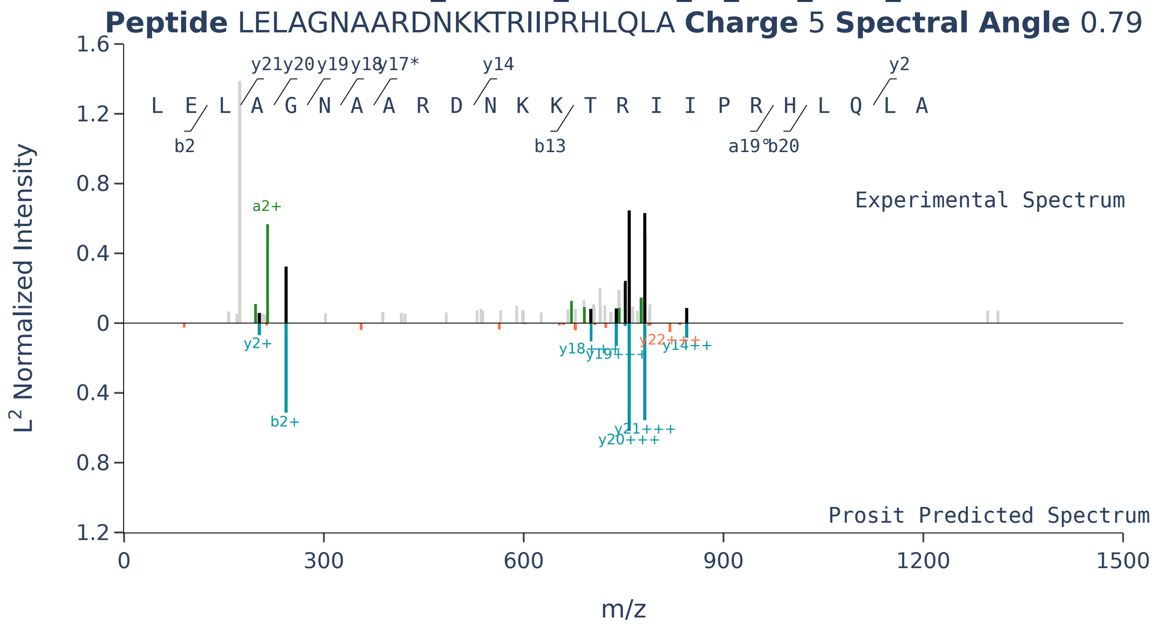


**Source** HRoetschke_291222_060123_Fu_H2A_b2R1_4h **Scan** 5741
**Peptide** PVGRVHRLLRKGNYAERVGA **Charge** 5 **Spectral Angle** 0.85

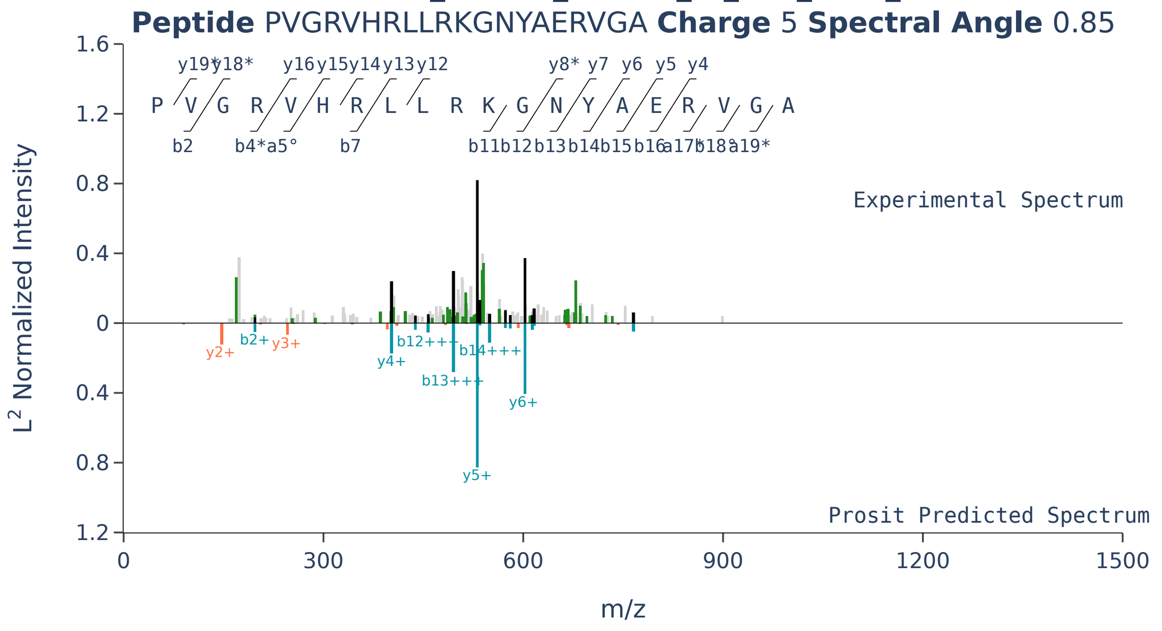


**Source** HRoetschke_291222_060123_Fu_H2A_b2R2_2h **Scan** 8572
**Peptide** KAKTRSSRAGLQFPVGRV **Charge** 5 **Spectral Angle** 0.69

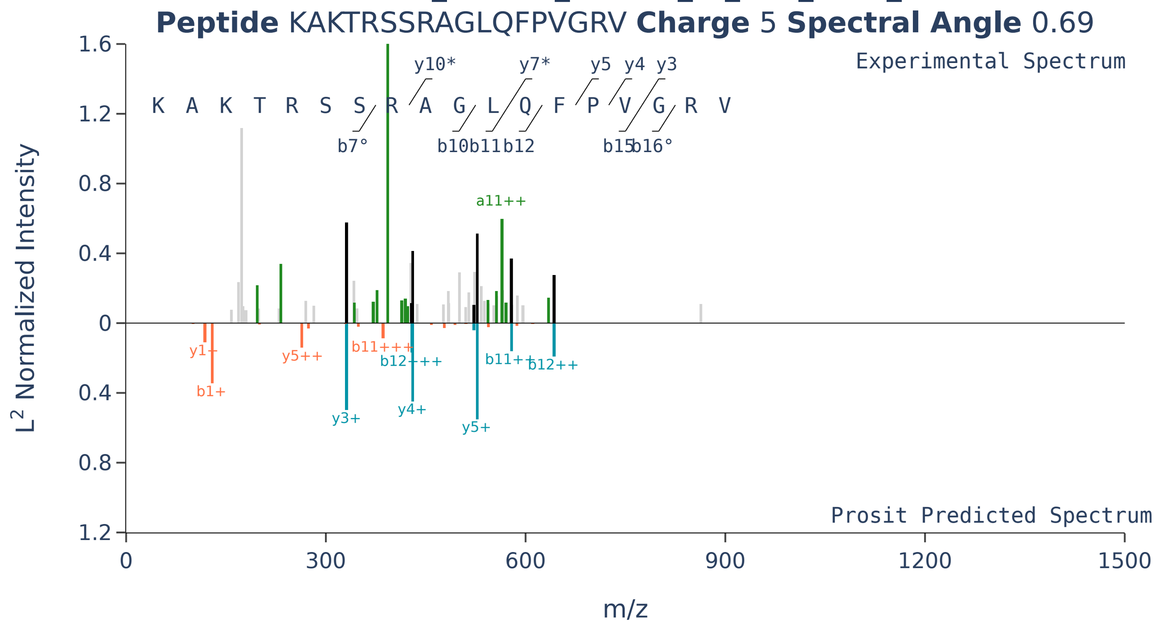


**Source** HRoetschke_291222_060123_Fu_H2A_b1R2_4h **Scan** 9541
**Peptide** RDNKKTRIIPRHLQLAV **Charge** 4 **Spectral Angle** 0.78

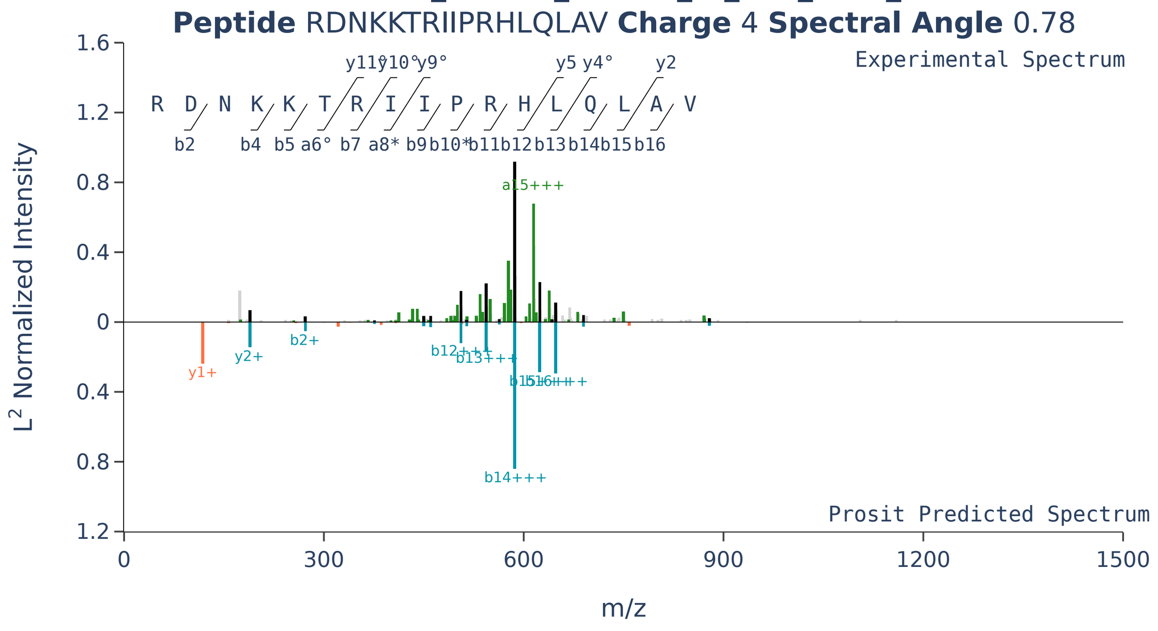


**Source** HRoetschke_291222_060123_Fu_H2A_b1R1_4h **Scan** 8111
**Peptide** LAGNAARDNKKTRIIPRHLQLAVR **Charge** 6 **Spectral Angle** 0.68

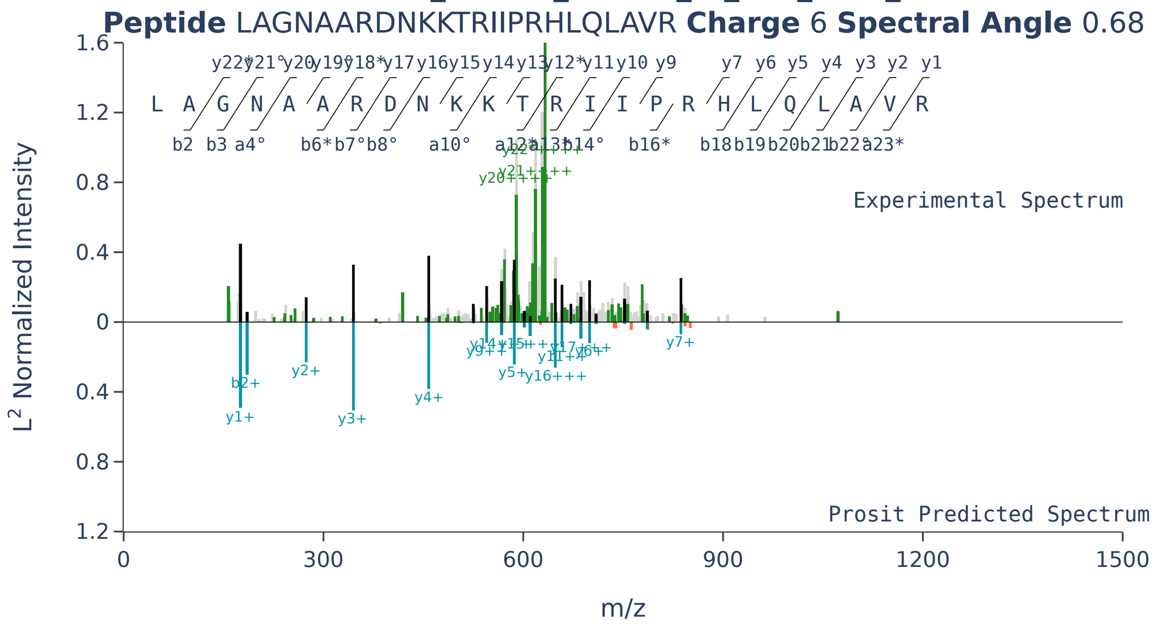


**Source** HRoetschke_291222_060123_Fu_H2A_b1R1_24h **Scan** 10224
**Peptide** ELLELAGNAARDNKKTRI **Charge** 4 **Spectral Angle** 0.68

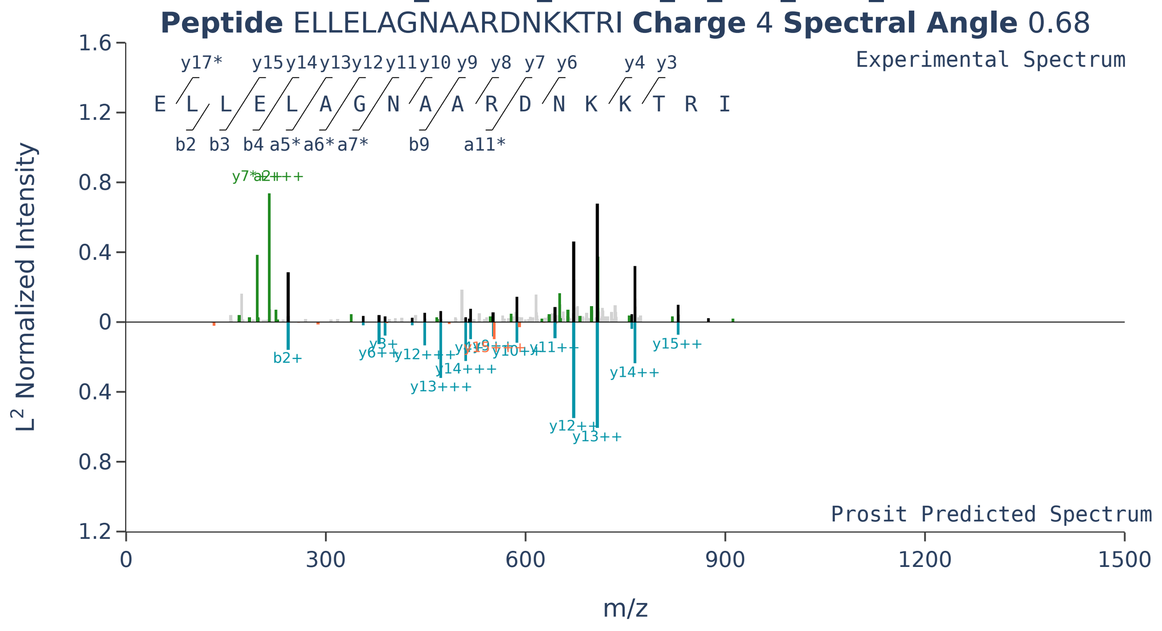


**Source** HRoetschke_291222_060123_Fu_H2A_b1R1_4h **Scan** 6422
**Peptide** QSVLLPKKT **Charge** 3 **Spectral Angle** 0.8

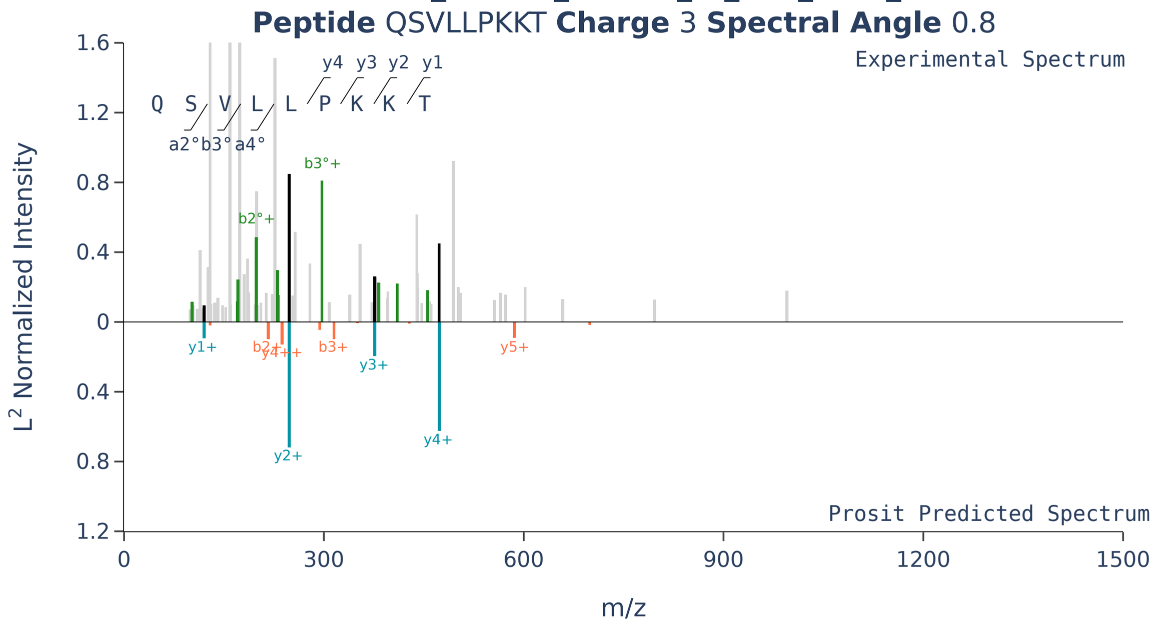


**Source** HRoetschke_291222_060123_Fu_H2A_b1R1_1h **Scan** 3752
**Peptide** NAARDNKKTRIIPRHL **Charge** 4 **Spectral Angle** 0.63

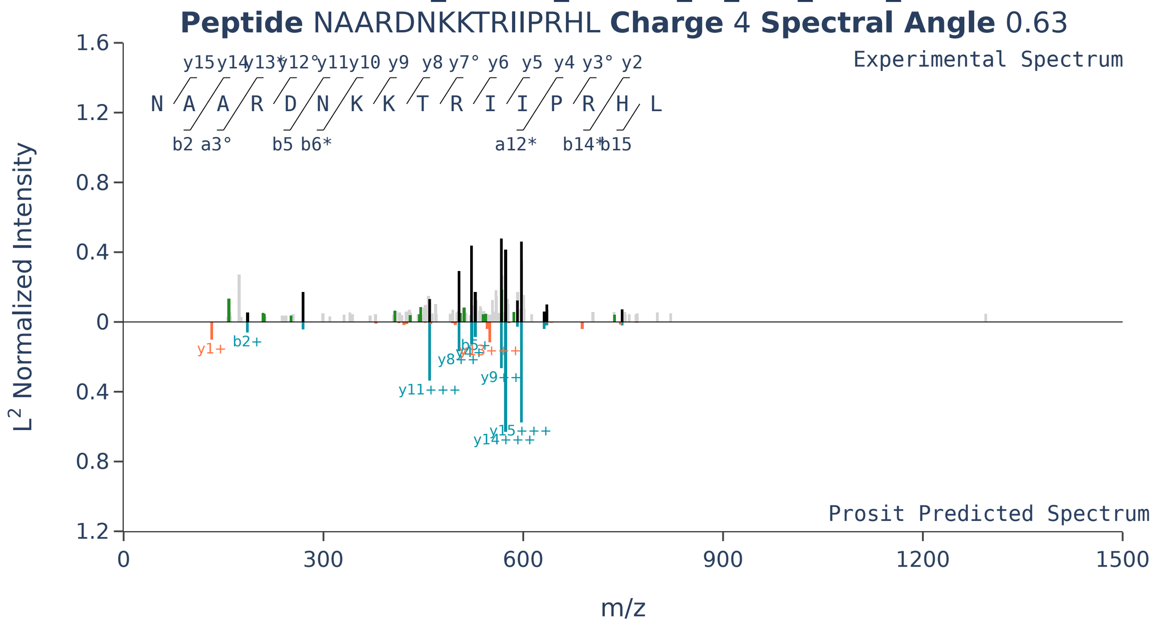


**Source** HRoetschke_291222_060123_Fu_H2A_b1R2_24h **Scan** 4765
**Peptide** KTRIIPRHL **Charge** 3 **Spectral Angle** 0.65

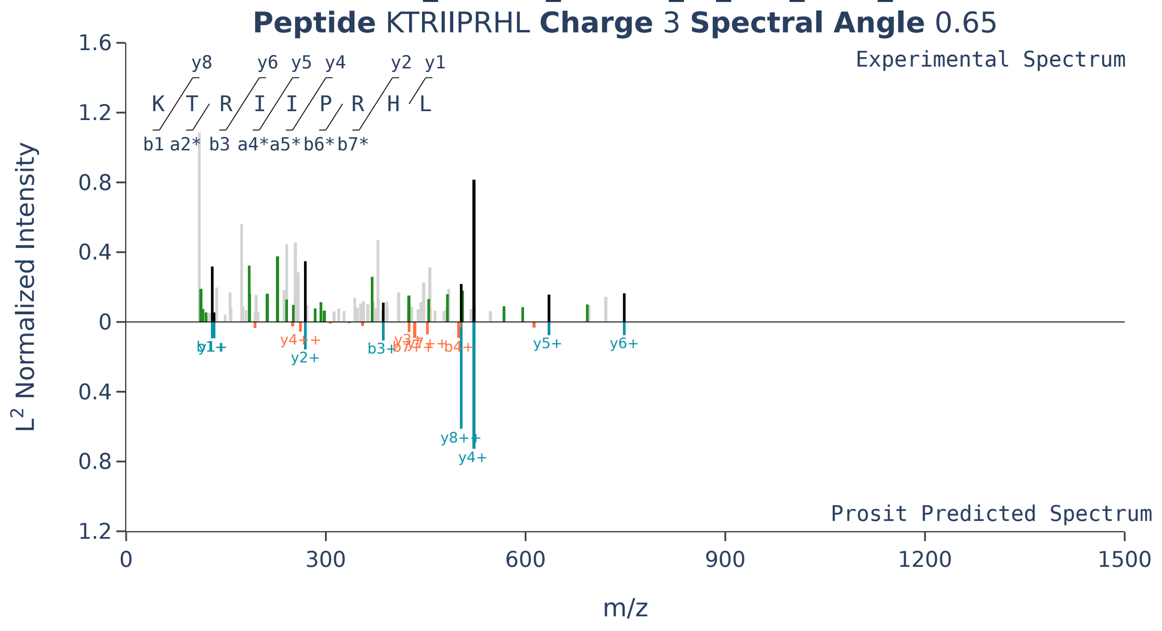

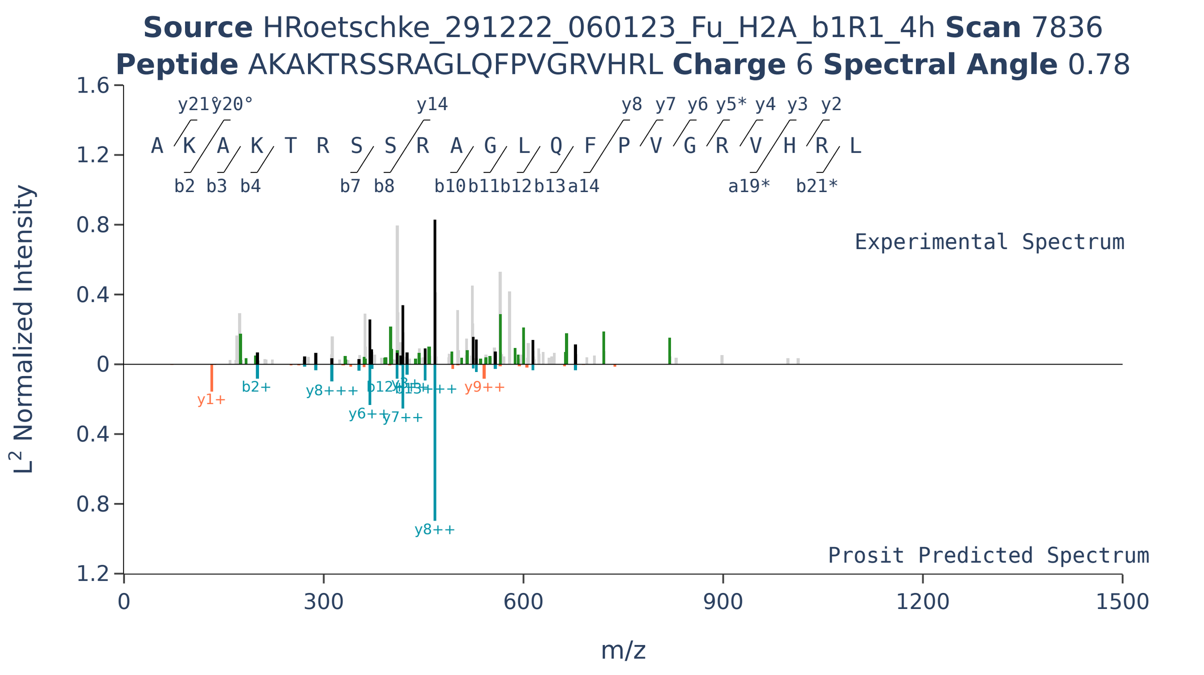
**Source** HRoetschke_291222_060123_Fu_H2A_b1R1_4h **Scan** 7836
**Peptide** AKAKTRSSRAGLQFPVGRVHRL **Charge** 6 **Spectral Angle** 0.78
Experimental Spectrum
Prosit Predicted Spectrum
L² Normalized Intensity
m/z

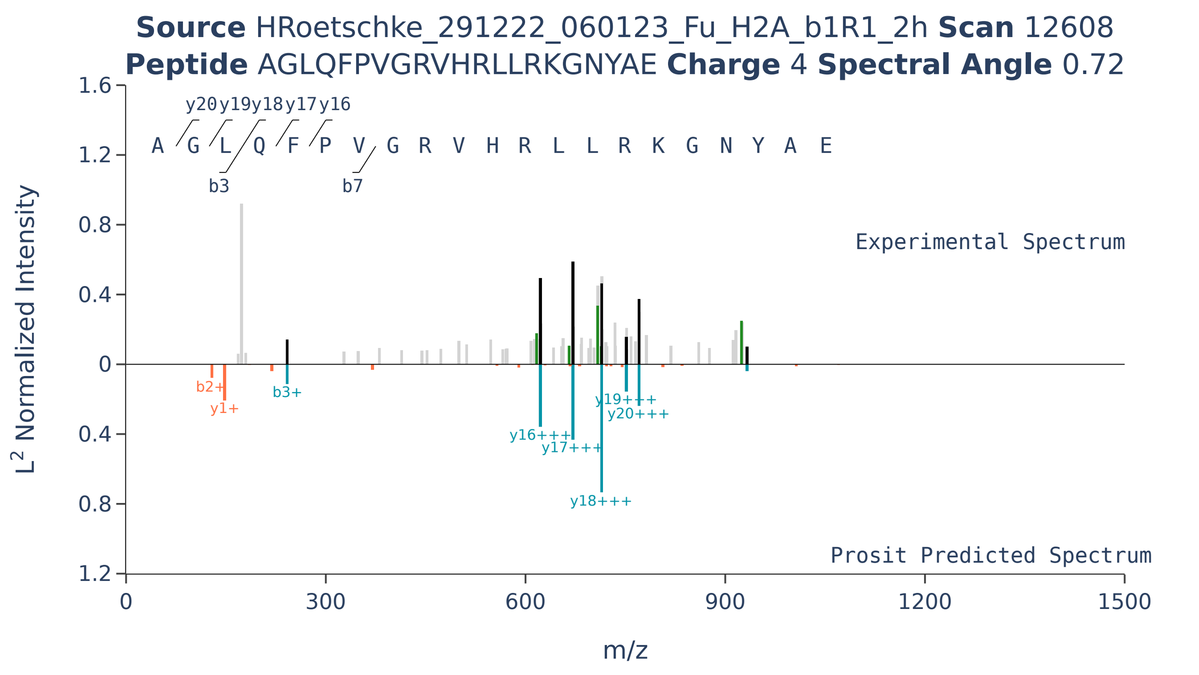
**Source** HRoetschke_291222_060123_Fu_H2A_b1R1_2h **Scan** 12608
**Peptide** AGLQFPVGRVHRLLRKGNYAE **Charge** 4 **Spectral Angle** 0.72
Experimental Spectrum
Prosit Predicted Spectrum
L² Normalized Intensity
m/z

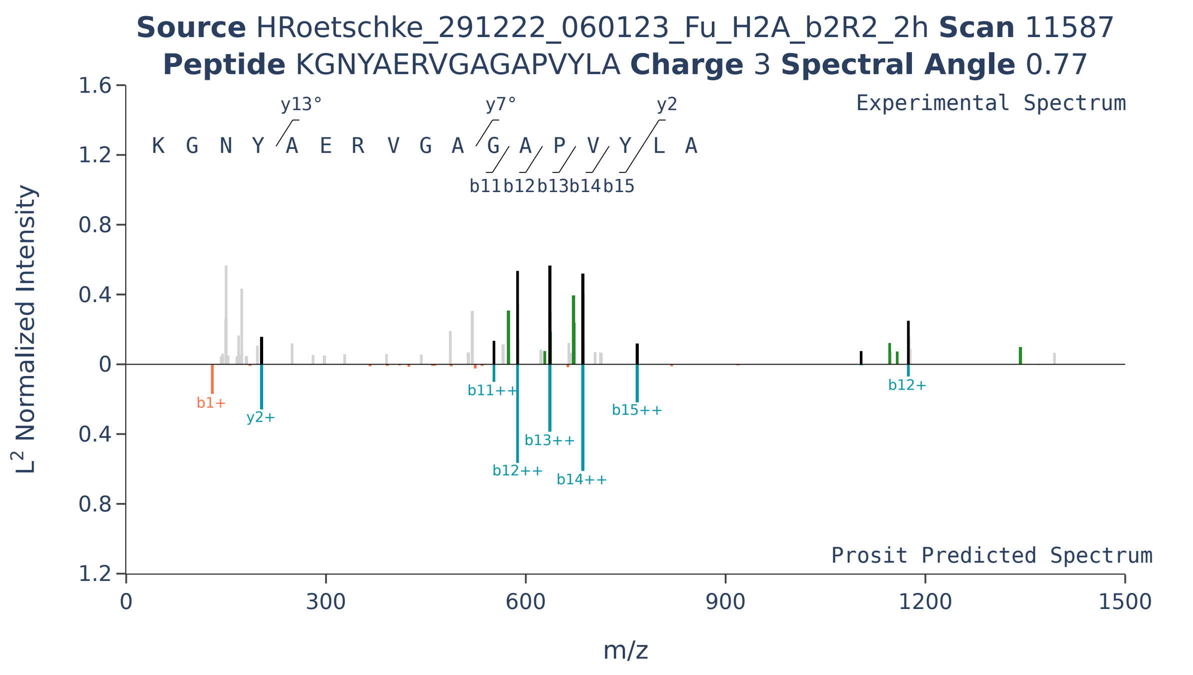
**Source** HRoetschke_291222_060123_Fu_H2A_b2R2_2h **Scan** 11587
**Peptide** KGNYAERVGAGAPVYLA **Charge** 3 **Spectral Angle** 0.77
Experimental Spectrum
Prosit Predicted Spectrum
L² Normalized Intensity
m/z

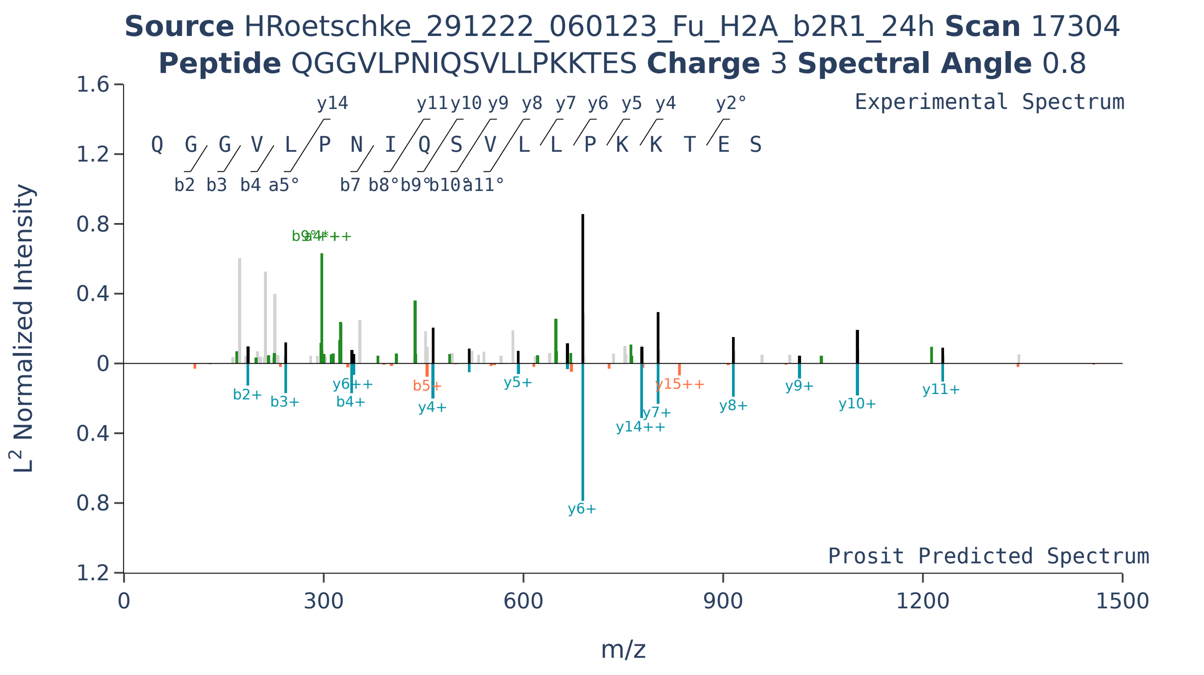
**Source** HRoetschke_291222_060123_Fu_H2A_b2R1_24h **Scan** 17304
**Peptide** QGGVLPNIQSVLLPKKTES **Charge** 3 **Spectral Angle** 0.8
Experimental Spectrum
Prosit Predicted Spectrum
L² Normalized Intensity
m/z

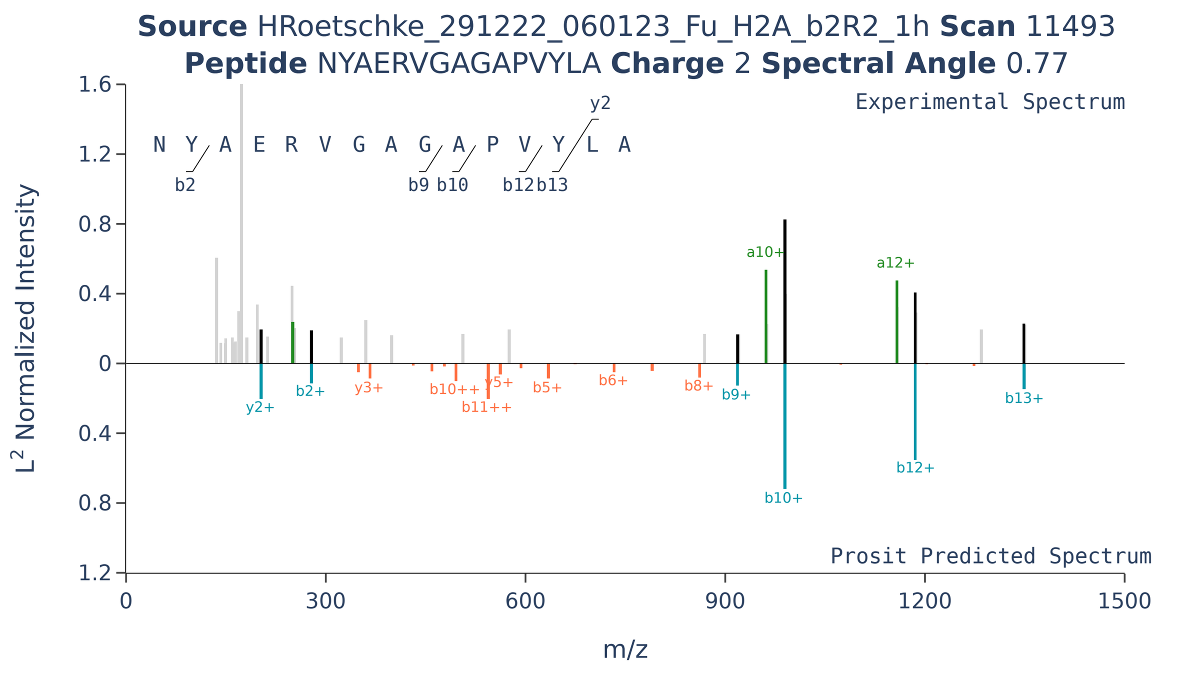
**Source** HRoetschke_291222_060123_Fu_H2A_b2R2_1h **Scan** 11493
**Peptide** NYAERVGAGAPVYLA **Charge** 2 **Spectral Angle** 0.77
Experimental Spectrum
Prosit Predicted Spectrum
L² Normalized Intensity
m/z

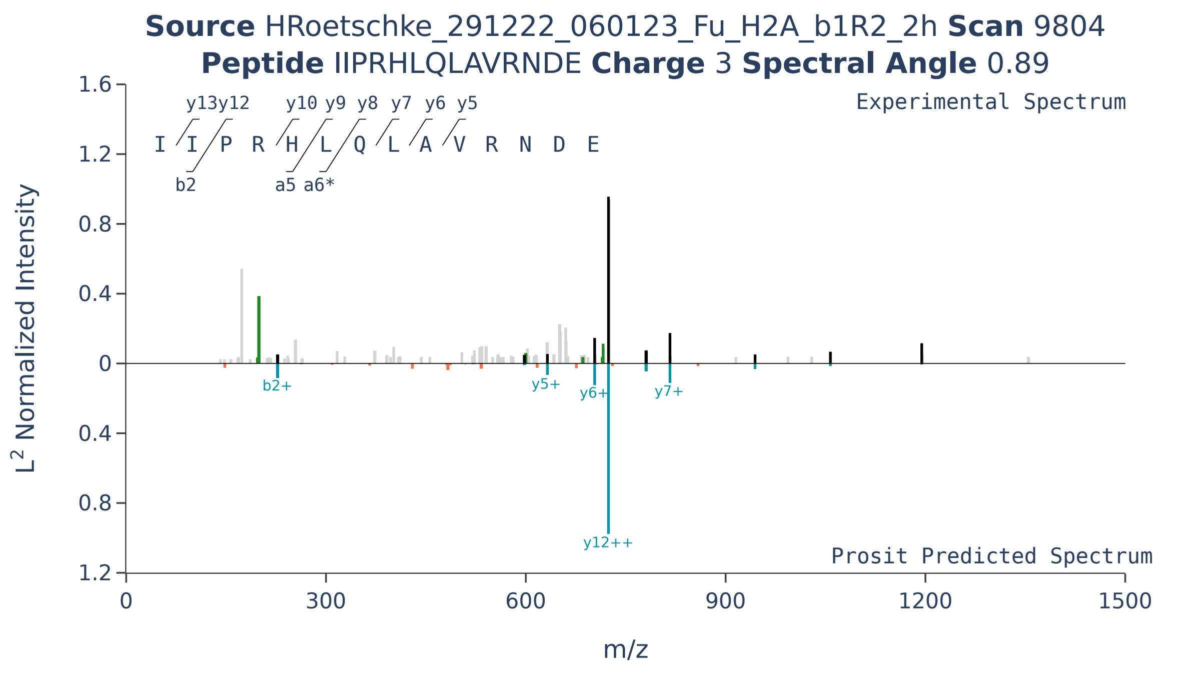
**Source** HRoetschke_291222_060123_Fu_H2A_b1R2_2h **Scan** 9804
**Peptide** IIPRHLQLAVRNDE **Charge** 3 **Spectral Angle** 0.89
Experimental Spectrum
Prosit Predicted Spectrum
L² Normalized Intensity
m/z

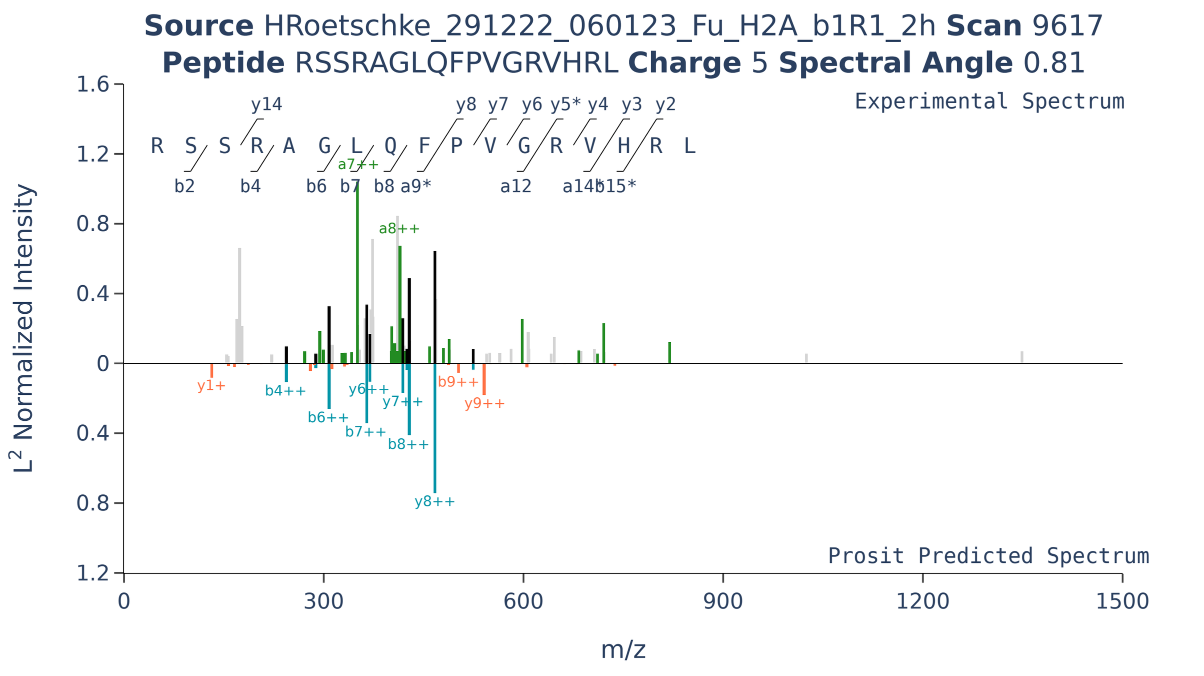
**Source** HRoetschke_291222_060123_Fu_H2A_b1R1_2h **Scan** 9617
**Peptide** RSSRAGLQFPVGRVHRL **Charge** 5 **Spectral Angle** 0.81
Experimental Spectrum
Prosit Predicted Spectrum
L² Normalized Intensity
m/z

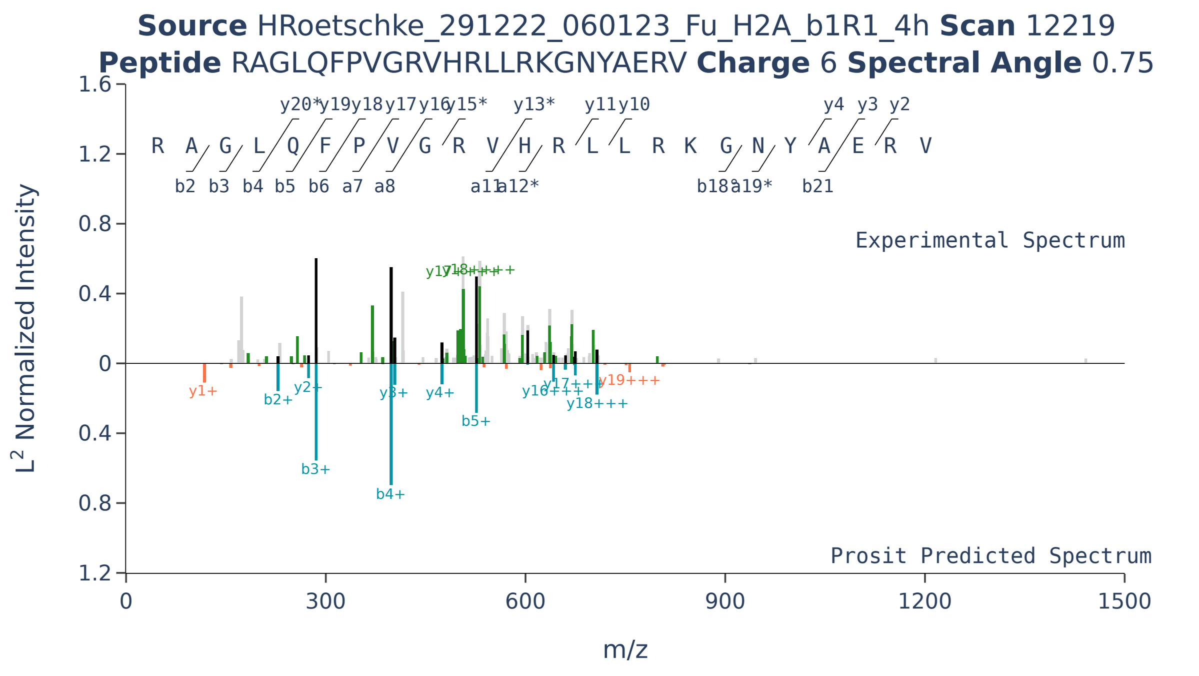
**Source** HRoetschke_291222_060123_Fu_H2A_b1R1_4h **Scan** 12219
**Peptide** RAGLQFPVGRVHRLLRKGNYAERV **Charge** 6 **Spectral Angle** 0.75
Experimental Spectrum
Prosit Predicted Spectrum
L² Normalized Intensity
m/z

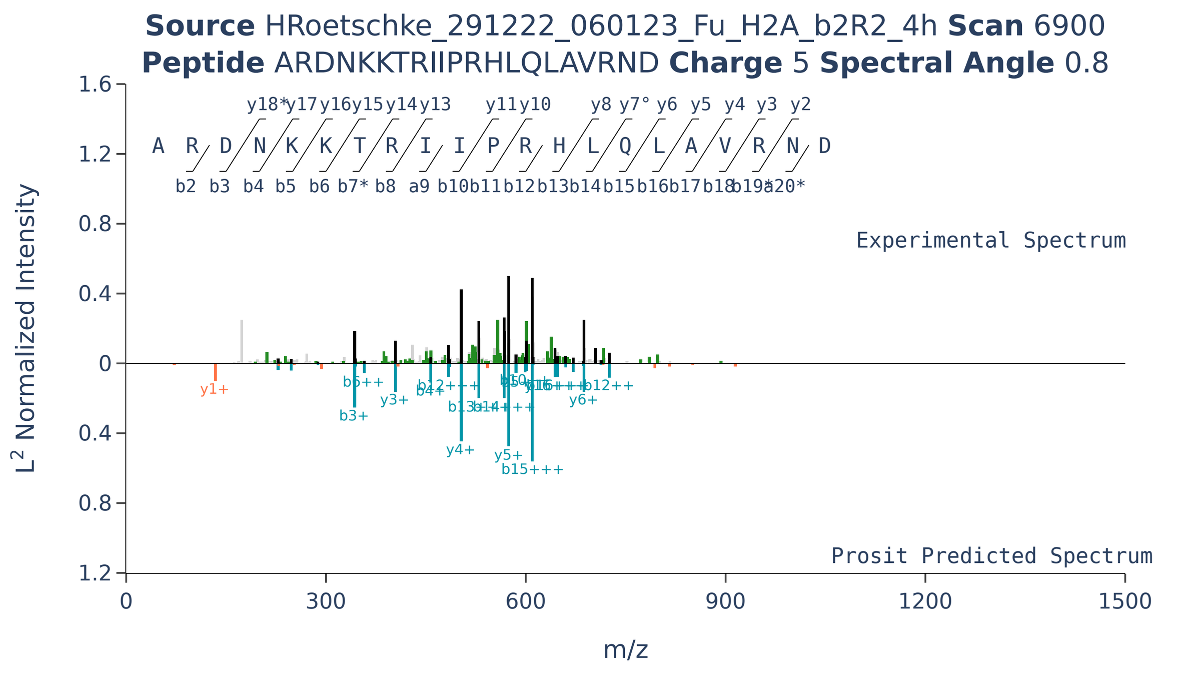
**Source** HRoetschke_291222_060123_Fu_H2A_b2R2_4h **Scan** 6900
**Peptide** ARDNKKTRIIPRHLQLAVRND **Charge** 5 **Spectral Angle** 0.8
Experimental Spectrum
Prosit Predicted Spectrum
L² Normalized Intensity
m/z

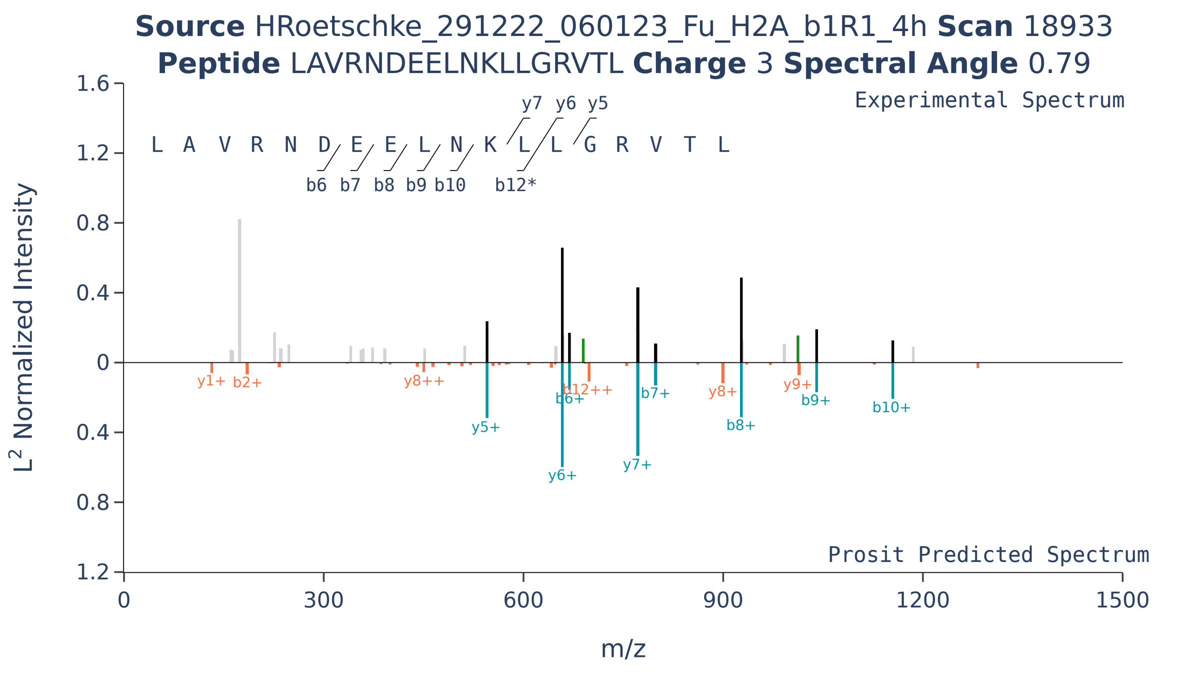
**Source** HRoetschke_291222_060123_Fu_H2A_b1R1_4h **Scan** 18933
**Peptide** LAVRNDEELNKLLGRVTL **Charge** 3 **Spectral Angle** 0.79
Experimental Spectrum
Prosit Predicted Spectrum
L² Normalized Intensity
m/z

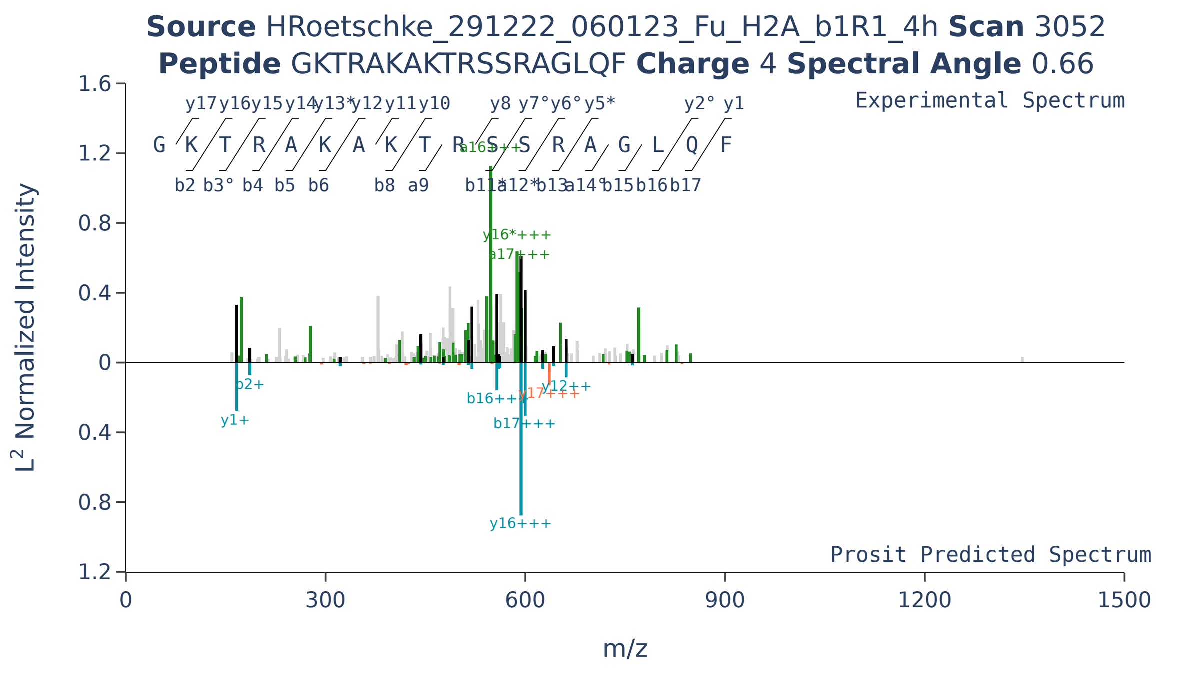
**Source** HRoetschke_291222_060123_Fu_H2A_b1R1_4h **Scan** 3052
**Peptide** GKTRAKAKTRSSRAGLQF **Charge** 4 **Spectral Angle** 0.66
Experimental Spectrum
Prosit Predicted Spectrum
L² Normalized Intensity
m/z

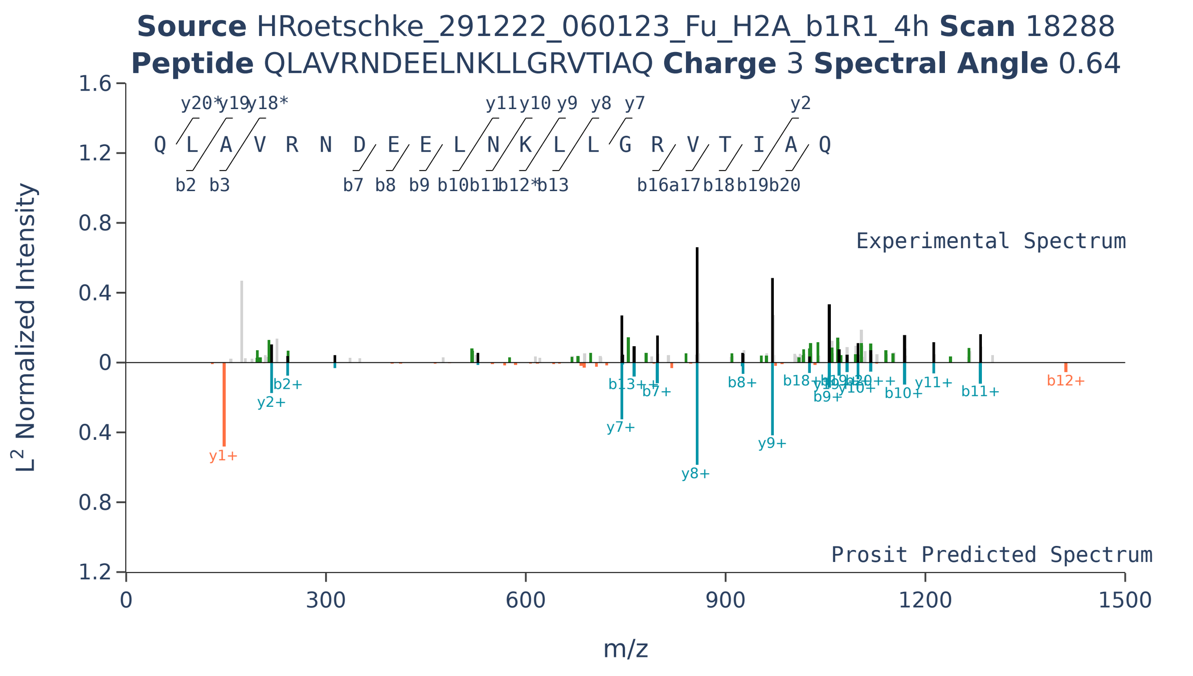
**Source** HRoetschke_291222_060123_Fu_H2A_b1R1_4h **Scan** 18288
**Peptide** QLAVRNDEELNKLLGRVTIAQ **Charge** 3 **Spectral Angle** 0.64
Experimental Spectrum
Prosit Predicted Spectrum
L² Normalized Intensity
m/z

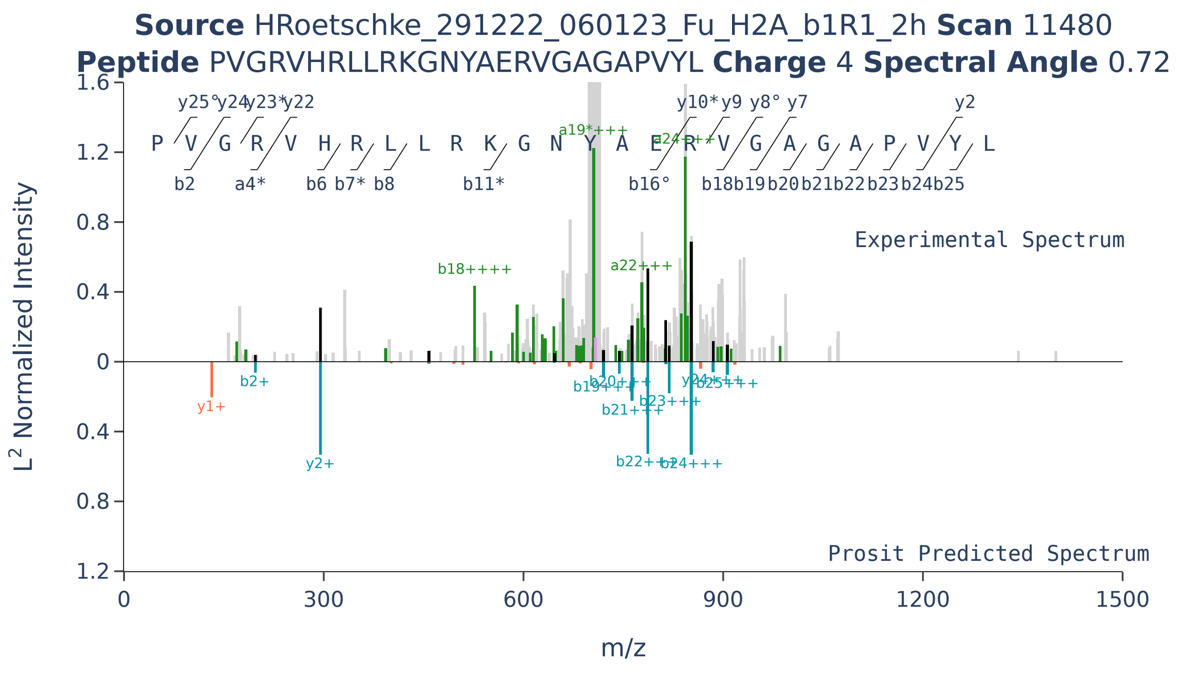
**Source** HRoetschke_291222_060123_Fu_H2A_b1R1_2h **Scan** 11480
**Peptide** PVGRVHRLLRKGNYAERVGAGAPVYL **Charge** 4 **Spectral Angle** 0.72
Experimental Spectrum
Prosit Predicted Spectrum
L² Normalized Intensity
m/z

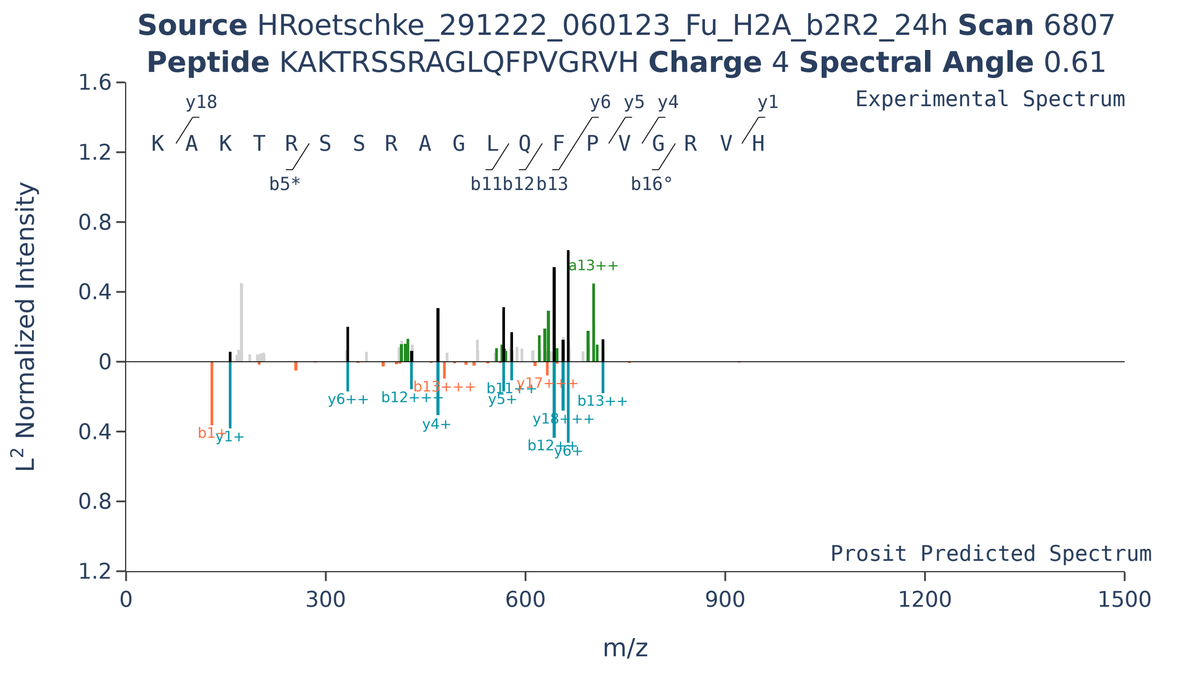
**Source** HRoetschke_291222_060123_Fu_H2A_b2R2_24h **Scan** 6807
**Peptide** KAKTRSSRAGLQFPVGRVH **Charge** 4 **Spectral Angle** 0.61
Experimental Spectrum
Prosit Predicted Spectrum
L² Normalized Intensity
m/z

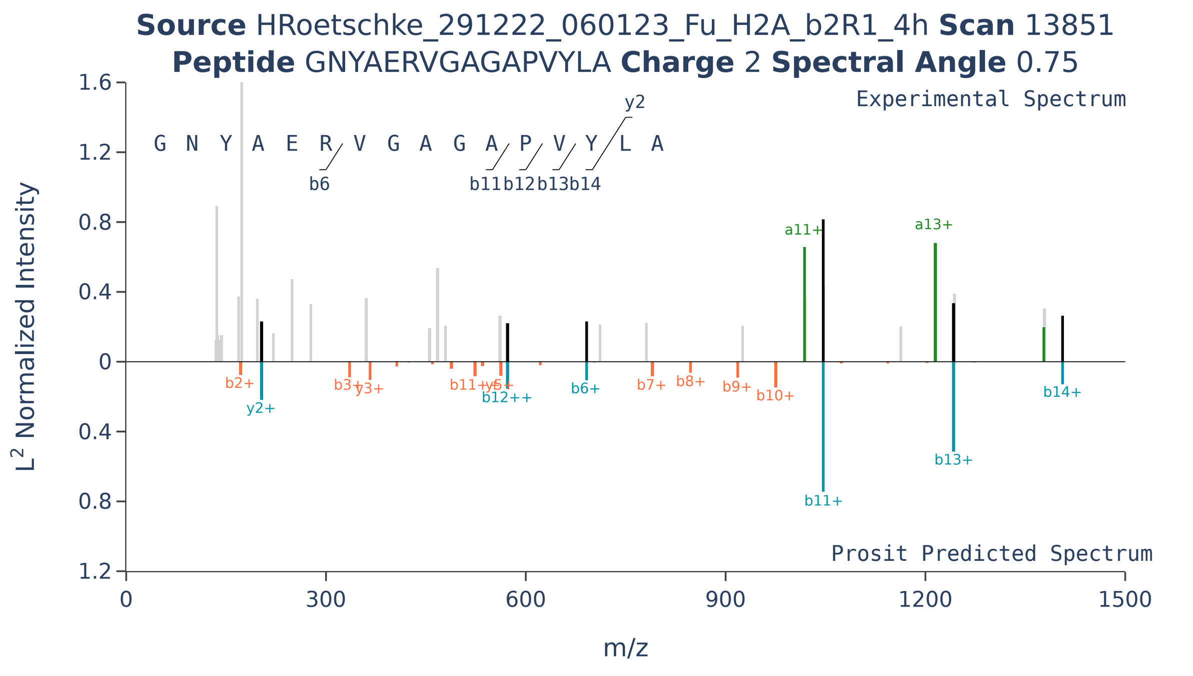
**Source** HRoetschke_291222_060123_Fu_H2A_b2R1_4h **Scan** 13851
**Peptide** GNYAERVGAGAPVYLA **Charge** 2 **Spectral Angle** 0.75
Experimental Spectrum
Prosit Predicted Spectrum
L² Normalized Intensity
m/z

**Source** HRoetschke_291222_060123_Fu_H2A_b2R1_24h **Scan** 7452
**Peptide** RDNKKTRIIPRHLQLAVRNDE **Charge** 5 **Spectral Angle** 0.73

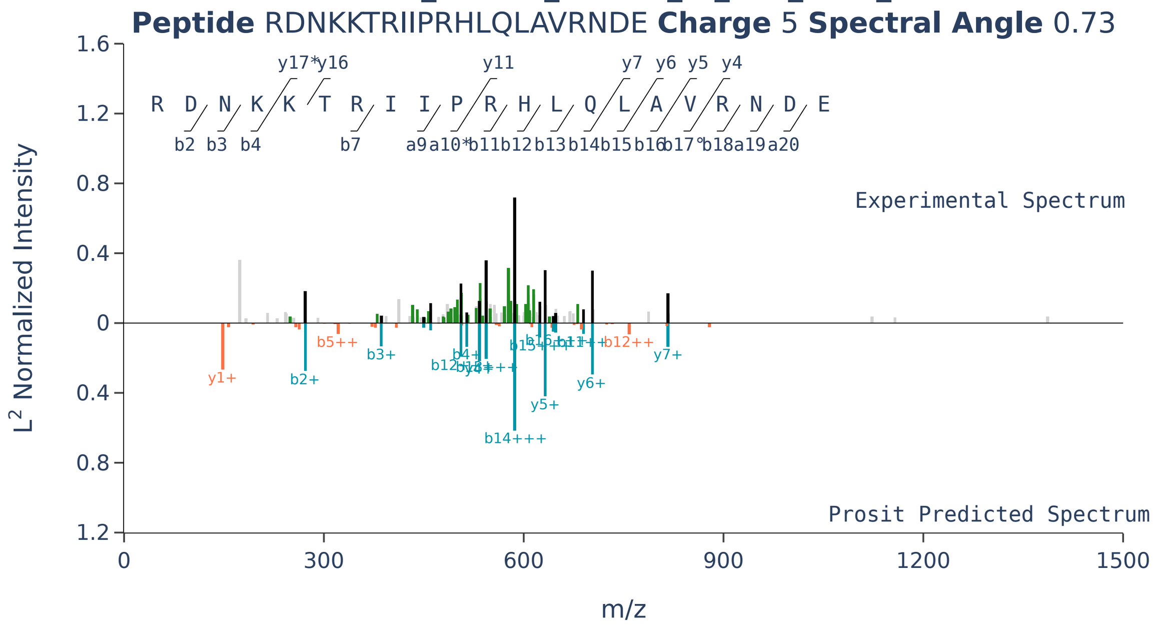


**Source** HRoetschke_291222_060123_Fu_H2A_b1R1_4h **Scan** 8019
**Peptide** LAGNAARDNKKTRIIPRHLQLAVRND **Charge** 6 **Spectral Angle** 0.7

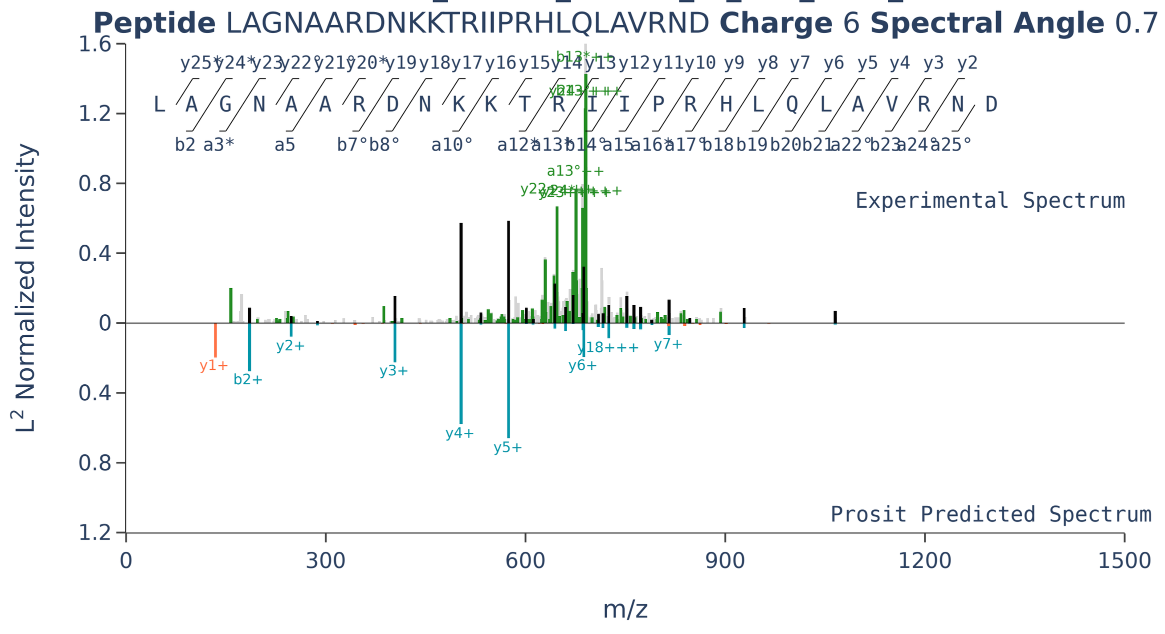


**Source** HRoetschke_291222_060123_Fu_H2A_b2R2_24h **Scan** 5877
**Peptide** TRAKAKTRSSRAGLQFPVGR **Charge** 5 **Spectral Angle** 0.82

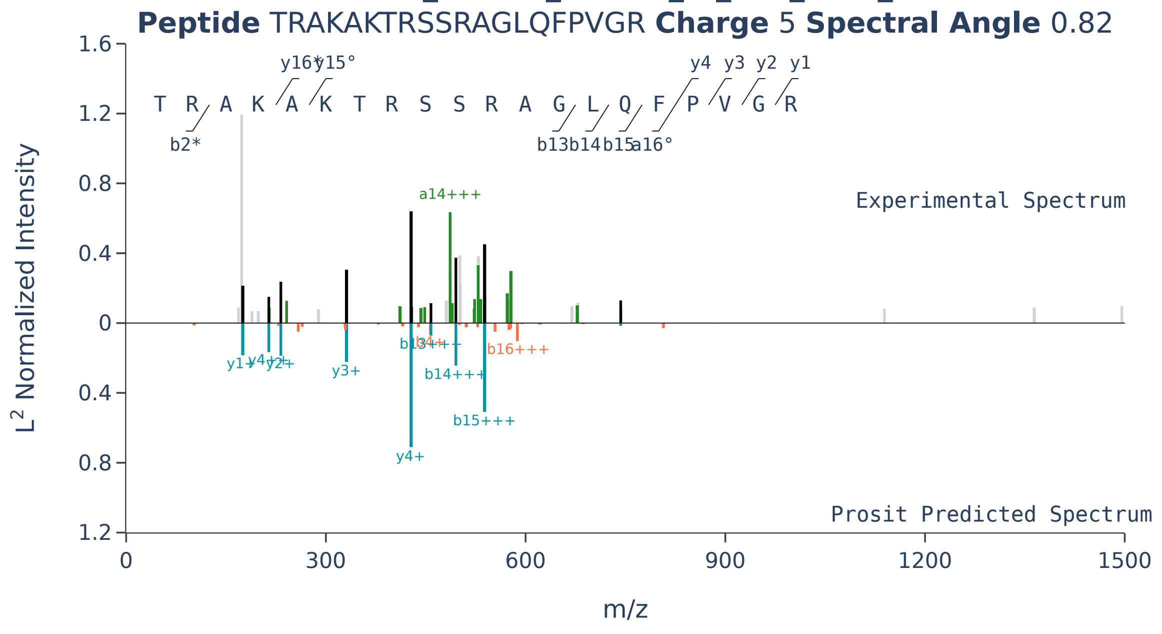


**Source** HRoetschke_291222_060123_Fu_H2A_b1R1_2h **Scan** 12728
**Peptide** KGNYAERVGAGAPVYLAA **Charge** 3 **Spectral Angle** 0.78

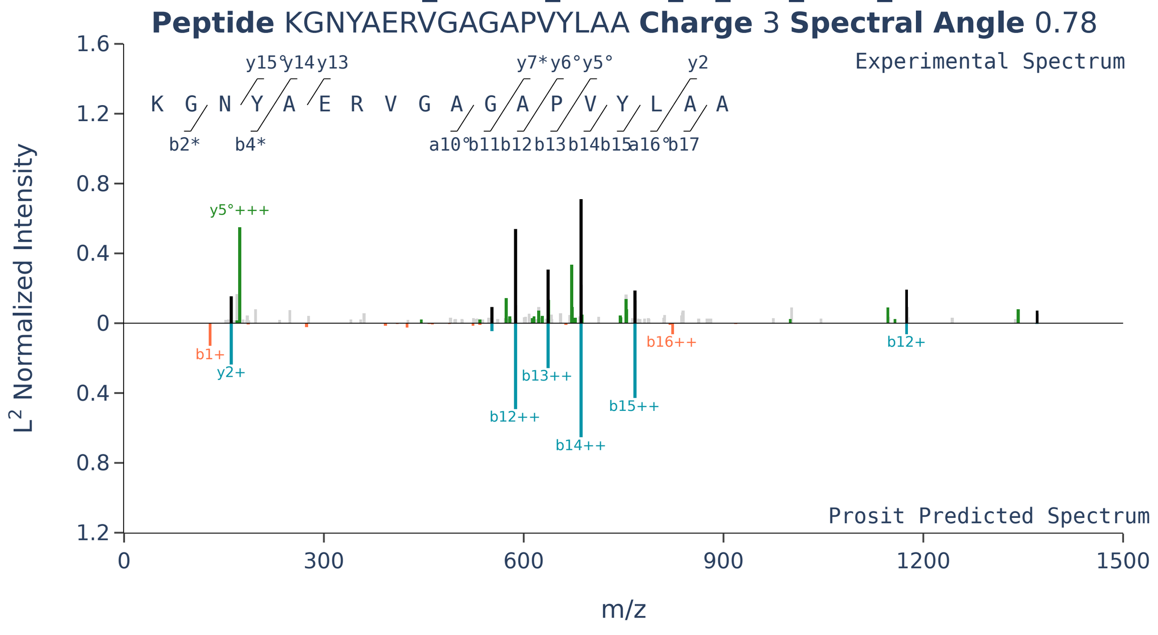


**Source** HRoetschke_291222_060123_Fu_H2A_b2R1_24h **Scan** 11968
**Peptide** GLQFPVGRVHRLLRK **Charge** 4 **Spectral Angle** 0.68

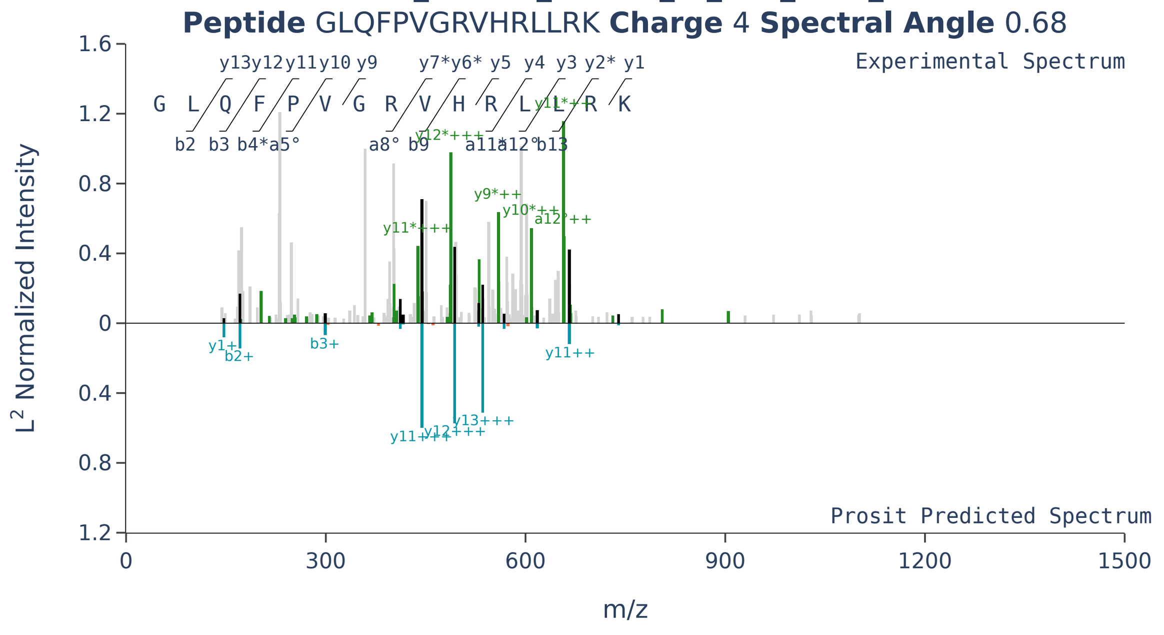


**Source** HRoetschke_291222_060123_Fu_H2A_b2R2_2h **Scan** 6923
**Peptide** RDNKKTRIIPRHLQL **Charge** 4 **Spectral Angle** 0.7

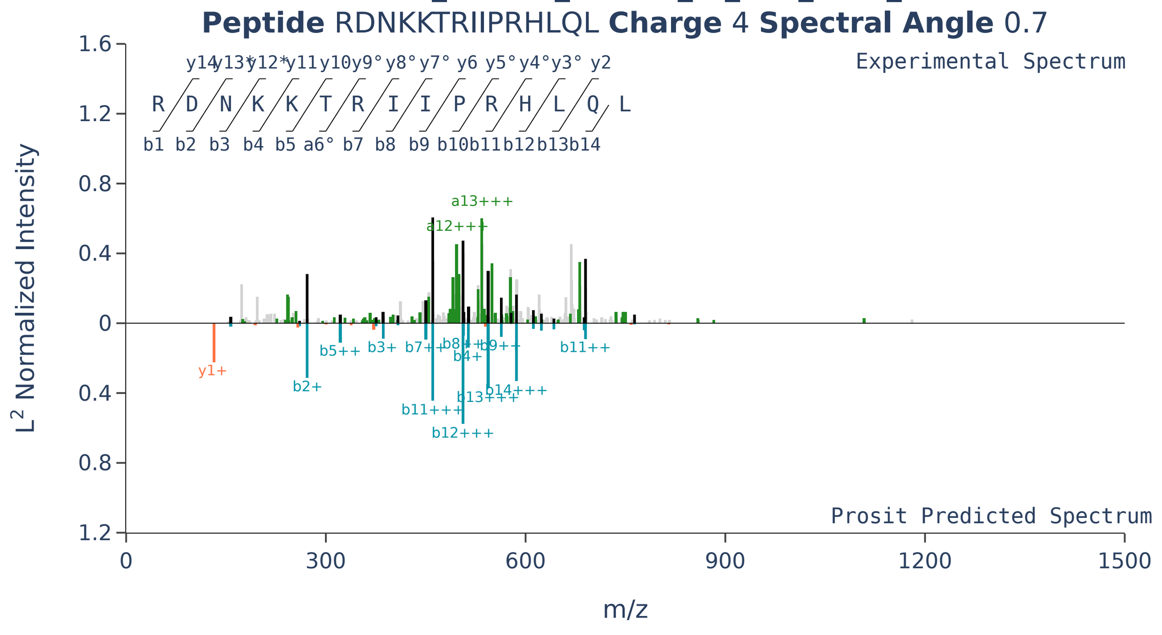


**Source** HRoetschke_291222_060123_Fu_H2A_b1R1_24h **Scan** 4587
**Peptide** VHRLLRKGNYAERVGA **Charge** 4 **Spectral Angle** 0.72

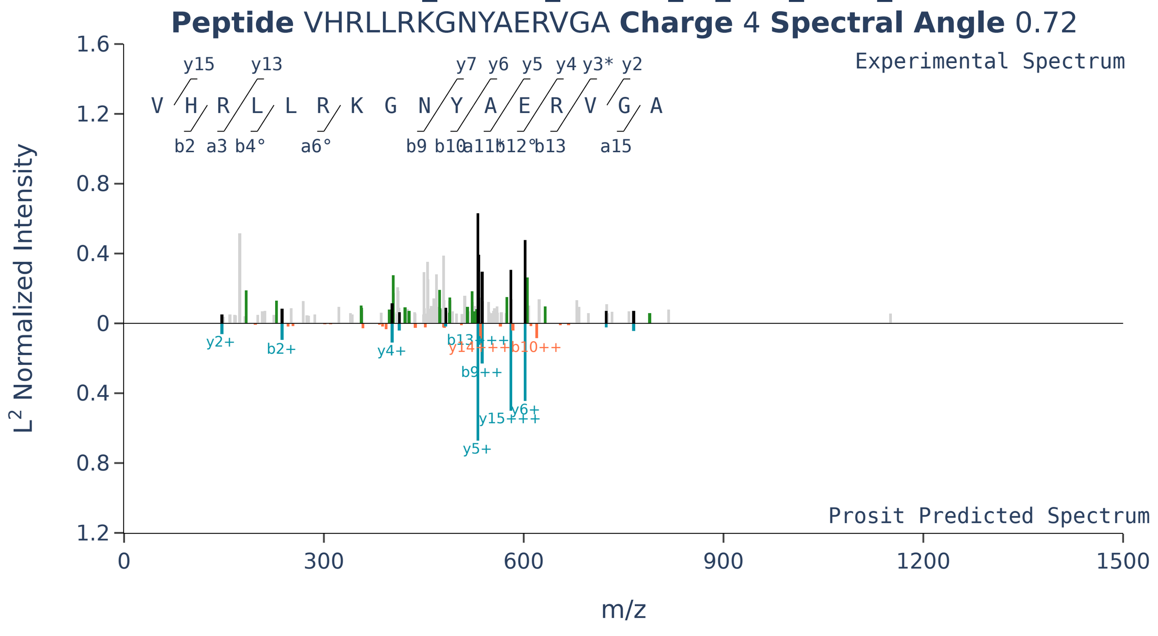


**Source** HRoetschke_291222_060123_Fu_H2A_b1R2_4h **Scan** 20228
**Peptide** AGAPVYLAAVLE **Charge** 2 **Spectral Angle** 0.8

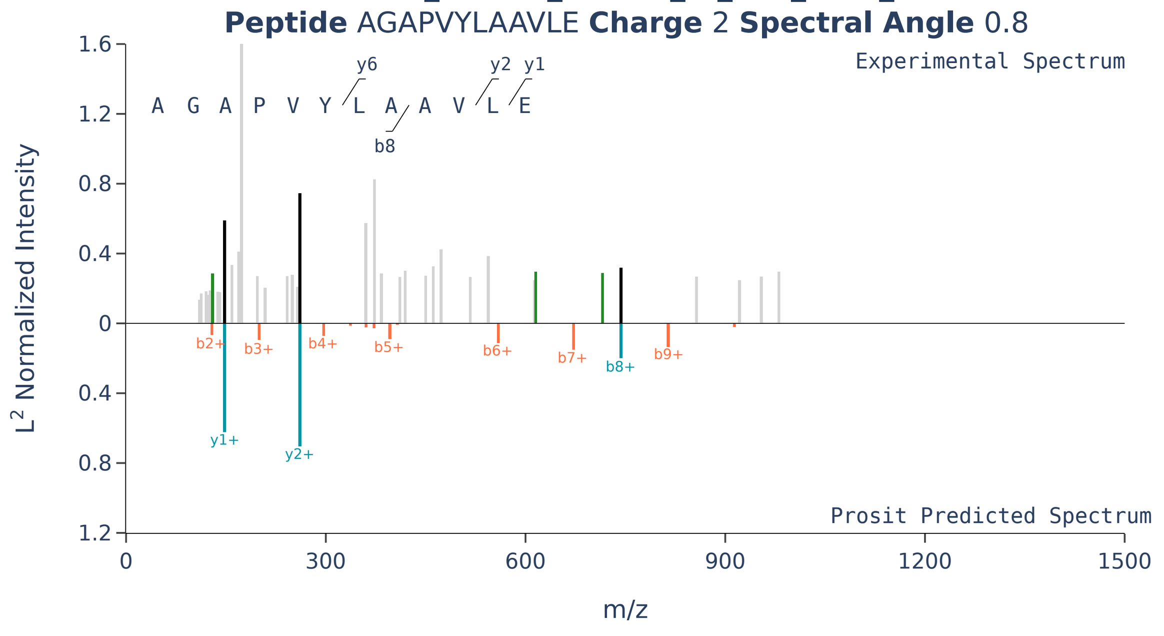


**Source** HRoetschke_291222_060123_Fu_H2A_b1R1_4h **Scan** 6350
**Peptide** PVGRVHRLL **Charge** 3 **Spectral Angle** 0.52

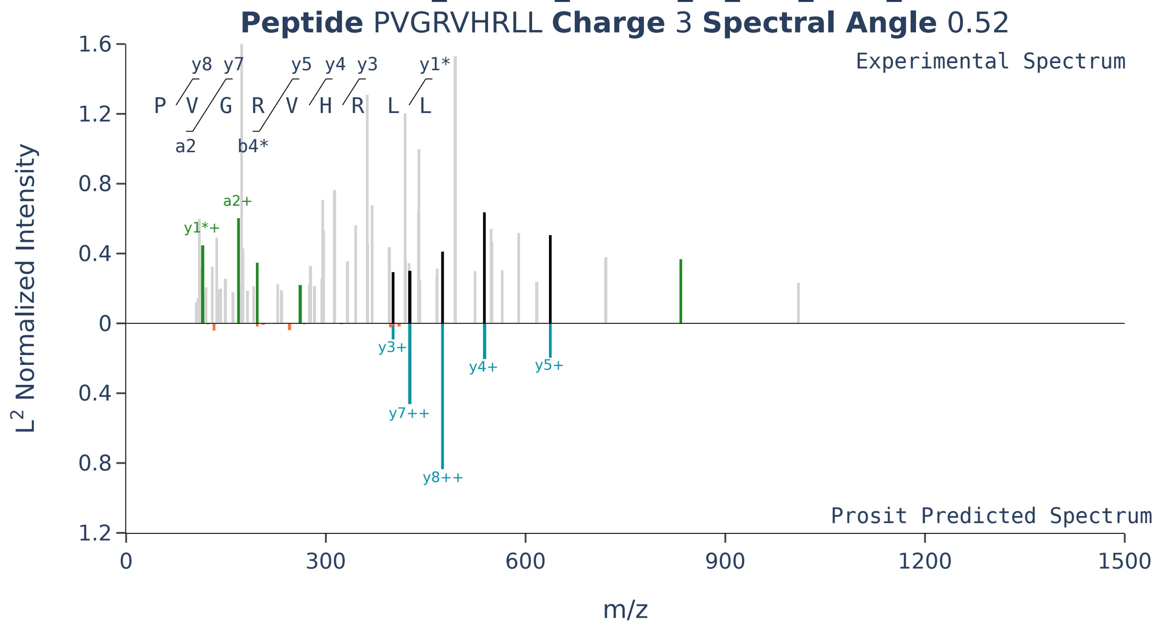


**Source** HRoetschke_291222_060123_Fu_H2A_b1R2_4h **Scan** 6056
**Peptide** RAKAKTRSSRAGLQFPVGRVH **Charge** 5 **Spectral Angle** 0.63

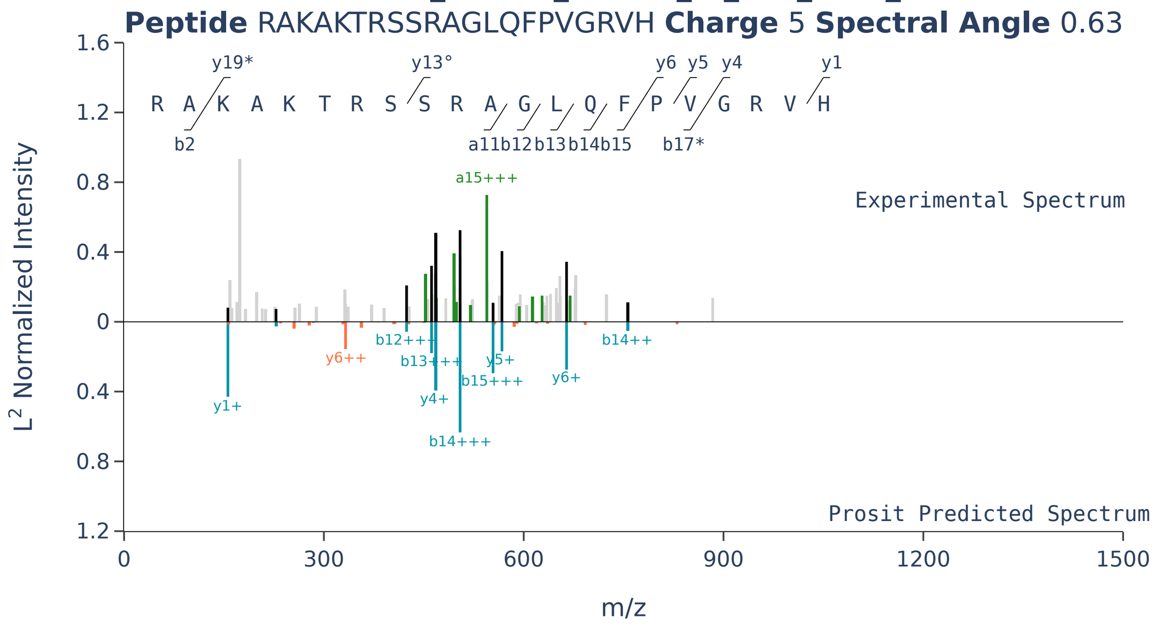


**Source** HRoetschke_291222_060123_Fu_H2A_b1R1_2h **Scan** 12058
**Peptide** AGLQFPVGRVHRLLRK **Charge** 4 **Spectral Angle** 0.68

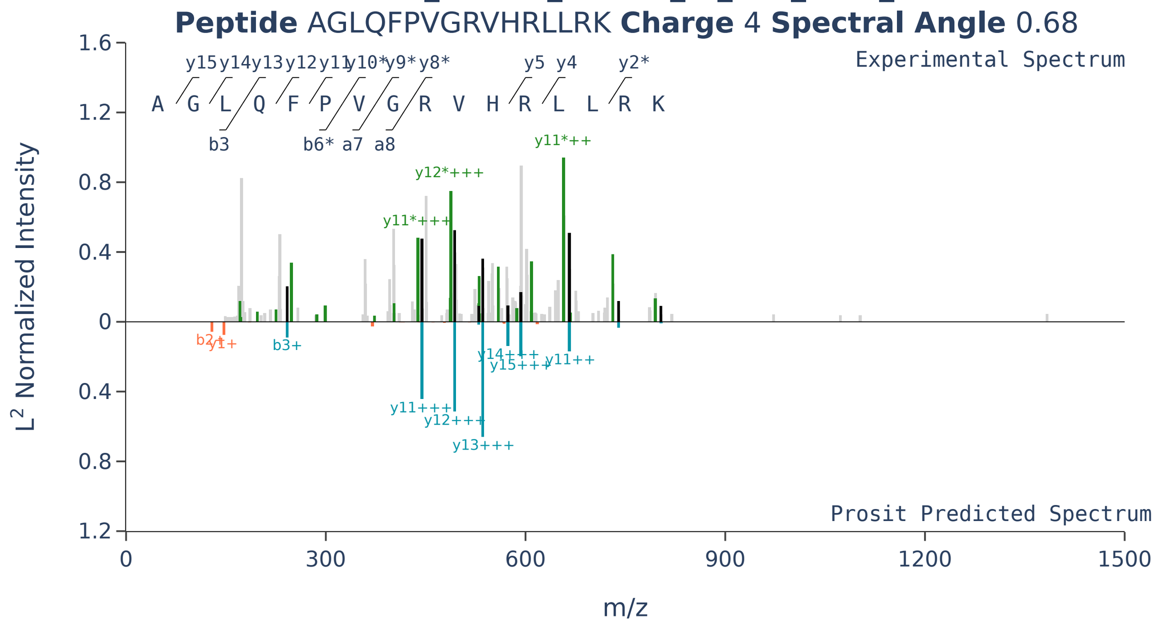


**Source** HRoetschke_291222_060123_Fu_H2A_b2R2_24h **Scan** 14788
**Peptide** TIAQGGVLPNLQ **Charge** 2 **Spectral Angle** 0.75

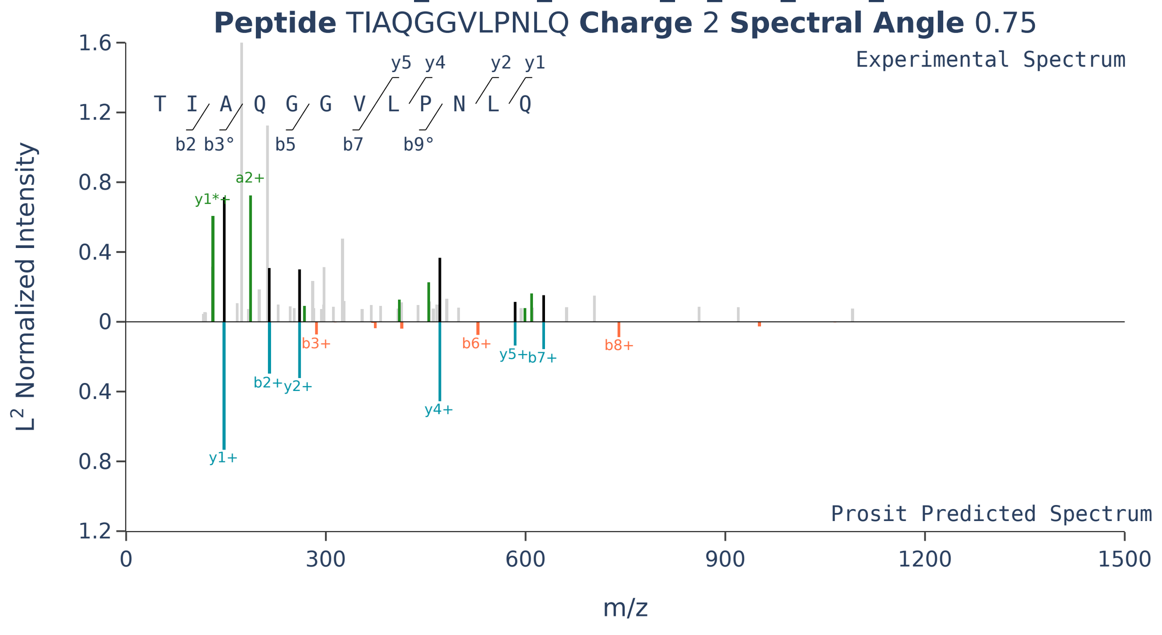


**Source** HRoetschke_291222_060123_Fu_H2A_b1R2_4h **Scan** 13341
**Peptide** KGNYAERVGAGAPVYL **Charge** 3 **Spectral Angle** 0.69

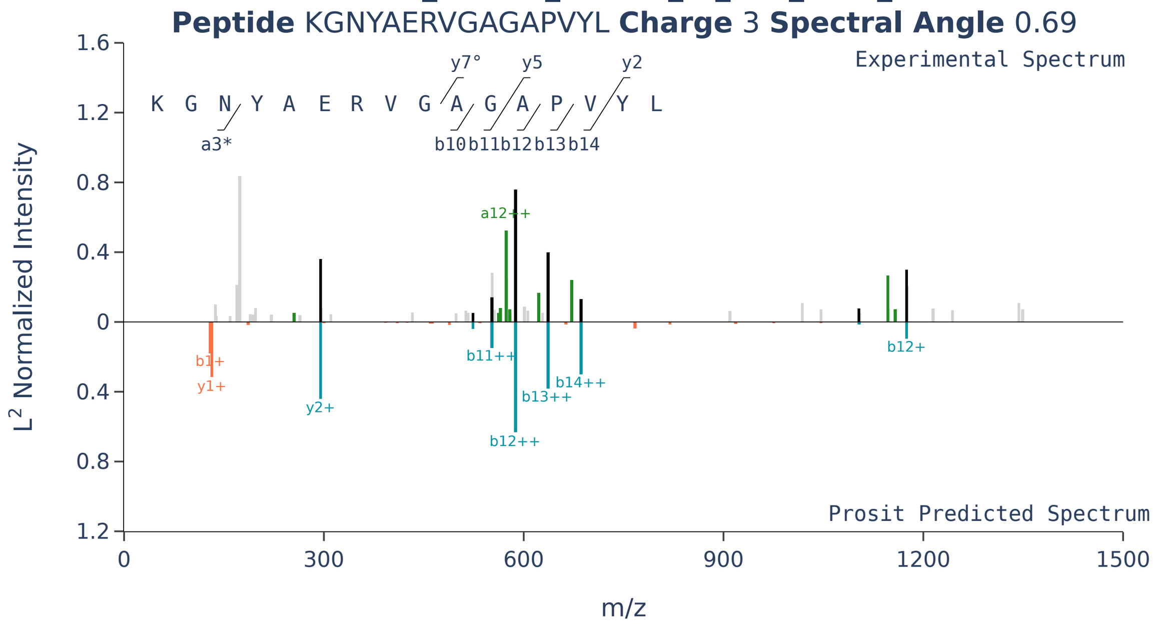


**Source** HRoetschke_291222_060123_Fu_H2A_b1R1_4h **Scan** 14479
**Peptide** LLELAGNAARDNKKTRIIPRHLQLAV **Charge** 5 **Spectral Angle** 0.65

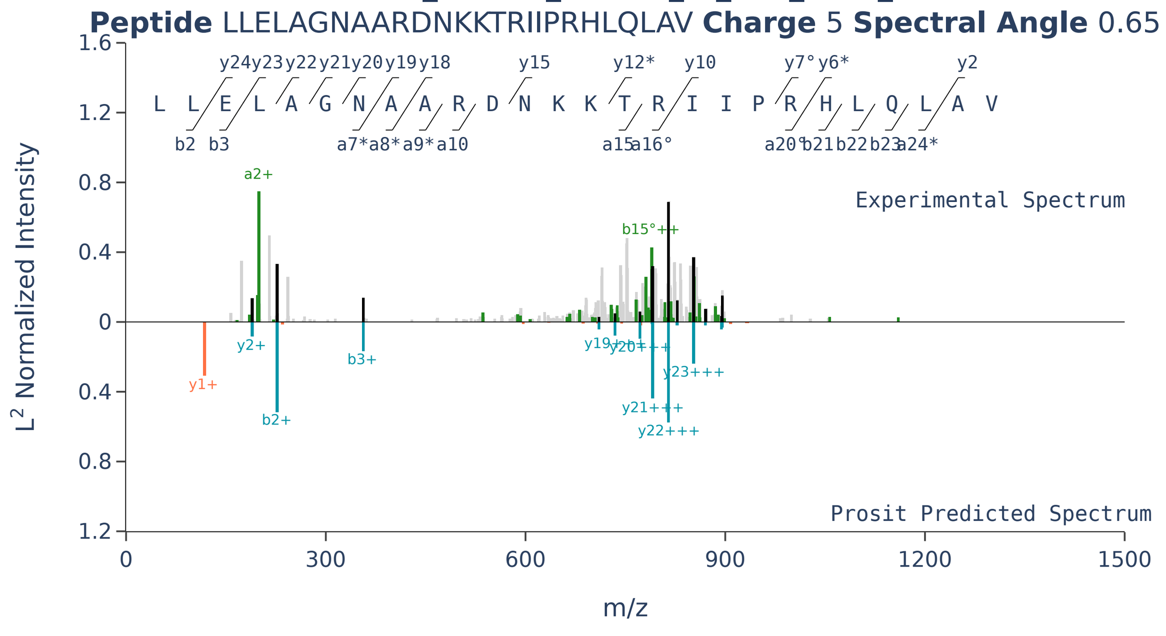


**Source** HRoetschke_291222_060123_Fu_H2A_b1R2_24h **Scan** 7624
**Peptide** TRSSRAGLQFPVGRVHR **Charge** 5 **Spectral Angle** 0.72

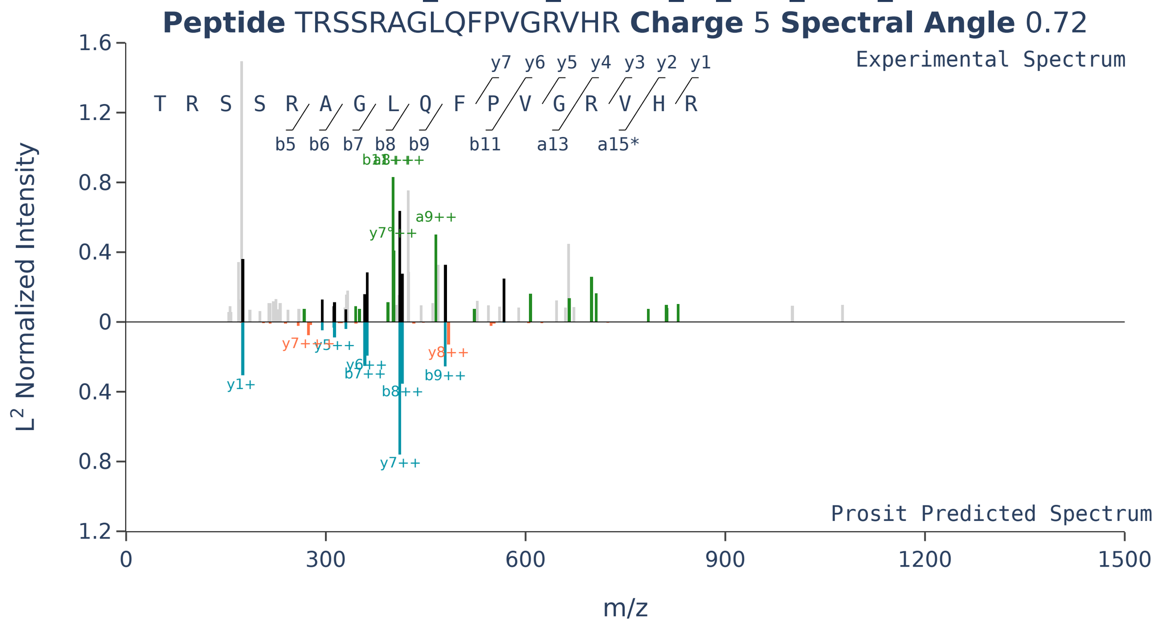

**Source** HRoetschke_291222_060123_Fu_H2A_b2R1_24h **Scan** 2797
**Peptide** ARDNKKTRLL **Charge** 3 **Spectral Angle** 0.87

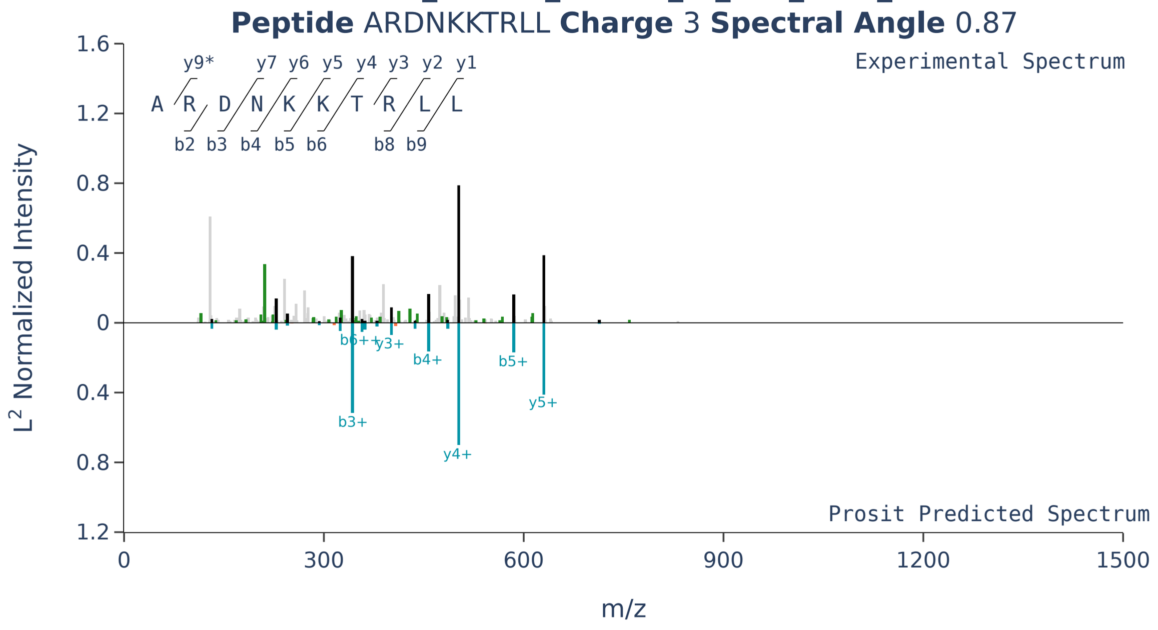


**Source** HRoetschke_291222_060123_Fu_H2A_b1R1_4h **Scan** 15289
**Peptide** GVLPNIQSVLLPKKTESSKSAKSK **Charge** 3 **Spectral Angle** 0.7

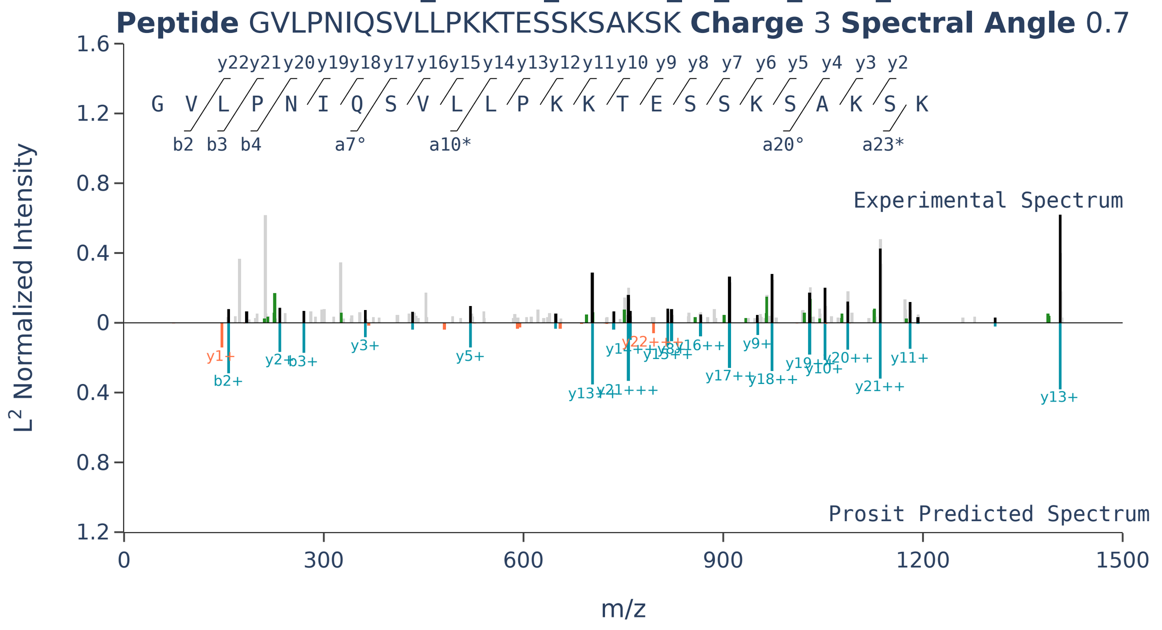


**Source** HRoetschke_291222_060123_Fu_H2A_b1R2_4h **Scan** 12748
**Peptide** LELAGNAARDNKKTRIIPRHLQLAV **Charge** 4 **Spectral Angle** 0.76

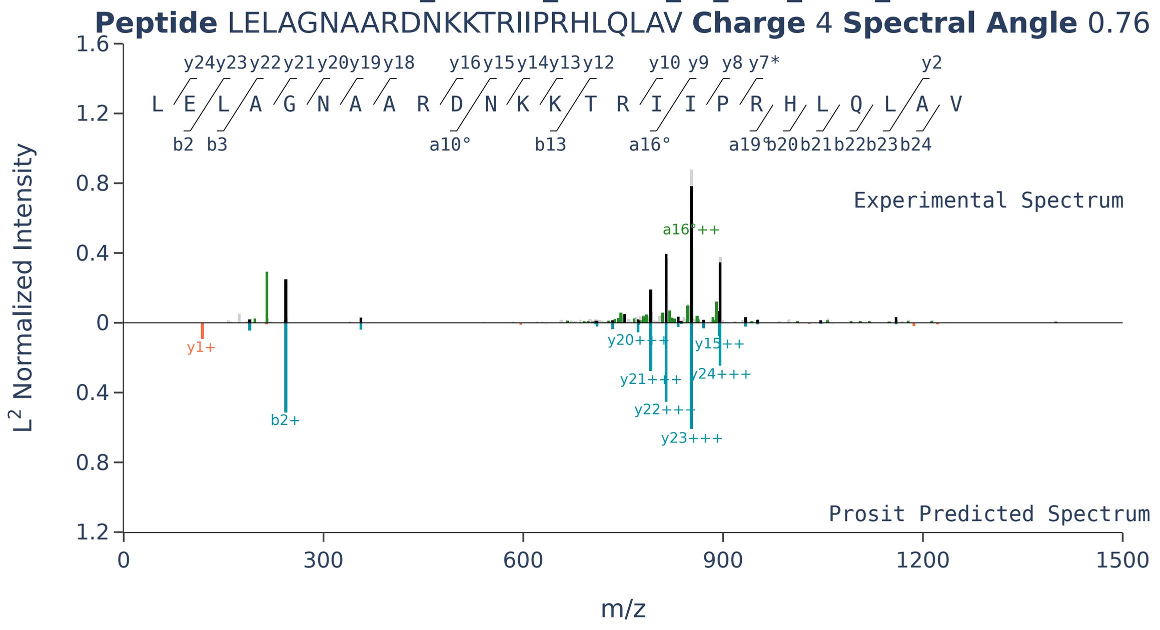


**Source** HRoetschke_291222_060123_Fu_H2A_b1R2_4h **Scan** 11936
**Peptide** SSRAGLQFPVGRVHRLLRKGNYAERV **Charge** 5 **Spectral Angle** 0.68

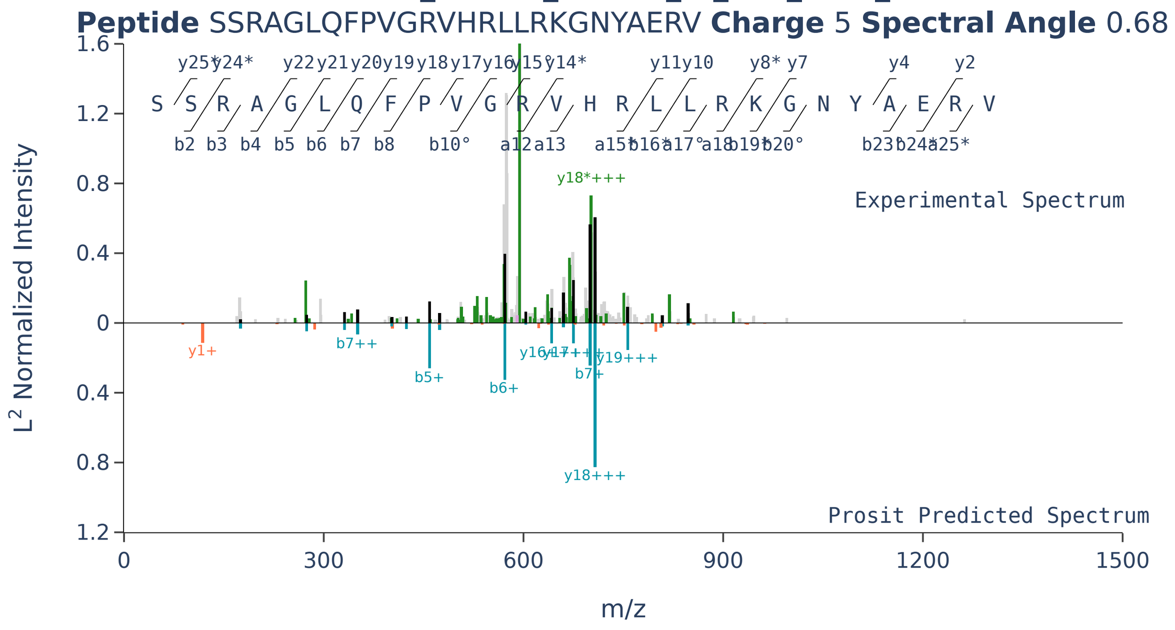


**Source** HRoetschke_291222_060123_Fu_H2A_b1R2_2h **Scan** 18368
**Peptide** EYLTAEILELAGNAARDNKKTRIIPRHL **Charge** 6 **Spectral Angle** 0.8

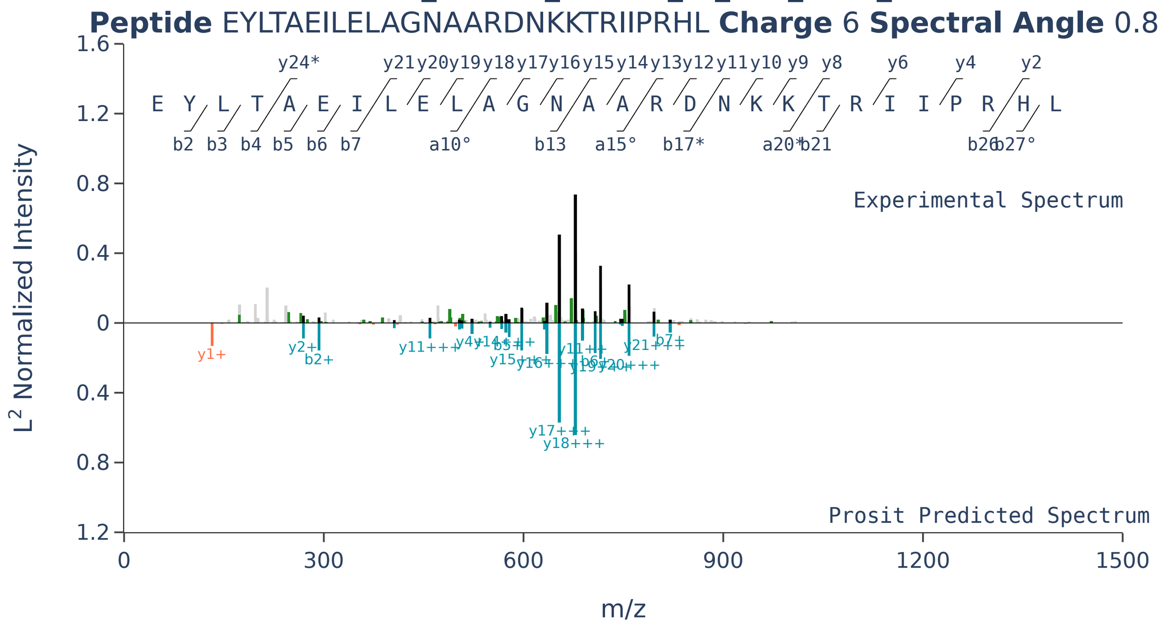


**Source** HRoetschke_291222_060123_Fu_H2A_b1R2_4h **Scan** 10712
**Peptide** SSRAGLQFPVGRVHRLLRKGN **Charge** 4 **Spectral Angle** 0.6

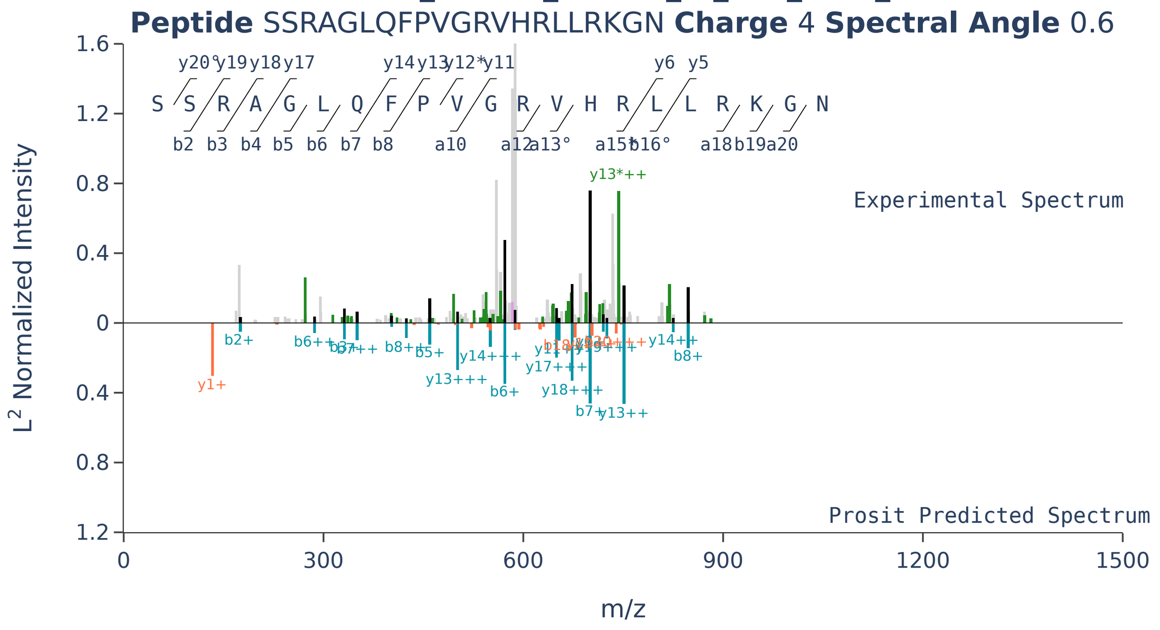


**Source** HRoetschke_291222_060123_Fu_H2A_b2R1_2h **Scan** 6437
**Peptide** NIQSVLLPKKTESSKSAKSK **Charge** 4 **Spectral Angle** 0.71

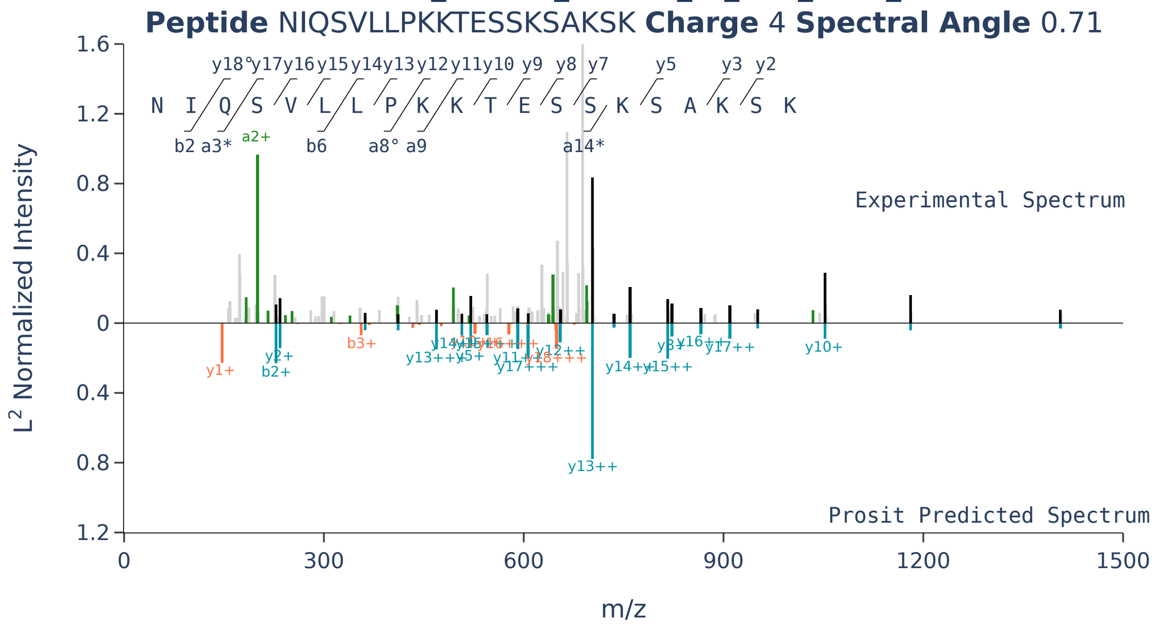


**Source** HRoetschke_291222_060123_Fu_H2A_b2R1_24h **Scan** 5194
**Peptide** QGGKTRAKAKTRSSRAGLQFPVGR **Charge** 5 **Spectral Angle** 0.81

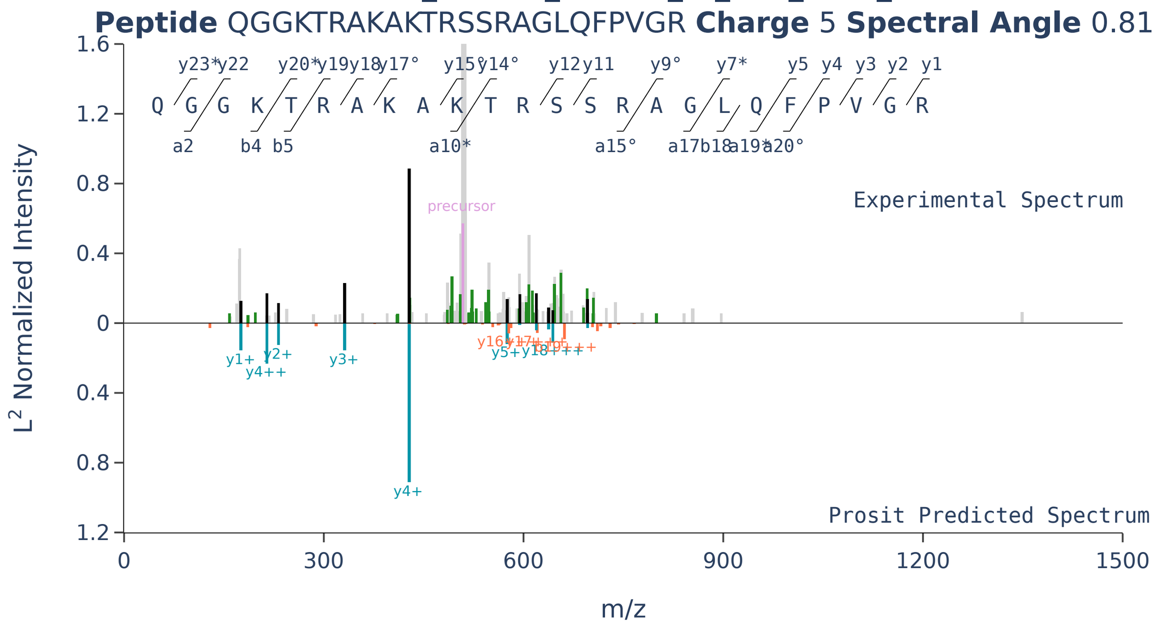


**Source** HRoetschke_291222_060123_Fu_H2A_b2R2_24h **Scan** 15439
**Peptide** LGRVTIAQGGVLPNIQS **Charge** 2 **Spectral Angle** 0.81

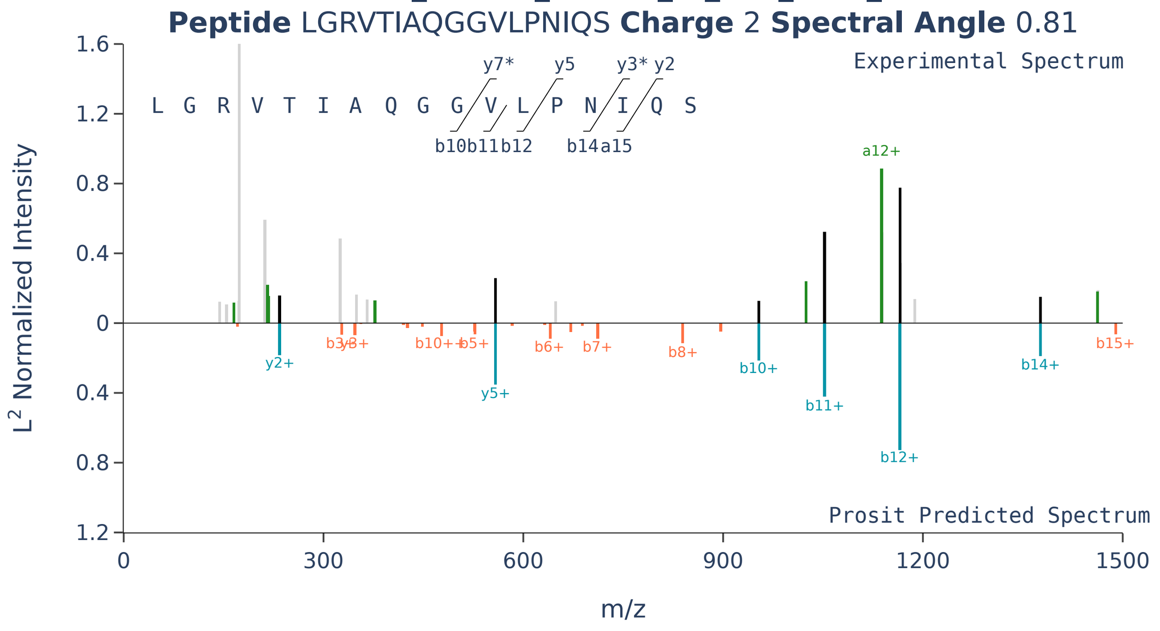


**Source** HRoetschke_291222_060123_Fu_H2A_b1R2_4h **Scan** 16436
**Peptide** VRNDEELNKLLGRVTIAQGG **Charge** 3 **Spectral Angle** 0.72

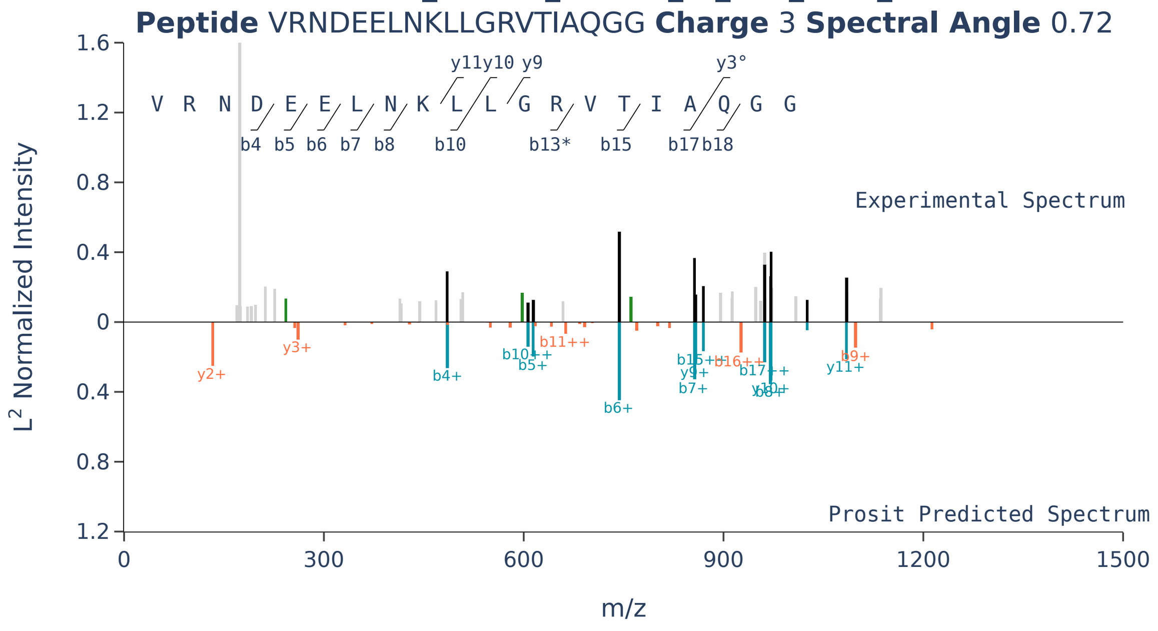


**Source** HRoetschke_291222_060123_Fu_H2A_b1R2_24h **Scan** 13710
**Peptide** ELNKLLGRVTIAQ **Charge** 2 **Spectral Angle** 0.81

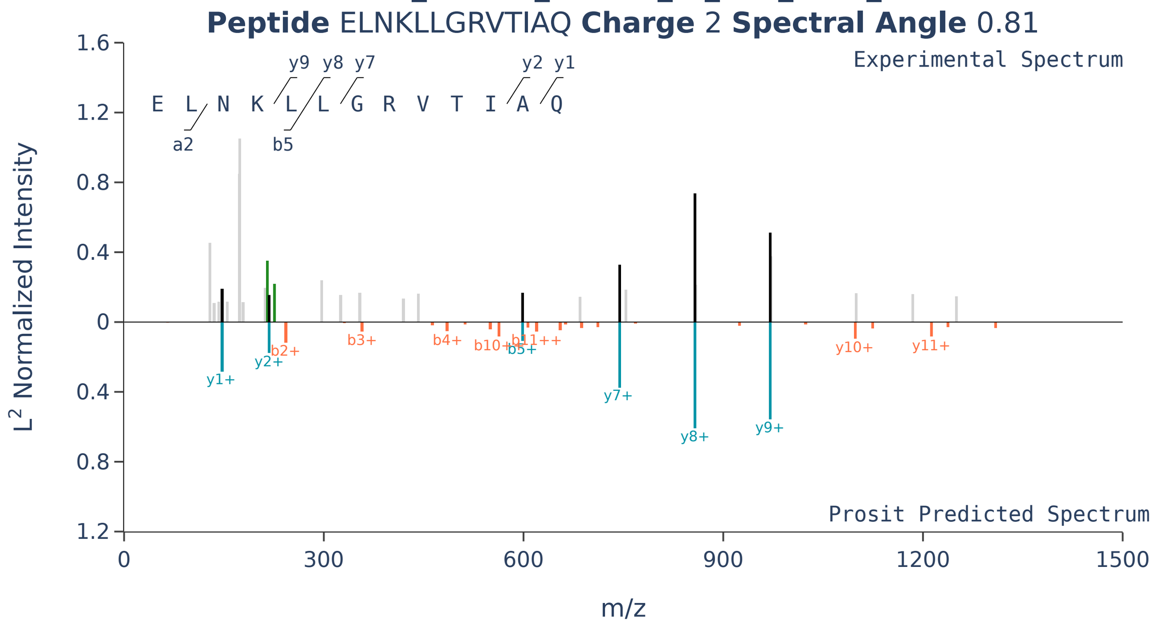


**Source** HRoetschke_291222_060123_Fu_H2A_b1R1_4h **Scan** 19513
**Peptide** KGNYAERVGAGAPVYLAAVLE **Charge** 3 **Spectral Angle** 0.75

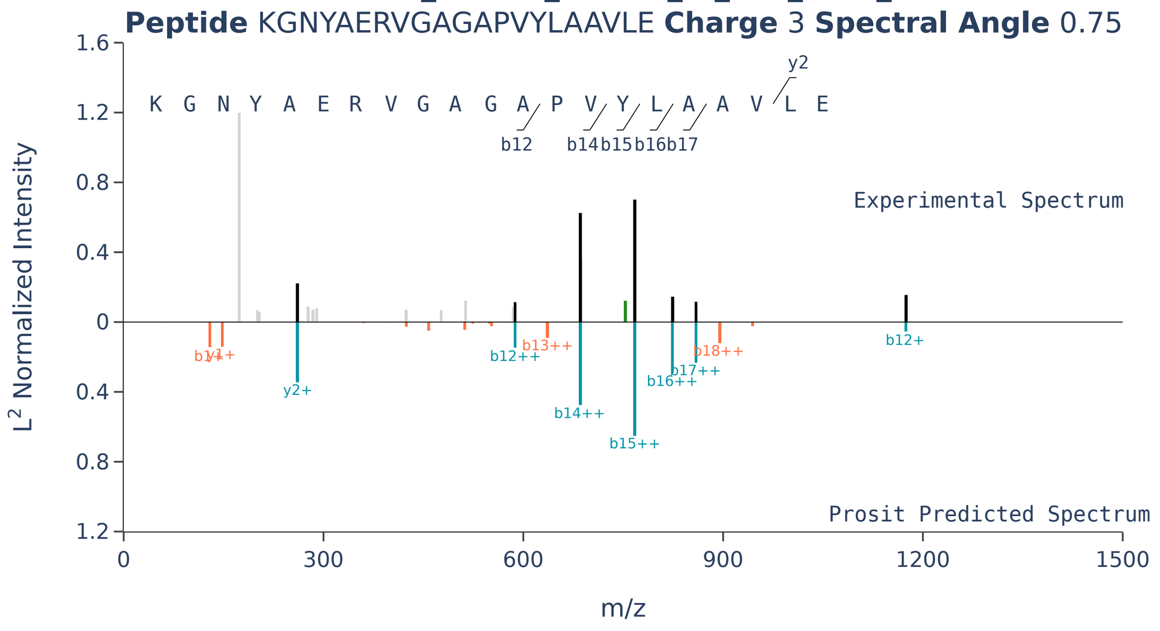


**Source** HRoetschke_291222_060123_Fu_H2A_b1R2_4h **Scan** 14186
**Peptide** LAVRNDEELNKLLGR **Charge** 4 **Spectral Angle** 0.81

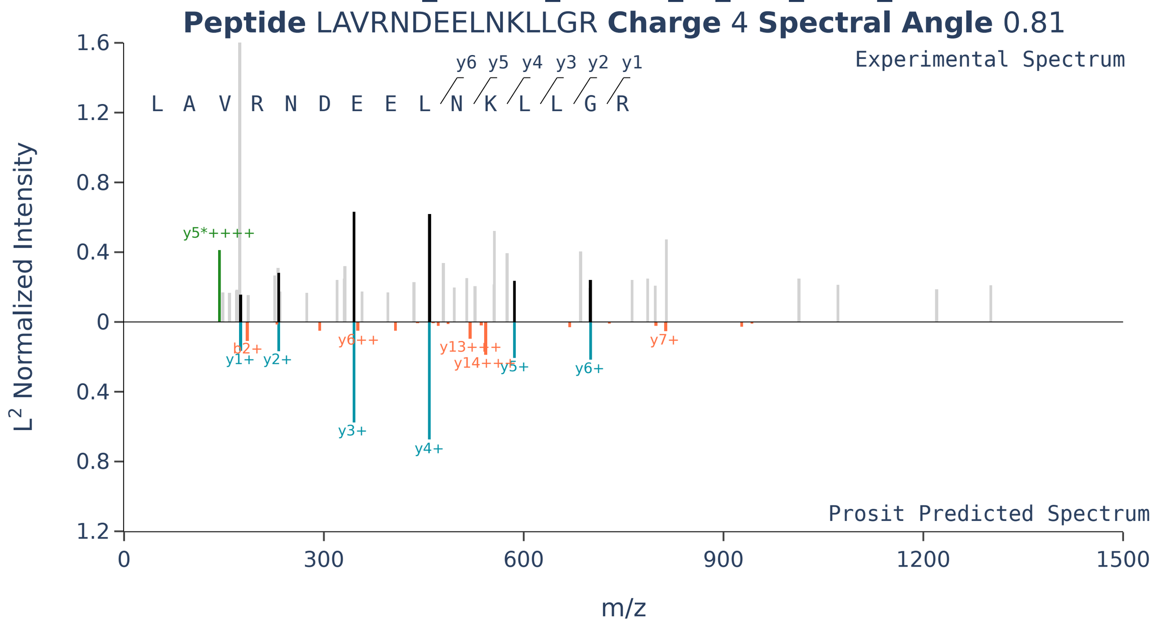


**Source** HRoetschke_291222_060123_Fu_H2A_b2R1_4h **Scan** 6232
**Peptide** TRAKAKTRSSRAGLQFPVGRVH **Charge** 5 **Spectral Angle** 0.67

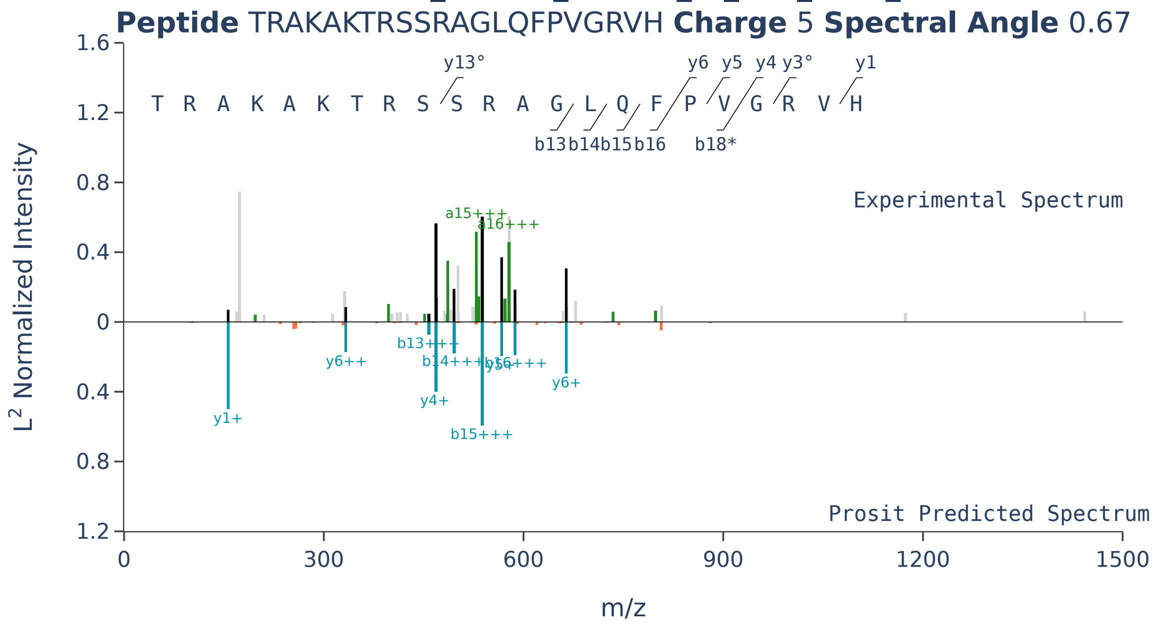


**Source** HRoetschke_291222_060123_Fu_H2A_b1R2_1h **Scan** 2888
**Peptide** QGGKTRAKAKTRSSRAGLQF **Charge** 4 **Spectral Angle** 0.8

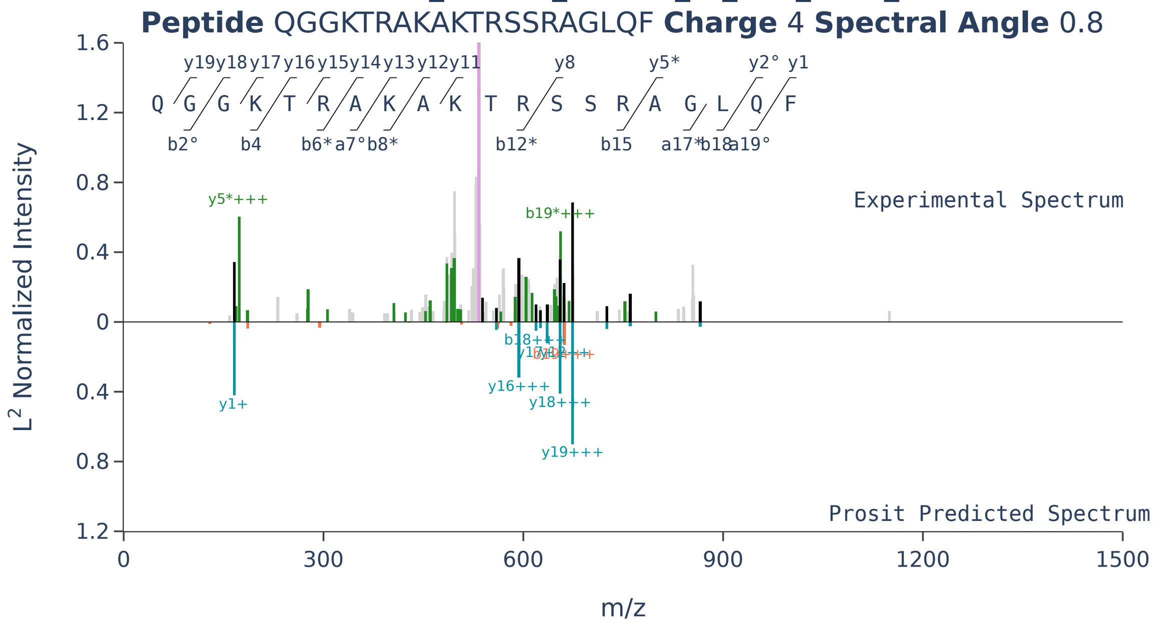

**Source** HRoetschke_291222_060123_Fu_H2A_b1R1_24h **Scan** 5179
**Peptide** DNKKTRIIPRHL **Charge** 3 **Spectral Angle** 0.73

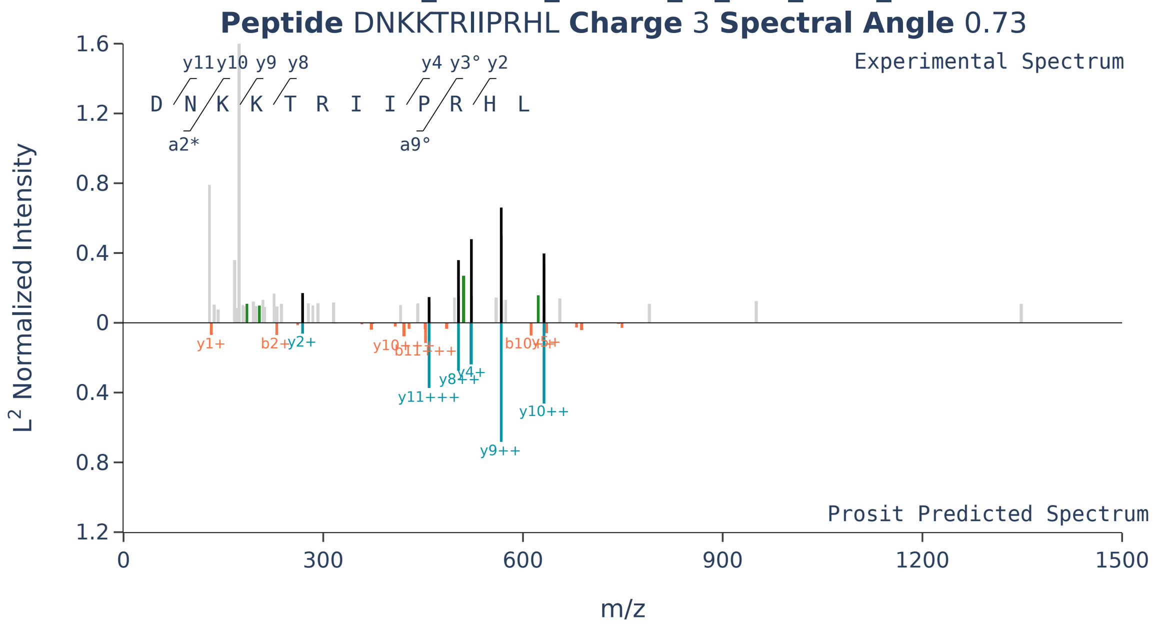


**Source** HRoetschke_291222_060123_Fu_H2A_b2R2_2h **Scan** 5271
**Peptide** GKTRAKAKTRSSRAGLQFPVGRVH **Charge** 5 **Spectral Angle** 0.64

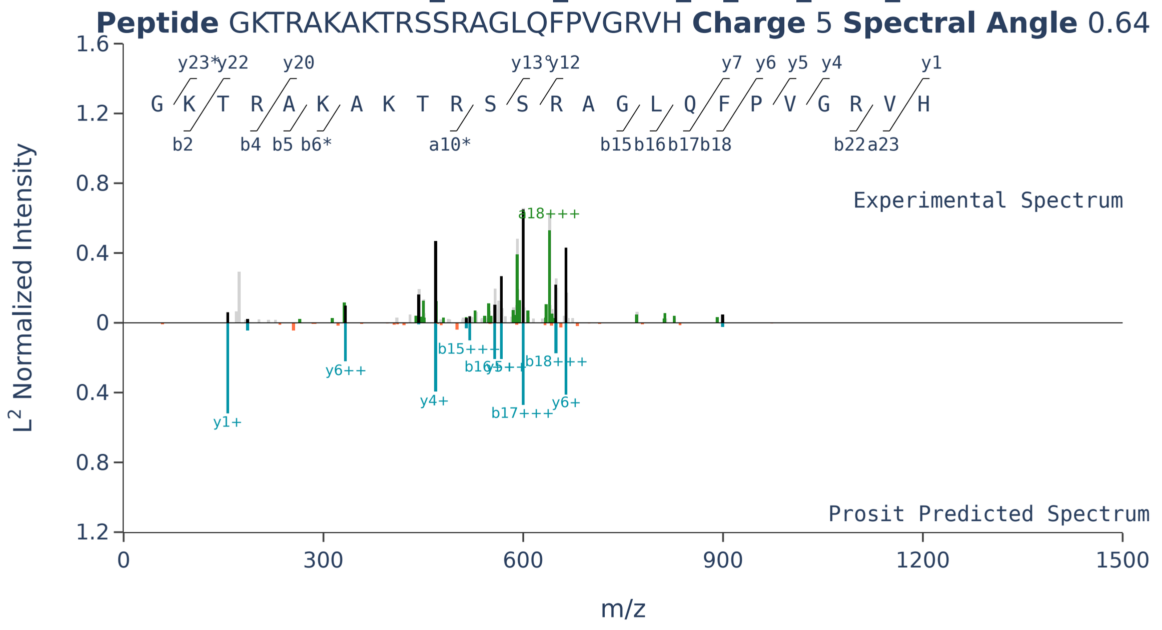


**Source** HRoetschke_291222_060123_Fu_H2A_b1R1_4h **Scan** 18413
**Peptide** YLTAEILELAGNAARDNKKTRIIPRH **Charge** 6 **Spectral Angle** 0.72

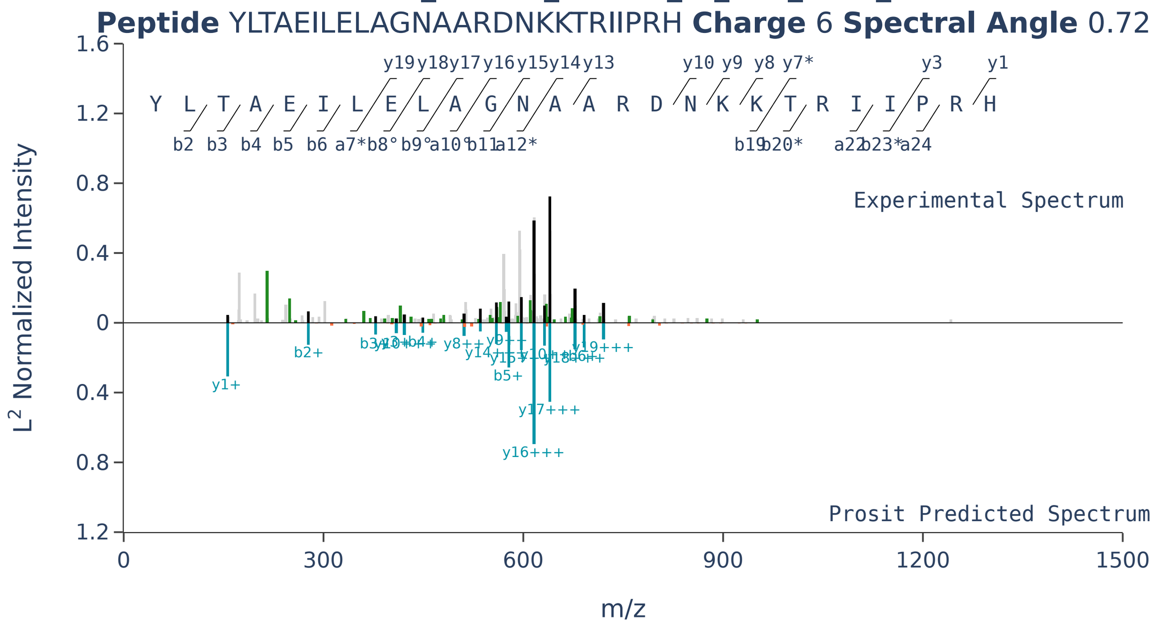


**Source** HRoetschke_291222_060123_Fu_H2A_b1R1_4h **Scan** 15072
**Peptide** GGVLPNIQSVLLPKKTESSKSAKSK **Charge** 4 **Spectral Angle** 0.63

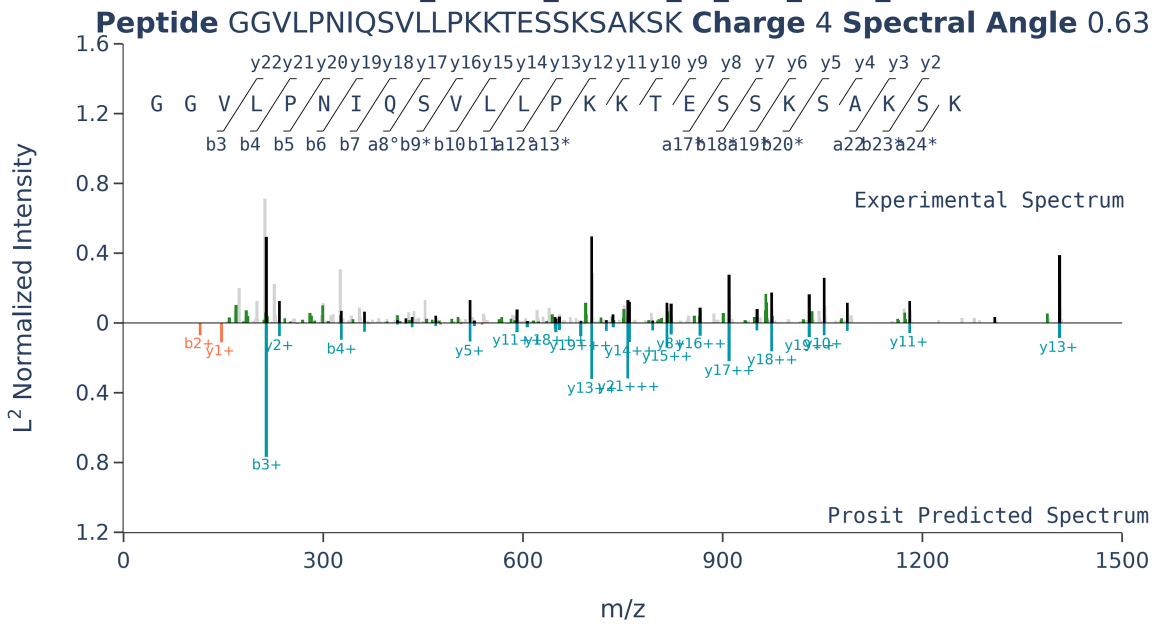


**Source** HRoetschke_291222_060123_Fu_H2A_b1R2_4h **Scan** 18410
**Peptide** LRKGNYAERVGAGAPVYLAAVL **Charge** 4 **Spectral Angle** 0.78

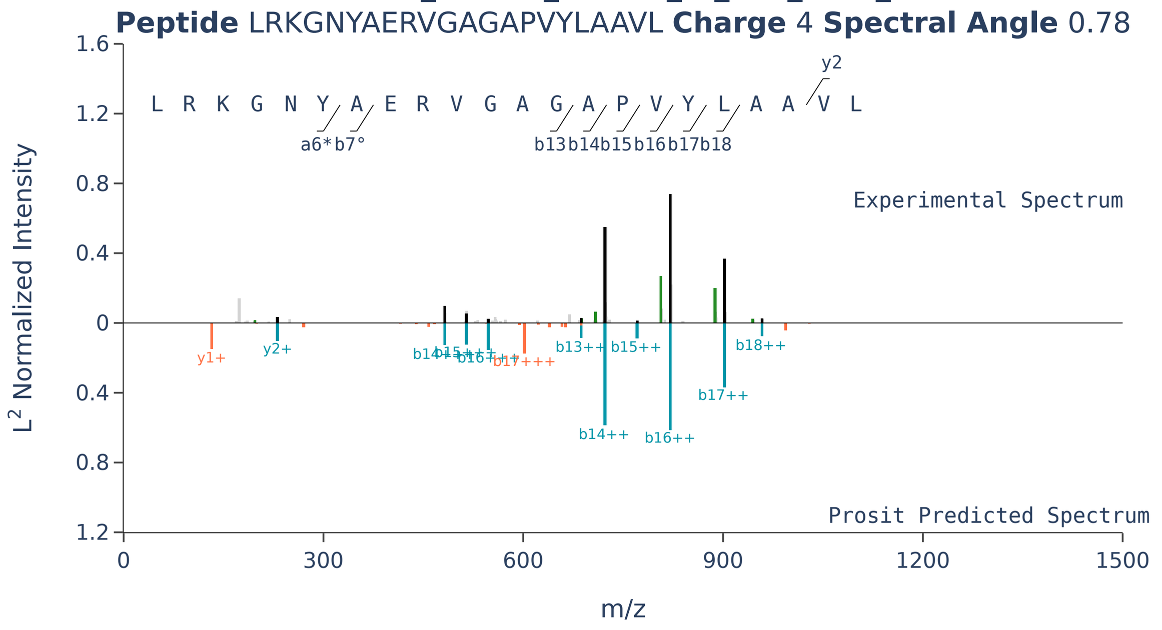


**Source** HRoetschke_291222_060123_Fu_H2A_b2R2_1h **Scan** 11666
**Peptide** YAERVGAGAPVYL **Charge** 2 **Spectral Angle** 0.8

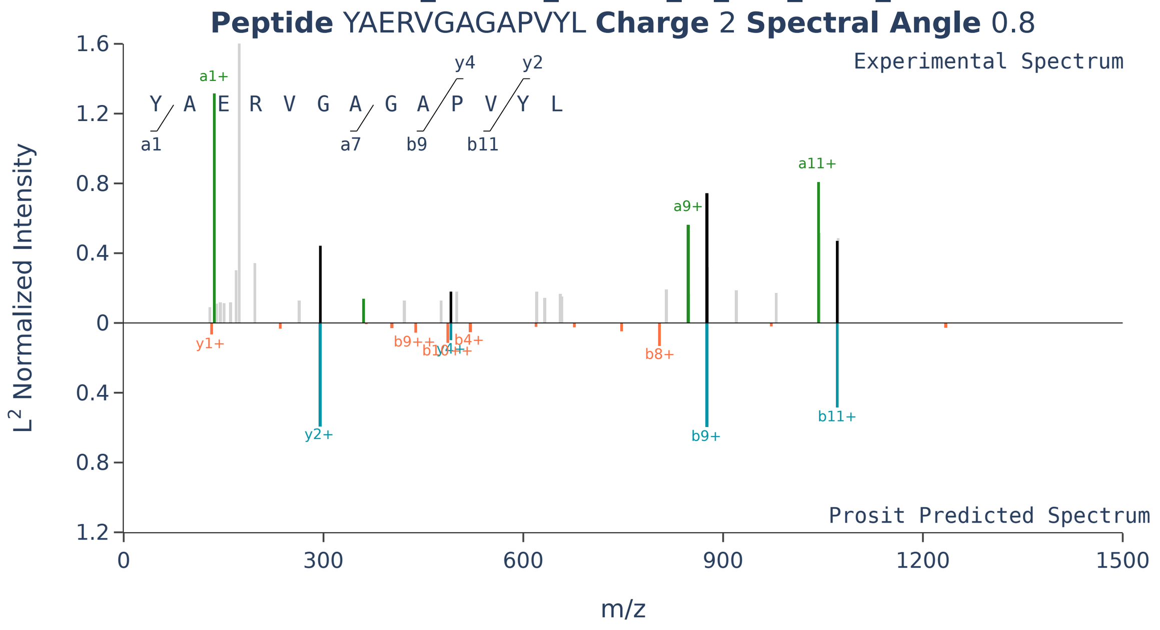


**Source** HRoetschke_291222_060123_Fu_H2A_b2R1_2h **Scan** 2838
**Peptide** VHRLLRKGNYA **Charge** 3 **Spectral Angle** 0.81

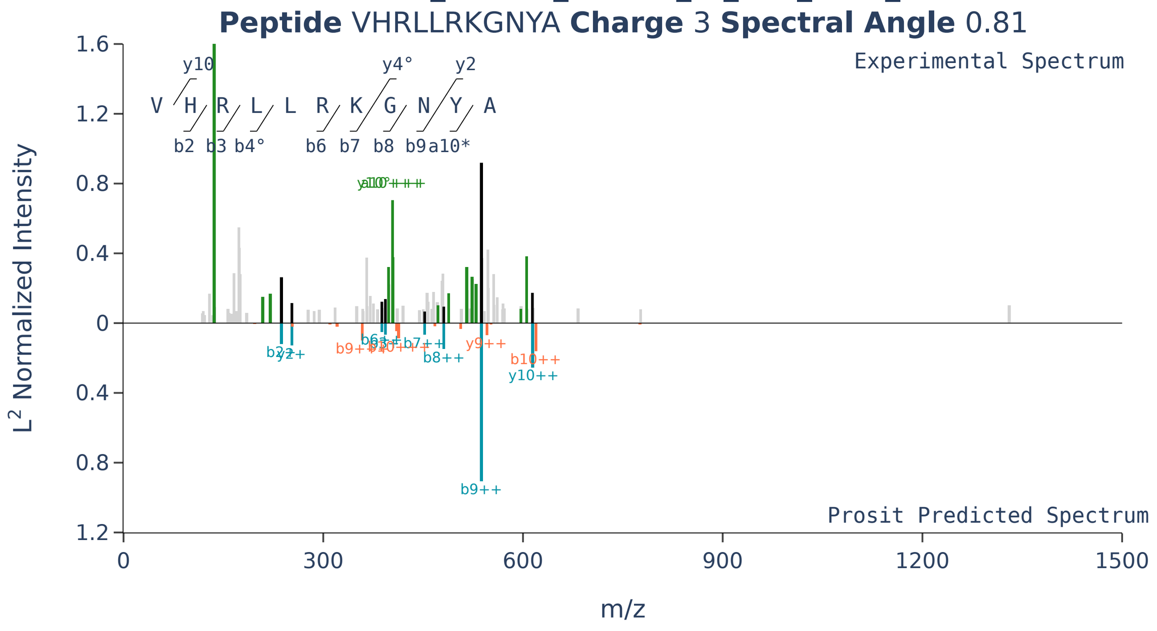


**Source** HRoetschke_291222_060123_Fu_H2A_b2R2_4h **Scan** 10323
**Peptide** EILELAGNAARDNKKTRIIPRHLQ **Charge** 5 **Spectral Angle** 0.74

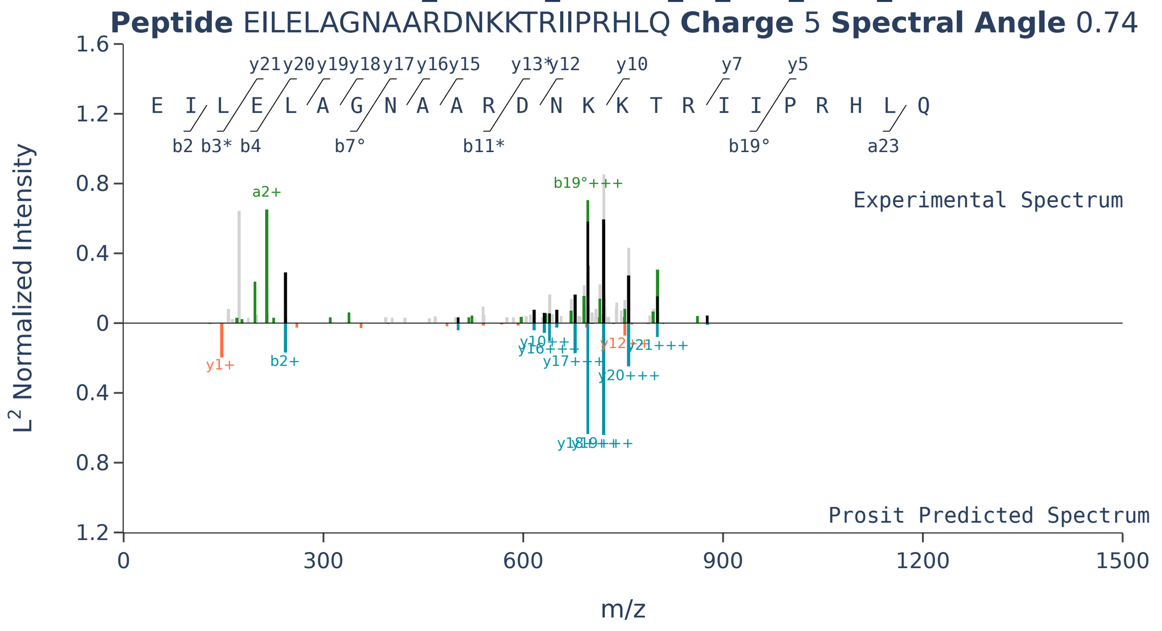


**Source** HRoetschke_291222_060123_Fu_H2A_b2R1_24h **Scan** 4497
**Peptide** PVGRVHRLLRKGNYA **Charge** 3 **Spectral Angle** 0.6

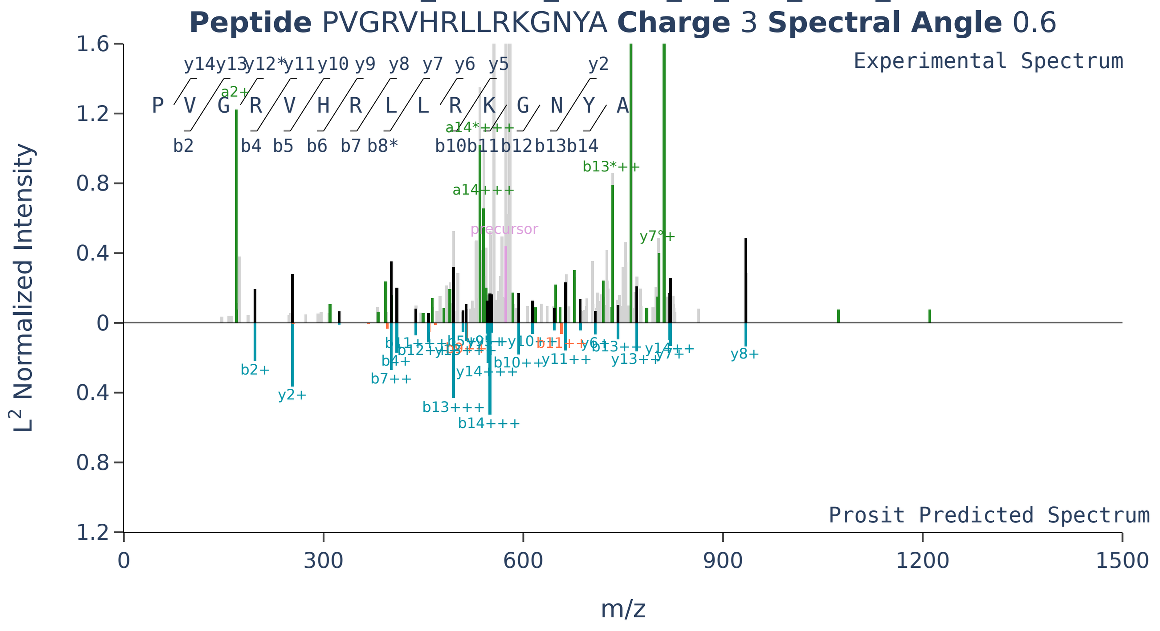


**Source** HRoetschke_291222_060123_Fu_H2A_b1R1_2h **Scan** 11108
**Peptide** PVGRVHRLLRKGNYAERVGAGAPVYLAA **Charge** 4 **Spectral Angle** 0.71

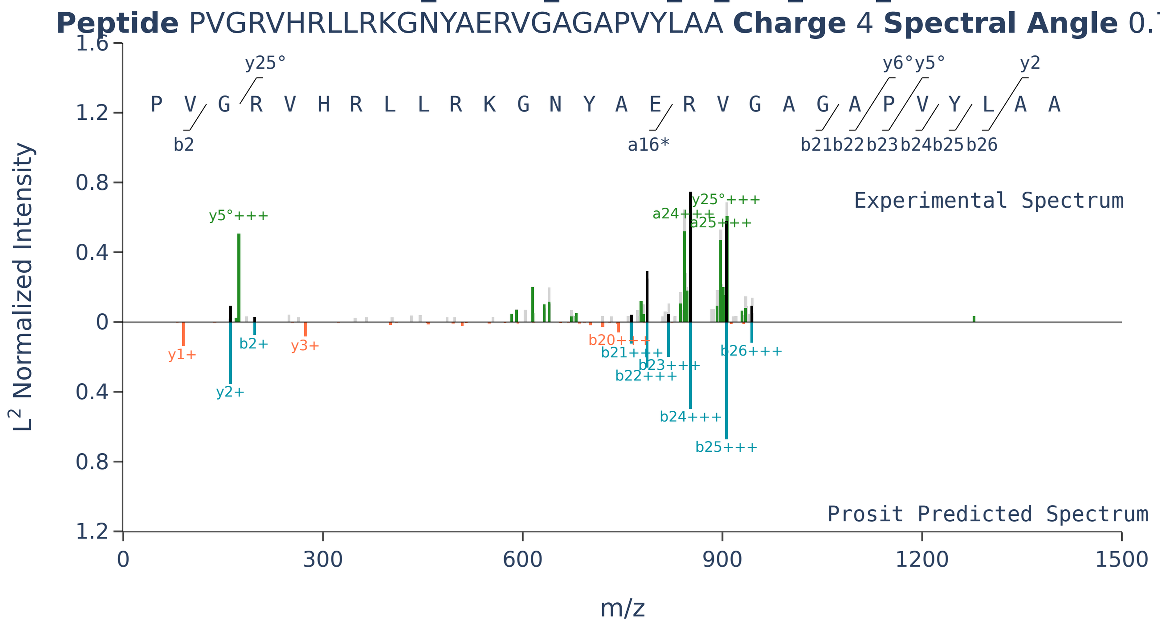


**Source** HRoetschke_291222_060123_Fu_H2A_b1R1_2h **Scan** 14891
**Peptide** AEILELAGNAARDNKKTRIIPRHLQL **Charge** 5 **Spectral Angle** 0.77

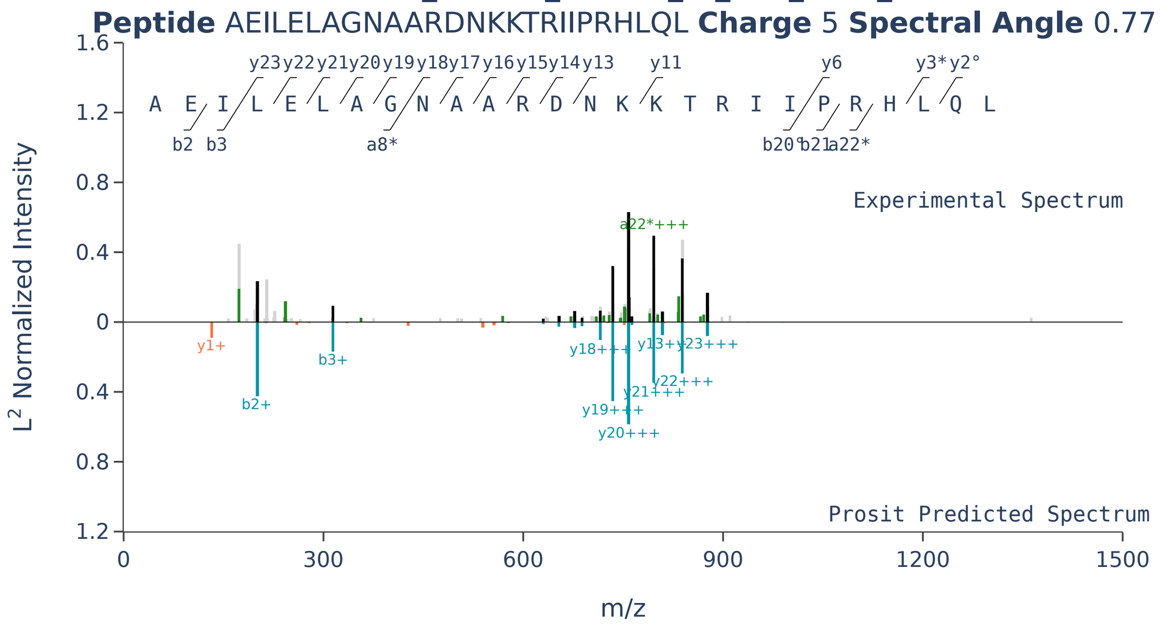


**Source** HRoetschke_291222_060123_Fu_H2A_b1R1_4h **Scan** 14609
**Peptide** SRAGLQFPVGRVHRLL **Charge** 4 **Spectral Angle** 0.76

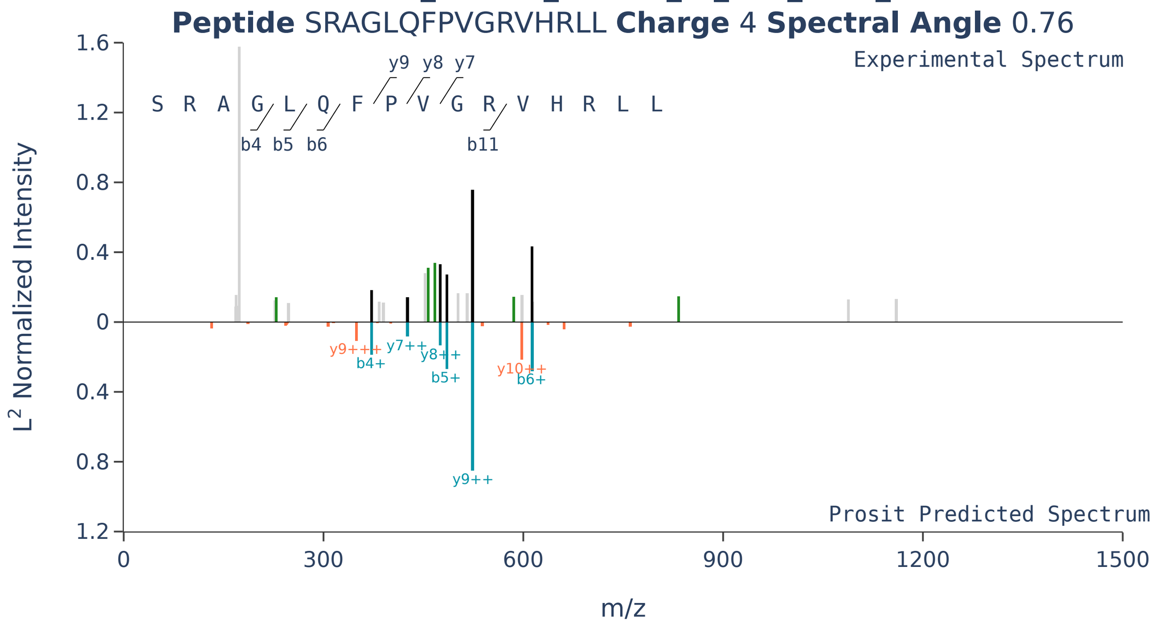


**Source** HRoetschke_291222_060123_Fu_H2A_b2R2_4h **Scan** 7214
**Peptide** AGNAARDNKKTRIIPRHLQLAVRND **Charge** 6 **Spectral Angle** 0.78

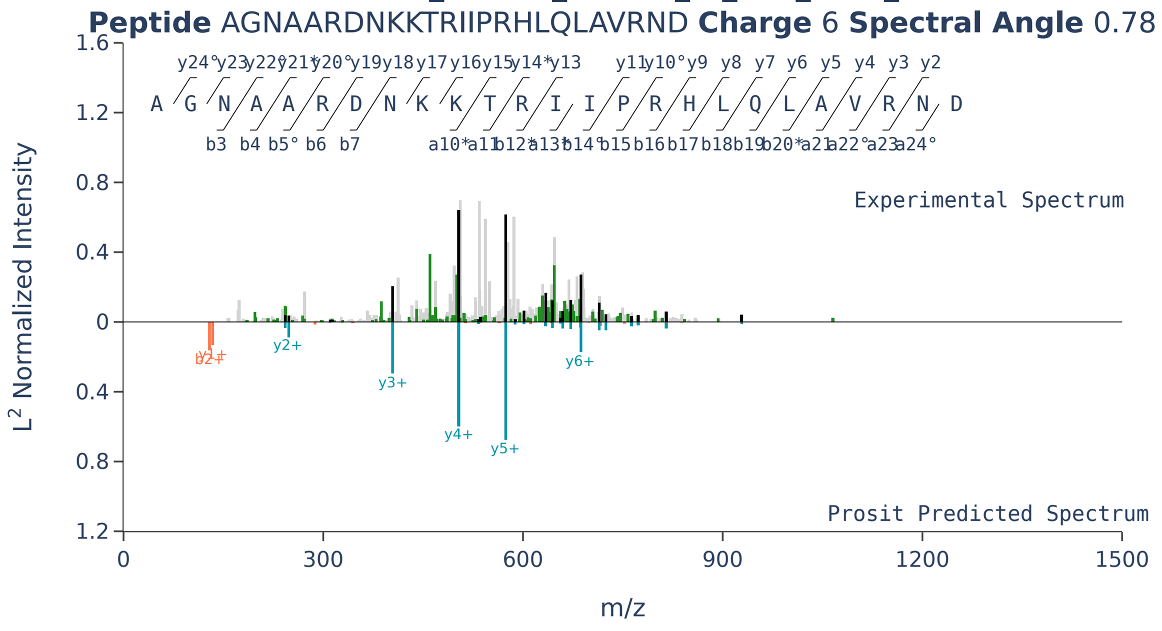


**Source** HRoetschke_291222_060123_Fu_H2A_b1R2_4h **Scan** 18277
**Peptide** NDEELNKLLGRVTLA **Charge** 3 **Spectral Angle** 0.84

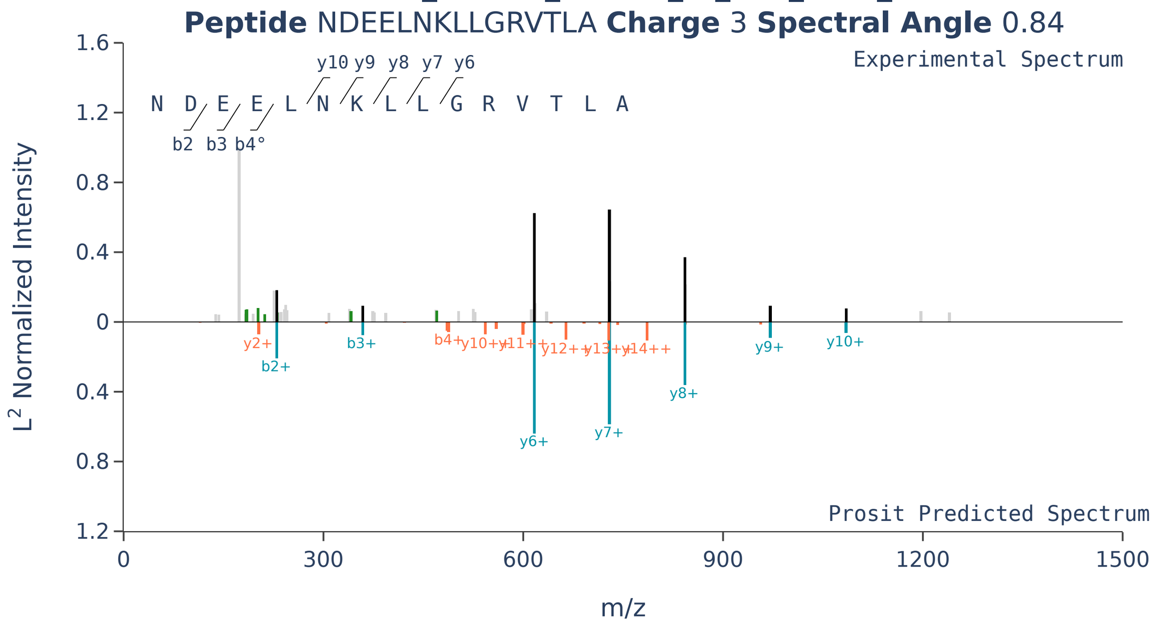


**Source** HRoetschke_291222_060123_Fu_H2A_b2R2_2h **Scan** 9241
**Peptide** RVHRLLRKGNYAERVGAGAPVYLA **Charge** 5 **Spectral Angle** 0.65

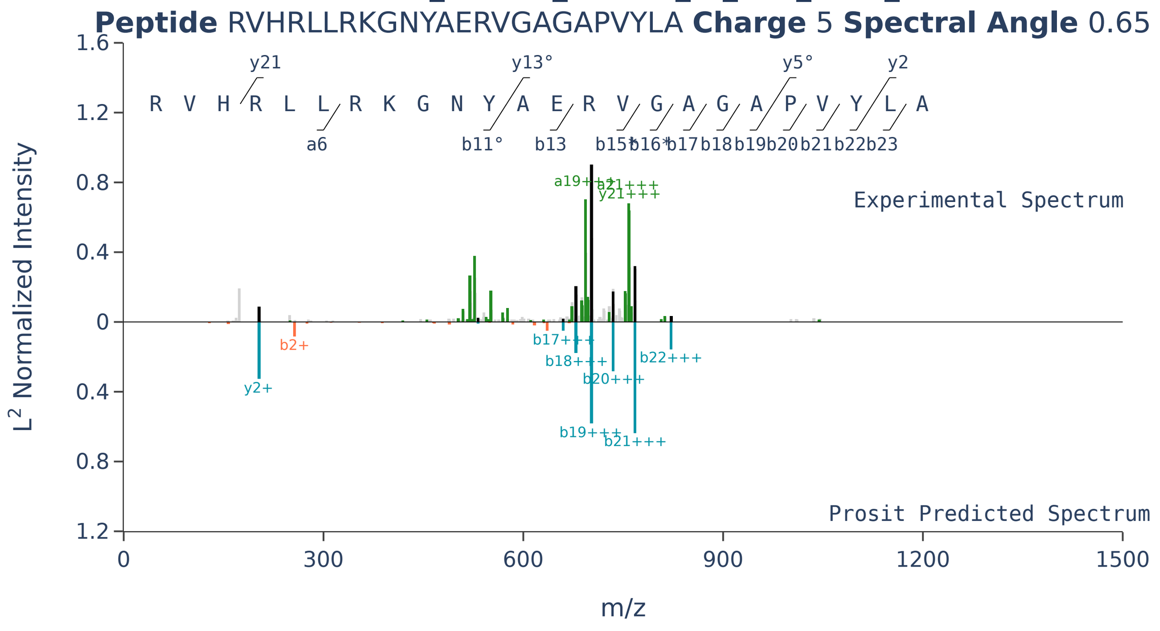

**Source** HRoetschke_291222_060123_Fu_H2A_b1R1_24h **Scan** 1939
**Peptide** ARDNKKTRIIPRH **Charge** 4 **Spectral Angle** 0.67

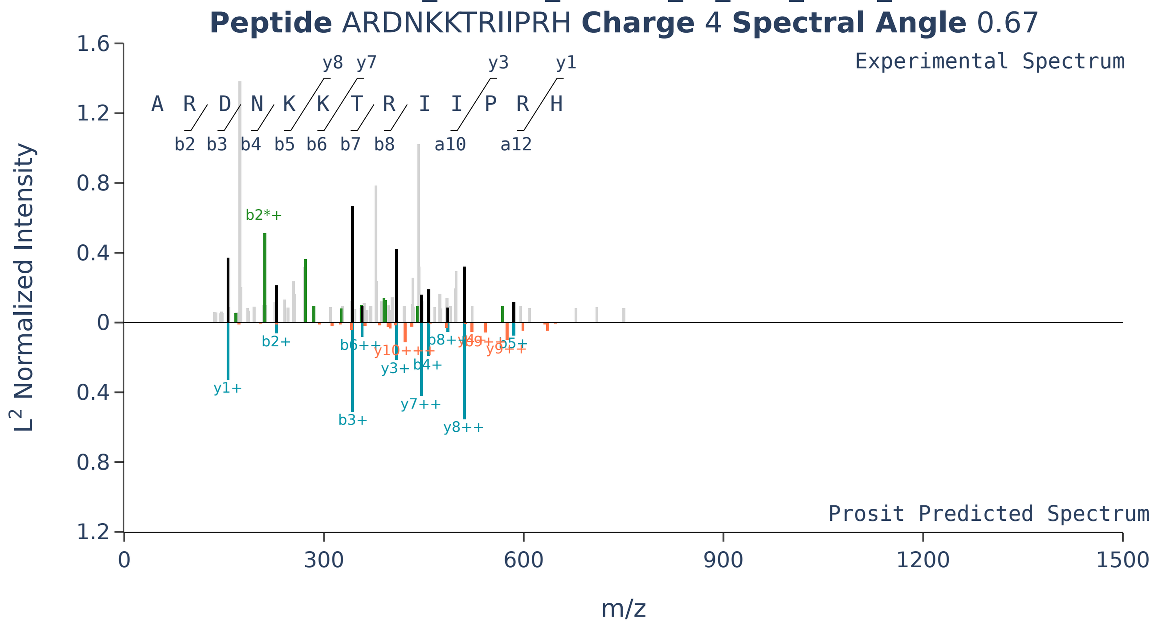


**Source** HRoetschke_291222_060123_Fu_H2A_b1R1_2h **Scan** 18954
**Peptide** EYLTAEILELAGNAARDNKKTR **Charge** 4 **Spectral Angle** 0.88

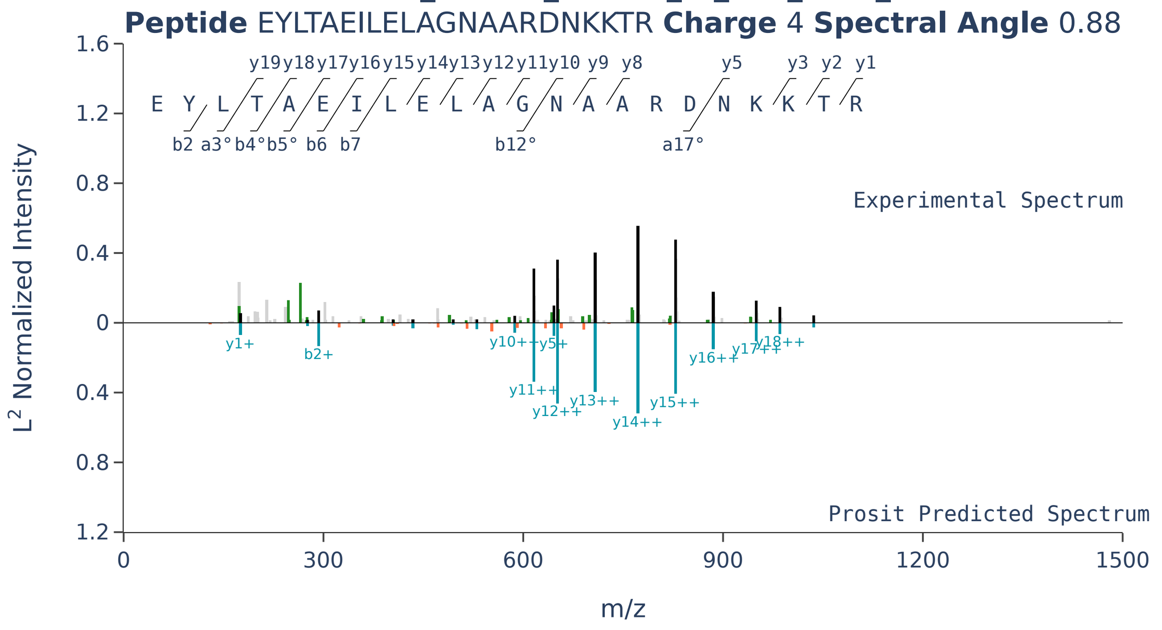


**Source** HRoetschke_291222_060123_Fu_H2A_b1R1_4h **Scan** 13203
**Peptide** TAEILELAGNAARDNKKT **Charge** 4 **Spectral Angle** 0.69

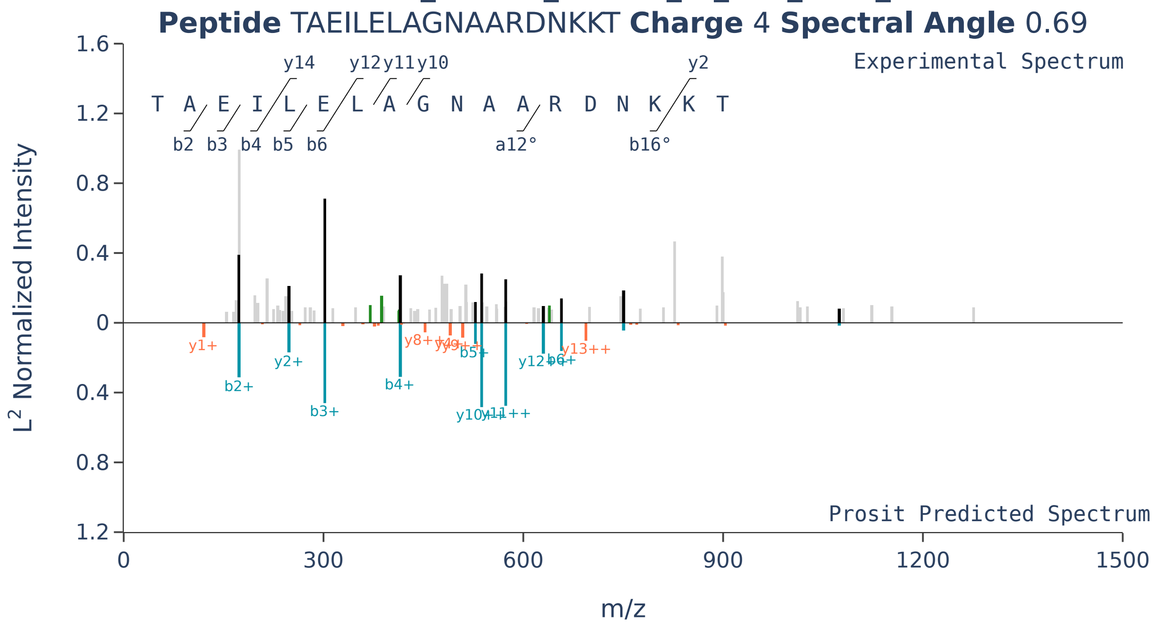


**Source** HRoetschke_291222_060123_Fu_H2A_b2R2_24h **Scan** 6942
**Peptide** AARDNKKTRIIPRHLQLA **Charge** 4 **Spectral Angle** 0.67

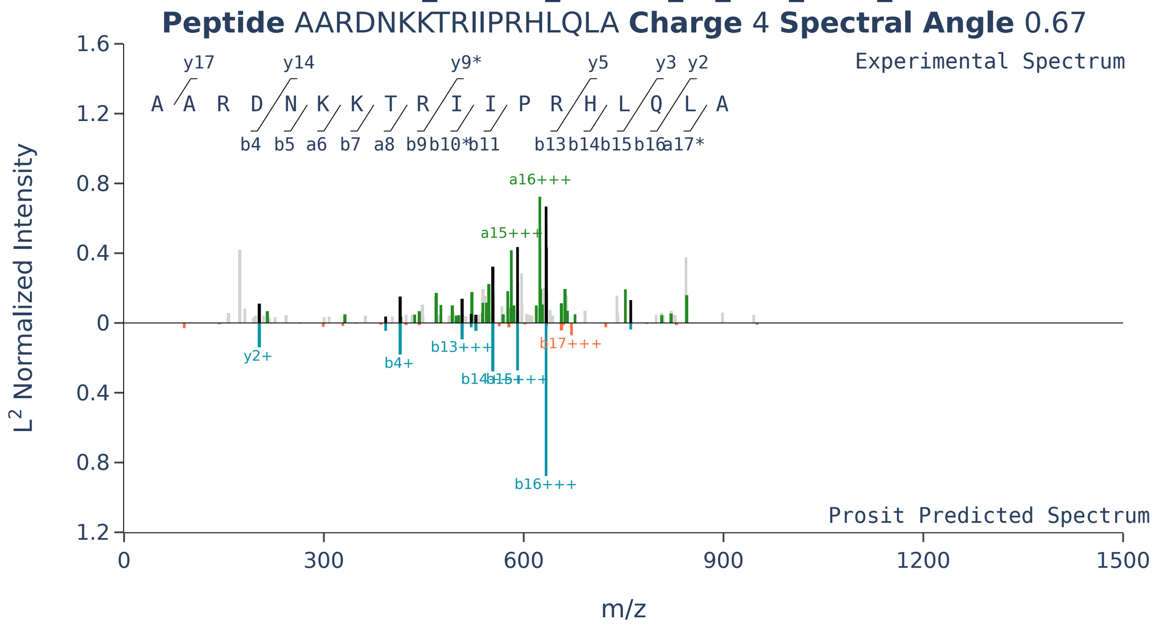


**Source** HRoetschke_291222_060123_Fu_H2A_b2R1_24h **Scan** 17427
**Peptide** QGGVLPNIQSVLLPKKT **Charge** 3 **Spectral Angle** 0.83

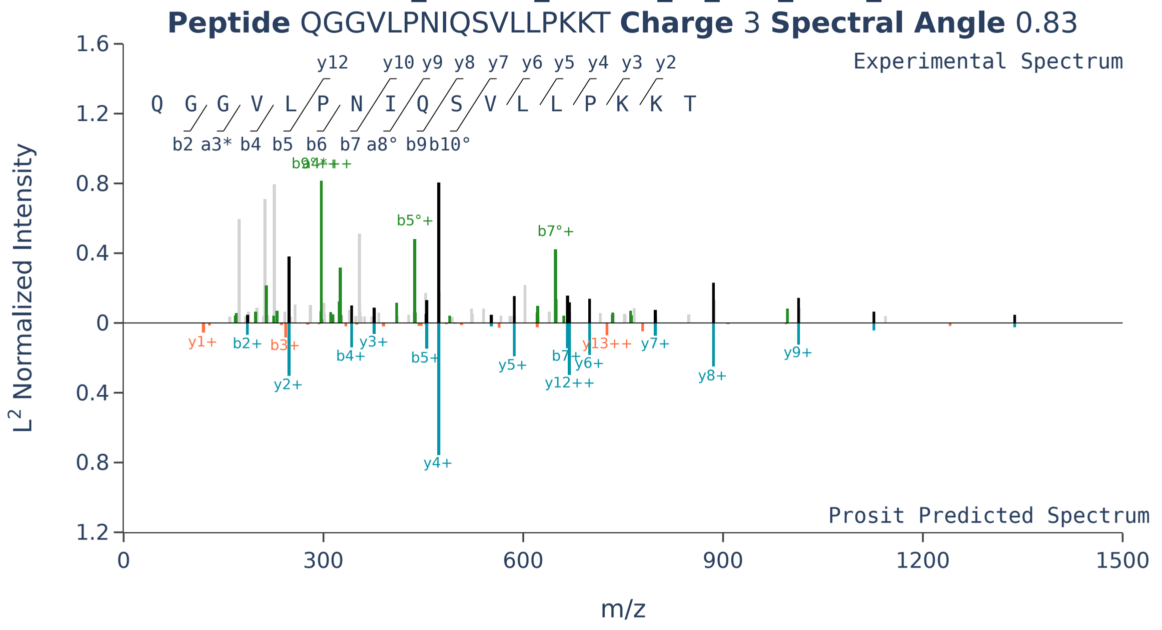


**Source** HRoetschke_291222_060123_Fu_H2A_b1R1_2h **Scan** 12257
**Peptide** RVHRLLRKGNYAERVGAGAPVYLAAV **Charge** 4 **Spectral Angle** 0.78

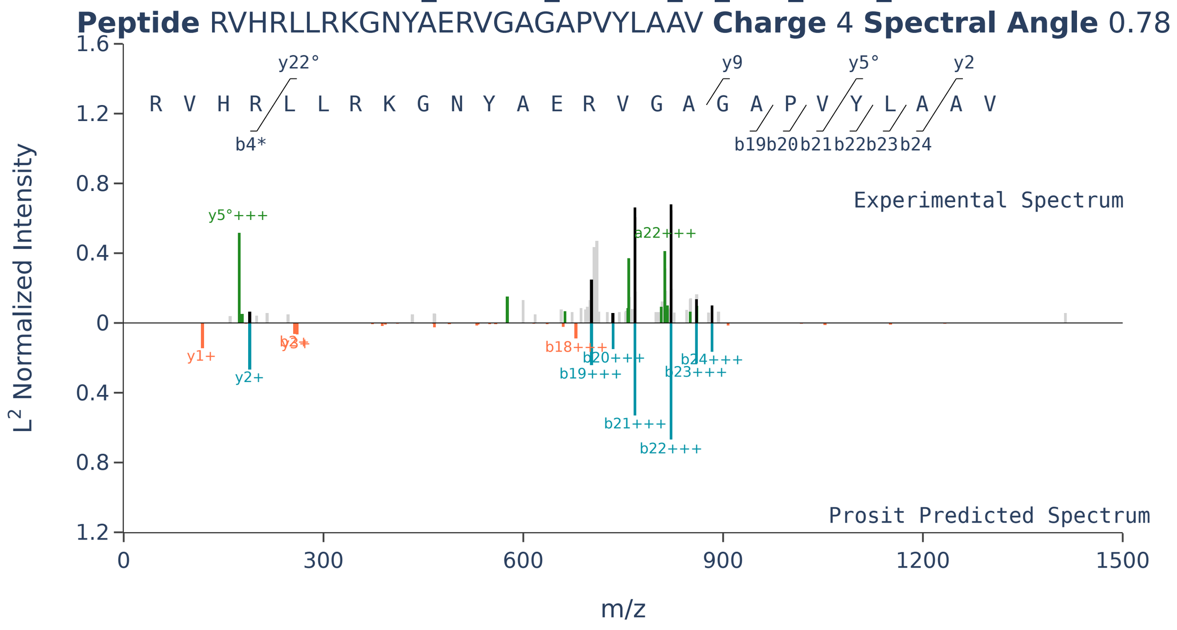


**Source** HRoetschke_291222_060123_Fu_H2A_b1R1_2h **Scan** 17293
**Peptide** QLAVRNDEELNKLLGRVTIAQGG **Charge** 3 **Spectral Angle** 0.74

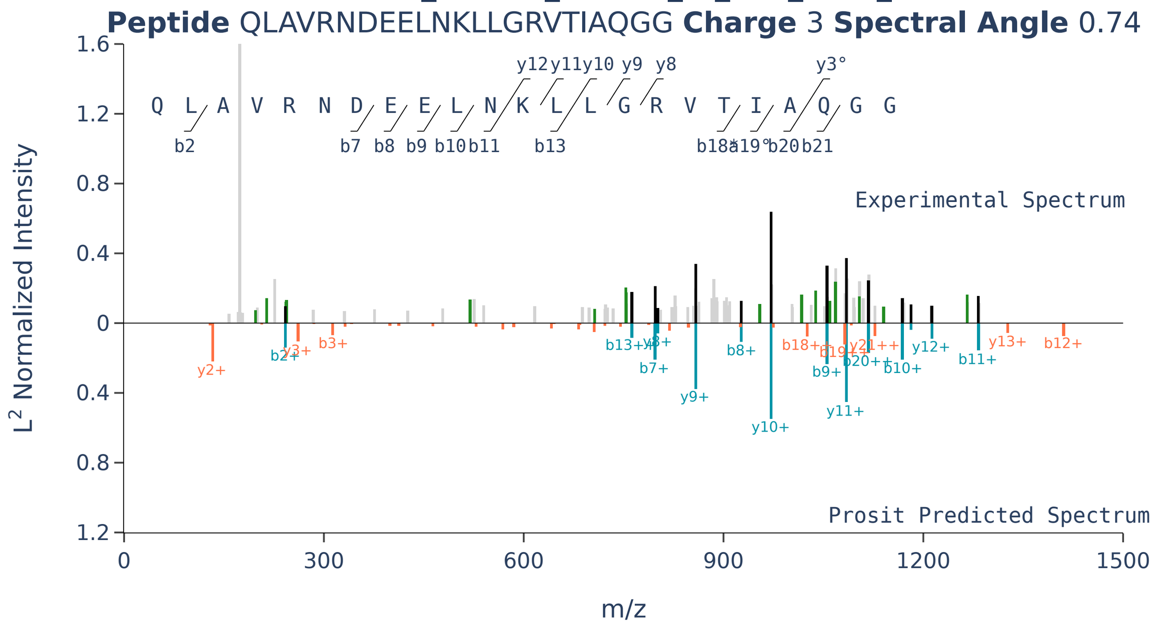


**Source** HRoetschke_291222_060123_Fu_H2A_b2R2_24h **Scan** 5697
**Peptide** QGGKTRAKAKTRSSRAGLQFPVGRVH **Charge** 6 **Spectral Angle** 0.64

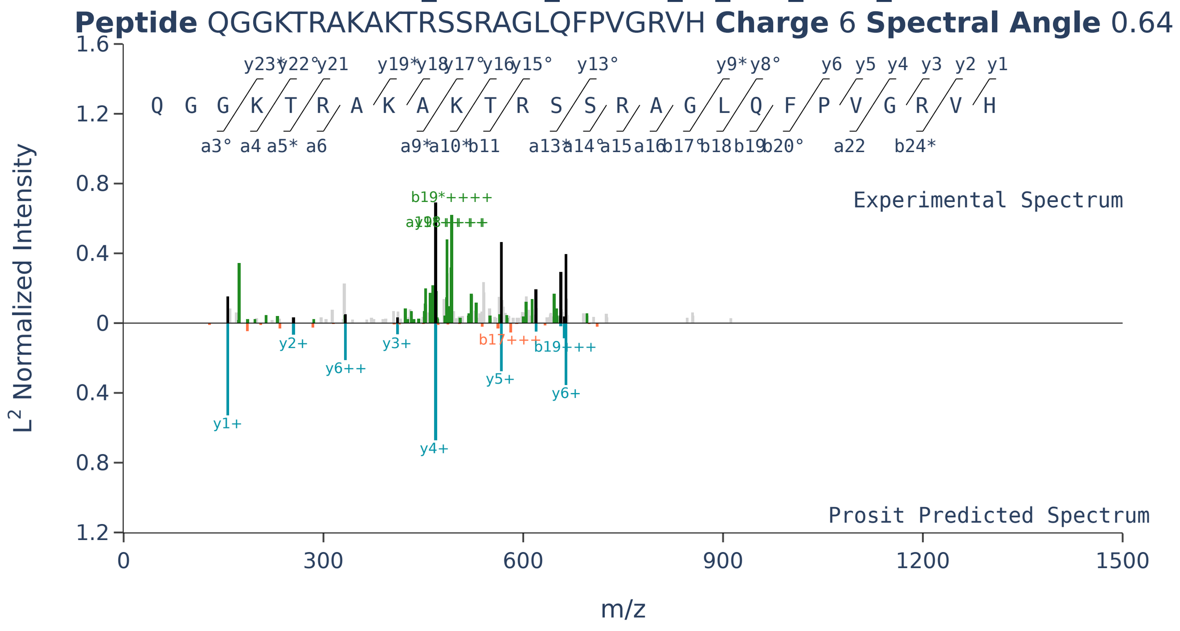


**Source** HRoetschke_291222_060123_Fu_H2A_b2R2_24h **Scan** 4116
**Peptide** VHRLLRKGNYAERV **Charge** 4 **Spectral Angle** 0.55

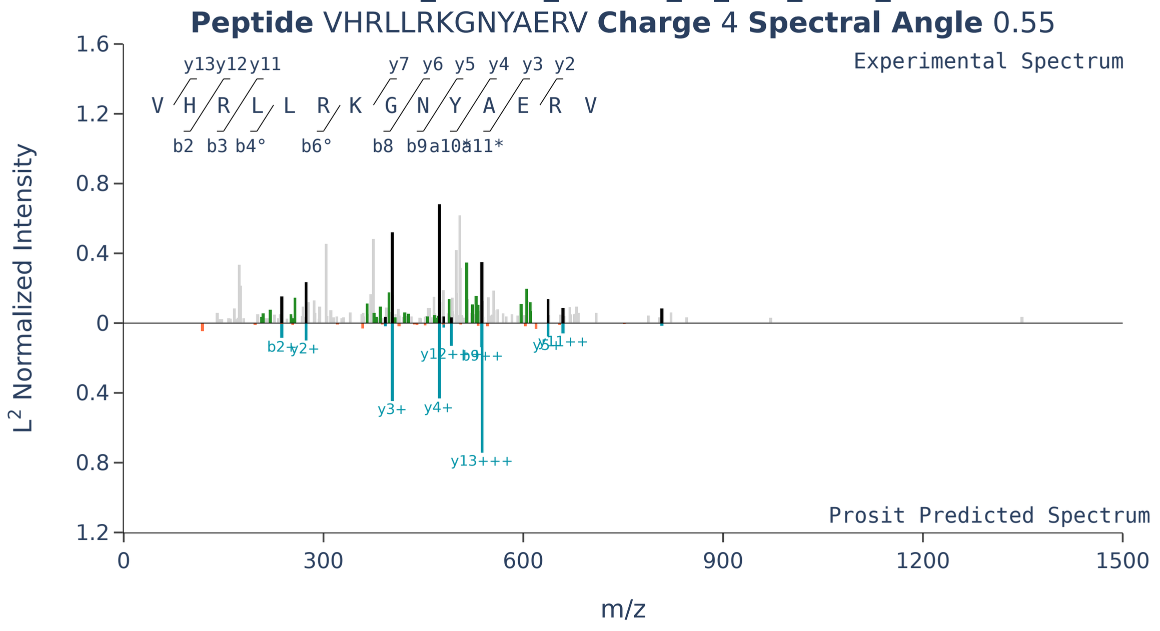


**Source** HRoetschke_291222_060123_Fu_H2A_b1R1_1h **Scan** 17889
**Peptide** EELNKLLGRVTIAQGGVLPNL **Charge** 3 **Spectral Angle** 0.71

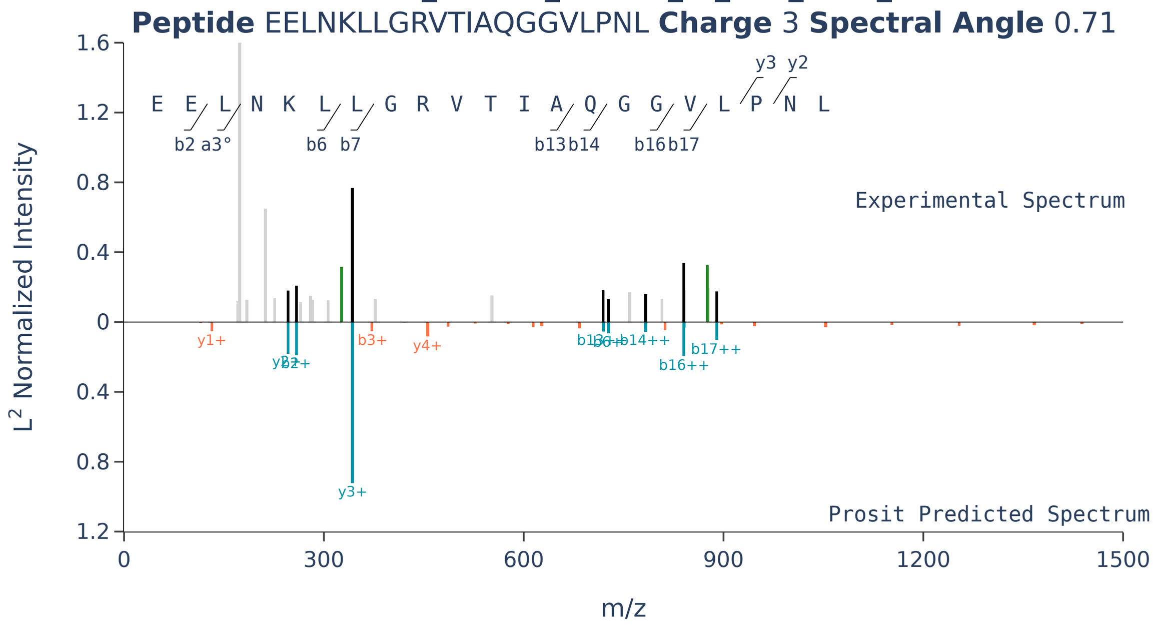


**Source** HRoetschke_291222_060123_Fu_H2A_b1R1_4h **Scan** 12082
**Peptide** GLQFPVGRVHRLLRKGN **Charge** 4 **Spectral Angle** 0.77

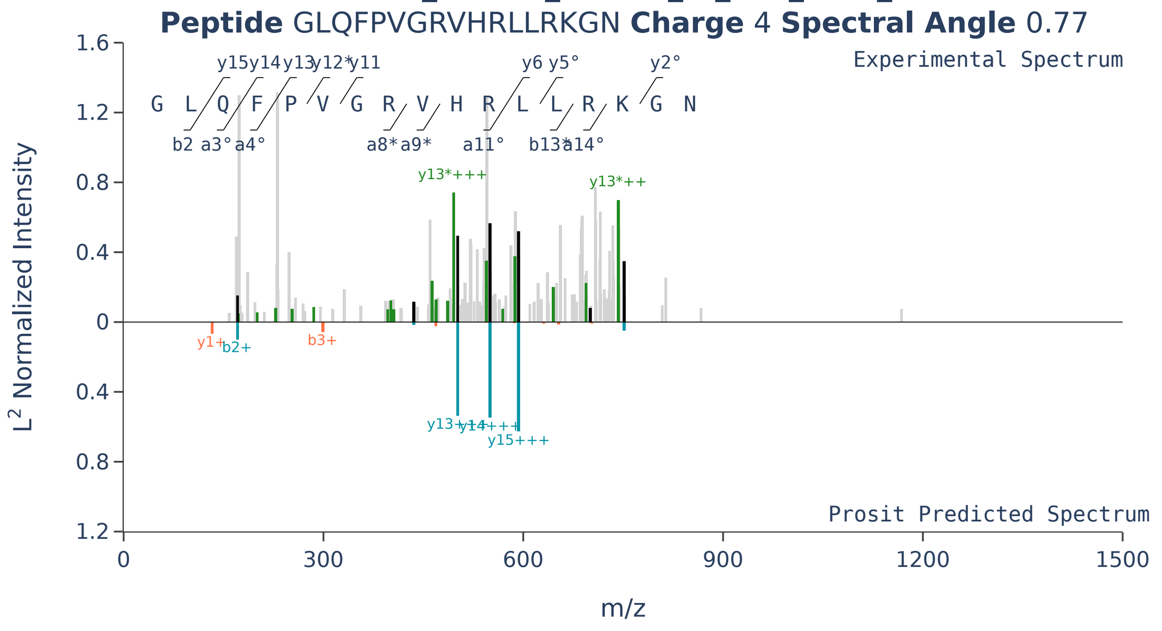


**Source** HRoetschke_291222_060123_Fu_H2A_b2R1_2h **Scan** 13939
**Peptide** EELNKLLGRVTIAQGG **Charge** 2 **Spectral Angle** 0.74

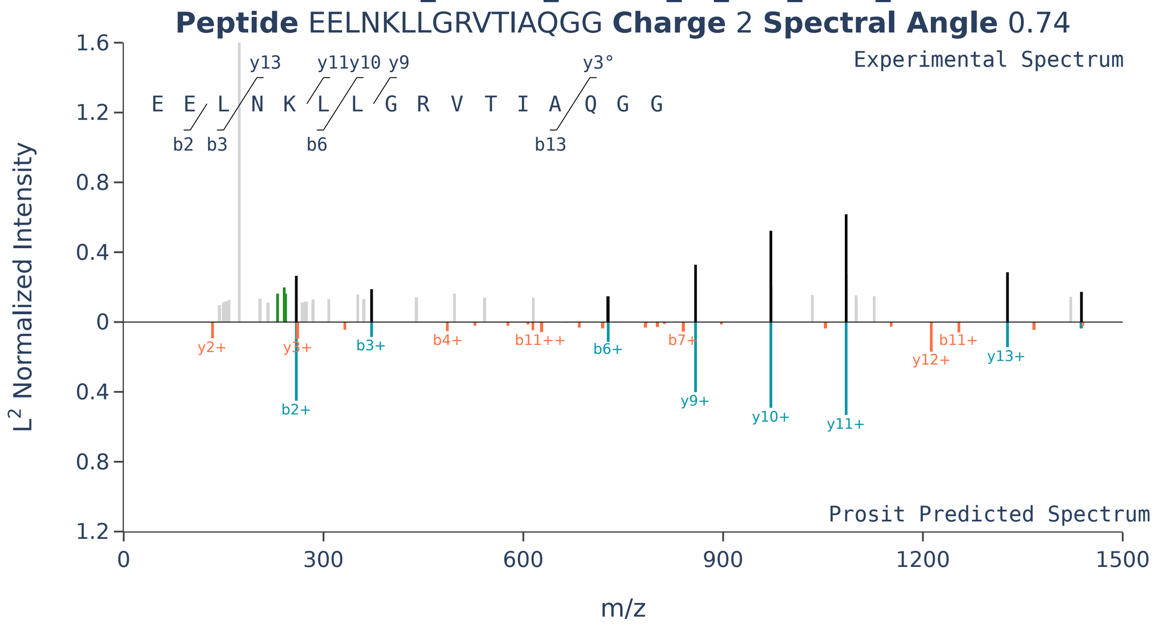


**Source** HRoetschke_291222_060123_Fu_H2A_b1R1_2h **Scan** 6937
**Peptide** QGGKTRAKAKTRSSRAGLQFPVGRV **Charge** 6 **Spectral Angle** 0.81

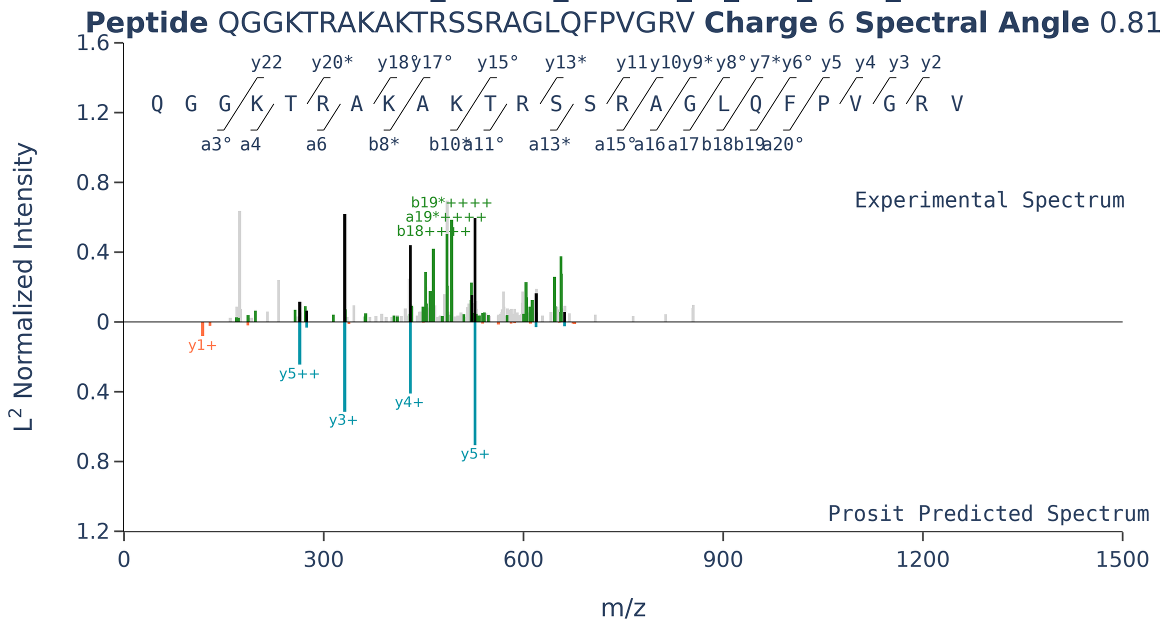


**Source** HRoetschke_291222_060123_Fu_H2A_b1R2_4h **Scan** 14124
**Peptide** AEILELAGNAARDNKKTRLL **Charge** 5 **Spectral Angle** 0.68

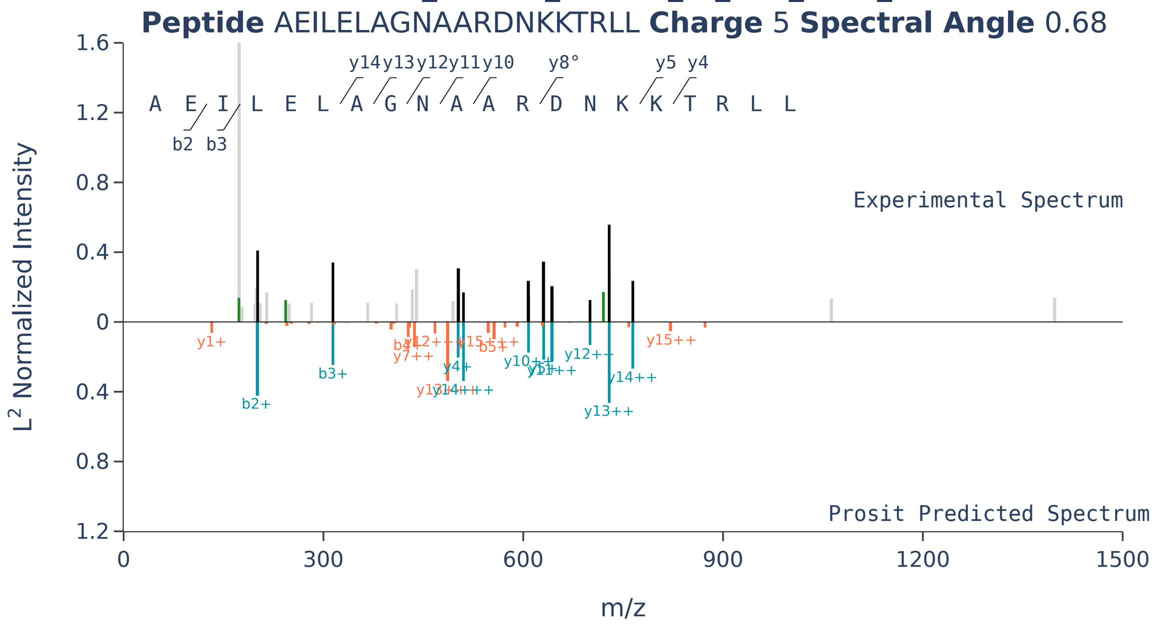


**Source** HRoetschke_291222_060123_Fu_H2A_b1R1_2h **Scan** 18610
**Peptide** EYLTAEILELAGNAARDNKKTRIIPRH **Charge** 5 **Spectral Angle** 0.83

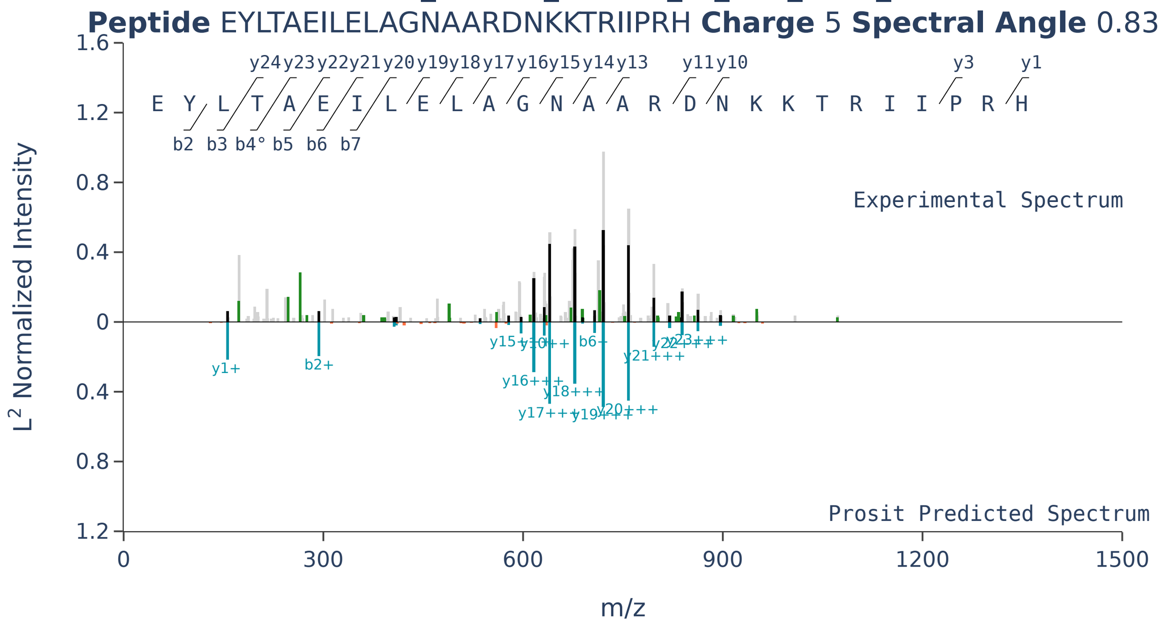

**Source** HRoetschke_291222_060123_Fu_H2A_b1R2_2h **Scan** 10619
**Peptide** PVGRVHRLLRKGNYAERVGAGAPVYLA **Charge** 5 **Spectral Angle** 0.61

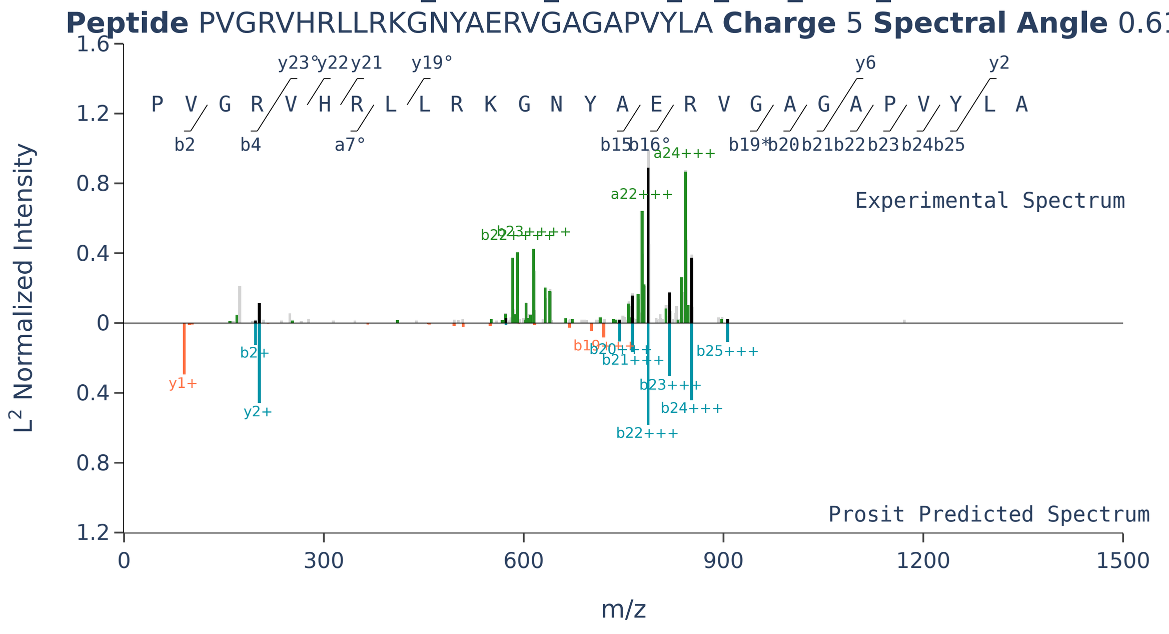


**Source** HRoetschke_291222_060123_Fu_H2A_b1R1_2h **Scan** 11413
**Peptide** SSRAGLQFPVGRVHRLLRKGNYAE **Charge** 5 **Spectral Angle** 0.62

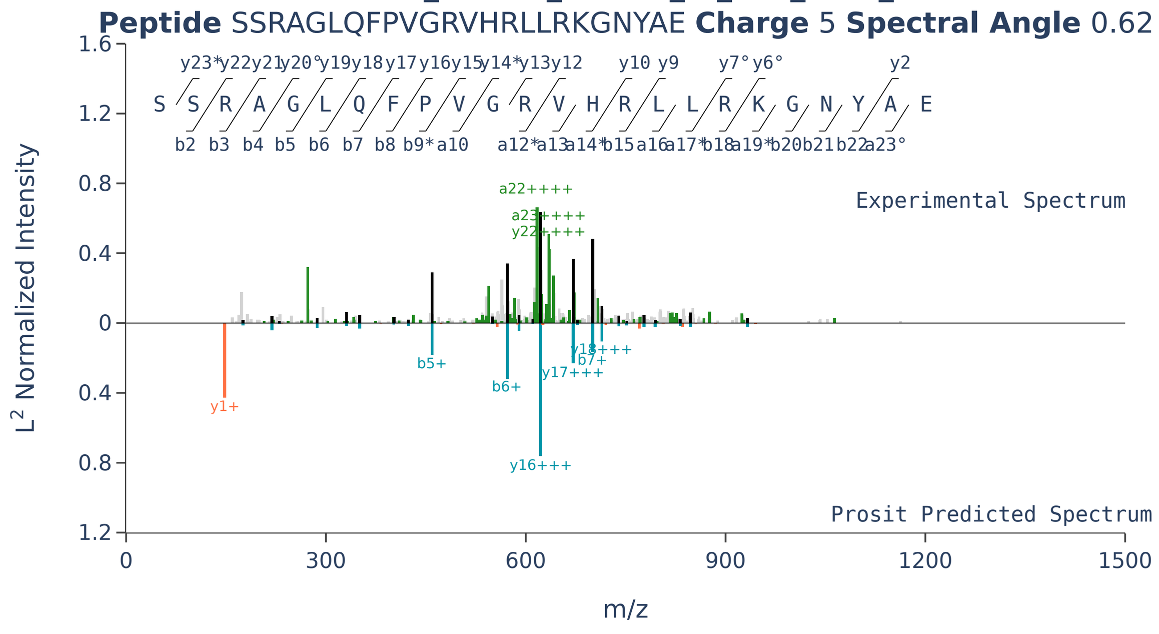


**Source** HRoetschke_291222_060123_Fu_H2A_b1R1_4h **Scan** 9456
**Peptide** NAARDNKKTRIIPRHLQLAV **Charge** 4 **Spectral Angle** 0.78

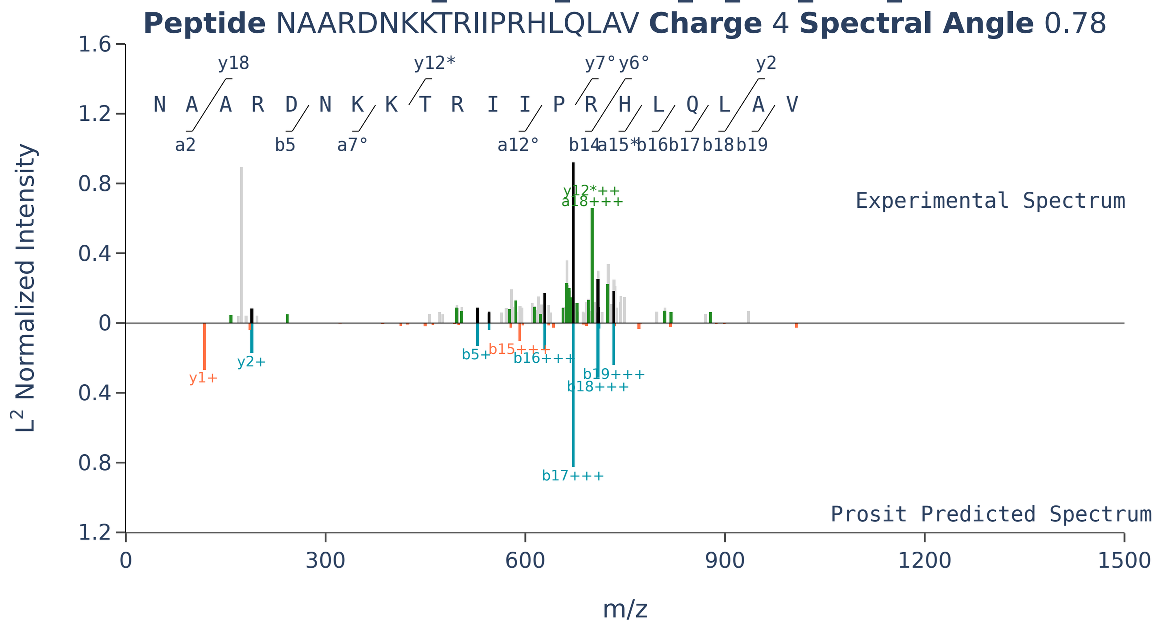


**Source** HRoetschke_291222_060123_Fu_H2A_b1R2_4h **Scan** 14537
**Peptide** LRKGNYAERVGAGAPVYLAAV **Charge** 4 **Spectral Angle** 0.79

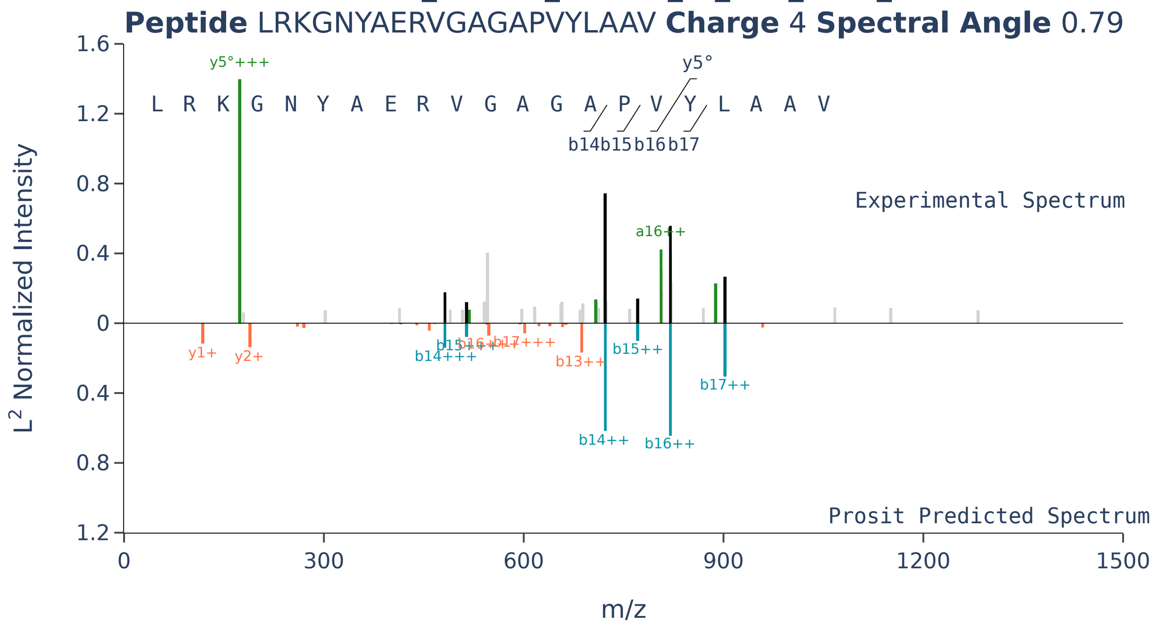


**Source** HRoetschke_291222_060123_Fu_H2A_b2R1_2h **Scan** 9352
**Peptide** RIIPRHLQL **Charge** 3 **Spectral Angle** 0.59

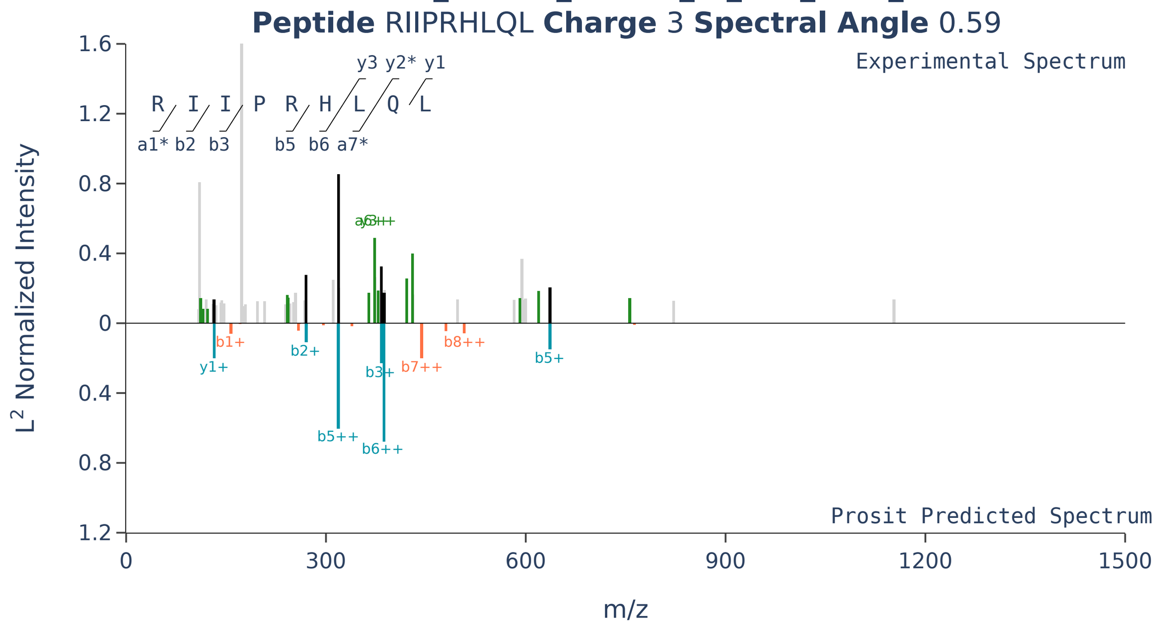


**Source** HRoetschke_291222_060123_Fu_H2A_b2R2_4h **Scan** 1563
**Peptide** SGRGKQGGKTRAKAKTRSSRAGL **Charge** 5 **Spectral Angle** 0.79

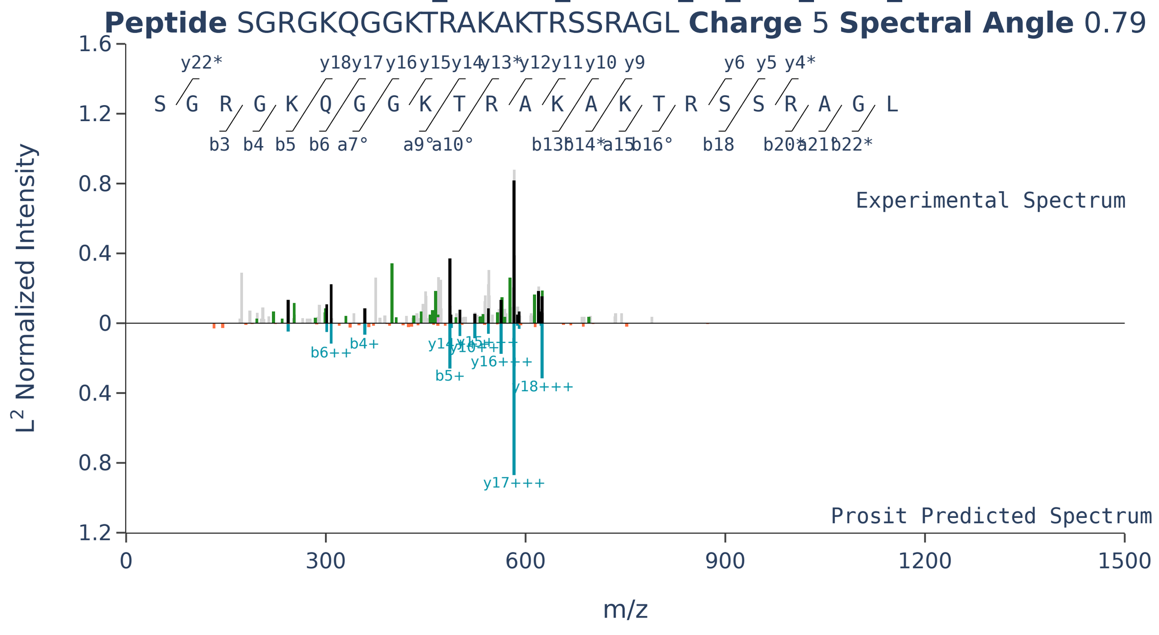


**Source** HRoetschke_291222_060123_Fu_H2A_b1R1_4h **Scan** 16288
**Peptide** TAEILELAGNAARDNKKTRIIPRHLQLA **Charge** 4 **Spectral Angle** 0.86

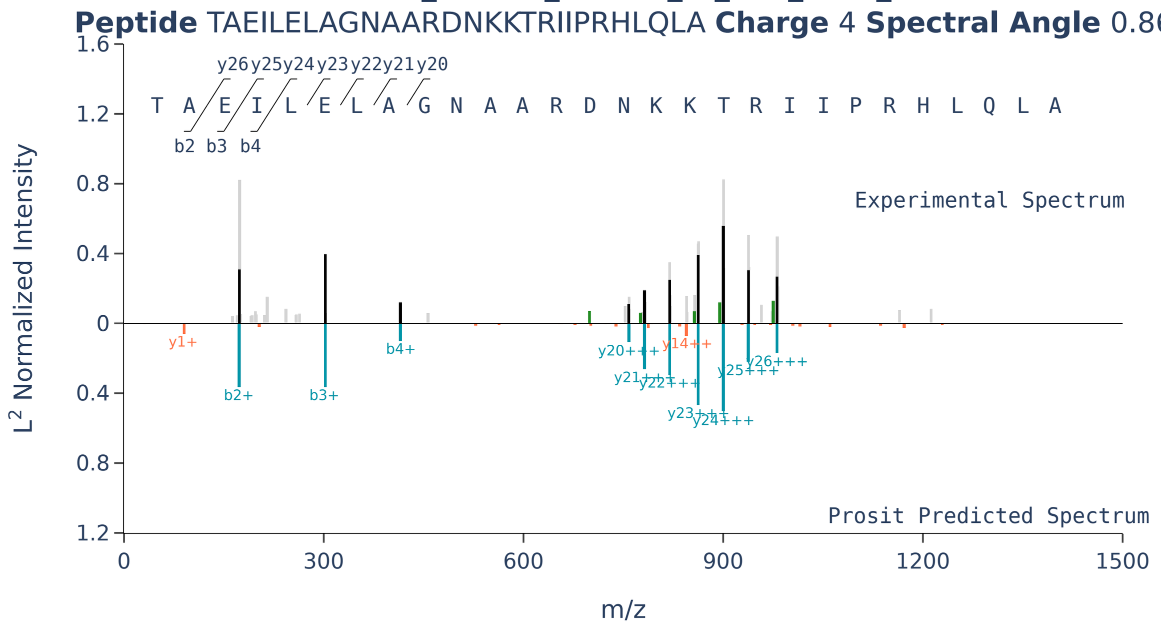


**Source** HRoetschke_291222_060123_Fu_H2A_b2R2_4h **Scan** 12643
**Peptide** GGVLPNIQS **Charge** 2 **Spectral Angle** 0.77

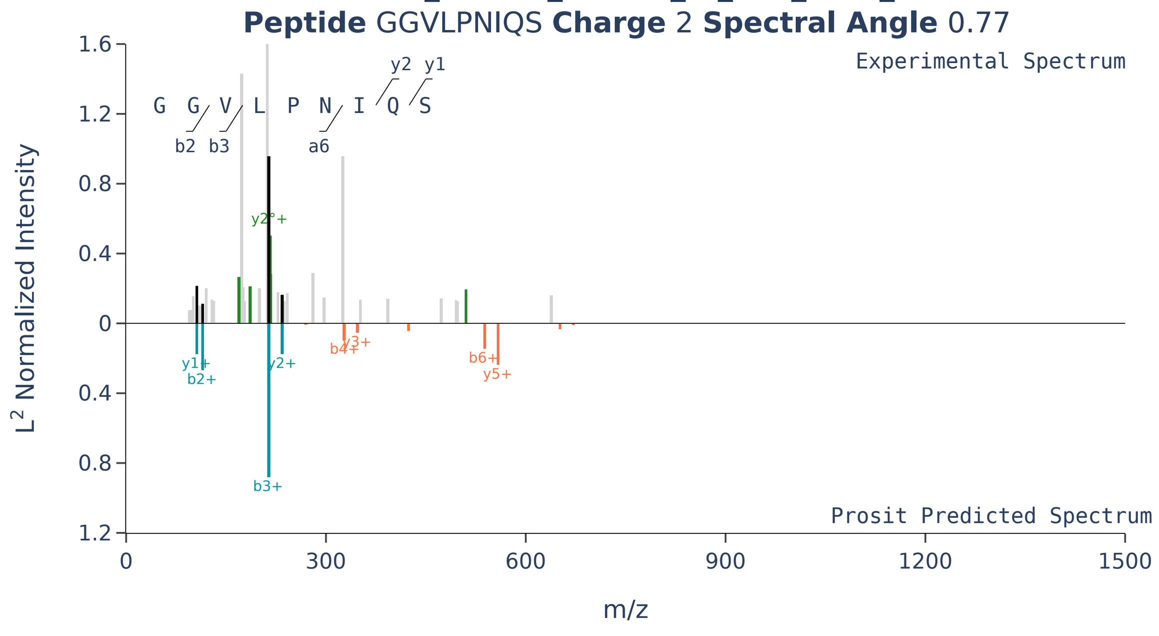


**Source** HRoetschke_291222_060123_Fu_H2A_b2R1_24h **Scan** 2948
**Peptide** VHRLLRKGNY **Charge** 3 **Spectral Angle** 0.64

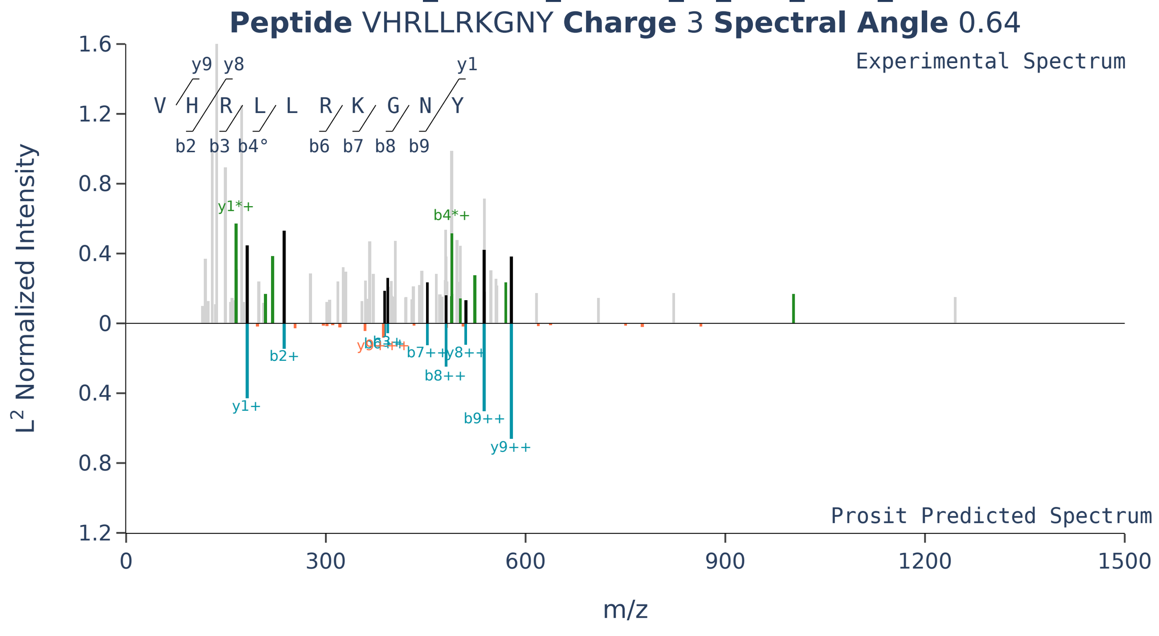


**Source** HRoetschke_291222_060123_Fu_H2A_b2R1_4h **Scan** 6818
**Peptide** RDNKKTRIIPRHLQLAVRN **Charge** 5 **Spectral Angle** 0.73

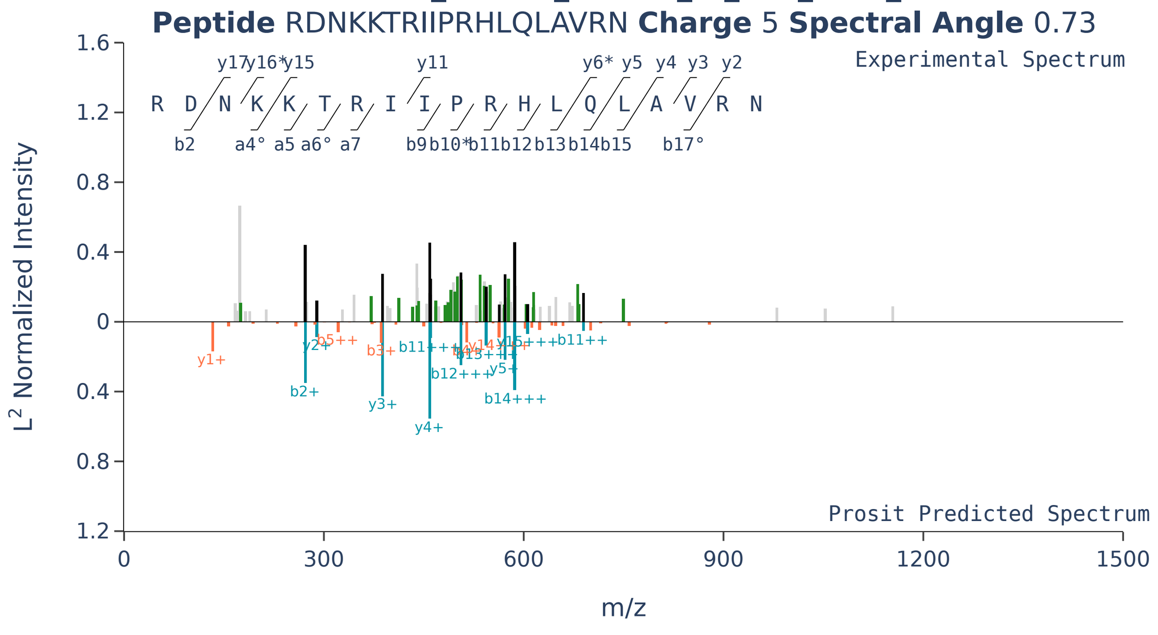


**Source** HRoetschke_291222_060123_Fu_H2A_b1R2_2h **Scan** 16931
**Peptide** DEELNKLLGRVTIAQ **Charge** 3 **Spectral Angle** 0.7

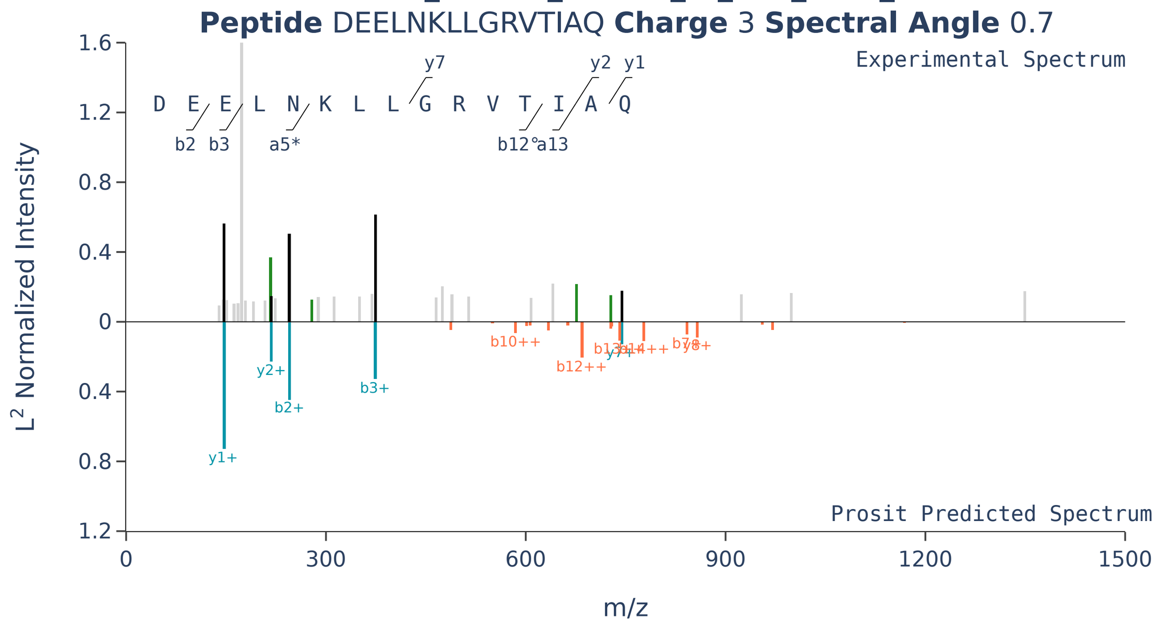


**Source** HRoetschke_291222_060123_Fu_H2A_b1R2_24h **Scan** 8799
**Peptide** LGRVTLA **Charge** 2 **Spectral Angle** 0.57

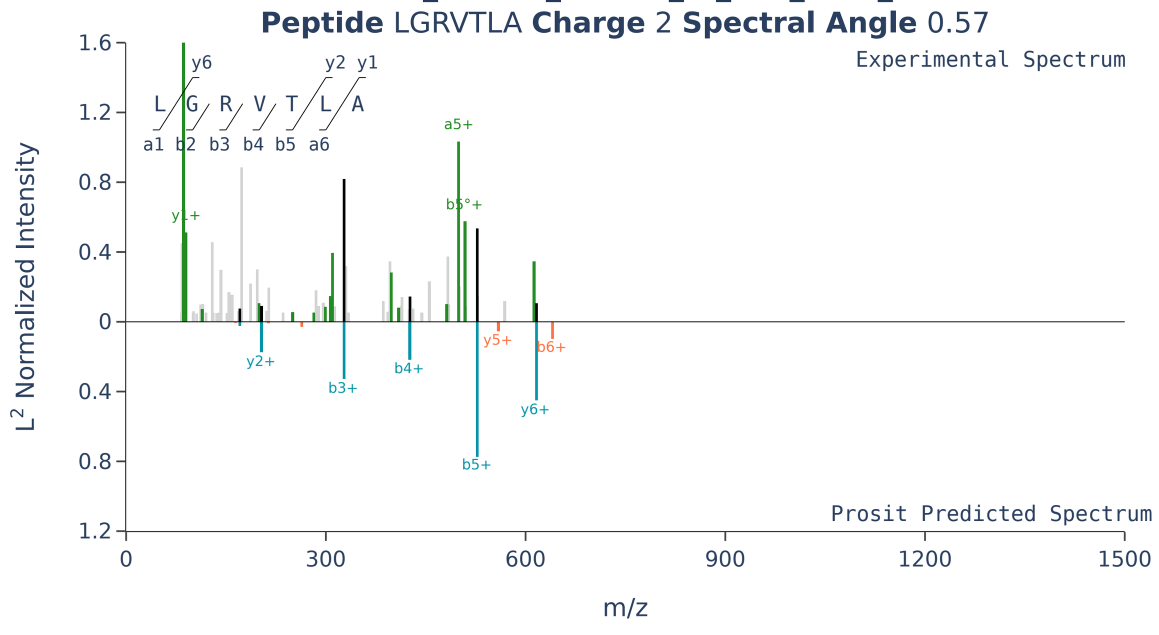


**Source** HRoetschke_291222_060123_Fu_H2A_b2R2_4h **Scan** 3625
**Peptide** RAKAKTRSSRAGLQF **Charge** 4 **Spectral Angle** 0.7

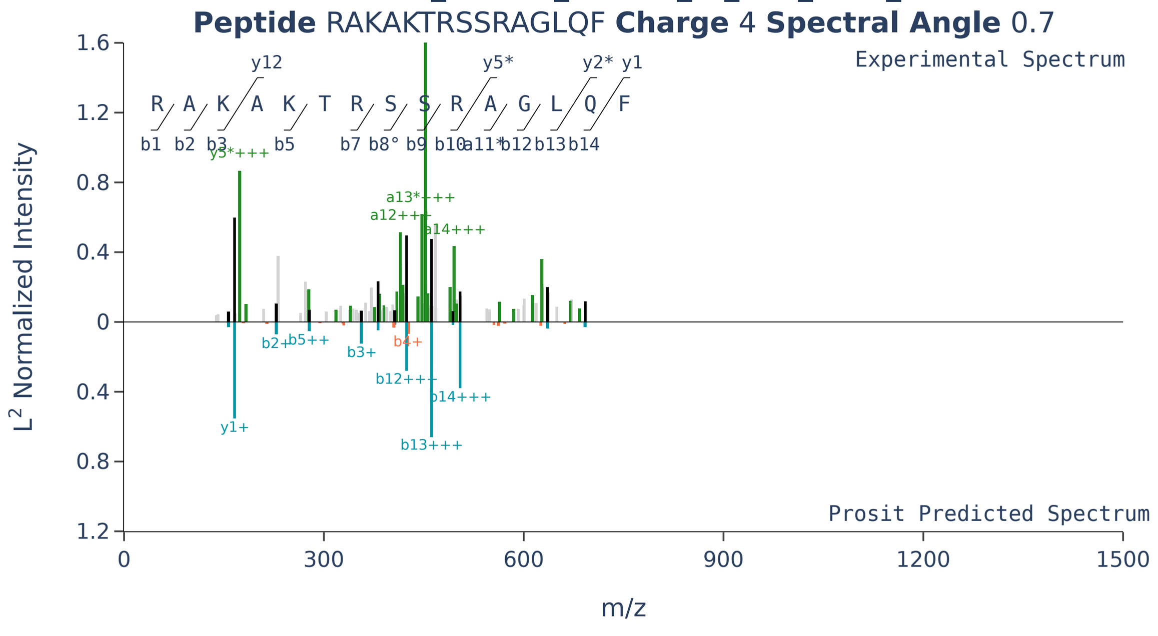


**Source** HRoetschke_291222_060123_Fu_H2A_b1R2_4h **Scan** 7091
**Peptide** ARDNKKTRIIPRHLQLAVR **Charge** 6 **Spectral Angle** 0.81

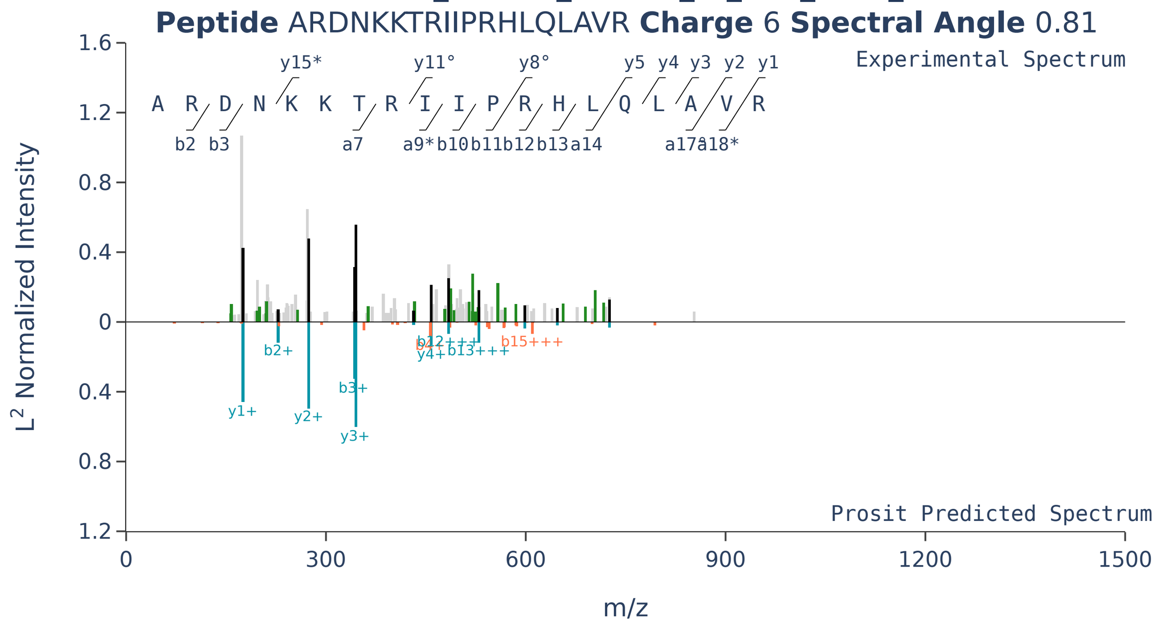


**Source** HRoetschke_291222_060123_Fu_H2A_b1R1_2h **Scan** 19111
**Peptide** AVRNDEELNKLLGRVTIAQGGVLPNLQ **Charge** 3 **Spectral Angle** 0.73

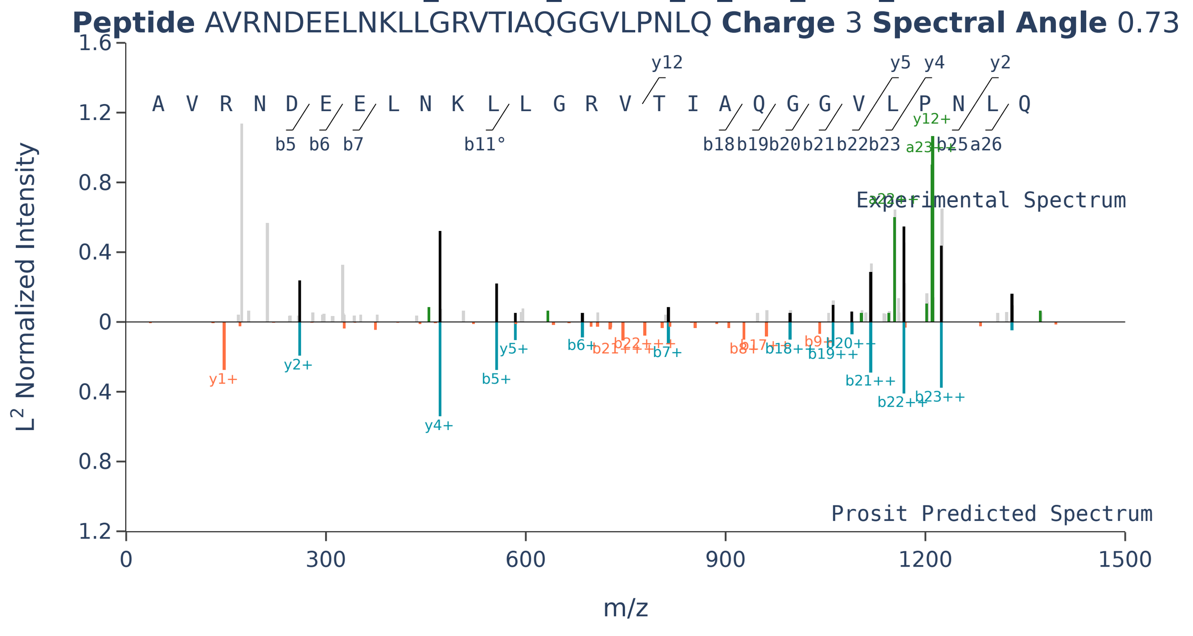

**Source** HRoetschke_291222_060123_Fu_H2A_b1R1_4h **Scan** 16580
**Peptide** AQGGVLPNIQSVLLPKKTESSKSAKS **Charge** 4 **Spectral Angle** 0.63

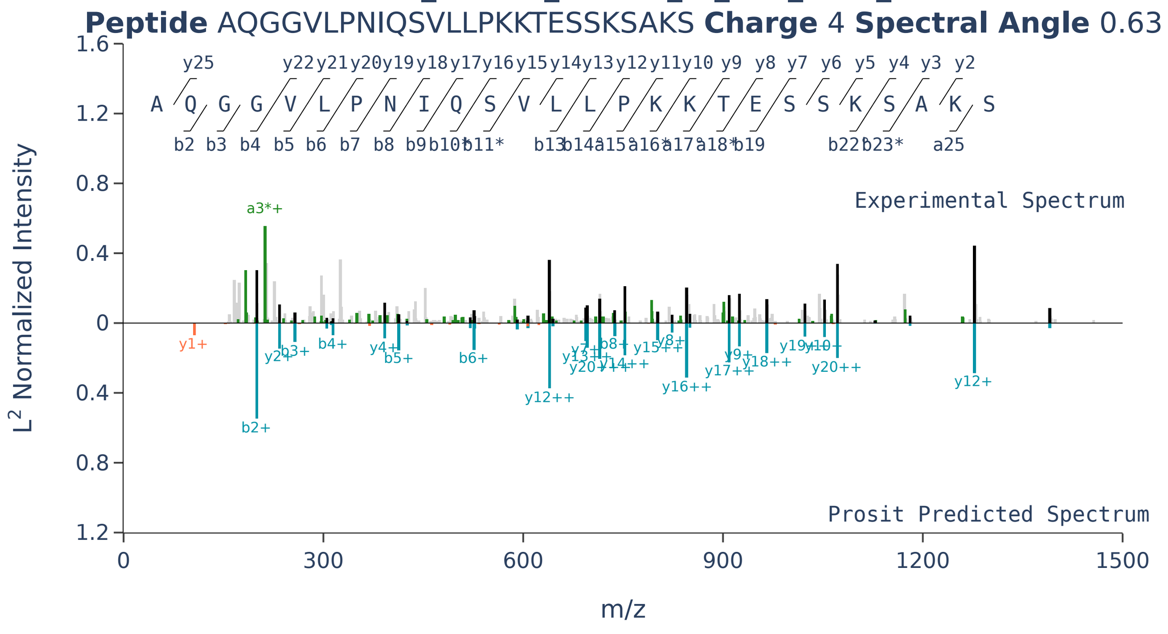


**Source** HRoetschke_291222_060123_Fu_H2A_b1R1_1h **Scan** 9886
**Peptide** VHRLLRKGNYAERVGAGAPVYL **Charge** 4 **Spectral Angle** 0.69

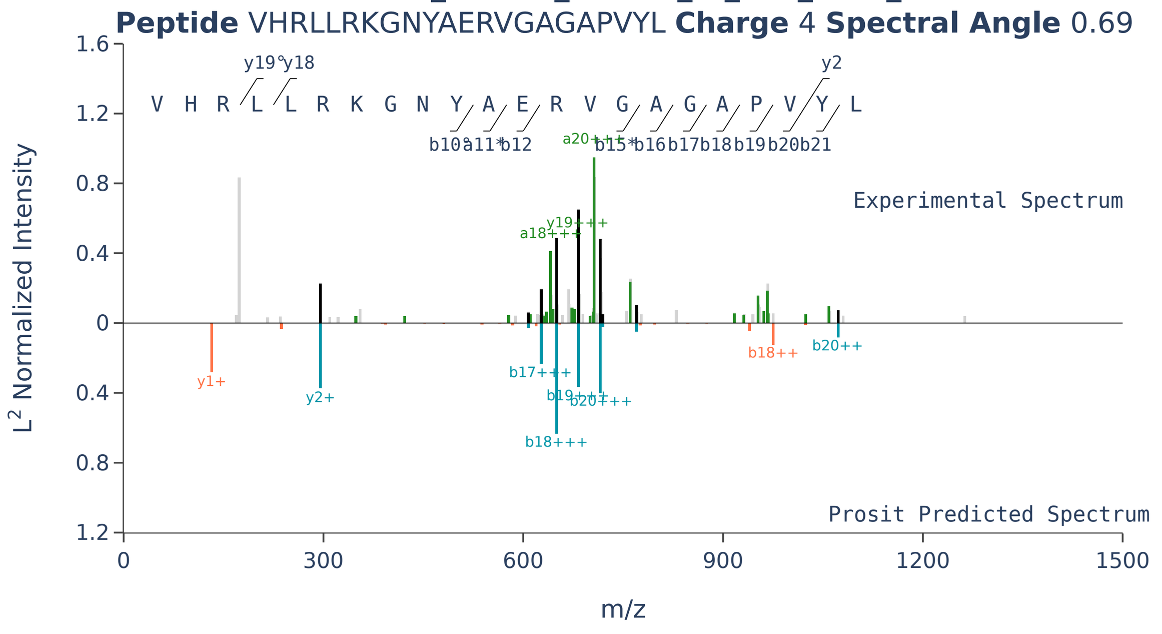


**Source** HRoetschke_291222_060123_Fu_H2A_b2R2_2h **Scan** 3305
**Peptide** NKKTRIIPRHL **Charge** 2 **Spectral Angle** 0.69

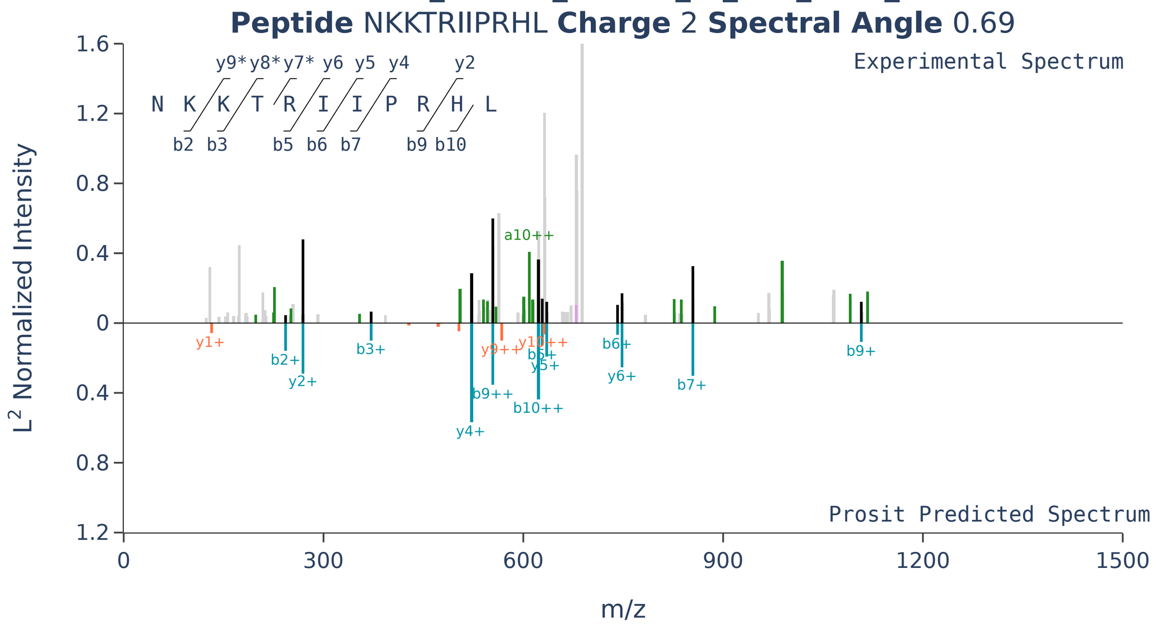


**Source** HRoetschke_291222_060123_Fu_H2A_b1R2_2h **Scan** 16826
**Peptide** RKGNYAERVGAGAPVYLAAVLE **Charge** 3 **Spectral Angle** 0.68

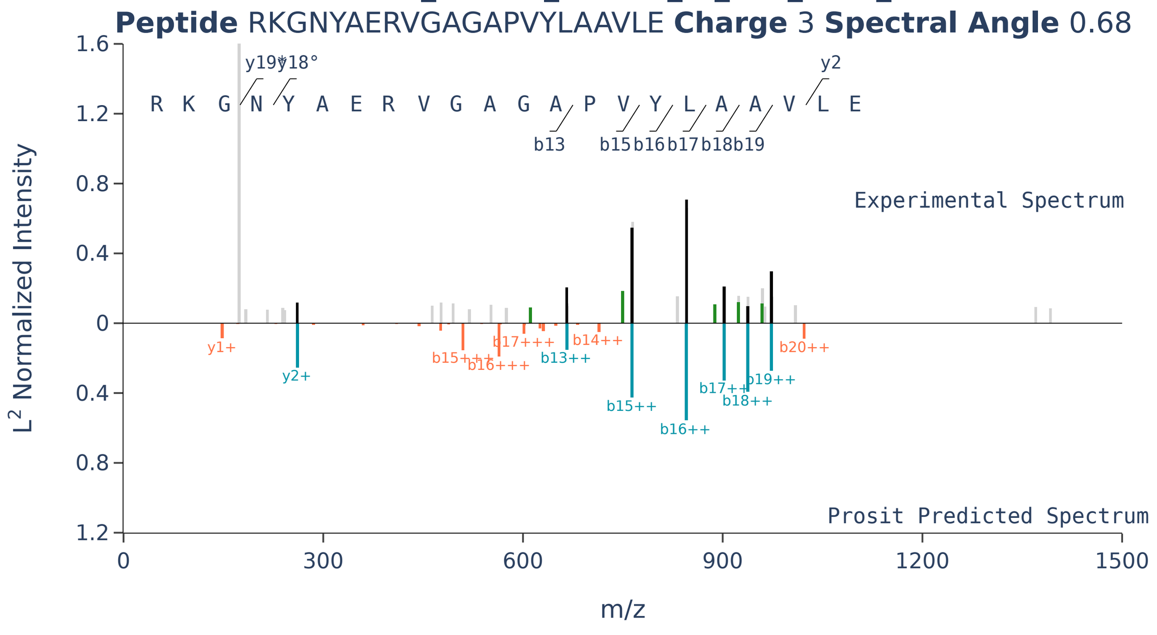


**Source** HRoetschke_291222_060123_Fu_H2A_b2R2_24h **Scan** 2792
**Peptide** ARDNKKTRII **Charge** 4 **Spectral Angle** 0.7

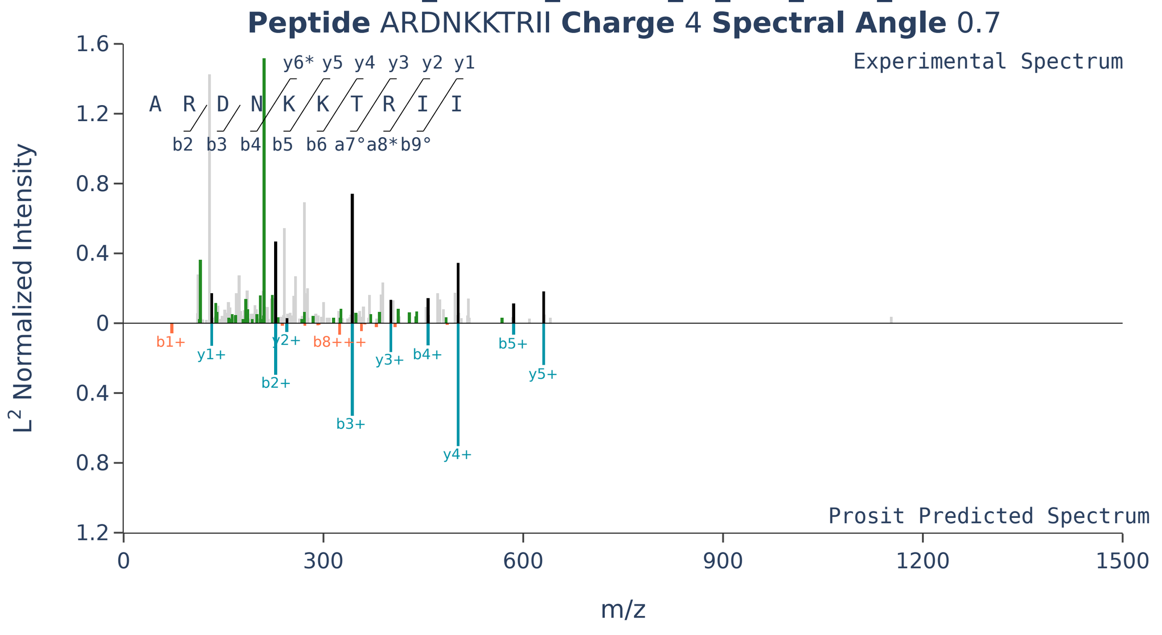


**Source** HRoetschke_291222_060123_Fu_H2A_b1R1_2h **Scan** 17561
**Peptide** RLLRKGNYAERVGAGAPVYLAAVLE **Charge** 4 **Spectral Angle** 0.66

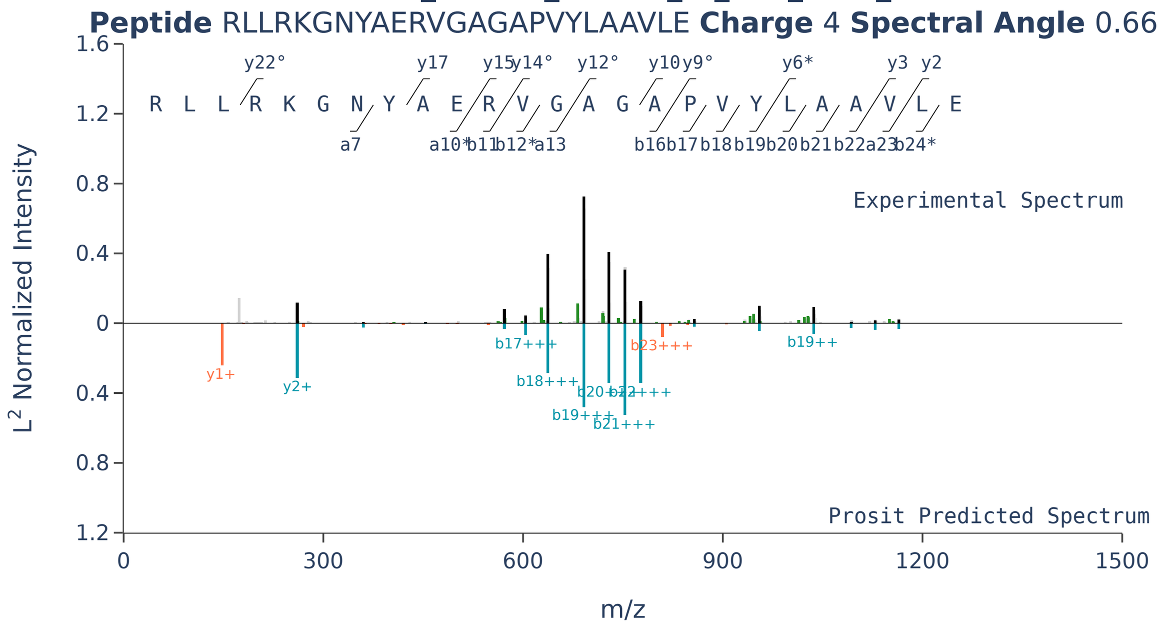


**Source** HRoetschke_291222_060123_Fu_H2A_b1R2_2h **Scan** 18233
**Peptide** EELNKLLGRVTIAQGGVLPNLQ **Charge** 3 **Spectral Angle** 0.46

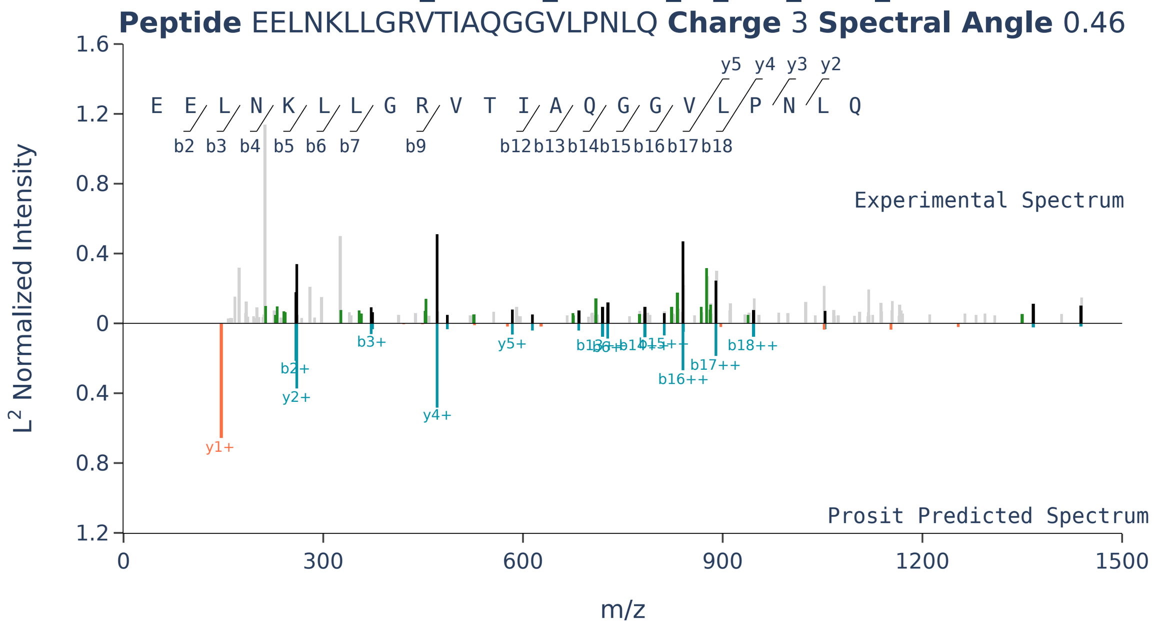


**Source** HRoetschke_291222_060123_Fu_H2A_b2R1_4h **Scan** 11433
**Peptide** SRAGLQFPVGRVHRLLRKGNYAERVG **Charge** 6 **Spectral Angle** 0.75

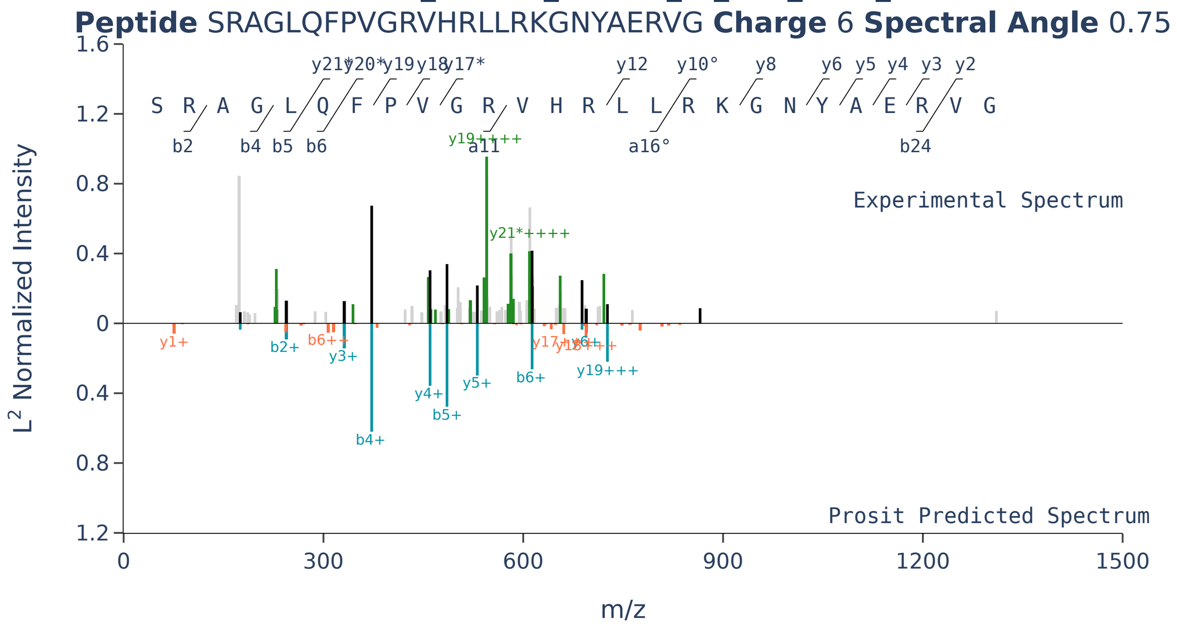


**Source** HRoetschke_291222_060123_Fu_H2A_b2R1_24h **Scan** 15303
**Peptide** QGGVLPNIQSVLLPKKTESSKSAKS **Charge** 4 **Spectral Angle** 0.7

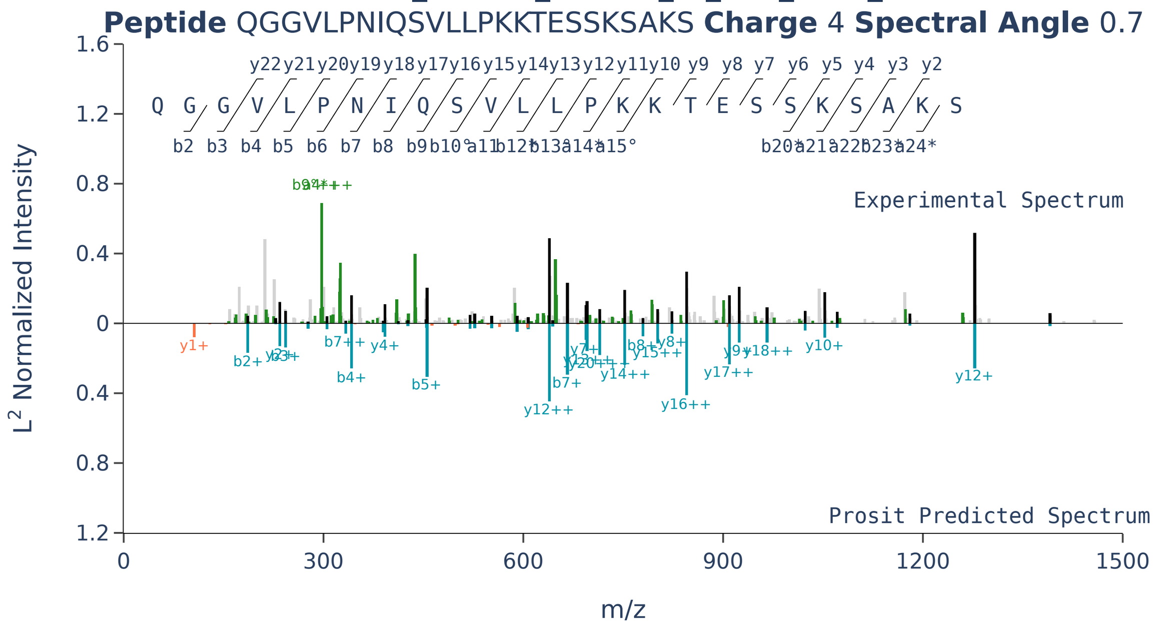


**Source** HRoetschke_291222_060123_Fu_H2A_b1R2_4h **Scan** 11402
**Peptide** VGRVHRLLRKGNYAERVGAGAPVYL **Charge** 4 **Spectral Angle** 0.78

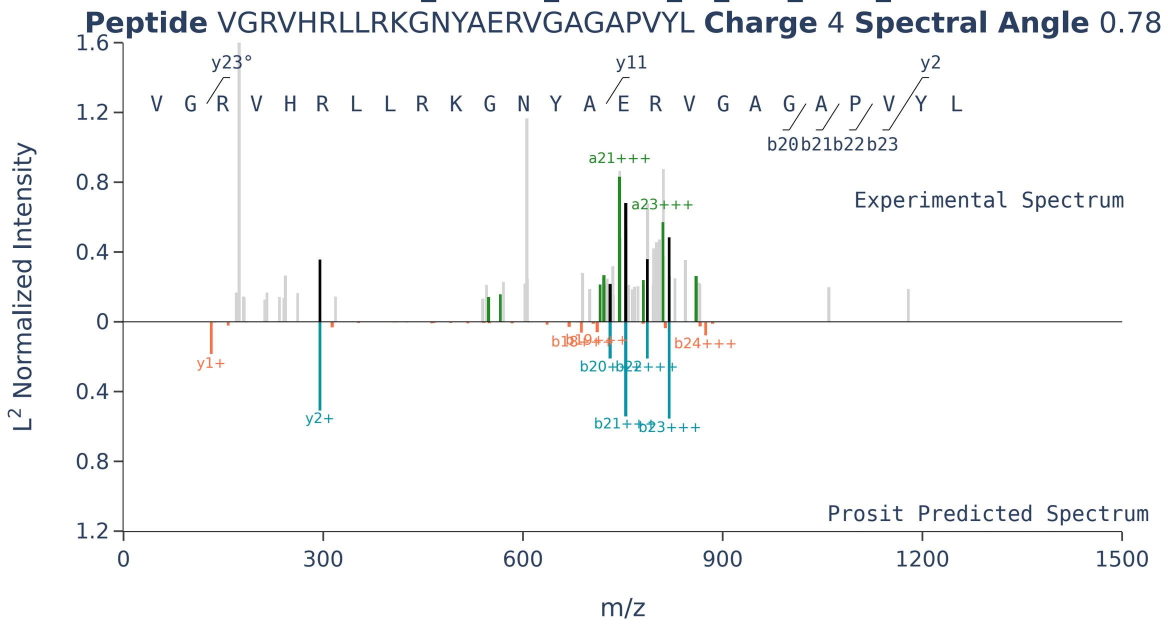


**Source** HRoetschke_291222_060123_Fu_H2A_b1R1_4h **Scan** 18665
**Peptide** LAVRNDEELNKLLGRVTLA **Charge** 3 **Spectral Angle** 0.75

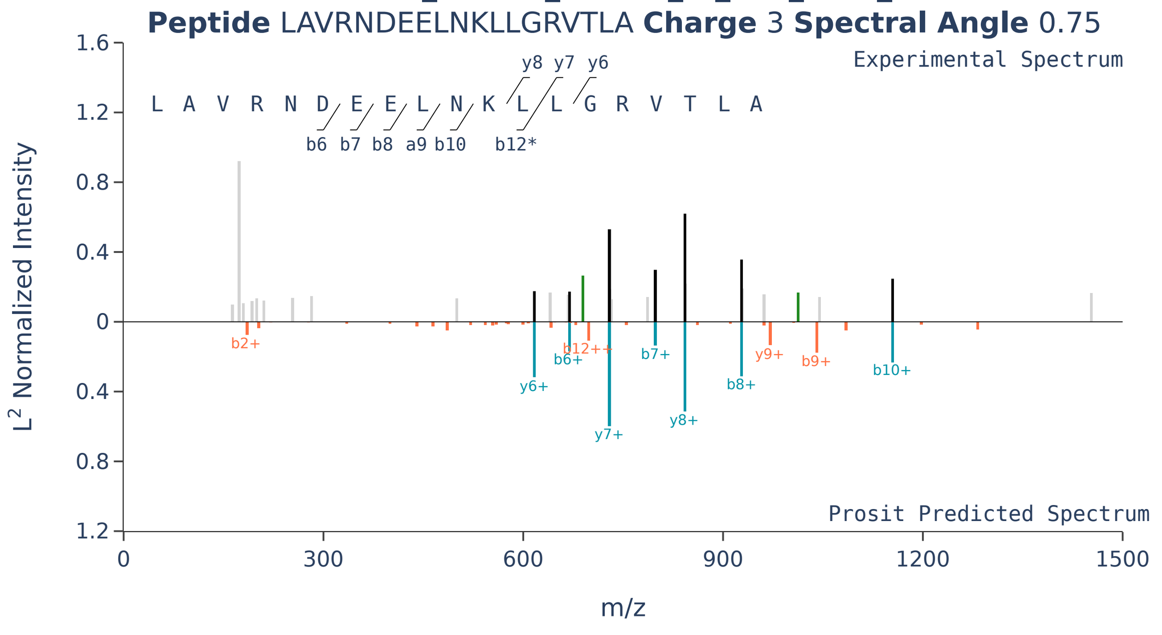


**Source** HRoetschke_291222_060123_Fu_H2A_b1R1_4h **Scan** 19936
**Peptide** TIAQGGVLPNIQSVLLPKKTE **Charge** 3 **Spectral Angle** 0.77

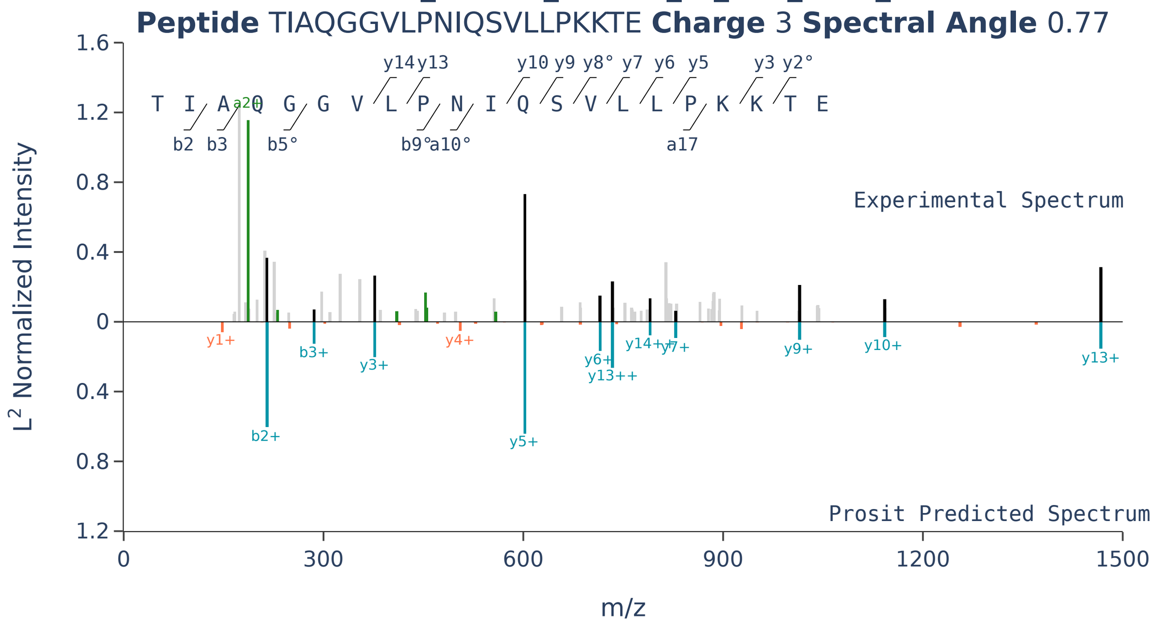


**Source** HRoetschke_291222_060123_Fu_H2A_b1R2_2h **Scan** 17571
**Peptide** YLTAEILELAGNAARDNKKTRIIPRHL **Charge** 5 **Spectral Angle** 0.86

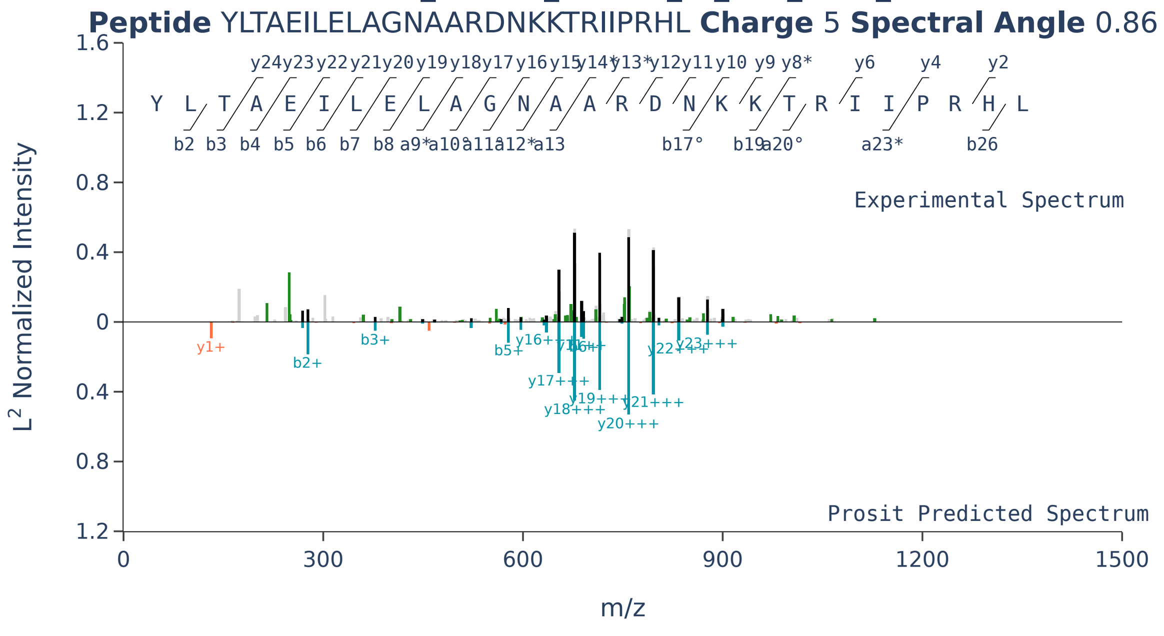


**Source** HRoetschke_291222_060123_Fu_H2A_b1R1_4h **Scan** 11082
**Peptide** FPVGRVH **Charge** 2 **Spectral Angle** 0.85

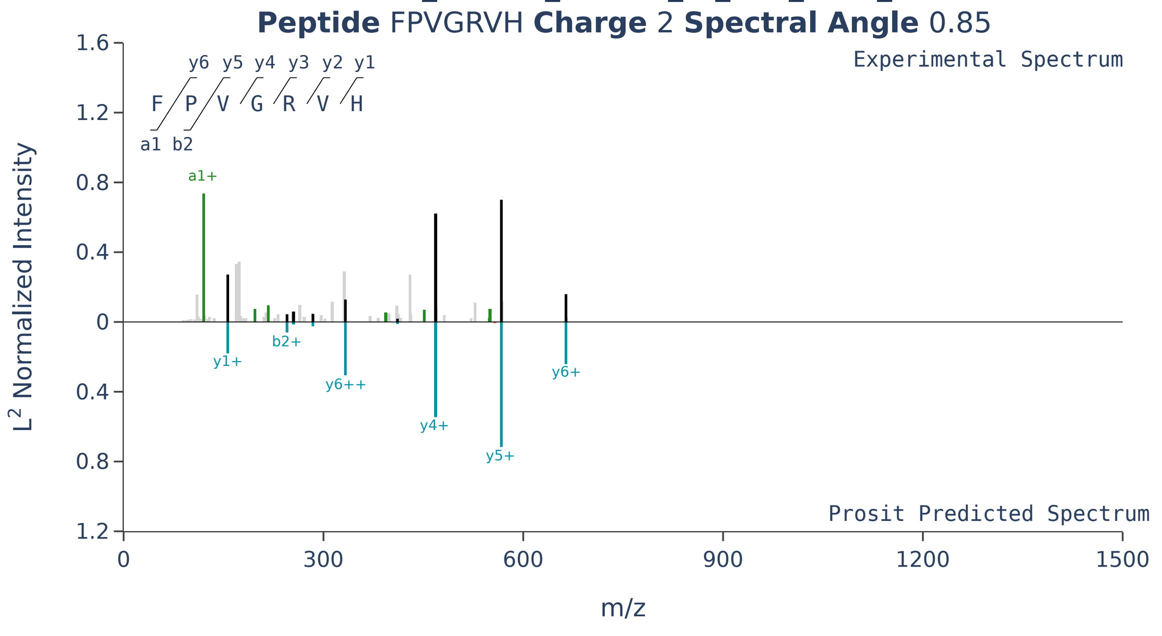


**Source** HRoetschke_291222_060123_Fu_H2A_b1R1_4h **Scan** 16888
**Peptide** TIAQGGVLPNIQSVLLPKKTESSKSAKSK **Charge** 4 **Spectral Angle** 0.7

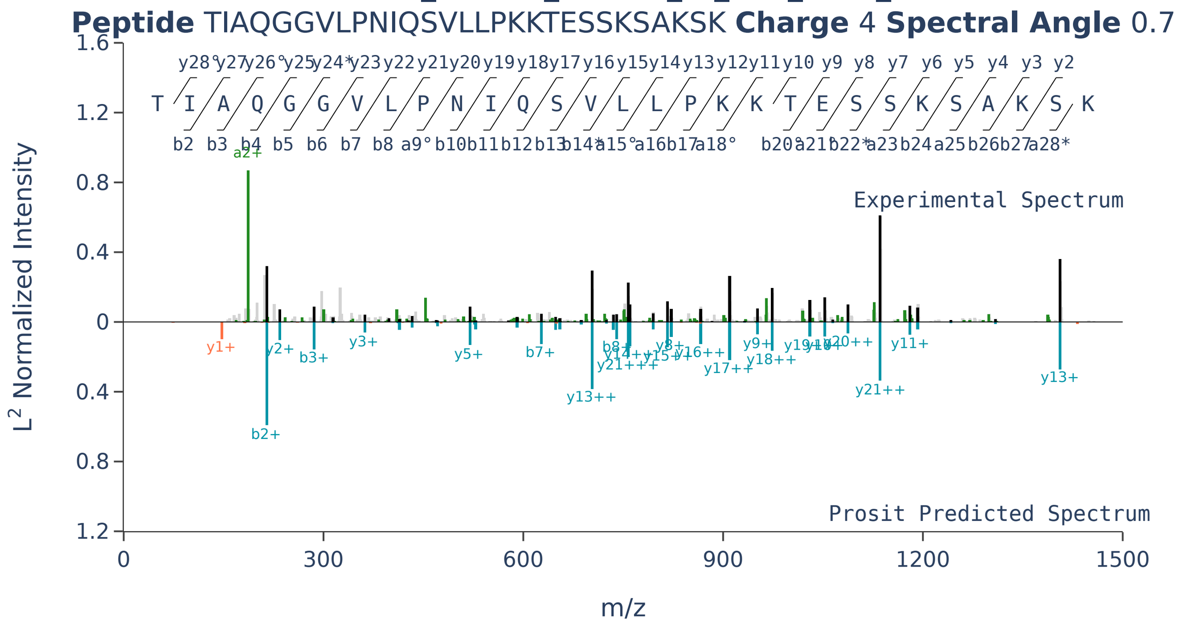

**Source** HRoetschke_291222_060123_Fu_H2A_b1R2_24h **Scan** 7253
**Peptide** AARDNKKTRIIPRHLQLAVRND **Charge** 5 **Spectral Angle** 0.75

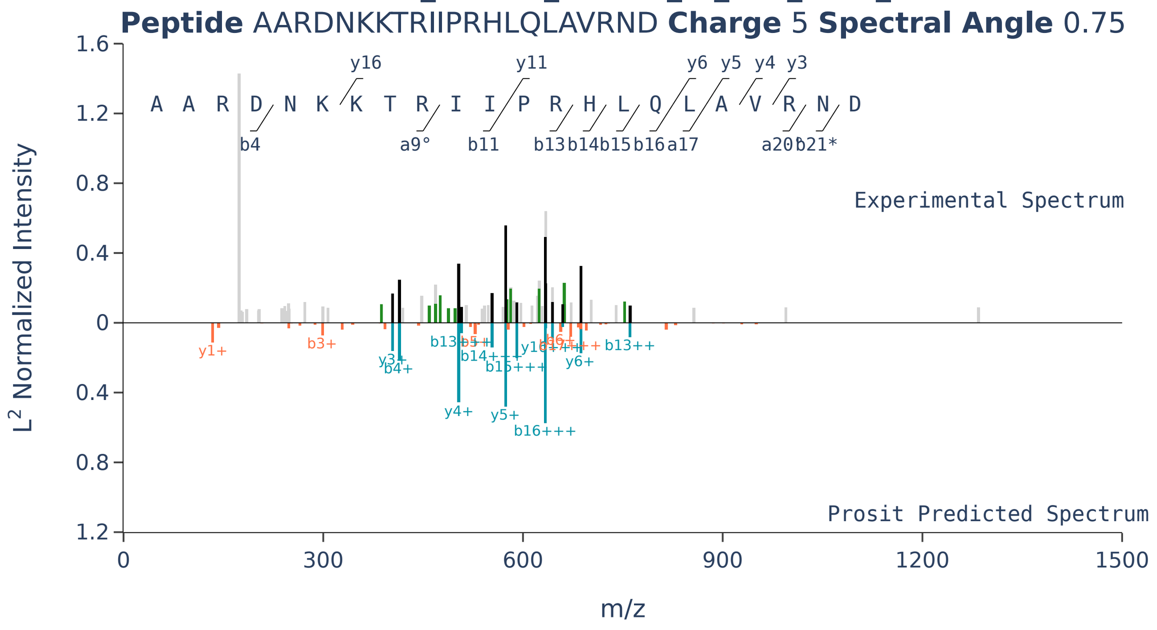


**Source** HRoetschke_291222_060123_Fu_H2A_b1R1_24h **Scan** 2613
**Peptide** AGNAARDNKKTRIIPR **Charge** 3 **Spectral Angle** 0.76

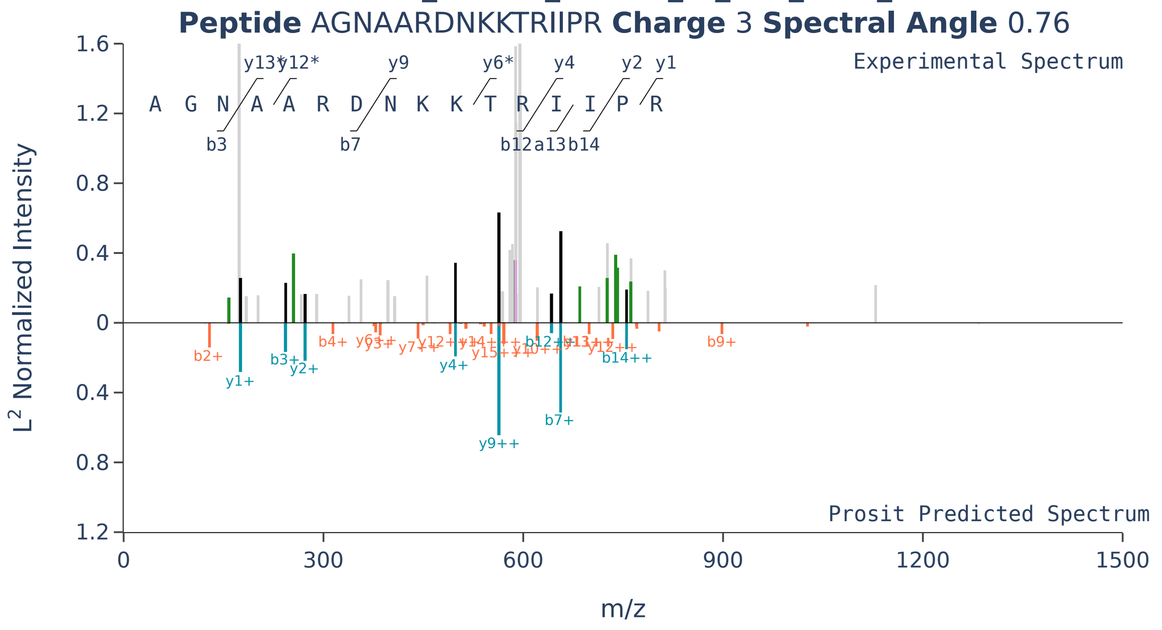


**Source** HRoetschke_291222_060123_Fu_H2A_b2R2_24h **Scan** 14605
**Peptide** ELNKLLGRVTLA **Charge** 2 **Spectral Angle** 0.72

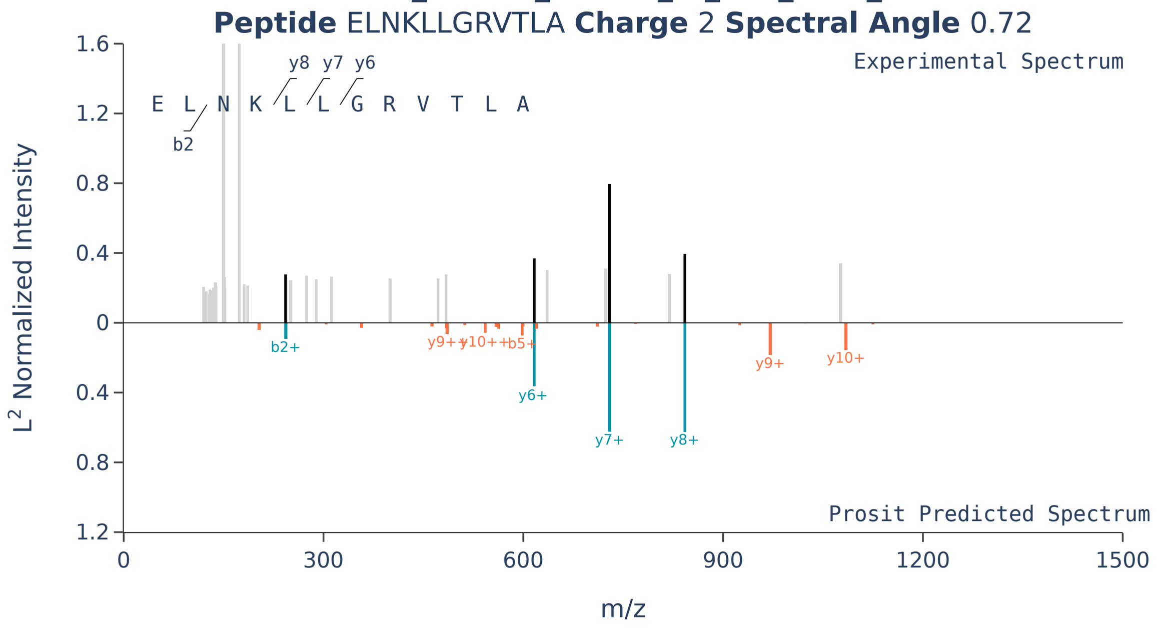


**Source** HRoetschke_291222_060123_Fu_H2A_b1R1_4h **Scan** 7259
**Peptide** AGNAARDNKKTRIIPRHLQLAVR **Charge** 6 **Spectral Angle** 0.65

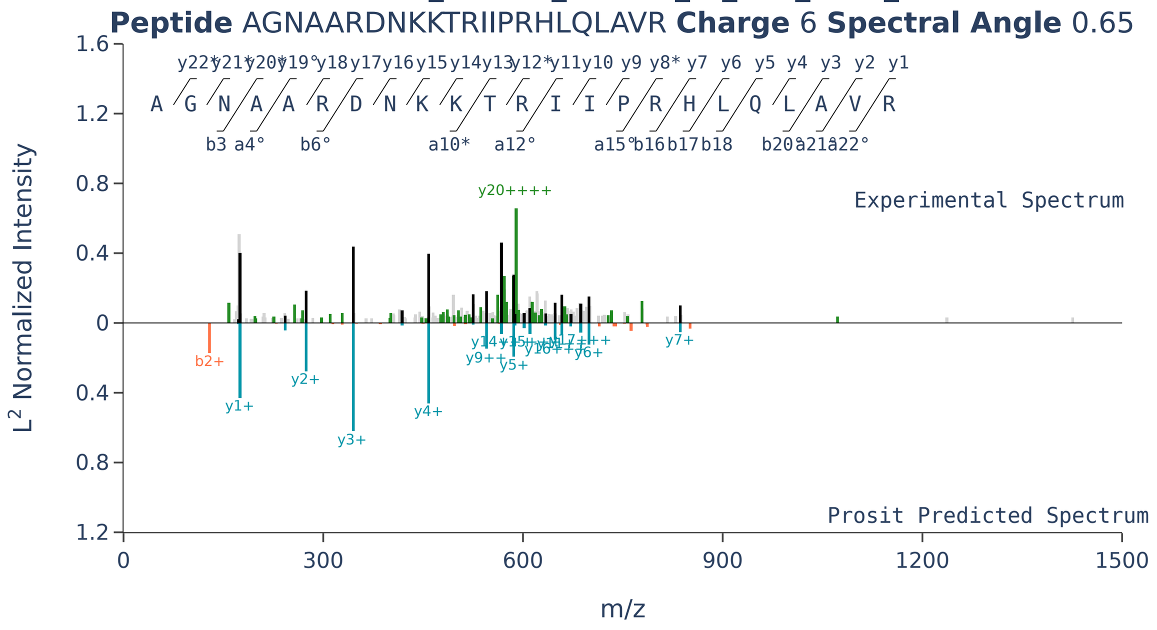


**Source** HRoetschke_291222_060123_Fu_H2A_b1R2_4h **Scan** 9562
**Peptide** AGNAARDNKKTRIIPRHLQLAV **Charge** 4 **Spectral Angle** 0.78

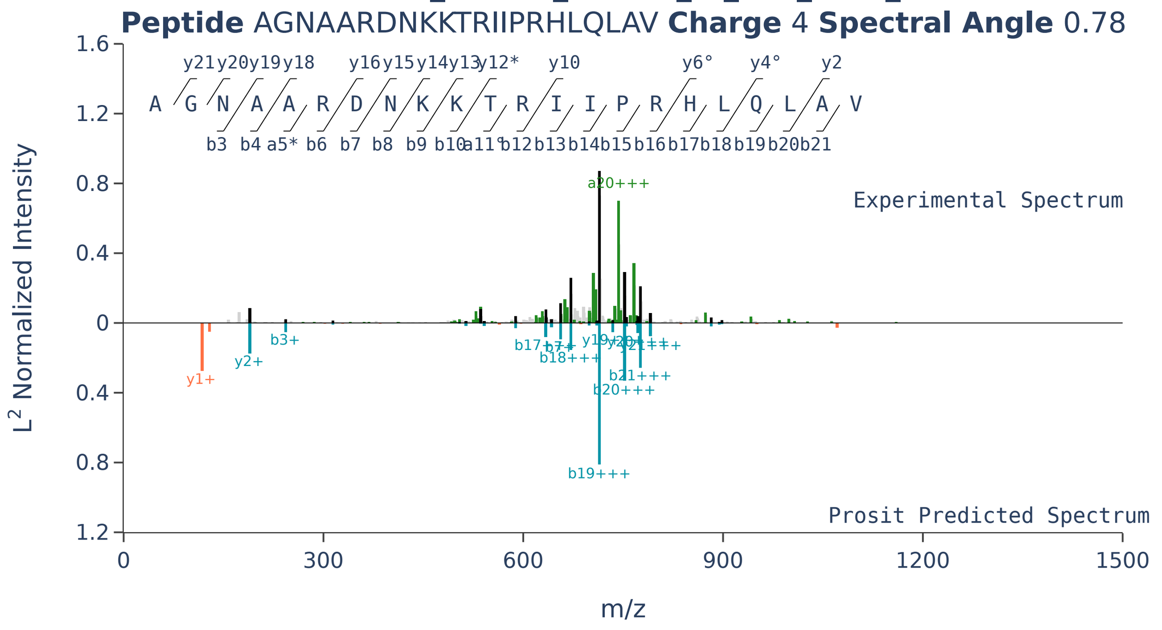


**Source** HRoetschke_291222_060123_Fu_H2A_b1R1_4h **Scan** 17673
**Peptide** QGGVLPNIQSVLLPKKTESSK **Charge** 4 **Spectral Angle** 0.7

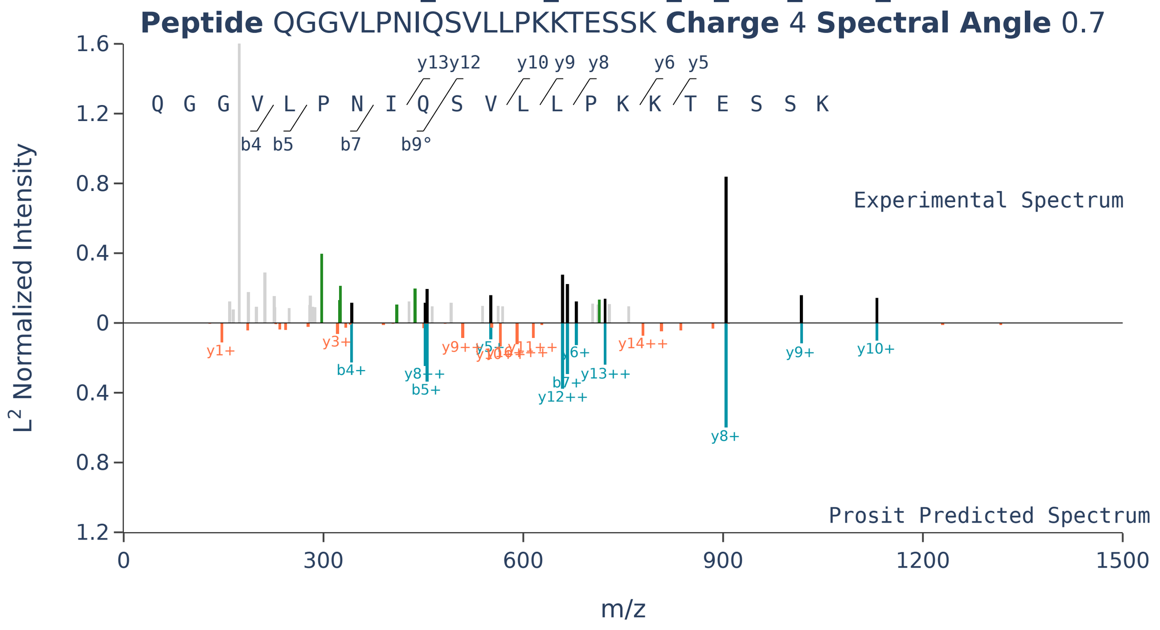


**Source** HRoetschke_291222_060123_Fu_H2A_b1R1_2h **Scan** 13364
**Peptide** VLPNIQSVLLPKKTESSKSAKSK **Charge** 4 **Spectral Angle** 0.66

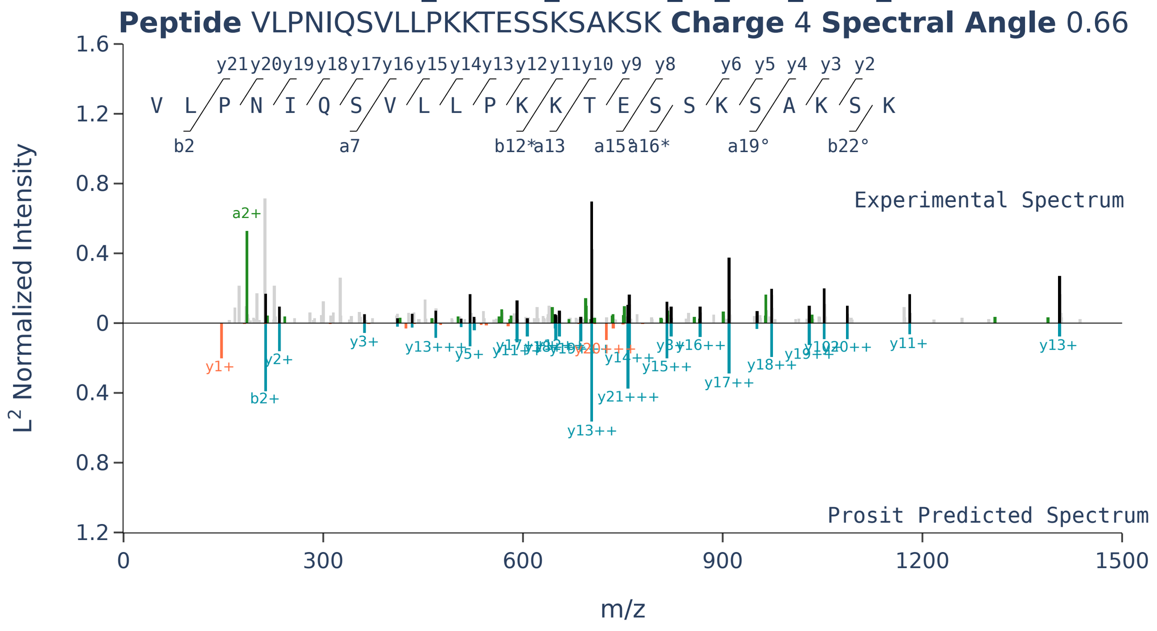


**Source** HRoetschke_291222_060123_Fu_H2A_b2R2_2h **Scan** 7284
**Peptide** VHRLLRKGNYAERVGAGAPVY **Charge** 4 **Spectral Angle** 0.63

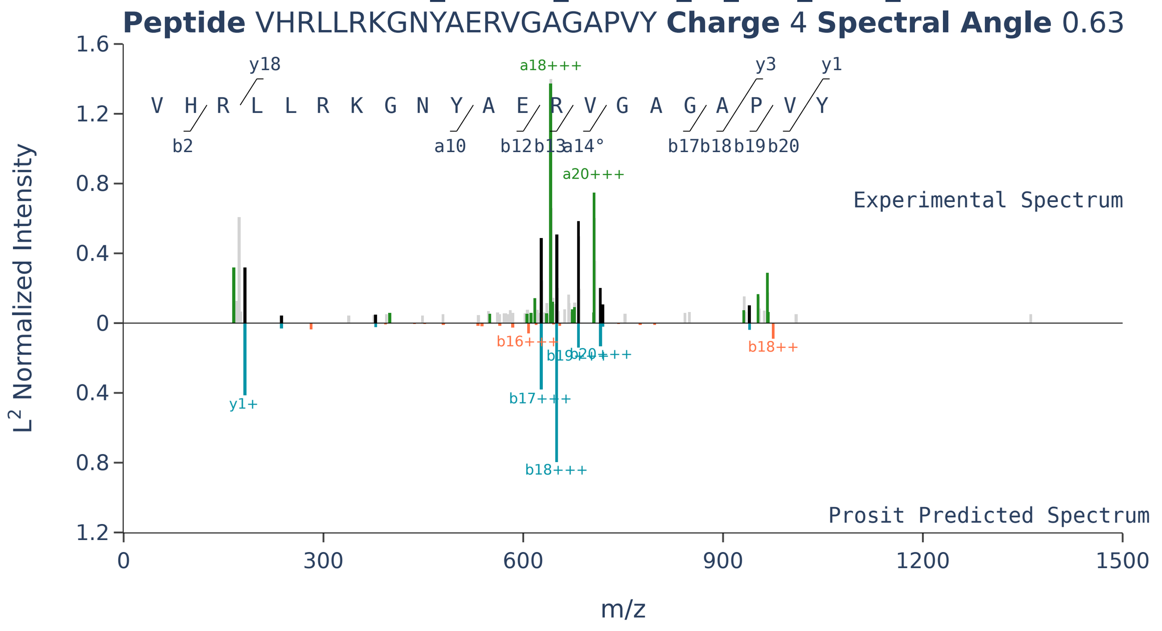


**Source** HRoetschke_291222_060123_Fu_H2A_b1R1_24h **Scan** 8846
**Peptide** KTRIIPRHLQL **Charge** 3 **Spectral Angle** 0.52

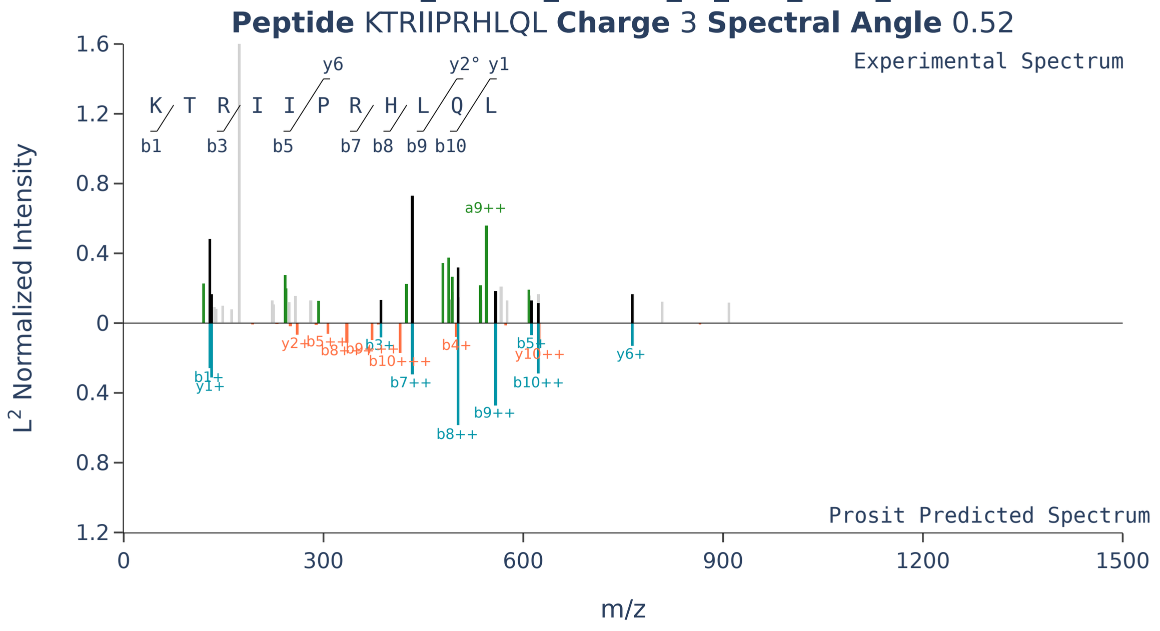


**Source** HRoetschke_291222_060123_Fu_H2A_b1R1_24h **Scan** 9078
**Peptide** SRAGLQFPVGRVHR **Charge** 4 **Spectral Angle** 0.68

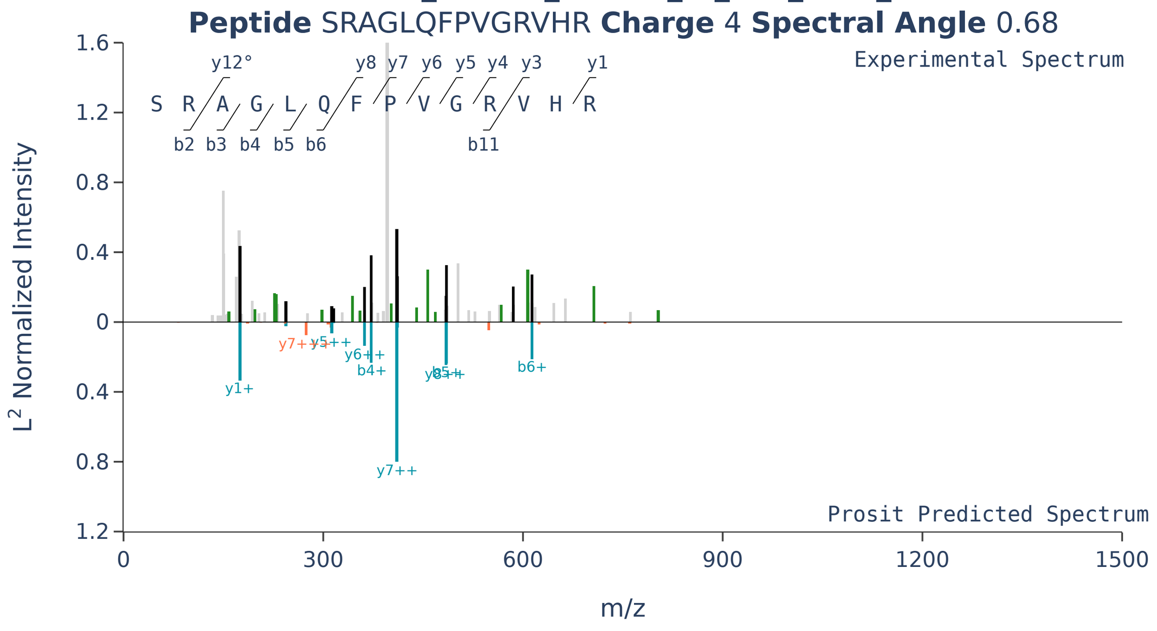


**Source** HRoetschke_291222_060123_Fu_H2A_b1R2_2h **Scan** 8852
**Peptide** LLRKGNYAERVGAGAPVY **Charge** 3 **Spectral Angle** 0.77

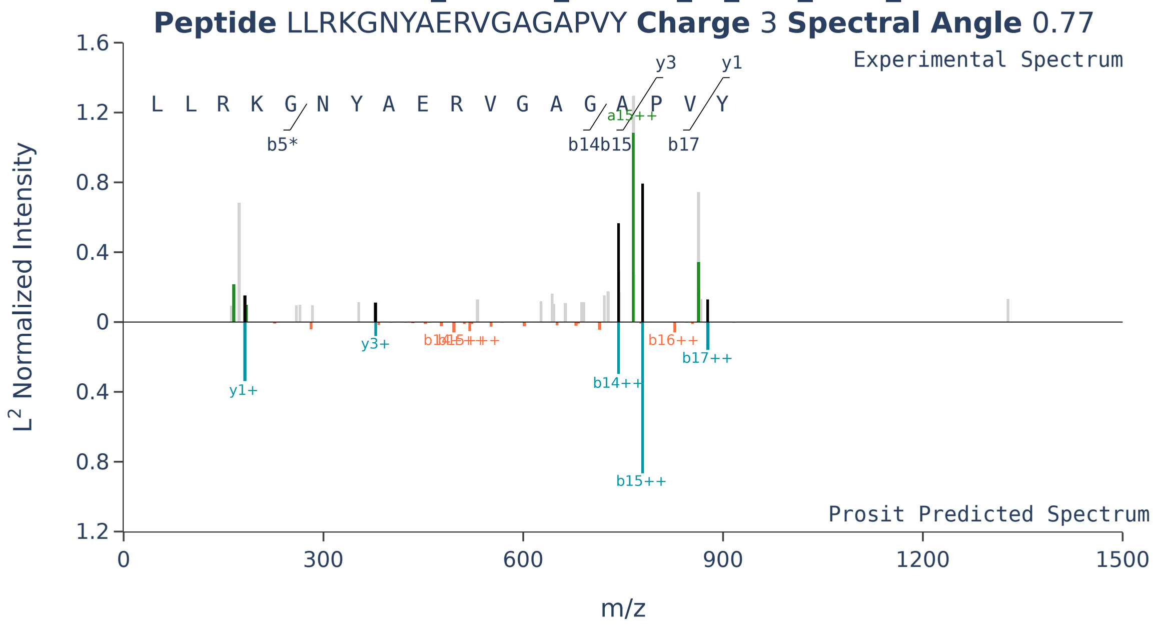


**Source** HRoetschke_291222_060123_Fu_H2A_b1R2_2h **Scan** 2734
**Peptide** SGRGKQGGKTRAKAKTRSSRAGLQF **Charge** 5 **Spectral Angle** 0.81

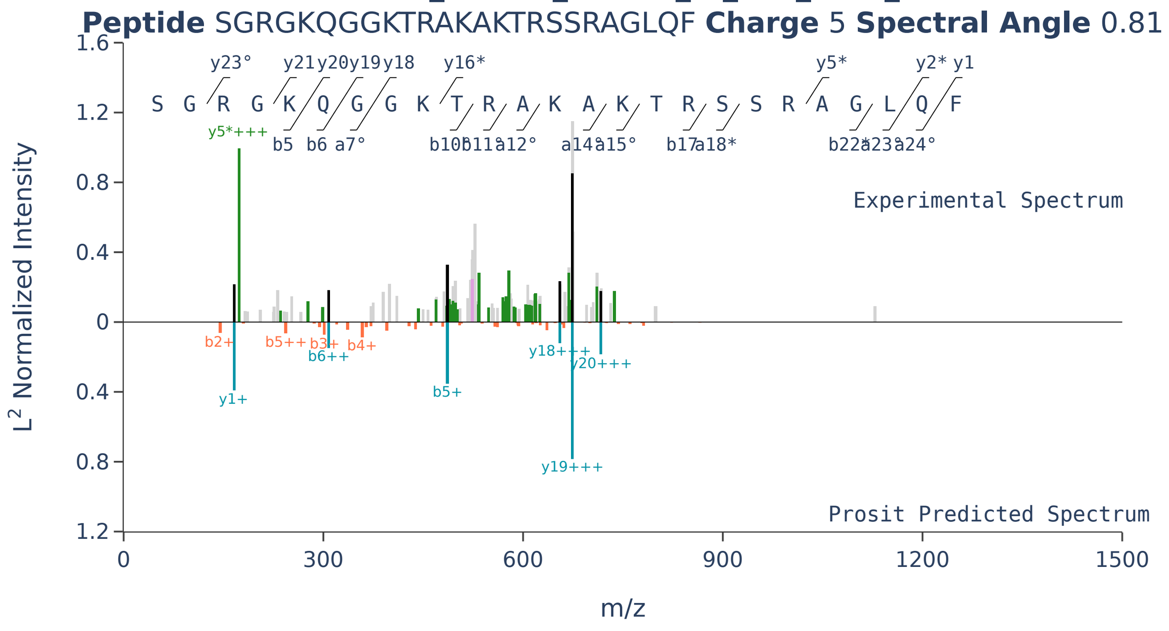


**Source** HRoetschke_291222_060123_Fu_H2A_b1R2_4h **Scan** 9651
**Peptide** TRSSRAGLQFPVGRVHRLLRKG **Charge** 5 **Spectral Angle** 0.63

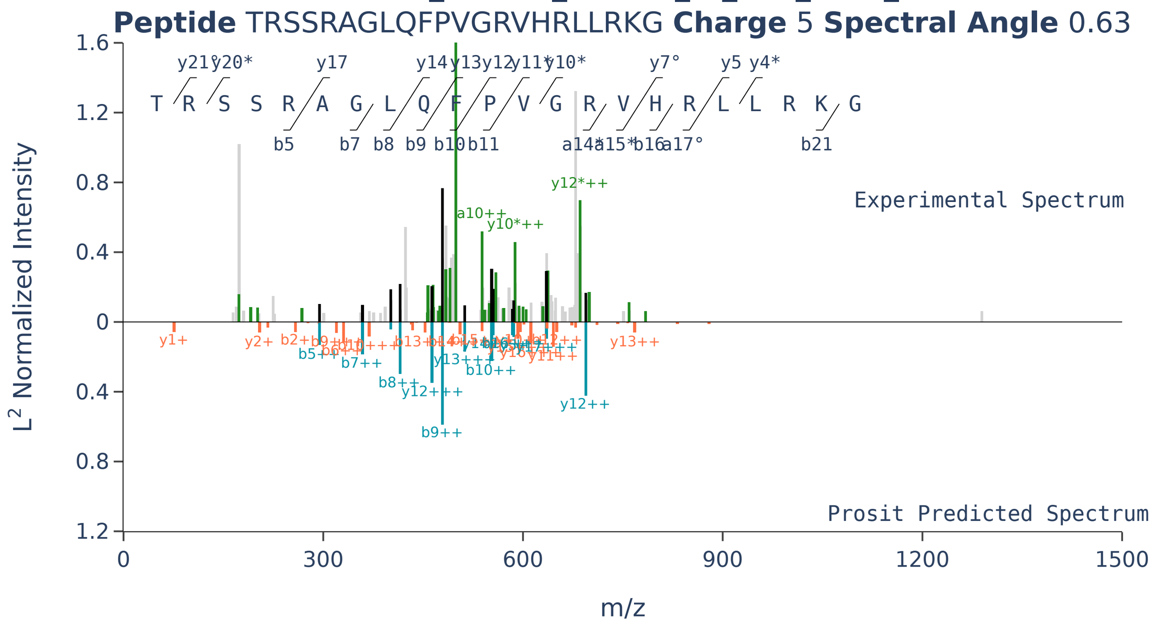


**Source** HRoetschke_291222_060123_Fu_H2A_b2R1_1h **Scan** 12483
**Peptide** AVRNDEELNKLLGRVTIAQGG **Charge** 3 **Spectral Angle** 0.72

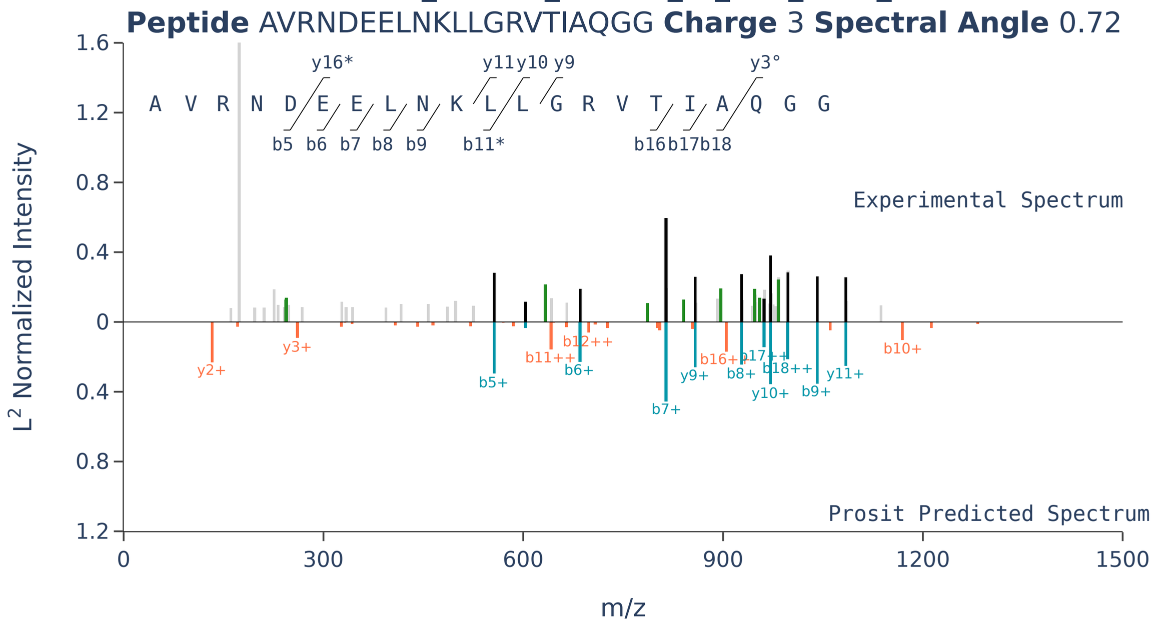


**Source** HRoetschke_291222_060123_Fu_H2A_b1R2_2h **Scan** 11220
**Peptide** RAGLQFPVGRVHRLLRKGNYAE **Charge** 5 **Spectral Angle** 0.64

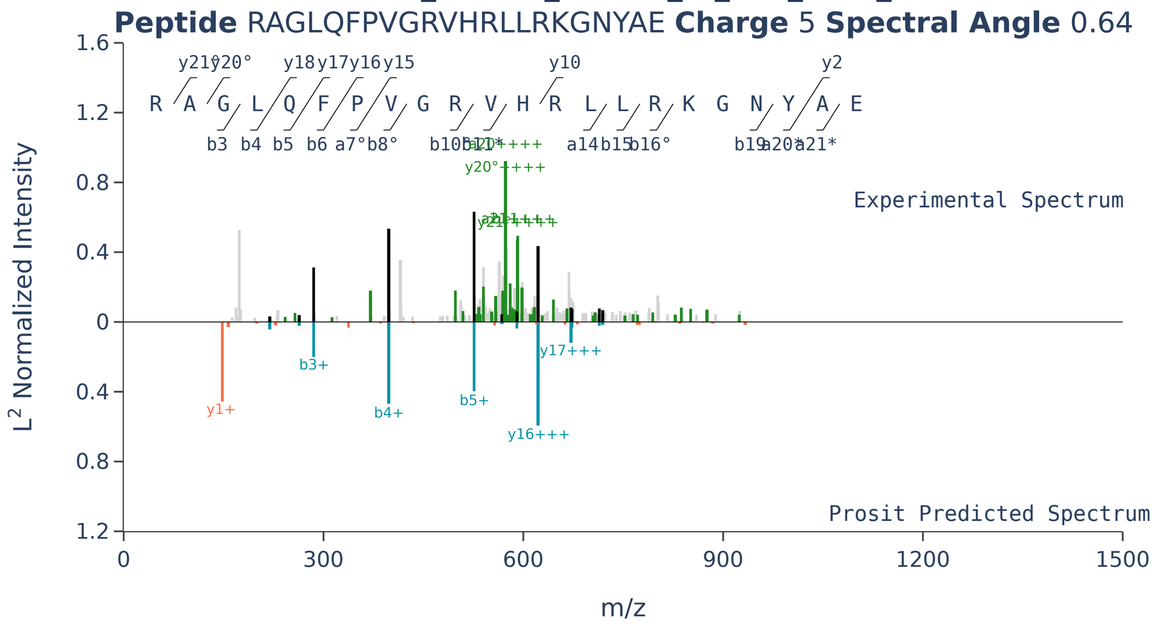

**Source** HRoetschke_291222_060123_Fu_H2A_b1R1_1h **Scan** 11898
**Peptide** TAEILELAGNAARDNKKTRL **Charge** 5 **Spectral Angle** 0.72

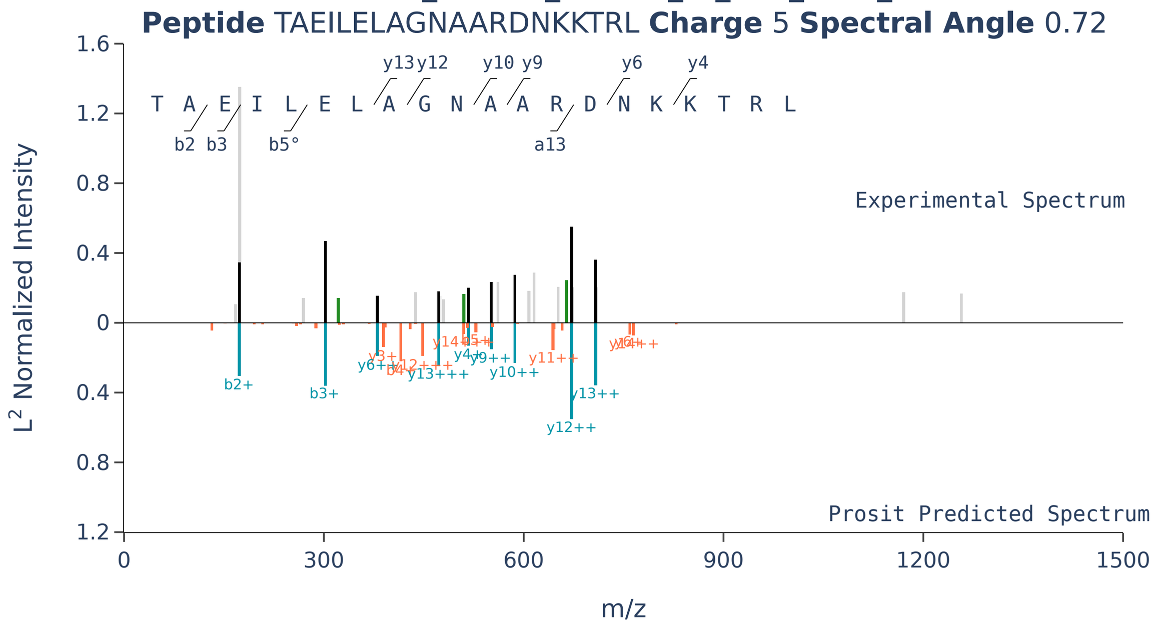


**Source** HRoetschke_291222_060123_Fu_H2A_b1R1_2h **Scan** 15613
**Peptide** GGVLPNIQSVLLPKKTESSKSAKS **Charge** 4 **Spectral Angle** 0.66

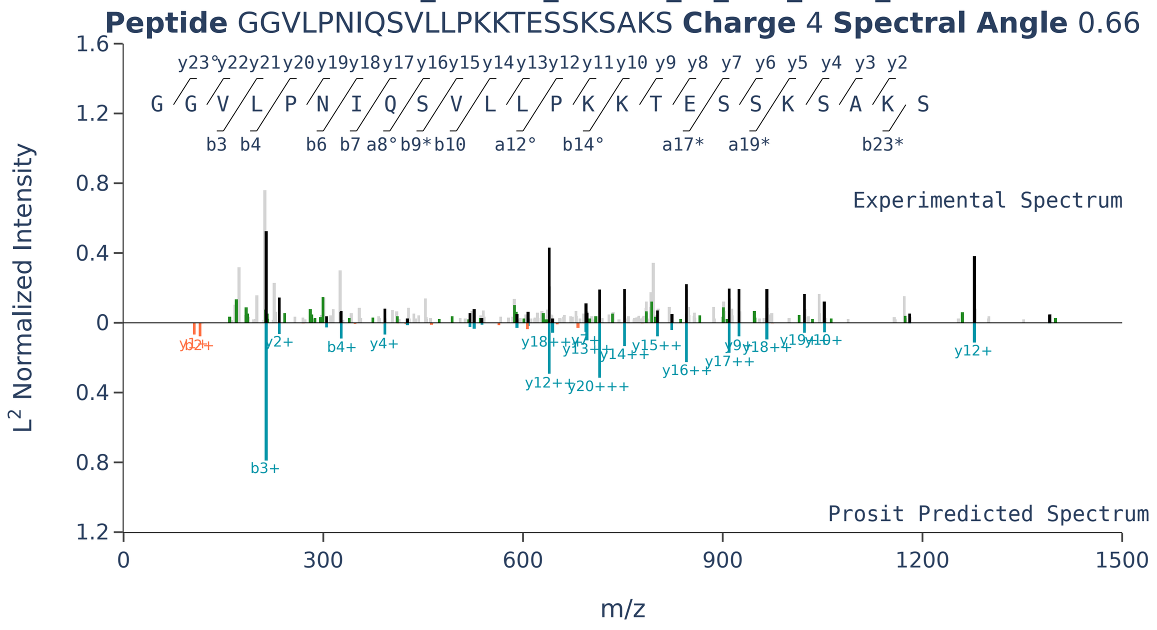


**Source** HRoetschke_291222_060123_Fu_H2A_b2R1_4h **Scan** 6831
**Peptide** ARDNKKTRIIPRHLQLAVRN **Charge** 5 **Spectral Angle** 0.72

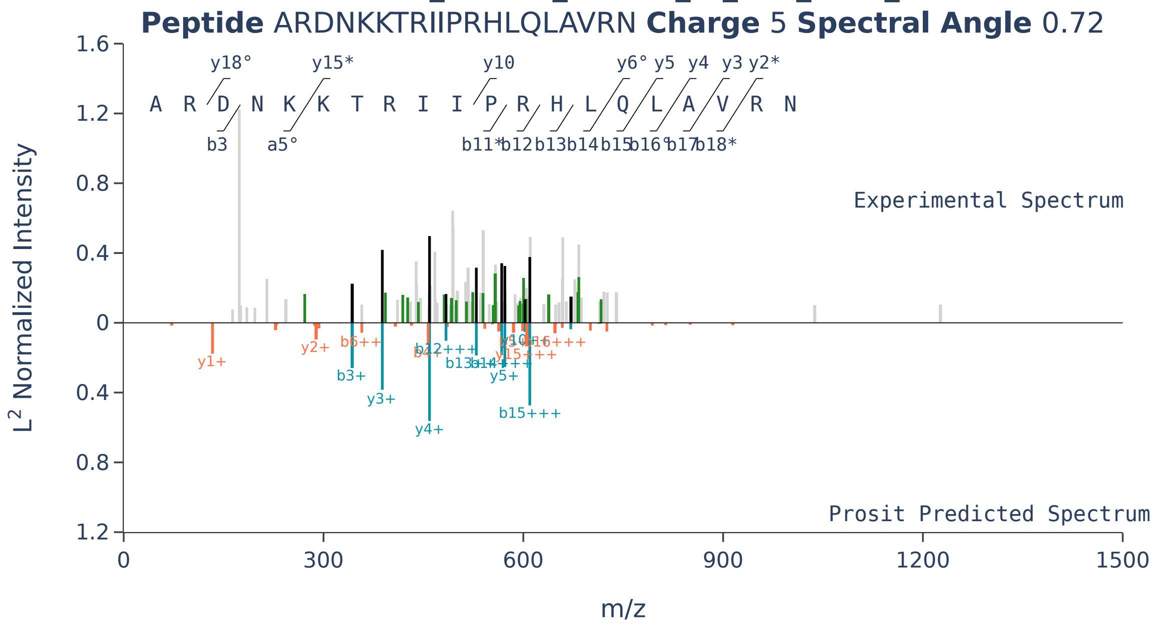


**Source** HRoetschke_291222_060123_Fu_H2A_b1R1_2h **Scan** 12563
**Peptide** AGLQFPVGRVHRLLRKGNYAERVG **Charge** 6 **Spectral Angle** 0.64

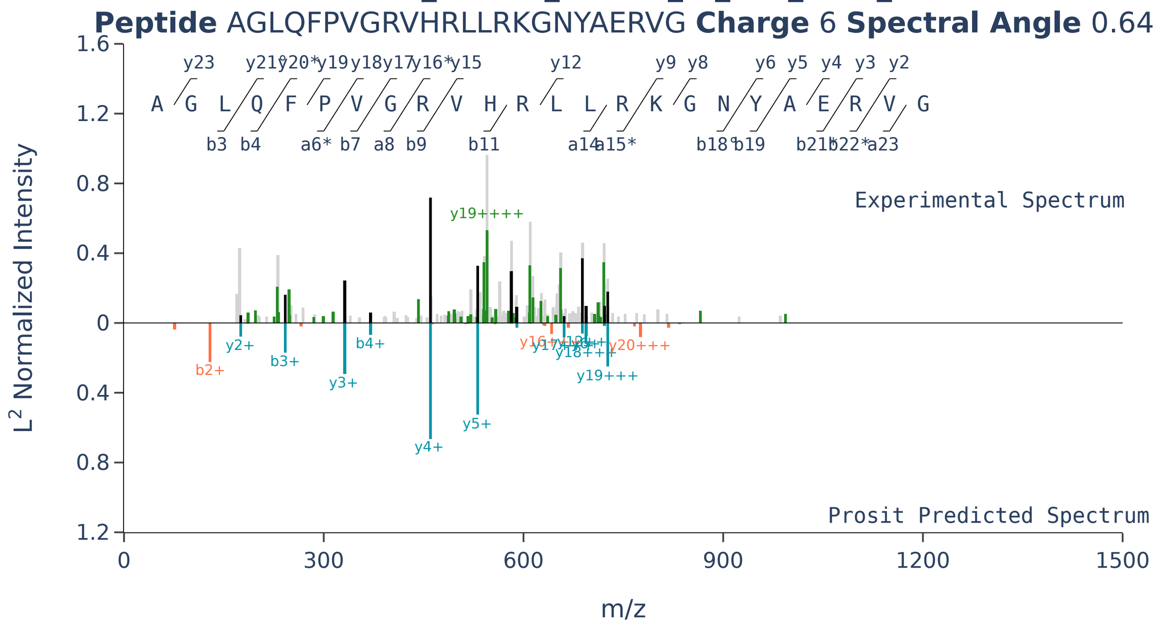


**Source** HRoetschke_291222_060123_Fu_H2A_b1R1_24h **Scan** 4786
**Peptide** LQSVLLPKKTESSKSAKSK **Charge** 5 **Spectral Angle** 0.6

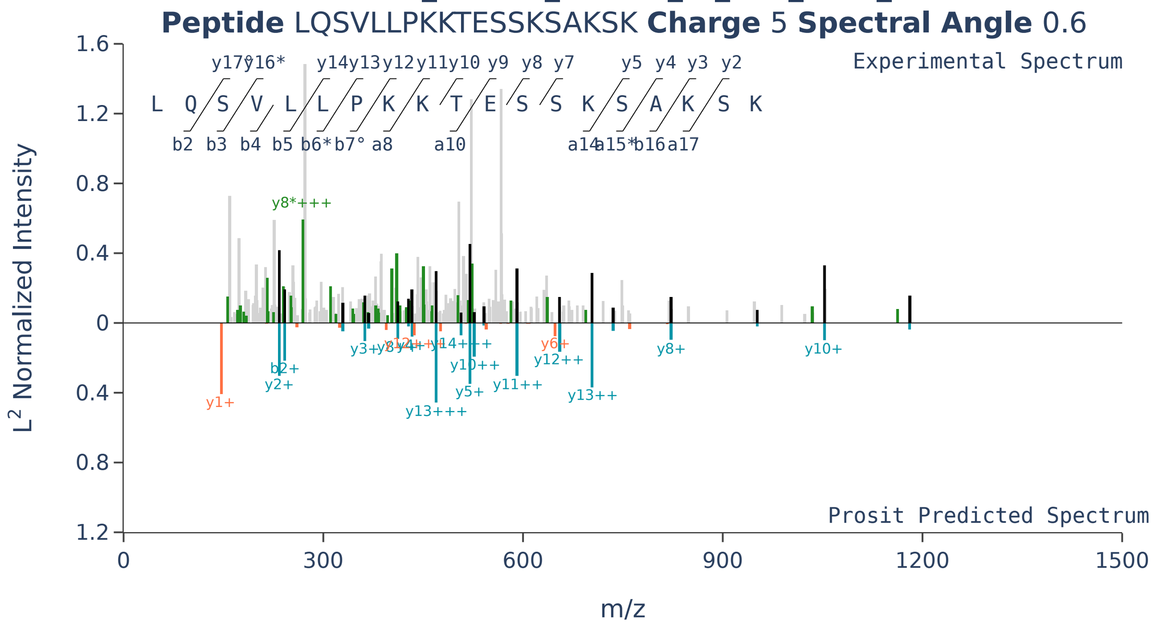


**Source** HRoetschke_291222_060123_Fu_H2A_b1R1_2h **Scan** 17868
**Peptide** LRKGNYAERVGAGAPVYLAAVLE **Charge** 4 **Spectral Angle** 0.64

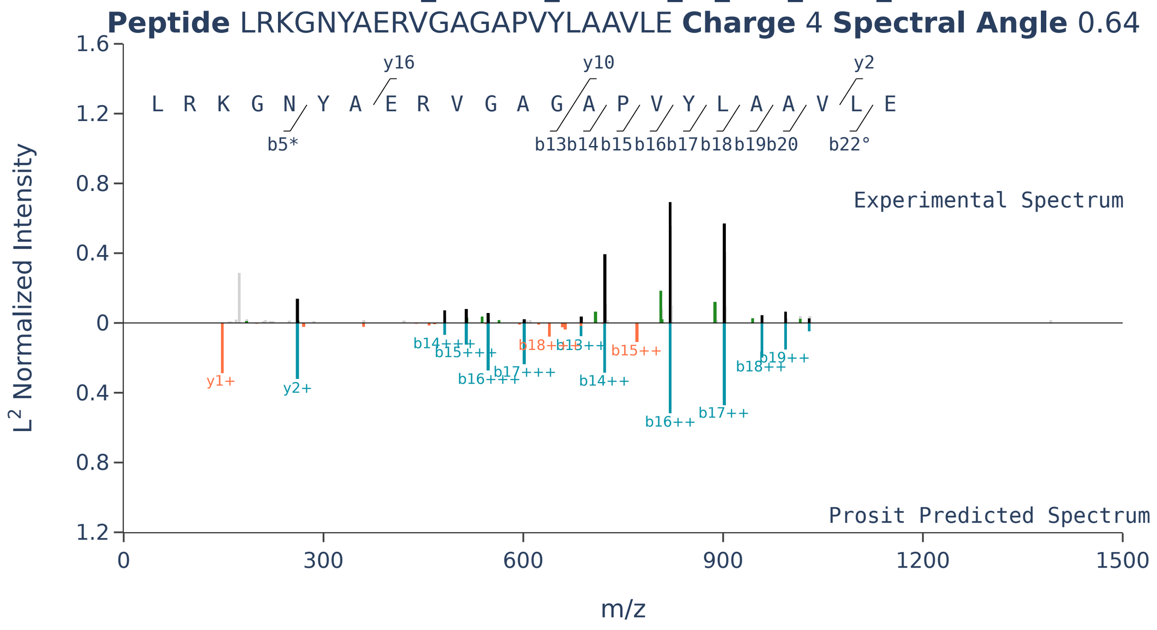


**Source** HRoetschke_291222_060123_Fu_H2A_b1R1_24h **Scan** 3287
**Peptide** GNAARDNKKTRIIPRHLQ **Charge** 4 **Spectral Angle** 0.69

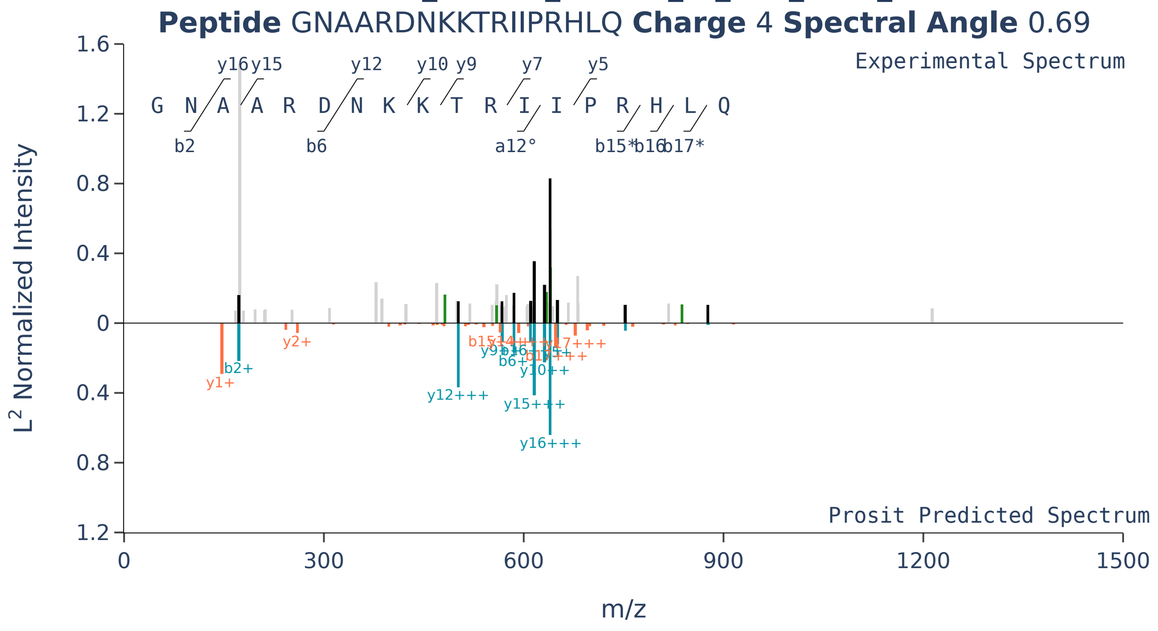


**Source** HRoetschke_291222_060123_Fu_H2A_b1R1_1h **Scan** 1904
**Peptide** NAARDNKKTRIIPRH **Charge** 4 **Spectral Angle** 0.61

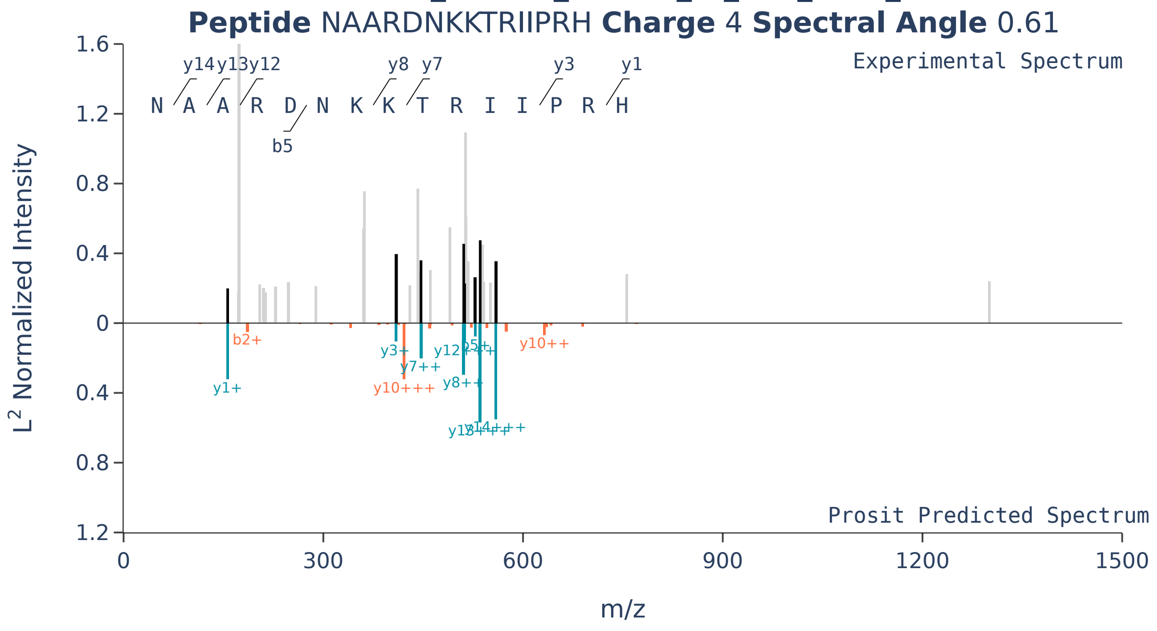


**Source** HRoetschke_291222_060123_Fu_H2A_b2R1_24h **Scan** 5636
**Peptide** PVGRVHRLLRKGNYAERV **Charge** 6 **Spectral Angle** 0.84

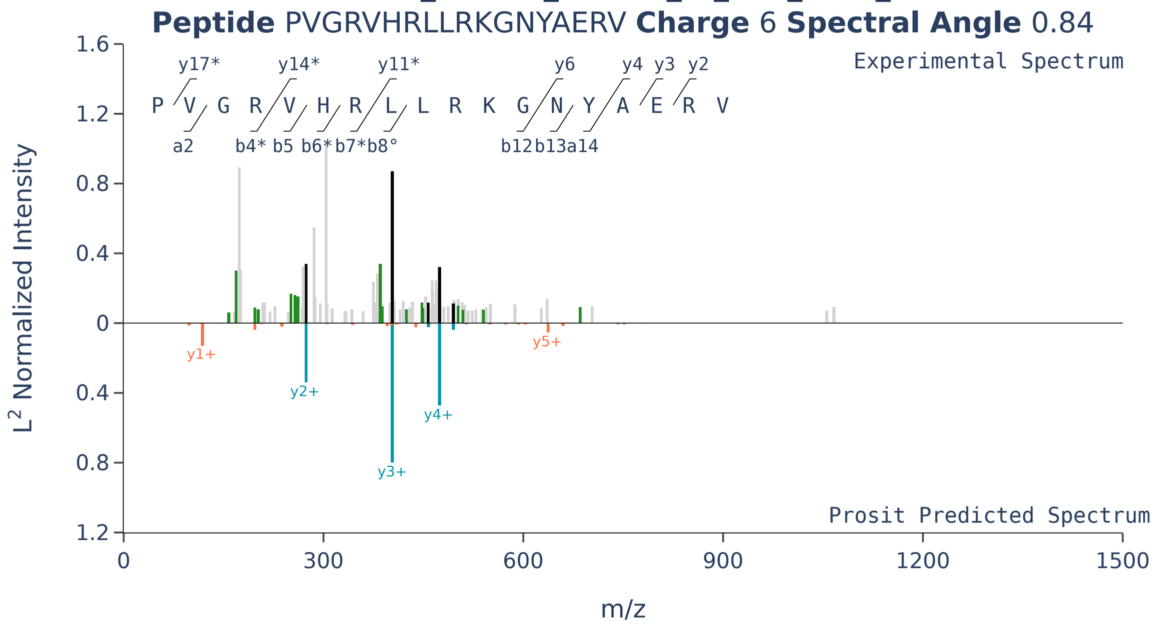


**Source** HRoetschke_291222_060123_Fu_H2A_b2R1_4h **Scan** 5649
**Peptide** GGKTRAKAKTRSSRAGLQFPVGRVH **Charge** 5 **Spectral Angle** 0.65

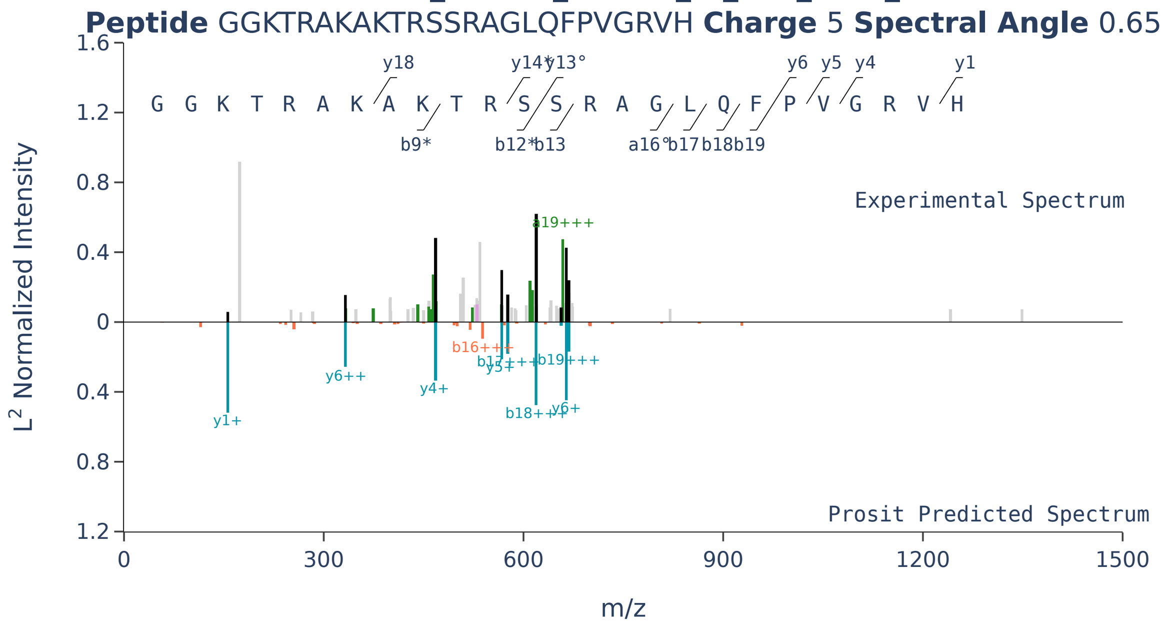


**Source** HRoetschke_291222_060123_Fu_H2A_b1R2_2h **Scan** 18544
**Peptide** EYLTAEILELAGNAARDNKKT **Charge** 4 **Spectral Angle** 0.72

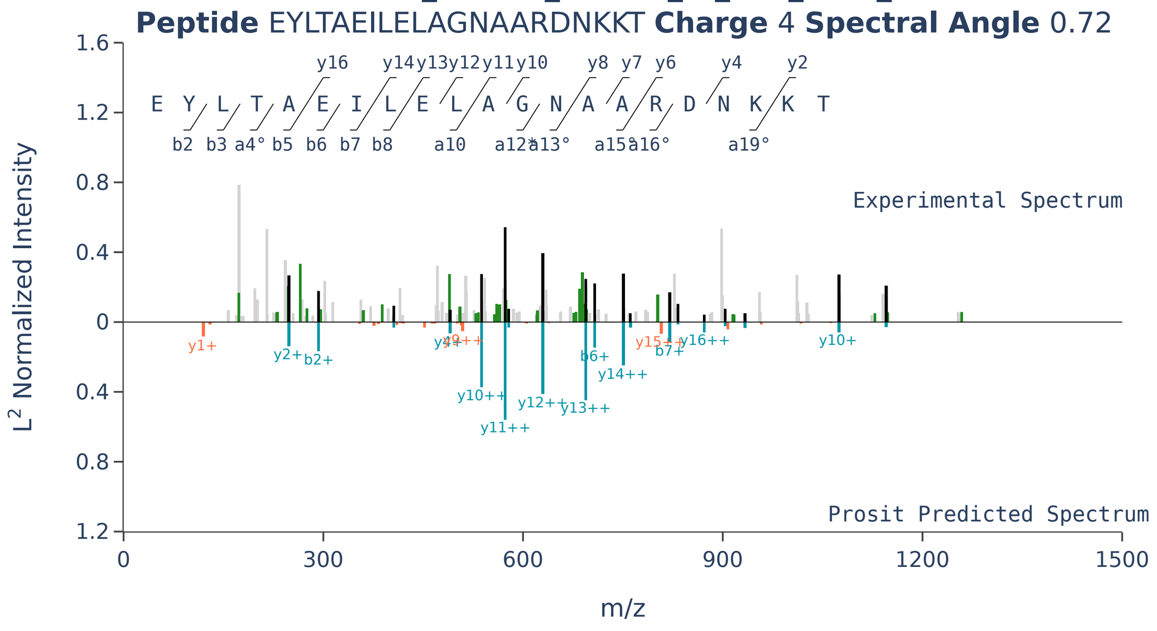


**Source** HRoetschke_291222_060123_Fu_H2A_b1R2_2h **Scan** 11509
**Peptide** SSRAGLQFPVGRVHRLLRKGNYAERVGA **Charge** 6 **Spectral Angle** 0.68

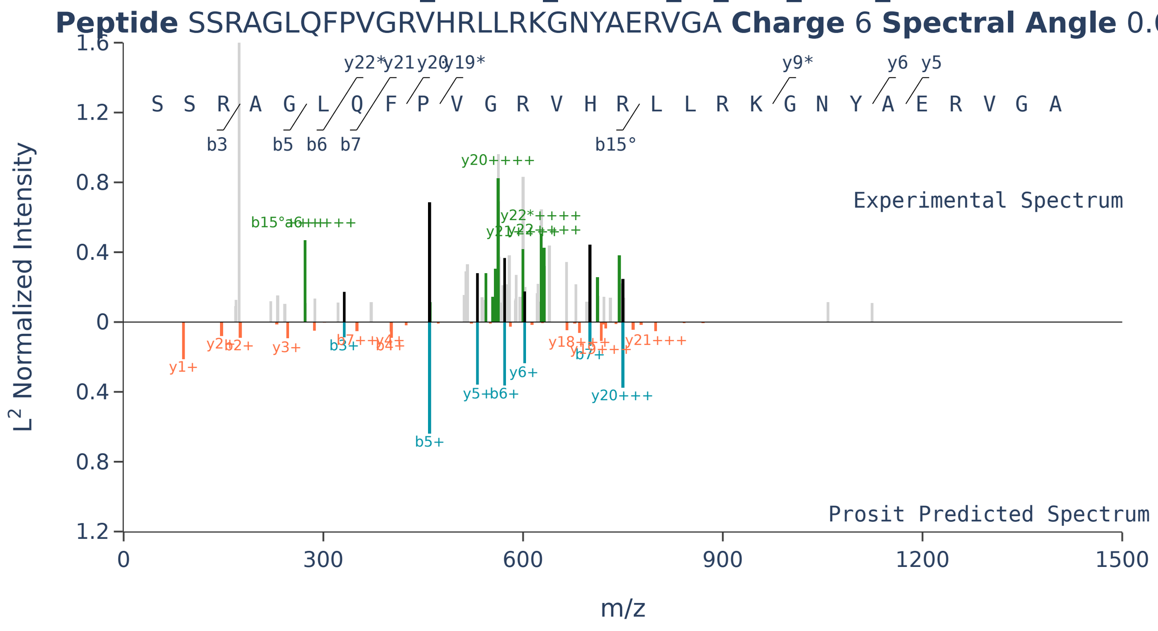


**Source** HRoetschke_291222_060123_Fu_H2A_b1R1_4h **Scan** 9276
**Peptide** ELLELAGNAARDNKKT **Charge** 3 **Spectral Angle** 0.48

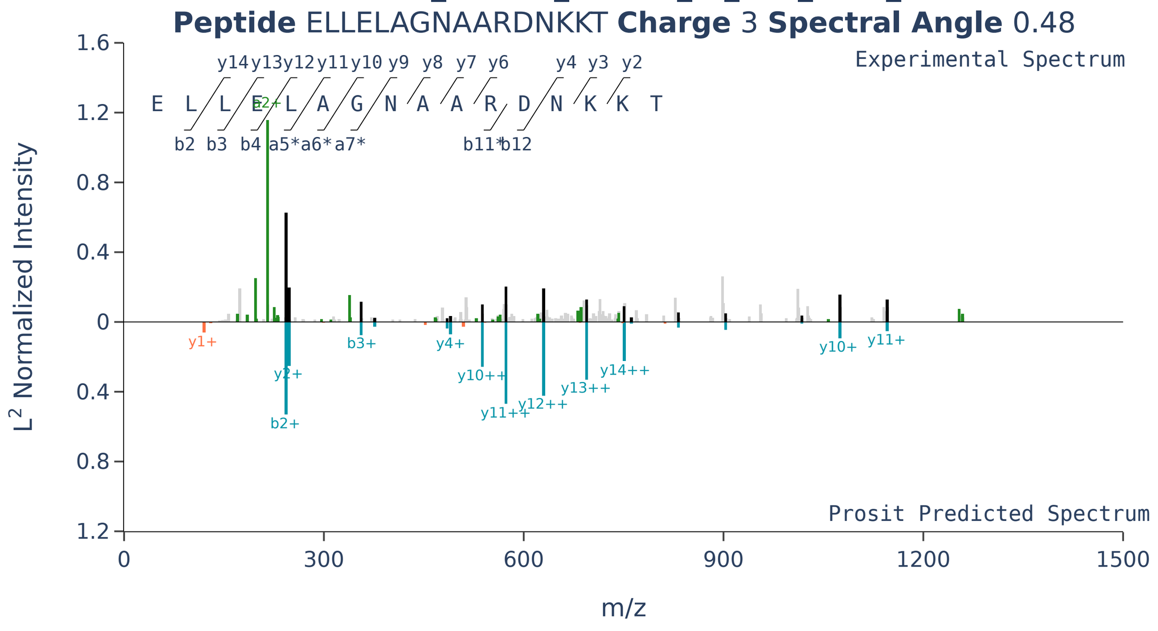


**Source** HRoetschke_291222_060123_Fu_H2A_b1R2_1h **Scan** 4195
**Peptide** QSVLLPKKTESSK **Charge** 4 **Spectral Angle** 0.67

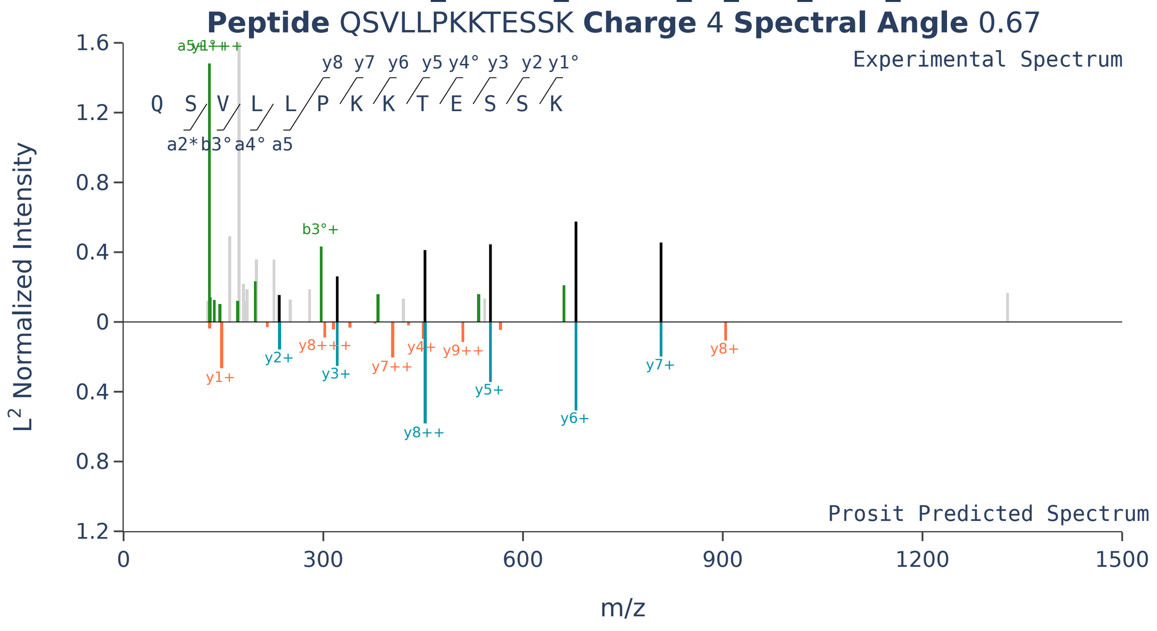


**Source** HRoetschke_291222_060123_Fu_H2A_b1R1_4h **Scan** 11023
**Peptide** LQLAVRNDEELNK **Charge** 3 **Spectral Angle** 0.42

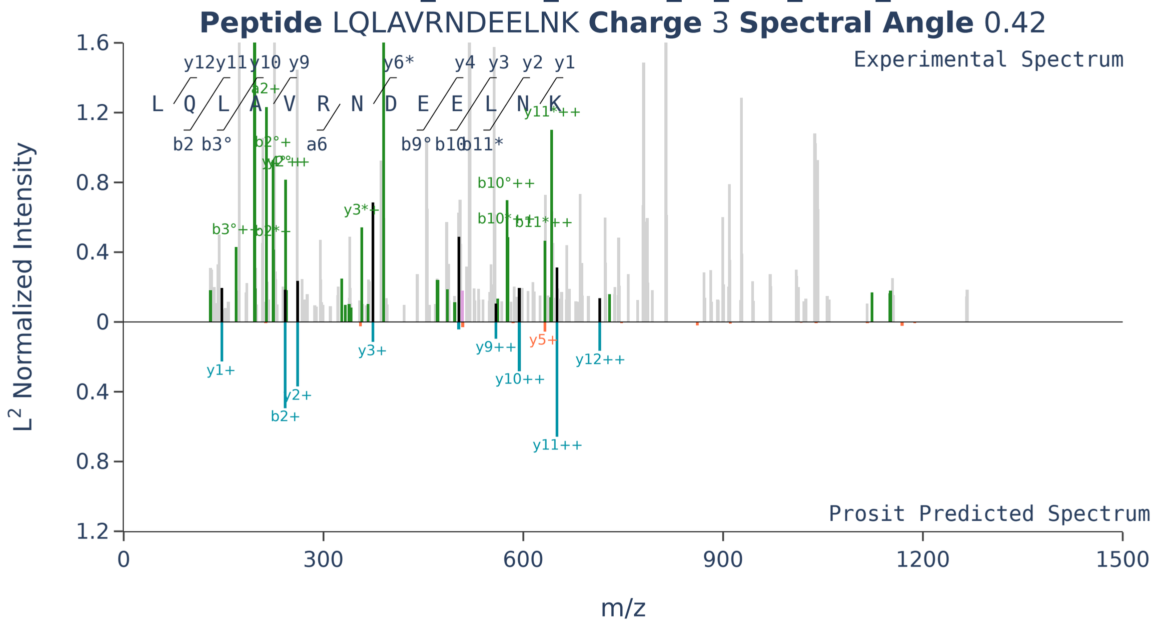

**Source** HRoetschke_291222_060123_Fu_H2A_b1R2_4h **Scan** 7818
**Peptide** KAKTRSSRAGLQFPVGRVHRL **Charge** 6 **Spectral Angle** 0.62

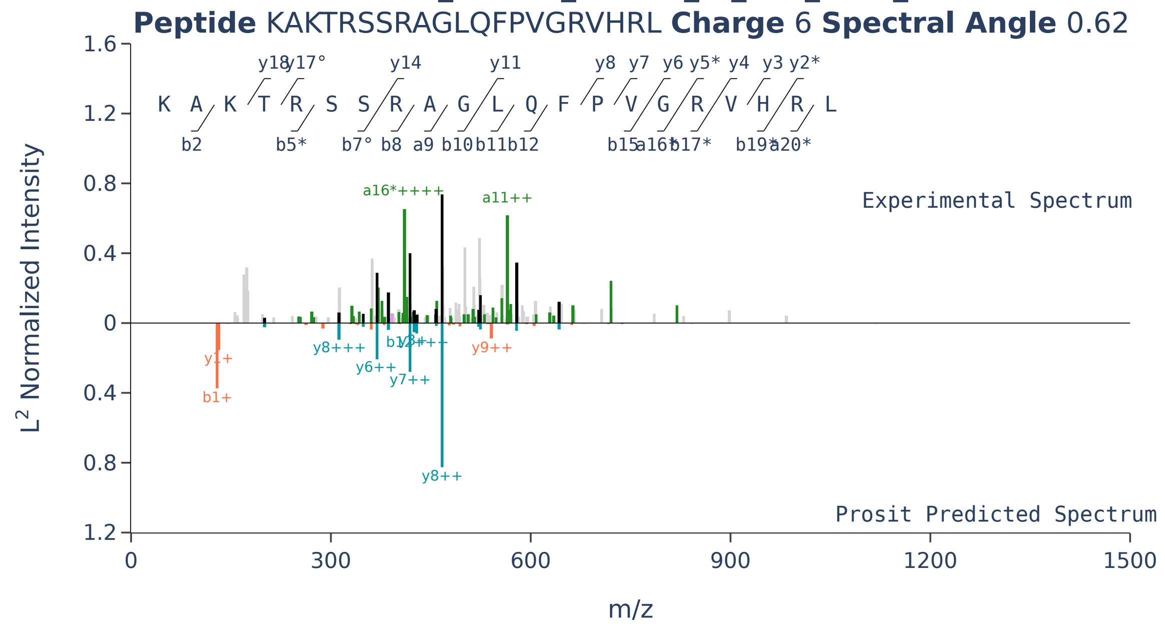


**Source** HRoetschke_291222_060123_Fu_H2A_b1R2_1h **Scan** 5760
**Peptide** QGGKTRAKAKTRSSRAGLQFPVG **Charge** 4 **Spectral Angle** 0.74

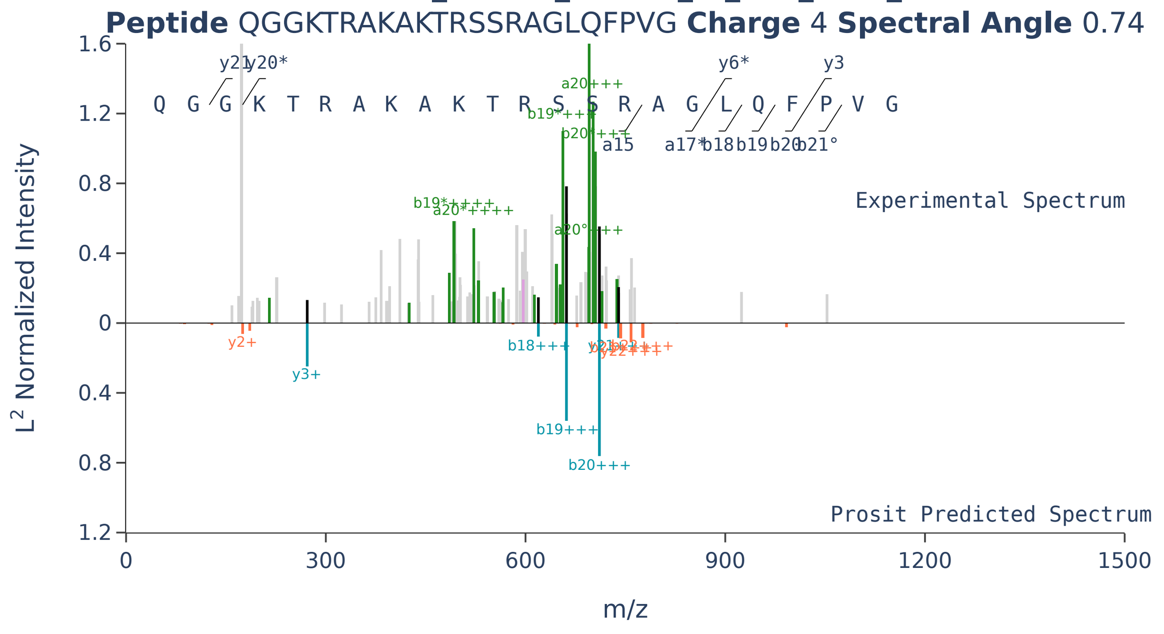


**Source** HRoetschke_291222_060123_Fu_H2A_b1R2_24h **Scan** 14444
**Peptide** GRVTIAQGGVLPNIQ **Charge** 2 **Spectral Angle** 0.78

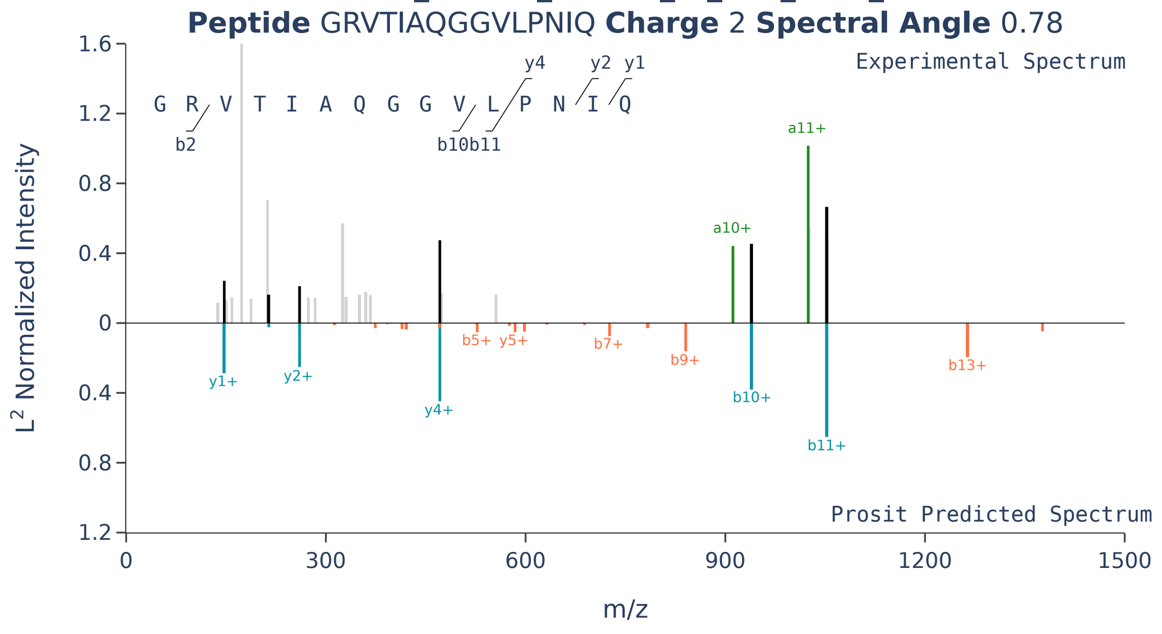


**Source** HRoetschke_291222_060123_Fu_H2A_b2R2_24h **Scan** 10871
**Peptide** ELLELAGNAARDNKKTRIIPR **Charge** 4 **Spectral Angle** 0.72

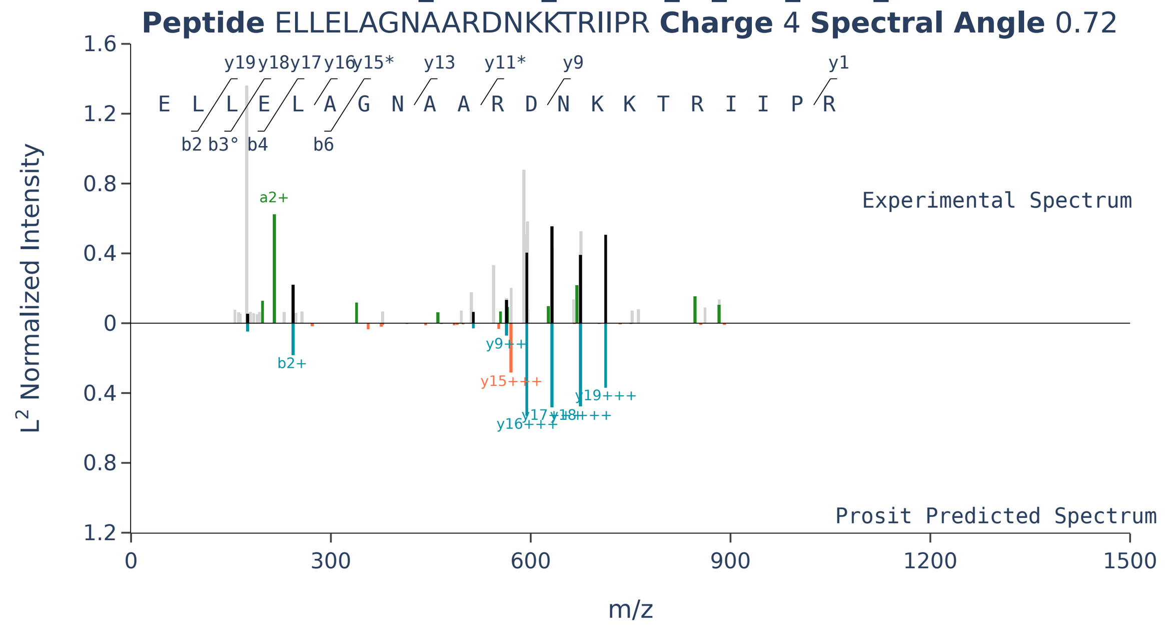


**Source** HRoetschke_291222_060123_Fu_H2A_b2R1_4h **Scan** 7101
**Peptide** GKTRAKAKTRSSRAGLQFPVGRV **Charge** 6 **Spectral Angle** 0.78

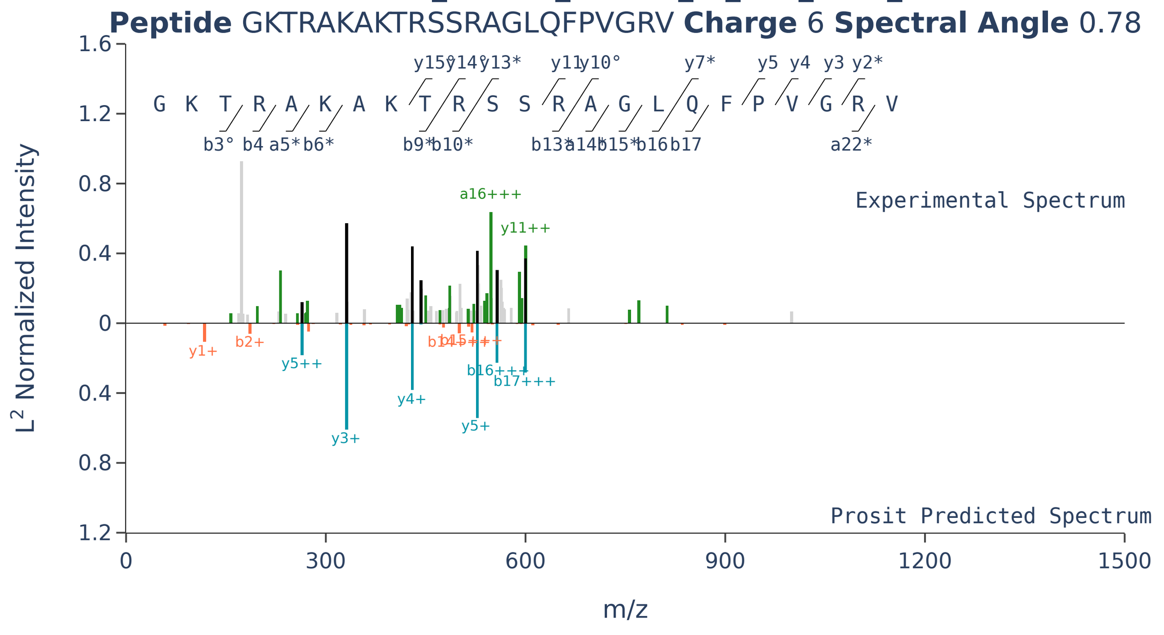


**Source** HRoetschke_291222_060123_Fu_H2A_b1R1_4h **Scan** 7225
**Peptide** ARDNKKTRIIPRHLQLAVRNDE **Charge** 5 **Spectral Angle** 0.7

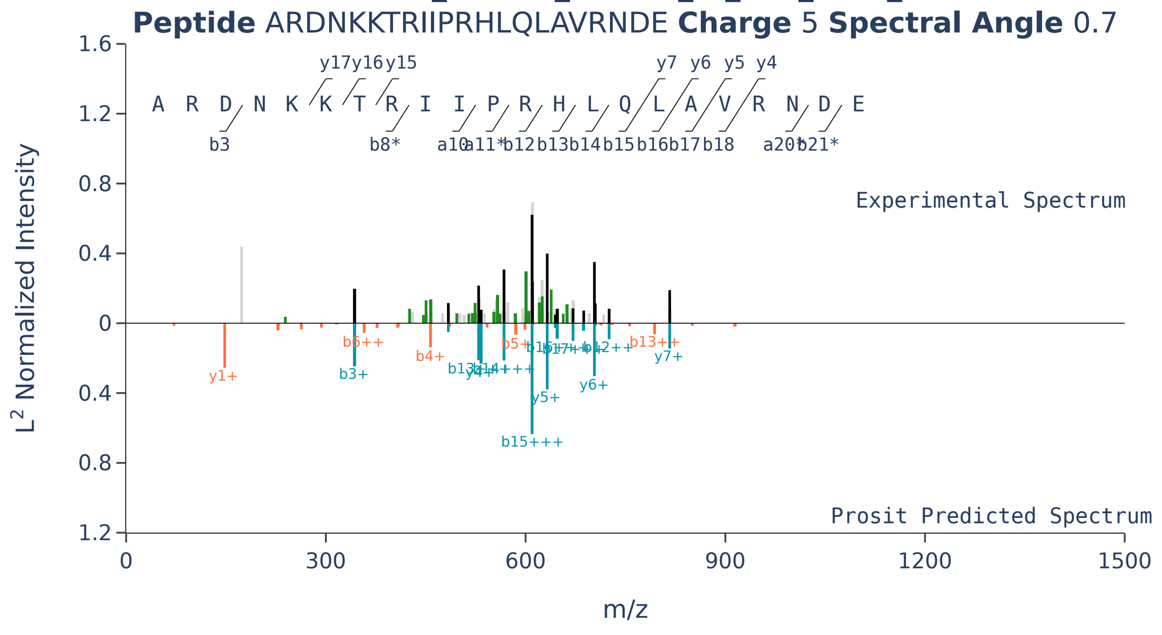


**Source** HRoetschke_291222_060123_Fu_H2A_b1R1_4h **Scan** 9485
**Peptide** GNAARDNKKTRIIPRHLQLAV **Charge** 4 **Spectral Angle** 0.77

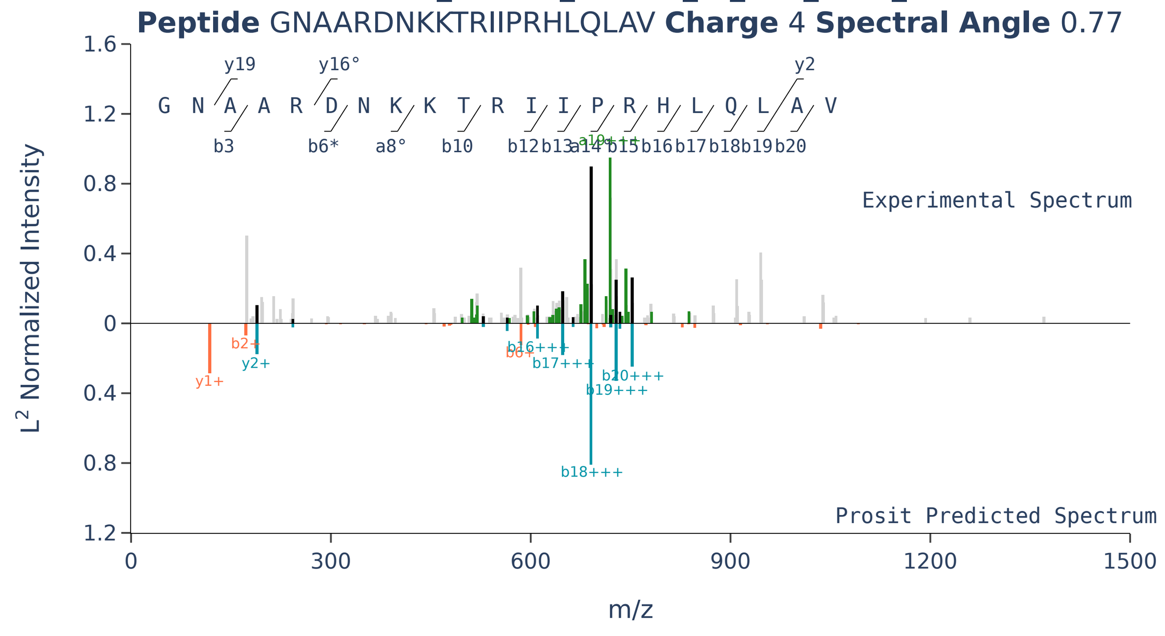


**Source** HRoetschke_291222_060123_Fu_H2A_b2R2_4h **Scan** 8659
**Peptide** DNKKTRIIPRHLQL **Charge** 3 **Spectral Angle** 0.69

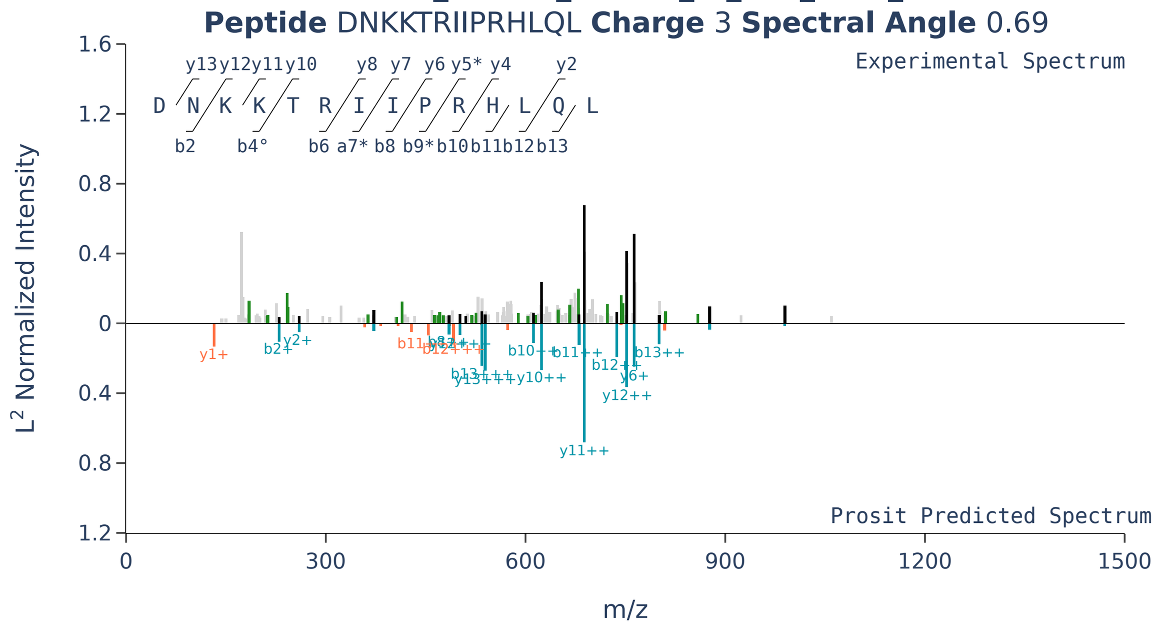


**Source** HRoetschke_291222_060123_Fu_H2A_b1R1_4h **Scan** 9041
**Peptide** AKTRSSRAGLQFPVGRVHRLLRKG **Charge** 6 **Spectral Angle** 0.76

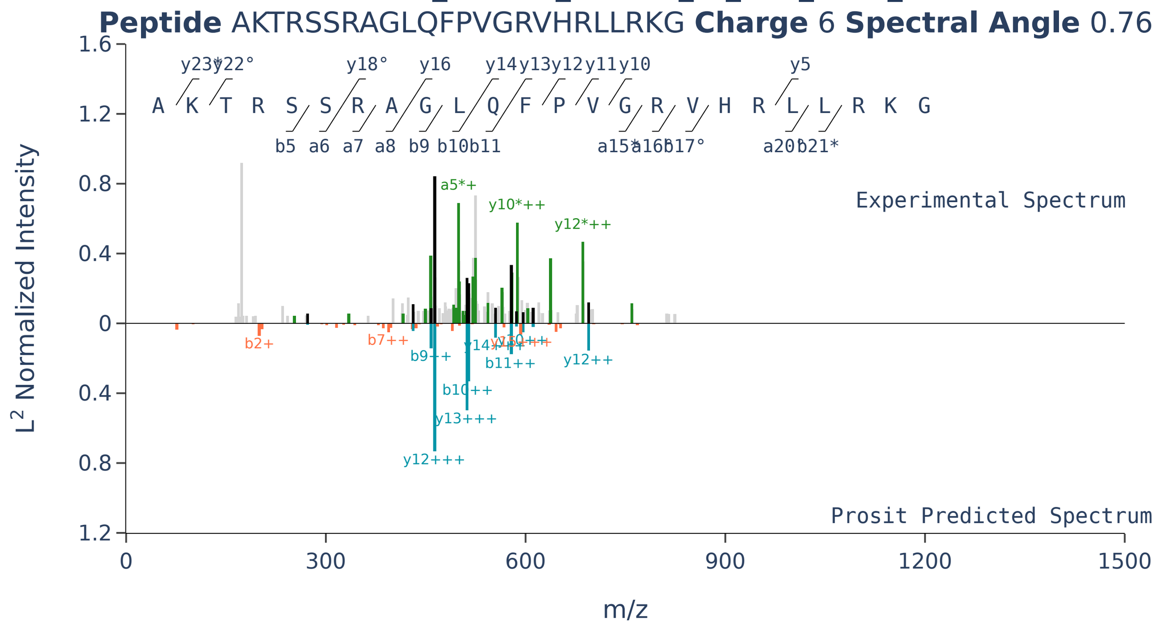


**Source** HRoetschke_291222_060123_Fu_H2A_b1R2_4h **Scan** 12993
**Peptide** GLQFPVGRVHRLLRKGNYAERV **Charge** 4 **Spectral Angle** 0.63

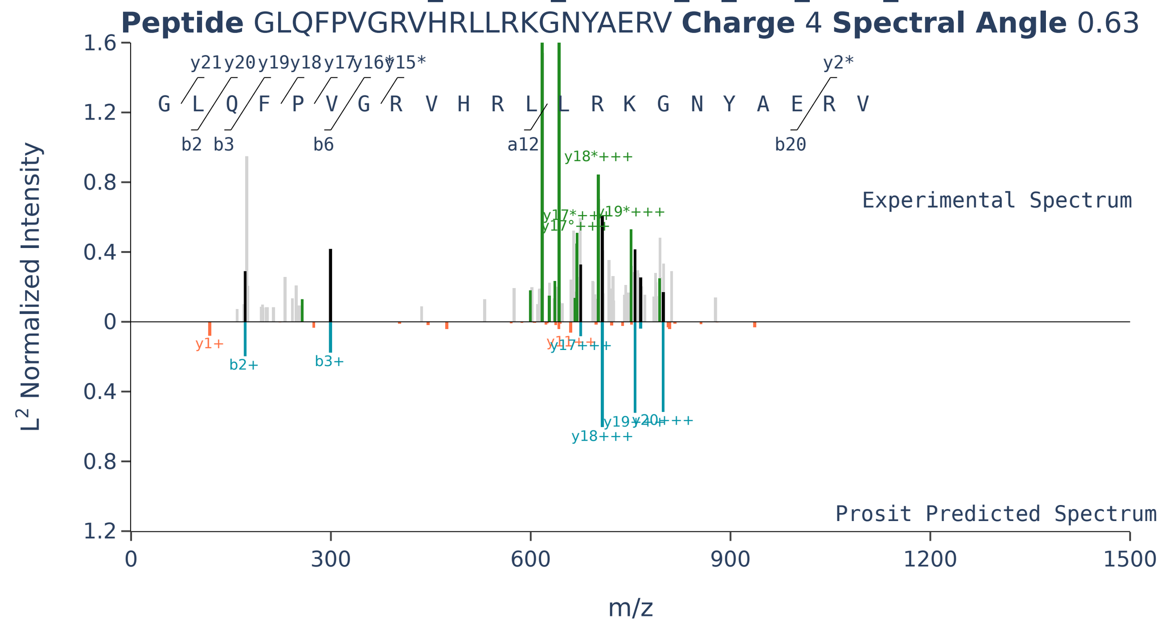


**Source** HRoetschke_291222_060123_Fu_H2A_b1R1_4h **Scan** 16381
**Peptide** IAQGGVLPNIQSVLLPKKTESSKSAKSK **Charge** 4 **Spectral Angle** 0.71

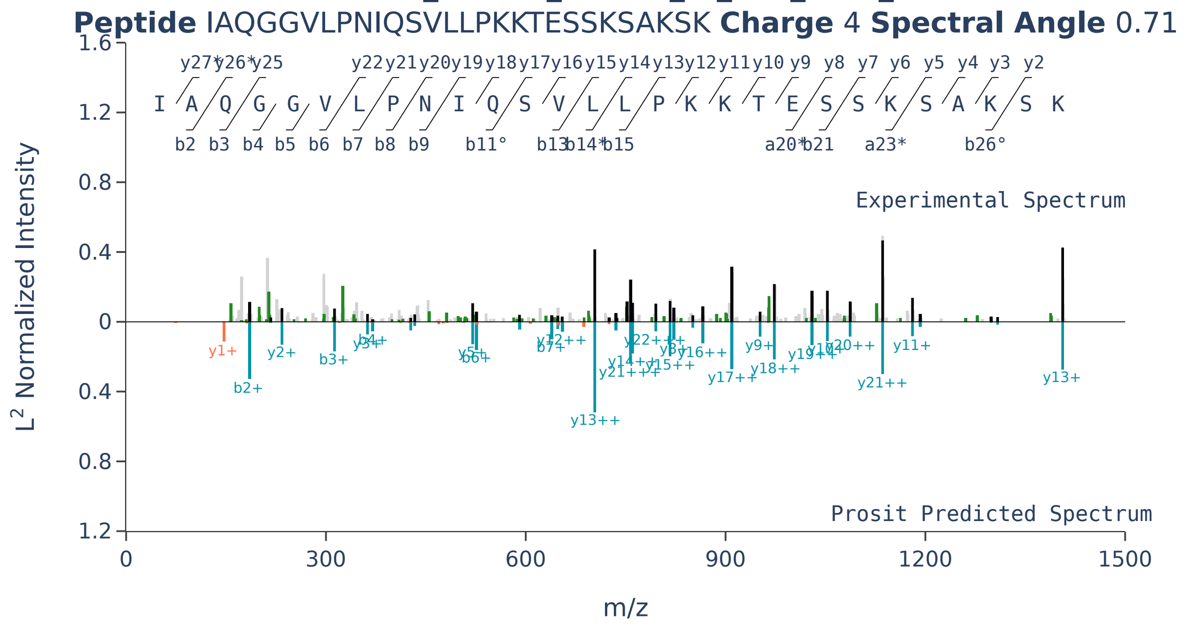


**Source** HRoetschke_291222_060123_Fu_H2A_b2R2_4h **Scan** 2928
**Peptide** NKKTRIIPRHLQ **Charge** 3 **Spectral Angle** 0.7

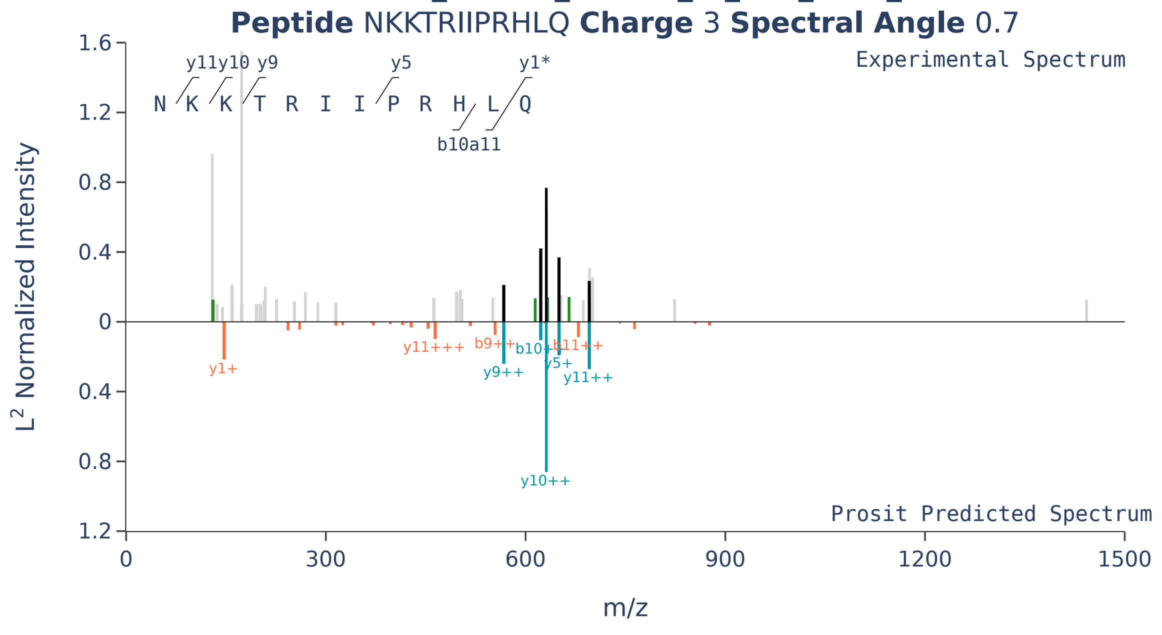


**Source** HRoetschke_291222_060123_Fu_H2A_b1R2_4h **Scan** 11965
**Peptide** GLQFPVGRVHRLLRKG **Charge** 5 **Spectral Angle** 0.6

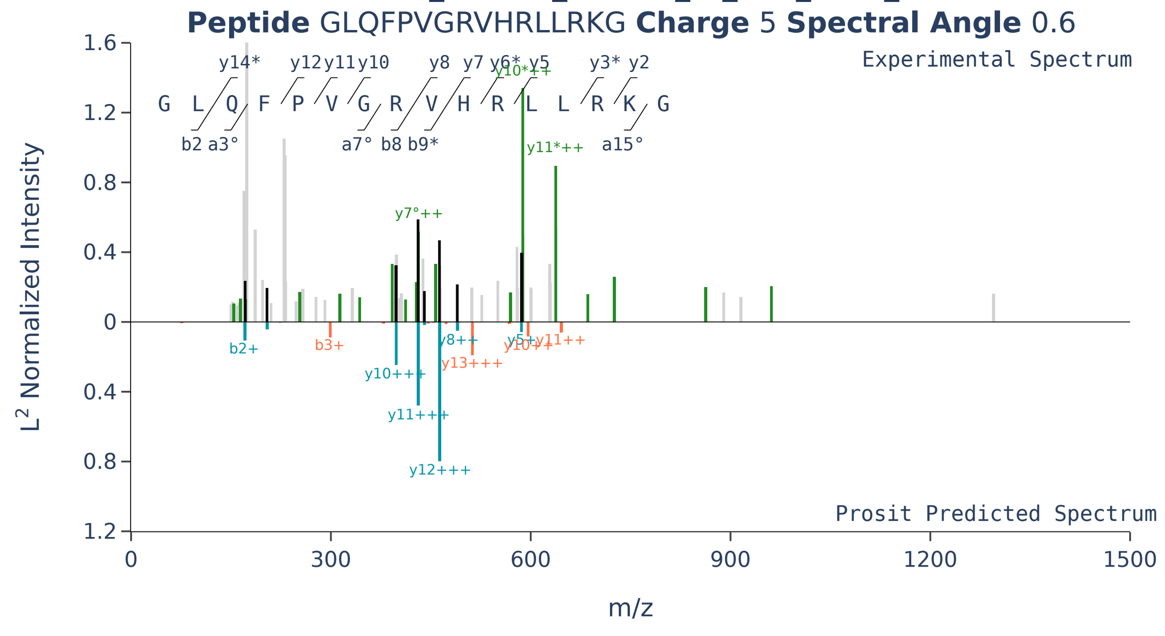


**Source** HRoetschke_291222_060123_Fu_H2A_b1R1_4h **Scan** 19309
**Peptide** YLTAEILELAGNAARDNKKT **Charge** 4 **Spectral Angle** 0.74

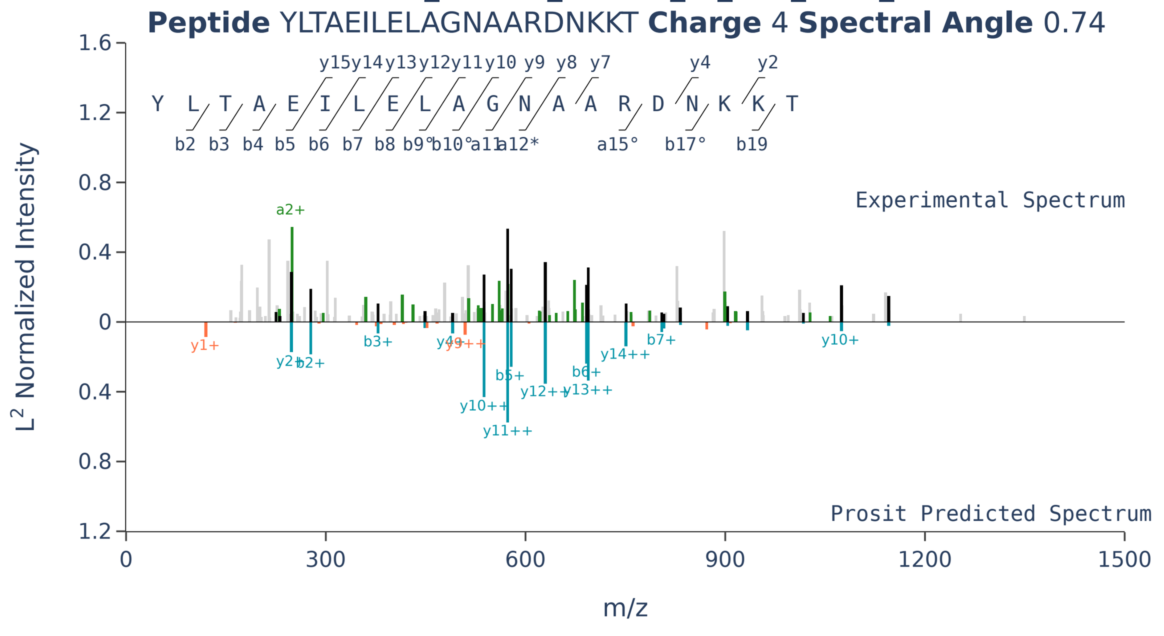


**Source** HRoetschke_291222_060123_Fu_H2A_b2R2_2h **Scan** 6728
**Peptide** GNAARDNKKTRIIPRHLQLAVRND **Charge** 5 **Spectral Angle** 0.63

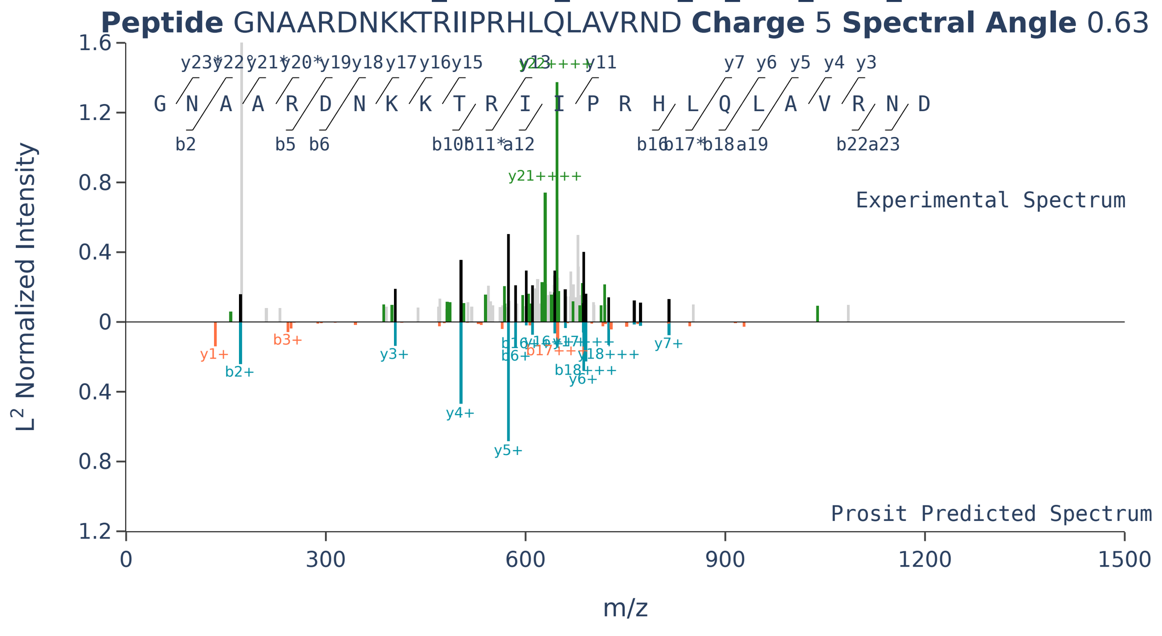

**Source** HRoetschke_291222_060123_Fu_H2A_b1R1_2h **Scan** 19330
**Peptide** ELNKLLGRVTIAQGGVLPNL **Charge** 3 **Spectral Angle** 0.79

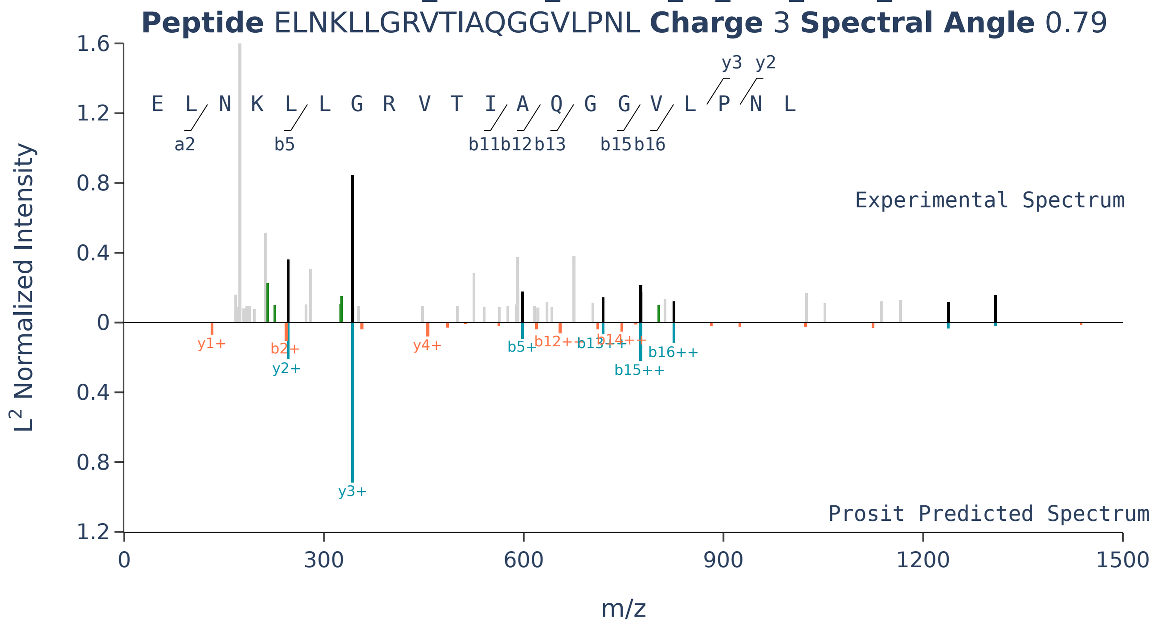


**Source** HRoetschke_291222_060123_Fu_H2A_b1R2_4h **Scan** 17305
**Peptide** GGVLPNIQSVLLPKKTESSKSA **Charge** 4 **Spectral Angle** 0.64

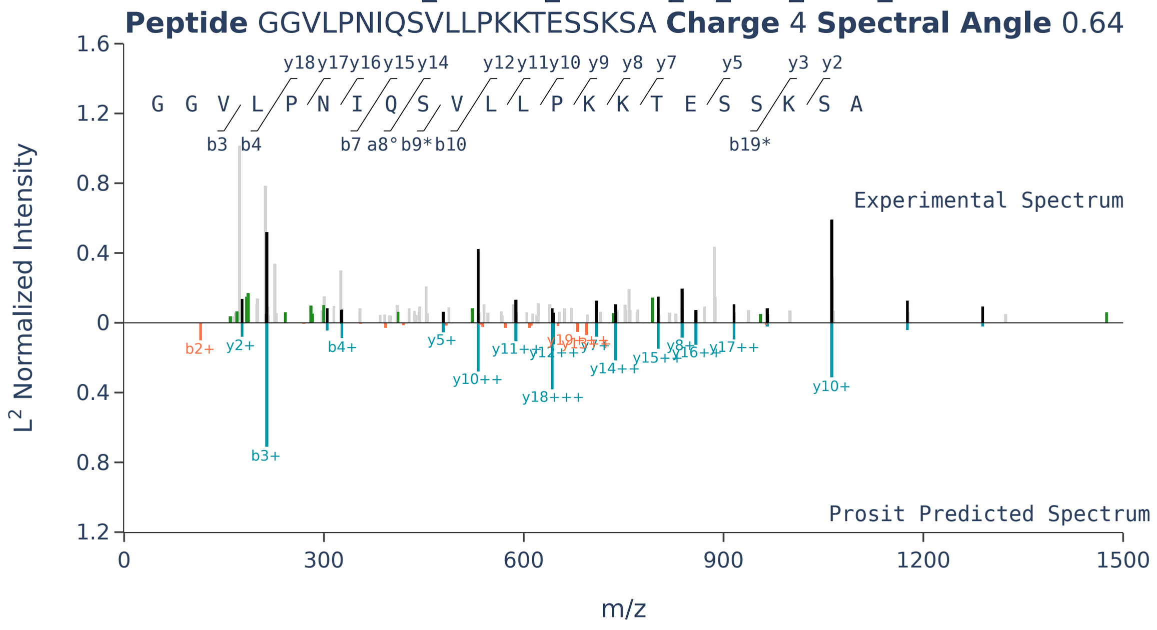


**Source** HRoetschke_291222_060123_Fu_H2A_b2R2_2h **Scan** 14477
**Peptide** LAQGGVLPNIQSVLLPKKTESSKSAKSK **Charge** 4 **Spectral Angle** 0.69

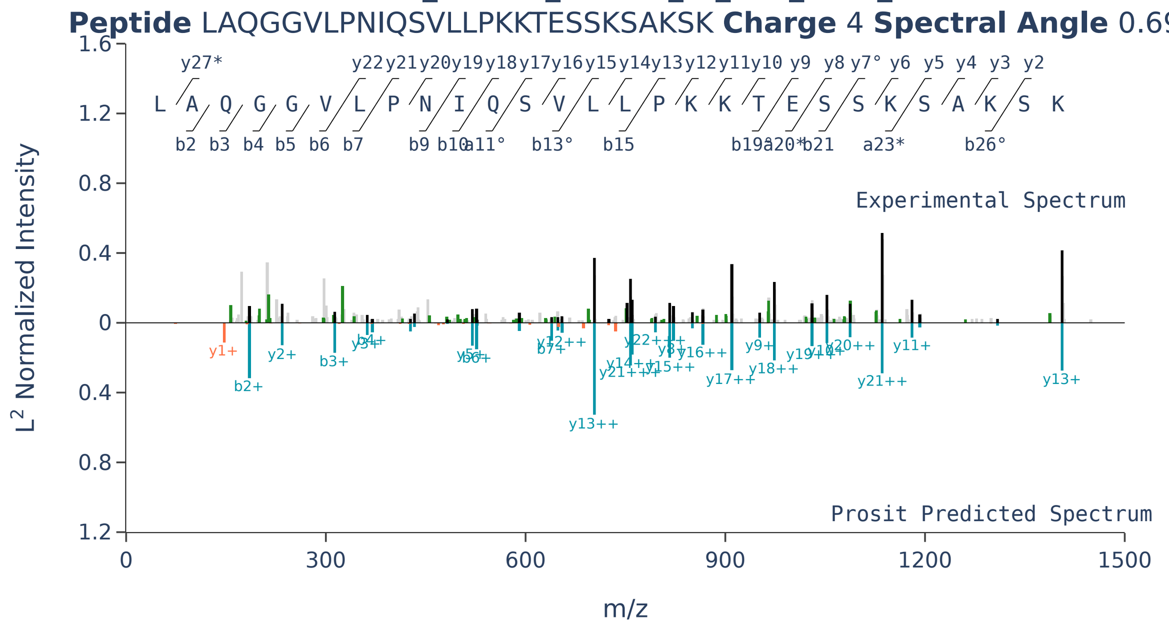


**Source** HRoetschke_291222_060123_Fu_H2A_b2R2_4h **Scan** 5536
**Peptide** KTRAKAKTRSSRAGLQFPVGRVH **Charge** 5 **Spectral Angle** 0.62

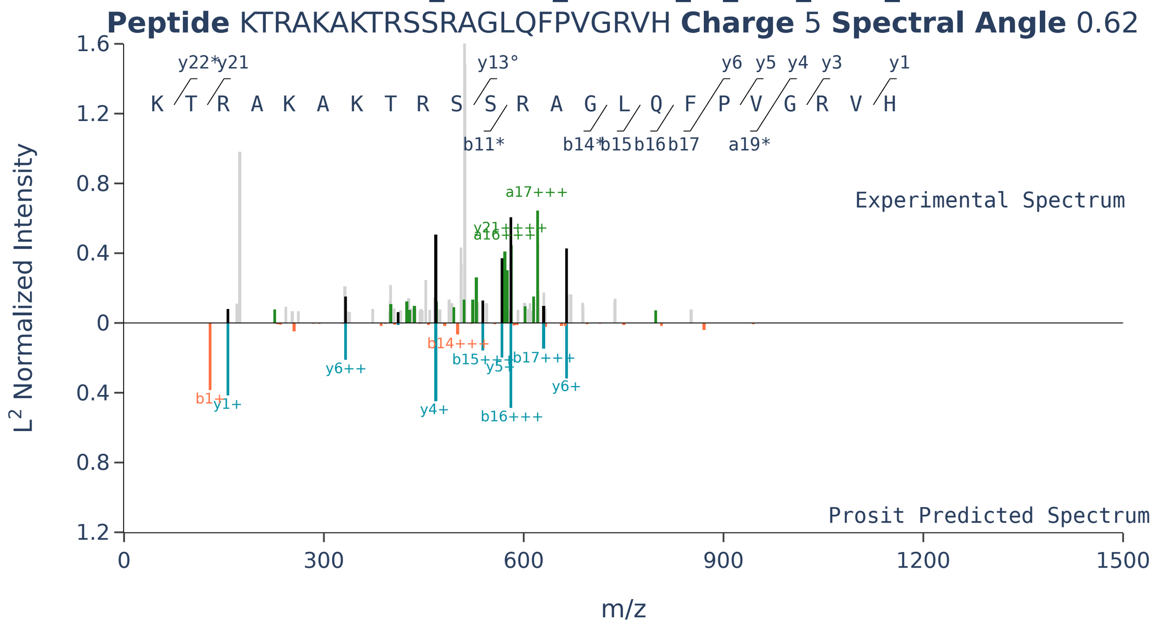


**Source** HRoetschke_291222_060123_Fu_H2A_b1R2_24h **Scan** 2550
**Peptide** LPKKTESSKSAKSK **Charge** 3 **Spectral Angle** 0.59

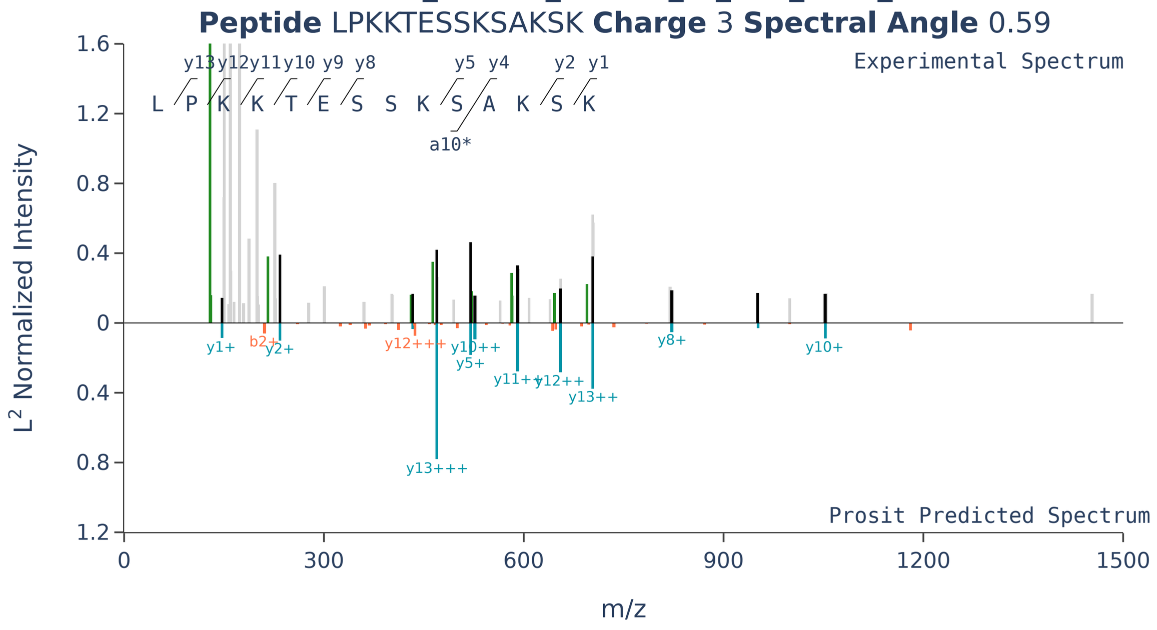


**Source** HRoetschke_291222_060123_Fu_H2A_b2R1_24h **Scan** 2047
**Peptide** GNAARDNKKTRIIPRH **Charge** 4 **Spectral Angle** 0.62

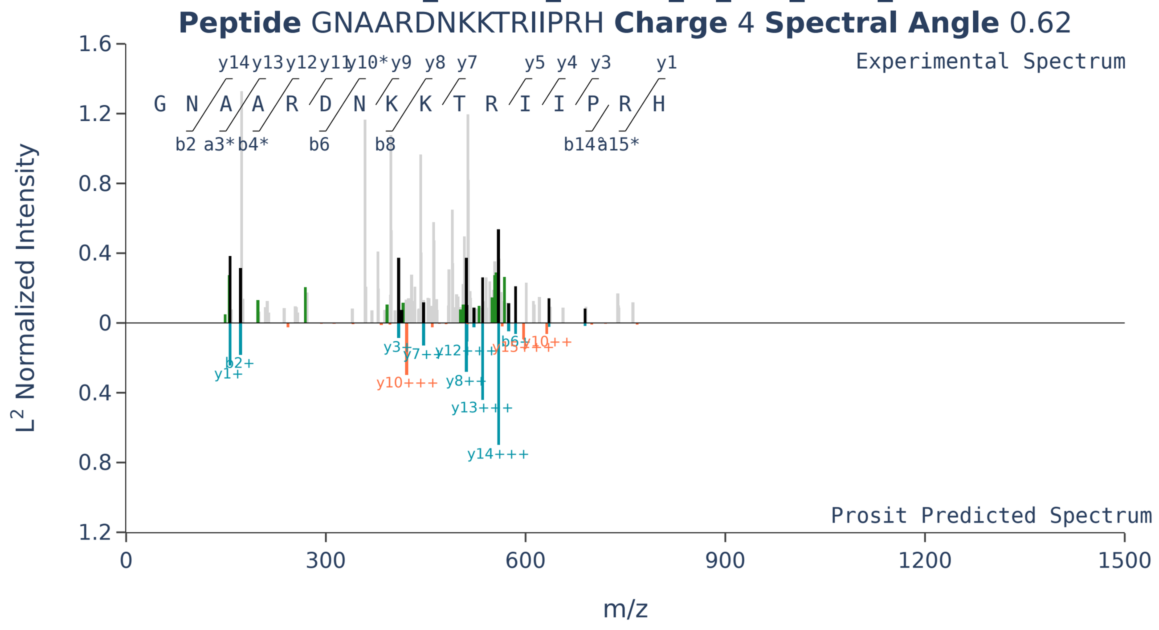


**Source** HRoetschke_291222_060123_Fu_H2A_b2R1_2h **Scan** 4241
**Peptide** RLLRKGNYAERVGAG **Charge** 4 **Spectral Angle** 0.54

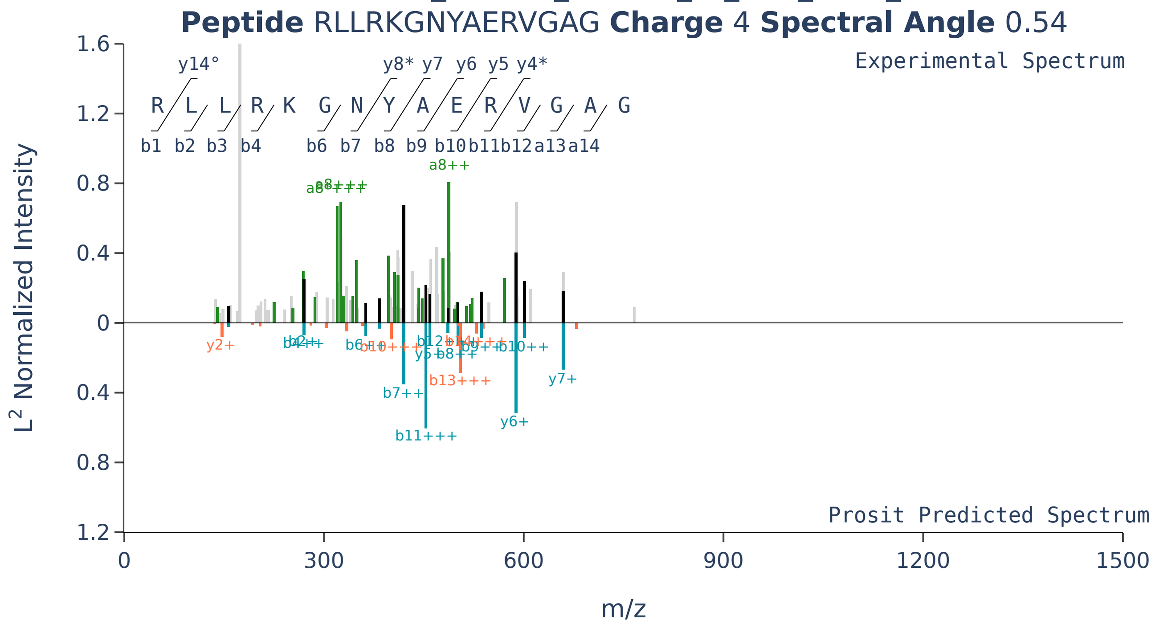


**Source** HRoetschke_291222_060123_Fu_H2A_b1R1_4h **Scan** 17847
**Peptide** VTIAQGGVLPNIQSVLLPKKTESSKSAKSK **Charge** 4 **Spectral Angle** 0.66

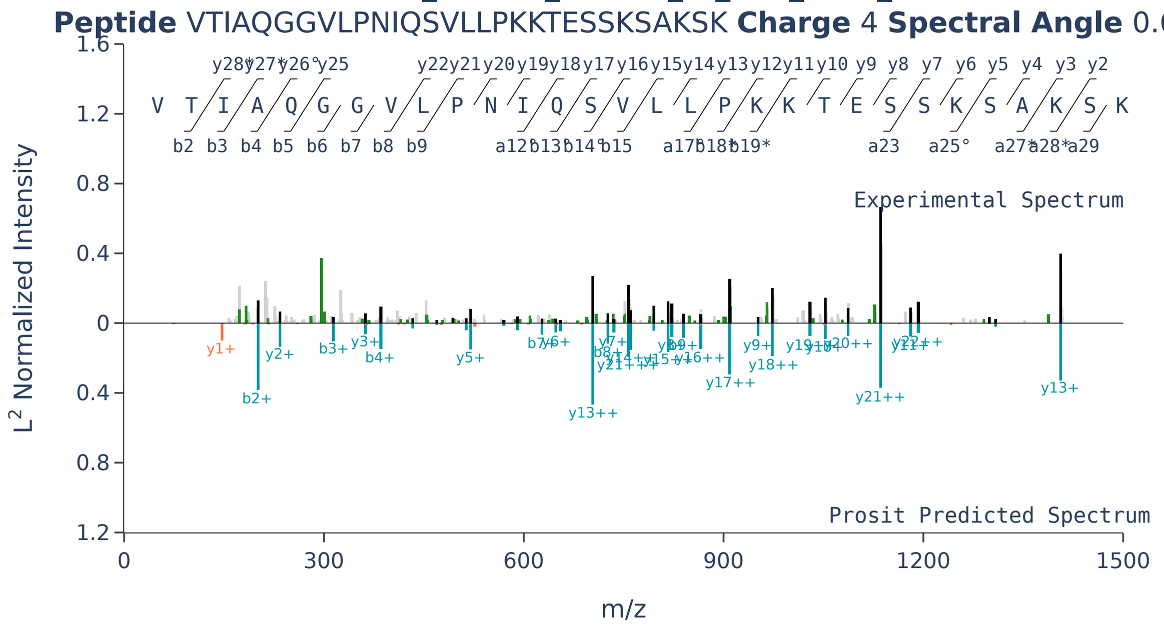


**Source** HRoetschke_291222_060123_Fu_H2A_b1R2_1h **Scan** 2558
**Peptide** PVGRVHRLLRKG **Charge** 3 **Spectral Angle** 0.83

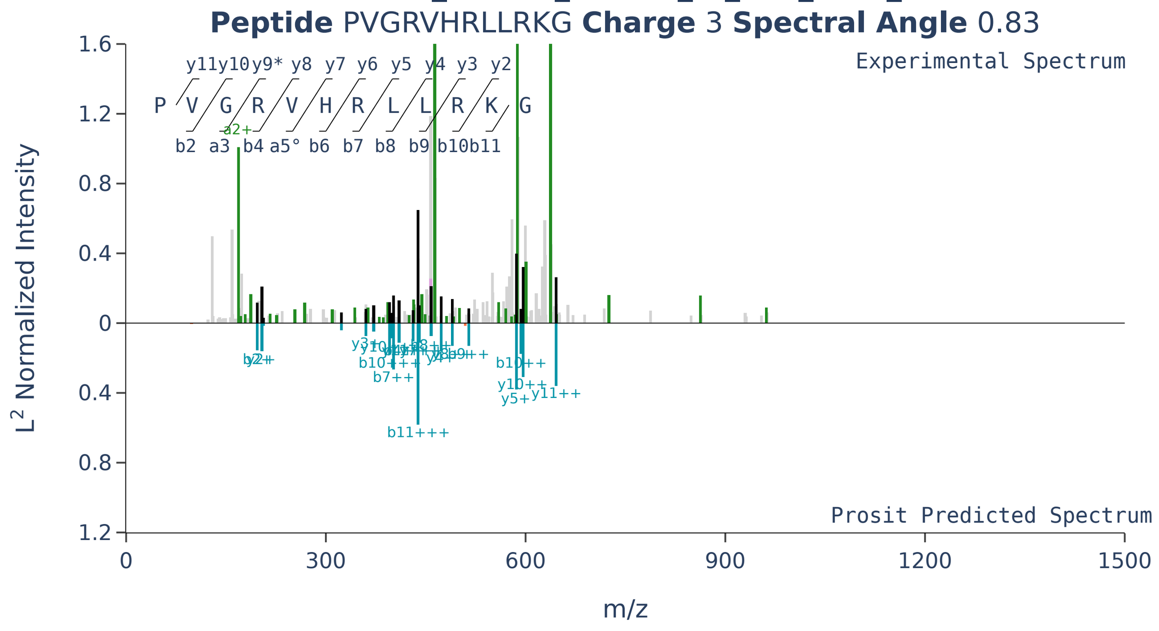


**Source** HRoetschke_291222_060123_Fu_H2A_b1R1_4h **Scan** 11197
**Peptide** PRHLQLAVRNDEELNKL **Charge** 4 **Spectral Angle** 0.62

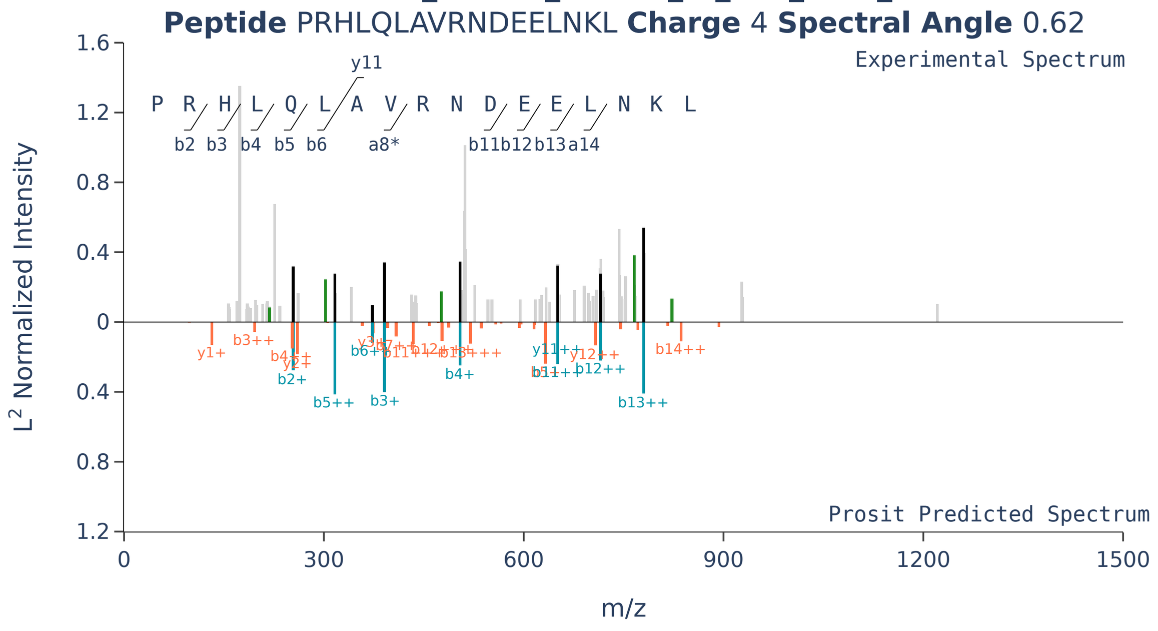


**Source** HRoetschke_291222_060123_Fu_H2A_b1R1_24h **Scan** 10693
**Peptide** ILELAGNAARDNKKTRIIPRHL **Charge** 4 **Spectral Angle** 0.85

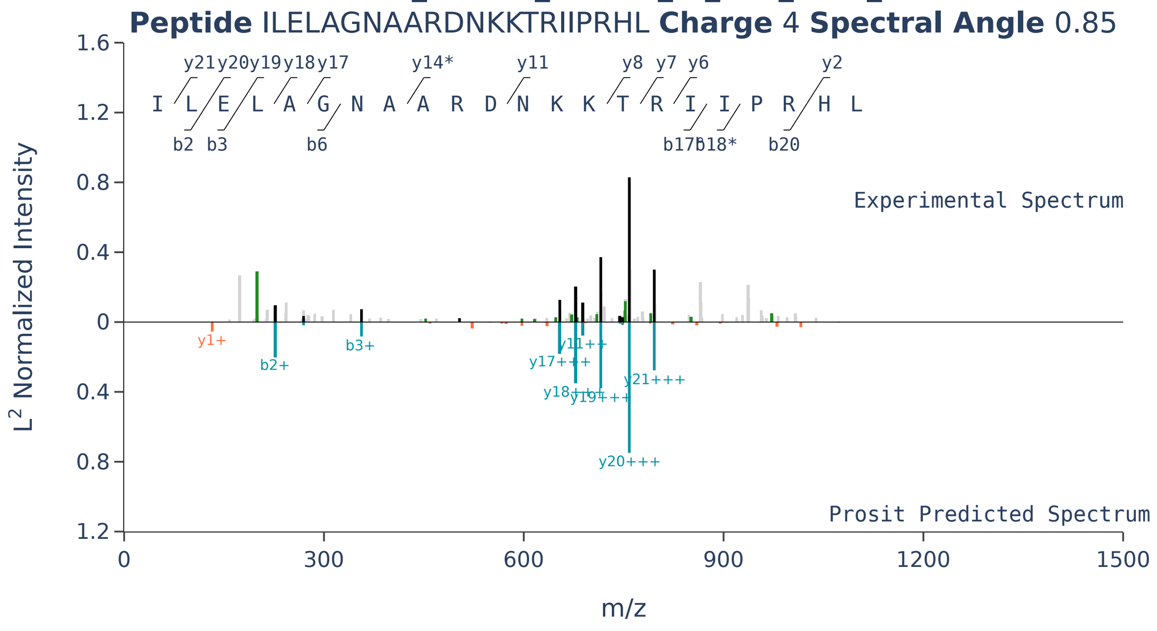


**Source** HRoetschke_291222_060123_Fu_H2A_b2R1_24h **Scan** 15494
**Peptide** VRNDEELNKLLGRVTIAQ **Charge** 3 **Spectral Angle** 0.63

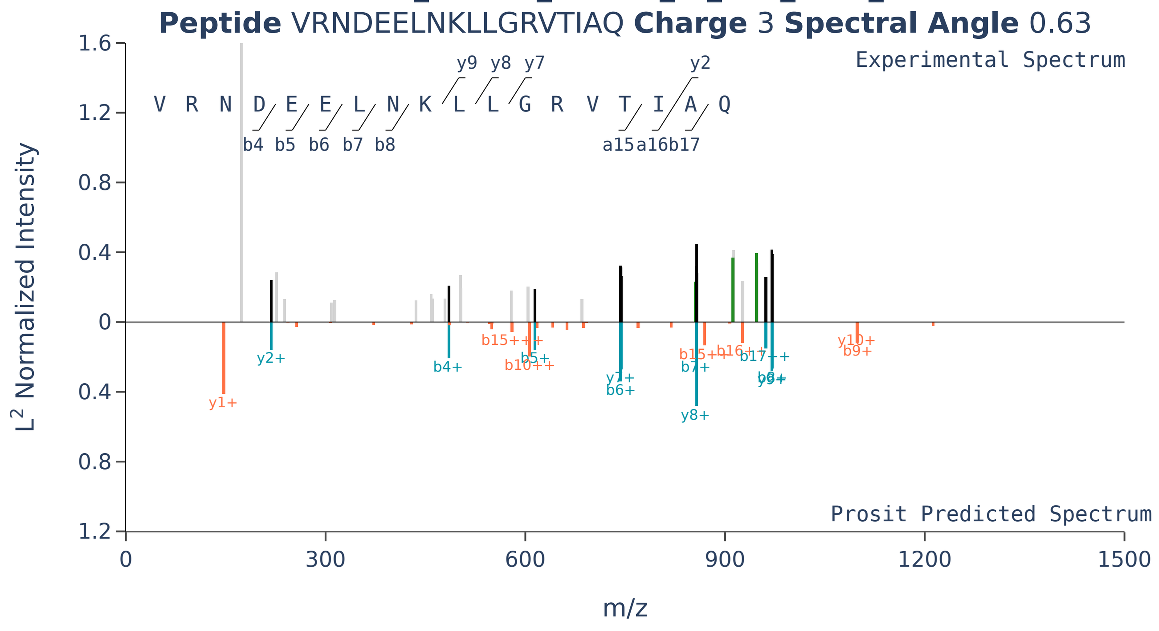


**Source** HRoetschke_291222_060123_Fu_H2A_b1R2_4h **Scan** 6815
**Peptide** AGNAARDNKKTRIIPRHLQLAVRN **Charge** 6 **Spectral Angle** 0.76

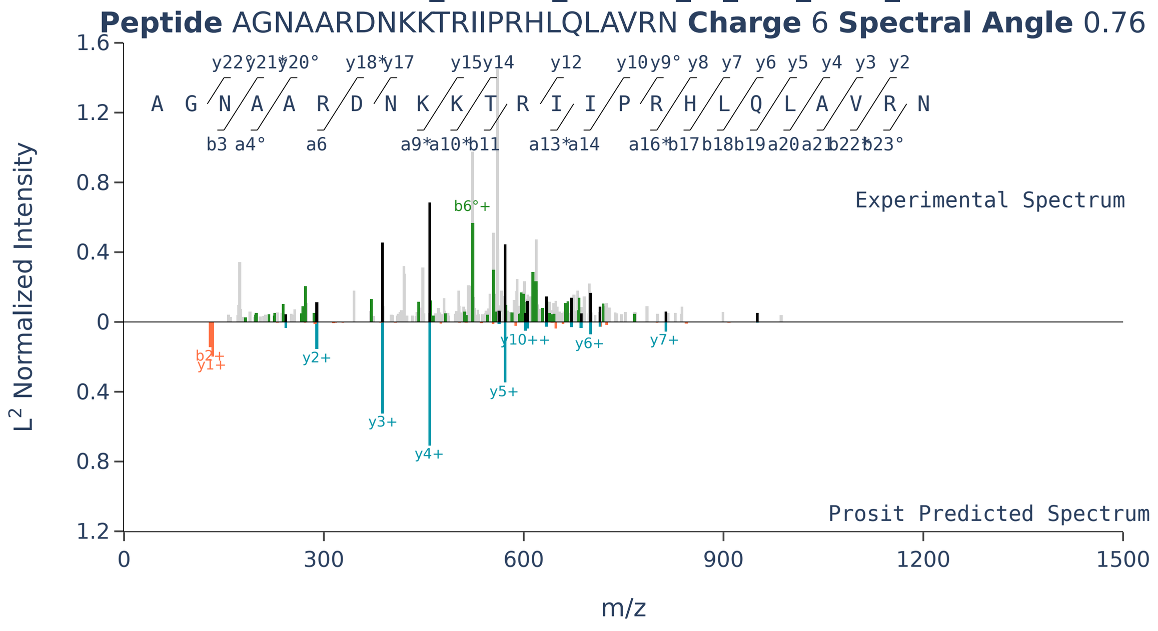


**Source** HRoetschke_291222_060123_Fu_H2A_b2R2_2h **Scan** 8043
**Peptide** PRHLQLAVRNDEELN **Charge** 4 **Spectral Angle** 0.42

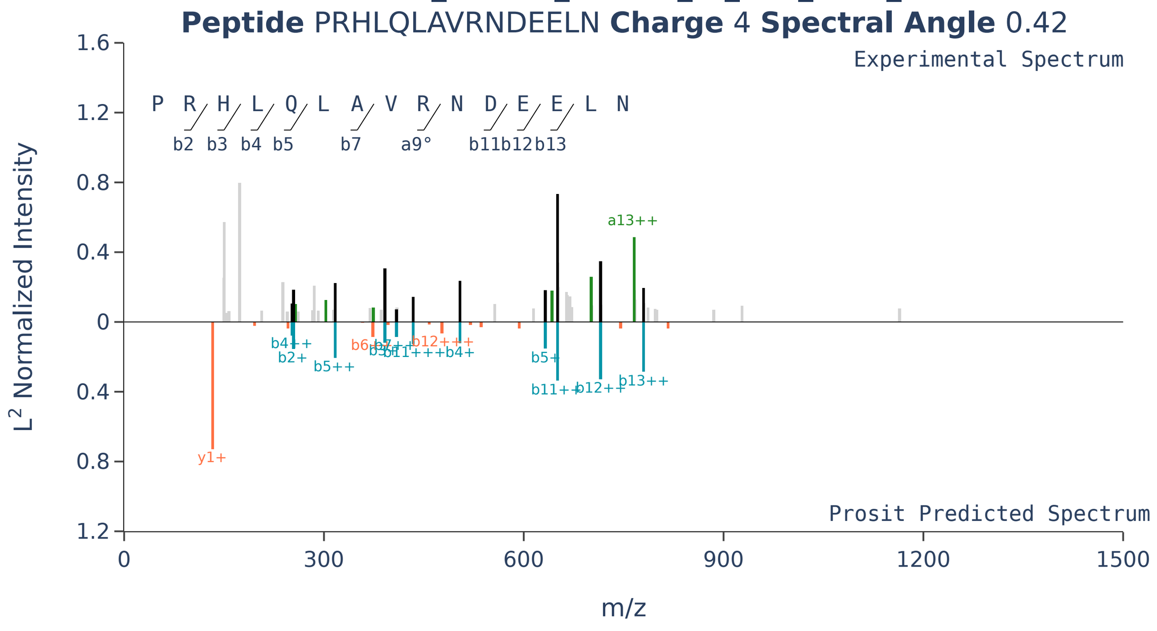


**Source** HRoetschke_291222_060123_Fu_H2A_b1R1_4h **Scan** 17940
**Peptide** NDEELNKLLGRVTIAQGG **Charge** 3 **Spectral Angle** 0.44

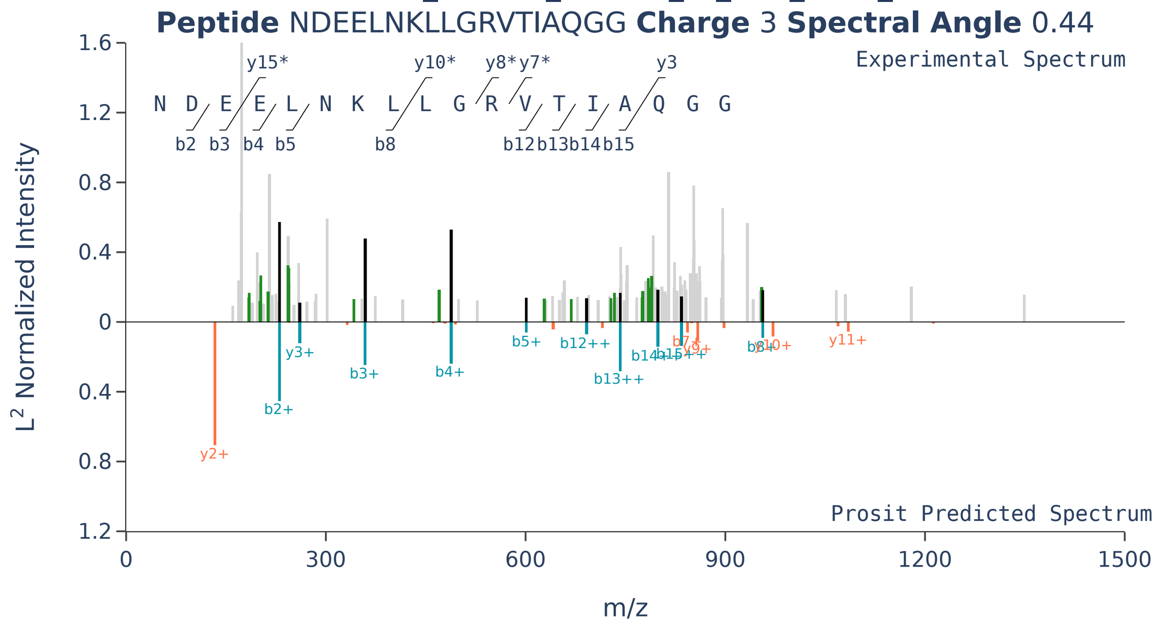

**Source** HRoetschke_291222_060123_Fu_H2A_b2R1_1h **Scan** 11122
**Peptide** ELNKLLGRVTIAQG **Charge** 2 **Spectral Angle** 0.73

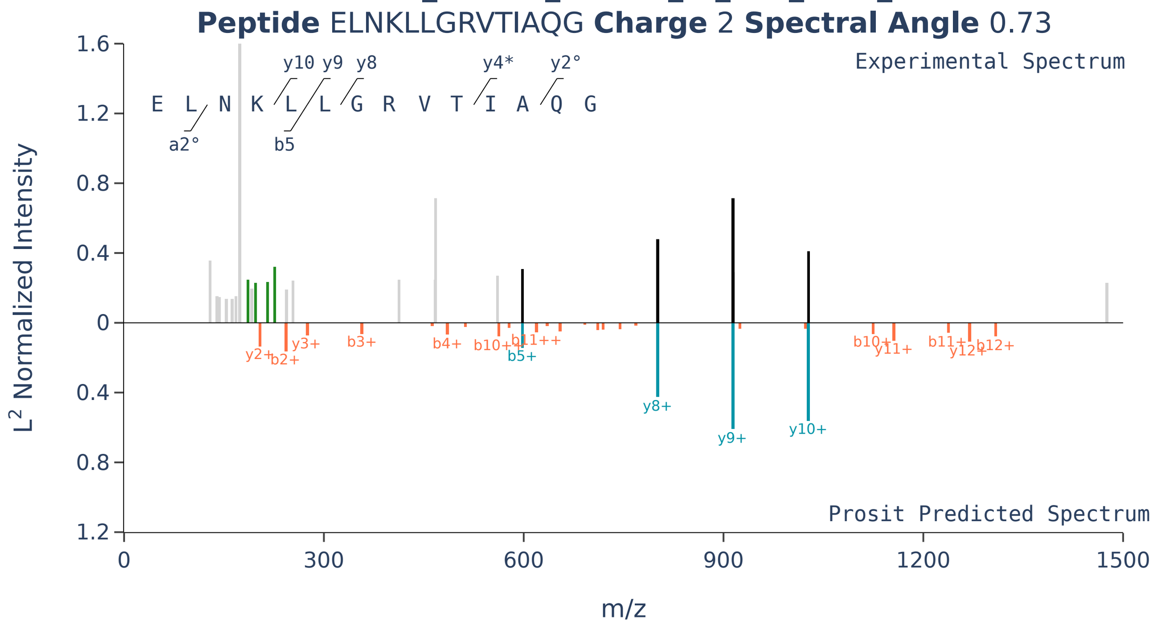


**Source** HRoetschke_291222_060123_Fu_H2A_b1R2_2h **Scan** 18629
**Peptide** AVRNDEELNKLLGRVTIAQGGVLPNL **Charge** 3 **Spectral Angle** 0.76

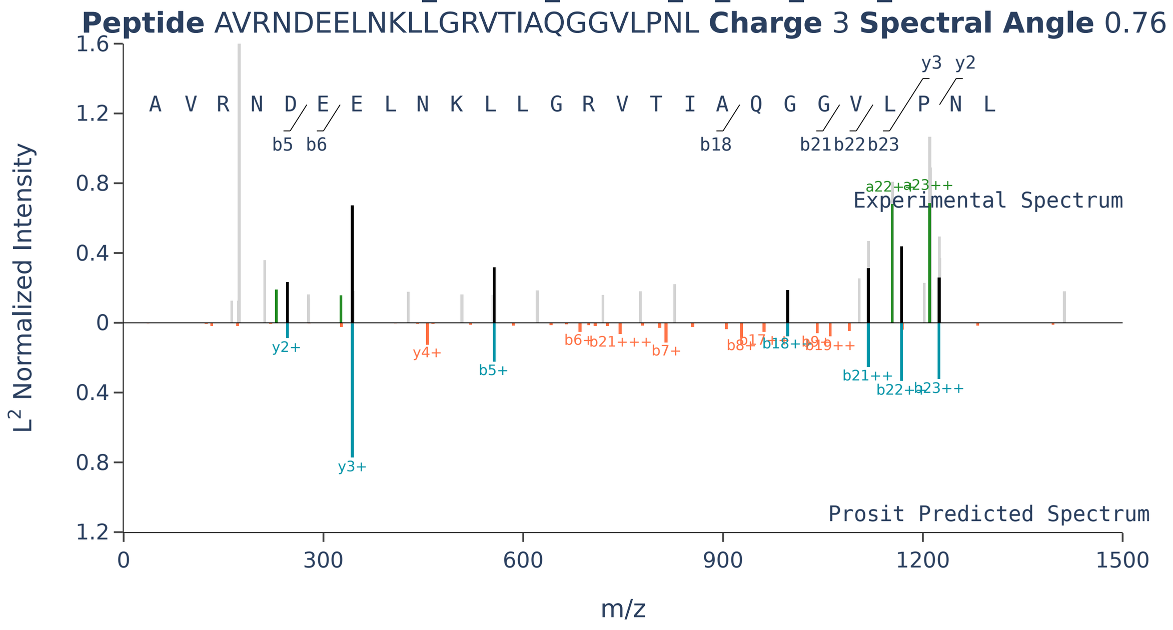


**Source** HRoetschke_291222_060123_Fu_H2A_b1R1_4h **Scan** 10154
**Peptide** LELAGNAARDNKKTRIIPRHLQLAVRND **Charge** 6 **Spectral Angle** 0.7

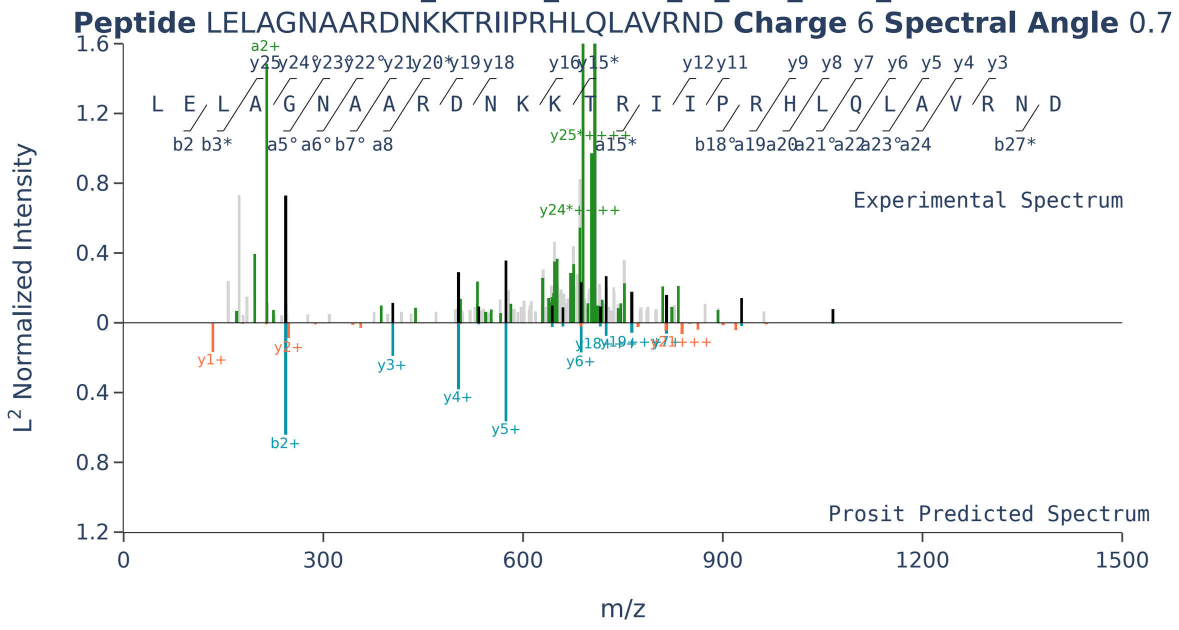


**Source** HRoetschke_291222_060123_Fu_H2A_b1R1_2h **Scan** 17502
**Peptide** LAQGGVLPNIQSVLLPKKTESSKSA **Charge** 4 **Spectral Angle** 0.66

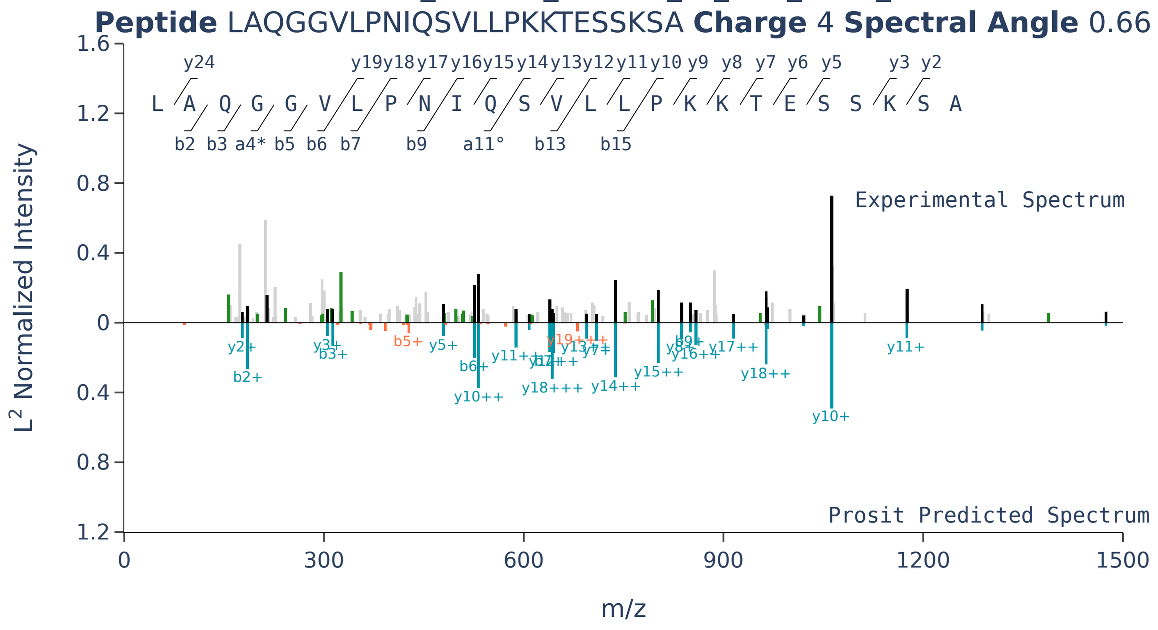


**Source** HRoetschke_291222_060123_Fu_H2A_b1R2_4h **Scan** 17502
**Peptide** TIAQGGVLPNIQSVLLPKKTESSKSAKS **Charge** 4 **Spectral Angle** 0.66

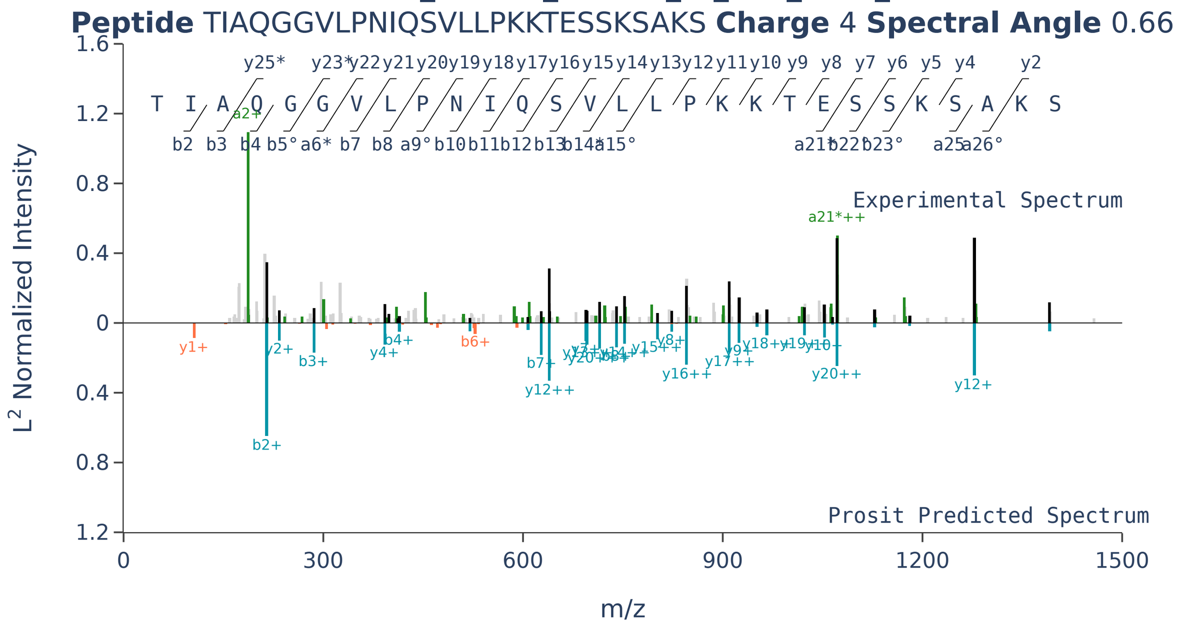


**Source** HRoetschke_291222_060123_Fu_H2A_b1R1_4h **Scan** 9649
**Peptide** RSSRAGLQFPVGRVHRLLRKG **Charge** 5 **Spectral Angle** 0.62

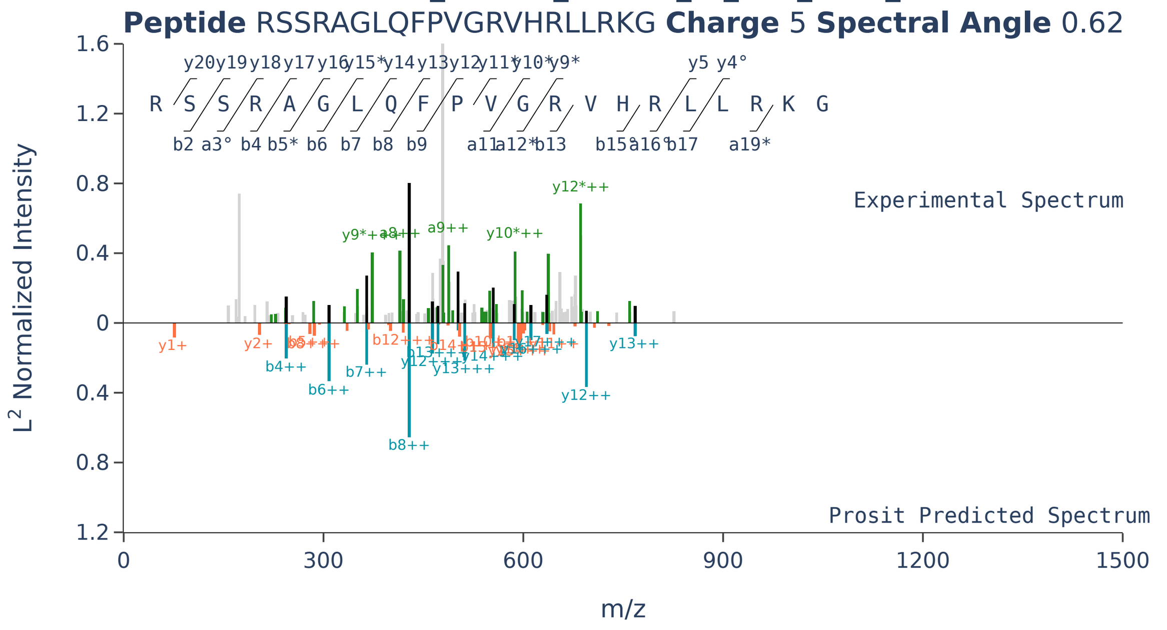


**Source** HRoetschke_291222_060123_Fu_H2A_b1R1_2h **Scan** 11336
**Peptide** SSRAGLQFPVGRVHRLLRKGNY **Charge** 6 **Spectral Angle** 0.71

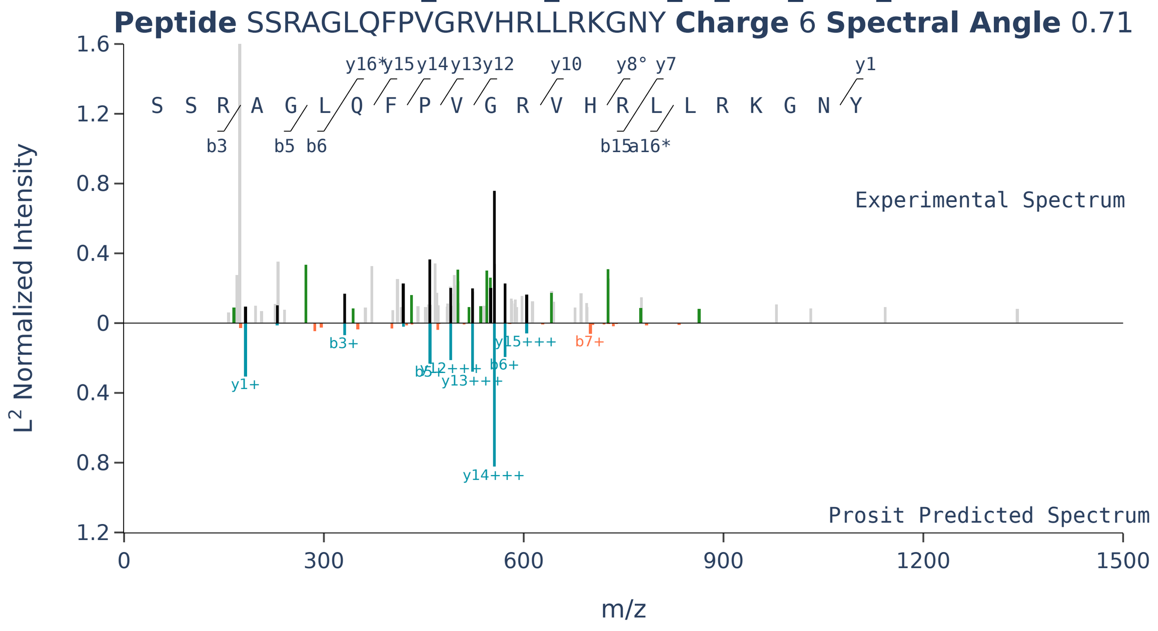


**Source** HRoetschke_291222_060123_Fu_H2A_b1R1_4h **Scan** 18890
**Peptide** TIAQGGVLPNIQSVLLPKKTESSKSA **Charge** 4 **Spectral Angle** 0.64

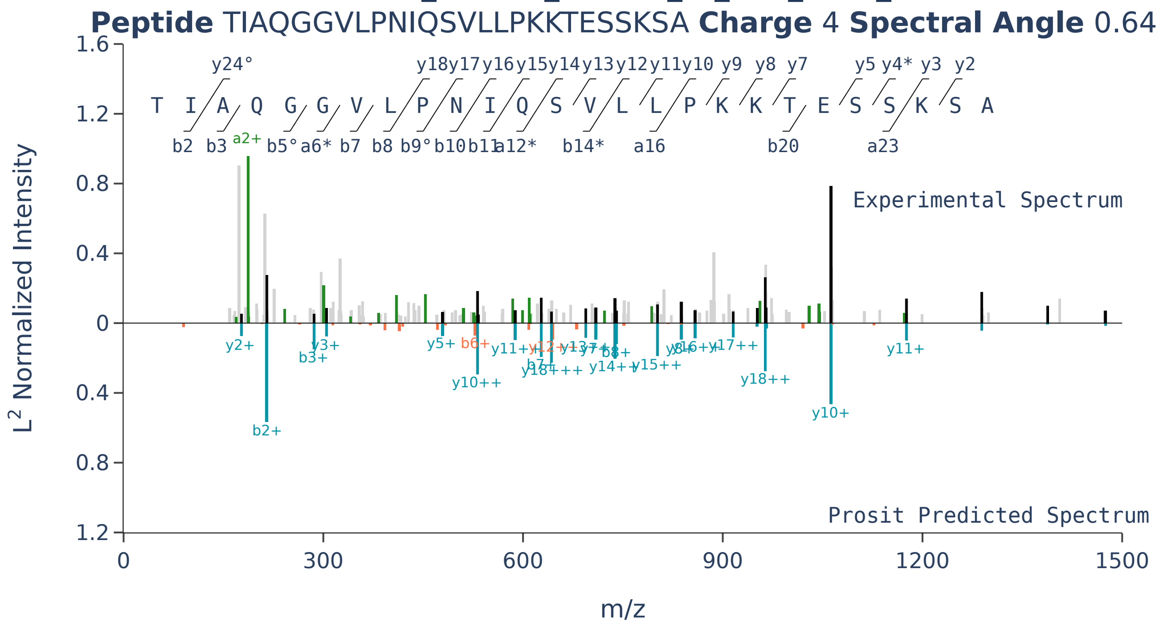


**Source** HRoetschke_291222_060123_Fu_H2A_b1R2_1h **Scan** 13256
**Peptide** GVLPNIQSVLLPKKTESSKSAKS **Charge** 4 **Spectral Angle** 0.58

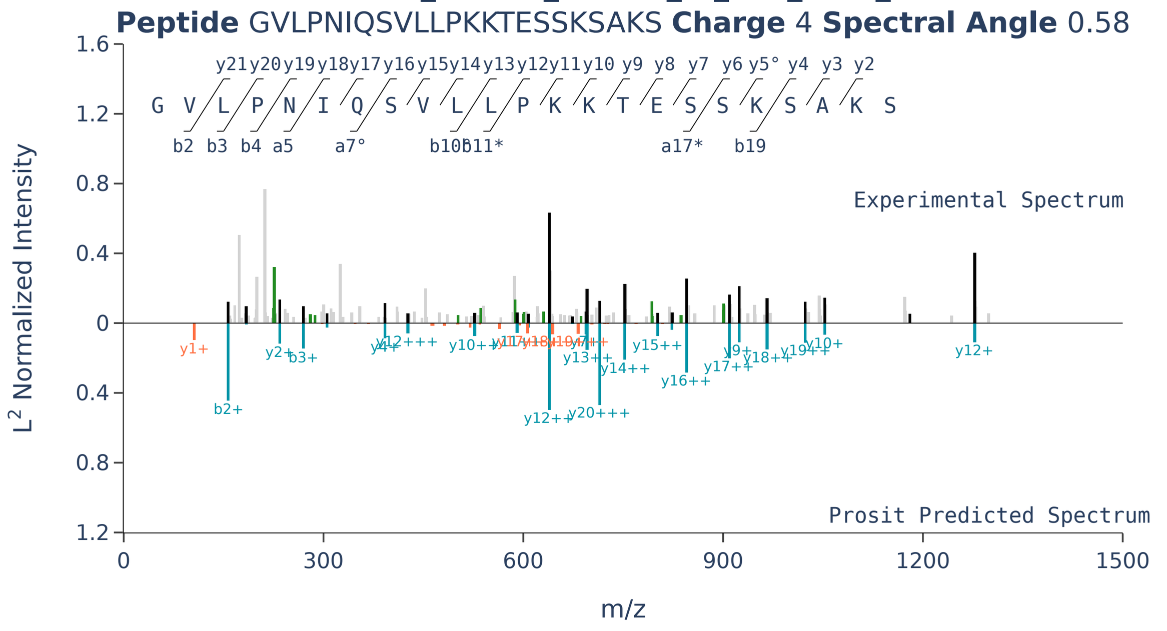


**Source** HRoetschke_291222_060123_Fu_H2A_b2R1_24h **Scan** 10954
**Peptide** AEILELAGNAARDNKKT **Charge** 3 **Spectral Angle** 0.73

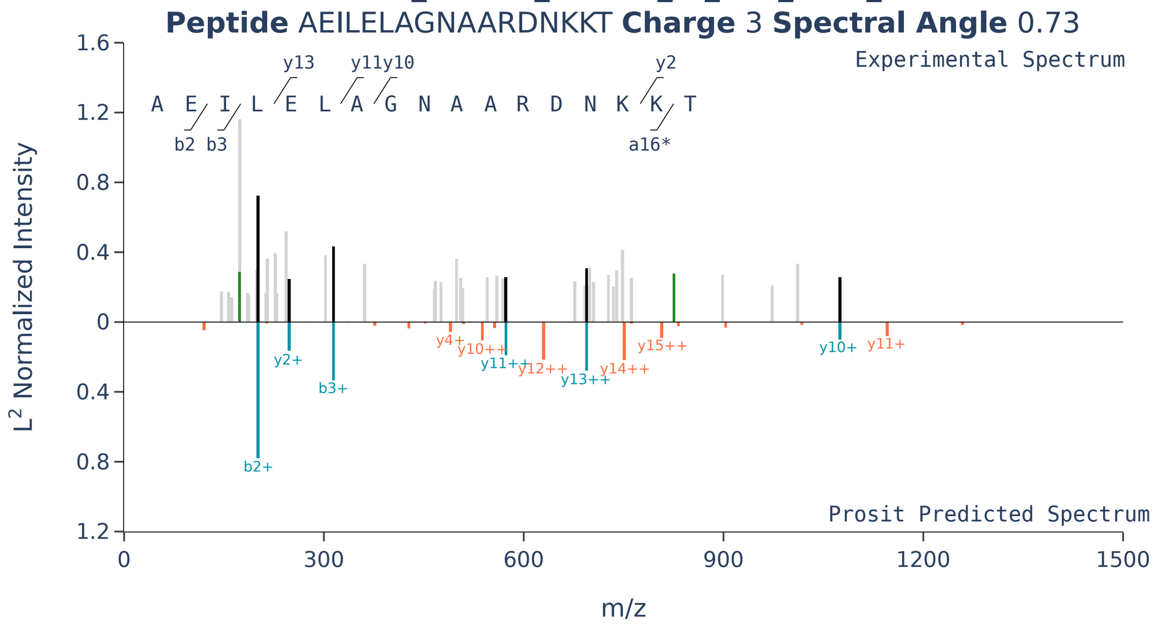


**Source** HRoetschke_291222_060123_Fu_H2A_b1R2_1h **Scan** 14052
**Peptide** KLLGRVTIAQGGVLPNLQ **Charge** 2 **Spectral Angle** 0.64

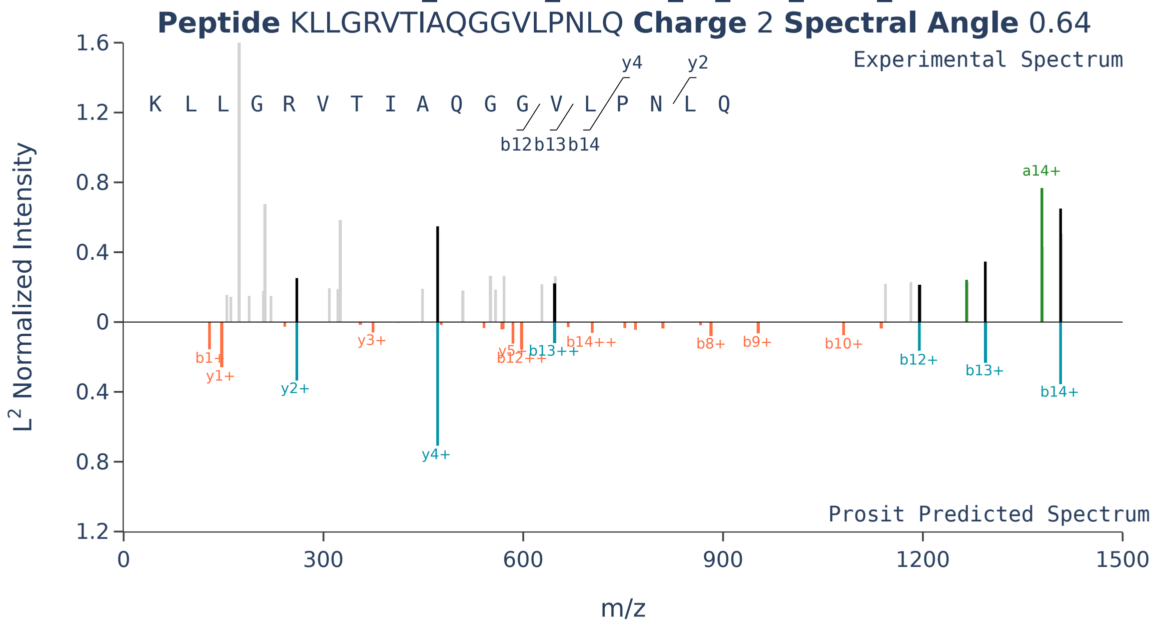


**Source** HRoetschke_291222_060123_Fu_H2A_b2R1_4h **Scan** 6715
**Peptide** GKTRAKAKTRSSRAGLQFPVGRVHRL **Charge** 6 **Spectral Angle** 0.78

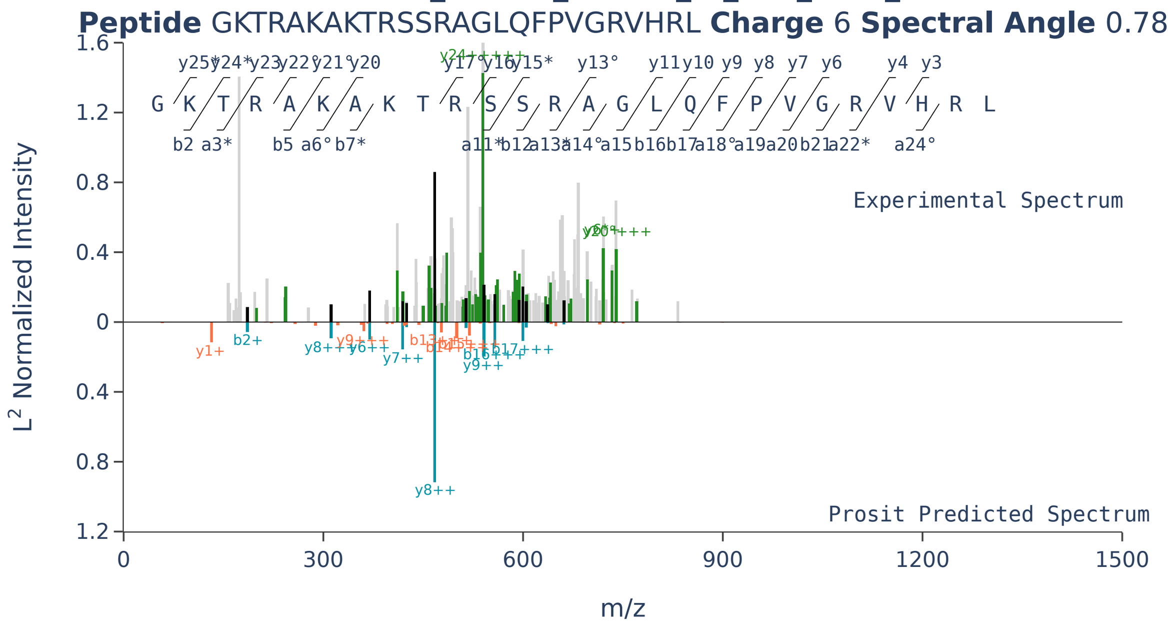


**Source** HRoetschke_291222_060123_Fu_H2A_b1R1_4h **Scan** 19493
**Peptide** RVTIAQGGVLPNIQSVLLPKKT **Charge** 4 **Spectral Angle** 0.75

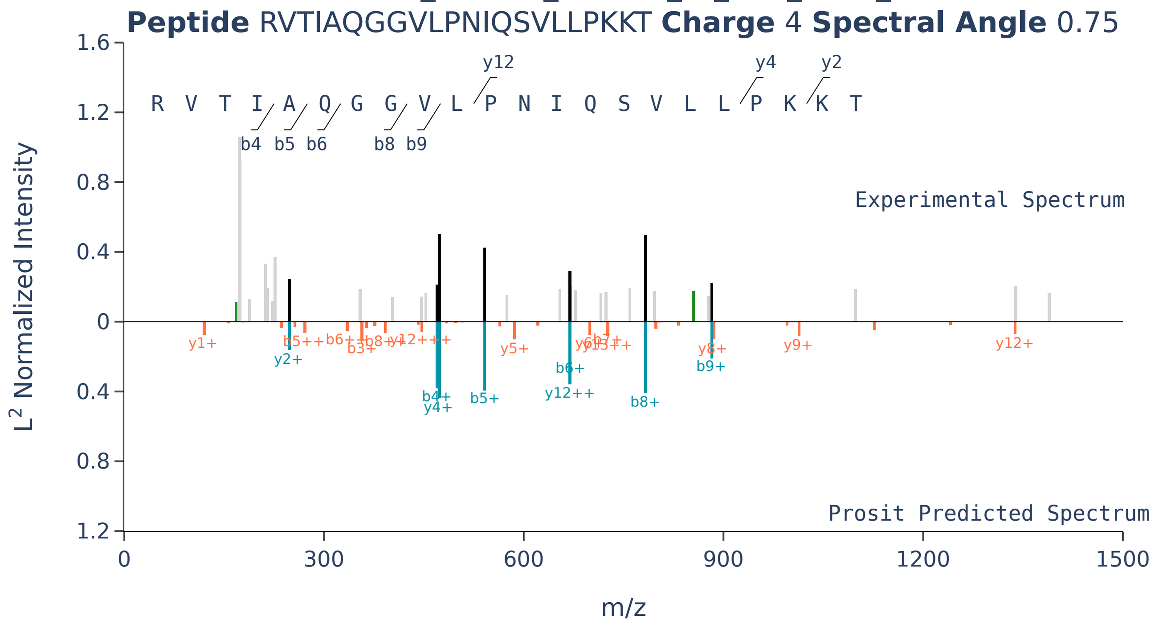


**Source** HRoetschke_291222_060123_Fu_H2A_b1R1_2h **Scan** 8018
**Peptide** LAGNAARDNKKTRIIPRHLQLAVRNDE **Charge** 6 **Spectral Angle** 0.61

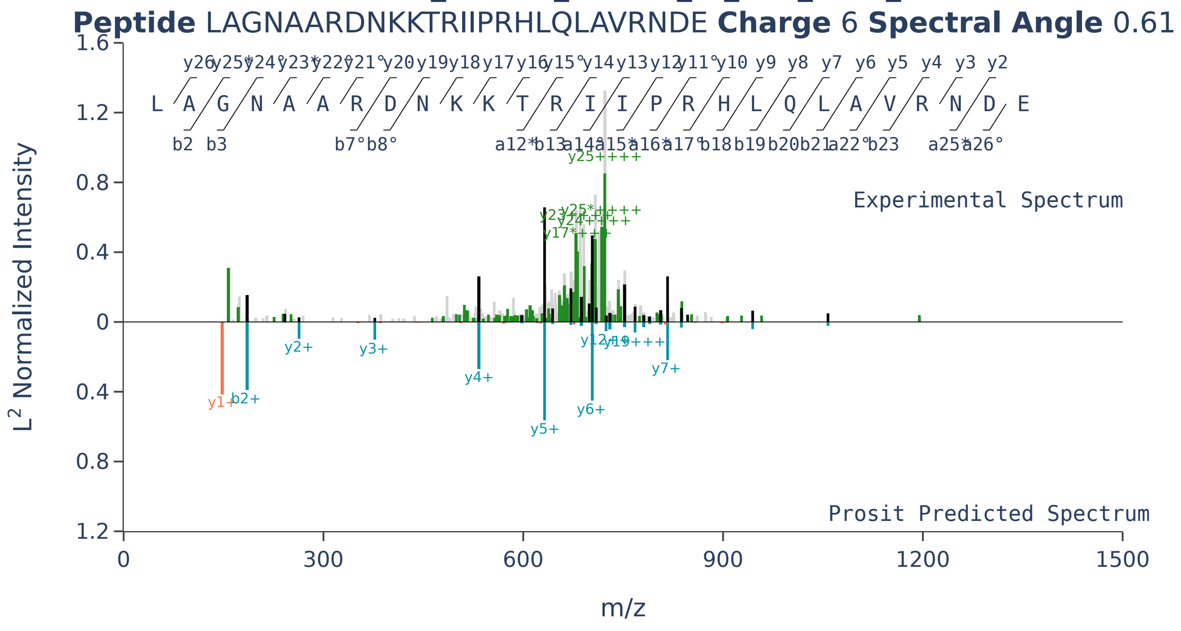


**Source** HRoetschke_291222_060123_Fu_H2A_b1R2_24h **Scan** 3568
**Peptide** LLRKGNYA **Charge** 3 **Spectral Angle** 0.52

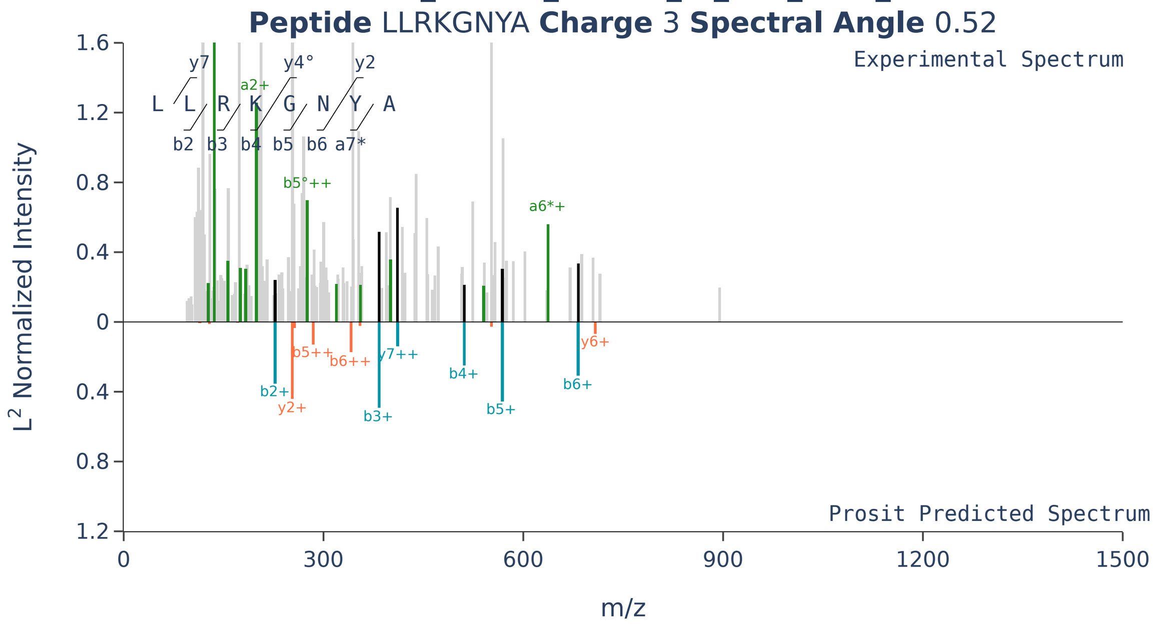

**Source** HRoetschke_291222_060123_Fu_H2A_b1R1_4h **Scan** 18877
**Peptide** LTAEILELAGNAARDNKKTRIIPRHLQL **Charge** 5 **Spectral Angle** 0.83

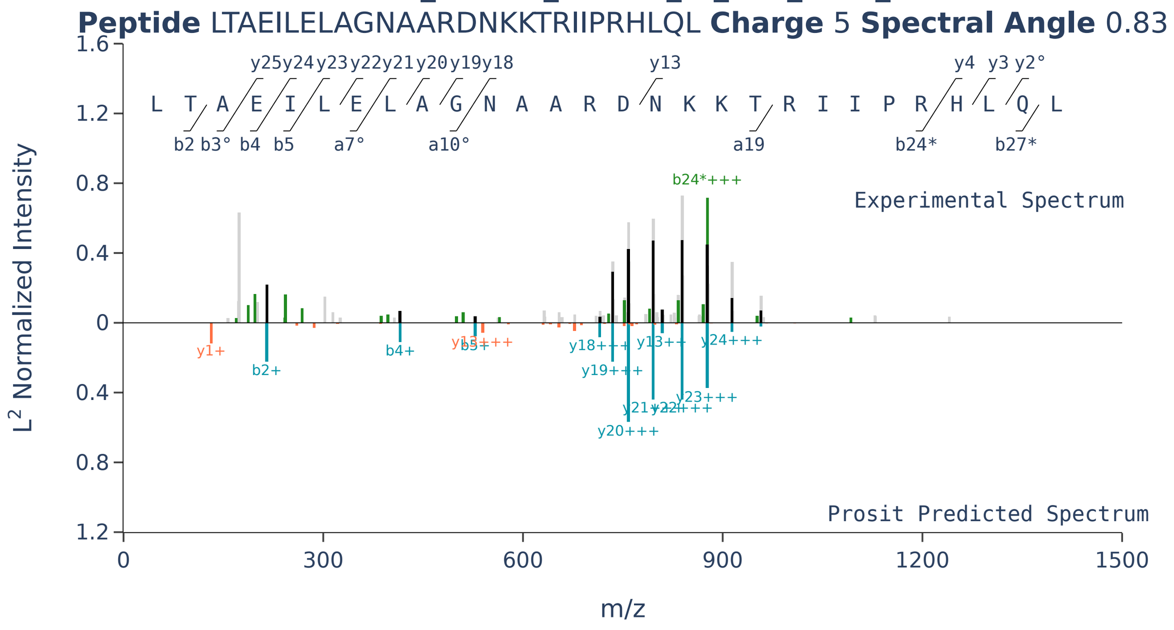


**Source** HRoetschke_291222_060123_Fu_H2A_b1R1_2h **Scan** 2455
**Peptide** RKGNYAERVGA **Charge** 2 **Spectral Angle** 0.35

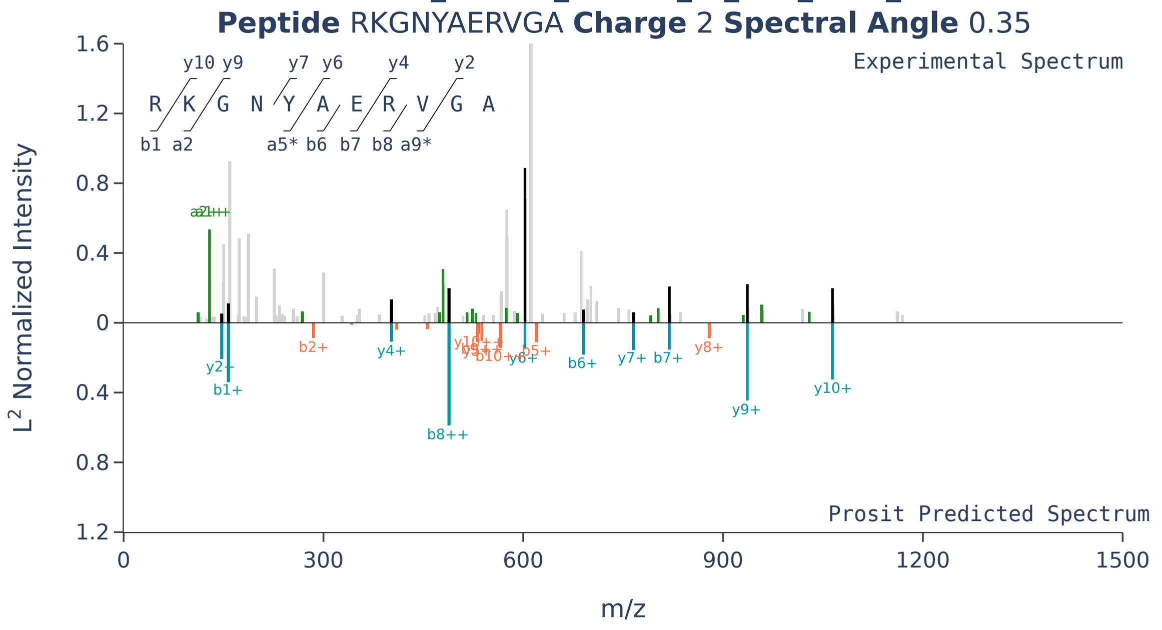


**Source** HRoetschke_291222_060123_Fu_H2A_b1R1_4h **Scan** 16701
**Peptide** LAVRNDEELNKLLGRV **Charge** 4 **Spectral Angle** 0.72

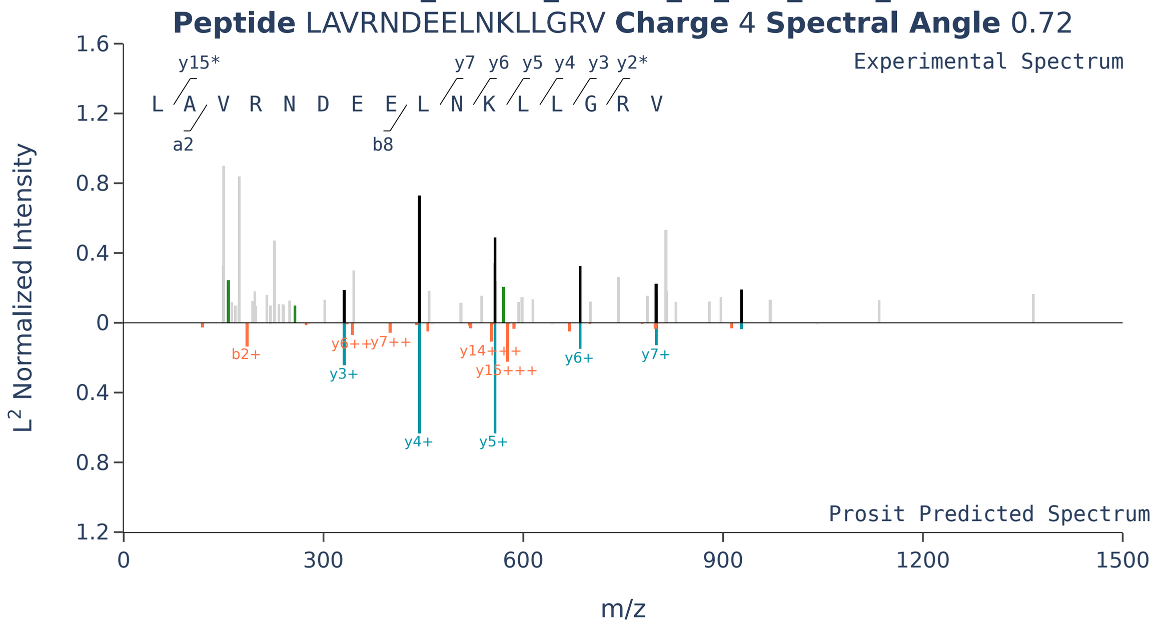


**Source** HRoetschke_291222_060123_Fu_H2A_b1R1_2h **Scan** 17884
**Peptide** PRHLQLAVRNDEELNKLLGRVTL **Charge** 5 **Spectral Angle** 0.75

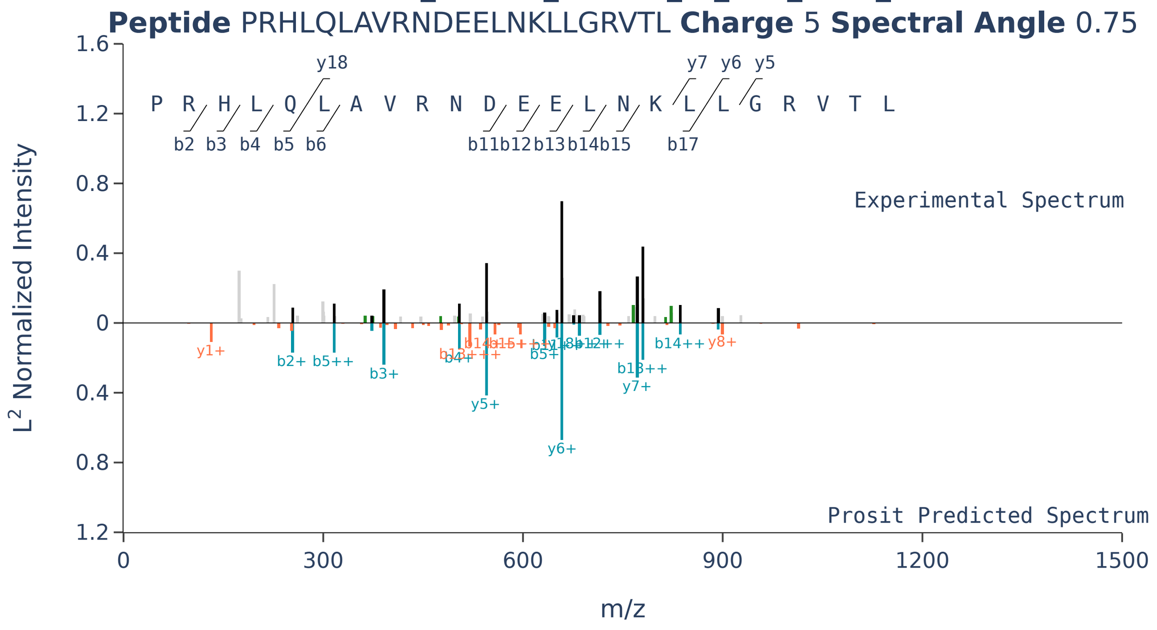


**Source** HRoetschke_291222_060123_Fu_H2A_b2R1_4h **Scan** 7693
**Peptide** LAGNAARDNKKTRIIPRHLQLAVRN **Charge** 6 **Spectral Angle** 0.59

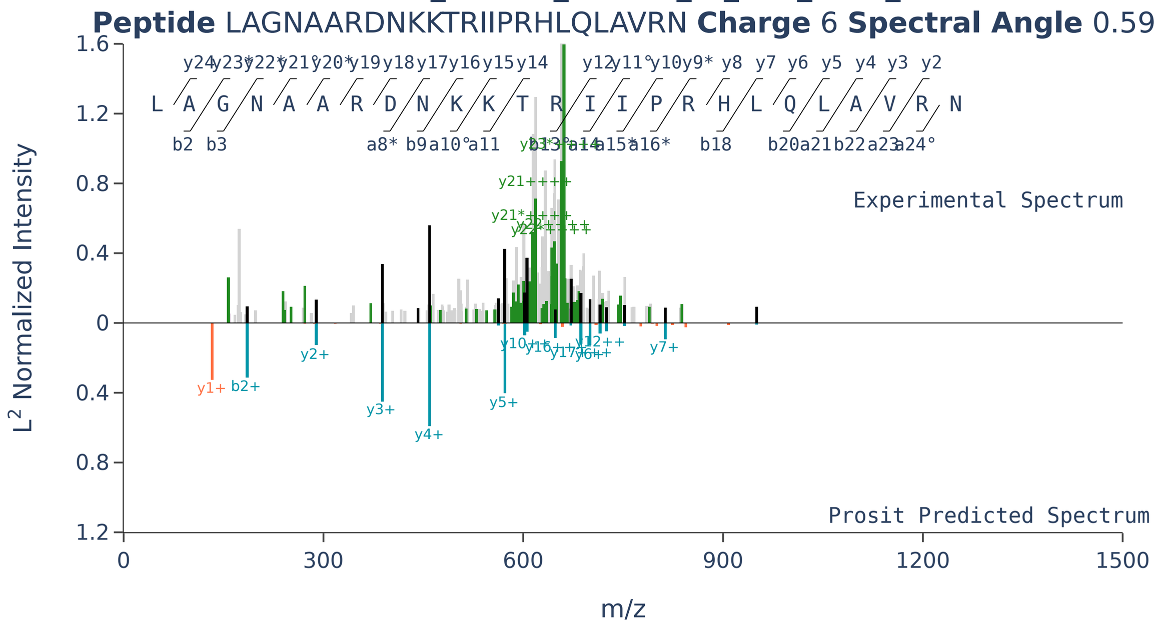


**Source** HRoetschke_291222_060123_Fu_H2A_b1R1_4h **Scan** 20205
**Peptide** YLTAEILELAGNAARDNKKTRLL **Charge** 5 **Spectral Angle** 0.71

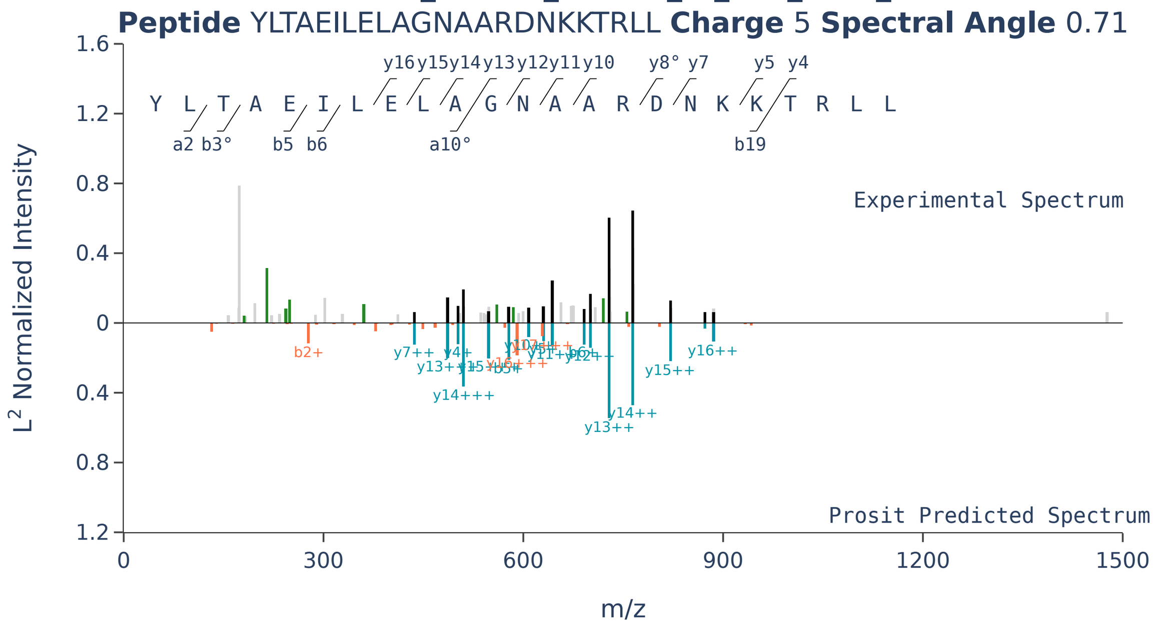


**Source** HRoetschke_291222_060123_Fu_H2A_b2R2_24h **Scan** 2680
**Peptide** PVGRVHRLLRKGN **Charge** 3 **Spectral Angle** 0.68

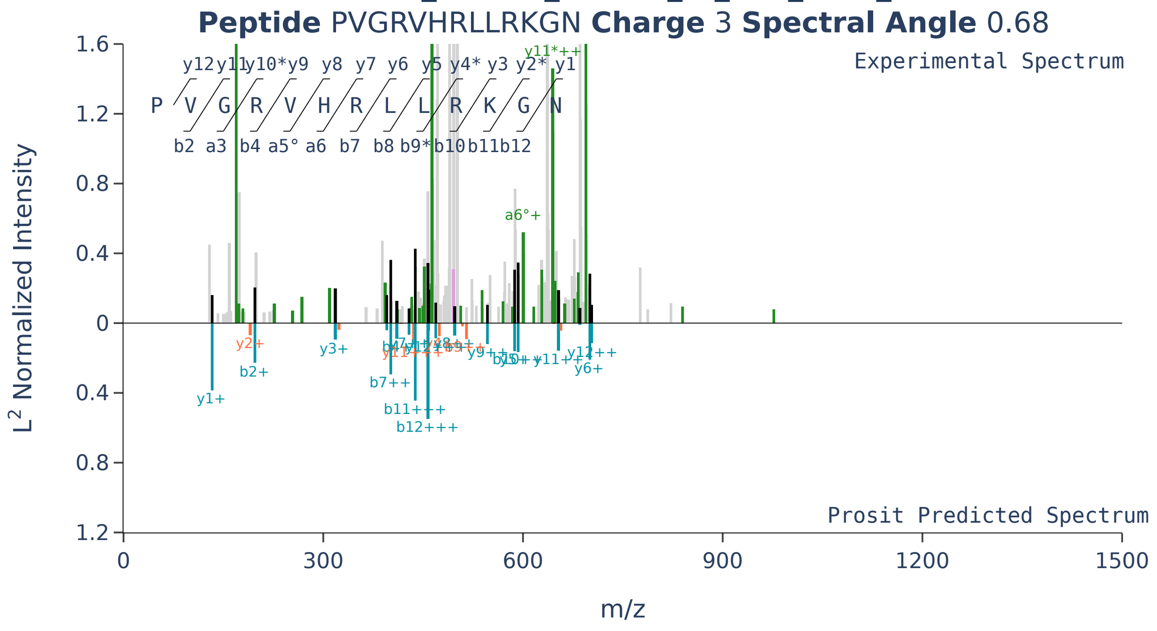


**Source** HRoetschke_291222_060123_Fu_H2A_b2R2_2h **Scan** 2244
**Peptide** RKGNYAERVGAG **Charge** 3 **Spectral Angle** 0.7

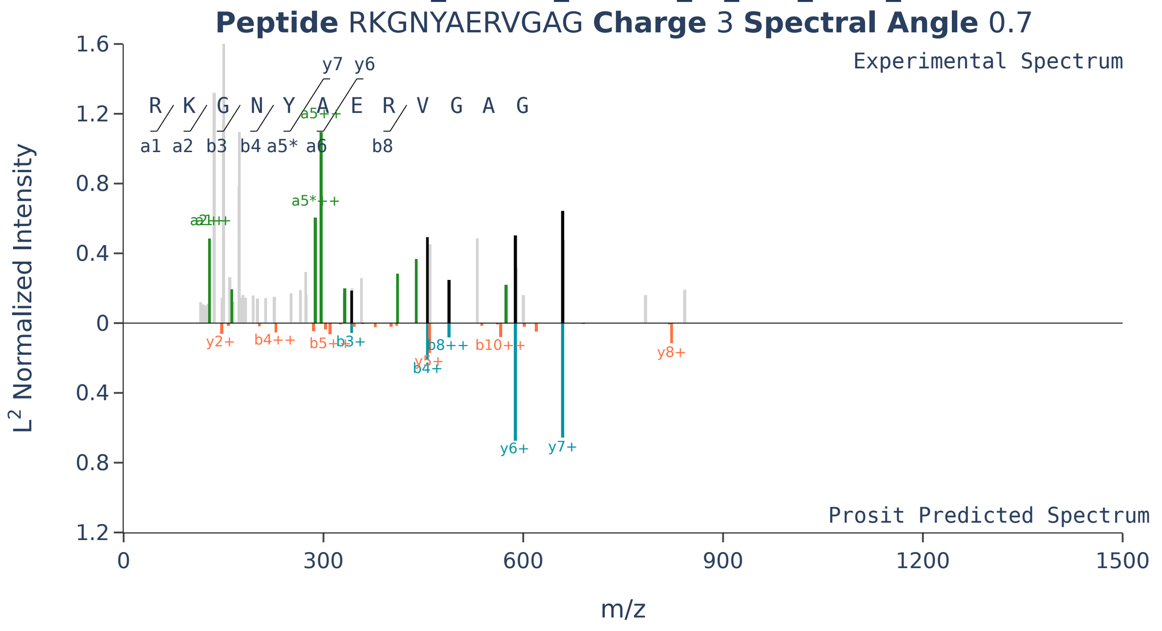


**Source** HRoetschke_291222_060123_Fu_H2A_b1R2_2h **Scan** 11788
**Peptide** LLELAGNAARDNKKTRIIPRHLQLAVR **Charge** 5 **Spectral Angle** 0.62

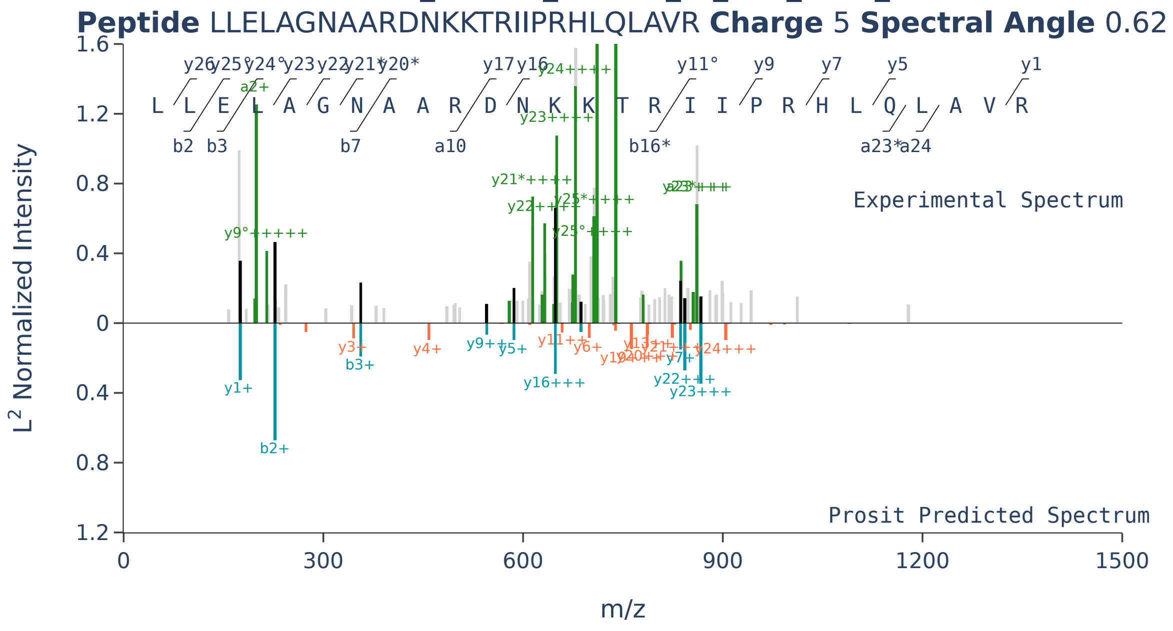


**Source** HRoetschke_291222_060123_Fu_H2A_b1R1_4h **Scan** 9636
**Peptide** RSSRAGLQFPVGRVHRLLRK **Charge** 6 **Spectral Angle** 0.77

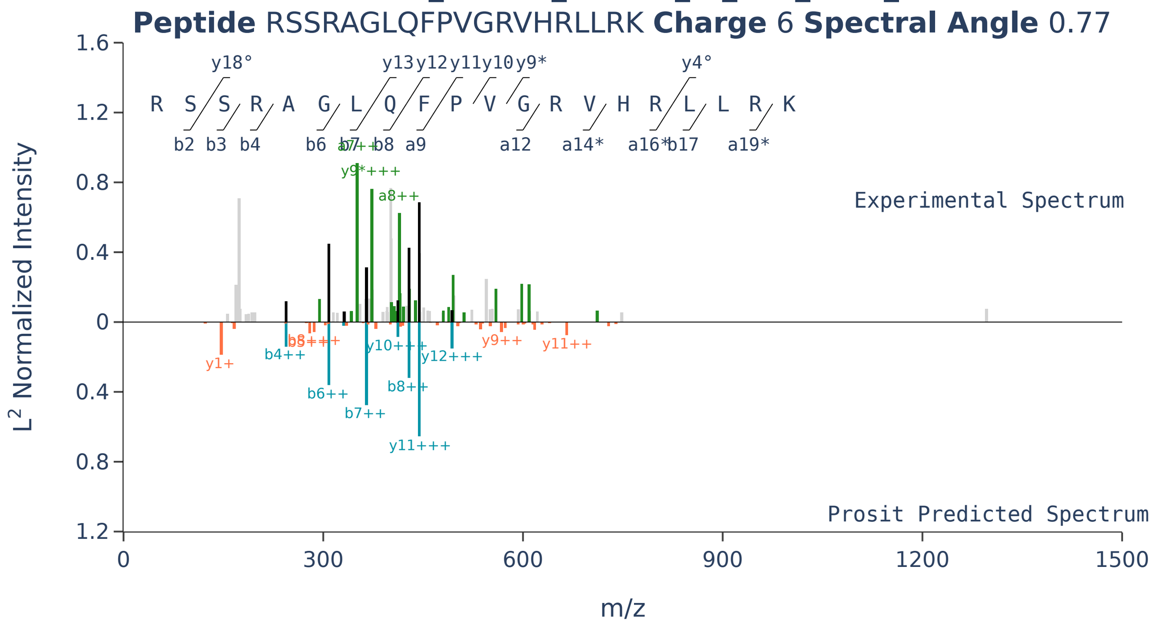


**Source** HRoetschke_291222_060123_Fu_H2A_b2R1_4h **Scan** 8741
**Peptide** ELAGNAARDNKKTRIIPRHLQLAVRND **Charge** 6 **Spectral Angle** 0.67

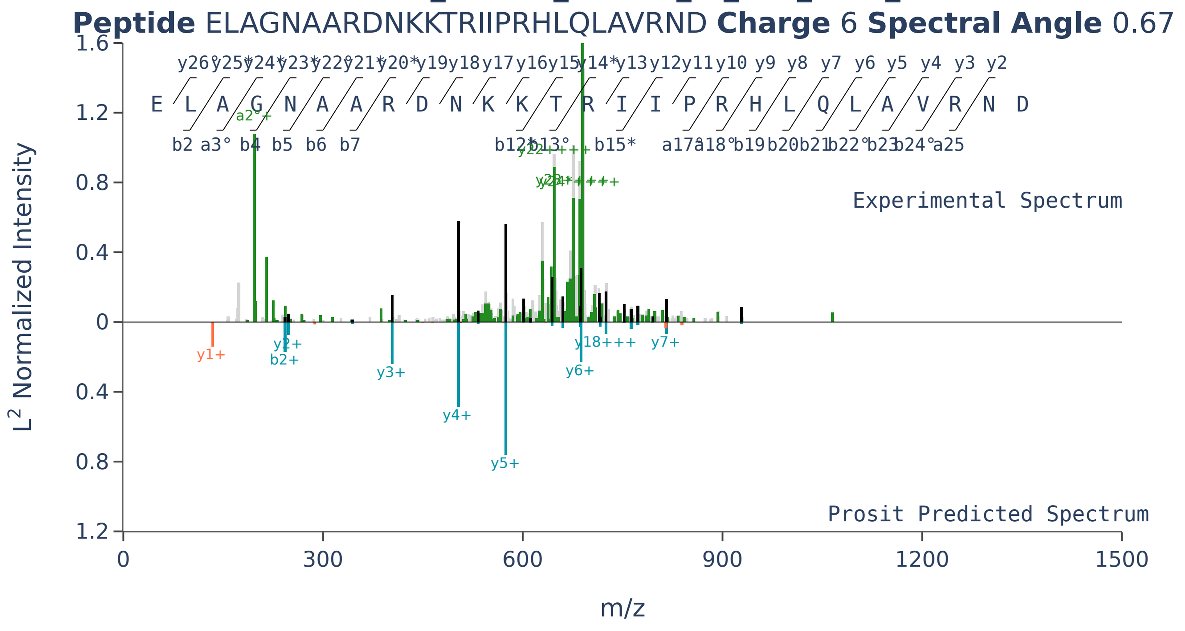


**Source** HRoetschke_291222_060123_Fu_H2A_b1R2_2h **Scan** 3900
**Peptide** PVGRVHRLLRKGNY **Charge** 4 **Spectral Angle** 0.66

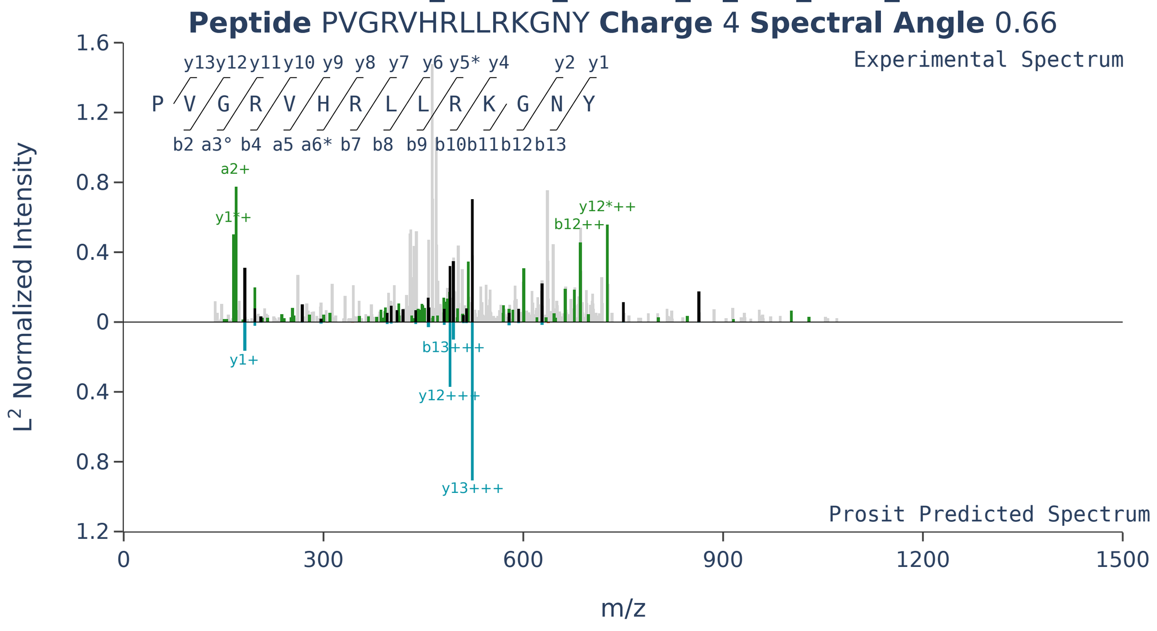


**Source** HRoetschke_291222_060123_Fu_H2A_b2R2_24h **Scan** 15710
**Peptide** GGVLPNIQSV **Charge** 2 **Spectral Angle** 0.72

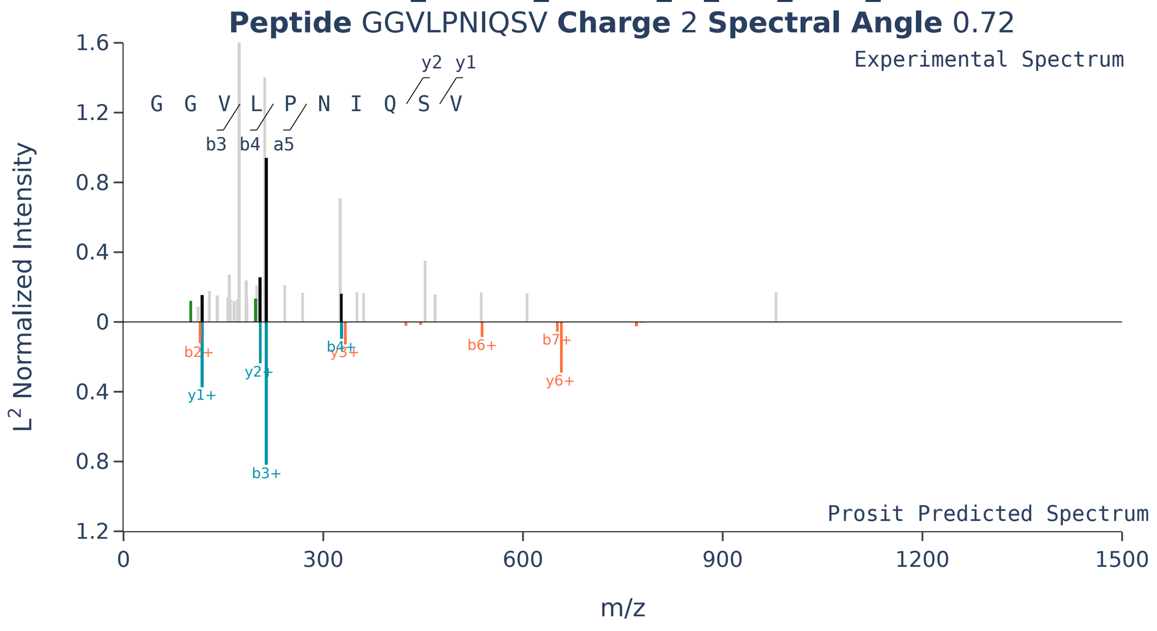


**Source** HRoetschke_291222_060123_Fu_H2A_b1R1_4h **Scan** 16662
**Peptide** PRHLQLAVRNDEELNKLLGRV **Charge** 5 **Spectral Angle** 0.65

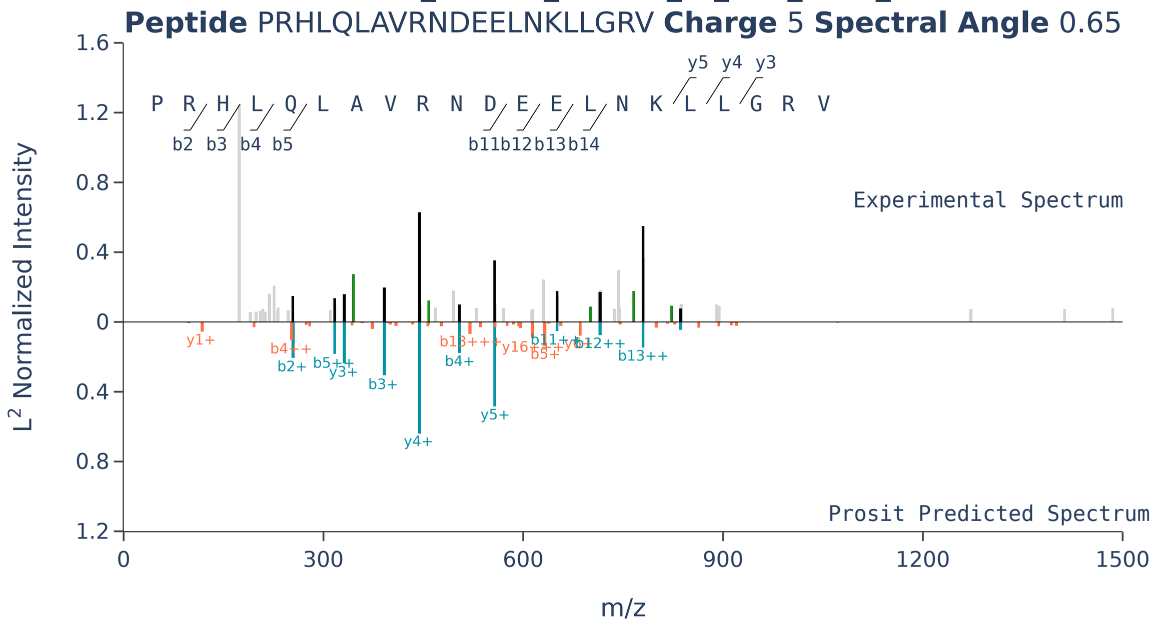


**Source** HRoetschke_291222_060123_Fu_H2A_b2R2_4h **Scan** 3647
**Peptide** SGRGKQGGKTRAKAKTRSSRAGLQFPVGR **Charge** 6 **Spectral Angle** 0.69

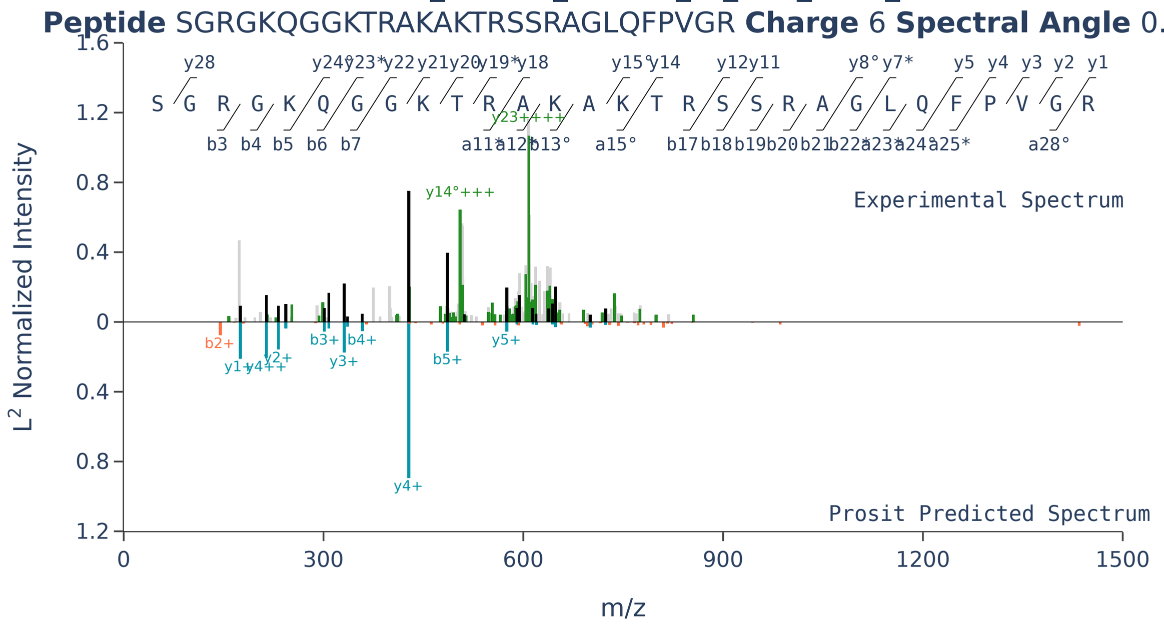

**Source** HRoetschke_291222_060123_Fu_H2A_b1R1_4h **Scan** 12229
**Peptide** RAGLQFPVGRVHRLLRKGNYAERVGA **Charge** 6 **Spectral Angle** 0.67

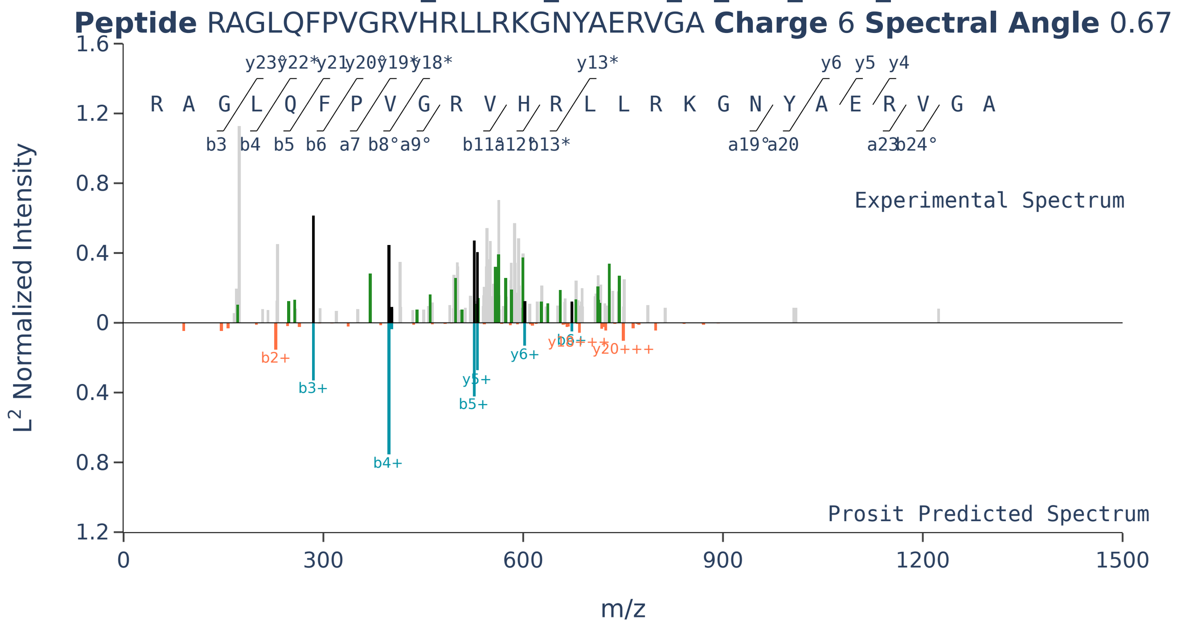


**Source** HRoetschke_291222_060123_Fu_H2A_b1R1_4h **Scan** 9433
**Peptide** AARDNKKTRIIPRHLQLAV **Charge** 4 **Spectral Angle** 0.65

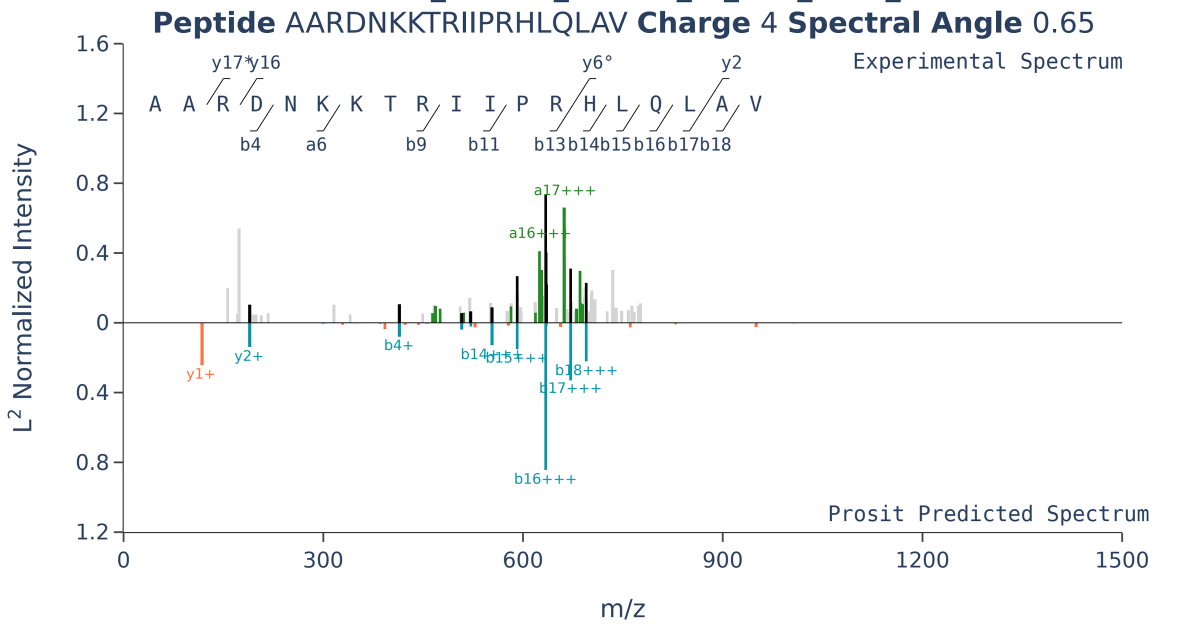


**Source** HRoetschke_291222_060123_Fu_H2A_b2R2_2h **Scan** 6135
**Peptide** KTRAKAKTRSSRAGLQFPVG **Charge** 4 **Spectral Angle** 0.7

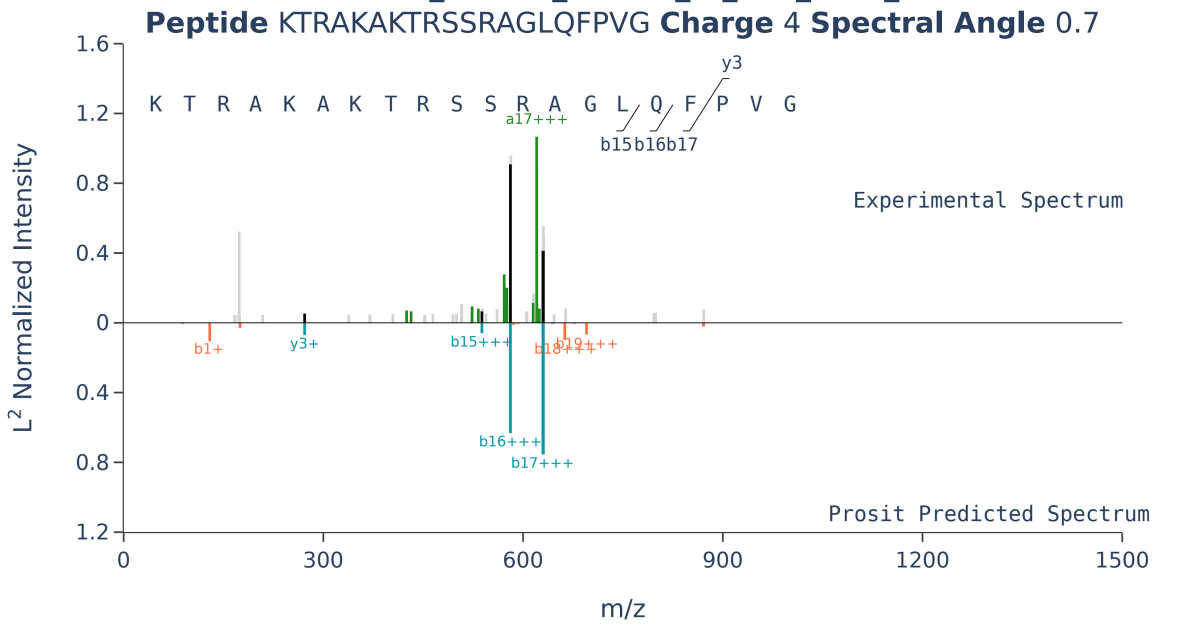


**Source** HRoetschke_291222_060123_Fu_H2A_b1R1_4h **Scan** 10627
**Peptide** SRAGLQFPVGRVHRLLRK **Charge** 5 **Spectral Angle** 0.63

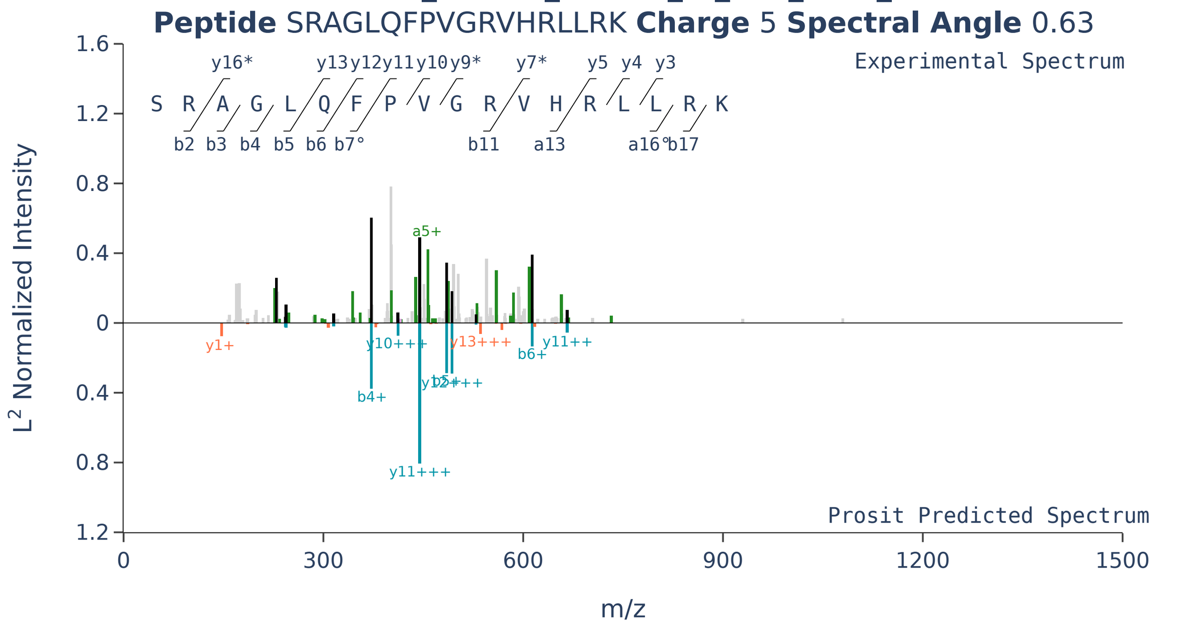


**Source** HRoetschke_291222_060123_Fu_H2A_b1R2_4h **Scan** 9469
**Peptide** KKTRIIPRHLQLAV **Charge** 4 **Spectral Angle** 0.54

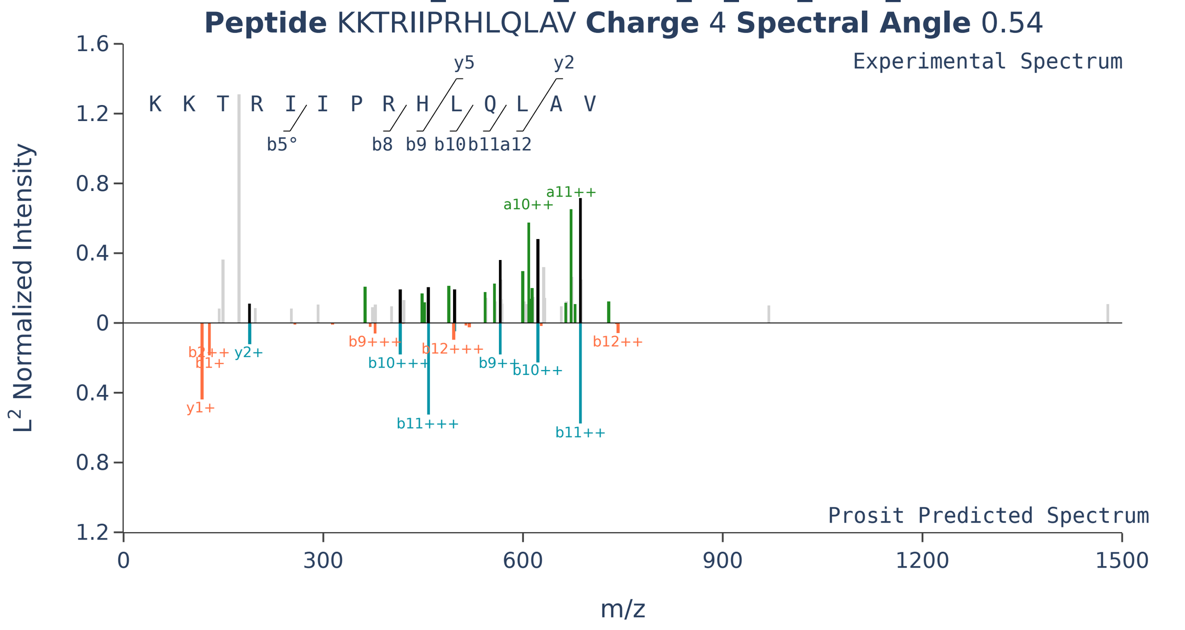


**Source** HRoetschke_291222_060123_Fu_H2A_b1R1_4h **Scan** 9657
**Peptide** KTRSSRAGLQFPVGRVHRLLR **Charge** 6 **Spectral Angle** 0.55

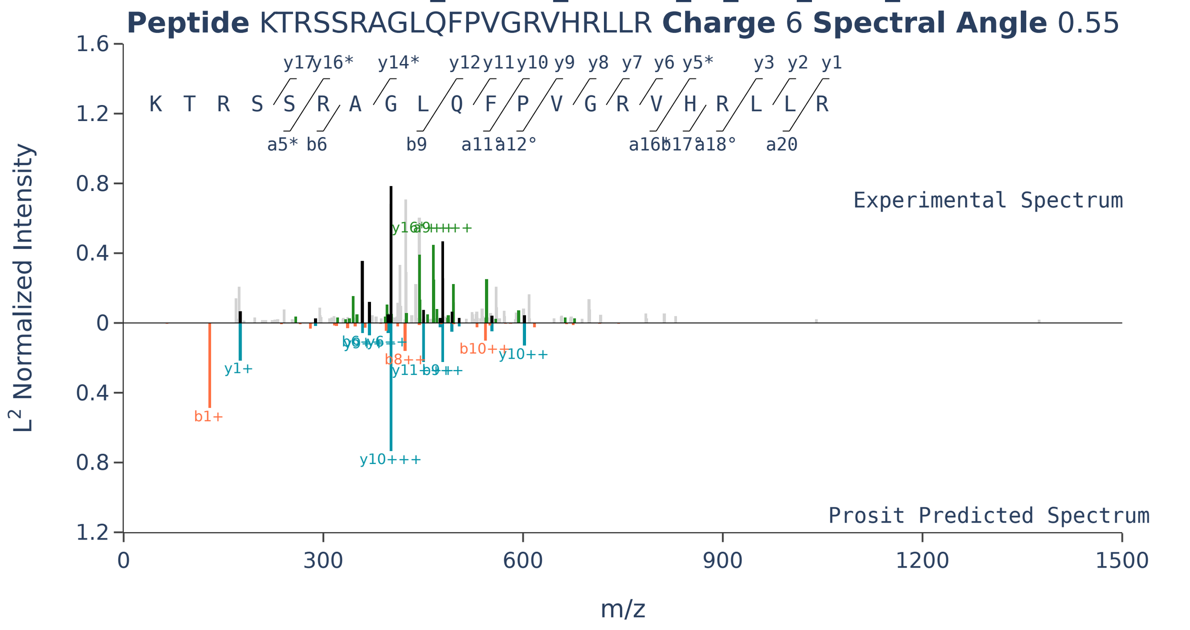


**Source** HRoetschke_291222_060123_Fu_H2A_b2R1_24h **Scan** 12134
**Peptide** ELLELAGNAARDN **Charge** 2 **Spectral Angle** 0.61

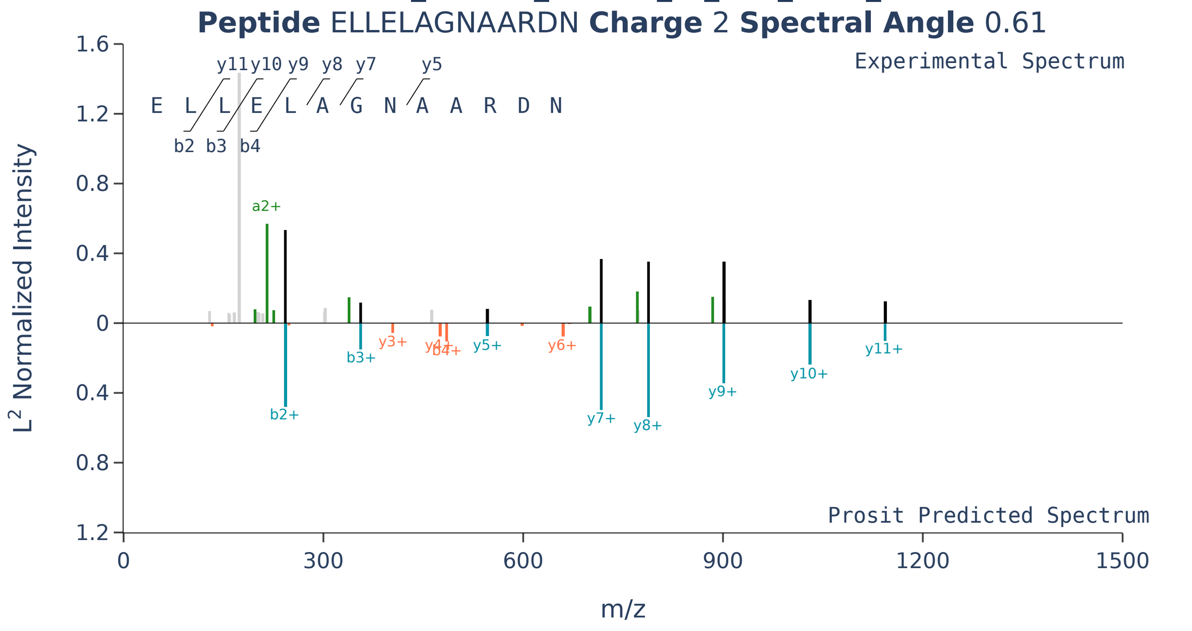


**Source** HRoetschke_291222_060123_Fu_H2A_b1R1_2h **Scan** 18544
**Peptide** LQLAVRNDEELNKLLGRVTIAQG **Charge** 4 **Spectral Angle** 0.72

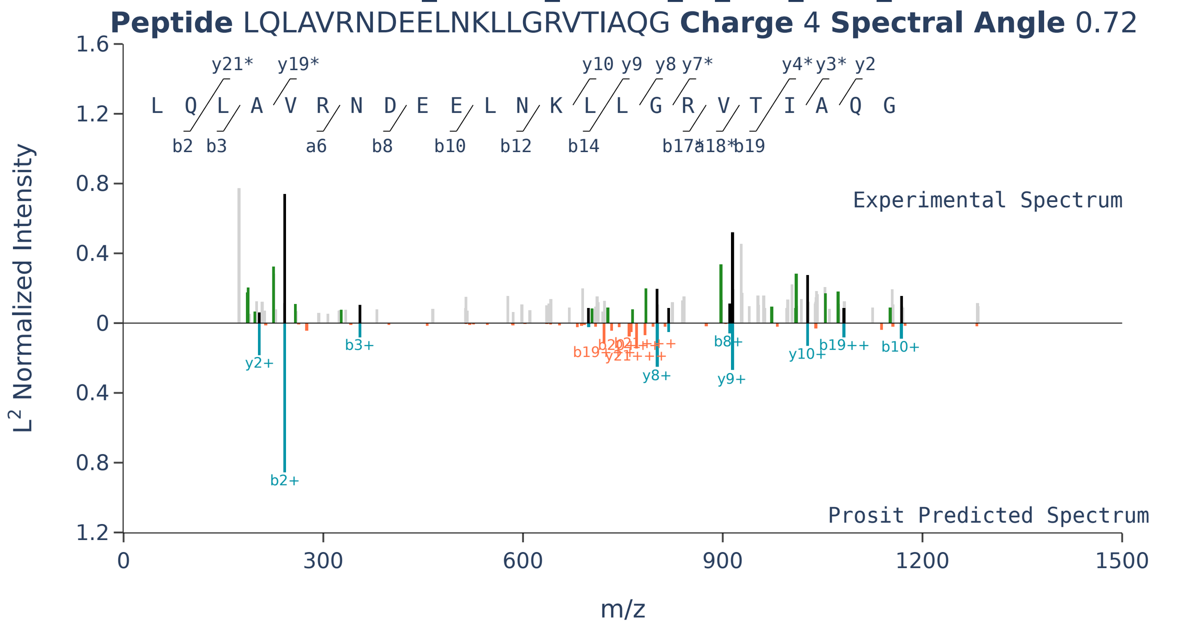


**Source** HRoetschke_291222_060123_Fu_H2A_b1R2_4h **Scan** 12438
**Peptide** SRAGLQFPVGRVHRLLR **Charge** 5 **Spectral Angle** 0.67

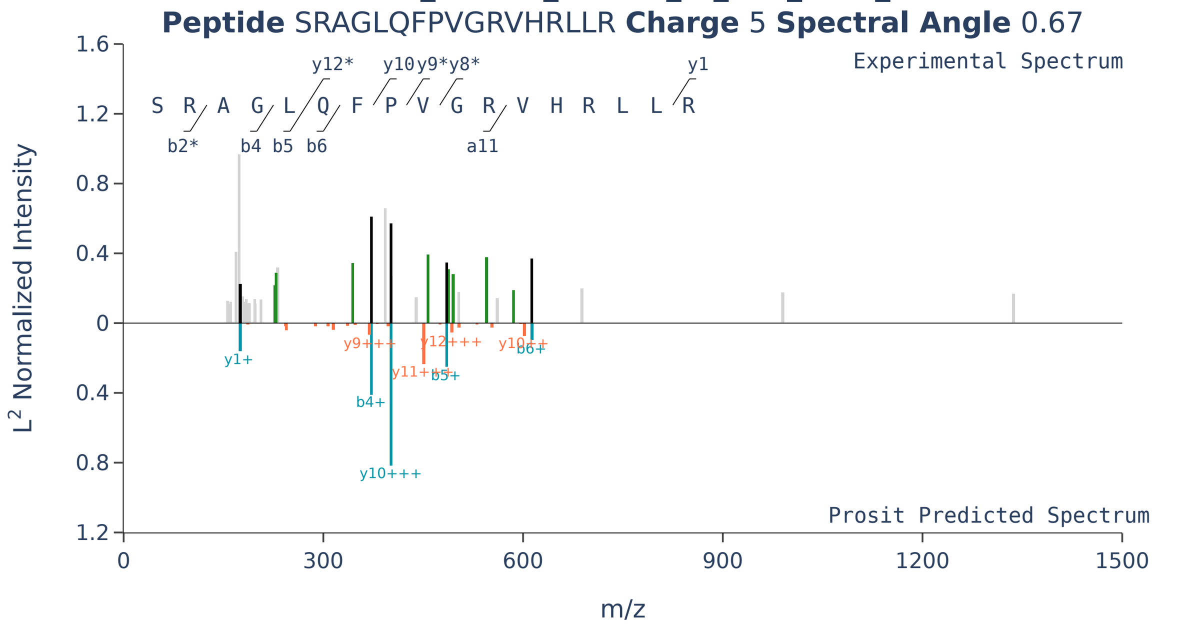


**Source** HRoetschke_291222_060123_Fu_H2A_b1R2_2h **Scan** 16182
**Peptide** VHRLLRKGNYAERVGAGAPVYLAAVL **Charge** 4 **Spectral Angle** 0.79

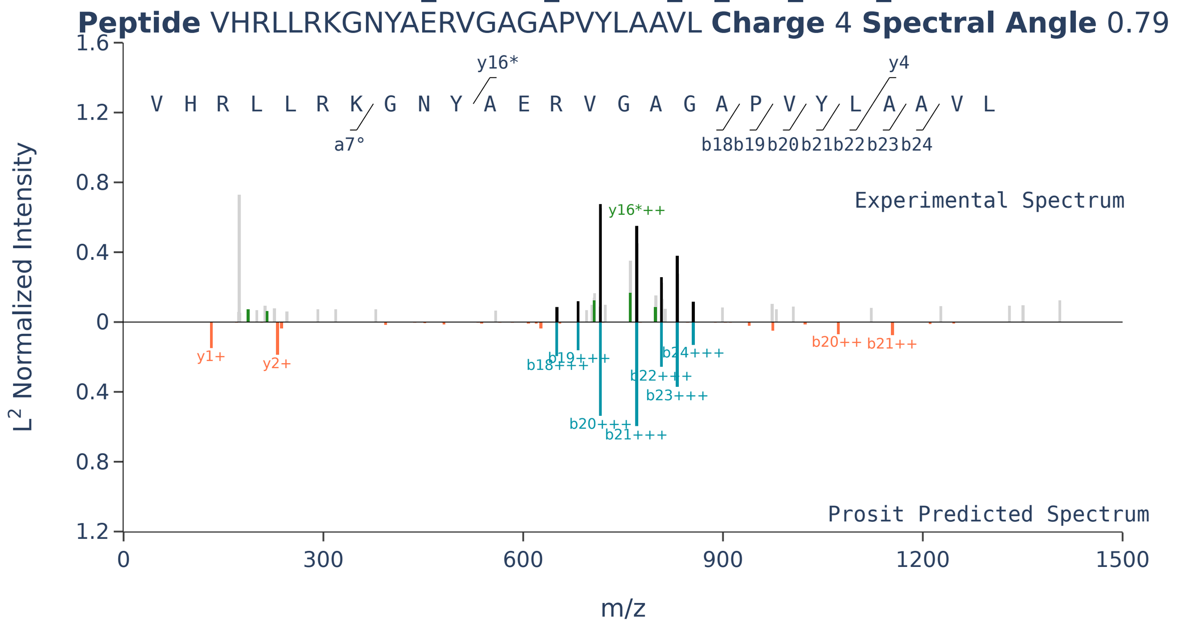


**Source** HRoetschke_291222_060123_Fu_H2A_b1R1_4h **Scan** 9609
**Peptide** TRSSRAGLQFPVGRVHRLLRKGNYAER **Charge** 6 **Spectral Angle** 0.52

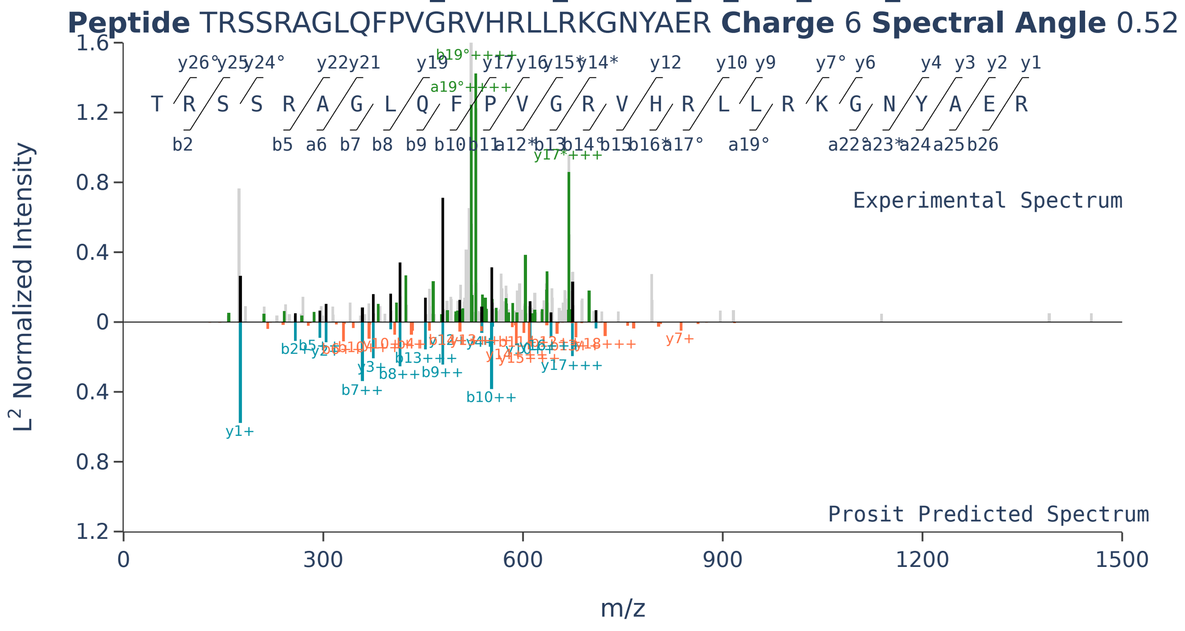


**Source** HRoetschke_291222_060123_Fu_H2A_b2R1_2h **Scan** 3114
**Peptide** NAARDNKKTRIIPRHLQ **Charge** 4 **Spectral Angle** 0.65

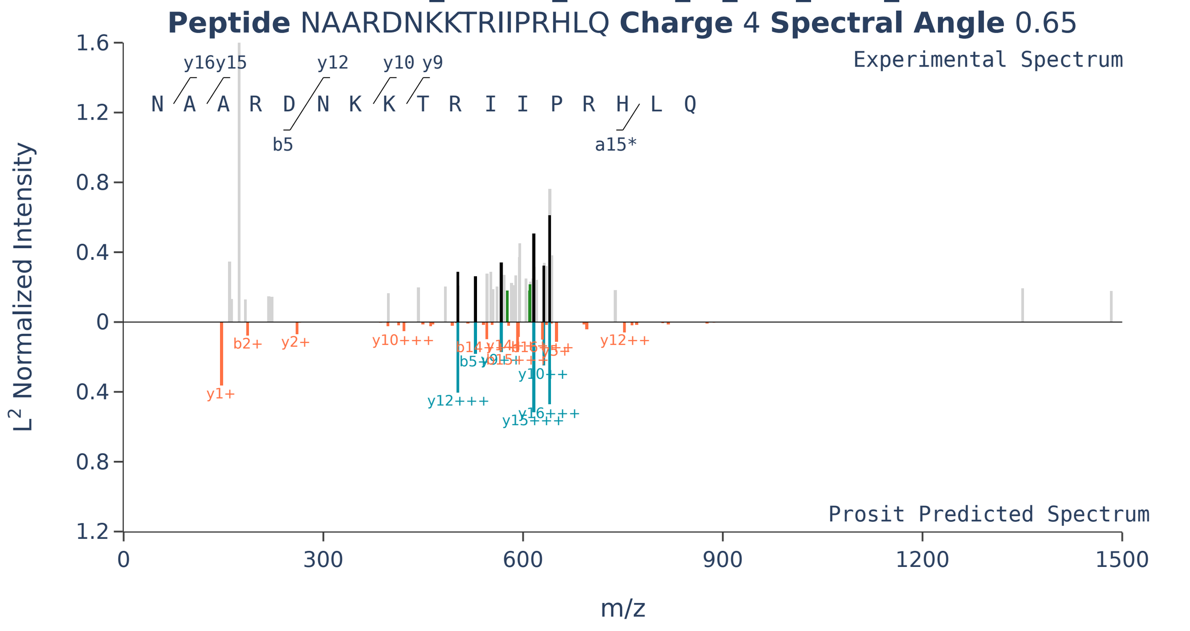


**Source** HRoetschke_291222_060123_Fu_H2A_b1R1_24h **Scan** 9172
**Peptide** ELLELAGNAARDNKK **Charge** 3 **Spectral Angle** 0.58

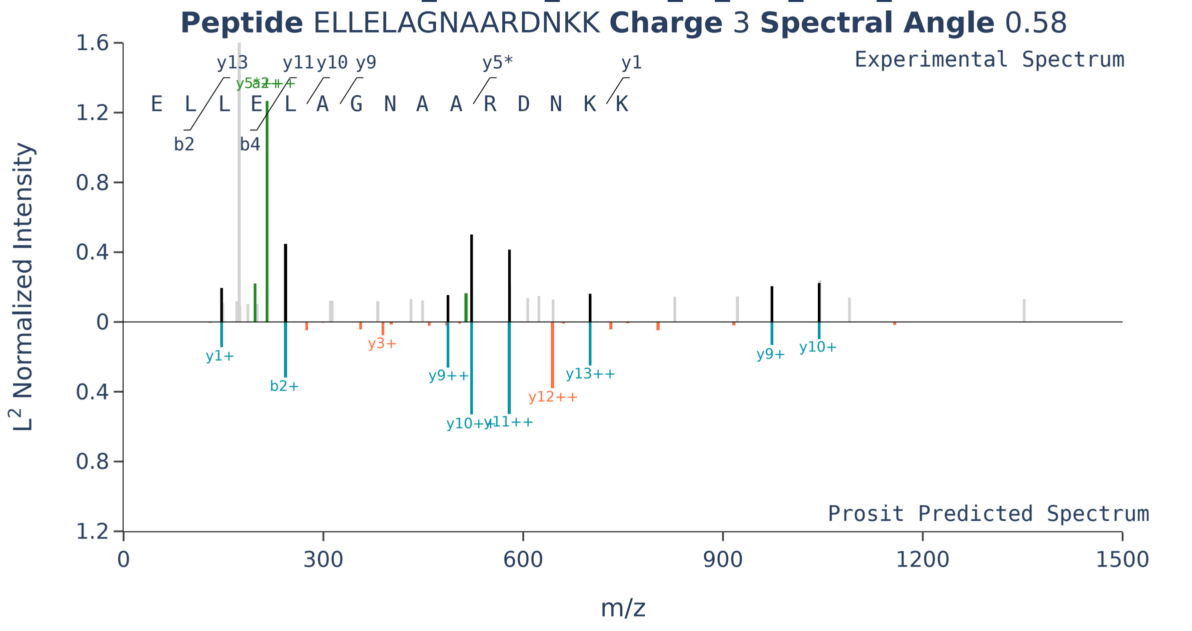


**Source** HRoetschke_291222_060123_Fu_H2A_b1R2_2h **Scan** 12033
**Peptide** LPNIQSVLLPKKTESSKSAKSK **Charge** 5 **Spectral Angle** 0.65

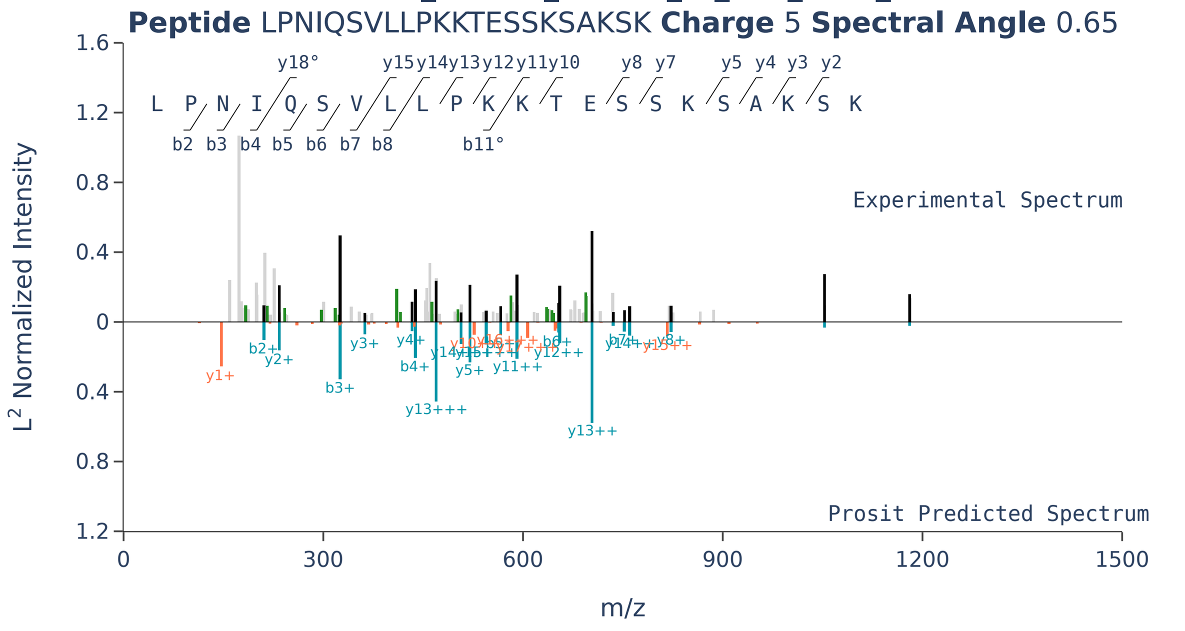


**Source** HRoetschke_291222_060123_Fu_H2A_b1R1_4h **Scan** 17143
**Peptide** KLLGRVTIAQGGVLPNIQS **Charge** 3 **Spectral Angle** 0.62

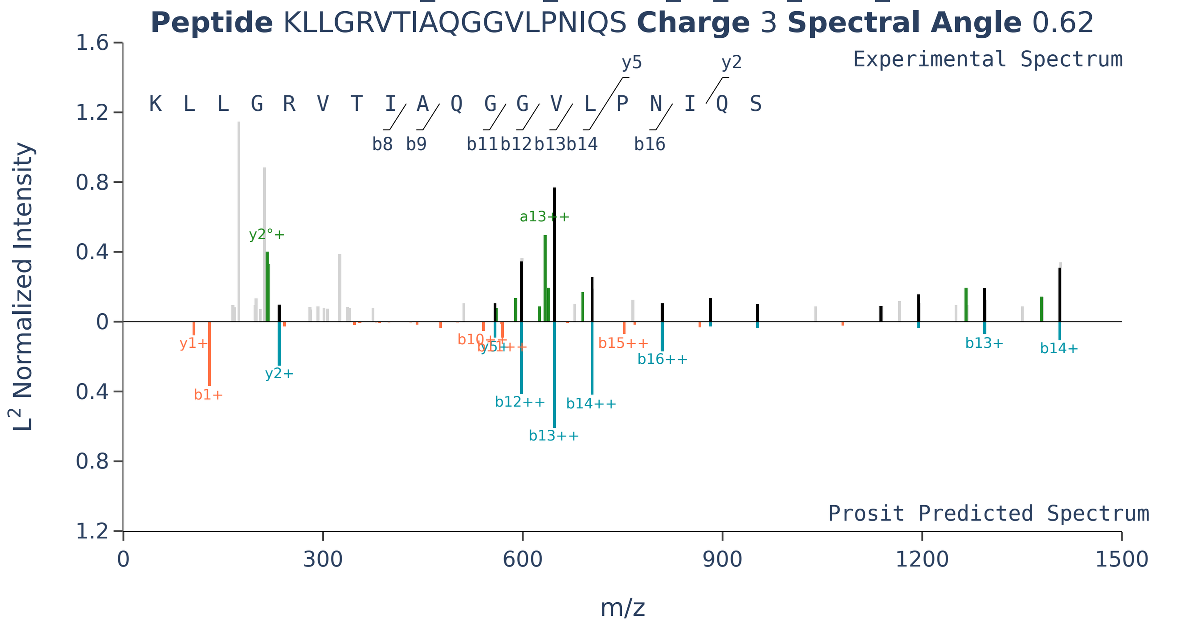

**Source** HRoetschke_291222_060123_Fu_H2A_b1R1_4h **Scan** 17013
**Peptide** RVHRLLRKGNYAERVGAGAPVYLAAVL **Charge** 4 **Spectral Angle** 0.66

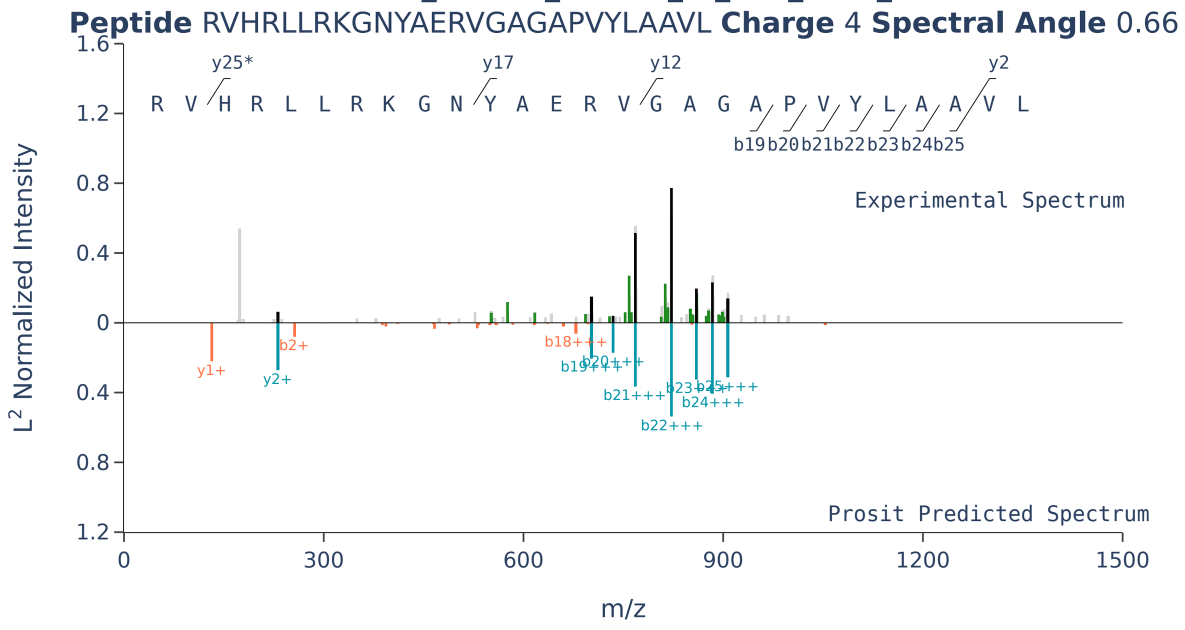


**Source** HRoetschke_291222_060123_Fu_H2A_b2R2_24h **Scan** 3180
**Peptide** GGKTRAKAKTRSSRAGLQF **Charge** 4 **Spectral Angle** 0.72

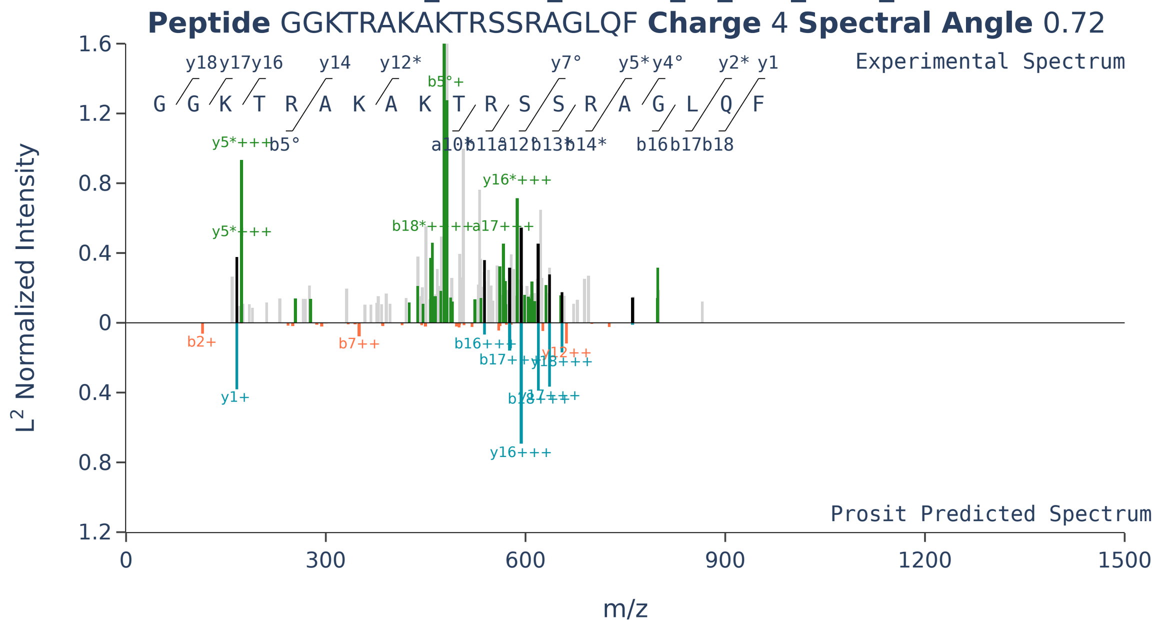


**Source** HRoetschke_291222_060123_Fu_H2A_b1R1_1h **Scan** 16011
**Peptide** KLLGRVTIAQGGVLPNL **Charge** 3 **Spectral Angle** 0.65

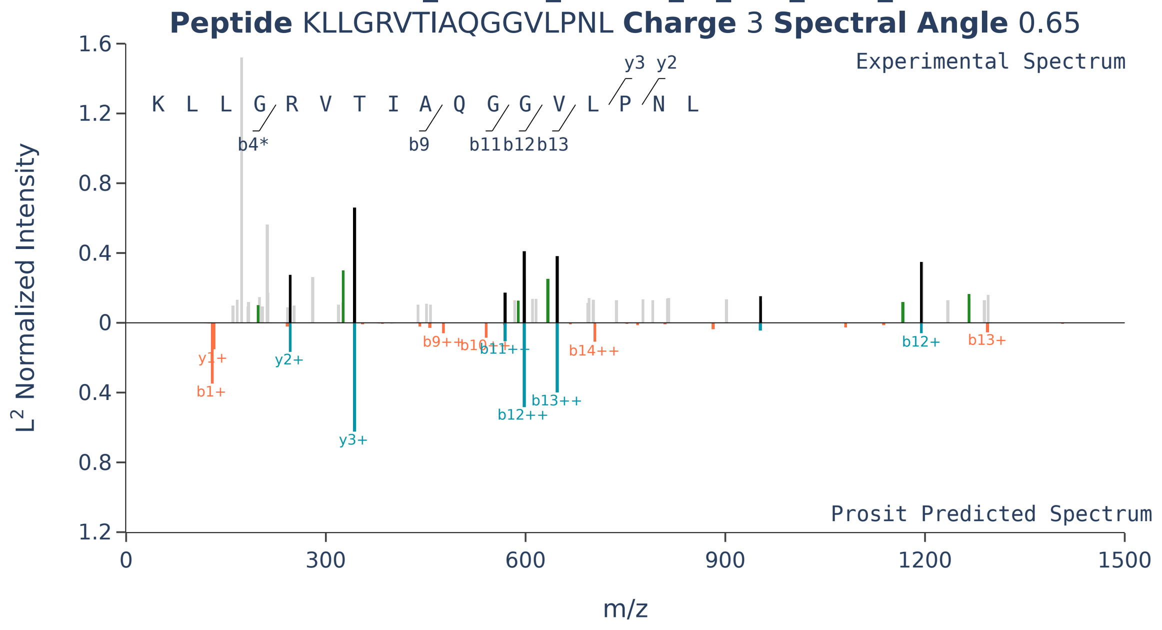


**Source** HRoetschke_291222_060123_Fu_H2A_b2R1_4h **Scan** 6990
**Peptide** KKTRIIPRHLQLAVRND **Charge** 4 **Spectral Angle** 0.59

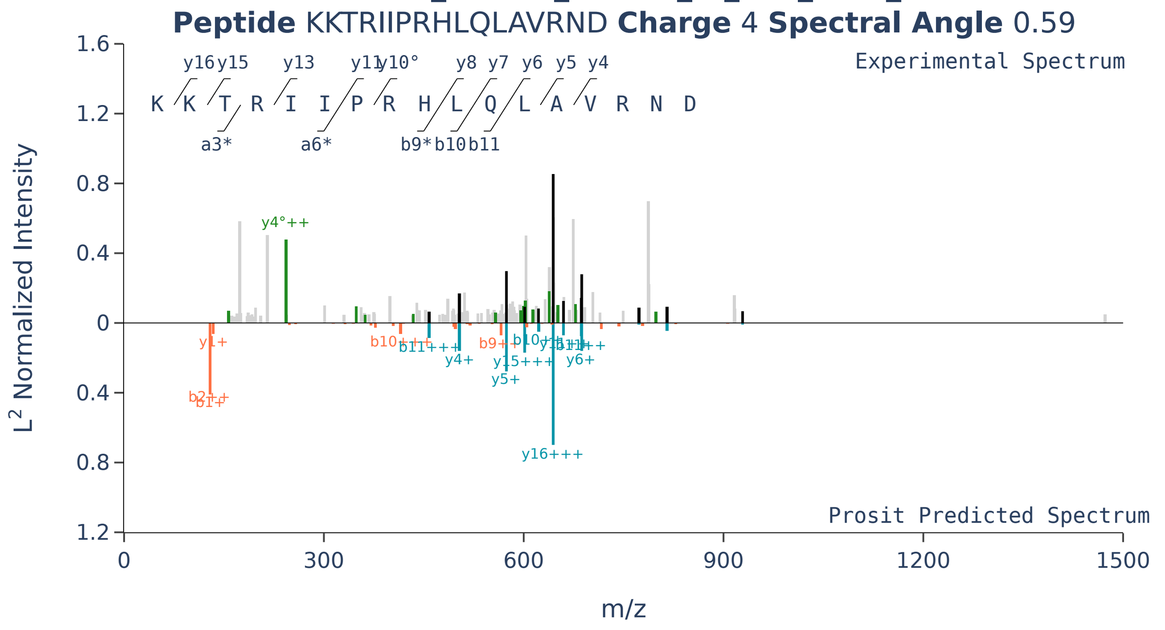


**Source** HRoetschke_291222_060123_Fu_H2A_b1R1_4h **Scan** 11739
**Peptide** SRAGLQFPVGRVHRLLRKGNY **Charge** 5 **Spectral Angle** 0.65

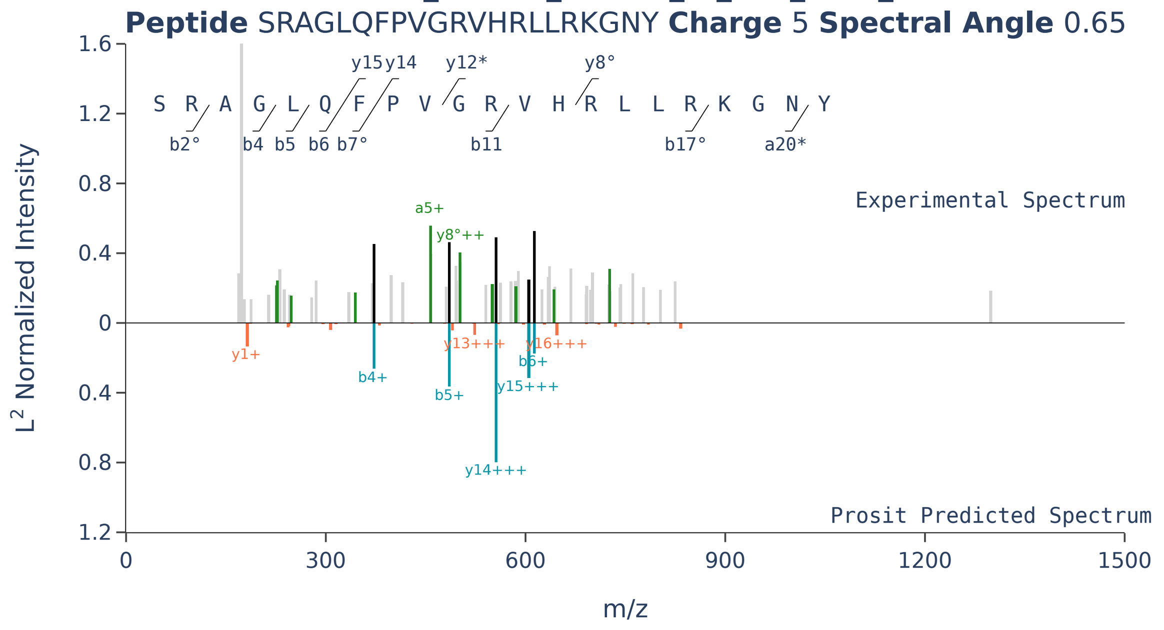


**Source** HRoetschke_291222_060123_Fu_H2A_b1R2_4h **Scan** 9340
**Peptide** ARDNKKTRIIPRHLQLAV **Charge** 4 **Spectral Angle** 0.75

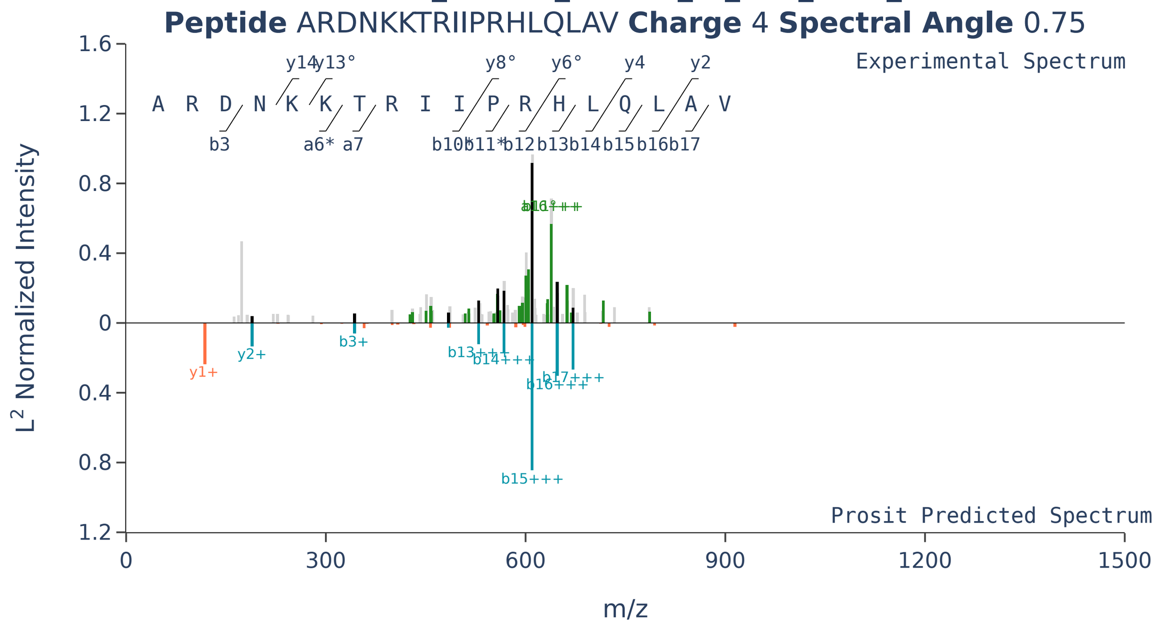


**Source** HRoetschke_291222_060123_Fu_H2A_b1R1_2h **Scan** 18822
**Peptide** RNDEELNKLLGRVTIAQGGVLPNLQ **Charge** 3 **Spectral Angle** 0.7

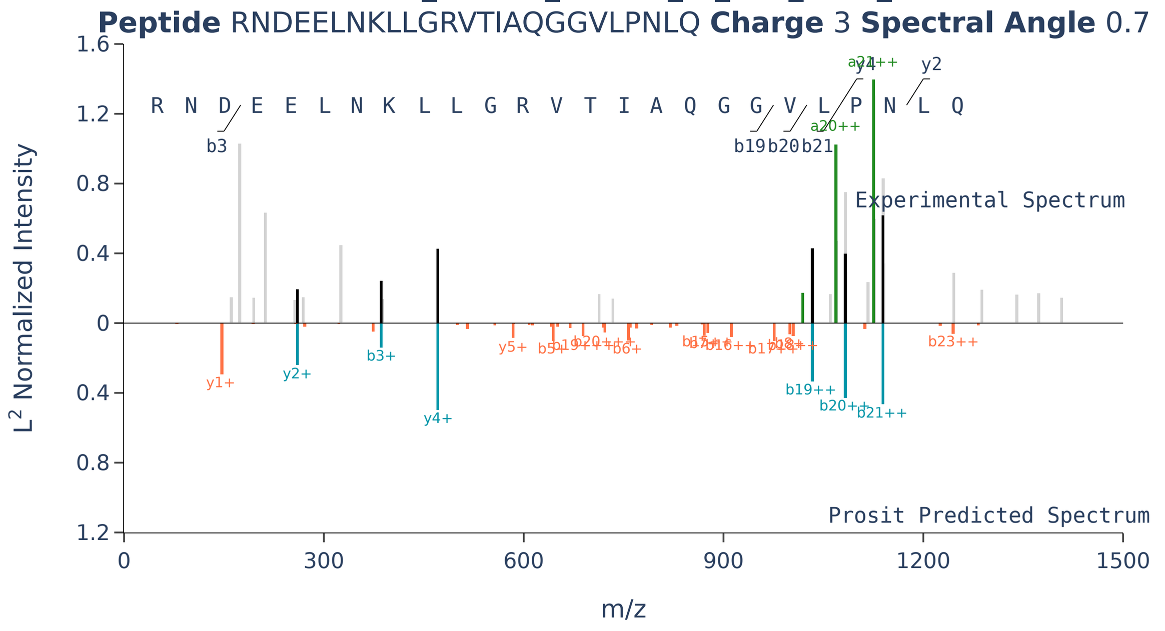


**Source** HRoetschke_291222_060123_Fu_H2A_b1R2_4h **Scan** 9160
**Peptide** AKTRSSRAGLQFPVGRVHRLLRK **Charge** 6 **Spectral Angle** 0.66

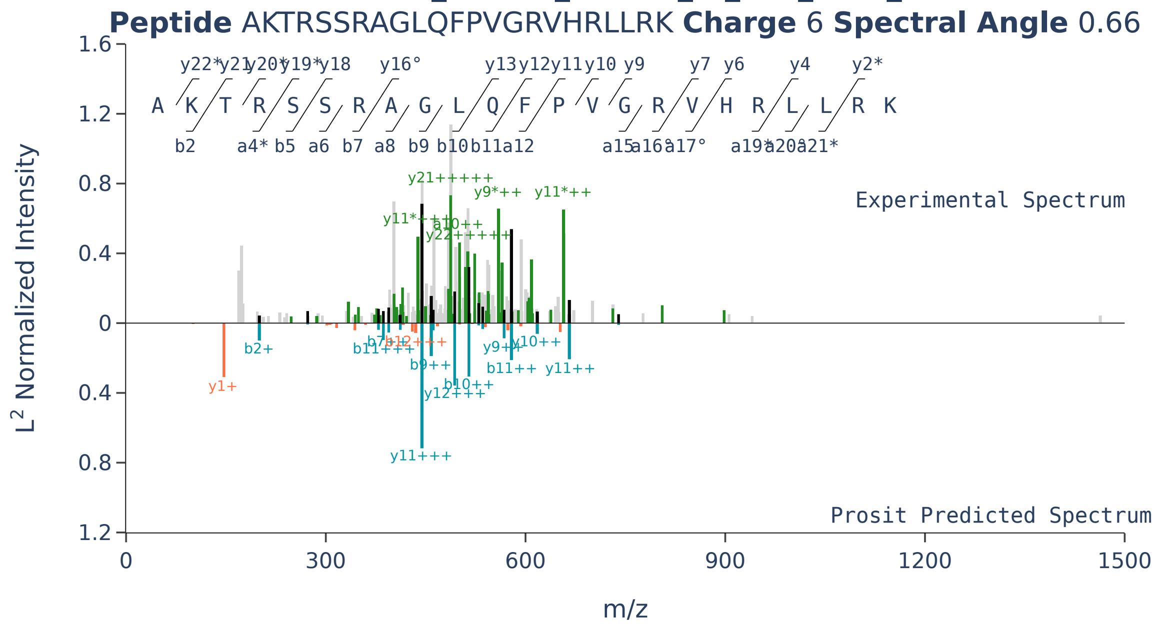


**Source** HRoetschke_291222_060123_Fu_H2A_b1R2_4h **Scan** 10180
**Peptide** LELAGNAARDNKKTRIIPRHLQLAVRNDE **Charge** 5 **Spectral Angle** 0.72

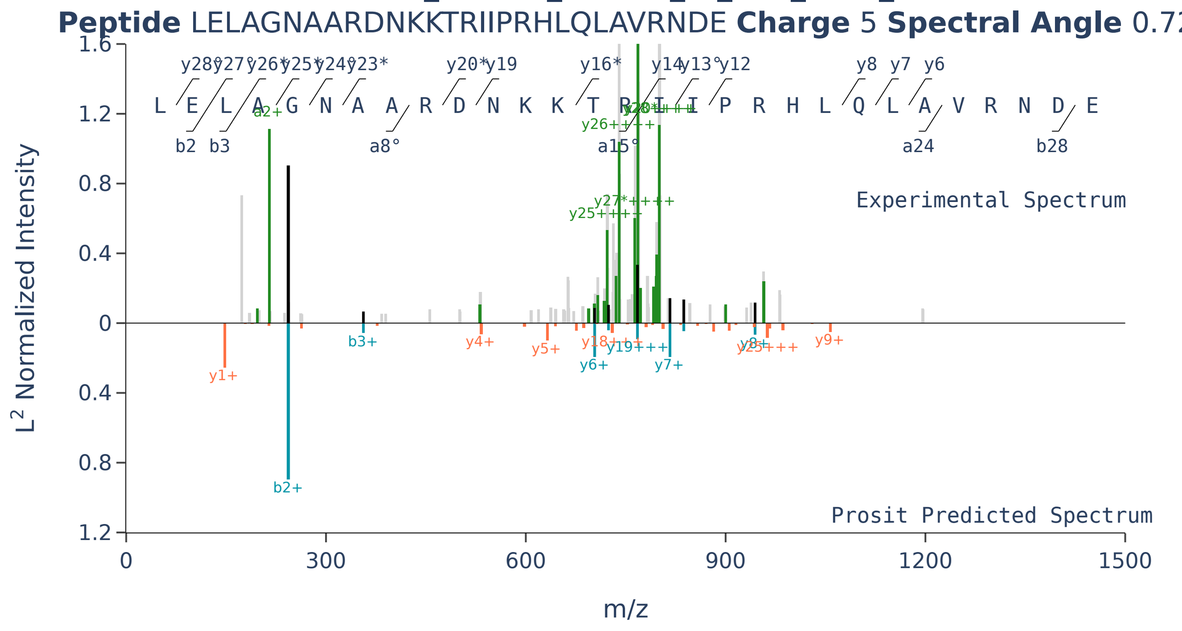


**Source** HRoetschke_291222_060123_Fu_H2A_b1R2_2h **Scan** 11767
**Peptide** AGLQFPVGRVHRLLRKGN **Charge** 4 **Spectral Angle** 0.61

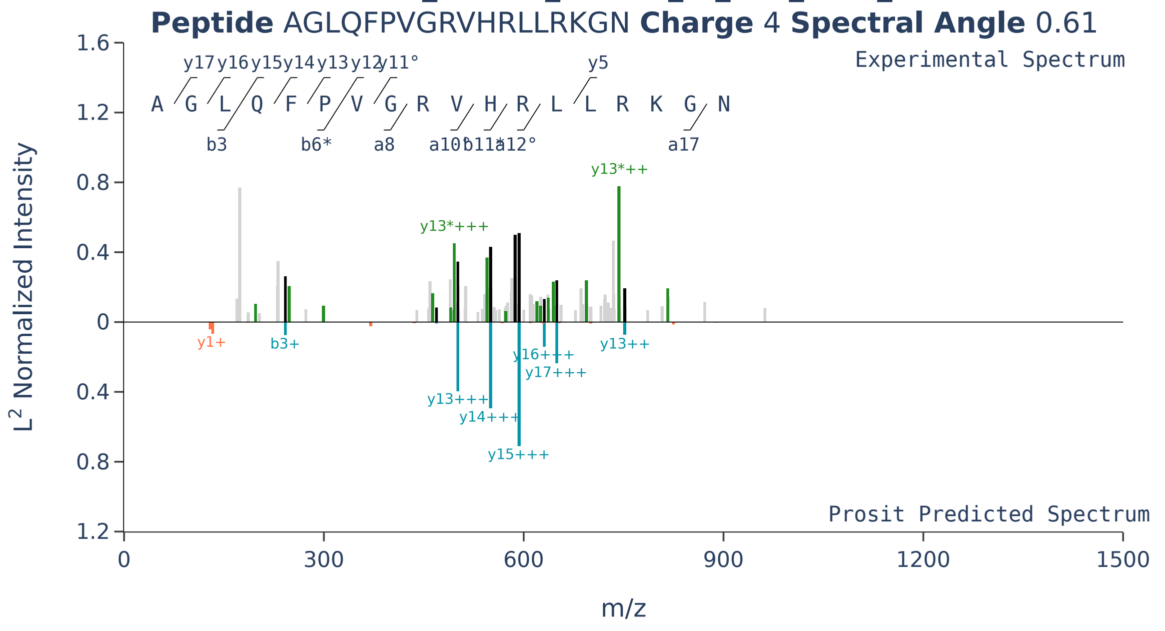


**Source** HRoetschke_291222_060123_Fu_H2A_b2R1_24h **Scan** 8427
**Peptide** DNKKTRIIPRHLQLA **Charge** 4 **Spectral Angle** 0.47

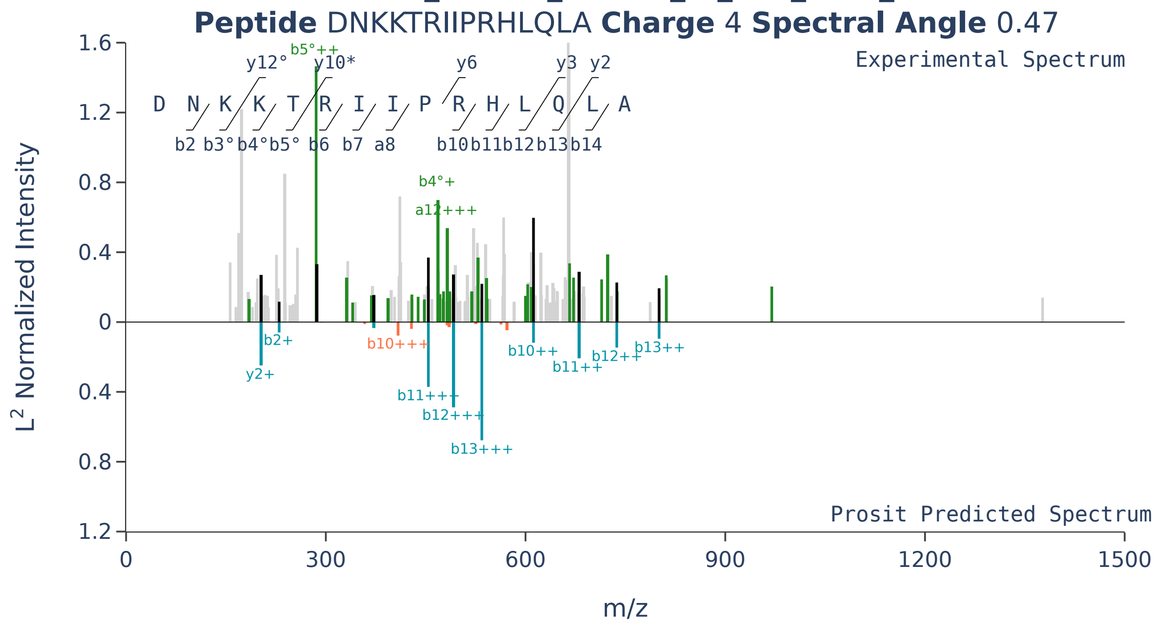


**Source** HRoetschke_291222_060123_Fu_H2A_b1R1_4h **Scan** 6944
**Peptide** KKTRIIPRHLQLAVR **Charge** 5 **Spectral Angle** 0.64

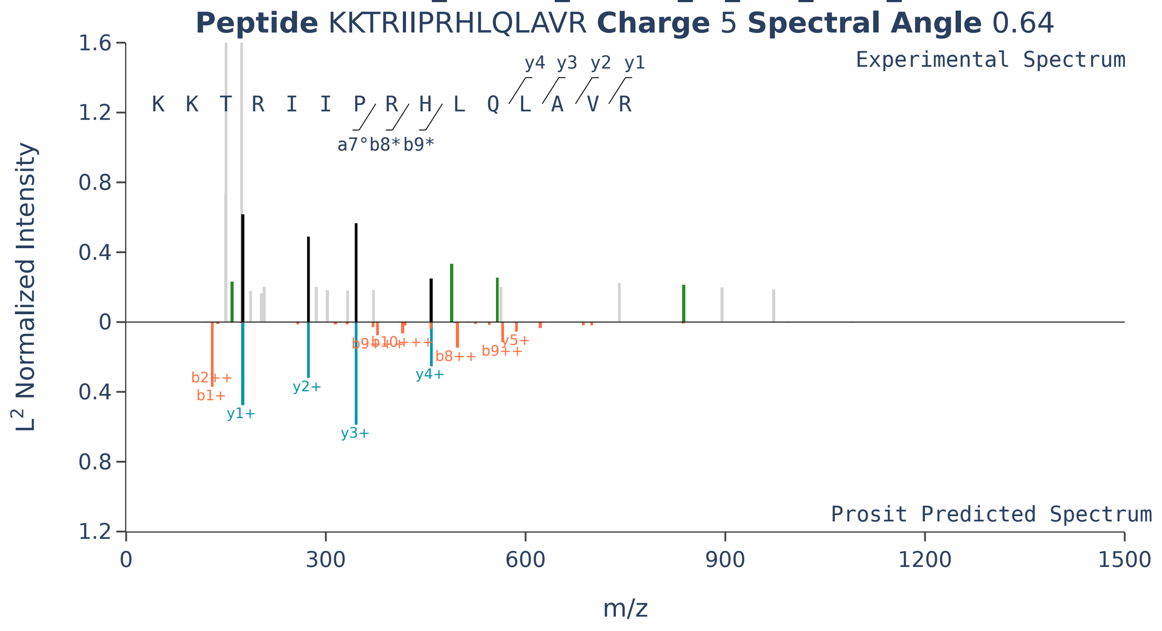


**Source** HRoetschke_291222_060123_Fu_H2A_b2R1_4h **Scan** 7513
**Peptide** RAKAKTRSSRAGLQFPVG **Charge** 4 **Spectral Angle** 0.7

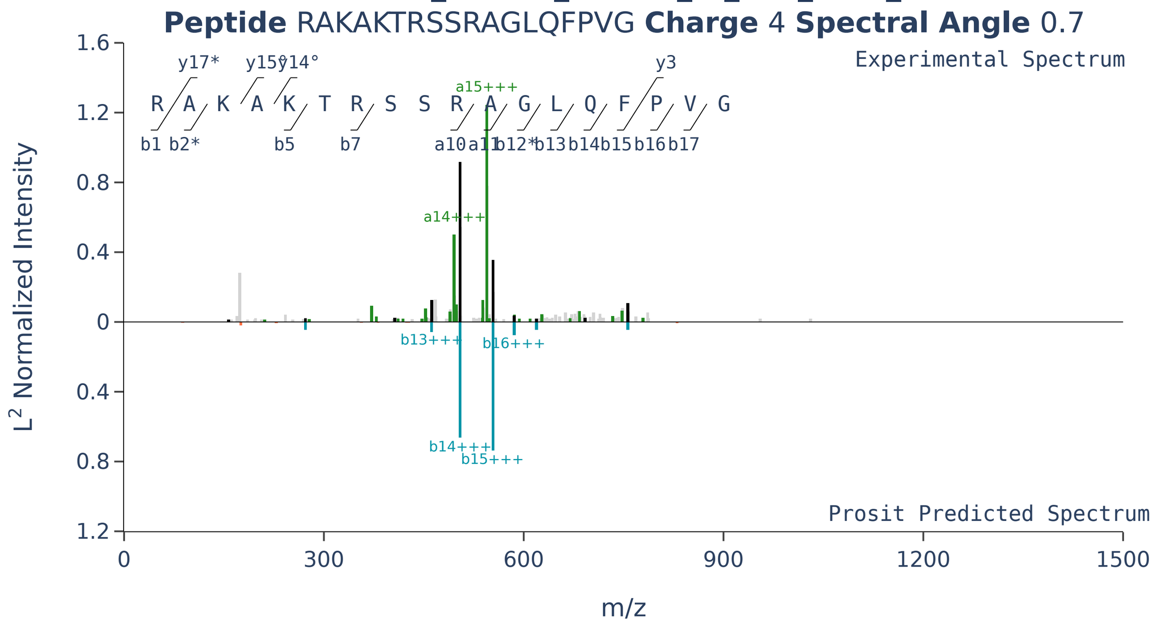


**Source** HRoetschke_291222_060123_Fu_H2A_b1R2_24h **Scan** 4112
**Peptide** RDNKKTRLLP **Charge** 4 **Spectral Angle** 0.72

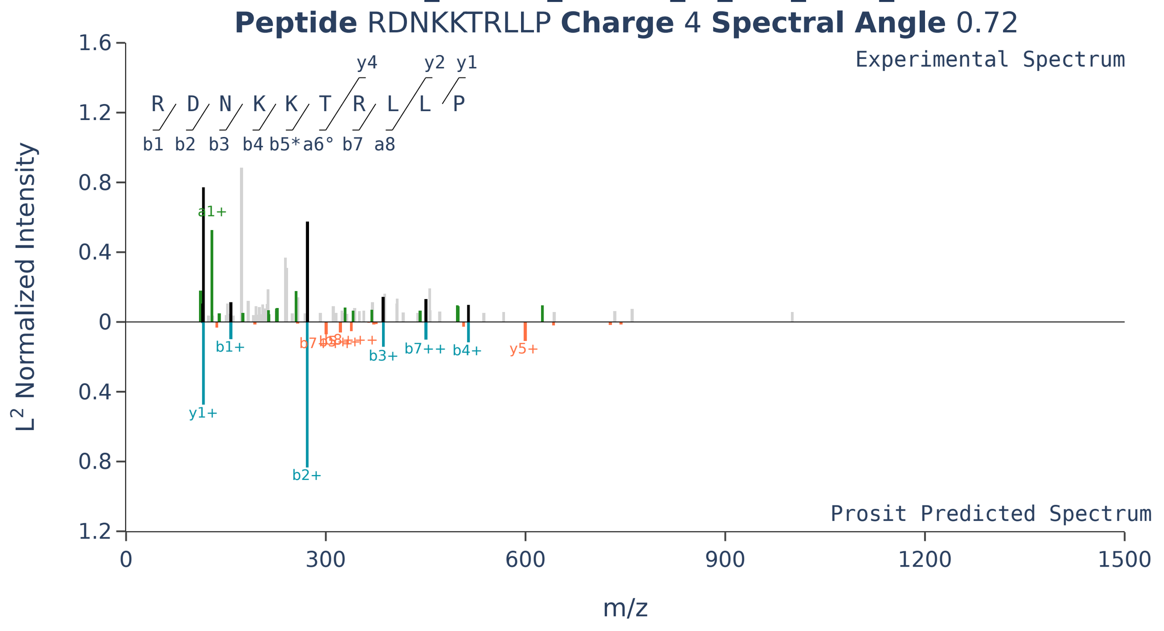


**Source** HRoetschke_291222_060123_Fu_H2A_b2R2_24h **Scan** 12373
**Peptide** TAEILELAGNAARDNKK **Charge** 3 **Spectral Angle** 0.64

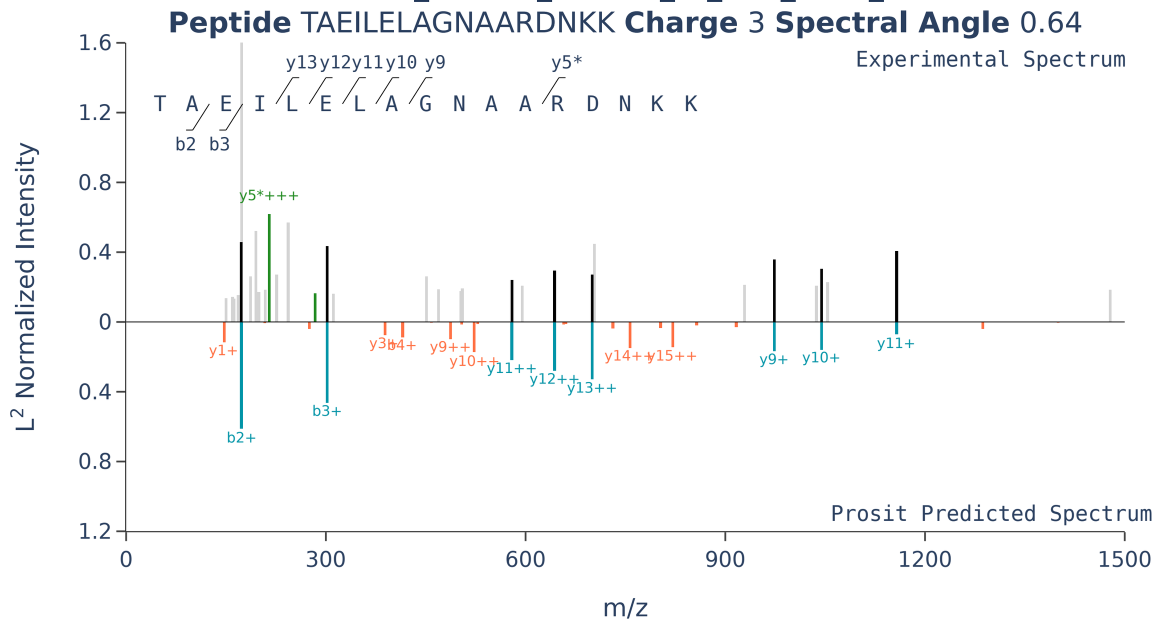

**Source** HRoetschke_291222_060123_Fu_H2A_b1R1_2h **Scan** 18589
**Peptide** ELNKLLGRVTIAQGGVLPNLQ **Charge** 3 **Spectral Angle** 0.47

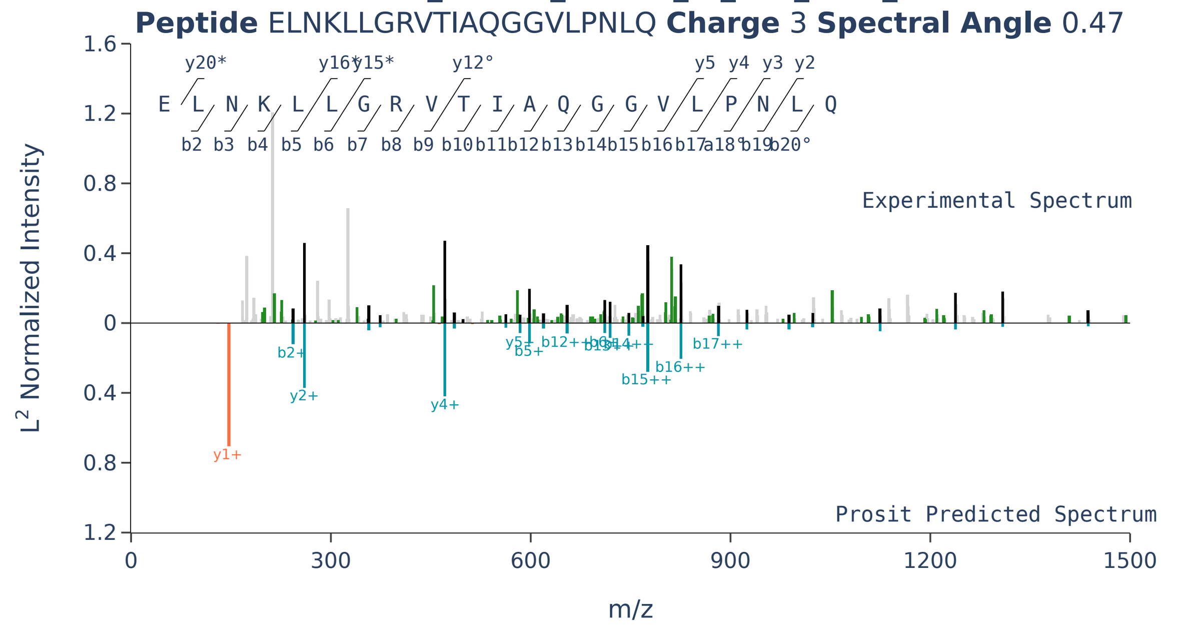


**Source** HRoetschke_291222_060123_Fu_H2A_b2R1_2h **Scan** 8312
**Peptide** ELAGNAARDNKKTRIIPRHLQLAVR **Charge** 5 **Spectral Angle** 0.6

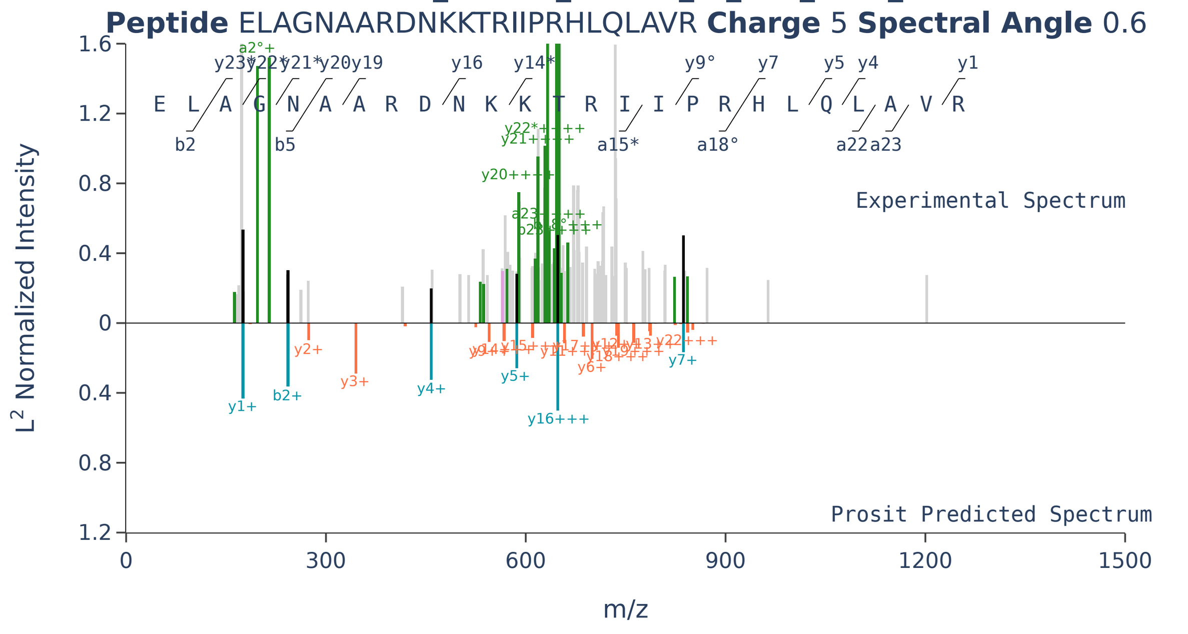


**Source** HRoetschke_291222_060123_Fu_H2A_b1R2_4h **Scan** 9389
**Peptide** QFPVGRVHRLLRKGNYAERV **Charge** 6 **Spectral Angle** 0.77

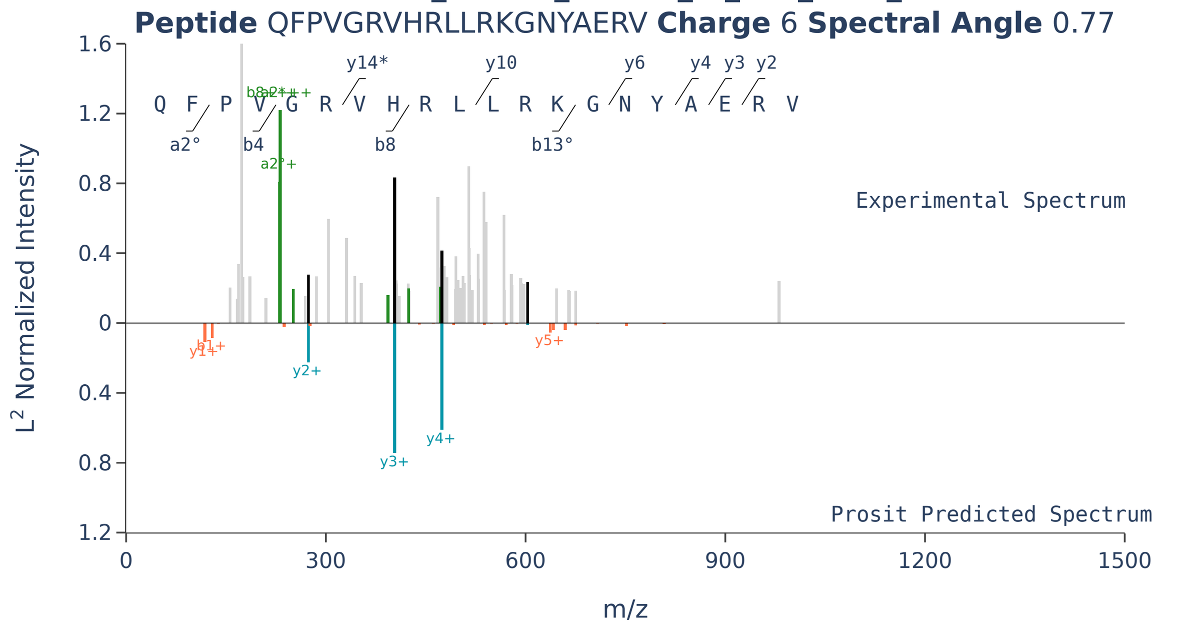


**Source** HRoetschke_291222_060123_Fu_H2A_b1R2_2h **Scan** 18552
**Peptide** RNDEELNKLLGRVTIAQGGVLPNL **Charge** 3 **Spectral Angle** 0.72

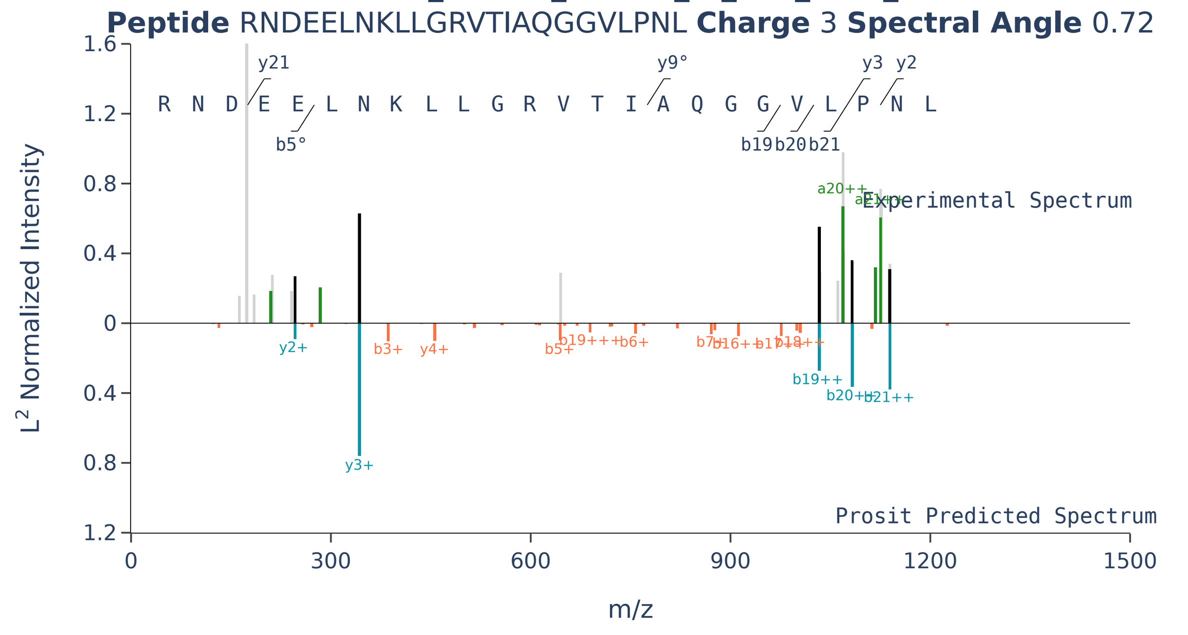


**Source** HRoetschke_291222_060123_Fu_H2A_b1R1_4h **Scan** 9667
**Peptide** TRSSRAGLQFPVGRVHRLLRK **Charge** 5 **Spectral Angle** 0.6

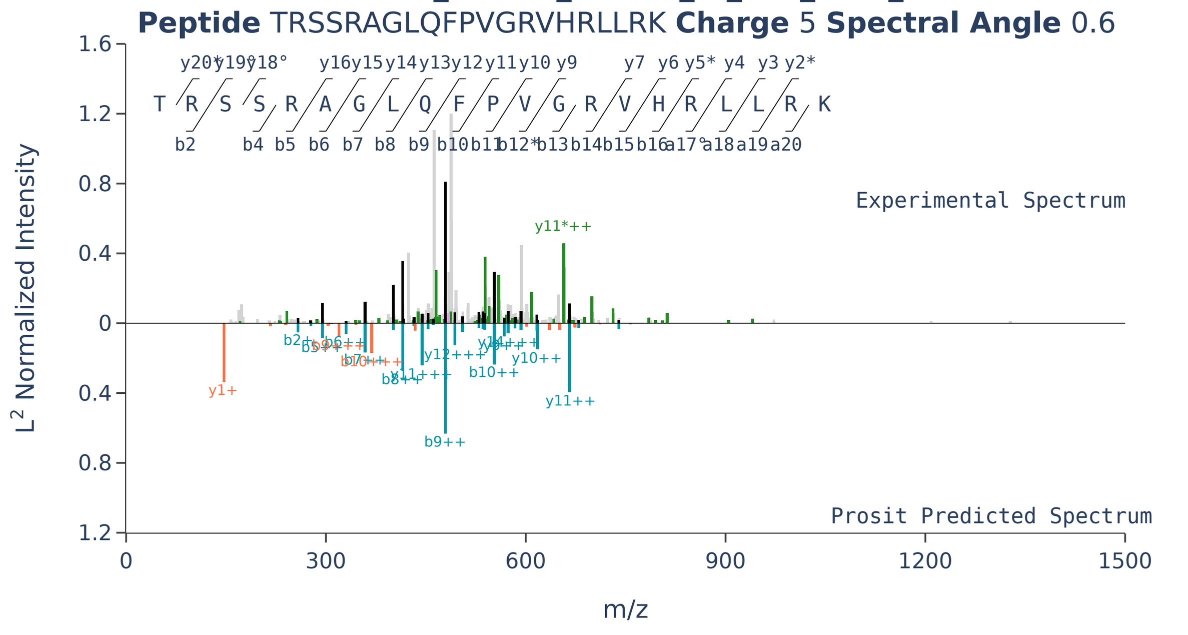


**Source** HRoetschke_291222_060123_Fu_H2A_b2R1_2h **Scan** 3649
**Peptide** TRAKAKTRSSRAGLQF **Charge** 4 **Spectral Angle** 0.61

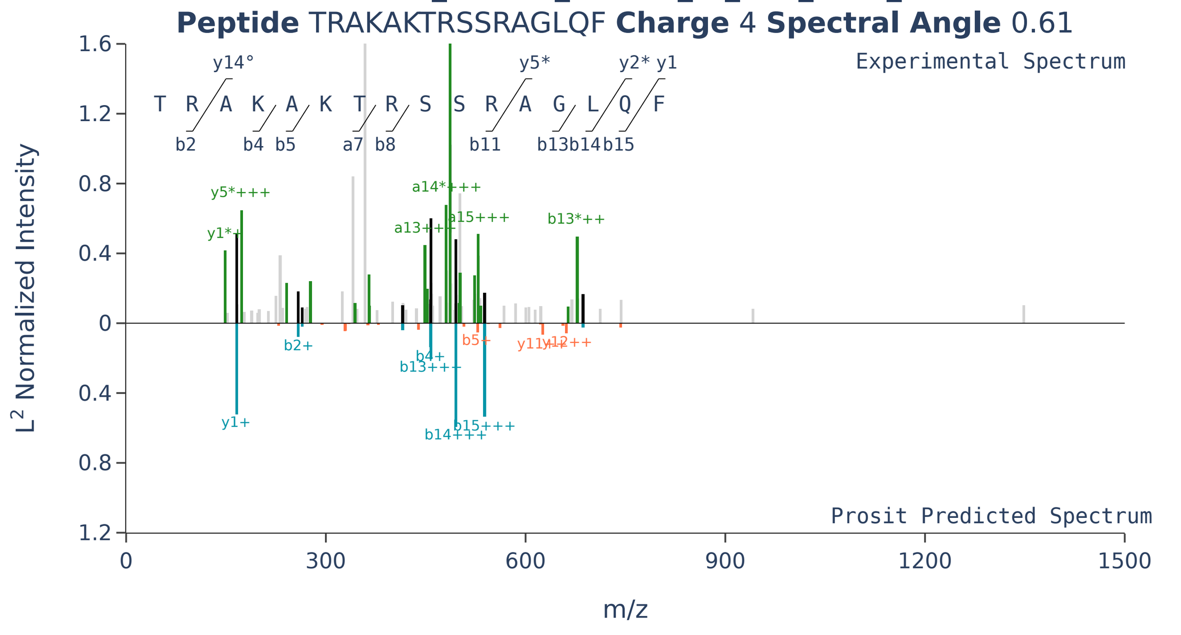


**Source** HRoetschke_291222_060123_Fu_H2A_b1R1_4h **Scan** 15801
**Peptide** ELLELAGNAARDNKKTRIIPRHLQLAV **Charge** 4 **Spectral Angle** 0.54

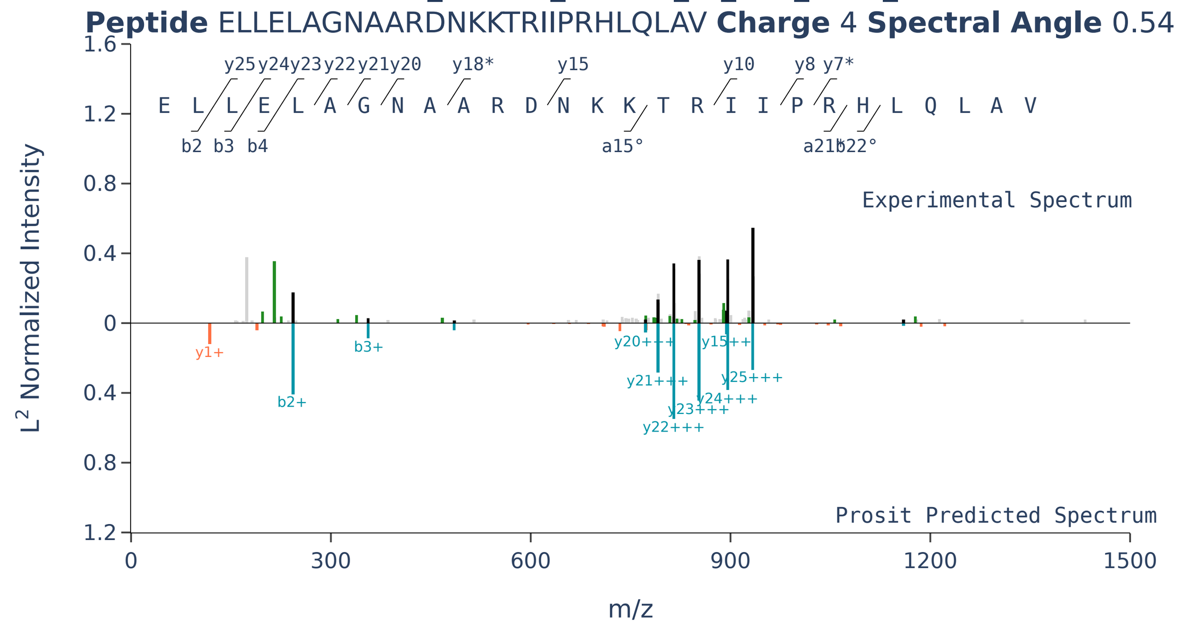


**Source** HRoetschke_291222_060123_Fu_H2A_b2R2_2h **Scan** 9938
**Peptide** VHRLLRKGNYAERVGAGAPVYLA **Charge** 4 **Spectral Angle** 0.47

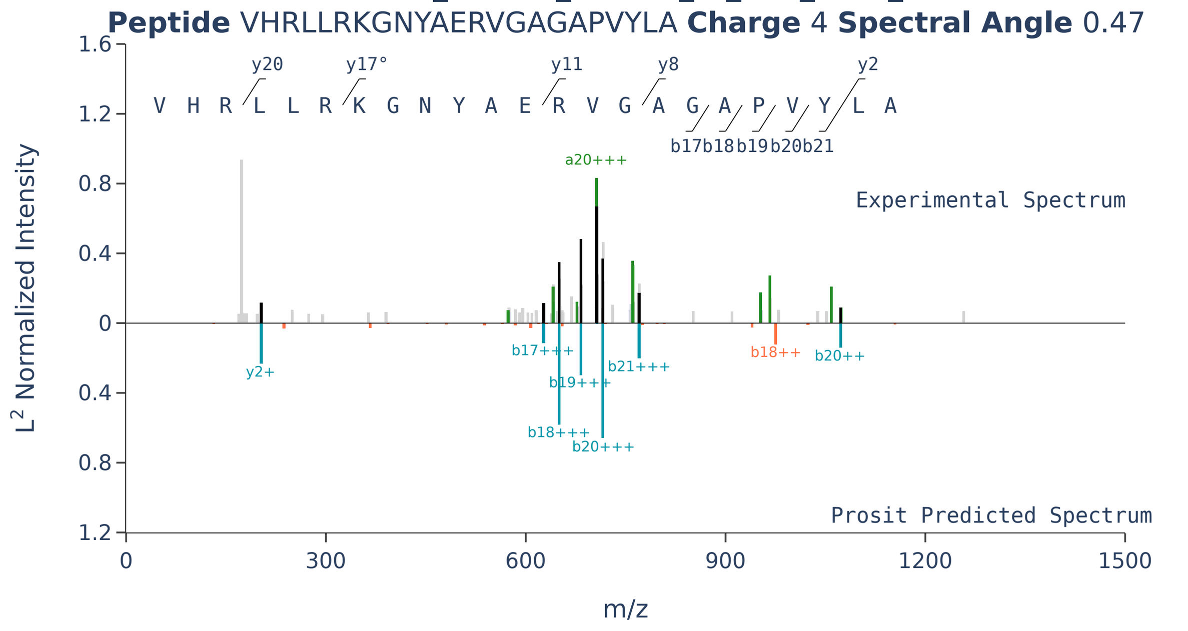


**Source** HRoetschke_291222_060123_Fu_H2A_b2R1_4h **Scan** 13932
**Peptide** ILELAGNAARDNKKTRIIPRHLQL **Charge** 5 **Spectral Angle** 0.81

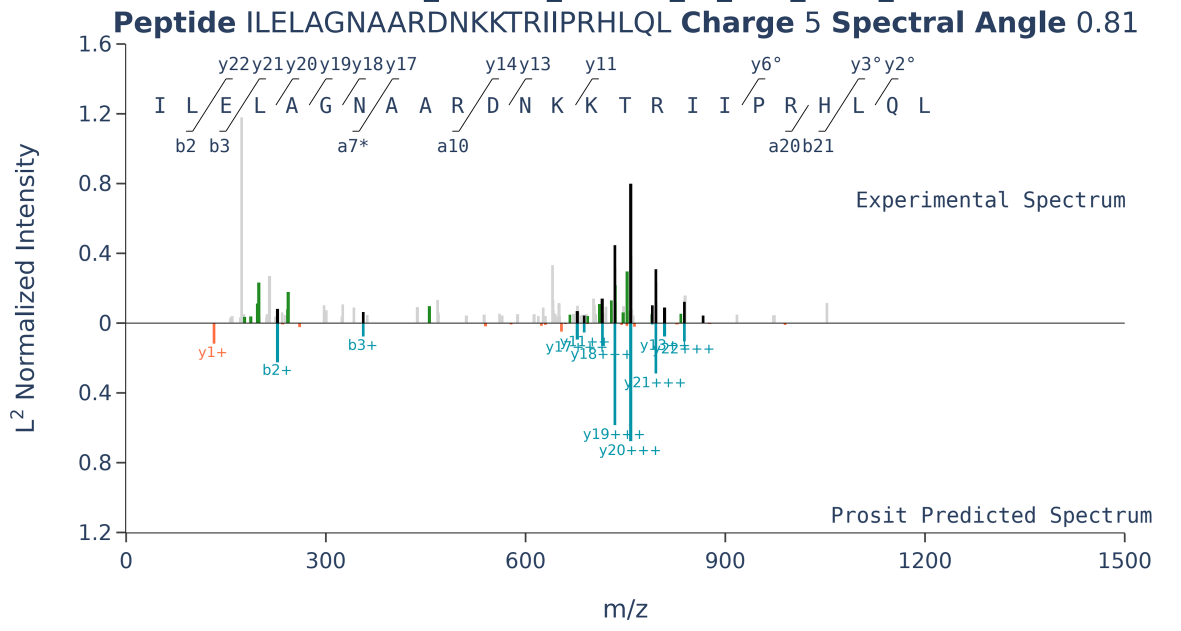


**Source** HRoetschke_291222_060123_Fu_H2A_b1R1_4h **Scan** 9831
**Peptide** KLLGRVTIAQ **Charge** 3 **Spectral Angle** 0.73

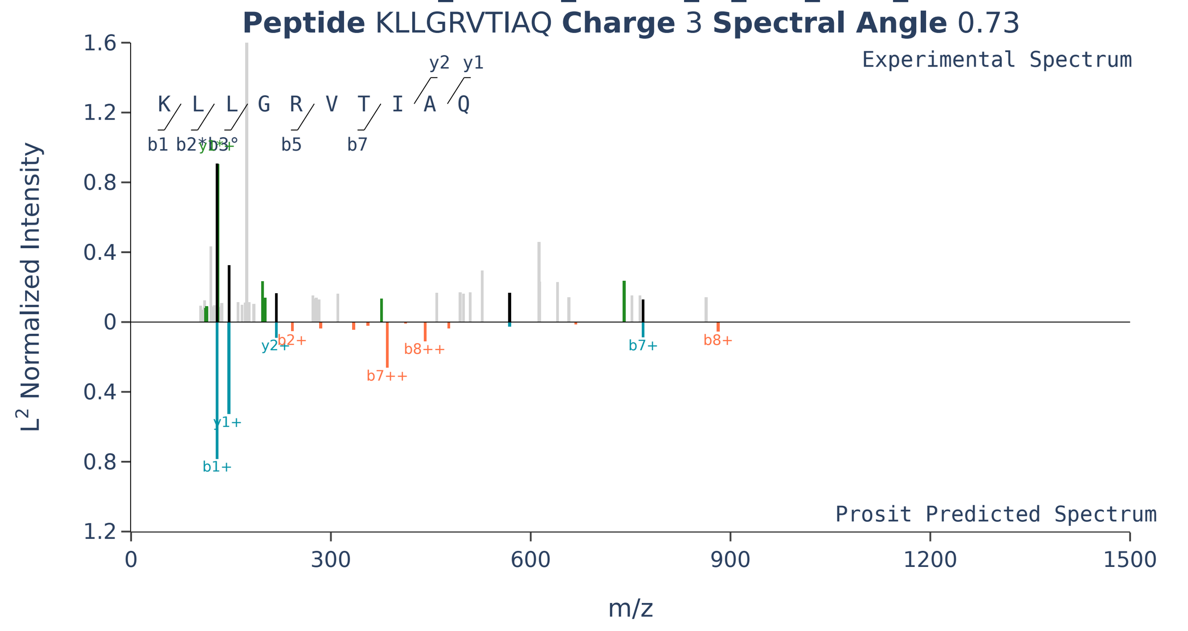


**Source** HRoetschke_291222_060123_Fu_H2A_b1R2_2h **Scan** 16750
**Peptide** LTAEILELAGNAARDNKKTRLL **Charge** 4 **Spectral Angle** 0.79

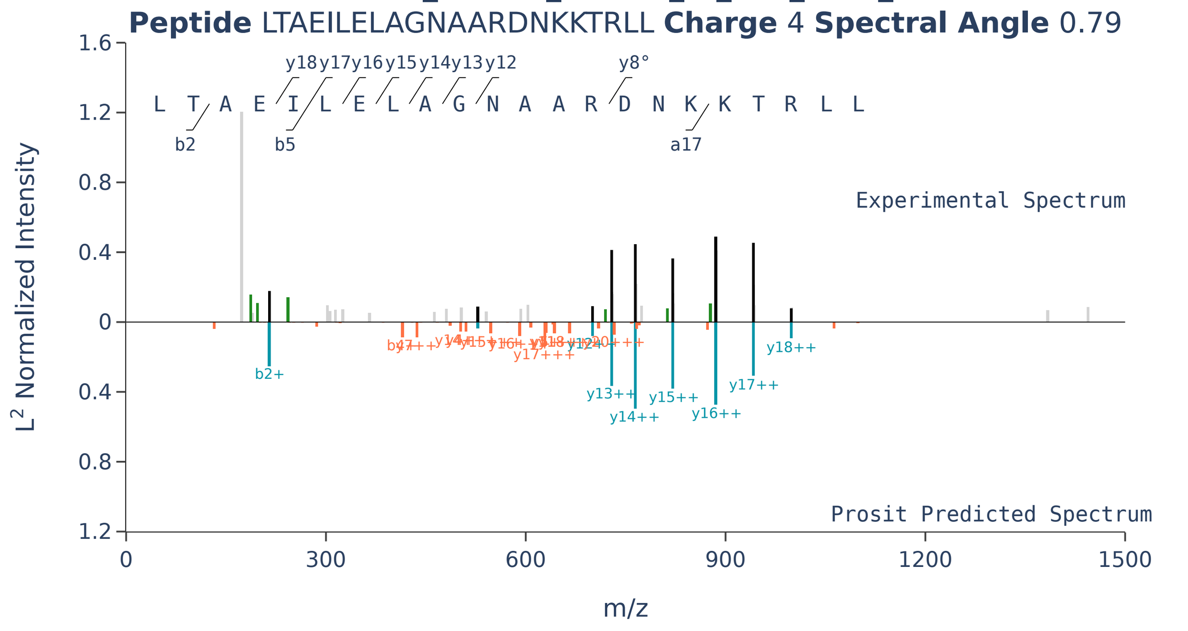


**Source** HRoetschke_291222_060123_Fu_H2A_b2R1_2h **Scan** 16089
**Peptide** QGGVLPNIQSVLLPKKTE **Charge** 3 **Spectral Angle** 0.7

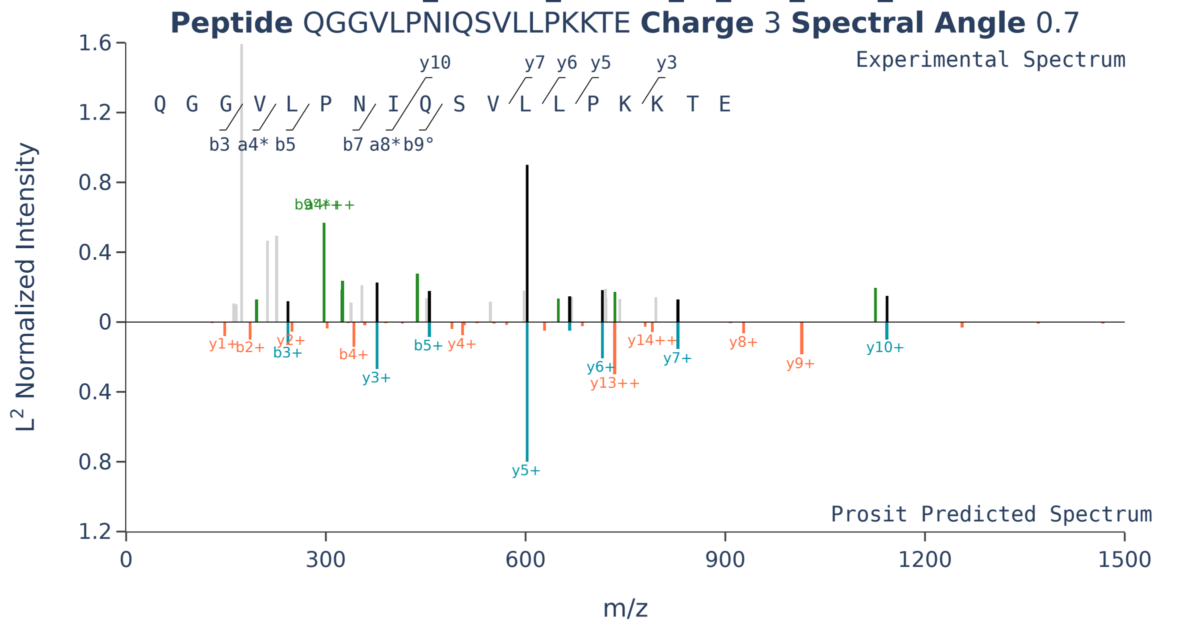


**Source** HRoetschke_291222_060123_Fu_H2A_b1R2_4h **Scan** 8902
**Peptide** QFPVGRVHRLLRKGNYAE **Charge** 4 **Spectral Angle** 0.72

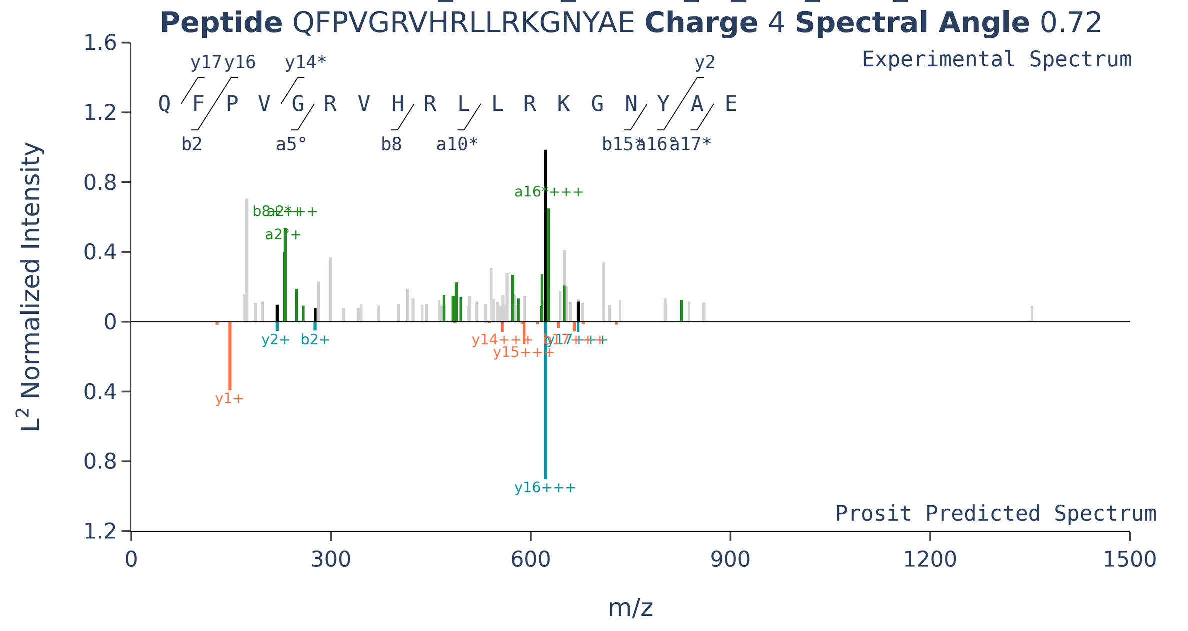


**Source** HRoetschke_291222_060123_Fu_H2A_b1R2_4h **Scan** 8709
**Peptide** ELAGNAARDNKKTRIIPRHLQLAVRNDE **Charge** 6 **Spectral Angle** 0.61

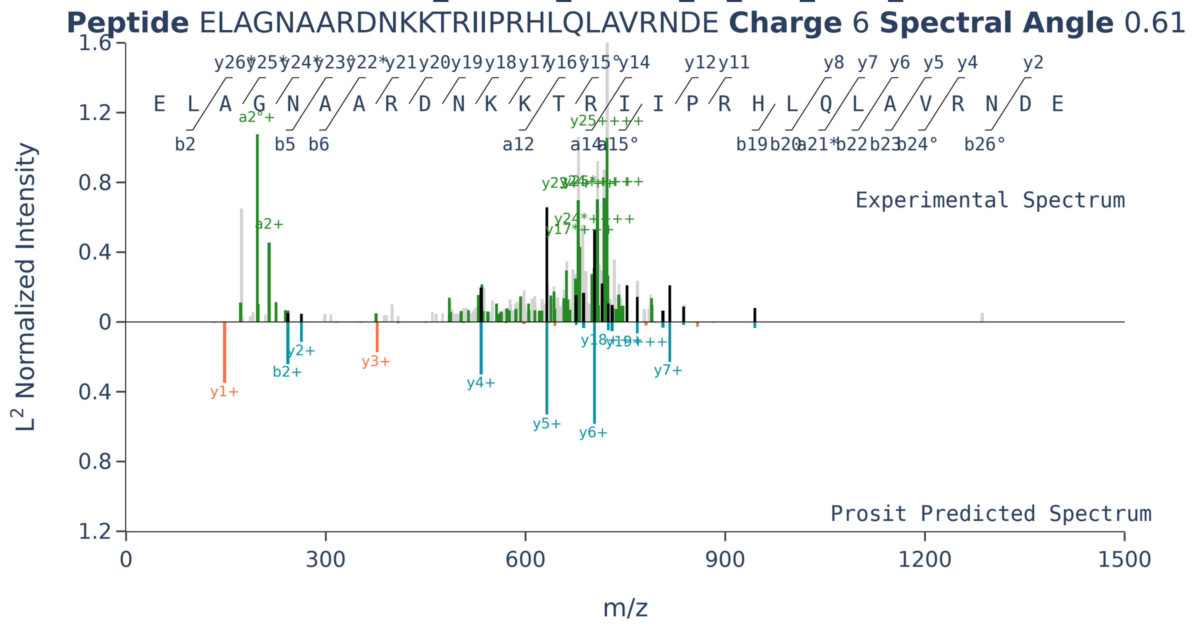


**Source** HRoetschke_291222_060123_Fu_H2A_b1R4_4h **Scan** 17609
**Peptide** QGGVLPNIQSVLLPKKTESSKS **Charge** 4 **Spectral Angle** 0.57

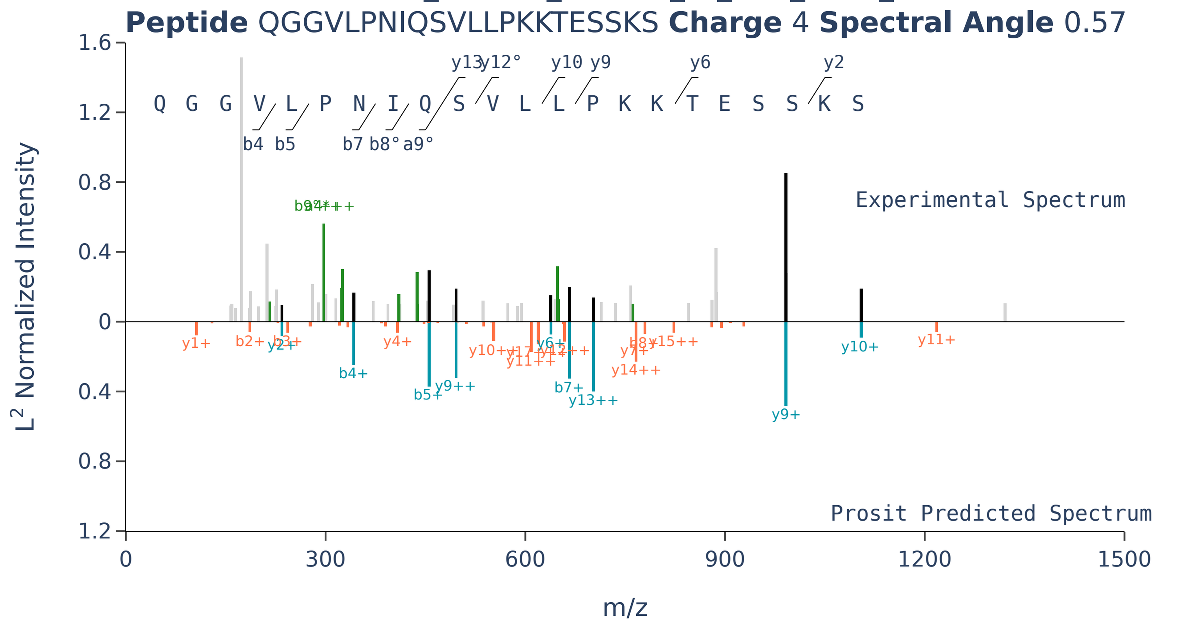

**Source** HRoetschke_291222_060123_Fu_H2A_b1R2_24h **Scan** 1979
**Peptide** AARDNKKTRIIPRH **Charge** 4 **Spectral Angle** 0.55

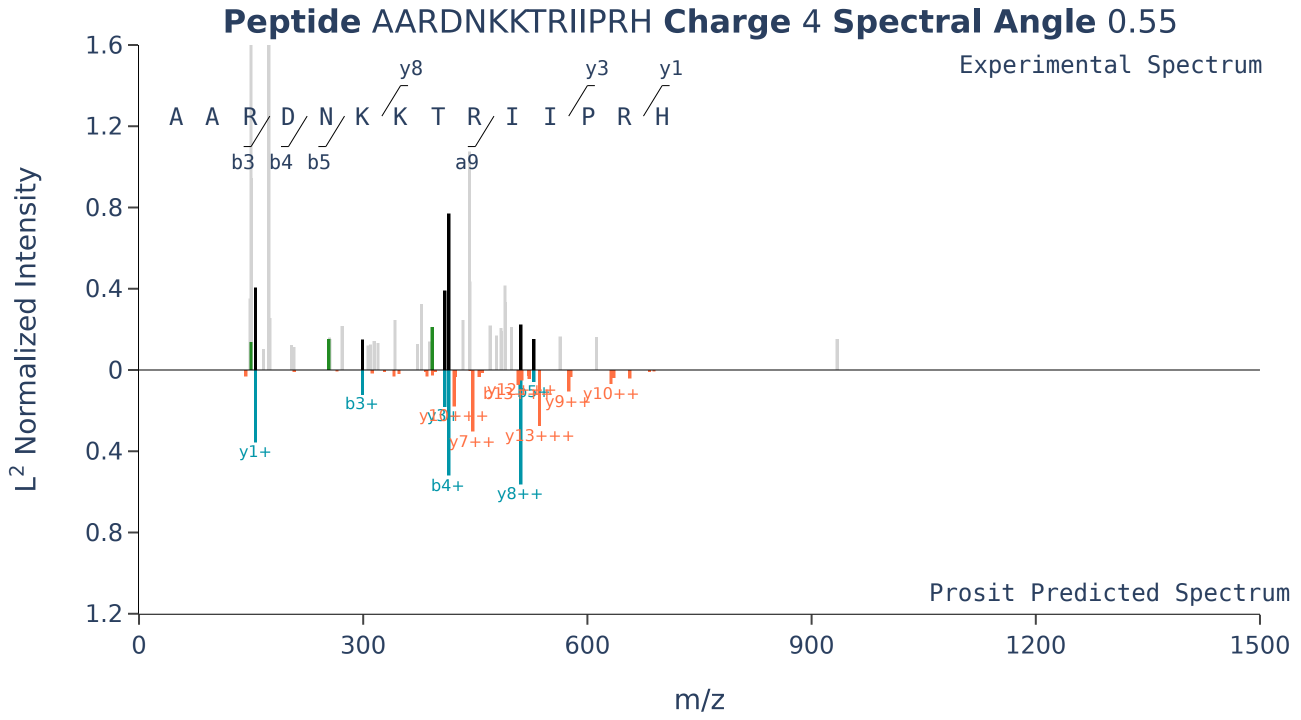


**Source** HRoetschke_291222_060123_Fu_H2A_b1R1_24h **Scan** 8640
**Peptide** DNKKTRIIPRHLQLAVR **Charge** 5 **Spectral Angle** 0.75

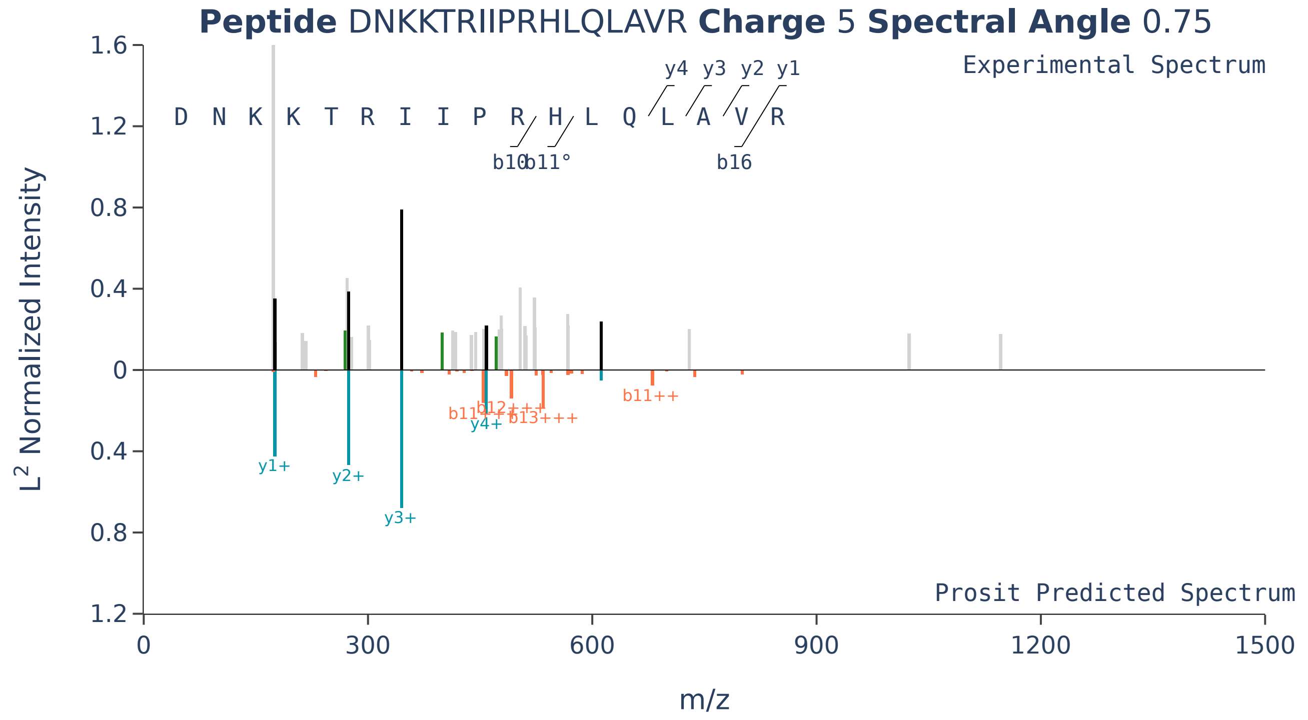


**Source** HRoetschke_291222_060123_Fu_H2A_b1R2_4h **Scan** 13922
**Peptide** TAEILELAGNAARDNKKTRIIPR **Charge** 4 **Spectral Angle** 0.67

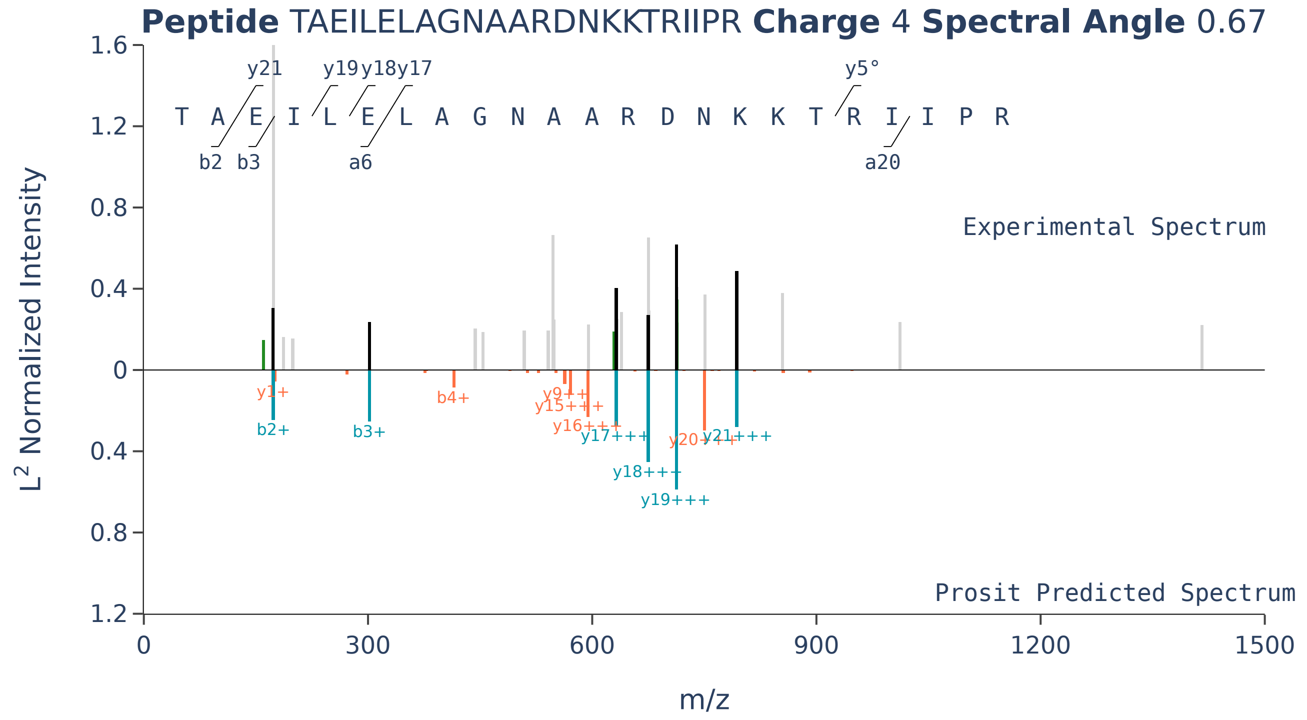


**Source** HRoetschke_291222_060123_Fu_H2A_b1R1_4h **Scan** 15075
**Peptide** VLPNIQSVLLPKKTESSKSAKS **Charge** 5 **Spectral Angle** 0.61

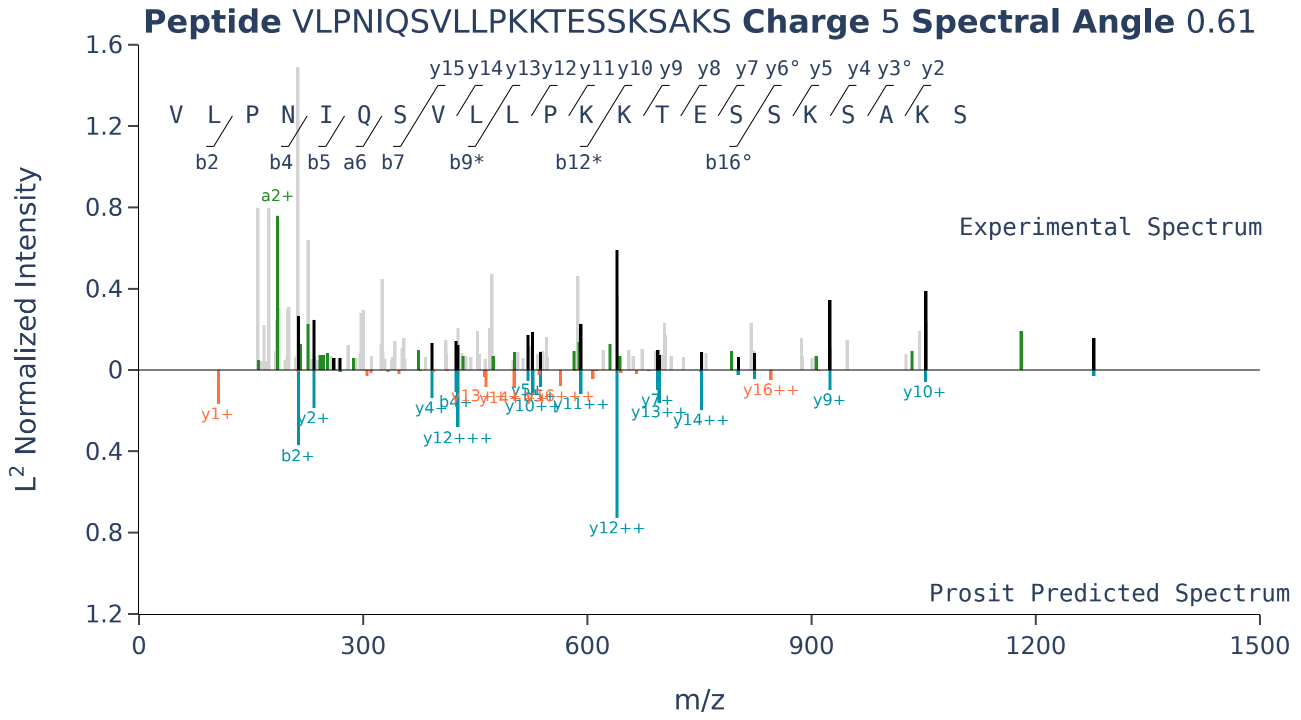


**Source** HRoetschke_291222_060123_Fu_H2A_b2R2_24h **Scan** 4430
**Peptide** PVGRVHRLLRKGNYAE **Charge** 3 **Spectral Angle** 0.48

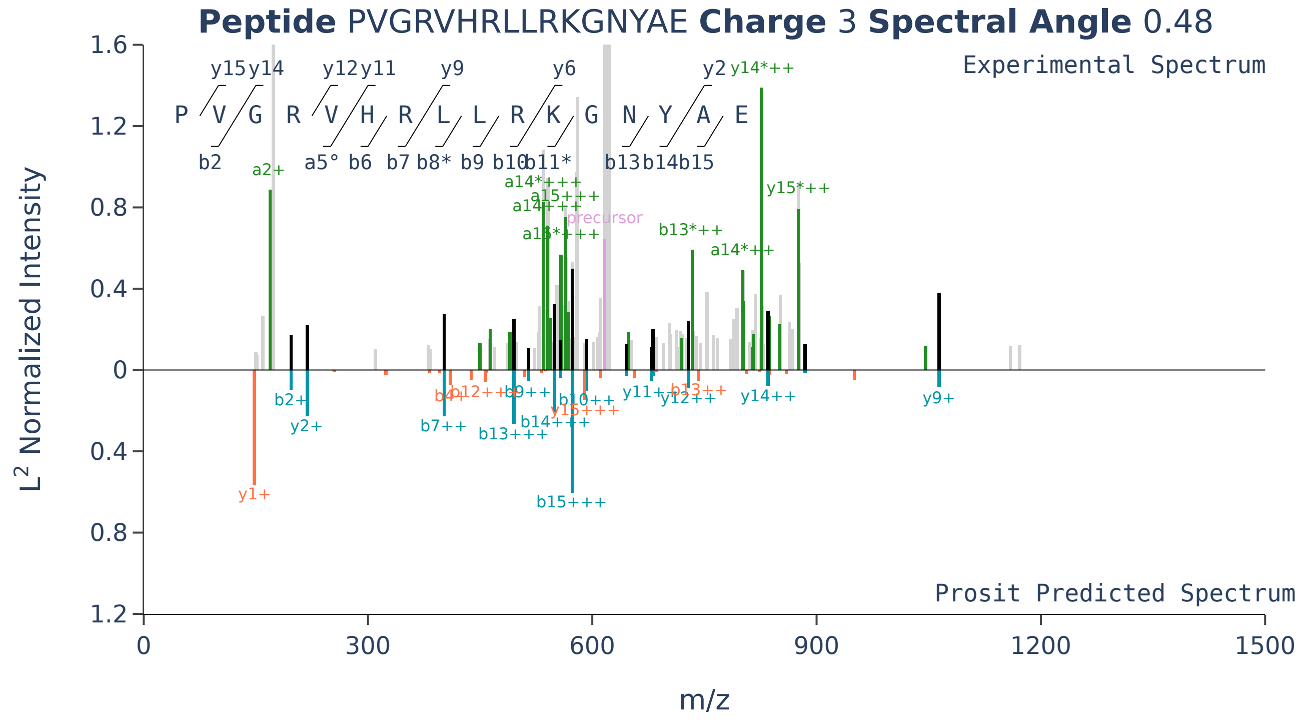


**Source** HRoetschke_291222_060123_Fu_H2A_b1R1_4h **Scan** 14999
**Peptide** AEILELAGNAARDNKKTRIIPRHLQLA **Charge** 5 **Spectral Angle** 0.65

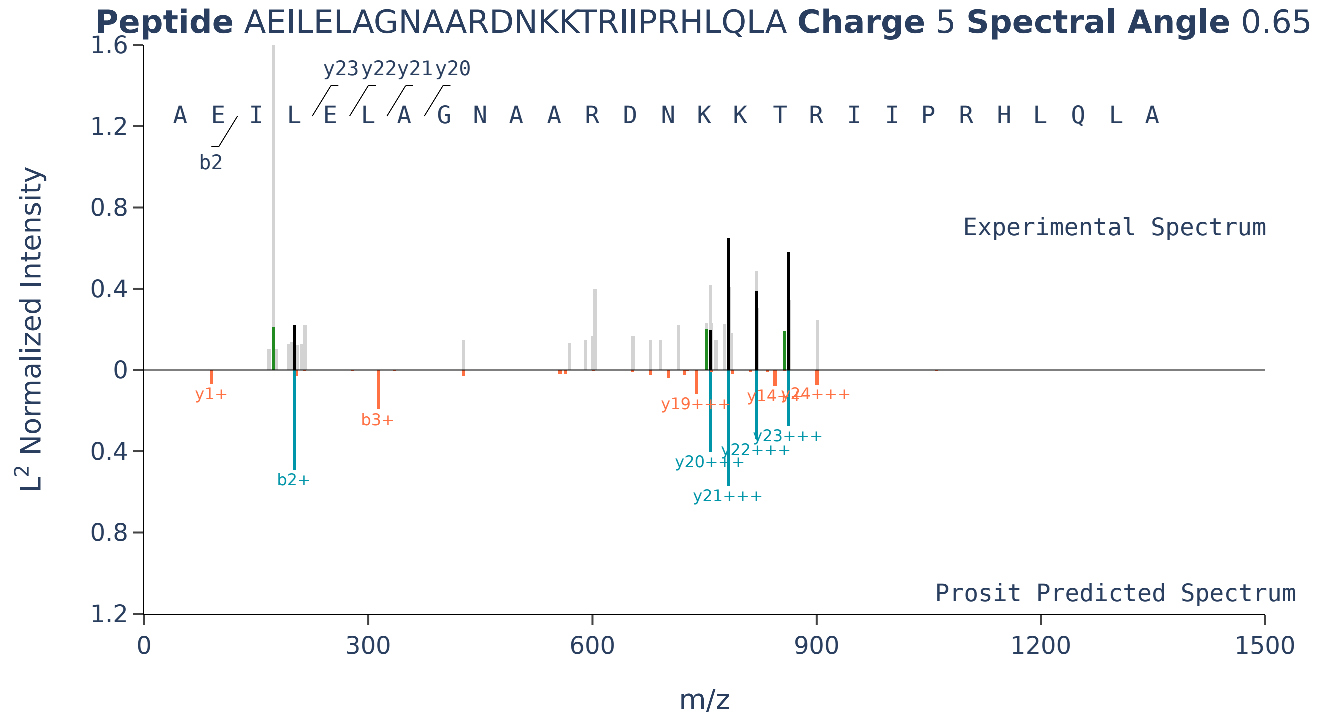


**Source** HRoetschke_291222_060123_Fu_H2A_b2R1_4h **Scan** 7613
**Peptide** AGNAARDNKKTRIIPRHLQLAVRNDE **Charge** 4 **Spectral Angle** 0.58

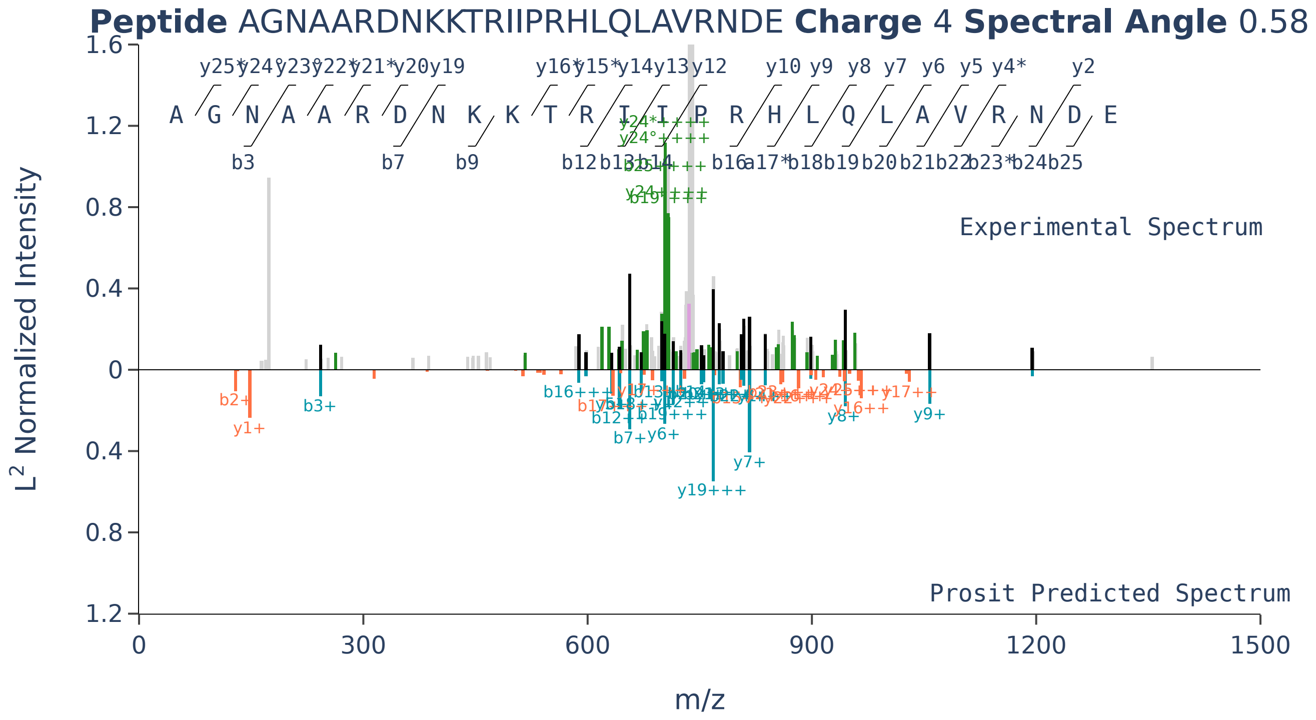


**Source** HRoetschke_291222_060123_Fu_H2A_b1R2_24h **Scan** 3793
**Peptide** HRLLRKGNYAERVGA **Charge** 4 **Spectral Angle** 0.72

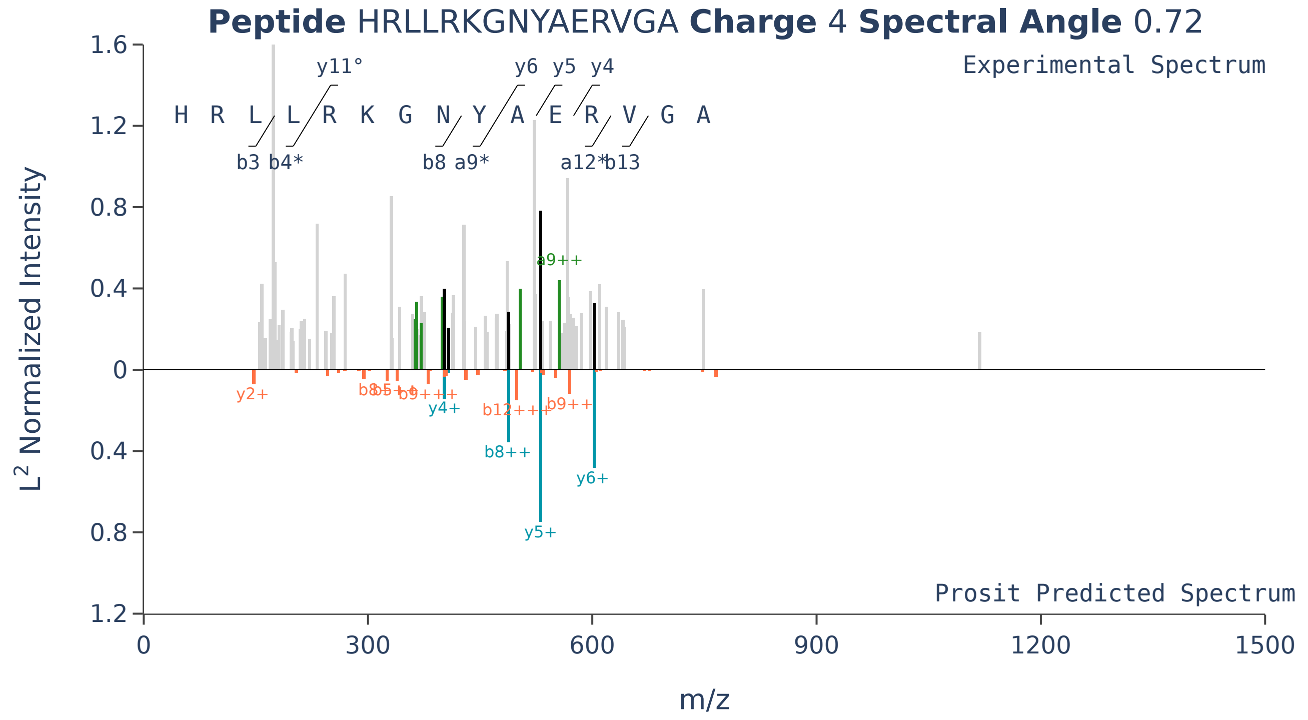


**Source** HRoetschke_291222_060123_Fu_H2A_b2R2_24h **Scan** 1689
**Peptide** ELAGNAARDNKKTR **Charge** 3 **Spectral Angle** 0.77

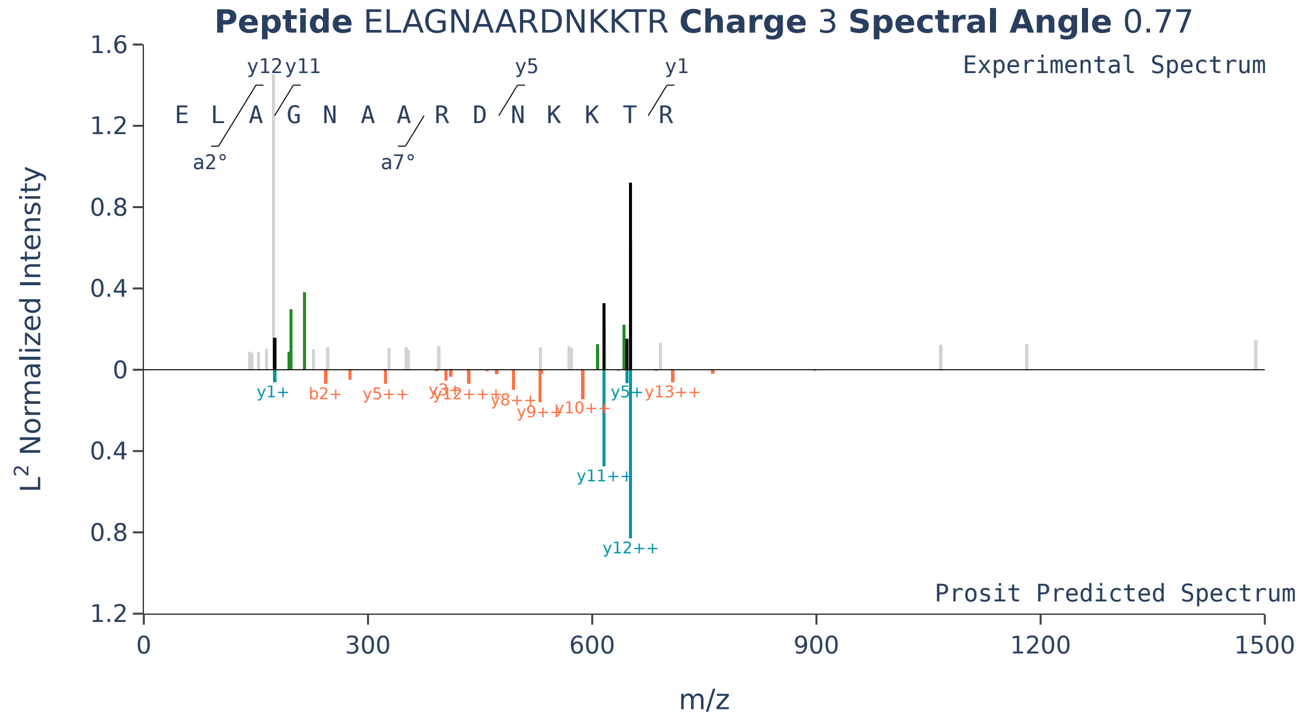


**Source** HRoetschke_291222_060123_Fu_H2A_b1R2_4h **Scan** 8866
**Peptide** LELAGNAARDNKKTRII **Charge** 4 **Spectral Angle** 0.63

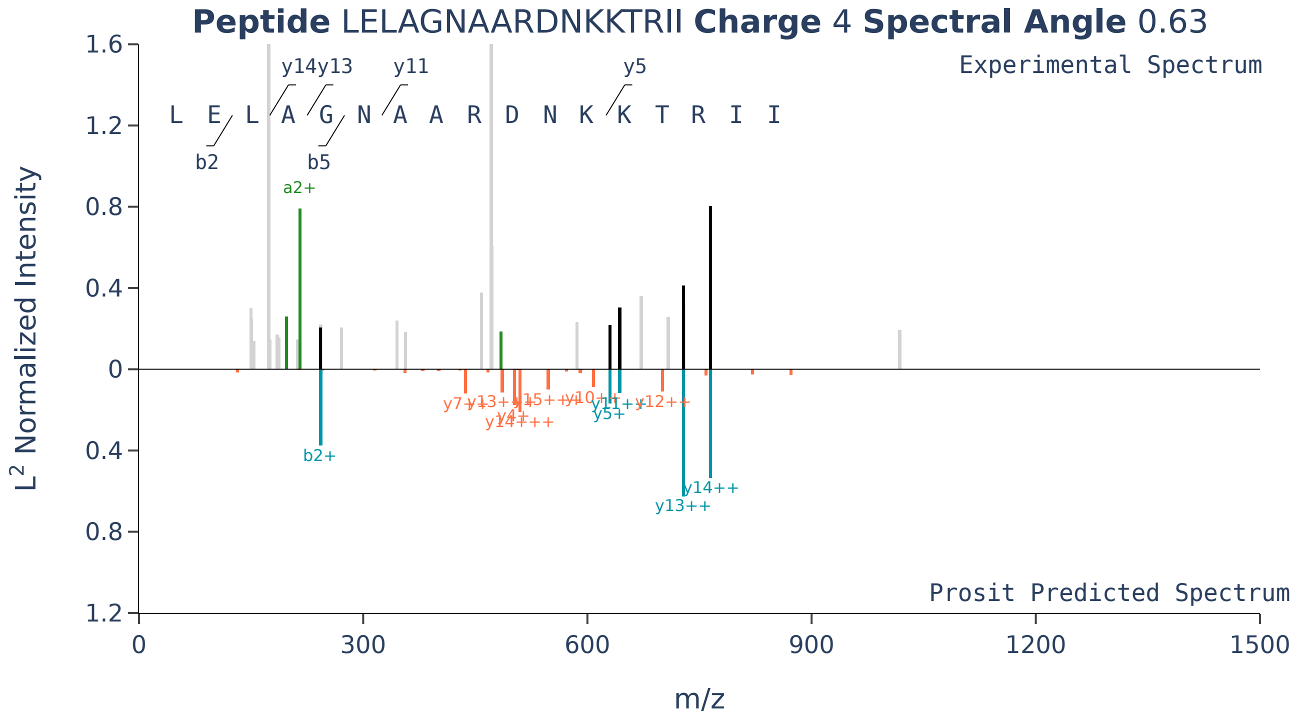


**Source** HRoetschke_291222_060123_Fu_H2A_b1R1_2h **Scan** 15623
**Peptide** PRHLQLAVRNDEELNKLLGRVT **Charge** 4 **Spectral Angle** 0.7

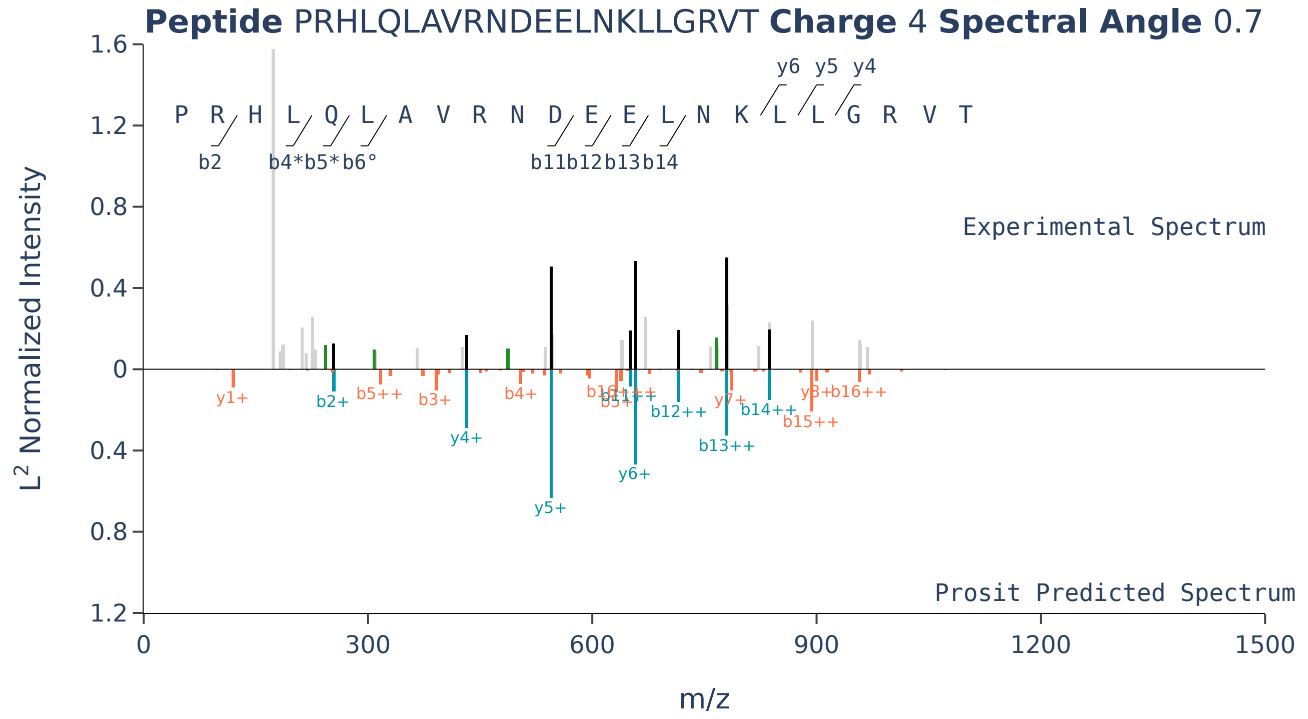

**Source** HRoetschke_291222_080123_Fu_H2B_b2R2_24h **Scan** 3543
**Peptide** SREIQTAVR **Charge** 2 **Spectral Angle** 0.95

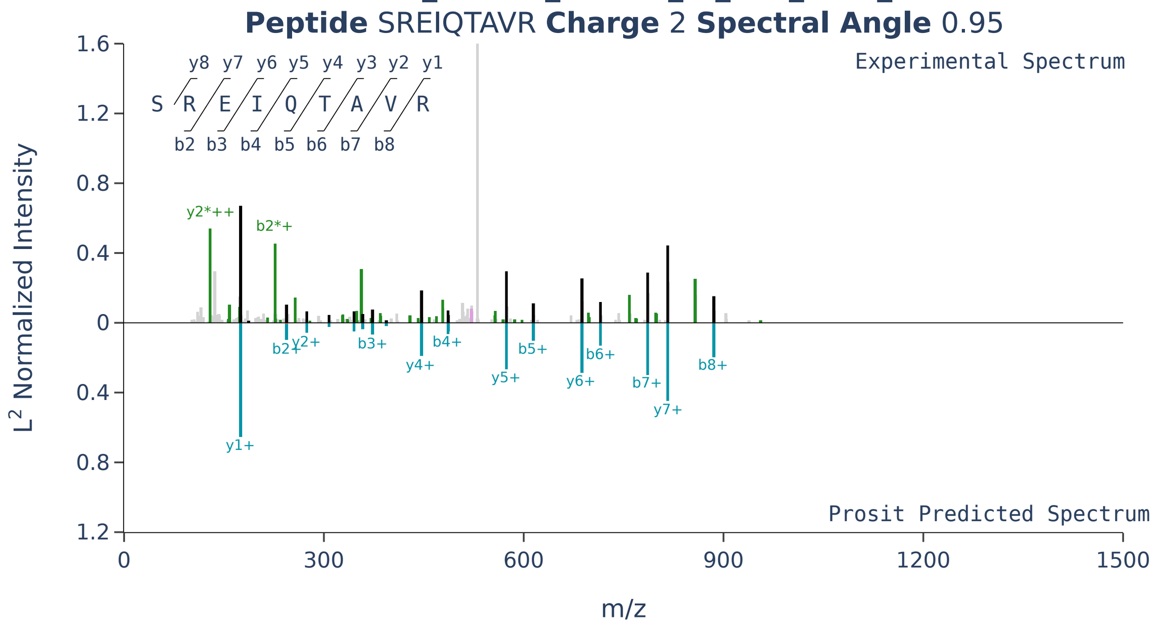


**Source** HRoetschke_291222_080123_Fu_H2B_b2R1_2h **Scan** 6657
**Peptide** LTSREIQTAVR **Charge** 2 **Spectral Angle** 0.93

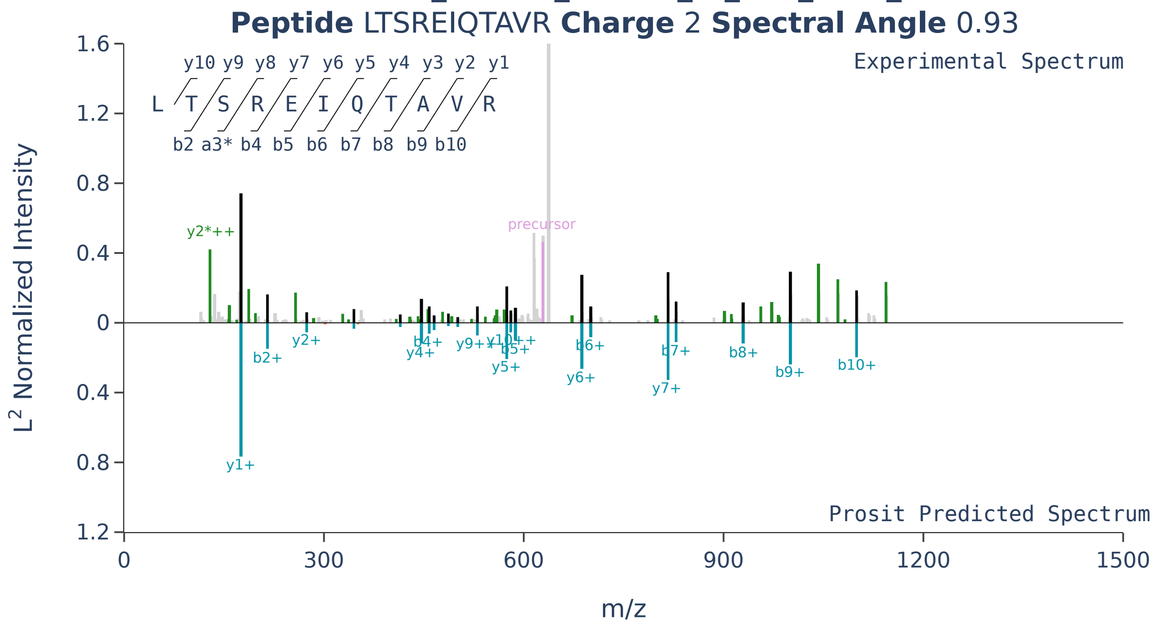


**Source** HRoetschke_291222_080123_Fu_H2B_b2R1_24h **Scan** 16251
**Peptide** AMSIMNSF **Charge** 2 **Spectral Angle** 0.9

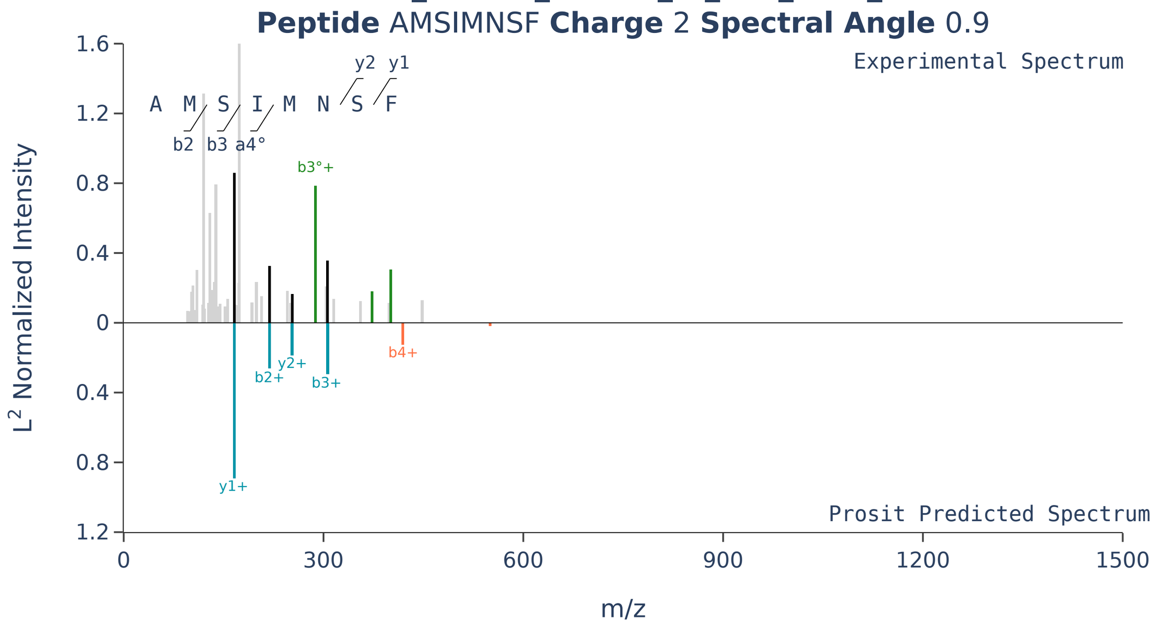


**Source** HRoetschke_291222_080123_Fu_H2B_b1R2_24h **Scan** 14117
**Peptide** VNDVFERIAG **Charge** 2 **Spectral Angle** 0.94

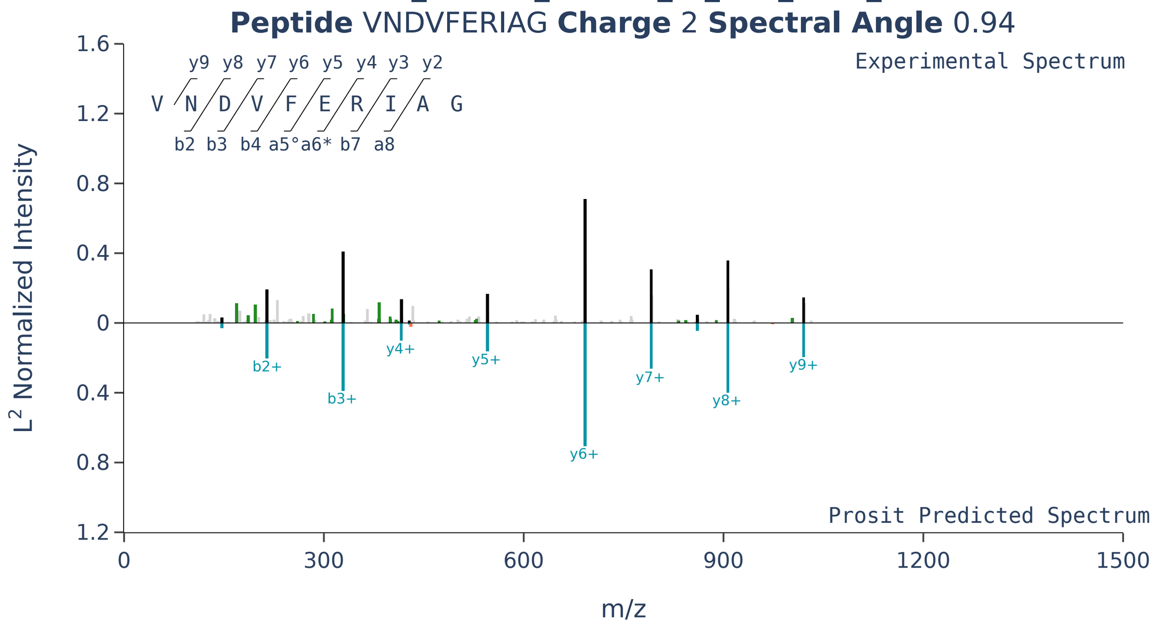


**Source** HRoetschke_291222_080123_Fu_H2B_b1R1_1h **Scan** 7587
**Peptide** STITSREIQTAVR **Charge** 3 **Spectral Angle** 0.92

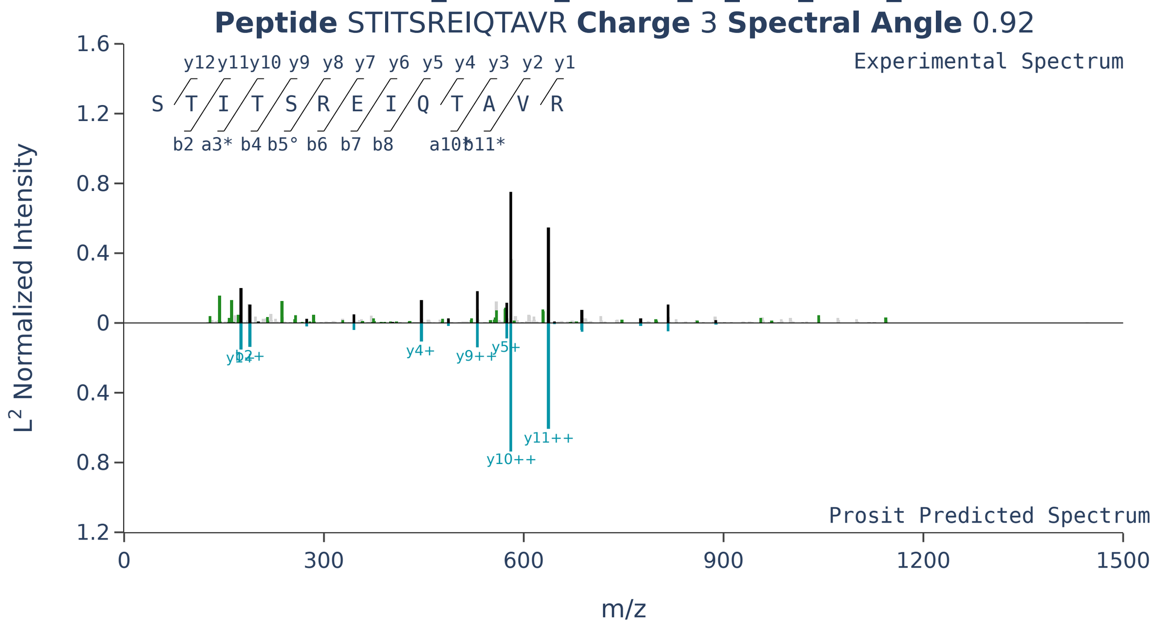


**Source** HRoetschke_291222_080123_Fu_H2B_b1R2_2h **Scan** 2762
**Peptide** REIQTAVR **Charge** 2 **Spectral Angle** 0.93

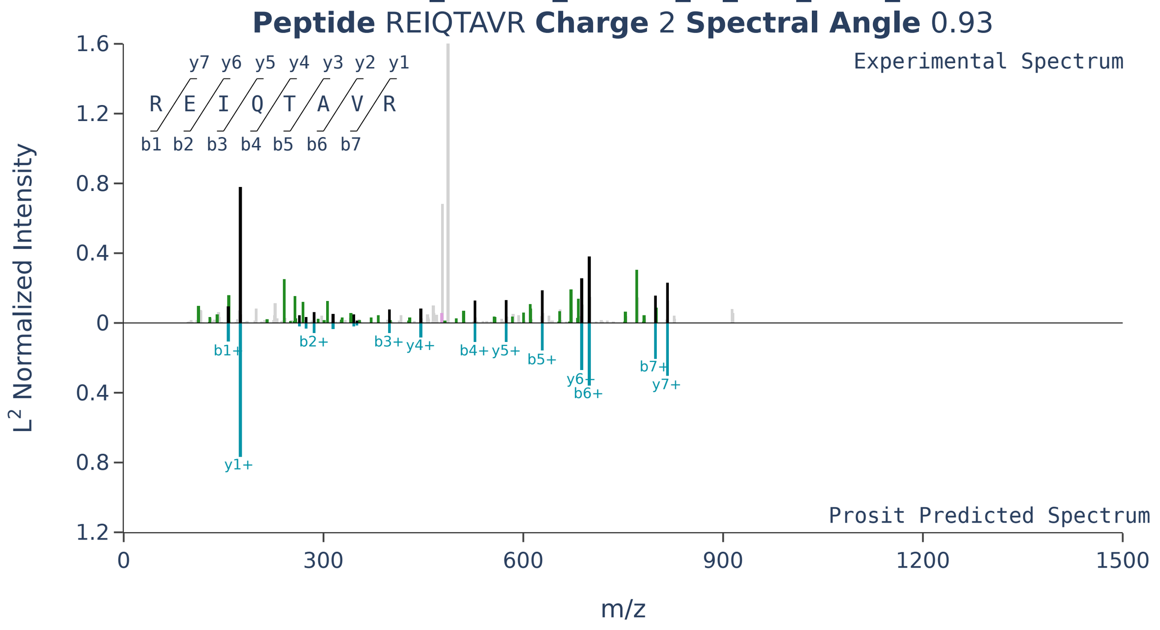


**Source** HRoetschke_291222_080123_Fu_H2B_b1R1_4h **Scan** 2478
**Peptide** EGTKAVTKYTSAK **Charge** 2 **Spectral Angle** 0.88

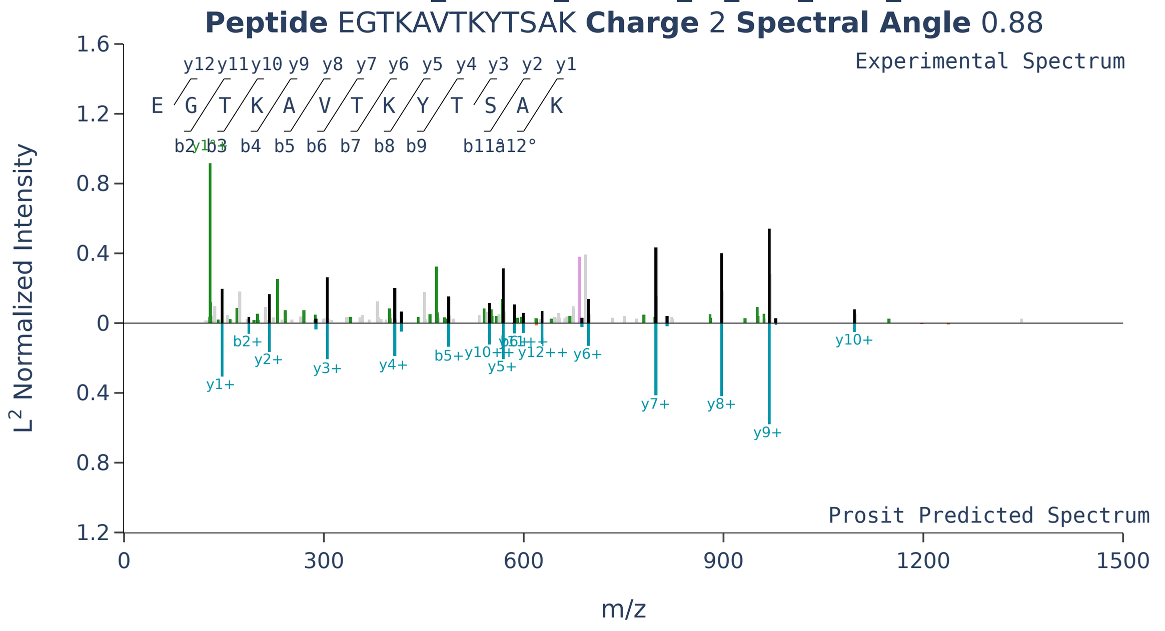


**Source** HRoetschke_291222_080123_Fu_H2B_b2R2_24h **Scan** 11264
**Peptide** SREIQTAVRL **Charge** 2 **Spectral Angle** 0.89

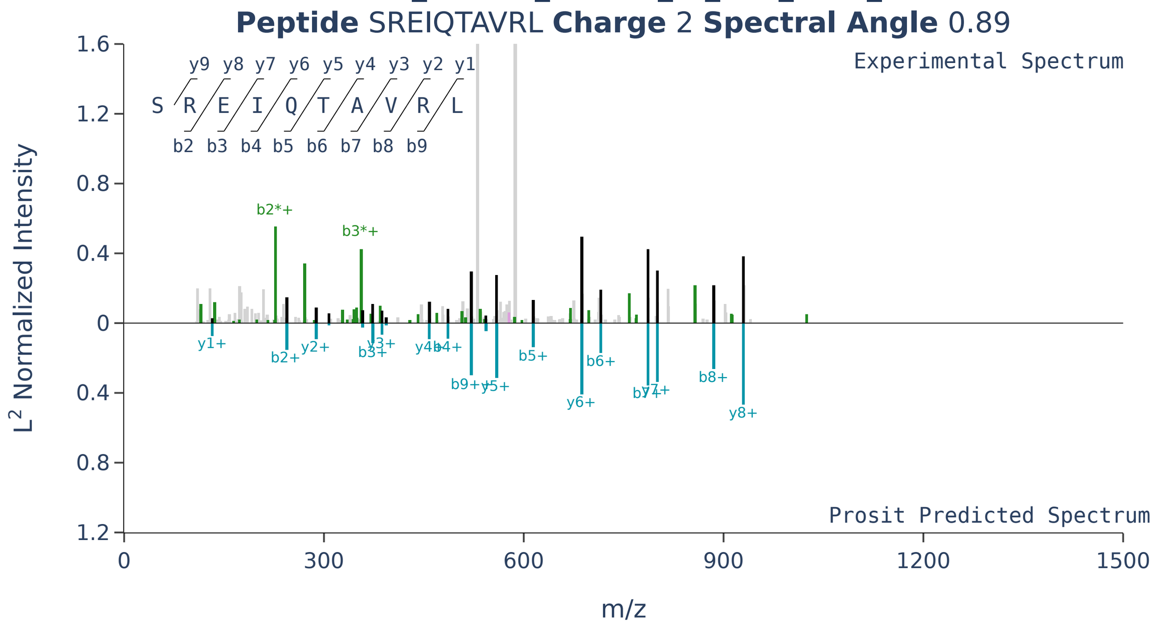


**Source** HRoetschke_291222_080123_Fu_H2B_b1R2_24h **Scan** 14312
**Peptide** VNDVFERIAGE **Charge** 2 **Spectral Angle** 0.9

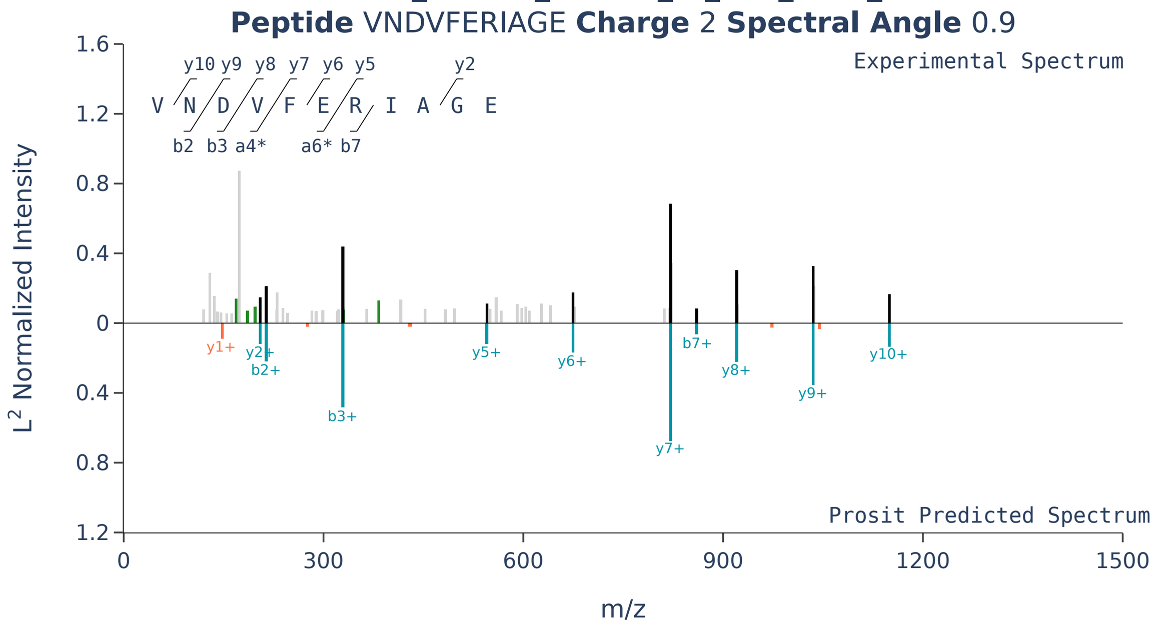


**Source** HRoetschke_291222_080123_Fu_H2B_b2R1_2h **Scan** 8294
**Peptide** VHPDTGISSKAMSL **Charge** 3 **Spectral Angle** 0.89

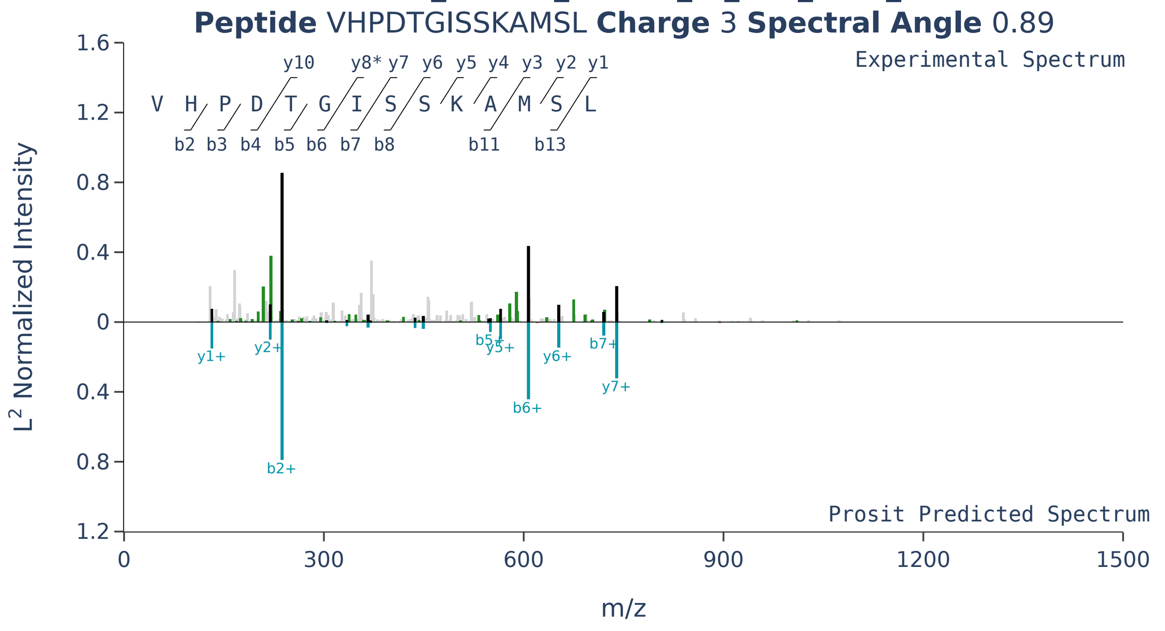


**Source** HRoetschke_291222_080123_Fu_H2B_b2R2_1h **Scan** 7772
**Peptide** VHPDTGISSKAMSI **Charge** 3 **Spectral Angle** 0.91

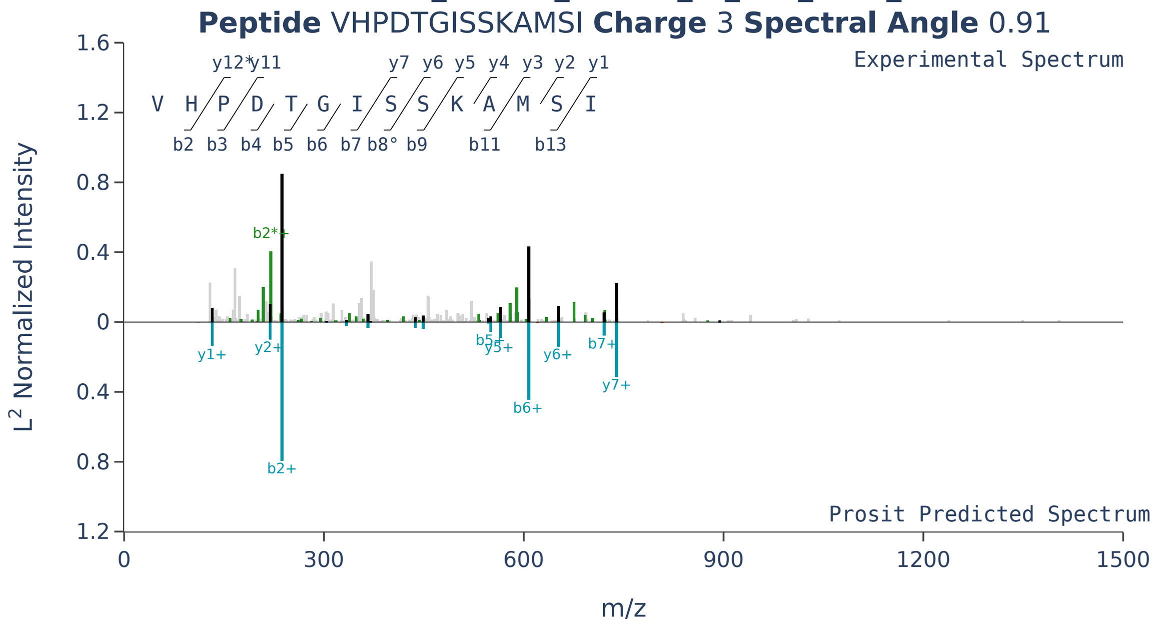


**Source** HRoetschke_291222_080123_Fu_H2B_b2R1_4h **Scan** 7415
**Peptide** HPDTGISSKAMSI **Charge** 3 **Spectral Angle** 0.86

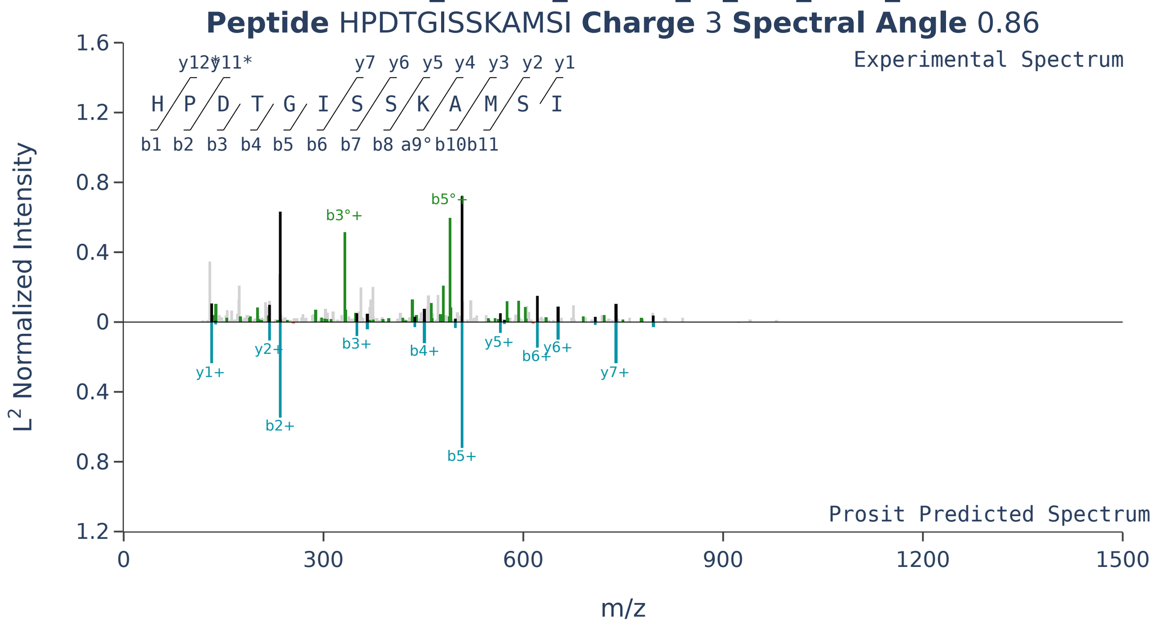


**Source** HRoetschke_291222_080123_Fu_H2B_b1R2_1h **Scan** 2963
**Peptide** VHPDTGISSKA **Charge** 3 **Spectral Angle** 0.89

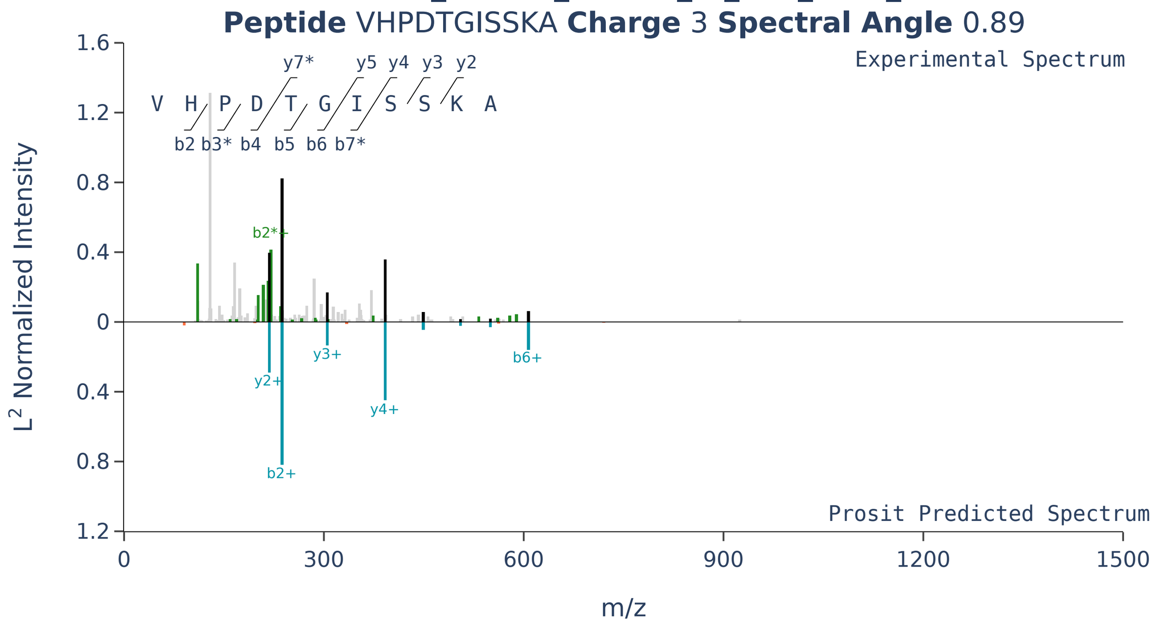


**Source** HRoetschke_291222_080123_Fu_H2B_b1R2_2h **Scan** 13864
**Peptide** KAMSIMNSF **Charge** 2 **Spectral Angle** 0.91

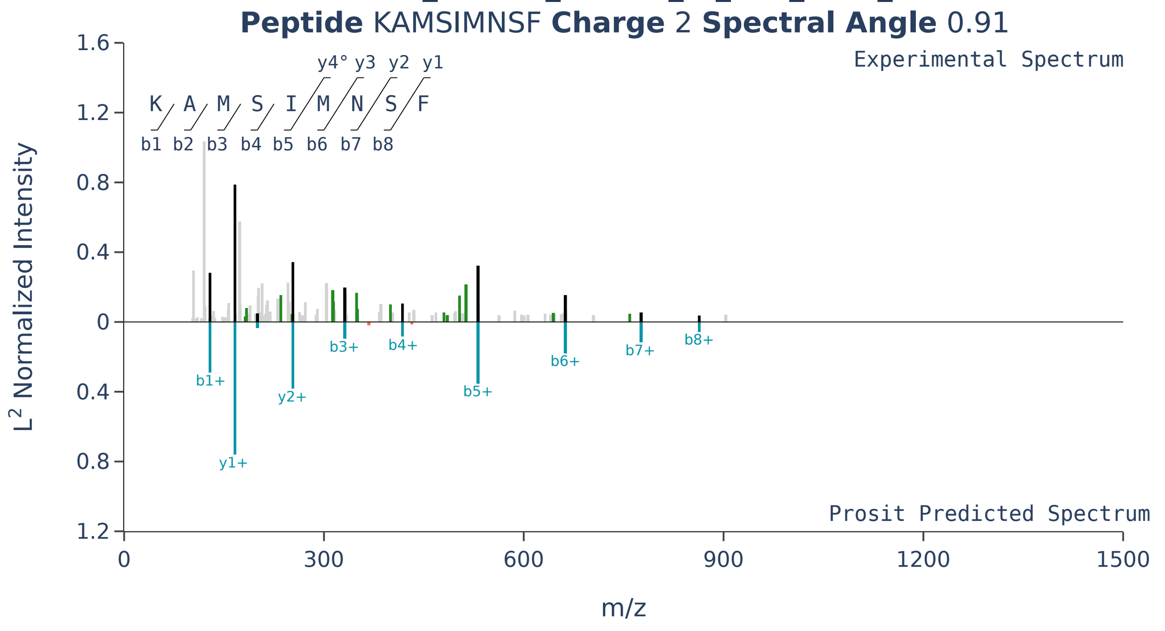


**Source** HRoetschke_291222_080123_Fu_H2B_b1R2_24h **Scan** 12880
**Peptide** ELQTAVRL **Charge** 2 **Spectral Angle** 0.9

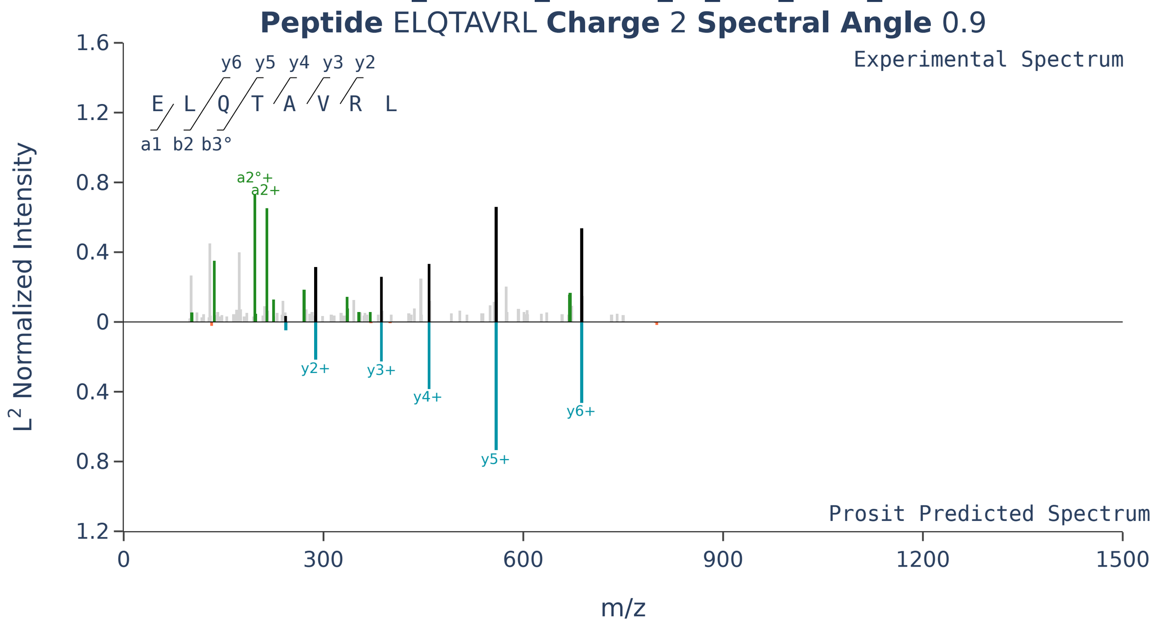

**Source** HRoetschke_291222_080123_Fu_H2B_b1R2_24h **Scan** 13408
**Peptide** RLLLPGE **Charge** 2 **Spectral Angle** 0.85

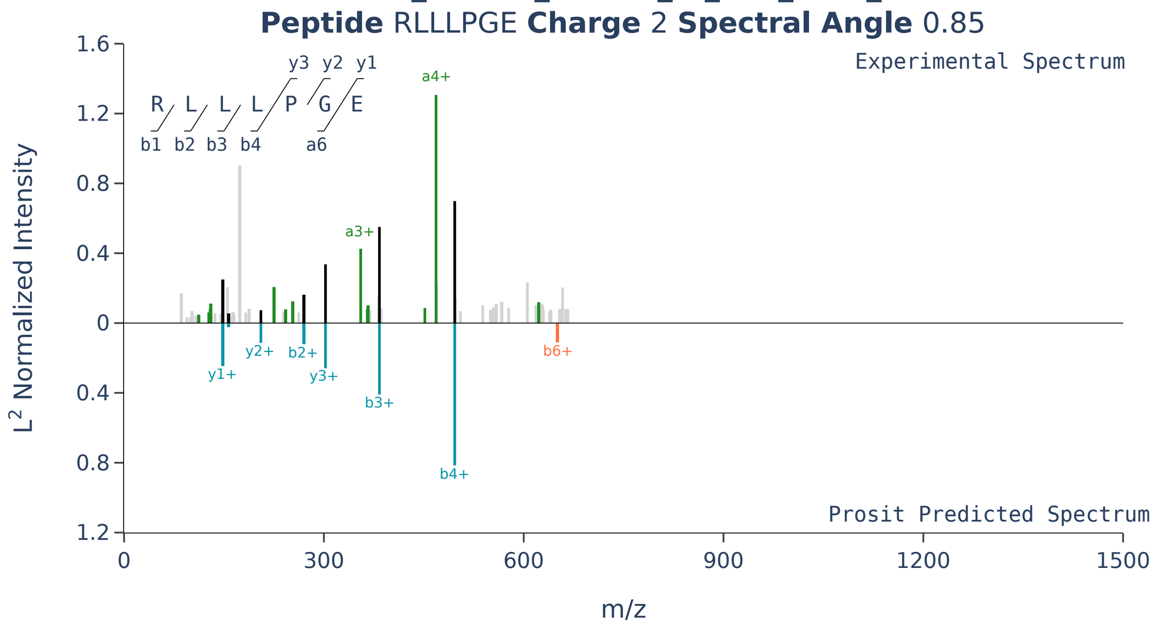


**Source** HRoetschke_291222_080123_Fu_H2B_b2R2_24h **Scan** 3166
**Peptide** SEGTKAVTKYTSAK **Charge** 2 **Spectral Angle** 0.88

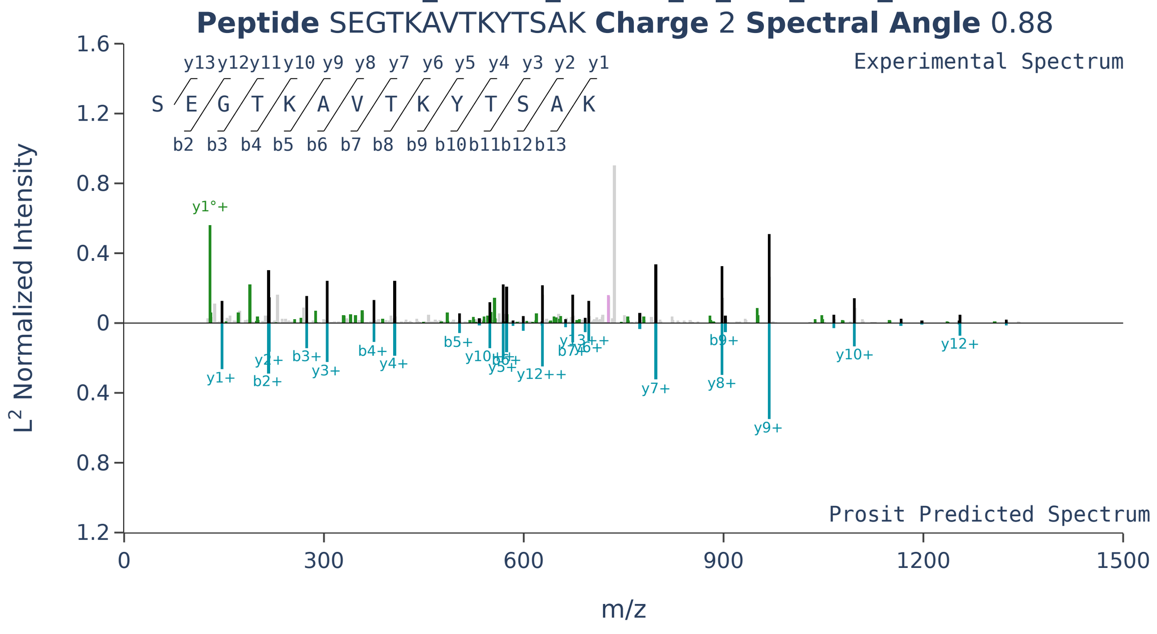


**Source** HRoetschke_291222_080123_Fu_H2B_b1R1_1h **Scan** 10679
**Peptide** VFERIAGEASRL **Charge** 2 **Spectral Angle** 0.87

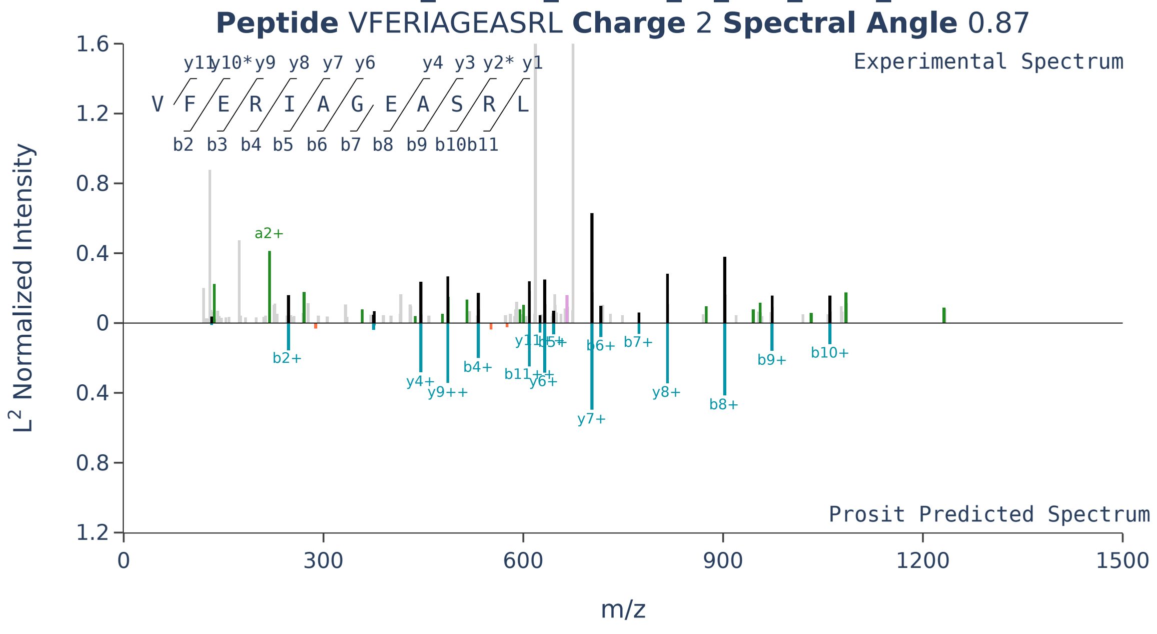


**Source** HRoetschke_291222_080123_Fu_H2B_b1R2_24h **Scan** 7465
**Peptide** STITSREIQT **Charge** 2 **Spectral Angle** 0.89

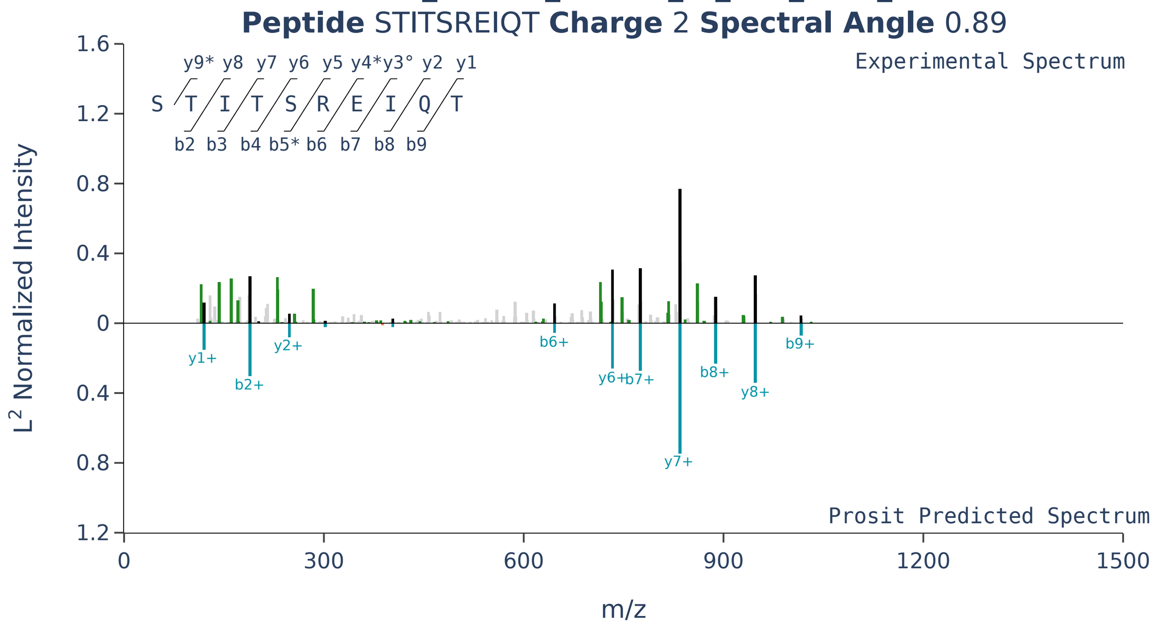


**Source** HRoetschke_291222_080123_Fu_H2B_b1R1_1h **Scan** 4687
**Peptide** LPGELAKH **Charge** 2 **Spectral Angle** 0.88

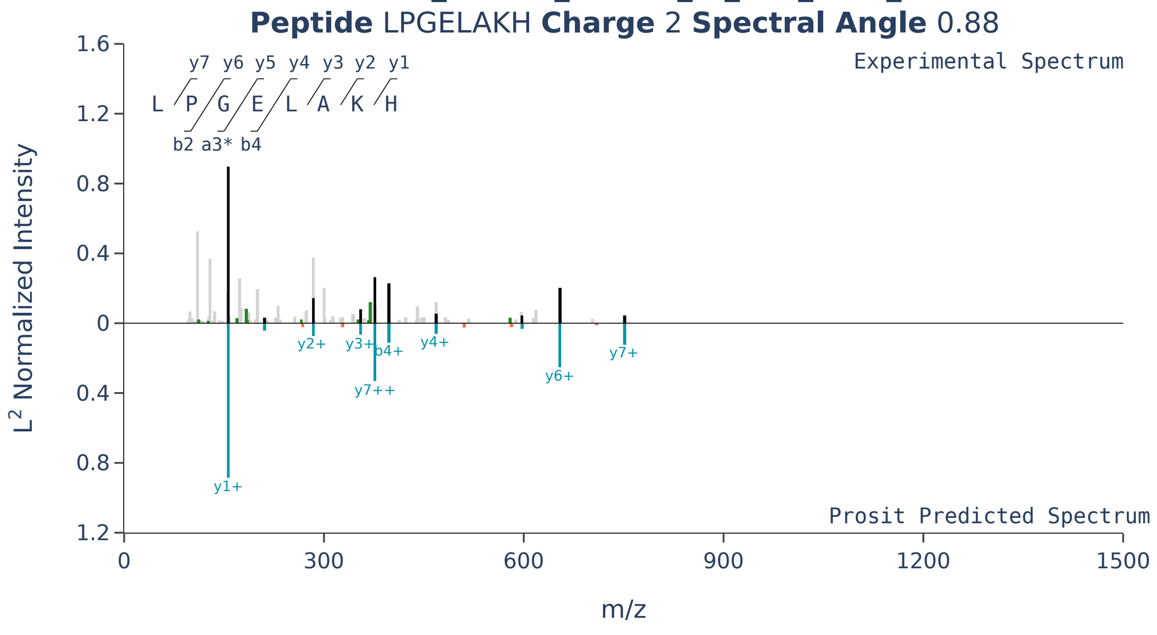


**Source** HRoetschke_291222_080123_Fu_H2B_b2R2_4h **Scan** 3550
**Peptide** PGELAKHAVSE **Charge** 2 **Spectral Angle** 0.86

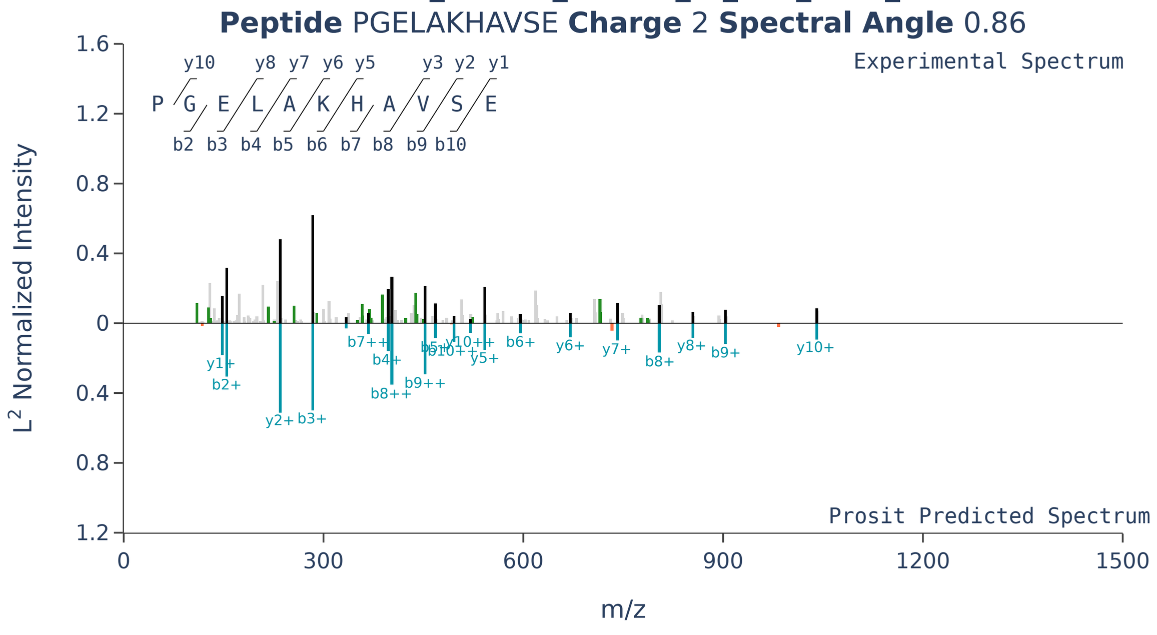


**Source** HRoetschke_291222_080123_Fu_H2B_b1R1_1h **Scan** 3081
**Peptide** VHPDTGISSKAM **Charge** 3 **Spectral Angle** 0.84

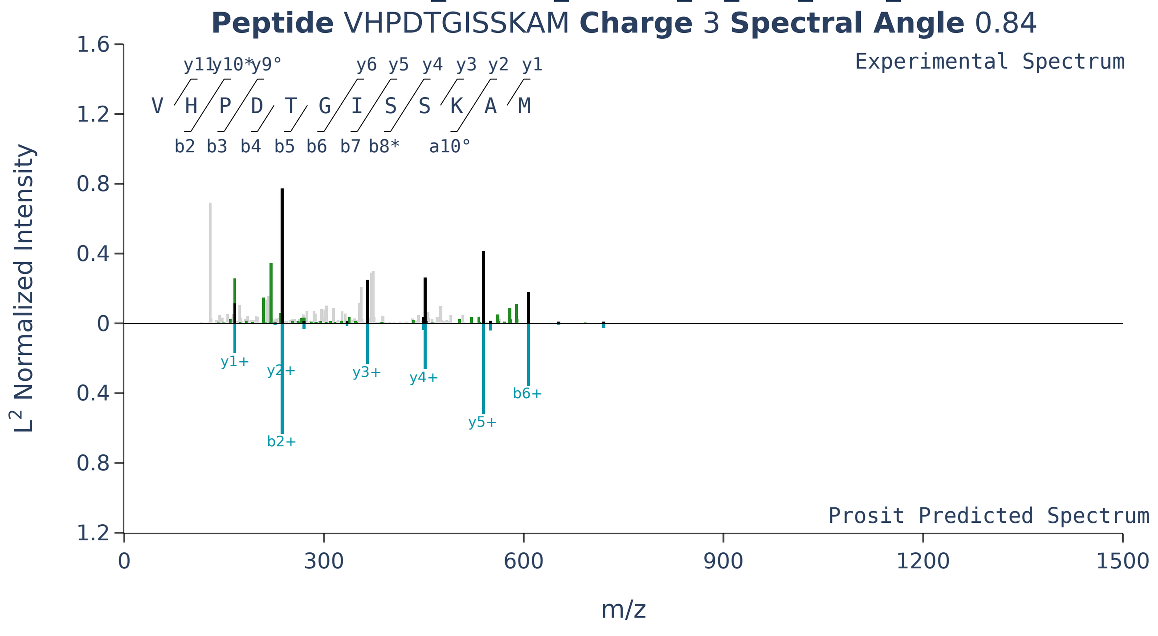


**Source** HRoetschke_291222_080123_Fu_H2B_b2R1_24h **Scan** 7621
**Peptide** VFERIAGEASR **Charge** 2 **Spectral Angle** 0.92

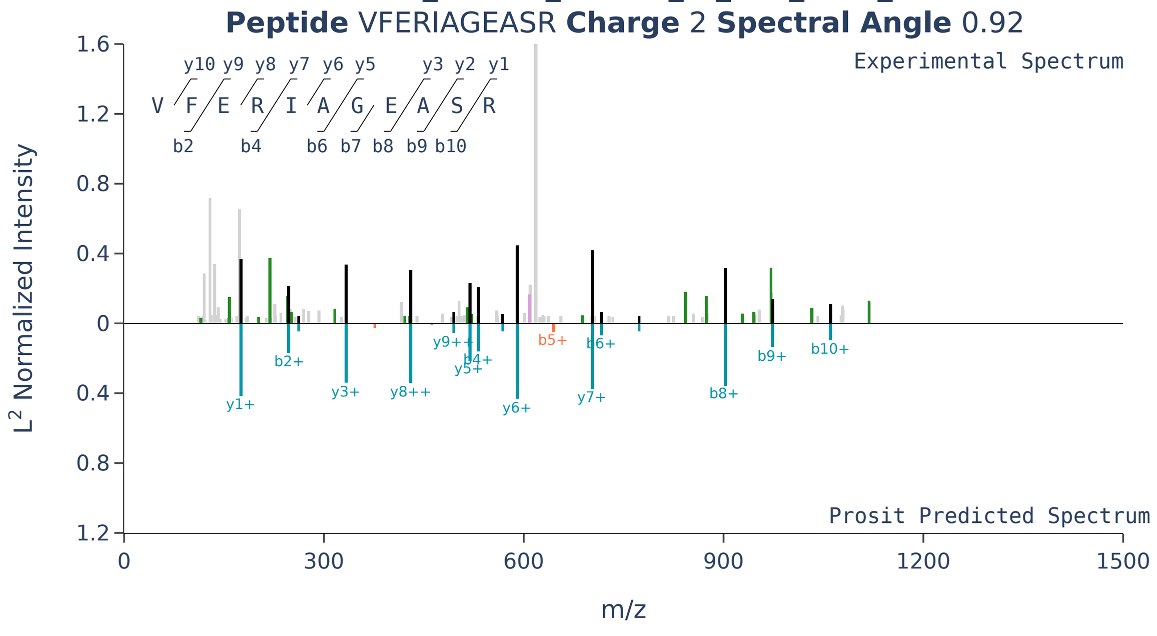


**Source** HRoetschke_291222_080123_Fu_H2B_b2R2_24h **Scan** 3848
**Peptide** LKQVHPDTGISSK **Charge** 2 **Spectral Angle** 0.9

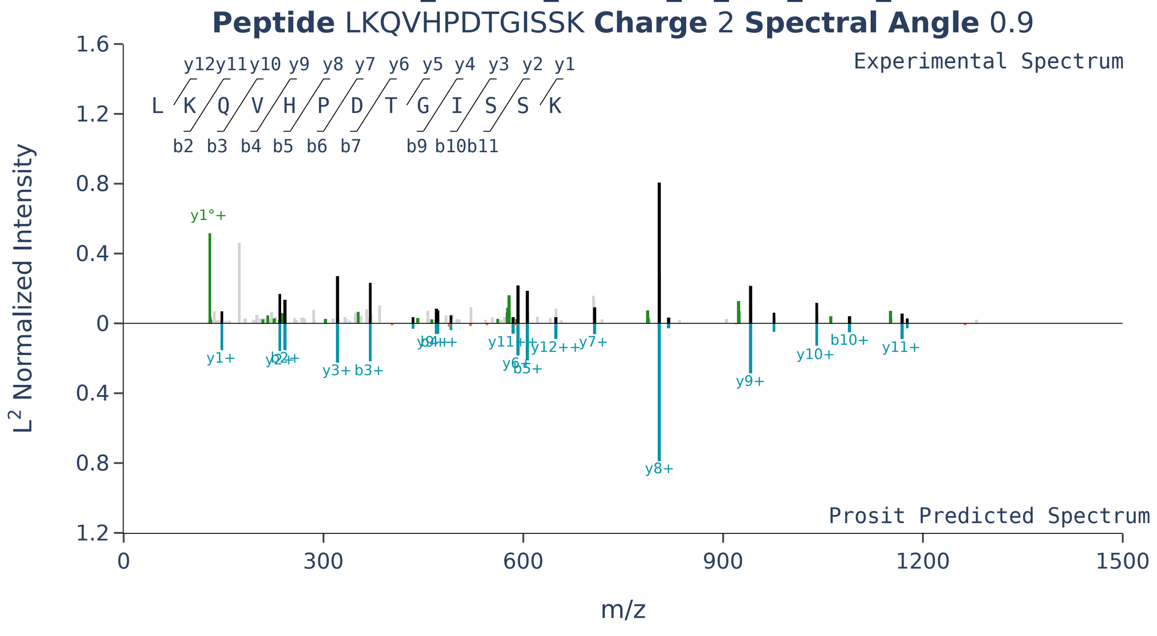


**Source** HRoetschke_291222_080123_Fu_H2B_b2R1_1h **Scan** 2787
**Peptide** GTKAVTKYTSA **Charge** 3 **Spectral Angle** 0.9

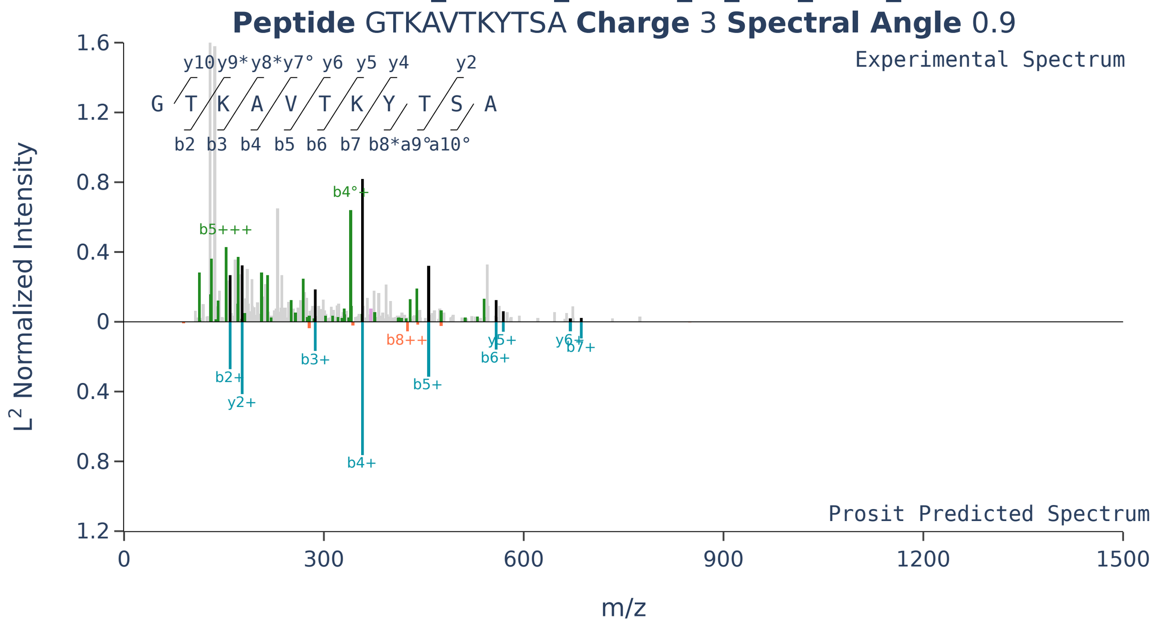


**Source** HRoetschke_291222_080123_Fu_H2B_b1R2_24h **Scan** 7025
**Peptide** TITSREIQTA **Charge** 2 **Spectral Angle** 0.94

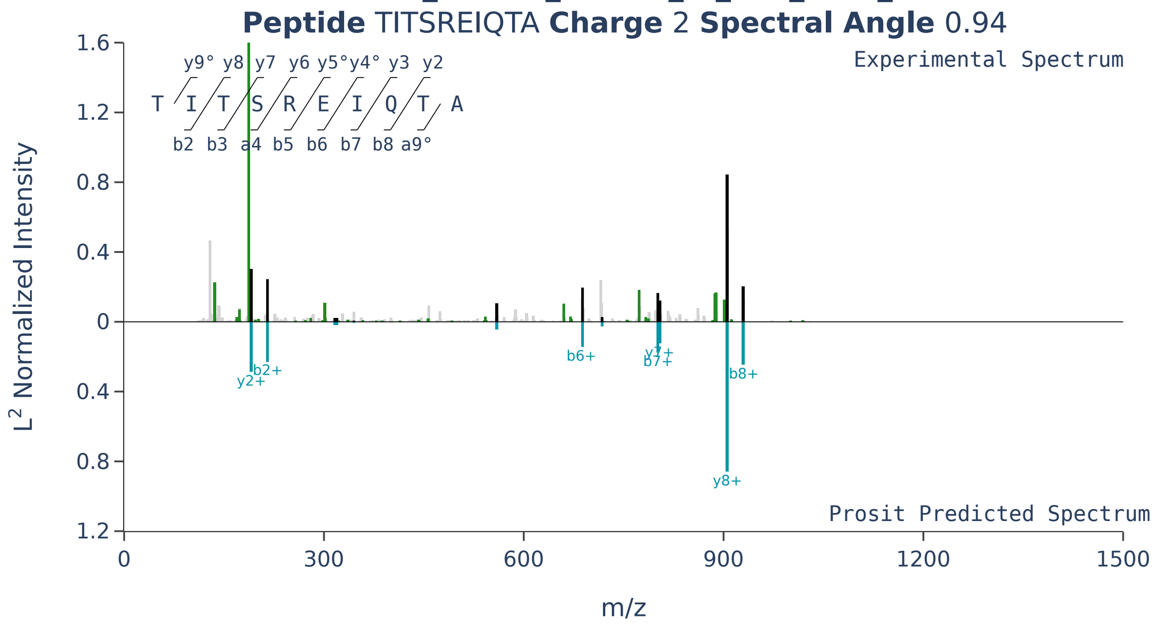


**Source** HRoetschke_291222_080123_Fu_H2B_b2R1_1h **Scan** 2347
**Peptide** VHPDTGISSK **Charge** 2 **Spectral Angle** 0.89

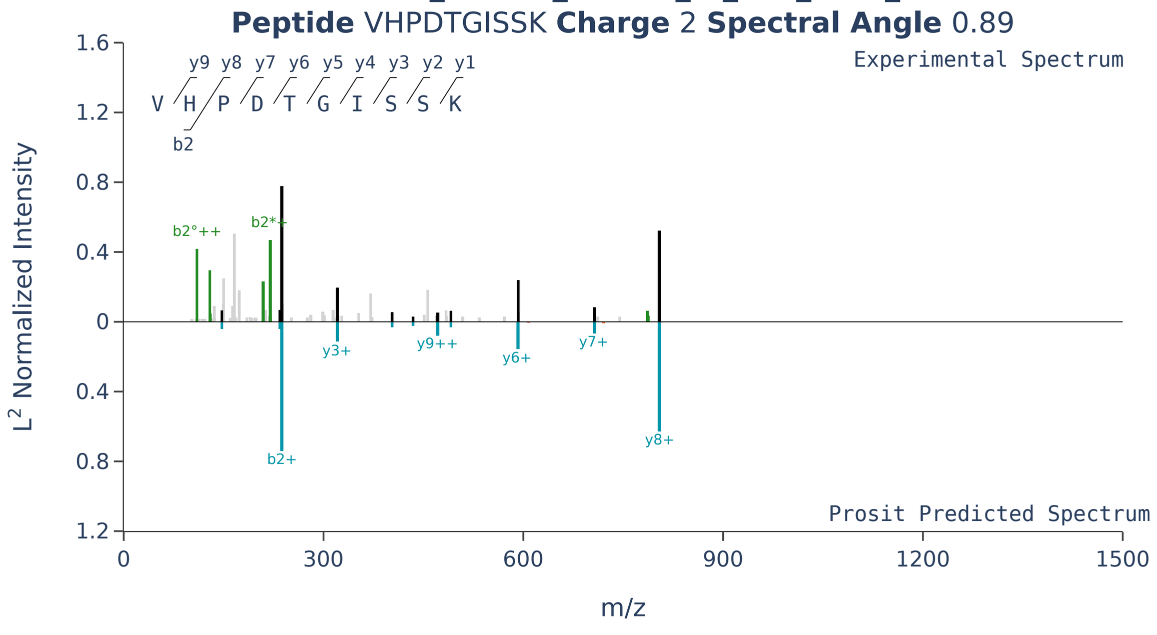


**Source** HRoetschke_291222_080123_Fu_H2B_b2R2_2h **Scan** 9974
**Peptide** TITSREIQTAV **Charge** 2 **Spectral Angle** 0.91

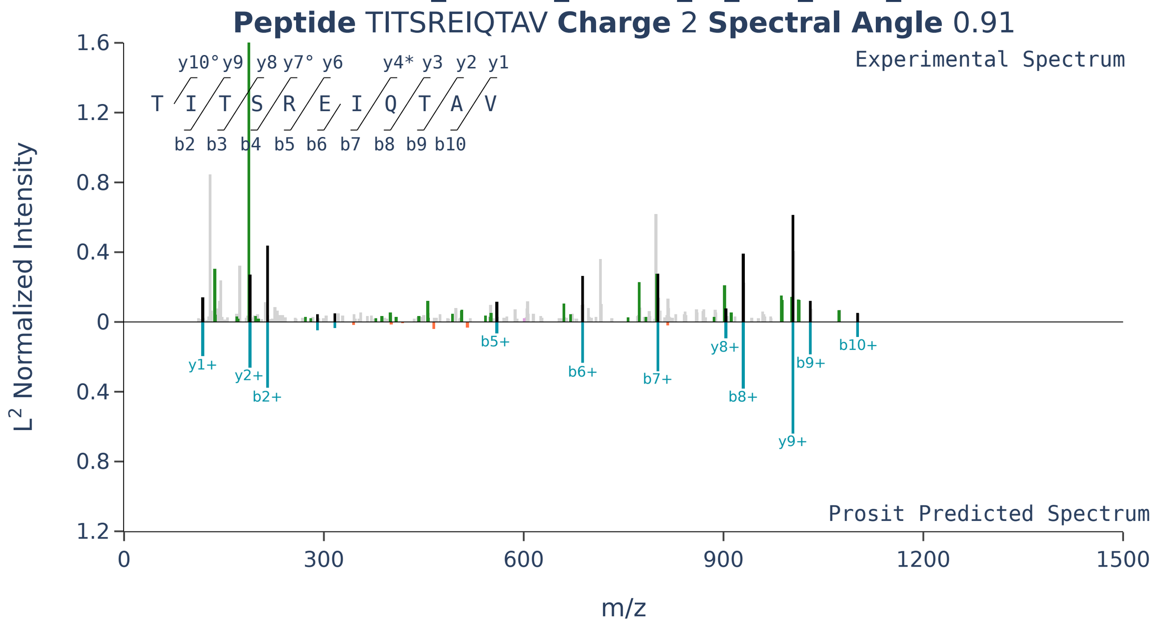


**Source** HRoetschke_291222_080123_Fu_H2B_b2R2_2h **Scan** 11663
**Peptide** RLLLPGELAKH **Charge** 3 **Spectral Angle** 0.91

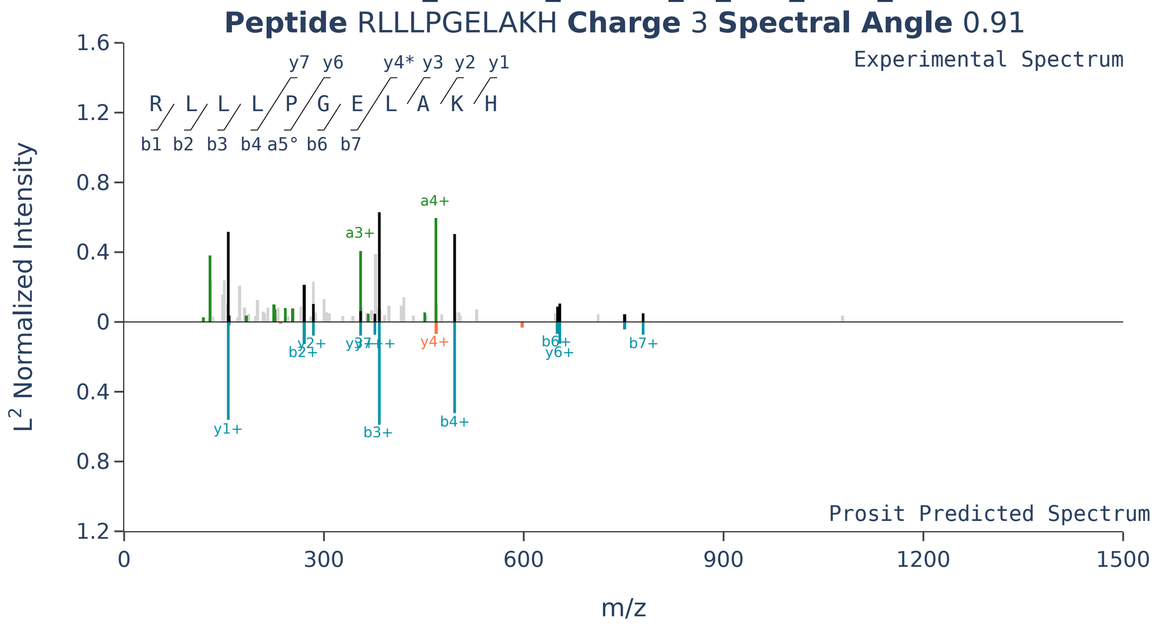


**Source** HRoetschke_291222_080123_Fu_H2B_b1R2_24h **Scan** 3636
**Peptide** SEGTKAVTKYTS **Charge** 2 **Spectral Angle** 0.89

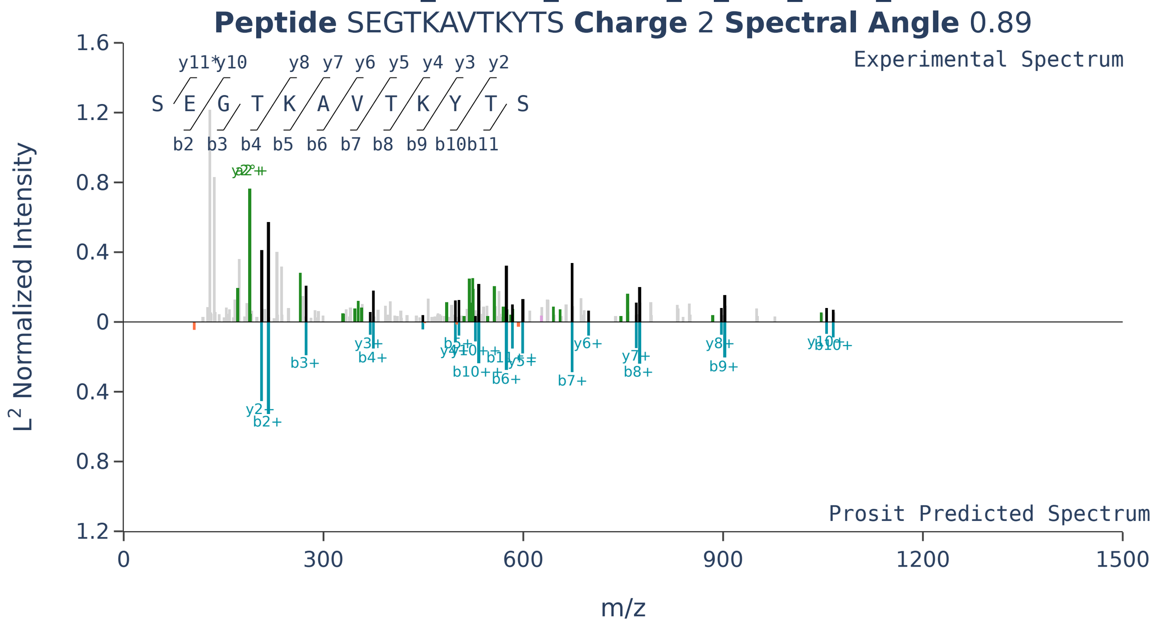

**Source** HRoetschke_291222_080123_Fu_H2B_b2R1_4h **Scan** 18937
**Peptide** MNSFVNDVFE **Charge** 2 **Spectral Angle** 0.92

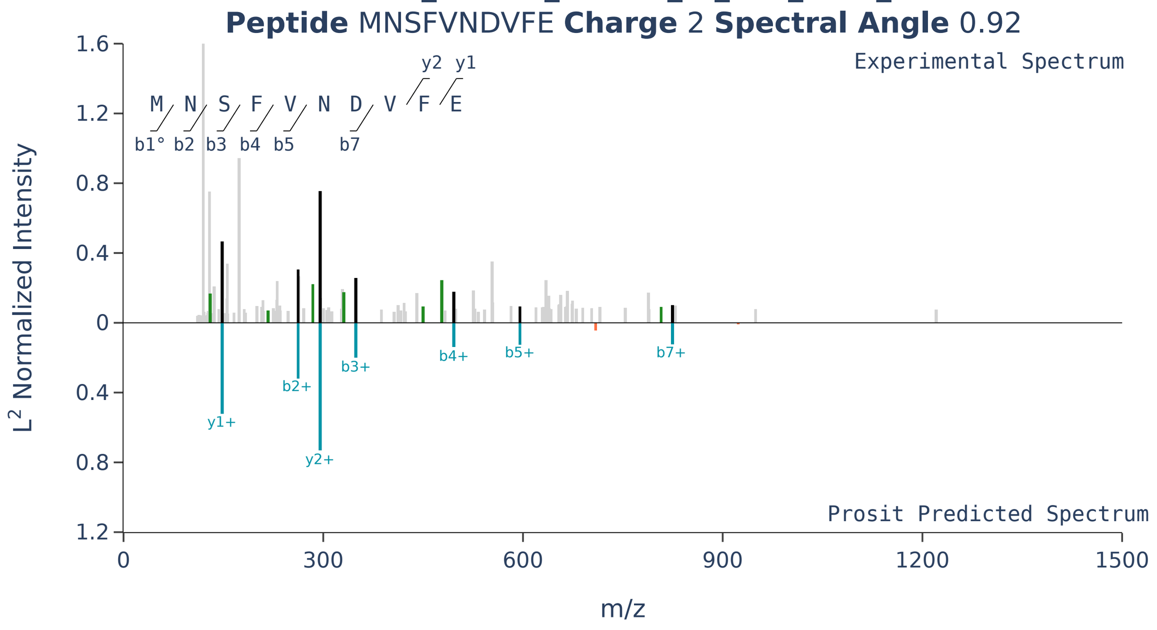


**Source** HRoetschke_291222_080123_Fu_H2B_b2R2_24h **Scan** 3925
**Peptide** TSREIQTAVR **Charge** 3 **Spectral Angle** 0.82

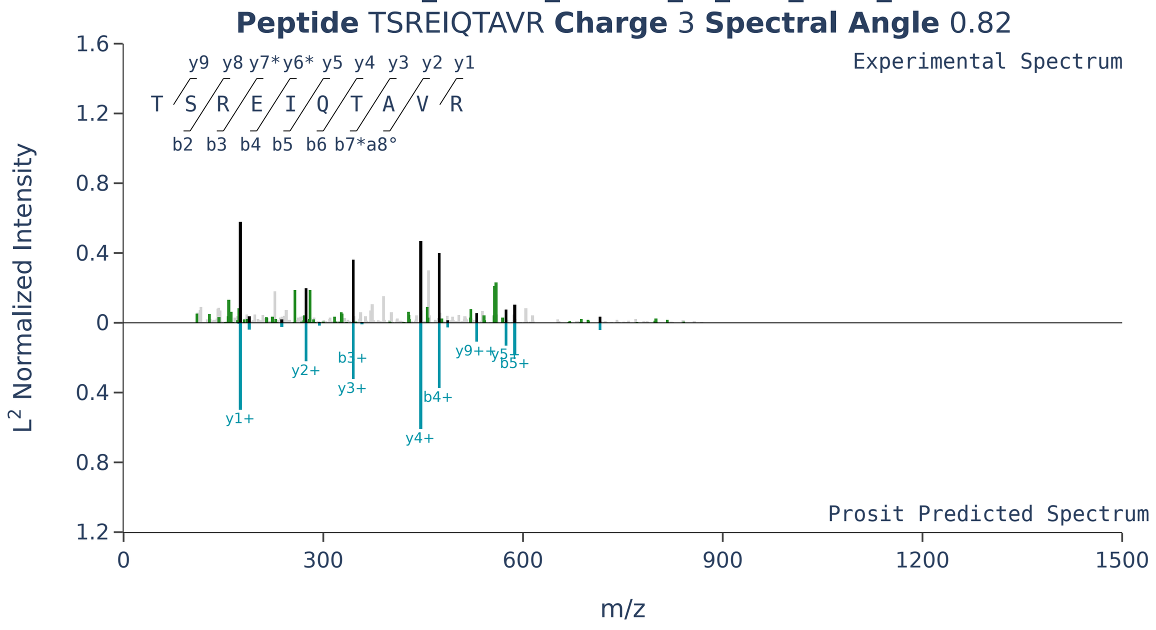


**Source** HRoetschke_291222_080123_Fu_H2B_b1R1_4h **Scan** 7607
**Peptide** MNSFVND **Charge** 1 **Spectral Angle** 0.93

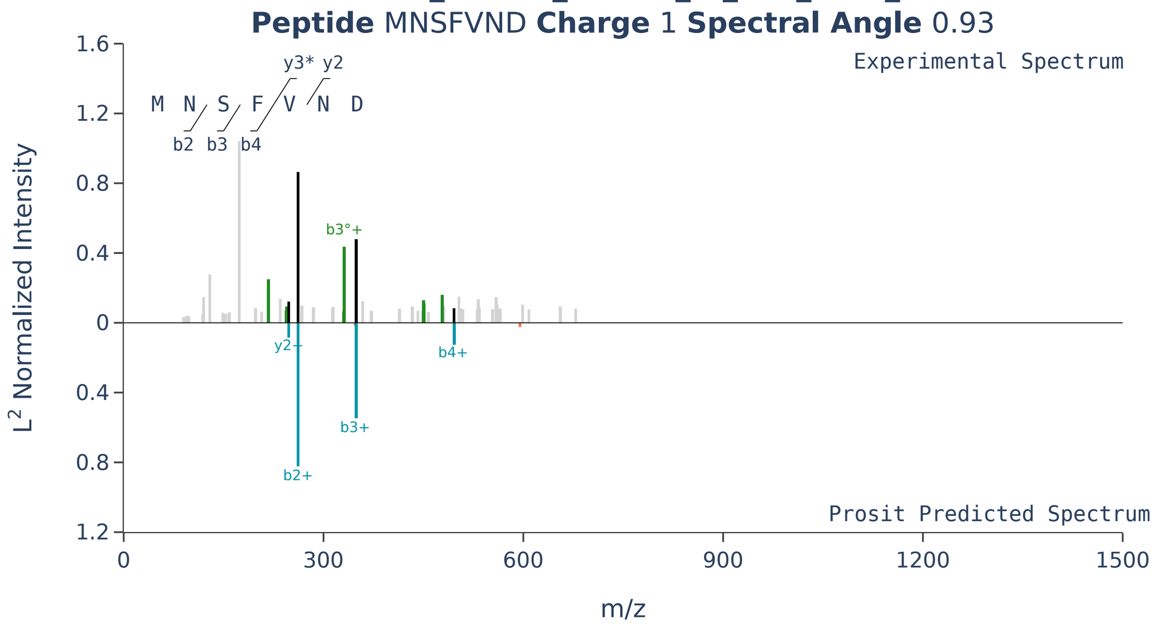


**Source** HRoetschke_291222_080123_Fu_H2B_b2R2_2h **Scan** 9544
**Peptide** KAMSIMNSFVND **Charge** 2 **Spectral Angle** 0.84

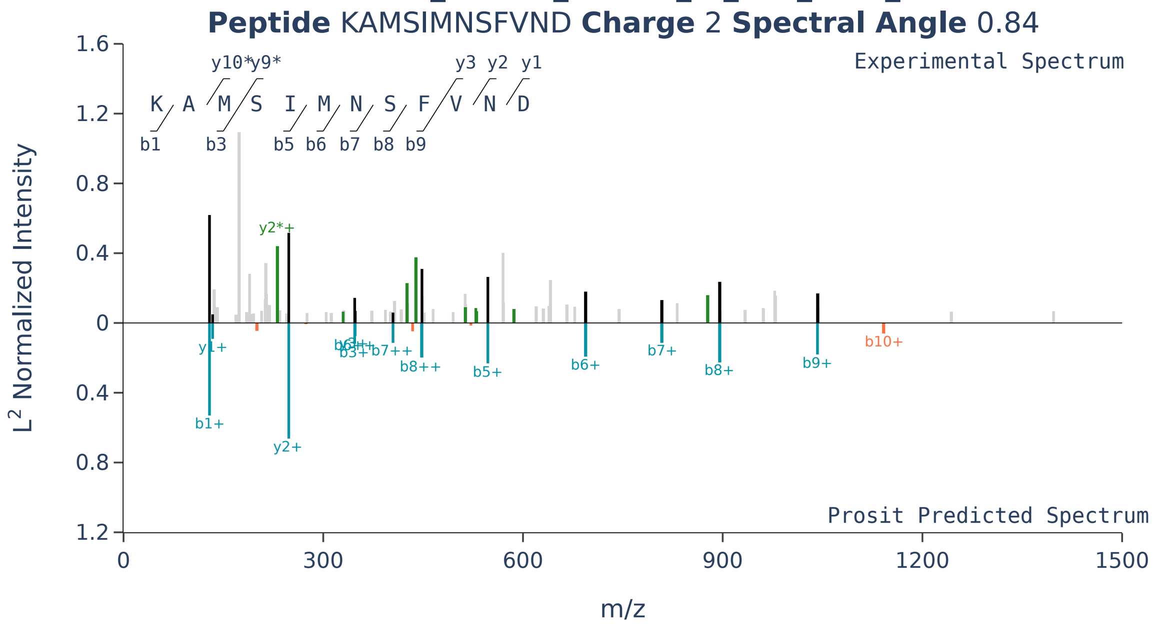


**Source** HRoetschke_291222_080123_Fu_H2B_b2R2_24h **Scan** 10897
**Peptide** REIQTAVRL **Charge** 2 **Spectral Angle** 0.85

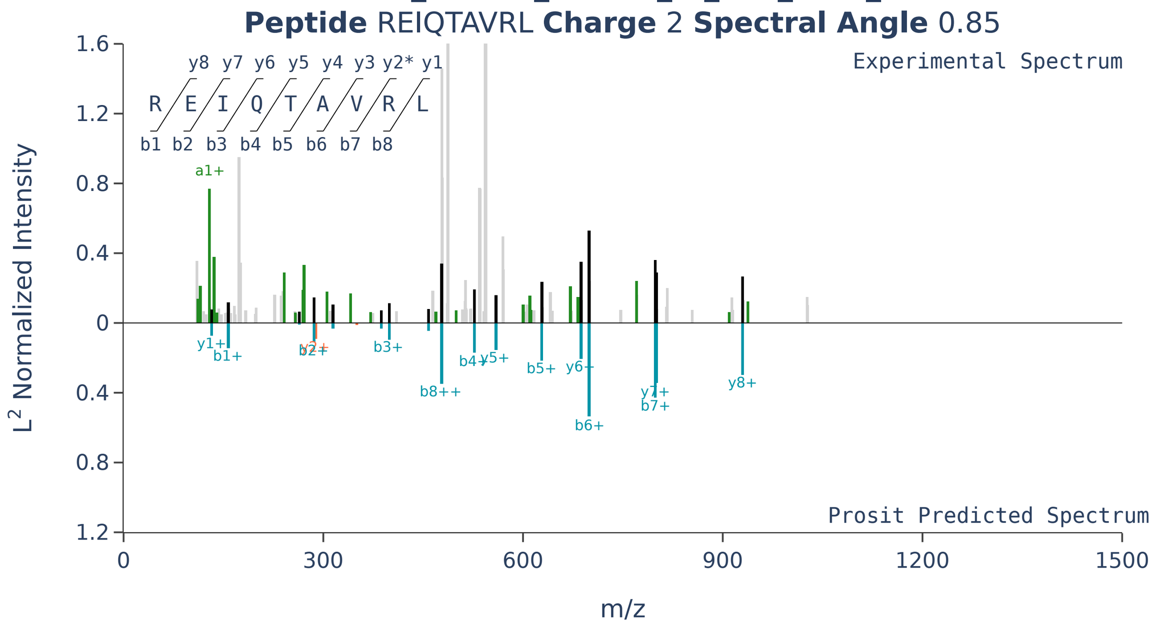


**Source** HRoetschke_291222_080123_Fu_H2B_b2R1_2h **Scan** 17337
**Peptide** RLLLPGEL **Charge** 2 **Spectral Angle** 0.91

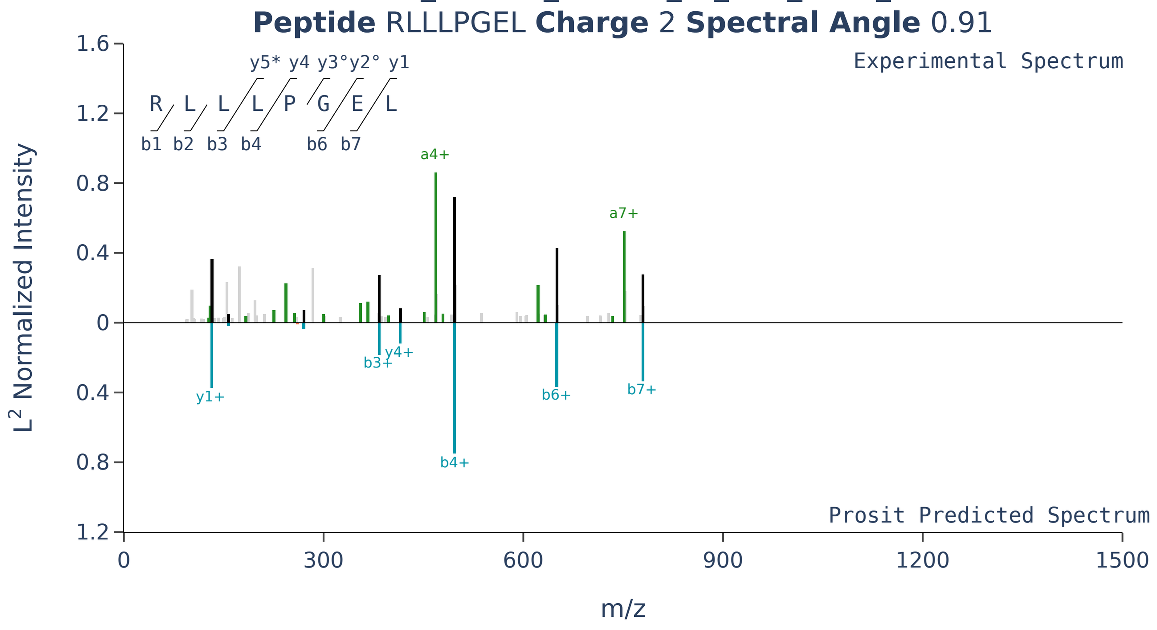


**Source** HRoetschke_291222_080123_Fu_H2B_b2R1_24h **Scan** 8947
**Peptide** VFERIAG **Charge** 2 **Spectral Angle** 0.9

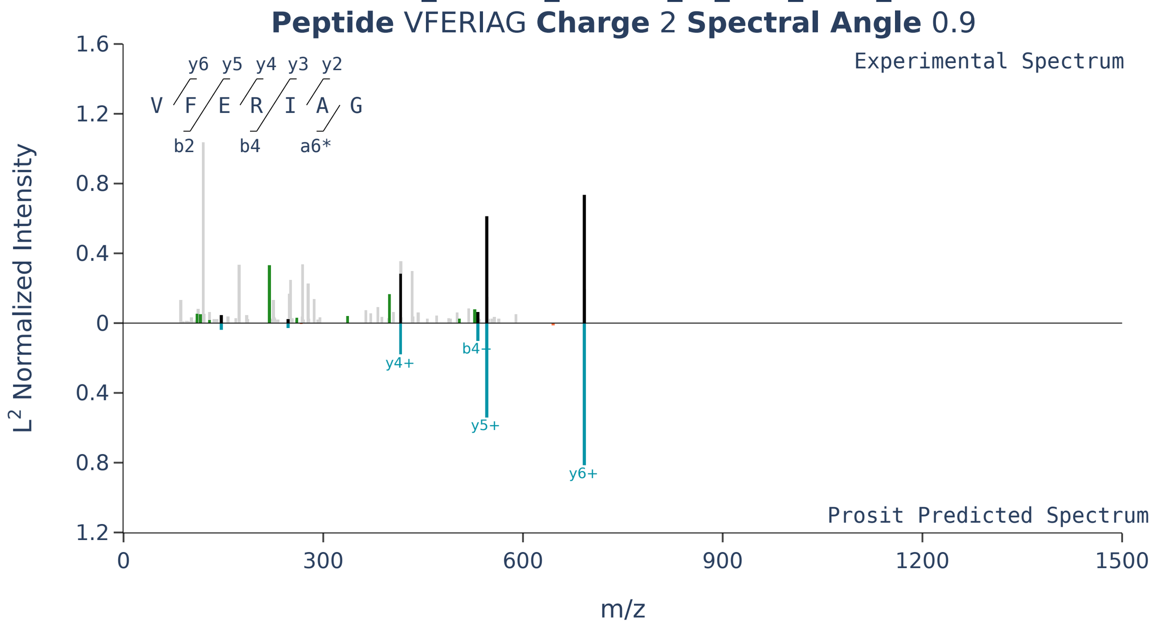


**Source** HRoetschke_291222_080123_Fu_H2B_b2R1_4h **Scan** 7225
**Peptide** ITSREIQTAVR **Charge** 3 **Spectral Angle** 0.78

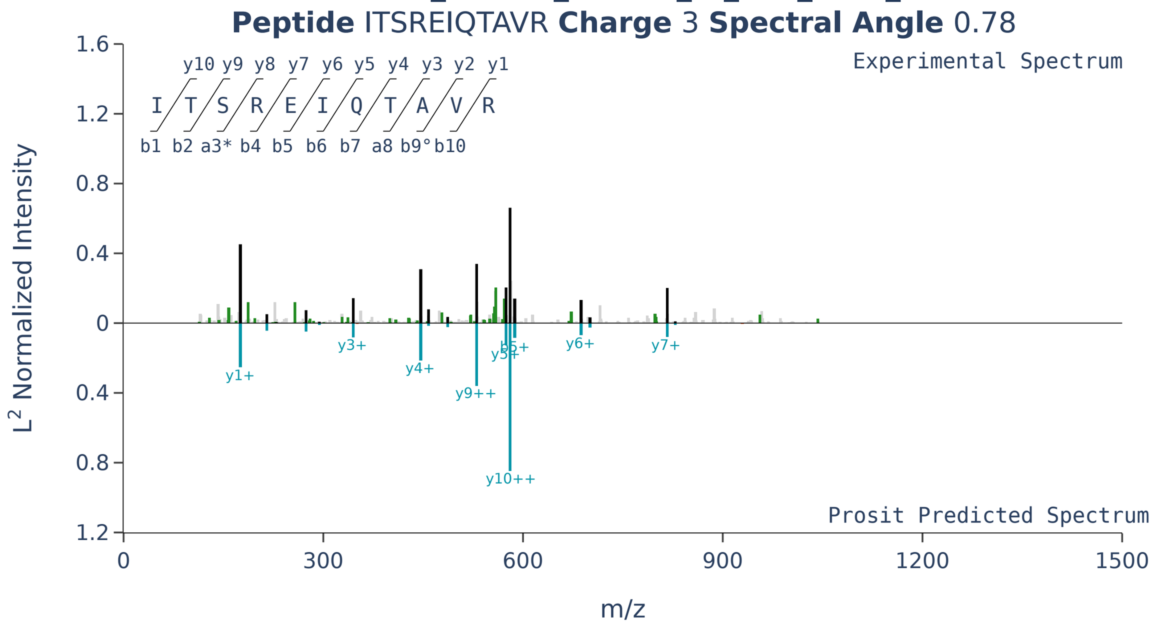


**Source** HRoetschke_291222_080123_Fu_H2B_b2R2_24h **Scan** 4080
**Peptide** VHPDTGISS **Charge** 2 **Spectral Angle** 0.81

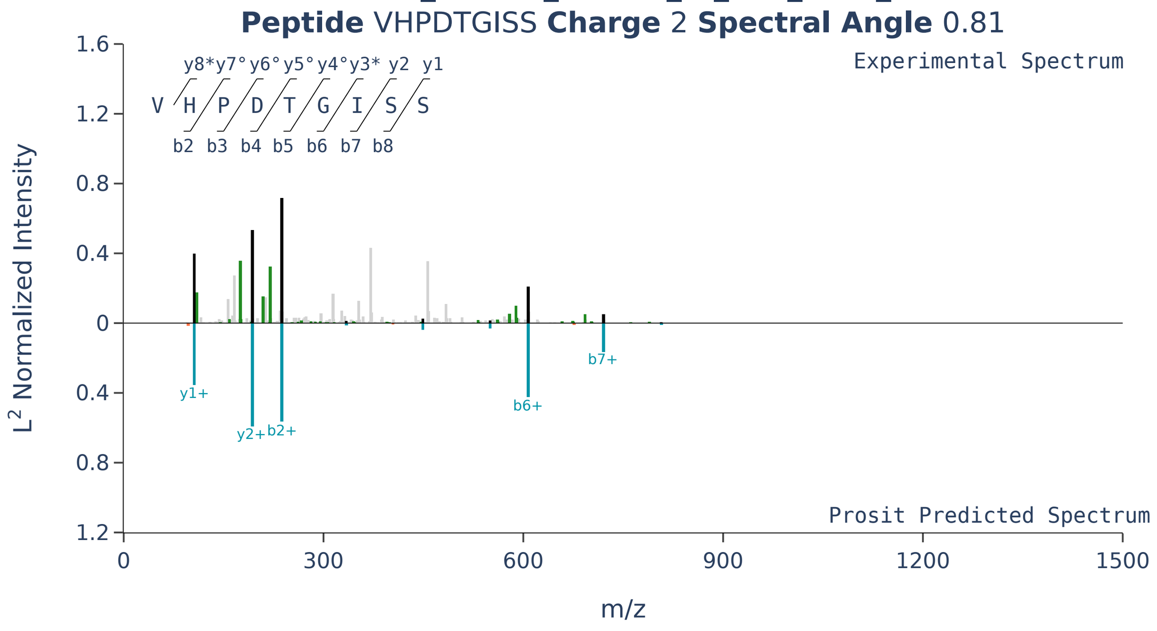


**Source** HRoetschke_291222_080123_Fu_H2B_b2R1_24h **Scan** 4026
**Peptide** AHYNKRSTITSREIQTA **Charge** 4 **Spectral Angle** 0.89

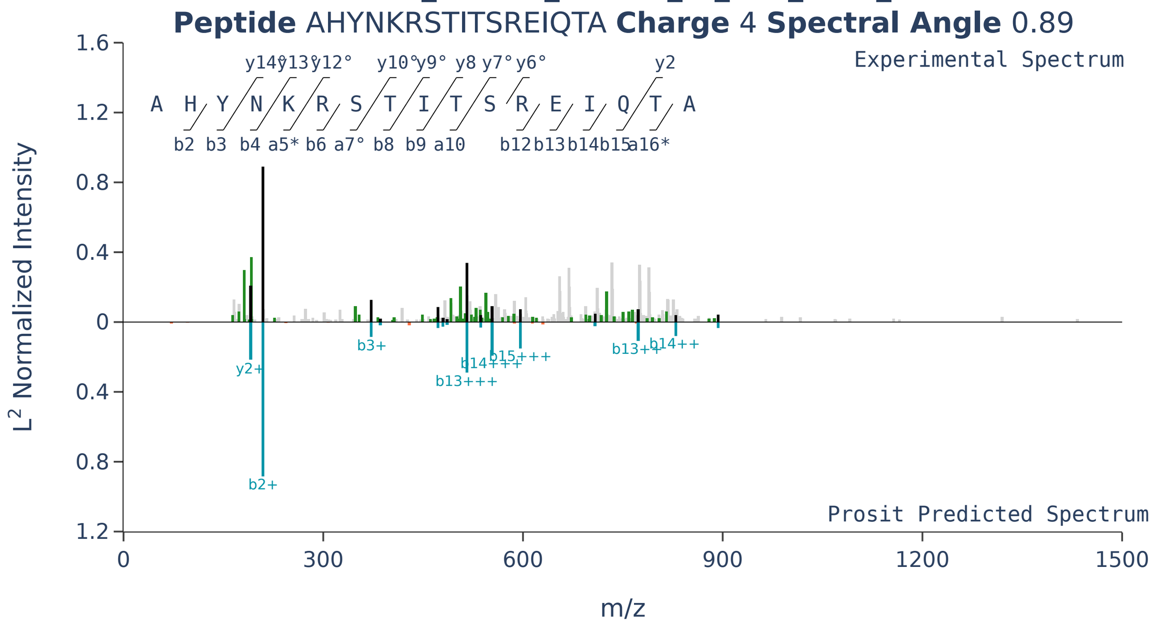


**Source** HRoetschke_291222_080123_Fu_H2B_b1R1_24h **Scan** 7043
**Peptide** ERIAGEASRLA **Charge** 3 **Spectral Angle** 0.81

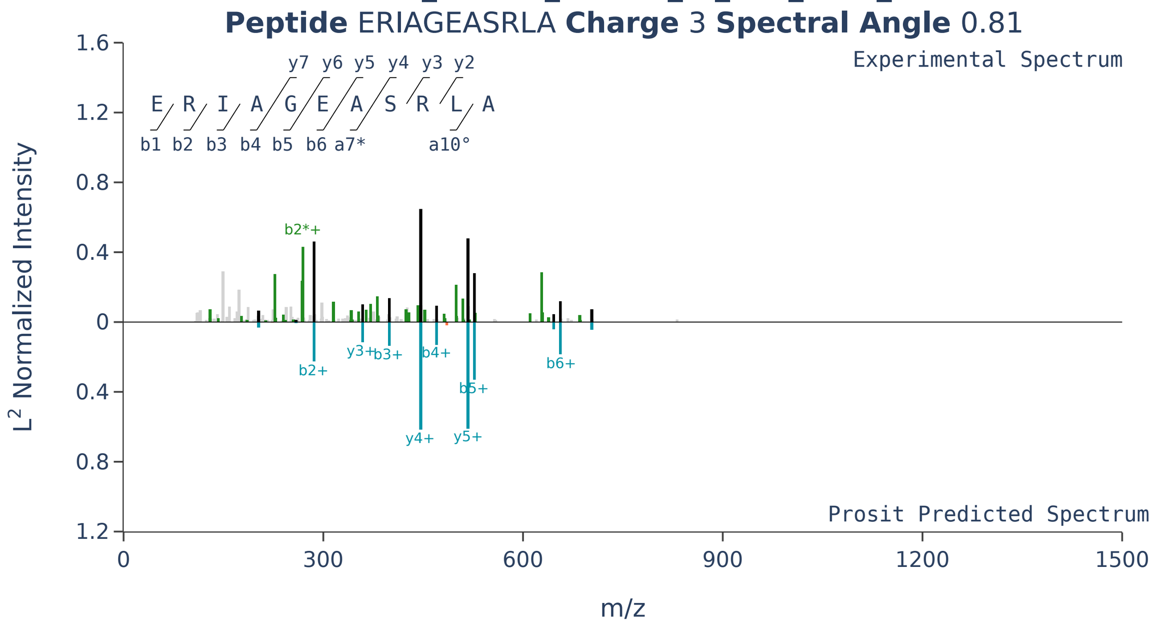


**Source** HRoetschke_291222_080123_Fu_H2B_b1R1_24h **Scan** 5805
**Peptide** AHYNKRSTITSREIQTAVR **Charge** 4 **Spectral Angle** 0.94

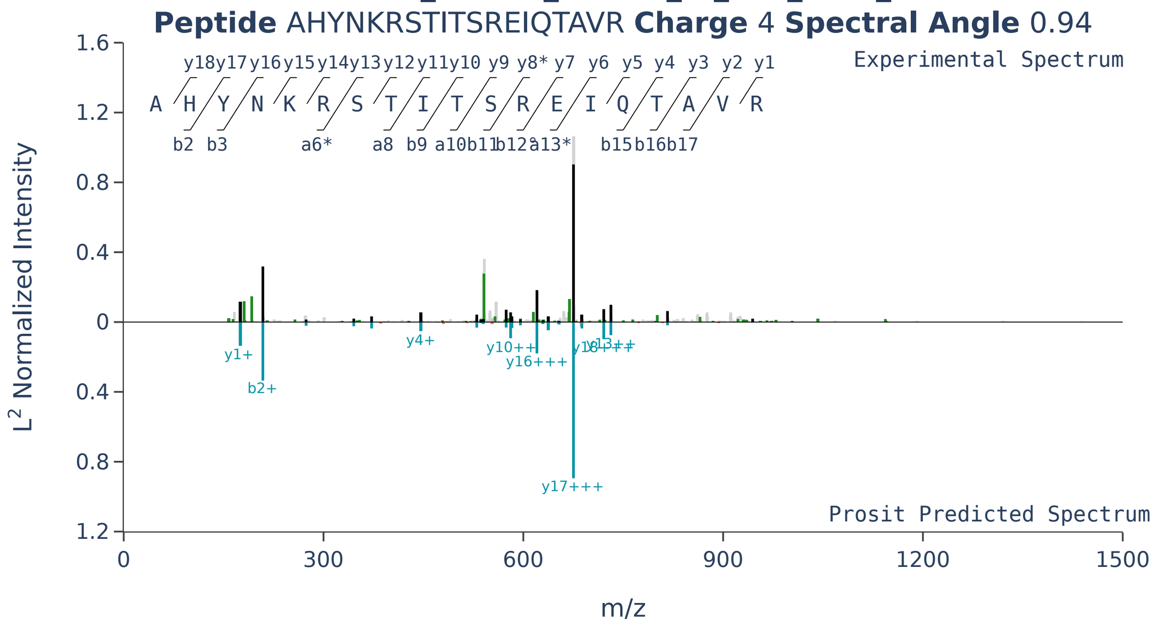


**Source** HRoetschke_291222_080123_Fu_H2B_b1R1_1h **Scan** 2446
**Peptide** AGEASRLAHYNKR **Charge** 3 **Spectral Angle** 0.89

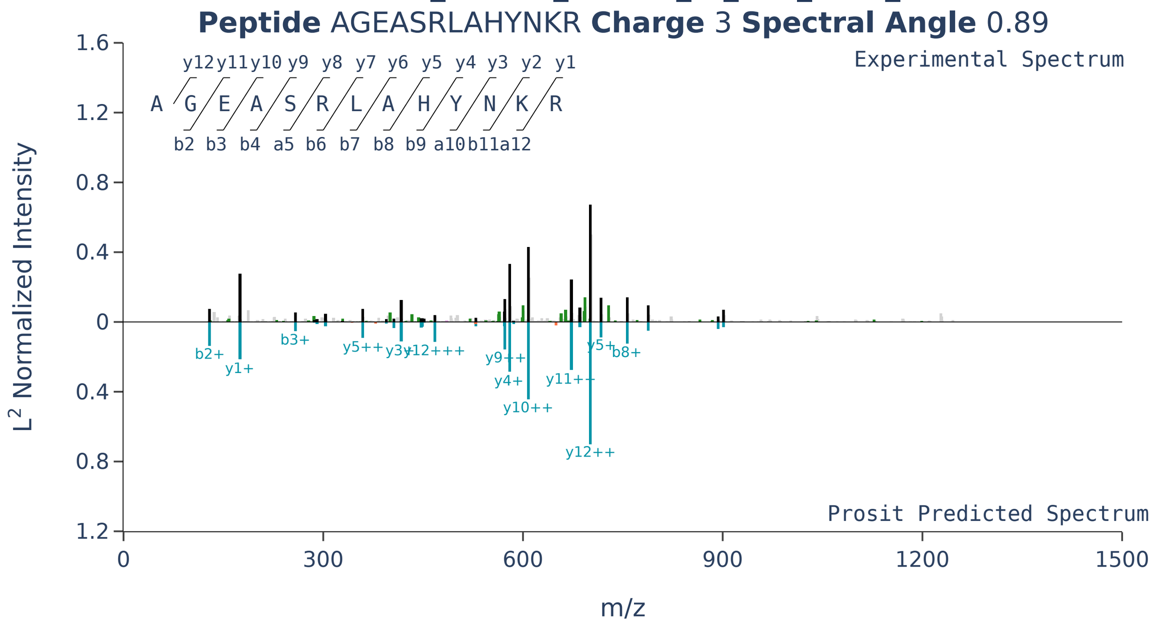


**Source** HRoetschke_291222_080123_Fu_H2B_b2R2_1h **Scan** 3707
**Peptide** ELQTAVR **Charge** 2 **Spectral Angle** 0.92

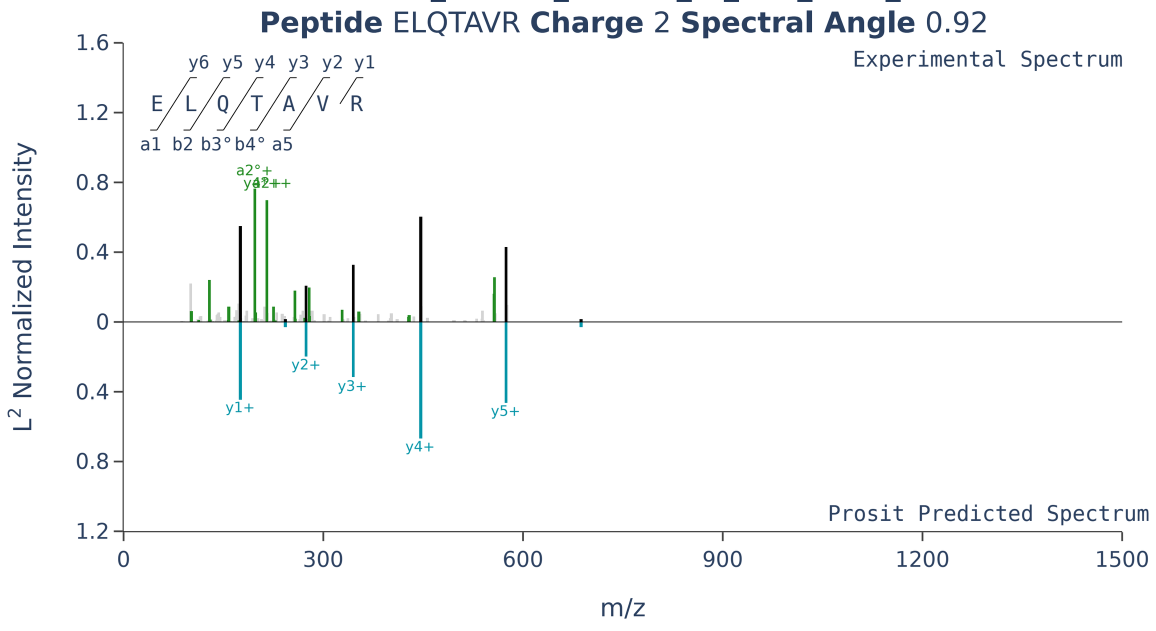


**Source** HRoetschke_291222_080123_Fu_H2B_b2R2_1h **Scan** 16074
**Peptide** NSFVNDVF **Charge** 2 **Spectral Angle** 0.89

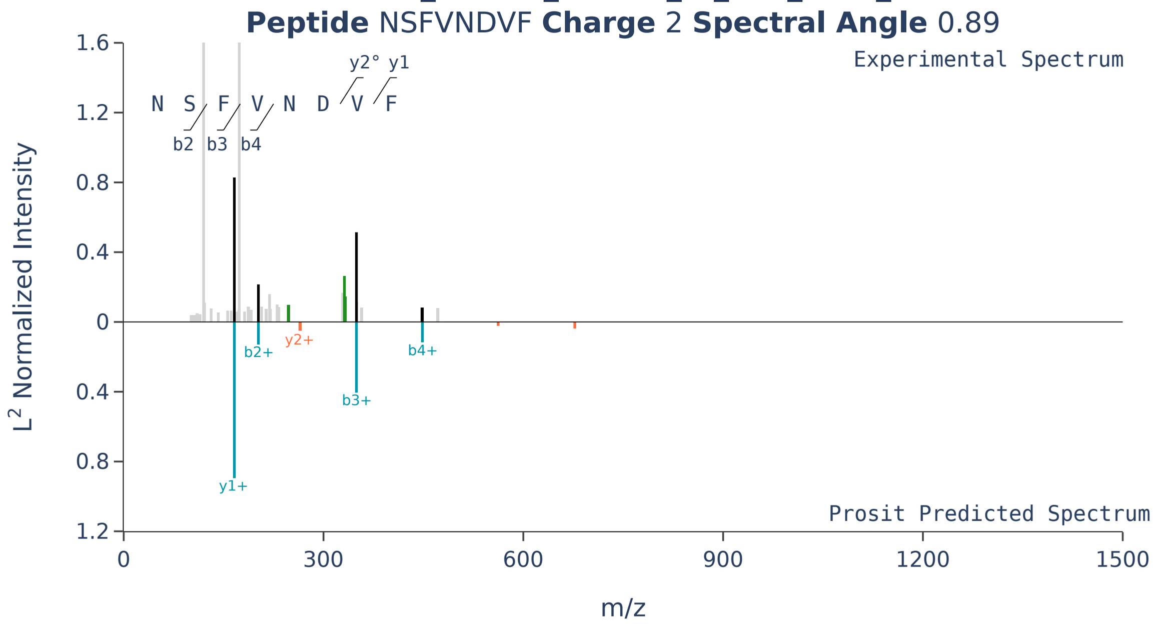

**Source** HRoetschke_291222_080123_Fu_H2B_b1R2_24h **Scan** 2105
**Peptide** AHYNKRSTL **Charge** 2 **Spectral Angle** 0.93

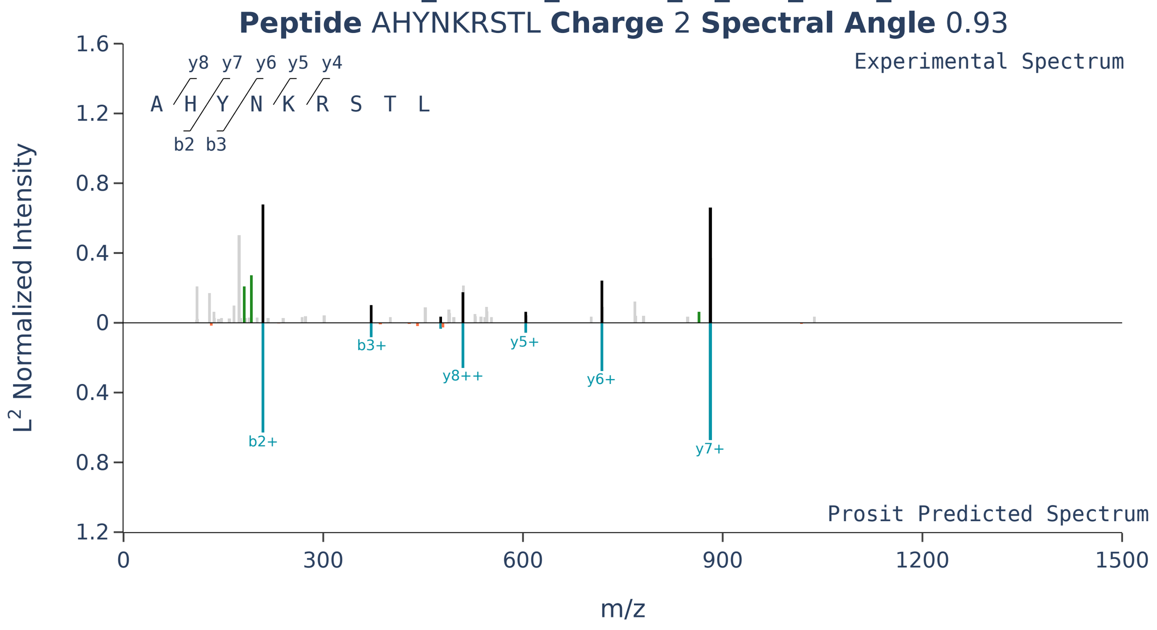


**Source** HRoetschke_291222_080123_Fu_H2B_b2R2_24h **Scan** 3218
**Peptide** EASRLAHYN **Charge** 2 **Spectral Angle** 0.86

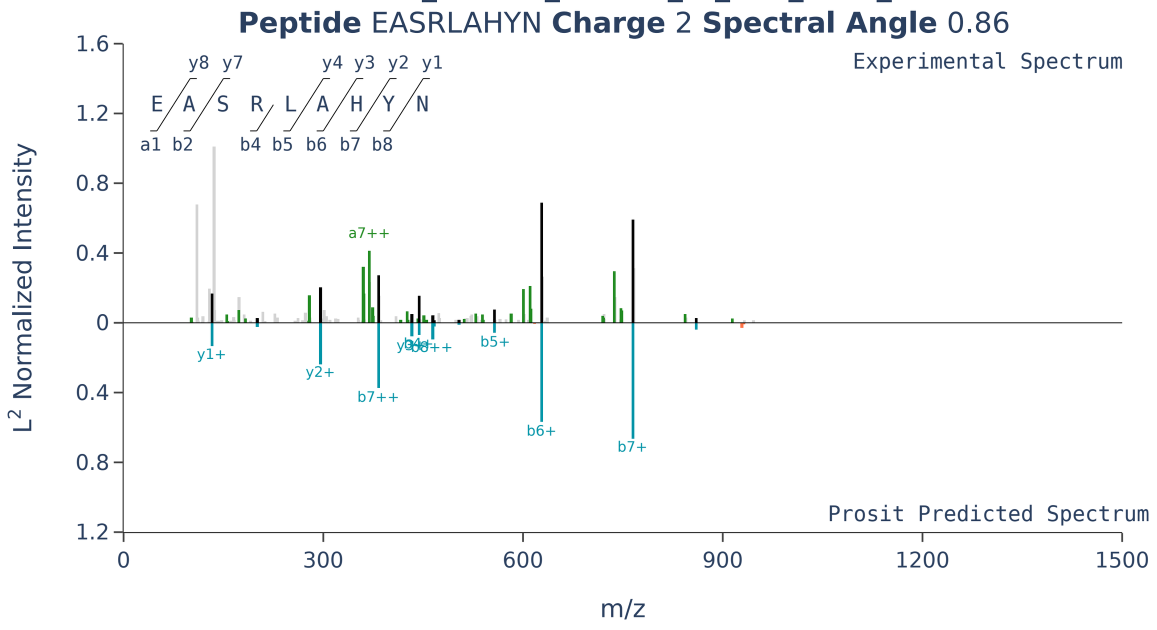


**Source** HRoetschke_291222_080123_Fu_H2B_b1R2_24h **Scan** 7581
**Peptide** HPDTGISSKAMSL **Charge** 3 **Spectral Angle** 0.86

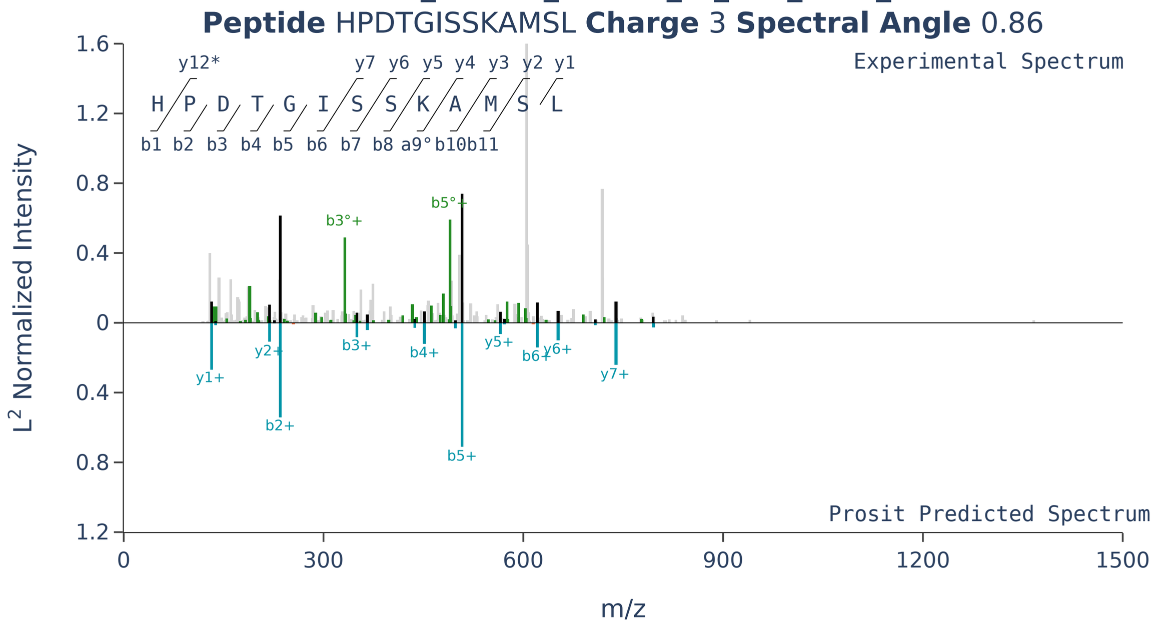


**Source** HRoetschke_291222_080123_Fu_H2B_b1R2_4h **Scan** 3618
**Peptide** SEGTKAVTKYT **Charge** 2 **Spectral Angle** 0.83

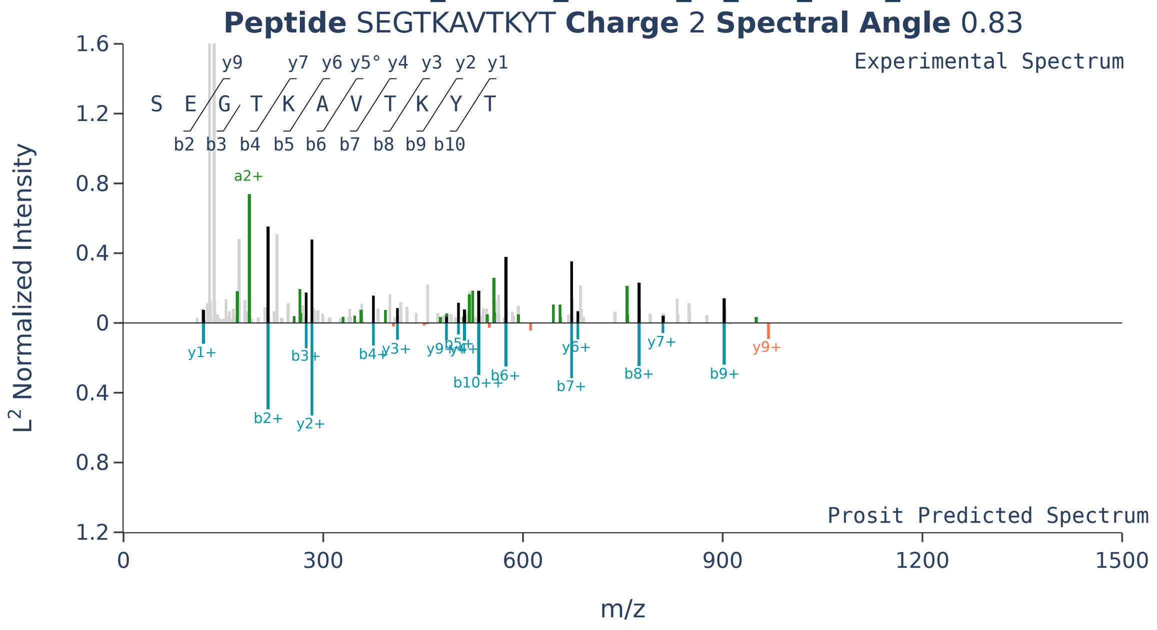


**Source** HRoetschke_291222_080123_Fu_H2B_b1R2_24h **Scan** 2728
**Peptide** KQVHPDTGISSKAM **Charge** 3 **Spectral Angle** 0.85

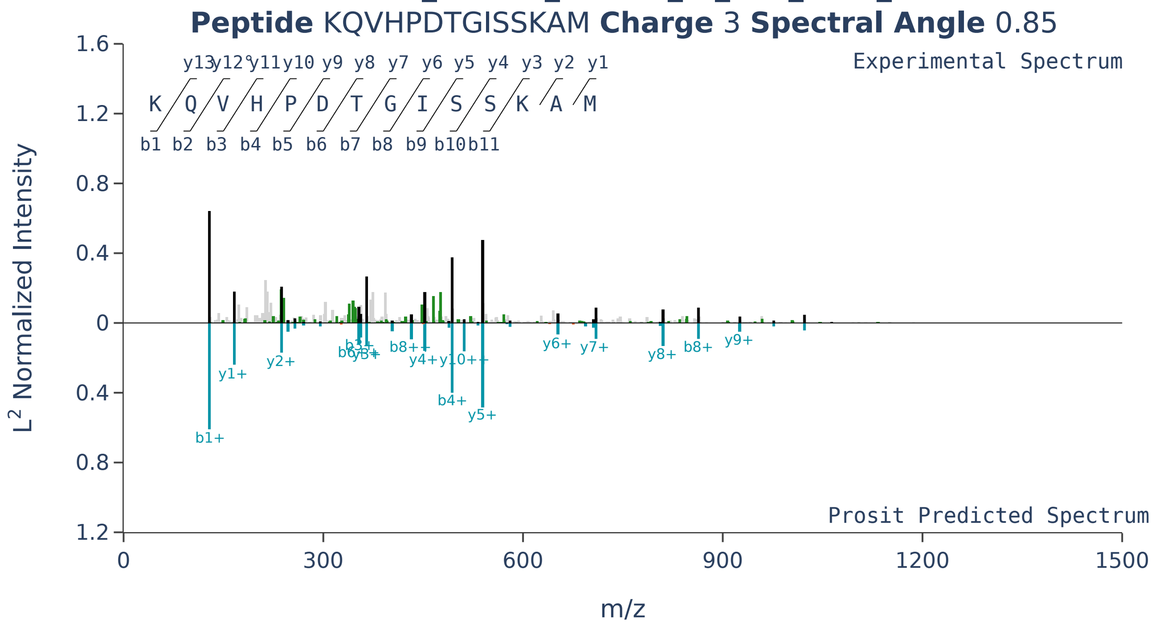


**Source** HRoetschke_291222_080123_Fu_H2B_b1R1_24h **Scan** 13623
**Peptide** LLLPGELAKH **Charge** 2 **Spectral Angle** 0.92

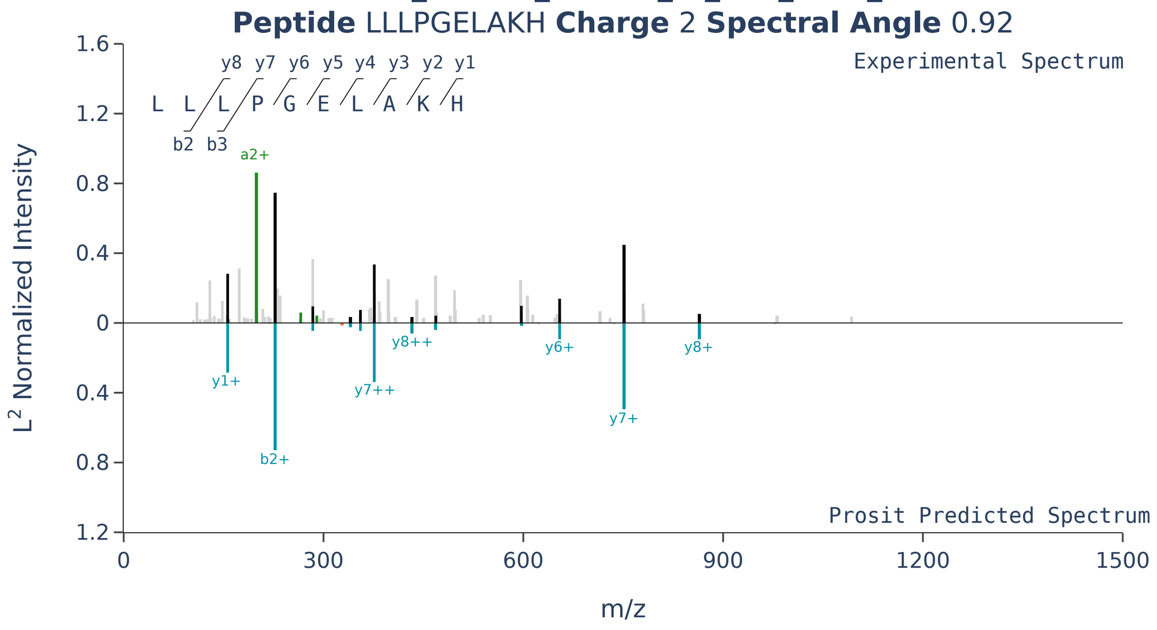


**Source** HRoetschke_291222_080123_Fu_H2B_b1R2_24h **Scan** 7824
**Peptide** STITSREIQTA **Charge** 2 **Spectral Angle** 0.93

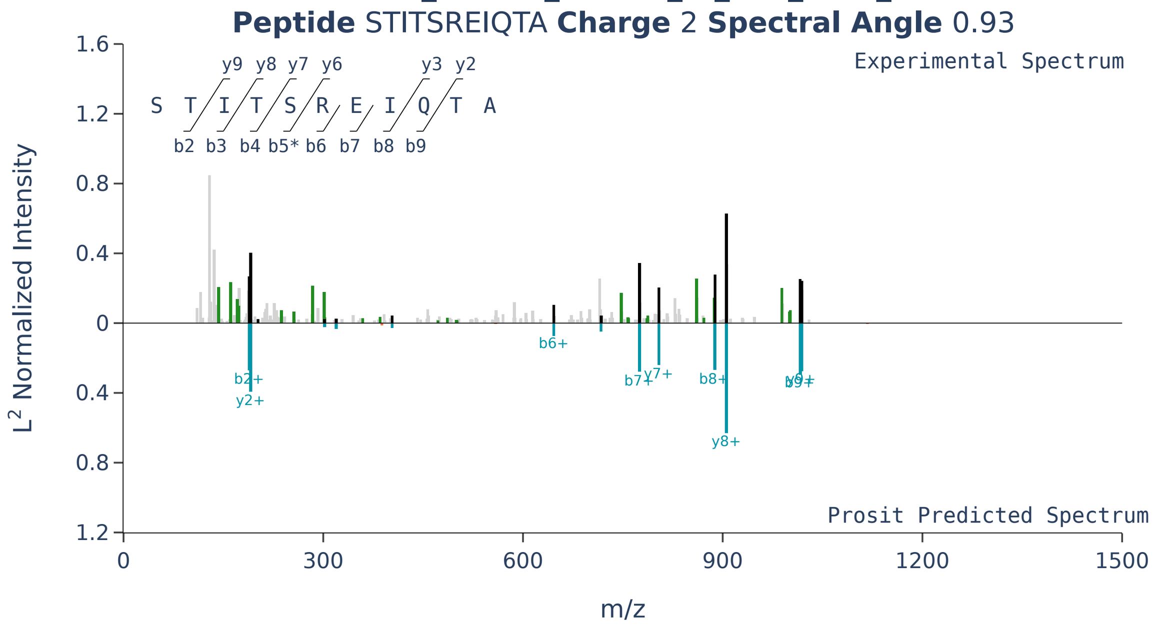


**Source** HRoetschke_291222_080123_Fu_H2B_b2R2_24h **Scan** 14103
**Peptide** RLLLPGELAKHA **Charge** 3 **Spectral Angle** 0.87

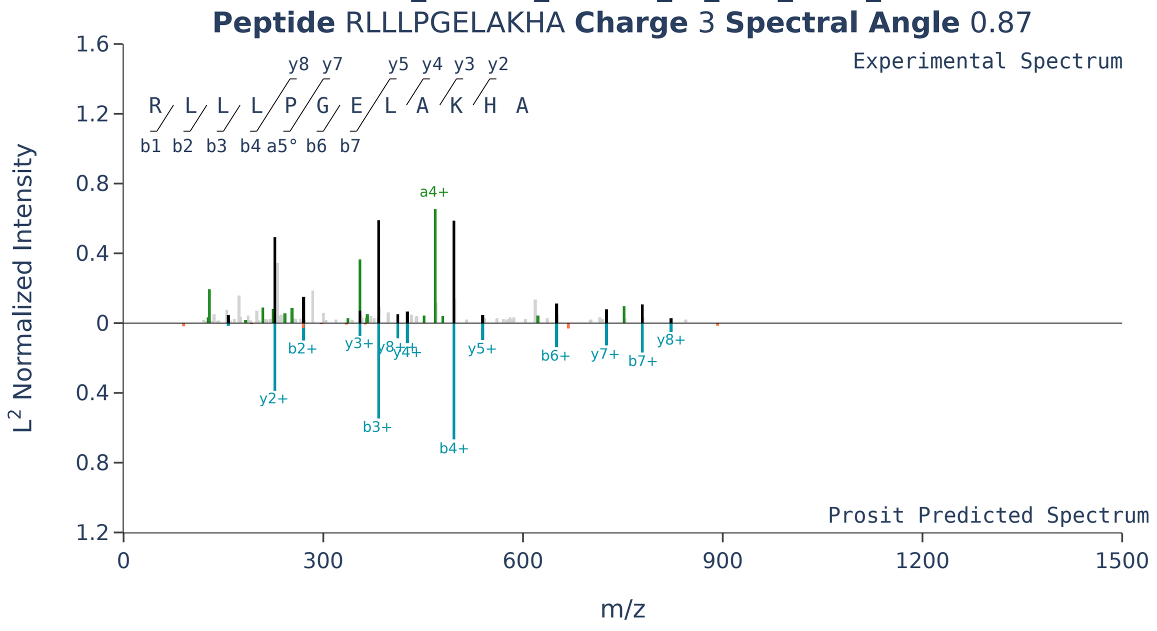


**Source** HRoetschke_291222_080123_Fu_H2B_b2R2_2h **Scan** 13442
**Peptide** KESYAIYVYKV **Charge** 3 **Spectral Angle** 0.9

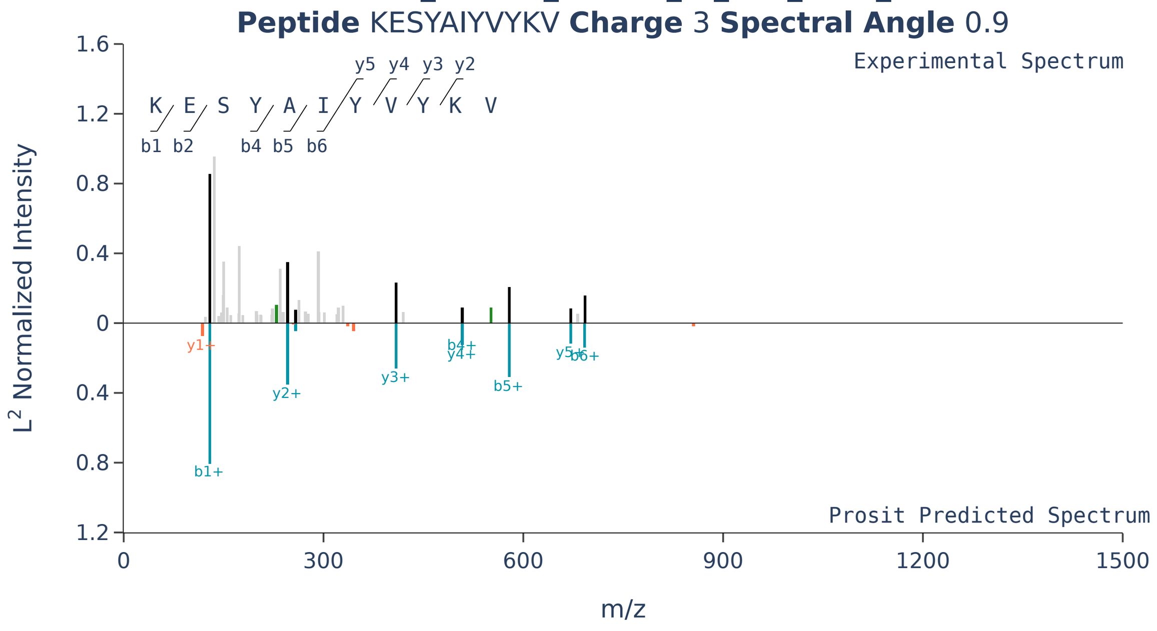


**Source** HRoetschke_291222_080123_Fu_H2B_b2R2_24h **Scan** 4087
**Peptide** VSEGTKAVTKYTSAK **Charge** 2 **Spectral Angle** 0.87

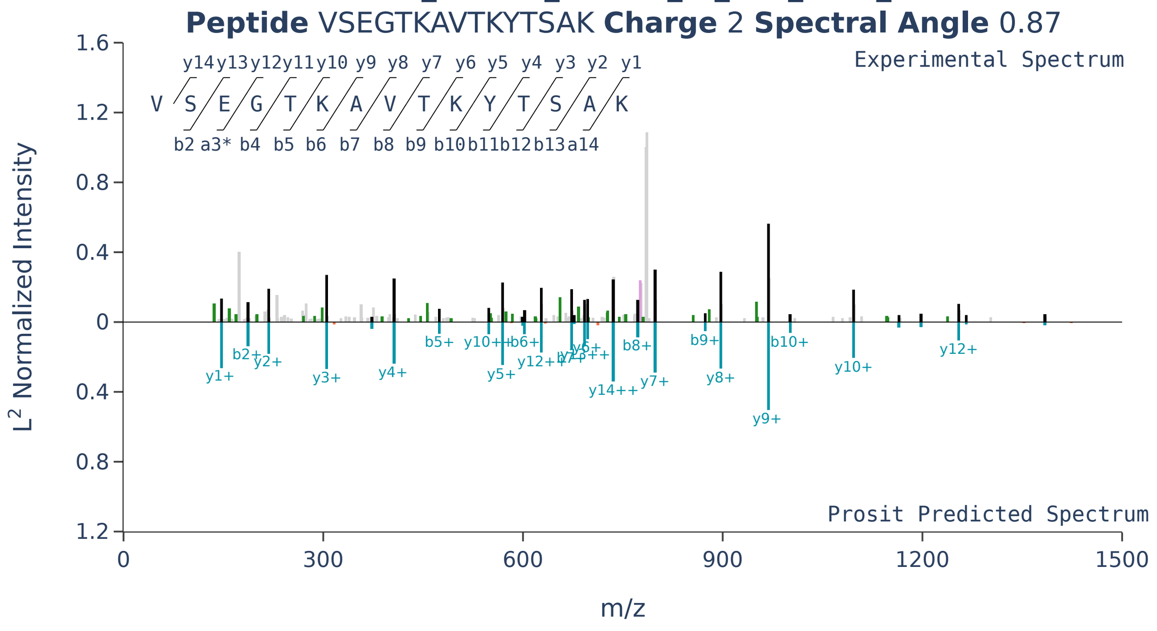


**Source** HRoetschke_291222_080123_Fu_H2B_b1R1_1h **Scan** 15049
**Peptide** MNSFVNDVF **Charge** 2 **Spectral Angle** 0.83

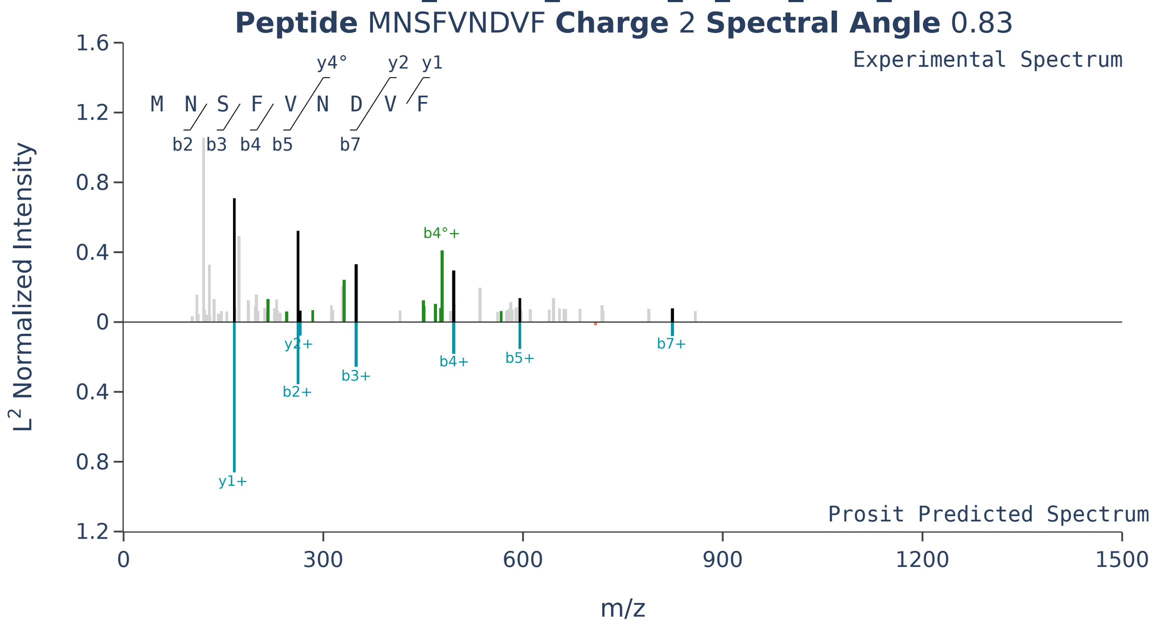


**Source** HRoetschke_291222_080123_Fu_H2B_b2R1_1h **Scan** 3064
**Peptide** VHPDTGISSKAMS **Charge** 3 **Spectral Angle** 0.84

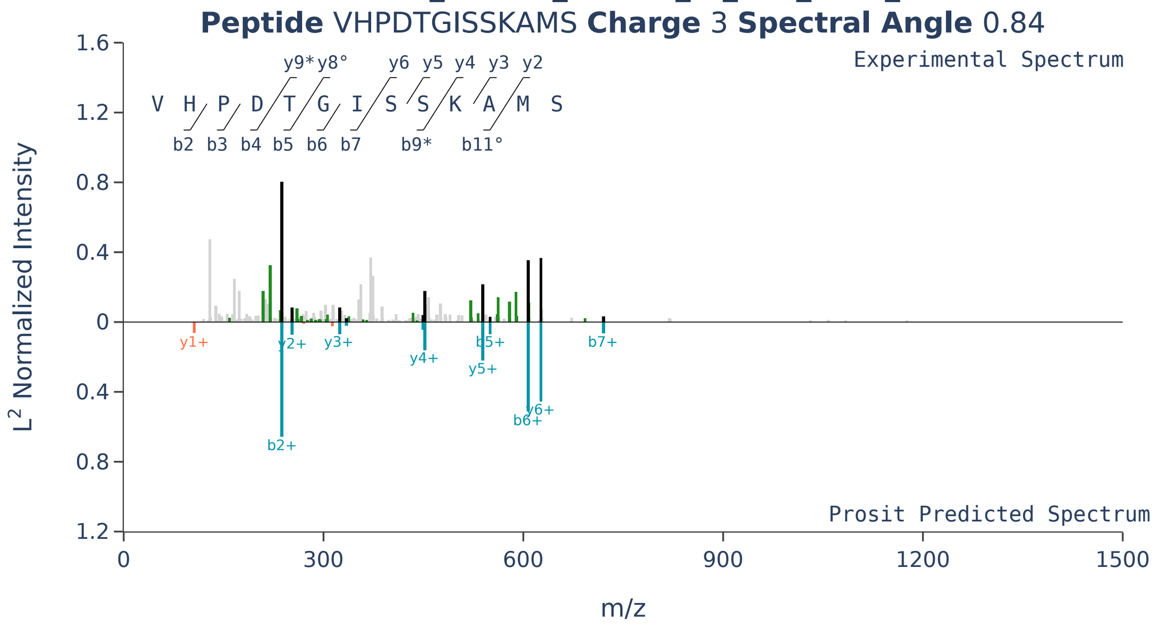


**Source** HRoetschke_291222_080123_Fu_H2B_b2R1_24h **Scan** 13798
**Peptide** NDVFERIAG **Charge** 2 **Spectral Angle** 0.91

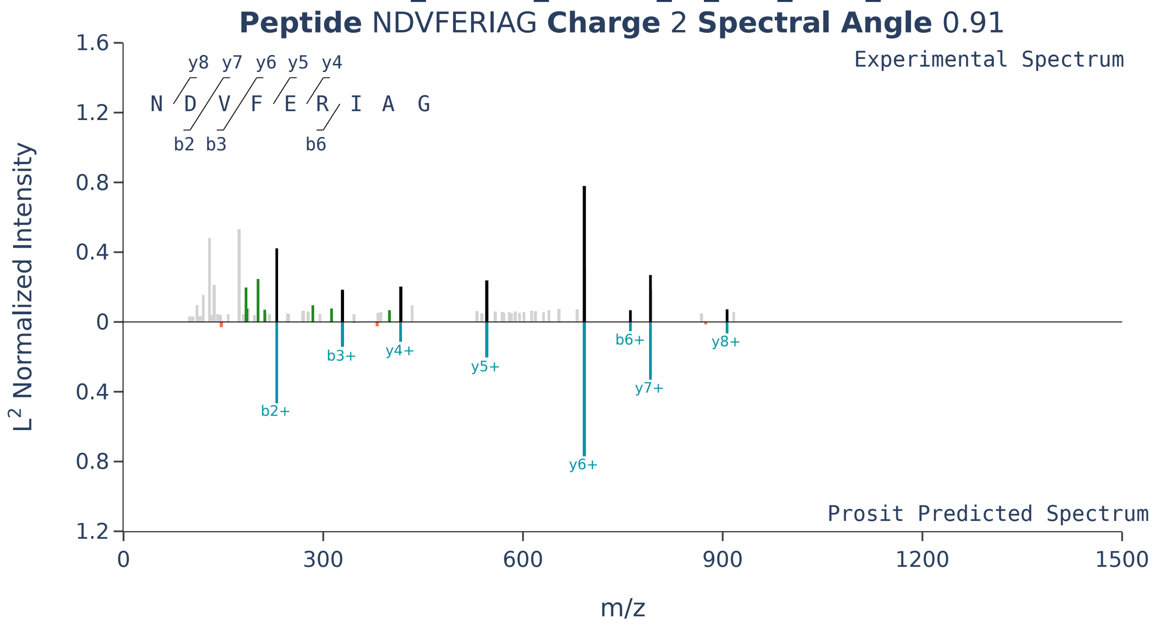


**Source** HRoetschke_291222_080123_Fu_H2B_b1R1_24h **Scan** 3122
**Peptide** AHYNKRSTITSREIQ **Charge** 3 **Spectral Angle** 0.91

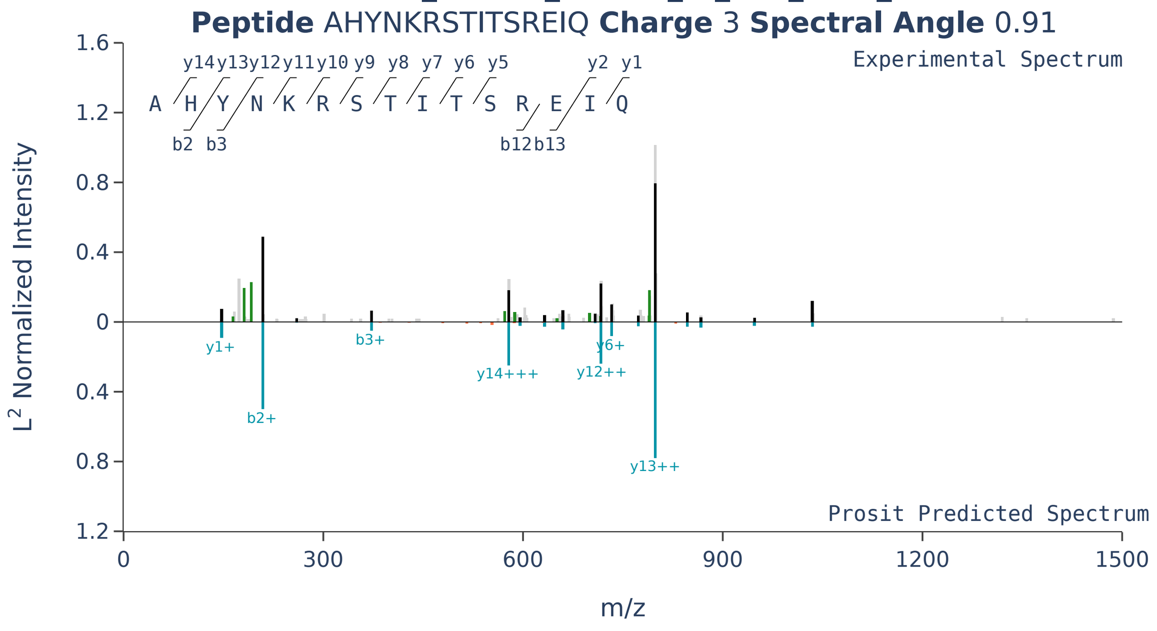


**Source** HRoetschke_291222_080123_Fu_H2B_b2R2_24h **Scan** 4136
**Peptide** QVHPDTGISSKA **Charge** 2 **Spectral Angle** 0.83

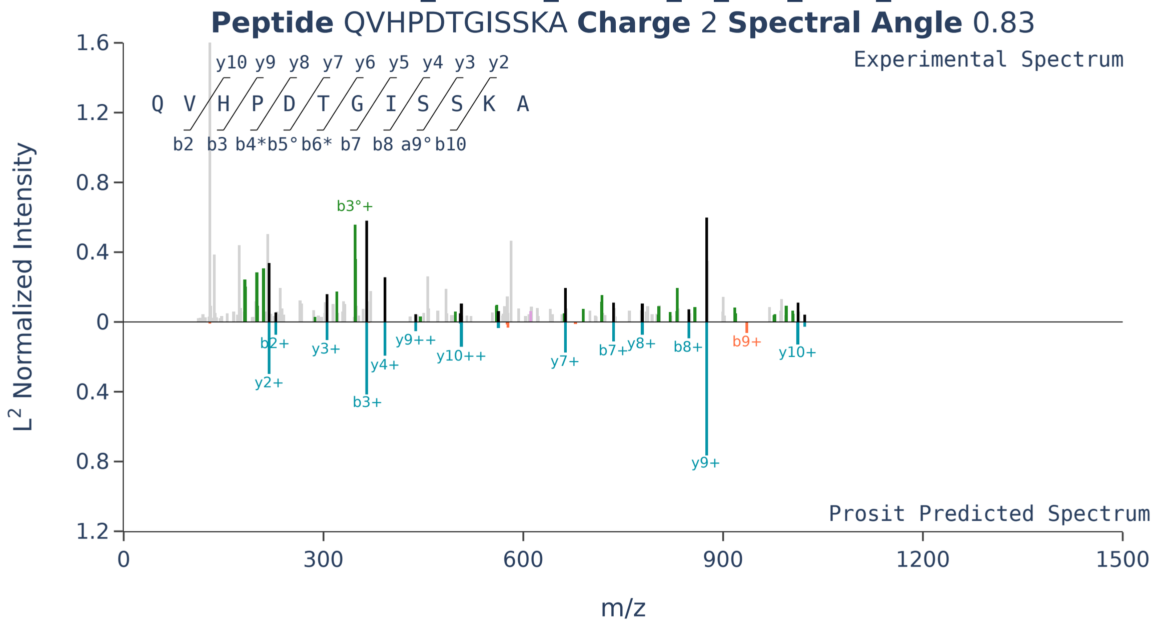

**Source** HRoetschke_291222_080123_Fu_H2B_b1R1_24h **Scan** 9334
**Peptide** SSKAMSIMNSF **Charge** 2 **Spectral Angle** 0.89

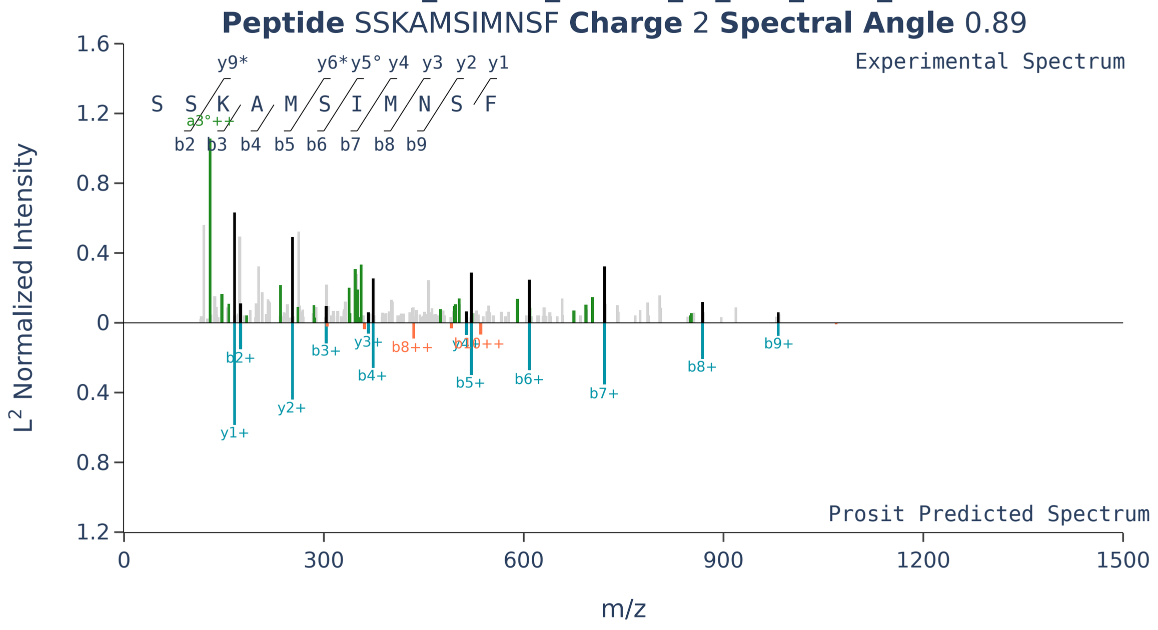


**Source** HRoetschke_291222_080123_Fu_H2B_b1R1_2h **Scan** 3765
**Peptide** HPDTGISSKAMS **Charge** 2 **Spectral Angle** 0.88

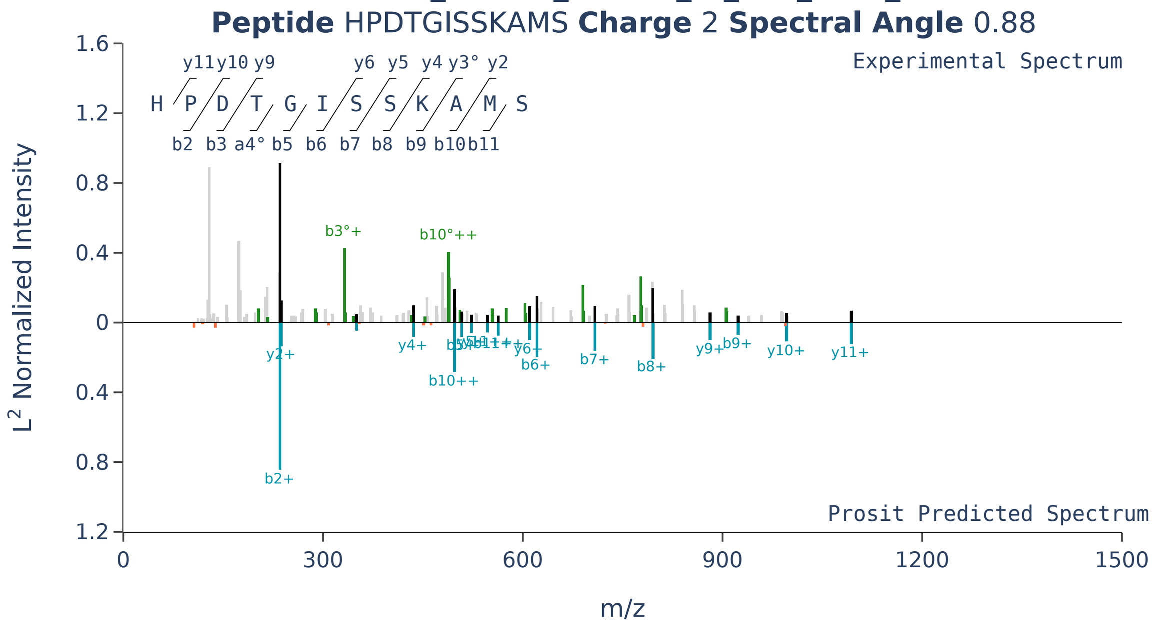


**Source** HRoetschke_291222_080123_Fu_H2B_b1R1_4h **Scan** 5426
**Peptide** STITSREIQ **Charge** 2 **Spectral Angle** 0.93

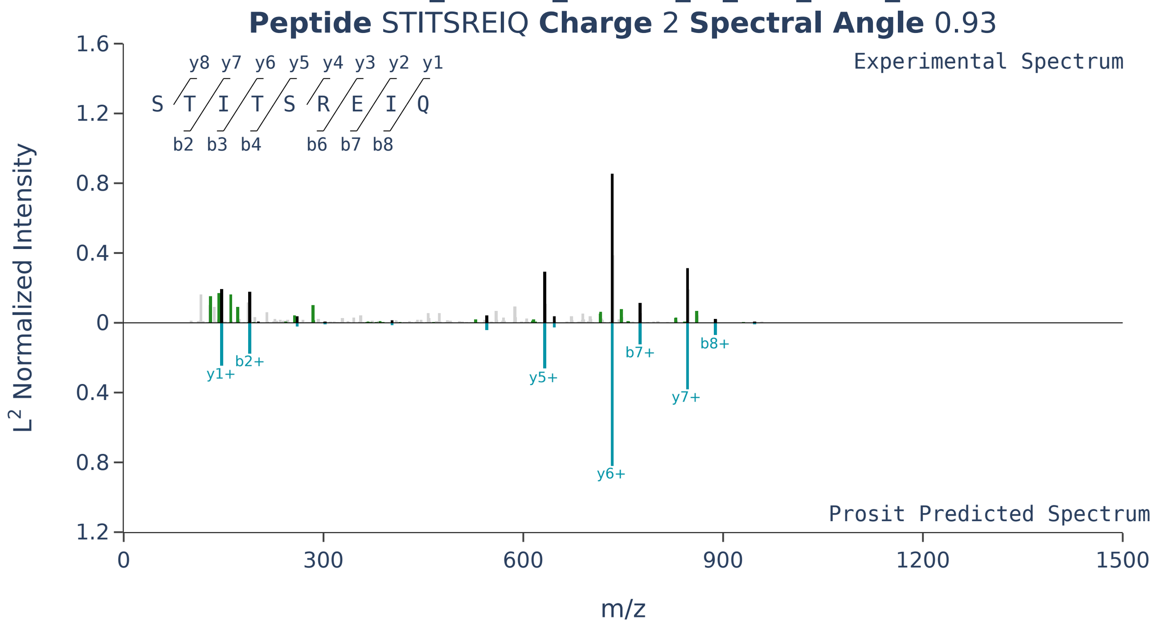


**Source** HRoetschke_291222_080123_Fu_H2B_b1R1_1h **Scan** 2134
**Peptide** GTKAVTKYTSAK **Charge** 3 **Spectral Angle** 0.85

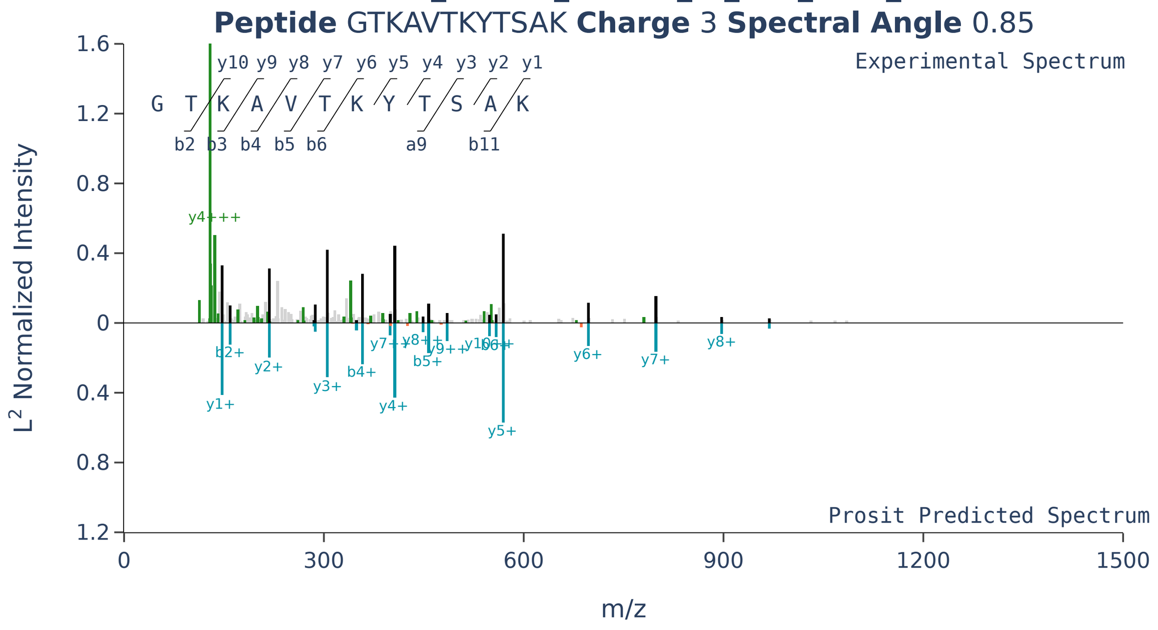


**Source** HRoetschke_291222_080123_Fu_H2B_b2R1_4h **Scan** 14986
**Peptide** LLLPGELAKHA **Charge** 2 **Spectral Angle** 0.9

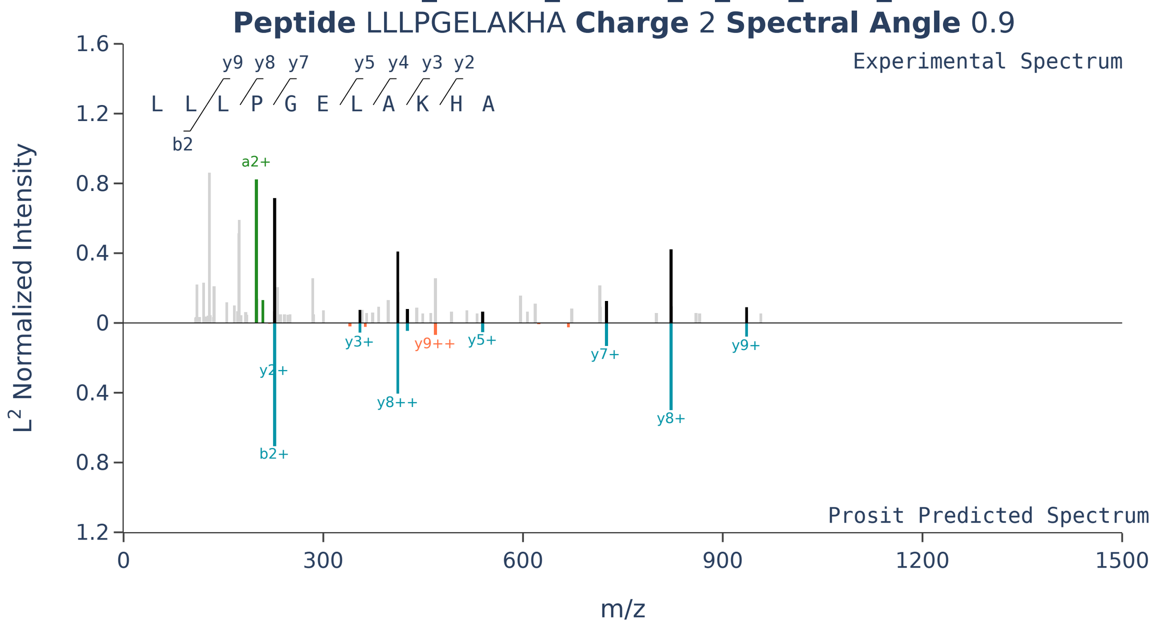


**Source** HRoetschke_291222_080123_Fu_H2B_b1R2_24h **Scan** 13986
**Peptide** VNDVFERL **Charge** 2 **Spectral Angle** 0.91

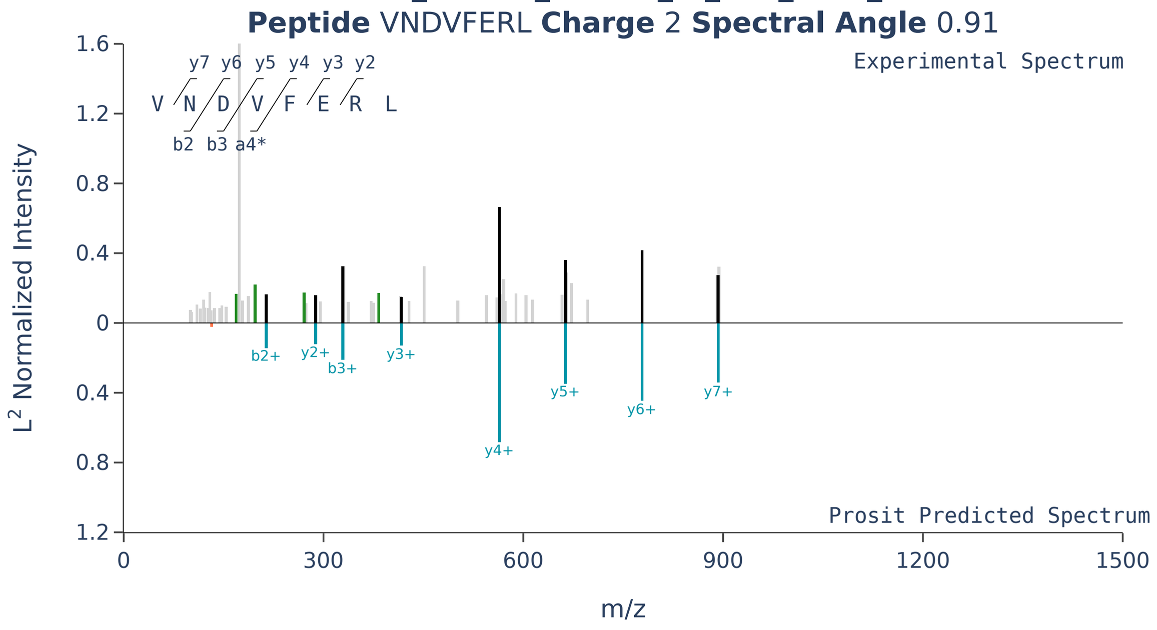


**Source** HRoetschke_291222_080123_Fu_H2B_b2R2_24h **Scan** 5063
**Peptide** VSEGTKAVTKYT **Charge** 2 **Spectral Angle** 0.85

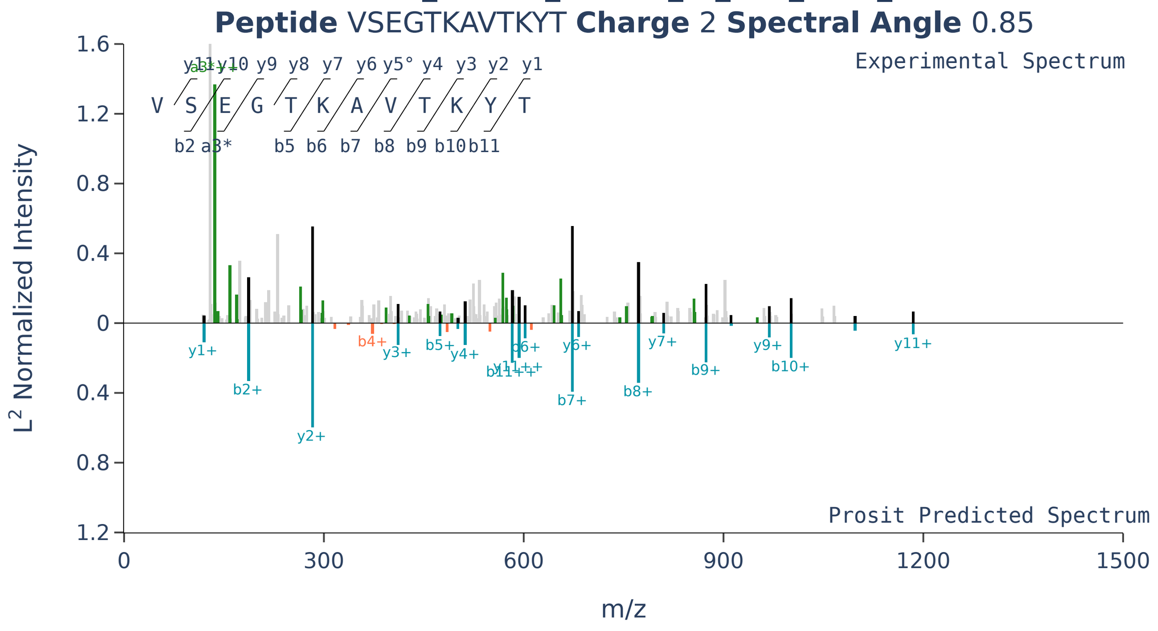


**Source** HRoetschke_291222_080123_Fu_H2B_b1R2_24h **Scan** 14735
**Peptide** VNDVFERIAGEAS **Charge** 2 **Spectral Angle** 0.89

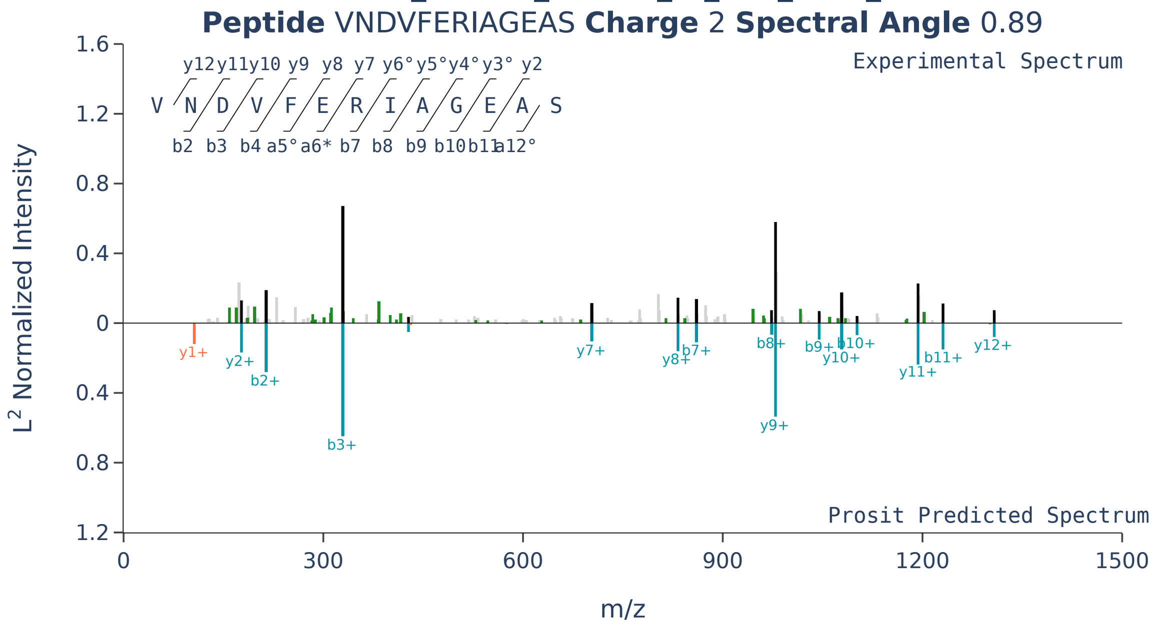


**Source** HRoetschke_291222_080123_Fu_H2B_b2R2_4h **Scan** 18926
**Peptide** VNDVFERIAGEASRLAHYNKR **Charge** 5 **Spectral Angle** 0.88

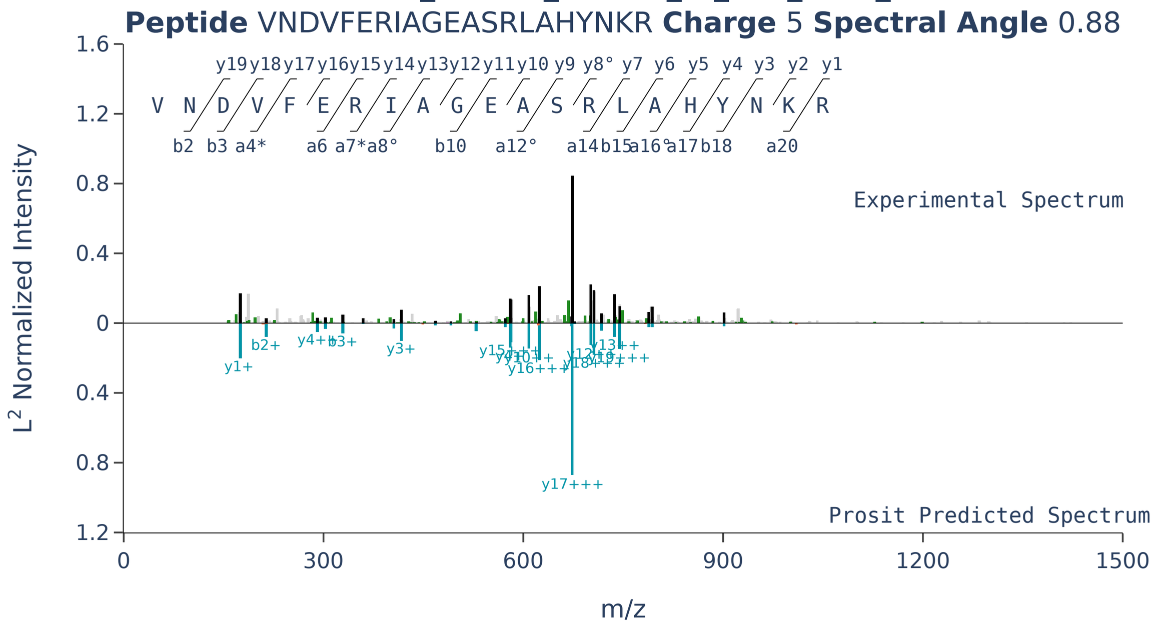


**Source** HRoetschke_291222_080123_Fu_H2B_b2R2_1h **Scan** 16447
**Peptide** STITSREIQTAVRLL **Charge** 3 **Spectral Angle** 0.93

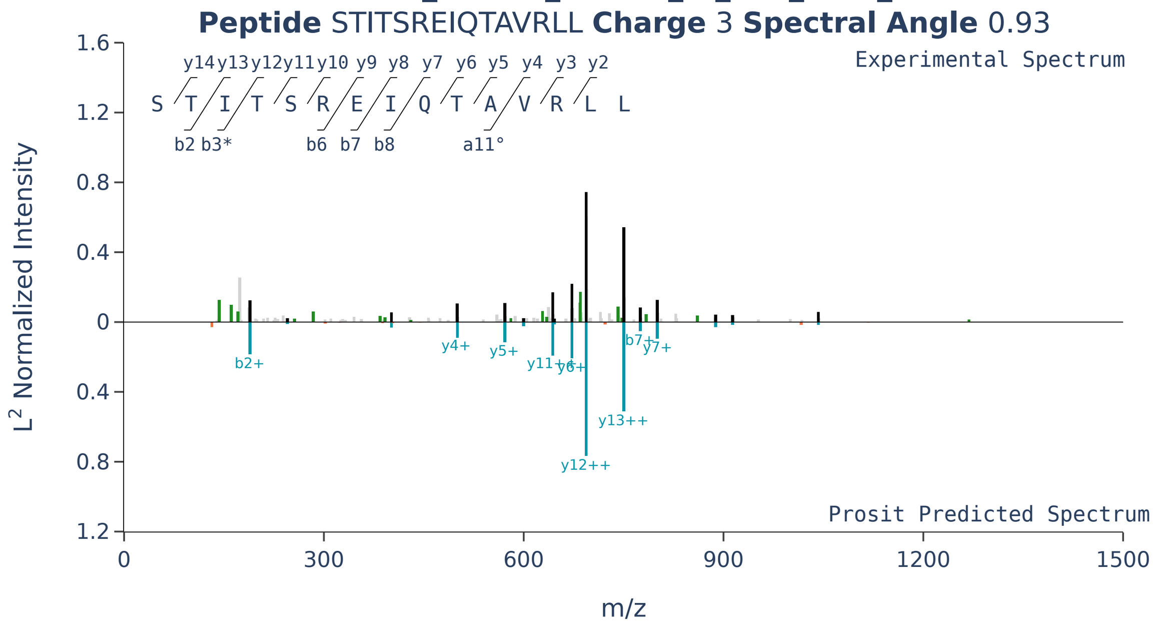


**Source** HRoetschke_291222_080123_Fu_H2B_b1R2_24h **Scan** 1844
**Peptide** AHYNKRSTITSR **Charge** 3 **Spectral Angle** 0.91

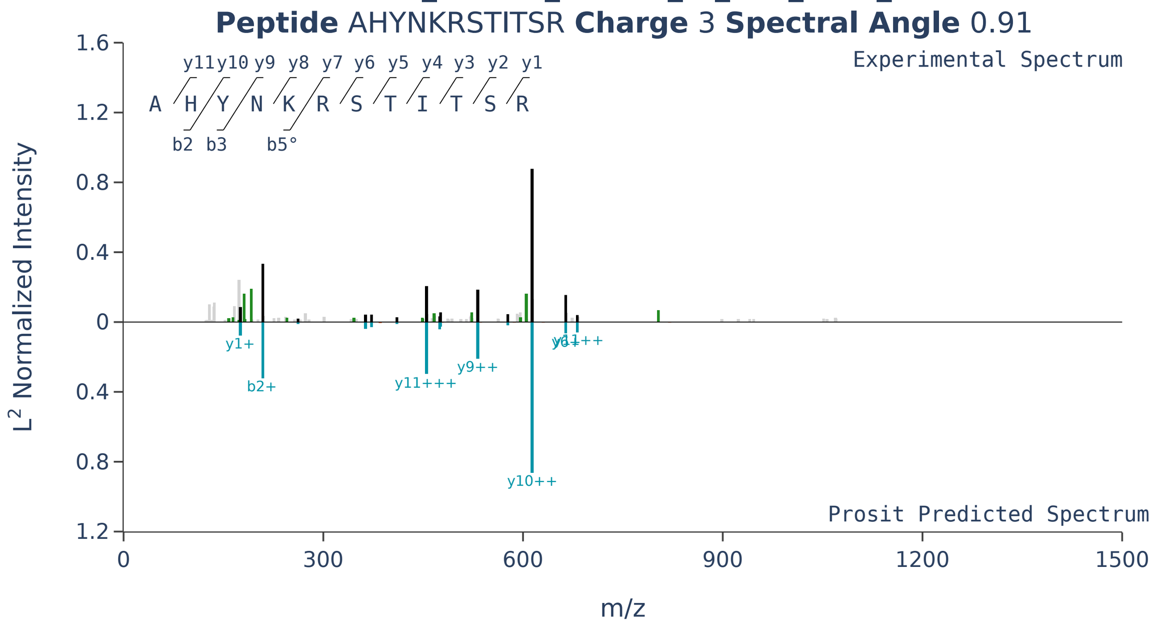


**Source** HRoetschke_291222_080123_Fu_H2B_b1R1_24h **Scan** 3691
**Peptide** KRSTITSREL **Charge** 3 **Spectral Angle** 0.97

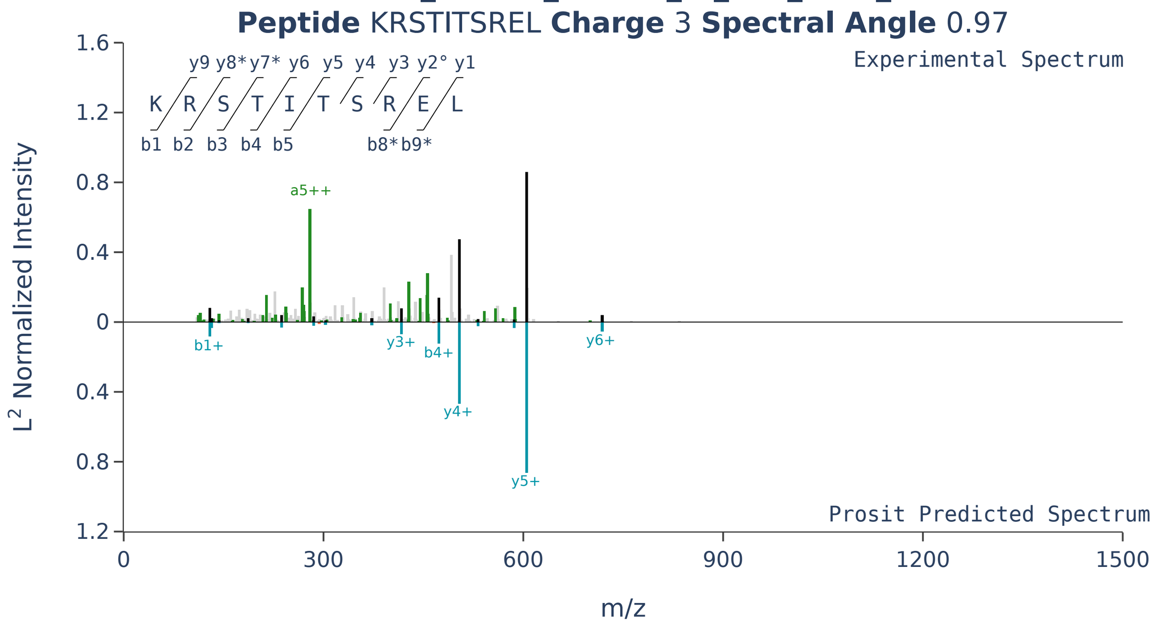


**Source** HRoetschke_291222_080123_Fu_H2B_b1R1_1h **Scan** 5312
**Peptide** TITSREIQT **Charge** 2 **Spectral Angle** 0.93

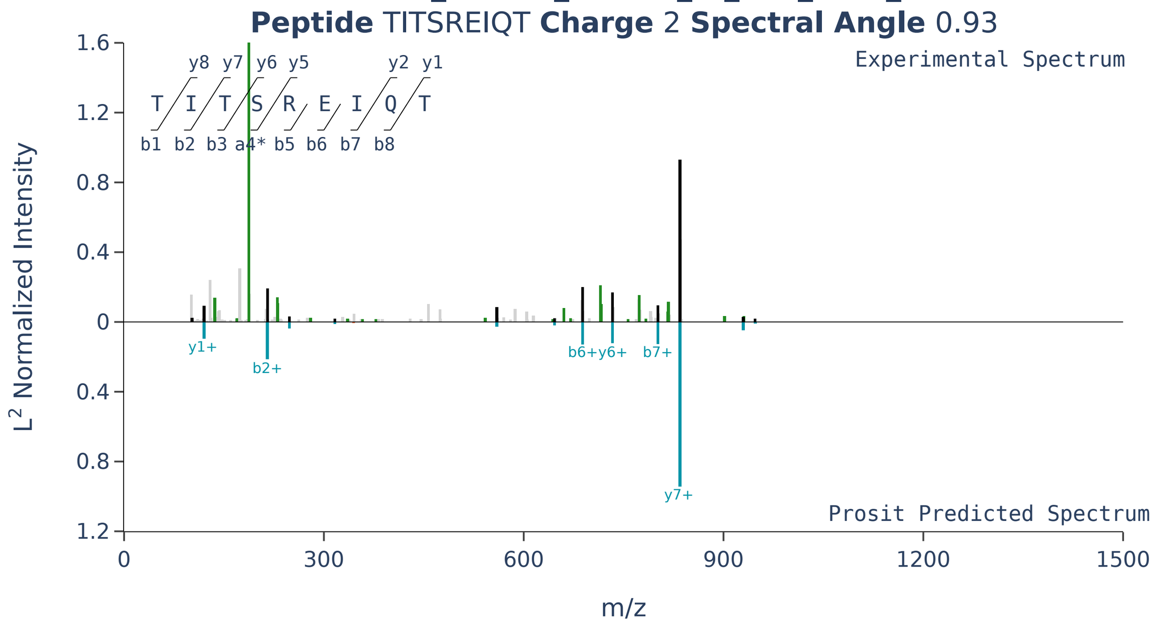


**Source** HRoetschke_291222_080123_Fu_H2B_b1R1_2h **Scan** 2049
**Peptide** KQVHPDTGISSK **Charge** 3 **Spectral Angle** 0.83

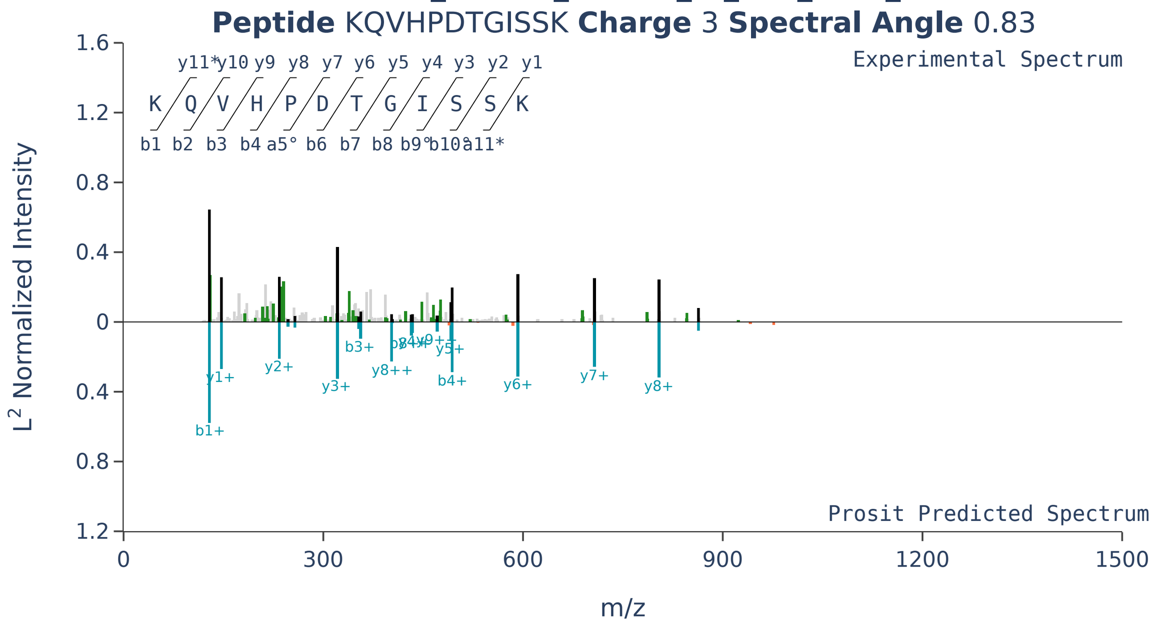


**Source** HRoetschke_291222_080123_Fu_H2B_b2R2_1h **Scan** 14754
**Peptide** DVFERIAGEASRLAHYNKR **Charge** 4 **Spectral Angle** 0.9

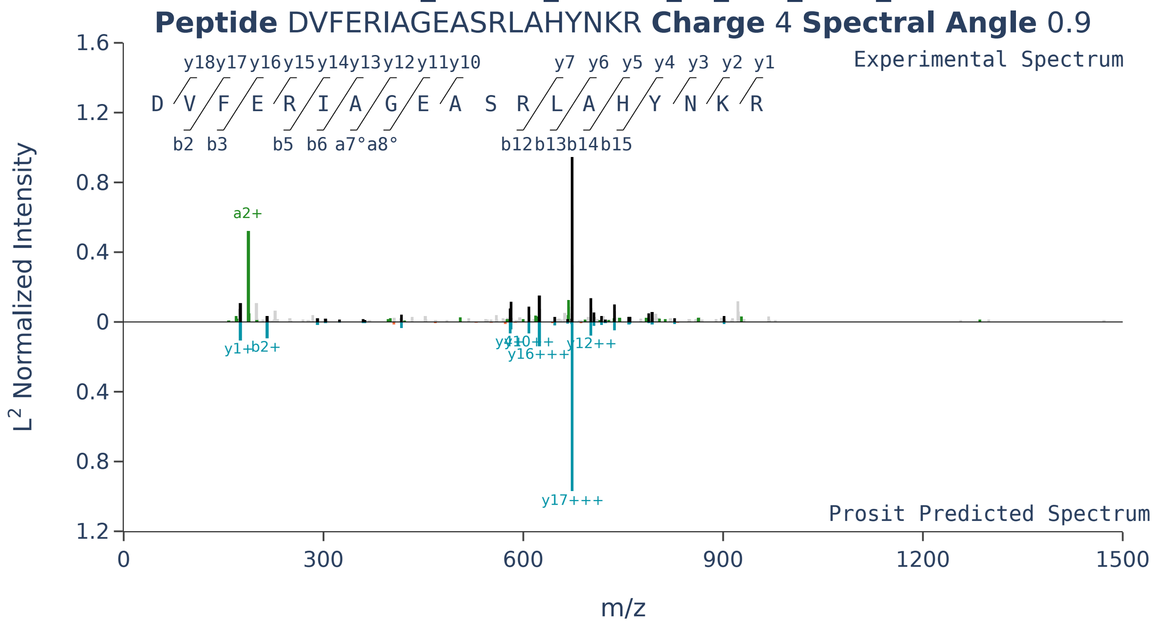

**Source** HRoetschke_291222_080123_Fu_H2B_b2R1_2h **Scan** 3529
**Peptide** EGTKAVTKYTSA **Charge** 2 **Spectral Angle** 0.85

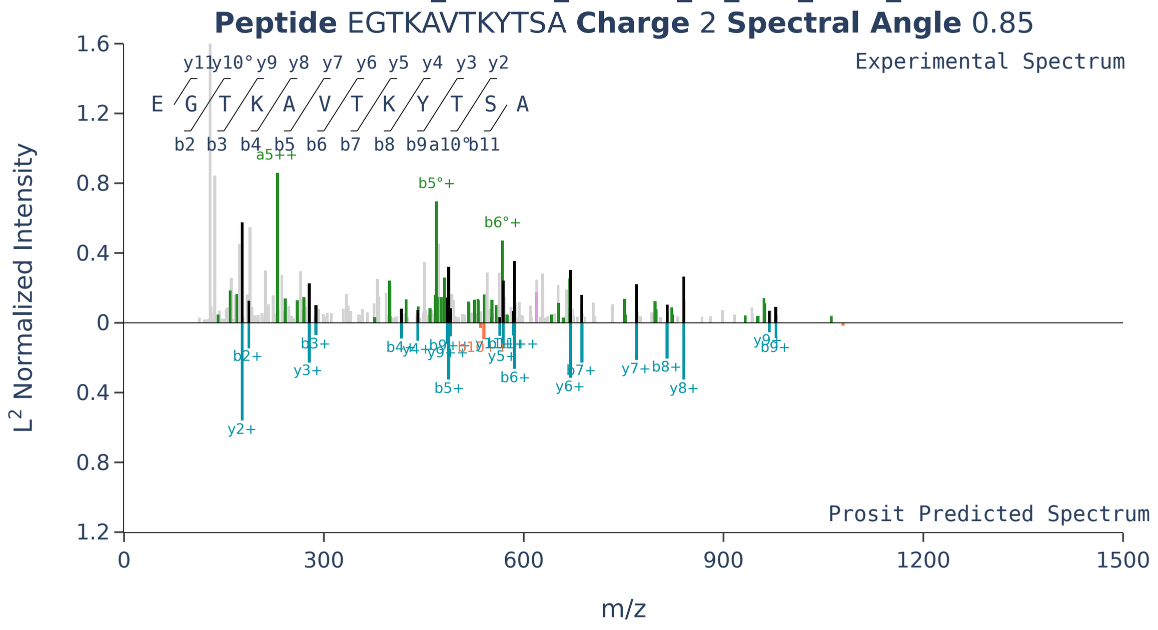


**Source** HRoetschke_291222_080123_Fu_H2B_b1R1_2h **Scan** 2238
**Peptide** HPDTGISSKAM **Charge** 3 **Spectral Angle** 0.87

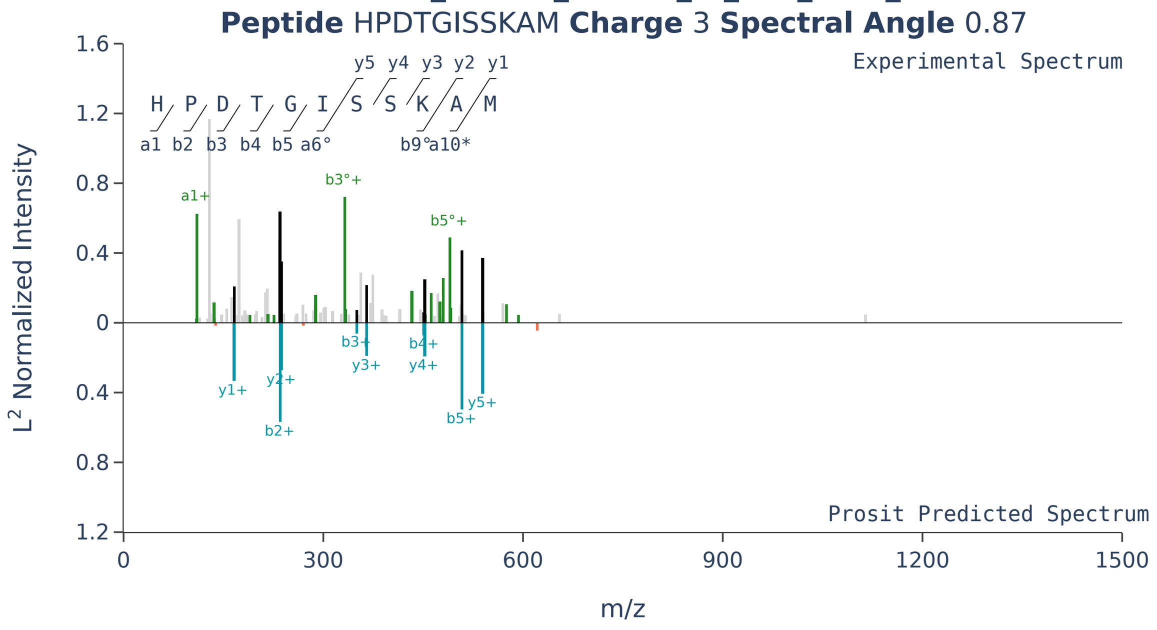


**Source** HRoetschke_291222_080123_Fu_H2B_b1R2_24h **Scan** 9342
**Peptide** VFERIAGEAS **Charge** 2 **Spectral Angle** 0.87

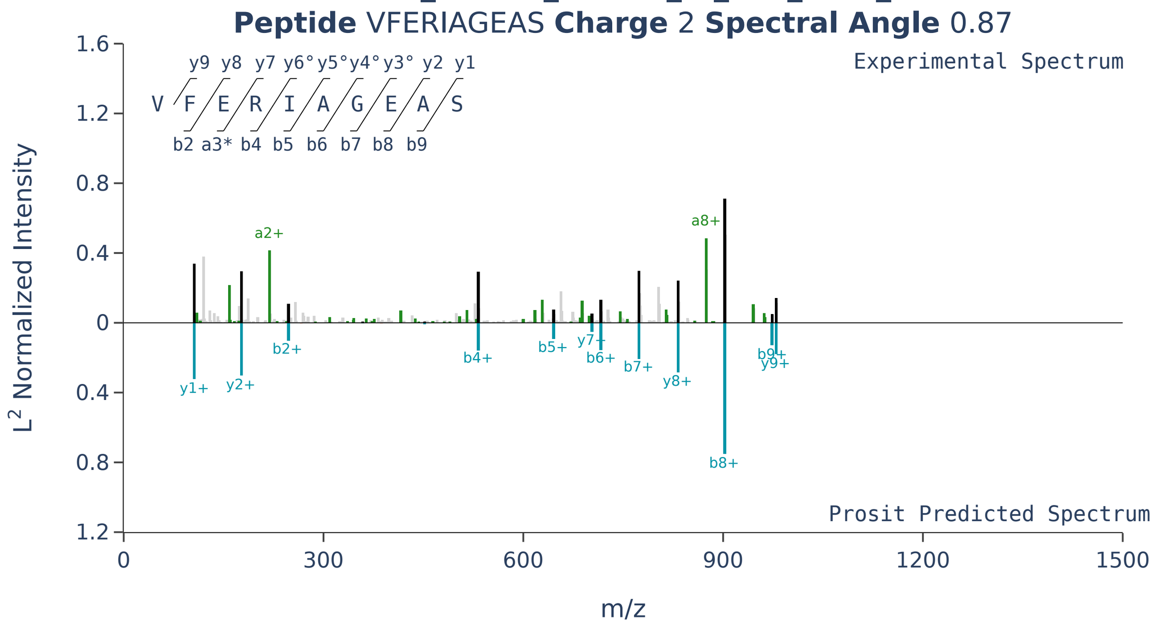


**Source** HRoetschke_291222_080123_Fu_H2B_b2R1_24h **Scan** 18354
**Peptide** NDVFERIAGEASRLAHYNKR **Charge** 4 **Spectral Angle** 0.86

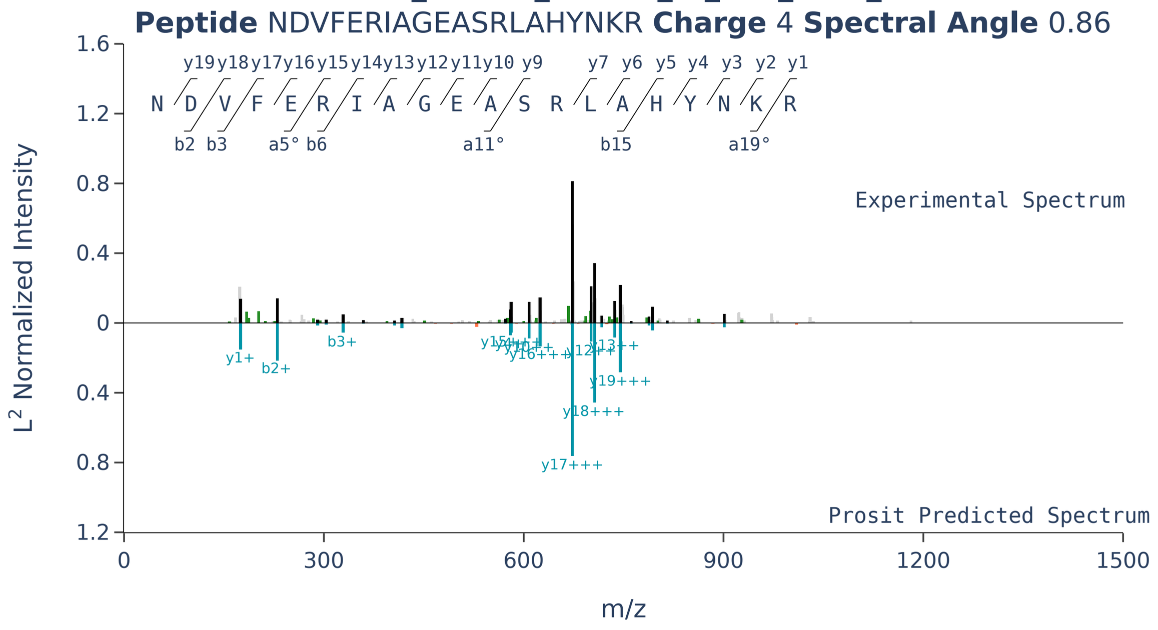


**Source** HRoetschke_291222_080123_Fu_H2B_b1R2_24h **Scan** 4084
**Peptide** VSEGTKAVTKY **Charge** 2 **Spectral Angle** 0.81

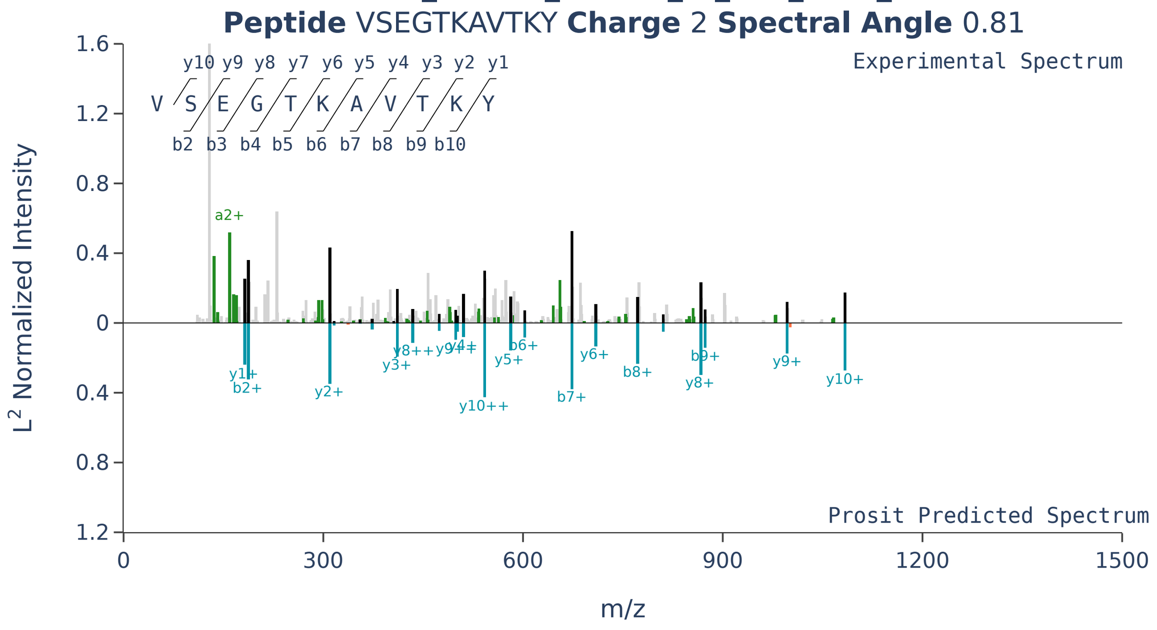


**Source** HRoetschke_291222_080123_Fu_H2B_b1R1_24h **Scan** 15330
**Peptide** RLLLPGELA **Charge** 2 **Spectral Angle** 0.86

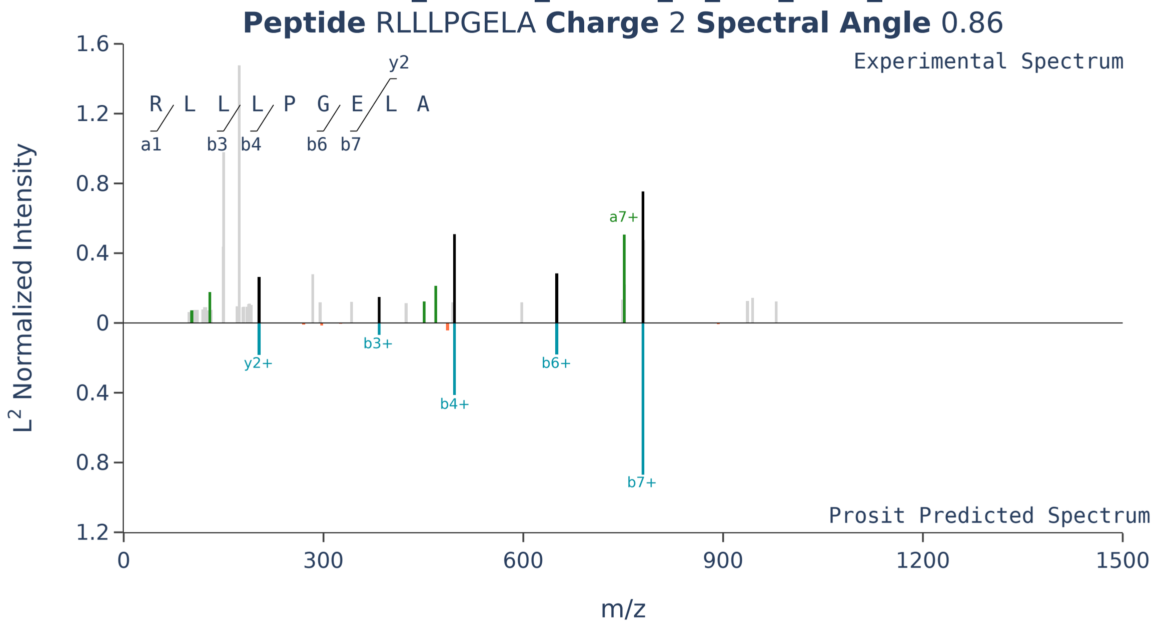


**Source** HRoetschke_291222_080123_Fu_H2B_b1R2_2h **Scan** 3888
**Peptide** KAMSIMN **Charge** 2 **Spectral Angle** 0.81

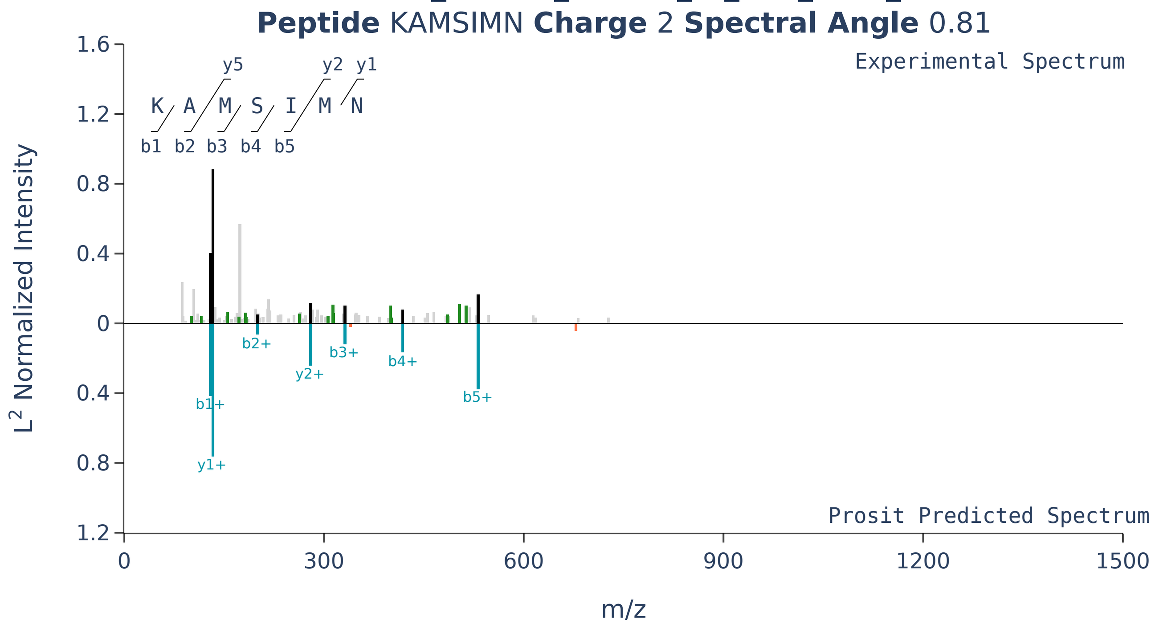


**Source** HRoetschke_291222_080123_Fu_H2B_b1R1_4h **Scan** 8182
**Peptide** LPGELAKHAVSE **Charge** 2 **Spectral Angle** 0.83

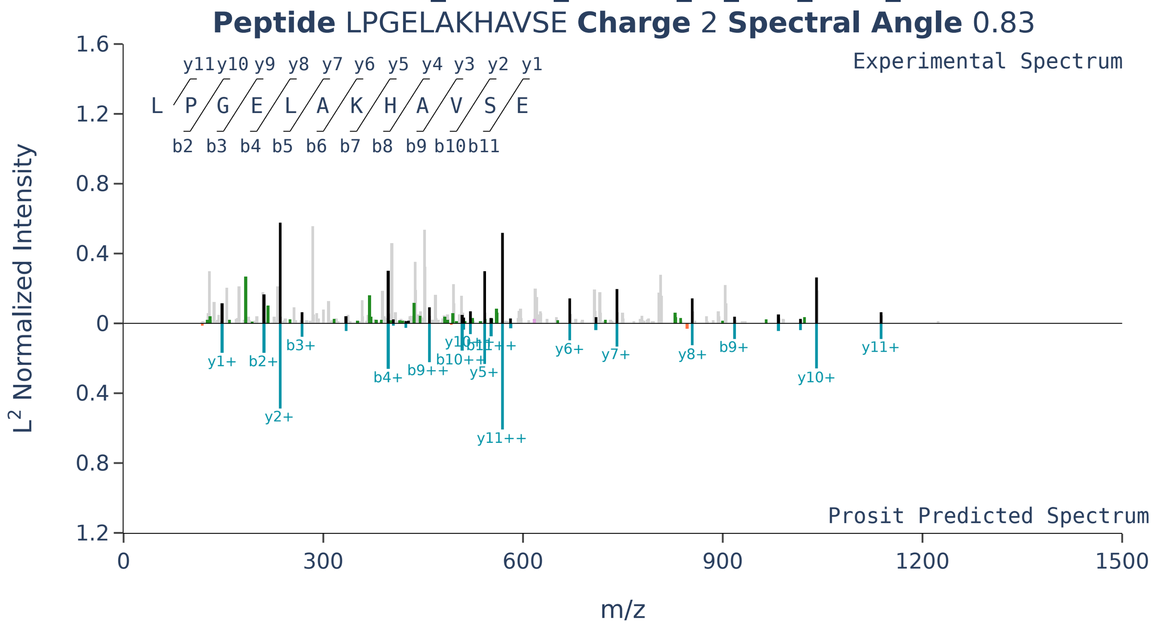


**Source** HRoetschke_291222_080123_Fu_H2B_b1R2_2h **Scan** 18452
**Peptide** SFVNDVFERI **Charge** 2 **Spectral Angle** 0.88

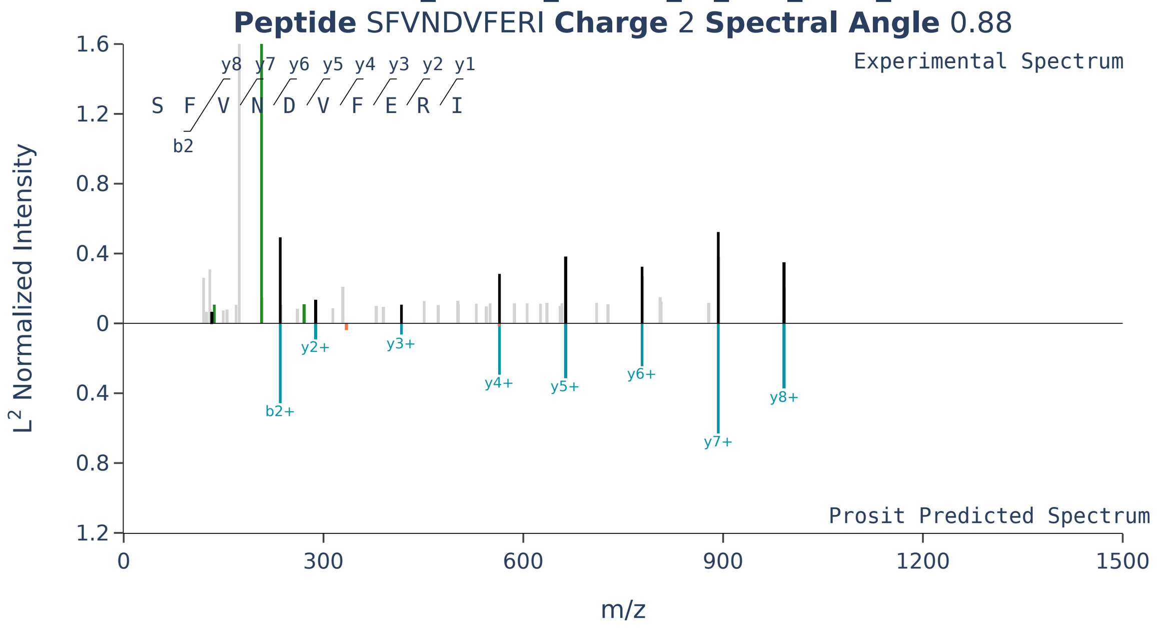


**Source** HRoetschke_291222_080123_Fu_H2B_b2R2_2h **Scan** 2151
**Peptide** GEASRLAHYNKR **Charge** 3 **Spectral Angle** 0.9

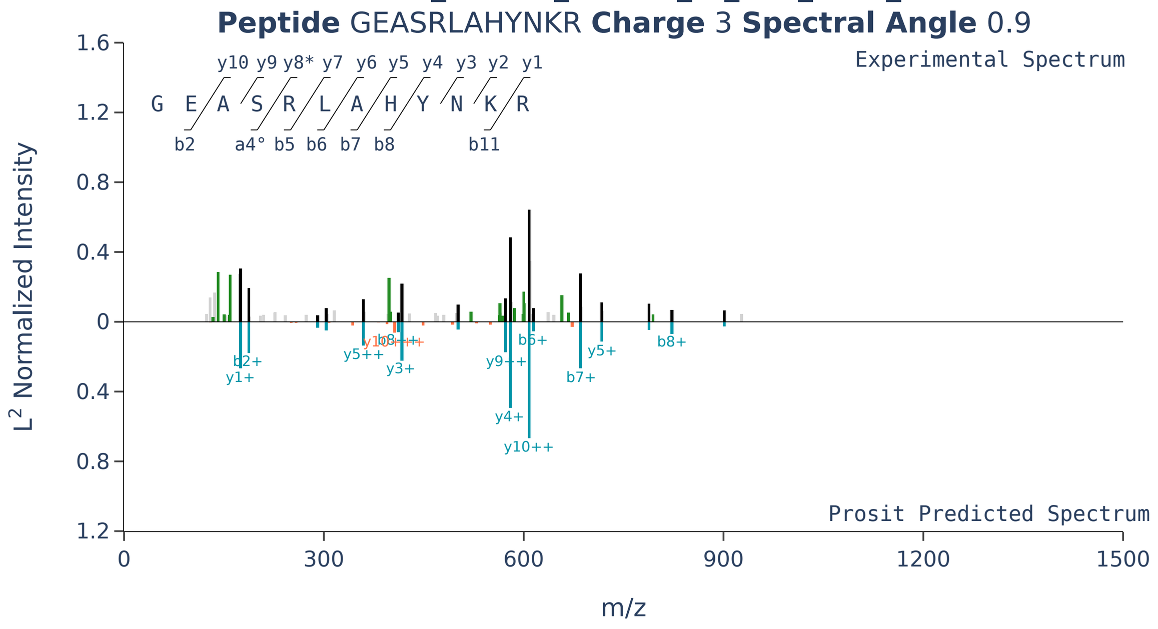


**Source** HRoetschke_291222_080123_Fu_H2B_b2R1_2h **Scan** 17301
**Peptide** LLLPGELAKHAVSEGTKAVTKYTSA **Charge** 4 **Spectral Angle** 0.91

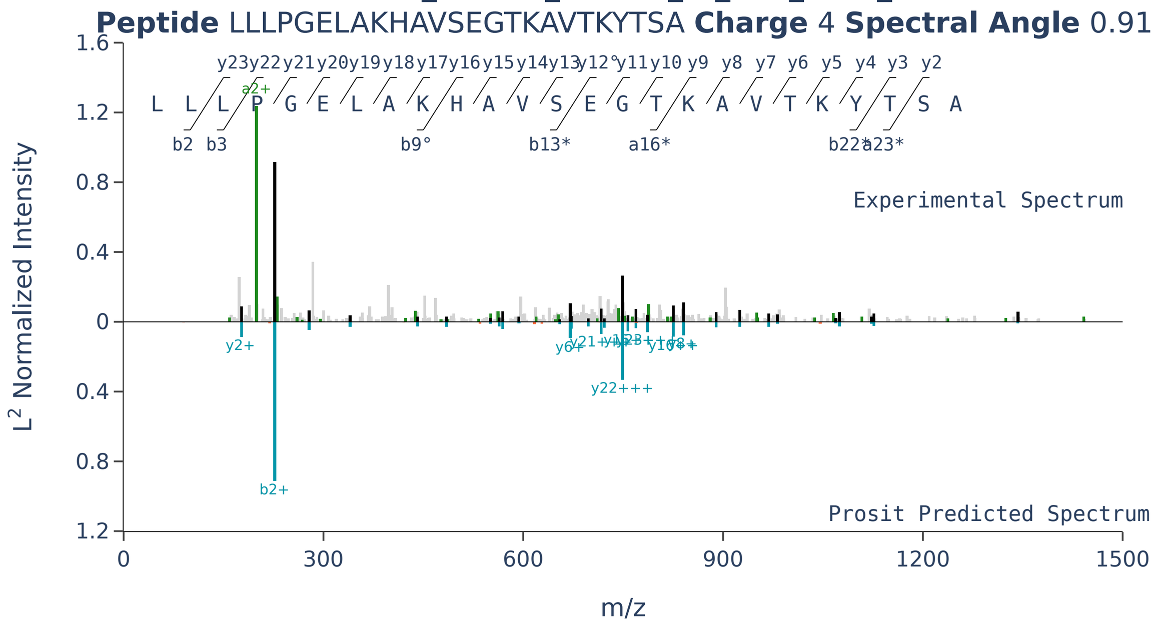


**Source** HRoetschke_291222_080123_Fu_H2B_b1R2_24h **Scan** 10174
**Peptide** LTSREIQTAV **Charge** 2 **Spectral Angle** 0.86

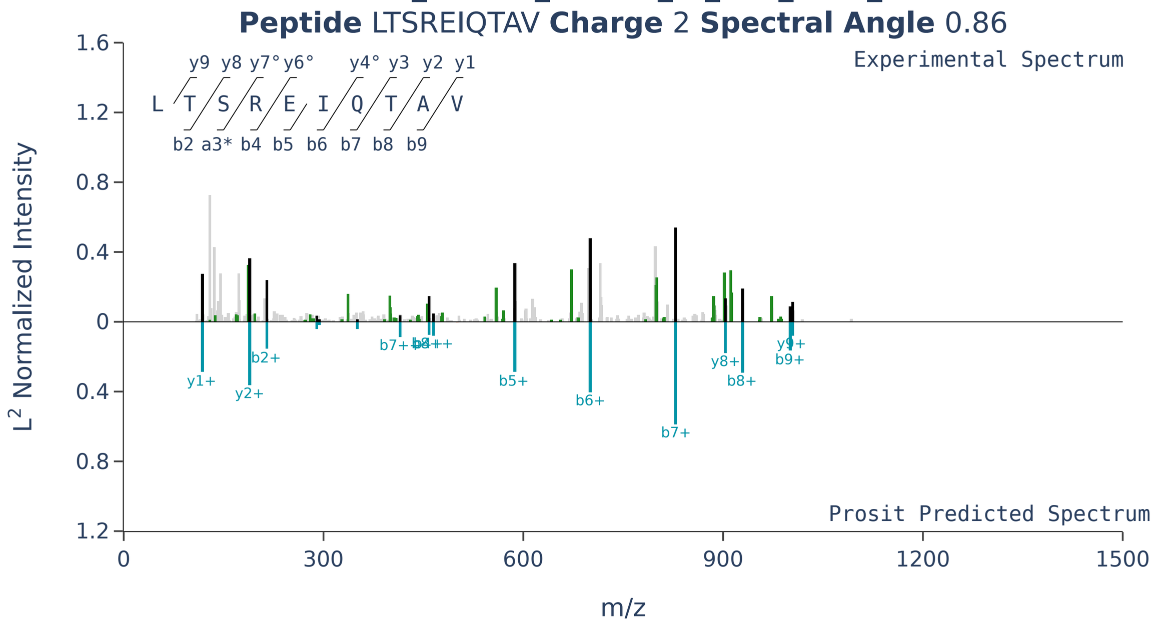


**Source** HRoetschke_291222_080123_Fu_H2B_b2R2_4h **Scan** 2531
**Peptide** KQVHPDTGISSKA **Charge** 2 **Spectral Angle** 0.88

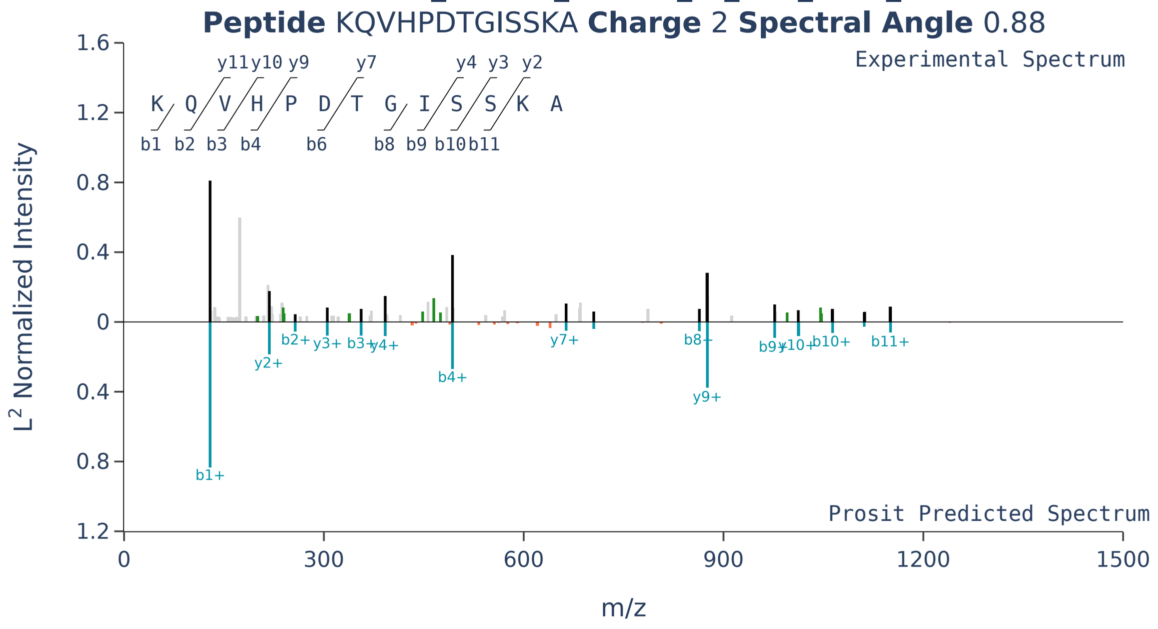


**Source** HRoetschke_291222_080123_Fu_H2B_b1R2_24h **Scan** 5051
**Peptide** LTSREIQT **Charge** 2 **Spectral Angle** 0.86

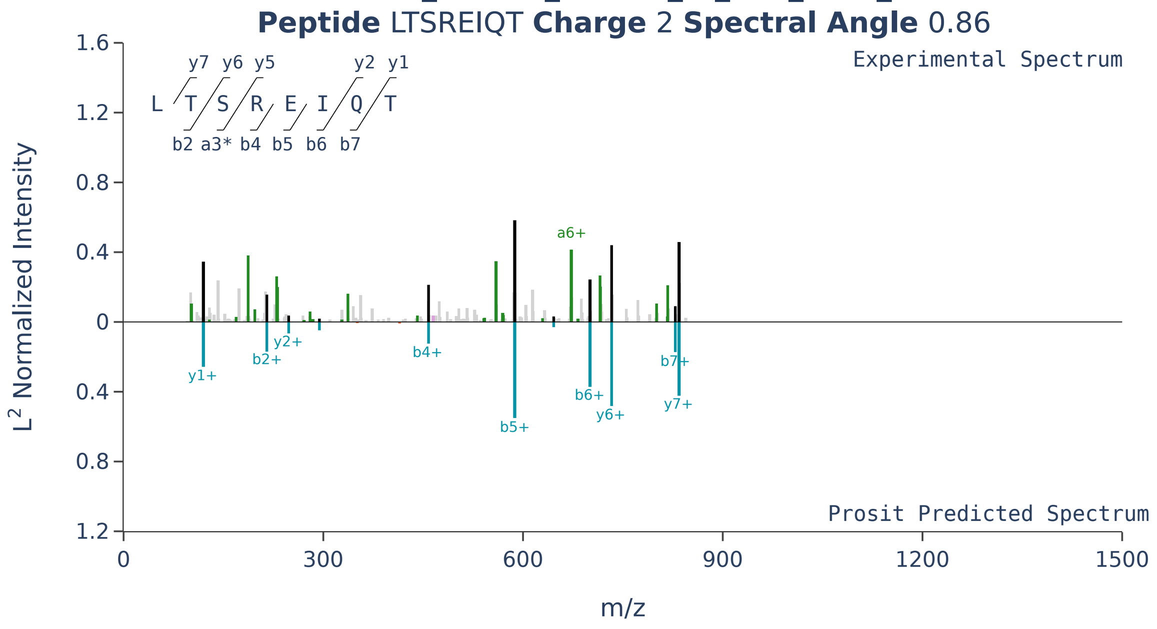


**Source** HRoetschke_291222_080123_Fu_H2B_b1R1_24h **Scan** 5404
**Peptide** LTSREIQTA **Charge** 2 **Spectral Angle** 0.87

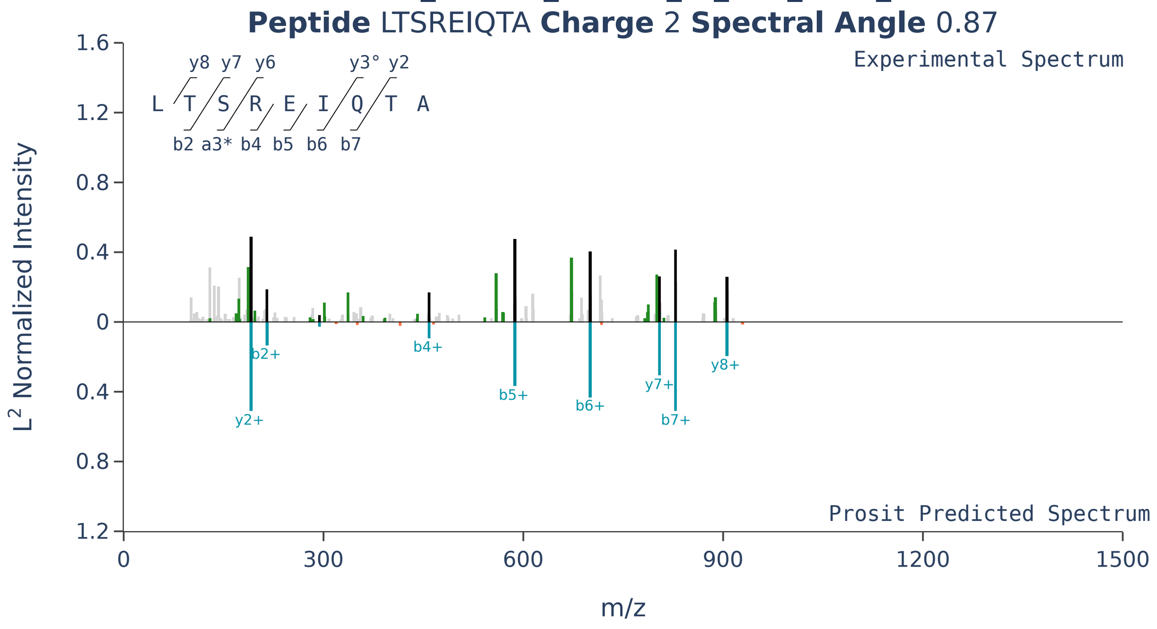

**Source** HRoetschke_291222_080123_Fu_H2B_b2R2_2h **Scan** 4893
**Peptide** AGEASRLAHY **Charge** 2 **Spectral Angle** 0.85

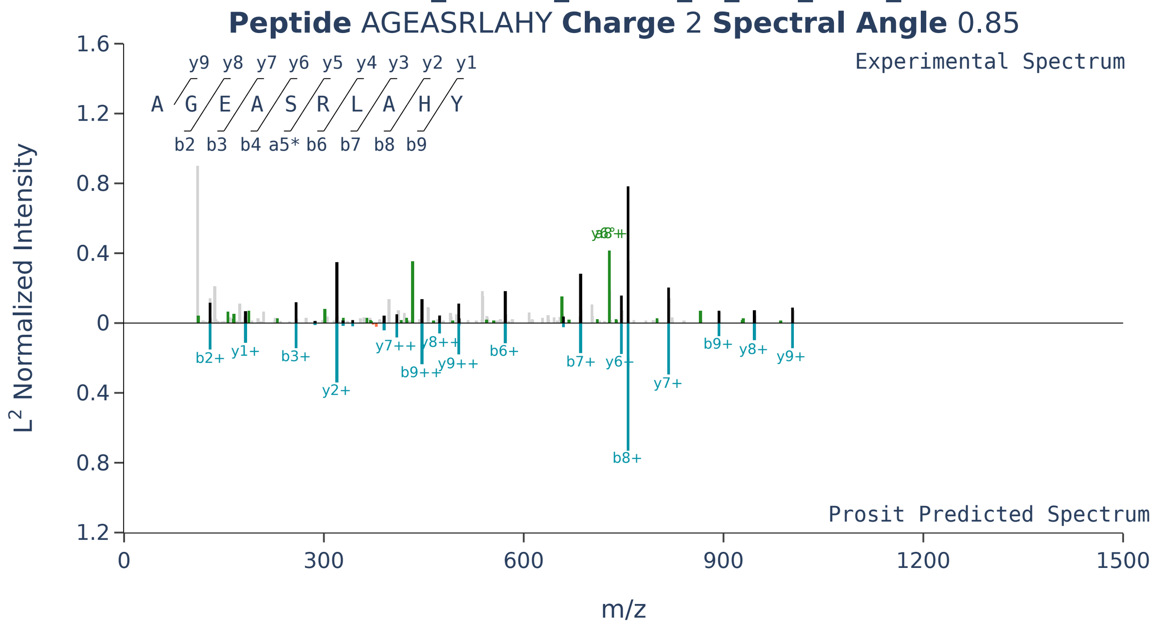


**Source** HRoetschke_291222_080123_Fu_H2B_b2R1_4h **Scan** 3479
**Peptide** KRSTITSREIQT **Charge** 2 **Spectral Angle** 0.75

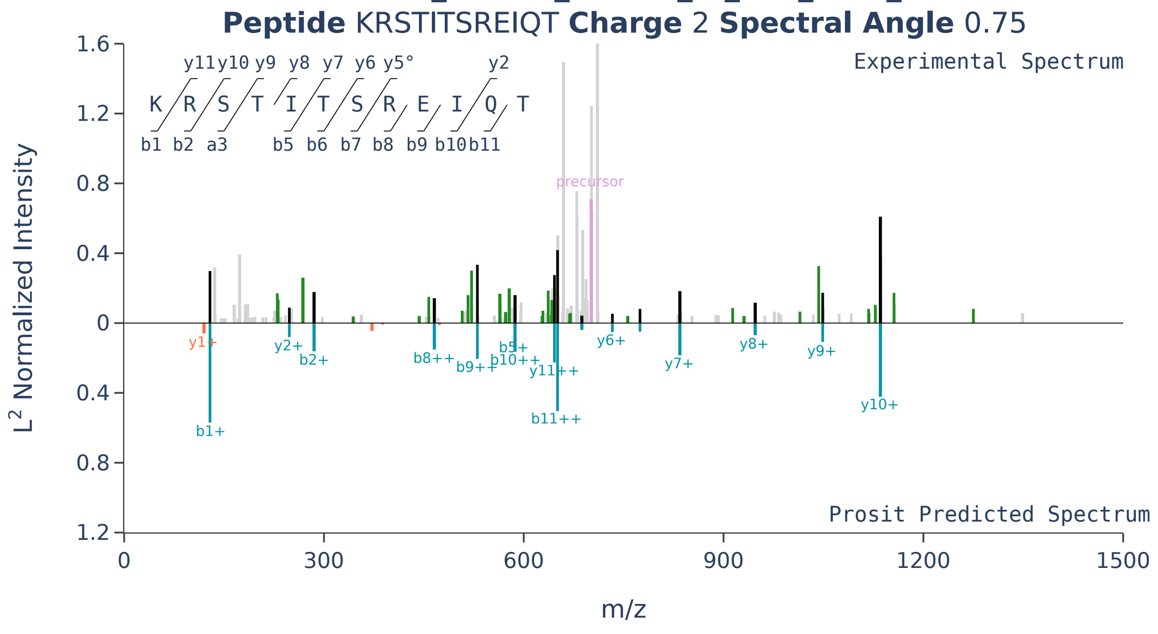


**Source** HRoetschke_291222_080123_Fu_H2B_b1R1_4h **Scan** 5405
**Peptide** RLAGEASRL **Charge** 2 **Spectral Angle** 0.87

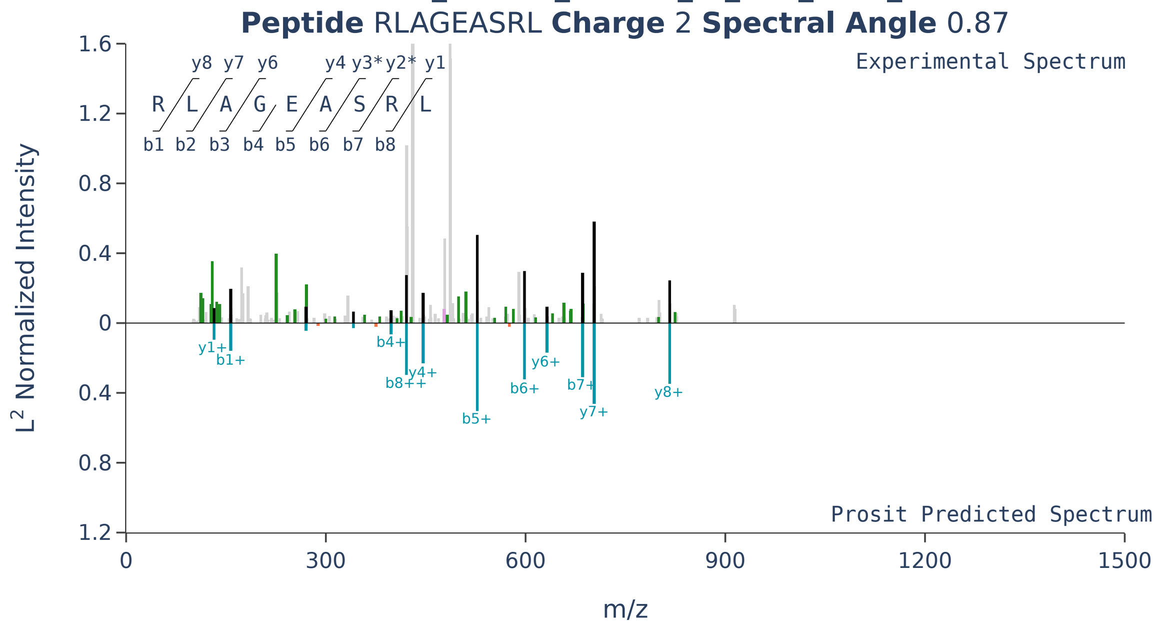


**Source** HRoetschke_291222_080123_Fu_H2B_b1R2_2h **Scan** 14302
**Peptide** VNDVFERIAGEA **Charge** 2 **Spectral Angle** 0.93

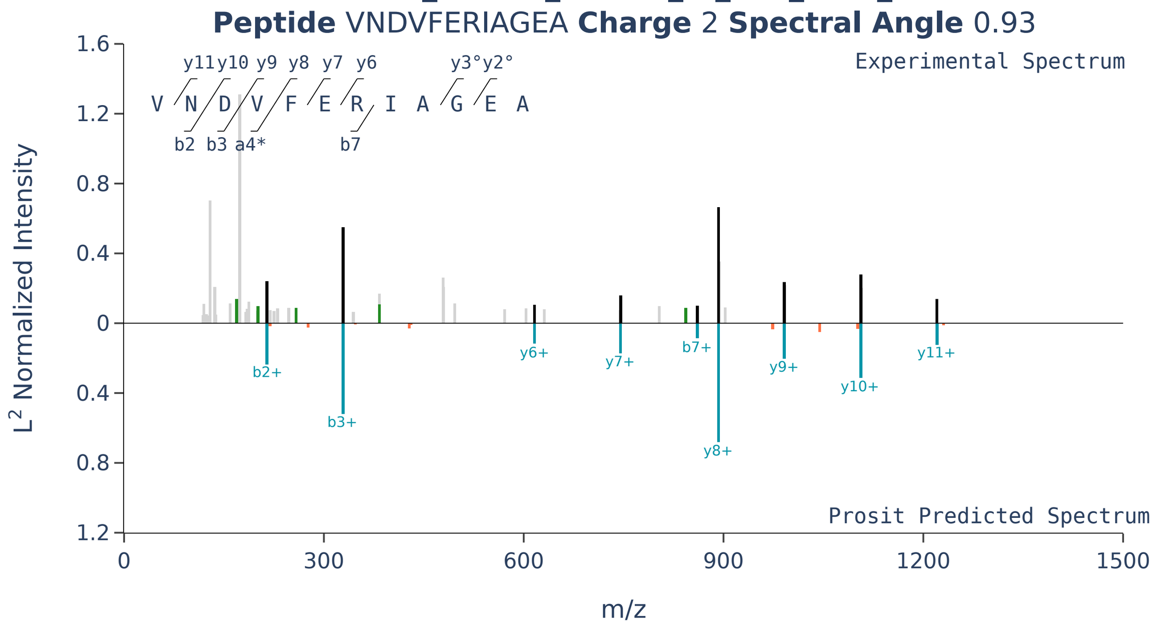


**Source** HRoetschke_291222_080123_Fu_H2B_b2R2_1h **Scan** 15153
**Peptide** QTAVRLLL **Charge** 2 **Spectral Angle** 0.91

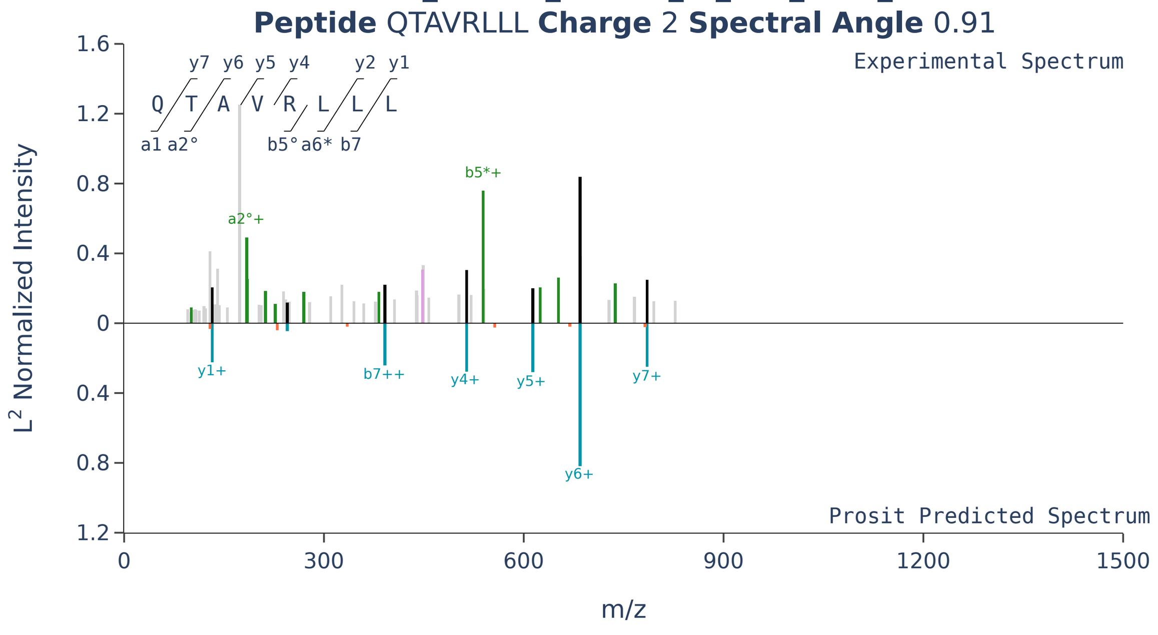


**Source** HRoetschke_291222_080123_Fu_H2B_b2R1_24h **Scan** 9995
**Peptide** VFERIAGEA **Charge** 2 **Spectral Angle** 0.9

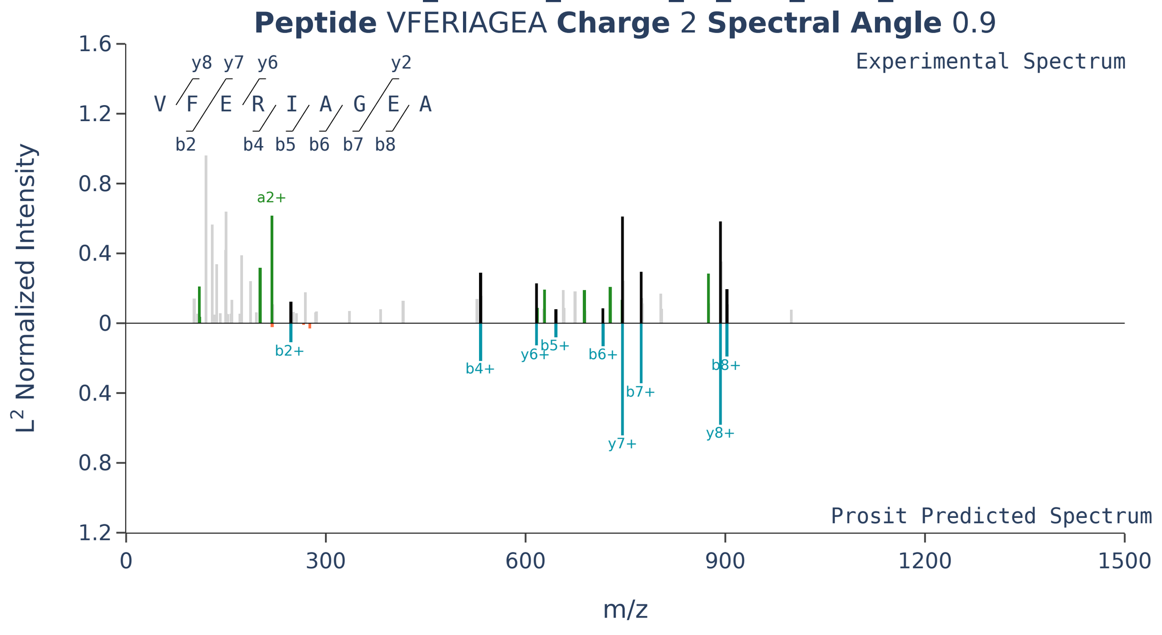


**Source** HRoetschke_291222_080123_Fu_H2B_b2R1_24h **Scan** 15257
**Peptide** VRLLLPGELAKH **Charge** 3 **Spectral Angle** 0.87

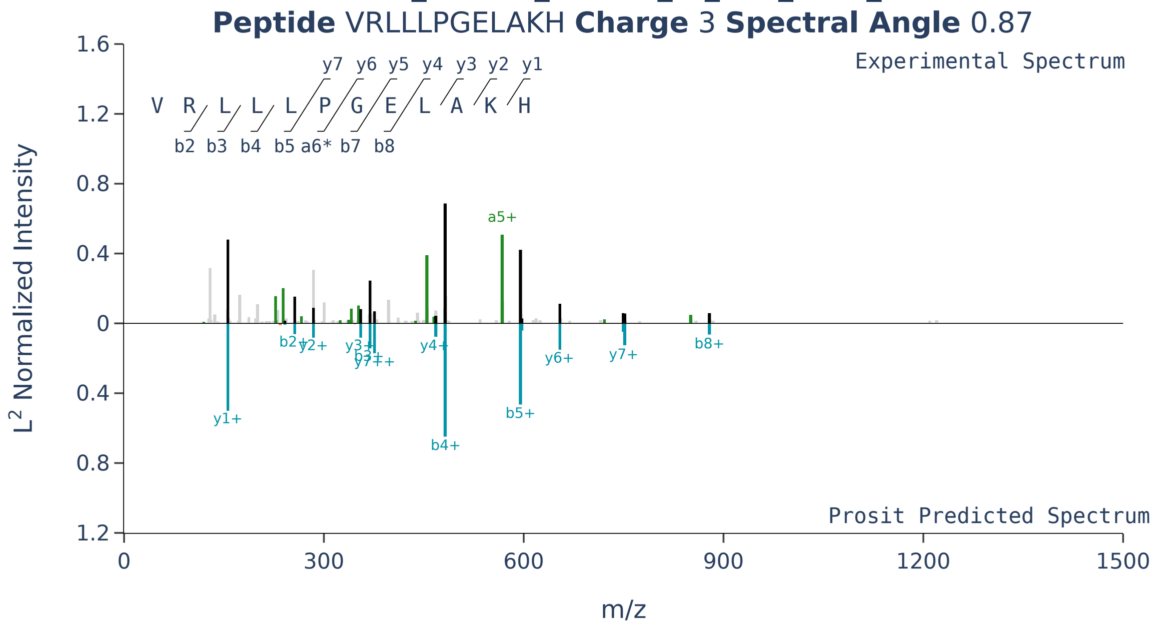


**Source** HRoetschke_291222_080123_Fu_H2B_b2R1_4h **Scan** 20196
**Peptide** VNDVFERIAGEASRL **Charge** 3 **Spectral Angle** 0.84

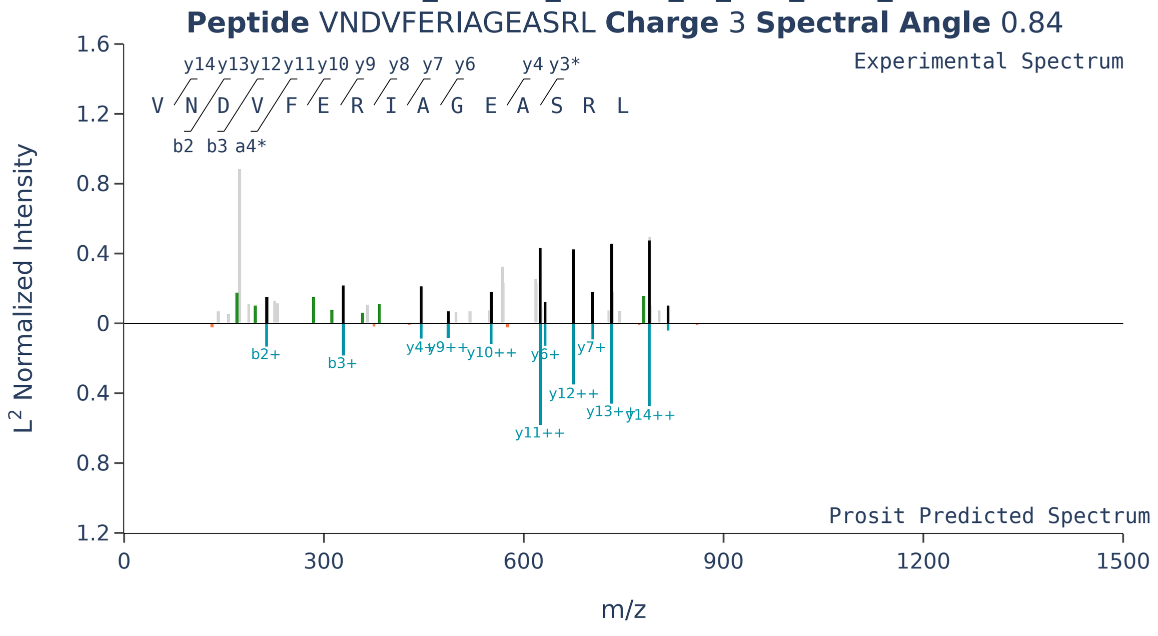


**Source** HRoetschke_291222_080123_Fu_H2B_b2R2_4h **Scan** 18376
**Peptide** KESYAIYVYKVL **Charge** 2 **Spectral Angle** 0.93

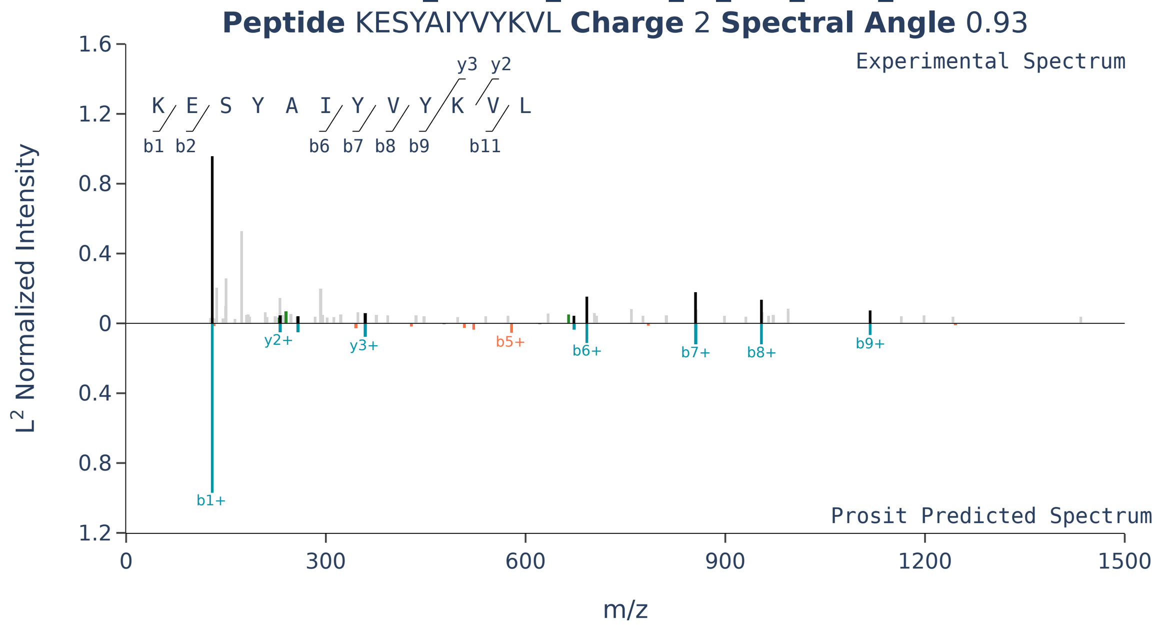


**Source** HRoetschke_291222_080123_Fu_H2B_b1R1_24h **Scan** 5438
**Peptide** RSTITSREL **Charge** 3 **Spectral Angle** 0.86

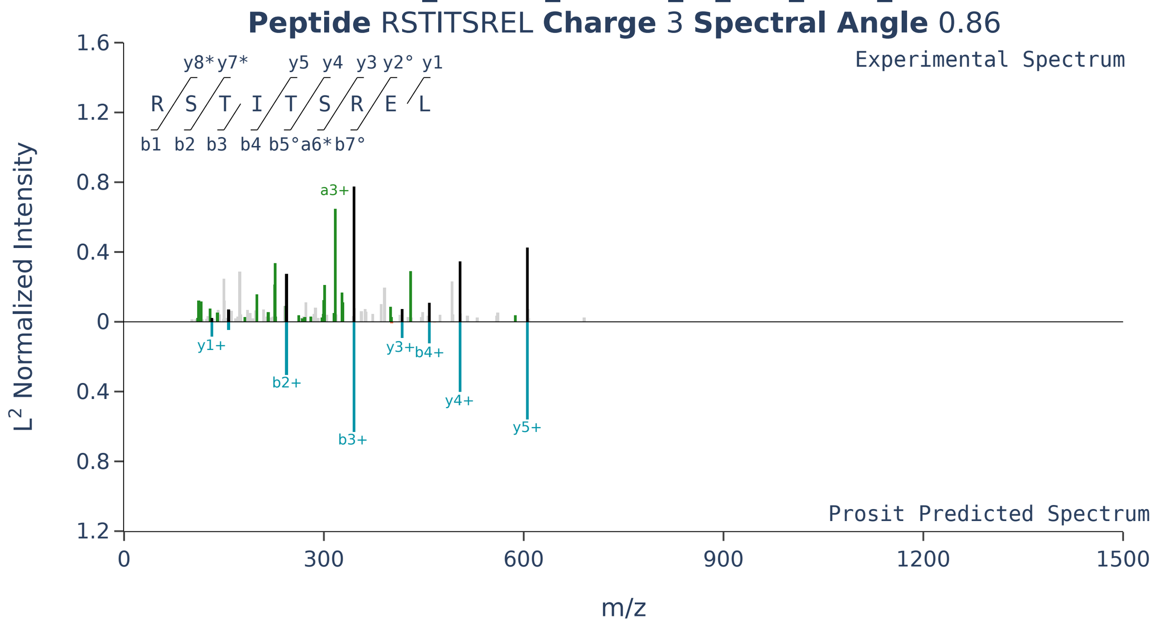


**Source** HRoetschke_291222_080123_Fu_H2B_b2R2_24h **Scan** 16291
**Peptide** AVRLLLPGELAKH **Charge** 3 **Spectral Angle** 0.83

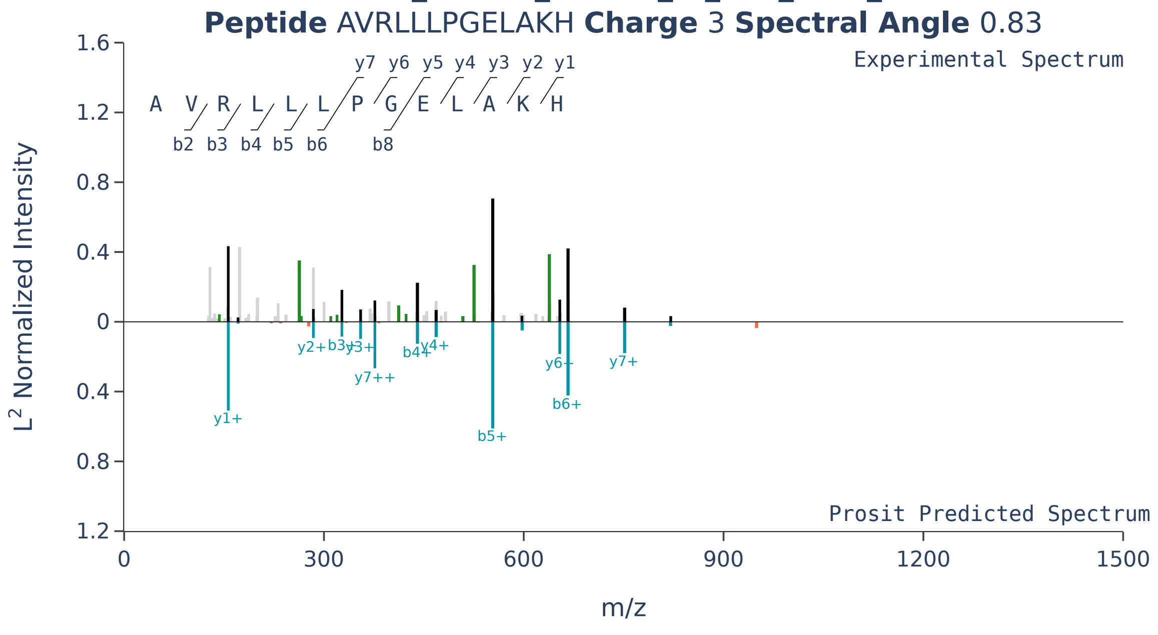


**Source** HRoetschke_291222_080123_Fu_H2B_b1R1_2h **Scan** 1876
**Peptide** EASRLAHYNKR **Charge** 3 **Spectral Angle** 0.9

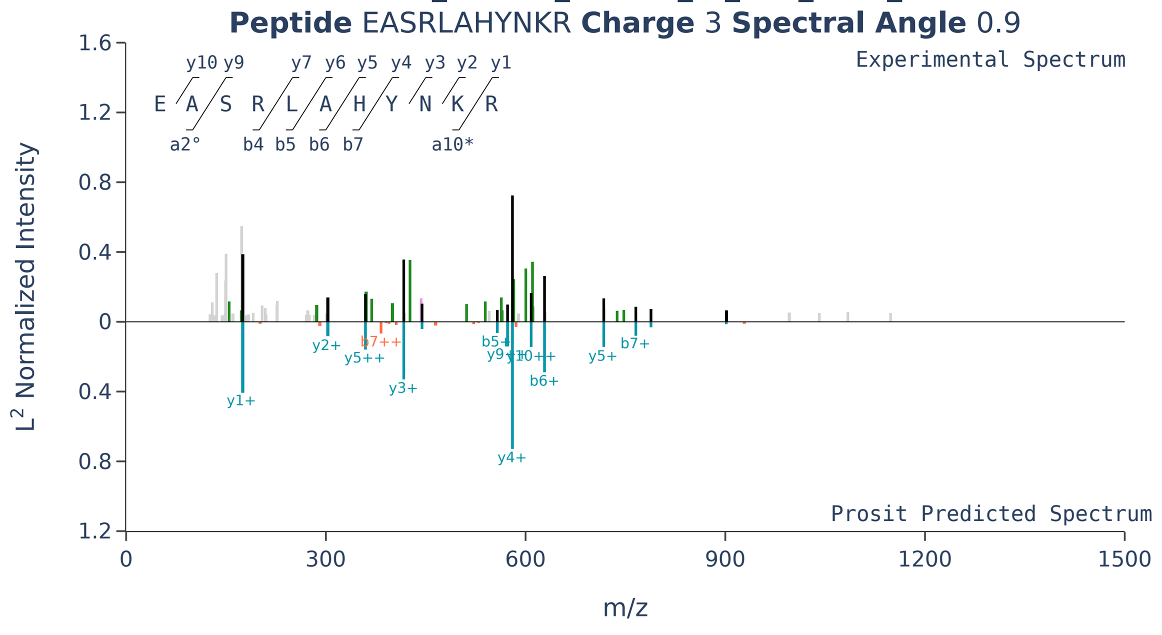


**Source** HRoetschke_291222_080123_Fu_H2B_b2R2_24h **Scan** 2858
**Peptide** KRSTITSREIQ **Charge** 3 **Spectral Angle** 0.77

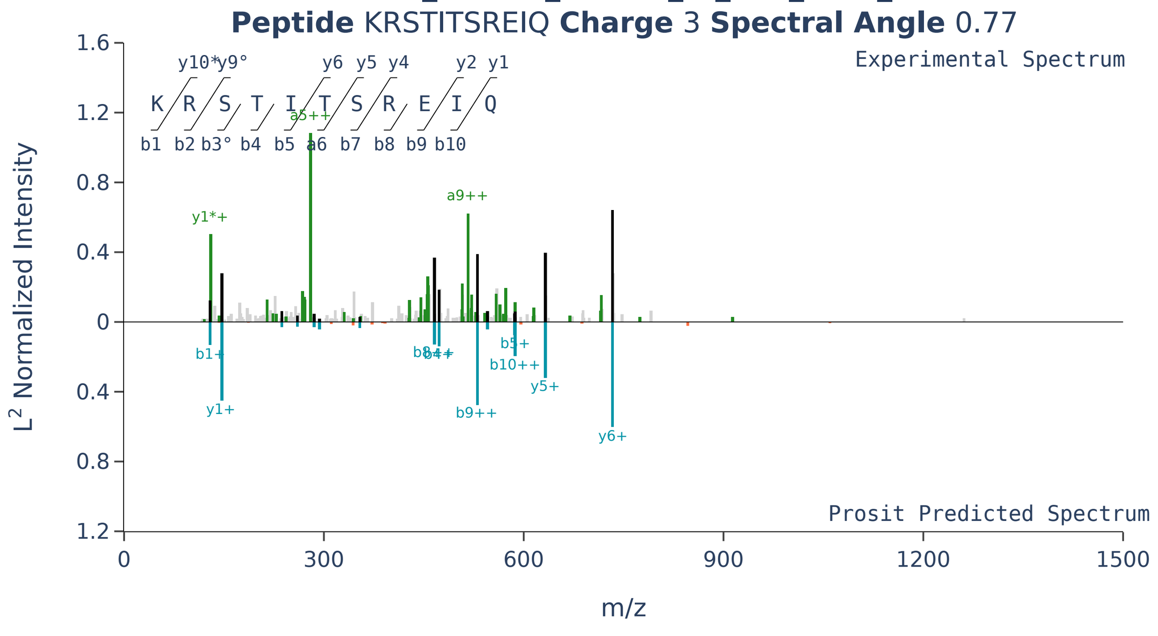


**Source** HRoetschke_291222_080123_Fu_H2B_b1R1_24h **Scan** 6764
**Peptide** ERIAGEASRL **Charge** 2 **Spectral Angle** 0.85

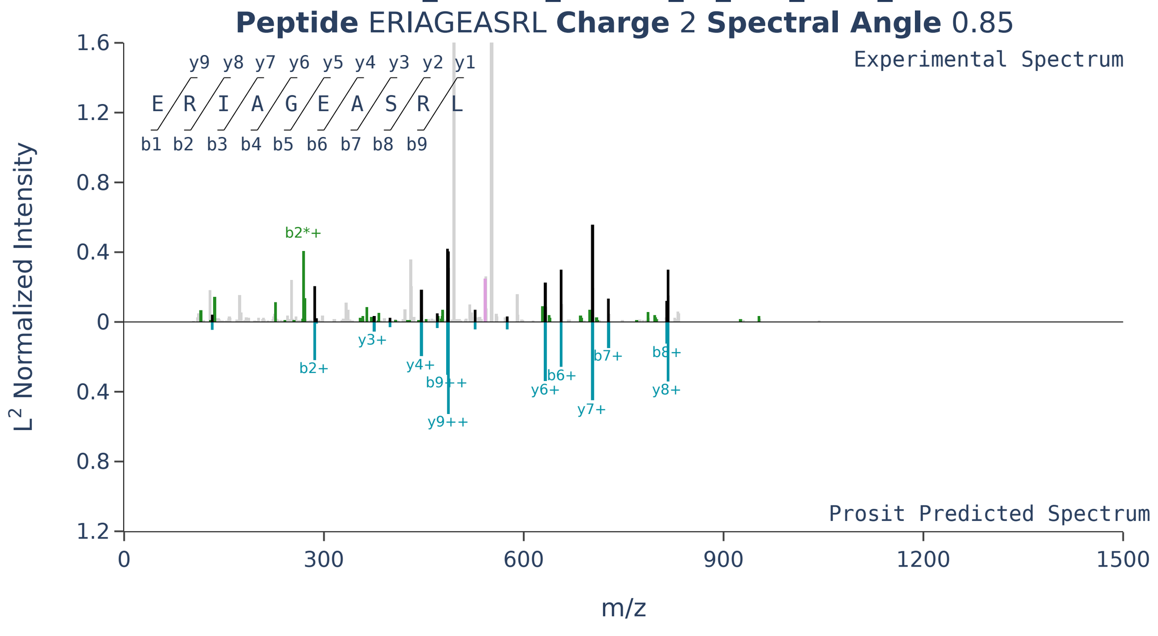


**Source** HRoetschke_291222_080123_Fu_H2B_b2R1_2h **Scan** 3701
**Peptide** RSTITSREIQ **Charge** 2 **Spectral Angle** 0.83

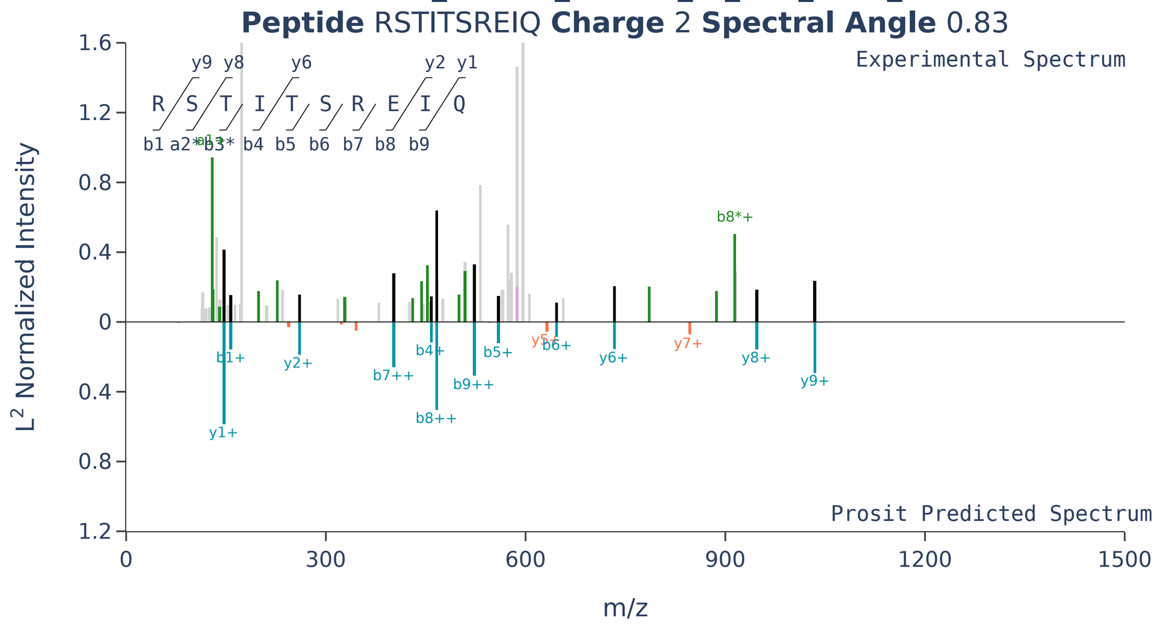

**Source** HRoetschke_291222_080123_Fu_H2B_b2R1_24h **Scan** 11928
**Peptide** RKESYAIYV **Charge** 2 **Spectral Angle** 0.9

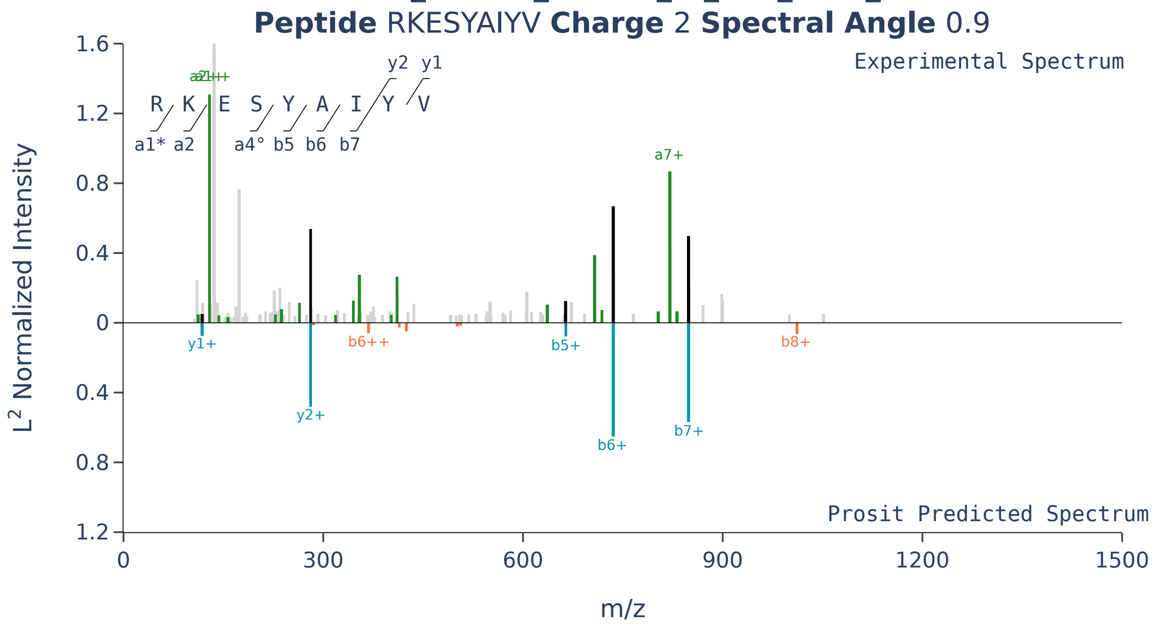


**Source** HRoetschke_291222_080123_Fu_H2B_b1R2_2h **Scan** 14647
**Peptide** ELQTAVRLL **Charge** 2 **Spectral Angle** 0.94

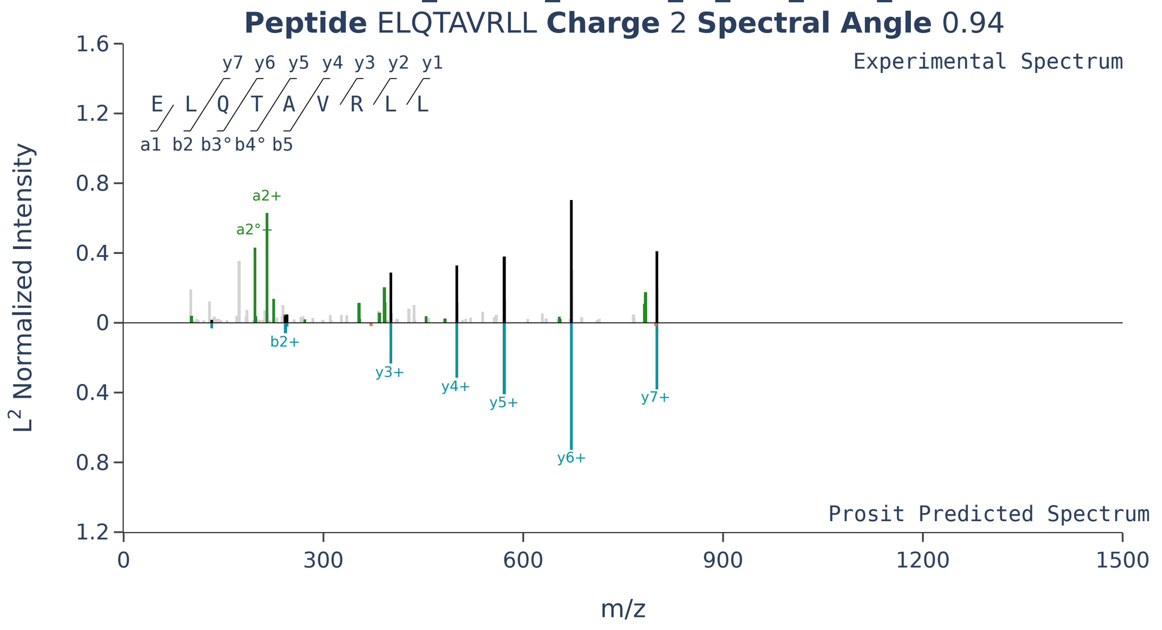


**Source** HRoetschke_291222_080123_Fu_H2B_b2R1_24h **Scan** 6472
**Peptide** LAHYNKRSTITSREIQTAVR **Charge** 4 **Spectral Angle** 0.88

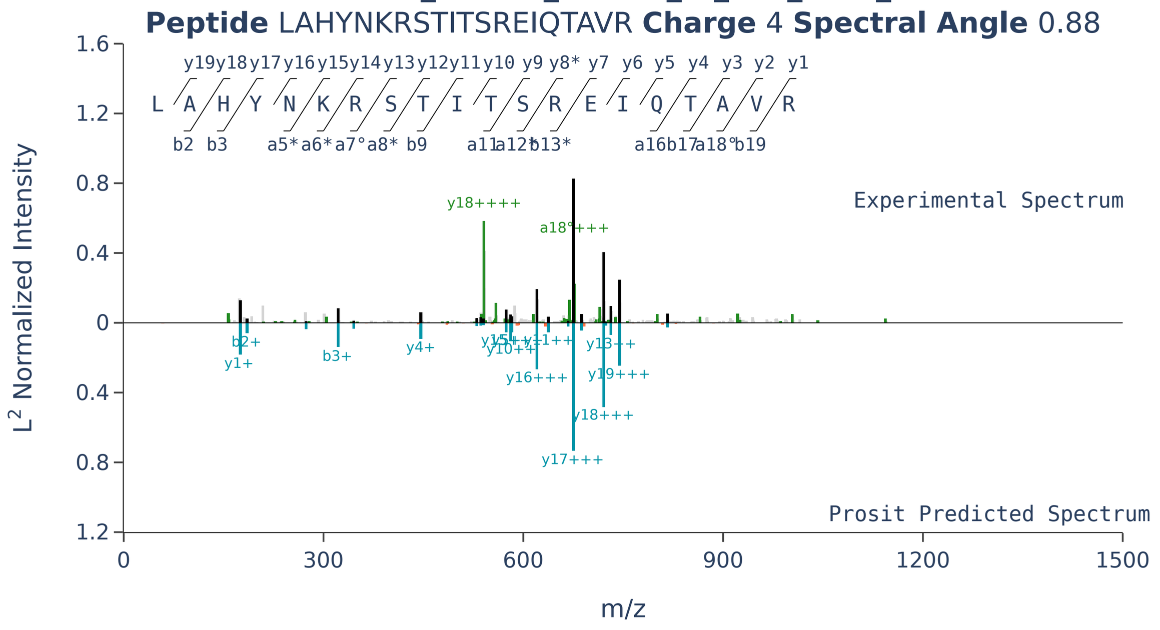


**Source** HRoetschke_291222_080123_Fu_H2B_b2R1_24h **Scan** 4799
**Peptide** KVLKQVHPDTGISS **Charge** 2 **Spectral Angle** 0.87

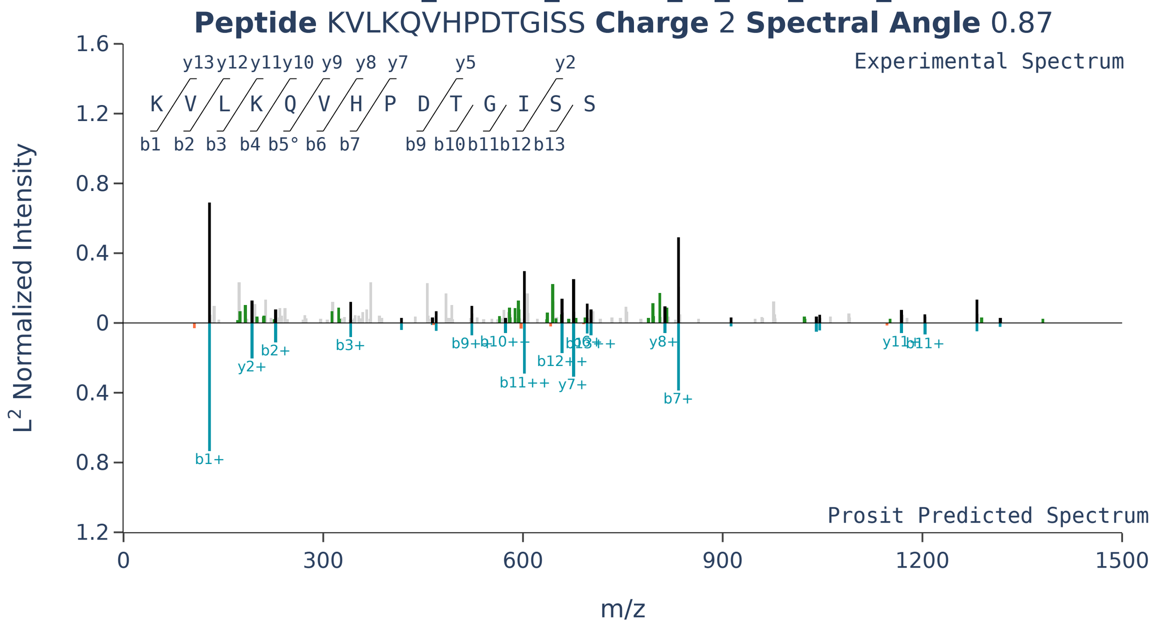


**Source** HRoetschke_291222_080123_Fu_H2B_b2R2_1h **Scan** 3403
**Peptide** GEASRLAHYN **Charge** 2 **Spectral Angle** 0.83

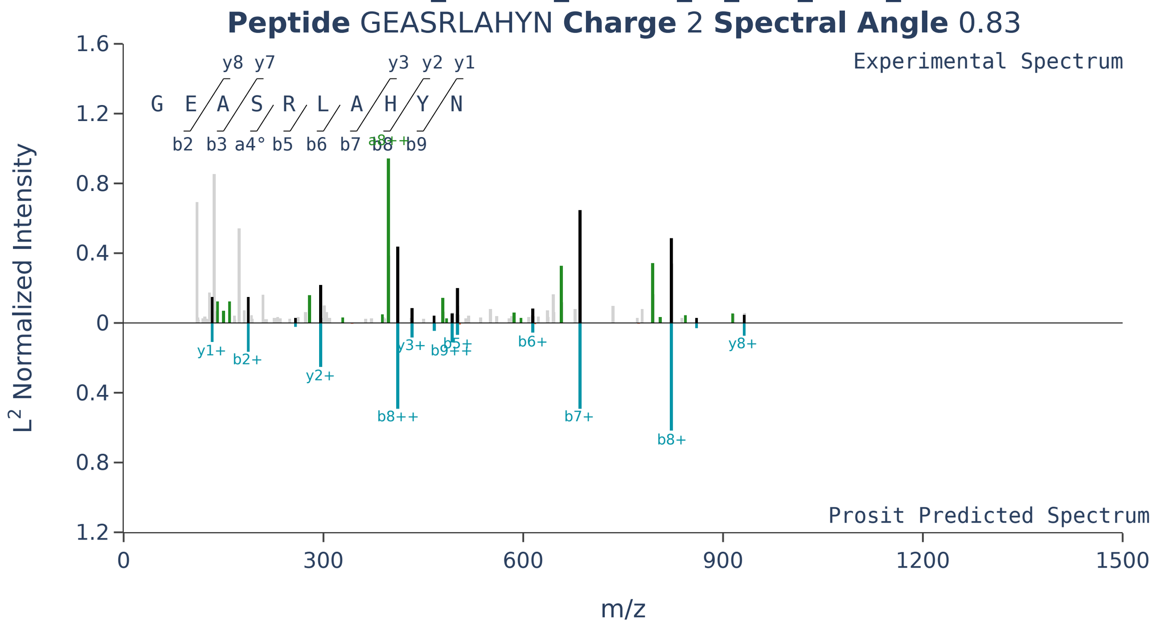


**Source** HRoetschke_291222_080123_Fu_H2B_b1R1_2h **Scan** 13760
**Peptide** SYAIYVYKV **Charge** 2 **Spectral Angle** 0.85

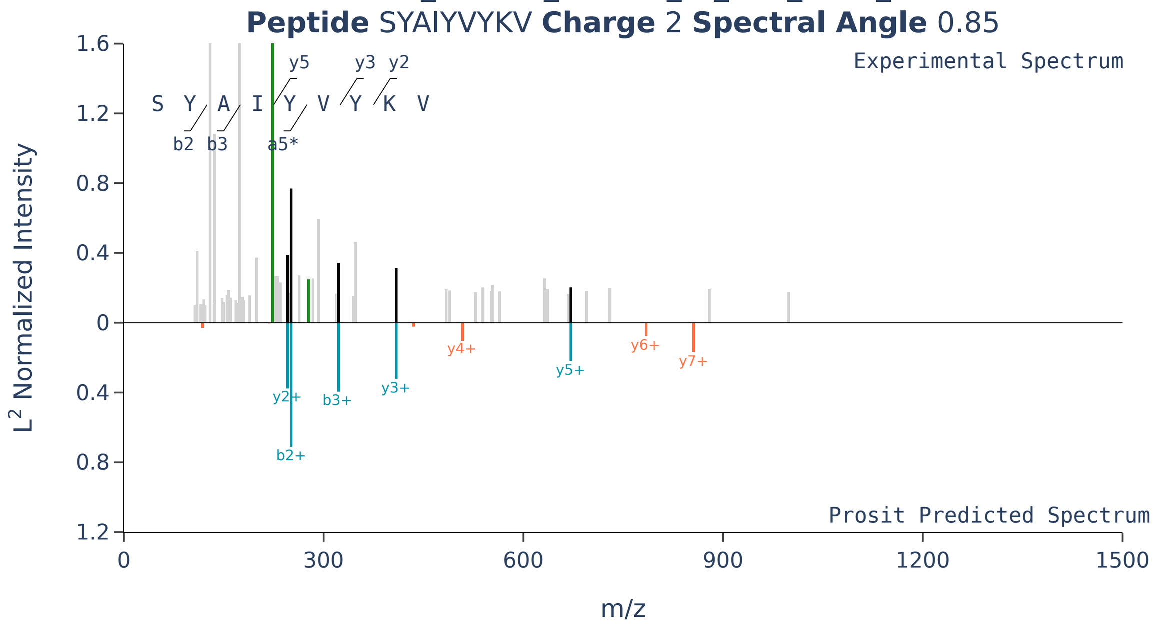


**Source** HRoetschke_291222_080123_Fu_H2B_b2R1_24h **Scan** 15088
**Peptide** STITSREIQTAVRL **Charge** 3 **Spectral Angle** 0.87

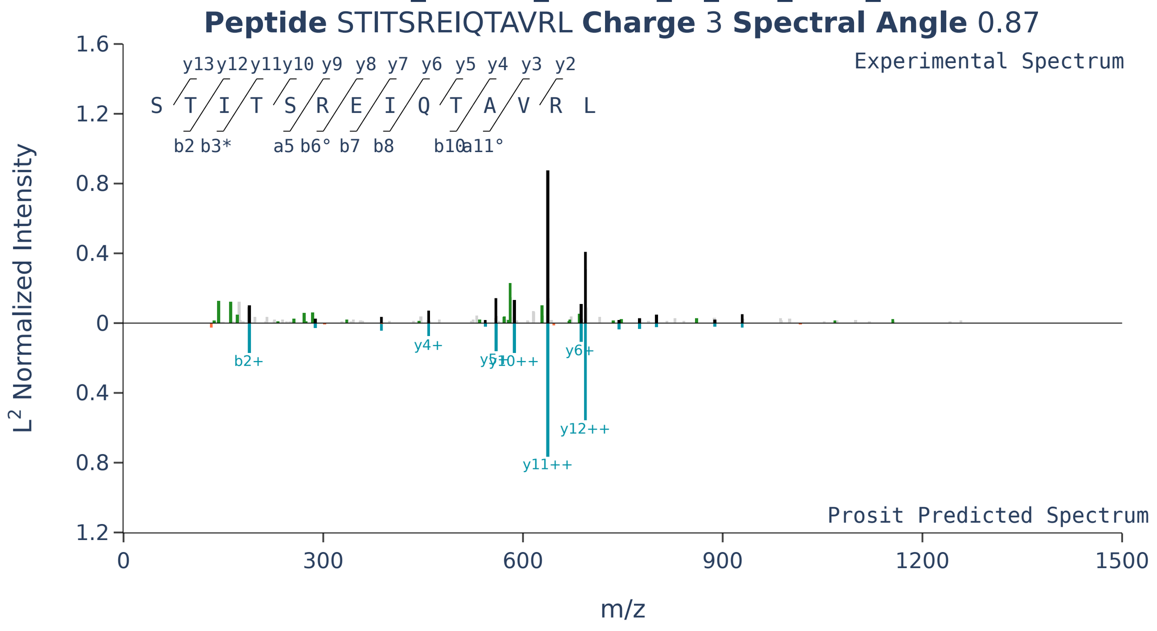


**Source** HRoetschke_291222_080123_Fu_H2B_b2R1_24h **Scan** 12682
**Peptide** LLPGELAKHAVSE **Charge** 2 **Spectral Angle** 0.87

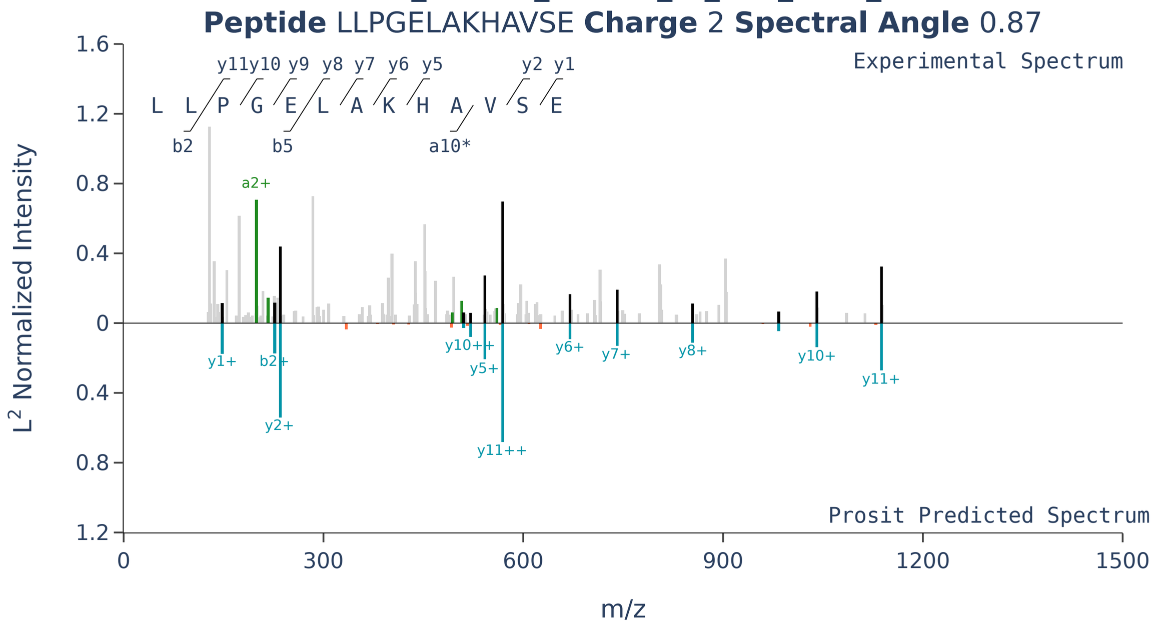


**Source** HRoetschke_291222_080123_Fu_H2B_b2R1_4h **Scan** 4261
**Peptide** LKQVHPDTGISSKAM **Charge** 4 **Spectral Angle** 0.81

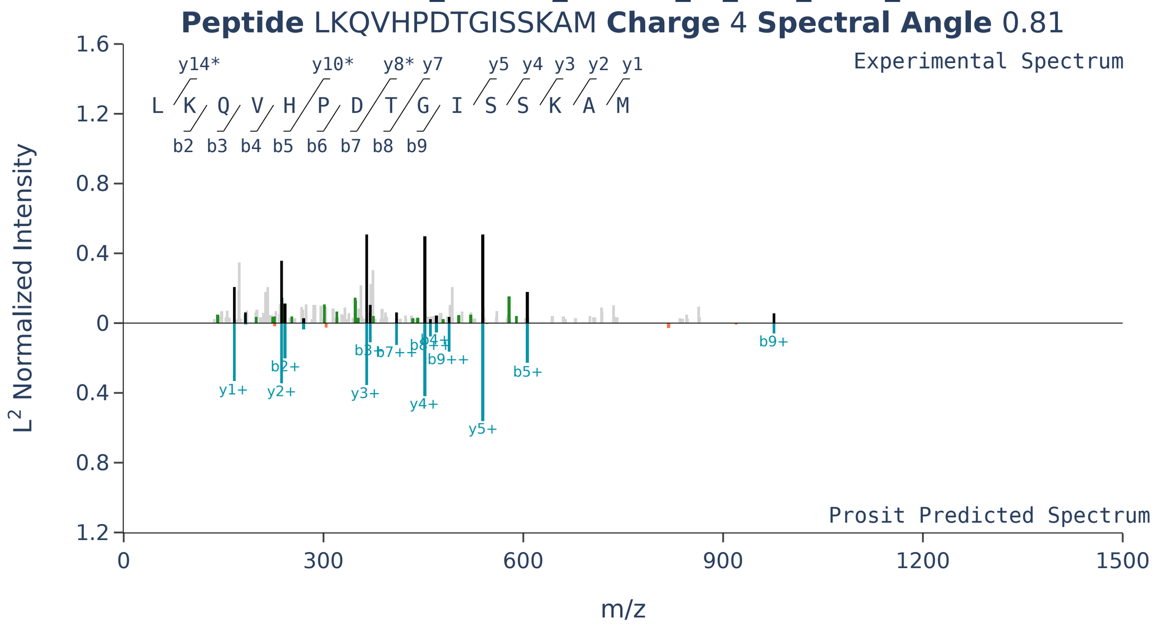


**Source** HRoetschke_291222_080123_Fu_H2B_b1R1_24h **Scan** 5753
**Peptide** NKRSTITSREIQTAVR **Charge** 4 **Spectral Angle** 0.83

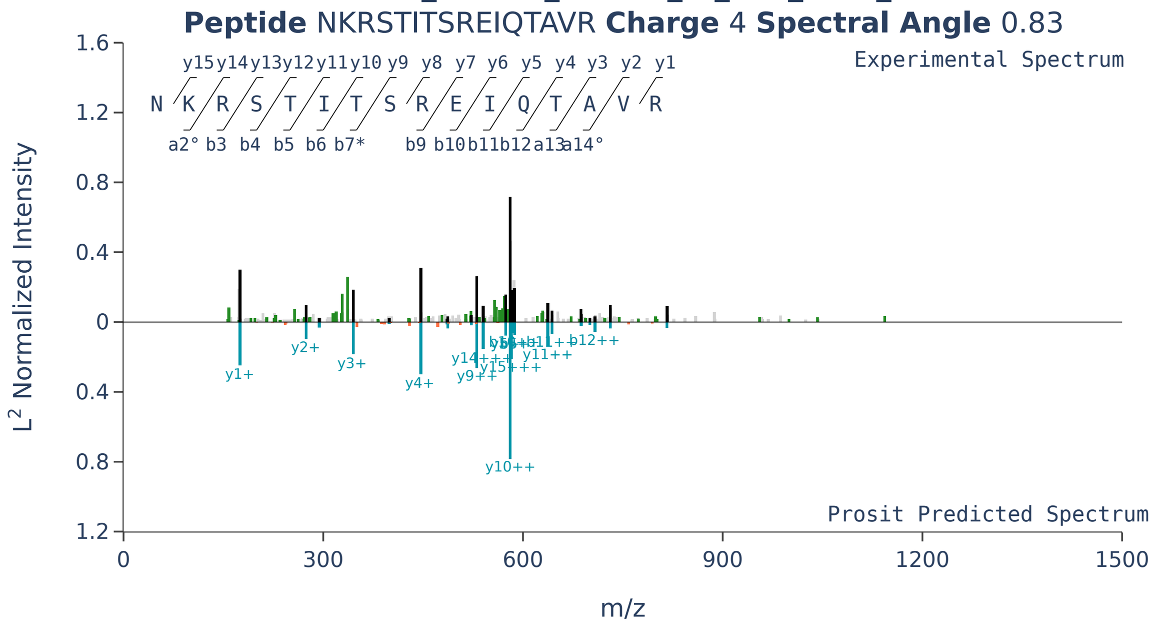


**Source** HRoetschke_291222_080123_Fu_H2B_b1R1_24h **Scan** 6776
**Peptide** AVSEGTKAVTKYTSA **Charge** 2 **Spectral Angle** 0.8

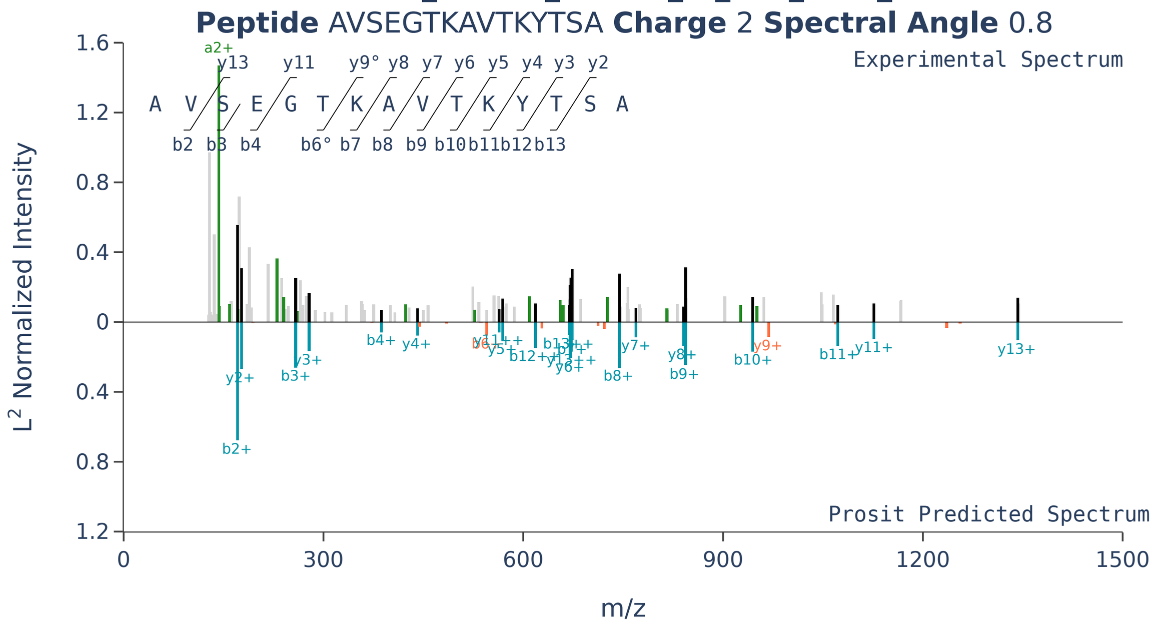


**Source** HRoetschke_291222_080123_Fu_H2B_b2R1_2h **Scan** 6395
**Peptide** RSTITSREIQTAVR **Charge** 4 **Spectral Angle** 0.77

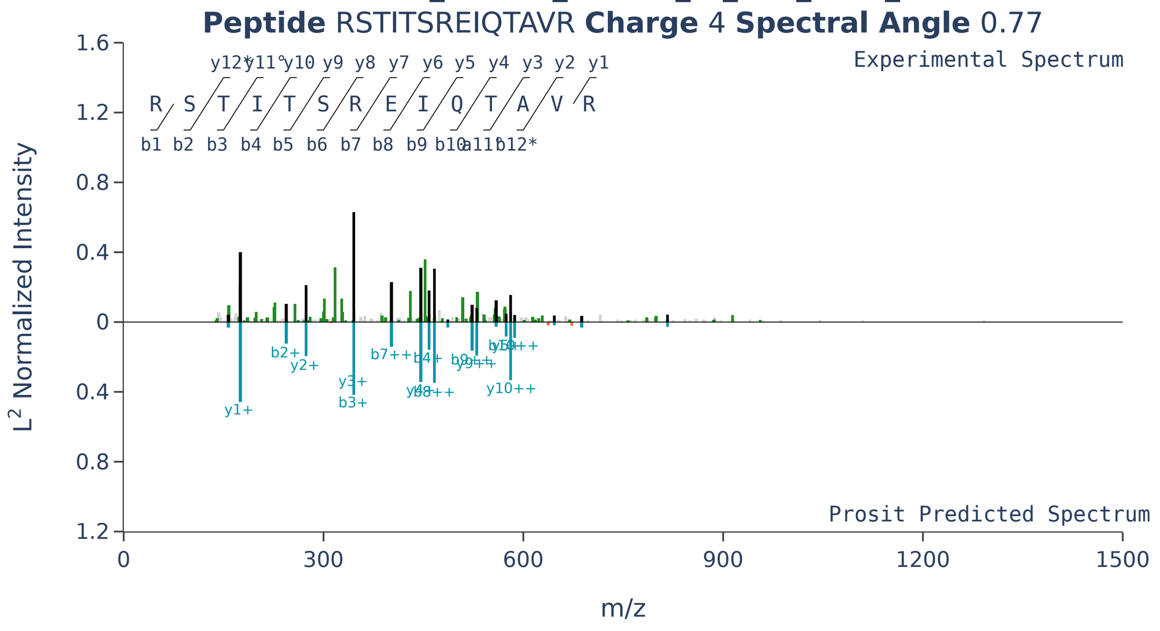


**Source** HRoetschke_291222_080123_Fu_H2B_b2R1_2h **Scan** 5367
**Peptide** KVLKQVHPDTGI **Charge** 3 **Spectral Angle** 0.88

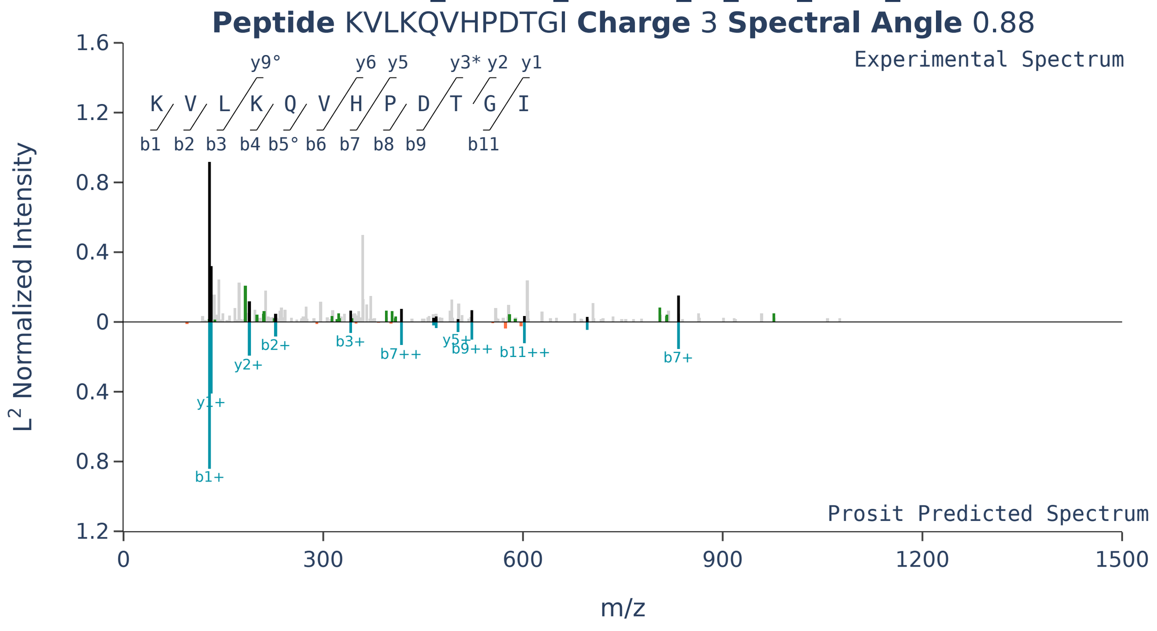


**Source** HRoetschke_291222_080123_Fu_H2B_b2R1_2h **Scan** 14993
**Peptide** MNSFVNDVFER **Charge** 2 **Spectral Angle** 0.88

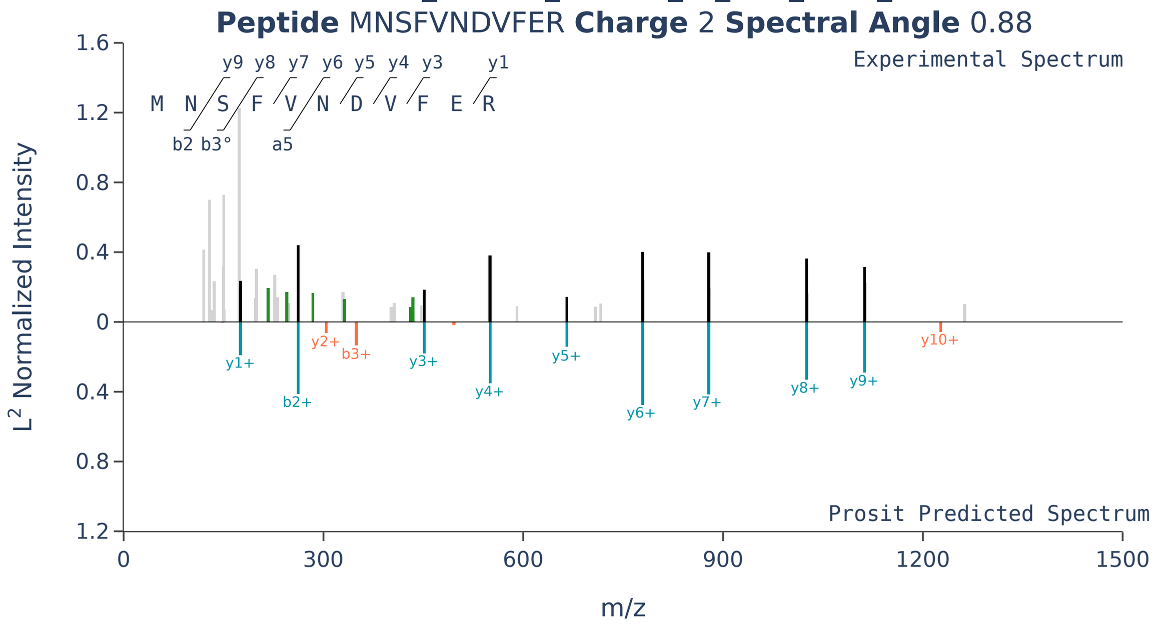


**Source** HRoetschke_291222_080123_Fu_H2B_b1R2_24h **Scan** 5476
**Peptide** HYNKRSTITSREIQTAVR **Charge** 5 **Spectral Angle** 0.84

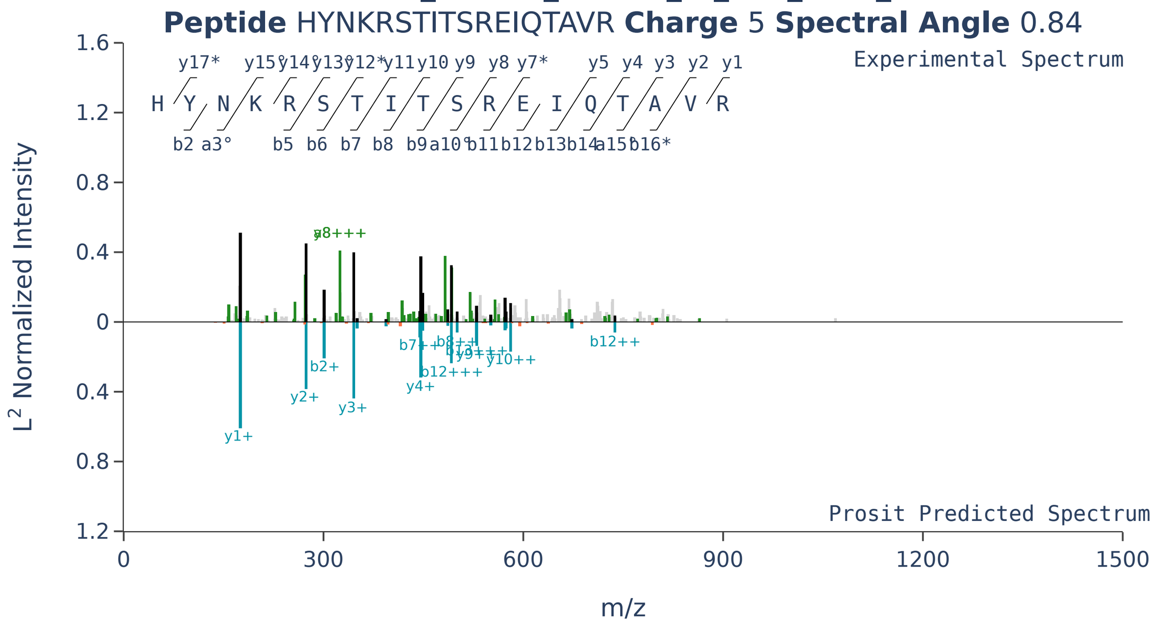

**Source** HRoetschke_291222_080123_Fu_H2B_b1R2_24h **Scan** 12741
**Peptide** MNSFVNDV **Charge** 2 **Spectral Angle** 0.73

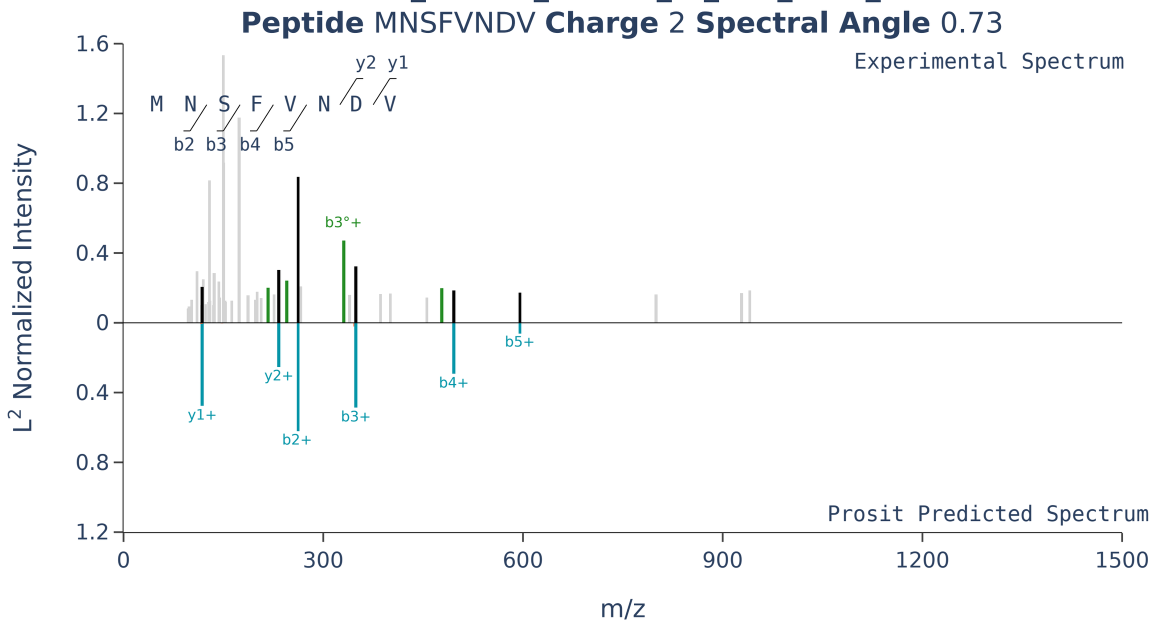


**Source** HRoetschke_291222_080123_Fu_H2B_b2R2_1h **Scan** 17770
**Peptide** MNSFVNDVFERL **Charge** 2 **Spectral Angle** 0.91

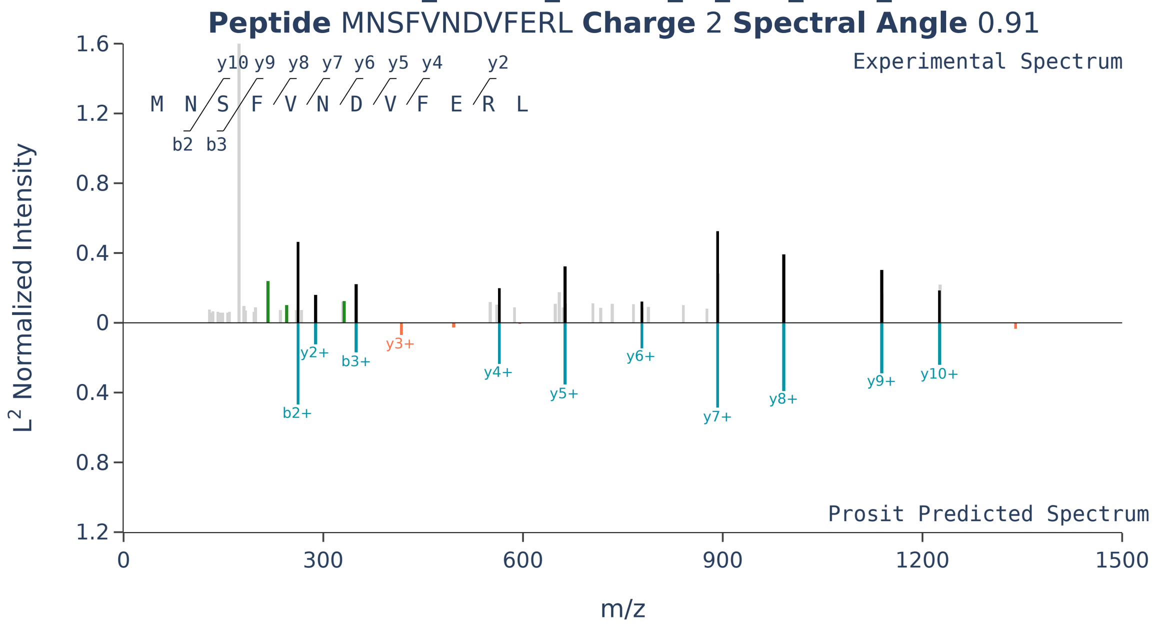


**Source** HRoetschke_291222_080123_Fu_H2B_b2R2_24h **Scan** 8893
**Peptide** TITSREIQTAVR **Charge** 2 **Spectral Angle** 0.88

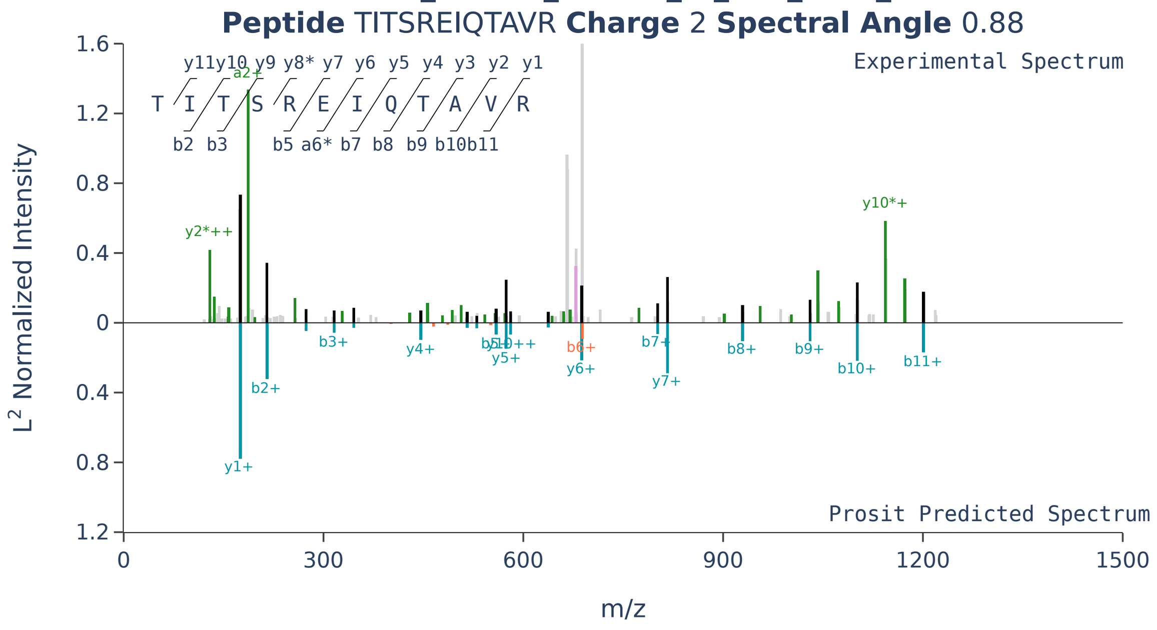


**Source** HRoetschke_291222_080123_Fu_H2B_b1R2_24h **Scan** 11027
**Peptide** MSIMNSF **Charge** 2 **Spectral Angle** 0.93

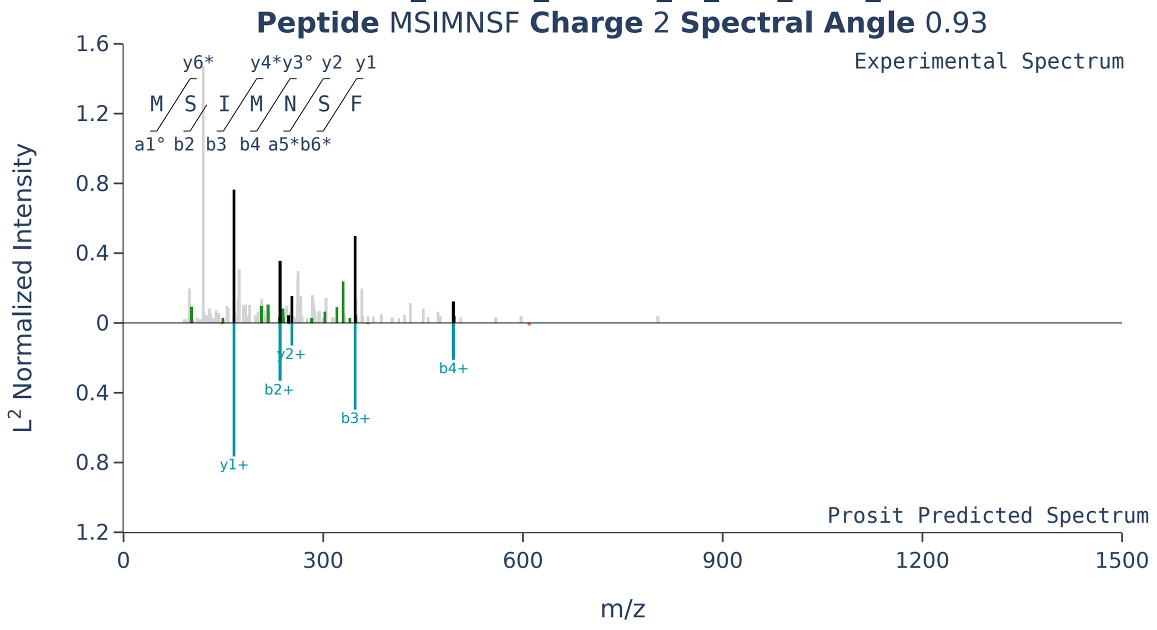


**Source** HRoetschke_291222_080123_Fu_H2B_b1R1_4h **Scan** 6097
**Peptide** YNKRSTITSREIQTAVR **Charge** 4 **Spectral Angle** 0.89

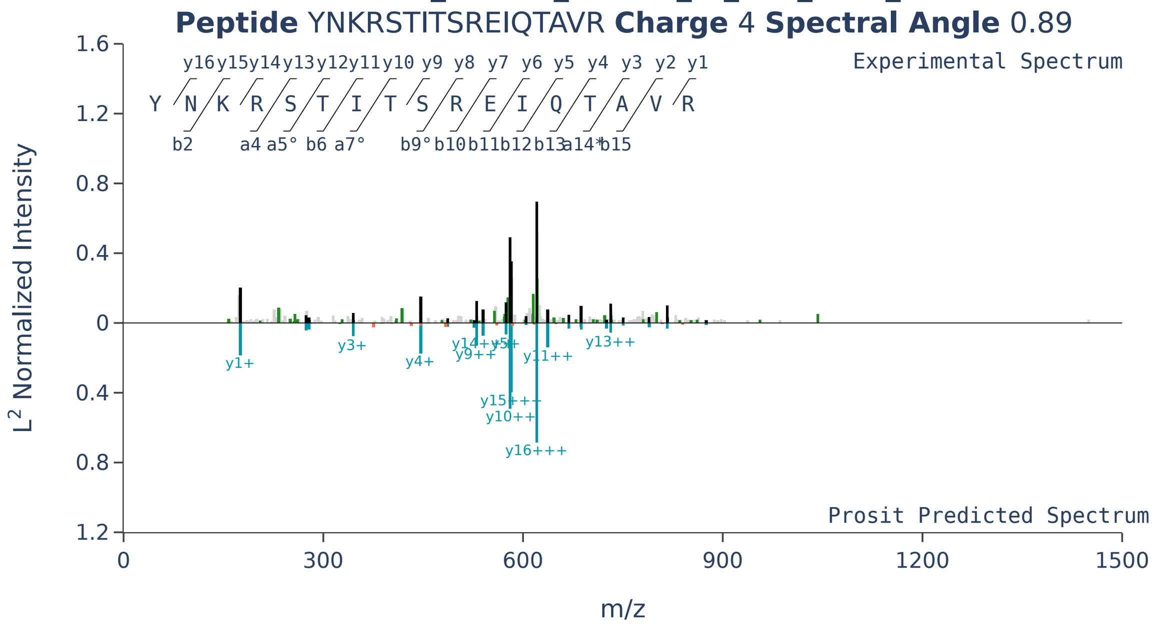


**Source** HRoetschke_291222_080123_Fu_H2B_b2R2_24h **Scan** 3033
**Peptide** SEGTKAVTKY **Charge** 2 **Spectral Angle** 0.87

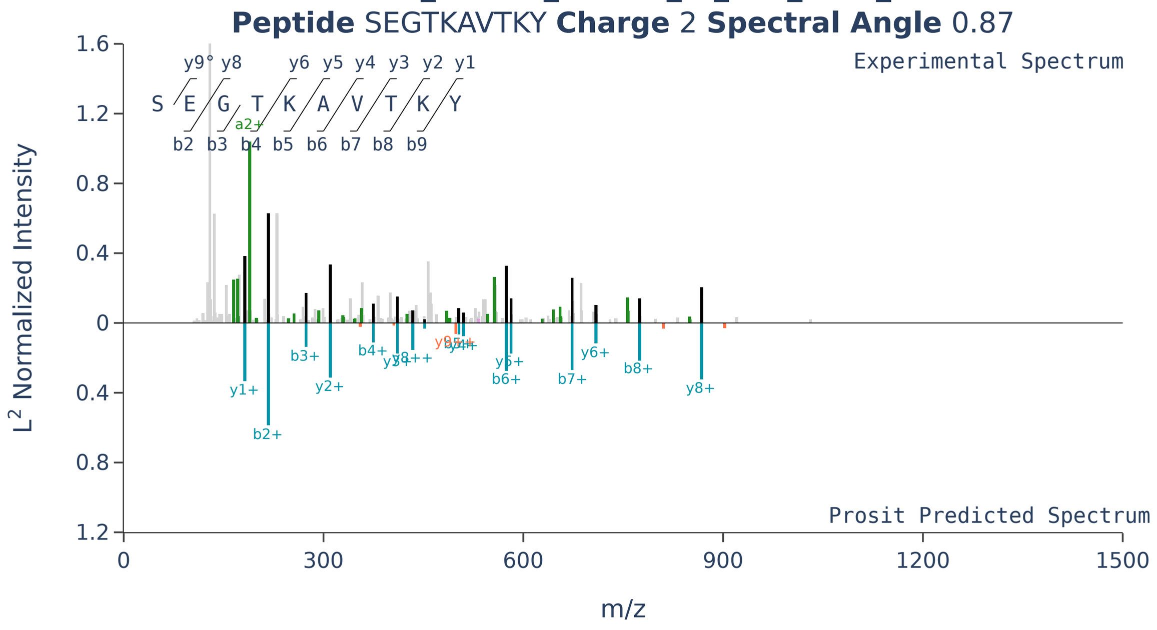


**Source** HRoetschke_291222_080123_Fu_H2B_b1R2_24h **Scan** 13439
**Peptide** NDVFERIAGE **Charge** 2 **Spectral Angle** 0.86

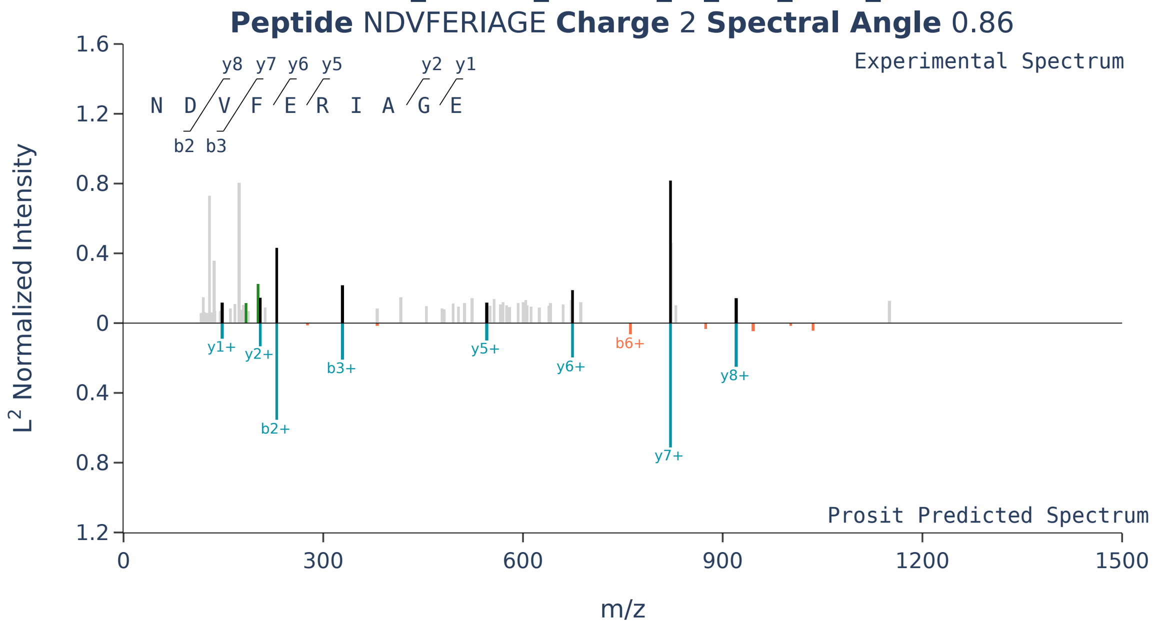


**Source** HRoetschke_291222_080123_Fu_H2B_b1R2_24h **Scan** 4399
**Peptide** HAVSEGTKAVTKYTSAK **Charge** 4 **Spectral Angle** 0.81

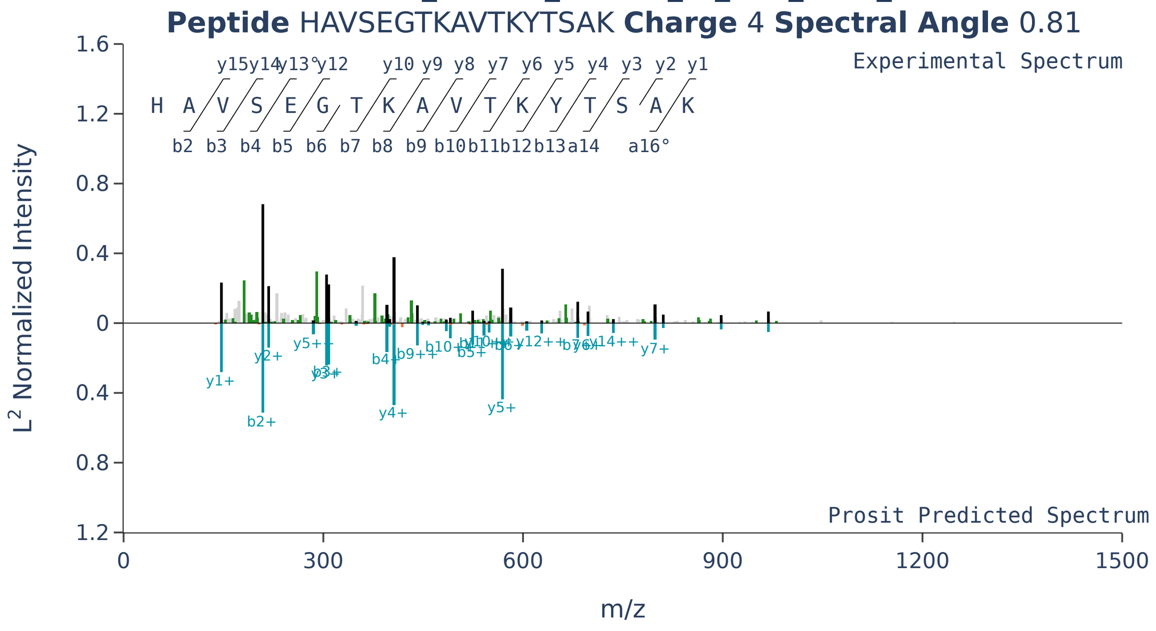


**Source** HRoetschke_291222_080123_Fu_H2B_b2R2_24h **Scan** 13683
**Peptide** RKESYAIYVY **Charge** 2 **Spectral Angle** 0.87

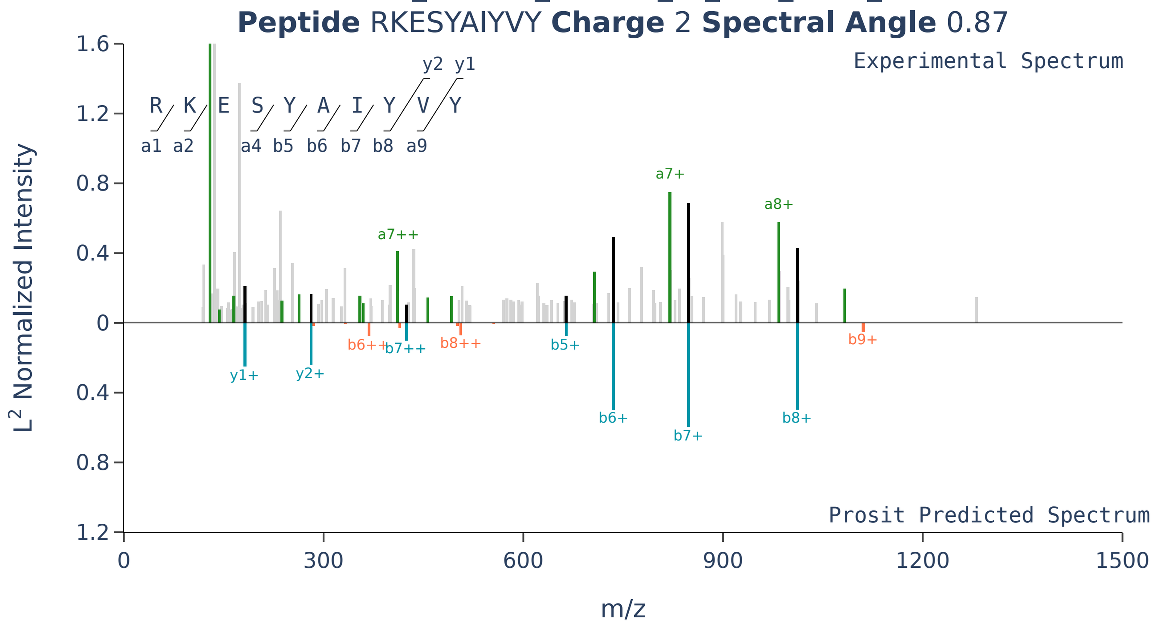


**Source** HRoetschke_291222_080123_Fu_H2B_b2R2_24h **Scan** 13076
**Peptide** HPDTGISSKAMSIMNSF **Charge** 3 **Spectral Angle** 0.88

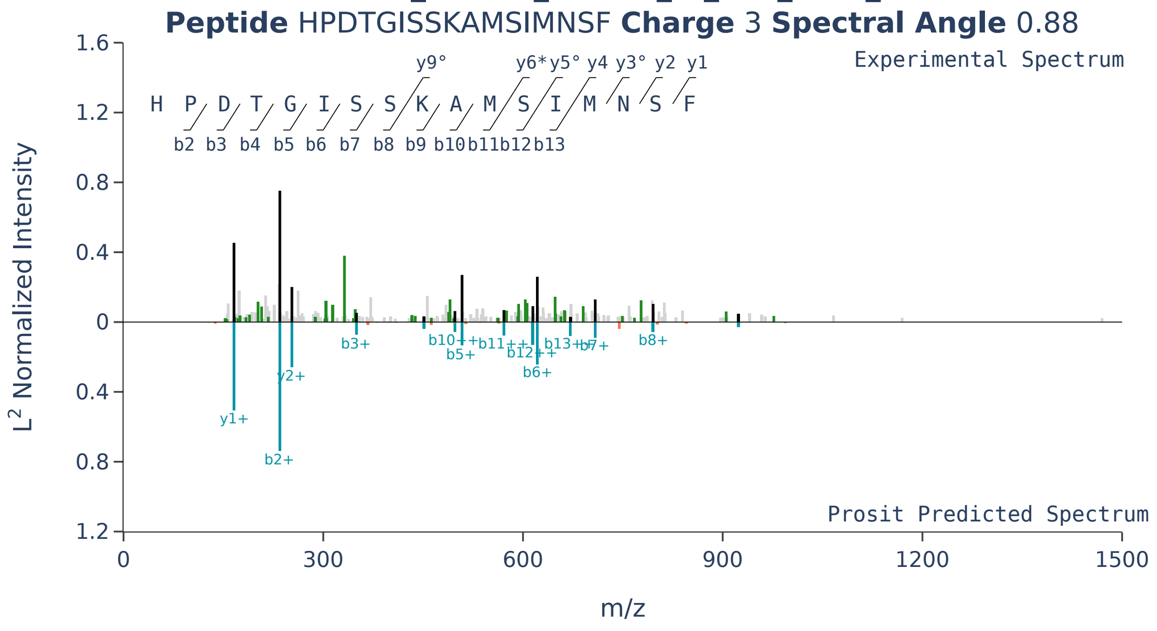


**Source** HRoetschke_291222_080123_Fu_H2B_b1R2_24h **Scan** 4686
**Peptide** SEGTKAVTKYTSA **Charge** 2 **Spectral Angle** 0.84

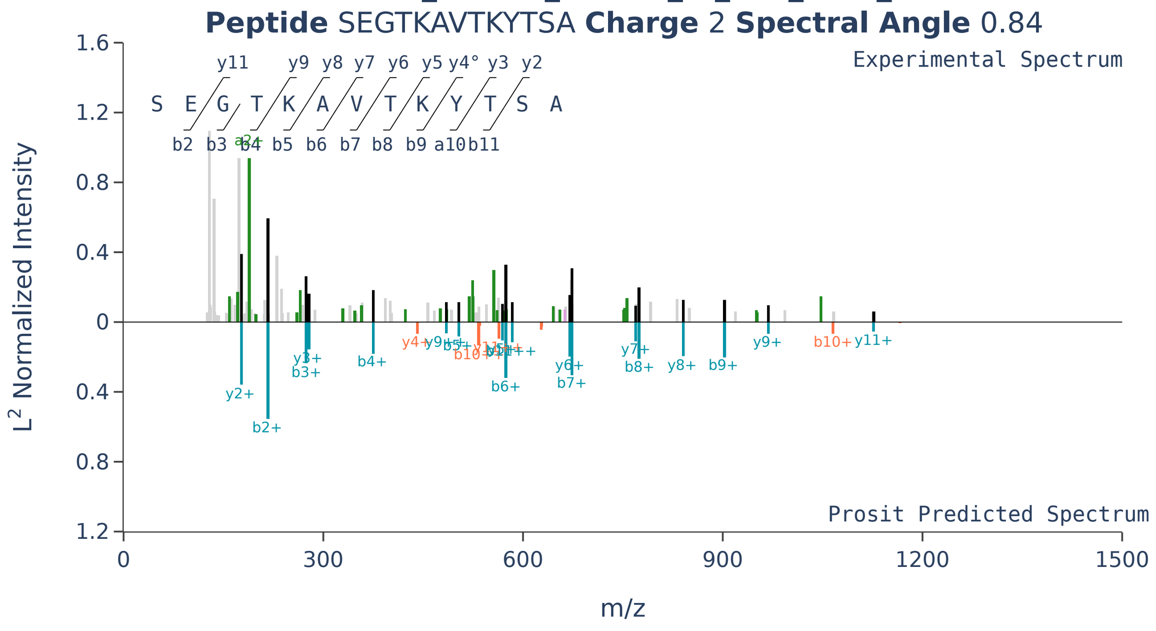


**Source** HRoetschke_291222_080123_Fu_H2B_b1R1_4h **Scan** 3952
**Peptide** EASRLAHY **Charge** 2 **Spectral Angle** 0.84

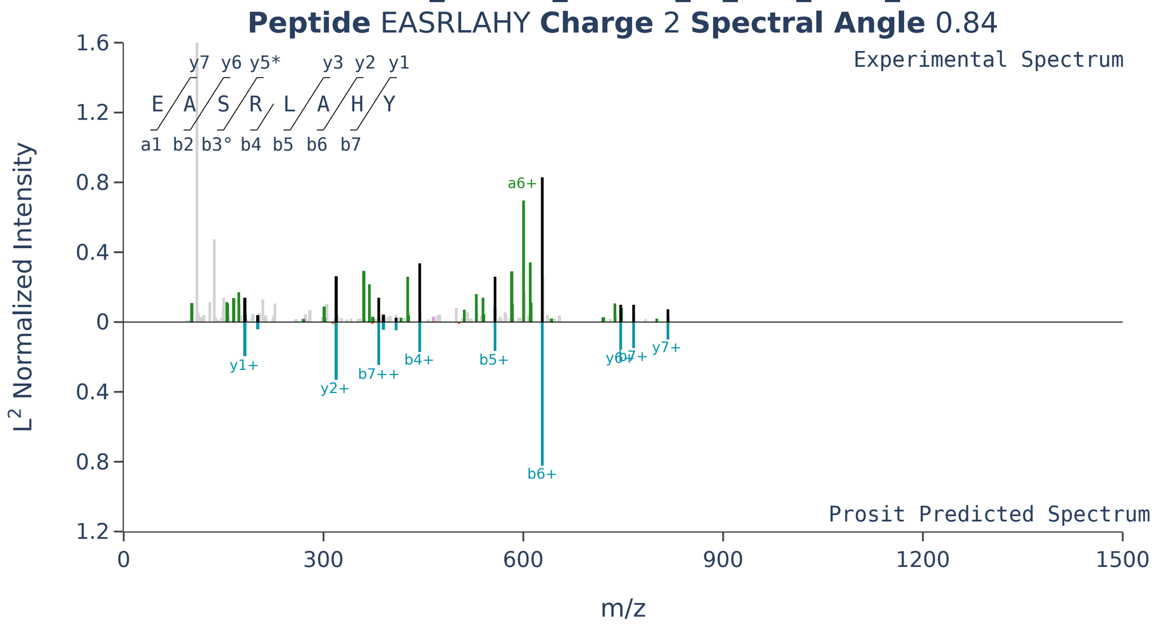


**Source** HRoetschke_291222_080123_Fu_H2B_b2R1_4h **Scan** 12313
**Peptide** VFERIAGEASRLAHYNKR **Charge** 4 **Spectral Angle** 0.89

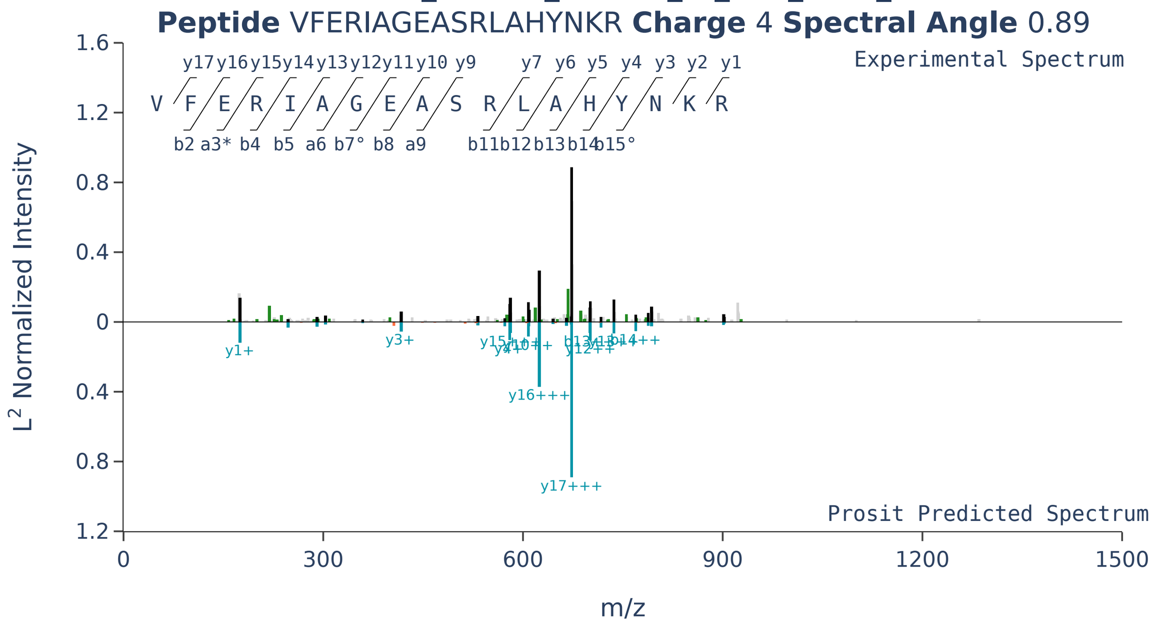


**Source** HRoetschke_291222_080123_Fu_H2B_b2R1_4h **Scan** 17154
**Peptide** AIYVYKVL **Charge** 2 **Spectral Angle** 0.83

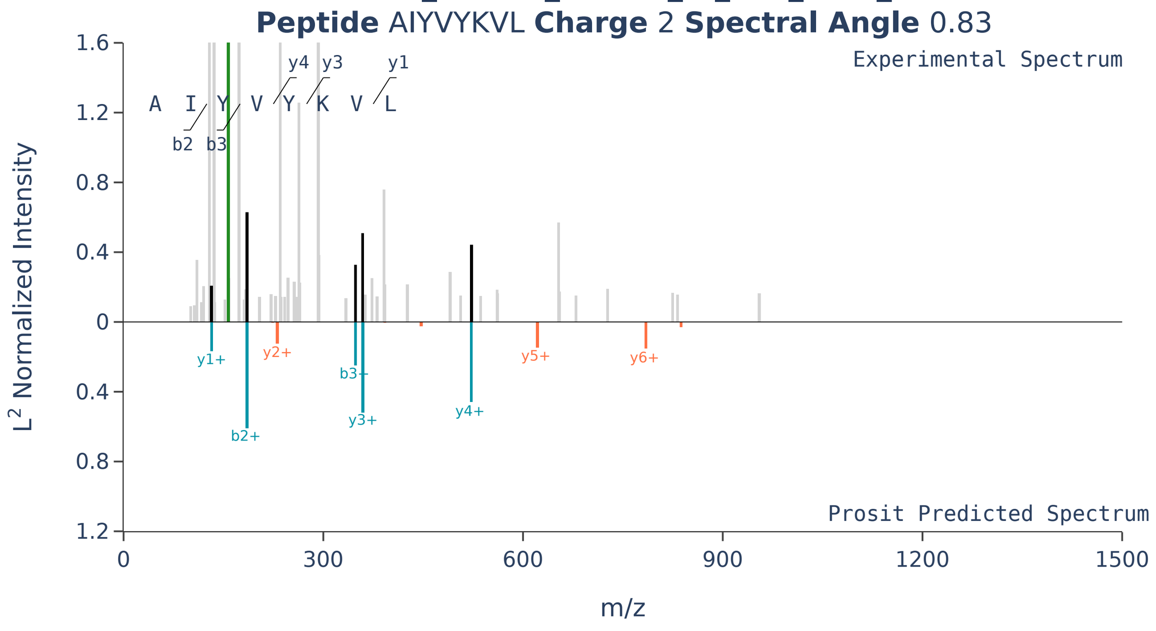


**Source** HRoetschke_291222_080123_Fu_H2B_b1R1_2h **Scan** 12682
**Peptide** VFERIAGEASRLA **Charge** 3 **Spectral Angle** 0.86

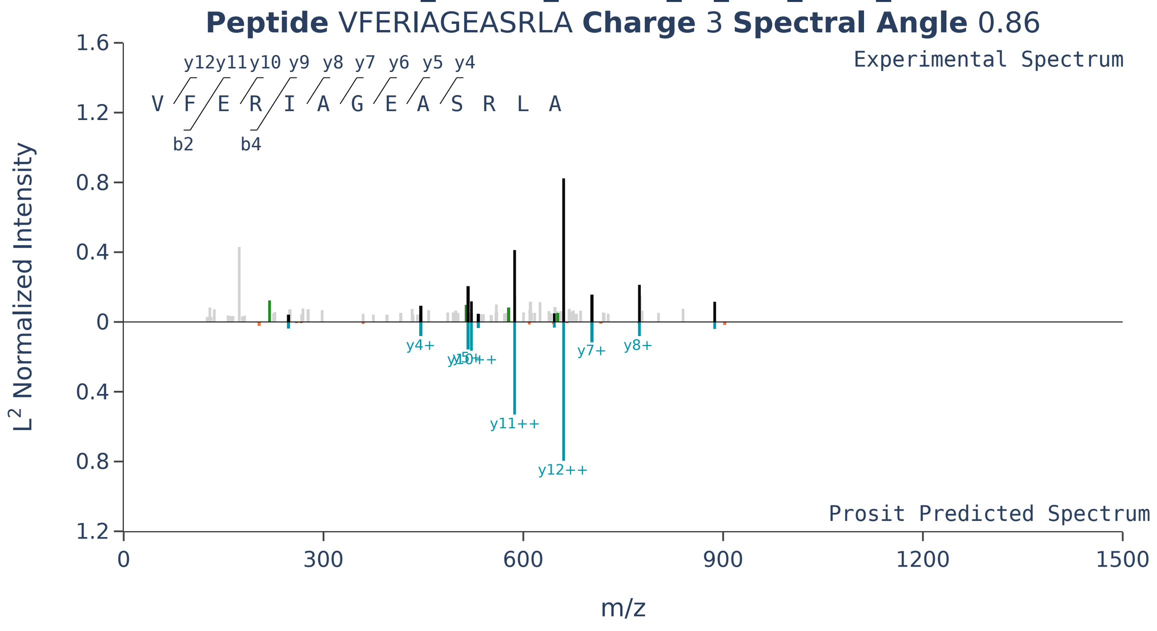

**Source** HRoetschke_291222_080123_Fu_H2B_b2R2_4h **Scan** 2645
**Peptide** EGTKAVTKY **Charge** 2 **Spectral Angle** 0.88

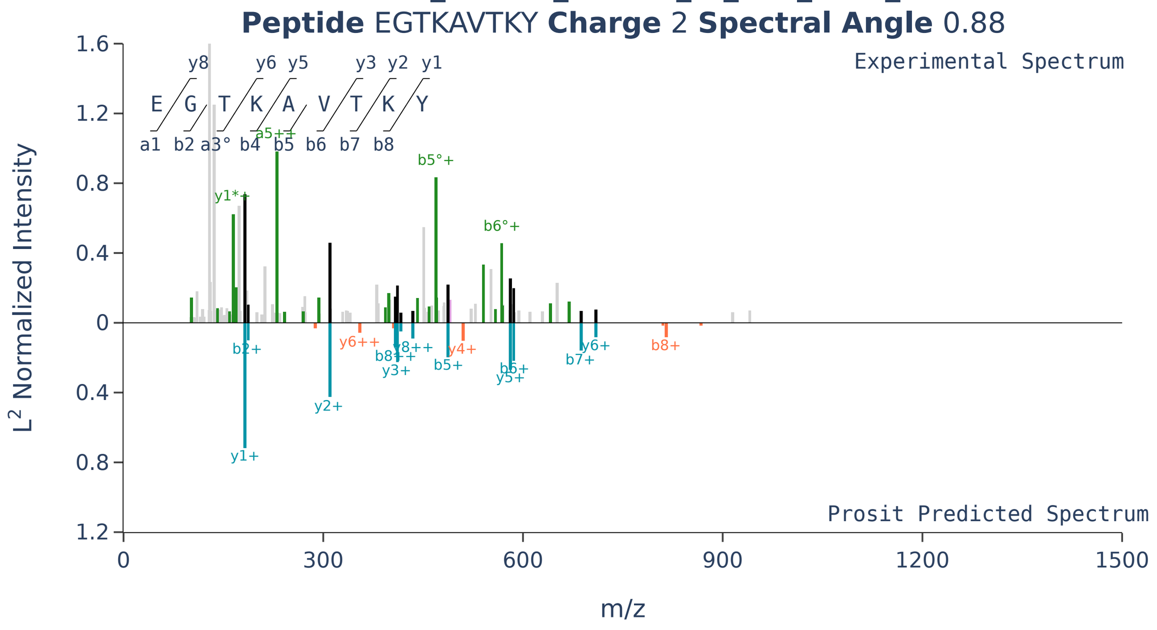


**Source** HRoetschke_291222_080123_Fu_H2B_b2R2_4h **Scan** 14488
**Peptide** TITSREIQTAVRL **Charge** 3 **Spectral Angle** 0.91

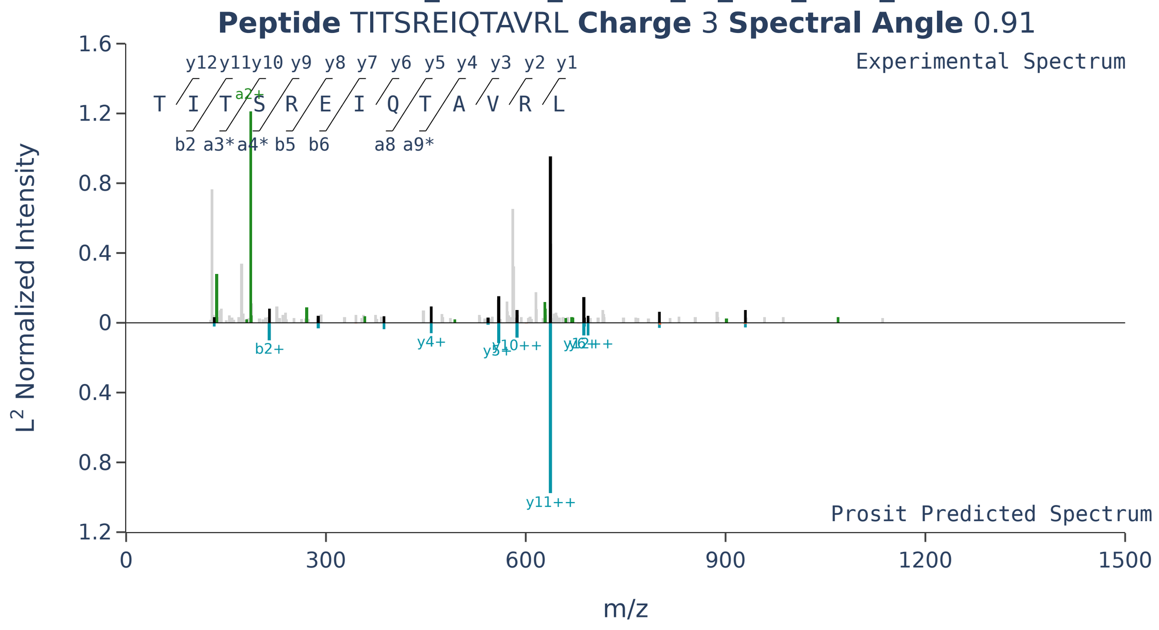


**Source** HRoetschke_291222_080123_Fu_H2B_b2R2_24h **Scan** 2846
**Peptide** EASRLAHYNKRSTITSR **Charge** 4 **Spectral Angle** 0.86

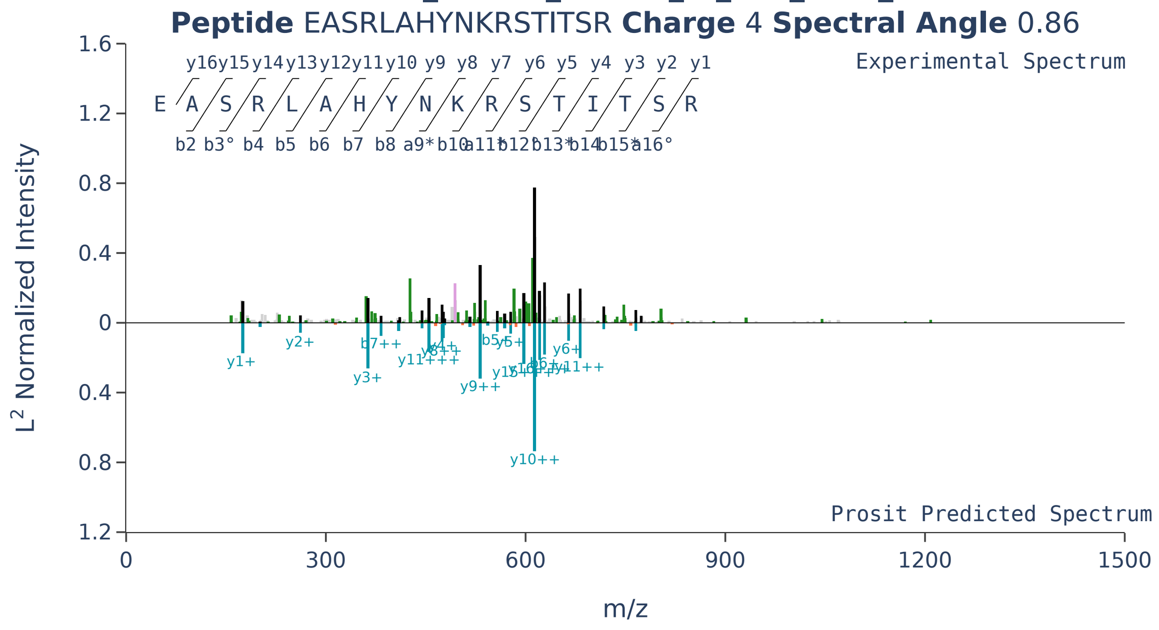


**Source** HRoetschke_291222_080123_Fu_H2B_b1R1_4h **Scan** 17911
**Peptide** AVRLLLPGEL **Charge** 2 **Spectral Angle** 0.87

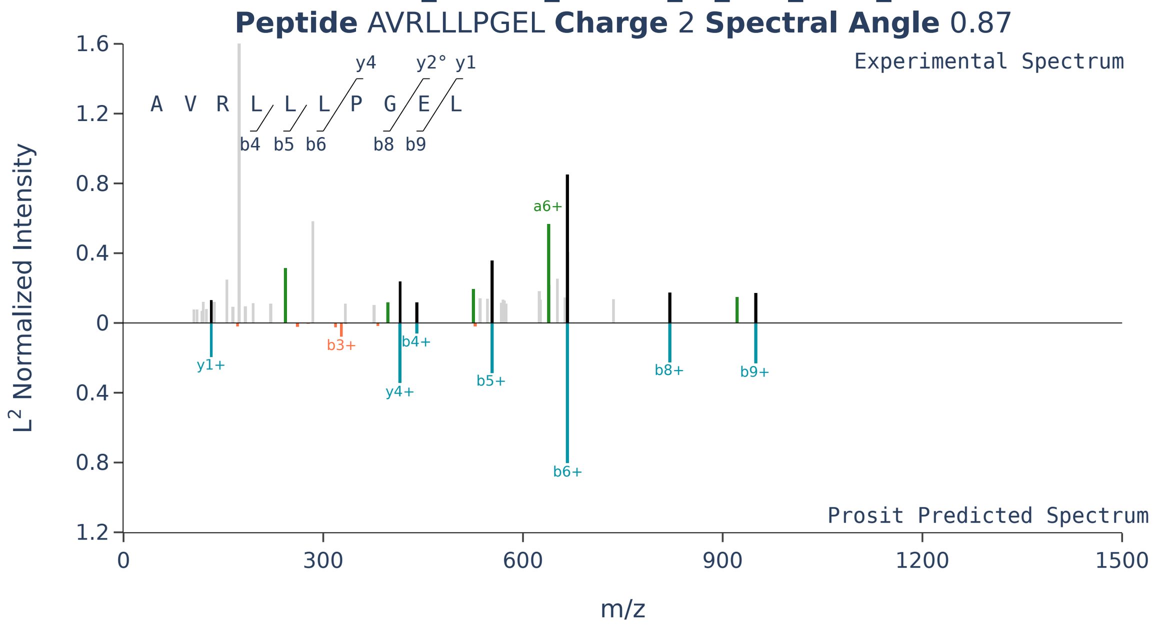


**Source** HRoetschke_291222_080123_Fu_H2B_b1R1_24h **Scan** 13725
**Peptide** SREIQTAVRLL **Charge** 3 **Spectral Angle** 0.84

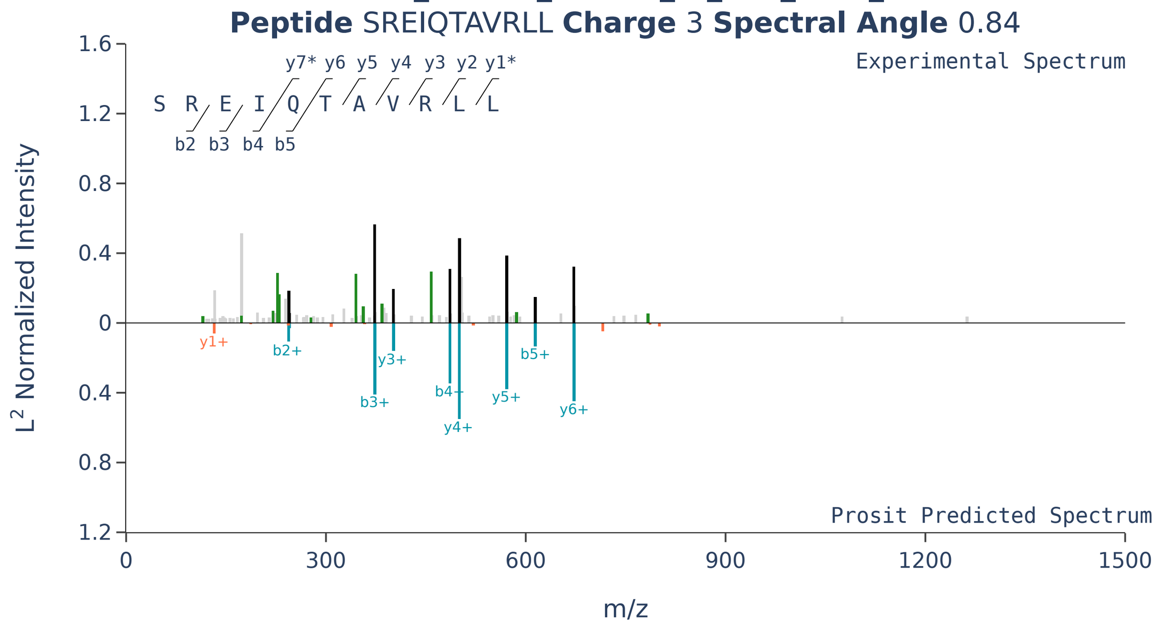


**Source** HRoetschke_291222_080123_Fu_H2B_b2R2_1h **Scan** 17677
**Peptide** SFVNDVFERL **Charge** 2 **Spectral Angle** 0.89

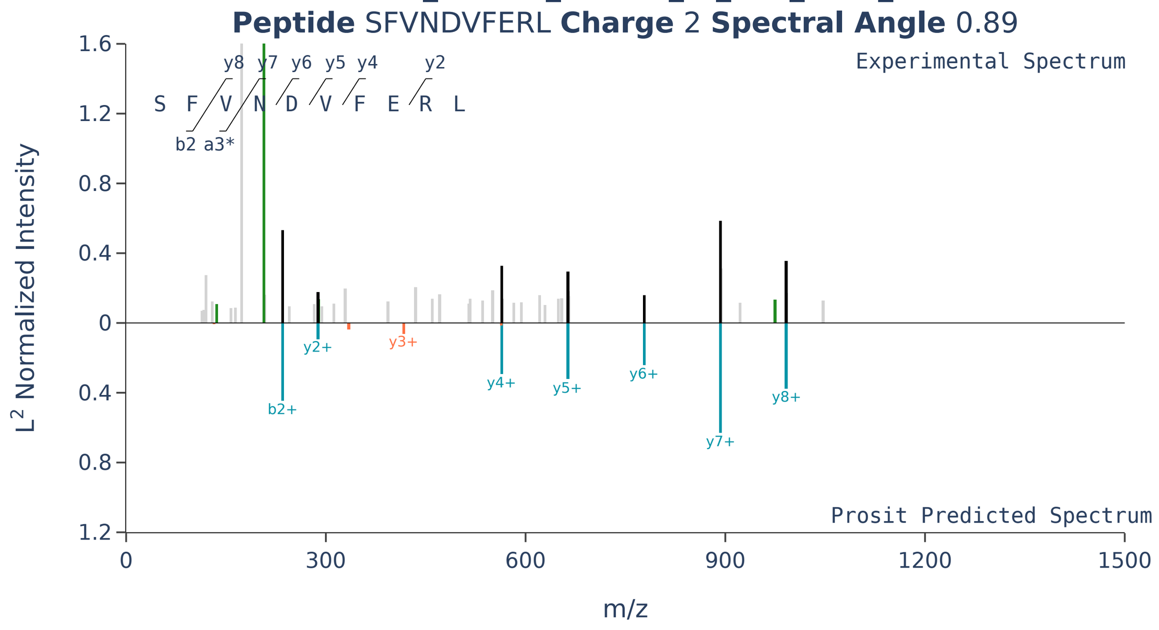


**Source** HRoetschke_291222_080123_Fu_H2B_b2R1_24h **Scan** 19326
**Peptide** SREIQTAVRLLL **Charge** 3 **Spectral Angle** 0.83

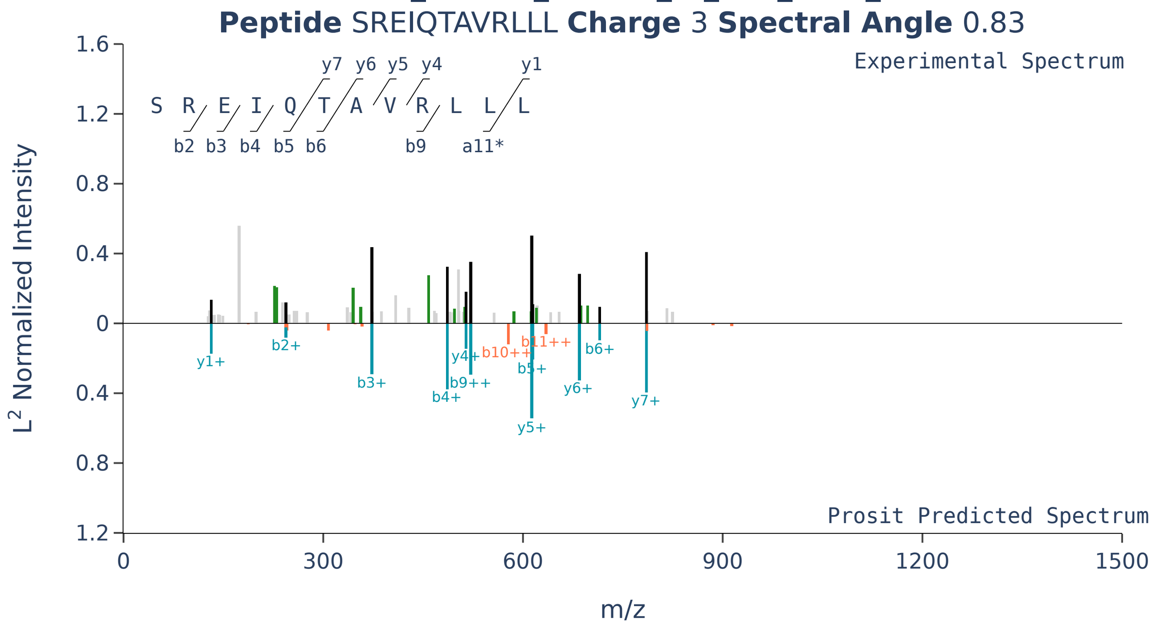


**Source** HRoetschke_291222_080123_Fu_H2B_b2R2_24h **Scan** 2567
**Peptide** KVLKQVHPDT **Charge** 2 **Spectral Angle** 0.88

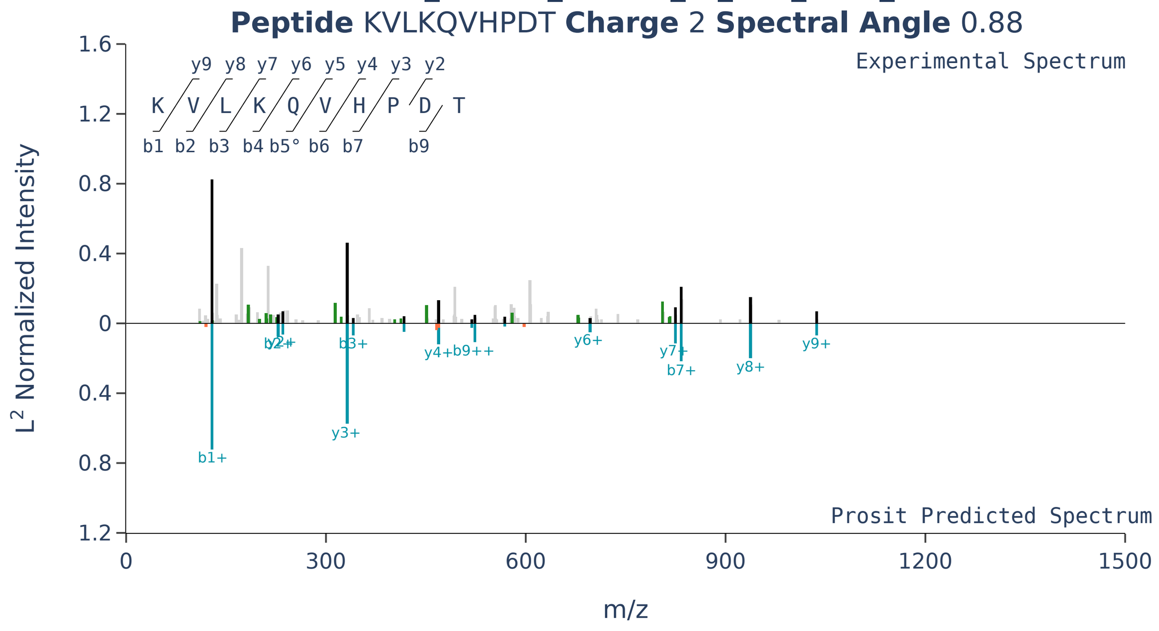


**Source** HRoetschke_291222_080123_Fu_H2B_b2R2_24h **Scan** 13526
**Peptide** RSTITSREIQTAVRL **Charge** 4 **Spectral Angle** 0.8

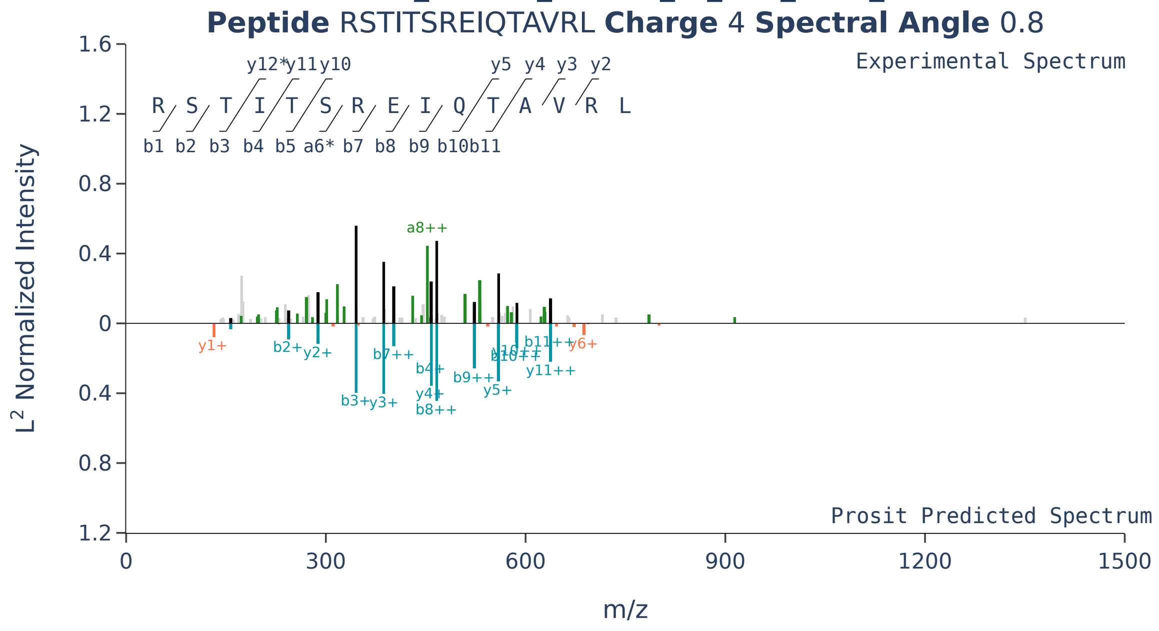


**Source** HRoetschke_291222_080123_Fu_H2B_b1R2_2h **Scan** 7912
**Peptide** VFERIAGE **Charge** 2 **Spectral Angle** 0.91

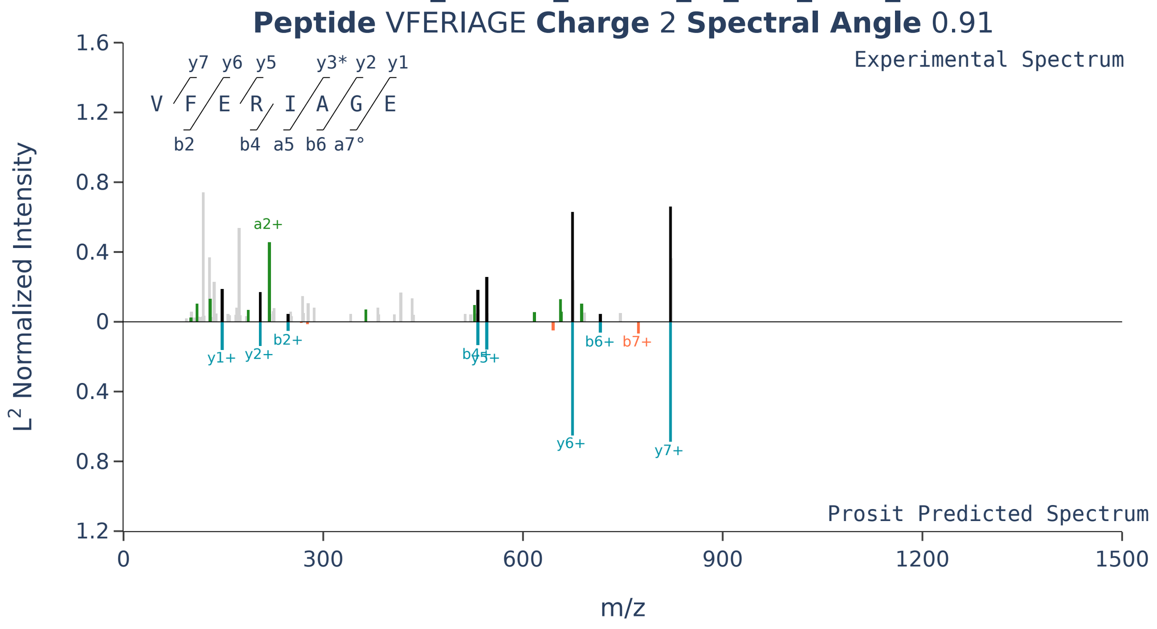


**Source** HRoetschke_291222_080123_Fu_H2B_b1R1_24h **Scan** 12439
**Peptide** VHPDTGISSKAMSIMNSF **Charge** 3 **Spectral Angle** 0.94

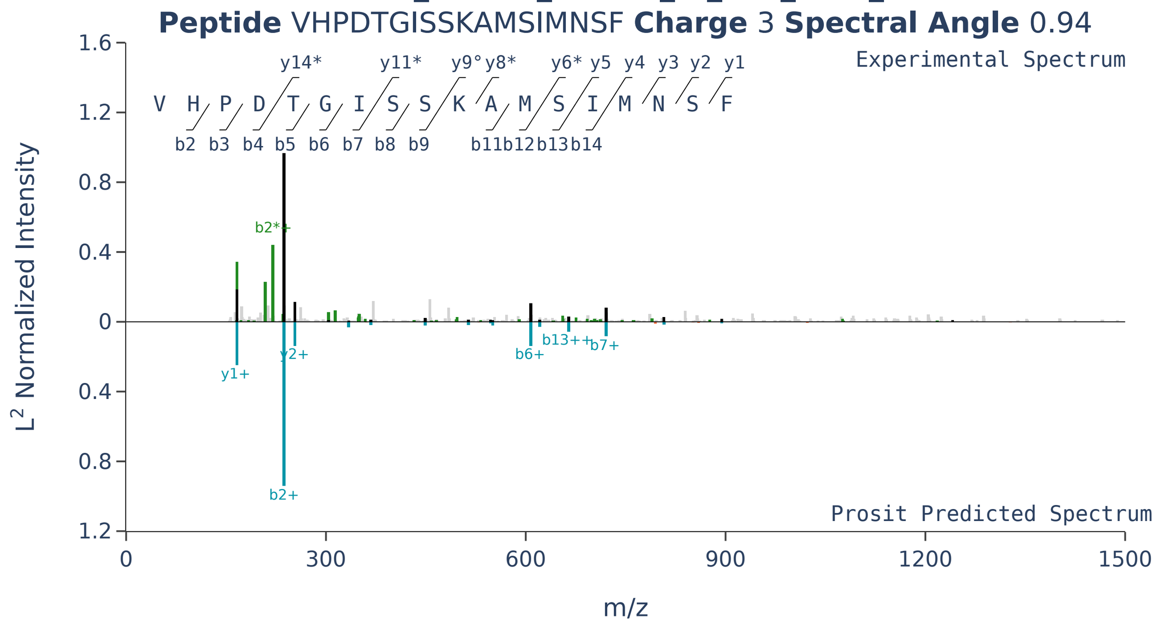


**Source** HRoetschke_291222_080123_Fu_H2B_b1R1_24h **Scan** 14690
**Peptide** LLLPGELAK **Charge** 2 **Spectral Angle** 0.86

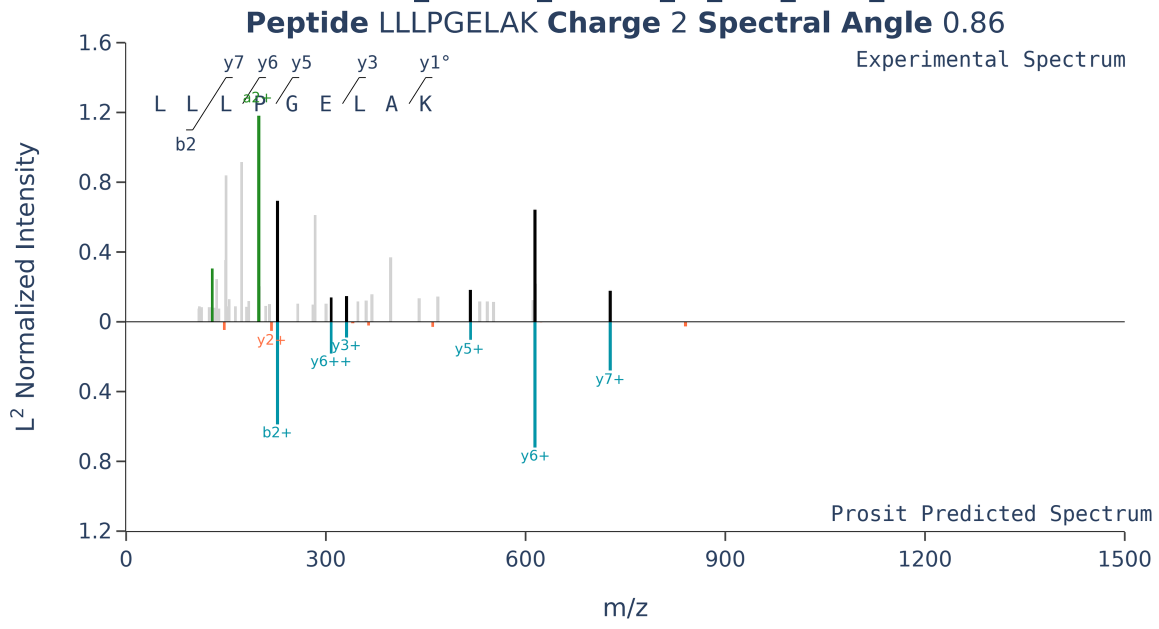


**Source** HRoetschke_291222_080123_Fu_H2B_b1R1_4h **Scan** 11984
**Peptide** PDTGISSKAMSL **Charge** 2 **Spectral Angle** 0.9

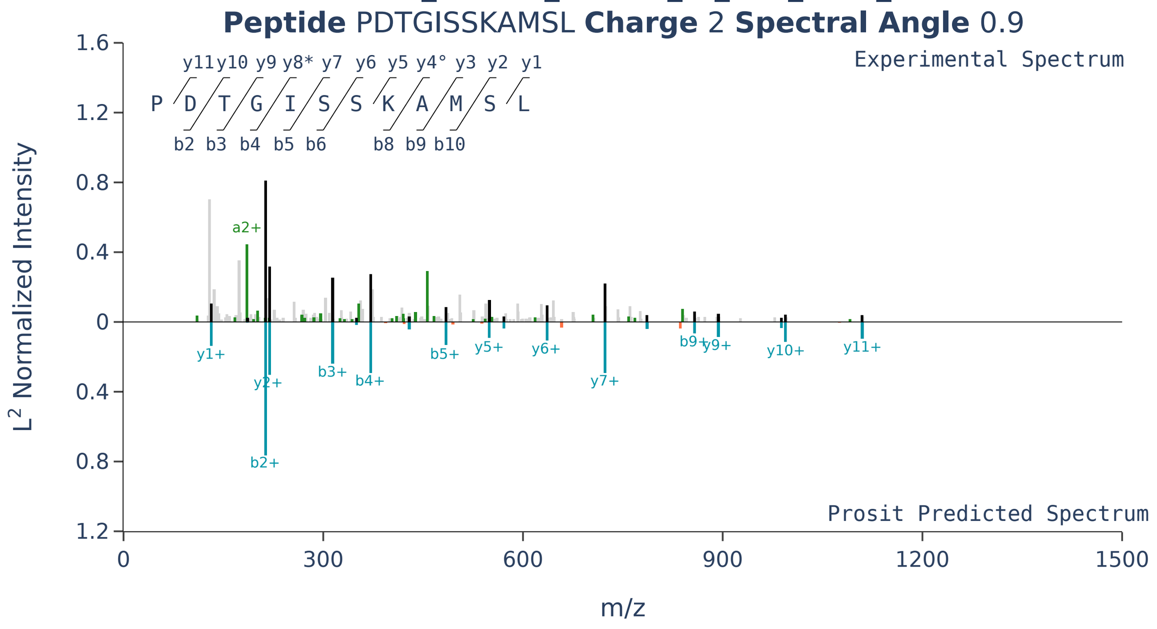


**Source** HRoetschke_291222_080123_Fu_H2B_b2R2_4h **Scan** 15118
**Peptide** LLLPGELAKHAVSEGTKA **Charge** 3 **Spectral Angle** 0.82

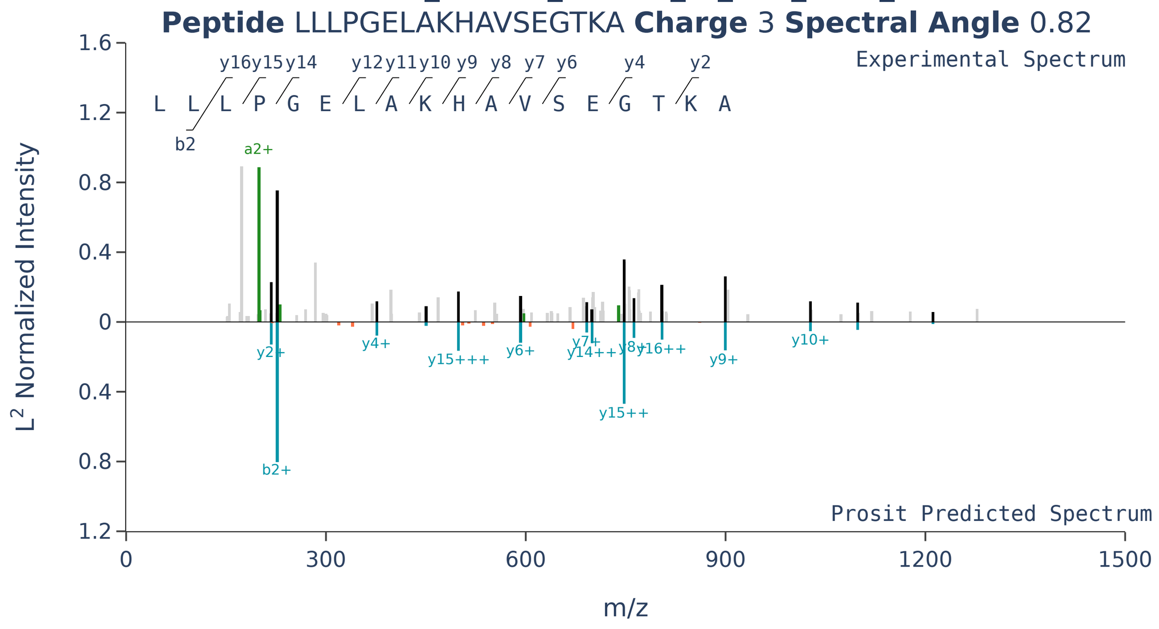


**Source** HRoetschke_291222_080123_Fu_H2B_b2R2_1h **Scan** 4064
**Peptide** VYKVLKQ **Charge** 2 **Spectral Angle** 0.77

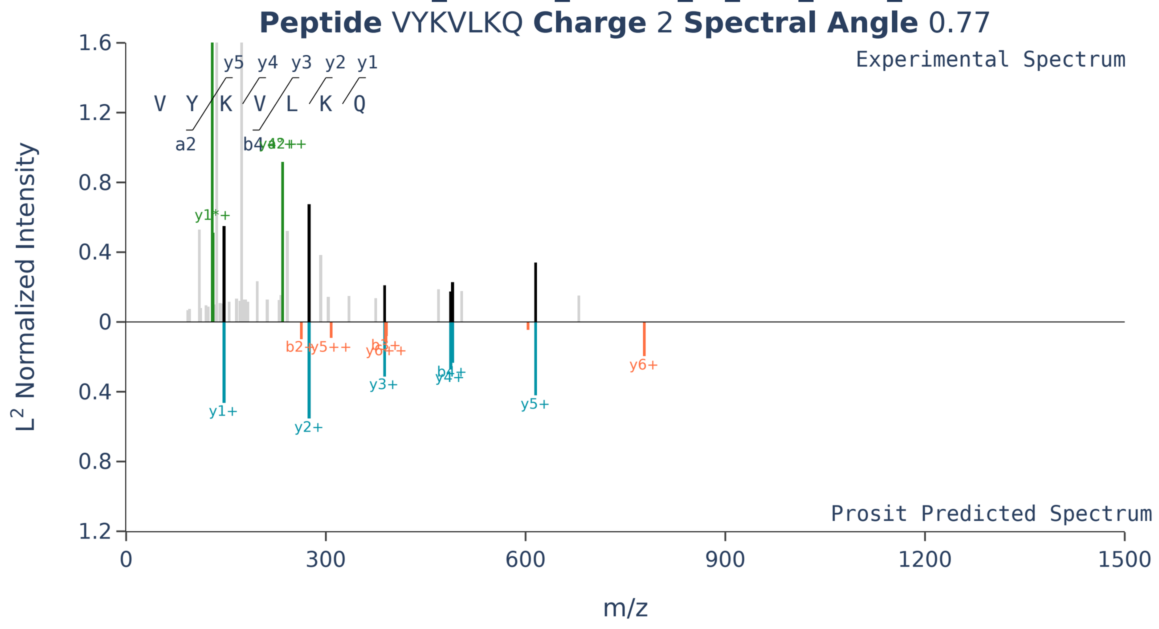

**Source** HRoetschke_291222_080123_Fu_H2B_b2R2_4h **Scan** 10792
**Peptide** TSREIQTAVRL **Charge** 3 **Spectral Angle** 0.83

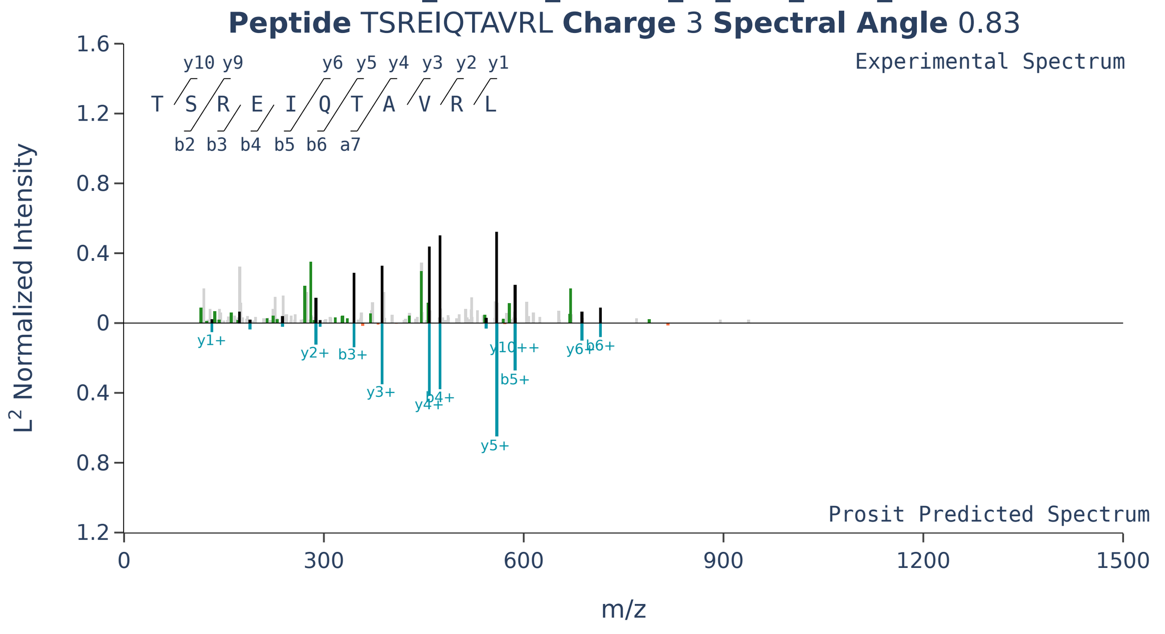


**Source** HRoetschke_291222_080123_Fu_H2B_b1R1_24h **Scan** 12839
**Peptide** AIYVYKVLKQ **Charge** 3 **Spectral Angle** 0.79

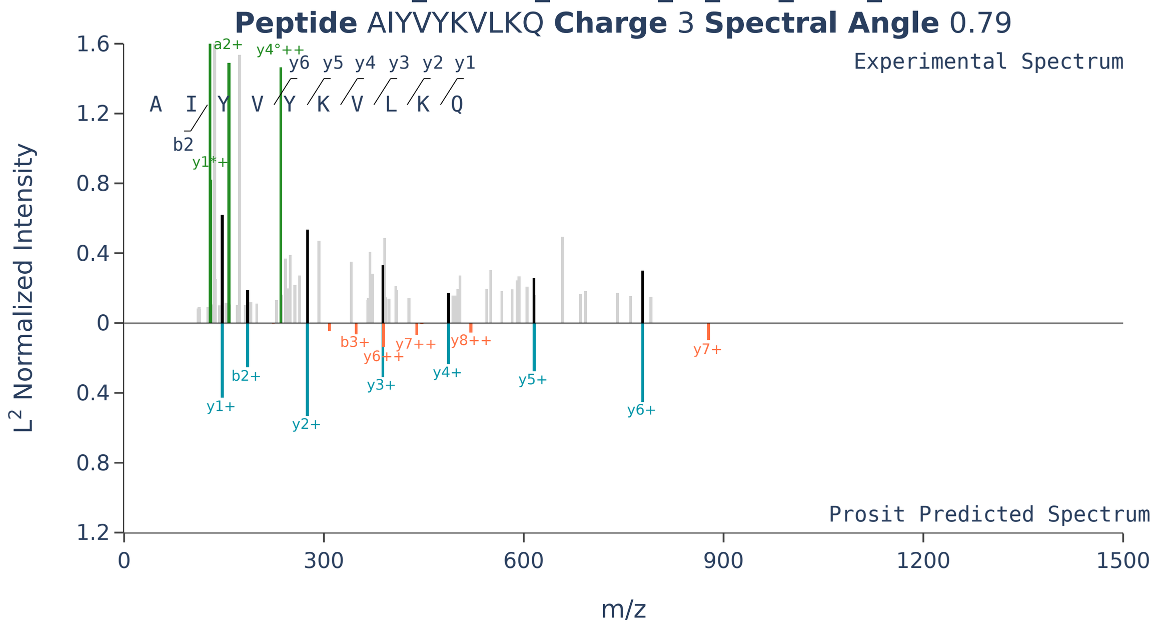


**Source** HRoetschke_291222_080123_Fu_H2B_b1R2_24h **Scan** 13963
**Peptide** VNDVFERLA **Charge** 2 **Spectral Angle** 0.89

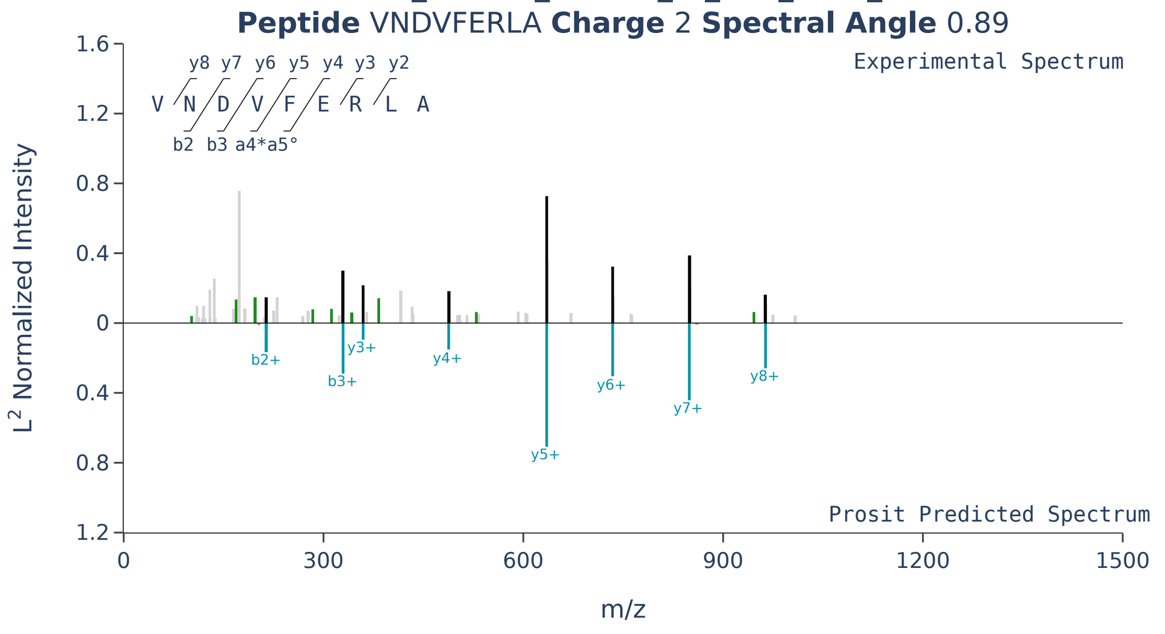


**Source** HRoetschke_291222_080123_Fu_H2B_b2R1_1h **Scan** 3180
**Peptide** AHYNKRSTITSREL **Charge** 3 **Spectral Angle** 0.92

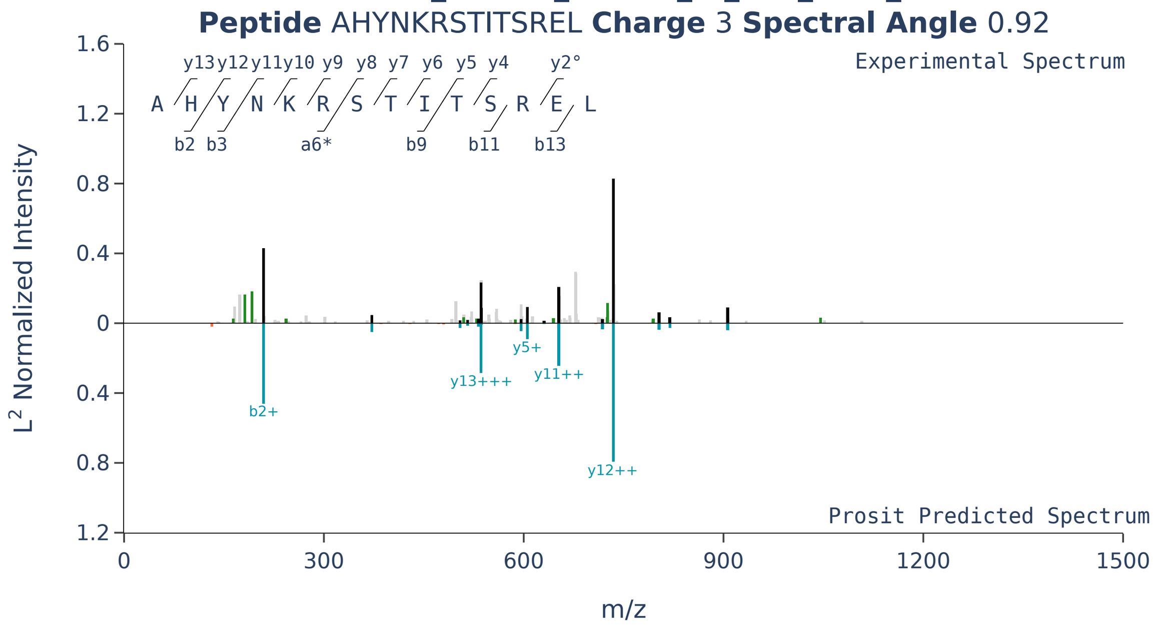


**Source** HRoetschke_291222_080123_Fu_H2B_b2R2_24h **Scan** 5999
**Peptide** AVSEGTKAVTKYT **Charge** 2 **Spectral Angle** 0.71

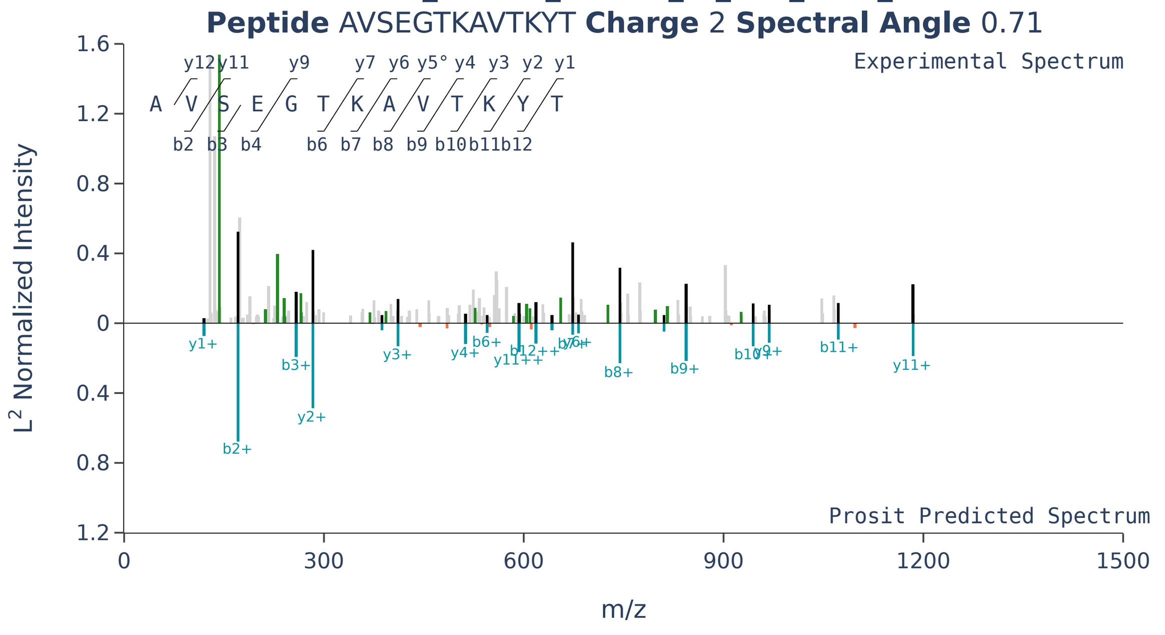


**Source** HRoetschke_291222_080123_Fu_H2B_b1R2_2h **Scan** 14962
**Peptide** SYAIYVYKVLKQ **Charge** 2 **Spectral Angle** 0.91

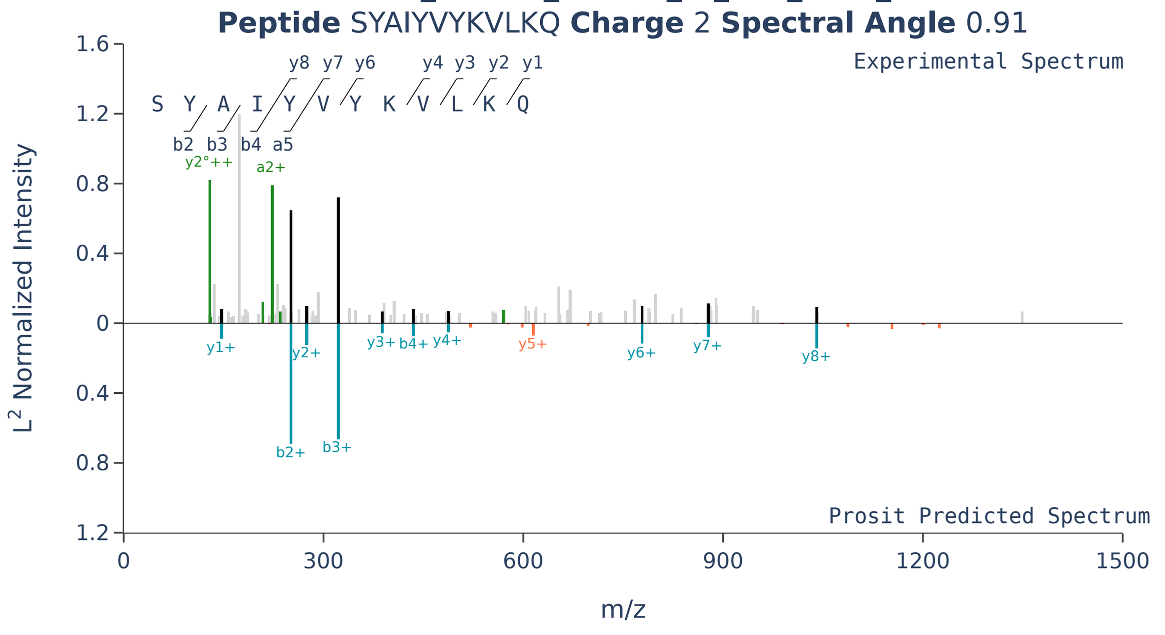


**Source** HRoetschke_291222_080123_Fu_H2B_b1R2_2h **Scan** 15509
**Peptide** LLLPGELAKHAVSEGTKAVTKYTS **Charge** 4 **Spectral Angle** 0.92

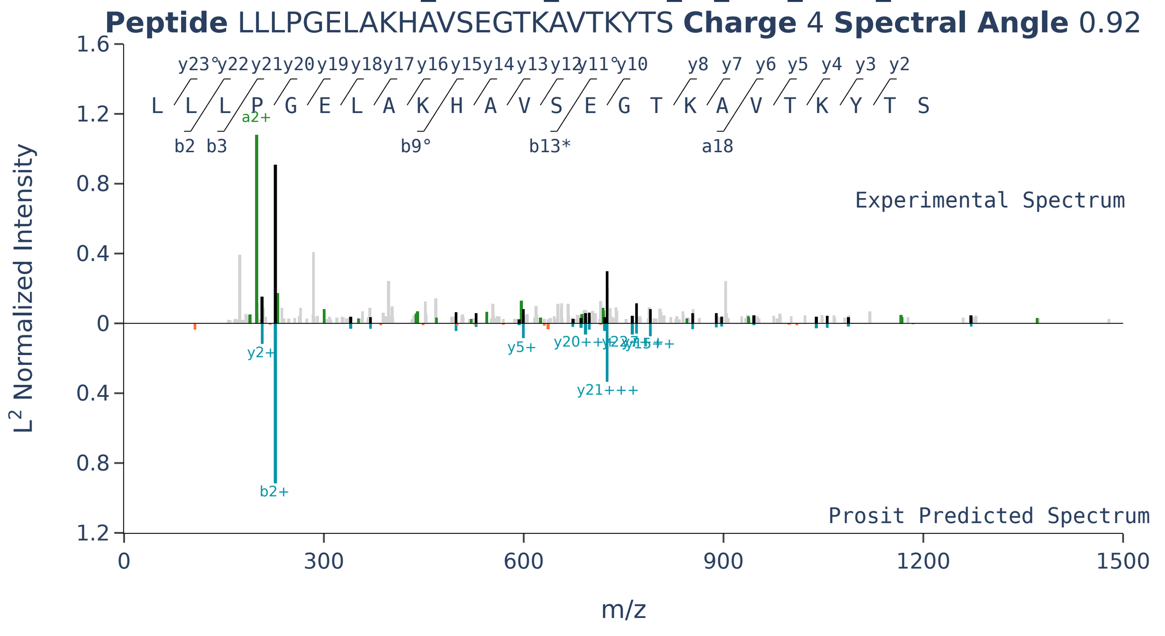


**Source** HRoetschke_291222_080123_Fu_H2B_b2R2_24h **Scan** 9313
**Peptide** LPGELAKHAV **Charge** 2 **Spectral Angle** 0.82

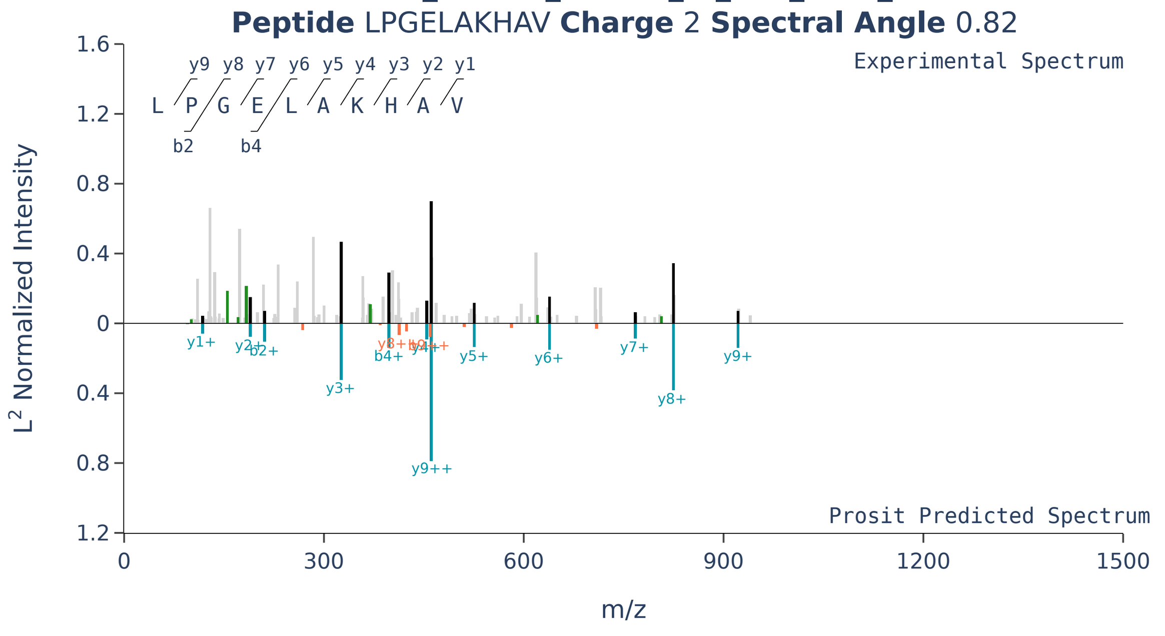


**Source** HRoetschke_291222_080123_Fu_H2B_b1R1_4h **Scan** 17709
**Peptide** VRLLLPGEL **Charge** 2 **Spectral Angle** 0.89

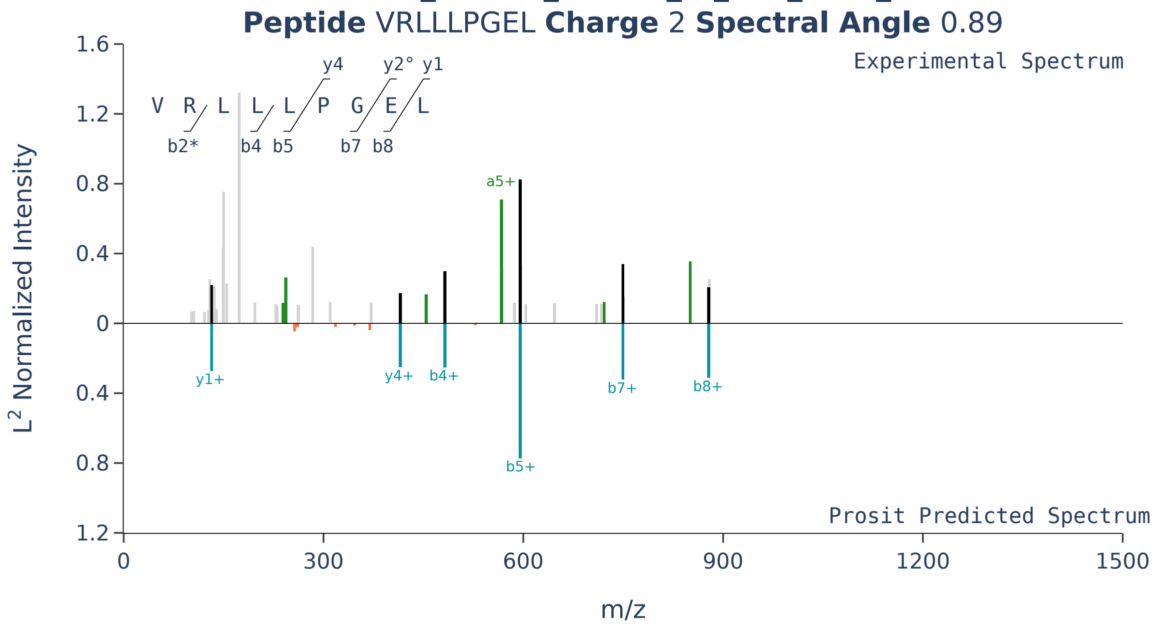


**Source** HRoetschke_291222_080123_Fu_H2B_b1R1_24h **Scan** 1982
**Peptide** AHYNKRSTIT **Charge** 2 **Spectral Angle** 0.9

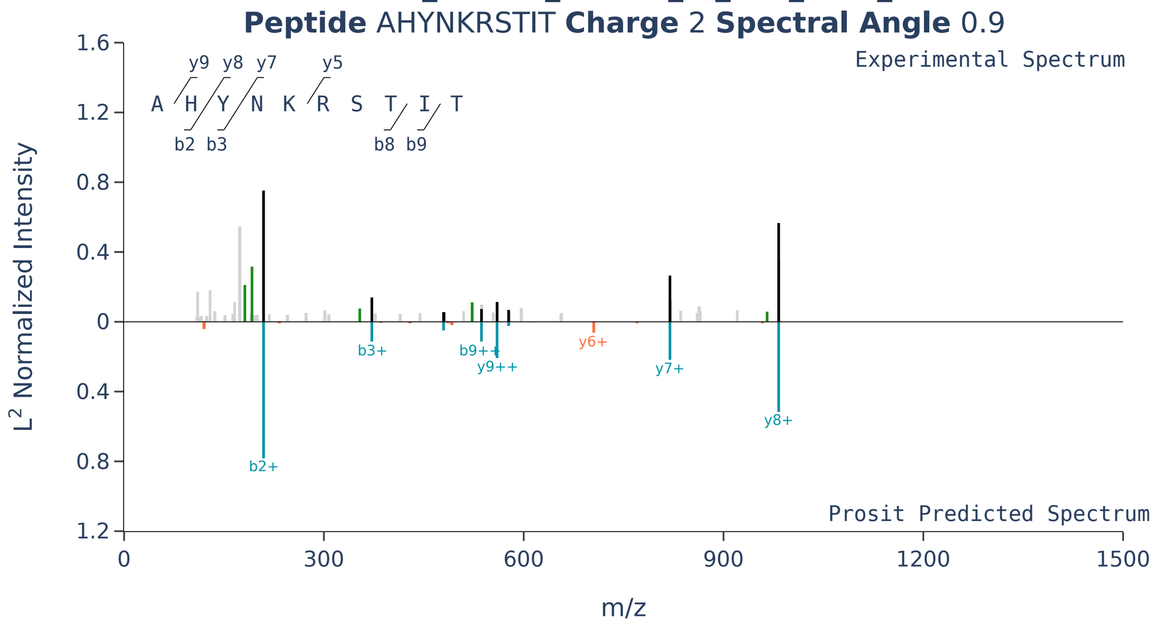


**Source** HRoetschke_291222_080123_Fu_H2B_b2R2_24h **Scan** 3435
**Peptide** GEASRLAHYNKRSTITSR **Charge** 4 **Spectral Angle** 0.83

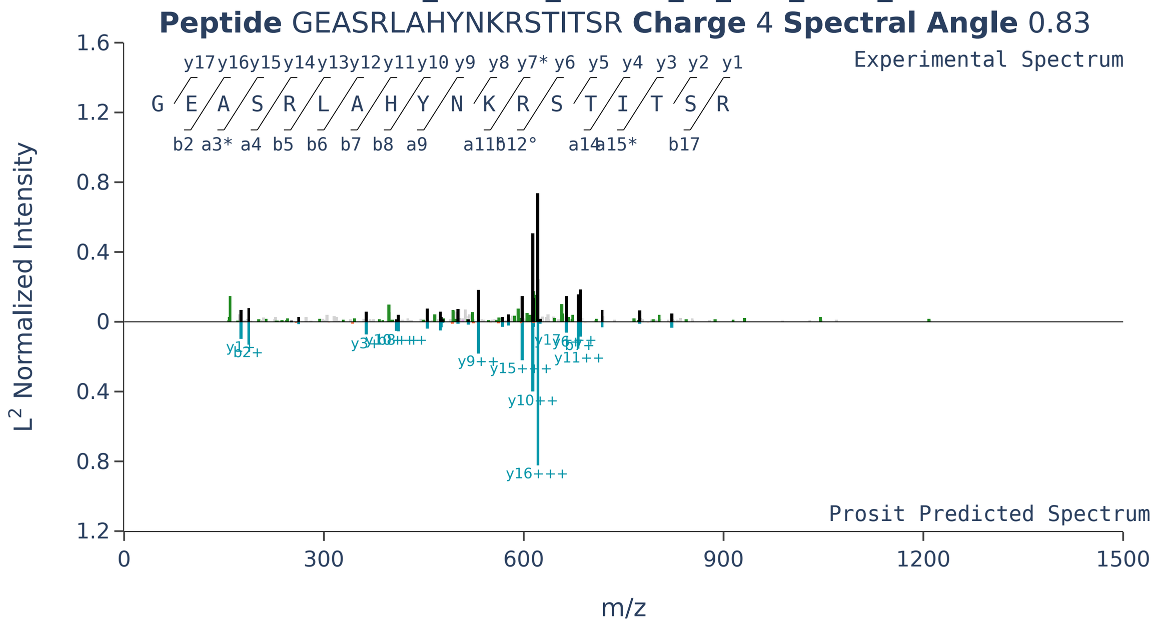


**Source** HRoetschke_291222_080123_Fu_H2B_b1R1_24h **Scan** 7994
**Peptide** LKQVHPDTGISSKAMSIM **Charge** 4 **Spectral Angle** 0.89

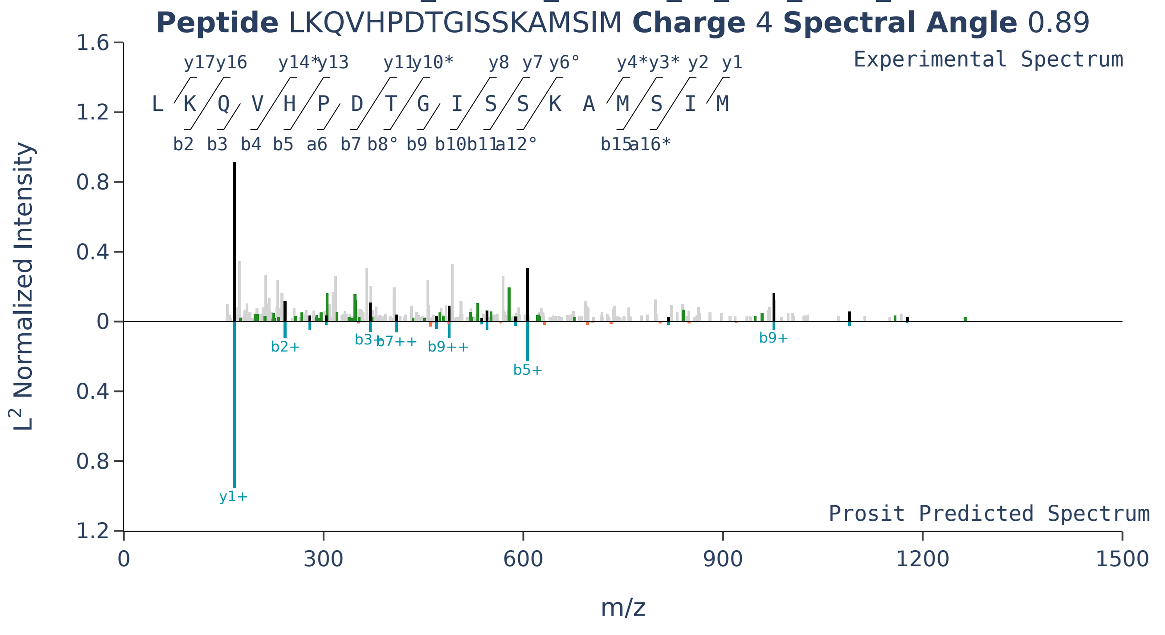


**Source** HRoetschke_291222_080123_Fu_H2B_b2R2_24h **Scan** 2788
**Peptide** AKHAVSEGTKAVTKY **Charge** 2 **Spectral Angle** 0.84

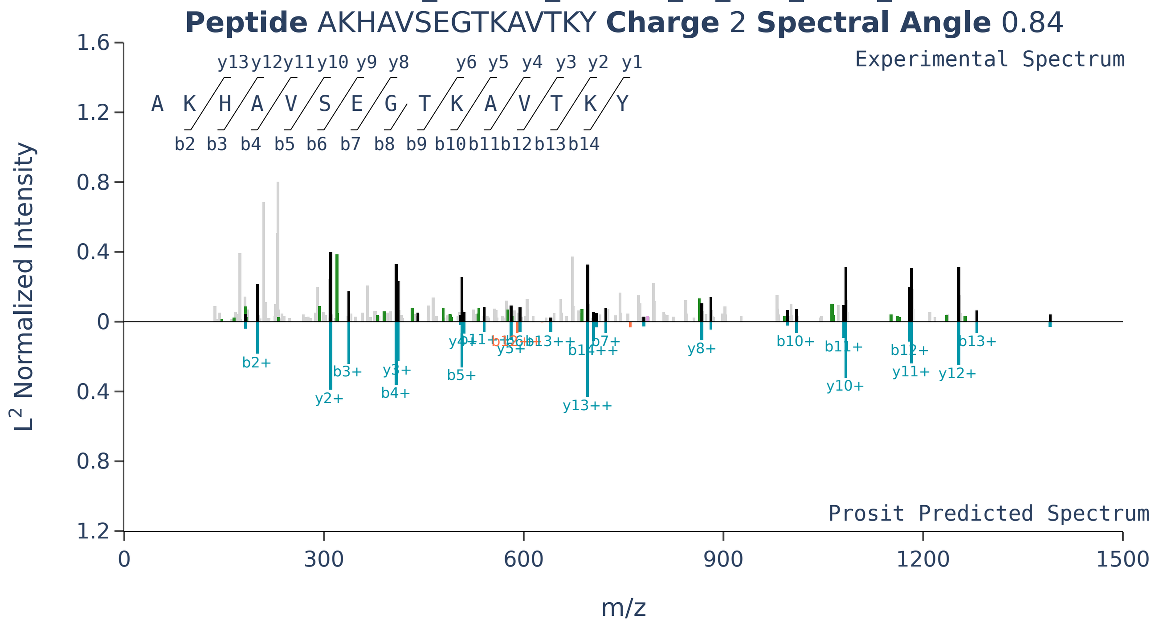


**Source** HRoetschke_291222_080123_Fu_H2B_b2R1_1h **Scan** 15860
**Peptide** SFVNDVFE **Charge** 1 **Spectral Angle** 0.94

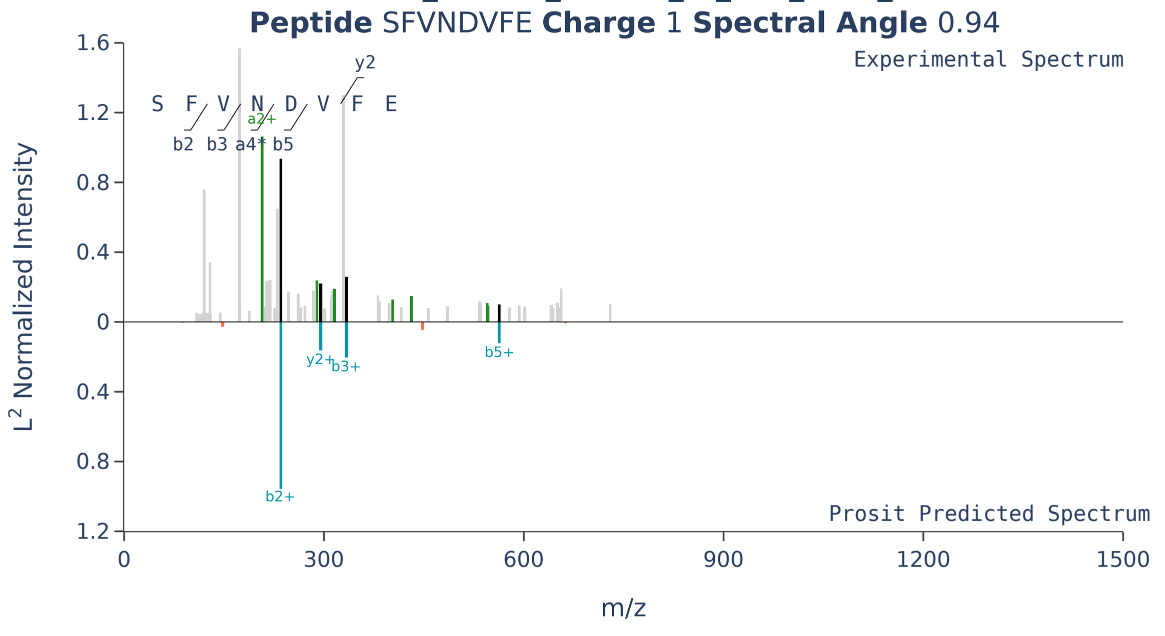


**Source** HRoetschke_291222_080123_Fu_H2B_b2R1_24h **Scan** 18508
**Peptide** REIQTAVRLLL **Charge** 3 **Spectral Angle** 0.82

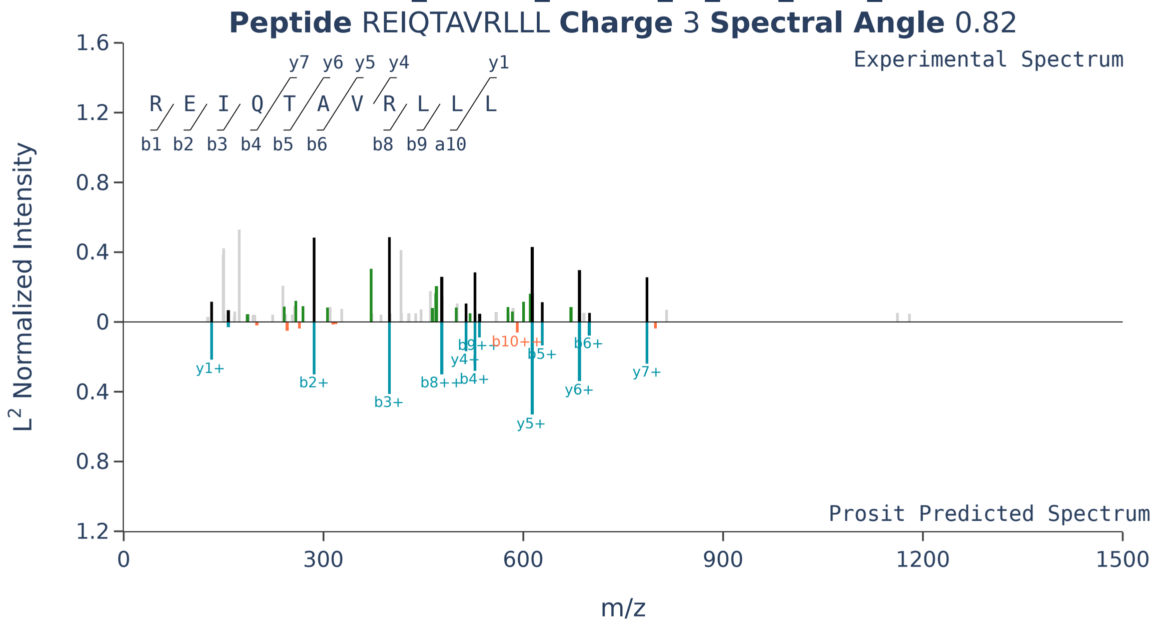

**Source** HRoetschke_291222_080123_Fu_H2B_b1R1_24h **Scan** 4734
**Peptide** LAGEASRLAHYNKR **Charge** 3 **Spectral Angle** 0.85

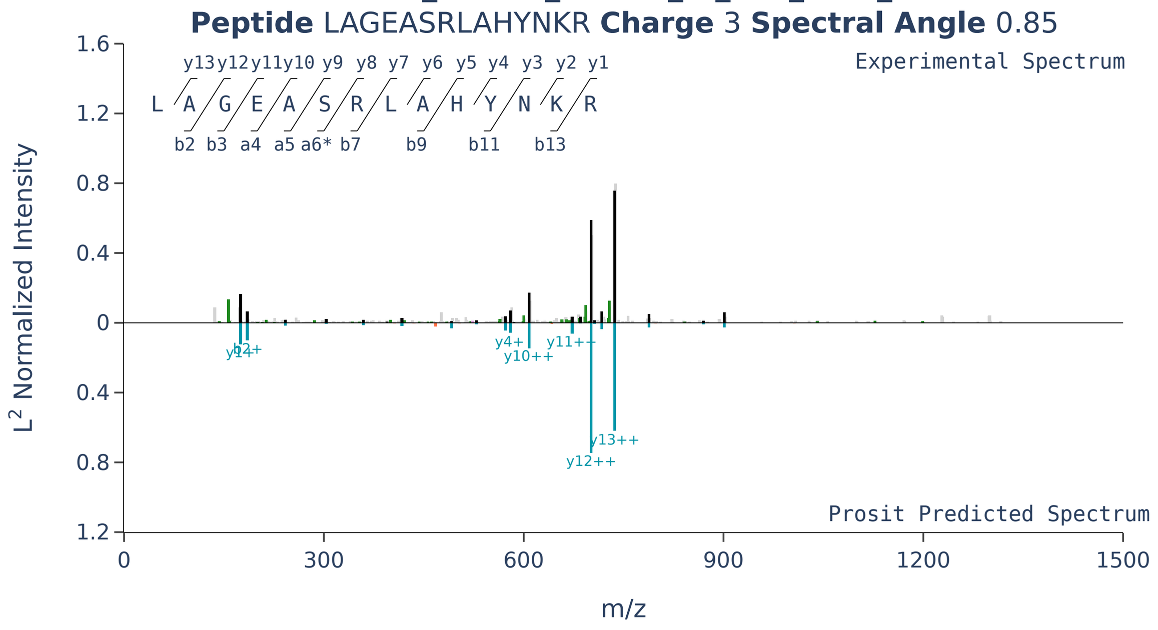


**Source** HRoetschke_291222_080123_Fu_H2B_b1R1_24h **Scan** 6203
**Peptide** GKKRRKTRKESYAIYVY **Charge** 4 **Spectral Angle** 0.76

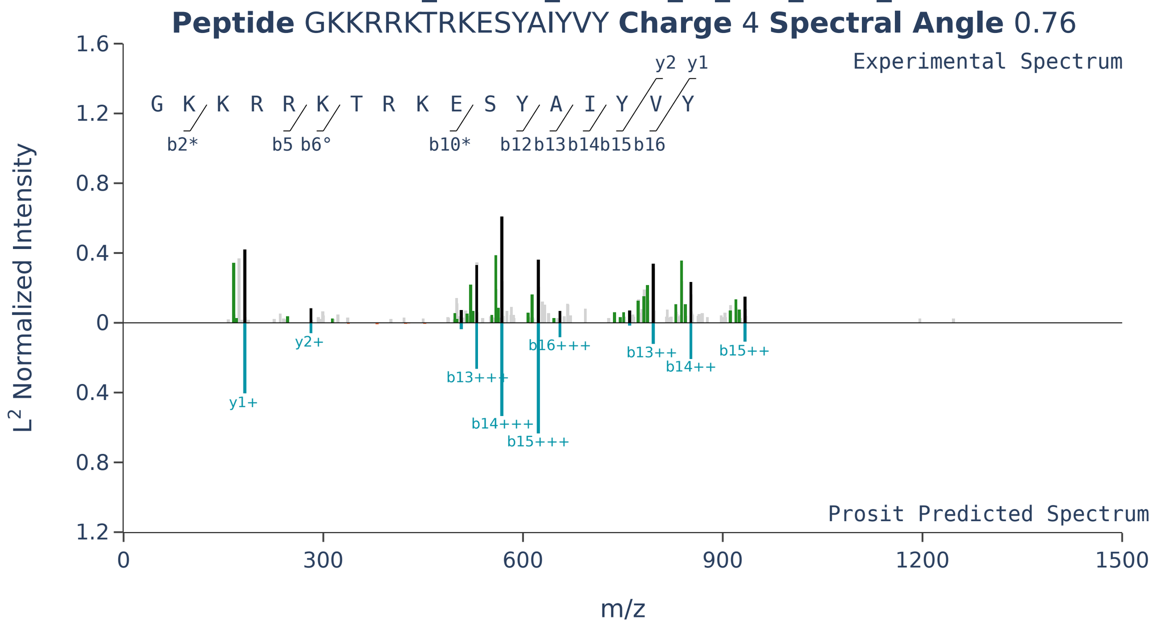


**Source** HRoetschke_291222_080123_Fu_H2B_b1R1_24h **Scan** 14776
**Peptide** LLLPGELAKHAV **Charge** 2 **Spectral Angle** 0.9

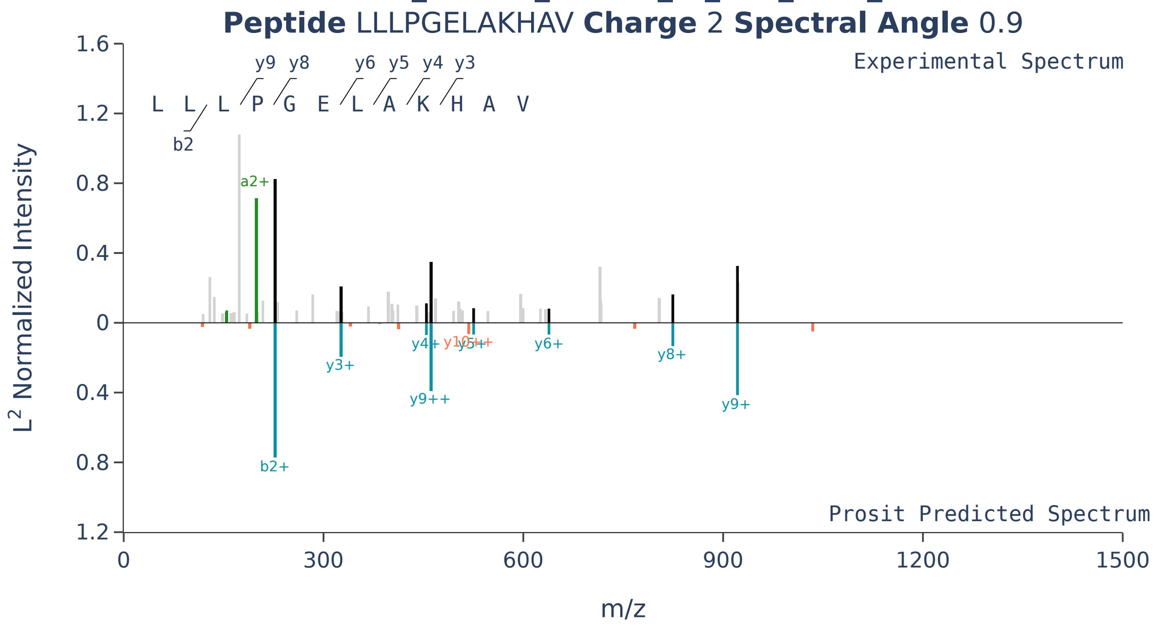


**Source** HRoetschke_291222_080123_Fu_H2B_b1R1_24h **Scan** 13322
**Peptide** REIQTAVRLL **Charge** 3 **Spectral Angle** 0.83

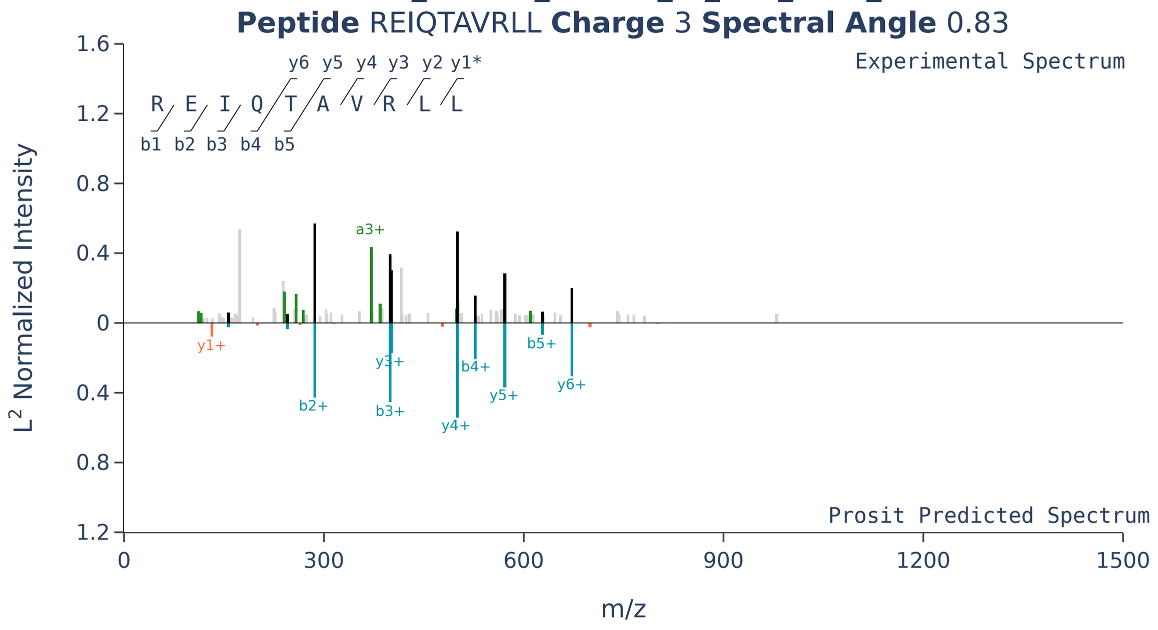


**Source** HRoetschke_291222_080123_Fu_H2B_b1R1_4h **Scan** 14725
**Peptide** VRLLLPGE **Charge** 2 **Spectral Angle** 0.78

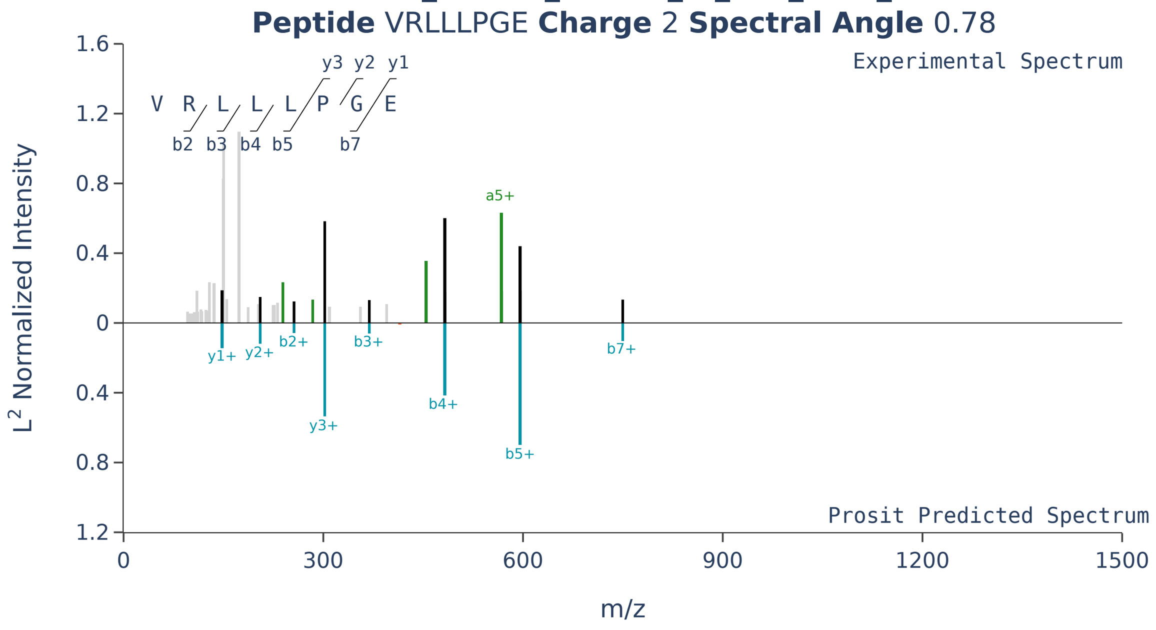


**Source** HRoetschke_291222_080123_Fu_H2B_b1R1_1h **Scan** 13415
**Peptide** REIQTAVRLLLPGELAKH **Charge** 4 **Spectral Angle** 0.79

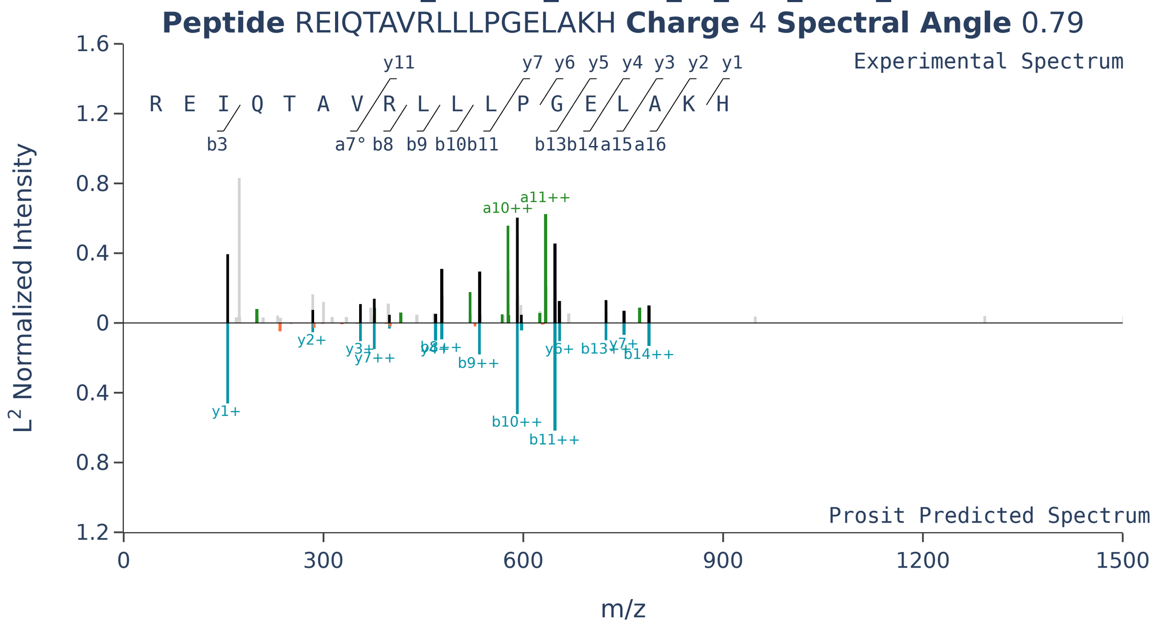


**Source** HRoetschke_291222_080123_Fu_H2B_b2R2_24h **Scan** 16610
**Peptide** FVNDVFE **Charge** 1 **Spectral Angle** 0.92

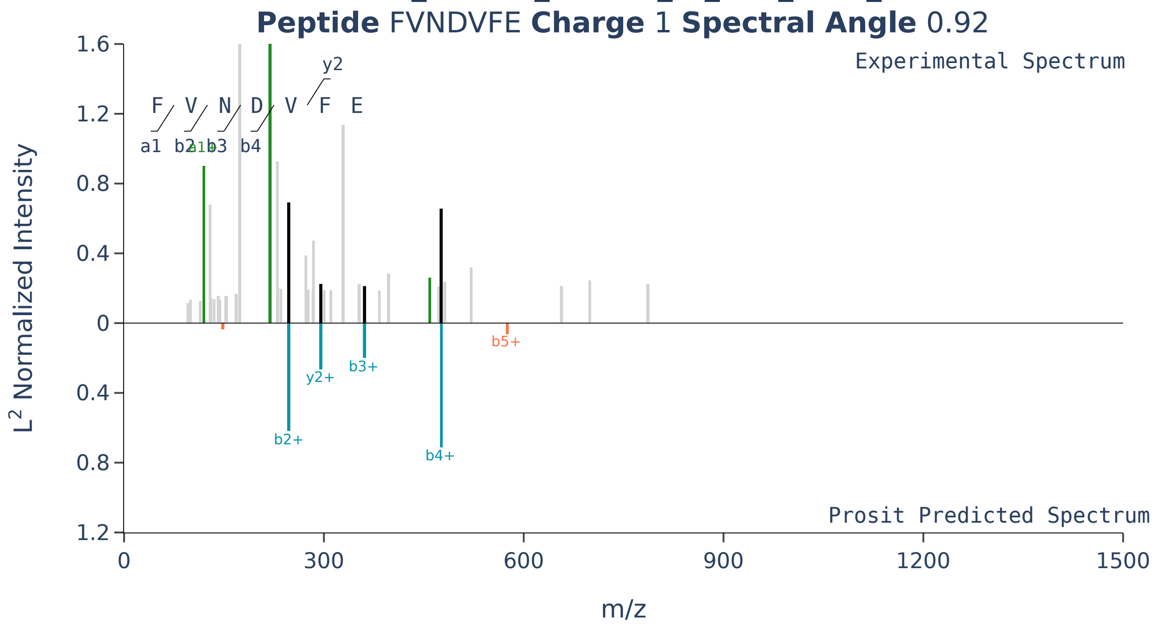


**Source** HRoetschke_291222_080123_Fu_H2B_b2R2_2h **Scan** 18332
**Peptide** TSREIQTAVRLLL **Charge** 3 **Spectral Angle** 0.84

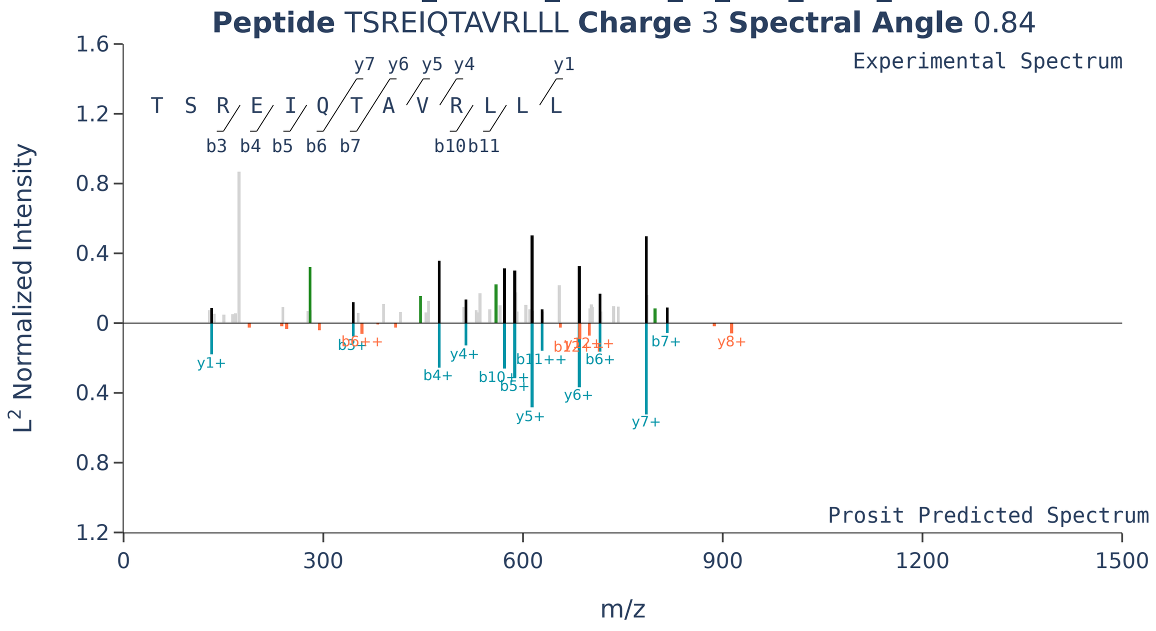


**Source** HRoetschke_291222_080123_Fu_H2B_b2R1_2h **Scan** 12214
**Peptide** QTAVRLL **Charge** 2 **Spectral Angle** 0.86

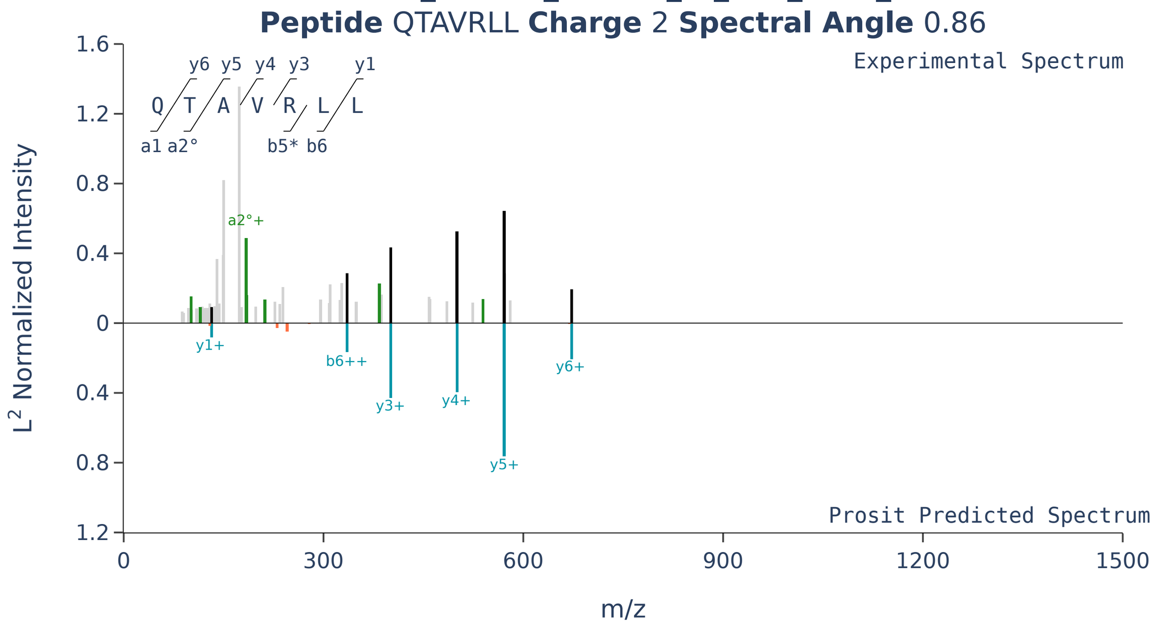


**Source** HRoetschke_291222_080123_Fu_H2B_b1R2_1h **Scan** 12141
**Peptide** VNDVFERIAGEASR **Charge** 3 **Spectral Angle** 0.89

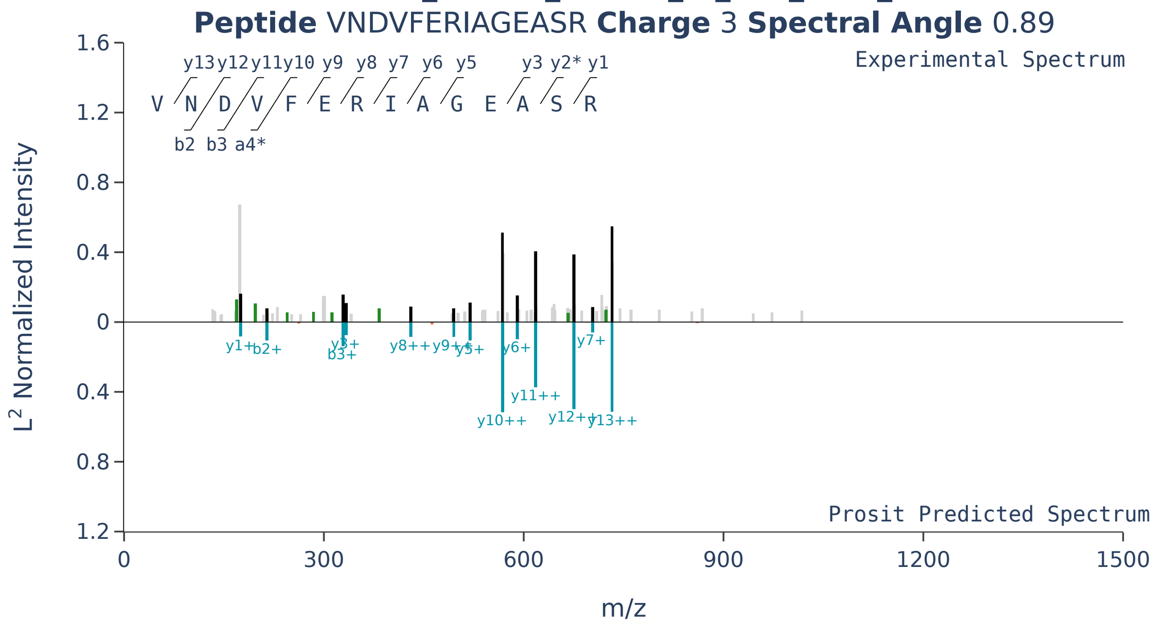


**Source** HRoetschke_291222_080123_Fu_H2B_b2R2_24h **Scan** 4626
**Peptide** QVHPDTGISS **Charge** 2 **Spectral Angle** 0.78

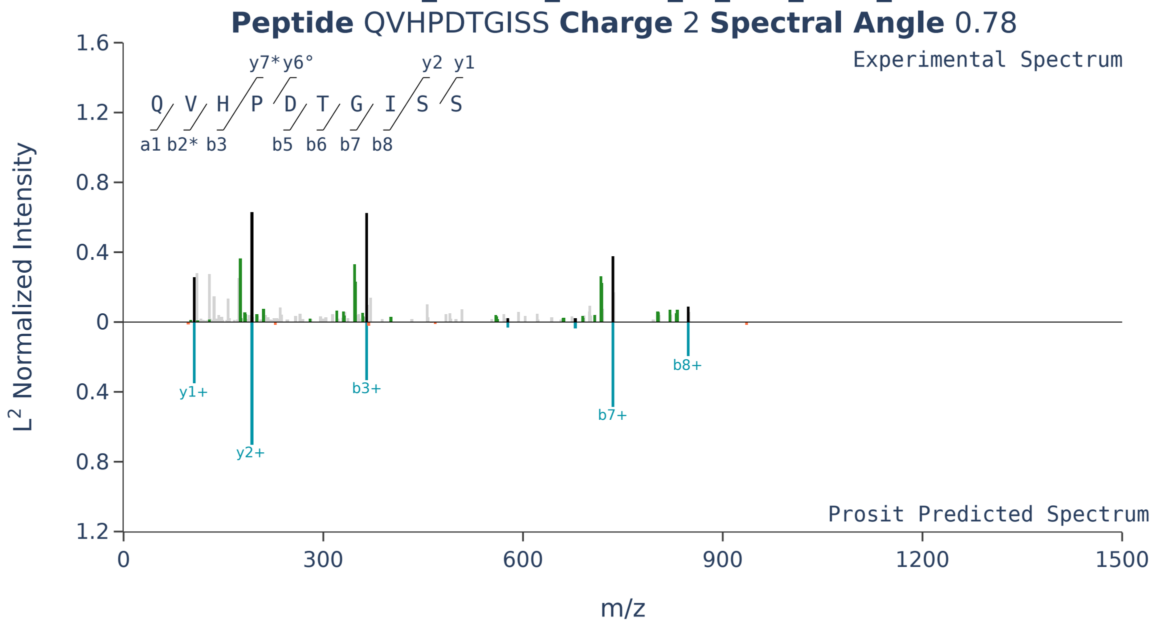


**Source** HRoetschke_291222_080123_Fu_H2B_b1R1_2h **Scan** 14865
**Peptide** LLLPGELAKHAVSEGTKAVTKYTSAK **Charge** 4 **Spectral Angle** 0.77

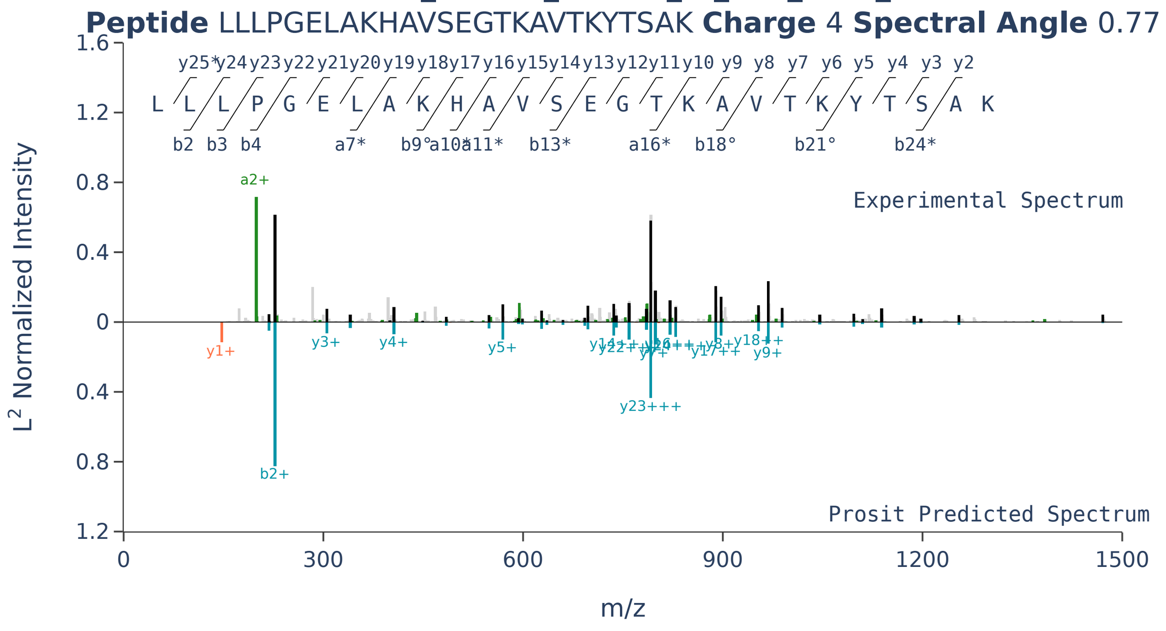


**Source** HRoetschke_291222_080123_Fu_H2B_b2R2_1h **Scan** 18297
**Peptide** MNSFVNDVFERIAG **Charge** 2 **Spectral Angle** 0.78

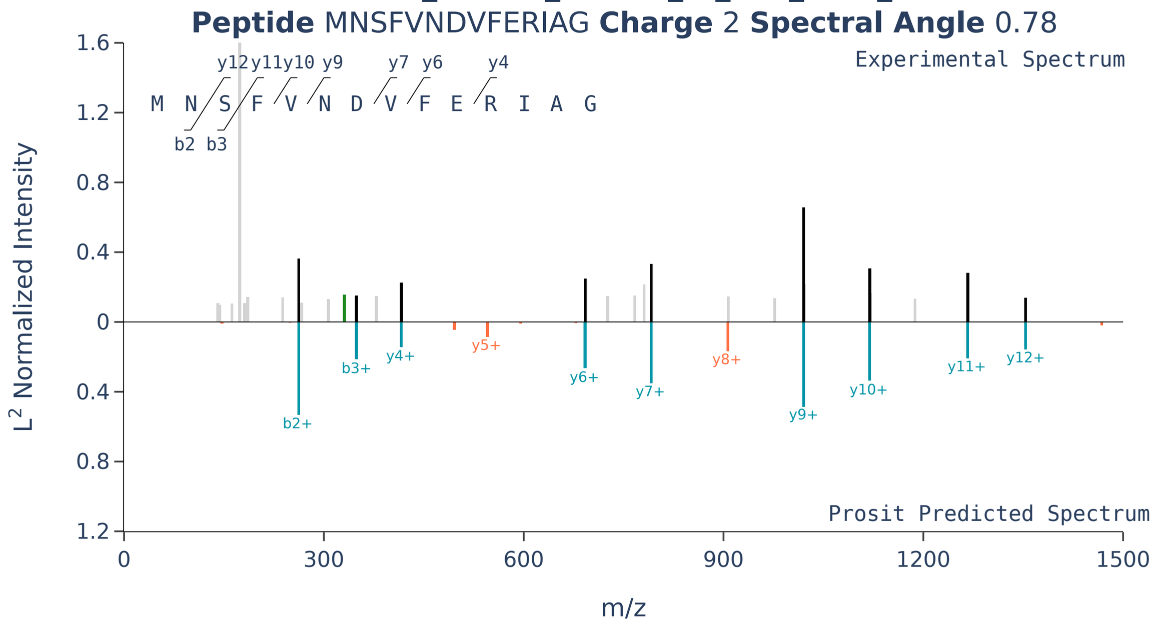


**Source** HRoetschke_291222_080123_Fu_H2B_b1R1_4h **Scan** 14374
**Peptide** SFVNDVFER **Charge** 2 **Spectral Angle** 0.84

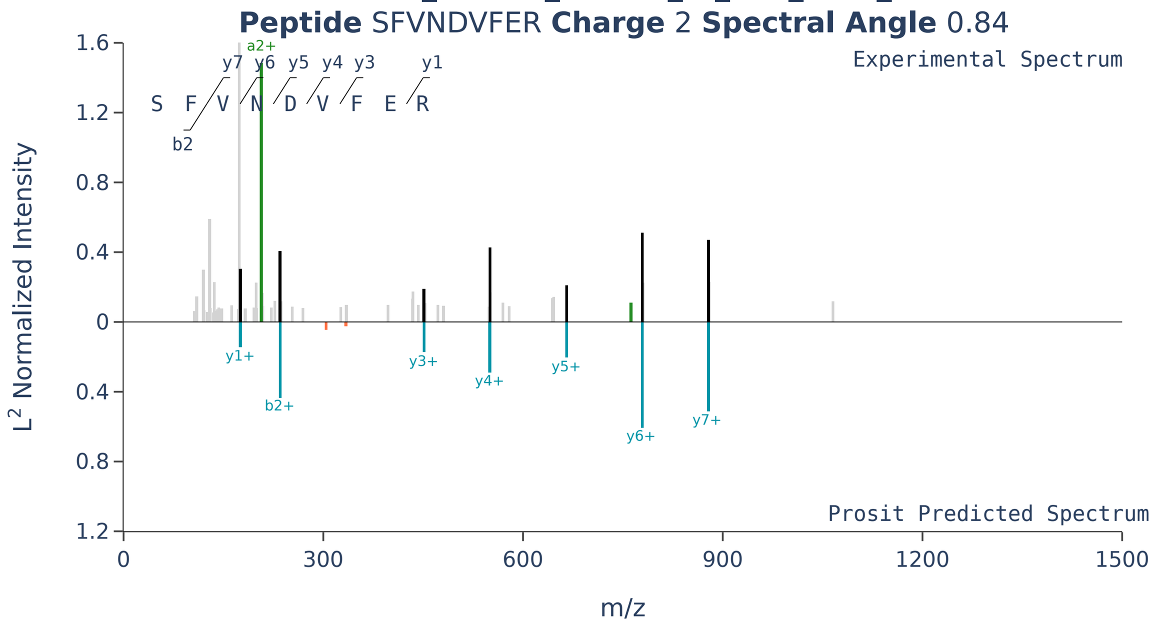


**Source** HRoetschke_291222_080123_Fu_H2B_b2R1_24h **Scan** 4193
**Peptide** PGELAKHAVSEGT **Charge** 2 **Spectral Angle** 0.83

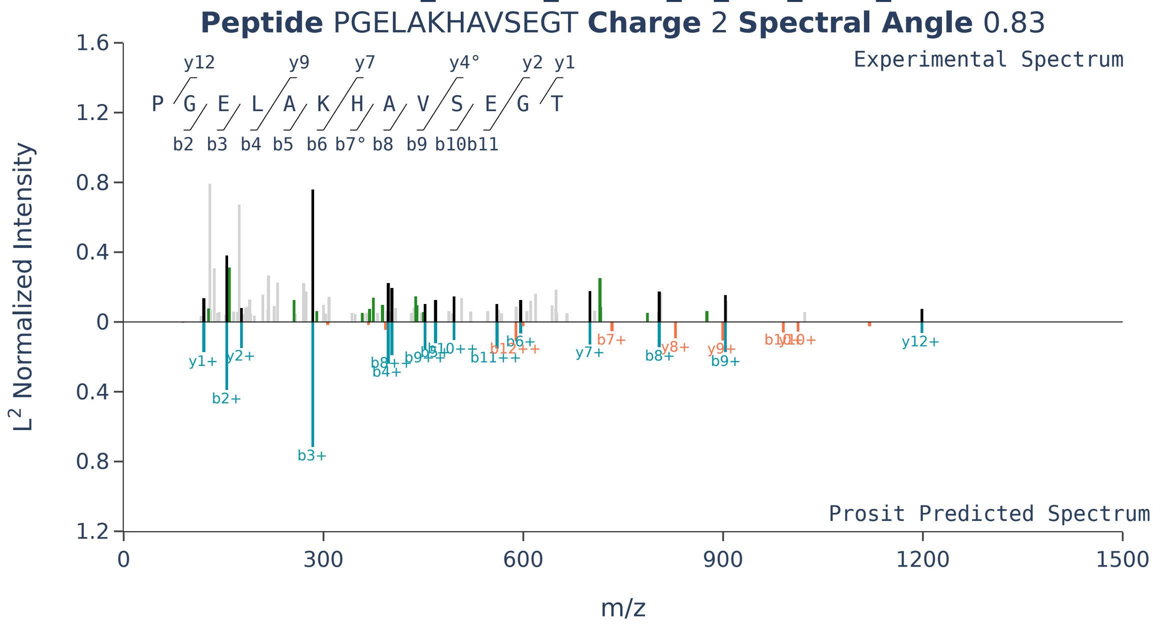

**Source** HRoetschke_291222_080123_Fu_H2B_b2R1_2h **Scan** 6673
**Peptide** STITSREL **Charge** 2 **Spectral Angle** 0.92

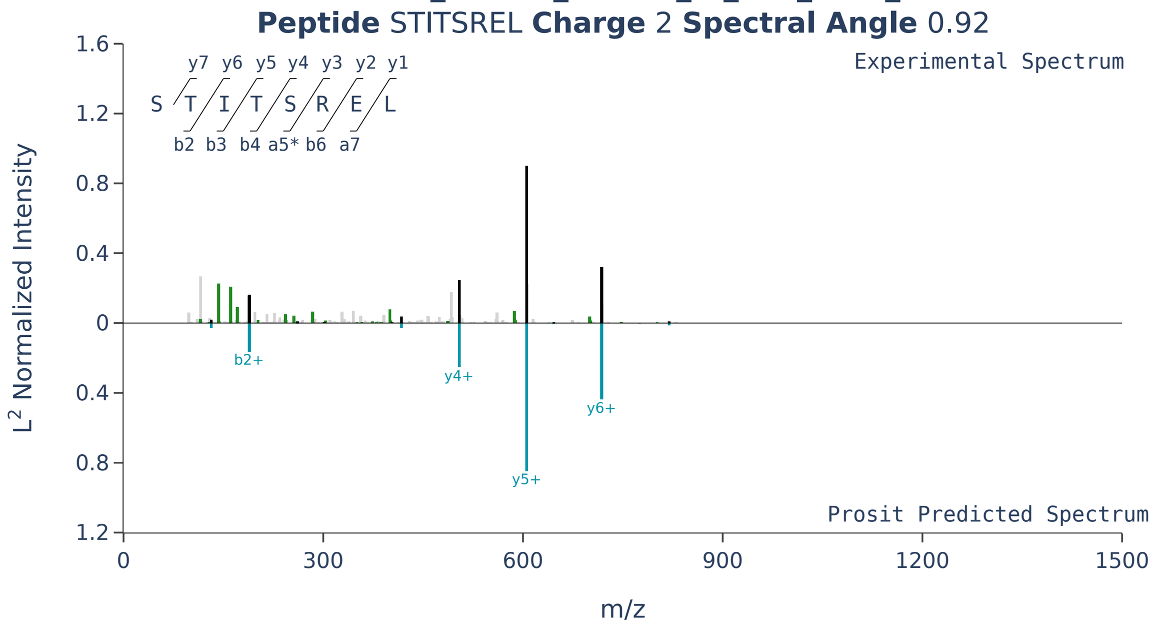


**Source** HRoetschke_291222_080123_Fu_H2B_b2R1_24h **Scan** 5335
**Peptide** AVSEGTKAVTKYTS **Charge** 2 **Spectral Angle** 0.75

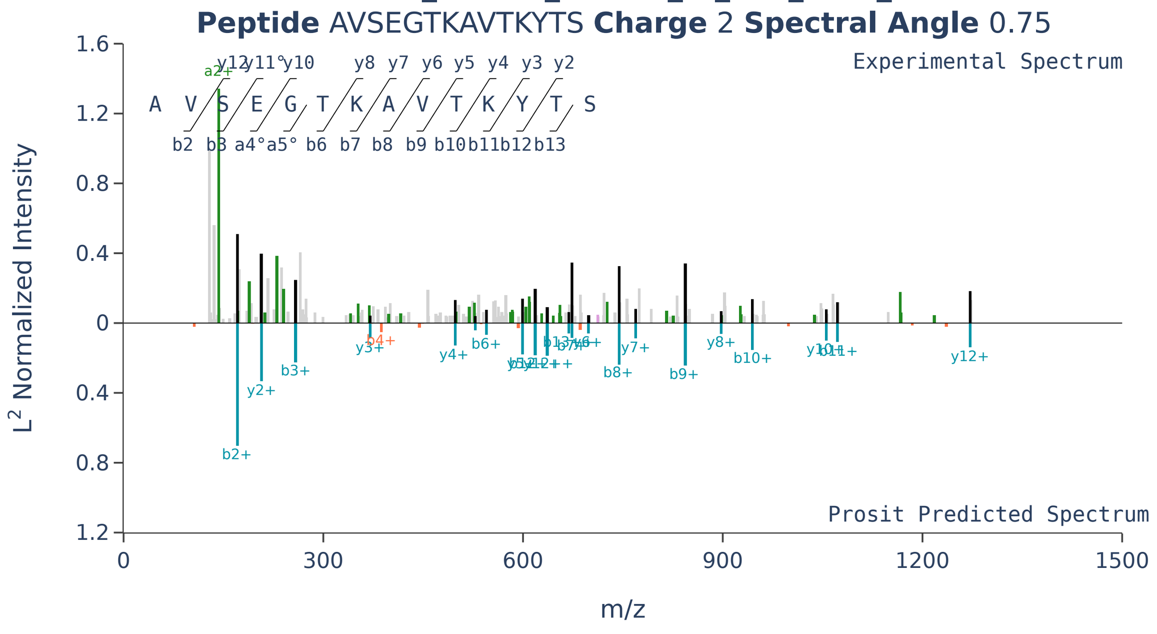


**Source** HRoetschke_291222_080123_Fu_H2B_b1R2_24h **Scan** 1923
**Peptide** AHYNKRSTITSRE **Charge** 3 **Spectral Angle** 0.89

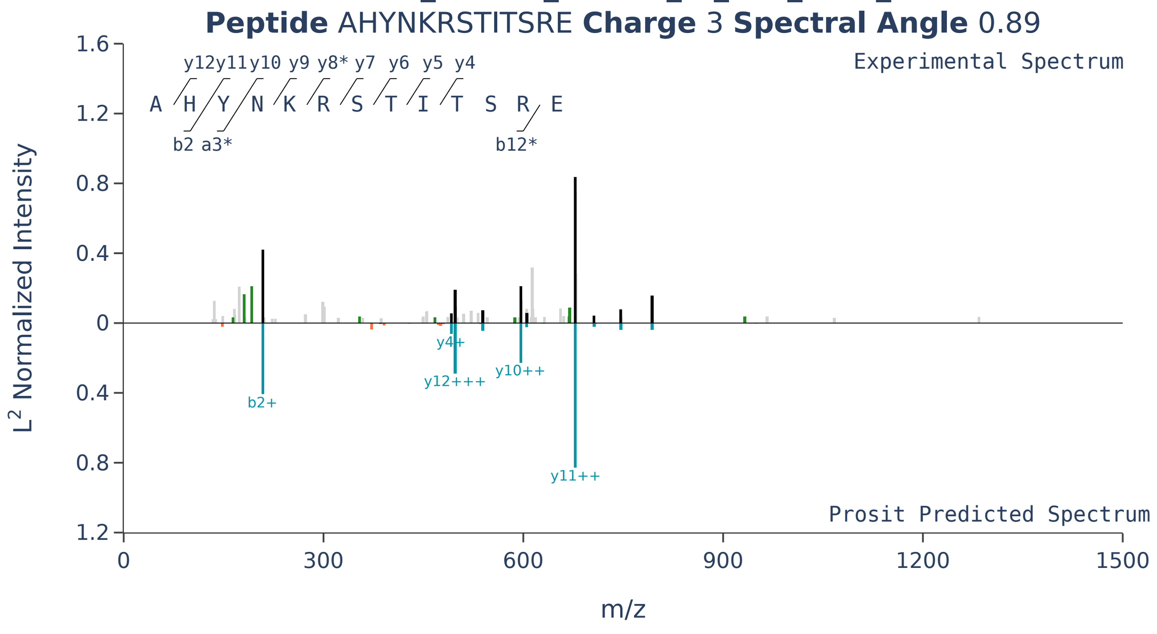


**Source** HRoetschke_291222_080123_Fu_H2B_b1R1_24h **Scan** 7254
**Peptide** RLAGEASRLAHY **Charge** 2 **Spectral Angle** 0.84

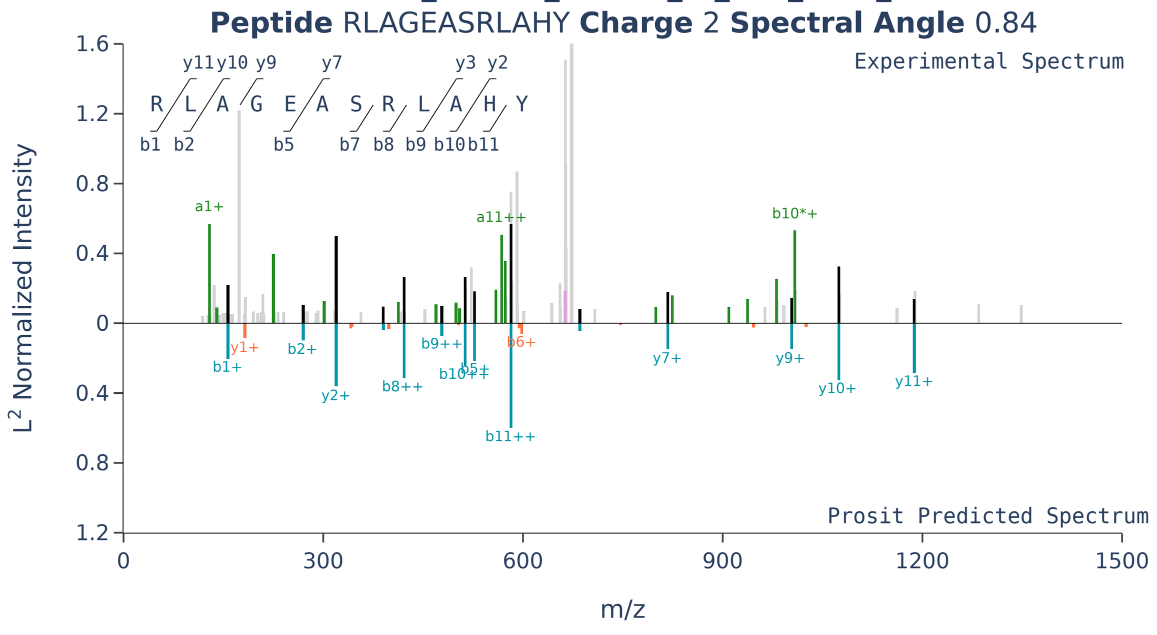


**Source** HRoetschke_291222_080123_Fu_H2B_b2R2_24h **Scan** 16912
**Peptide** QTAVRLLLPGELAKH **Charge** 3 **Spectral Angle** 0.9

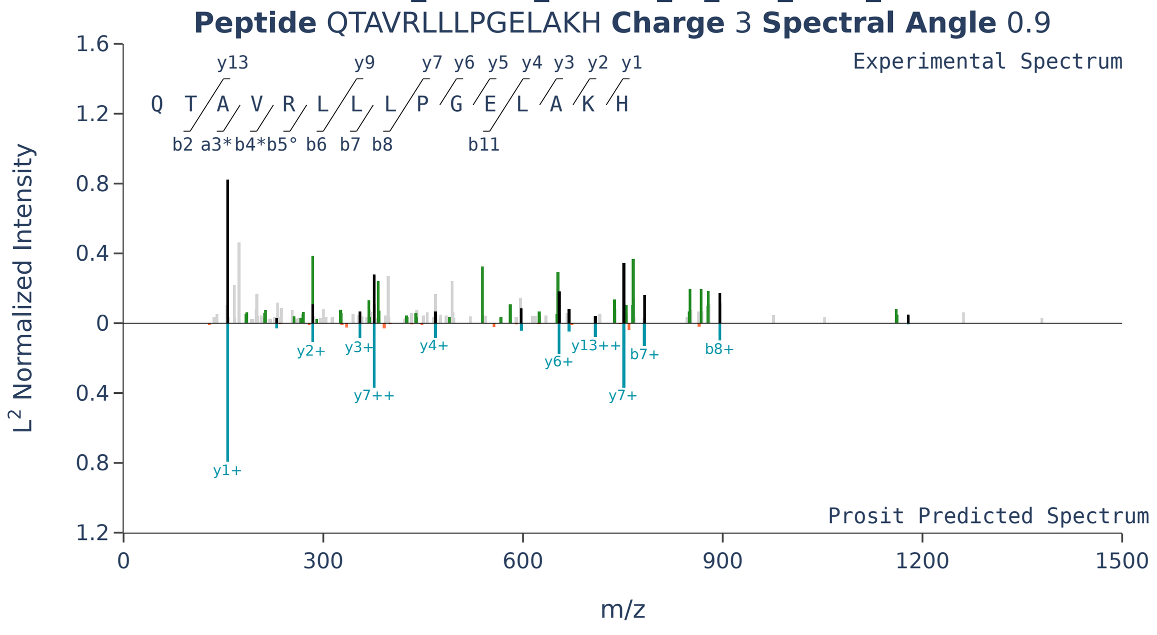


**Source** HRoetschke_291222_080123_Fu_H2B_b1R2_2h **Scan** 2729
**Peptide** EGTKAVTKYTS **Charge** 2 **Spectral Angle** 0.83

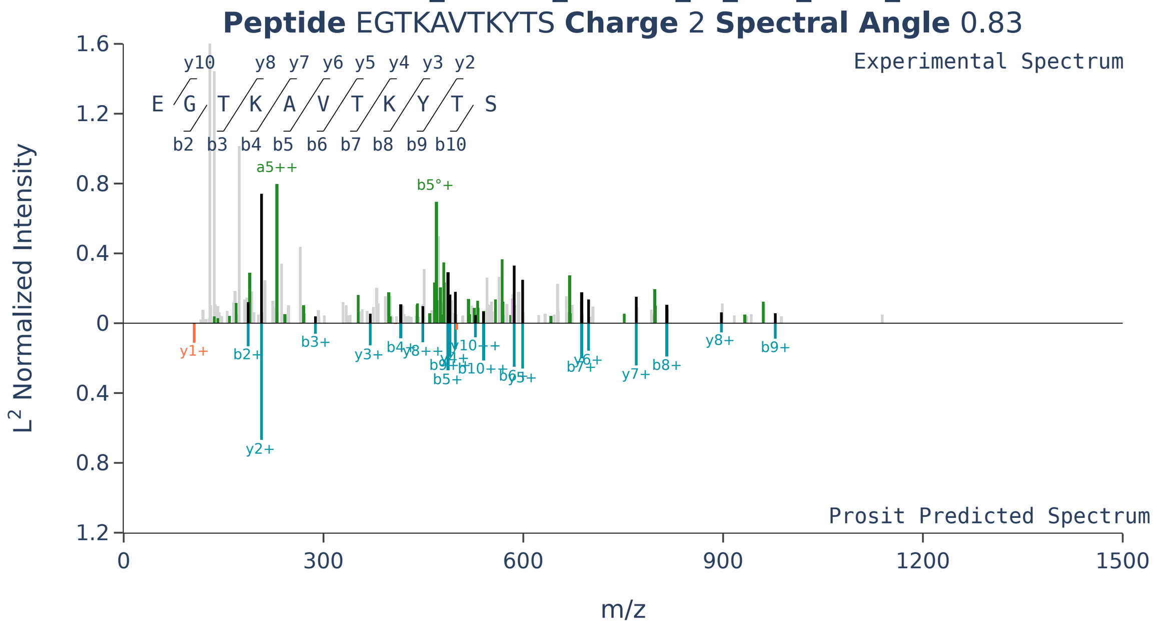


**Source** HRoetschke_291222_080123_Fu_H2B_b2R2_4h **Scan** 6467
**Peptide** QVHPDTGISSKAM **Charge** 2 **Spectral Angle** 0.86

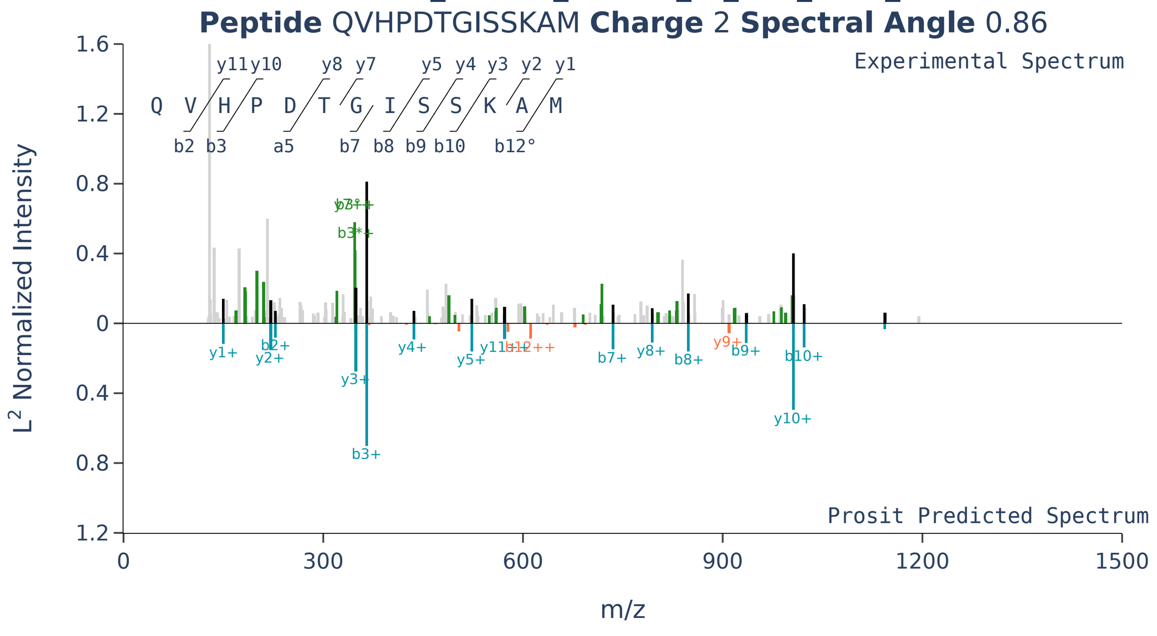


**Source** HRoetschke_291222_080123_Fu_H2B_b1R2_4h **Scan** 6217
**Peptide** LKQVHPDTGL **Charge** 3 **Spectral Angle** 0.83

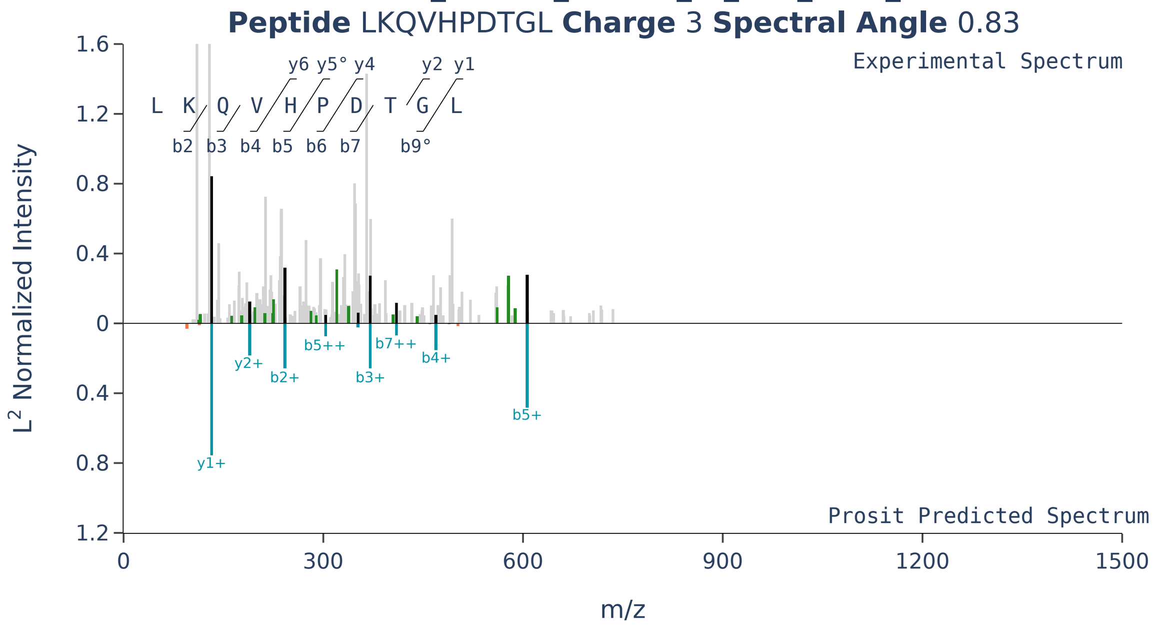


**Source** HRoetschke_291222_080123_Fu_H2B_b1R1_2h **Scan** 5092
**Peptide** ERIAGEASRLAHYNKR **Charge** 3 **Spectral Angle** 0.78

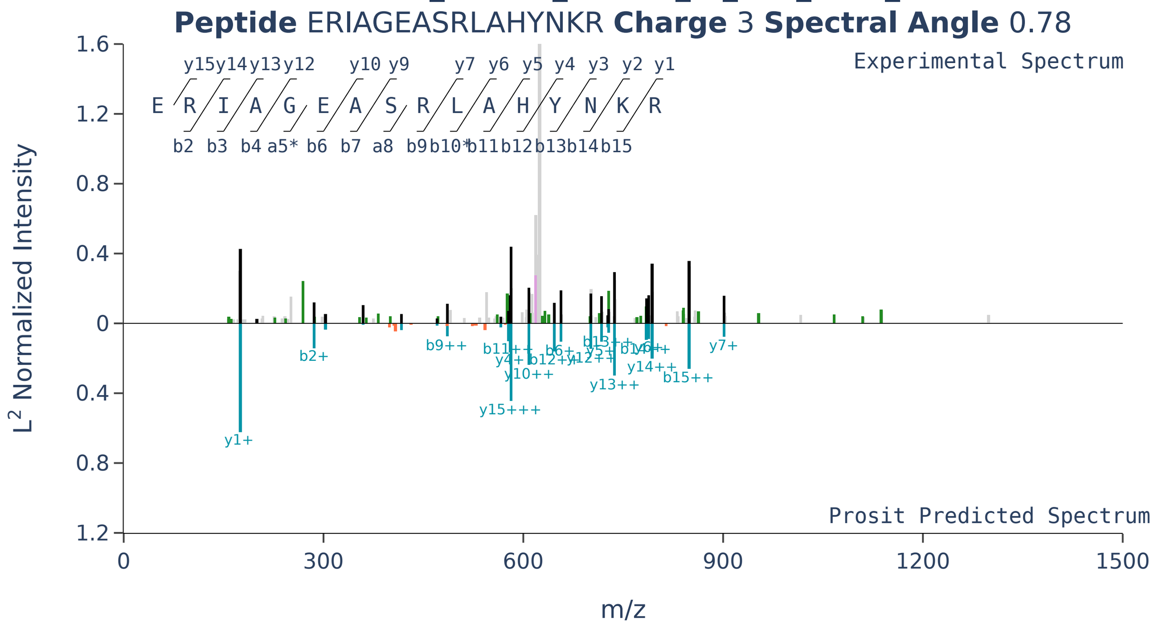


**Source** HRoetschke_291222_080123_Fu_H2B_b2R2_4h **Scan** 6047
**Peptide** LPGELAKHA **Charge** 2 **Spectral Angle** 0.74

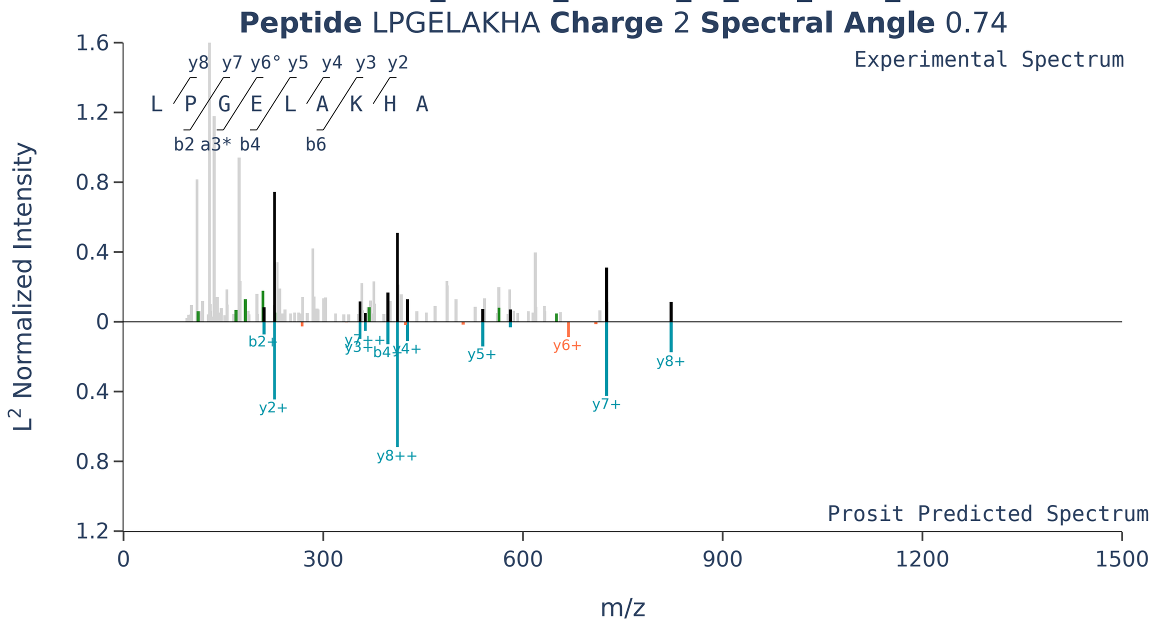


**Source** HRoetschke_291222_080123_Fu_H2B_b2R1_24h **Scan** 4529
**Peptide** LKQVHPDTGISSKA **Charge** 2 **Spectral Angle** 0.84

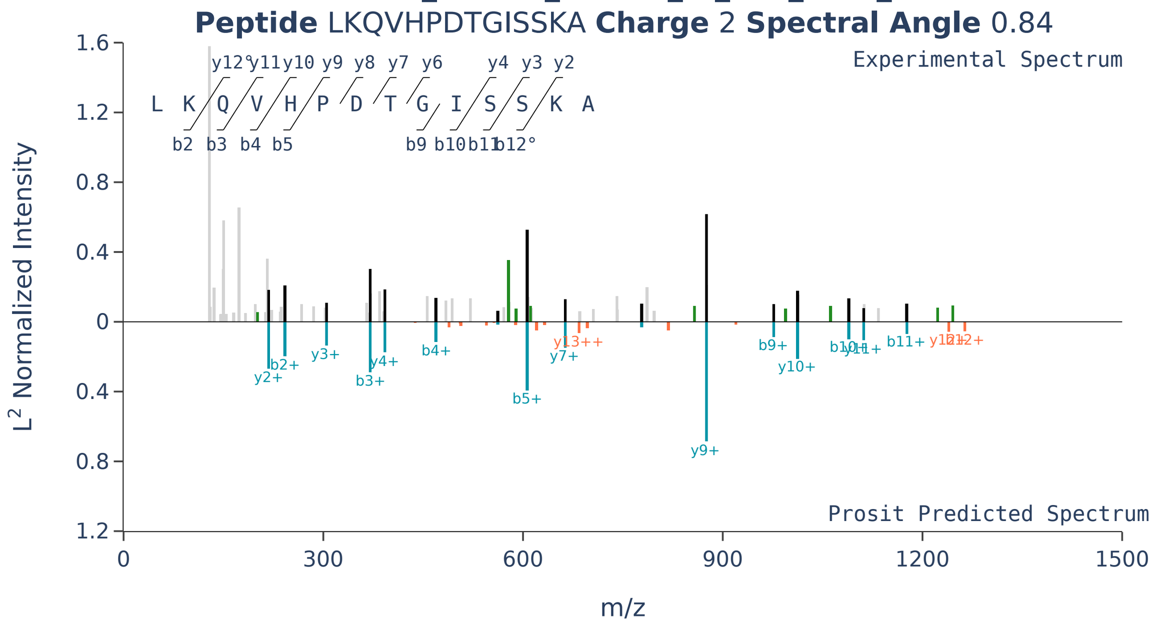


**Source** HRoetschke_291222_080123_Fu_H2B_b1R2_24h **Scan** 5098
**Peptide** AVSEGTKAVTKYTSAK **Charge** 2 **Spectral Angle** 0.81

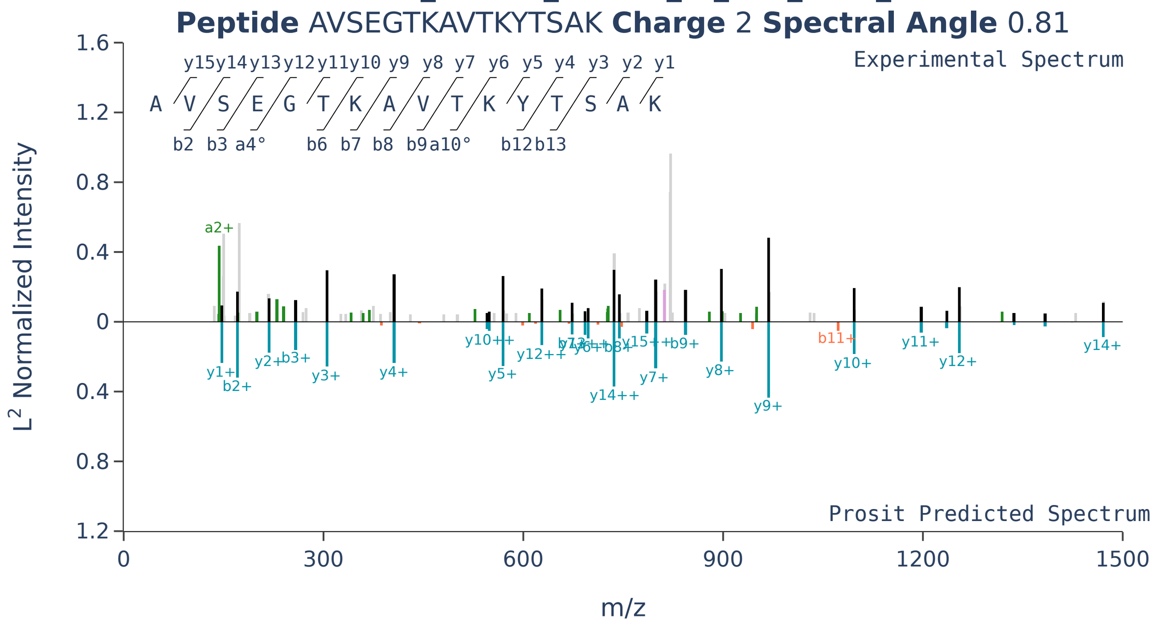


**Source** HRoetschke_291222_080123_Fu_H2B_b1R2_24h **Scan** 3533
**Peptide** EGTKAVTKYT **Charge** 2 **Spectral Angle** 0.78

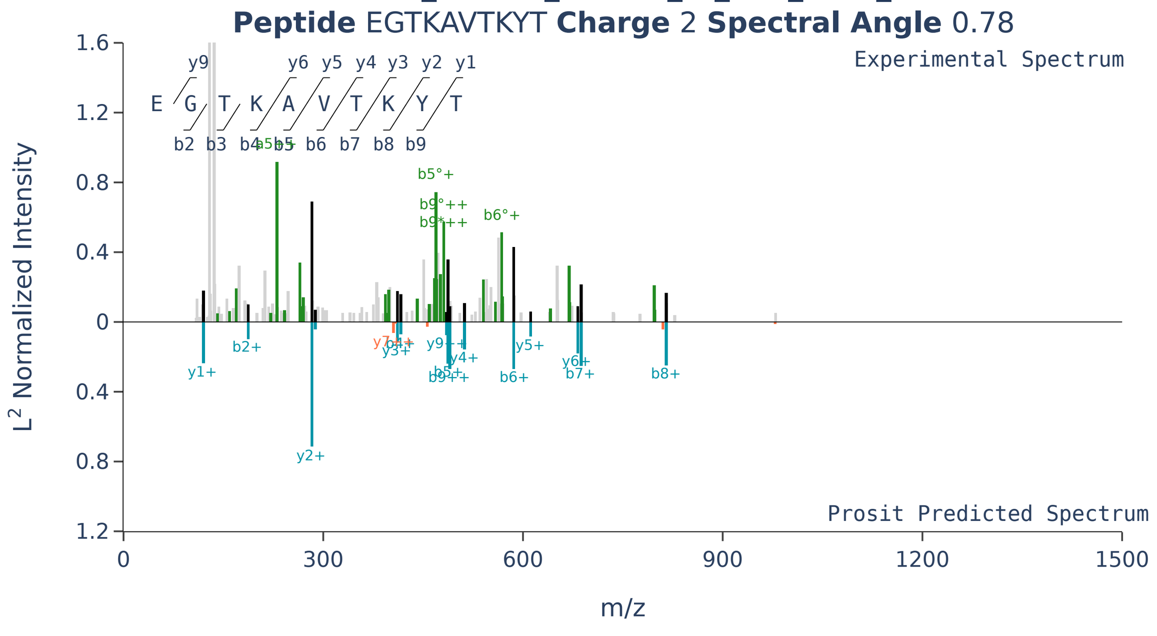


**Source** HRoetschke_291222_080123_Fu_H2B_b2R1_2h **Scan** 16288
**Peptide** QTAVRLLLPGE **Charge** 2 **Spectral Angle** 0.88

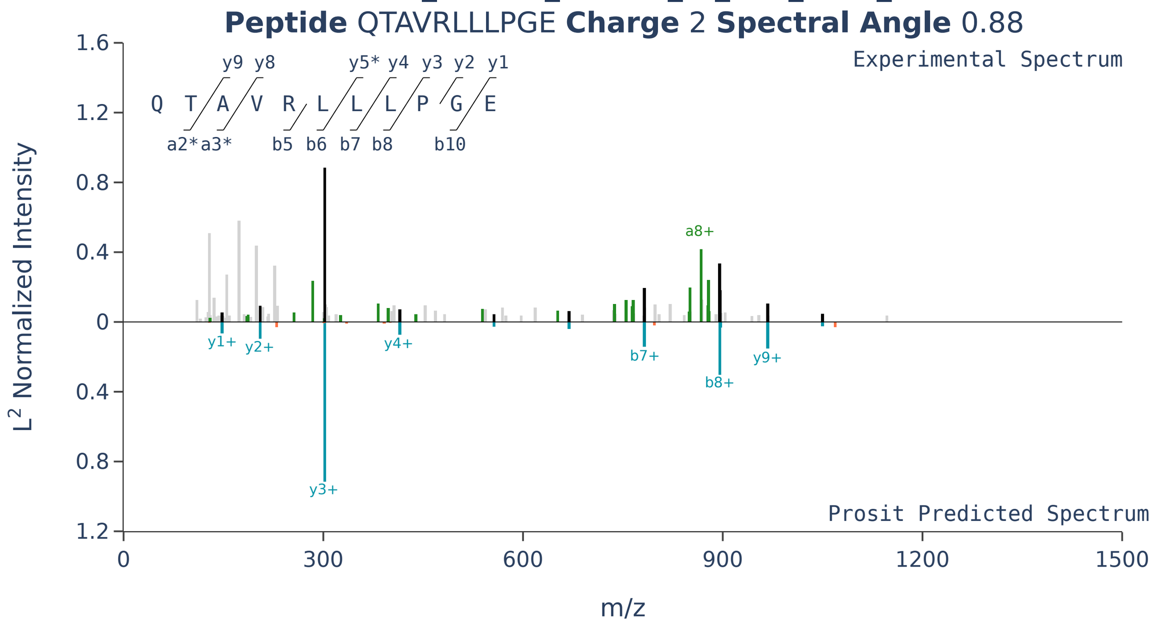


**Source** HRoetschke_291222_080123_Fu_H2B_b1R2_1h **Scan** 4523
**Peptide** ITSREIQTA **Charge** 2 **Spectral Angle** 0.86

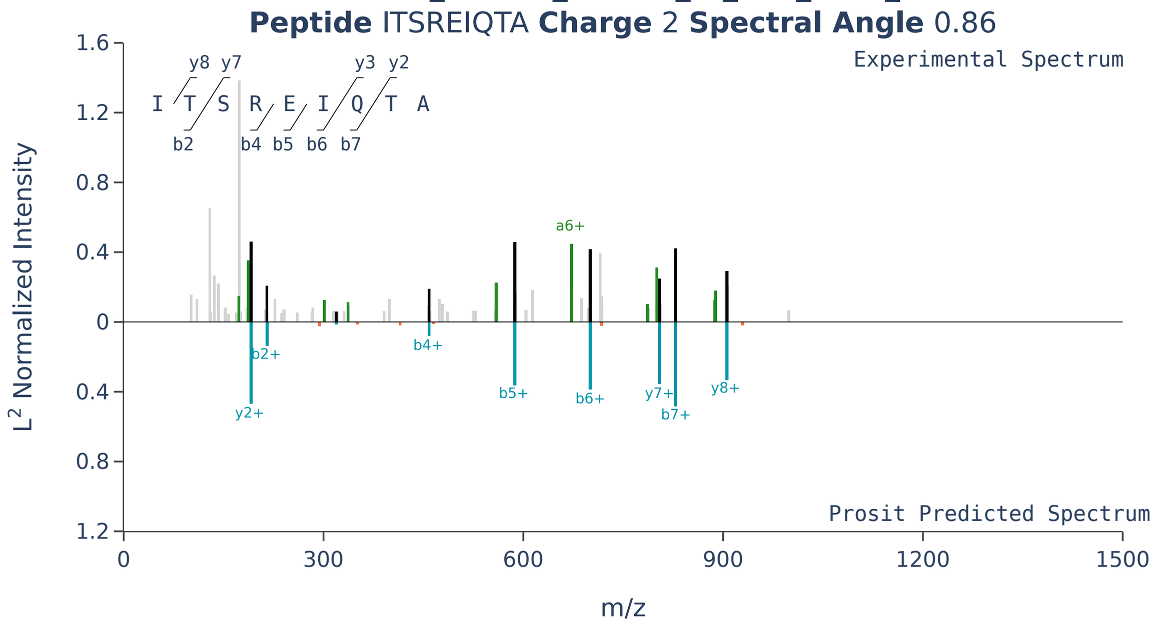

**Source** HRoetschke_291222_080123_Fu_H2B_b2R1_24h **Scan** 2700
**Peptide** AGEASRLAHYNKRST **Charge** 2 **Spectral Angle** 0.78

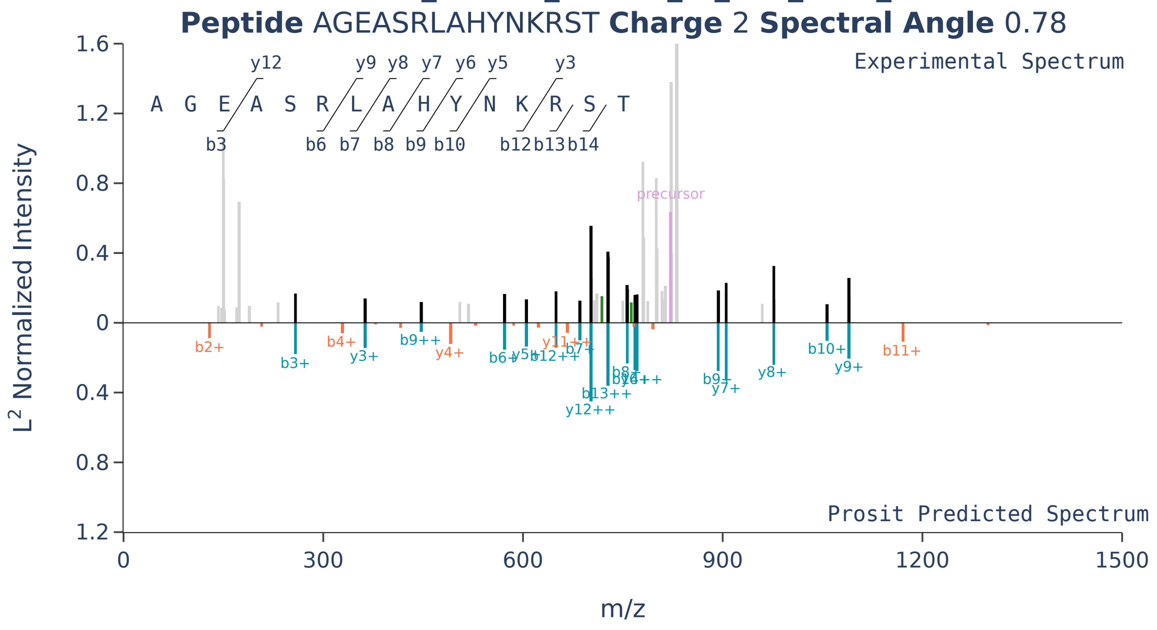


**Source** HRoetschke_291222_080123_Fu_H2B_b1R1_1h **Scan** 14035
**Peptide** LKQVHPDTGISSKAMSIMNSF **Charge** 4 **Spectral Angle** 0.78

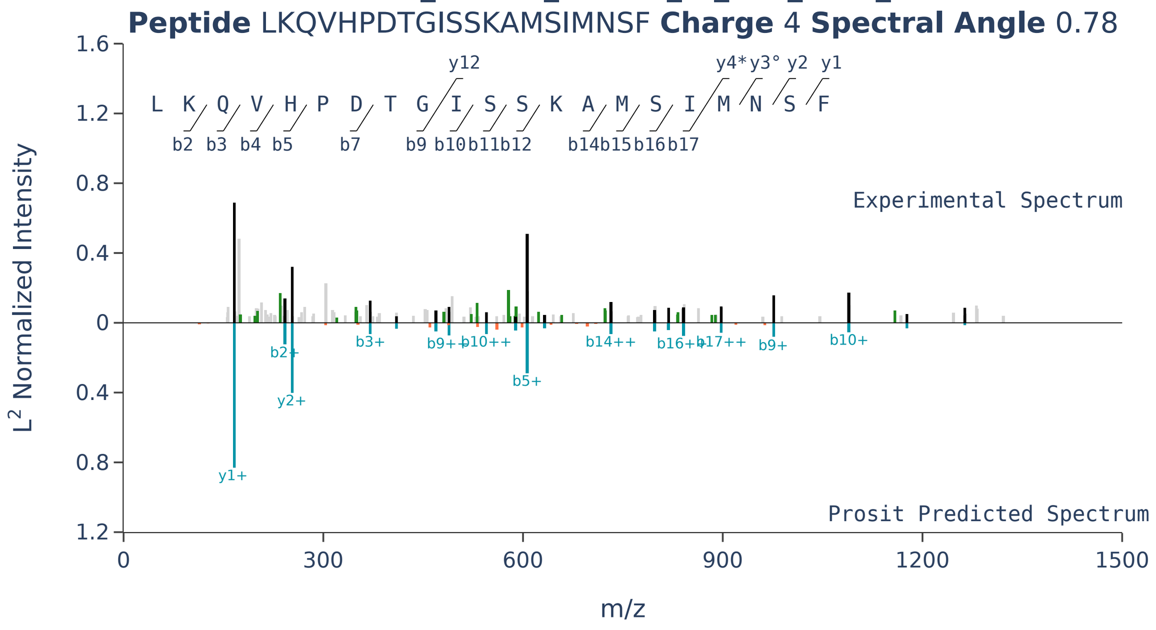


**Source** HRoetschke_291222_080123_Fu_H2B_b1R2_2h **Scan** 4667
**Peptide** RLAGEASRLA **Charge** 3 **Spectral Angle** 0.8

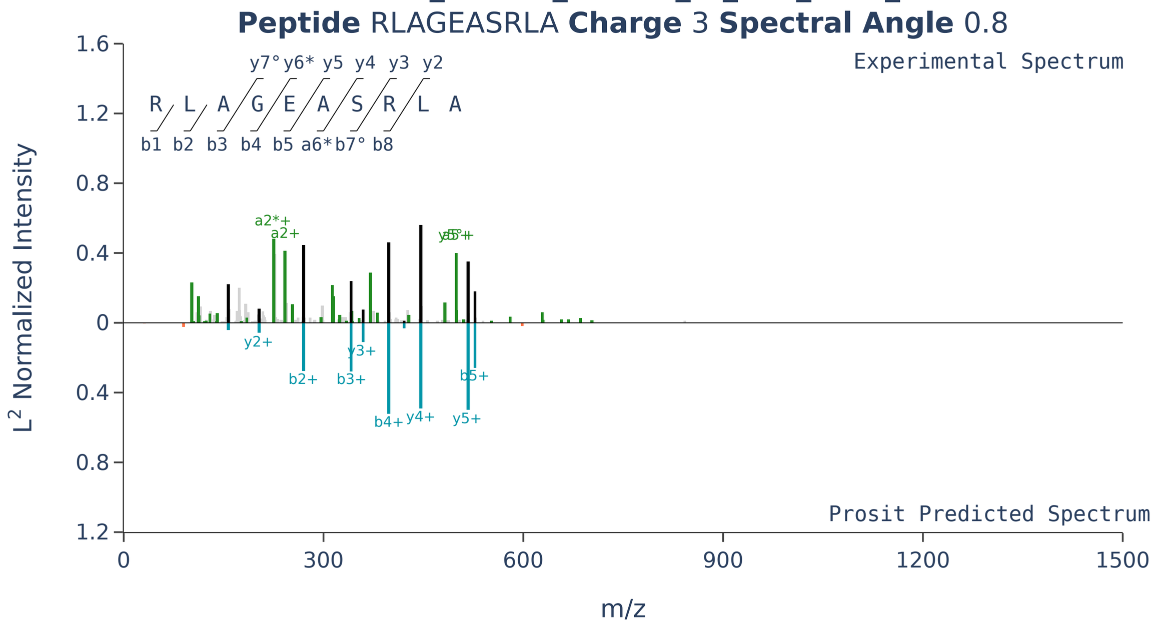


**Source** HRoetschke_291222_080123_Fu_H2B_b2R1_24h **Scan** 10250
**Peptide** RKTRKESYAIYVYKV **Charge** 4 **Spectral Angle** 0.89

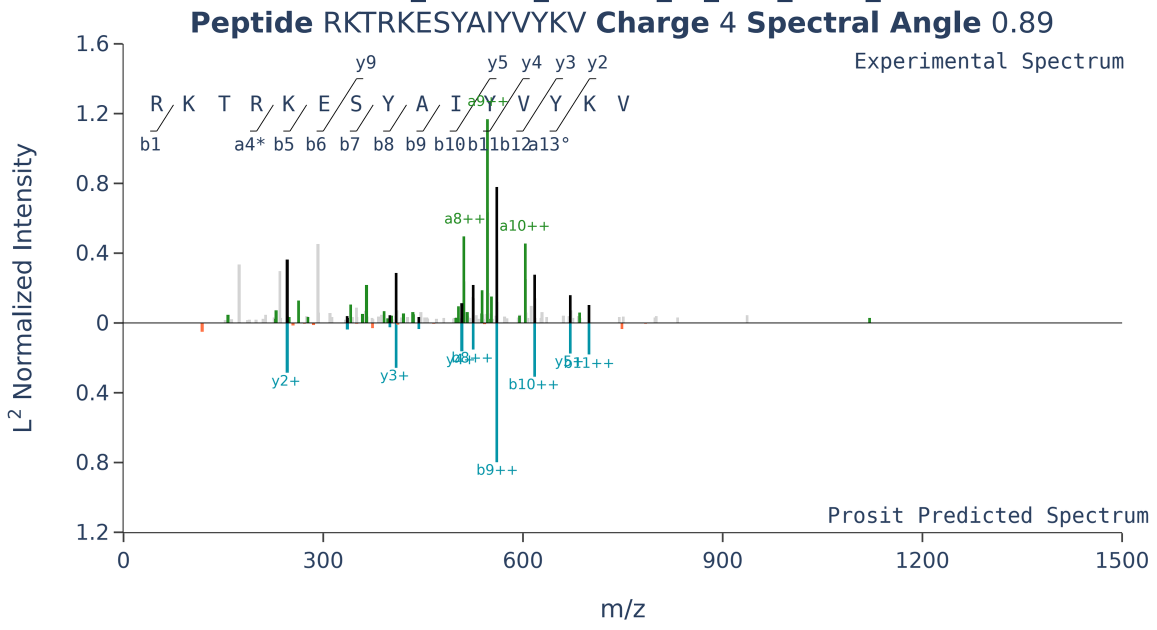


**Source** HRoetschke_291222_080123_Fu_H2B_b2R2_24h **Scan** 3945
**Peptide** AGEASRLAHYNKRSTITSR **Charge** 4 **Spectral Angle** 0.82

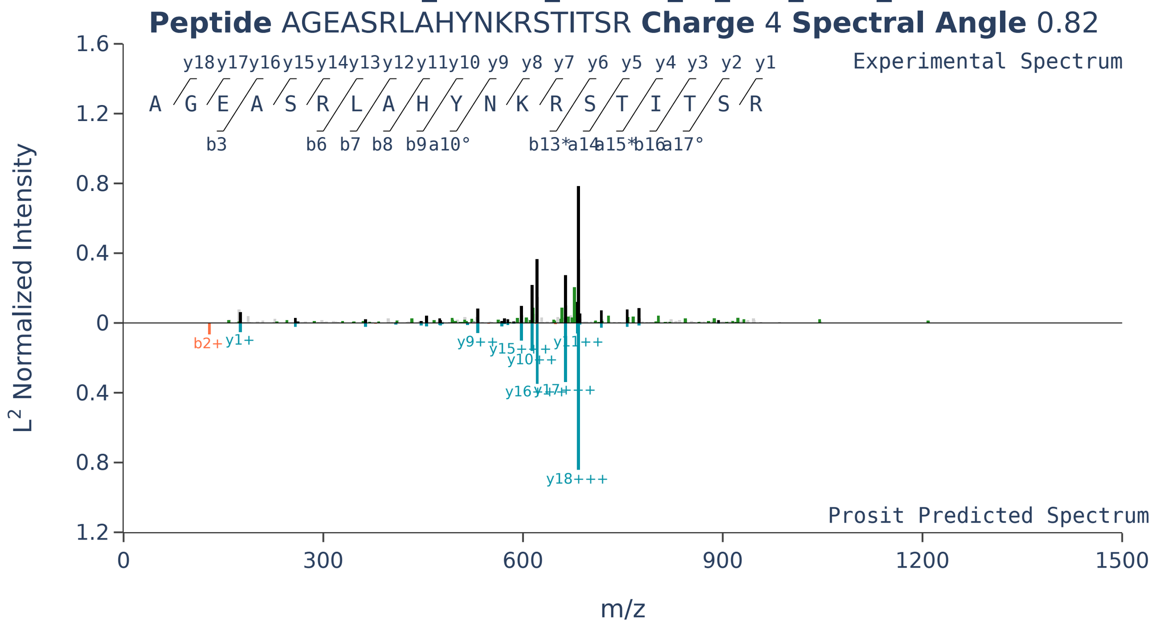


**Source** HRoetschke_291222_080123_Fu_H2B_b1R1_24h **Scan** 6991
**Peptide** DGKKRRKTRKESYAIYVY **Charge** 4 **Spectral Angle** 0.78

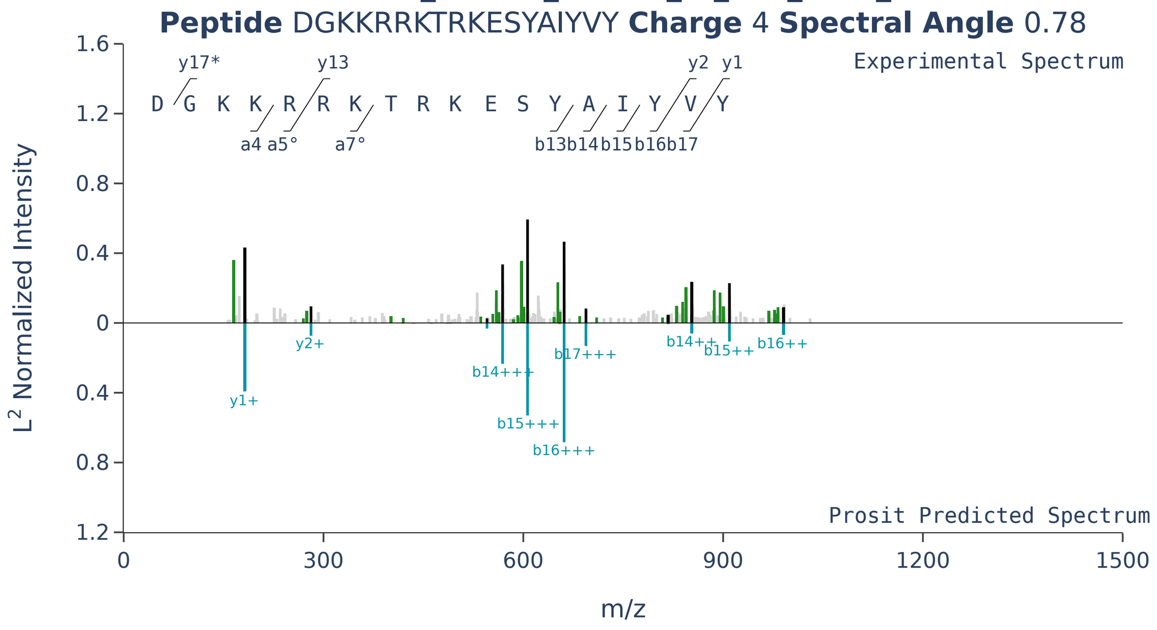


**Source** HRoetschke_291222_080123_Fu_H2B_b1R1_2h **Scan** 2668
**Peptide** KQVHPDTGISS **Charge** 2 **Spectral Angle** 0.81

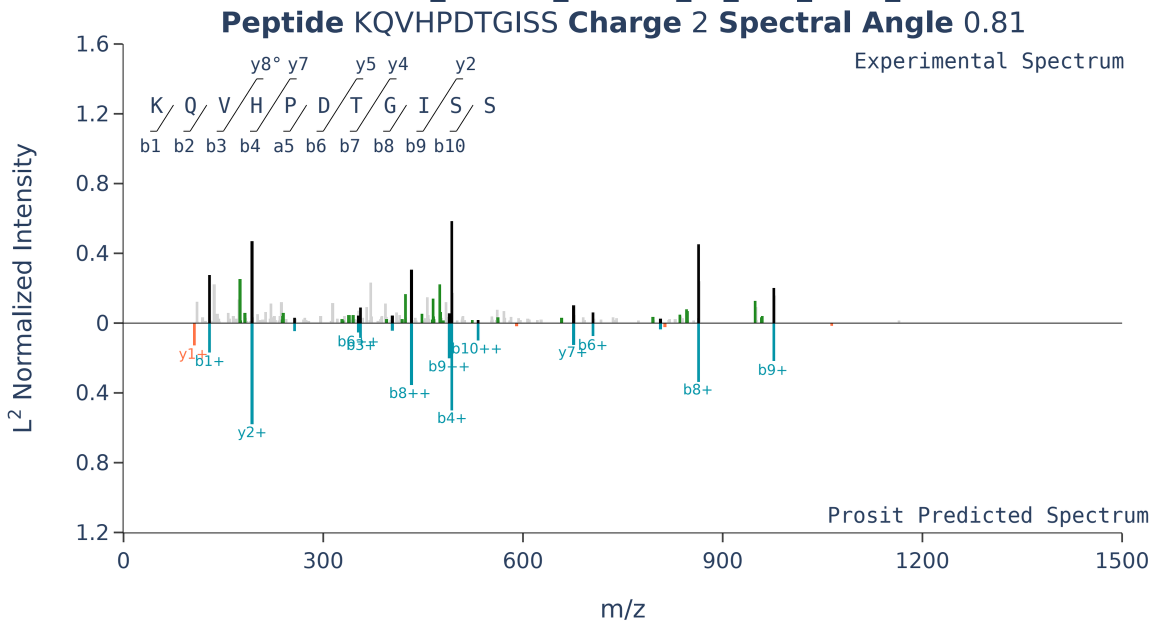


**Source** HRoetschke_291222_080123_Fu_H2B_b2R1_4h **Scan** 20443
**Peptide** VNDVFERIAGEASRLA **Charge** 3 **Spectral Angle** 0.83

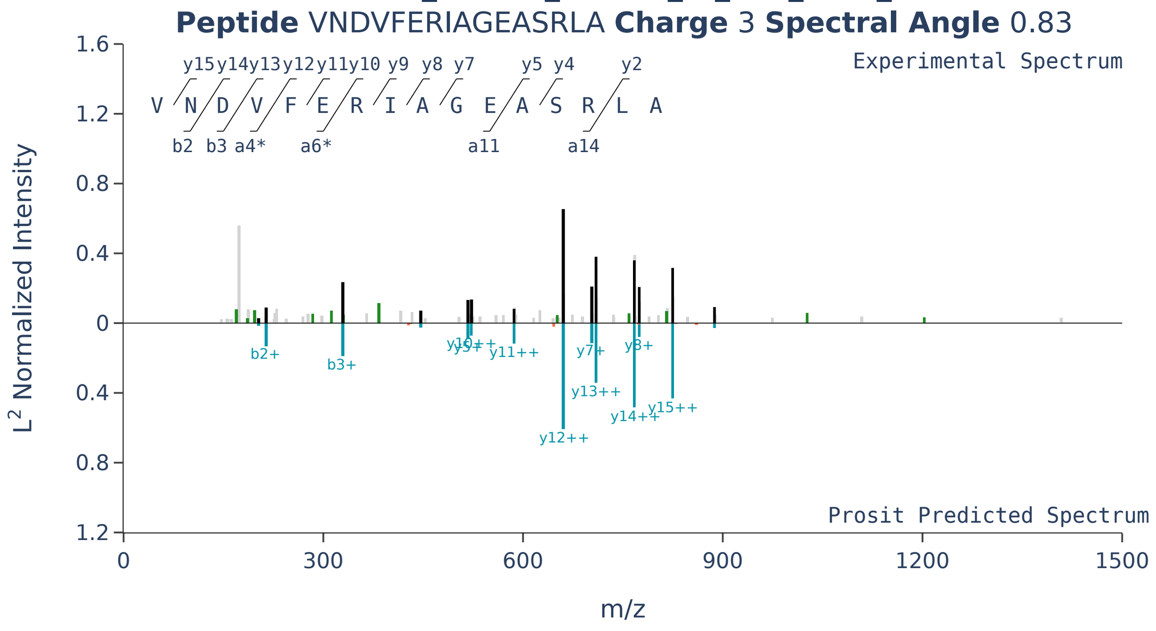


**Source** HRoetschke_291222_080123_Fu_H2B_b1R2_24h **Scan** 11570
**Peptide** STITSREIQTAV **Charge** 2 **Spectral Angle** 0.86

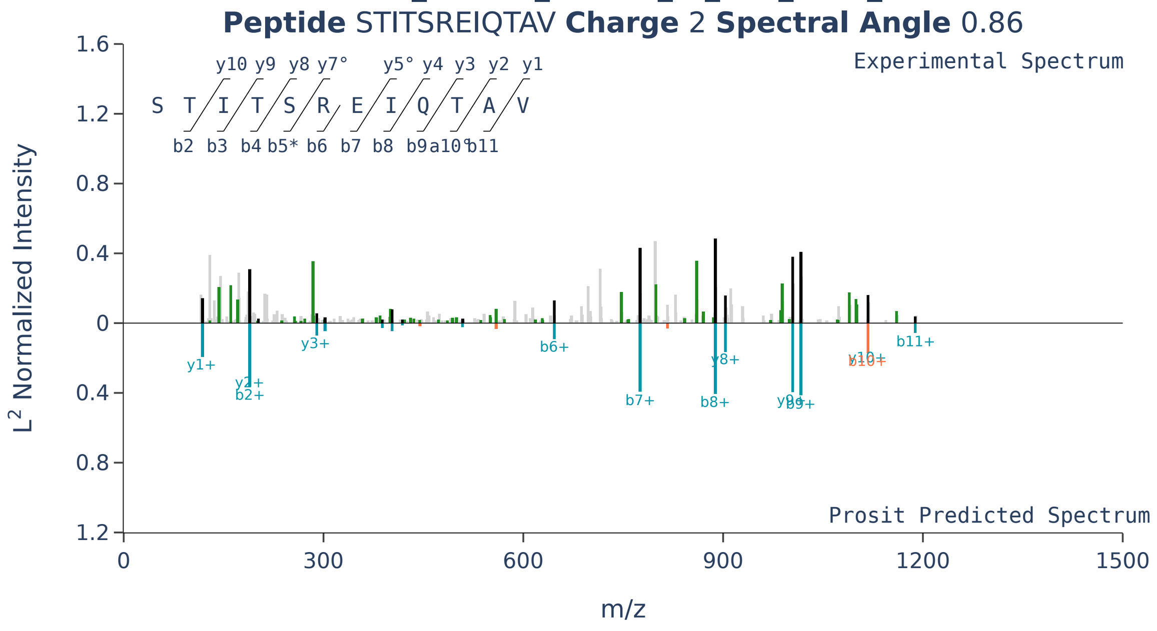


**Source** HRoetschke_291222_080123_Fu_H2B_b2R1_4h **Scan** 18368
**Peptide** AVRLLLPGELAKHAVSEGTKAVTKYTSA **Charge** 5 **Spectral Angle** 0.88

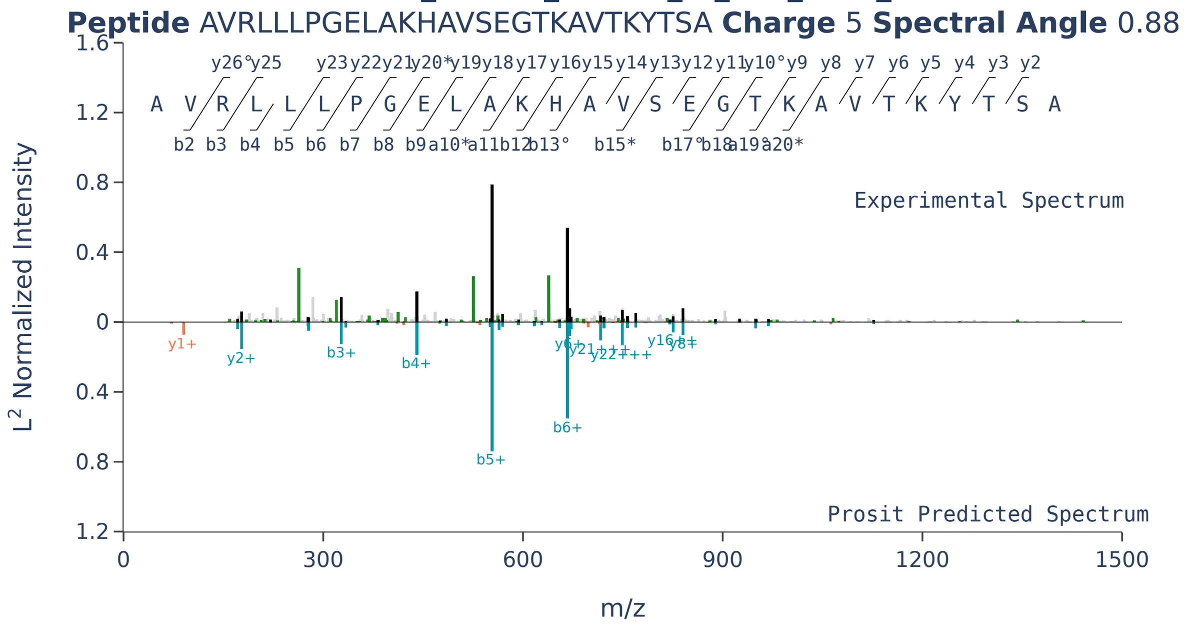


**Source** HRoetschke_291222_080123_Fu_H2B_b1R1_24h **Scan** 6042
**Peptide** LAGEASRLAHYNKRSTITSR **Charge** 4 **Spectral Angle** 0.84

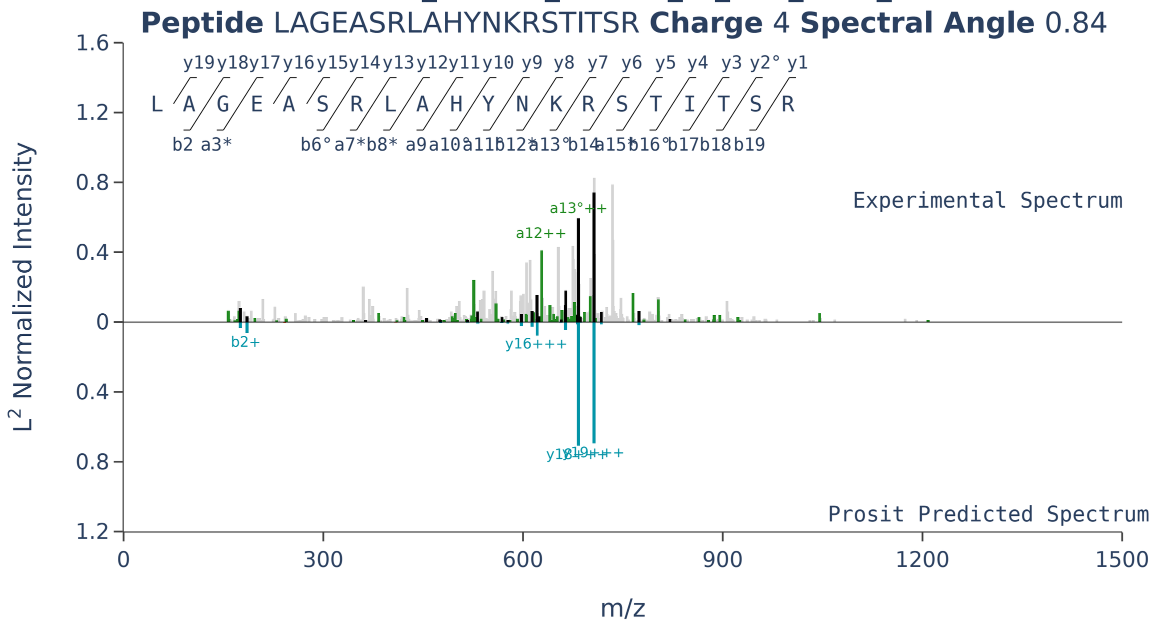


**Source** HRoetschke_291222_080123_Fu_H2B_b1R2_1h **Scan** 13855
**Peptide** REIQTAVRLLLPGELAKHA **Charge** 4 **Spectral Angle** 0.72

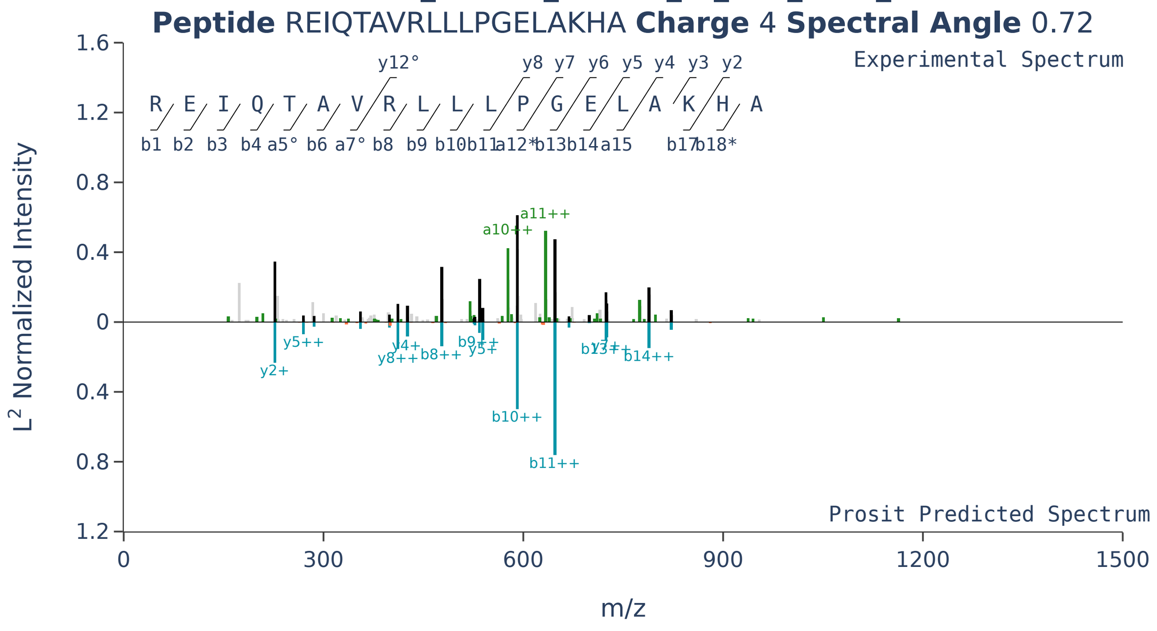


**Source** HRoetschke_291222_080123_Fu_H2B_b1R2_2h **Scan** 14604
**Peptide** AVRLLLPGE **Charge** 2 **Spectral Angle** 0.87

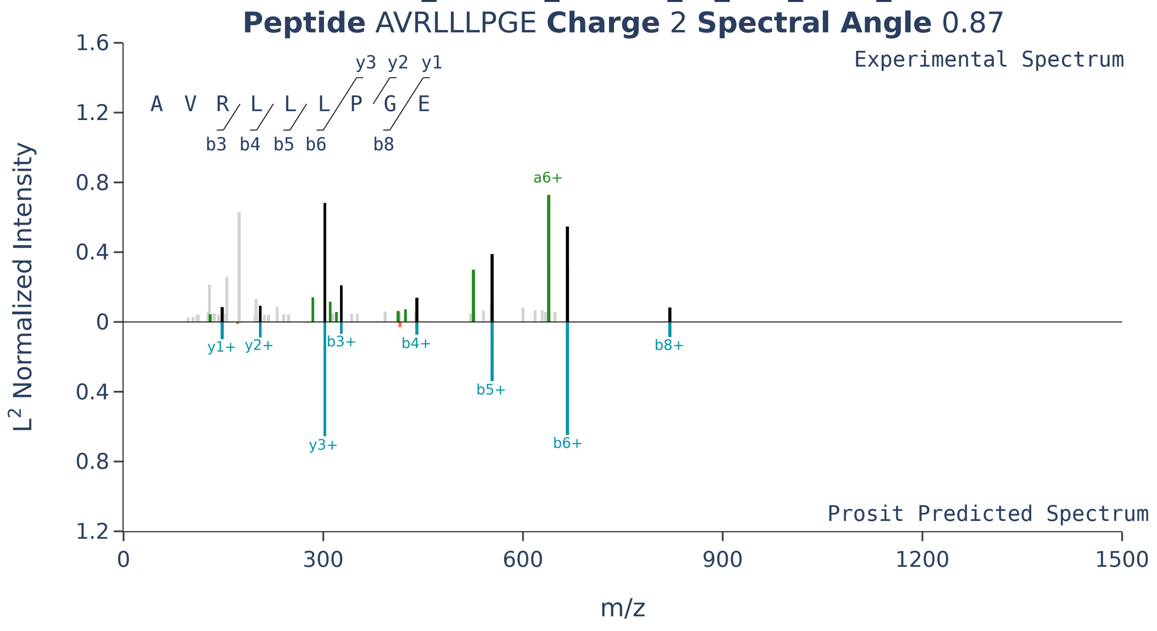


**Source** HRoetschke_291222_080123_Fu_H2B_b2R2_24h **Scan** 6989
**Peptide** ERIAGEASRLAHYNKRSTITSR **Charge** 5 **Spectral Angle** 0.73

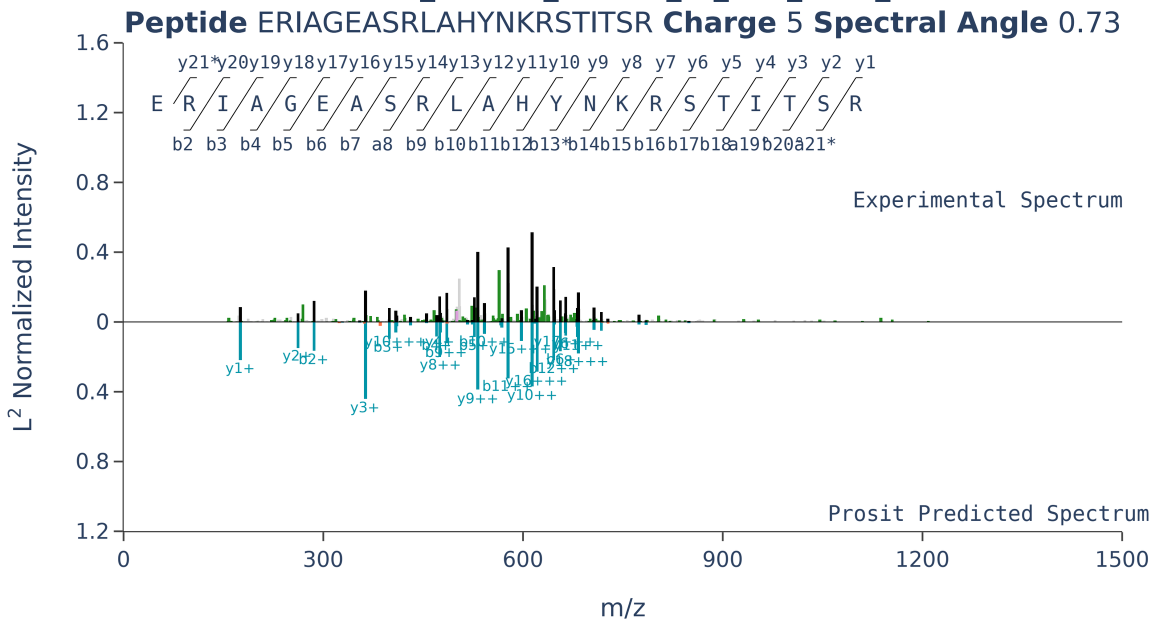


**Source** HRoetschke_291222_080123_Fu_H2B_b2R2_4h **Scan** 1874
**Peptide** AHYNKRSTITS **Charge** 2 **Spectral Angle** 0.91

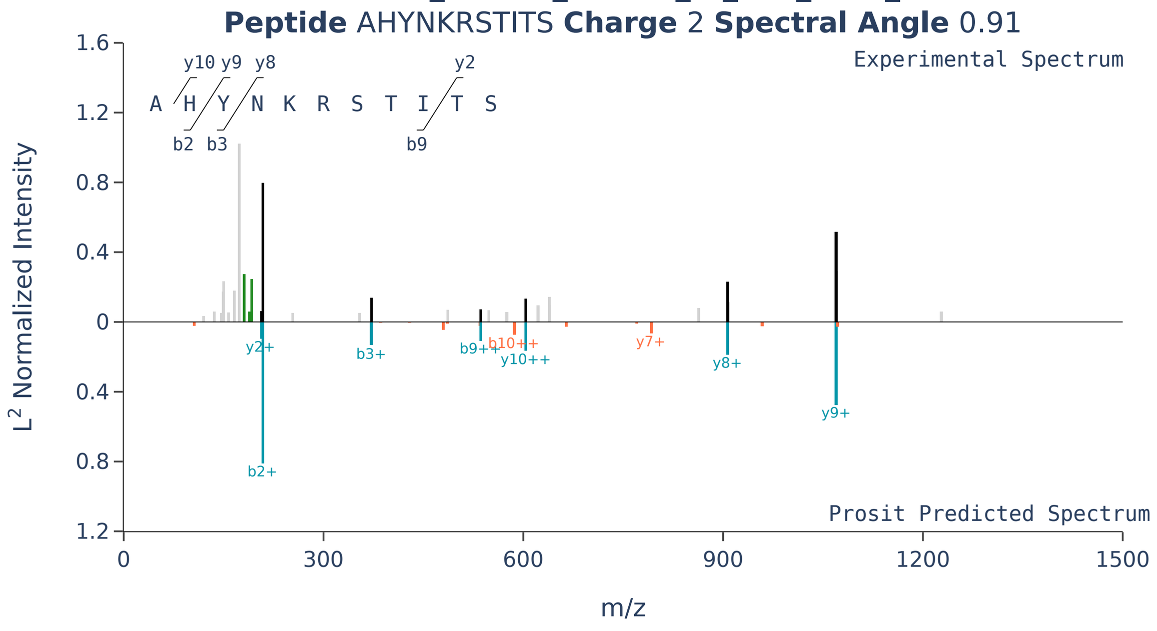

**Source** HRoetschke_291222_080123_Fu_H2B_b2R2_4h **Scan** 18904
**Peptide** VNDVFERIAGEASRLAHYNKRS **Charge** 5 **Spectral Angle** 0.84

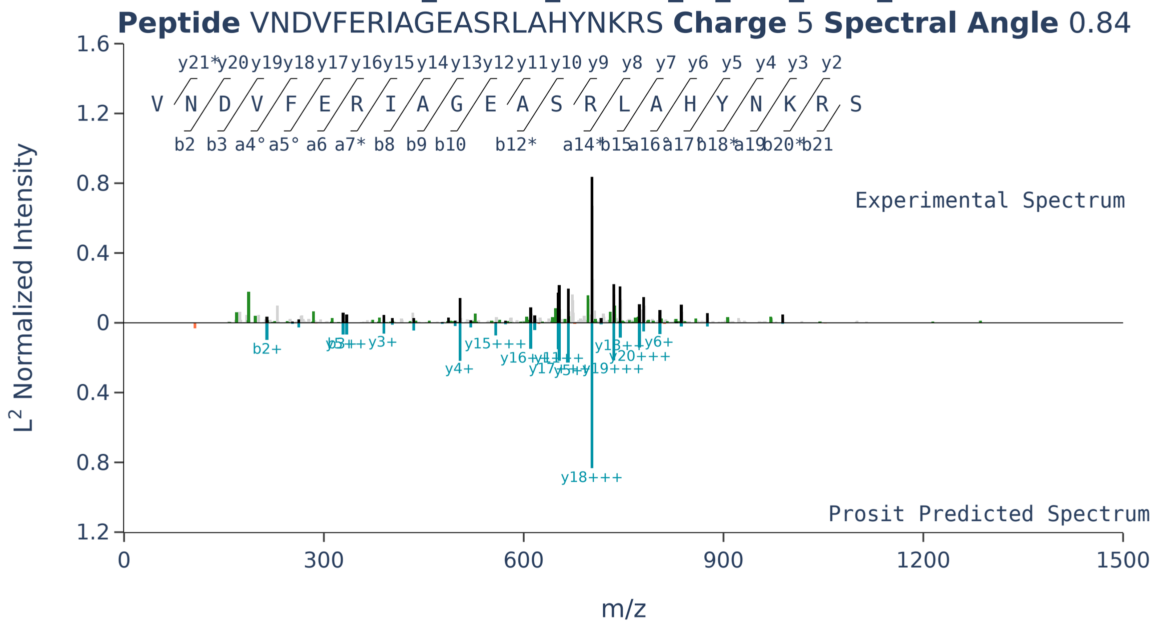


**Source** HRoetschke_291222_080123_Fu_H2B_b1R1_1h **Scan** 3916
**Peptide** AGEASRLAHYN **Charge** 2 **Spectral Angle** 0.8

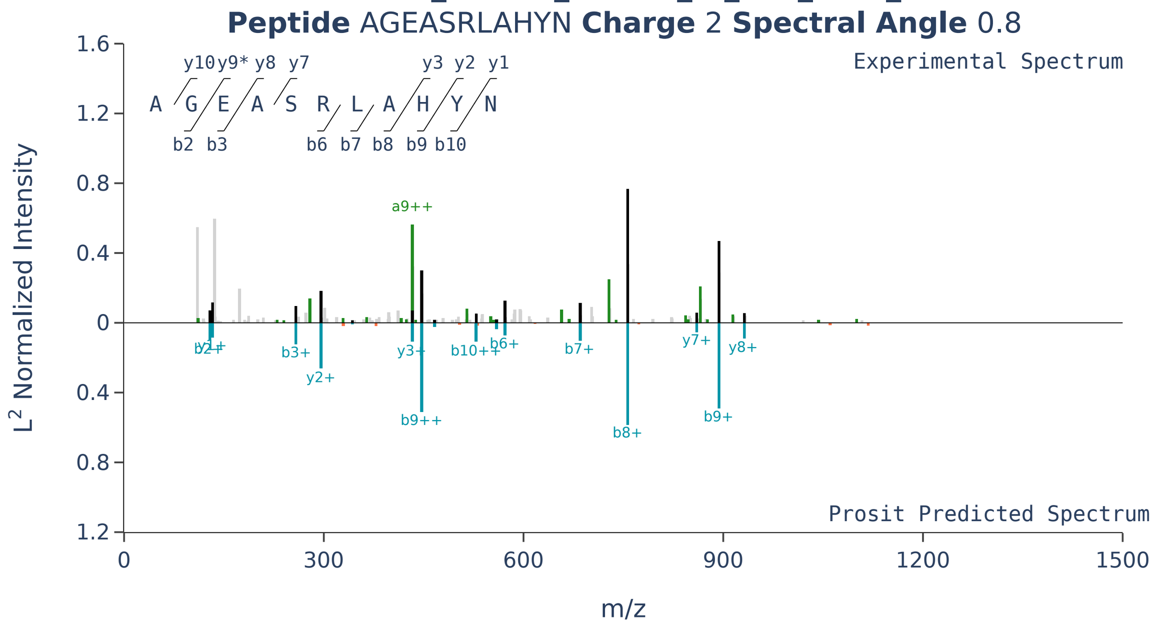


**Source** HRoetschke_291222_080123_Fu_H2B_b1R2_4h **Scan** 12507
**Peptide** KESYAIYV **Charge** 2 **Spectral Angle** 0.74

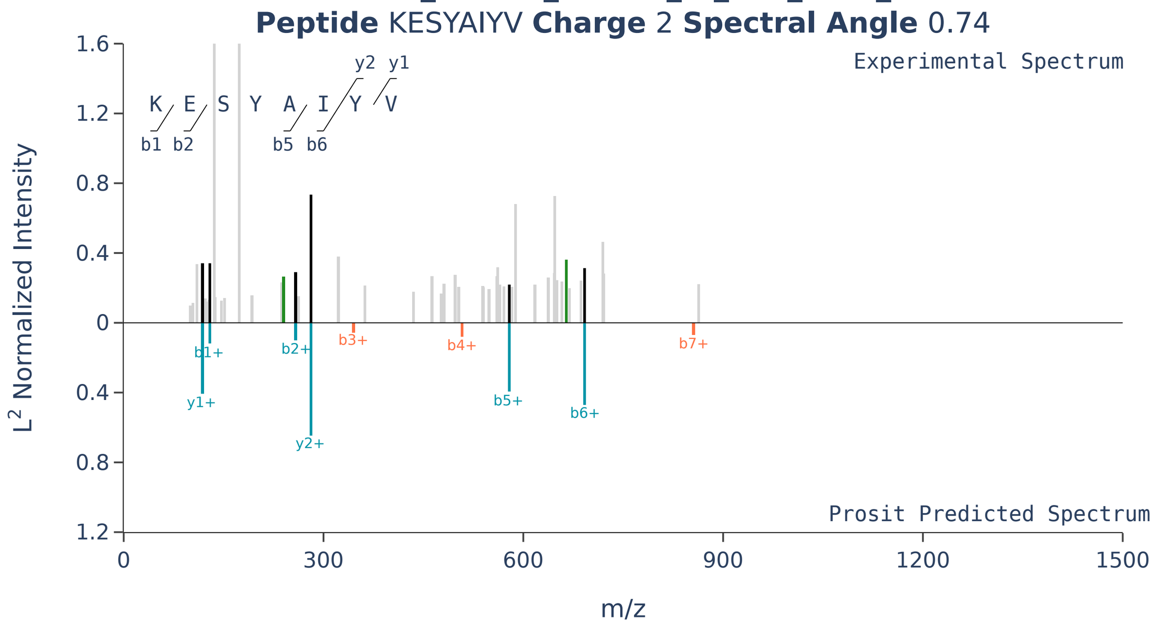


**Source** HRoetschke_291222_080123_Fu_H2B_b2R2_24h **Scan** 18590
**Peptide** VRLLLPGELAKHAVSEGTKAVTKYTS **Charge** 5 **Spectral Angle** 0.89

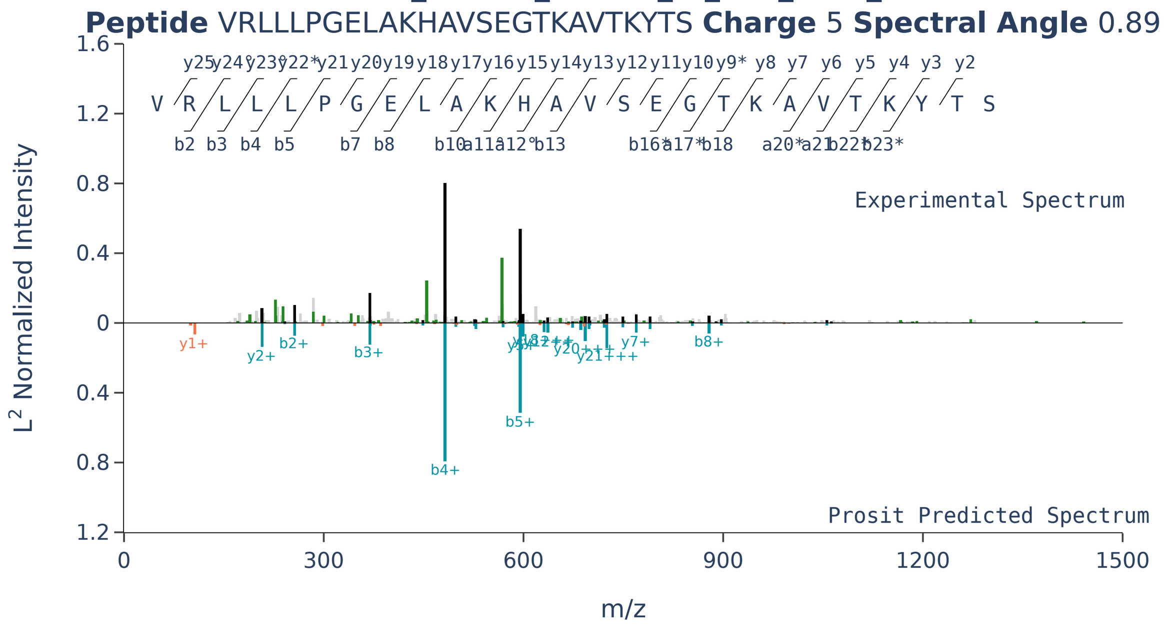


**Source** HRoetschke_291222_080123_Fu_H2B_b1R1_1h **Scan** 11554
**Peptide** RLLLPGELAKHAVSE **Charge** 3 **Spectral Angle** 0.81

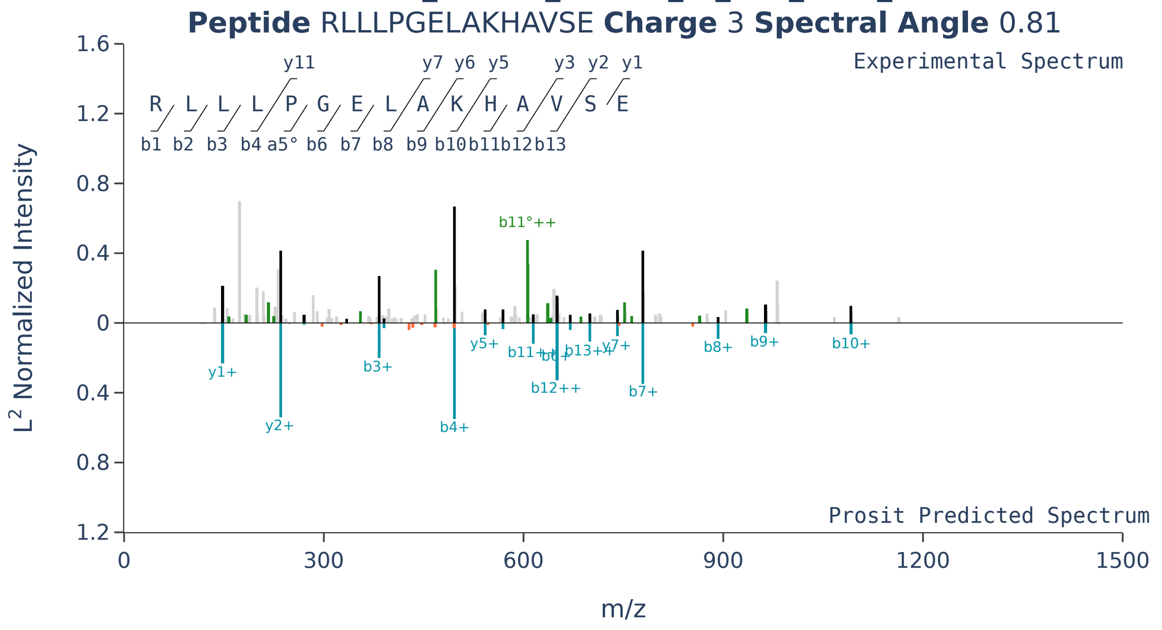


**Source** HRoetschke_291222_080123_Fu_H2B_b2R1_1h **Scan** 5137
**Peptide** SREIQTAV **Charge** 2 **Spectral Angle** 0.8

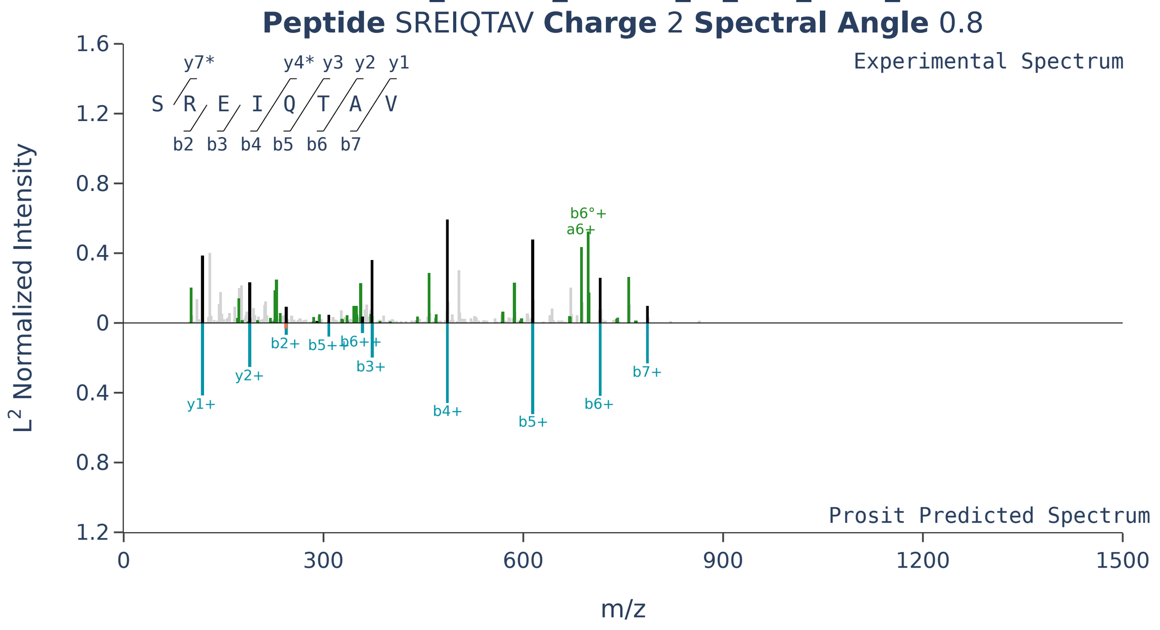


**Source** HRoetschke_291222_080123_Fu_H2B_b2R1_2h **Scan** 2141
**Peptide** LAHYNKRSTITSR **Charge** 3 **Spectral Angle** 0.82

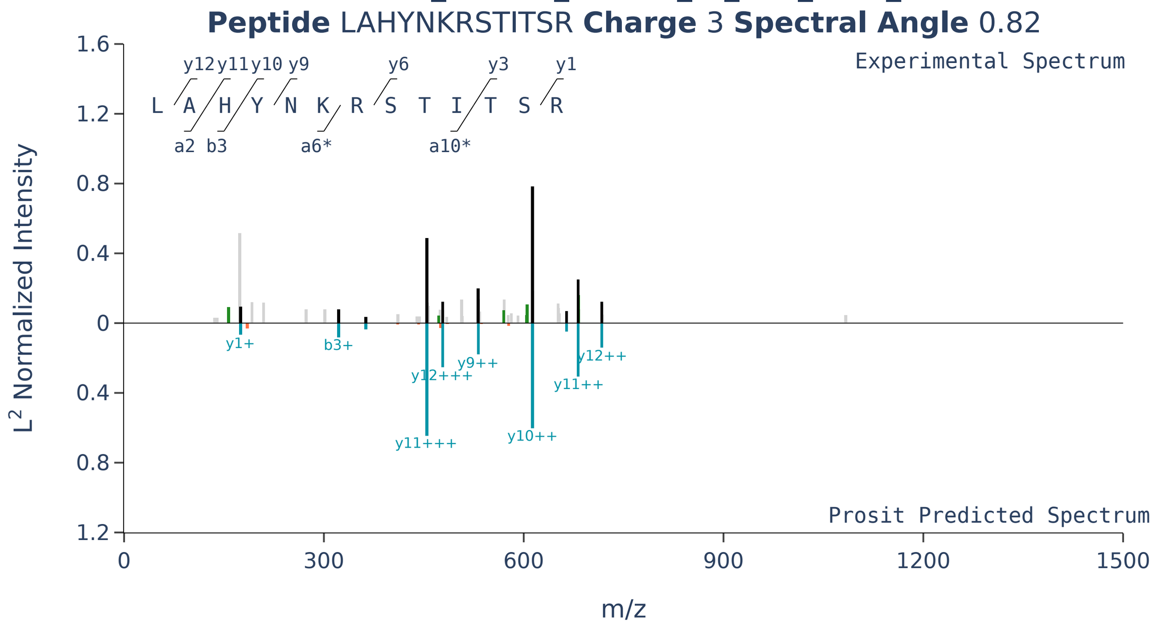


**Source** HRoetschke_291222_080123_Fu_H2B_b2R2_2h **Scan** 4524
**Peptide** RSTITSREIQT **Charge** 3 **Spectral Angle** 0.68

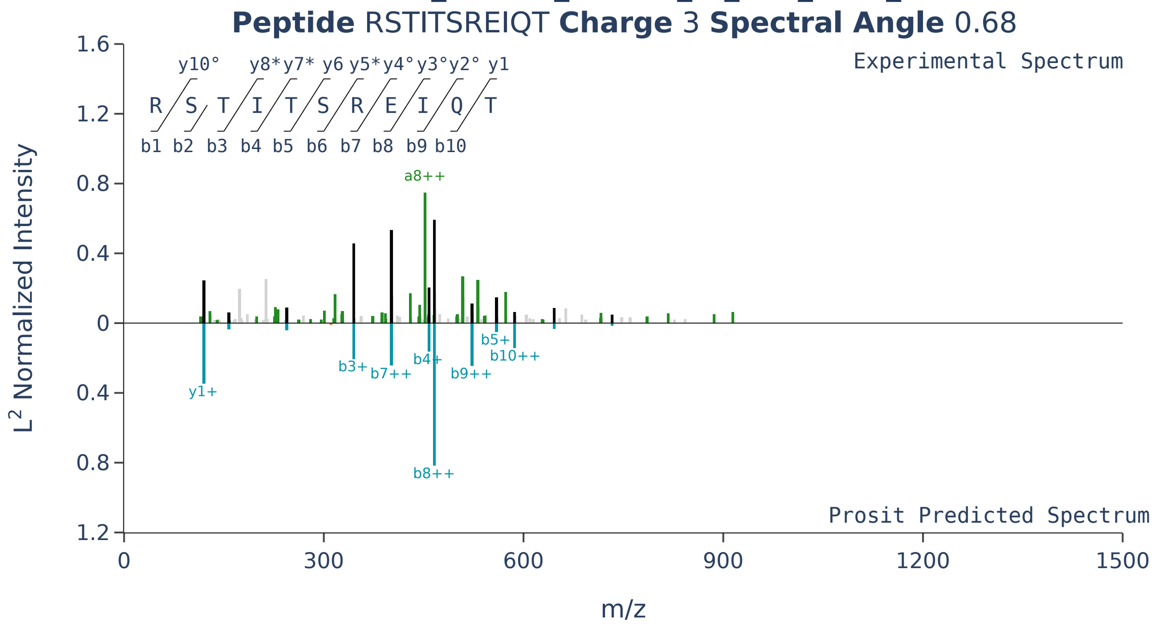


**Source** HRoetschke_291222_080123_Fu_H2B_b1R1_2h **Scan** 15039
**Peptide** SREIQTAVRLLLPGELAKH **Charge** 4 **Spectral Angle** 0.75

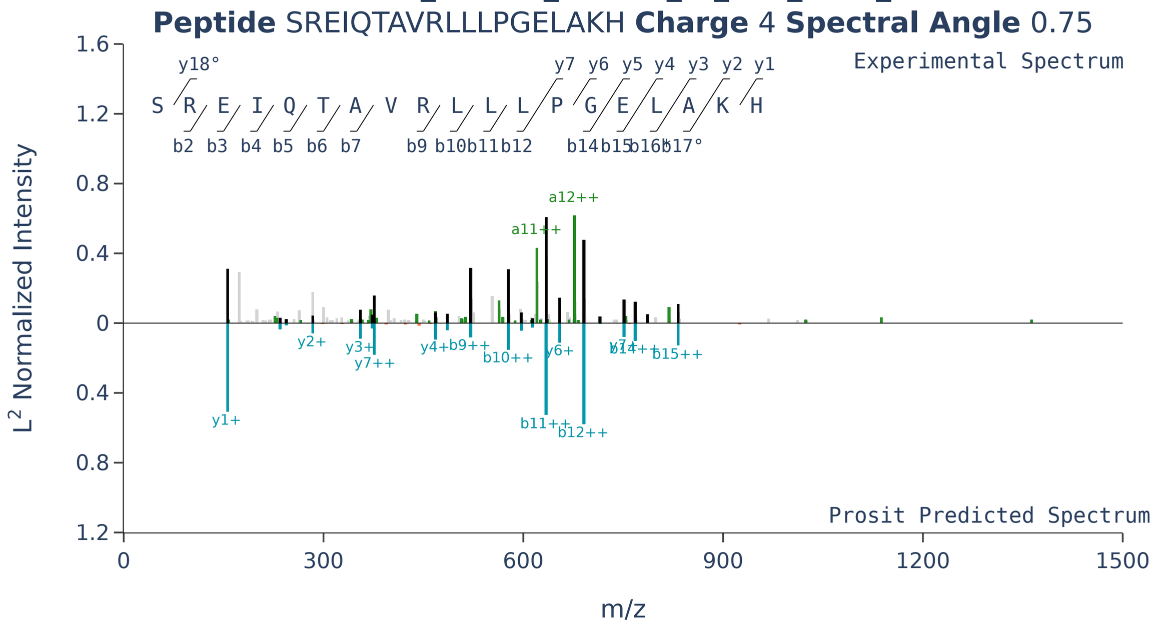


**Source** HRoetschke_291222_080123_Fu_H2B_b1R1_24h **Scan** 4538
**Peptide** AKHAVSEGTKAVTKYTSA **Charge** 3 **Spectral Angle** 0.77

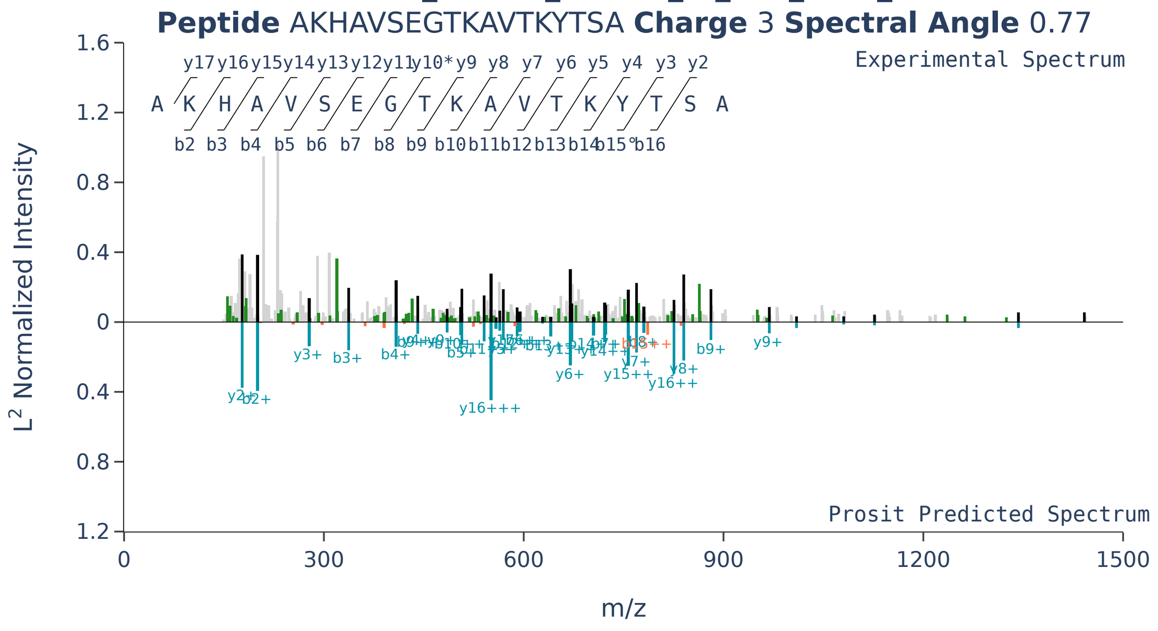


**Source** HRoetschke_291222_080123_Fu_H2B_b1R1_24h **Scan** 7534
**Peptide** IAGEASRLAHYN **Charge** 2 **Spectral Angle** 0.79

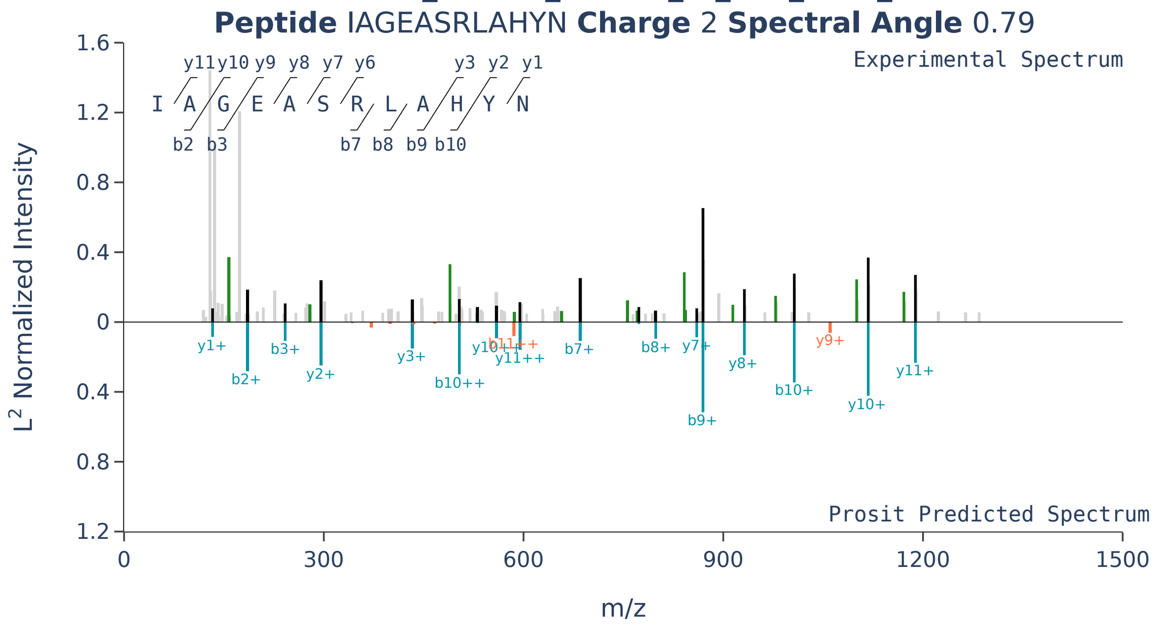


**Source** HRoetschke_291222_080123_Fu_H2B_b2R2_24h **Scan** 5926
**Peptide** KKRRKTRKESYAIYVY **Charge** 4 **Spectral Angle** 0.76

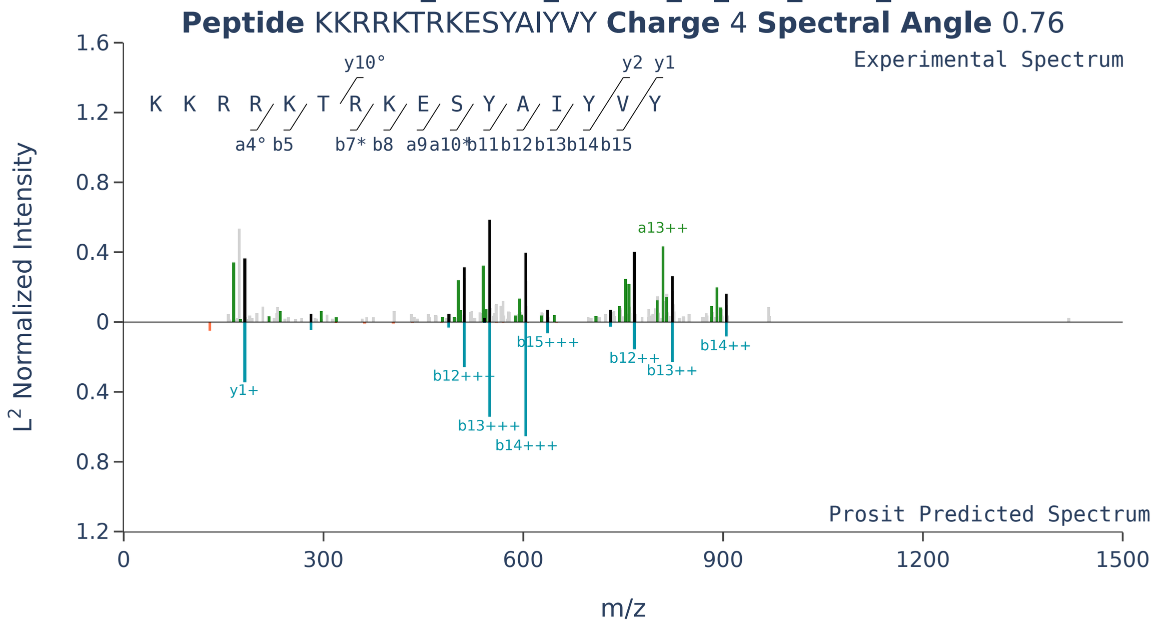


**Source** HRoetschke_291222_080123_Fu_H2B_b1R2_2h **Scan** 17903
**Peptide** SYAIYVYKVL **Charge** 2 **Spectral Angle** 0.92

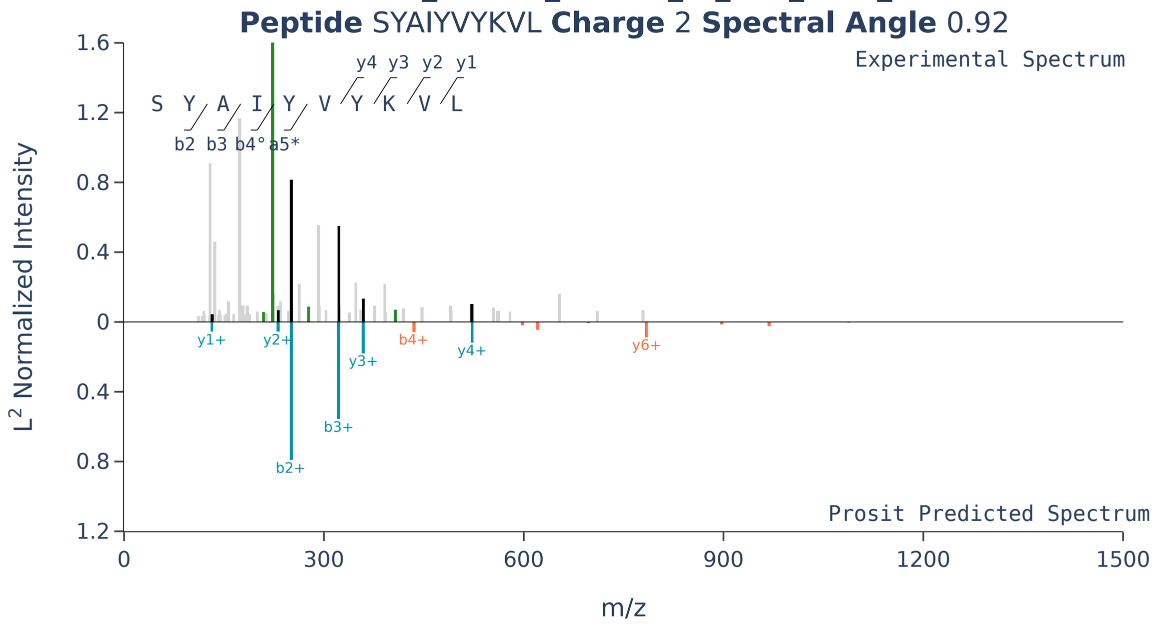


**Source** HRoetschke_291222_080123_Fu_H2B_b2R2_1h **Scan** 18177
**Peptide** SFVNDVFERIAGEASR **Charge** 3 **Spectral Angle** 0.87

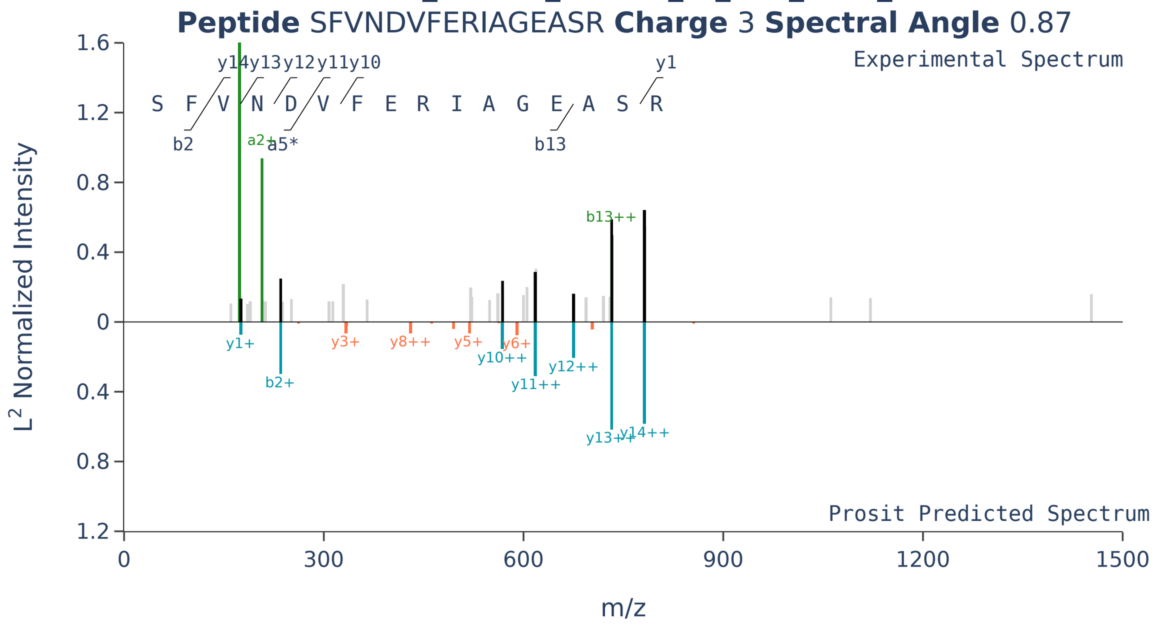


**Source** HRoetschke_291222_080123_Fu_H2B_b2R1_24h **Scan** 3846
**Peptide** PGELAKHAV **Charge** 3 **Spectral Angle** 0.91

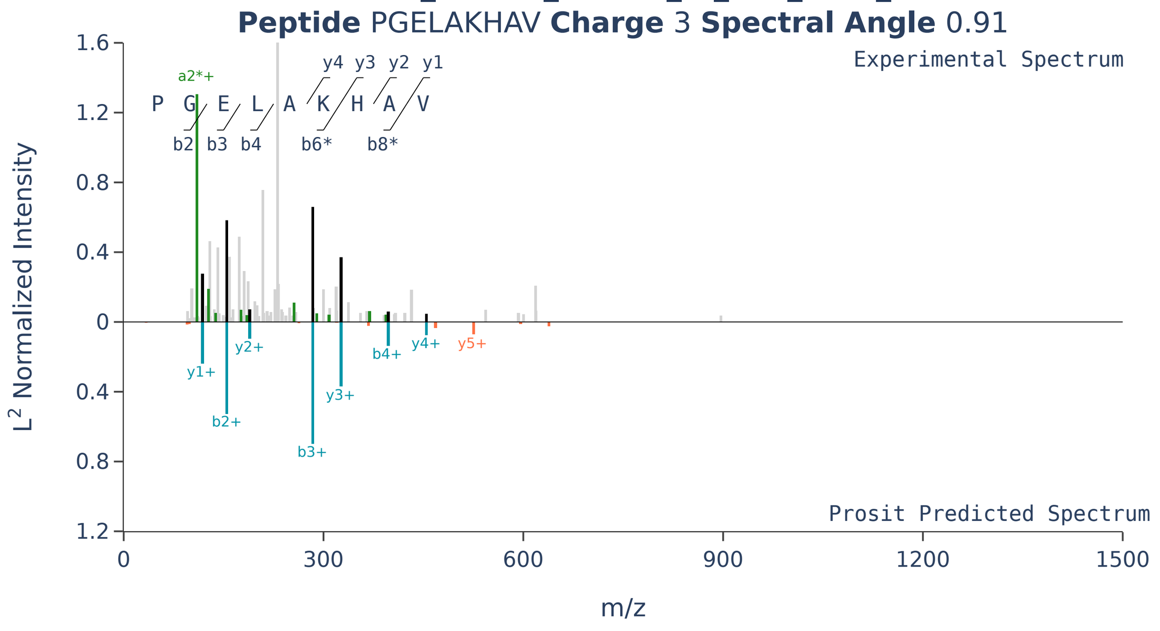

**Source** HRoetschke_291222_080123_Fu_H2B_b1R1_24h **Scan** 5939
**Peptide** VLKQVHPDTGISSKAM **Charge** 4 **Spectral Angle** 0.82

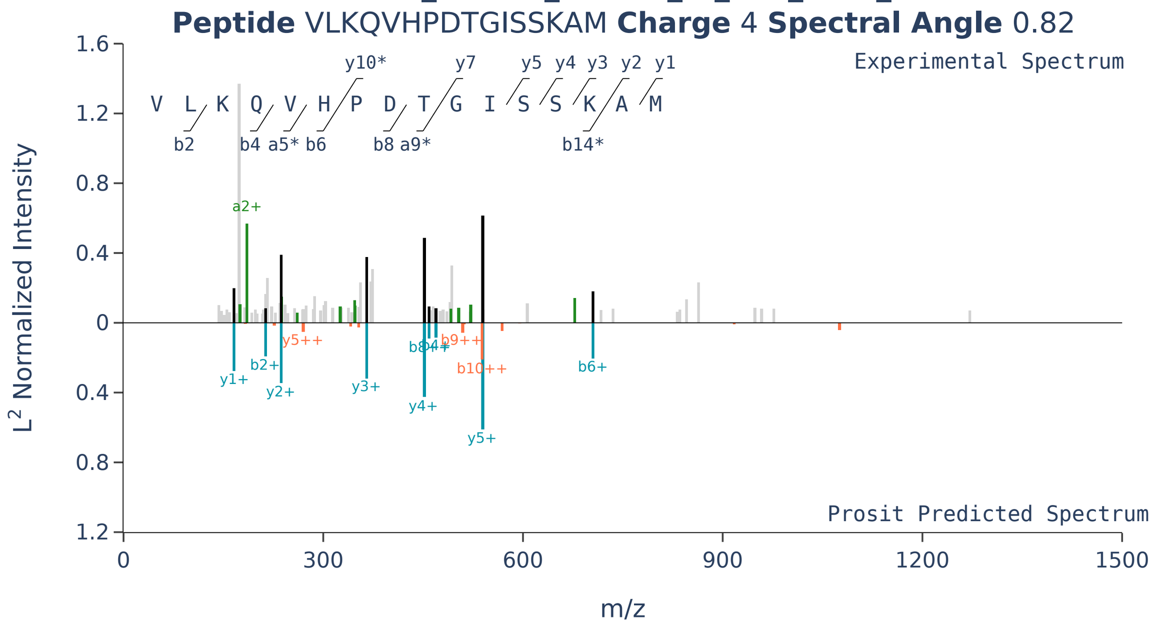


**Source** HRoetschke_291222_080123_Fu_H2B_b1R2_24h **Scan** 7220
**Peptide** AHYNKRSTITSREIQTAV **Charge** 3 **Spectral Angle** 0.83

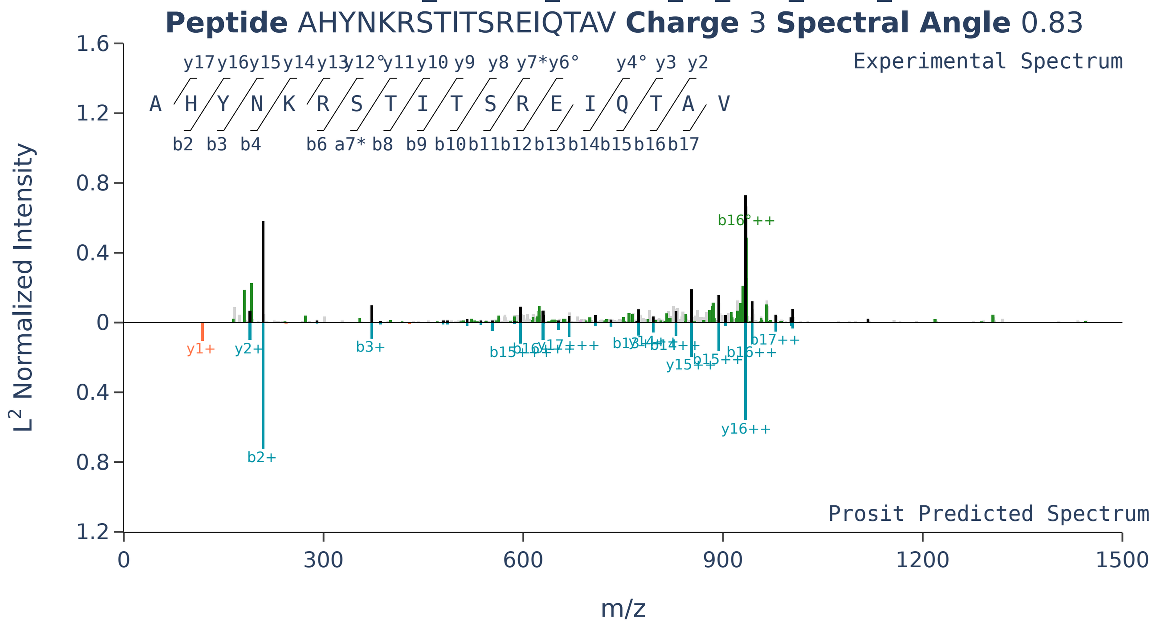


**Source** HRoetschke_291222_080123_Fu_H2B_b1R2_4h **Scan** 3623
**Peptide** KQVHPDTGL **Charge** 3 **Spectral Angle** 0.79

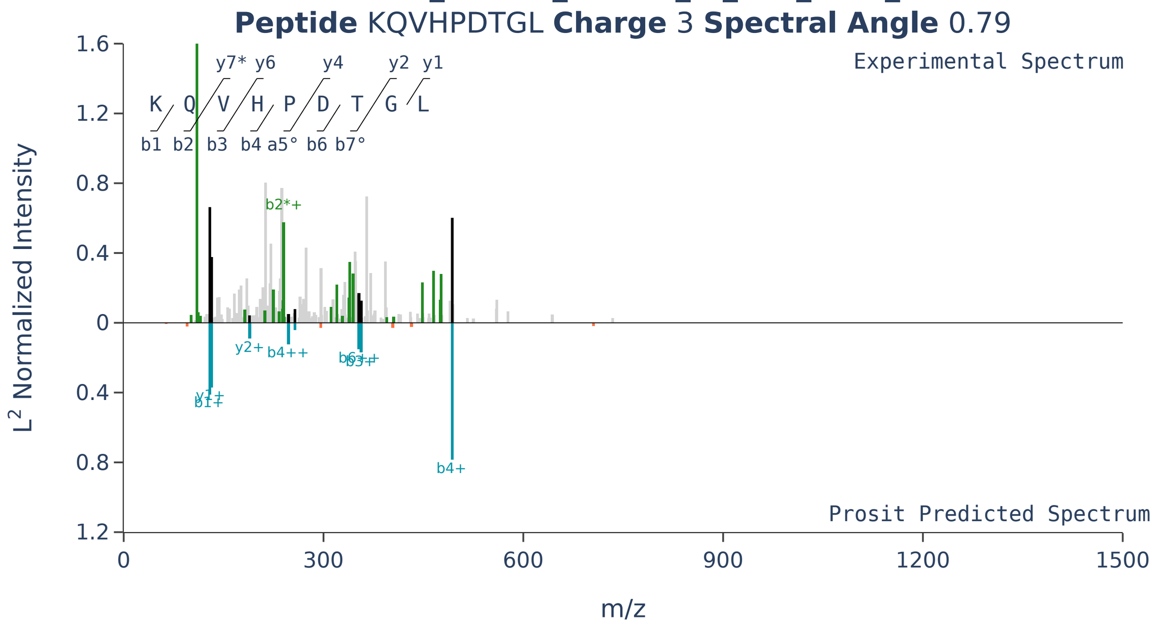


**Source** HRoetschke_291222_080123_Fu_H2B_b2R2_24h **Scan** 18018
**Peptide** LLLPGELAKHAVSEGTKAVTKY **Charge** 4 **Spectral Angle** 0.79

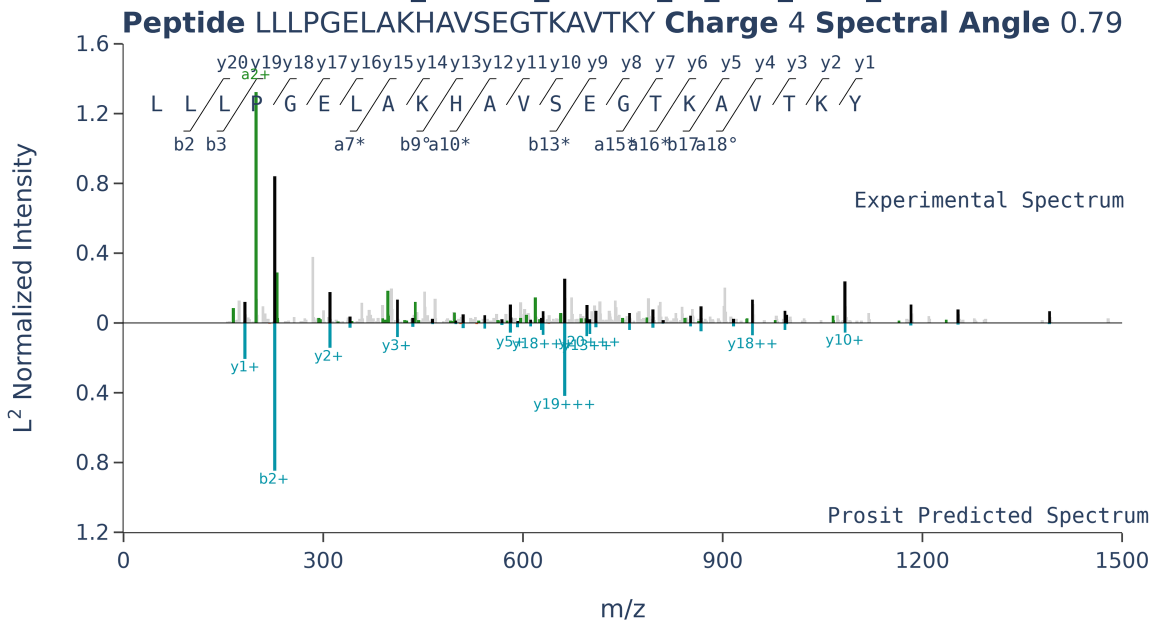


**Source** HRoetschke_291222_080123_Fu_H2B_b2R1_24h **Scan** 3665
**Peptide** EASRLAHYNKRSTL **Charge** 3 **Spectral Angle** 0.85

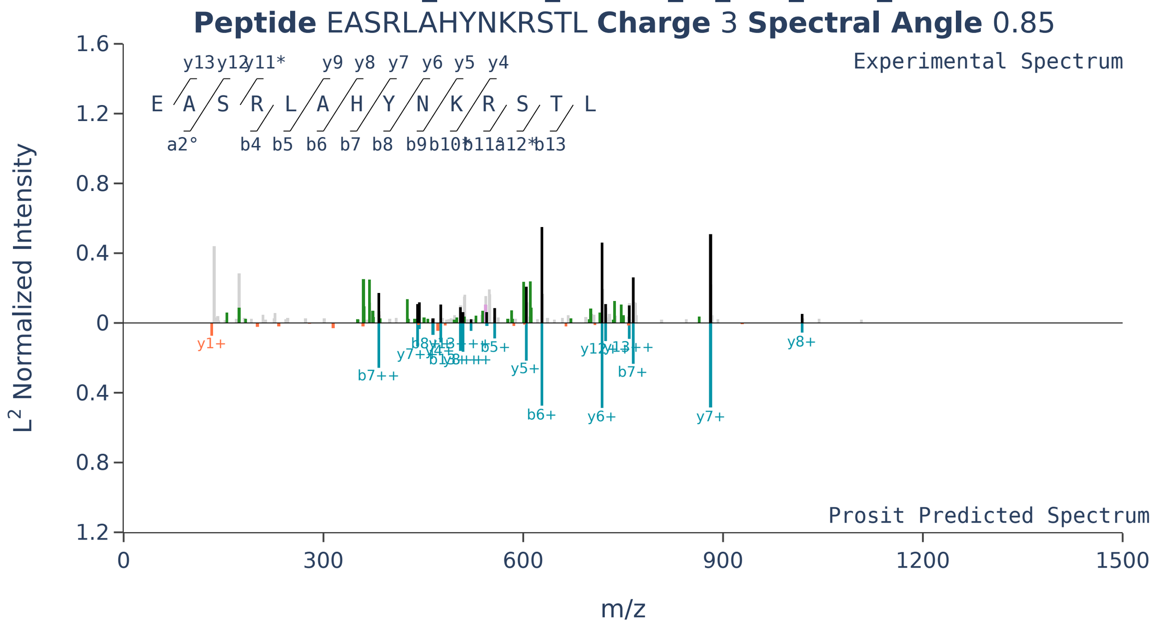


**Source** HRoetschke_291222_080123_Fu_H2B_b1R1_24h **Scan** 12084
**Peptide** HPDTGISSKAMSIMN **Charge** 3 **Spectral Angle** 0.89

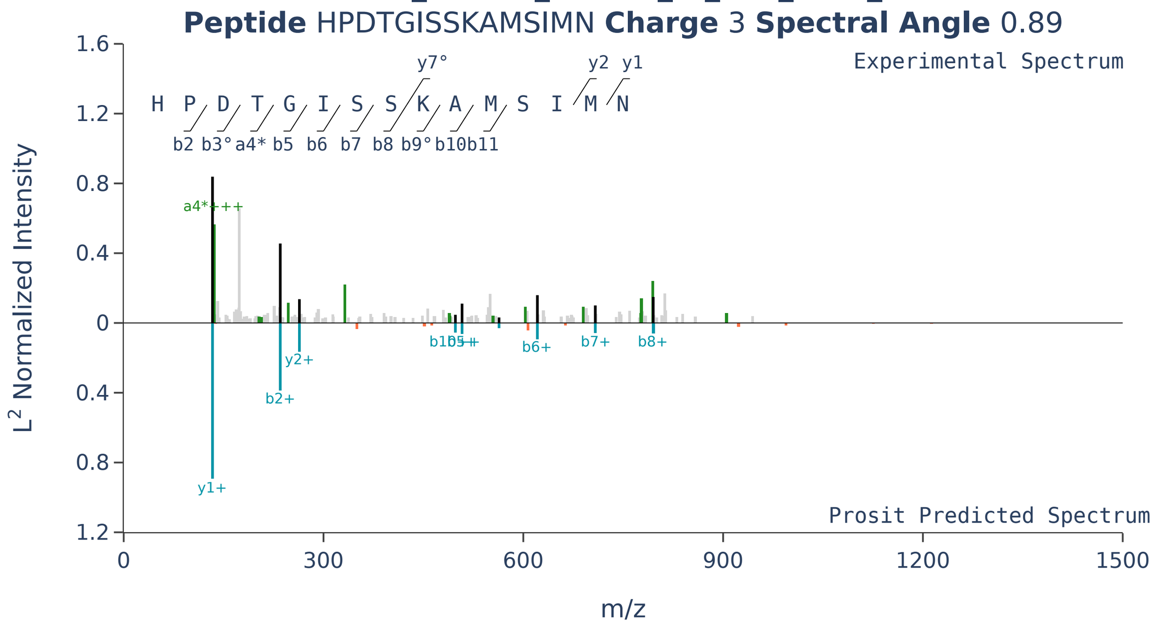


**Source** HRoetschke_291222_080123_Fu_H2B_b1R1_4h **Scan** 2204
**Peptide** SRLAHYNKRSTITSR **Charge** 4 **Spectral Angle** 0.8

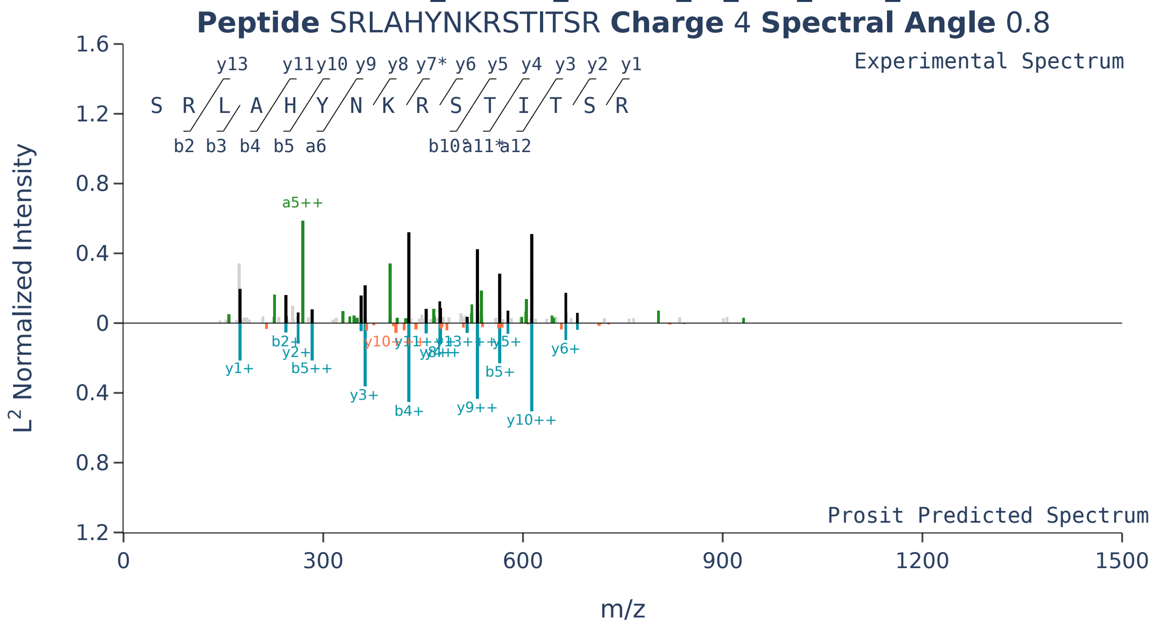


**Source** HRoetschke_291222_080123_Fu_H2B_b1R2_2h **Scan** 2451
**Peptide** GTKAVTKYT **Charge** 2 **Spectral Angle** 0.81

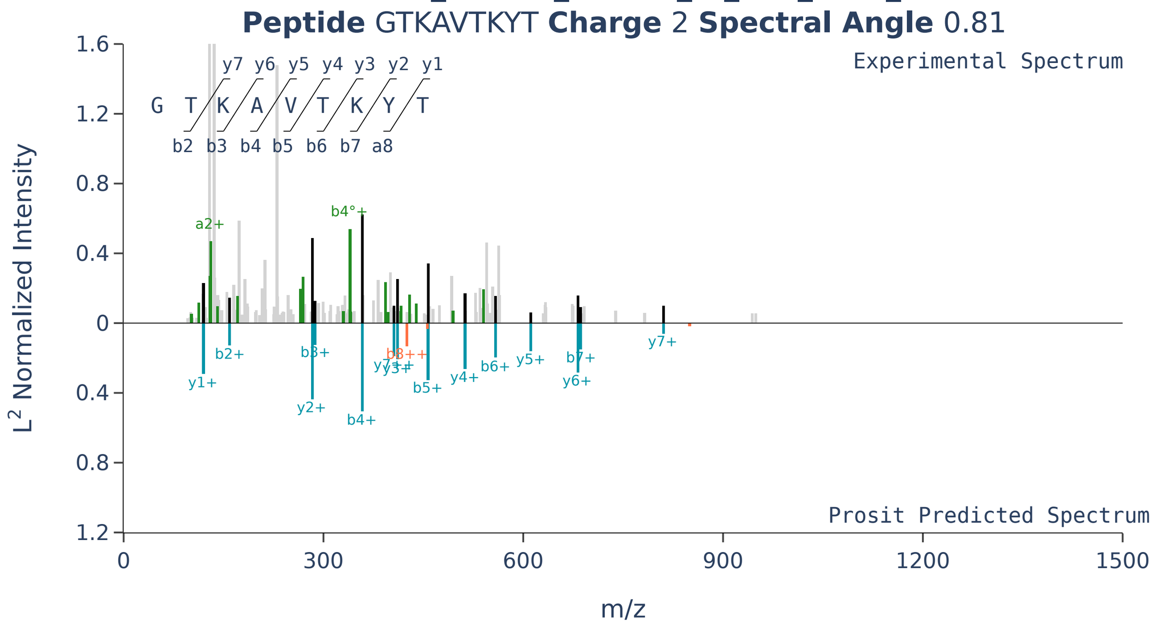


**Source** HRoetschke_291222_080123_Fu_H2B_b2R2_2h **Scan** 3811
**Peptide** RLAGEASRLAHYNKR **Charge** 3 **Spectral Angle** 0.79

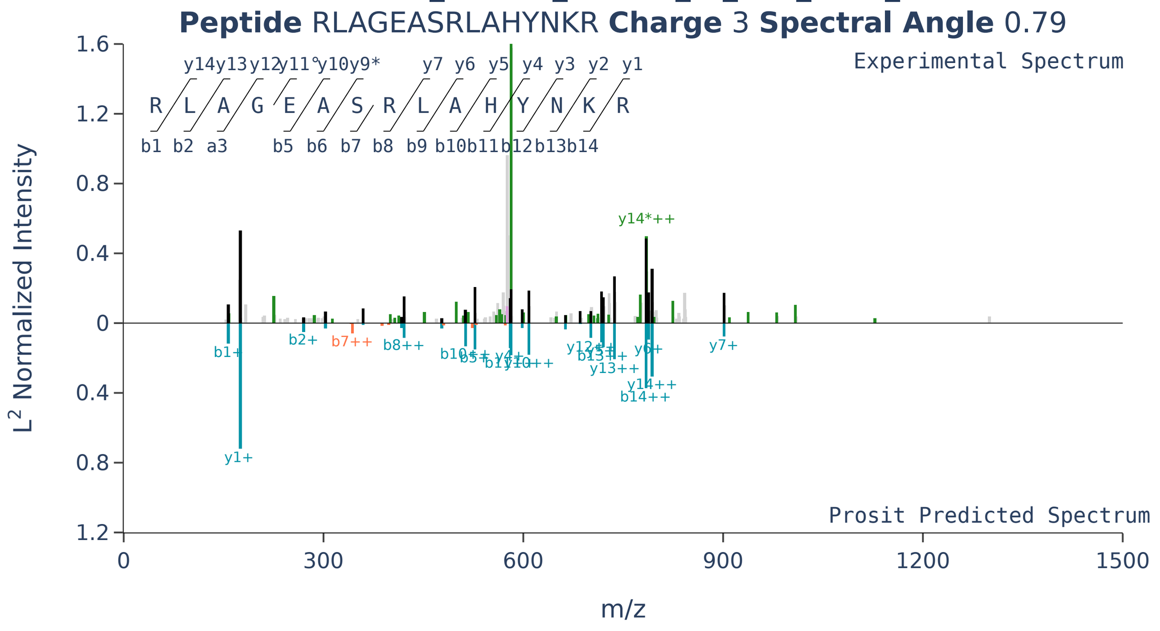


**Source** HRoetschke_291222_080123_Fu_H2B_b1R1_24h **Scan** 3791
**Peptide** HAVSEGTKAVTKY **Charge** 3 **Spectral Angle** 0.77

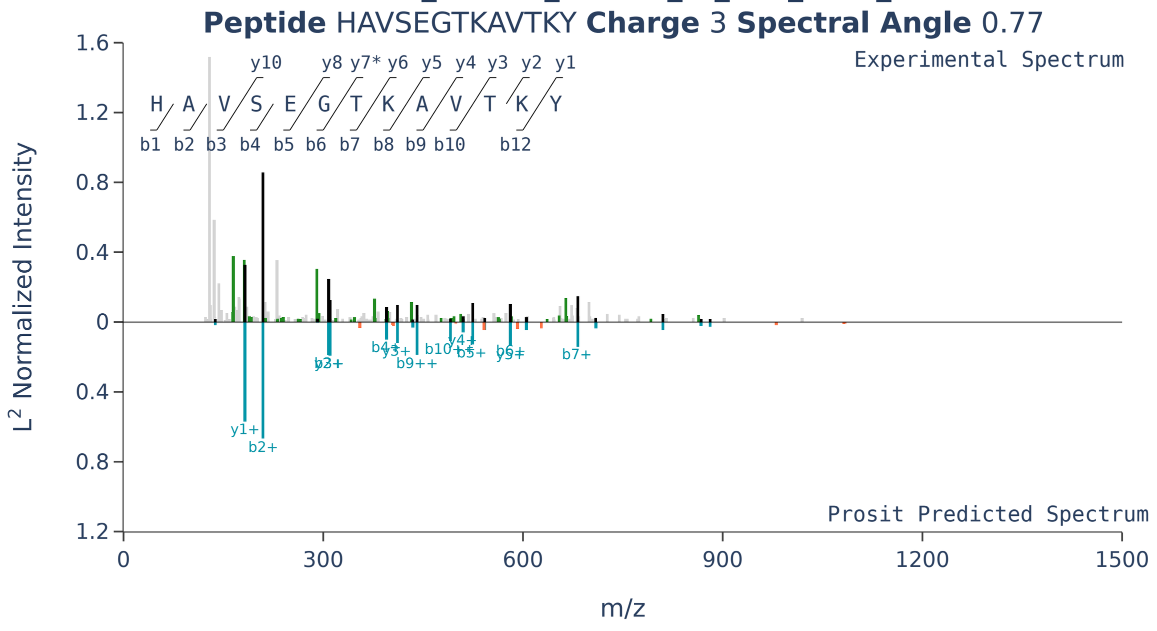


**Source** HRoetschke_291222_080123_Fu_H2B_b1R1_4h **Scan** 14046
**Peptide** TSREIQTAVRLL **Charge** 3 **Spectral Angle** 0.81

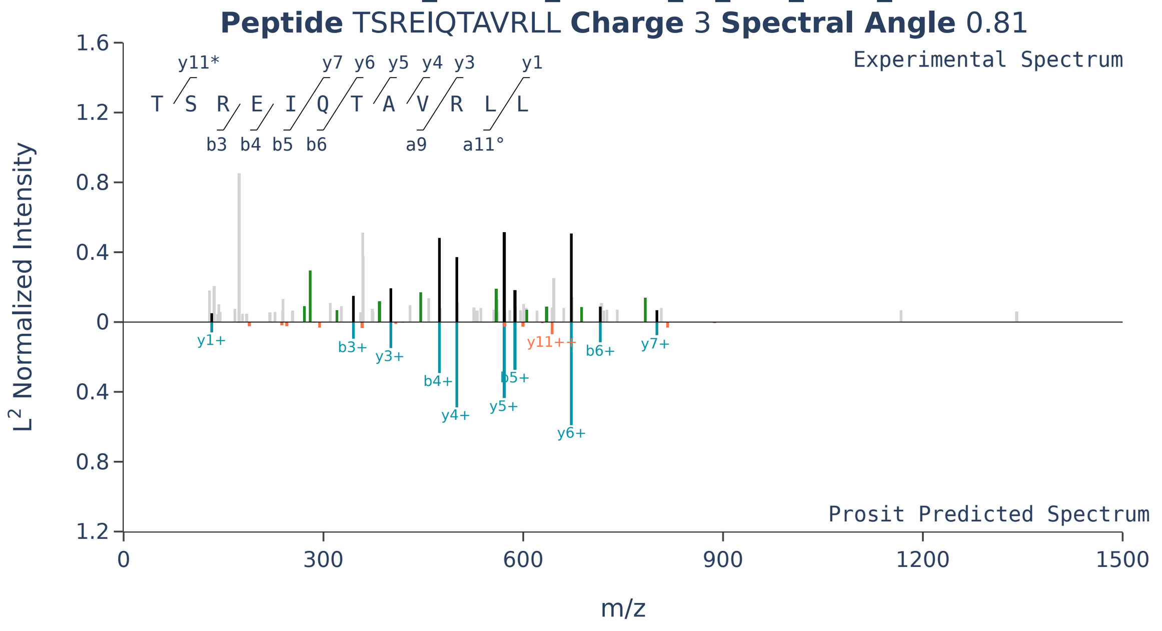


**Source** HRoetschke_291222_080123_Fu_H2B_b2R1_1h **Scan** 2761
**Peptide** EASRLAHYNKRSTITSRE **Charge** 4 **Spectral Angle** 0.8

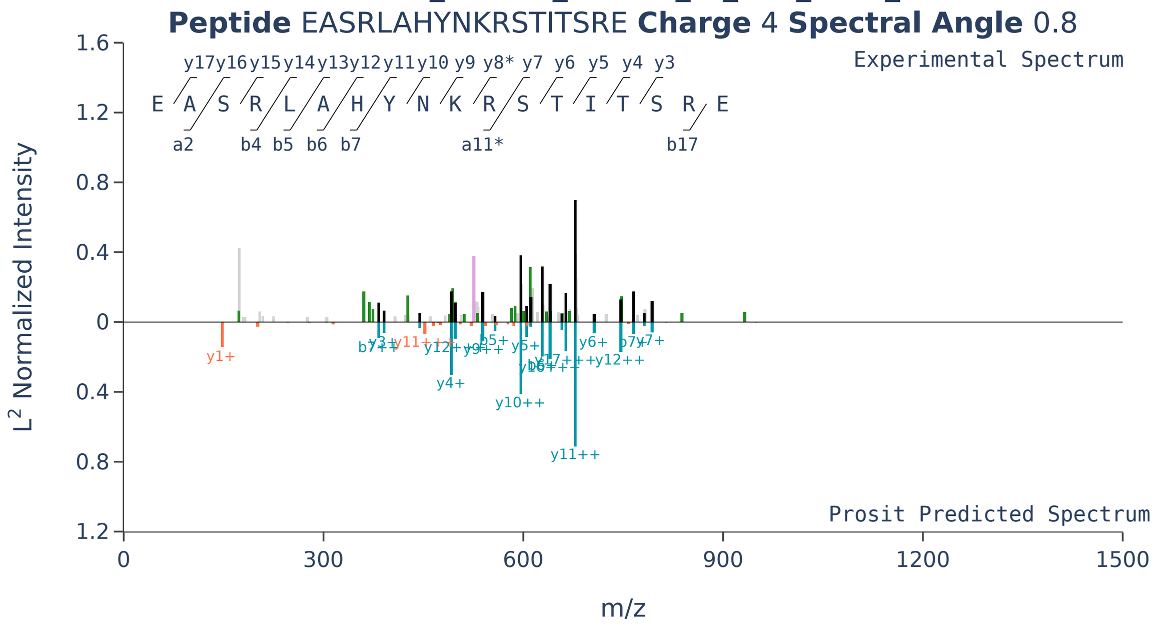


**Source** HRoetschke_291222_080123_Fu_H2B_b1R2_1h **Scan** 14262
**Peptide** AVRLLLPGELAKHAVSEGTKAVTKYTSAK **Charge** 5 **Spectral Angle** 0.85

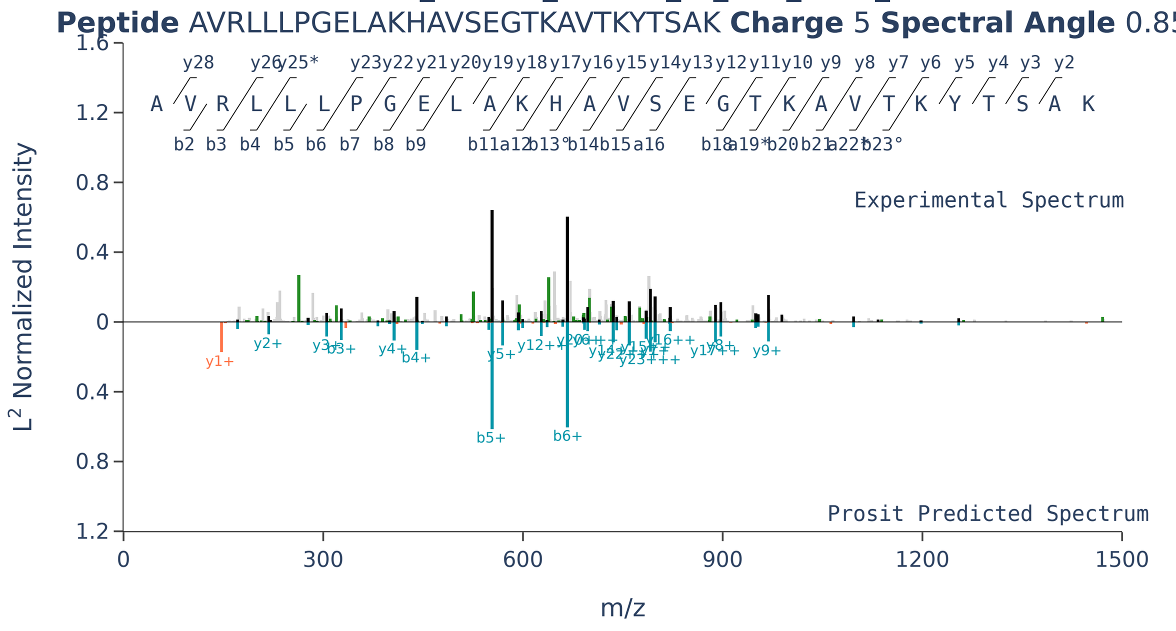


**Source** HRoetschke_291222_080123_Fu_H2B_b2R2_4h **Scan** 15509
**Peptide** AVRLLLPGELAKHAVSEGTKA **Charge** 4 **Spectral Angle** 0.78

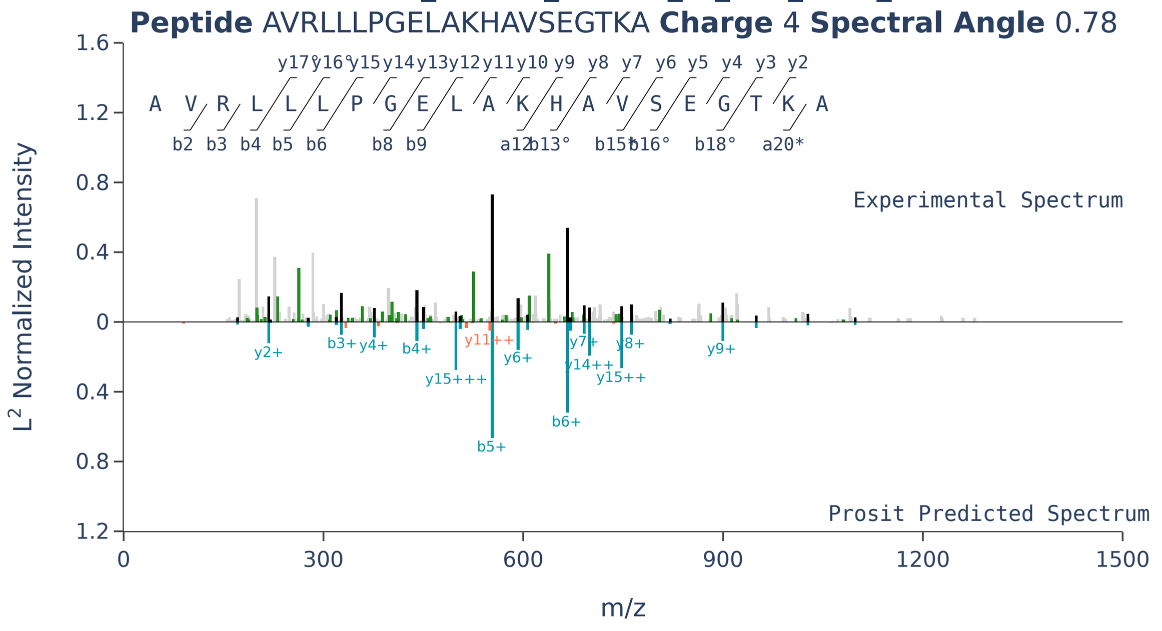


**Source** HRoetschke_291222_080123_Fu_H2B_b2R2_1h **Scan** 4662
**Peptide** REIQTAV **Charge** 2 **Spectral Angle** 0.77

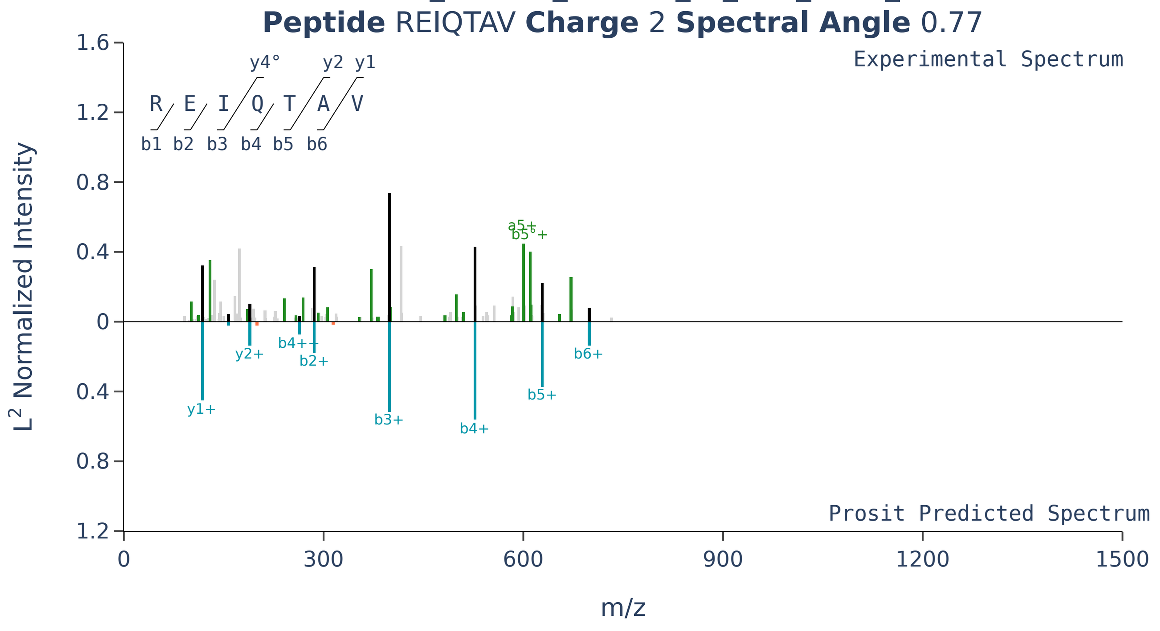

**Source** HRoetschke_291222_080123_Fu_H2B_b2R2_2h **Scan** 15030
**Peptide** AMSIMNSFVND **Charge** 2 **Spectral Angle** 0.83

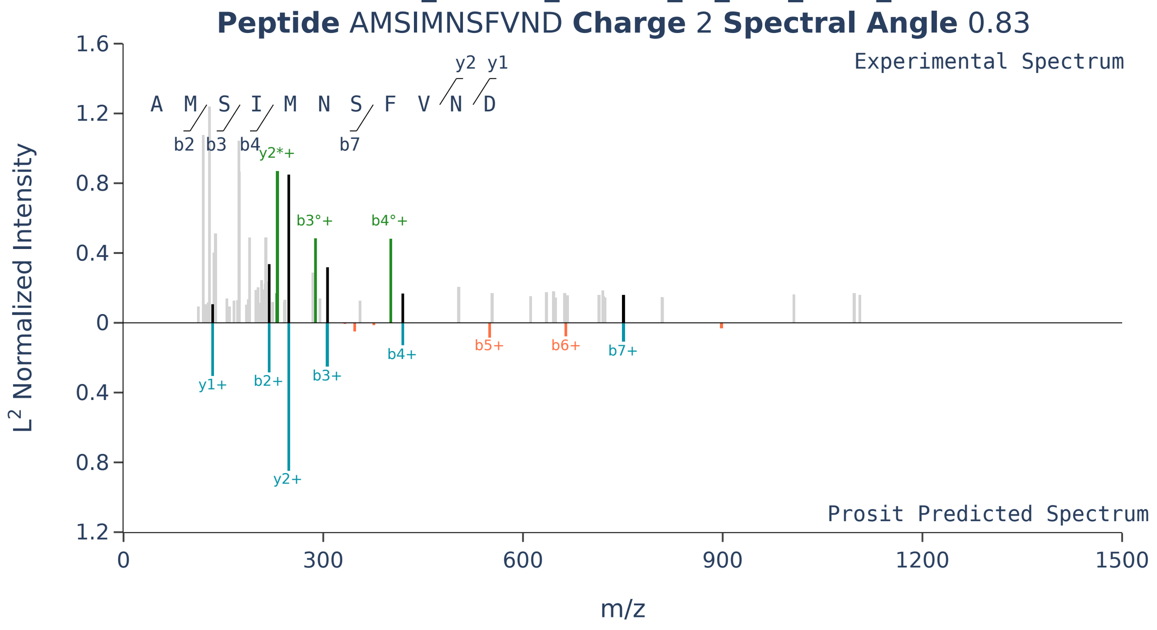


**Source** HRoetschke_291222_080123_Fu_H2B_b2R2_24h **Scan** 2616
**Peptide** KHAVSEGTKAVTKY **Charge** 2 **Spectral Angle** 0.8

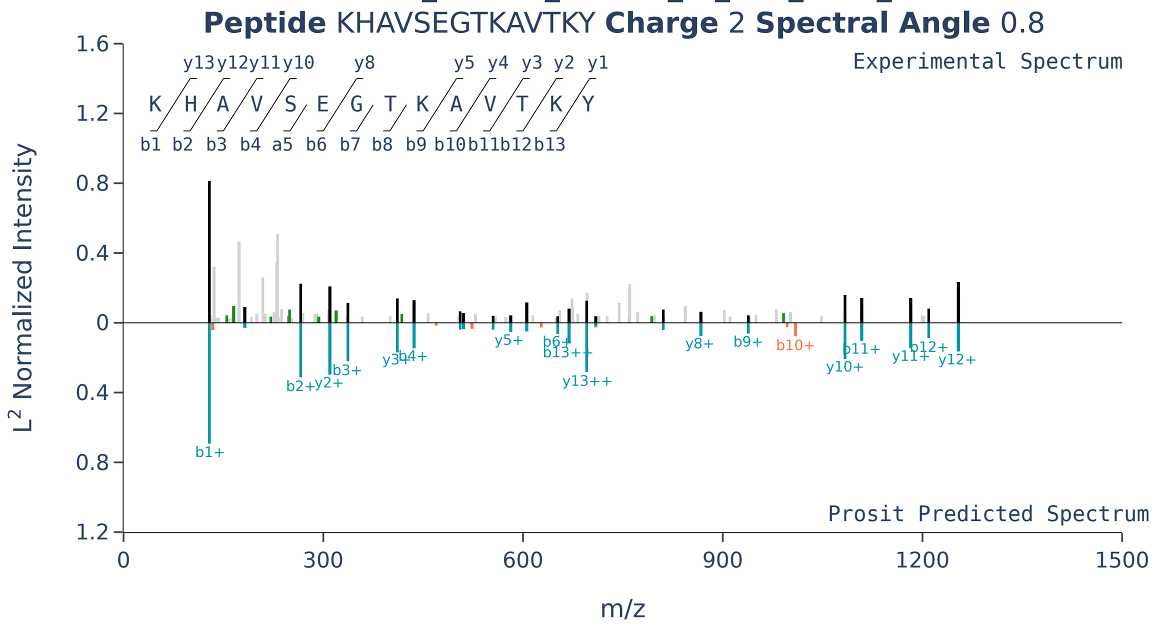


**Source** HRoetschke_291222_080123_Fu_H2B_b1R1_2h **Scan** 4031
**Peptide** AVSEGTKAVTKY **Charge** 2 **Spectral Angle** 0.71

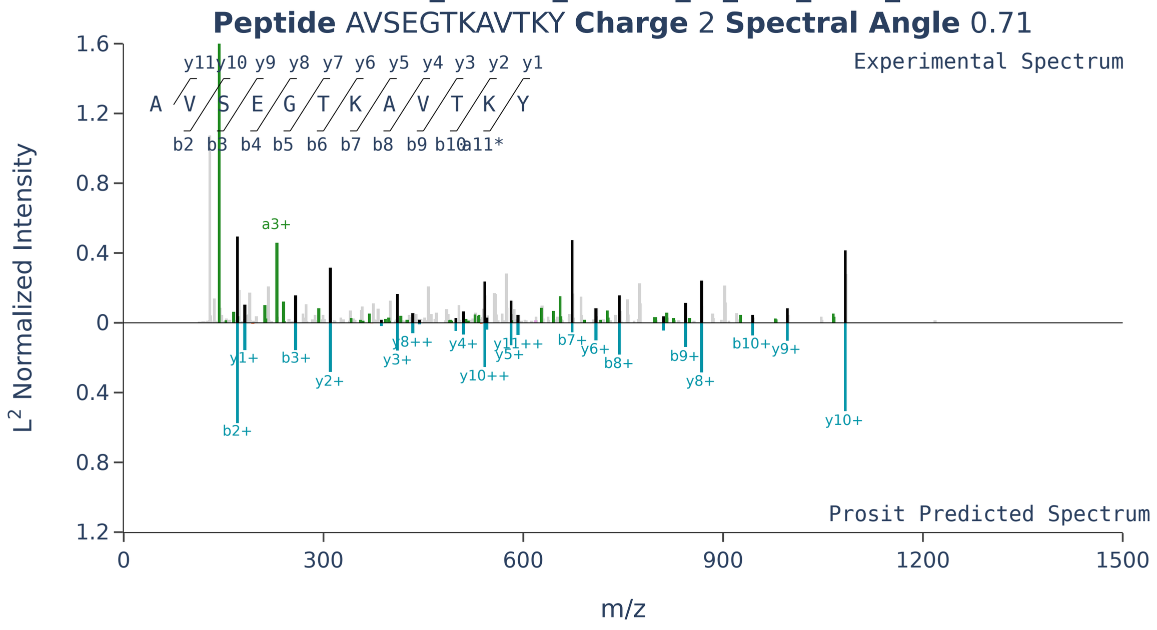


**Source** HRoetschke_291222_080123_Fu_H2B_b2R1_2h **Scan** 11252
**Peptide** VFERIAGEASRLAH **Charge** 3 **Spectral Angle** 0.82

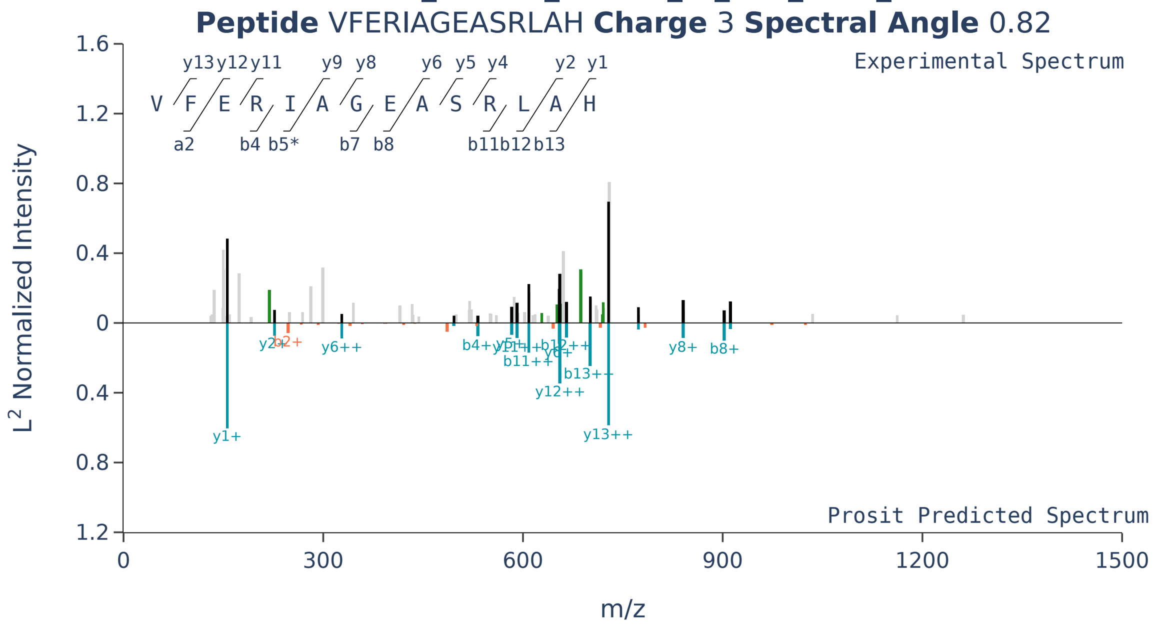


**Source** HRoetschke_291222_080123_Fu_H2B_b1R2_24h **Scan** 5555
**Peptide** LAHYNKRSTITSREIQTA **Charge** 3 **Spectral Angle** 0.85

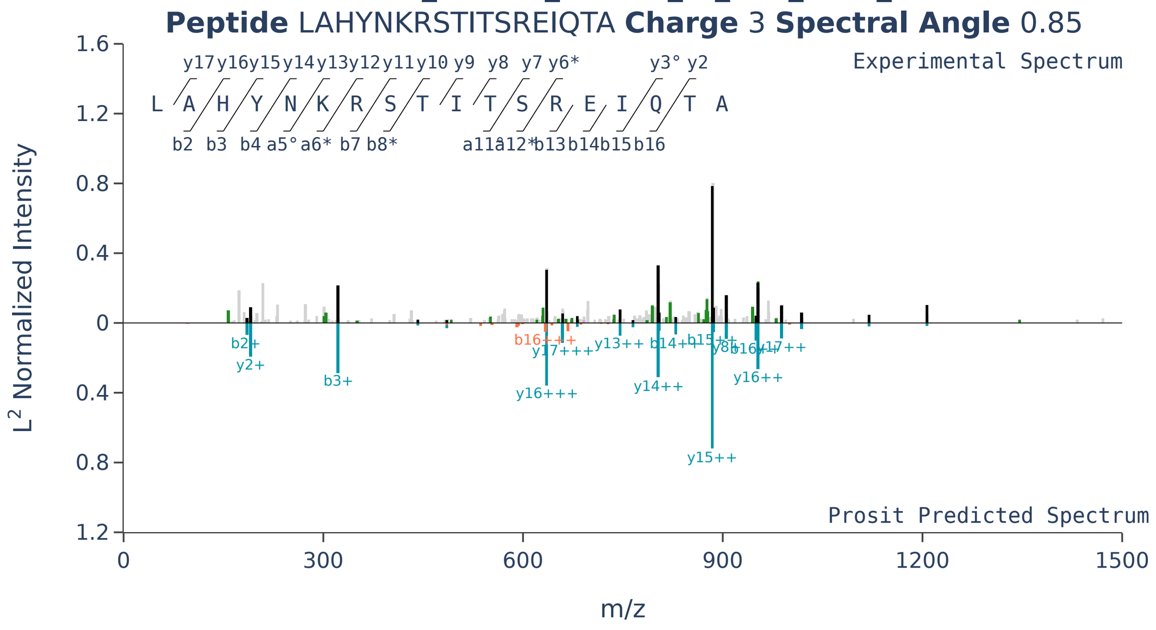


**Source** HRoetschke_291222_080123_Fu_H2B_b1R2_24h **Scan** 4796
**Peptide** LAGEASRLAHYNKRST **Charge** 3 **Spectral Angle** 0.85

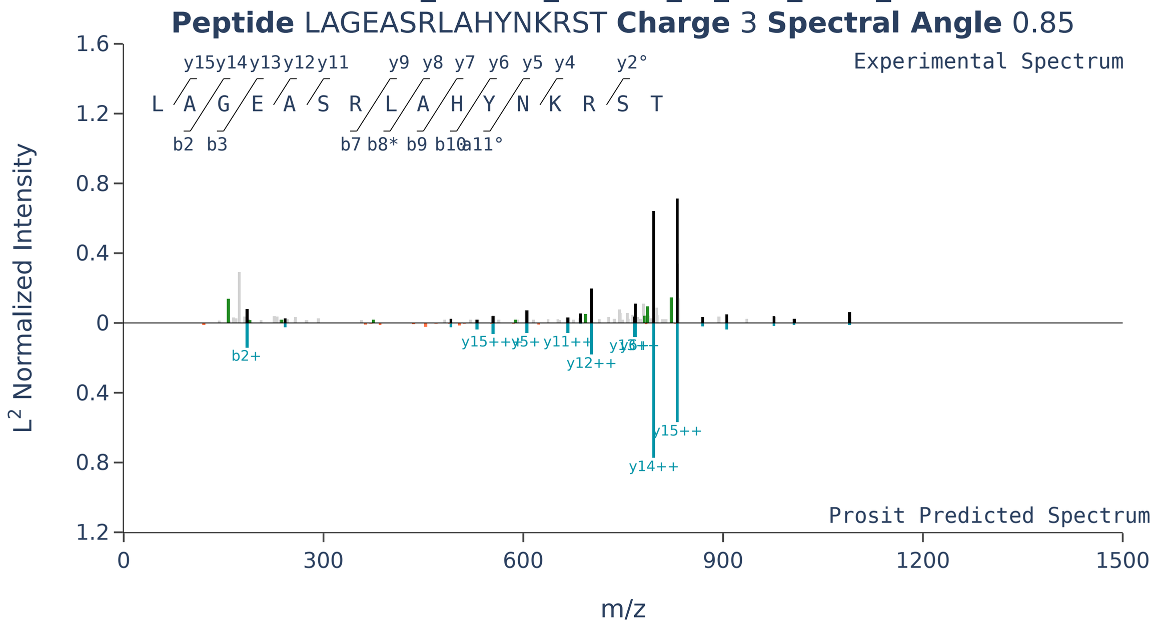


**Source** HRoetschke_291222_080123_Fu_H2B_b2R1_24h **Scan** 19900
**Peptide** VRLLLPGELA **Charge** 2 **Spectral Angle** 0.89

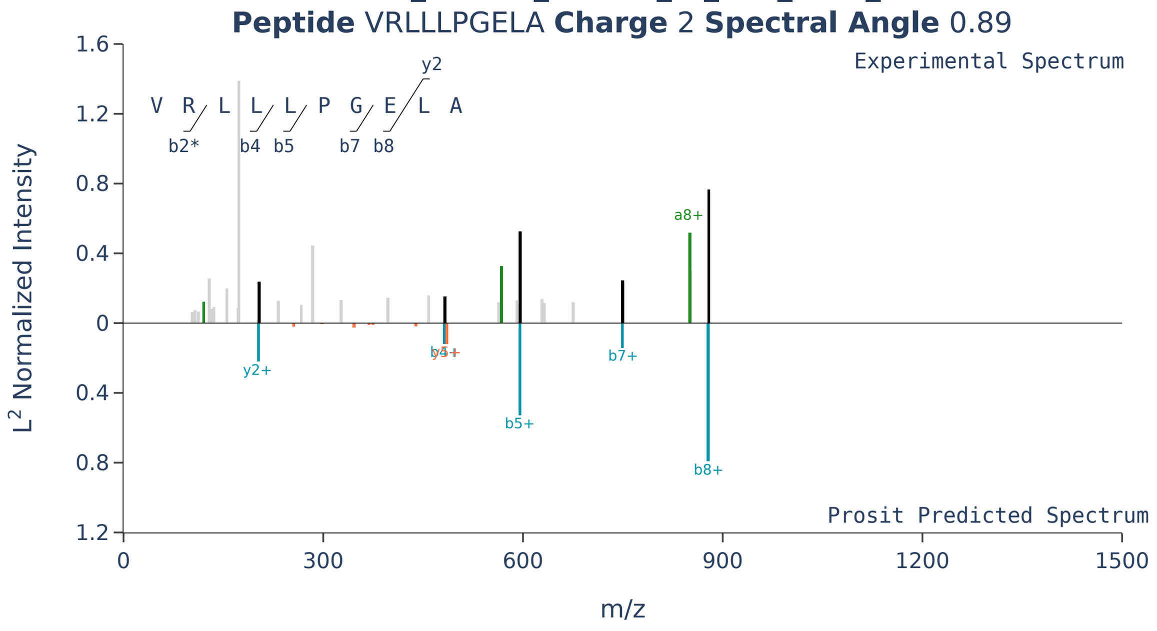


**Source** HRoetschke_291222_080123_Fu_H2B_b1R1_24h **Scan** 8308
**Peptide** ERIAGEASRLAHYN **Charge** 2 **Spectral Angle** 0.76

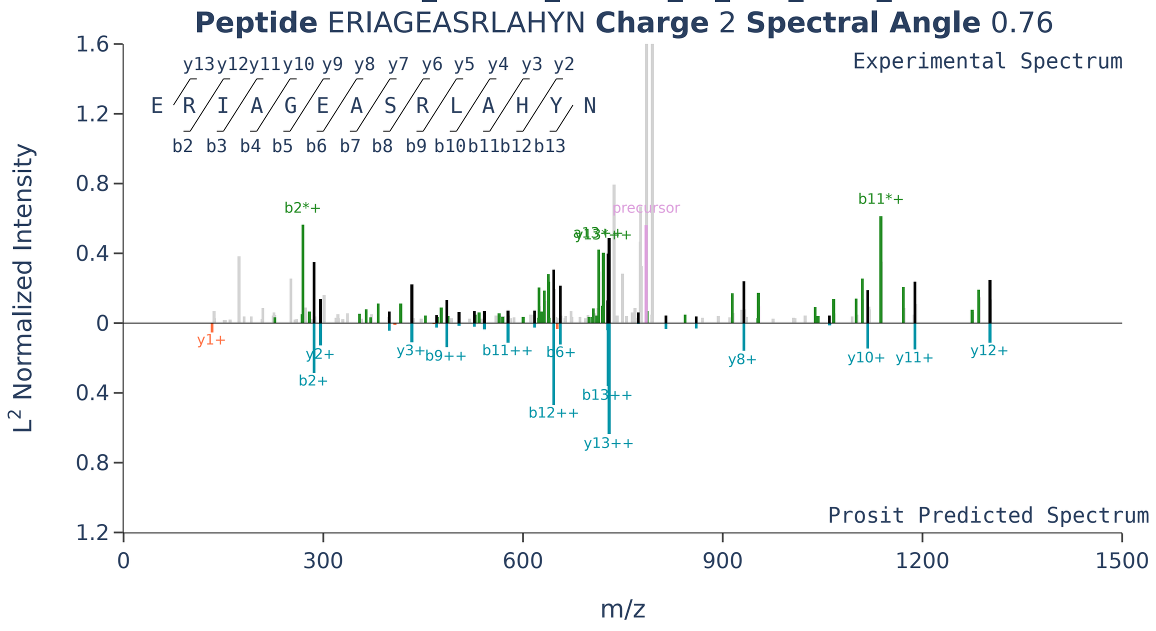


**Source** HRoetschke_291222_080123_Fu_H2B_b2R1_24h **Scan** 6782
**Peptide** KRRKTRKESYAIYVY **Charge** 4 **Spectral Angle** 0.82

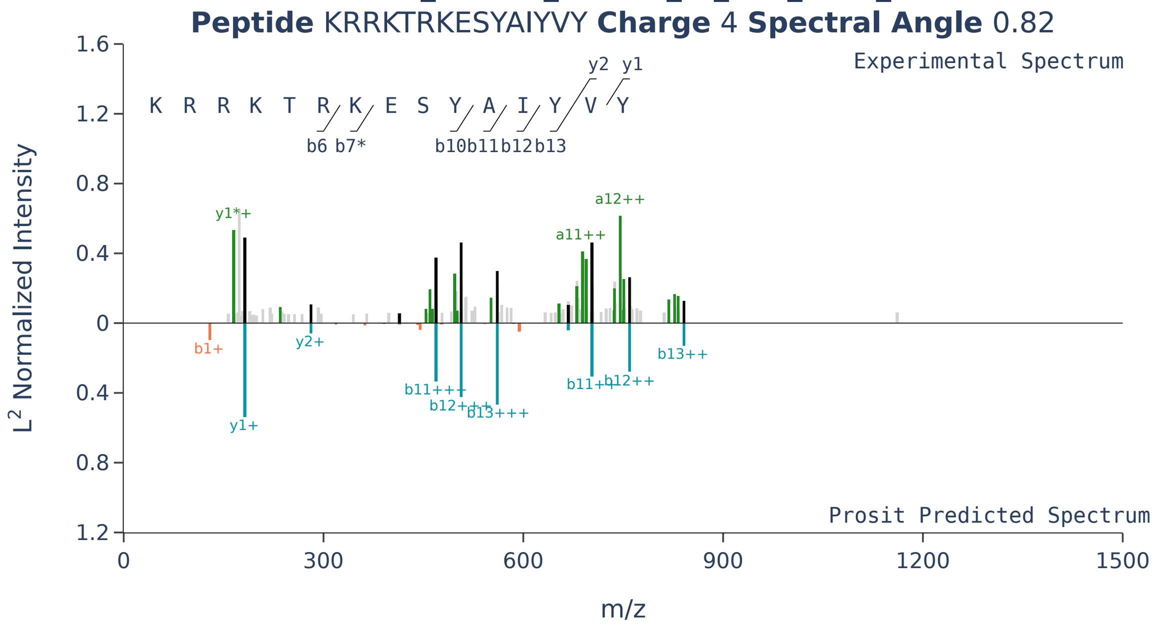


**Source** HRoetschke_291222_080123_Fu_H2B_b2R2_4h **Scan** 13797
**Peptide** LTSREIQTAVRL **Charge** 3 **Spectral Angle** 0.79

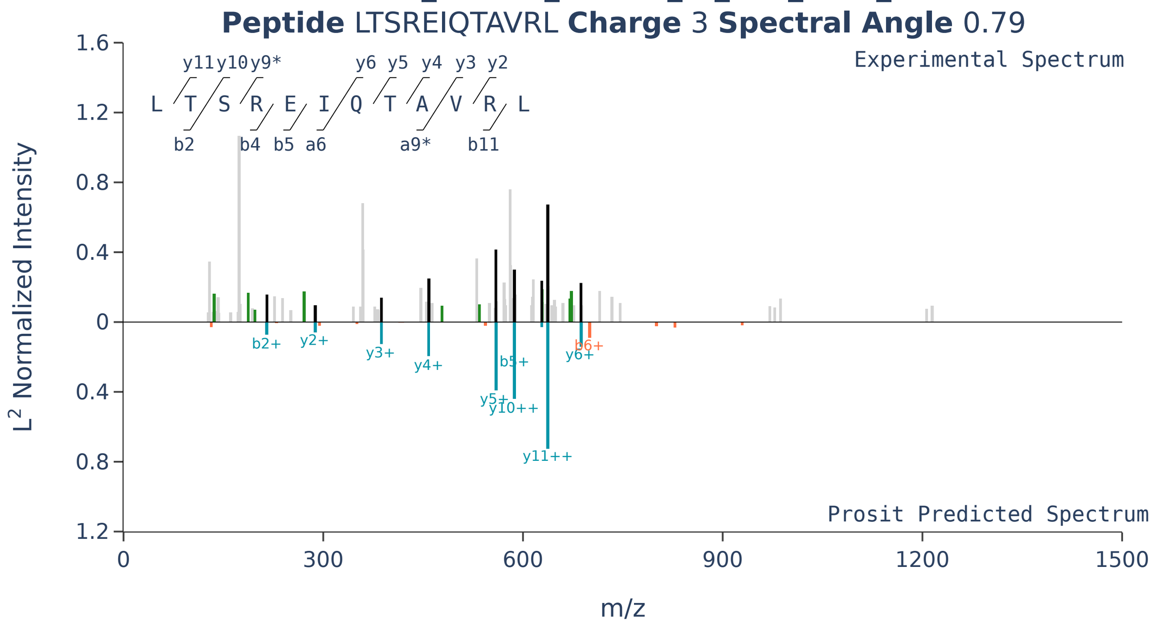


**Source** HRoetschke_291222_080123_Fu_H2B_b2R2_24h **Scan** 7351
**Peptide** LAGEASRLAHYN **Charge** 2 **Spectral Angle** 0.79

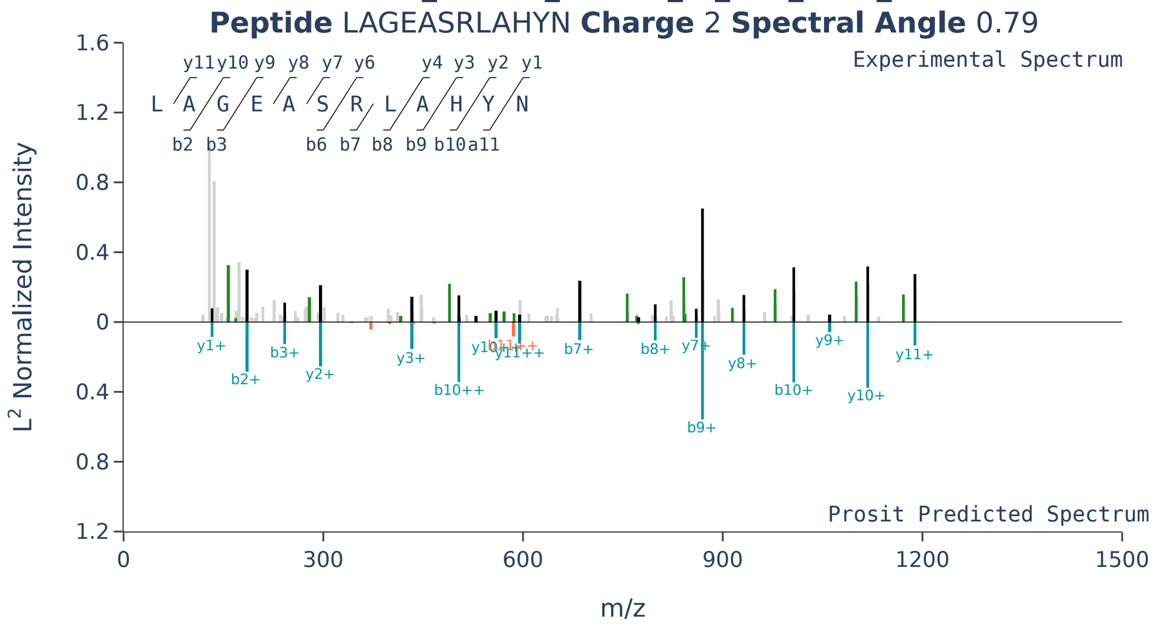


**Source** HRoetschke_291222_080123_Fu_H2B_b2R2_24h **Scan** 2862
**Peptide** LAHYNKRSTITS **Charge** 2 **Spectral Angle** 0.81

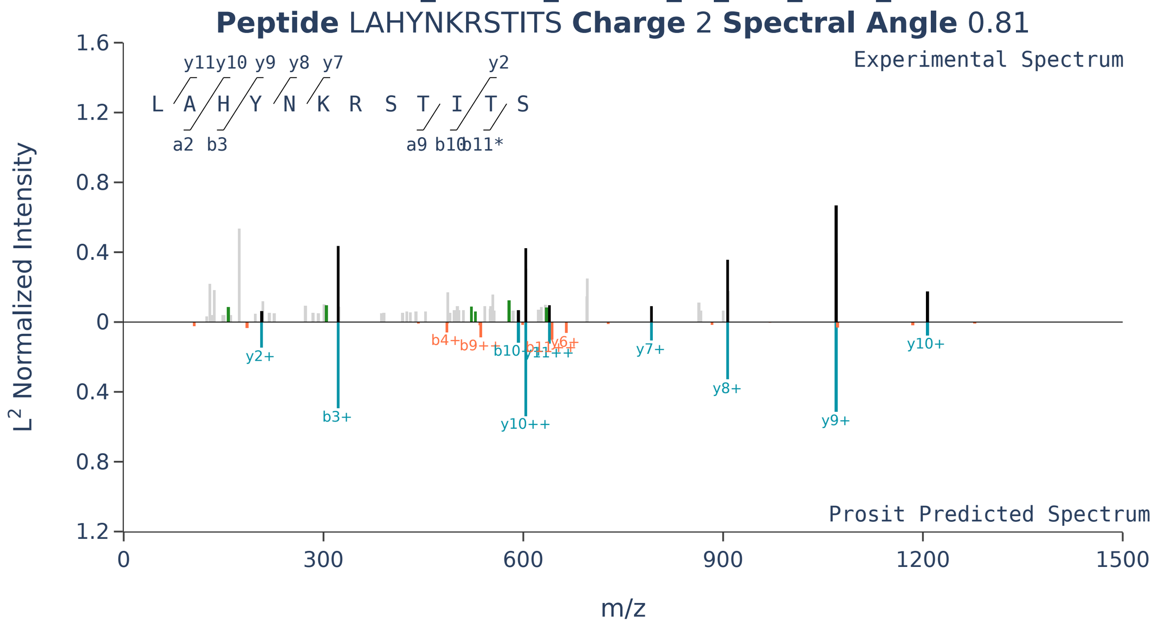


**Source** HRoetschke_291222_080123_Fu_H2B_b2R2_24h **Scan** 4755
**Peptide** TQKKDGKKRRKTRKESYAIYVY **Charge** 4 **Spectral Angle** 0.86

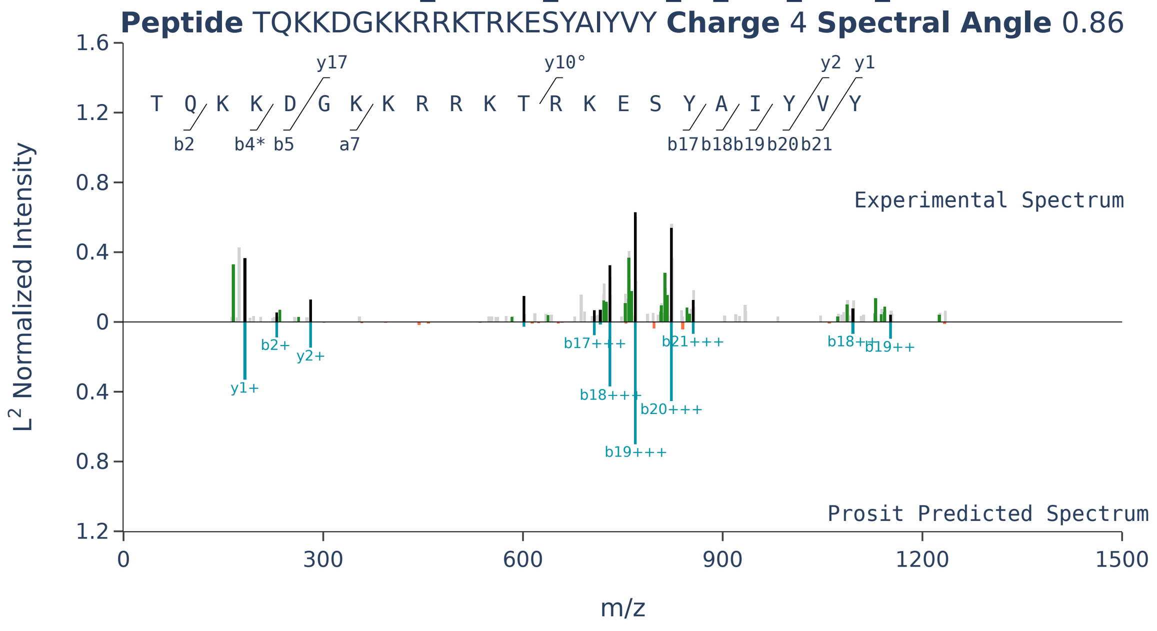


**Source** HRoetschke_291222_080123_Fu_H2B_b2R1_4h **Scan** 20795
**Peptide** NSFVNDVFERL **Charge** 2 **Spectral Angle** 0.92

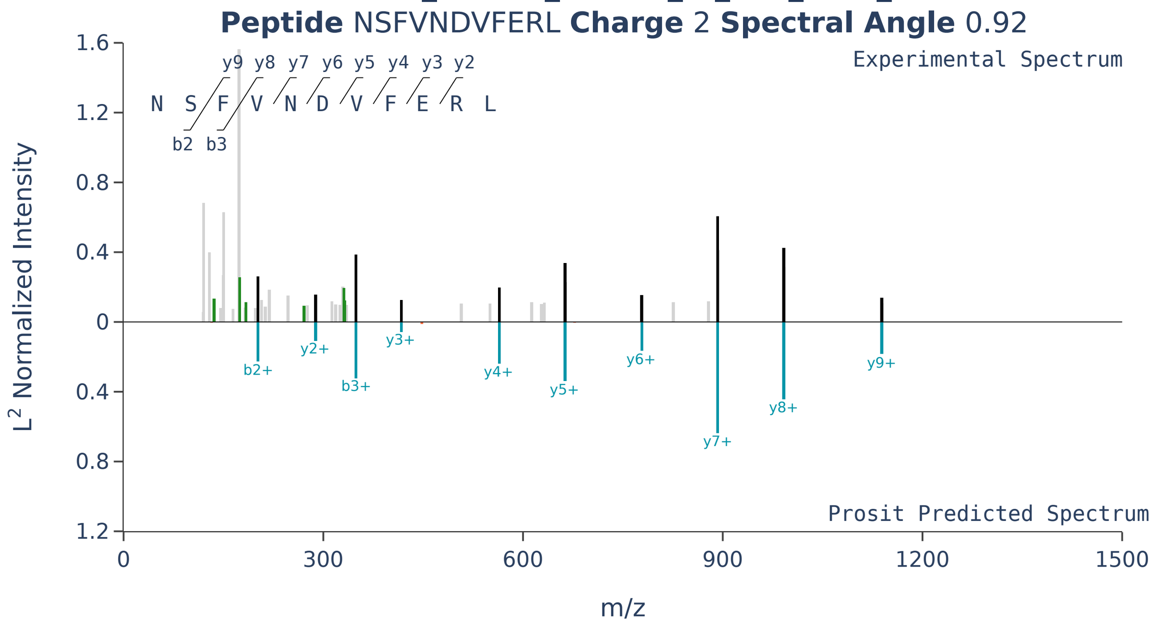


**Source** HRoetschke_291222_080123_Fu_H2B_b2R2_24h **Scan** 19142
**Peptide** SREIQTAVRLLLPGELAKHA **Charge** 4 **Spectral Angle** 0.75

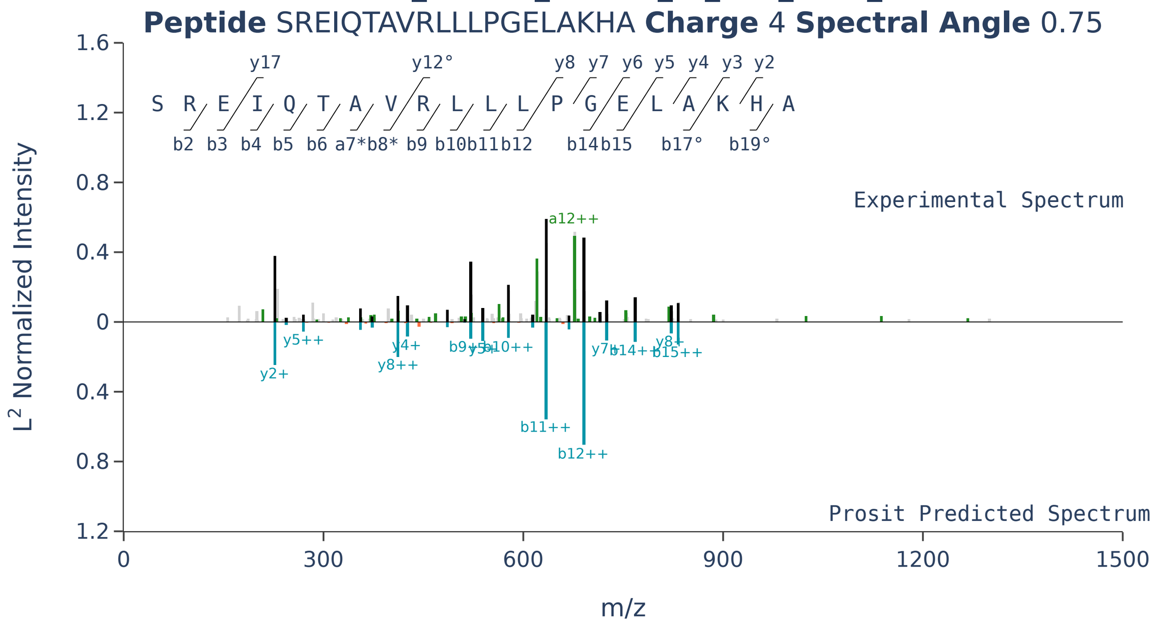

**Source** HRoetschke_291222_080123_Fu_H2B_b2R2_24h **Scan** 4993
**Peptide** ERIAGEASRLAH **Charge** 4 **Spectral Angle** 0.83

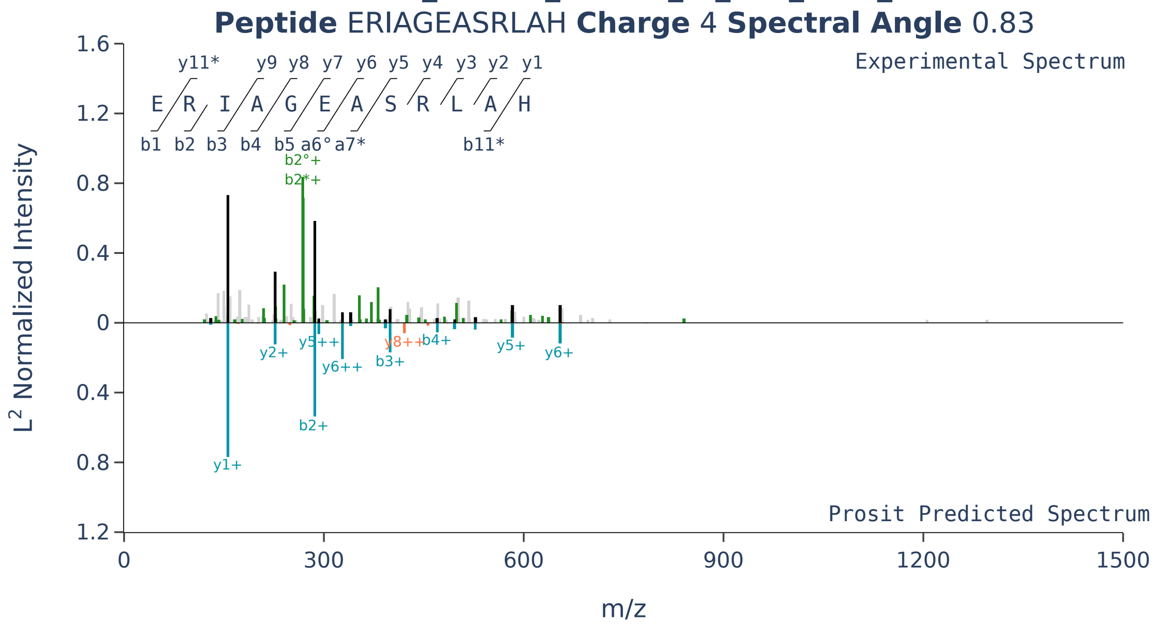


**Source** HRoetschke_291222_080123_Fu_H2B_b1R1_1h **Scan** 4901
**Peptide** IAGEASRLAHYNKRSTITSR **Charge** 4 **Spectral Angle** 0.79

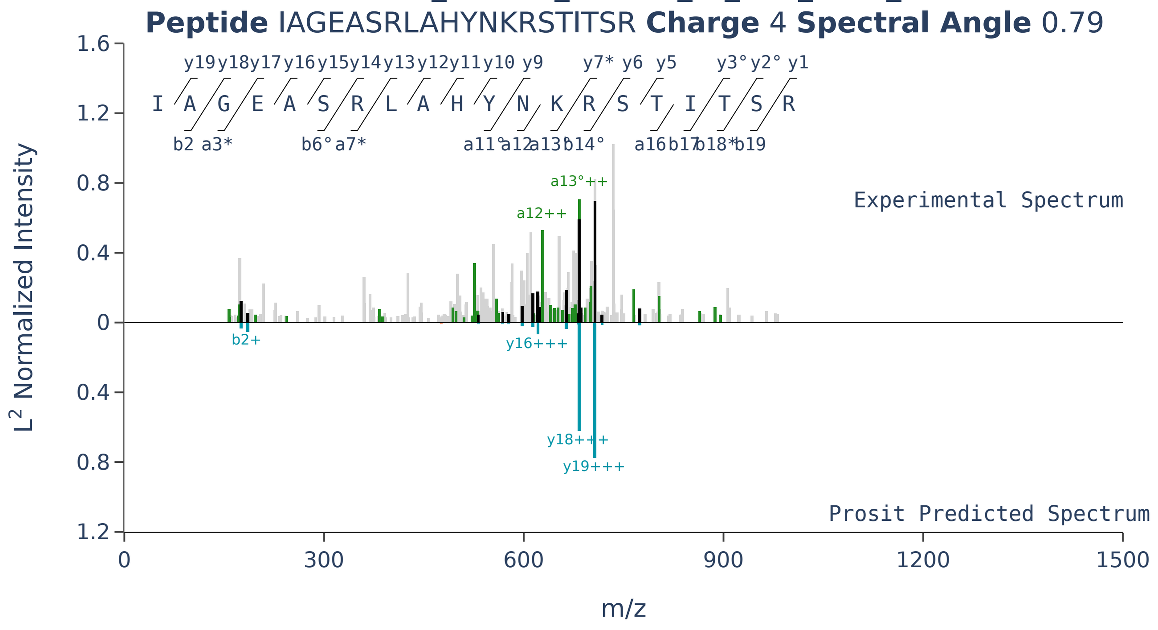


**Source** HRoetschke_291222_080123_Fu_H2B_b2R1_24h **Scan** 16522
**Peptide** VRLLLPGELAKHAVSE **Charge** 3 **Spectral Angle** 0.8

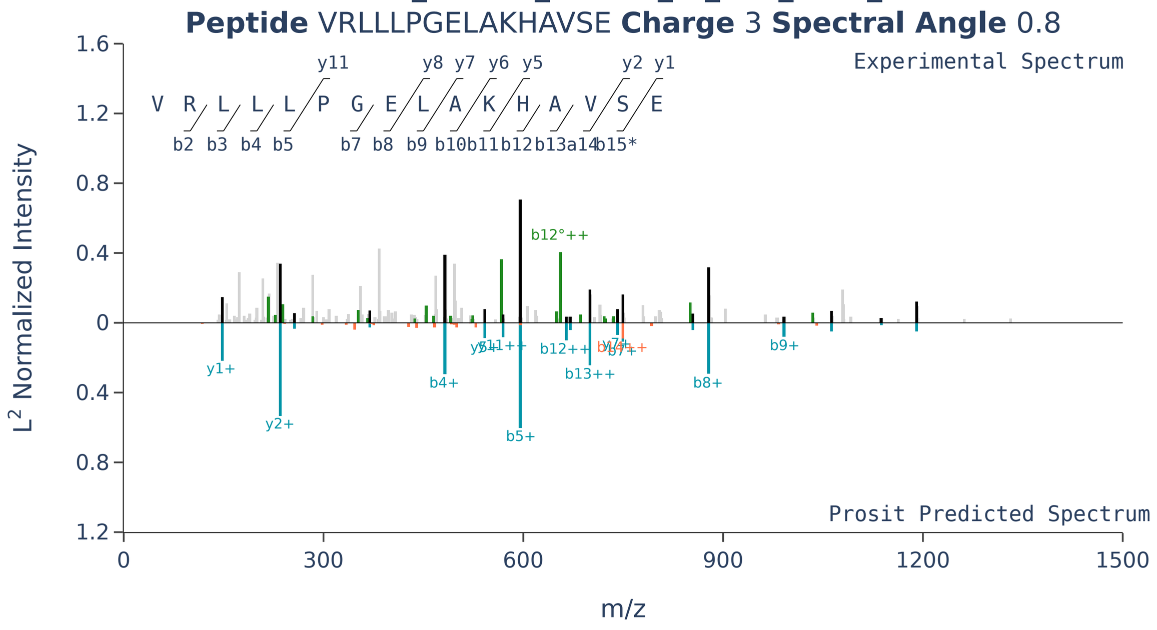


**Source** HRoetschke_291222_080123_Fu_H2B_b1R1_4h **Scan** 2344
**Peptide** HPDTGISSKA **Charge** 2 **Spectral Angle** 0.72

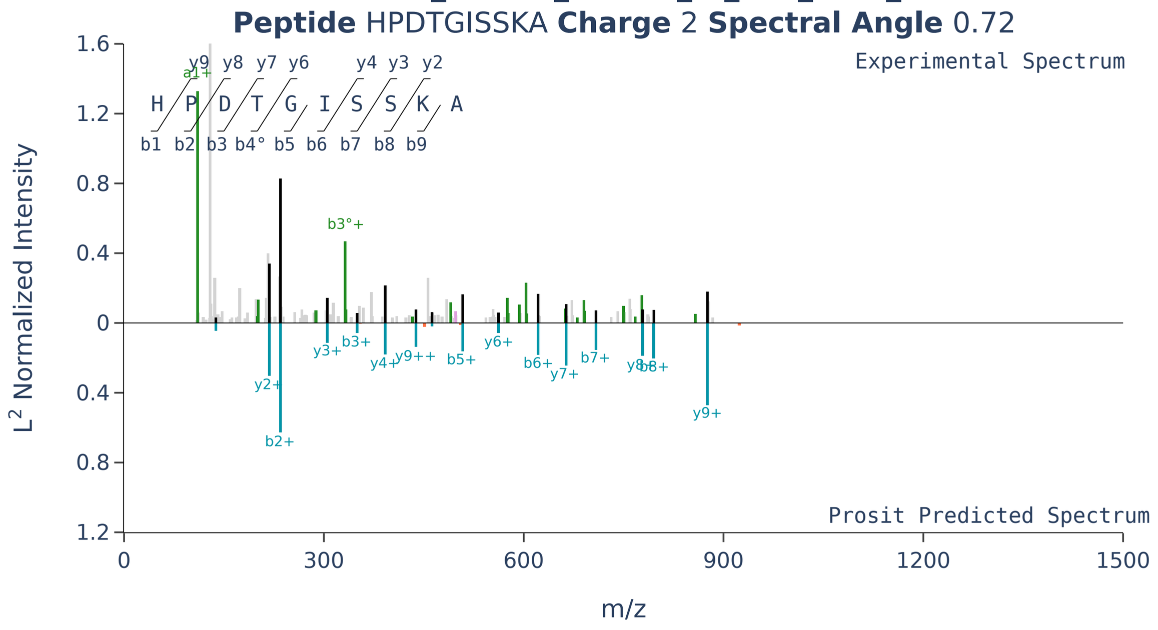


**Source** HRoetschke_291222_080123_Fu_H2B_b1R1_24h **Scan** 4443
**Peptide** AGEASRLAHYNKRSTITSRE **Charge** 4 **Spectral Angle** 0.86

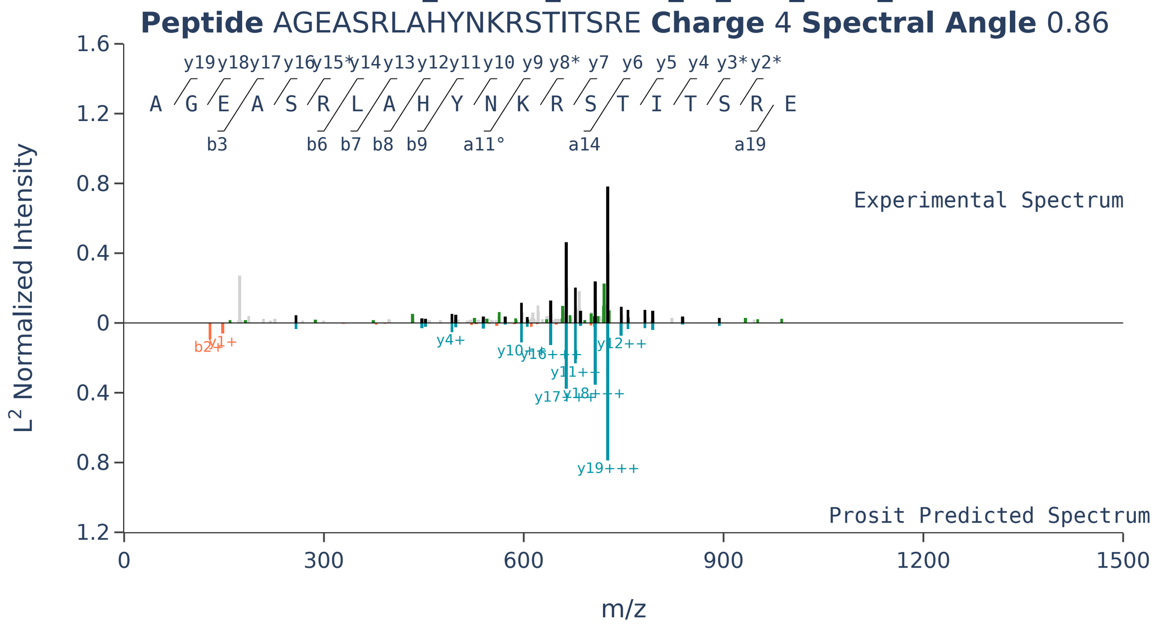


**Source** HRoetschke_291222_080123_Fu_H2B_b2R2_1h **Scan** 17689
**Peptide** ELQTAVRLLL **Charge** 2 **Spectral Angle** 0.87

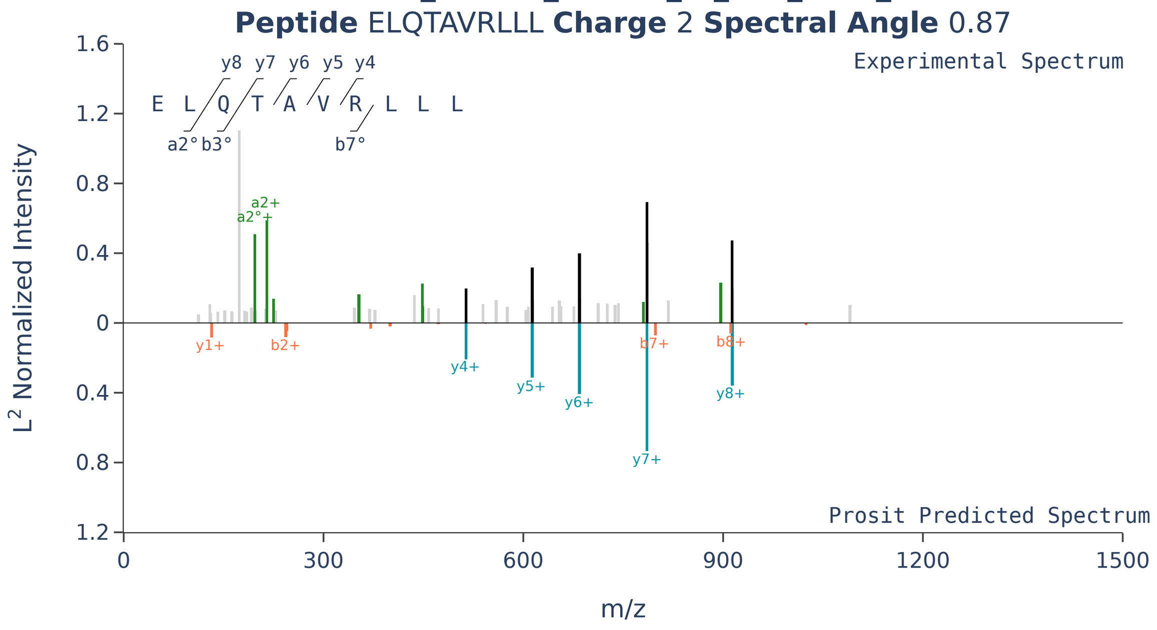


**Source** HRoetschke_291222_080123_Fu_H2B_b2R2_24h **Scan** 5433
**Peptide** LKQVHPDTGISS **Charge** 2 **Spectral Angle** 0.84

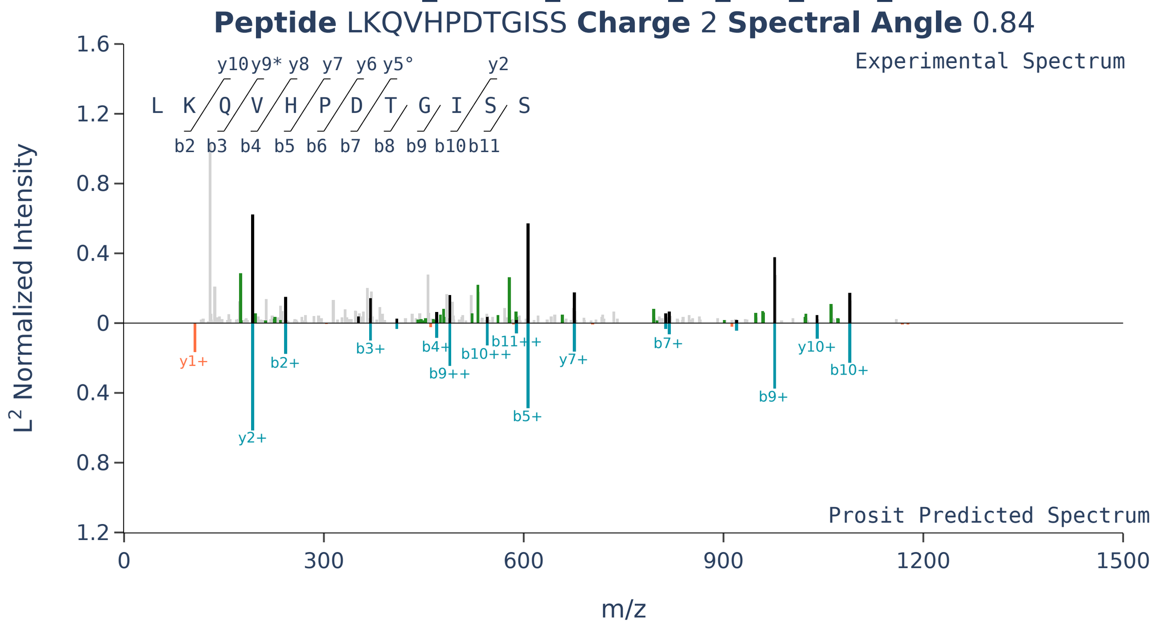


**Source** HRoetschke_291222_080123_Fu_H2B_b1R2_4h **Scan** 11983
**Peptide** TRKESYAIYVY **Charge** 3 **Spectral Angle** 0.87

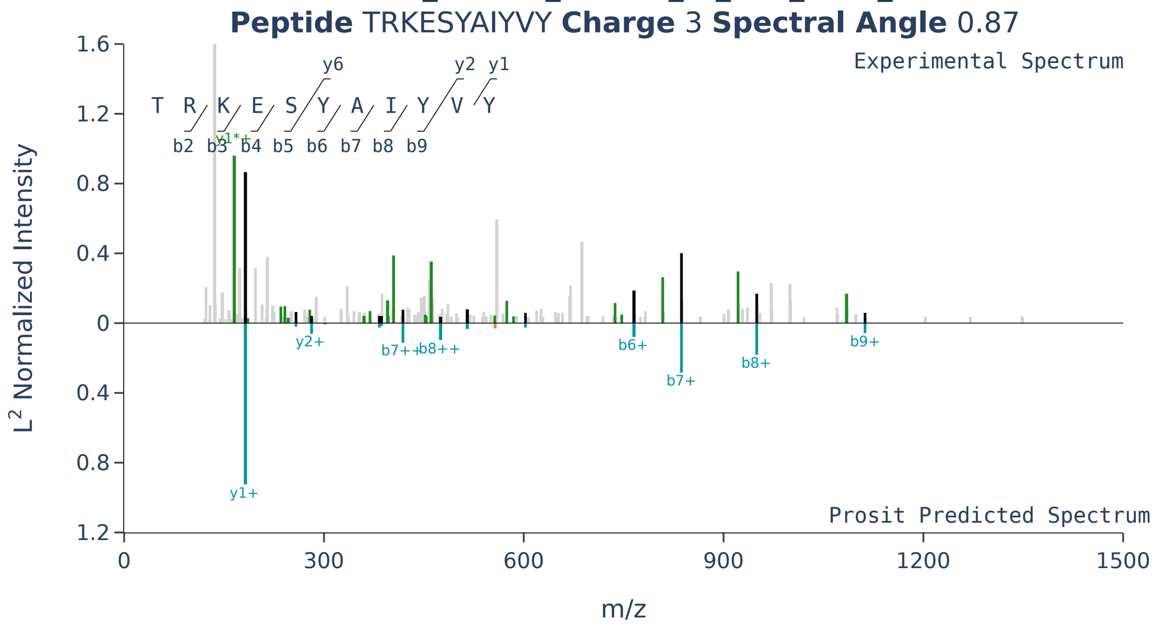


**Source** HRoetschke_291222_080123_Fu_H2B_b2R1_2h **Scan** 14747
**Peptide** SIMNSFVND **Charge** 2 **Spectral Angle** 0.73

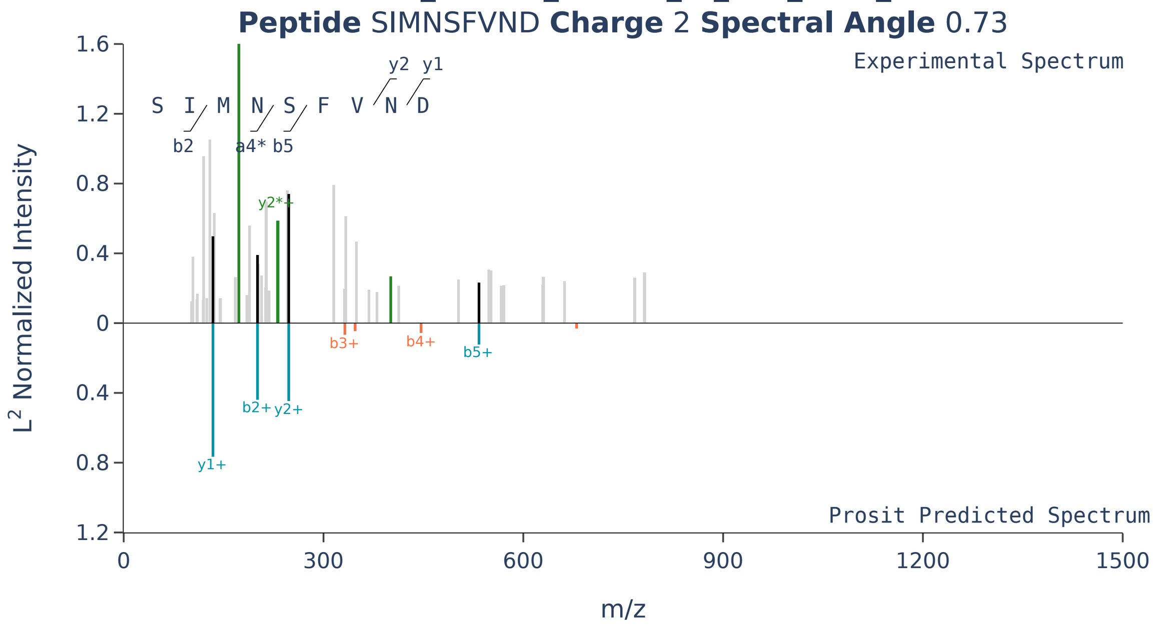


**Source** HRoetschke_291222_080123_Fu_H2B_b1R1_2h **Scan** 2873
**Peptide** QVHPDTGISSK **Charge** 3 **Spectral Angle** 0.77

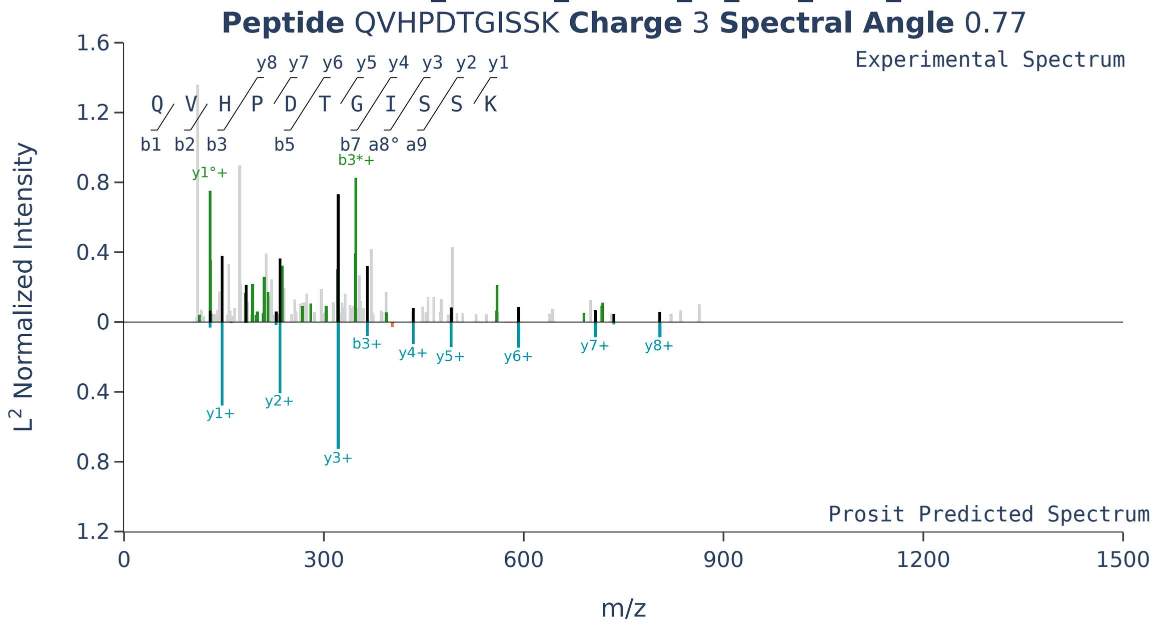


**Source** HRoetschke_291222_080123_Fu_H2B_b1R1_24h **Scan** 3065
**Peptide** NKRSTITSREIQ **Charge** 3 **Spectral Angle** 0.7

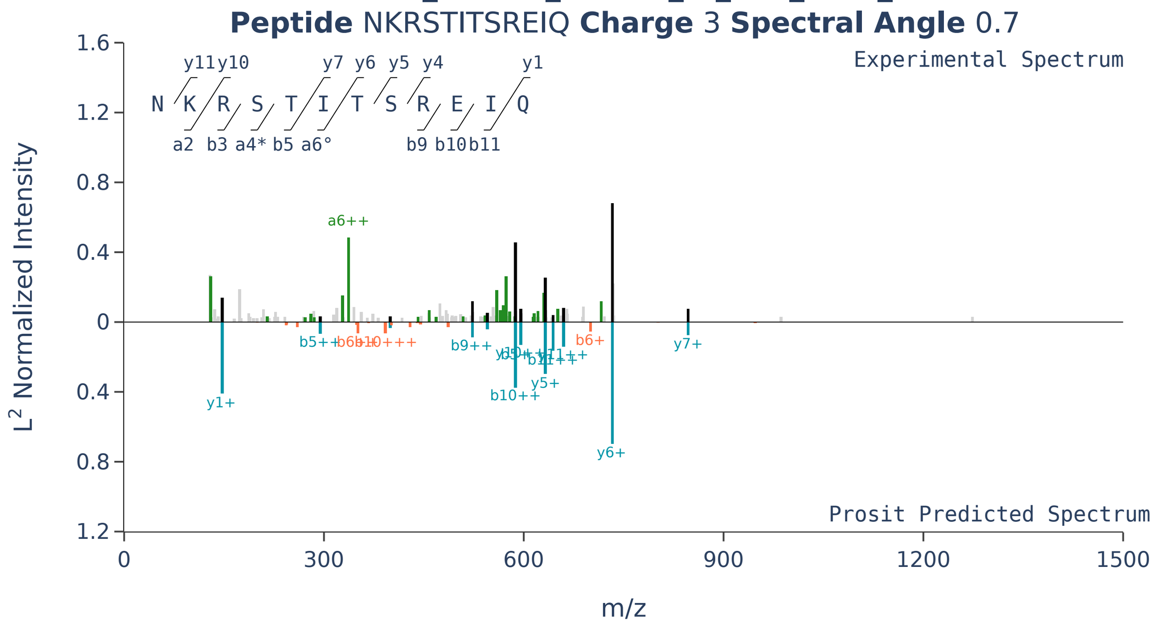


**Source** HRoetschke_291222_080123_Fu_H2B_b2R2_4h **Scan** 17563
**Peptide** VRLLLPGELAKHAVSEGTKAVTKYTSAK **Charge** 5 **Spectral Angle** 0.82

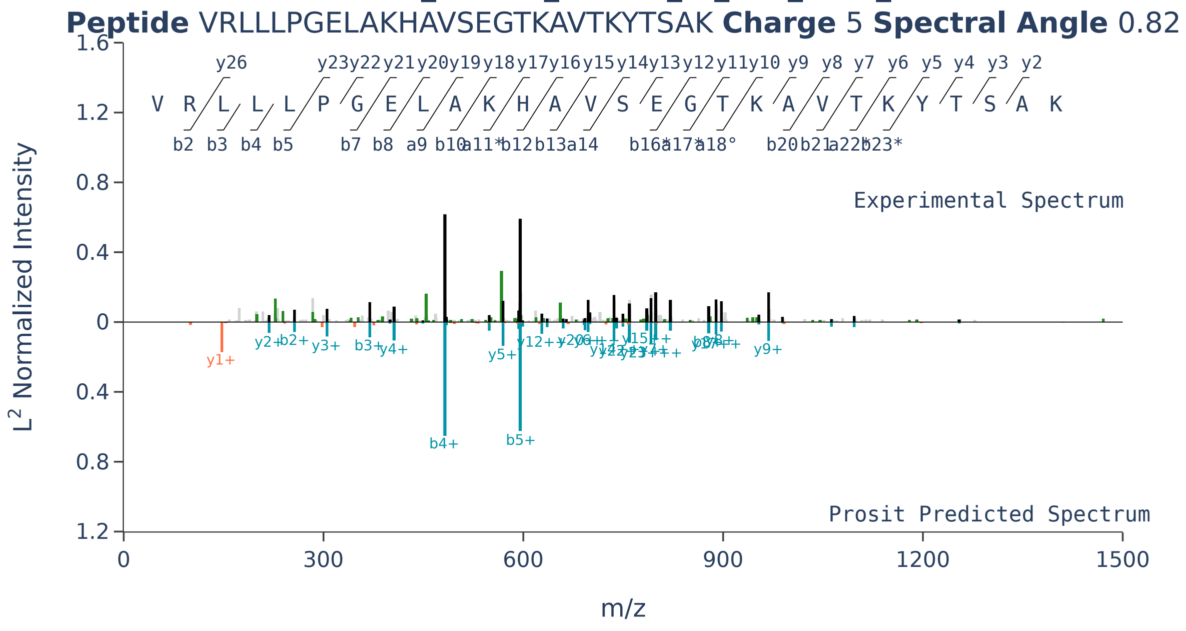


**Source** HRoetschke_291222_080123_Fu_H2B_b2R2_4h **Scan** 21565
**Peptide** NSFVNDVFERIAG **Charge** 2 **Spectral Angle** 0.83

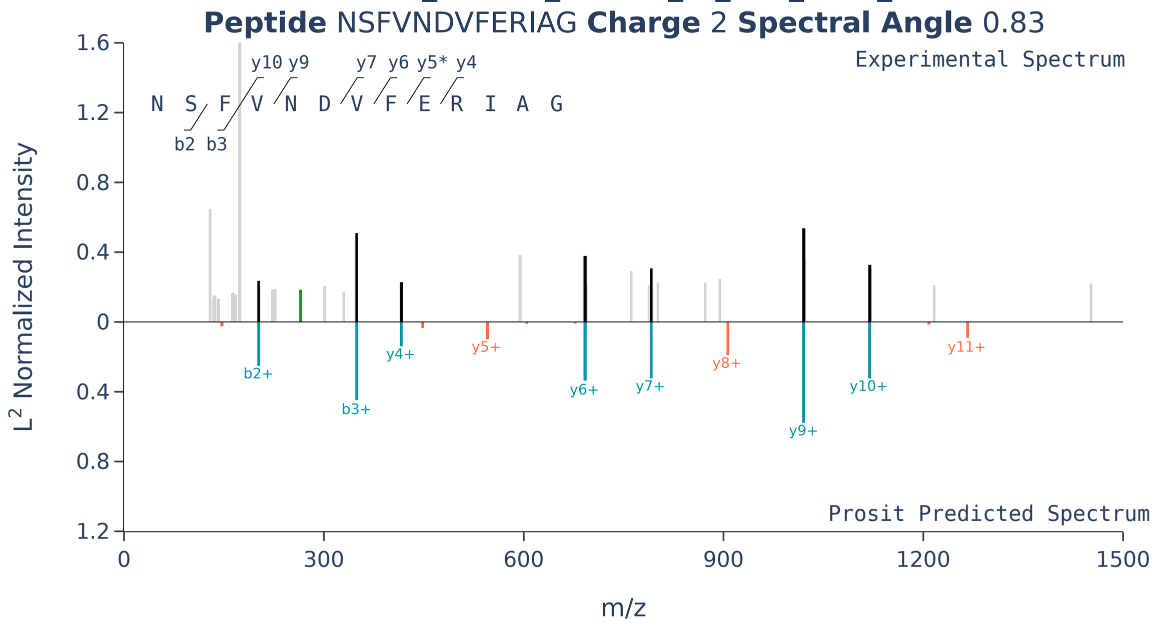


**Source** HRoetschke_291222_080123_Fu_H2B_b2R2_4h **Scan** 19037
**Peptide** VNDVFERIAGEASRLAHYNKRST **Charge** 5 **Spectral Angle** 0.78

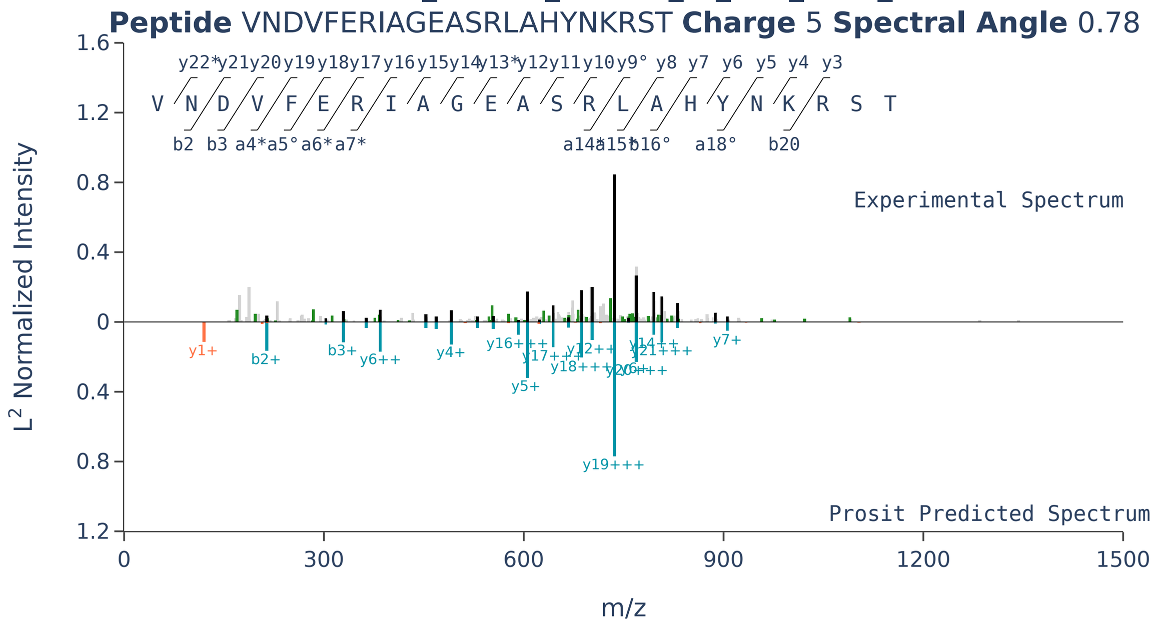


**Source** HRoetschke_291222_080123_Fu_H2B_b1R1_24h **Scan** 6575
**Peptide** YKVLKQVHPDTGISSK **Charge** 4 **Spectral Angle** 0.79

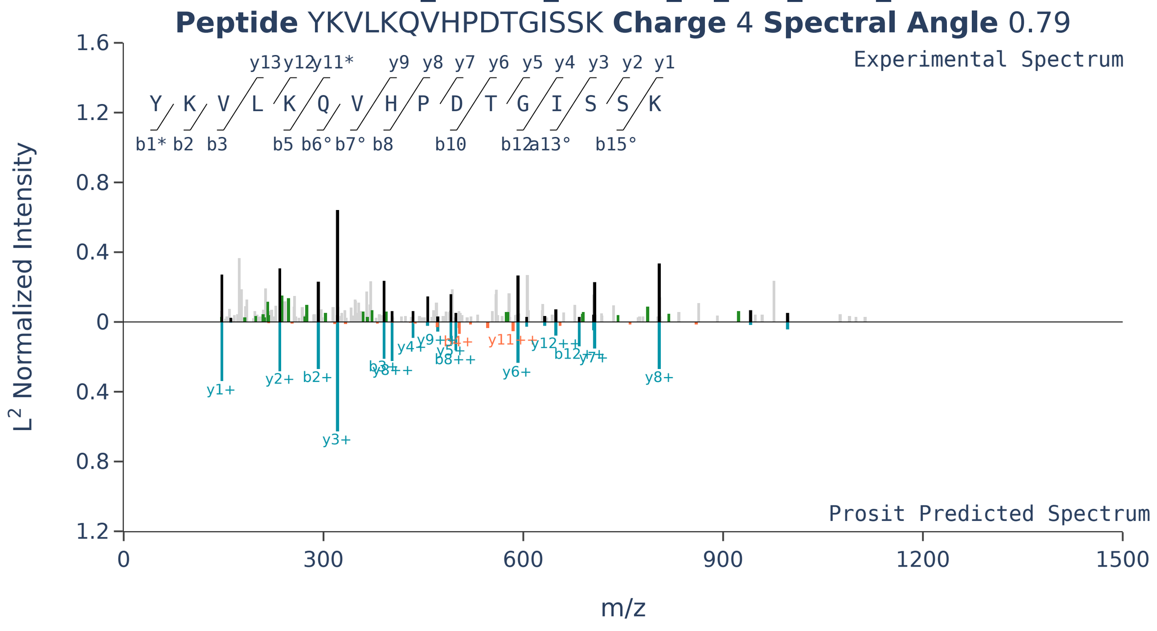

**Source** HRoetschke_291222_080123_Fu_H2B_b1R1_24h **Scan** 8737
**Peptide** DTGISSKAMSL **Charge** 2 **Spectral Angle** 0.88

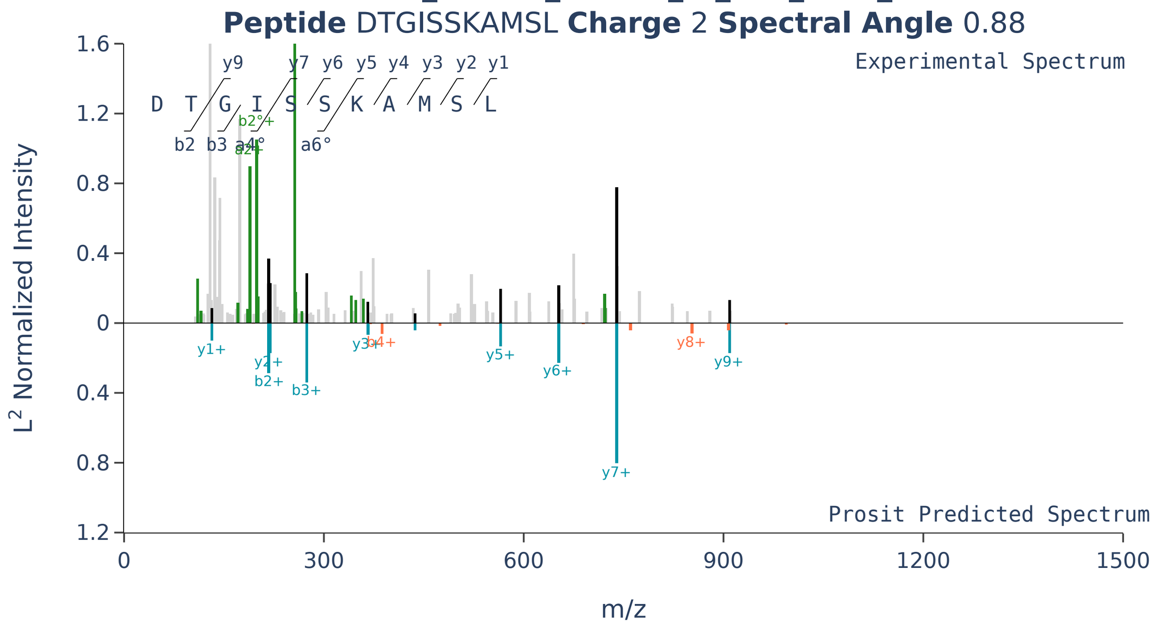


**Source** HRoetschke_291222_080123_Fu_H2B_b1R1_1h **Scan** 12750
**Peptide** LLLPGELAKHAVSE **Charge** 2 **Spectral Angle** 0.86

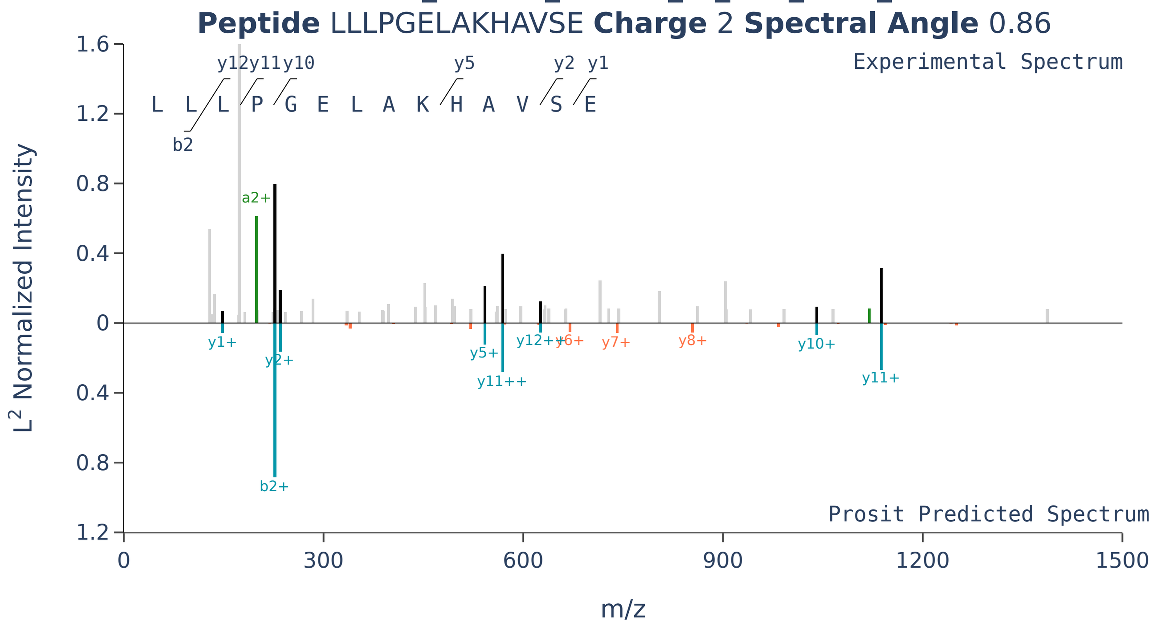


**Source** HRoetschke_291222_080123_Fu_H2B_b2R2_4h **Scan** 1774
**Peptide** ASRLAHYNKR **Charge** 3 **Spectral Angle** 0.76

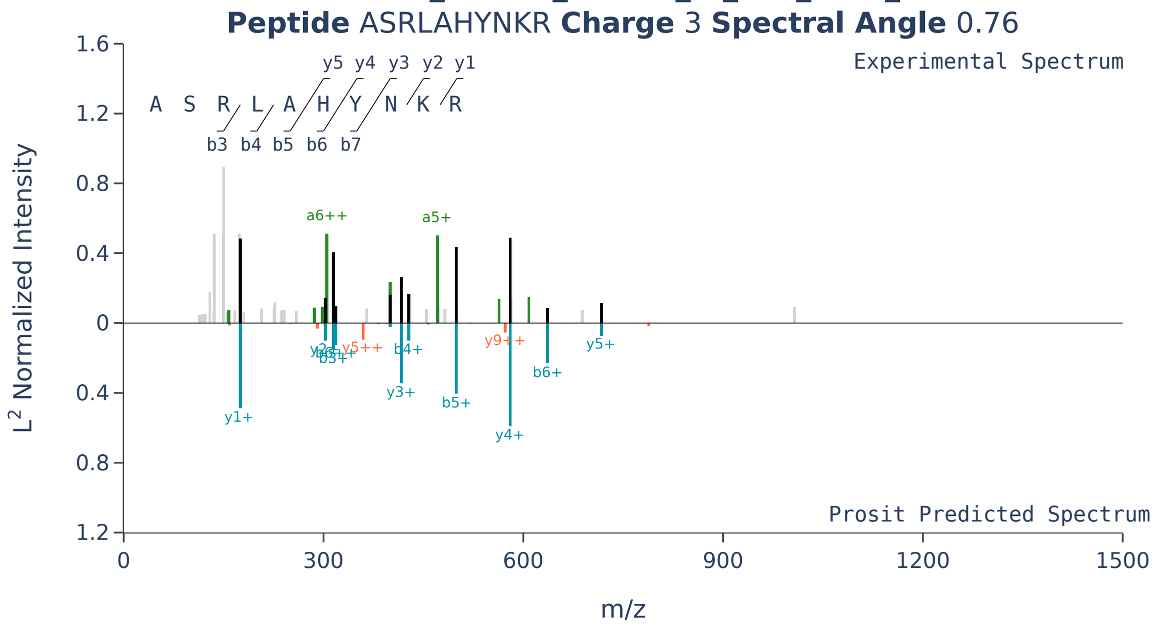


**Source** HRoetschke_291222_080123_Fu_H2B_b2R2_24h **Scan** 8834
**Peptide** QVHPDTGISSKAMSL **Charge** 2 **Spectral Angle** 0.89

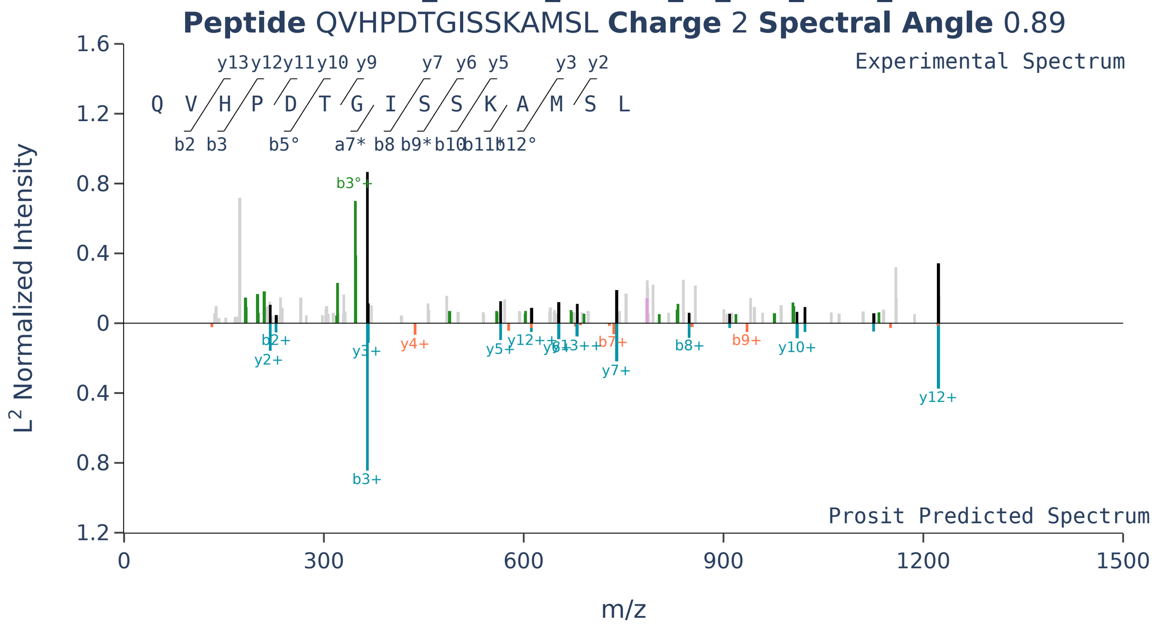


**Source** HRoetschke_291222_080123_Fu_H2B_b2R1_1h **Scan** 6640
**Peptide** GEASRLAHYNKRSTITSREIQTAVR **Charge** 5 **Spectral Angle** 0.72

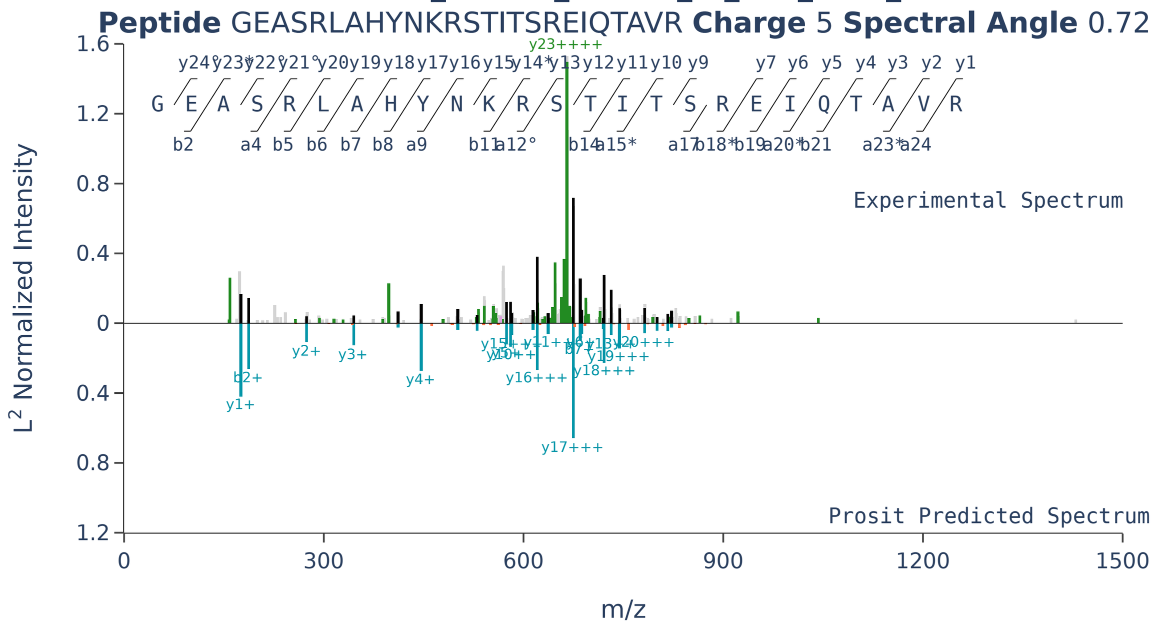


**Source** HRoetschke_291222_080123_Fu_H2B_b2R2_24h **Scan** 2891
**Peptide** GELAKHAVSE **Charge** 2 **Spectral Angle** 0.83

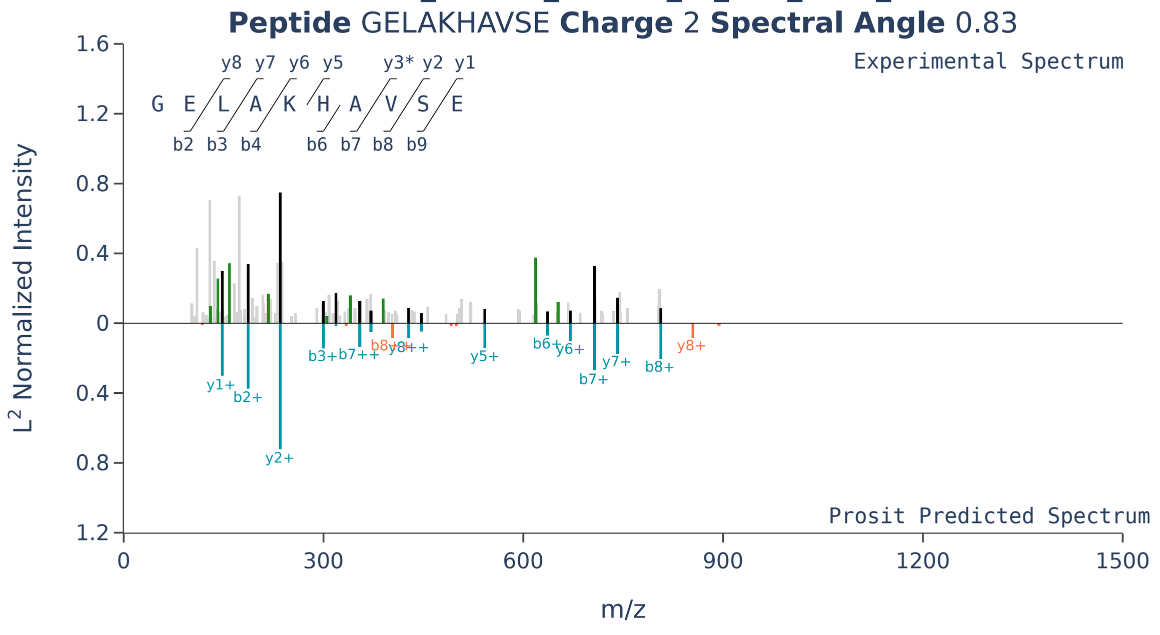


**Source** HRoetschke_291222_080123_Fu_H2B_b2R1_4h **Scan** 16199
**Peptide** RKESYAIYVYKVL **Charge** 3 **Spectral Angle** 0.78

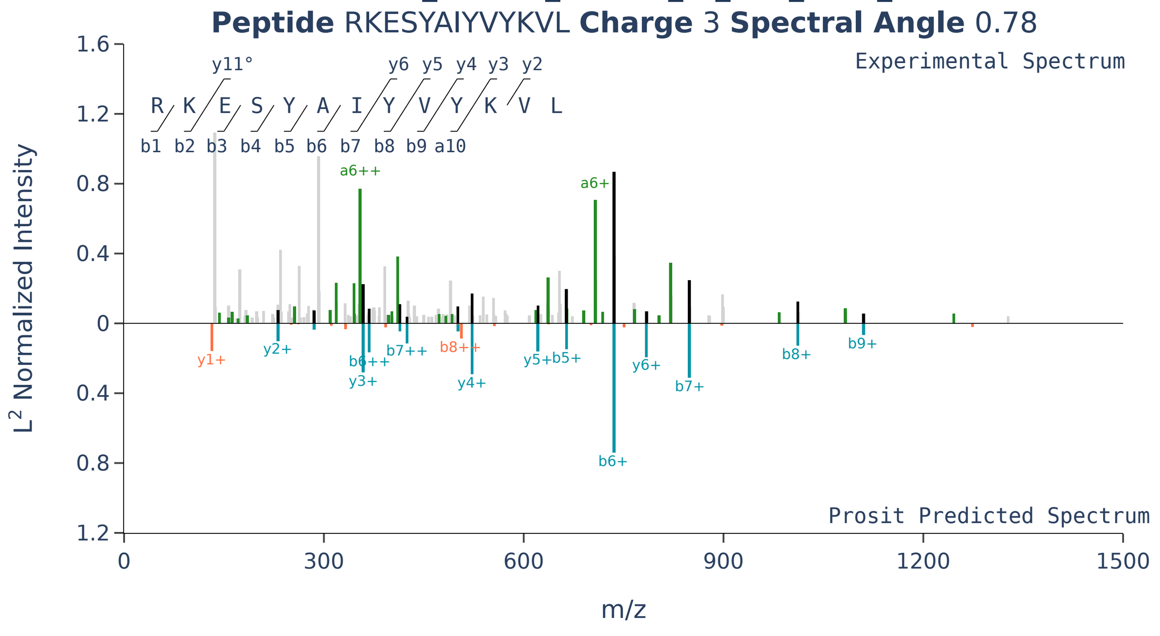


**Source** HRoetschke_291222_080123_Fu_H2B_b2R1_4h **Scan** 13769
**Peptide** VFERIAGEASRLAHYNKRSTL **Charge** 4 **Spectral Angle** 0.79

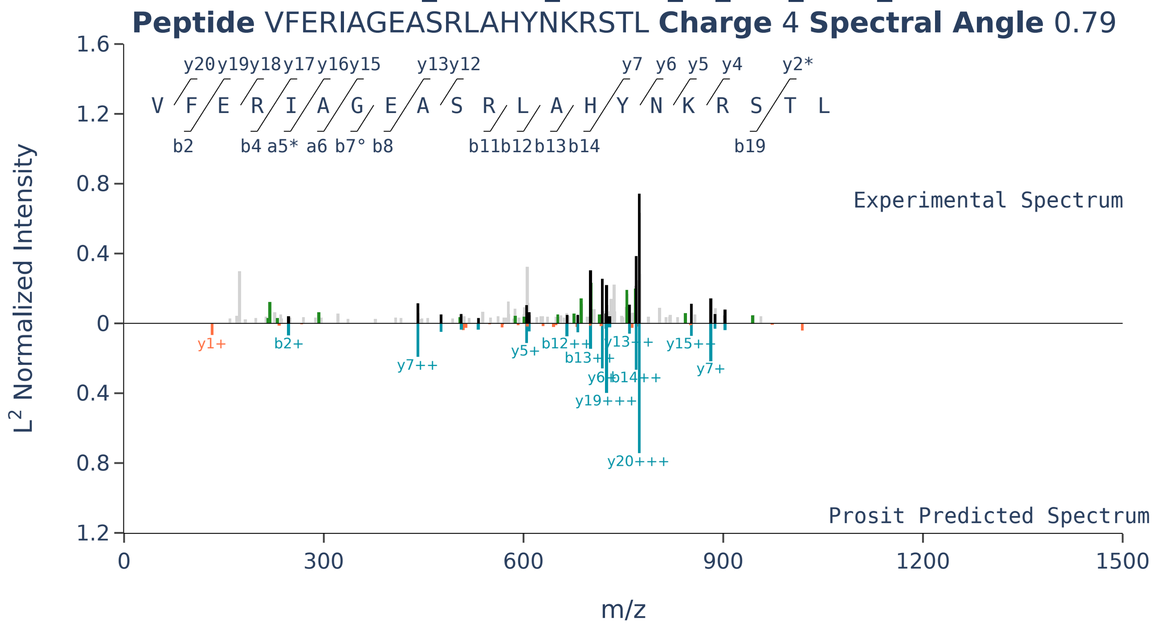


**Source** HRoetschke_291222_080123_Fu_H2B_b2R2_4h **Scan** 2914
**Peptide** LAHYNKRSTL **Charge** 3 **Spectral Angle** 0.79

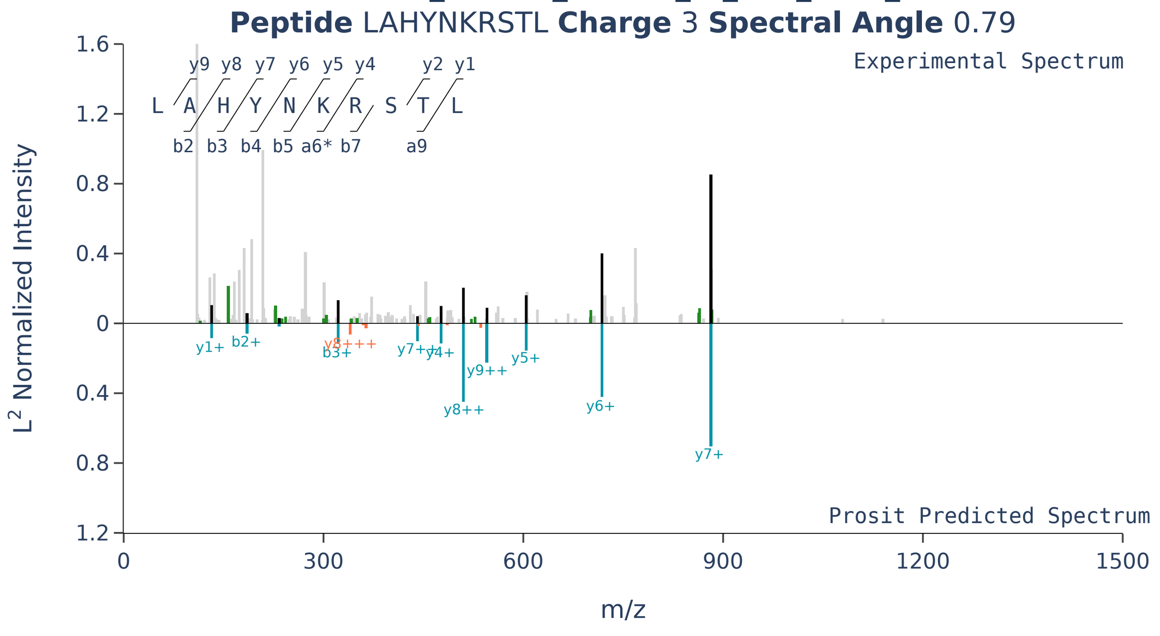


**Source** HRoetschke_291222_080123_Fu_H2B_b2R2_24h **Scan** 14104
**Peptide** AIYVYKV **Charge** 2 **Spectral Angle** 0.81

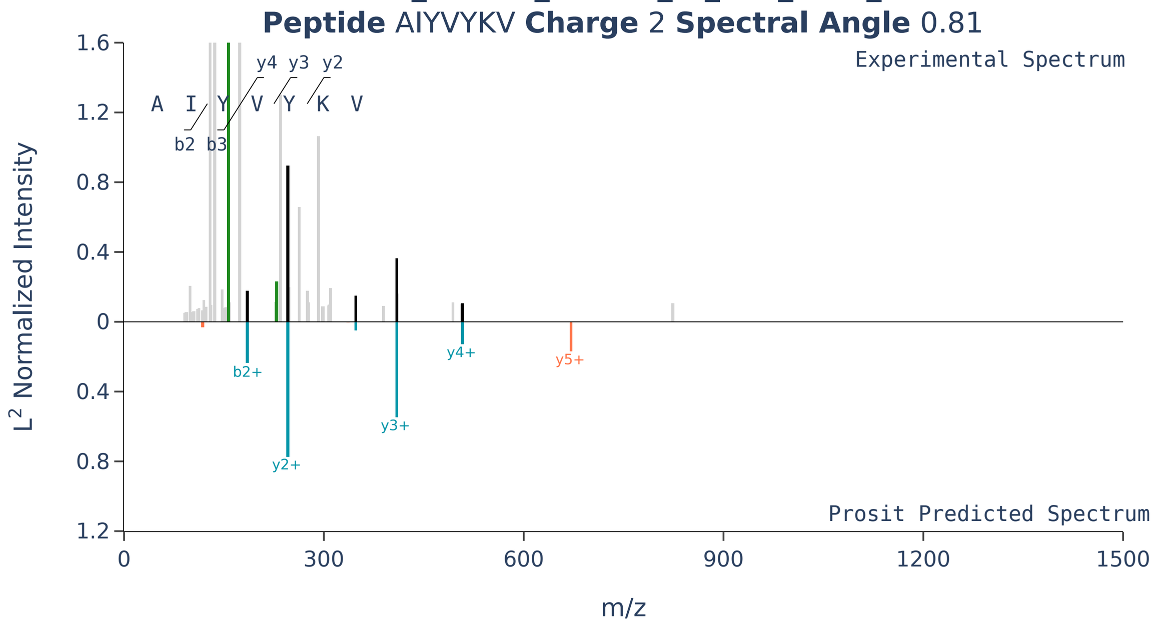


**Source** HRoetschke_291222_080123_Fu_H2B_b2R2_24h **Scan** 4514
**Peptide** LKQVHPDTGISSKAMS **Charge** 4 **Spectral Angle** 0.75

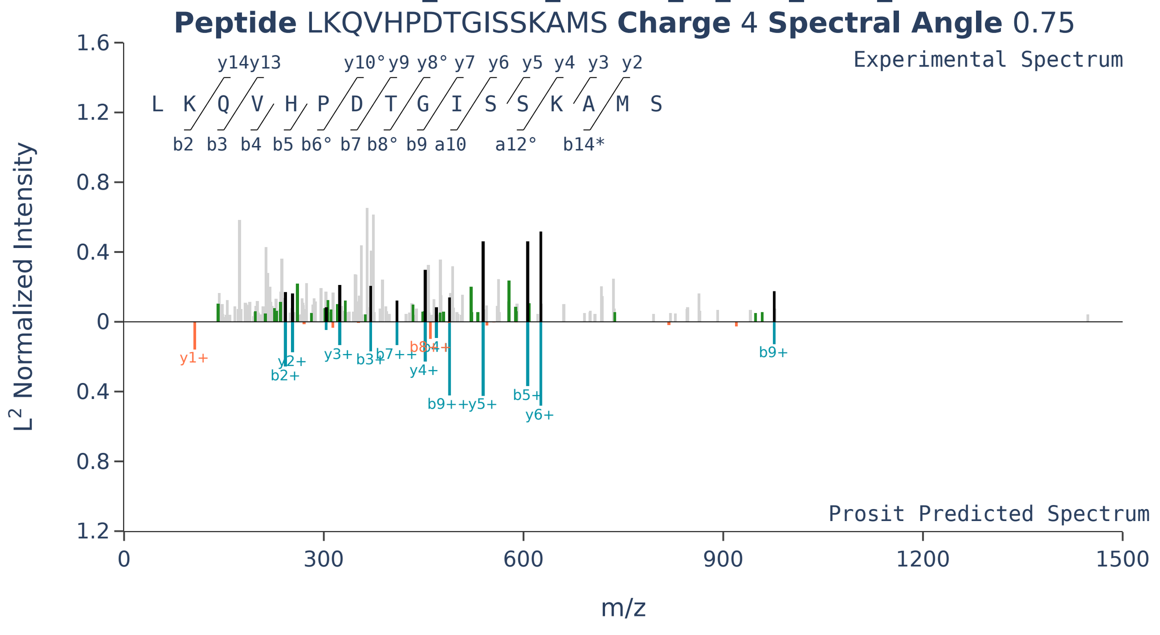


**Source** HRoetschke_291222_080123_Fu_H2B_b2R2_4h **Scan** 19818
**Peptide** VNDVFERIAGEASRLAHYNKRSTL **Charge** 4 **Spectral Angle** 0.86

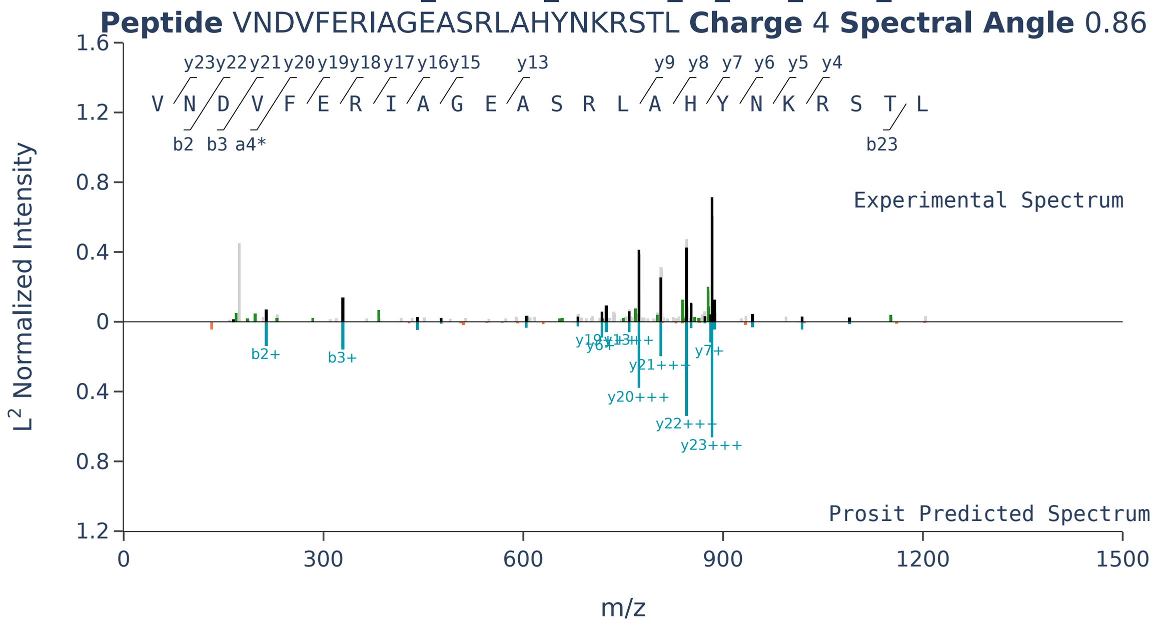


**Source** HRoetschke_291222_080123_Fu_H2B_b1R1_4h **Scan** 8296
**Peptide** LKQVHPDTGISSKAMSL **Charge** 3 **Spectral Angle** 0.77

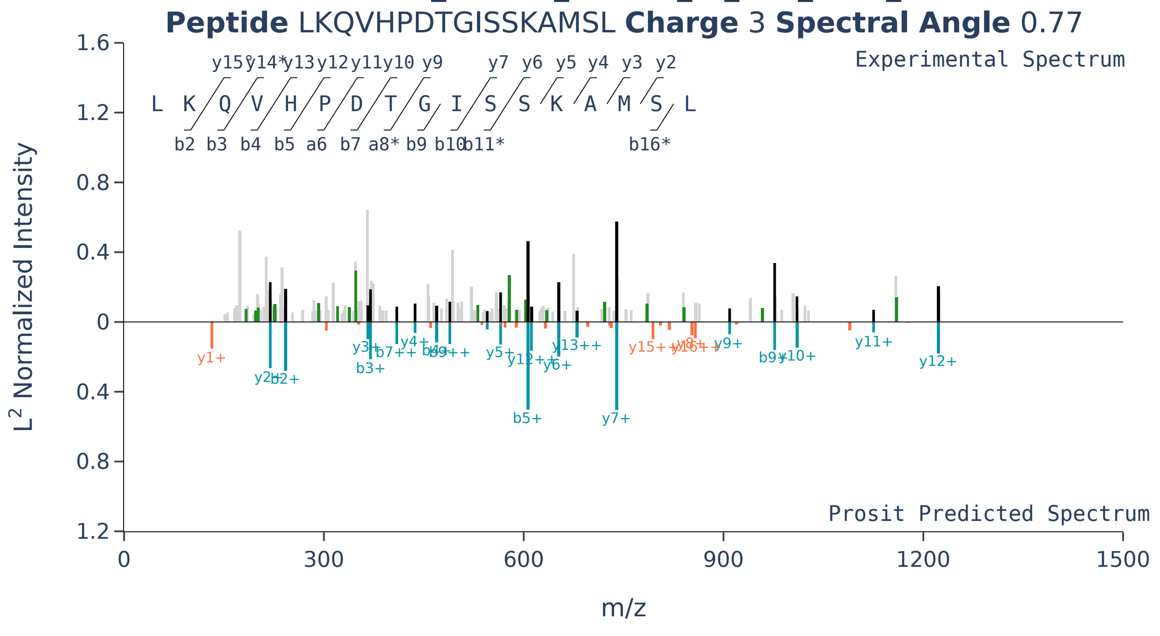


**Source** HRoetschke_291222_080123_Fu_H2B_b2R1_1h **Scan** 14649
**Peptide** RSTITSREIQTAVRLL **Charge** 4 **Spectral Angle** 0.77

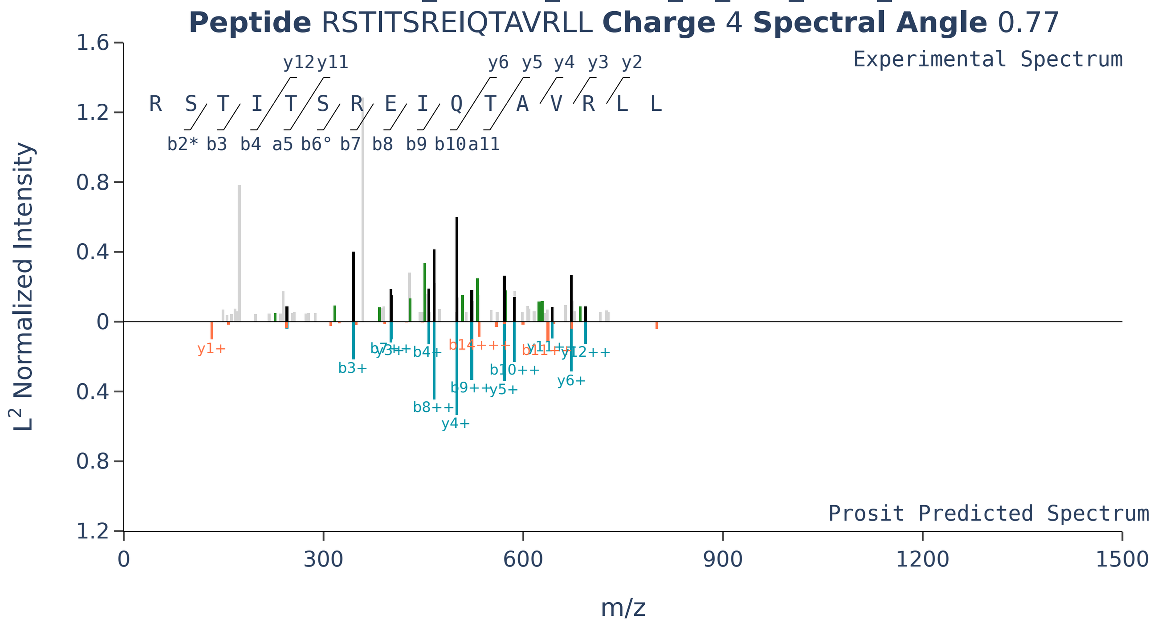


**Source** HRoetschke_291222_080123_Fu_H2B_b2R1_24h **Scan** 15221
**Peptide** KESYAIYVY **Charge** 2 **Spectral Angle** 0.83

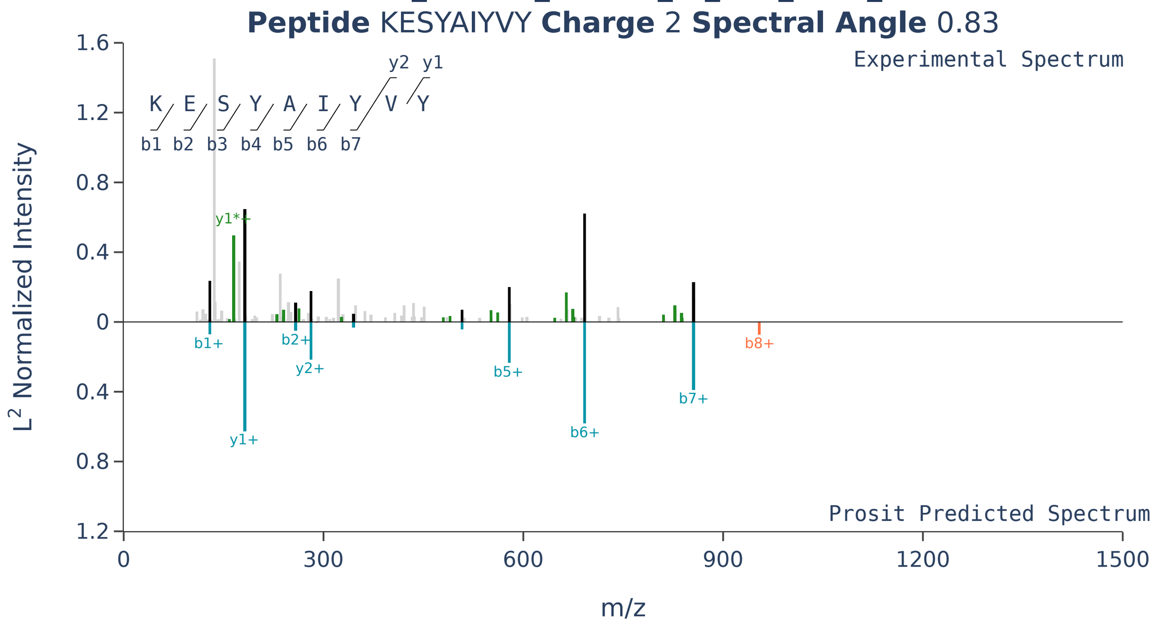

**Source** HRoetschke_291222_080123_Fu_H2B_b1R1_2h **Scan** 13365
**Peptide** LLPGELAKHAVSEGTKAVTKYTSAK **Charge** 4 **Spectral Angle** 0.74

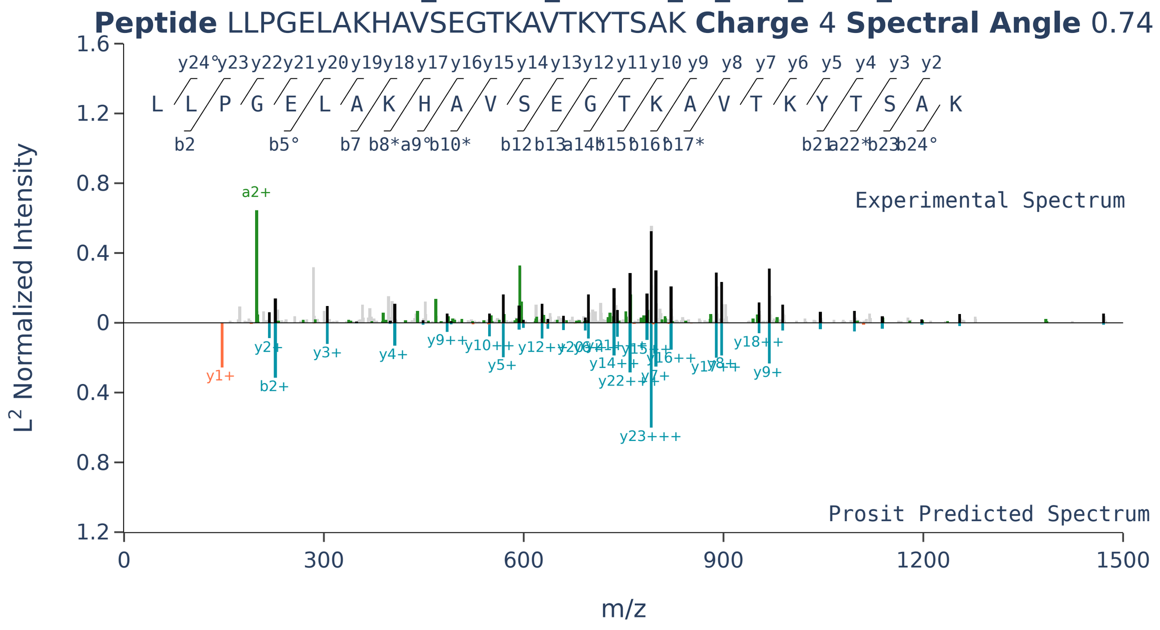


**Source** HRoetschke_291222_080123_Fu_H2B_b1R1_4h **Scan** 6722
**Peptide** PGELAKHAVSEGTKAVT **Charge** 2 **Spectral Angle** 0.81

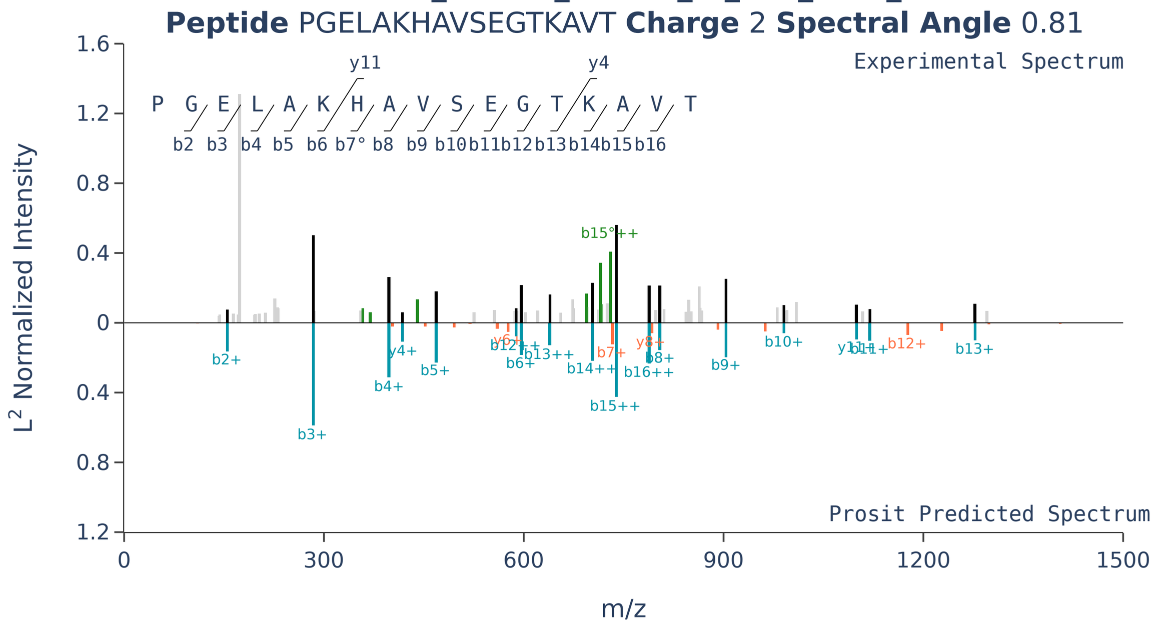


**Source** HRoetschke_291222_080123_Fu_H2B_b2R2_4h **Scan** 12508
**Peptide** VFERIAGEASRLAHYNKRSTITSR **Charge** 6 **Spectral Angle** 0.75

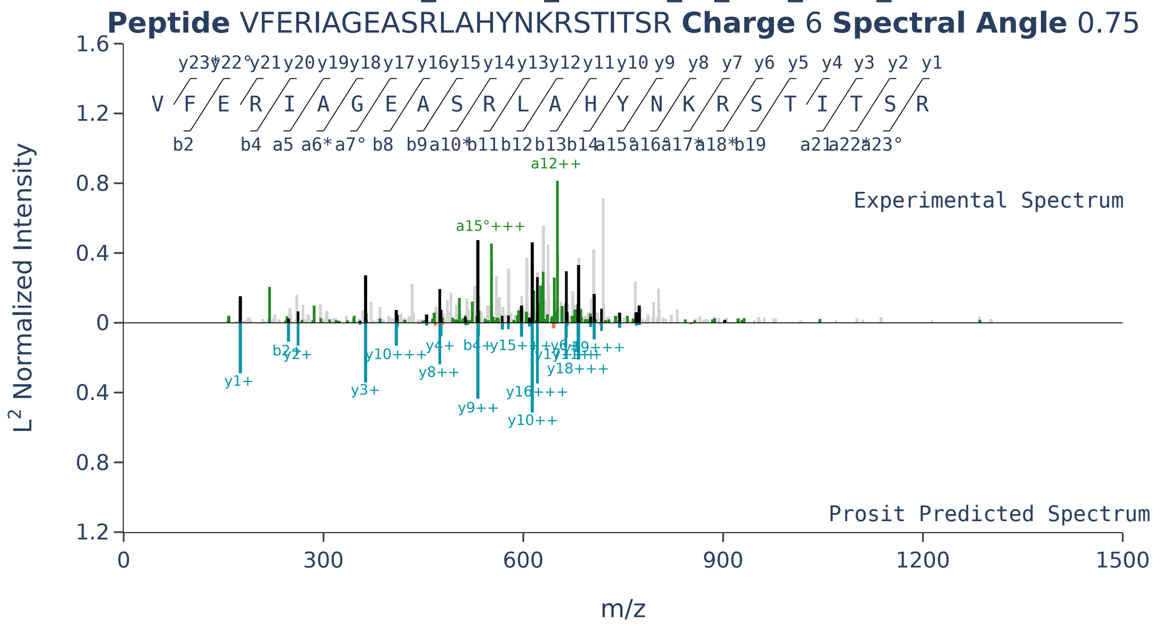


**Source** HRoetschke_291222_080123_Fu_H2B_b1R2_1h **Scan** 2996
**Peptide** RLAGEASRLAH **Charge** 3 **Spectral Angle** 0.82

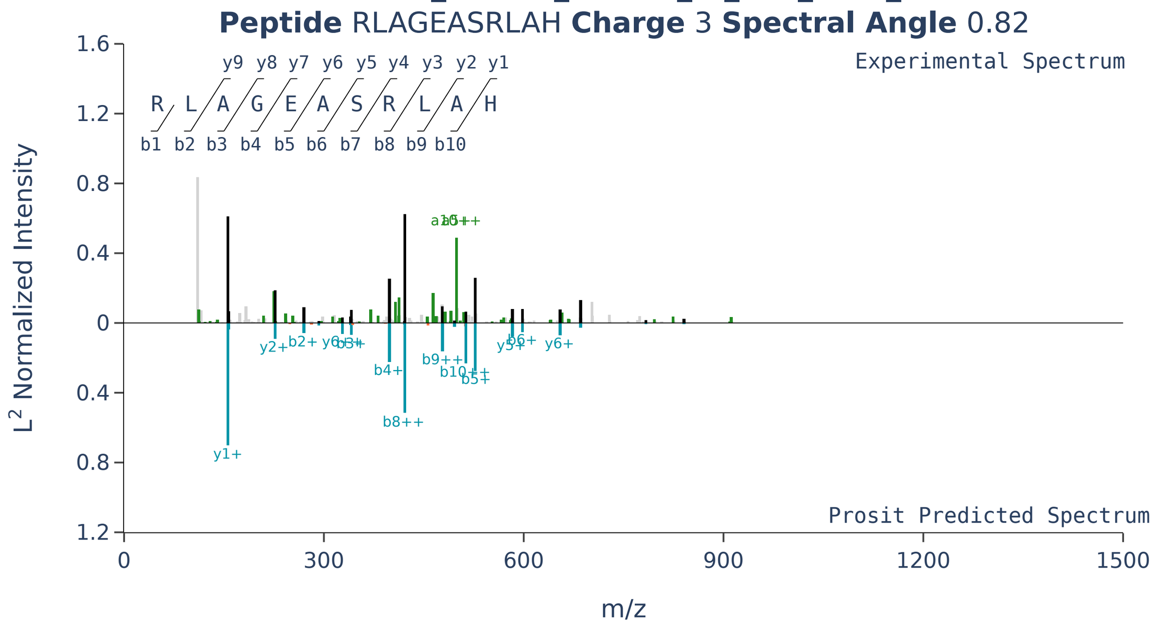


**Source** HRoetschke_291222_080123_Fu_H2B_b2R2_24h **Scan** 18680
**Peptide** NDVFERIAGEASRLAHYNKRST **Charge** 4 **Spectral Angle** 0.84

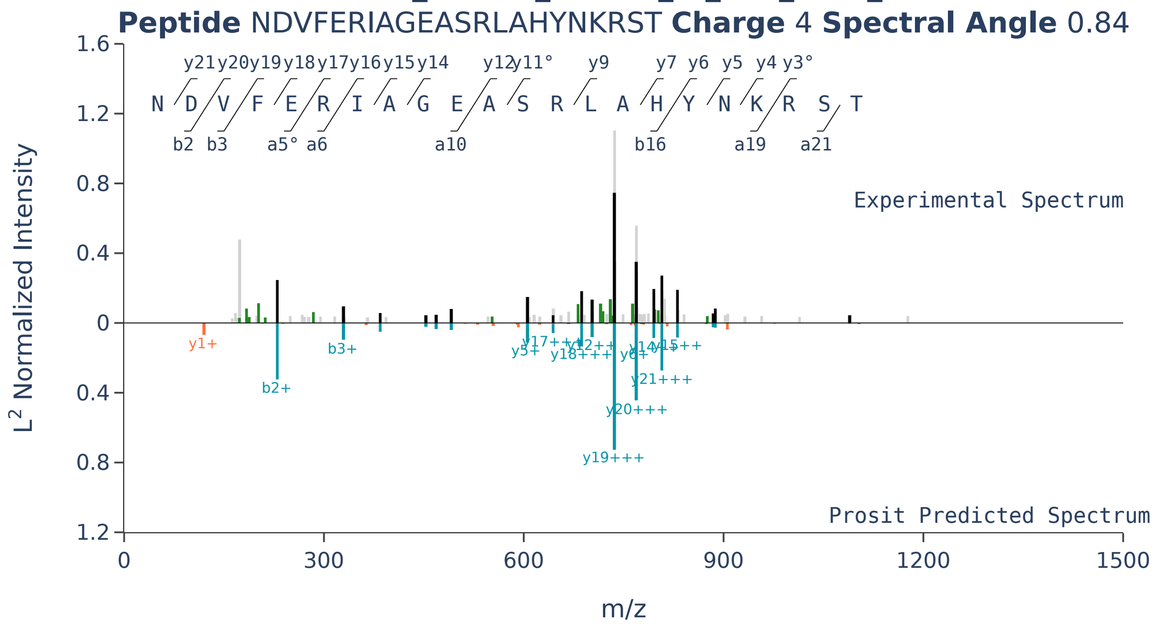


**Source** HRoetschke_291222_080123_Fu_H2B_b2R2_1h **Scan** 4638
**Peptide** RLAGEASRLAHYNKRSTITSR **Charge** 5 **Spectral Angle** 0.75

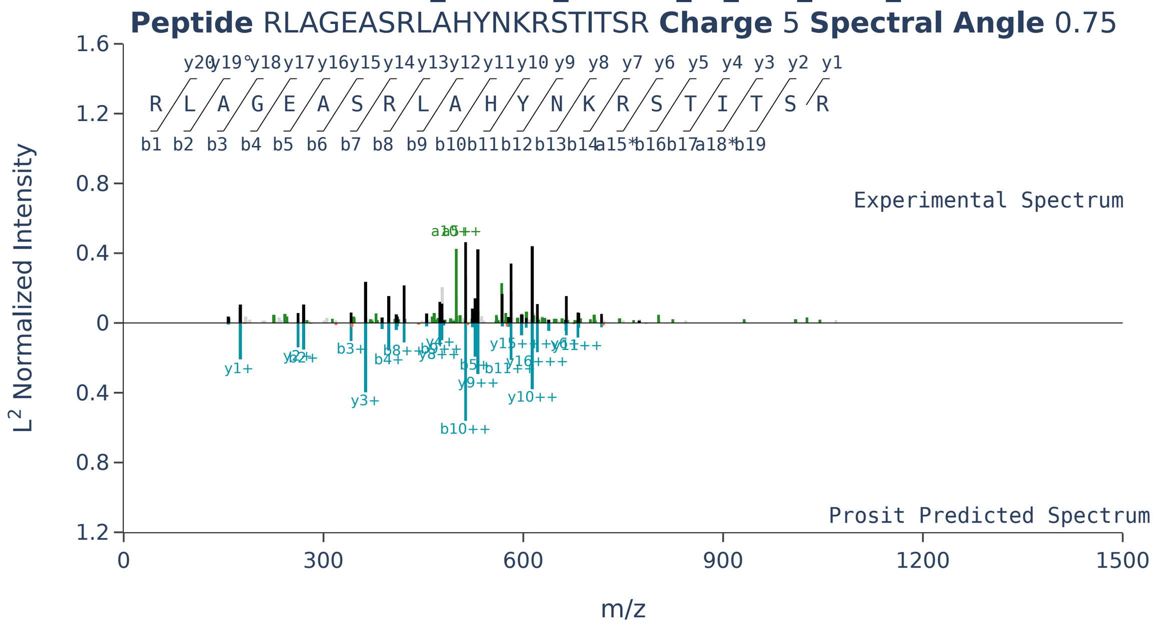


**Source** HRoetschke_291222_080123_Fu_H2B_b2R1_24h **Scan** 18888
**Peptide** TSREIQTAVRLLLPGELAKH **Charge** 4 **Spectral Angle** 0.73

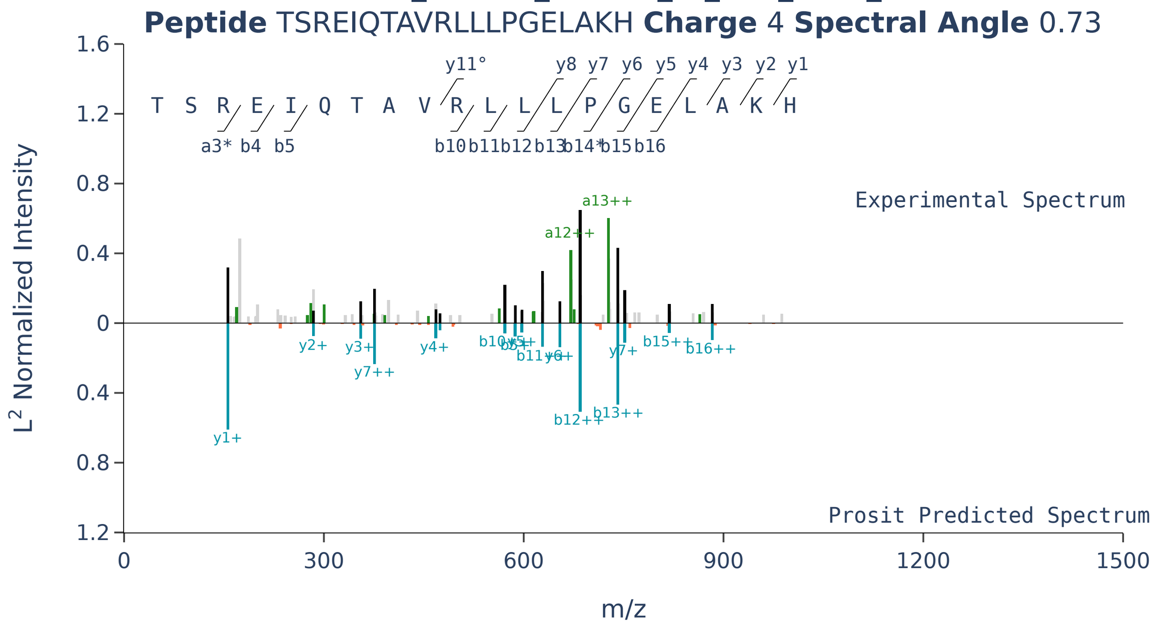


**Source** HRoetschke_291222_080123_Fu_H2B_b1R1_24h **Scan** 5162
**Peptide** RLAGEASRLAHYNK **Charge** 3 **Spectral Angle** 0.84

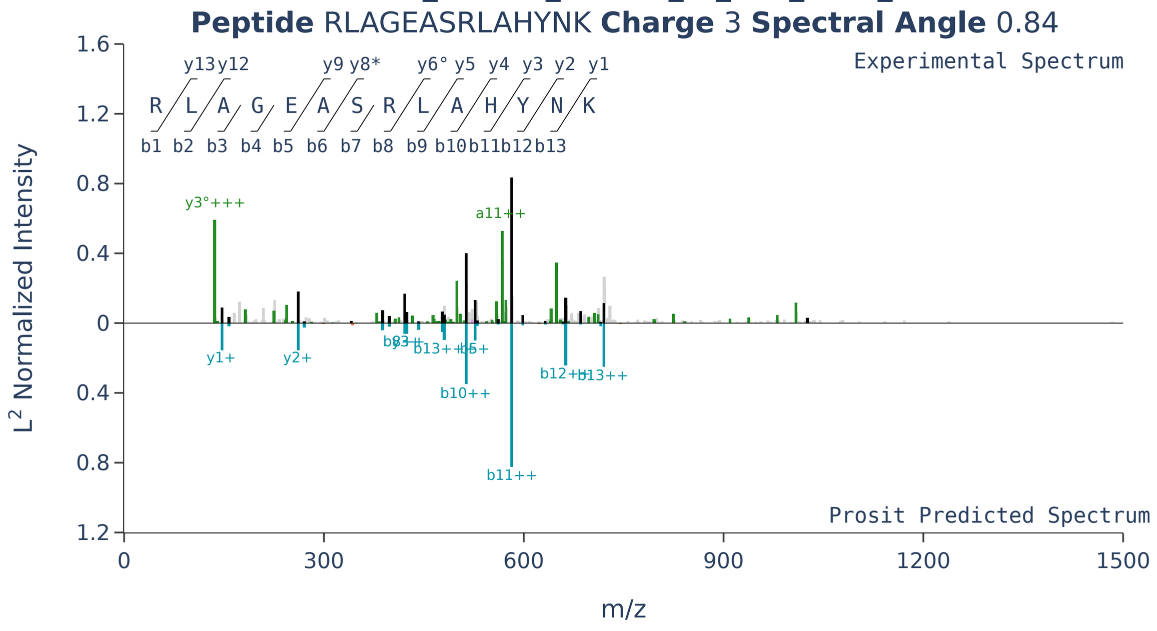


**Source** HRoetschke_291222_080123_Fu_H2B_b2R2_4h **Scan** 16627
**Peptide** VRLLLPGELAKHAVSEGTKAVTKY **Charge** 5 **Spectral Angle** 0.83

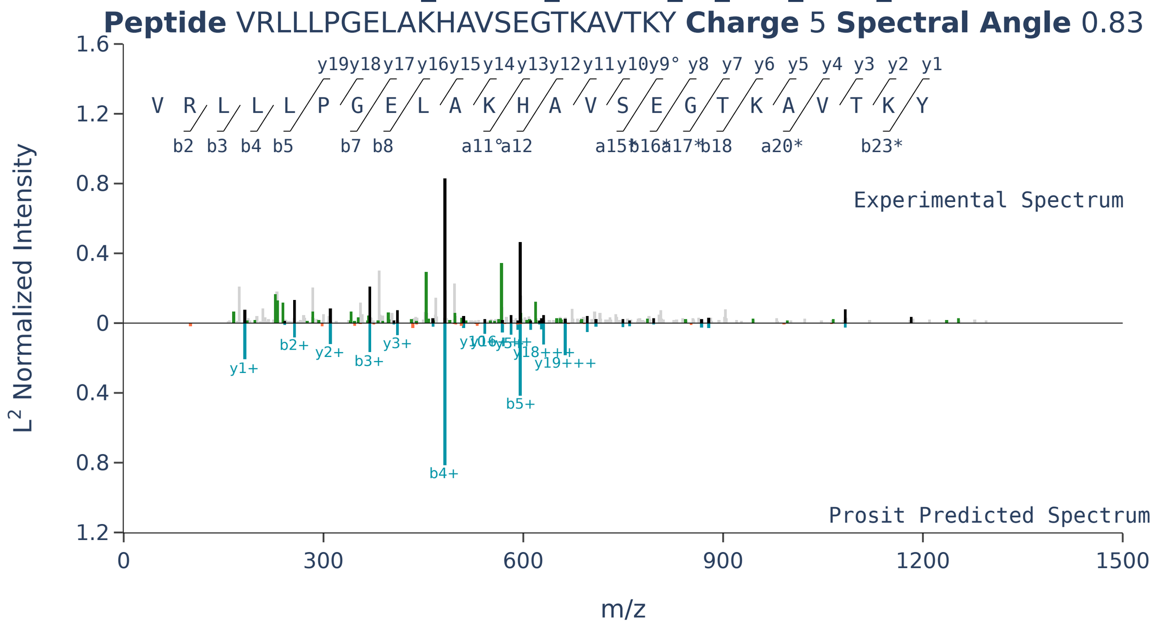


**Source** HRoetschke_291222_080123_Fu_H2B_b2R1_24h **Scan** 17638
**Peptide** AVRLLLPGELAKHAVSEGTKAVTKY **Charge** 5 **Spectral Angle** 0.78

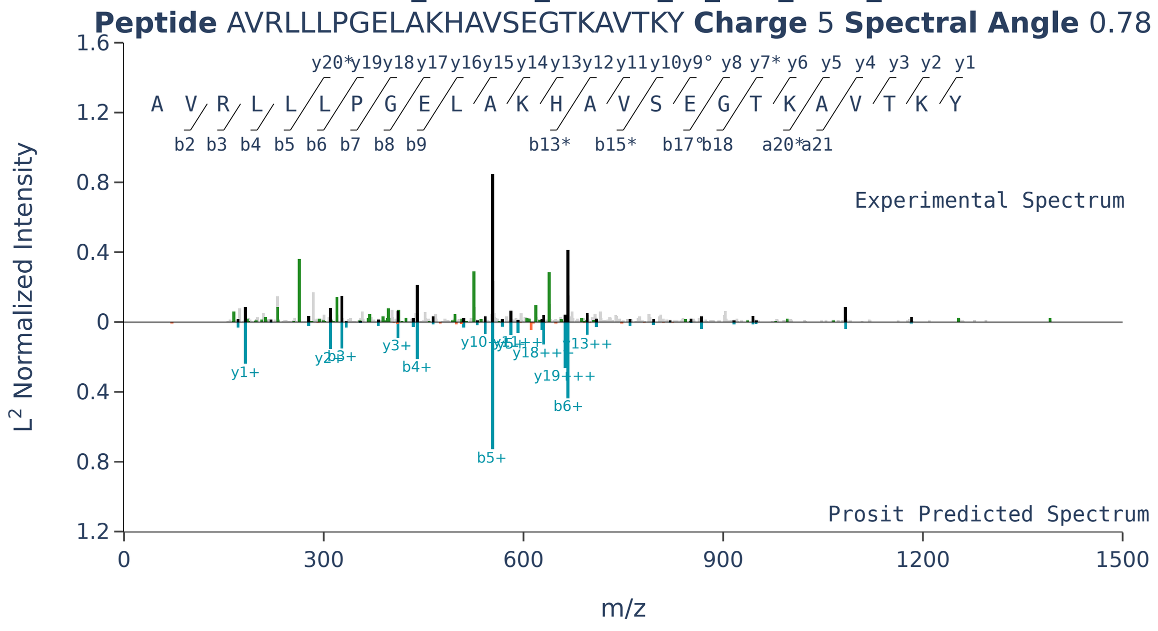


**Source** HRoetschke_291222_080123_Fu_H2B_b2R2_2h **Scan** 3973
**Peptide** RIAGEASRLAHYNKR **Charge** 4 **Spectral Angle** 0.74

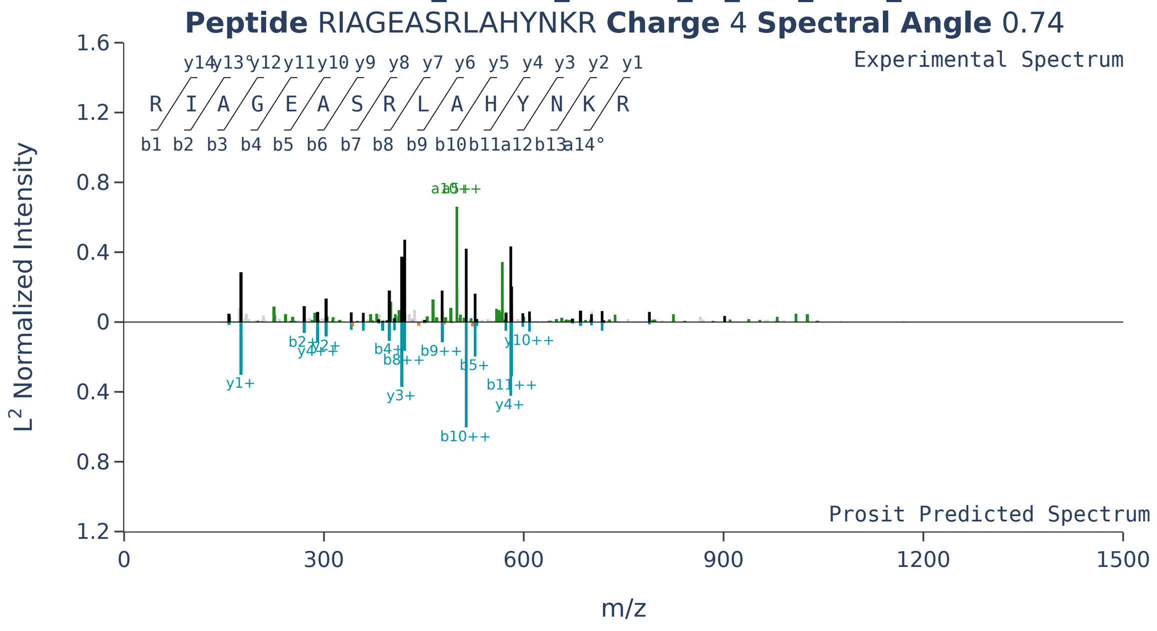


**Source** HRoetschke_291222_080123_Fu_H2B_b2R2_24h **Scan** 17454
**Peptide** AVRLLLPGELAKHAVSE **Charge** 3 **Spectral Angle** 0.79

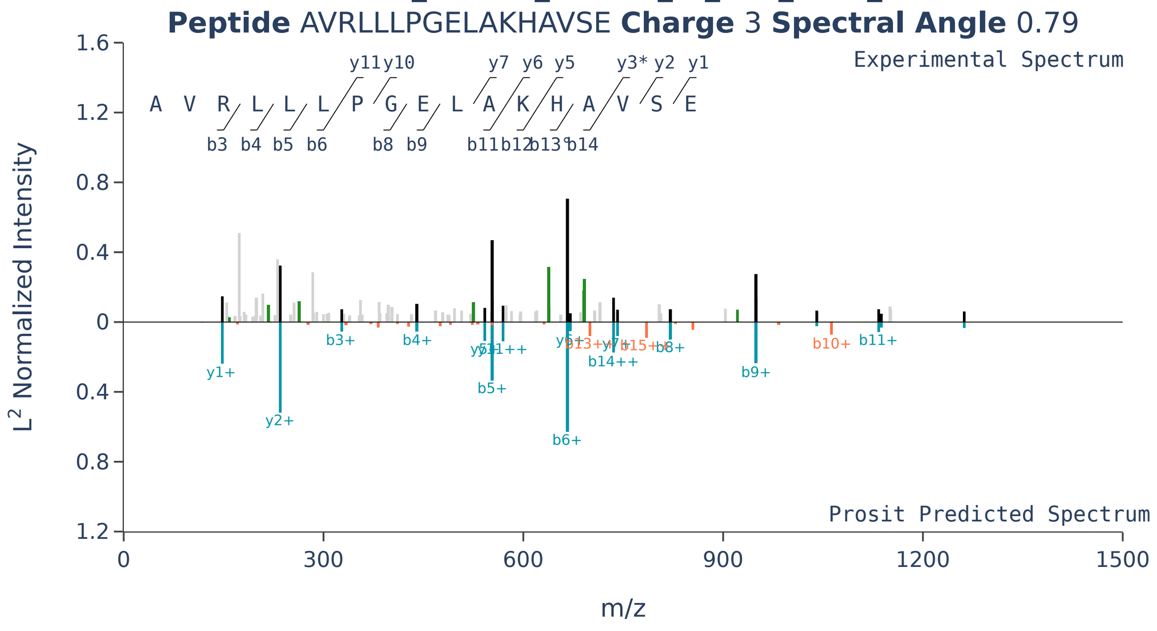


**Source** HRoetschke_291222_080123_Fu_H2B_b2R2_4h **Scan** 15821
**Peptide** AVRLLLPGELAKHAVSEGTKAVTK **Charge** 4 **Spectral Angle** 0.82

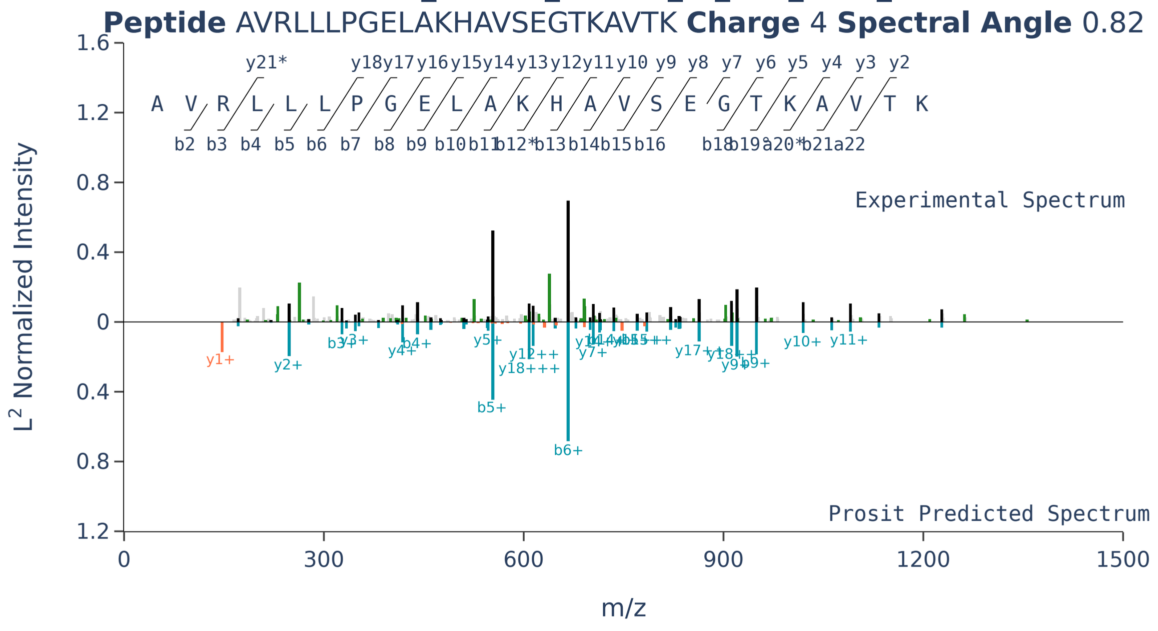


**Source** HRoetschke_291222_080123_Fu_H2B_b2R1_24h **Scan** 13465
**Peptide** VHPDTGISSKAMSIMNSFVND **Charge** 3 **Spectral Angle** 0.93

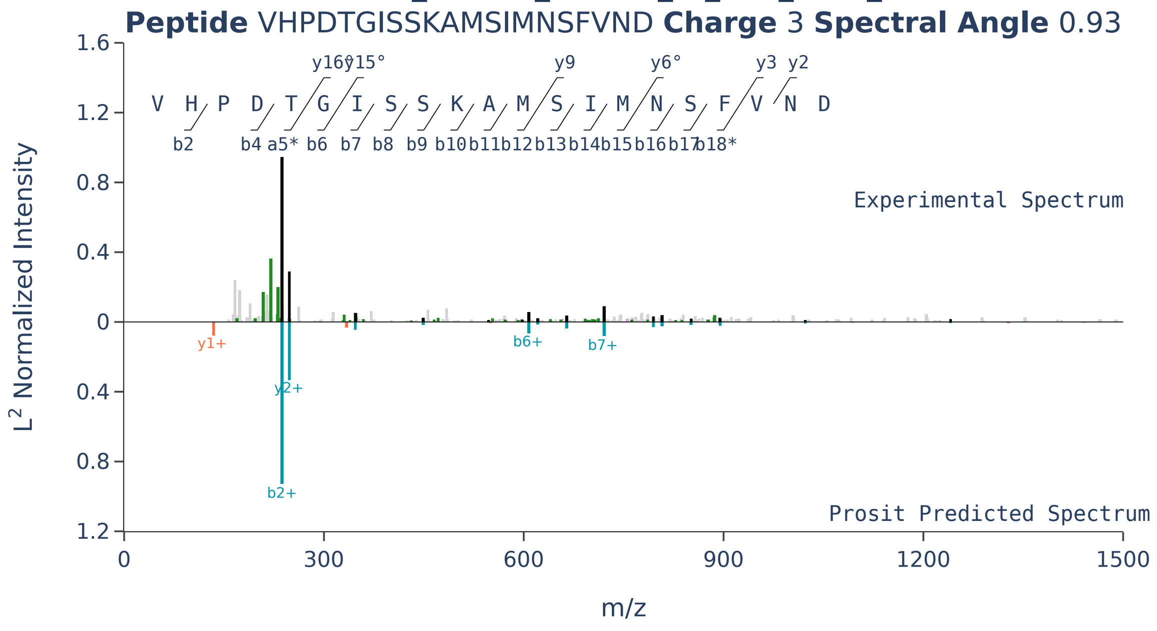


**Source** HRoetschke_291222_080123_Fu_H2B_b2R1_1h **Scan** 16921
**Peptide** VNDVFERIAGEASRLAHYNKRSTIT **Charge** 4 **Spectral Angle** 0.82

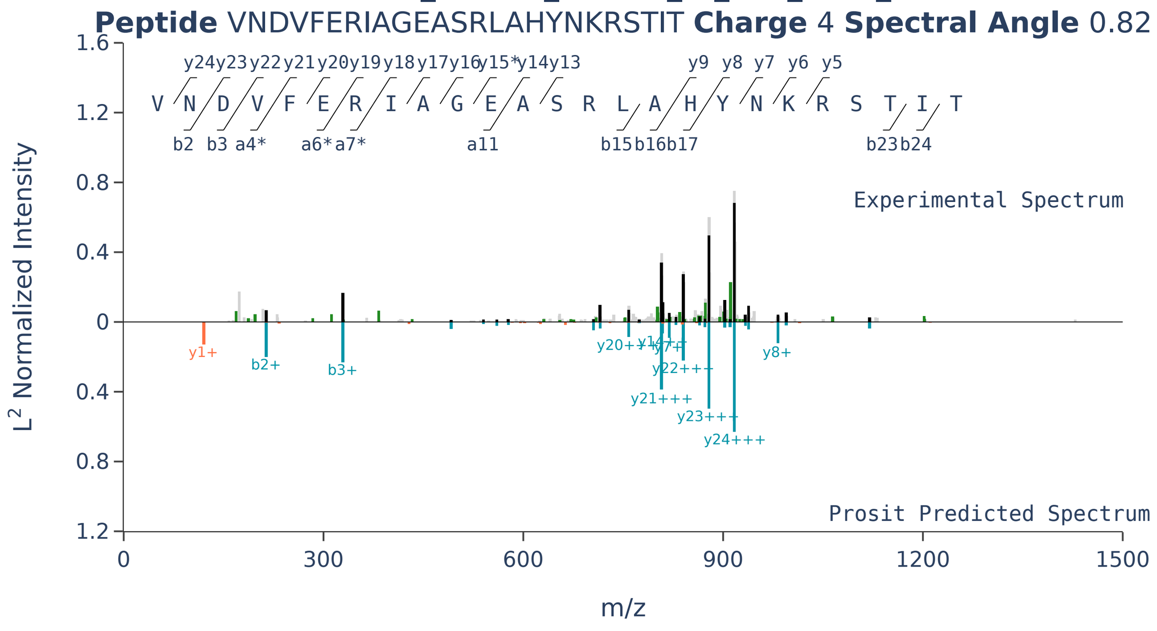

**Source** HRoetschke_291222_080123_Fu_H2B_b2R1_2h **Scan** 7261
**Peptide** LPGELAKHAVS **Charge** 2 **Spectral Angle** 0.73

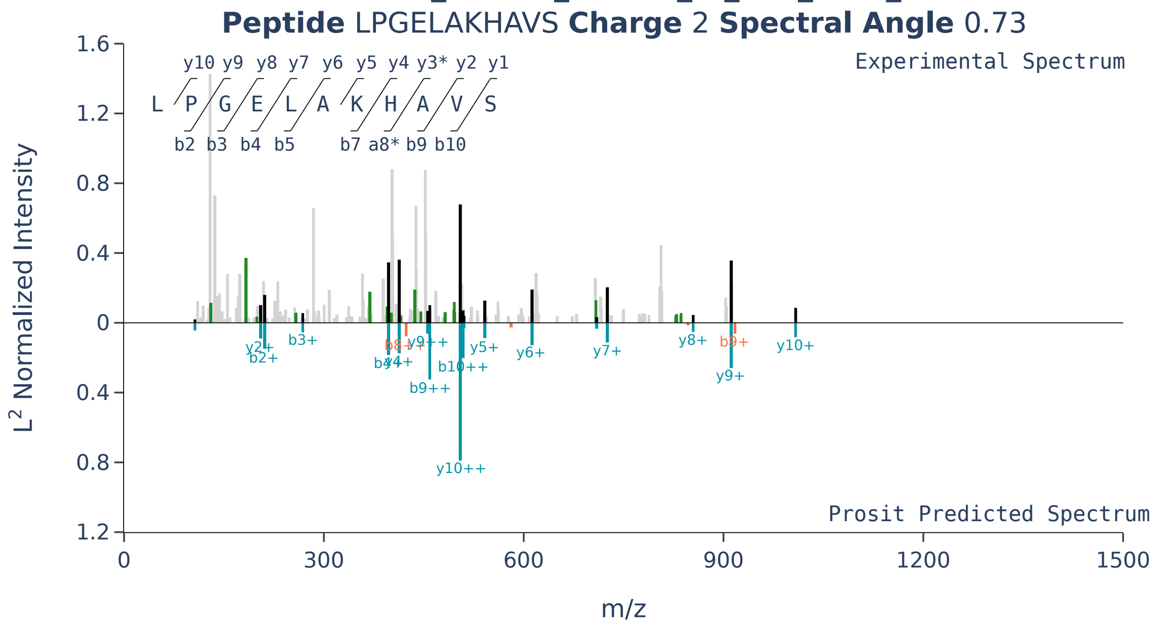


**Source** HRoetschke_291222_080123_Fu_H2B_b2R2_24h **Scan** 8085
**Peptide** RKTRKESYAIYV **Charge** 3 **Spectral Angle** 0.83

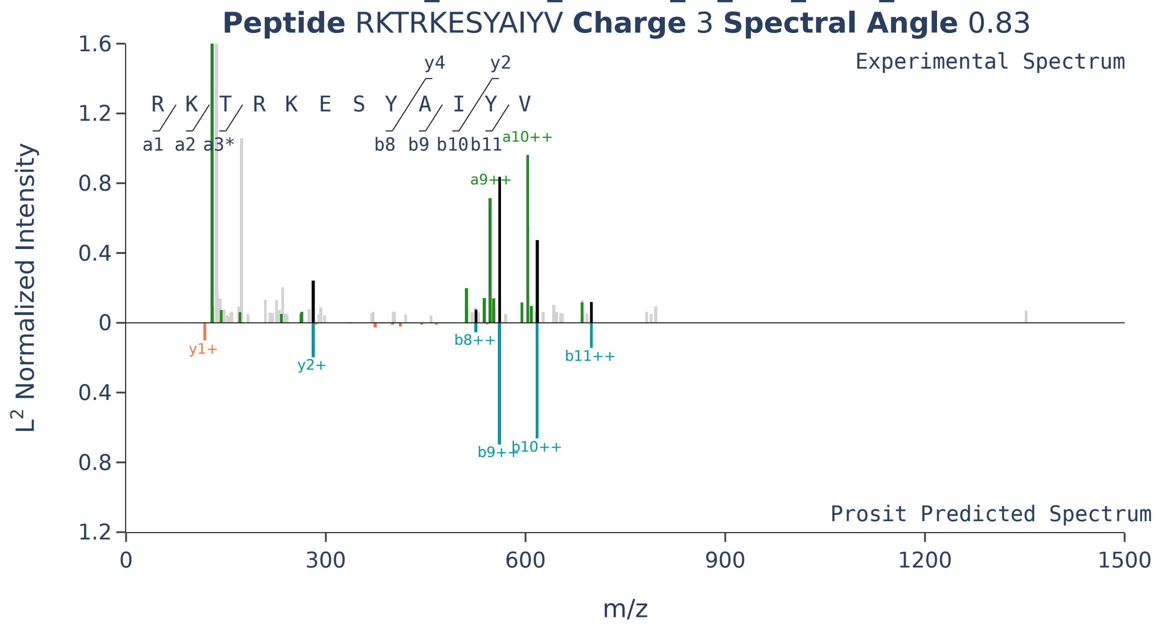


**Source** HRoetschke_291222_080123_Fu_H2B_b2R2_2h **Scan** 5403
**Peptide** PGELAKHAVSEGTKAVTK **Charge** 3 **Spectral Angle** 0.78

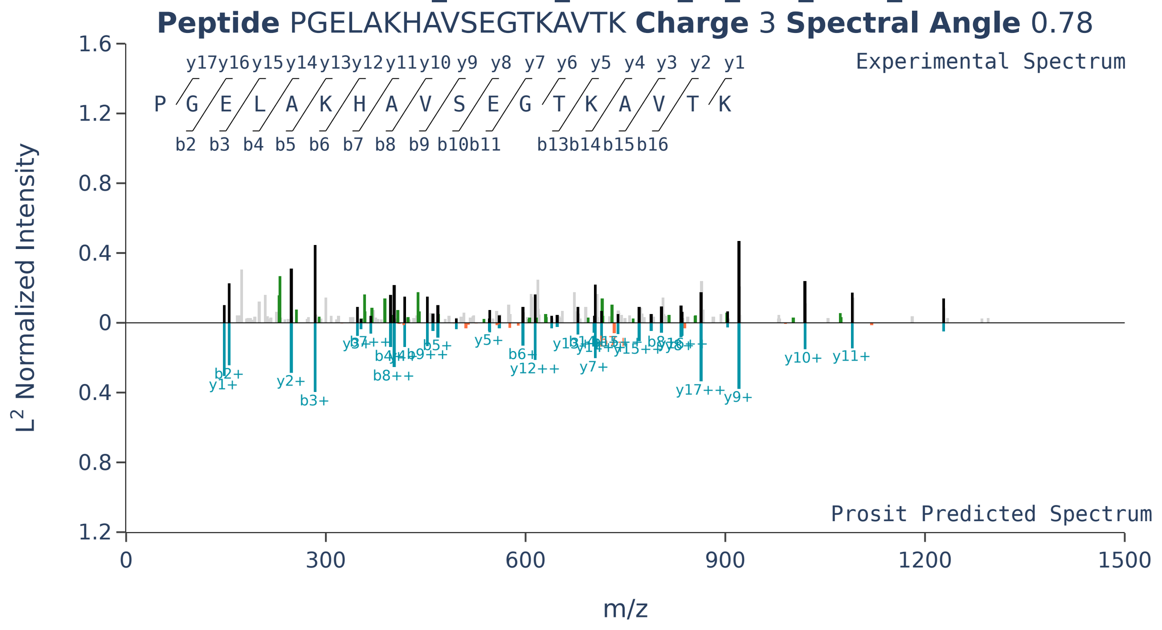


**Source** HRoetschke_291222_080123_Fu_H2B_b2R2_24h **Scan** 6058
**Peptide** KVLKQVHPDTGL **Charge** 2 **Spectral Angle** 0.78

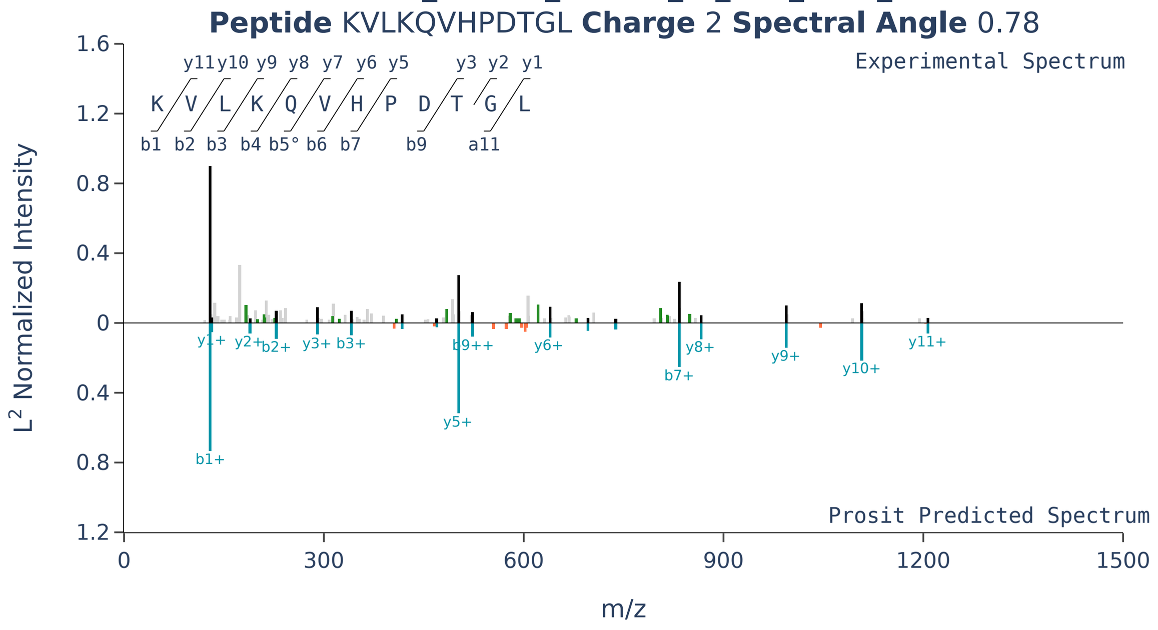


**Source** HRoetschke_291222_080123_Fu_H2B_b2R1_1h **Scan** 3431
**Peptide** PGELAKHAVSEGTKA **Charge** 2 **Spectral Angle** 0.76

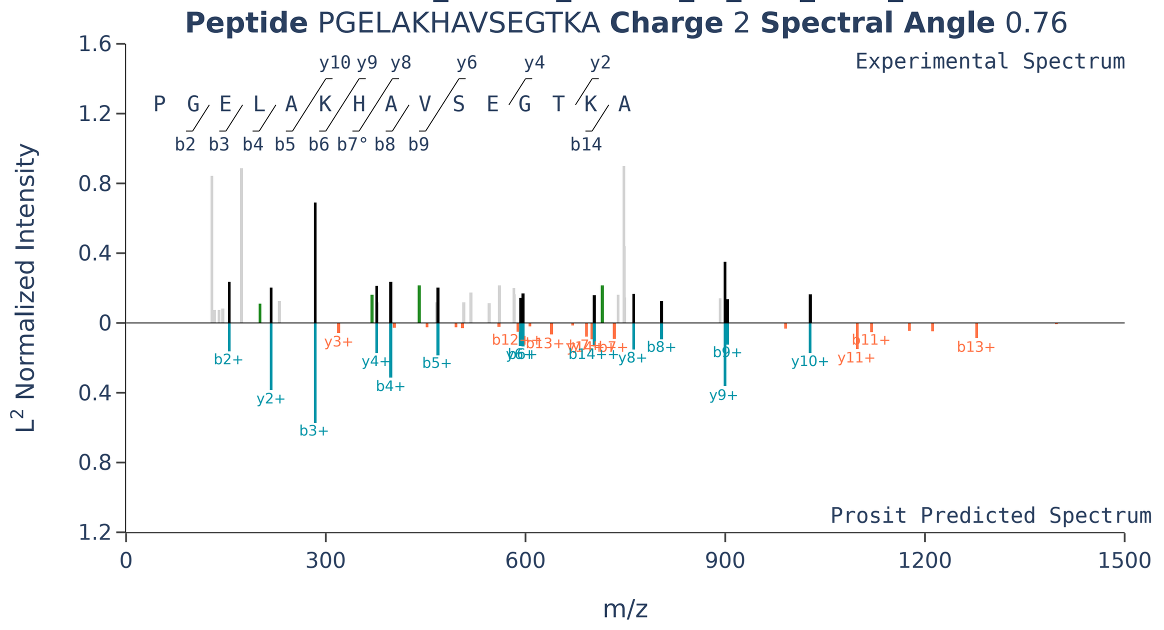


**Source** HRoetschke_291222_080123_Fu_H2B_b1R2_1h **Scan** 6344
**Peptide** RLAGEASRLAHYN **Charge** 3 **Spectral Angle** 0.78

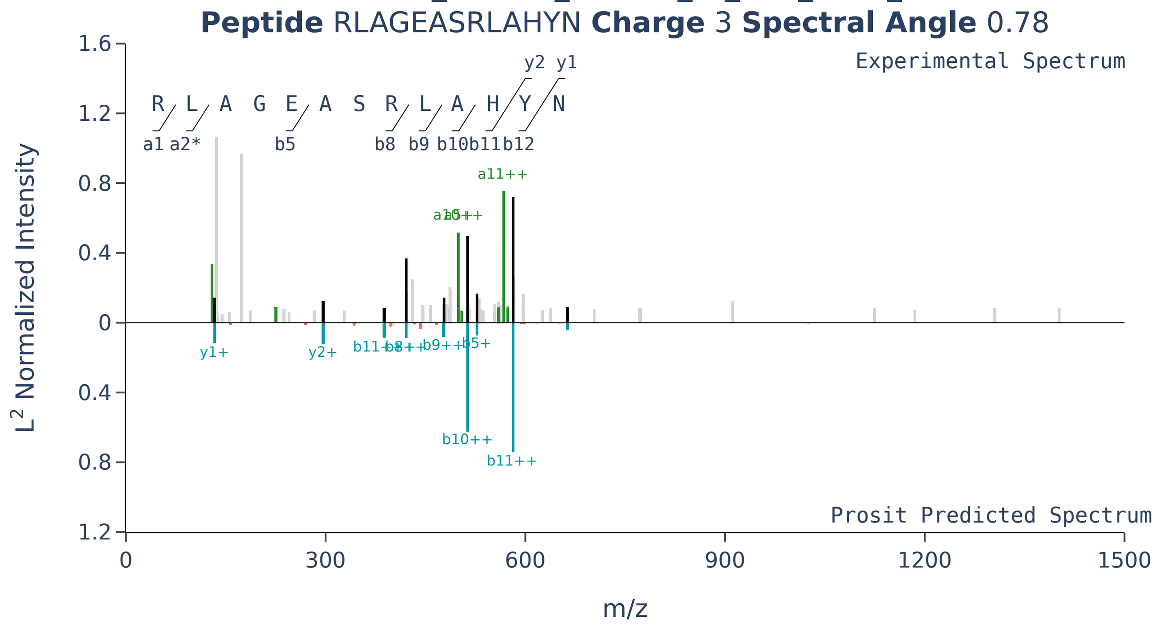


**Source** HRoetschke_291222_080123_Fu_H2B_b1R2_24h **Scan** 5870
**Peptide** KDGKKRRKTRKESYAIYVY **Charge** 4 **Spectral Angle** 0.83

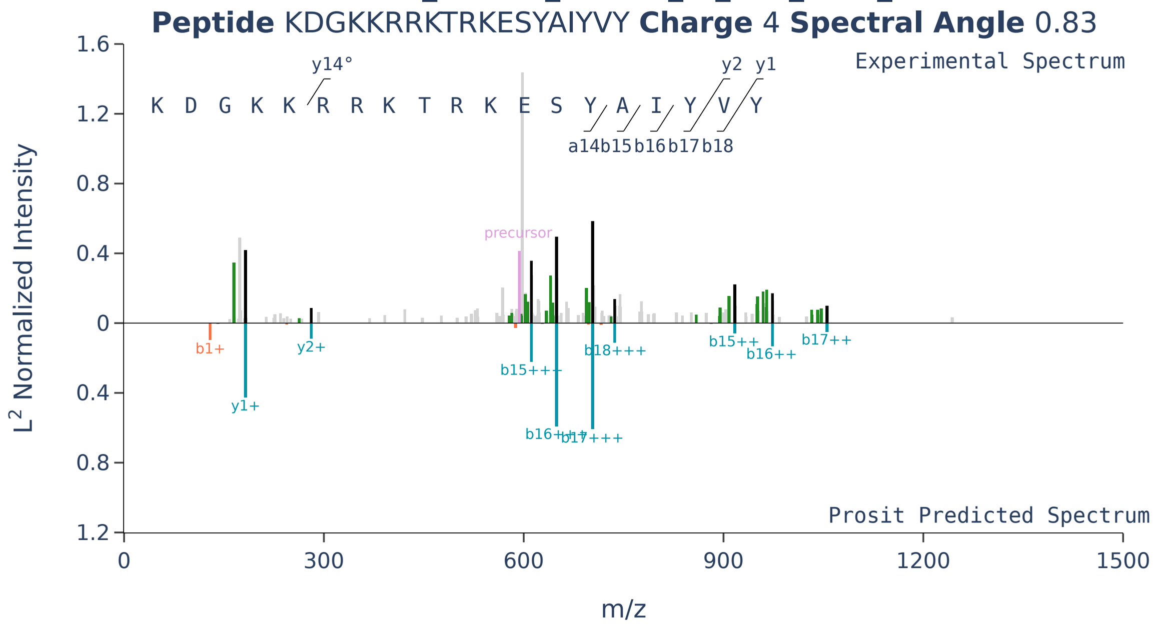


**Source** HRoetschke_291222_080123_Fu_H2B_b2R1_24h **Scan** 12321
**Peptide** LPGELAKHAVSEGTKAVTKY **Charge** 3 **Spectral Angle** 0.7

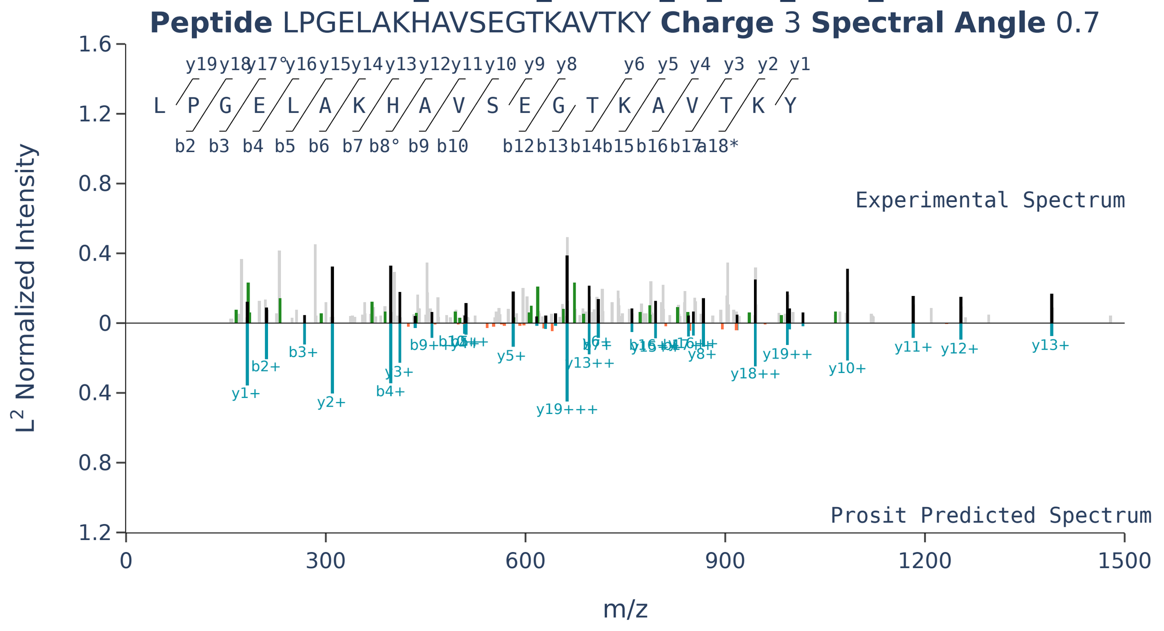


**Source** HRoetschke_291222_080123_Fu_H2B_b1R2_4h **Scan** 2263
**Peptide** GEASRLAHYNKRST **Charge** 3 **Spectral Angle** 0.78

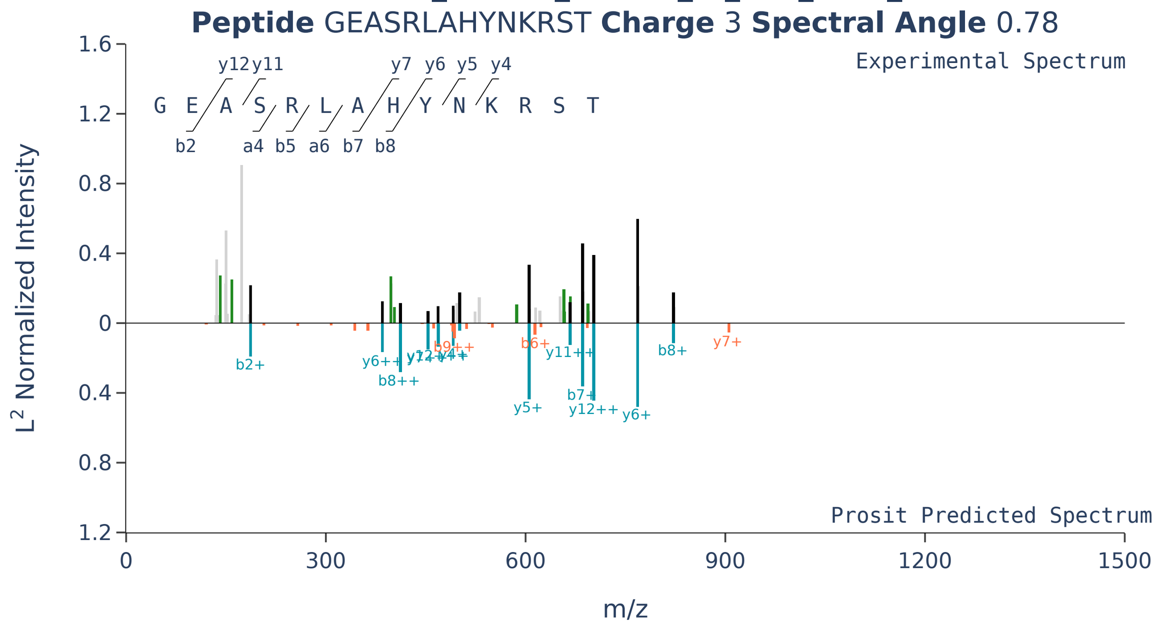


**Source** HRoetschke_291222_080123_Fu_H2B_b1R2_1h **Scan** 2189
**Peptide** RLAHYNKRSTL **Charge** 3 **Spectral Angle** 0.91

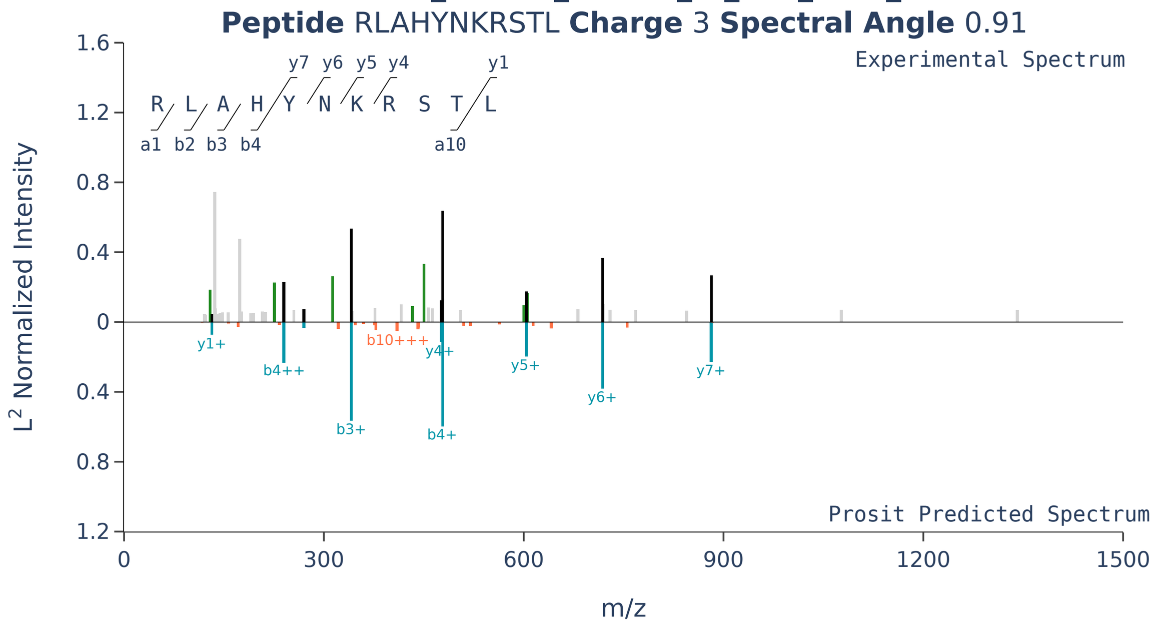


**Source** HRoetschke_291222_080123_Fu_H2B_b2R1_4h **Scan** 13108
**Peptide** NDVFERL **Charge** 2 **Spectral Angle** 0.92

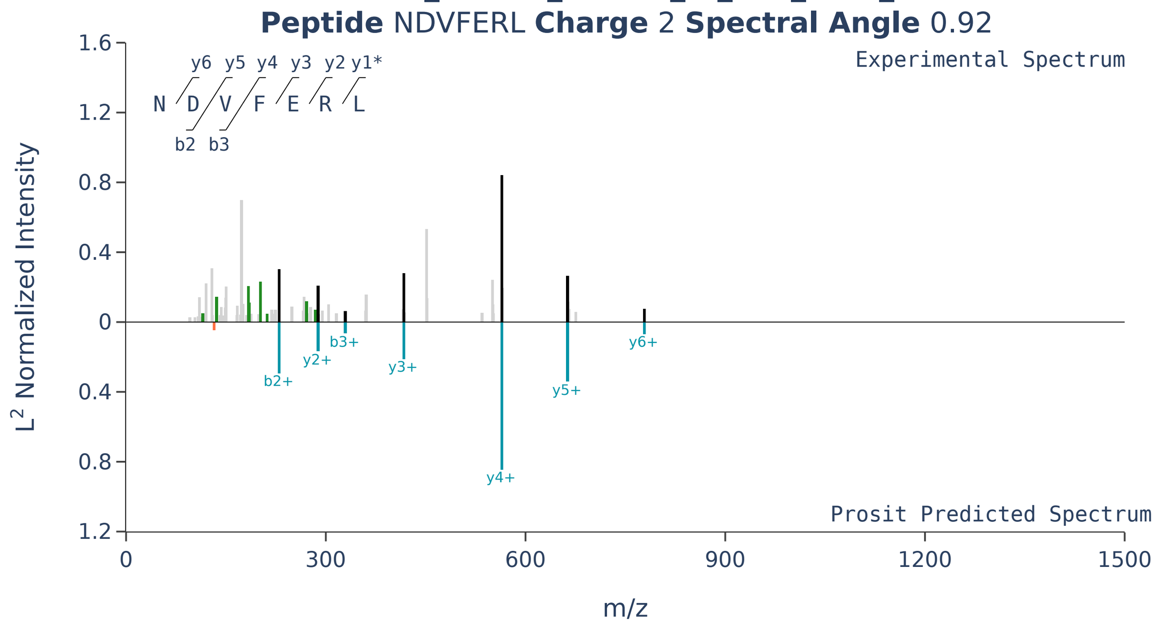


**Source** HRoetschke_291222_080123_Fu_H2B_b1R2_24h **Scan** 6019
**Peptide** ERIAGEASRLAHYNKRS **Charge** 4 **Spectral Angle** 0.81

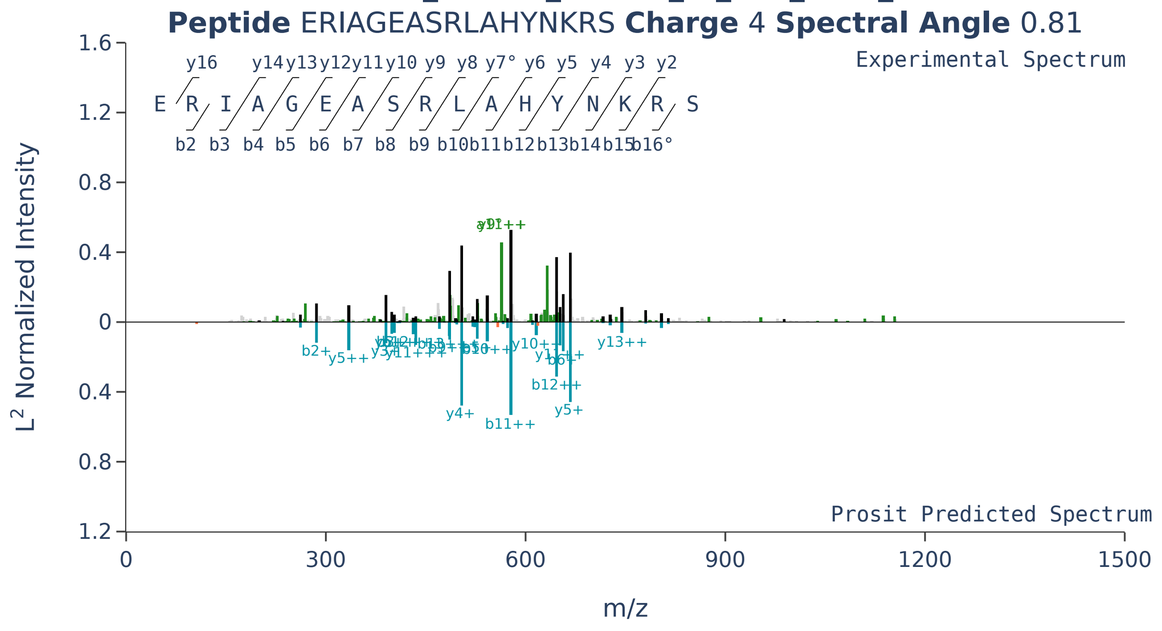


**Source** HRoetschke_291222_080123_Fu_H2B_b1R1_2h **Scan** 15344
**Peptide** TAVRLLLPGELAKHAVSEGTKAVTKYTSAK **Charge** 6 **Spectral Angle** 0.74

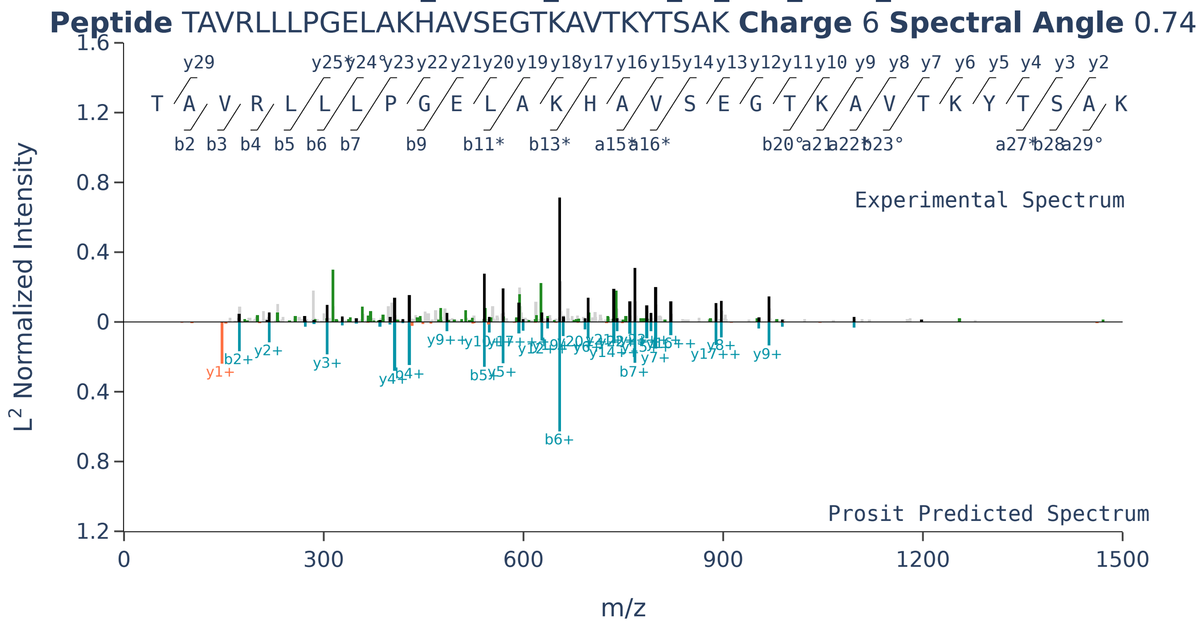


**Source** HRoetschke_291222_080123_Fu_H2B_b1R2_24h **Scan** 7463
**Peptide** AGEASRLAHYNKRSTITSREL **Charge** 4 **Spectral Angle** 0.87

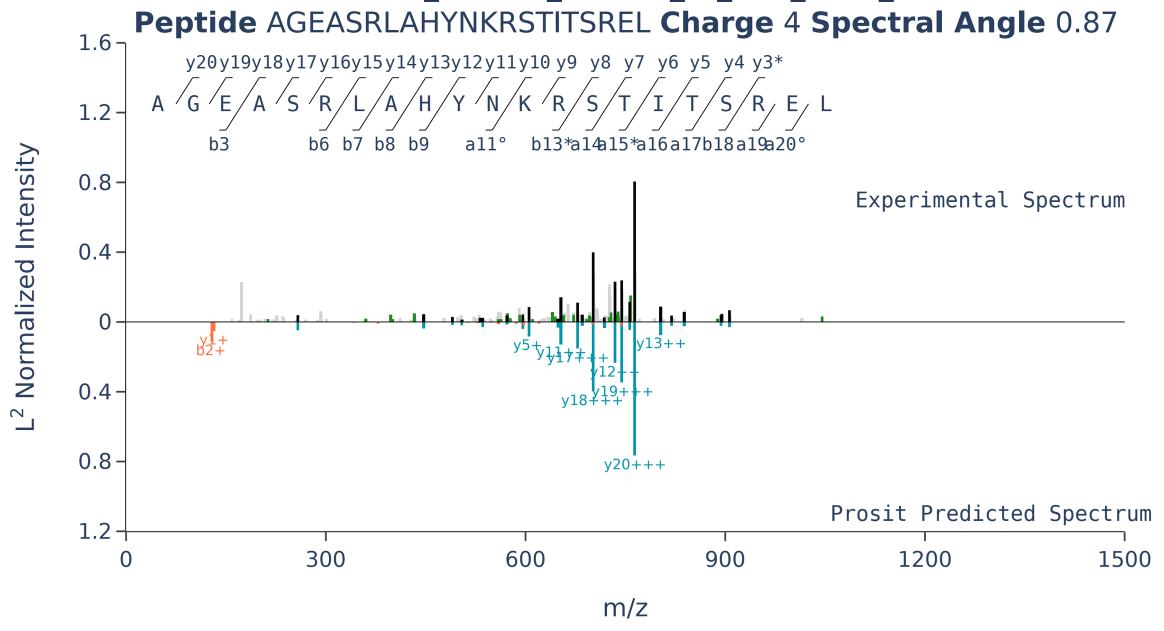


**Source** HRoetschke_291222_080123_Fu_H2B_b2R1_2h **Scan** 7325
**Peptide** LPGELAKHAVSEGTKA **Charge** 3 **Spectral Angle** 0.65

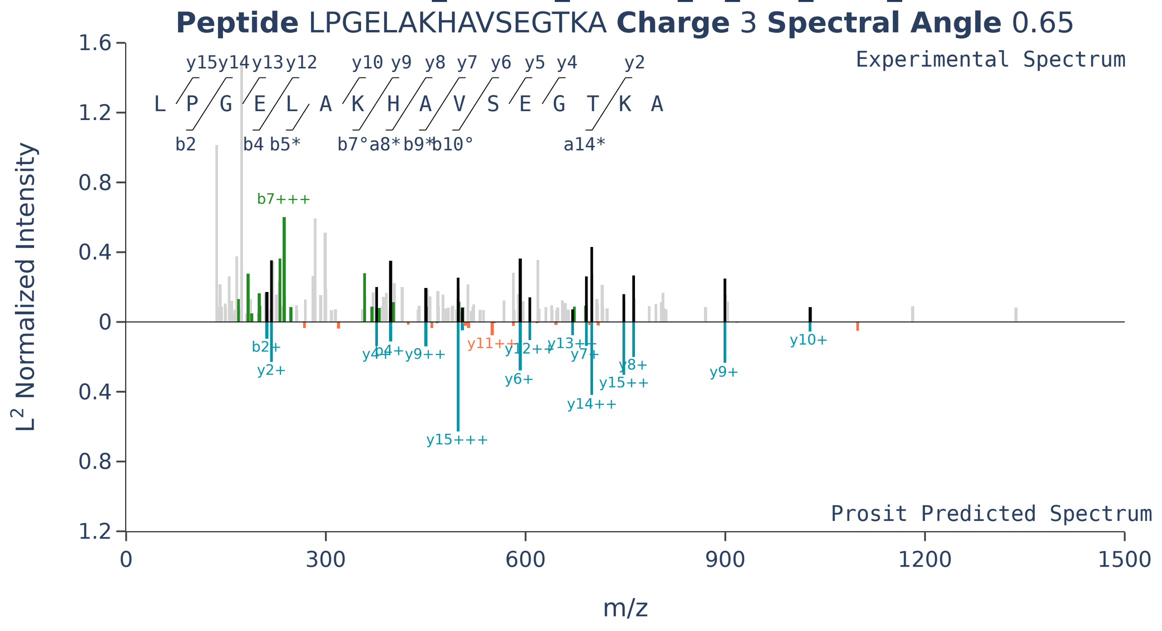

**Source** HRoetschke_291222_080123_Fu_H2B_b2R1_24h **Scan** 11940
**Peptide** YNKRSTITSREIQTAVRL **Charge** 4 **Spectral Angle** 0.84

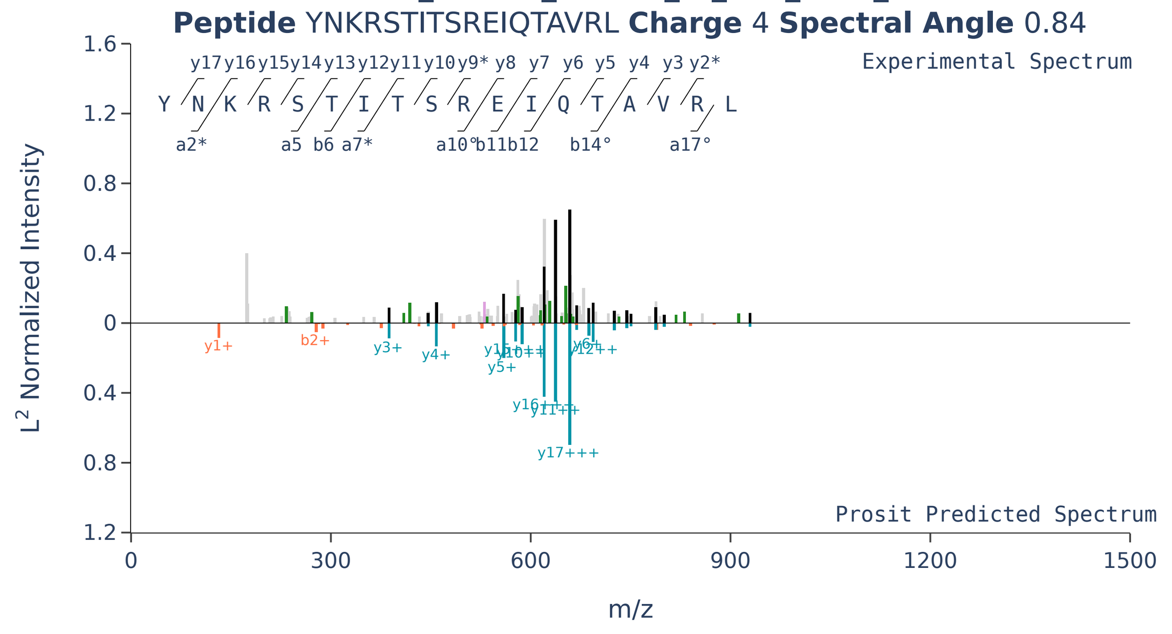


**Source** HRoetschke_291222_080123_Fu_H2B_b2R1_4h **Scan** 20947
**Peptide** FVNDVFERIAGEASRLAHYNKR **Charge** 5 **Spectral Angle** 0.86

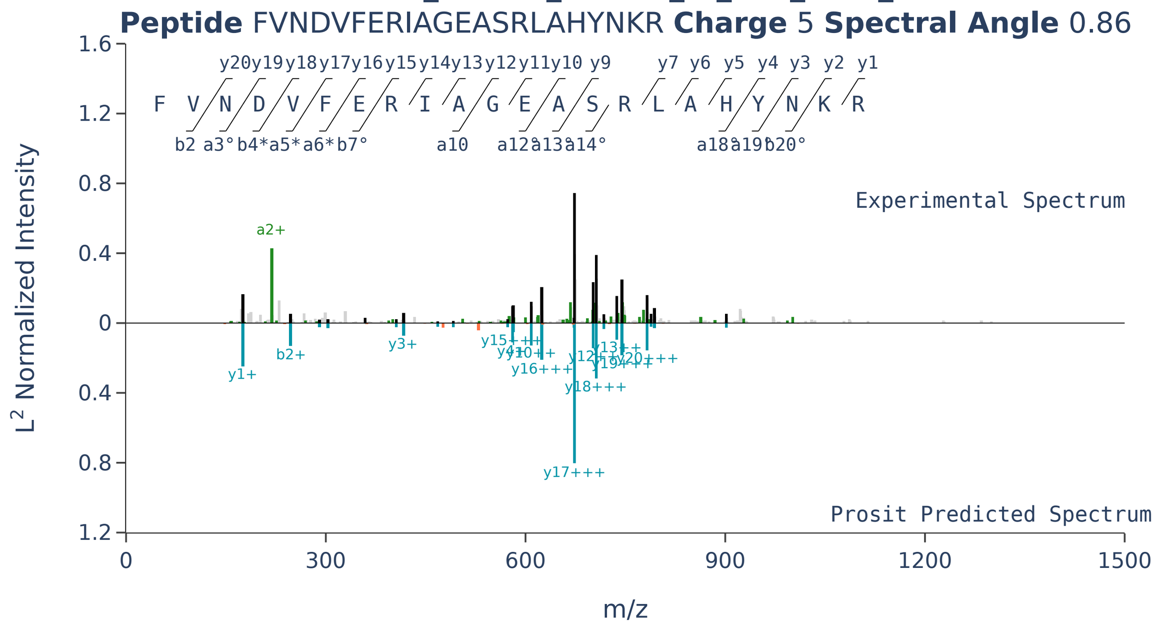


**Source** HRoetschke_291222_080123_Fu_H2B_b2R1_4h **Scan** 1795
**Peptide** ASRLAHYNKRST **Charge** 3 **Spectral Angle** 0.82

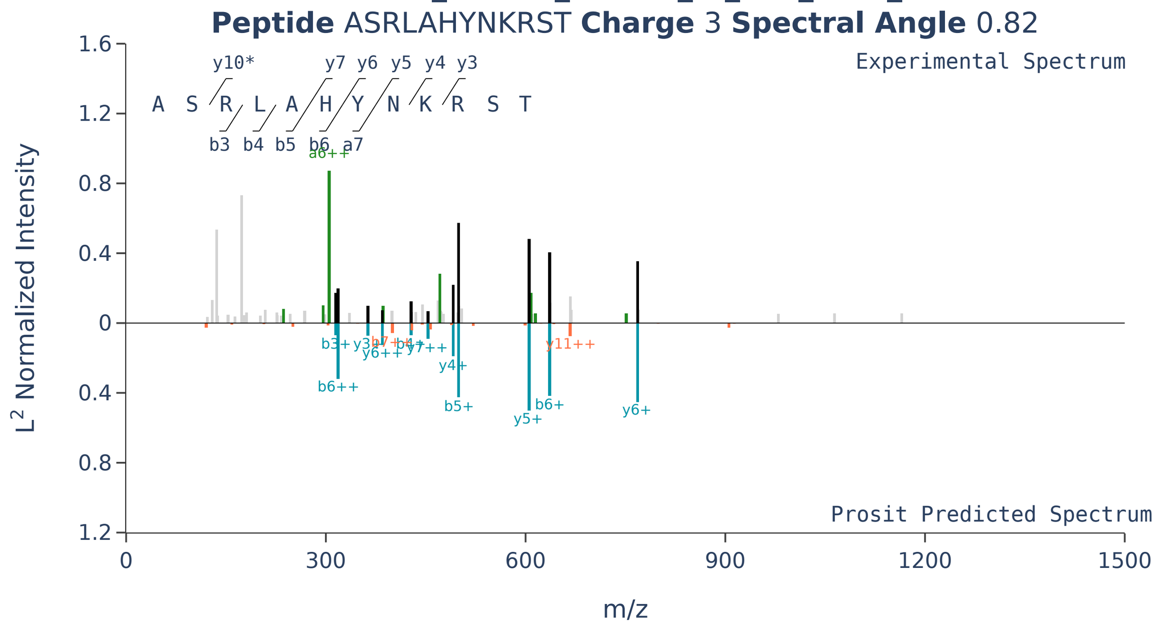


**Source** HRoetschke_291222_080123_Fu_H2B_b2R2_24h **Scan** 18982
**Peptide** AVRLLLPGELAKHAVSEGTKAVTKYTS **Charge** 5 **Spectral Angle** 0.85

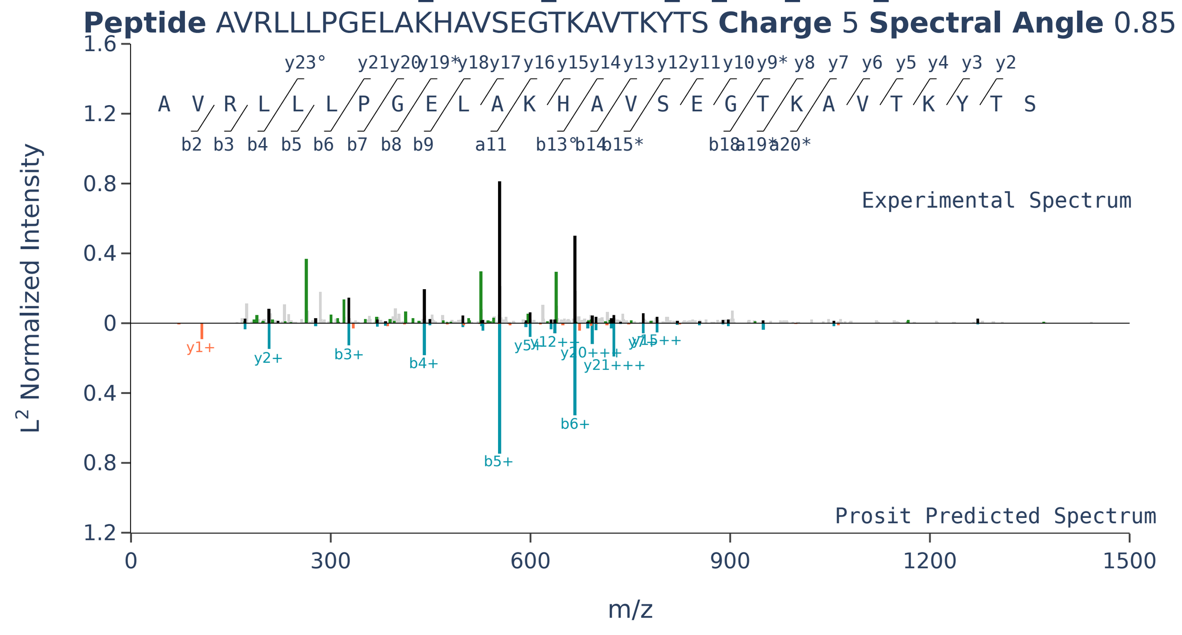


**Source** HRoetschke_291222_080123_Fu_H2B_b1R2_1h **Scan** 14400
**Peptide** TAVRLLL **Charge** 2 **Spectral Angle** 0.78

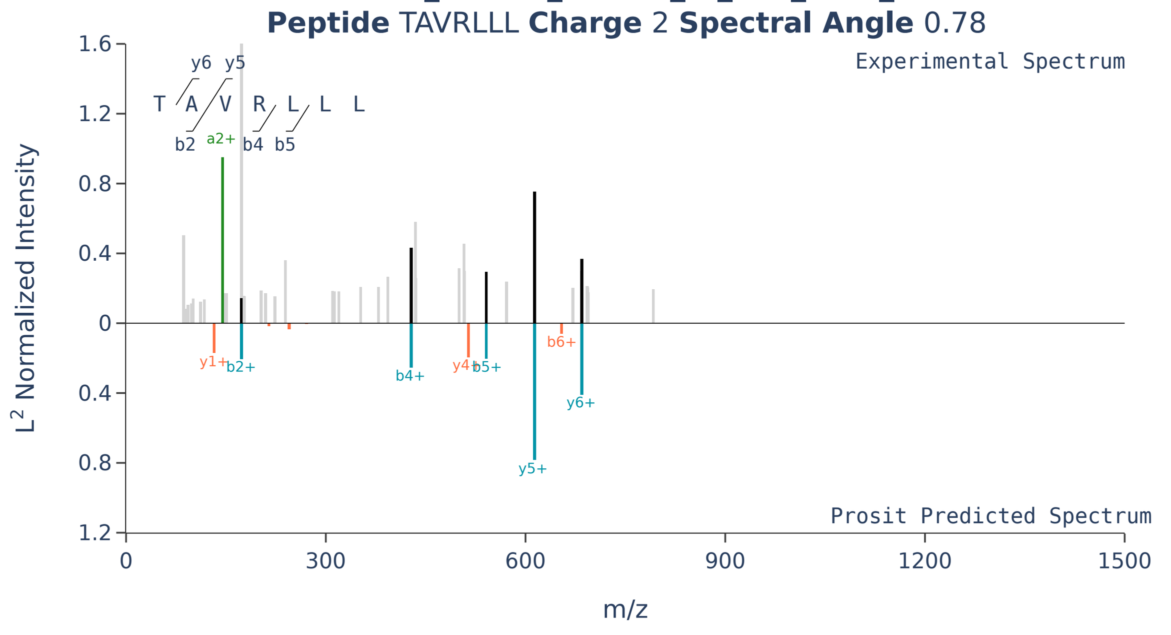


**Source** HRoetschke_291222_080123_Fu_H2B_b2R1_1h **Scan** 13561
**Peptide** RLLLPGELAKHAVSEGTKAVTKY **Charge** 5 **Spectral Angle** 0.86

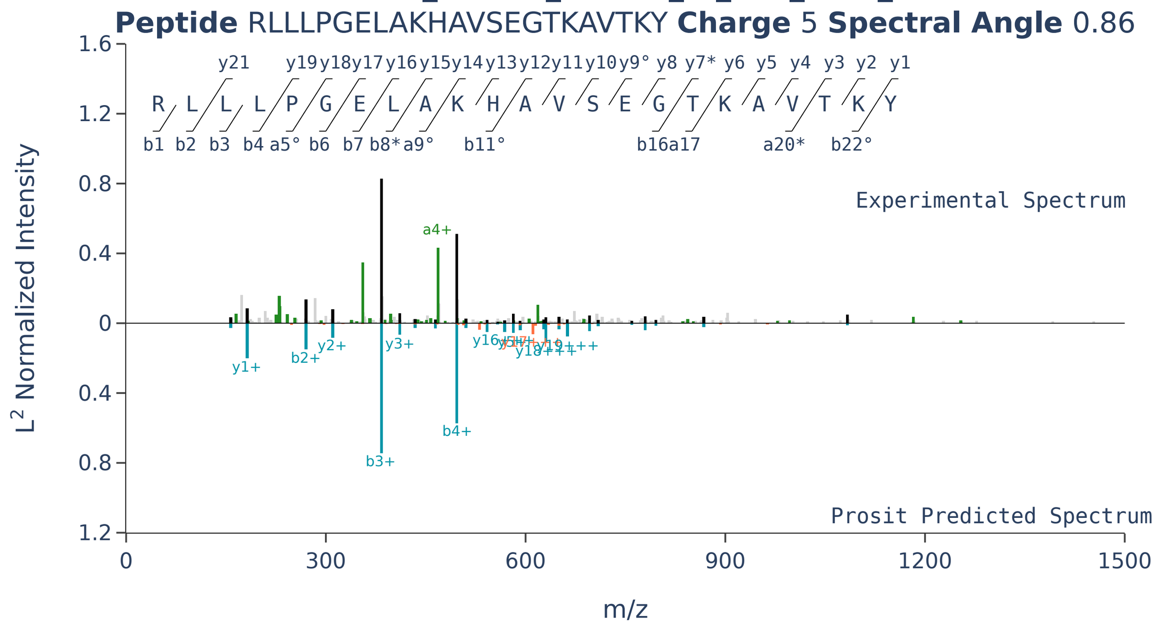


**Source** HRoetschke_291222_080123_Fu_H2B_b1R2_24h **Scan** 2757
**Peptide** AGEASRLAHYNKRS **Charge** 3 **Spectral Angle** 0.8

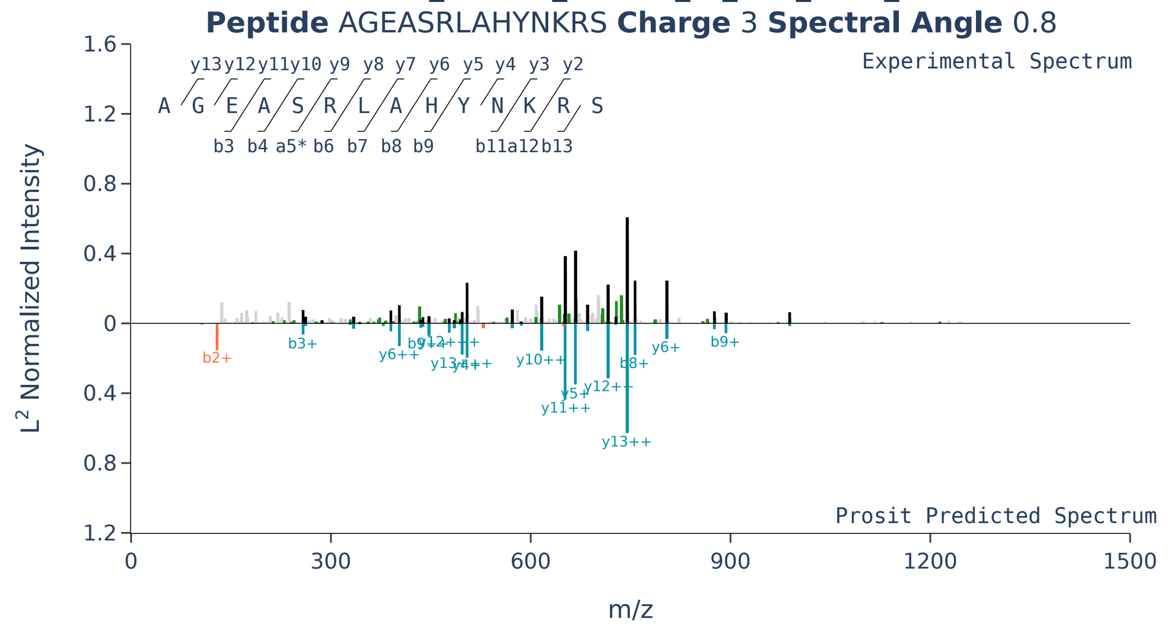


**Source** HRoetschke_291222_080123_Fu_H2B_b1R2_4h **Scan** 5056
**Peptide** VHPDTGI **Charge** 2 **Spectral Angle** 0.9

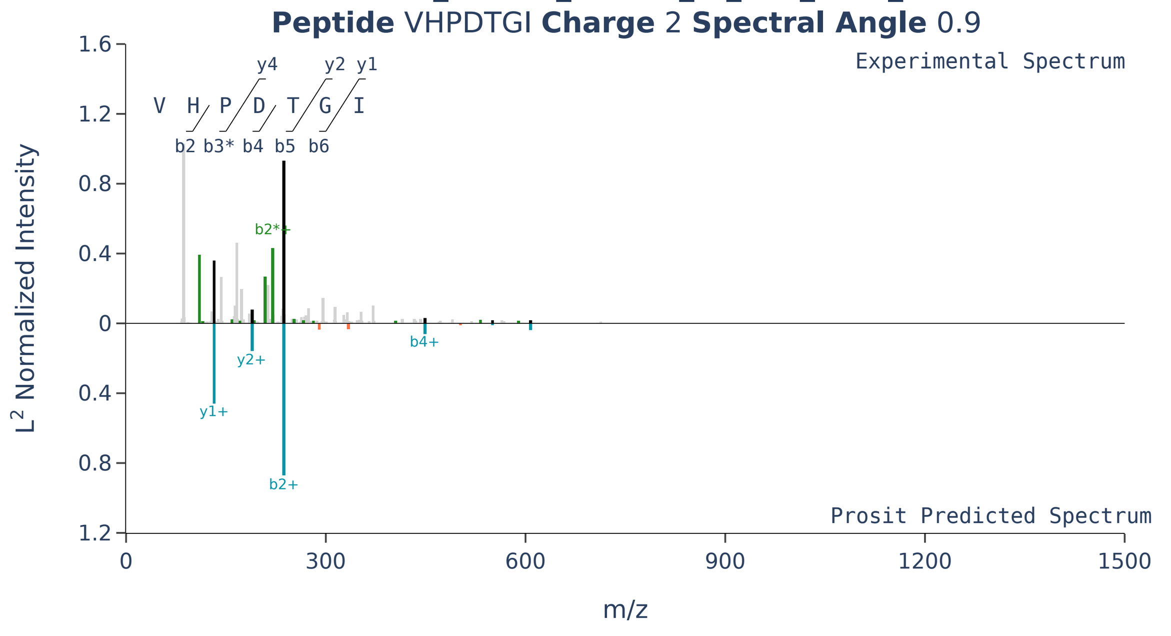


**Source** HRoetschke_291222_080123_Fu_H2B_b1R1_1h **Scan** 9693
**Peptide** LLPGELAKHAVS **Charge** 2 **Spectral Angle** 0.8

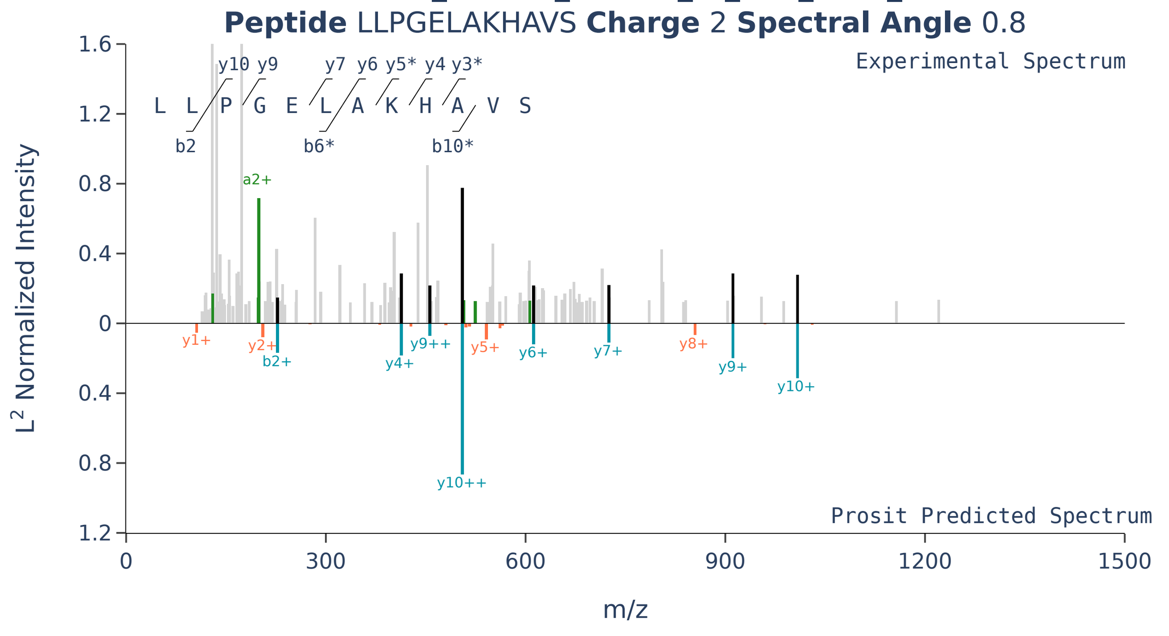


**Source** HRoetschke_291222_080123_Fu_H2B_b1R1_24h **Scan** 6548
**Peptide** ASRLAHYNKRSTITSREIQTAVR **Charge** 5 **Spectral Angle** 0.72

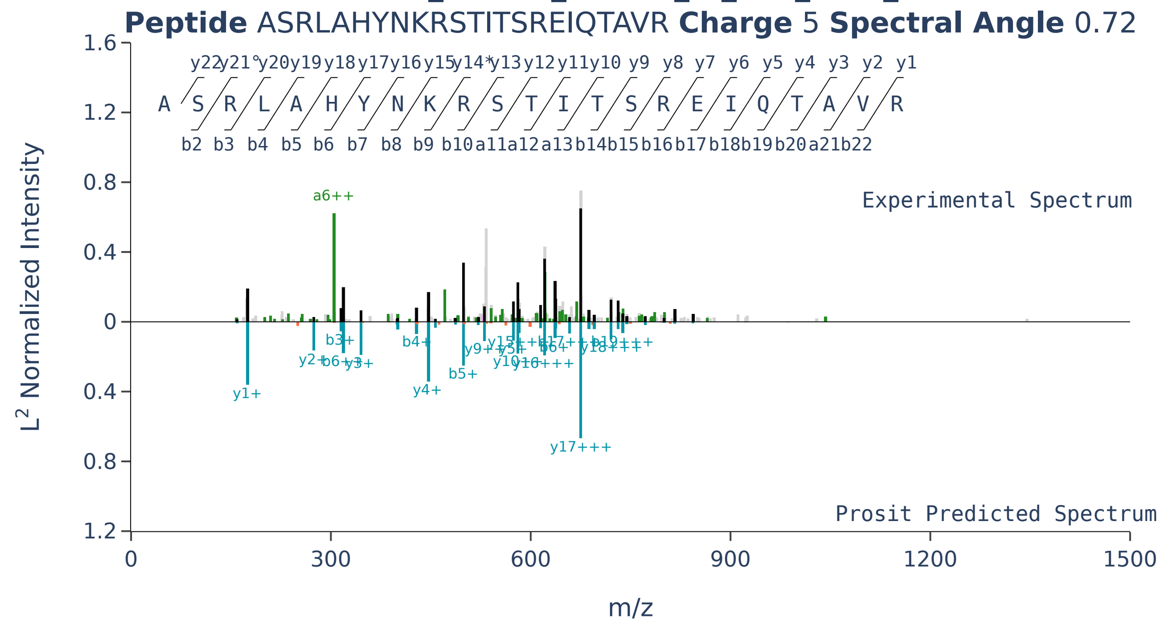


**Source** HRoetschke_291222_080123_Fu_H2B_b1R1_4h **Scan** 5486
**Peptide** RLAHYNKRSTITSREIQTAVR **Charge** 5 **Spectral Angle** 0.67

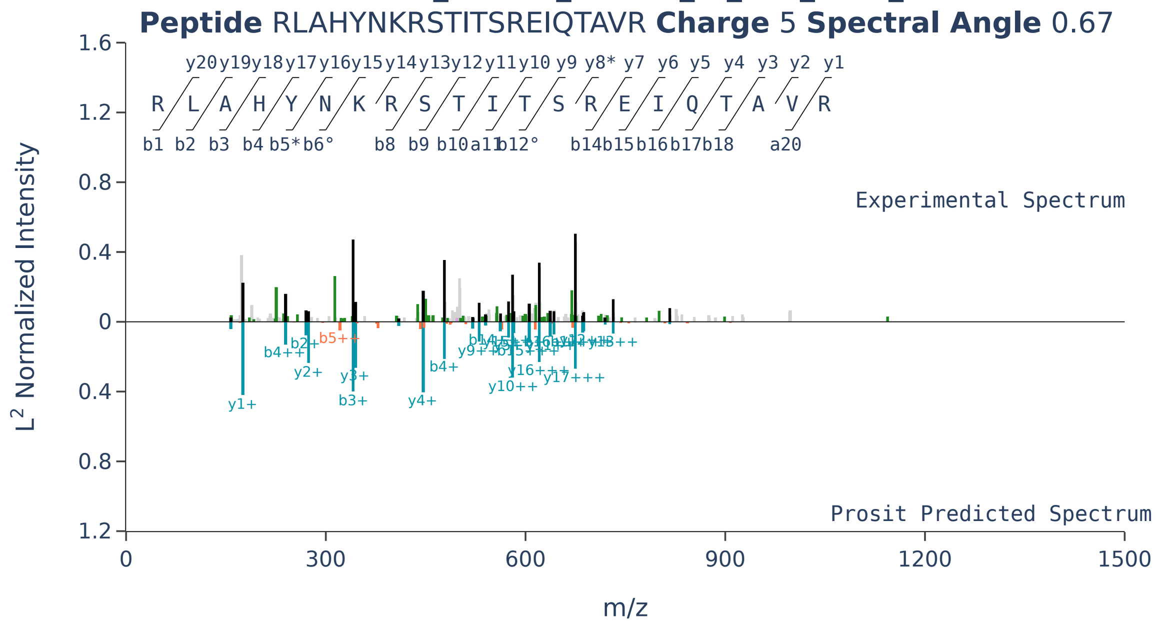


**Source** HRoetschke_291222_080123_Fu_H2B_b2R2_24h **Scan** 3823
**Peptide** NKRSTITSREIQT **Charge** 2 **Spectral Angle** 0.78

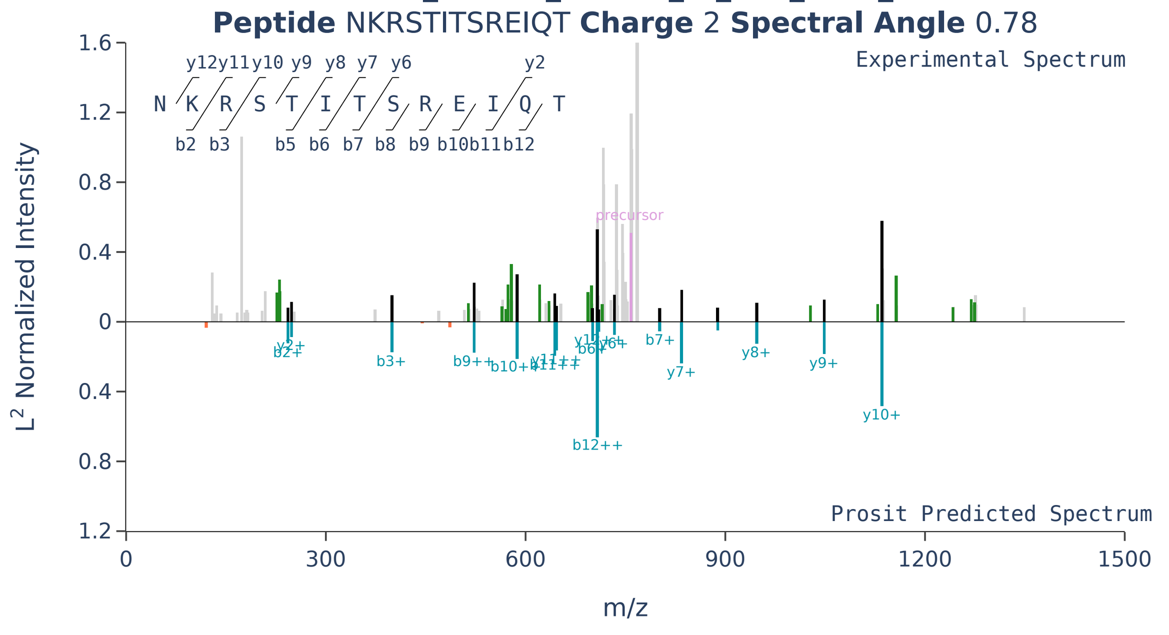


**Source** HRoetschke_291222_080123_Fu_H2B_b2R1_2h **Scan** 10974
**Peptide** LLPGELAKHAVSEGTKA **Charge** 3 **Spectral Angle** 0.79

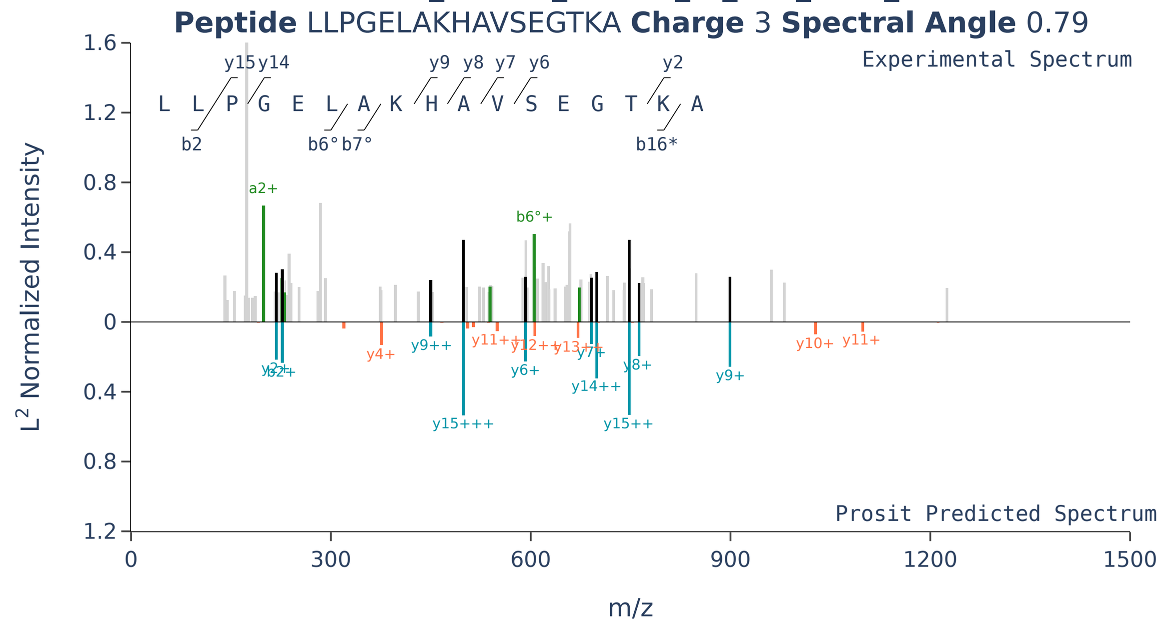


**Source** HRoetschke_291222_080123_Fu_H2B_b2R2_4h **Scan** 3318
**Peptide** HYNKRSTITSREL **Charge** 3 **Spectral Angle** 0.89

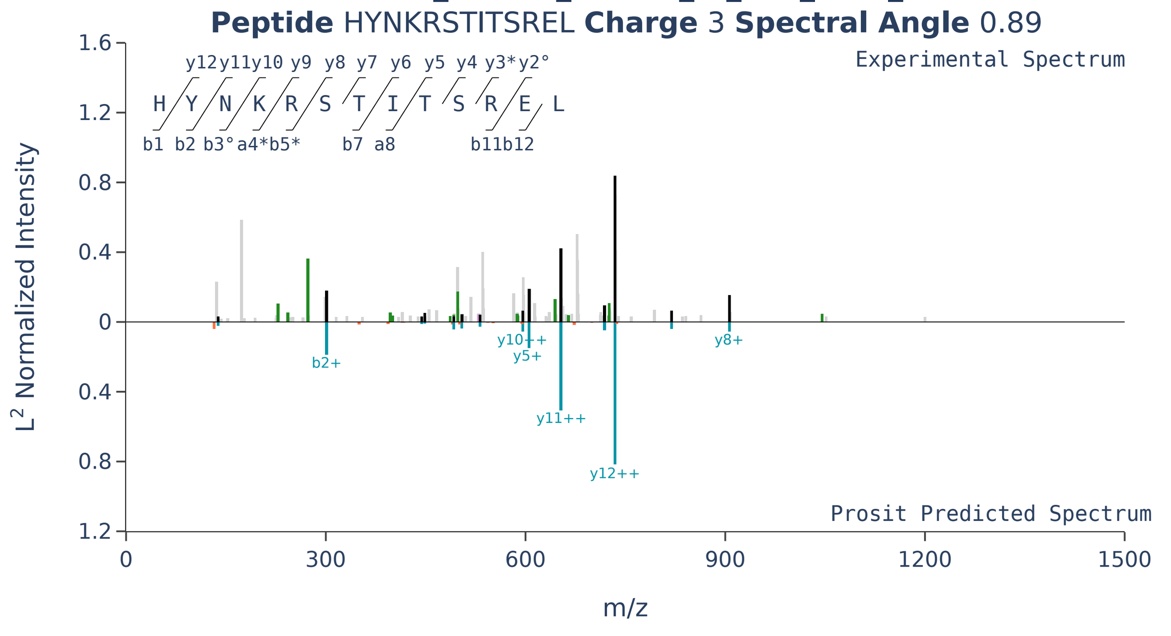


**Source** HRoetschke_291222_080123_Fu_H2B_b1R2_4h **Scan** 12991
**Peptide** NDVFERIAGEAS **Charge** 2 **Spectral Angle** 0.87

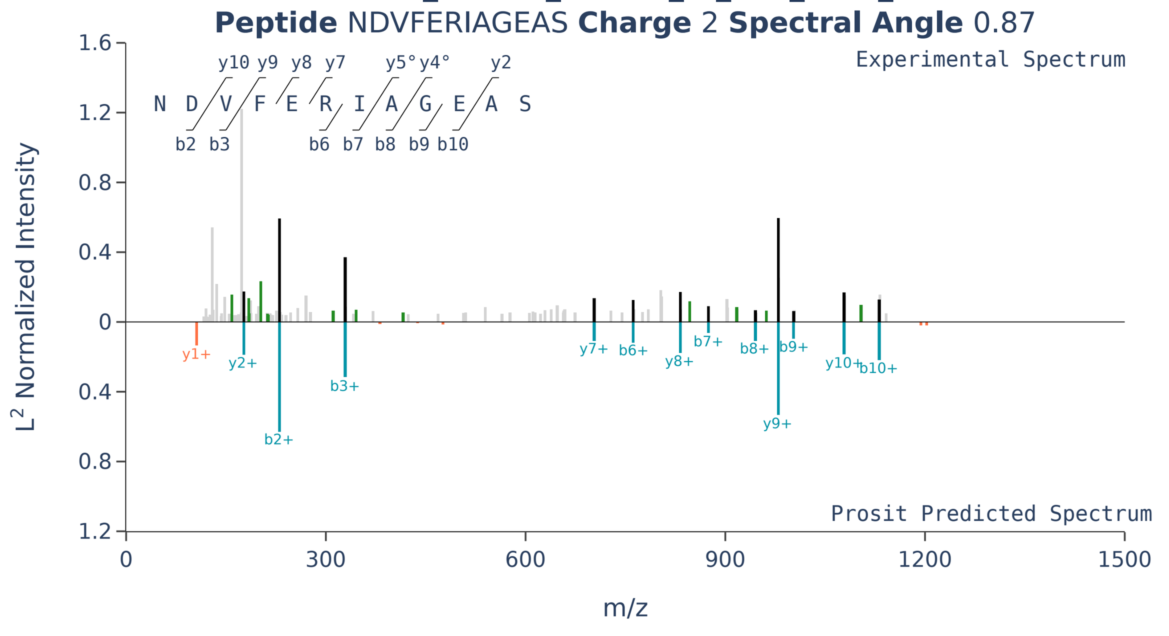

**Source** HRoetschke_291222_080123_Fu_H2B_b2R2_4h **Scan** 15524
**Peptide** LLLPGELAKHAVSEGTKAVTK **Charge** 3 **Spectral Angle** 0.84

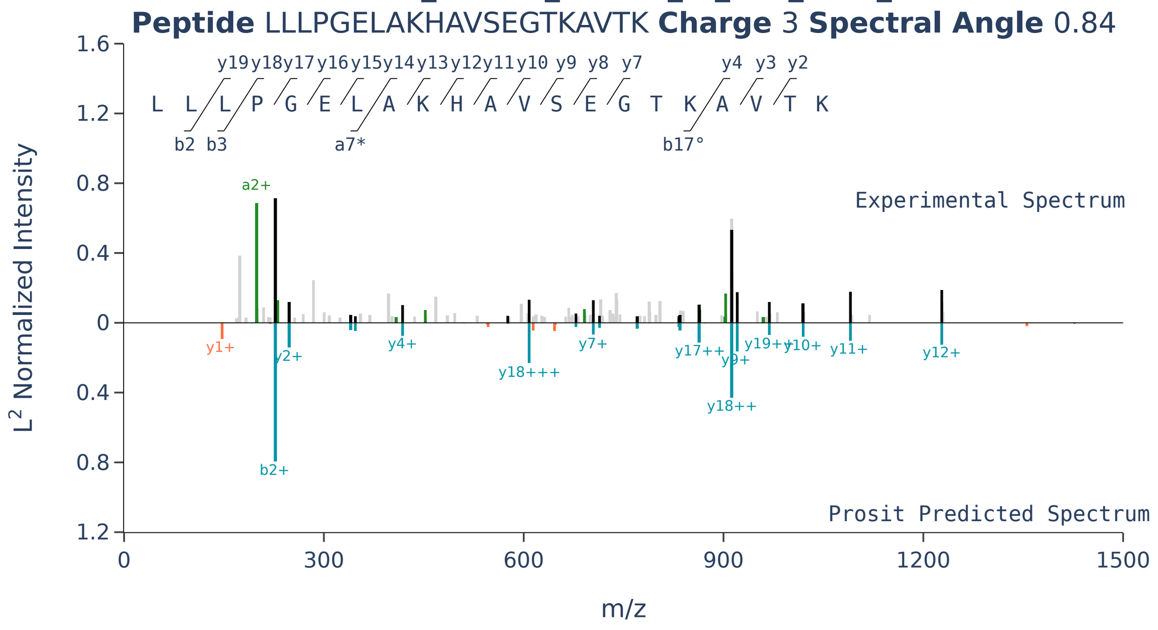


**Source** HRoetschke_291222_080123_Fu_H2B_b1R1_24h **Scan** 4897
**Peptide** RLAHYNKRSTITSREIQTA **Charge** 4 **Spectral Angle** 0.77

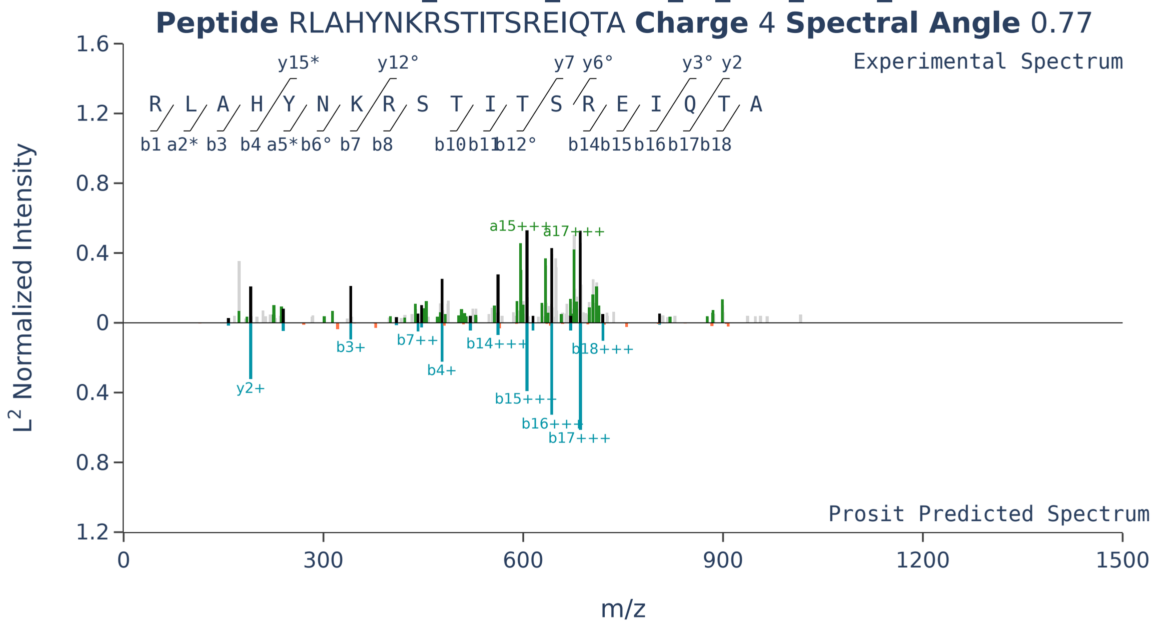


**Source** HRoetschke_291222_080123_Fu_H2B_b2R1_24h **Scan** 19433
**Peptide** VNDVFERIAGEASRLAH **Charge** 3 **Spectral Angle** 0.84

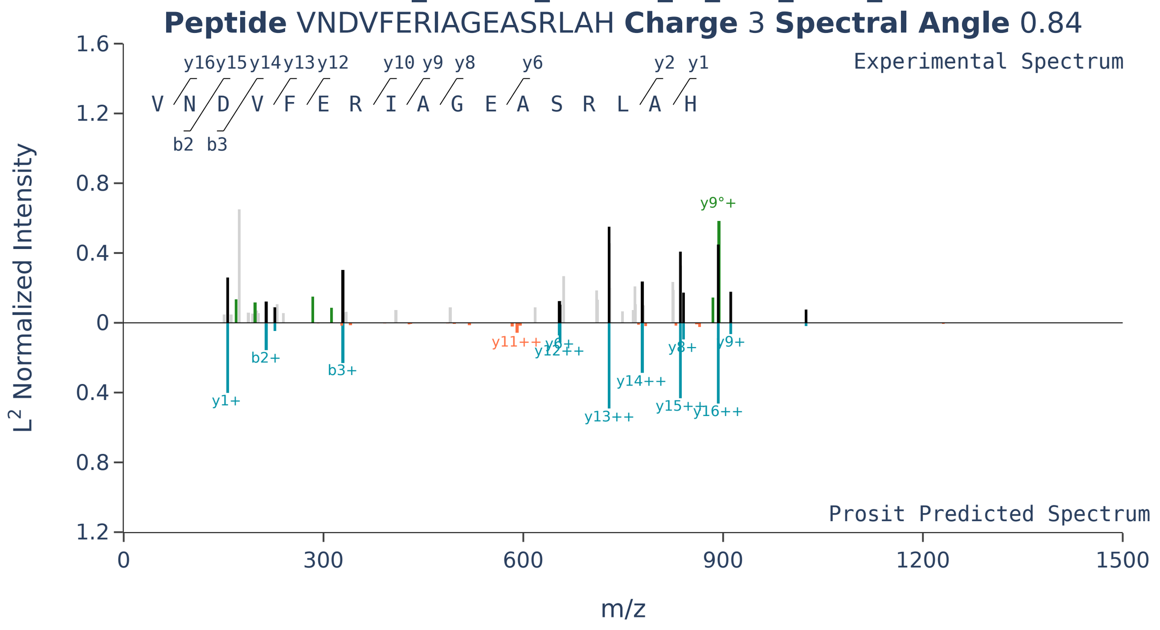


**Source** HRoetschke_291222_080123_Fu_H2B_b2R1_24h **Scan** 5867
**Peptide** SRLAHYNKRSTITSREIQTAVR **Charge** 5 **Spectral Angle** 0.72

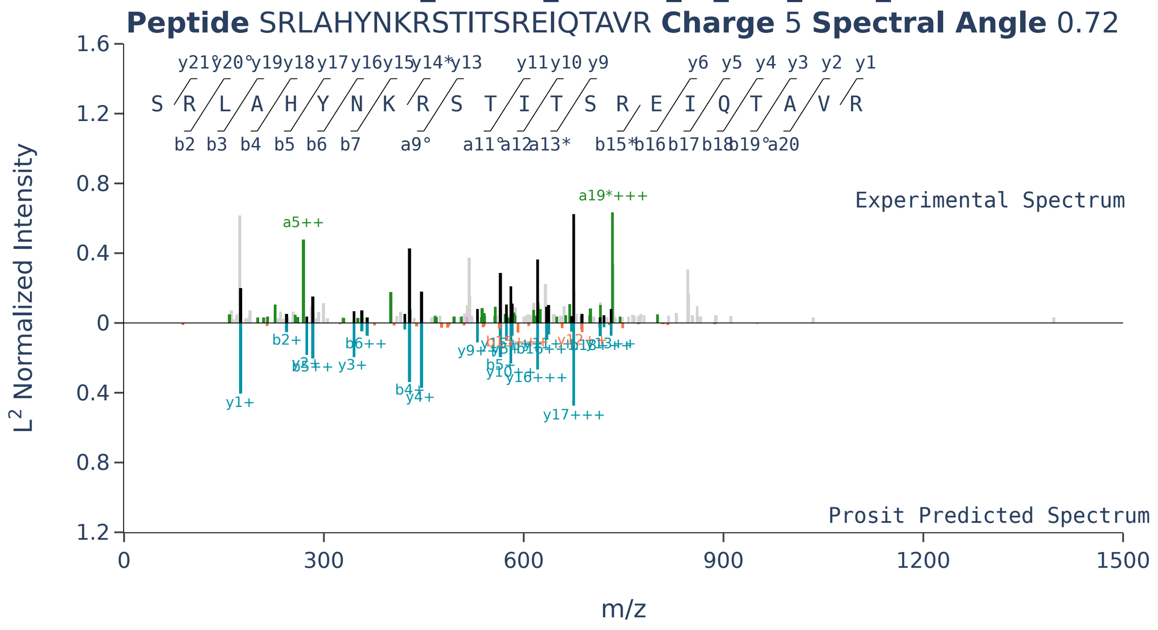


**Source** HRoetschke_291222_080123_Fu_H2B_b2R1_24h **Scan** 6779
**Peptide** PDTGISSKAMSIMN **Charge** 2 **Spectral Angle** 0.86

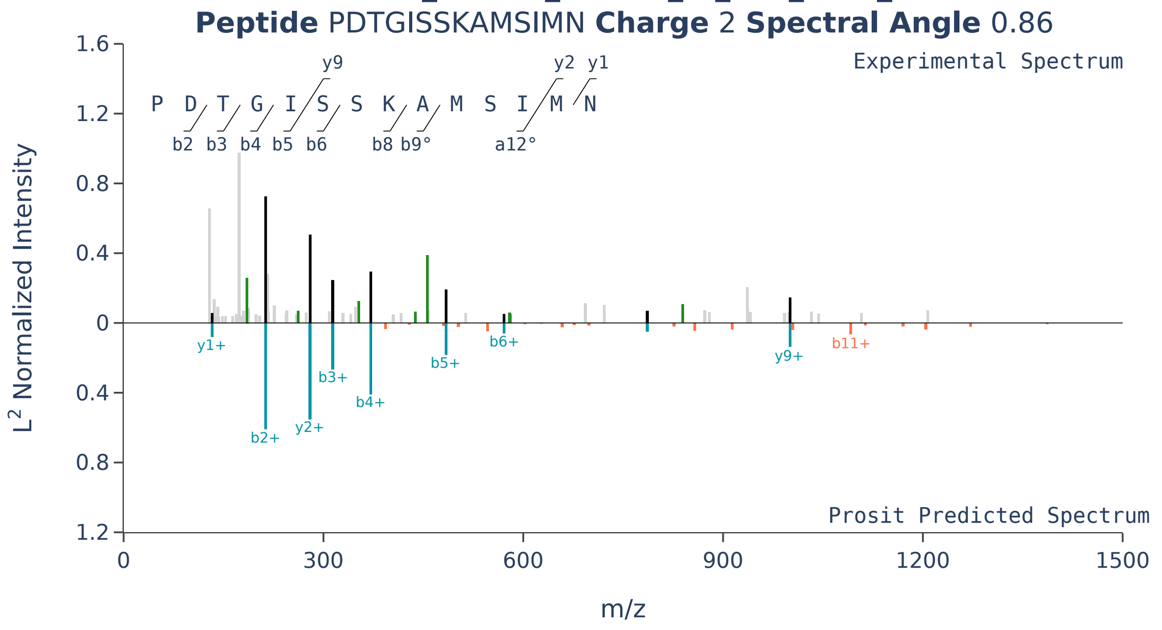


**Source** HRoetschke_291222_080123_Fu_H2B_b2R2_2h **Scan** 4789
**Peptide** VHPDTGL **Charge** 2 **Spectral Angle** 0.88

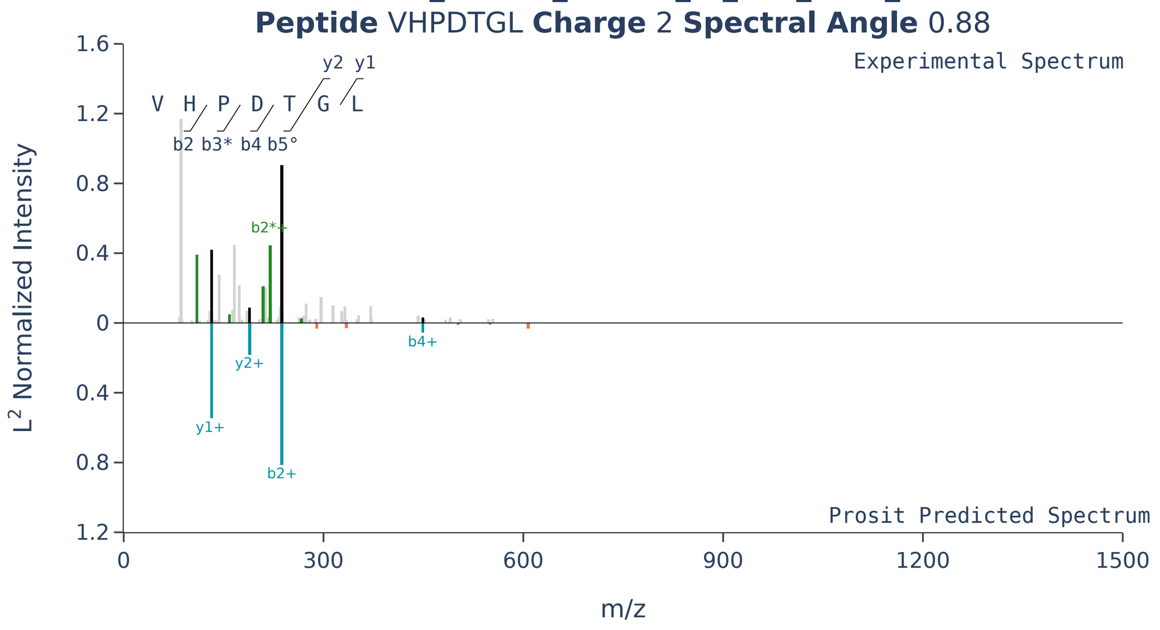


**Source** HRoetschke_291222_080123_Fu_H2B_b2R1_24h **Scan** 5733
**Peptide** ERIAGEASRLAHYNKRST **Charge** 3 **Spectral Angle** 0.73

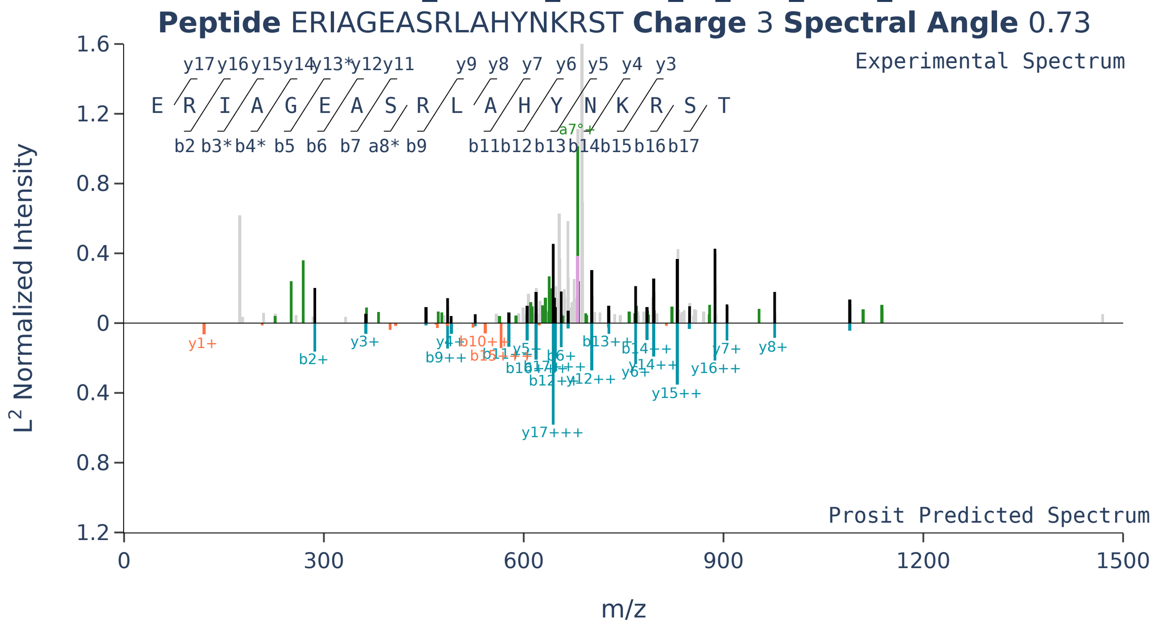


**Source** HRoetschke_291222_080123_Fu_H2B_b2R1_2h **Scan** 11580
**Peptide** VFERIAGEASRLAHYNKRST **Charge** 4 **Spectral Angle** 0.84

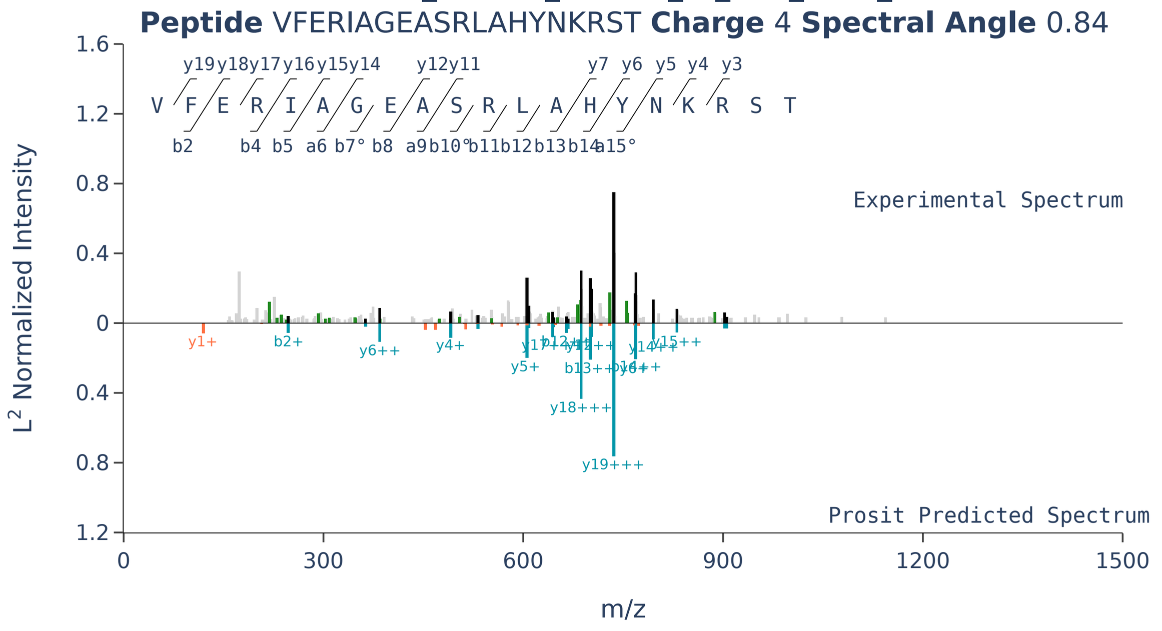


**Source** HRoetschke_291222_080123_Fu_H2B_b2R1_24h **Scan** 14000
**Peptide** NDVFERIAGEASR **Charge** 3 **Spectral Angle** 0.8

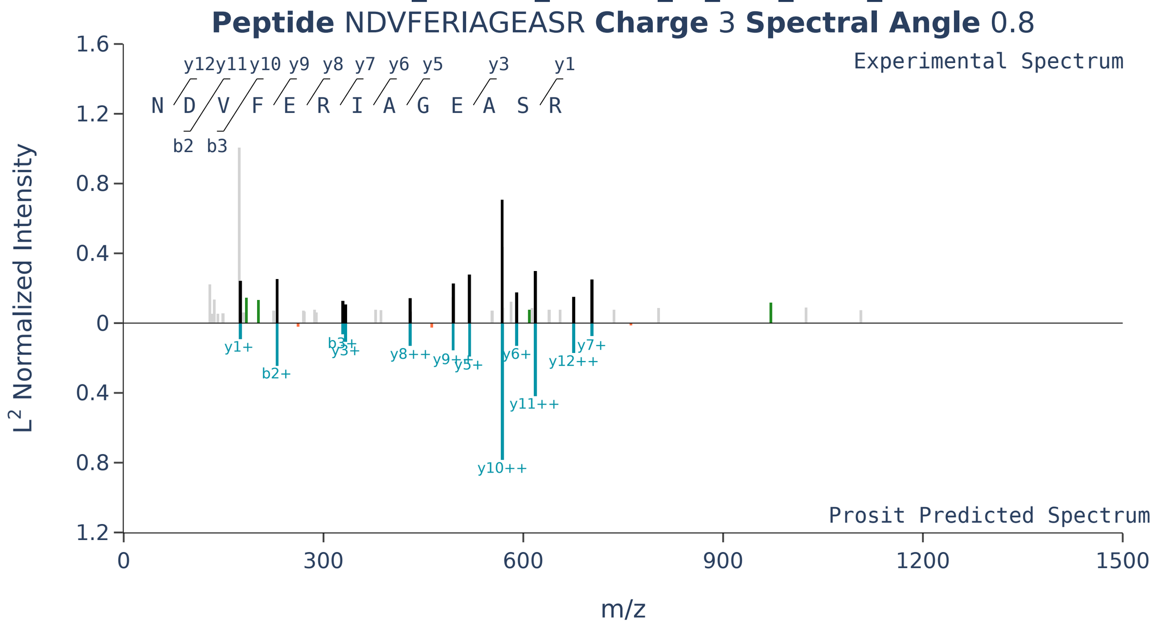


**Source** HRoetschke_291222_080123_Fu_H2B_b2R1_24h **Scan** 10619
**Peptide** AHYNKRSTITSREIQTAVRL **Charge** 5 **Spectral Angle** 0.8

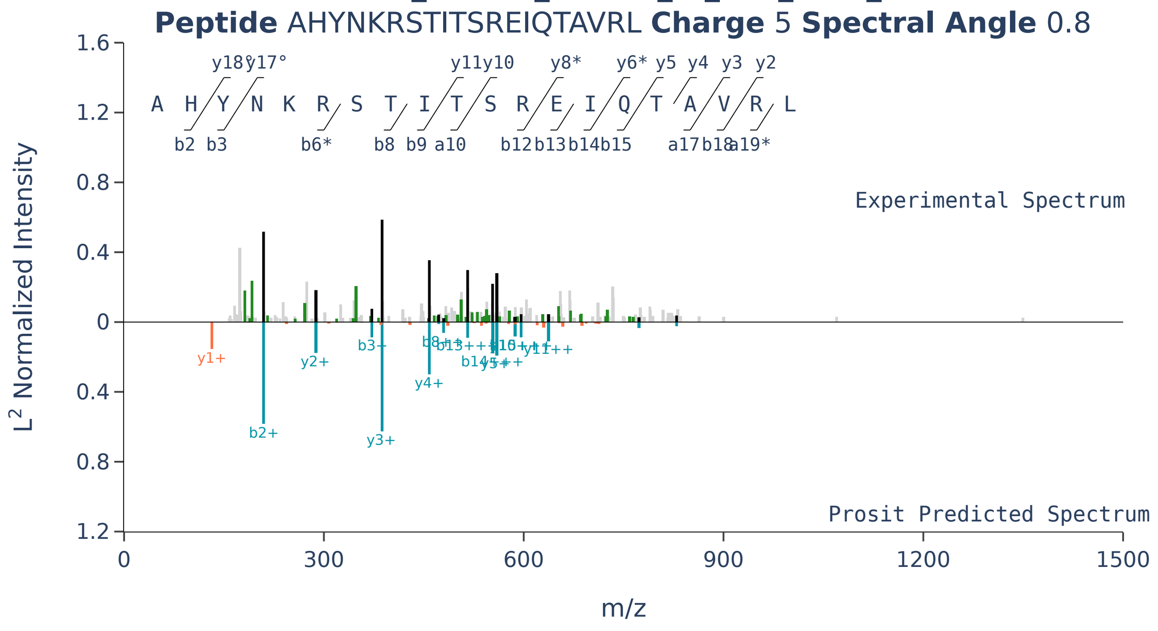


**Source** HRoetschke_291222_080123_Fu_H2B_b1R1_4h **Scan** 4412
**Peptide** YNKRSTITSREL **Charge** 3 **Spectral Angle** 0.87

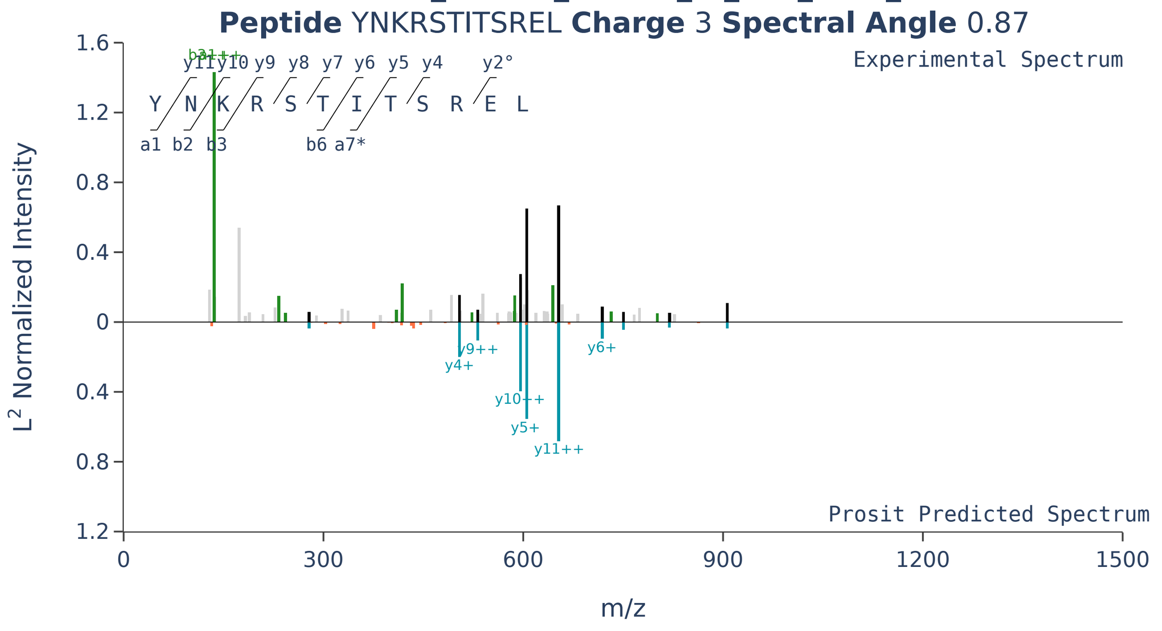


**Source** HRoetschke_291222_080123_Fu_H2B_b1R2_1h **Scan** 3902
**Peptide** RLAGEASRLAHYNKRST **Charge** 4 **Spectral Angle** 0.8

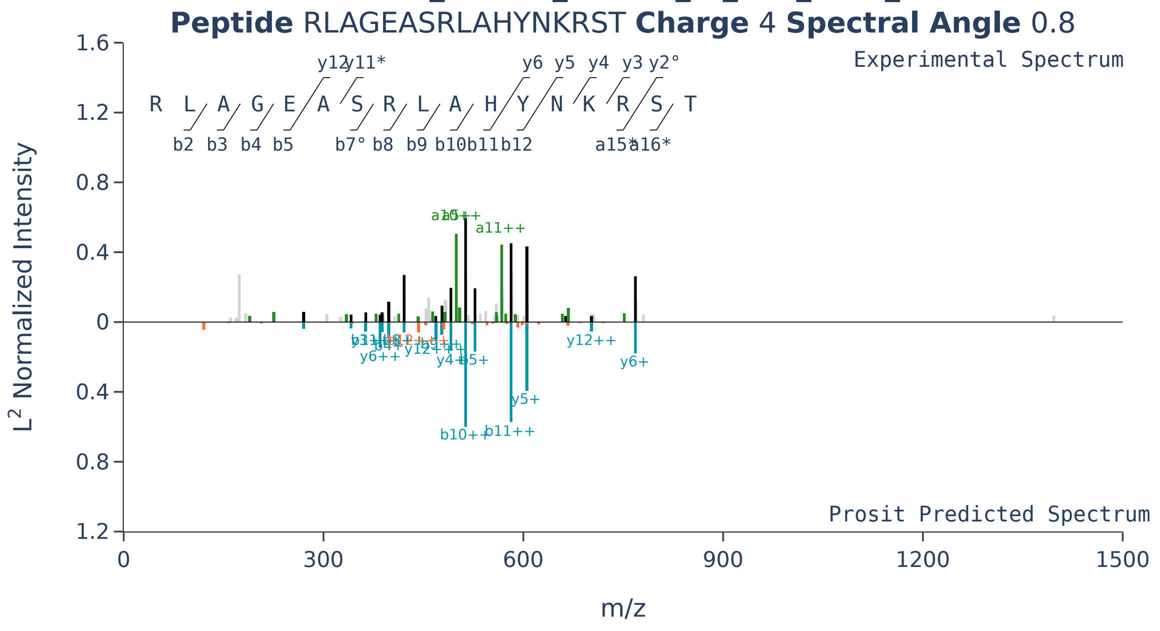


**Source** HRoetschke_291222_080123_Fu_H2B_b2R2_24h **Scan** 15645
**Peptide** AHYNKRSTITSREIQTAVRLL **Charge** 5 **Spectral Angle** 0.79

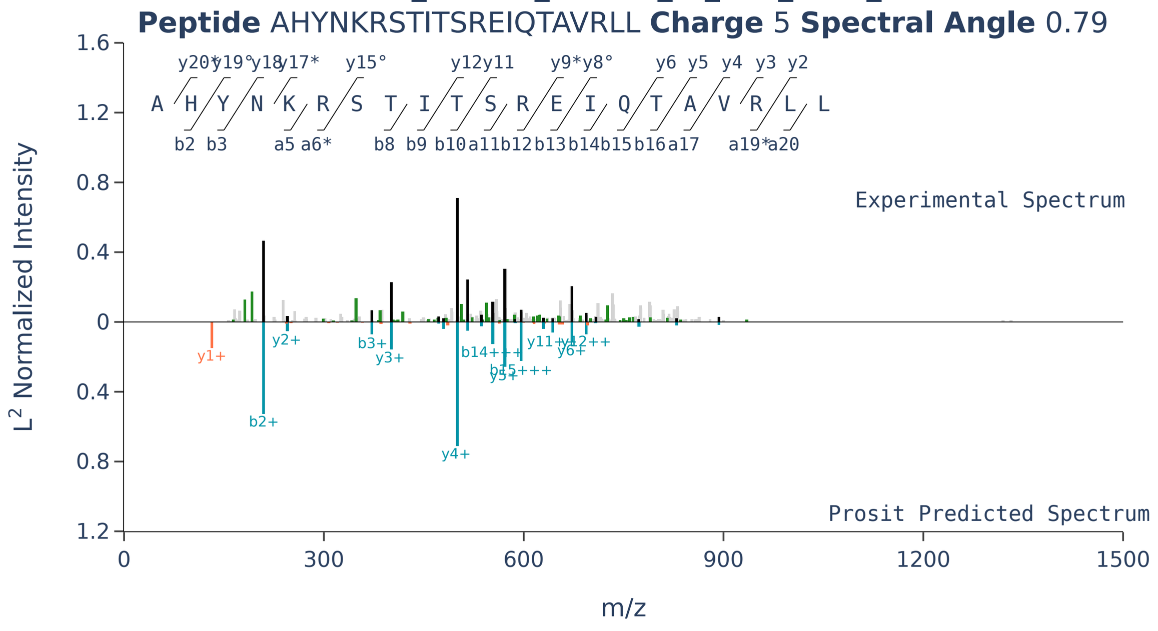


**Source** HRoetschke_291222_080123_Fu_H2B_b2R1_24h **Scan** 16255
**Peptide** LLLPGELAKHAVS **Charge** 2 **Spectral Angle** 0.87

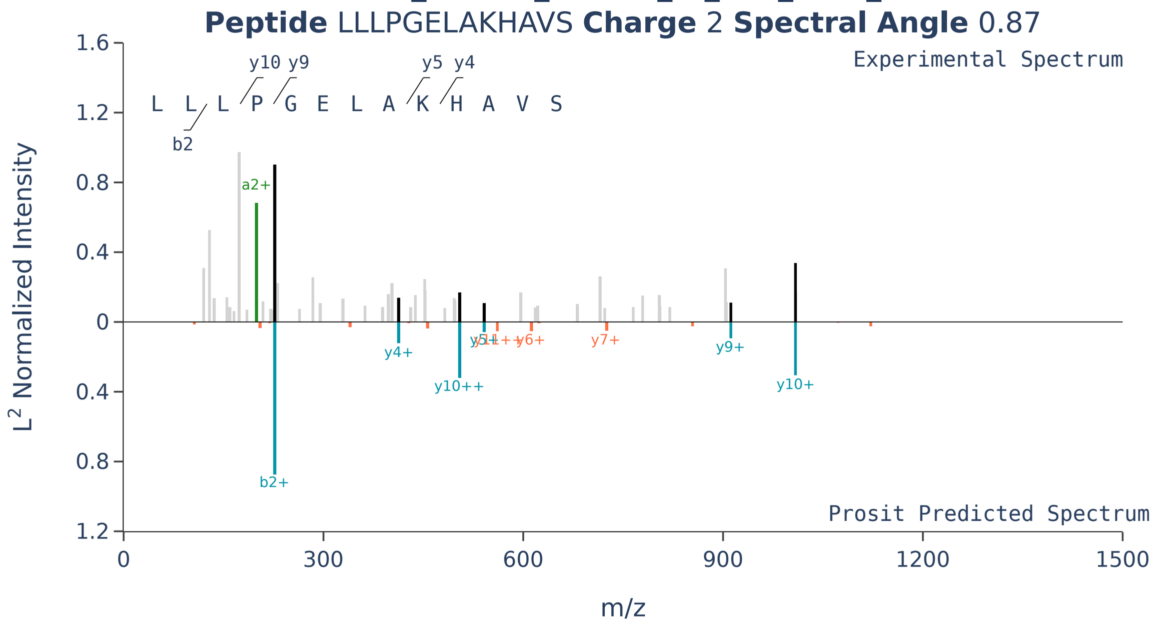


**Source** HRoetschke_291222_080123_Fu_H2B_b1R1_24h **Scan** 9876
**Peptide** RKTRKESYAIYVY **Charge** 4 **Spectral Angle** 0.77

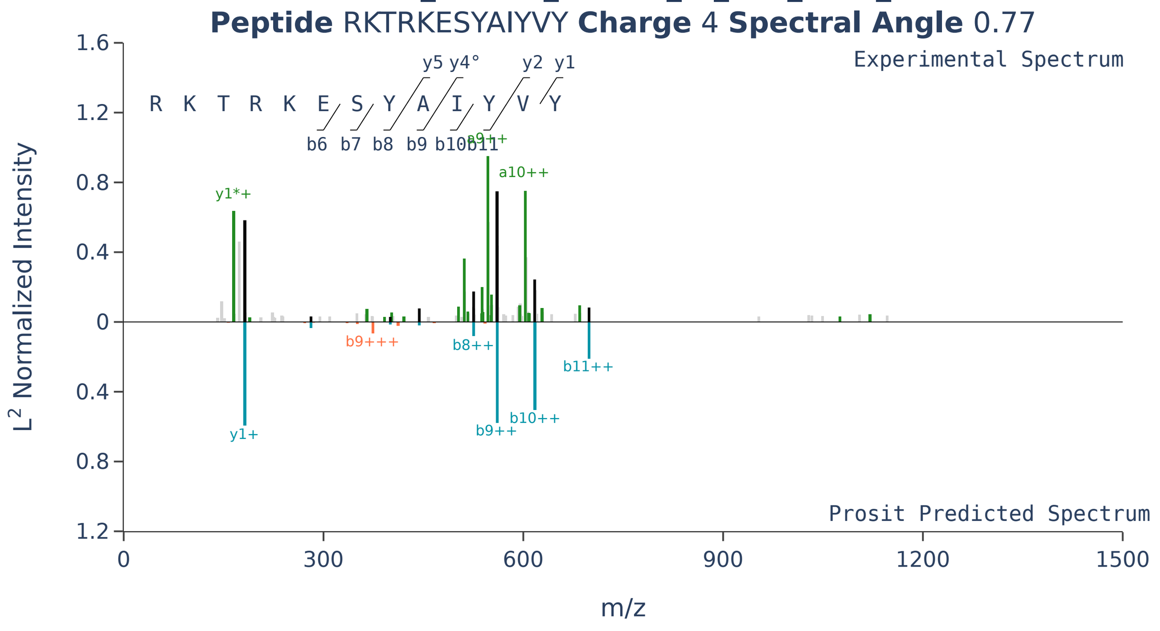

**Source** HRoetschke_291222_080123_Fu_H2B_b1R1_4h **Scan** 16374
**Peptide** KAMSIMNSFVNDVFE **Charge** 2 **Spectral Angle** 0.76

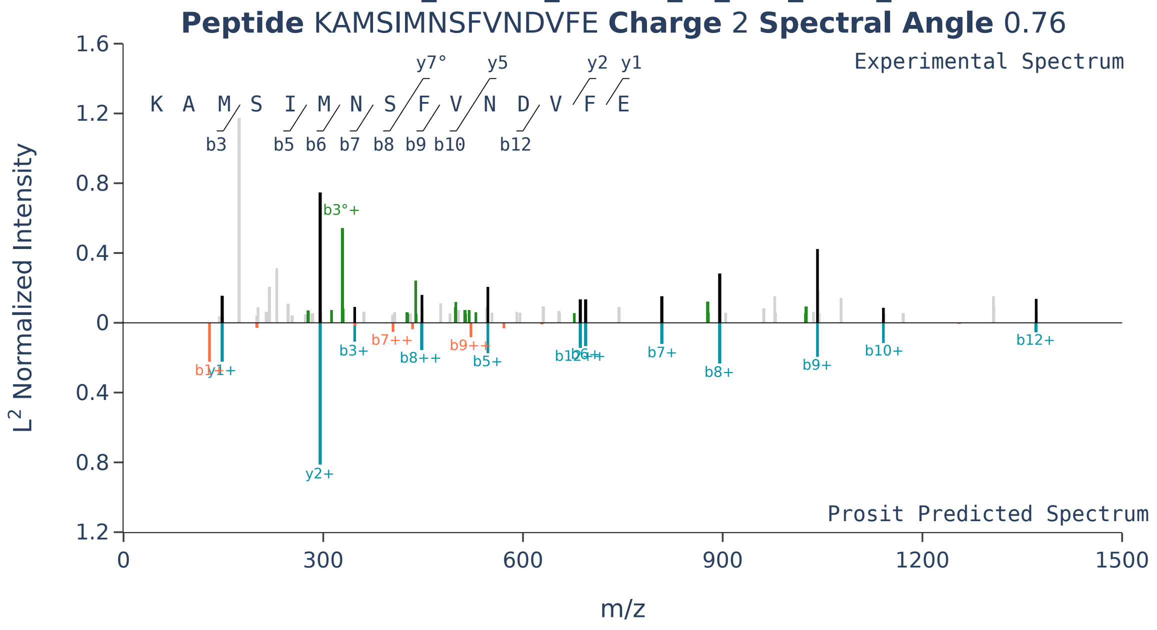


**Source** HRoetschke_291222_080123_Fu_H2B_b1R1_1h **Scan** 6659
**Peptide** ASRLAHYNKRSTITSREIQTAV **Charge** 4 **Spectral Angle** 0.74

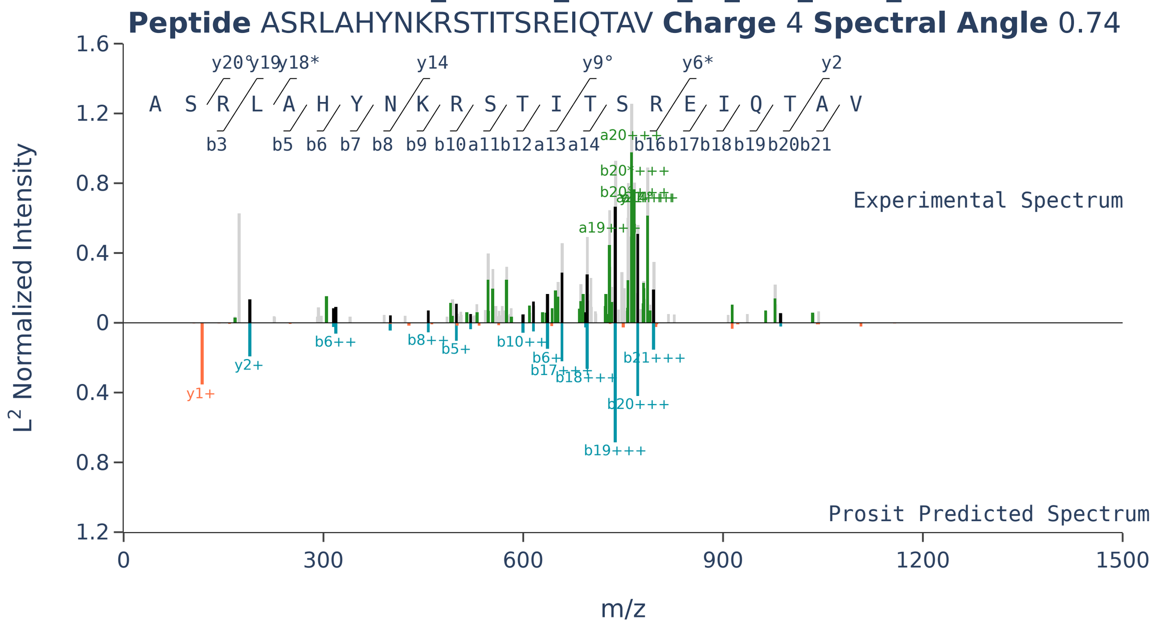


**Source** HRoetschke_291222_080123_Fu_H2B_b1R1_24h **Scan** 5050
**Peptide** YNKRSTITSREIQTA **Charge** 4 **Spectral Angle** 0.69

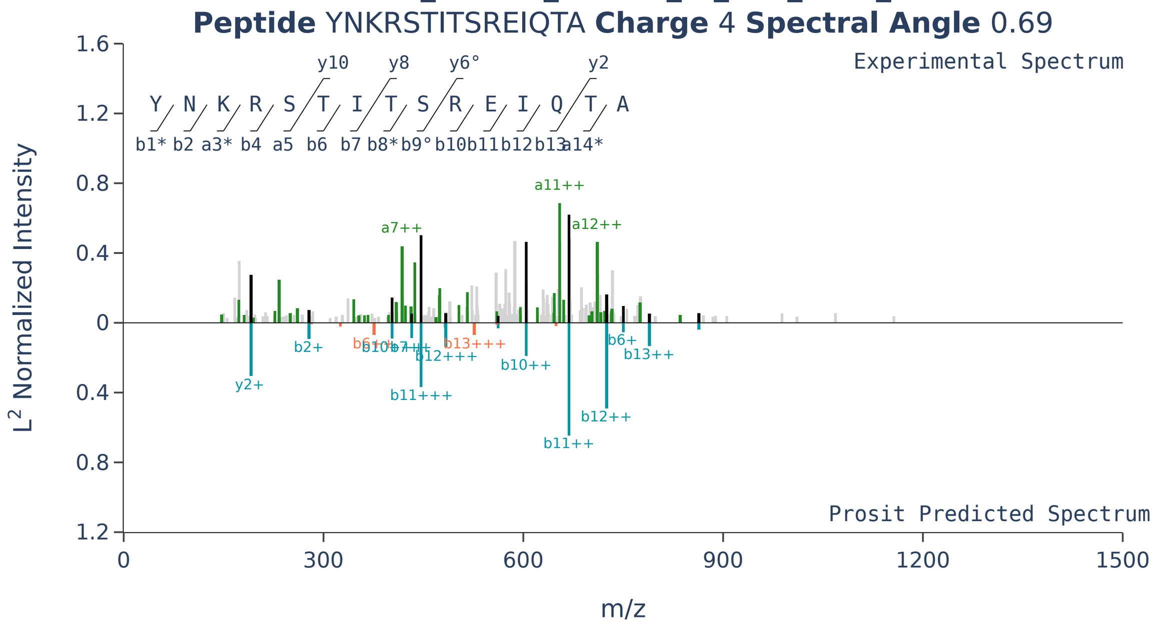


**Source** HRoetschke_291222_080123_Fu_H2B_b1R1_4h **Scan** 11948
**Peptide** VFERIAGEASRLAHYNKRS **Charge** 4 **Spectral Angle** 0.85

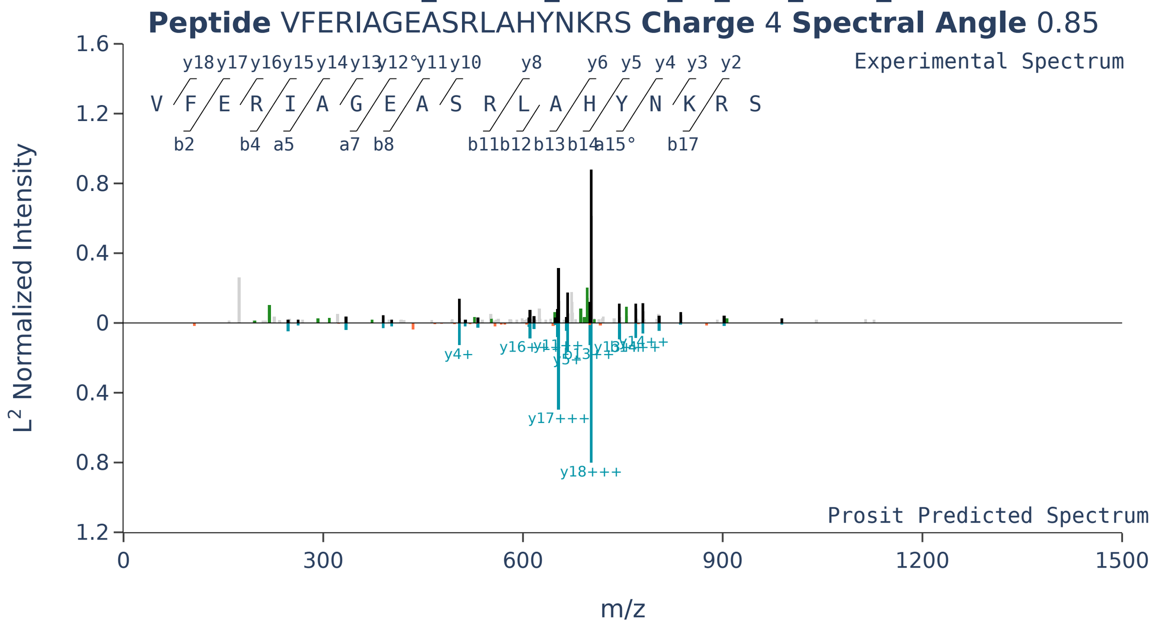


**Source** HRoetschke_291222_080123_Fu_H2B_b1R2_24h **Scan** 2925
**Peptide** SRLAHYNKRSTL **Charge** 3 **Spectral Angle** 0.85

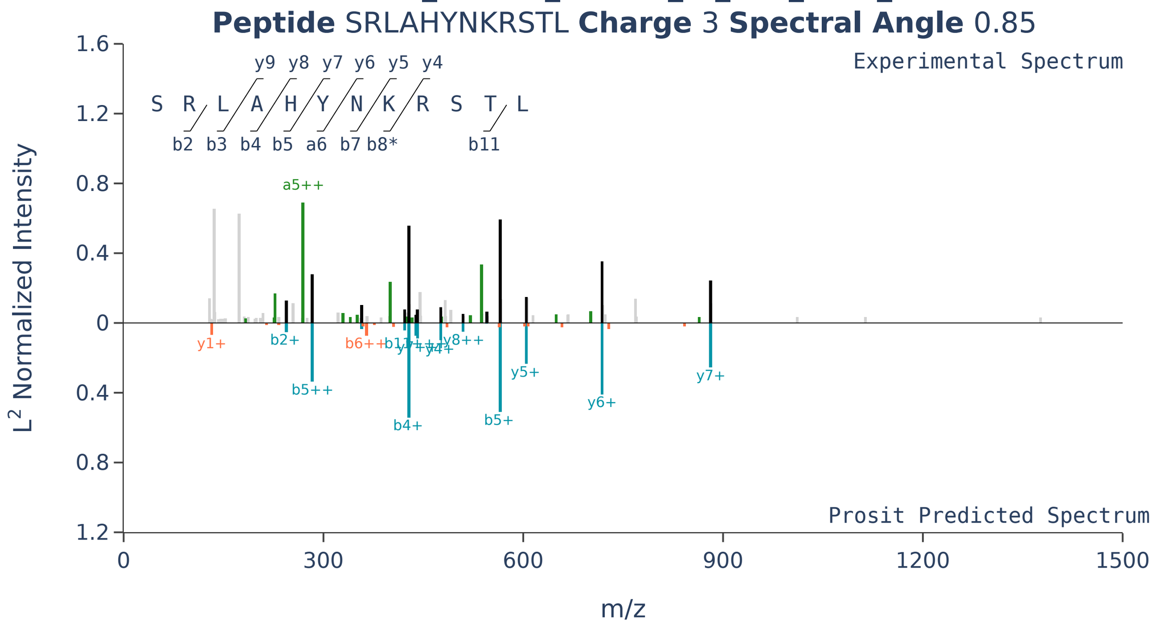


**Source** HRoetschke_291222_080123_Fu_H2B_b2R1_4h **Scan** 17509
**Peptide** NDVFERIAGEASRLAHYNKRS **Charge** 5 **Spectral Angle** 0.74

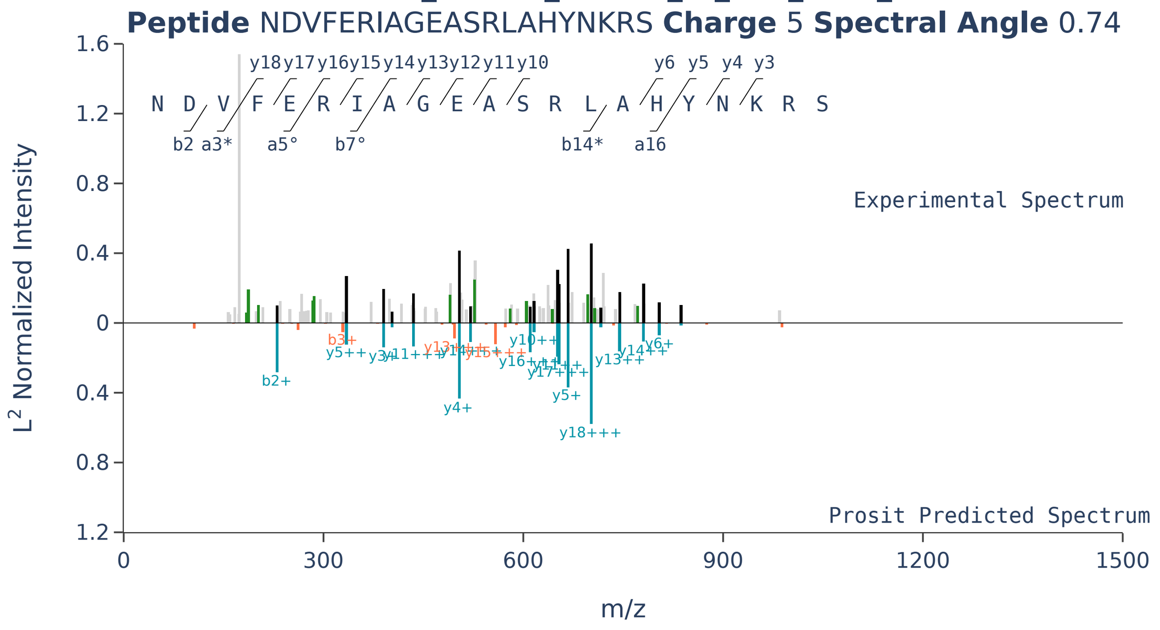


**Source** HRoetschke_291222_080123_Fu_H2B_b1R1_4h **Scan** 16920
**Peptide** SREIQTAVRLLLPGE **Charge** 3 **Spectral Angle** 0.7

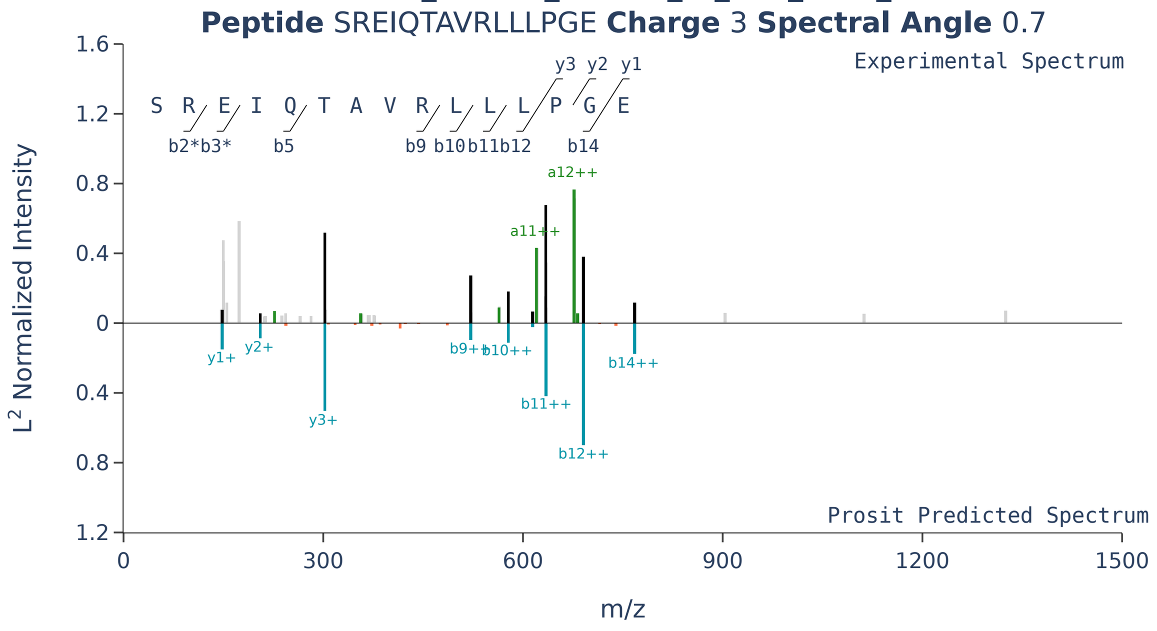


**Source** HRoetschke_291222_080123_Fu_H2B_b2R1_1h **Scan** 15735
**Peptide** VRLLLPGELAKHAVSEGTKAVTKYTSA **Charge** 5 **Spectral Angle** 0.88

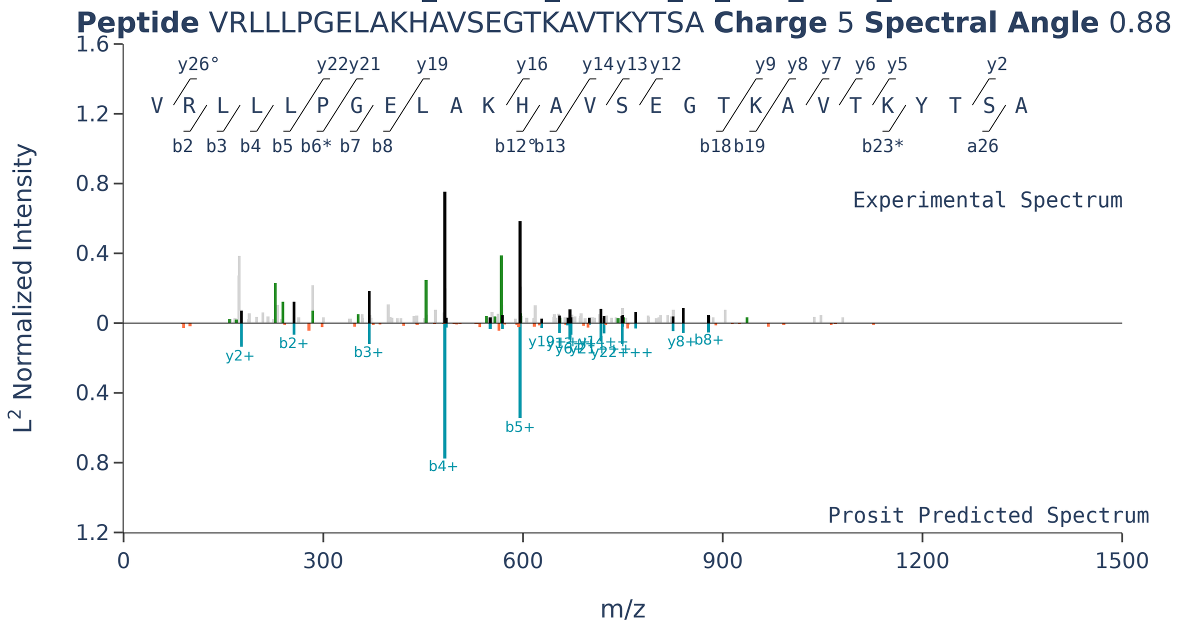


**Source** HRoetschke_291222_080123_Fu_H2B_b1R1_4h **Scan** 5049
**Peptide** RKESYAL **Charge** 2 **Spectral Angle** 0.83

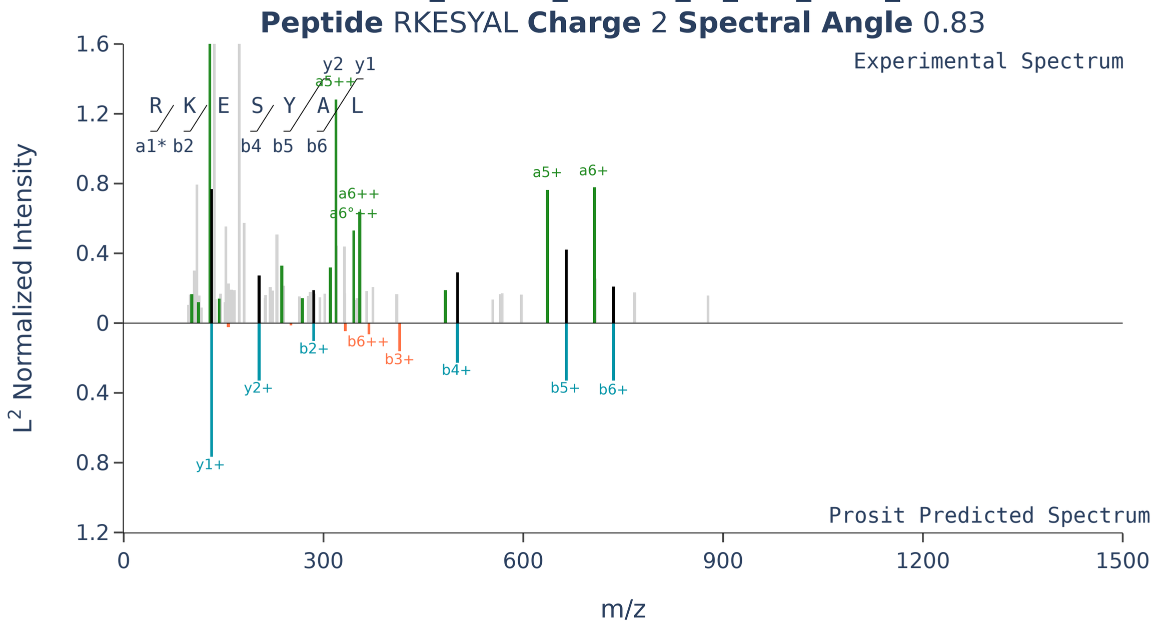


**Source** HRoetschke_291222_080123_Fu_H2B_b2R2_4h **Scan** 4224
**Peptide** LAGEASRLAHYNKRS **Charge** 3 **Spectral Angle** 0.9

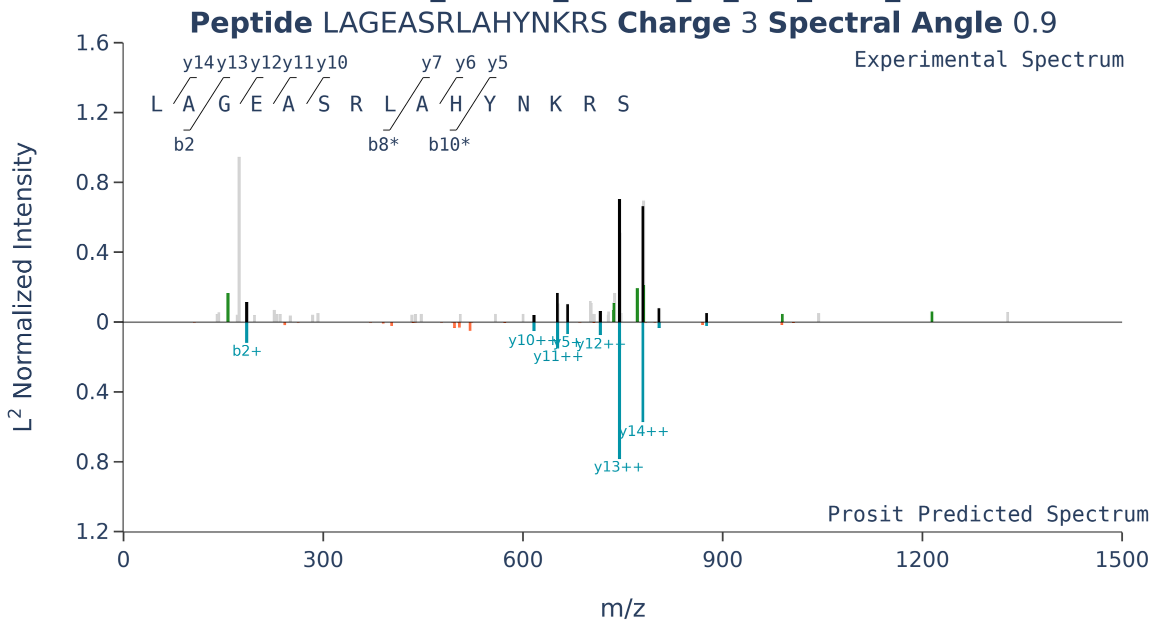


**Source** HRoetschke_291222_080123_Fu_H2B_b1R2_24h **Scan** 3466
**Peptide** AGEASRLAHYNK **Charge** 3 **Spectral Angle** 0.56

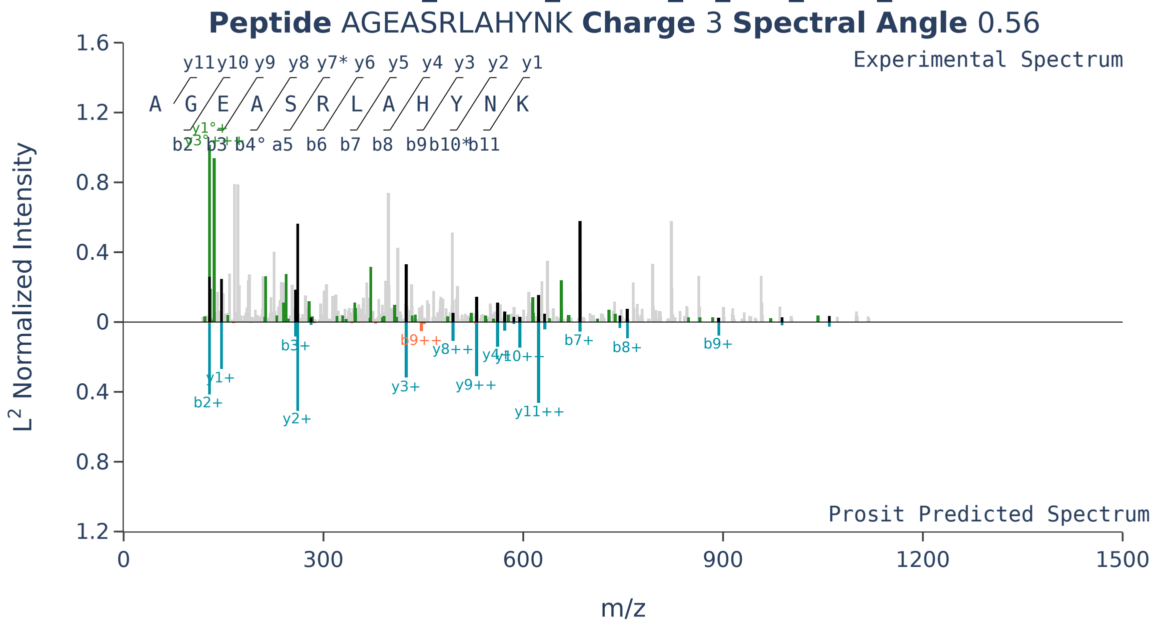


**Source** HRoetschke_291222_080123_Fu_H2B_b2R2_24h **Scan** 3333
**Peptide** AKHAVSEGTKAVTKYTSAK **Charge** 3 **Spectral Angle** 0.71

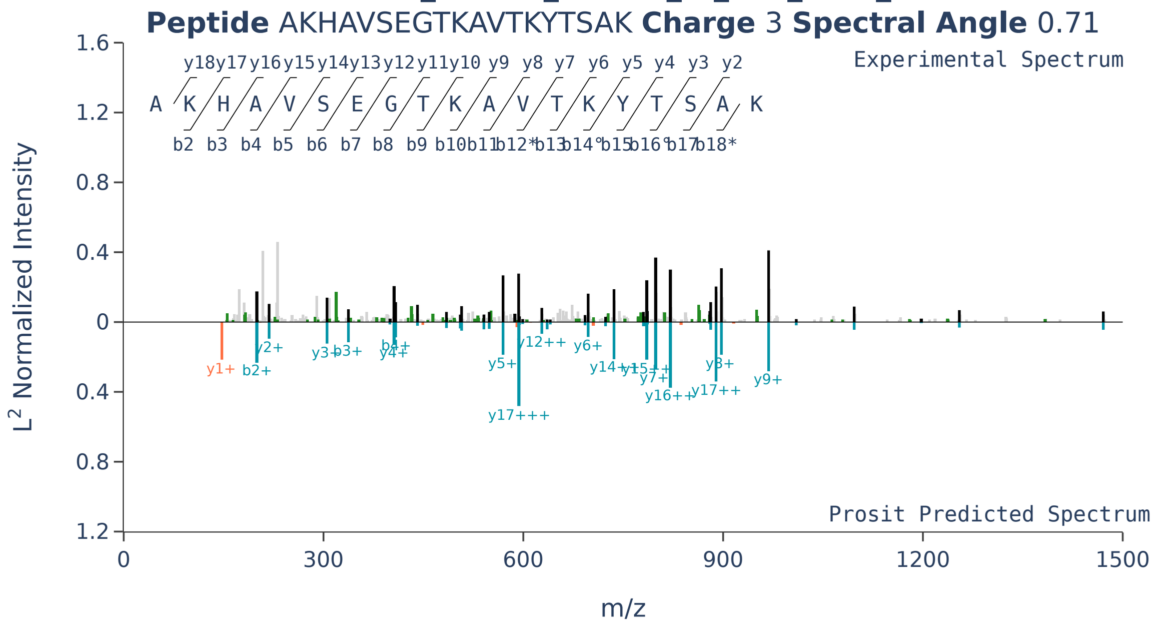


**Source** HRoetschke_291222_080123_Fu_H2B_b1R1_2h **Scan** 19792
**Peptide** MNSFVNDVFERIAGEASRLAHYNKR **Charge** 5 **Spectral Angle** 0.85

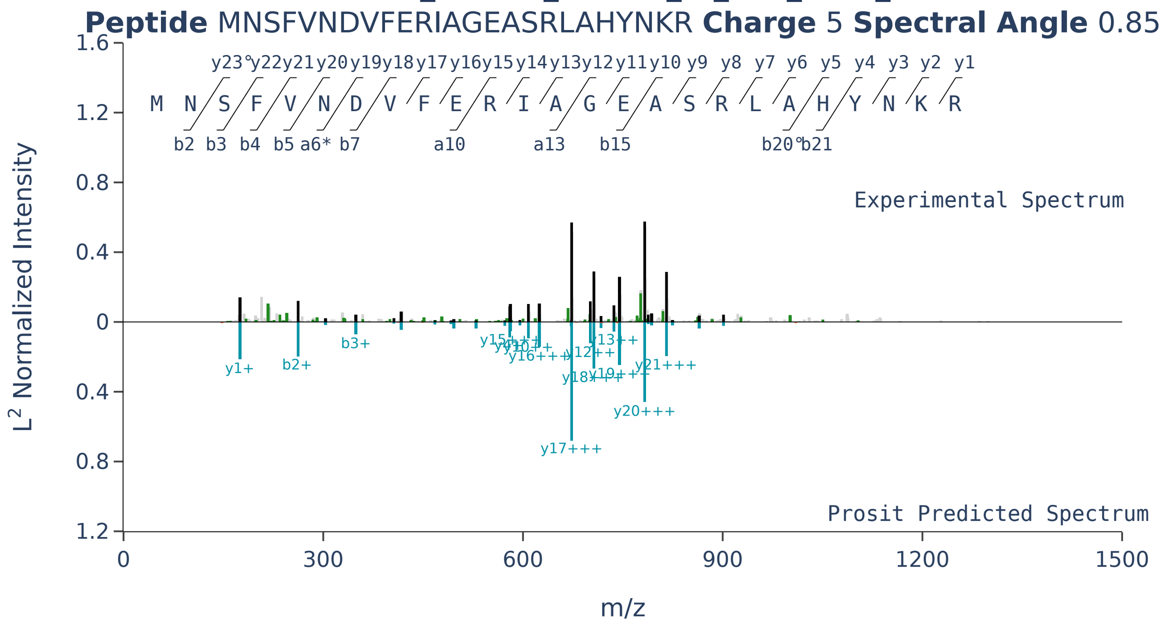


**Source** HRoetschke_291222_080123_Fu_H2B_b2R1_24h **Scan** 13213
**Peptide** RKESYAIYVYKV **Charge** 3 **Spectral Angle** 0.79

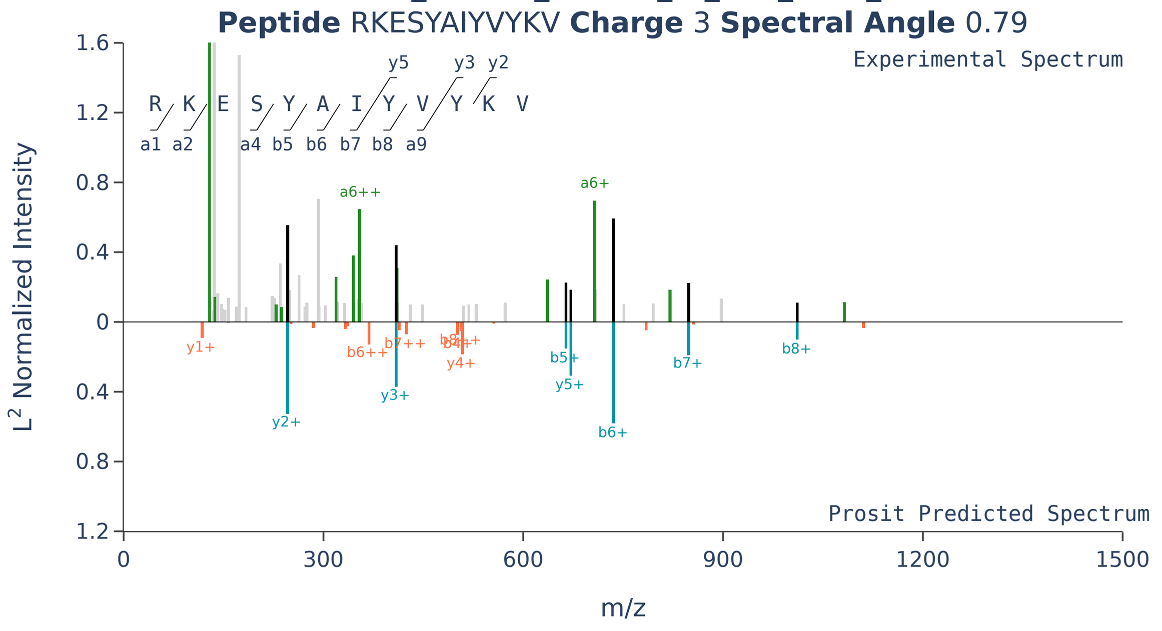


**Source** HRoetschke_291222_080123_Fu_H2B_b1R1_4h **Scan** 4145
**Peptide** GEASRLAHYNKRSTITS **Charge** 3 **Spectral Angle** 0.71

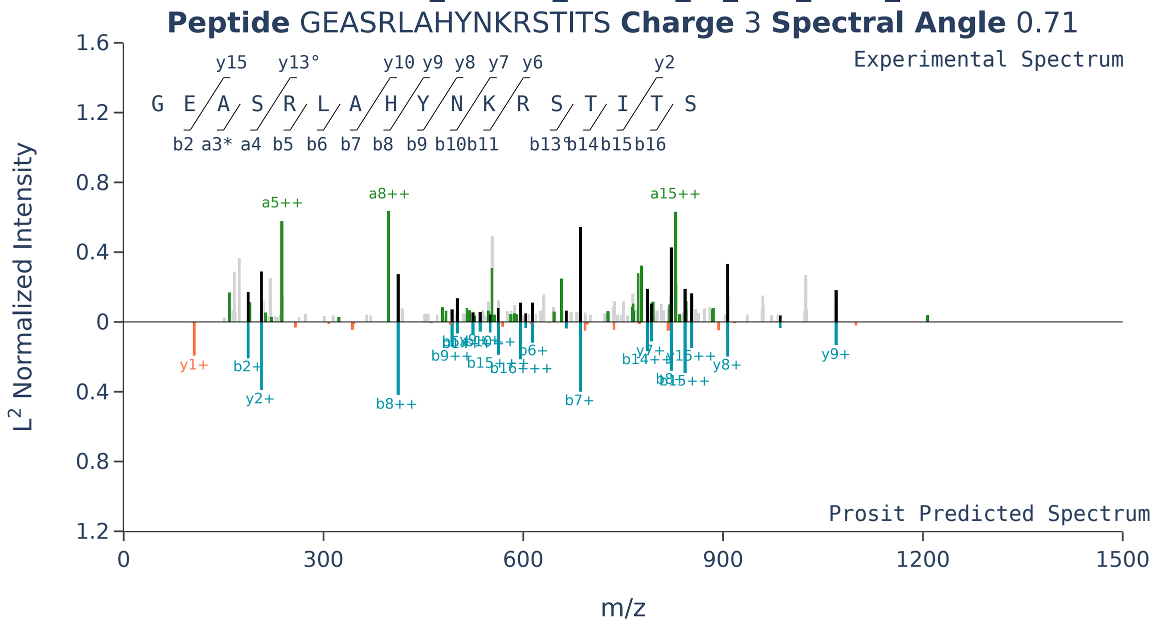

**Source** HRoetschke_291222_080123_Fu_H2B_b1R2_2h **Scan** 2212
**Peptide** ASRLAHYNKRSTITSR **Charge** 4 **Spectral Angle** 0.77

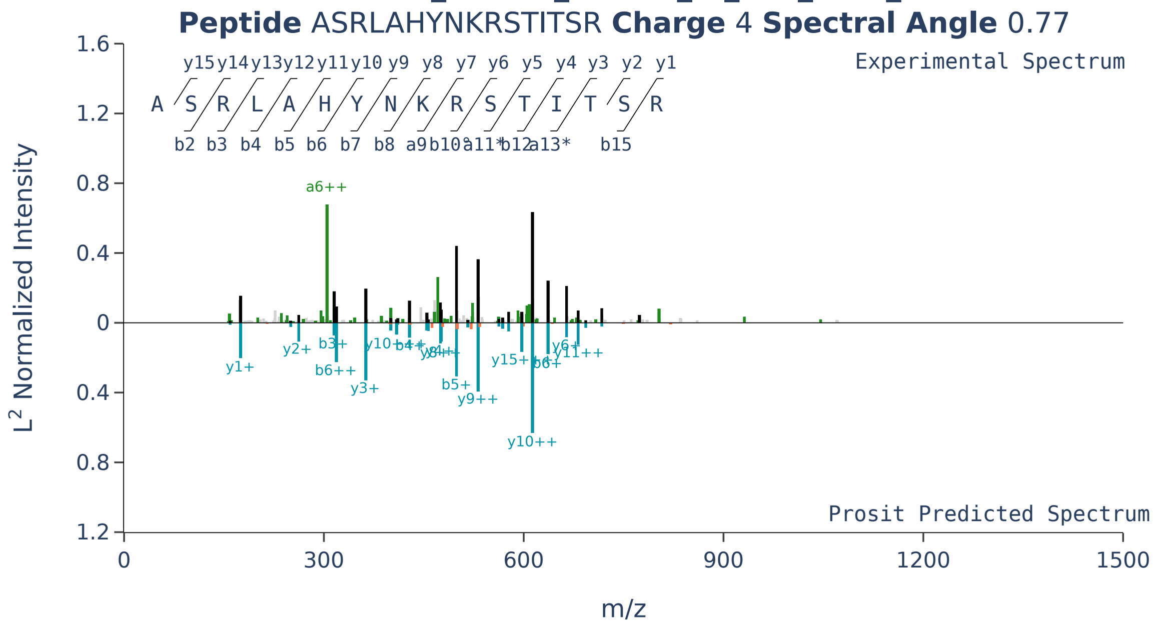


**Source** HRoetschke_291222_080123_Fu_H2B_b2R1_24h **Scan** 15719
**Peptide** VRLLLPGELAKHAVSEGTKA **Charge** 4 **Spectral Angle** 0.8

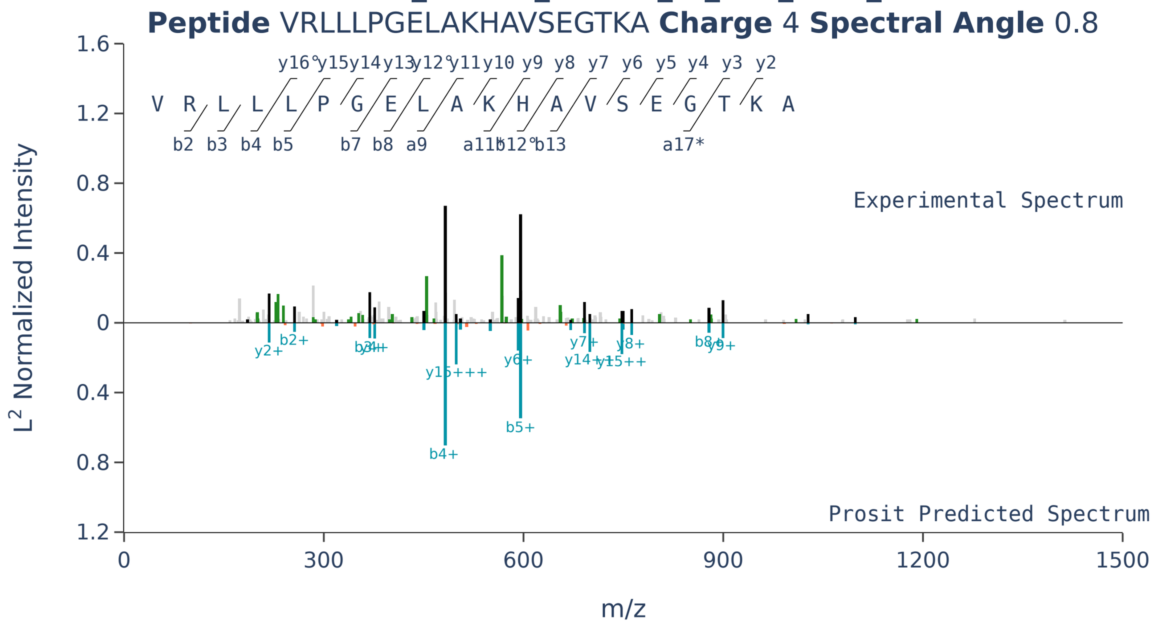


**Source** HRoetschke_291222_080123_Fu_H2B_b2R1_2h **Scan** 9046
**Peptide** LMNSFVND **Charge** 2 **Spectral Angle** 0.76

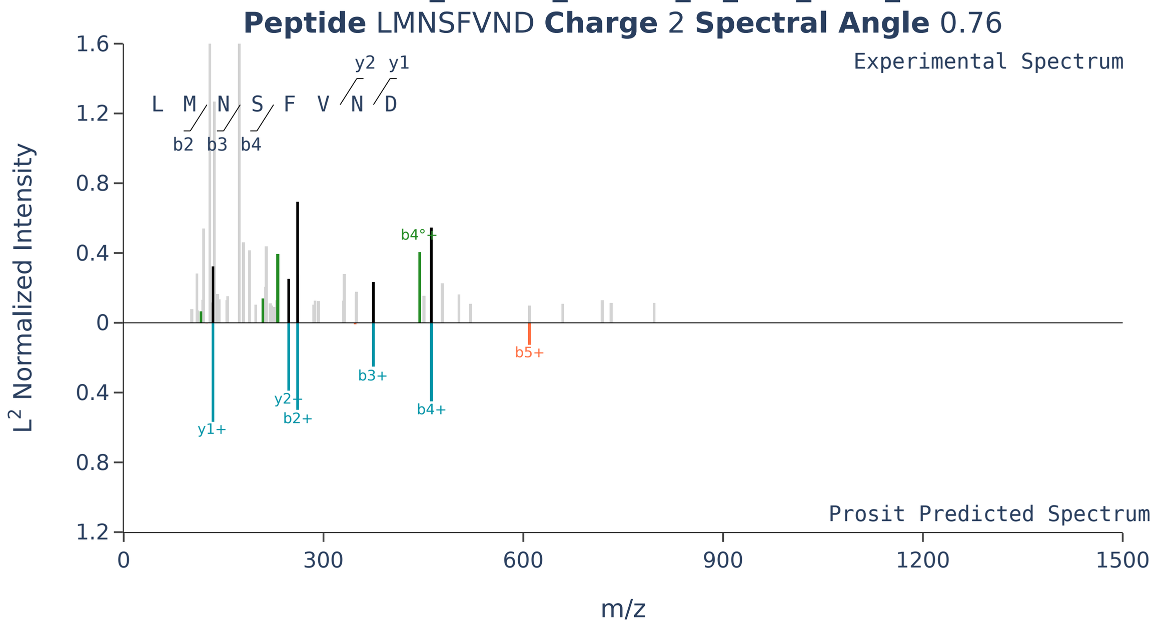


**Source** HRoetschke_291222_080123_Fu_H2B_b1R2_24h **Scan** 3562
**Peptide** AKHAVSEGTKAVTKYTS **Charge** 2 **Spectral Angle** 0.71

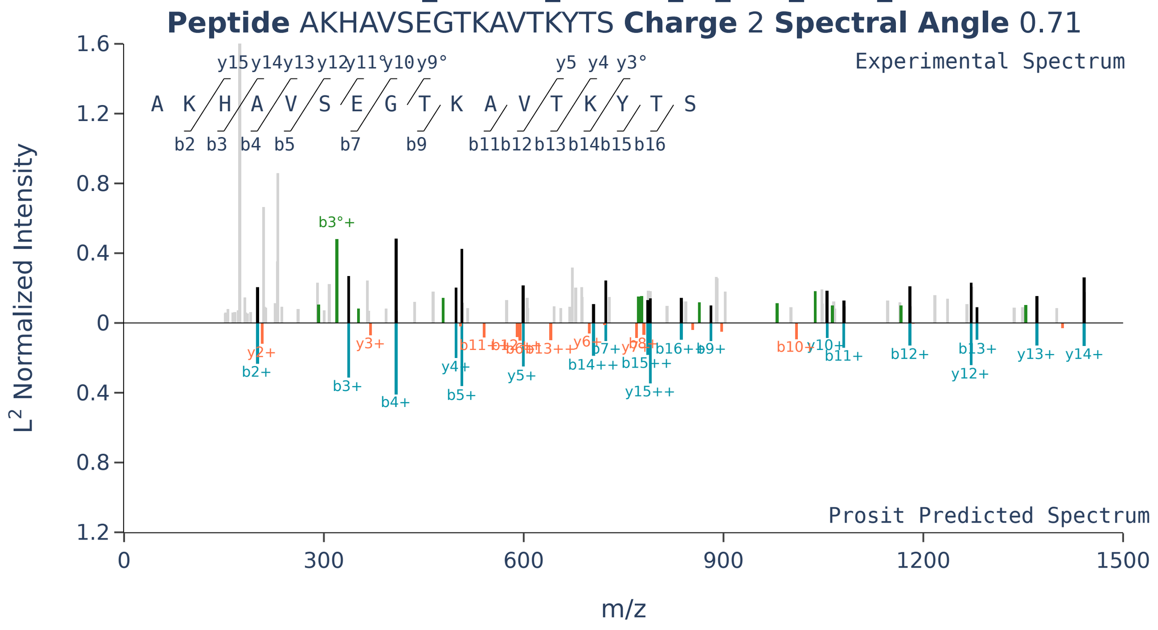


**Source** HRoetschke_291222_080123_Fu_H2B_b2R1_4h **Scan** 5352
**Peptide** VSEGTKAVTKYTSA **Charge** 2 **Spectral Angle** 0.78

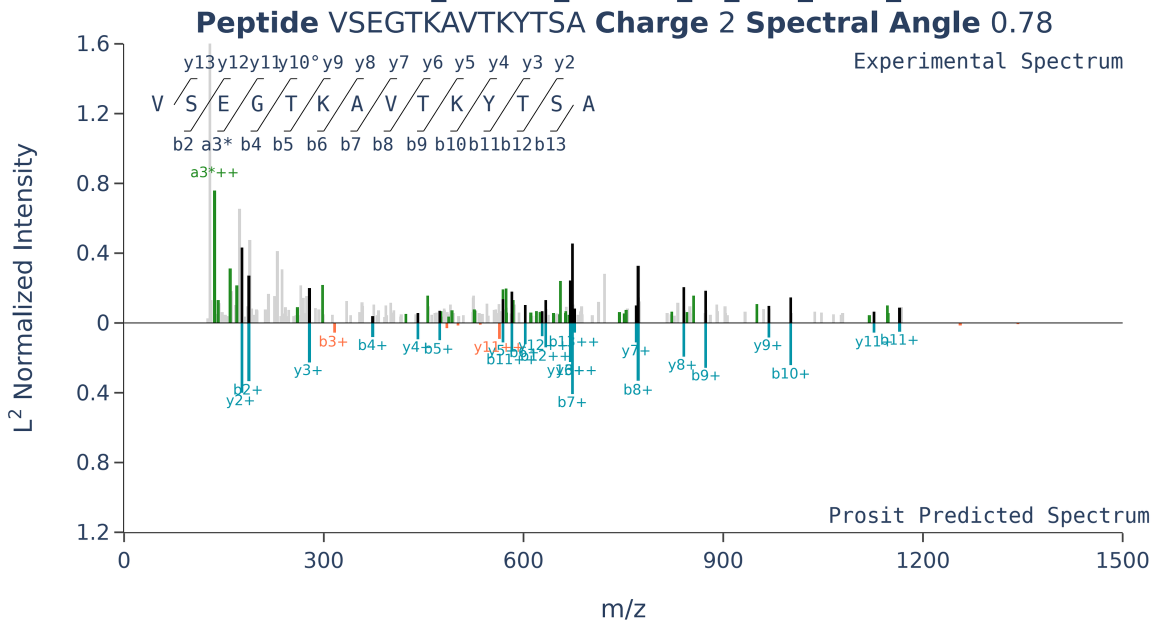


**Source** HRoetschke_291222_080123_Fu_H2B_b2R1_24h **Scan** 13712
**Peptide** LPGELAKHAVSEGTKAVTKYTSAK **Charge** 3 **Spectral Angle** 0.74

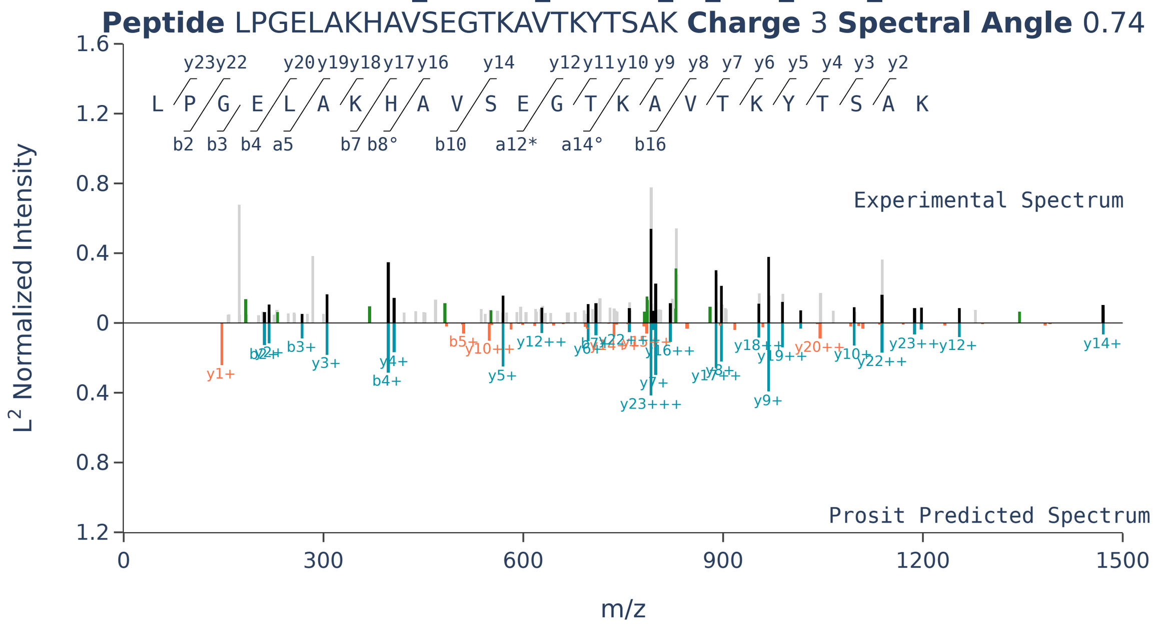


**Source** HRoetschke_291222_080123_Fu_H2B_b2R1_1h **Scan** 2248
**Peptide** RLAHYNKRSTI **Charge** 3 **Spectral Angle** 0.86

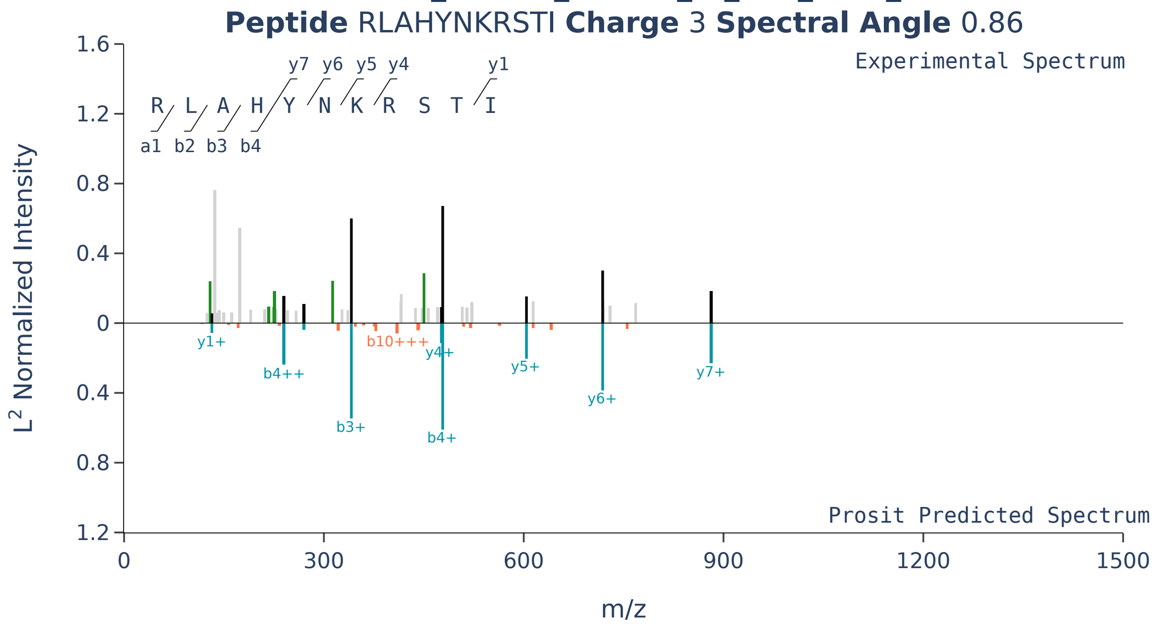


**Source** HRoetschke_291222_080123_Fu_H2B_b2R1_4h **Scan** 7928
**Peptide** LAGEASRLAHYNKRSTITSREIQT **Charge** 4 **Spectral Angle** 0.87

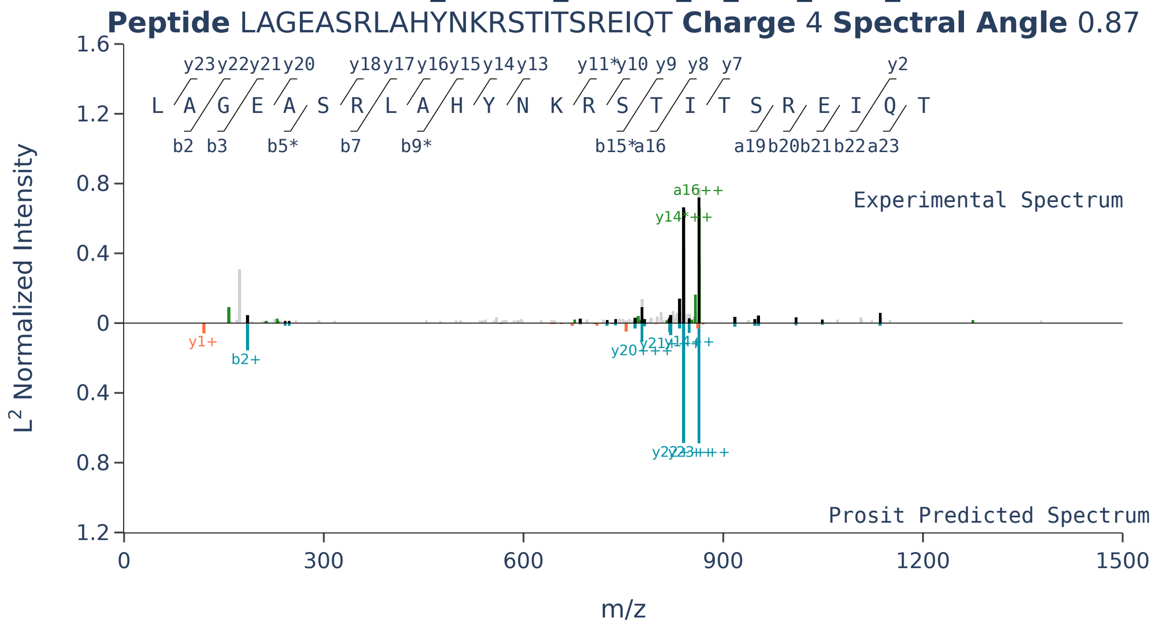


**Source** HRoetschke_291222_080123_Fu_H2B_b2R1_4h **Scan** 16323
**Peptide** REIQTAVRLLLPGELAKHAVSEGTKA **Charge** 5 **Spectral Angle** 0.81

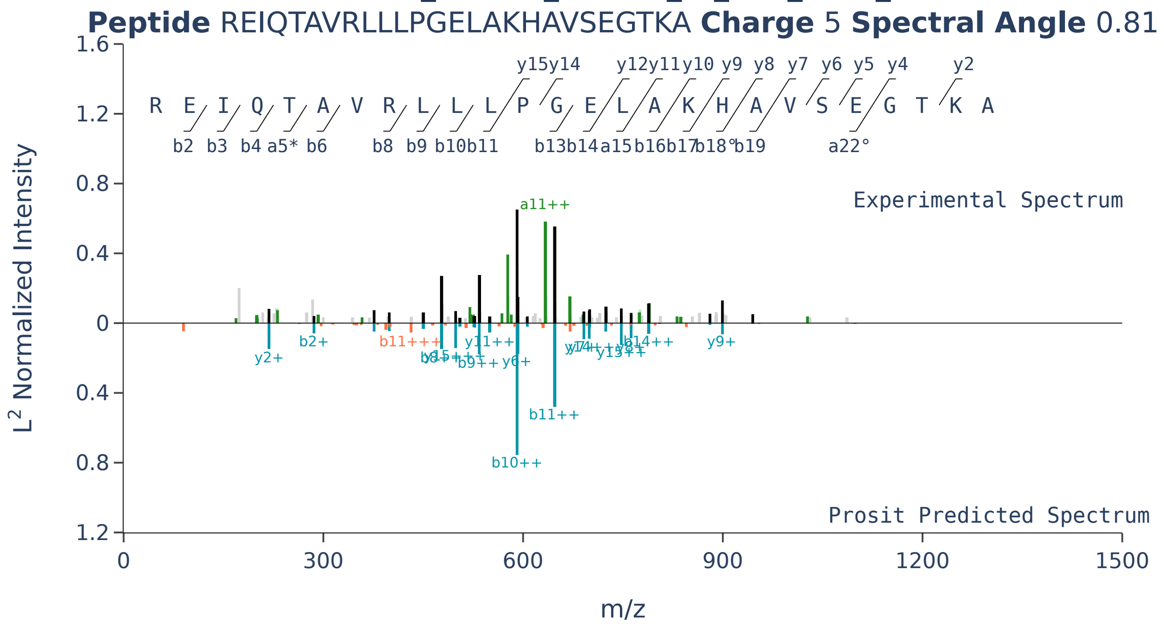


**Source** HRoetschke_291222_080123_Fu_H2B_b1R1_2h **Scan** 7043
**Peptide** KAMSIMNS **Charge** 2 **Spectral Angle** 0.69

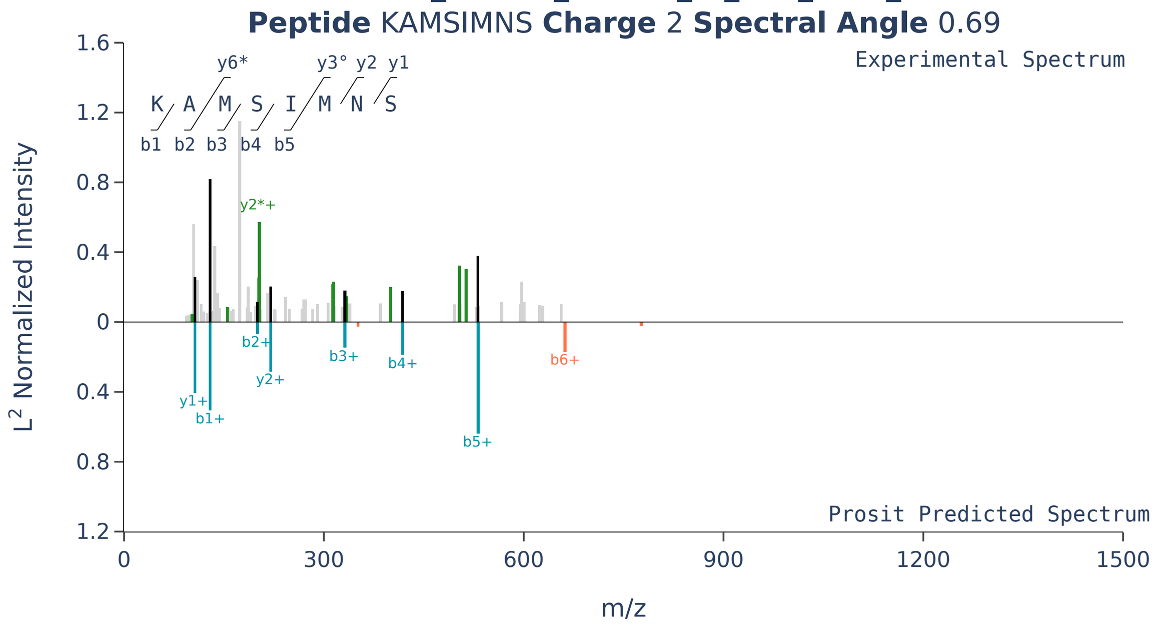


**Source** HRoetschke_291222_080123_Fu_H2B_b1R1_24h **Scan** 7183
**Peptide** HYNKRSTITSREIQTAV **Charge** 3 **Spectral Angle** 0.71

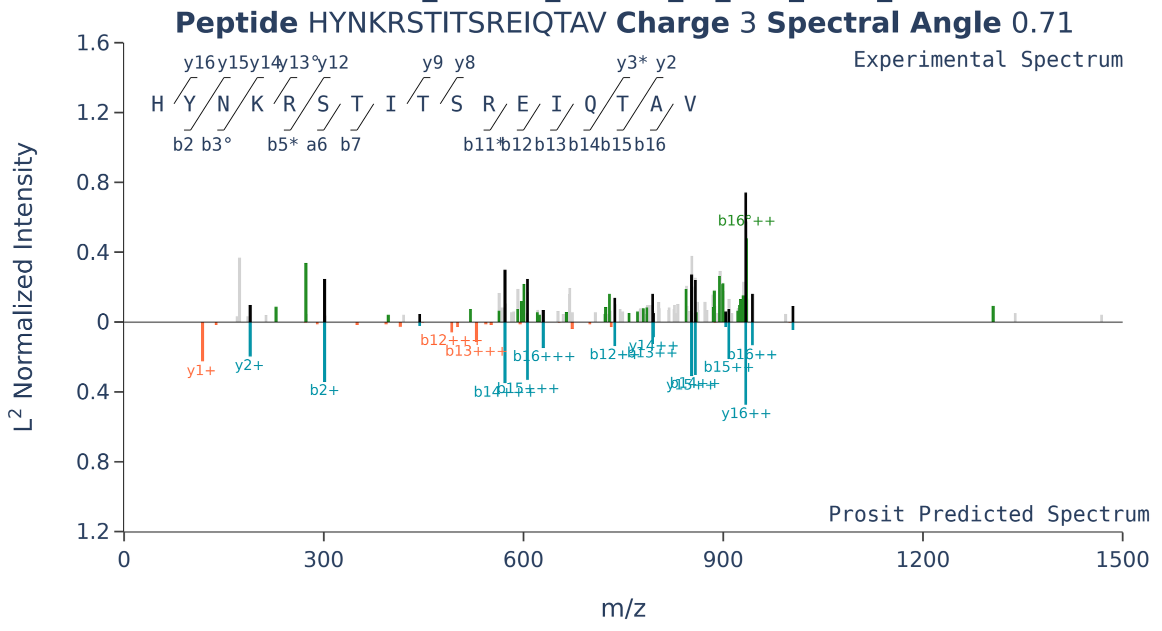


**Source** HRoetschke_291222_080123_Fu_H2B_b1R2_1h **Scan** 11169
**Peptide** RLLLPGELAKHAVSEGTKA **Charge** 4 **Spectral Angle** 0.86

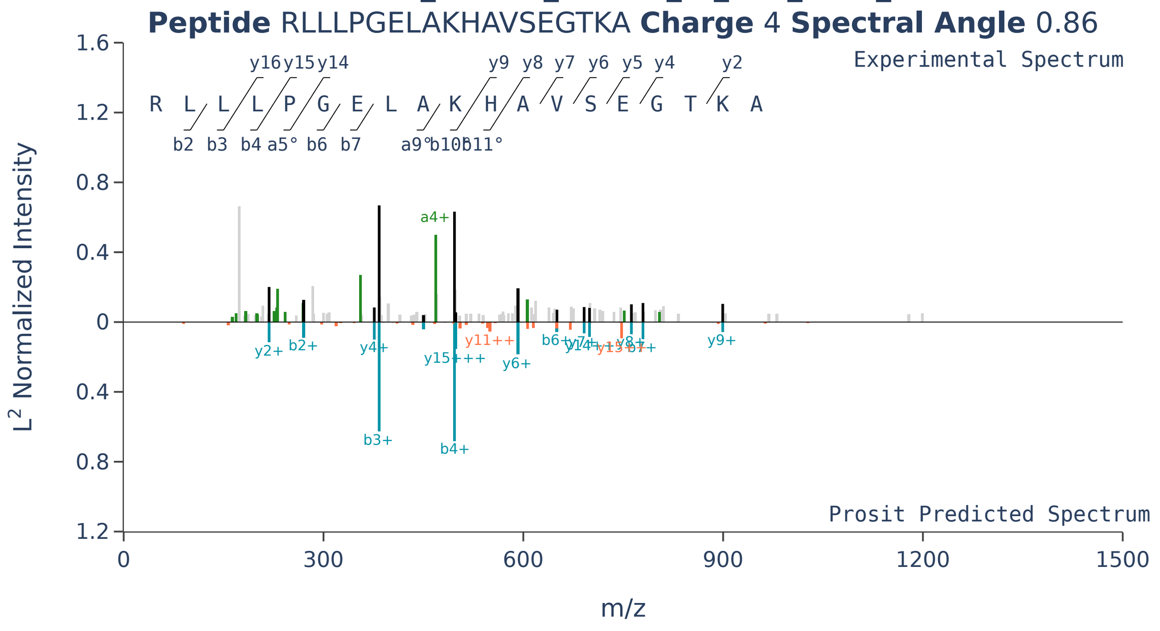


**Source** HRoetschke_291222_080123_Fu_H2B_b2R2_24h **Scan** 19855
**Peptide** ELQTAVRLLLPGELAKH **Charge** 3 **Spectral Angle** 0.83

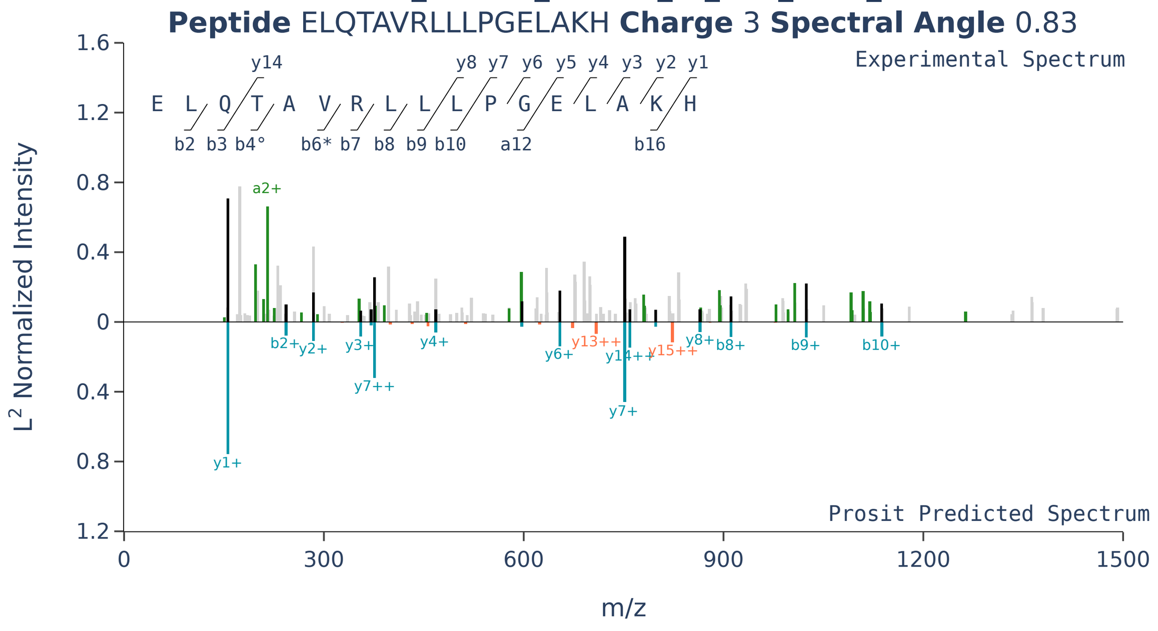


**Source** HRoetschke_291222_080123_Fu_H2B_b2R2_24h **Scan** 16802
**Peptide** LLPGELAKHAVSEGTKAVTKYTS **Charge** 4 **Spectral Angle** 0.68

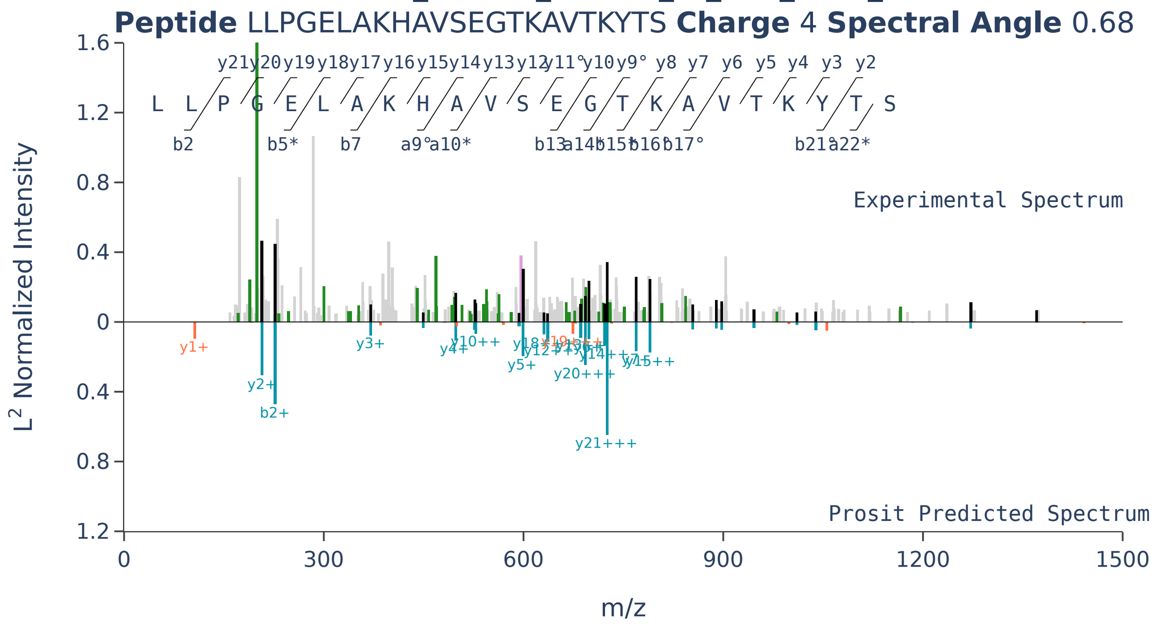


**Source** HRoetschke_291222_080123_Fu_H2B_b2R1_1h **Scan** 2137
**Peptide** GEASRLAHYNKRS **Charge** 3 **Spectral Angle** 0.76

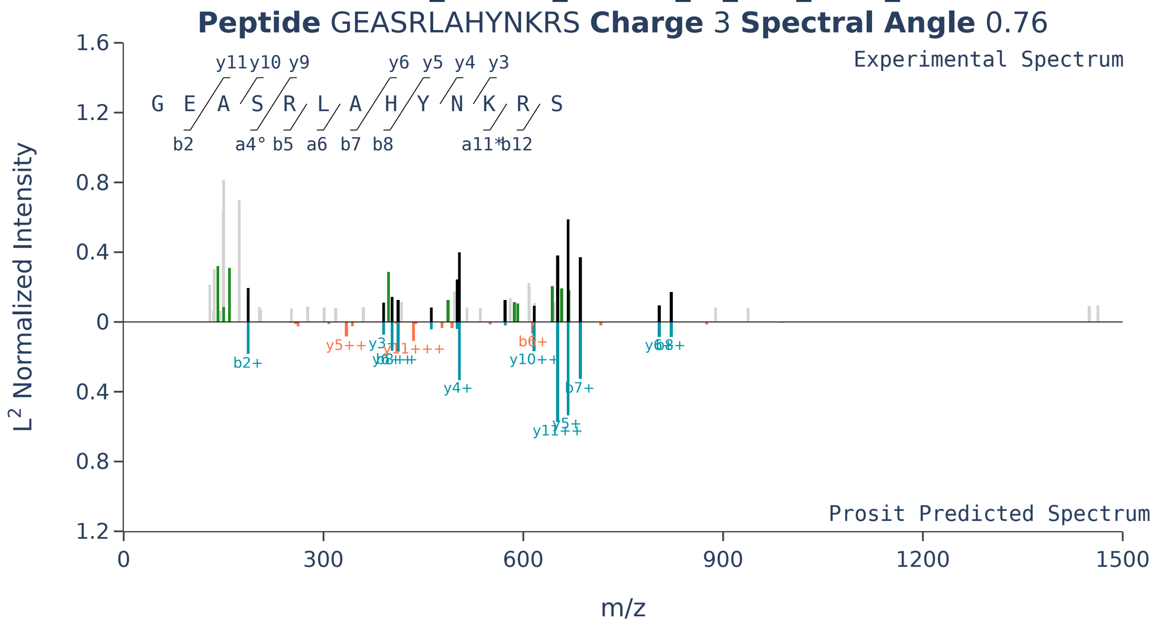

**Source** HRoetschke_291222_080123_Fu_H2B_b1R1_24h **Scan** 2630
**Peptide** ASRLAHYNKRSTITSRE **Charge** 4 **Spectral Angle** 0.8

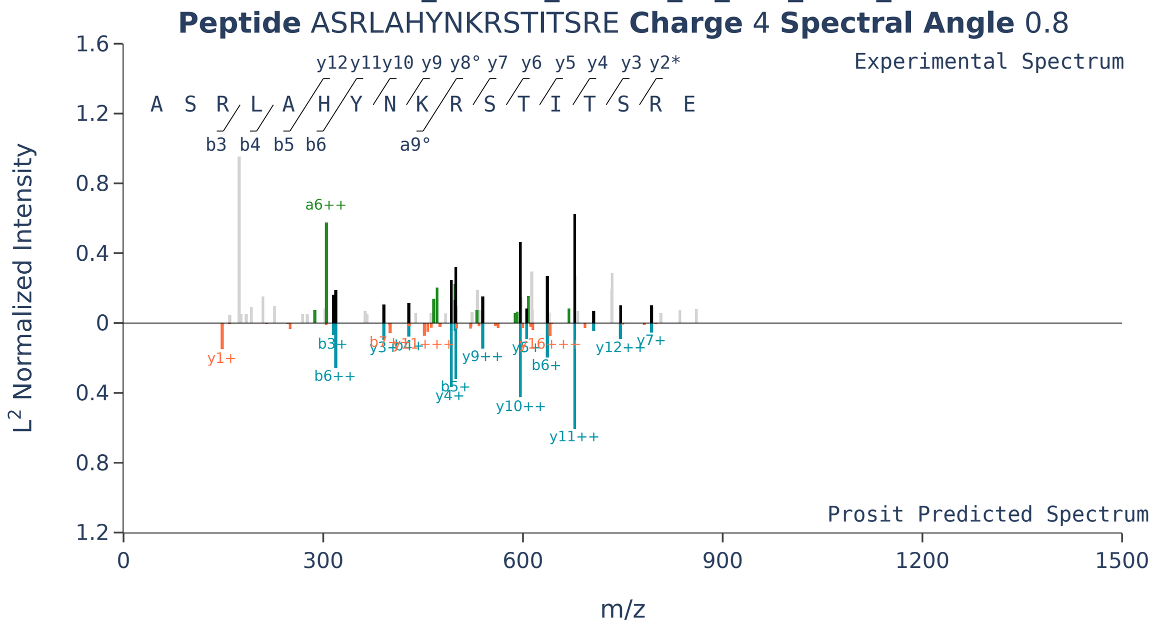


**Source** HRoetschke_291222_080123_Fu_H2B_b1R2_24h **Scan** 10275
**Peptide** IMNSFVND **Charge** 2 **Spectral Angle** 0.76

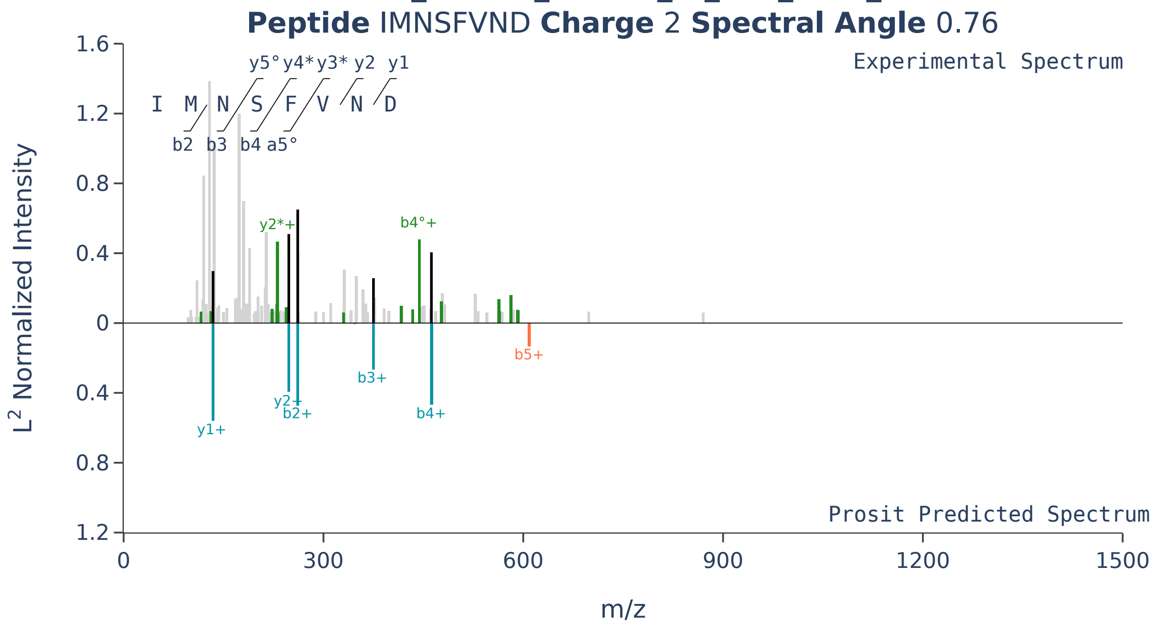


**Source** HRoetschke_291222_080123_Fu_H2B_b2R1_24h **Scan** 3686
**Peptide** KRSTITSREIQTA **Charge** 2 **Spectral Angle** 0.66

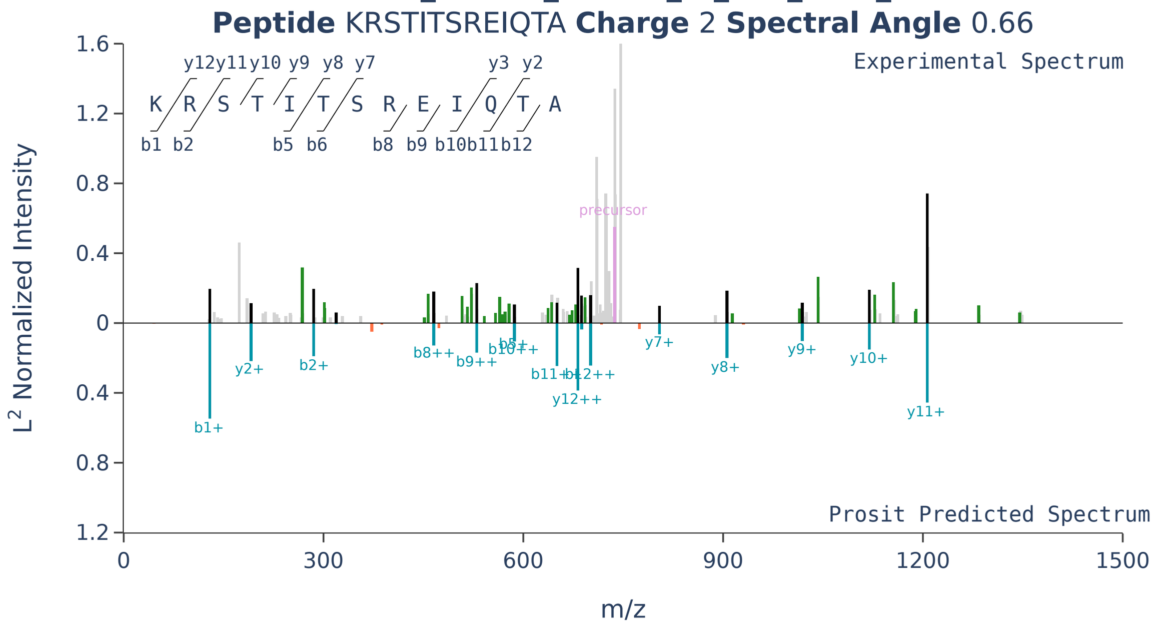


**Source** HRoetschke_291222_080123_Fu_H2B_b2R2_4h **Scan** 16461
**Peptide** RLLLPGELAKHAVSEGTKAVTKYTSAK **Charge** 6 **Spectral Angle** 0.81

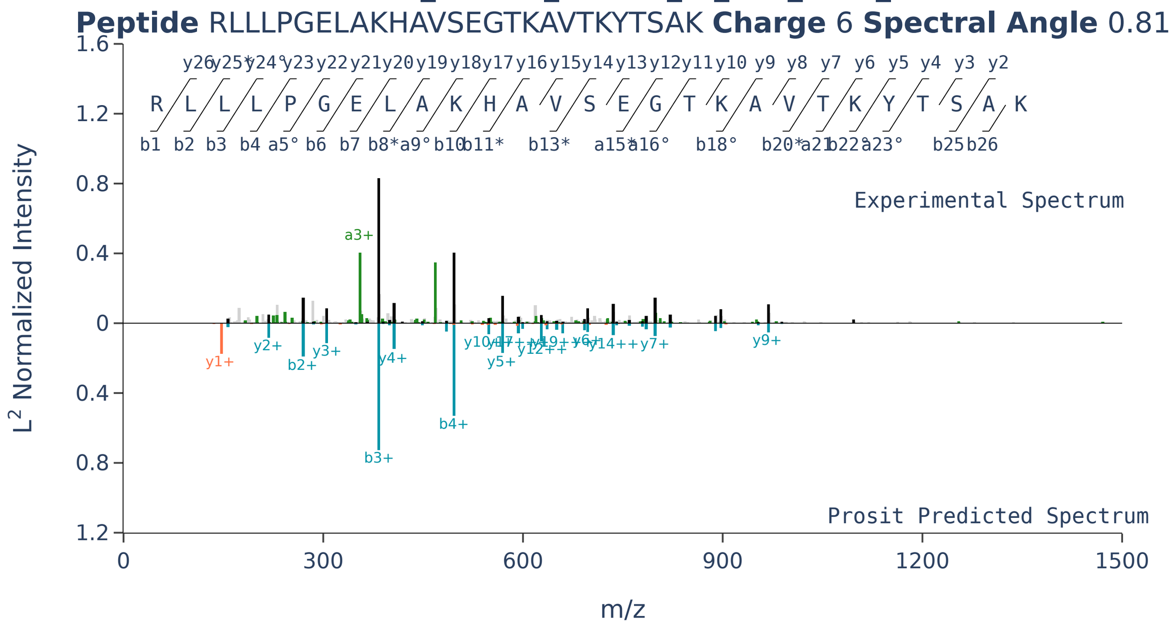


**Source** HRoetschke_291222_080123_Fu_H2B_b2R1_1h **Scan** 13960
**Peptide** AVRLLLPGELAKHAVS **Charge** 3 **Spectral Angle** 0.83

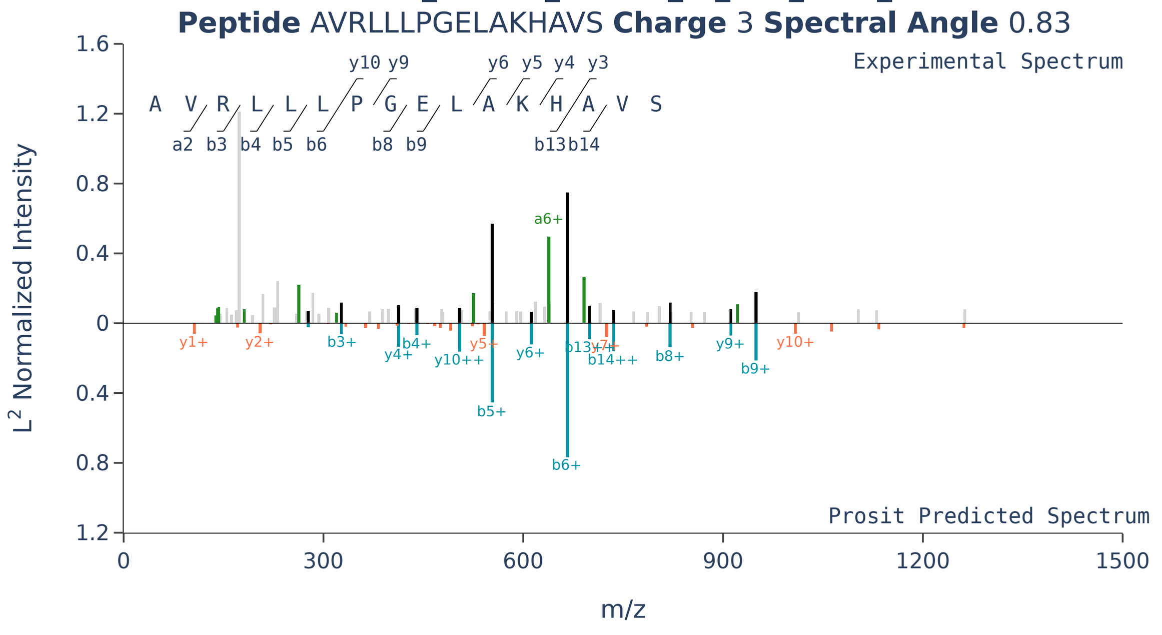


**Source** HRoetschke_291222_080123_Fu_H2B_b2R2_4h **Scan** 9515
**Peptide** LPGELAKHAVSEGTKAVTK **Charge** 3 **Spectral Angle** 0.76

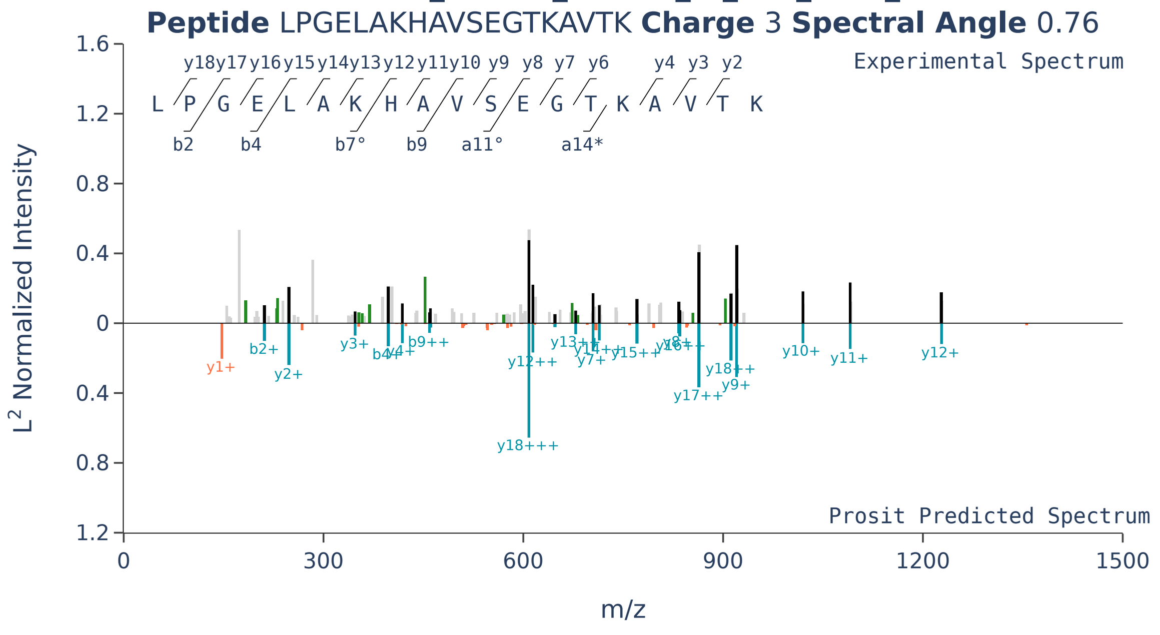


**Source** HRoetschke_291222_080123_Fu_H2B_b2R1_1h **Scan** 2039
**Peptide** LAKHAVSEGTKAVTK **Charge** 3 **Spectral Angle** 0.74

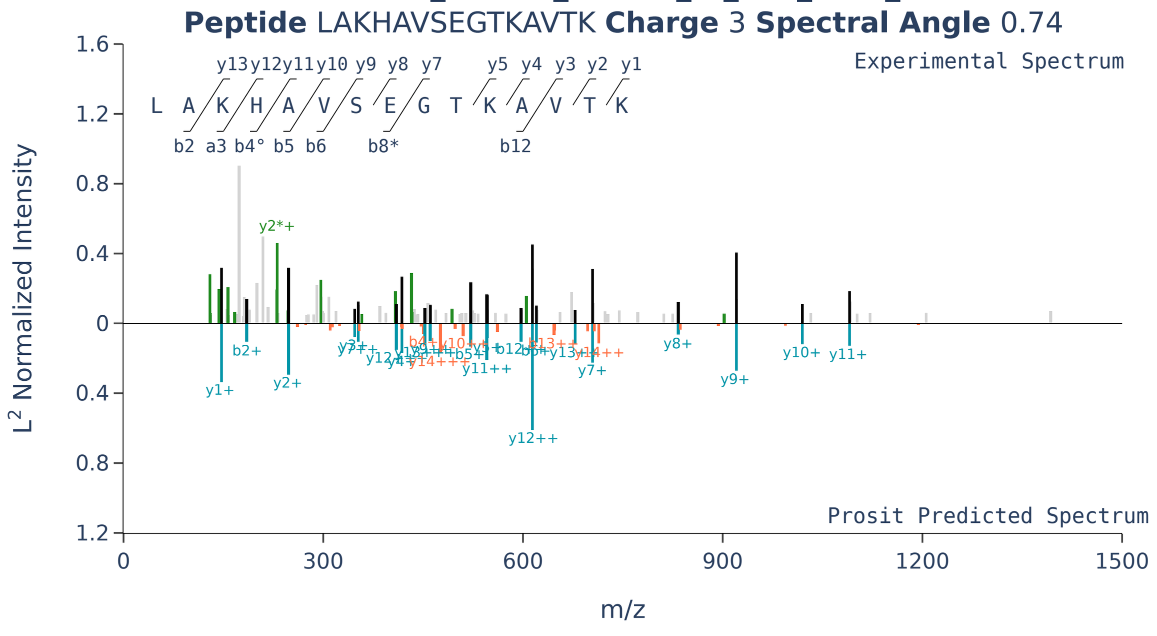


**Source** HRoetschke_291222_080123_Fu_H2B_b2R1_4h **Scan** 13328
**Peptide** DVFERIAGEASR **Charge** 3 **Spectral Angle** 0.78

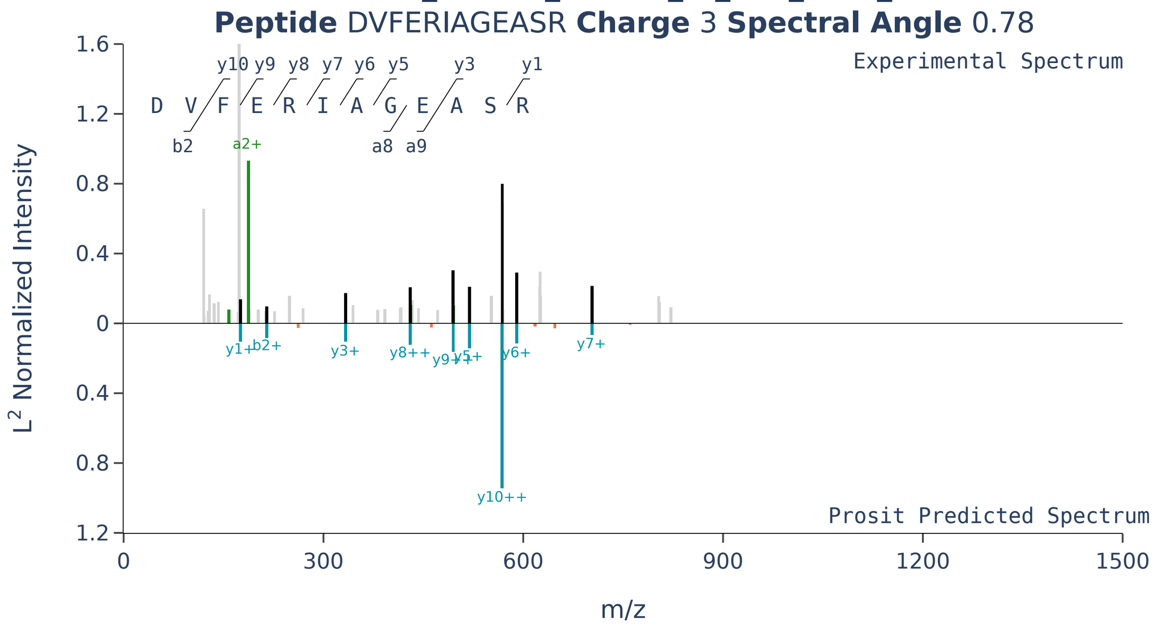


**Source** HRoetschke_291222_080123_Fu_H2B_b1R1_2h **Scan** 4647
**Peptide** AGEASRLAHYNKRSTL **Charge** 3 **Spectral Angle** 0.76

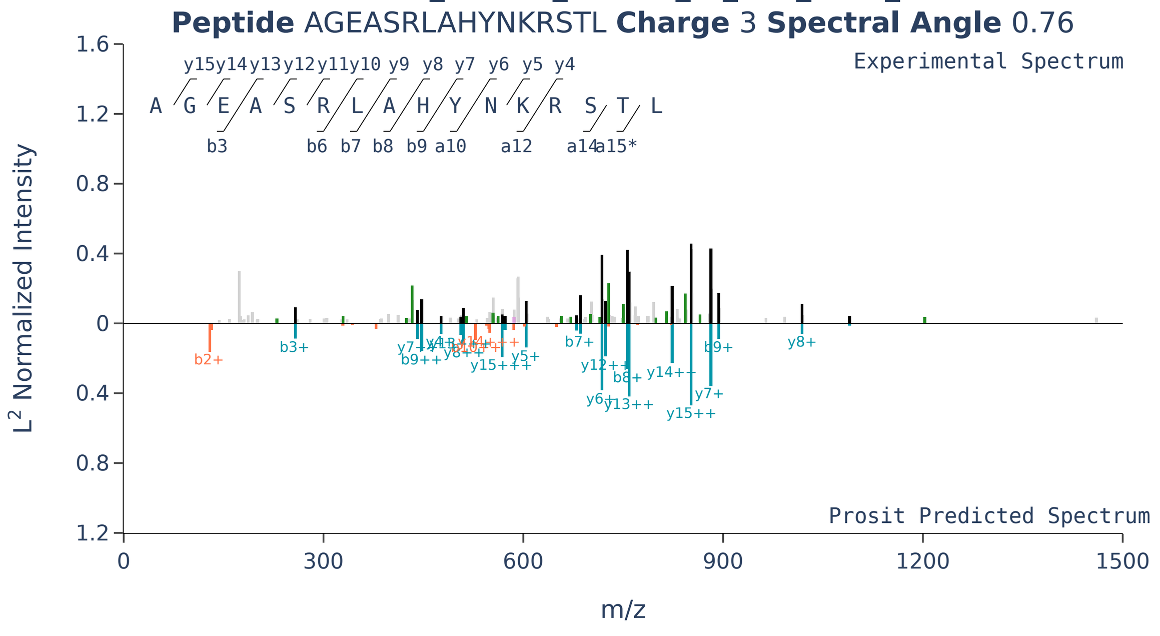


**Source** HRoetschke_291222_080123_Fu_H2B_b2R1_2h **Scan** 1916
**Peptide** EASRLAHYNKRST **Charge** 3 **Spectral Angle** 0.8

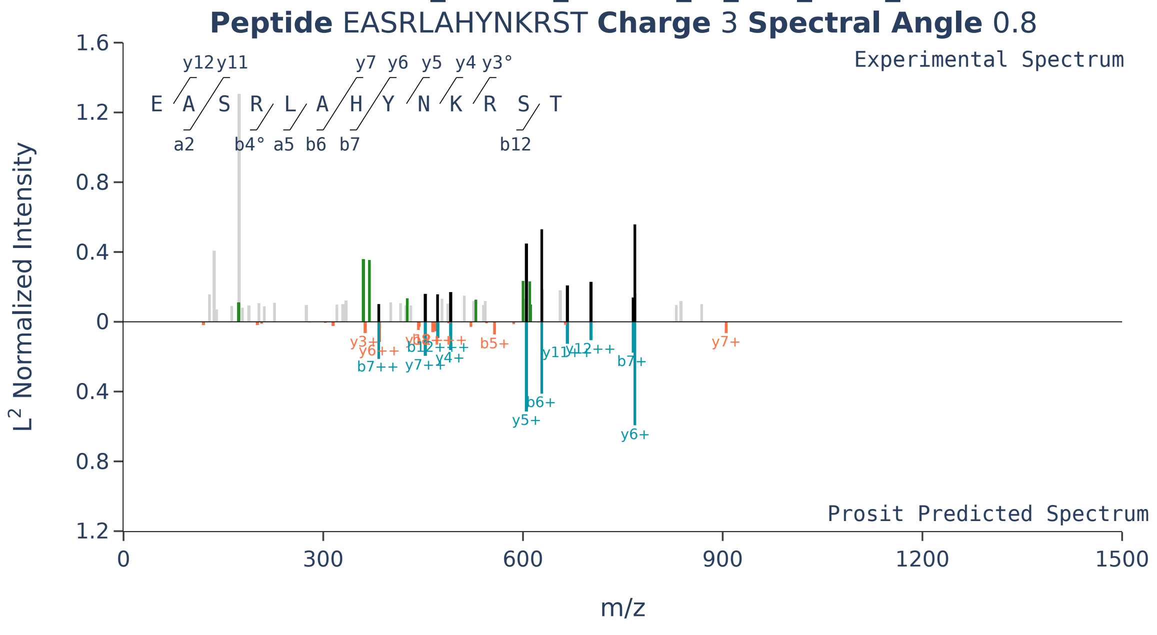


**Source** HRoetschke_291222_080123_Fu_H2B_b2R1_1h **Scan** 1923
**Peptide** RLAHYNKRSTITSR **Charge** 4 **Spectral Angle** 0.72

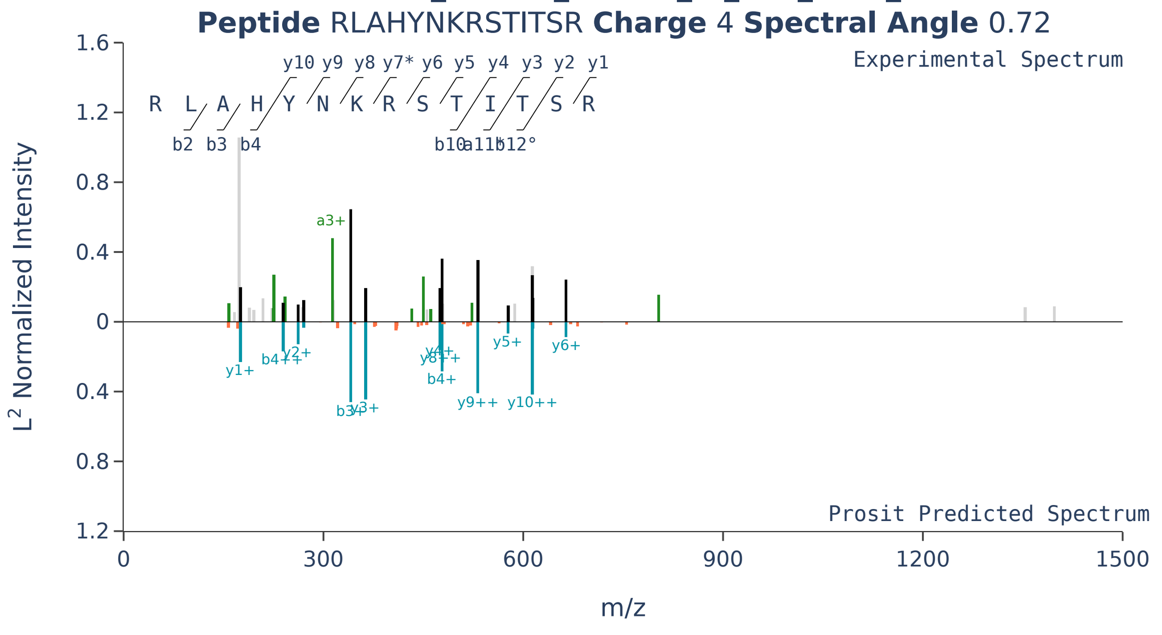


**Source** HRoetschke_291222_080123_Fu_H2B_b2R2_24h **Scan** 7857
**Peptide** QVHPDTGISSKAMSIM **Charge** 3 **Spectral Angle** 0.81

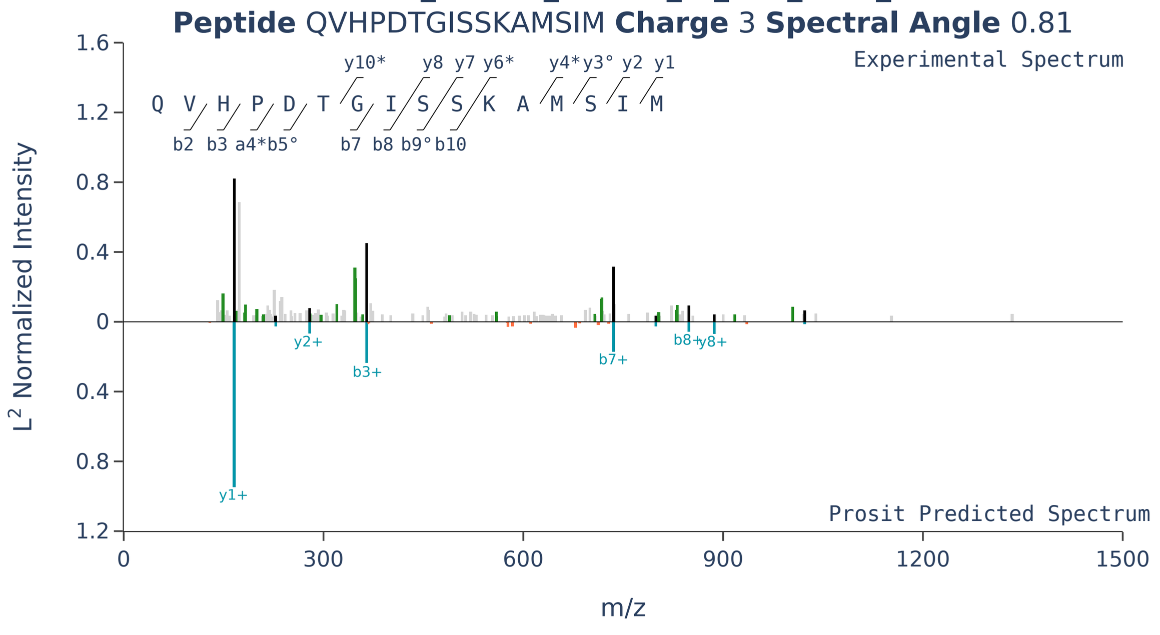


**Source** HRoetschke_291222_080123_Fu_H2B_b2R1_24h **Scan** 11600
**Peptide** LAHYNKRSTITSREIQTAVRL **Charge** 5 **Spectral Angle** 0.74

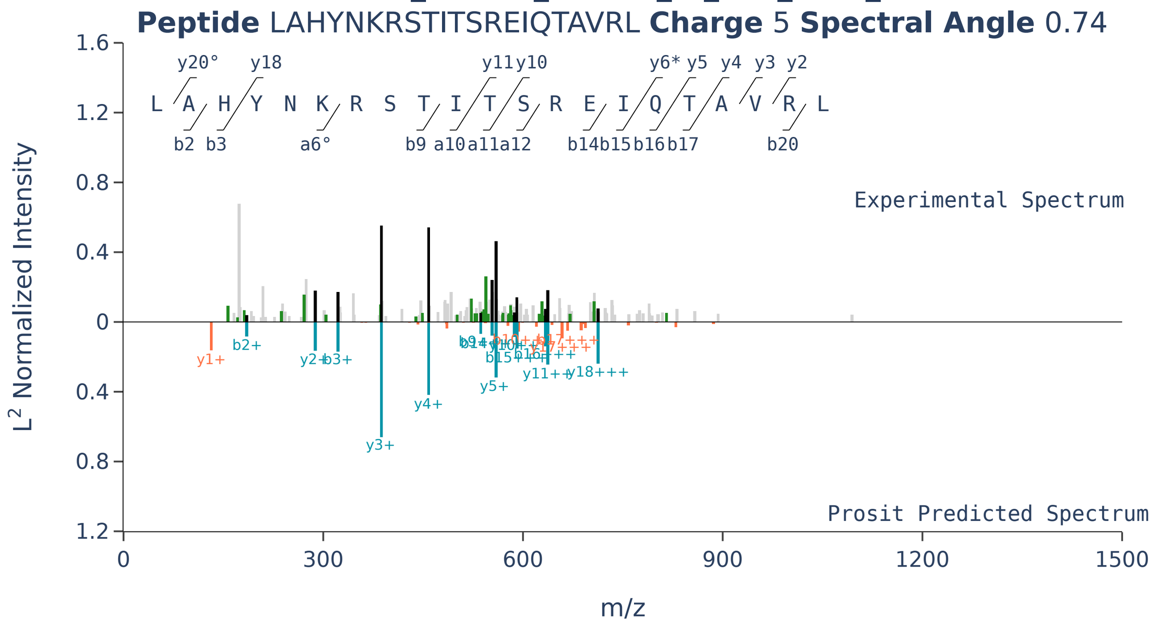


**Source** HRoetschke_291222_080123_Fu_H2B_b2R2_24h **Scan** 8561
**Peptide** LAHYNKRSTITSREIQTAV **Charge** 3 **Spectral Angle** 0.77

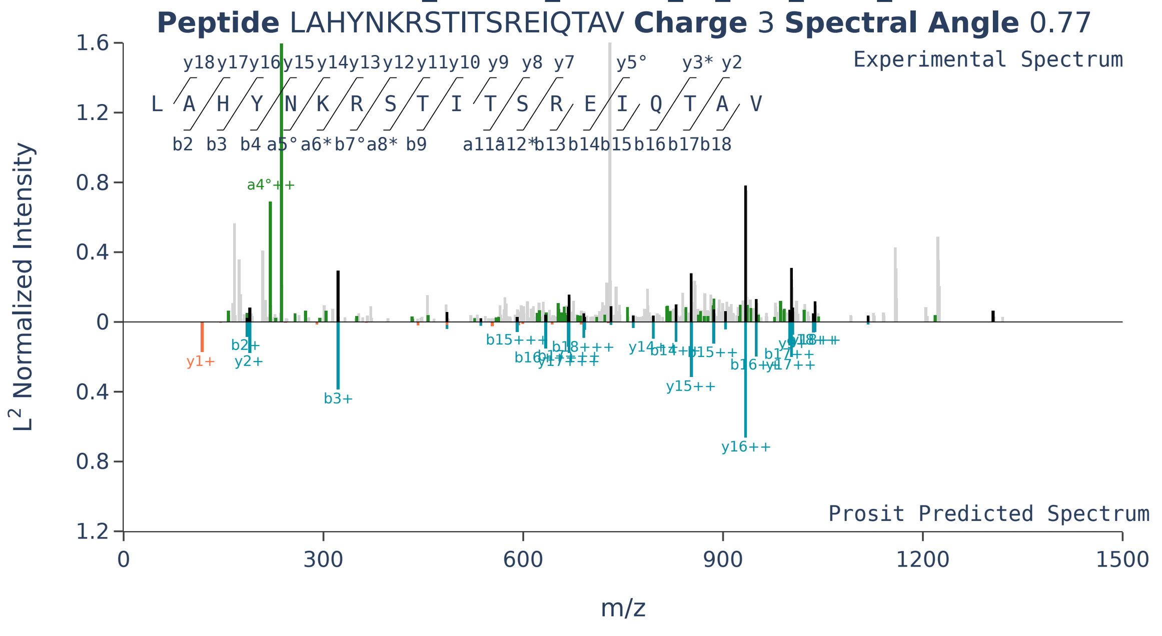


**Source** HRoetschke_291222_080123_Fu_H2B_b2R2_24h **Scan** 18011
**Peptide** RLLLPGELAKHAVSEGTKAVTKYTSA **Charge** 5 **Spectral Angle** 0.87

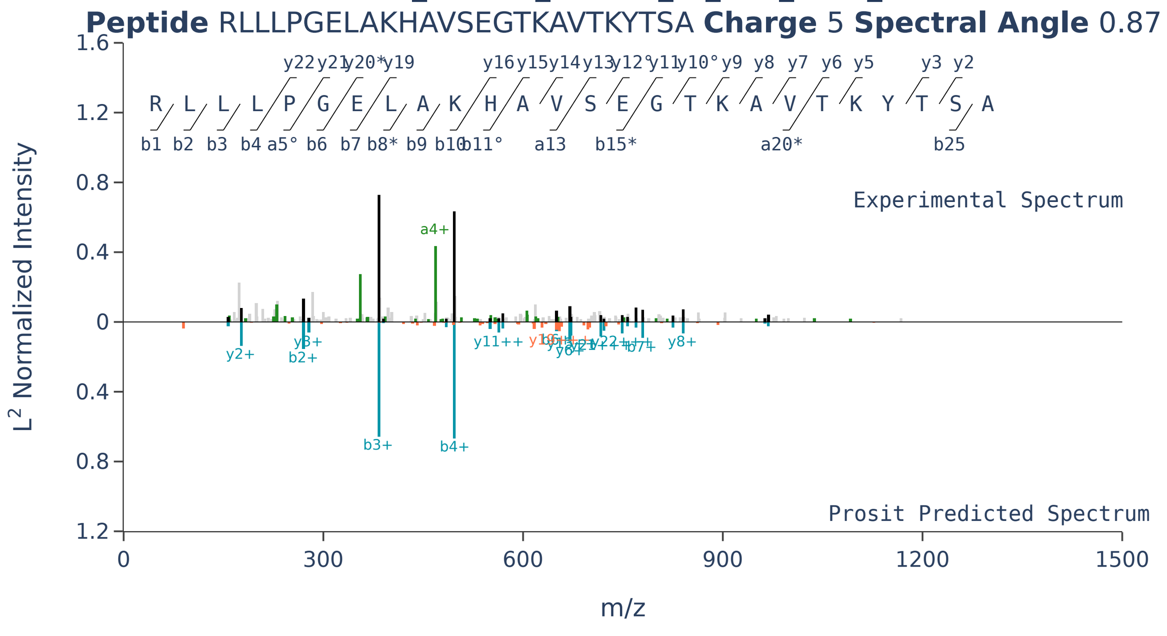

**Source** HRoetschke_291222_080123_Fu_H2B_b1R2_4h **Scan** 6216
**Peptide** VHPDTGISSKAMSIMN **Charge** 3 **Spectral Angle** 0.79

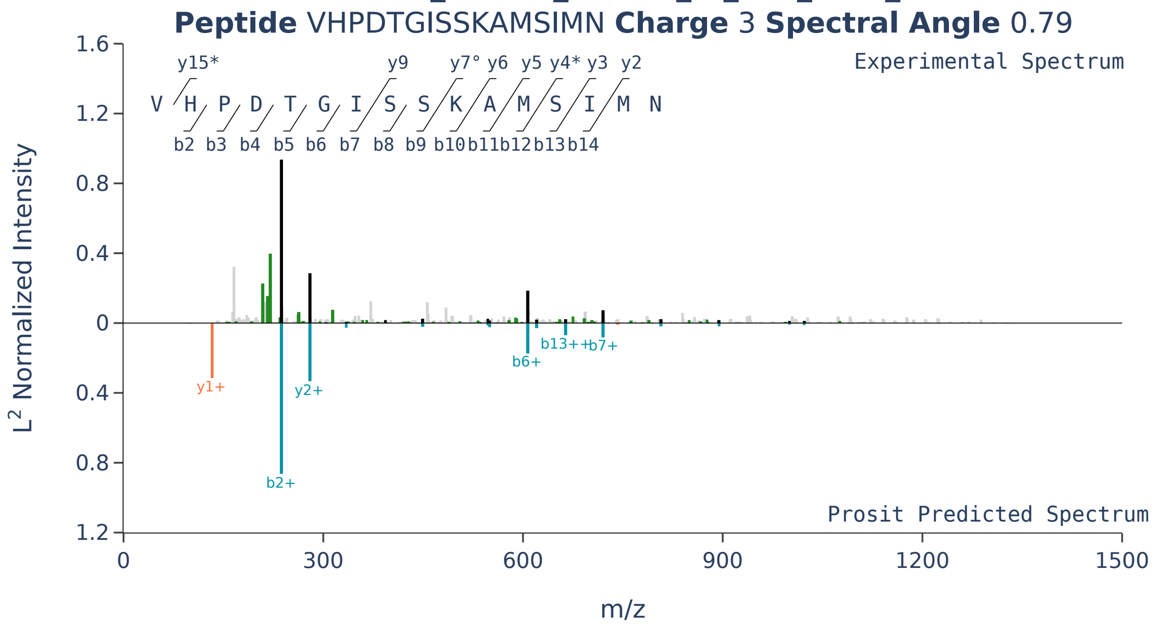


**Source** HRoetschke_291222_080123_Fu_H2B_b2R1_4h **Scan** 16778
**Peptide** DVFERIAGEASRLAHYNKRSTITSR **Charge** 6 **Spectral Angle** 0.7

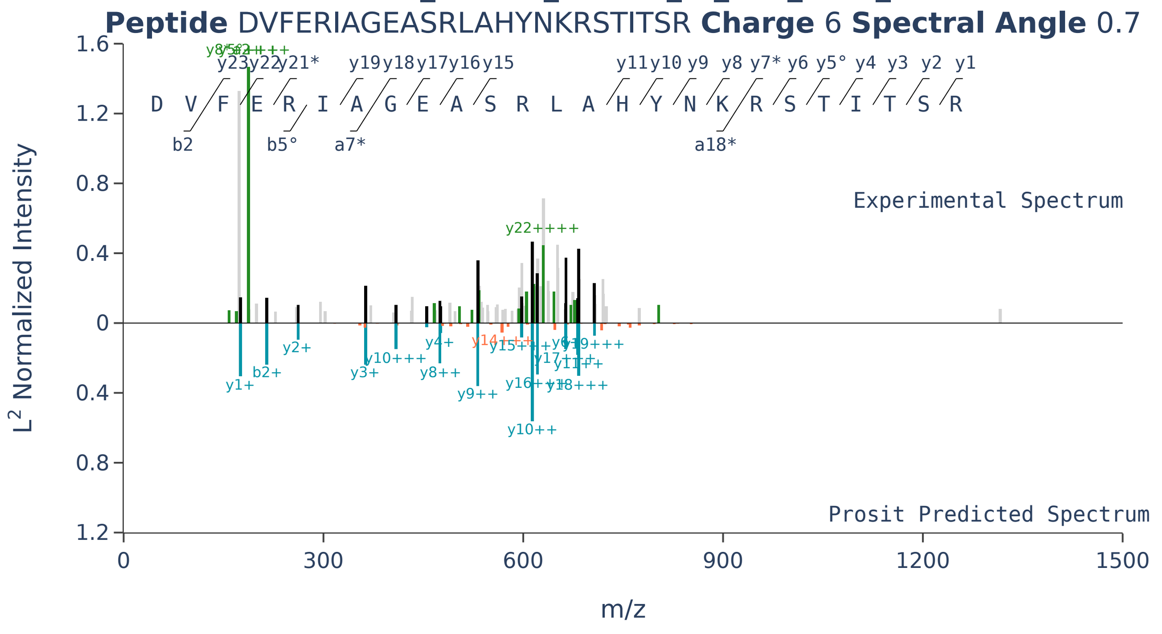


**Source** HRoetschke_291222_080123_Fu_H2B_b1R1_24h **Scan** 2971
**Peptide** ASRLAHYNKRSTITS **Charge** 3 **Spectral Angle** 0.63

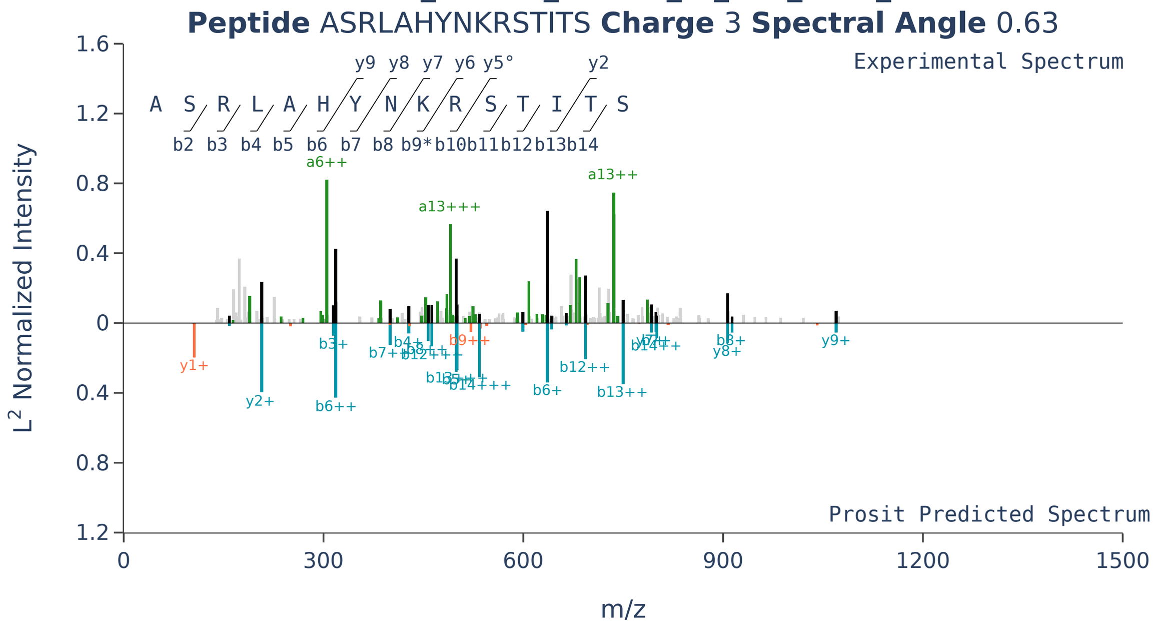


**Source** HRoetschke_291222_080123_Fu_H2B_b2R1_24h **Scan** 3486
**Peptide** KVLKQVHPDTGISSK **Charge** 2 **Spectral Angle** 0.73

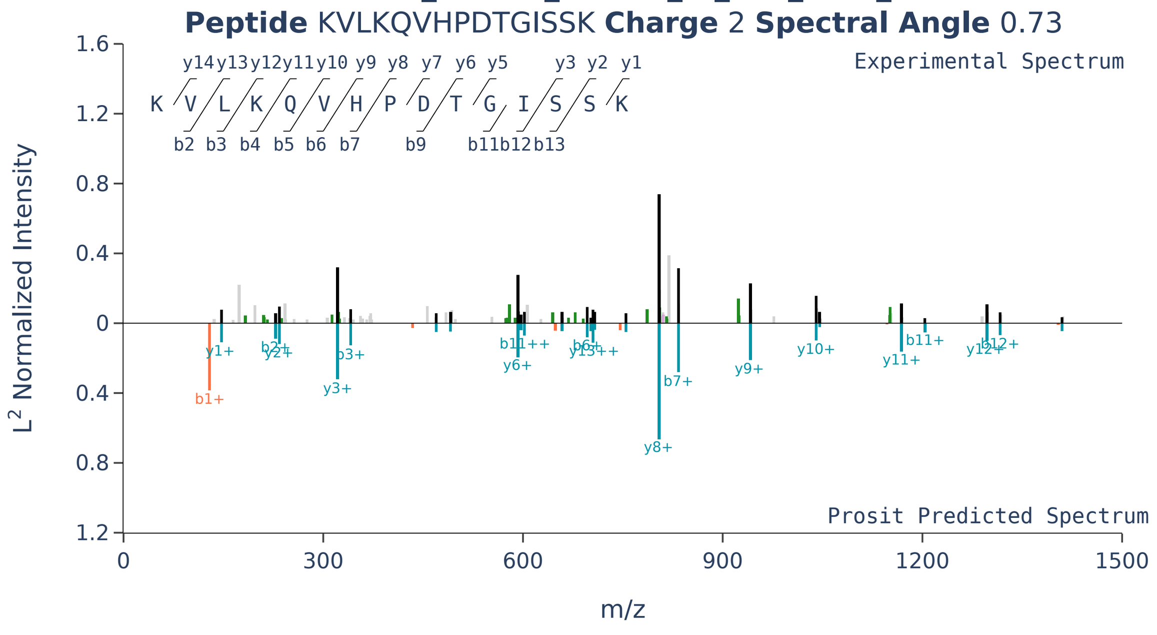


**Source** HRoetschke_291222_080123_Fu_H2B_b1R2_1h **Scan** 4411
**Peptide** LAHYNKRSTITSREL **Charge** 3 **Spectral Angle** 0.86

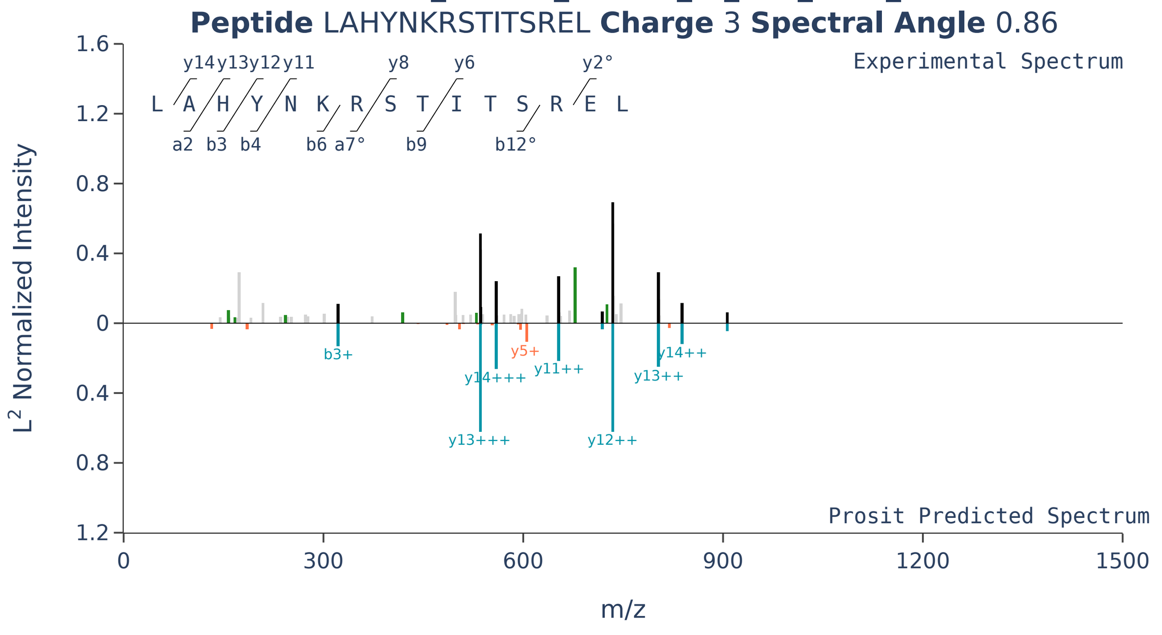


**Source** HRoetschke_291222_080123_Fu_H2B_b2R2_1h **Scan** 17626
**Peptide** VNDVFERIAGEASRLAHYN **Charge** 3 **Spectral Angle** 0.8

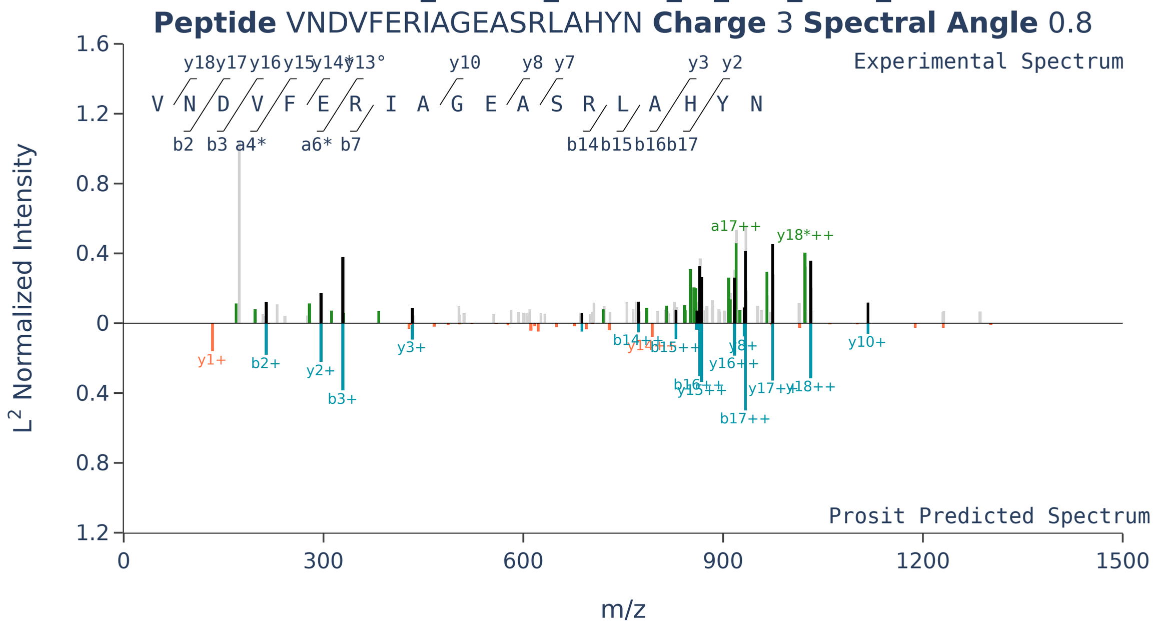


**Source** HRoetschke_291222_080123_Fu_H2B_b1R1_24h **Scan** 8605
**Peptide** RRKTRKESYAIYVY **Charge** 4 **Spectral Angle** 0.69

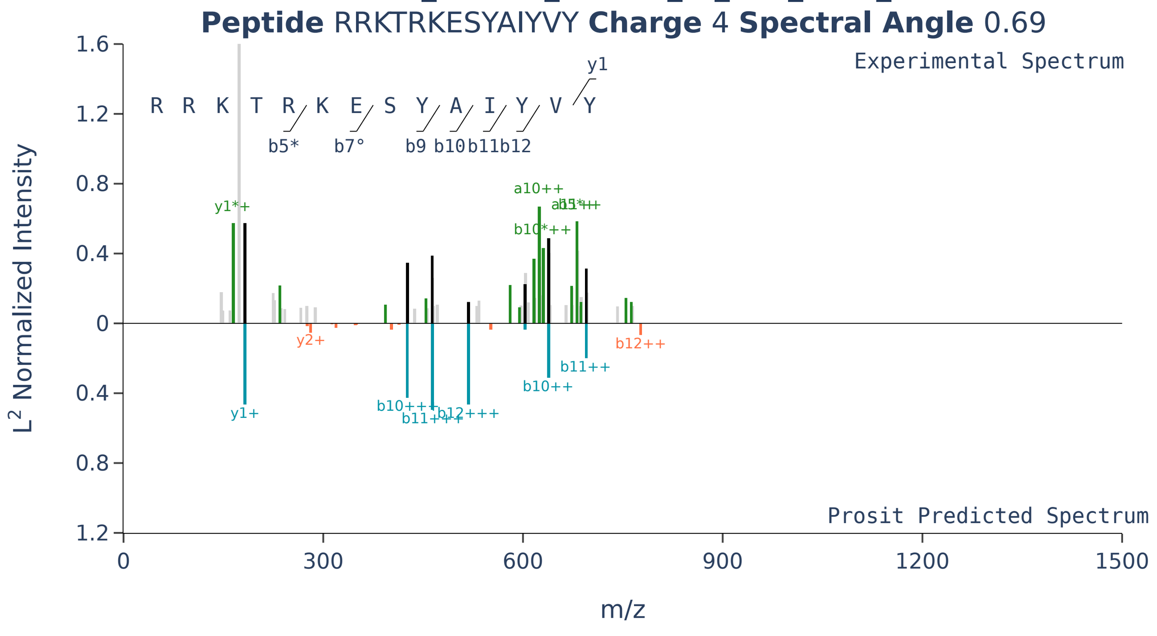


**Source** HRoetschke_291222_080123_Fu_H2B_b1R2_24h **Scan** 3833
**Peptide** NKRSTITSREL **Charge** 2 **Spectral Angle** 0.74

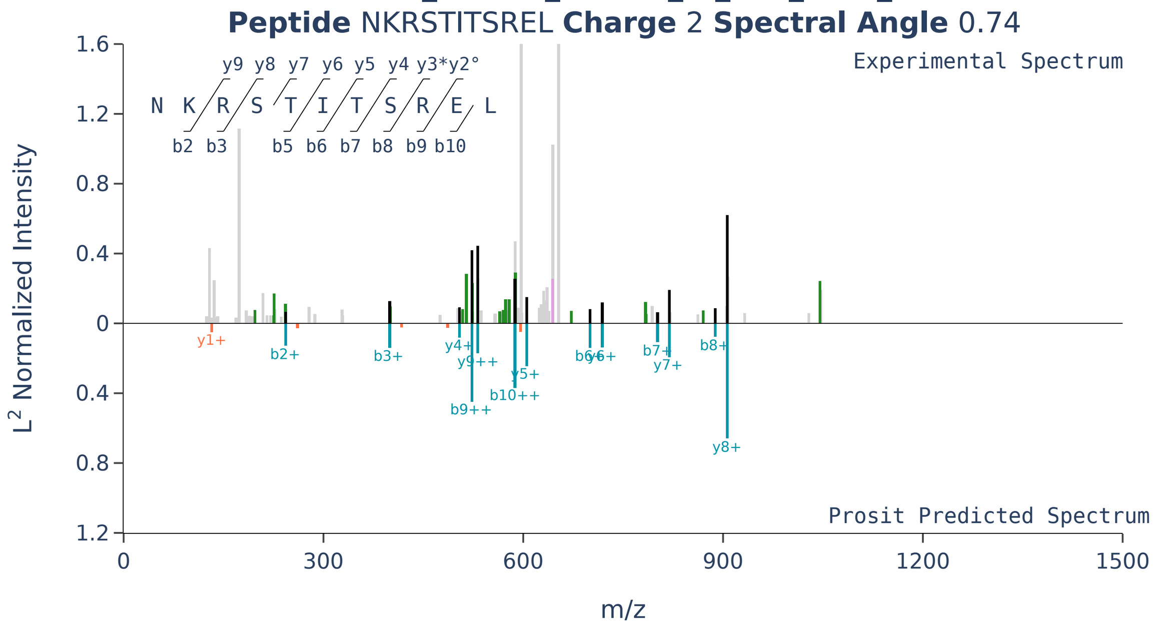


**Source** HRoetschke_291222_080123_Fu_H2B_b2R2_24h **Scan** 19450
**Peptide** EIQTAVRLLLPGELAKHAVSEGTKA **Charge** 4 **Spectral Angle** 0.73

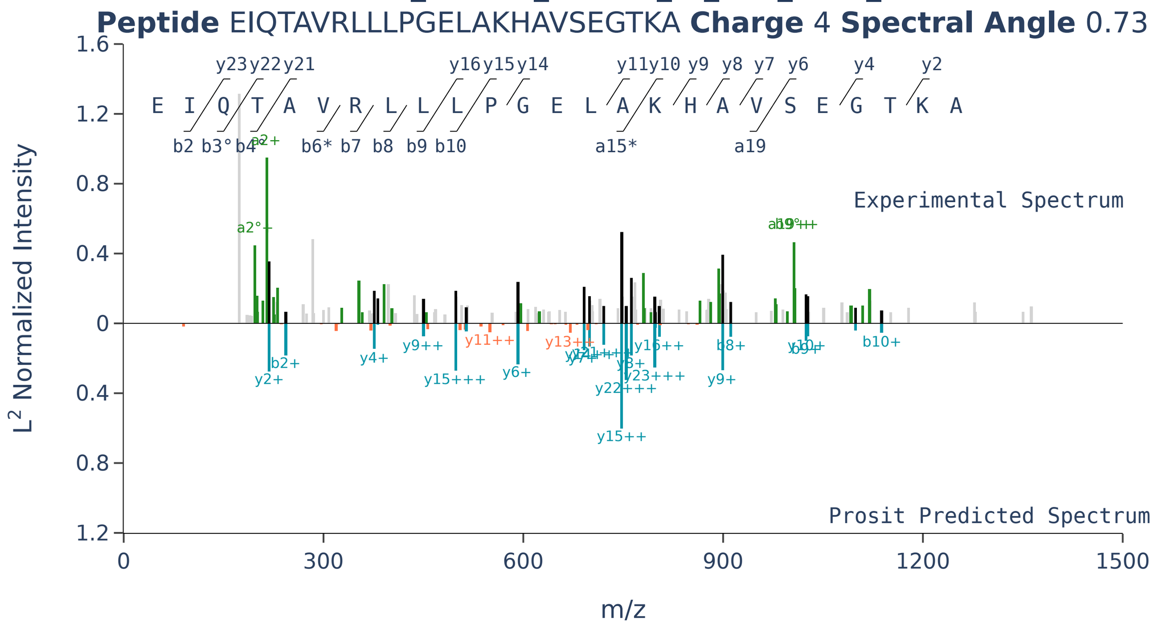


**Source** HRoetschke_291222_080123_Fu_H2B_b1R1_1h **Scan** 3738
**Peptide** RLAGEASRLAHYNKRS **Charge** 4 **Spectral Angle** 0.75

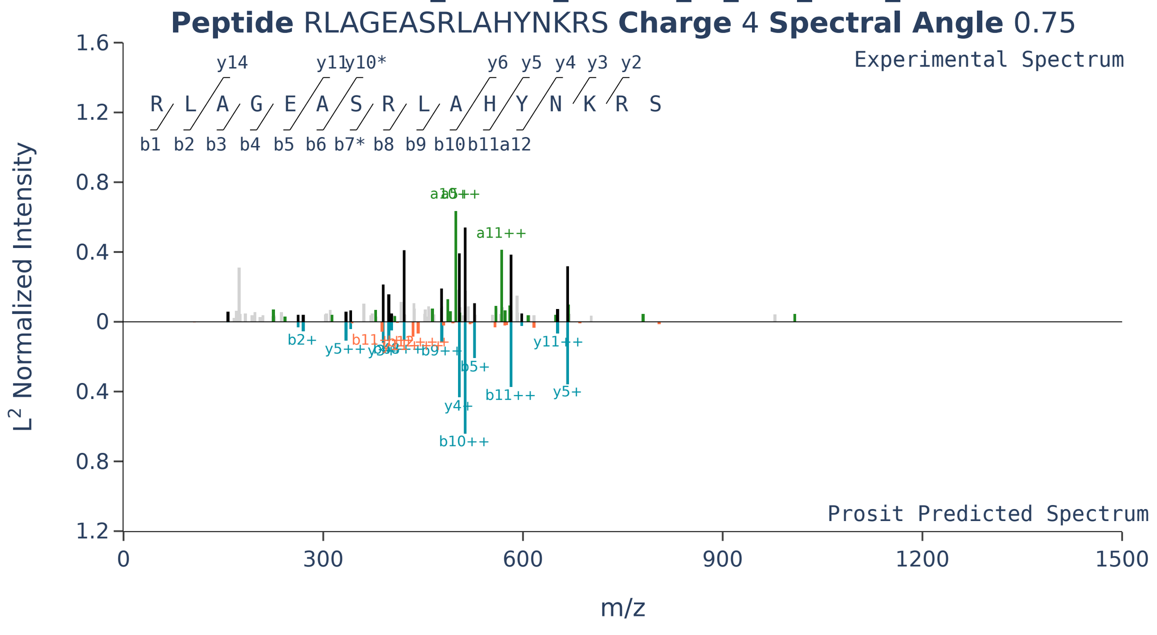


**Source** HRoetschke_291222_080123_Fu_H2B_b2R1_4h **Scan** 21395
**Peptide** MNSFVNDVFERIAGEASR **Charge** 3 **Spectral Angle** 0.82

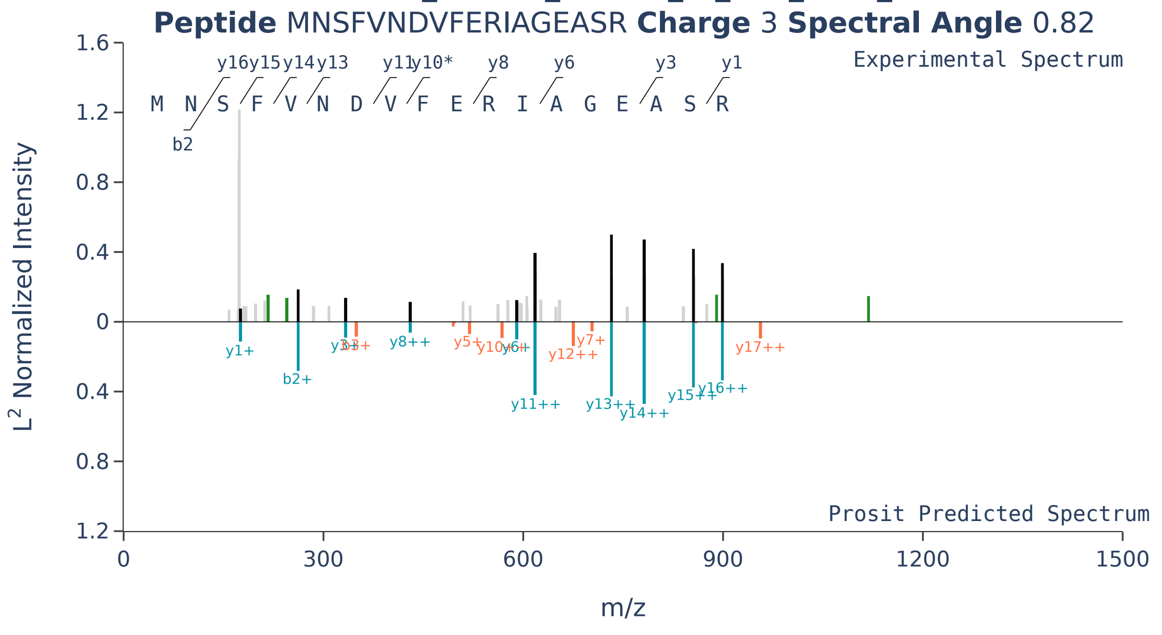


**Source** HRoetschke_291222_080123_Fu_H2B_b1R1_24h **Scan** 7465
**Peptide** VHPDTGISSKAMSIM **Charge** 3 **Spectral Angle** 0.8

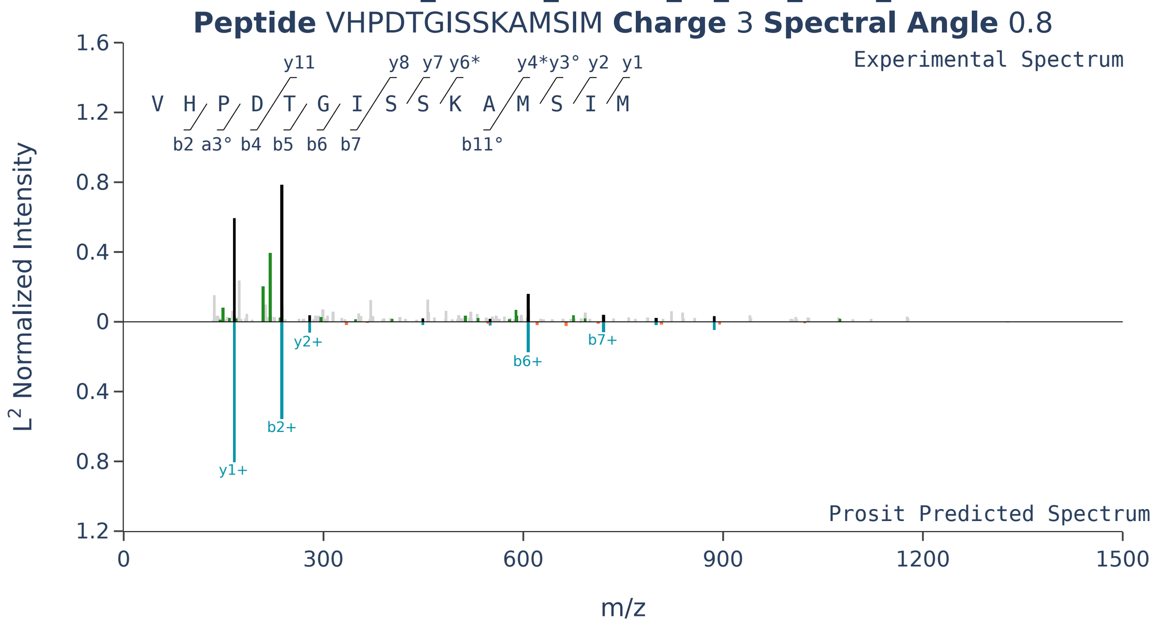


**Source** HRoetschke_291222_080123_Fu_H2B_b1R1_24h **Scan** 8608
**Peptide** RLAGEASRLAHYNKRSTITSREI **Charge** 5 **Spectral Angle** 0.78

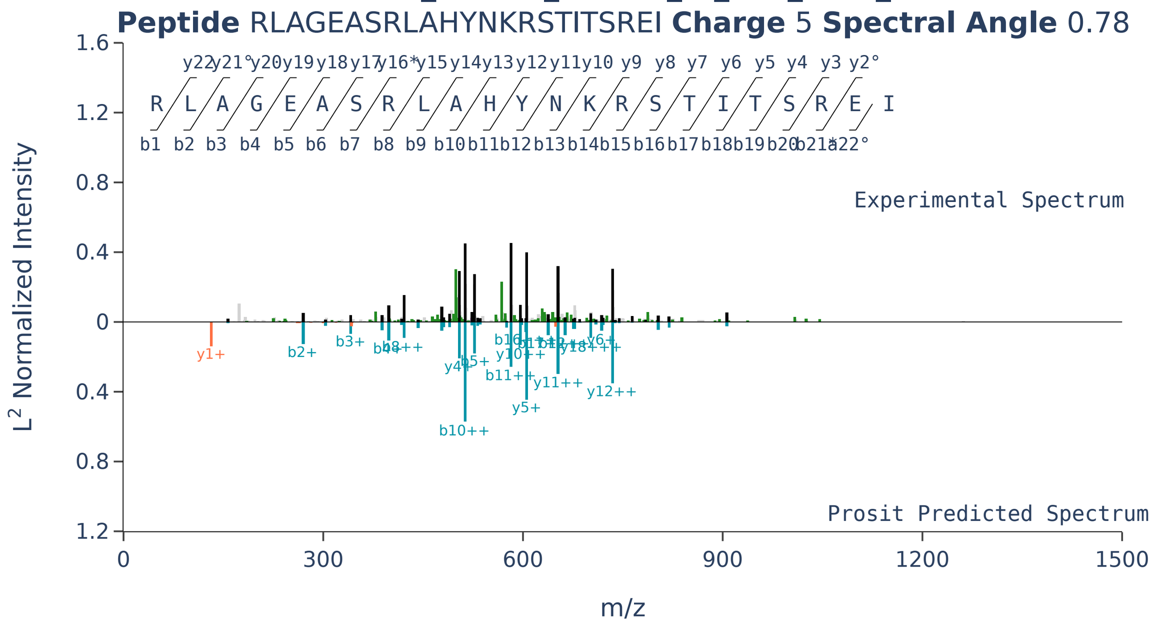


**Source** HRoetschke_291222_080123_Fu_H2B_b2R2_24h **Scan** 16114
**Peptide** VRLLLPGELAKHA **Charge** 4 **Spectral Angle** 0.75

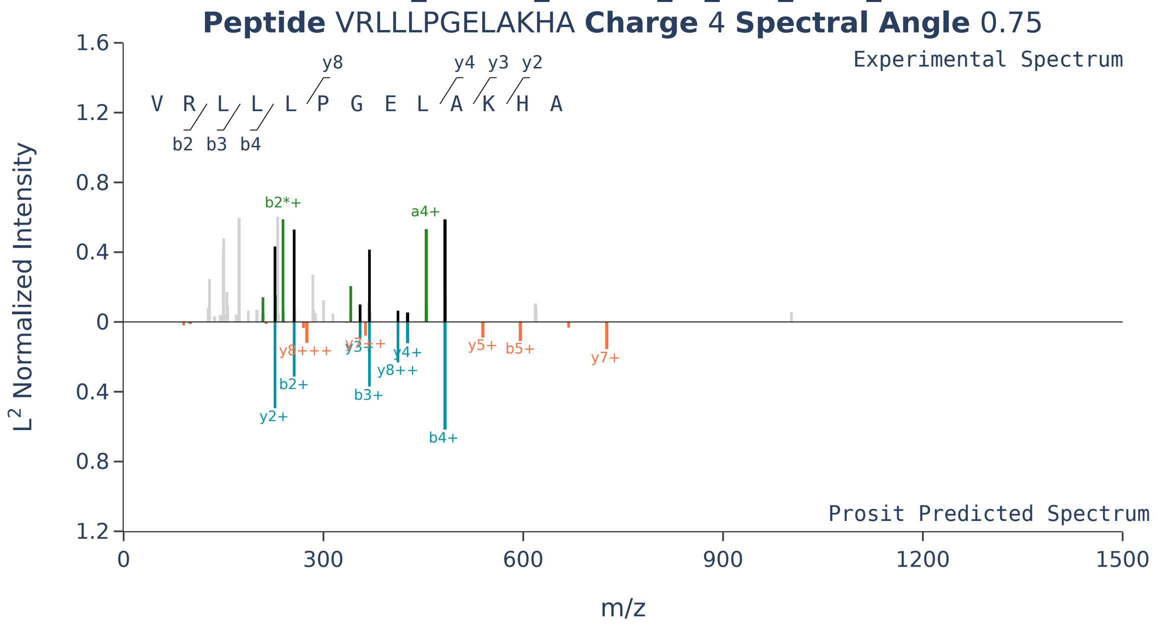


**Source** HRoetschke_291222_080123_Fu_H2B_b2R2_24h **Scan** 4879
**Peptide** SRLAHYNKRSTITSREIQTA **Charge** 5 **Spectral Angle** 0.71

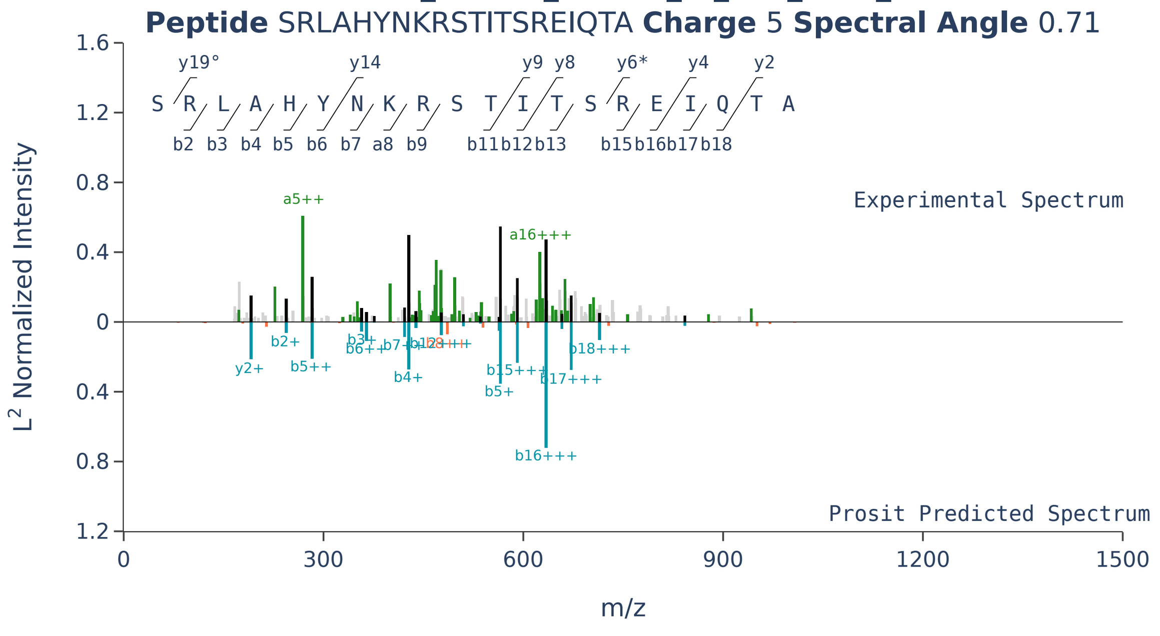

**Source** HRoetschke_291222_080123_Fu_H2B_b1R2_24h **Scan** 5046
**Peptide** AGEASRLAHYNKRSTITS **Charge** 3 **Spectral Angle** 0.64

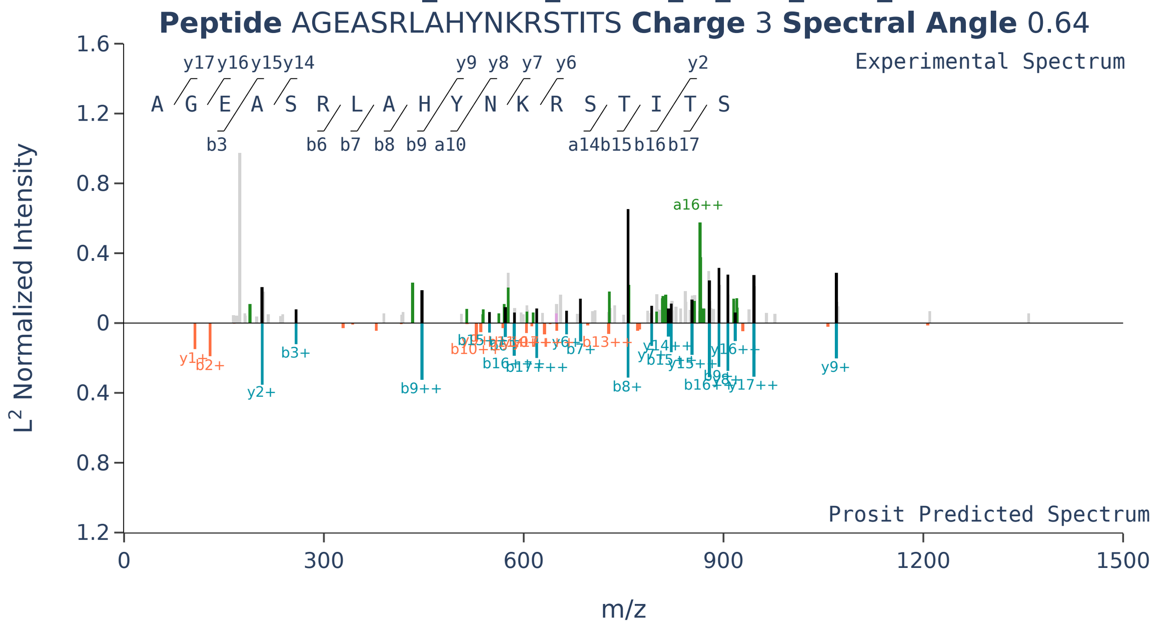


**Source** HRoetschke_291222_080123_Fu_H2B_b2R2_1h **Scan** 15237
**Peptide** LLLPGELAKHAVSEGTKAVTKYT **Charge** 4 **Spectral Angle** 0.83

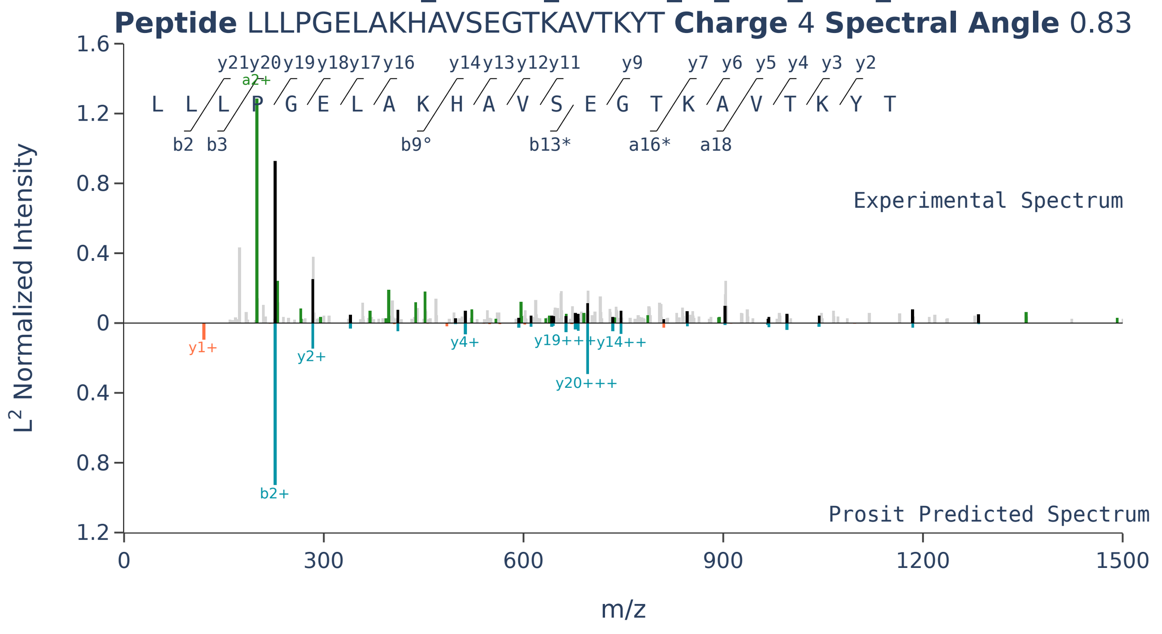


**Source** HRoetschke_291222_080123_Fu_H2B_b1R2_24h **Scan** 1798
**Peptide** HYNKRSTITSR **Charge** 3 **Spectral Angle** 0.81

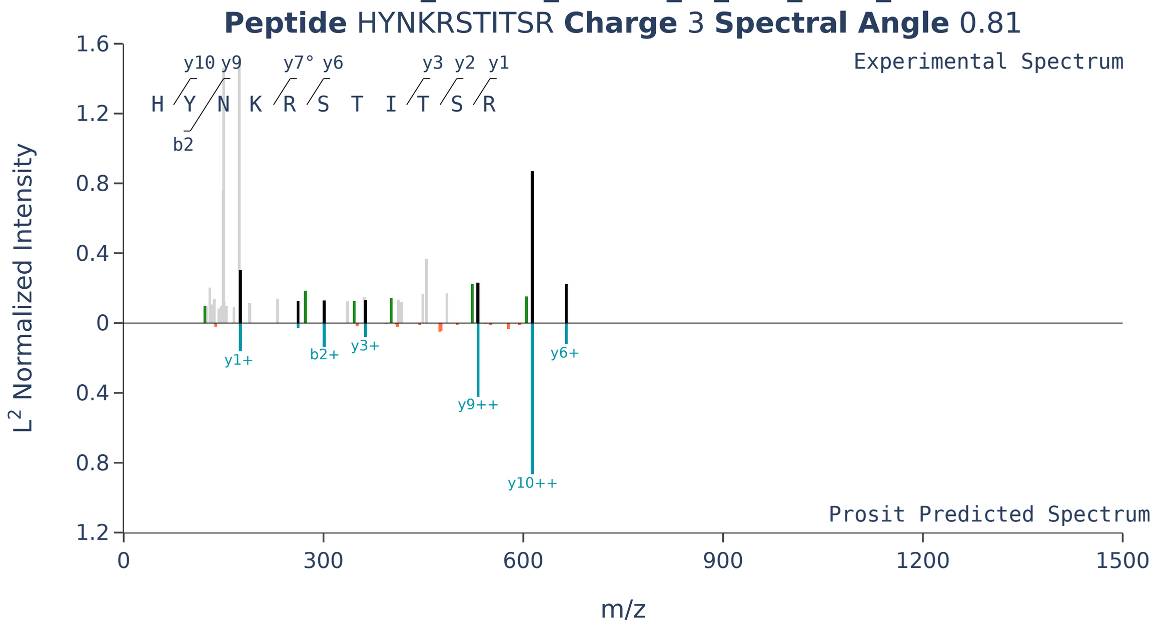


**Source** HRoetschke_291222_080123_Fu_H2B_b2R1_4h **Scan** 20540
**Peptide** ELQTAVRLLLPGE **Charge** 2 **Spectral Angle** 0.87

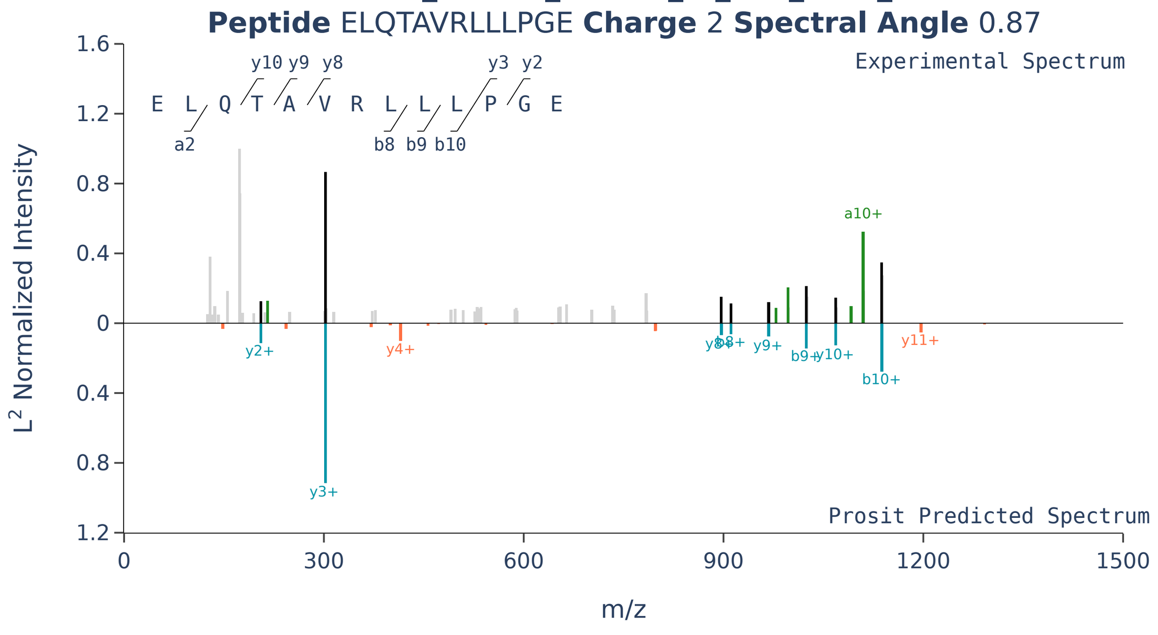


**Source** HRoetschke_291222_080123_Fu_H2B_b2R2_4h **Scan** 19459
**Peptide** VNDVFERIAGEASRLAHYNKRSTITS **Charge** 4 **Spectral Angle** 0.78

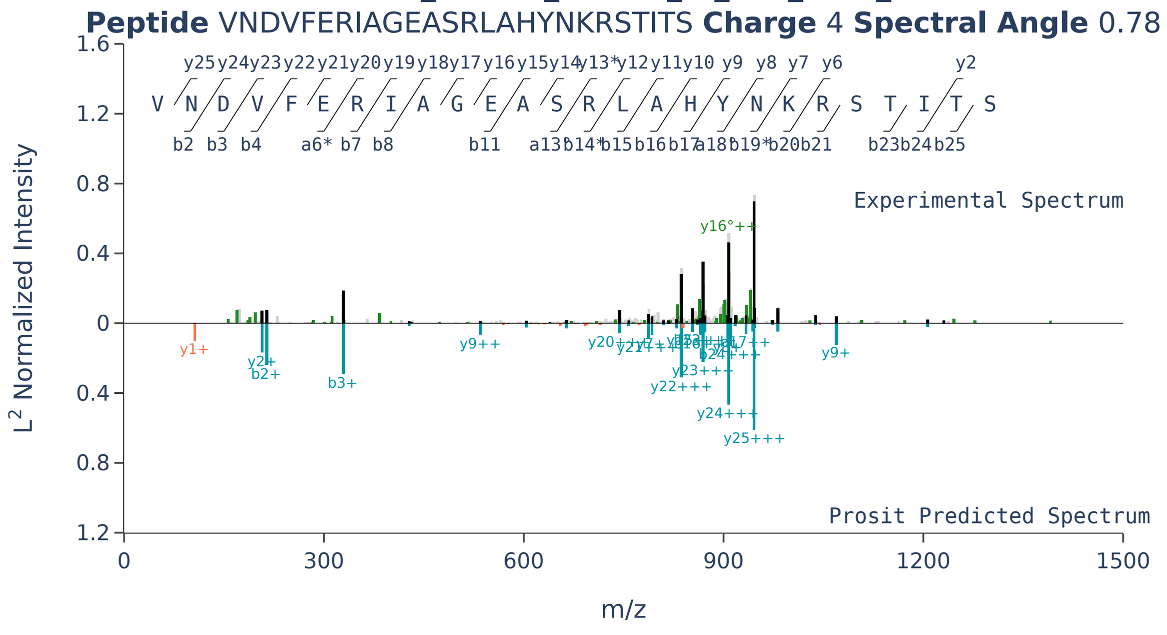


**Source** HRoetschke_291222_080123_Fu_H2B_b1R1_24h **Scan** 7903
**Peptide** PDTGISSKAMSIM **Charge** 2 **Spectral Angle** 0.86

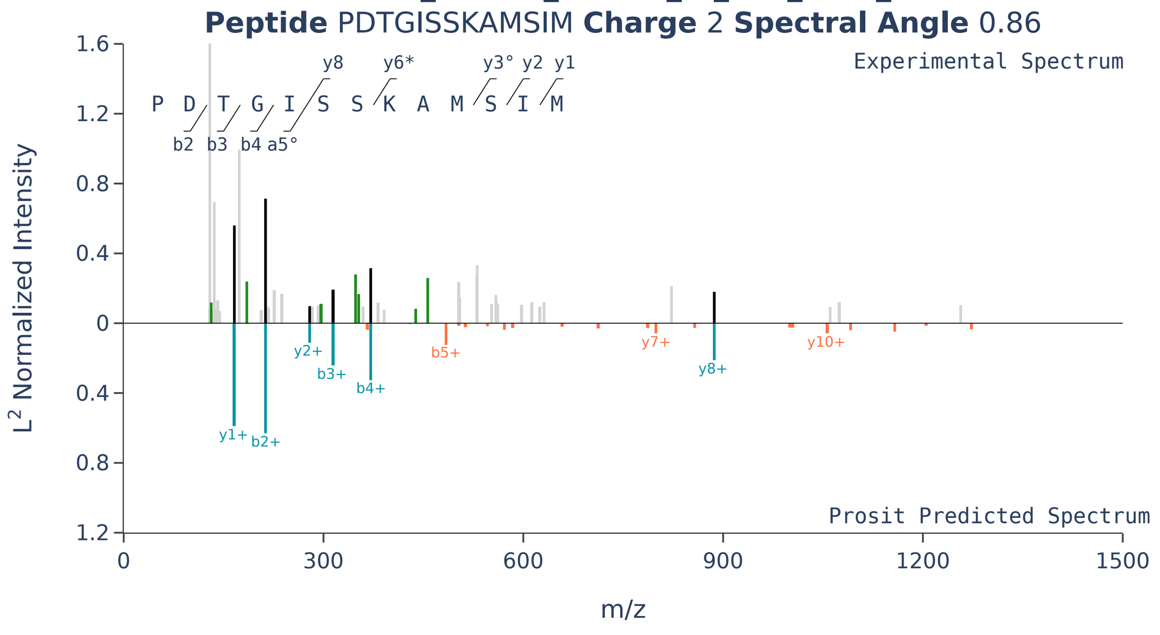


**Source** HRoetschke_291222_080123_Fu_H2B_b2R1_1h **Scan** 14609
**Peptide** QTAVRLLLPGELAKHAVSE **Charge** 3 **Spectral Angle** 0.69

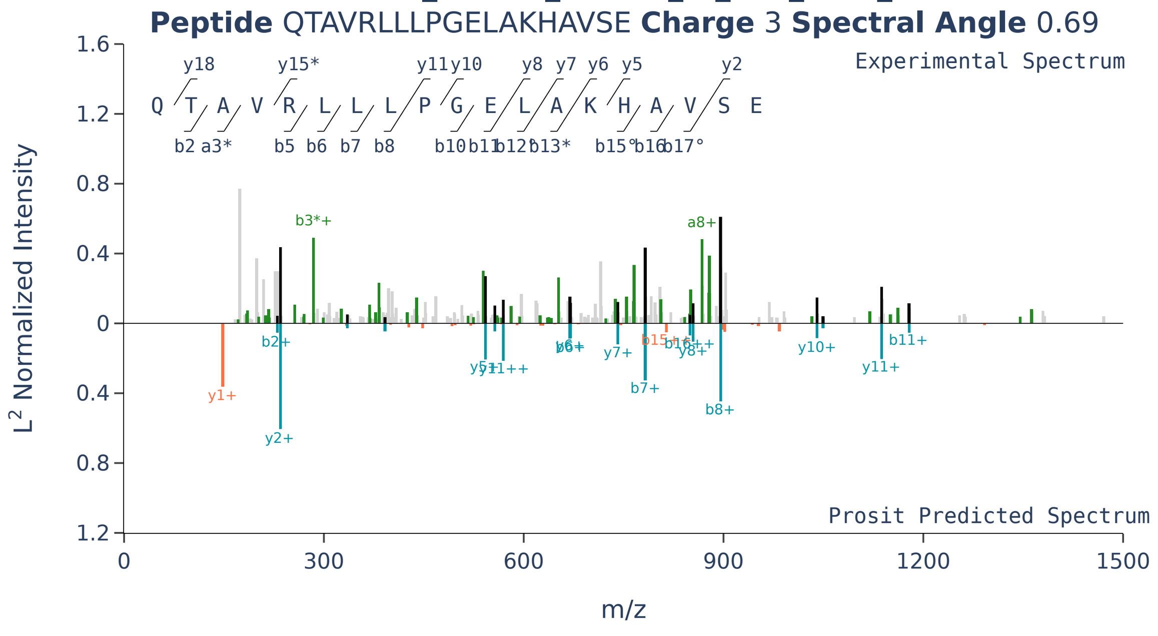


**Source** HRoetschke_291222_080123_Fu_H2B_b2R1_4h **Scan** 10899
**Peptide** GKKRRKTRKESYAIYVYKVL **Charge** 5 **Spectral Angle** 0.82

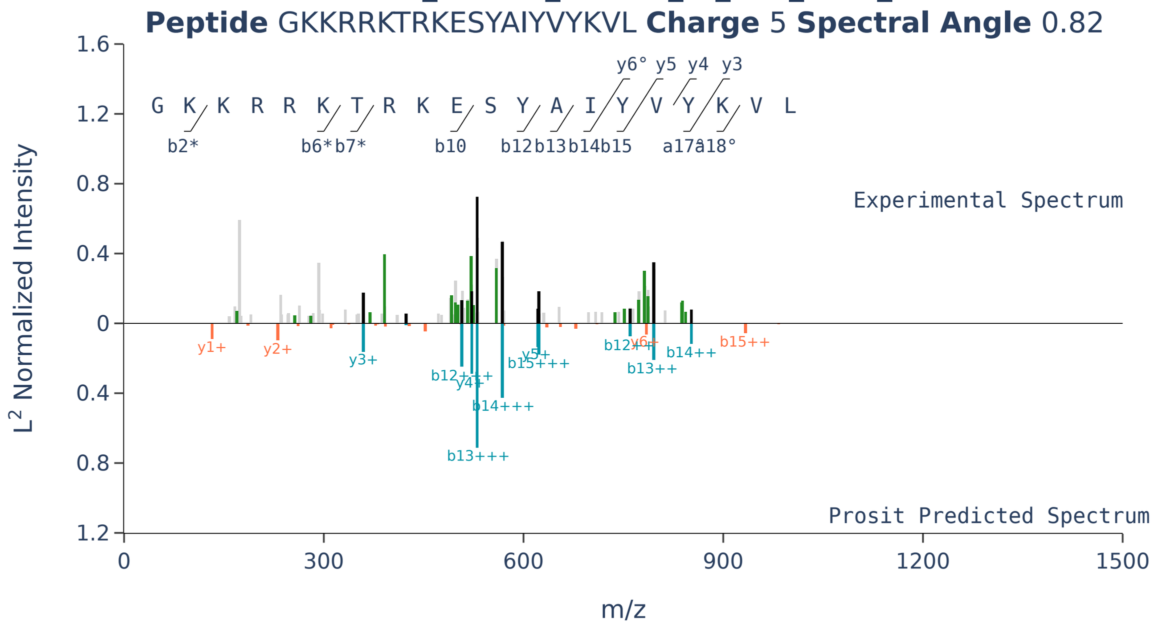


**Source** HRoetschke_291222_080123_Fu_H2B_b1R1_24h **Scan** 7476
**Peptide** RLAHYNKRSTITSREIQTAV **Charge** 4 **Spectral Angle** 0.67

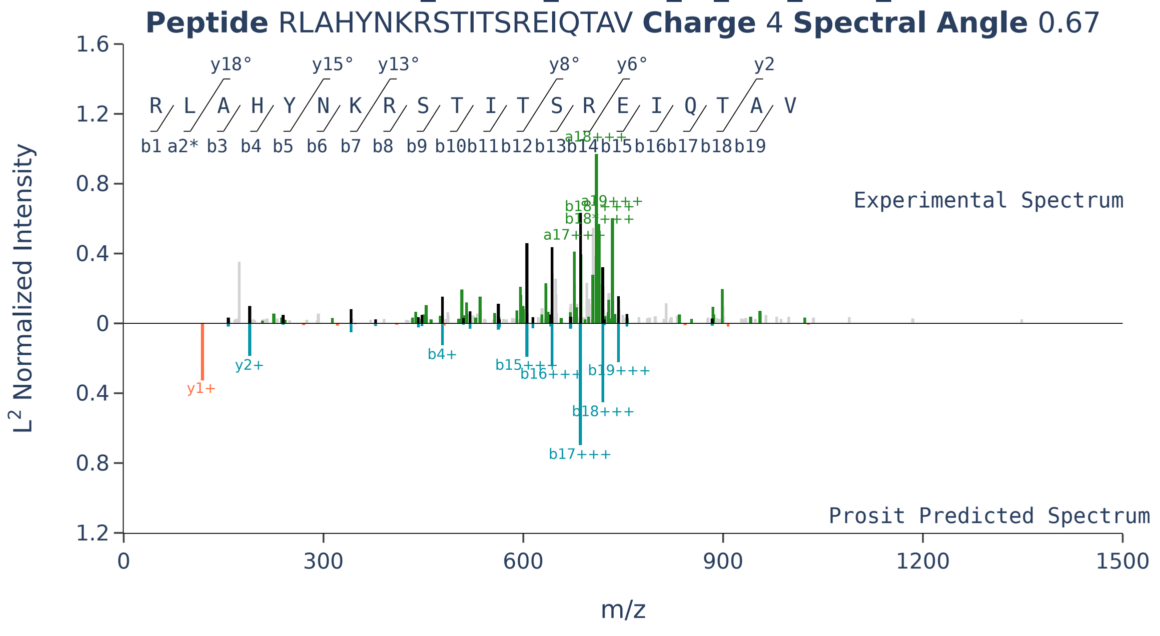


**Source** HRoetschke_291222_080123_Fu_H2B_b1R1_1h **Scan** 15760
**Peptide** TSREIQTAVRLLLPGE **Charge** 3 **Spectral Angle** 0.75

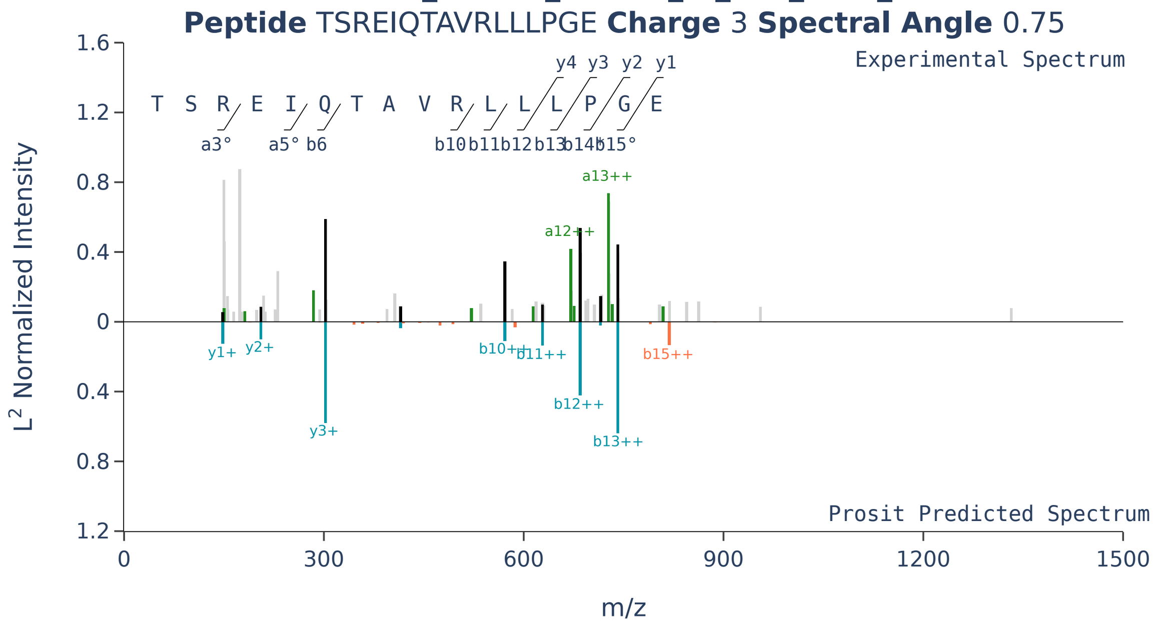


**Source** HRoetschke_291222_080123_Fu_H2B_b2R2_24h **Scan** 3790
**Peptide** AHYNKRSTITSREIQT **Charge** 4 **Spectral Angle** 0.72

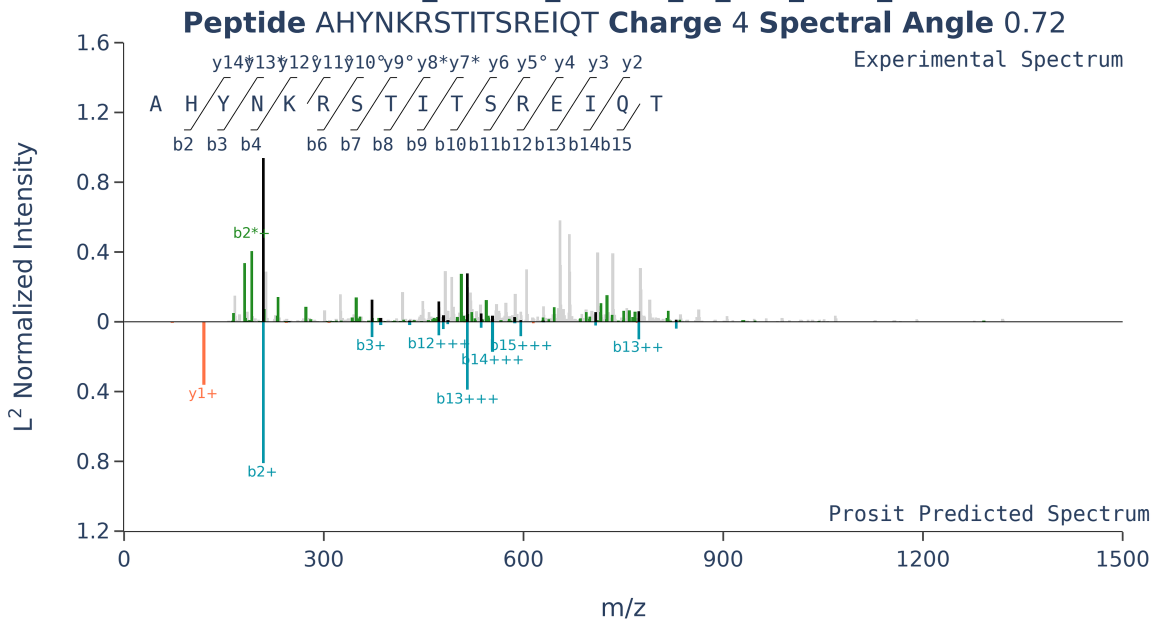


**Source** HRoetschke_291222_080123_Fu_H2B_b1R1_4h **Scan** 1969
**Peptide** TKAVTKYTSAK **Charge** 2 **Spectral Angle** 0.79

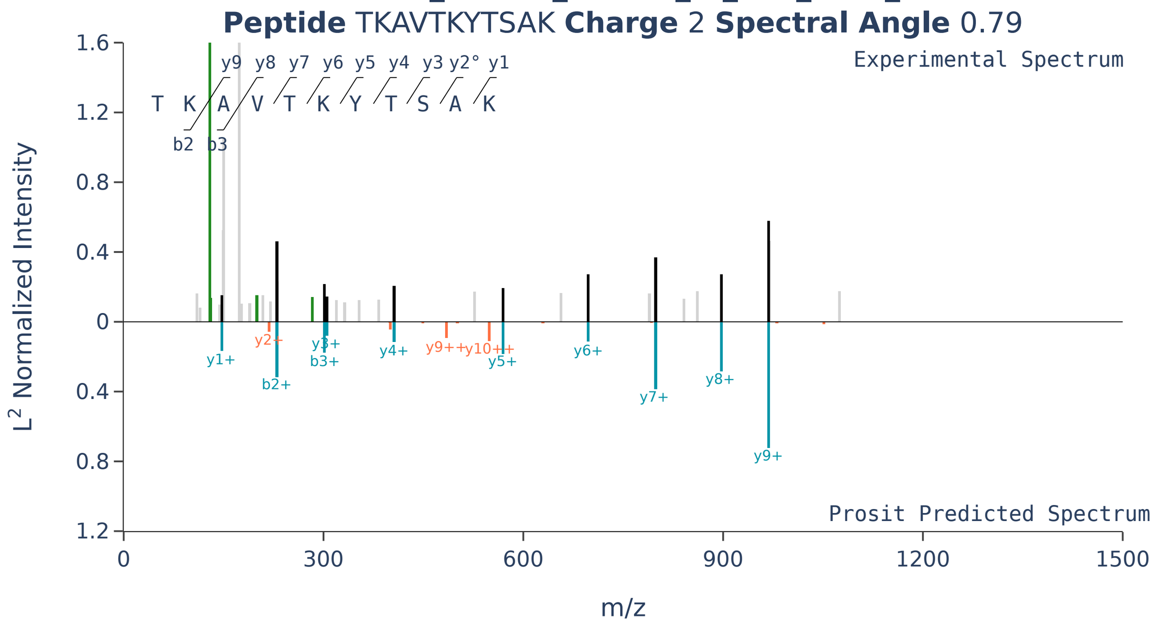


**Source** HRoetschke_291222_080123_Fu_H2B_b1R2_1h **Scan** 5291
**Peptide** GEASRLAHYNKRSTITSREL **Charge** 4 **Spectral Angle** 0.84

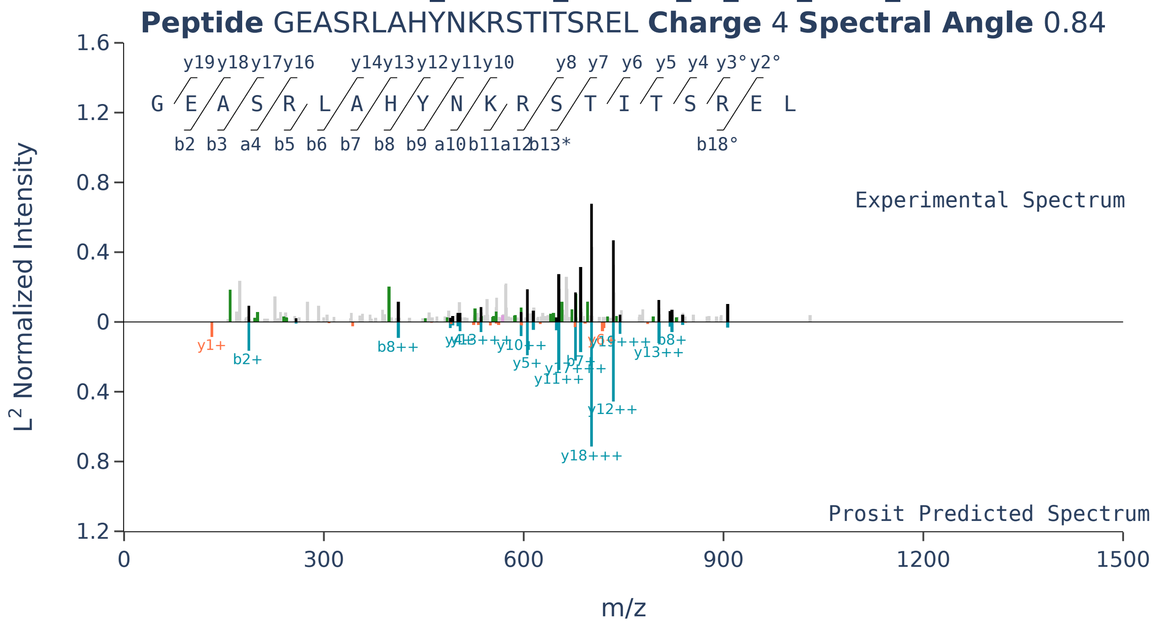


**Source** HRoetschke_291222_080123_Fu_H2B_b2R2_24h **Scan** 10758
**Peptide** RLAHYNKRSTITSREIQTAVRL **Charge** 6 **Spectral Angle** 0.8

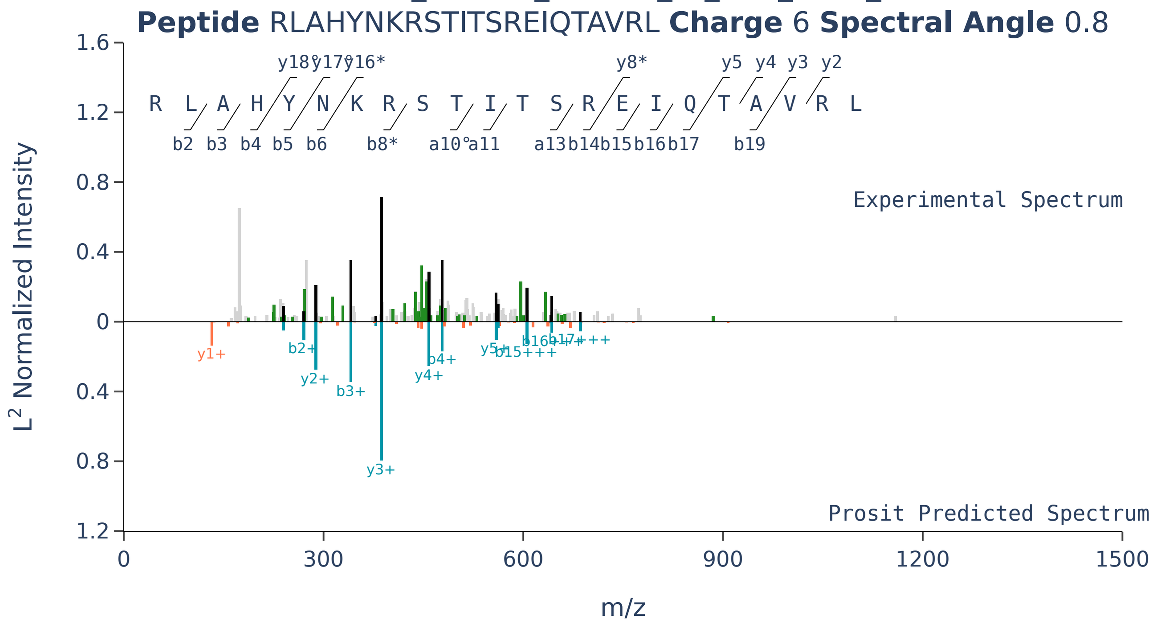


**Source** HRoetschke_291222_080123_Fu_H2B_b2R2_4h **Scan** 13336
**Peptide** VFERIAGEASRLAHYNK **Charge** 3 **Spectral Angle** 0.79

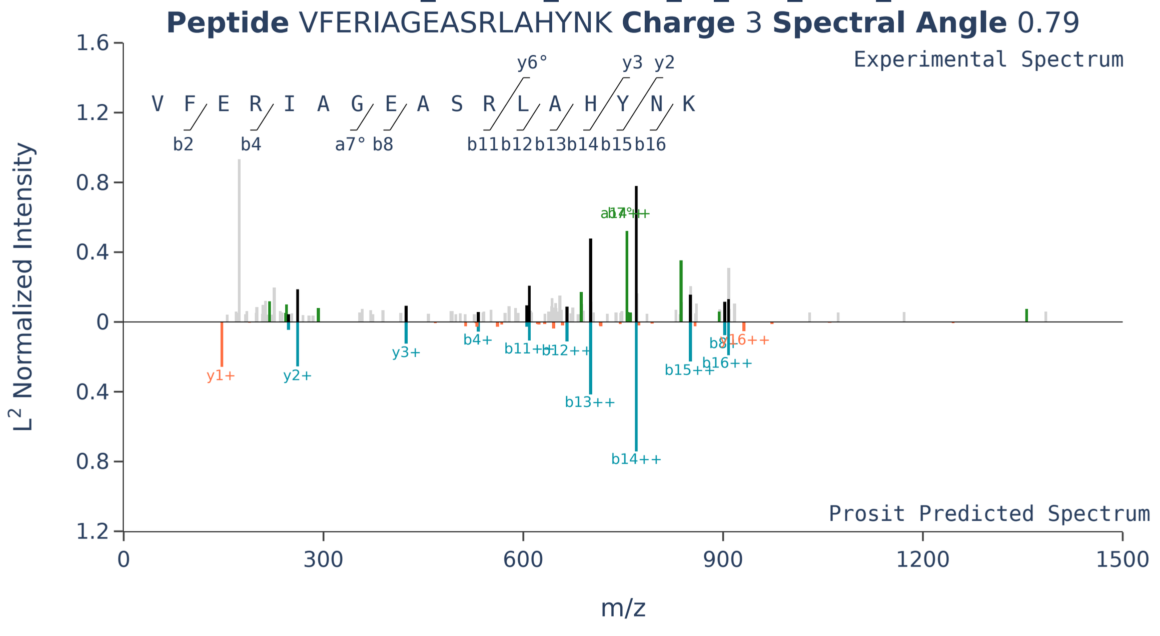

**Source** HRoetschke_291222_080123_Fu_H2B_b2R2_24h **Scan** 2490
**Peptide** GTKAVTKYTS **Charge** 2 **Spectral Angle** 0.8

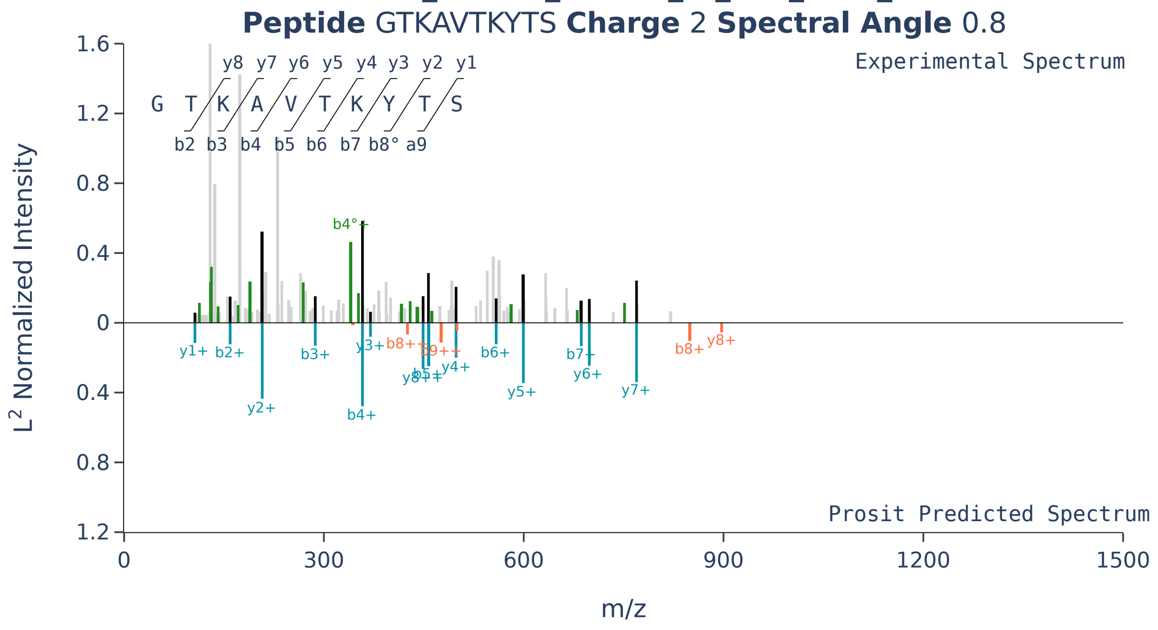


**Source** HRoetschke_291222_080123_Fu_H2B_b1R2_2h **Scan** 12528
**Peptide** VFERIAGEASRLAHYN **Charge** 3 **Spectral Angle** 0.82

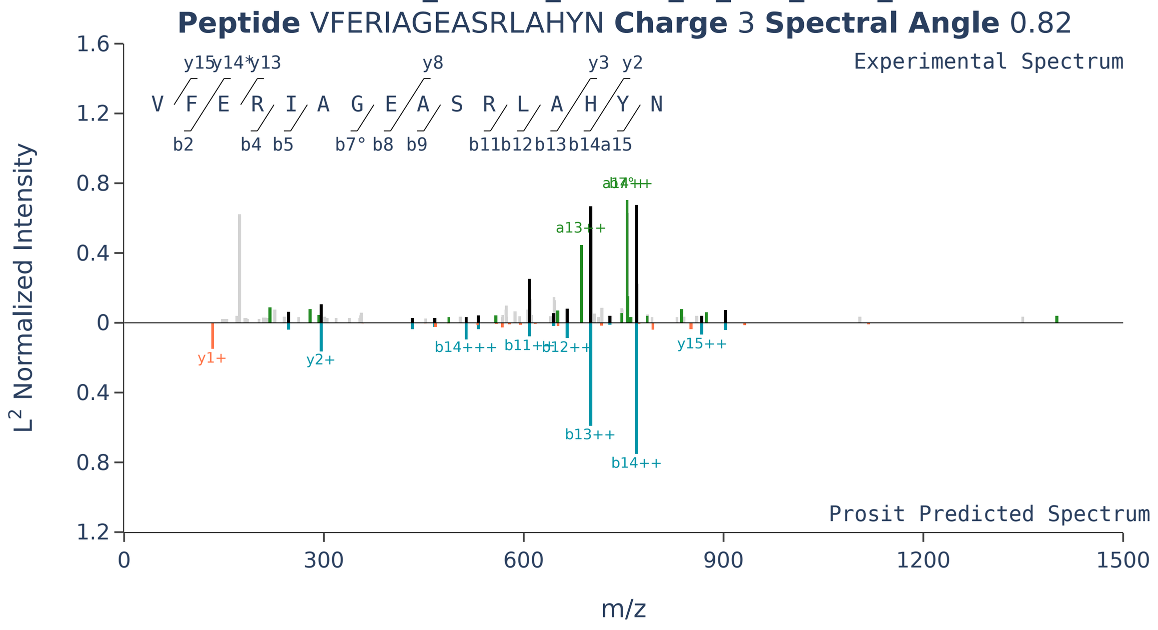


**Source** HRoetschke_291222_080123_Fu_H2B_b2R2_2h **Scan** 13665
**Peptide** VFERIAGEASRLAHY **Charge** 3 **Spectral Angle** 0.78

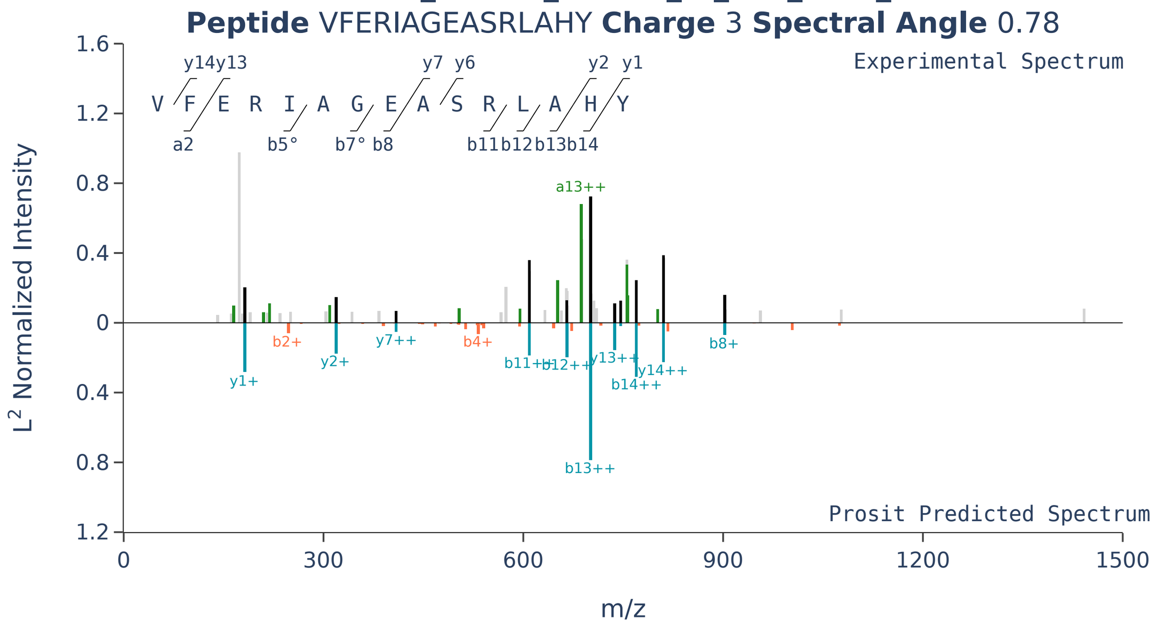


**Source** HRoetschke_291222_080123_Fu_H2B_b1R2_4h **Scan** 5717
**Peptide** AGEASRLAHYNKRSTITSREIQ **Charge** 4 **Spectral Angle** 0.76

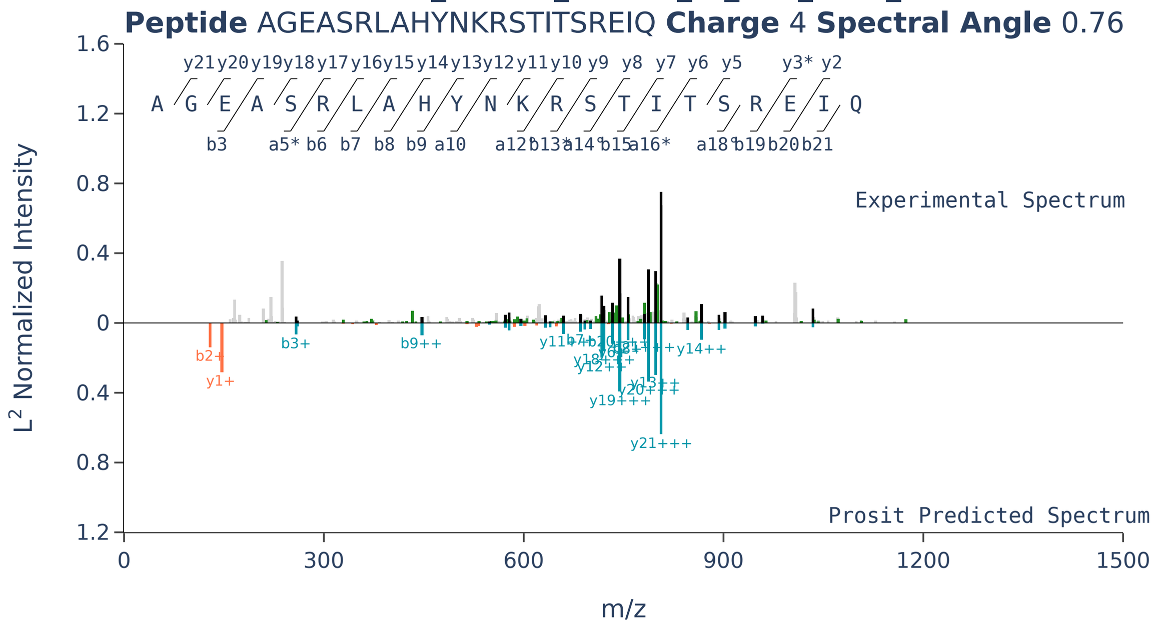


**Source** HRoetschke_291222_080123_Fu_H2B_b2R2_24h **Scan** 3903
**Peptide** QVHPDTGISSKAMS **Charge** 3 **Spectral Angle** 0.79

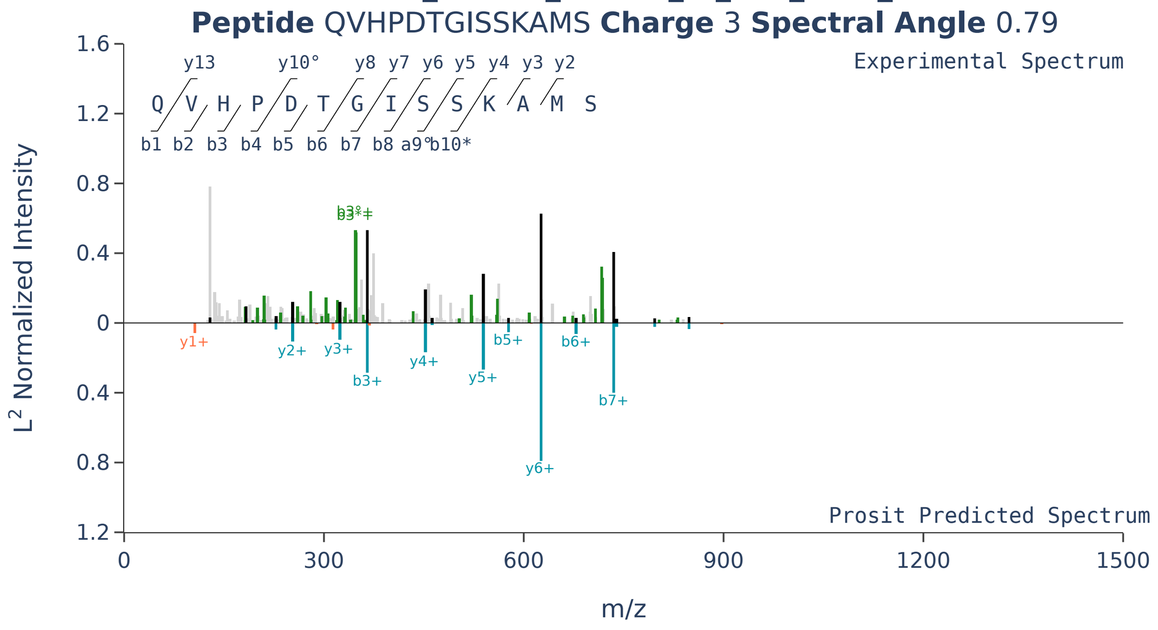


**Source** HRoetschke_291222_080123_Fu_H2B_b1R1_1h **Scan** 5945
**Peptide** RLAGEASRLAHYNKRSTI **Charge** 4 **Spectral Angle** 0.83

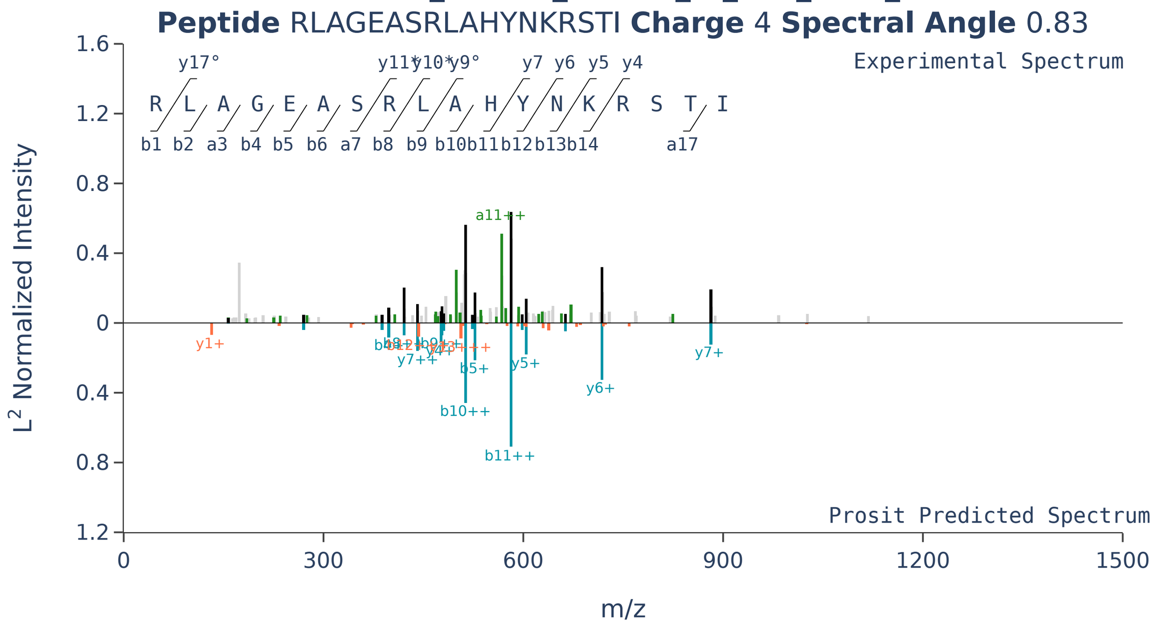


**Source** HRoetschke_291222_080123_Fu_H2B_b2R1_2h **Scan** 17109
**Peptide** LTSREIQTAVRLL **Charge** 3 **Spectral Angle** 0.8

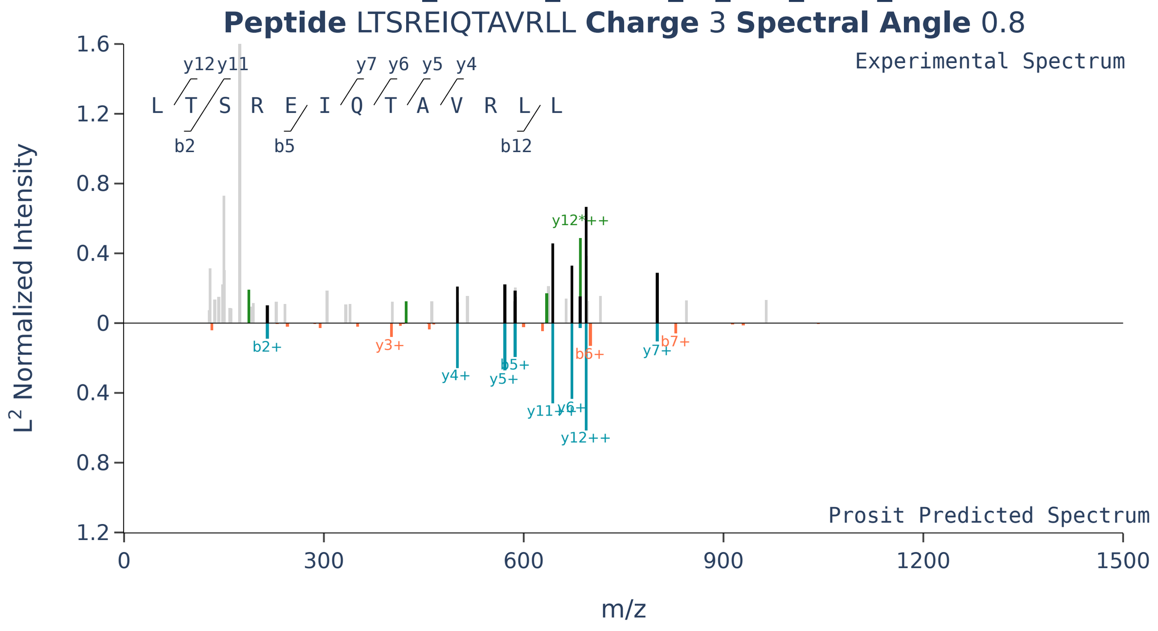


**Source** HRoetschke_291222_080123_Fu_H2B_b1R2_24h **Scan** 4651
**Peptide** HAVSEGTKAVTKYTS **Charge** 3 **Spectral Angle** 0.79

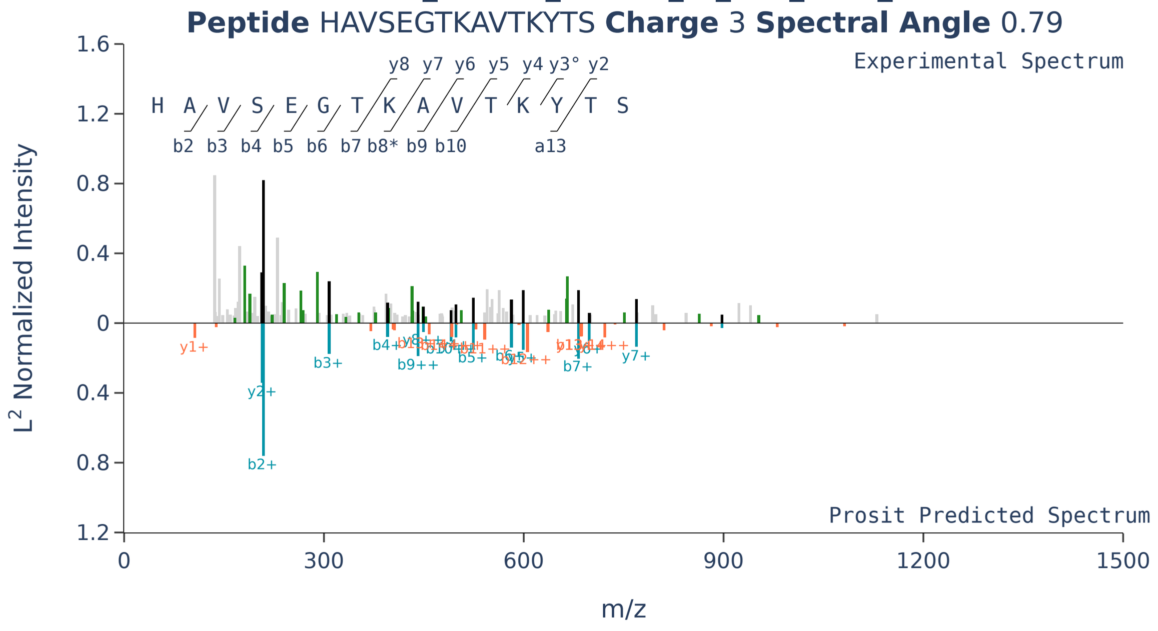


**Source** HRoetschke_291222_080123_Fu_H2B_b1R1_4h **Scan** 2116
**Peptide** RLAHYNKRSTITSRE **Charge** 4 **Spectral Angle** 0.74

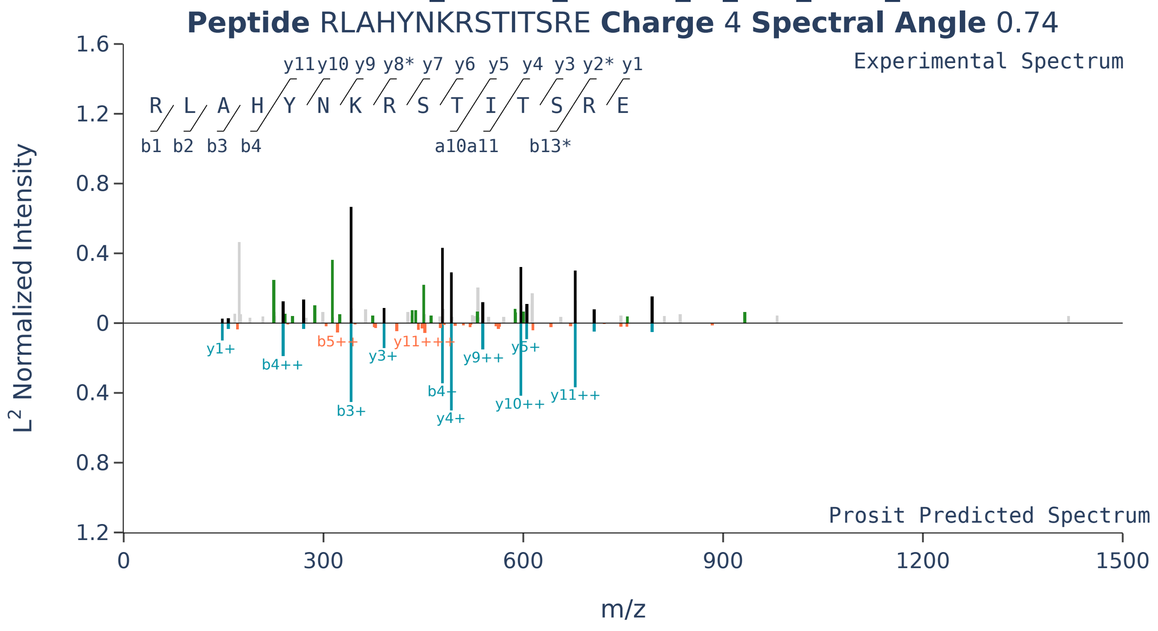


**Source** HRoetschke_291222_080123_Fu_H2B_b2R2_4h **Scan** 8867
**Peptide** ERIAGEASRLAHYNKRSTITSREL **Charge** 5 **Spectral Angle** 0.71

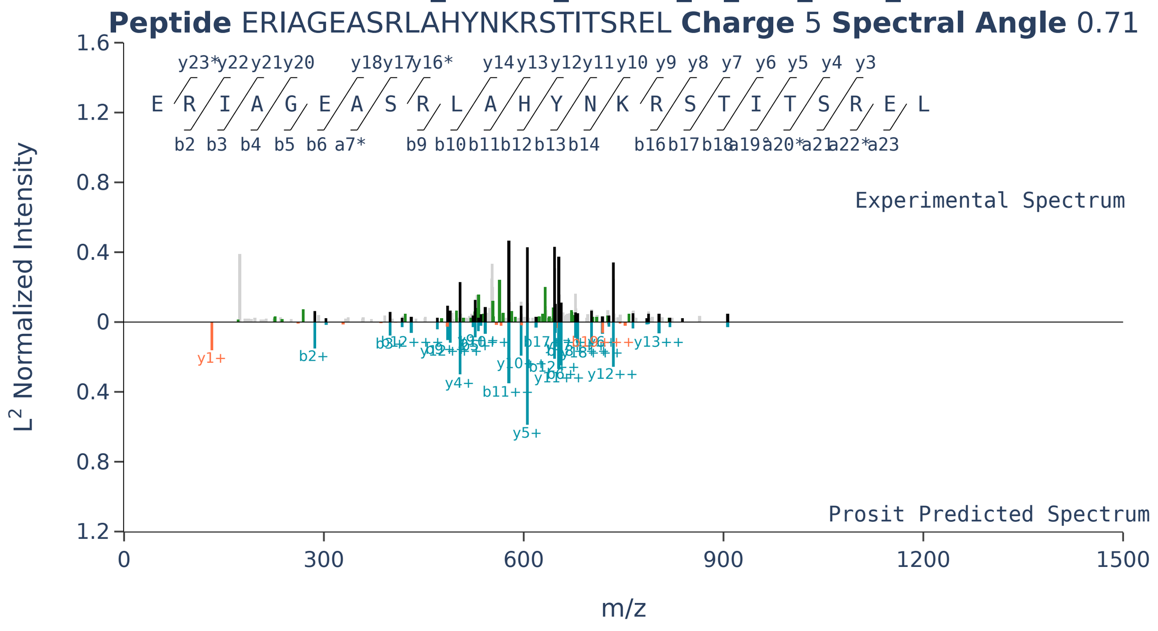


**Source** HRoetschke_291222_080123_Fu_H2B_b2R1_24h **Scan** 18106
**Peptide** REIQTAVRLLLPGELAKHAVSE **Charge** 3 **Spectral Angle** 0.67

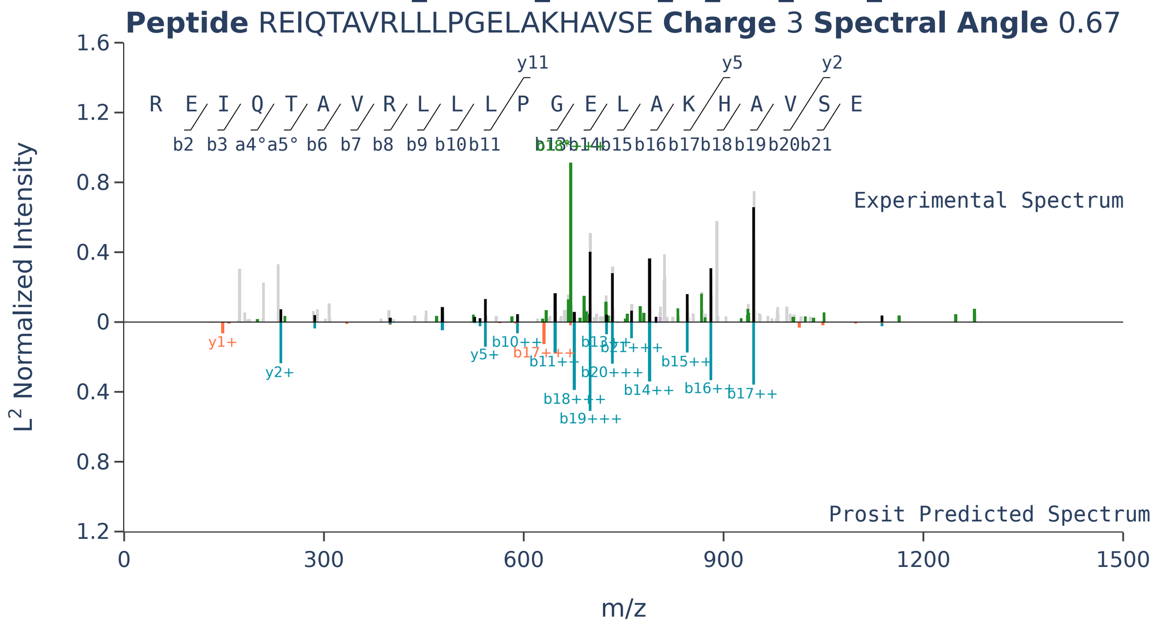


**Source** HRoetschke_291222_080123_Fu_H2B_b2R2_24h **Scan** 7292
**Peptide** EASRLAHYNKRSTITSREIQTAVR **Charge** 5 **Spectral Angle** 0.71

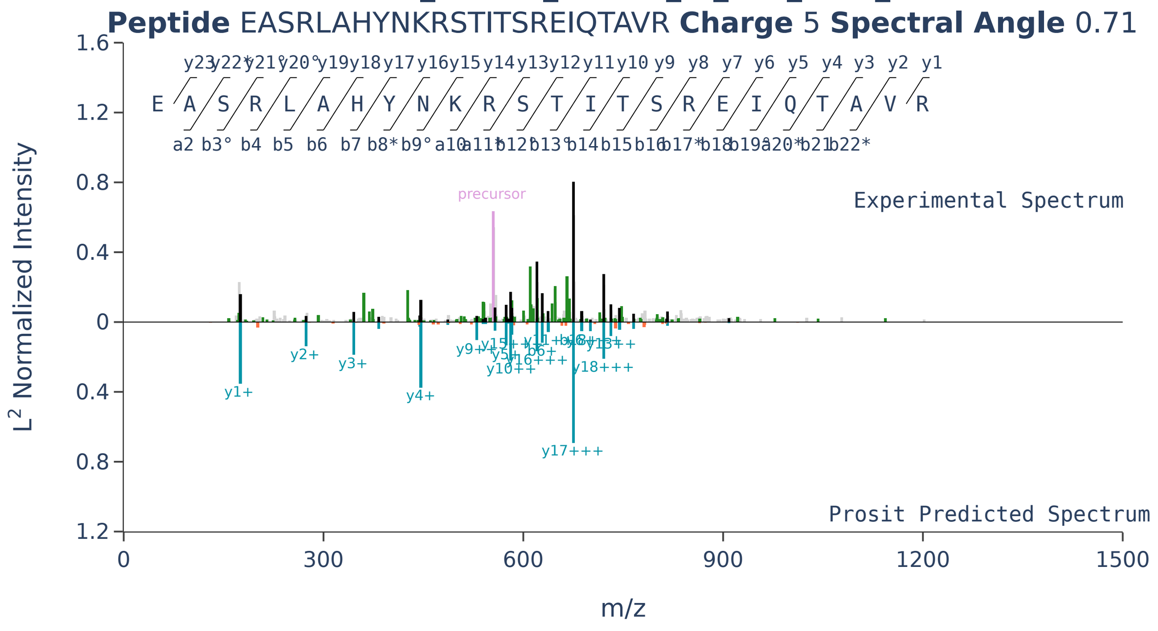


**Source** HRoetschke_291222_080123_Fu_H2B_b2R2_24h **Scan** 4070
**Peptide** TKTQKKDGKKRRKTRKESYAIYVY **Charge** 5 **Spectral Angle** 0.73

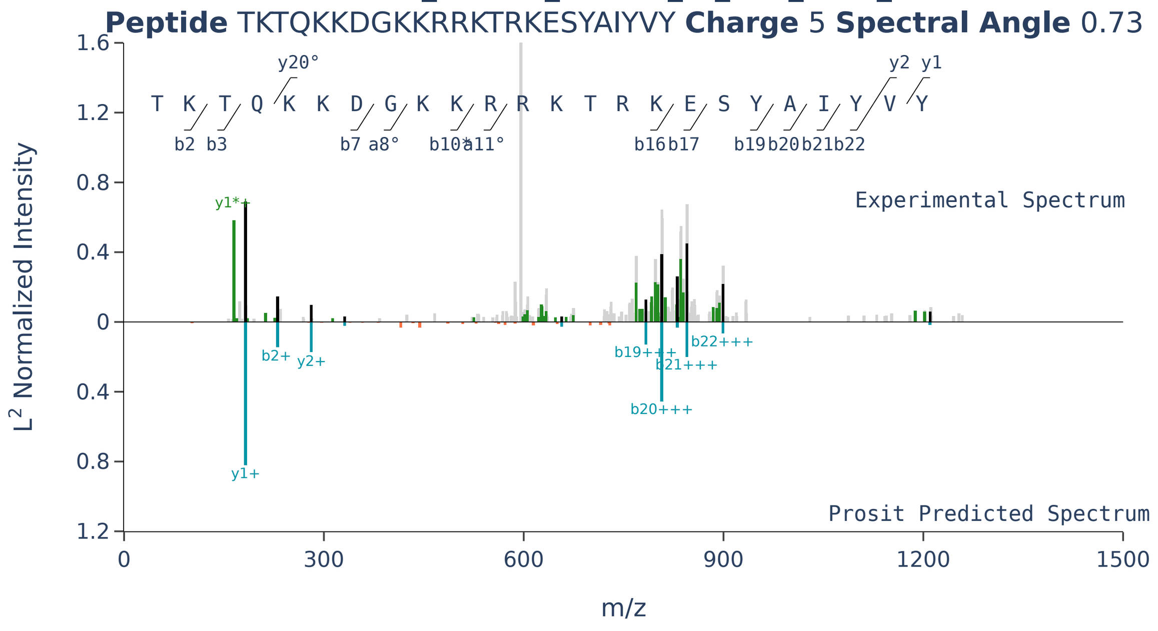


**Source** HRoetschke_291222_080123_Fu_H2B_b1R2_24h **Scan** 8911
**Peptide** LAGEASRLAHYNKRSTITSREI **Charge** 5 **Spectral Angle** 0.78

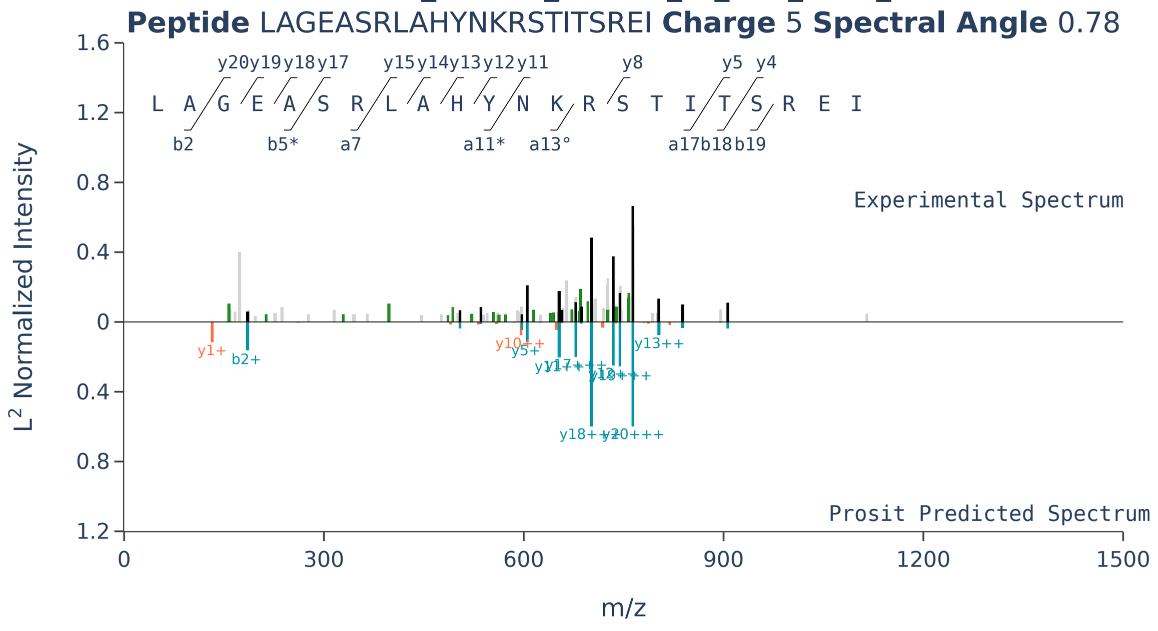


**Source** HRoetschke_291222_080123_Fu_H2B_b2R1_4h **Scan** 21705
**Peptide** STITSREIQTAVRLLLPGELAKHA **Charge** 4 **Spectral Angle** 0.82

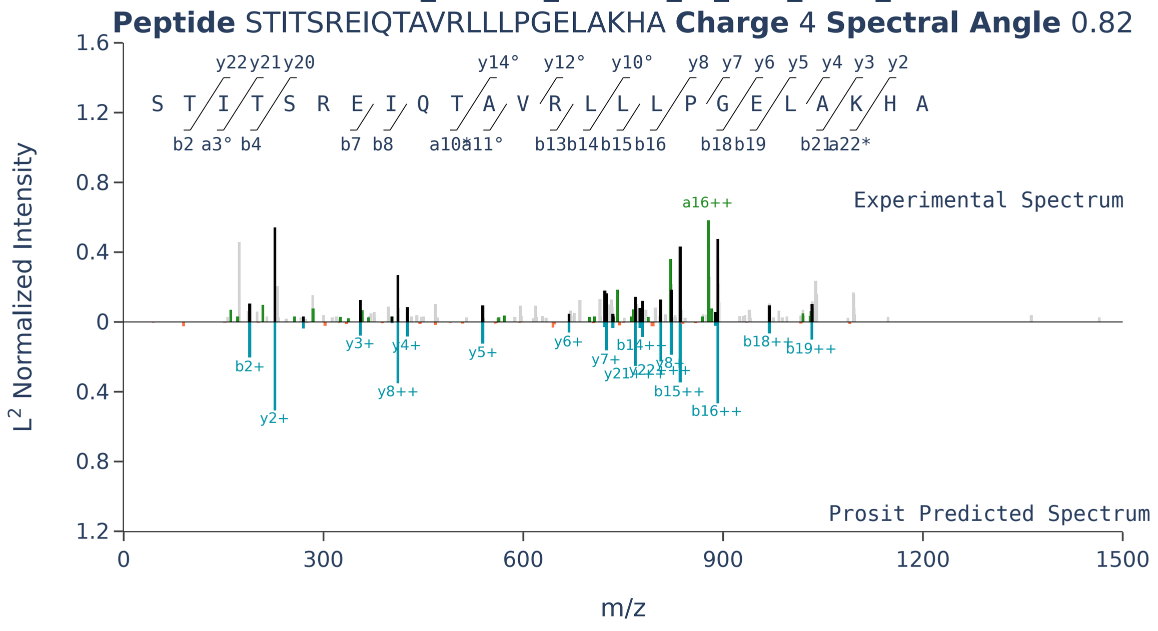

**Source** HRoetschke_291222_080123_Fu_H2B_b1R2_4h **Scan** 5100
**Peptide** RIAGEASRLAHYNKRSTITSR **Charge** 5 **Spectral Angle** 0.73

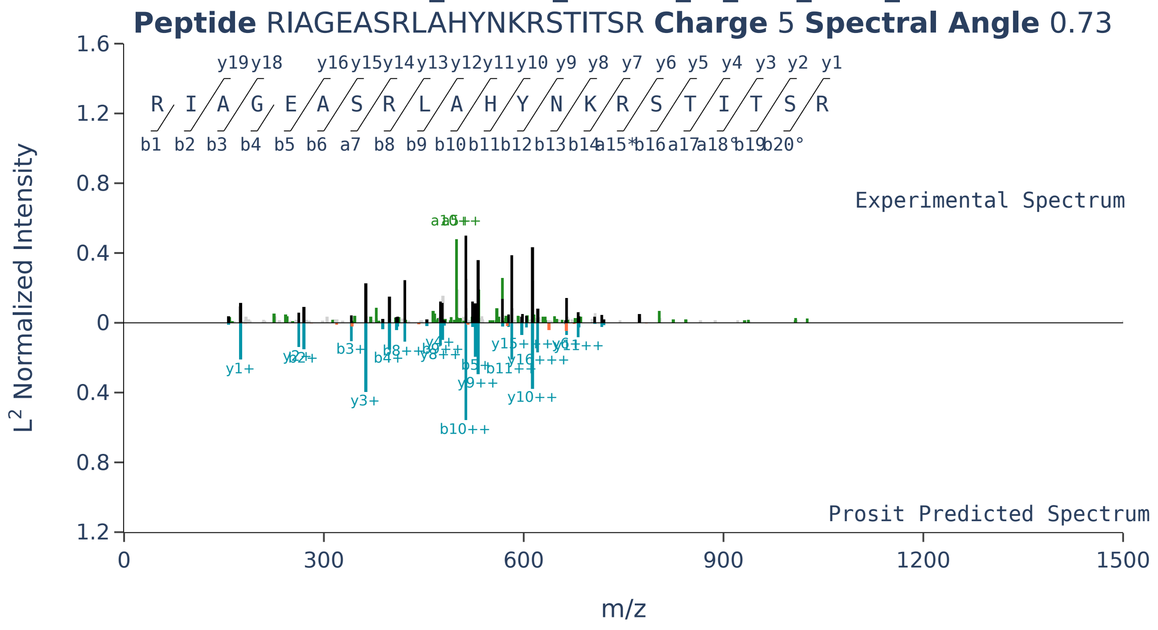


**Source** HRoetschke_291222_080123_Fu_H2B_b2R2_24h **Scan** 2802
**Peptide** LAKHAVSEGTKAV **Charge** 3 **Spectral Angle** 0.71

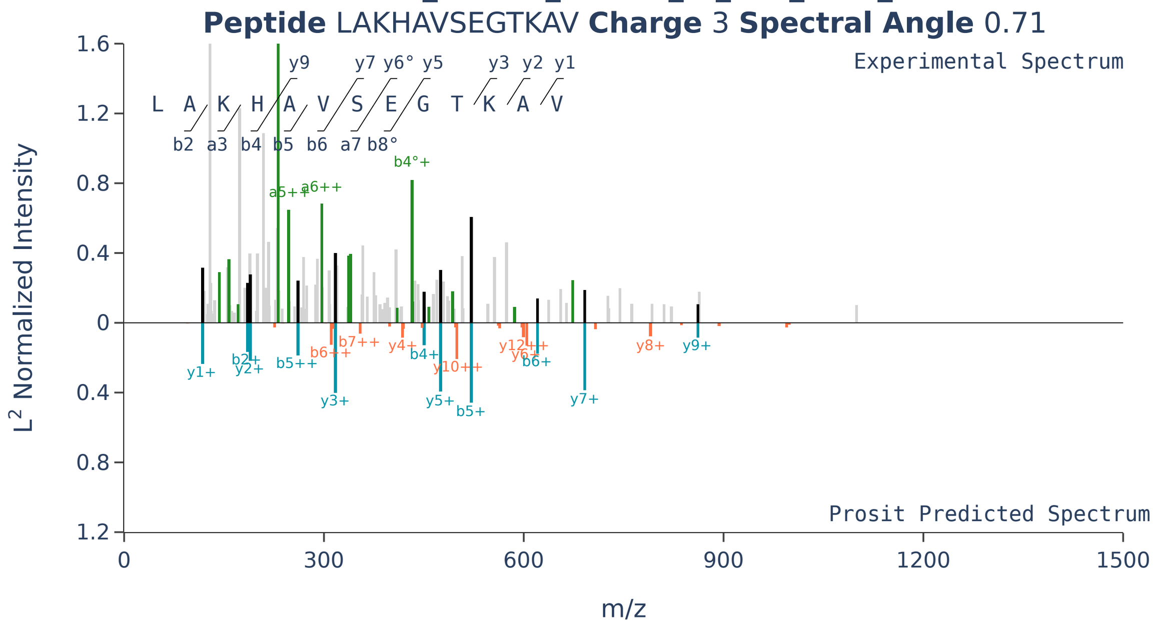


**Source** HRoetschke_291222_080123_Fu_H2B_b2R1_4h **Scan** 16200
**Peptide** RLLLPGELAKHAVSEGTKAVTKYTS **Charge** 5 **Spectral Angle** 0.85

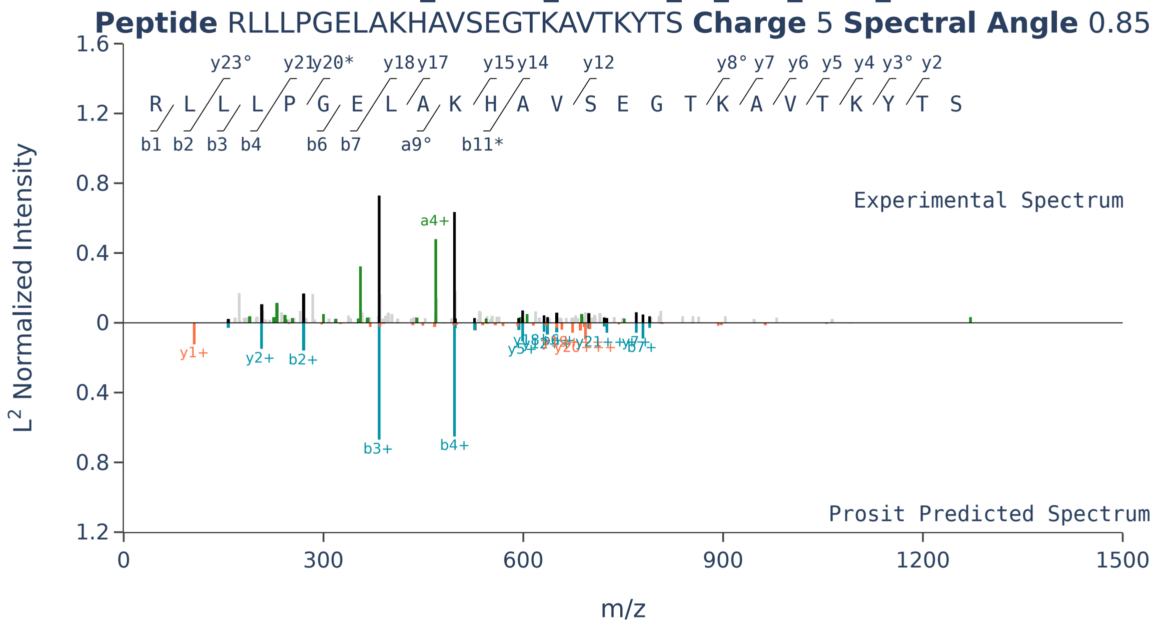


**Source** HRoetschke_291222_080123_Fu_H2B_b2R2_4h **Scan** 11490
**Peptide** TRKESYAIYV **Charge** 3 **Spectral Angle** 0.77

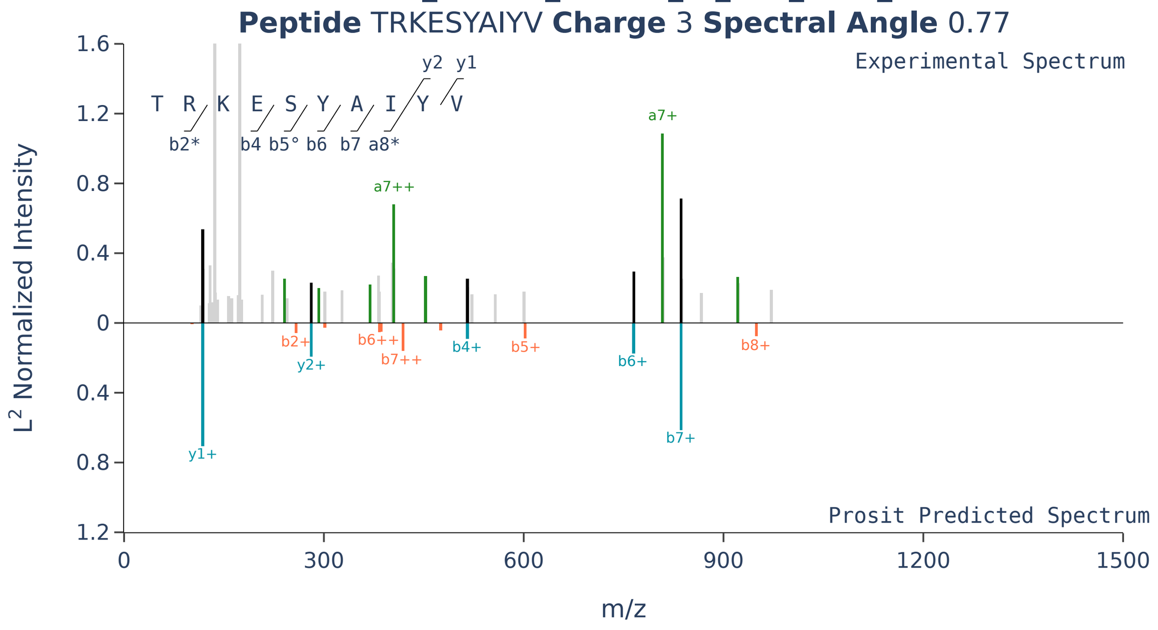


**Source** HRoetschke_291222_080123_Fu_H2B_b1R2_24h **Scan** 2718
**Peptide** KQVHPDTGISSKAMS **Charge** 2 **Spectral Angle** 0.67

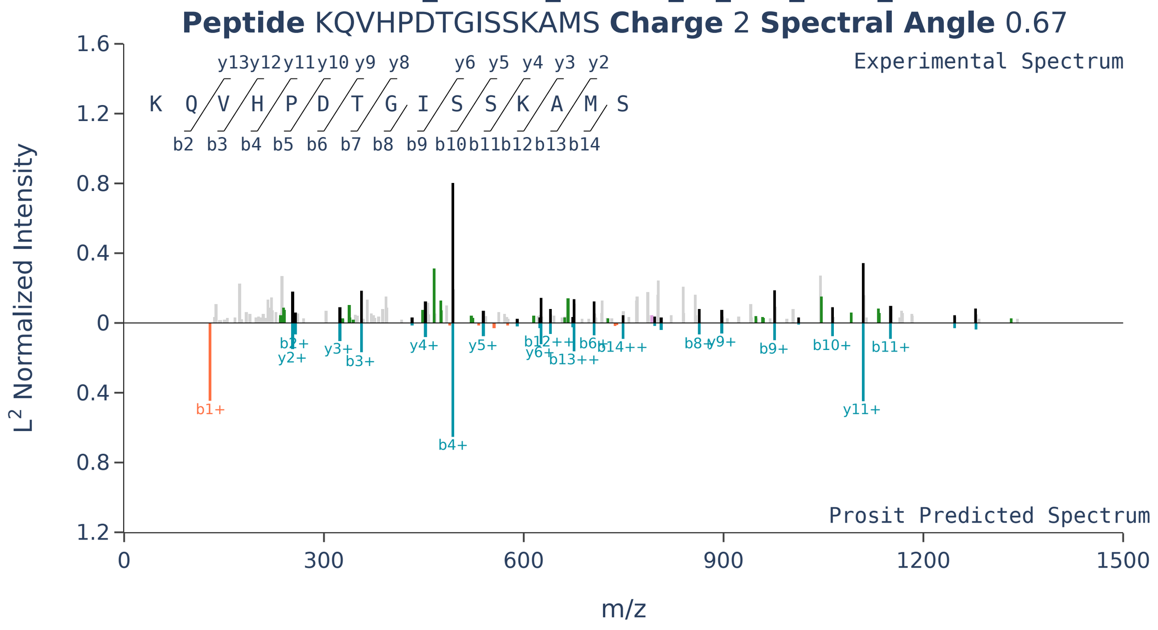


**Source** HRoetschke_291222_080123_Fu_H2B_b1R2_1h **Scan** 16184
**Peptide** TSREIQTAVRLLLPGELAKHAV **Charge** 4 **Spectral Angle** 0.78

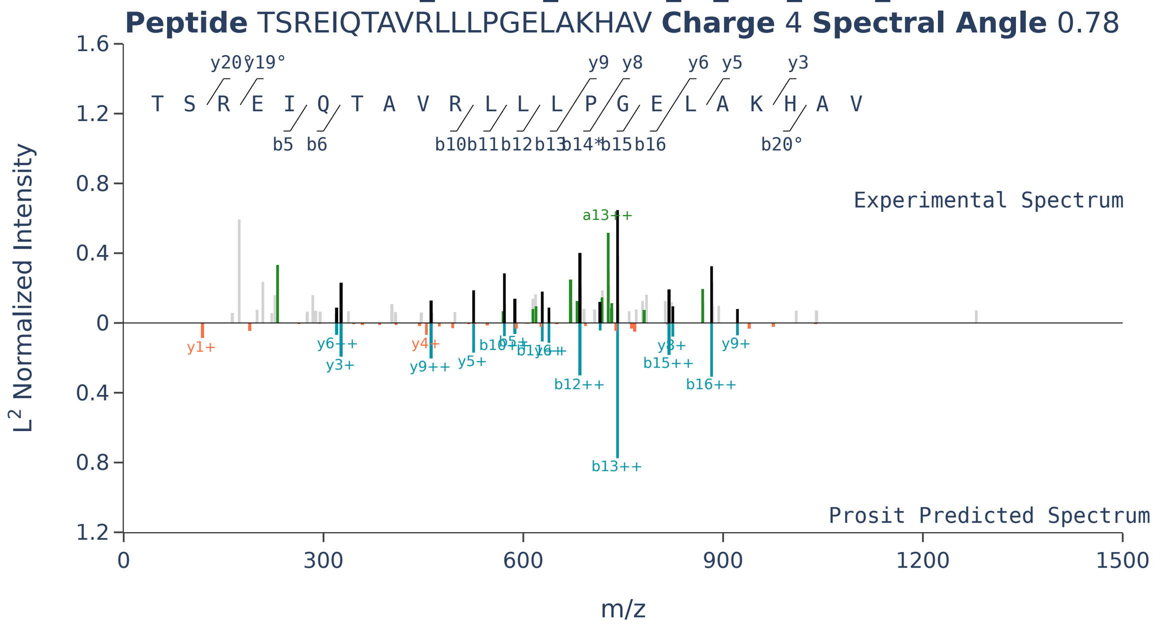


**Source** HRoetschke_291222_080123_Fu_H2B_b2R2_24h **Scan** 15800
**Peptide** LLPGELAKHAVSEGTKAVTKY **Charge** 4 **Spectral Angle** 0.62

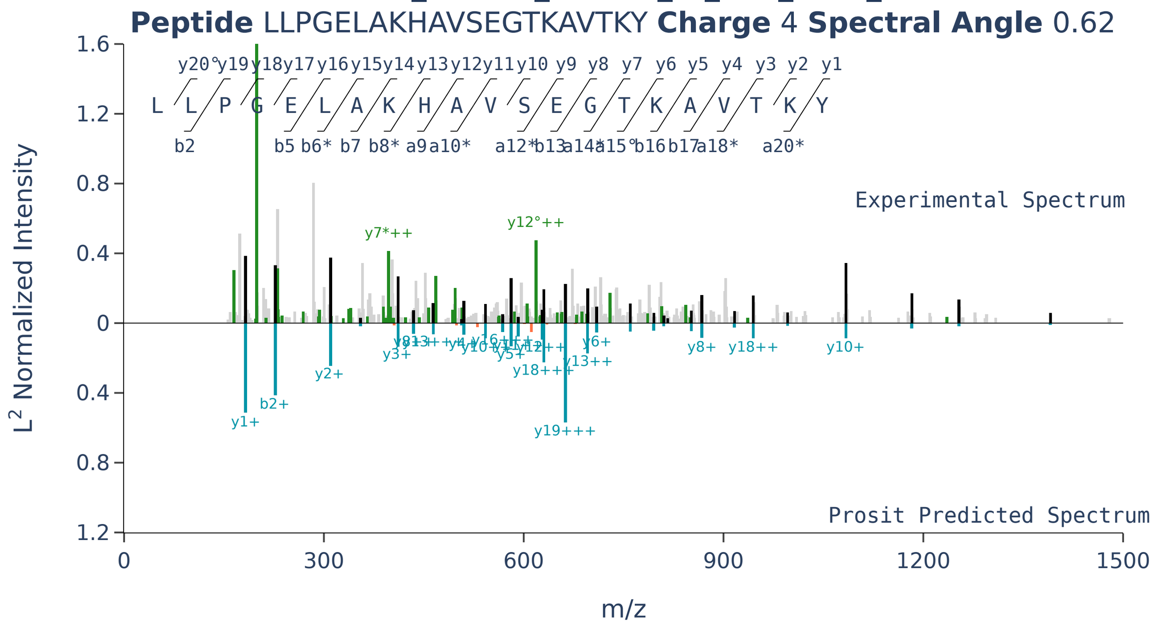


**Source** HRoetschke_291222_080123_Fu_H2B_b1R1_24h **Scan** 2998
**Peptide** LAHYNKRSTIT **Charge** 3 **Spectral Angle** 0.78

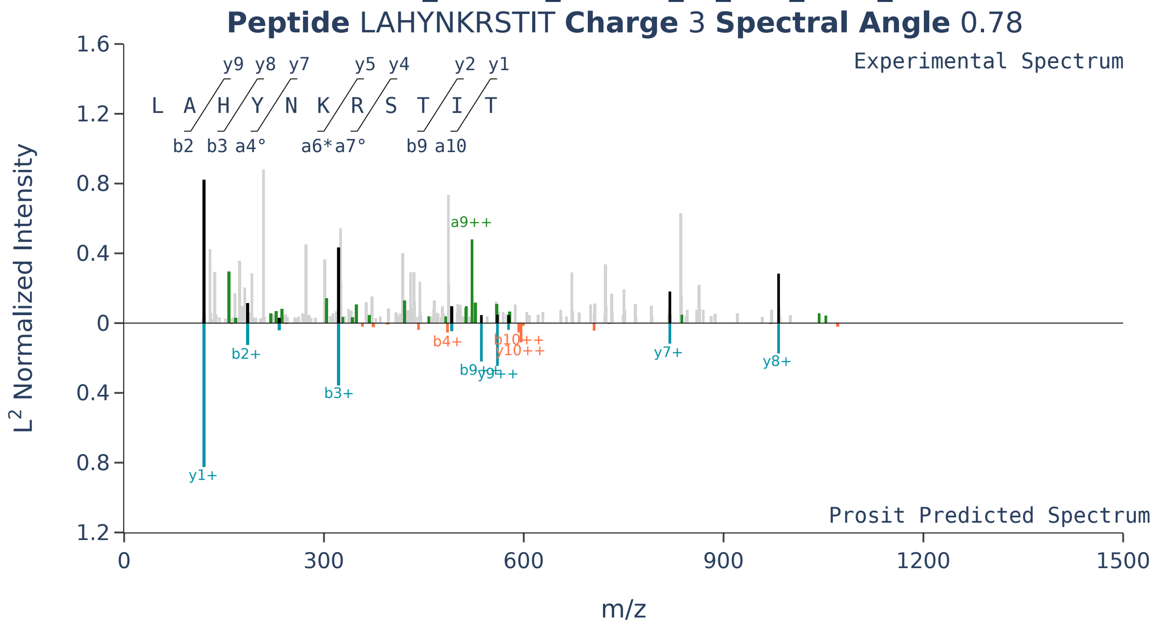


**Source** HRoetschke_291222_080123_Fu_H2B_b1R1_4h **Scan** 16246
**Peptide** REIQTAVRLLLPGE **Charge** 3 **Spectral Angle** 0.61

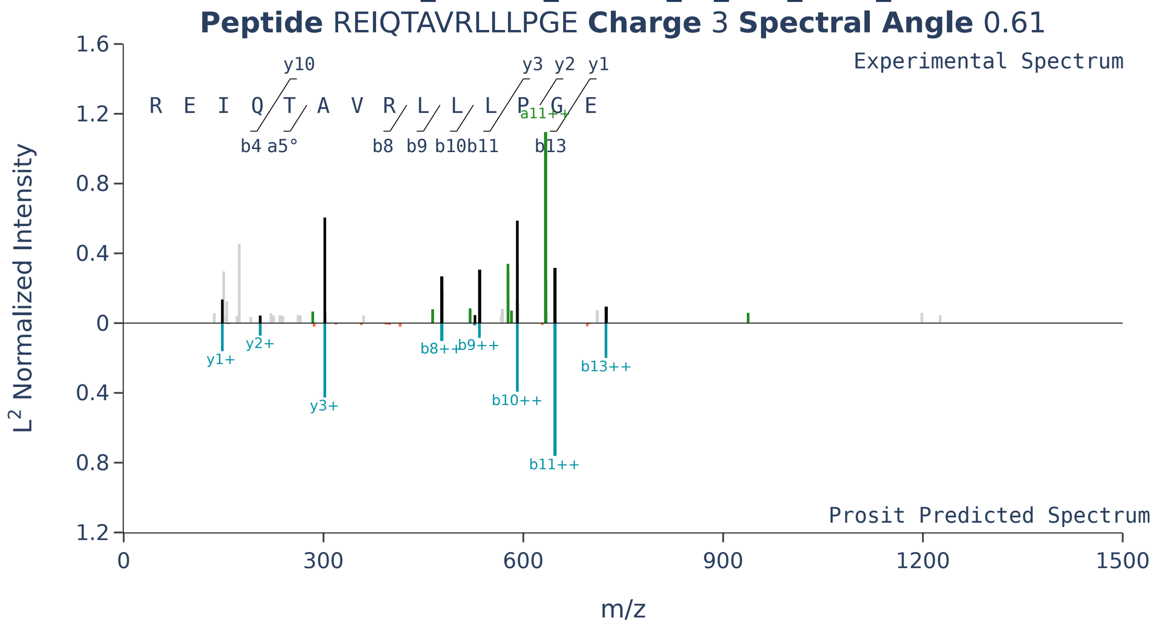


**Source** HRoetschke_291222_080123_Fu_H2B_b2R1_4h **Scan** 18843
**Peptide** QVHPDTGISSKAMSIMNSF **Charge** 3 **Spectral Angle** 0.81

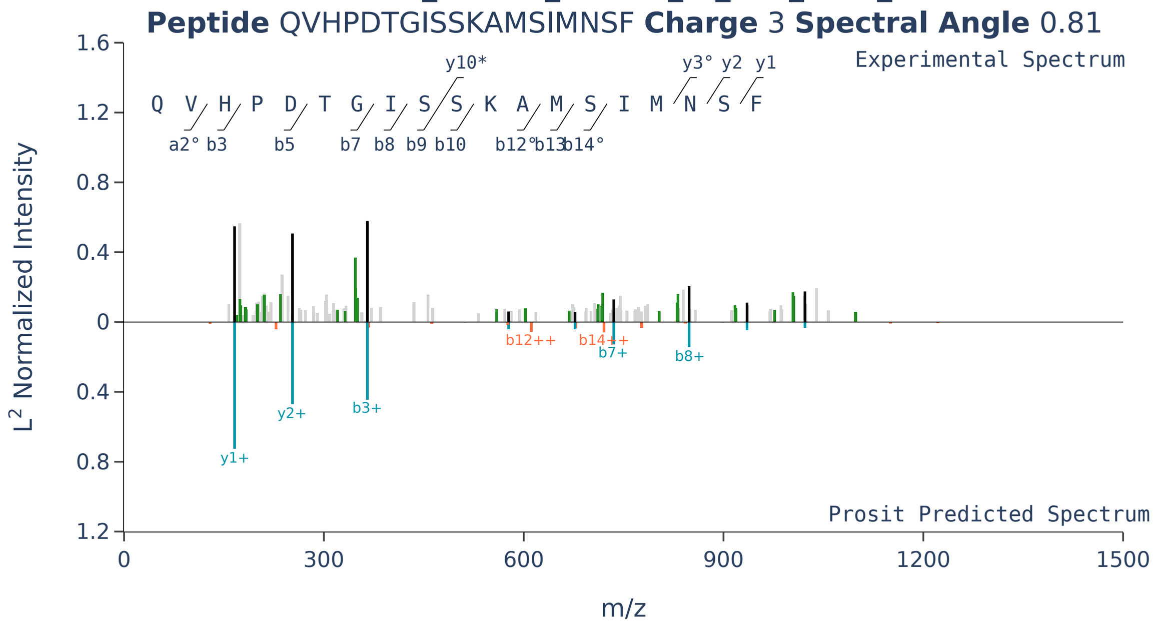


**Source** HRoetschke_291222_080123_Fu_H2B_b2R1_4h **Scan** 16170
**Peptide** AIYVYKVLKQV **Charge** 3 **Spectral Angle** 0.68

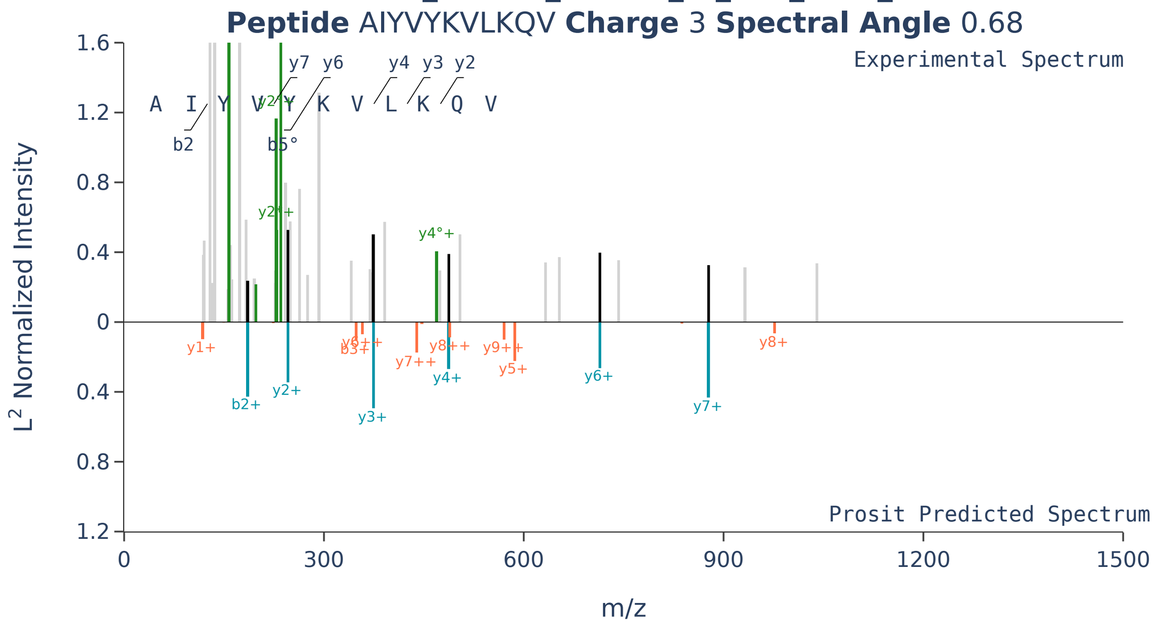


**Source** HRoetschke_291222_080123_Fu_H2B_b2R2_1h **Scan** 4225
**Peptide** ASRLAHYNKRSTITSREL **Charge** 4 **Spectral Angle** 0.83

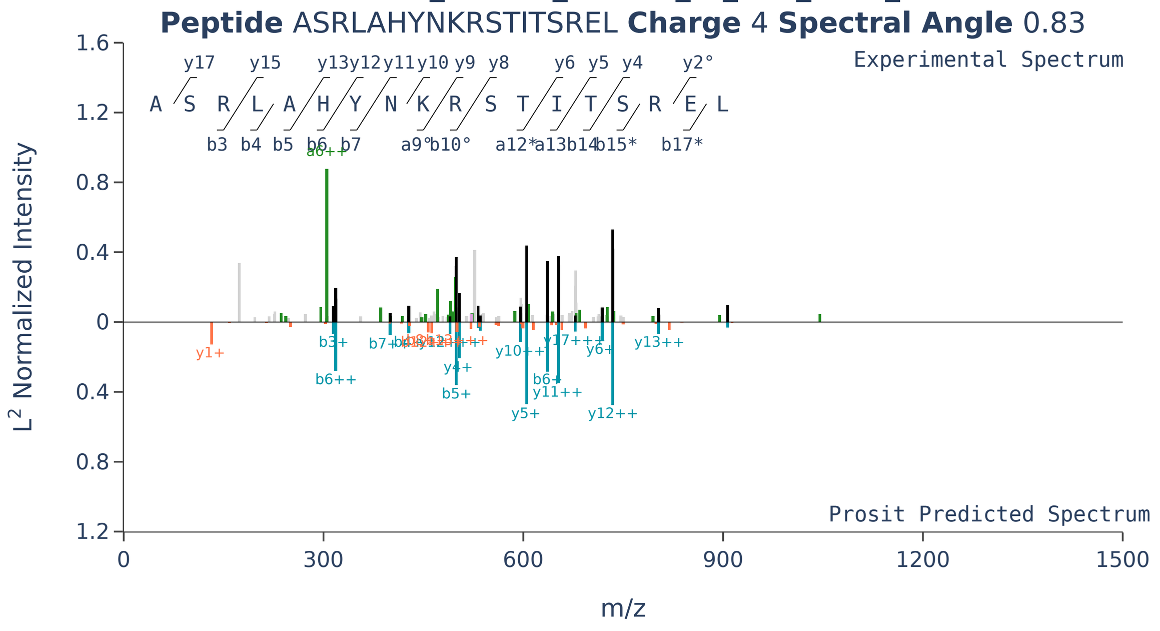


**Source** HRoetschke_291222_080123_Fu_H2B_b2R1_1h **Scan** 19629
**Peptide** STITSREIQTAVRLLL **Charge** 3 **Spectral Angle** 0.91

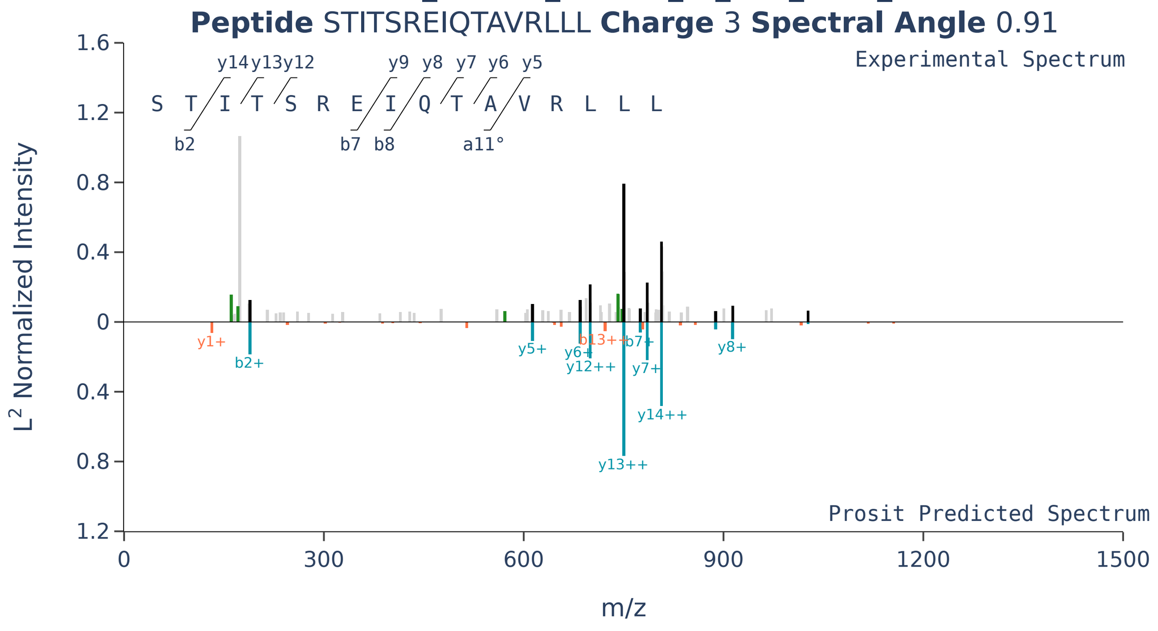


**Source** HRoetschke_291222_080123_Fu_H2B_b2R2_24h **Scan** 1768
**Peptide** SRLAHYNKR **Charge** 3 **Spectral Angle** 0.76

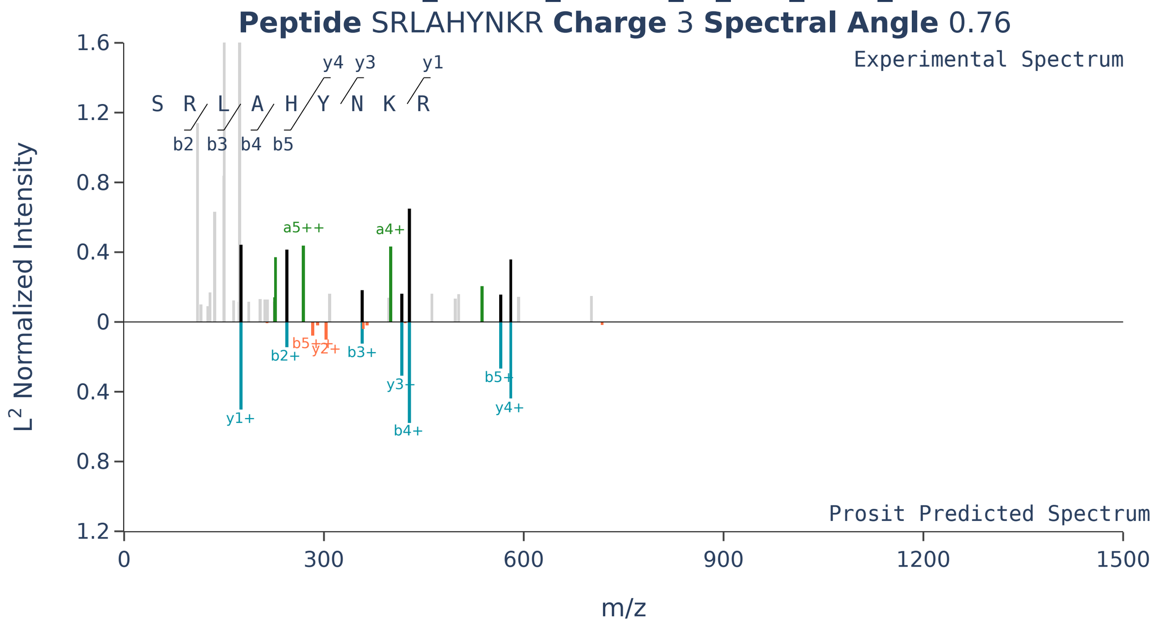


**Source** HRoetschke_291222_080123_Fu_H2B_b2R2_24h **Scan** 7985
**Peptide** ERIAGEASRLAHYNKRSTITS **Charge** 4 **Spectral Angle** 0.6

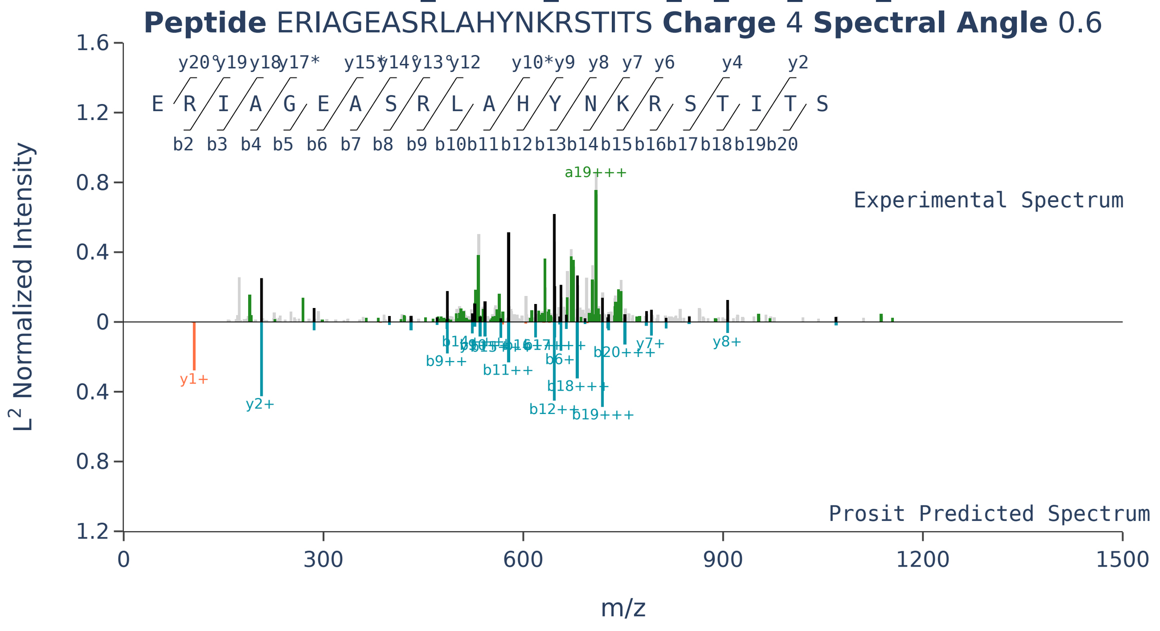

**Source** HRoetschke_291222_080123_Fu_H2B_b2R2_4h **Scan** 20885
**Peptide** KAMSIMNSFVNDVFERI **Charge** 3 **Spectral Angle** 0.72

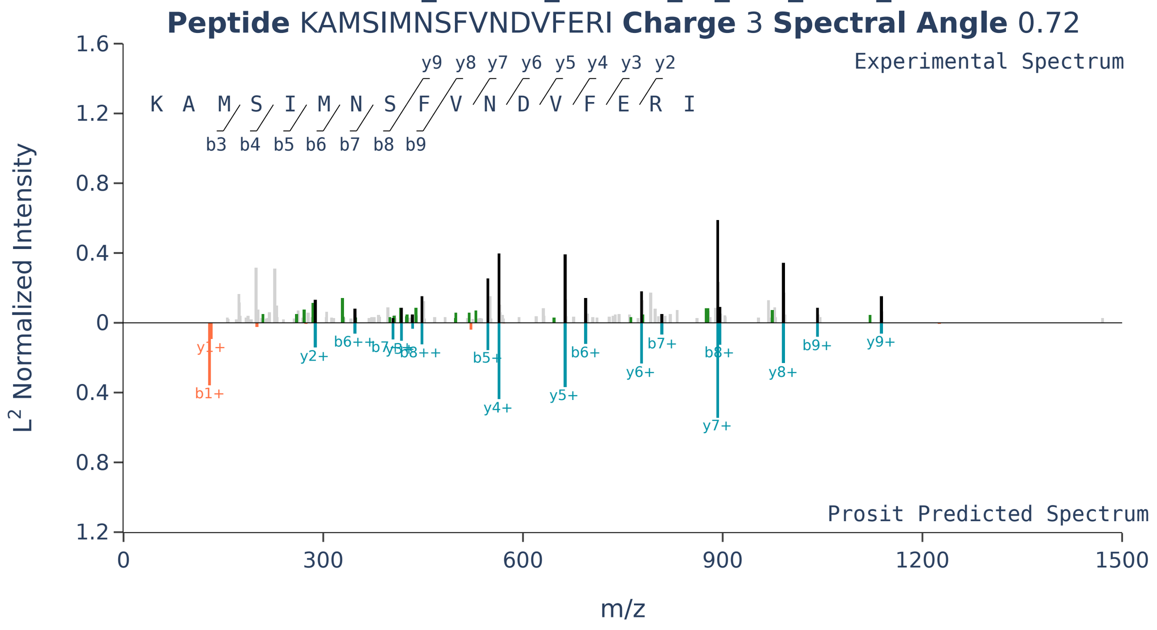


**Source** HRoetschke_291222_080123_Fu_H2B_b2R1_24h **Scan** 13709
**Peptide** HPDTGISSKAMSIM **Charge** 3 **Spectral Angle** 0.82

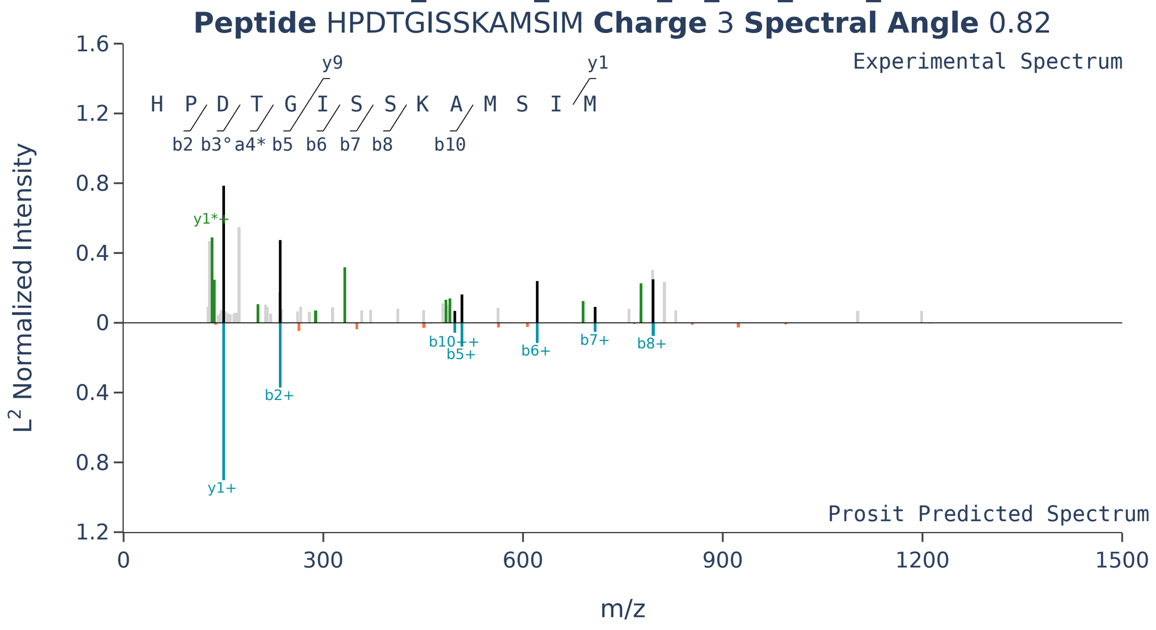


**Source** HRoetschke_291222_080123_Fu_H2B_b1R2_24h **Scan** 9102
**Peptide** FERIAGEASRLAHYNKR **Charge** 4 **Spectral Angle** 0.74

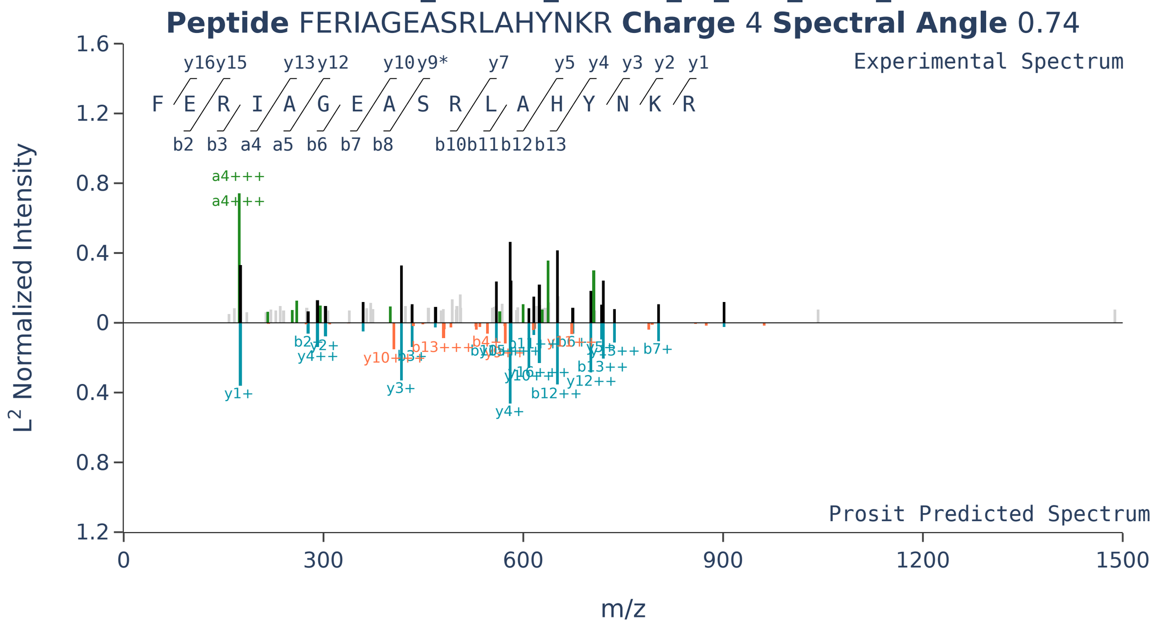


**Source** HRoetschke_291222_080123_Fu_H2B_b2R2_24h **Scan** 16246
**Peptide** KRSTITSREIQTAVRLL **Charge** 4 **Spectral Angle** 0.77

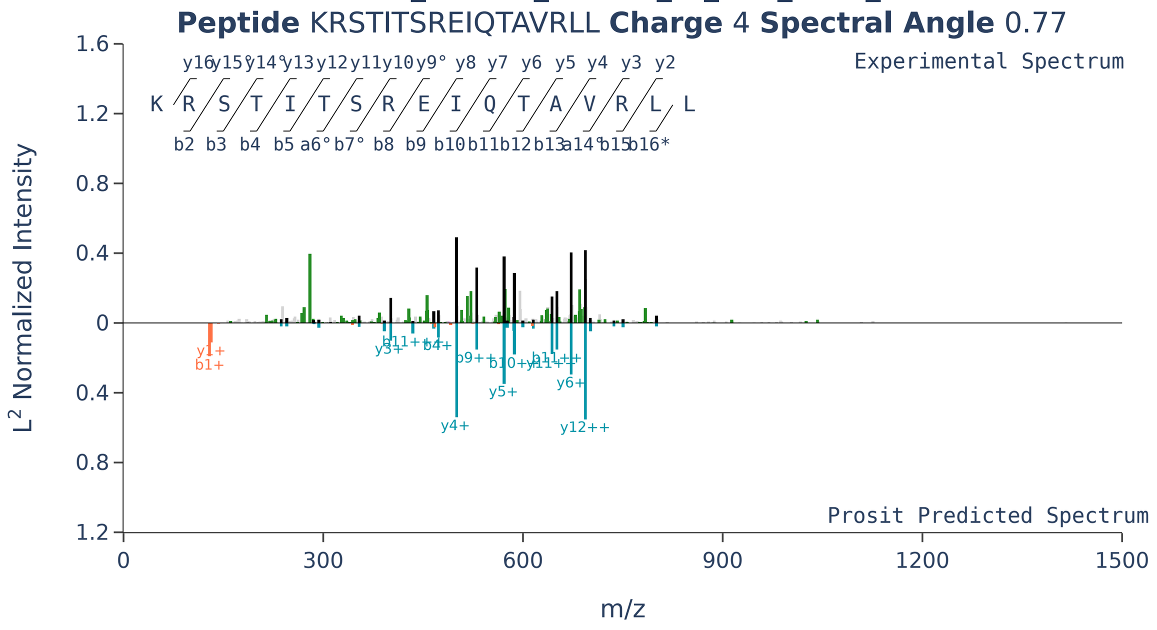


**Source** HRoetschke_291222_080123_Fu_H2B_b2R1_X **Scan** 1628
**Peptide** AKSAPAPKKGSKKAVTKTQKKDGKKRR **Charge** 5 **Spectral Angle** 0.76

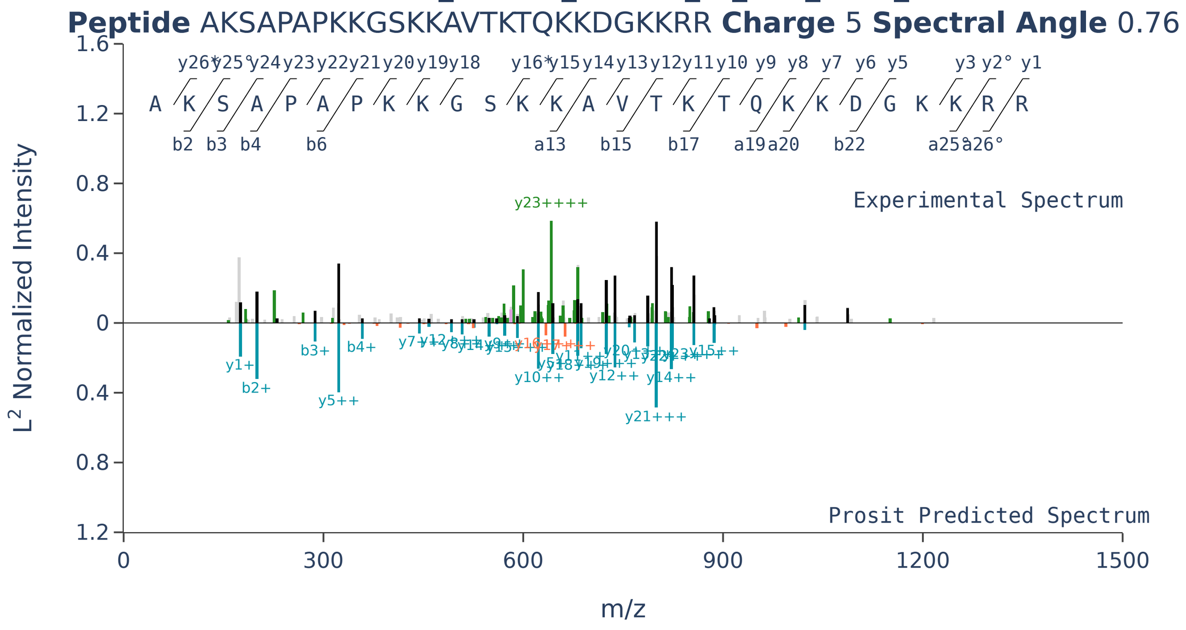


**Source** HRoetschke_291222_080123_Fu_H2B_b2R1_2h **Scan** 5249
**Peptide** EASRLAHYNKRSTITSREIQTA **Charge** 4 **Spectral Angle** 0.72

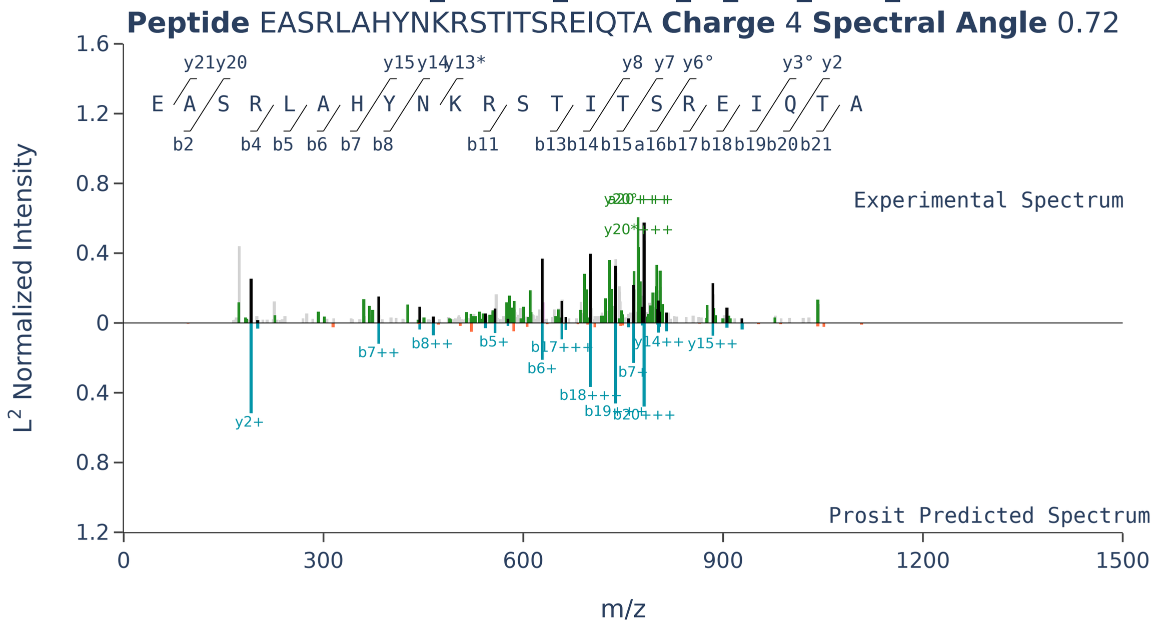


**Source** HRoetschke_291222_080123_Fu_H2B_b2R1_24h **Scan** 4962
**Peptide** ASRLAHYNKRSTITSREIQTA **Charge** 5 **Spectral Angle** 0.65

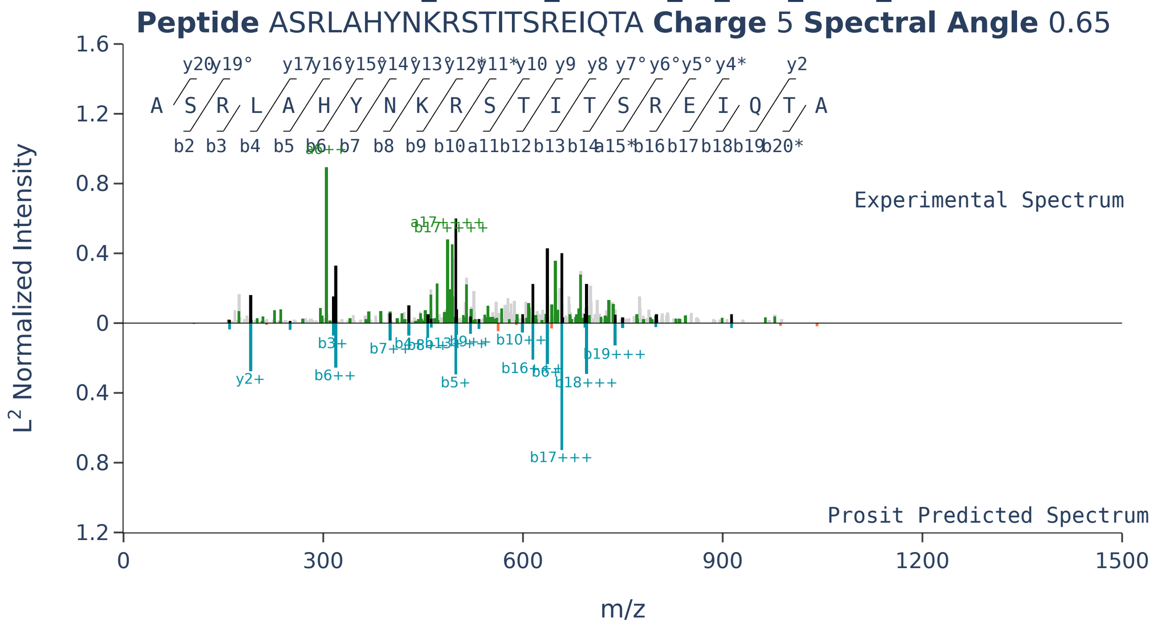


**Source** HRoetschke_291222_080123_Fu_H2B_b2R1_X **Scan** 1604
**Peptide** AKSAPAPKKGSKKAVTKTQKKDGKKR **Charge** 5 **Spectral Angle** 0.66

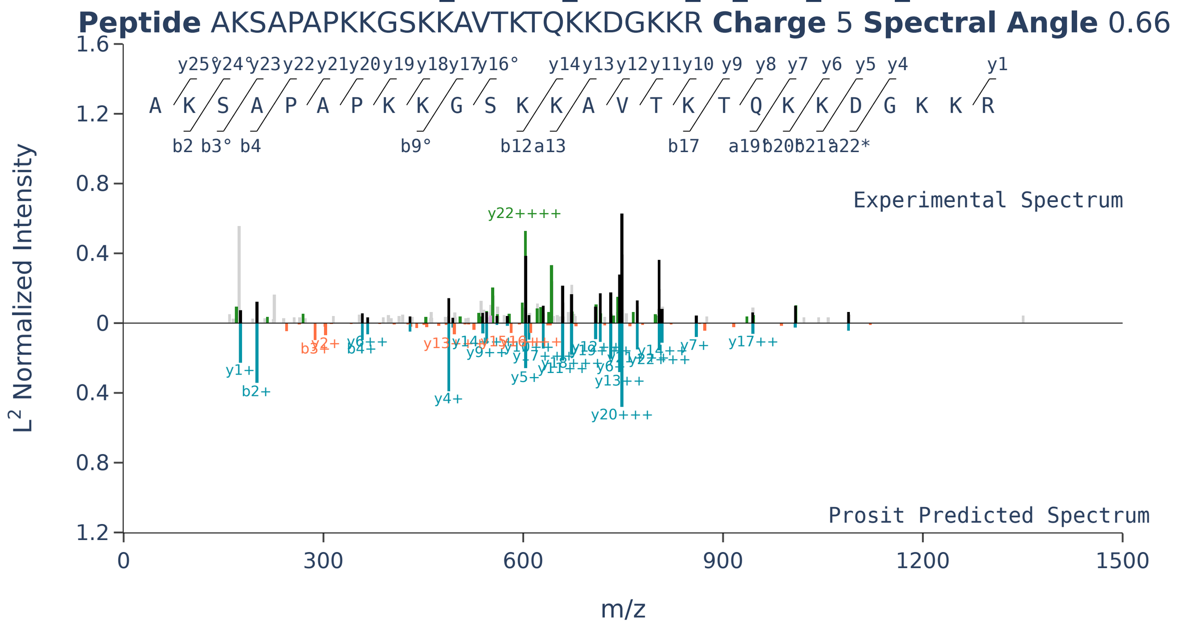


**Source** HRoetschke_291222_080123_Fu_H2B_b1R2_24h **Scan** 11057
**Peptide** VHPDTGISSKAMSIMNS **Charge** 3 **Spectral Angle** 0.9

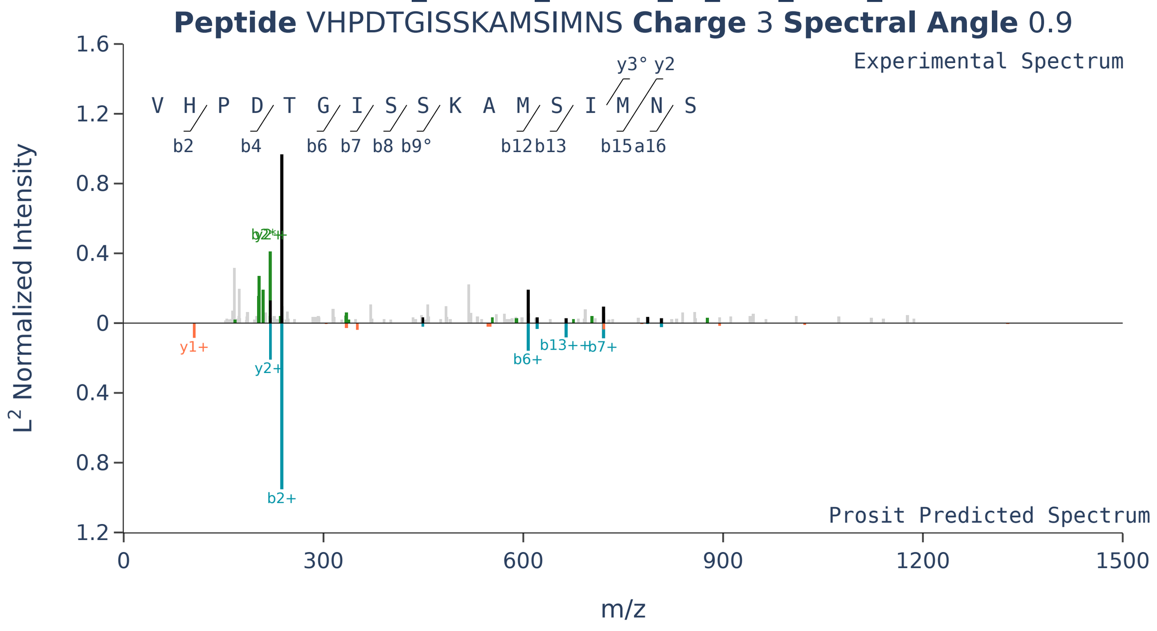


**Source** HRoetschke_291222_080123_Fu_H2B_b2R1_24h **Scan** 14011
**Peptide** VFERIAGEASRLAHYNKRSTITS **Charge** 4 **Spectral Angle** 0.65

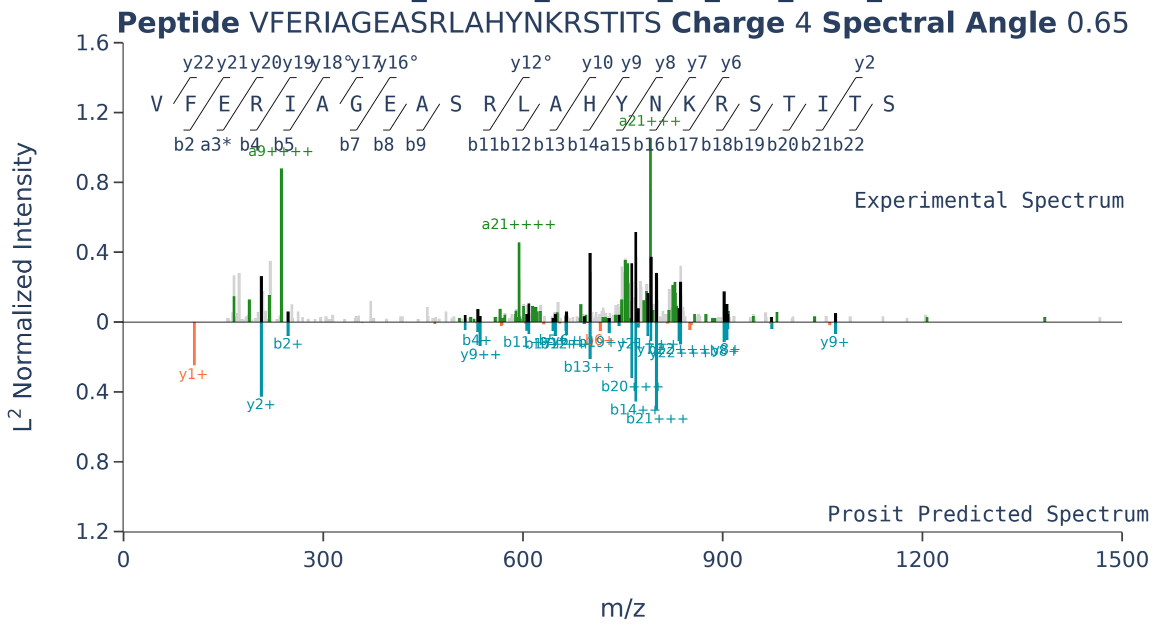


**Source** HRoetschke_291222_080123_Fu_H2B_b2R2_24h **Scan** 16895
**Peptide** QTAVRLLLPGELAKHAVSEGTKA **Charge** 4 **Spectral Angle** 0.67

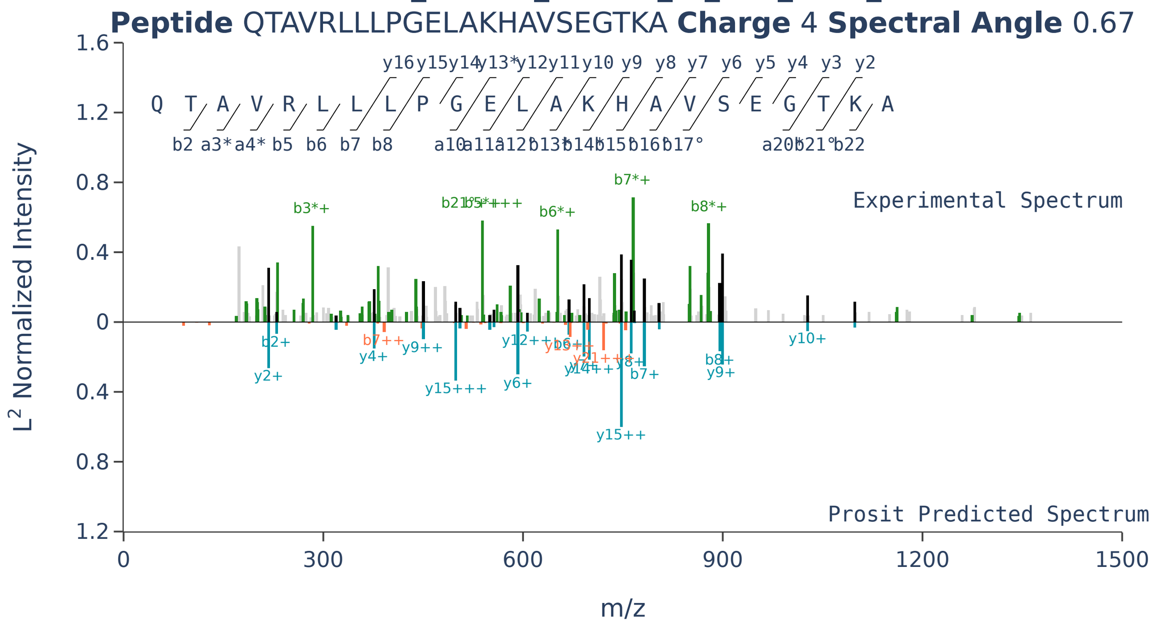


**Source** HRoetschke_291222_080123_Fu_H2B_b2R2_1h **Scan** 16251
**Peptide** VHPDTGISSKAMSIMNSFVNDVFE **Charge** 3 **Spectral Angle** 0.88

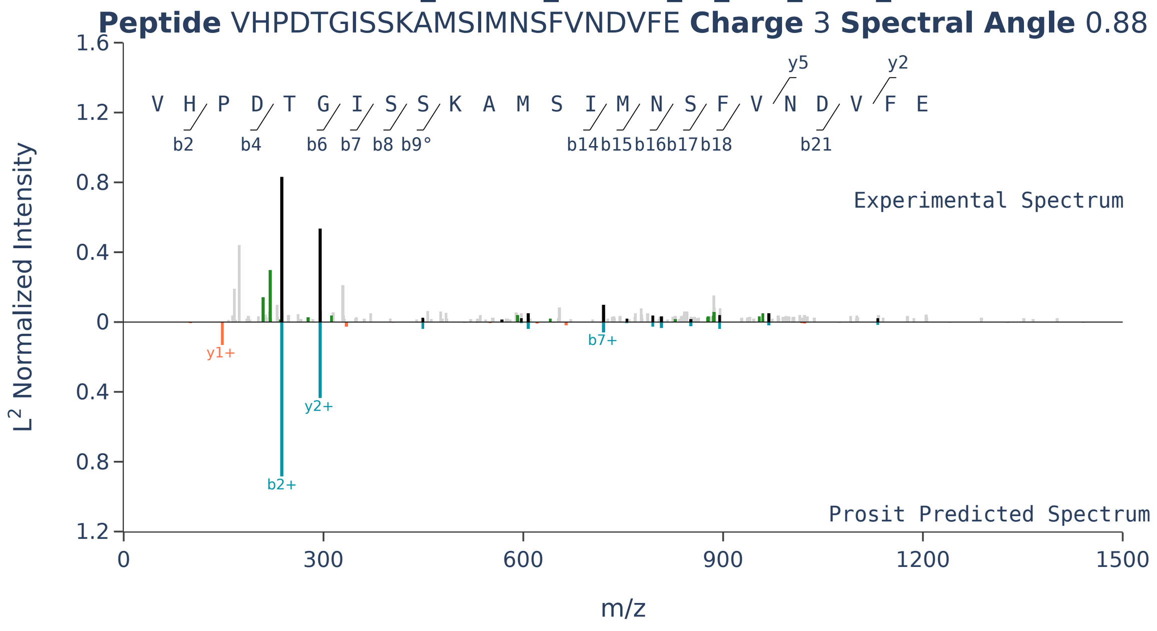


**Source** HRoetschke_291222_080123_Fu_H2B_b1R1_24h **Scan** 7471
**Peptide** LKQVHPDTGISSKAMSIMN **Charge** 3 **Spectral Angle** 0.66

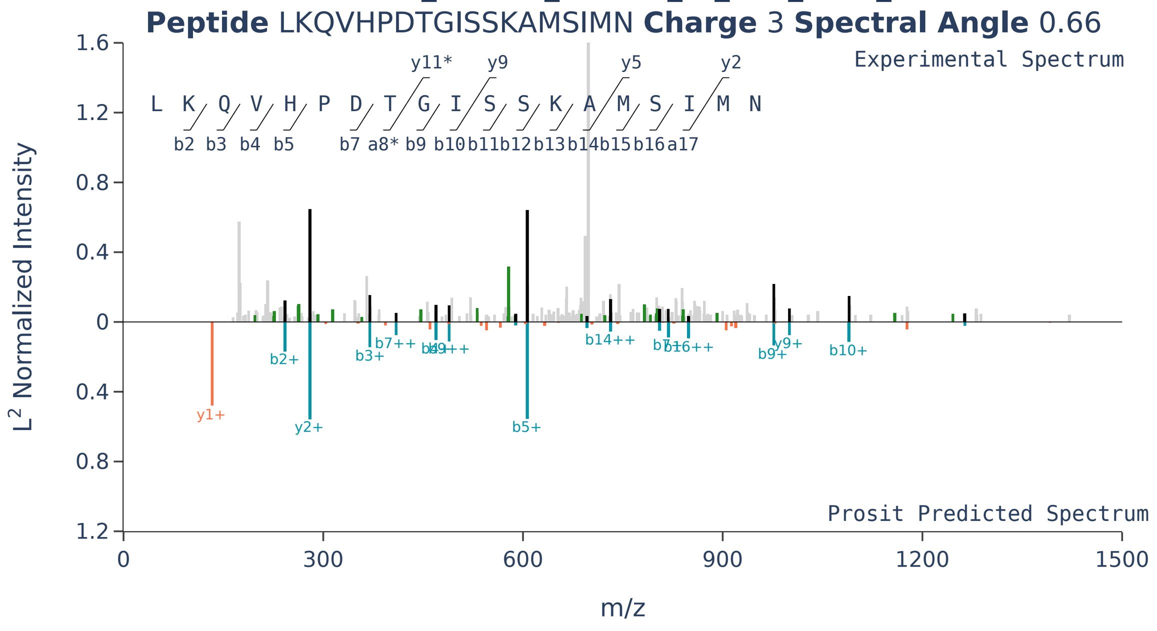


**Source** HRoetschke_291222_080123_Fu_H2B_b2R2_24h **Scan** 13985
**Peptide** LLPGELAKHAVSEGTKAVTK **Charge** 3 **Spectral Angle** 0.8

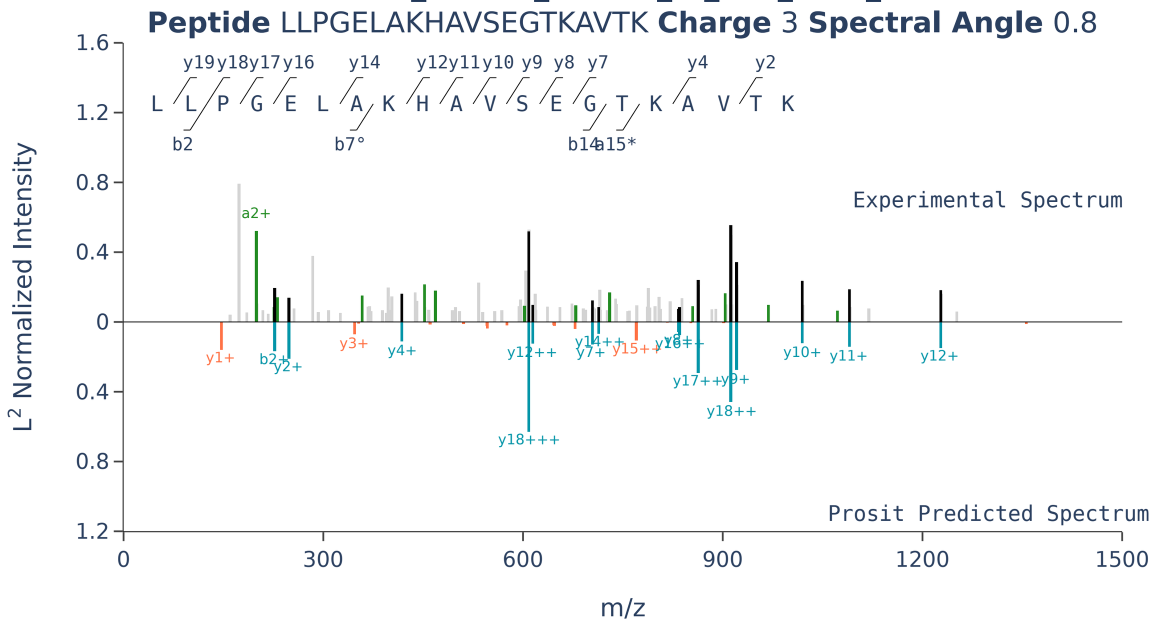


**Source** HRoetschke_291222_080123_Fu_H2B_b2R1_2h **Scan** 5224
**Peptide** LAKHAVSEGTKAVTKYTSAK **Charge** 4 **Spectral Angle** 0.66

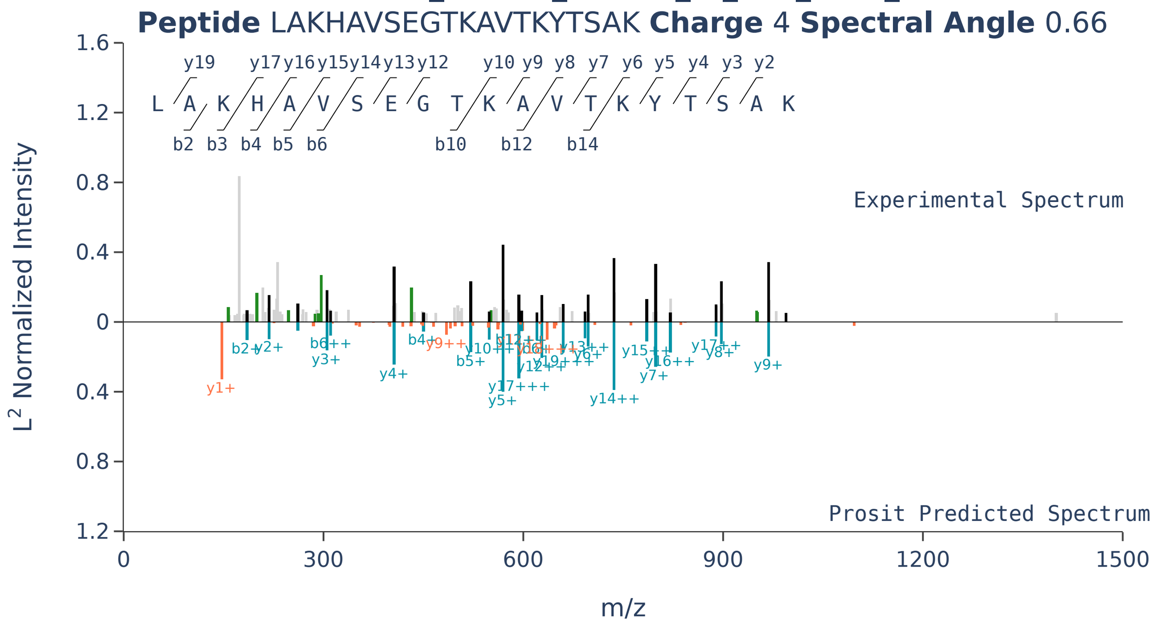

**Source** HRoetschke_291222_080123_Fu_H2B_b2R1_1h **Scan** 14956
**Peptide** DVFERIAGEASRLAHYNKRST **Charge** 4 **Spectral Angle** 0.86

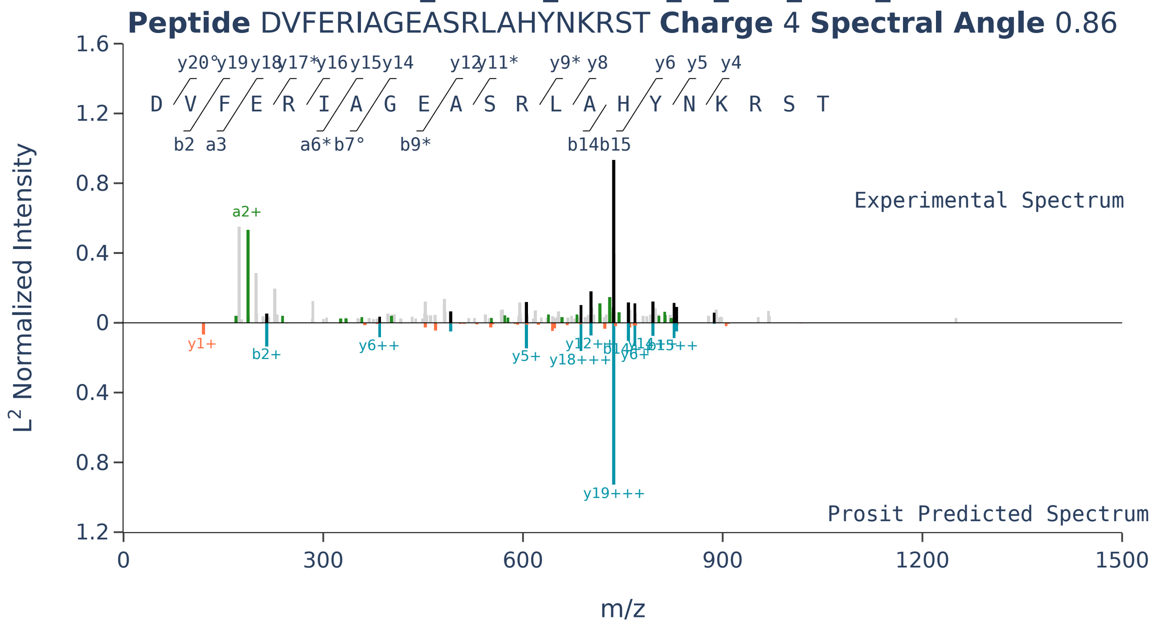


**Source** HRoetschke_291222_080123_Fu_H2B_b1R1_2h **Scan** 19921
**Peptide** SFVNDVFERIAGEASRLAHYNKR **Charge** 5 **Spectral Angle** 0.86

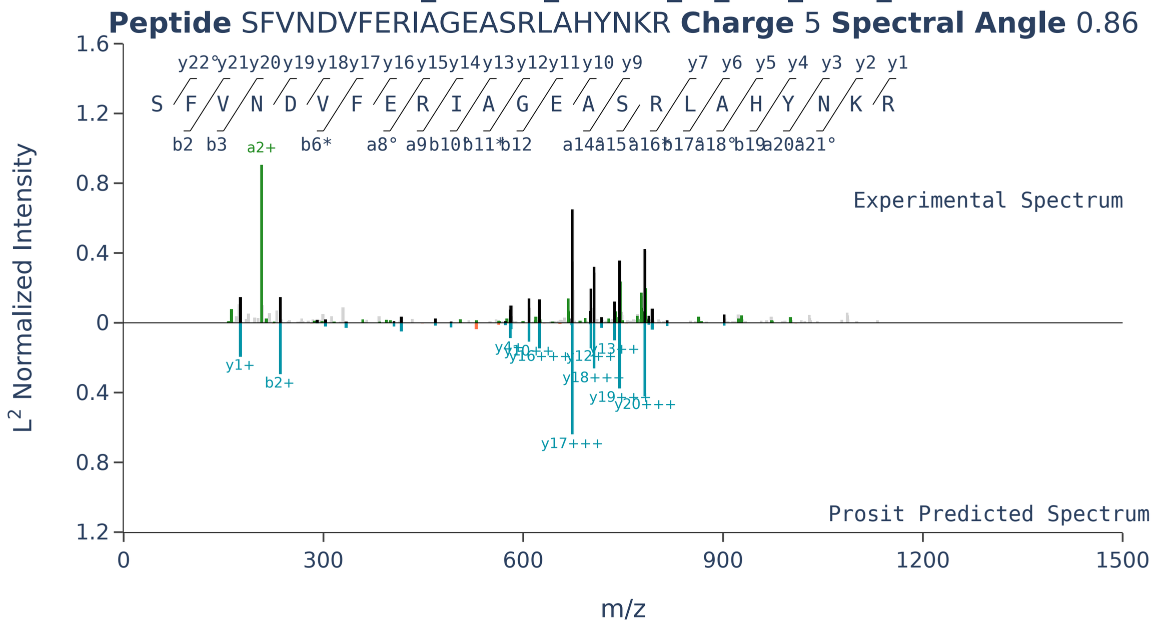


**Source** HRoetschke_291222_080123_Fu_H2B_b2R1_4h **Scan** 6572
**Peptide** RIAGEASRLAHYNKRSTI **Charge** 4 **Spectral Angle** 0.82

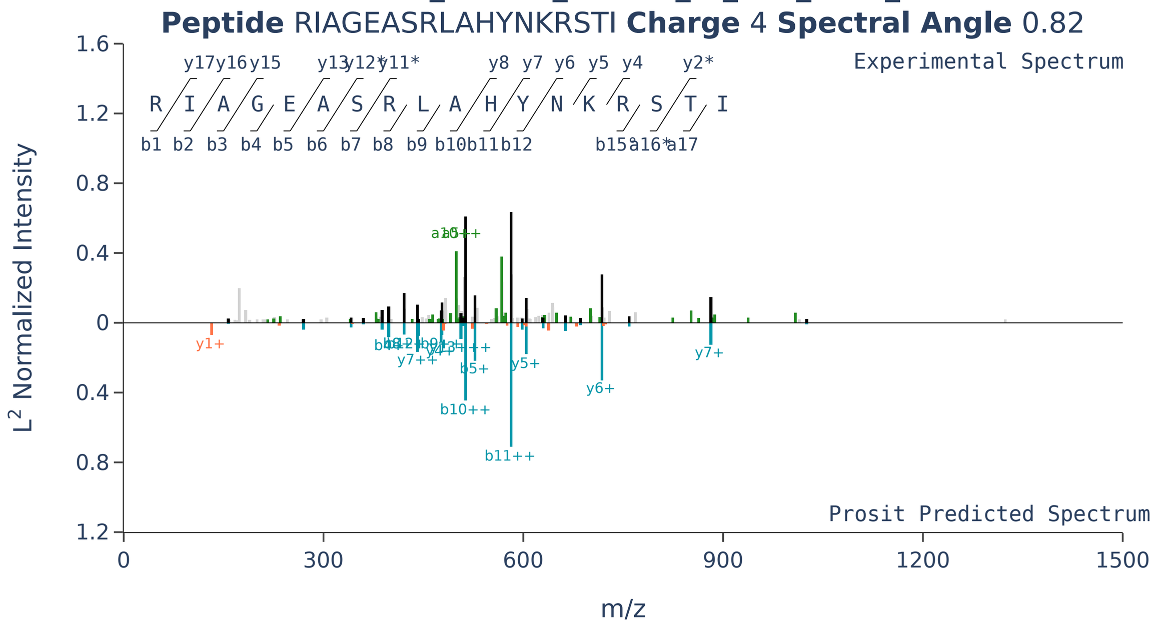


**Source** HRoetschke_291222_080123_Fu_H2B_b1R1_24h **Scan** 11403
**Peptide** KQVHPDTGISSKAMSIMNSF **Charge** 3 **Spectral Angle** 0.68

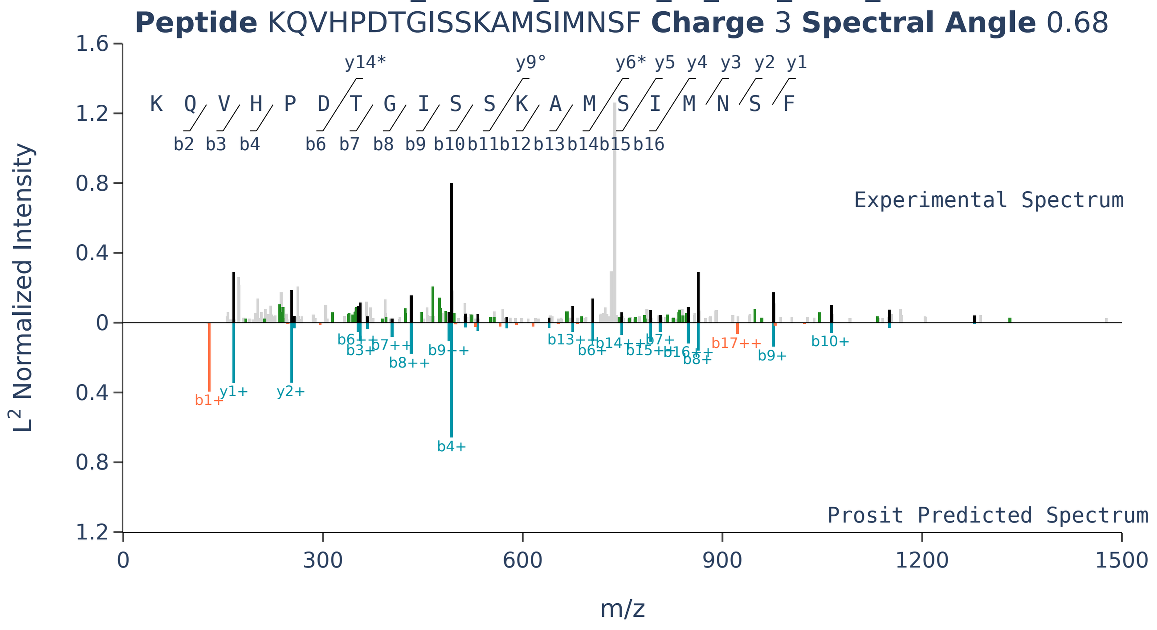


**Source** HRoetschke_291222_080123_Fu_H2B_b2R2_24h **Scan** 3684
**Peptide** YNKRSTITSREIQ **Charge** 3 **Spectral Angle** 0.74

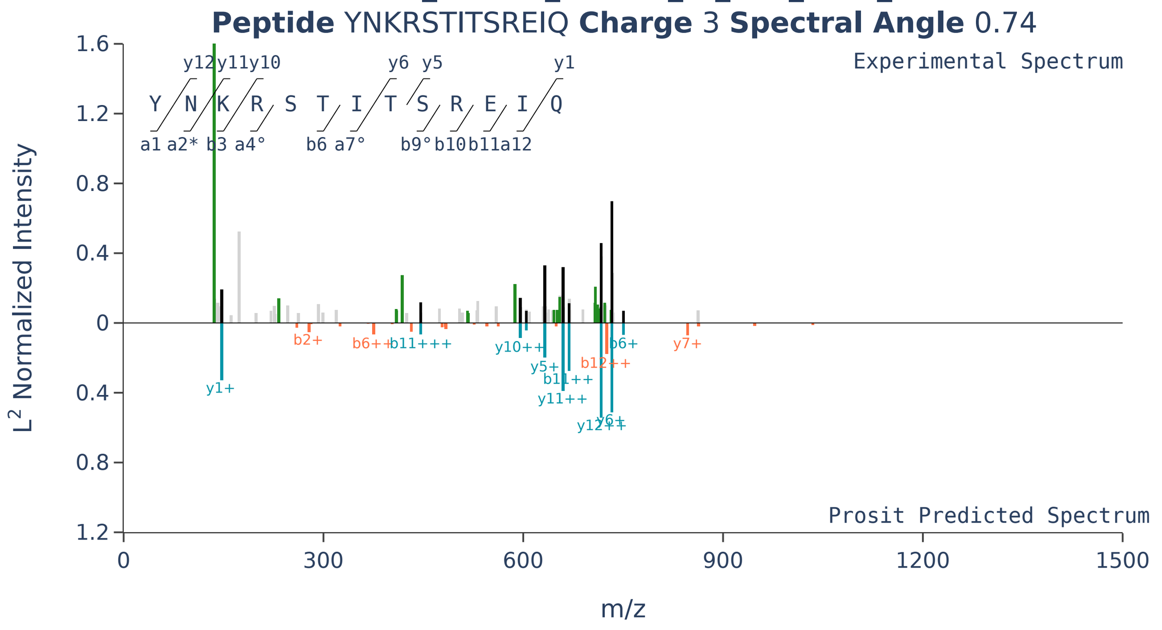


**Source** HRoetschke_291222_080123_Fu_H2B_b1R2_2h **Scan** 16042
**Peptide** ELQTAVRLLLPGELAKHAVSEGTKAVTK **Charge** 4 **Spectral Angle** 0.69

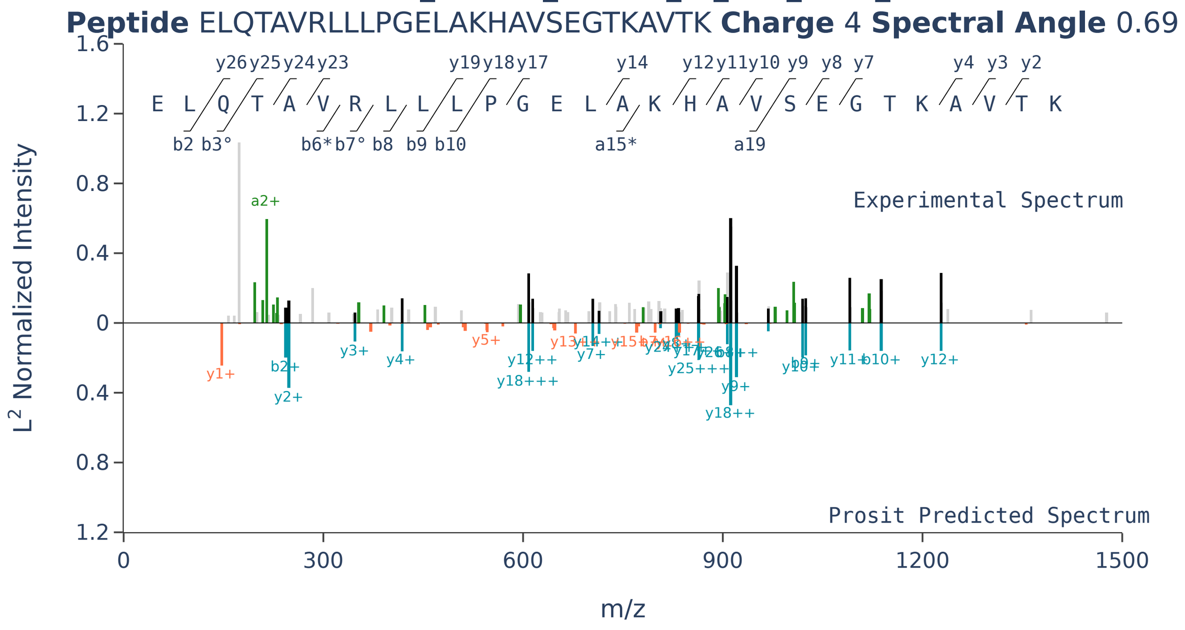


**Source** HRoetschke_291222_080123_Fu_H2B_b1R1_2h **Scan** 4732
**Peptide** RSTITSREIQTA **Charge** 3 **Spectral Angle** 0.69

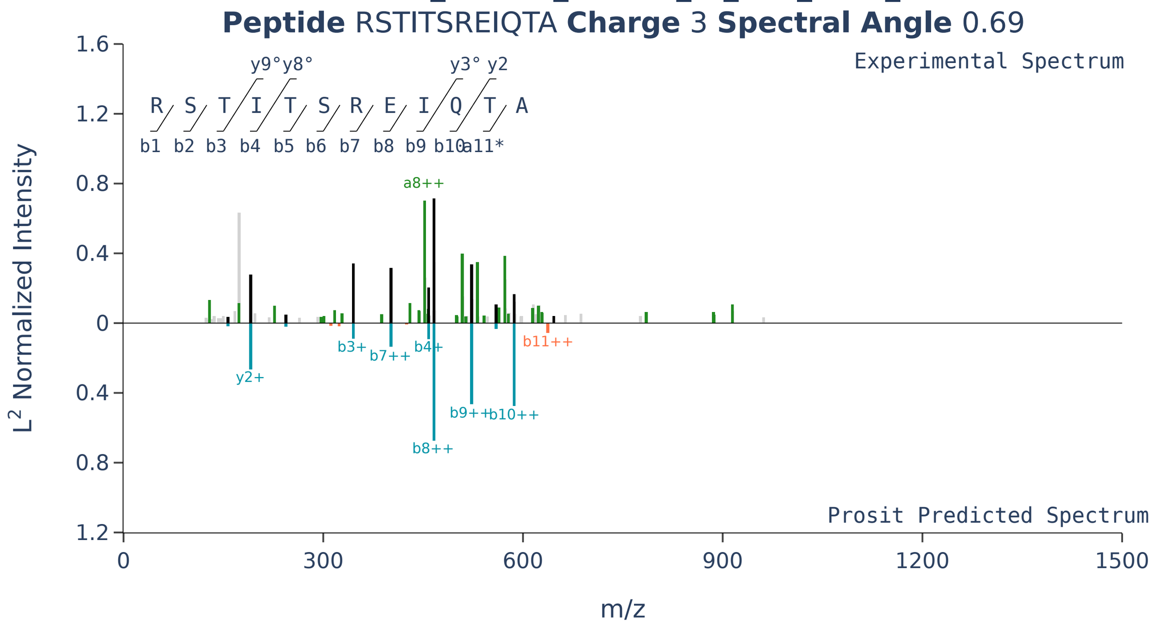


**Source** HRoetschke_291222_080123_Fu_H2B_b2R1_1h **Scan** 4613
**Peptide** KRSTITSREIQTAVR **Charge** 4 **Spectral Angle** 0.74

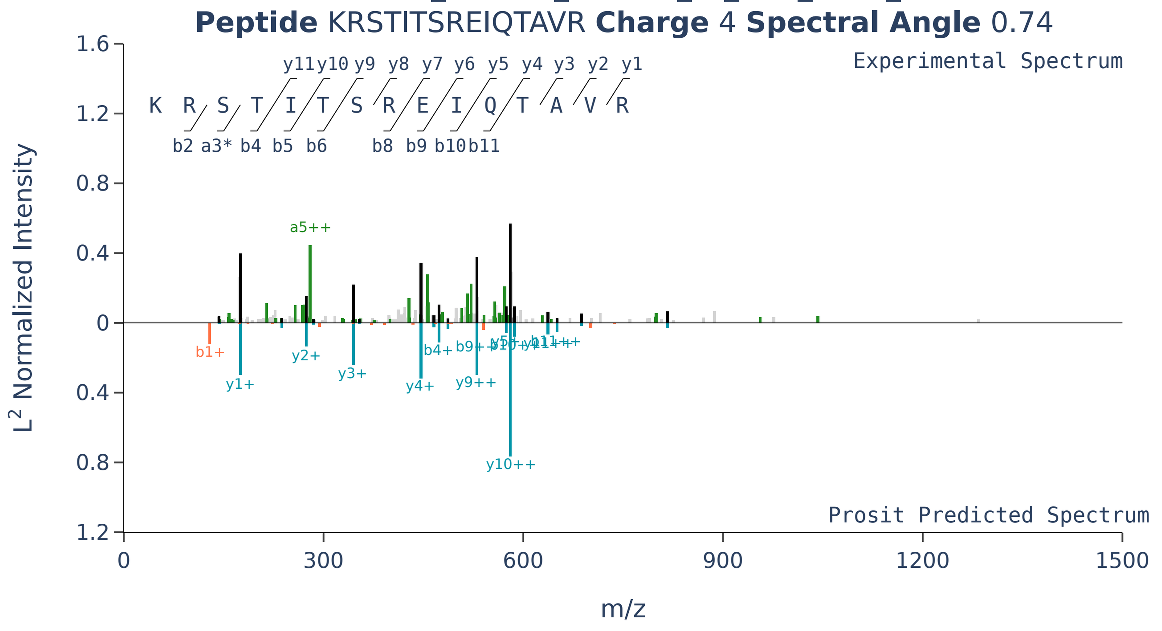


**Source** HRoetschke_291222_080123_Fu_H2B_b1R2_24h **Scan** 4716
**Peptide** VSEGTKAVTKYTS **Charge** 2 **Spectral Angle** 0.69

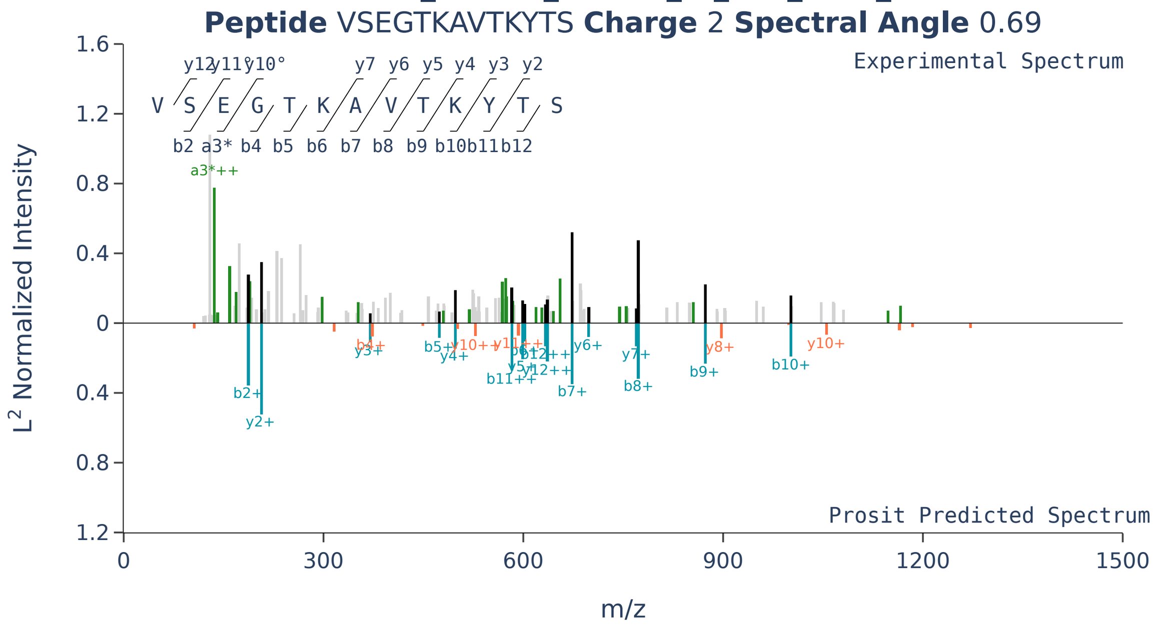


**Source** HRoetschke_291222_080123_Fu_H2B_b1R1_24h **Scan** 8888
**Peptide** IAGEASRLAHYNKRSTITSREI **Charge** 5 **Spectral Angle** 0.8

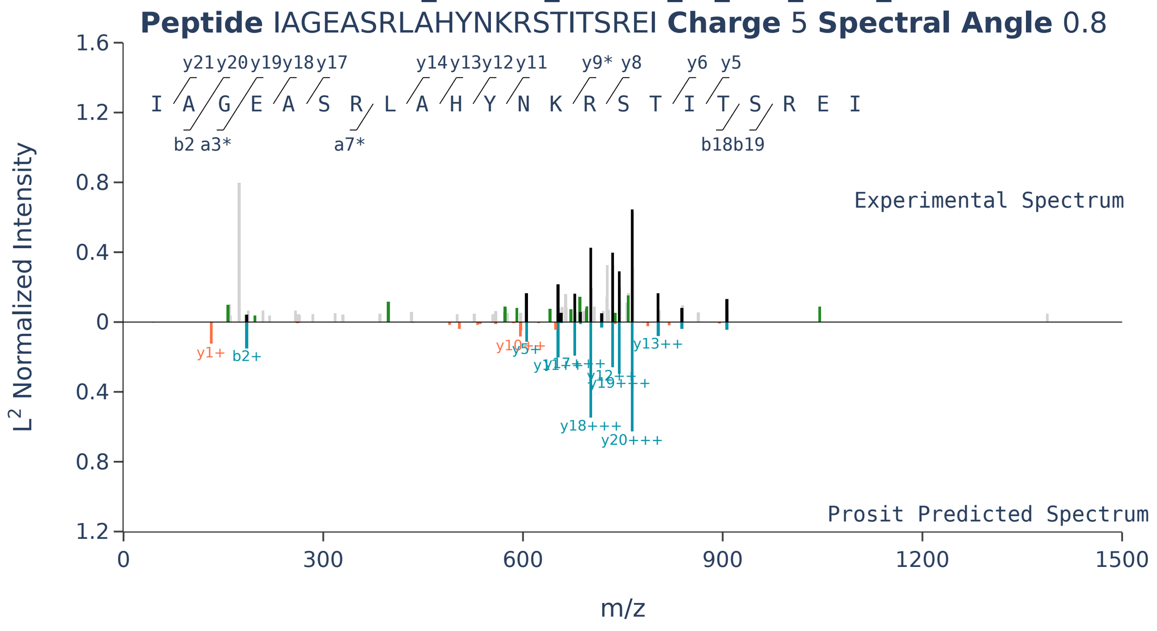


**Source** HRoetschke_291222_080123_Fu_H2B_b1R2_4h **Scan** 13218
**Peptide** RLLLPGELAKHAV **Charge** 3 **Spectral Angle** 0.81

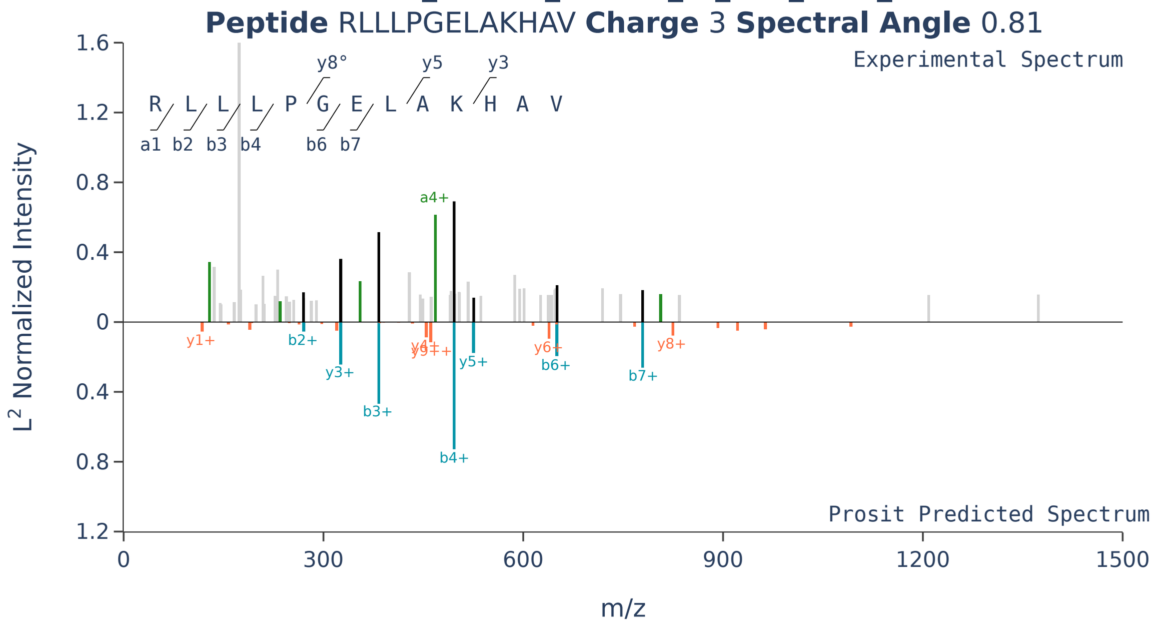


**Source** HRoetschke_291222_080123_Fu_H2B_b1R2_24h **Scan** 4400
**Peptide** RLAHYNKRSTITSREL **Charge** 3 **Spectral Angle** 0.77

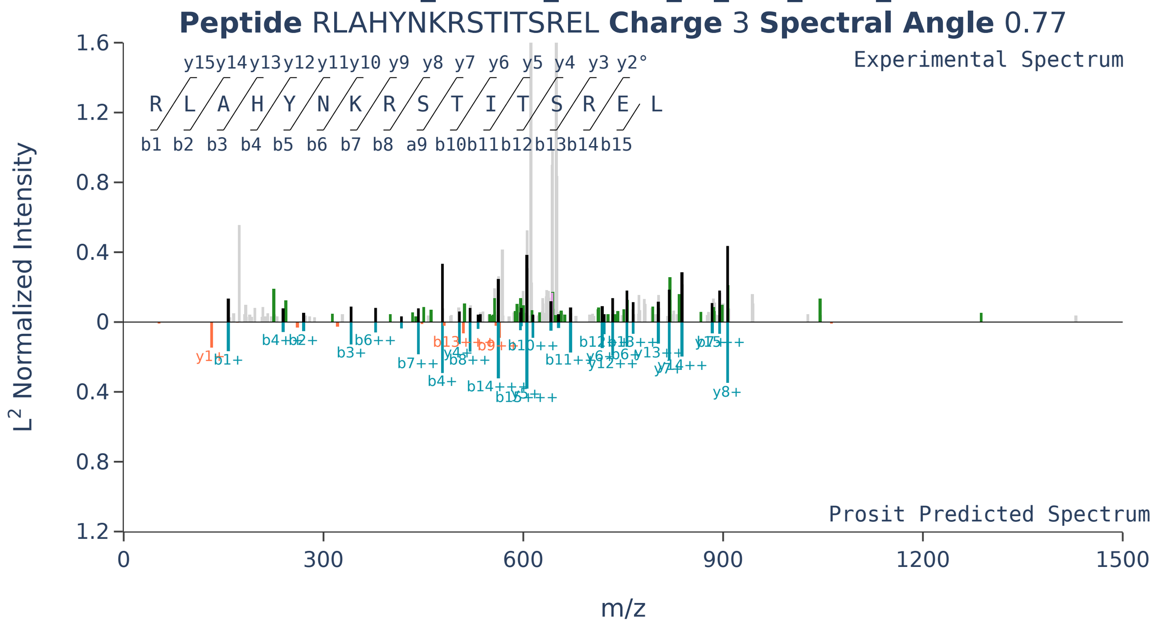


**Source** HRoetschke_291222_080123_Fu_H2B_b1R1_24h **Scan** 7980
**Peptide** SRLAHYNKRSTITSREIQTAV **Charge** 4 **Spectral Angle** 0.74

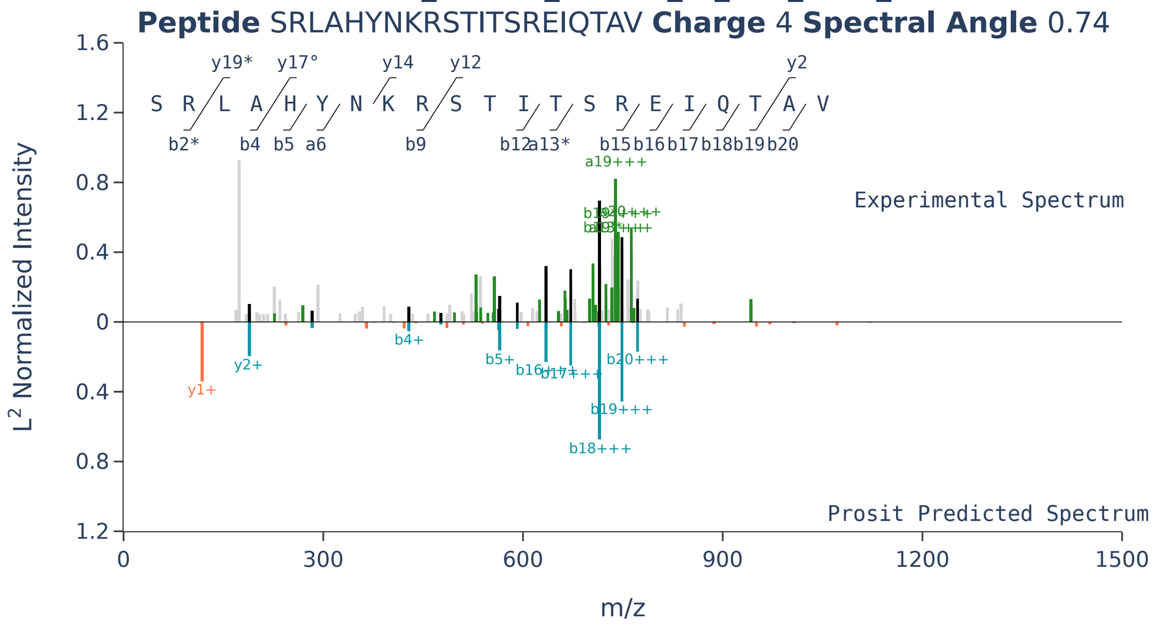


**Source** HRoetschke_291222_080123_Fu_H2B_b2R1_24h **Scan** 19361
**Peptide** SFVNDVF **Charge** 2 **Spectral Angle** 0.93

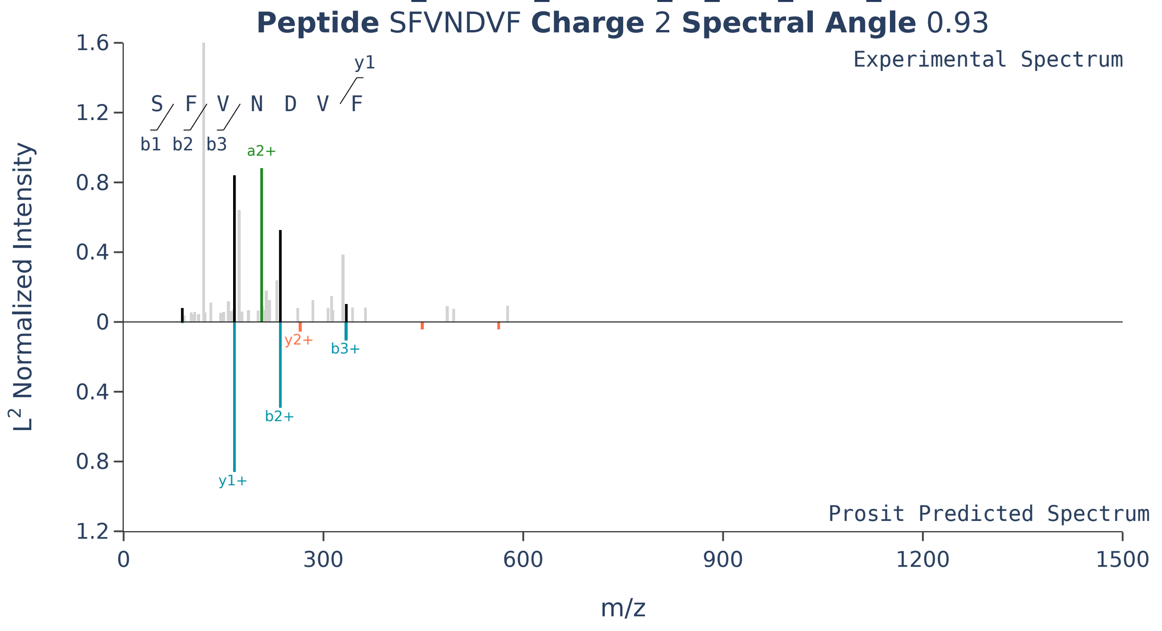


**Source** HRoetschke_291222_080123_Fu_H2B_b2R1_24h **Scan** 2499
**Peptide** SRLAHYNKRSTITS **Charge** 3 **Spectral Angle** 0.68

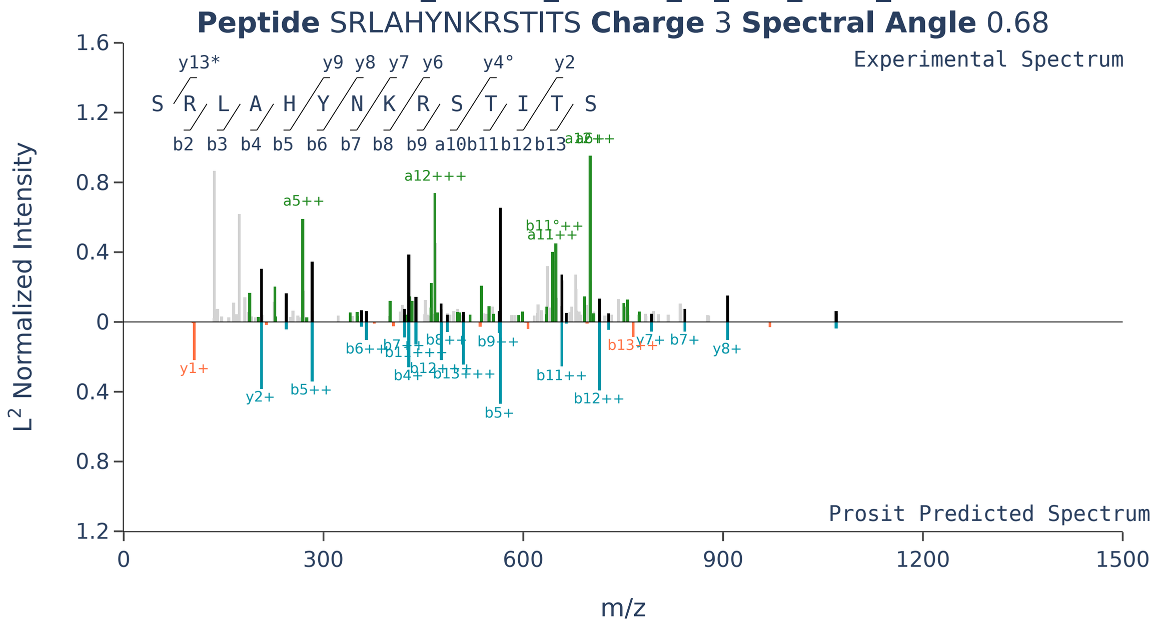

**Source** HRoetschke_291222_080123_Fu_H2B_b2R2_4h **Scan** 15732
**Peptide** AVRLLLPGELAKHA **Charge** 4 **Spectral Angle** 0.69

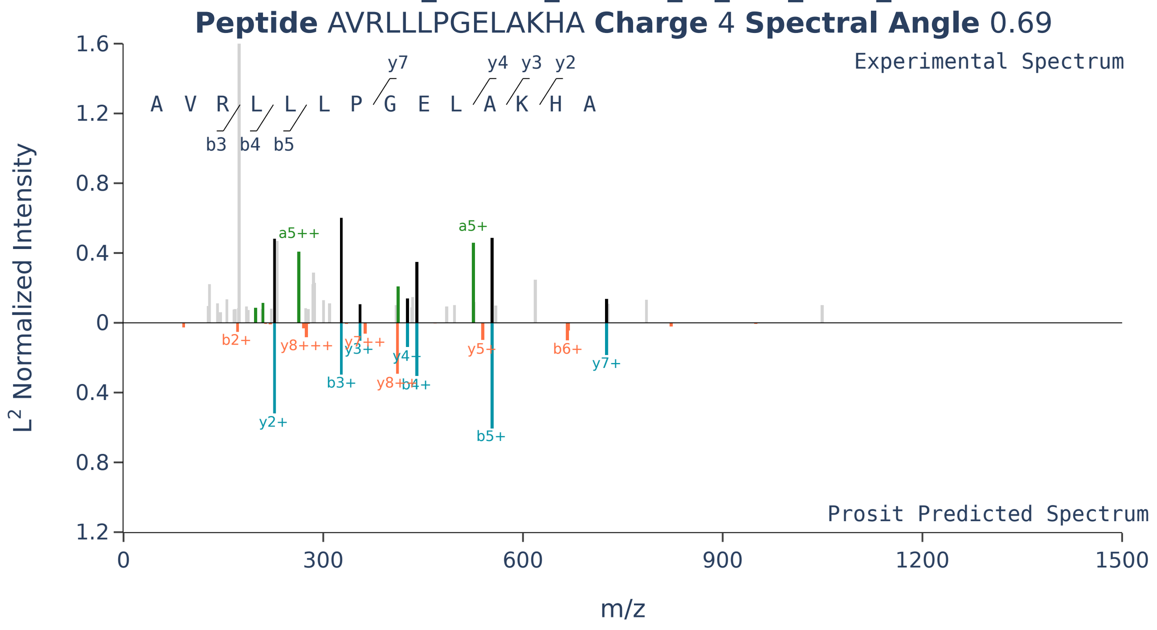


**Source** HRoetschke_291222_080123_Fu_H2B_b1R1_24h **Scan** 4314
**Peptide** LAKHAVSEGTKAVTKY **Charge** 2 **Spectral Angle** 0.69

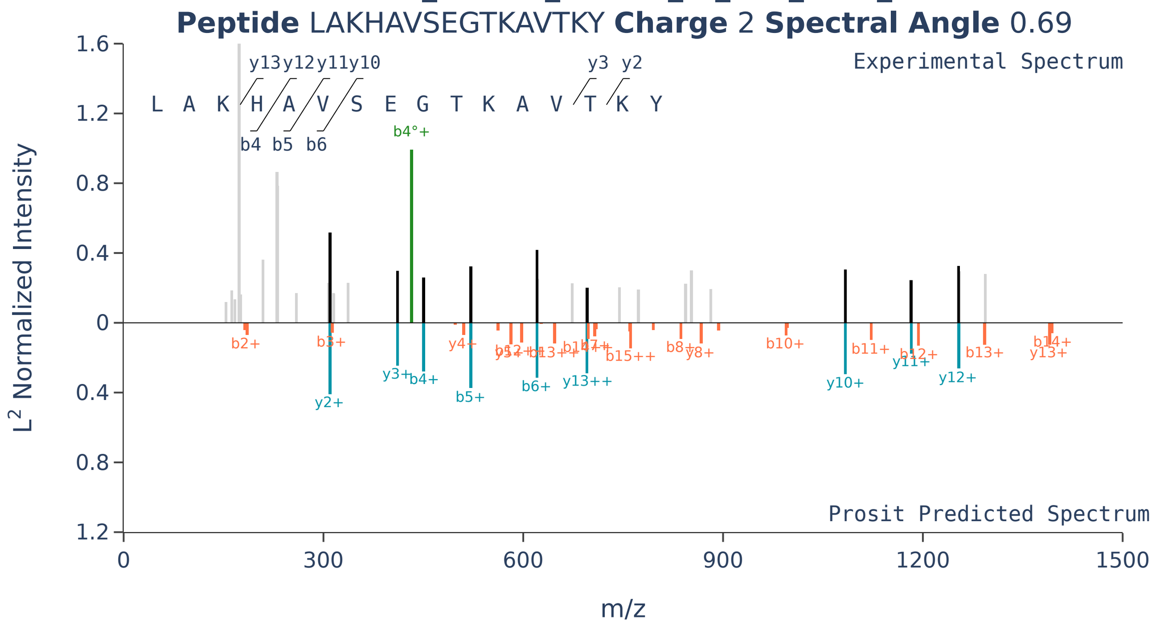


**Source** HRoetschke_291222_080123_Fu_H2B_b2R1_1h **Scan** 2102
**Peptide** EASRLAHYNK **Charge** 2 **Spectral Angle** 0.7

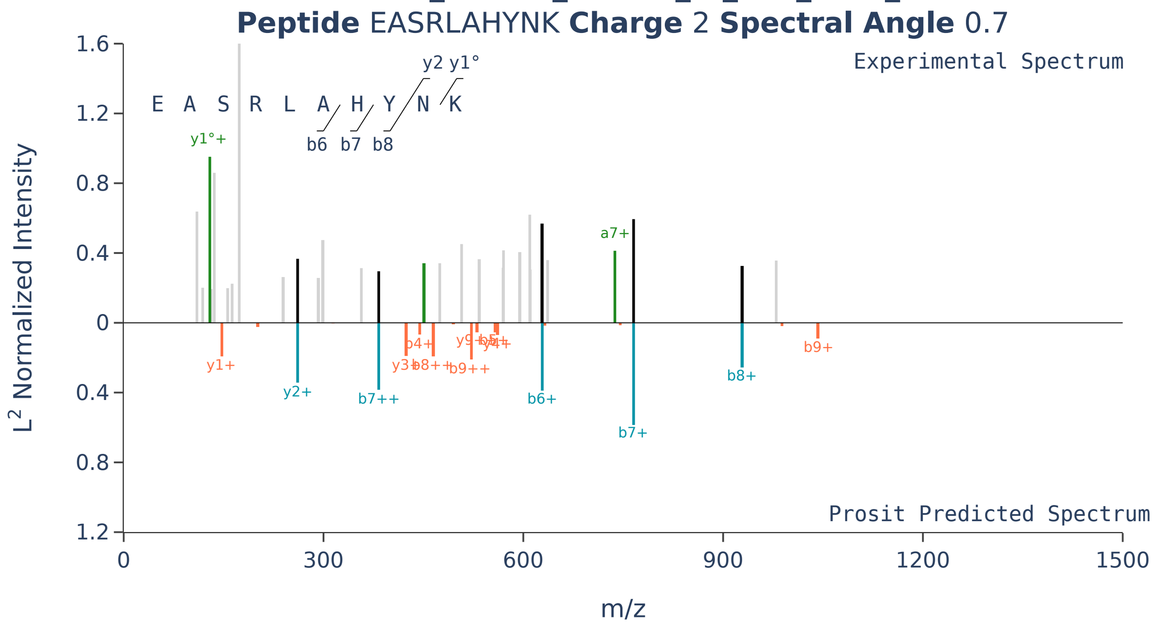


**Source** HRoetschke_291222_080123_Fu_H2B_b2R2_24h **Scan** 9377
**Peptide** PGELAKHAVSEGTKAVTKY **Charge** 3 **Spectral Angle** 0.69

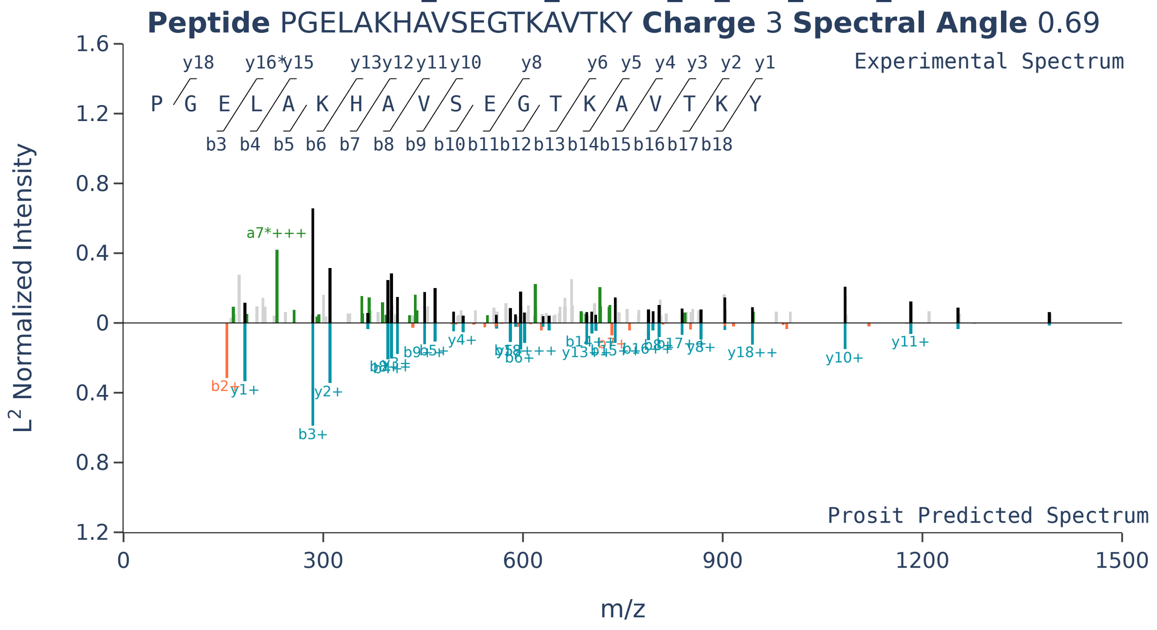


**Source** HRoetschke_291222_080123_Fu_H2B_b2R2_4h **Scan** 18242
**Peptide** VNDVFERIAGEASRLAHYNKRSTITSR **Charge** 6 **Spectral Angle** 0.59

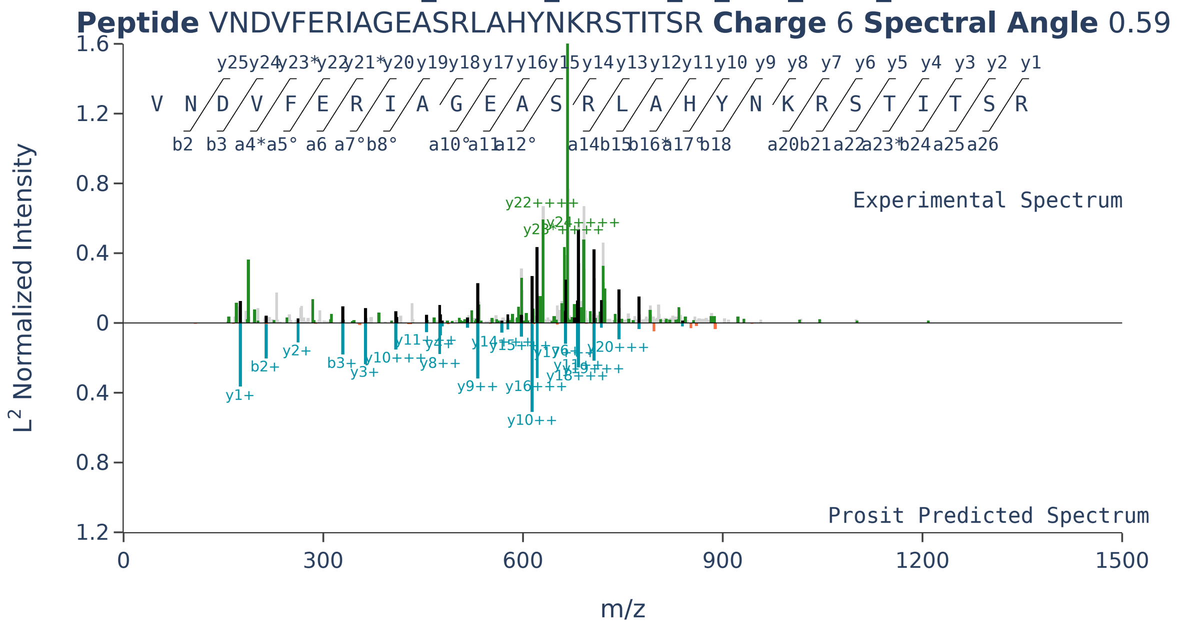


**Source** HRoetschke_291222_080123_Fu_H2B_b2R1_24h **Scan** 15213
**Peptide** AIYVYKVLKQVHPDTGISSKAM **Charge** 4 **Spectral Angle** 0.63

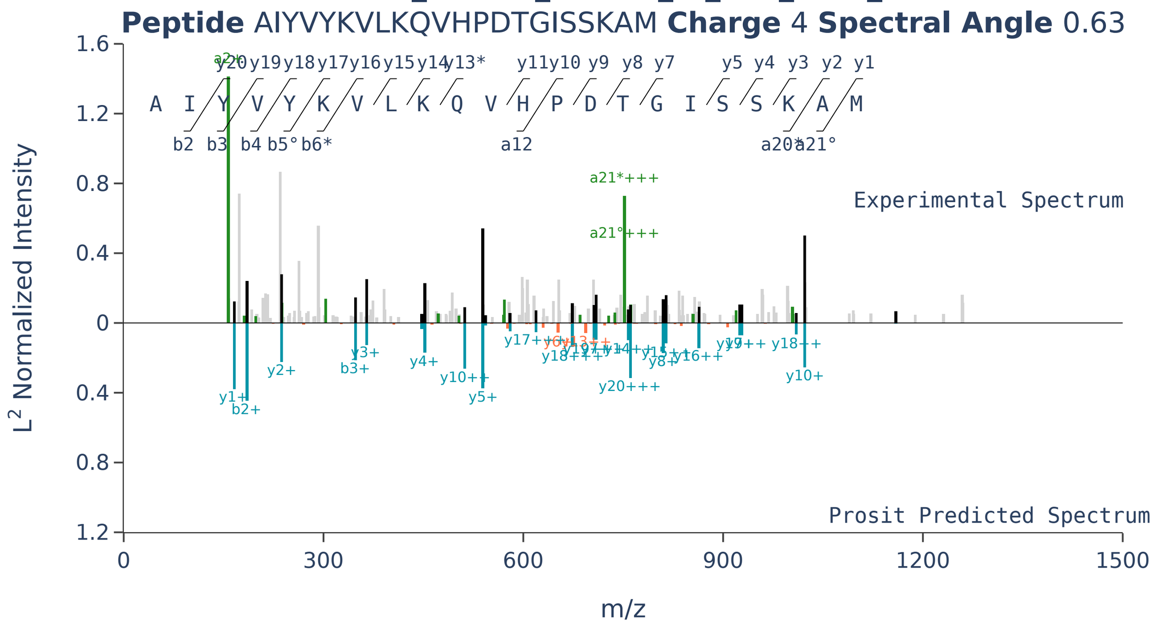


**Source** HRoetschke_291222_080123_Fu_H2B_b1R1_4h **Scan** 4904
**Peptide** TITSREIQ **Charge** 2 **Spectral Angle** 0.91

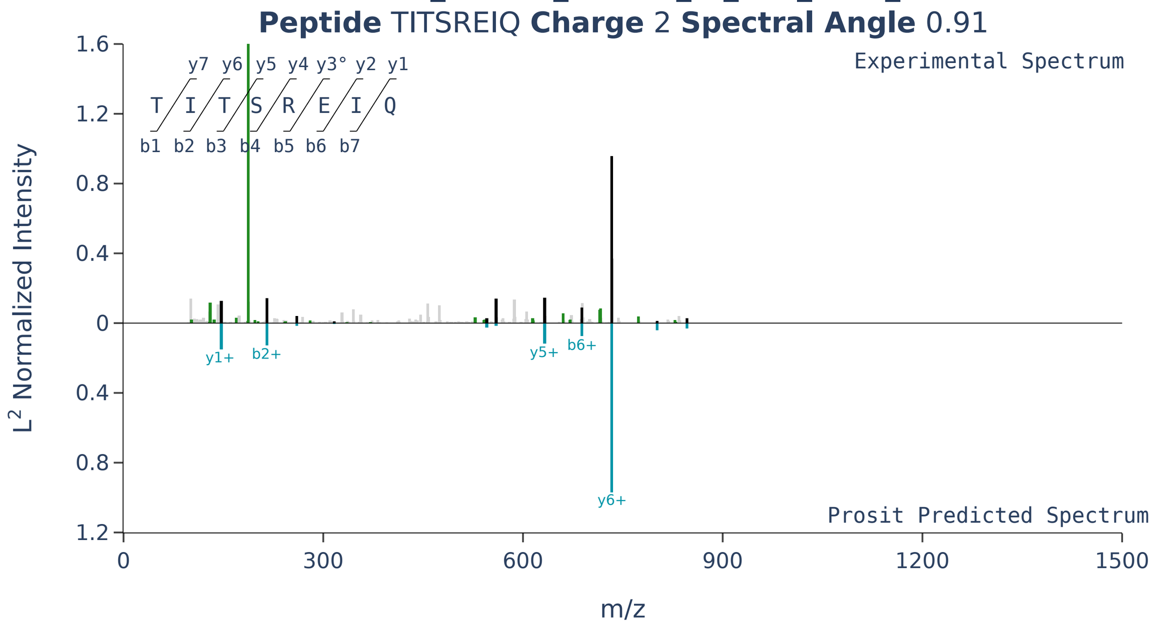


**Source** HRoetschke_291222_080123_Fu_H2B_b2R1_24h **Scan** 17891
**Peptide** QTAVRLLLPGELAKHAVSEGTKAVTKY **Charge** 5 **Spectral Angle** 0.67

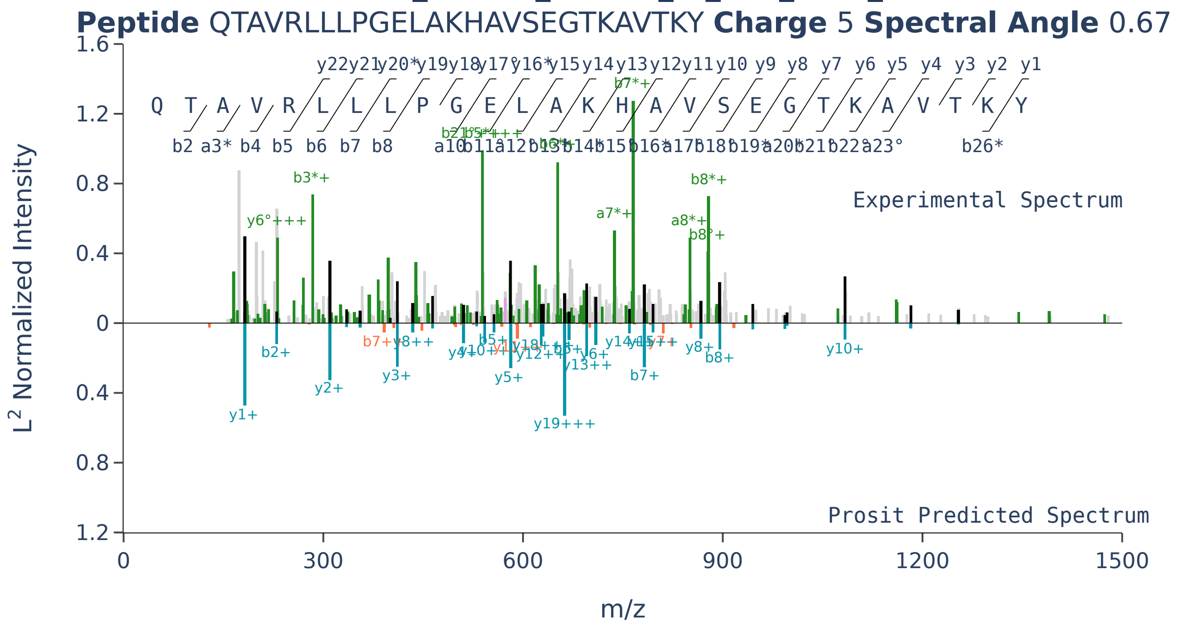


**Source** HRoetschke_291222_080123_Fu_H2B_b1R1_24h **Scan** 6718
**Peptide** GEASRLAHYNKRSTITSREIQTA **Charge** 4 **Spectral Angle** 0.69

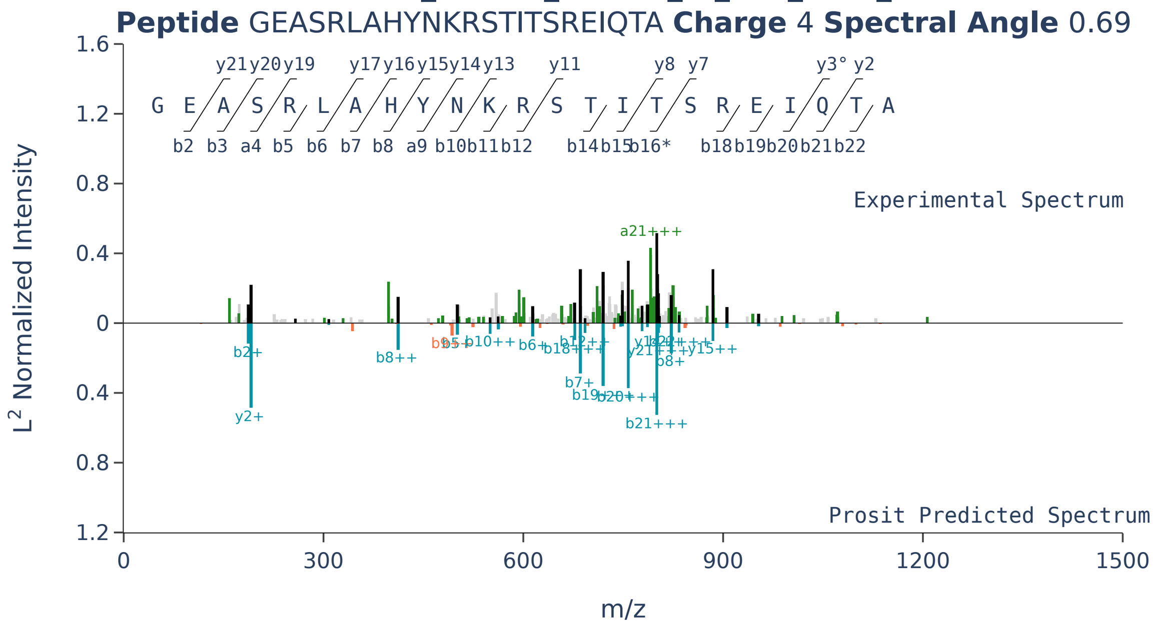


**Source** HRoetschke_291222_080123_Fu_H2B_b2R2_4h **Scan** 19918
**Peptide** VNDVFERIAGEASRLAHYNK **Charge** 4 **Spectral Angle** 0.57

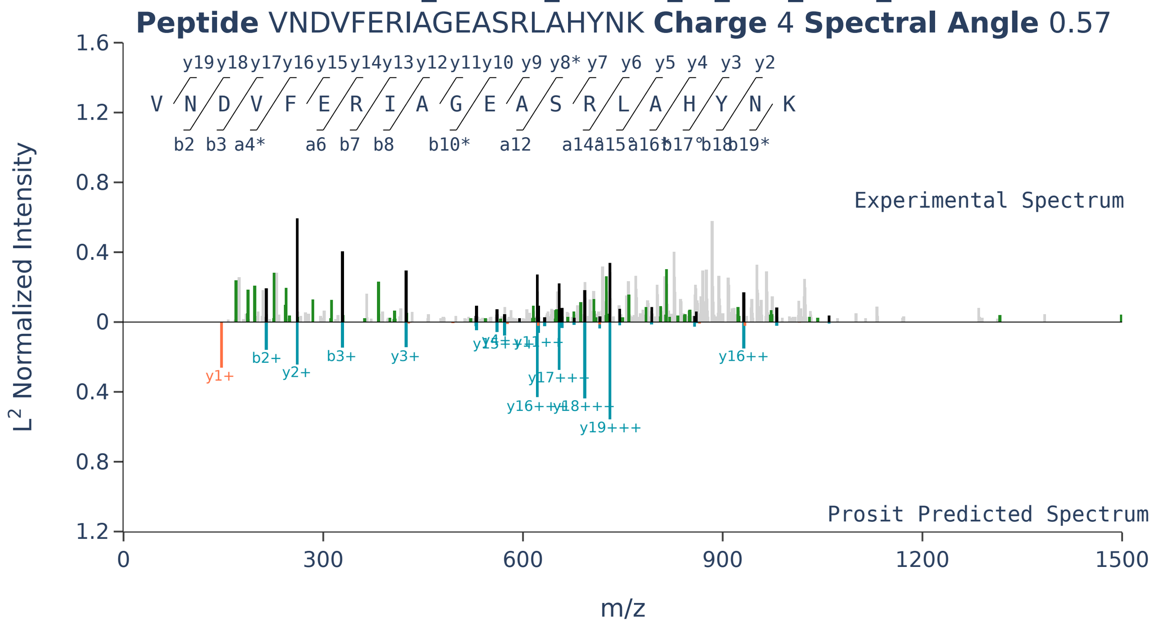


**Source** HRoetschke_291222_080123_Fu_H2B_b2R1_4h **Scan** 3829
**Peptide** NKRSTITSREIQTA **Charge** 4 **Spectral Angle** 0.52

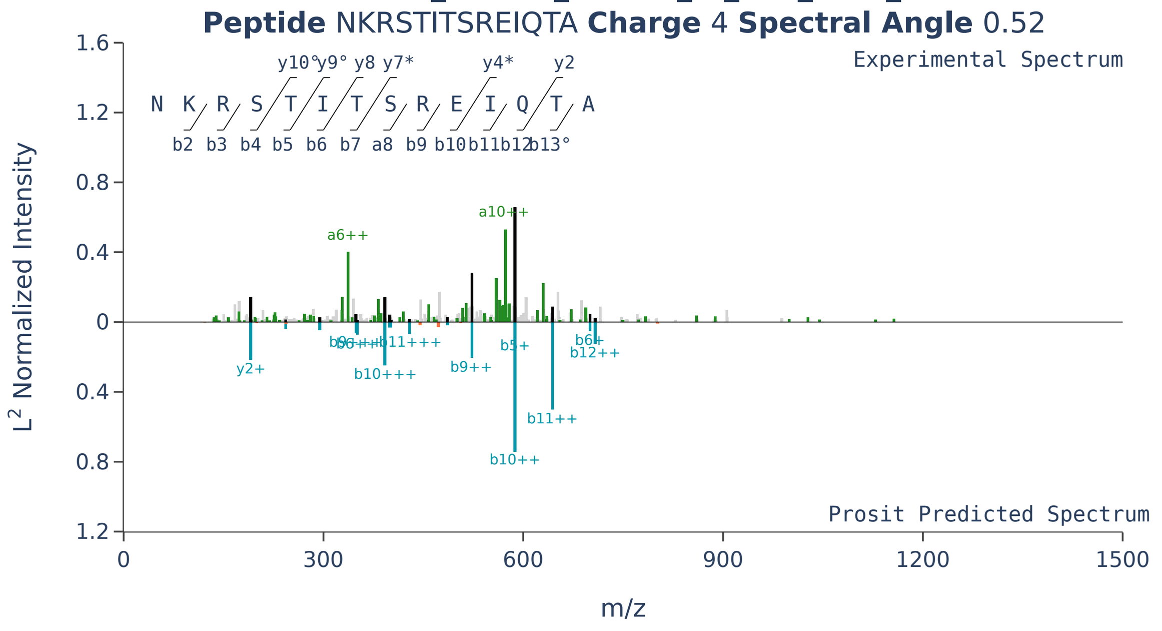


**Source** HRoetschke_291222_080123_Fu_H2B_b2R1_4h **Scan** 15974
**Peptide** QTAVRLLLPGELAKHAVSEGTKAVTK **Charge** 4 **Spectral Angle** 0.74

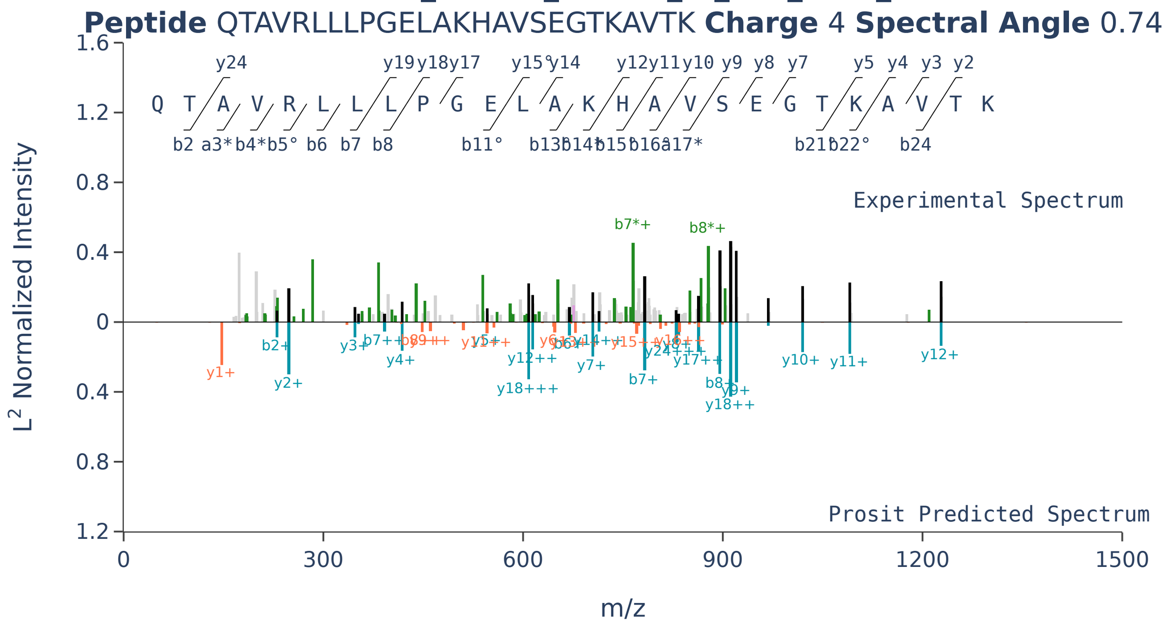


**Source** HRoetschke_291222_080123_Fu_H2B_b1R2_1h **Scan** 14588
**Peptide** REIQTAVRLLLPGELAKHAV **Charge** 4 **Spectral Angle** 0.8

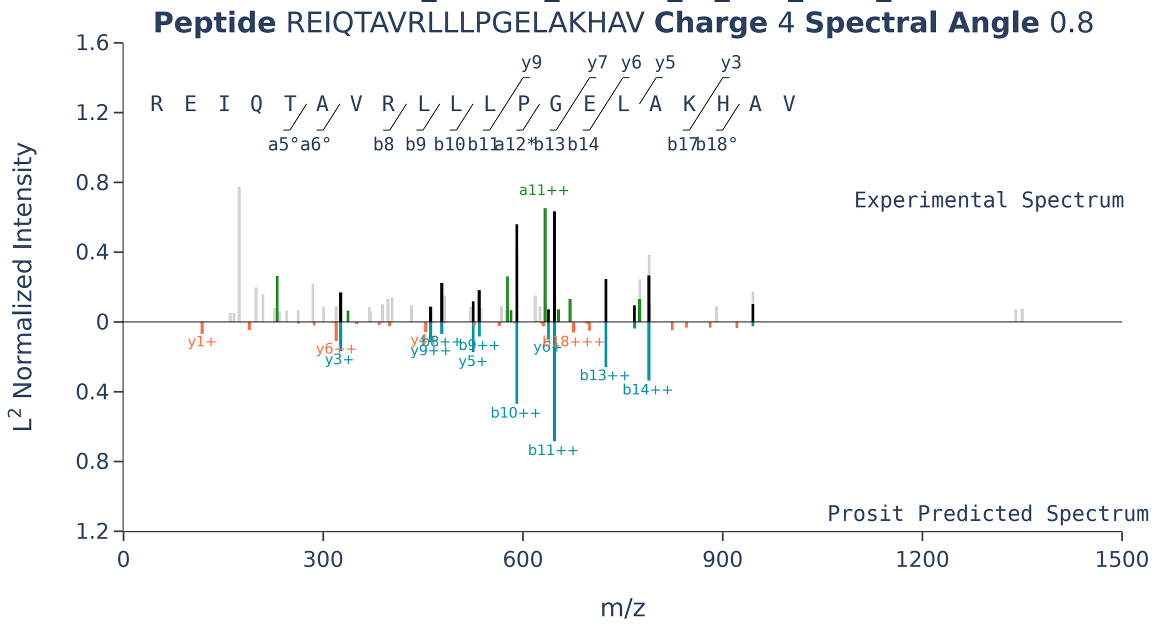


**Source** HRoetschke_291222_080123_Fu_H2B_b1R2_24h **Scan** 6072
**Peptide** KQVHPDTGISSKAMSIM **Charge** 3 **Spectral Angle** 0.71

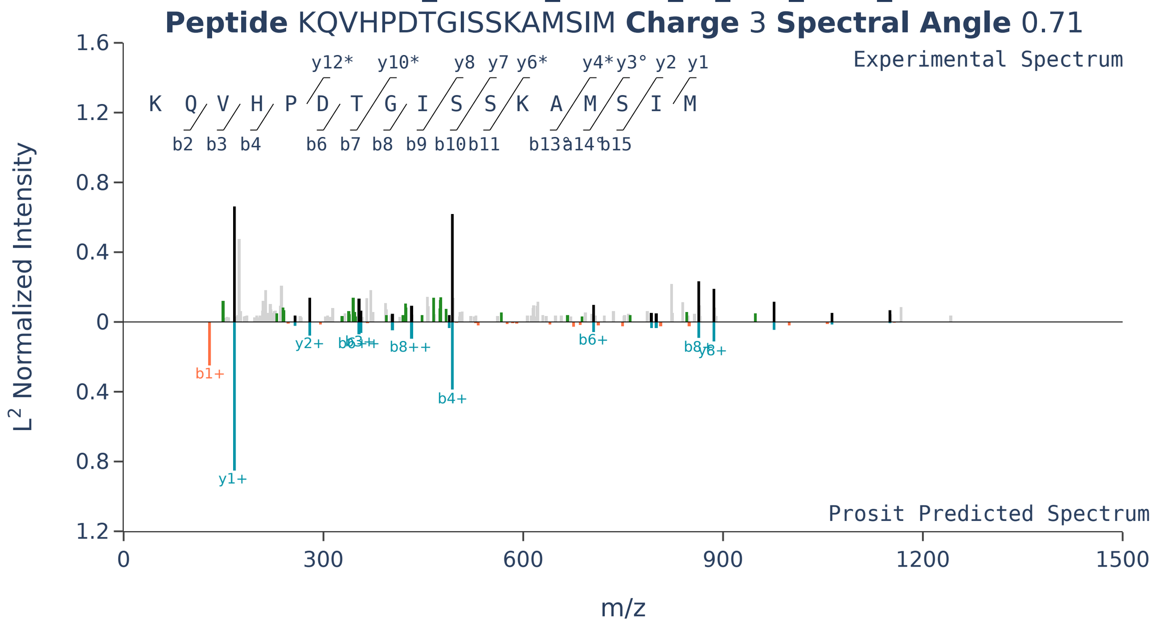


**Source** HRoetschke_291222_080123_Fu_H2B_b2R1_2h **Scan** 9715
**Peptide** PGELAKHAVSEGTKAVTKYTSAK **Charge** 3 **Spectral Angle** 0.72

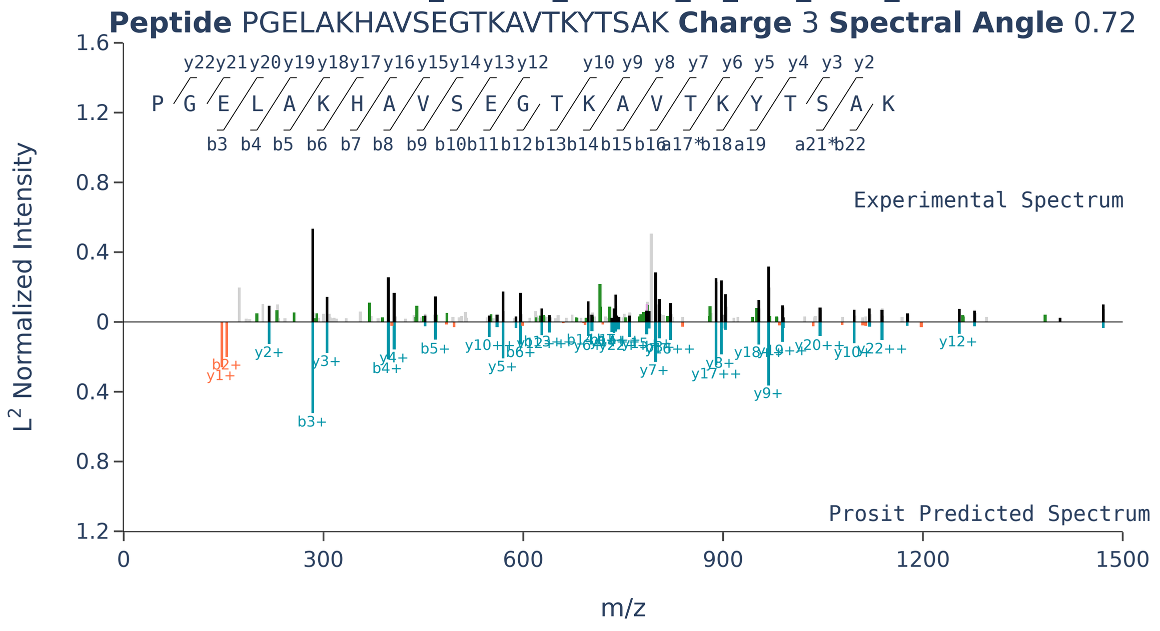

**Source** HRoetschke_291222_080123_Fu_H2B_b2R1_4h **Scan** 10844
**Peptide** KTRKESYAIYVY **Charge** 3 **Spectral Angle** 0.55

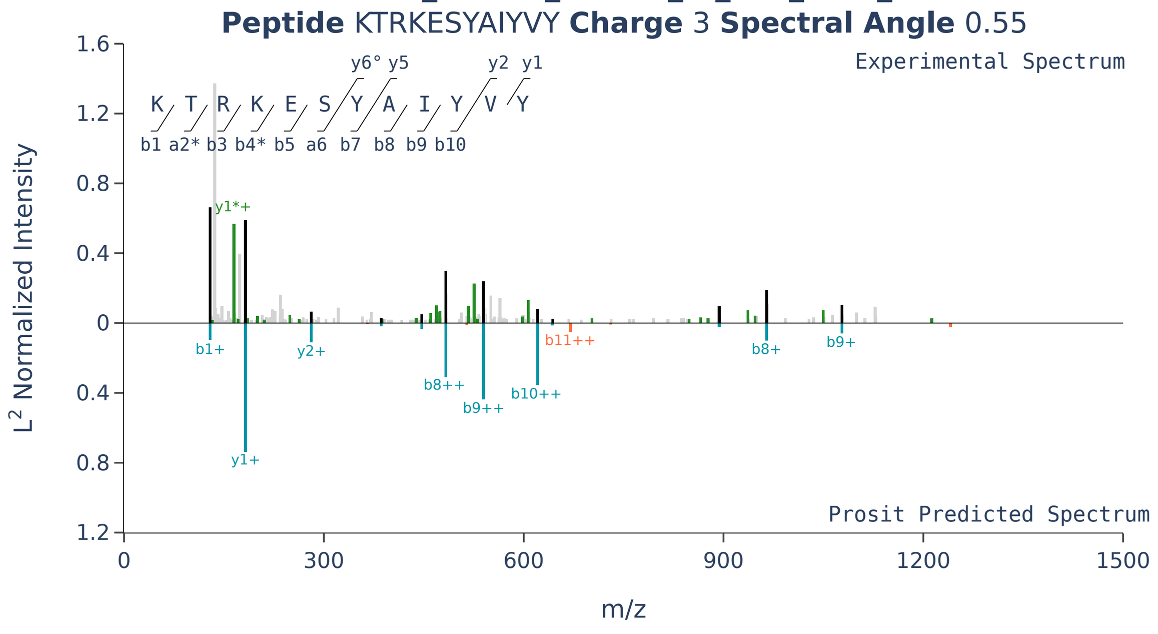

**Source** HRoetschke_291222_080123_Fu_H2B_b1R2_24h **Scan** 5315
**Peptide** LAHYNKRSTITSREIQT **Charge** 3 **Spectral Angle** 0.79

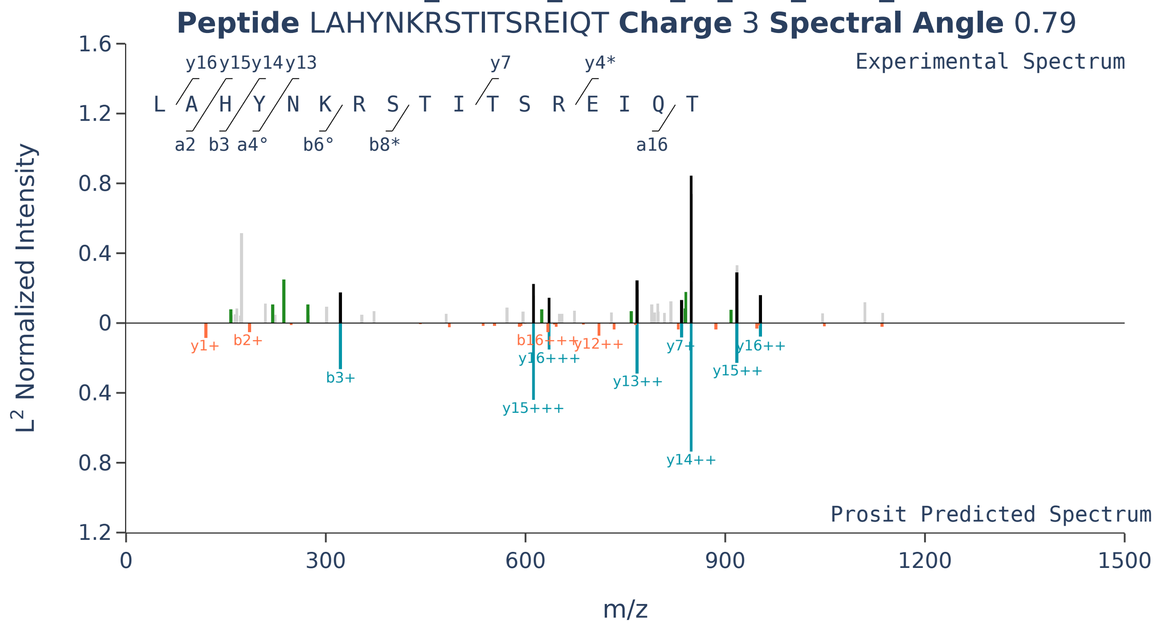

**Source** HRoetschke_291222_080123_Fu_H2B_b1R1_1h **Scan** 6373
**Peptide** NKRSTITSREIQTAV **Charge** 3 **Spectral Angle** 0.68

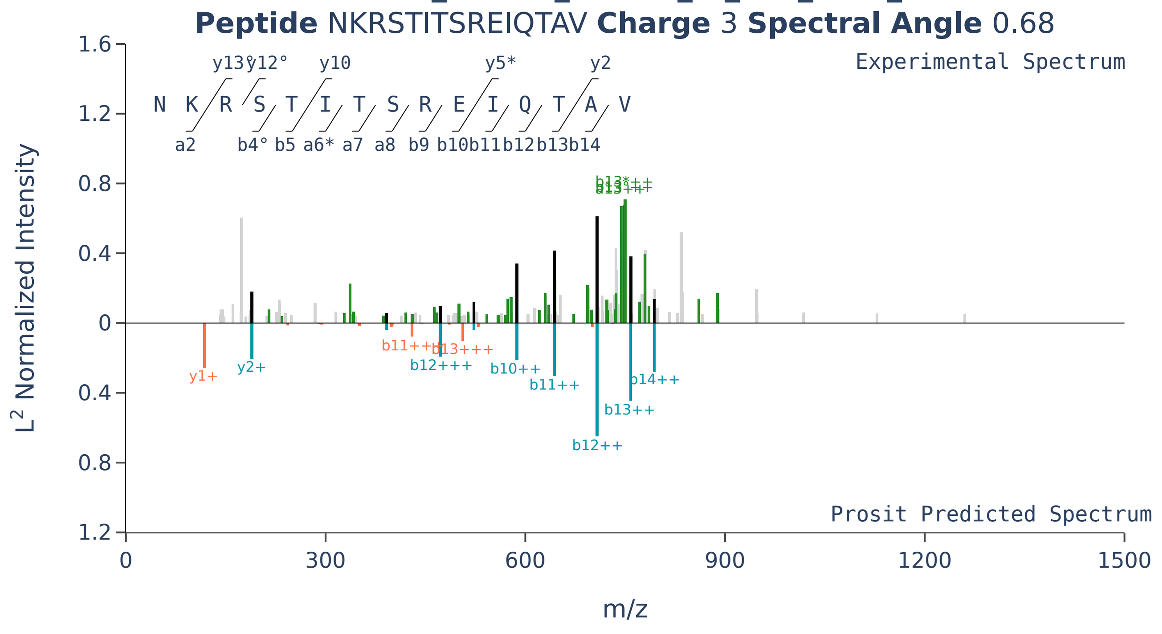

**Source** HRoetschke_291222_080123_Fu_H2B_b2R2_24h **Scan** 16529
**Peptide** VRLLLPGELAKHAVSEGTKAVTK **Charge** 5 **Spectral Angle** 0.81

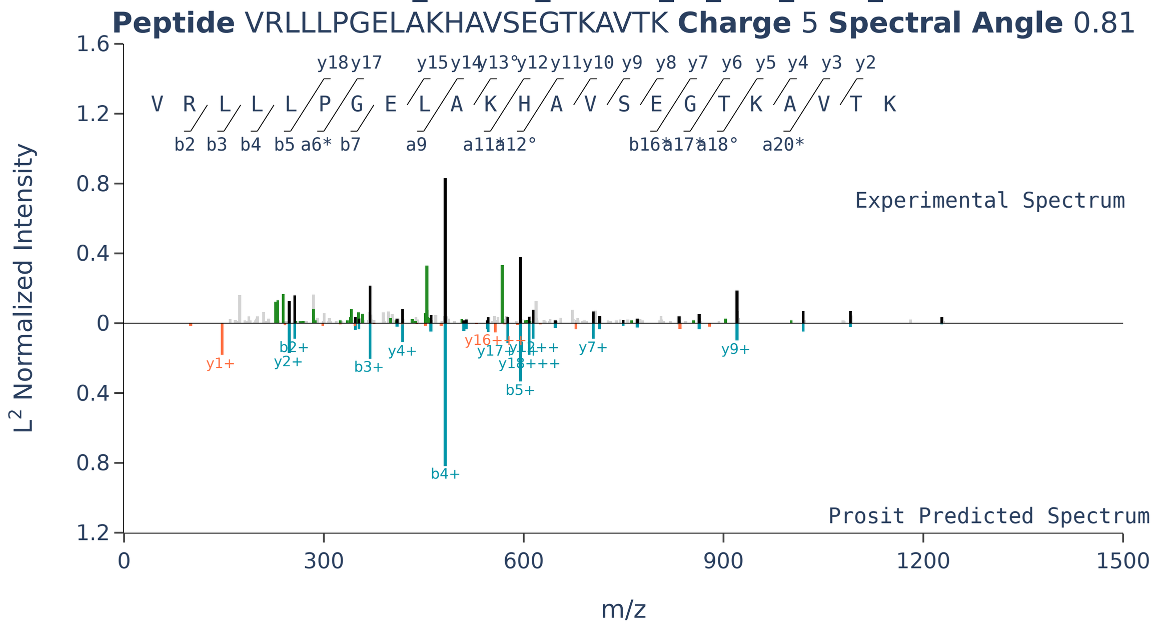

**Source** HRoetschke_291222_080123_Fu_H2B_b1R2_24h **Scan** 6463
**Peptide** ASRLAHYN **Charge** 2 **Spectral Angle** 0.79

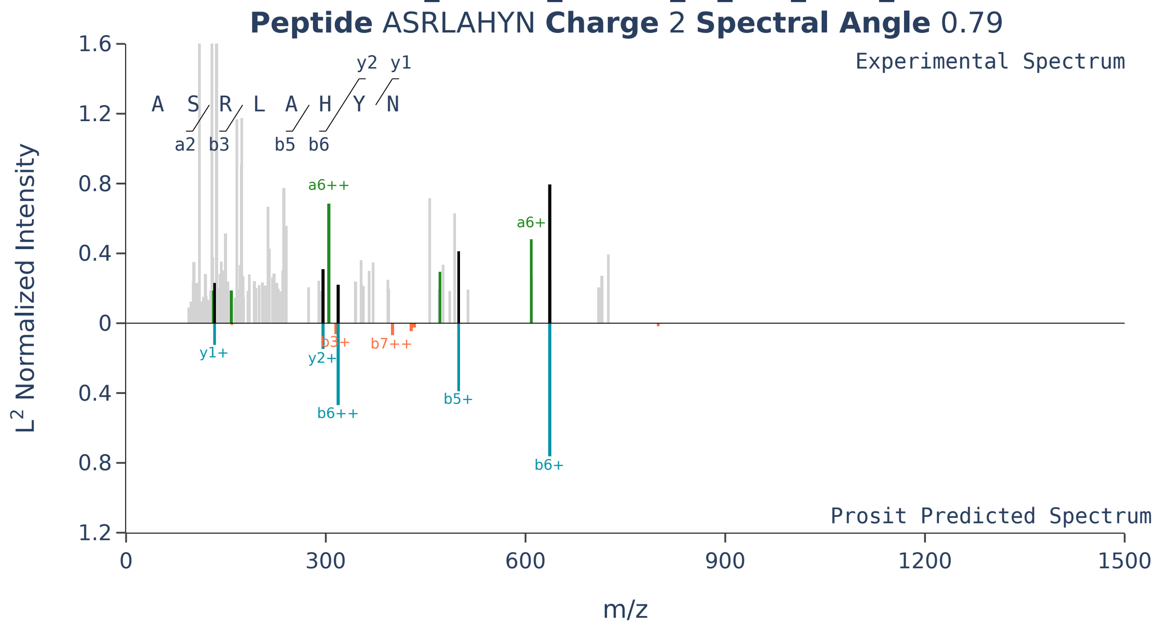

**Source** HRoetschke_291222_080123_Fu_H2B_b2R1_24h **Scan** 13530
**Peptide** LPGELAKHAVSEGTKAVTKYTS **Charge** 3 **Spectral Angle** 0.72

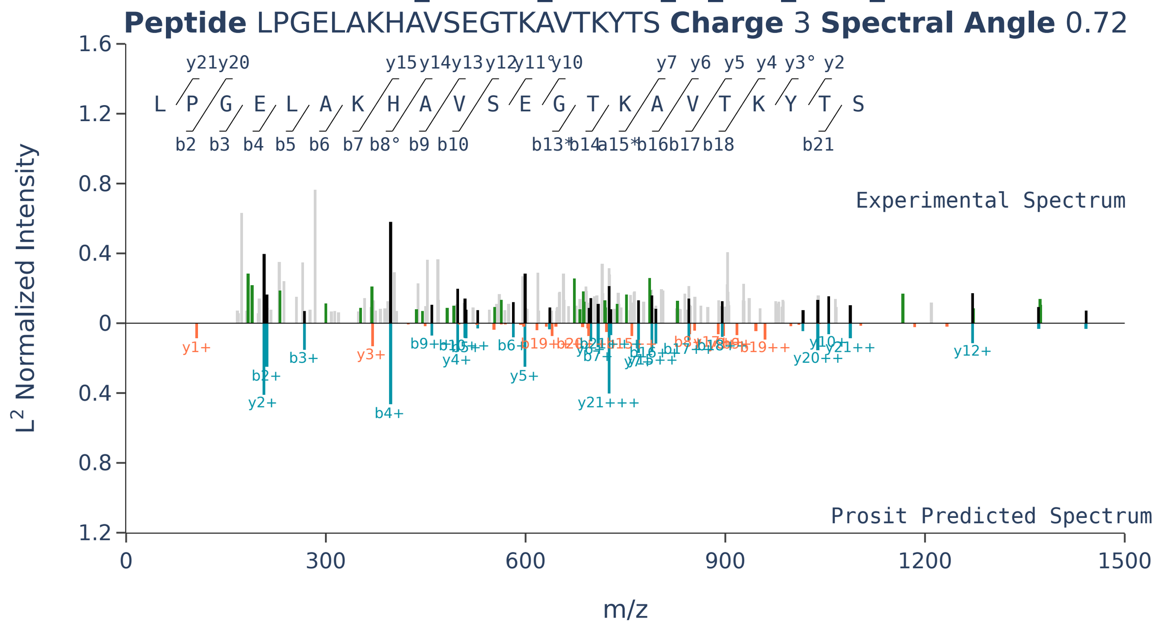

**Source** HRoetschke_291222_080123_Fu_H2B_b2R2_4h **Scan** 21675
**Peptide** STITSREIQTAVRLLLPGELAKH **Charge** 4 **Spectral Angle** 0.7

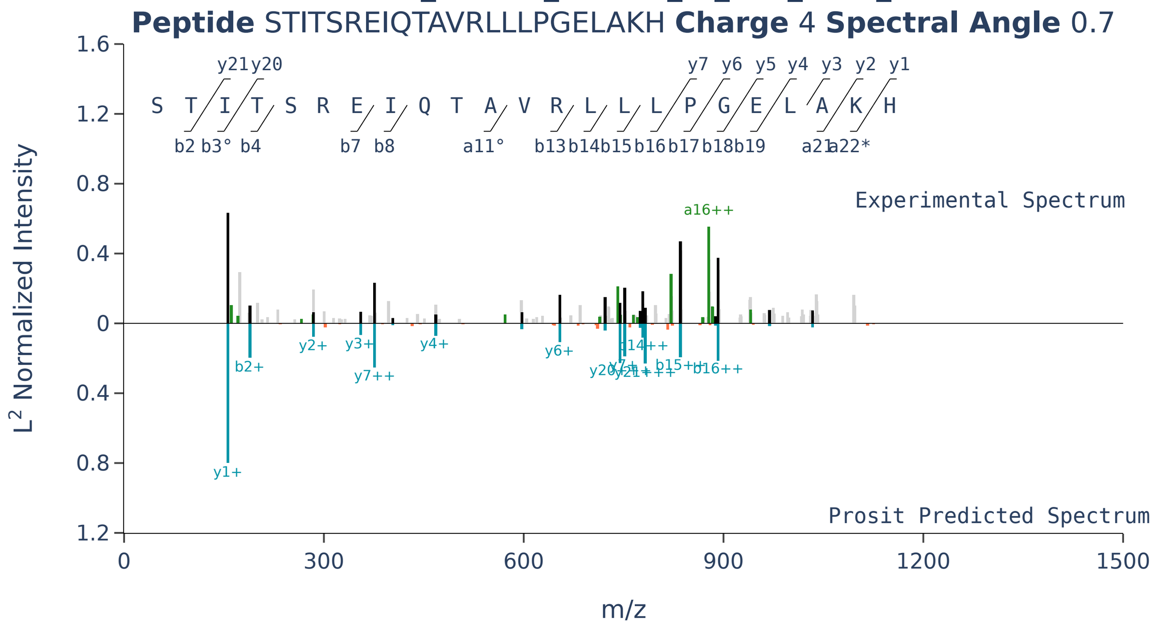

**Source** HRoetschke_291222_080123_Fu_H2B_b2R2_2h **Scan** 19129
**Peptide** REIQTAVRLLLPGEL **Charge** 3 **Spectral Angle** 0.69

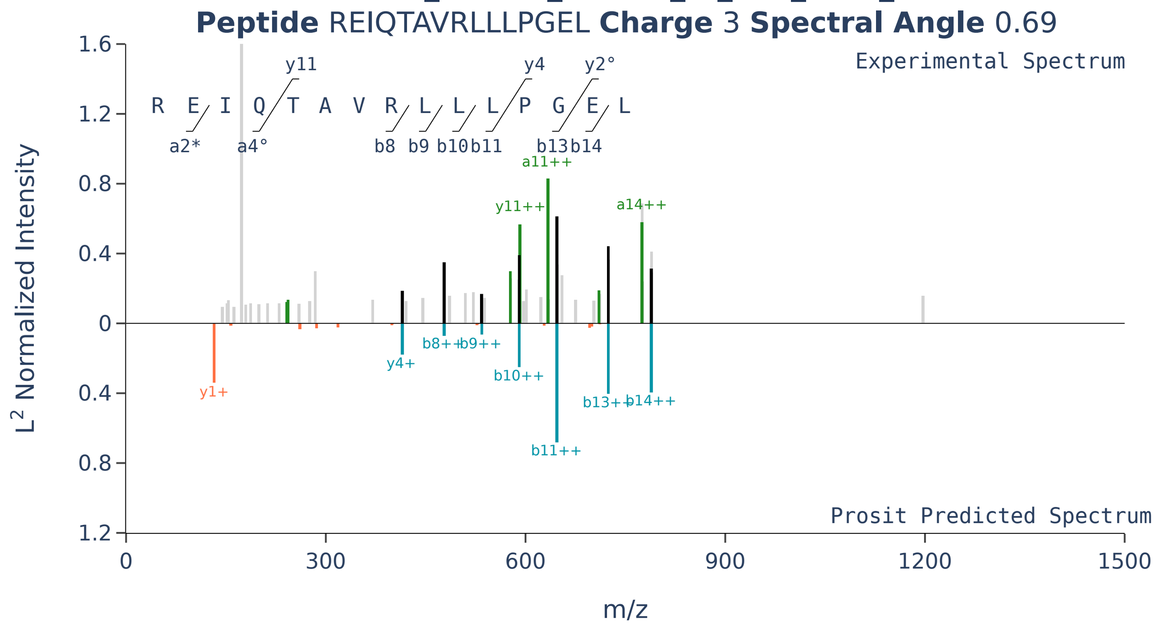

**Source** HRoetschke_291222_080123_Fu_H2B_b1R2_1h **Scan** 6958
**Peptide** ERIAGEASRLAHYNKRSTL **Charge** 4 **Spectral Angle** 0.78

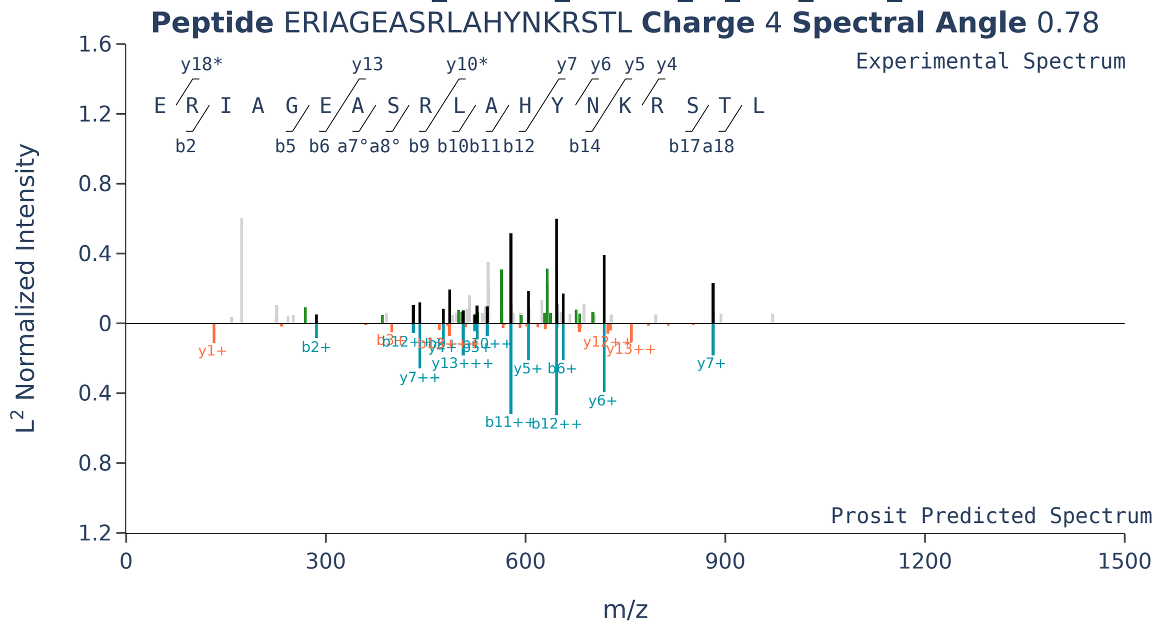

**Source** HRoetschke_291222_080123_Fu_H2B_b2R2_4h **Scan** 12723
**Peptide** HPDTGISSKAMSIMNSFVND **Charge** 3 **Spectral Angle** 0.83

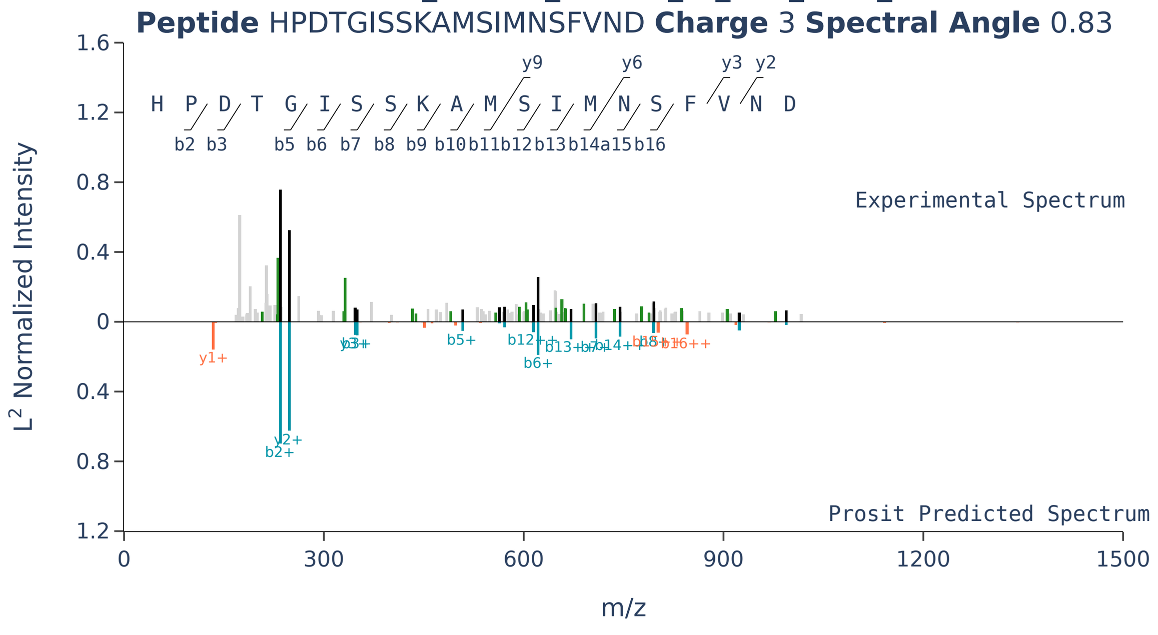

**Source** HRoetschke_291222_080123_Fu_H2B_b2R1_4h **Scan** 16146
**Peptide** QTAVRLLLPGELAKHA **Charge** 4 **Spectral Angle** 0.74

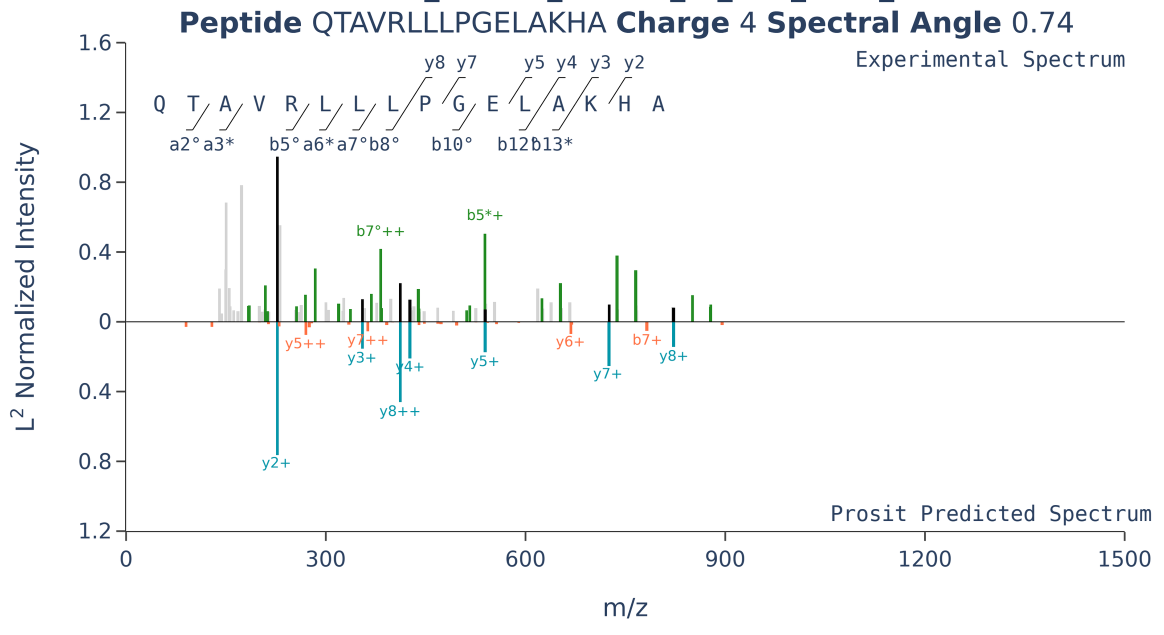

**Source** HRoetschke_291222_080123_Fu_H2B_b2R1_24h **Scan** 16193
**Peptide** YNKRSTITSREIQTAVRLL **Charge** 4 **Spectral Angle** 0.85

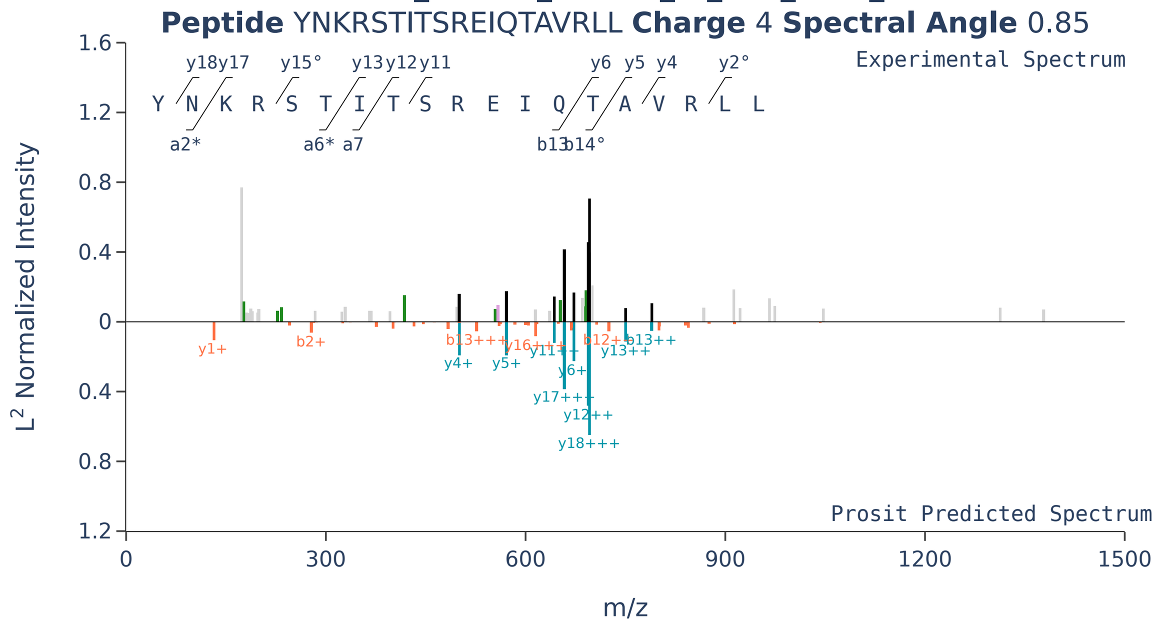

**Source** HRoetschke_291222_080123_Fu_H2B_b1R1_2h **Scan** 19837
**Peptide** NSFVNDVFERIAGEASRLAHYNKR **Charge** 4 **Spectral Angle** 0.84

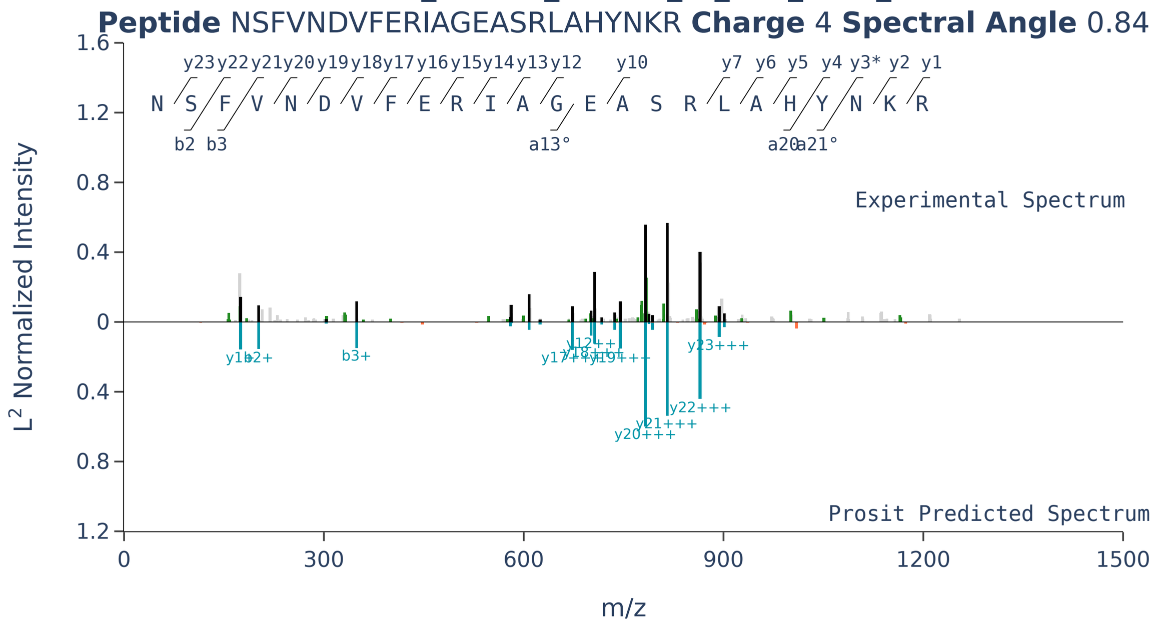

**Source** HRoetschke_291222_080123_Fu_H2B_b1R1_2h **Scan** 3520
**Peptide** KRSTITSREI **Charge** 3 **Spectral Angle** 0.74

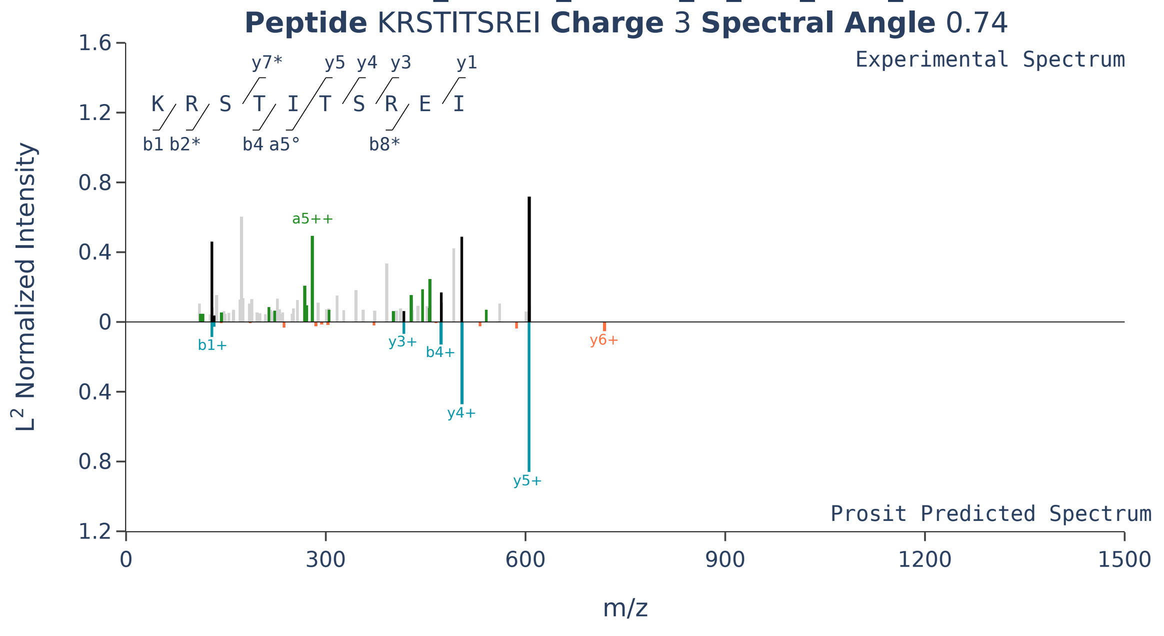

**Source** HRoetschke_291222_080123_Fu_H2B_b2R2_4h **Scan** 21509
**Peptide** KAMSIMNSFVNDVFERIAGEASR **Charge** 4 **Spectral Angle** 0.79

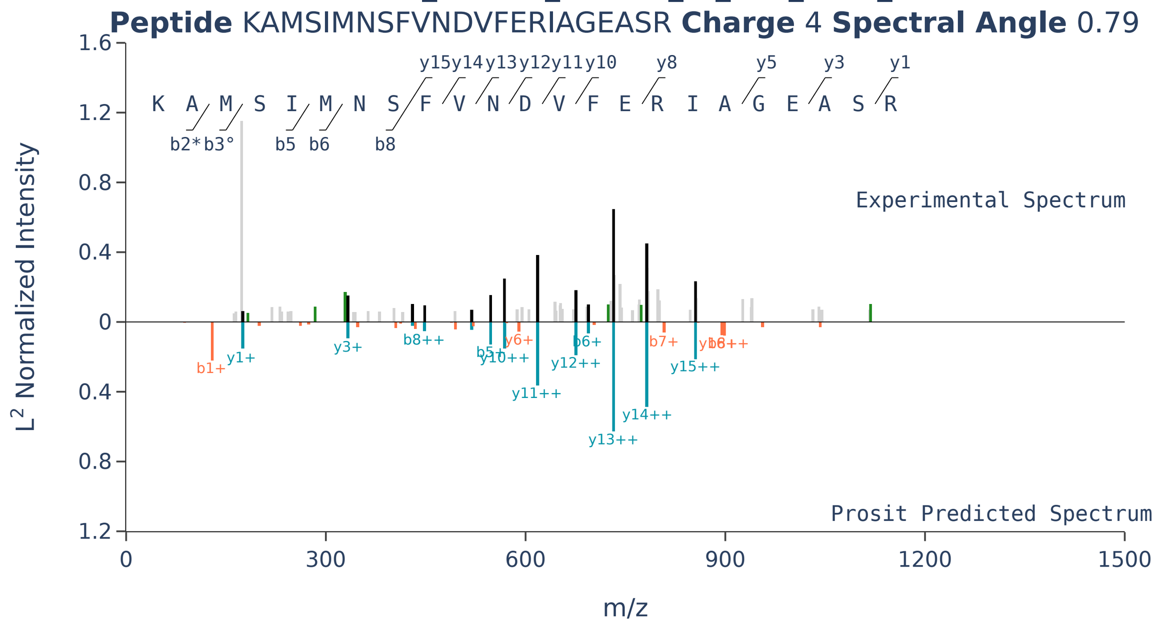

**Source** HRoetschke_291222_080123_Fu_H2B_b2R1_1h **Scan** 19526
**Peptide** STITSREIQTAVRLLLPGE **Charge** 3 **Spectral Angle** 0.69

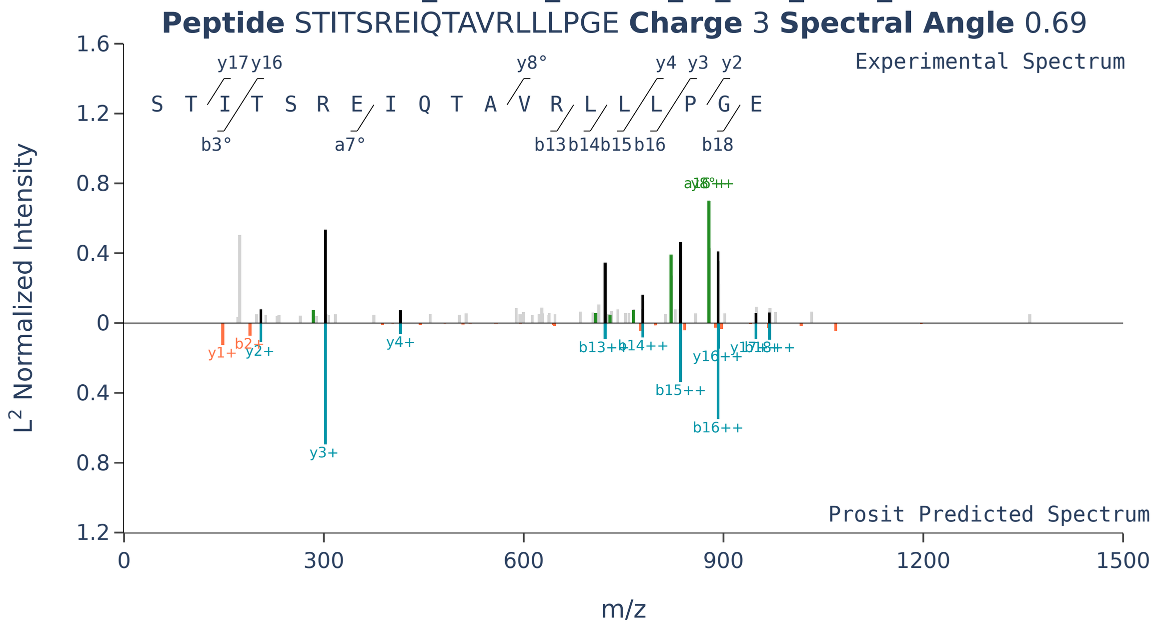


**Source** HRoetschke_291222_080123_Fu_H2B_b2R1_24h **Scan** 7915
**Peptide** VYKVLKQVHPDTGISSK **Charge** 3 **Spectral Angle** 0.7

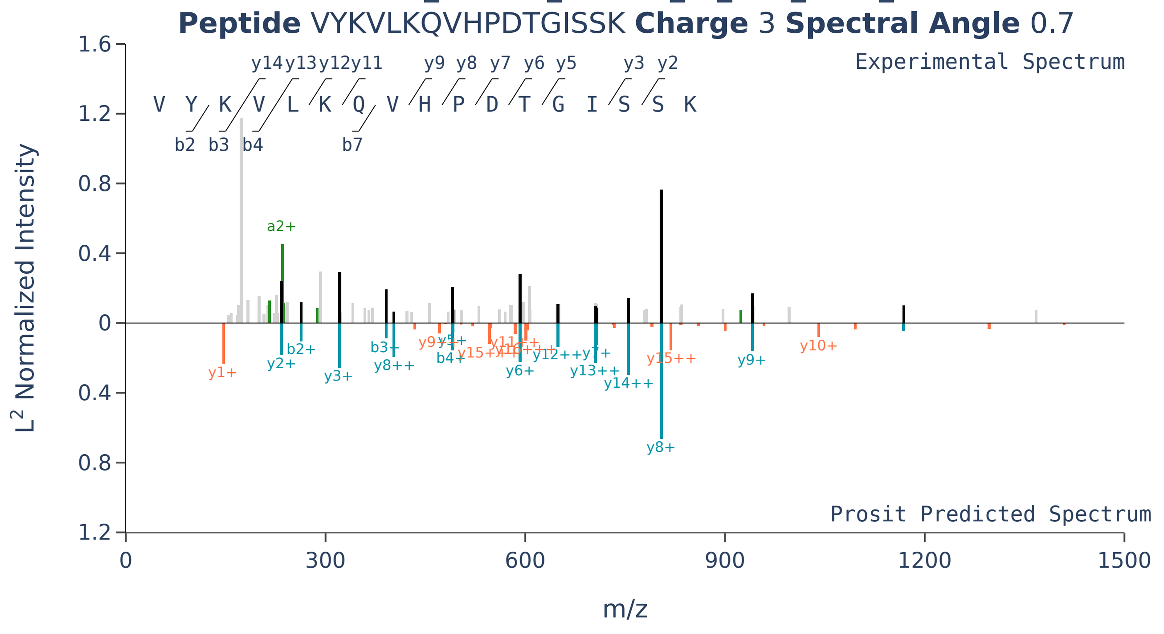


**Source** HRoetschke_291222_080123_Fu_H2B_b2R1_2h **Scan** 10847
**Peptide** QVHPDTGISSKAMSI **Charge** 3 **Spectral Angle** 0.73

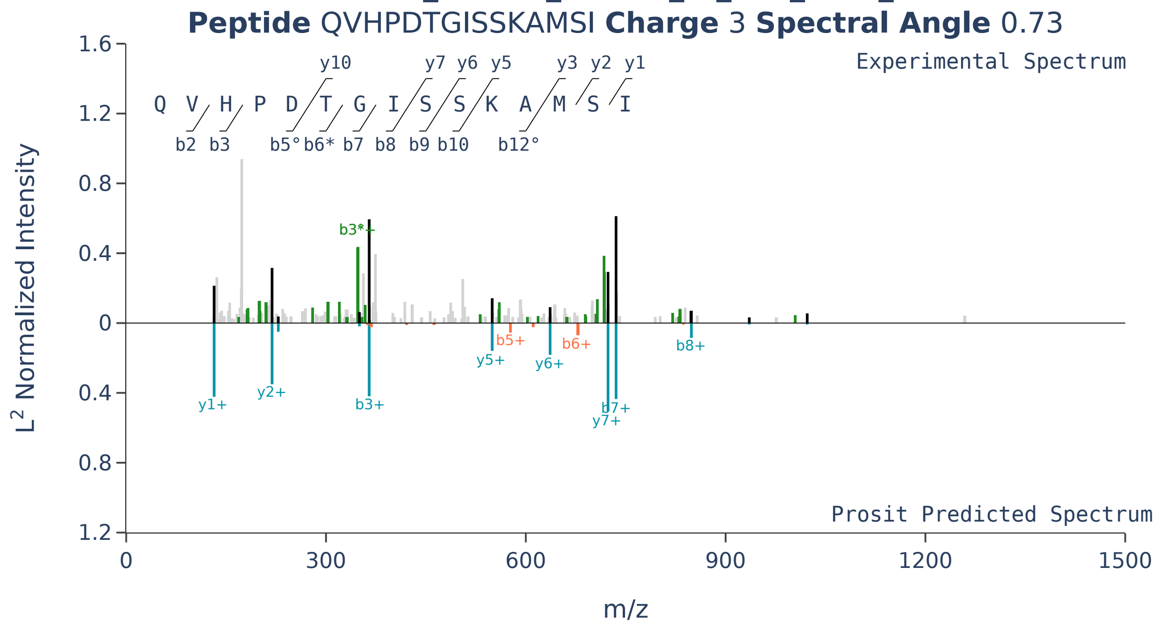


**Source** HRoetschke_291222_080123_Fu_H2B_b2R1_24h **Scan** 16013
**Peptide** YAIYVYKVLKQ **Charge** 3 **Spectral Angle** 0.68

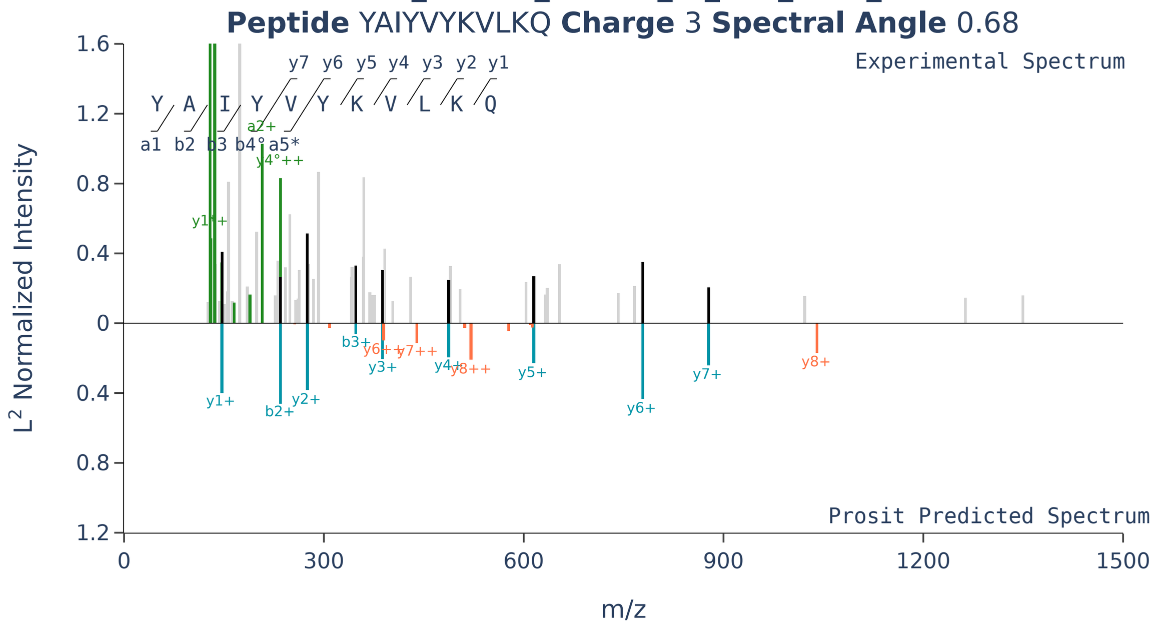


**Source** HRoetschke_291222_080123_Fu_H2B_b2R1_24h **Scan** 4112
**Peptide** LAGEASRLAH **Charge** 3 **Spectral Angle** 0.77

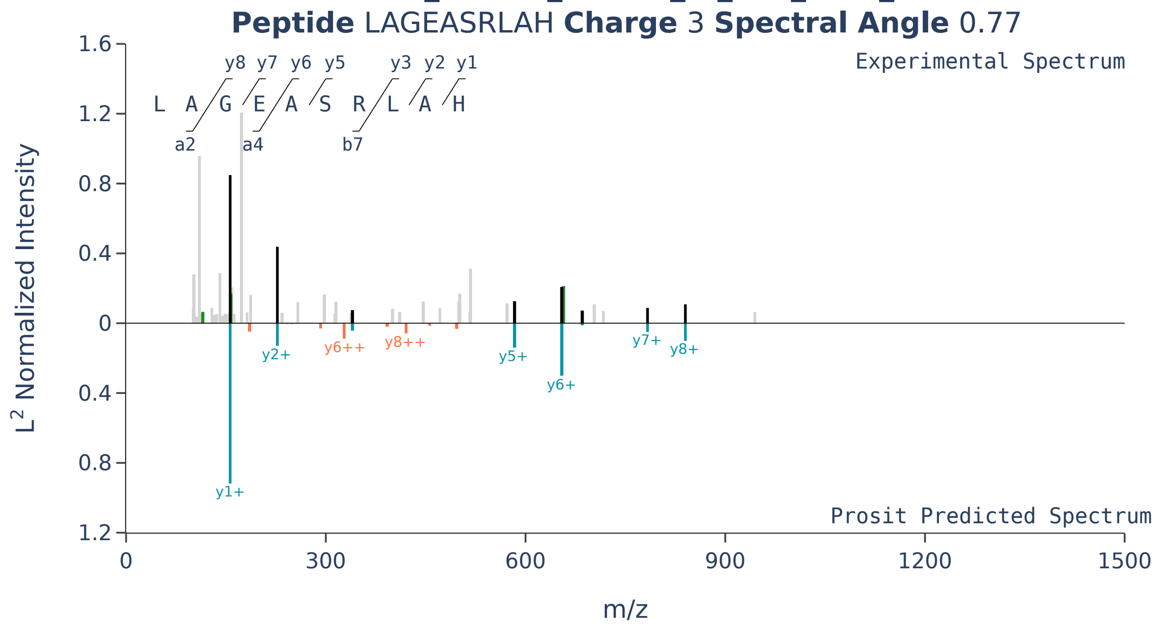


**Source** HRoetschke_291222_080123_Fu_H2B_b2R1_24h **Scan** 17964
**Peptide** DVFERIAGEASRLAHYNKRS **Charge** 4 **Spectral Angle** 0.83

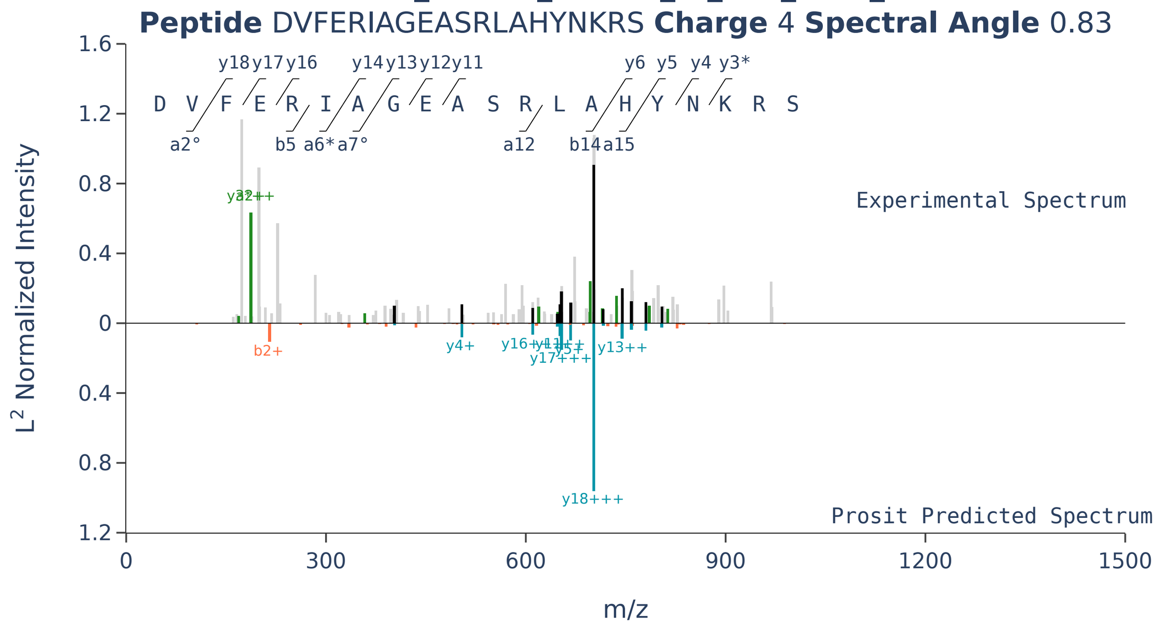


**Source** HRoetschke_291222_080123_Fu_H2B_b2R2_2h **Scan** 6427
**Peptide** LAGEASRLAHYNKRSTI **Charge** 4 **Spectral Angle** 0.54

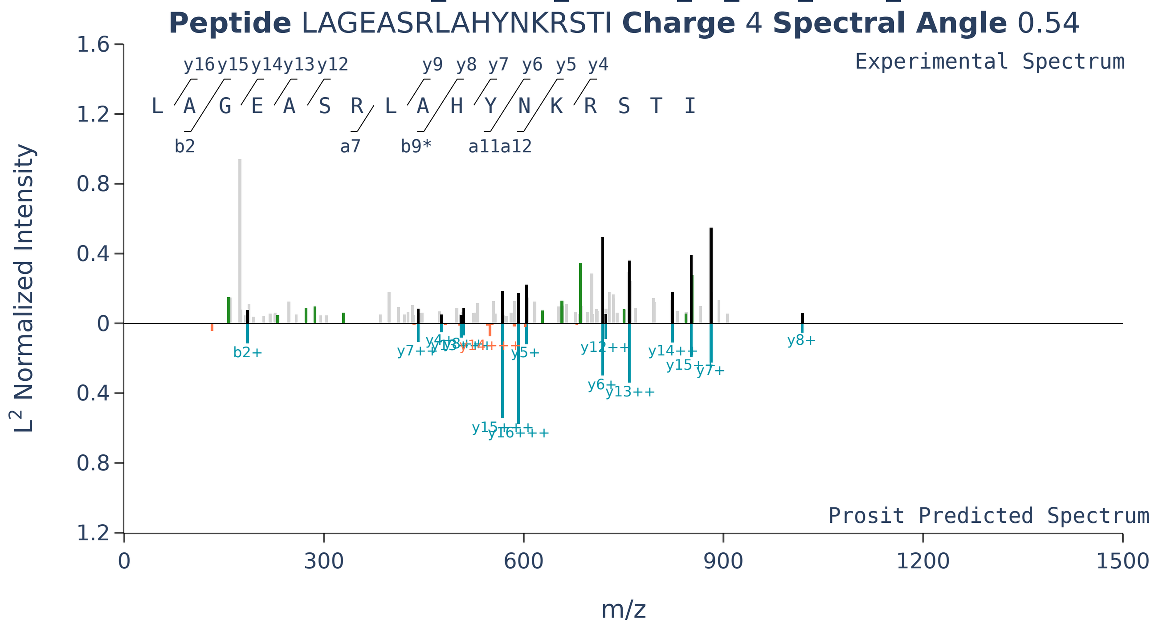


**Source** HRoetschke_291222_080123_Fu_H2B_b1R2_2h **Scan** 1919
**Peptide** AKHAVSEGTKAV **Charge** 3 **Spectral Angle** 0.67

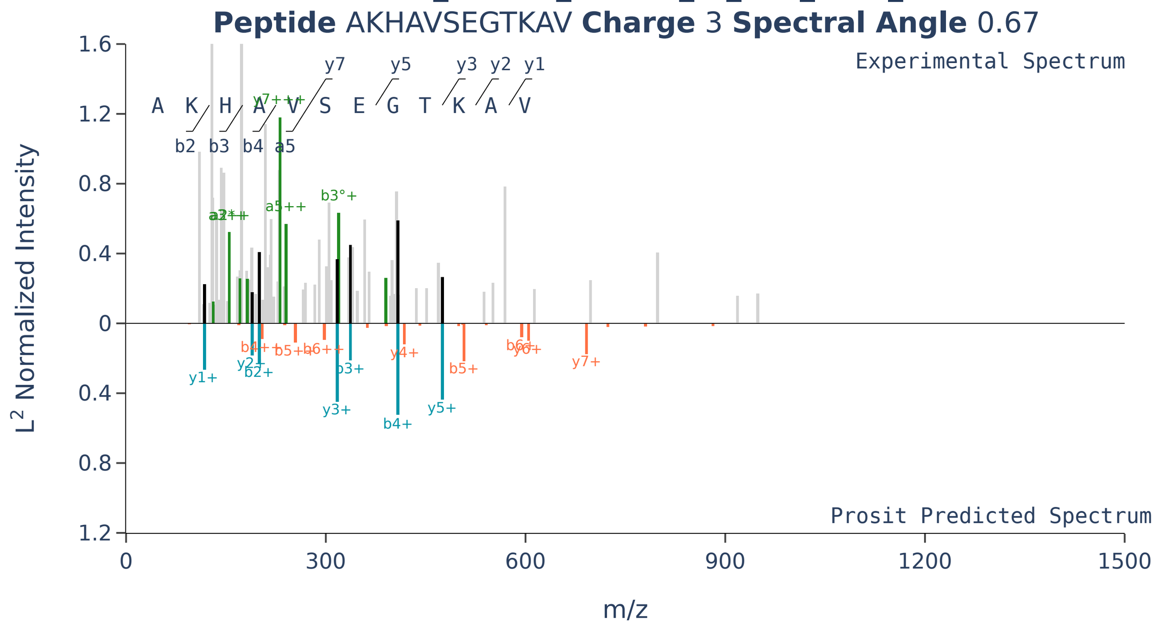


**Source** HRoetschke_291222_080123_Fu_H2B_b1R1_1h **Scan** 5116
**Peptide** LAKHAVSEGTKAVTKYTSA **Charge** 4 **Spectral Angle** 0.71

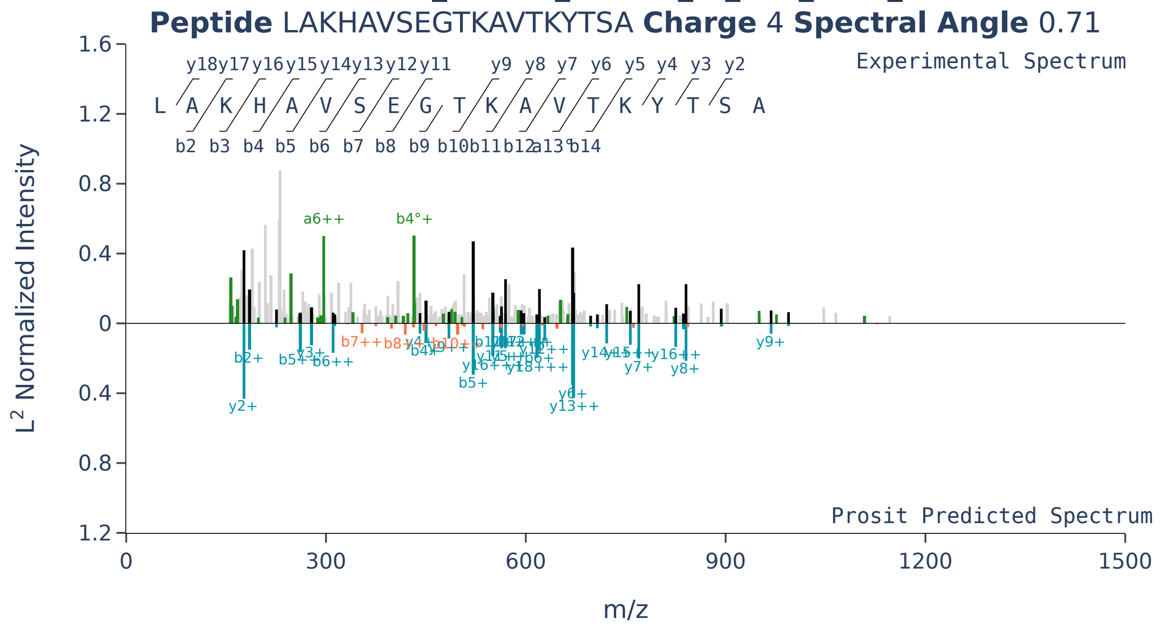


**Source** HRoetschke_291222_080123_Fu_H2B_b2R1_4h **Scan** 16123
**Peptide** LLPGELAKHAVSEGTKAVTKYTSA **Charge** 3 **Spectral Angle** 0.72

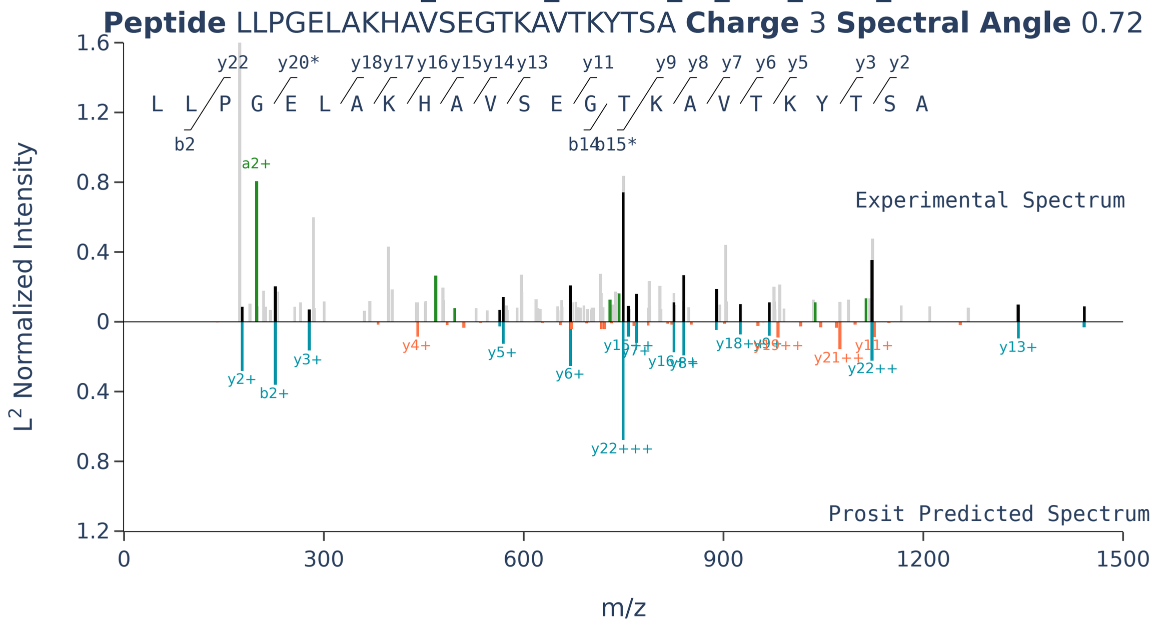


**Source** HRoetschke_291222_080123_Fu_H2B_b1R1_2h **Scan** 2829
**Peptide** PGELAKHAVS **Charge** 3 **Spectral Angle** 0.69

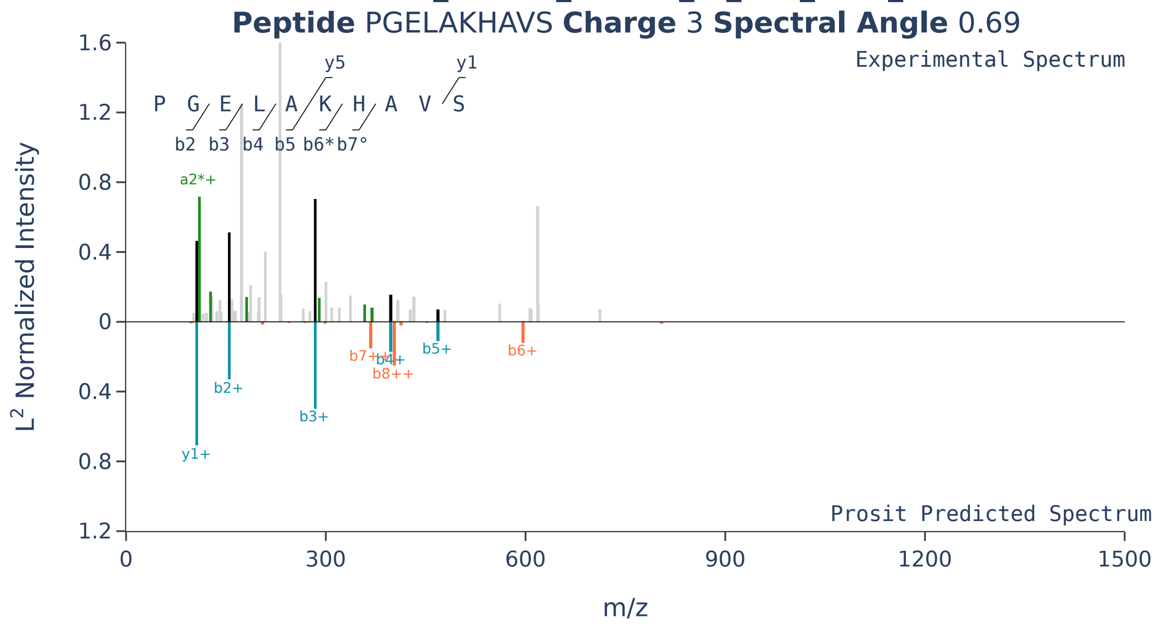


**Source** HRoetschke_291222_080123_Fu_H2B_b1R1_24h **Scan** 4552
**Peptide** LAHYNKRSTITSREIQ **Charge** 3 **Spectral Angle** 0.79

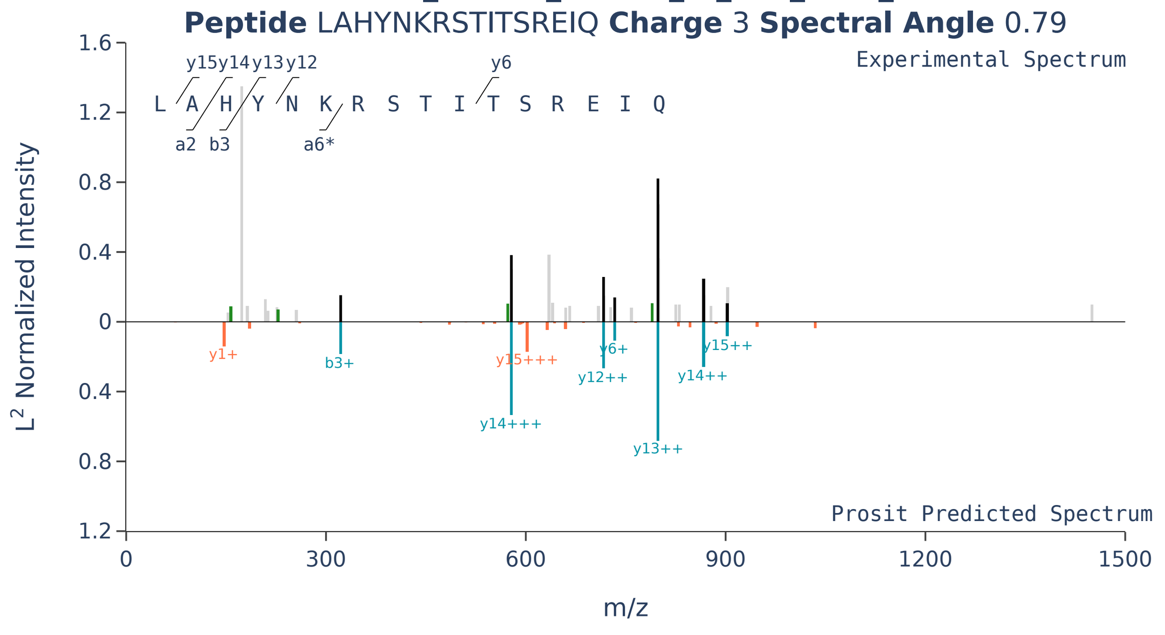


**Source** HRoetschke_291222_080123_Fu_H2B_b2R1_24h **Scan** 4445
**Peptide** GEASRLAHYNKRSTL **Charge** 3 **Spectral Angle** 0.79

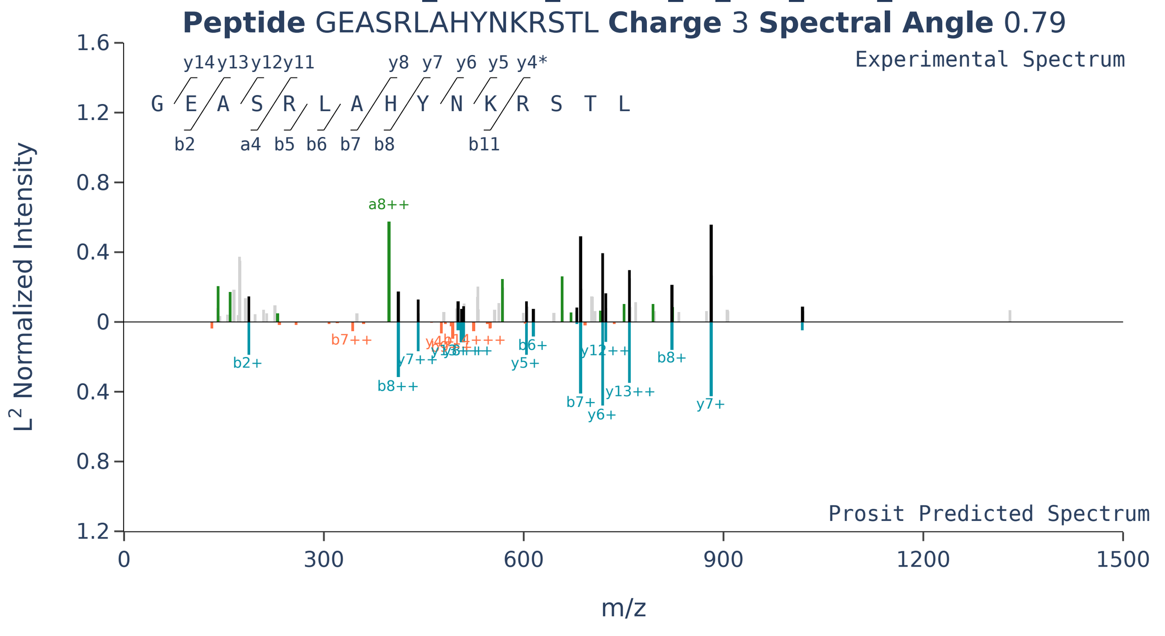


**Source** HRoetschke_291222_080123_Fu_H2B_b2R2_1h **Scan** 16822
**Peptide** ELQTAVRLLLPGELAKHAVSE **Charge** 3 **Spectral Angle** 0.74

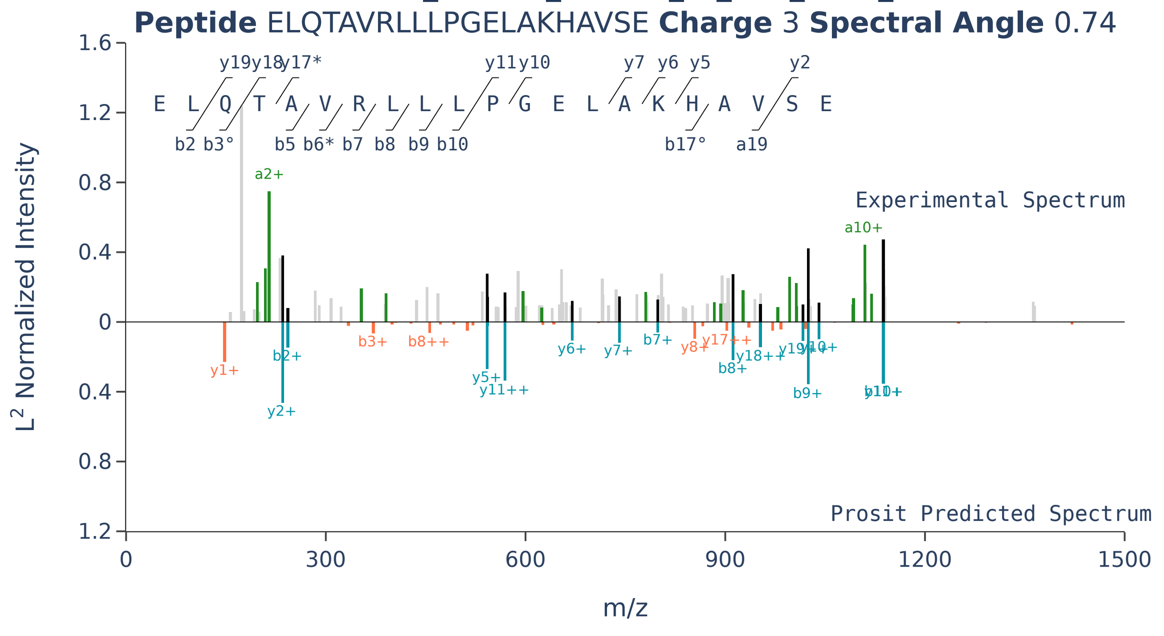


**Source** HRoetschke_291222_080123_Fu_H2B_b1R2_1h **Scan** 3142
**Peptide** NKRSTITSREI **Charge** 3 **Spectral Angle** 0.85

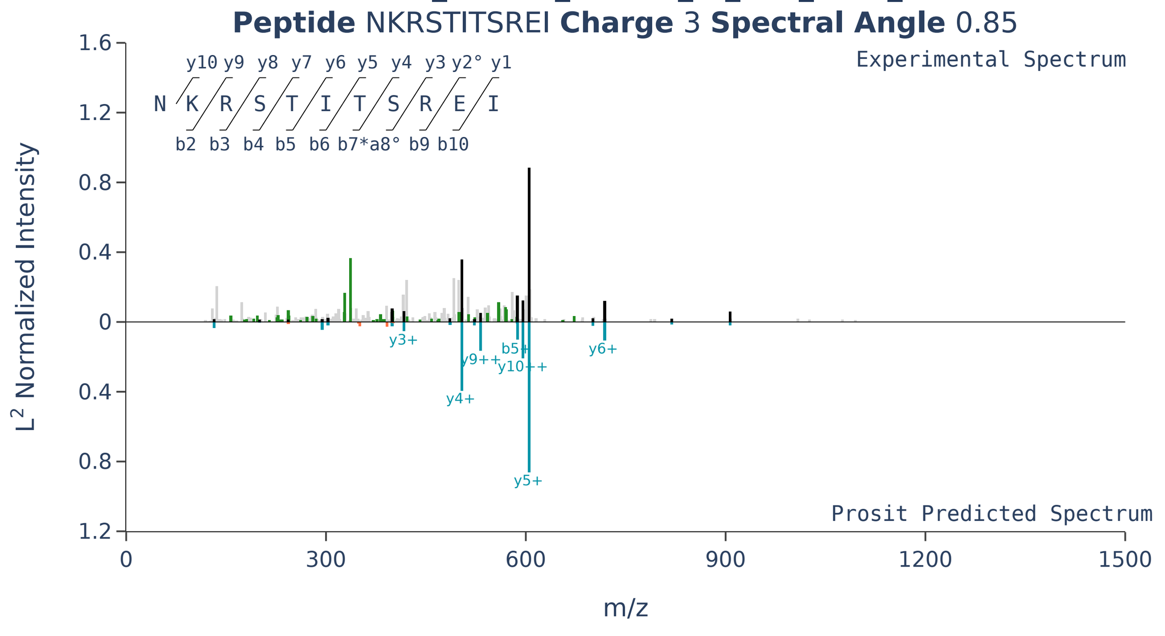

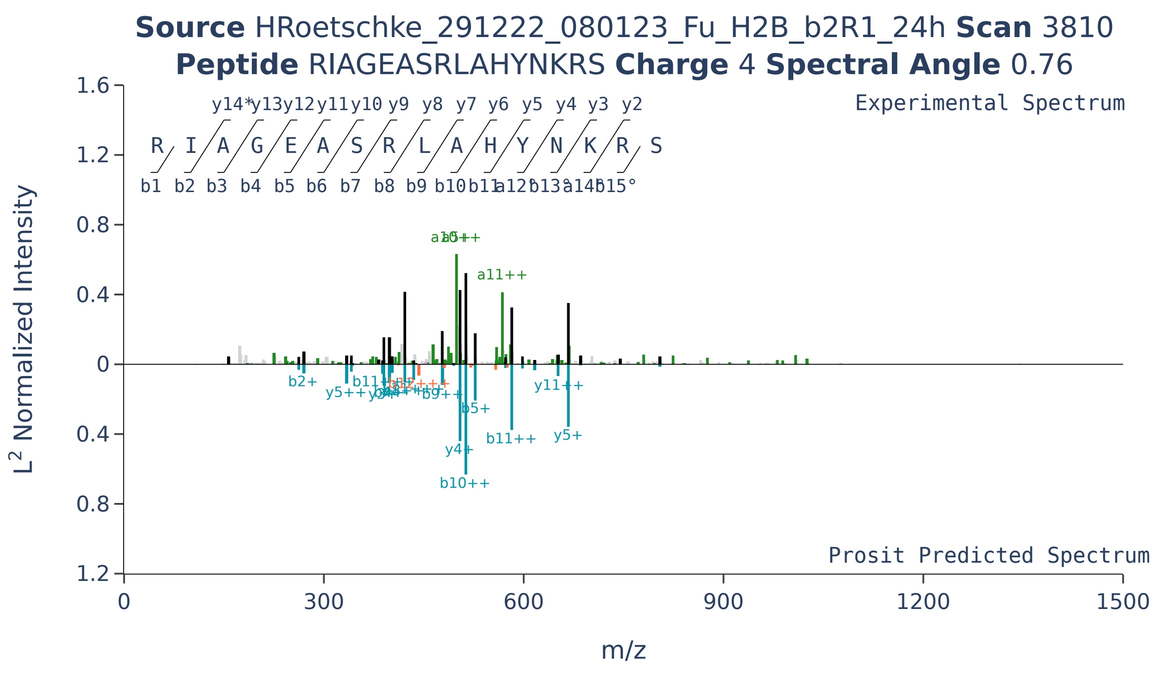
Source HRoetschke_291222_080123_Fu_H2B_b2R1_24h Scan 3810
Peptide RIAGEASRLAHYNKRS Charge 4 Spectral Angle 0.76
Experimental Spectrum
Prosit Predicted Spectrum
L² Normalized Intensity
m/z
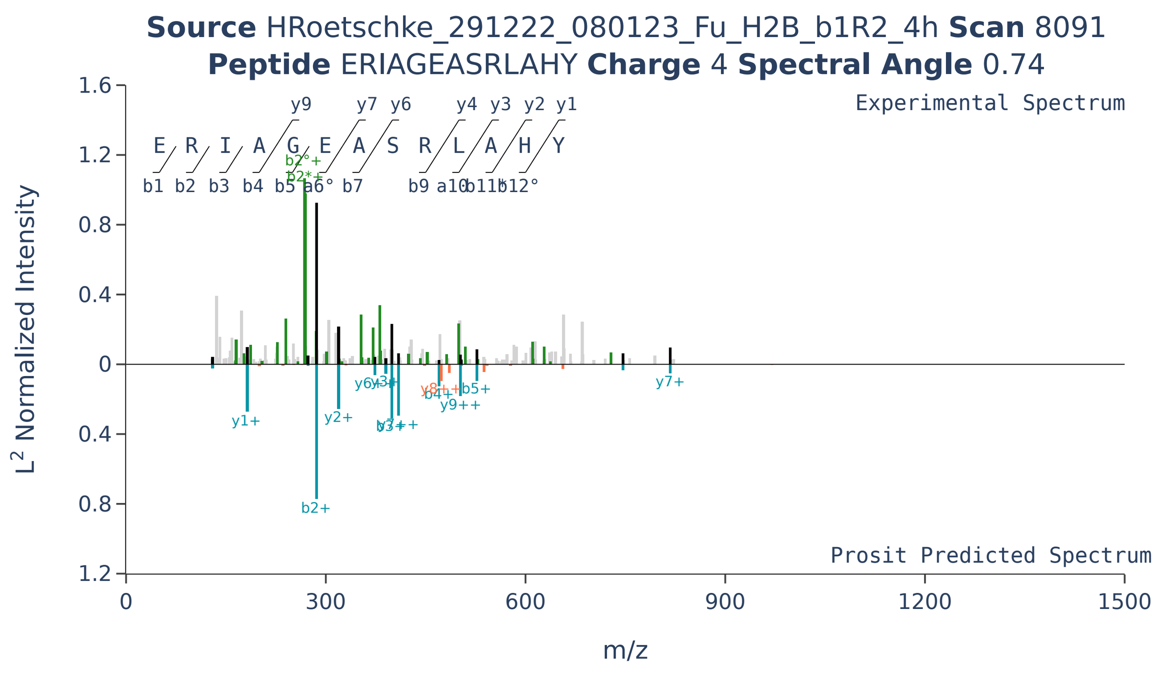
Source HRoetschke_291222_080123_Fu_H2B_b1R2_4h Scan 8091
Peptide ERIAGEASRLAHY Charge 4 Spectral Angle 0.74
Experimental Spectrum
Prosit Predicted Spectrum
L² Normalized Intensity
m/z
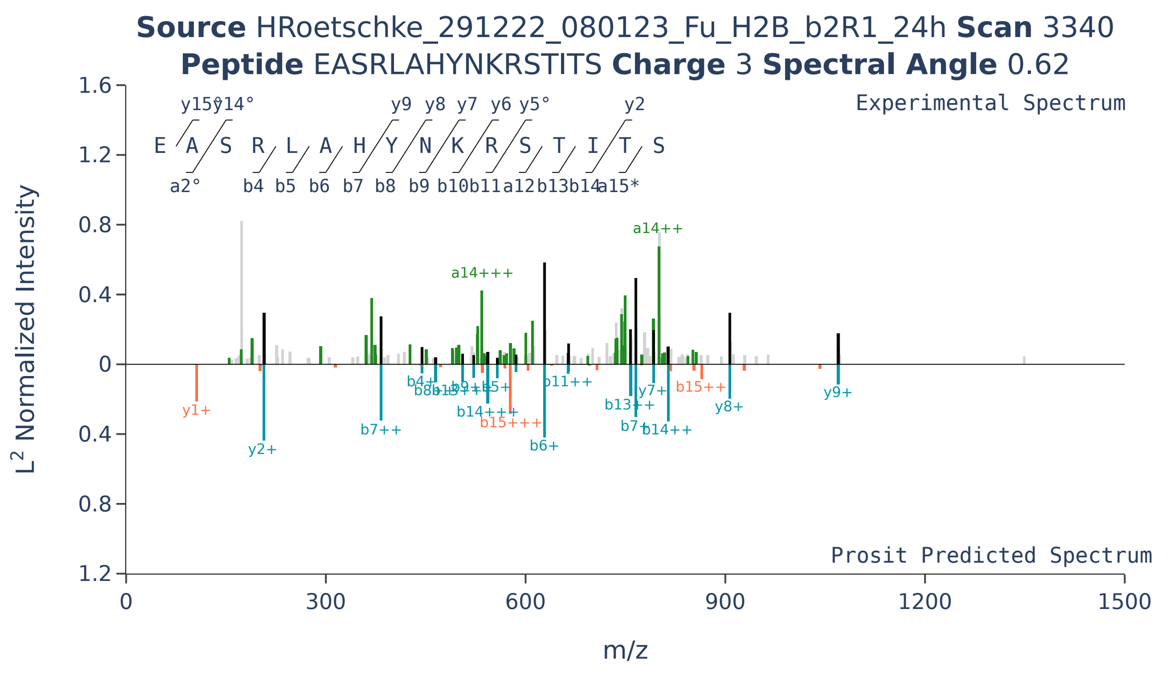
Source HRoetschke_291222_080123_Fu_H2B_b2R1_24h Scan 3340
Peptide EASRLAHYNKRSTITS Charge 3 Spectral Angle 0.62
Experimental Spectrum
Prosit Predicted Spectrum
L² Normalized Intensity
m/z
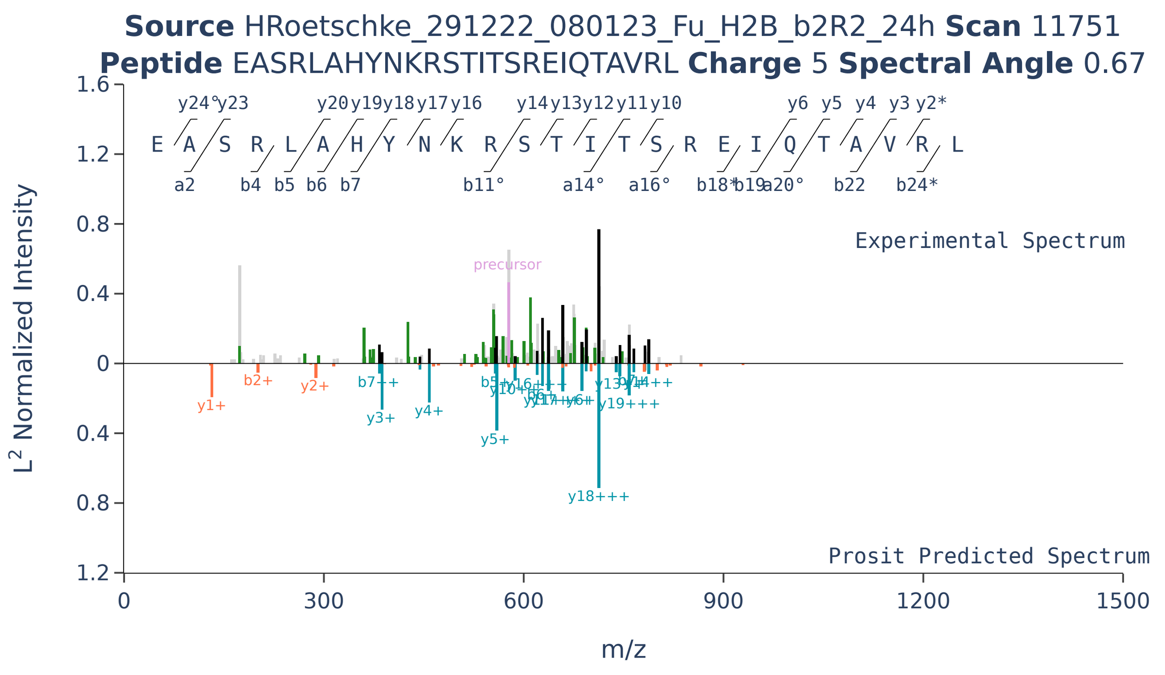
Source HRoetschke_291222_080123_Fu_H2B_b2R2_24h Scan 11751
Peptide EASRLAHYNKRSTITSREIQTAVRL Charge 5 Spectral Angle 0.67
Experimental Spectrum
Prosit Predicted Spectrum
L² Normalized Intensity
m/z
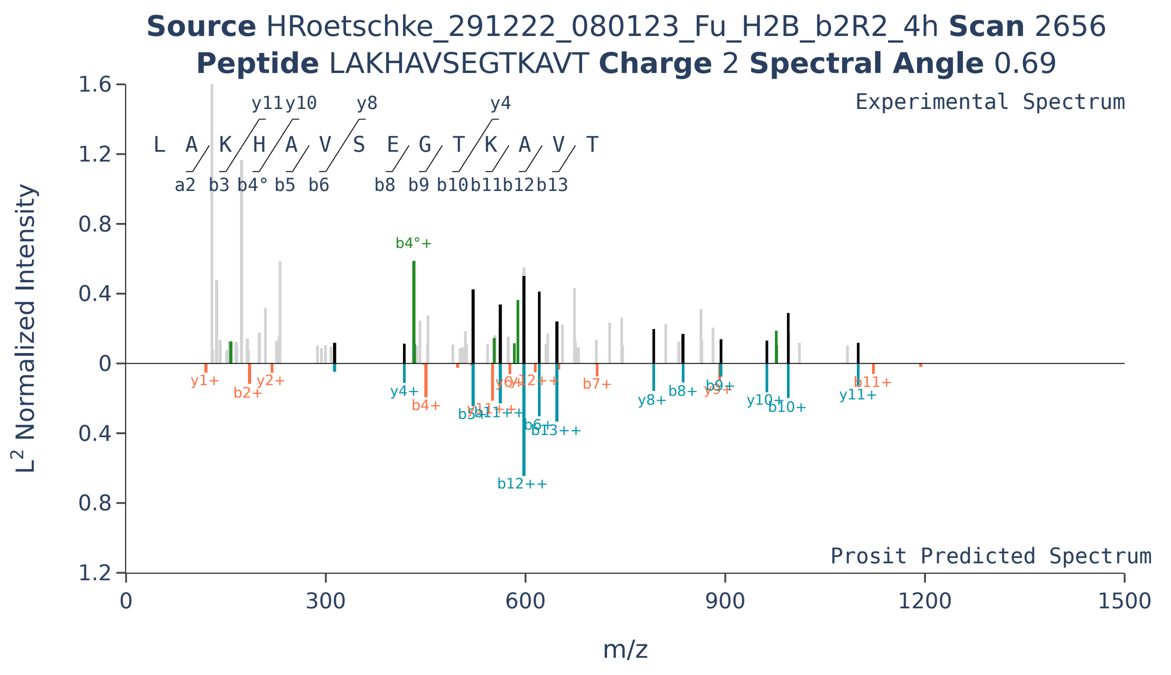
Source HRoetschke_291222_080123_Fu_H2B_b2R2_4h Scan 2656
Peptide LAKHAVSEGTKAVT Charge 2 Spectral Angle 0.69
Experimental Spectrum
Prosit Predicted Spectrum
L² Normalized Intensity
m/z
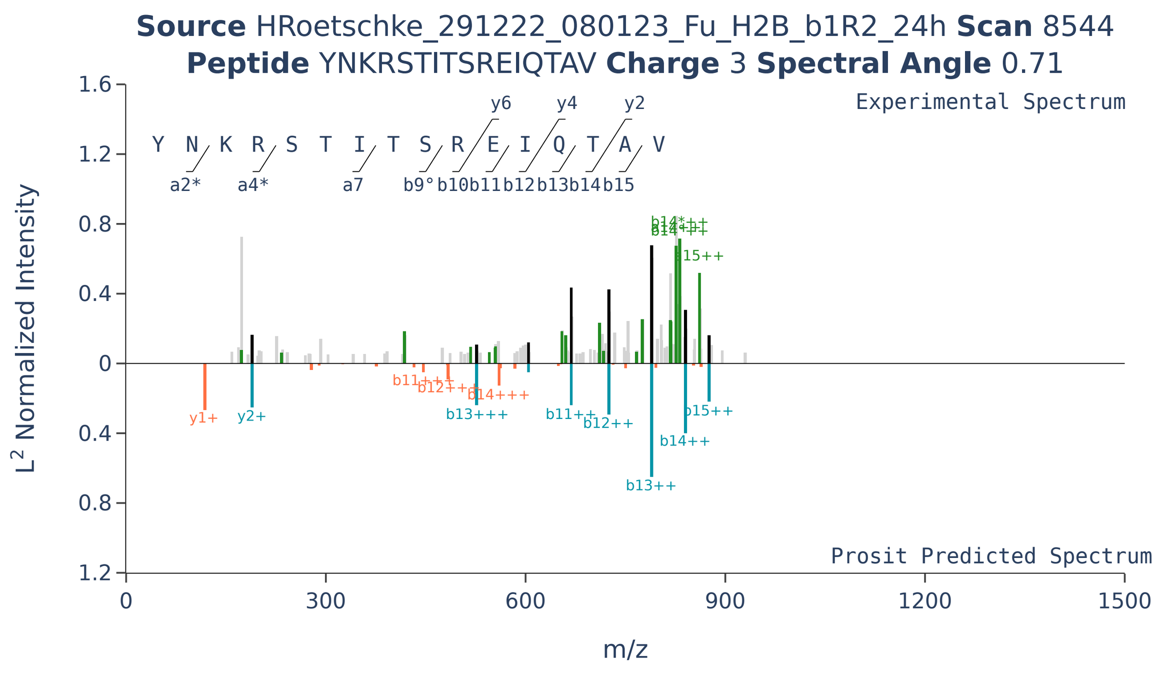
Source HRoetschke_291222_080123_Fu_H2B_b1R2_24h Scan 8544
Peptide YNKRSTITSREIQTAV Charge 3 Spectral Angle 0.71
Experimental Spectrum
Prosit Predicted Spectrum
L² Normalized Intensity
m/z
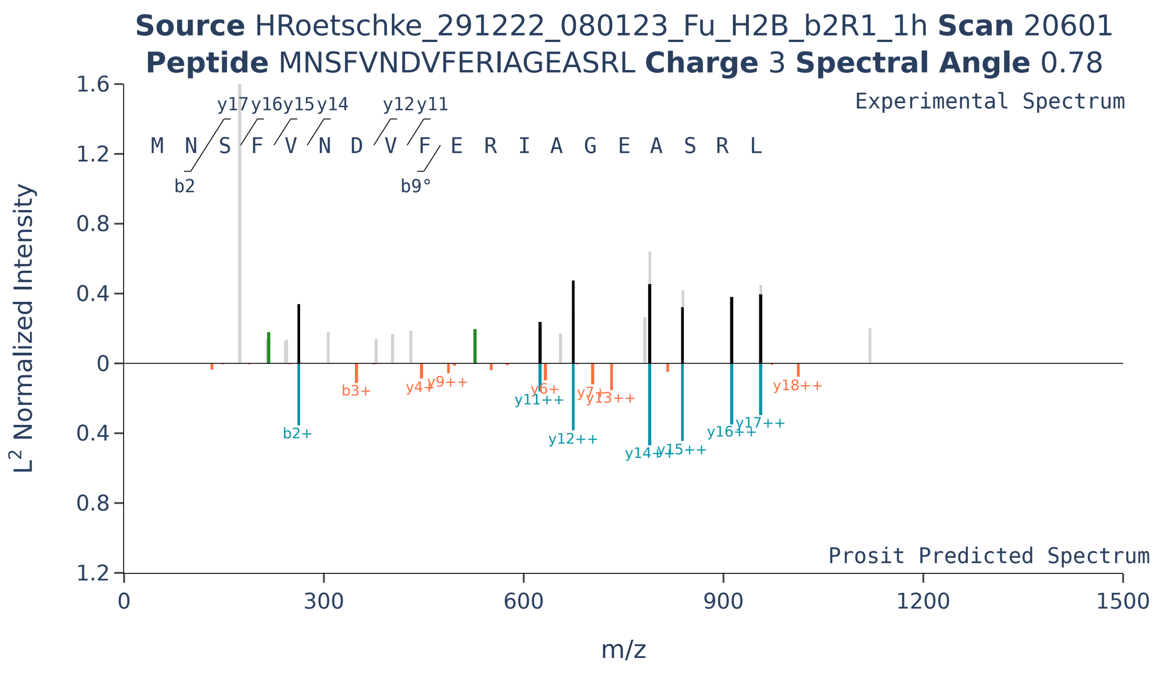
Source HRoetschke_291222_080123_Fu_H2B_b2R1_1h Scan 20601
Peptide MNSFVNDVFERIAGEASRL Charge 3 Spectral Angle 0.78
Experimental Spectrum
Prosit Predicted Spectrum
L² Normalized Intensity
m/z
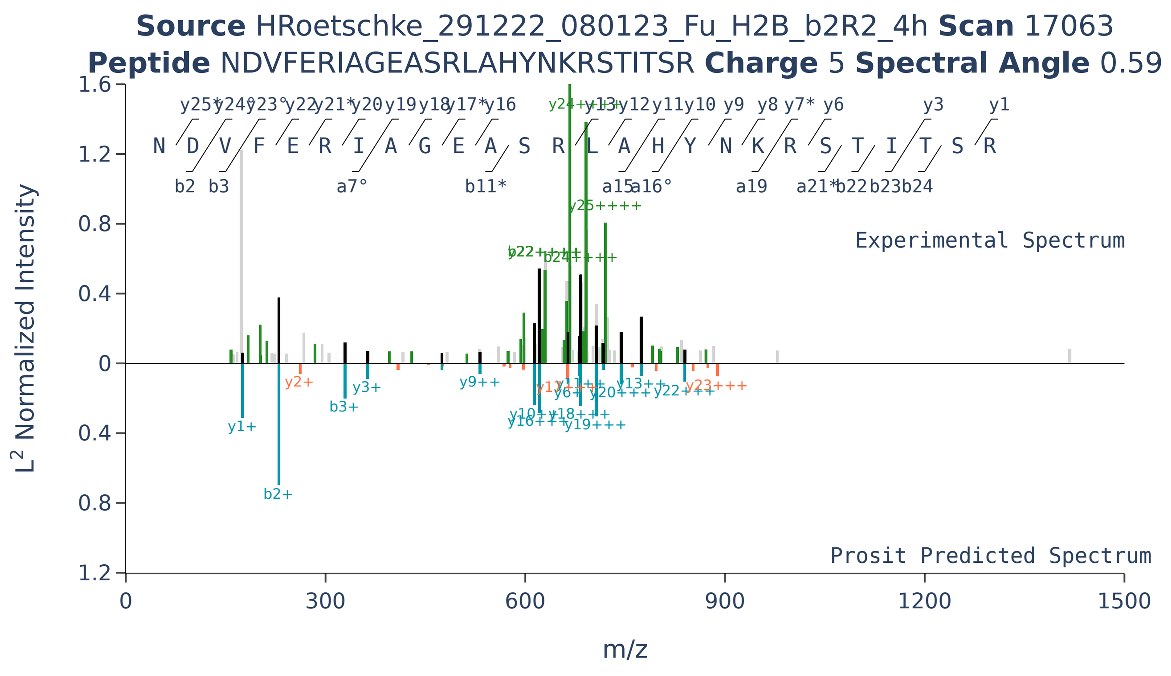
Source HRoetschke_291222_080123_Fu_H2B_b2R2_4h Scan 17063
Peptide NDVFERIAGEASRLAHYNKRSTITSR Charge 5 Spectral Angle 0.59
Experimental Spectrum
Prosit Predicted Spectrum
L² Normalized Intensity
m/z
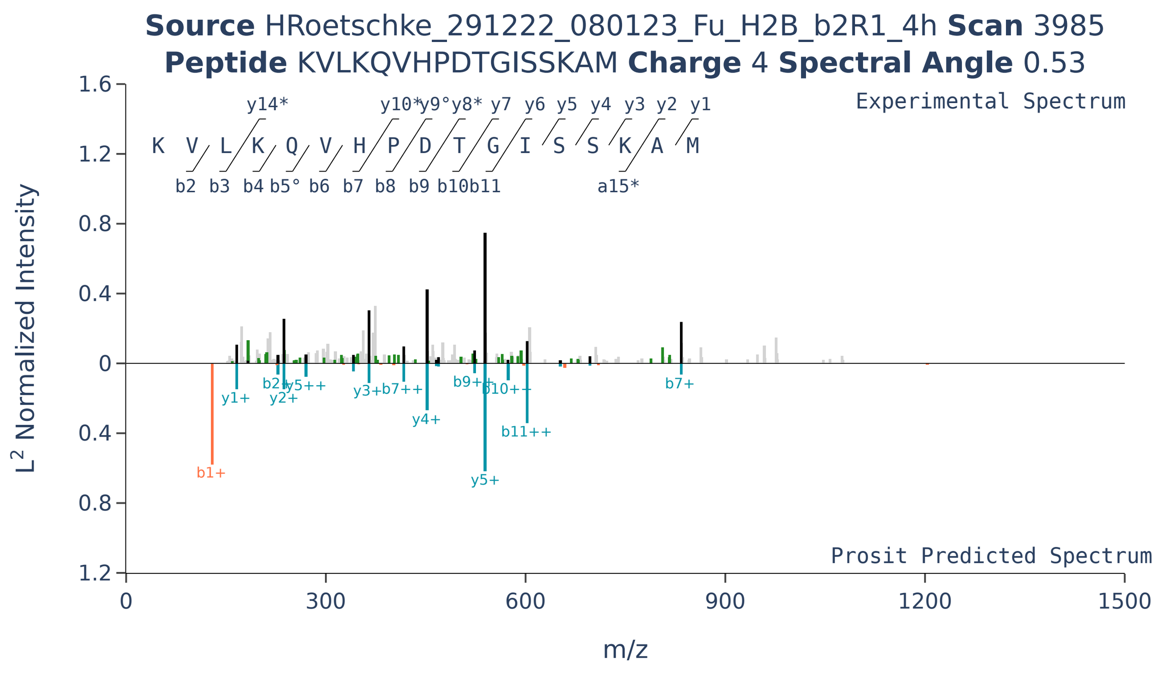
Source HRoetschke_291222_080123_Fu_H2B_b2R1_4h Scan 3985
Peptide KVLKQVHPDTGISSKAM Charge 4 Spectral Angle 0.53
Experimental Spectrum
Prosit Predicted Spectrum
L² Normalized Intensity
m/z
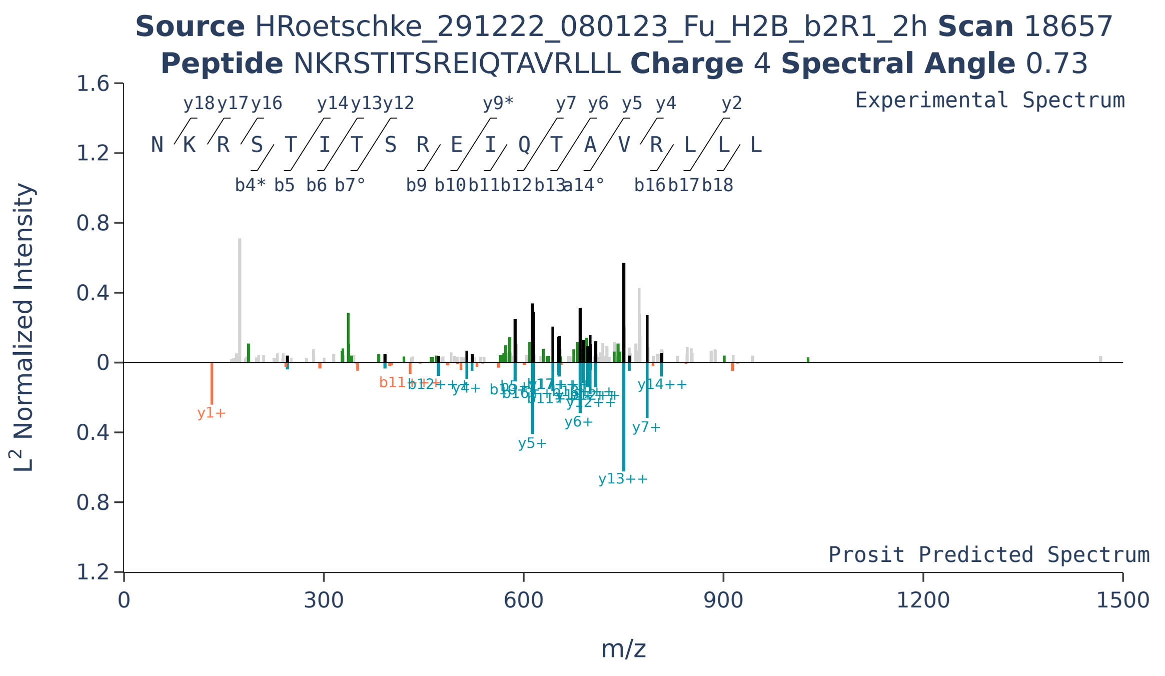
Source HRoetschke_291222_080123_Fu_H2B_b2R1_2h Scan 18657
Peptide NKRSTITSREIQTAVRLLL Charge 4 Spectral Angle 0.73
Experimental Spectrum
Prosit Predicted Spectrum
L² Normalized Intensity
m/z
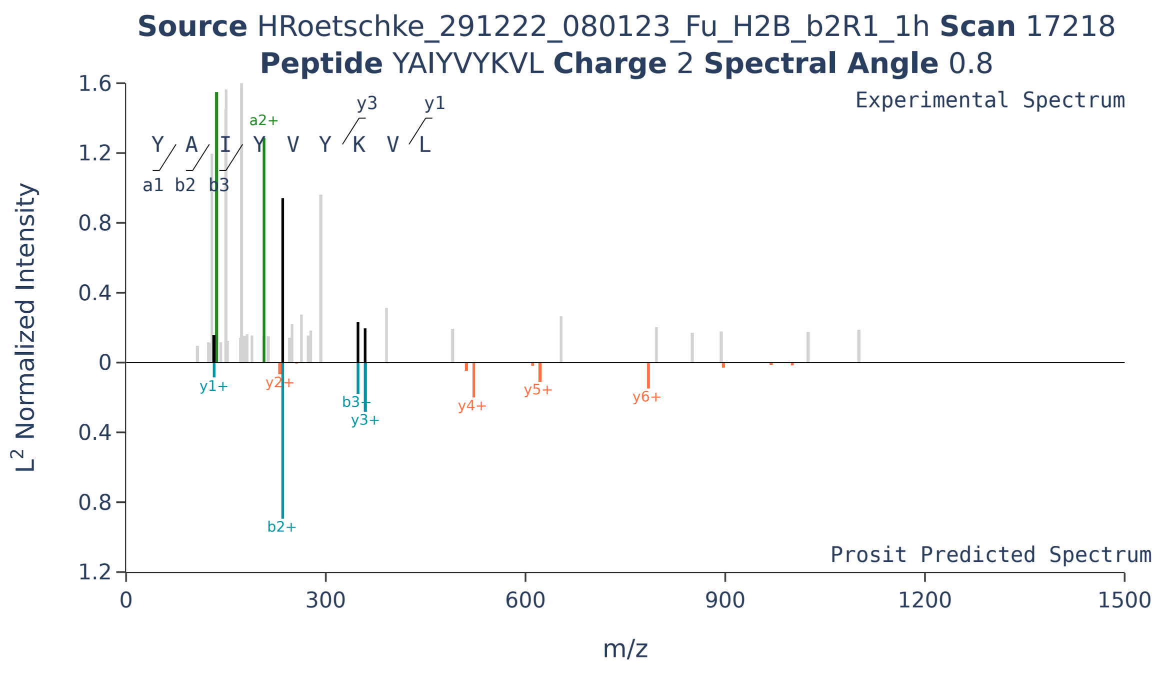
Source HRoetschke_291222_080123_Fu_H2B_b2R1_1h Scan 17218
Peptide YAIYVYKVL Charge 2 Spectral Angle 0.8
Experimental Spectrum
Prosit Predicted Spectrum
L² Normalized Intensity
m/z
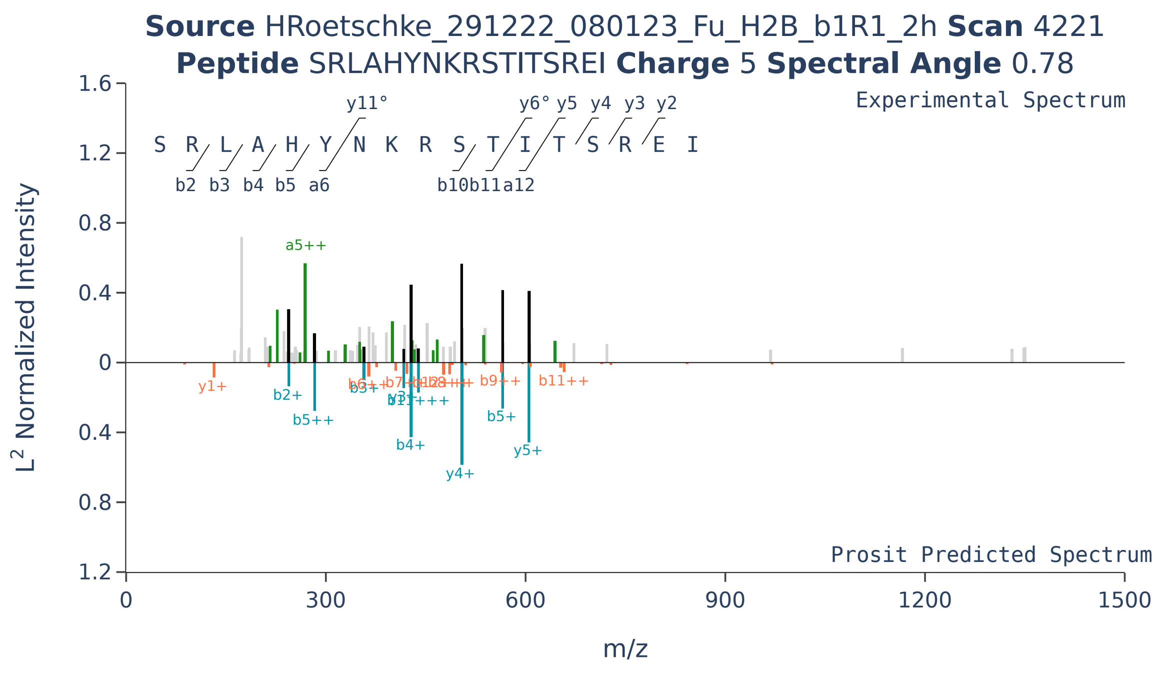
Source HRoetschke_291222_080123_Fu_H2B_b1R1_2h Scan 4221
Peptide SRLAHYNKRSTITSREI Charge 5 Spectral Angle 0.78
Experimental Spectrum
Prosit Predicted Spectrum
L² Normalized Intensity
m/z
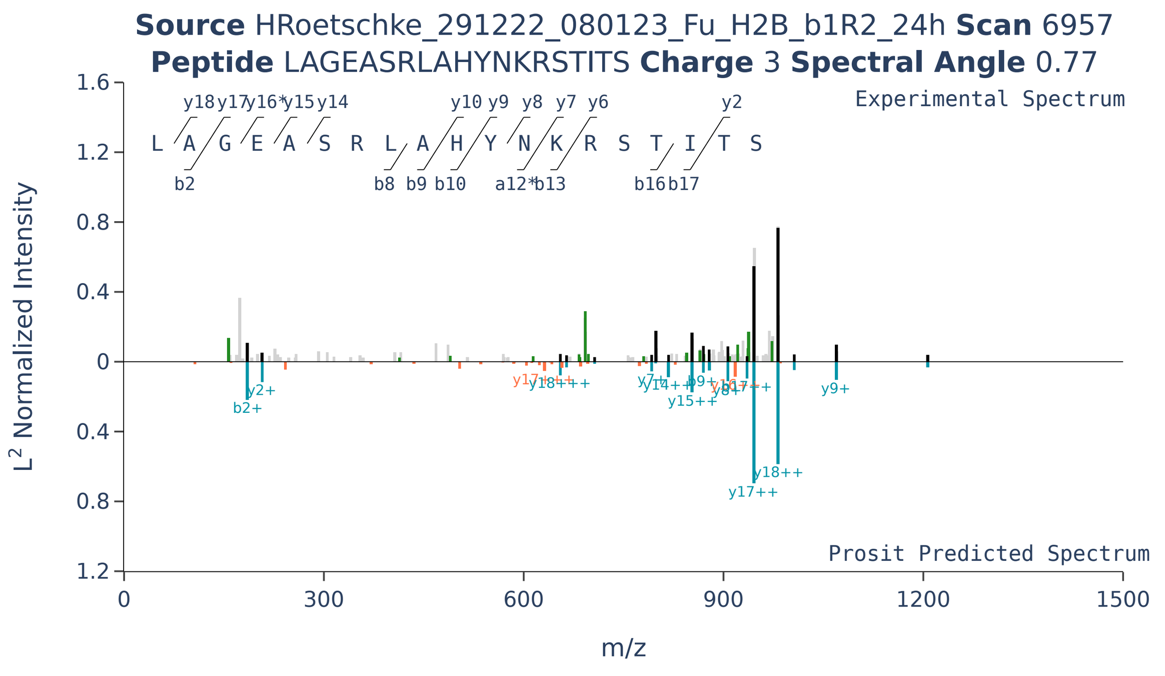
Source HRoetschke_291222_080123_Fu_H2B_b1R2_24h Scan 6957
Peptide LAGEASRLAHYNKRSTITS Charge 3 Spectral Angle 0.77
Experimental Spectrum
Prosit Predicted Spectrum
L² Normalized Intensity
m/z
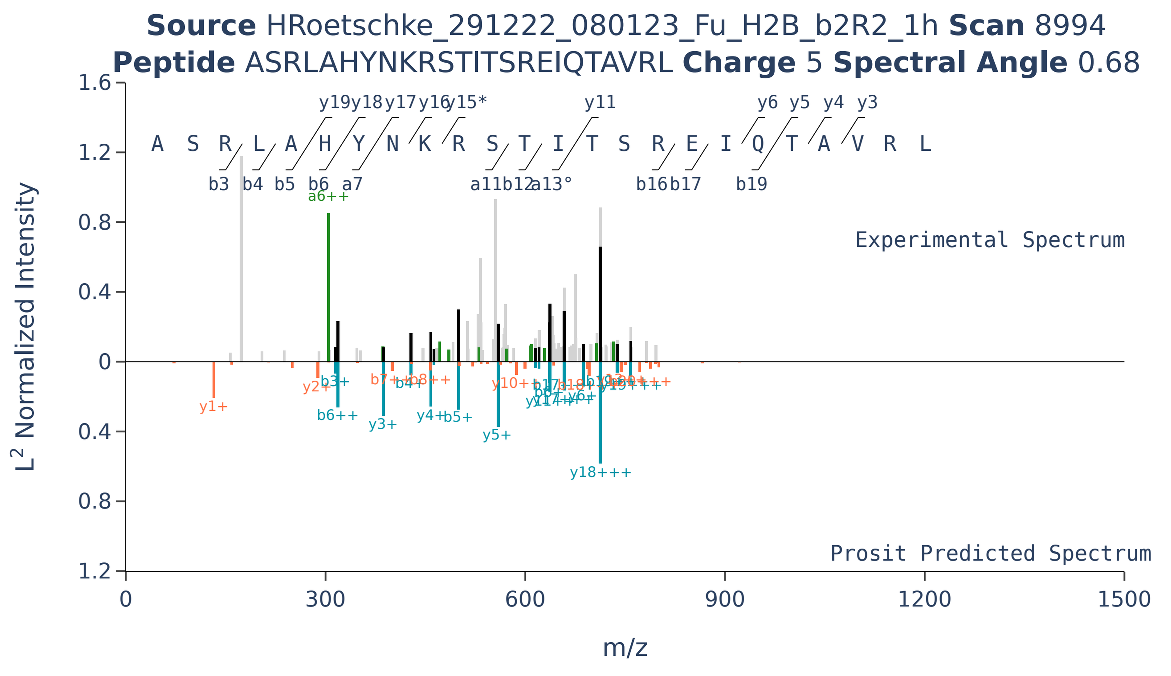
Source HRoetschke_291222_080123_Fu_H2B_b2R2_1h Scan 8994
Peptide ASRLAHYNKRSTITSREIQTAVRL Charge 5 Spectral Angle 0.68
Experimental Spectrum
Prosit Predicted Spectrum
L² Normalized Intensity
m/z
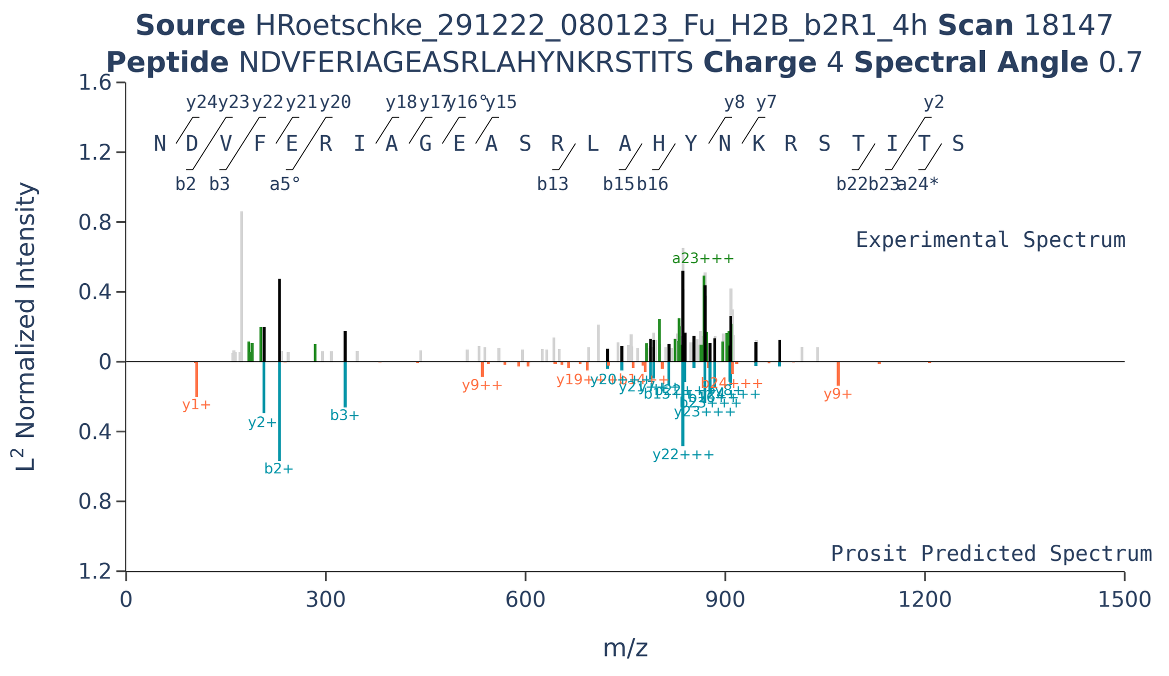
Source HRoetschke_291222_080123_Fu_H2B_b2R1_4h Scan 18147
Peptide NDVFERIAGEASRLAHYNKRSTITS Charge 4 Spectral Angle 0.7
Experimental Spectrum
Prosit Predicted Spectrum
L² Normalized Intensity
m/z

**Source** HRoetschke_291222_080123_Fu_H2B_b2R1_1h **Scan** 16877
**Peptide** ELQTAVRLLLPGELAKHAVSEGTKAVTKY **Charge** 4 **Spectral Angle** 0.6

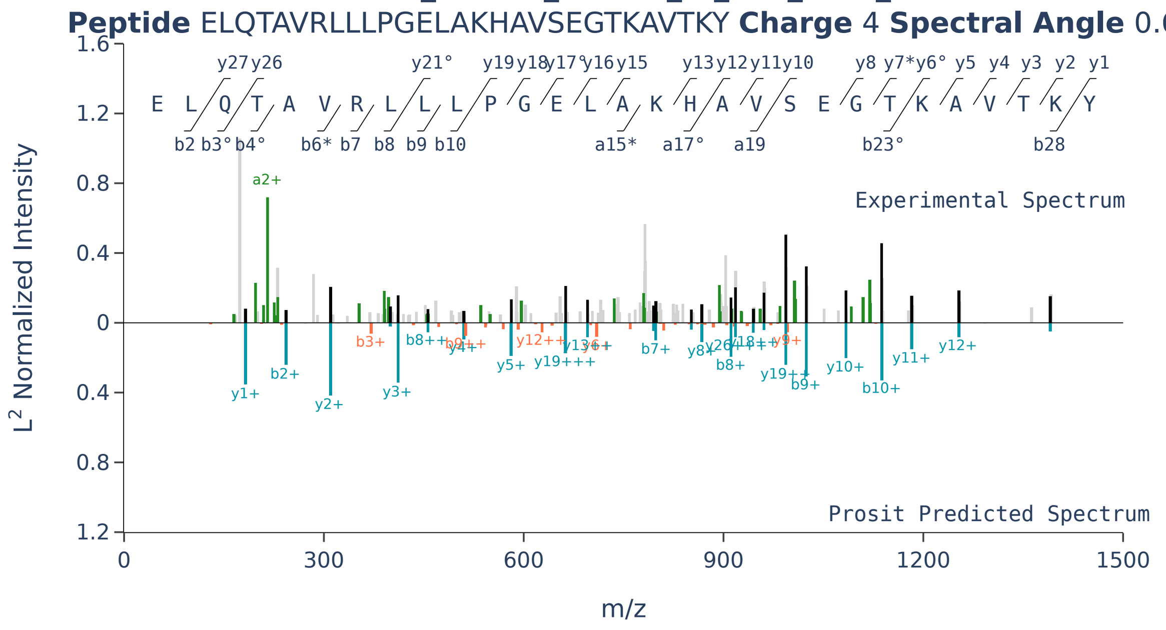


**Source** HRoetschke_291222_080123_Fu_H2B_b2R1_4h **Scan** 16360
**Peptide** TRKESYAIYVYKVL **Charge** 3 **Spectral Angle** 0.68

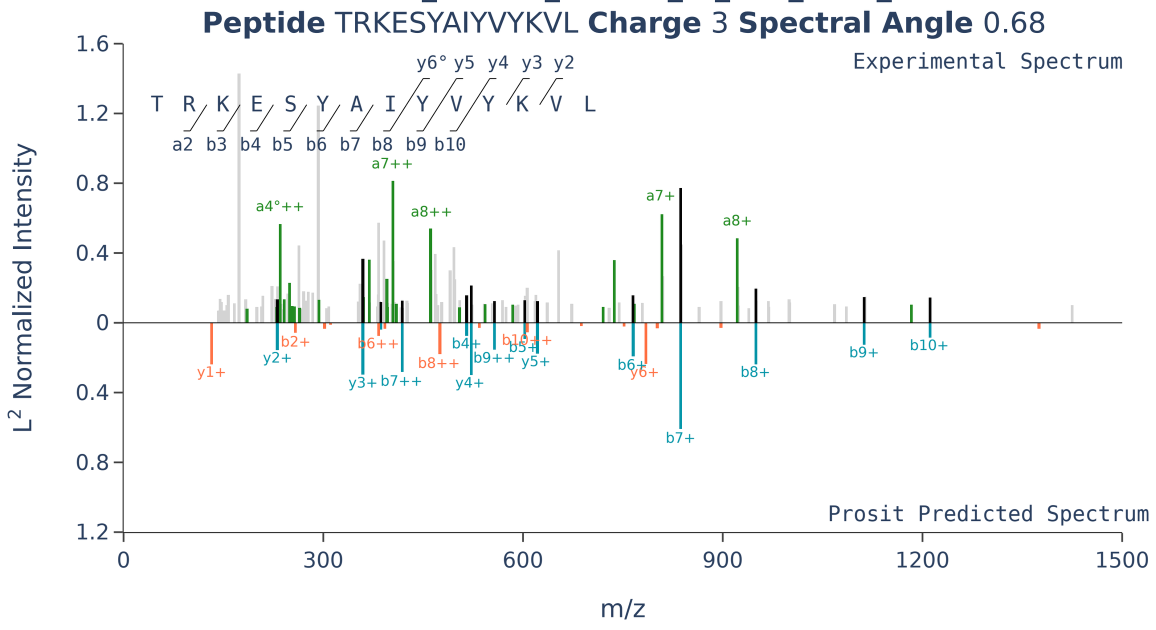


**Source** HRoetschke_291222_080123_Fu_H2B_b1R2_24h **Scan** 7332
**Peptide** KVLKQVHPDTGISSKAMSIM **Charge** 4 **Spectral Angle** 0.62

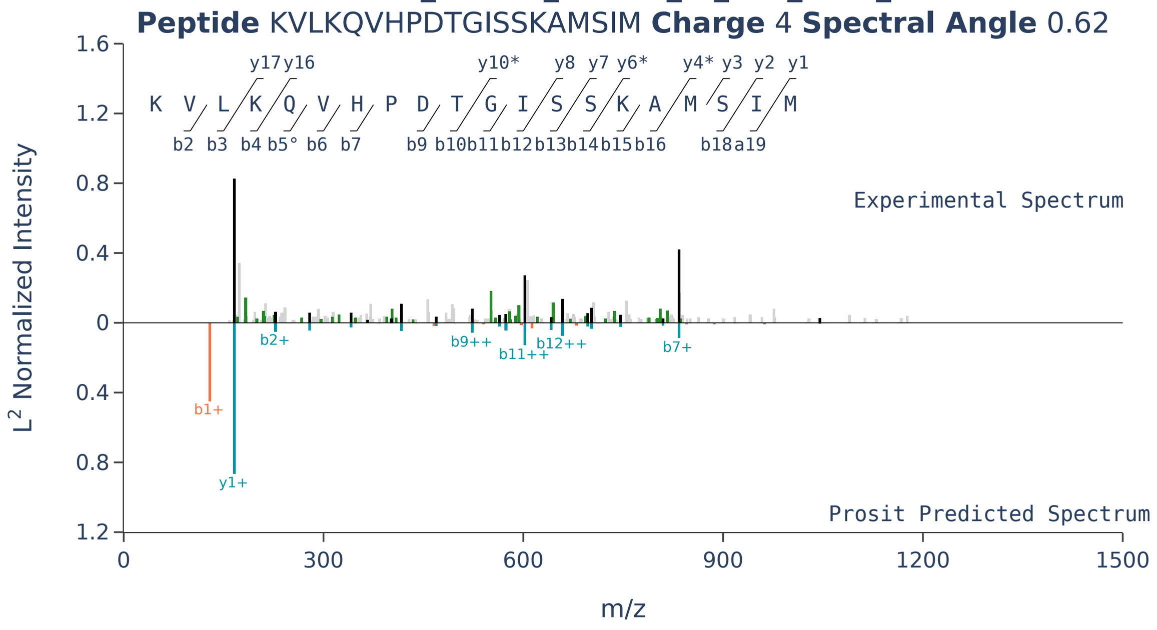


**Source** HRoetschke_291222_080123_Fu_H2B_b1R1_4h **Scan** 5291
**Peptide** RLAGEASRLAHYNKRSTITSRE **Charge** 5 **Spectral Angle** 0.79

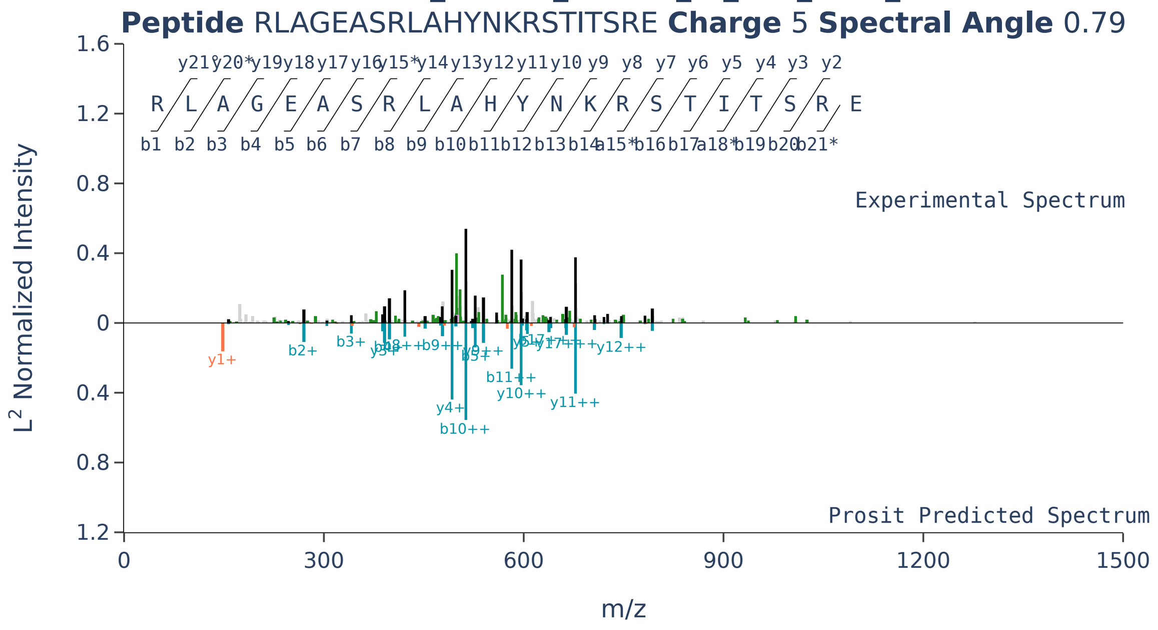


**Source** HRoetschke_291222_080123_Fu_H2B_b2R1_4h **Scan** 10053
**Peptide** PGELAKHAVSEGTKAVTKYTS **Charge** 3 **Spectral Angle** 0.66

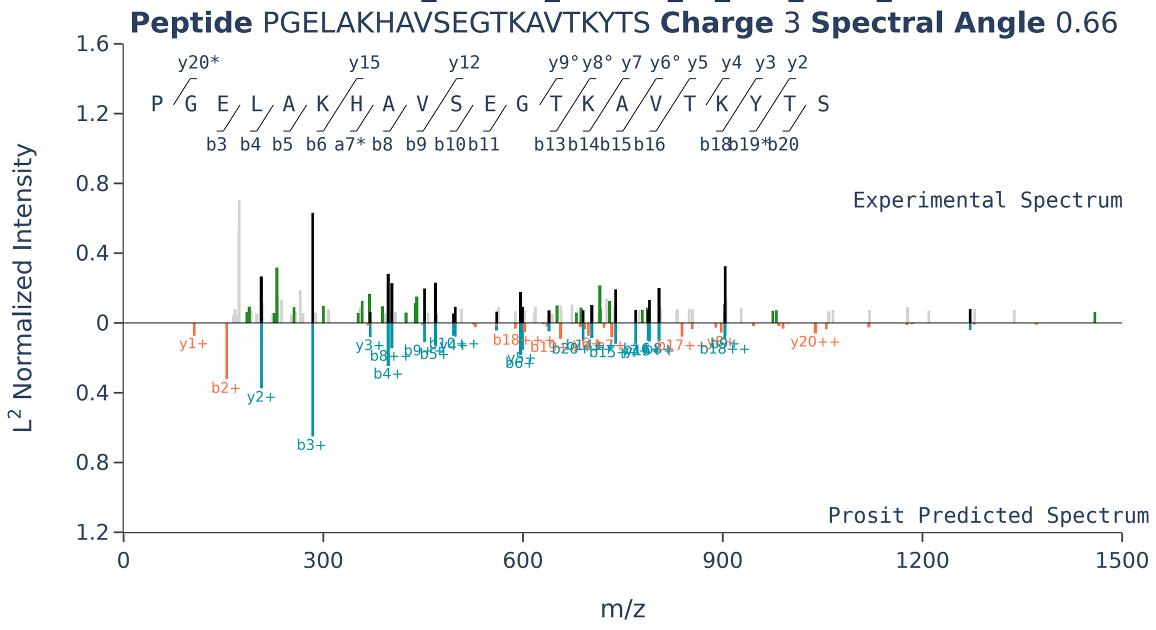


**Source** HRoetschke_291222_080123_Fu_H2B_b2R2_24h **Scan** 18775
**Peptide** REIQTAVRLLLPGELAKHAVSEGTKAV **Charge** 4 **Spectral Angle** 0.63

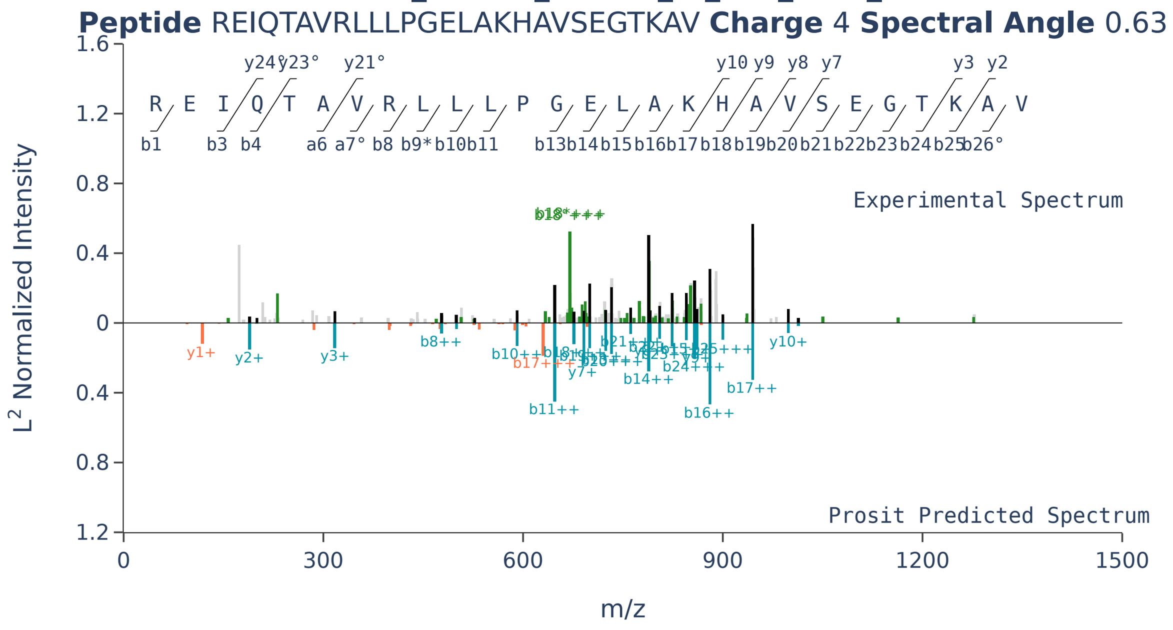


**Source** HRoetschke_291222_080123_Fu_H2B_b2R1_1h **Scan** 5807
**Peptide** AGEASRLAHYNKRSTITSREIQT **Charge** 5 **Spectral Angle** 0.62

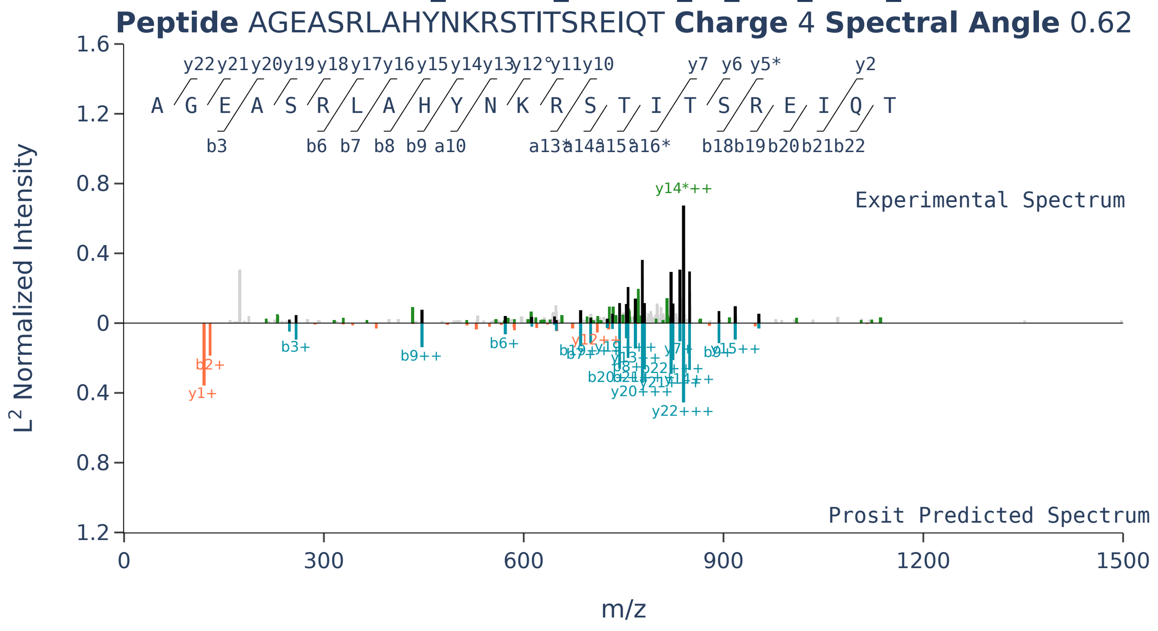


**Source** HRoetschke_291222_080123_Fu_H2B_b1R2_24h **Scan** 8634
**Peptide** IAGEASRLAHYNKRSTITSREIQT **Charge** 4 **Spectral Angle** 0.9

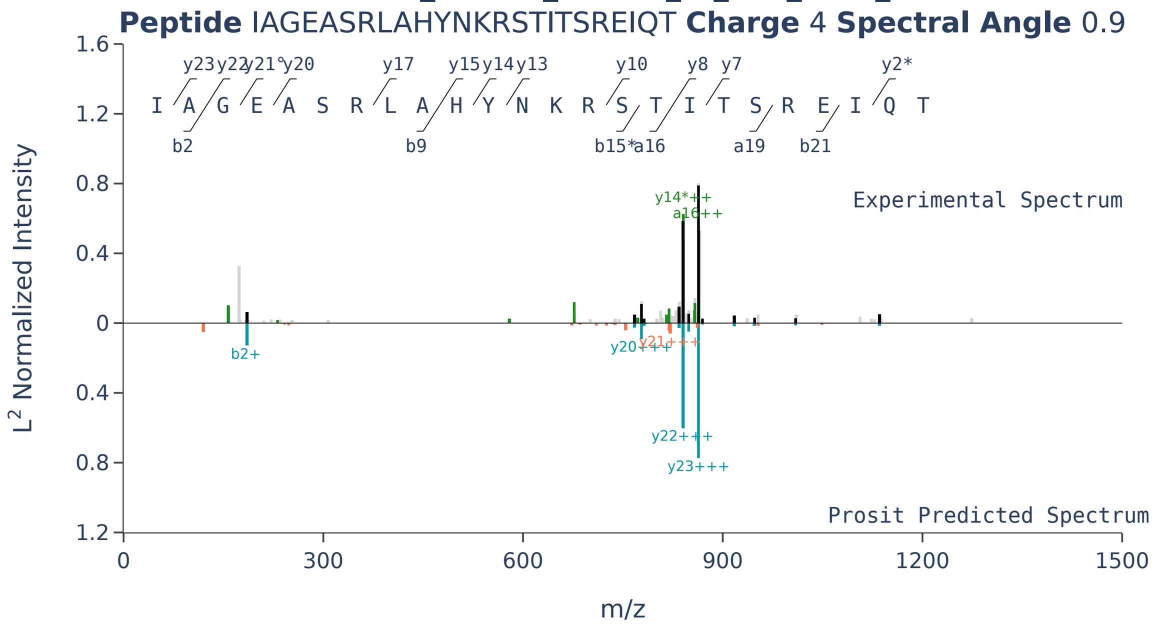


**Source** HRoetschke_291222_080123_Fu_H2B_b1R1_1h **Scan** 4710
**Peptide** RIAGEASRLAHYNKRSTITSRE **Charge** 5 **Spectral Angle** 0.77

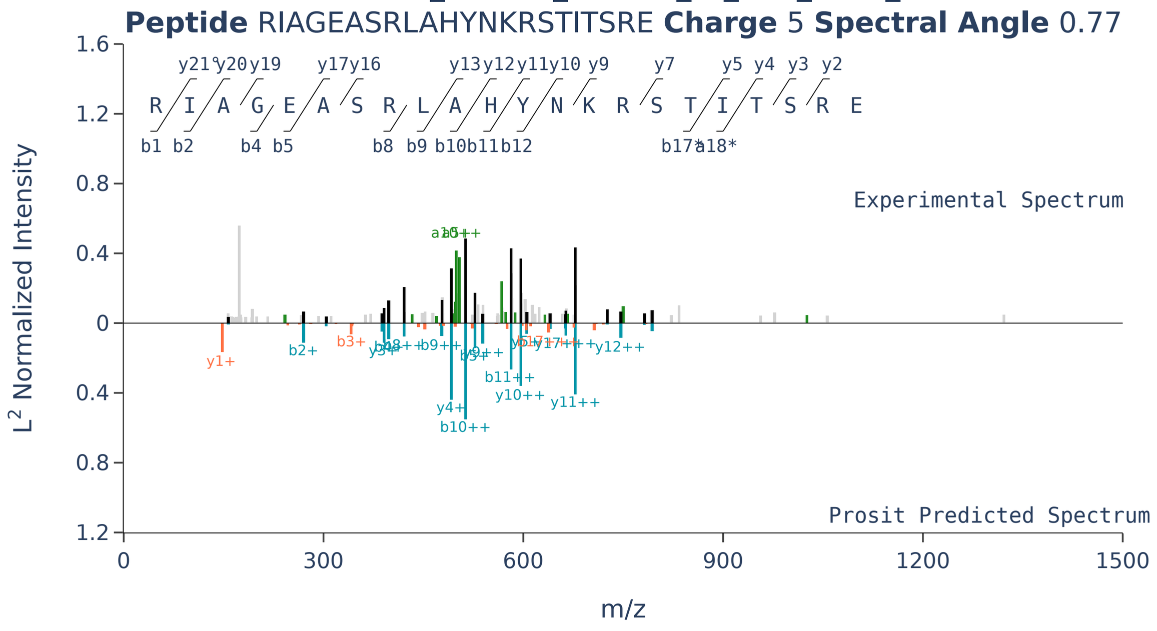


**Source** HRoetschke_291222_080123_Fu_H2B_b2R2_24h **Scan** 4151
**Peptide** KTQKKDGKKRRKTRKESYAIYVY **Charge** 4 **Spectral Angle** 0.72

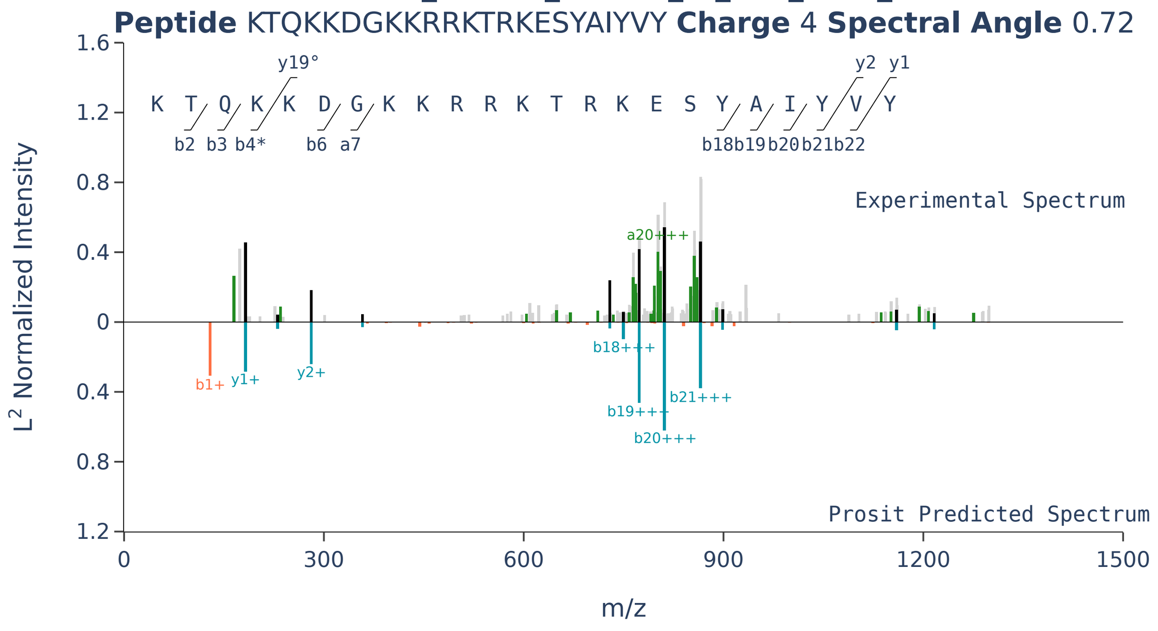


**Source** HRoetschke_291222_080123_Fu_H2B_b1R1_4h **Scan** 4544
**Peptide** SRLAHYNKRSTITSREL **Charge** 4 **Spectral Angle** 0.83

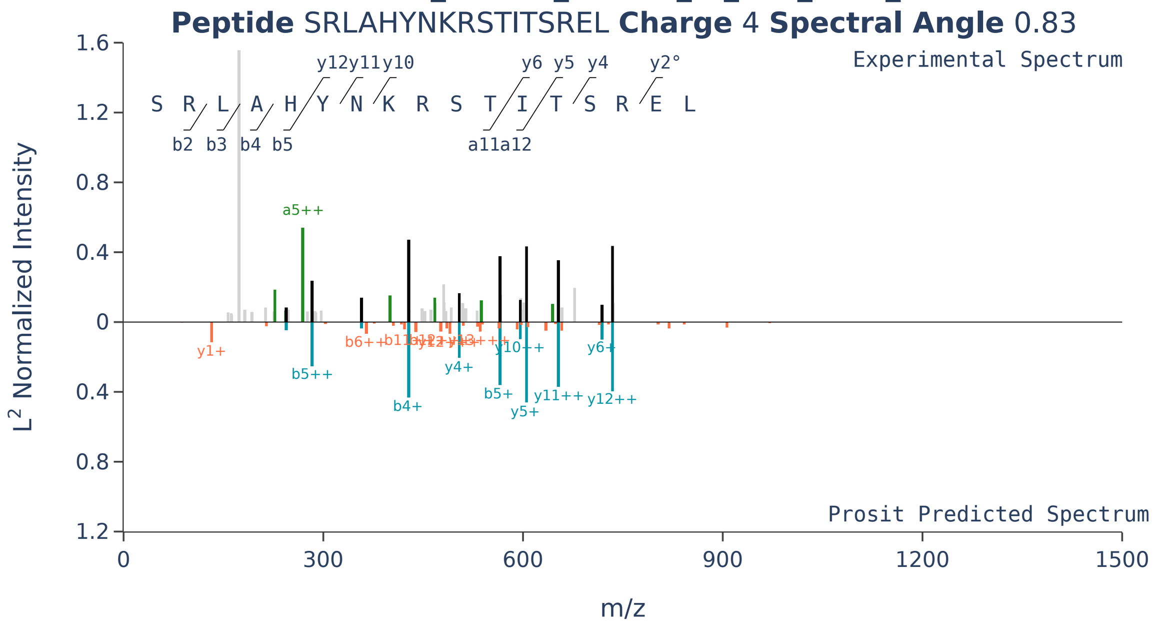


**Source** HRoetschke_291222_080123_Fu_H2B_b2R1_24h **Scan** 9271
**Peptide** GELAKHAVSEGTKAVTKYTSAK **Charge** 4 **Spectral Angle** 0.65

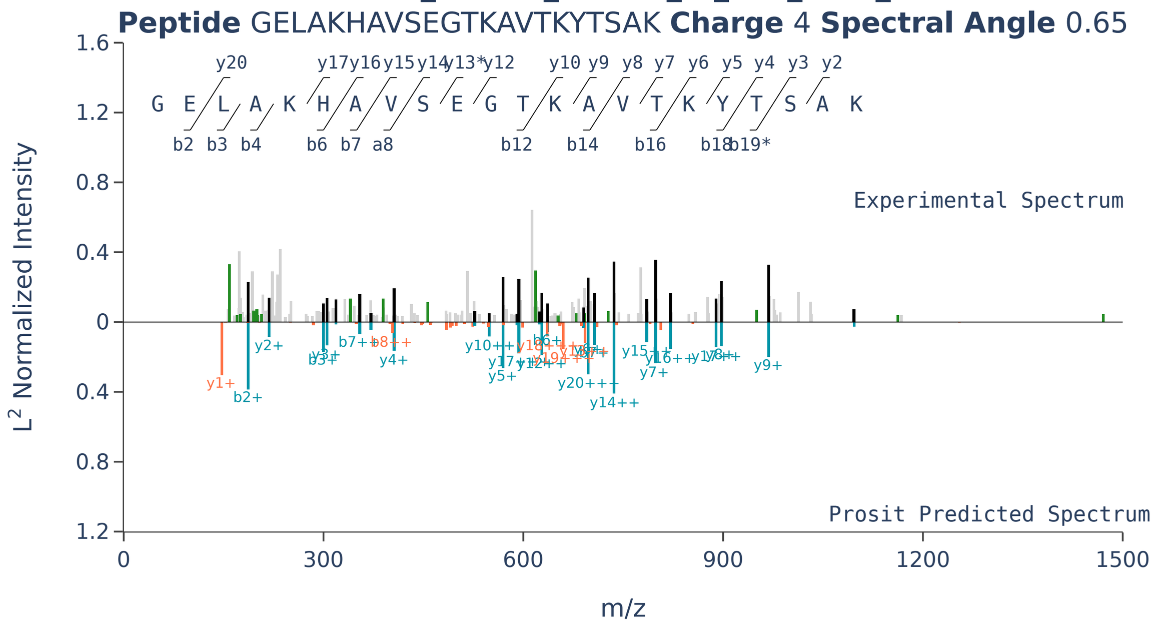


**Source** HRoetschke_291222_080123_Fu_H2B_b1R2_4h **Scan** 7618
**Peptide** AGEASRLAHYNKRSTITSREIQTAVR **Charge** 5 **Spectral Angle** 0.64

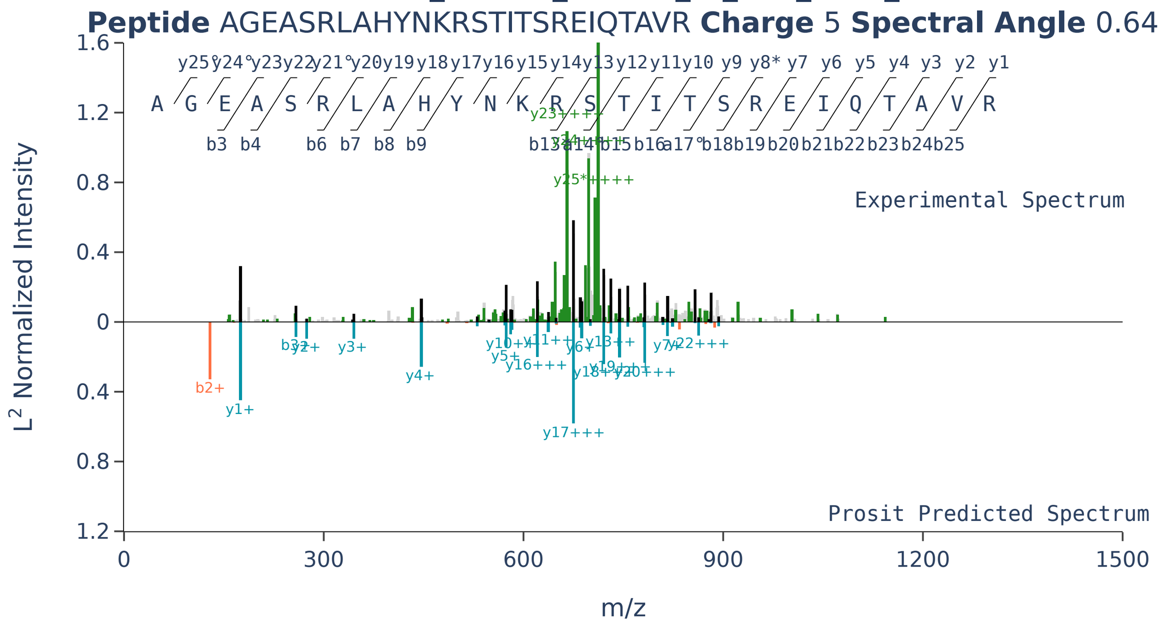


**Source** HRoetschke_291222_080123_Fu_H2B_b2R1_24h **Scan** 11555
**Peptide** NKRSTITSREIQTAVRL **Charge** 4 **Spectral Angle** 0.79

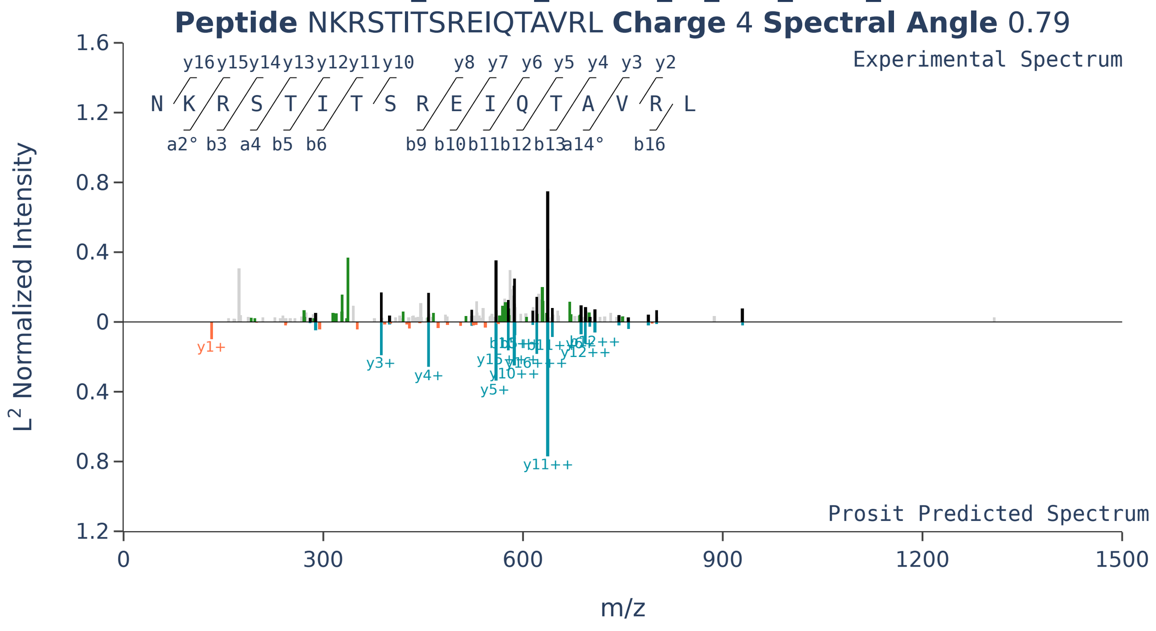


**Source** HRoetschke_291222_080123_Fu_H2B_b1R2_1h **Scan** 12867
**Peptide** VRLLLPGELAKHAVS **Charge** 4 **Spectral Angle** 0.78

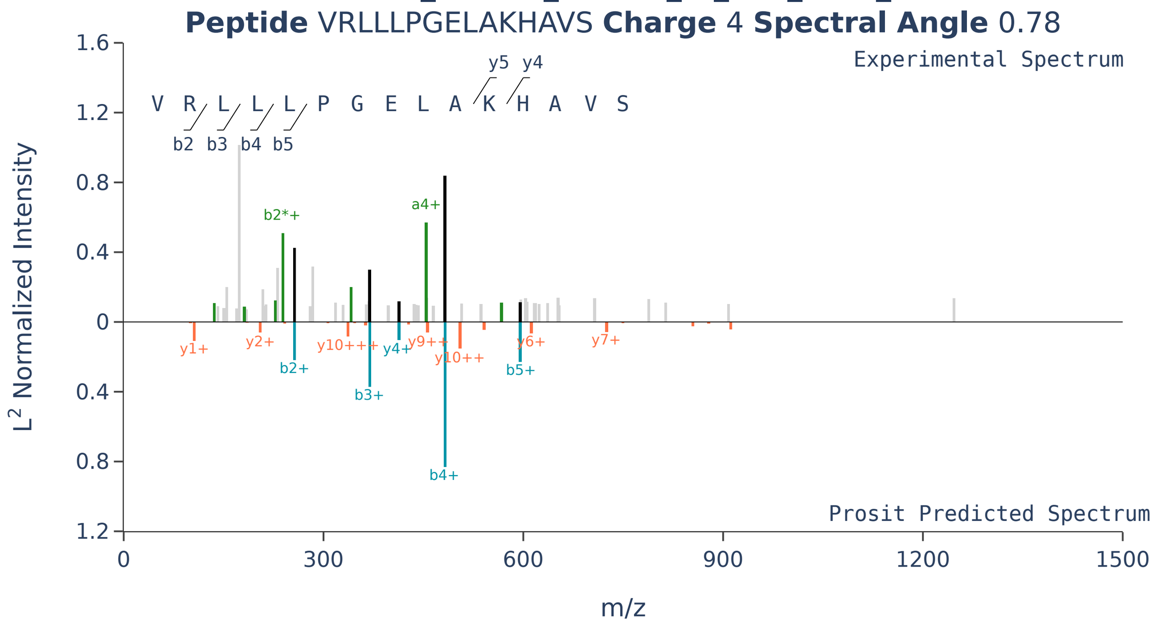

**Source** HRoetschke_291222_080123_Fu_H2B_b2R2_4h **Scan** 14208
**Peptide** RLAHYNKRSTITSREIQTAVRLL **Charge** 5 **Spectral Angle** 0.58

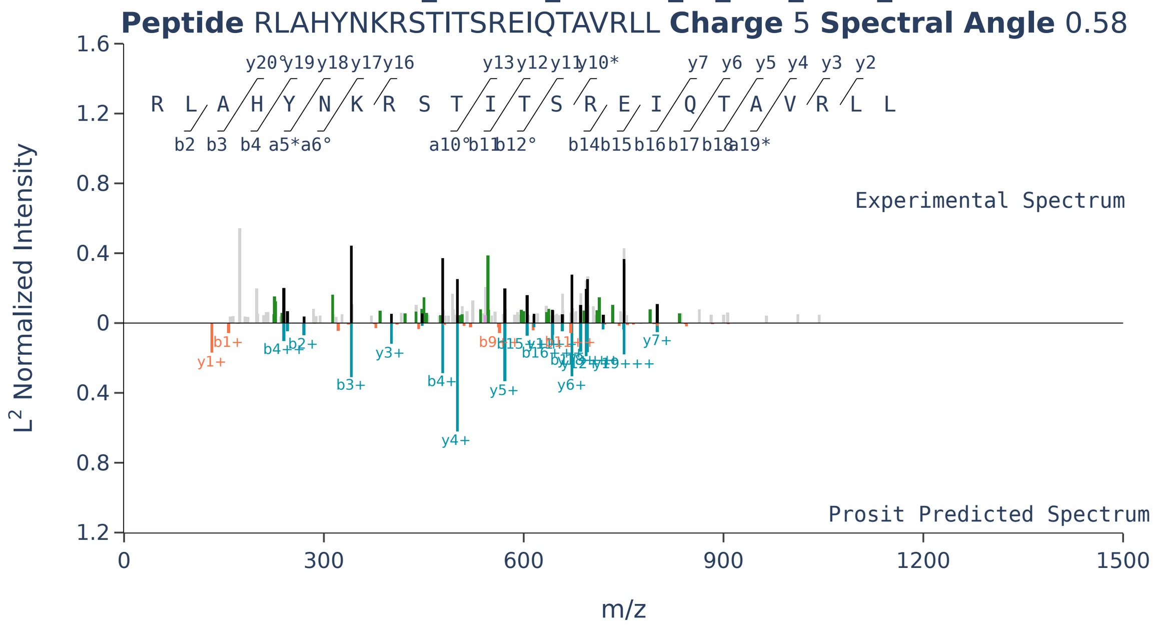


**Source** HRoetschke_291222_080123_Fu_H2B_b2R2_24h **Scan** 3857
**Peptide** AKHAVSEGTKAVTKYT **Charge** 3 **Spectral Angle** 0.64

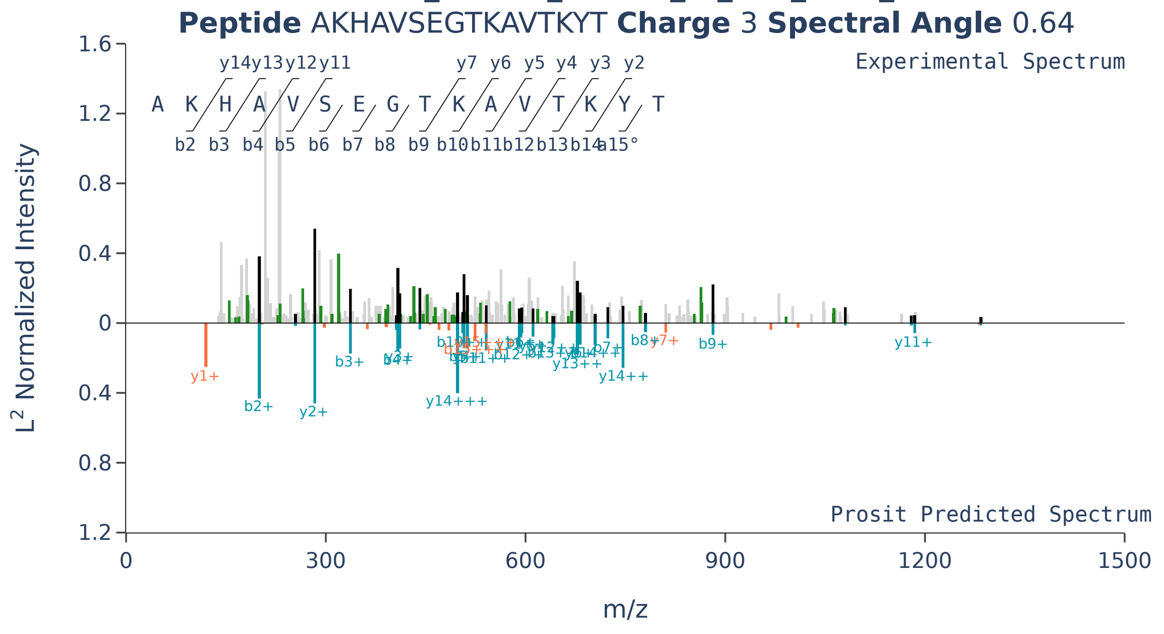


**Source** HRoetschke_291222_080123_Fu_H2B_b1R1_4h **Scan** 9095
**Peptide** ERIAGEASRLAHYNKRSTITSREI **Charge** 5 **Spectral Angle** 0.7

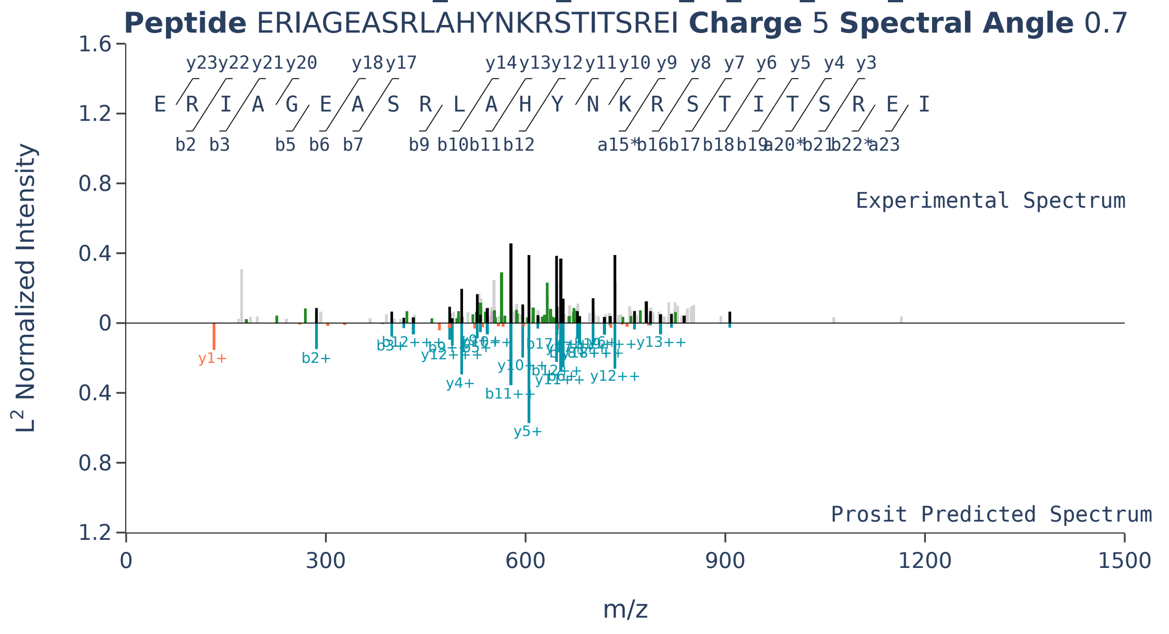


**Source** HRoetschke_291222_080123_Fu_H2B_b2R1_1h **Scan** 16873
**Peptide** TSREIQTAVRLLLPGELAKHAVS **Charge** 4 **Spectral Angle** 0.82

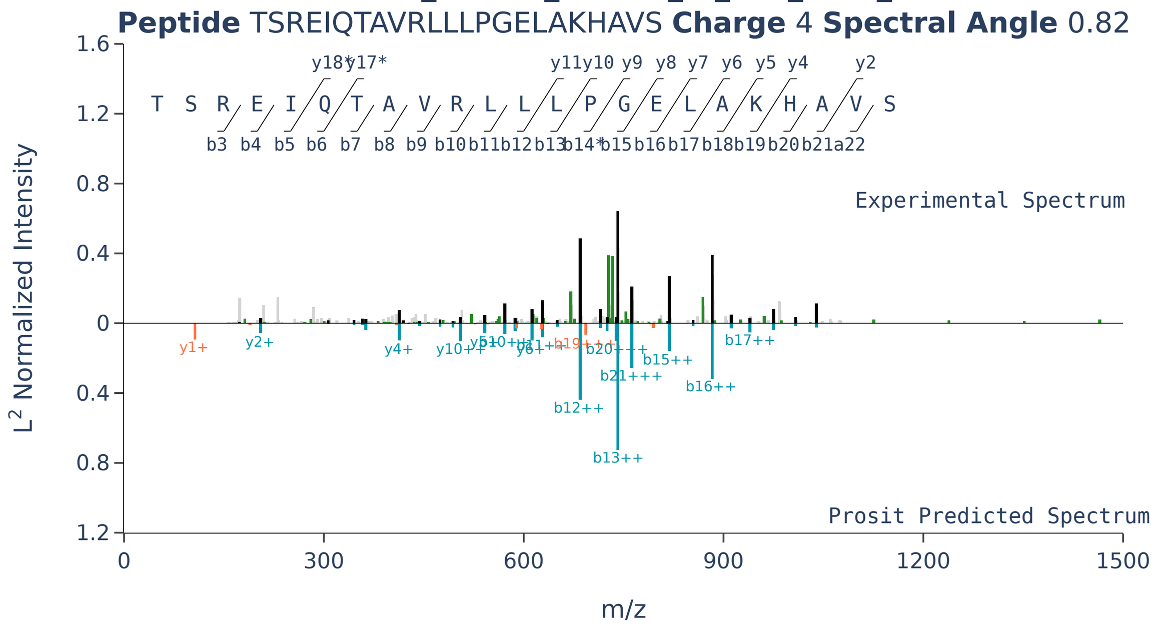


**Source** HRoetschke_291222_080123_Fu_H2B_b2R2_24h **Scan** 5821
**Peptide** PDTGISSKAMS **Charge** 2 **Spectral Angle** 0.78

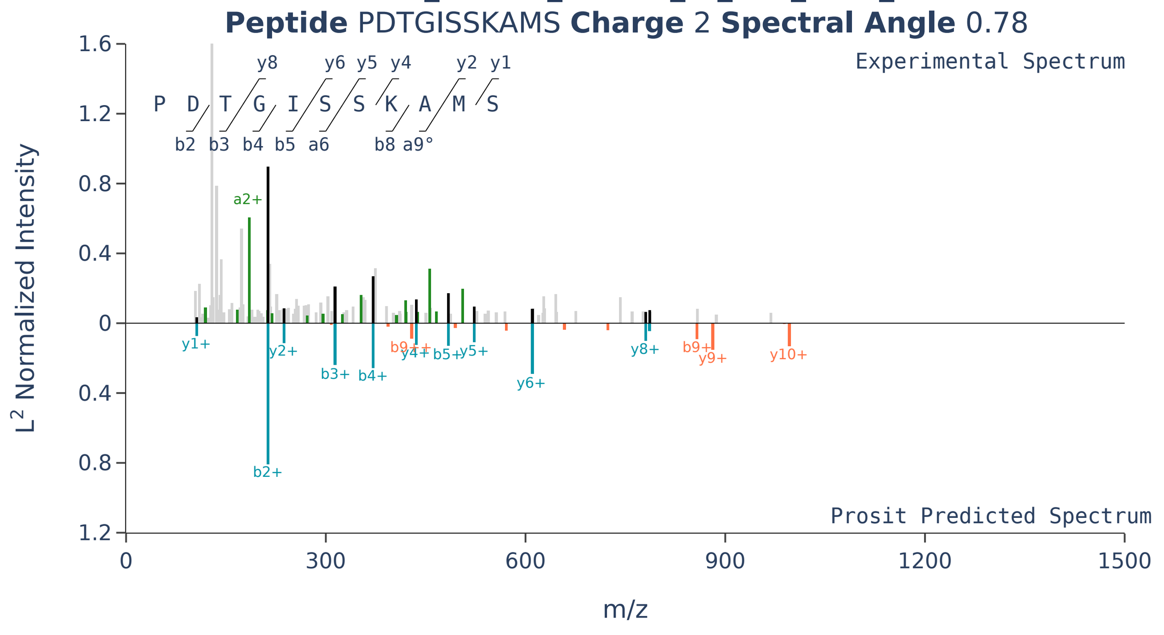


**Source** HRoetschke_291222_080123_Fu_H2B_b1R1_24h **Scan** 10061
**Peptide** YKVLKQVHPDTGISSKAMSL **Charge** 4 **Spectral Angle** 0.66

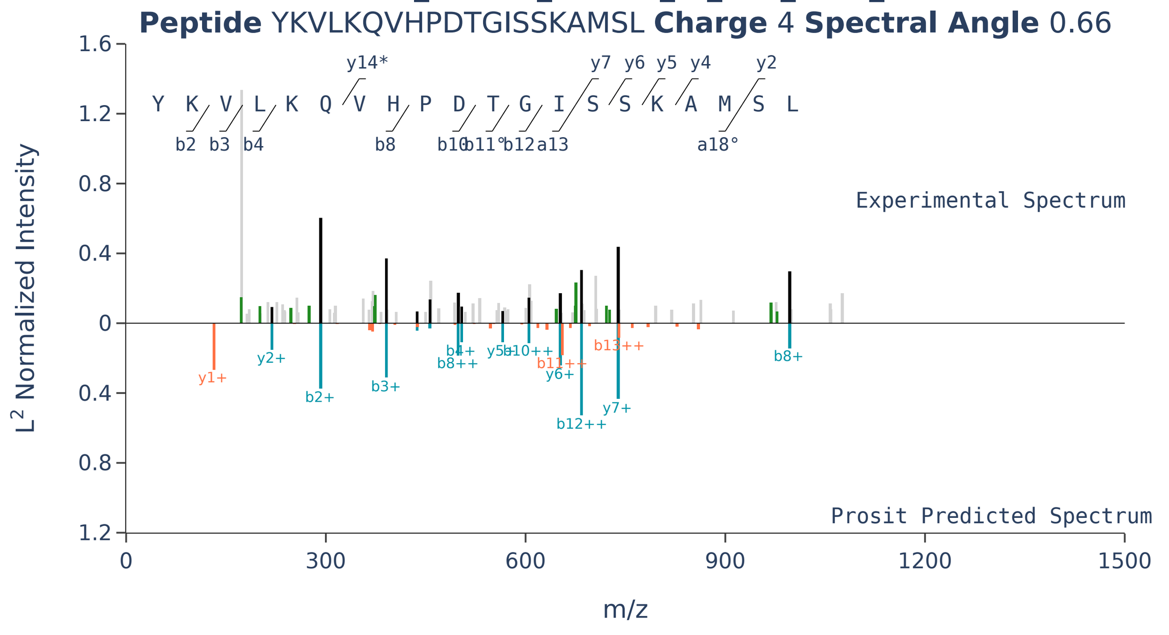


**Source** HRoetschke_291222_080123_Fu_H2B_b2R2_24h **Scan** 18453
**Peptide** SREIQTAVRLLLPGELAKHAVSEGTKA **Charge** 4 **Spectral Angle** 0.58

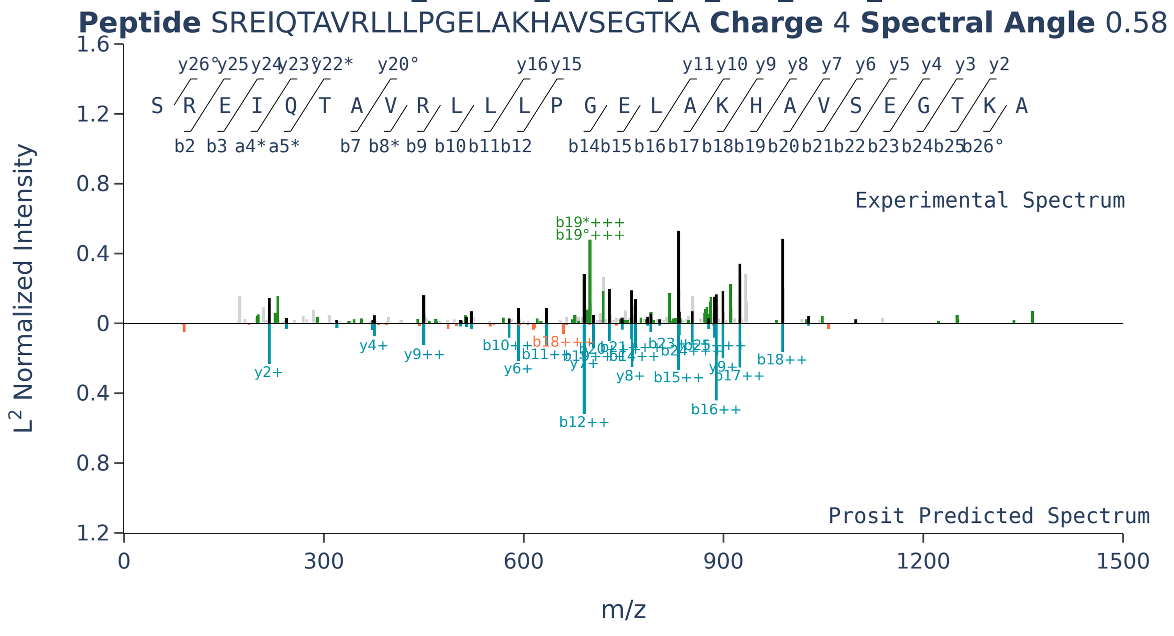


**Source** HRoetschke_291222_080123_Fu_H2B_b2R1_24h **Scan** 15604
**Peptide** LAHYNKRSTITSREIQTAVRLL **Charge** 5 **Spectral Angle** 0.77

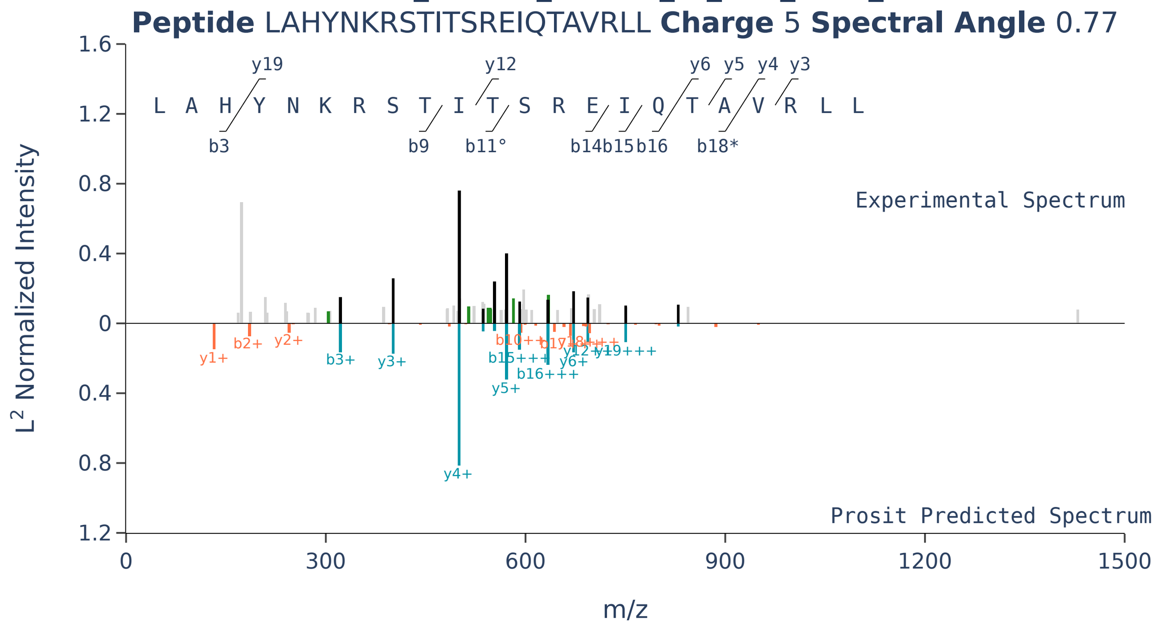


**Source** HRoetschke_291222_080123_Fu_H2B_b1R2_24h **Scan** 4475
**Peptide** GEASRLAHYNKRSTIT **Charge** 3 **Spectral Angle** 0.67

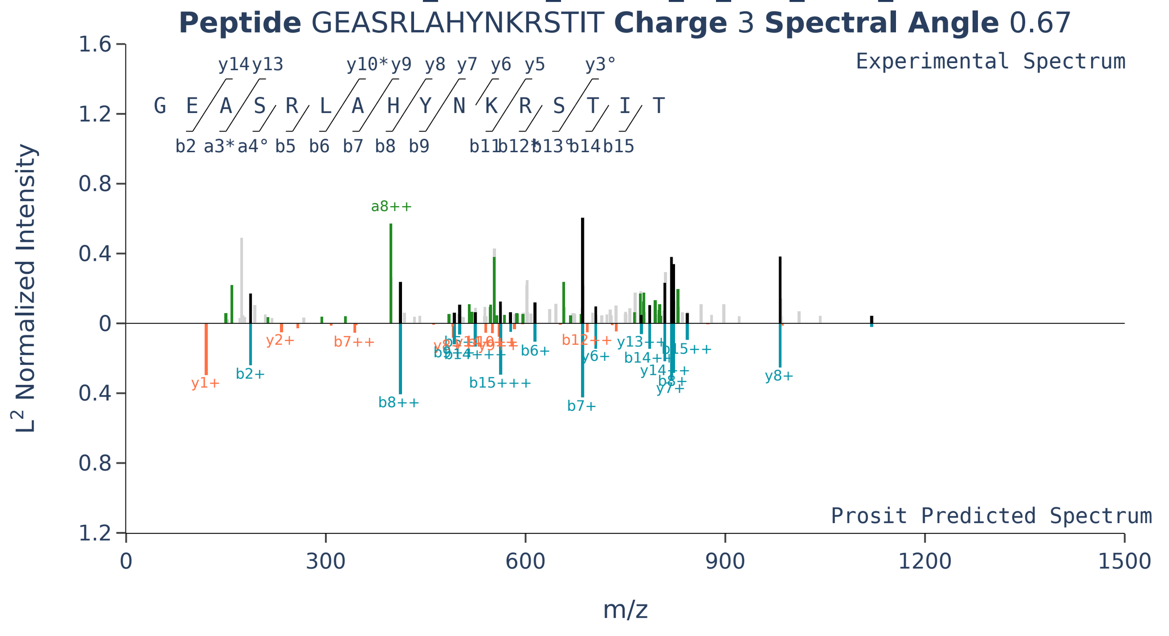


**Source** HRoetschke_291222_080123_Fu_H2B_b2R1_4h **Scan** 6874
**Peptide** IAGEASRLAHYNKRSTI **Charge** 4 **Spectral Angle** 0.55

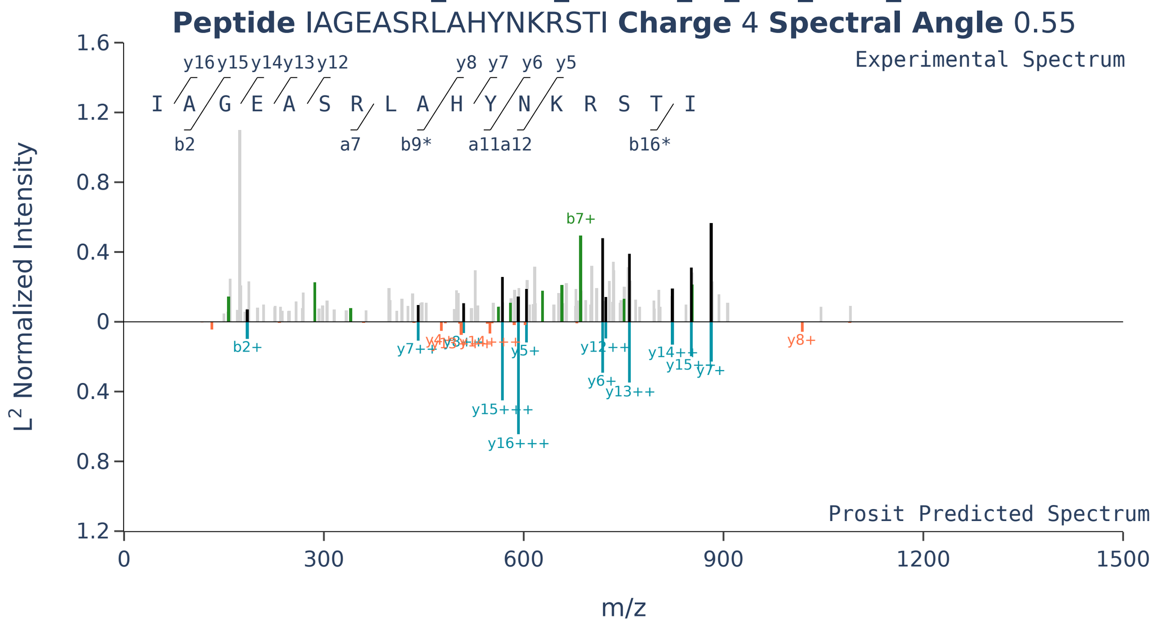


**Source** HRoetschke_291222_080123_Fu_H2B_b1R2_24h **Scan** 6729
**Peptide** KQVHPDTGISSKAMSL **Charge** 2 **Spectral Angle** 0.71

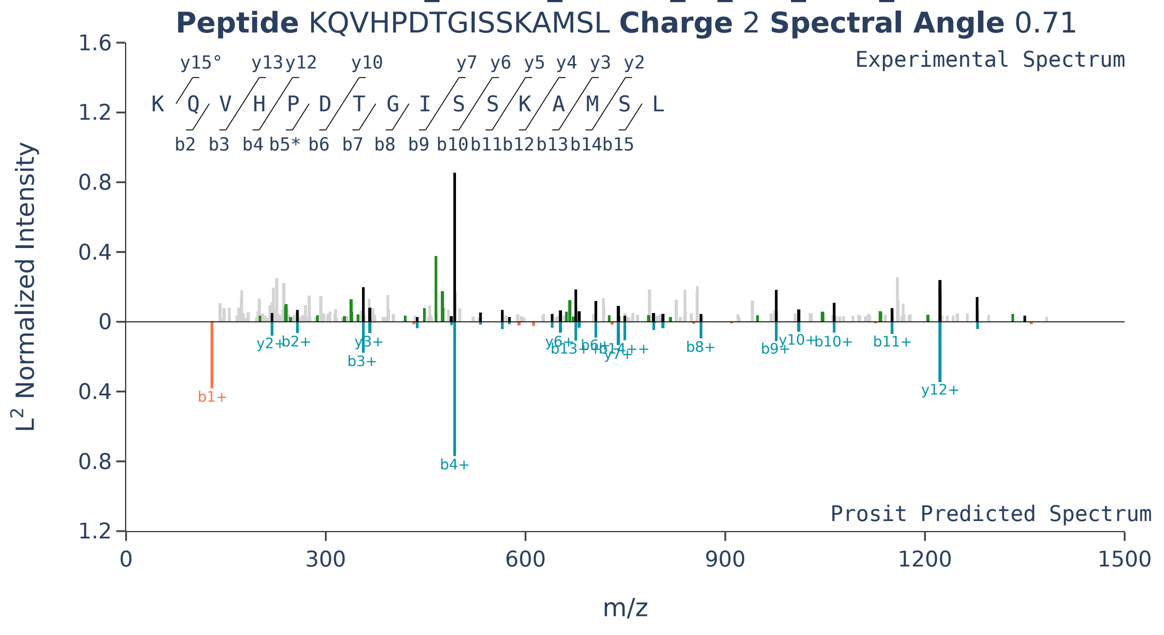


**Source** HRoetschke_291222_080123_Fu_H2B_b2R1_4h **Scan** 15933
**Peptide** TAVRLLLPGELAKHAVSEGTKAVTK **Charge** 4 **Spectral Angle** 0.79

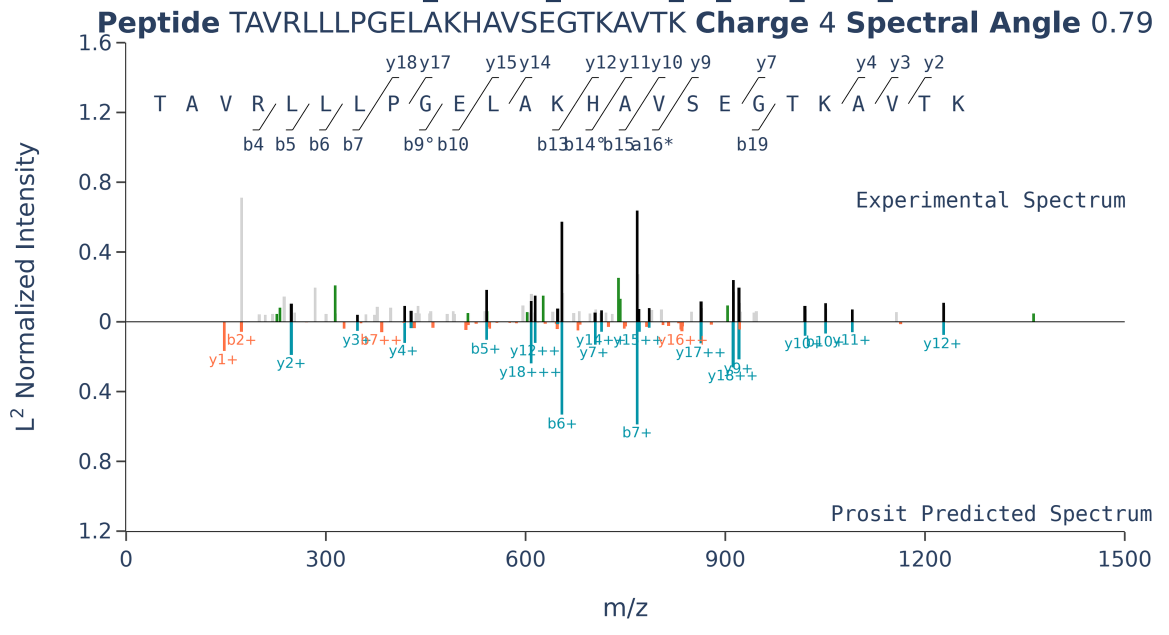


**Source** HRoetschke_291222_080123_Fu_H2B_b1R1_24h **Scan** 5290
**Peptide** EASRLAHYNKRSTITSREIQ **Charge** 4 **Spectral Angle** 0.64

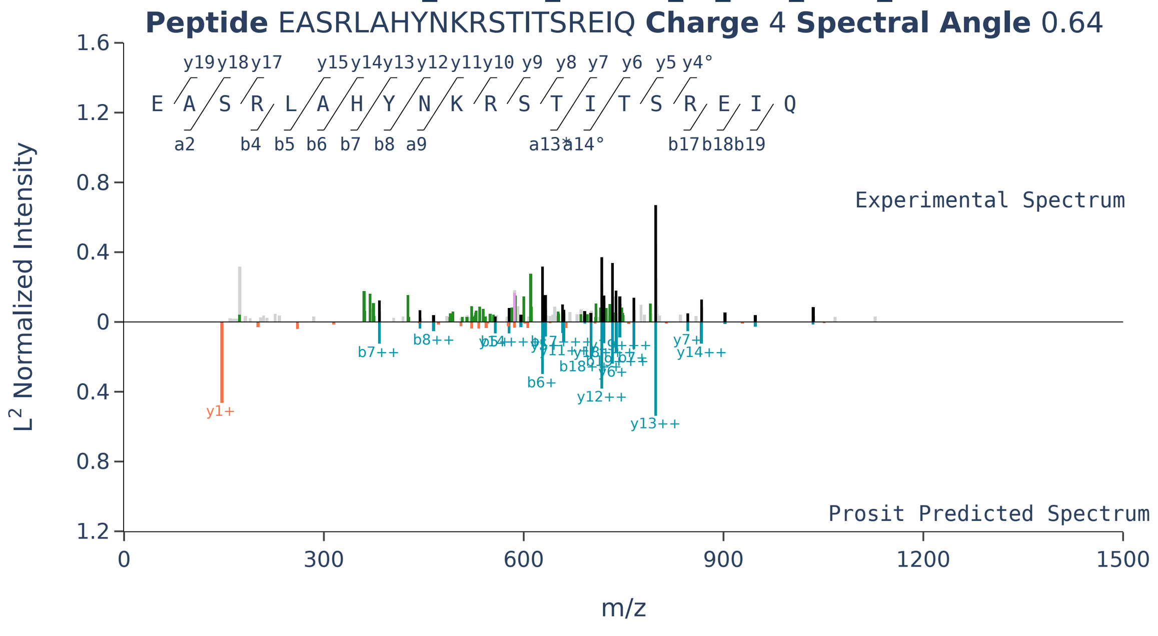


**Source** HRoetschke_291222_080123_Fu_H2B_b1R2_4h **Scan** 4612
**Peptide** AGEASRLAHYNKRSTIT **Charge** 3 **Spectral Angle** 0.66

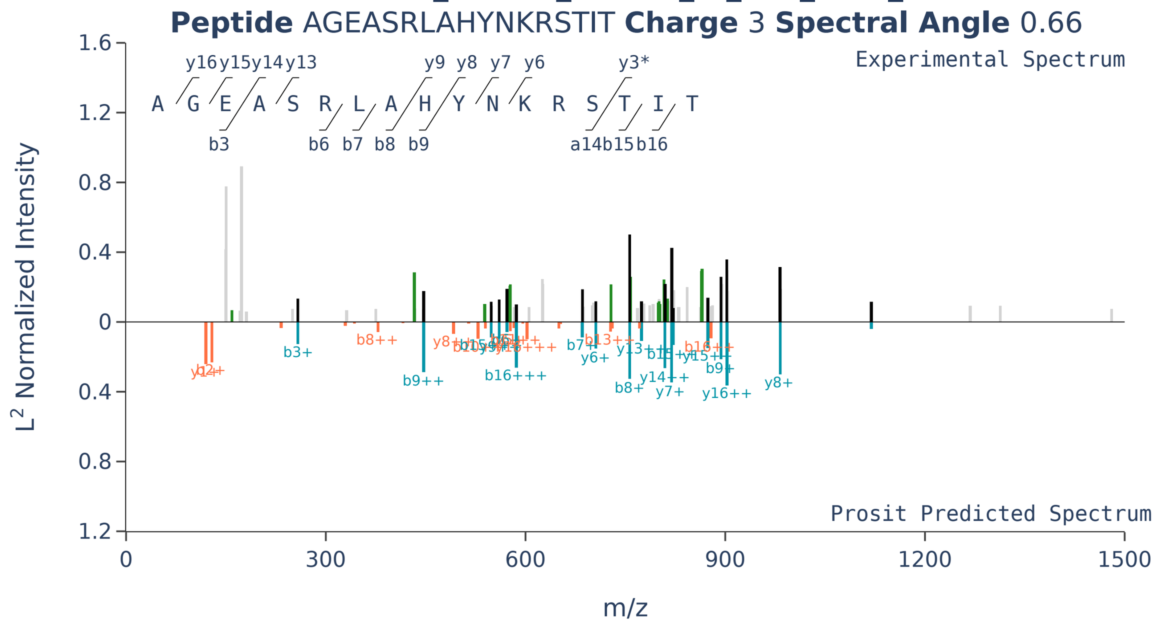


**Source** HRoetschke_291222_080123_Fu_H2B_b2R2_4h **Scan** 22011
**Peptide** MNSFVNDVFERIAGEAS **Charge** 2 **Spectral Angle** 0.79

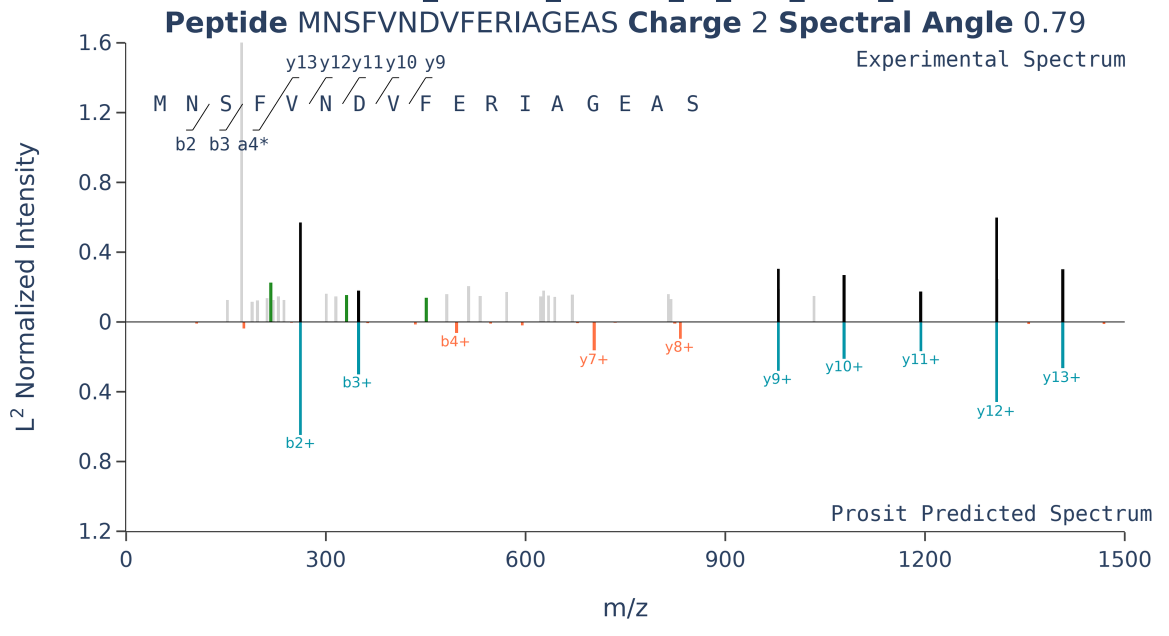

**Source** HRoetschke_291222_080123_Fu_H2B_b2R1_4h **Scan** 19711
**Peptide** KRSTITSREIQTAVRLLLPGE **Charge** 4 **Spectral Angle** 0.66

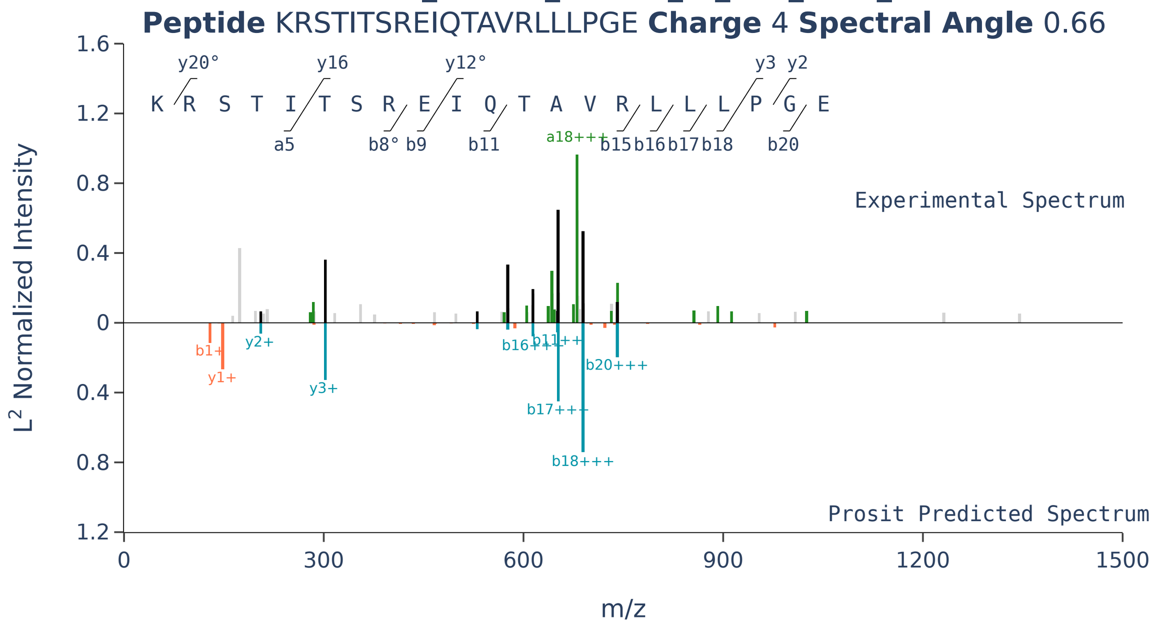


**Source** HRoetschke_291222_080123_Fu_H2B_b2R1_4h **Scan** 19906
**Peptide** NKRSTITSREIQTAVRLLLPGE **Charge** 4 **Spectral Angle** 0.76

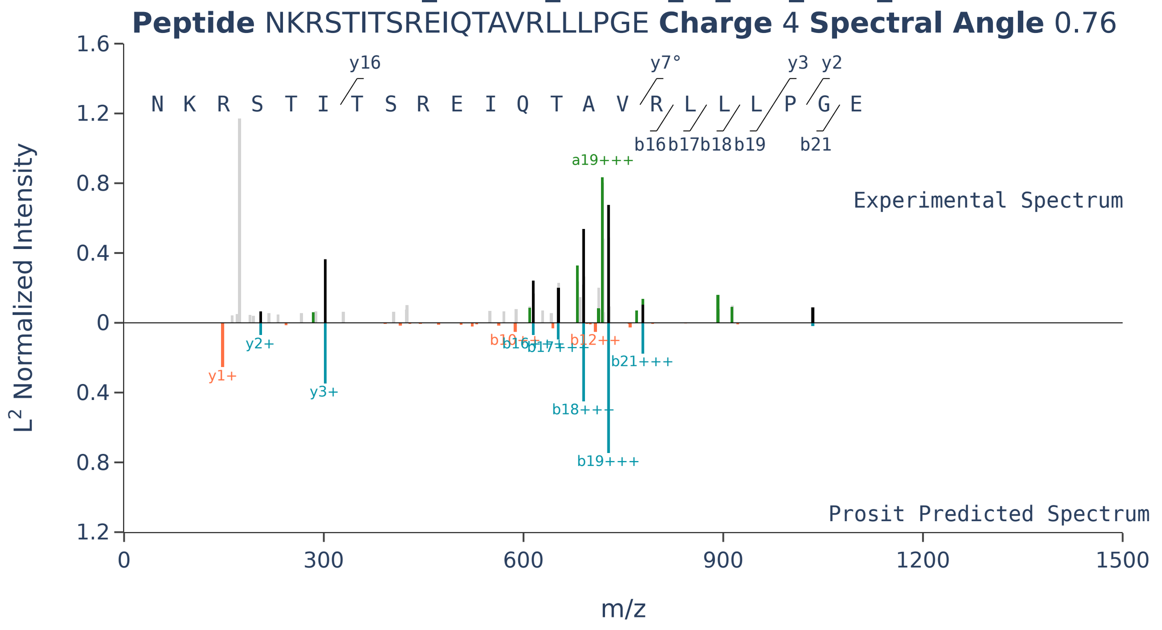


**Source** HRoetschke_291222_080123_Fu_H2B_b2R2_24h **Scan** 5884
**Peptide** TQKKDGKKRRKTRKESYAIYVYKV **Charge** 5 **Spectral Angle** 0.61

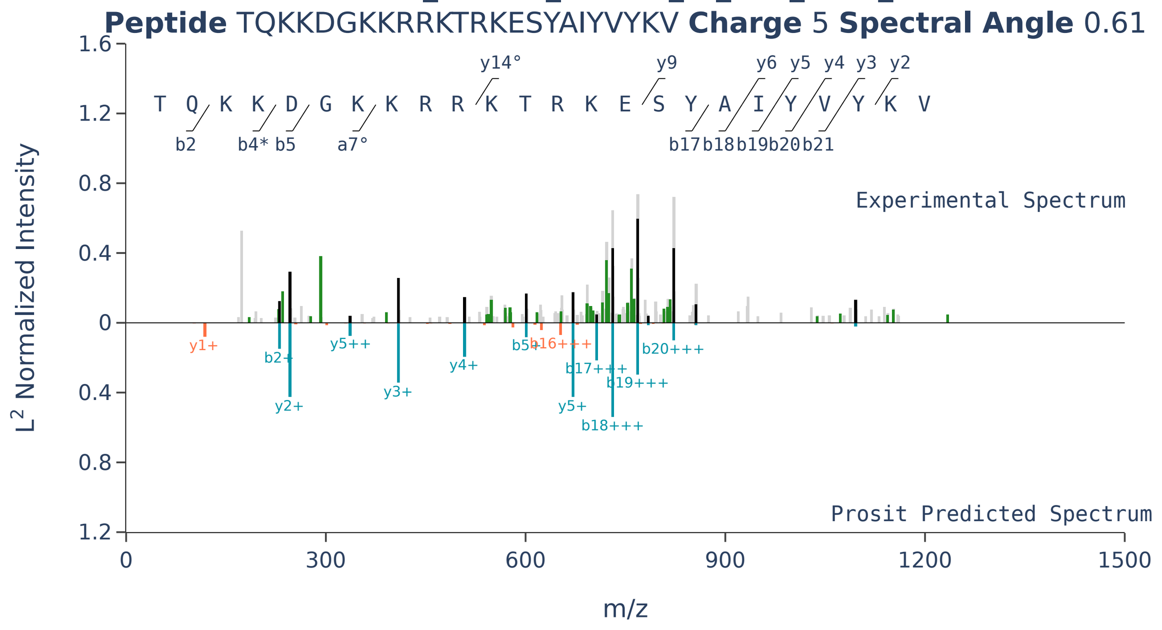


**Source** HRoetschke_291222_080123_Fu_H2B_b2R2_4h **Scan** 13940
**Peptide** VFERIAGEASRLAHYNKRSTITSREL **Charge** 5 **Spectral Angle** 0.67

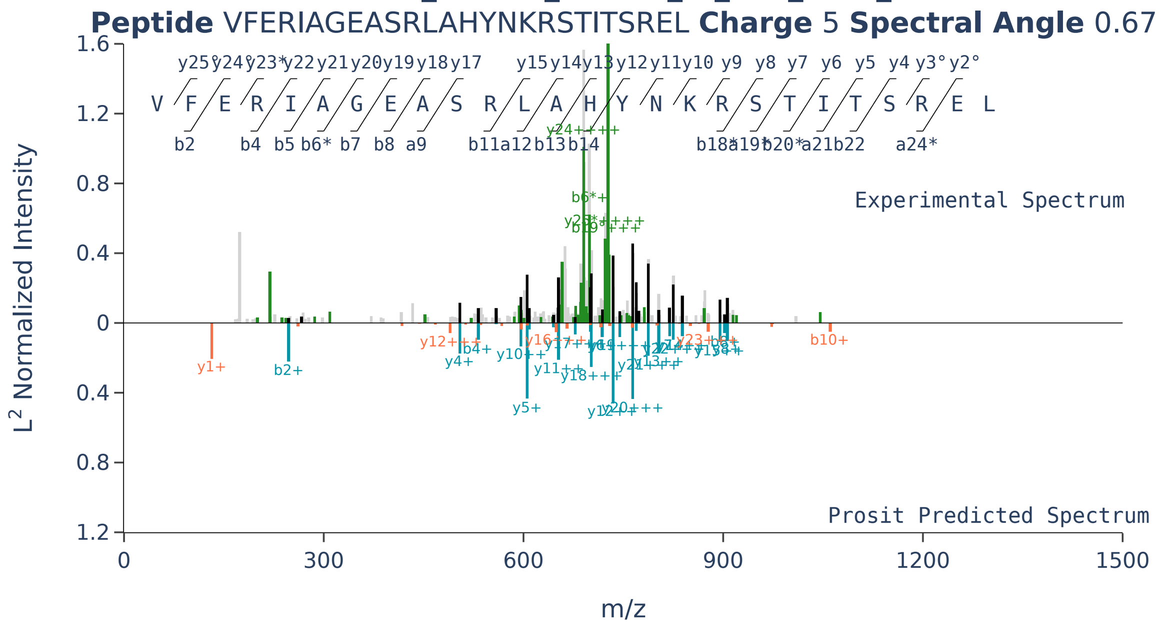


**Source** HRoetschke_291222_080123_Fu_H2B_b1R1_4h **Scan** 2739
**Peptide** SRLAHYNKRSTI **Charge** 3 **Spectral Angle** 0.82

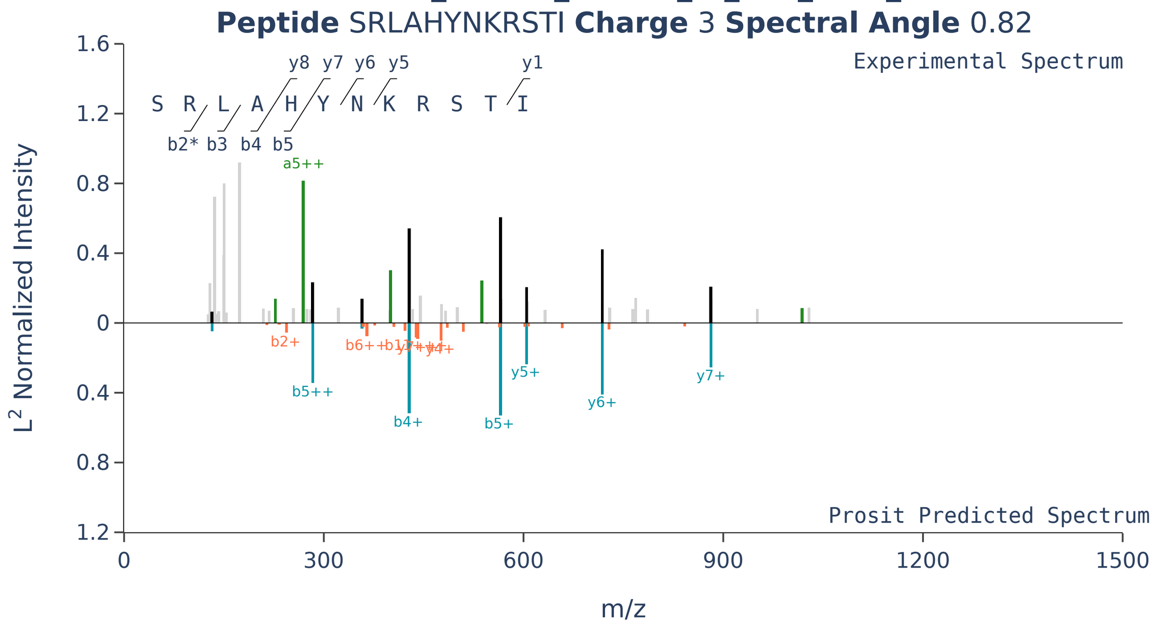


**Source** HRoetschke_291222_080123_Fu_H2B_b2R1_24h **Scan** 2237
**Peptide** RLAHYNKRSTIT **Charge** 2 **Spectral Angle** 0.56

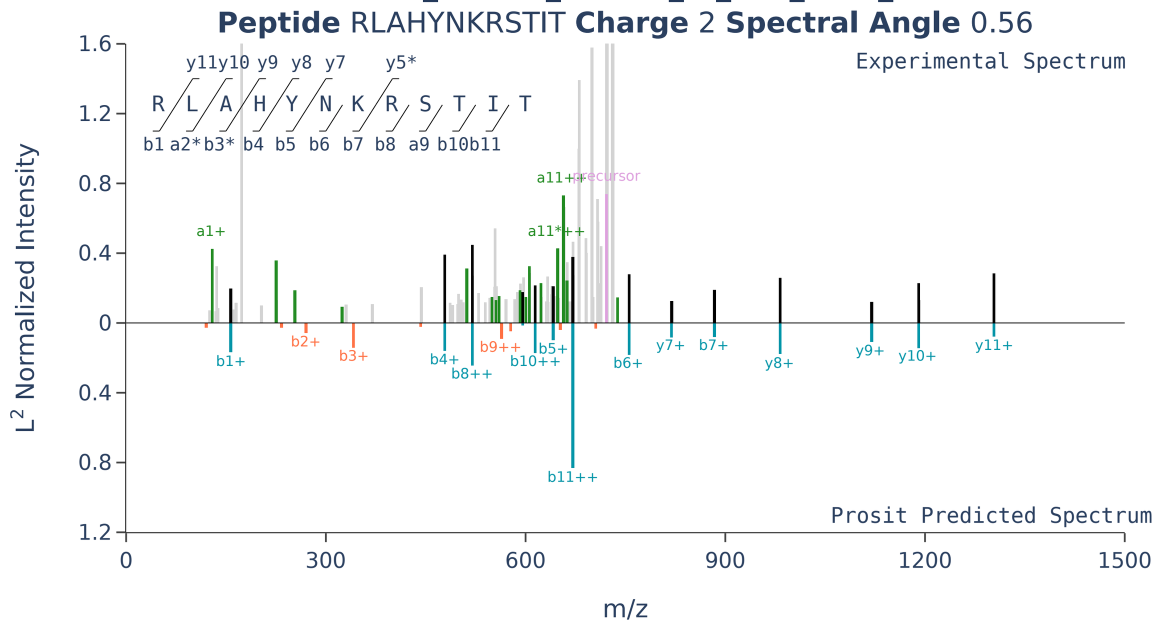


**Source** HRoetschke_291222_080123_Fu_H2B_b2R2_4h **Scan** 19422
**Peptide** VNDVFERIAGEASRLAHYNKRSTITSREL **Charge** 6 **Spectral Angle** 0.54

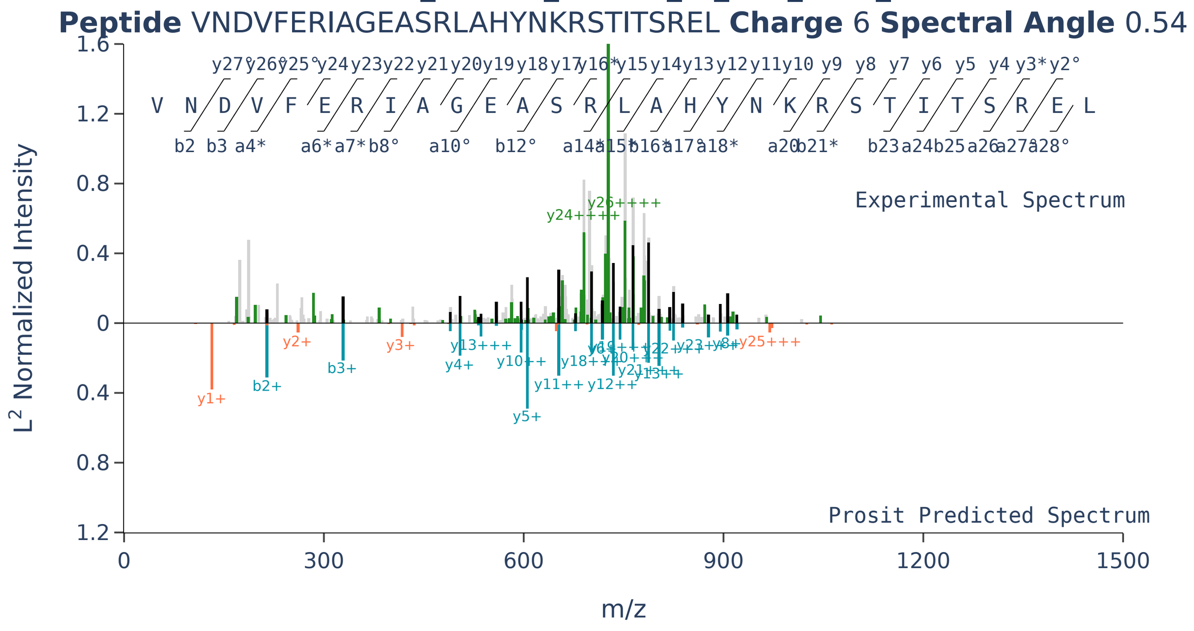


**Source** HRoetschke_291222_080123_Fu_H2B_b2R1_4h **Scan** 16314
**Peptide** VRLLLPGELAKHAV **Charge** 4 **Spectral Angle** 0.74

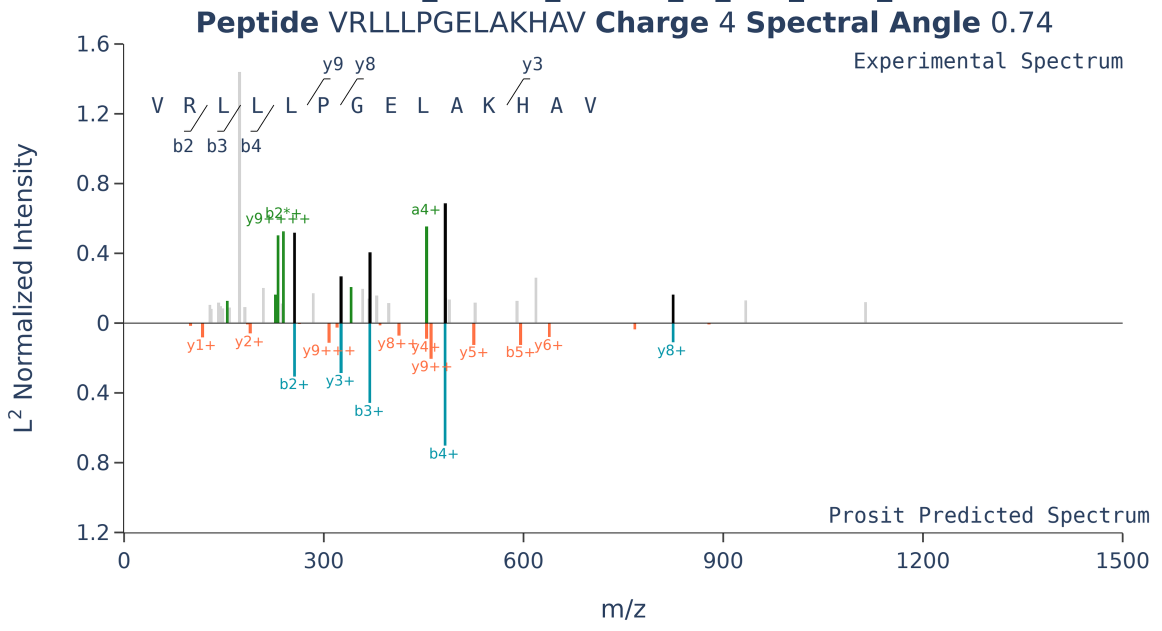


**Source** HRoetschke_291222_080123_Fu_H2B_b2R2_24h **Scan** 17365
**Peptide** VRLLLPGELAKHAVSEGTKAV **Charge** 4 **Spectral Angle** 0.81

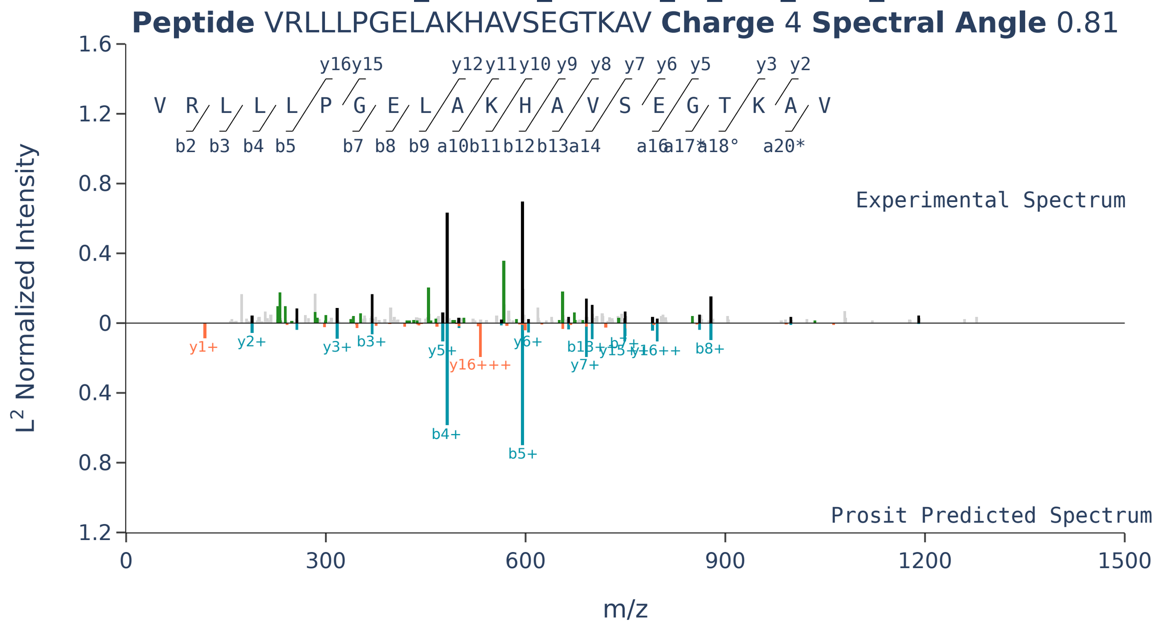


**Source** HRoetschke_291222_080123_Fu_H2B_b1R2_24h **Scan** 4985
**Peptide** QKKDGKKRRKTRKESYAIYVY **Charge** 5 **Spectral Angle** 0.89

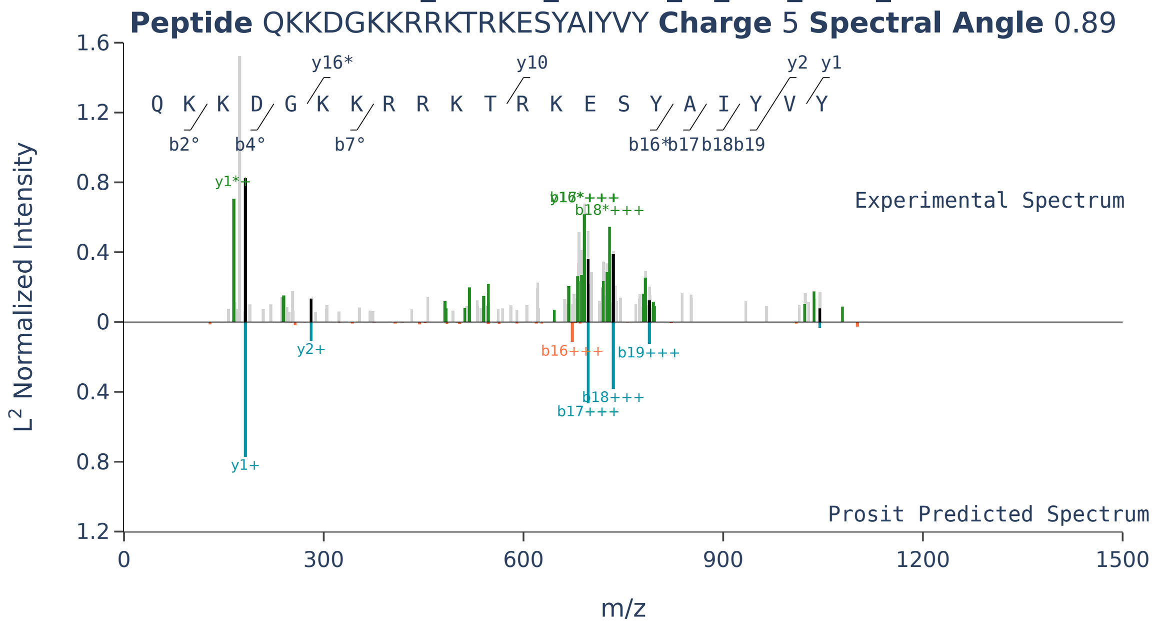


**Source** HRoetschke_291222_080123_Fu_H2B_b1R1_24h **Scan** 4755
**Peptide** GISSKAMSIMN **Charge** 2 **Spectral Angle** 0.8

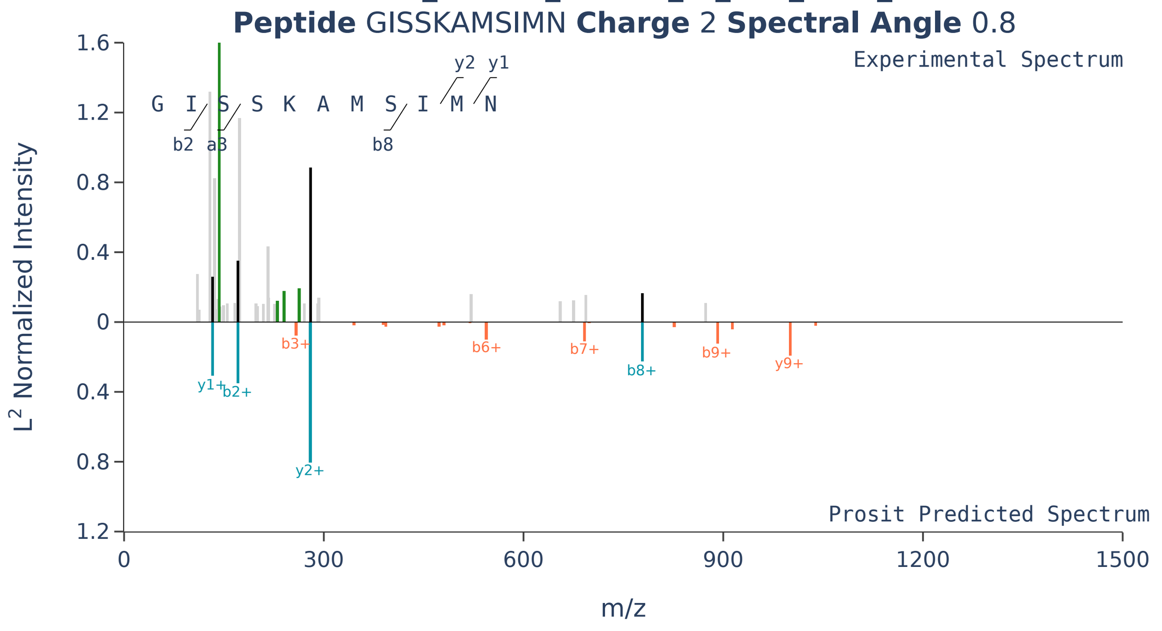


**Source** HRoetschke_291222_080123_Fu_H2B_b1R2_24h **Scan** 13719
**Peptide** RLLLPGELAKHAVS **Charge** 3 **Spectral Angle** 0.8

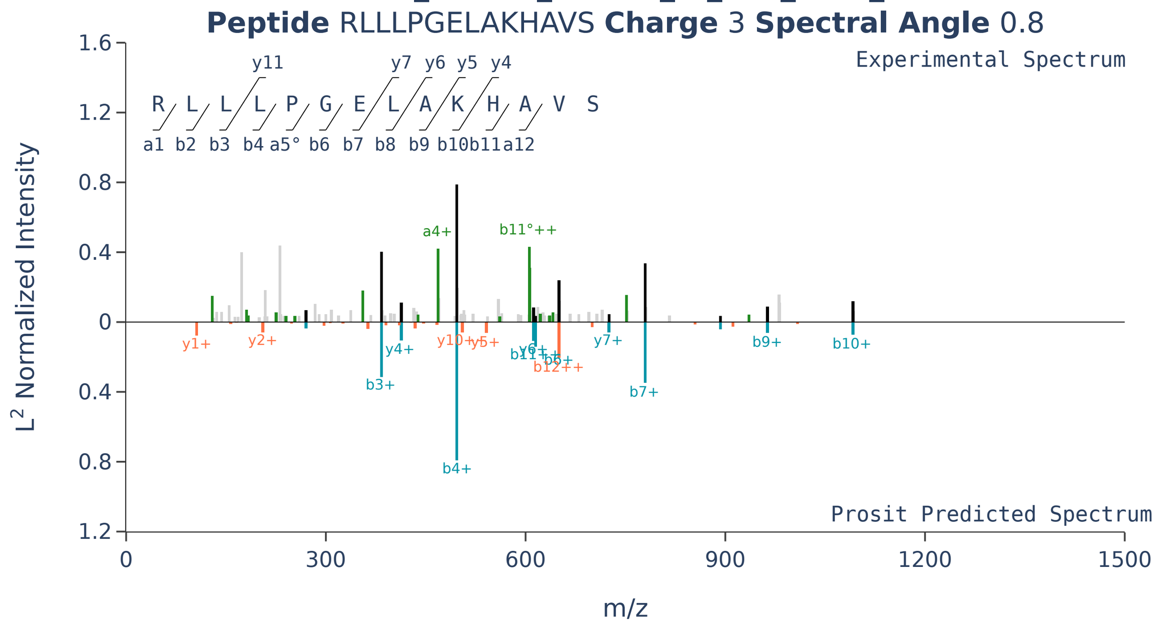


**Source** HRoetschke_291222_080123_Fu_H2B_b2R2_24h **Scan** 9677
**Peptide** VLKQVHPDTGISSKAMSL **Charge** 4 **Spectral Angle** 0.61

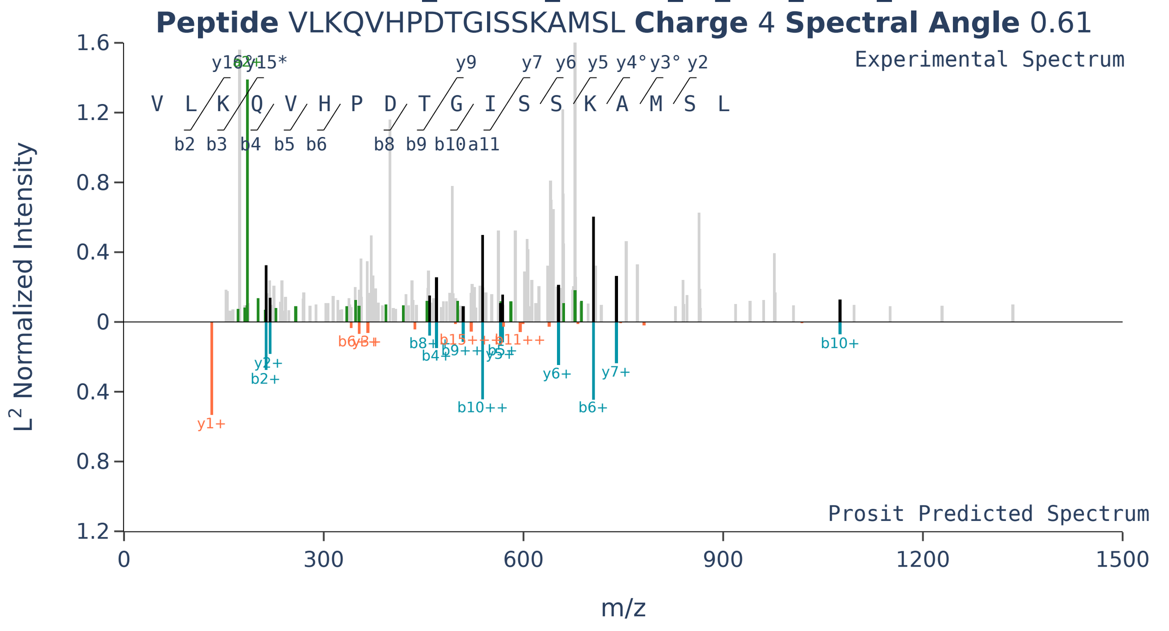


**Source** HRoetschke_291222_080123_Fu_H2B_b2R1_4h **Scan** 21719
**Peptide** MNSFVNDVFERIAGE **Charge** 2 **Spectral Angle** 0.72

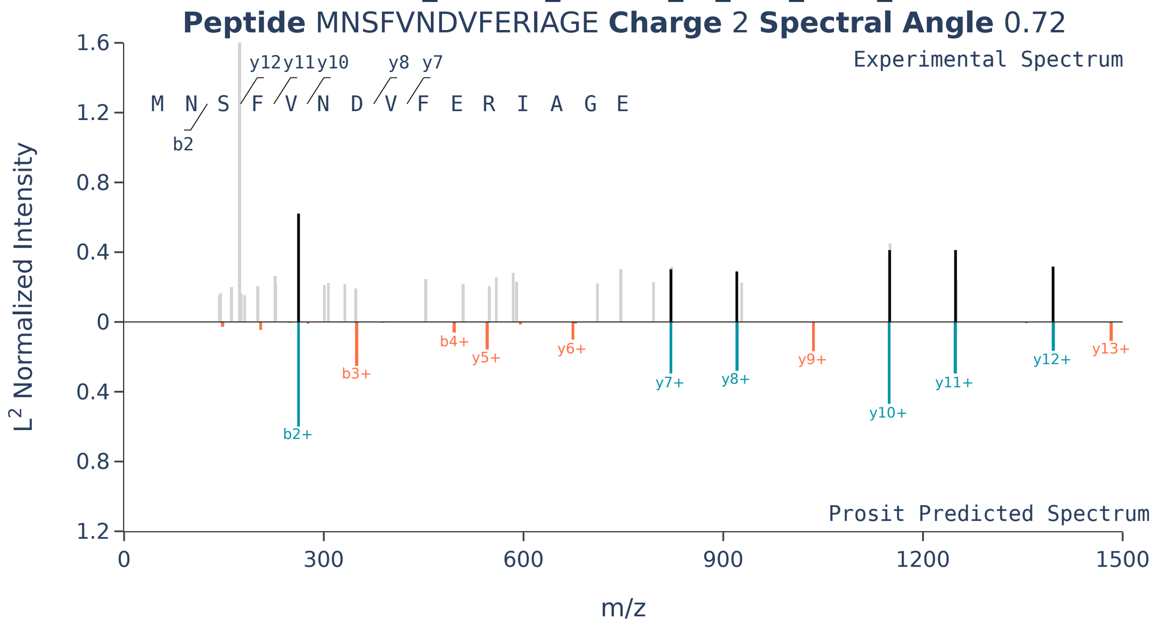


**Source** HRoetschke_291222_080123_Fu_H2B_b2R2_24h **Scan** 14096
**Peptide** RLLLPGELAKHAVSEGTK **Charge** 4 **Spectral Angle** 0.76

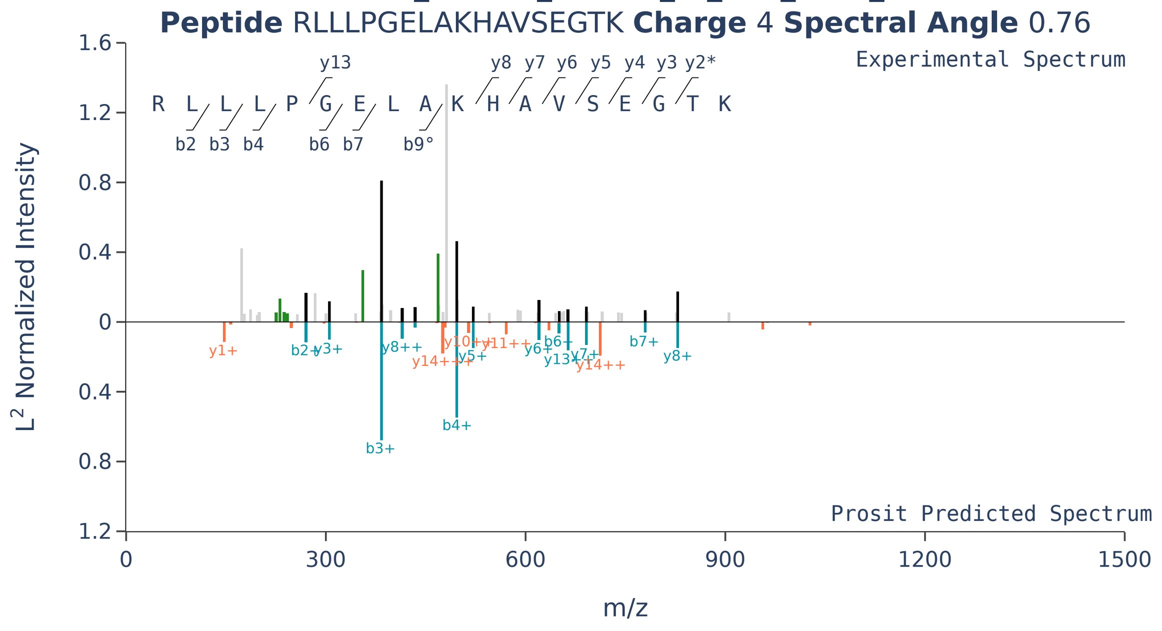

**Source** HRoetschke_291222_080123_Fu_H2B_b2R2_1h **Scan** 18233
**Peptide** NSFVNDVFERIAGEASR **Charge** 3 **Spectral Angle** 0.76

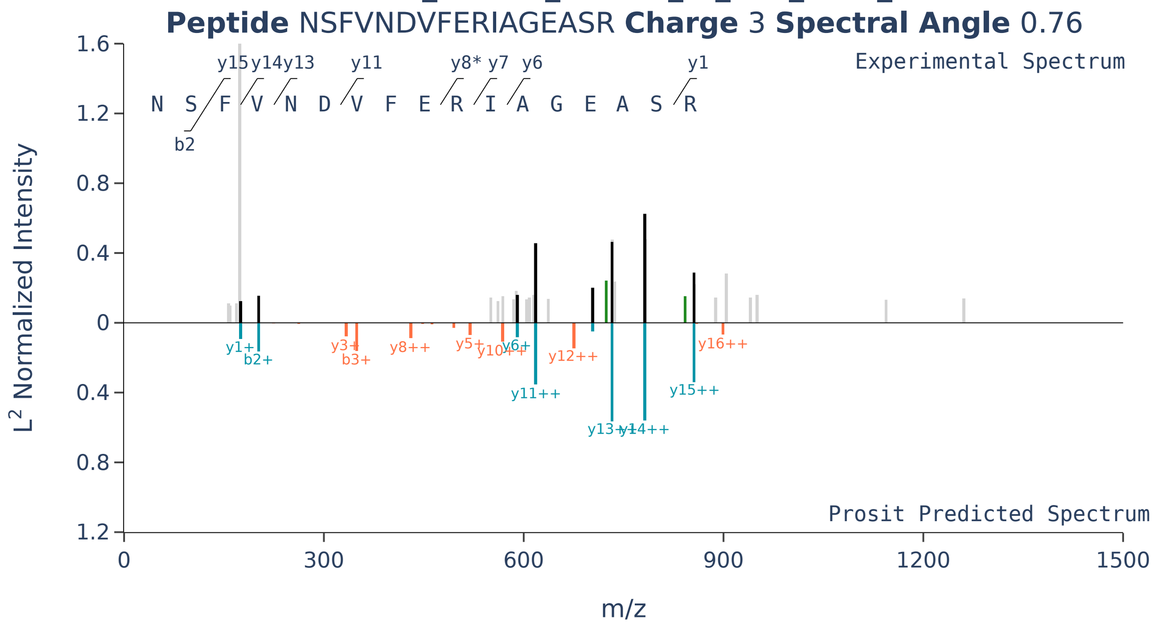


**Source** HRoetschke_291222_080123_Fu_H2B_b1R1_24h **Scan** 12397
**Peptide** LLPGELAKHAV **Charge** 2 **Spectral Angle** 0.68

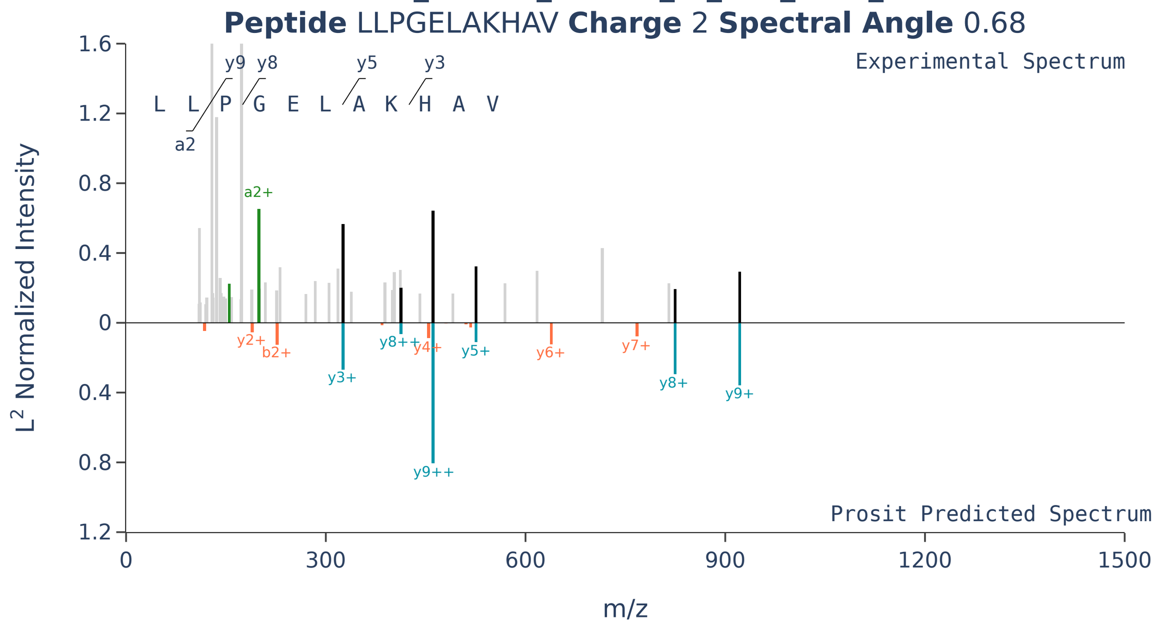


**Source** HRoetschke_291222_080123_Fu_H2B_b1R2_24h **Scan** 1737
**Peptide** RLAHYNKRST **Charge** 3 **Spectral Angle** 0.73

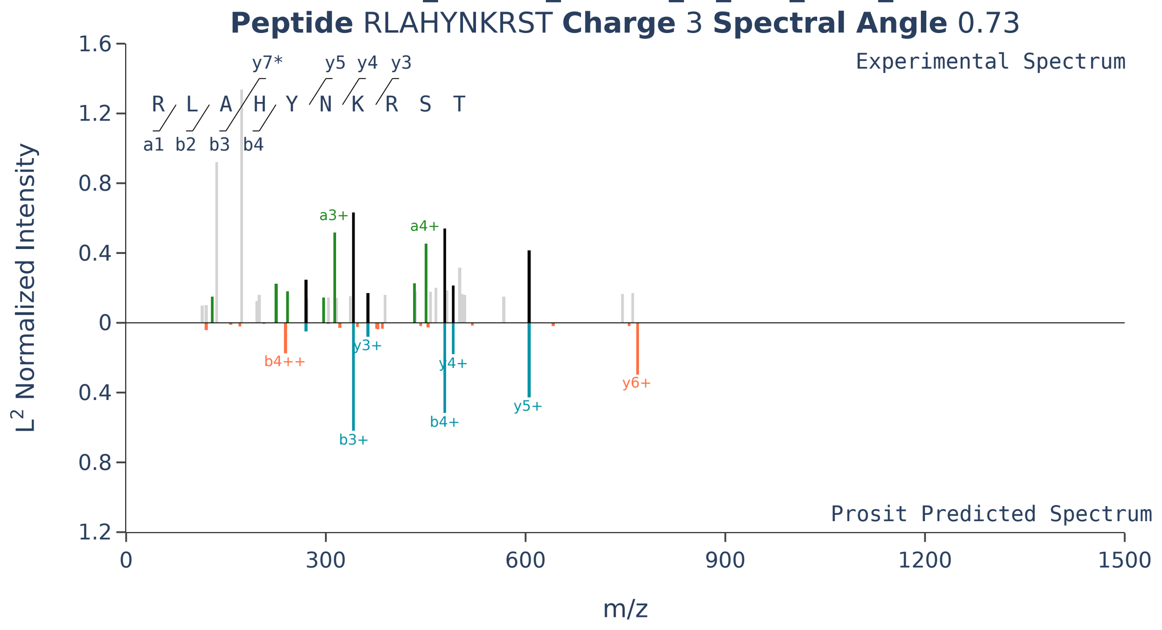


**Source** HRoetschke_291222_080123_Fu_H2B_b2R1_24h **Scan** 3356
**Peptide** TQKKDGKKRRKTRKESYAIYV **Charge** 4 **Spectral Angle** 0.81

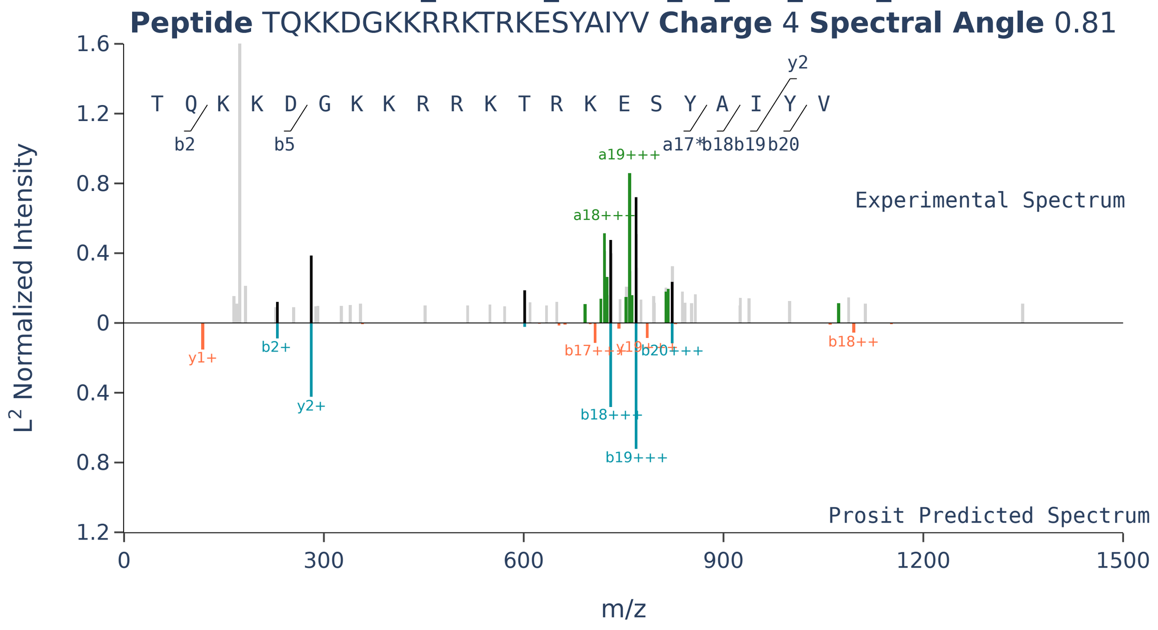


**Source** HRoetschke_291222_080123_Fu_H2B_b1R1_24h **Scan** 7749
**Peptide** RLAGEASRLAHYNKRSTITSREIQ **Charge** 5 **Spectral Angle** 0.6

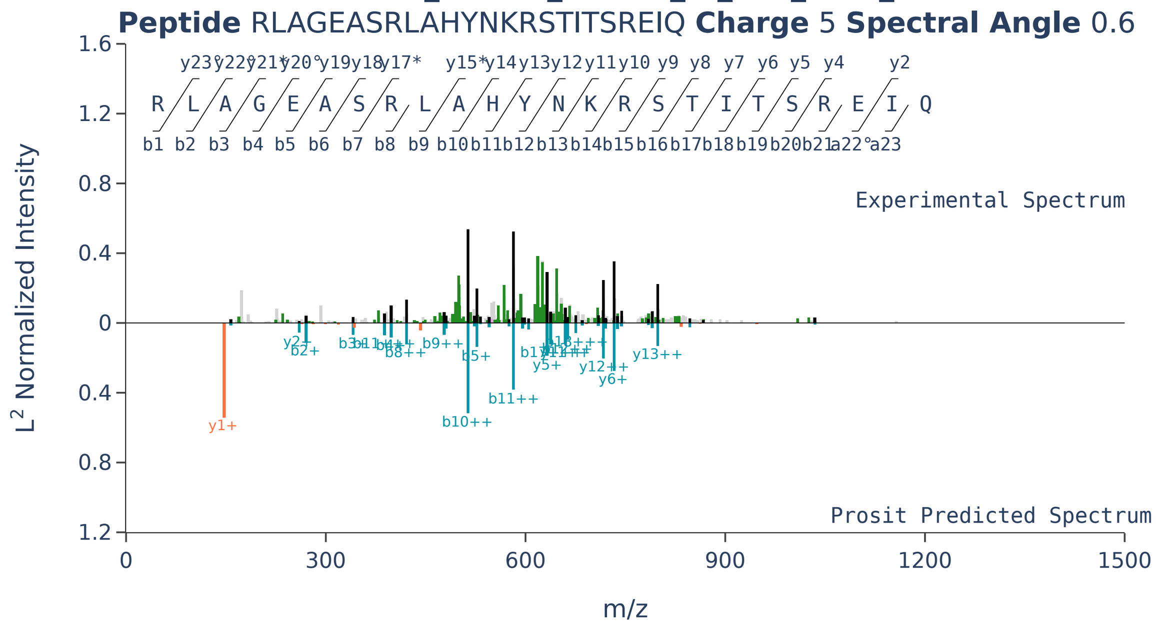


**Source** HRoetschke_291222_080123_Fu_H2B_b2R2_24h **Scan** 7405
**Peptide** AGEASRLAHYNKRSTITSREIQTA **Charge** 4 **Spectral Angle** 0.57

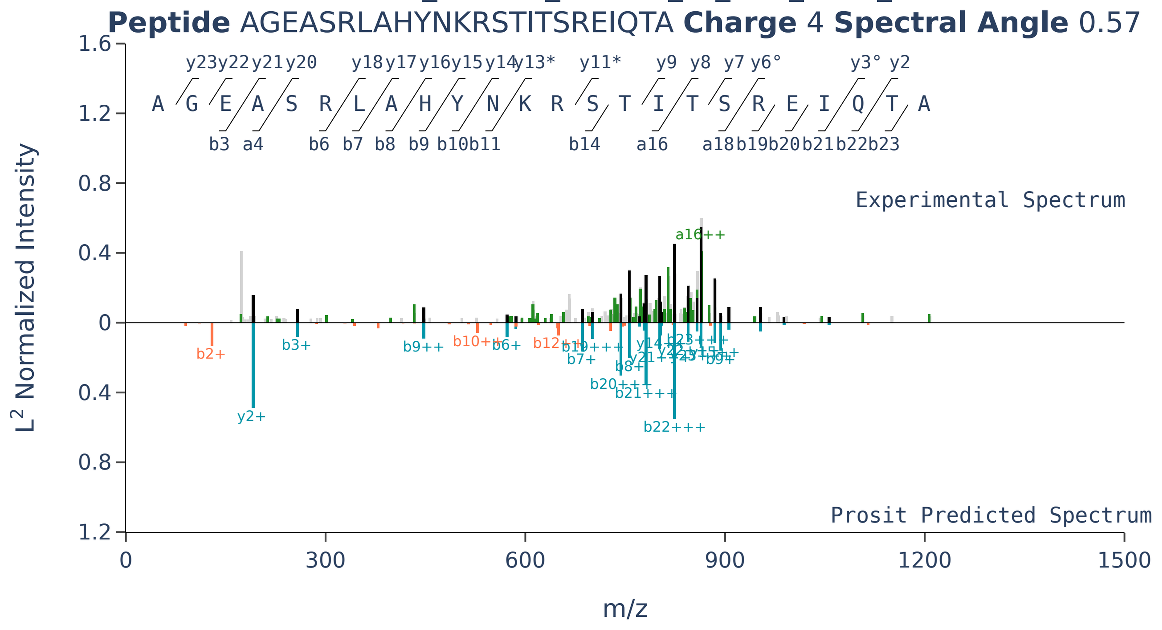


**Source** HRoetschke_291222_080123_Fu_H2B_b1R1_4h **Scan** 12069
**Peptide** HPDTGISSKAMSIMNS **Charge** 3 **Spectral Angle** 0.72

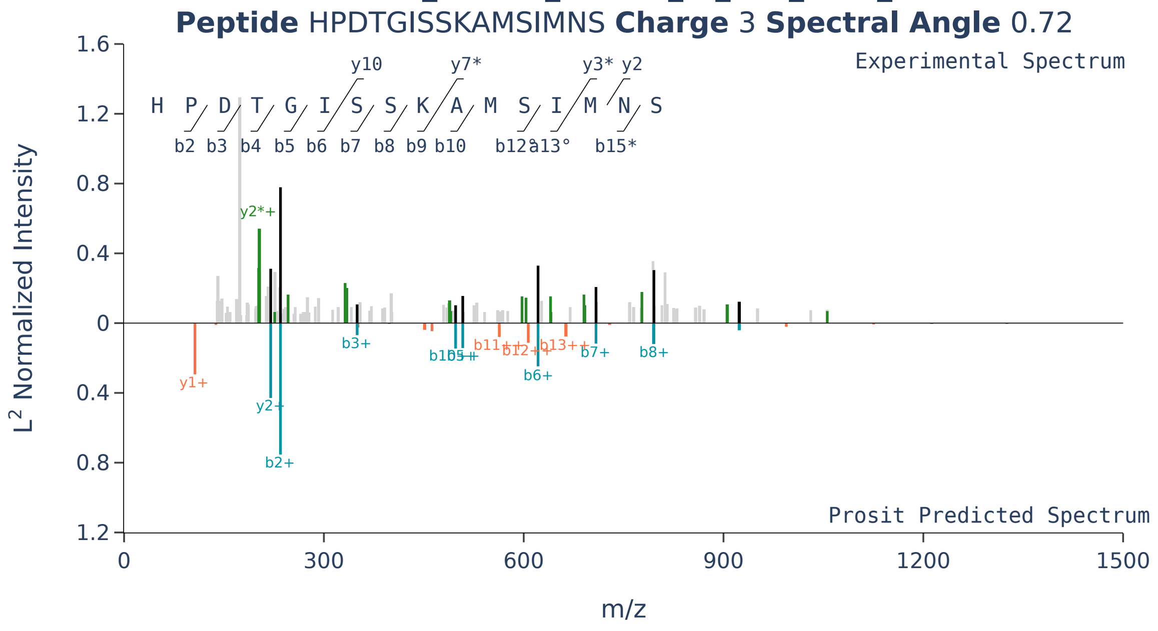


**Source** HRoetschke_291222_080123_Fu_H2B_b2R2_1h **Scan** 7821
**Peptide** LAGEASRLAHYNKRSTITSREIQTAVR **Charge** 6 **Spectral Angle** 0.54

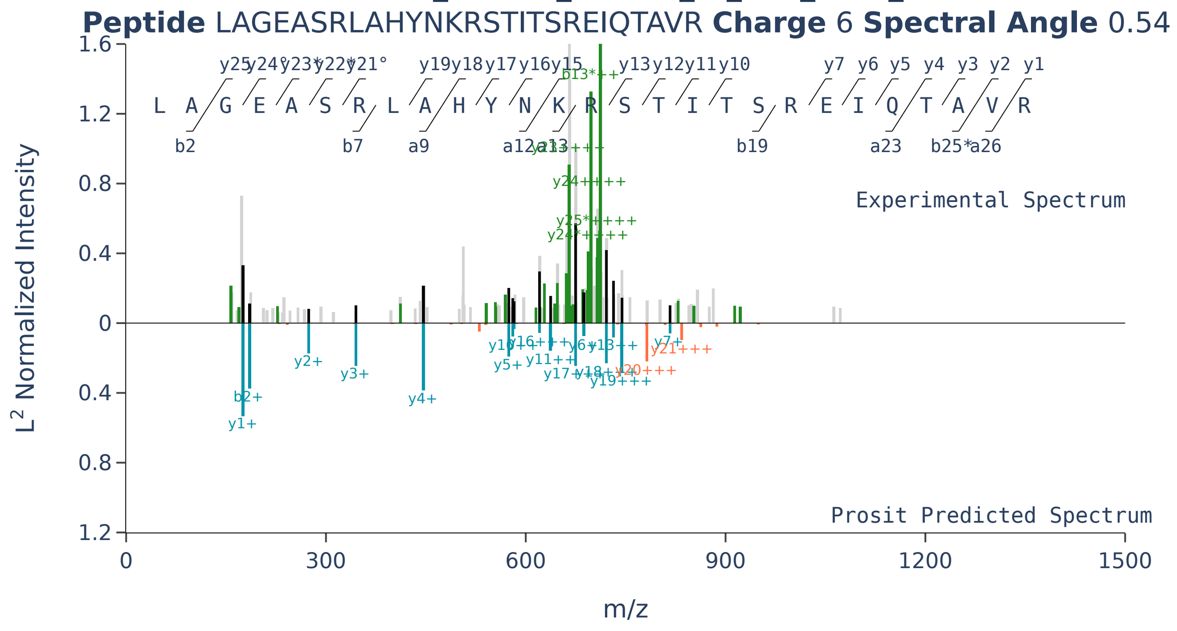


**Source** HRoetschke_291222_080123_Fu_H2B_b2R2_4h **Scan** 19056
**Peptide** AHYNKRSTITSREIQTAVRLLL **Charge** 4 **Spectral Angle** 0.86

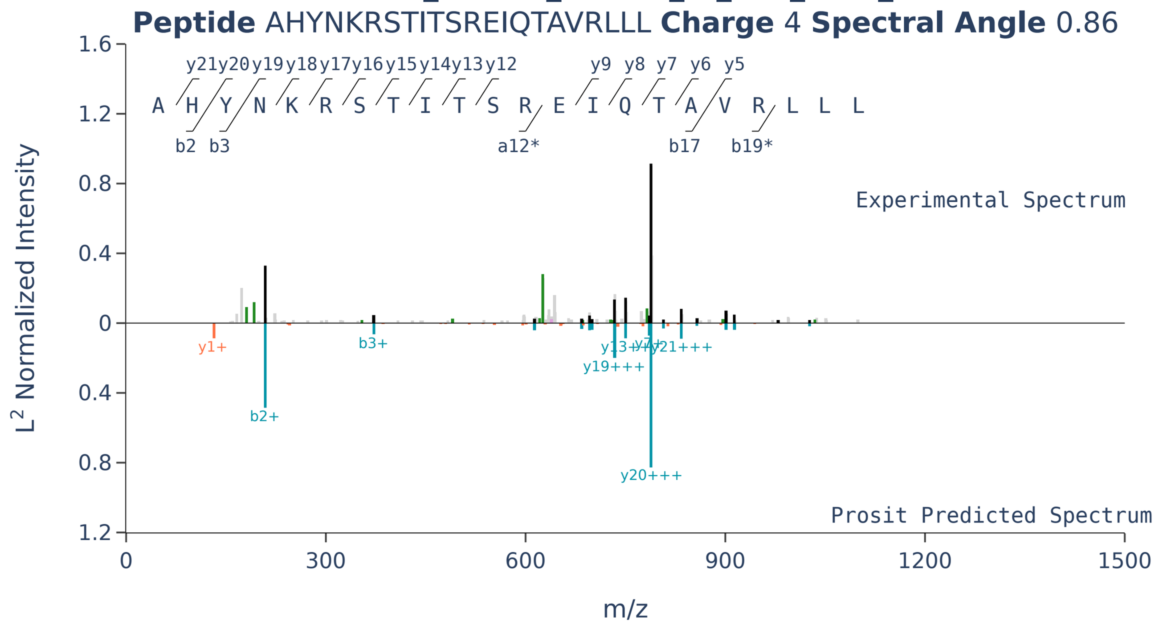


**Source** HRoetschke_291222_080123_Fu_H2B_b2R2_24h **Scan** 21593
**Peptide** SREIQTAVRLLLPGEL **Charge** 3 **Spectral Angle** 0.74

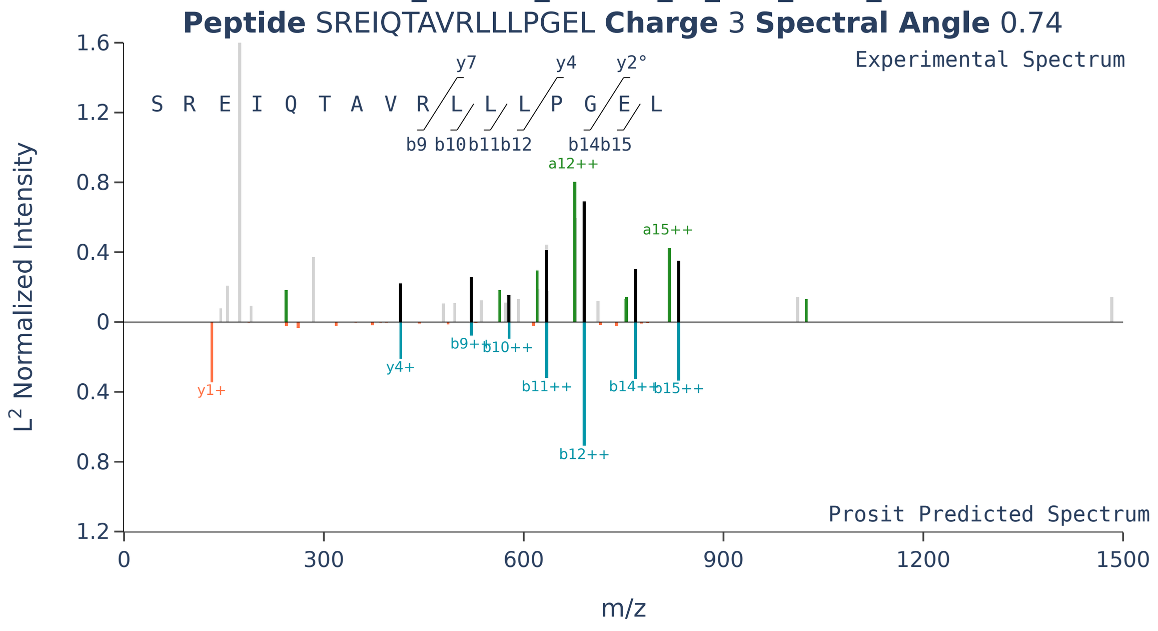


**Source** HRoetschke_291222_080123_Fu_H2B_b2R1_24h **Scan** 3098
**Peptide** PDTGISSKAM **Charge** 2 **Spectral Angle** 0.75

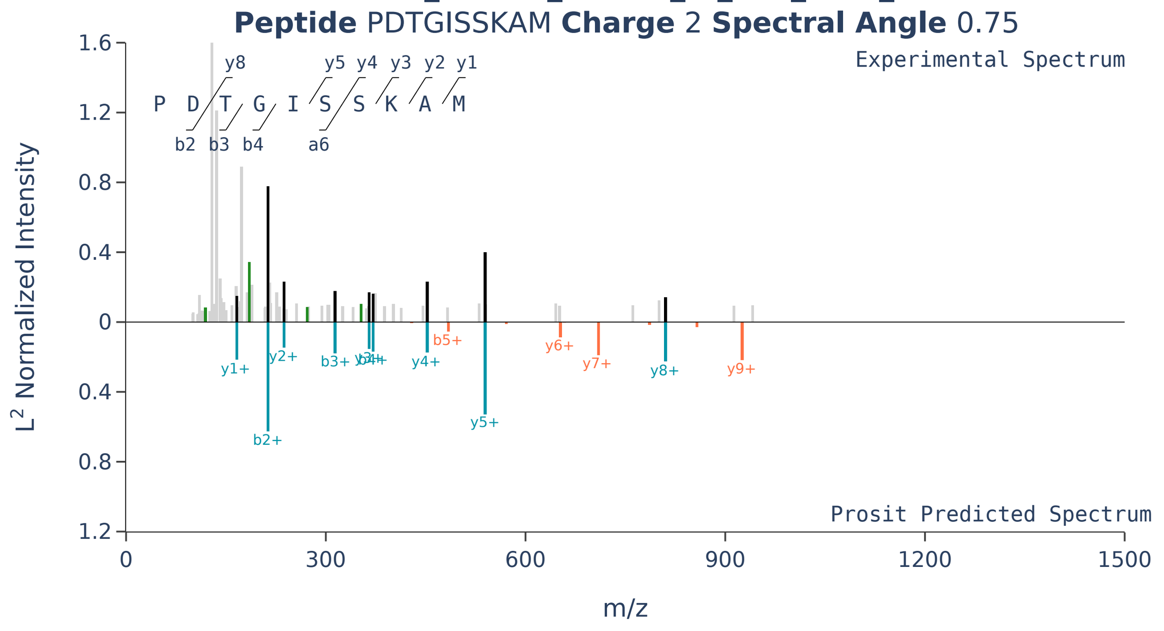


**Source** HRoetschke_291222_080123_Fu_H2B_b2R2_24h **Scan** 10958
**Peptide** SRLAHYNKRSTITSREIQTAVRL **Charge** 5 **Spectral Angle** 0.66

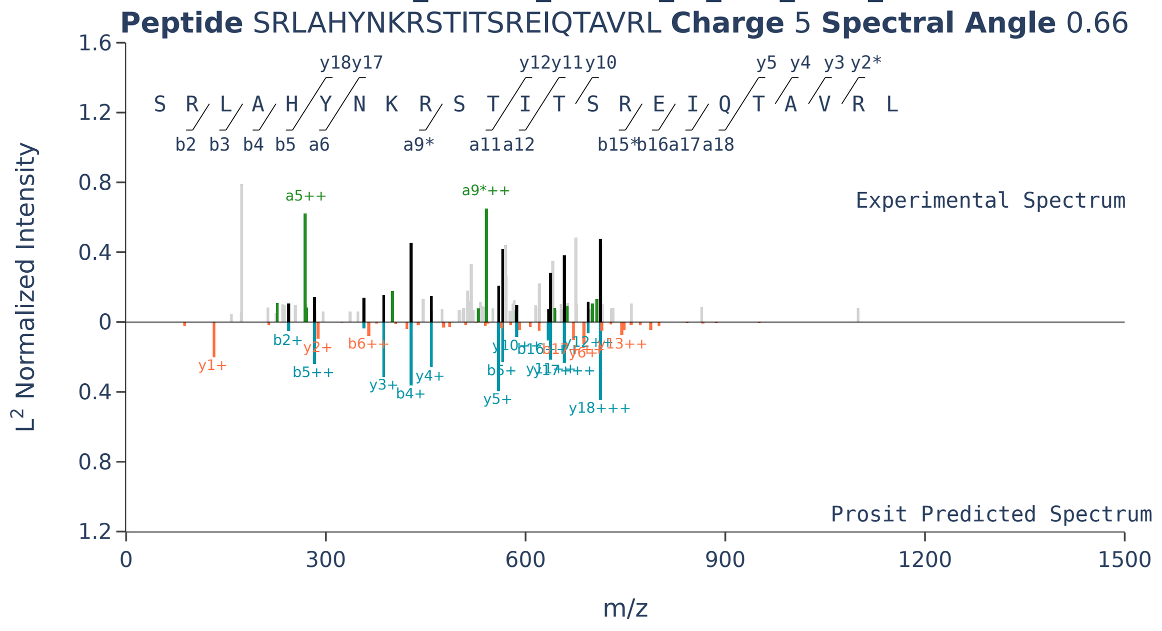


**Source** HRoetschke_291222_080123_Fu_H2B_b1R2_24h **Scan** 8838
**Peptide** LAGEASRLAHYNKRSTITSREIQTA **Charge** 5 **Spectral Angle** 0.59

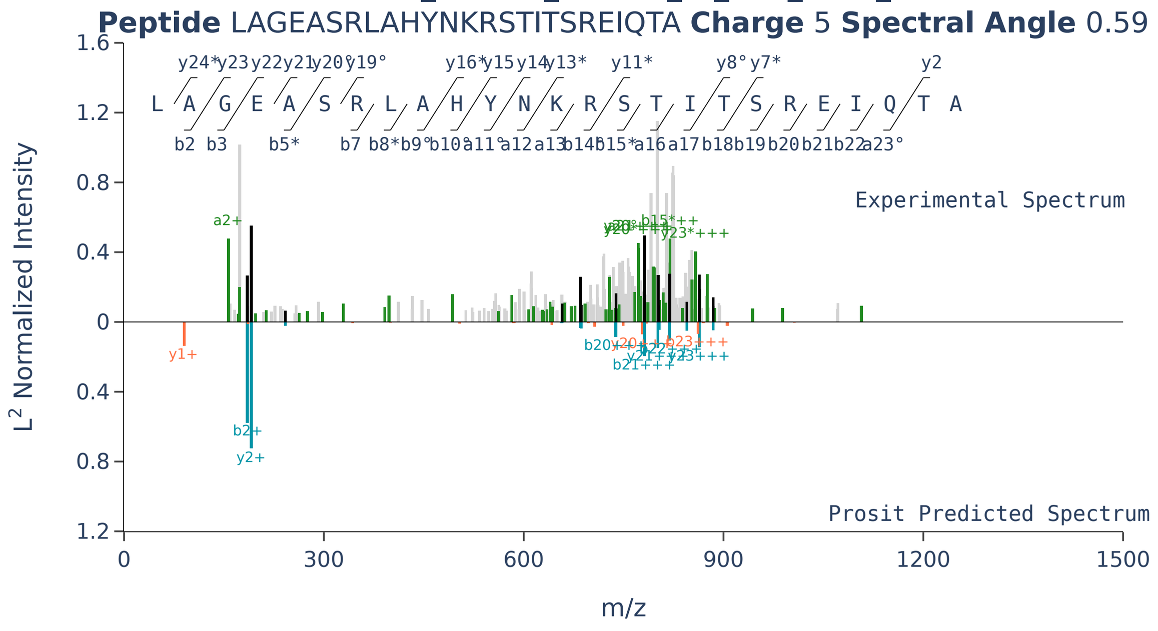


**Source** HRoetschke_291222_080123_Fu_H2B_b1R1_24h **Scan** 5028
**Peptide** GEASRLAHY **Charge** 3 **Spectral Angle** 0.73

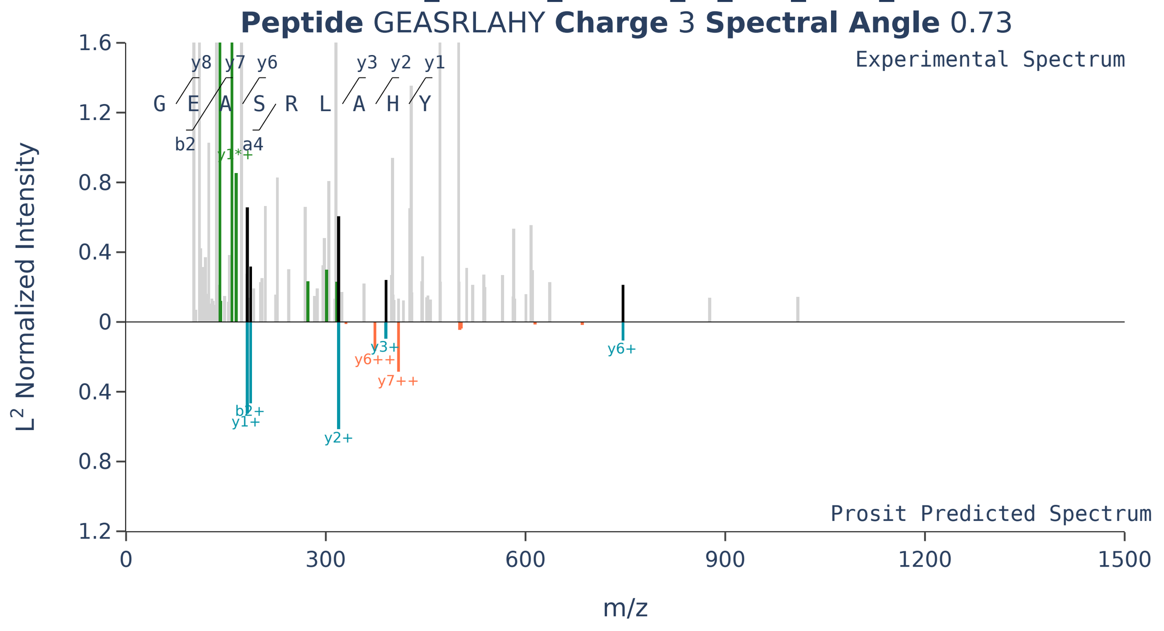


**Source** HRoetschke_291222_080123_Fu_H2B_b1R2_24h **Scan** 5425
**Peptide** LAKHAVSEGTKAVTKYTS **Charge** 3 **Spectral Angle** 0.68

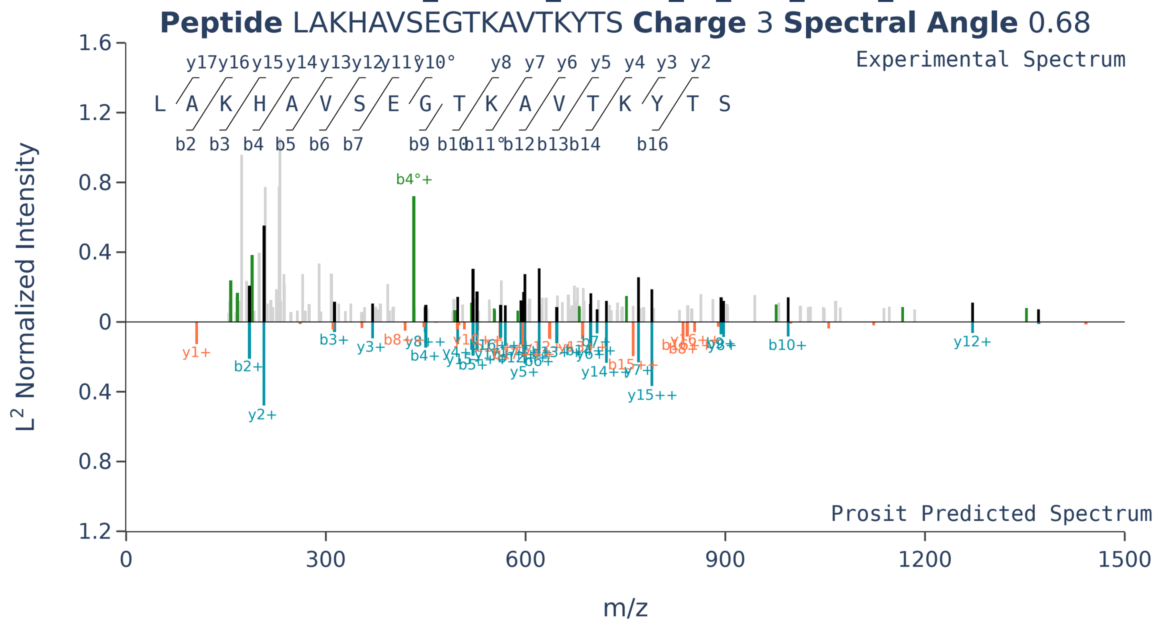

**Source** HRoetschke_291222_080123_Fu_H2B_b2R2_24h **Scan** 6740
**Peptide** ERIAGEASRLAHYNK **Charge** 5 **Spectral Angle** 0.85

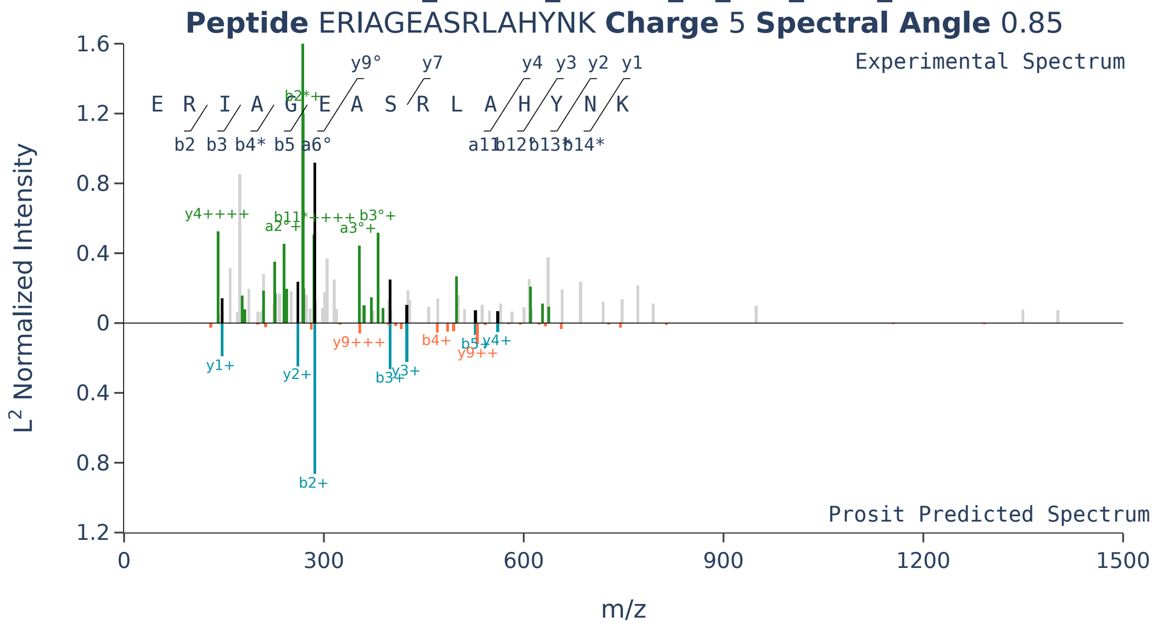


**Source** HRoetschke_291222_080123_Fu_H2B_b2R1_4h **Scan** 14208
**Peptide** RLLLPGELAKHAVSEGTKAVTK **Charge** 4 **Spectral Angle** 0.74

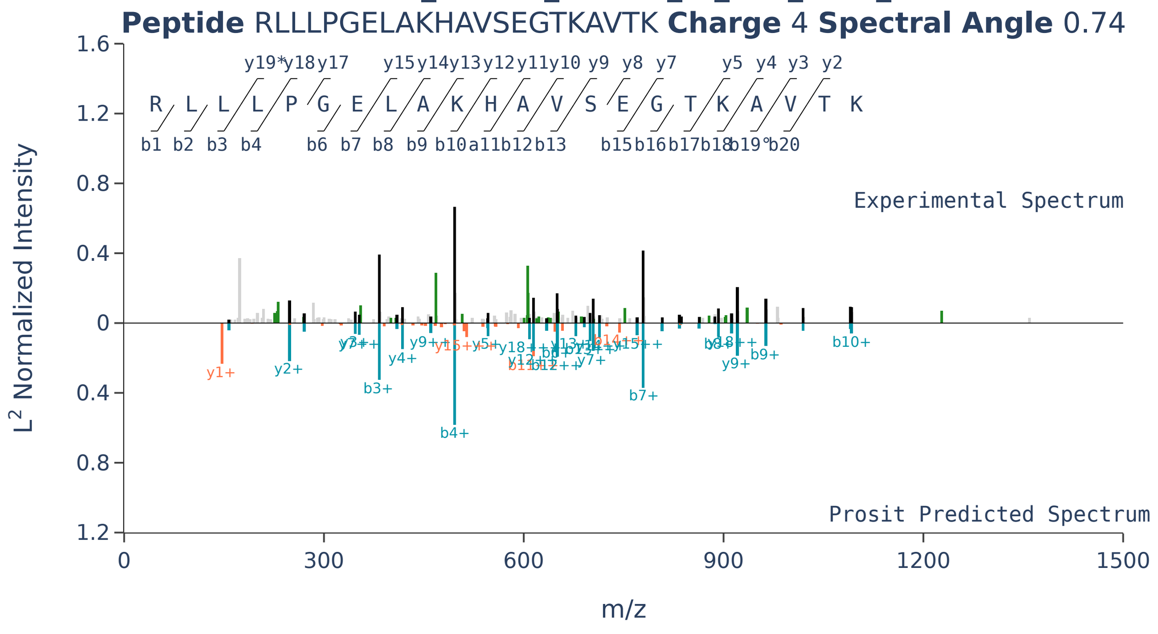


**Source** HRoetschke_291222_080123_Fu_H2B_b2R2_4h **Scan** 17646
**Peptide** VRLLLPGELAKHAVSEGTKAVTKYT **Charge** 5 **Spectral Angle** 0.86

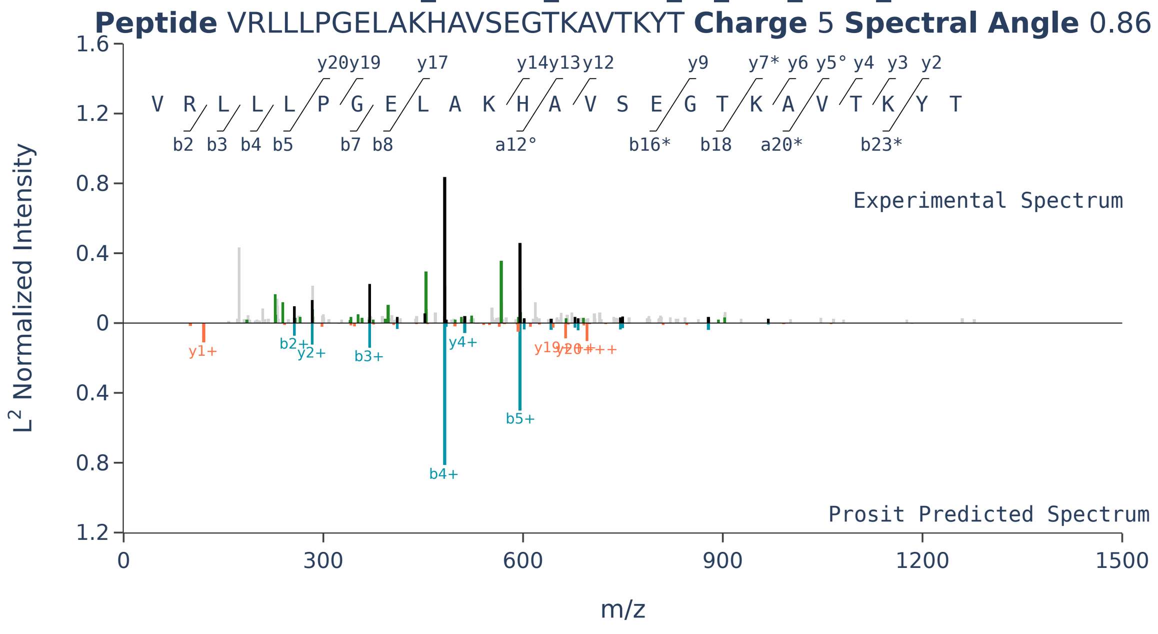


**Source** HRoetschke_291222_080123_Fu_H2B_b1R2_24h **Scan** 7077
**Peptide** LAGEASRLAHYNKRSTIT **Charge** 3 **Spectral Angle** 0.81

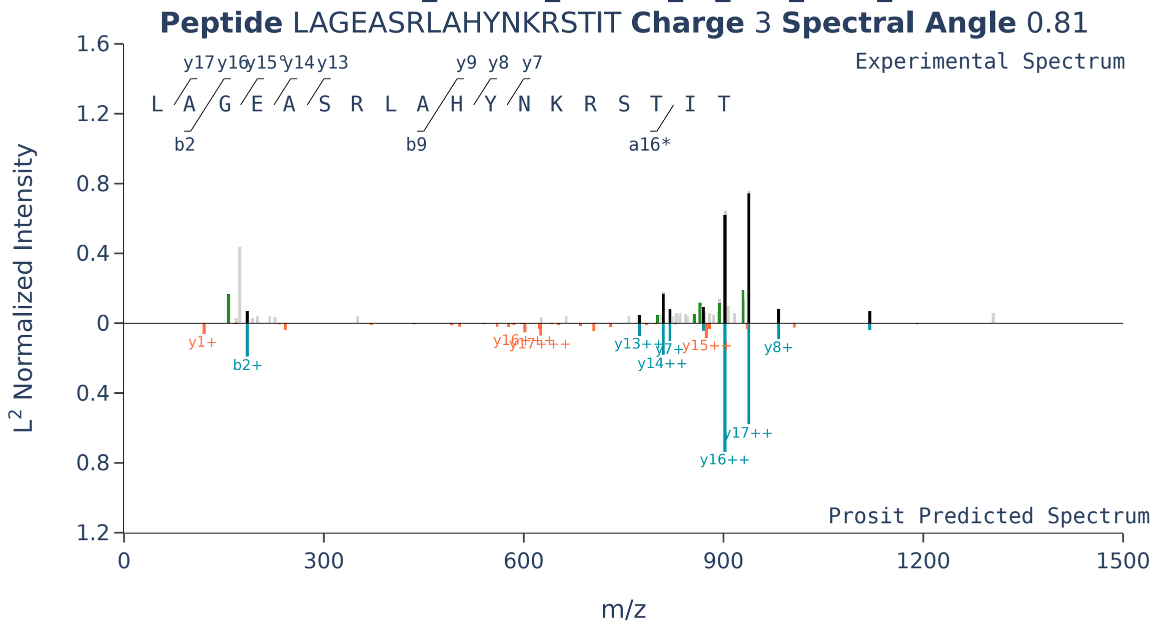


**Source** HRoetschke_291222_080123_Fu_H2B_b2R2_24h **Scan** 9629
**Peptide** LLPGELAKH **Charge** 3 **Spectral Angle** 0.92

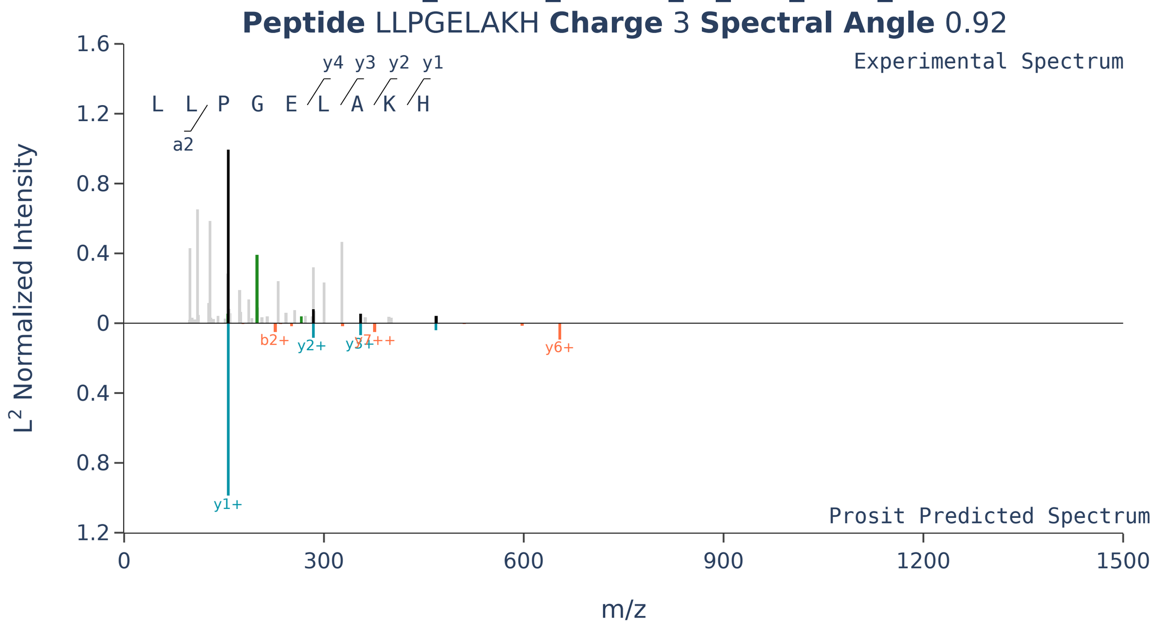


**Source** HRoetschke_291222_080123_Fu_H2B_b2R2_24h **Scan** 2884
**Peptide** ASRLAHYNKRSTIT **Charge** 3 **Spectral Angle** 0.58

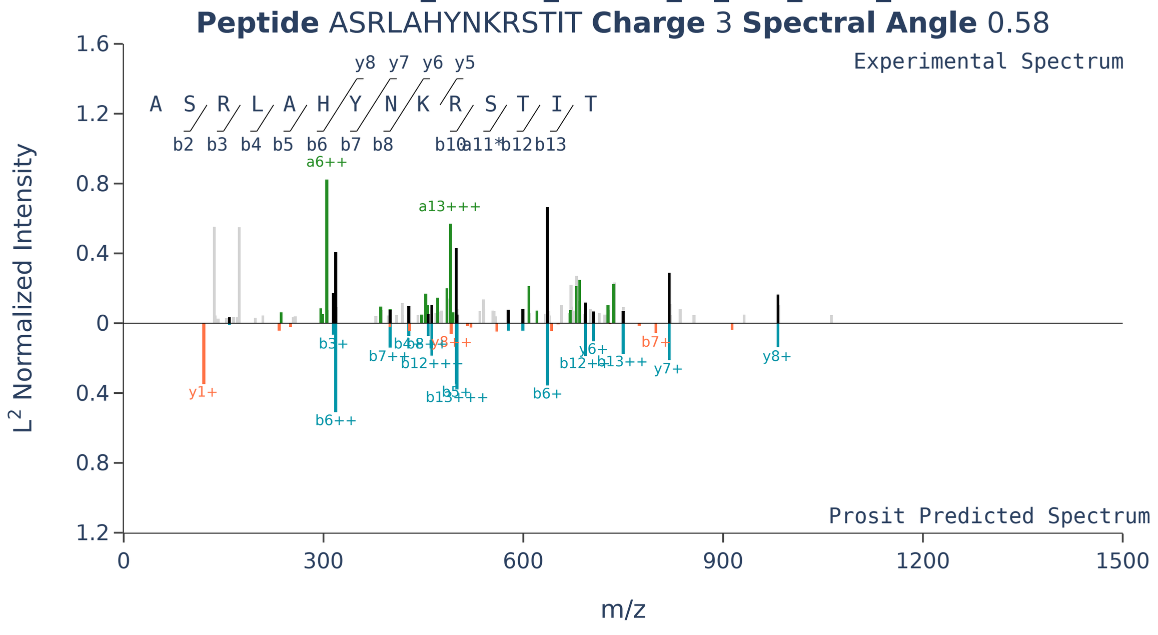


**Source** HRoetschke_291222_080123_Fu_H2B_b2R1_1h **Scan** 4631
**Peptide** HAVSEGTKAVTKYTSA **Charge** 3 **Spectral Angle** 0.73

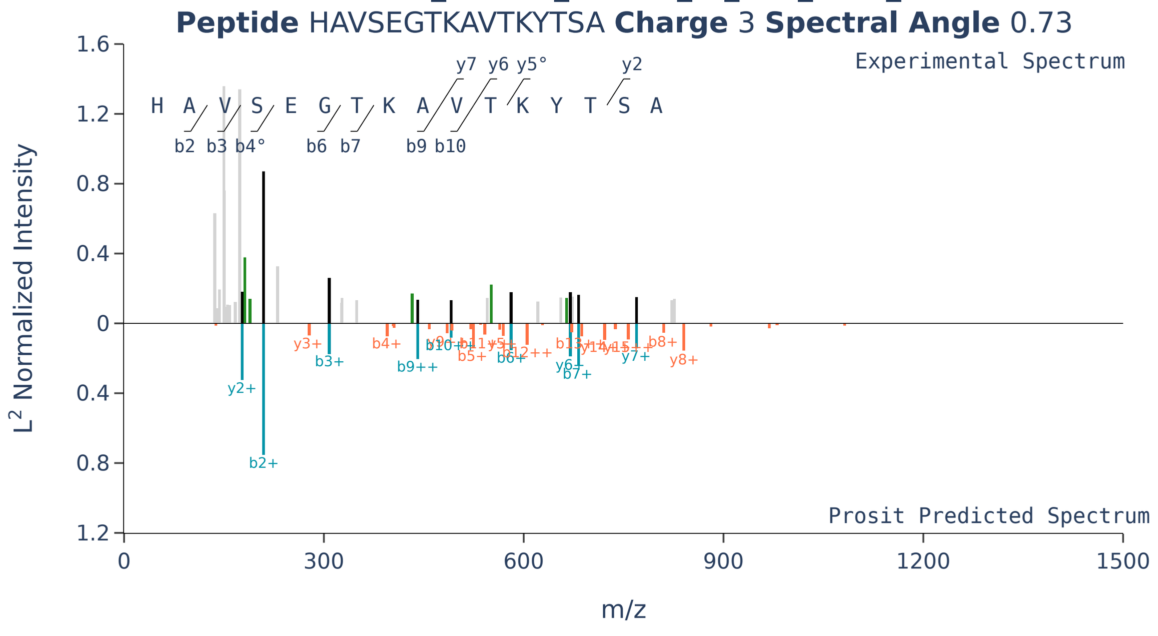


**Source** HRoetschke_291222_080123_Fu_H2B_b2R2_4h **Scan** 18679
**Peptide** TAVRLLLPGELAKHAVSEGTKAVTKYTSA **Charge** 5 **Spectral Angle** 0.82

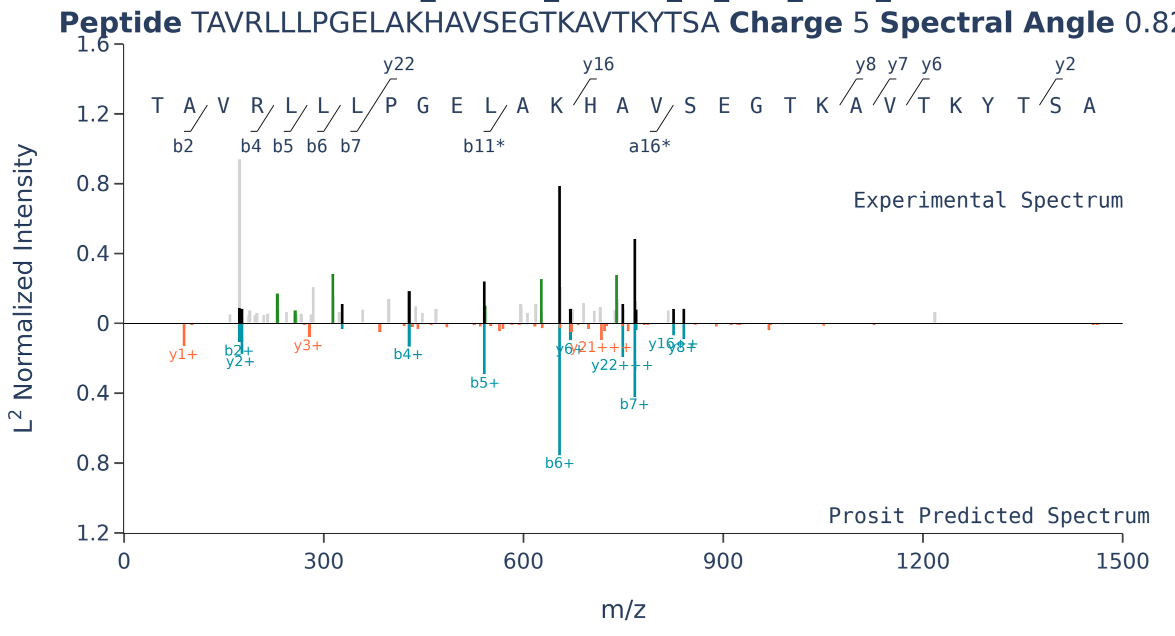


**Source** HRoetschke_291222_080123_Fu_H2B_b2R1_4h **Scan** 18322
**Peptide** SREIQTAVRLLLPGELAKHAVS **Charge** 4 **Spectral Angle** 0.83

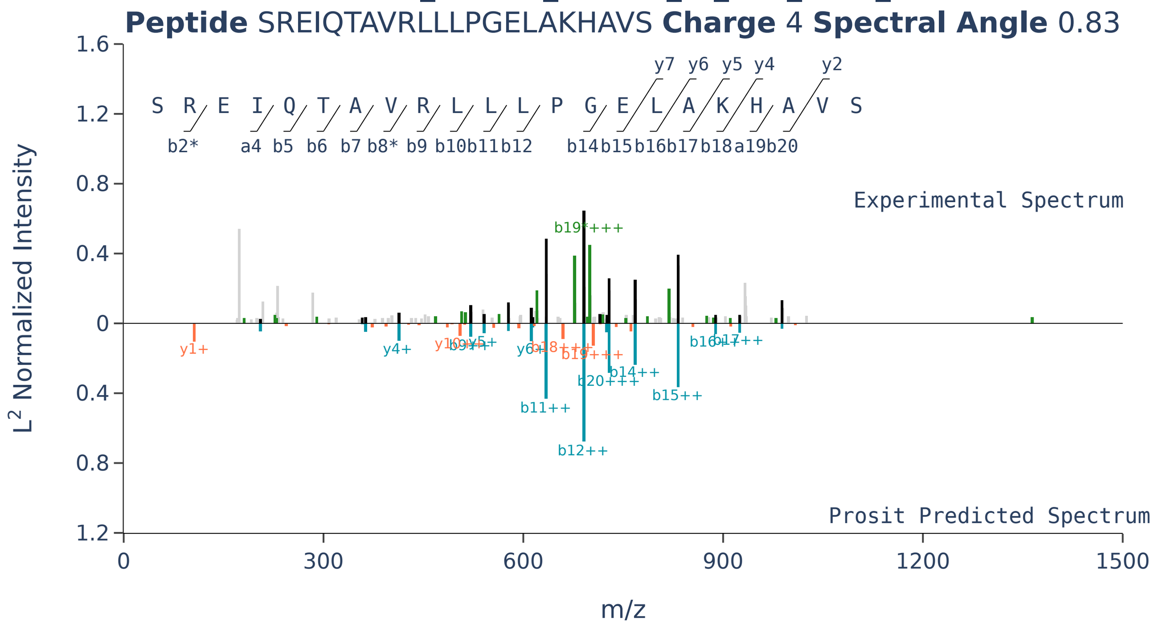


**Source** HRoetschke_291222_080123_Fu_H2B_b2R2_24h **Scan** 17894
**Peptide** AVRLLLPGELAKHAV **Charge** 3 **Spectral Angle** 0.72

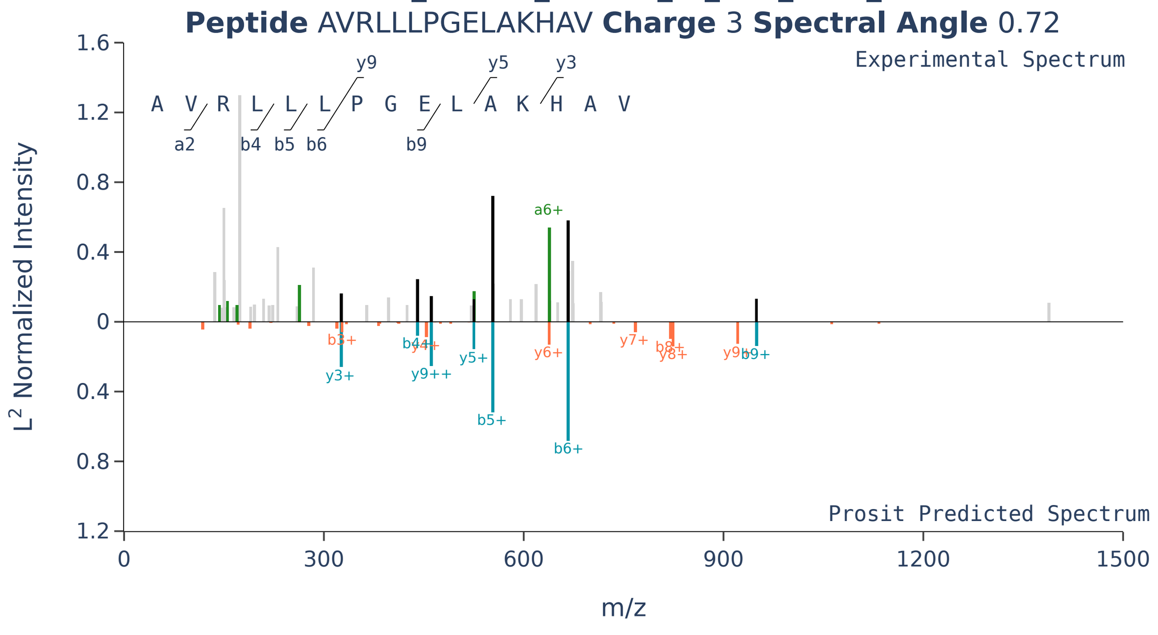


**Source** RIAGEASRLAHYNKRST_291222_080123_Fu_H2B_b2R1_4h **Scan** 4078
**Peptide** RIAGEASRLAHYNKRST **Charge** 4 **Spectral Angle** 0.78

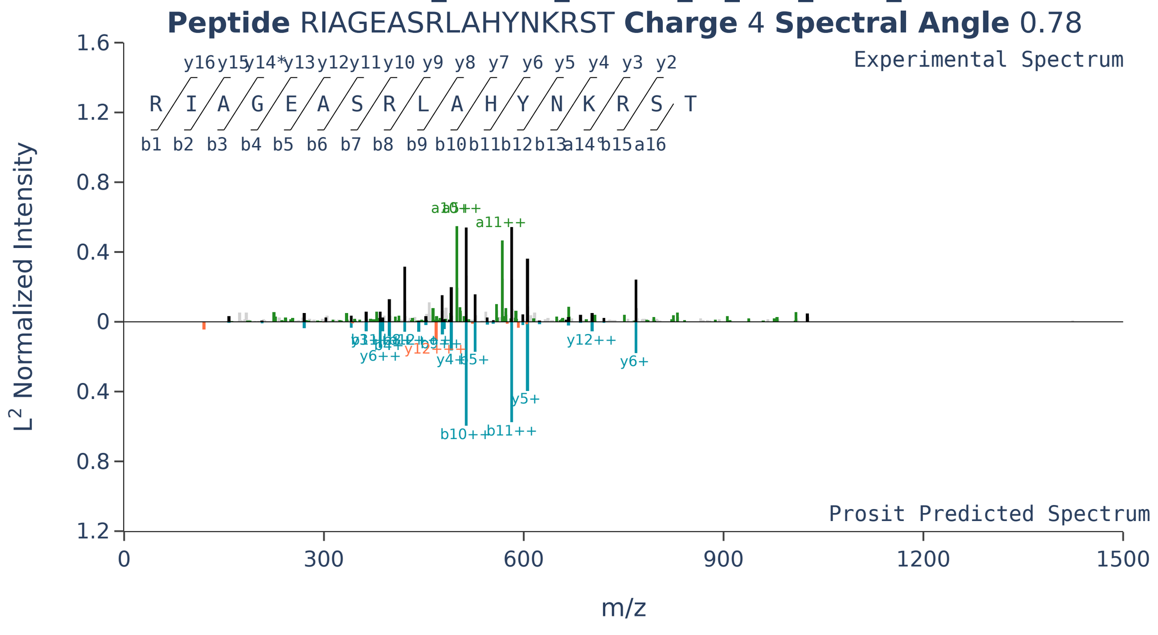


**Source** HRoetschke_291222_080123_Fu_H2B_b1R2_24h **Scan** 10999
**Peptide** HYNKRSTITSREIQTAVRL **Charge** 4 **Spectral Angle** 0.85

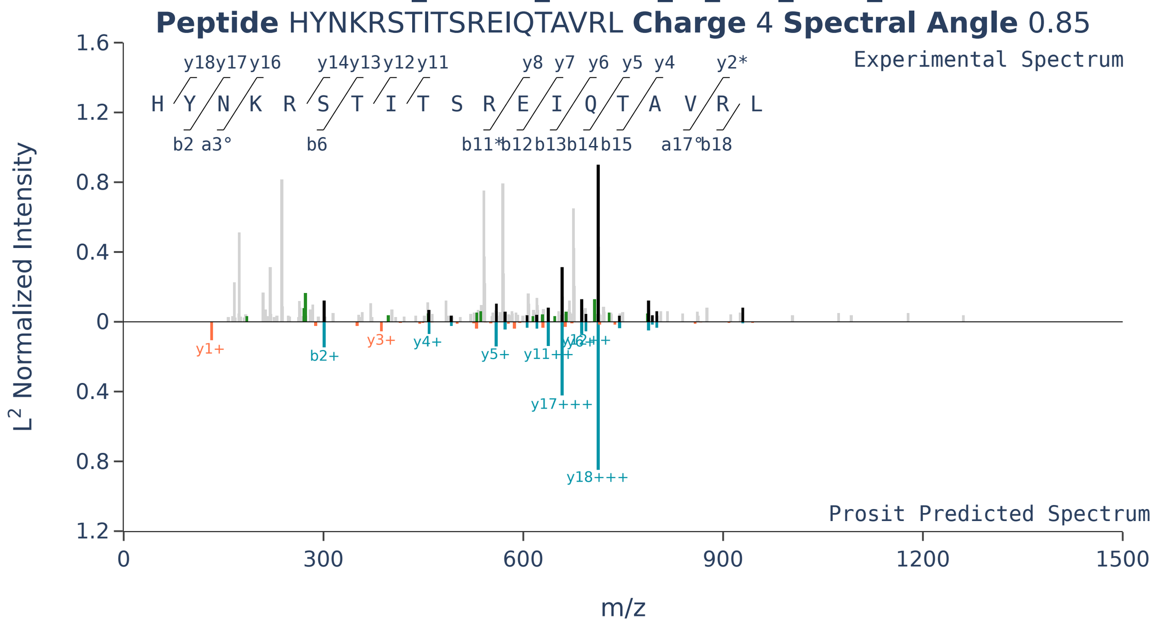


**Source** HRoetschke_291222_080123_Fu_H2B_b2R1_24h **Scan** 18695
**Peptide** ELQTAVRLLLPGELAKHAVSEGTKA **Charge** 4 **Spectral Angle** 0.68

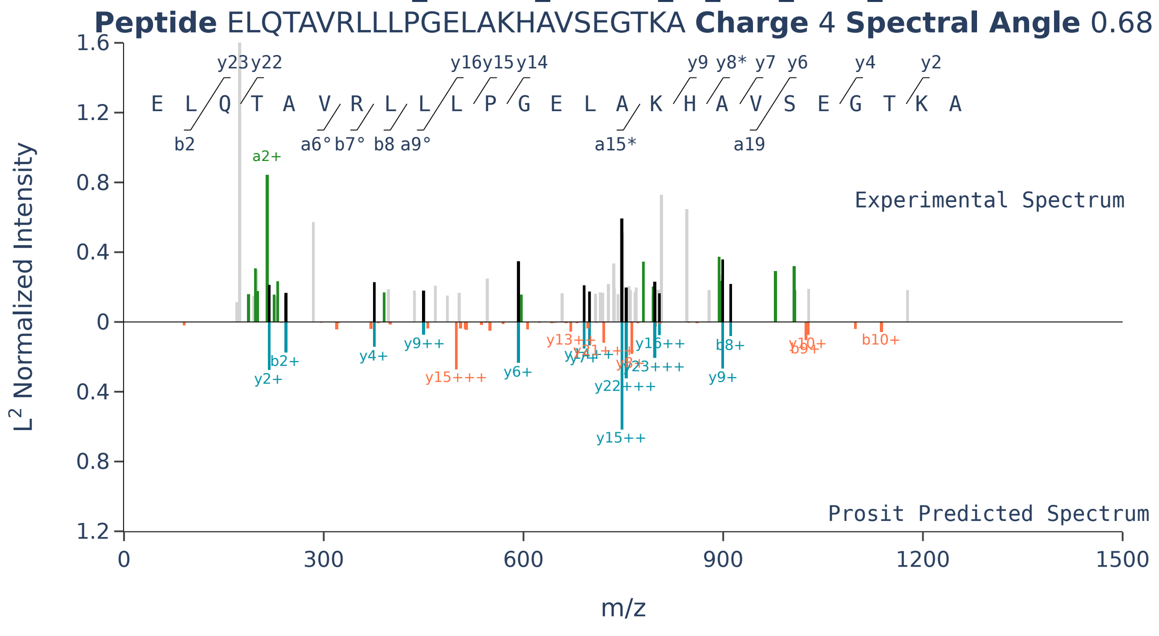


**Source** HRoetschke_291222_080123_Fu_H2B_b2R1_4h **Scan** 21806
**Peptide** STITSREIQTAVRLLLPGELAKHAVS **Charge** 4 **Spectral Angle** 0.78

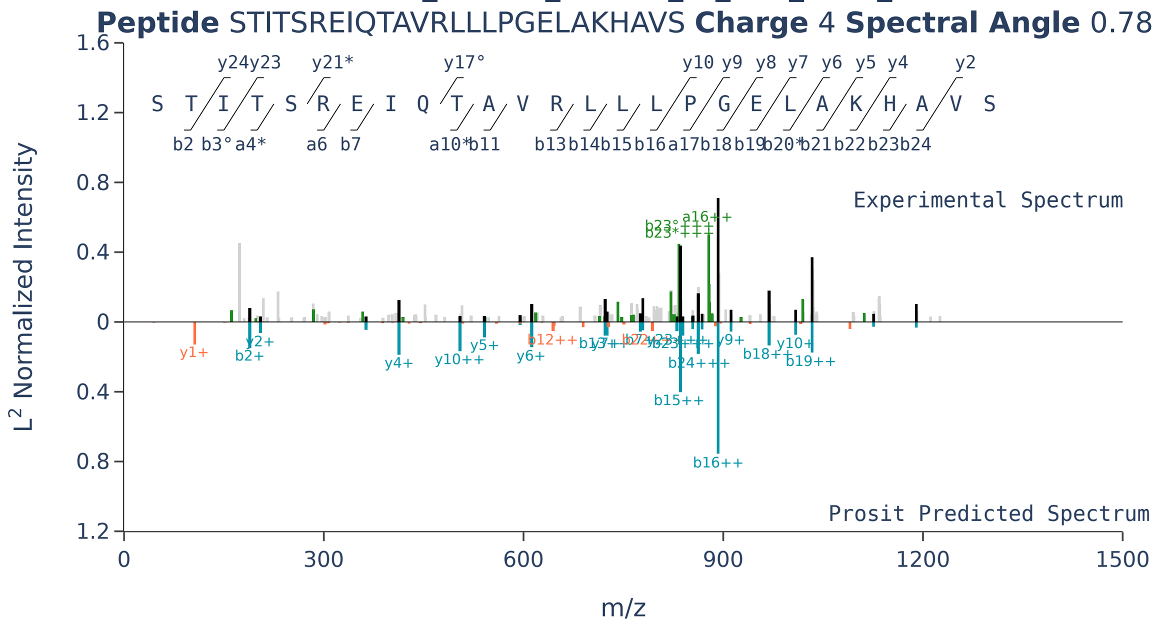


**Source** HRoetschke_291222_080123_Fu_H2B_b2R2_24h **Scan** 19667
**Peptide** TSREIQTAVRLLLPGELAKHA **Charge** 4 **Spectral Angle** 0.77

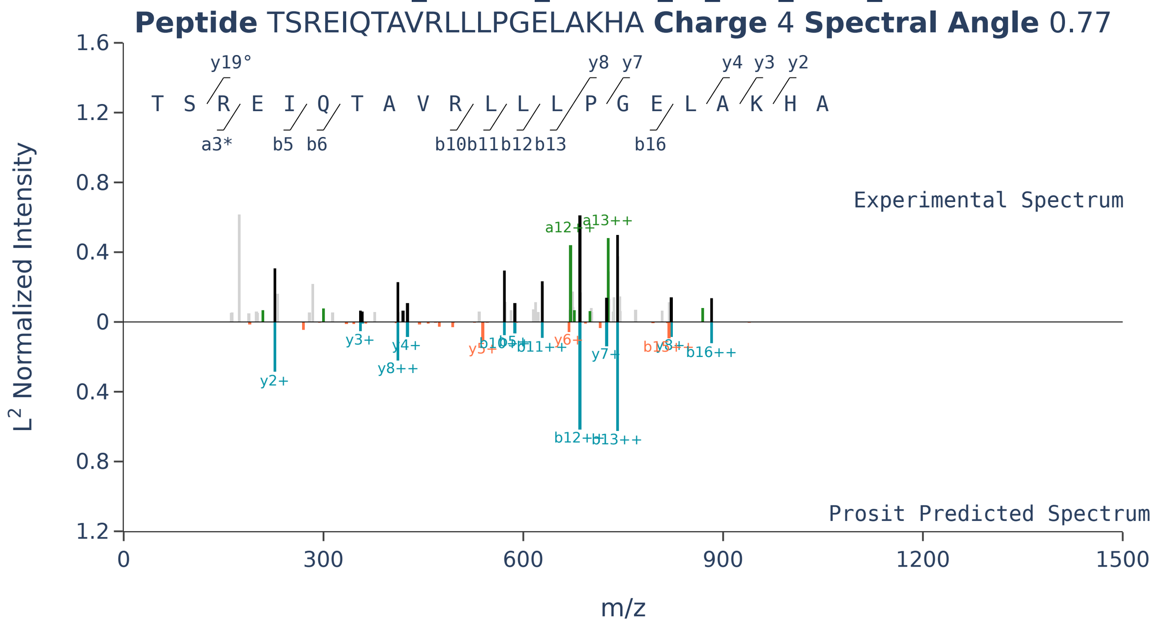

**Source** HRoetschke_291222_080123_Fu_H2B_b2R2_24h **Scan** 18544
**Peptide** REIQTAVRLLLPGELAKHAVS **Charge** 4 **Spectral Angle** 0.77

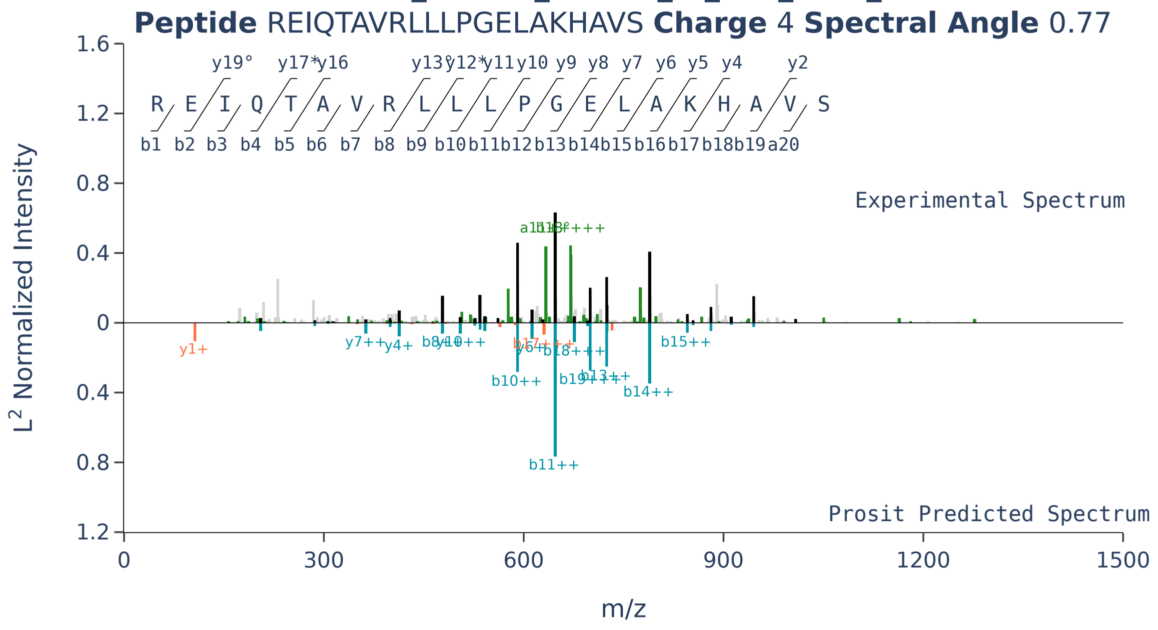


**Source** HRoetschke_291222_080123_Fu_H2B_b2R1_24h **Scan** 11483
**Peptide** LKQVHPDTGISSKAMSI **Charge** 3 **Spectral Angle** 0.72

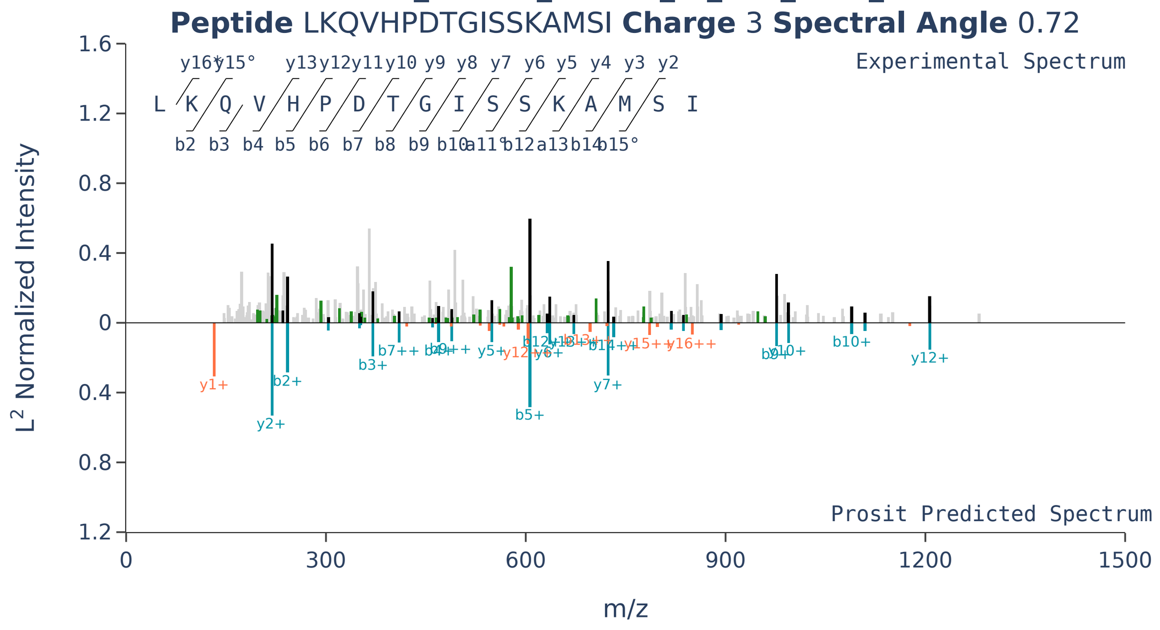


**Source** HRoetschke_291222_080123_Fu_H2B_b2R2_4h **Scan** 19019
**Peptide** NDVFERIAGEASRLAHYNKRSTL **Charge** 5 **Spectral Angle** 0.66

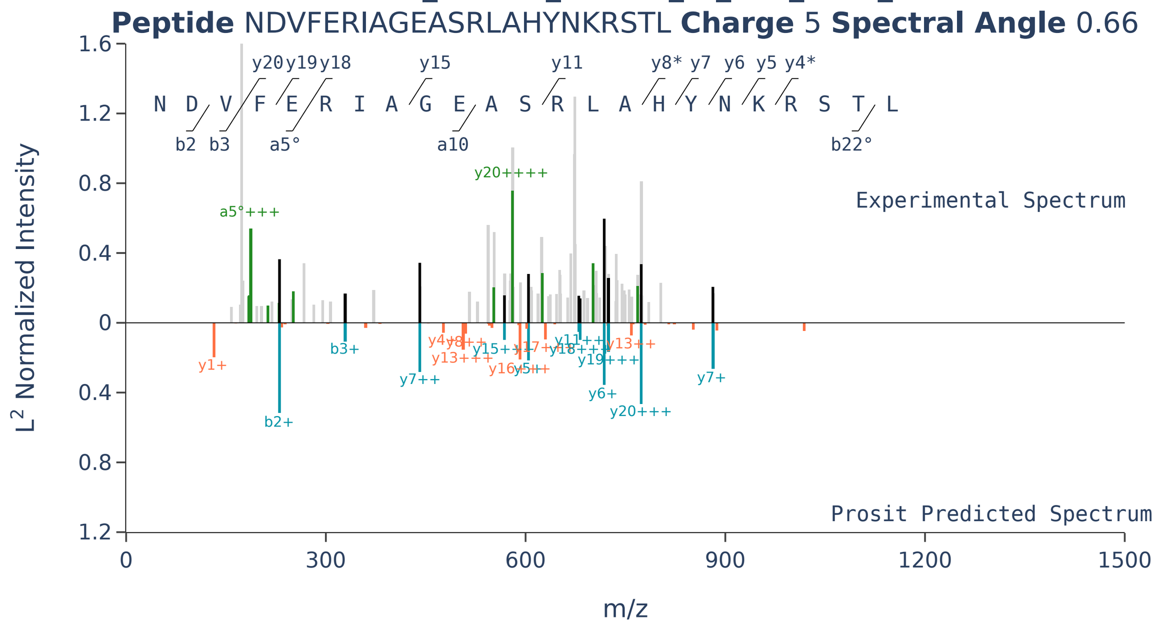


**Source** HRoetschke_291222_080123_Fu_H2B_b2R2_24h **Scan** 17225
**Peptide** KESYAIYVYKVLKQ **Charge** 3 **Spectral Angle** 0.35

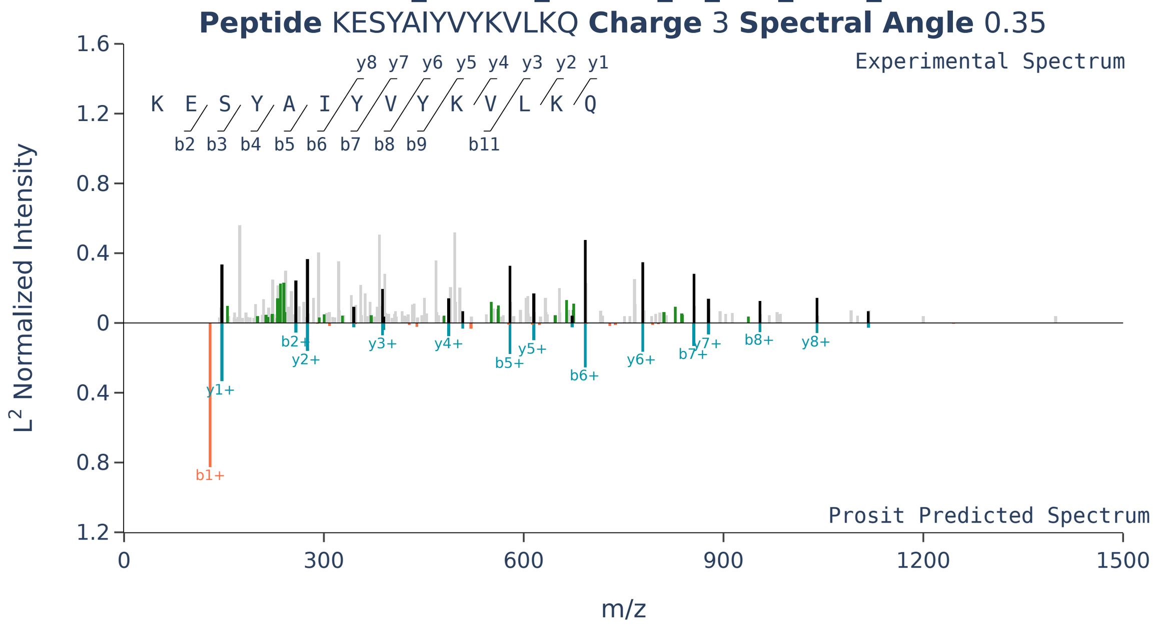


**Source** HRoetschke_291222_080123_Fu_H2B_b2R2_1h **Scan** 18624
**Peptide** SFVNDVFERIAGEAS **Charge** 2 **Spectral Angle** 0.76

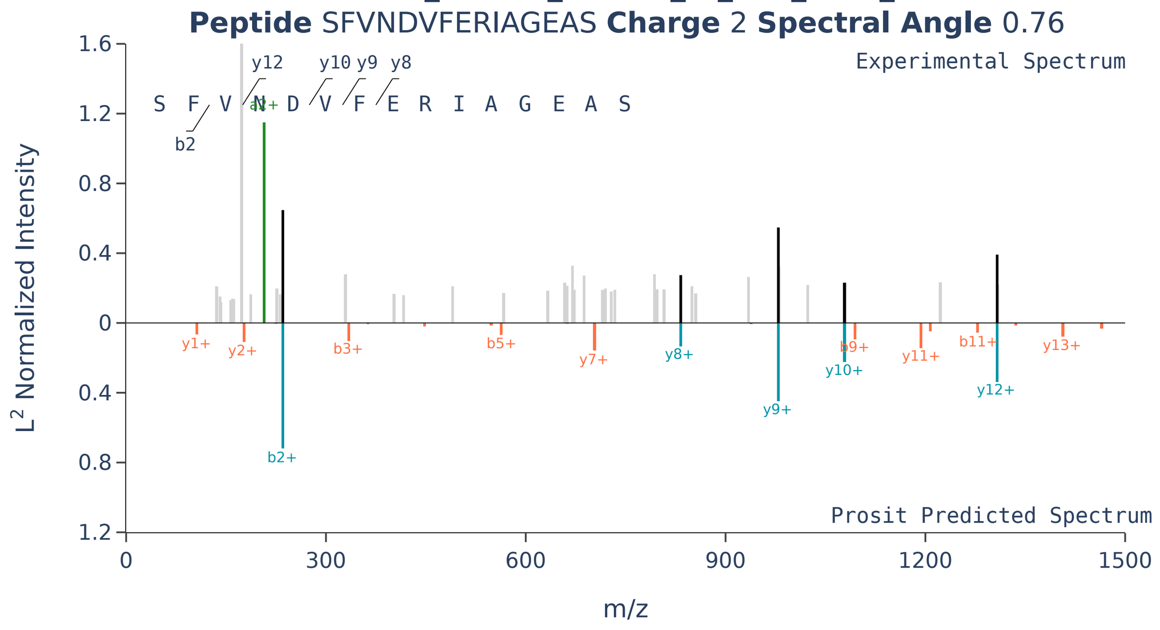


**Source** HRoetschke_291222_080123_Fu_H2B_b1R1_4h **Scan** 12925
**Peptide** LPGELAKHAVSEGTKAVTKYTSA **Charge** 4 **Spectral Angle** 0.62

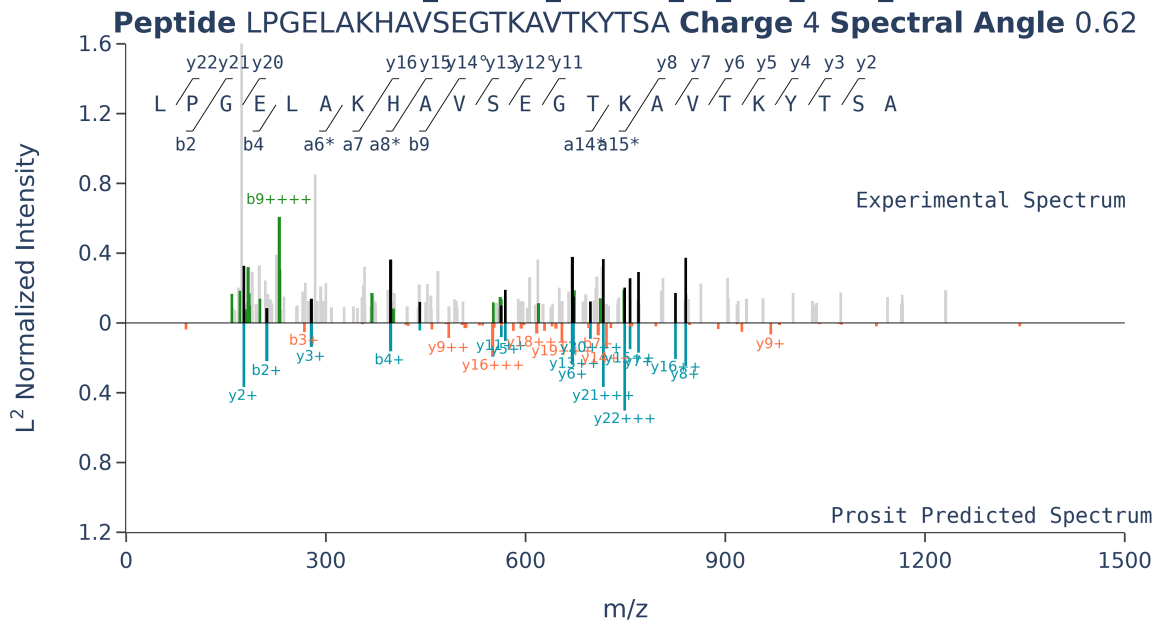


**Source** HRoetschke_291222_080123_Fu_H2B_b2R2_24h **Scan** 10763
**Peptide** GKKRKTRKESYAIYVYKVLKQ **Charge** 4 **Spectral Angle** 0.63

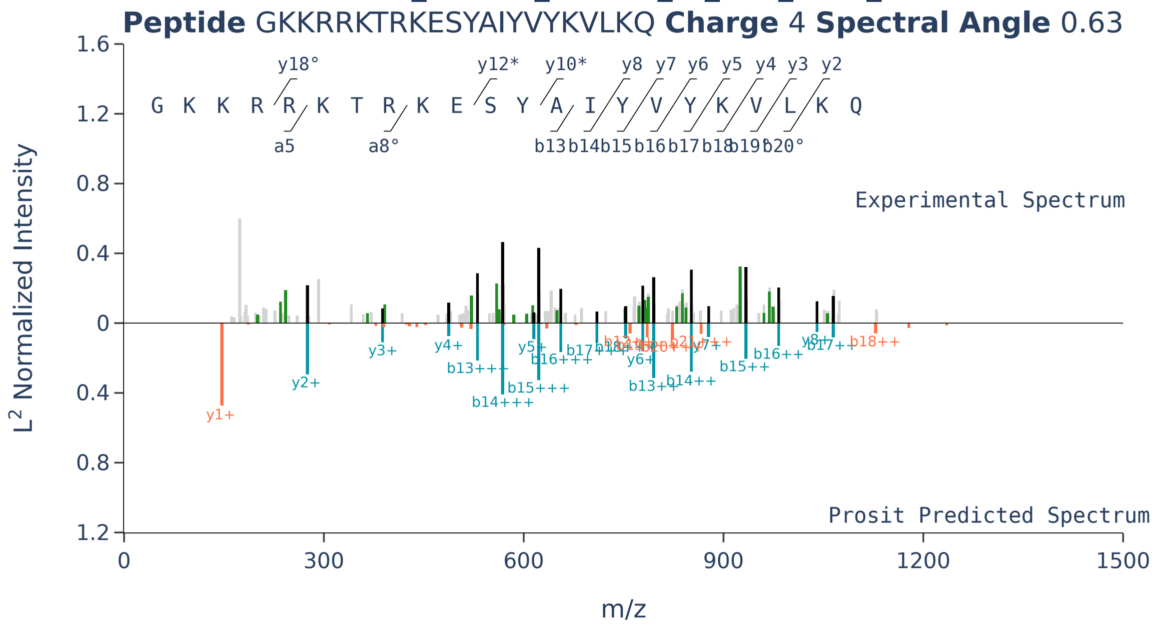


**Source** HRoetschke_291222_080123_Fu_H2B_b2R1_4h **Scan** 14689
**Peptide** GEASRLAHYNKRSTITSREIQTAVRLL **Charge** 5 **Spectral Angle** 0.54

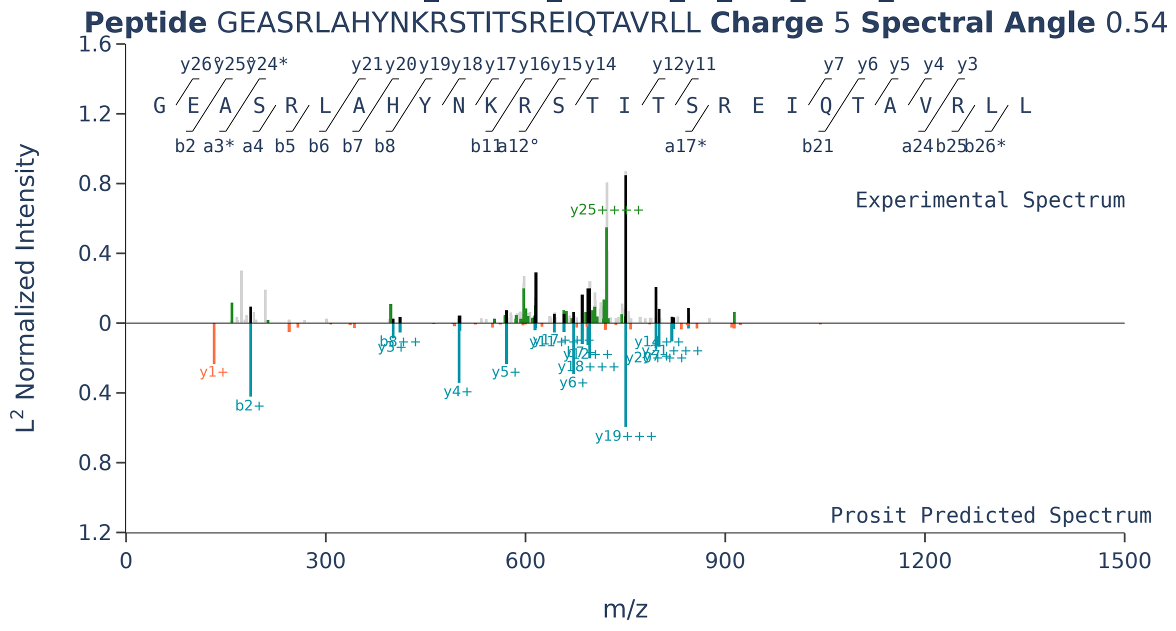


**Source** HRoetschke_291222_080123_Fu_H2B_b2R2_24h **Scan** 6421
**Peptide** GEASRLAHYNKRSTITSREIQT **Charge** 4 **Spectral Angle** 0.6

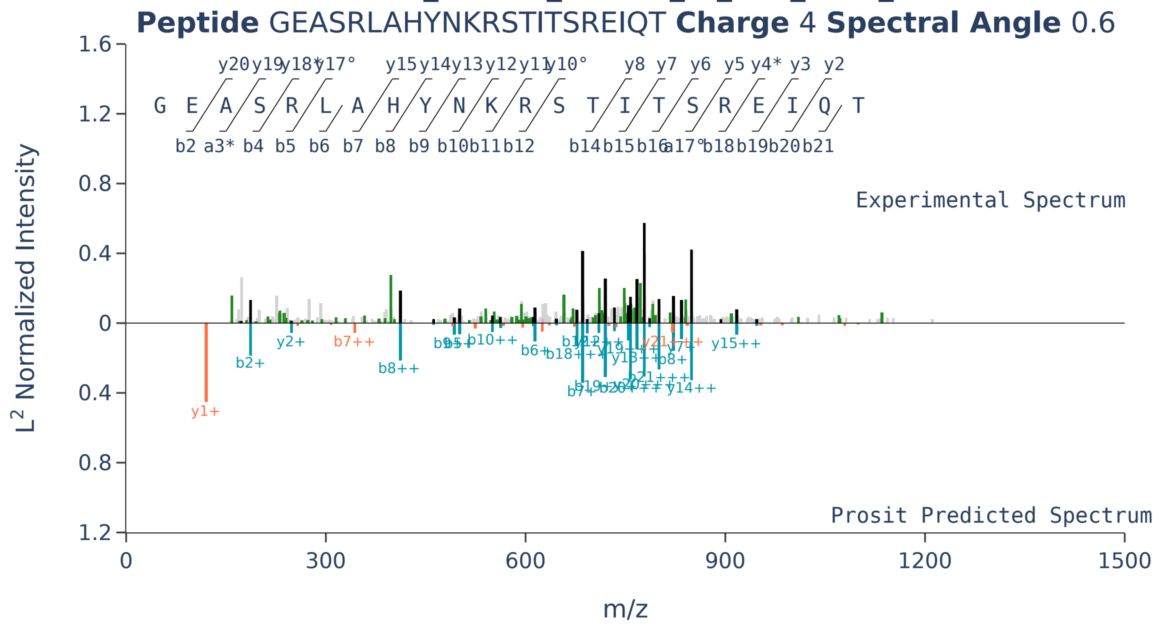


**Source** HRoetschke_291222_080123_Fu_H2B_b2R1_24h **Scan** 19940
**Peptide** NDVFERIAGEASRLAHYN **Charge** 3 **Spectral Angle** 0.8

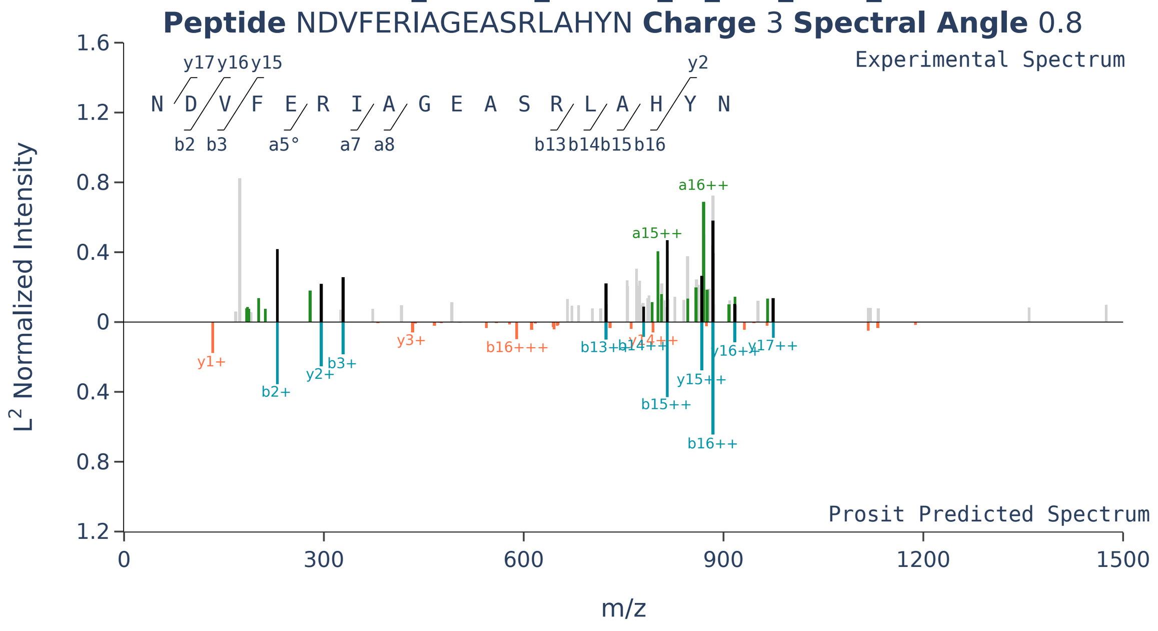


**Source** HRoetschke_291222_080123_Fu_H2B_b2R1_24h **Scan** 15346
**Peptide** VRLLLPGELAKHAVSEGTK **Charge** 4 **Spectral Angle** 0.72

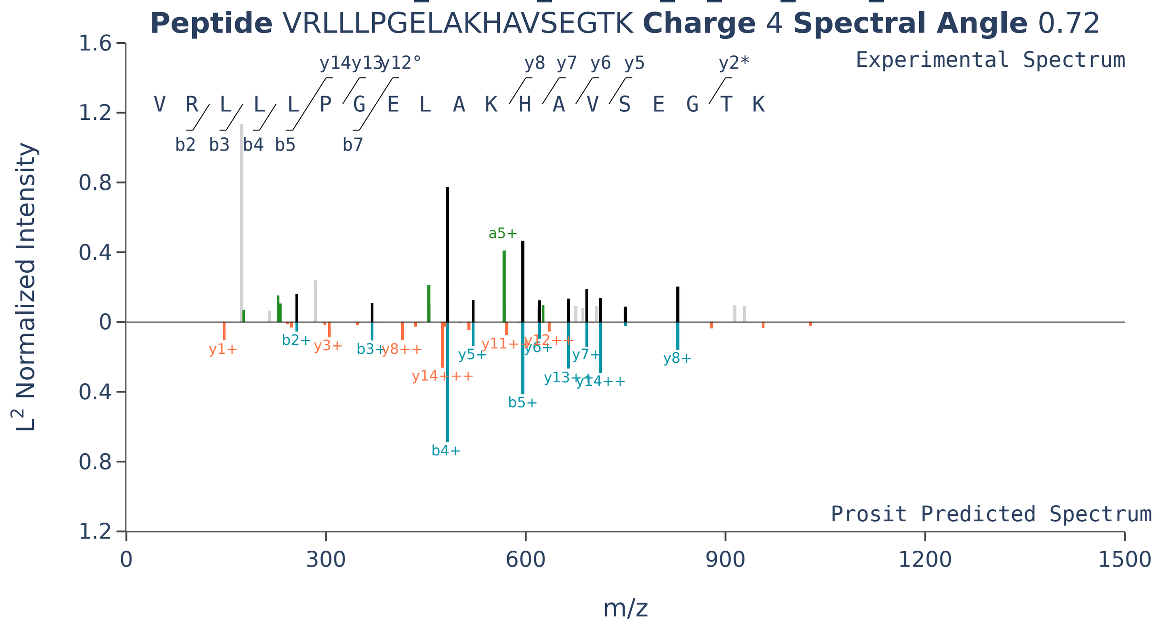


**Source** HRoetschke_291222_080123_Fu_H2B_b2R1_2h **Scan** 16079
**Peptide** TAVRLLLPGE **Charge** 2 **Spectral Angle** 0.83

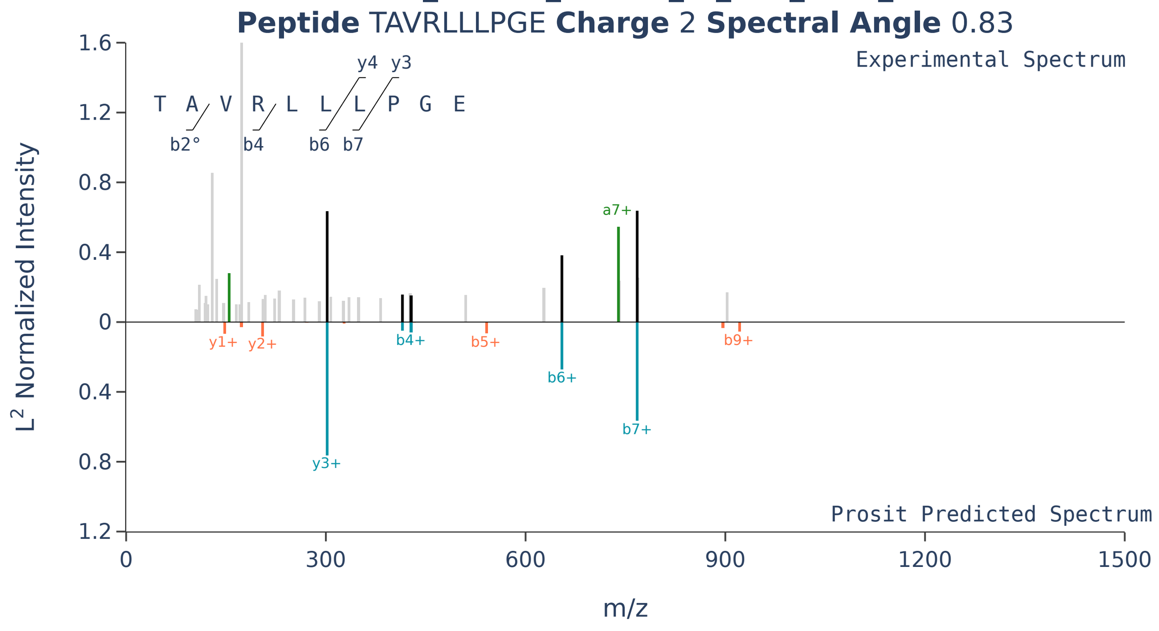


**Source** HRoetschke_291222_080123_Fu_H2B_b2R1_2h **Scan** 14035
**Peptide** SSKAMSIMNSFVND **Charge** 2 **Spectral Angle** 0.72

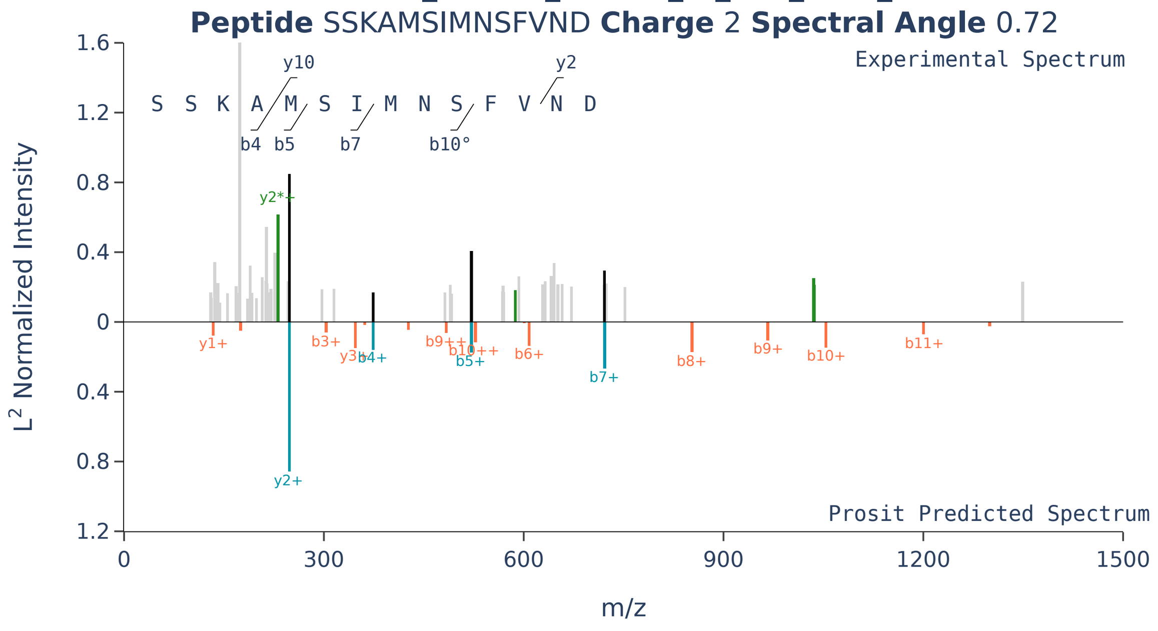


**Source** HRoetschke_291222_080123_Fu_H2B_b1R1_24h **Scan** 13432
**Peptide** DVFERIAGEAS **Charge** 2 **Spectral Angle** 0.7

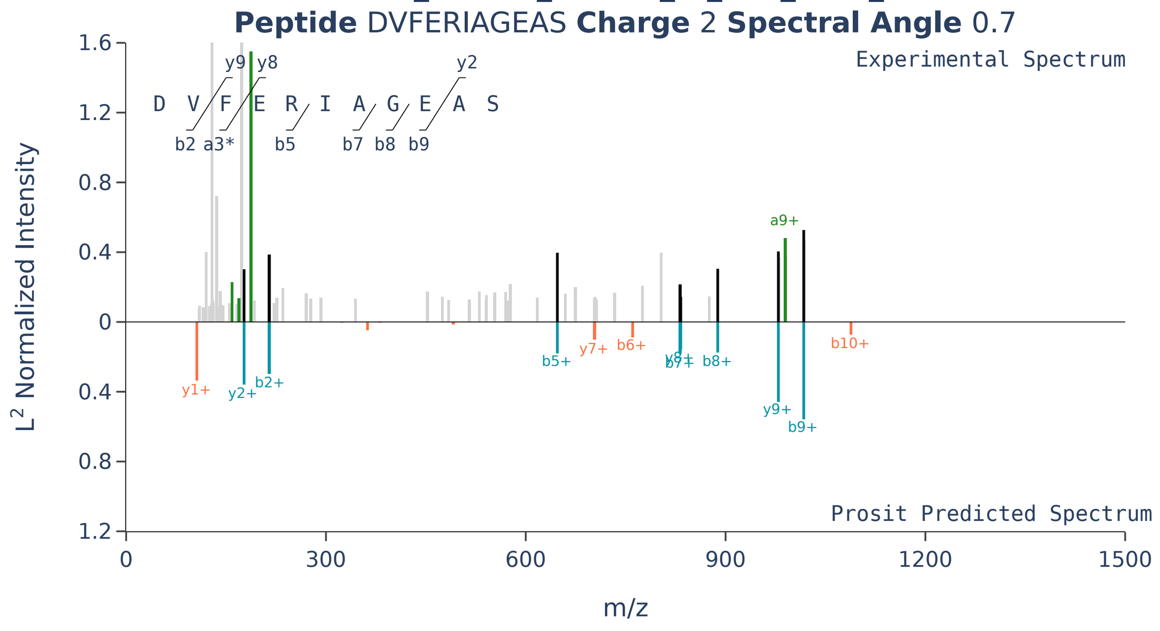


**Source** HRoetschke_291222_080123_Fu_H2B_b2R1_4h **Scan** 15159
**Peptide** NKRSTITSREIQTAVRLL **Charge** 4 **Spectral Angle** 0.73

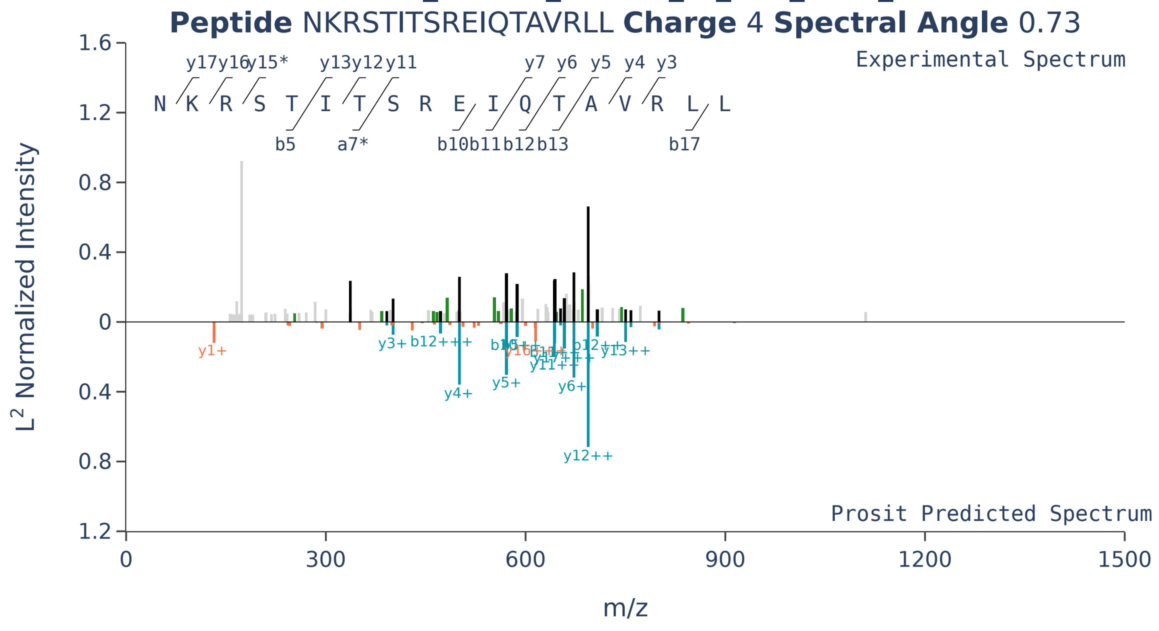

**Source** HRoetschke_291222_080123_Fu_H2B_b1R1_2h **Scan** 2751
**Peptide** ASRLAHYNKRSTL **Charge** 4 **Spectral Angle** 0.62

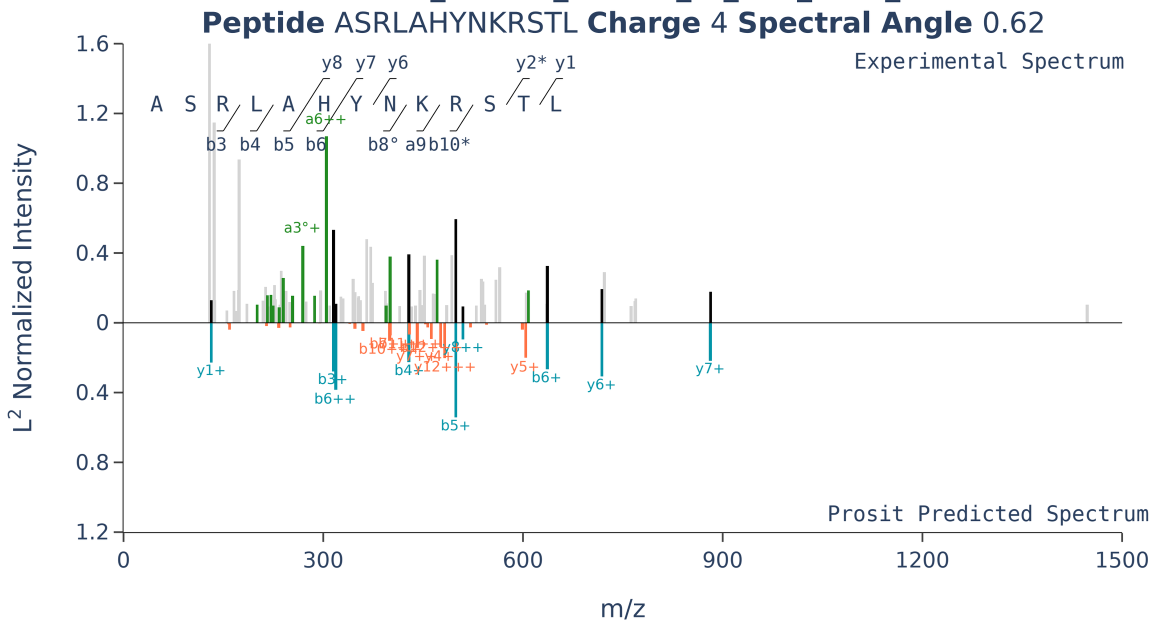


**Source** HRoetschke_291222_080123_Fu_H2B_b1R1_4h **Scan** 2306
**Peptide** GTKAVTKY **Charge** 2 **Spectral Angle** 0.75

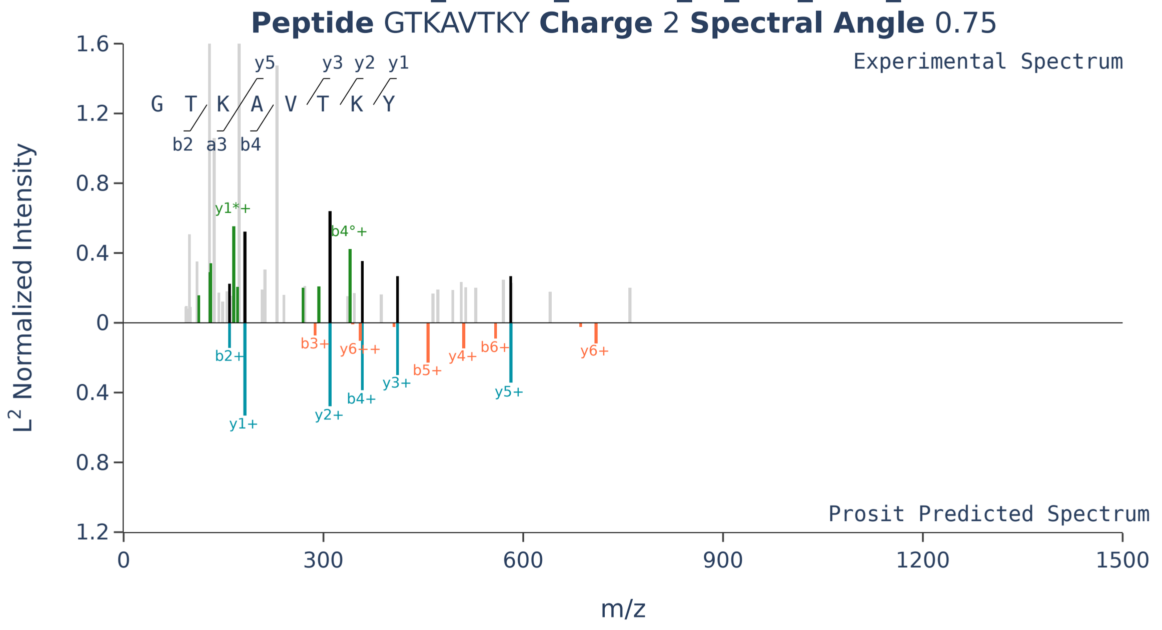


**Source** HRoetschke_291222_080123_Fu_H2B_b1R1_24h **Scan** 8280
**Peptide** ERIAGEASRLAHYNKRSTIT **Charge** 4 **Spectral Angle** 0.54

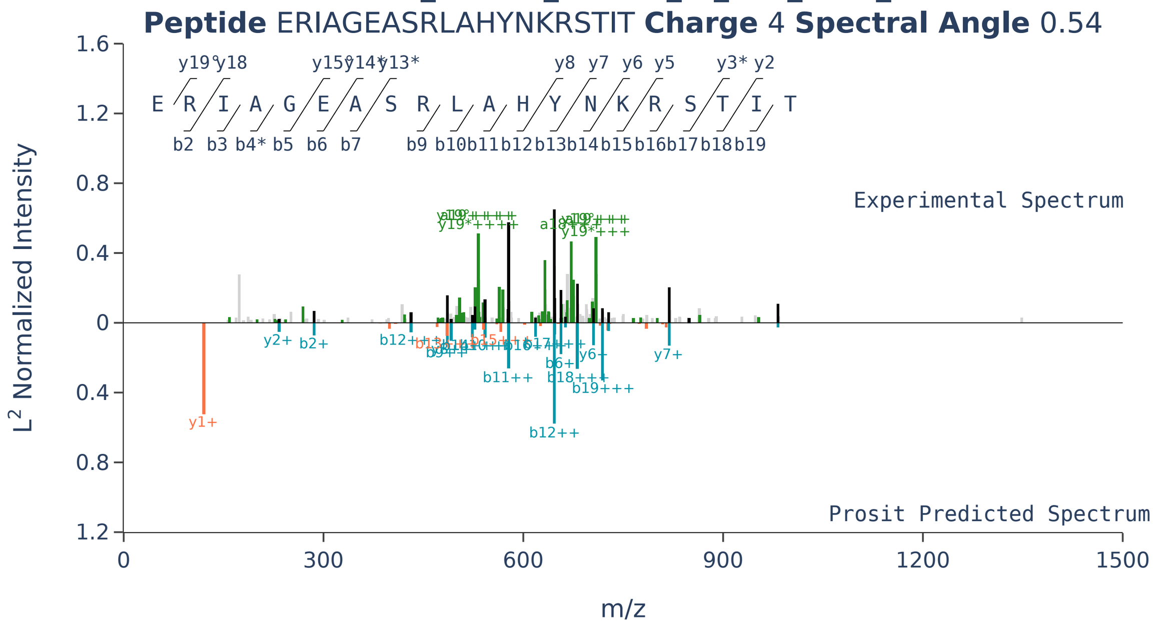


**Source** HRoetschke_291222_080123_Fu_H2B_b1R2_24h **Scan** 8558
**Peptide** RLAGEASRLAHYNKRSTITSREIQTA **Charge** 5 **Spectral Angle** 0.66

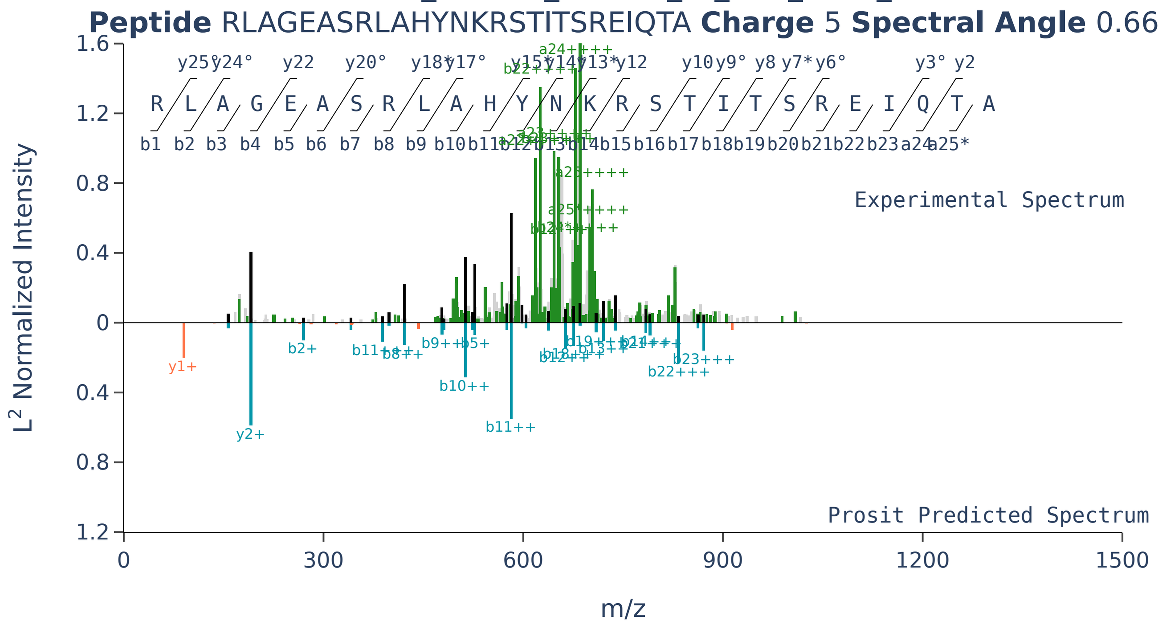


**Source** HRoetschke_291222_080123_Fu_H2B_b2R1_4h **Scan** 19114
**Peptide** TITSREIQTAVRLL **Charge** 3 **Spectral Angle** 0.9

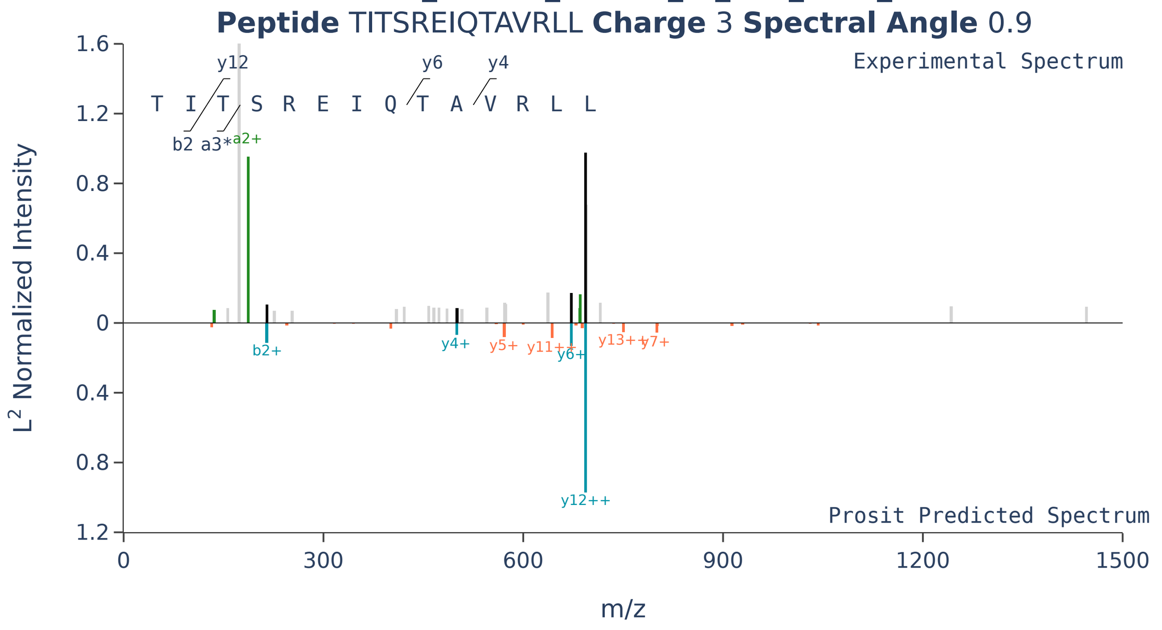


**Source** HRoetschke_291222_080123_Fu_H2B_b1R2_24h **Scan** 4451
**Peptide** VTKTQKKDGKKRRKTRKESYAIYVY **Charge** 4 **Spectral Angle** 0.77

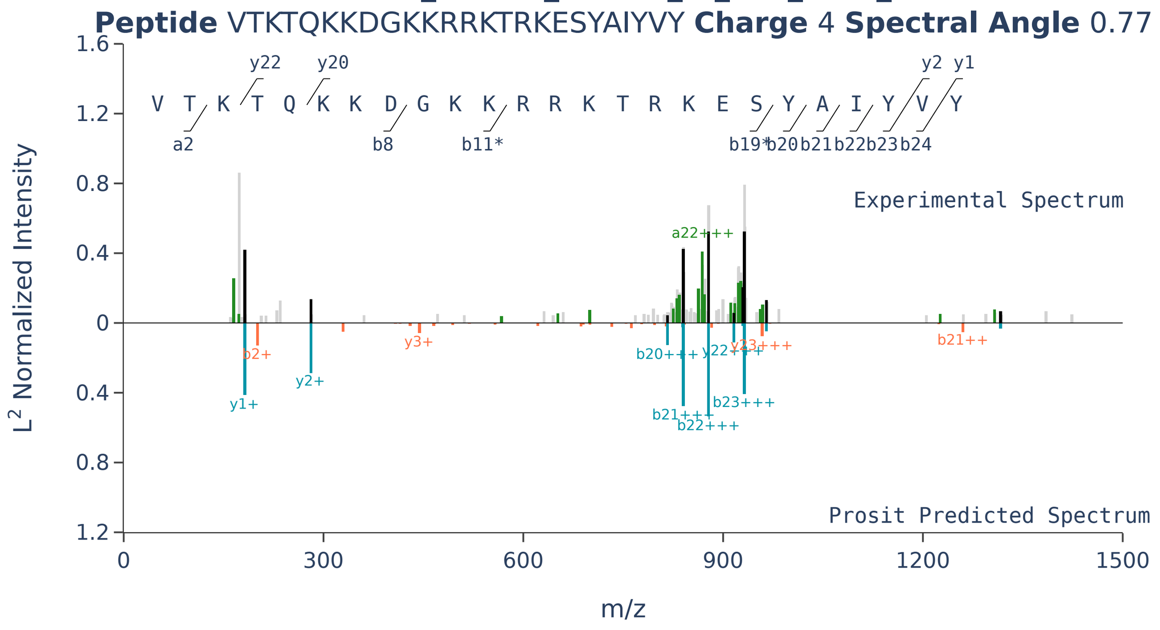


**Source** HRoetschke_291222_080123_Fu_H2B_b2R2_4h **Scan** 2776
**Peptide** HYNKRSTITSREIQ **Charge** 4 **Spectral Angle** 0.58

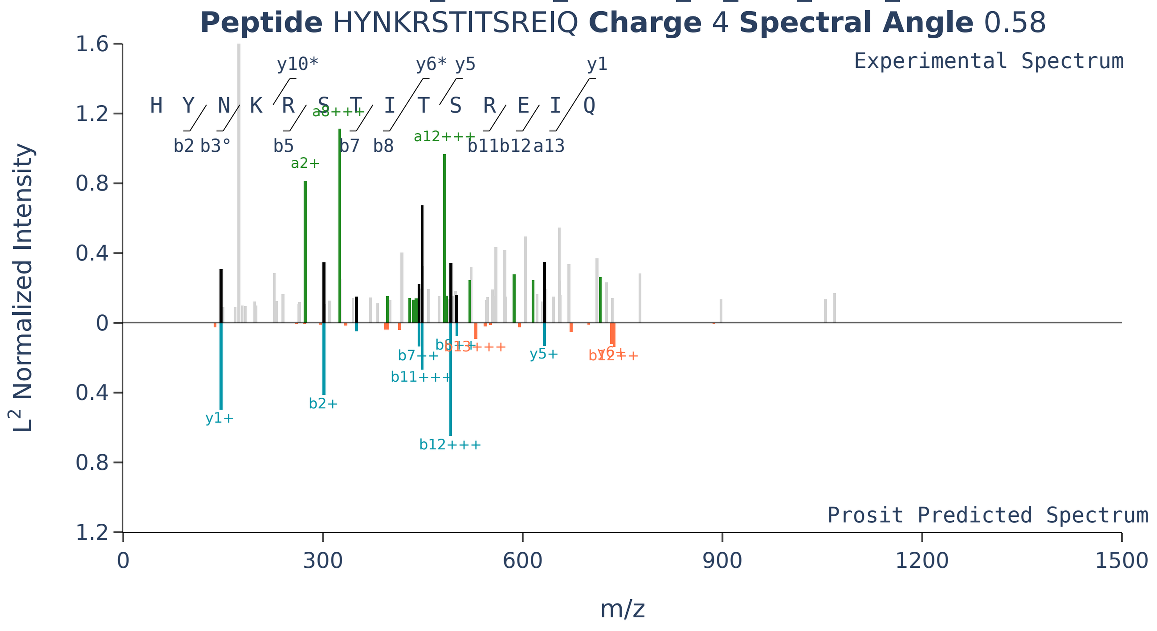


**Source** HRoetschke_291222_080123_Fu_H2B_b1R2_1h **Scan** 7541
**Peptide** RLAGEASRLAHYNKRSTITSREIQTAVR **Charge** 6 **Spectral Angle** 0.34

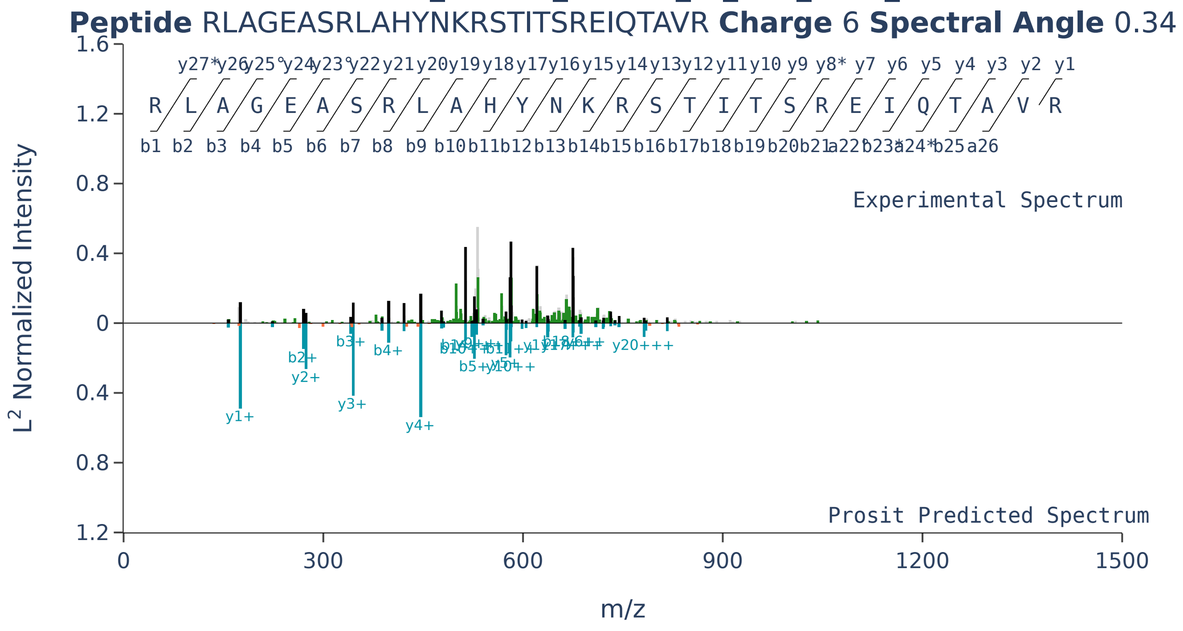


**Source** HRoetschke_291222_080123_Fu_H2B_b2R2_24h **Scan** 19463
**Peptide** SREIQTAVRLLLPGELAKHAVSE **Charge** 4 **Spectral Angle** 0.67

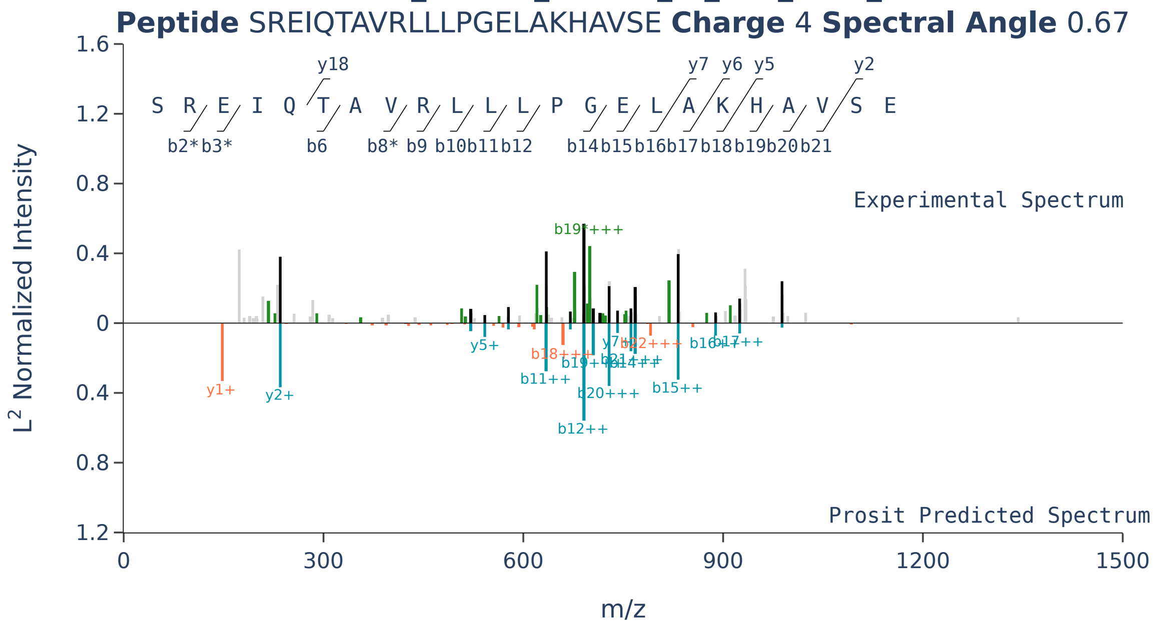


**Source** HRoetschke_291222_080123_Fu_H2B_b1R2_24h **Scan** 1811
**Peptide** AKHAVSEGTKAVTK **Charge** 2 **Spectral Angle** 0.65

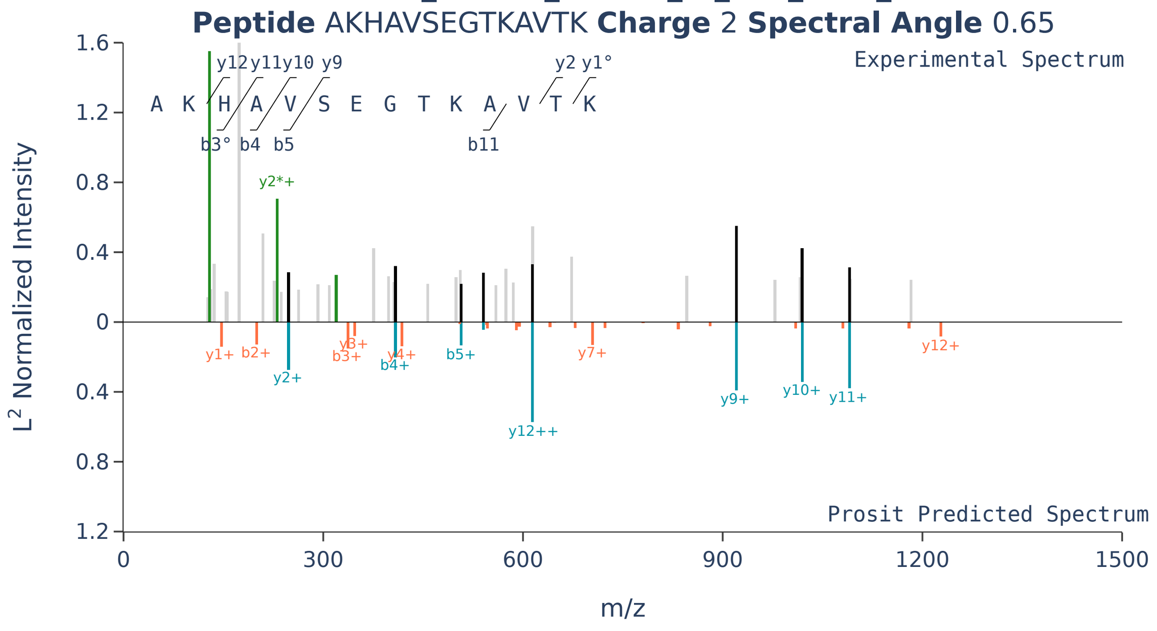


**Source** HRoetschke_291222_080123_Fu_H2B_b2R1_2h **Scan** 4145
**Peptide** YNKRSTITSREIQT **Charge** 3 **Spectral Angle** 0.62

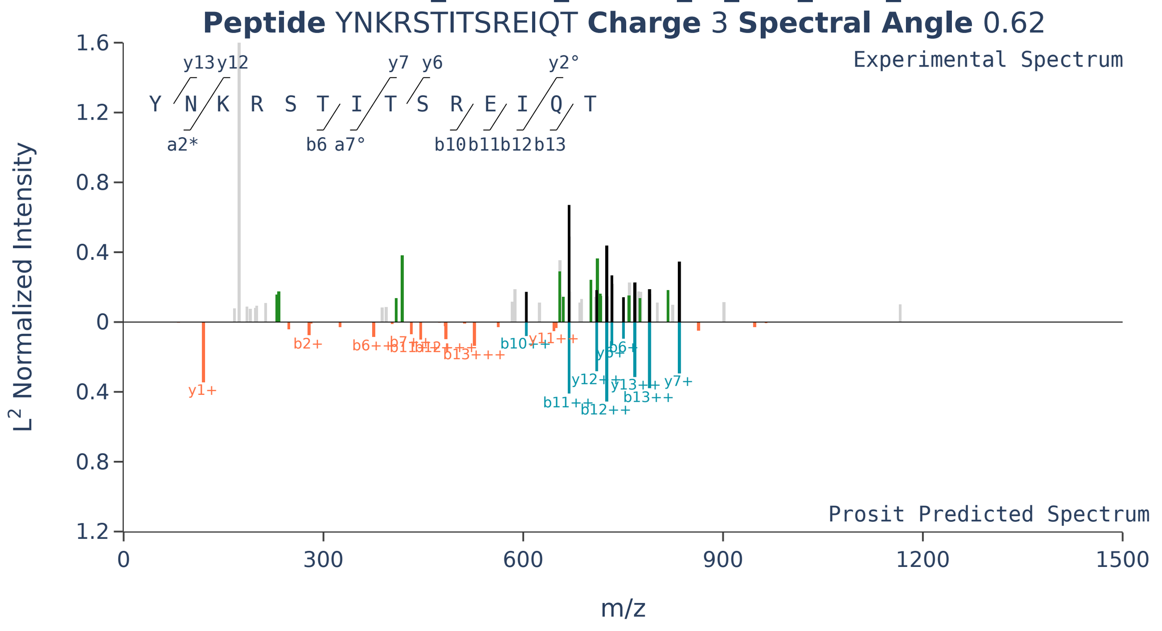


**Source** HRoetschke_291222_080123_Fu_H2B_b2R1_4h **Scan** 14257
**Peptide** ASRLAHYNKRSTITSREIQTAVRLL **Charge** 6 **Spectral Angle** 0.8

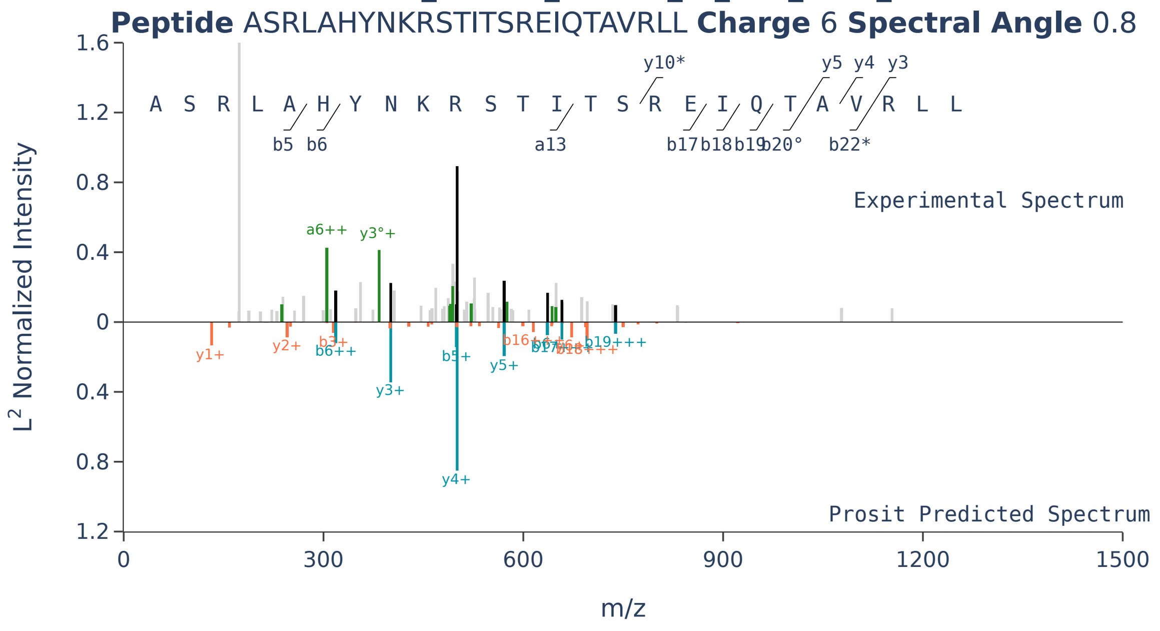


**Source** HRoetschke_291222_080123_Fu_H2B_b2R1_4h **Scan** 19428
**Peptide** FVNDVFERIAGEAS **Charge** 2 **Spectral Angle** 0.73

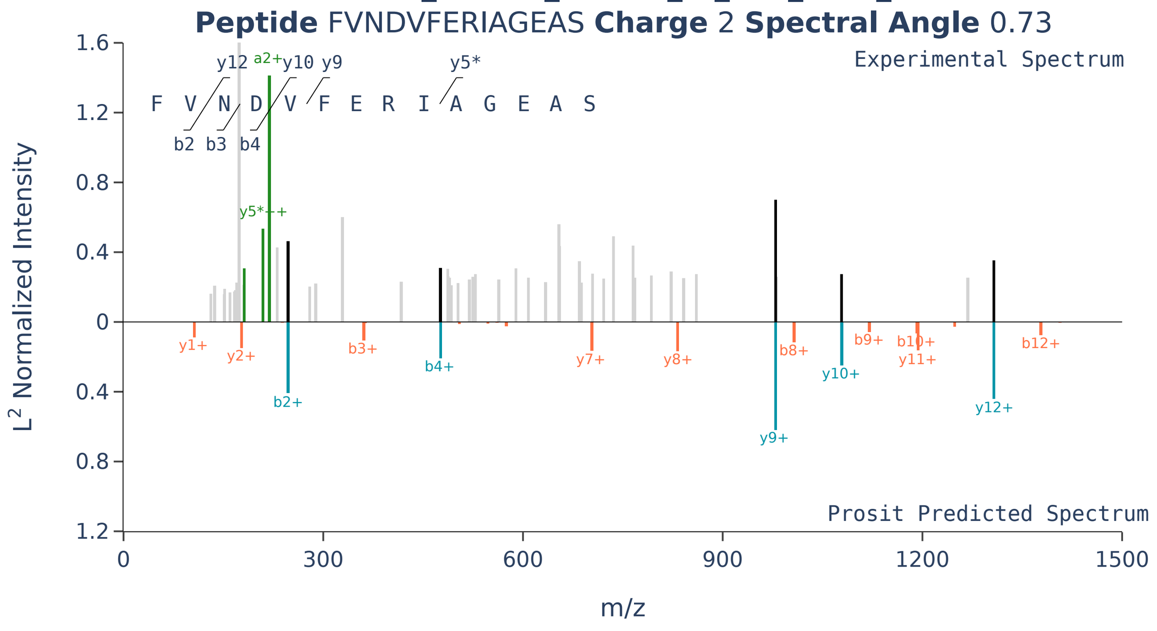


**Source** HRoetschke_291222_080123_Fu_H2B_b2R2_24h **Scan** 11610
**Peptide** KRSTITSREIQTAVRL **Charge** 5 **Spectral Angle** 0.74

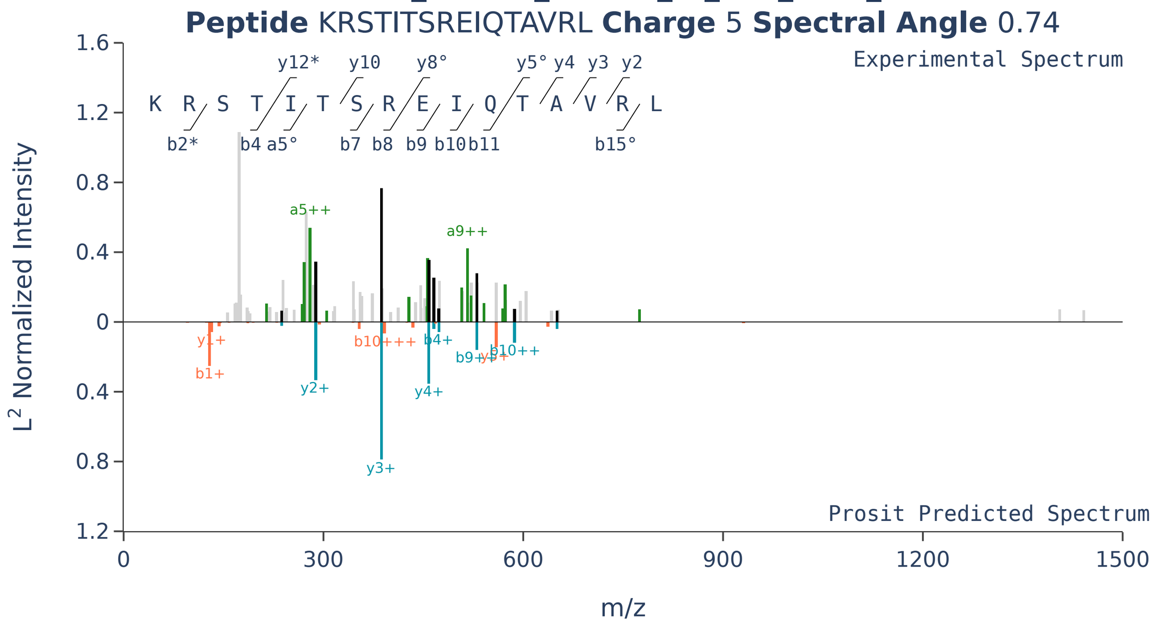


**Source** HRoetschke_291222_080123_Fu_H2B_b1R1_4h **Scan** 14978
**Peptide** KAMSIMNSFVNDVFER **Charge** 3 **Spectral Angle** 0.65

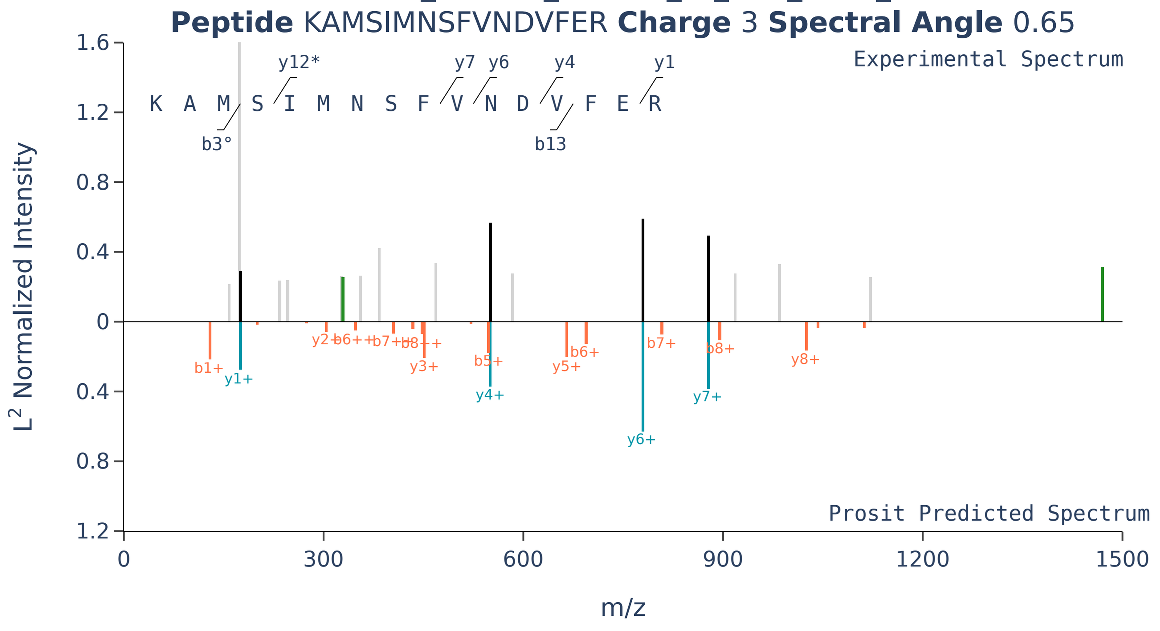

**Source** HRoetschke_291222_080123_Fu_H2B_b2R1_1h **Scan** 19926
**Peptide** MNSFVNDVFERIAGEASRLAHYNKRS **Charge** 5 **Spectral Angle** 0.87

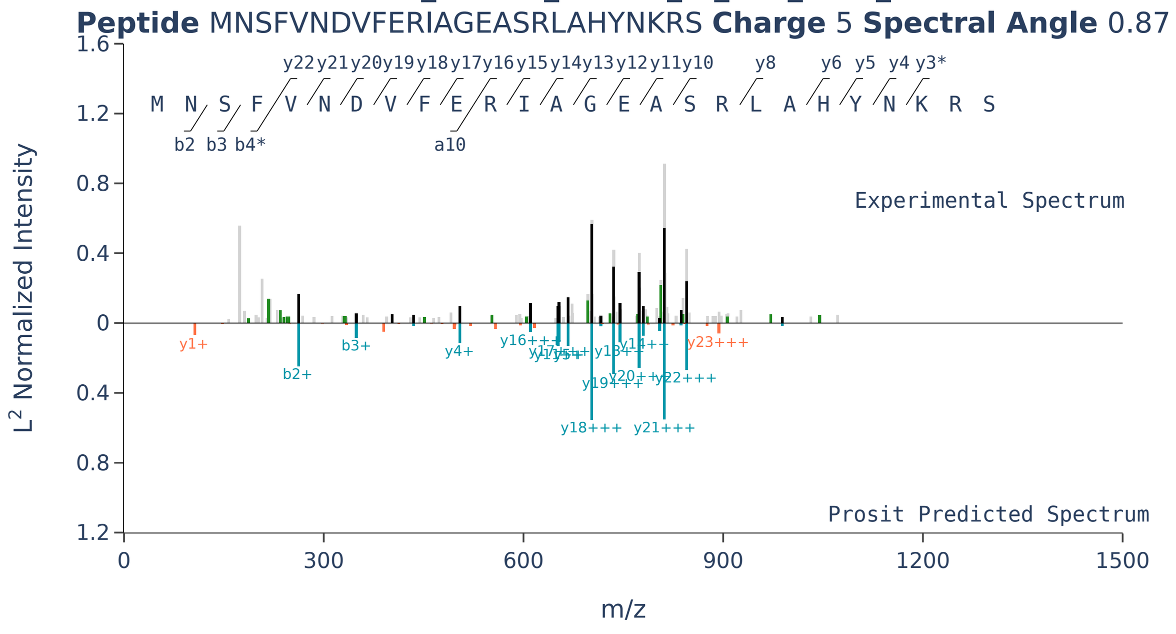


**Source** HRoetschke_291222_080123_Fu_H2B_b2R1_24h **Scan** 11548
**Peptide** PGELAKHAVSEGTKAVTKYTSA **Charge** 4 **Spectral Angle** 0.59

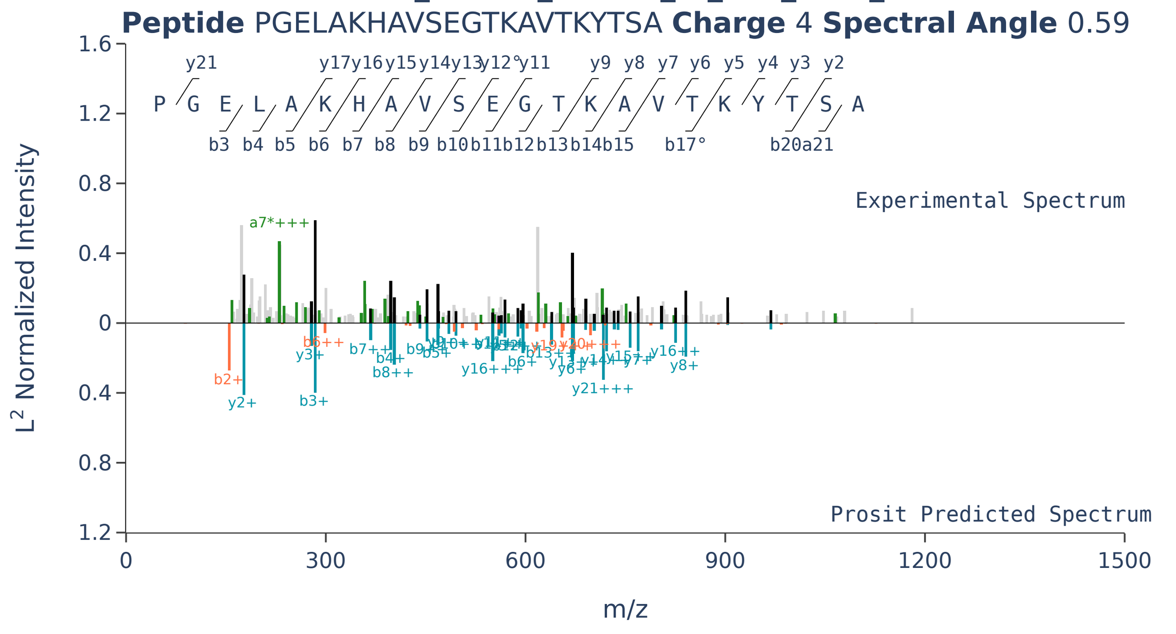


**Source** HRoetschke_291222_080123_Fu_H2B_b2R2_2h **Scan** 5527
**Peptide** GEASRLAHYNKRSTITSREI **Charge** 4 **Spectral Angle** 0.82

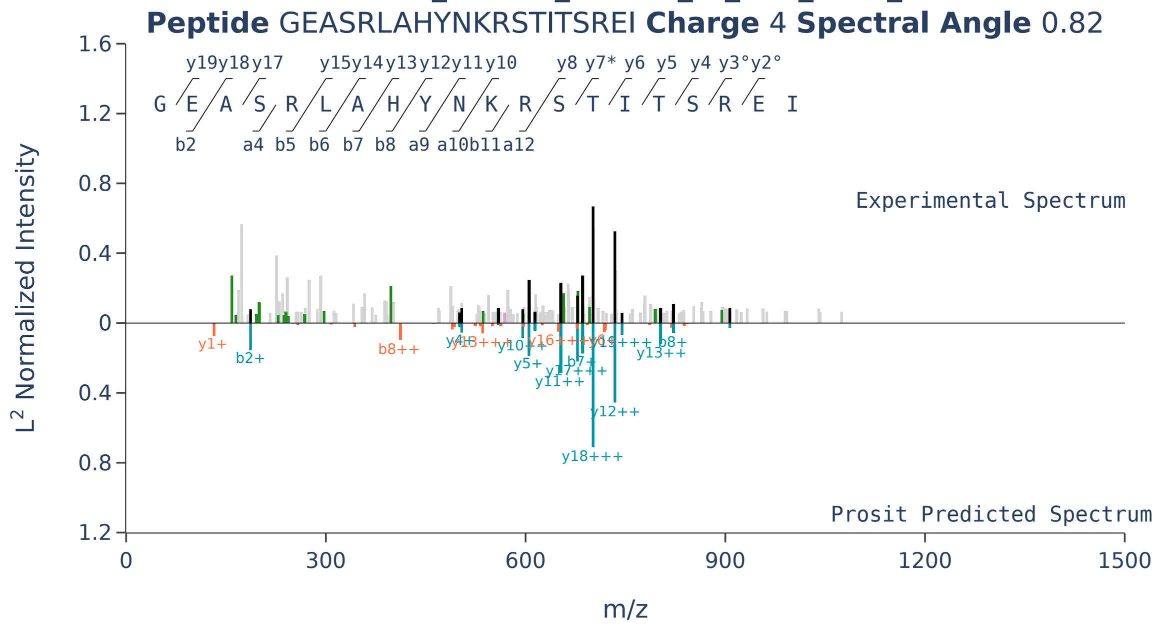


**Source** HRoetschke_291222_080123_Fu_H2B_b2R1_2h **Scan** 6135
**Peptide** PGELAKHAVSEGTKAV **Charge** 4 **Spectral Angle** 0.62

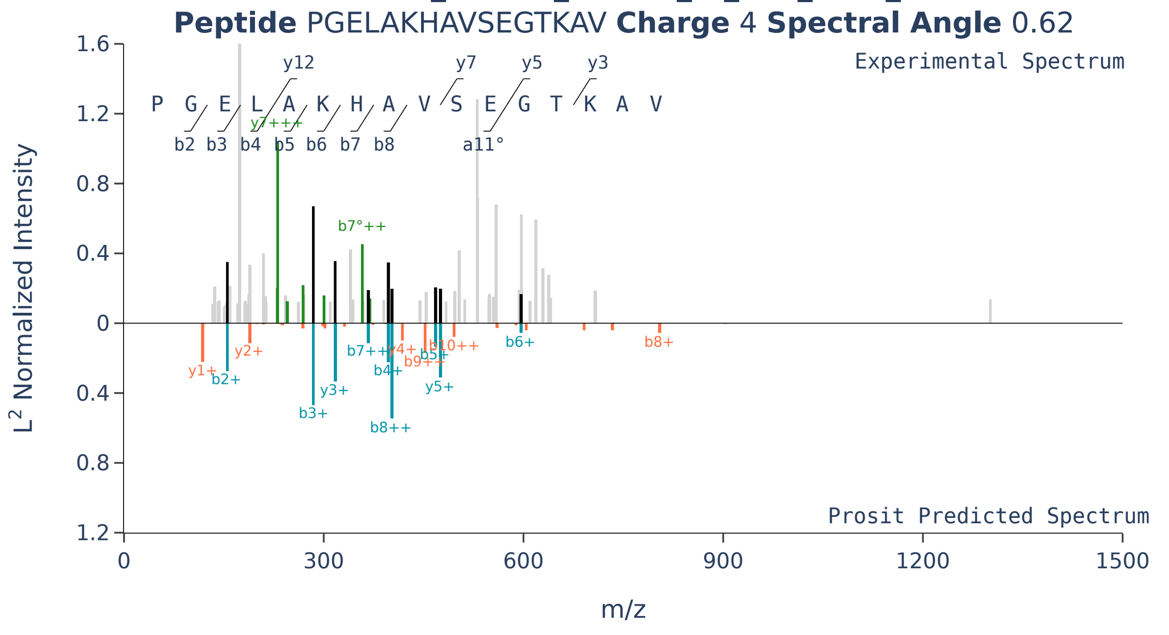


**Source** HRoetschke_291222_080123_Fu_H2B_b1R1_24h **Scan** 2444
**Peptide** SRLAHYNKRSTITSRE **Charge** 4 **Spectral Angle** 0.72

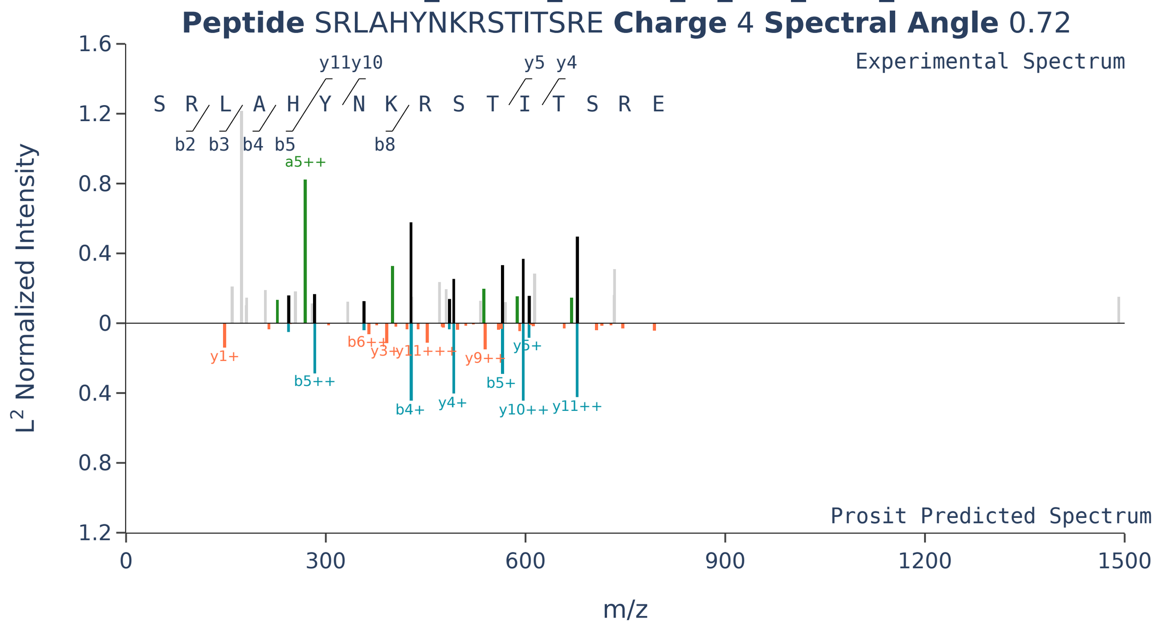


**Source** HRoetschke_291222_080123_Fu_H2B_b1R2_24h **Scan** 3886
**Peptide** HYNKRSTITSREIQT **Charge** 3 **Spectral Angle** 0.77

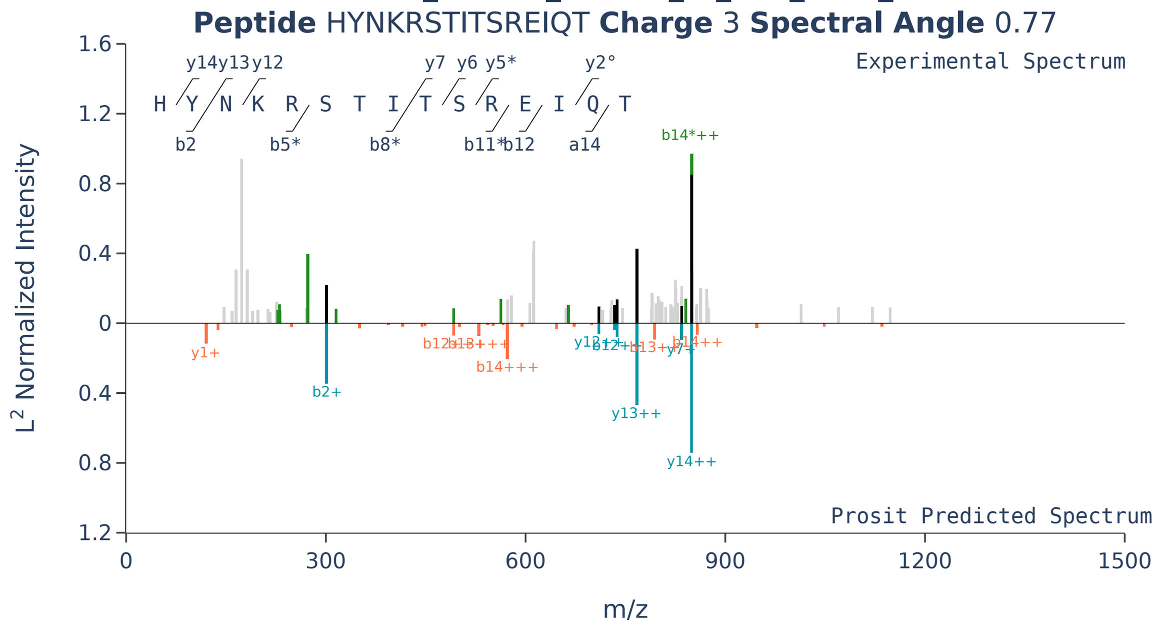


**Source** HRoetschke_291222_080123_Fu_H2B_b2R1_2h **Scan** 5073
**Peptide** EASRLAHYNKRSTITSREIQT **Charge** 4 **Spectral Angle** 0.56

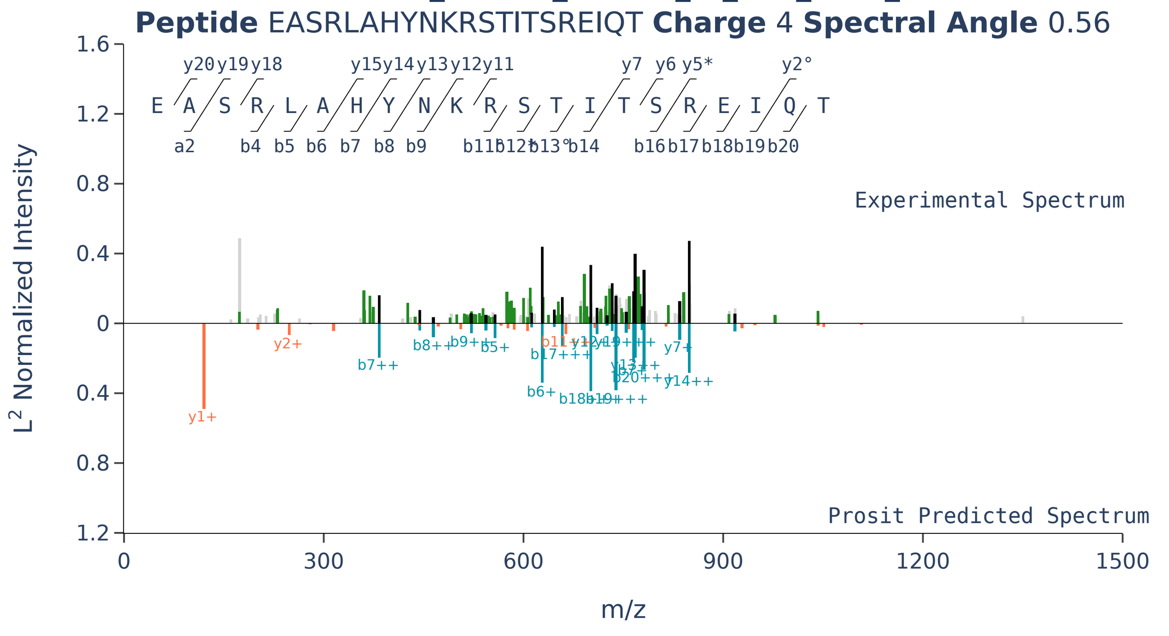


**Source** HRoetschke_291222_080123_Fu_H2B_b2R2_4h **Scan** 18591
**Peptide** HPDTGISSKAMSIMNSFVNDVFE **Charge** 3 **Spectral Angle** 0.78

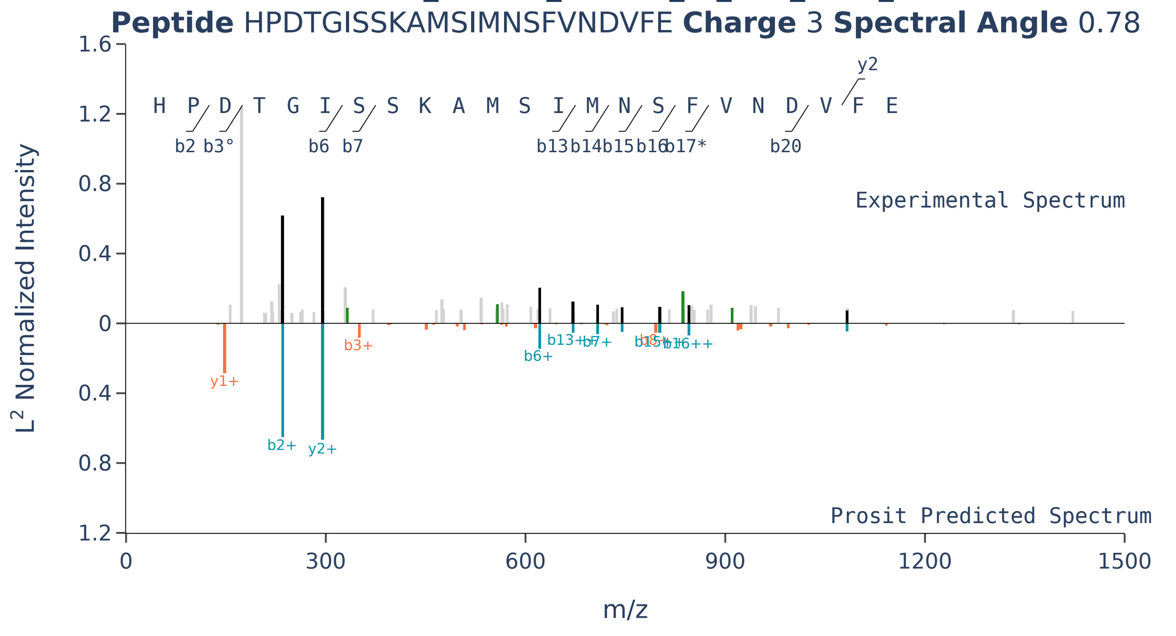


**Source** HRoetschke_291222_080123_Fu_H2B_b2R1_4h **Scan** 20891
**Peptide** FVNDVFERIAGEASRLAHYNKRST **Charge** 5 **Spectral Angle** 0.74

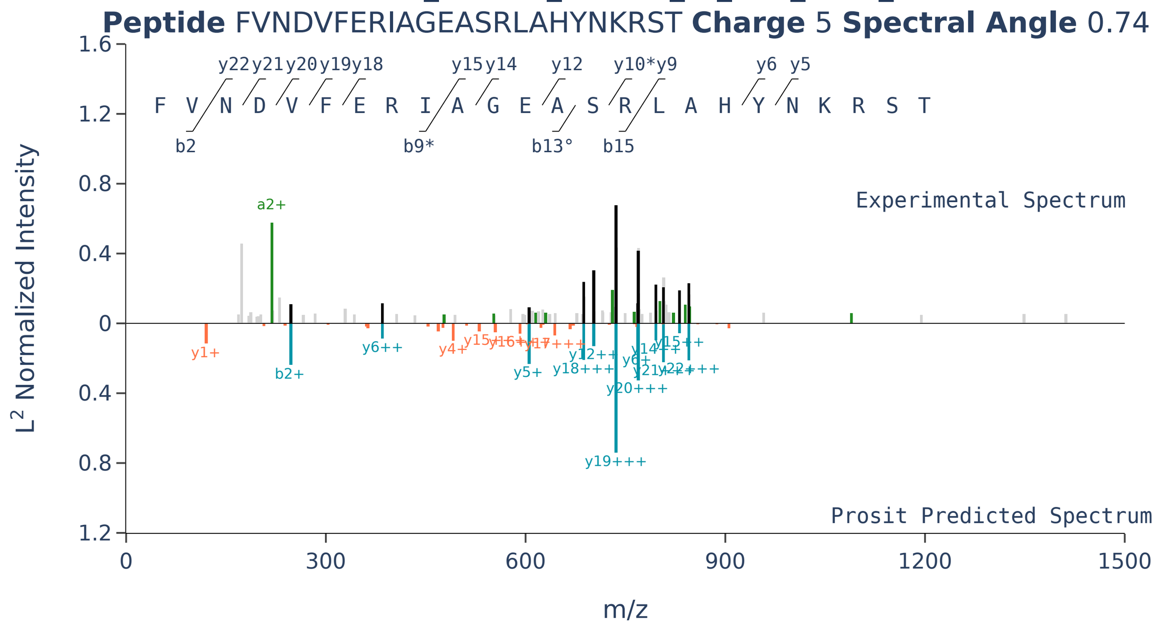


**Source** HRoetschke_291222_080123_Fu_H2B_b2R1_4h **Scan** 21917
**Peptide** NSFVNDVFERIAGEAS **Charge** 2 **Spectral Angle** 0.78

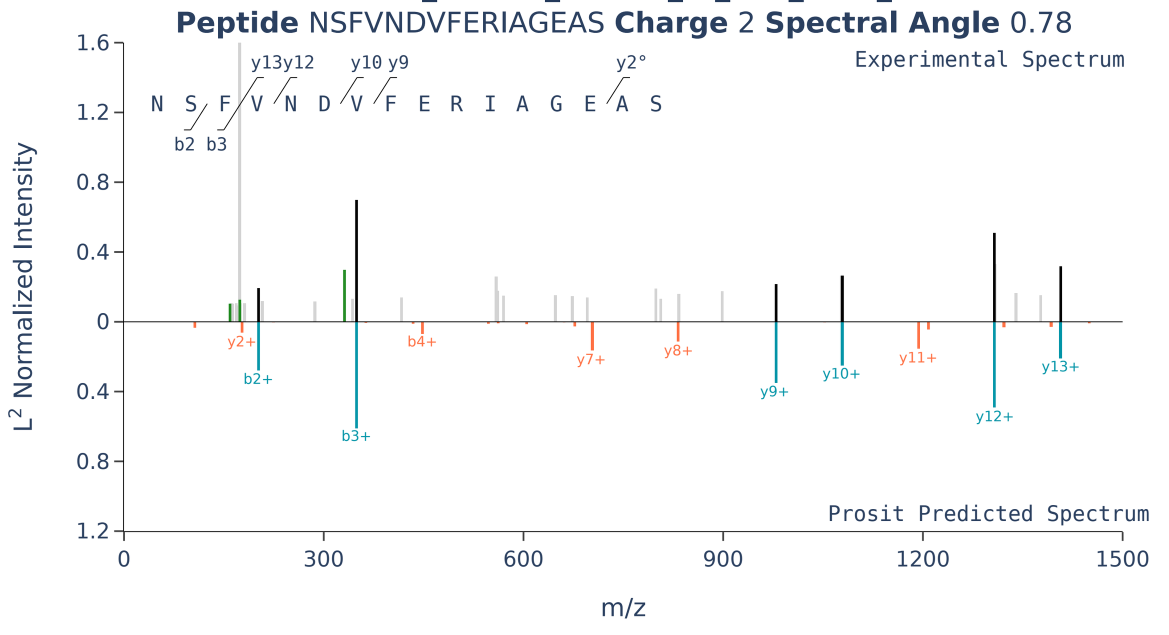


**Source** HRoetschke_291222_080123_Fu_H2B_b1R2_24h **Scan** 7564
**Peptide** KRSTITSREIQTAV **Charge** 3 **Spectral Angle** 0.64

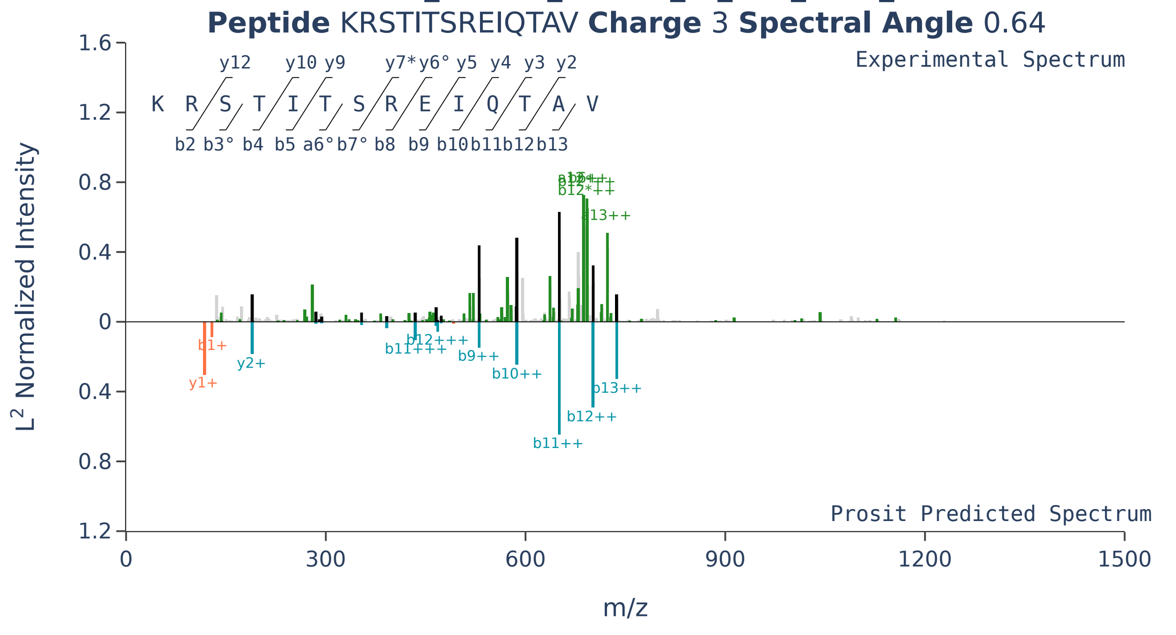


**Source** HRoetschke_291222_080123_Fu_H2B_b2R1_24h **Scan** 16346
**Peptide** MSIMNSFVND **Charge** 2 **Spectral Angle** 0.63

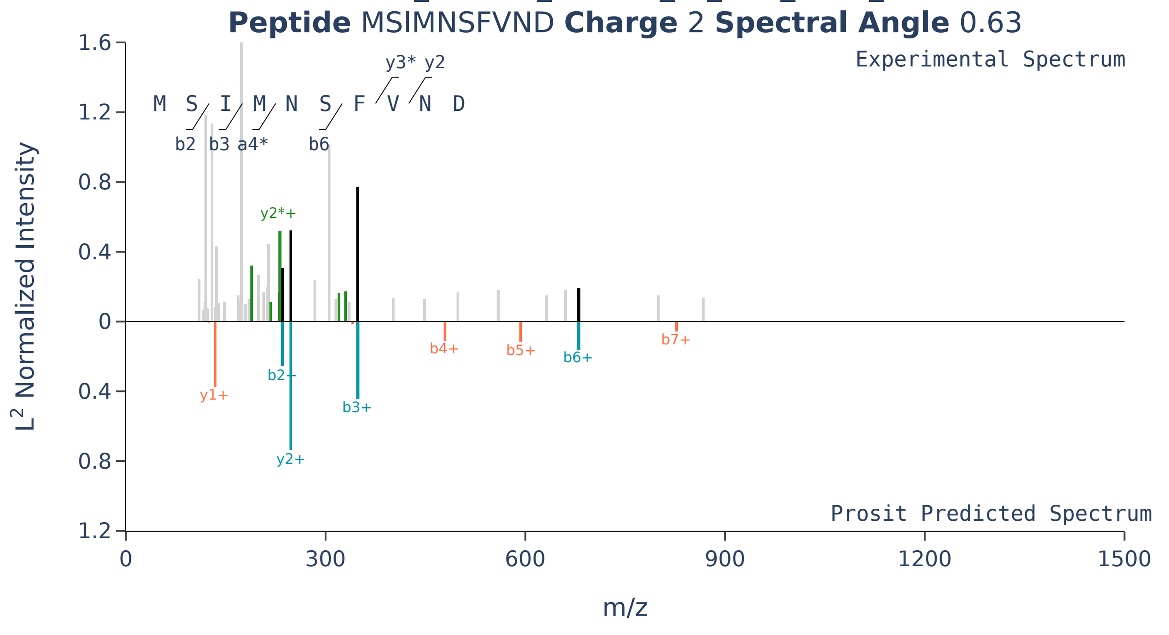


**Source** HRoetschke_291222_080123_Fu_H2B_b2R1_2h **Scan** 17829
**Peptide** AHYNKRSTITSREIQTAVRLLLPGE **Charge** 4 **Spectral Angle** 0.71

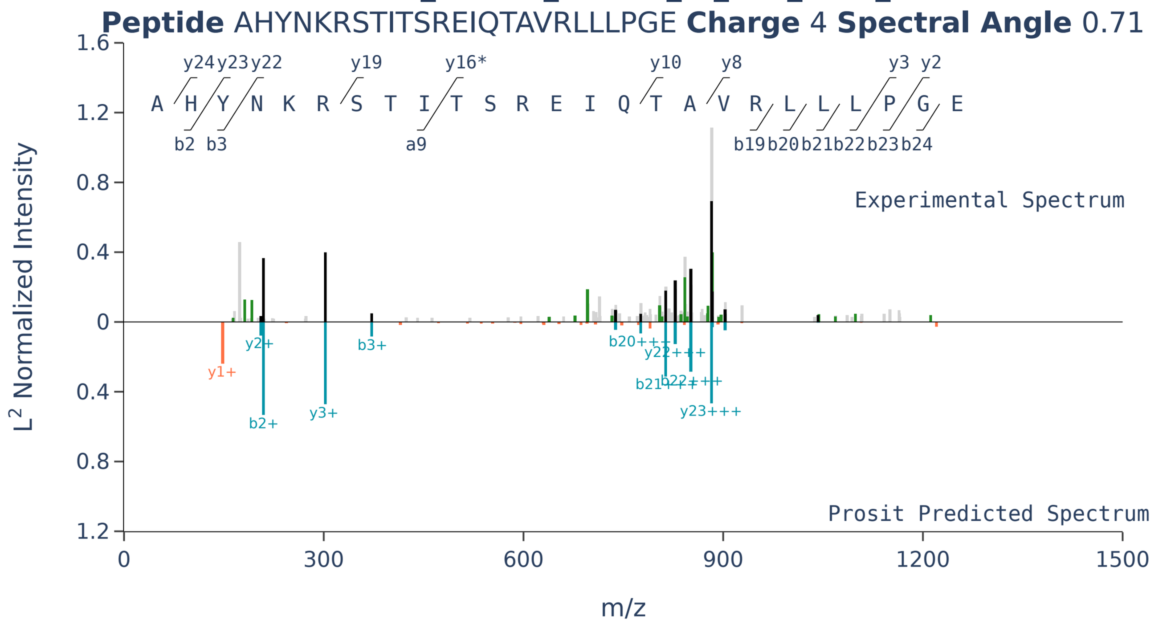


**Source** HRoetschke_291222_080123_Fu_H2B_b1R2_4h **Scan** 13352
**Peptide** KVLKQVHPDTGISSKAMSIMNSF **Charge** 4 **Spectral Angle** 0.45

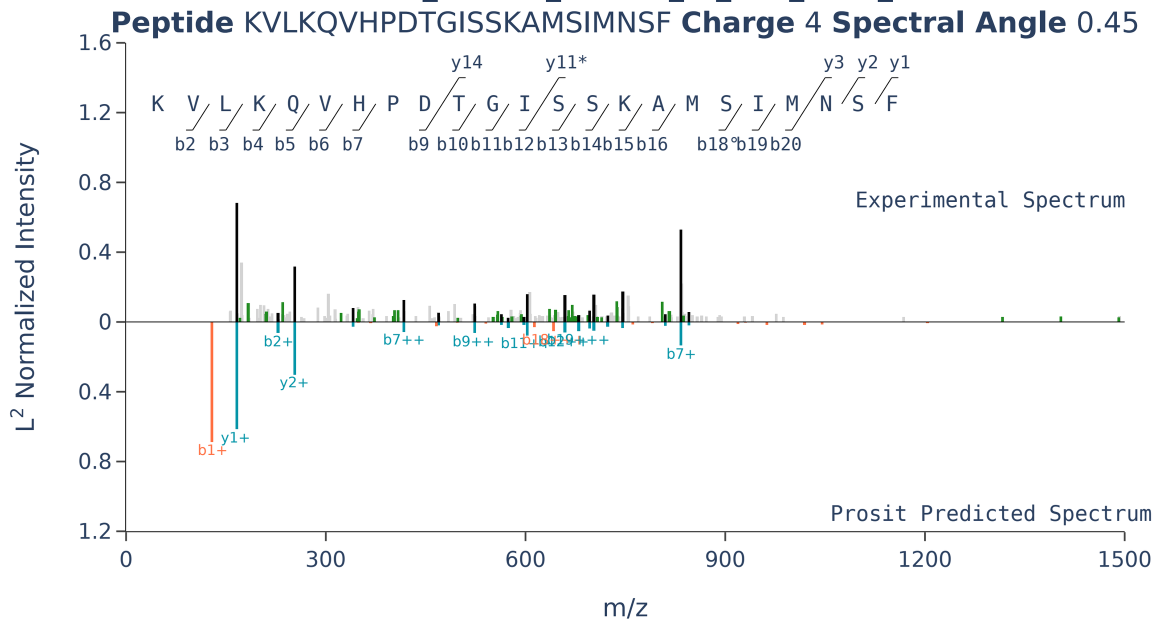


**Source** HRoetschke_291222_080123_Fu_H2B_b2R2_24h **Scan** 15509
**Peptide** AIYVYKVLKQVHPDTGISSK **Charge** 5 **Spectral Angle** 0.56

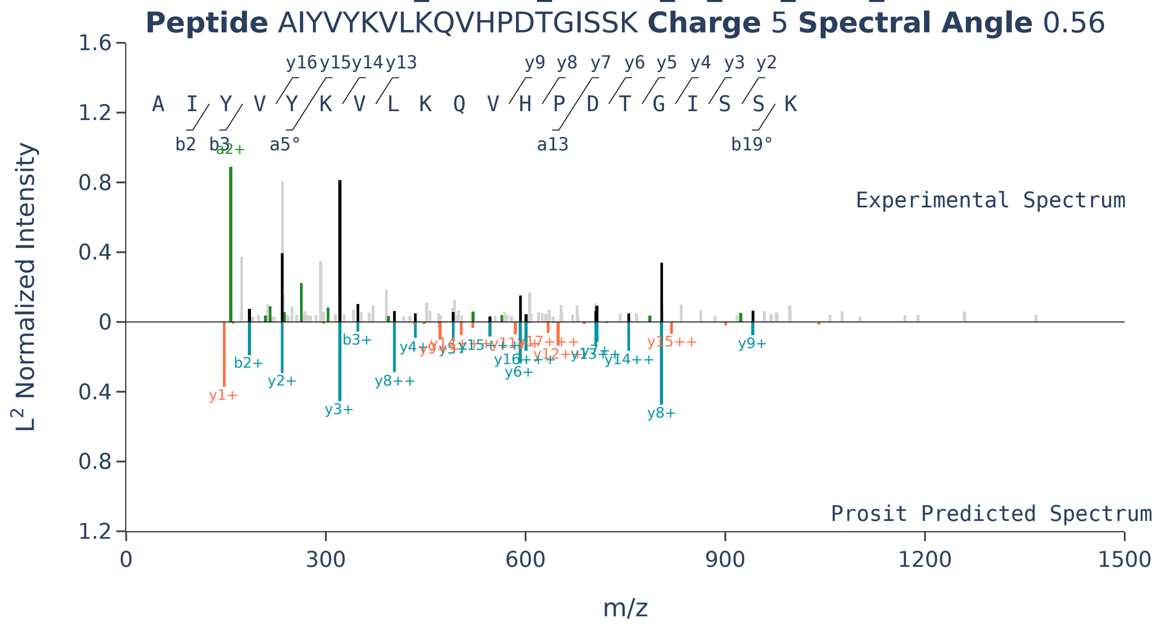

**Source** HRoetschke_291222_080123_Fu_H2B_b2R2_24h **Scan** 5676
**Peptide** GEASRLAHYNKRSTITSREIQ **Charge** 4 **Spectral Angle** 0.67

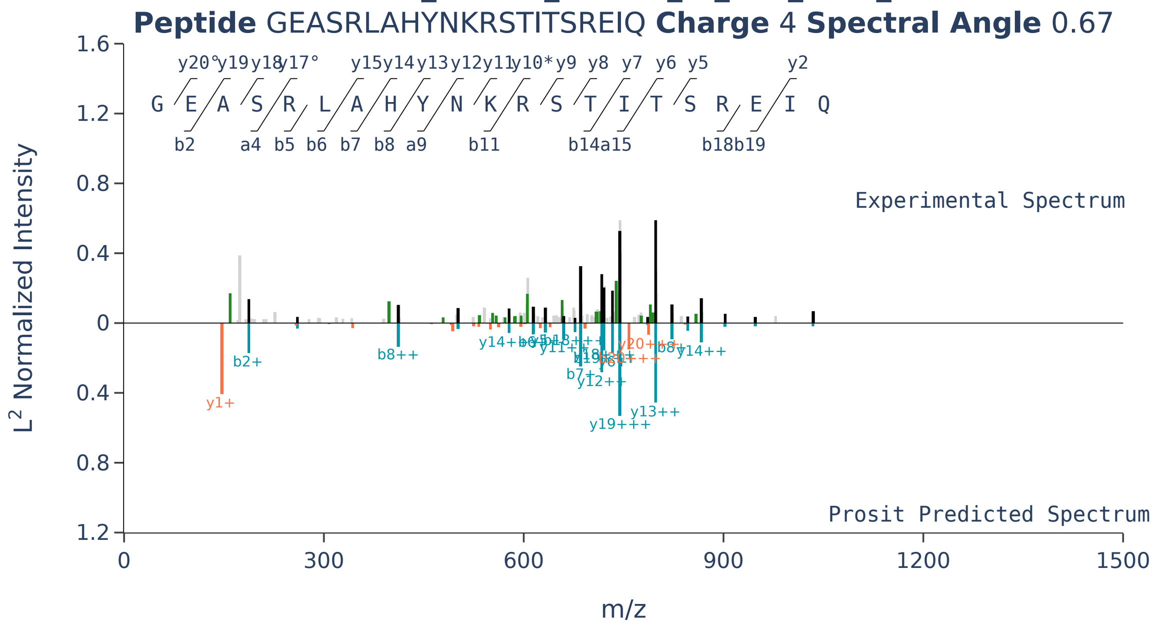


**Source** HRoetschke_291222_080123_Fu_H2B_b2R2_24h **Scan** 10851
**Peptide** LLPGELAKHA **Charge** 3 **Spectral Angle** 0.79

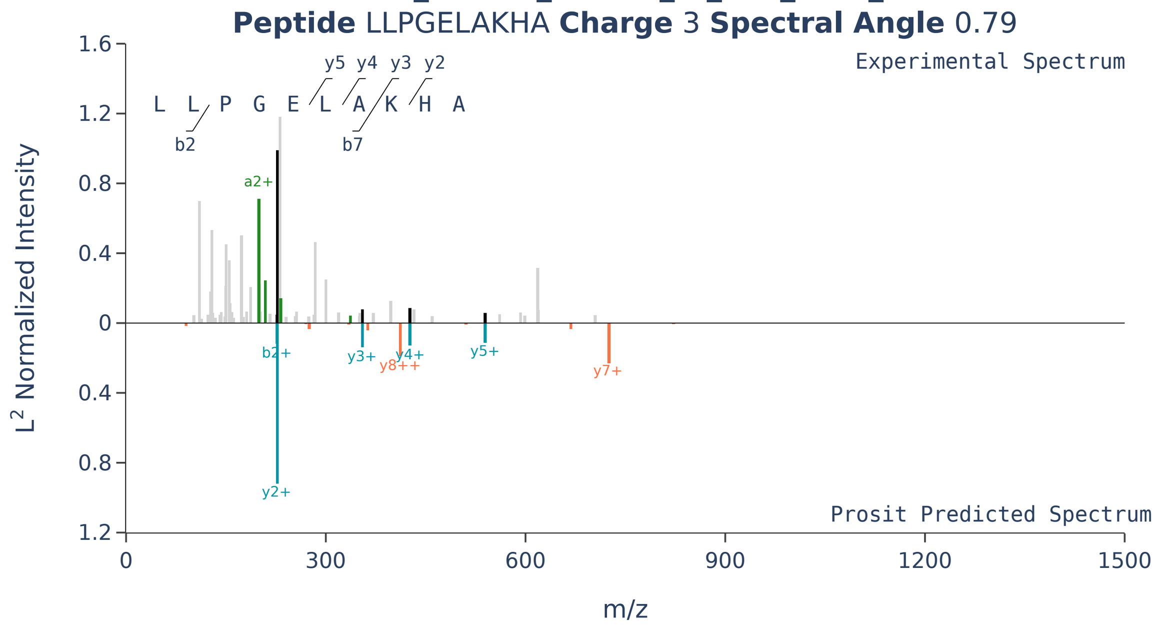


**Source** HRoetschke_291222_080123_Fu_H2B_b2R1_2h **Scan** 7644
**Peptide** RIAGEASRLAHYNKRSTITSREI **Charge** 6 **Spectral Angle** 0.57

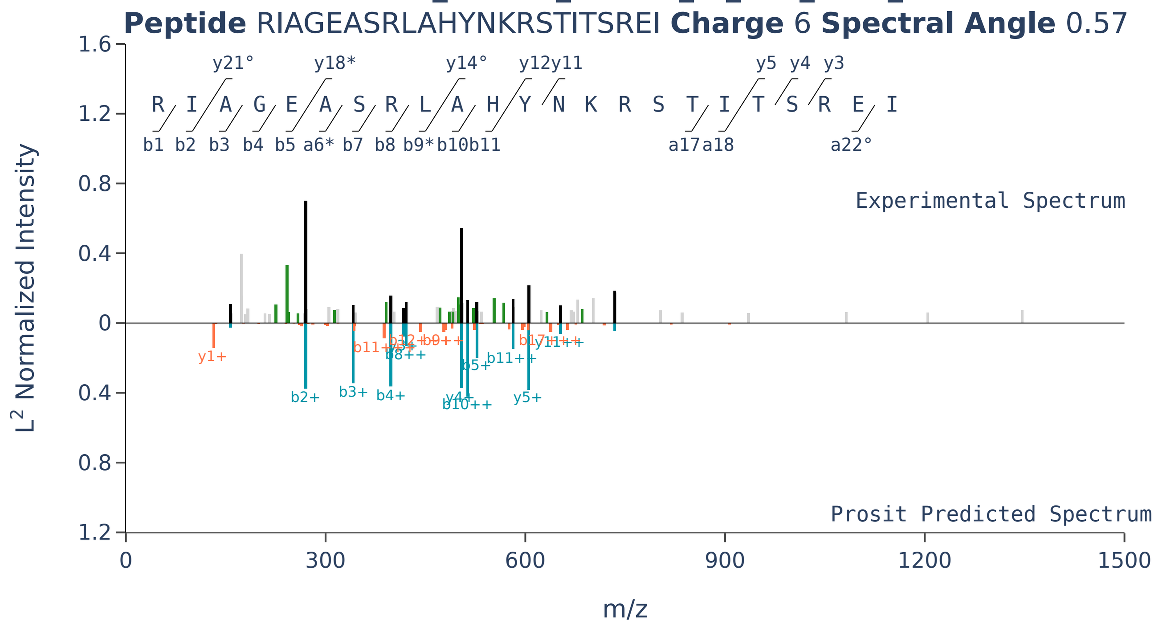


**Source** HRoetschke_291222_080123_Fu_H2B_b1R2_2h **Scan** 3261
**Peptide** RLAHYNKRSTITSREIQ **Charge** 4 **Spectral Angle** 0.61

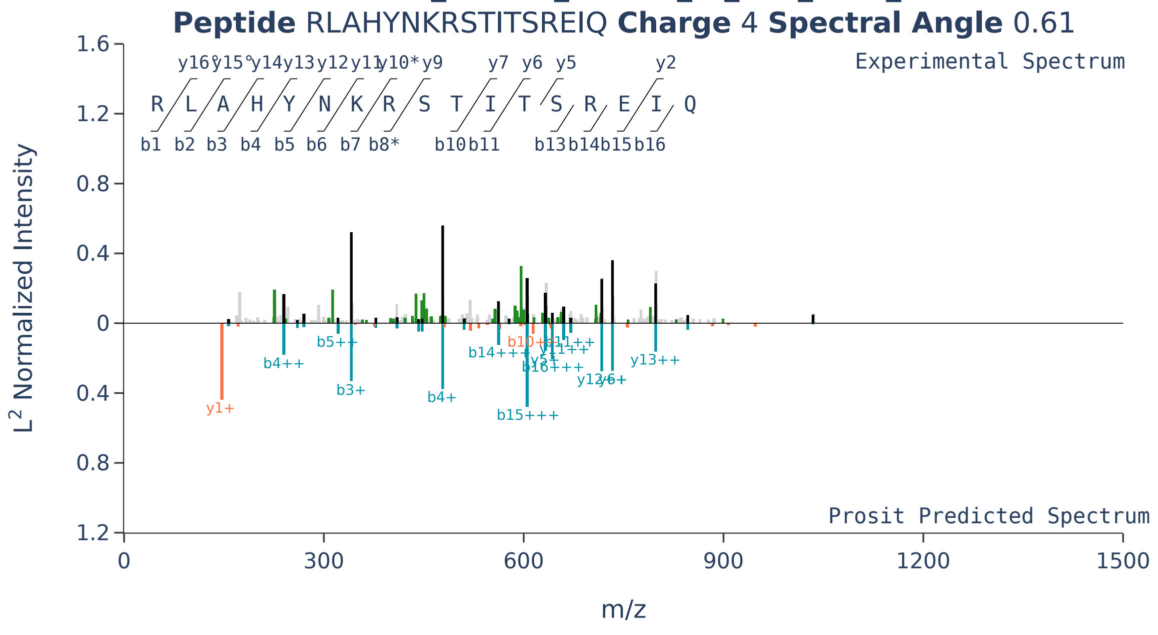


**Source** HRoetschke_291222_080123_Fu_H2B_b2R1_2h **Scan** 2849
**Peptide** KHAVSEGTKAVTKYTSAK **Charge** 3 **Spectral Angle** 0.41

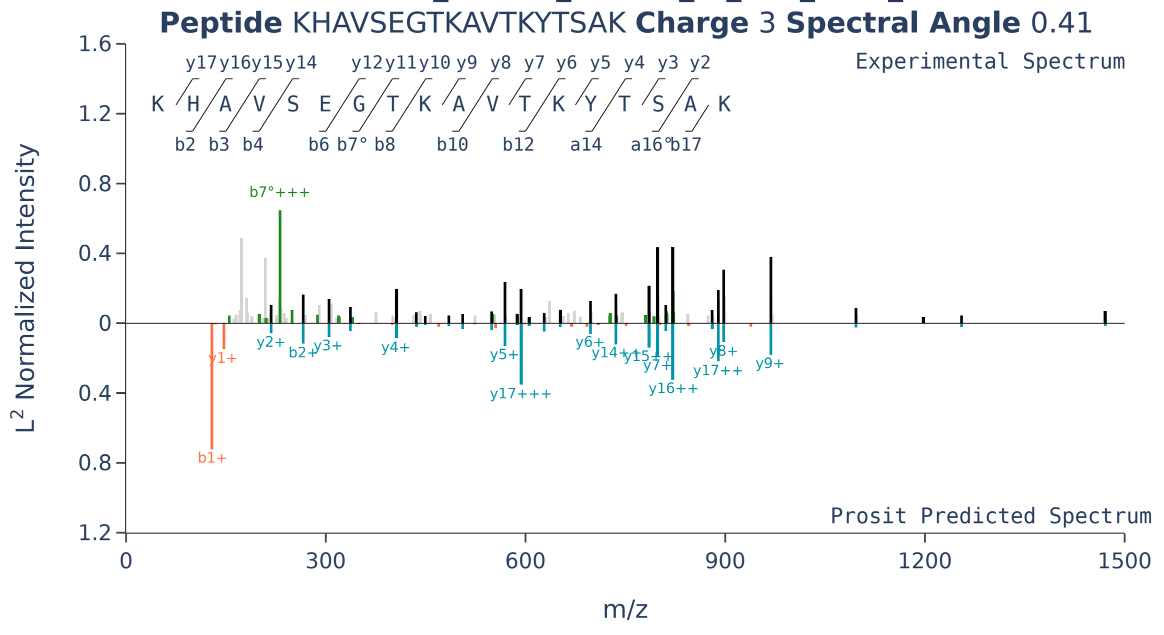


**Source** HRoetschke_291222_080123_Fu_H2B_b2R1_4h **Scan** 21687
**Peptide** NSFVNDVFERIAGE **Charge** 2 **Spectral Angle** 0.71

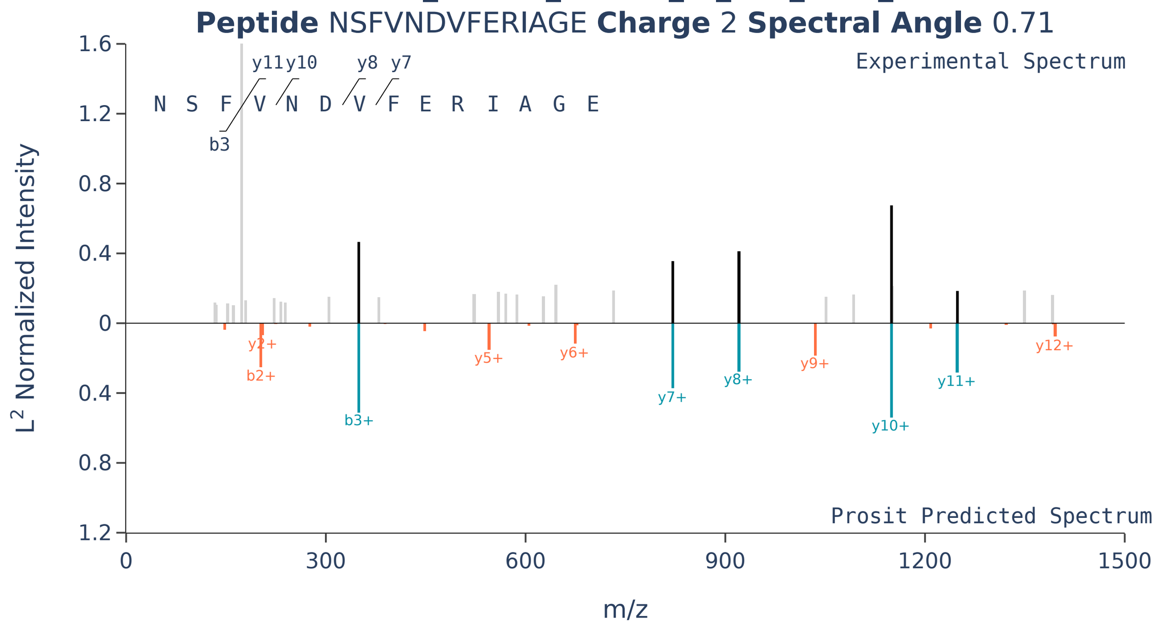


**Source** HRoetschke_291222_080123_Fu_H2B_b2R2_24h **Scan** 9777
**Peptide** VYKVLKQVHPDTGISS **Charge** 3 **Spectral Angle** 0.55

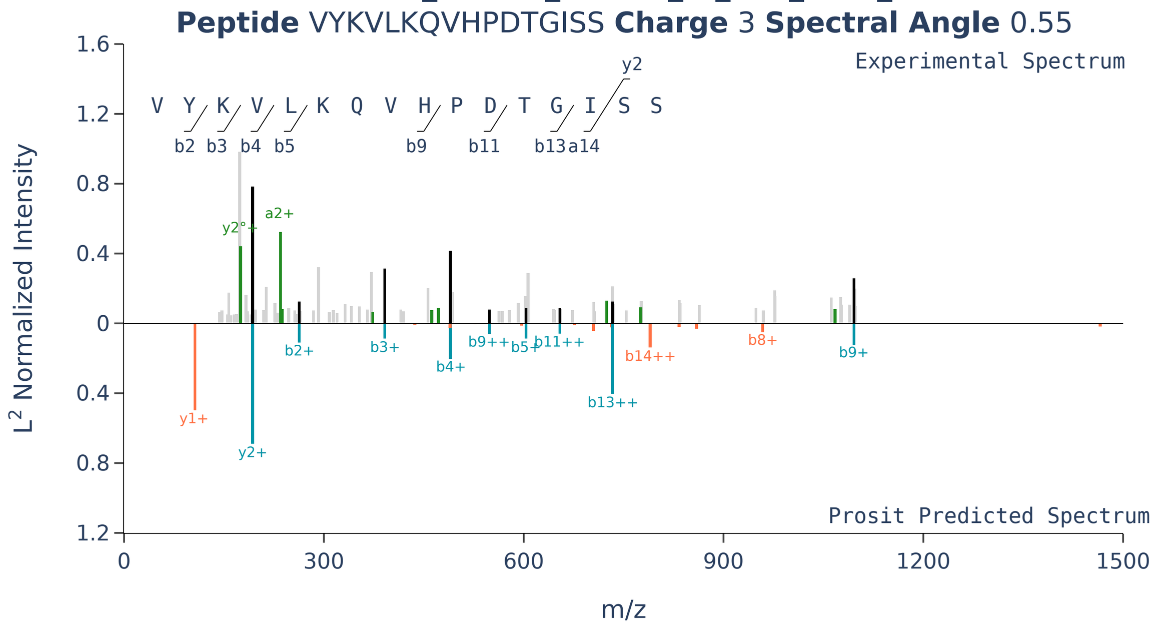


**Source** HRoetschke_291222_080123_Fu_H2B_b2R2_24h **Scan** 16743
**Peptide** QTAVRLLLPGELAKHAVSEGTK **Charge** 4 **Spectral Angle** 0.63

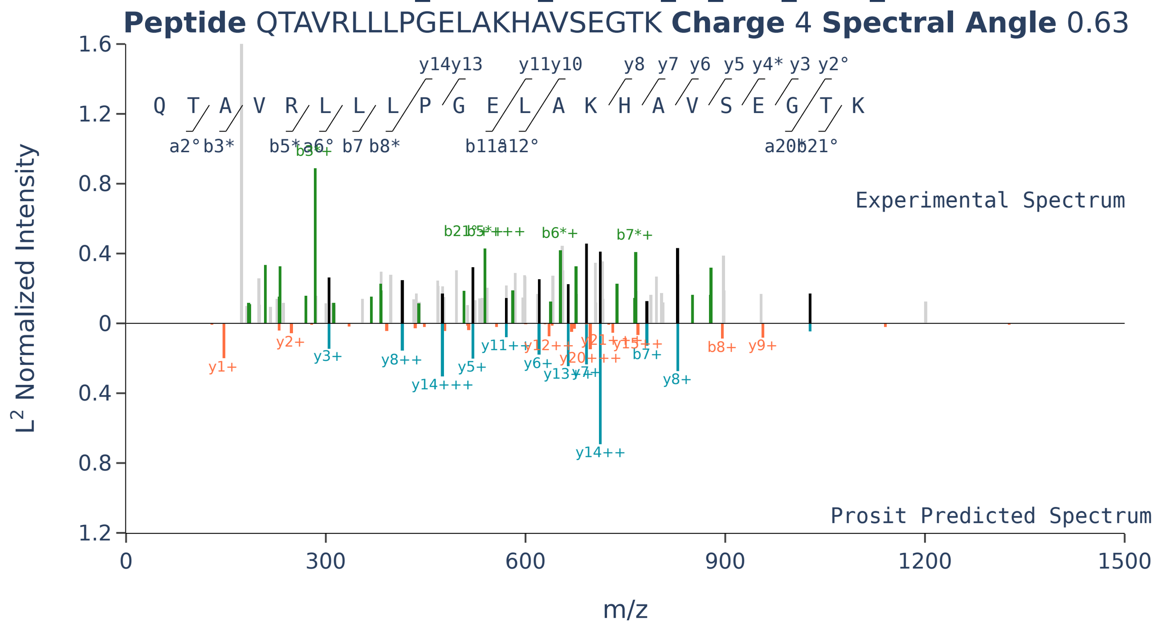


**Source** HRoetschke_291222_080123_Fu_H2B_b1R2_24h **Scan** 5101
**Peptide** KKDGKKRRKTRKESYAIYVY **Charge** 4 **Spectral Angle** 0.78

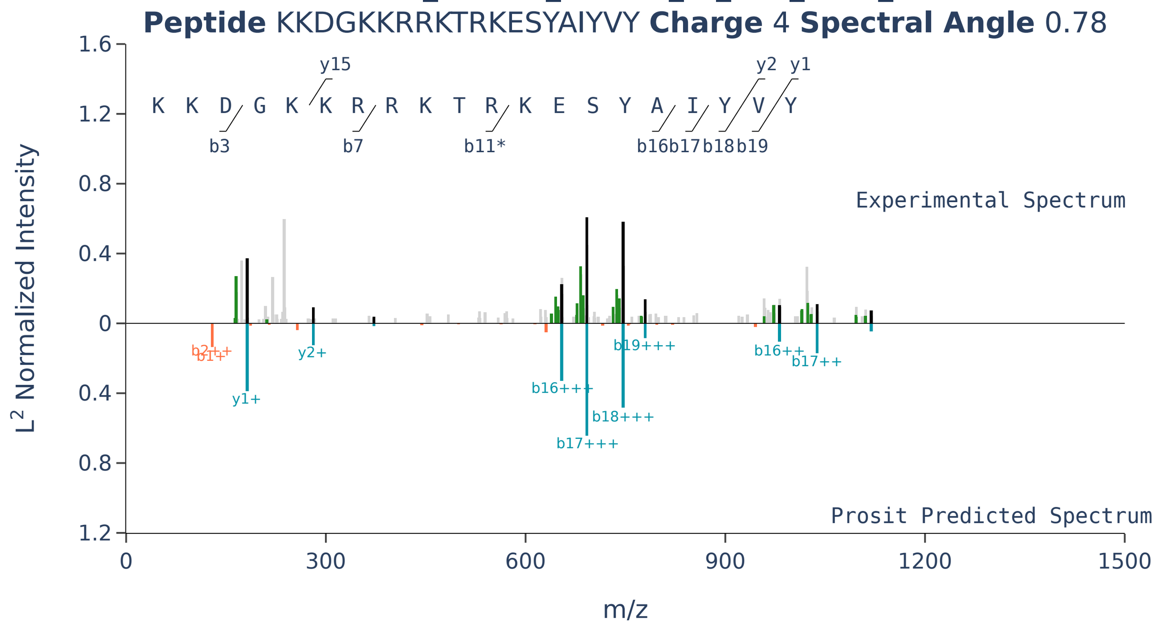


**Source** HRoetschke_291222_080123_Fu_H2B_b2R1_1h **Scan** 16923
**Peptide** TSREIQTAVRLLLPGELAKHAVSE **Charge** 3 **Spectral Angle** 0.64

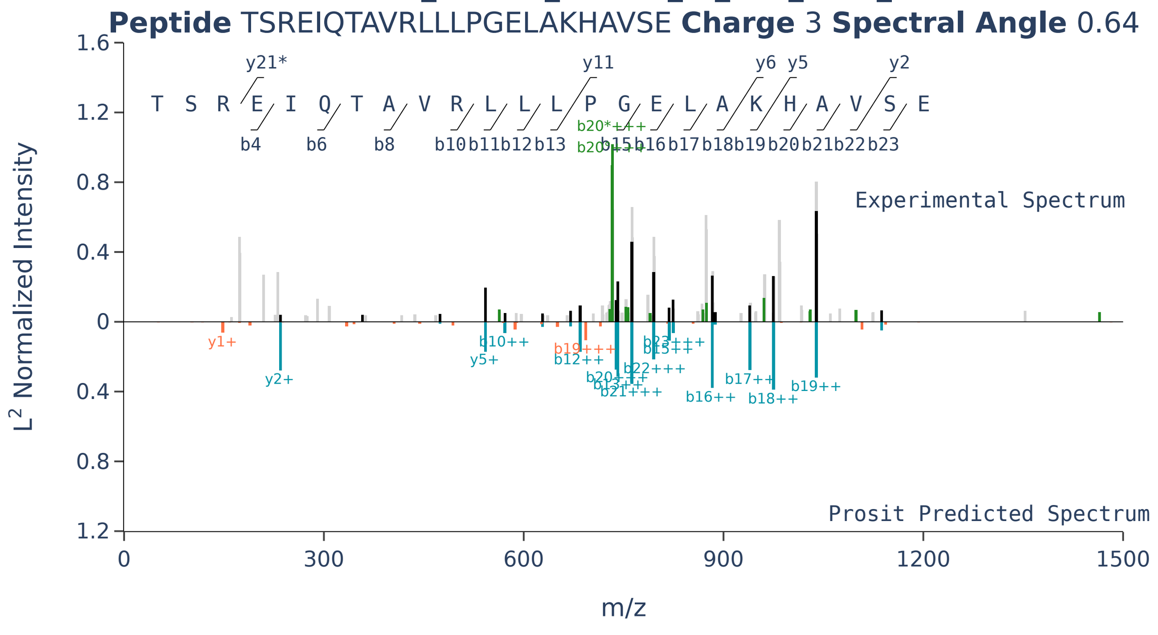


**Source** HRoetschke_291222_080123_Fu_H2B_b1R1_1h **Scan** 13304
**Peptide** AVRLLLPGELAKHAVSEGTKAV **Charge** 4 **Spectral Angle** 0.81

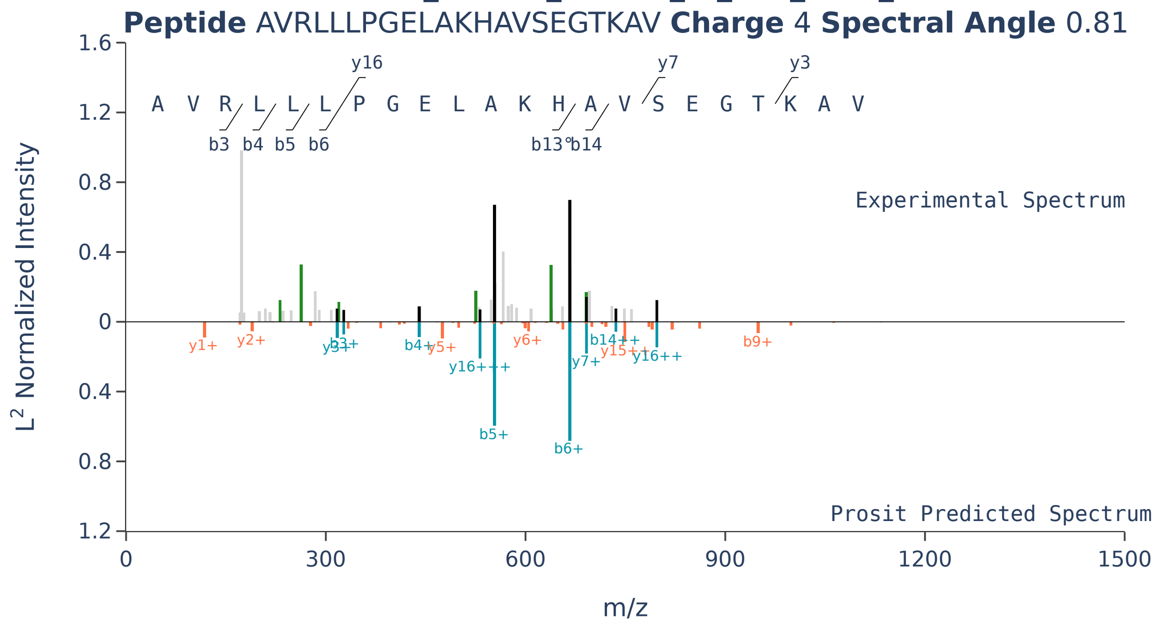


**Source** HRoetschke_291222_080123_Fu_H2B_b2R1_4h **Scan** 21396
**Peptide** KAMSIMNSFVNDVFERIAG **Charge** 3 **Spectral Angle** 0.64

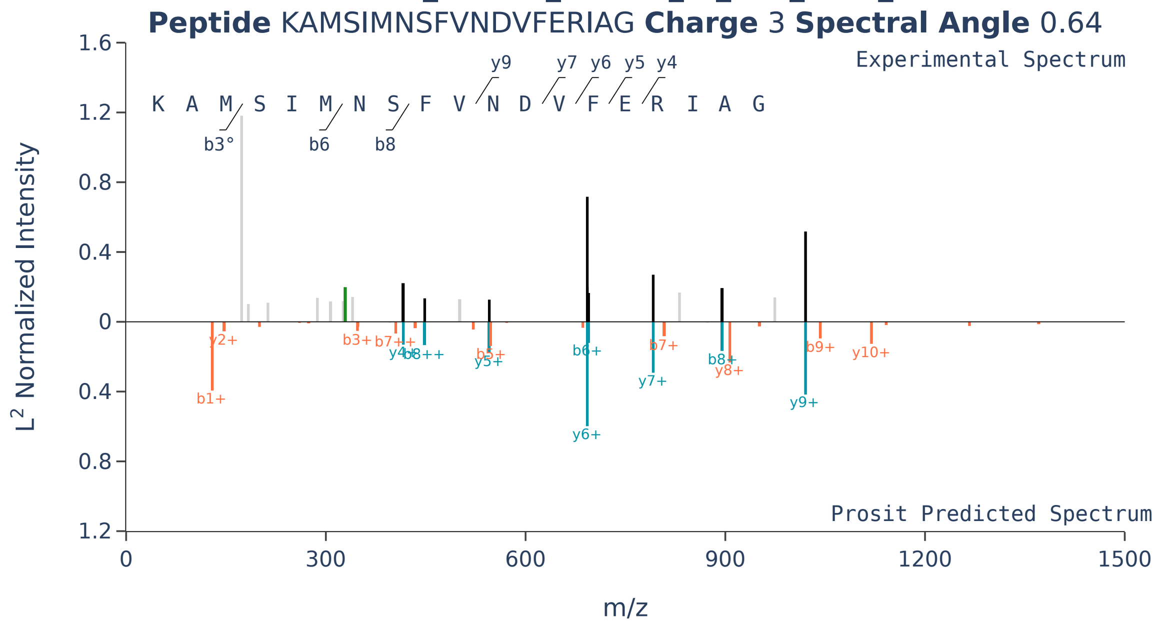


**Source** HRoetschke_291222_080123_Fu_H2B_b2R2_1h **Scan** 14490
**Peptide** TAVRLLLPGELAKHAVSEGTKAVTKY **Charge** 5 **Spectral Angle** 0.73

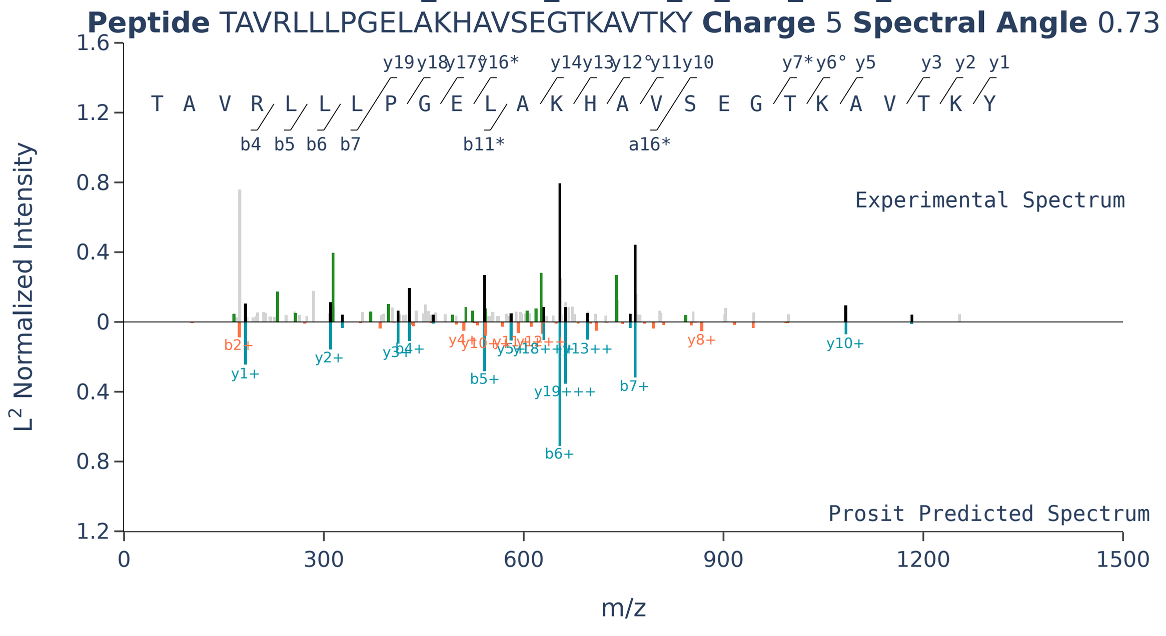


**Source** HRoetschke_291222_080123_Fu_H2B_b2R1_1h **Scan** 11729
**Peptide** VFERIAGEASRLAHYNKRSTIT **Charge** 4 **Spectral Angle** 0.63

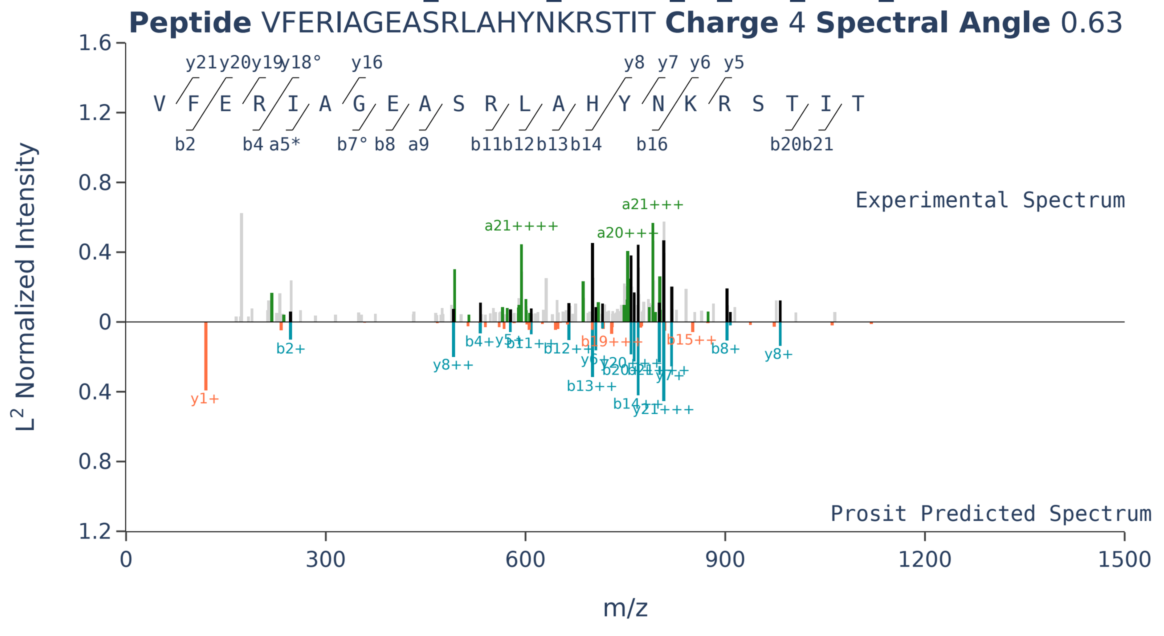


**Source** HRoetschke_291222_080123_Fu_H2B_b2R2_4h **Scan** 16402
**Peptide** VRLLLPGELAKHAVSEGTKAVT **Charge** 3 **Spectral Angle** 0.65

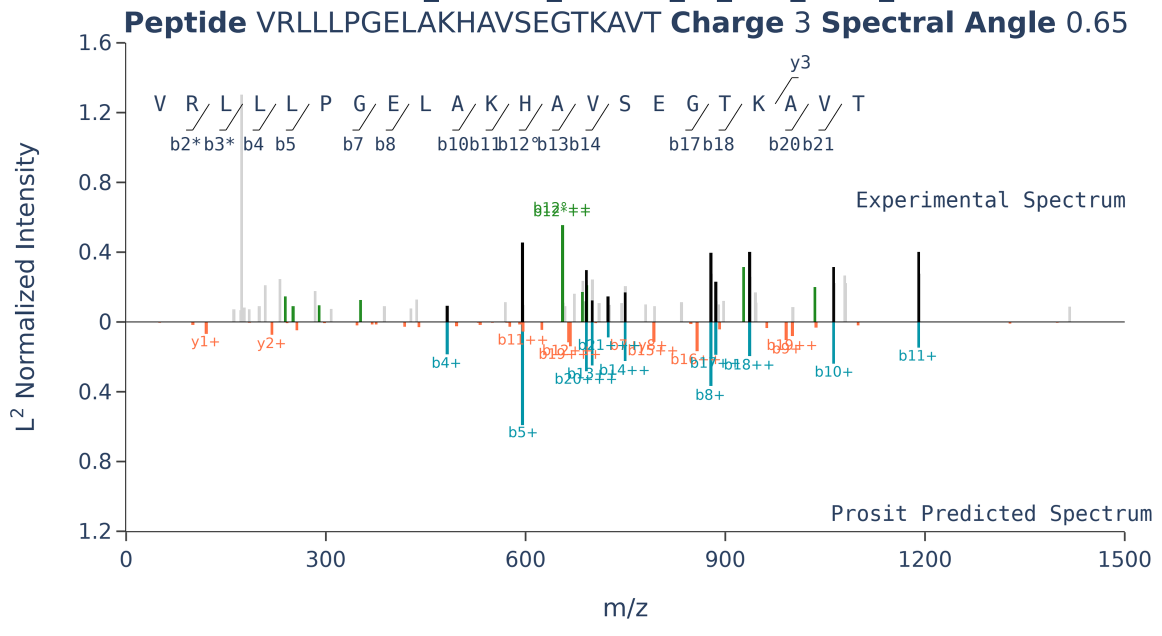

**Source** HRoetschke_291222_080123_Fu_H2B_b2R1_1h **Scan** 18356
**Peptide** VNDVFERIAGEASRLAHY **Charge** 3 **Spectral Angle** 0.69

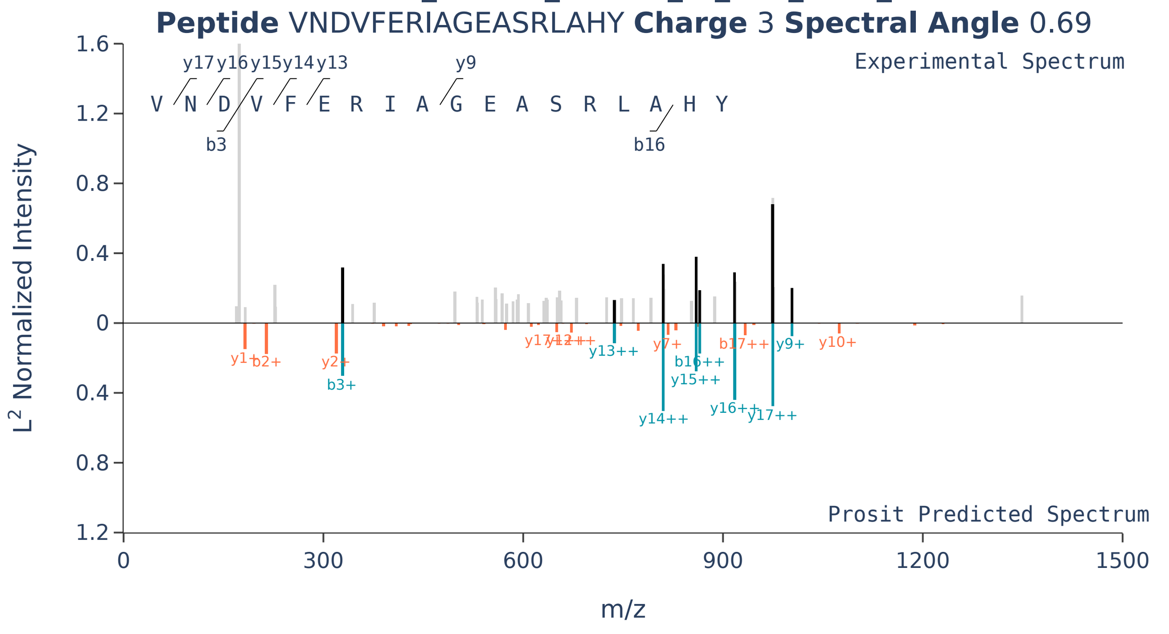


**Source** HRoetschke_291222_080123_Fu_H2B_b2R2_24h **Scan** 5347
**Peptide** VTKTQKKDGKKRRKTRKESYAIYVYKV **Charge** 6 **Spectral Angle** 0.74

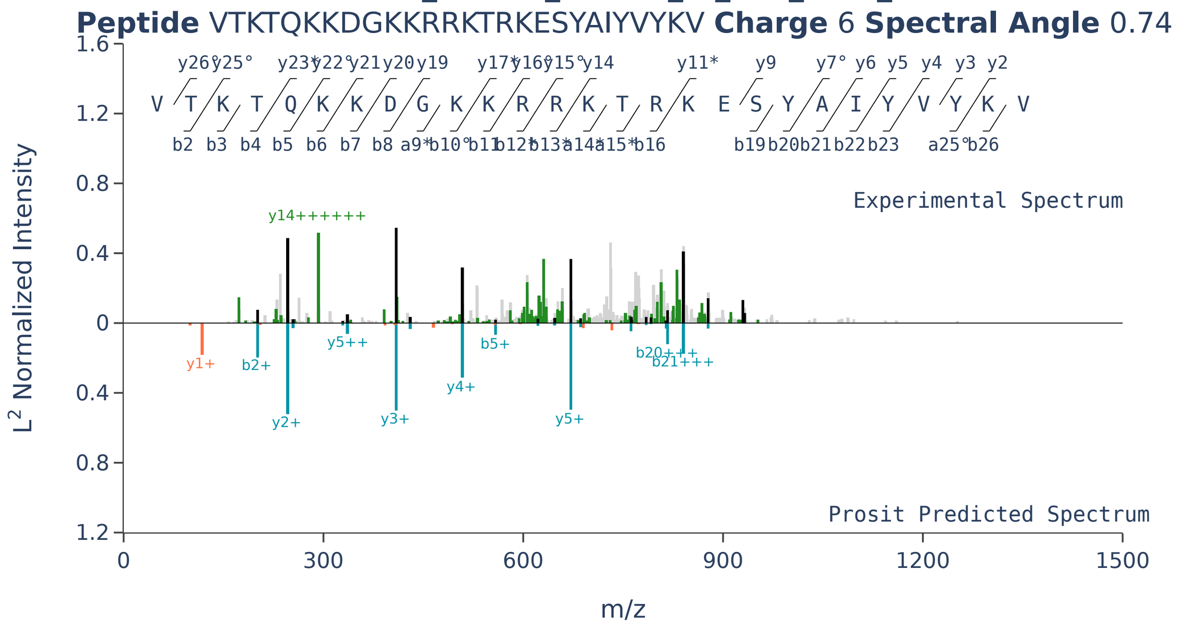


**Source** HRoetschke_291222_080123_Fu_H2B_b2R1_4h **Scan** 19713
**Peptide** KRSTITSREIQTAVRLLL **Charge** 4 **Spectral Angle** 0.64

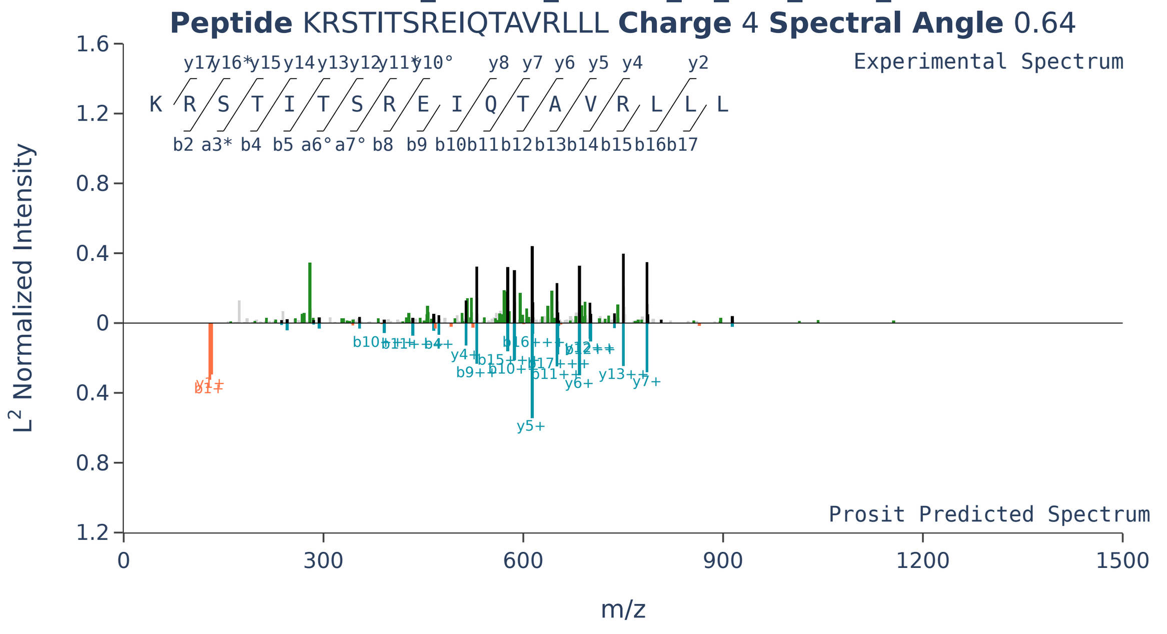


**Source** HRoetschke_291222_080123_Fu_H2B_b1R1_1h **Scan** 4280
**Peptide** ASRLAHYNKRSTITSREIQT **Charge** 4 **Spectral Angle** 0.65

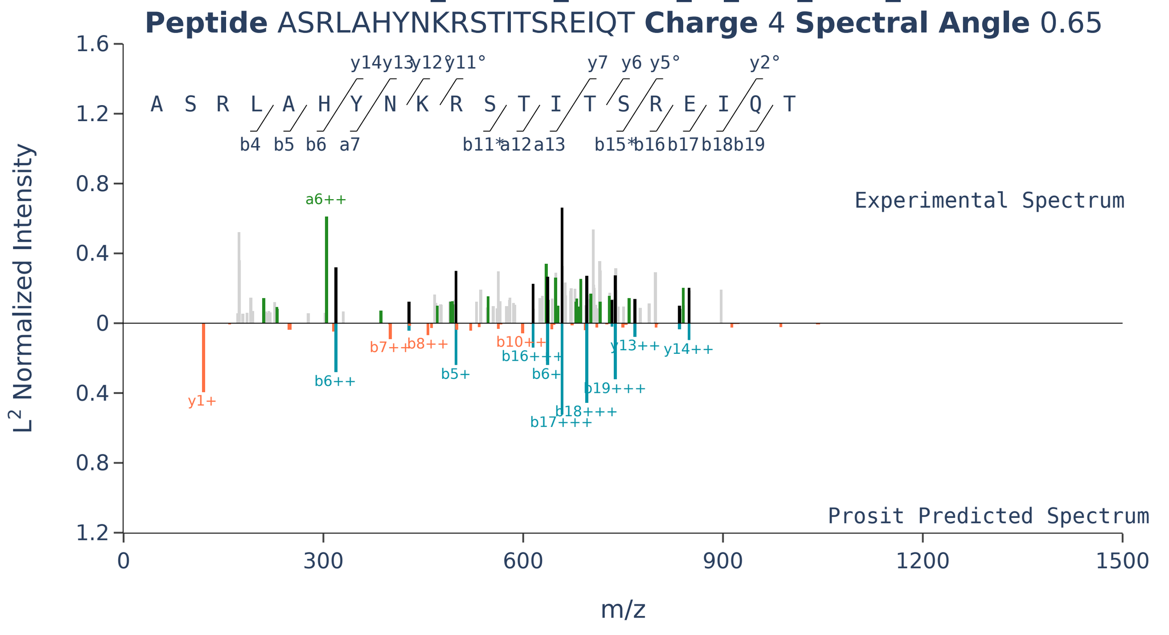


**Source** HRoetschke_291222_080123_Fu_H2B_b2R1_1h **Scan** 20549
**Peptide** SIMNSFVNDVFERIAGEASRLAHYNKR **Charge** 5 **Spectral Angle** 0.75

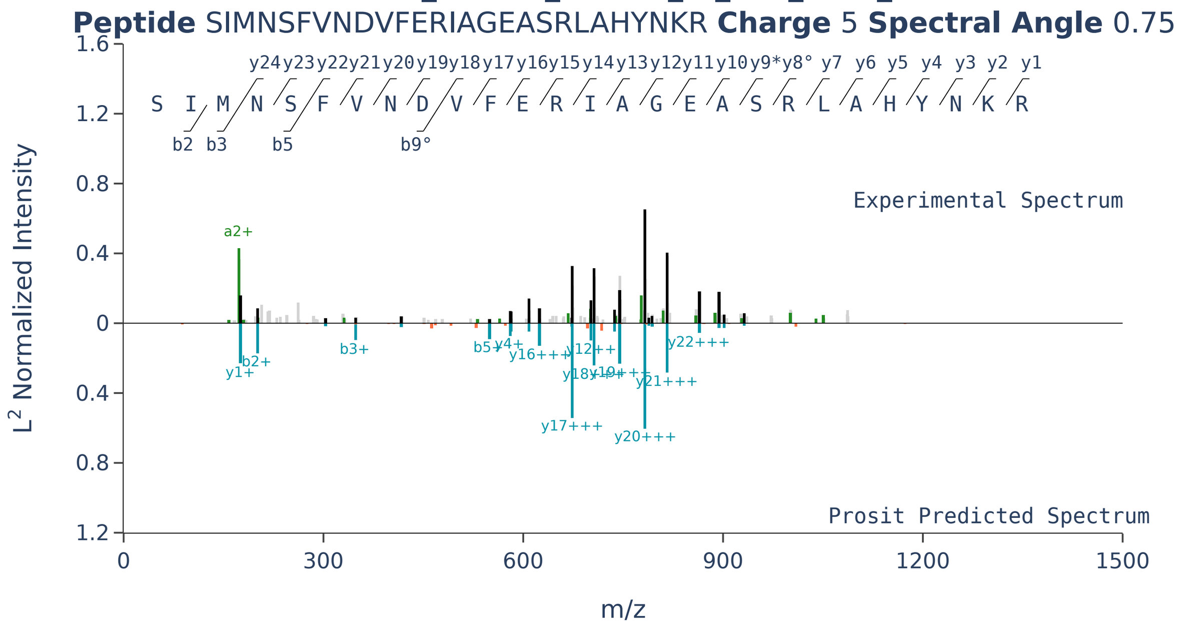


**Source** HRoetschke_291222_080123_Fu_H2B_b2R2_4h **Scan** 9933
**Peptide** TQKKDGKKRRKTRKESYAIYVYKVL **Charge** 5 **Spectral Angle** 0.69

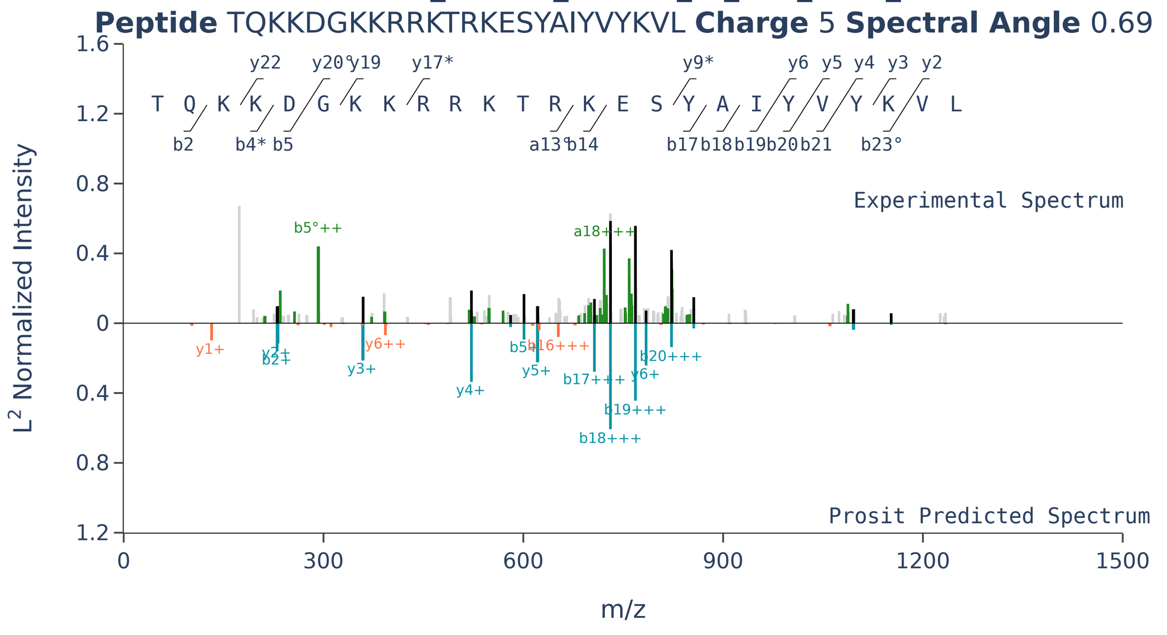


**Source** HRoetschke_291222_080123_Fu_H2B_b2R2_24h **Scan** 16840
**Peptide** AIYVYKVLKQVHPDTGISS **Charge** 3 **Spectral Angle** 0.68

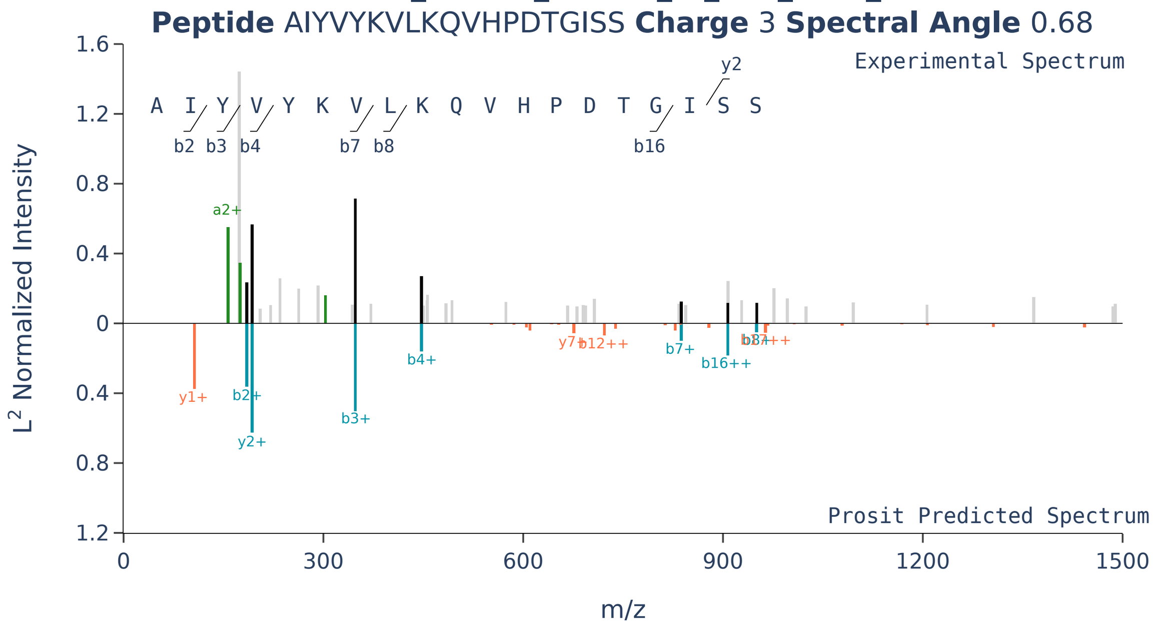


**Source** HRoetschke_291222_080123_Fu_H2B_b2R1_4h **Scan** 18522
**Peptide** VNDVFERIAGEASRLAHYNKRSTITSREIQ **Charge** 5 **Spectral Angle** 0.49

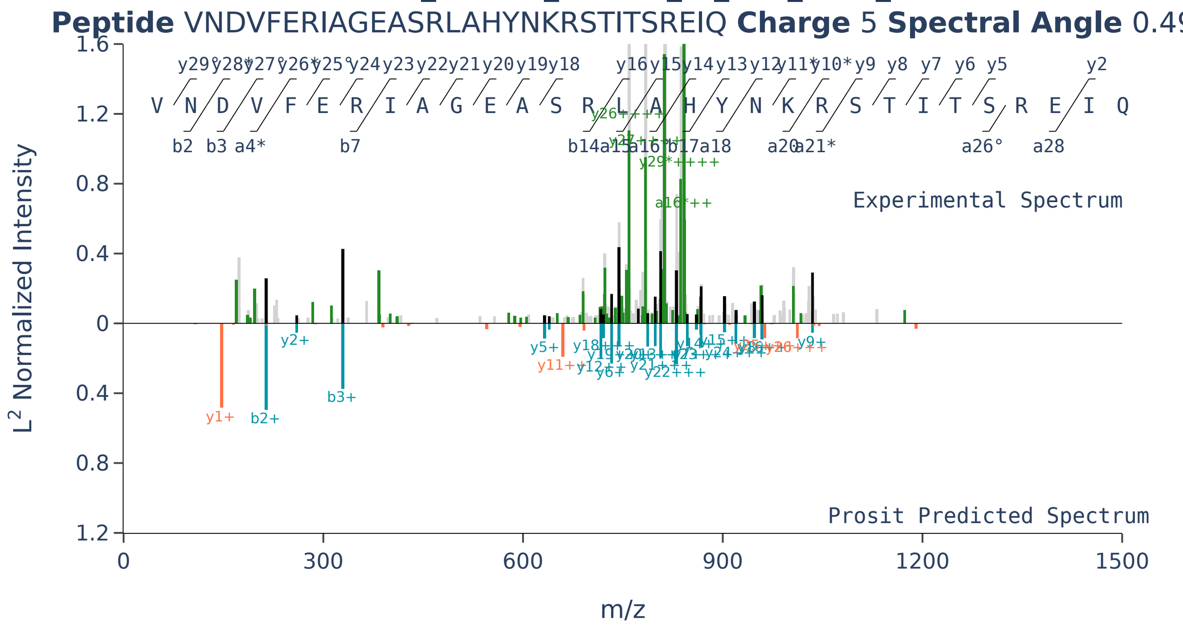


**Source** HRoetschke_291222_080123_Fu_H2B_b2R1_24h **Scan** 17546
**Peptide** QTAVRLLLPGELAKHAVSEGTKAV **Charge** 4 **Spectral Angle** 0.65

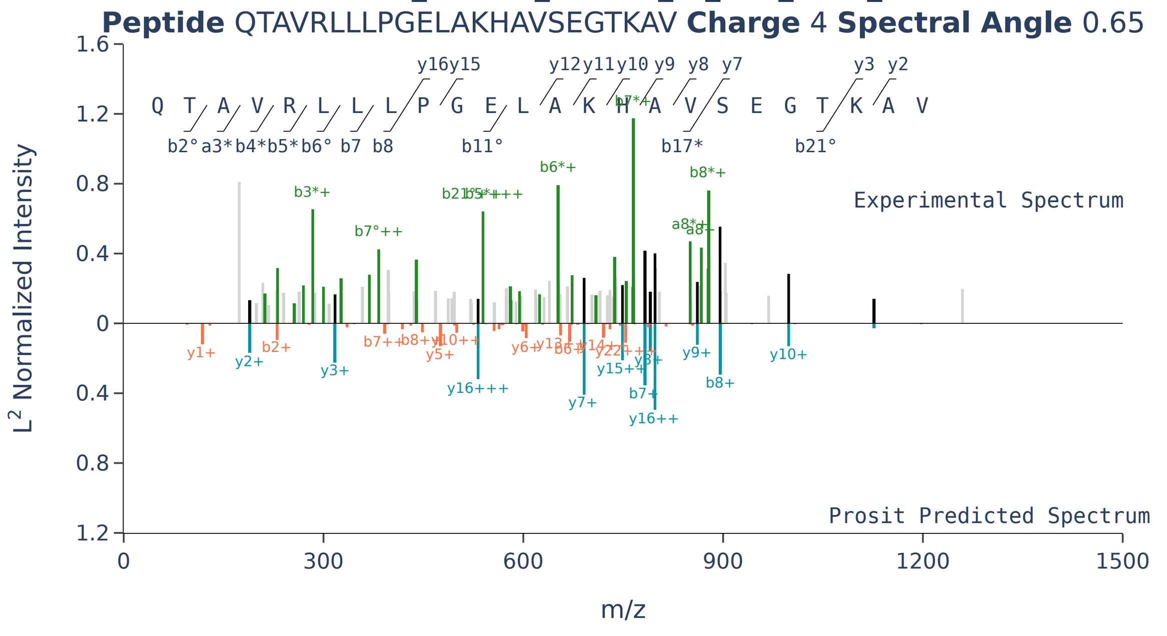


**Source** HRoetschke_291222_080123_Fu_H2B_b2R1_4h **Scan** 20842
**Peptide** FVNDVFERIAGEASRLAHYNKRS **Charge** 5 **Spectral Angle** 0.76

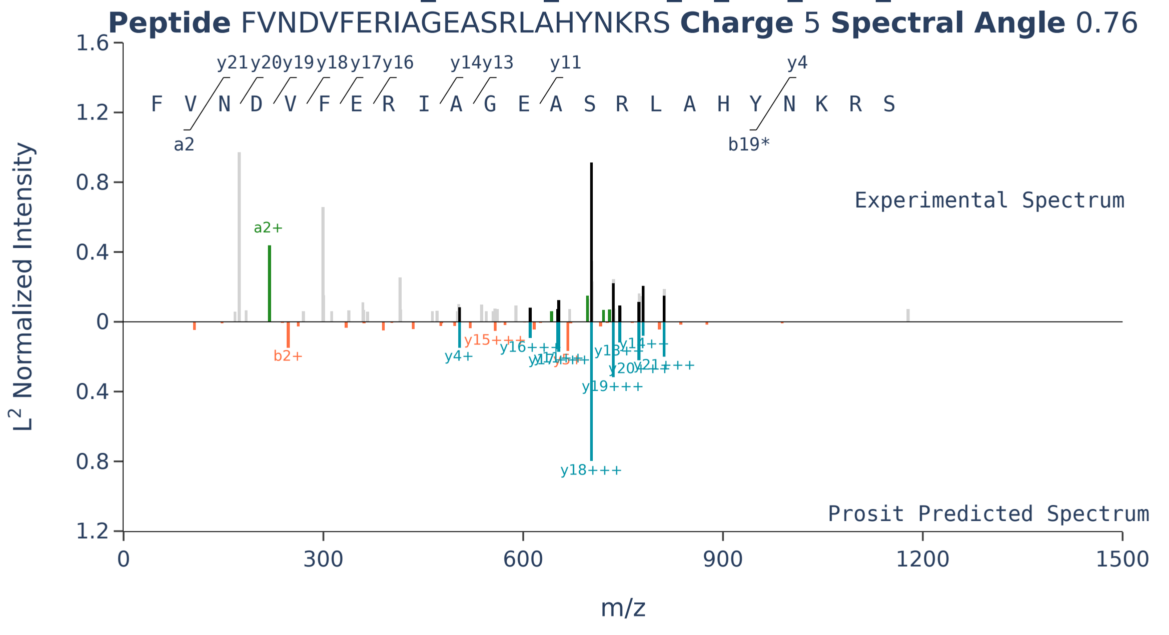


**Source** HRoetschke_291222_080123_Fu_H2B_b1R1_4h **Scan** 2909
**Peptide** AVSEGTKAVT **Charge** 2 **Spectral Angle** 0.68

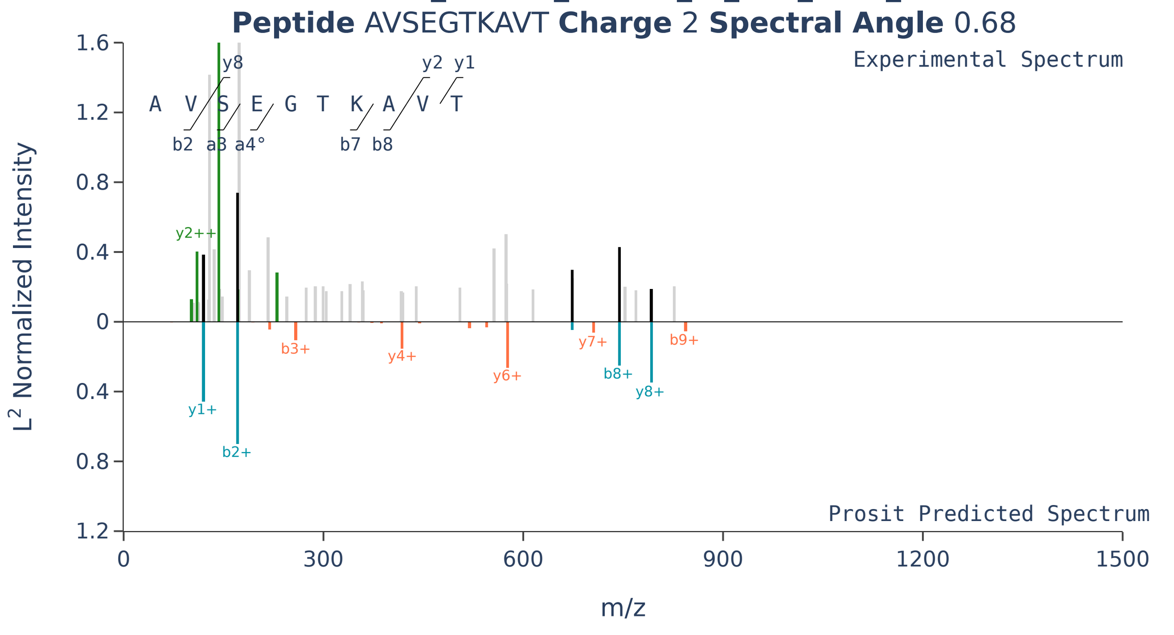


**Source** HRoetschke_291222_080123_Fu_H2B_b2R2_4h **Scan** 18913
**Peptide** SYAIYVYKVLKQVHPDTGISSK **Charge** 4 **Spectral Angle** 0.67

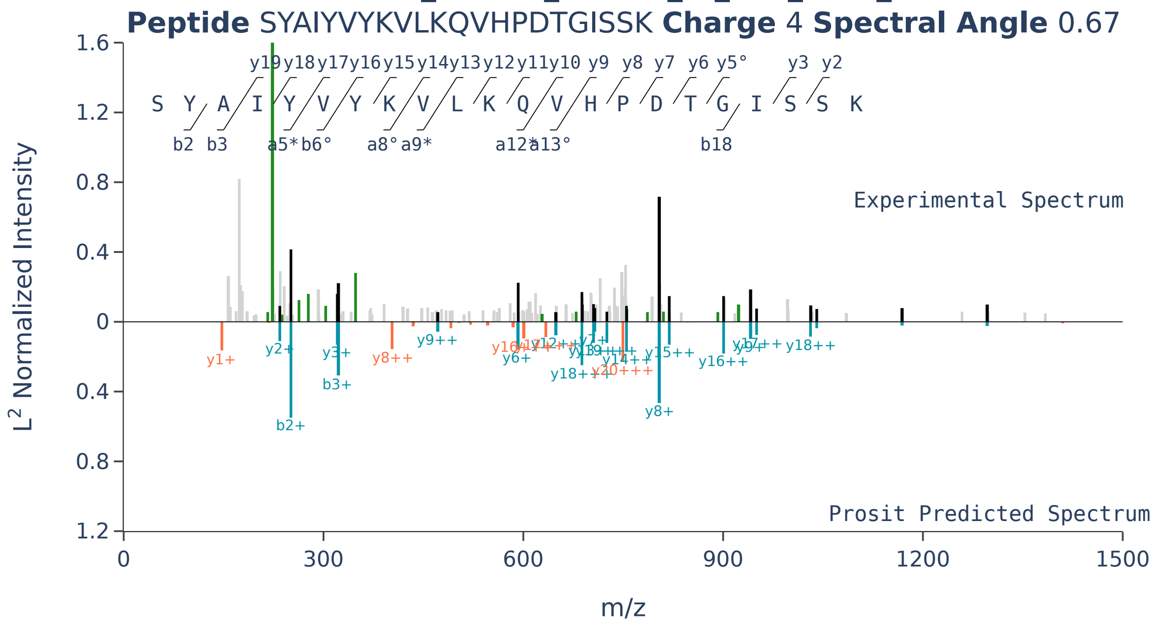


**Source** HRoetschke_291222_080123_Fu_H2B_b2R2_24h **Scan** 13673
**Peptide** TRKESYAIYVYKV **Charge** 3 **Spectral Angle** 0.66

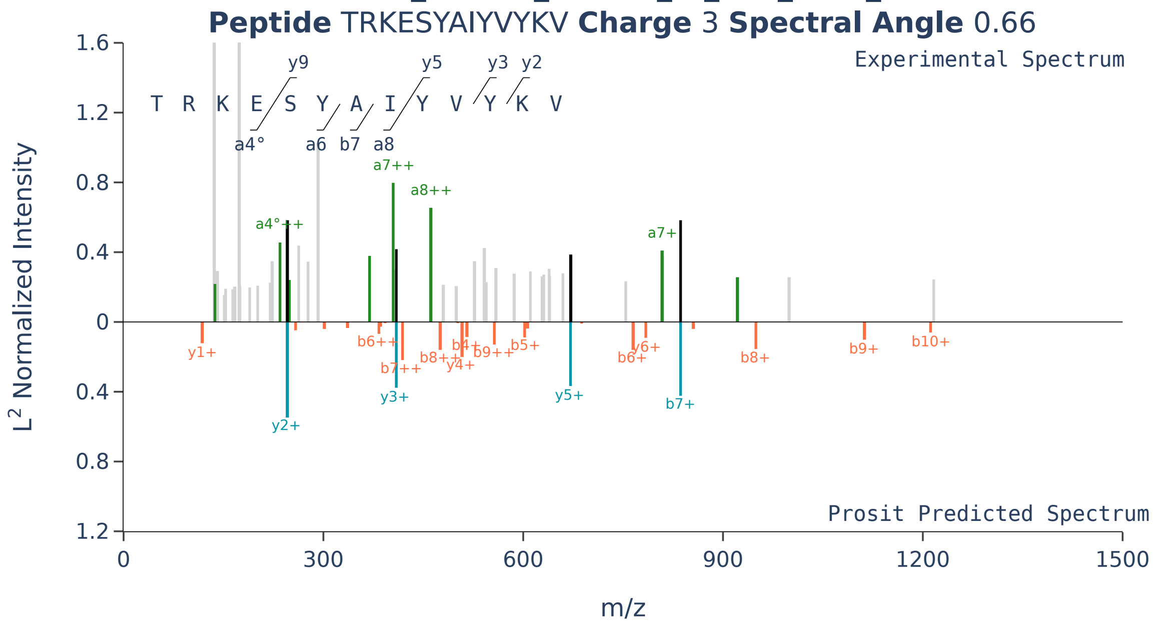


**Source** HRoetschke_291222_080123_Fu_H2B_b1R1_2h **Scan** 8142
**Peptide** AGEASRLAHYNKRSTITSREIQTAV **Charge** 4 **Spectral Angle** 0.59

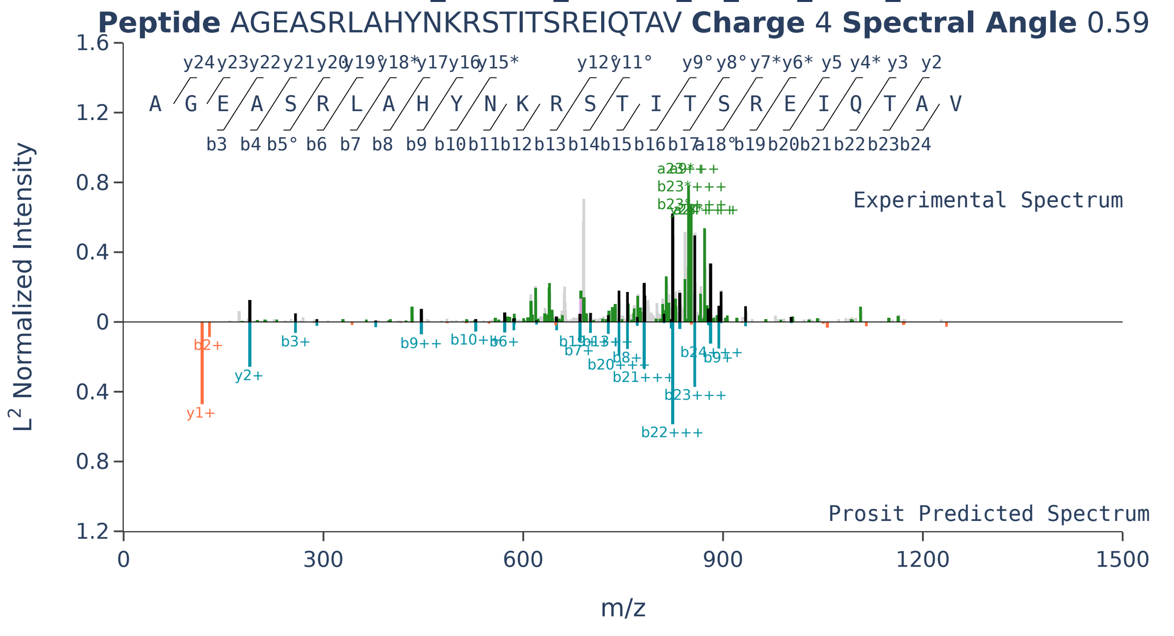


**Source** HRoetschke_291222_080123_Fu_H2B_b2R1_24h **Scan** 20713
**Peptide** QTAVRLLLPGEL **Charge** 2 **Spectral Angle** 0.79

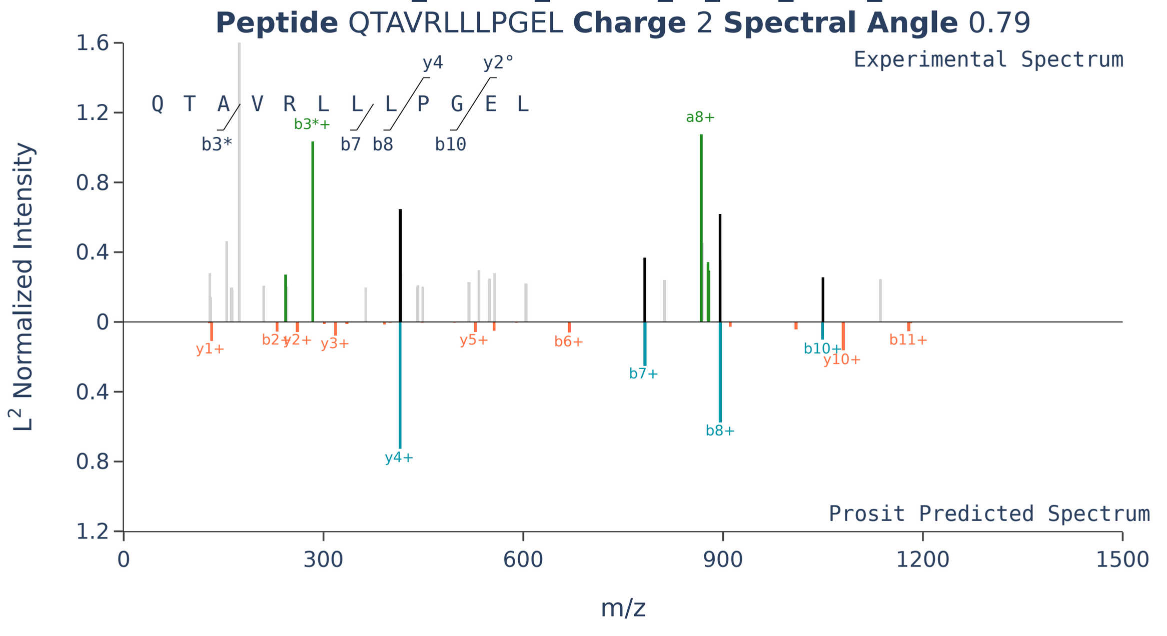

**Source** HRoetschke_291222_080123_Fu_H2B_b1R1_2h **Scan** 19737
**Peptide** MNSFVNDVFERIAGEASRLAHYNKRST **Charge** 5 **Spectral Angle** 0.74

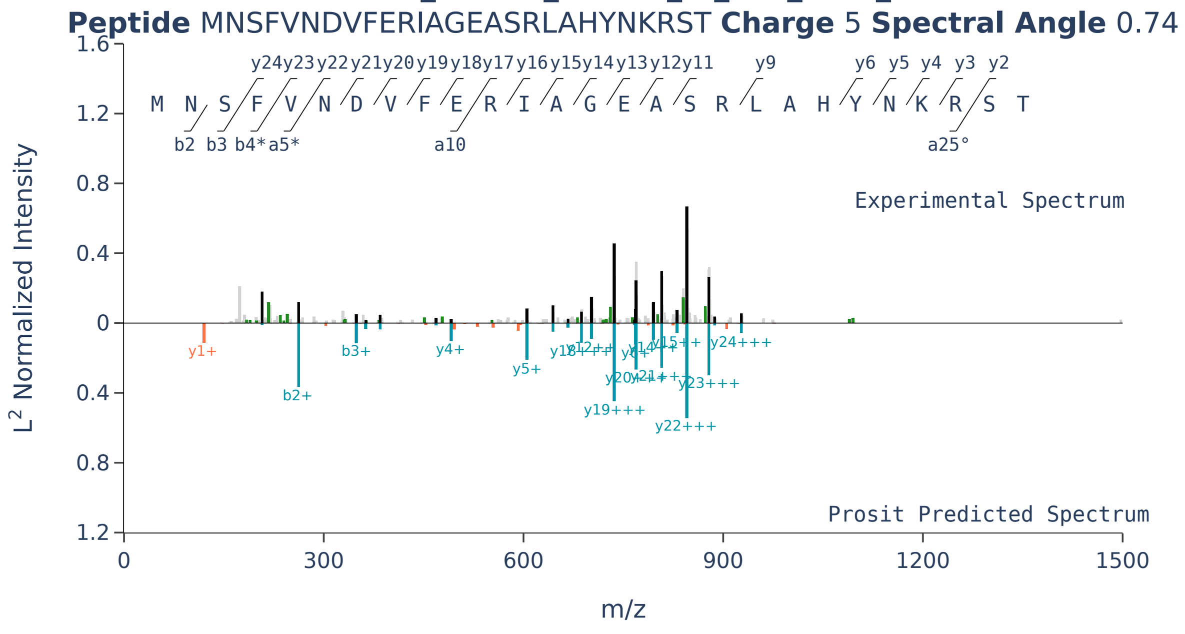


**Source** HRoetschke_291222_080123_Fu_H2B_b2R2_24h **Scan** 4116
**Peptide** HYNKRSTITSREIQTA **Charge** 4 **Spectral Angle** 0.58

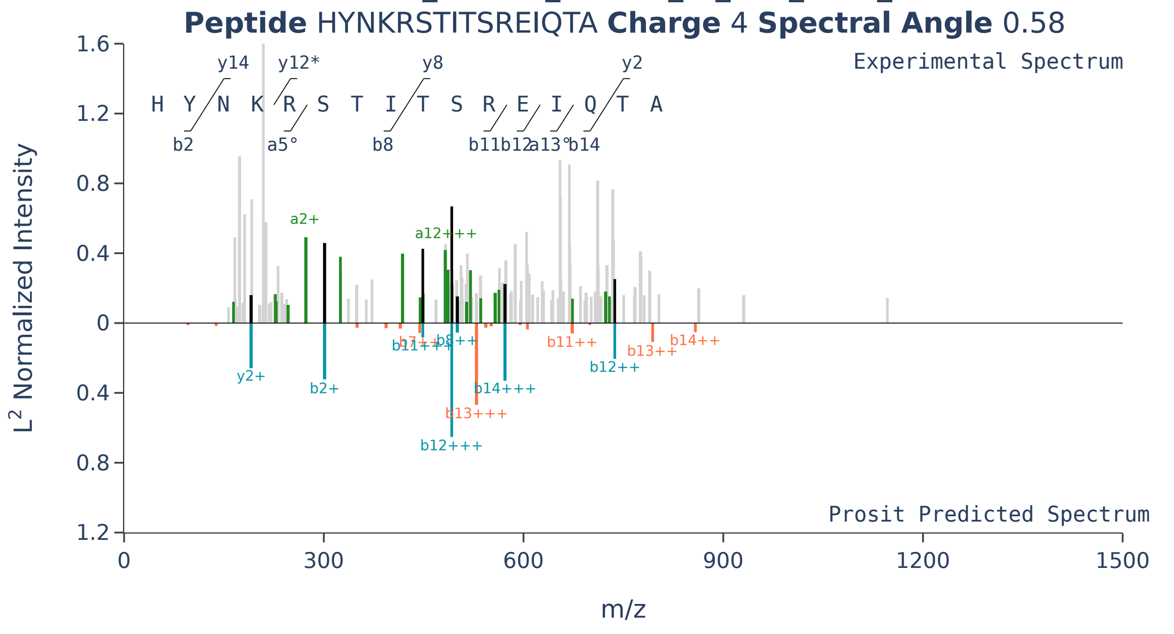


**Source** HRoetschke_291222_080123_Fu_H2B_b2R2_2h **Scan** 3826
**Peptide** RLAHYNKRSTITSREIQT **Charge** 4 **Spectral Angle** 0.56

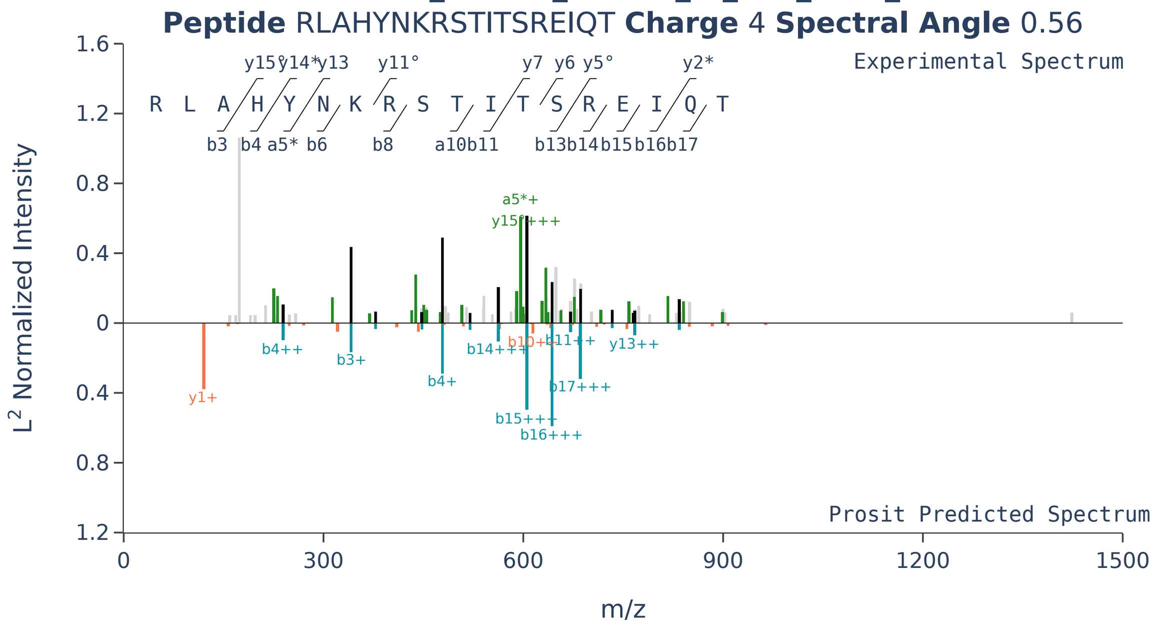


**Source** HRoetschke_291222_080123_Fu_H2B_b1R1_4h **Scan** 5097
**Peptide** IAGEASRLAHYNKRST **Charge** 4 **Spectral Angle** 0.63

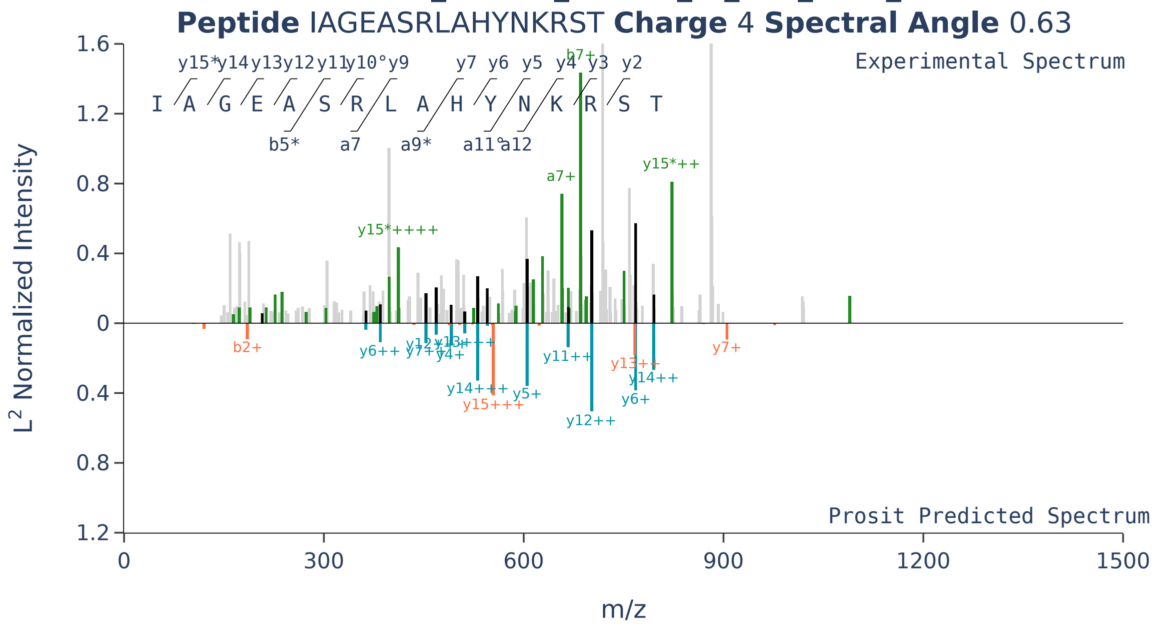


**Source** HRoetschke_291222_080123_Fu_H2B_b2R1_1h **Scan** 14775
**Peptide** KQVHPDTGISSKAMSIMNSFVNDVFE **Charge** 4 **Spectral Angle** 0.61

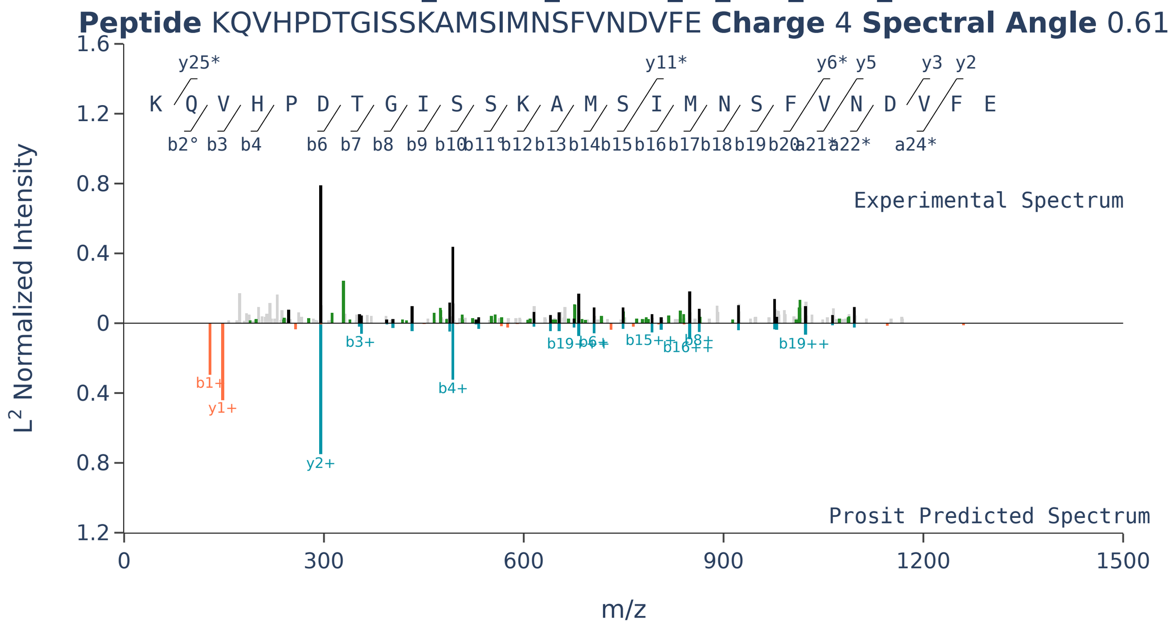


**Source** HRoetschke_291222_080123_Fu_H2B_b2R1_1h **Scan** 18454
**Peptide** RSTITSREIQTAVRLLL **Charge** 4 **Spectral Angle** 0.71

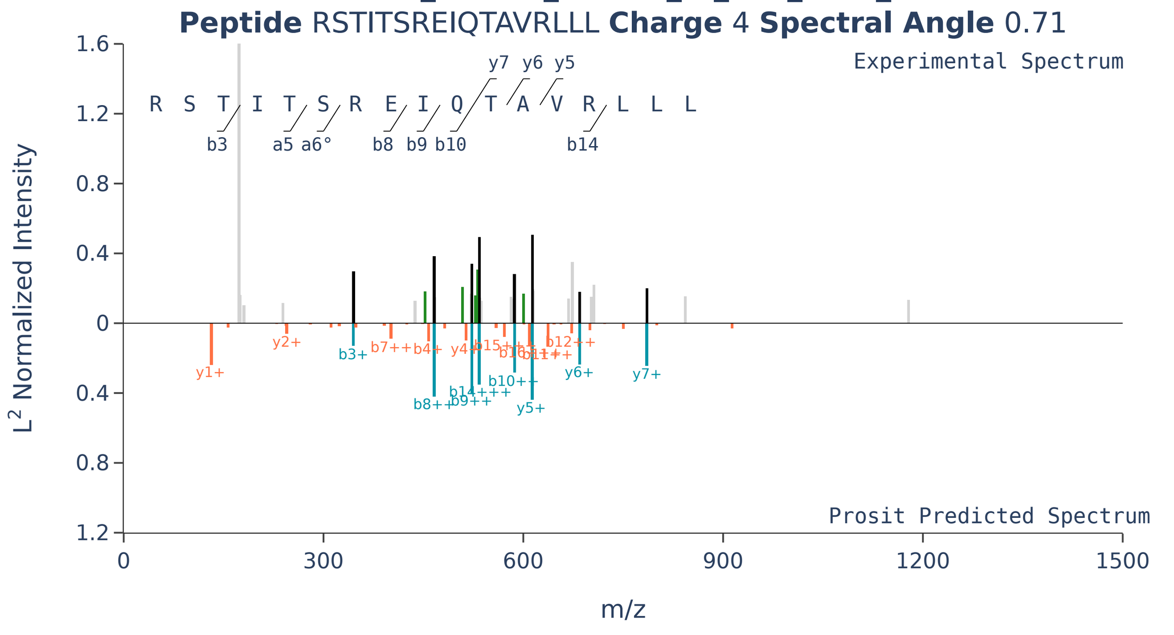


**Source** HRoetschke_291222_080123_Fu_H2B_b2R2_24h **Scan** 4152
**Peptide** RLAHYNKRSTITSREI **Charge** 4 **Spectral Angle** 0.69

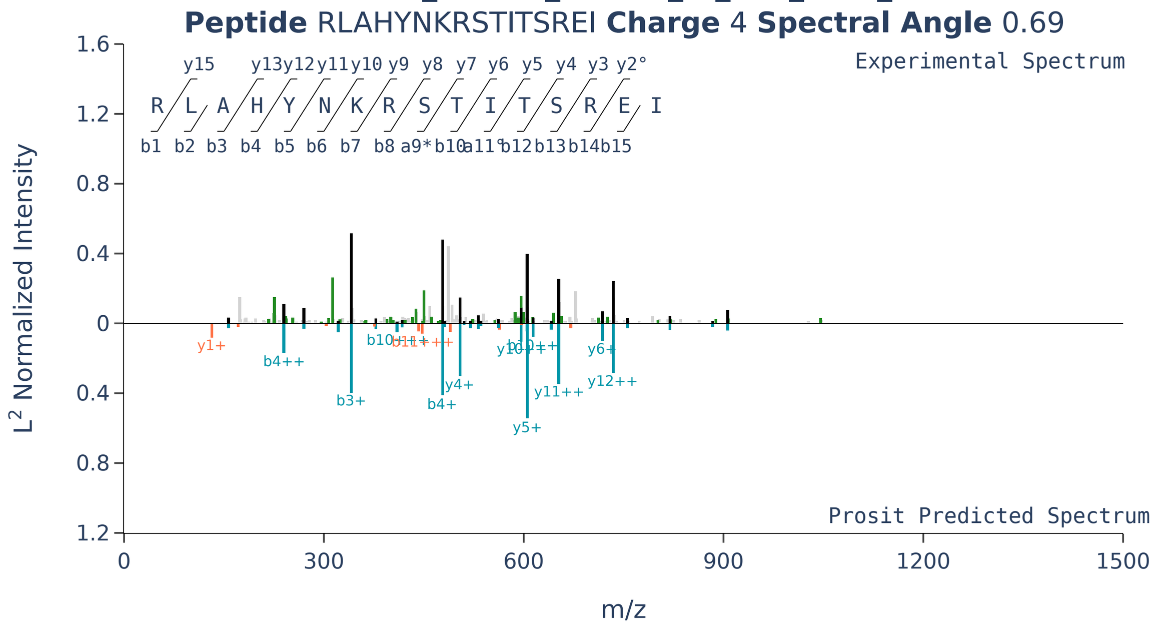


**Source** HRoetschke_291222_080123_Fu_H2B_b2R2_4h **Scan** 13590
**Peptide** QVHPDTGISSKAMSIMNS **Charge** 3 **Spectral Angle** 0.75

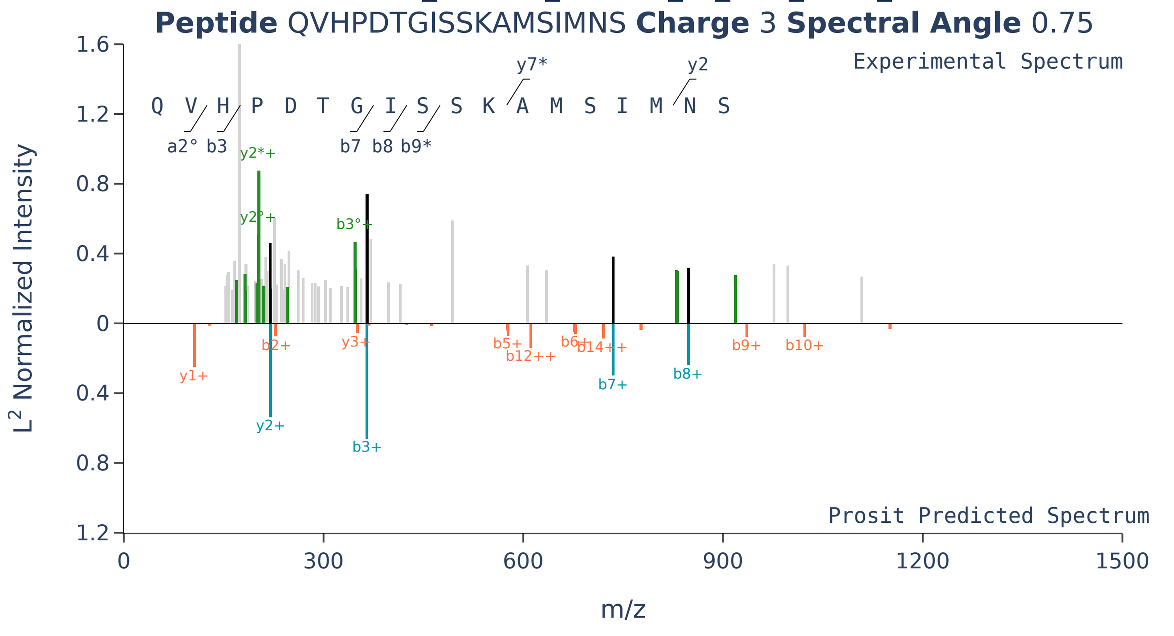


**Source** HRoetschke_291222_080123_Fu_H2B_b2R1_24h **Scan** 9024
**Peptide** GEASRLAHYNKRSTITSREIQTAV **Charge** 5 **Spectral Angle** 0.32

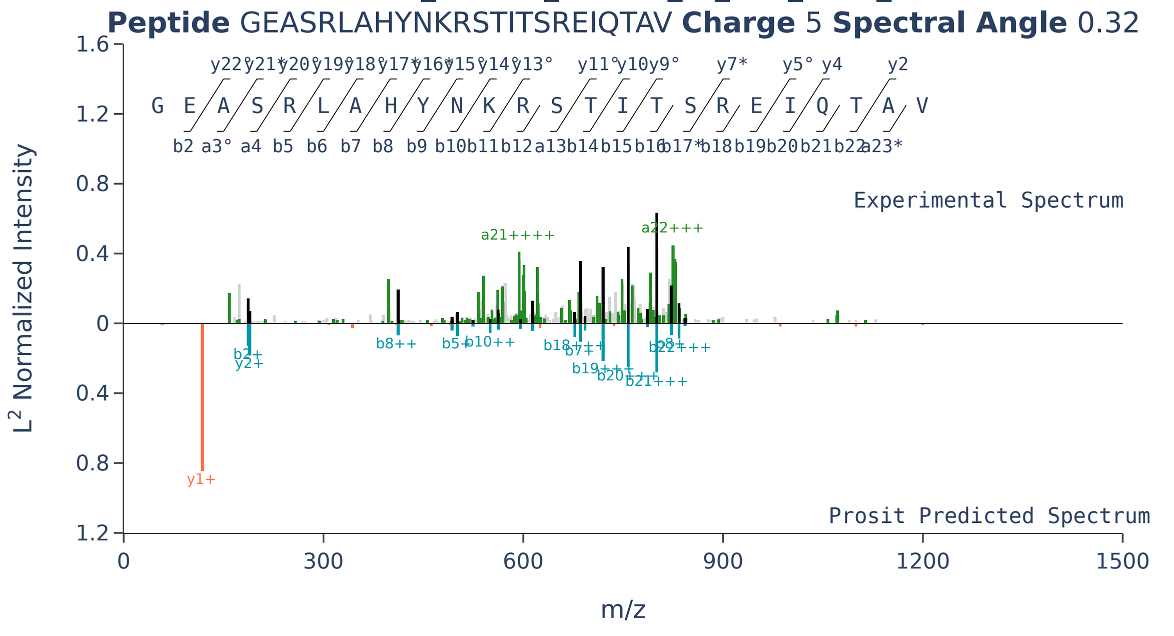


**Source** HRoetschke_291222_080123_Fu_H2B_b2R2_1h **Scan** 14253
**Peptide** SYAIYVYKVLK **Charge** 3 **Spectral Angle** 0.66

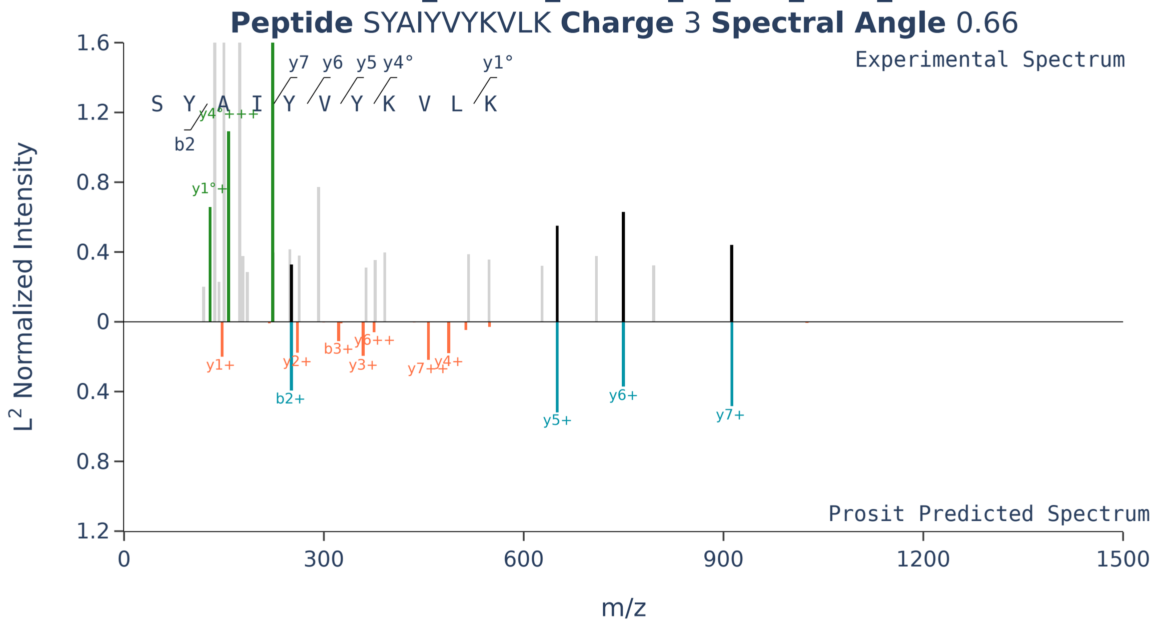


**Source** HRoetschke_291222_080123_Fu_H2B_b2R1_4h **Scan** 17989
**Peptide** TAVRLLLPGELAKHAVSEGTKAVTKYTS **Charge** 5 **Spectral Angle** 0.74

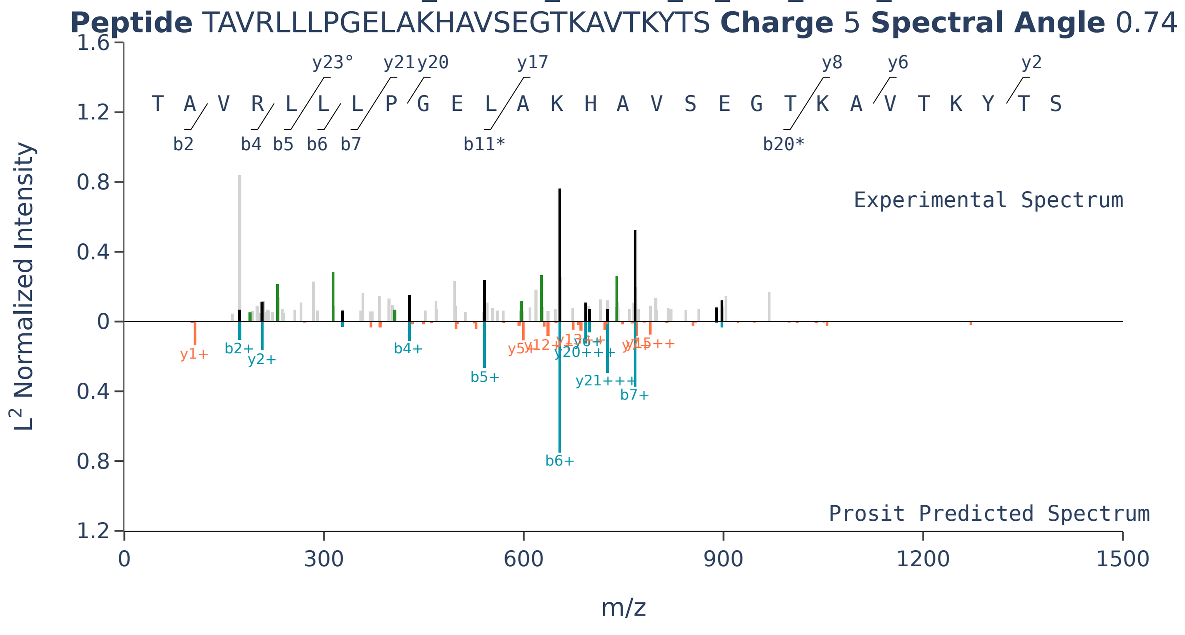


**Source** HRoetschke_291222_080123_Fu_H2B_b1R1_24h **Scan** 6936
**Peptide** VLKQVHPDTGISS **Charge** 3 **Spectral Angle** 0.46

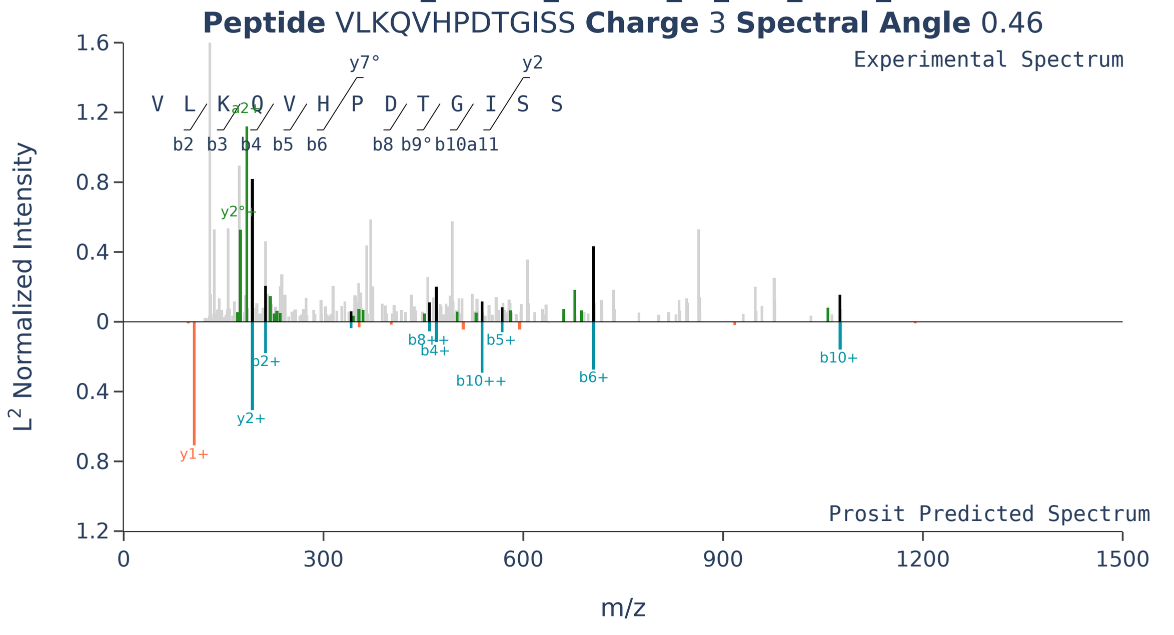


**Source** HRoetschke_291222_080123_Fu_H2B_b1R1_24h **Scan** 8225
**Peptide** KVLKQVHPDTGISSKAMSI **Charge** 3 **Spectral Angle** 0.22

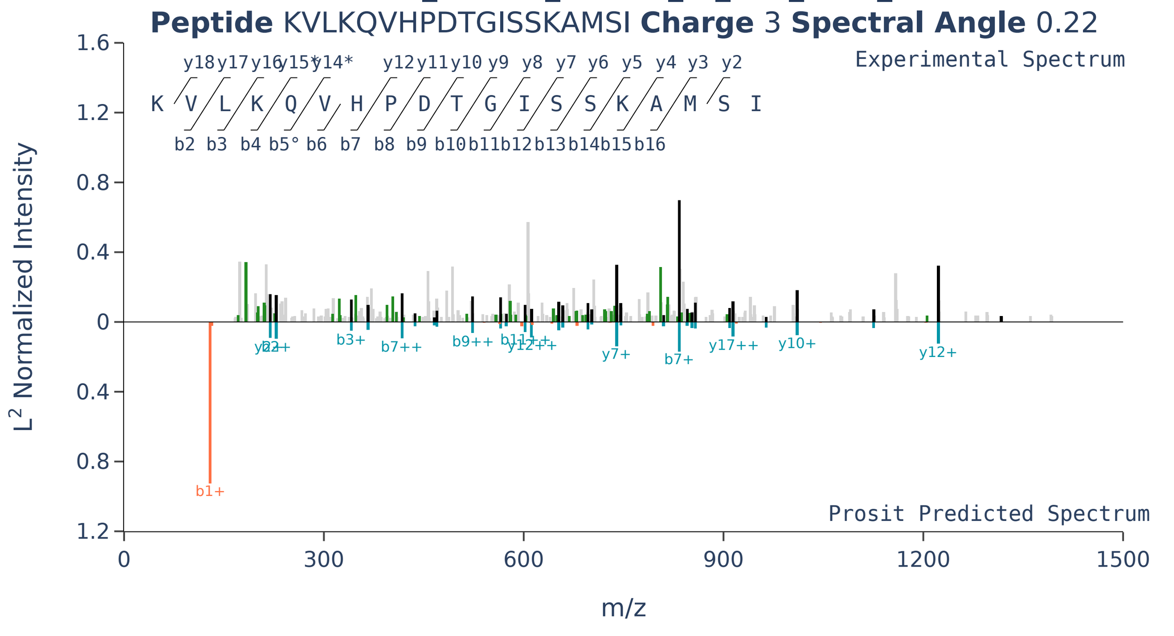


**Source** HRoetschke_291222_080123_Fu_H2B_b2R2_24h **Scan** 18343
**Peptide** SREIQTAVRLLLPGELAKHAVSEGTK **Charge** 5 **Spectral Angle** 0.69

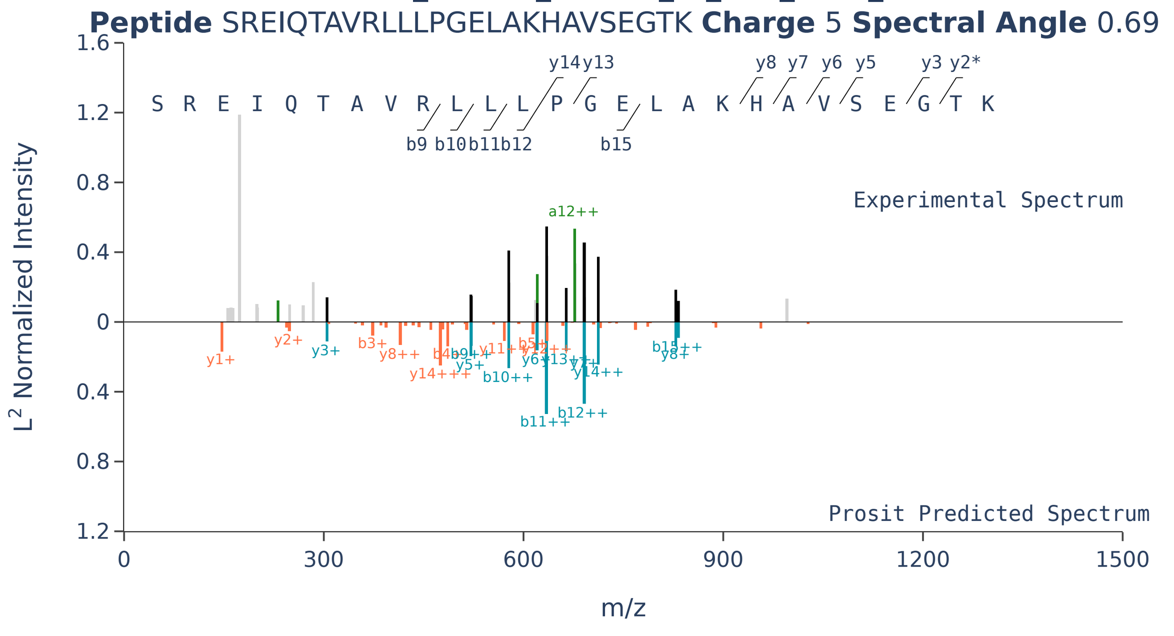


**Source** HRoetschke_291222_080123_Fu_H2B_b2R2_2h **Scan** 17273
**Peptide** ESYAIYVYKVLKQ **Charge** 3 **Spectral Angle** 0.56

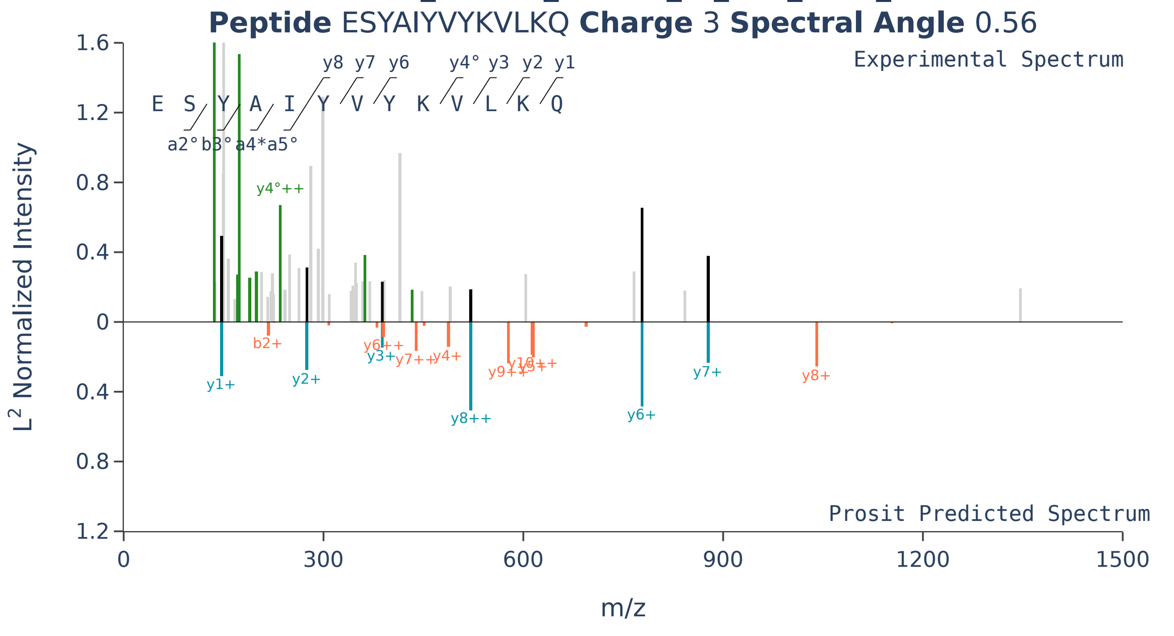

**Source** HRoetschke_291222_080123_Fu_H2B_b2R1_1h **Scan** 15471
**Peptide** AVRLLLPGELAKHAVSEGTKAVTKYT **Charge** 5 **Spectral Angle** 0.81

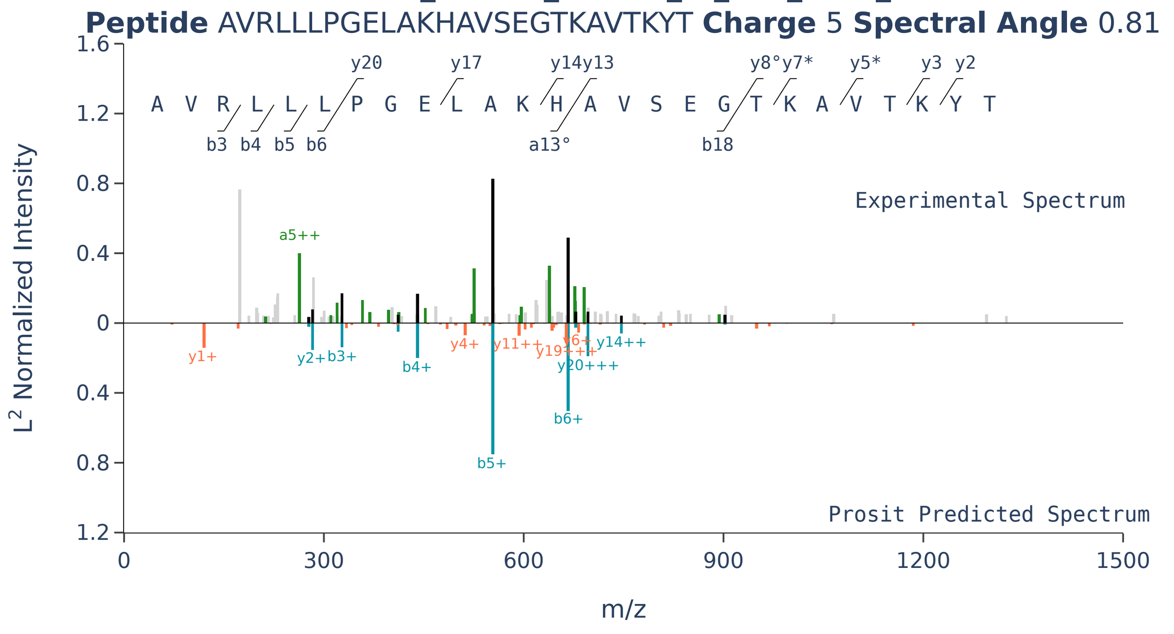


**Source** HRoetschke_291222_080123_Fu_H2B_b1R2_2h **Scan** 1804
**Peptide** LAKHAVSEGTKA **Charge** 2 **Spectral Angle** 0.63

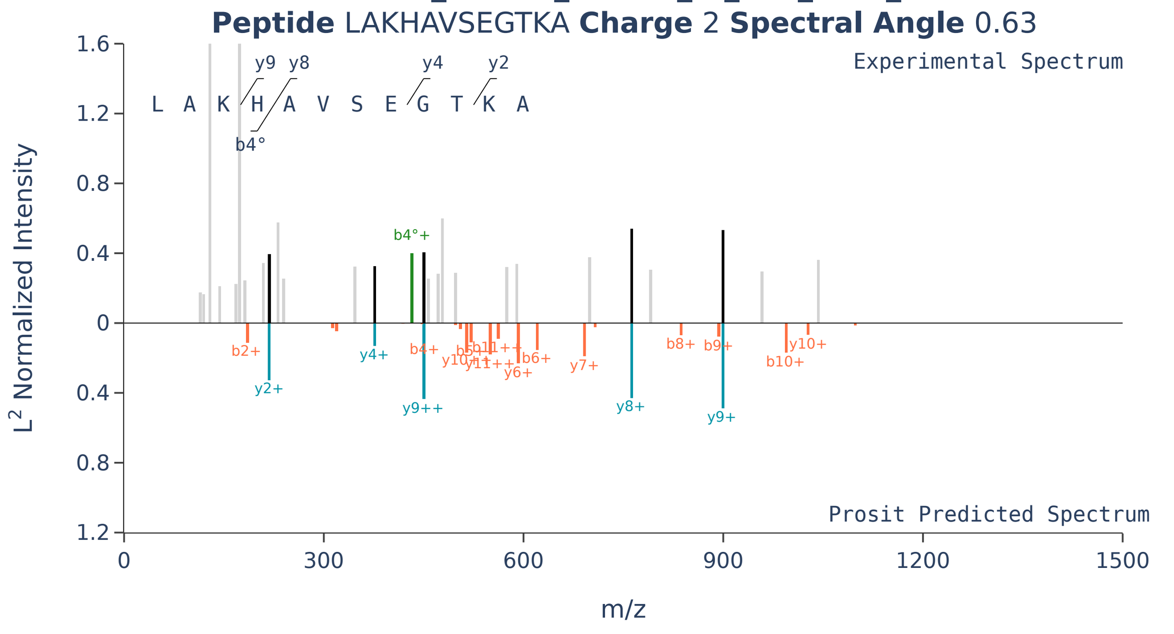


**Source** HRoetschke_291222_080123_Fu_H2B_b1R1_2h **Scan** 4880
**Peptide** KQVHPDTGISSKAMSIMN **Charge** 3 **Spectral Angle** 0.56

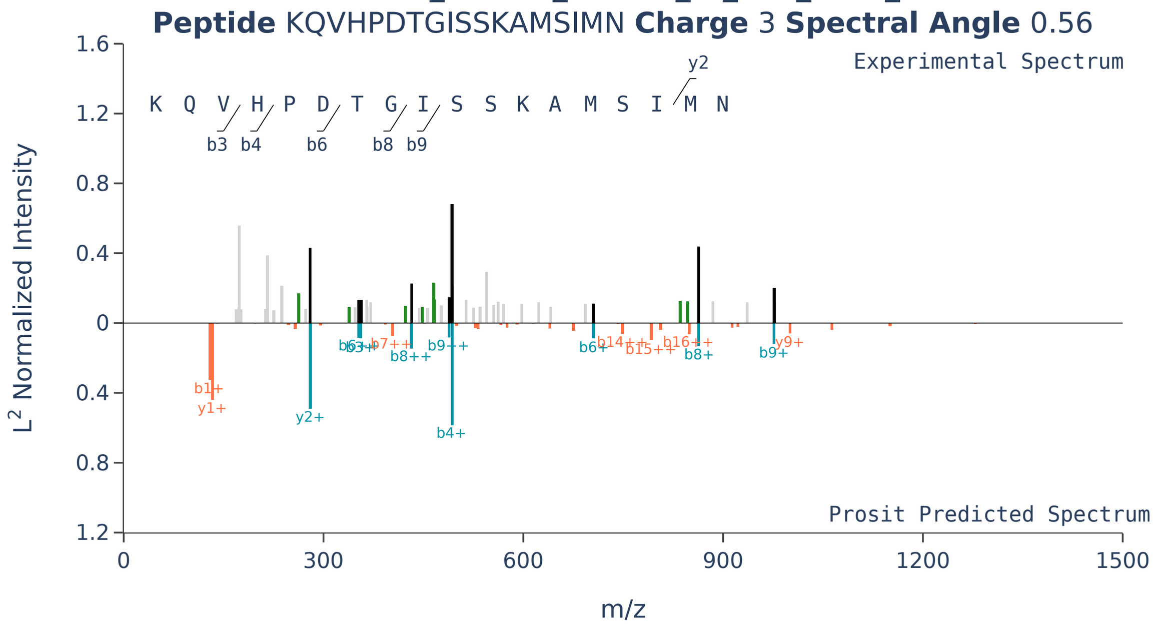


**Source** HRoetschke_291222_080123_Fu_H2B_b2R2_4h **Scan** 18384
**Peptide** NDVFERIAGEASRLAHYNKRSTIT **Charge** 4 **Spectral Angle** 0.71

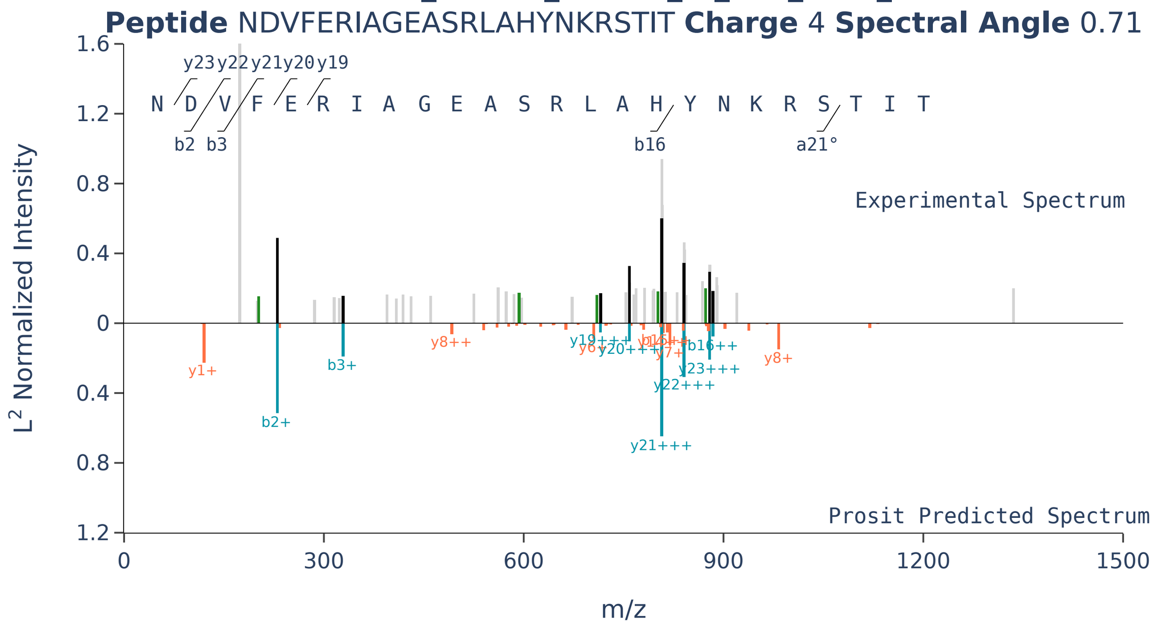


**Source** HRoetschke_291222_080123_Fu_H2B_b2R2_24h **Scan** 18728
**Peptide** REIQTAVRLLLPGELAKHAVSEGT **Charge** 4 **Spectral Angle** 0.58

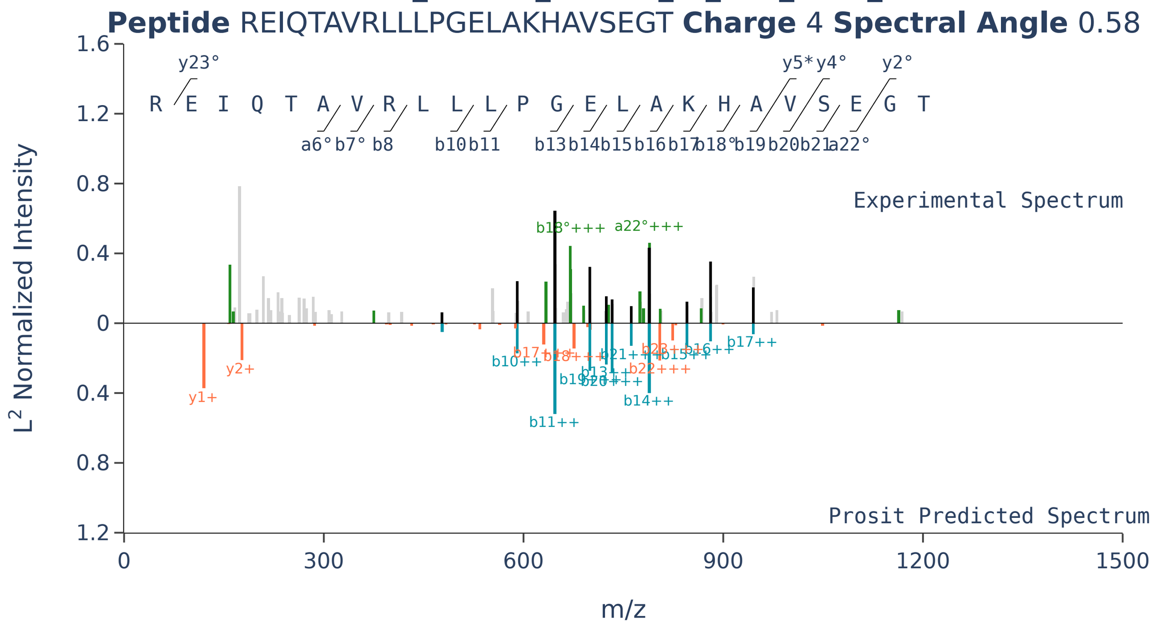


**Source** HRoetschke_291222_080123_Fu_H2B_b1R2_24h **Scan** 9972
**Peptide** KTRKESYAIYV **Charge** 3 **Spectral Angle** 0.5

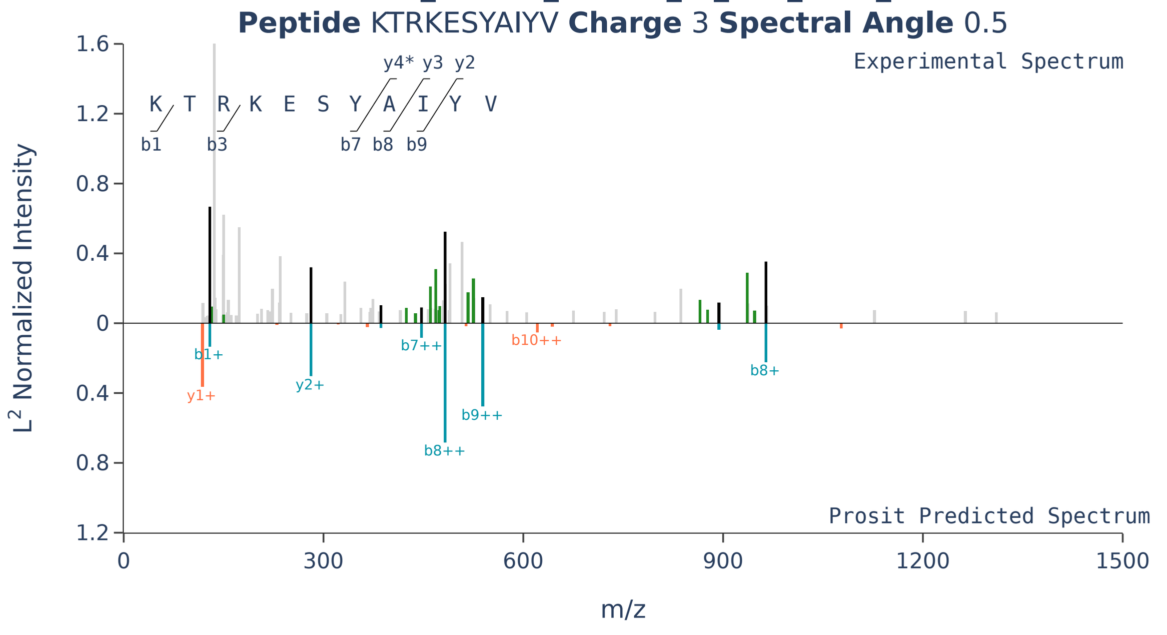


**Source** HRoetschke_291222_080123_Fu_H2B_b1R2_24h **Scan** 11784
**Peptide** KAMSIMNSFV **Charge** 2 **Spectral Angle** 0.41

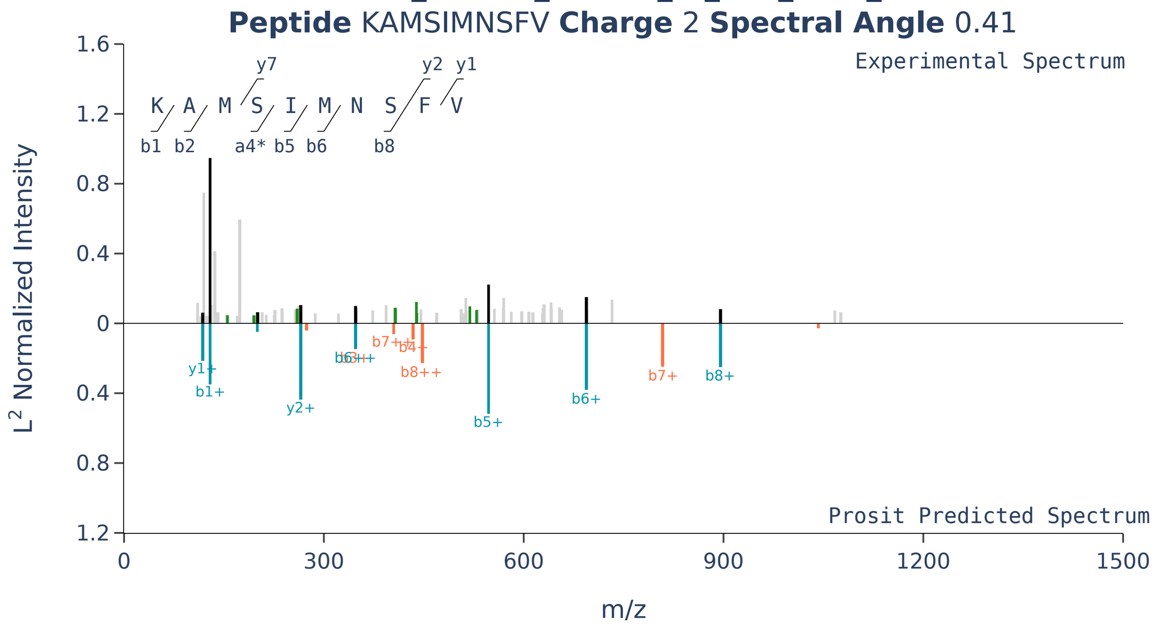


**Source** HRoetschke_291222_080123_Fu_H2B_b1R2_2h **Scan** 13901
**Peptide** LLPGELAKHAVSEGTKAVTKYT **Charge** 4 **Spectral Angle** 0.59

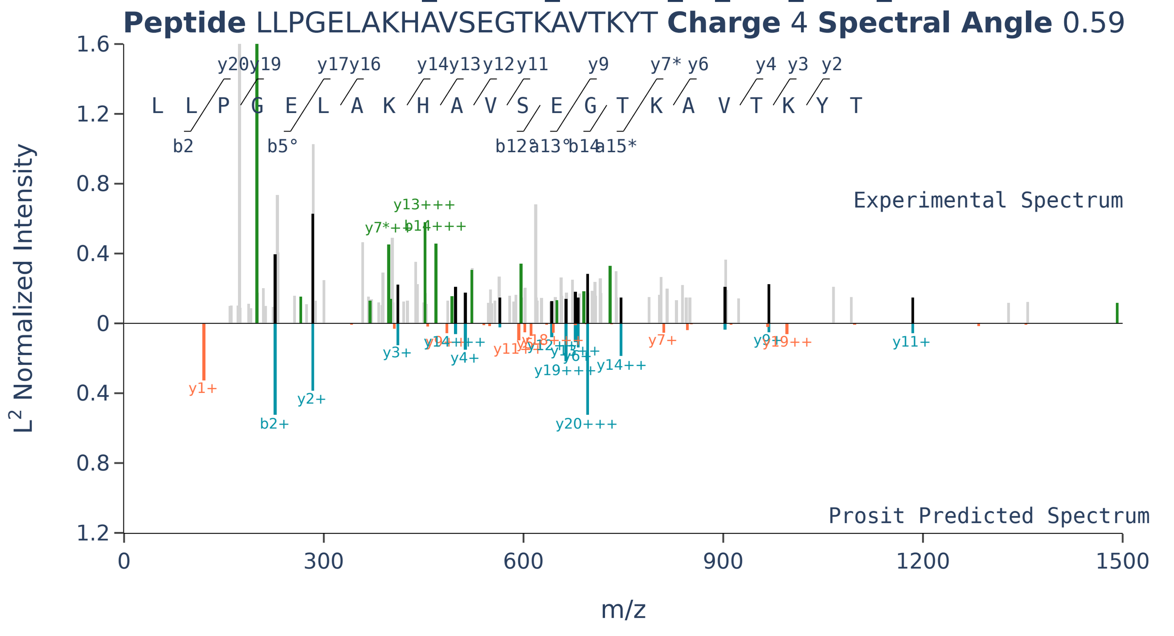


**Source** HRoetschke_291222_080123_Fu_H2B_b2R1_4h **Scan** 15648
**Peptide** KESYAIYVYKVLK **Charge** 3 **Spectral Angle** 0.6

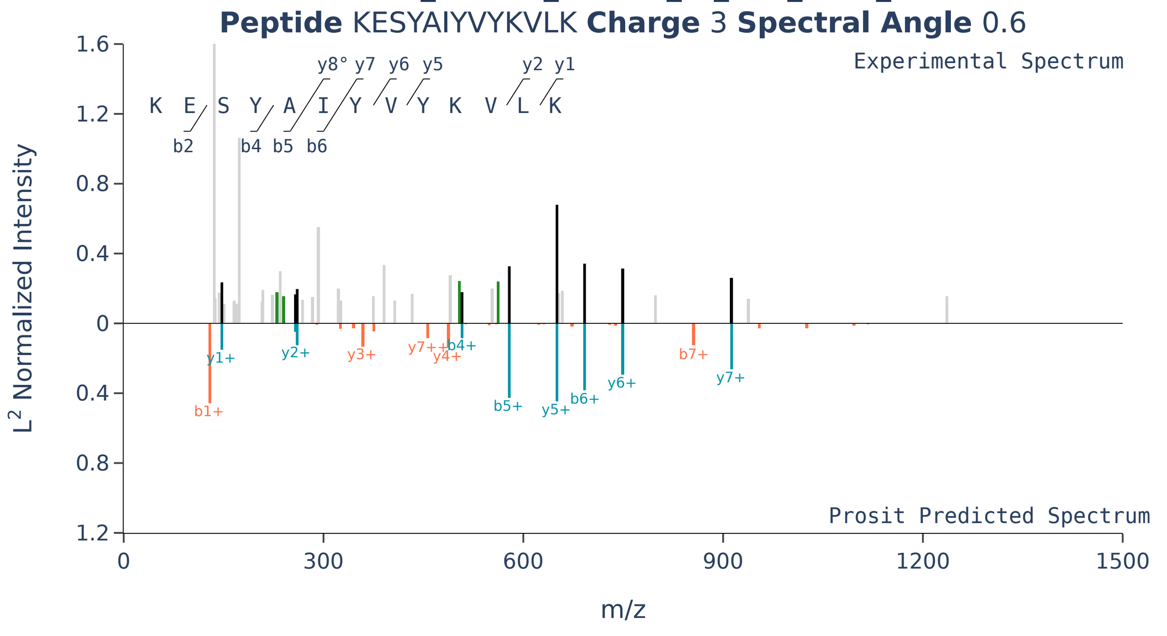


**Source** HRoetschke_291222_080123_Fu_H2B_b2R1_24h **Scan** 8966
**Peptide** YKVLKQVHPDTGISSKAM **Charge** 4 **Spectral Angle** 0.69

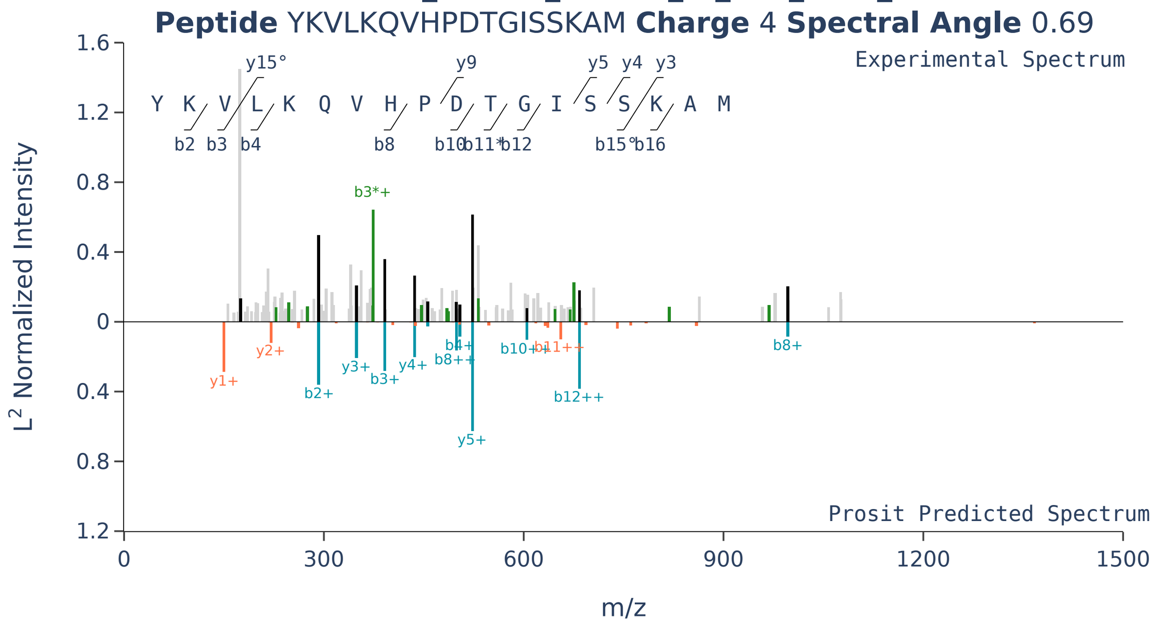


**Source** HRoetschke_291222_080123_Fu_H2B_b2R2_24h **Scan** 11387
**Peptide** VYKVLKQVHPDTGISSKAMSL **Charge** 4 **Spectral Angle** 0.63

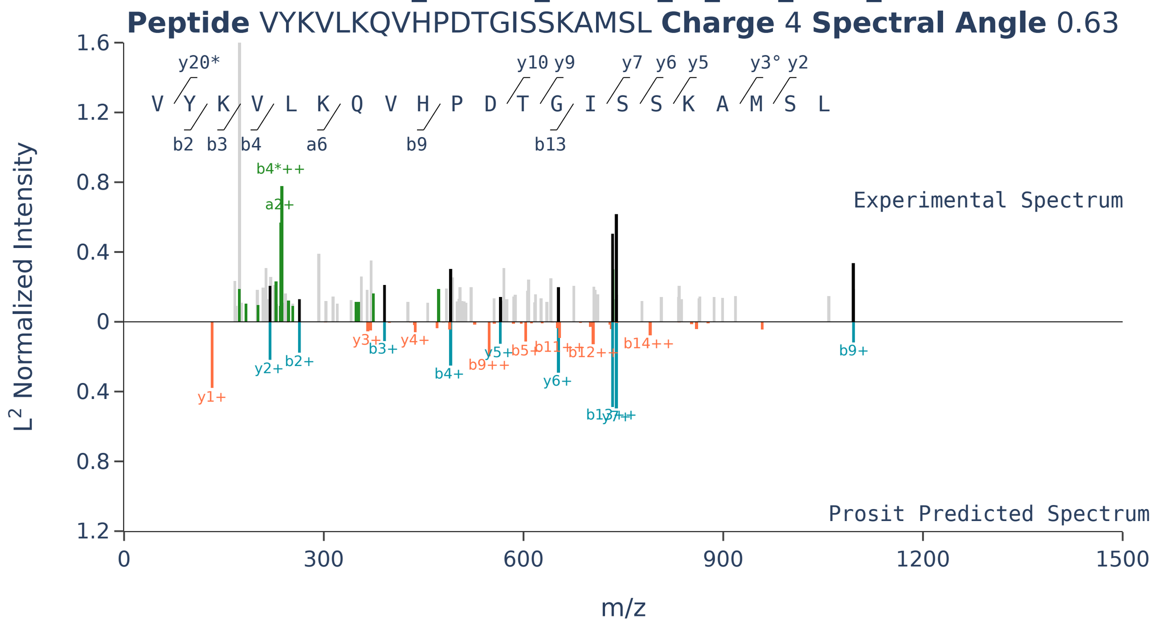


**Source** HRoetschke_291222_080123_Fu_H2B_b2R2_4h **Scan** 12804
**Peptide** LKQVHPDTGISSKAMSIMNSFVND **Charge** 4 **Spectral Angle** 0.67

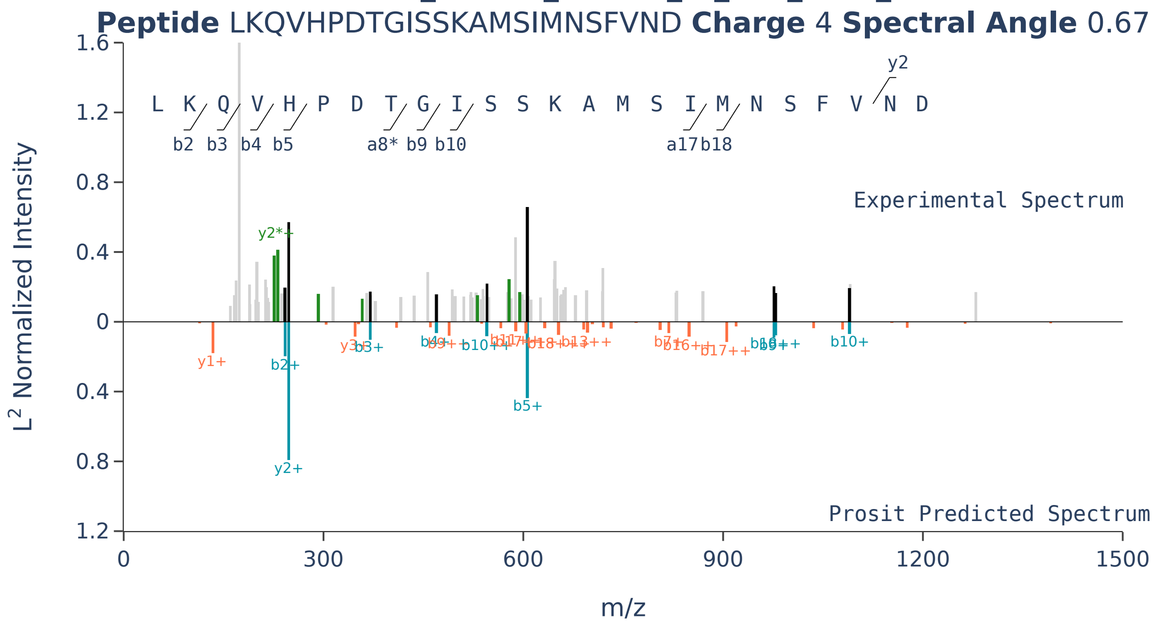


**Source** HRoetschke_291222_080123_Fu_H2B_b2R2_4h **Scan** 17392
**Peptide** AIYVYKVLKQVHPDTGISSKAMSI **Charge** 4 **Spectral Angle** 0.62

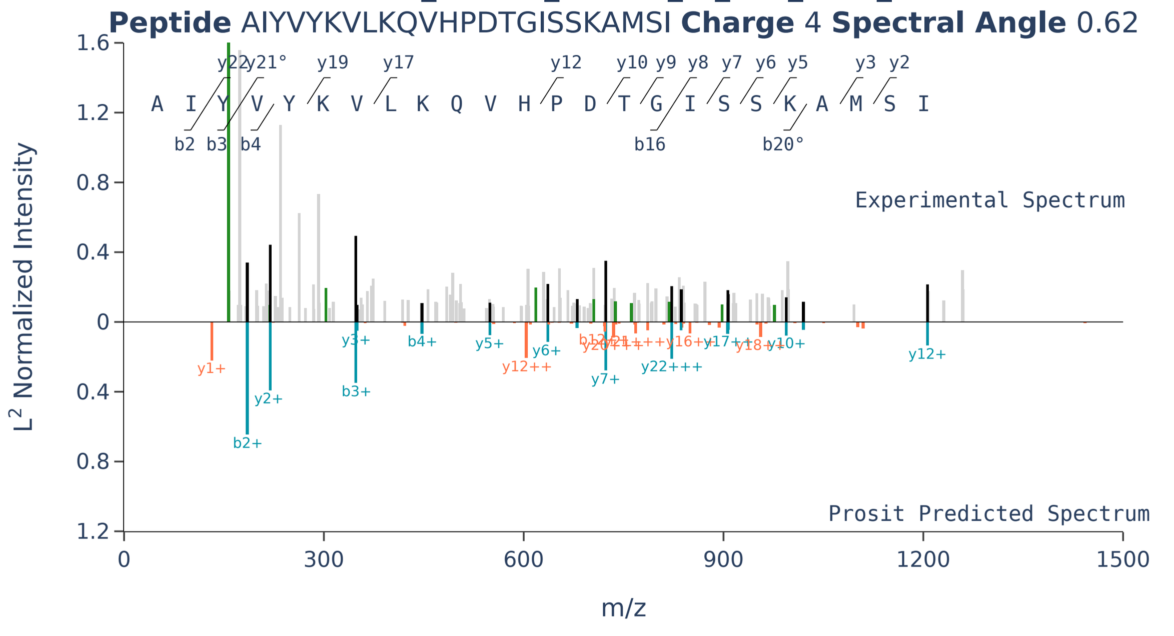


**Source** HRoetschke_291222_080123_Fu_H2B_b2R1_4h **Scan** 16482
**Peptide** AVRLLLPGELAKHAVSEGTKAVT **Charge** 4 **Spectral Angle** 0.78

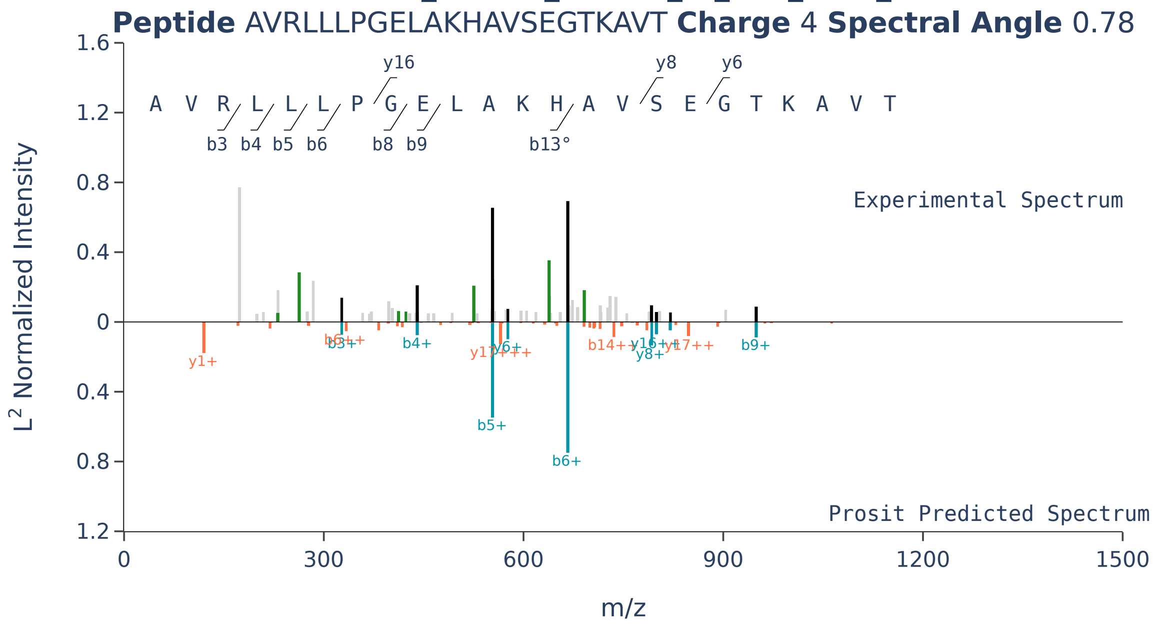


**Source** HRoetschke_291222_080123_Fu_H2B_b2R2_2h **Scan** 3829
**Peptide** ASRLAHYNKRSTITSREIQ **Charge** 4 **Spectral Angle** 0.56

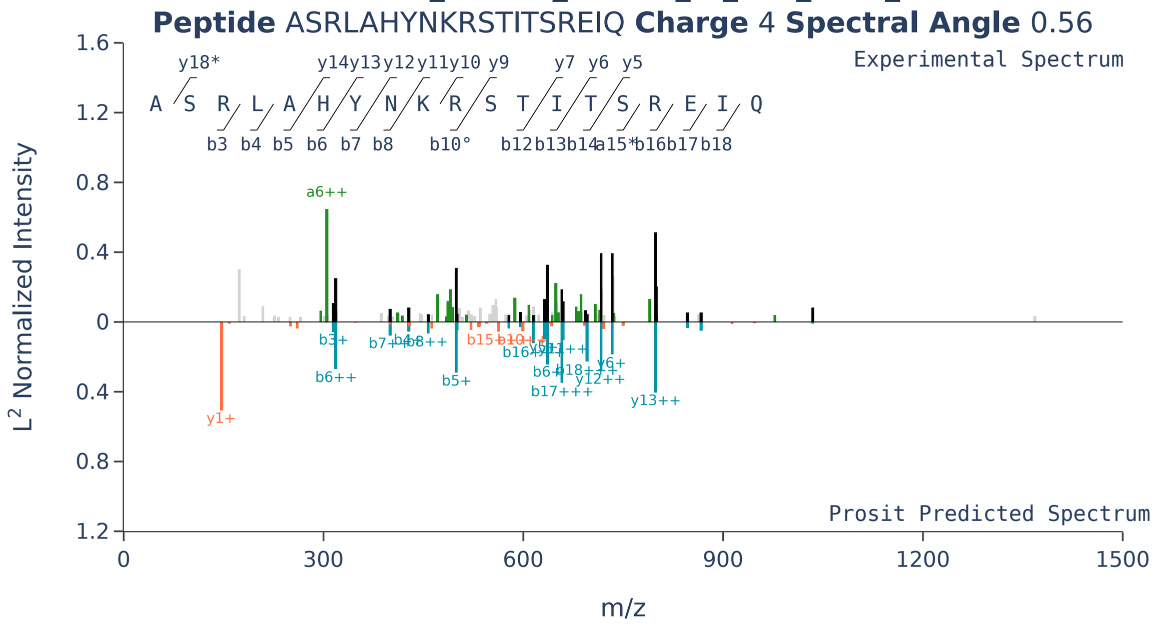

**Source** HRoetschke_291222_080123_Fu_H2B_b1R1_2h **Scan** 19840
**Peptide** NSFVNDVFERIAGEASRLAHYNKRST **Charge** 5 **Spectral Angle** 0.77

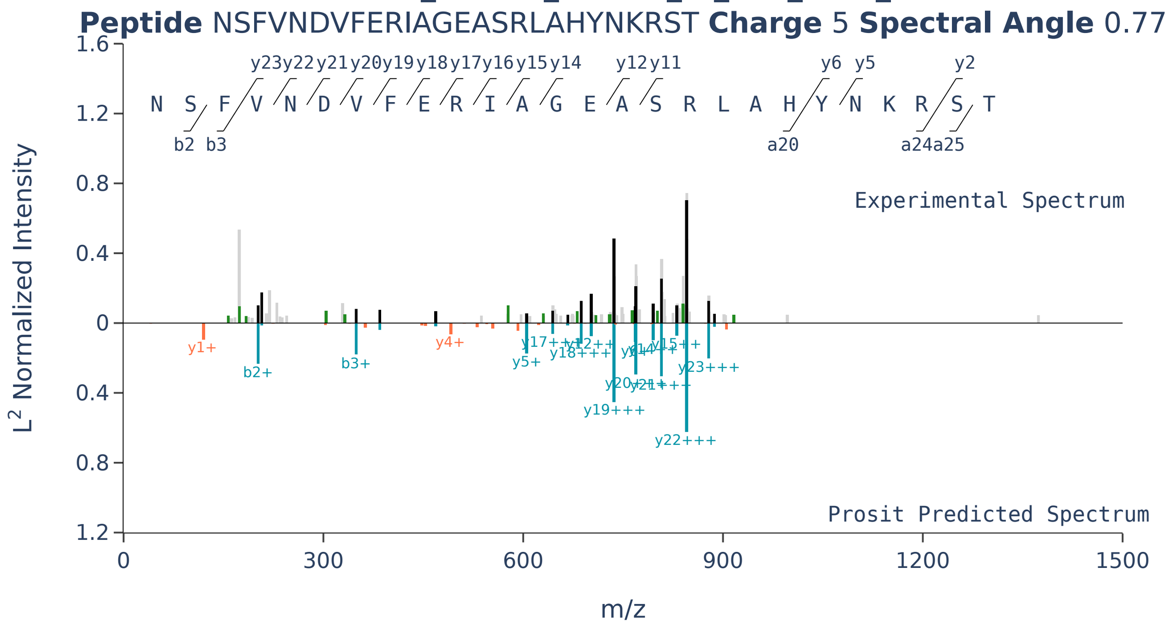


**Source** HRoetschke_291222_080123_Fu_H2B_b2R2_24h **Scan** 8917
**Peptide** KVLKQVHPDTGISSKAMSL **Charge** 4 **Spectral Angle** 0.4

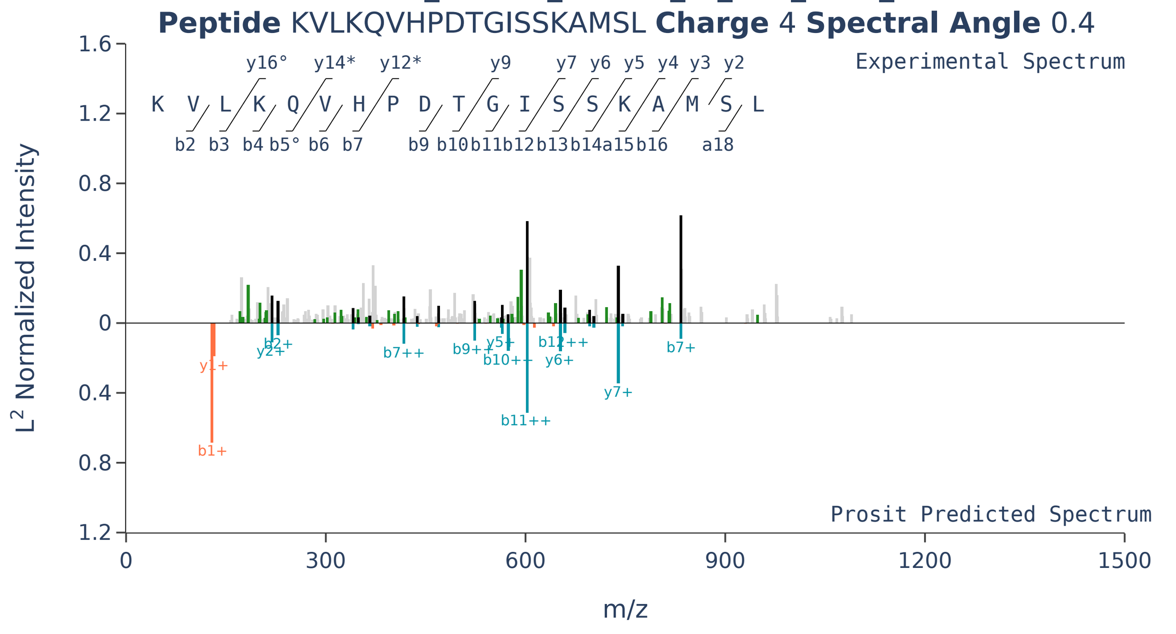


**Source** HRoetschke_291222_080123_Fu_H2B_b2R1_4h **Scan** 8074
**Peptide** IAGEASRLAHYNKRSTITSREIQTA **Charge** 5 **Spectral Angle** 0.52

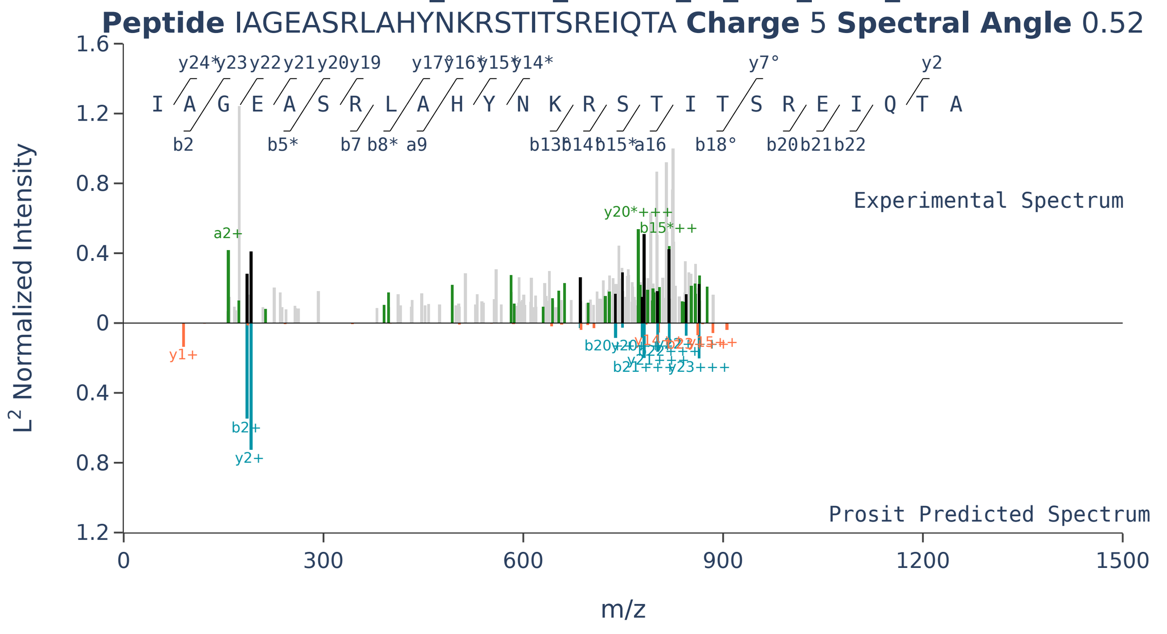


**Source** HRoetschke_291222_080123_Fu_H2B_b1R2_2h **Scan** 13335
**Peptide** AGEASRLAHYNKRSTITSREIQTAVRLL **Charge** 5 **Spectral Angle** 0.47

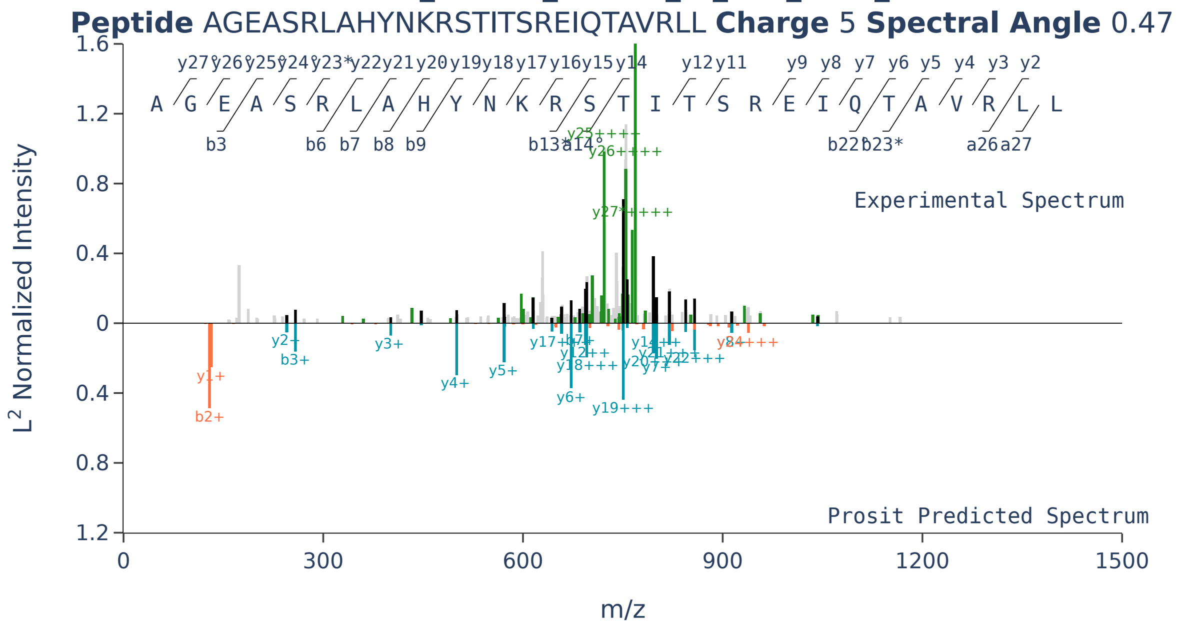


**Source** HRoetschke_291222_080123_Fu_H2B_b2R2_4h **Scan** 14159
**Peptide** VFERIAGEASRLAHYNKRSTITSREI **Charge** 5 **Spectral Angle** 0.59

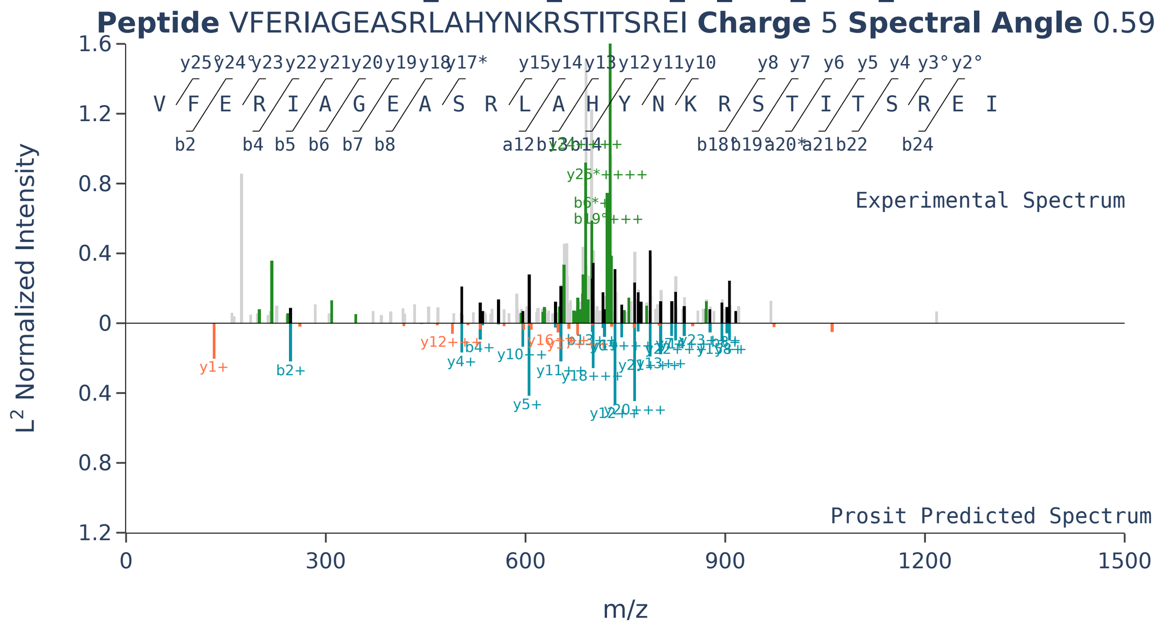


**Source** HRoetschke_291222_080123_Fu_H2B_b1R1_4h **Scan** 1564
**Peptide** VTKTQKKDGKKRRKTRKESY **Charge** 4 **Spectral Angle** 0.77

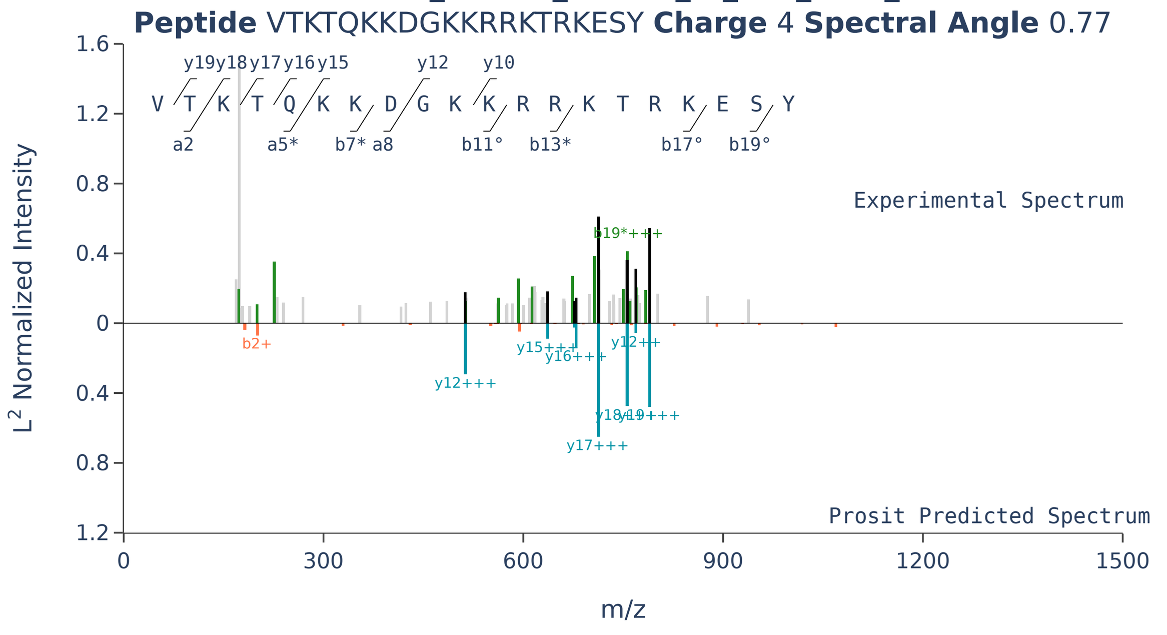


**Source** HRoetschke_291222_080123_Fu_H2B_b2R1_24h **Scan** 11750
**Peptide** GEASRLAHYNKRSTITSREIQTAVRL **Charge** 5 **Spectral Angle** 0.36

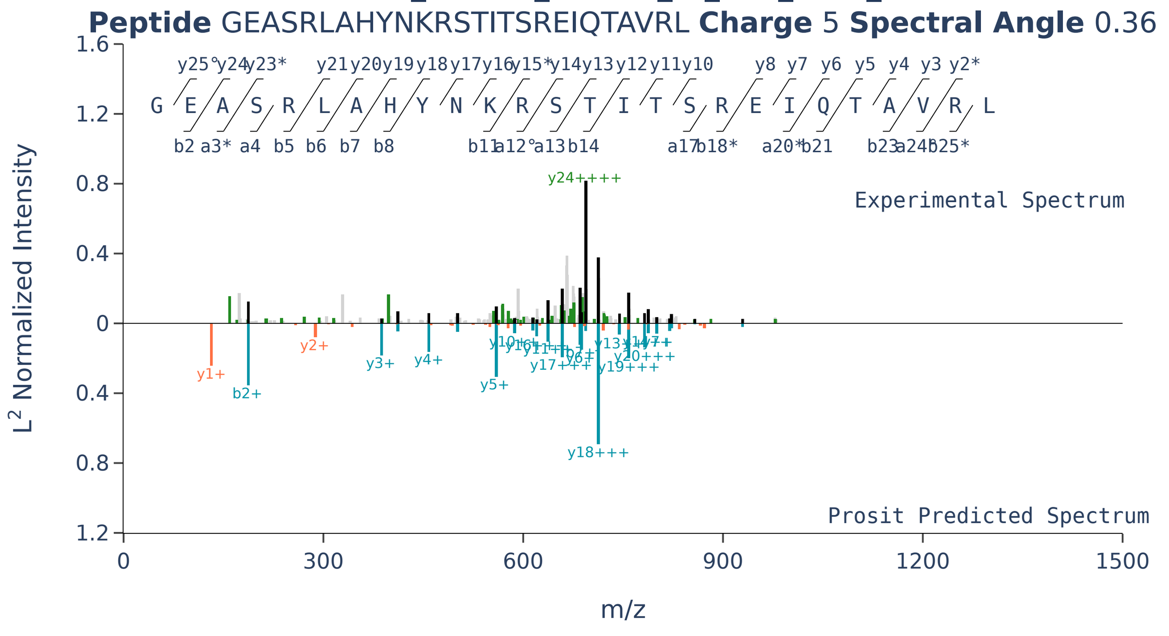


**Source** HRoetschke_291222_080123_Fu_H2B_b1R1_24h **Scan** 10393
**Peptide** ERIAGEASRLAHYNKRSTITSREIQTAVR **Charge** 5 **Spectral Angle** 0.56

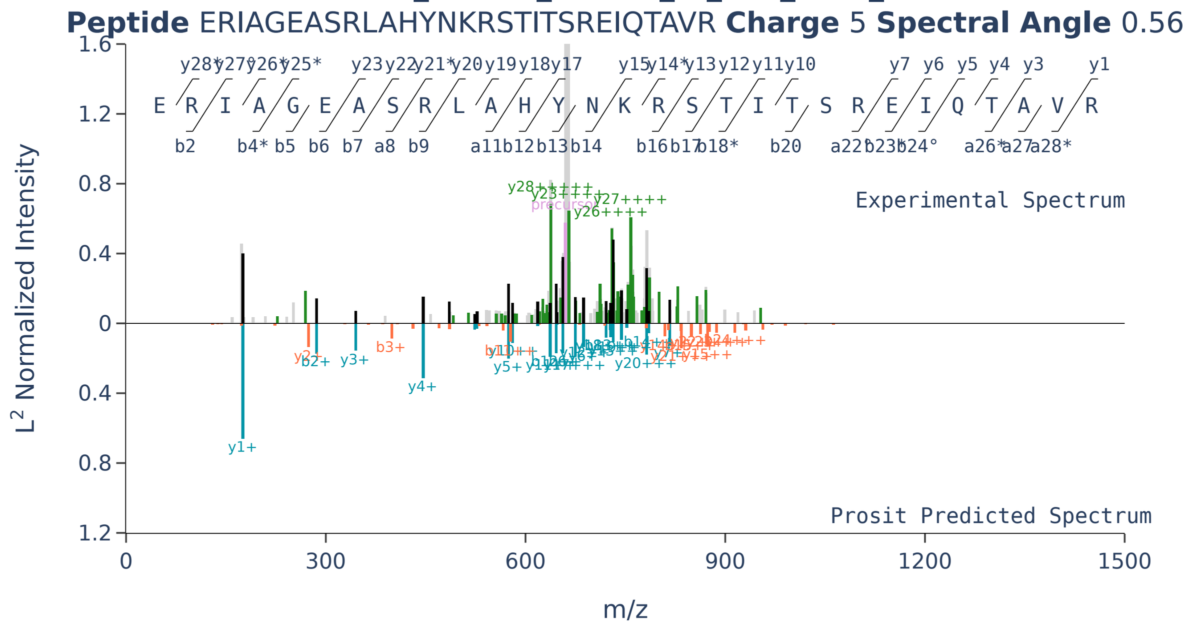


**Source** HRoetschke_291222_080123_Fu_H2B_b2R1_1h **Scan** 19957
**Peptide** SFVNDVFERIAGEASRLAHYNKRS **Charge** 5 **Spectral Angle** 0.81

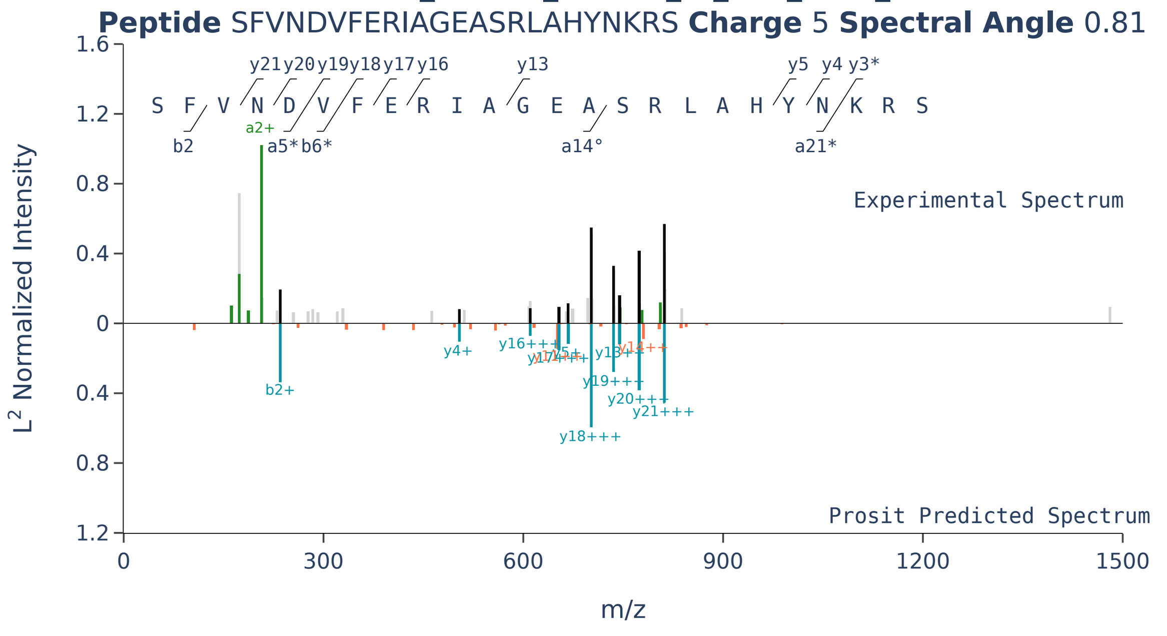


**Source** HRoetschke_291222_080123_Fu_H2B_b1R1_4h **Scan** 1827
**Peptide** KAVTKYTSAK **Charge** 3 **Spectral Angle** 0.75

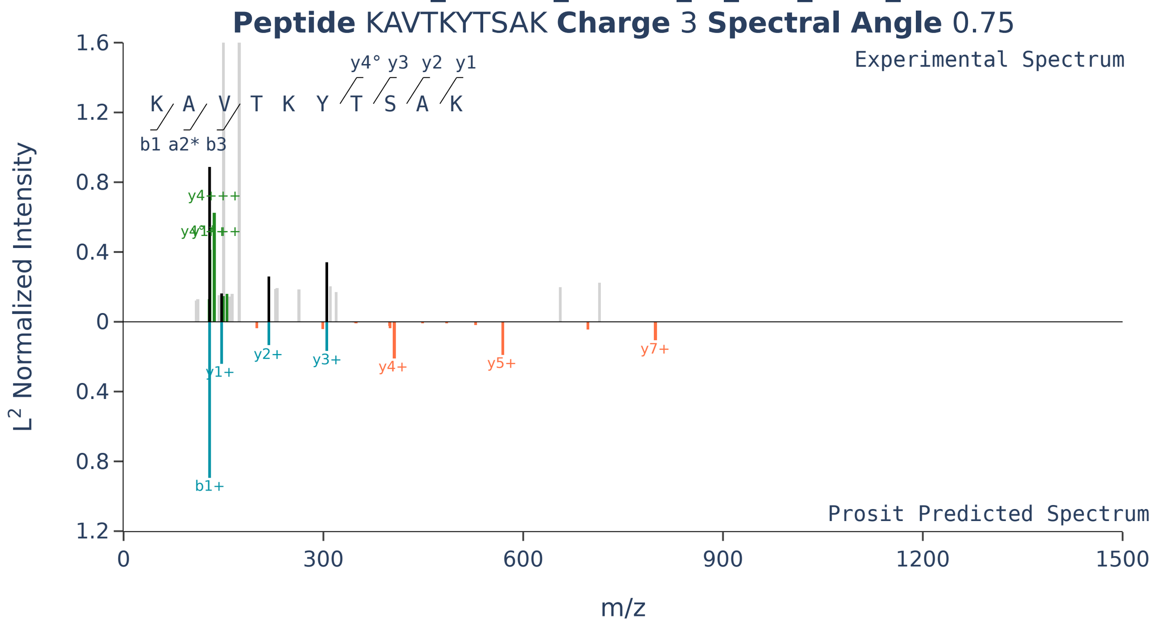


**Source** HRoetschke_291222_080123_Fu_H2B_b1R2_1h **Scan** 15966
**Peptide** NKRSTITSREIQTAVRLLLPGELAKHA **Charge** 5 **Spectral Angle** 0.69

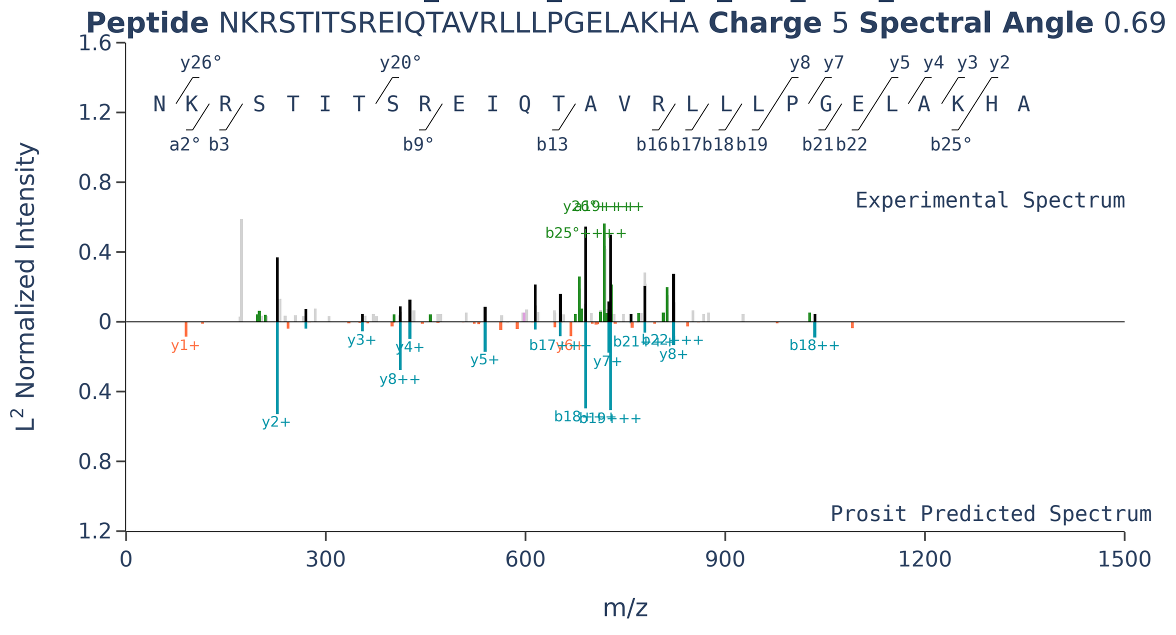


**Source** HRoetschke_291222_080123_Fu_H2B_b1R2_24h **Scan** 6232
**Peptide** KVLKQVHPDTGISSKAMS **Charge** 4 **Spectral Angle** 0.37

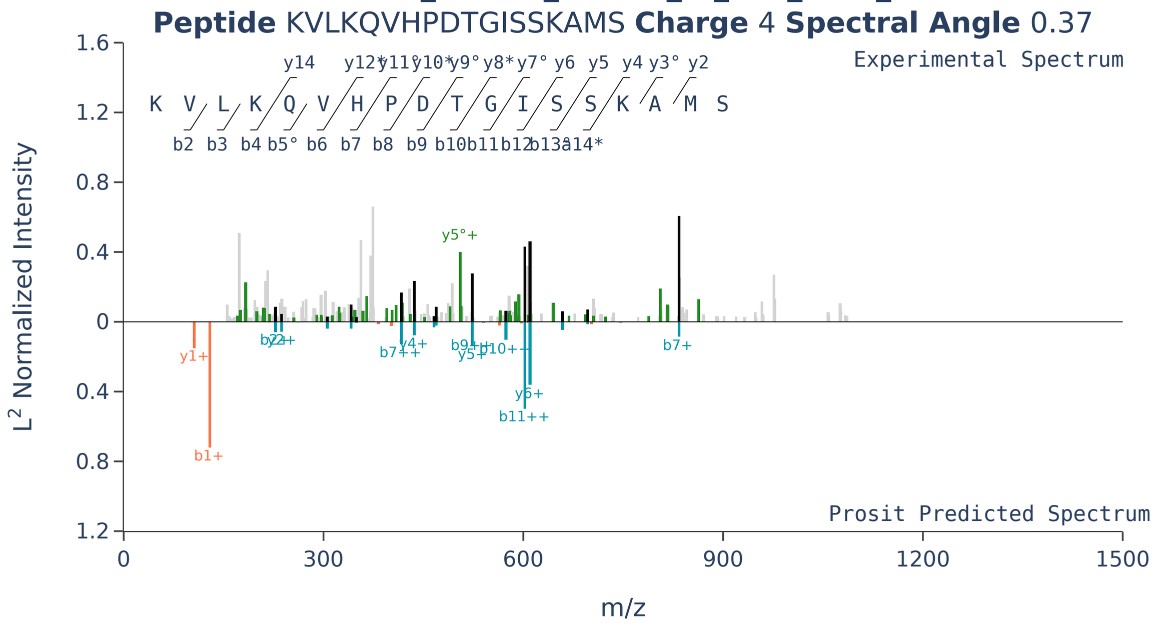


**Source** HRoetschke_291222_080123_Fu_H2B_b2R1_2h **Scan** 21837
**Peptide** AMSIMNSFVNDVFERIAGEASRLAHYNKR **Charge** 5 **Spectral Angle** 0.81

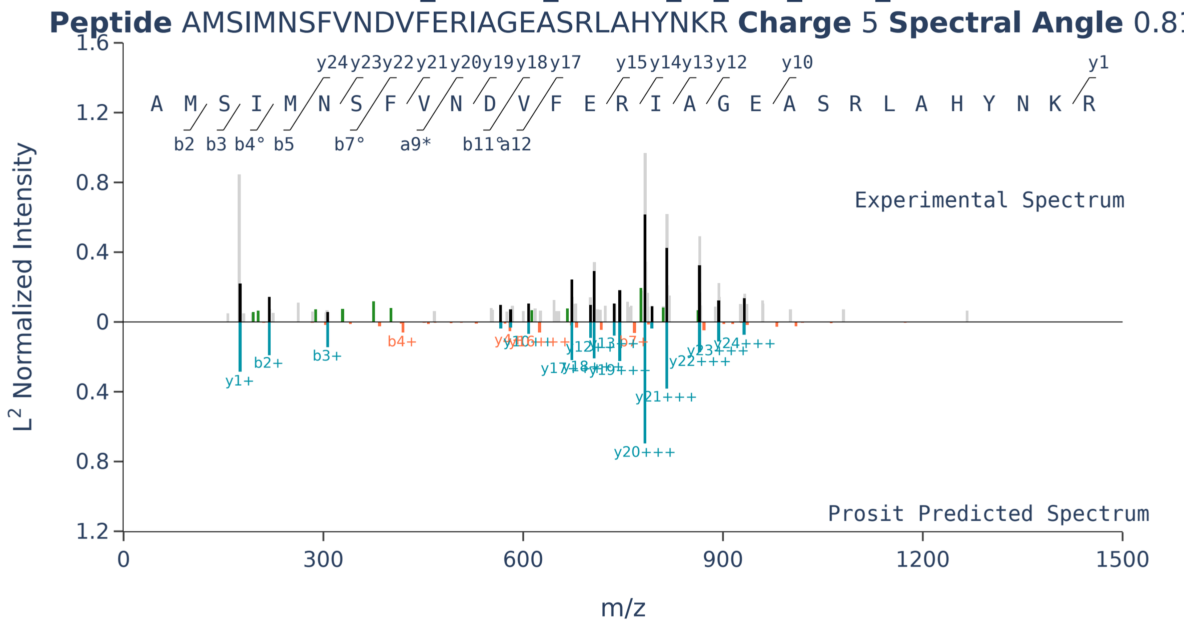


**Source** HRoetschke_291222_080123_Fu_H2B_b1R1_24h **Scan** 10444
**Peptide** FERIAGEASRL **Charge** 3 **Spectral Angle** 0.62

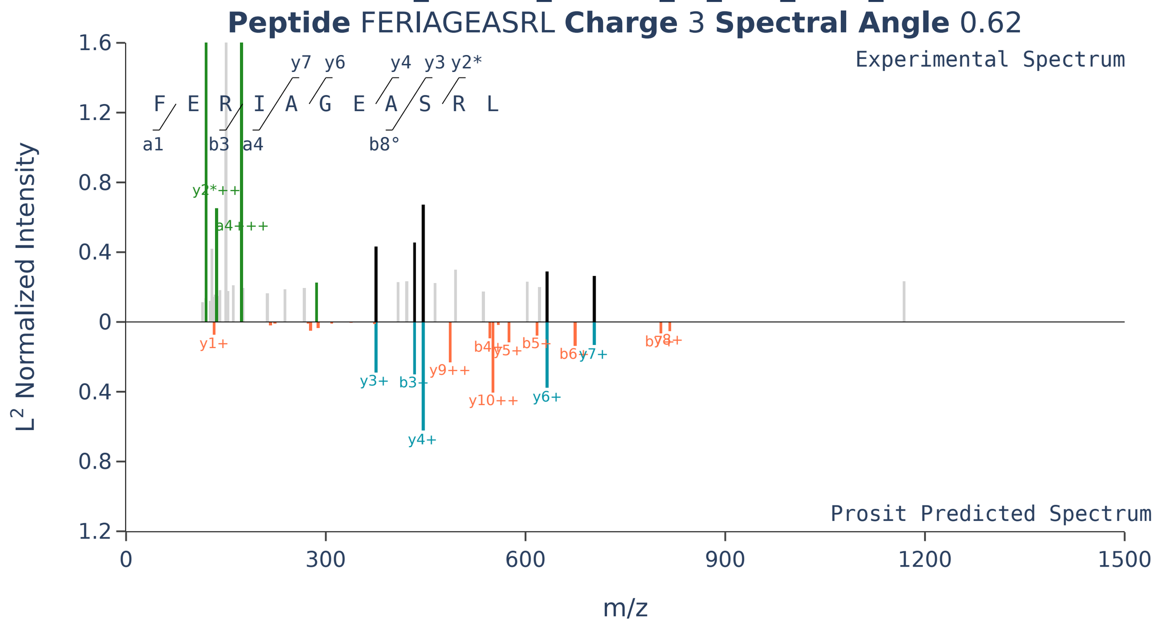


**Source** HRoetschke_291222_080123_Fu_H2B_b2R2_2h **Scan** 14021
**Peptide** RLAGEASRLAHYNKRSTITSREIQTAVRLL **Charge** 5 **Spectral Angle** 0.48

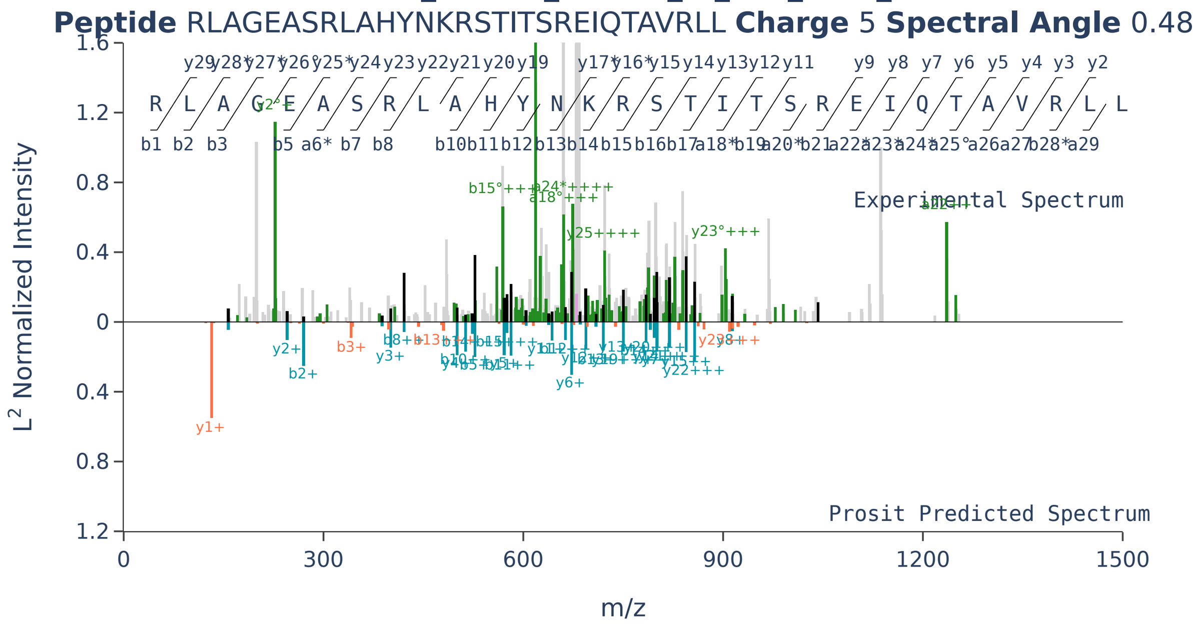

**Source** HRoetschke_291222_080123_Fu_H2B_b1R2_24h **Scan** 2028
**Peptide** AKHAVSEGTKAVT **Charge** 3 **Spectral Angle** 0.68

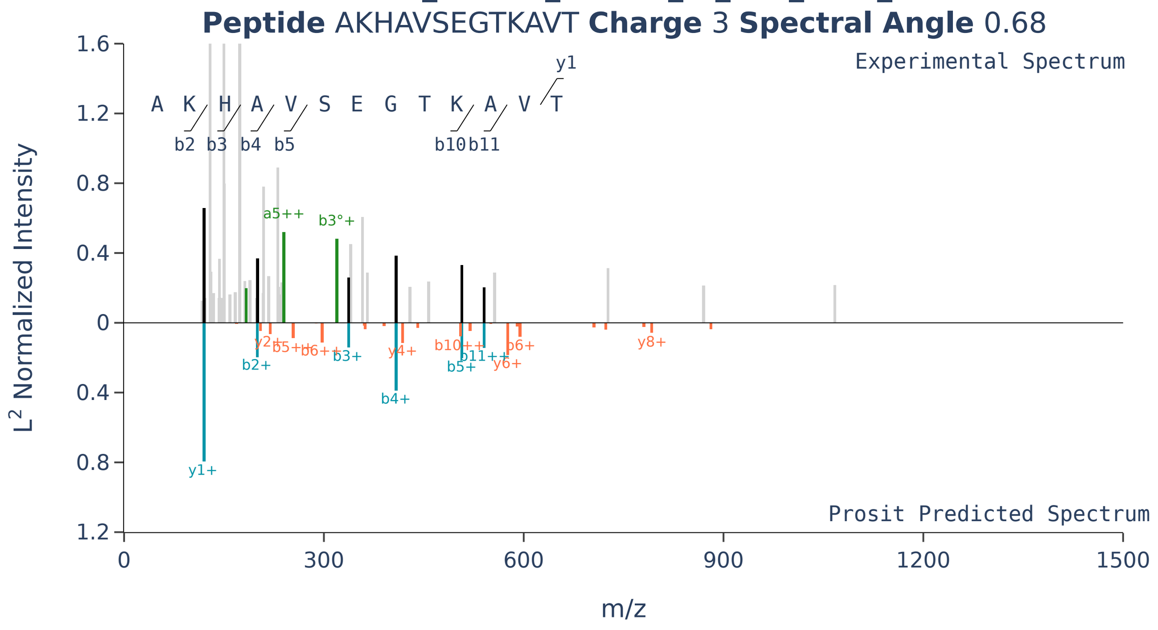


**Source** HRoetschke_291222_080123_Fu_H2B_b1R2_2h **Scan** 12996
**Peptide** EASRLAHYNKRSTITSREIQTAVRLL **Charge** 5 **Spectral Angle** 0.57

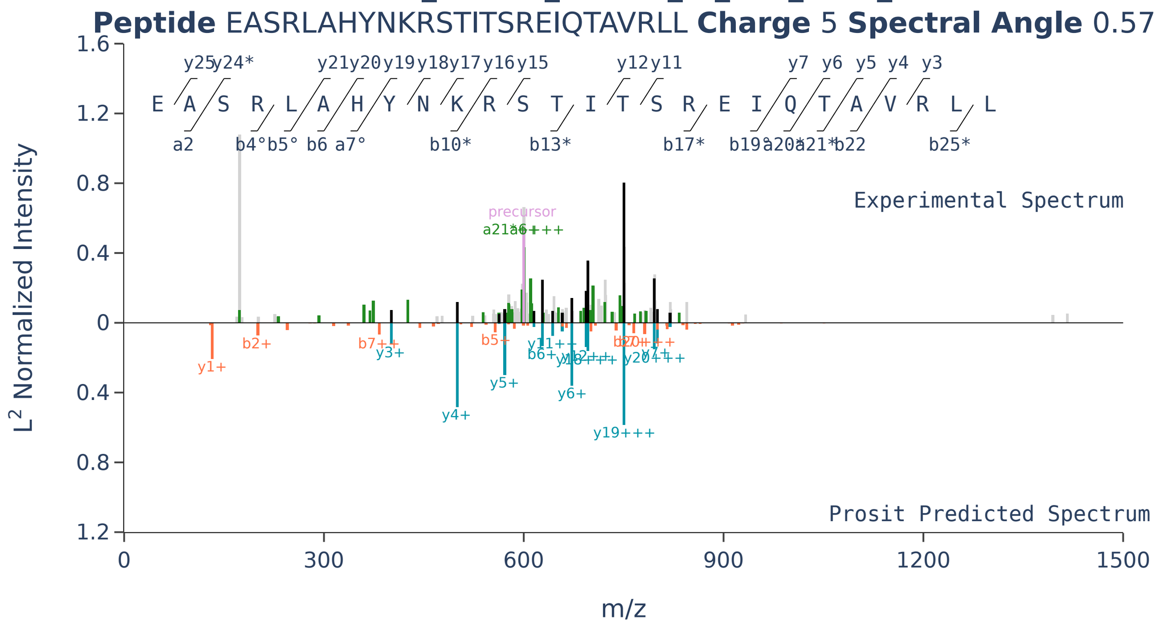


**Source** HRoetschke_291222_080123_Fu_H2B_b2R1_24h **Scan** 4588
**Peptide** SRLAHYNKRSTITSREIQT **Charge** 5 **Spectral Angle** 0.42

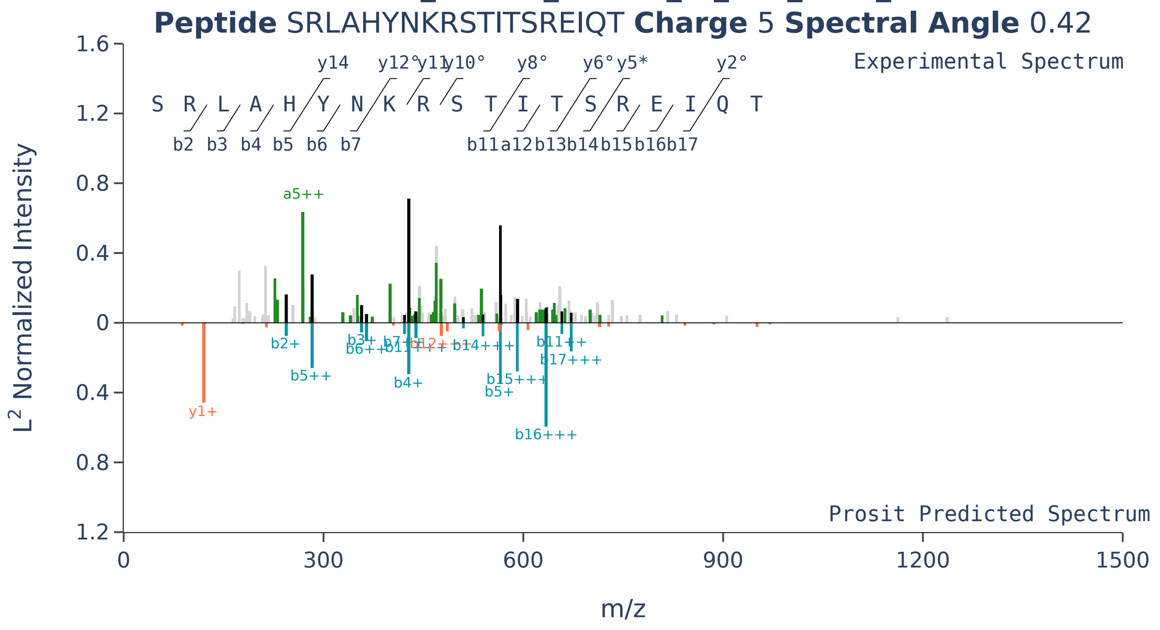


**Source** HRoetschke_291222_080123_Fu_H2B_b1R2_24h **Scan** 3109
**Peptide** ASRLAHYNKRSTI **Charge** 4 **Spectral Angle** 0.59

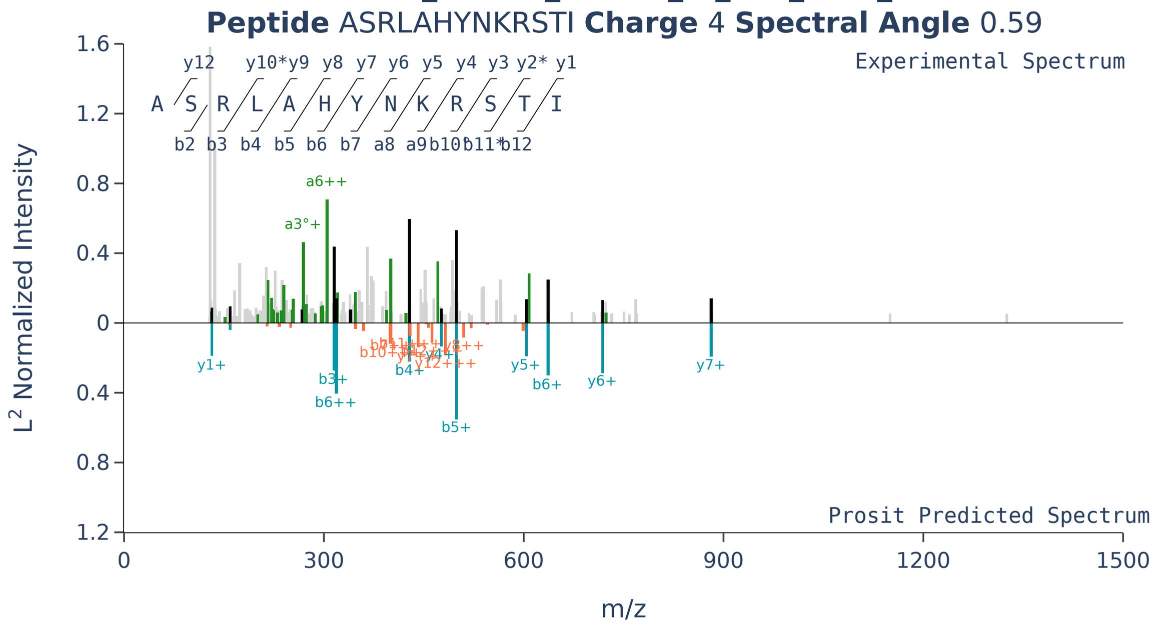


**Source** HRoetschke_291222_080123_Fu_H2B_b2R2_2h **Scan** 17317
**Peptide** QTAVRLLLPGELAKHAVSEGTKAVTKYTSA **Charge** 5 **Spectral Angle** 0.52

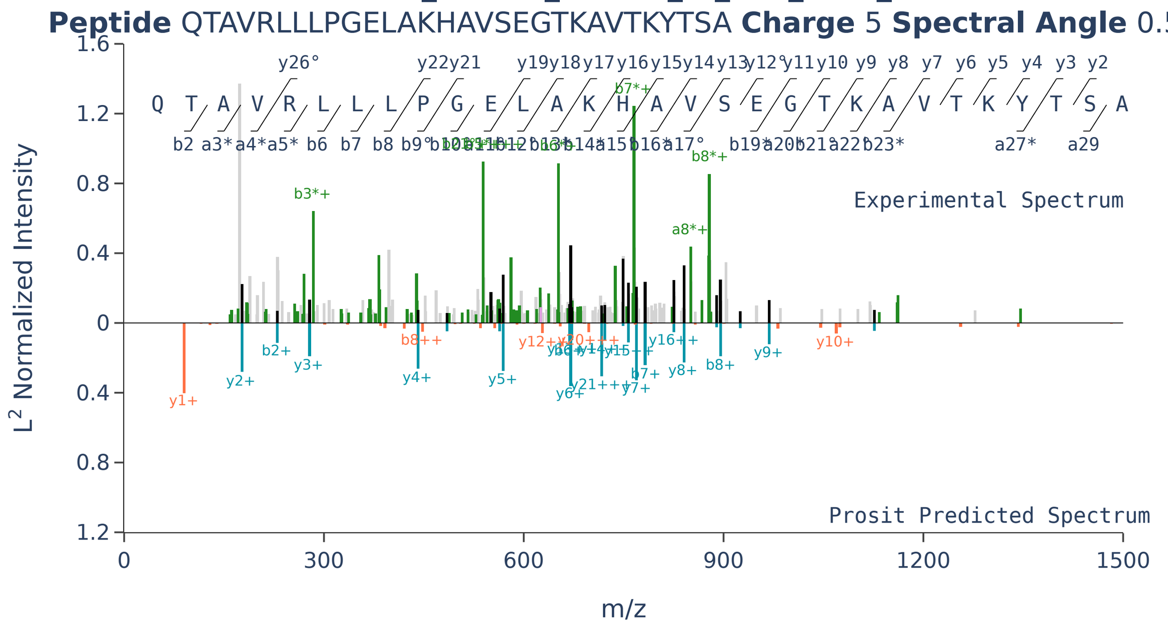


**Source** HRoetschke_291222_080123_Fu_H2B_b1R2_24h **Scan** 10722
**Peptide** LAGEASRLAHYNKRSTITSREIQTAV **Charge** 4 **Spectral Angle** 0.5

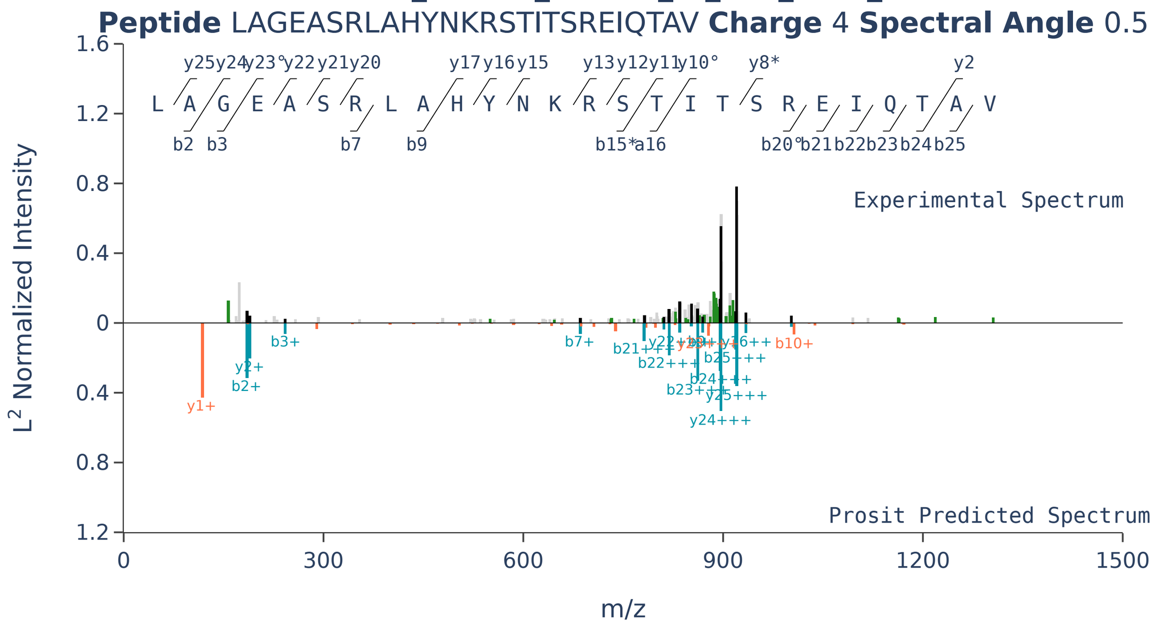


**Source** HRoetschke_291222_080123_Fu_H2B_b2R1_24h **Scan** 11716
**Peptide** YVYKVLKQVHPDTGISS **Charge** 3 **Spectral Angle** 0.63

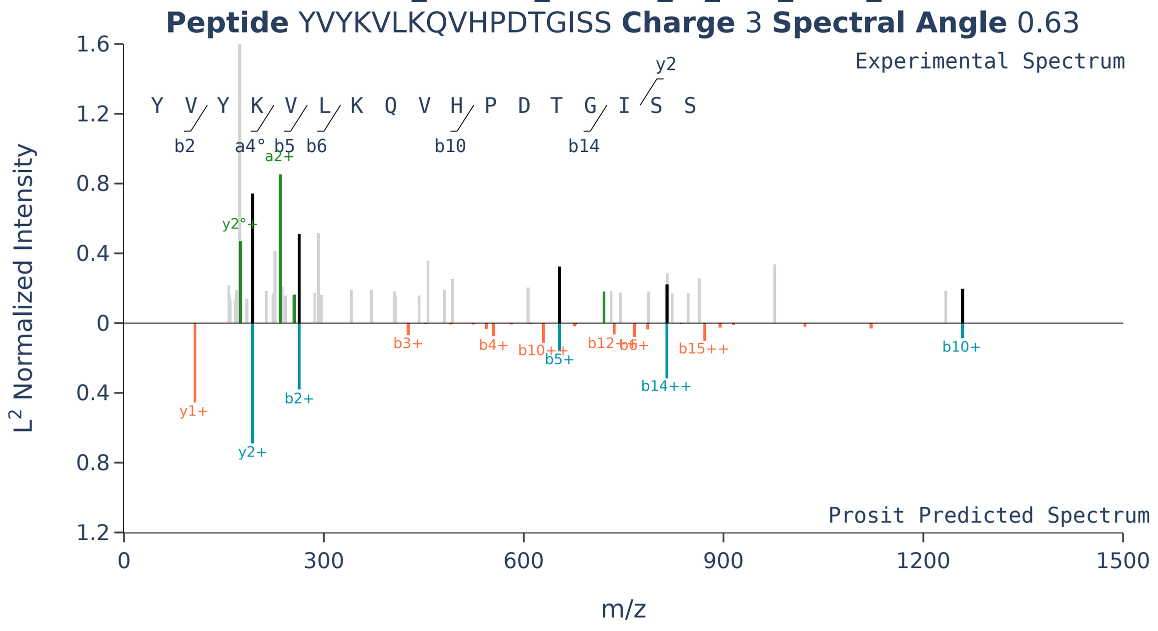


**Source** HRoetschke_291222_080123_Fu_H2B_b2R2_4h **Scan** 3739
**Peptide** SRLAHYNKRSTITSREIQ **Charge** 4 **Spectral Angle** 0.53

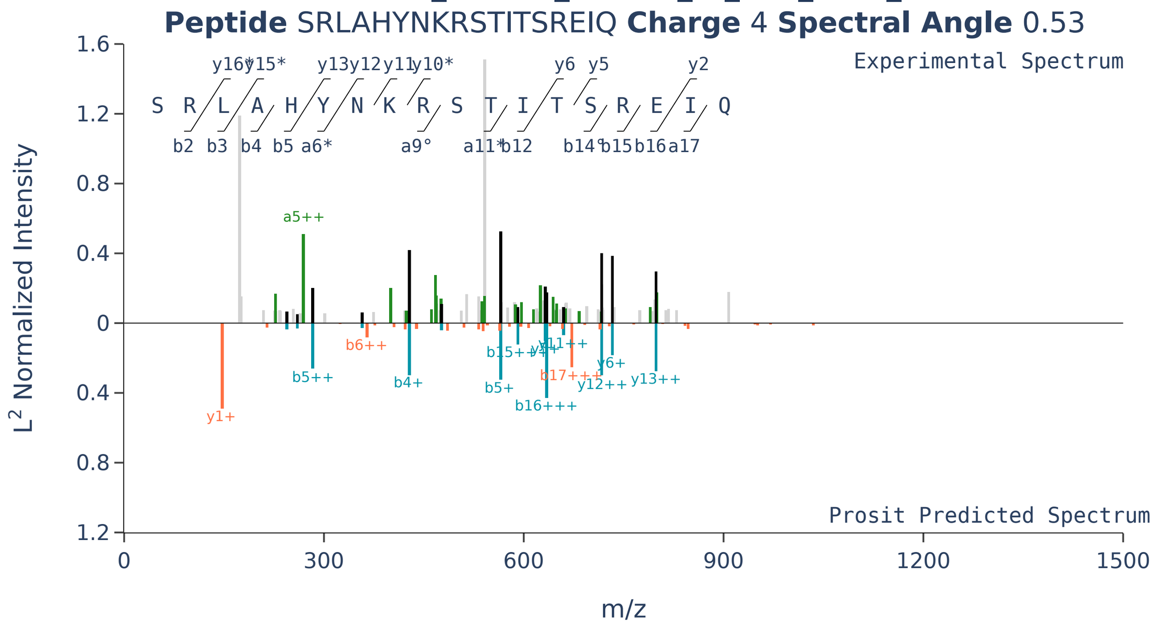


**Source** HRoetschke_291222_080123_Fu_H2B_b2R2_24h **Scan** 19451
**Peptide** KAMSIMNSFVNDVF **Charge** 2 **Spectral Angle** 0.53

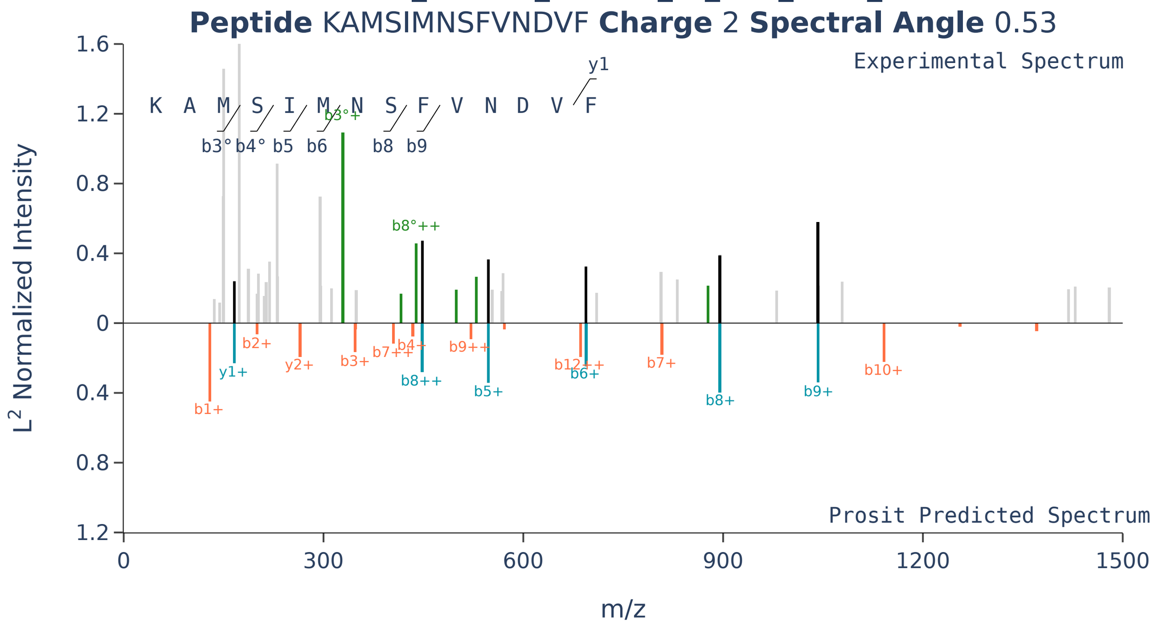


**Source** HRoetschke_291222_080123_Fu_H2B_b2R1_1h **Scan** 20314
**Peptide** LMNSFVNDVFERIAGEASRLAHYNKR **Charge** 5 **Spectral Angle** 0.78

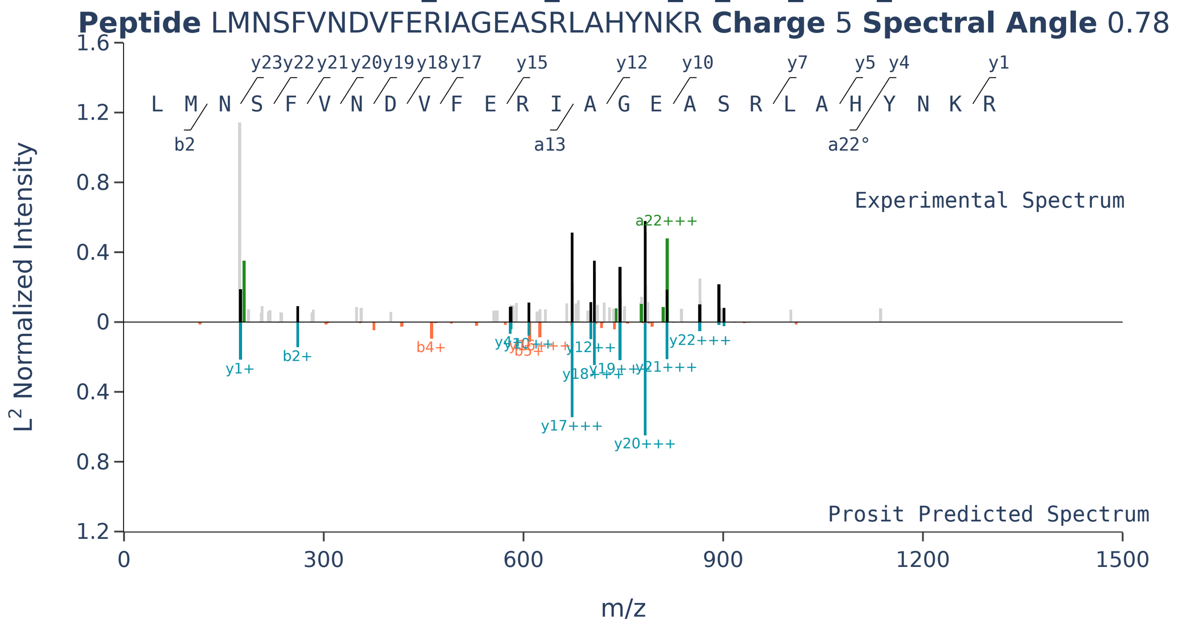


**Source** HRoetschke_291222_080123_Fu_H2B_b2R2_4h **Scan** 13313
**Peptide** PDTGISSKAMSIMNSF **Charge** 2 **Spectral Angle** 0.73

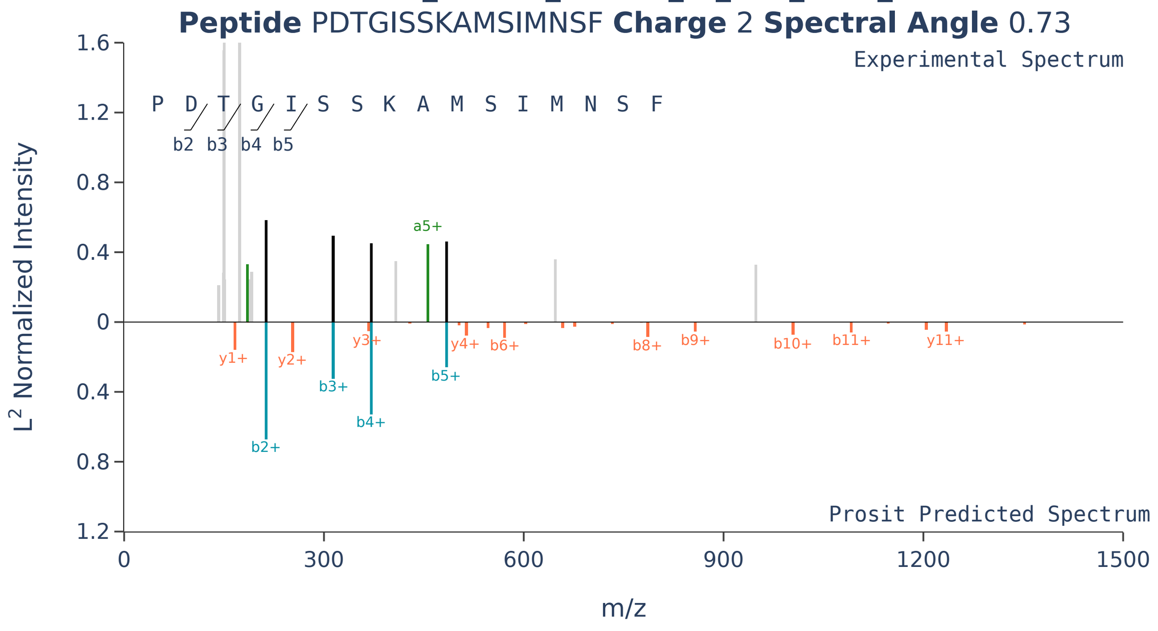


**Source** HRoetschke_291222_080123_Fu_H2B_b1R1_2h **Scan** 11709
**Peptide** VFERIAGEASRLAHYNKRSTITSREIQ **Charge** 5 **Spectral Angle** 0.42

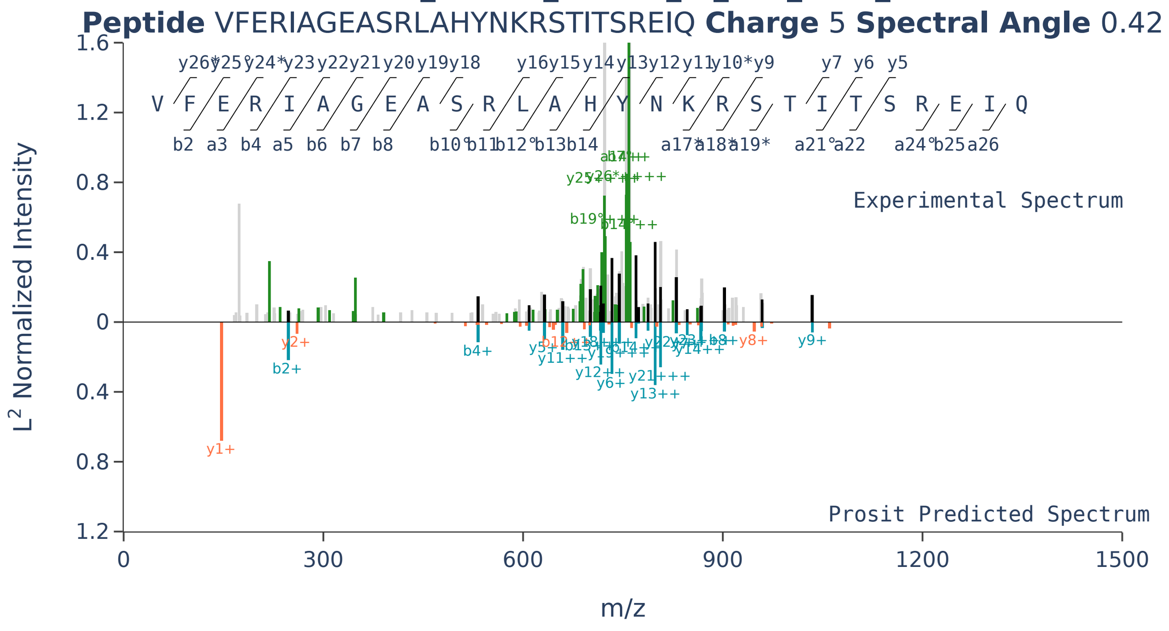


**Source** HRoetschke_291222_080123_Fu_H2B_b2R2_24h **Scan** 15600
**Peptide** RKESYAIYVYKVLKQ **Charge** 4 **Spectral Angle** 0.51

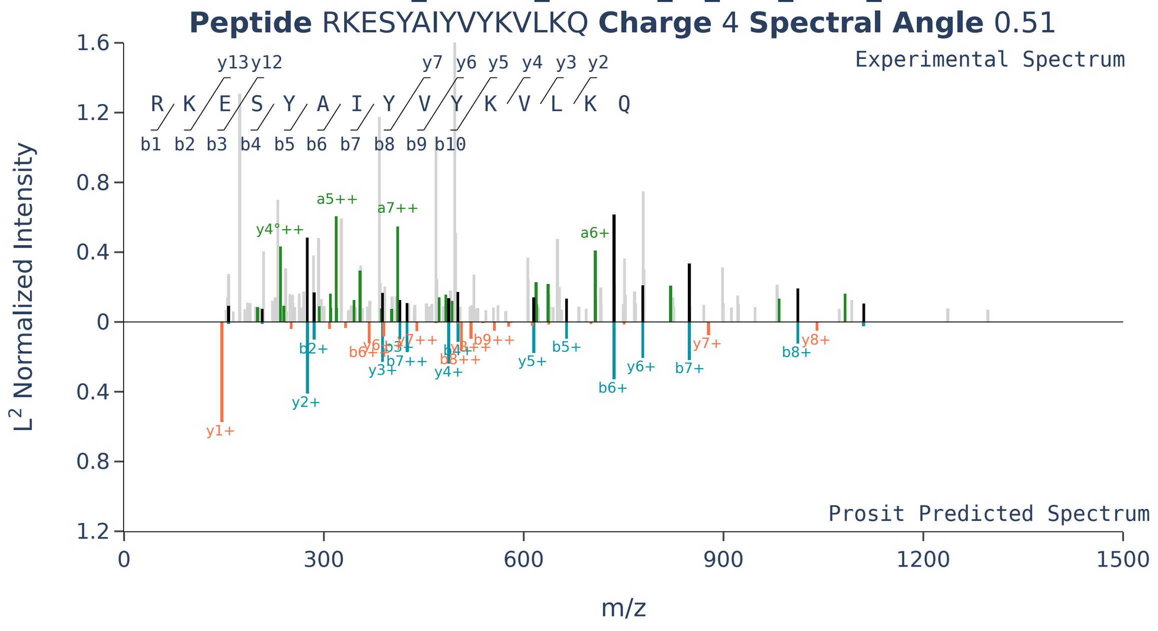


**Source** HRoetschke_291222_080123_Fu_H2B_b2R2_24h **Scan** 15632
**Peptide** HYNKRSTITSREIQTAVRLL **Charge** 5 **Spectral Angle** 0.69

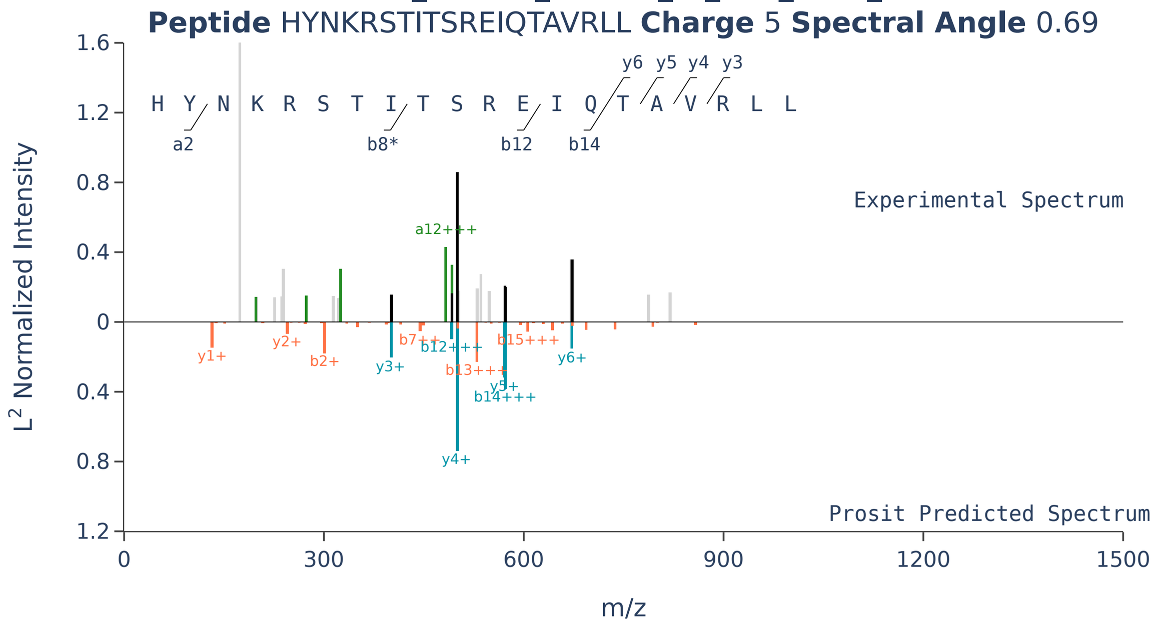


**Source** HRoetschke_291222_080123_Fu_H2B_b2R1_4h **Scan** 17230
**Peptide** AIYVYKVLKQVHPDTGISSKAMSL **Charge** 4 **Spectral Angle** 0.58

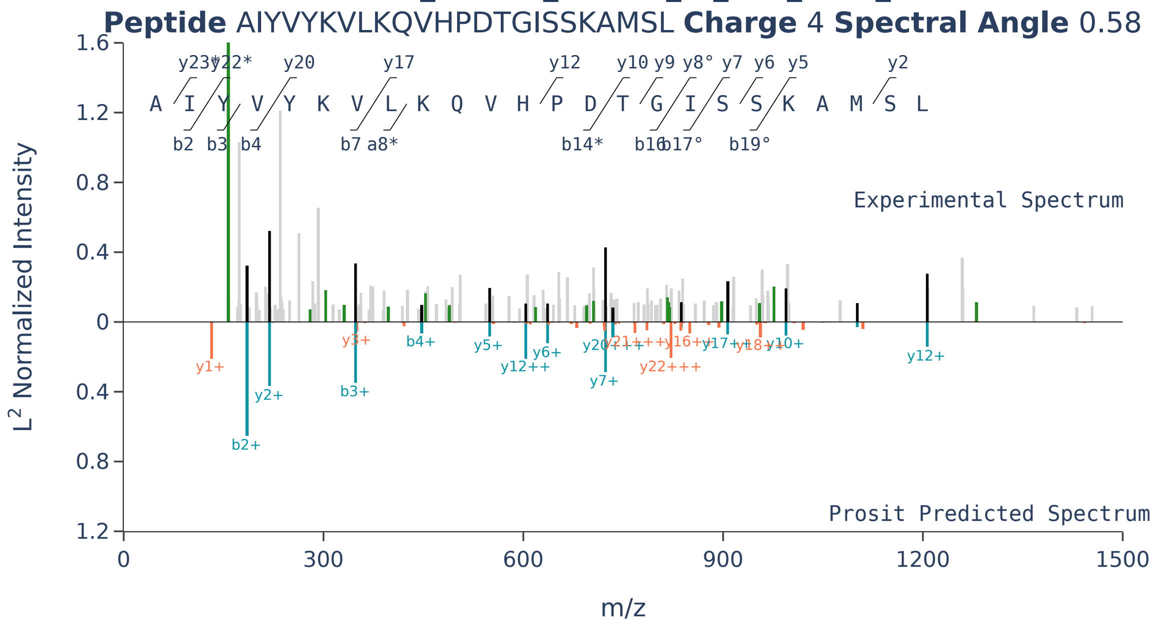

**Source** HRoetschke_291222_080123_Fu_H2B_b2R2_2h **Scan** 2445
**Peptide** SRLAHYNKRSTIT **Charge** 3 **Spectral Angle** 0.58

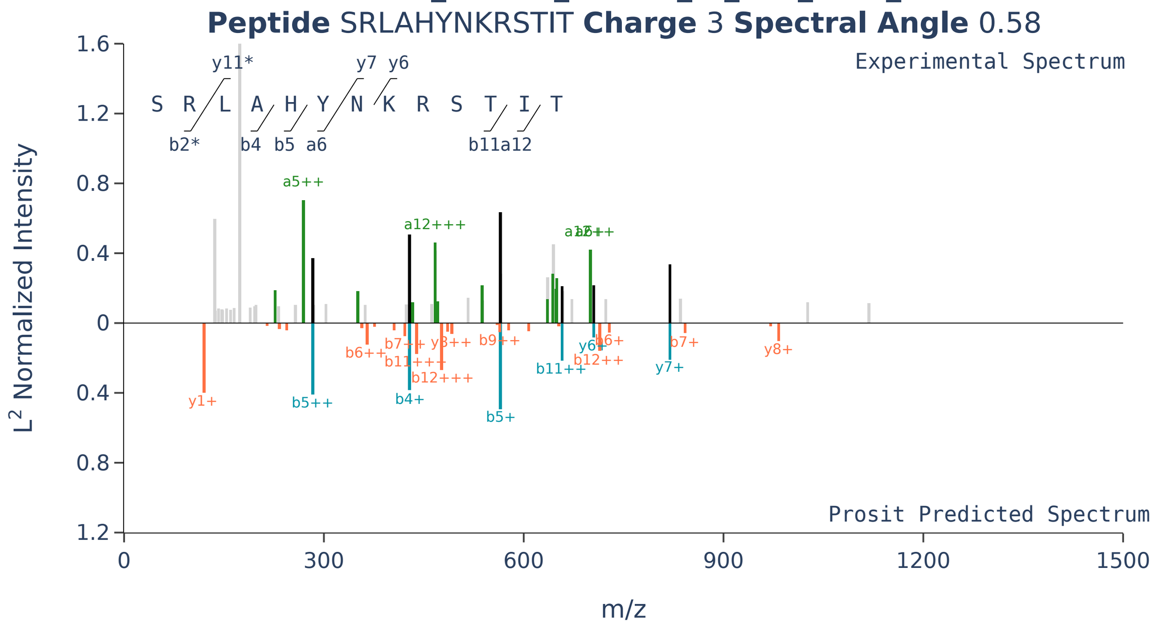


**Source** HRoetschke_291222_080123_Fu_H2B_b2R1_24h **Scan** 3060
**Peptide** SKKAVTKTQKKDGKKRRKTRKESYAIYVY **Charge** 6 **Spectral Angle** 0.85

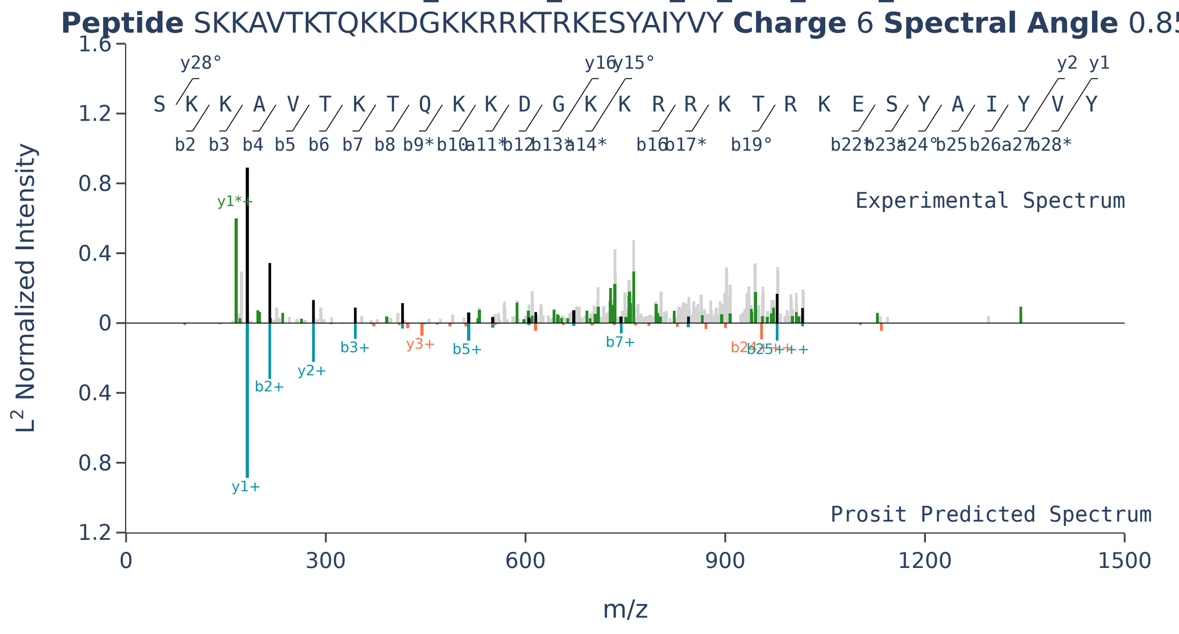


**Source** HRoetschke_291222_080123_Fu_H2B_b1R2_2h **Scan** 5081
**Peptide** EASRLAHYNKRSTITSREL **Charge** 5 **Spectral Angle** 0.78

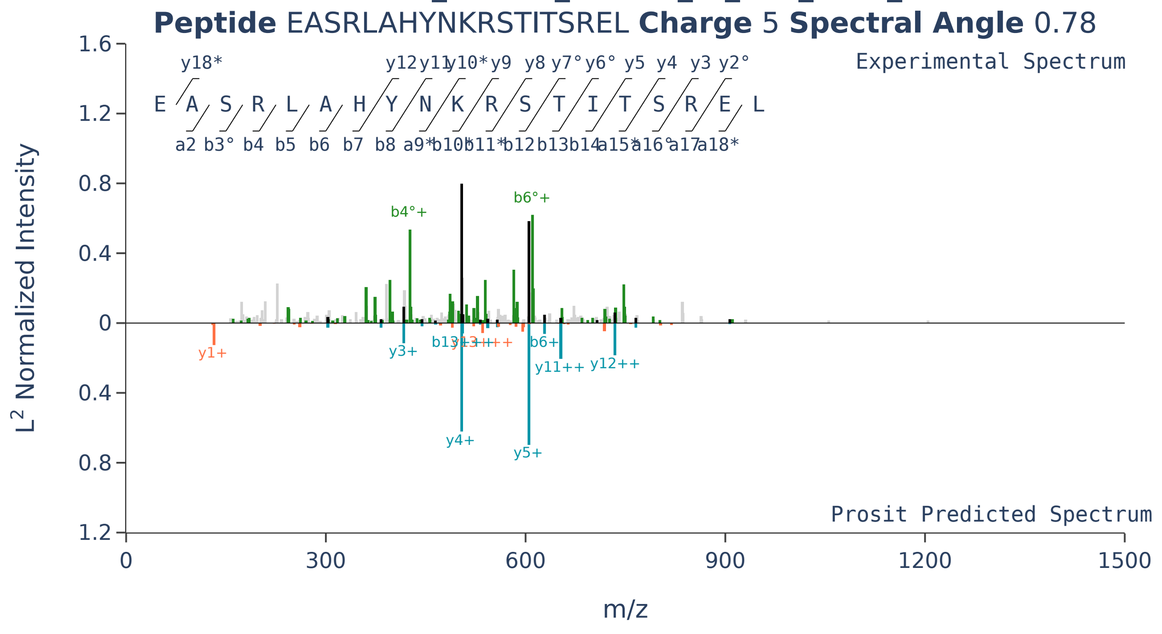


**Source** HRoetschke_291222_080123_Fu_H2B_b2R2_X **Scan** 3967
**Peptide** ELAKHAVSEGTKAVTKYTSAK **Charge** 4 **Spectral Angle** 0.66

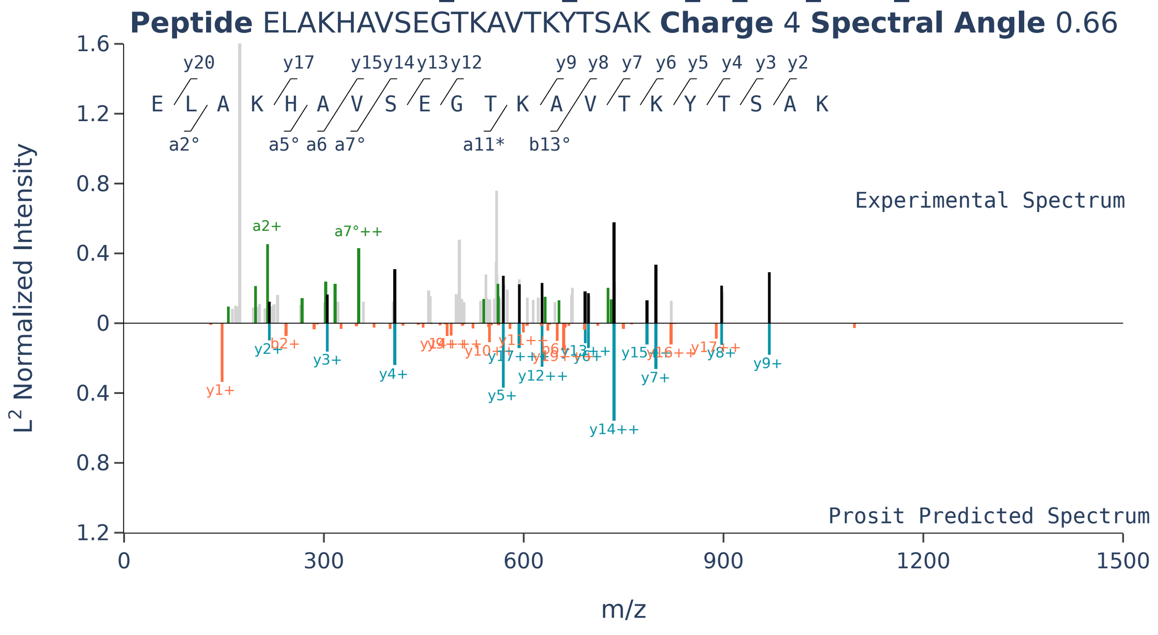


**Source** HRoetschke_291222_080123_Fu_H2B_b1R1_1h **Scan** 9846
**Peptide** KQVHPDTGISSKAMSIMNSFVND **Charge** 4 **Spectral Angle** 0.49

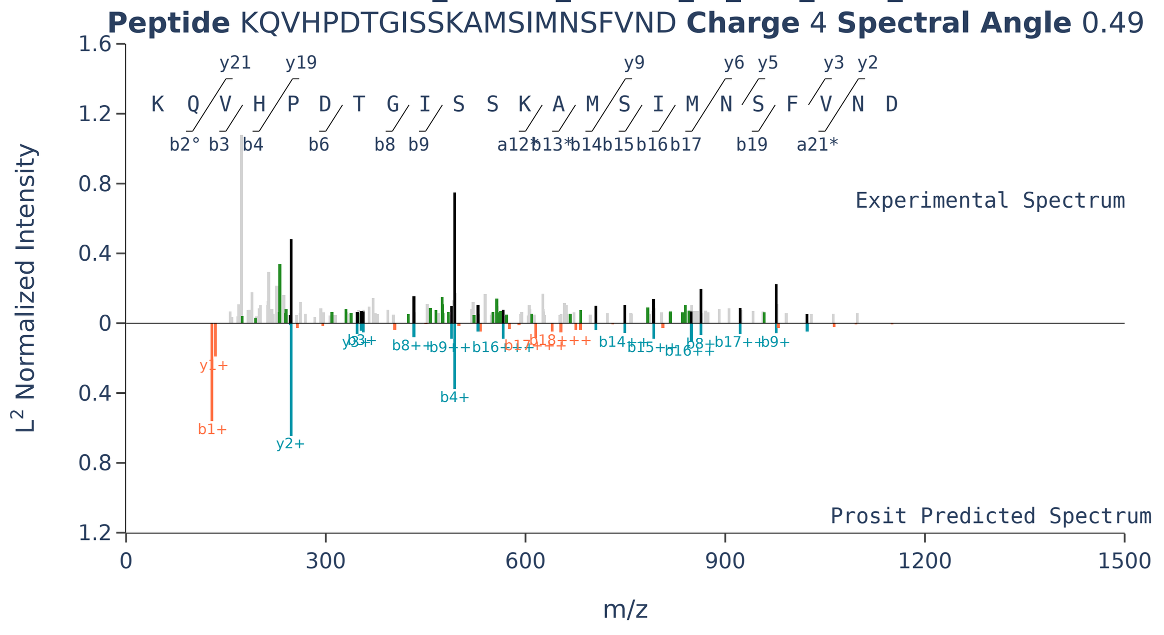


**Source** HRoetschke_291222_080123_Fu_H2B_b2R1_24h **Scan** 19724
**Peptide** LMNSFVNDVFE **Charge** 2 **Spectral Angle** 0.73

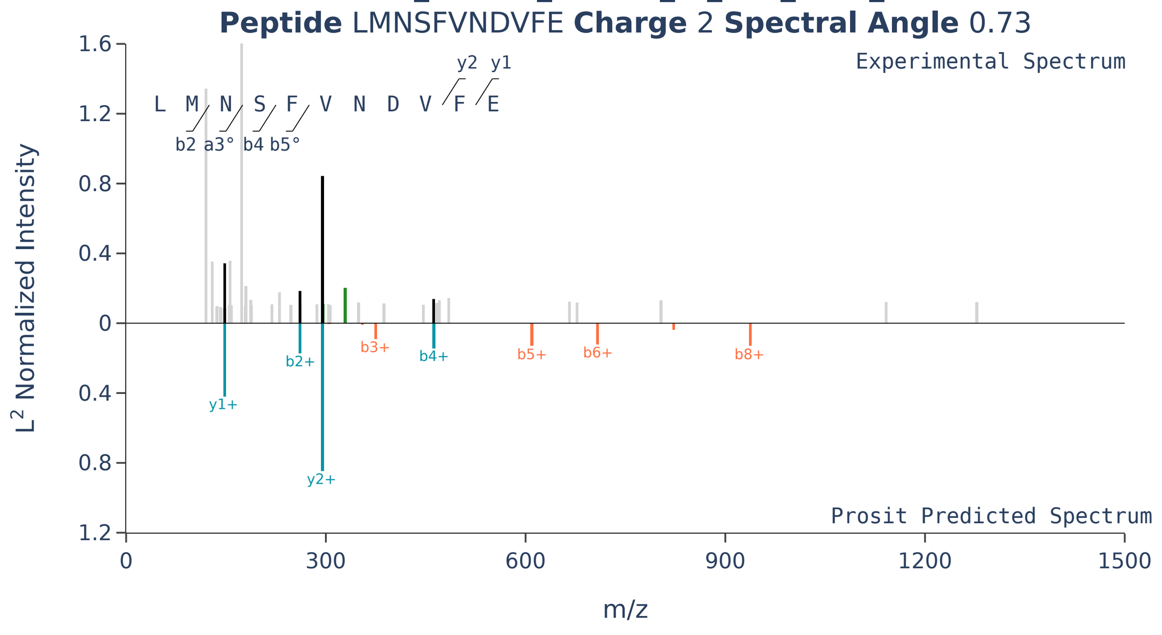


**Source** HRoetschke_291222_080123_Fu_H2B_b2R1_4h **Scan** 18714
**Peptide** NDVFERIAGEASRLAHYNK **Charge** 4 **Spectral Angle** 0.66

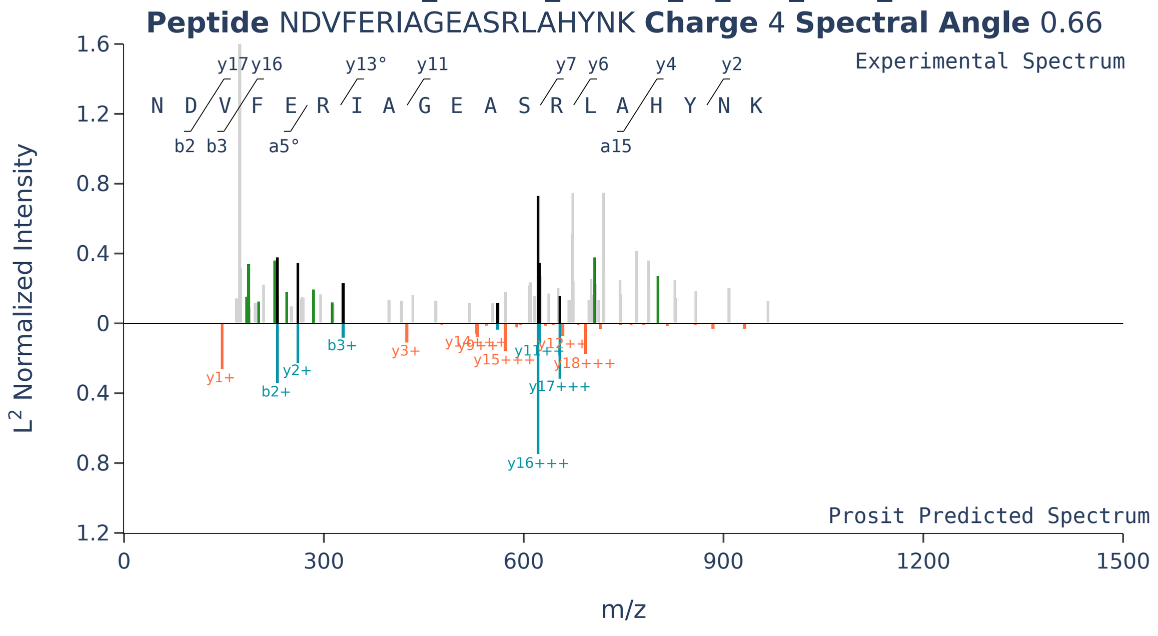


**Source** HRoetschke_291222_080123_Fu_H2B_b1R1_24h **Scan** 7209
**Peptide** YKVLKQVHPDTGISSKA **Charge** 4 **Spectral Angle** 0.7

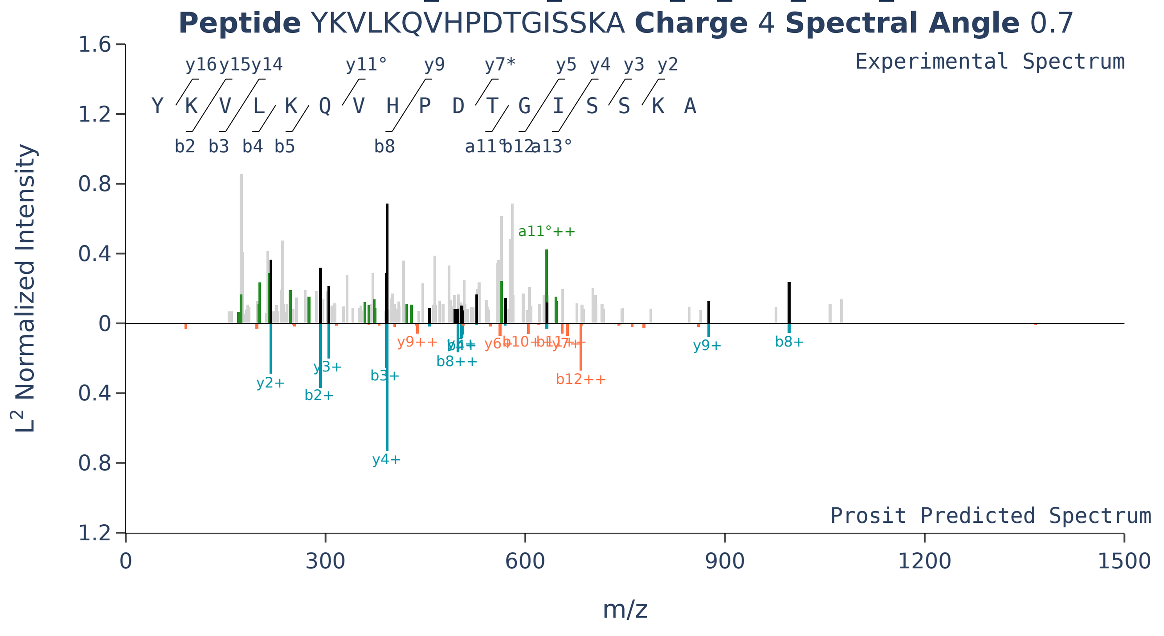


**Source** HRoetschke_291222_080123_Fu_H2B_b2R1_24h **Scan** 19416
**Peptide** SREIQTAVRLLLPGELAKHAV **Charge** 4 **Spectral Angle** 0.7

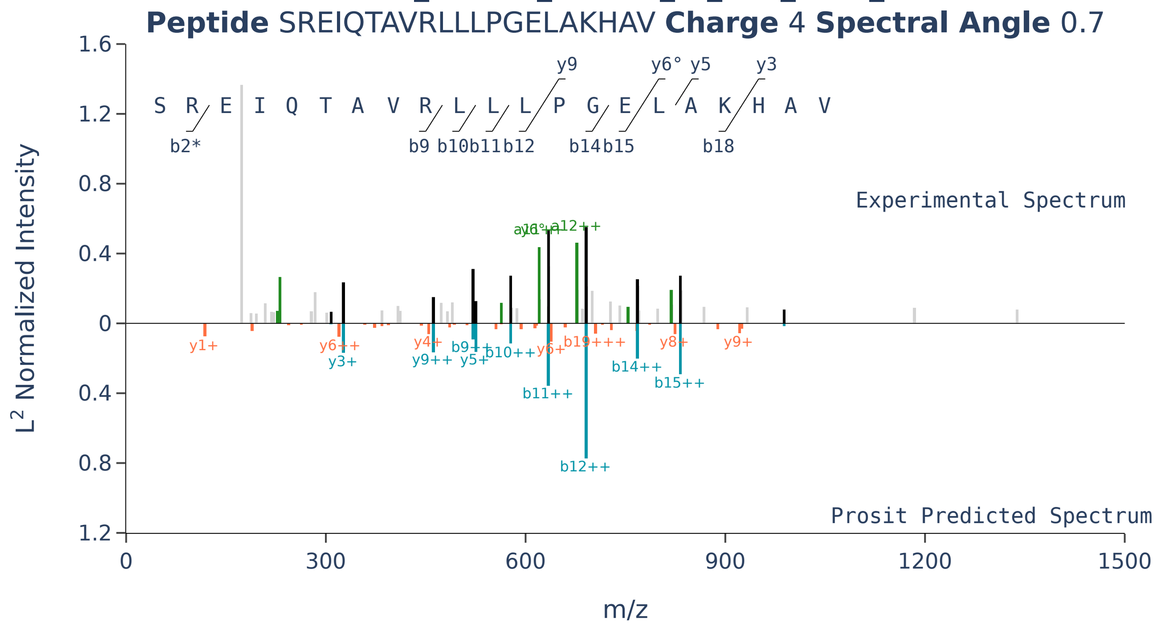


**Source** HRoetschke_291222_080123_Fu_H2B_b2R2_24h **Scan** 20460
**Peptide** NKRSTITSREIQTAVRLLLPGELAKH **Charge** 5 **Spectral Angle** 0.57

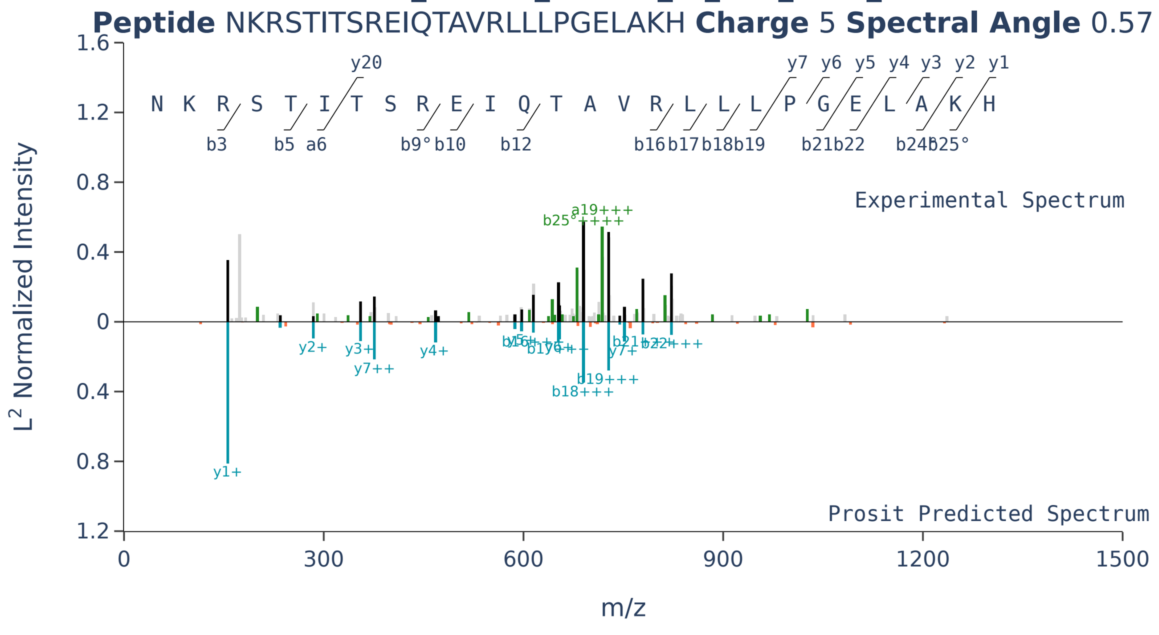


**Source** HRoetschke_291222_080123_Fu_H2B_b2R1_24h **Scan** 16330
**Peptide** TAVRLLLPGELAKHAVSEGTKA **Charge** 4 **Spectral Angle** 0.67

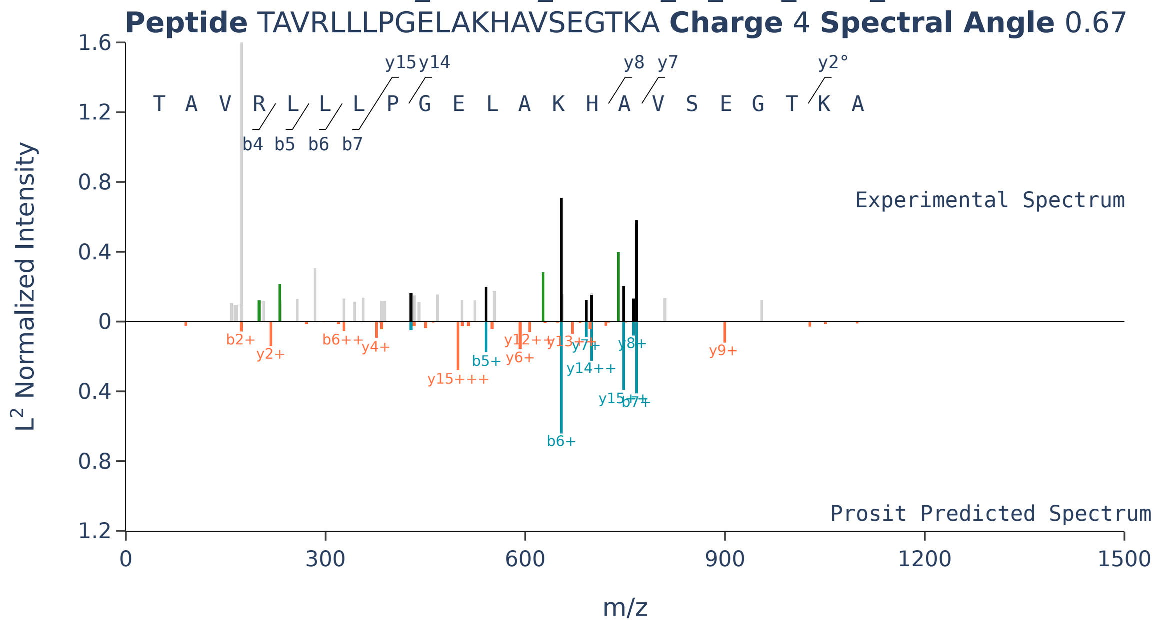


**Source** HRoetschke_291222_080123_Fu_H2B_b2R1_1h **Scan** 19916
**Peptide** NSFVNDVFERIAGEASRLAHYNKRS **Charge** 5 **Spectral Angle** 0.79

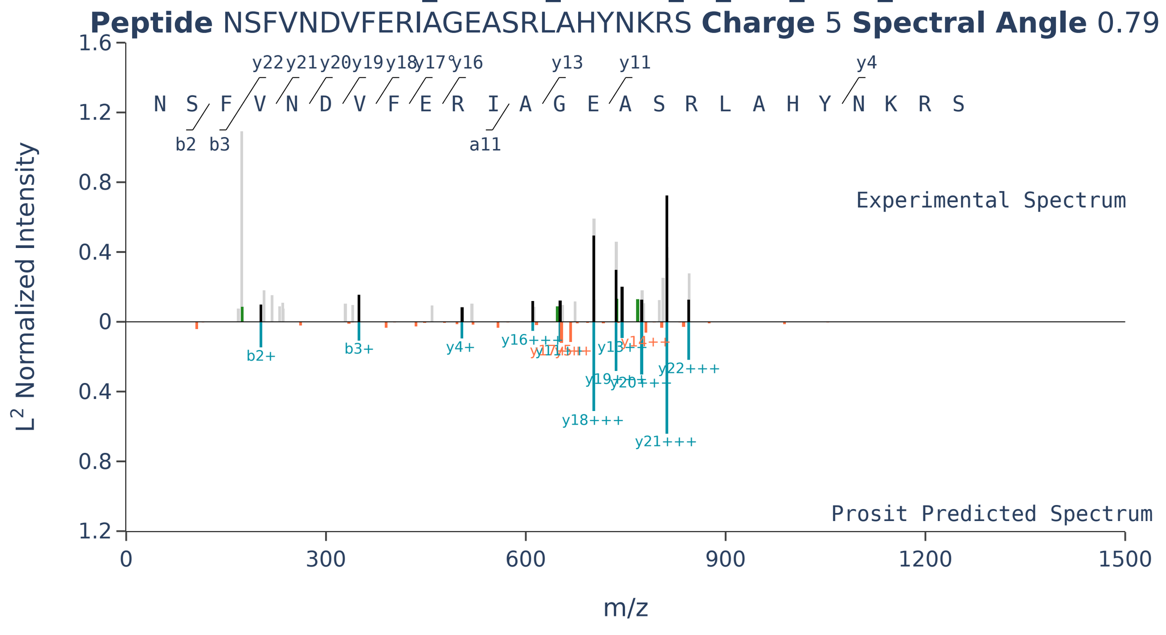


**Source** HRoetschke_291222_080123_Fu_H2B_b1R1_24h **Scan** 5539
**Peptide** AGEASRLAHYNKRSTI **Charge** 5 **Spectral Angle** 0.73

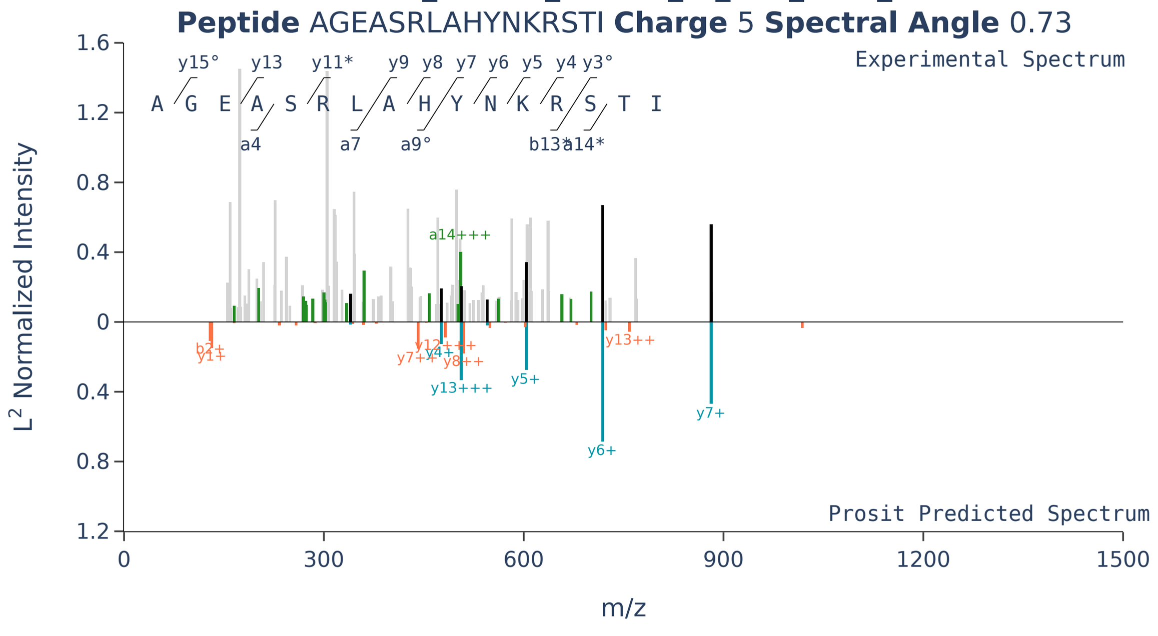


**Source** HRoetschke_291222_080123_Fu_H2B_b2R2_24h **Scan** 14783
**Peptide** LLPGELAKHAVSEGTKAV **Charge** 3 **Spectral Angle** 0.54

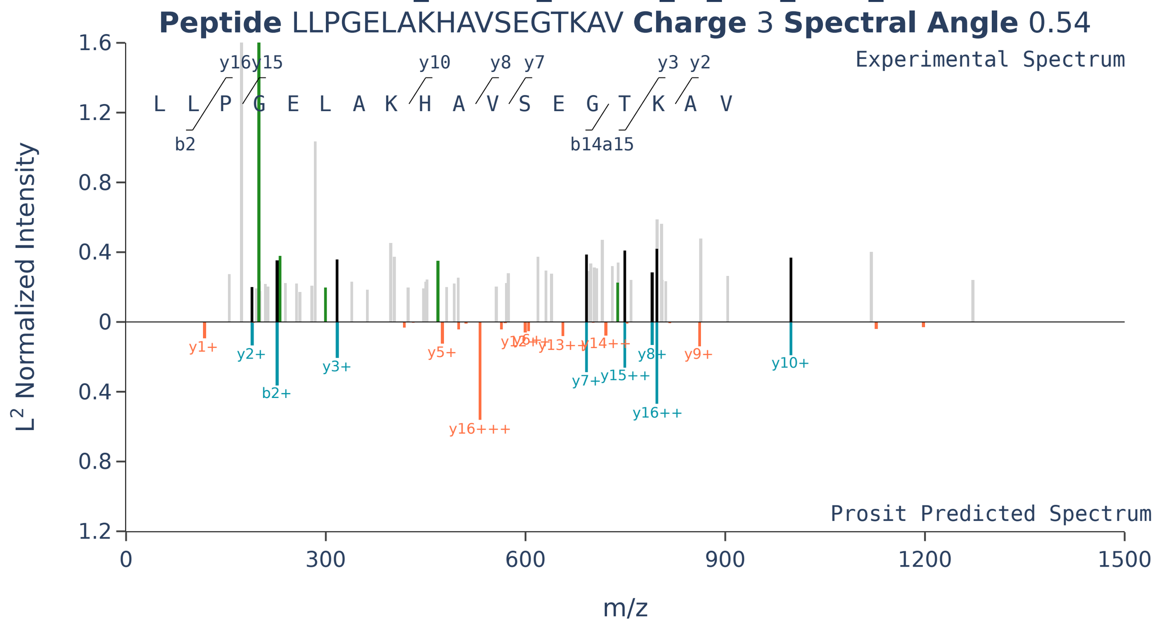


**Source** HRoetschke_291222_080123_Fu_H2B_b2R2_24h **Scan** 17545
**Peptide** LLLPGELAKHAVSEGTKAV **Charge** 4 **Spectral Angle** 0.72

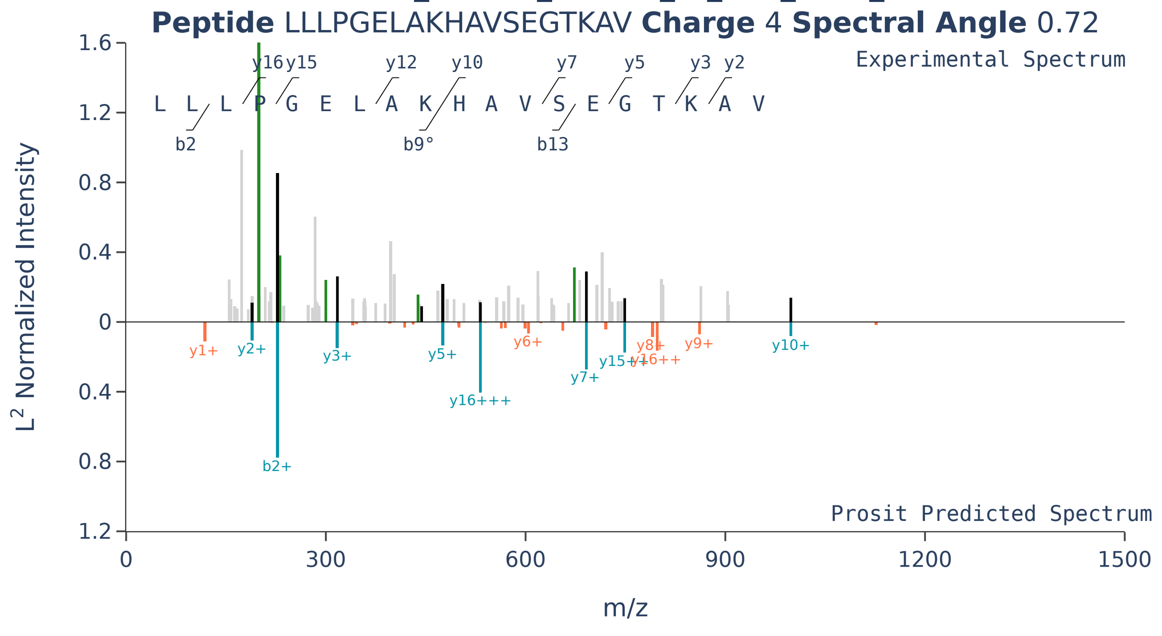

**Source** HRoetschke_291222_080123_Fu_H2B_b1R2_4h **Scan** 8554
**Peptide** ERIAGEASRLAHYNKRSTITSREIQTA **Charge** 5 **Spectral Angle** 0.55

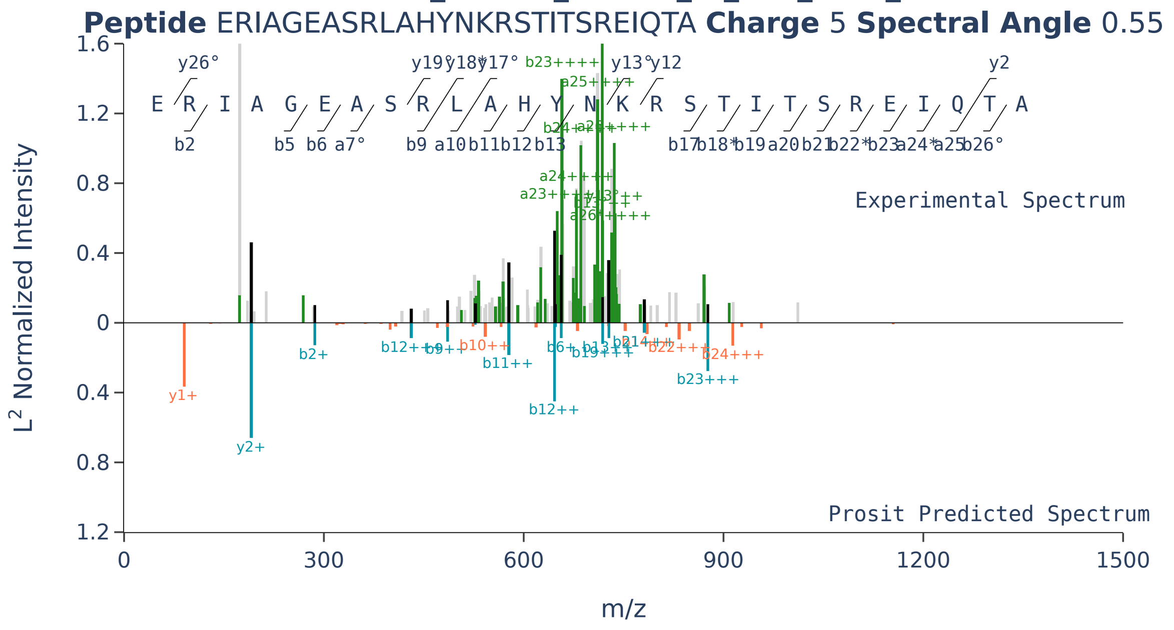


**Source** HRoetschke_291222_080123_Fu_H2B_b2R1_24h **Scan** 6762
**Peptide** IAGEASRLAHYNKRSTIT **Charge** 4 **Spectral Angle** 0.41

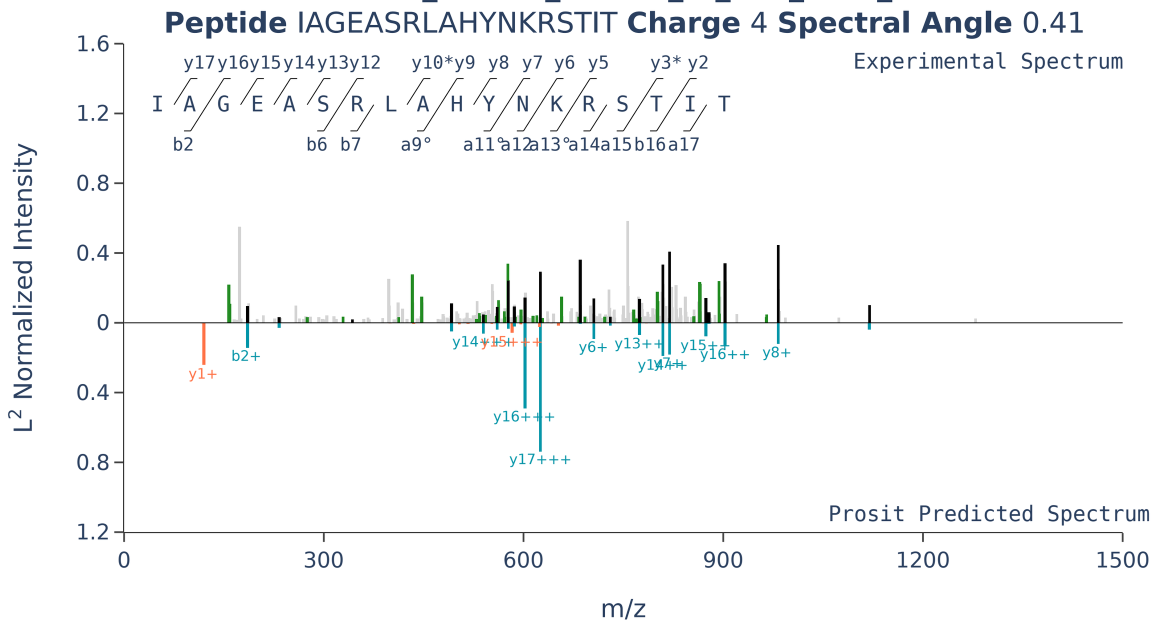


**Source** HRoetschke_291222_080123_Fu_H2B_b2R2_2h **Scan** 6338
**Peptide** QVHPDTGISSKAMSIMN **Charge** 3 **Spectral Angle** 0.58

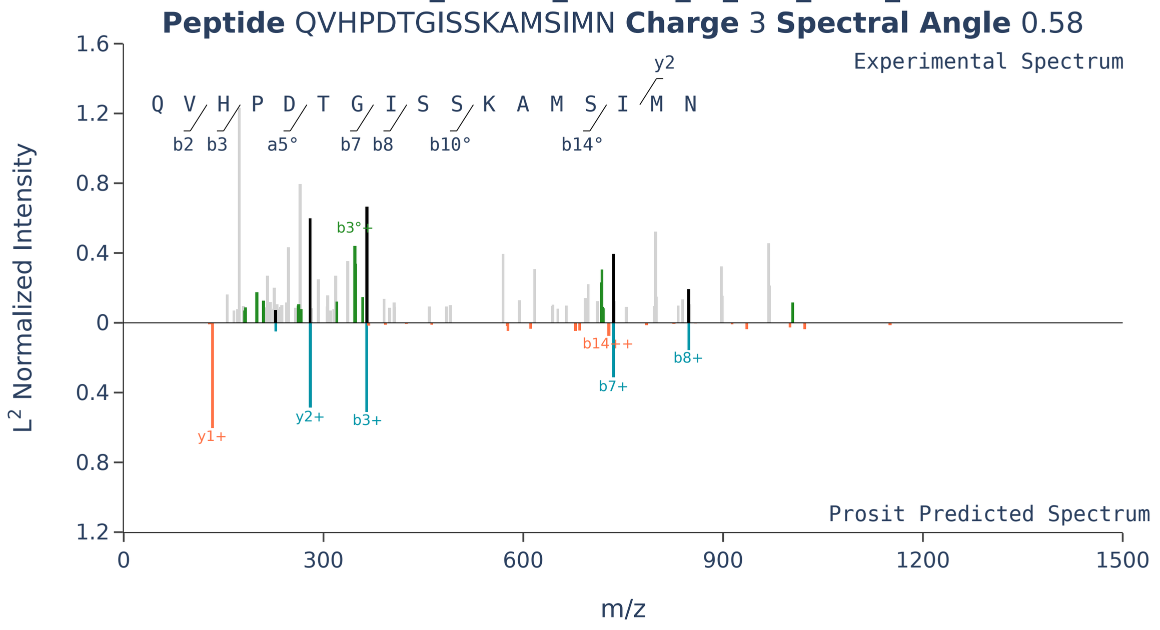


**Source** HRoetschke_291222_080123_Fu_H2B_b2R1_1h **Scan** 20009
**Peptide** SFVNDVFERIAGEASRLAHYNKRST **Charge** 4 **Spectral Angle** 0.83

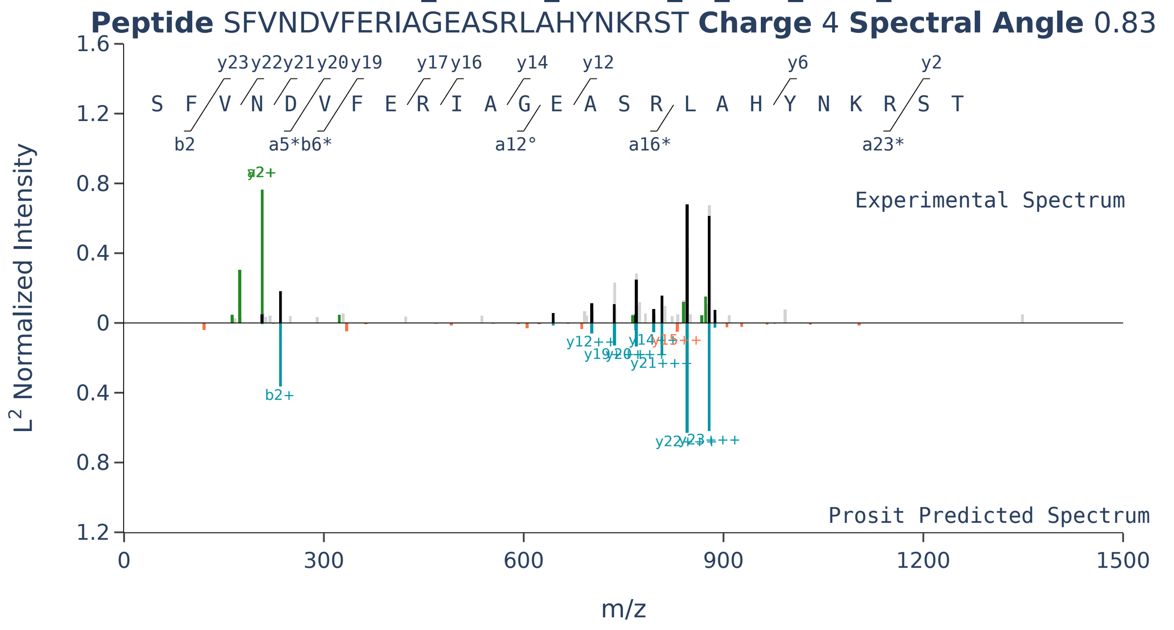


**Source** HRoetschke_291222_080123_Fu_H2B_b2R1_24h **Scan** 8697
**Peptide** ERIAGEASRLAHYNKRSTITSREIQ **Charge** 5 **Spectral Angle** 0.4

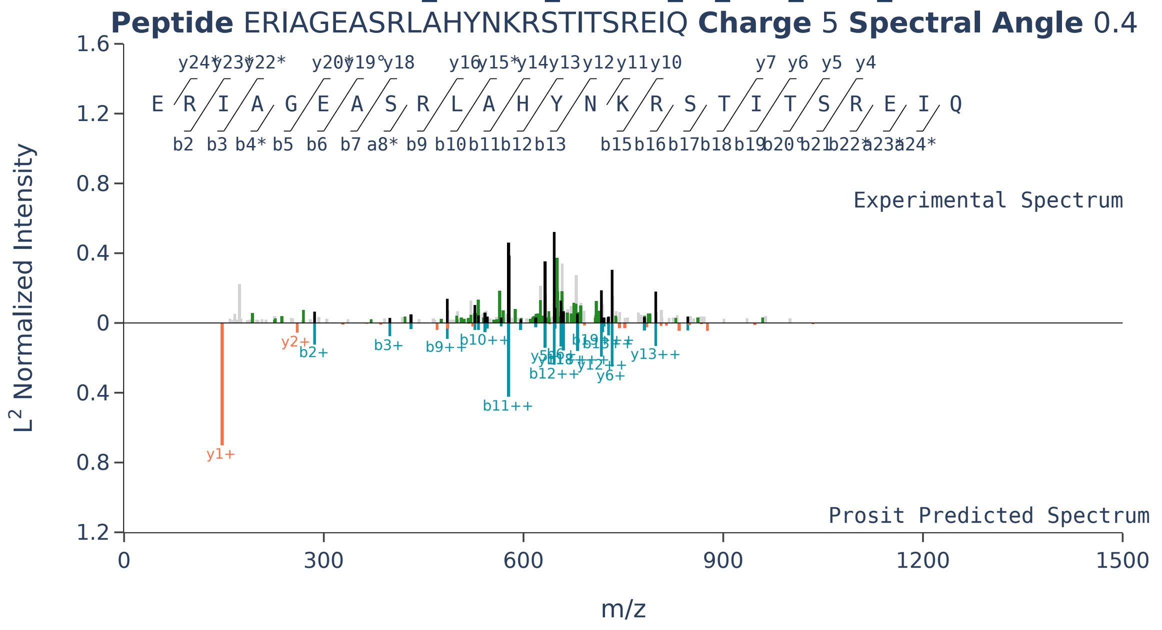


**Source** HRoetschke_291222_080123_Fu_H2B_b2R1_24h **Scan** 14310
**Peptide** AIYVYKVLKQVH **Charge** 3 **Spectral Angle** 0.61

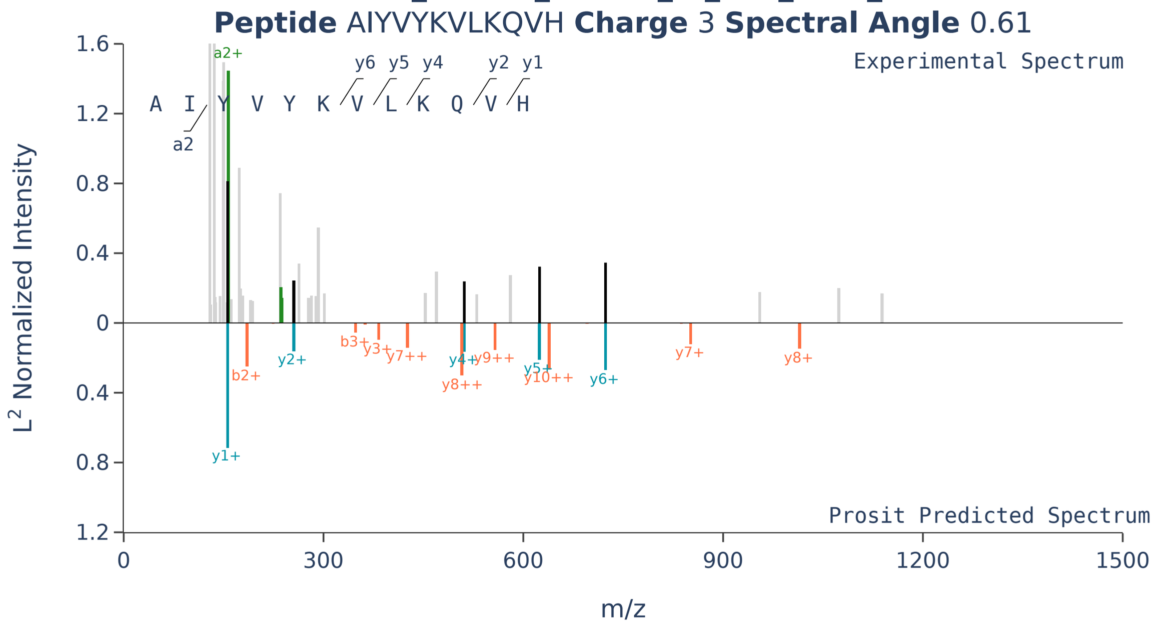


**Source** HRoetschke_291222_080123_Fu_H2B_b1R1_24h **Scan** 6613
**Peptide** RLAGEASRLAHYNKRSTITS **Charge** 5 **Spectral Angle** 0.42

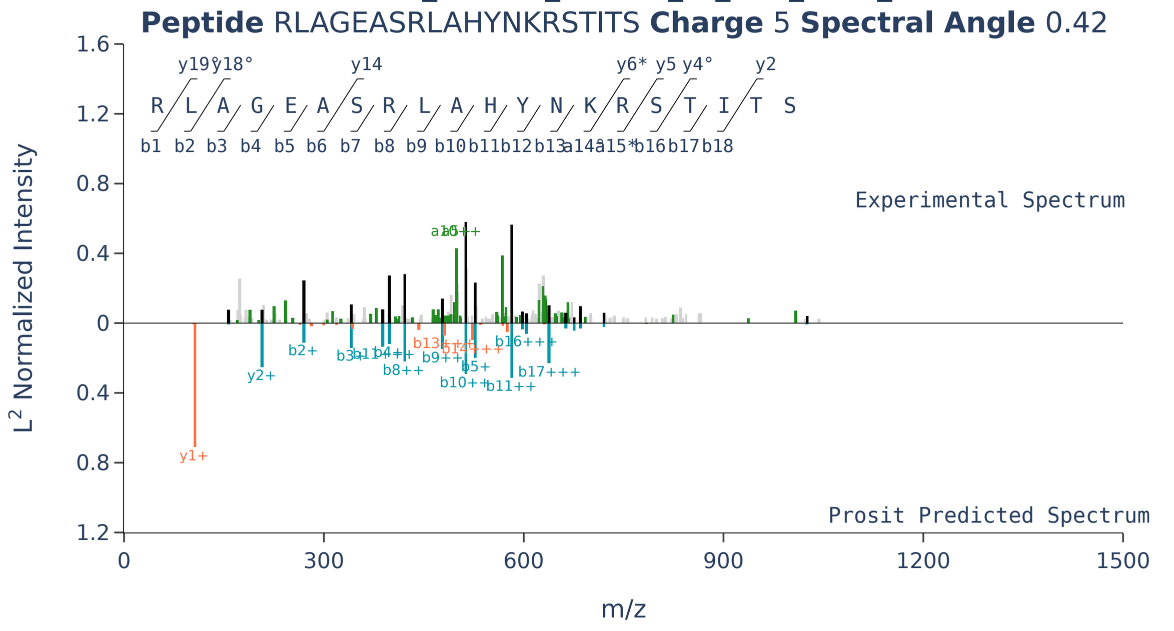


**Source** HRoetschke_291222_080123_Fu_H2B_b2R2_24h **Scan** 4218
**Peptide** AVTKTQKKDGKKRRKTRKESYAIYVY **Charge** 5 **Spectral Angle** 0.7

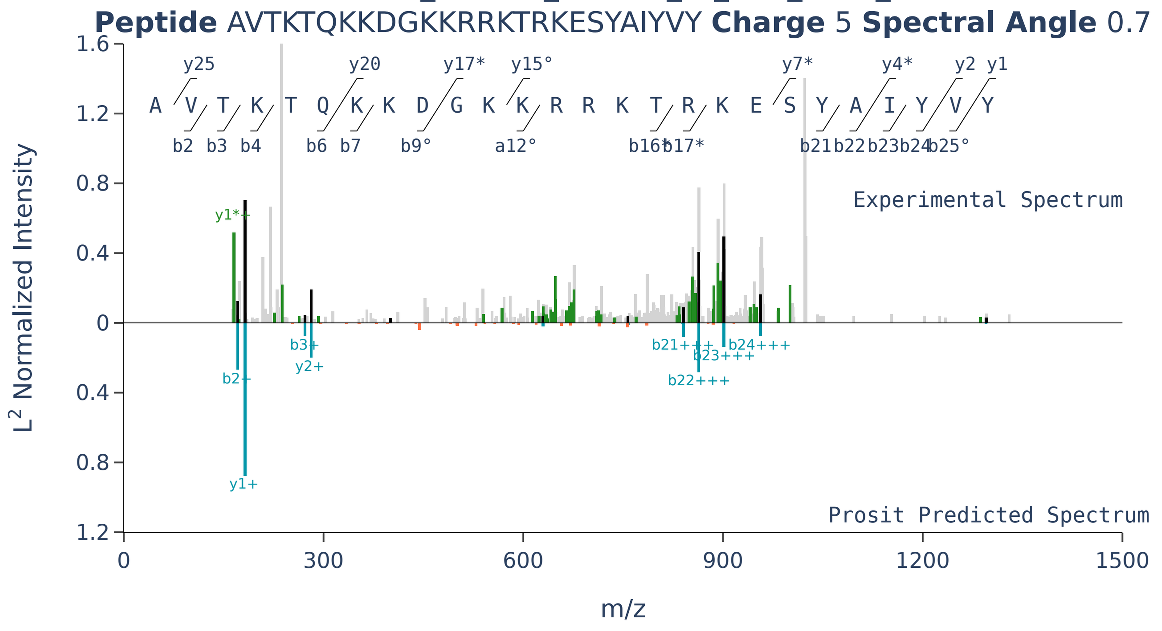


**Source** HRoetschke_291222_080123_Fu_H2B_b2R2_4h **Scan** 6563
**Peptide** RIAGEASRLAHY **Charge** 4 **Spectral Angle** 0.7

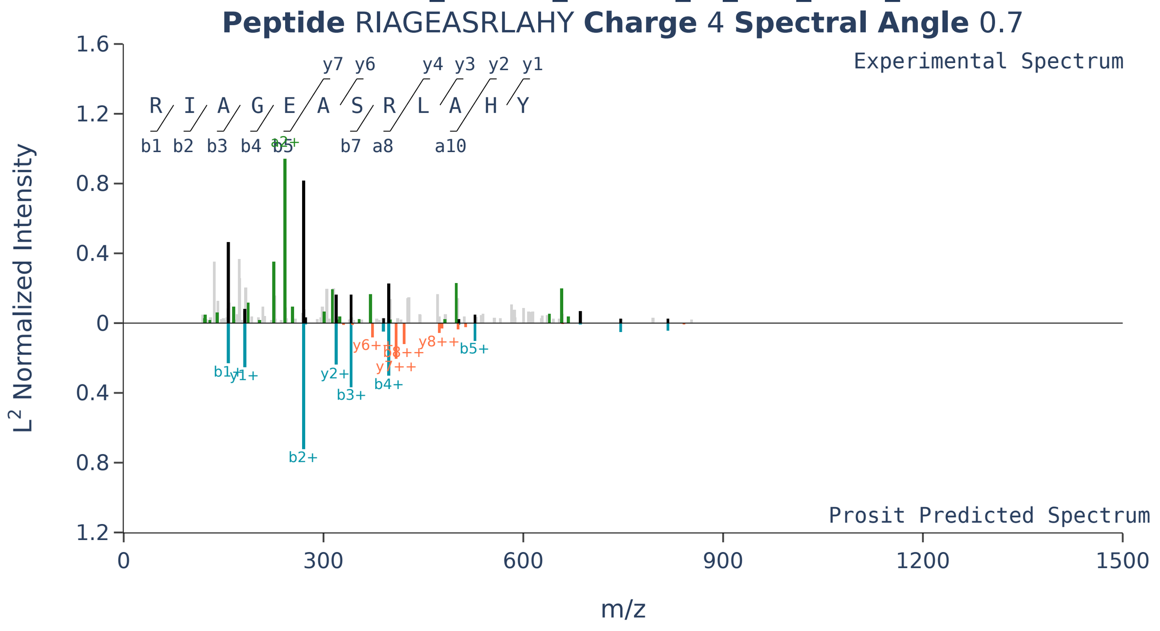


**Source** HRoetschke_291222_080123_Fu_H2B_b2R1_1h **Scan** 18493
**Peptide** VHPDTGISSKAMSIMNSFVNDVFERI **Charge** 4 **Spectral Angle** 0.76

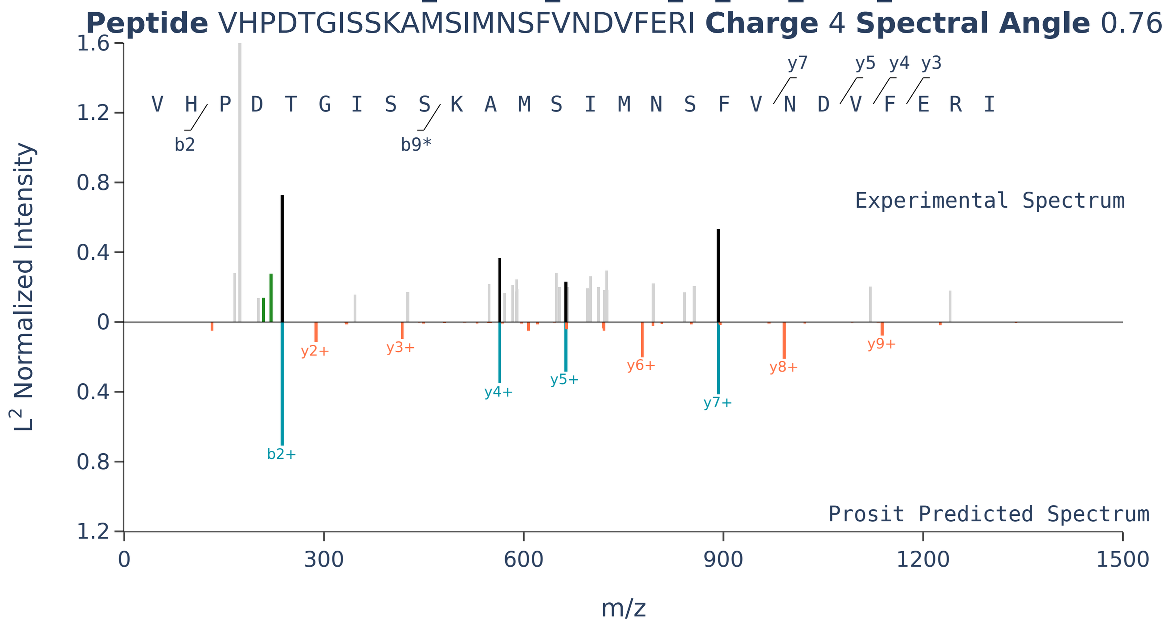


**Source** HRoetschke_291222_080123_Fu_H2B_b2R1_1h **Scan** 16617
**Peptide** KRSTITSREIQTAVRLLLPGELAKH **Charge** 5 **Spectral Angle** 0.45

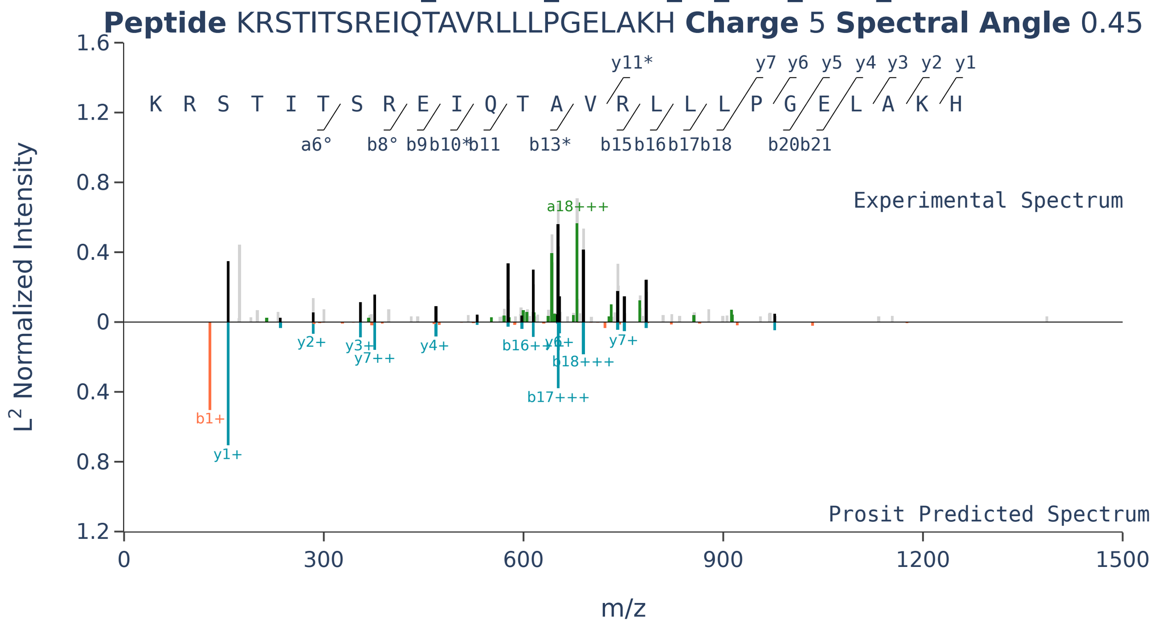


**Source** HRoetschke_291222_080123_Fu_H2B_b2R1_4h **Scan** 6740
**Peptide** ERIAGEASRLAHYNKRSTITSRE **Charge** 4 **Spectral Angle** 0.51

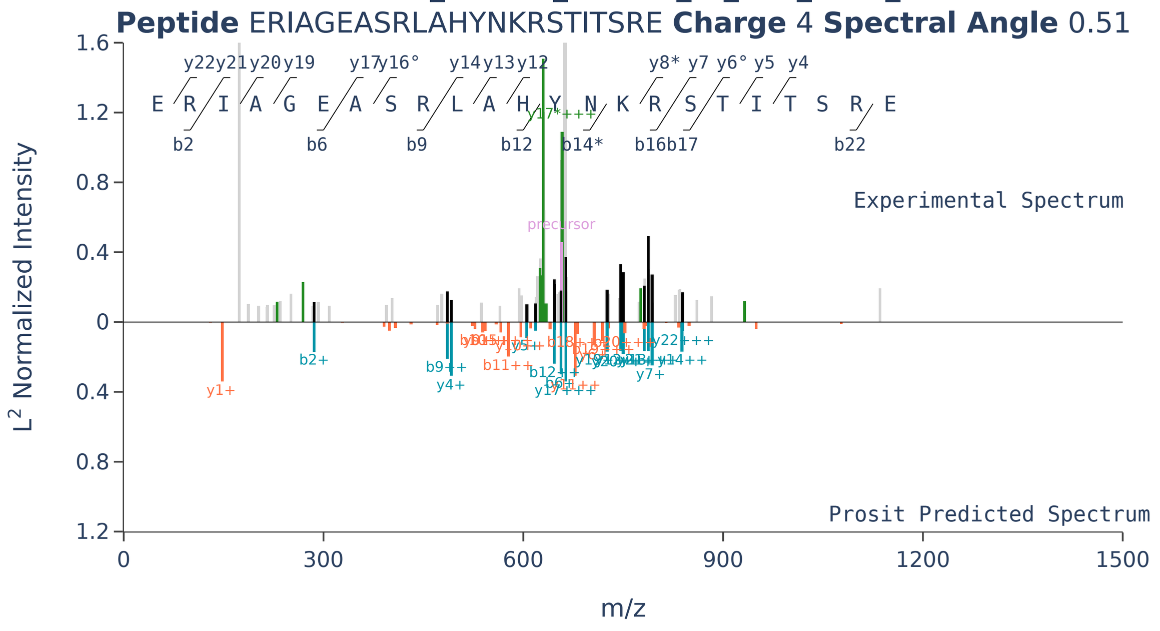


**Source** HRoetschke_291222_080123_Fu_H2B_b2R2_24h **Scan** 6428
**Peptide** RLAGEASRLAHYNKRSTIT **Charge** 4 **Spectral Angle** 0.59

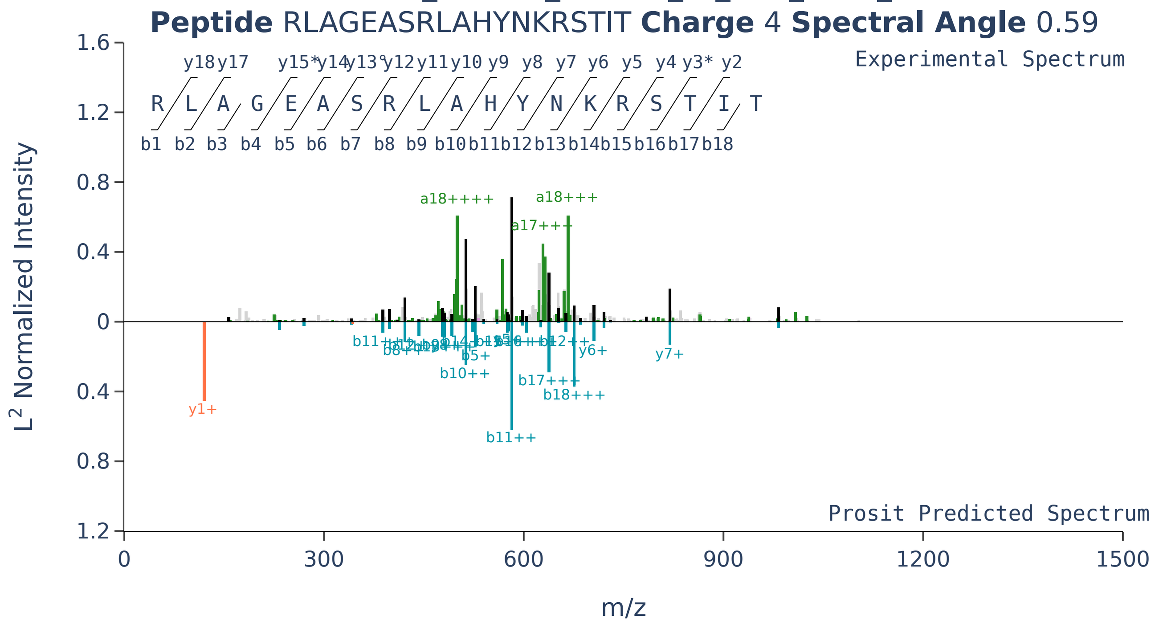


**Source** HRoetschke_291222_080123_Fu_H2B_b2R2_24h **Scan** 15399
**Peptide** SRLAHYNKRSTITSREIQTAVRLL **Charge** 5 **Spectral Angle** 0.53

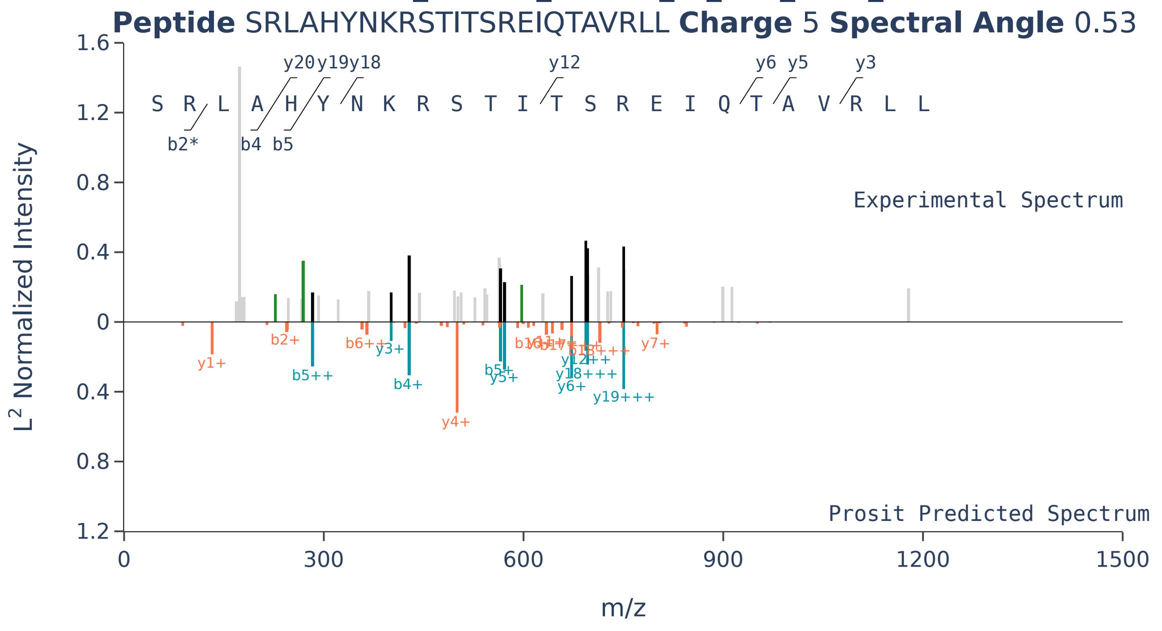


**Source** HRoetschke_291222_080123_Fu_H2B_b2R2_4h **Scan** 19736
**Peptide** KRSTITSREIQTAVRLLLPGELAKHA **Charge** 5 **Spectral Angle** 0.53

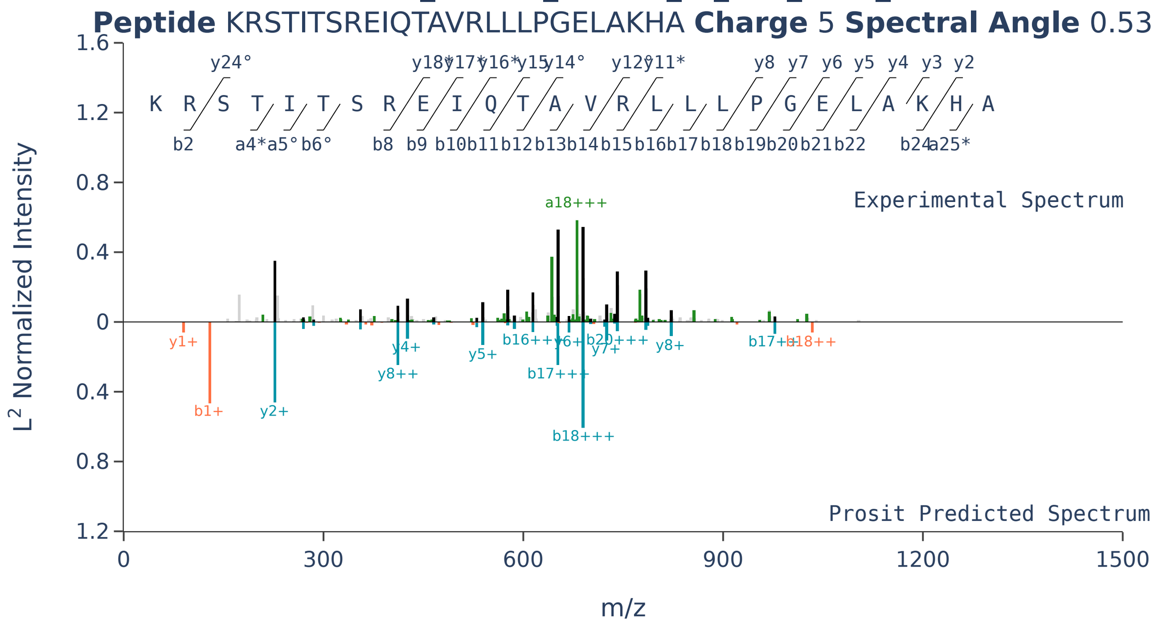

**Source** HRoetschke_291222_080123_Fu_H2B_b2R2_4h **Scan** 19967
**Peptide** SYAIYVYKVLKQVHPDTGISSKAMSL **Charge** 4 **Spectral Angle** 0.79

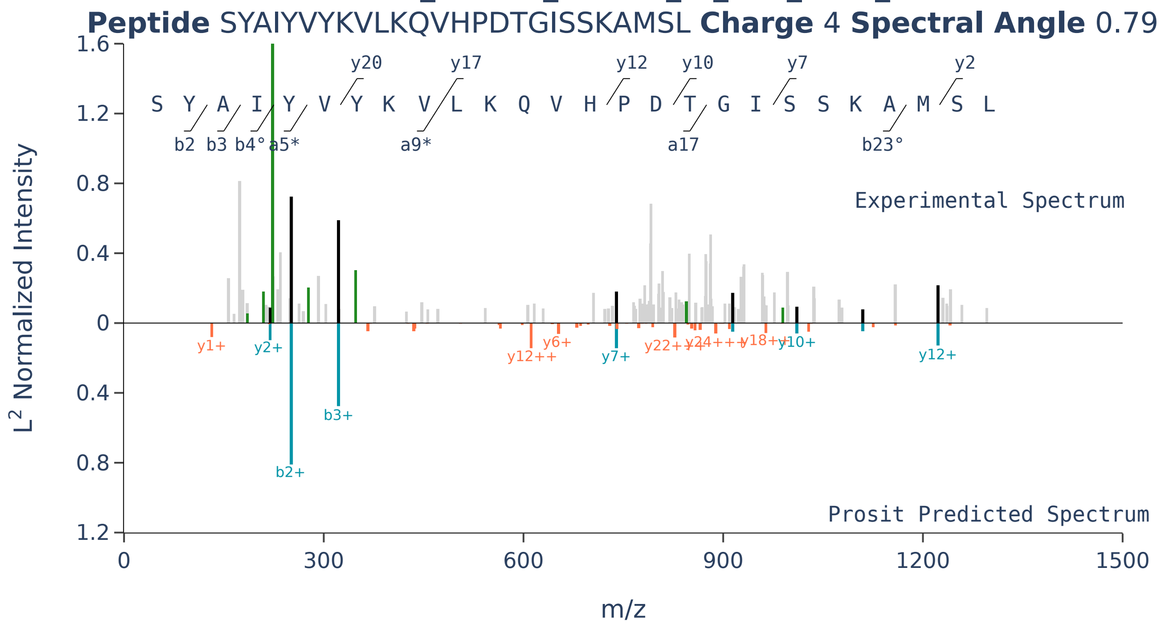


**Source** HRoetschke_291222_080123_Fu_H2B_b2R1_2h **Scan** 7100
**Peptide** RLAGEASRLAHYNKRSTITSREIQT **Charge** 6 **Spectral Angle** 0.36

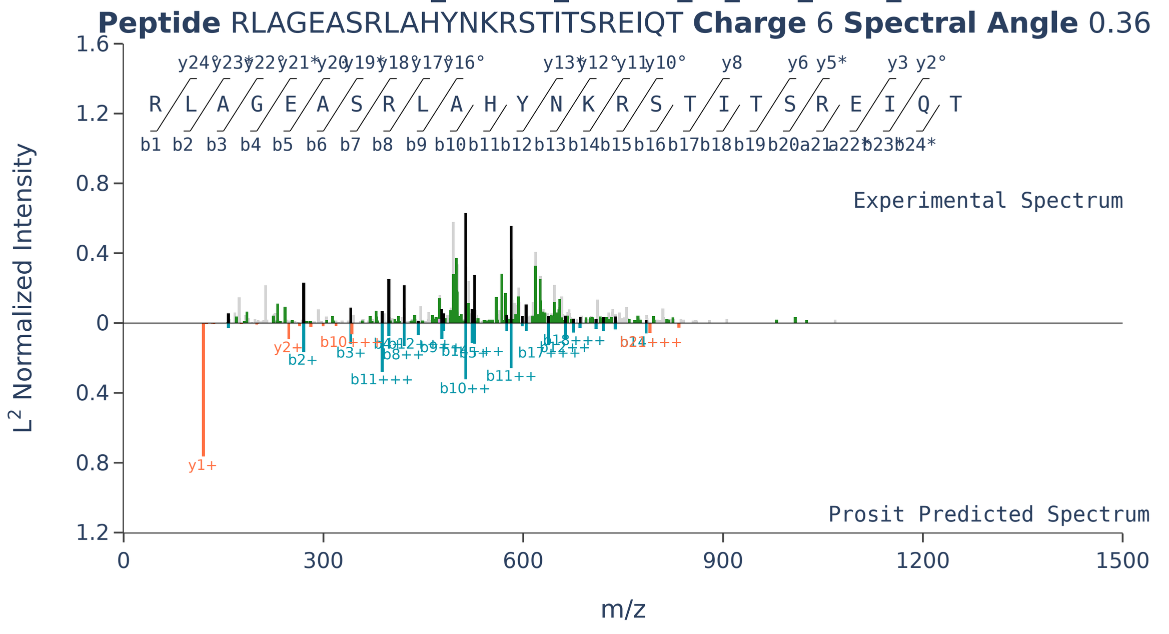


**Source** HRoetschke_291222_080123_Fu_H2B_b2R1_1h **Scan** 19923
**Peptide** KAMSIMNSFVNDVFERIAGEASRLAHYNKR **Charge** 5 **Spectral Angle** 0.6

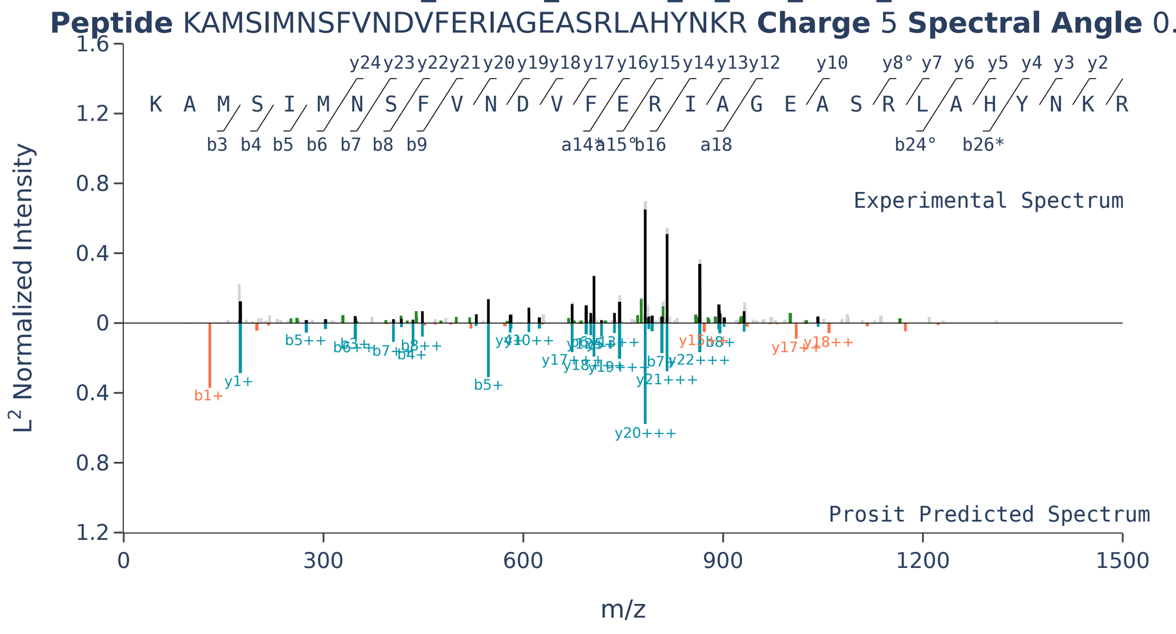


**Source** HRoetschke_291222_080123_Fu_H2B_b2R1_24h **Scan** 11065
**Peptide** FERIAGEASRLAHYN **Charge** 3 **Spectral Angle** 0.62

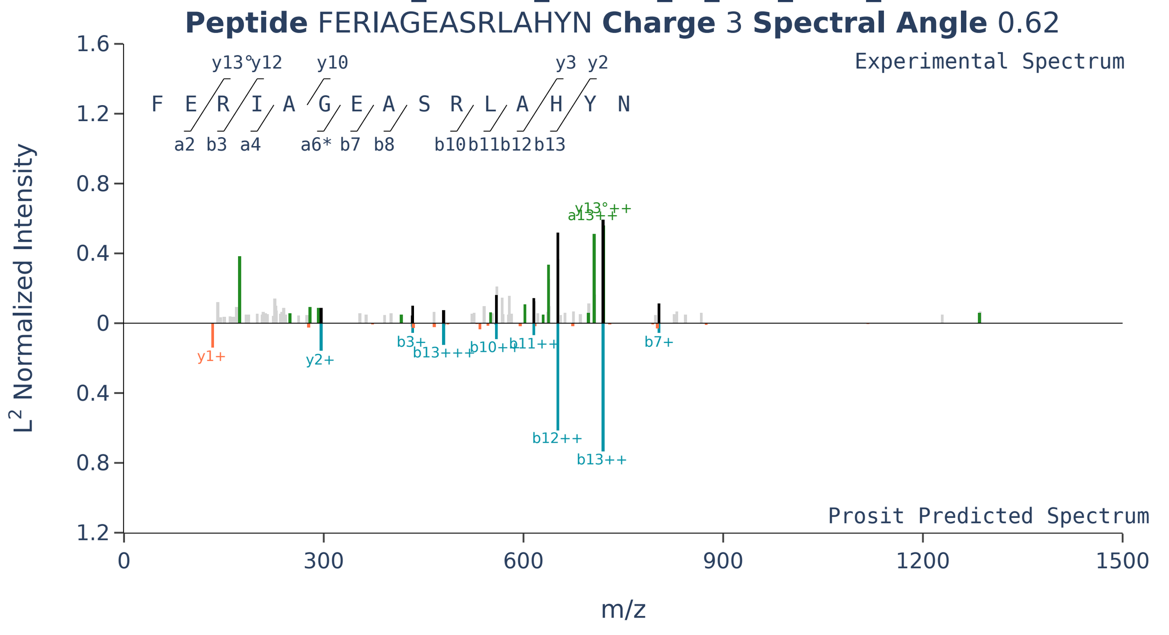


**Source** HRoetschke_291222_080123_Fu_H2B_b2R2_4h **Scan** 16618
**Peptide** REIQTAVRLLLPGELAKHAVSEGTKAVTK **Charge** 6 **Spectral Angle** 0.62

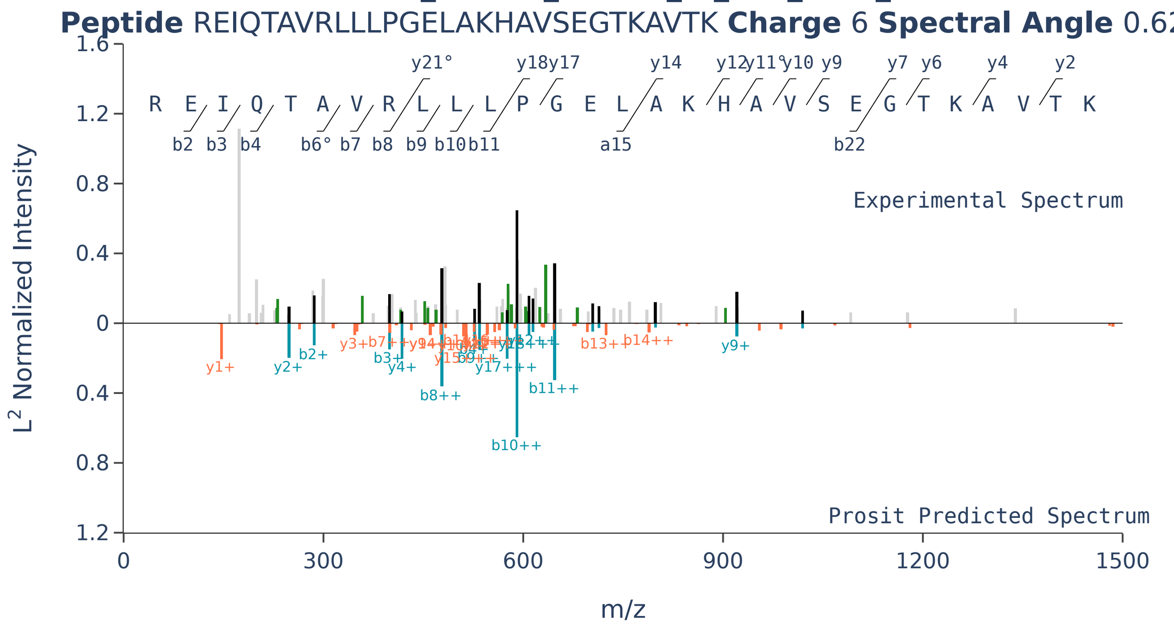


**Source** HRoetschke_291222_080123_Fu_H2B_b2R1_4h **Scan** 13764
**Peptide** GKKRKKTRKESYAIYVYKVLKQV **Charge** 5 **Spectral Angle** 0.71

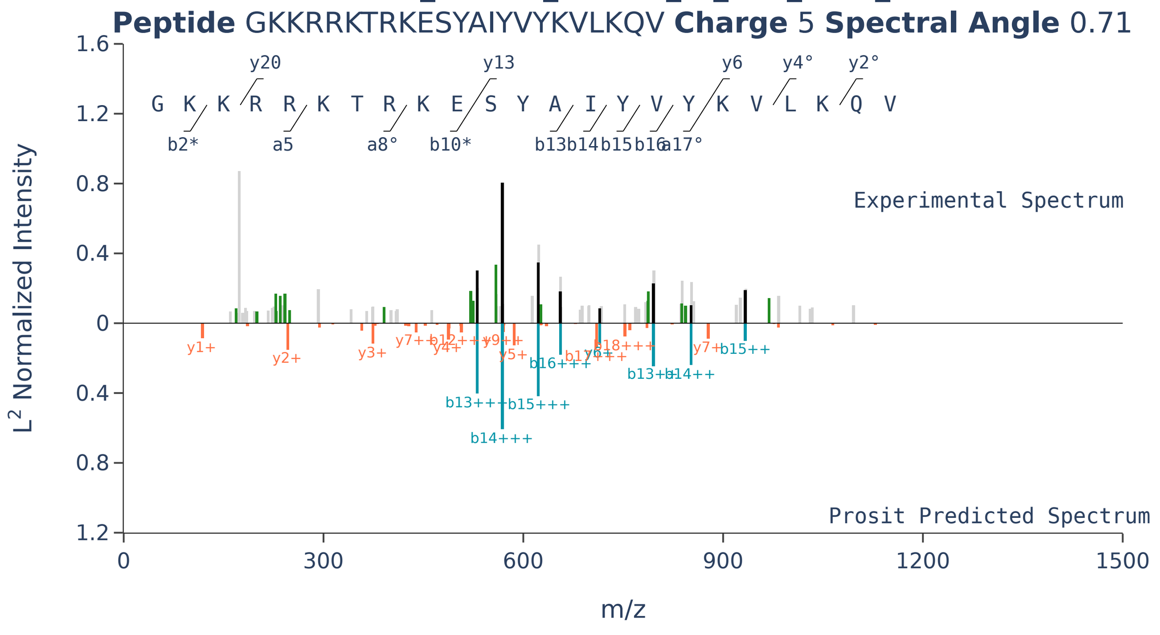


**Source** HRoetschke_291222_080123_Fu_H2B_b1R1_1h **Scan** 5363
**Peptide** RIAGEASRLAHYNKRSTITS **Charge** 5 **Spectral Angle** 0.4

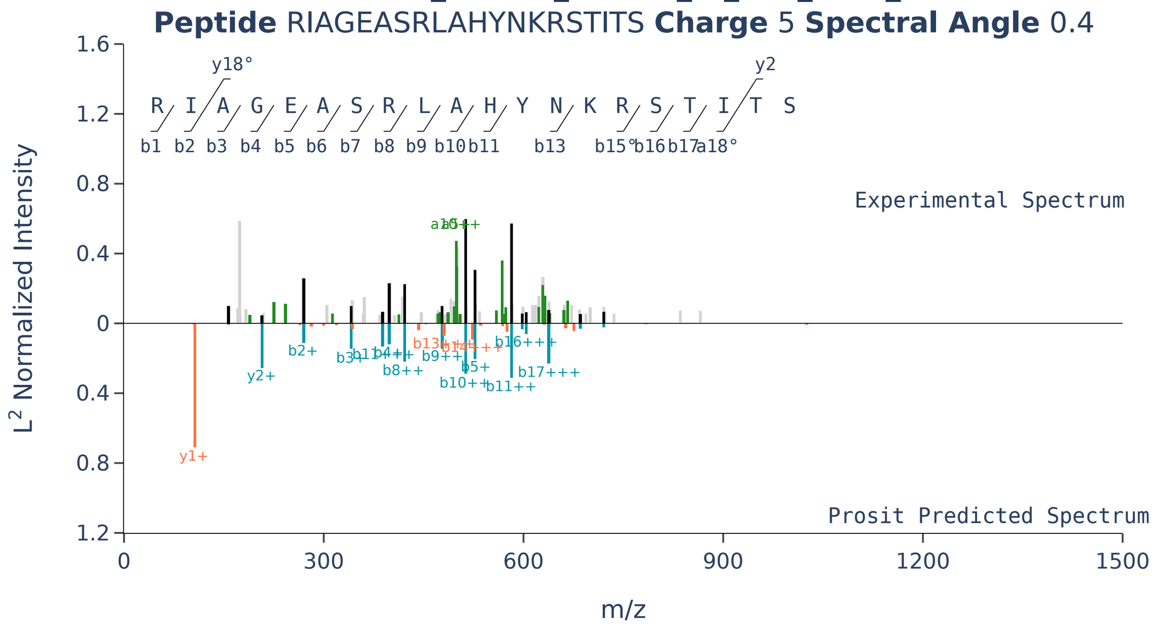


**Source** HRoetschke_291222_080123_Fu_H2B_b2R1_4h **Scan** 5828
**Peptide** QVHPDTGL **Charge** 2 **Spectral Angle** 0.74

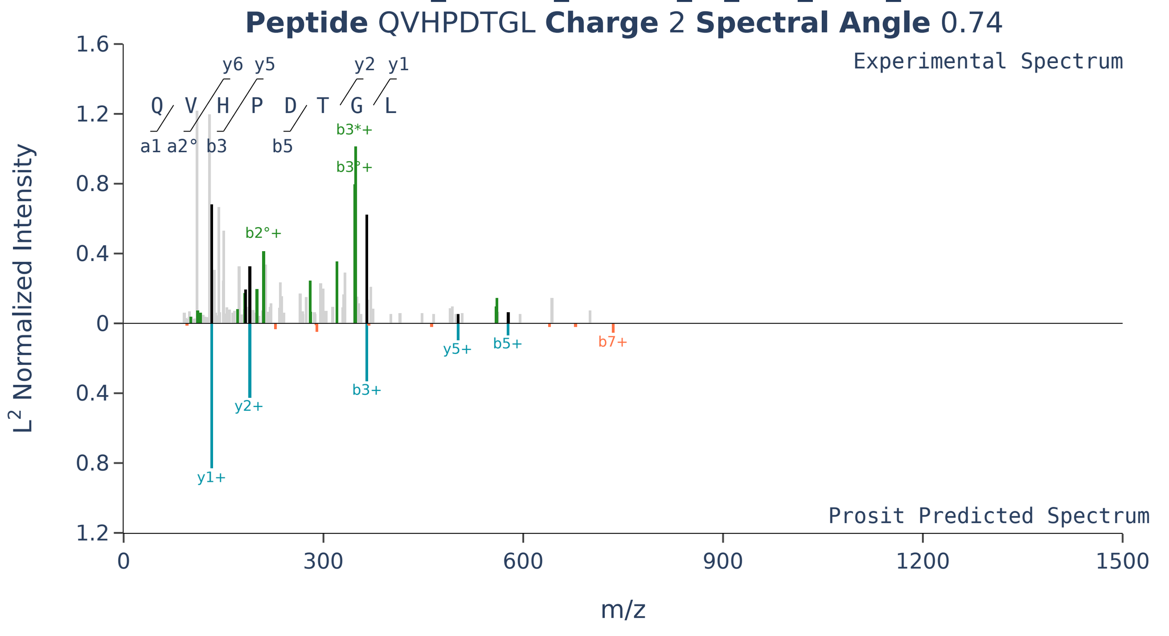


**Source** HRoetschke_291222_080123_Fu_H2B_b1R1_24h **Scan** 6698
**Peptide** RIAGEASRLAHYNKRSTIT **Charge** 4 **Spectral Angle** 0.56

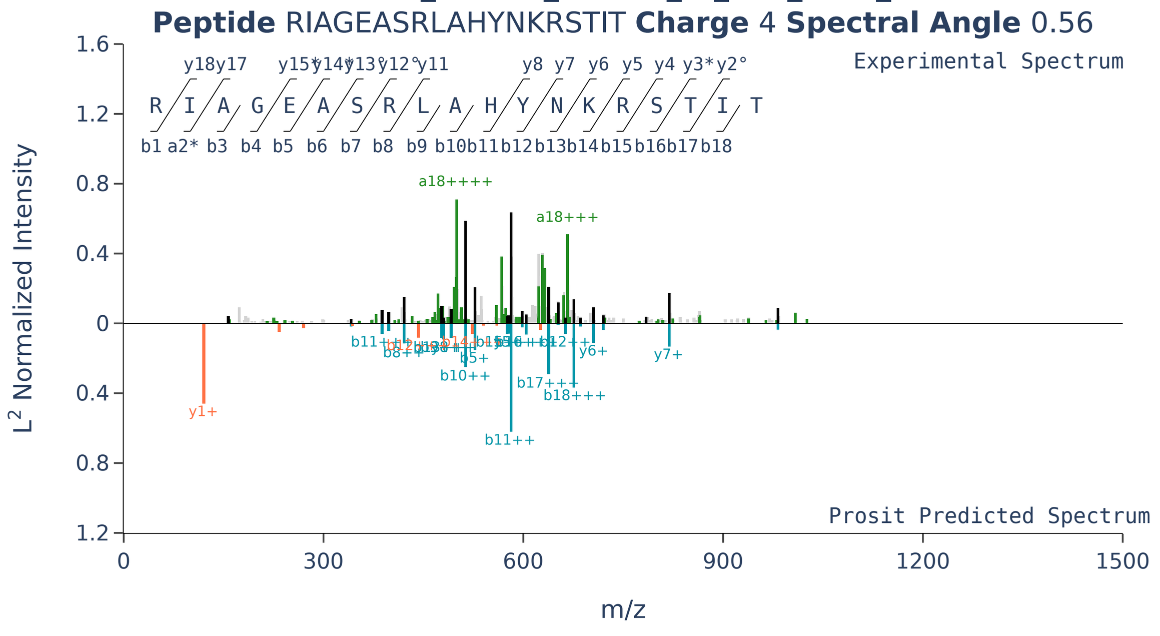


**Source** HRoetschke_291222_080123_Fu_H2B_b2R1_24h **Scan** 10877
**Peptide** LKQVHPDTGISSKAMSIMNS **Charge** 4 **Spectral Angle** 0.53

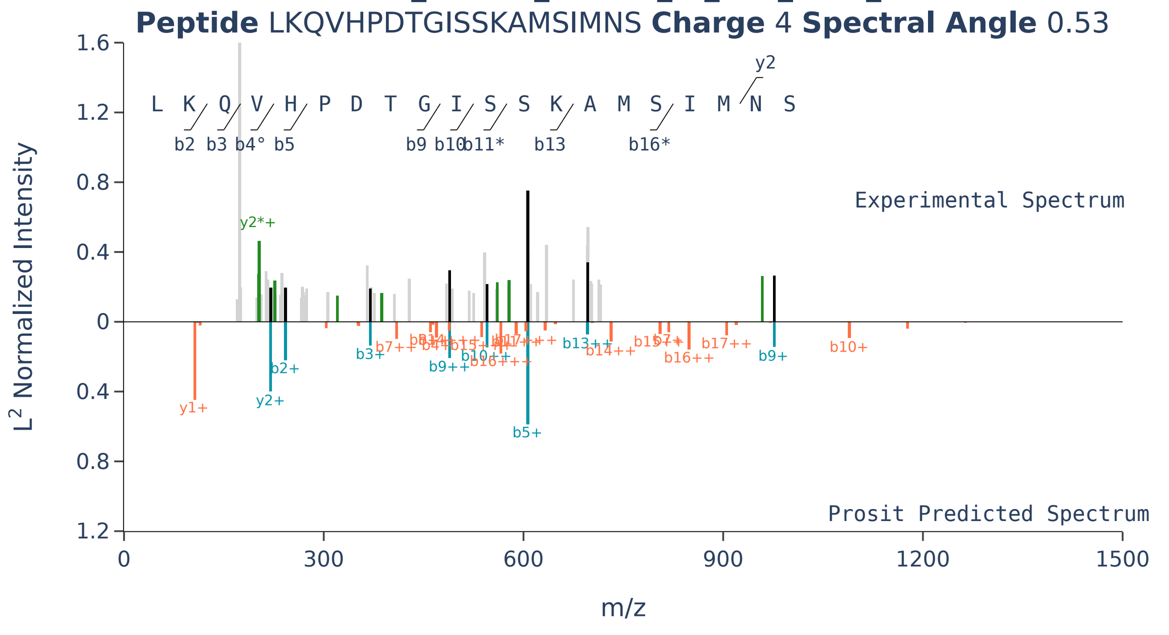


**Source** HRoetschke_291222_080123_Fu_H2B_b1R1_24h **Scan** 9632
**Peptide** KQVHPDTGISSKAMSIMNS **Charge** 3 **Spectral Angle** 0.45

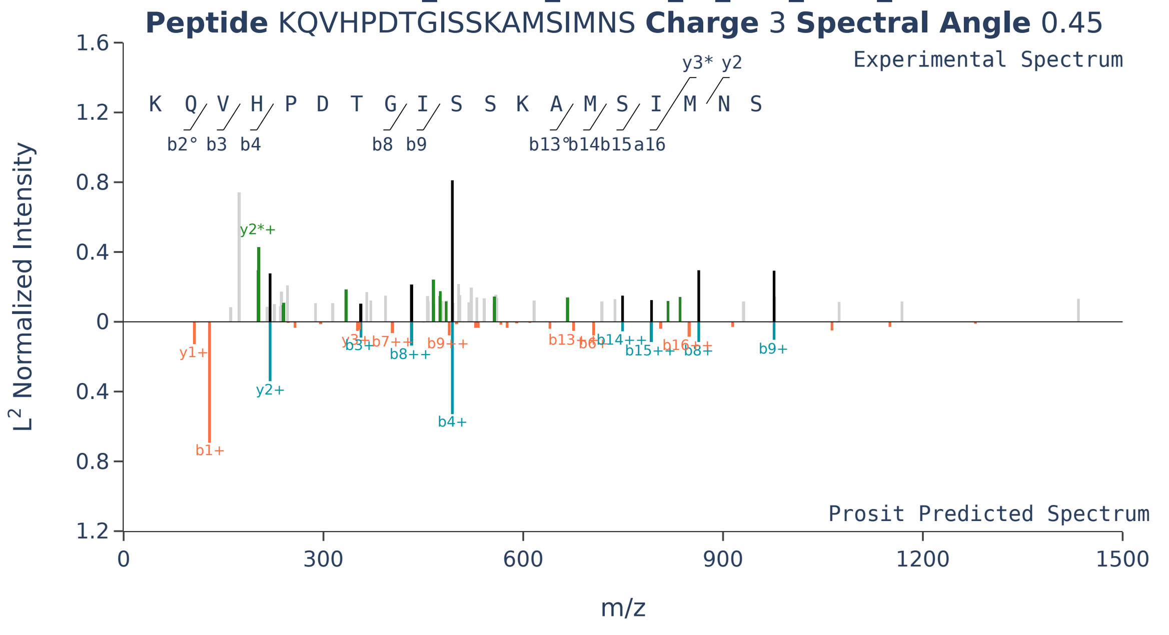


**Source** HRoetschke_291222_080123_Fu_H2B_b2R2_1h **Scan** 18461
**Peptide** STITSREIQTAVRLLLPGELAKHAVSE **Charge** 4 **Spectral Angle** 0.66

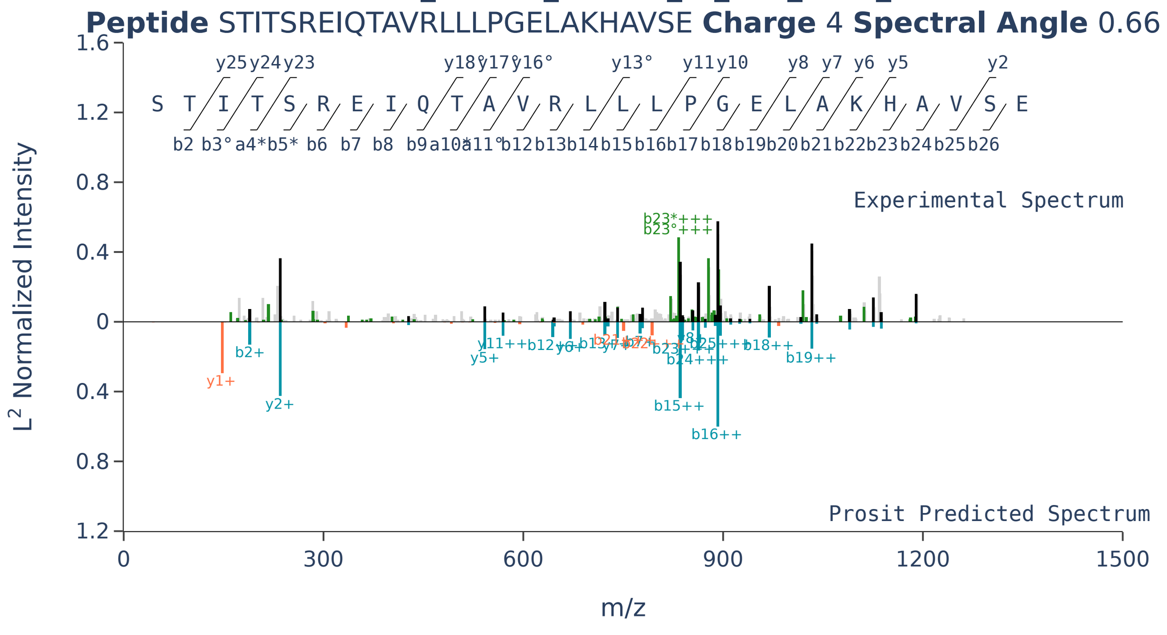


**Source** HRoetschke_291222_080123_Fu_H2B_b2R2_4h **Scan** 11458
**Peptide** AGEASRLAHYNKRSTITSREIQTAVRL **Charge** 6 **Spectral Angle** 0.3

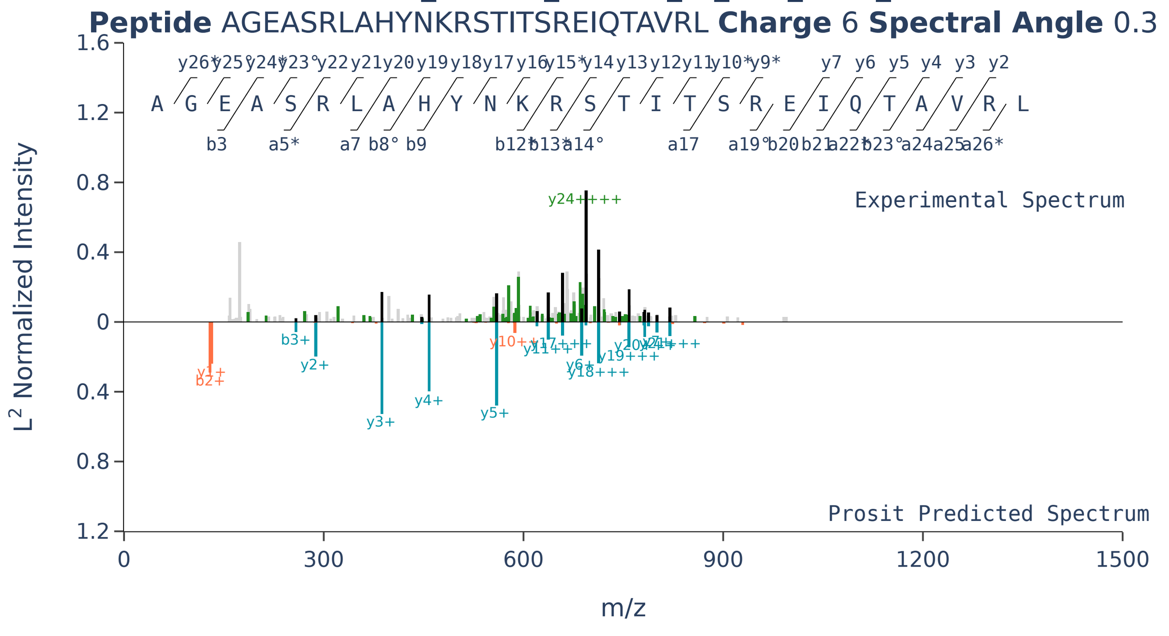


**Source** HRoetschke_291222_080123_Fu_H2B_b2R1_24h **Scan** 17447
**Peptide** AIYVYKVLKQVHPDTGL **Charge** 3 **Spectral Angle** 0.55

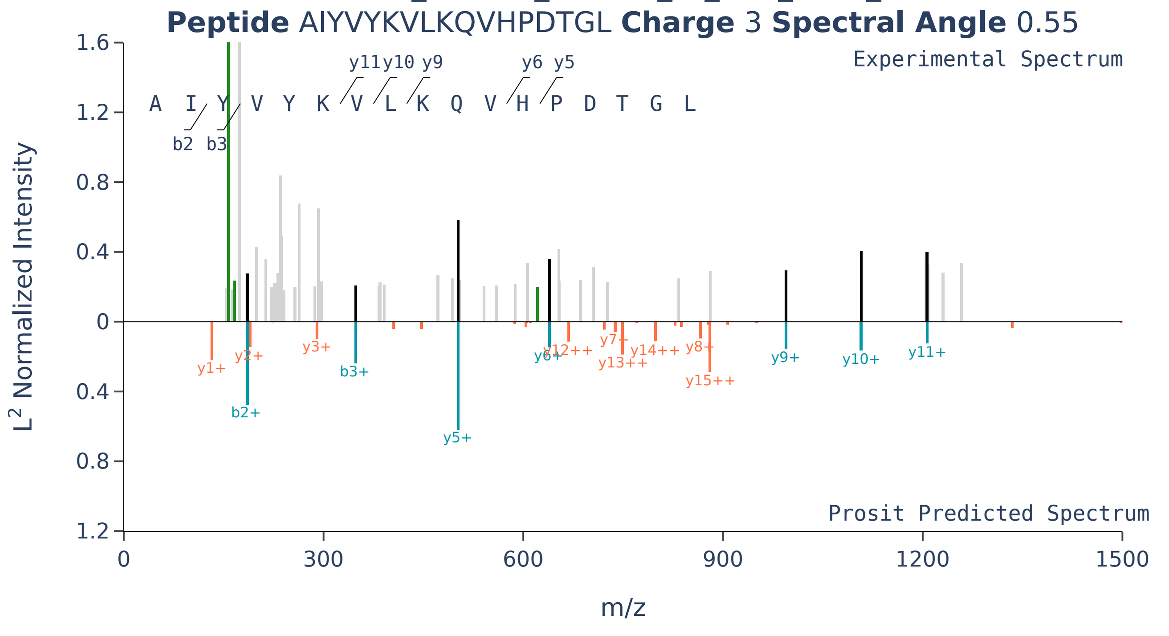


**Source** HRoetschke_291222_080123_Fu_H2B_b1R1_2h **Scan** 14287
**Peptide** QTAVRLLLPGELAKHAVS **Charge** 4 **Spectral Angle** 0.57

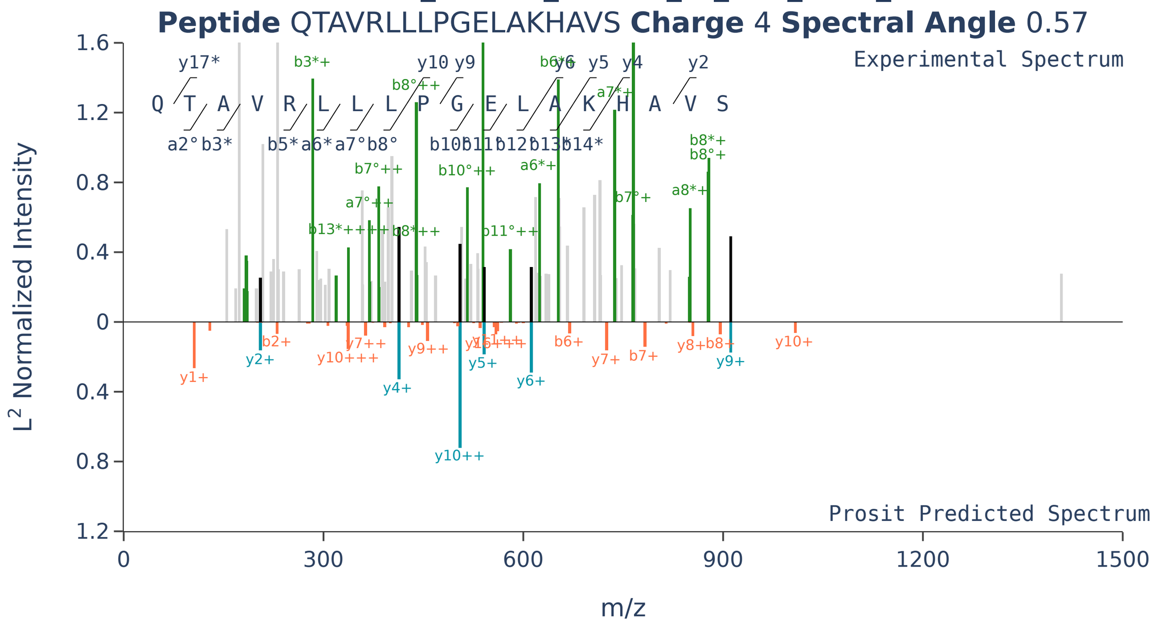

**Source** HRoetschke_291222_080123_Fu_H2B_b1R2_24h **Scan** 5756
**Peptide** AVTKTQKKDGKKRRKTRKESYAIYVYKV **Charge** 6 **Spectral Angle** 0.71

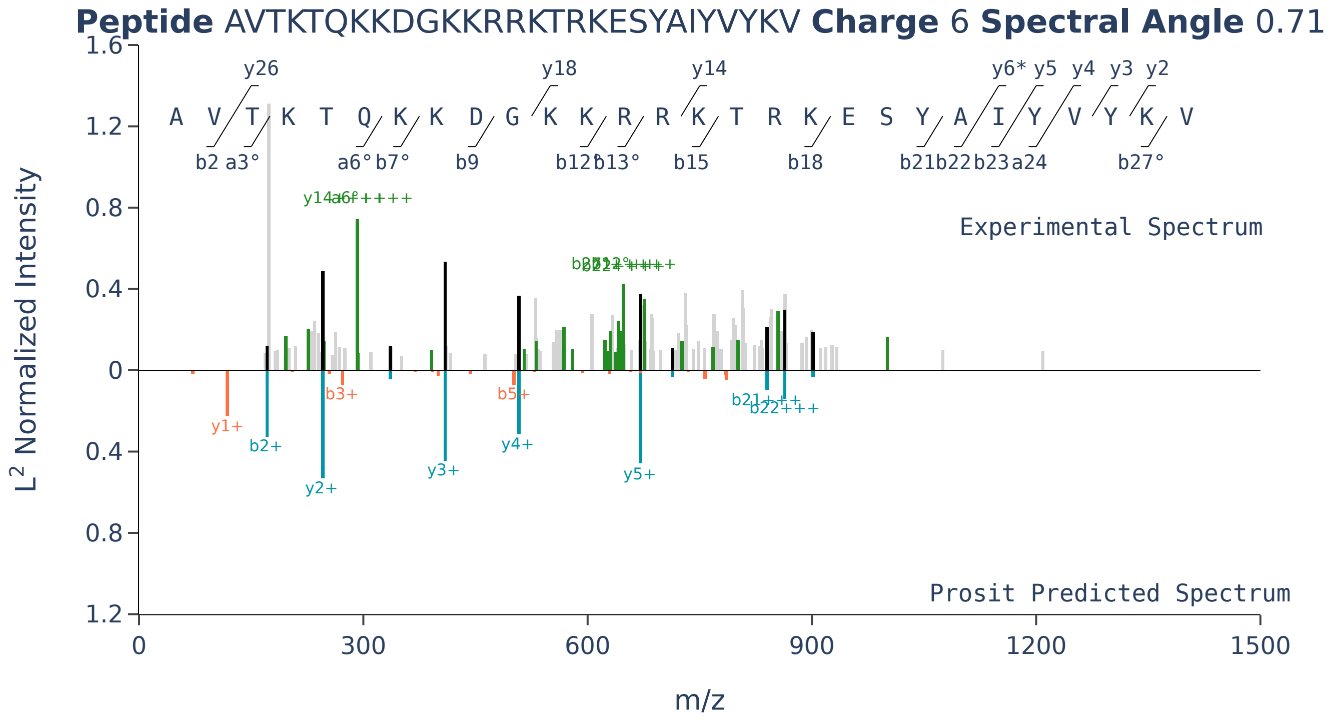


**Source** HRoetschke_291222_080123_Fu_H2B_b1R1_2h **Scan** 15643
**Peptide** ASRLAHYNKRSTITSREIQTAVRLLL **Charge** 5 **Spectral Angle** 0.53

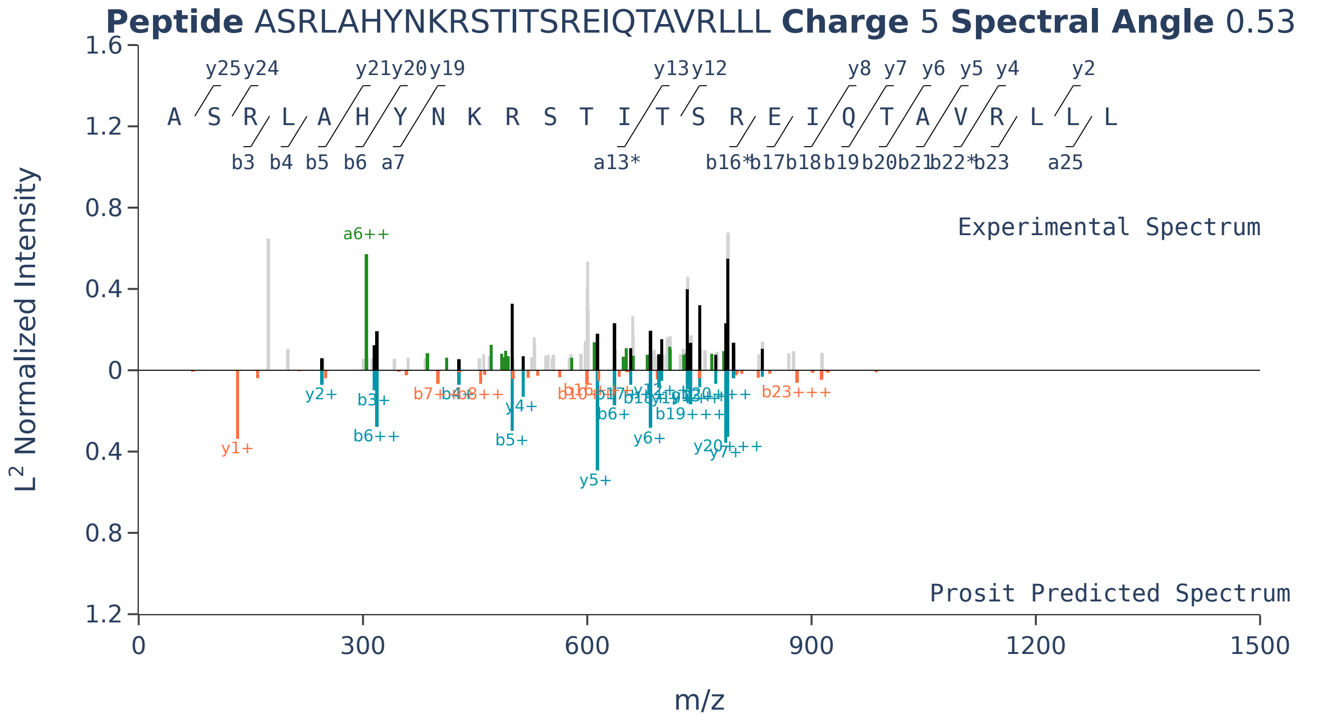


**Source** HRoetschke_291222_080123_Fu_H2B_b2R1_4h **Scan** 21361
**Peptide** NKRSTITSREIQTAVRLLLPGEL **Charge** 4 **Spectral Angle** 0.69

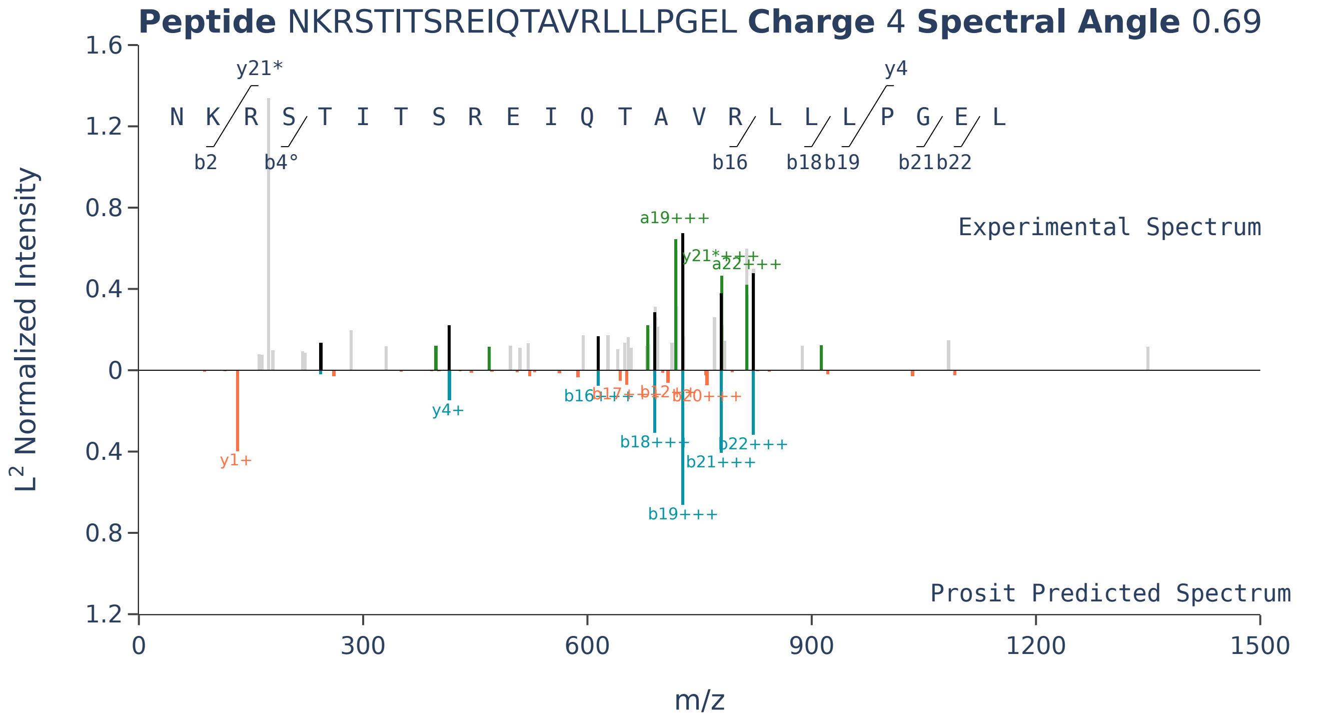


**Source** HRoetschke_291222_080123_Fu_H2B_b2R1_4h **Scan** 17916
**Peptide** VNDVFERIAGEASRLAHYNKRSTITSRE **Charge** 6 **Spectral Angle** 0.43

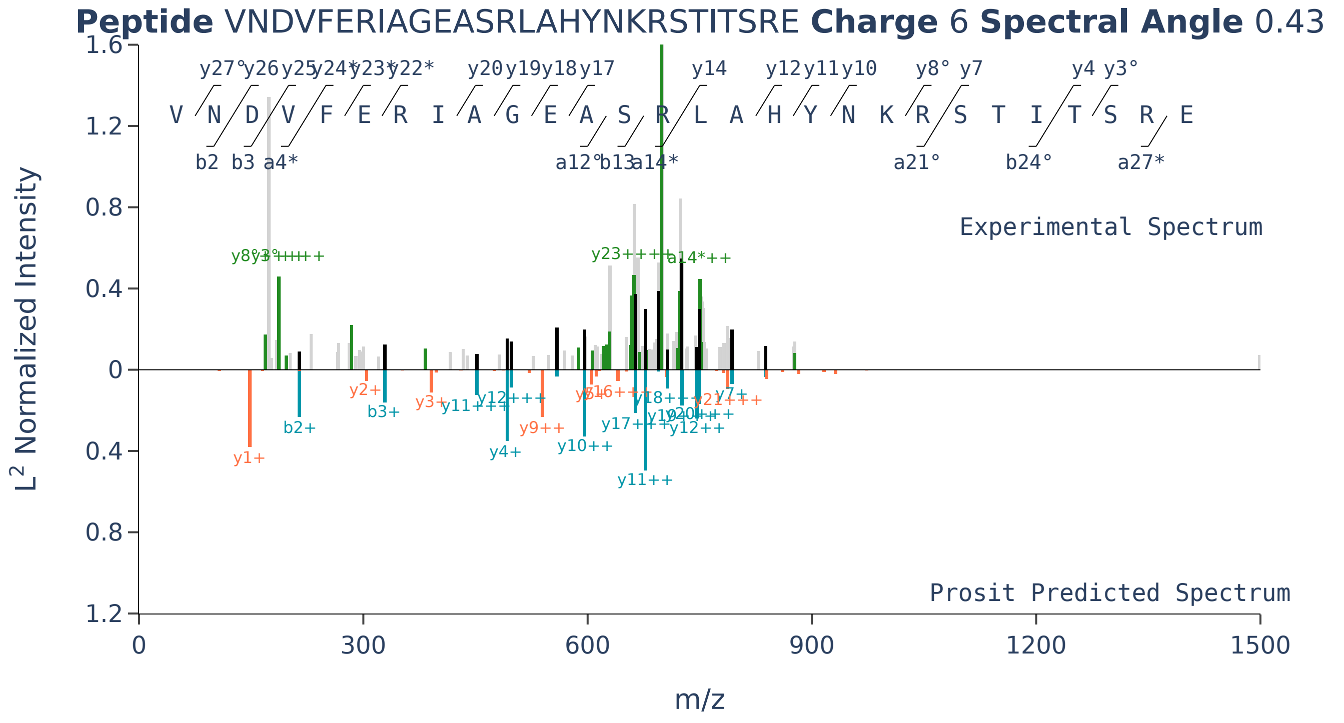


**Source** HRoetschke_291222_080123_Fu_H2B_b2R2_24h **Scan** 13337
**Peptide** RKTRKESYAIYVYKVLKQ **Charge** 5 **Spectral Angle** 0.61

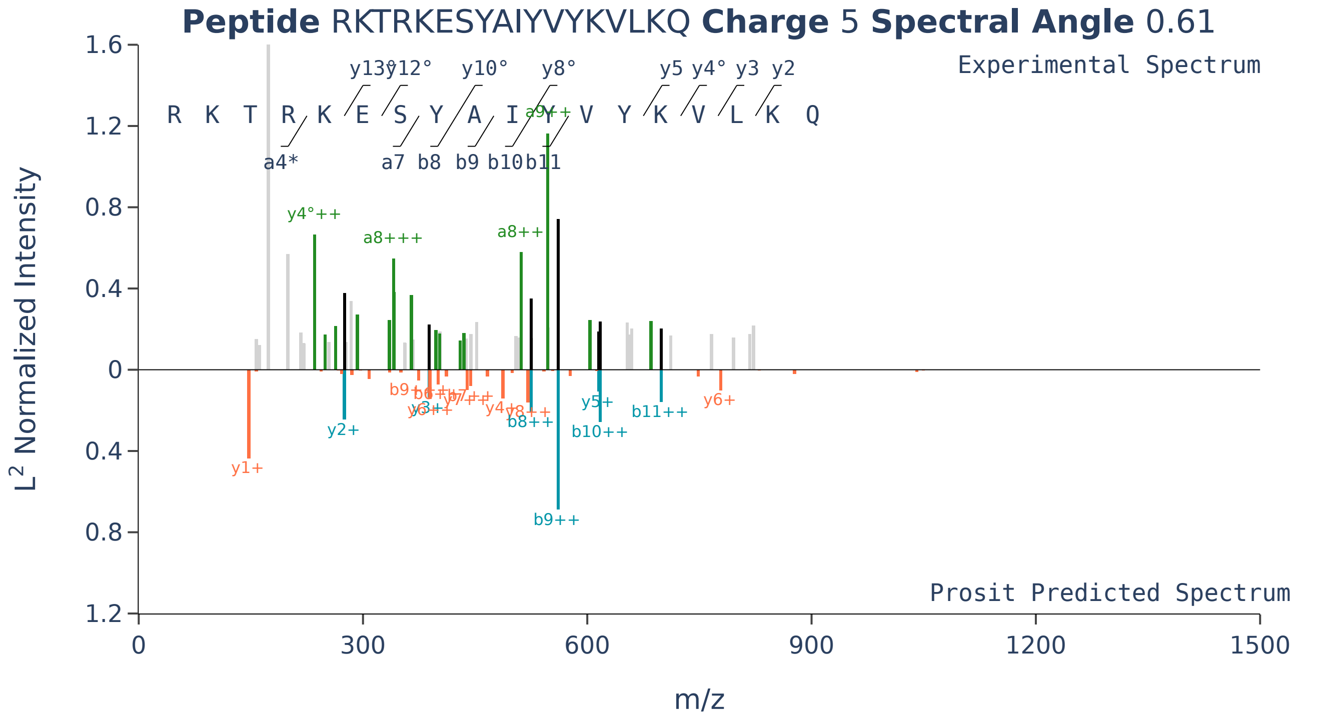


**Source** HRoetschke_291222_080123_Fu_H2B_b1R1_24h **Scan** 6349
**Peptide** QKKDGKKRRKTRKESYAIYVYKV **Charge** 5 **Spectral Angle** 0.64

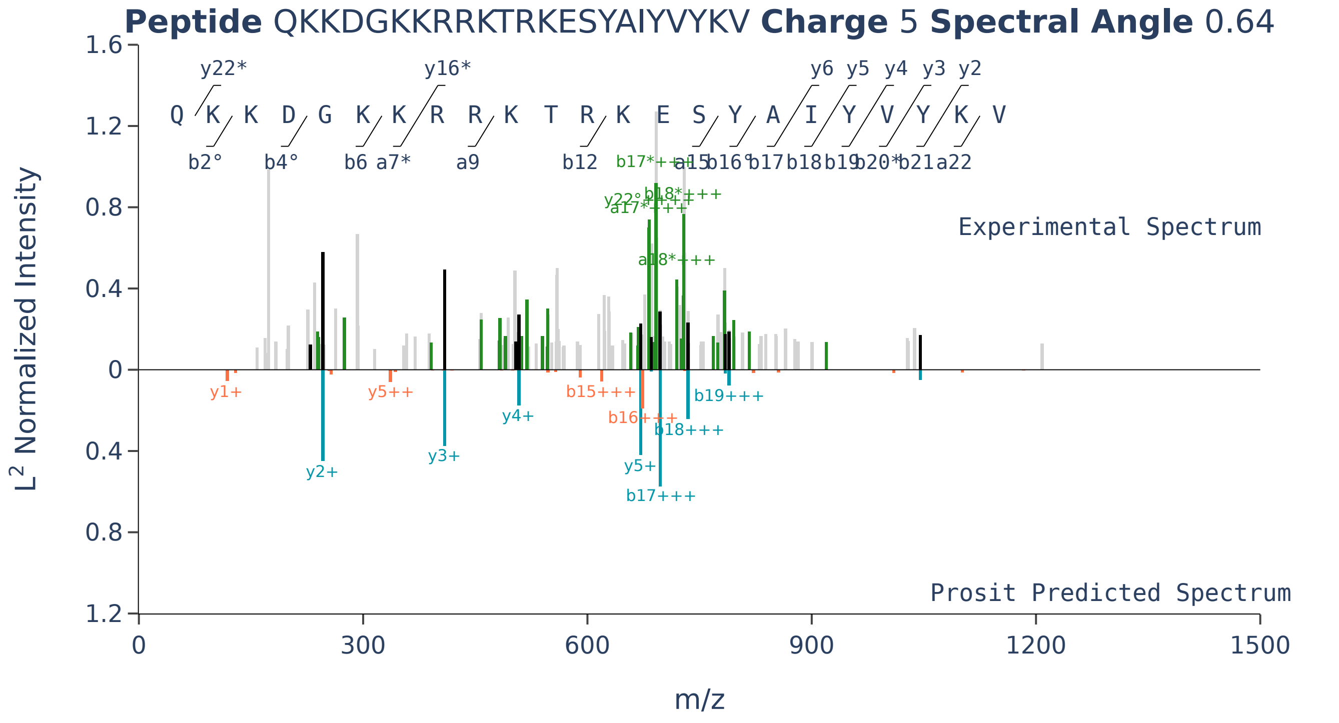


**Source** HRoetschke_291222_080123_Fu_H2B_b2R1_2h **Scan** 18526
**Peptide** EIQTAVRLLLPGELAKHAVSE **Charge** 4 **Spectral Angle** 0.69

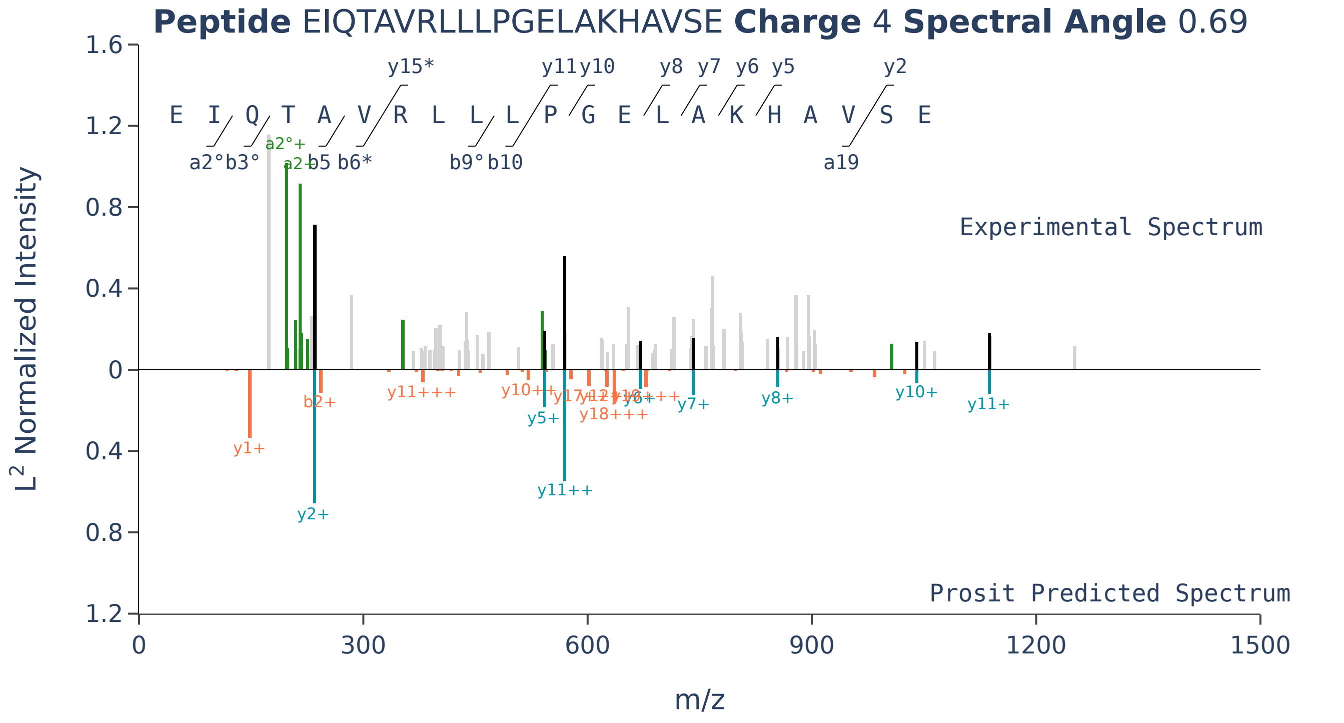


**Source** HRoetschke_291222_080123_Fu_H2B_b2R2_4h **Scan** 18513
**Peptide** AHYNKRSTITSREIQTAVRLLLPGELAKH **Charge** 6 **Spectral Angle** 0.66

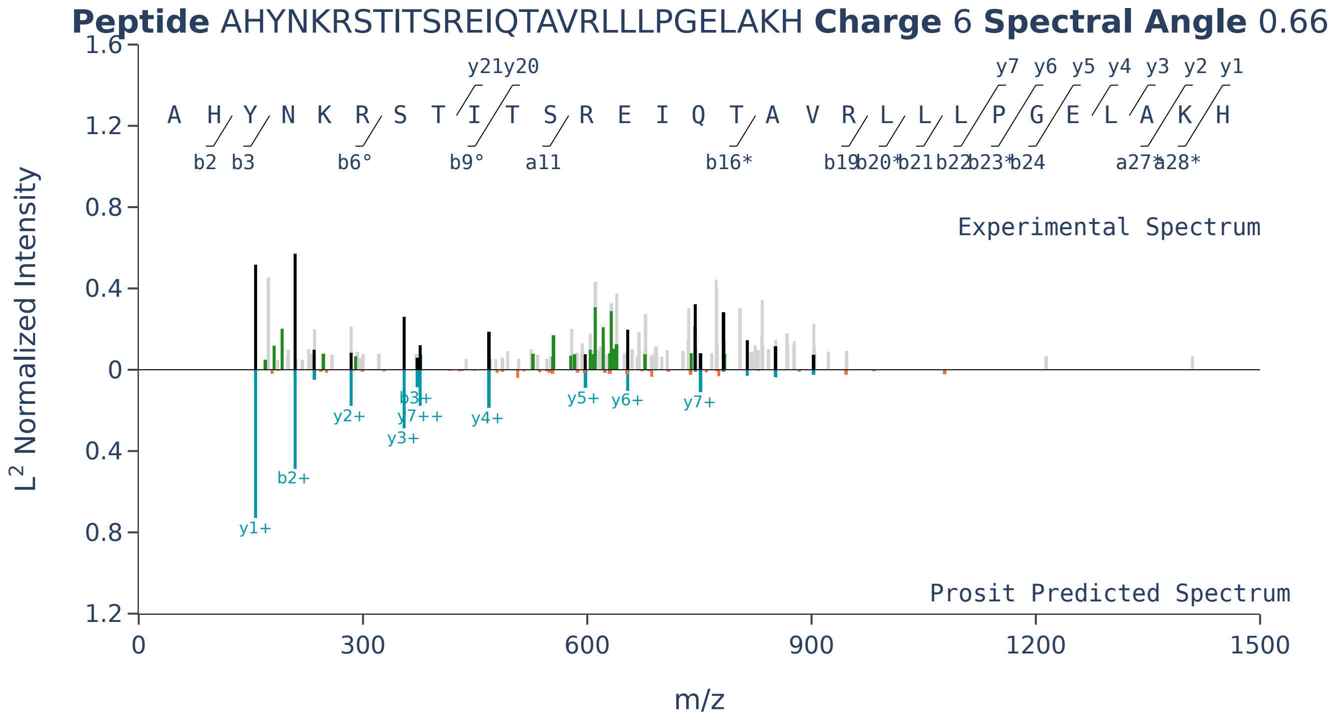


**Source** HRoetschke_291222_080123_Fu_H2B_b2R1_1h **Scan** 18000
**Peptide** SYAIYVYKVLKQV **Charge** 3 **Spectral Angle** 0.6

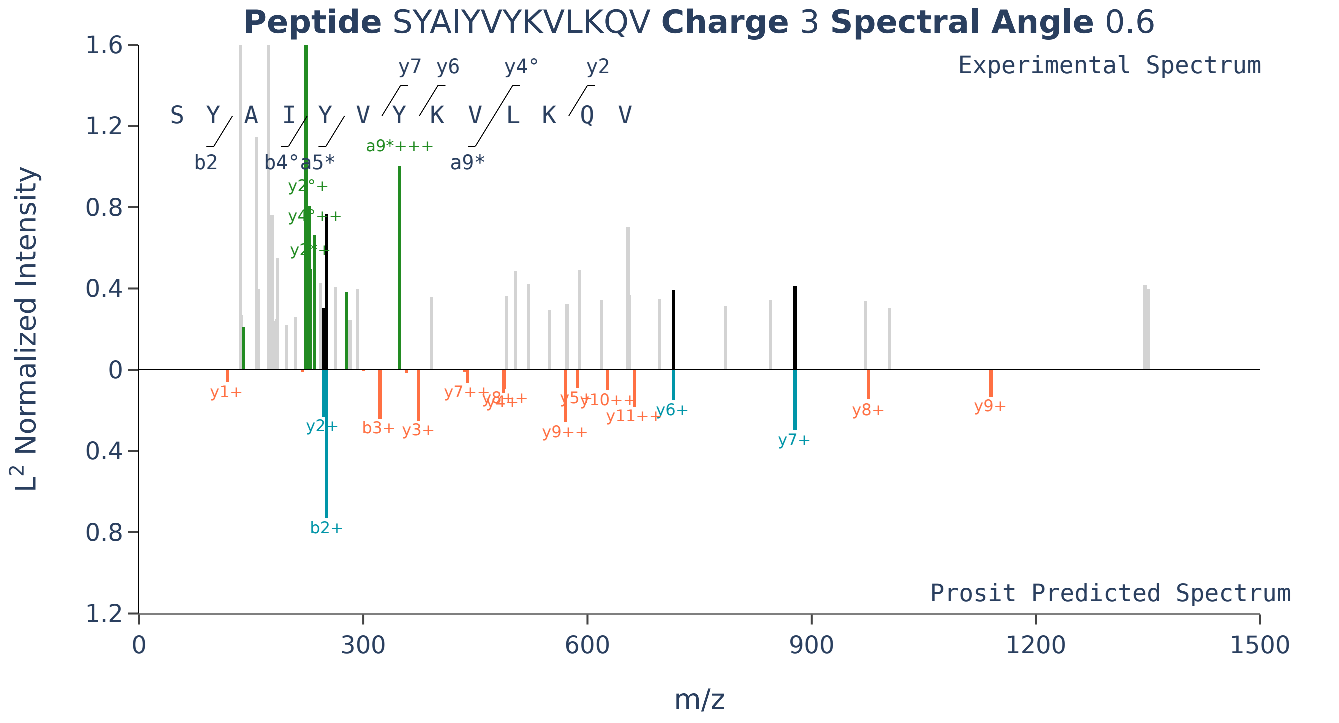


**Source** HRoetschke_291222_080123_Fu_H2B_b1R2_1h **Scan** 17120
**Peptide** AHYNKRSTITSREIQTAVRLLLPGEL **Charge** 4 **Spectral Angle** 0.66

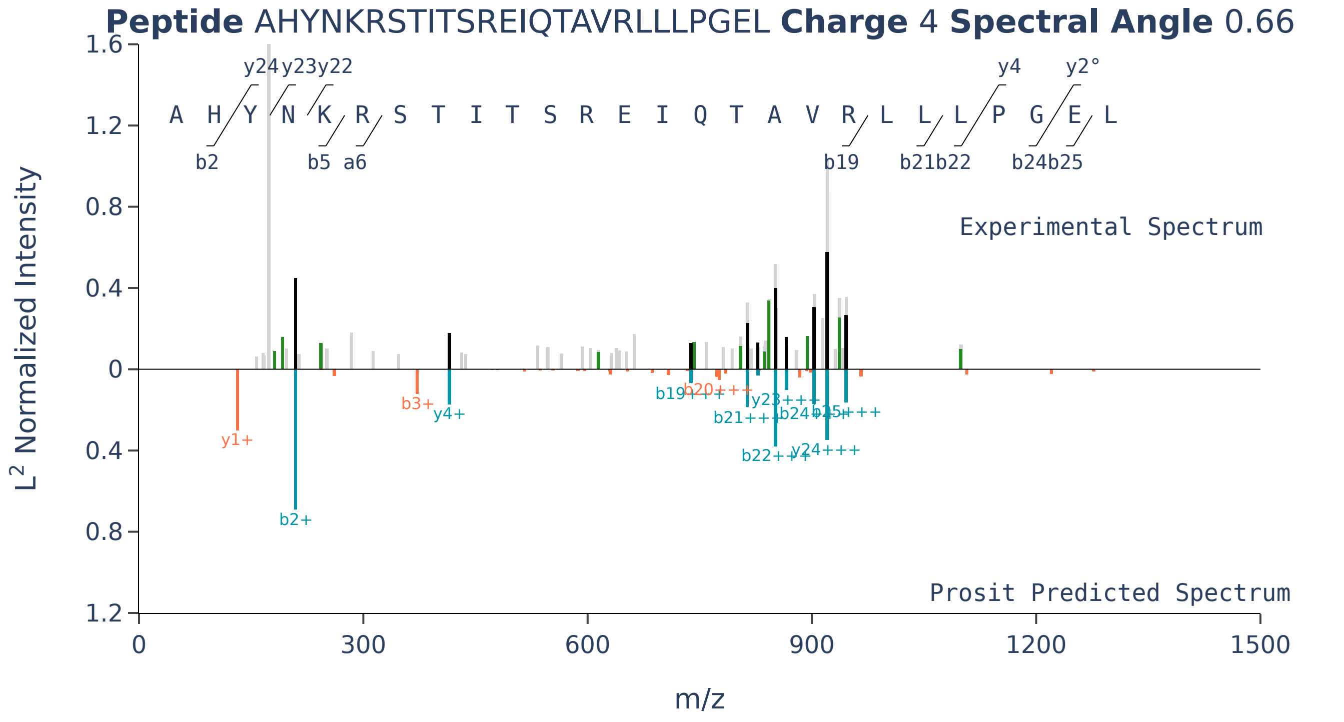

# inSPIRE Spectral Plotting for H3

## Experimental Spectrum Colour Code:

- Experimental peak matched to a Prosit predicted peak.
- Possible ion unknown to Prosit.
- Precursor matched peak.
- Experimental peak not matched to any potential ion.

## Prosit Spectrum Colour Code:

- Prosit predicted peak matched to experimental spectrum.
- Prosit predicted peak not matched to experimental spectrum.

Loading [MathJax]/extensions/MathMenu.js

**Source** HRoetschke_271222_120123_HFGoe_H3_b2R2_24h **Scan** 14095
**Peptide** FEDTNLC **Charge** 2 **Spectral Angle** 0.97

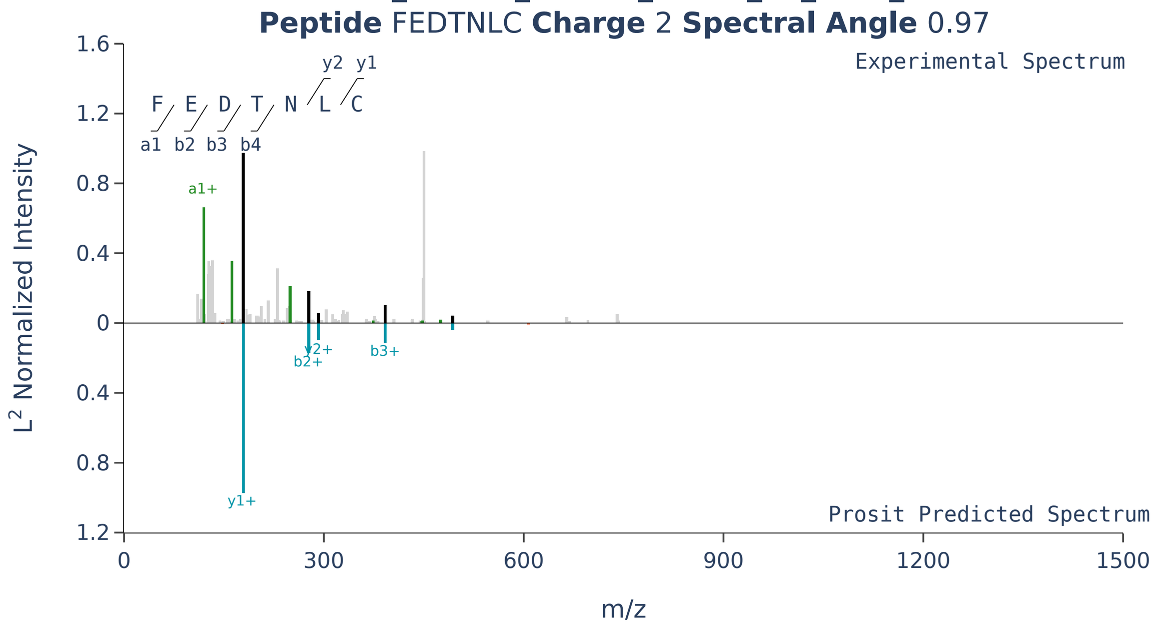


**Source** HRoetschke_271222_120123_HFGoe_H3_b2R1_4h **Scan** 13105
**Peptide** VTIMPKD **Charge** 2 **Spectral Angle** 0.96

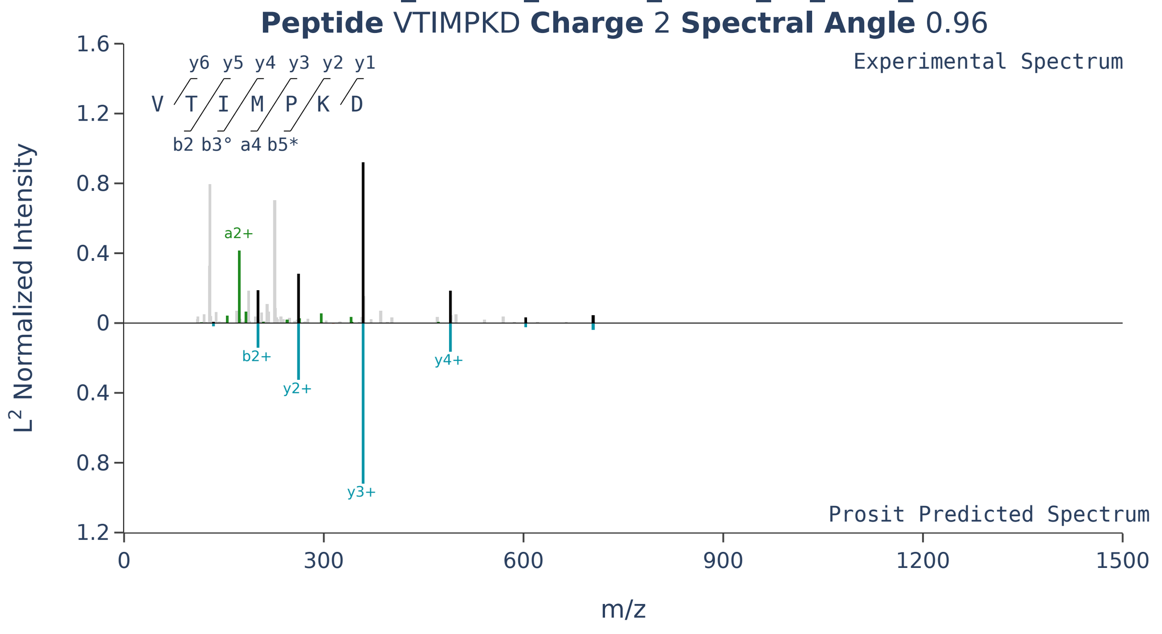


**Source** HRoetschke_271222_120123_HFGoe_H3_b1R2_24h **Scan** 13259
**Peptide** LAQDFKTDLR **Charge** 3 **Spectral Angle** 0.92

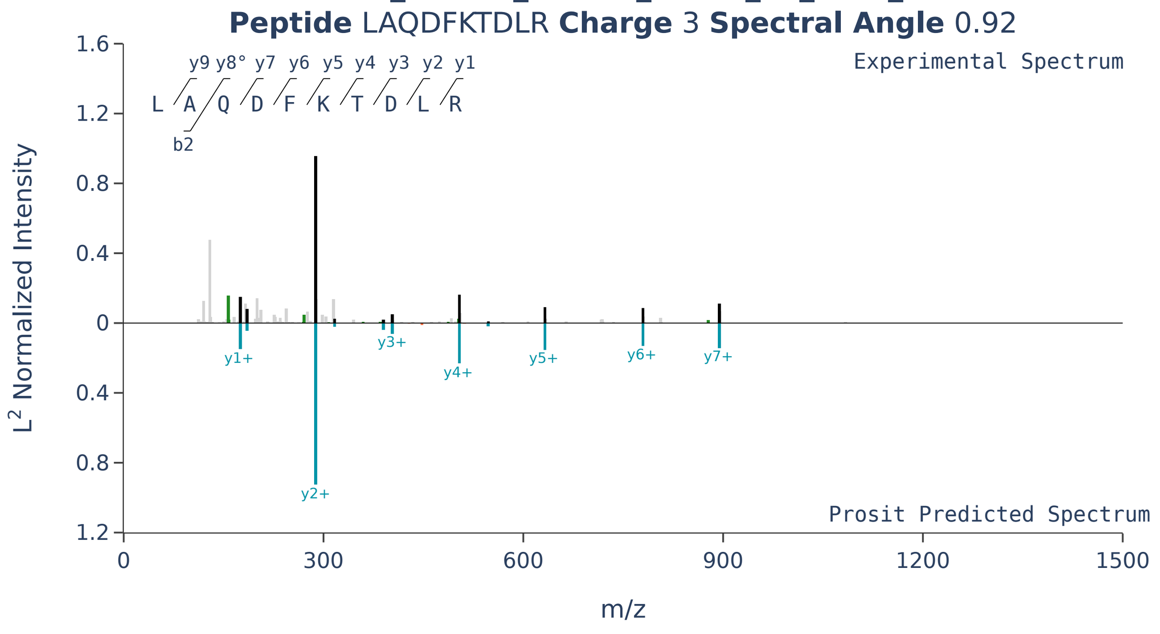


**Source** HRoetschke_271222_120123_HFGoe_H3_b2R2_4h **Scan** 12428
**Peptide** VREIAQDFKT **Charge** 3 **Spectral Angle** 0.94

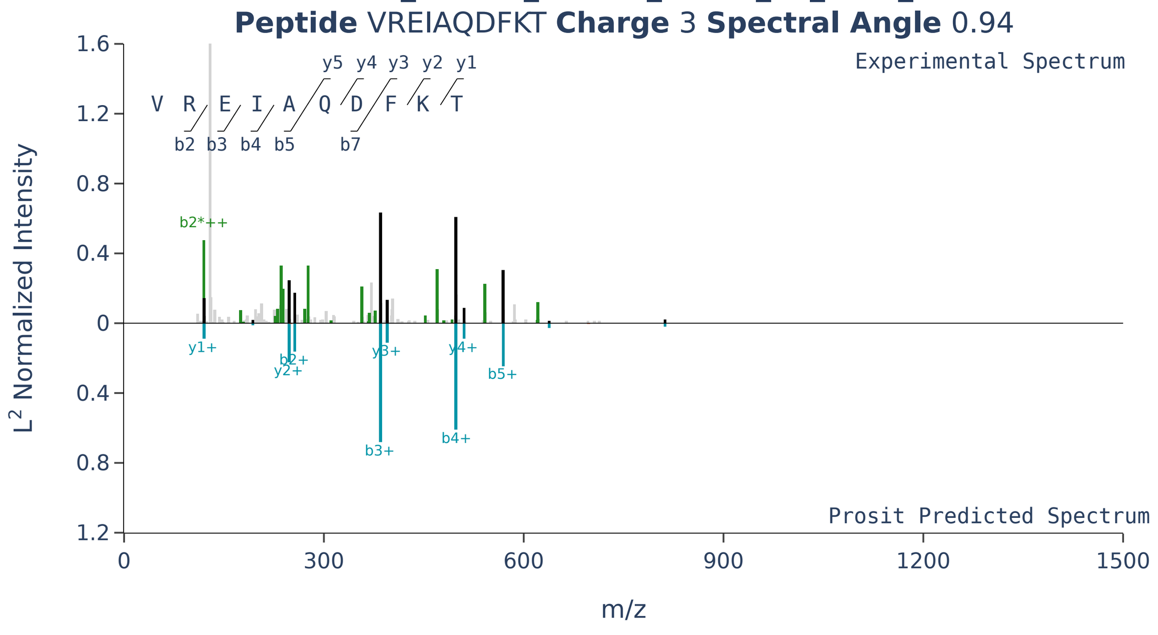


**Source** HRoetschke_271222_120123_HFGoe_H3_b1R1_24h **Scan** 9797
**Peptide** AQDFKTDLR **Charge** 3 **Spectral Angle** 0.96

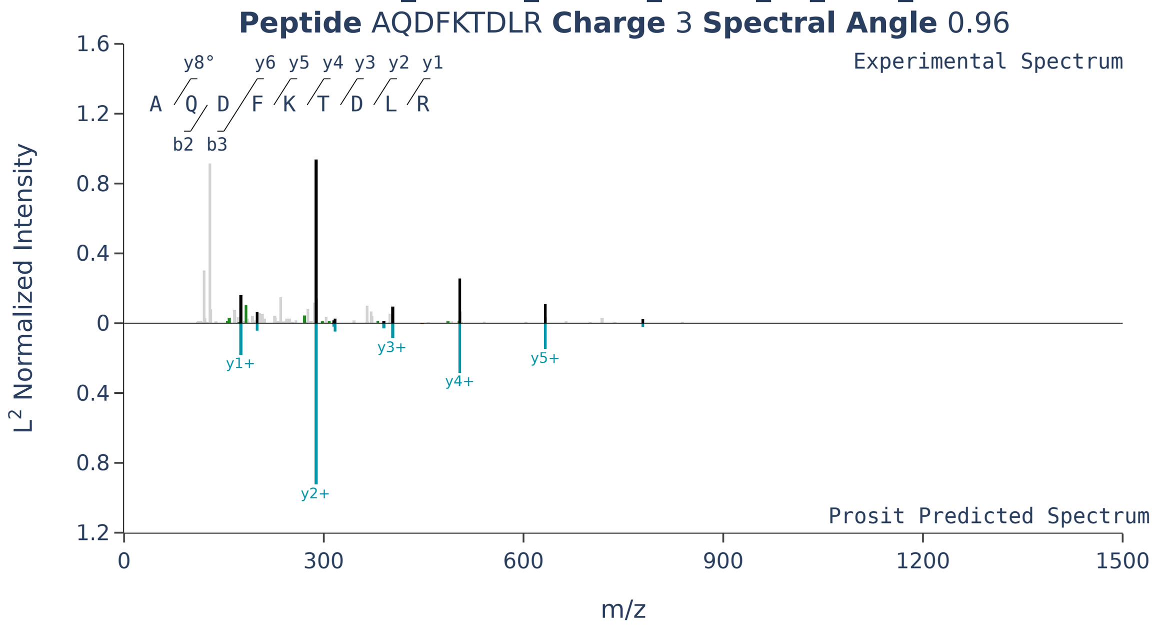


**Source** HRoetschke_271222_120123_HFGoe_H3_b1R2_1h **Scan** 8667
**Peptide** PGTVALR **Charge** 2 **Spectral Angle** 0.86

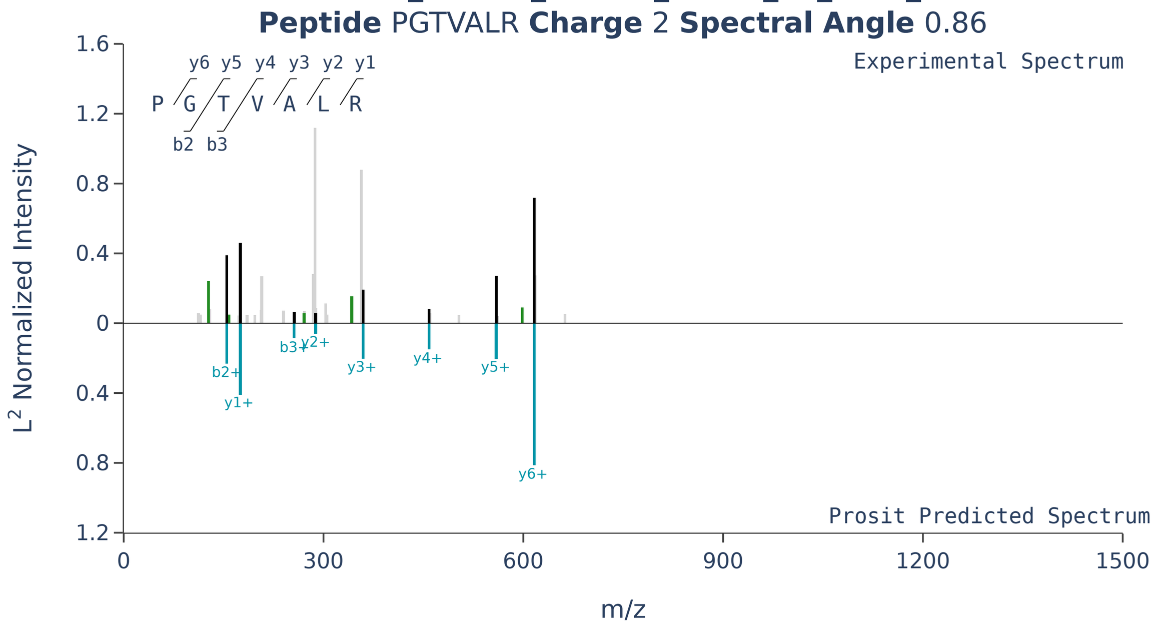


**Source** HRoetschke_271222_120123_HFGoe_H3_b2R1_4h **Scan** 13141
**Peptide** NLCAIHA **Charge** 2 **Spectral Angle** 0.96

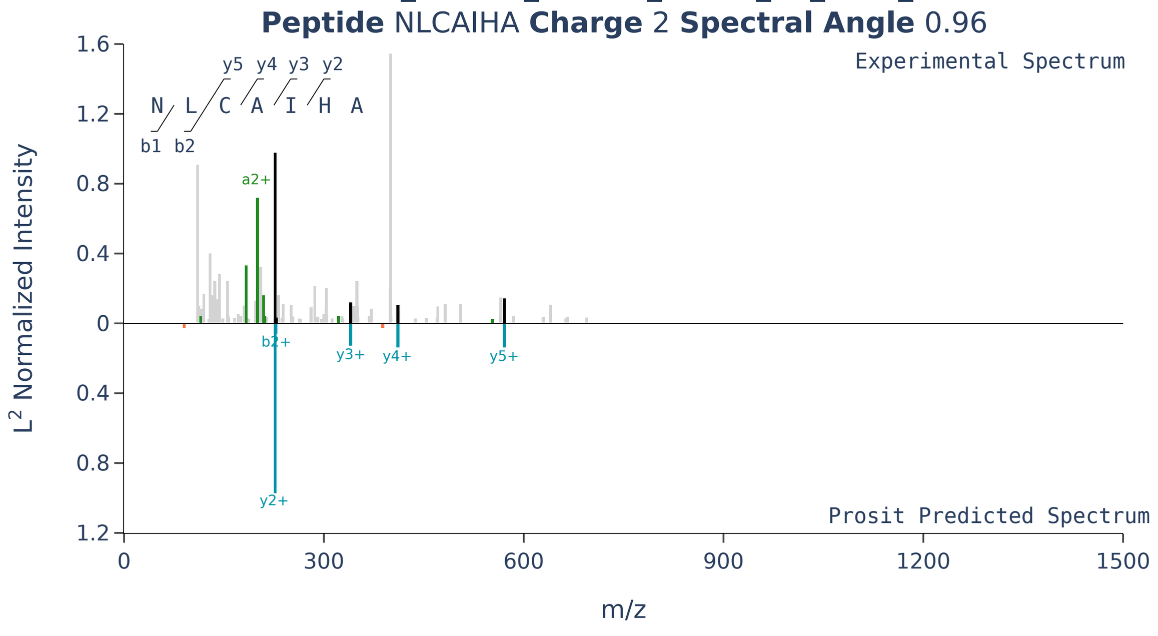


**Source** HRoetschke_271222_120123_HFGoe_H3_b2R1_1h **Scan** 7551
**Peptide** RFQSSAVM **Charge** 2 **Spectral Angle** 0.87

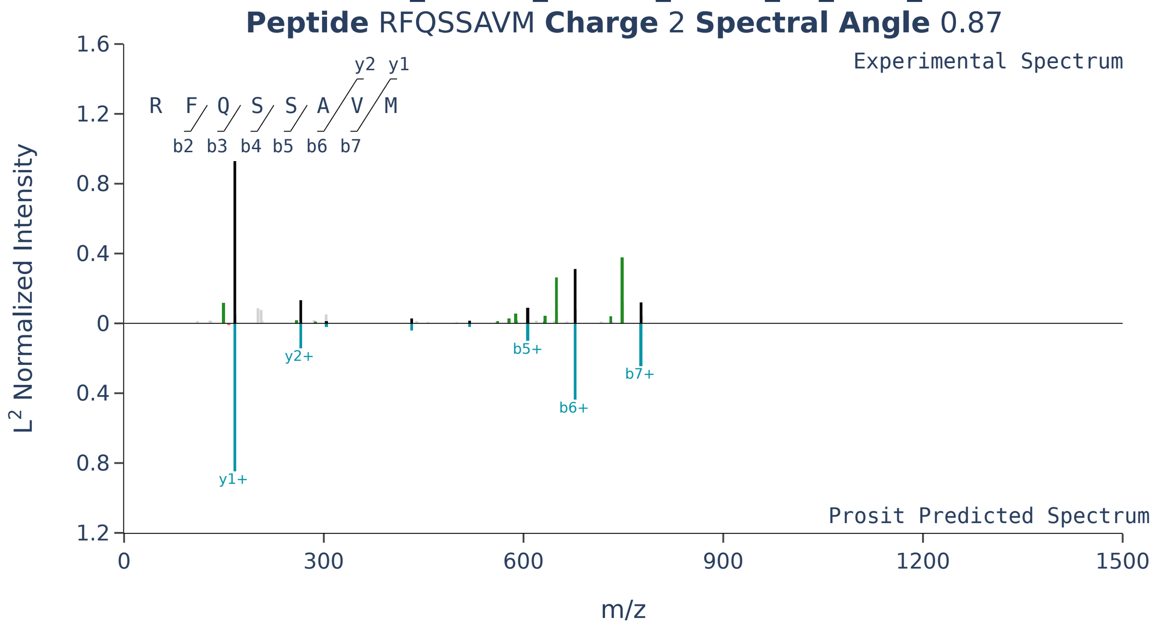


**Source** HRoetschke_271222_120123_HFGoe_H3_b1R1_1h **Scan** 16631
**Peptide** ASEAYLVA **Charge** 1 **Spectral Angle** 0.91

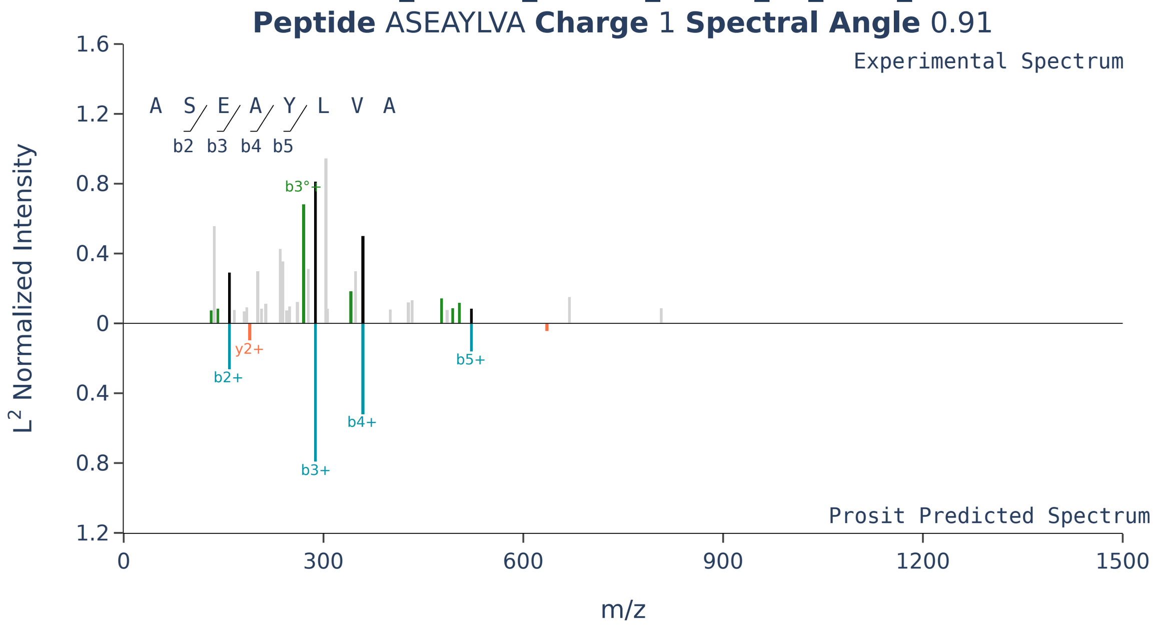


**Source** HRoetschke_271222_120123_HFGoe_H3_b2R2_2h **Scan** 12833
**Peptide** IAQDFKTDLR **Charge** 3 **Spectral Angle** 0.92

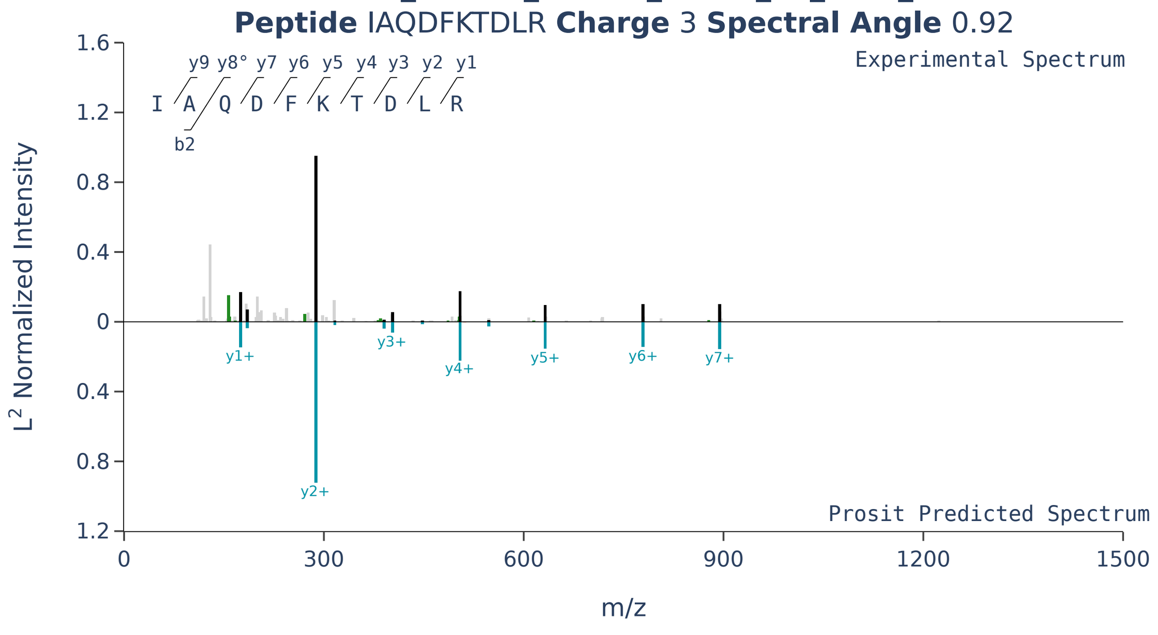


**Source** HRoetschke_271222_120123_HFGoe_H3_b2R1_1h **Scan** 12090
**Peptide** REIAQDF **Charge** 2 **Spectral Angle** 0.85

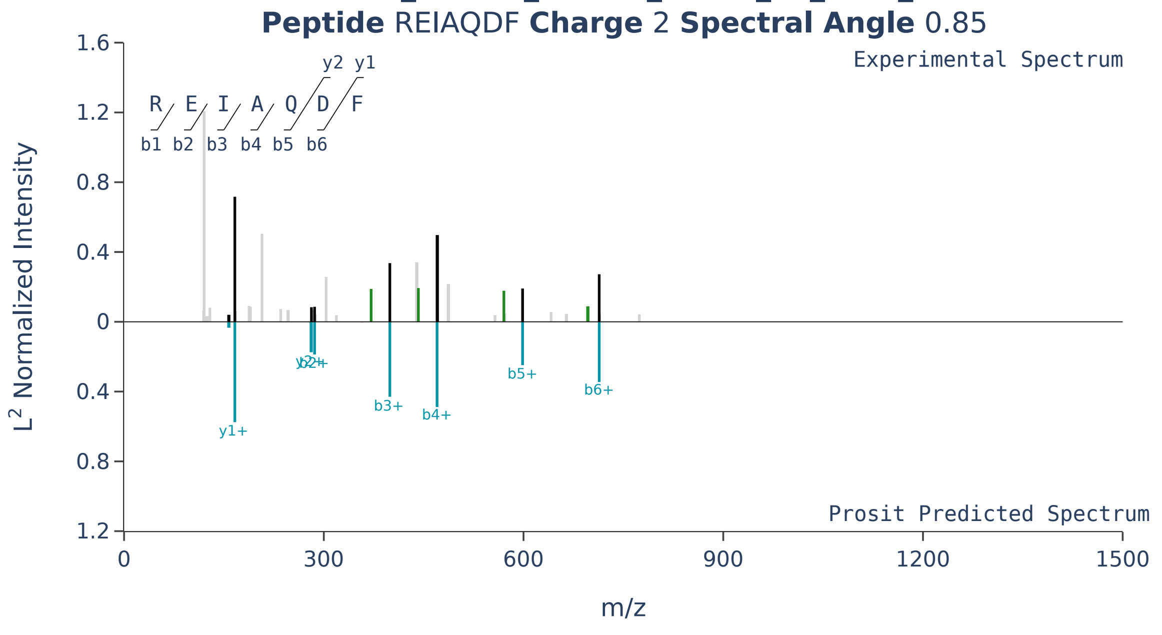


**Source** HRoetschke_271222_120123_HFGoe_H3_b2R2_4h **Scan** 14725
**Peptide** AQDFKTDL **Charge** 1 **Spectral Angle** 0.85

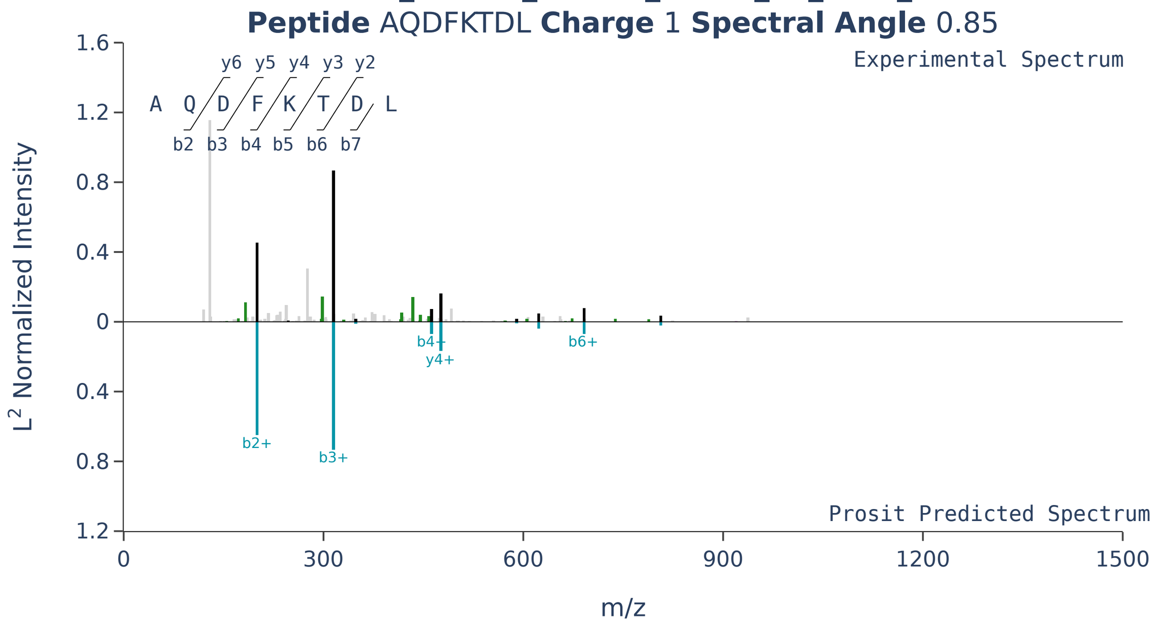


**Source** HRoetschke_271222_120123_HFGoe_H3_b2R2_1h **Scan** 9383
**Peptide** REIAQDFKT **Charge** 3 **Spectral Angle** 0.87

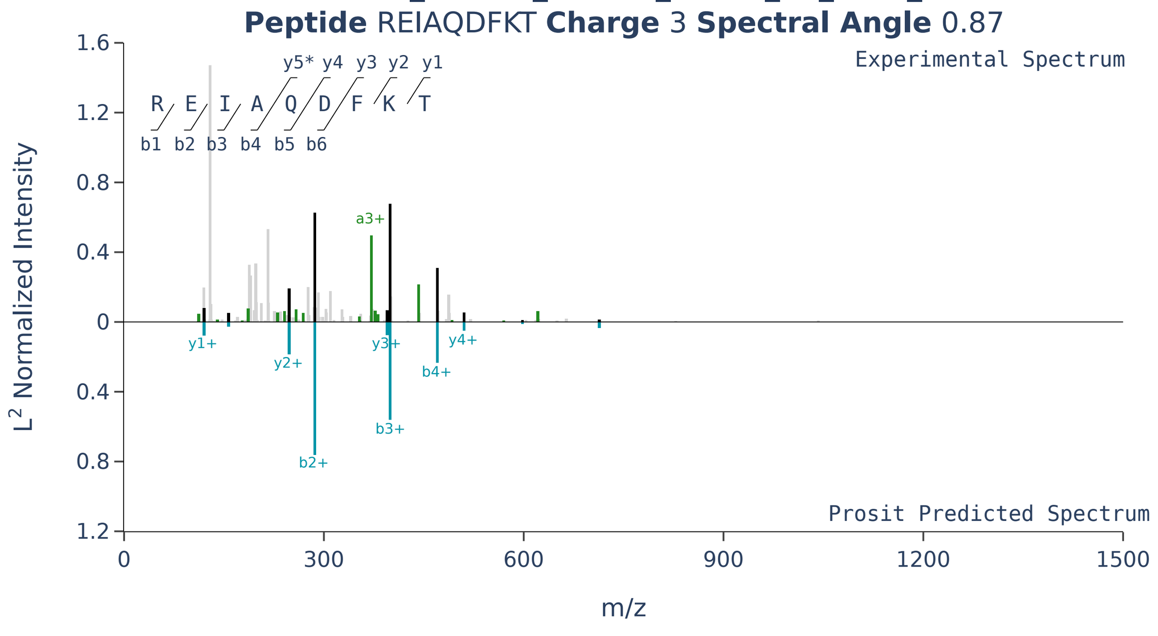


**Source** HRoetschke_271222_120123_HFGoe_H3_b2R1_2h **Scan** 12227
**Peptide** TNLCAIHA **Charge** 2 **Spectral Angle** 0.91

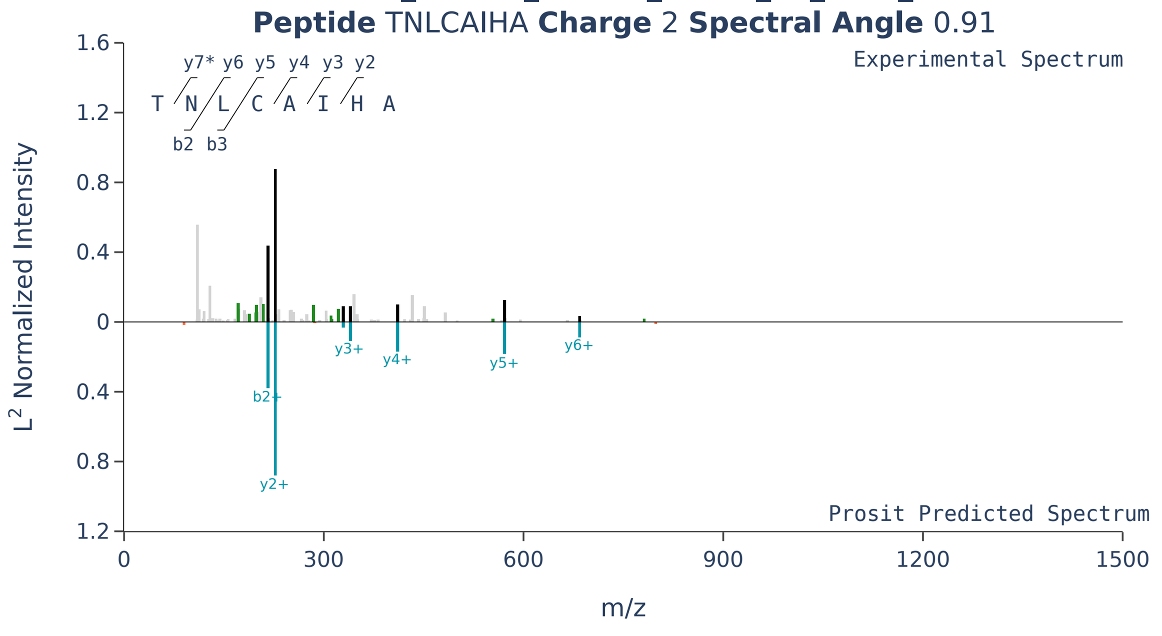


**Source** HRoetschke_271222_120123_HFGoe_H3_b2R2_2h **Scan** 10779
**Peptide** EIAQDFK **Charge** 2 **Spectral Angle** 0.92

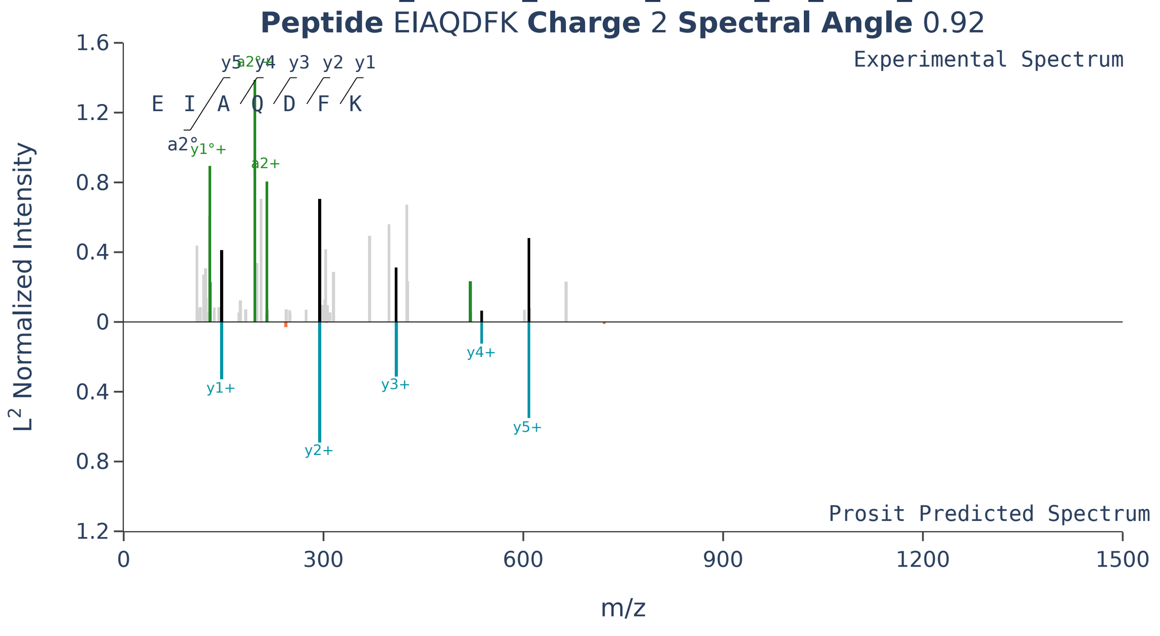

**Source** HRoetschke_271222_120123_HFGoe_H3_b2R2_2h **Scan** 16517
**Peptide** AVMALQE **Charge** 1 **Spectral Angle** 0.88

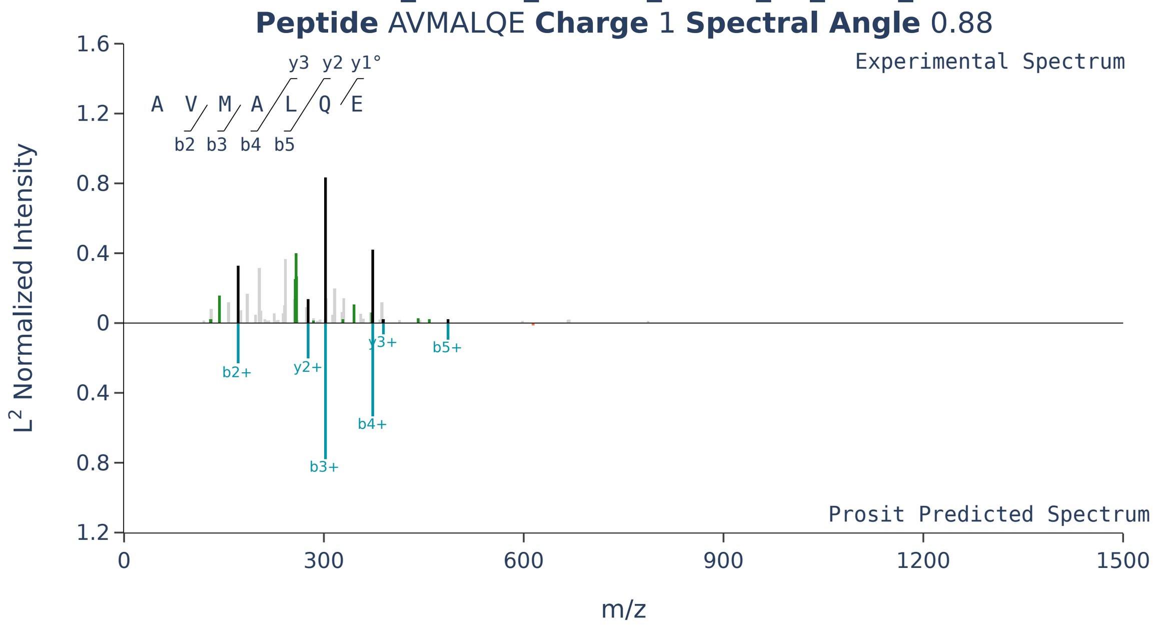

**Source** HRoetschke_271222_120123_HFGoe_H3_b1R1_24h **Scan** 12119
**Peptide** SAVMALQE **Charge** 1 **Spectral Angle** 0.89

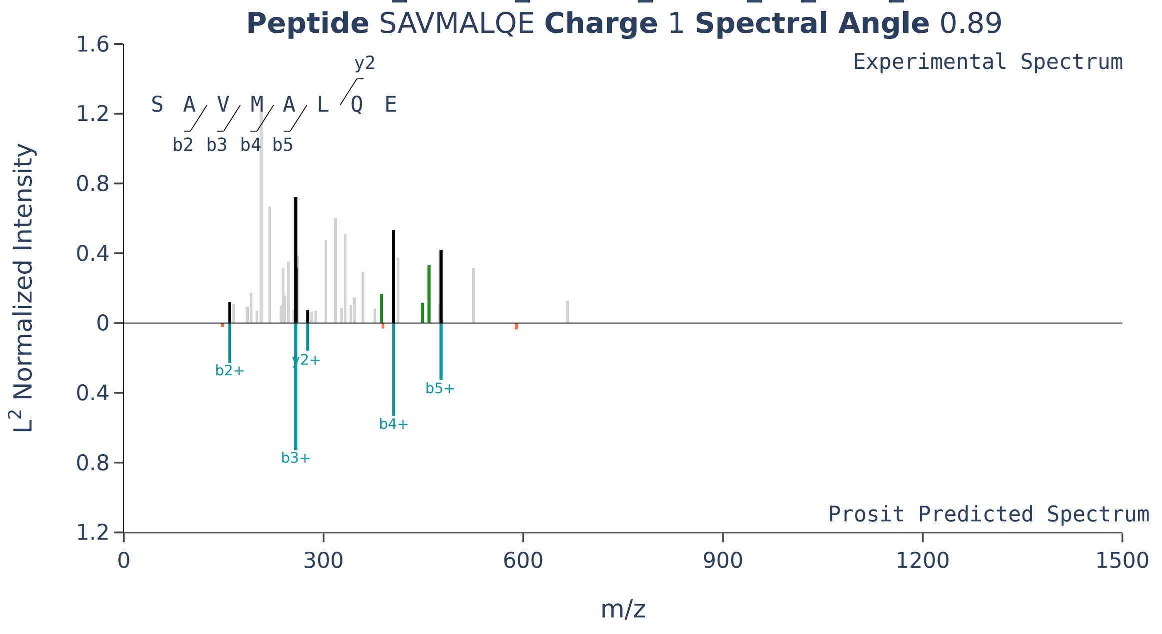

**Source** HRoetschke_271222_120123_HFGoe_H3_b1R1_4h **Scan** 15838
**Peptide** REIAQDFKTDL **Charge** 3 **Spectral Angle** 0.92

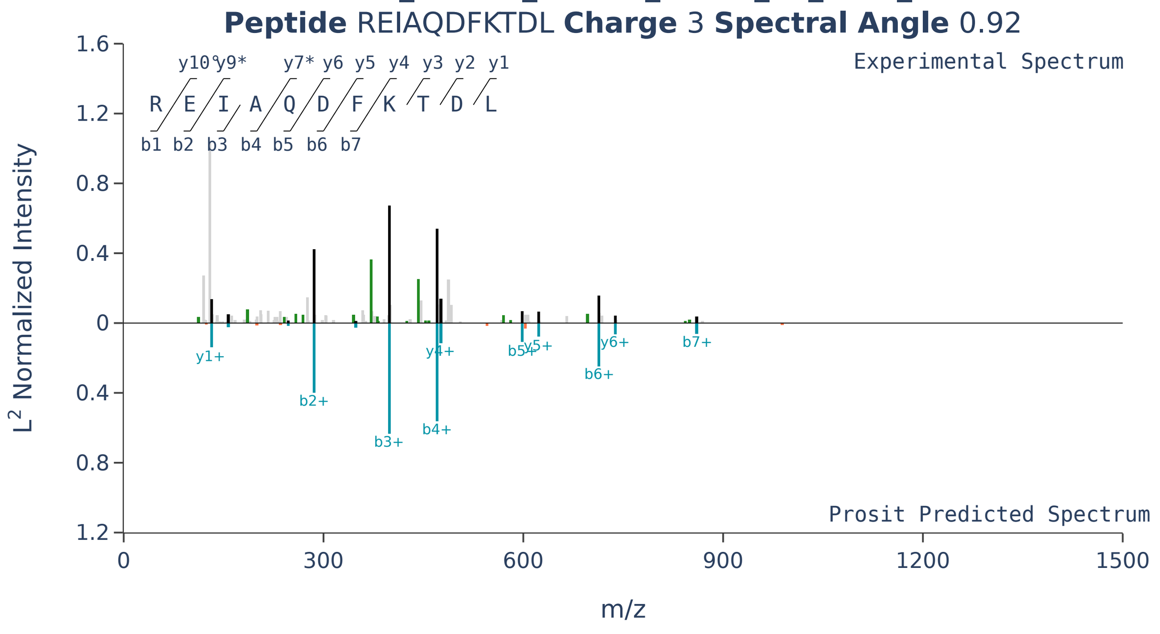

**Source** HRoetschke_271222_120123_HFGoe_H3_b1R1_24h **Scan** 12264
**Peptide** RKLPFQRL **Charge** 3 **Spectral Angle** 0.91

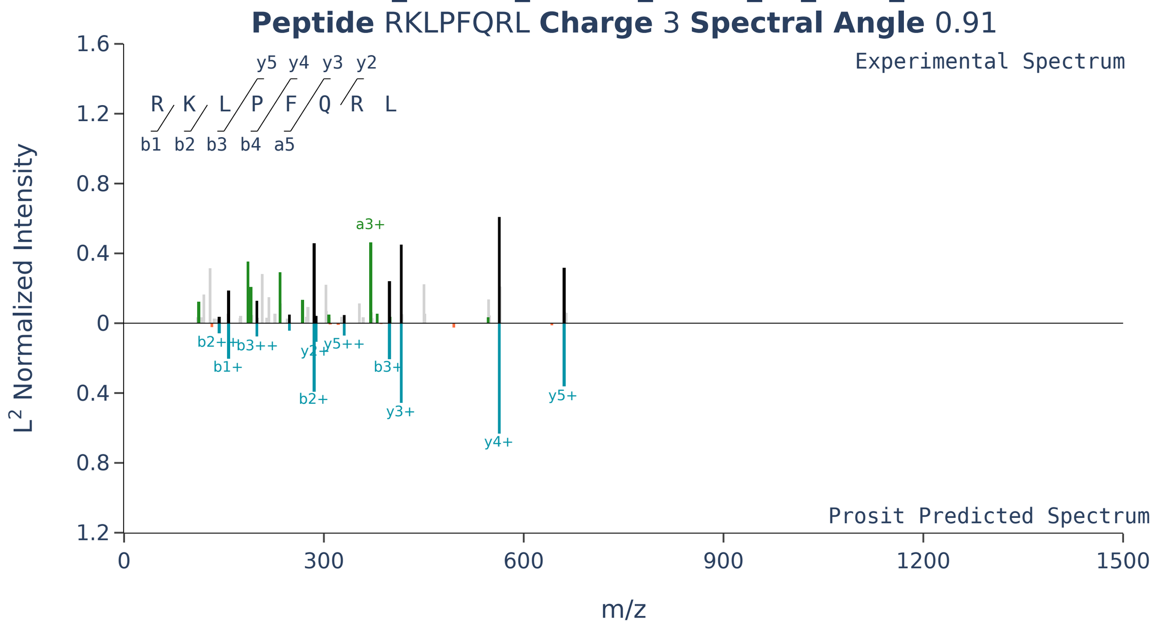

**Source** HRoetschke_271222_120123_HFGoe_H3_b2R1_24h **Scan** 12346
**Peptide** PKDIQLA **Charge** 1 **Spectral Angle** 0.88

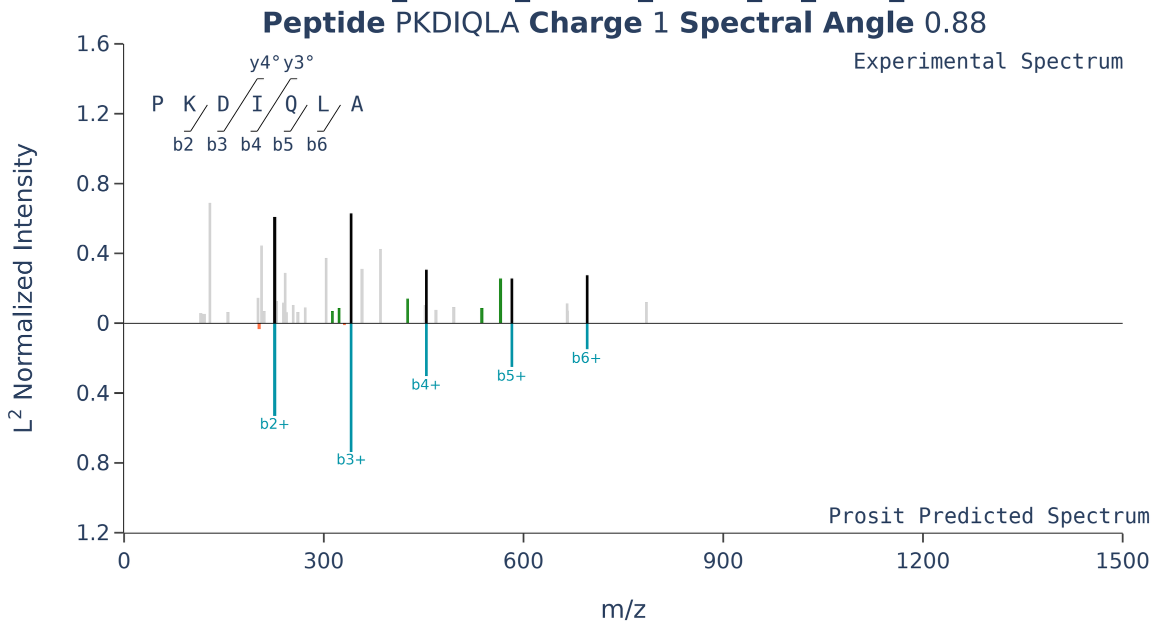

**Source** HRoetschke_271222_120123_HFGoe_H3_b1R1_24h **Scan** 16298
**Peptide** ELLIRKL **Charge** 2 **Spectral Angle** 0.92

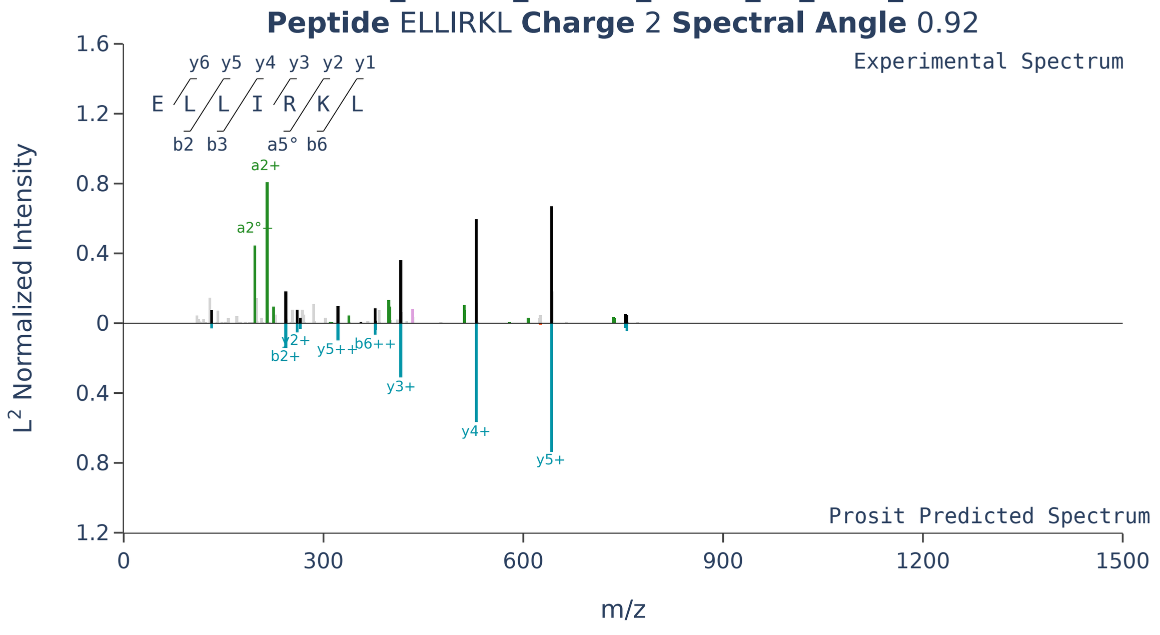

**Source** HRoetschke_271222_120123_HFGoe_H3_b1R1_4h **Scan** 7110
**Peptide** KRVTIMPKD **Charge** 3 **Spectral Angle** 0.93

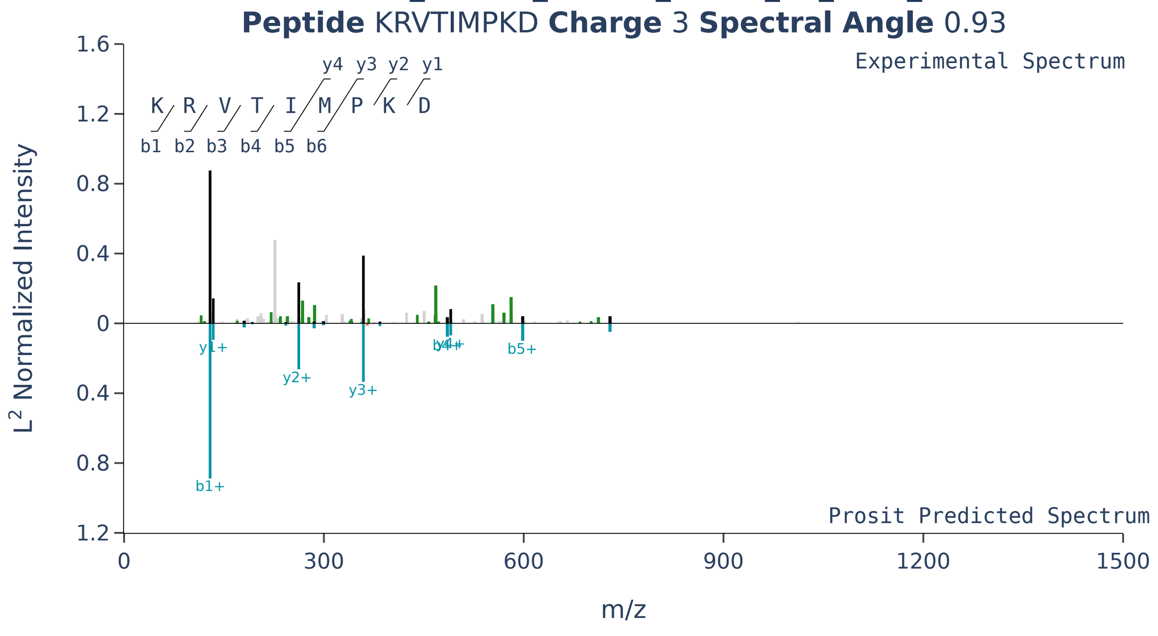

**Source** HRoetschke_271222_120123_HFGoe_H3_b2R2_4h **Scan** 15186
**Peptide** DTNLCAIHA **Charge** 2 **Spectral Angle** 0.83

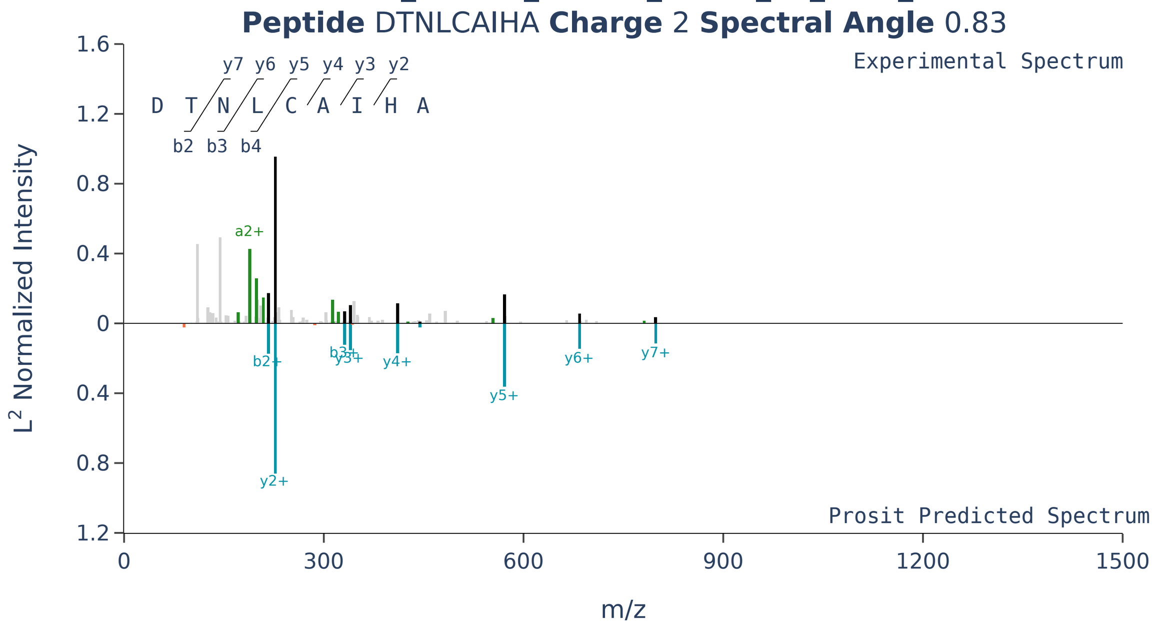

**Source** HRoetschke_271222_120123_HFGoe_H3_b2R2_4h **Scan** 14645
**Peptide** VREIAQDFKTDLR **Charge** 4 **Spectral Angle** 0.89

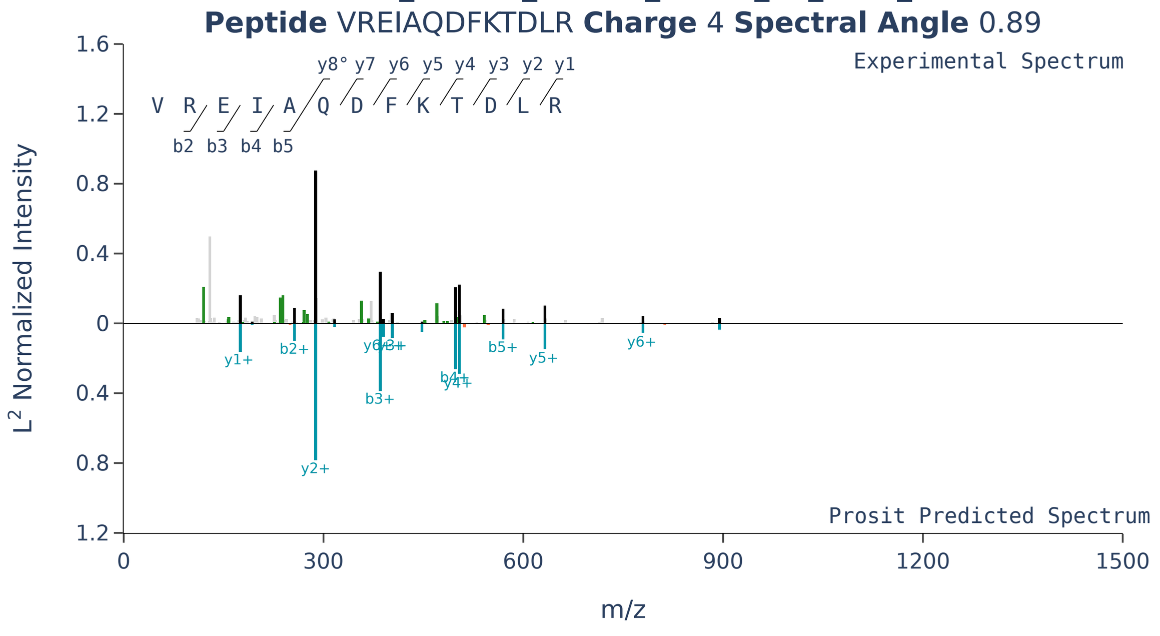

**Source** HRoetschke_271222_120123_HFGoe_H3_b2R2_4h **Scan** 15885
**Peptide** VREIAQDF **Charge** 2 **Spectral Angle** 0.81

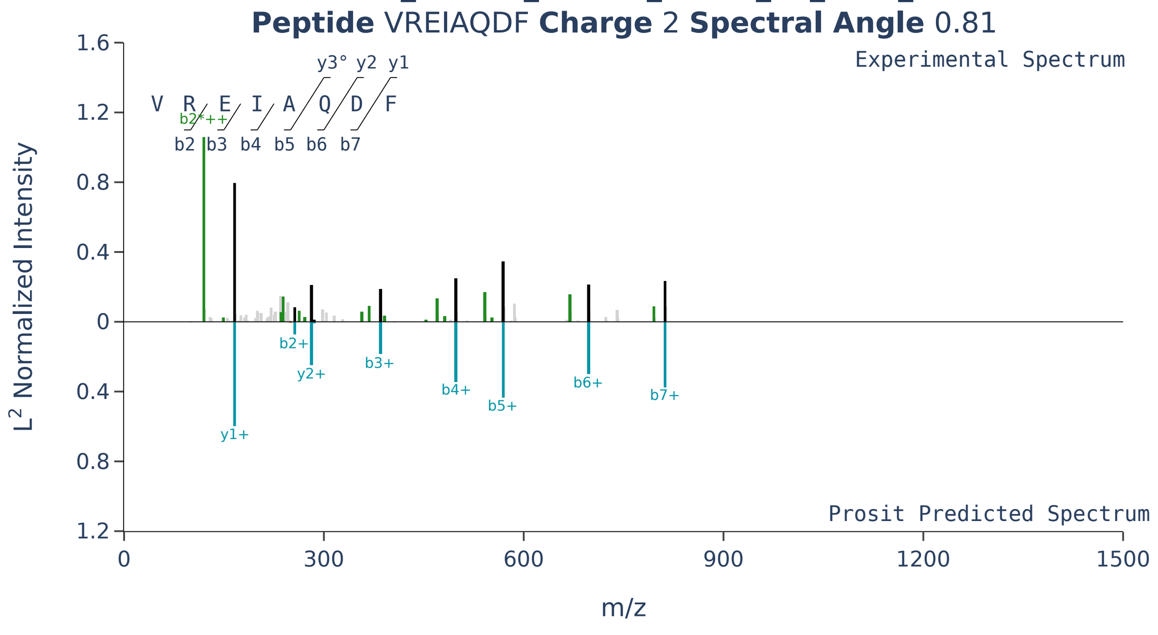

**Source** HRoetschke_271222_120123_HFGoe_H3_b1R1_4h **Scan** 17237
**Peptide** VREIAQDFKTDL **Charge** 2 **Spectral Angle** 0.89

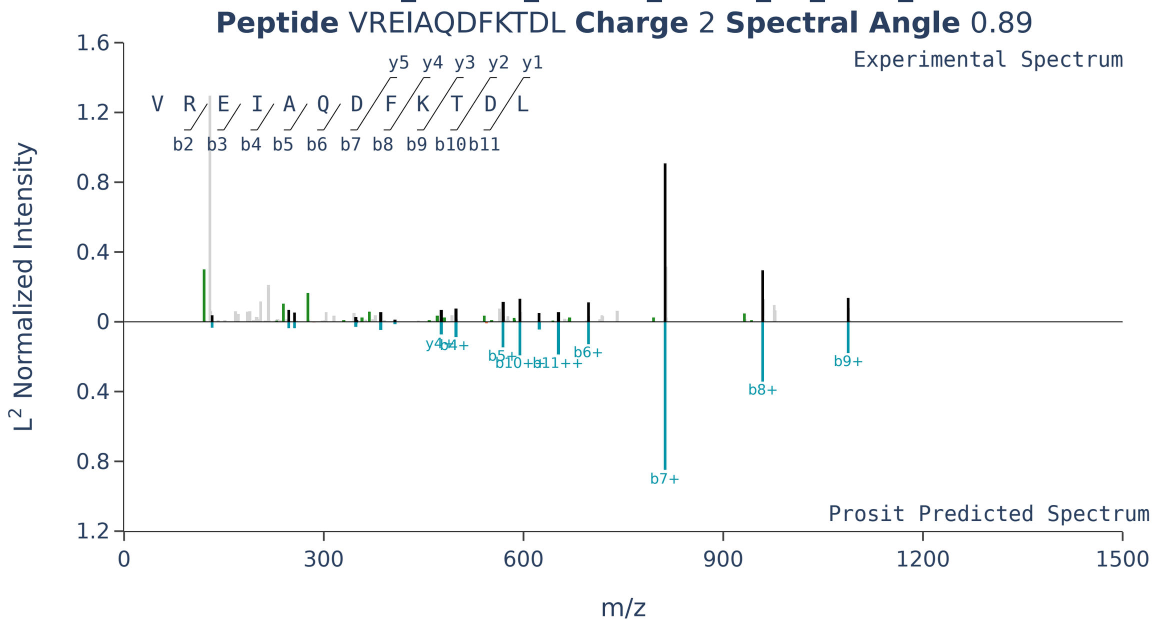

**Source** HRoetschke_271222_120123_HFGoe_H3_b1R1_24h **Scan** 15174
**Peptide** TVALREI **Charge** 2 **Spectral Angle** 0.89

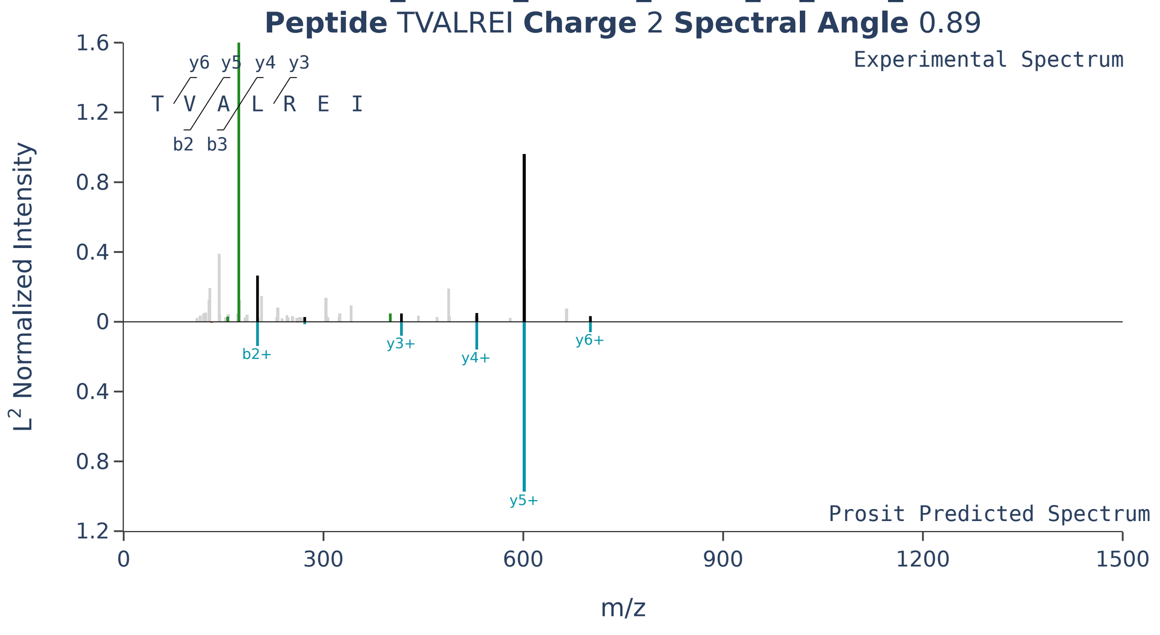

**Source** HRoetschke_271222_120123_HFGoe_H3_b1R1_1h **Scan** 13769
**Peptide** IRKLPFQRL **Charge** 3 **Spectral Angle** 0.91

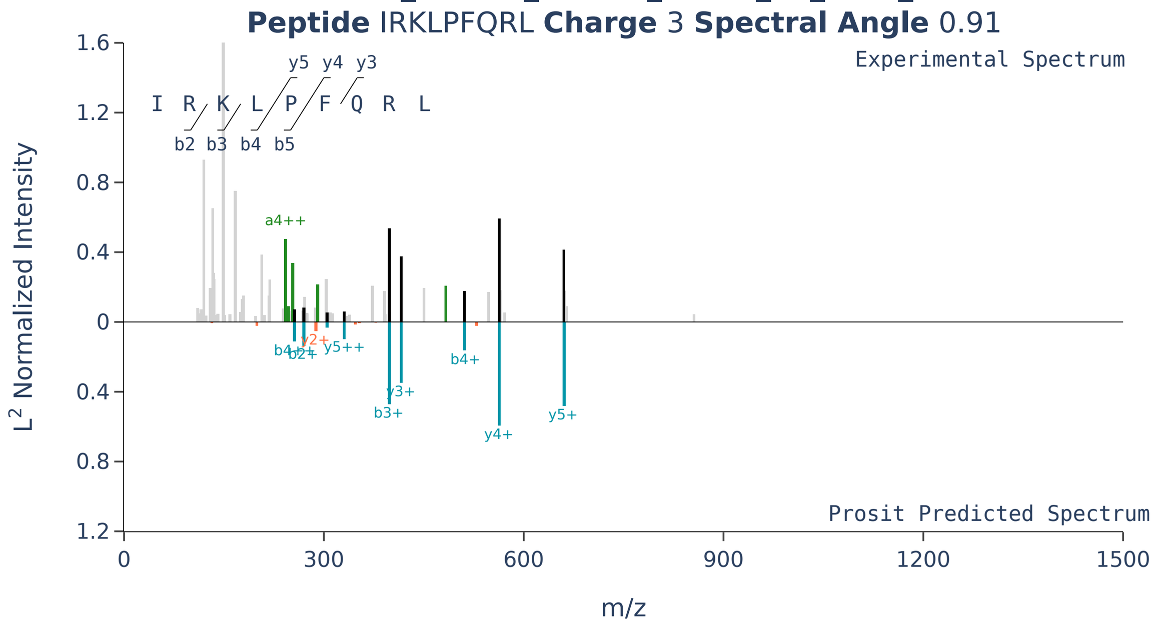

**Source** HRoetschke_271222_120123_HFGoe_H3_b2R1_4h **Scan** 12761
**Peptide** TIMPKDI **Charge** 1 **Spectral Angle** 0.85

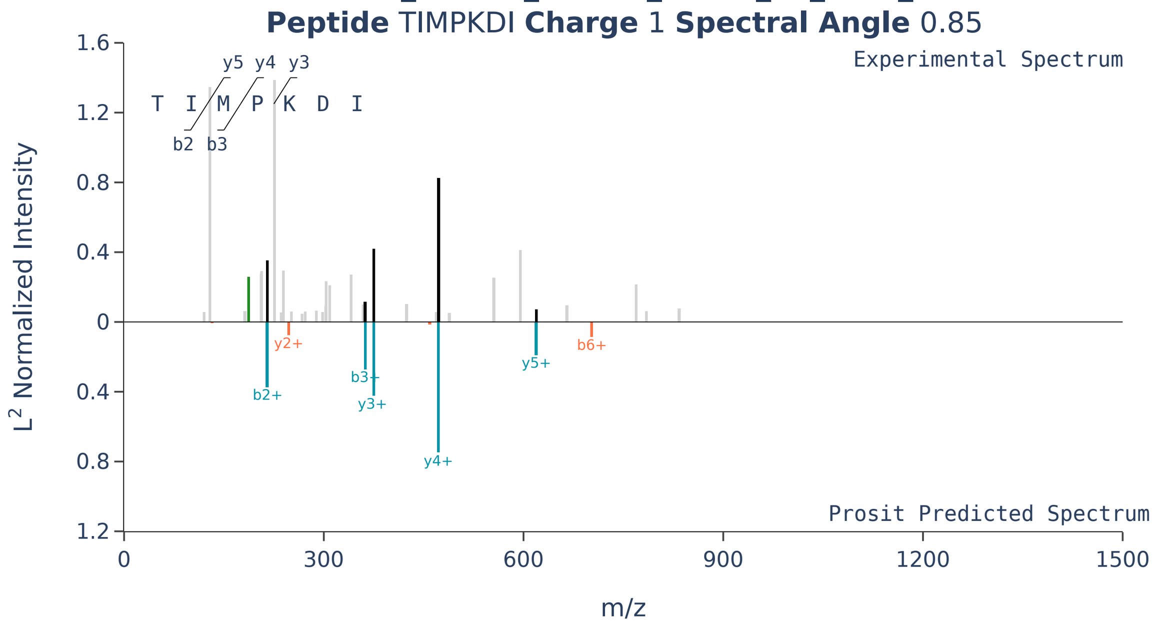

**Source** HRoetschke_271222_120123_HFGoe_H3_b1R1_24h **Scan** 17837
**Peptide** AQDFKTDLRF **Charge** 3 **Spectral Angle** 0.89

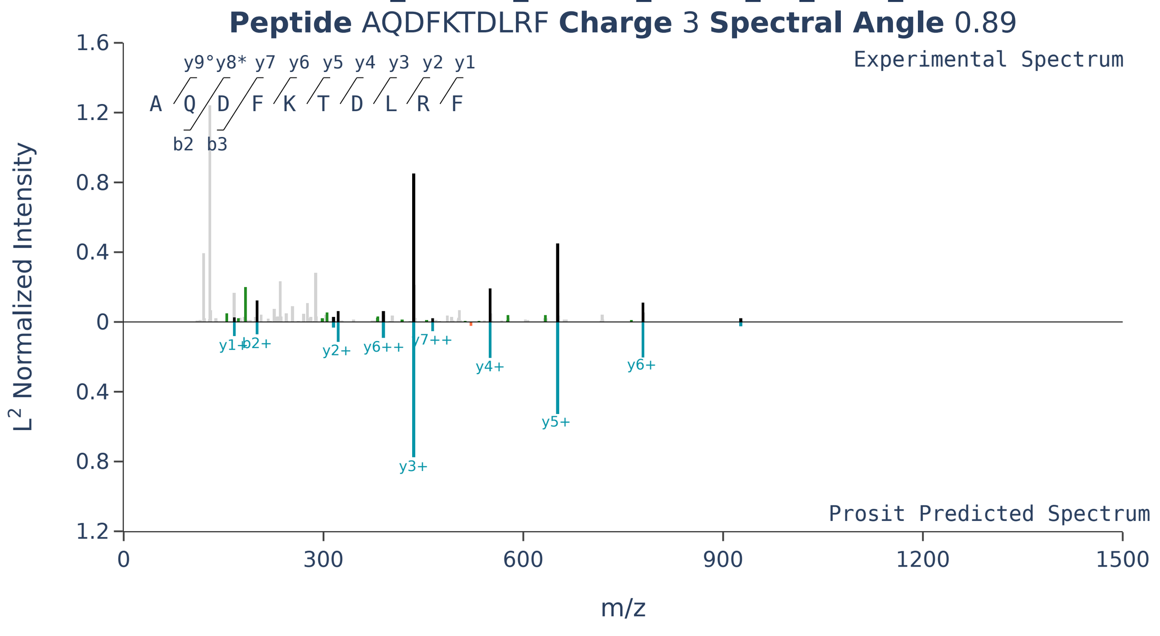

**Source** HRoetschke_271222_120123_HFGoe_H3_b2R2_4h **Scan** 14524
**Peptide** RPGTVALREI **Charge** 3 **Spectral Angle** 0.88

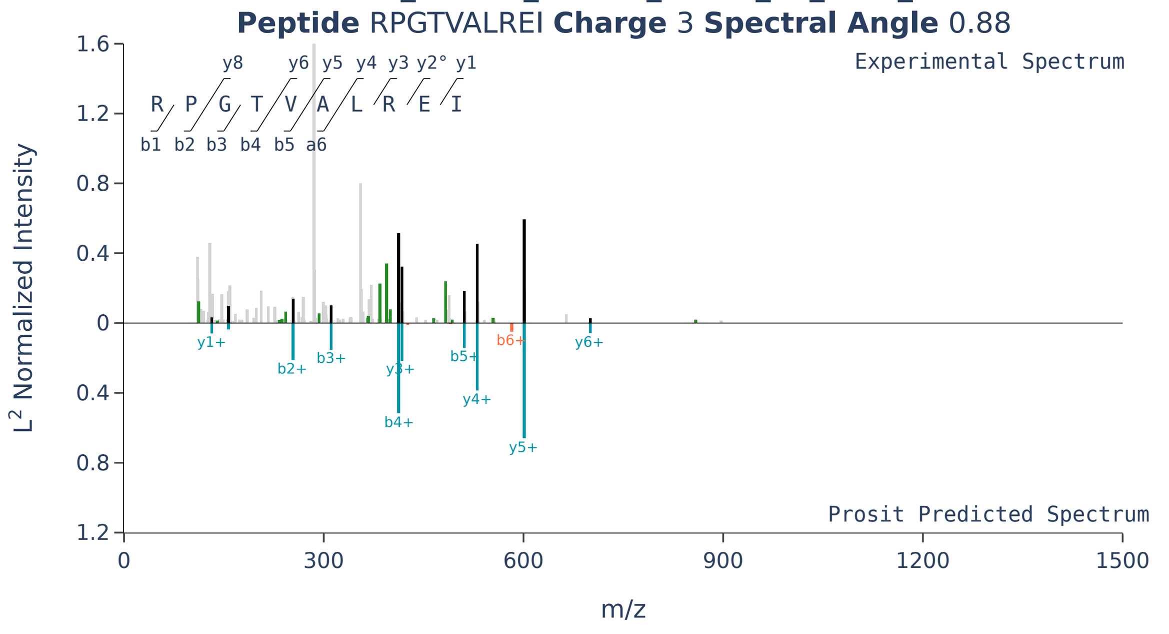


**Source** HRoetschke_271222_120123_HFGoe_H3_b2R2_4h **Scan** 20040
**Peptide** LFEDTNLC **Charge** 2 **Spectral Angle** 0.97

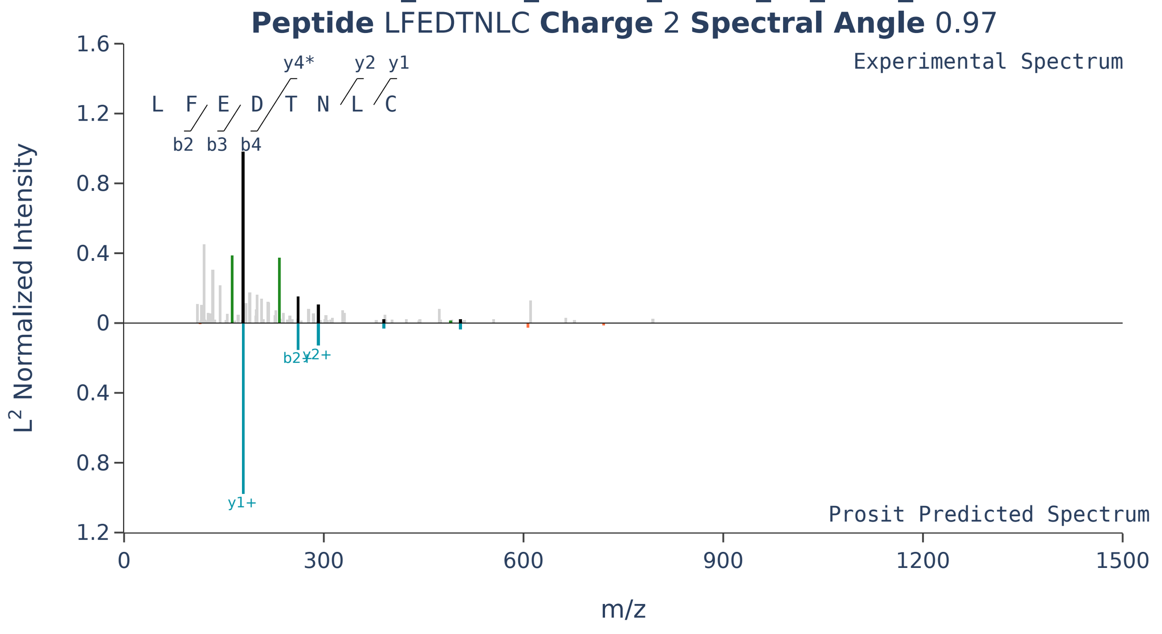


**Source** HRoetschke_271222_120123_HFGoe_H3_b1R1_1h **Scan** 15912
**Peptide** PGTVALREI **Charge** 2 **Spectral Angle** 0.82

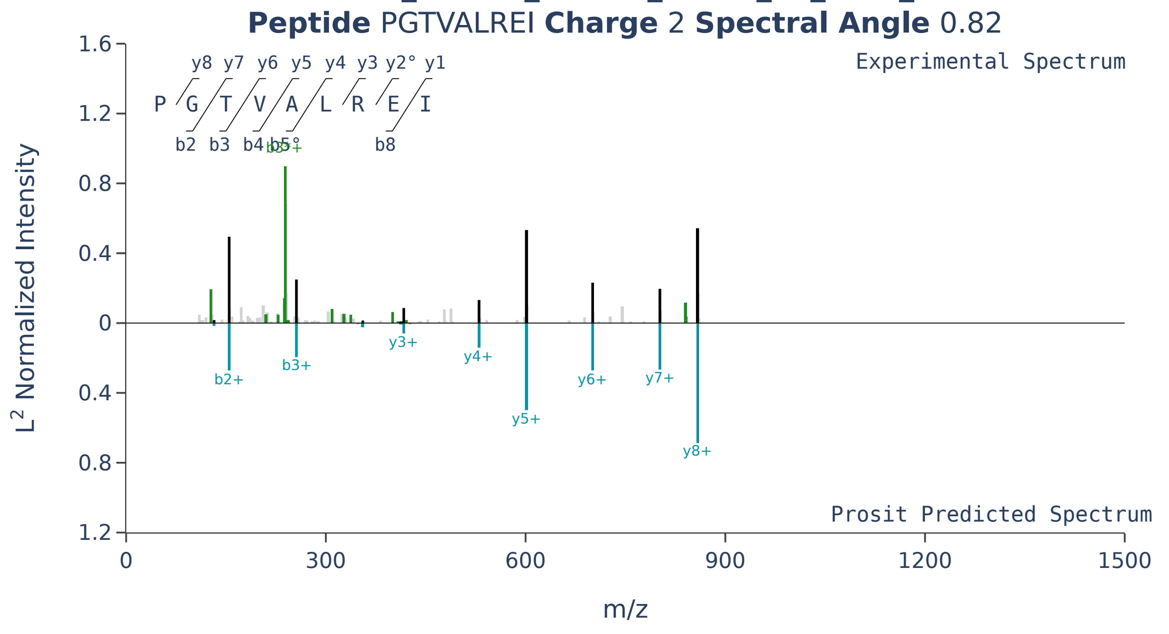


**Source** HRoetschke_271222_120123_HFGoe_H3_b1R2_2h **Scan** 10343
**Peptide** DFKTDLR **Charge** 2 **Spectral Angle** 0.9

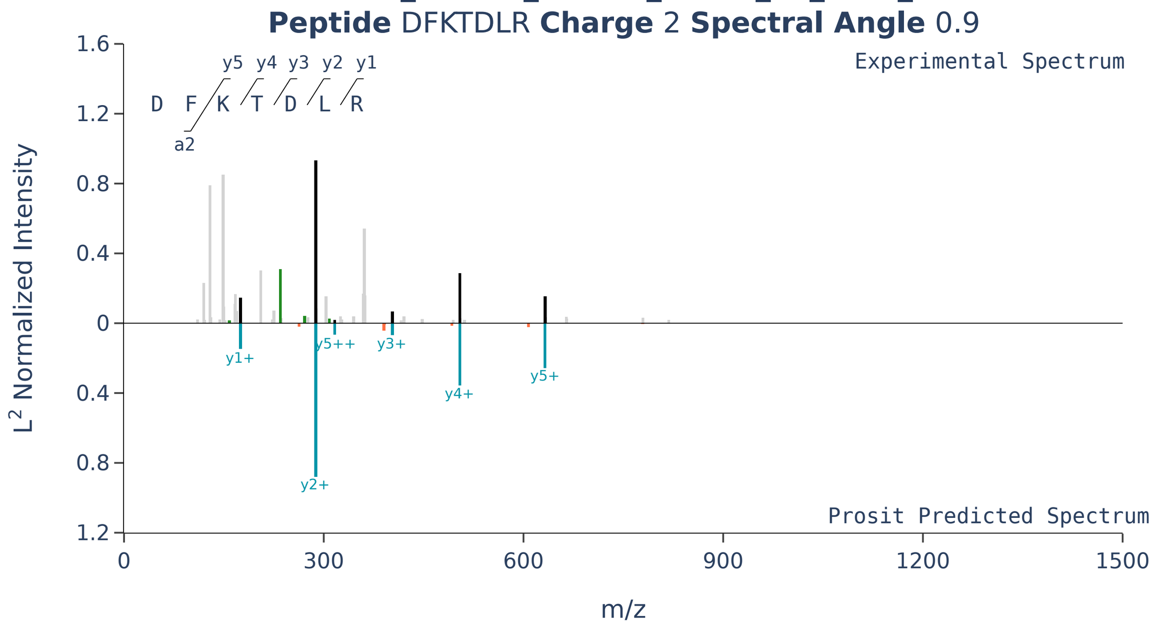


**Source** HRoetschke_271222_120123_HFGoe_H3_b2R2_2h **Scan** 15138
**Peptide** EIAQDFKTDLR **Charge** 3 **Spectral Angle** 0.88

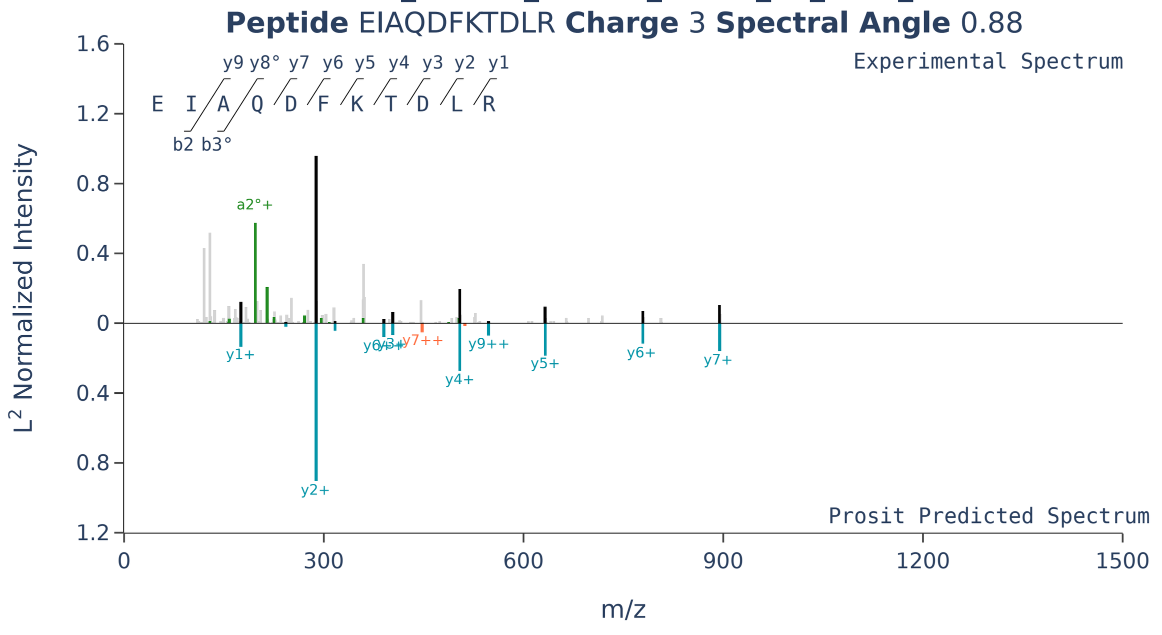


**Source** HRoetschke_271222_120123_HFGoe_H3_b2R1_24h **Scan** 12222
**Peptide** VREIAQDFKTD **Charge** 3 **Spectral Angle** 0.93

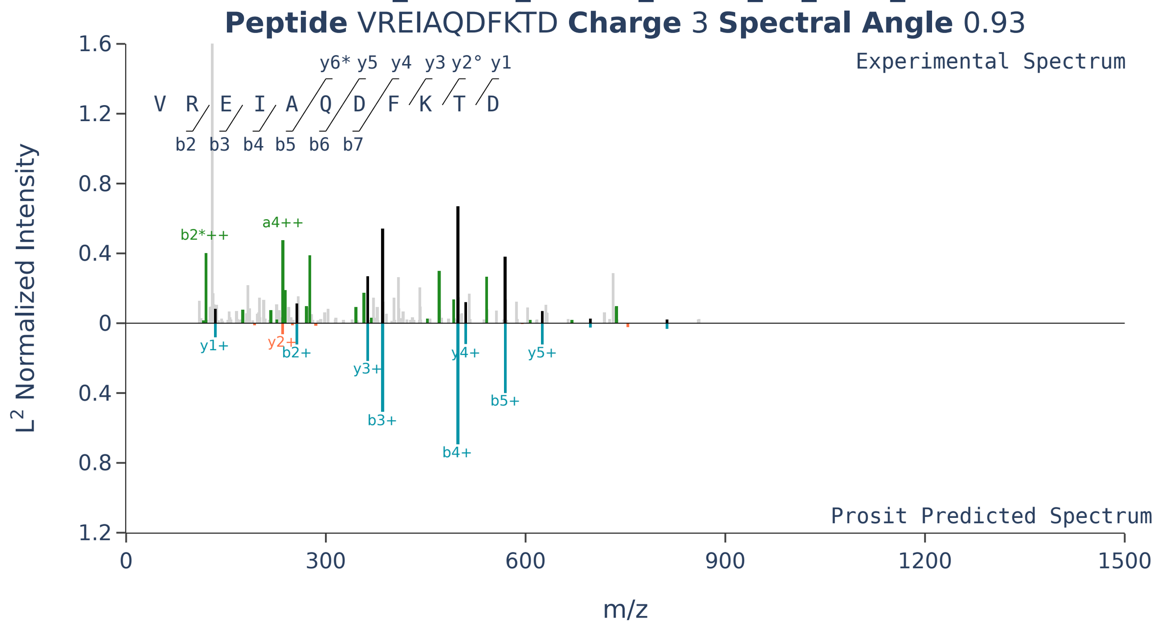


**Source** HRoetschke_271222_120123_HFGoe_H3_b1R2_24h **Scan** 12765
**Peptide** KSTELLLR **Charge** 2 **Spectral Angle** 0.82

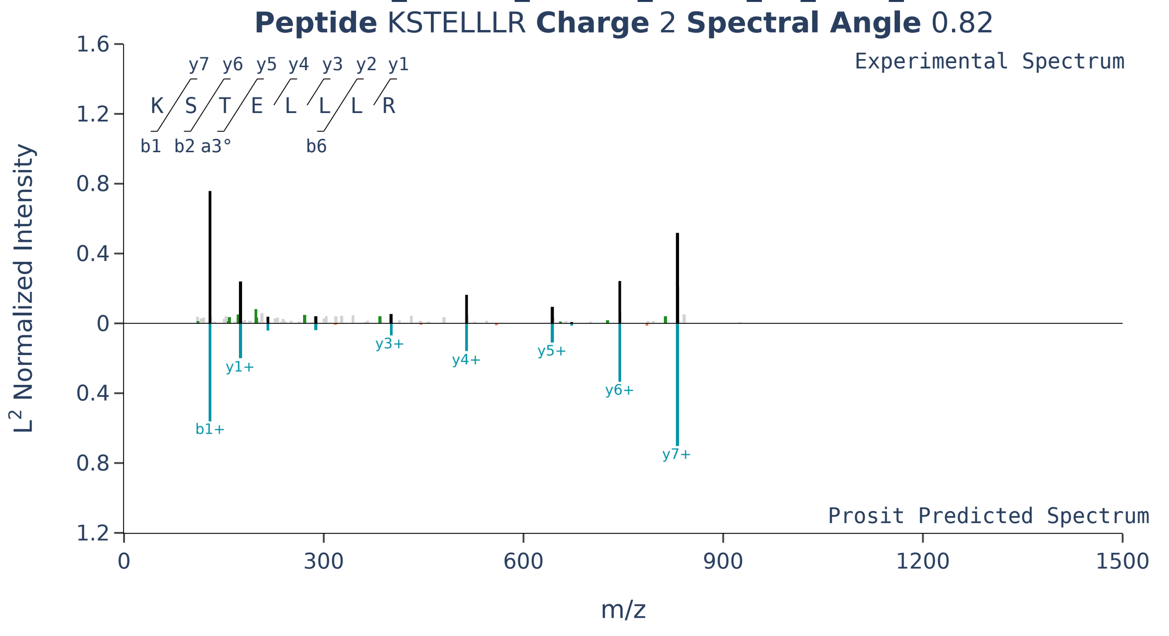


**Source** HRoetschke_271222_120123_HFGoe_H3_b1R1_1h **Scan** 23788
**Peptide** ASEAYLVAL **Charge** 1 **Spectral Angle** 0.85

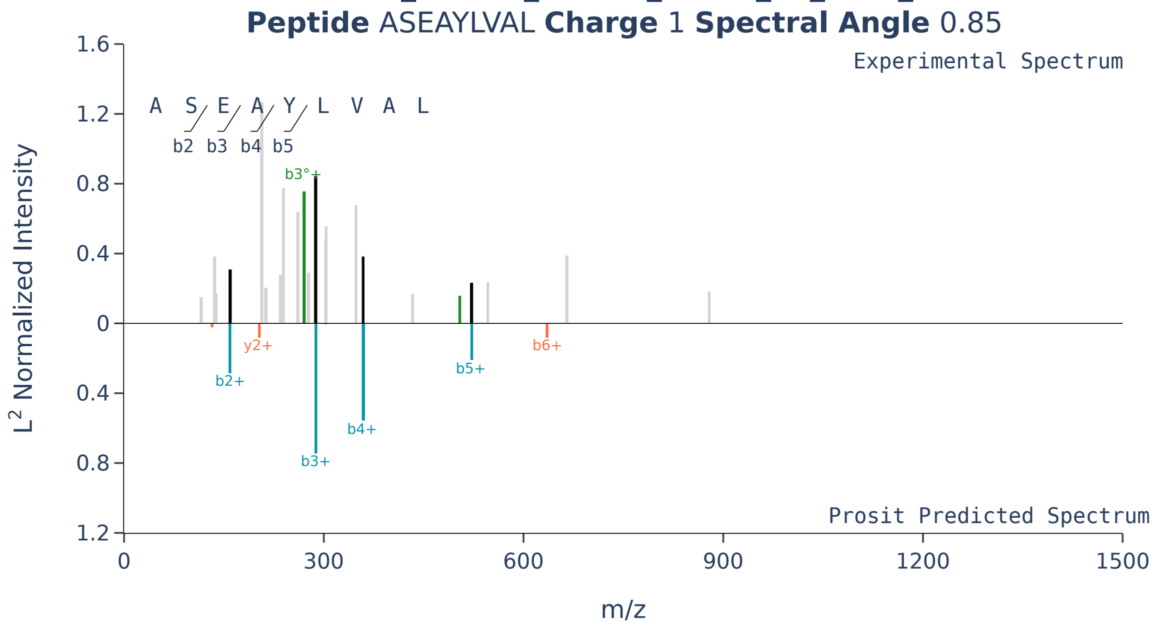


**Source** HRoetschke_271222_120123_HFGoe_H3_b1R2_4h **Scan** 13484
**Peptide** QDFKTDL **Charge** 2 **Spectral Angle** 0.9

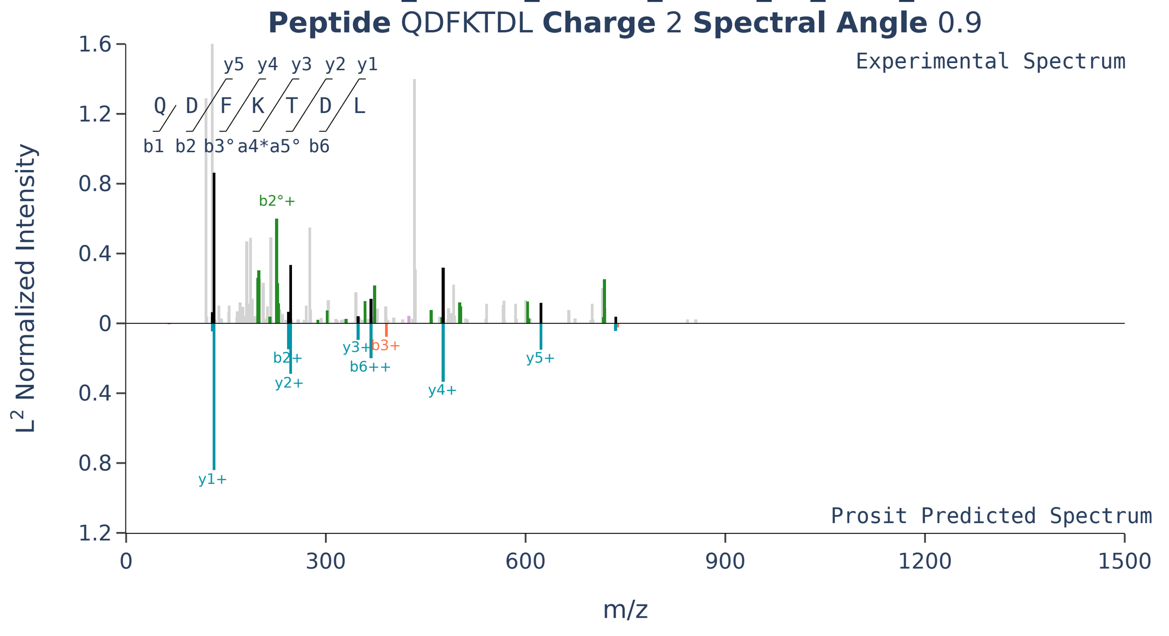


**Source** HRoetschke_271222_120123_HFGoe_H3_b1R1_24h **Scan** 17749
**Peptide** QDFKTDLRF **Charge** 3 **Spectral Angle** 0.91

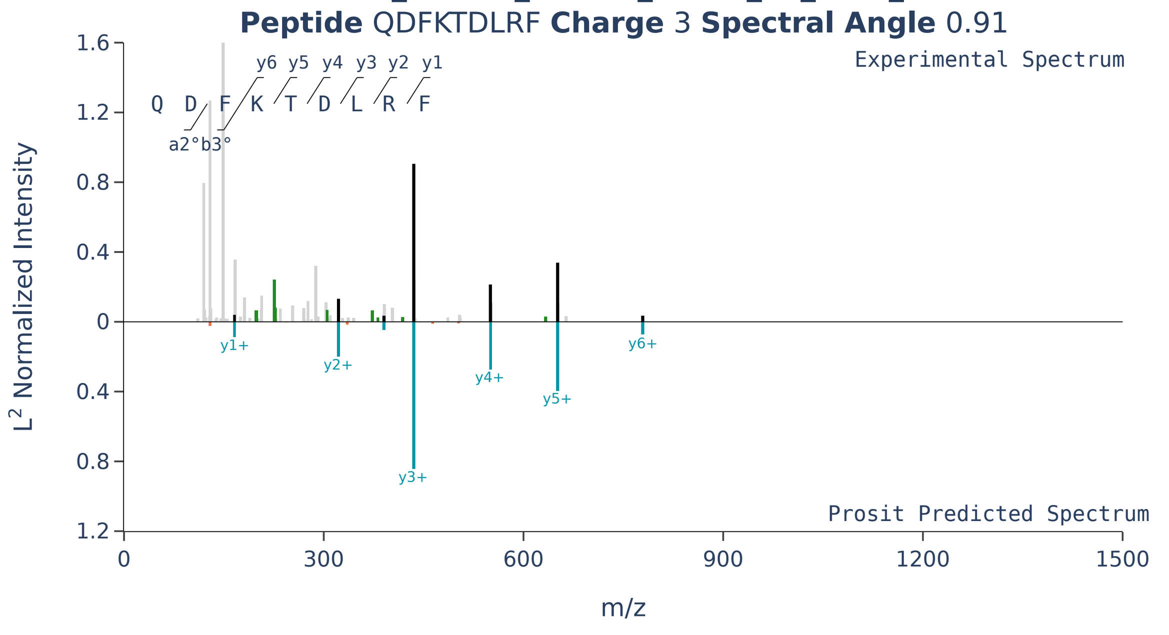


**Source** HRoetschke_271222_120123_HFGoe_H3_b1R1_4h **Scan** 12799
**Peptide** SSAVMALQE **Charge** 2 **Spectral Angle** 0.95

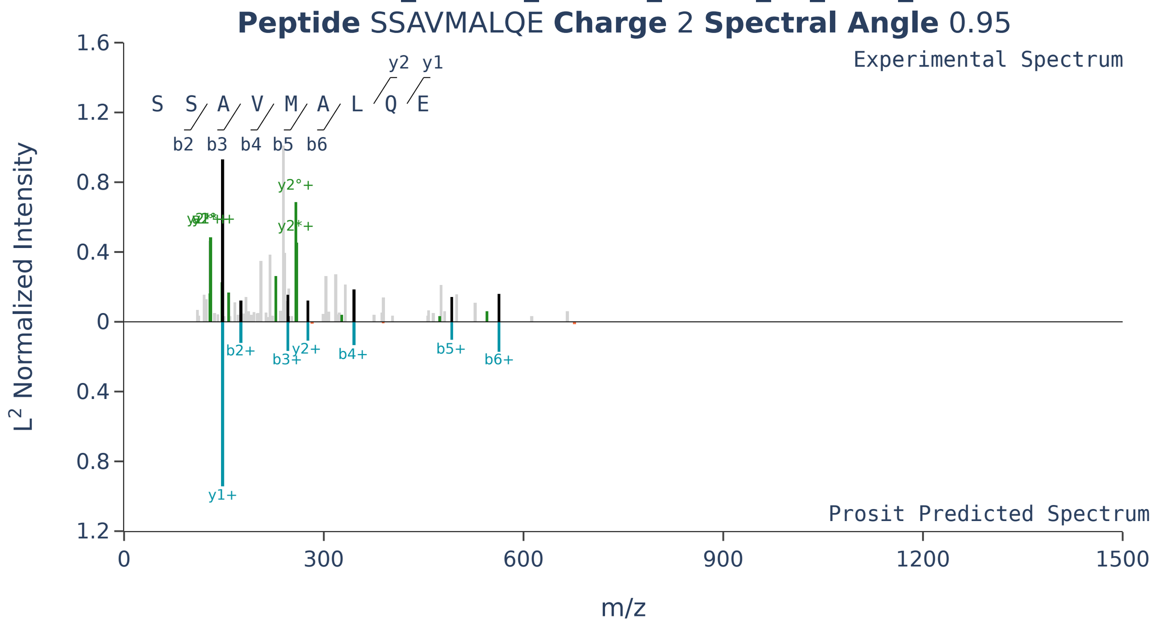


**Source** HRoetschke_271222_120123_HFGoe_H3_b1R1_4h **Scan** 16371
**Peptide** PFQRLVREI **Charge** 3 **Spectral Angle** 0.96

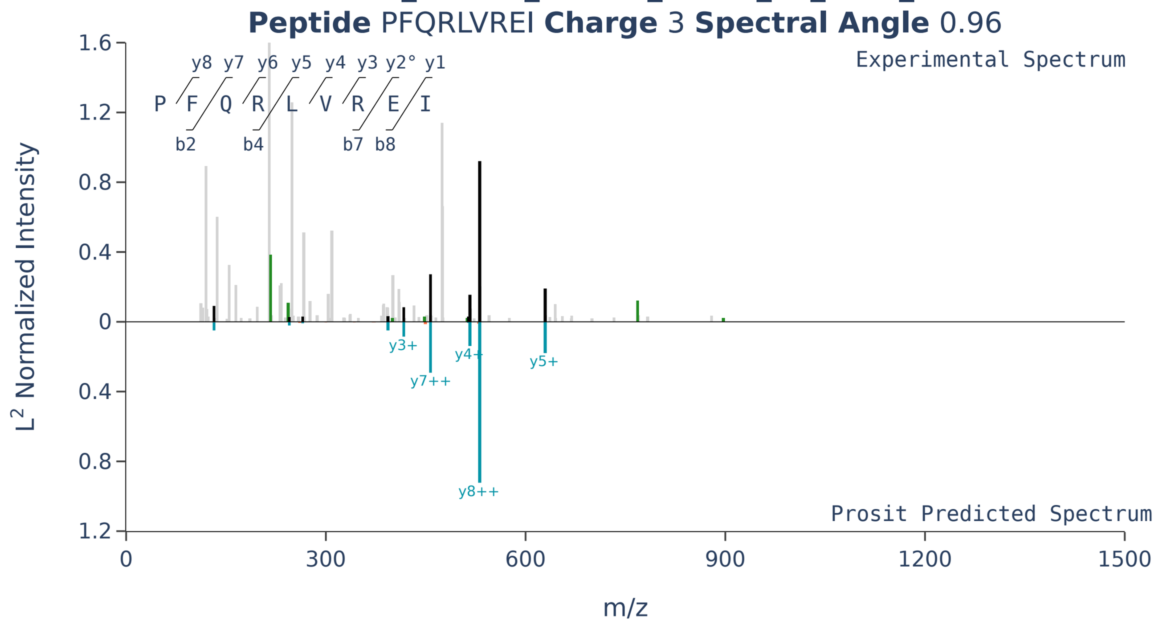


**Source** HRoetschke_271222_120123_HFGoe_H3_b2R2_2h **Scan** 12102
**Peptide** QSSAVMALQ **Charge** 2 **Spectral Angle** 0.93

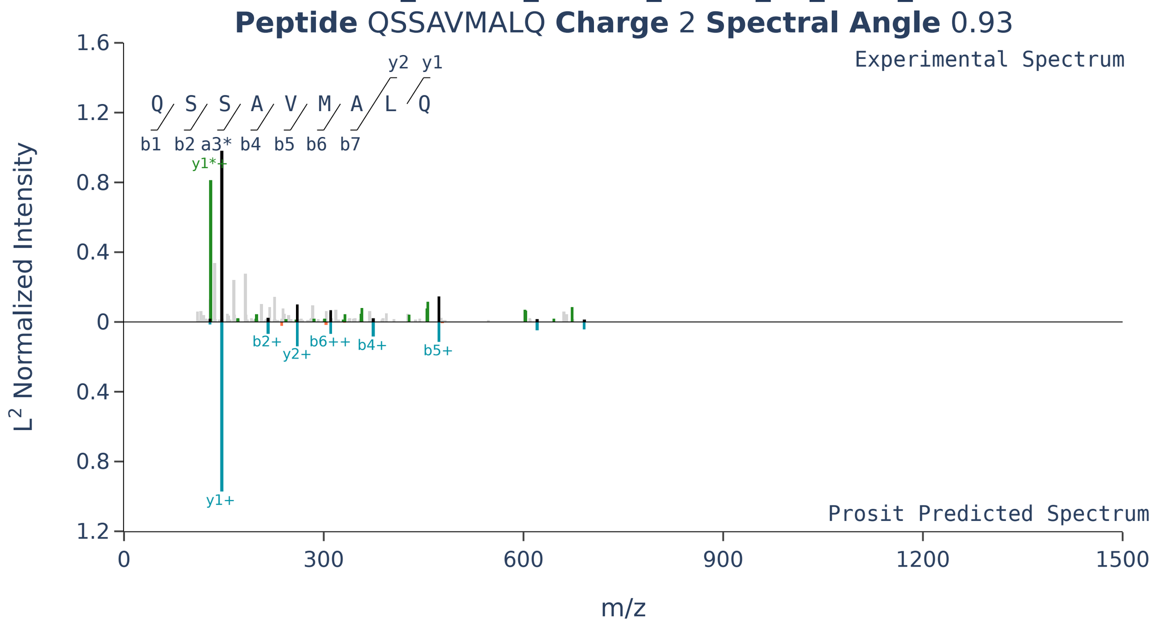


**Source** HRoetschke_271222_120123_HFGoe_H3_b2R1_2h **Scan** 14068
**Peptide** QKSTELLIRKL **Charge** 3 **Spectral Angle** 0.92

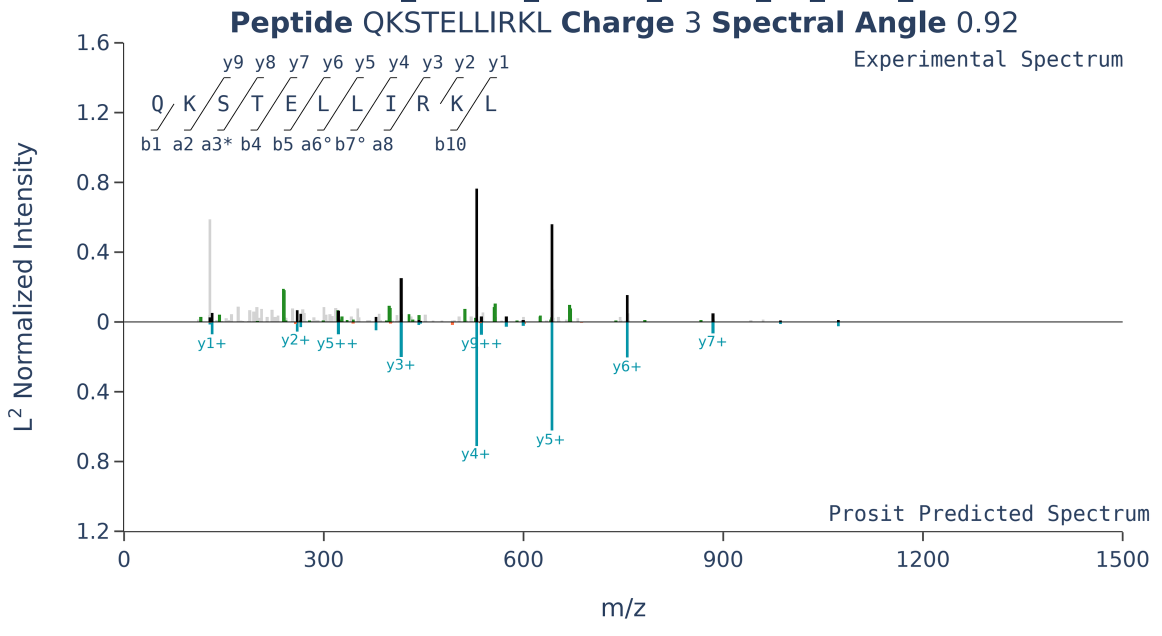


**Source** HRoetschke_271222_120123_HFGoe_H3_b2R1_4h **Scan** 13696
**Peptide** REIAQDFKTDLR **Charge** 4 **Spectral Angle** 0.89

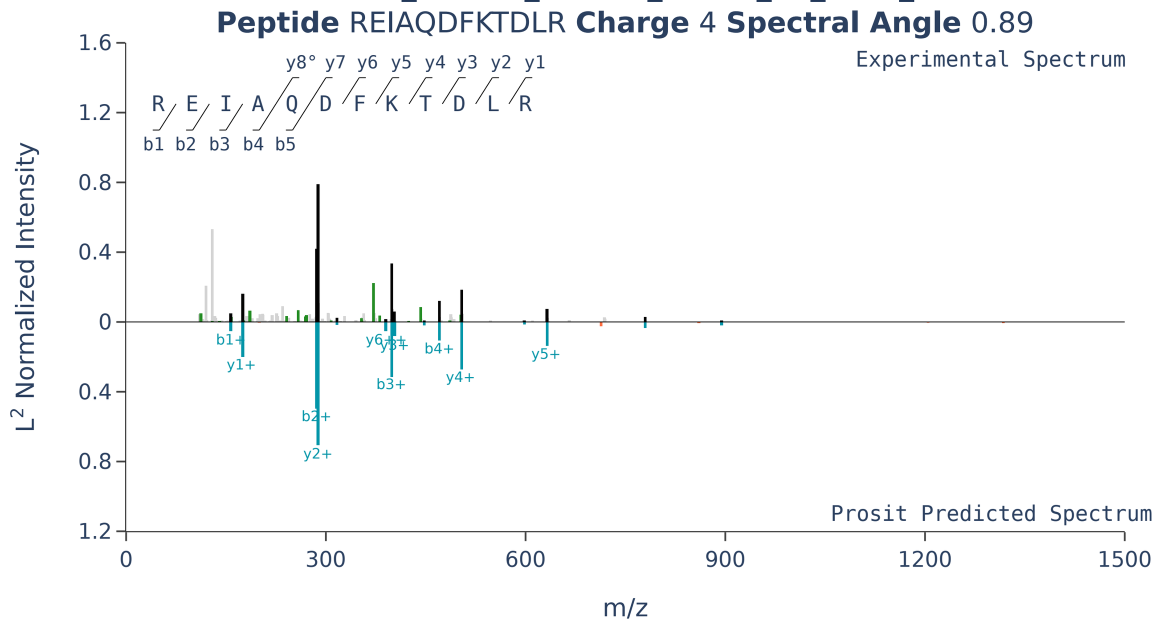

**Source** HRoetschke_271222_120123_HFGoe_H3_b2R2_2h **Scan** 12580
**Peptide** QKSTELLIR **Charge** 2 **Spectral Angle** 0.86

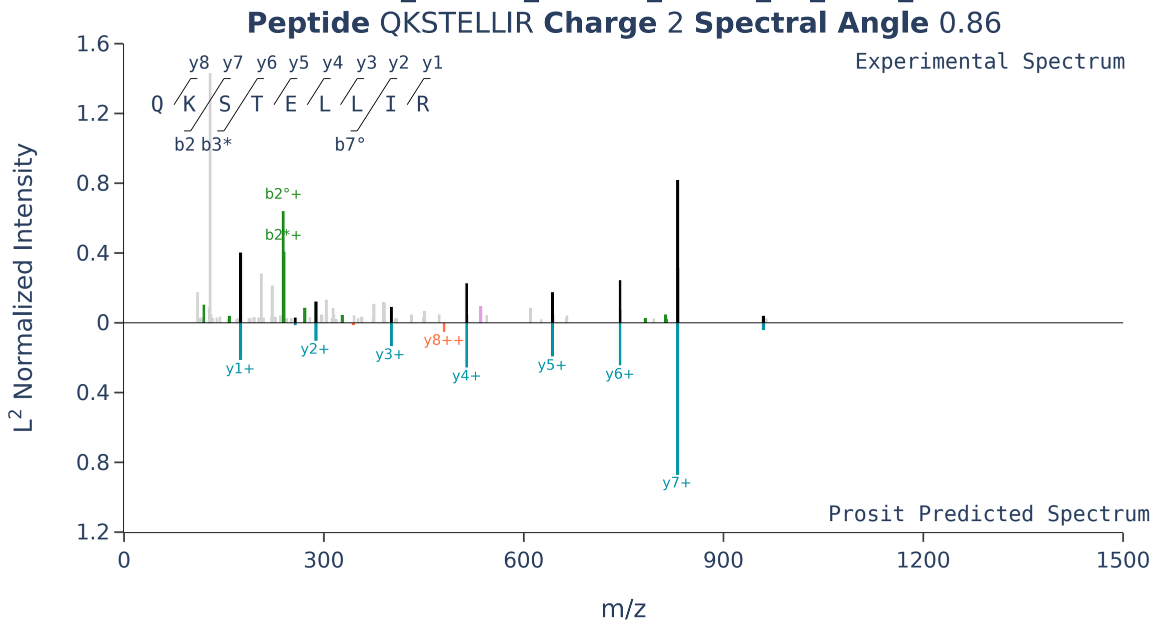


**Source** HRoetschke_271222_120123_HFGoe_H3_b2R1_2h **Scan** 9238
**Peptide** NLCAIHAKRV **Charge** 3 **Spectral Angle** 0.87

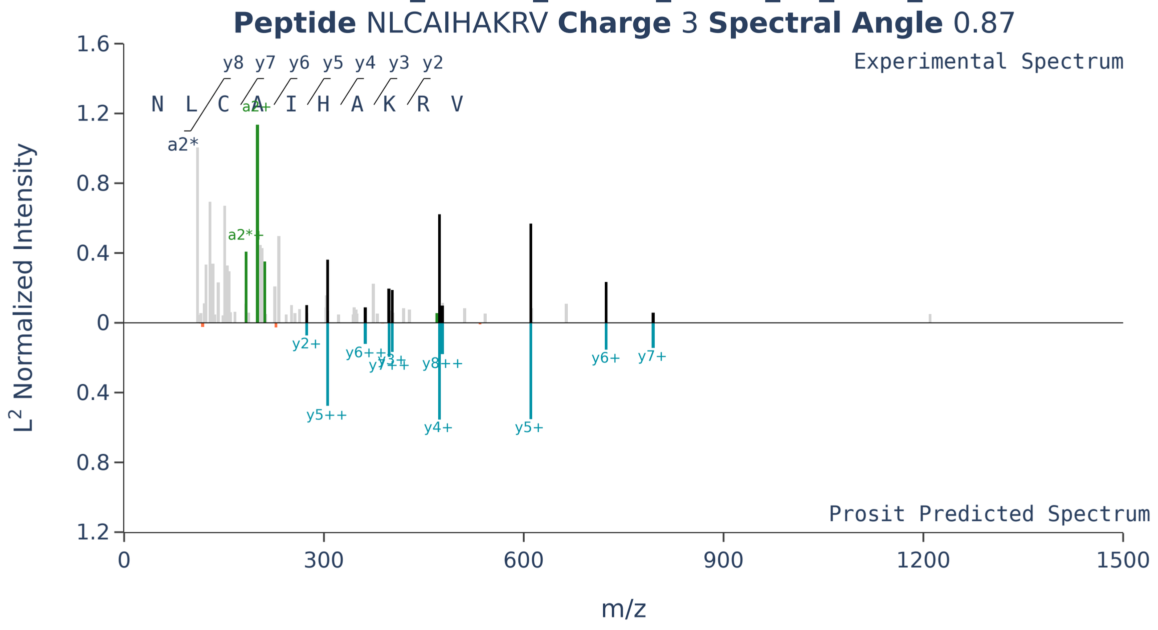


**Source** HRoetschke_271222_120123_HFGoe_H3_b1R2_24h **Scan** 9810
**Peptide** RFQSSAVMA **Charge** 2 **Spectral Angle** 0.88

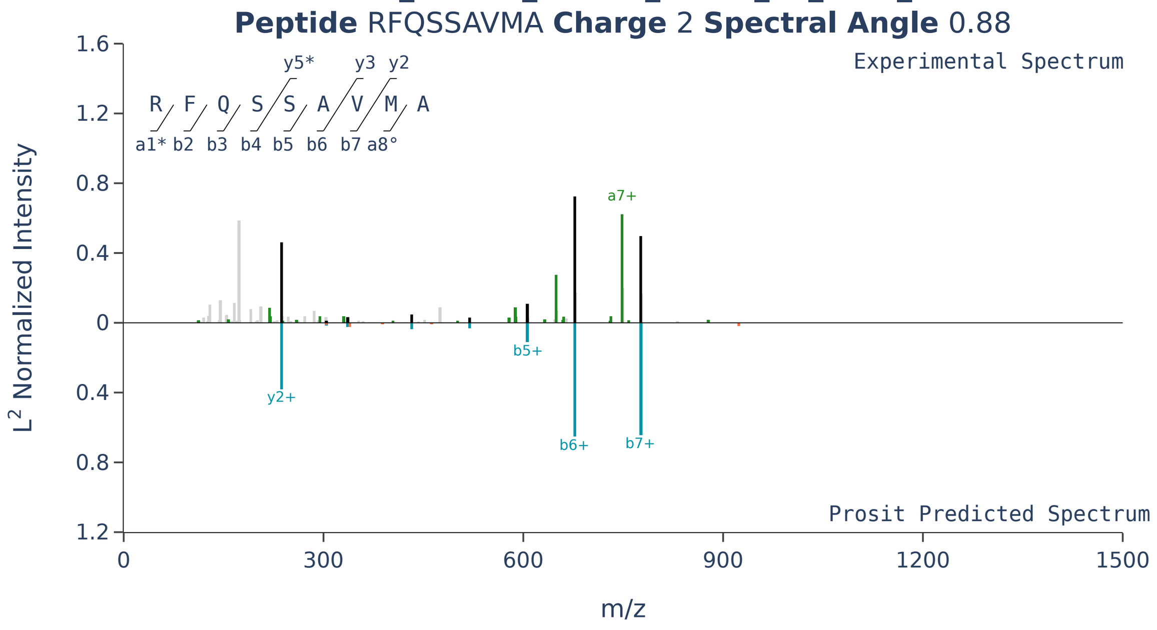


**Source** HRoetschke_271222_120123_HFGoe_H3_b1R1_24h **Scan** 14340
**Peptide** KSTELLIRKL **Charge** 3 **Spectral Angle** 0.78

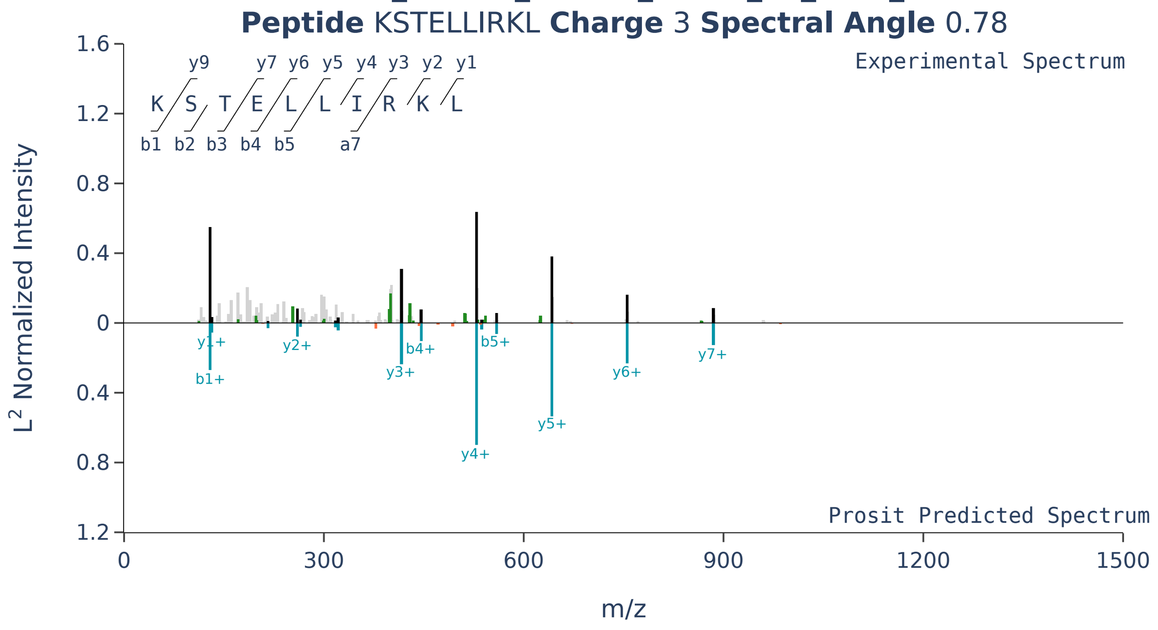


**Source** HRoetschke_271222_120123_HFGoe_H3_b2R2_1h **Scan** 28706
**Peptide** AYLVALFE **Charge** 1 **Spectral Angle** 0.95

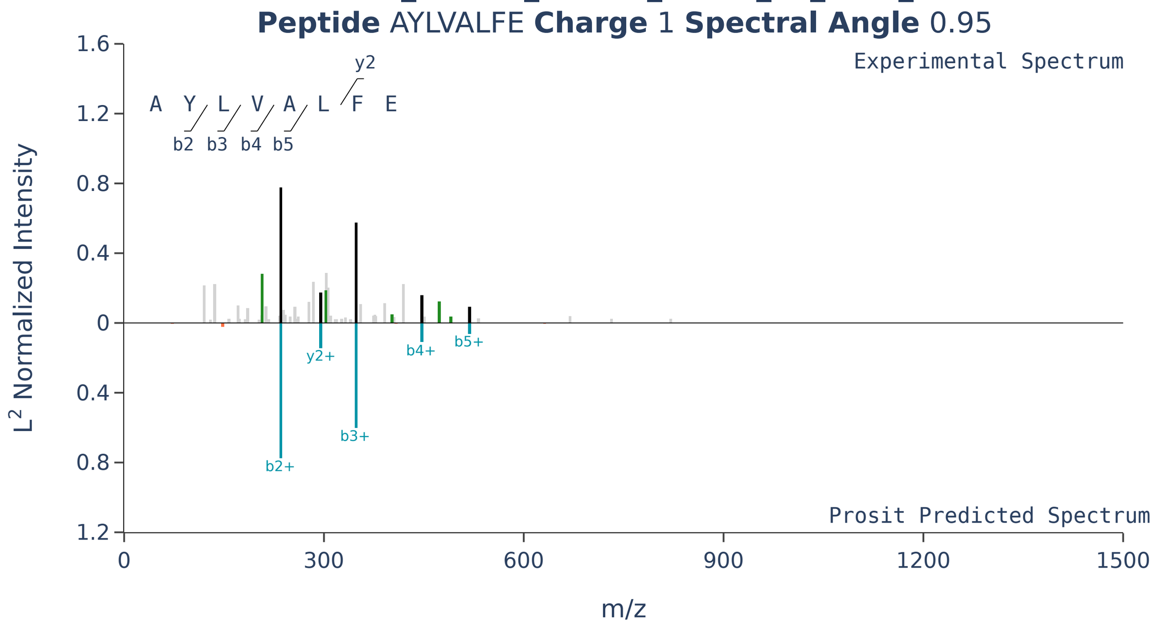


**Source** HRoetschke_271222_120123_HFGoe_H3_b1R2_2h **Scan** 14739
**Peptide** STELLIR **Charge** 2 **Spectral Angle** 0.86

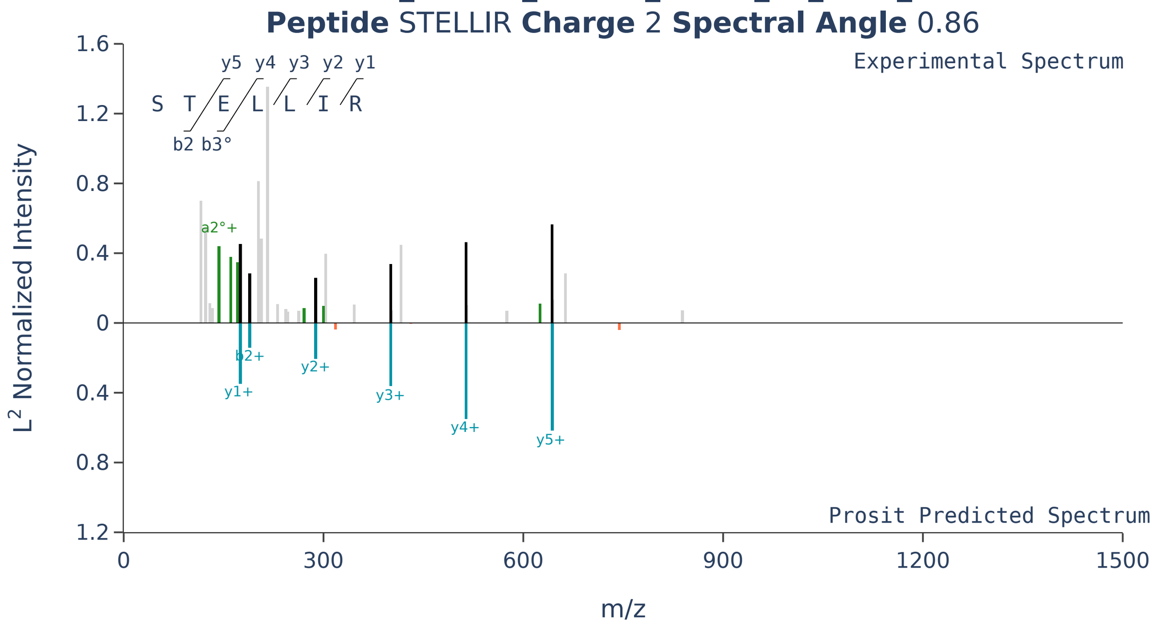


**Source** HRoetschke_271222_120123_HFGoe_H3_b2R1_4h **Scan** 14073
**Peptide** FEDTNLCAIHAKRV **Charge** 4 **Spectral Angle** 0.84

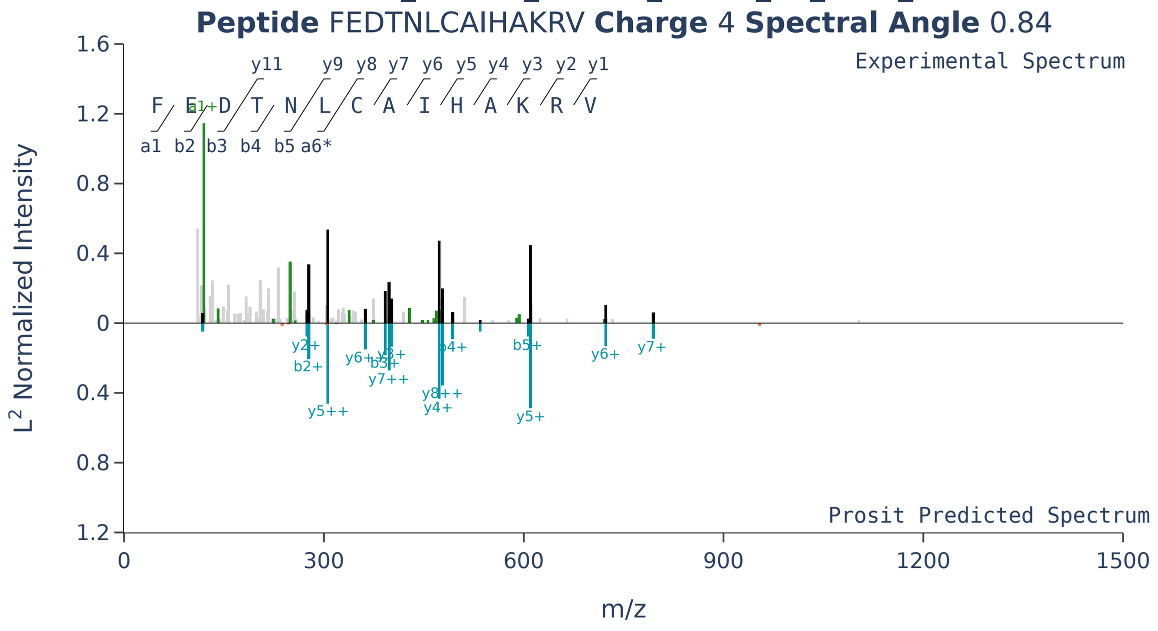


**Source** HRoetschke_271222_120123_HFGoe_H3_b2R2_4h **Scan** 14278
**Peptide** YQKSTELLIR **Charge** 3 **Spectral Angle** 0.88

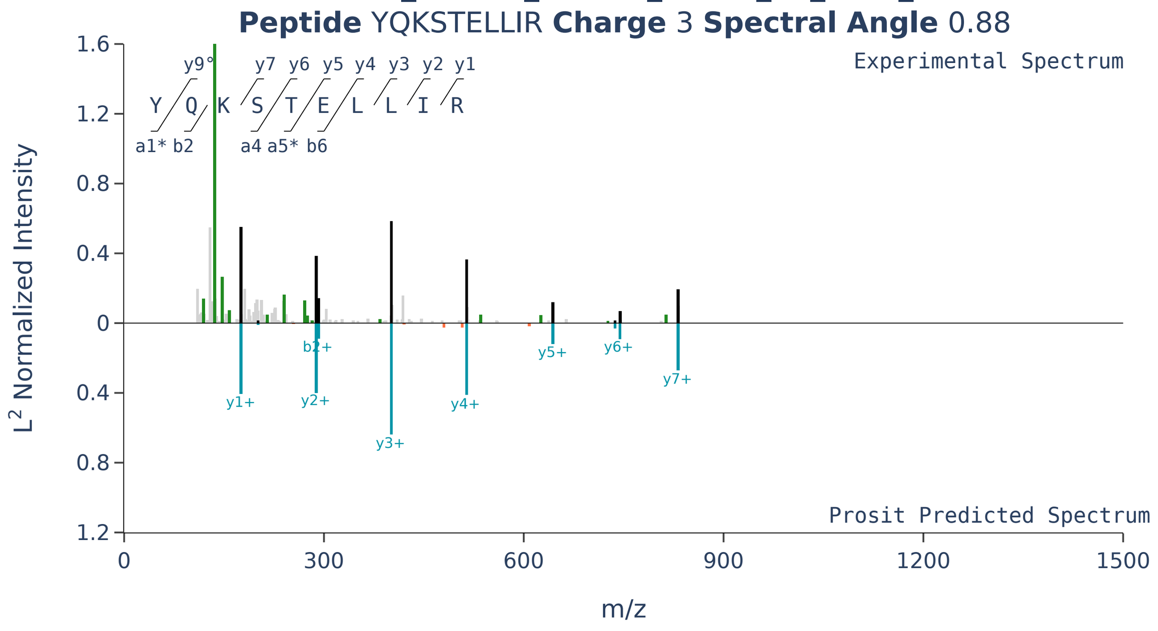


**Source** HRoetschke_271222_120123_HFGoe_H3_b2R2_4h **Scan** 14993
**Peptide** TIMPKDIQLARR **Charge** 2 **Spectral Angle** 0.82

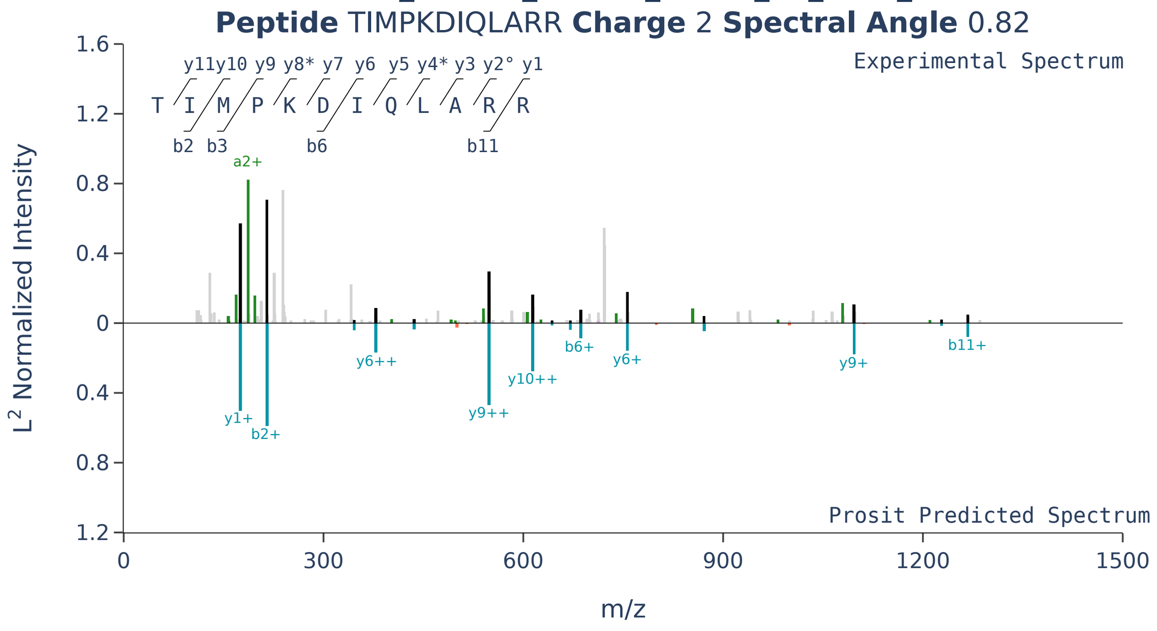


**Source** HRoetschke_271222_120123_HFGoe_H3_b2R2_2h **Scan** 7908
**Peptide** TNLCAIHAKR **Charge** 3 **Spectral Angle** 0.84

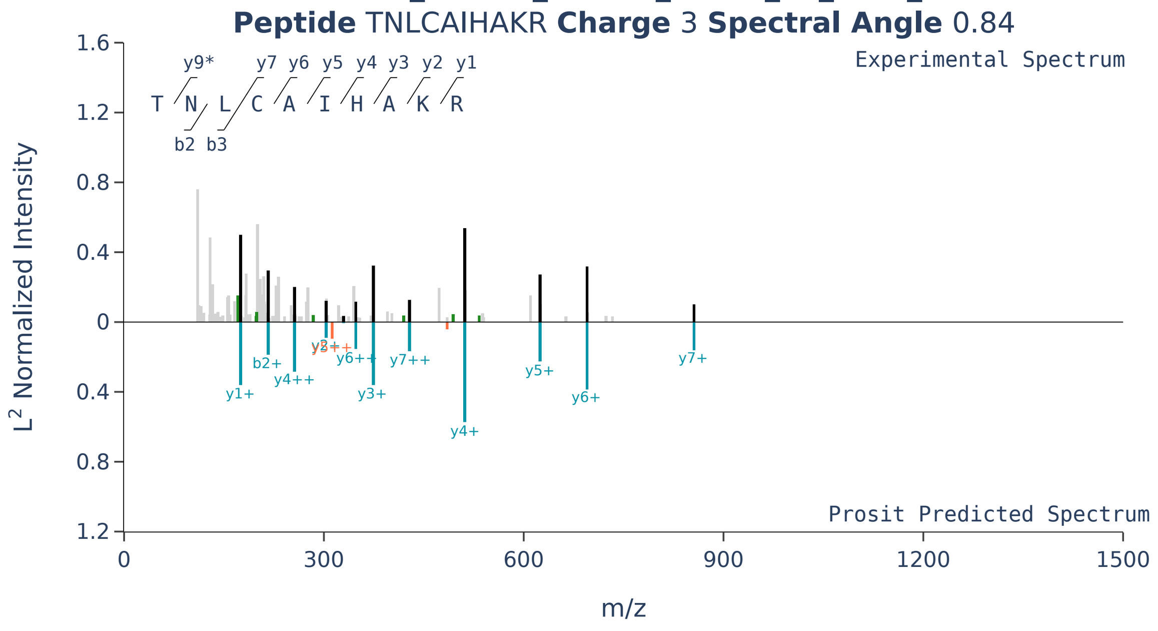


**Source** HRoetschke_271222_120123_HFGoe_H3_b2R2_4h **Scan** 16006
**Peptide** DLRFQSSAVM **Charge** 2 **Spectral Angle** 0.85

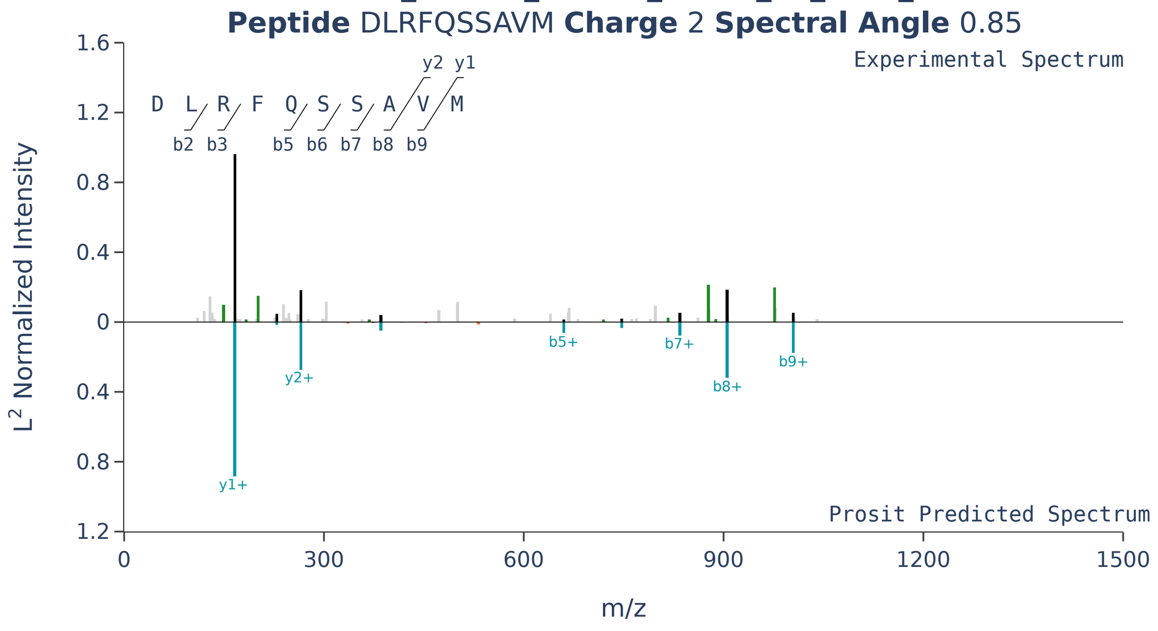


**Source** HRoetschke_271222_120123_HFGoe_H3_b2R2_4h **Scan** 10702
**Peptide** VREIAQDFK **Charge** 3 **Spectral Angle** 0.85

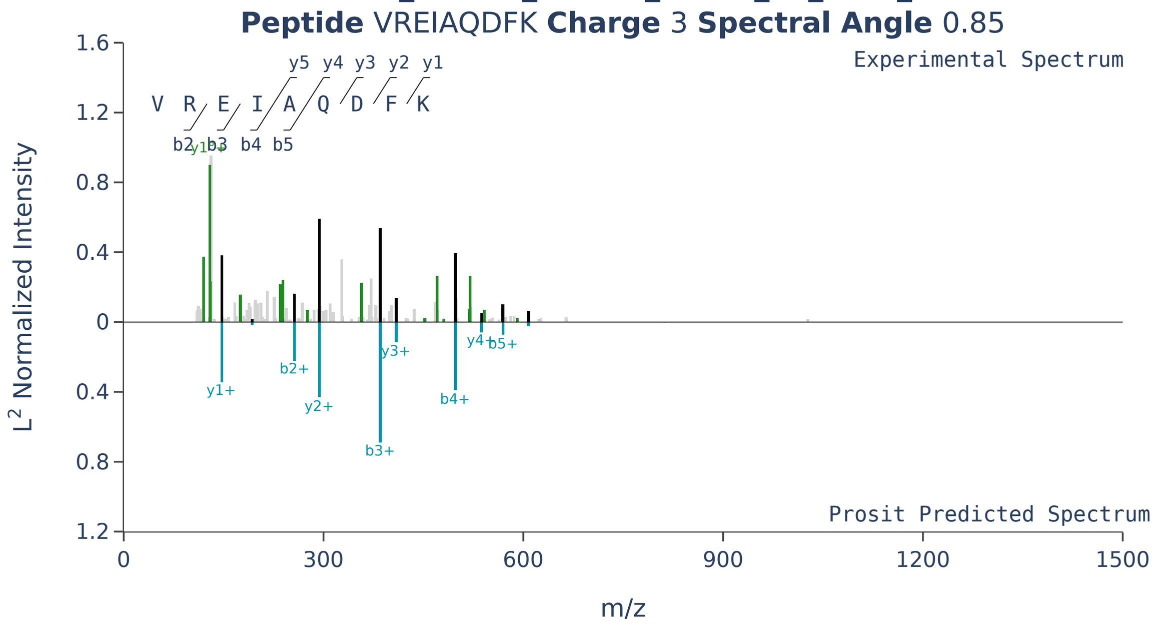


**Source** HRoetschke_271222_120123_HFGoe_H3_b2R2_2h **Scan** 8626
**Peptide** REIAQDFK **Charge** 2 **Spectral Angle** 0.88

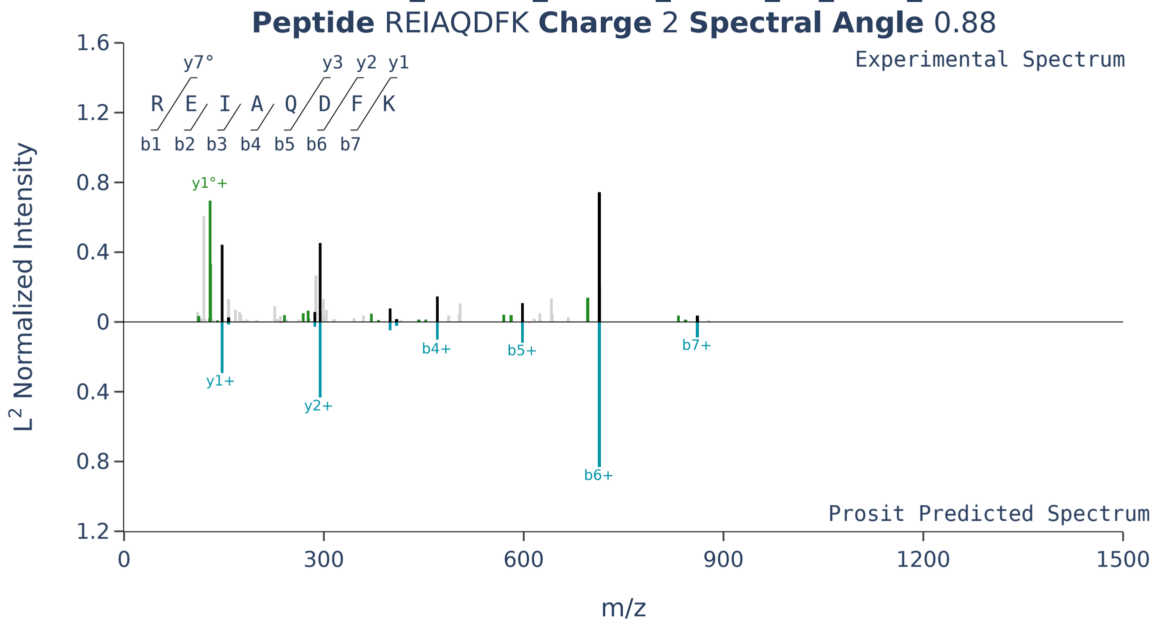


**Source** HRoetschke_271222_120123_HFGoe_H3_b2R2_4h **Scan** 17419
**Peptide** IAQDFKTDL **Charge** 1 **Spectral Angle** 0.84

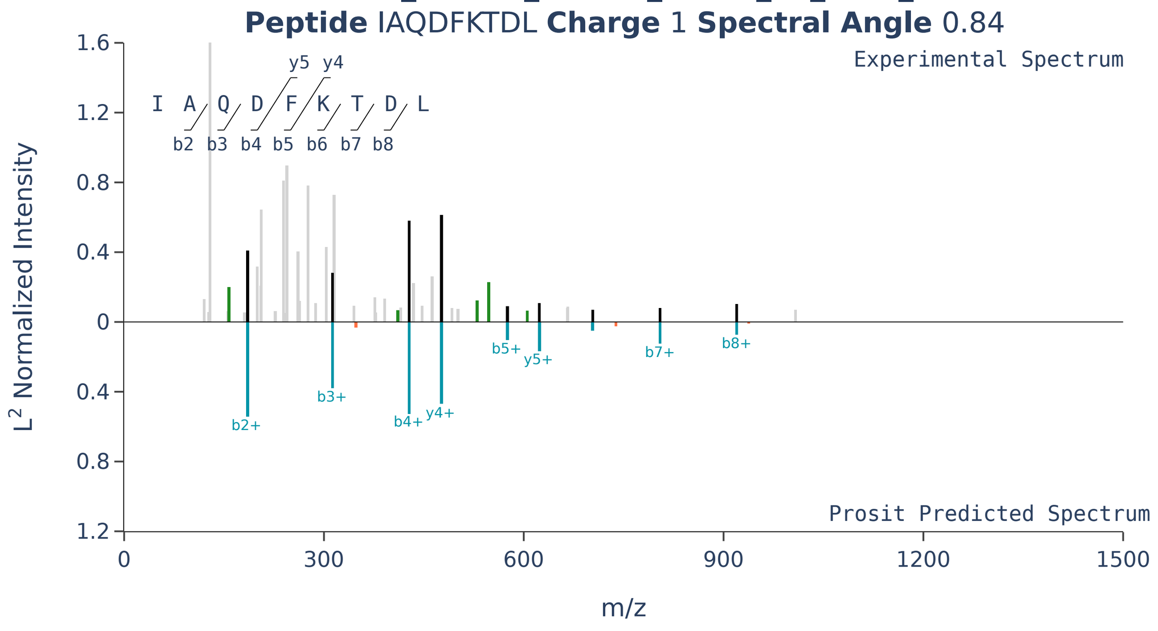


**Source** HRoetschke_271222_120123_HFGoe_H3_b2R2_2h **Scan** 7559
**Peptide** RPGTVALR **Charge** 2 **Spectral Angle** 0.85

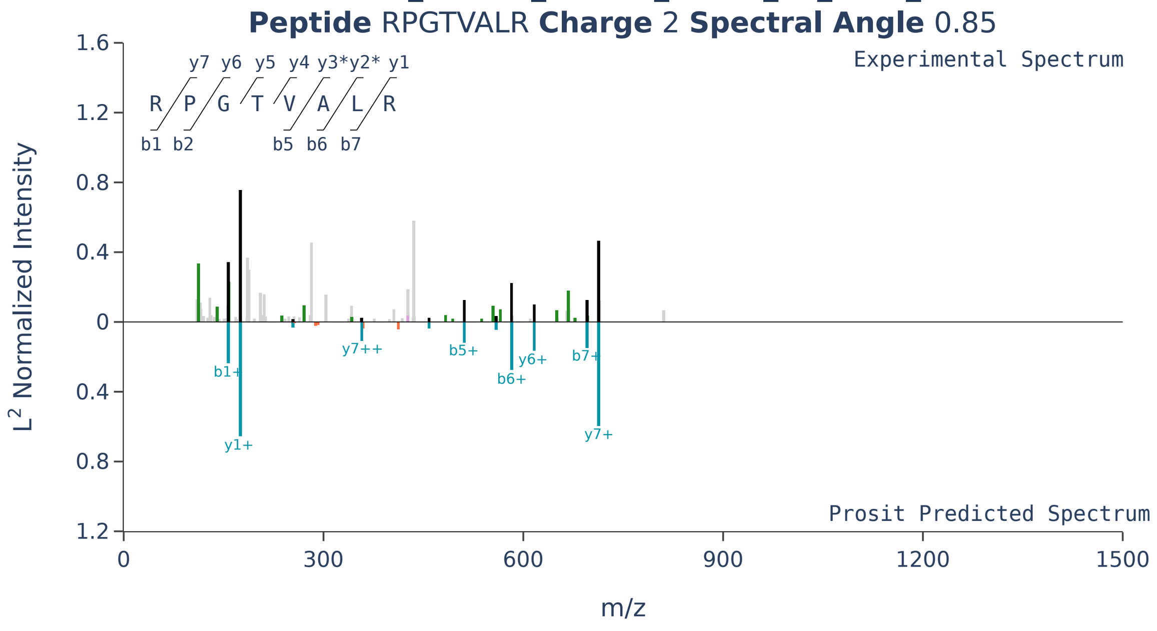

**Source** HRoetschke_271222_120123_HFGoe_H3_b1R1_24h **Scan** 16096
**Peptide** AQDFKTDLRFQ **Charge** 3 **Spectral Angle** 0.84

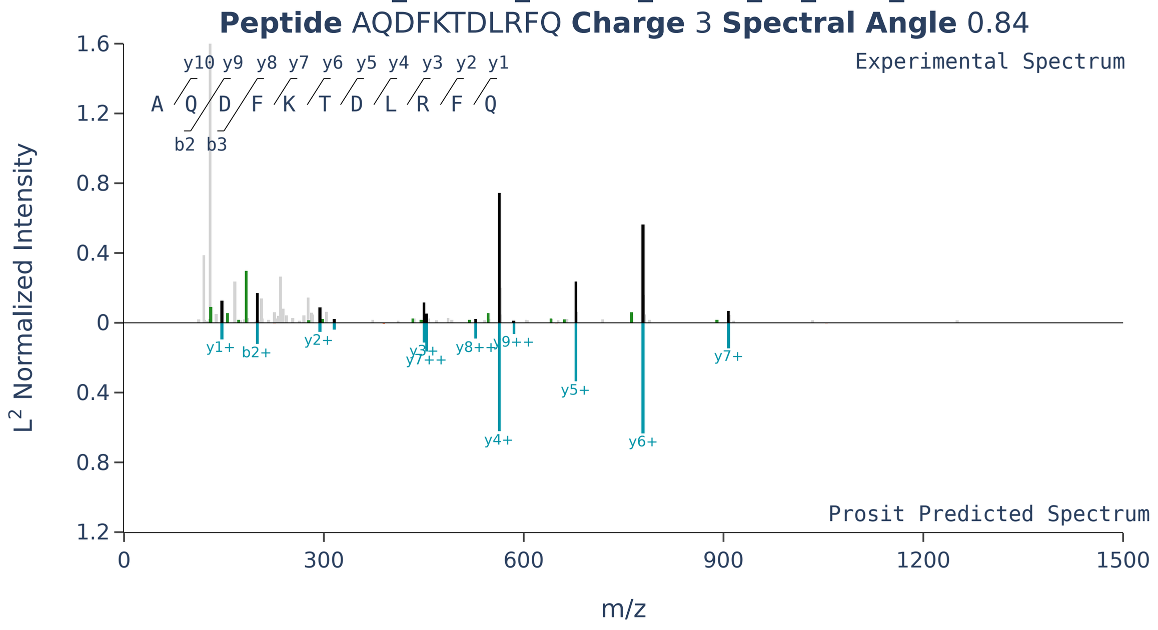


**Source** HRoetschke_271222_120123_HFGoe_H3_b1R1_4h **Scan** 16928
**Peptide** AQDFKTDLRFQSSAVM **Charge** 3 **Spectral Angle** 0.93

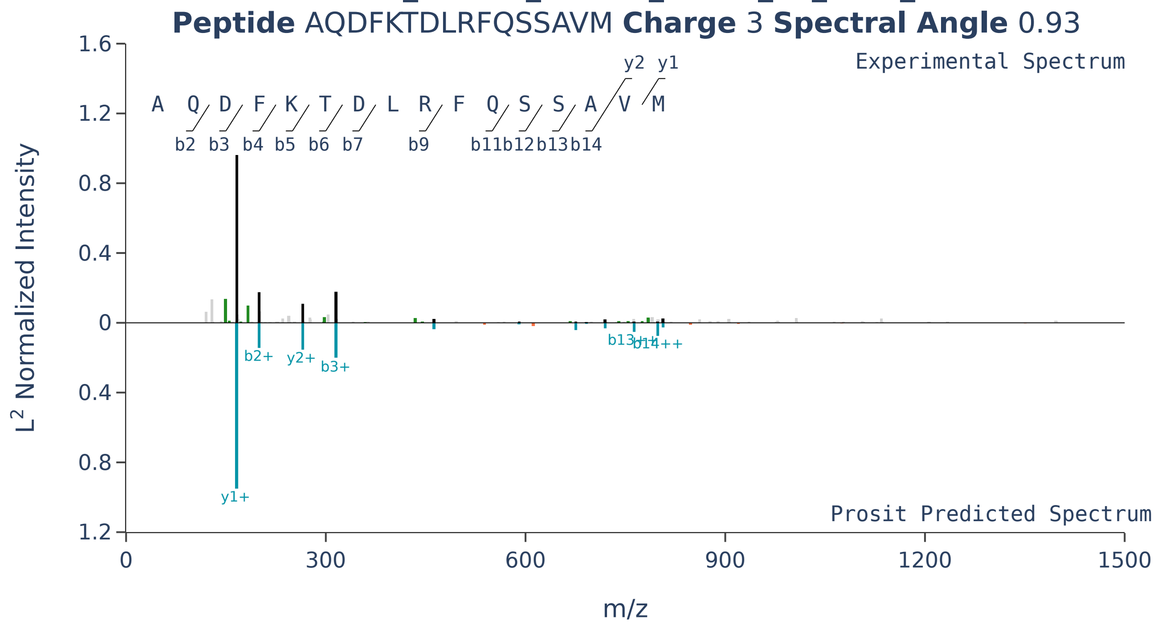


**Source** HRoetschke_271222_120123_HFGoe_H3_b1R2_2h **Scan** 12171
**Peptide** QSSAVMALQE **Charge** 2 **Spectral Angle** 0.85

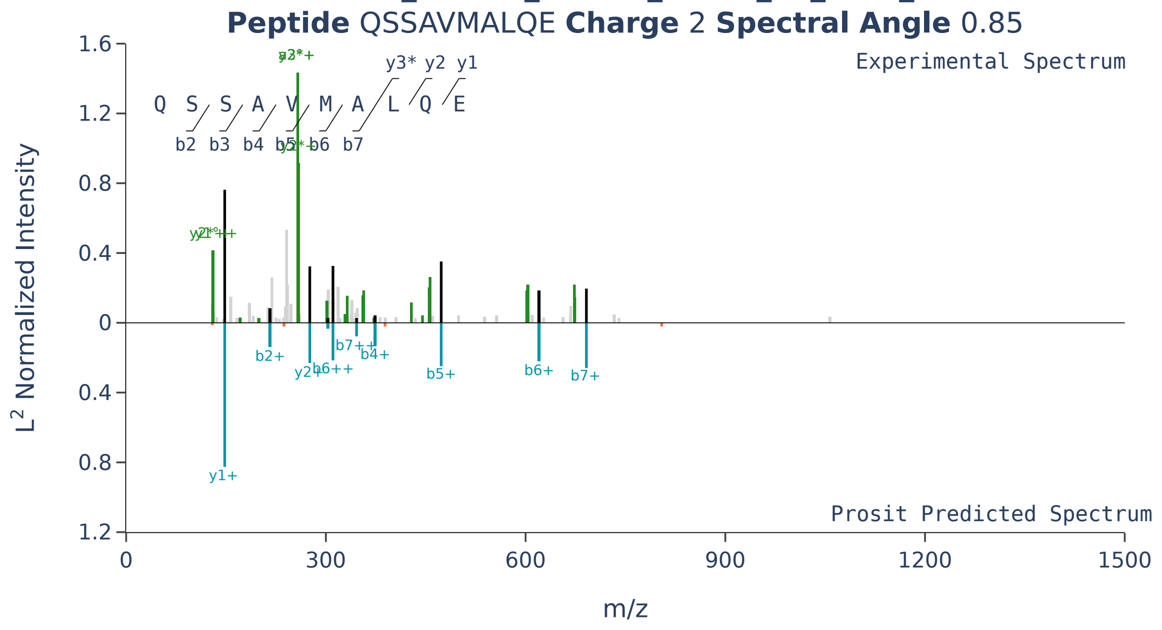


**Source** HRoetschke_271222_120123_HFGoe_H3_b2R1_4h **Scan** 13082
**Peptide** RPGTVAL **Charge** 2 **Spectral Angle** 0.81

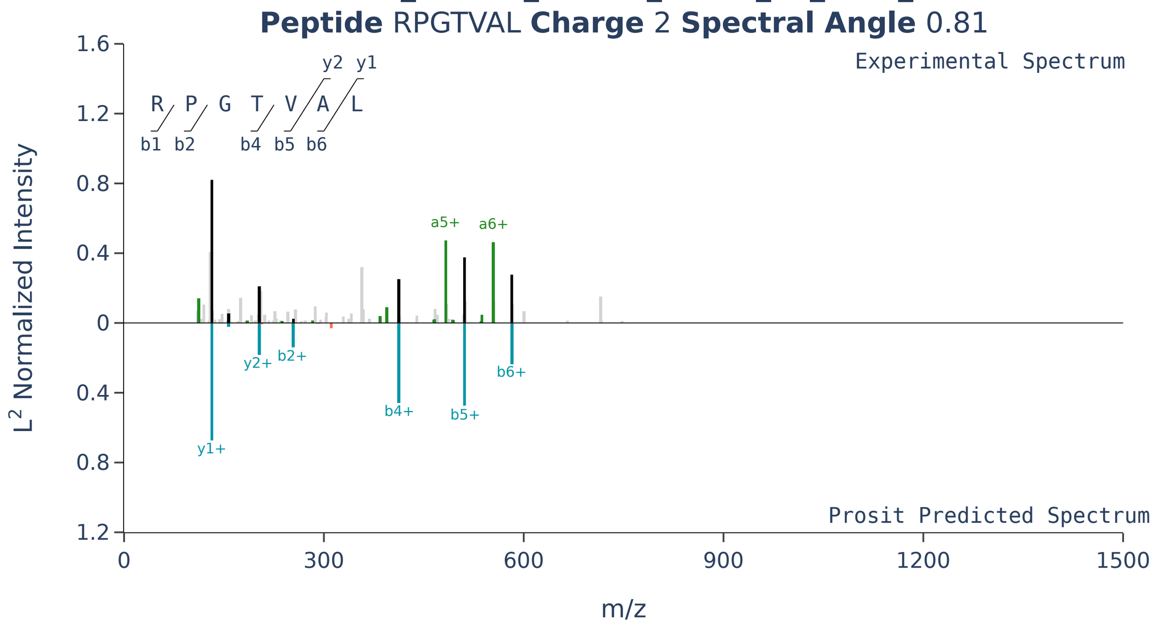


**Source** HRoetschke_271222_120123_HFGoe_H3_b1R1_4h **Scan** 14647
**Peptide** RFQSSAVMALQE **Charge** 2 **Spectral Angle** 0.82

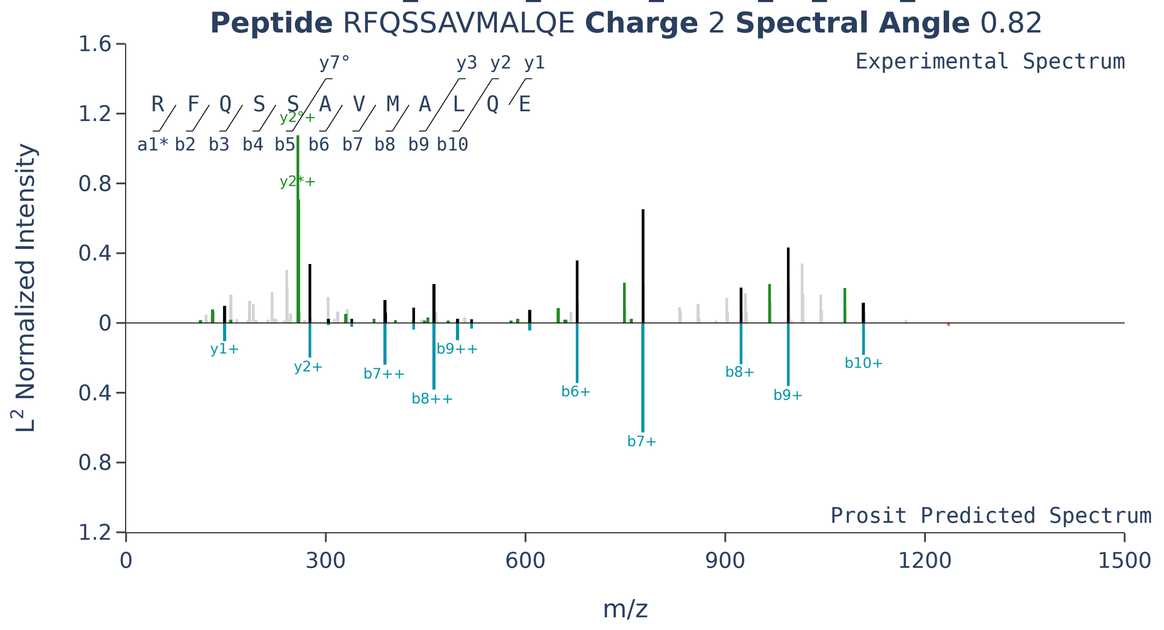


**Source** HRoetschke_271222_120123_HFGoe_H3_b2R1_4h **Scan** 14922
**Peptide** YQKSTELLLR **Charge** 3 **Spectral Angle** 0.85

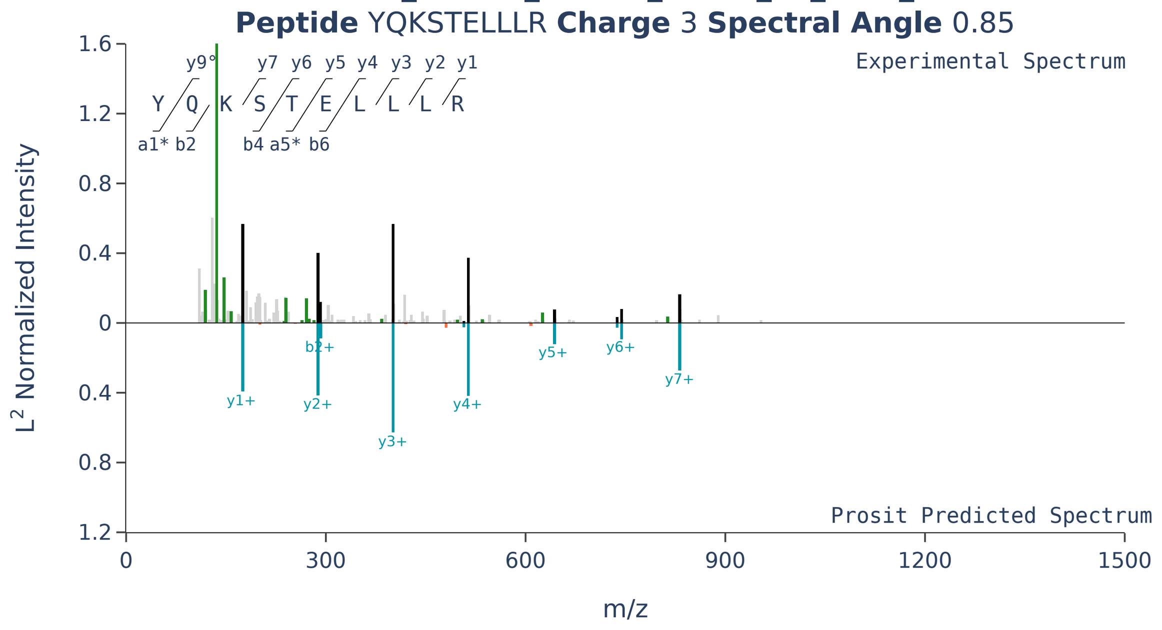


**Source** HRoetschke_271222_120123_HFGoe_H3_b1R1_24h **Scan** 17584
**Peptide** SEAYLVA **Charge** 1 **Spectral Angle** 0.81

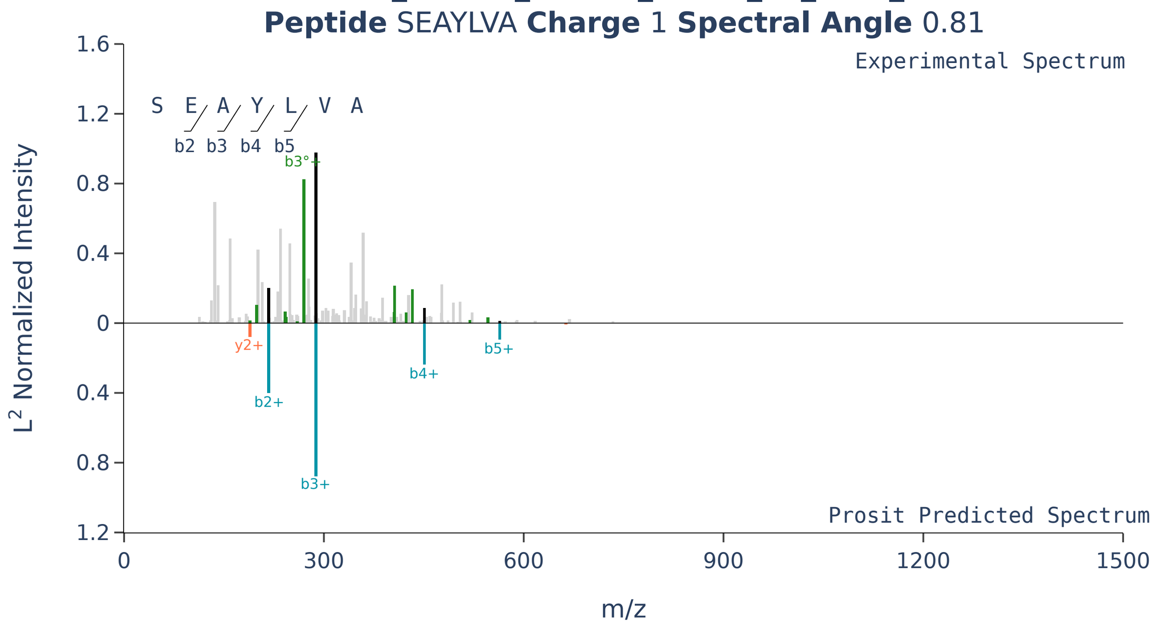


**Source** HRoetschke_271222_120123_HFGoe_H3_b2R1_2h **Scan** 10754
**Peptide** IRKLPFQR **Charge** 3 **Spectral Angle** 0.89

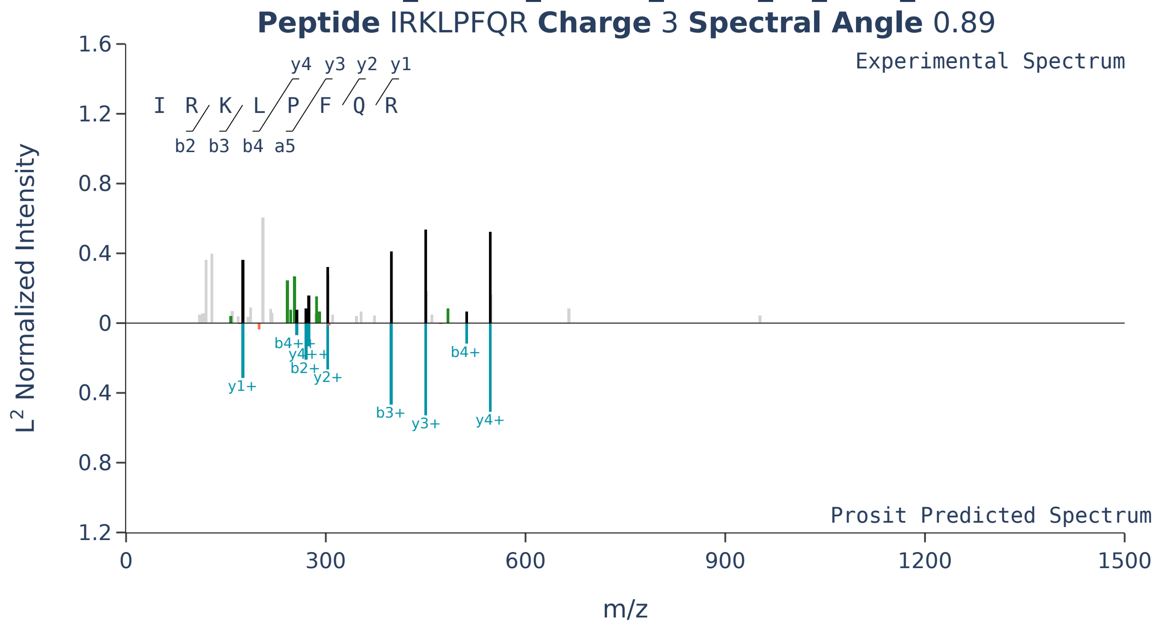


**Source** HRoetschke_271222_120123_HFGoe_H3_b1R1_4h **Scan** 17632
**Peptide** STELLIRKL **Charge** 3 **Spectral Angle** 0.93

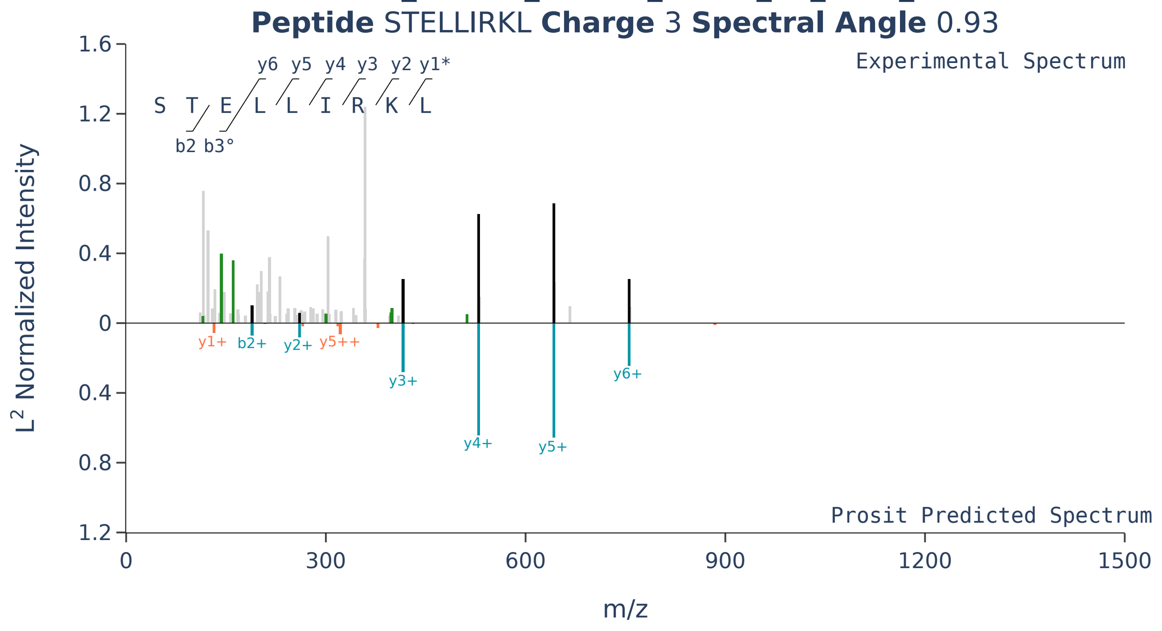


**Source** HRoetschke_271222_120123_HFGoe_H3_b1R1_24h **Scan** 16454
**Peptide** GTVALREI **Charge** 1 **Spectral Angle** 0.9

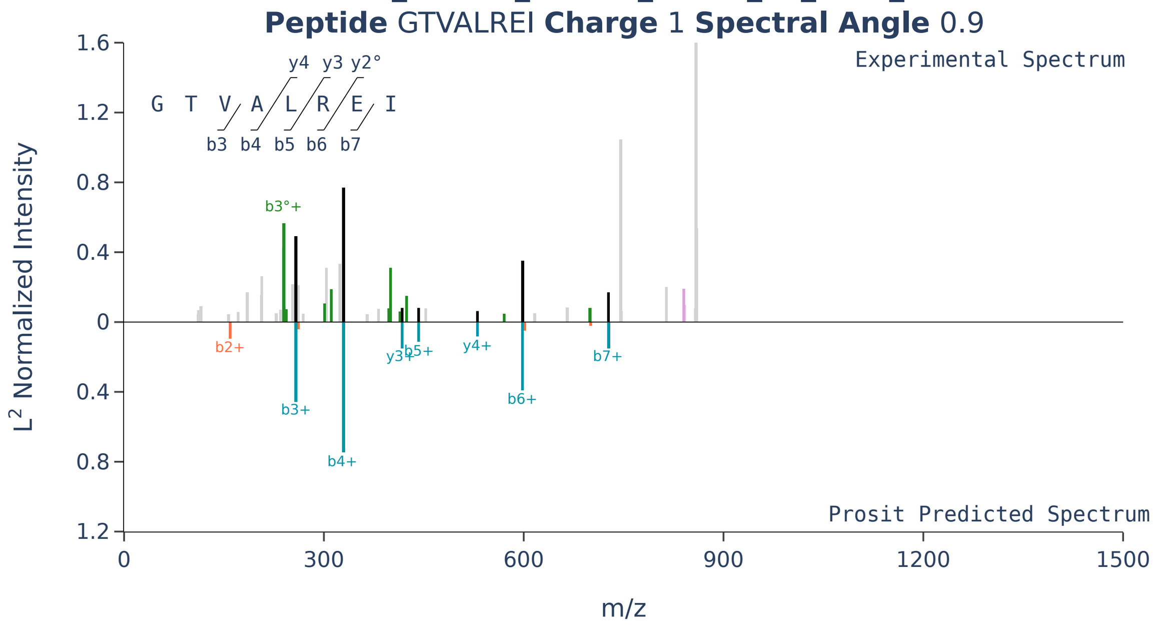


**Source** HRoetschke_271222_120123_HFGoe_H3_b1R1_24h **Scan** 8487
**Peptide** QKSTELLIRK **Charge** 3 **Spectral Angle** 0.93

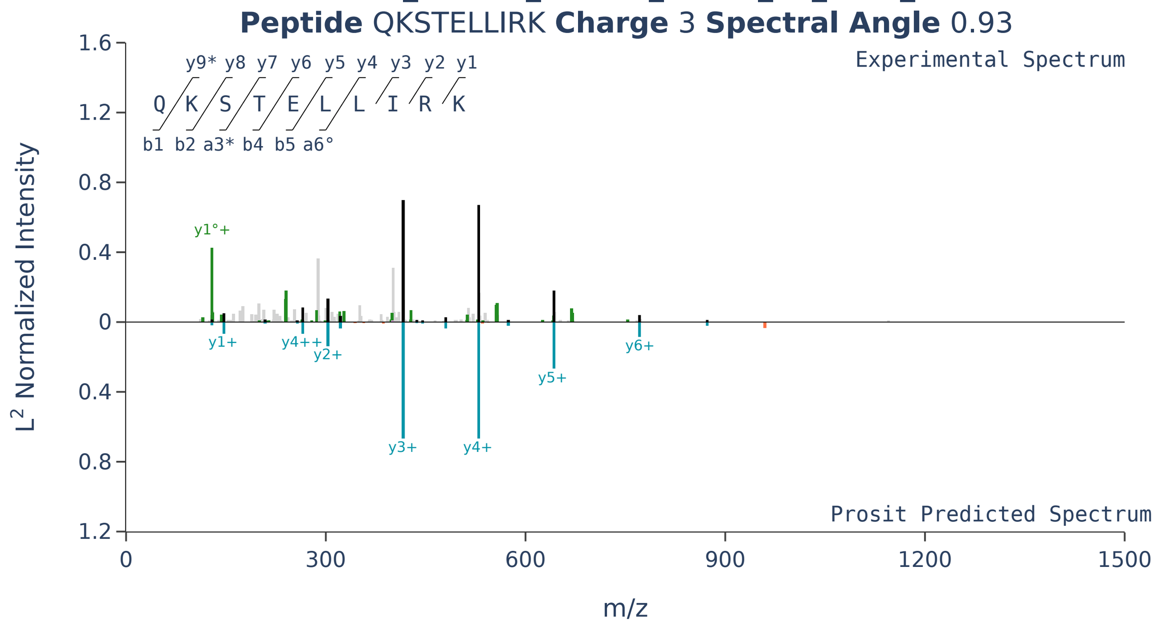


**Source** HRoetschke_271222_120123_HFGoe_H3_b1R2_4h **Scan** 15218
**Peptide** LVREIAQDFKT **Charge** 2 **Spectral Angle** 0.92

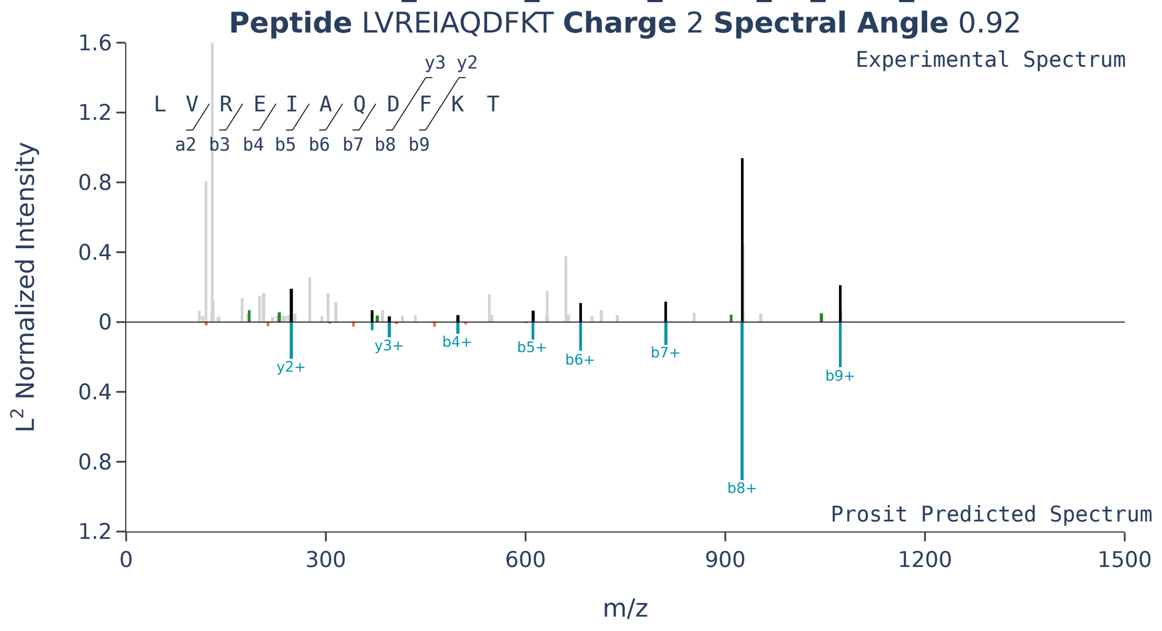


**Source** HRoetschke_271222_120123_HFGoe_H3_b2R2_2h **Scan** 15470
**Peptide** QEASEAYL **Charge** 2 **Spectral Angle** 0.84

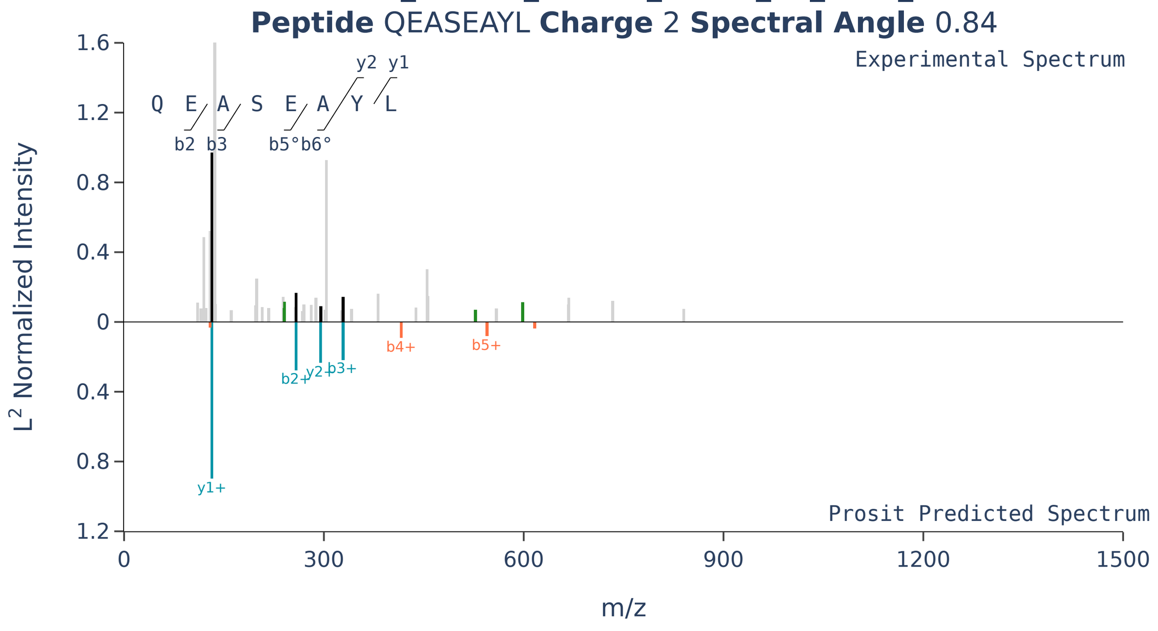


**Source** HRoetschke_271222_120123_HFGoe_H3_b1R1_4h **Scan** 15643
**Peptide** AQDFKTDLRFQSS **Charge** 2 **Spectral Angle** 0.84

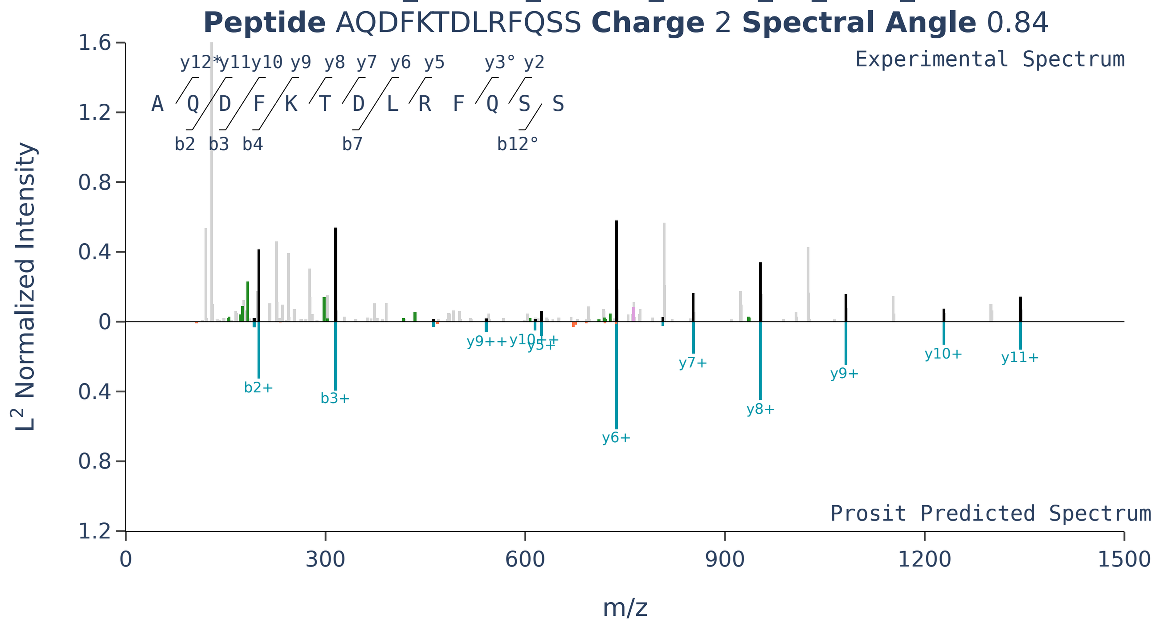


**Source** HRoetschke_271222_120123_HFGoe_H3_b1R2_4h **Scan** 14173
**Peptide** FKTDLRFQSS **Charge** 2 **Spectral Angle** 0.83

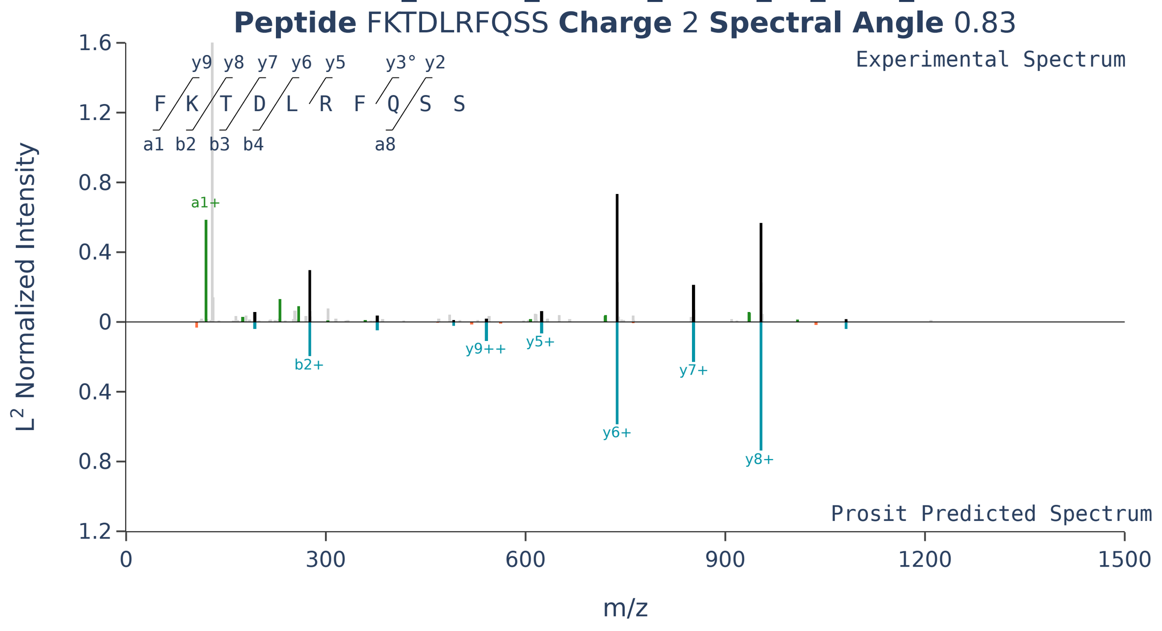

**Source** HRoetschke_271222_120123_HFGoe_H3_b1R1_24h **Scan** 8119
**Peptide** KSTELLIRK **Charge** 3 **Spectral Angle** 0.82

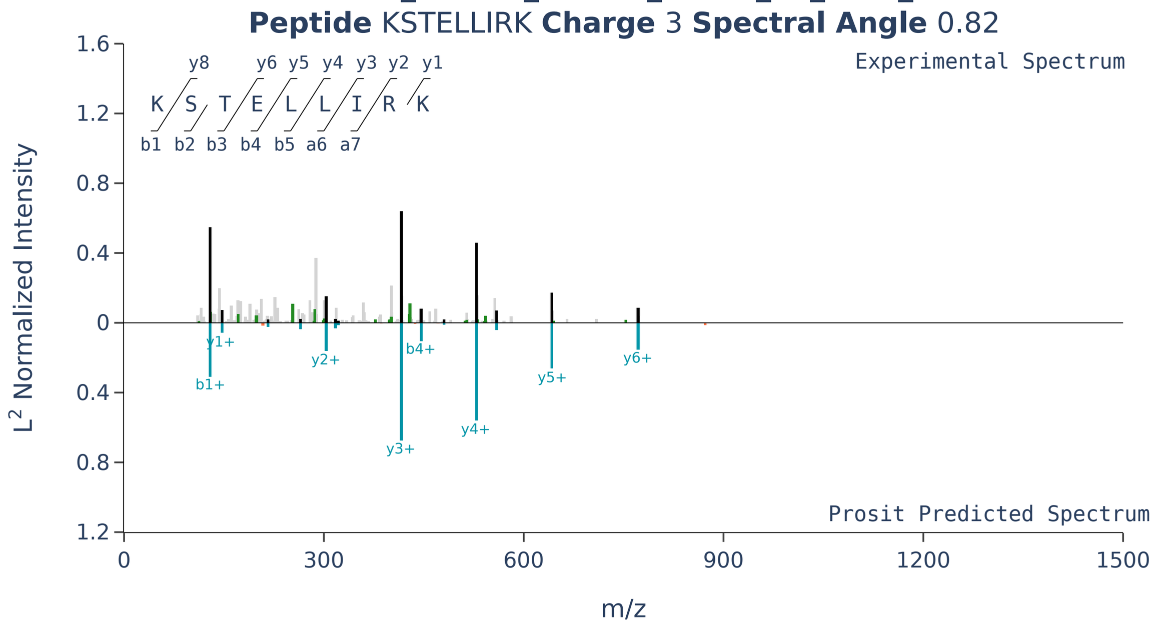


**Source** HRoetschke_271222_120123_HFGoe_H3_b2R1_24h **Scan** 10276
**Peptide** REIAQDFKTD **Charge** 3 **Spectral Angle** 0.92

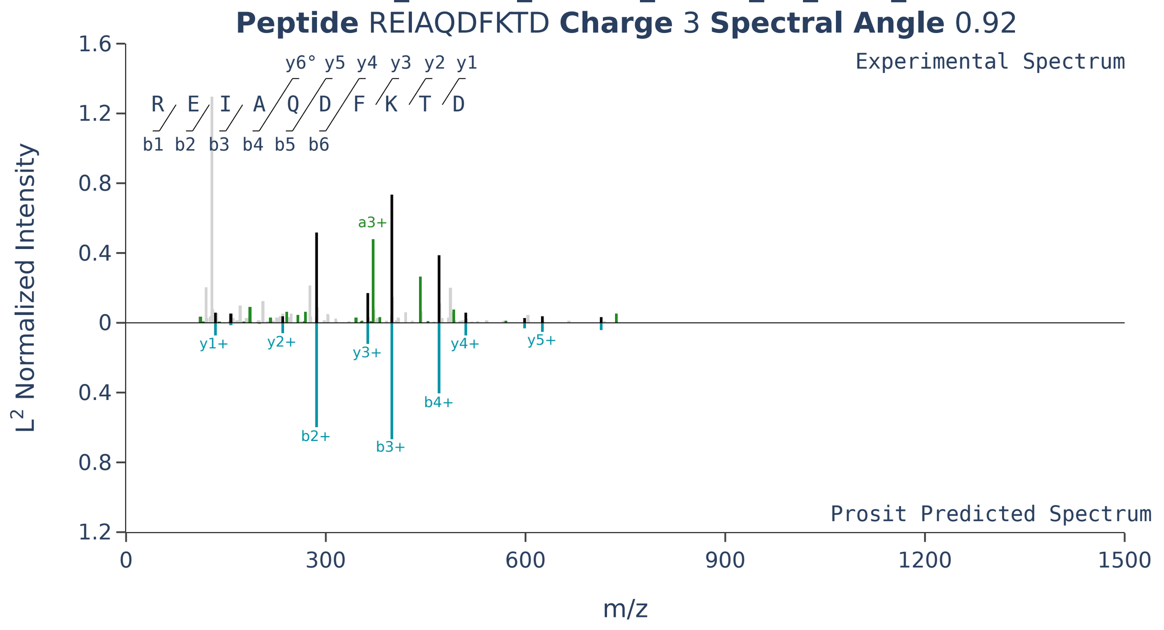


**Source** HRoetschke_271222_120123_HFGoe_H3_b2R1_24h **Scan** 11862
**Peptide** STELLIRK **Charge** 2 **Spectral Angle** 0.86

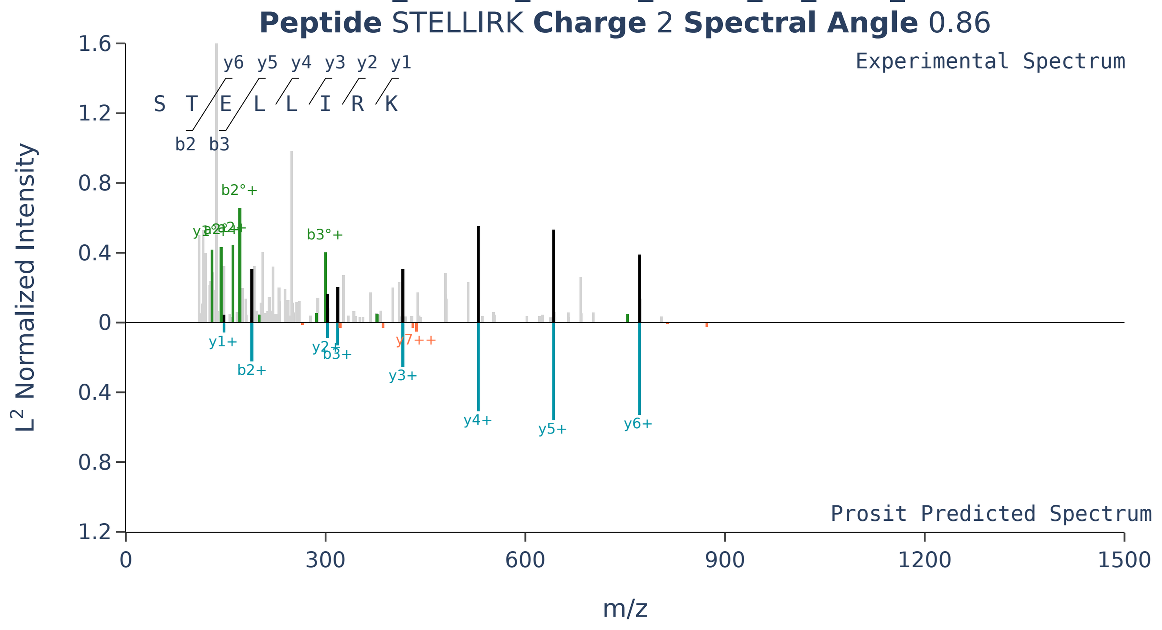


**Source** HRoetschke_271222_120123_HFGoe_H3_b2R1_4h **Scan** 12744
**Peptide** SSAVMALQ **Charge** 1 **Spectral Angle** 0.81

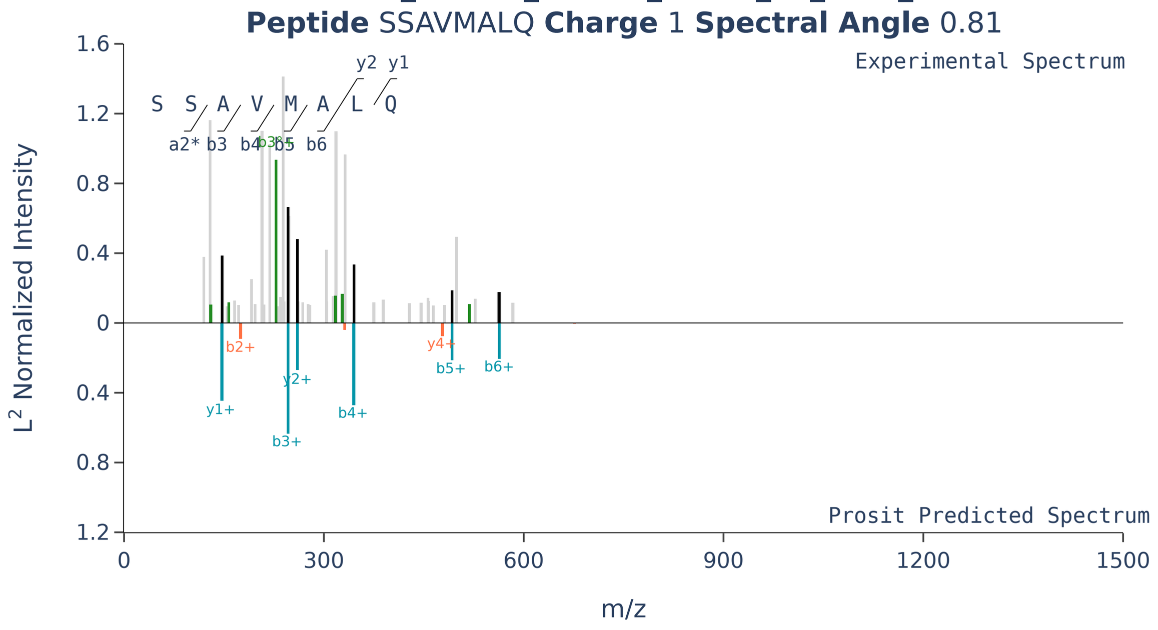


**Source** HRoetschke_271222_120123_HFGoe_H3_b1R2_24h **Scan** 12966
**Peptide** TIMPKDL **Charge** 2 **Spectral Angle** 0.78

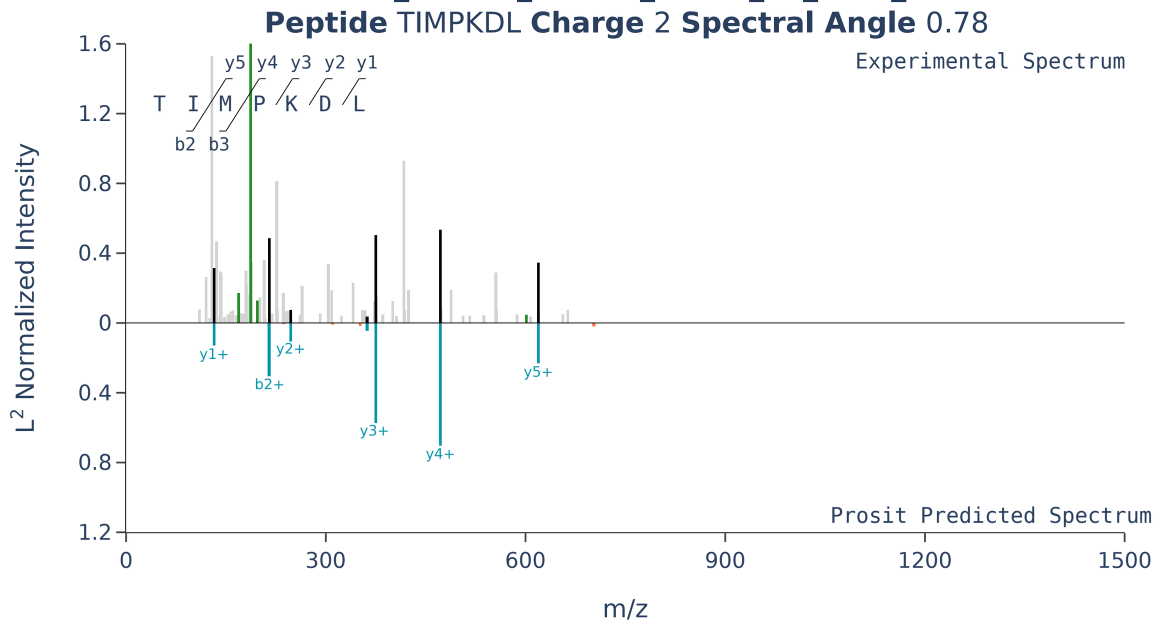


**Source** HRoetschke_271222_120123_HFGoe_H3_b2R2_2h **Scan** 17003
**Peptide** DLRFQSSAVMA **Charge** 2 **Spectral Angle** 0.82

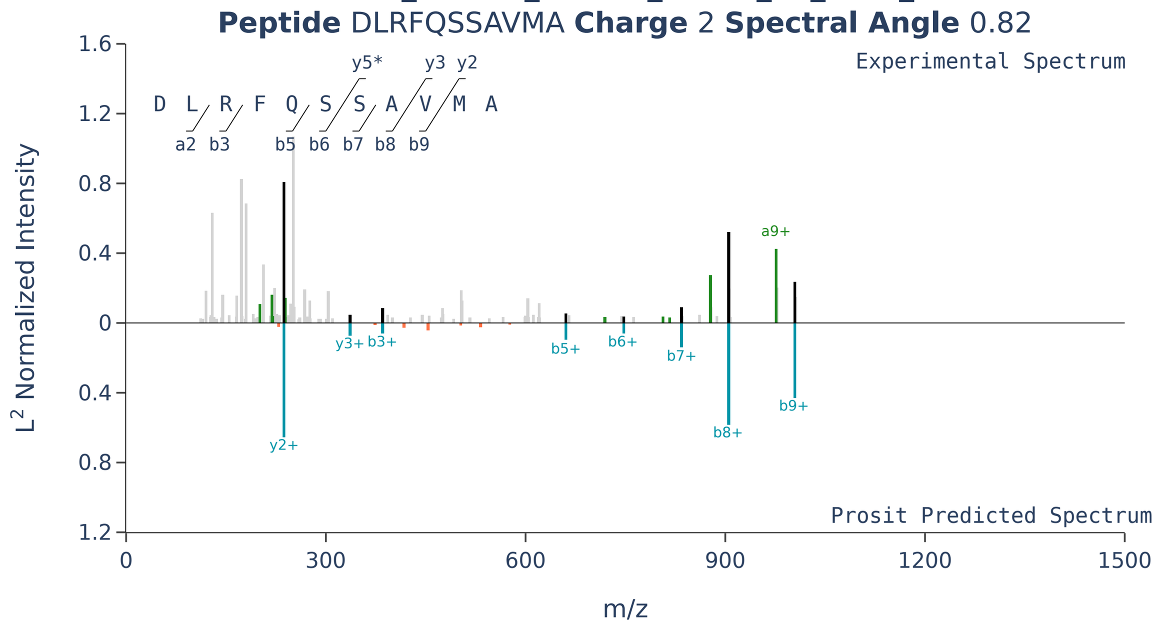


**Source** HRoetschke_271222_120123_HFGoe_H3_b1R2_24h **Scan** 13265
**Peptide** KRVTIMPKDI **Charge** 3 **Spectral Angle** 0.87

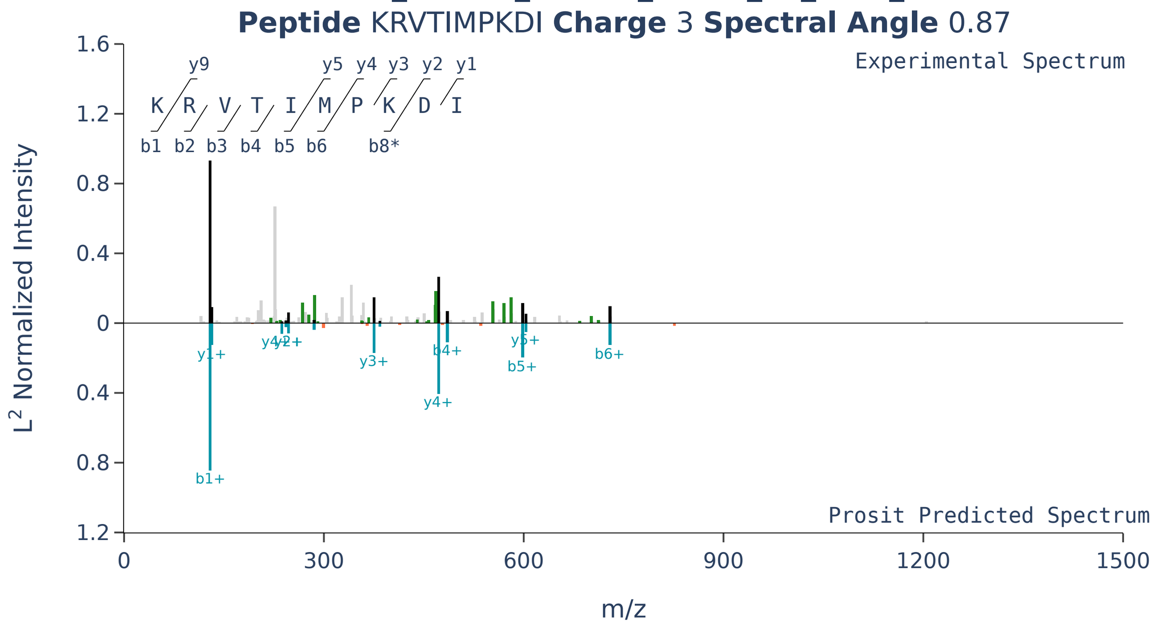


**Source** HRoetschke_271222_120123_HFGoe_H3_b2R2_2h **Scan** 14528
**Peptide** YQKSTELL **Charge** 2 **Spectral Angle** 0.76

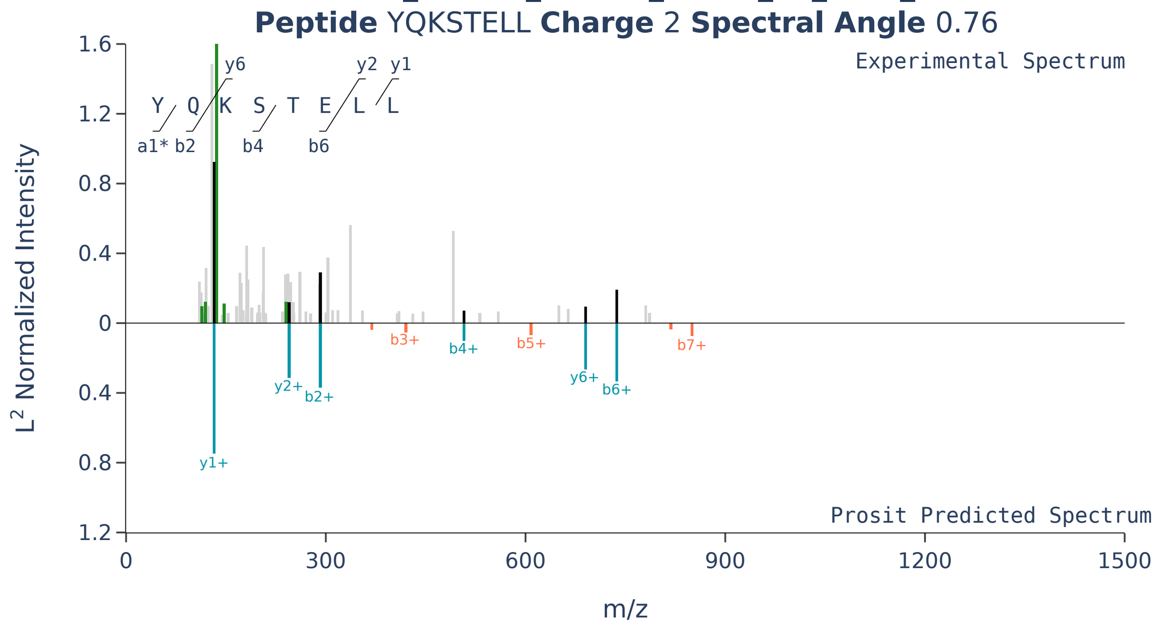


**Source** HRoetschke_271222_120123_HFGoe_H3_b2R2_2h **Scan** 9221
**Peptide** RKLPFQR **Charge** 2 **Spectral Angle** 0.77

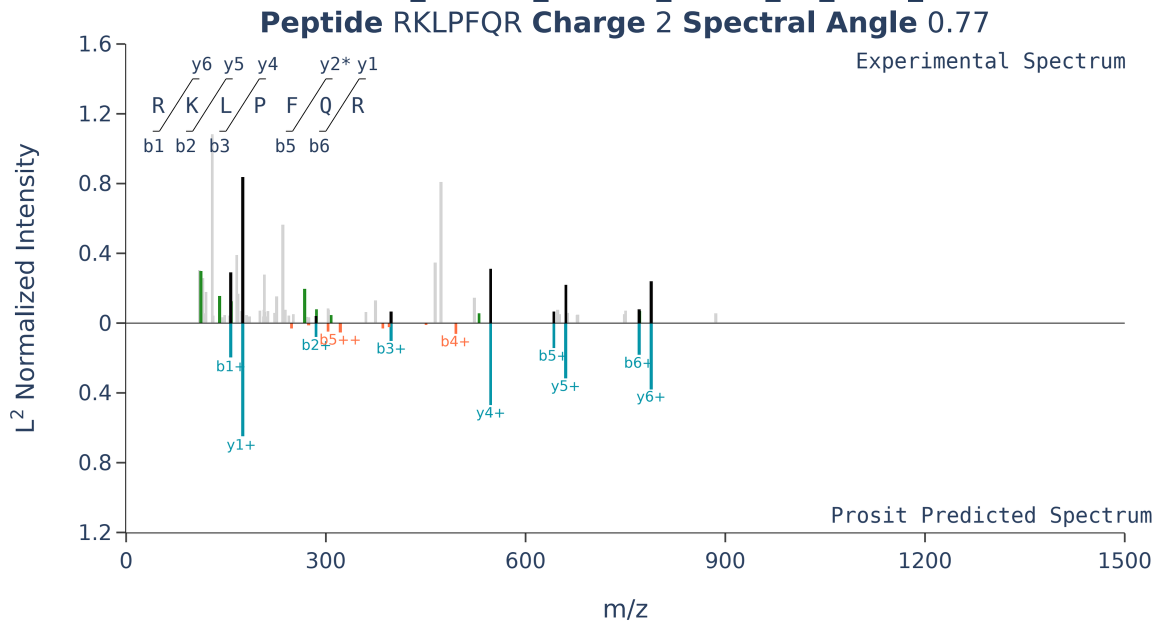


**Source** HRoetschke_271222_120123_HFGoe_H3_b2R1_2h **Scan** 12280
**Peptide** DLRFQSSA **Charge** 2 **Spectral Angle** 0.78

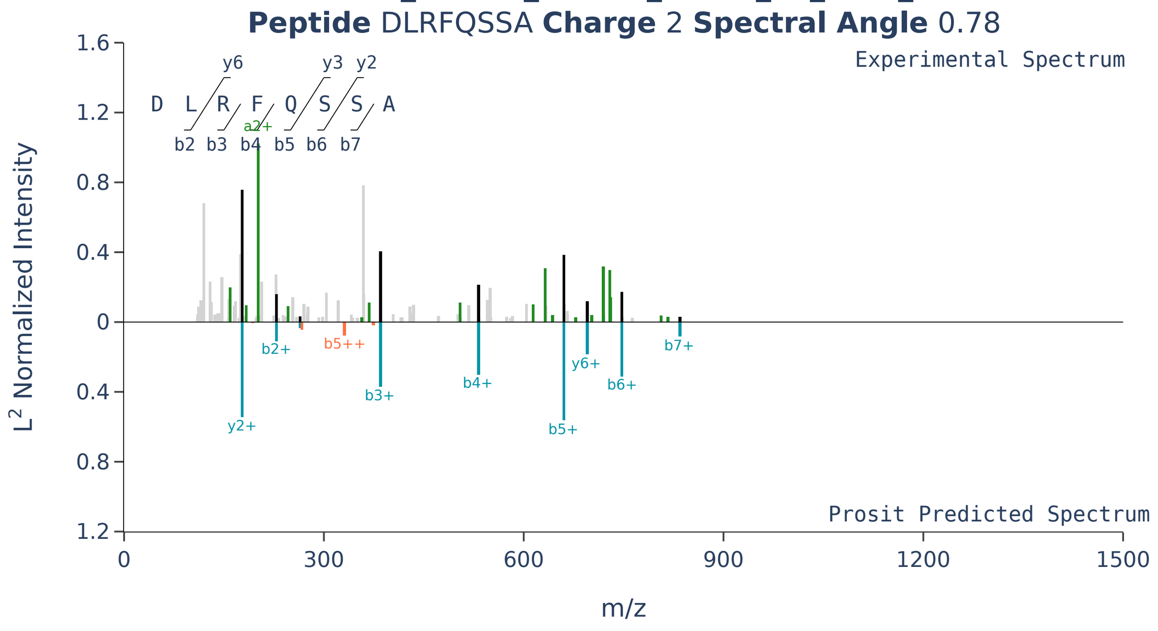


**Source** HRoetschke_271222_120123_HFGoe_H3_b1R1_4h **Scan** 20654
**Peptide** LFEDTNL **Charge** 1 **Spectral Angle** 0.91

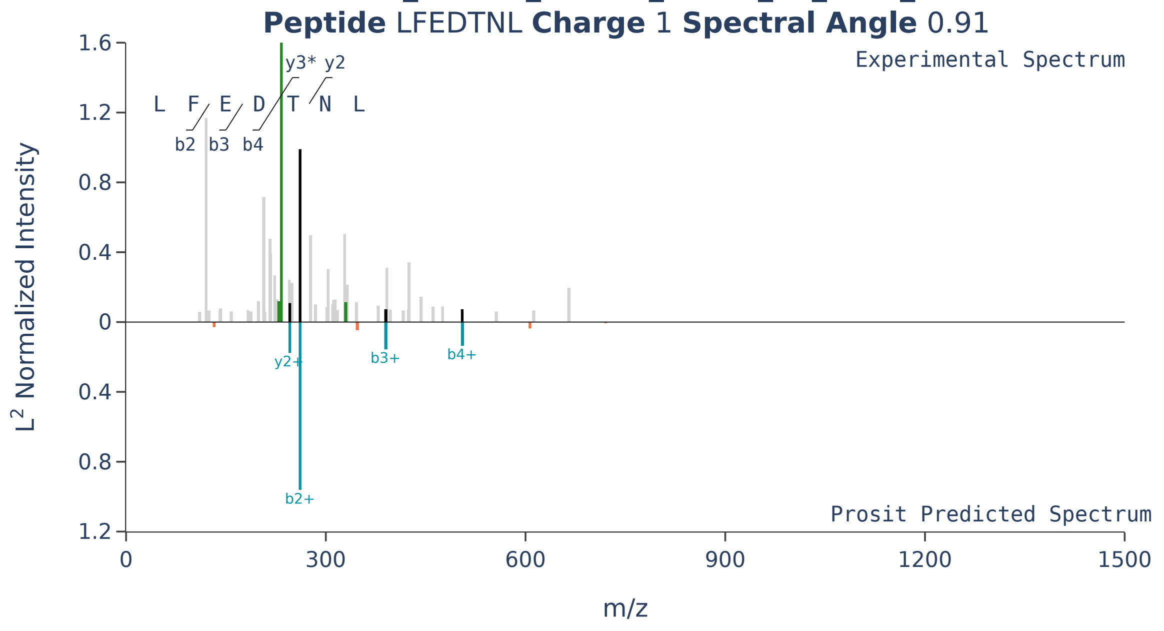


**Source** HRoetschke_271222_120123_HFGoe_H3_b1R2_24h **Scan** 14623
**Peptide** RPGTVALREL **Charge** 3 **Spectral Angle** 0.9

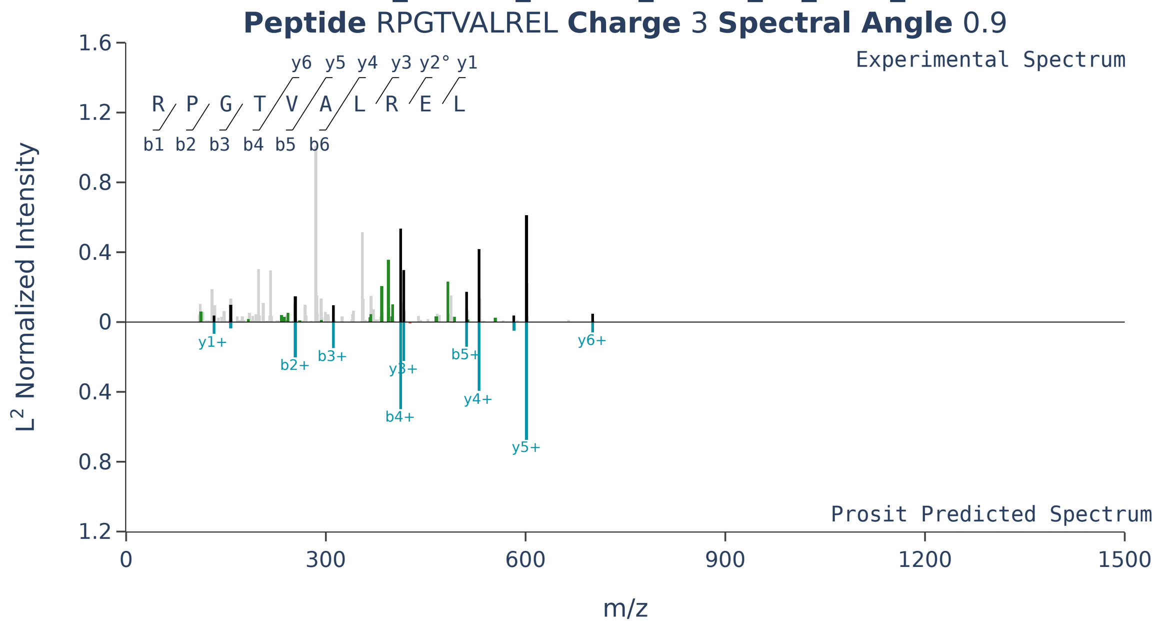


**Source** HRoetschke_271222_120123_HFGoe_H3_b1R1_24h **Scan** 14737
**Peptide** YRPGTVAL **Charge** 2 **Spectral Angle** 0.83

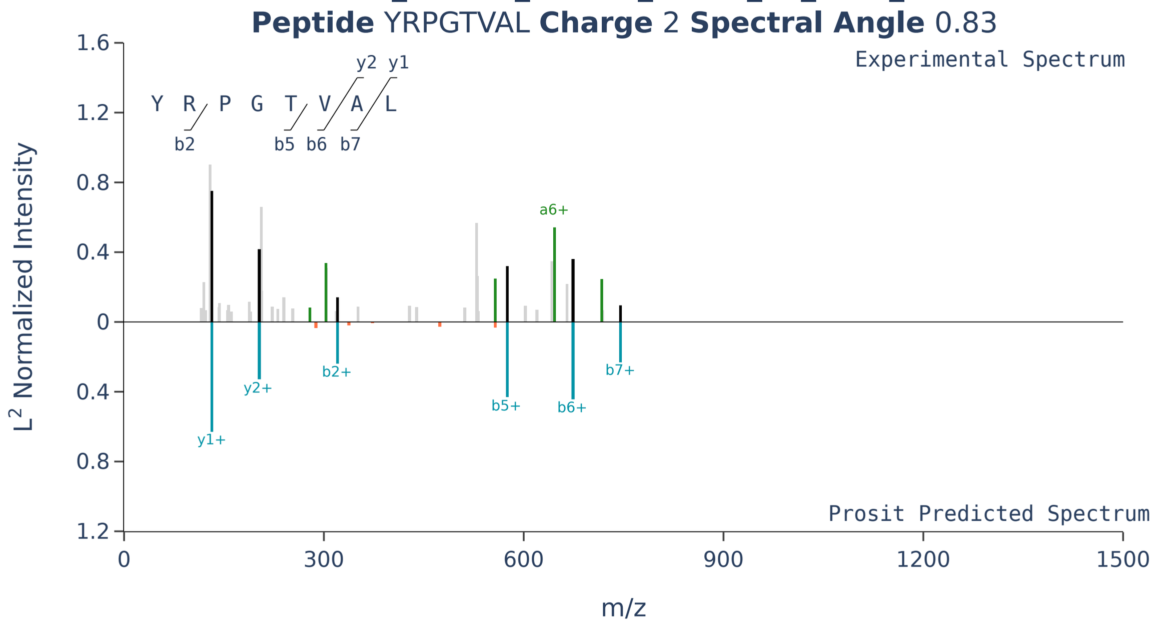


**Source** HRoetschke_271222_120123_HFGoe_H3_b2R1_4h **Scan** 10286
**Peptide** LCAIHAKRV **Charge** 2 **Spectral Angle** 0.82

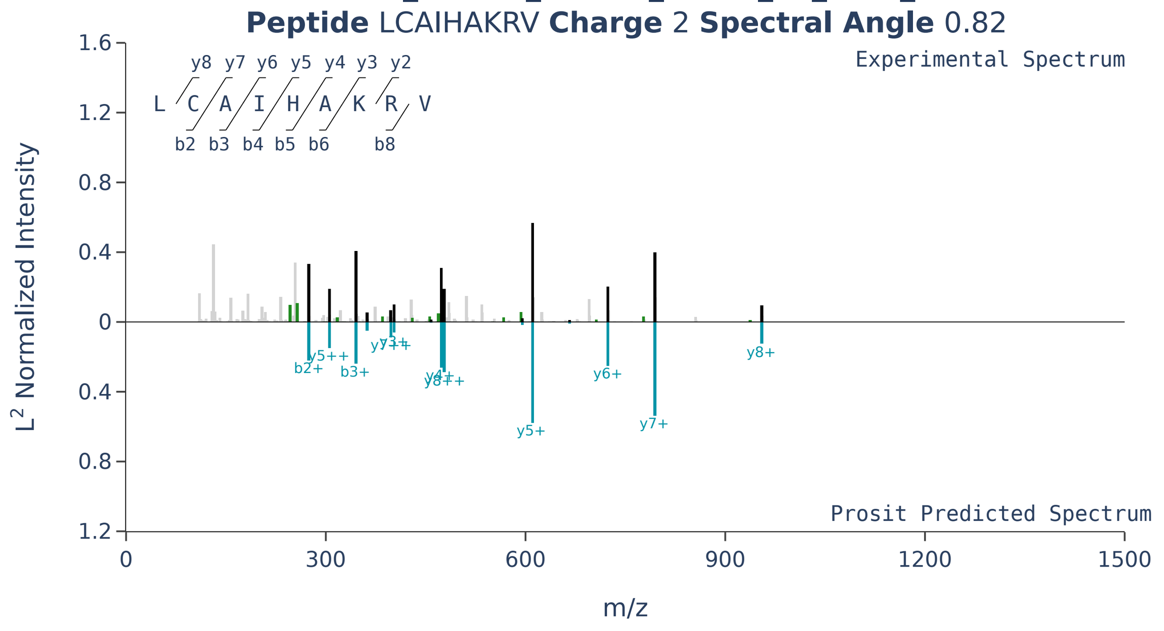


**Source** HRoetschke_271222_120123_HFGoe_H3_b1R2_2h **Scan** 6949
**Peptide** RVTIMPKD **Charge** 2 **Spectral Angle** 0.87

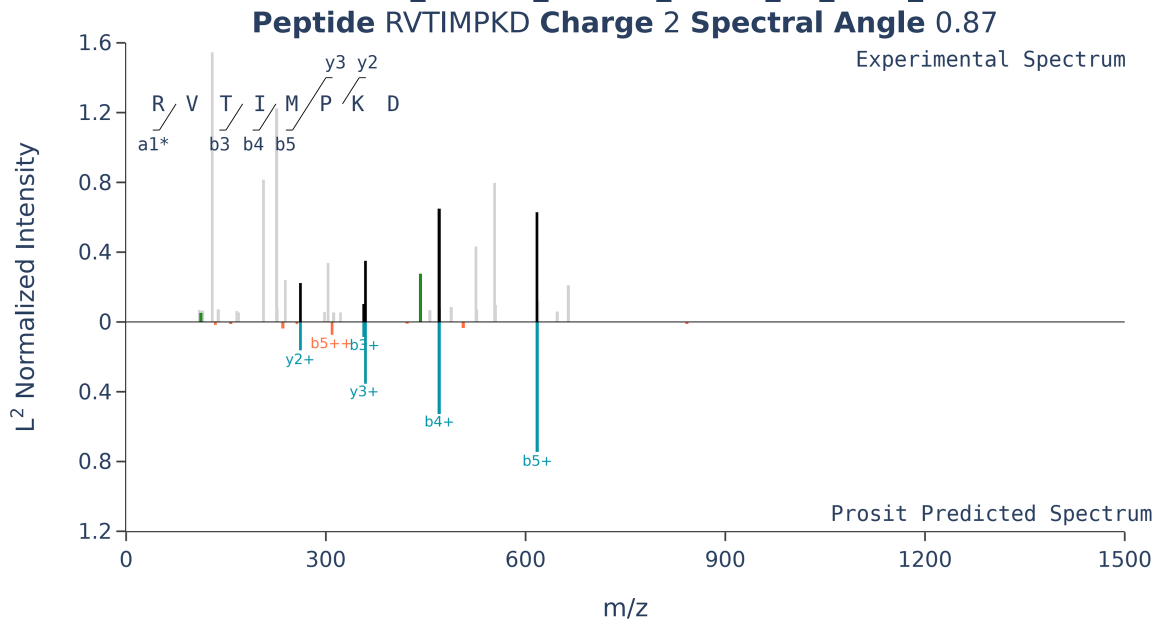

**Source** HRoetschke_271222_120123_HFGoe_H3_b1R1_4h **Scan** 18795
**Peptide** ELAQDFKTDL **Charge** 2 **Spectral Angle** 0.81

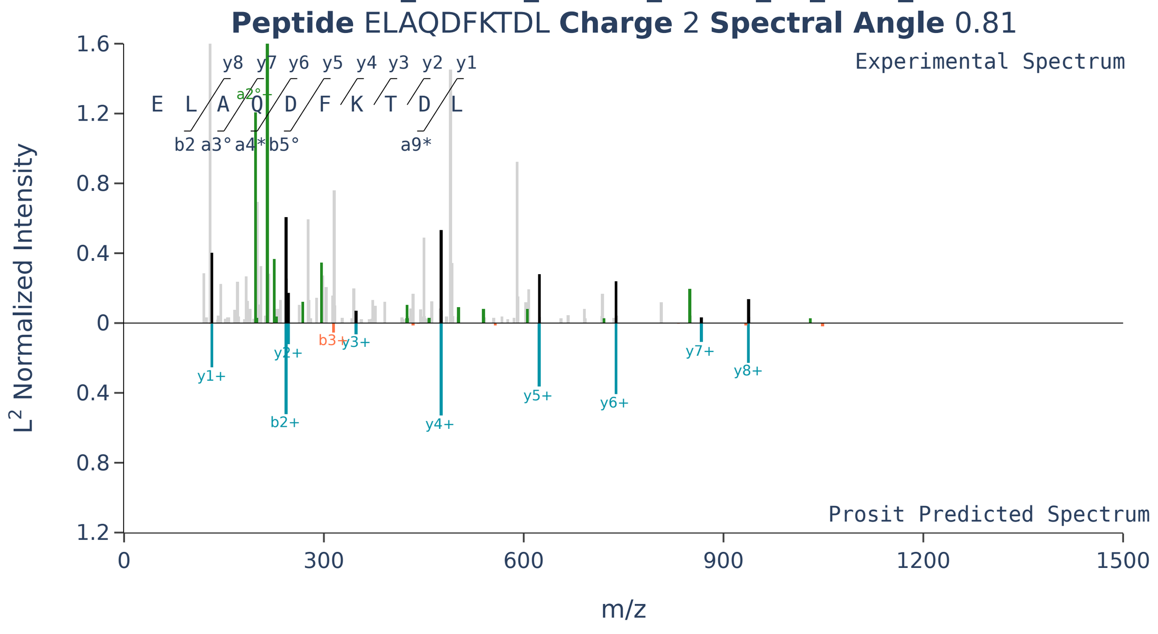


**Source** HRoetschke_271222_120123_HFGoe_H3_b2R1_24h **Scan** 10326
**Peptide** TNLCAIHAKRV **Charge** 3 **Spectral Angle** 0.79

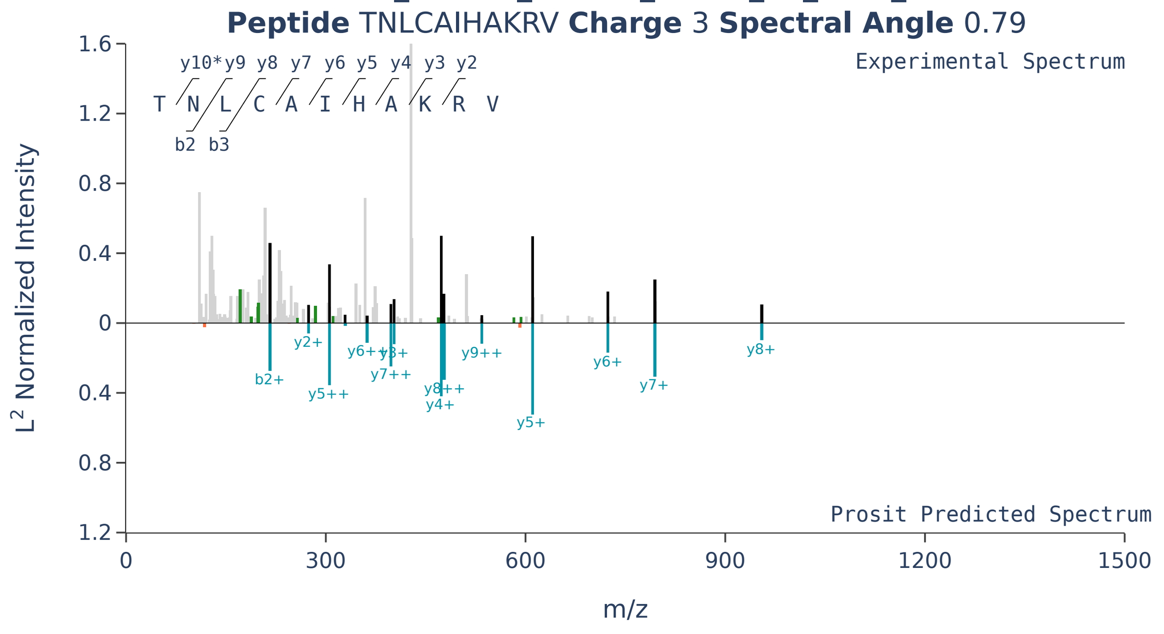


**Source** HRoetschke_271222_120123_HFGoe_H3_b2R1_4h **Scan** 13048
**Peptide** ALQEASEAY **Charge** 1 **Spectral Angle** 0.82

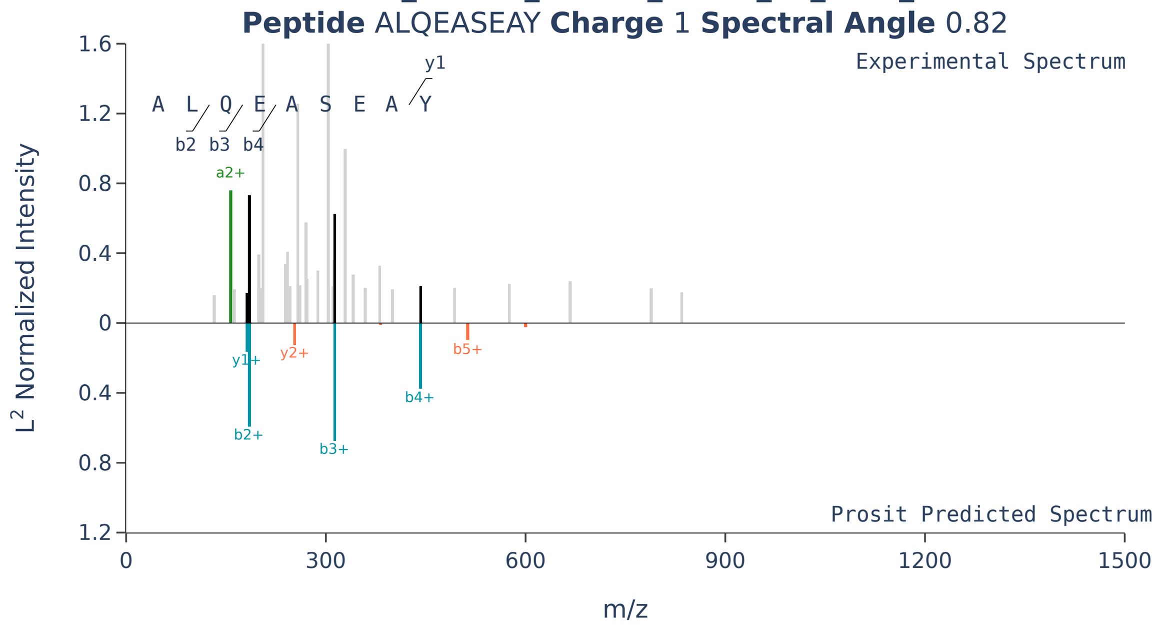


**Source** HRoetschke_271222_120123_HFGoe_H3_b2R2_24h **Scan** 12878
**Peptide** KSTELLIR **Charge** 2 **Spectral Angle** 0.83

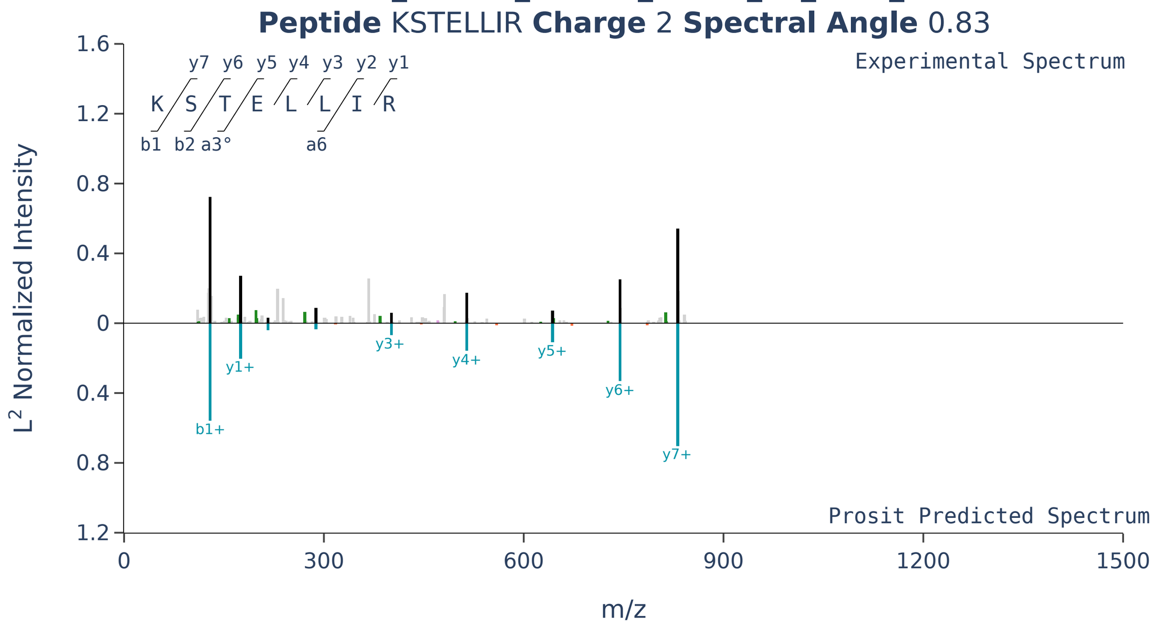


**Source** HRoetschke_271222_120123_HFGoe_H3_b1R1_1h **Scan** 16207
**Peptide** FKTDLRF **Charge** 2 **Spectral Angle** 0.79

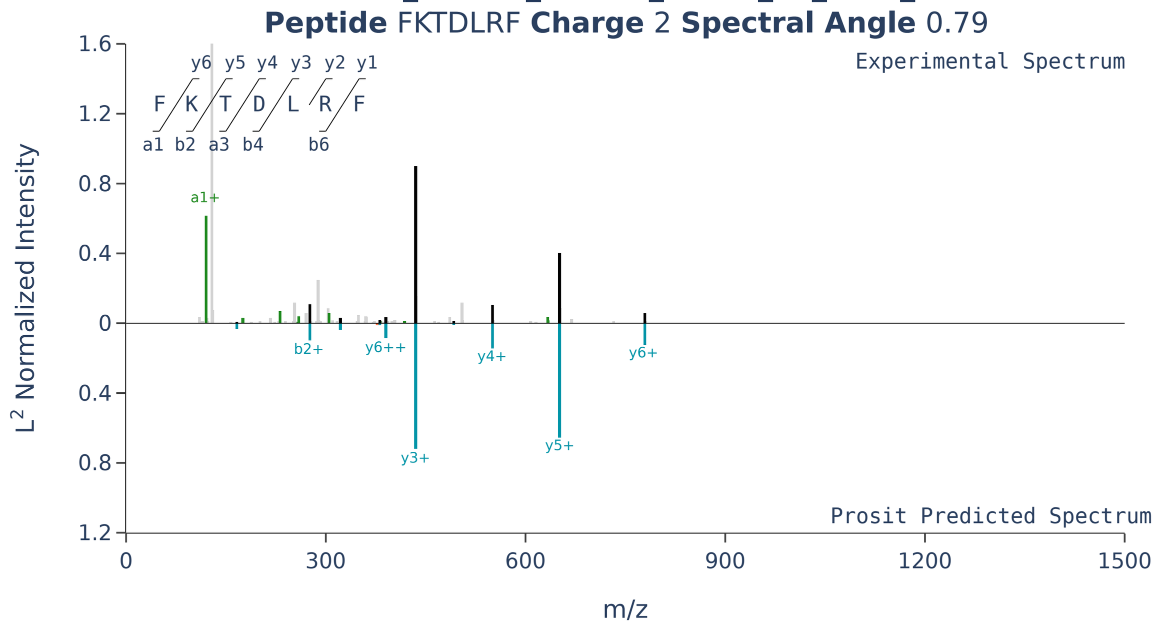


**Source** HRoetschke_271222_120123_HFGoe_H3_b1R1_4h **Scan** 15424
**Peptide** YQKSTELLIRKL **Charge** 4 **Spectral Angle** 0.91

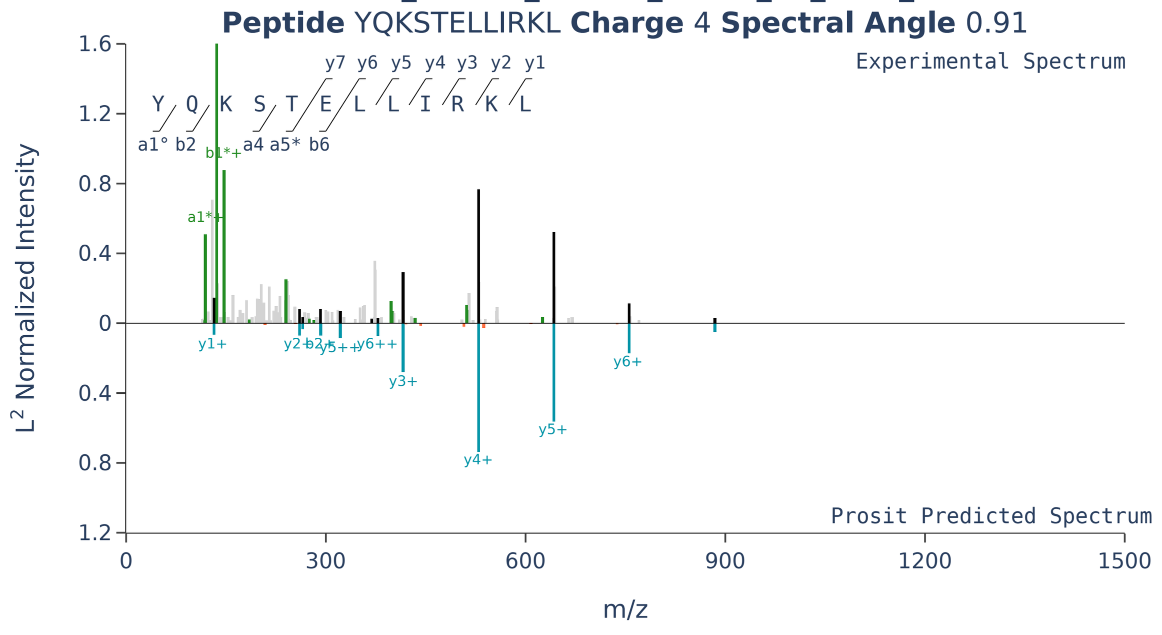


**Source** HRoetschke_271222_120123_HFGoe_H3_b1R1_4h **Scan** 13320
**Peptide** LRFQSSAV **Charge** 2 **Spectral Angle** 0.78

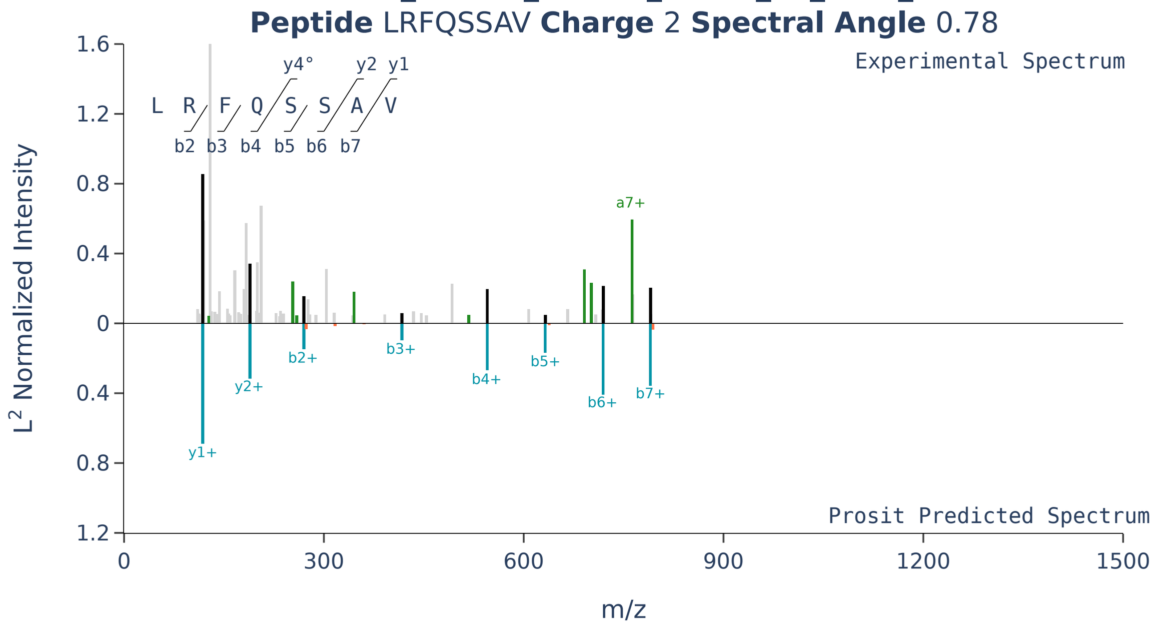


**Source** HRoetschke_271222_120123_HFGoe_H3_b1R1_4h **Scan** 19068
**Peptide** REIAQDFKTDLRF **Charge** 3 **Spectral Angle** 0.82

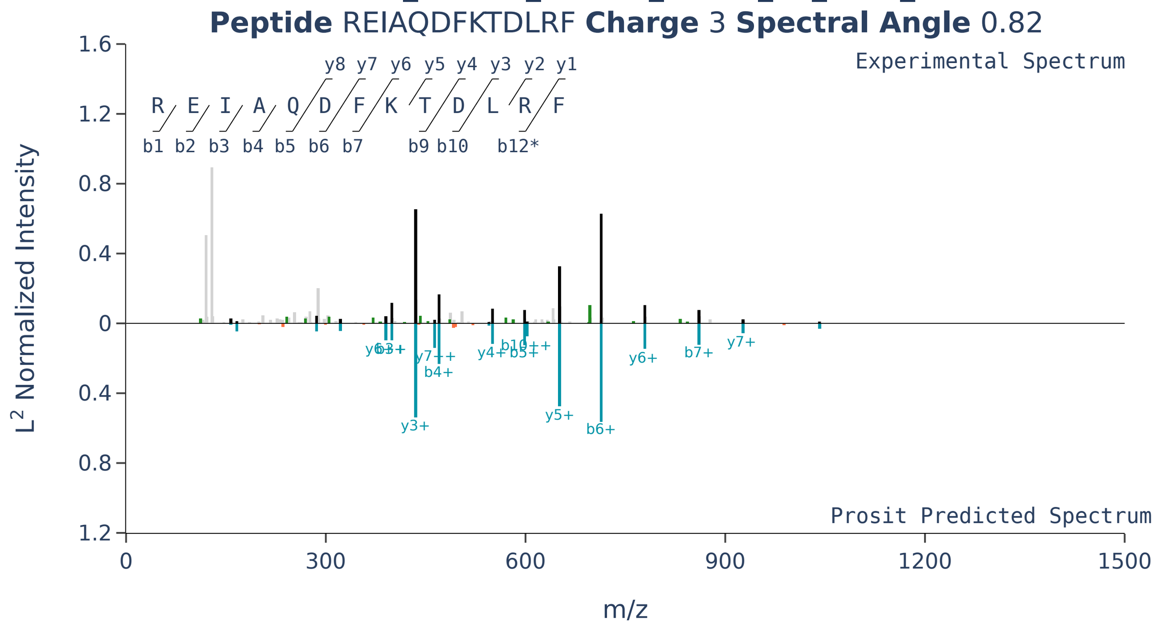


**Source** HRoetschke_271222_120123_HFGoe_H3_b1R1_1h **Scan** 9507
**Peptide** YRPGTVALR **Charge** 2 **Spectral Angle** 0.82

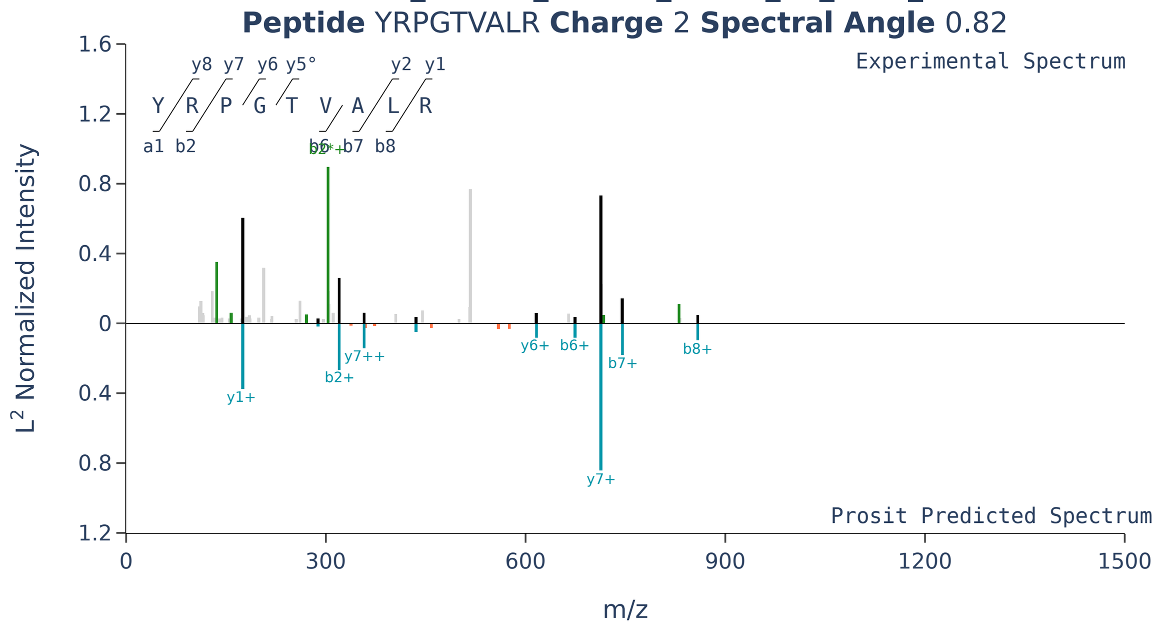


**Source** HRoetschke_271222_120123_HFGoe_H3_b2R2_1h **Scan** 25478
**Peptide** LVALFEDTNLC **Charge** 2 **Spectral Angle** 0.9

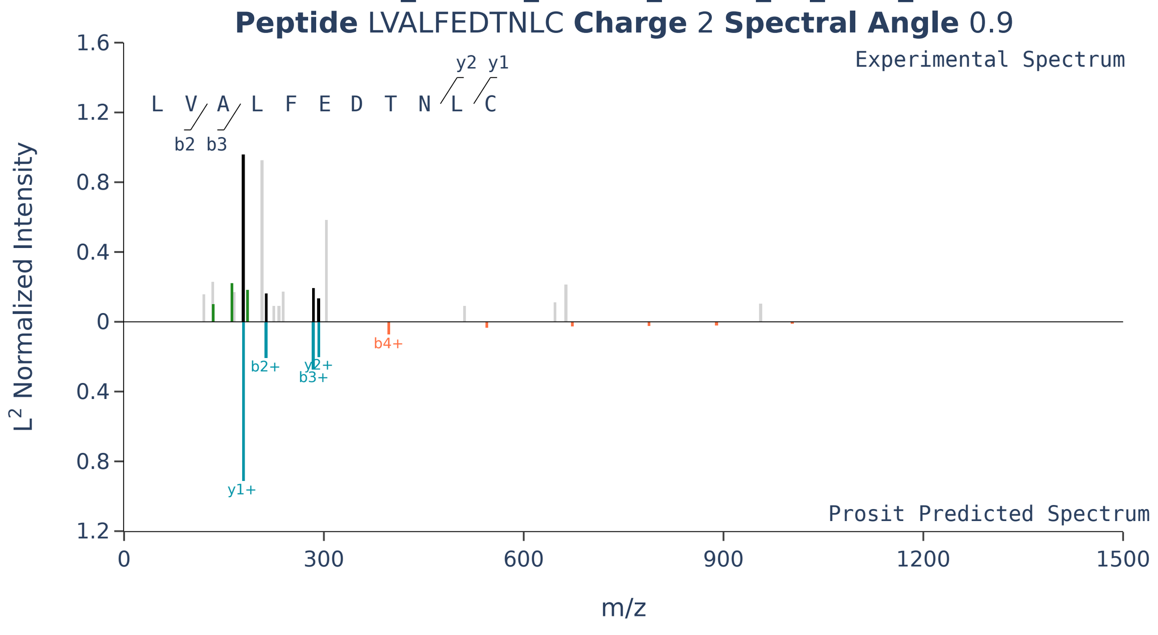


**Source** HRoetschke_271222_120123_HFGoe_H3_b2R1_4h **Scan** 12947
**Peptide** DLRFQSS **Charge** 1 **Spectral Angle** 0.84

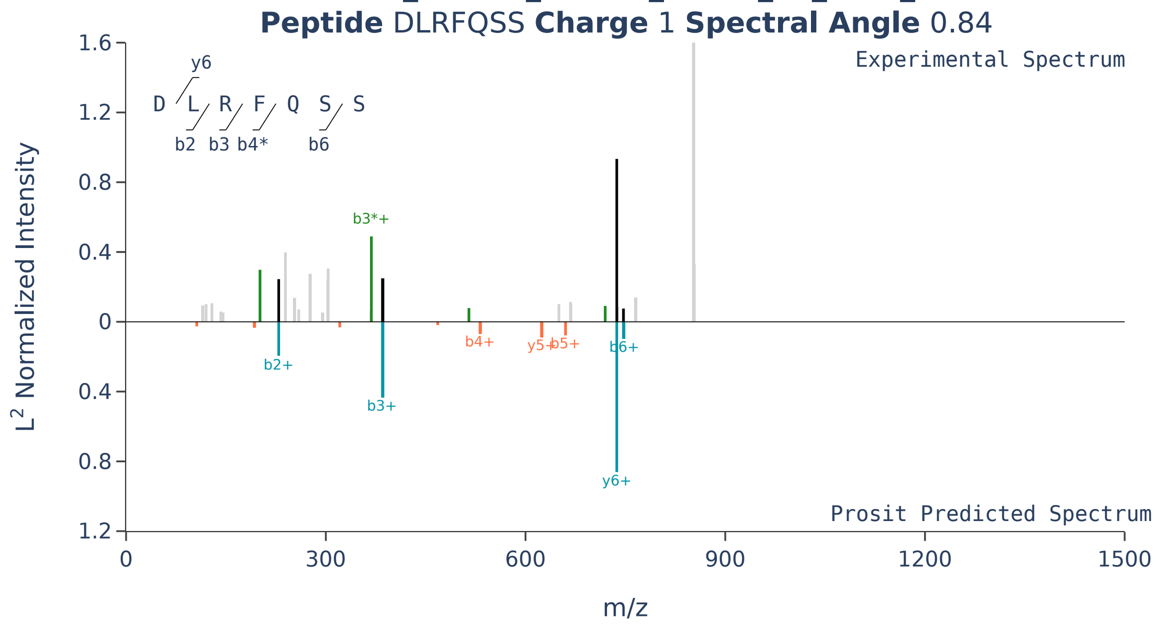


**Source** HRoetschke_271222_120123_HFGoe_H3_b2R1_4h **Scan** 12582
**Peptide** DTNLCAIH **Charge** 2 **Spectral Angle** 0.9

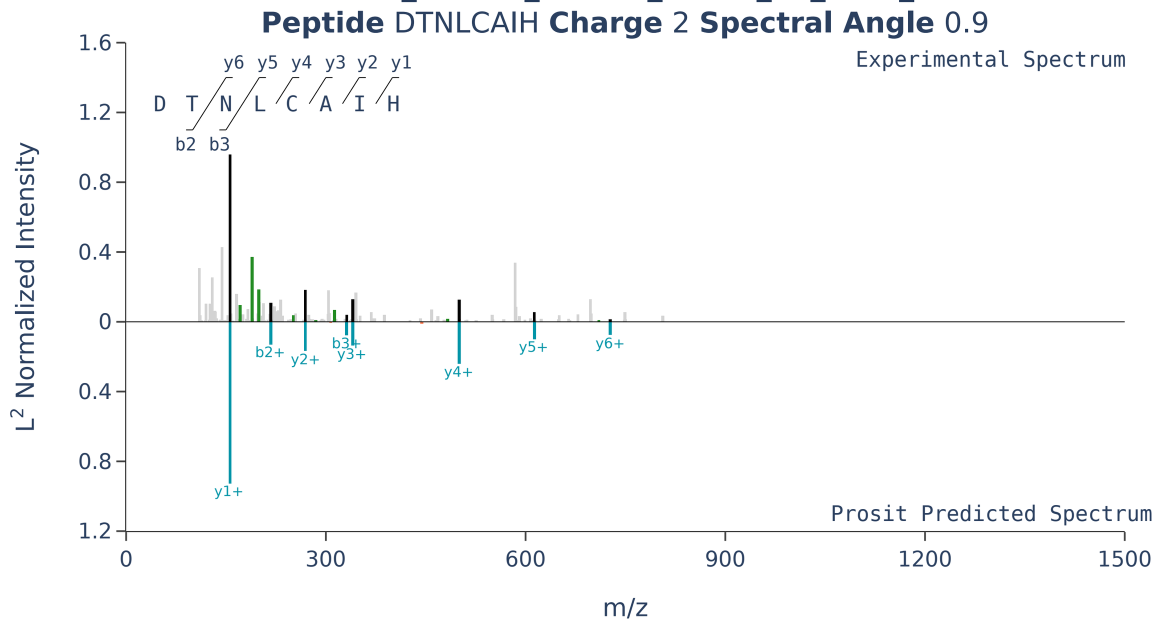


**Source** HRoetschke_271222_120123_HFGoe_H3_b1R1_4h **Scan** 19884
**Peptide** VREIAQDFKTDLRF **Charge** 4 **Spectral Angle** 0.75

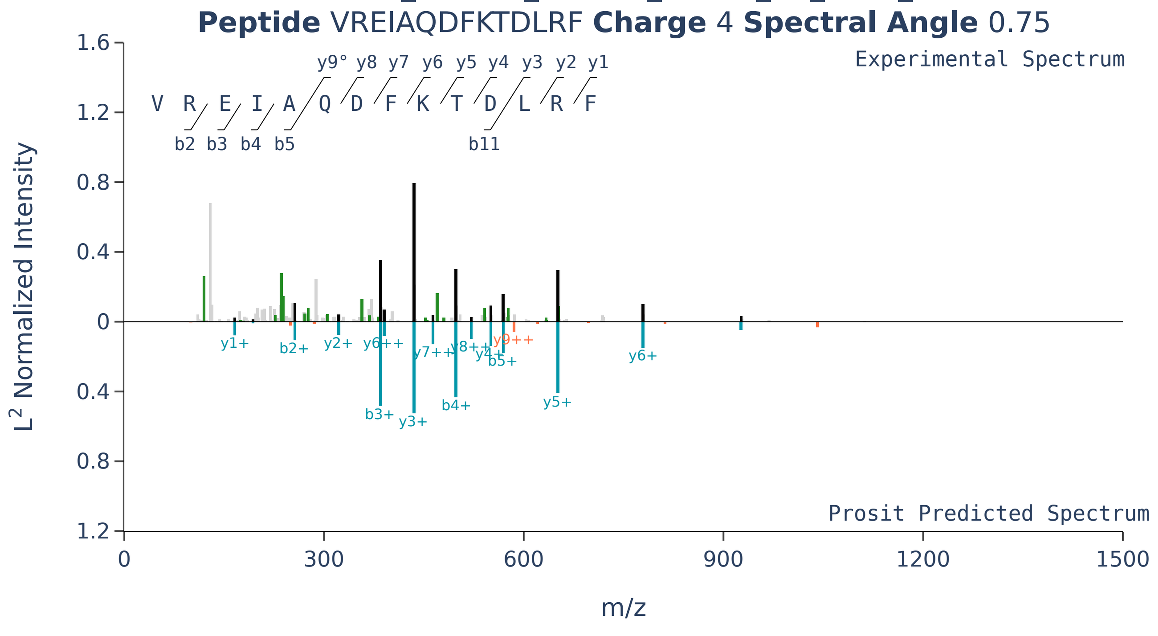


**Source** HRoetschke_271222_120123_HFGoe_H3_b2R2_2h **Scan** 10168
**Peptide** DTNLCAIHAKRV **Charge** 4 **Spectral Angle** 0.9

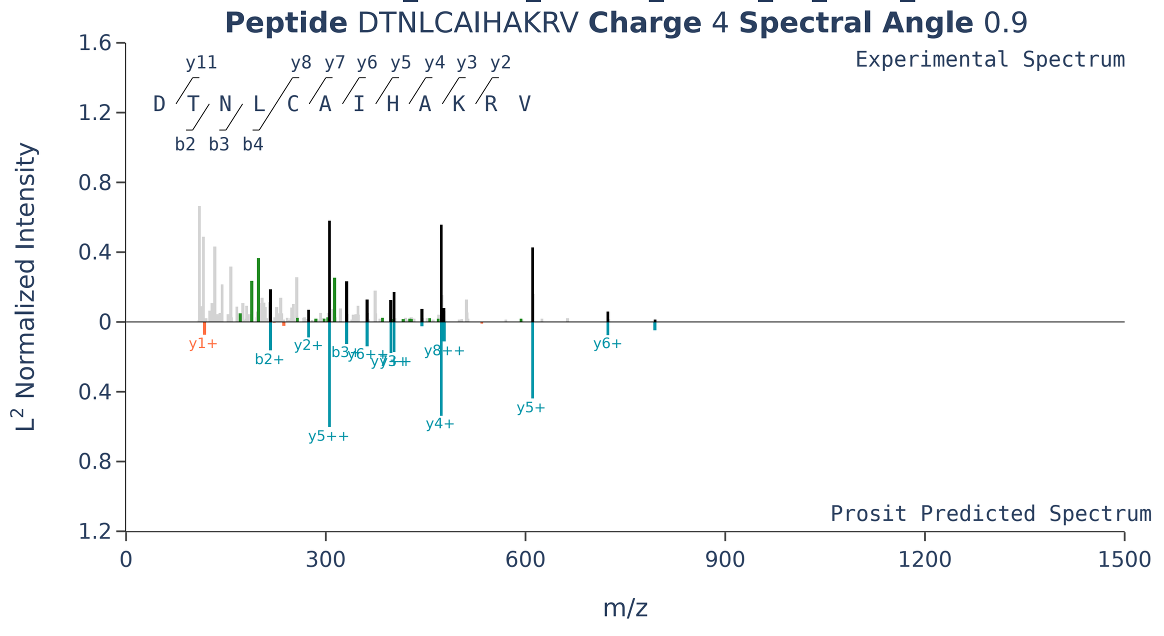


**Source** HRoetschke_271222_120123_HFGoe_H3_b1R1_2h **Scan** 16695
**Peptide** ASEAYLV **Charge** 1 **Spectral Angle** 0.78

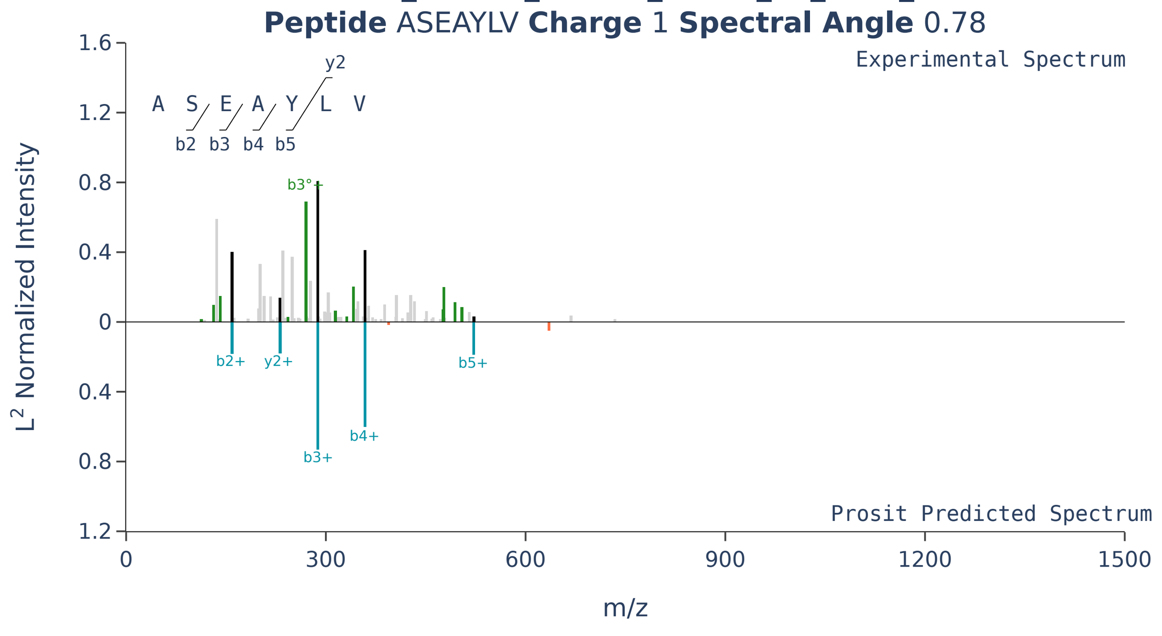

**Source** HRoetschke_271222_120123_HFGoe_H3_b2R2_1h **Scan** 29572
**Peptide** ASEAYLVALFE **Charge** 2 **Spectral Angle** 0.88

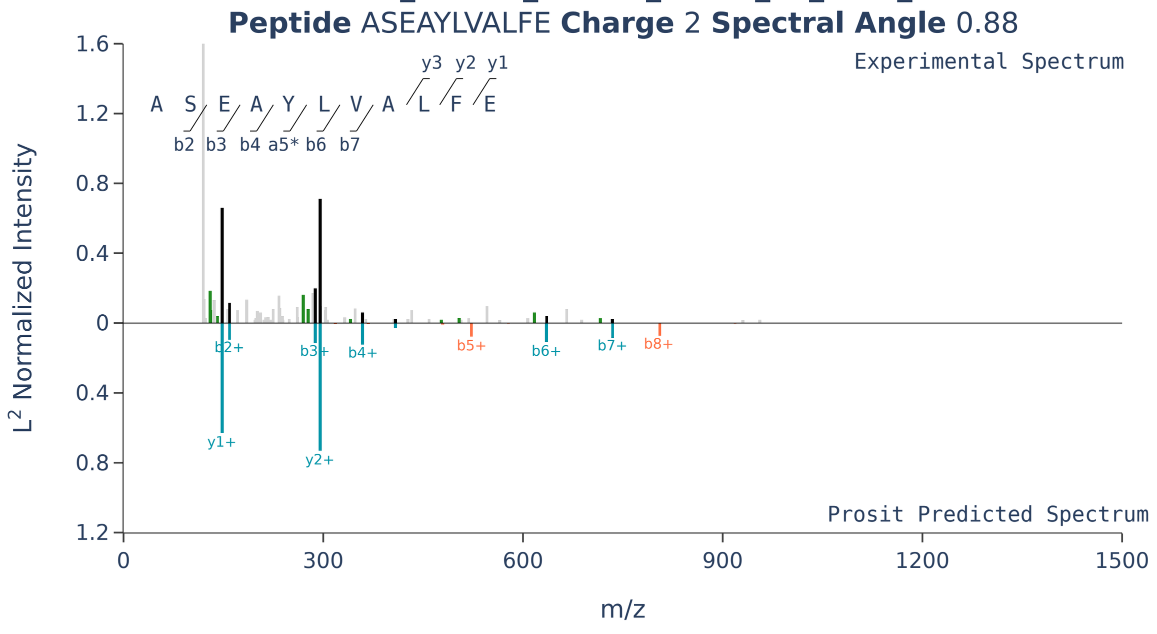


**Source** HRoetschke_271222_120123_HFGoe_H3_b2R1_2h **Scan** 15311
**Peptide** KLPFQRL **Charge** 2 **Spectral Angle** 0.81

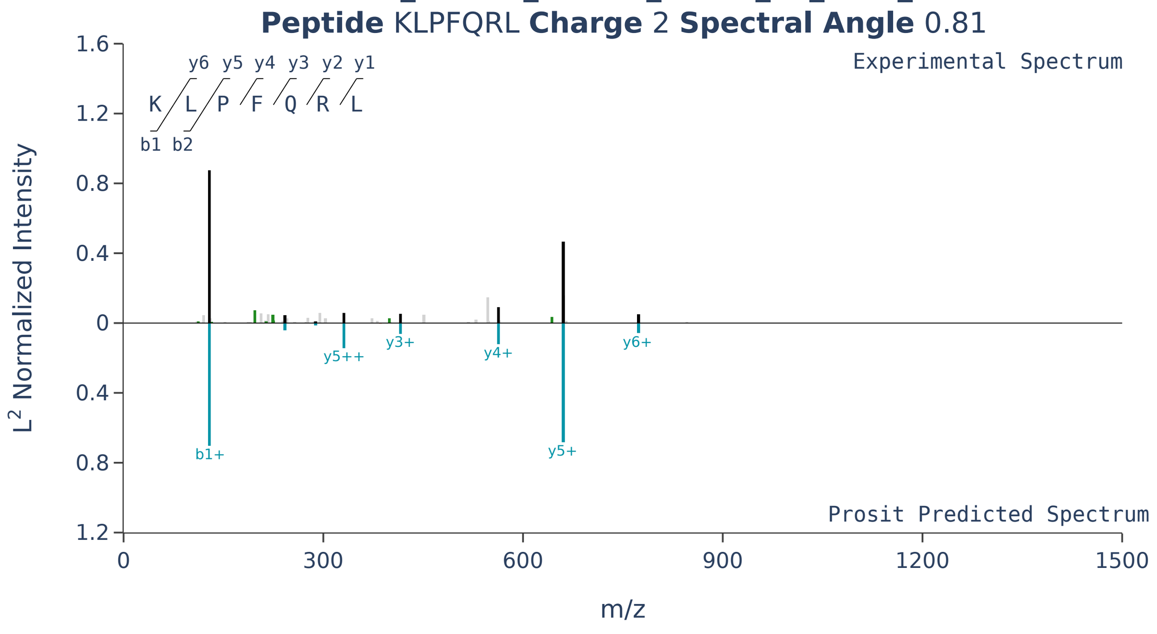


**Source** HRoetschke_271222_120123_HFGoe_H3_b2R2_1h **Scan** 9293
**Peptide** QDFKTDLR **Charge** 2 **Spectral Angle** 0.86

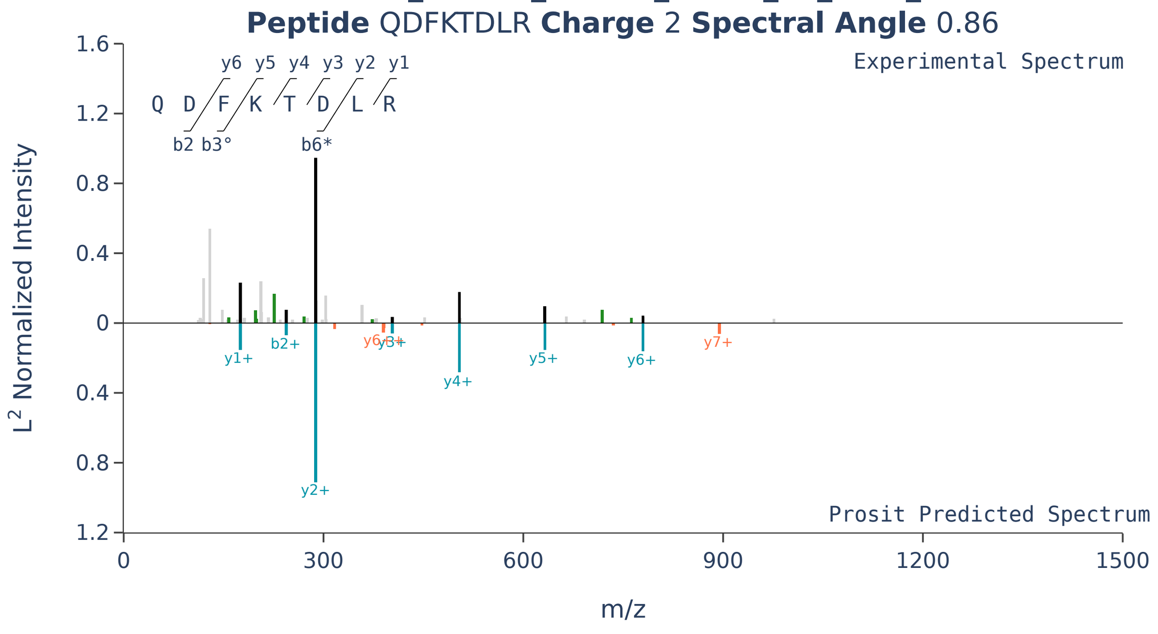


**Source** HRoetschke_271222_120123_HFGoe_H3_b1R2_4h **Scan** 15607
**Peptide** AQDFKTDLRFQS **Charge** 2 **Spectral Angle** 0.81

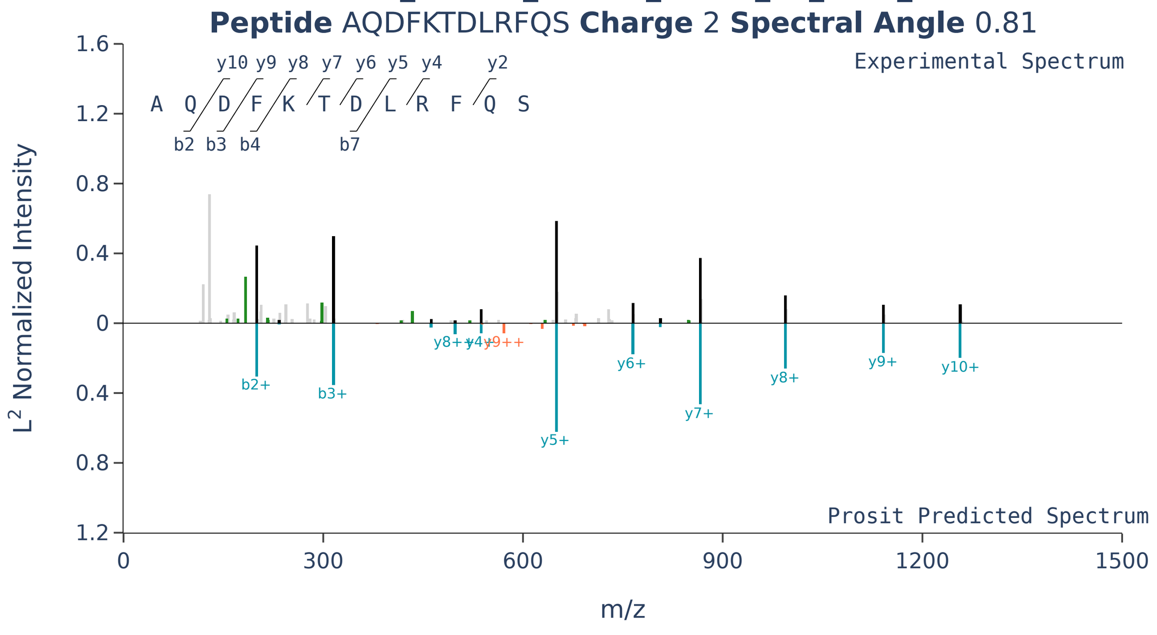


**Source** HRoetschke_271222_120123_HFGoe_H3_b1R2_2h **Scan** 8944
**Peptide** LVREIAQD **Charge** 2 **Spectral Angle** 0.76

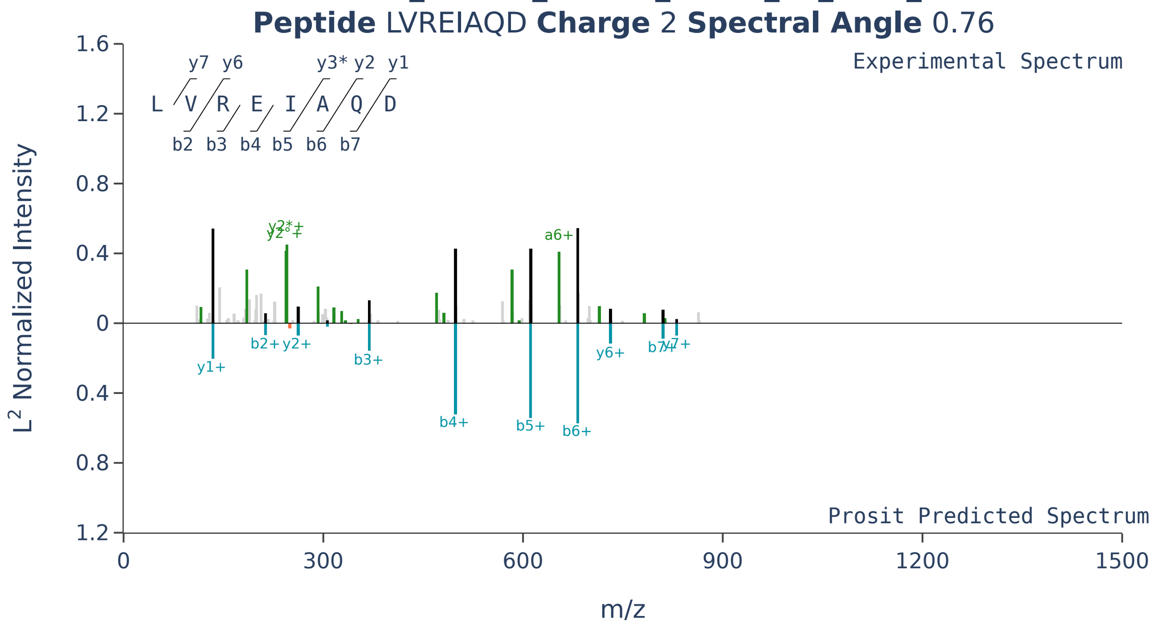


**Source** HRoetschke_271222_120123_HFGoe_H3_b2R2_4h **Scan** 14652
**Peptide** LLRKLPFQR **Charge** 3 **Spectral Angle** 0.86

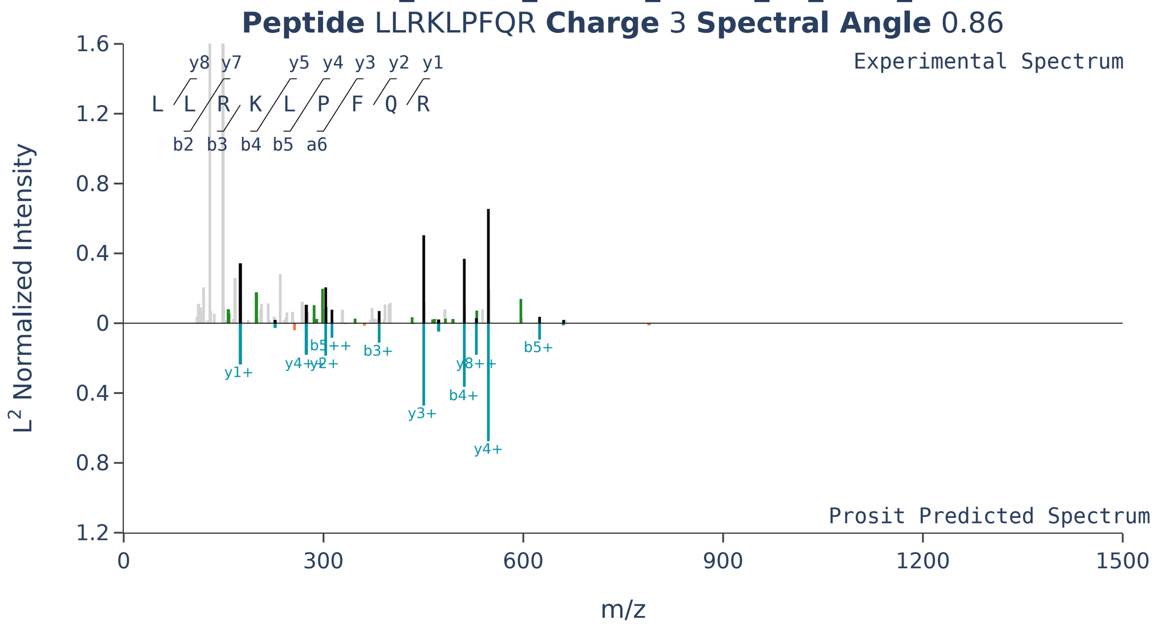


**Source** HRoetschke_271222_120123_HFGoe_H3_b1R2_4h **Scan** 15754
**Peptide** QDFKTDLRFQS **Charge** 2 **Spectral Angle** 0.86

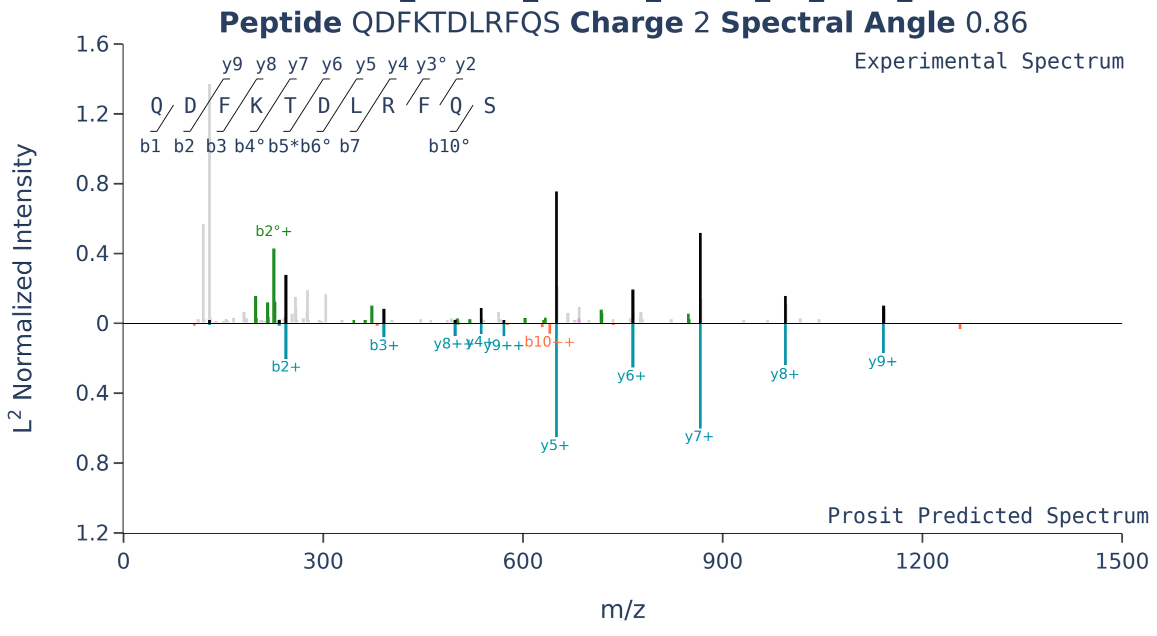


**Source** HRoetschke_271222_120123_HFGoe_H3_b2R2_4h **Scan** 17237
**Peptide** LFEDTNLCAIHAKRV **Charge** 4 **Spectral Angle** 0.78

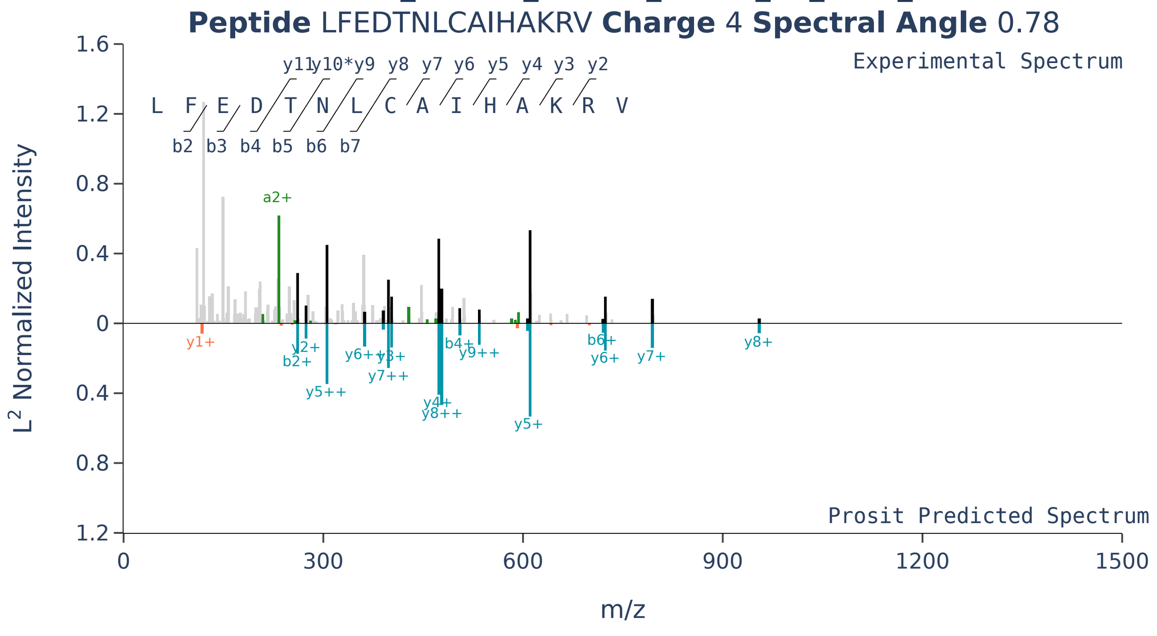


**Source** HRoetschke_271222_120123_HFGoe_H3_b2R1_4h **Scan** 16267
**Peptide** ELAQDFKTDLR **Charge** 3 **Spectral Angle** 0.9

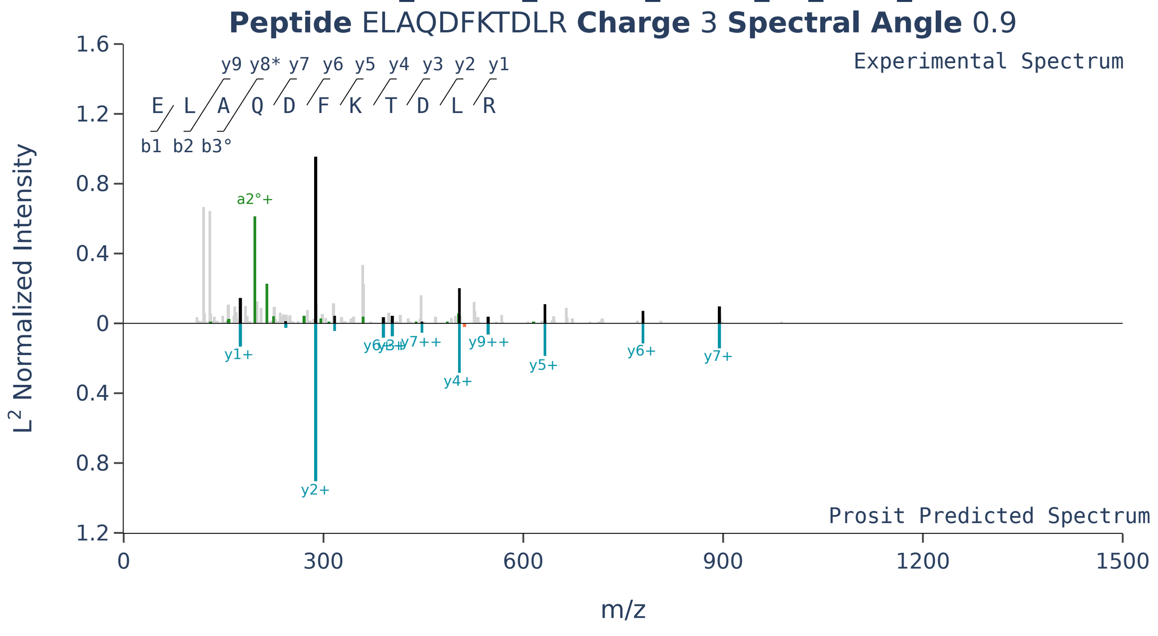


**Source** HRoetschke_271222_120123_HFGoe_H3_b2R2_2h **Scan** 15032
**Peptide** LRKLPFQRL **Charge** 3 **Spectral Angle** 0.87

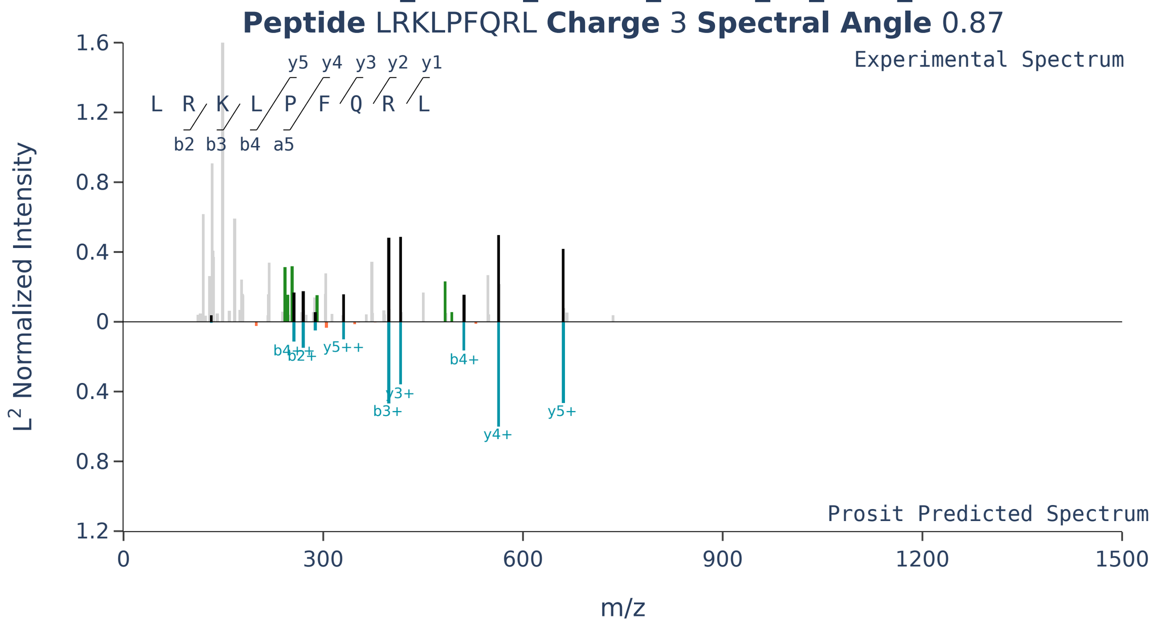


**Source** HRoetschke_271222_120123_HFGoe_H3_b2R2_4h **Scan** 17846
**Peptide** PGTVALREL **Charge** 2 **Spectral Angle** 0.77

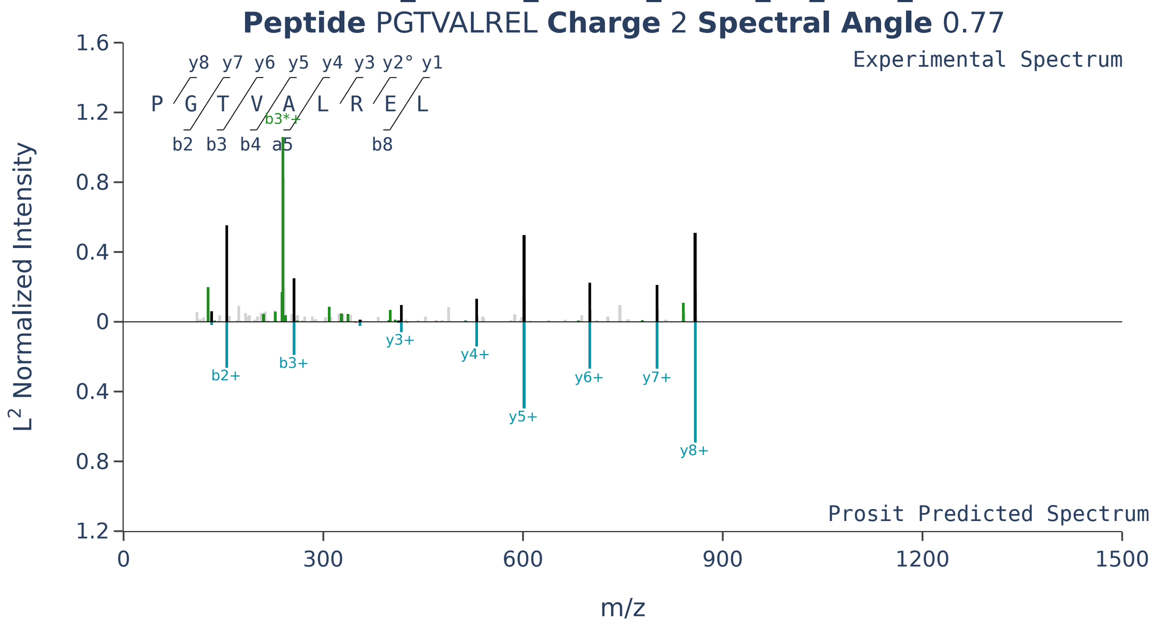


**Source** HRoetschke_271222_120123_HFGoe_H3_b1R2_4h **Scan** 16432
**Peptide** LAQDFKTDL **Charge** 2 **Spectral Angle** 0.72

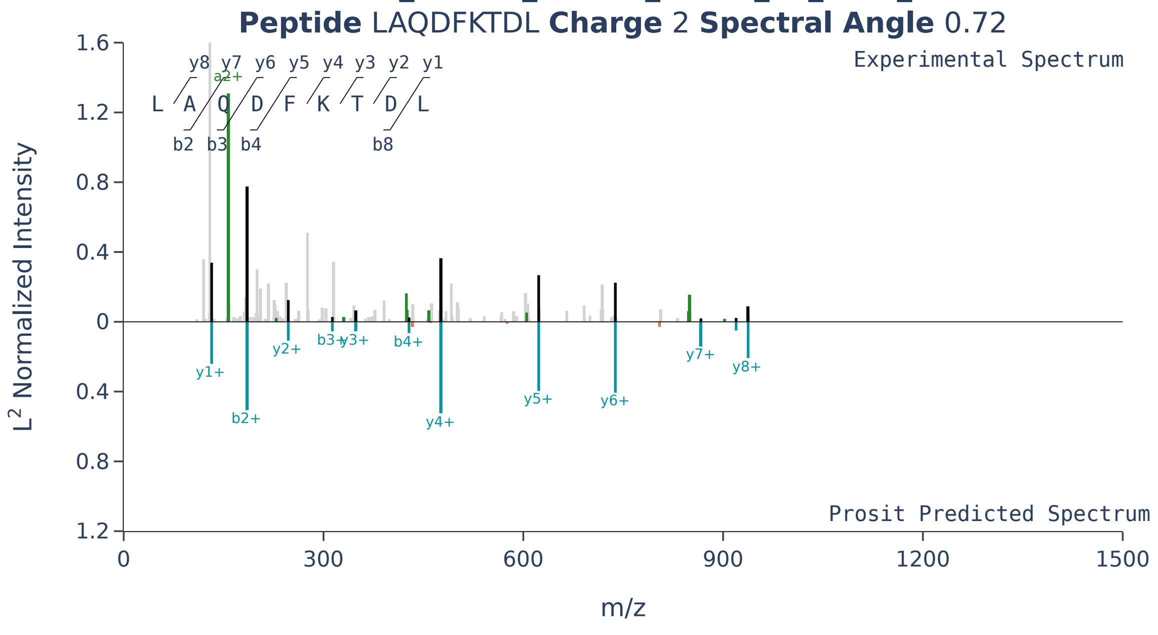


**Source** HRoetschke_271222_120123_HFGoe_H3_b2R1_1h **Scan** 19515
**Peptide** ALFEDTNLC **Charge** 1 **Spectral Angle** 0.85

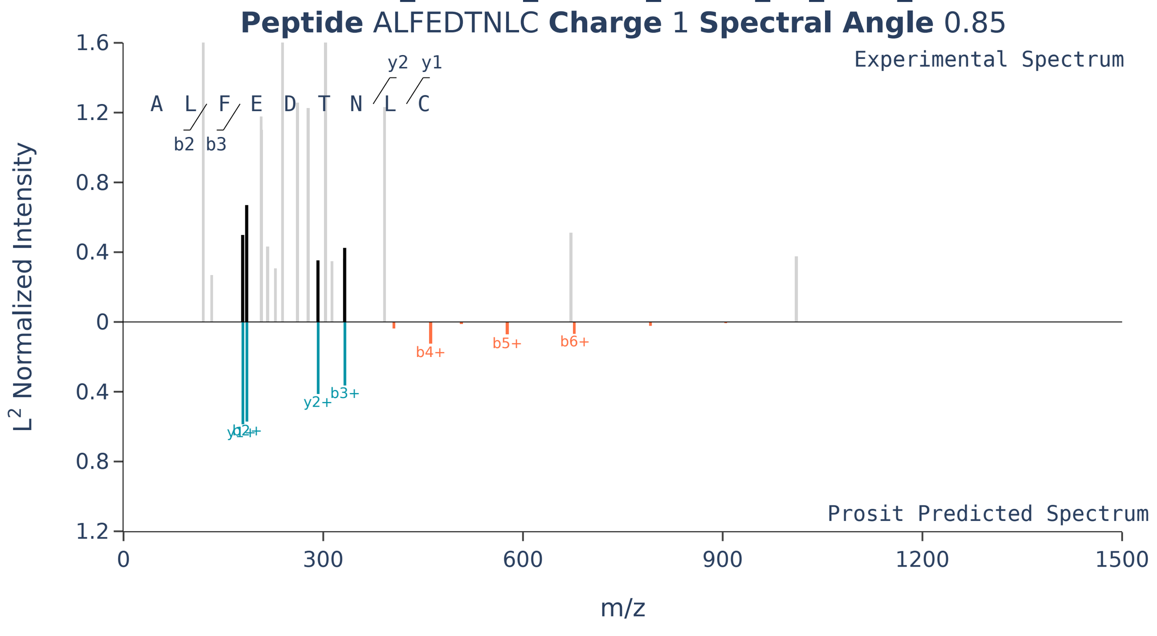


**Source** HRoetschke_271222_120123_HFGoe_H3_b2R1_2h **Scan** 13210
**Peptide** EDTNLCAIHA **Charge** 2 **Spectral Angle** 0.84

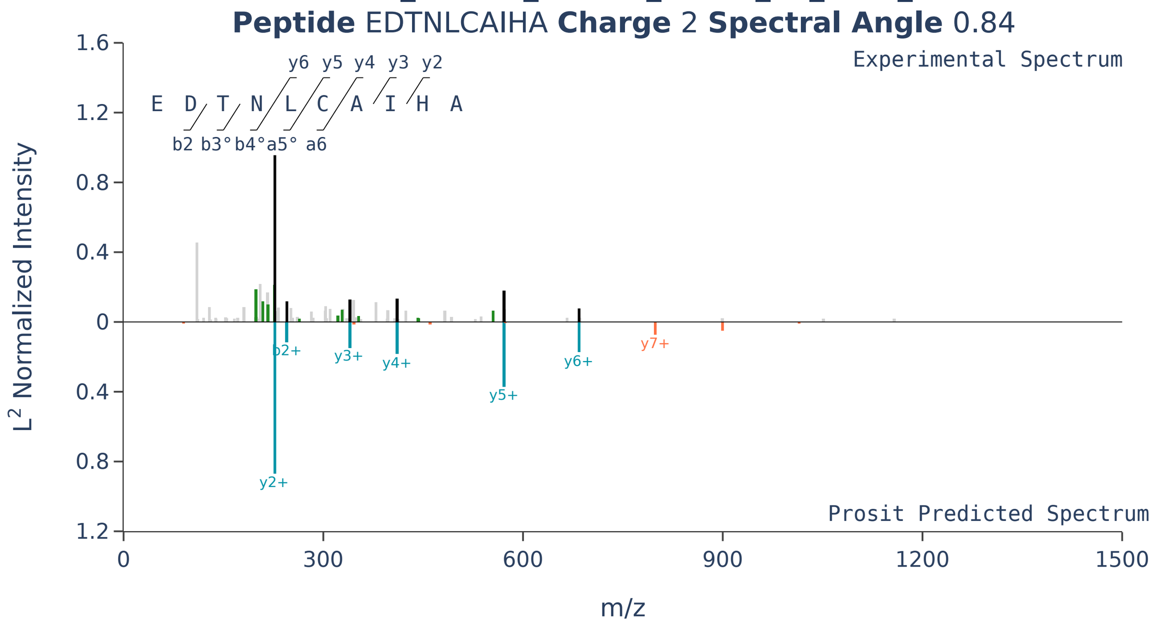


**Source** HRoetschke_271222_120123_HFGoe_H3_b1R1_1h **Scan** 16911
**Peptide** DFKTDLRFQ **Charge** 3 **Spectral Angle** 0.86

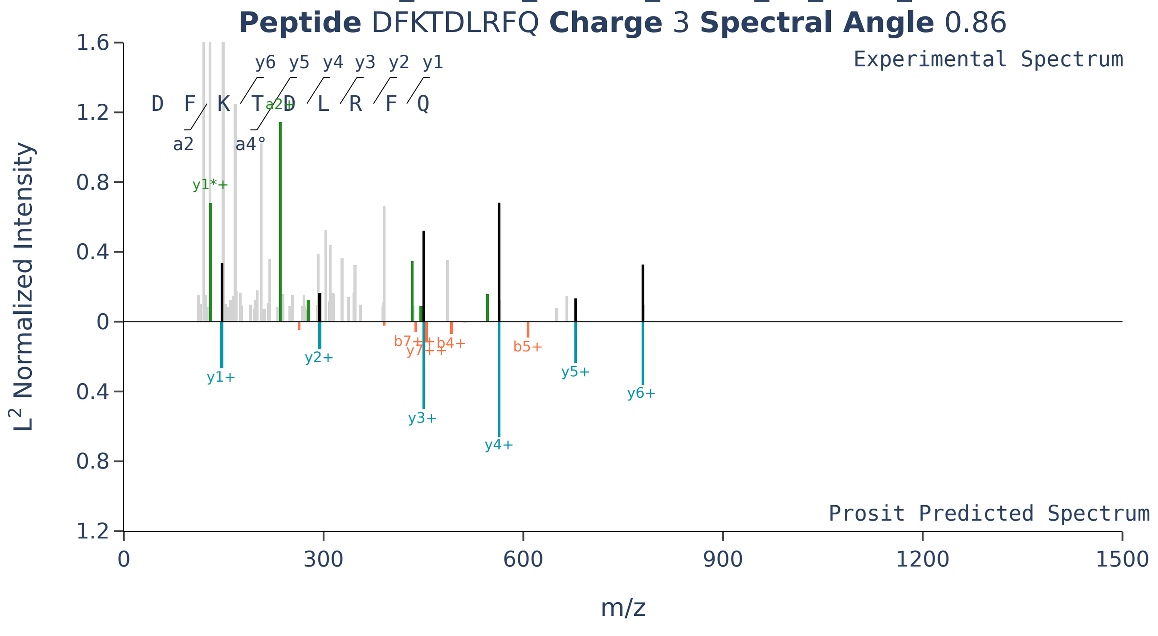

**Source** HRoetschke_271222_120123_HFGoe_H3_b1R1_4h **Scan** 17194
**Peptide** TIMPKDIQLA **Charge** 2 **Spectral Angle** 0.75

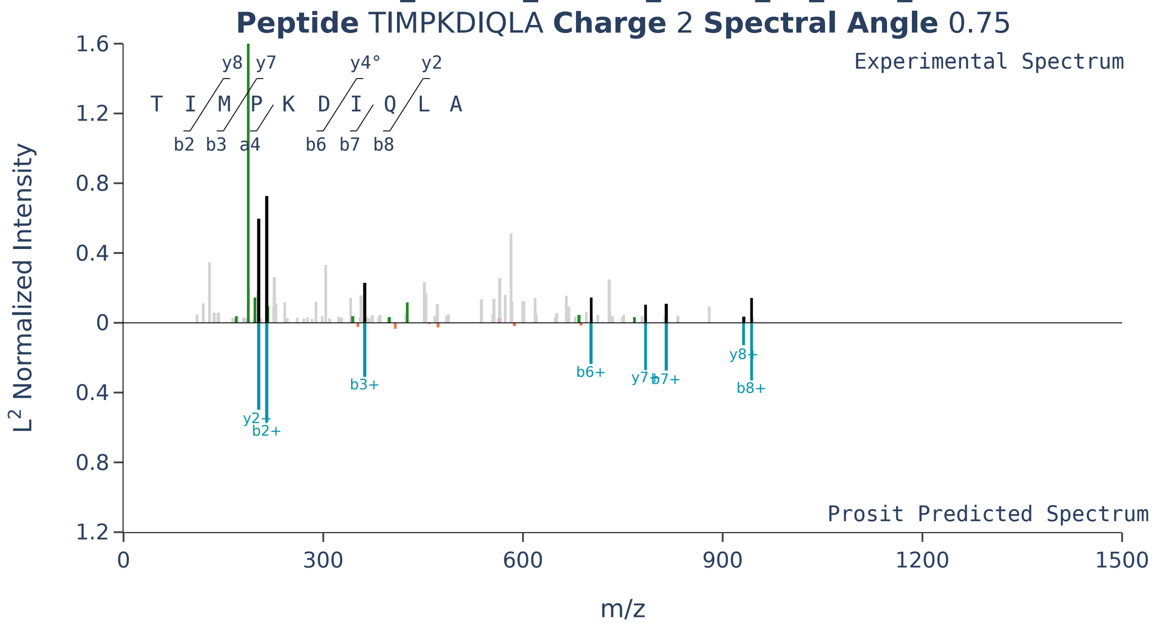


**Source** HRoetschke_271222_120123_HFGoe_H3_b2R2_1h **Scan** 12962
**Peptide** LRFQSSAVMA **Charge** 2 **Spectral Angle** 0.79

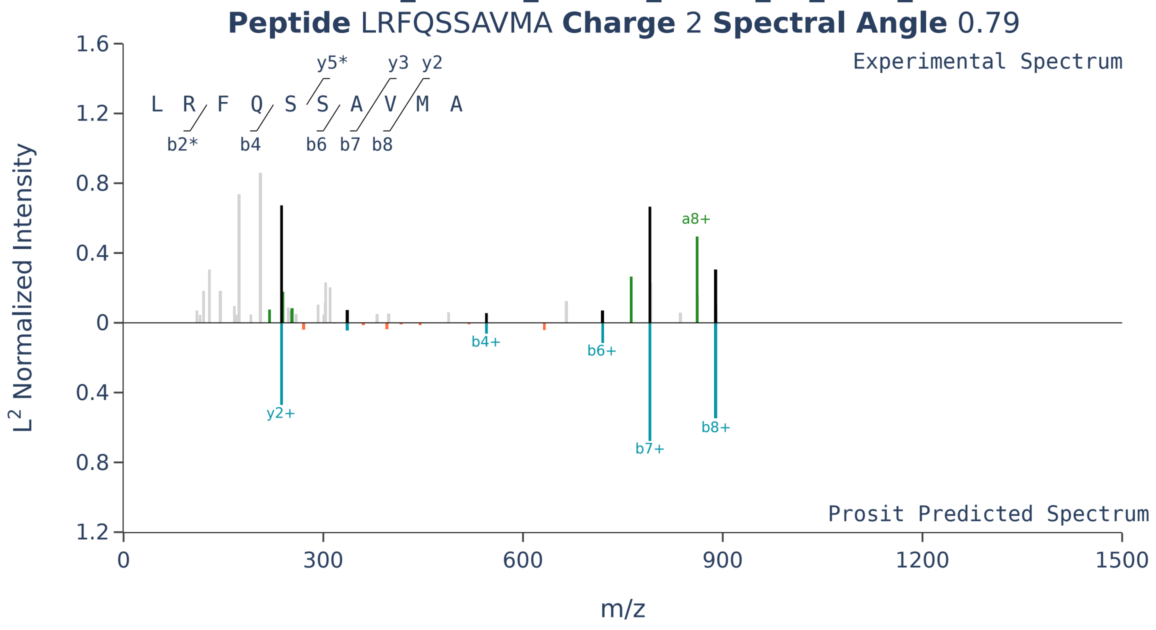


**Source** HRoetschke_271222_120123_HFGoe_H3_b2R1_1h **Scan** 14121
**Peptide** QDFKTDLRFQSS **Charge** 2 **Spectral Angle** 0.84

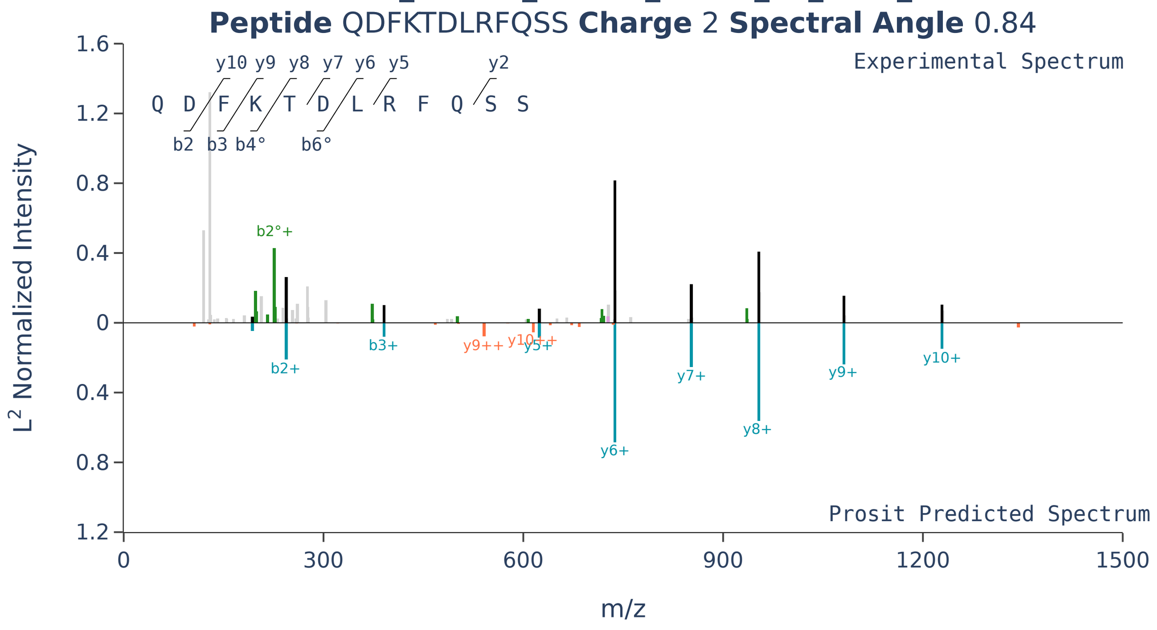


**Source** HRoetschke_271222_120123_HFGoe_H3_b2R2_2h **Scan** 15974
**Peptide** RFQSSAVMAL **Charge** 2 **Spectral Angle** 0.81

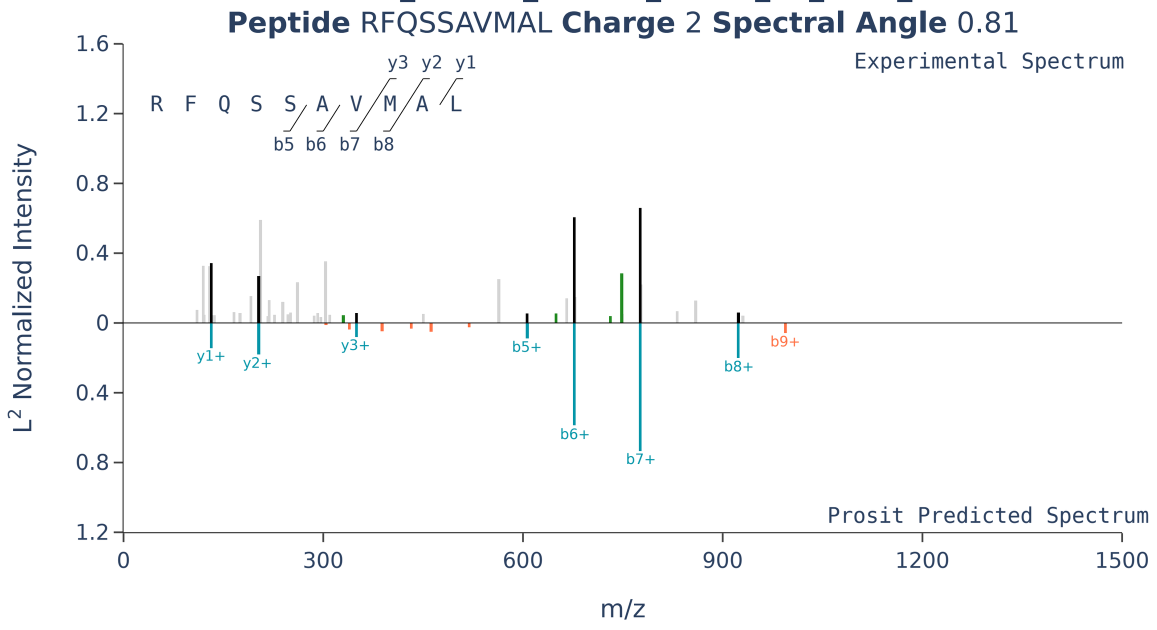


**Source** HRoetschke_271222_120123_HFGoe_H3_b2R1_4h **Scan** 10318
**Peptide** IAQDFKT **Charge** 2 **Spectral Angle** 0.84

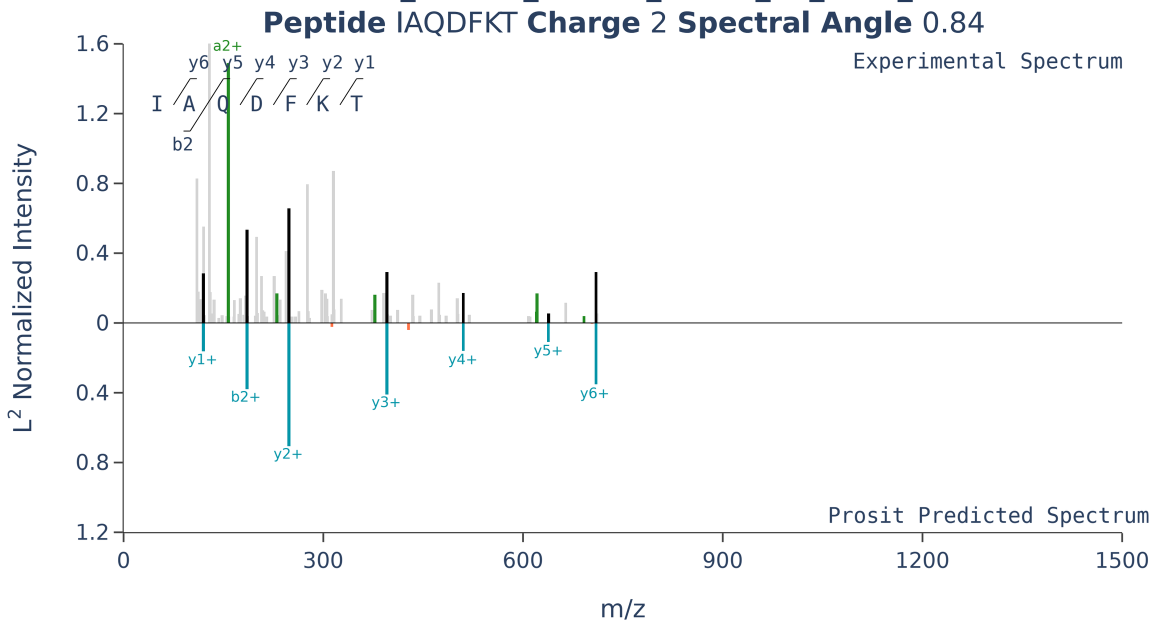


**Source** HRoetschke_271222_120123_HFGoe_H3_b2R2_2h **Scan** 10458
**Peptide** PFQRLVR **Charge** 2 **Spectral Angle** 0.84

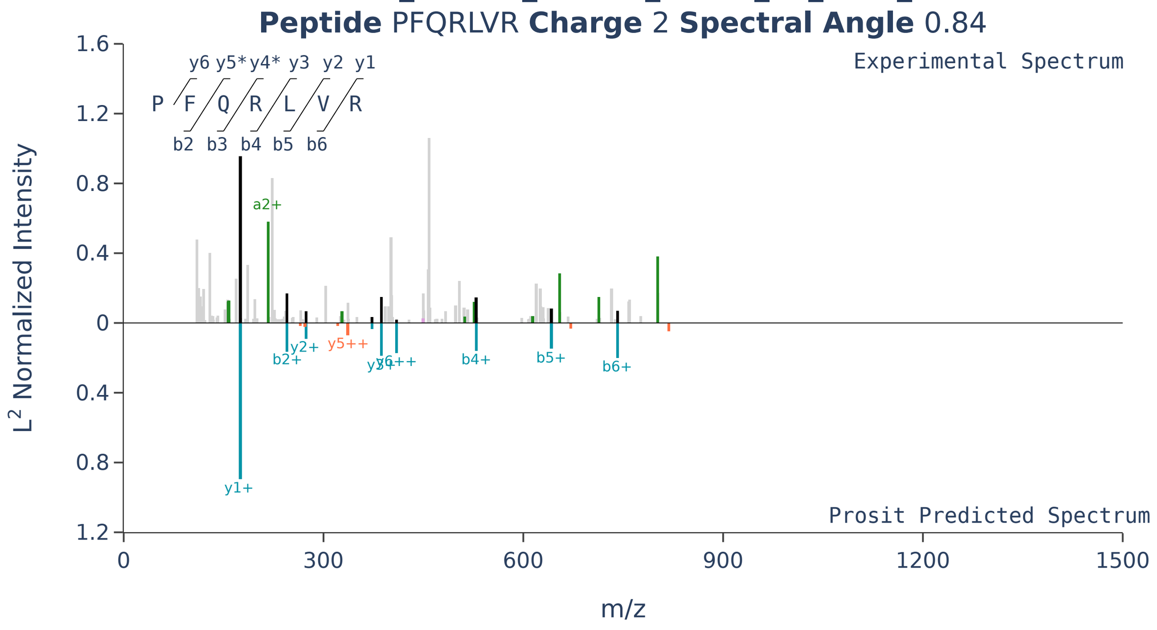


**Source** HRoetschke_271222_120123_HFGoe_H3_b2R1_4h **Scan** 10908
**Peptide** LRFQSSA **Charge** 2 **Spectral Angle** 0.84

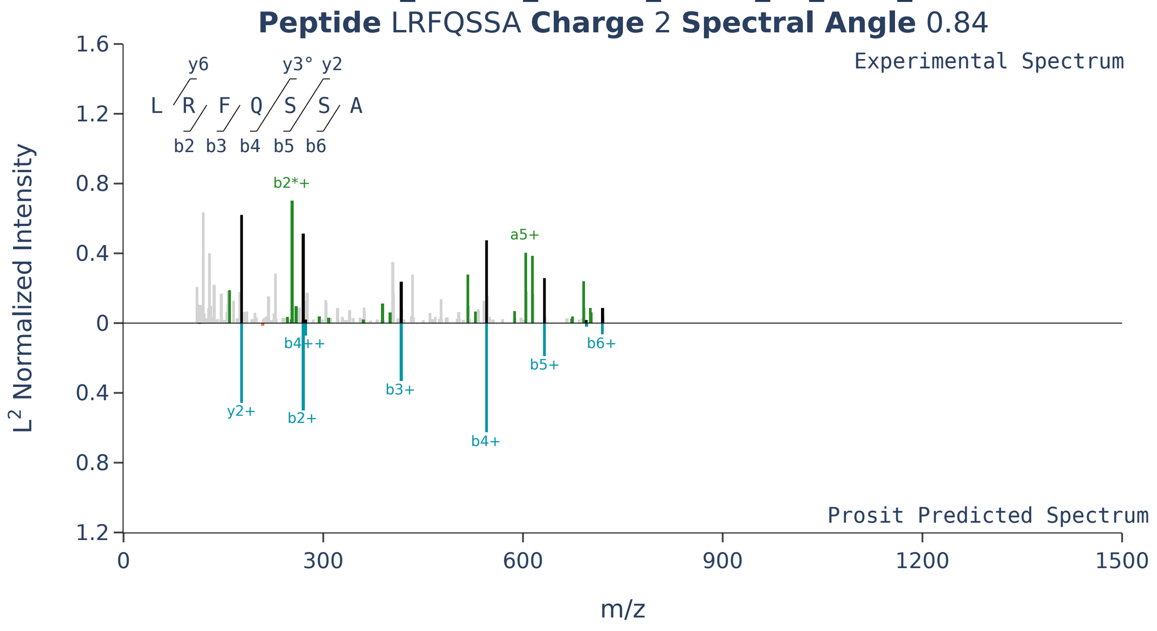


**Source** HRoetschke_271222_120123_HFGoe_H3_b1R2_4h **Scan** 9000
**Peptide** IAQDFKTD **Charge** 2 **Spectral Angle** 0.79

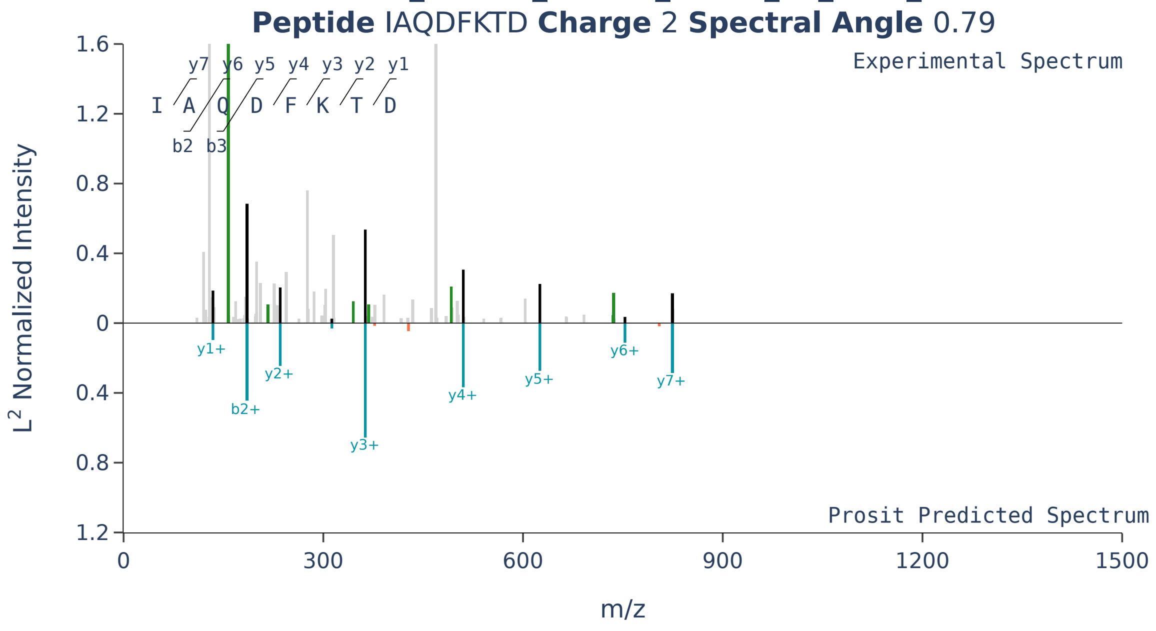


**Source** HRoetschke_271222_120123_HFGoe_H3_b2R1_4h **Scan** 13369
**Peptide** AVMALQEA **Charge** 1 **Spectral Angle** 0.75

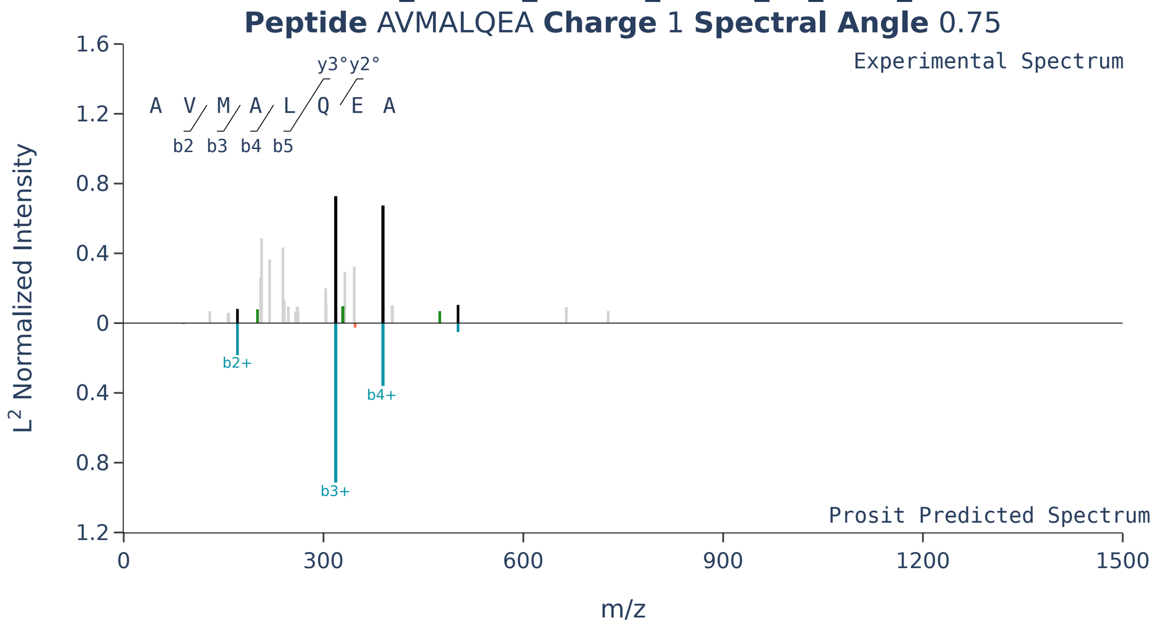


**Source** HRoetschke_271222_120123_HFGoe_H3_b2R1_4h **Scan** 12059
**Peptide** FEDTNLCAIHAKR **Charge** 4 **Spectral Angle** 0.83

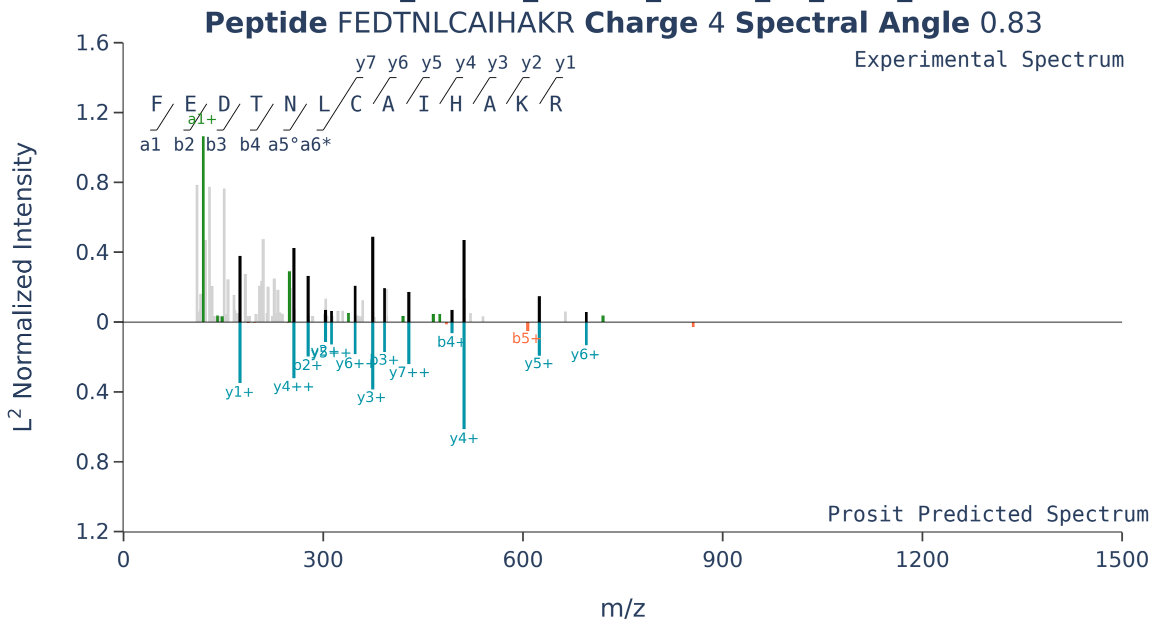


**Source** HRoetschke_271222_120123_HFGoe_H3_b1R1_24h **Scan** 14424
**Peptide** FKTDLRFQSSA **Charge** 3 **Spectral Angle** 0.85

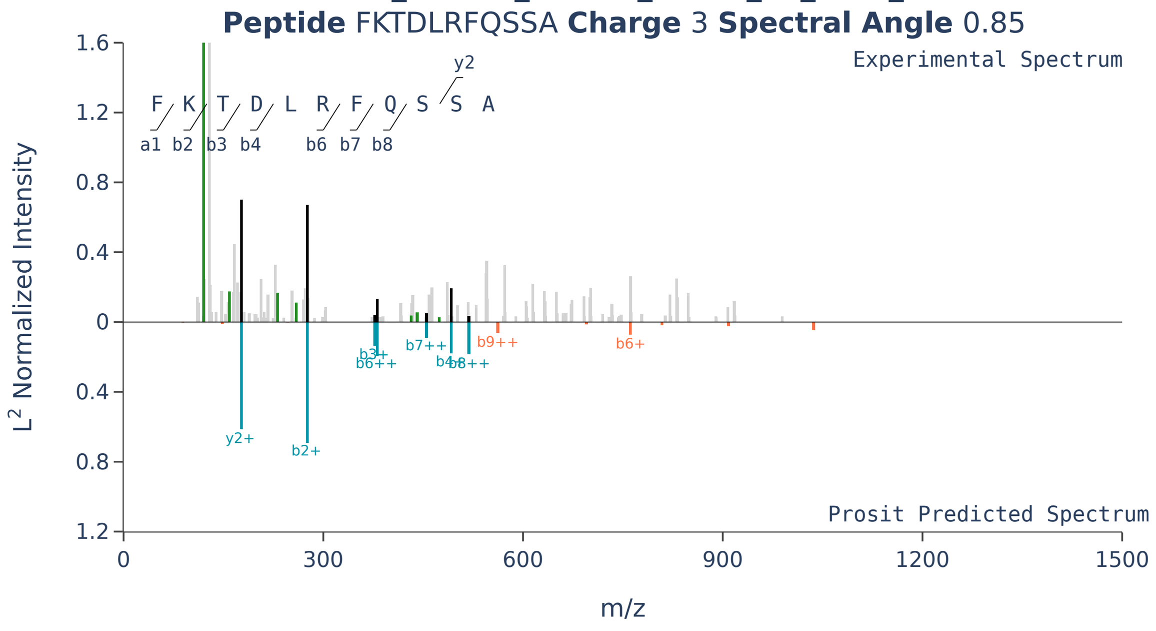


**Source** HRoetschke_271222_120123_HFGoe_H3_b1R1_4h **Scan** 9169
**Peptide** EDTNLCAIHAKRV **Charge** 4 **Spectral Angle** 0.9

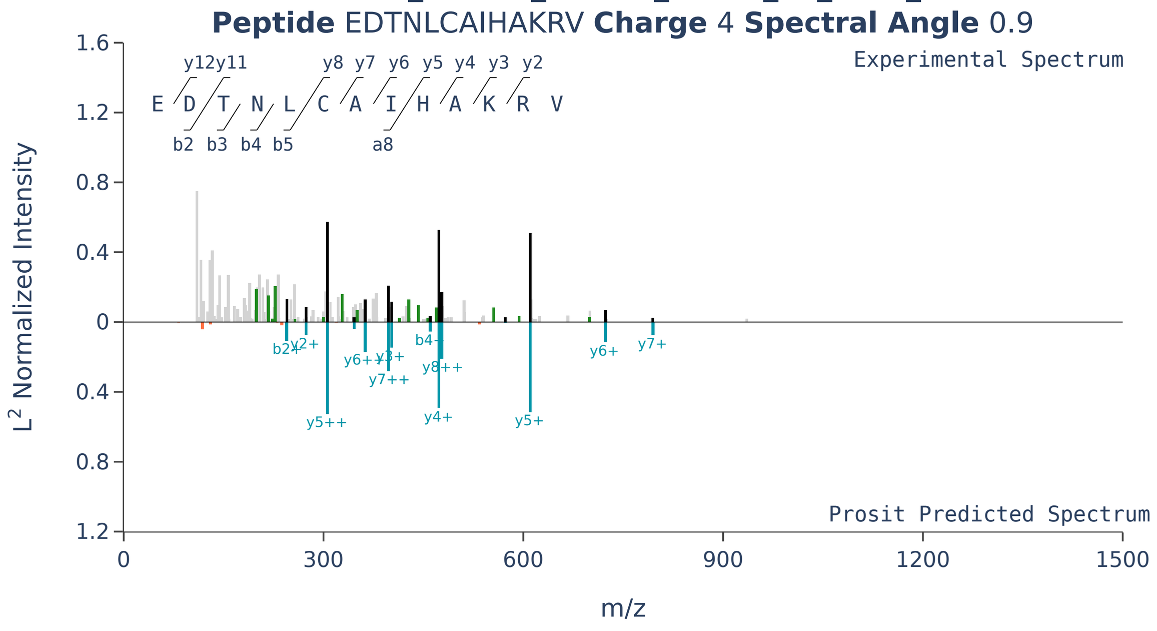


**Source** HRoetschke_271222_120123_HFGoe_H3_b2R2_2h **Scan** 6863
**Peptide** AIHAKRVTIMPKD **Charge** 2 **Spectral Angle** 0.79

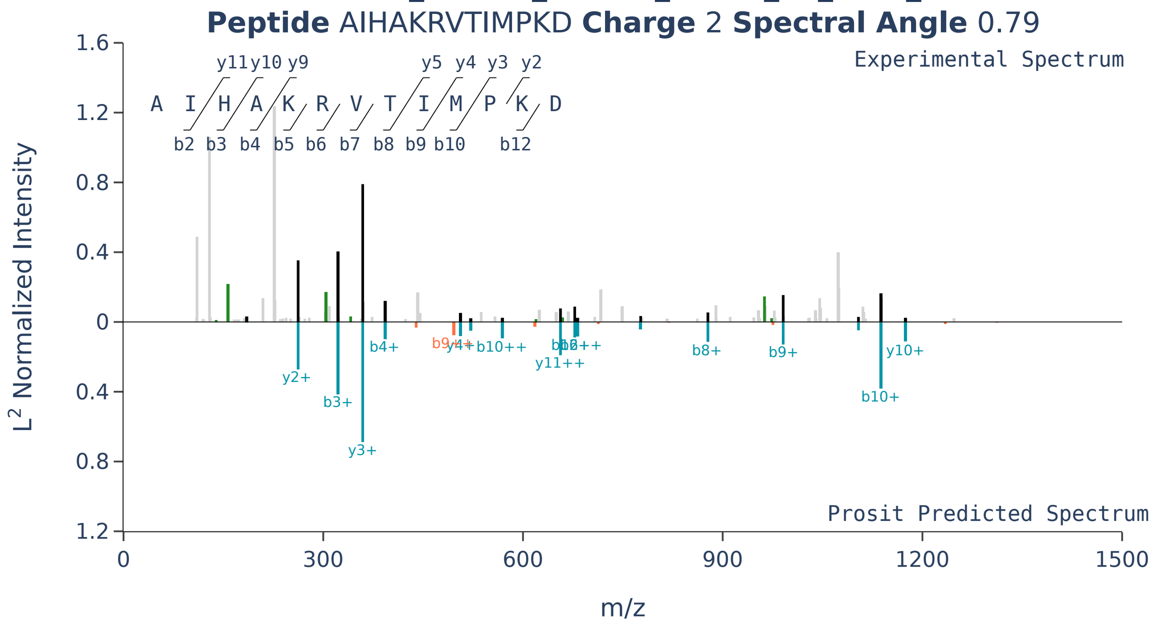


**Source** HRoetschke_271222_120123_HFGoe_H3_b1R1_4h **Scan** 11748
**Peptide** KTDLRFQSSAVM **Charge** 3 **Spectral Angle** 0.89

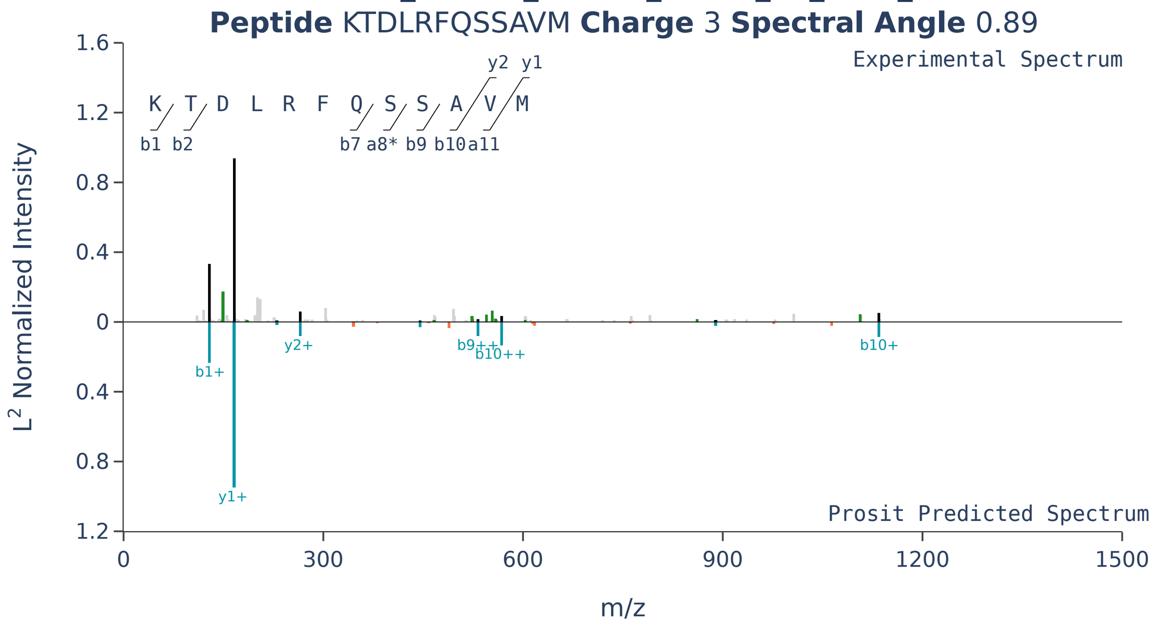


**Source** HRoetschke_271222_120123_HFGoe_H3_b2R2_2h **Scan** 9818
**Peptide** DTNLCAIHAK **Charge** 3 **Spectral Angle** 0.89

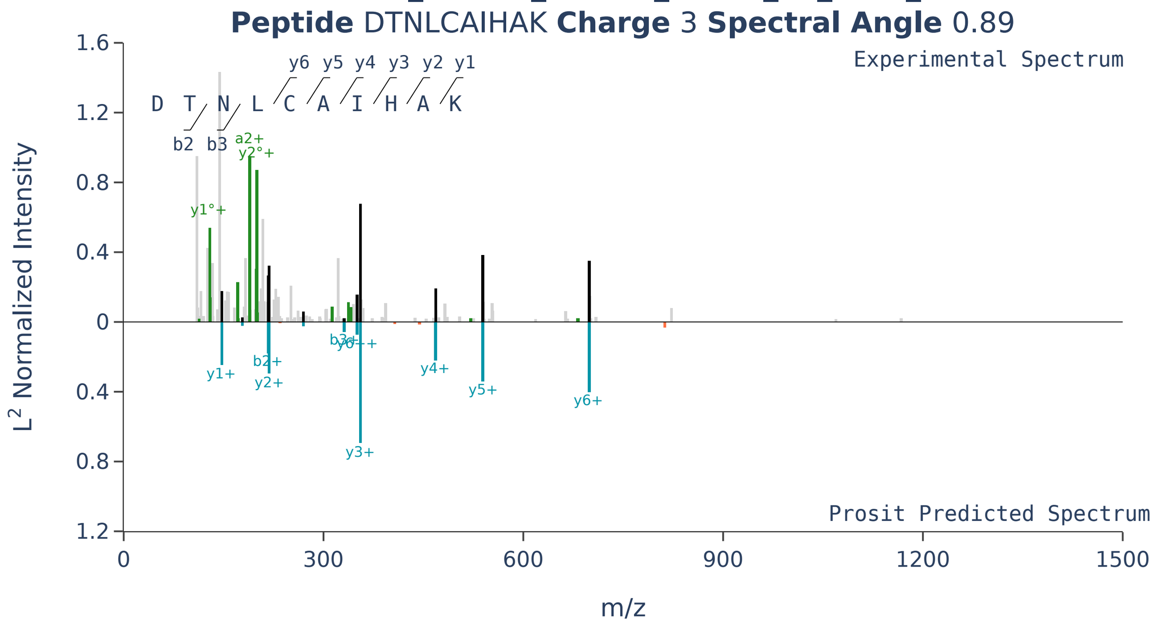

**Source** HRoetschke_271222_120123_HFGoe_H3_b1R1_4h **Scan** 14522
**Peptide** FKTDLRFQS **Charge** 2 **Spectral Angle** 0.82

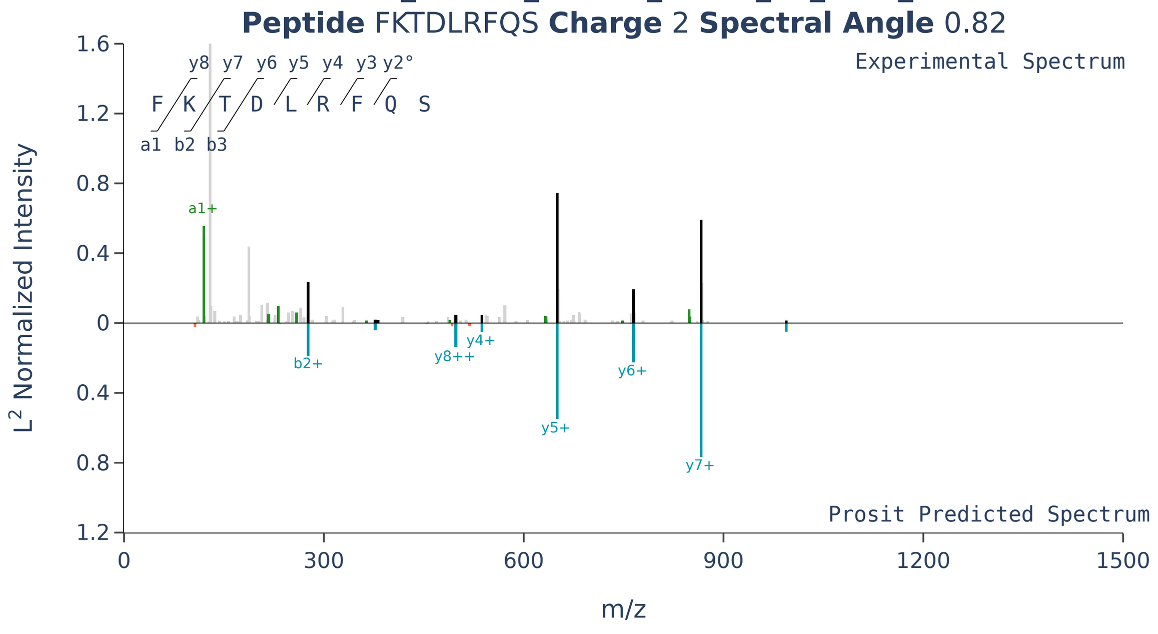


**Source** HRoetschke_271222_120123_HFGoe_H3_b2R1_24h **Scan** 11476
**Peptide** PFQRLVRE **Charge** 2 **Spectral Angle** 0.71

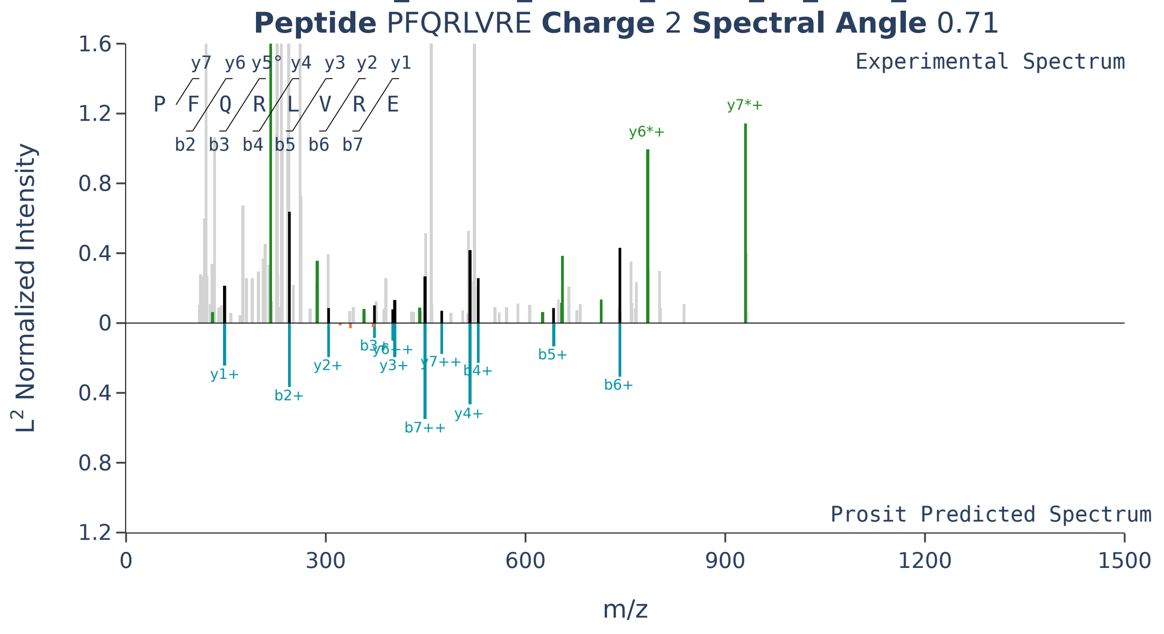


**Source** HRoetschke_271222_120123_HFGoe_H3_b2R1_2h **Scan** 8509
**Peptide** IQLARRIRGERA **Charge** 4 **Spectral Angle** 0.87

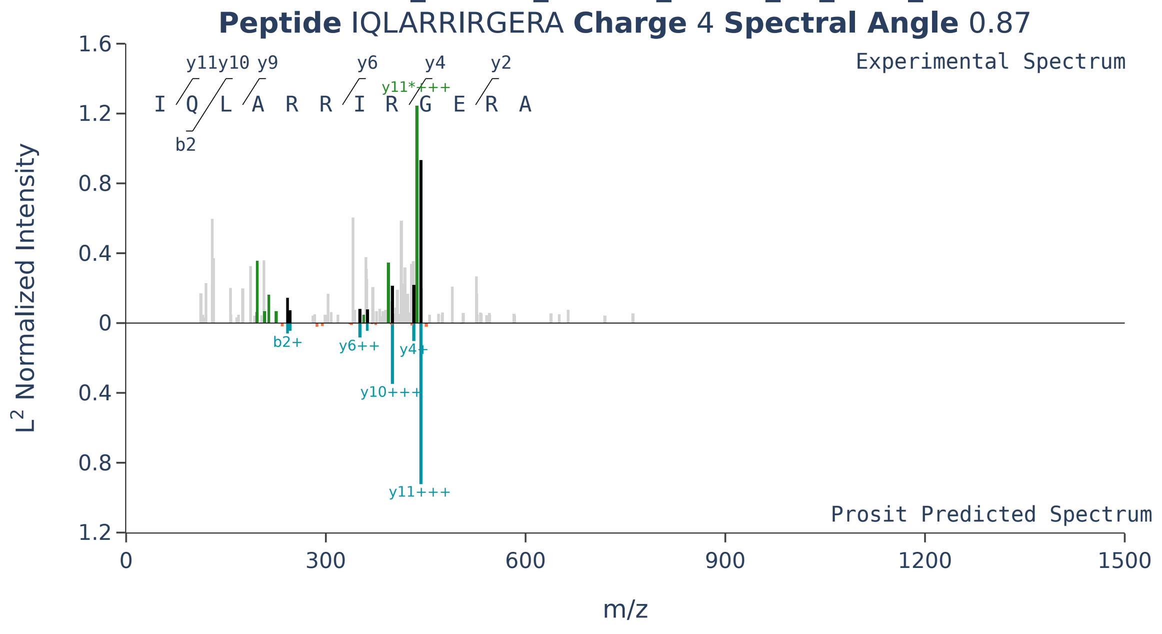


**Source** HRoetschke_271222_120123_HFGoe_H3_b2R1_4h **Scan** 12604
**Peptide** KRVTIMPKDIQLAR **Charge** 3 **Spectral Angle** 0.85

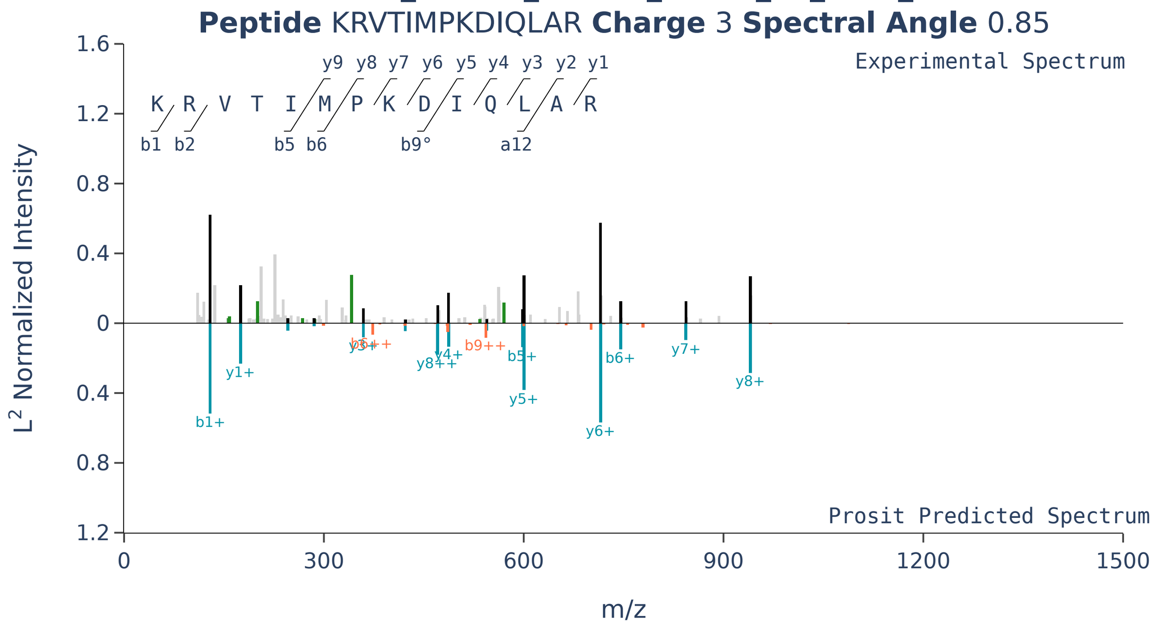


**Source** HRoetschke_271222_120123_HFGoe_H3_b2R2_2h **Scan** 10521
**Peptide** TNLCAIH **Charge** 2 **Spectral Angle** 0.89

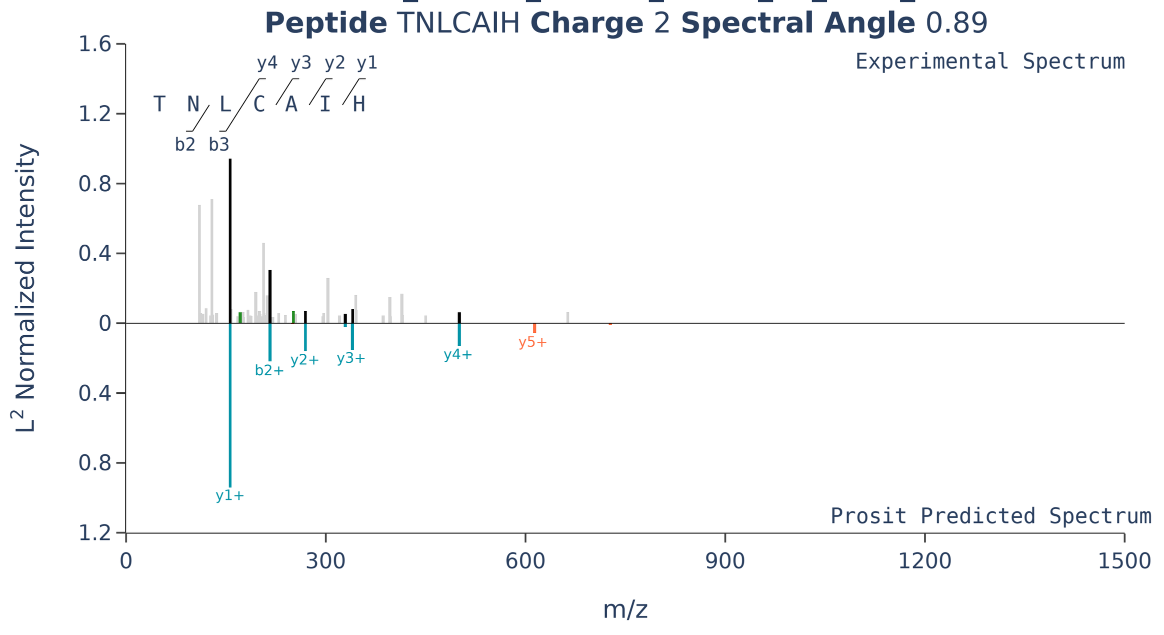


**Source** HRoetschke_271222_120123_HFGoe_H3_b1R2_4h **Scan** 15709
**Peptide** AQDFKTDLRFQSSA **Charge** 2 **Spectral Angle** 0.77

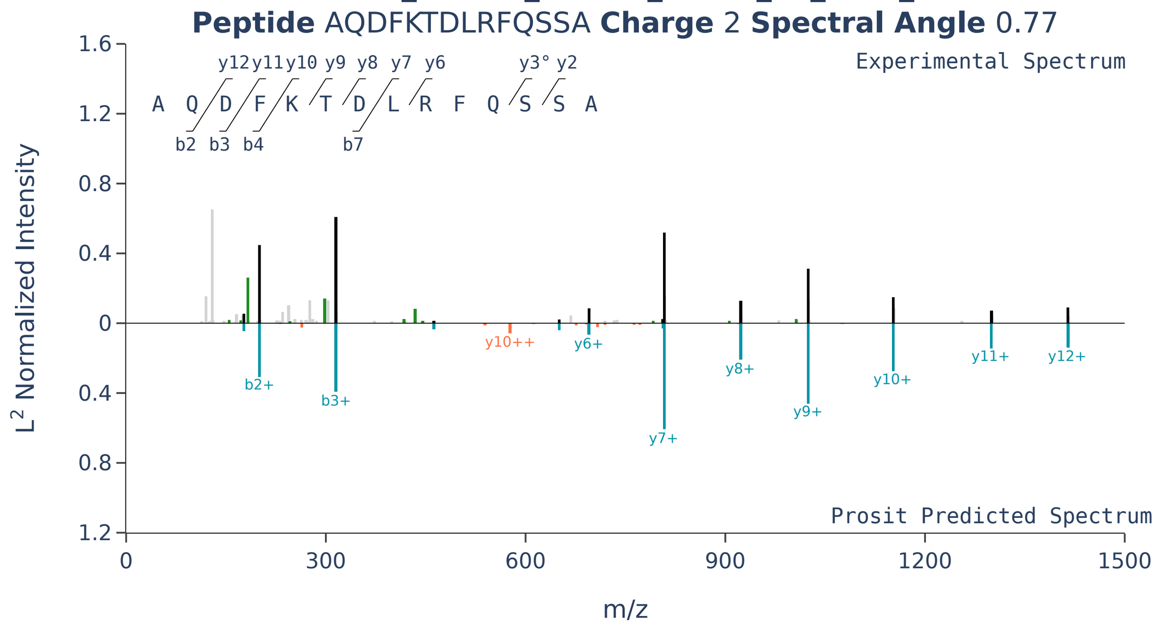


**Source** HRoetschke_271222_120123_HFGoe_H3_b2R2_2h **Scan** 10025
**Peptide** AIHAKRVTIM **Charge** 3 **Spectral Angle** 0.8

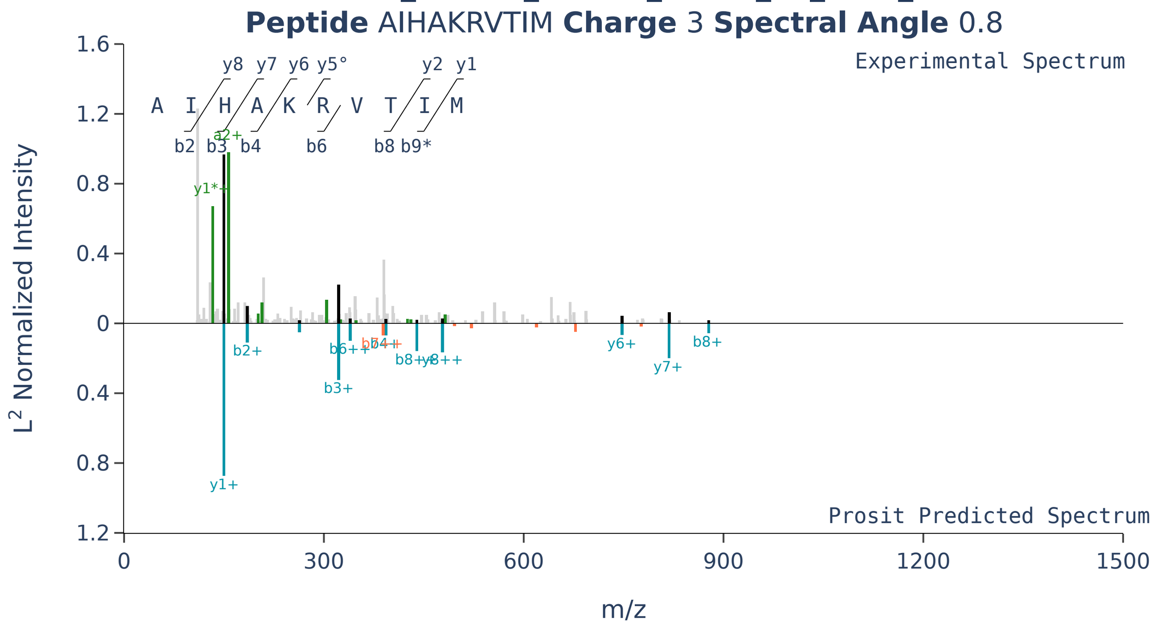


**Source** HRoetschke_271222_120123_HFGoe_H3_b1R2_4h **Scan** 16036
**Peptide** QDFKTDLRFQ **Charge** 2 **Spectral Angle** 0.78

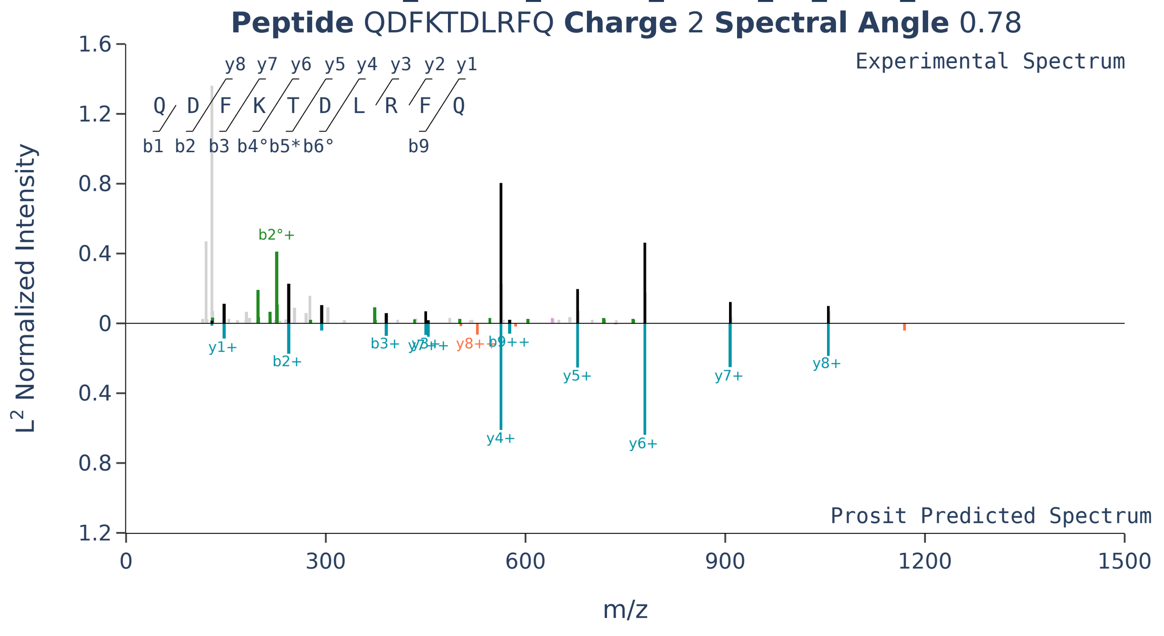


**Source** HRoetschke_271222_120123_HFGoe_H3_b2R2_4h **Scan** 18287
**Peptide** DTNLCAI **Charge** 1 **Spectral Angle** 0.85

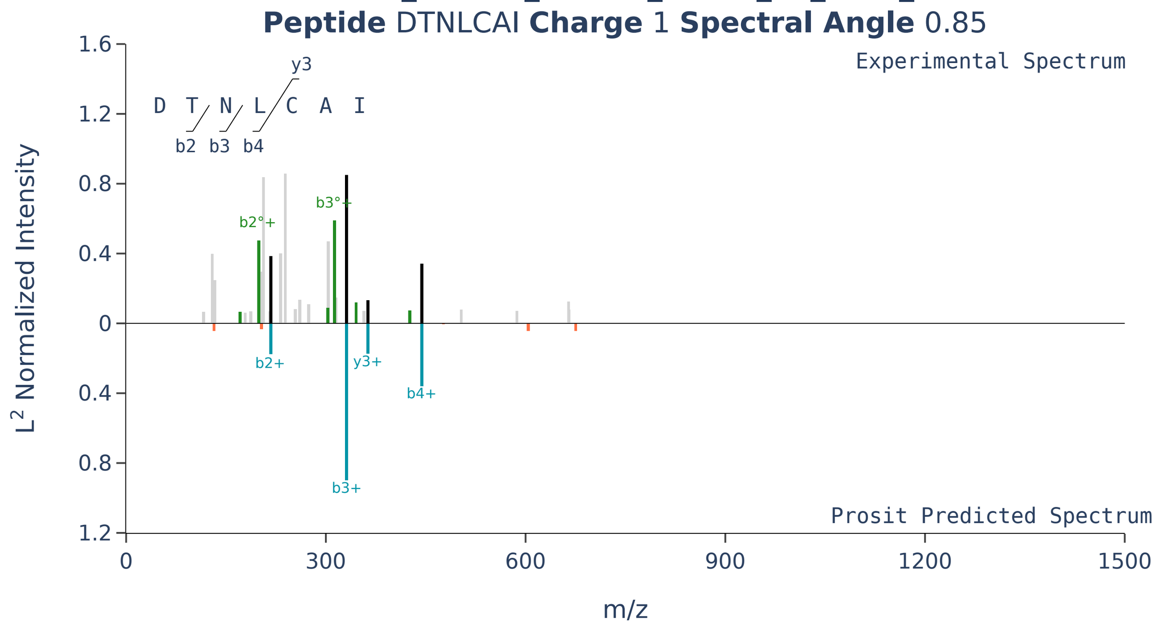


**Source** HRoetschke_271222_120123_HFGoe_H3_b2R2_4h **Scan** 14718
**Peptide** RFQSSAVMALQ **Charge** 2 **Spectral Angle** 0.69

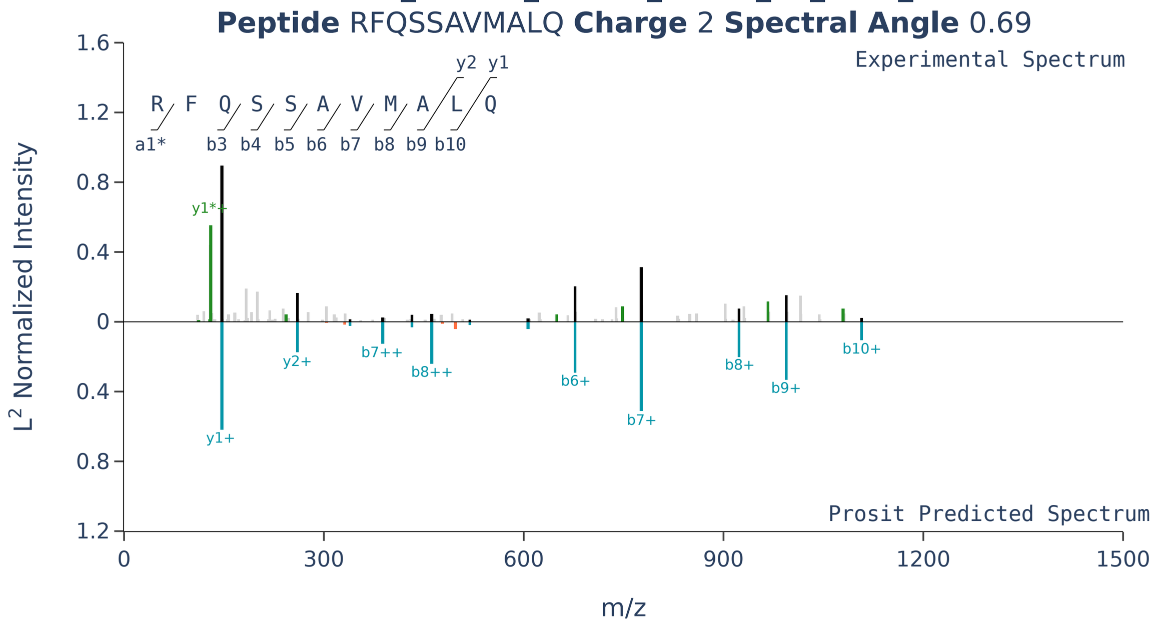


**Source** HRoetschke_271222_120123_HFGoe_H3_b2R1_4h **Scan** 12919
**Peptide** RRYQKSTELLIRKL **Charge** 4 **Spectral Angle** 0.87

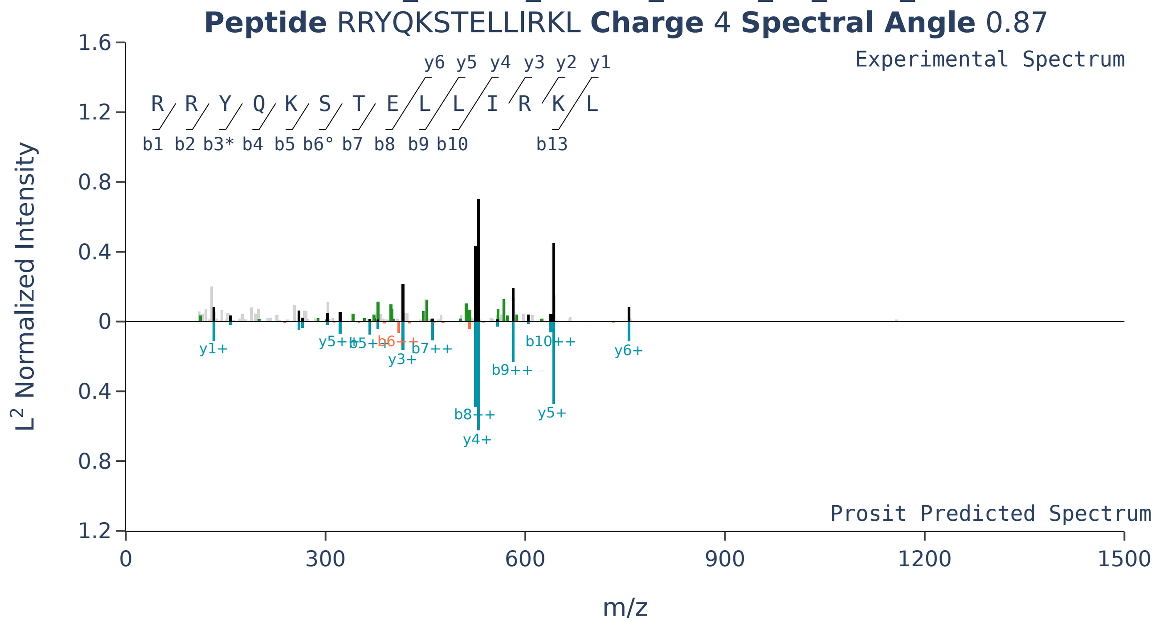


**Source** HRoetschke_271222_120123_HFGoe_H3_b2R2_4h **Scan** 9391
**Peptide** LVREIAQ **Charge** 2 **Spectral Angle** 0.74

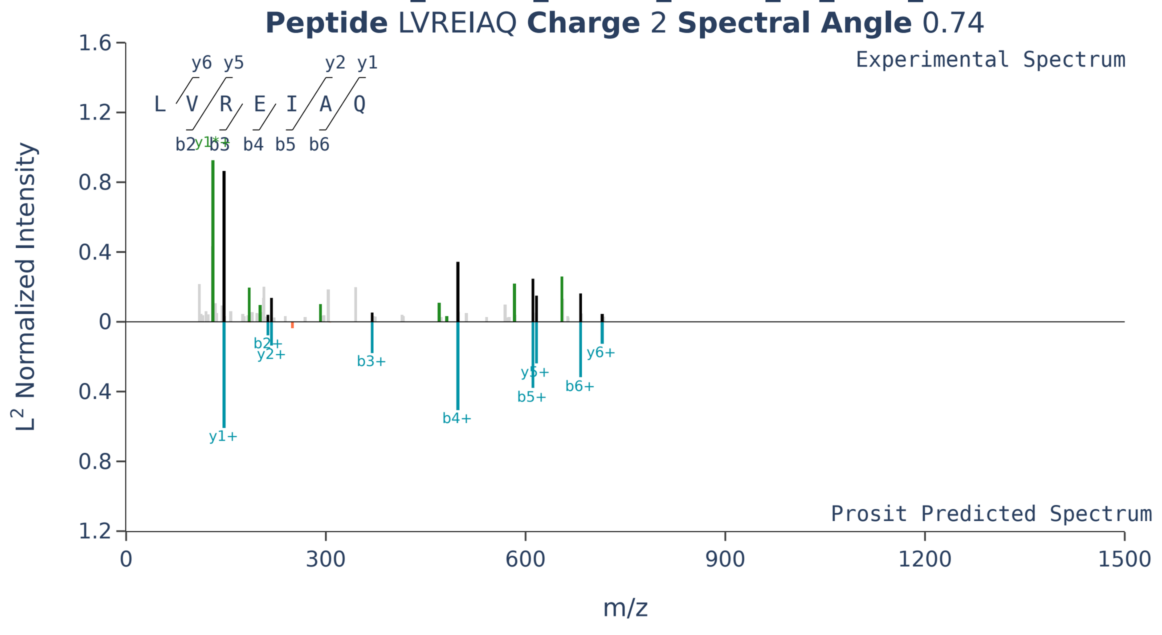


**Source** HRoetschke_271222_120123_HFGoe_H3_b1R1_24h **Scan** 17132
**Peptide** TELLIRKL **Charge** 2 **Spectral Angle** 0.81

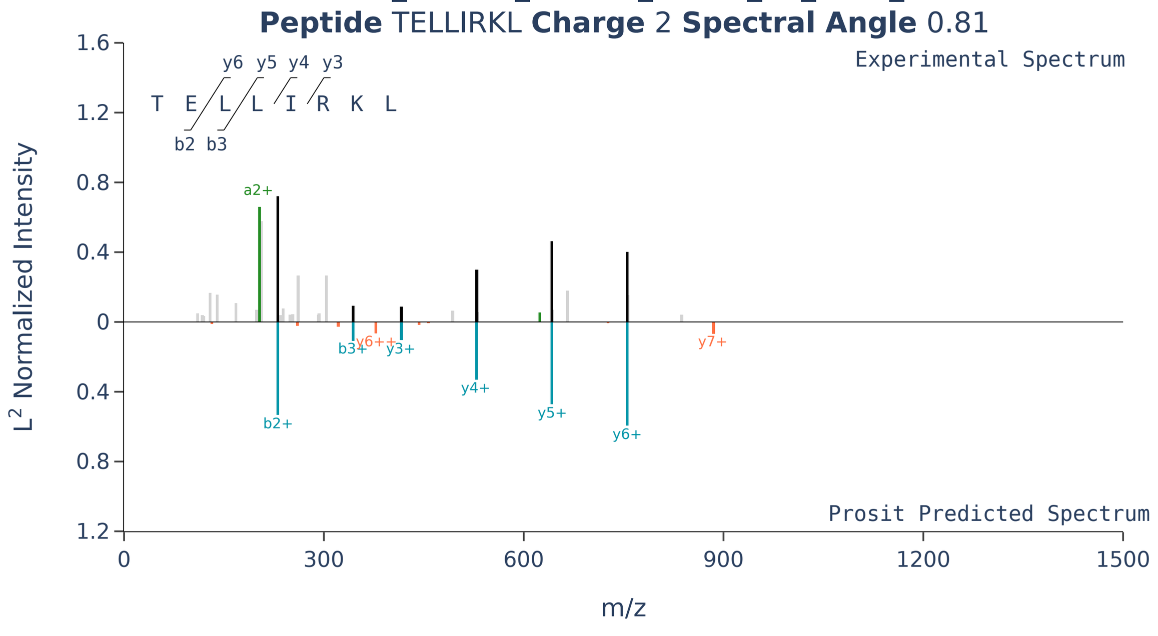


**Source** HRoetschke_271222_120123_HFGoe_H3_b1R2_4h **Scan** 14941
**Peptide** FKTDLRFQ **Charge** 3 **Spectral Angle** 0.72

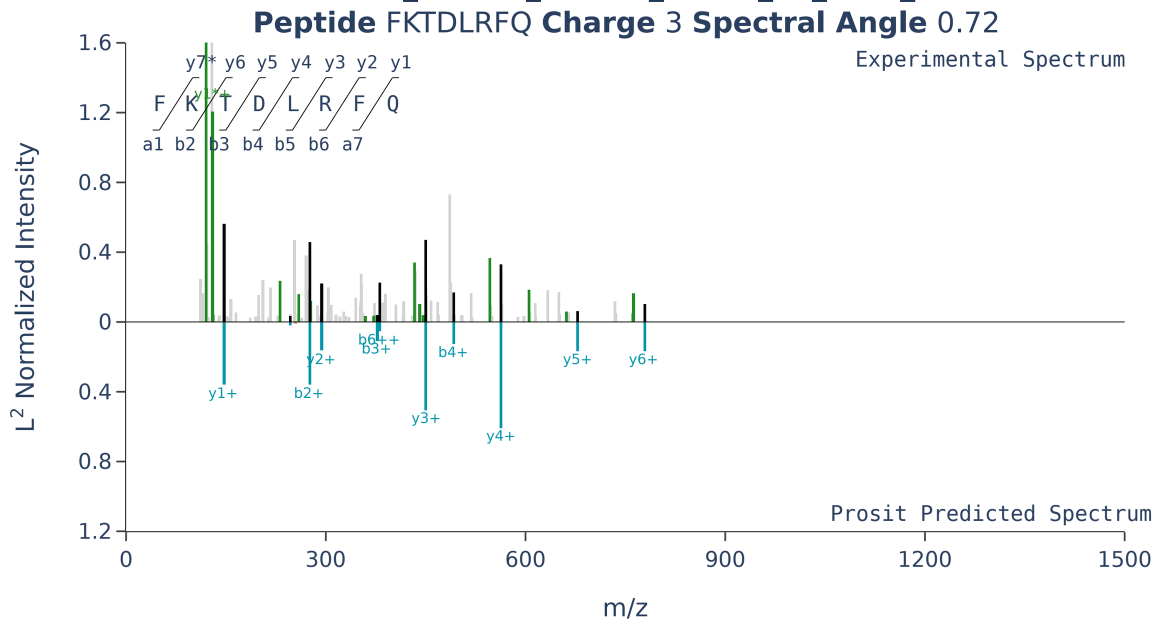


**Source** HRoetschke_271222_120123_HFGoe_H3_b1R2_4h **Scan** 13407
**Peptide** TIMPKDIQLAR **Charge** 3 **Spectral Angle** 0.8

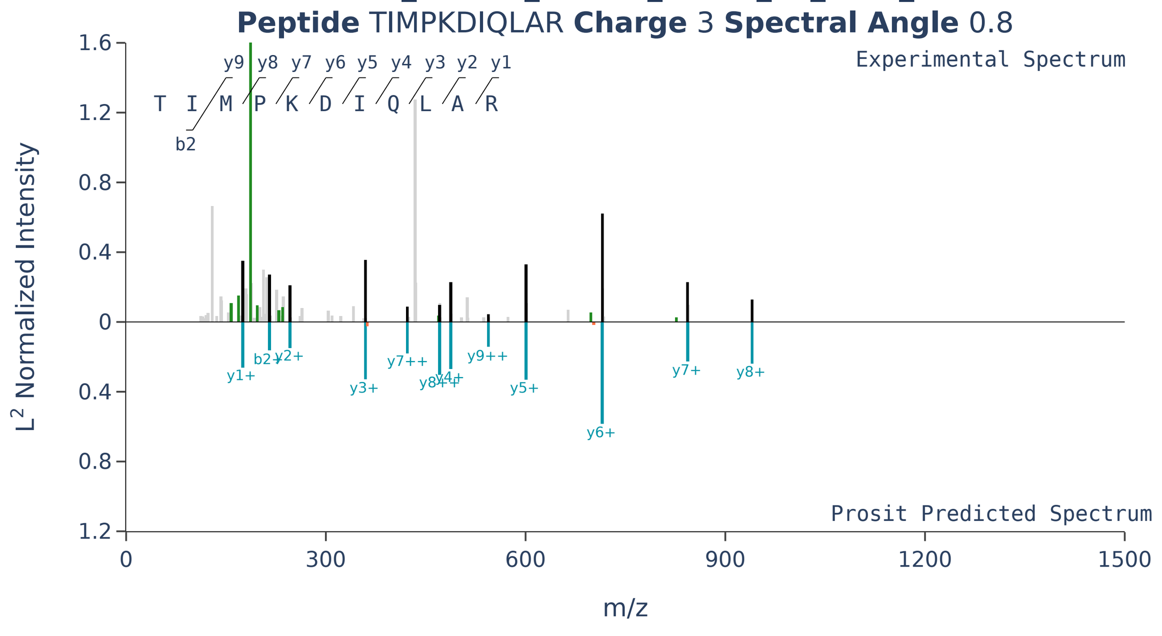

**Source** HRoetschke_271222_120123_HFGoe_H3_b1R1_24h **Scan** 17968
**Peptide** TIMPKDIQL **Charge** 2 **Spectral Angle** 0.77

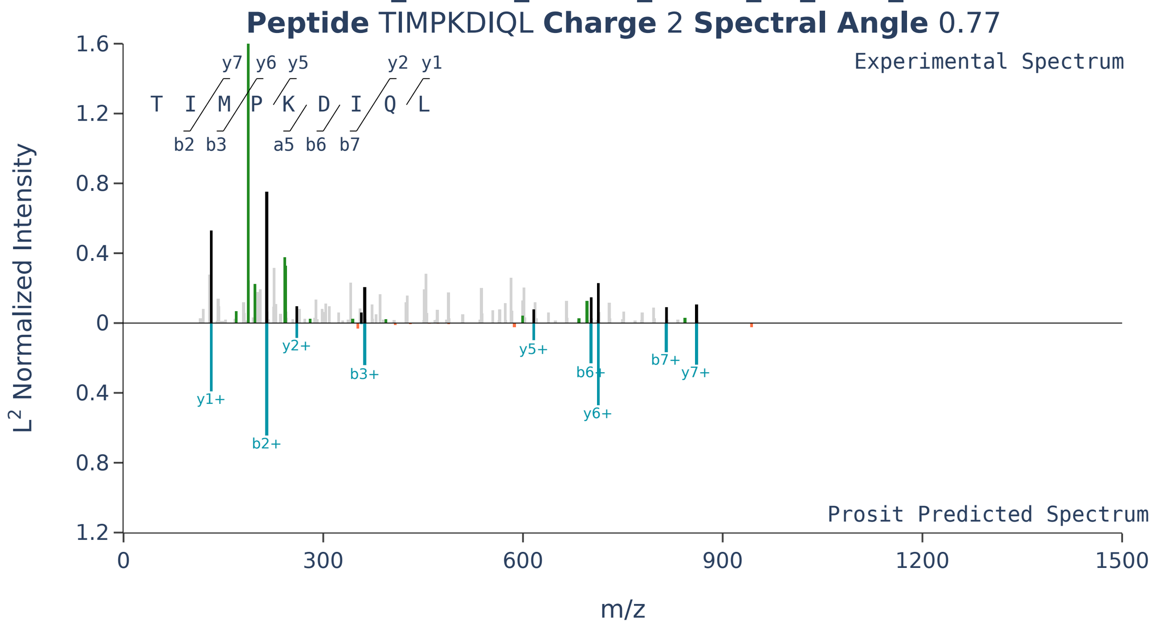


**Source** HRoetschke_271222_120123_HFGoe_H3_b1R1_1h **Scan** 9100
**Peptide** KTDLRFQSSA **Charge** 3 **Spectral Angle** 0.83

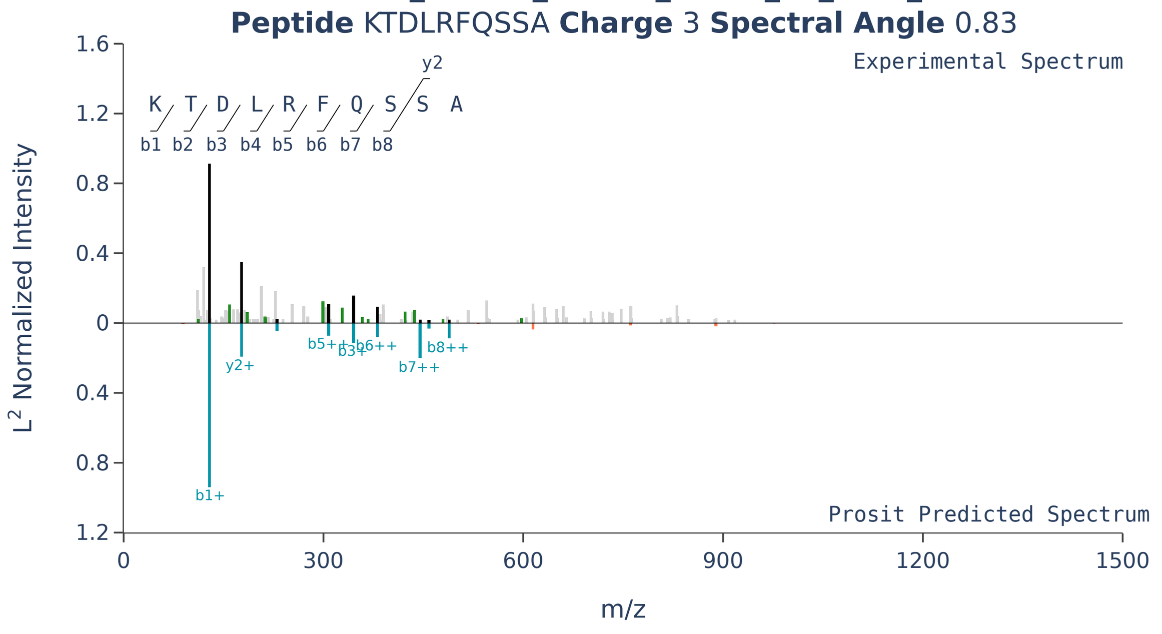


**Source** HRoetschke_271222_120123_HFGoe_H3_b1R1_1h **Scan** 14984
**Peptide** ALFEDTN **Charge** 1 **Spectral Angle** 0.82

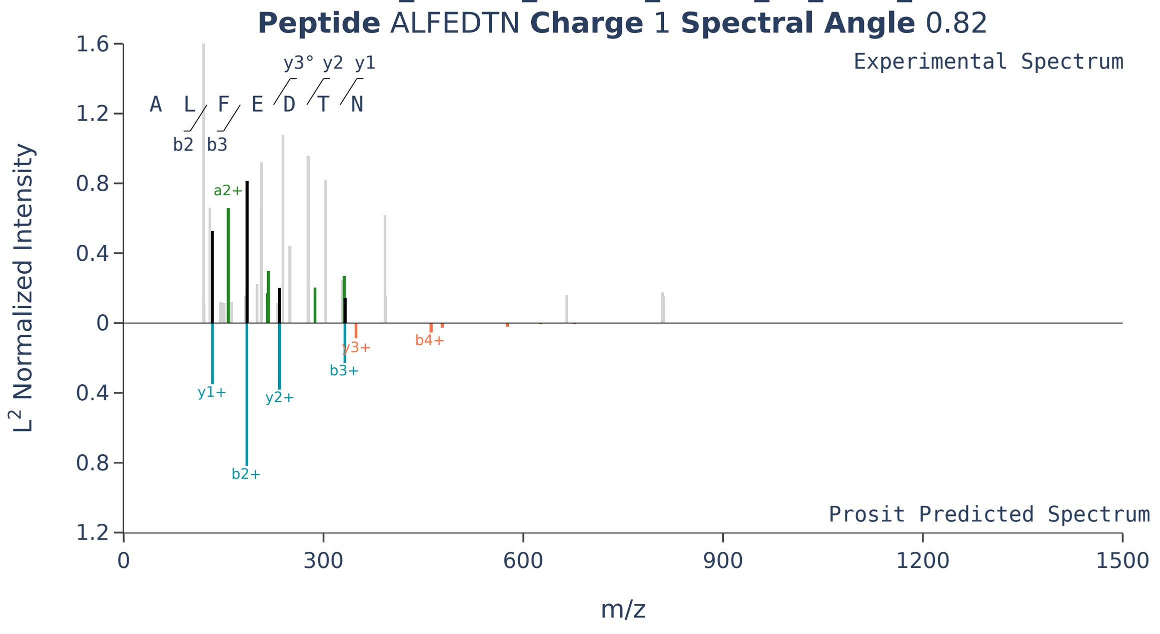


**Source** HRoetschke_271222_120123_HFGoe_H3_b2R2_4h **Scan** 13660
**Peptide** TIMPKDIQLARRLR **Charge** 4 **Spectral Angle** 0.67

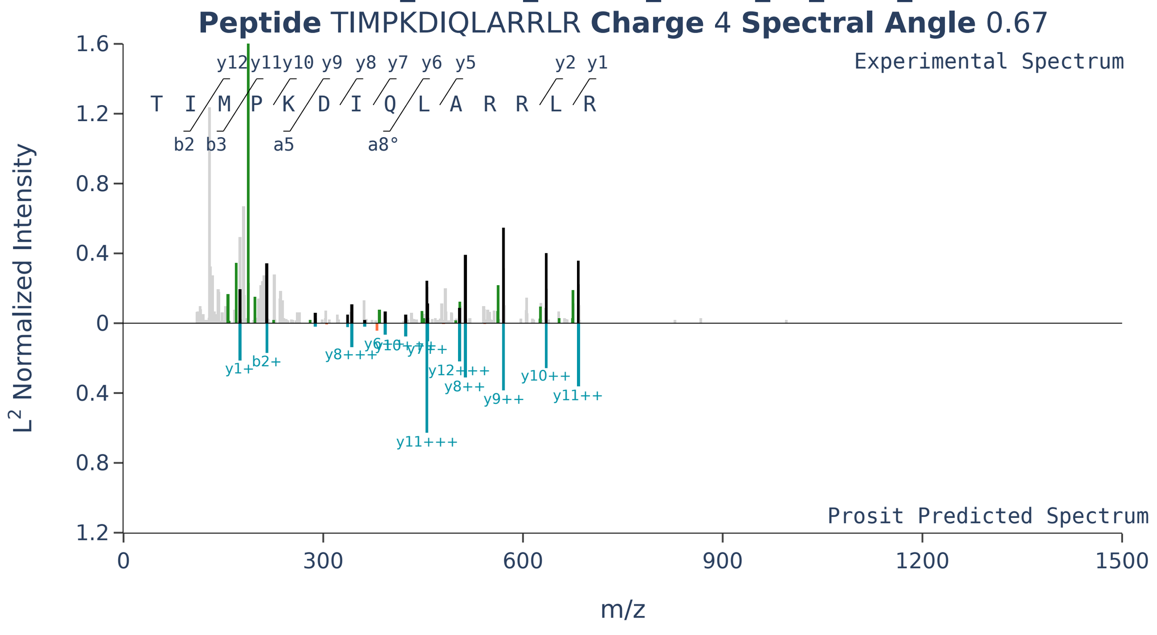


**Source** HRoetschke_271222_120123_HFGoe_H3_b2R2_4h **Scan** 17584
**Peptide** DLRFQSSAV **Charge** 2 **Spectral Angle** 0.72

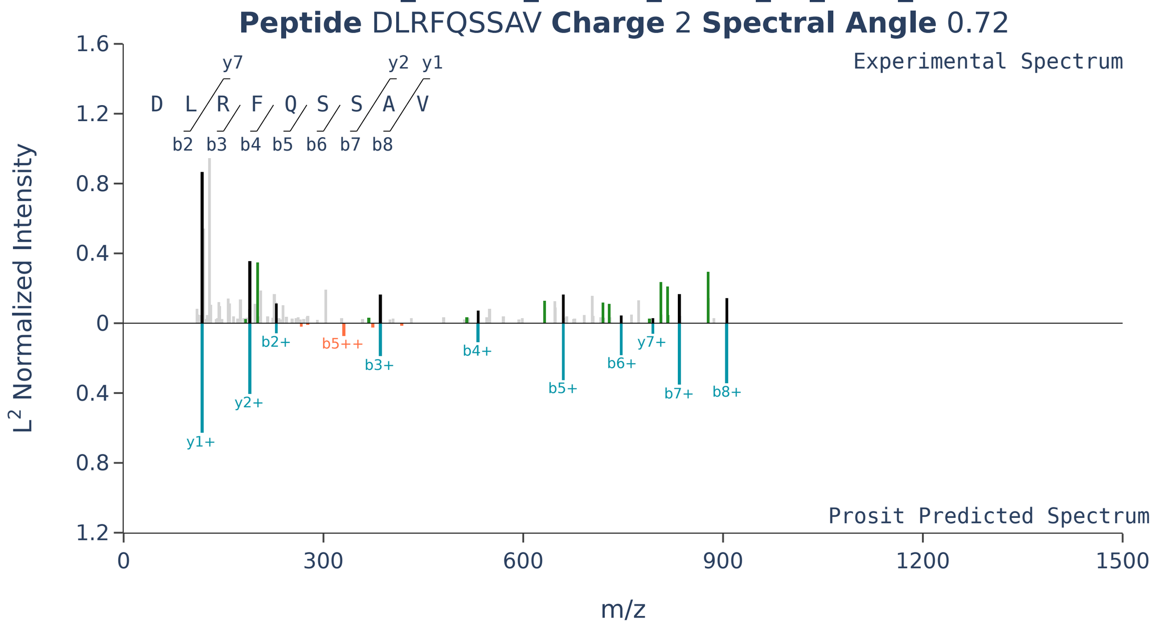


**Source** HRoetschke_271222_120123_HFGoe_H3_b1R1_1h **Scan** 11954
**Peptide** TIMPKDIQLARRIR **Charge** 4 **Spectral Angle** 0.72

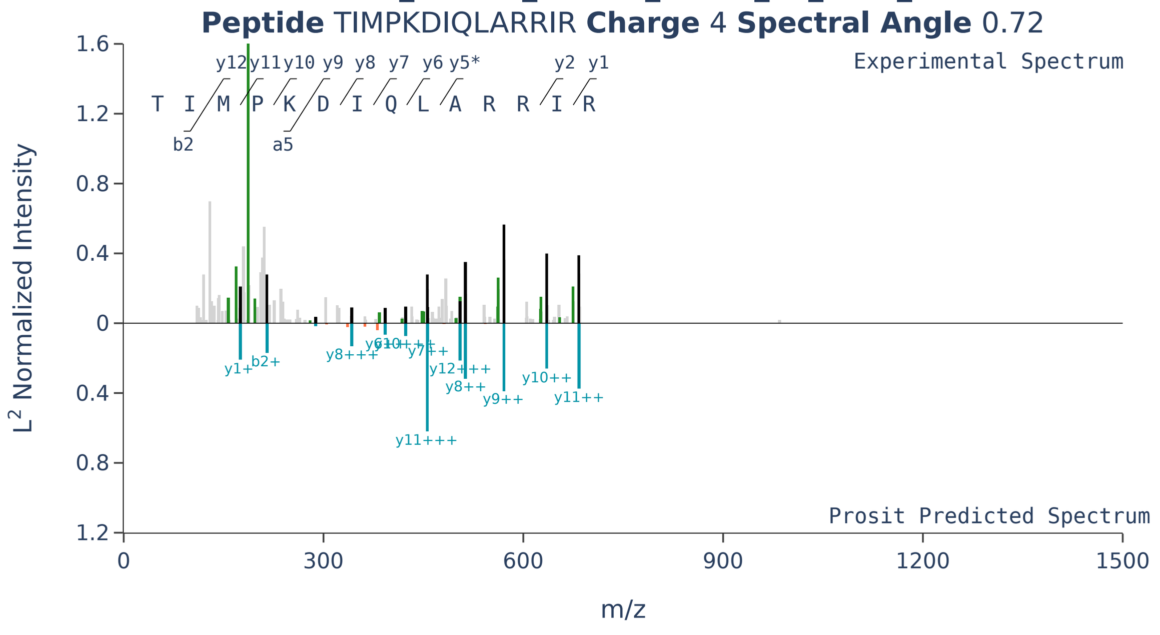


**Source** HRoetschke_271222_120123_HFGoe_H3_b1R2_2h **Scan** 9209
**Peptide** KTDLRFQ **Charge** 2 **Spectral Angle** 0.81

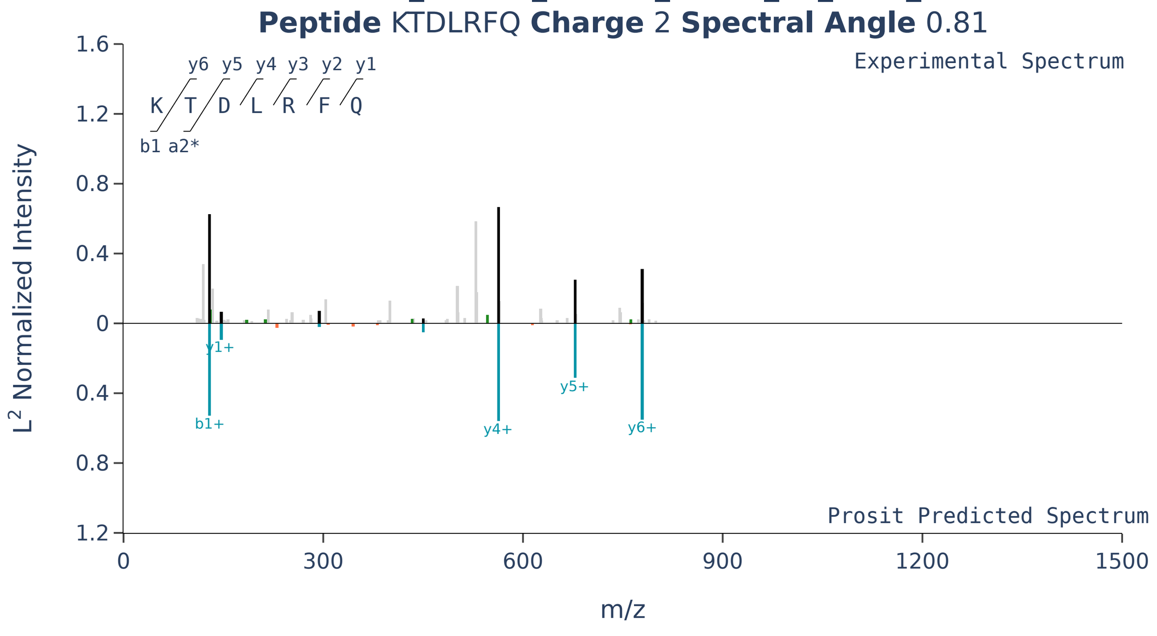


**Source** HRoetschke_271222_120123_HFGoe_H3_b2R2_4h **Scan** 19886
**Peptide** EIAQDFKTDL **Charge** 2 **Spectral Angle** 0.73

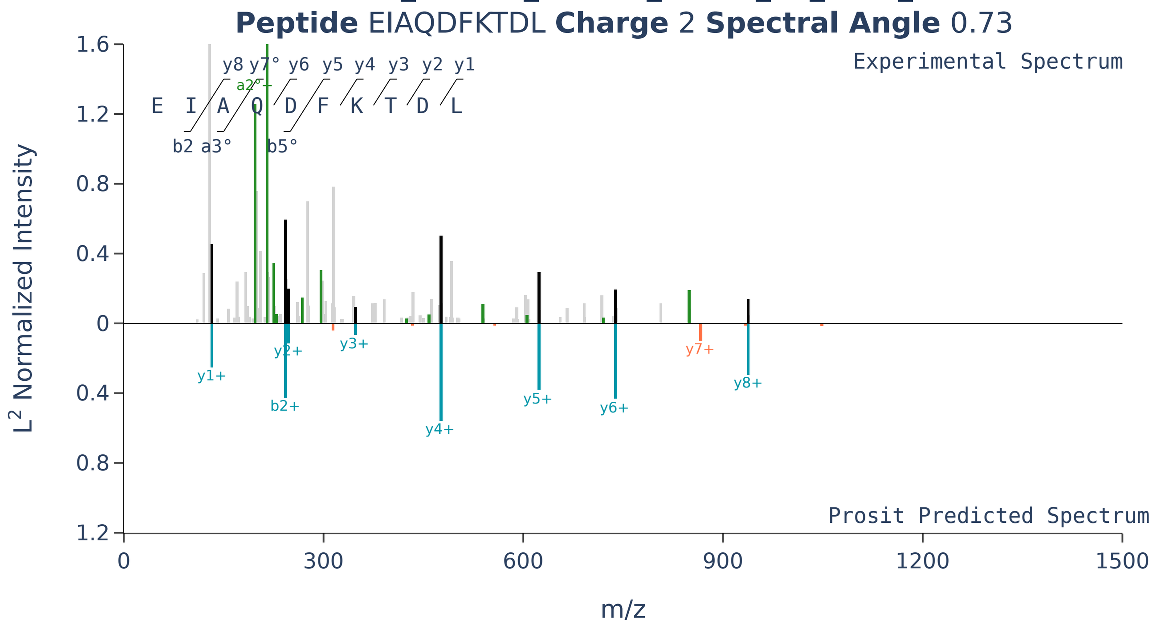


**Source** HRoetschke_271222_120123_HFGoe_H3_b2R1_2h **Scan** 7892
**Peptide** HAKRVTIMPKD **Charge** 3 **Spectral Angle** 0.76

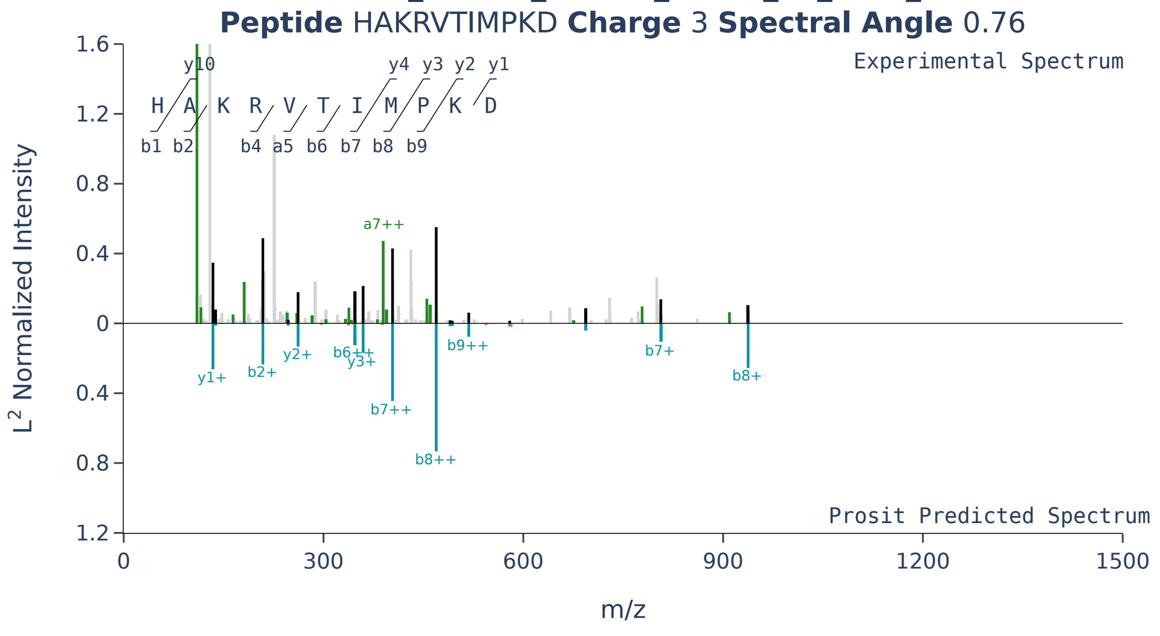


**Source** HRoetschke_271222_120123_HFGoe_H3_b1R1_24h **Scan** 15789
**Peptide** IMPKDIQLA **Charge** 2 **Spectral Angle** 0.7

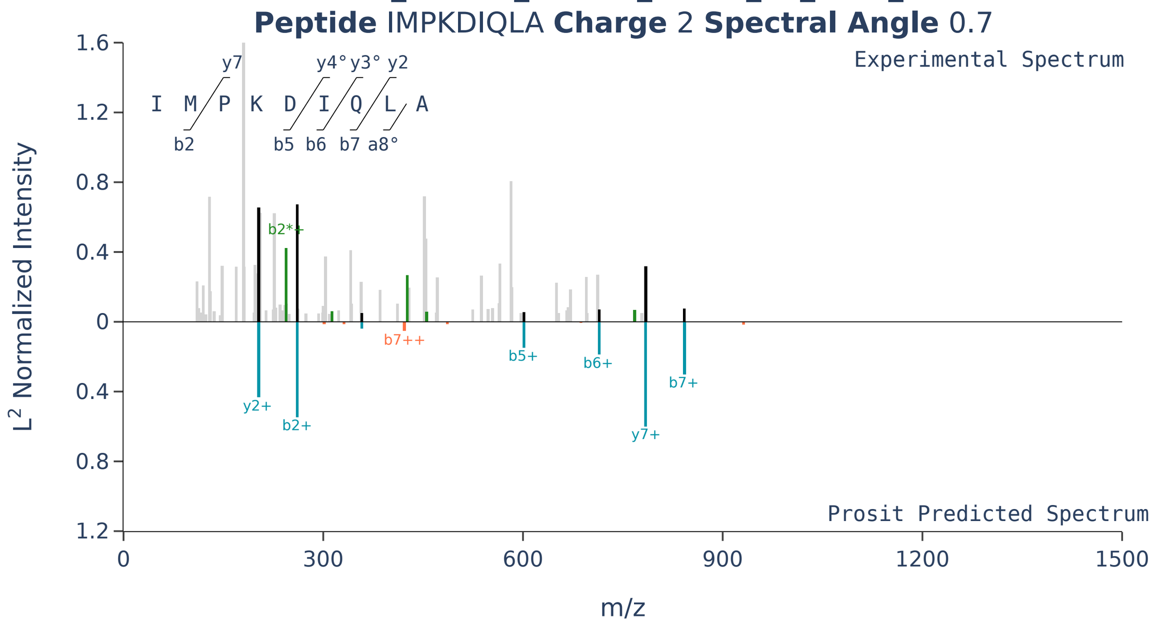


**Source** HRoetschke_271222_120123_HFGoe_H3_b1R1_4h **Scan** 15256
**Peptide** TIMPKDIQLARRI **Charge** 4 **Spectral Angle** 0.86

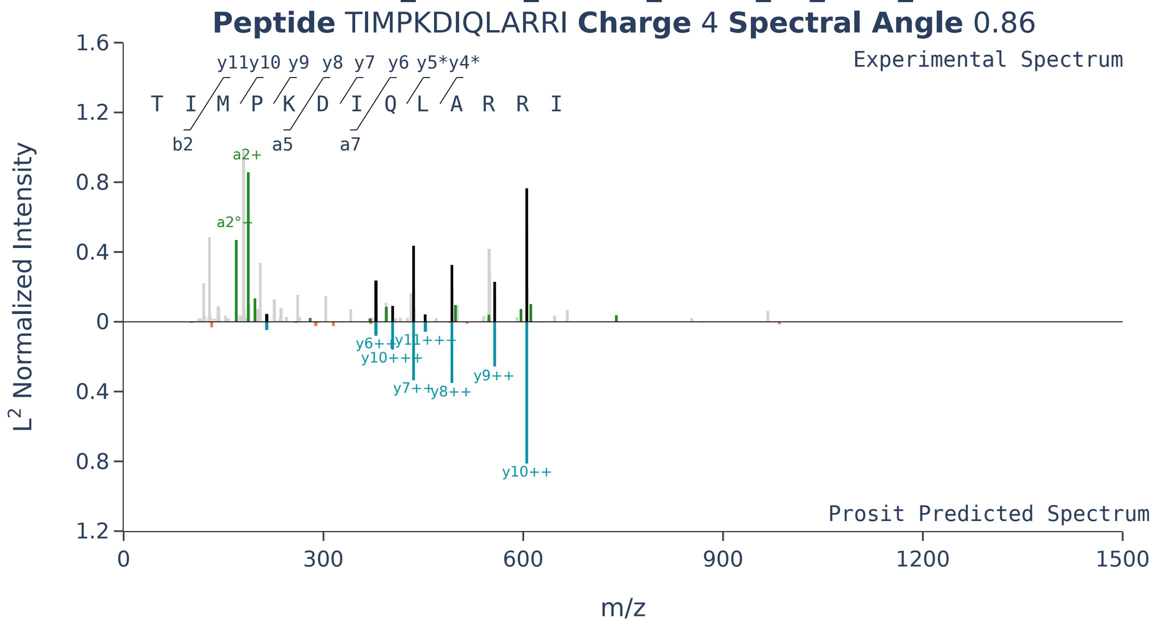


**Source** HRoetschke_271222_120123_HFGoe_H3_b1R2_2h **Scan** 11548
**Peptide** ELAQDFKT **Charge** 2 **Spectral Angle** 0.8

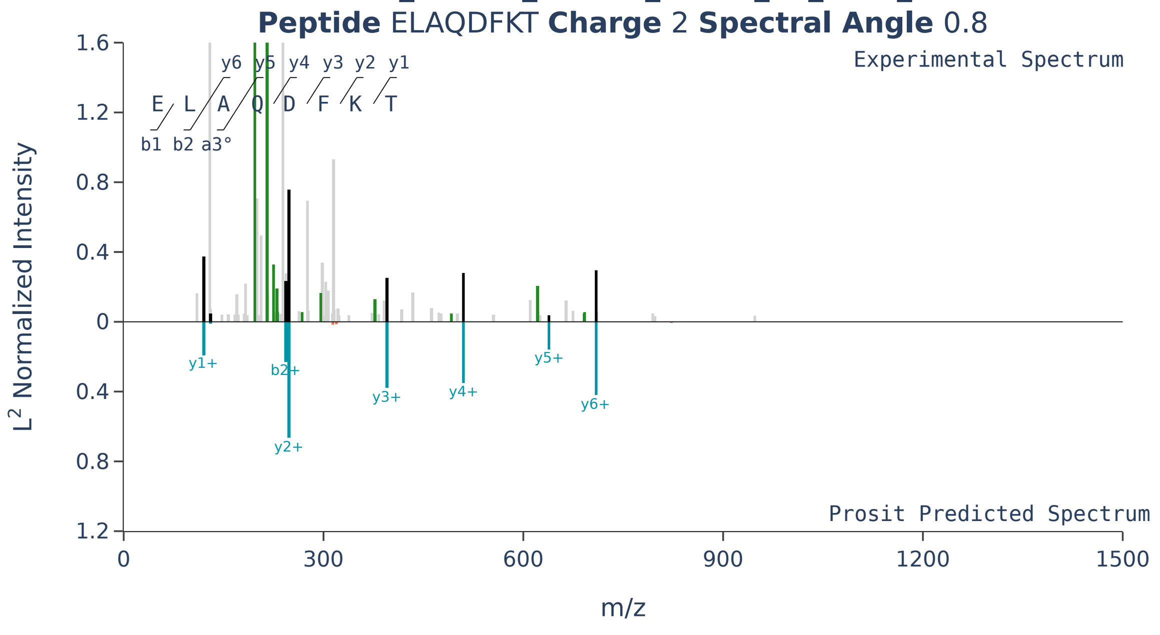


**Source** HRoetschke_271222_120123_HFGoe_H3_b1R1_4h **Scan** 17361
**Peptide** REIAQDFKTDLRFQ **Charge** 3 **Spectral Angle** 0.87

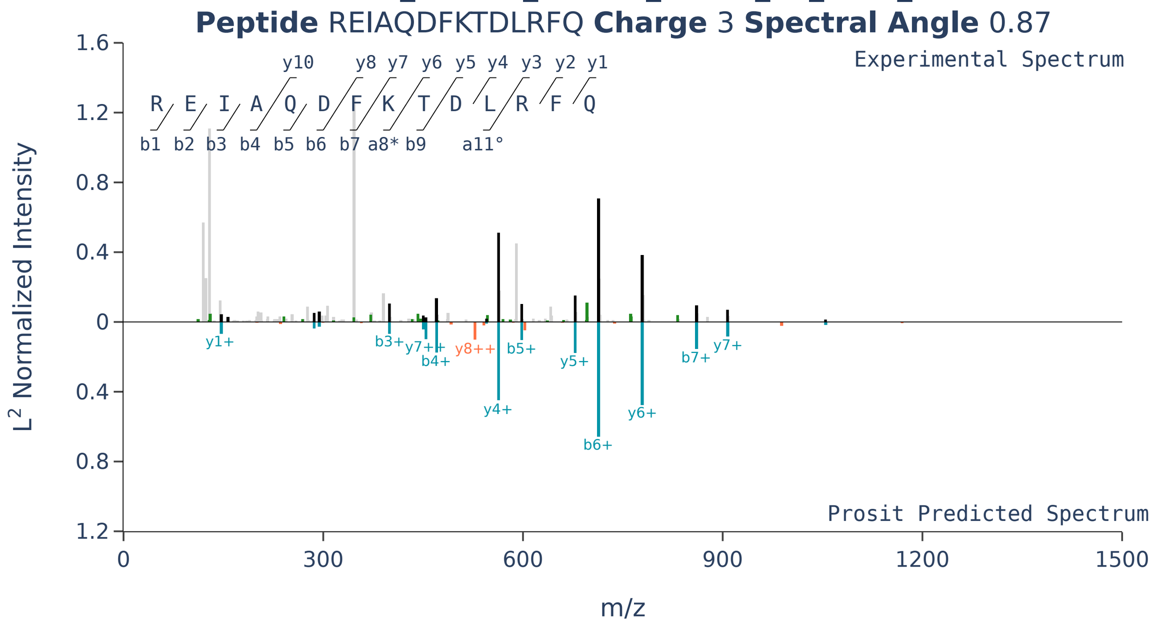


**Source** HRoetschke_271222_120123_HFGoe_H3_b2R1_4h **Scan** 13411
**Peptide** GTVALREIRRY **Charge** 3 **Spectral Angle** 0.76

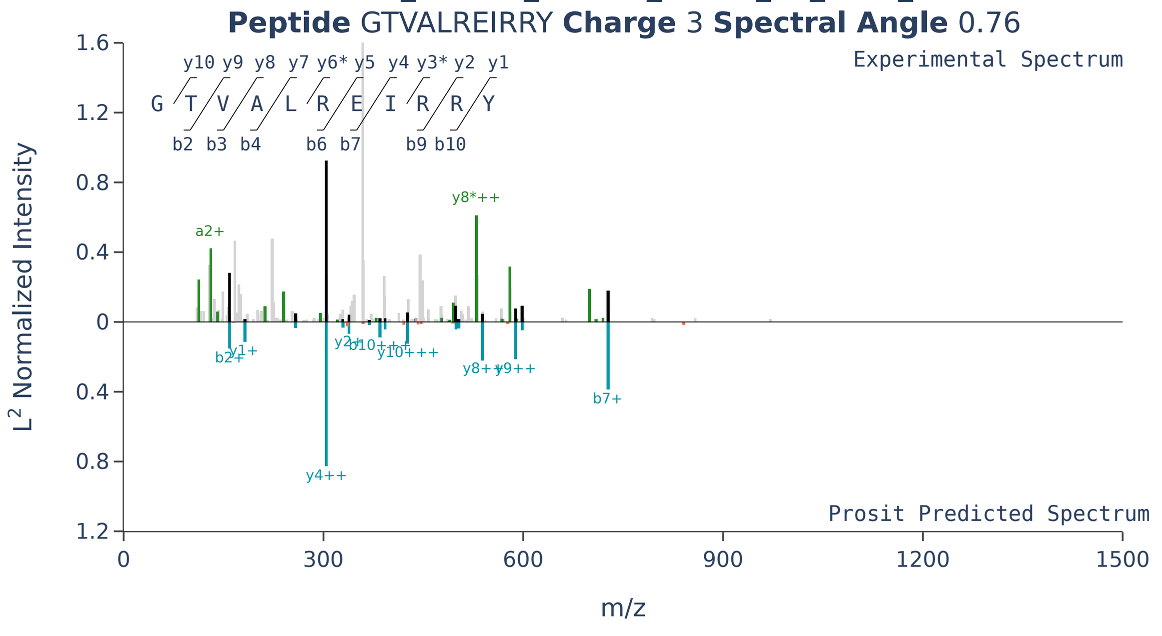


**Source** HRoetschke_271222_120123_HFGoe_H3_b1R1_24h **Scan** 14954
**Peptide** RKLPFQRLV **Charge** 3 **Spectral Angle** 0.79

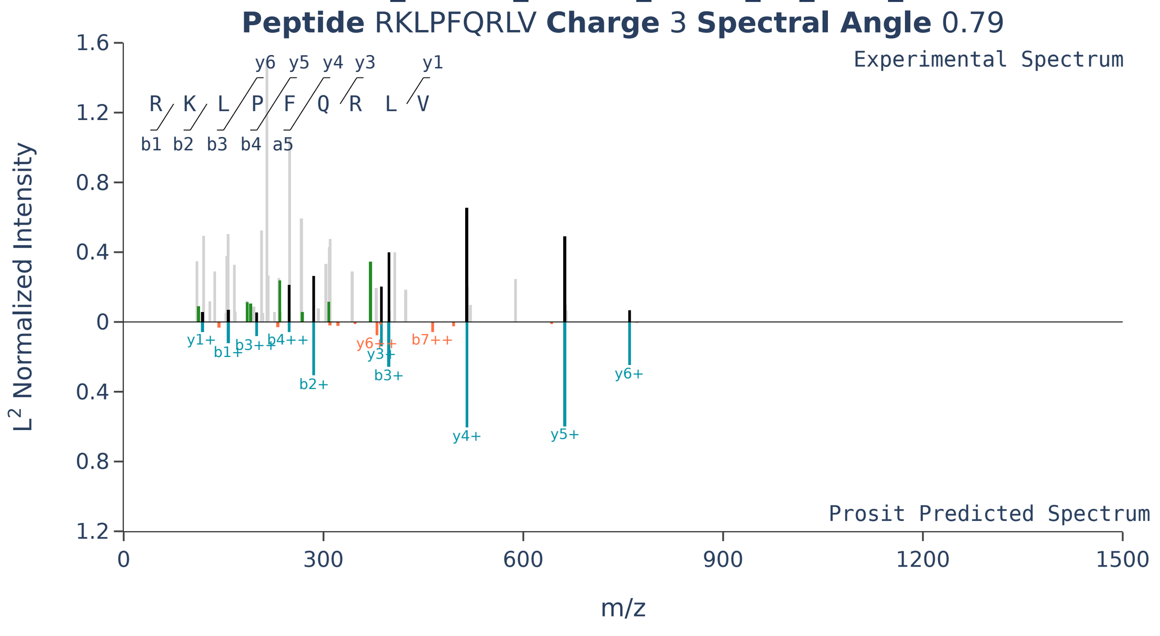

**Source** HRoetschke_271222_120123_HFGoe_H3_b2R1_2h **Scan** 17137
**Peptide** DFKTDLRF **Charge** 2 **Spectral Angle** 0.71

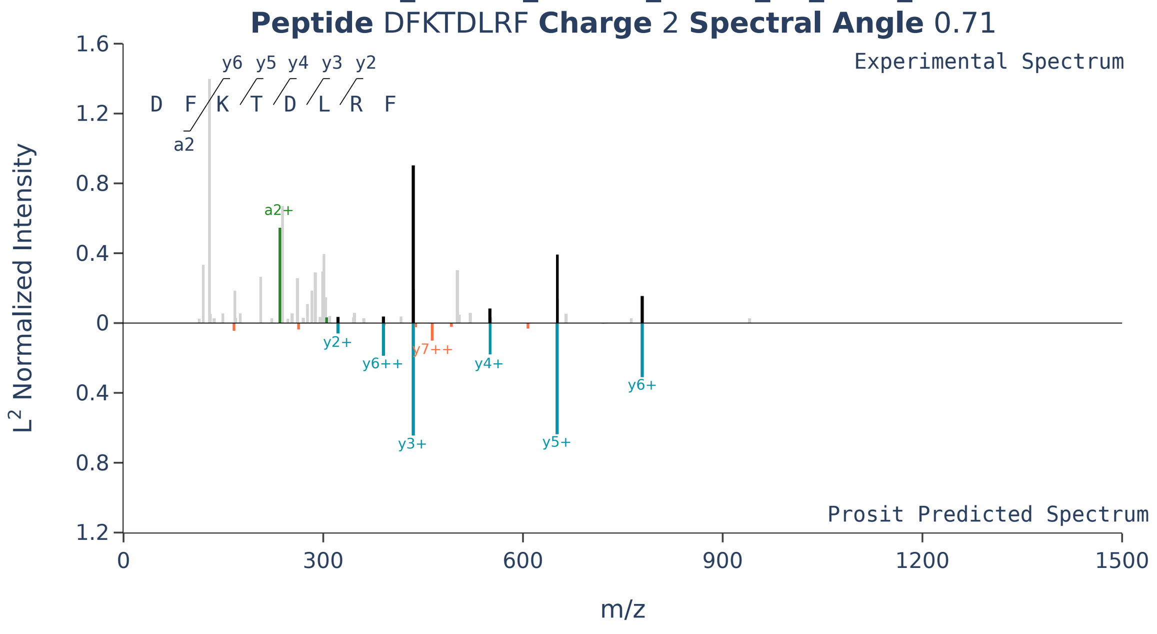


**Source** HRoetschke_271222_120123_HFGoe_H3_b1R2_24h **Scan** 18444
**Peptide** DTNLCAL **Charge** 2 **Spectral Angle** 0.87

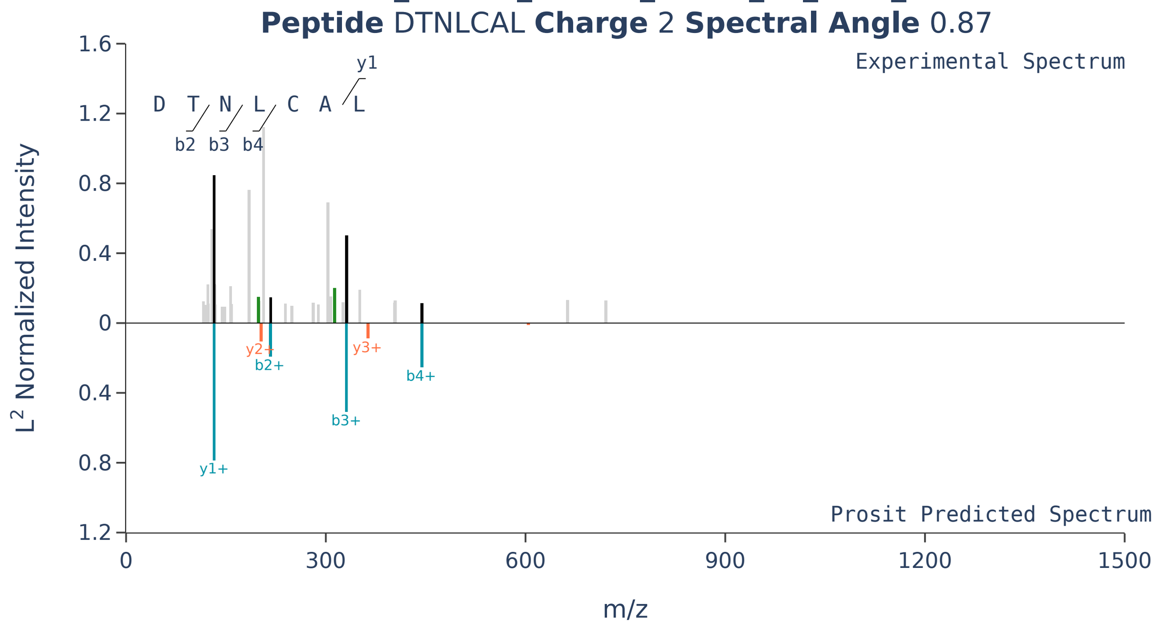


**Source** HRoetschke_271222_120123_HFGoe_H3_b1R1_24h **Scan** 6892
**Peptide** ALREIRRY **Charge** 3 **Spectral Angle** 0.79

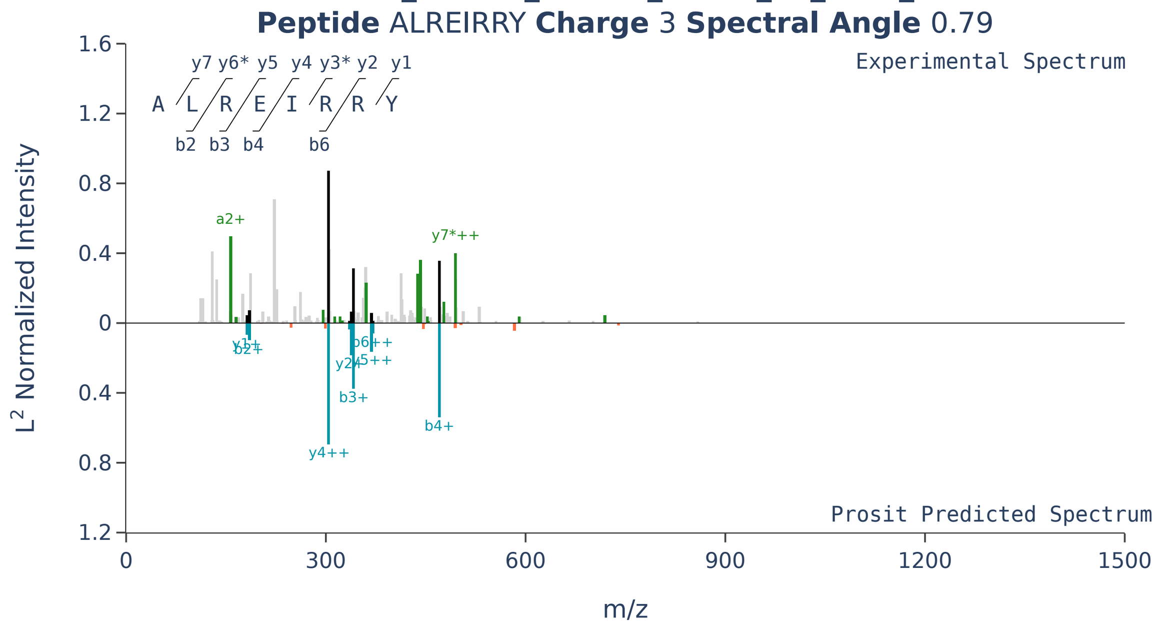


**Source** HRoetschke_271222_120123_HFGoe_H3_b2R1_4h **Scan** 13435
**Peptide** EIAQDFKT **Charge** 1 **Spectral Angle** 0.84

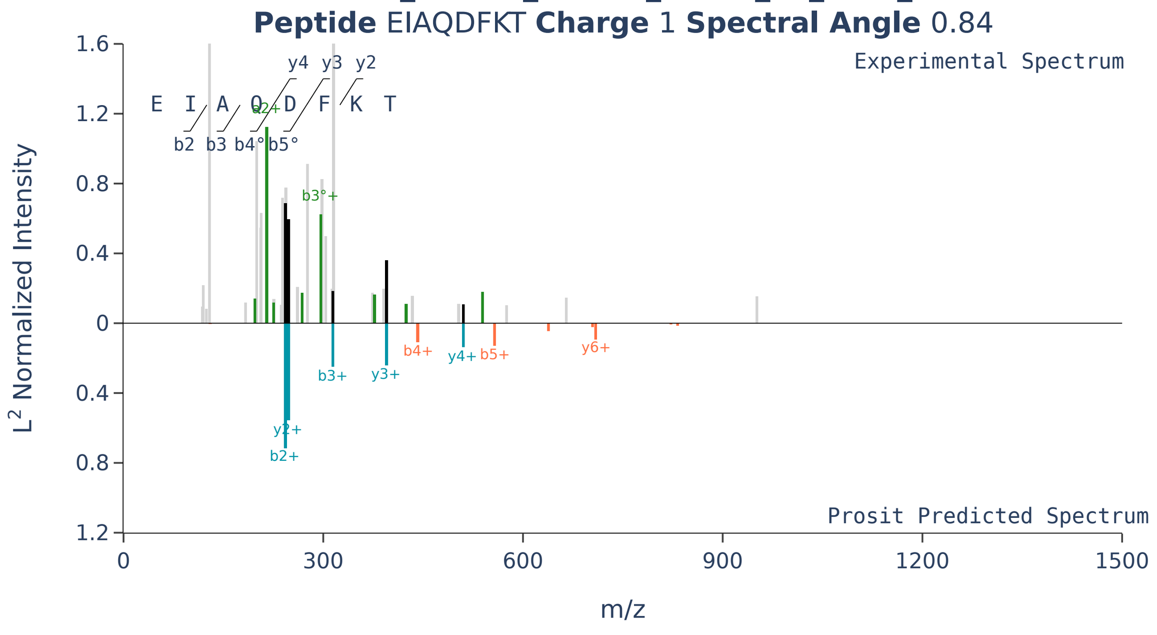


**Source** HRoetschke_271222_120123_HFGoe_H3_b1R1_24h **Scan** 7577
**Peptide** DTNLCAIHAKR **Charge** 3 **Spectral Angle** 0.81

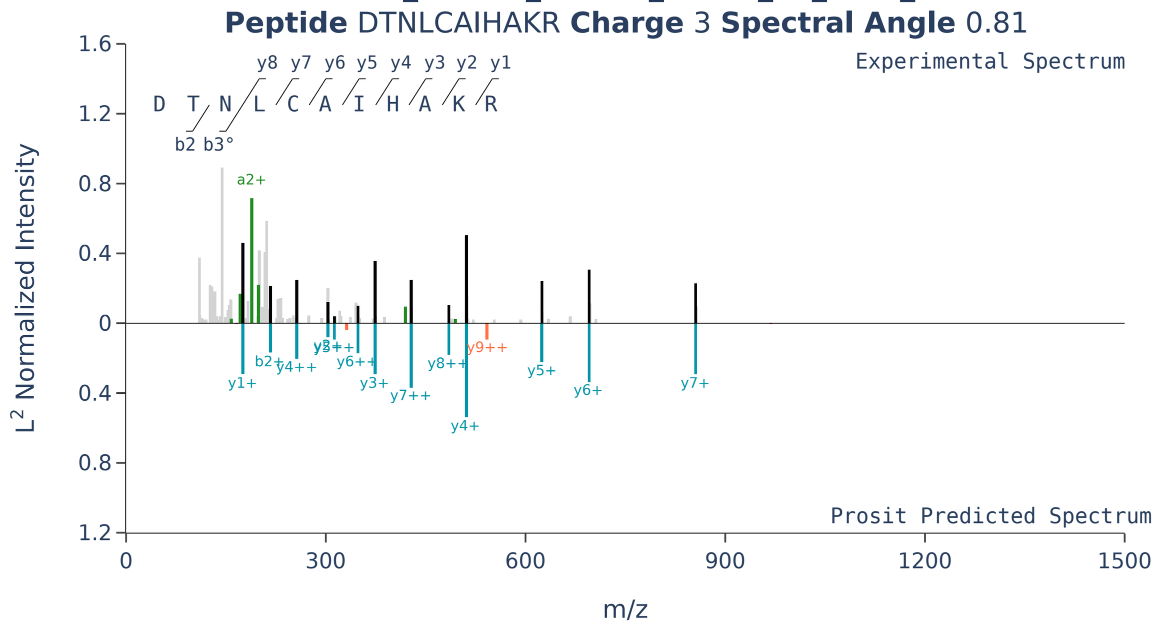


**Source** HRoetschke_271222_120123_HFGoe_H3_b1R2_4h **Scan** 12417
**Peptide** KTDLRFQSSAV **Charge** 3 **Spectral Angle** 0.88

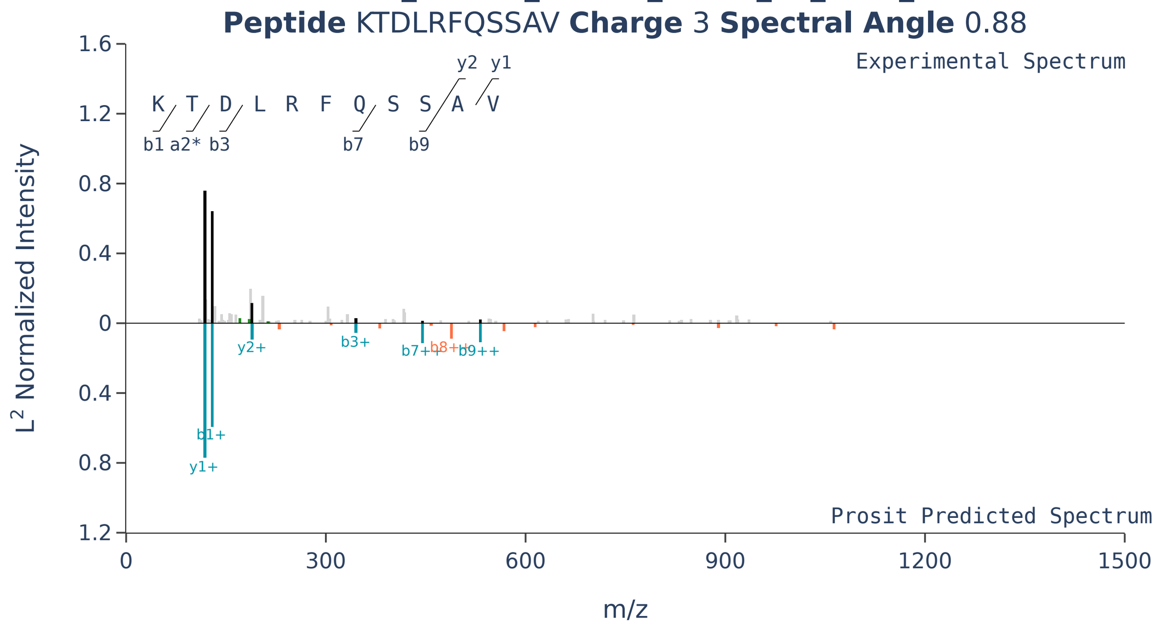


**Source** HRoetschke_271222_120123_HFGoe_H3_b2R2_4h **Scan** 4986
**Peptide** PATGGVKKPHRYR **Charge** 4 **Spectral Angle** 0.77

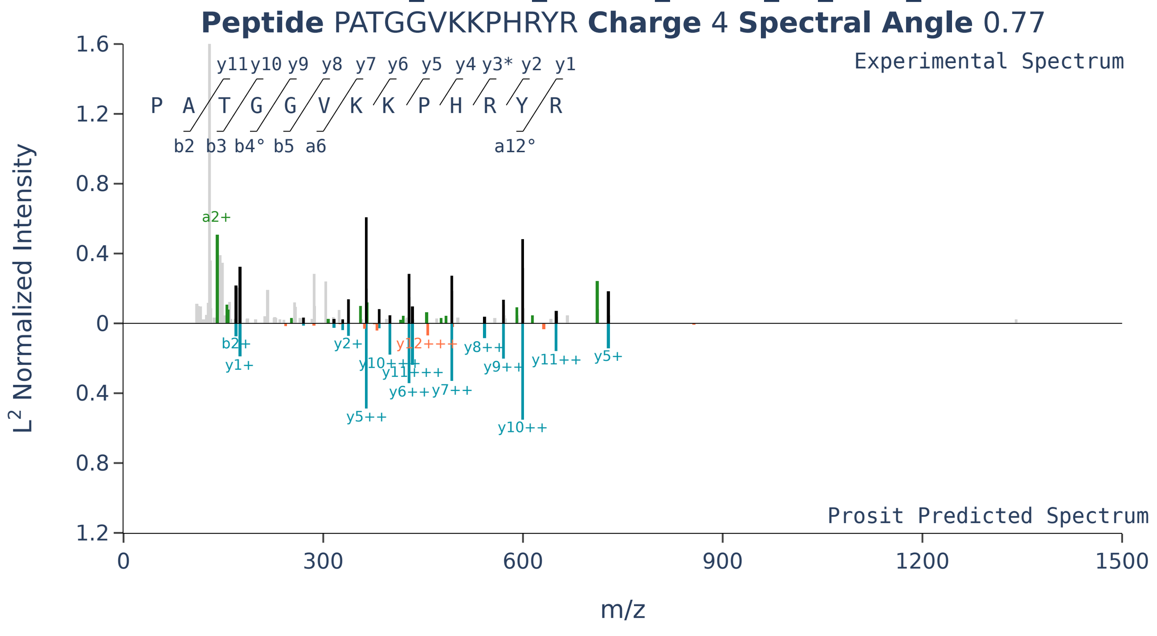


**Source** HRoetschke_271222_120123_HFGoe_H3_b2R1_1h **Scan** 7096
**Peptide** LQLARRIRGERA **Charge** 4 **Spectral Angle** 0.88

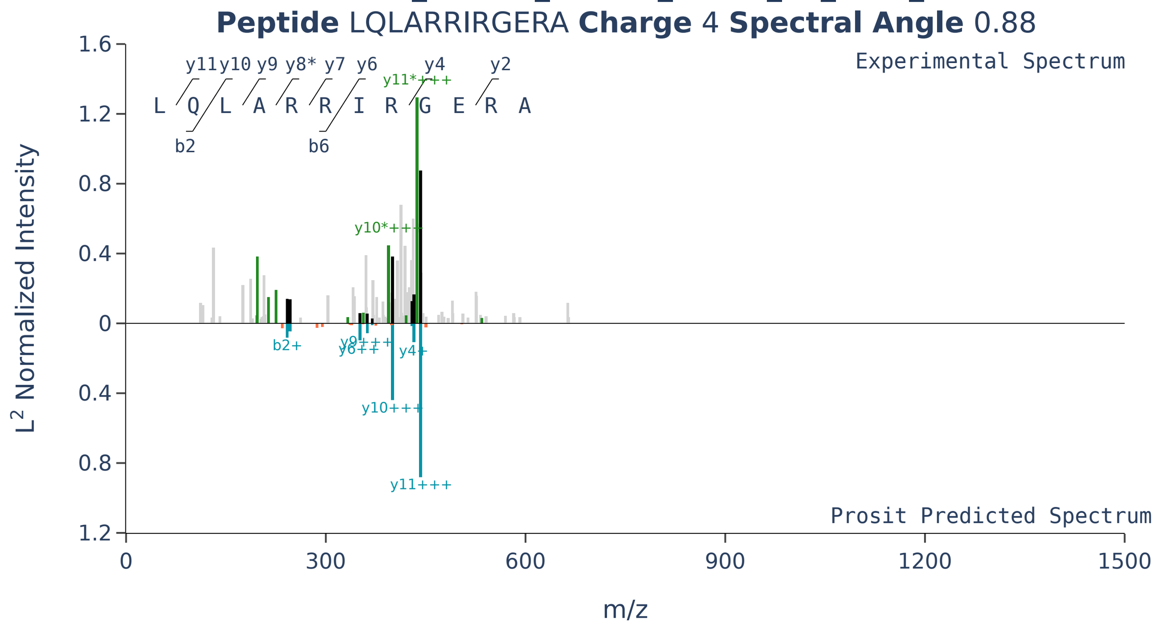


**Source** HRoetschke_271222_120123_HFGoe_H3_b2R2_4h **Scan** 12521
**Peptide** KRVTIMPKDIQLARR **Charge** 5 **Spectral Angle** 0.72

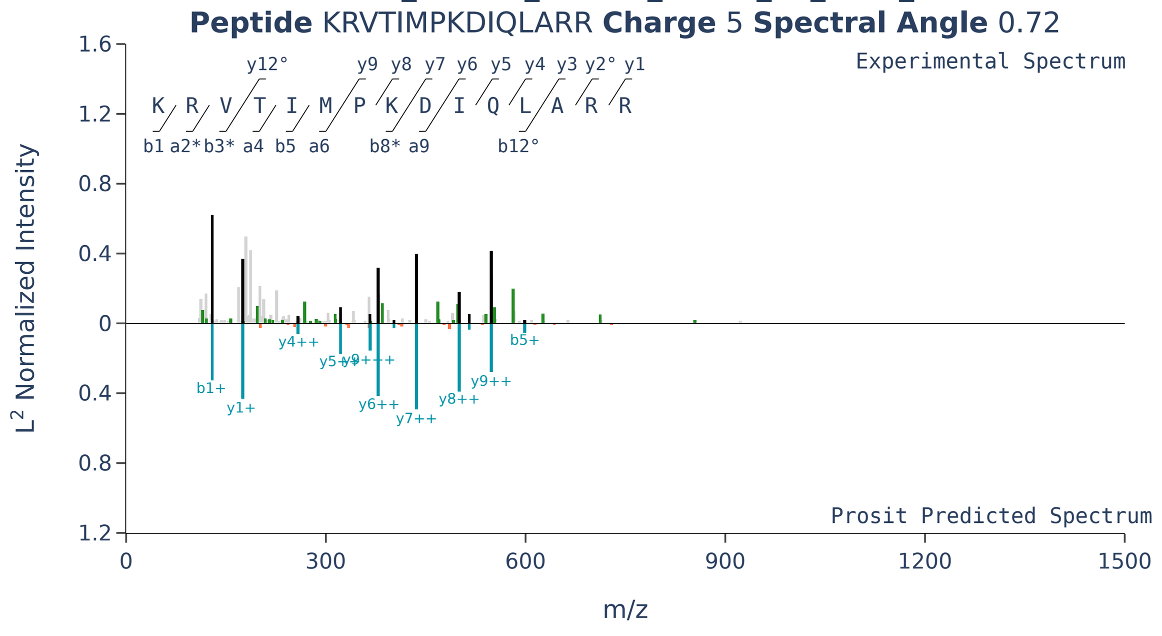


**Source** HRoetschke_271222_120123_HFGoe_H3_b1R1_24h **Scan** 9260
**Peptide** KTDLRFQS **Charge** 2 **Spectral Angle** 0.8

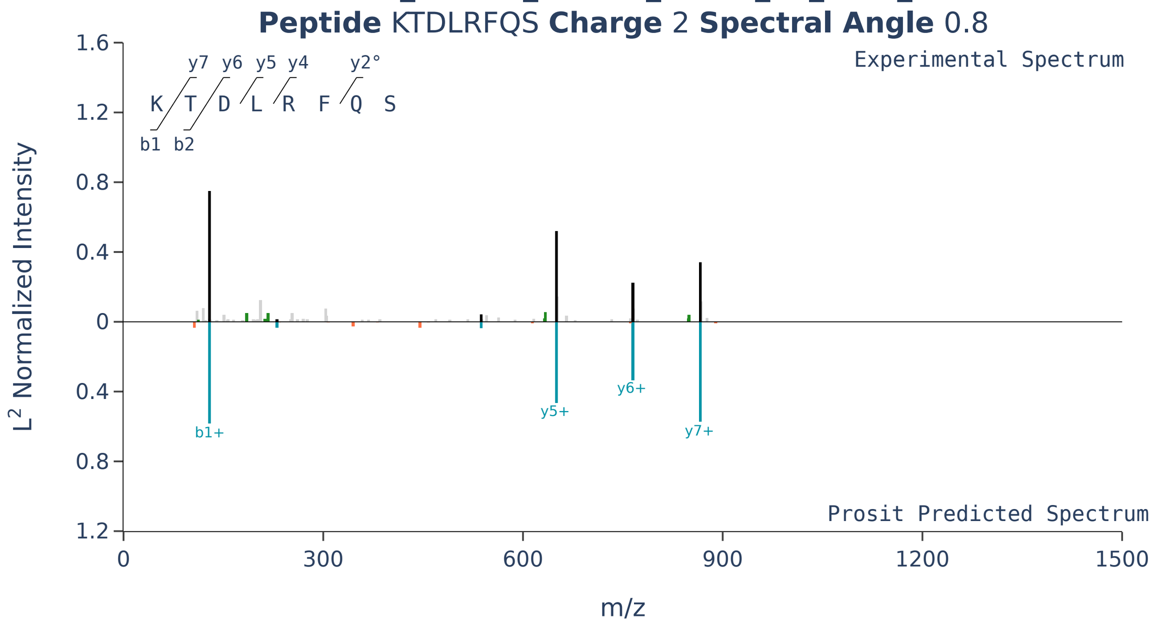


**Source** HRoetschke_271222_120123_HFGoe_H3_b1R1_24h **Scan** 5795
**Peptide** APATGGVKKPHRYRPGTV **Charge** 4 **Spectral Angle** 0.7

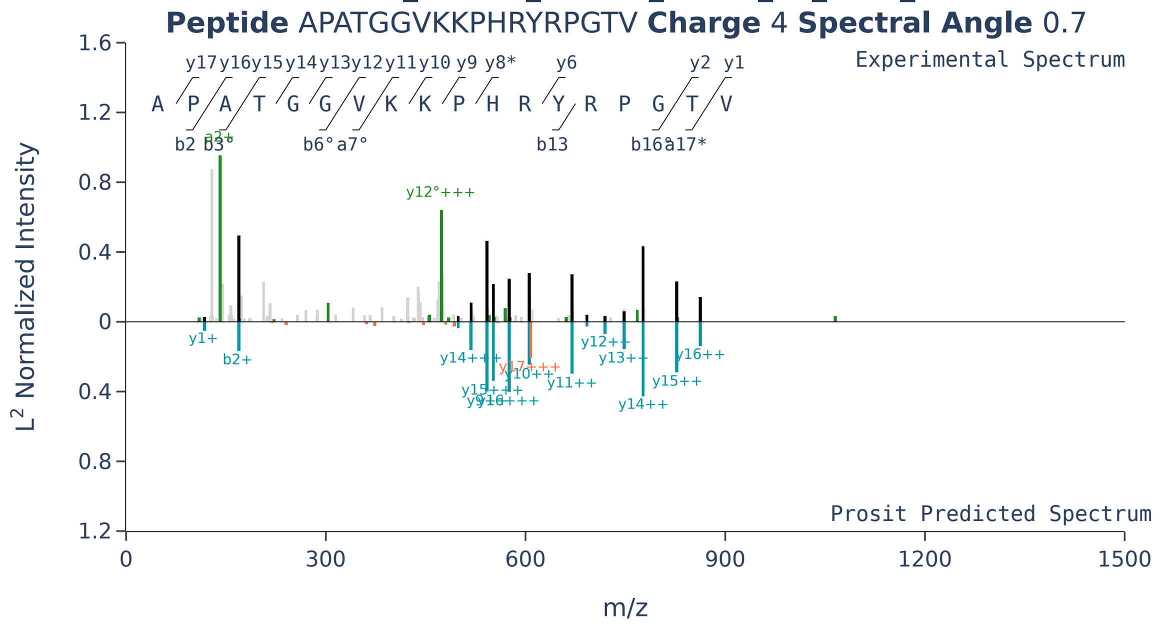


**Source** HRoetschke_271222_120123_HFGoe_H3_b1R2_2h **Scan** 11321
**Peptide** AVMALQEASE **Charge** 2 **Spectral Angle** 0.88

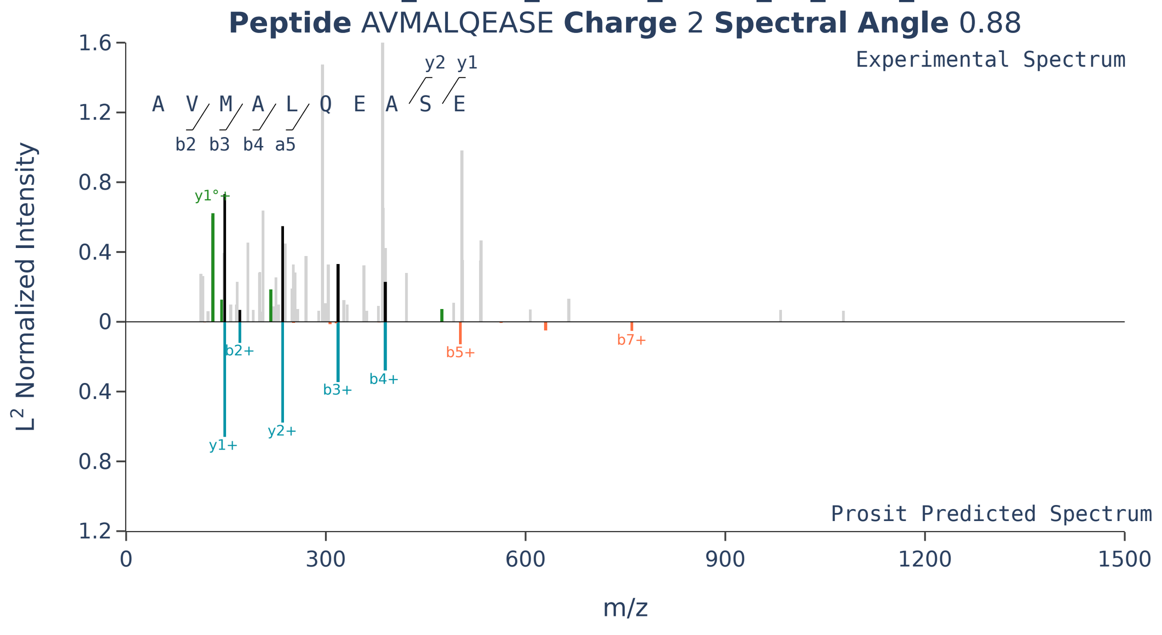


**Source** HRoetschke_271222_120123_HFGoe_H3_b1R1_4h **Scan** 17976
**Peptide** IAQDFKTDLRFQ **Charge** 3 **Spectral Angle** 0.75

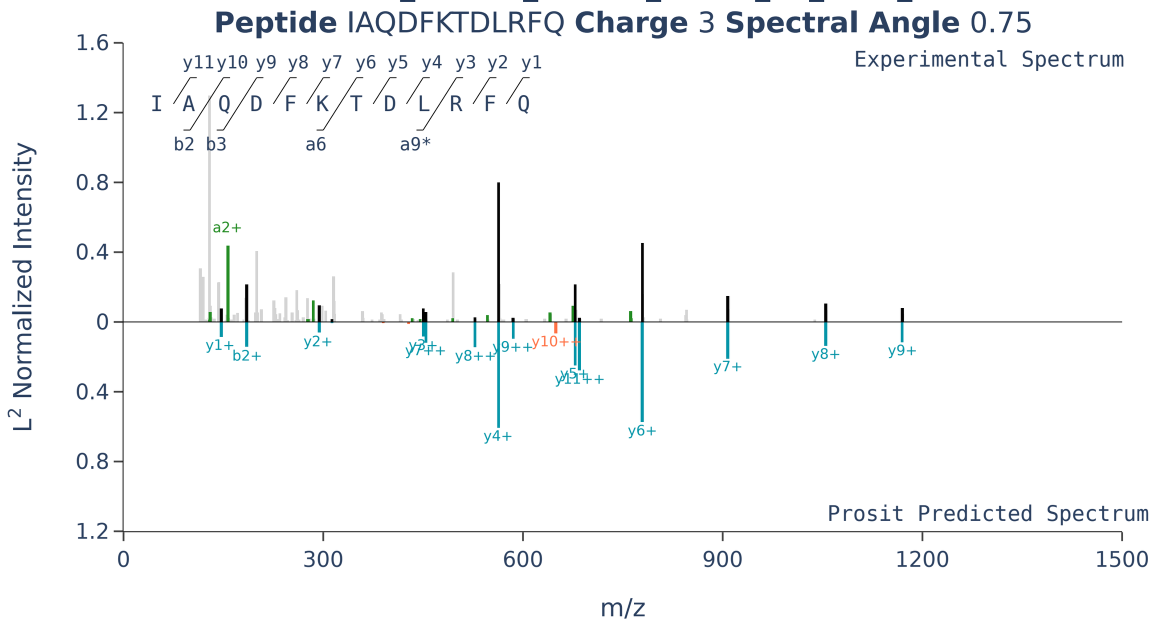


**Source** HRoetschke_271222_120123_HFGoe_H3_b2R2_24h **Scan** 12456
**Peptide** EIAQDFKTD **Charge** 2 **Spectral Angle** 0.8

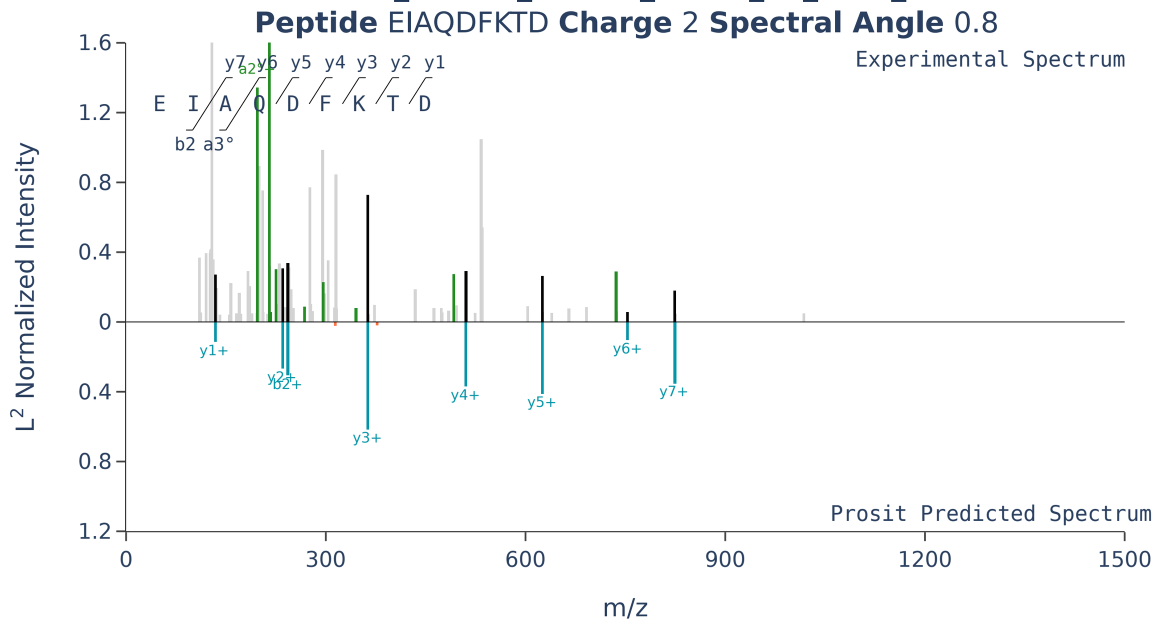


**Source** HRoetschke_271222_120123_HFGoe_H3_b1R2_4h **Scan** 18179
**Peptide** LVREIAQDF **Charge** 2 **Spectral Angle** 0.66

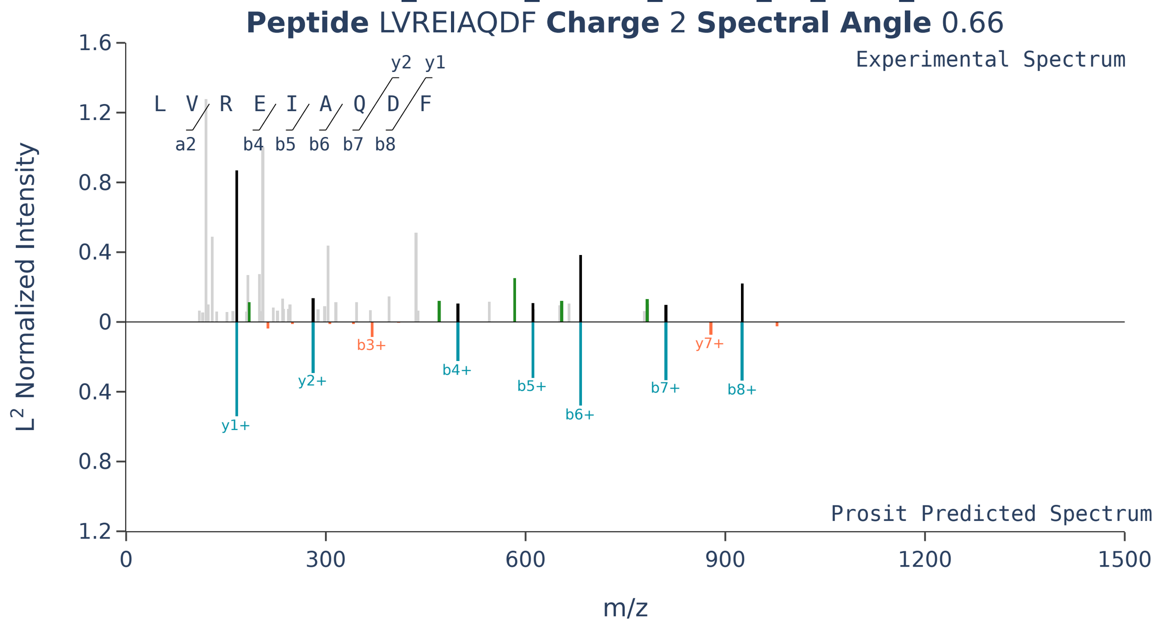

**Source** HRoetschke_271222_120123_HFGoe_H3_b2R2_4h **Scan** 11556
**Peptide** MPKDIQLARRIR **Charge** 4 **Spectral Angle** 0.83

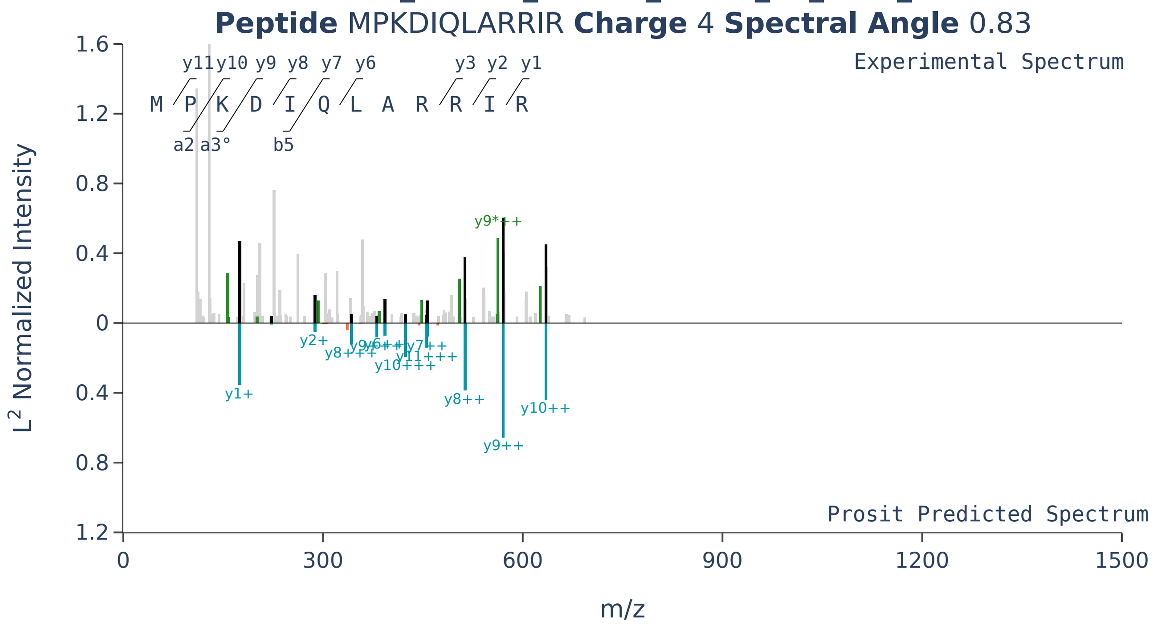


**Source** HRoetschke_271222_120123_HFGoe_H3_b2R2_1h **Scan** 8644
**Peptide** KTDLRFQSS **Charge** 2 **Spectral Angle** 0.74

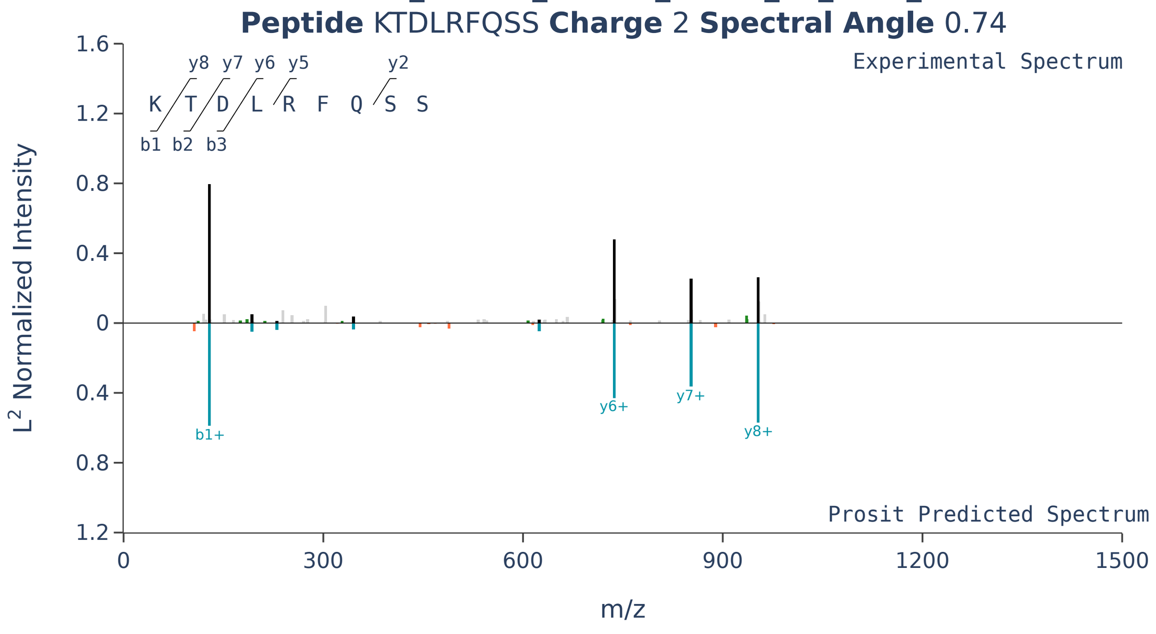


**Source** HRoetschke_271222_120123_HFGoe_H3_b2R2_4h **Scan** 9980
**Peptide** MPKDIQLARR **Charge** 3 **Spectral Angle** 0.8

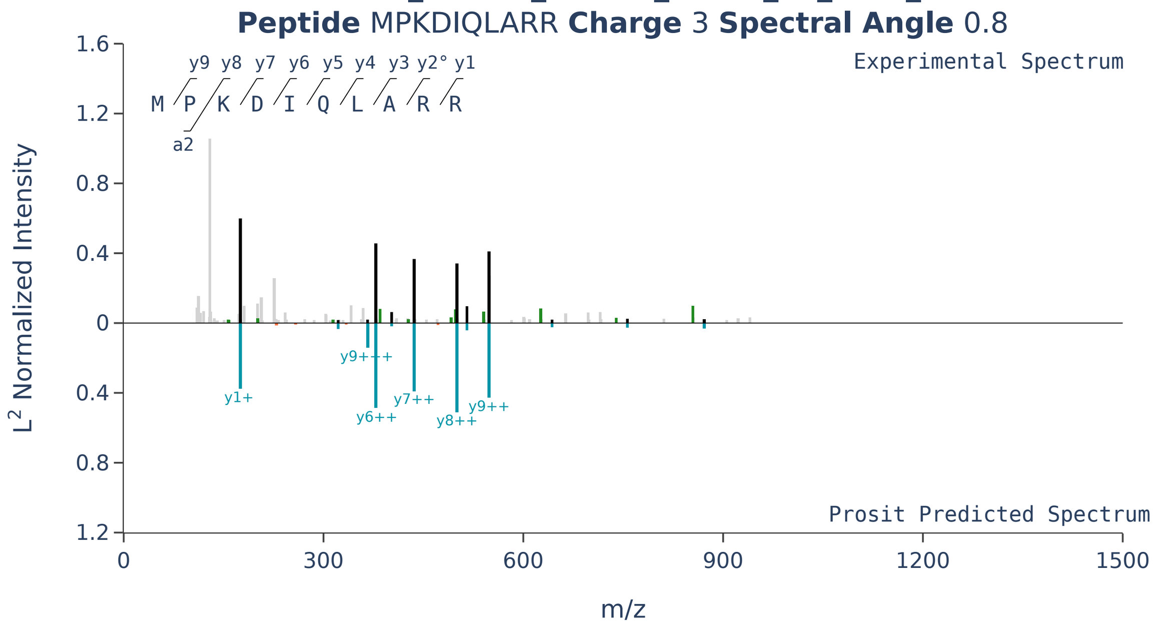


**Source** HRoetschke_271222_120123_HFGoe_H3_b1R1_4h **Scan** 12076
**Peptide** LRFQSSAVM **Charge** 2 **Spectral Angle** 0.85

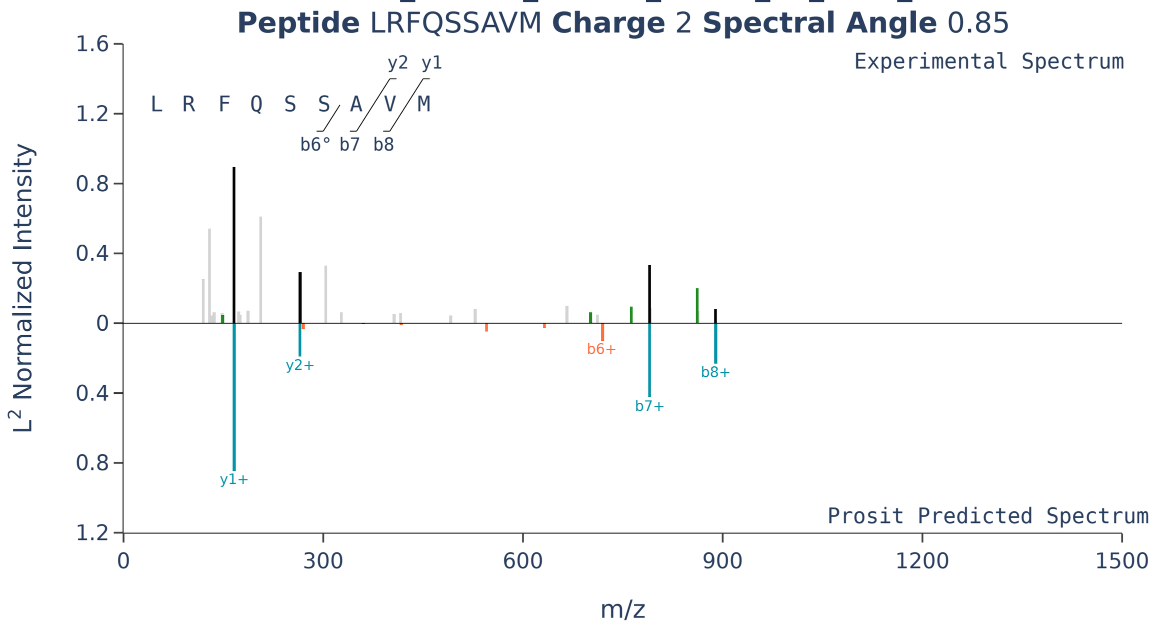


**Source** HRoetschke_271222_120123_HFGoe_H3_b2R2_4h **Scan** 15365
**Peptide** DTNLCAIHAKRVTI **Charge** 2 **Spectral Angle** 0.77

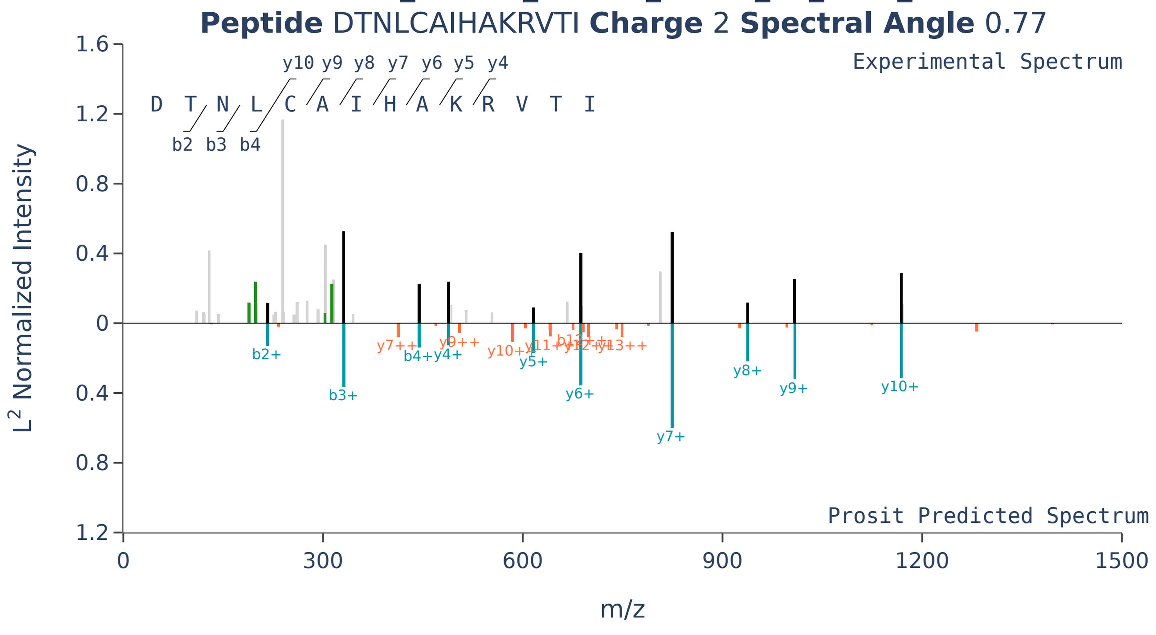


**Source** HRoetschke_271222_120123_HFGoe_H3_b2R2_24h **Scan** 13098
**Peptide** MPKDIQLA **Charge** 2 **Spectral Angle** 0.8

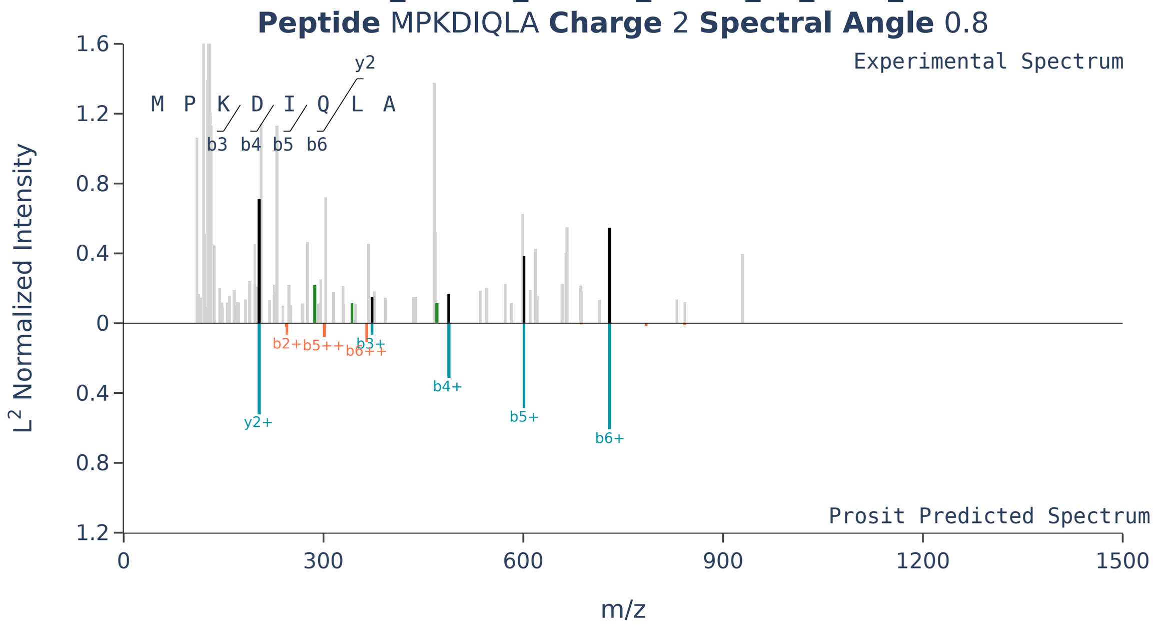


**Source** HRoetschke_271222_120123_HFGoe_H3_b1R1_4h **Scan** 16710
**Peptide** LIRKLPFQRL **Charge** 3 **Spectral Angle** 0.82

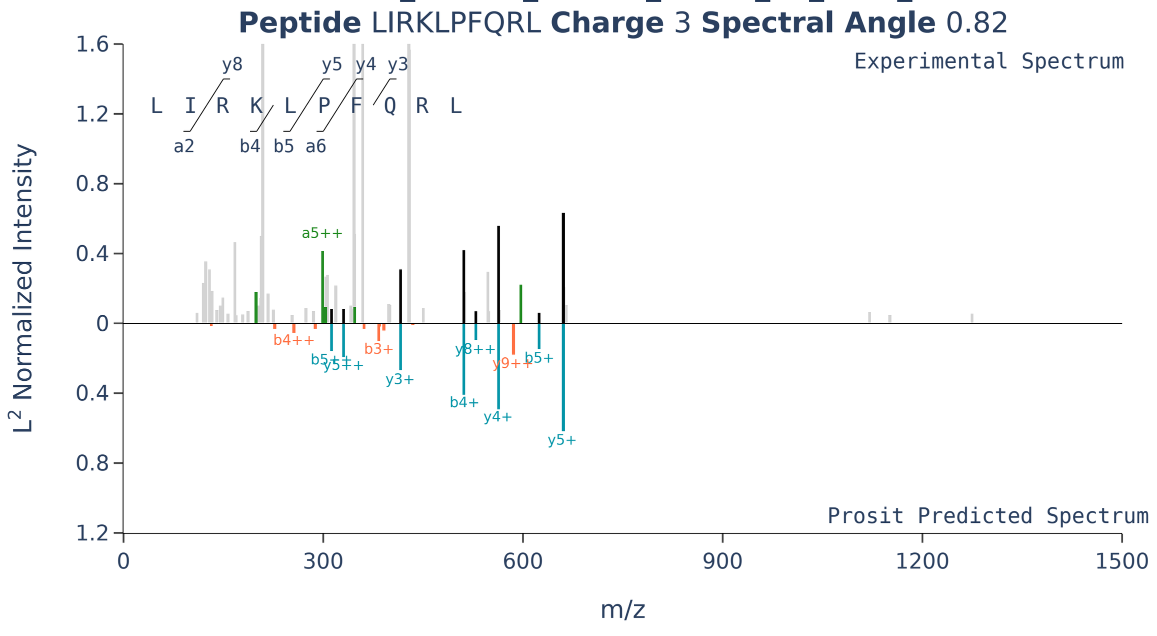


**Source** HRoetschke_271222_120123_HFGoe_H3_b2R1_2h **Scan** 11333
**Peptide** MPKDIQLAR **Charge** 3 **Spectral Angle** 0.78

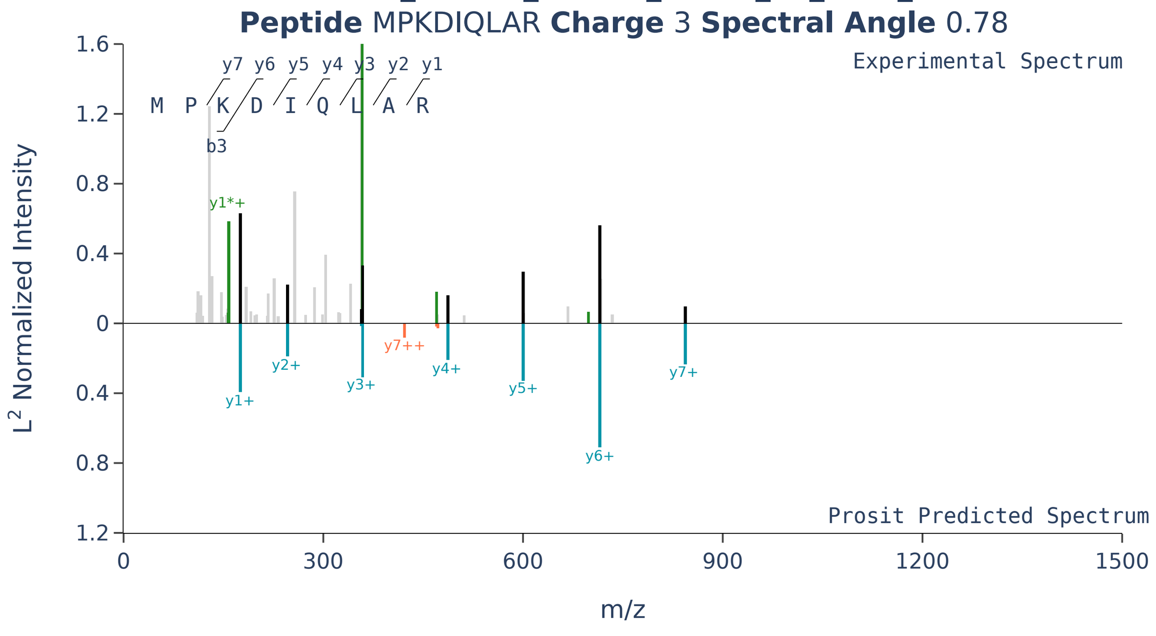


**Source** HRoetschke_271222_120123_HFGoe_H3_b2R1_2h **Scan** 7323
**Peptide** AKRVTIMPKD **Charge** 3 **Spectral Angle** 0.8

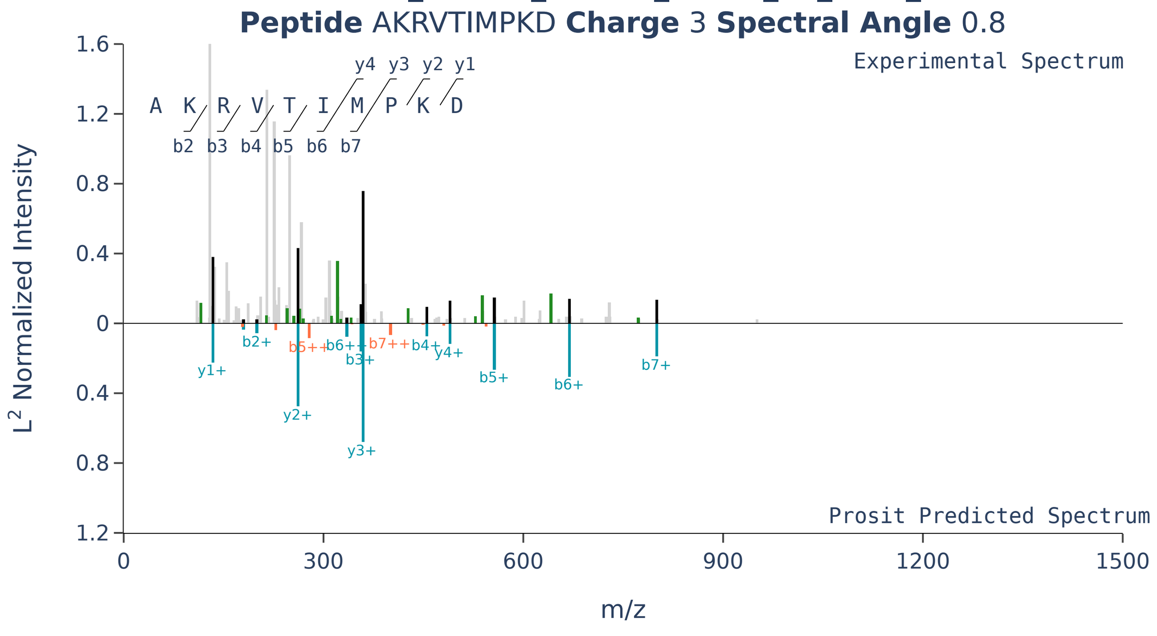


**Source** HRoetschke_271222_120123_HFGoe_H3_b1R1_4h **Scan** 7853
**Peptide** PKDIQLARRIR **Charge** 3 **Spectral Angle** 0.73

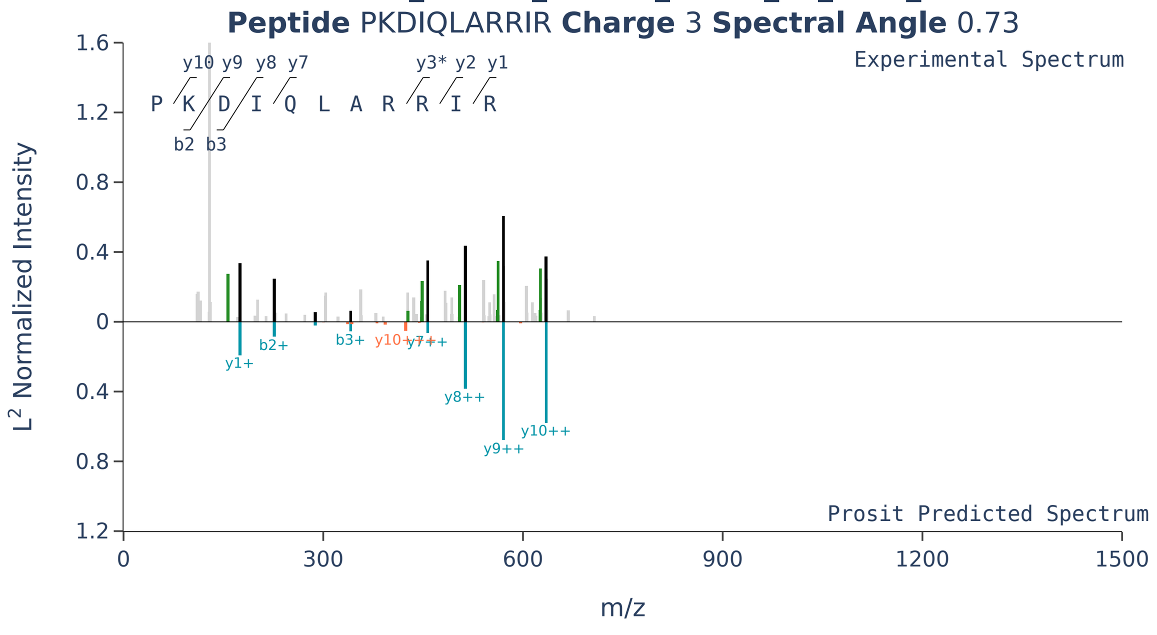


**Source** HRoetschke_271222_120123_HFGoe_H3_b1R1_4h **Scan** 8469
**Peptide** MPKDIQLARRIRGERA **Charge** 4 **Spectral Angle** 0.83

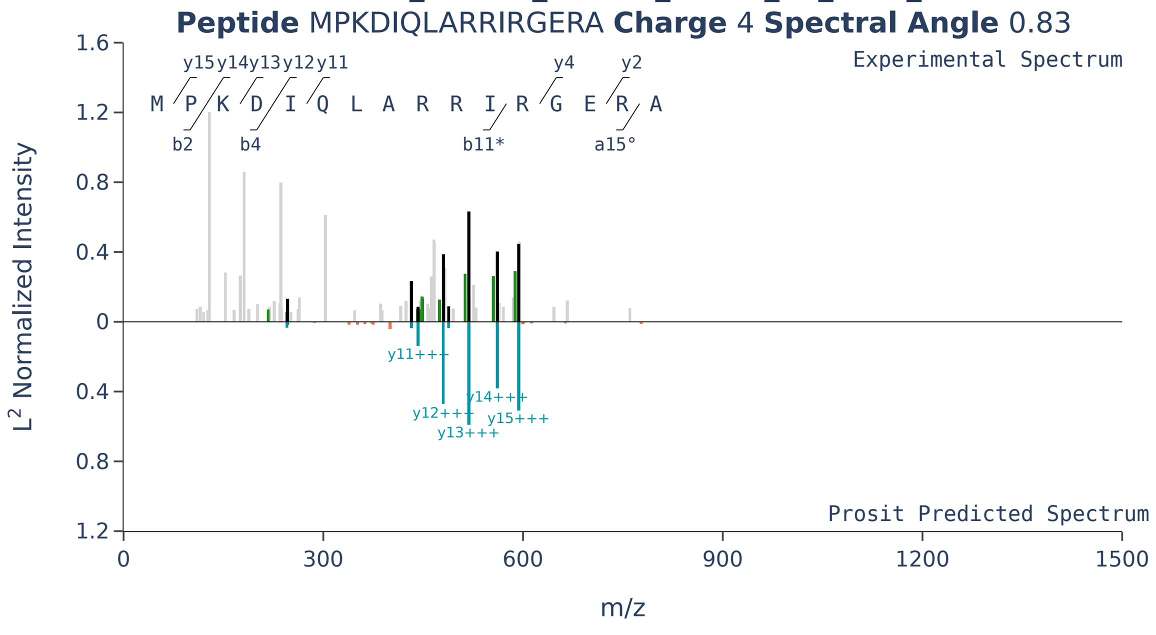


**Source** HRoetschke_271222_120123_HFGoe_H3_b1R1_24h **Scan** 5691
**Peptide** PHRYRPGTV **Charge** 3 **Spectral Angle** 0.75

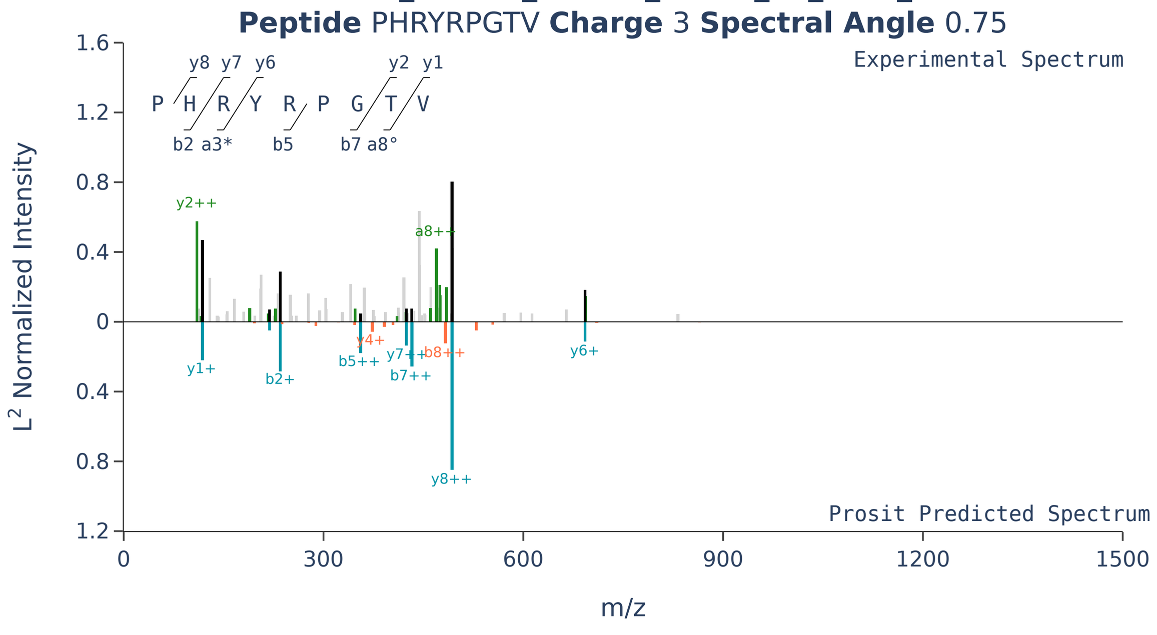


**Source** HRoetschke_271222_120123_HFGoe_H3_b1R1_4h **Scan** 16709
**Peptide** DFKTDLRFQSS **Charge** 2 **Spectral Angle** 0.8

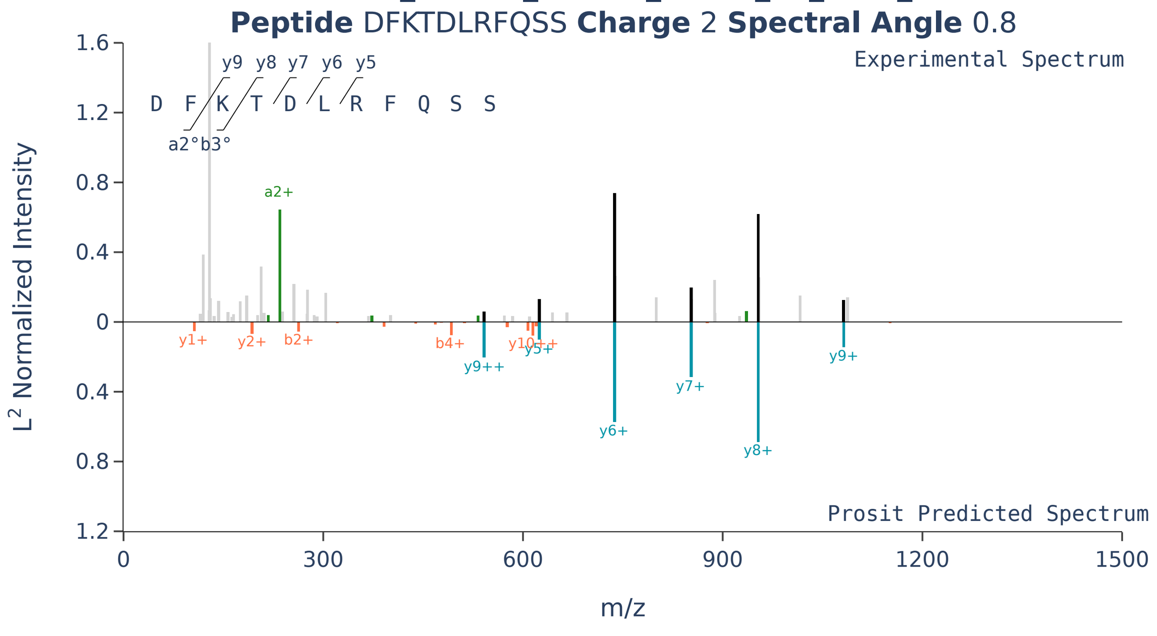


**Source** HRoetschke_271222_120123_HFGoe_H3_b2R2_4h **Scan** 8352
**Peptide** KDIQLARRIR **Charge** 3 **Spectral Angle** 0.76

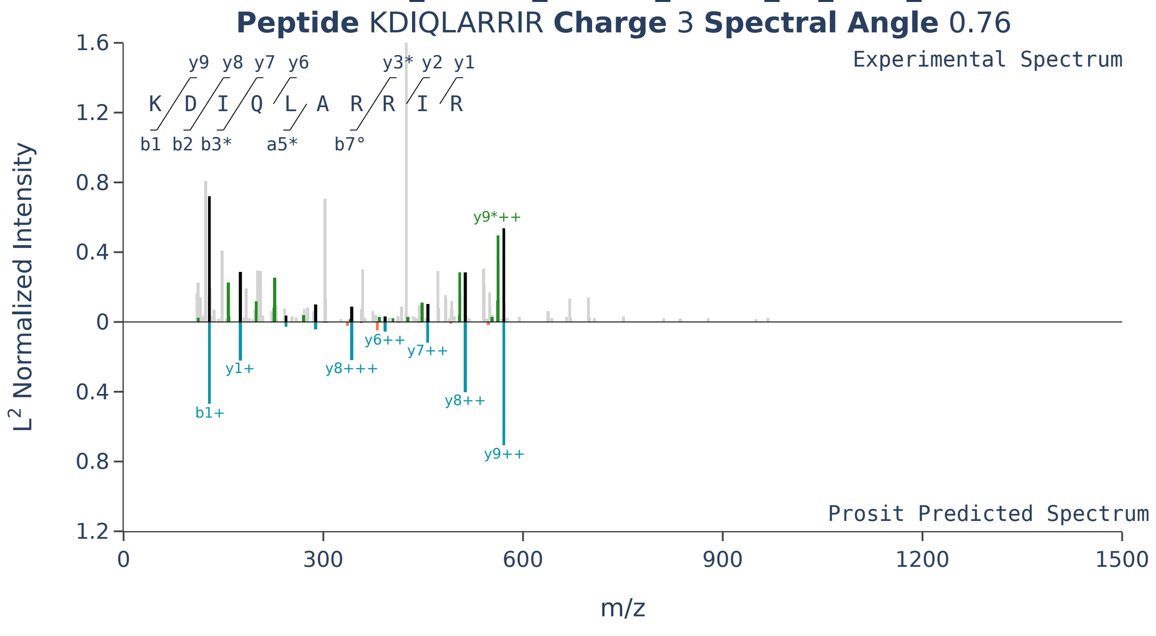


**Source** HRoetschke_271222_120123_HFGoe_H3_b1R1_4h **Scan** 16390
**Peptide** RFQSSAVMALQEA **Charge** 2 **Spectral Angle** 0.75

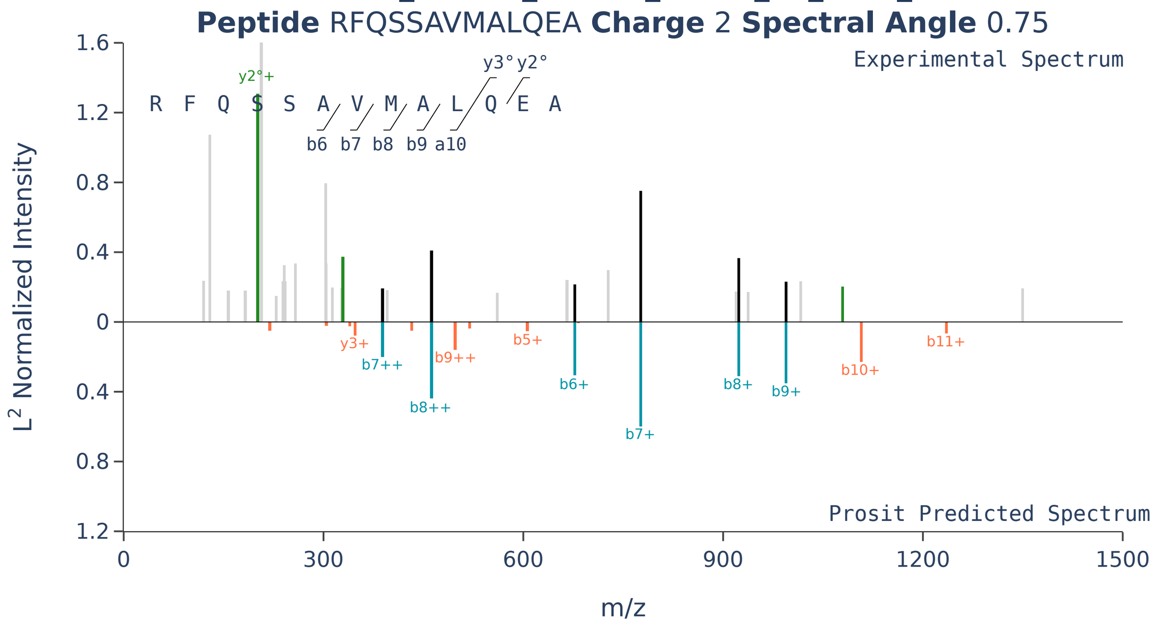

**Source** HRoetschke_271222_120123_HFGoe_H3_b2R2_4h **Scan** 9030
**Peptide** QSSAVMA **Charge** 1 **Spectral Angle** 0.77

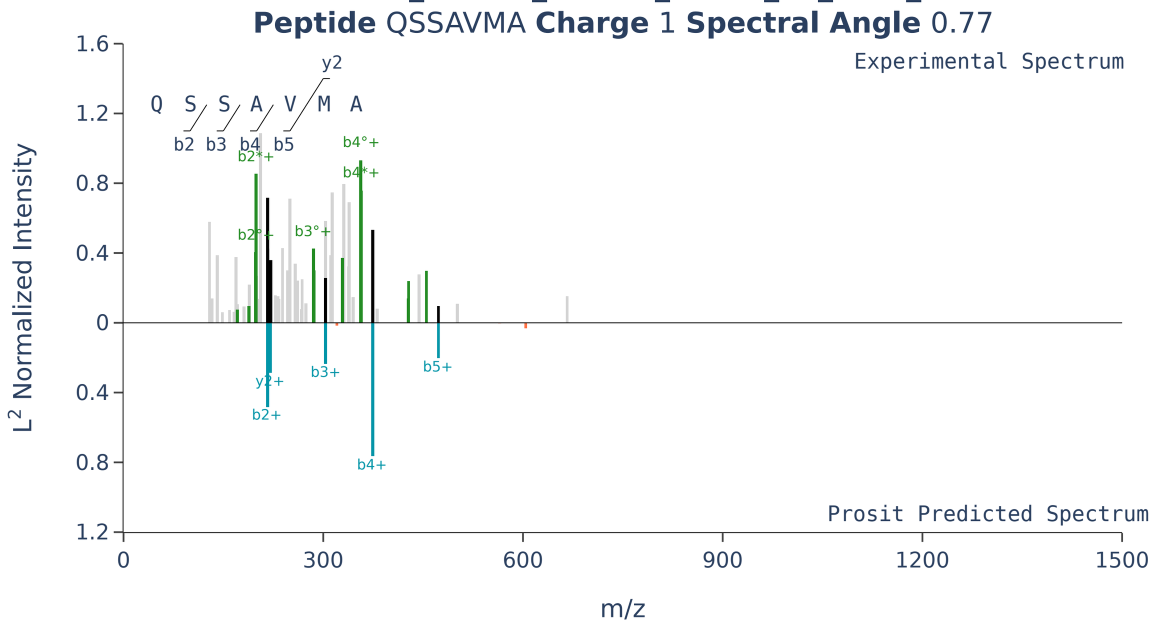


**Source** HRoetschke_271222_120123_HFGoe_H3_b2R2_1h **Scan** 14899
**Peptide** FKTDLRFQSSAVM **Charge** 3 **Spectral Angle** 0.94

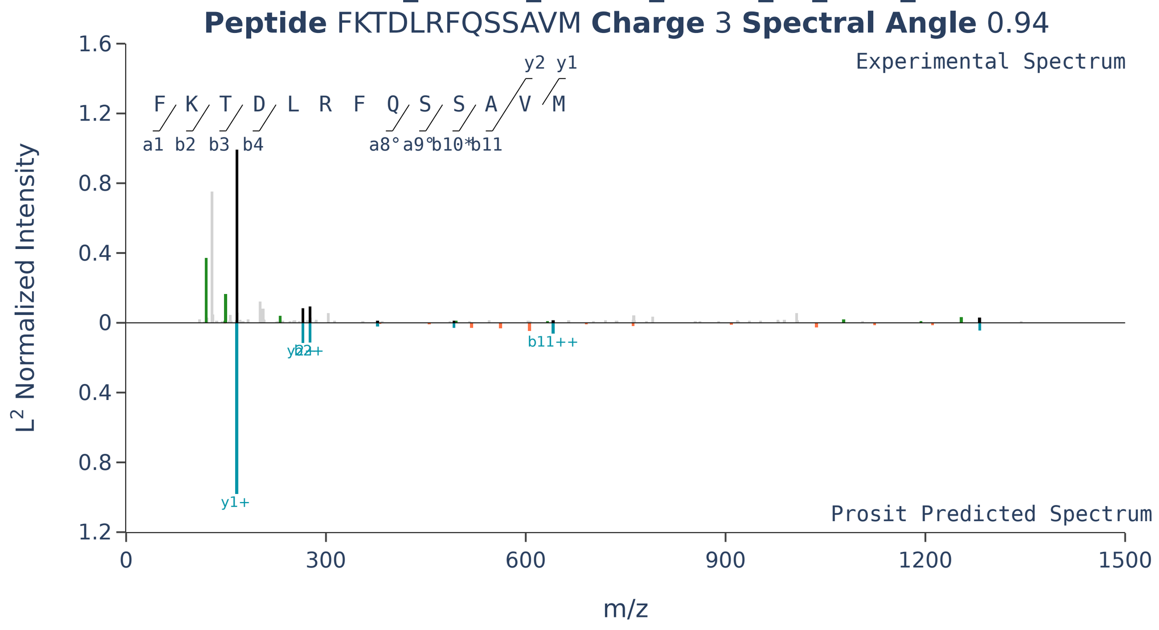


**Source** HRoetschke_271222_120123_HFGoe_H3_b1R1_4h **Scan** 18668
**Peptide** VREIAQDFKTDLRFQ **Charge** 3 **Spectral Angle** 0.84

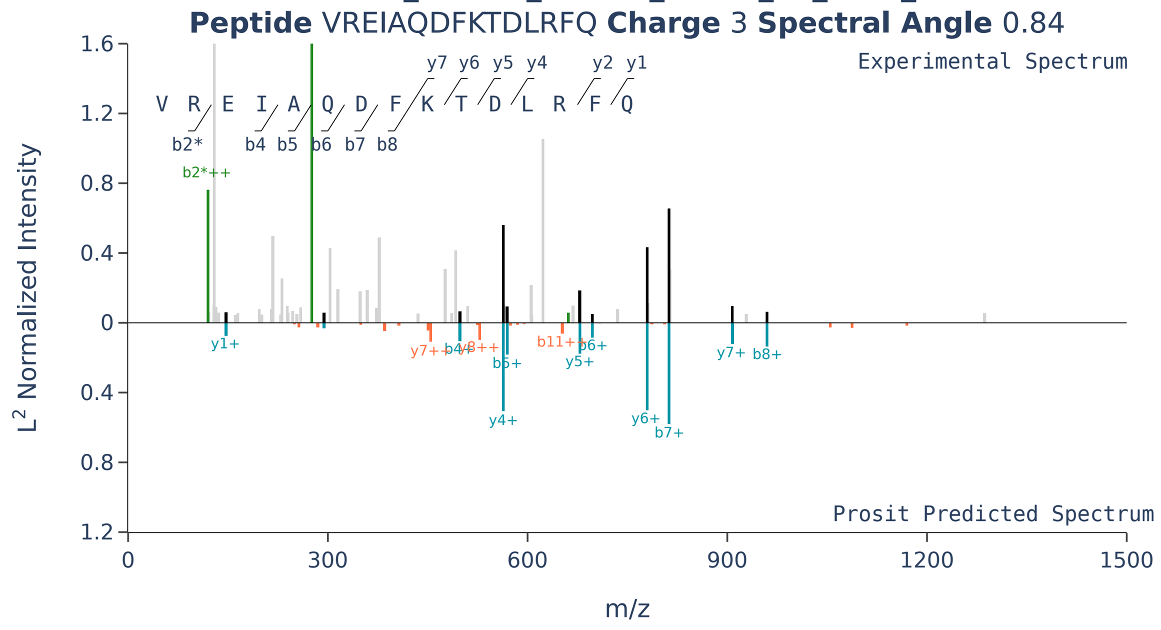


**Source** HRoetschke_271222_120123_HFGoe_H3_b2R1_2h **Scan** 15053
**Peptide** DFKTDLRFQS **Charge** 2 **Spectral Angle** 0.77

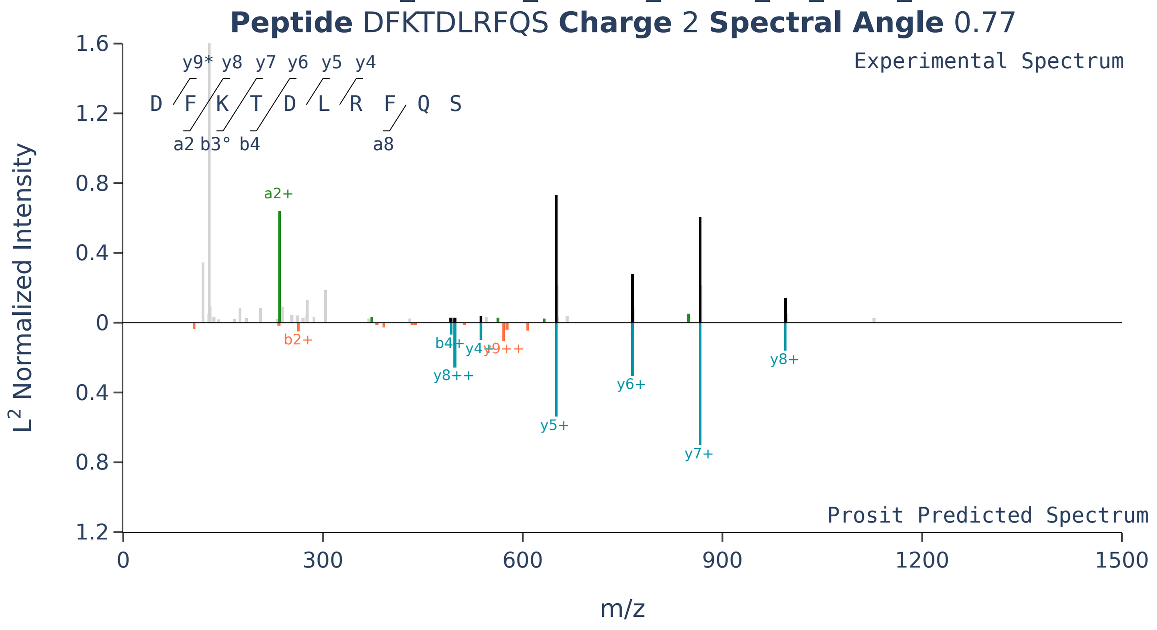


**Source** HRoetschke_271222_120123_HFGoe_H3_b2R2_2h **Scan** 9349
**Peptide** RRYQKSTELLIR **Charge** 4 **Spectral Angle** 0.85

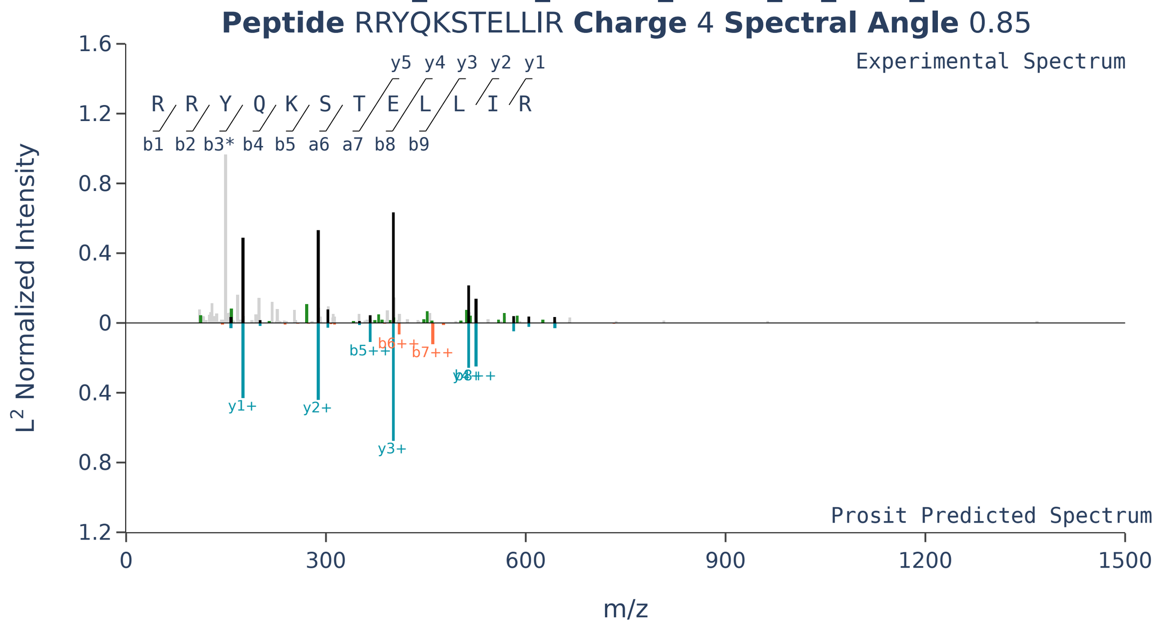


**Source** HRoetschke_271222_120123_HFGoe_H3_b2R1_4h **Scan** 13648
**Peptide** NLCAIHAKRVTI **Charge** 3 **Spectral Angle** 0.74

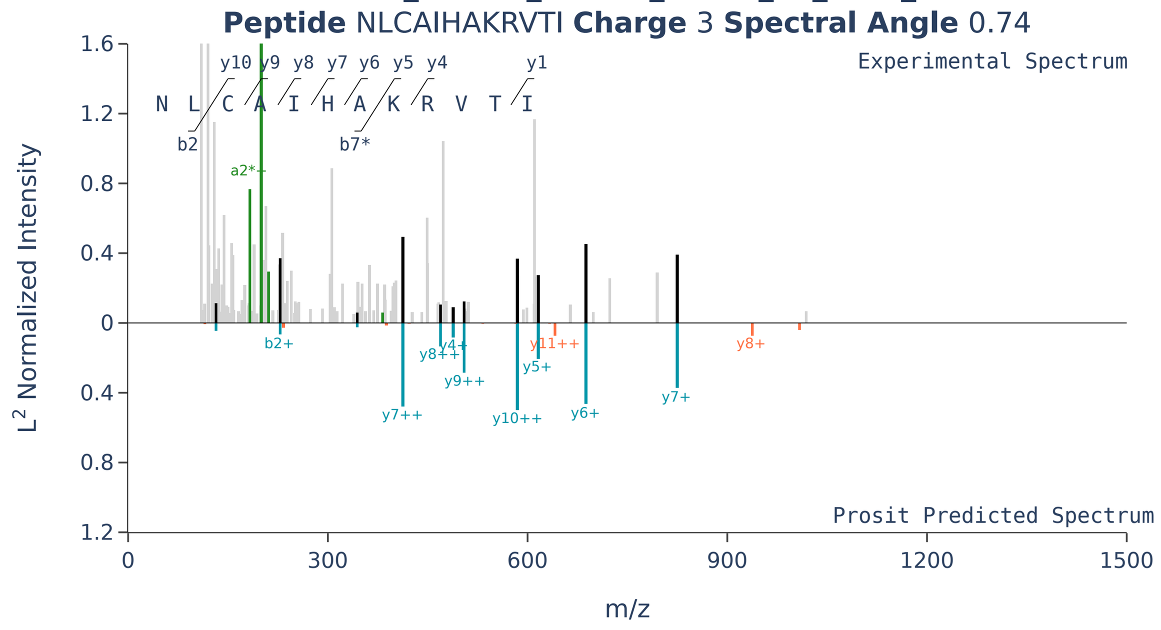


**Source** HRoetschke_271222_120123_HFGoe_H3_b1R1_4h **Scan** 17644
**Peptide** VREIAQDFKTDLRFQSS **Charge** 3 **Spectral Angle** 0.82

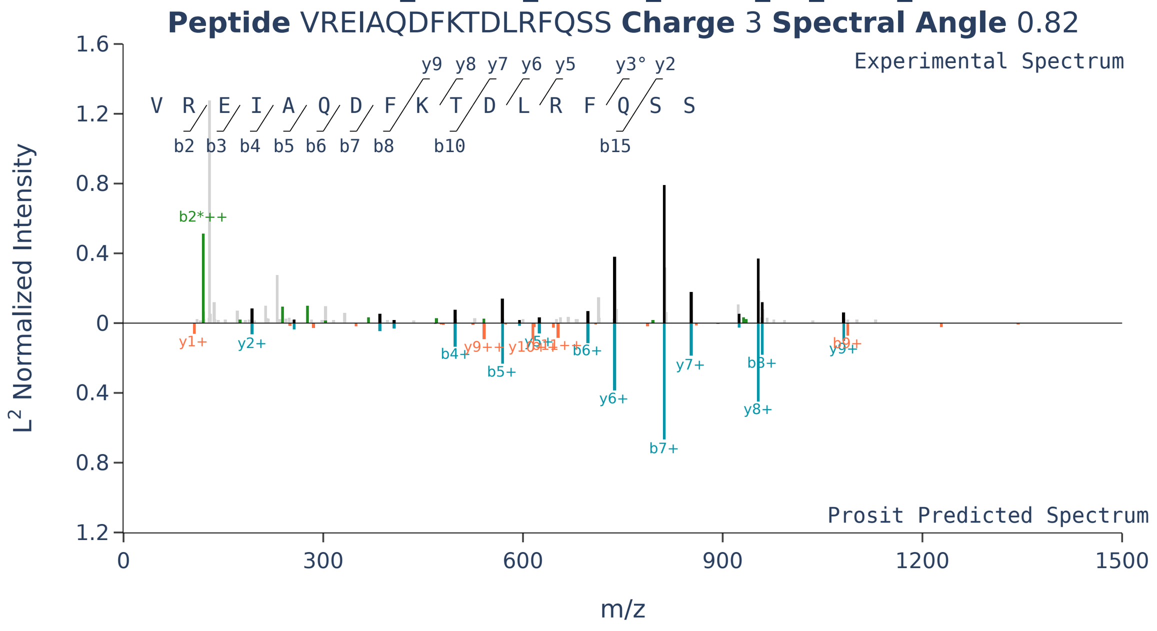


**Source** HRoetschke_271222_120123_HFGoe_H3_b1R1_4h **Scan** 19817
**Peptide** IAQDFKTDLRF **Charge** 3 **Spectral Angle** 0.81

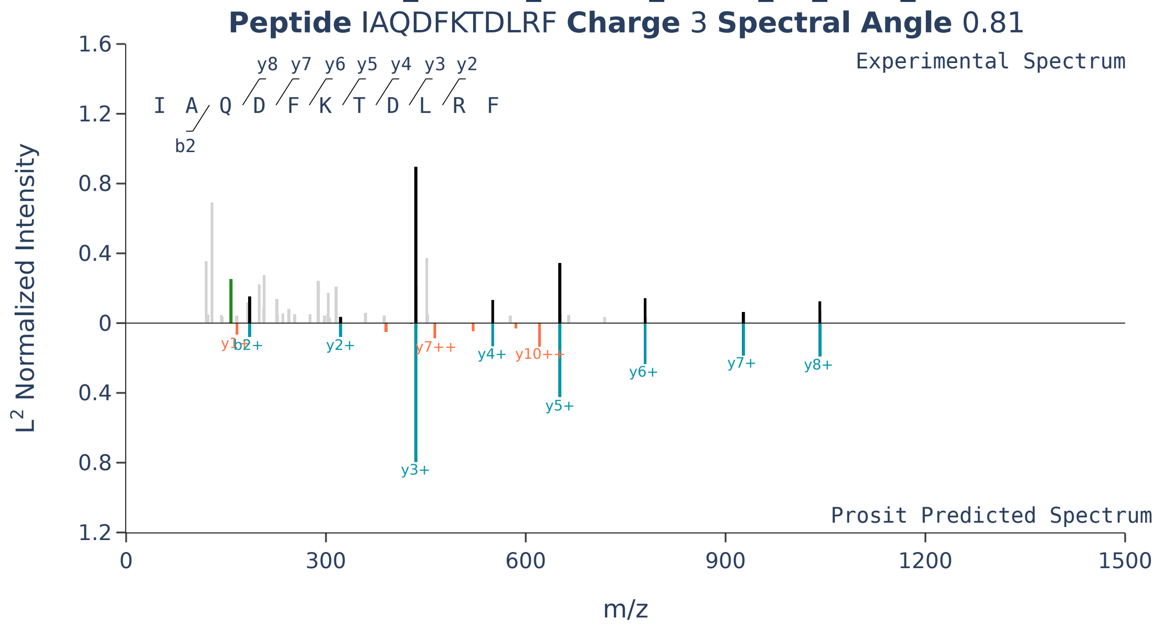


**Source** HRoetschke_271222_120123_HFGoe_H3_b2R2_4h **Scan** 18274
**Peptide** PFQRLVREIAQD **Charge** 2 **Spectral Angle** 0.69

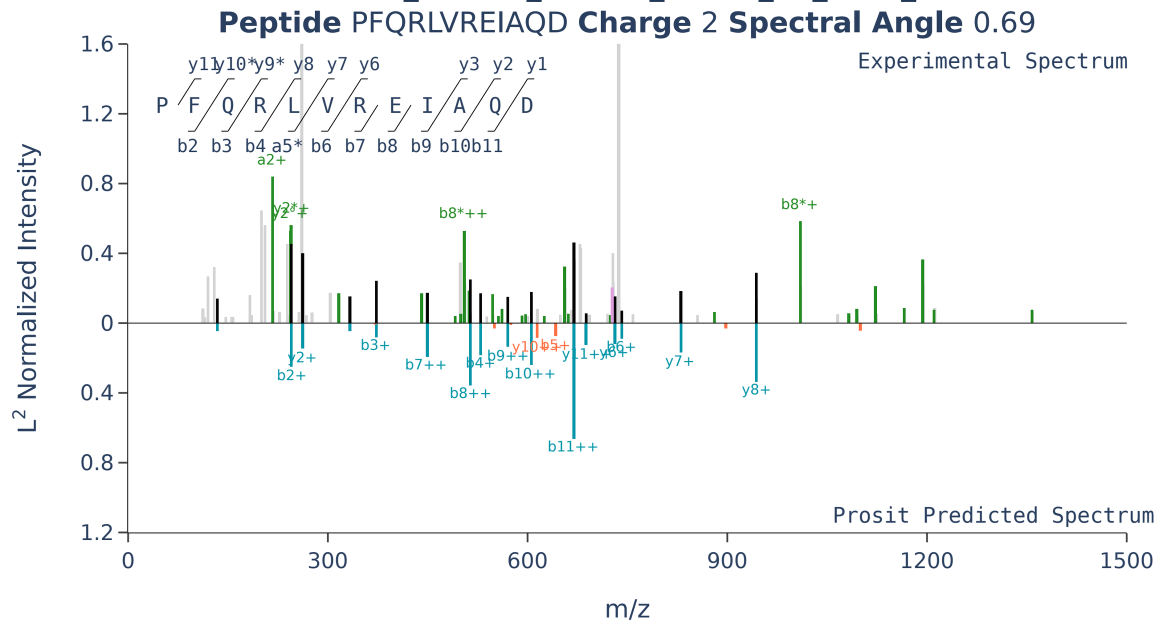


**Source** HRoetschke_271222_120123_HFGoe_H3_b1R1_24h **Scan** 9430
**Peptide** LAQDFKT **Charge** 2 **Spectral Angle** 0.74

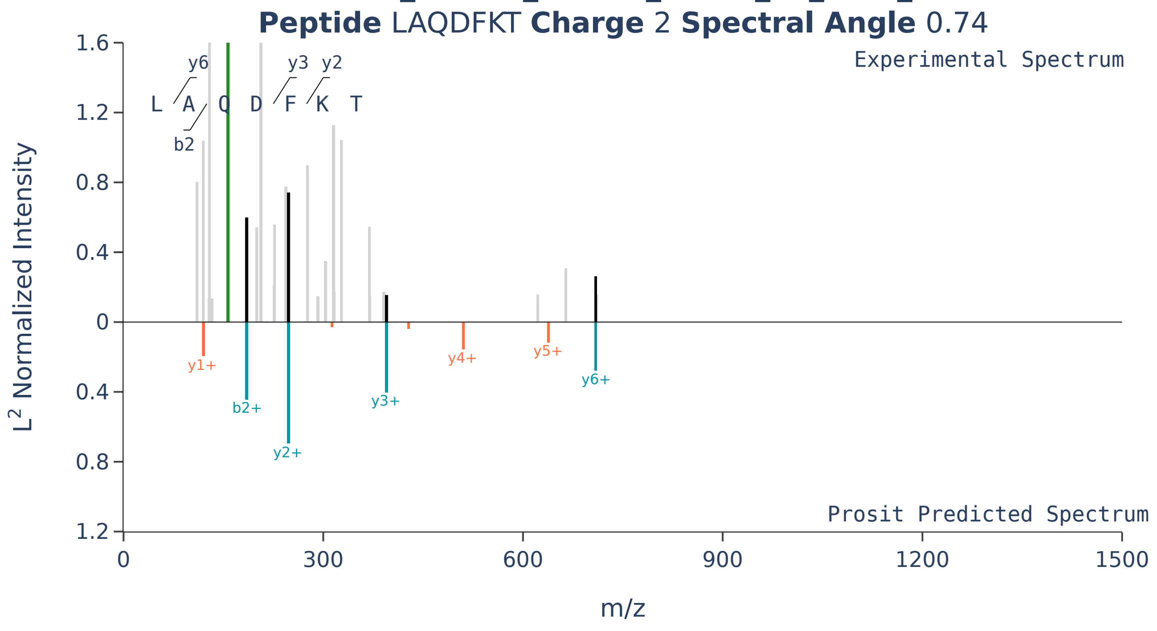


**Source** HRoetschke_271222_120123_HFGoe_H3_b2R1_4h **Scan** 13045
**Peptide** MPKDIQLARRIRGE **Charge** 4 **Spectral Angle** 0.74

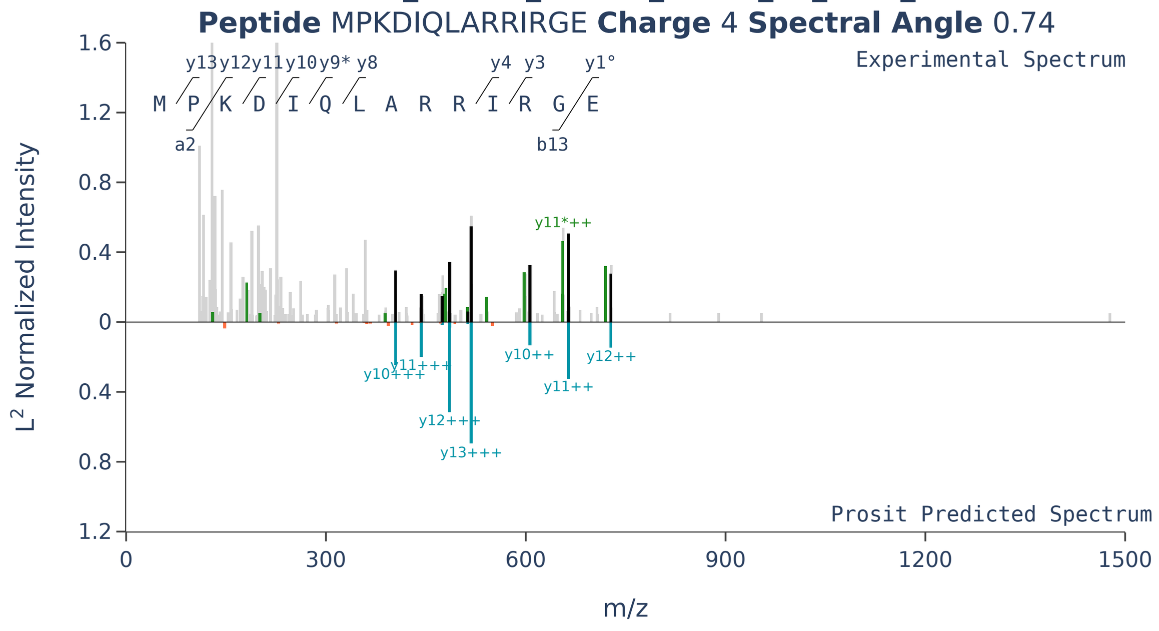


**Source** HRoetschke_271222_120123_HFGoe_H3_b1R1_24h **Scan** 9507
**Peptide** GTVALREIRR **Charge** 3 **Spectral Angle** 0.75

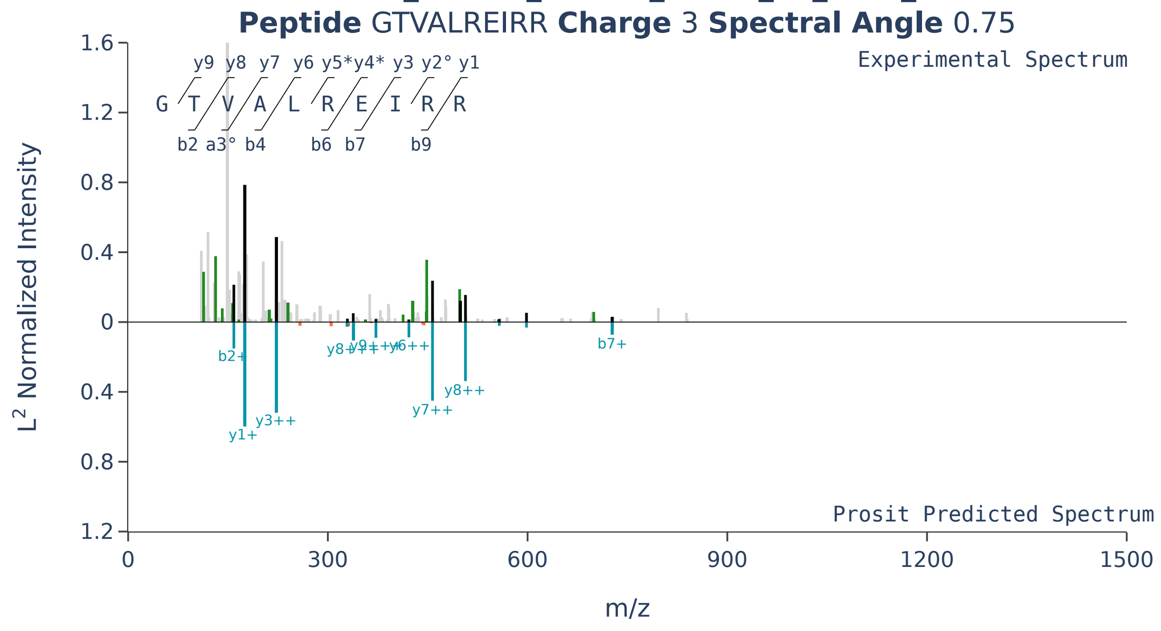


**Source** HRoetschke_271222_120123_HFGoe_H3_b1R2_2h **Scan** 7775
**Peptide** RFQSSAV **Charge** 2 **Spectral Angle** 0.79

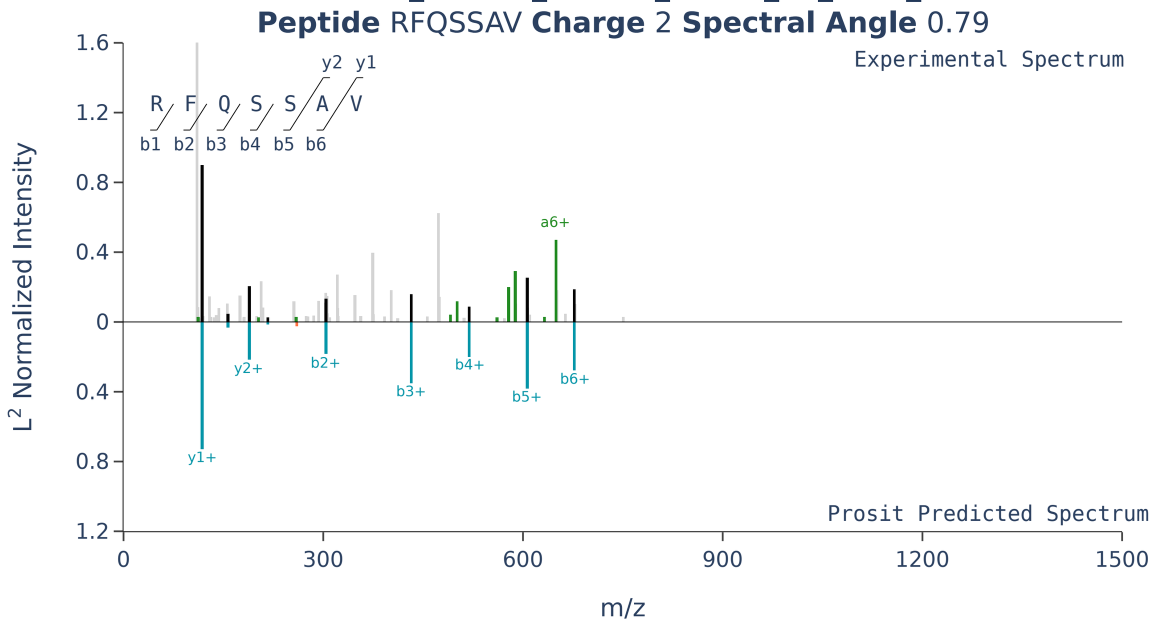


**Source** HRoetschke_271222_120123_HFGoe_H3_b1R1_24h **Scan** 5949
**Peptide** ALREIRRYQKST **Charge** 4 **Spectral Angle** 0.75

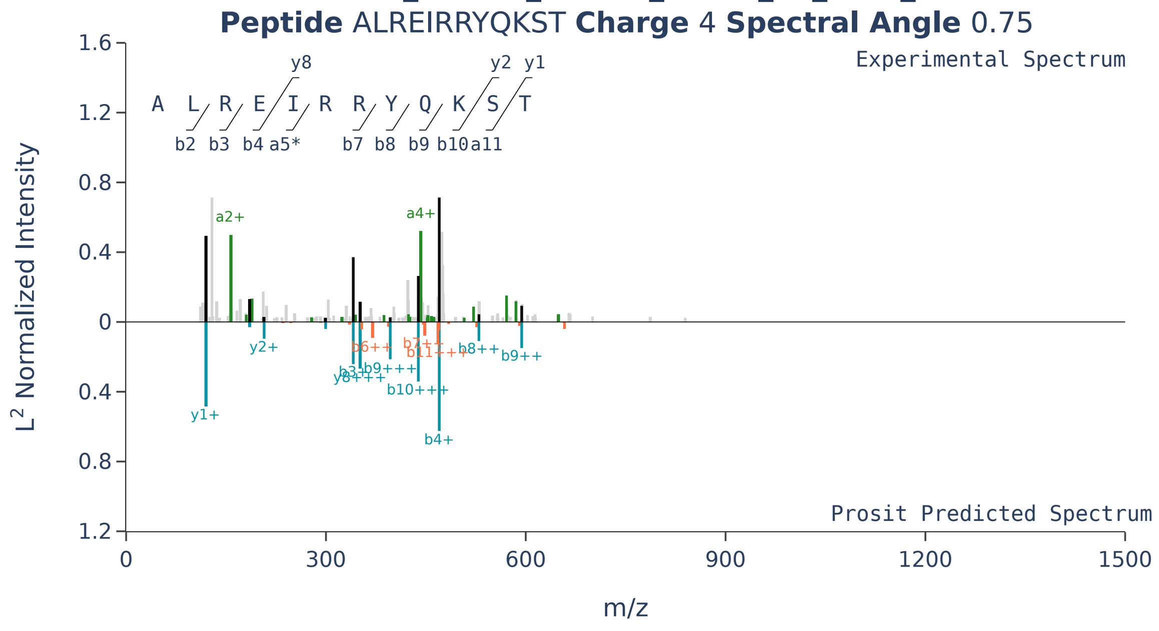


**Source** HRoetschke_271222_120123_HFGoe_H3_b1R1_24h **Scan** 15510
**Peptide** QDFKTDLRFQSSA **Charge** 2 **Spectral Angle** 0.78

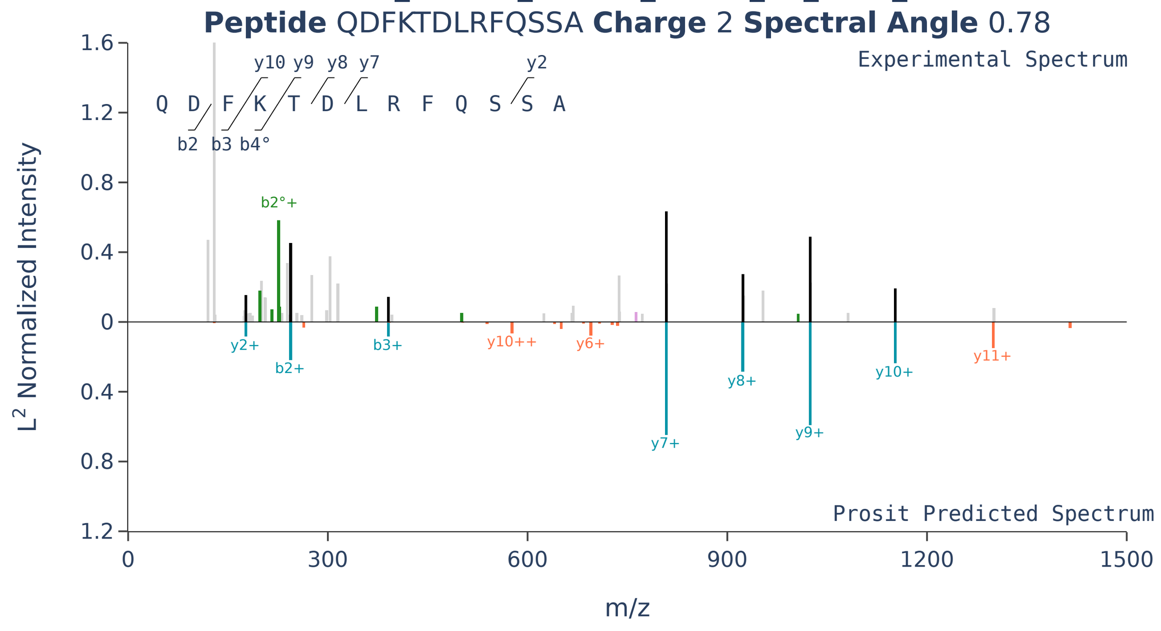

**Source** HRoetschke_271222_120123_HFGoe_H3_b1R1_4h **Scan** 13256
**Peptide** RRYQKSTELLI **Charge** 3 **Spectral Angle** 0.76

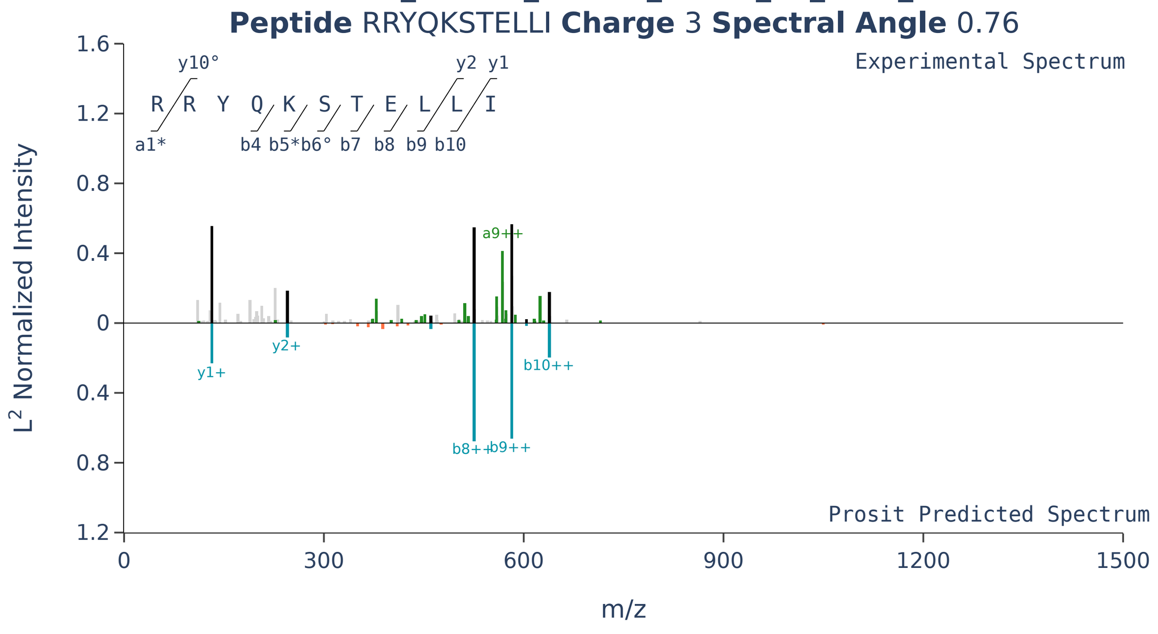


**Source** HRoetschke_271222_120123_HFGoe_H3_b1R1_4h **Scan** 16845
**Peptide** REIAQDFKTDLRFQSS **Charge** 3 **Spectral Angle** 0.77

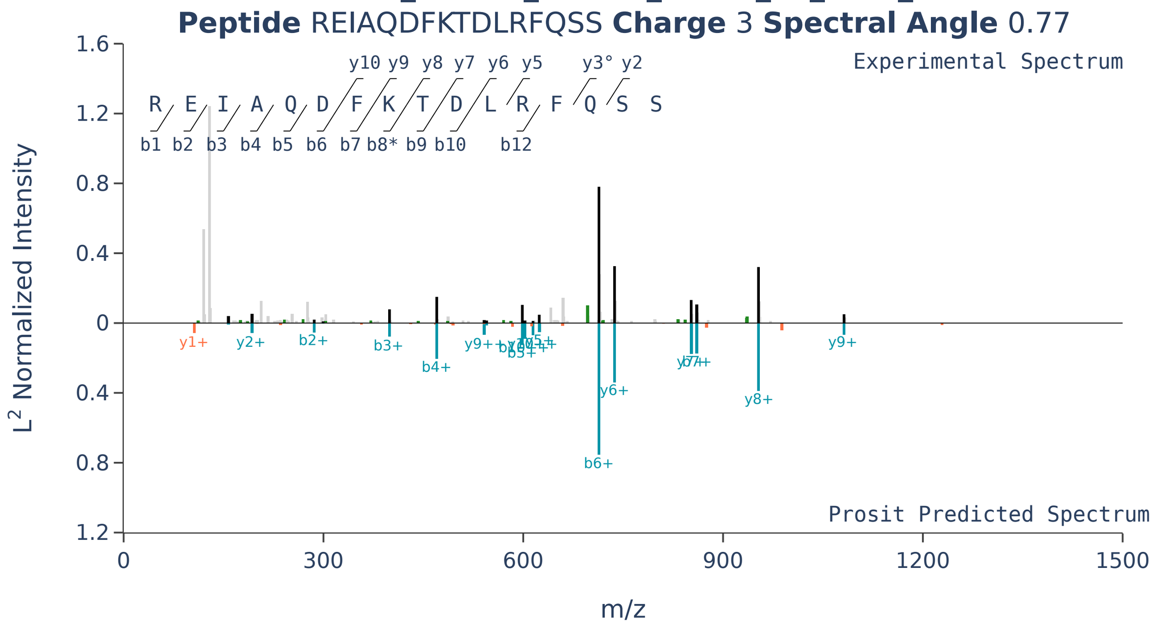


**Source** HRoetschke_271222_120123_HFGoe_H3_b2R1_4h **Scan** 13373
**Peptide** PGTVALREIRRY **Charge** 4 **Spectral Angle** 0.75

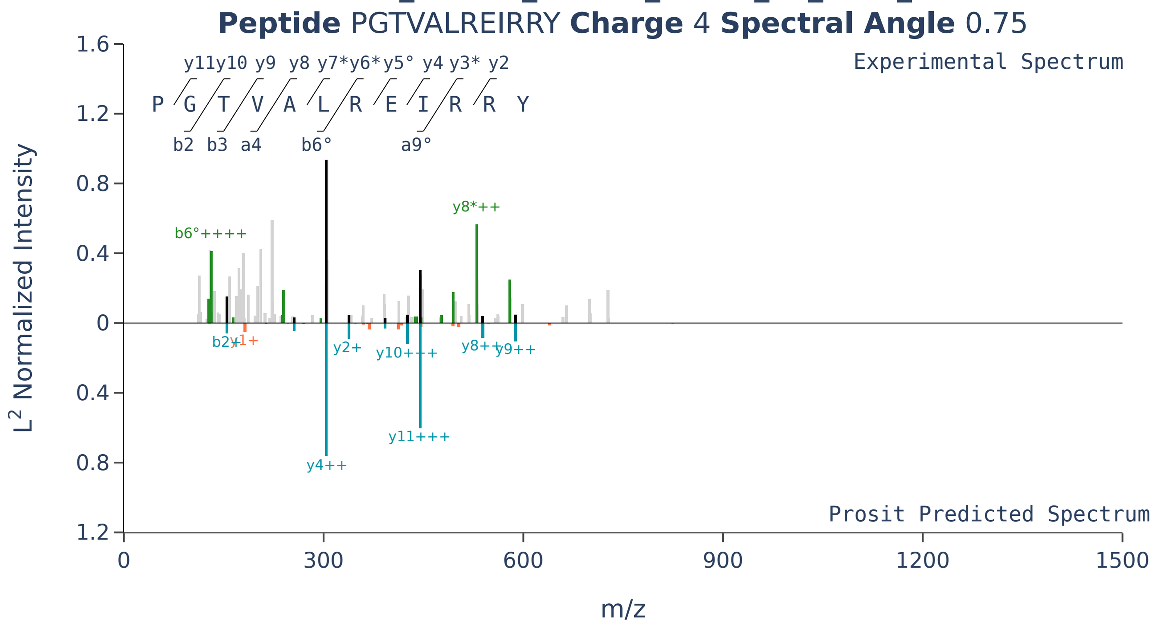


**Source** HRoetschke_271222_120123_HFGoe_H3_b2R2_2h **Scan** 5027
**Peptide** APATGGVKKPHRYR **Charge** 4 **Spectral Angle** 0.69

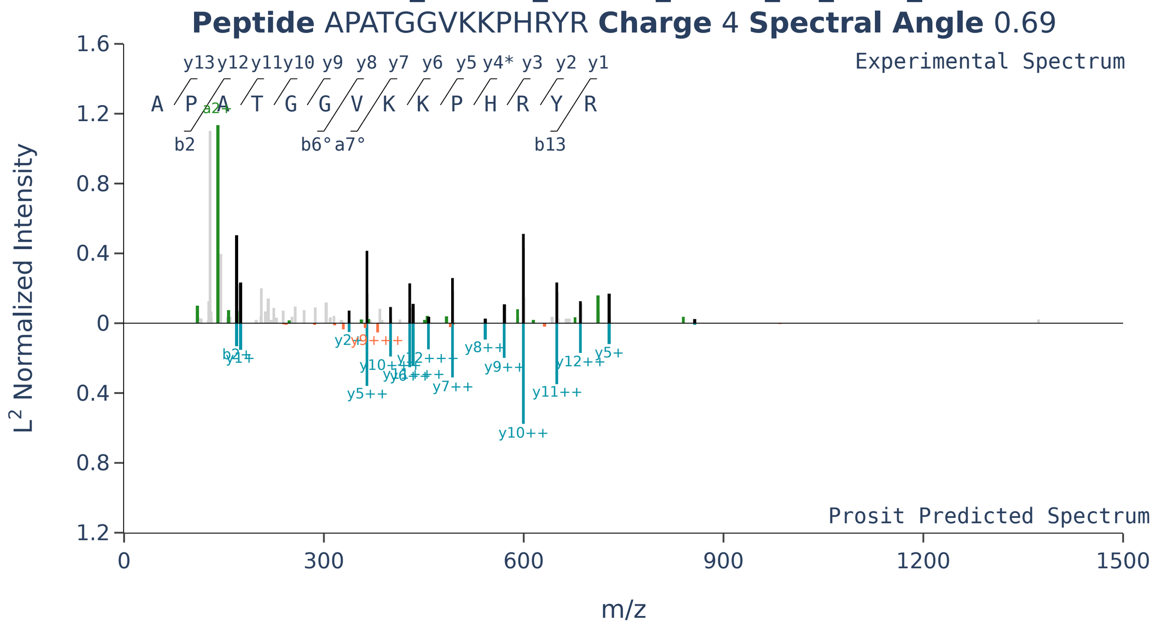


**Source** HRoetschke_271222_120123_HFGoe_H3_b2R2_4h **Scan** 7102
**Peptide** RRYQKSTELLIRK **Charge** 4 **Spectral Angle** 0.86

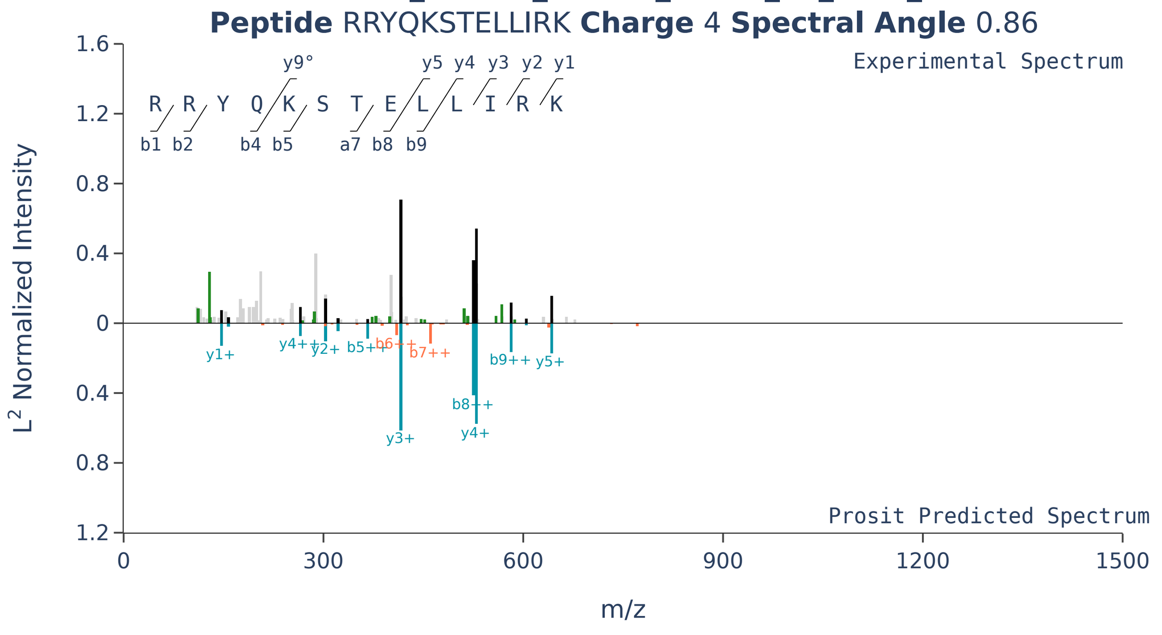


**Source** HRoetschke_271222_120123_HFGoe_H3_b1R1_4h **Scan** 15174
**Peptide** TIMPKDIQLARRIRGE **Charge** 4 **Spectral Angle** 0.64

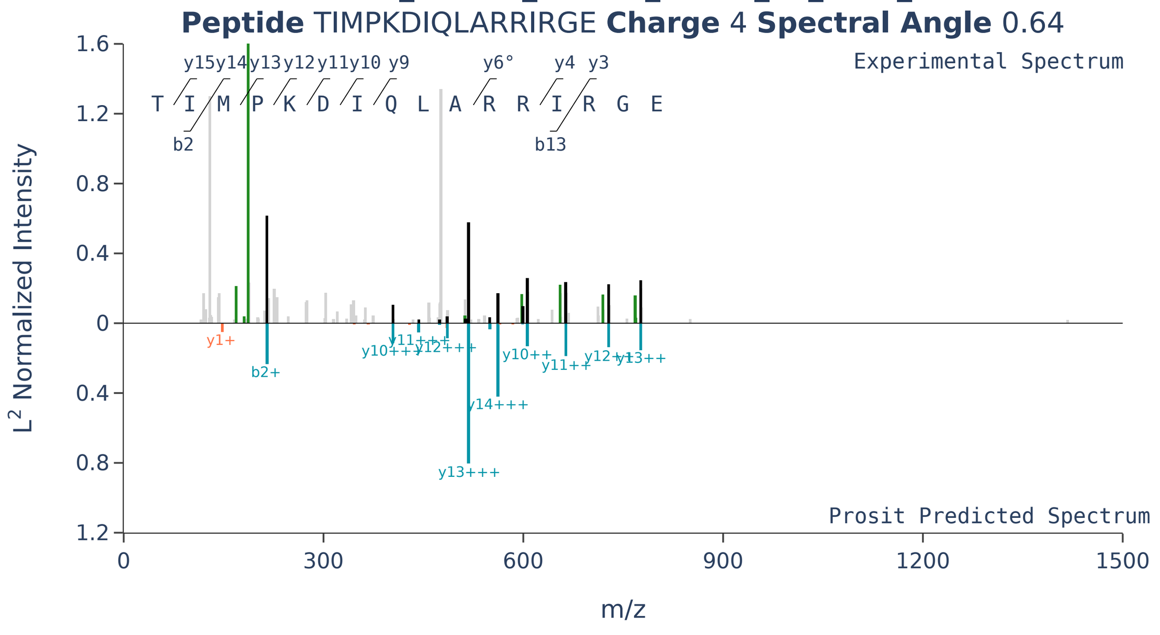


**Source** HRoetschke_271222_120123_HFGoe_H3_b2R2_4h **Scan** 9894
**Peptide** DTNLCAIHAKRVT **Charge** 2 **Spectral Angle** 0.84

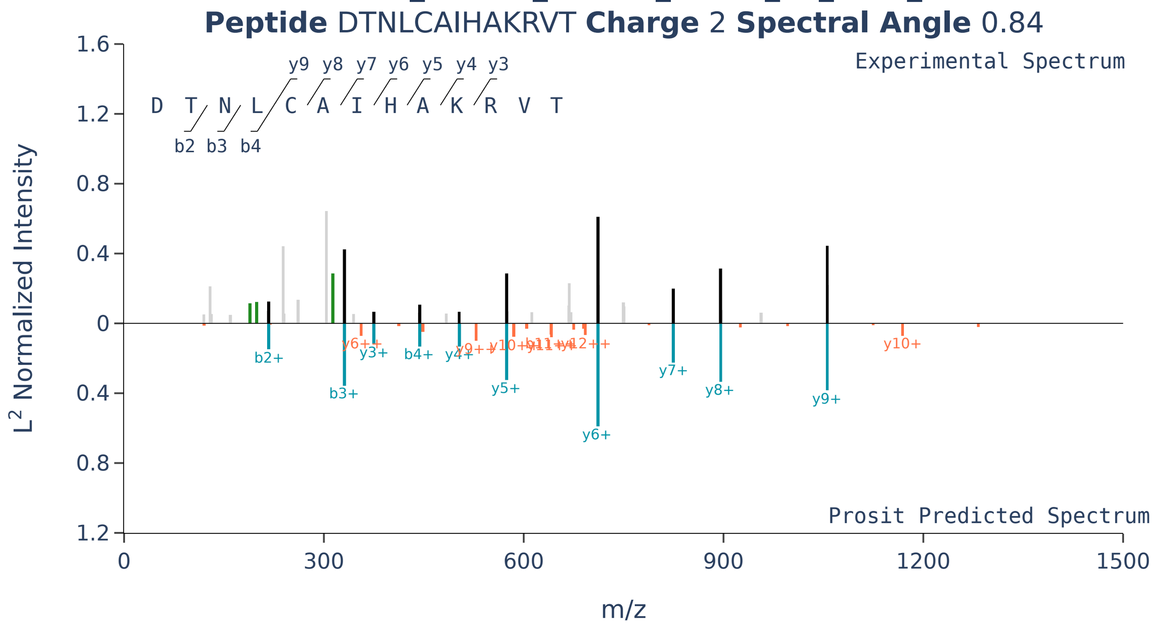


**Source** HRoetschke_271222_120123_HFGoe_H3_b1R1_24h **Scan** 8460
**Peptide** APATGGVKKPHRYRPGTVAL **Charge** 4 **Spectral Angle** 0.63

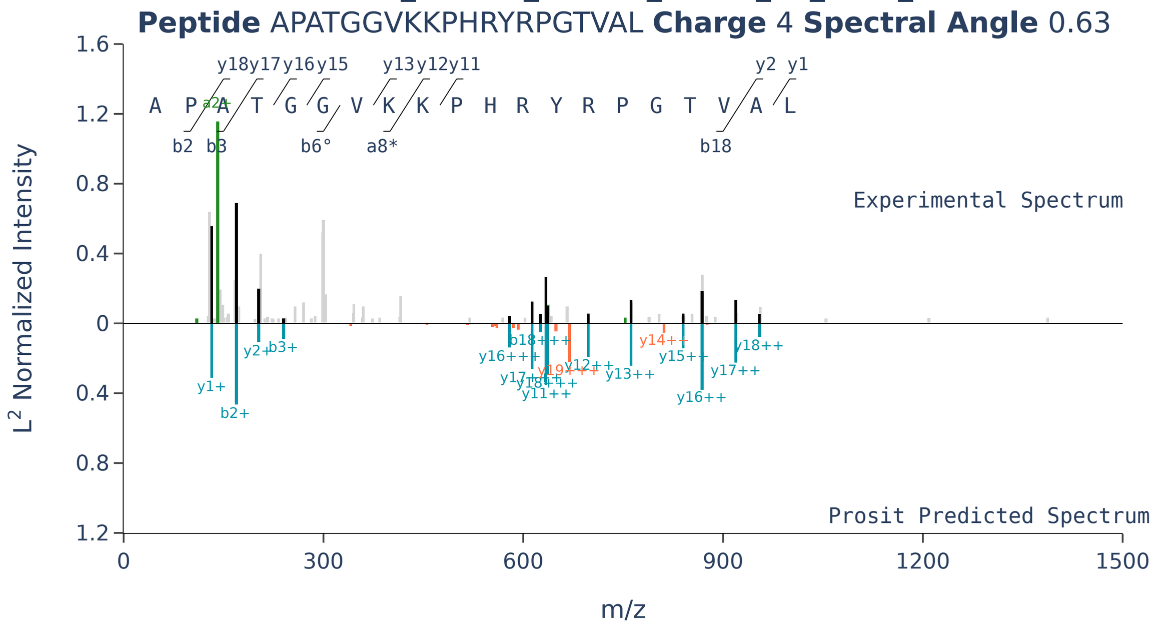


**Source** HRoetschke_271222_120123_HFGoe_H3_b1R1_24h **Scan** 14924
**Peptide** KRVTIMPKDIQL **Charge** 3 **Spectral Angle** 0.62

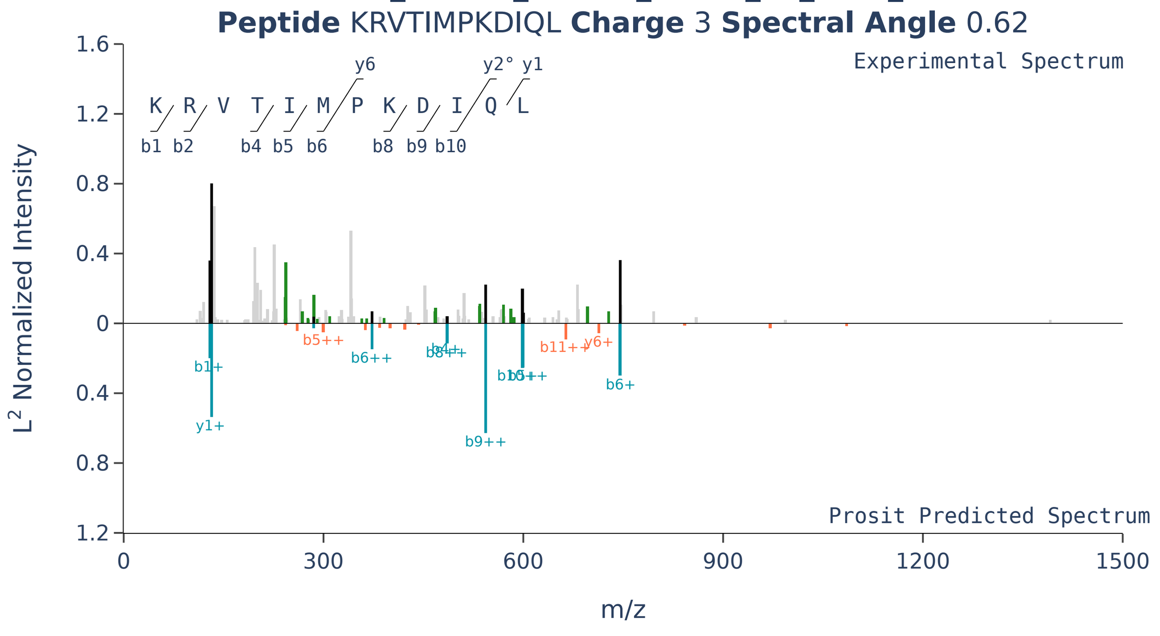


**Source** HRoetschke_271222_120123_HFGoe_H3_b2R1_1h **Scan** 16770
**Peptide** QEASEAYLV **Charge** 2 **Spectral Angle** 0.72

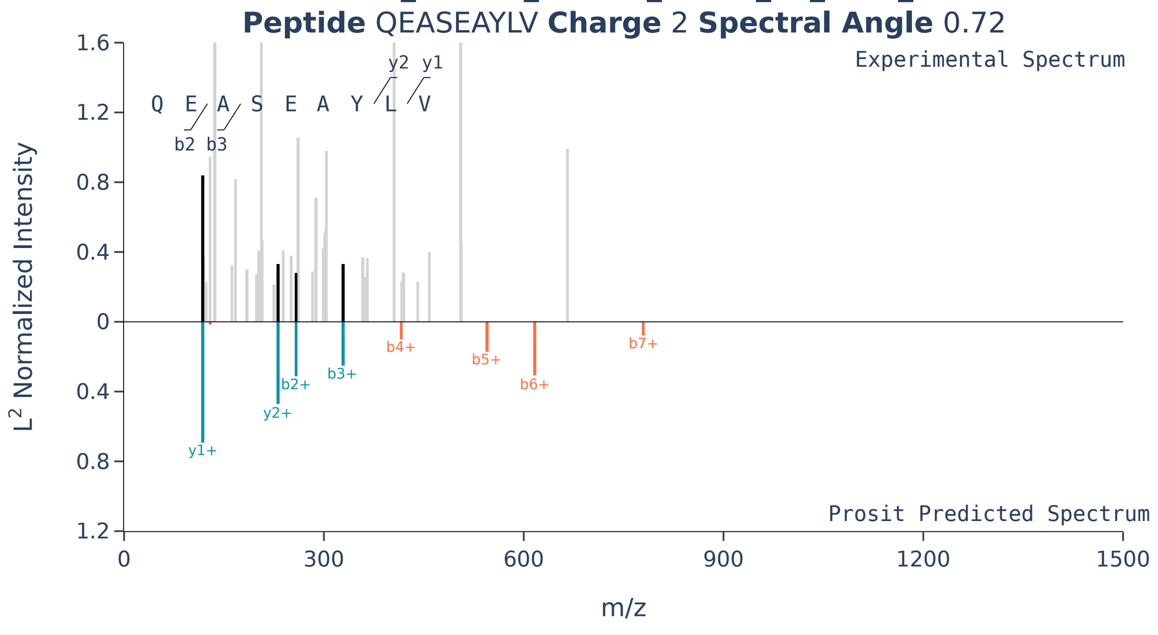


**Source** HRoetschke_271222_120123_HFGoe_H3_b1R2_4h **Scan** 16648
**Peptide** RVTIMPKDIQLA **Charge** 3 **Spectral Angle** 0.76

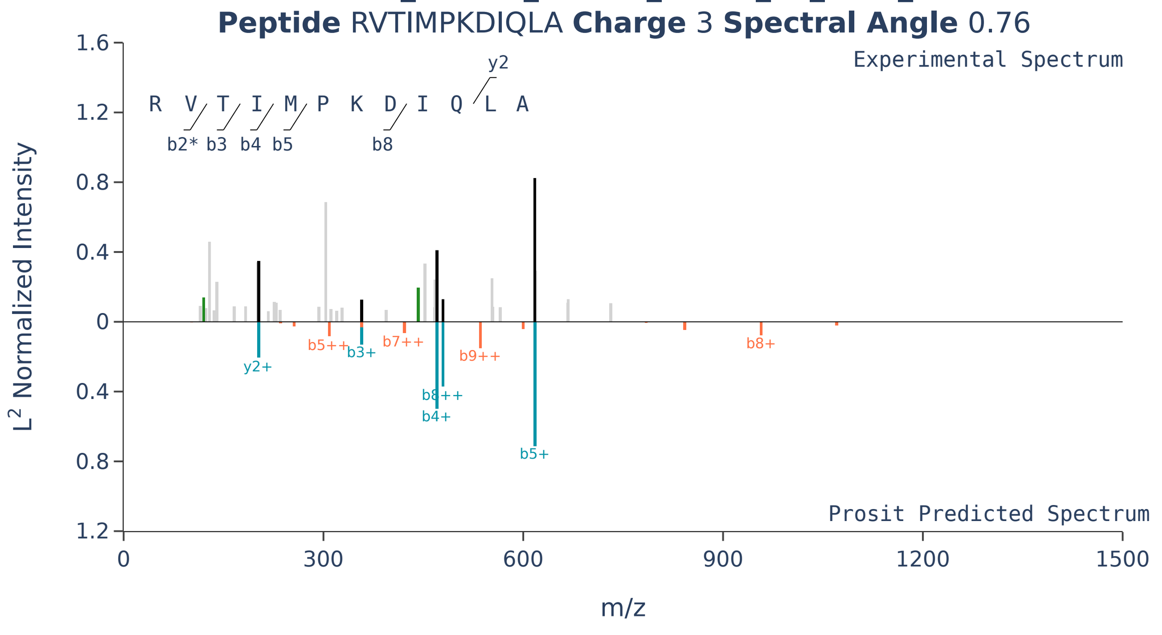


**Source** HRoetschke_271222_120123_HFGoe_H3_b1R1_4h **Scan** 13109
**Peptide** RYQKSTELLIRKL **Charge** 4 **Spectral Angle** 0.82

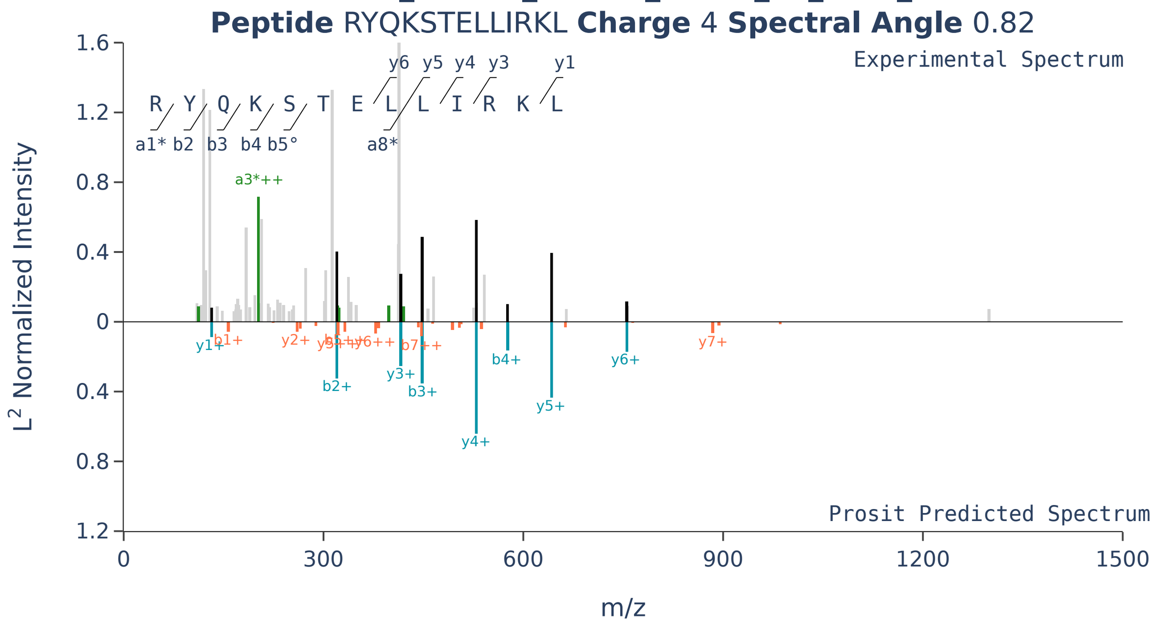


**Source** HRoetschke_271222_120123_HFGoe_H3_b2R2_2h **Scan** 13642
**Peptide** FEDTNLCAIHAK **Charge** 3 **Spectral Angle** 0.74

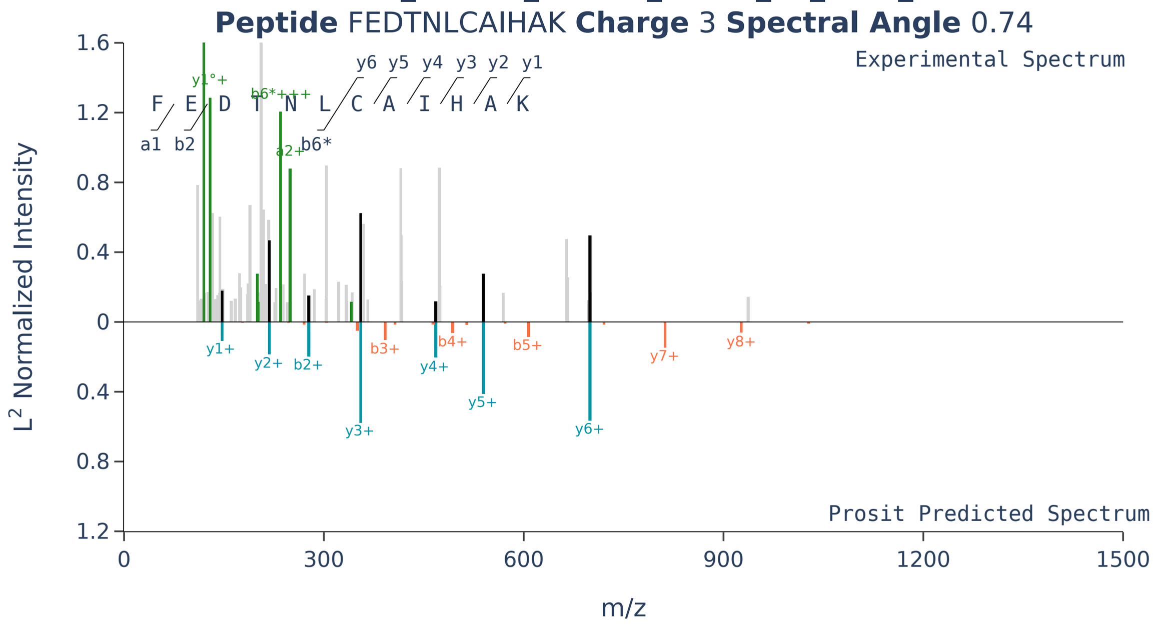


**Source** HRoetschke_271222_120123_HFGoe_H3_b2R2_4h **Scan** 14782
**Peptide** IMPKDIQLARR **Charge** 3 **Spectral Angle** 0.79

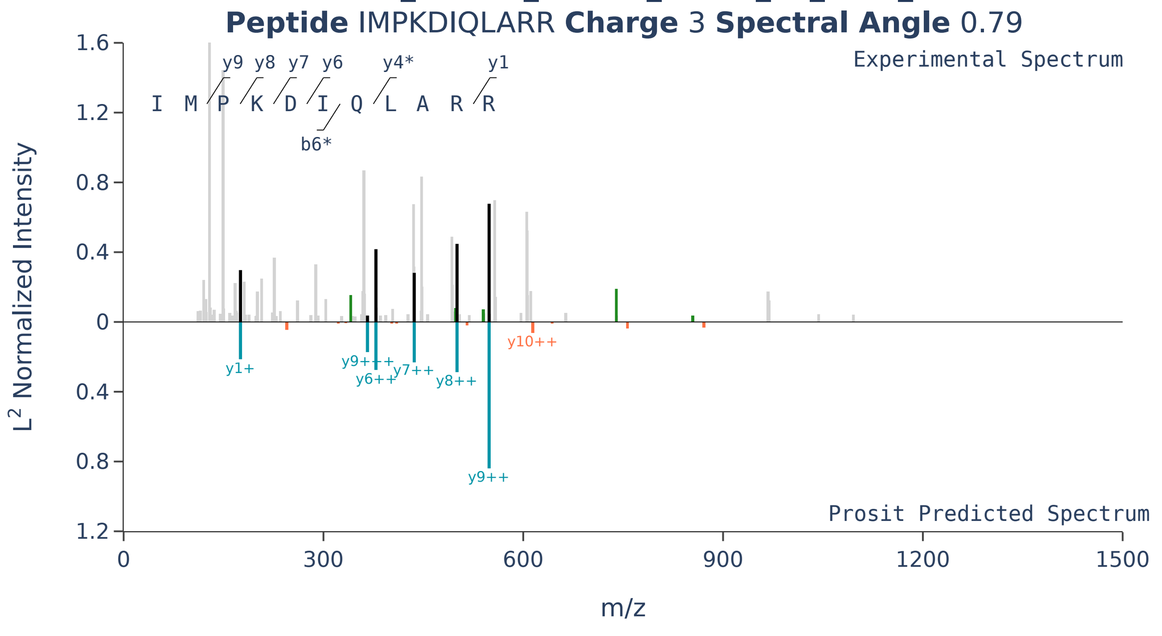


**Source** HRoetschke_271222_120123_HFGoe_H3_b2R2_1h **Scan** 16639
**Peptide** AQDFKTDLRFQSSAV **Charge** 2 **Spectral Angle** 0.7

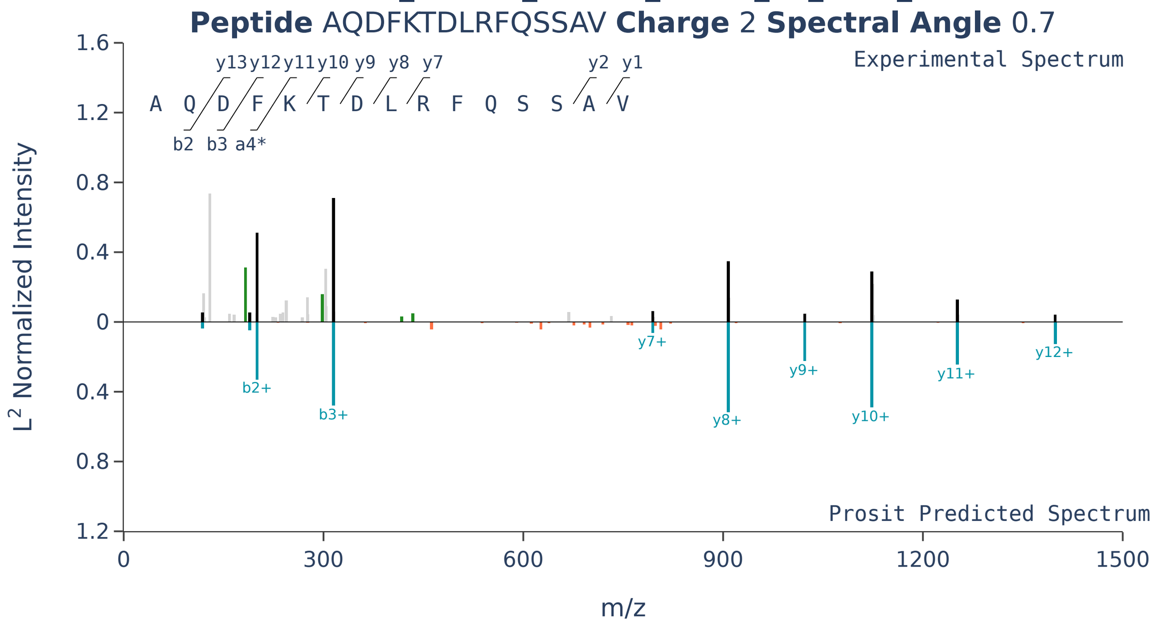

**Source** HRoetschke_271222_120123_HFGoe_H3_b1R1_24h **Scan** 8486
**Peptide** IQLARRIRGE **Charge** 3 **Spectral Angle** 0.81

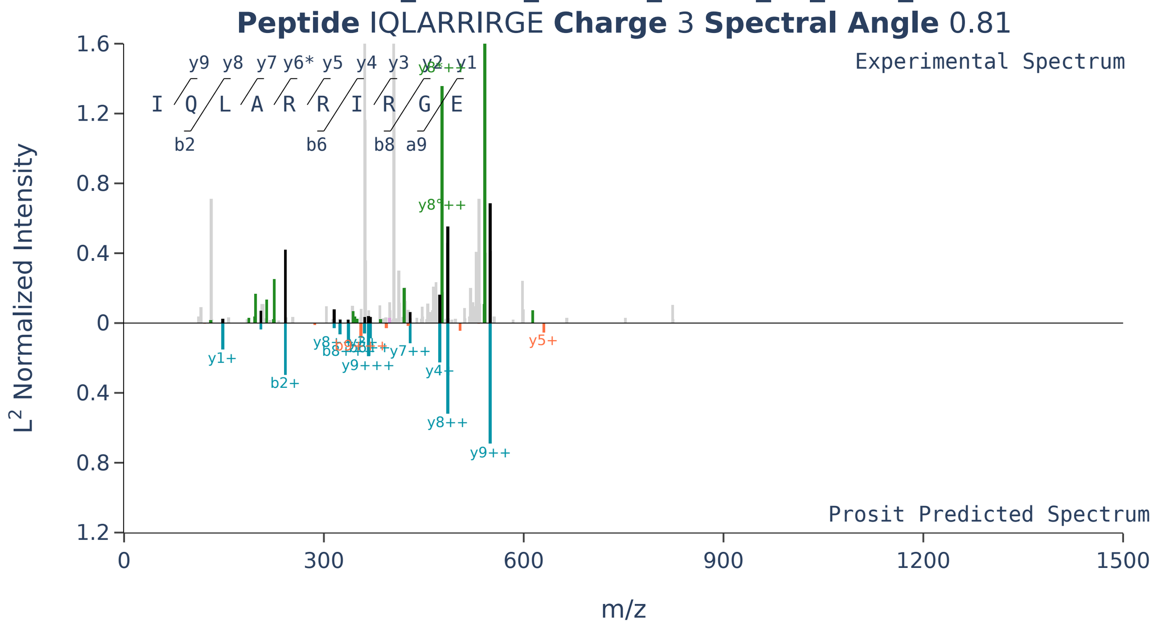


**Source** HRoetschke_271222_120123_HFGoe_H3_b2R2_2h **Scan** 17167
**Peptide** ALFEDTNLCAIHAKR **Charge** 4 **Spectral Angle** 0.72

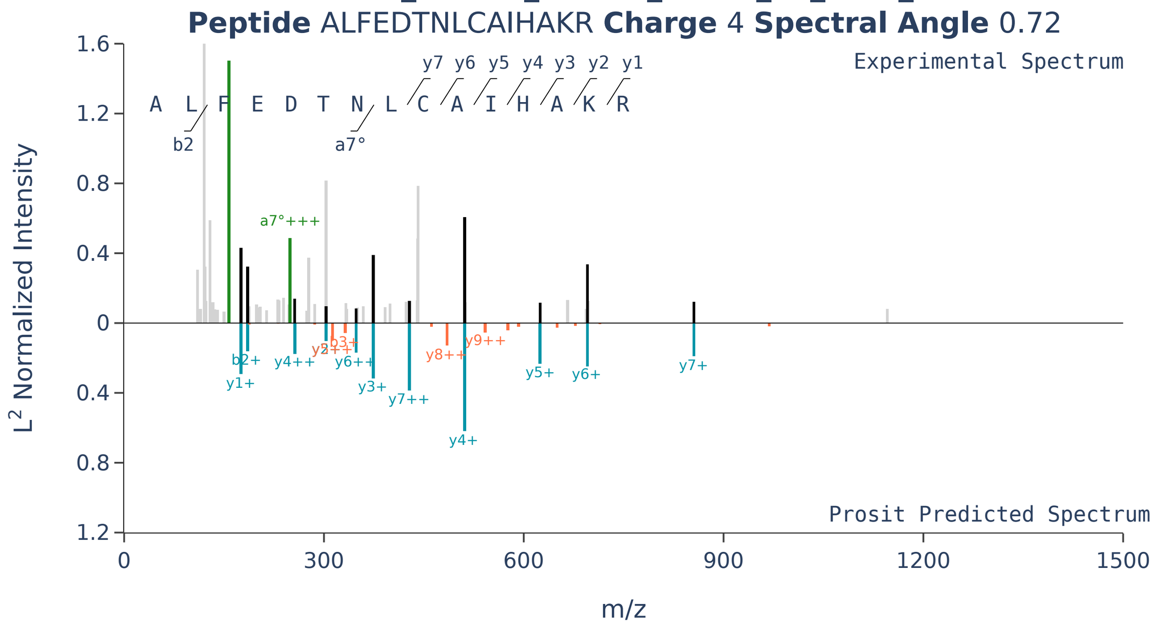


**Source** HRoetschke_271222_120123_HFGoe_H3_b2R1_1h **Scan** 27574
**Peptide** AYLVALFED **Charge** 1 **Spectral Angle** 0.94

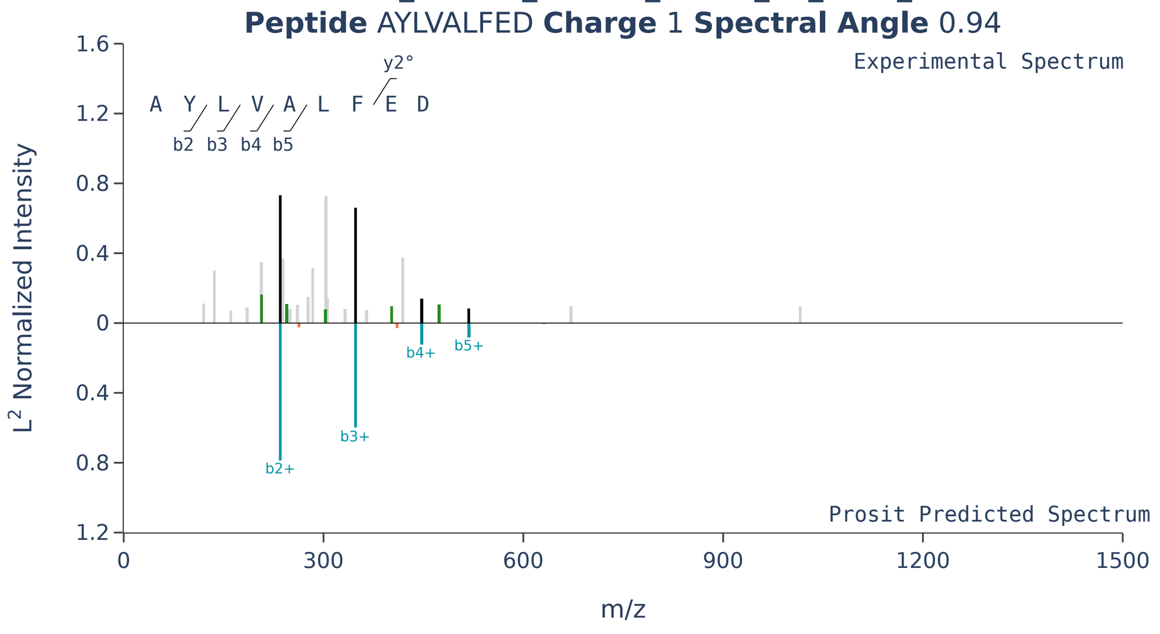


**Source** HRoetschke_271222_120123_HFGoe_H3_b1R1_24h **Scan** 7966
**Peptide** RPGTVALREIRR **Charge** 4 **Spectral Angle** 0.8

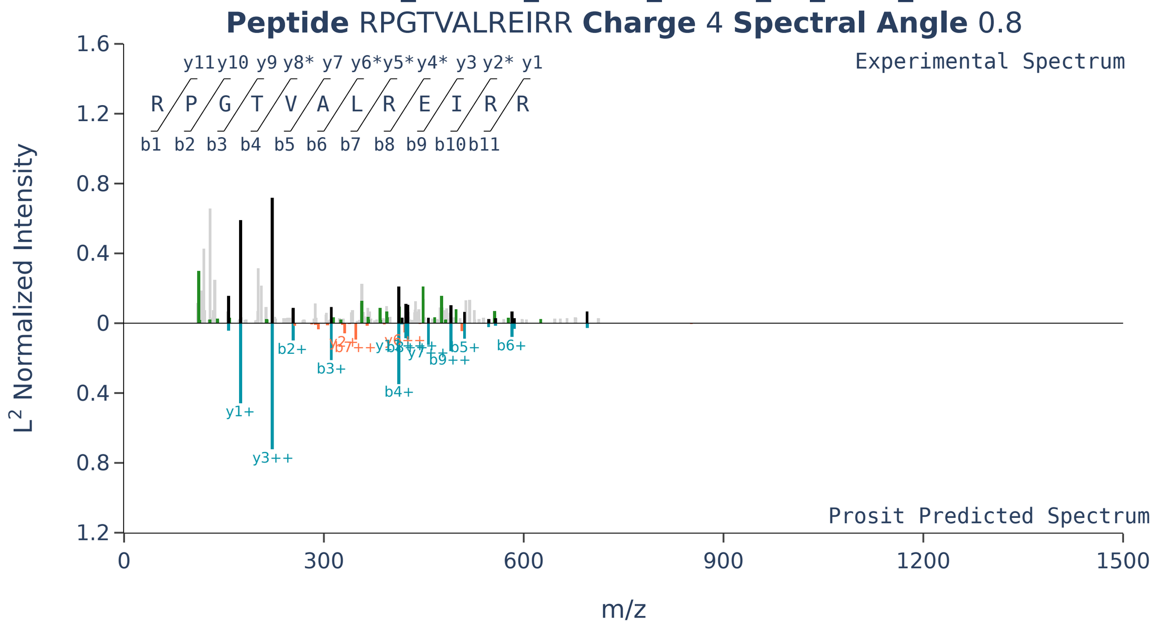


**Source** HRoetschke_271222_120123_HFGoe_H3_b2R2_2h **Scan** 10027
**Peptide** VALREIRRY **Charge** 3 **Spectral Angle** 0.76

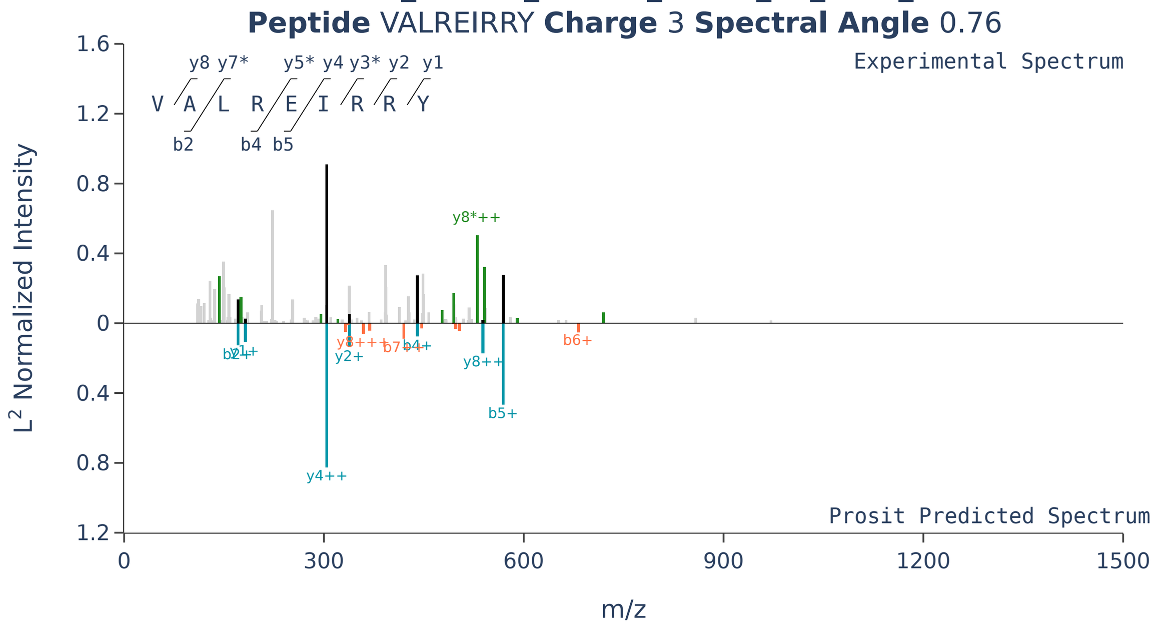


**Source** HRoetschke_271222_120123_HFGoe_H3_b2R2_24h **Scan** 13086
**Peptide** QKSTELLLR **Charge** 3 **Spectral Angle** 0.91

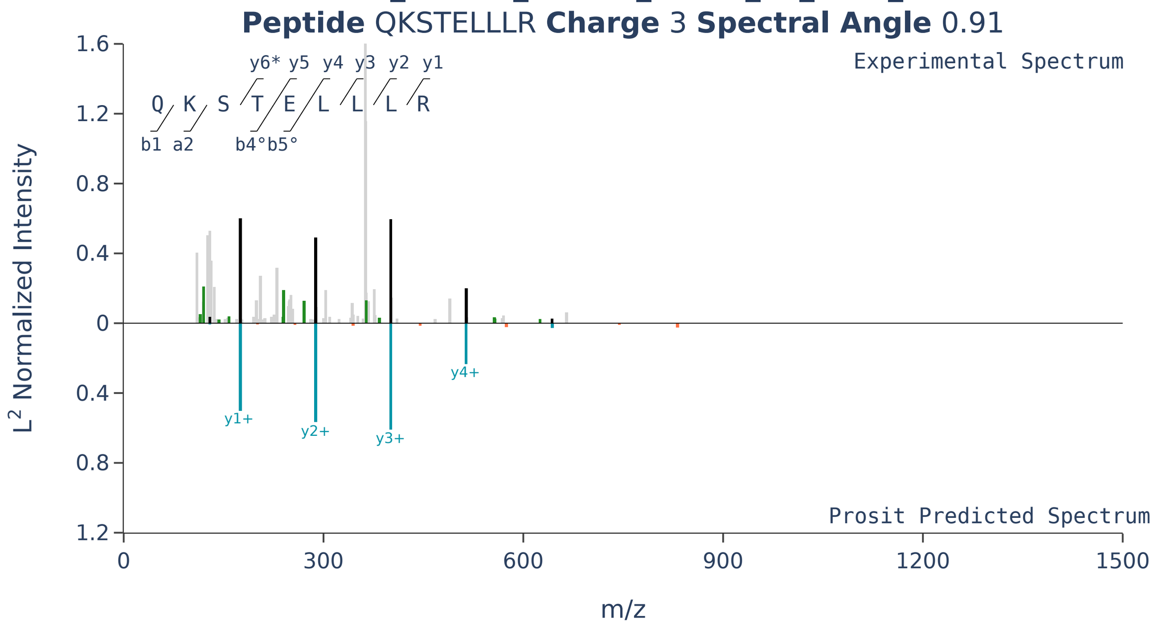


**Source** HRoetschke_271222_120123_HFGoe_H3_b1R1_4h **Scan** 8677
**Peptide** PKDIQLARRIRGE **Charge** 4 **Spectral Angle** 0.69

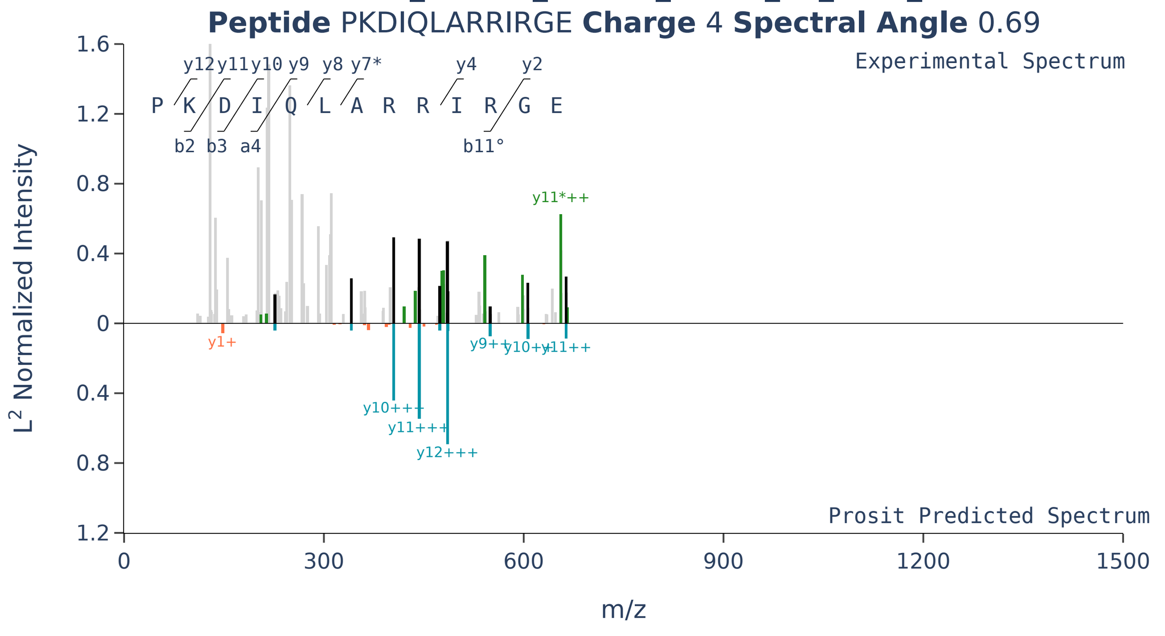


**Source** HRoetschke_271222_120123_HFGoe_H3_b1R1_4h **Scan** 16557
**Peptide** KRVTIMPKDIQLA **Charge** 3 **Spectral Angle** 0.64

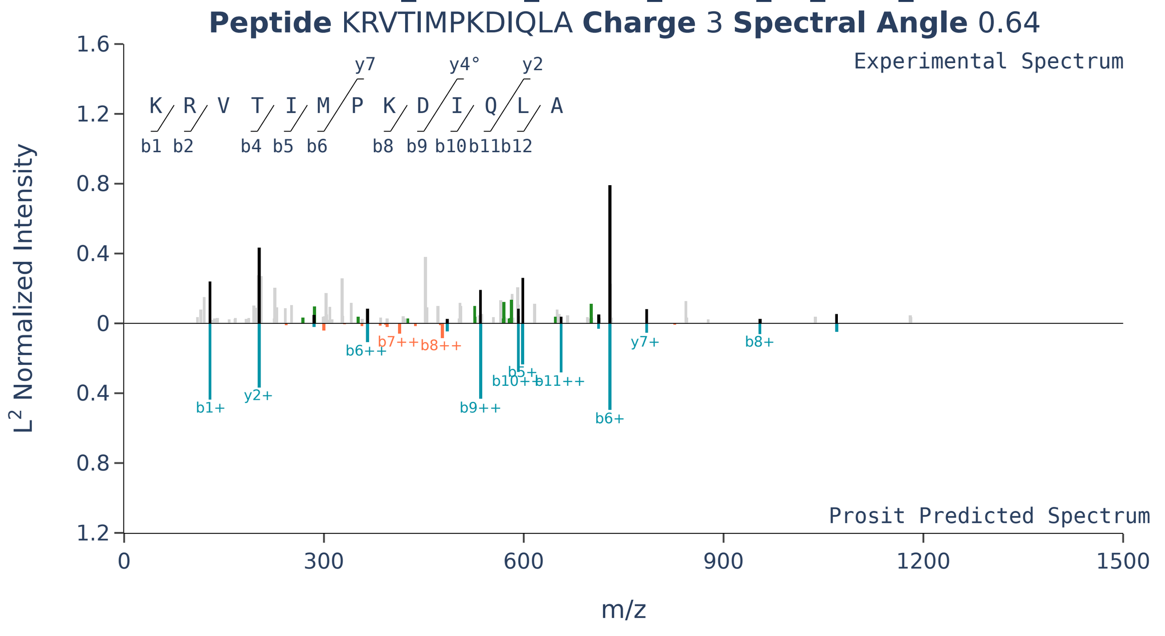


**Source** HRoetschke_271222_120123_HFGoe_H3_b2R1_1h **Scan** 11362
**Peptide** TIMPKDIQLARRIRGERA **Charge** 5 **Spectral Angle** 0.78

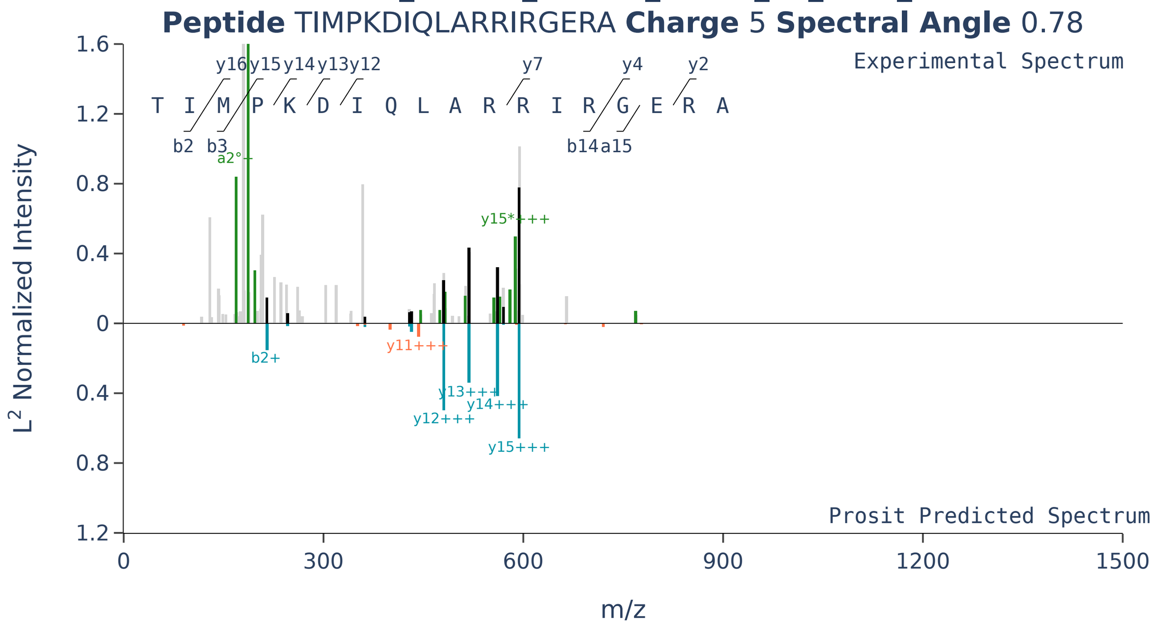


**Source** HRoetschke_271222_120123_HFGoe_H3_b1R2_4h **Scan** 6861
**Peptide** ALREIRRYQ **Charge** 3 **Spectral Angle** 0.82

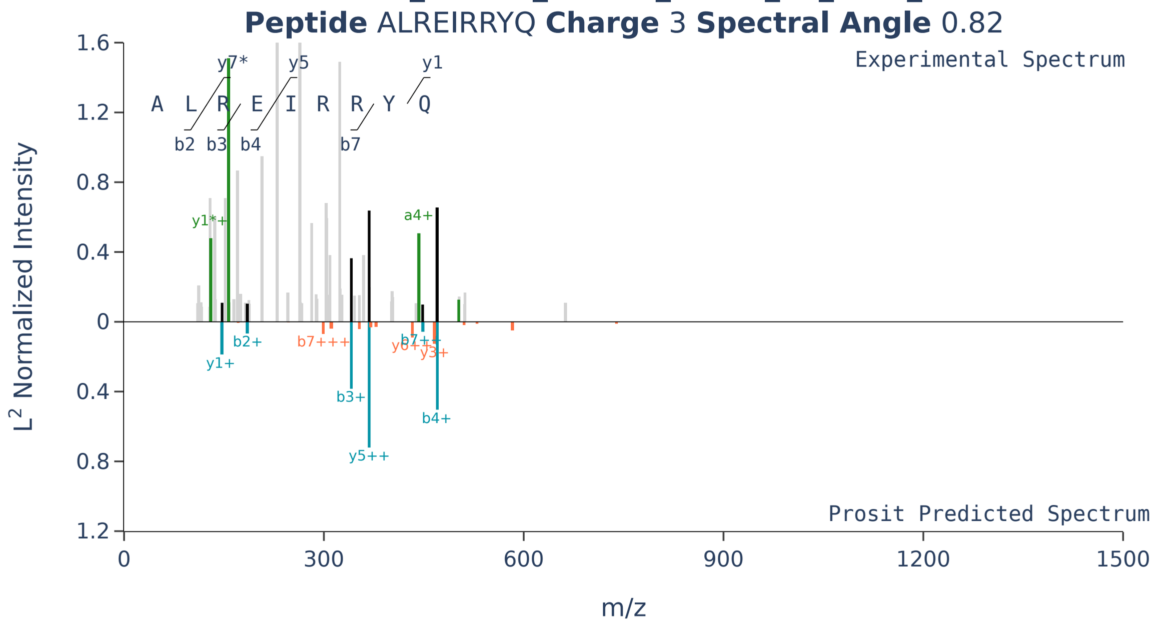


**Source** HRoetschke_271222_120123_HFGoe_H3_b1R1_24h **Scan** 6036
**Peptide** REIRRYQKSTEL **Charge** 4 **Spectral Angle** 0.78

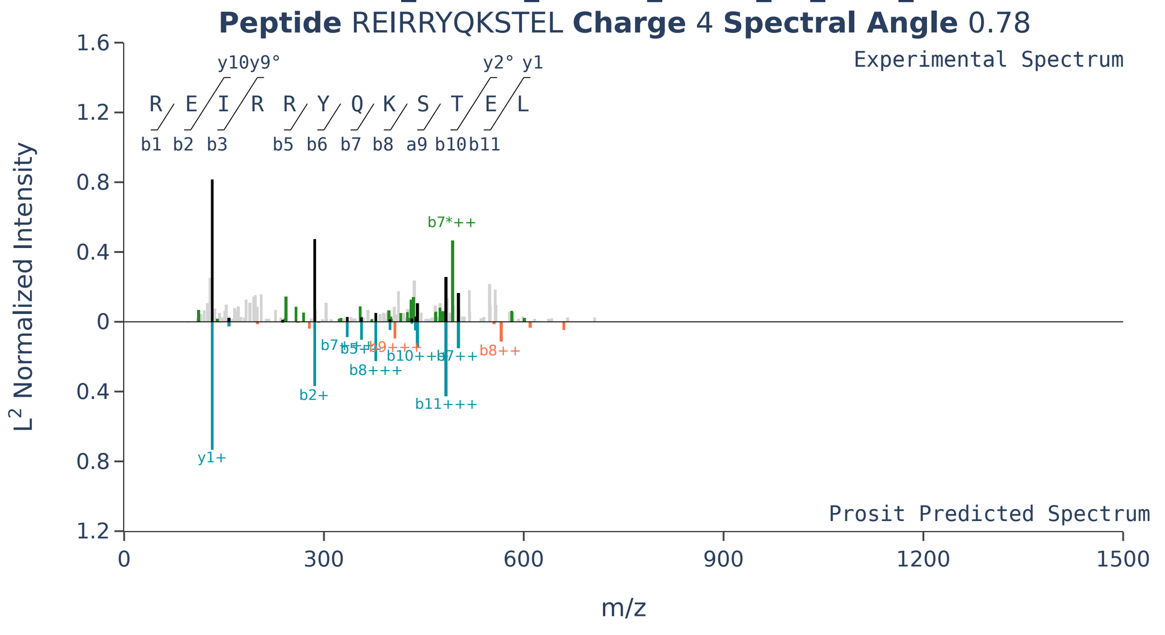


**Source** HRoetschke_271222_120123_HFGoe_H3_b2R2_4h **Scan** 24149
**Peptide** ALFEDTNL **Charge** 1 **Spectral Angle** 0.96

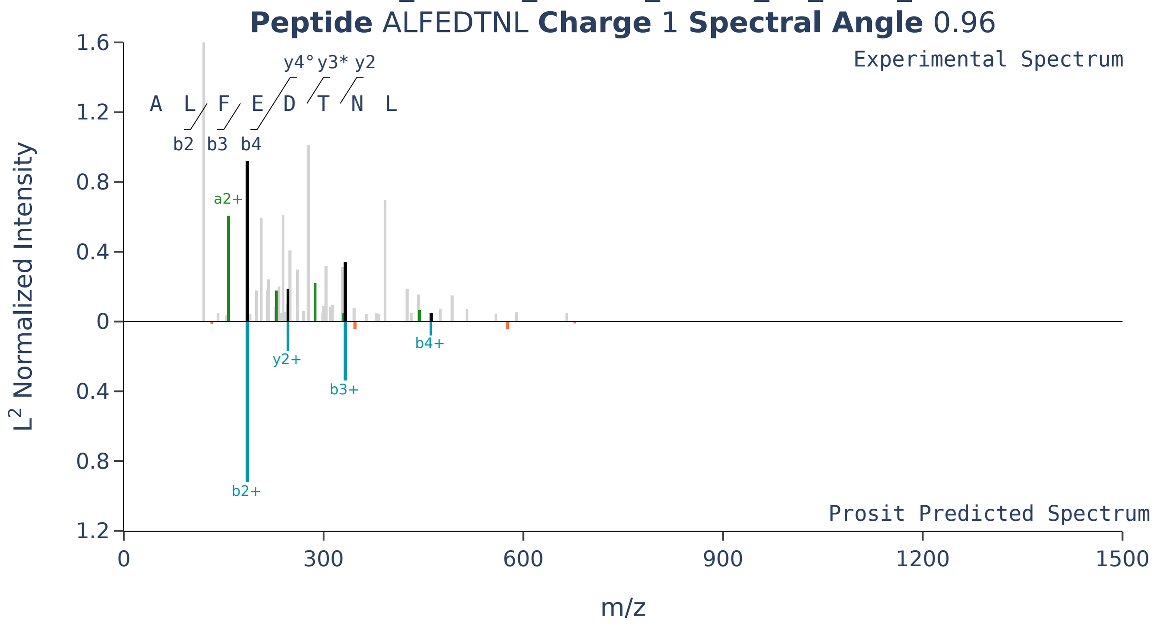


**Source** HRoetschke_271222_120123_HFGoe_H3_b1R1_4h **Scan** 8603
**Peptide** RRYQKSTELLLR **Charge** 4 **Spectral Angle** 0.84

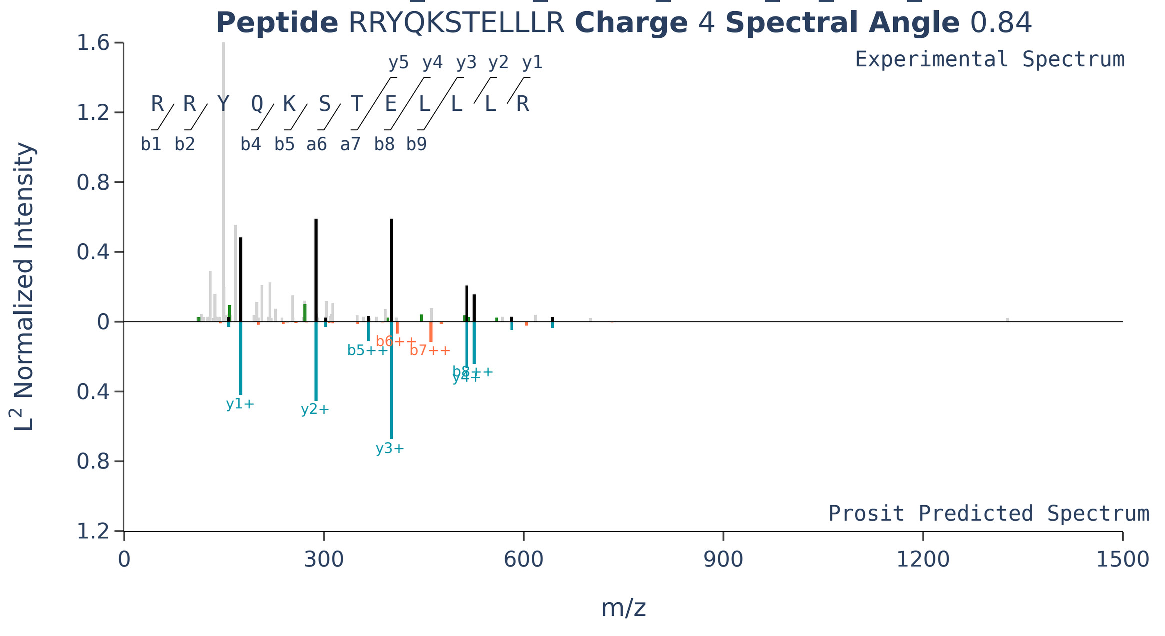


**Source** HRoetschke_271222_120123_HFGoe_H3_b2R2_2h **Scan** 19724
**Peptide** KLPFQRLV **Charge** 2 **Spectral Angle** 0.74

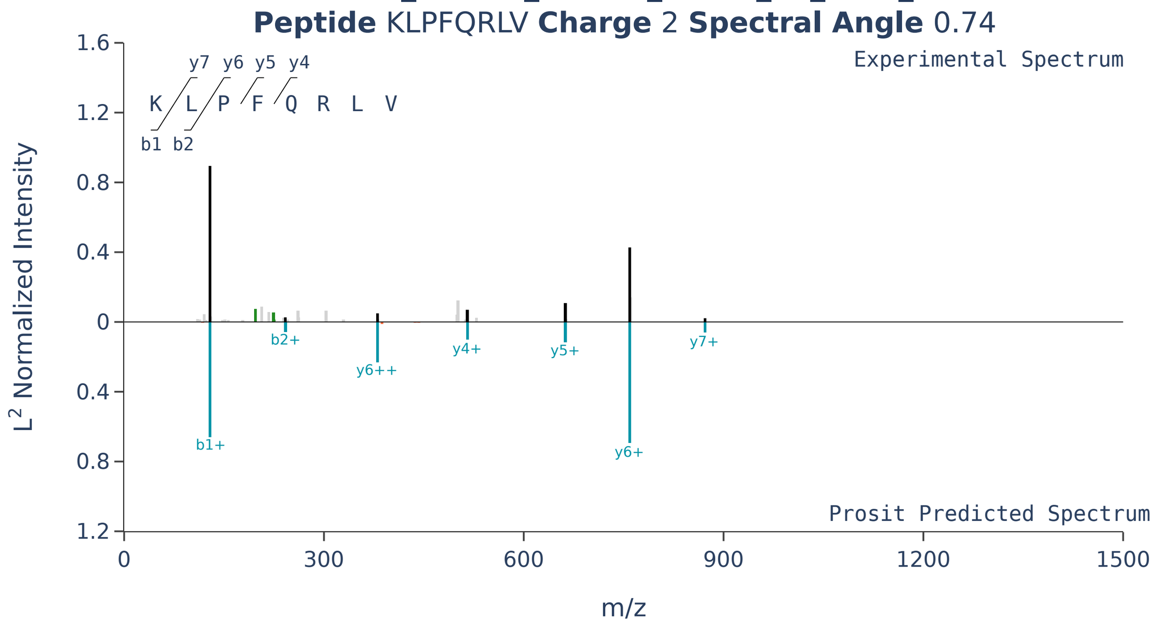


**Source** HRoetschke_271222_120123_HFGoe_H3_b1R1_4h **Scan** 18345
**Peptide** IAQDFKTDLRFQSSAVM **Charge** 3 **Spectral Angle** 0.92

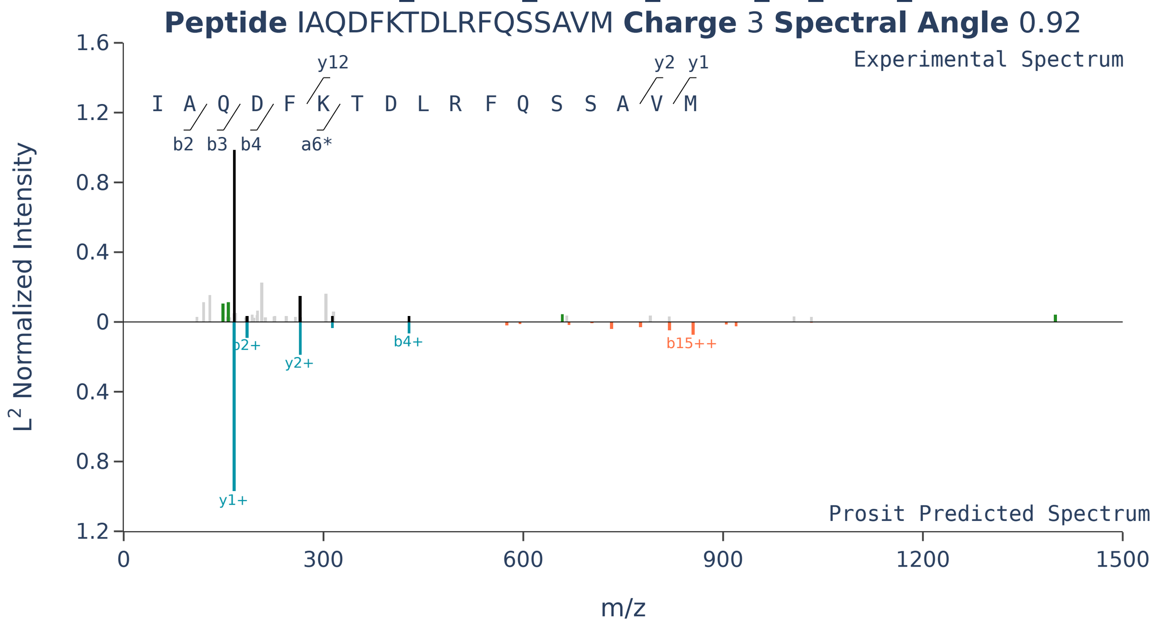

**Source** HRoetschke_271222_120123_HFGoe_H3_b1R1_24h **Scan** 7557
**Peptide** AIHAKRVTI **Charge** 2 **Spectral Angle** 0.8

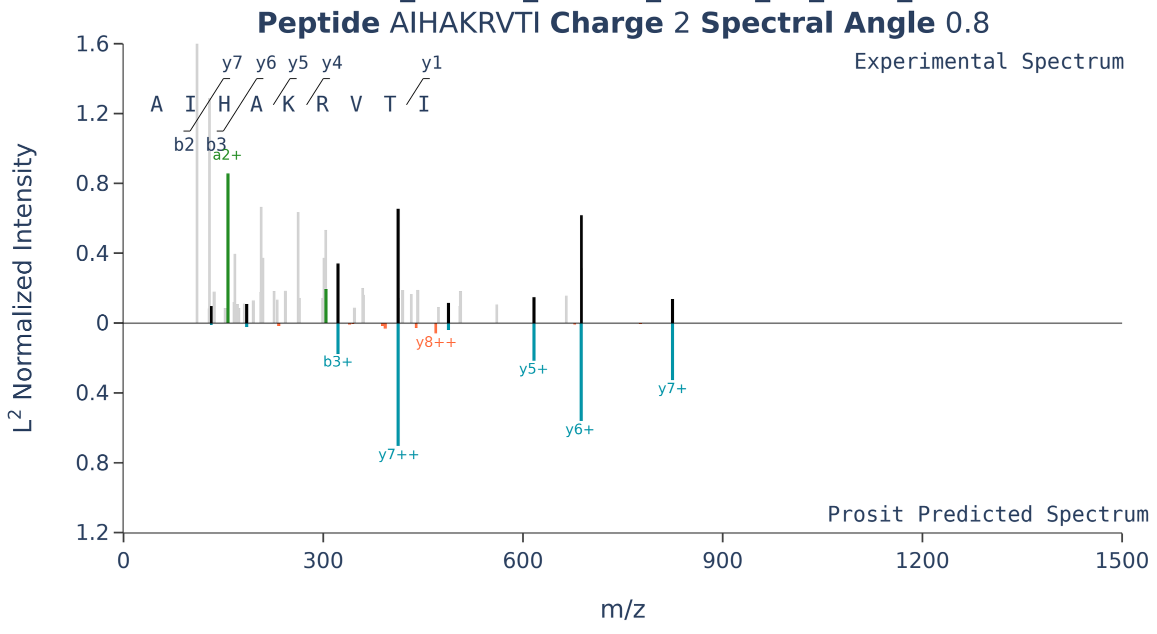


**Source** HRoetschke_271222_120123_HFGoe_H3_b1R2_4h **Scan** 15537
**Peptide** DFKTDLRFQSSA **Charge** 2 **Spectral Angle** 0.74

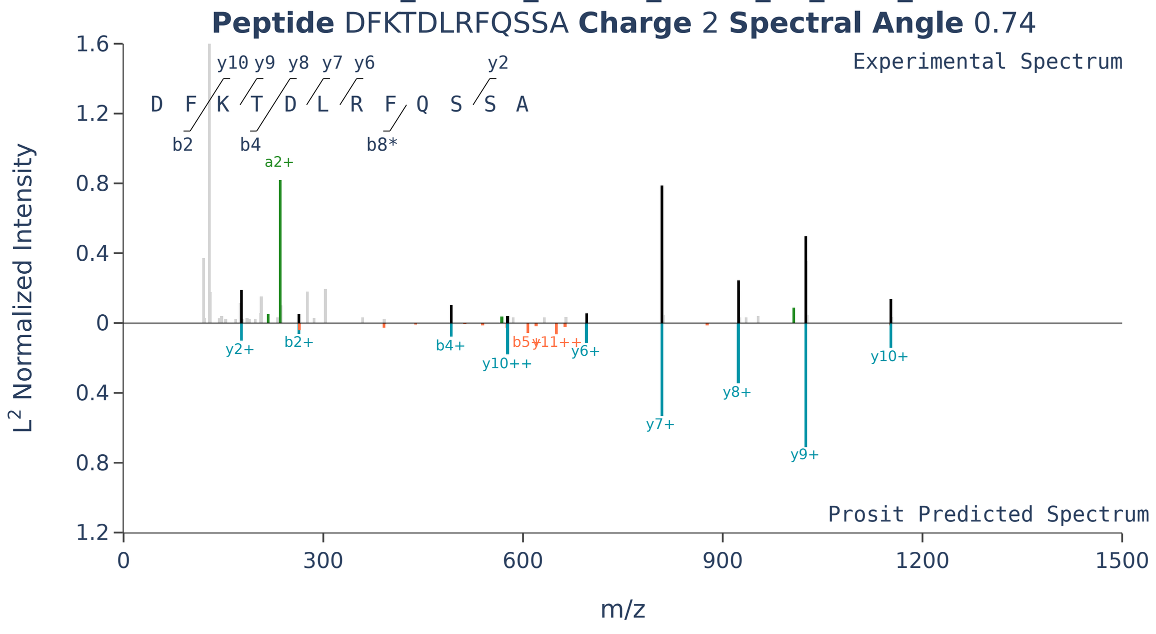


**Source** HRoetschke_271222_120123_HFGoe_H3_b2R1_4h **Scan** 12015
**Peptide** DIQLARRIR **Charge** 3 **Spectral Angle** 0.8

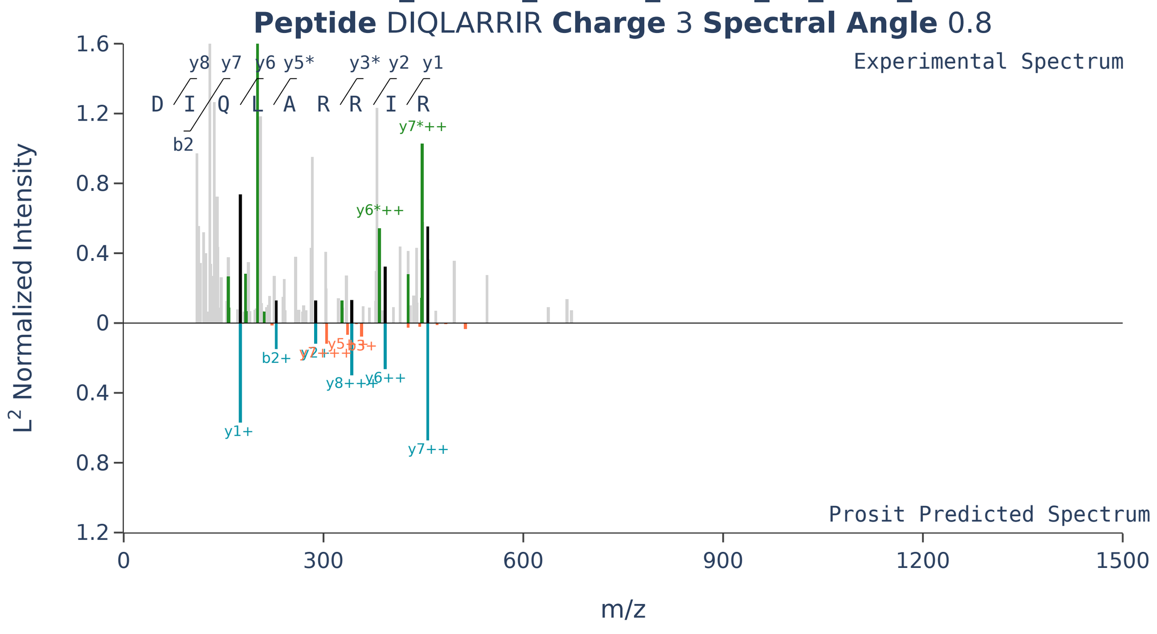


**Source** HRoetschke_271222_120123_HFGoe_H3_b2R1_24h **Scan** 12830
**Peptide** RYRPGTVAL **Charge** 2 **Spectral Angle** 0.61

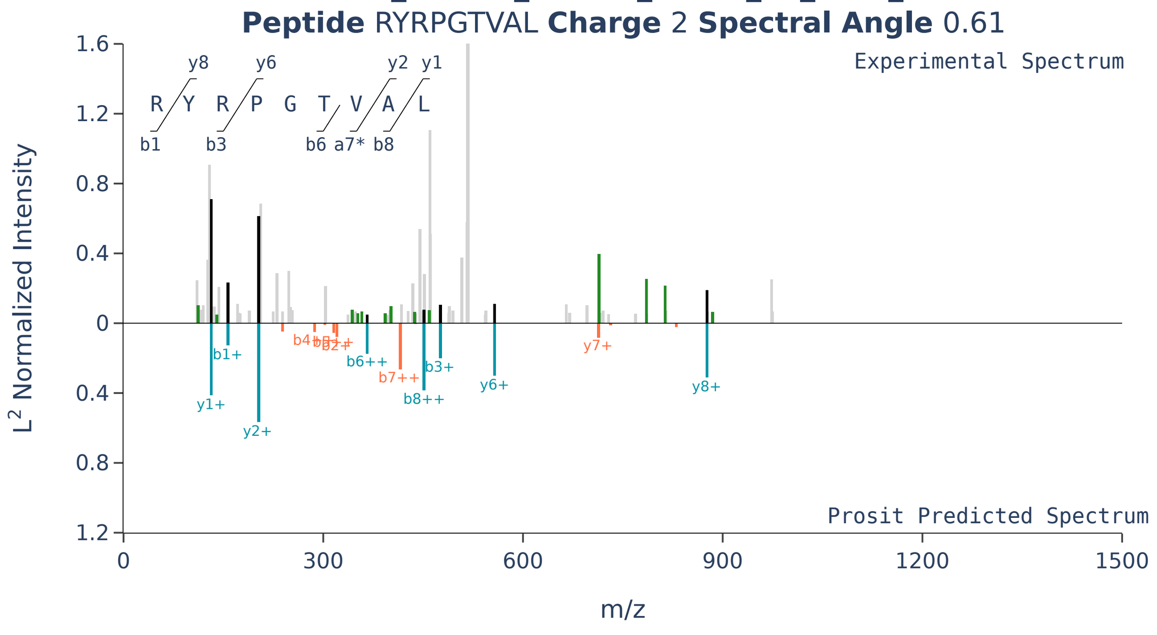


**Source** HRoetschke_271222_120123_HFGoe_H3_b1R2_1h **Scan** 15894
**Peptide** FEDTNLCAIHA **Charge** 2 **Spectral Angle** 0.76

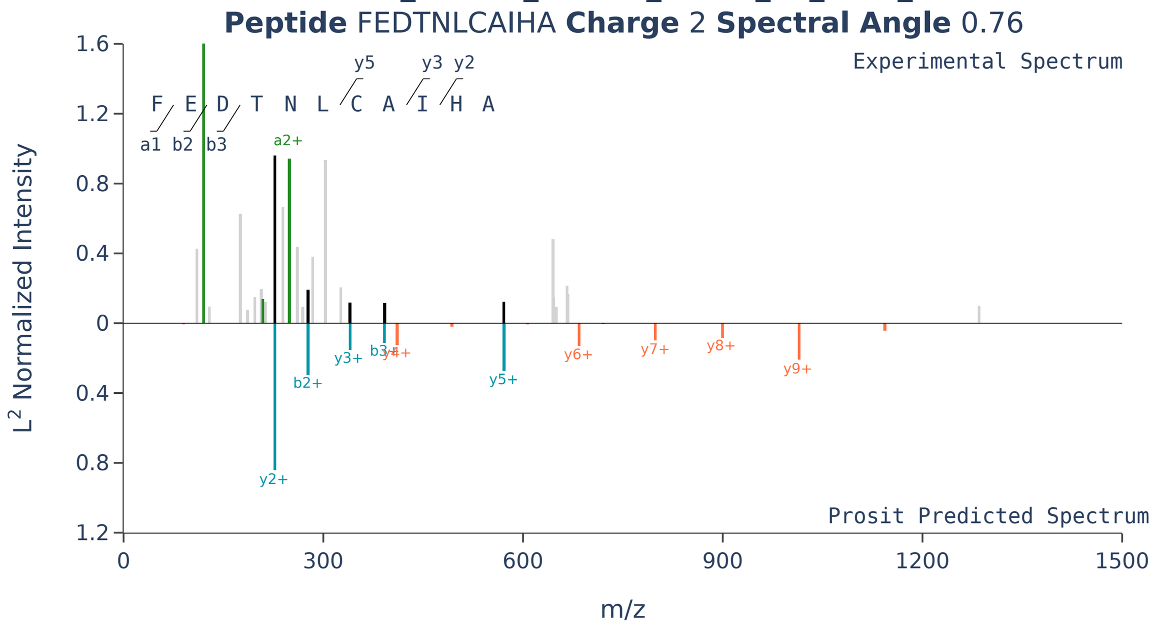


**Source** HRoetschke_271222_120123_HFGoe_H3_b2R1_1h **Scan** 7684
**Peptide** IQLARRIRG **Charge** 3 **Spectral Angle** 0.67

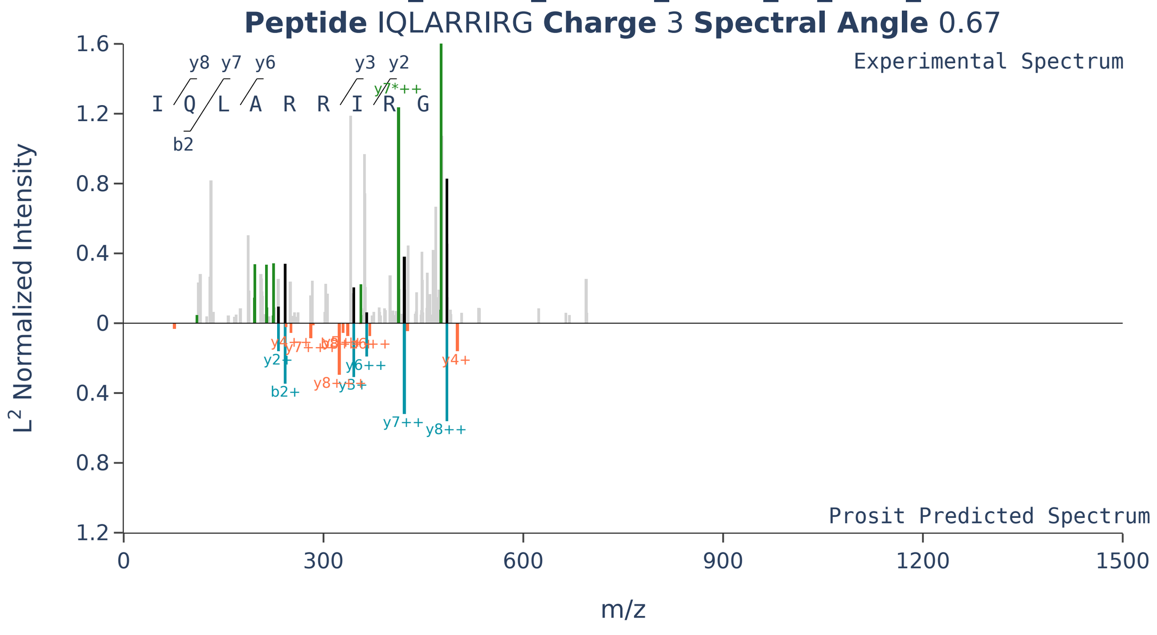


**Source** HRoetschke_271222_120123_HFGoe_H3_b2R1_1h **Scan** 15678
**Peptide** IAQDFKTDLRFQSS **Charge** 2 **Spectral Angle** 0.77

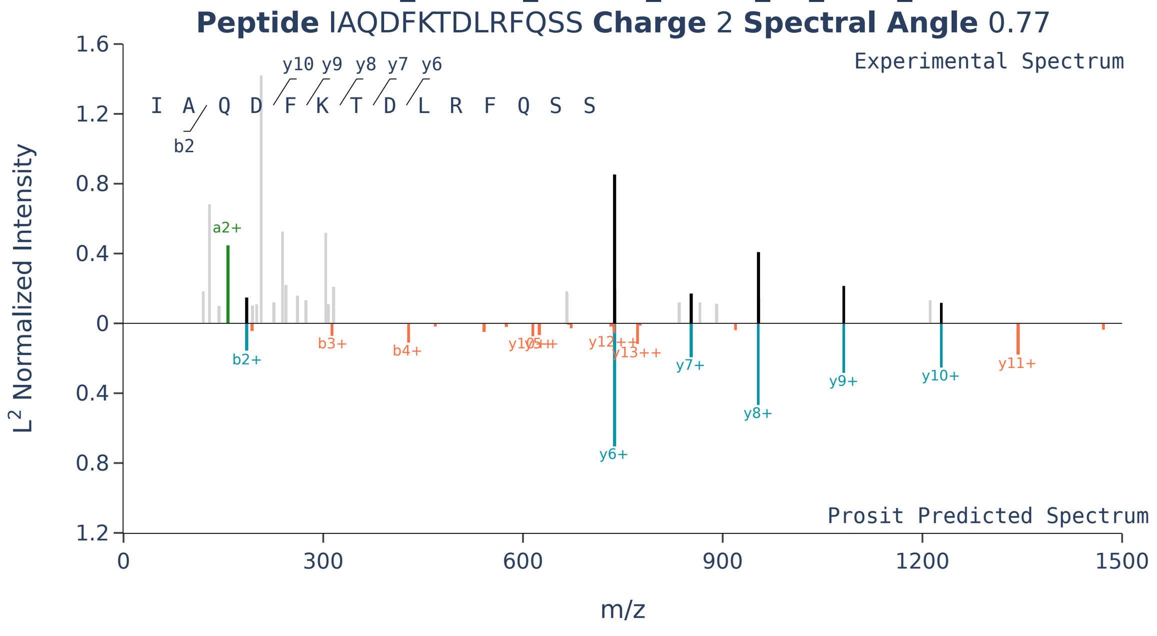


**Source** HRoetschke_271222_120123_HFGoe_H3_b2R1_1h **Scan** 16394
**Peptide** AQDFKTDLRFQSSAVMA **Charge** 3 **Spectral Angle** 0.74

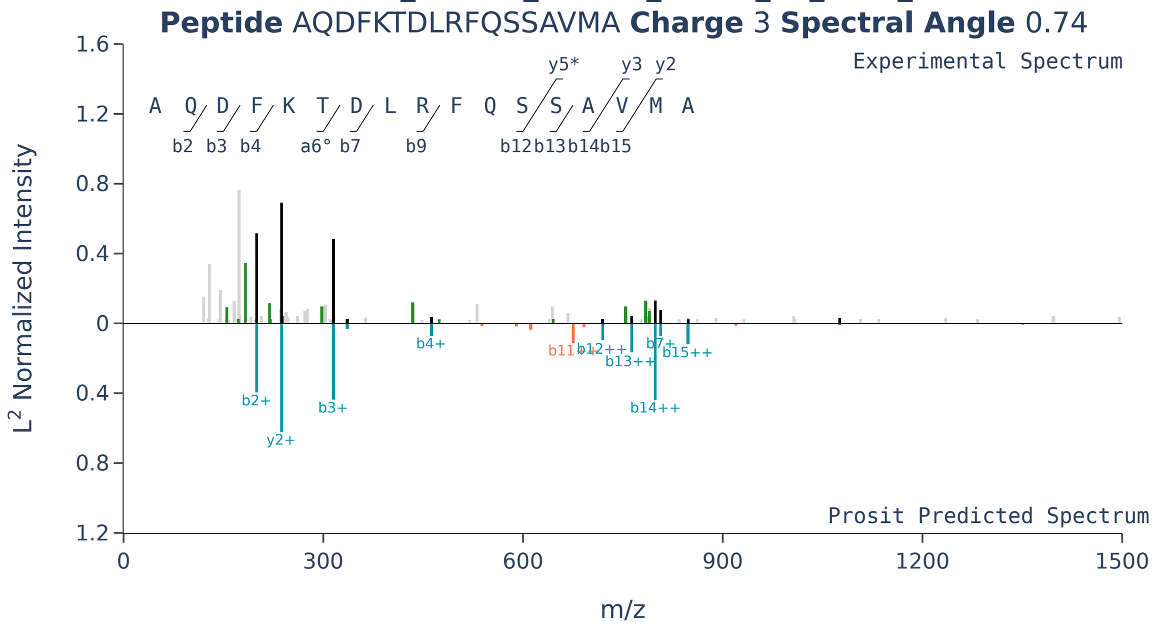


**Source** HRoetschke_271222_120123_HFGoe_H3_b1R1_4h **Scan** 17032
**Peptide** REIAQDFKTDLRFQS **Charge** 3 **Spectral Angle** 0.85

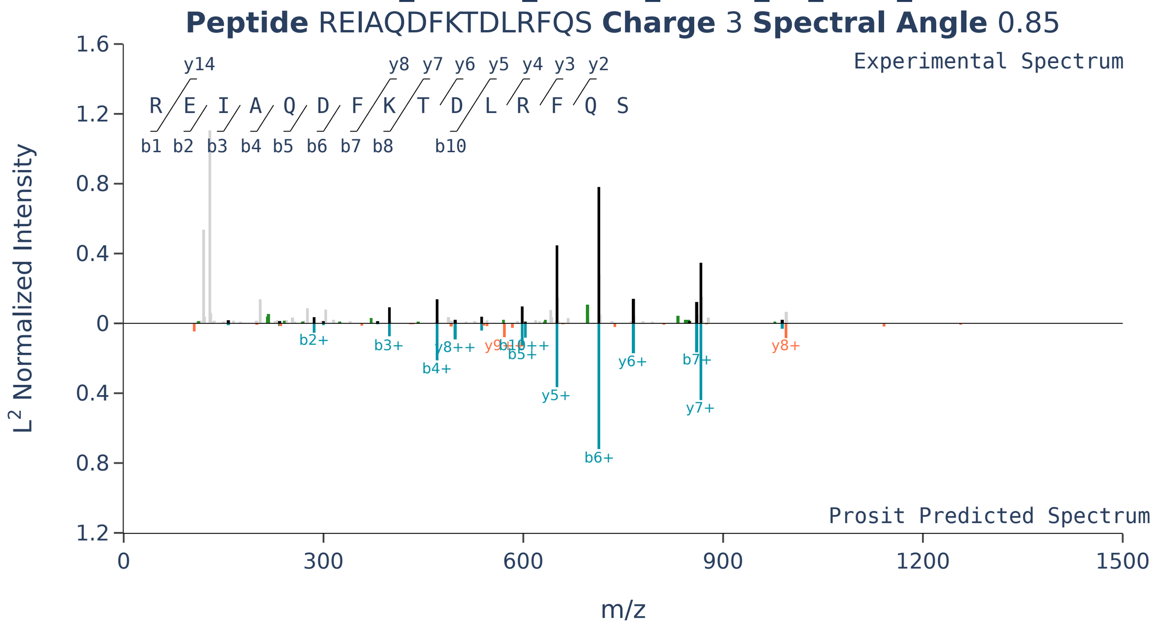


**Source** HRoetschke_271222_120123_HFGoe_H3_b2R1_1h **Scan** 7675
**Peptide** PKDIQLAR **Charge** 2 **Spectral Angle** 0.58

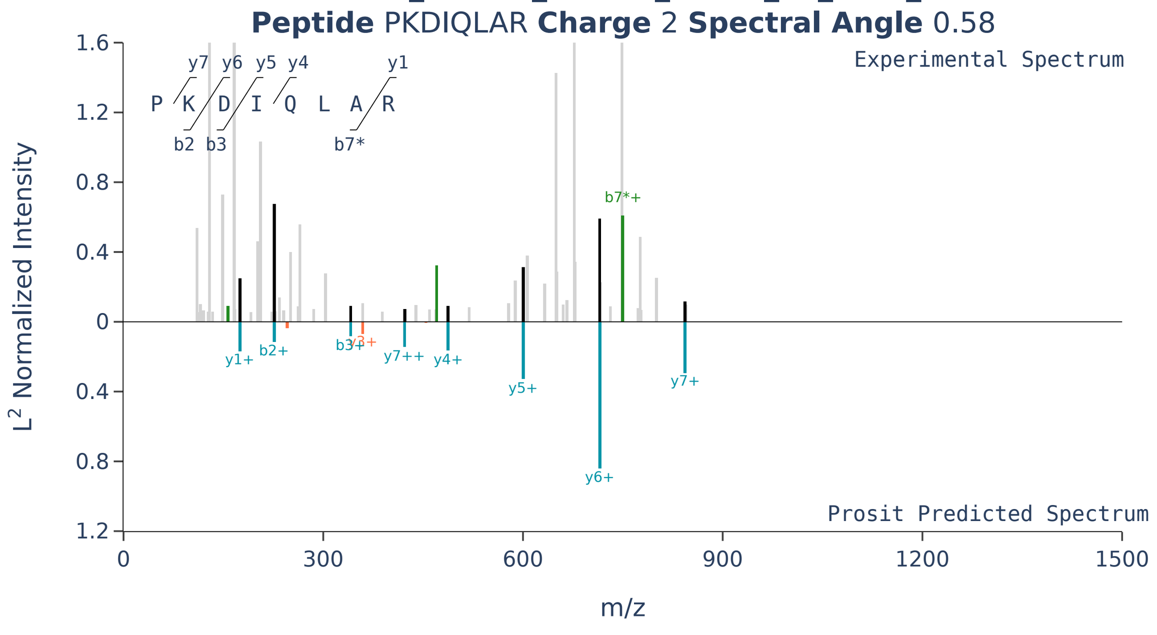


**Source** HRoetschke_271222_120123_HFGoe_H3_b1R1_4h **Scan** 16874
**Peptide** REIAQDFKTDLRFQSSA **Charge** 3 **Spectral Angle** 0.73

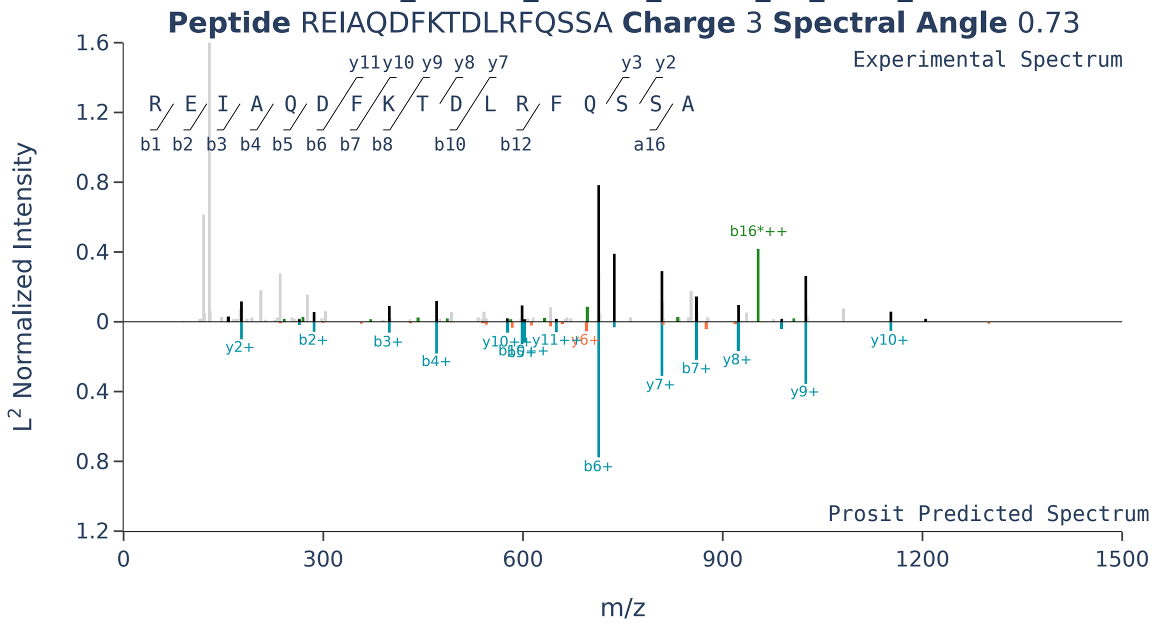


**Source** HRoetschke_271222_120123_HFGoe_H3_b1R1_24h **Scan** 16845
**Peptide** LMPKDIQL **Charge** 2 **Spectral Angle** 0.67

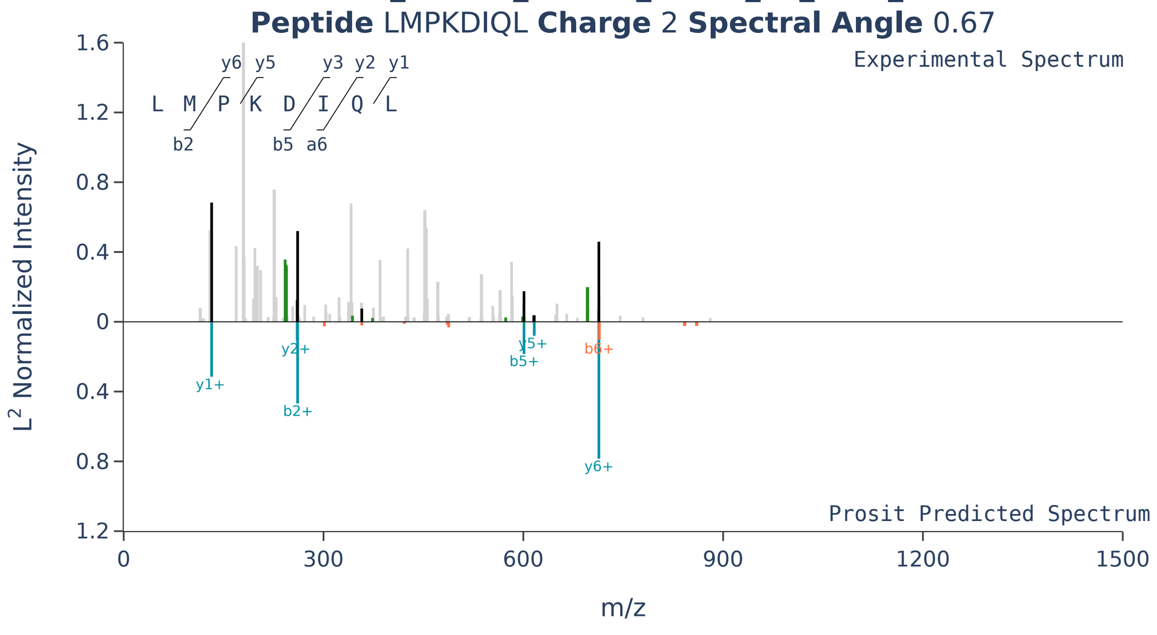


**Source** HRoetschke_271222_120123_HFGoe_H3_b1R1_24h **Scan** 18499
**Peptide** QKSTELLI **Charge** 2 **Spectral Angle** 0.8

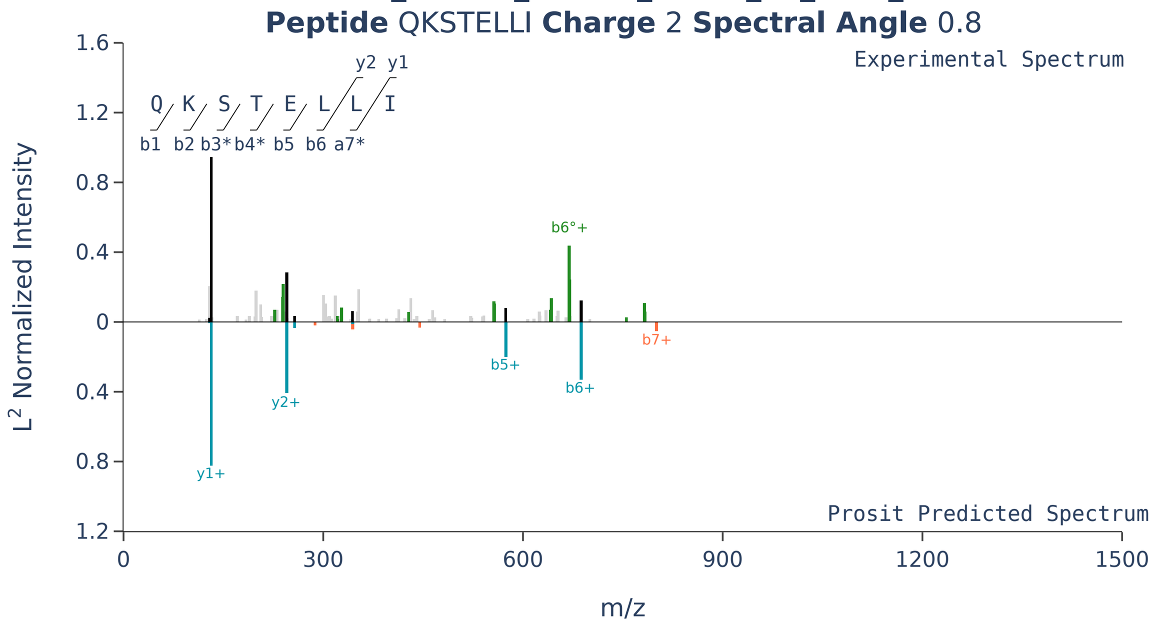


**Source** HRoetschke_271222_120123_HFGoe_H3_b2R2_4h **Scan** 9214
**Peptide** ALHAKRVTIMPKD **Charge** 4 **Spectral Angle** 0.71

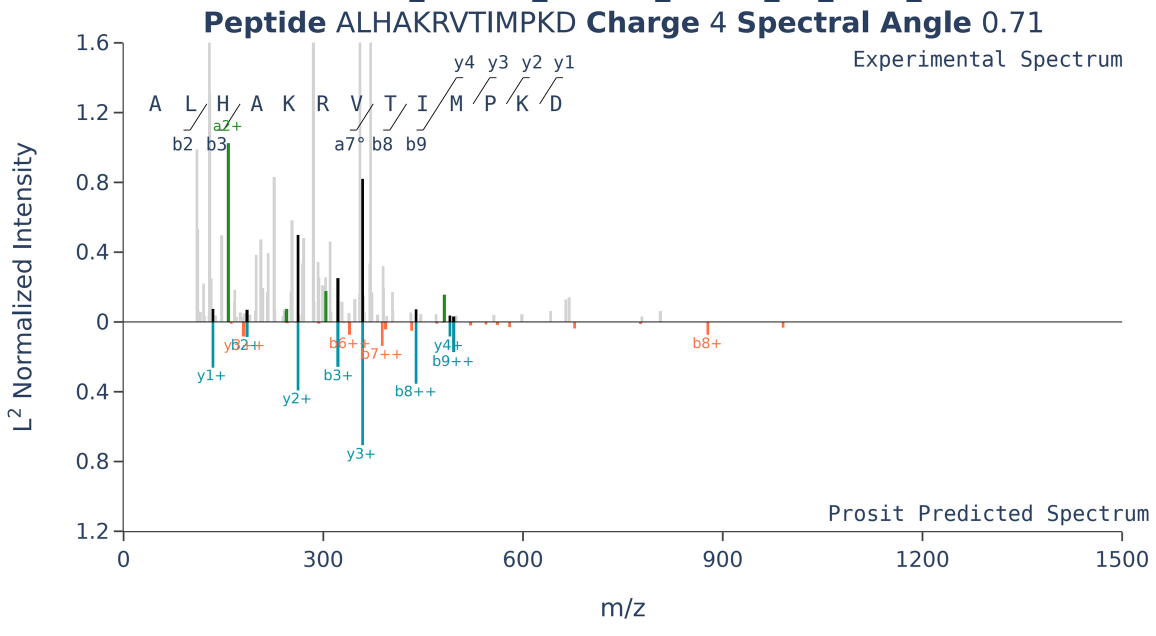


**Source** HRoetschke_271222_120123_HFGoe_H3_b1R1_24h **Scan** 17084
**Peptide** IMPKDIQL **Charge** 2 **Spectral Angle** 0.63

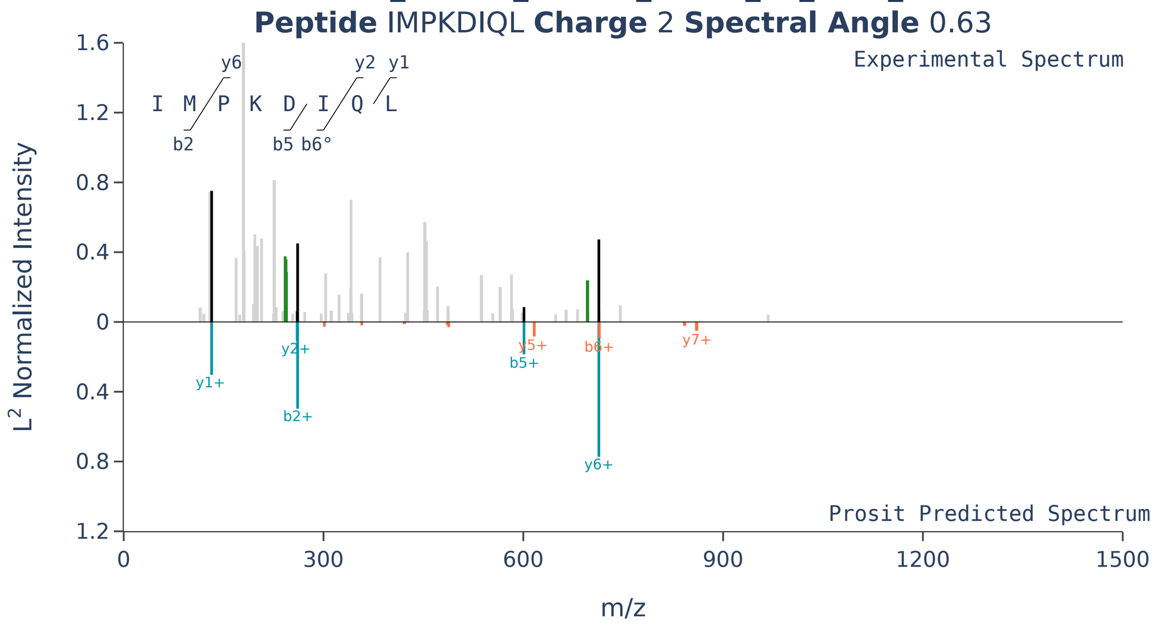

**Source** HRoetschke_271222_120123_HFGoe_H3_b2R2_4h **Scan** 14118
**Peptide** KRVTIMPKDIQLARRL **Charge** 5 **Spectral Angle** 0.72

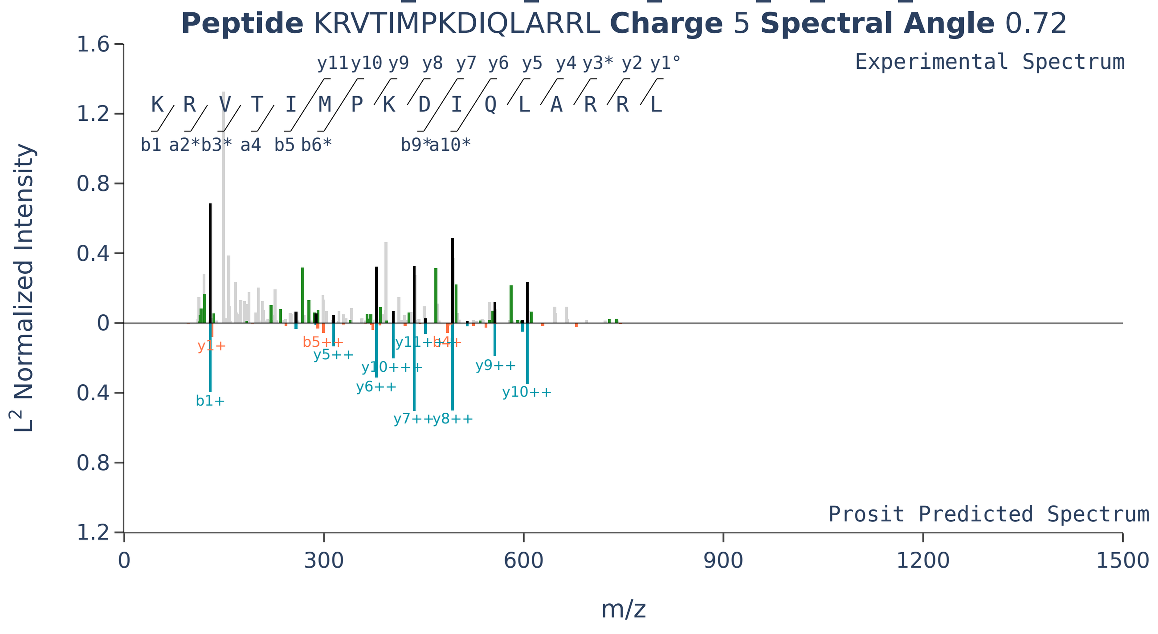

**Source** HRoetschke_271222_120123_HFGoe_H3_b2R2_2h **Scan** 11801
**Peptide** MPKDIQLARRI **Charge** 3 **Spectral Angle** 0.76

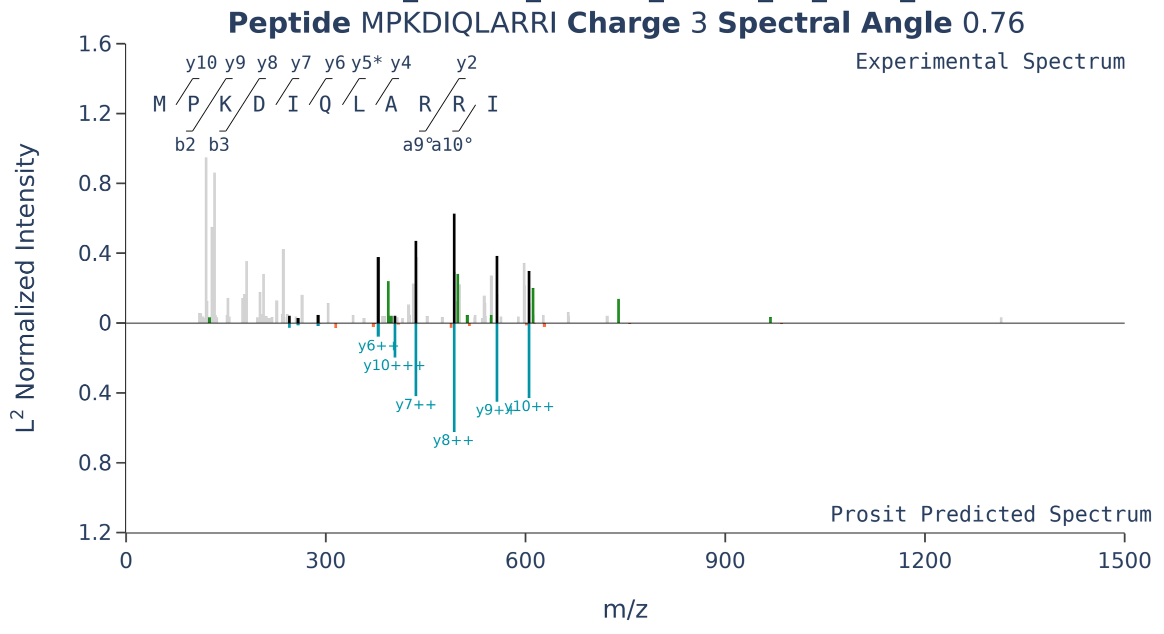

**Source** HRoetschke_271222_120123_HFGoe_H3_b2R2_4h **Scan** 11225
**Peptide** PKDIQLARRI **Charge** 3 **Spectral Angle** 0.68

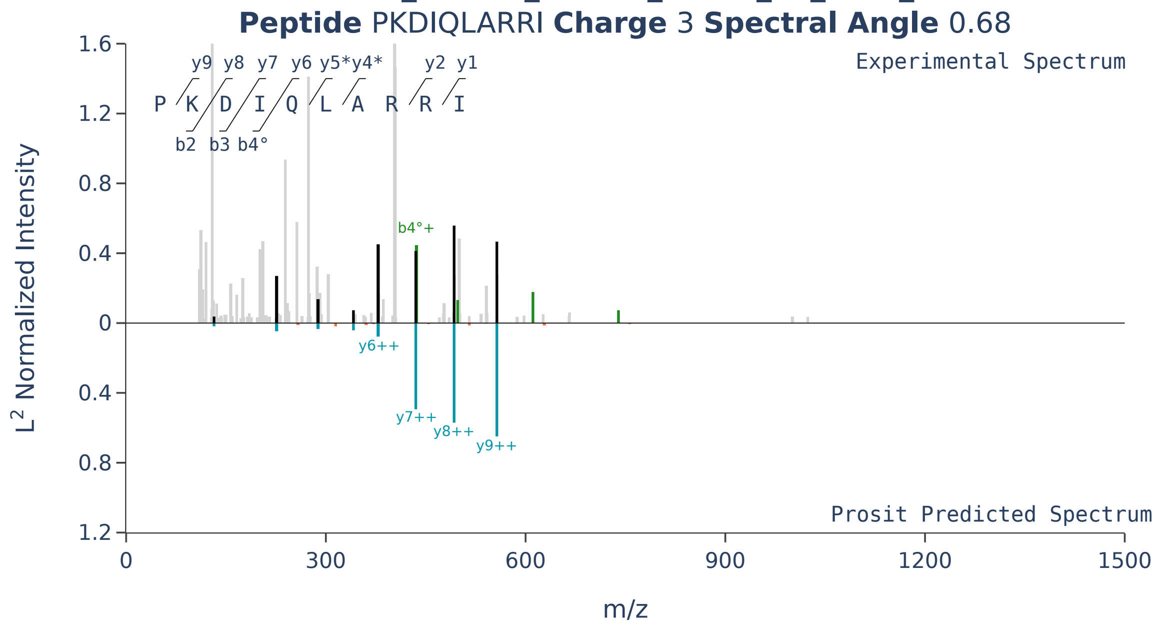

**Source** HRoetschke_271222_120123_HFGoe_H3_b1R1_1h **Scan** 12509
**Peptide** TIMPKDIQ **Charge** 2 **Spectral Angle** 0.74

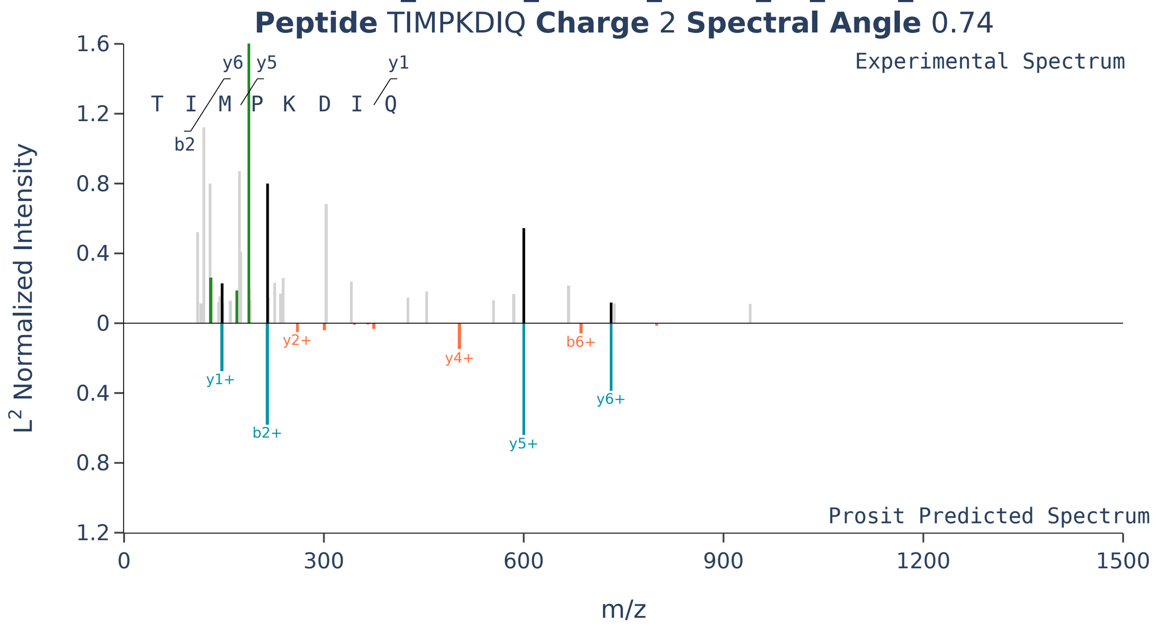

**Source** HRoetschke_271222_120123_HFGoe_H3_b2R1_1h **Scan** 18033
**Peptide** EIAQDFKTDLRFQ **Charge** 3 **Spectral Angle** 0.75

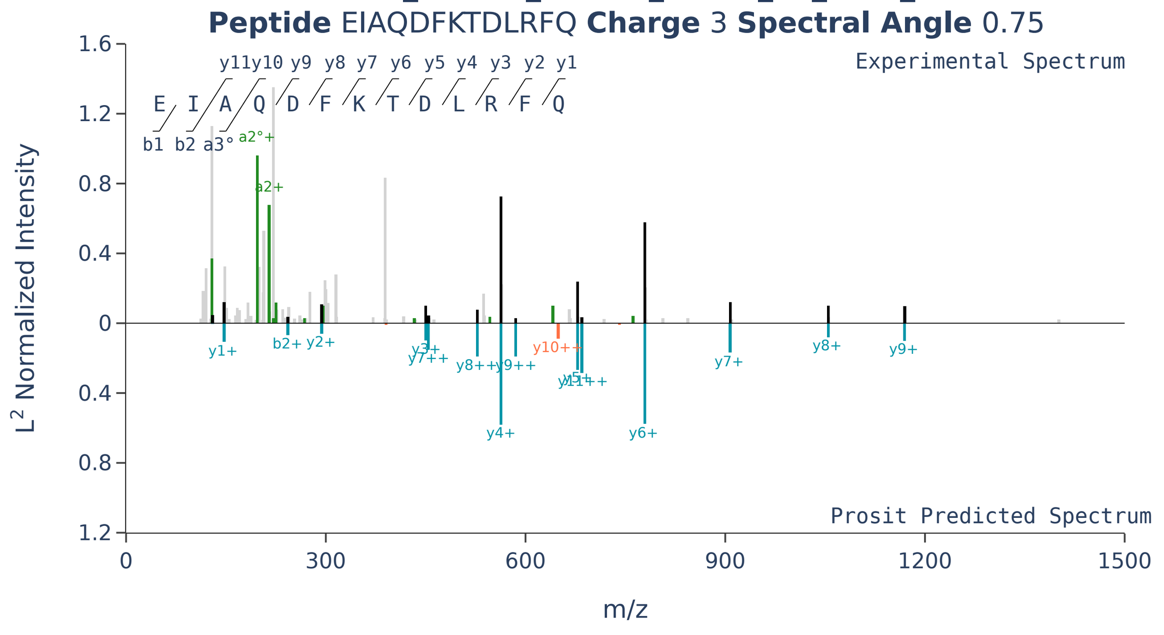

**Source** HRoetschke_271222_120123_HFGoe_H3_b1R2_24h **Scan** 10550
**Peptide** YQKSTELLIRK **Charge** 3 **Spectral Angle** 0.81

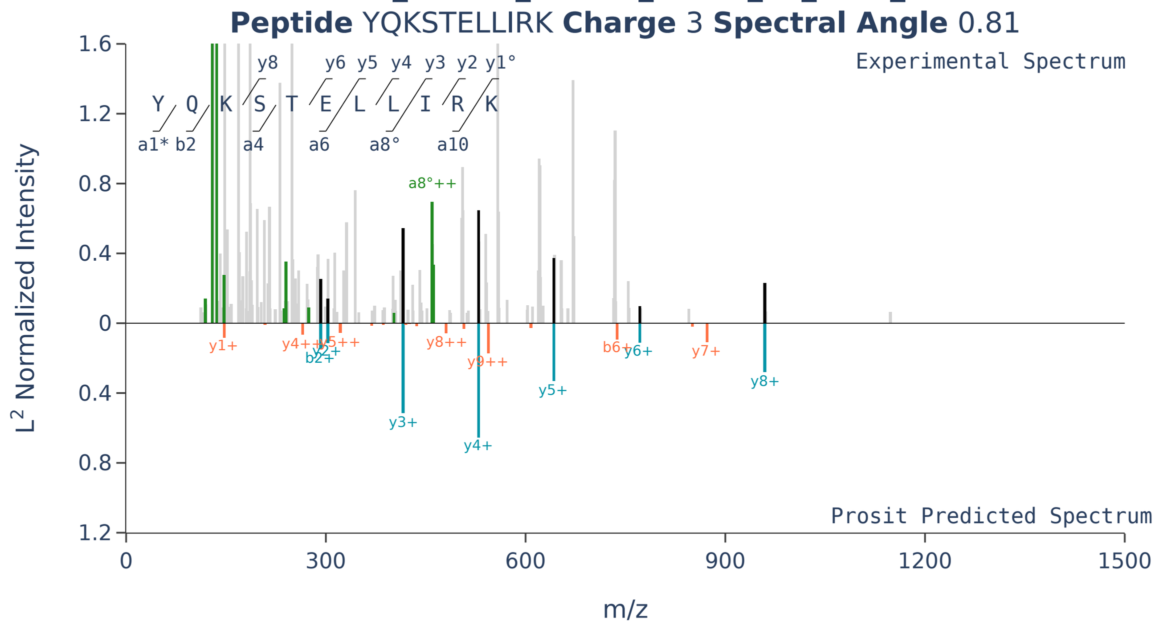

**Source** HRoetschke_271222_120123_HFGoe_H3_b2R2_4h **Scan** 5193
**Peptide** LATKAARKSAPAT **Charge** 3 **Spectral Angle** 0.71

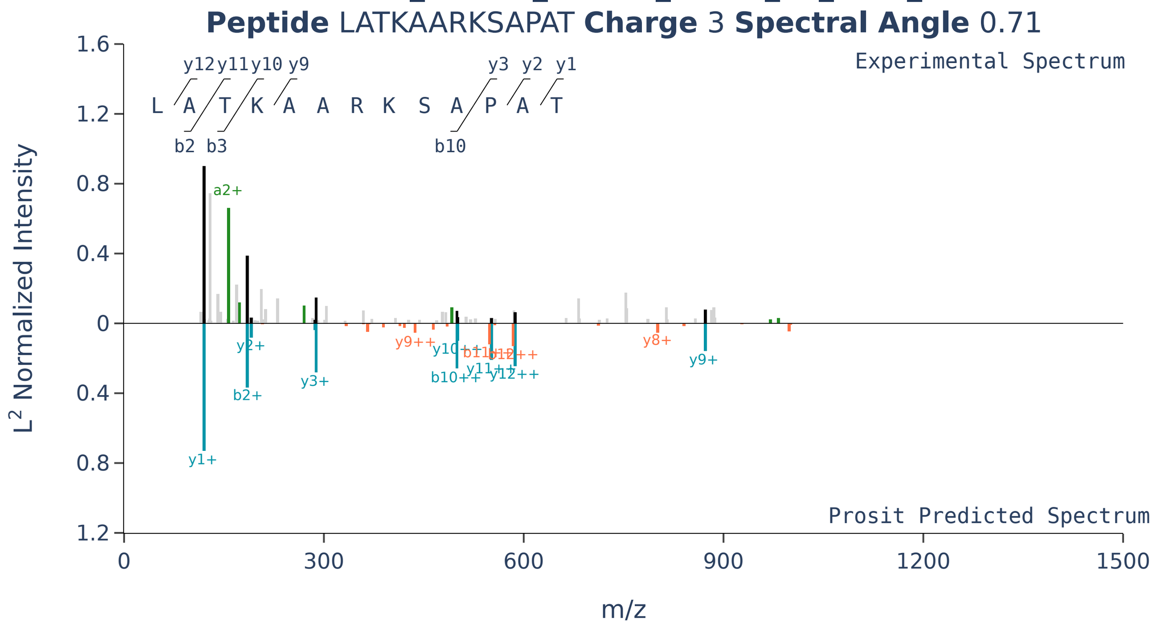

**Source** HRoetschke_271222_120123_HFGoe_H3_b1R1_1h **Scan** 16689
**Peptide** IAQDFKTDLRFQS **Charge** 2 **Spectral Angle** 0.79

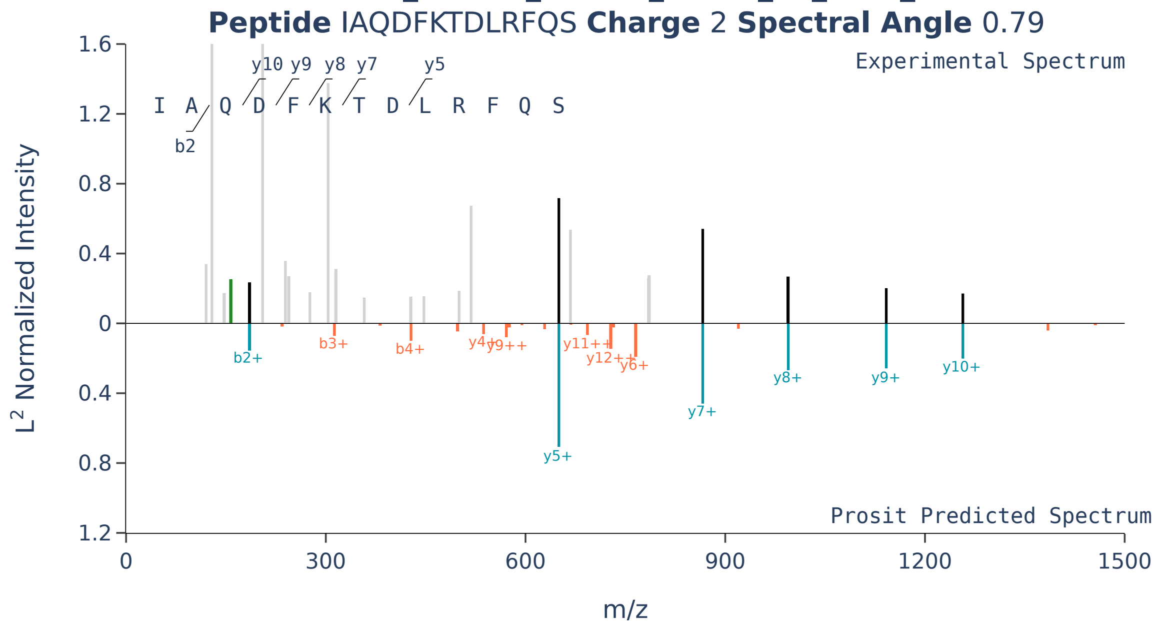

**Source** HRoetschke_271222_120123_HFGoe_H3_b2R1_2h **Scan** 10364
**Peptide** KRVTIMPKDIQ **Charge** 3 **Spectral Angle** 0.79

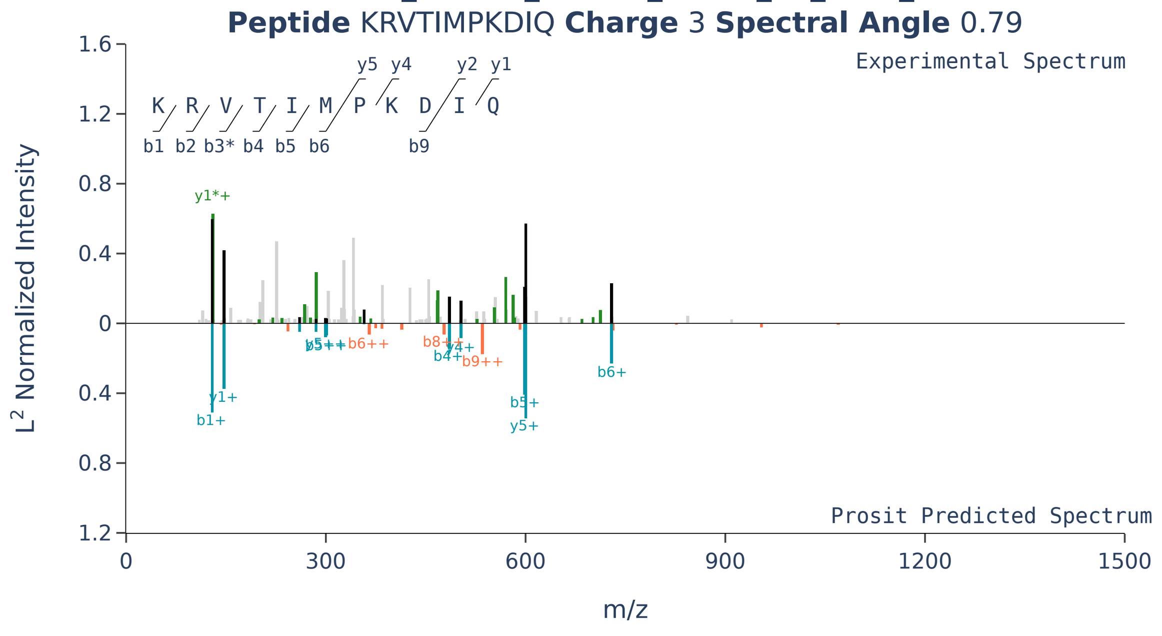

**Source** HRoetschke_271222_120123_HFGoe_H3_b1R1_4h **Scan** 17712
**Peptide** QKSTELLIRKLPFQR **Charge** 3 **Spectral Angle** 0.74

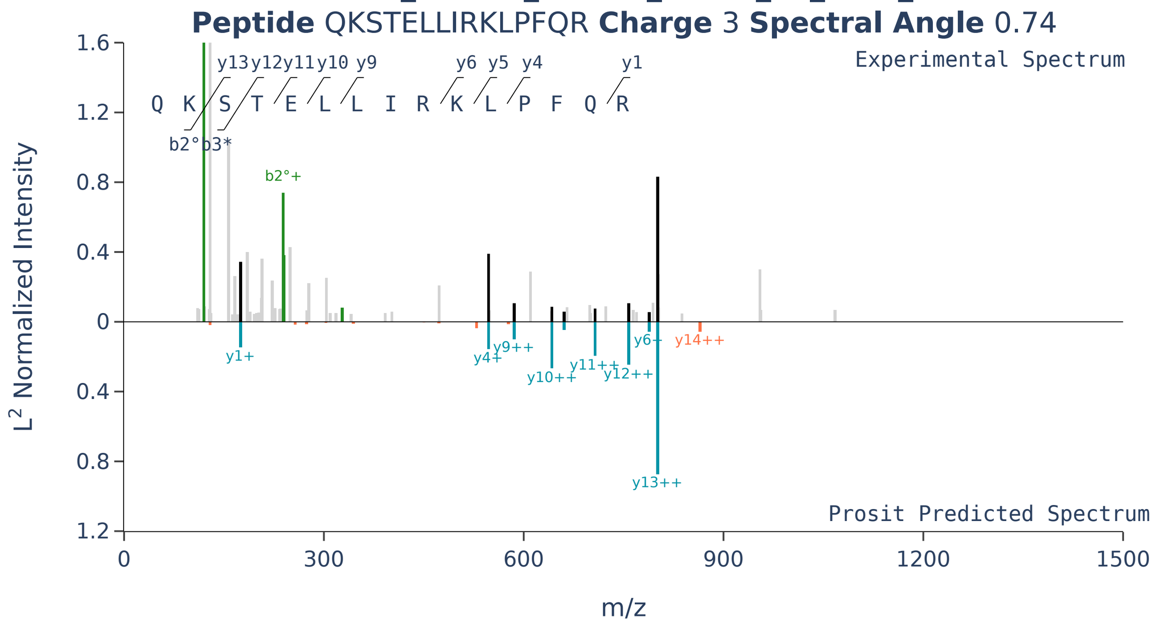

**Source** HRoetschke_271222_120123_HFGoe_H3_b1R1_4h **Scan** 15258
**Peptide** TIMPKDIQLARRL **Charge** 3 **Spectral Angle** 0.65

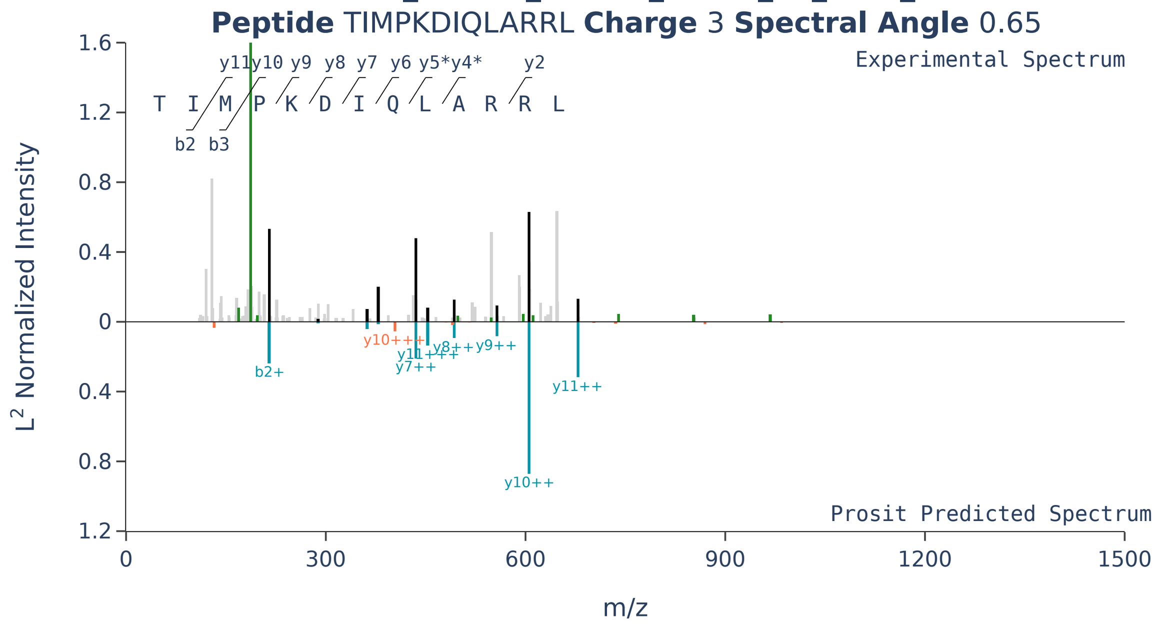

**Source** HRoetschke_271222_120123_HFGoe_H3_b2R2_4h **Scan** 5222
**Peptide** ATGGVKKPHRY **Charge** 3 **Spectral Angle** 0.74

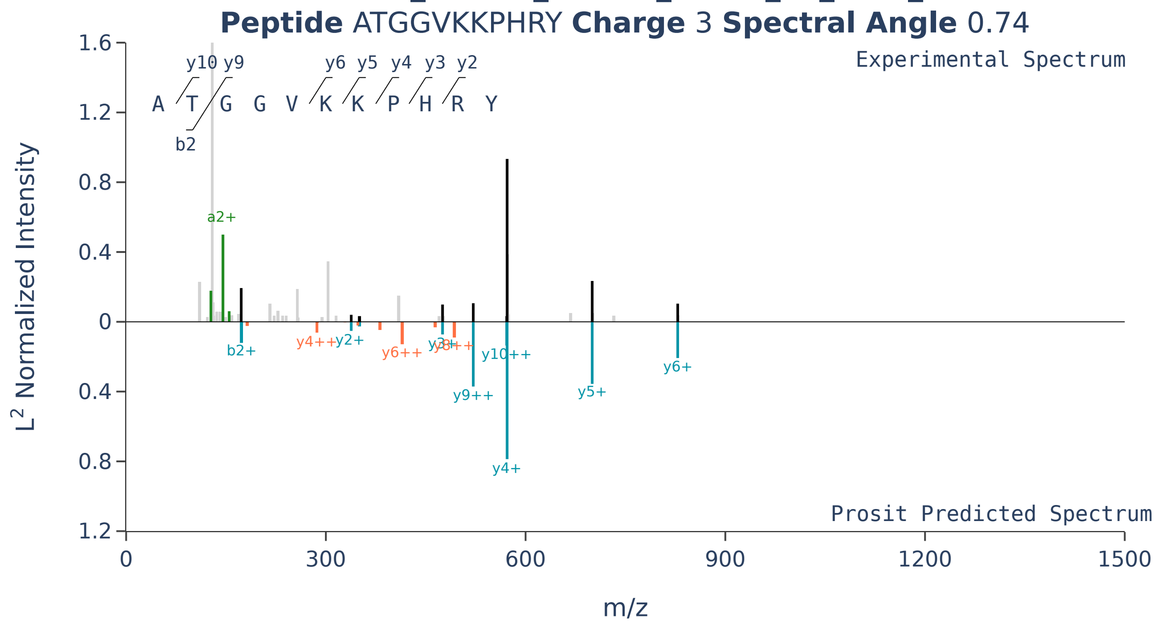

**Source** HRoetschke_271222_120123_HFGoe_H3_b2R2_2h **Scan** 7834
**Peptide** IHAKRVTIMPKD **Charge** 4 **Spectral Angle** 0.76

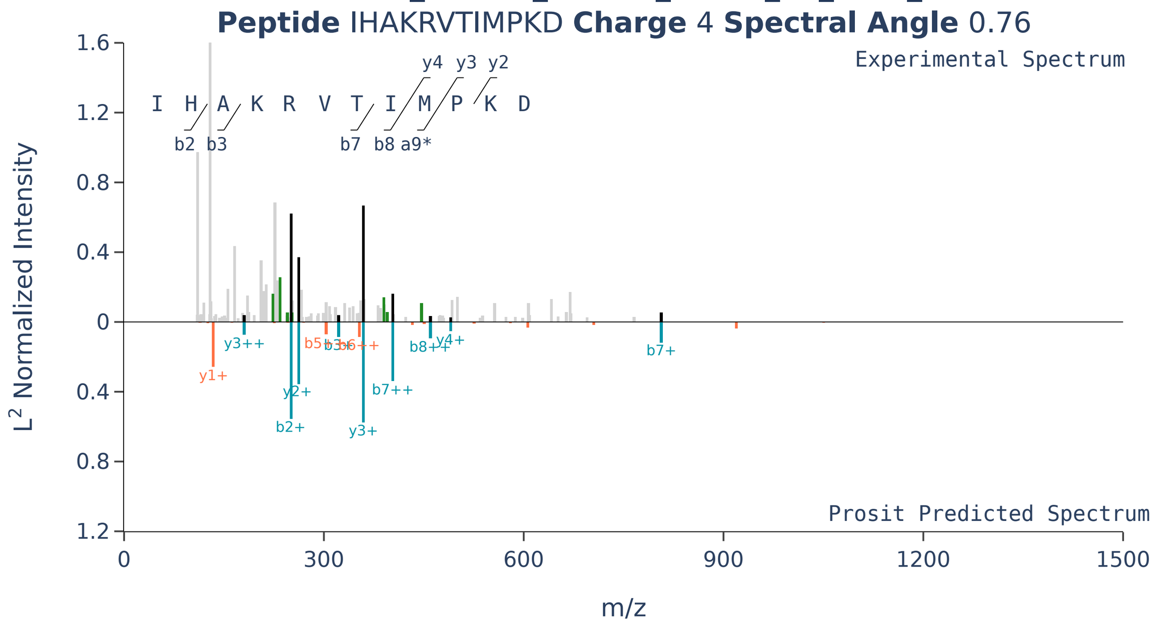

**Source** HRoetschke_271222_120123_HFGoe_H3_b1R2_2h **Scan** 7635
**Peptide** YQKSTEL **Charge** 2 **Spectral Angle** 0.85

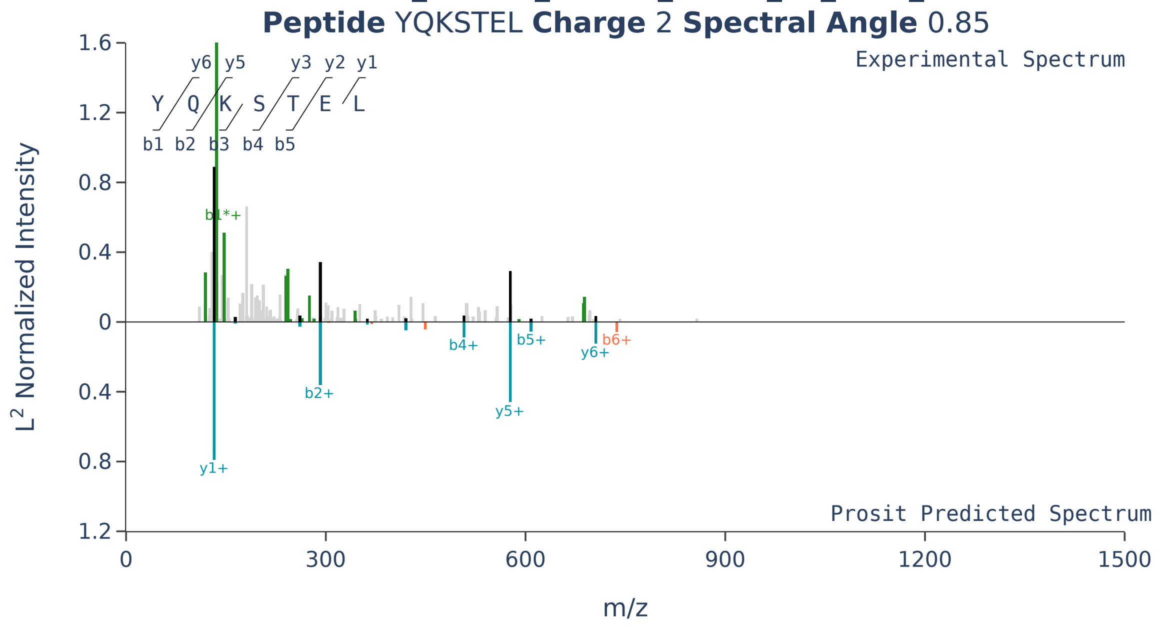

**Source** HRoetschke_271222_120123_HFGoe_H3_b1R1_4h **Scan** 16861
**Peptide** FKTDLRFQSSAV **Charge** 3 **Spectral Angle** 0.88

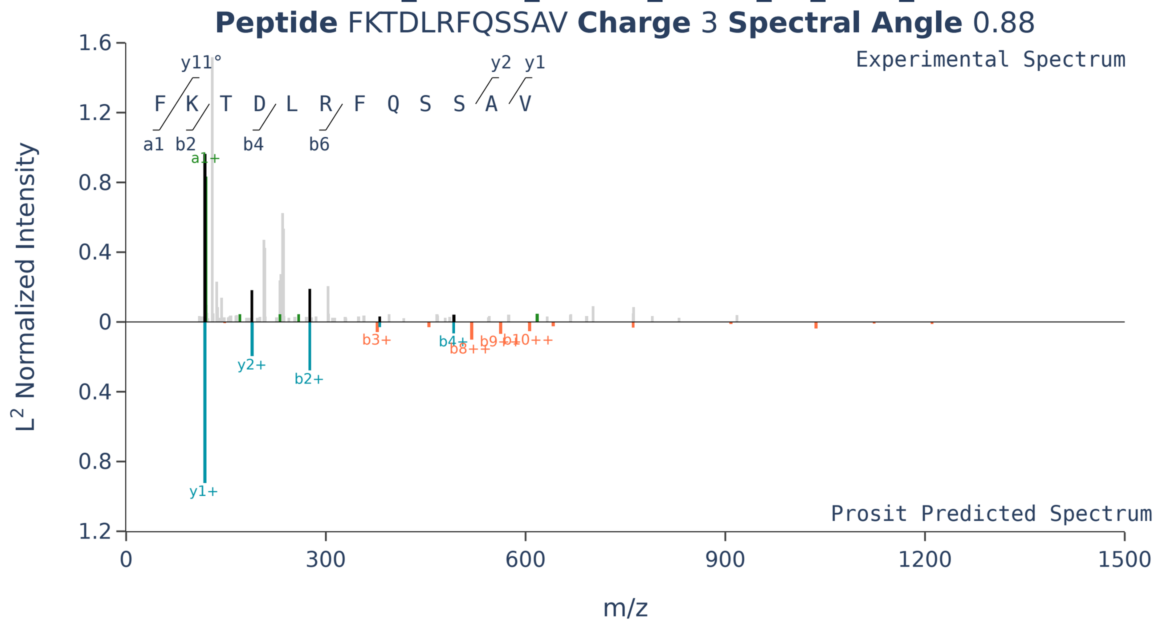

**Source** HRoetschke_271222_120123_HFGoe_H3_b1R1_4h **Scan** 17577
**Peptide** LVREIAQDFKTDLR **Charge** 3 **Spectral Angle** 0.77

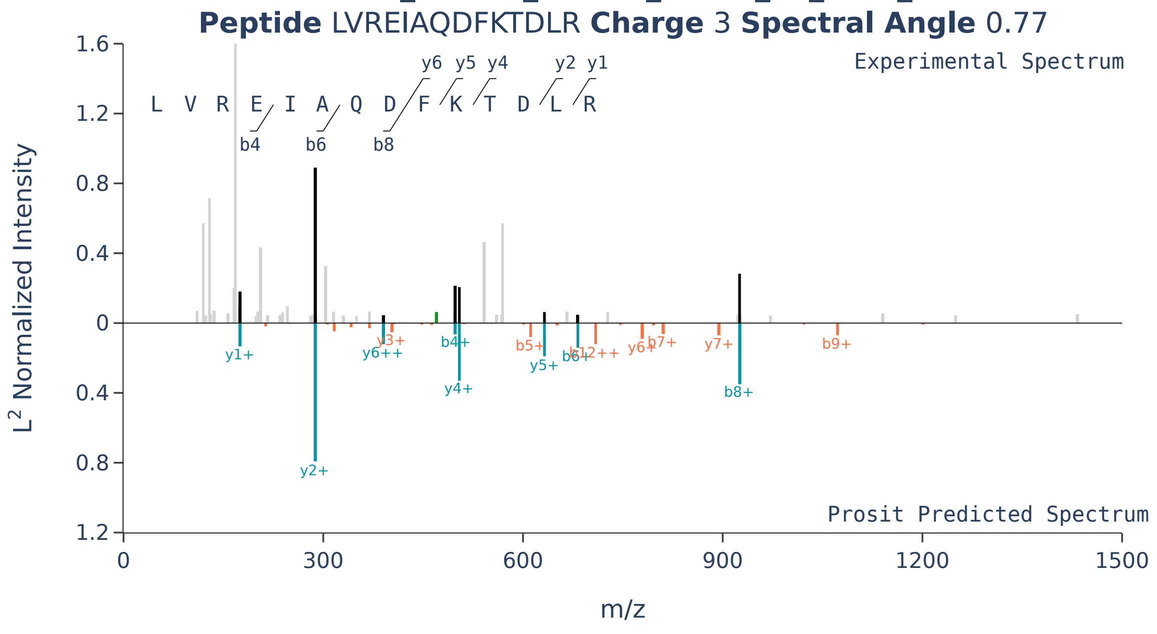


**Source** HRoetschke_271222_120123_HFGoe_H3_b1R2_4h **Scan** 6313
**Peptide** VREIAQD **Charge** 2 **Spectral Angle** 0.76

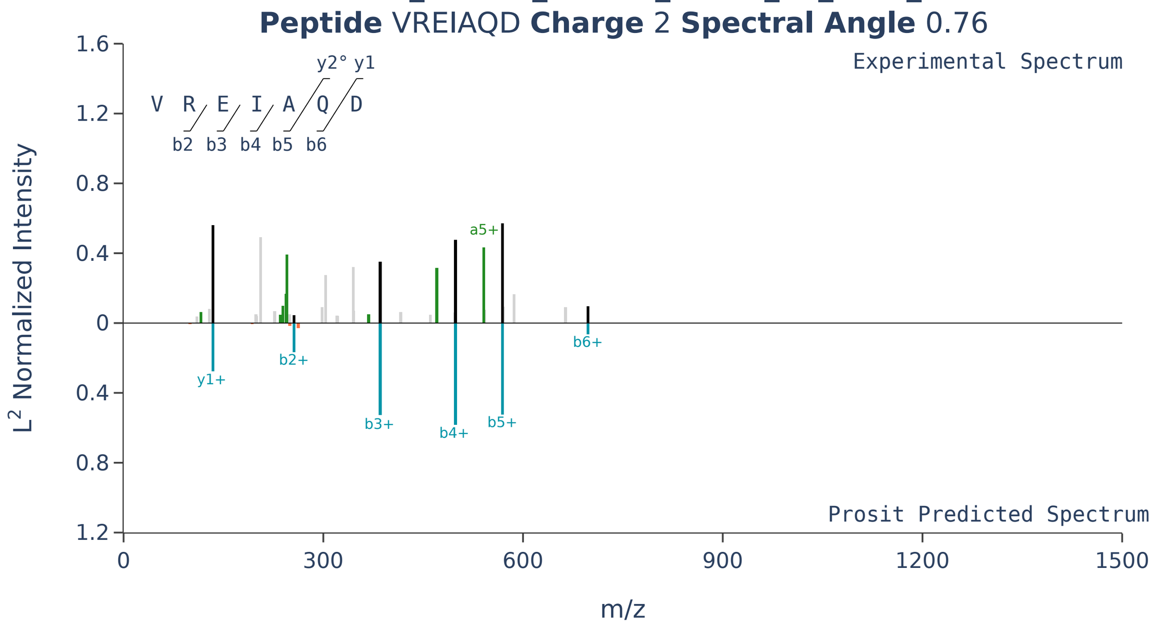


**Source** HRoetschke_271222_120123_HFGoe_H3_b2R1_4h **Scan** 13705
**Peptide** AIHAKRVTIMPKDI **Charge** 4 **Spectral Angle** 0.61

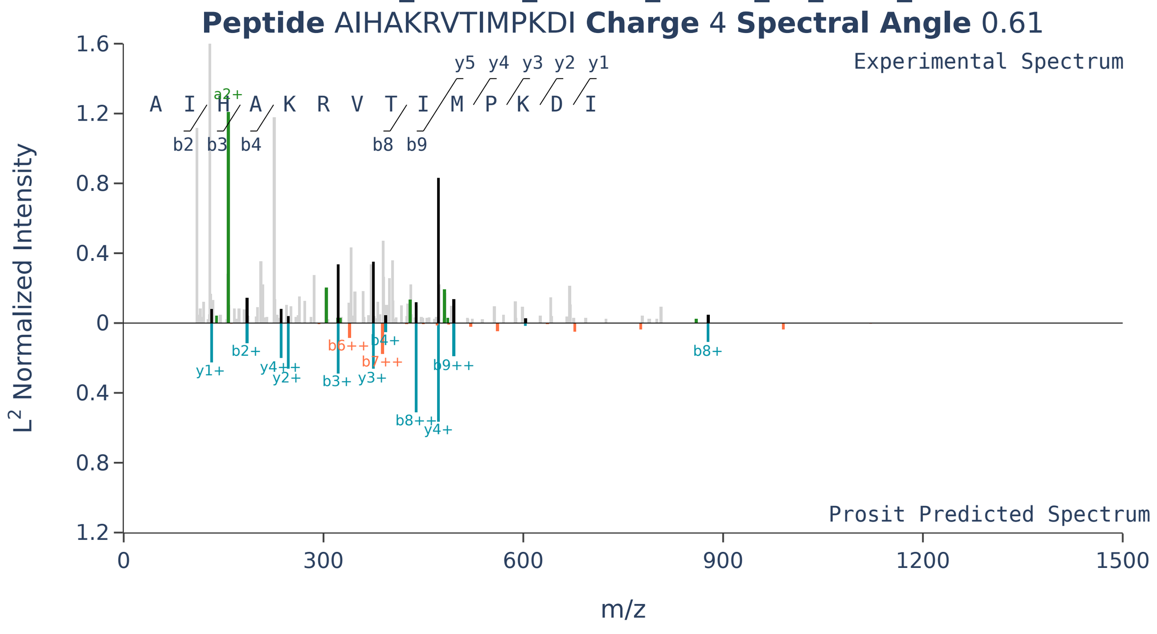


**Source** HRoetschke_271222_120123_HFGoe_H3_b2R1_4h **Scan** 12828
**Peptide** IQLARRI **Charge** 2 **Spectral Angle** 0.92

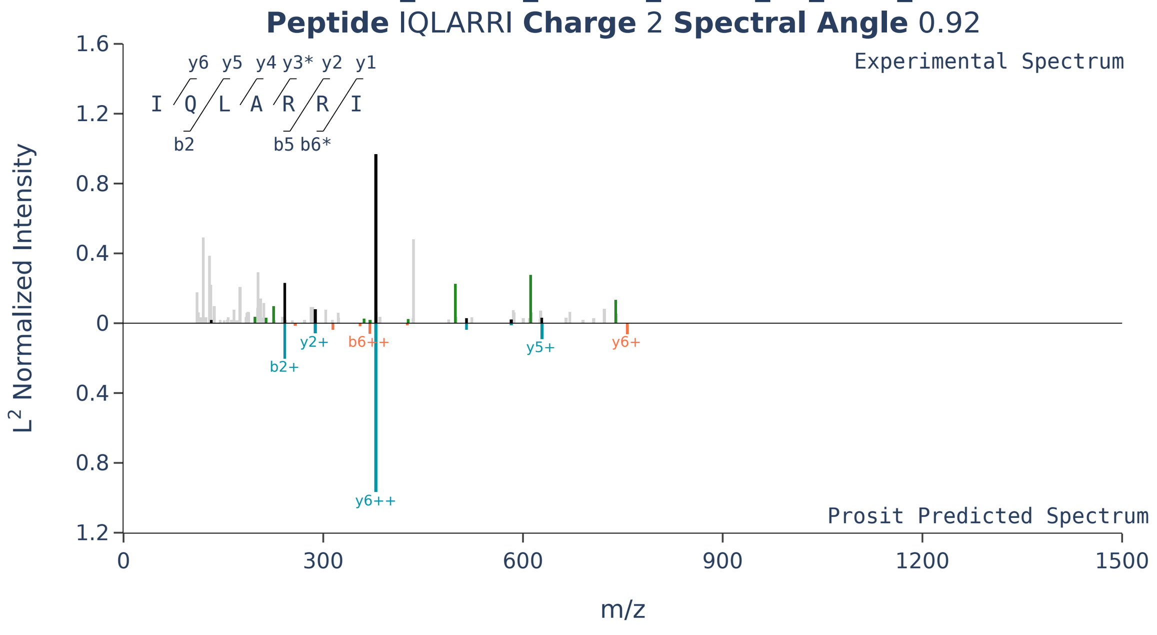


**Source** HRoetschke_271222_120123_HFGoe_H3_b2R2_2h **Scan** 6709
**Peptide** PKDIQLARR **Charge** 3 **Spectral Angle** 0.91

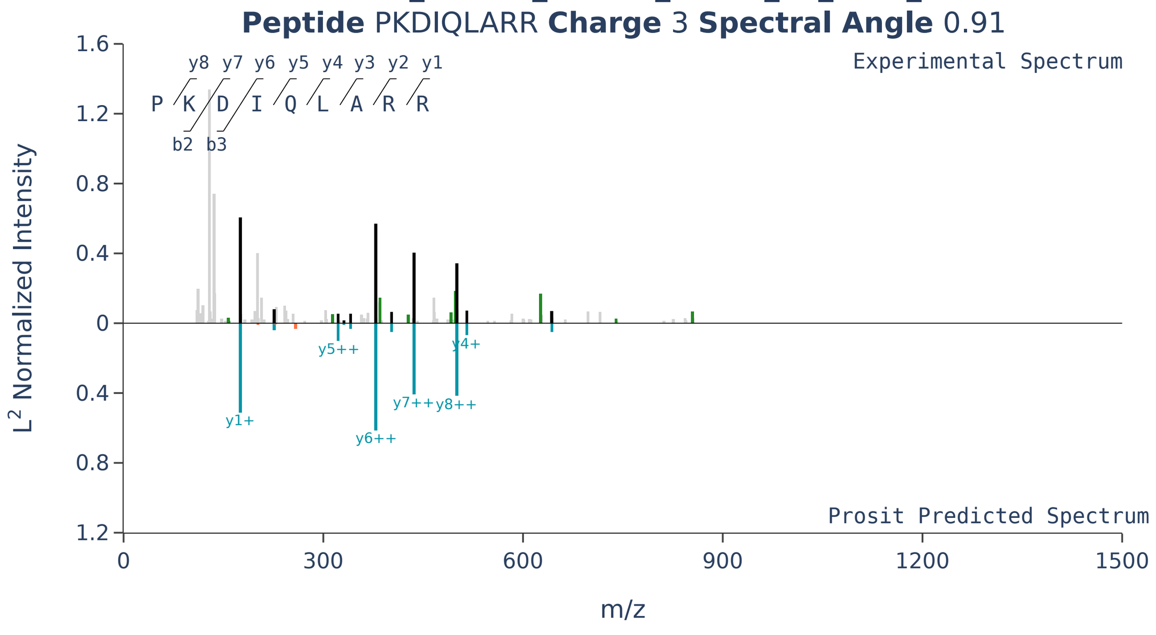


**Source** HRoetschke_271222_120123_HFGoe_H3_b2R1_4h **Scan** 12407
**Peptide** MPKDIQLARRIRG **Charge** 4 **Spectral Angle** 0.67

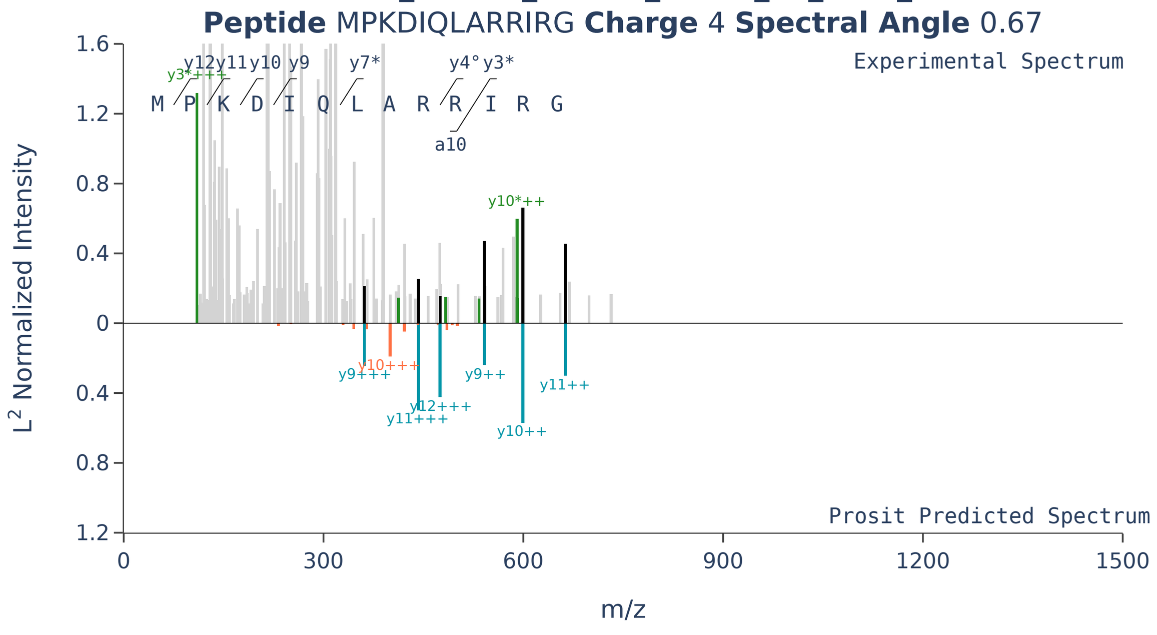


**Source** HRoetschke_271222_120123_HFGoe_H3_b2R2_2h **Scan** 13115
**Peptide** QKSTELL **Charge** 2 **Spectral Angle** 0.94

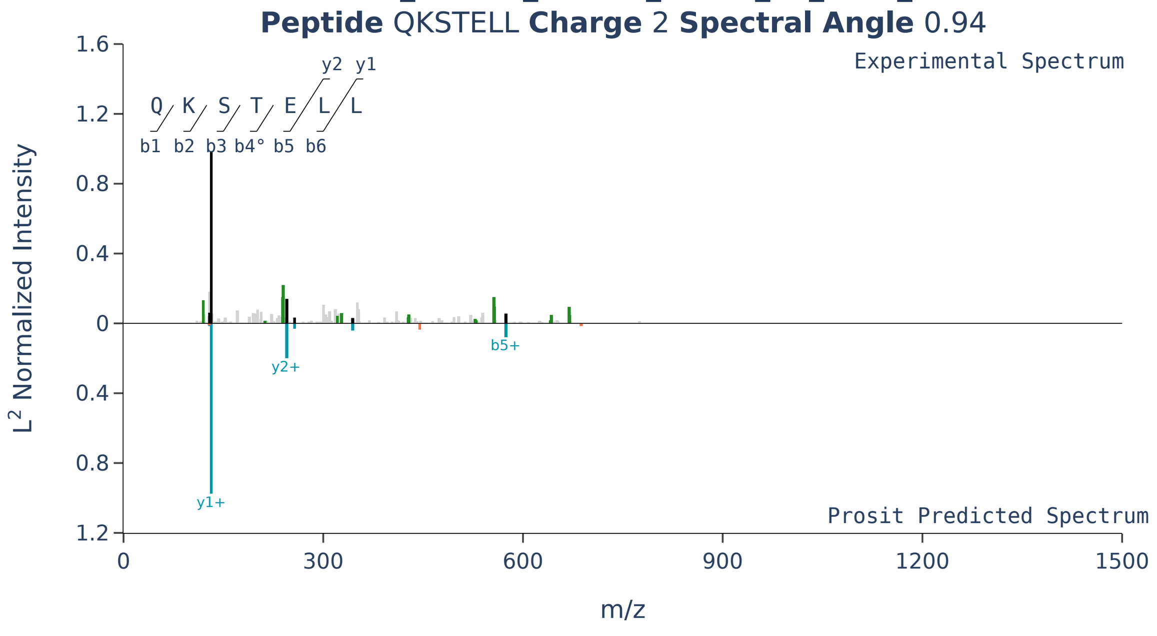


**Source** HRoetschke_271222_120123_HFGoe_H3_b1R1_4h **Scan** 12580
**Peptide** KTDLRFQSSAVMA **Charge** 3 **Spectral Angle** 0.57

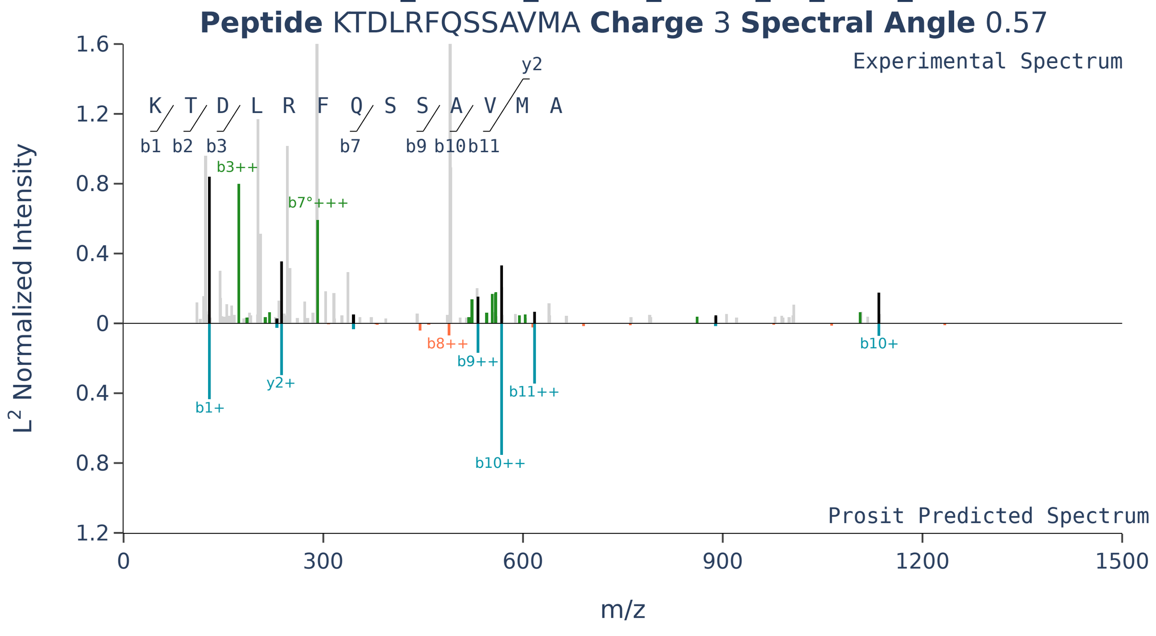


**Source** HRoetschke_271222_120123_HFGoe_H3_b2R2_4h **Scan** 4957
**Peptide** REIRRYQKSTE **Charge** 4 **Spectral Angle** 0.8

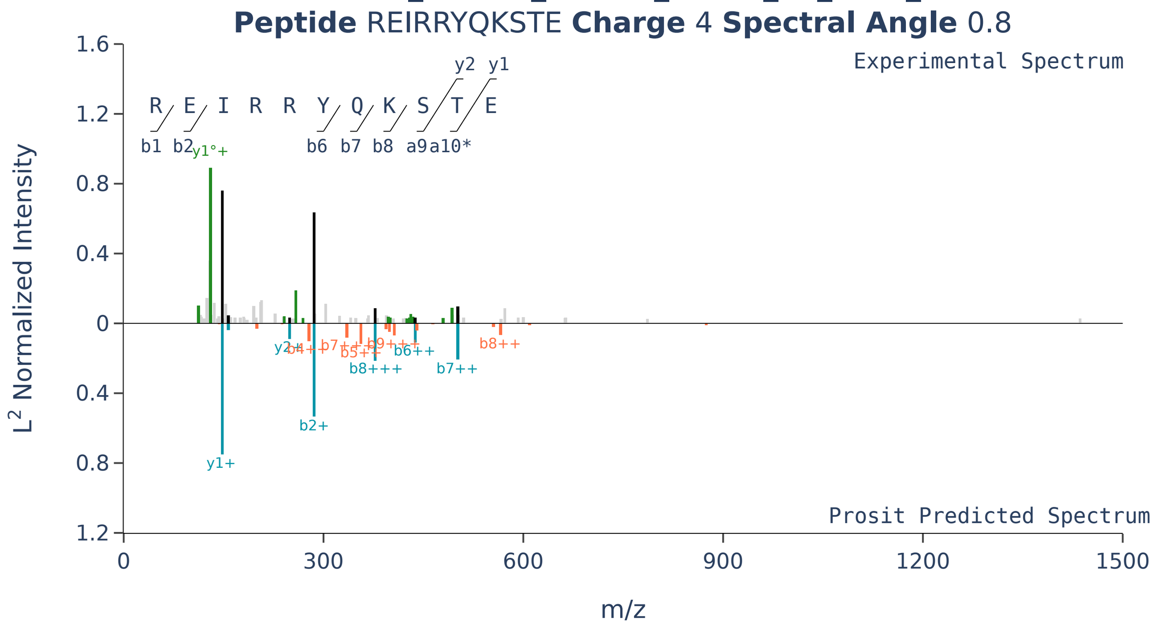


**Source** HRoetschke_271222_120123_HFGoe_H3_b2R2_2h **Scan** 9946
**Peptide** PGTVALREIRR **Charge** 2 **Spectral Angle** 0.78

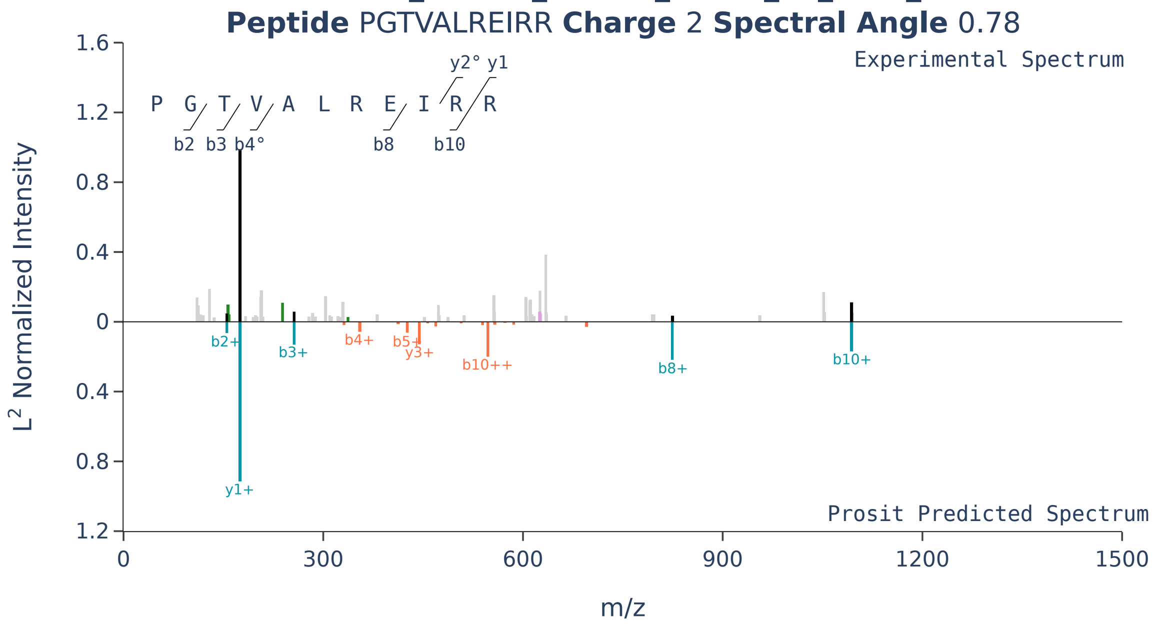


**Source** HRoetschke_271222_120123_HFGoe_H3_b2R2_4h **Scan** 5294
**Peptide** SAPATGGVKKPHRY **Charge** 4 **Spectral Angle** 0.7

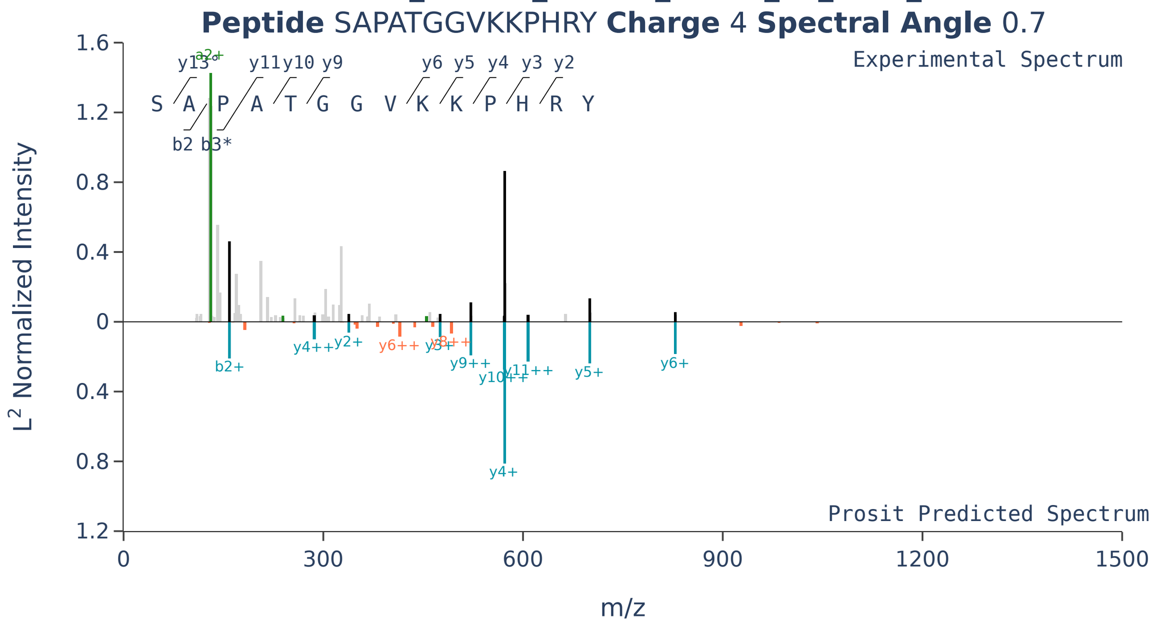


**Source** HRoetschke_271222_120123_HFGoe_H3_b1R1_4h **Scan** 18086
**Peptide** VREIAQDFKTDLRFQS **Charge** 4 **Spectral Angle** 0.73

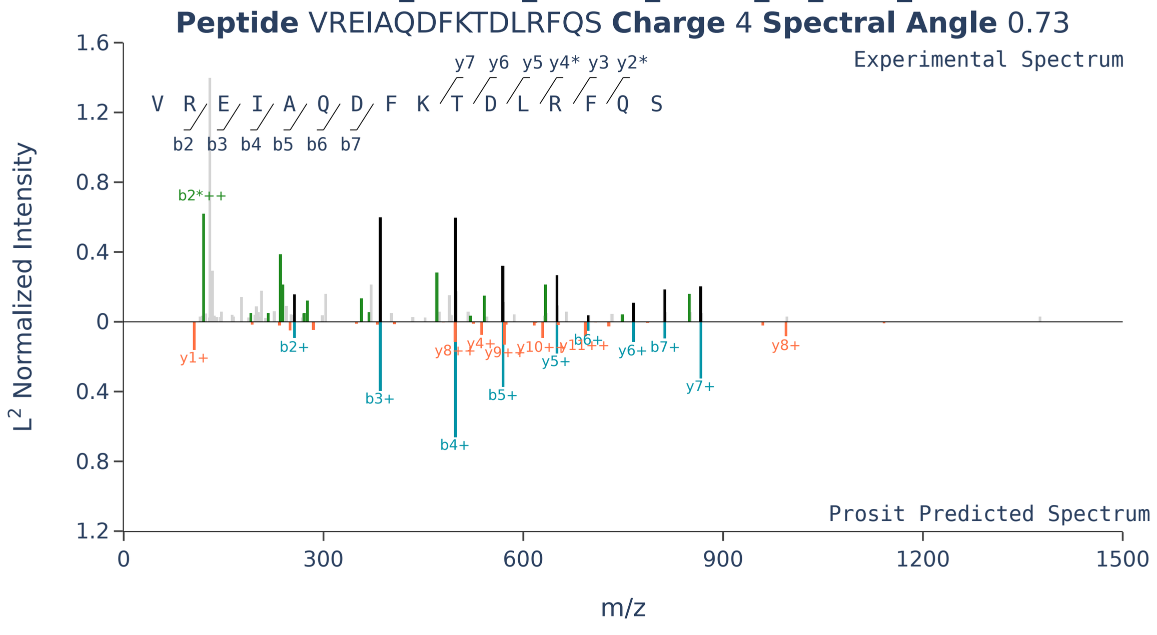


**Source** HRoetschke_271222_120123_HFGoe_H3_b1R1_4h **Scan** 14363
**Peptide** AIHAKRVTIMPKDIQL **Charge** 4 **Spectral Angle** 0.69

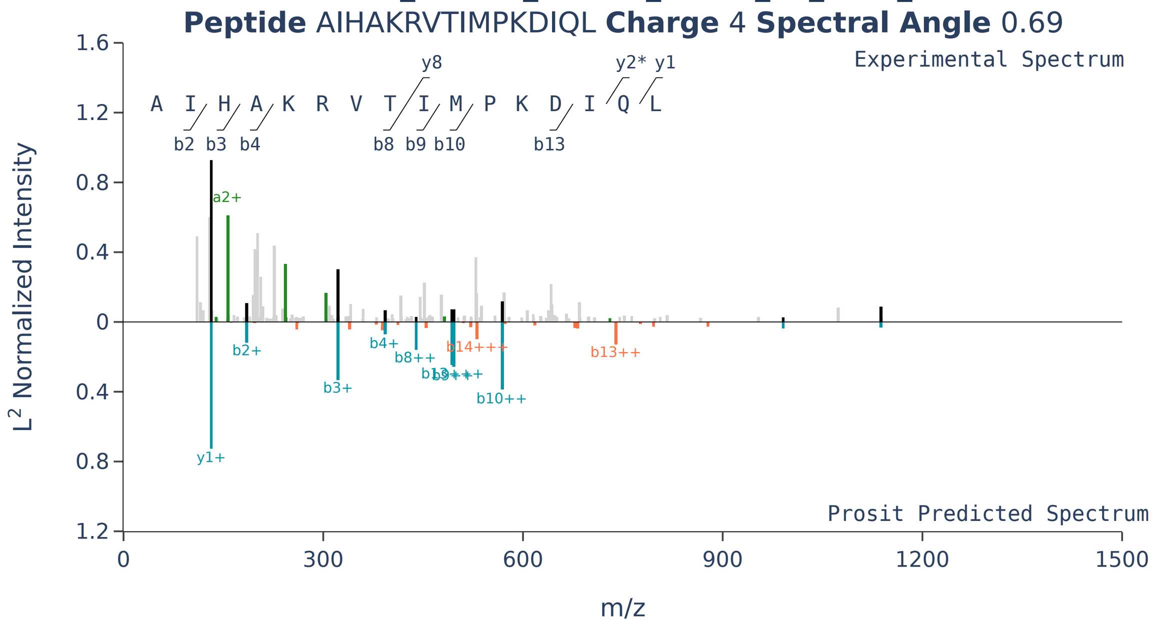


**Source** HRoetschke_271222_120123_HFGoe_H3_b2R1_4h **Scan** 14236
**Peptide** REIRRYQKSTELLI **Charge** 3 **Spectral Angle** 0.55

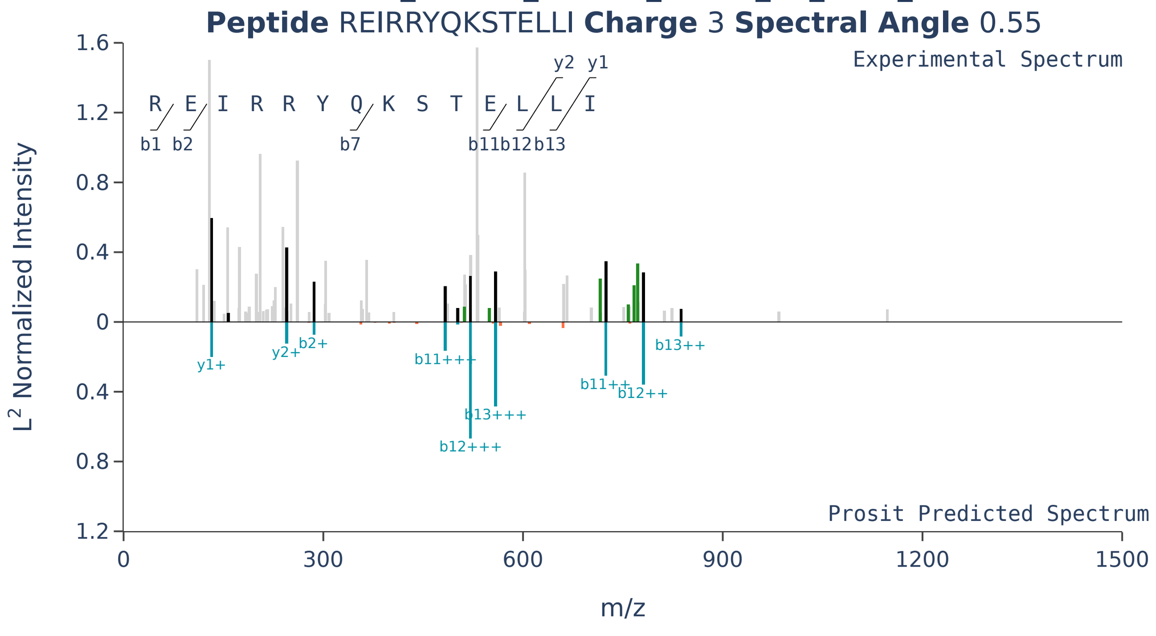


**Source** HRoetschke_271222_120123_HFGoe_H3_b2R1_2h **Scan** 14244
**Peptide** LFEDTNLCAIHAKR **Charge** 4 **Spectral Angle** 0.72

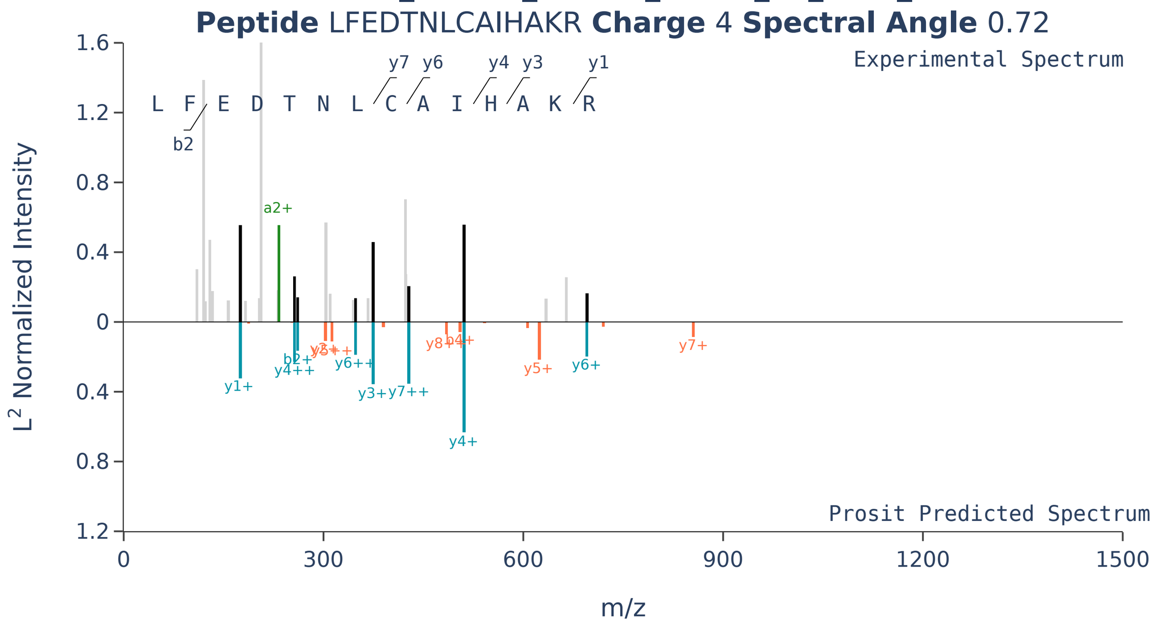

**Source** HRoetschke_271222_120123_HFGoe_H3_b1R2_4h **Scan** 20301
**Peptide** LVREIAQDFKTDL **Charge** 3 **Spectral Angle** 0.72

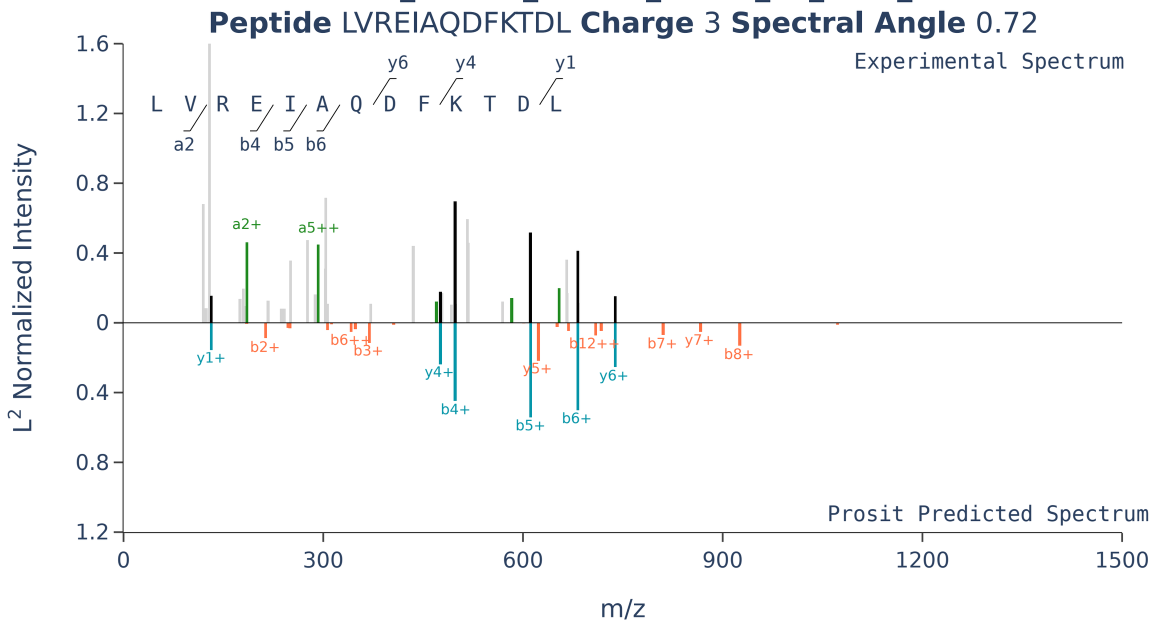


**Source** HRoetschke_271222_120123_HFGoe_H3_b1R1_4h **Scan** 8323
**Peptide** RRYQKSTELL **Charge** 3 **Spectral Angle** 0.62

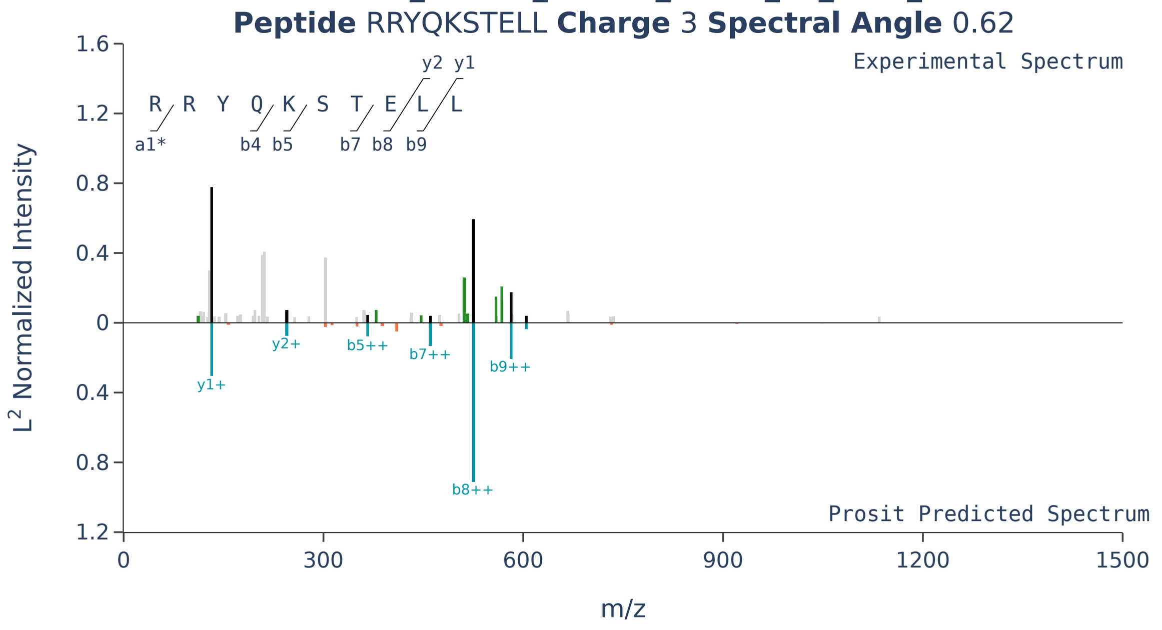


**Source** HRoetschke_271222_120123_HFGoe_H3_b2R1_1h **Scan** 20932
**Peptide** ALFEDTNLCAIHA **Charge** 2 **Spectral Angle** 0.71

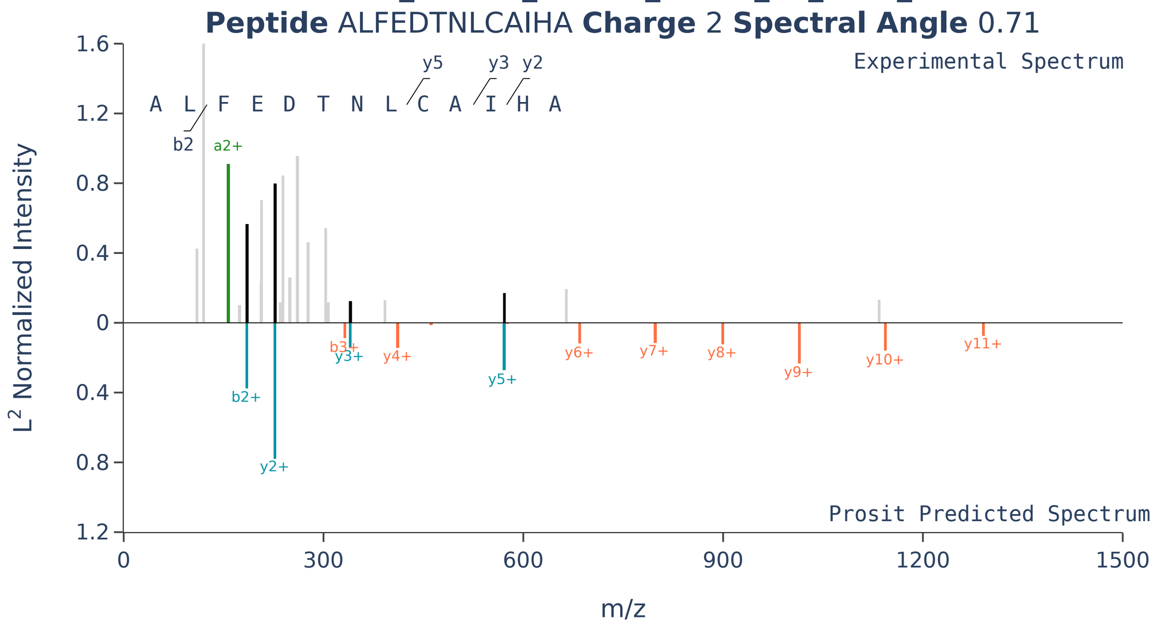


**Source** HRoetschke_271222_120123_HFGoe_H3_b1R2_24h **Scan** 14184
**Peptide** MPKDIQL **Charge** 2 **Spectral Angle** 0.73

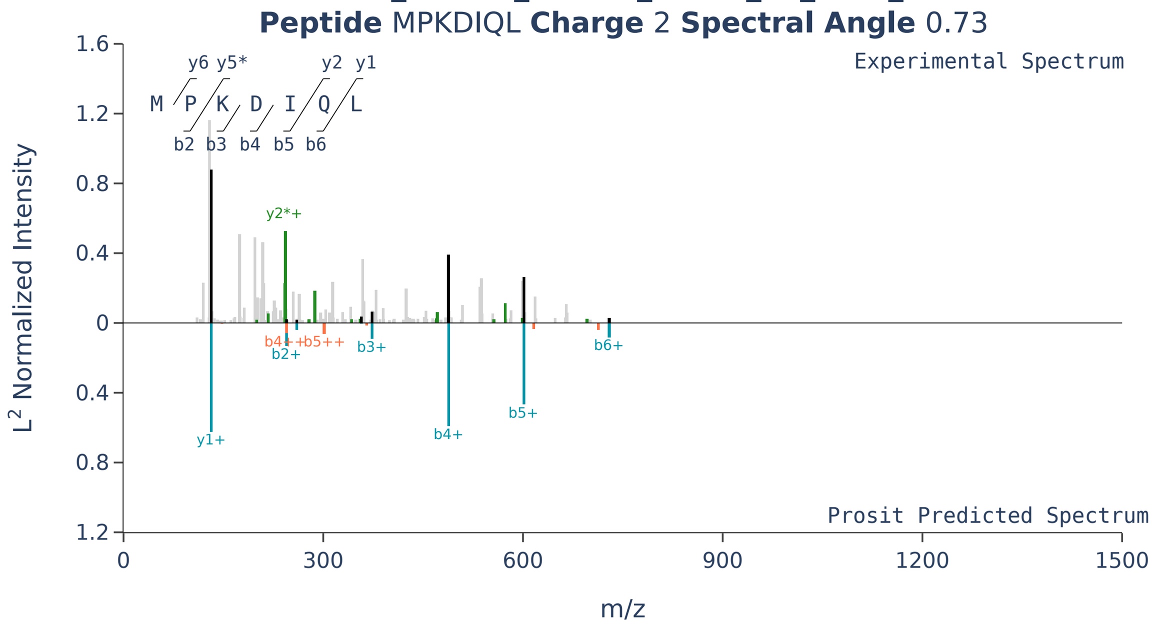


**Source** HRoetschke_271222_120123_HFGoe_H3_b2R1_4h **Scan** 13374
**Peptide** RKLPFQRLVRE **Charge** 3 **Spectral Angle** 0.64

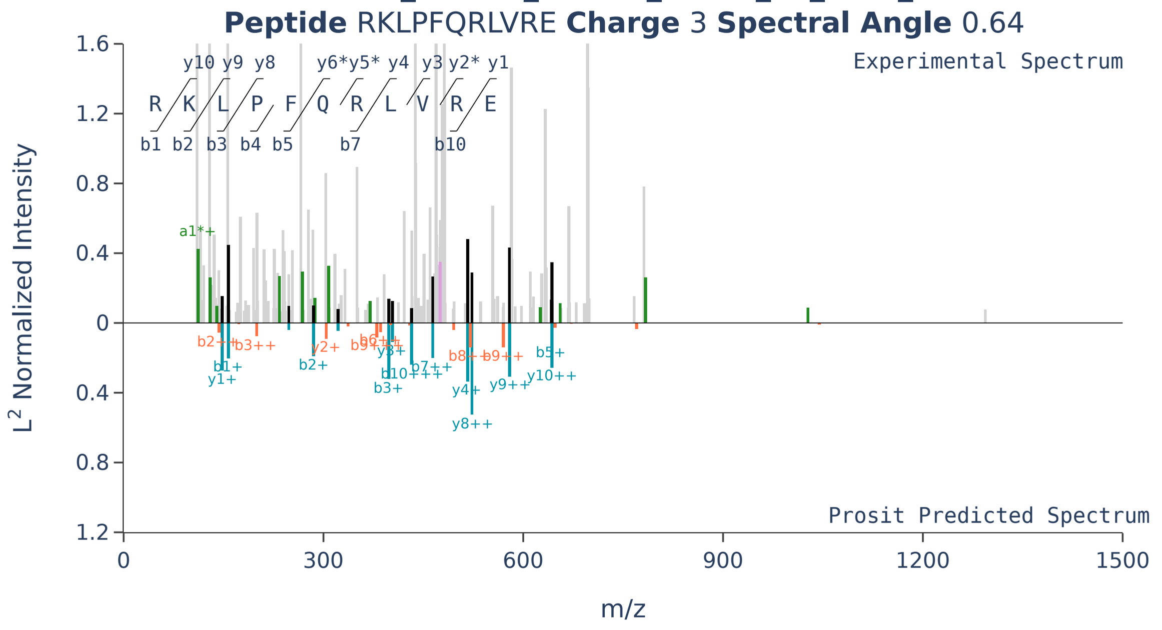


**Source** HRoetschke_271222_120123_HFGoe_H3_b1R1_4h **Scan** 14124
**Peptide** EIRRYQKSTELLIRKL **Charge** 4 **Spectral Angle** 0.78

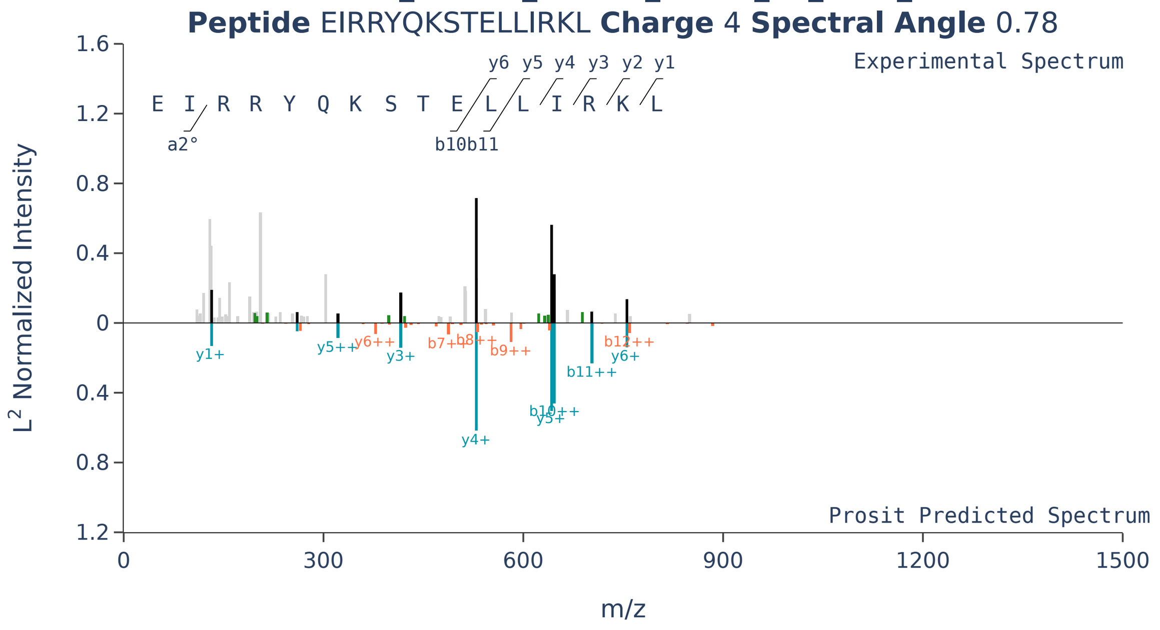


**Source** HRoetschke_271222_120123_HFGoe_H3_b1R1_4h **Scan** 19780
**Peptide** YQKSTELLI **Charge** 2 **Spectral Angle** 0.59

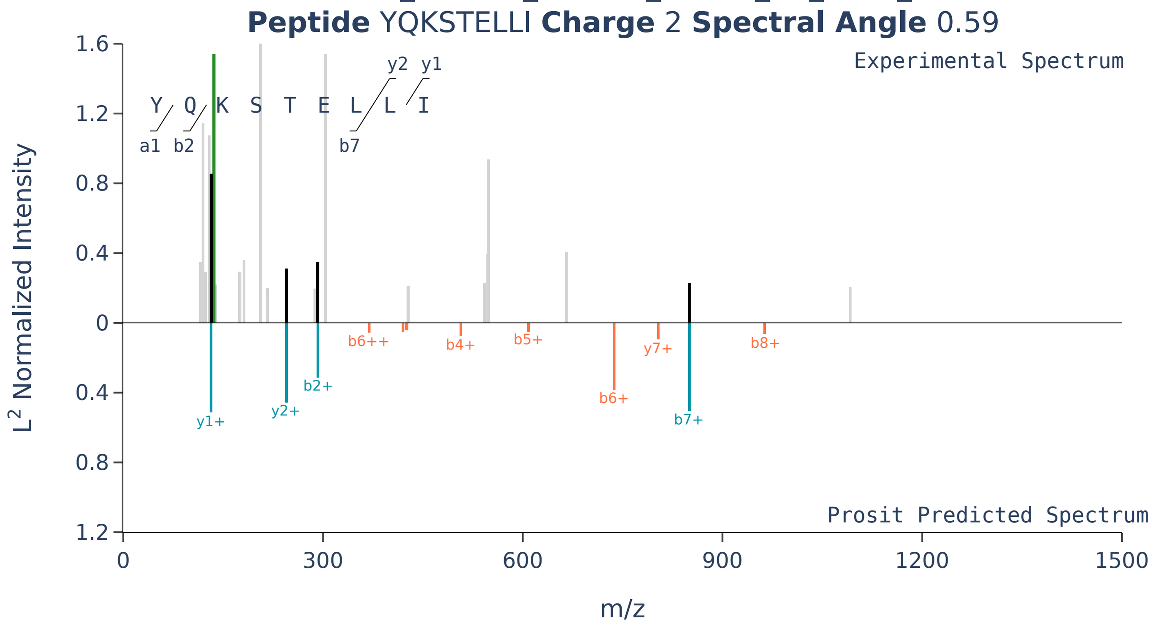


**Source** HRoetschke_271222_120123_HFGoe_H3_b2R2_1h **Scan** 19808
**Peptide** LFEDTNLCAIHA **Charge** 2 **Spectral Angle** 0.73

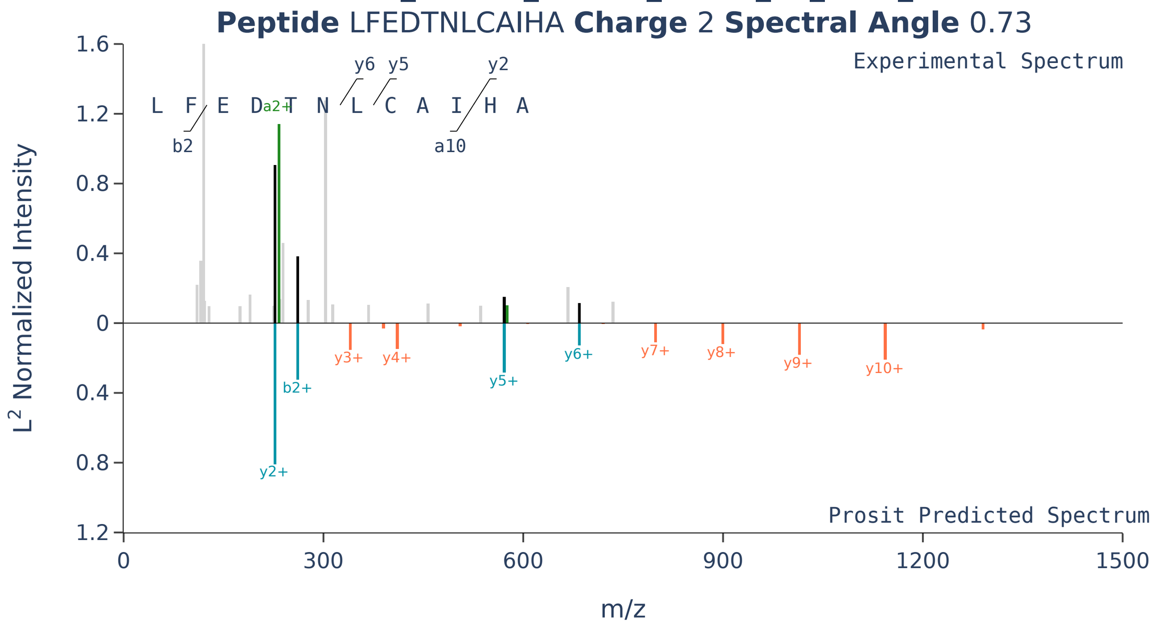


**Source** HRoetschke_271222_120123_HFGoe_H3_b1R1_4h **Scan** 12911
**Peptide** KRVTIMPKDIQLARRI **Charge** 5 **Spectral Angle** 0.7

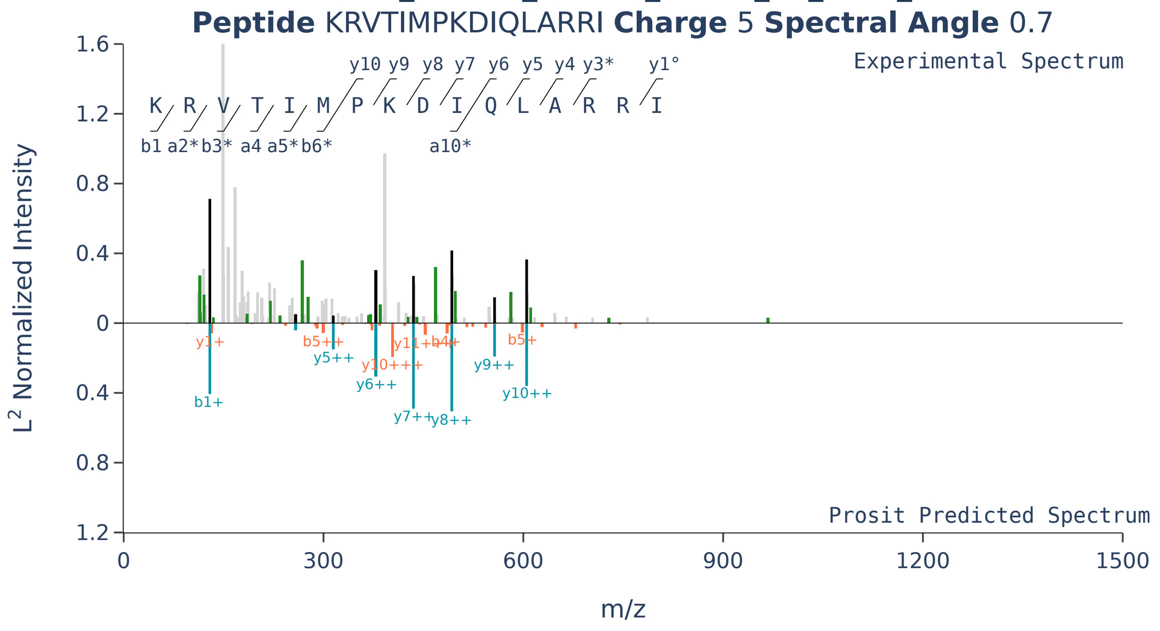


**Source** HRoetschke_271222_120123_HFGoe_H3_b2R2_4h **Scan** 8239
**Peptide** PKDIQLARRIRGERA **Charge** 4 **Spectral Angle** 0.79

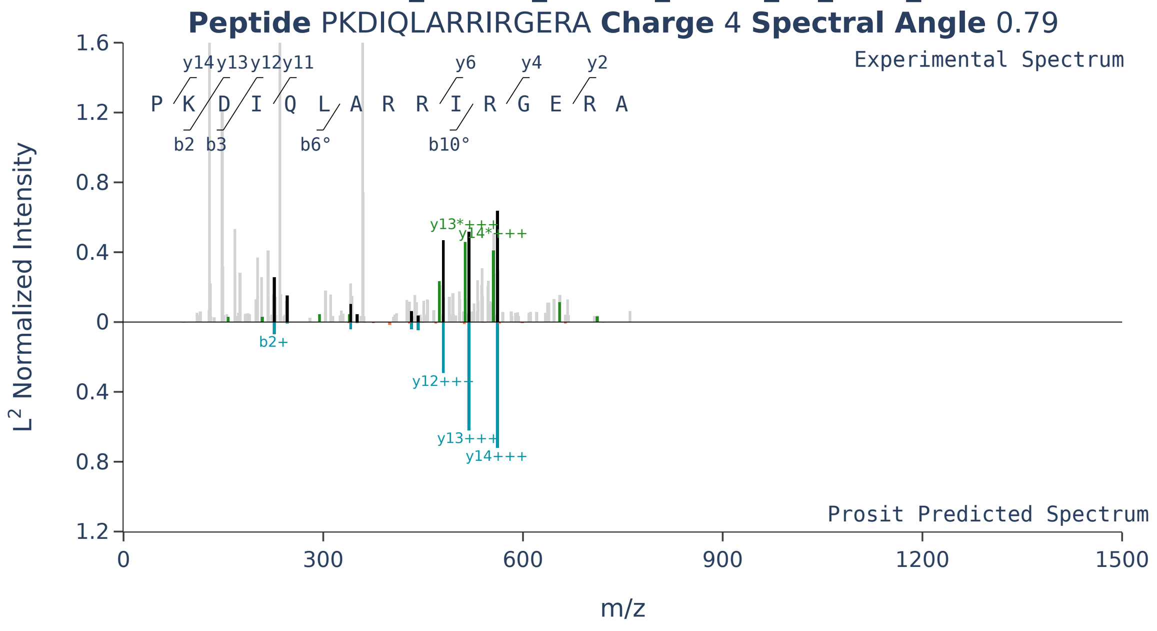


**Source** HRoetschke_271222_120123_HFGoe_H3_b1R1_1h **Scan** 18670
**Peptide** LAQDFKTDLRF **Charge** 3 **Spectral Angle** 0.76

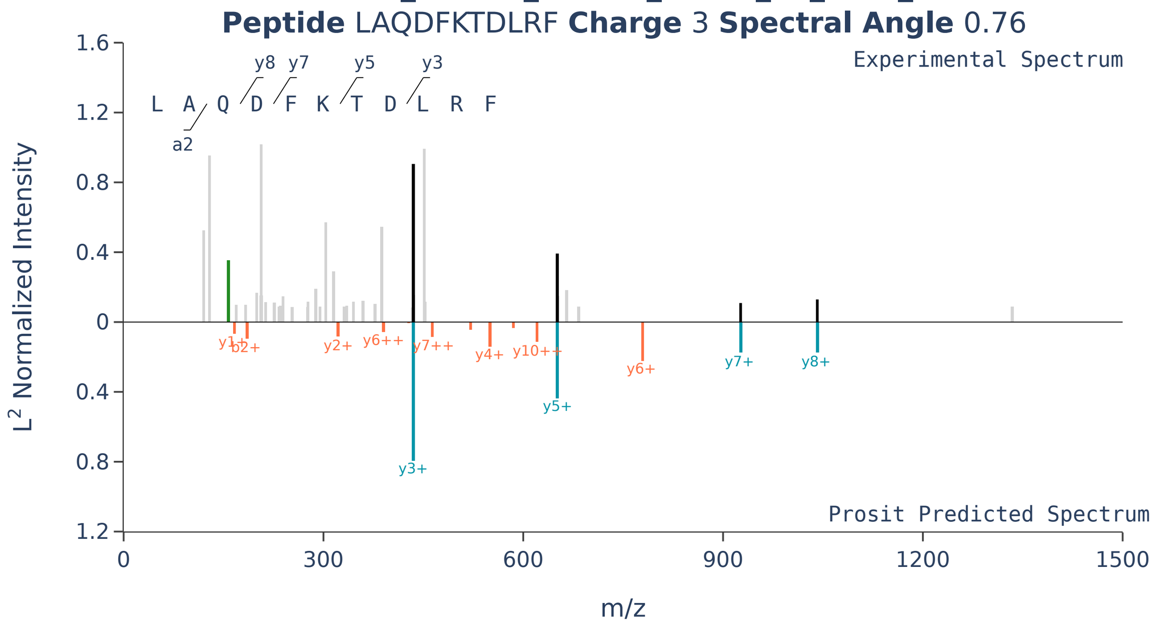


**Source** HRoetschke_271222_120123_HFGoe_H3_b2R2_24h **Scan** 14576
**Peptide** RRYQKSTELLL **Charge** 3 **Spectral Angle** 0.66

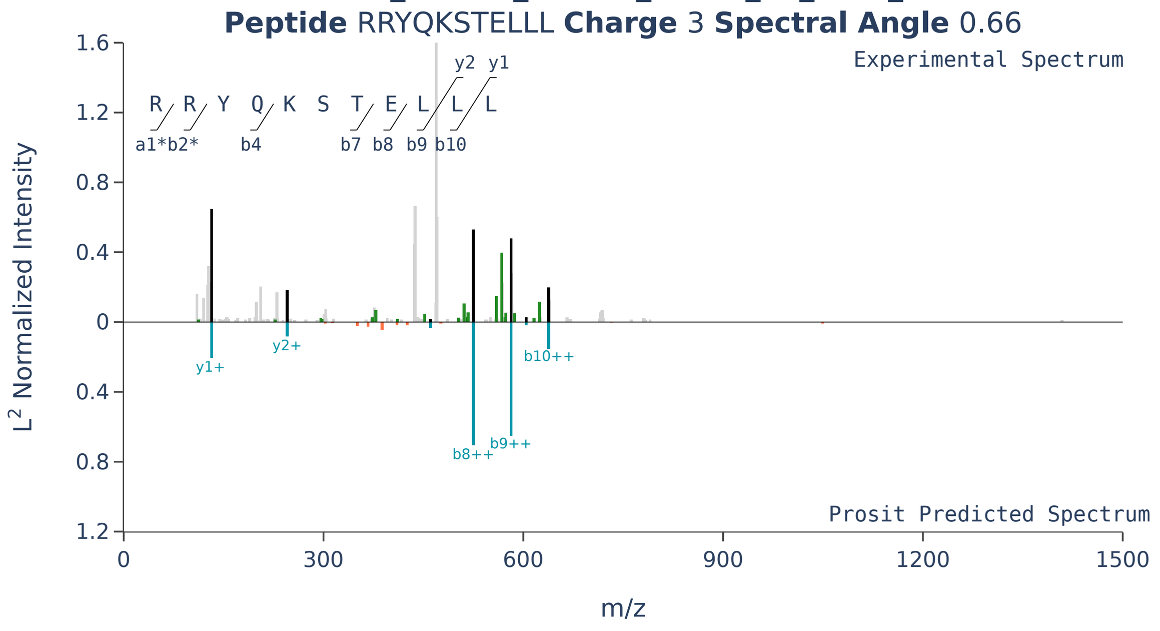


**Source** HRoetschke_271222_120123_HFGoe_H3_b2R2_2h **Scan** 6661
**Peptide** LHAKRVTIMPKD **Charge** 3 **Spectral Angle** 0.57

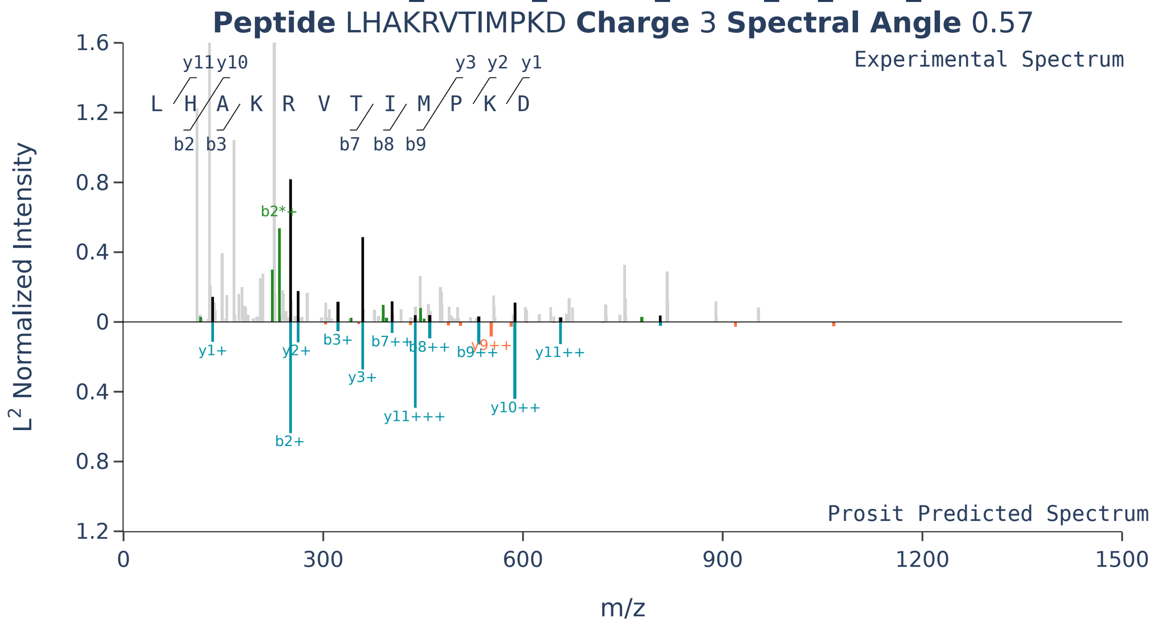


**Source** HRoetschke_271222_120123_HFGoe_H3_b2R2_4h **Scan** 7911
**Peptide** EDTNLCAIHAKR **Charge** 4 **Spectral Angle** 0.77

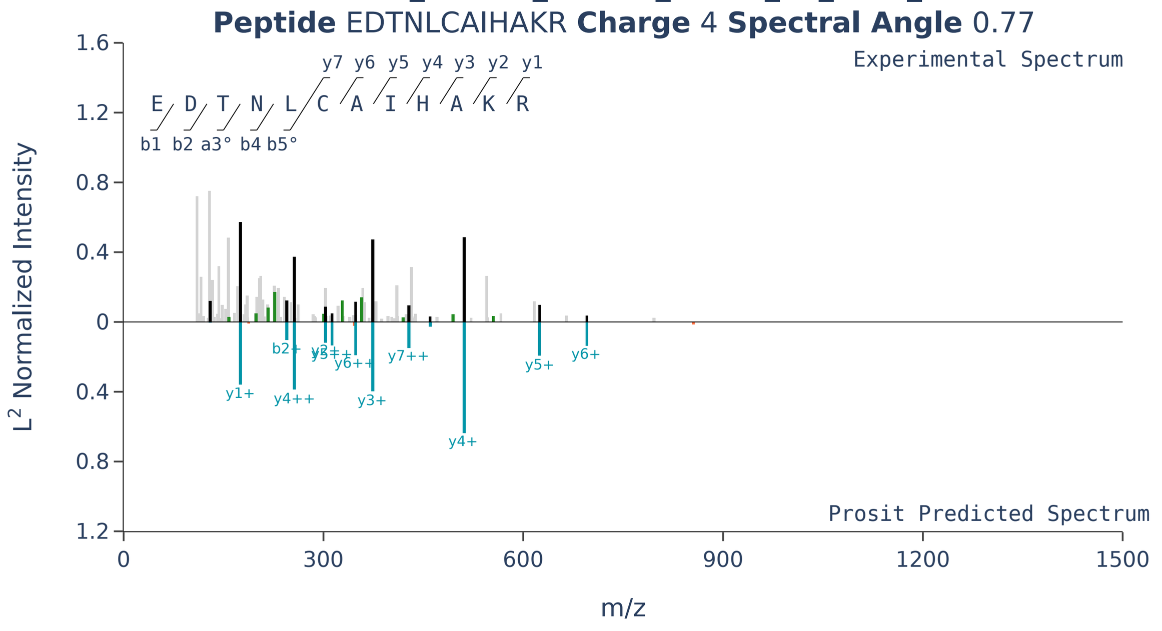


**Source** HRoetschke_271222_120123_HFGoe_H3_b2R2_4h **Scan** 5959
**Peptide** ALREIRRYQK **Charge** 3 **Spectral Angle** 0.76

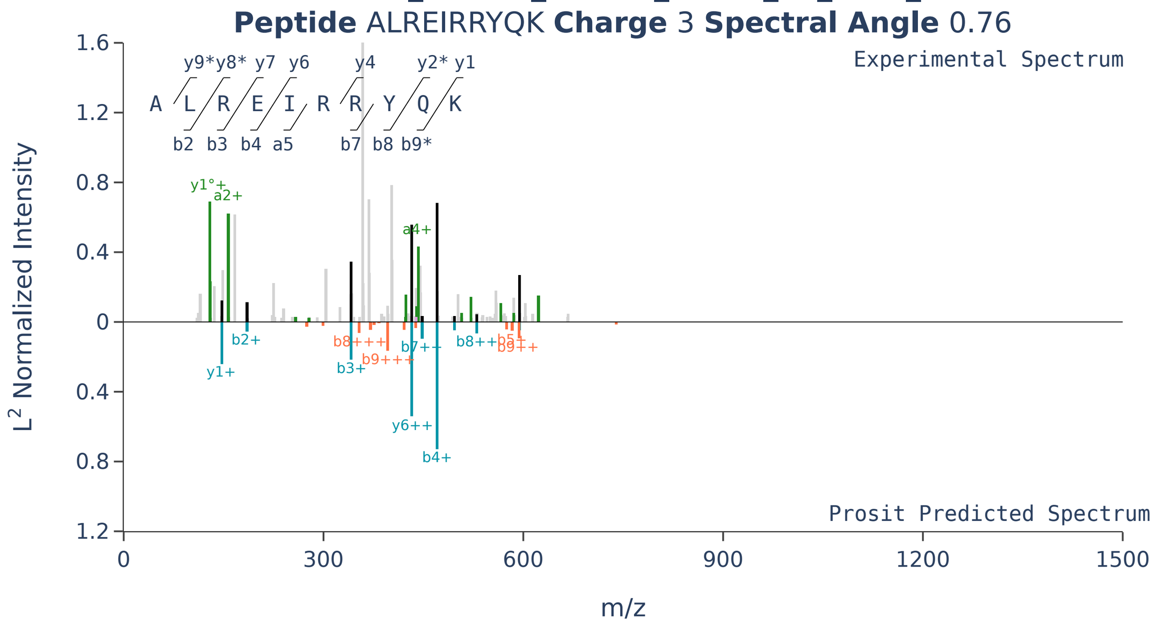

**Source** HRoetschke_271222_120123_HFGoe_H3_b1R1_4h **Scan** 13897
**Peptide** AIHAKRVTIMPKDIQLA **Charge** 3 **Spectral Angle** 0.67

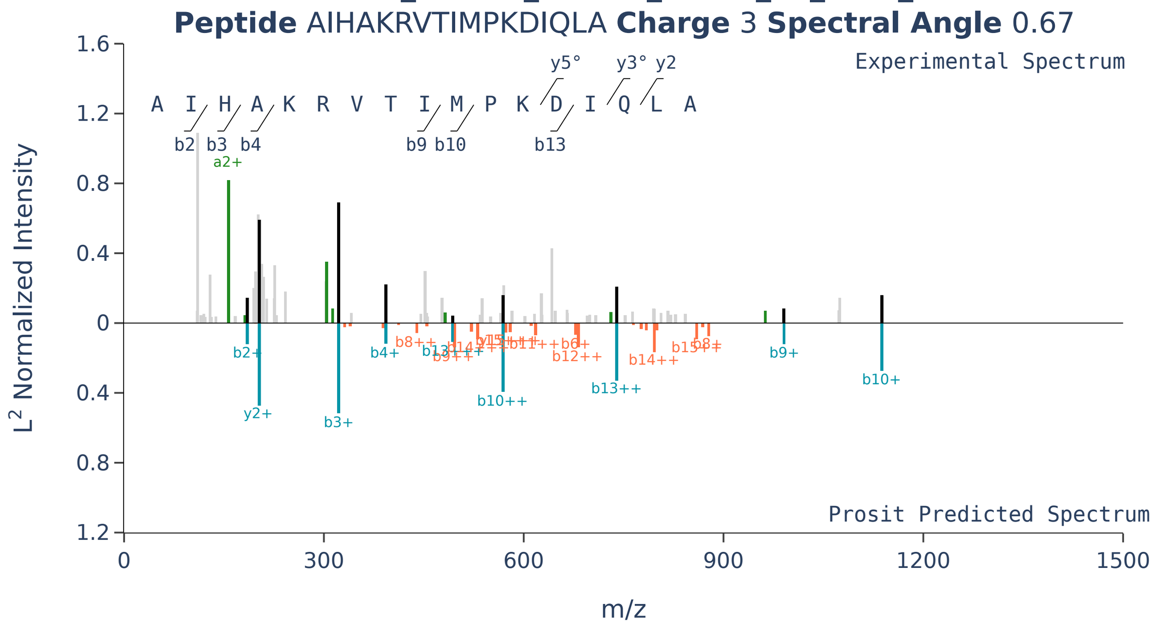


**Source** HRoetschke_271222_120123_HFGoe_H3_b2R1_4h **Scan** 13071
**Peptide** DTNLCAIHAKRVTIMPKD **Charge** 2 **Spectral Angle** 0.77

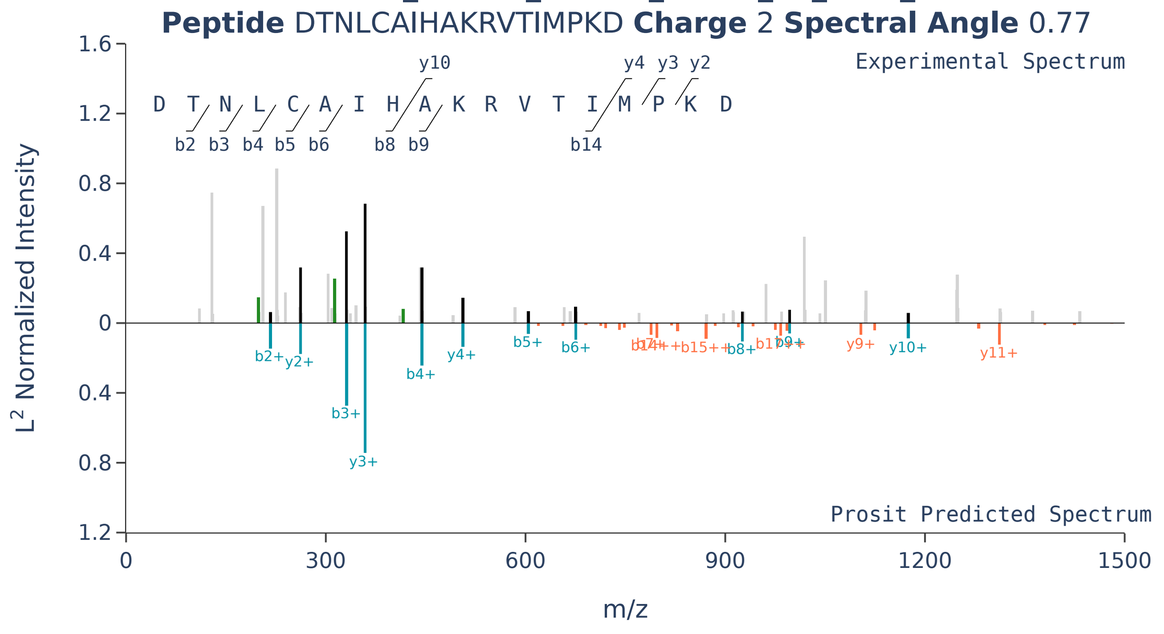


**Source** HRoetschke_271222_120123_HFGoe_H3_b1R1_4h **Scan** 17181
**Peptide** KTDLRFQSSAVMALQE **Charge** 3 **Spectral Angle** 0.62

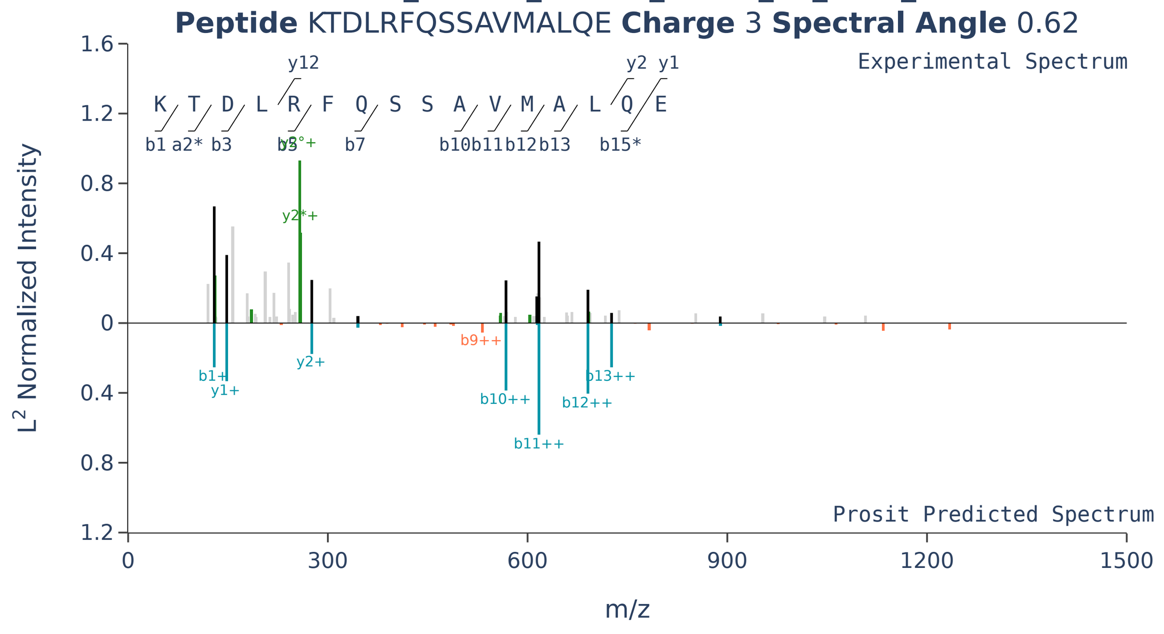


**Source** HRoetschke_271222_120123_HFGoe_H3_b2R1_24h **Scan** 10520
**Peptide** PHRYRPGTVAL **Charge** 3 **Spectral Angle** 0.57

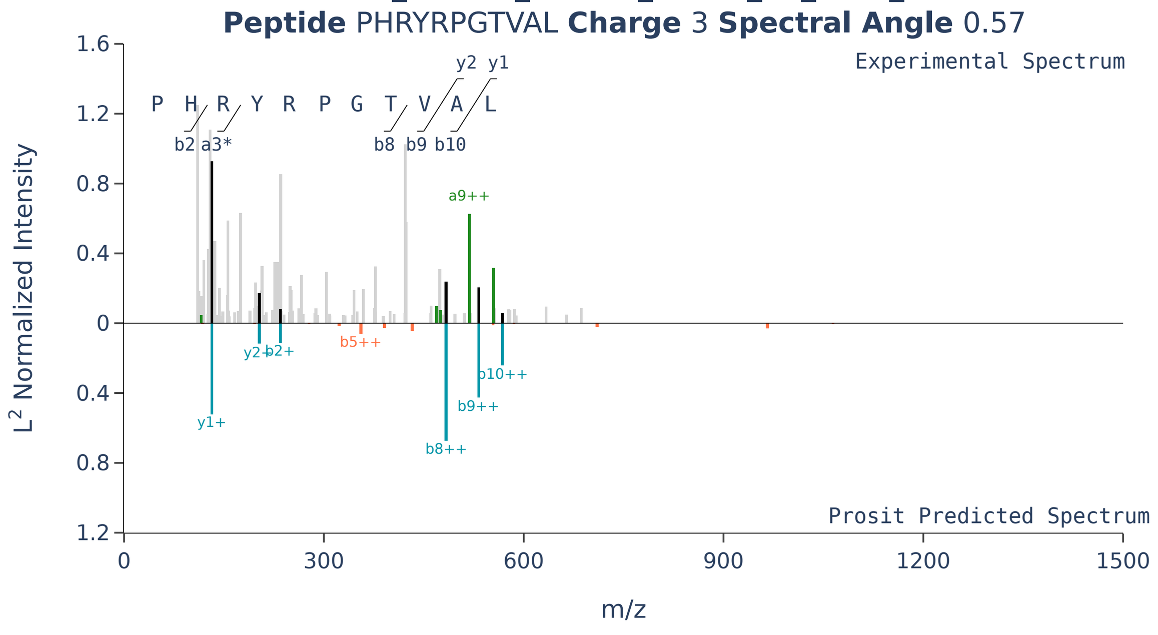


**Source** HRoetschke_271222_120123_HFGoe_H3_b2R2_2h **Scan** 7631
**Peptide** NLCAIHAK **Charge** 2 **Spectral Angle** 0.76

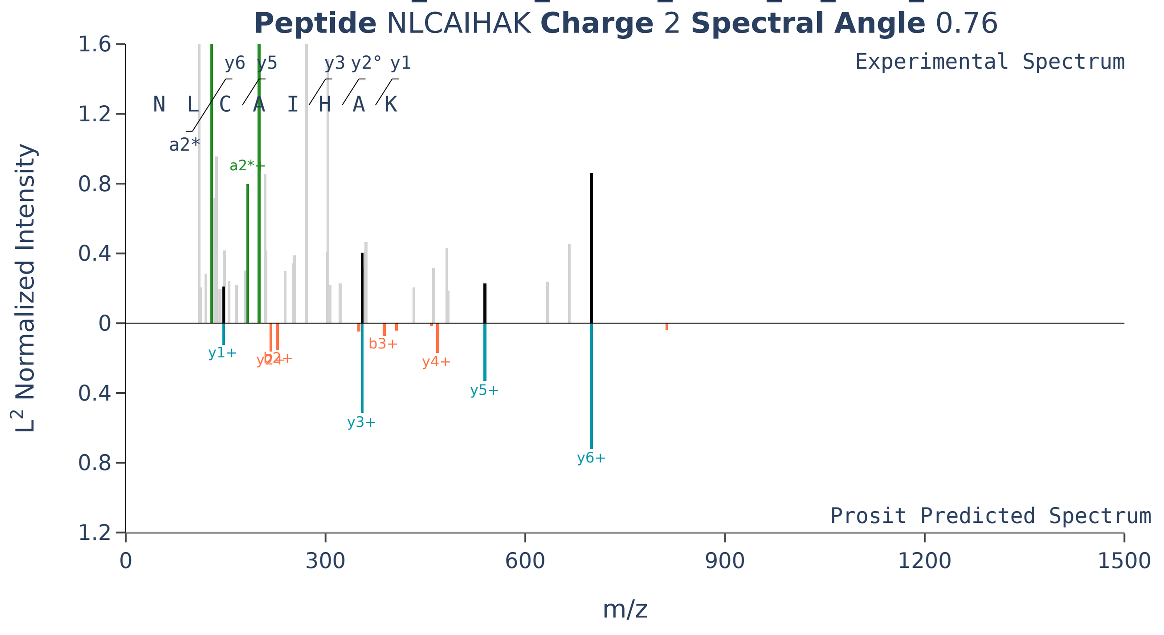


**Source** HRoetschke_271222_120123_HFGoe_H3_b2R1_2h **Scan** 14769
**Peptide** FEDTNLCAIH **Charge** 2 **Spectral Angle** 0.71

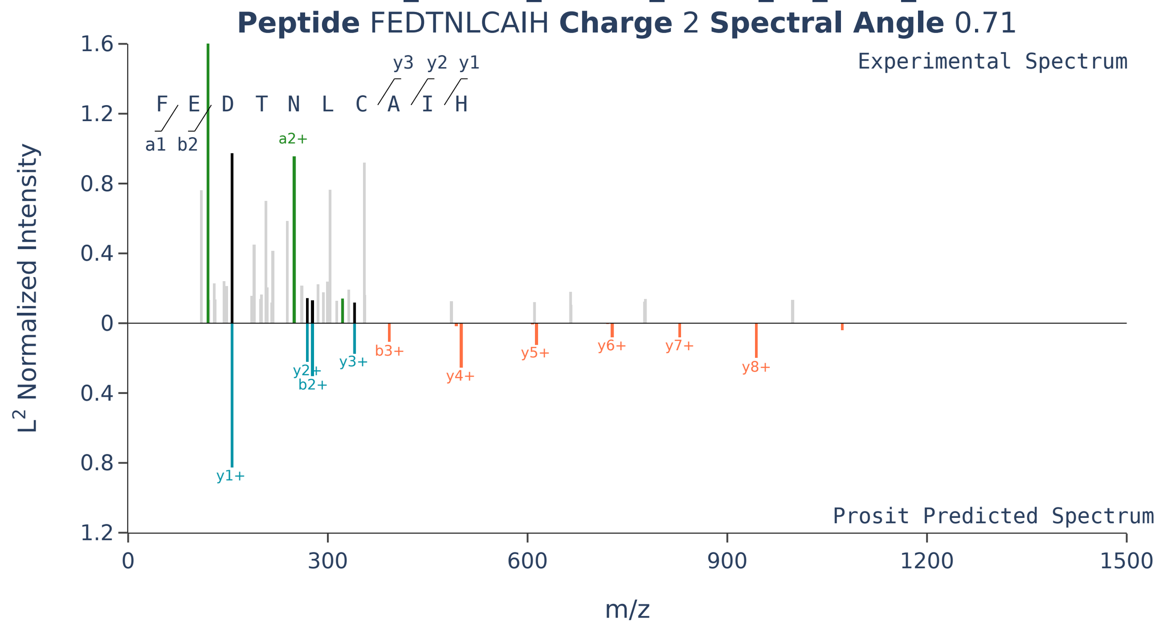


**Source** HRoetschke_271222_120123_HFGoe_H3_b2R1_4h **Scan** 11628
**Peptide** PGTVALREIRRYQKST **Charge** 4 **Spectral Angle** 0.74

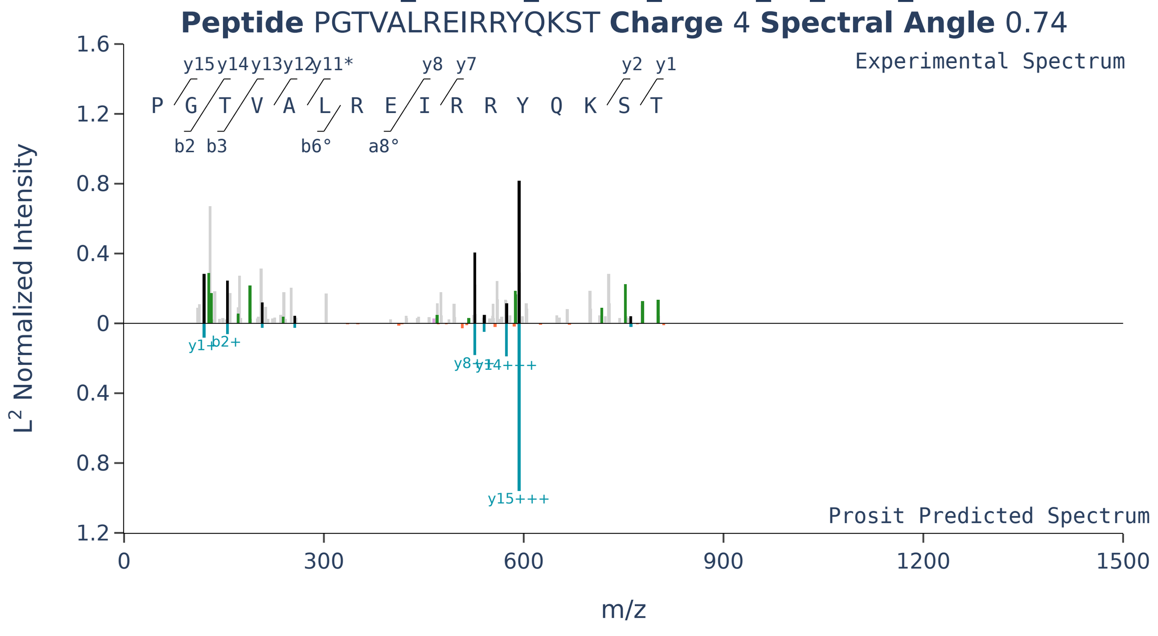


**Source** HRoetschke_271222_120123_HFGoe_H3_b2R2_2h **Scan** 11714
**Peptide** EDTNLCAIH **Charge** 2 **Spectral Angle** 0.78

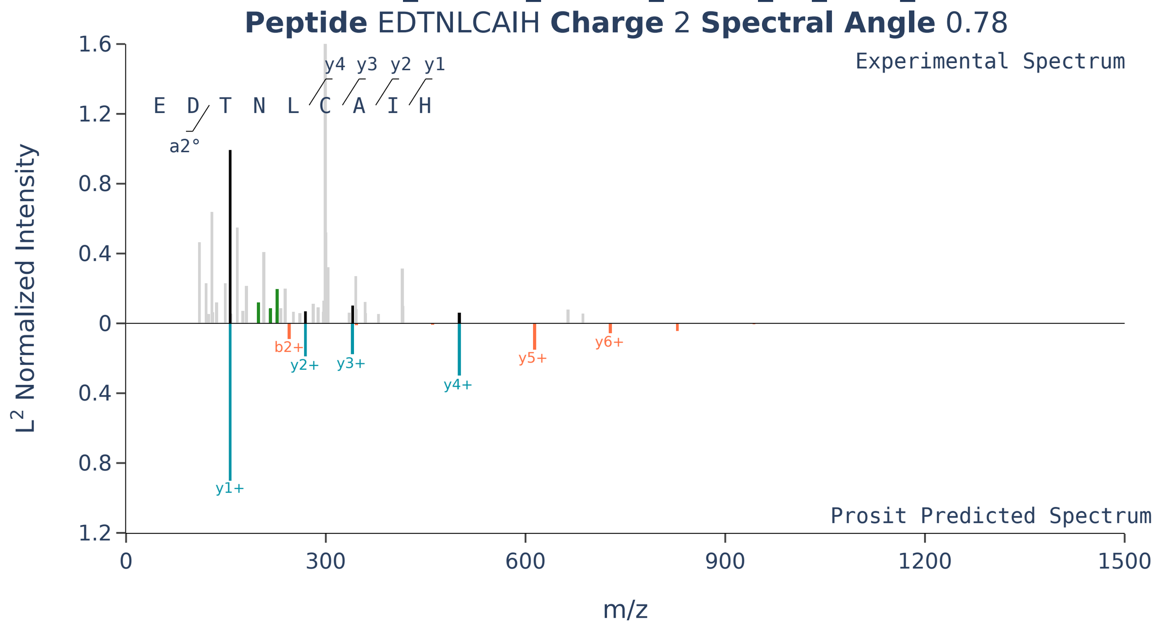


**Source** HRoetschke_271222_120123_HFGoe_H3_b2R1_4h **Scan** 13406
**Peptide** PGTVALREIRRYQ **Charge** 3 **Spectral Angle** 0.64

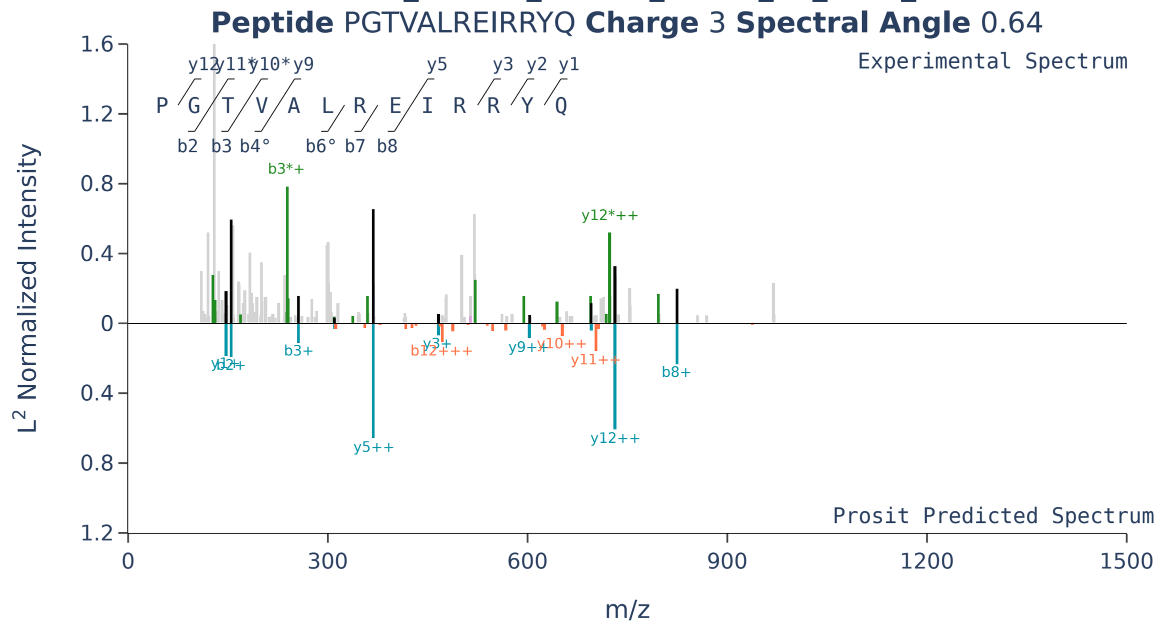


**Source** HRoetschke_271222_120123_HFGoe_H3_b2R2_4h **Scan** 5383
**Peptide** GGVKKPHRYRPGTV **Charge** 4 **Spectral Angle** 0.59

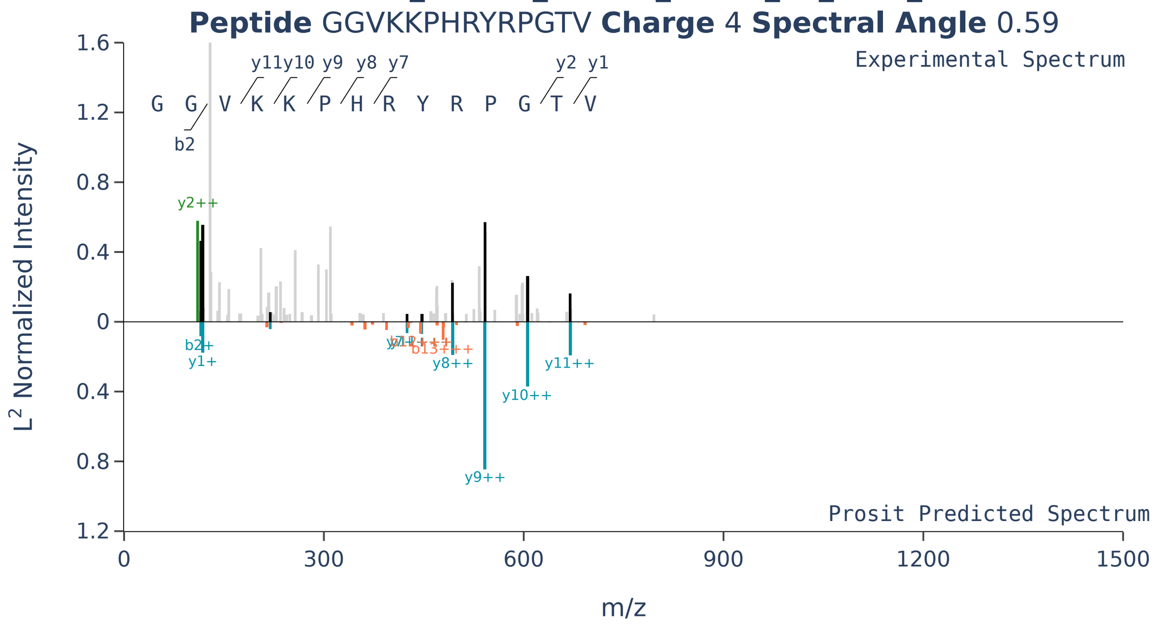


**Source** HRoetschke_271222_120123_HFGoe_H3_b2R2_4h **Scan** 5044
**Peptide** TGGKAPRKQLATKA **Charge** 4 **Spectral Angle** 0.75

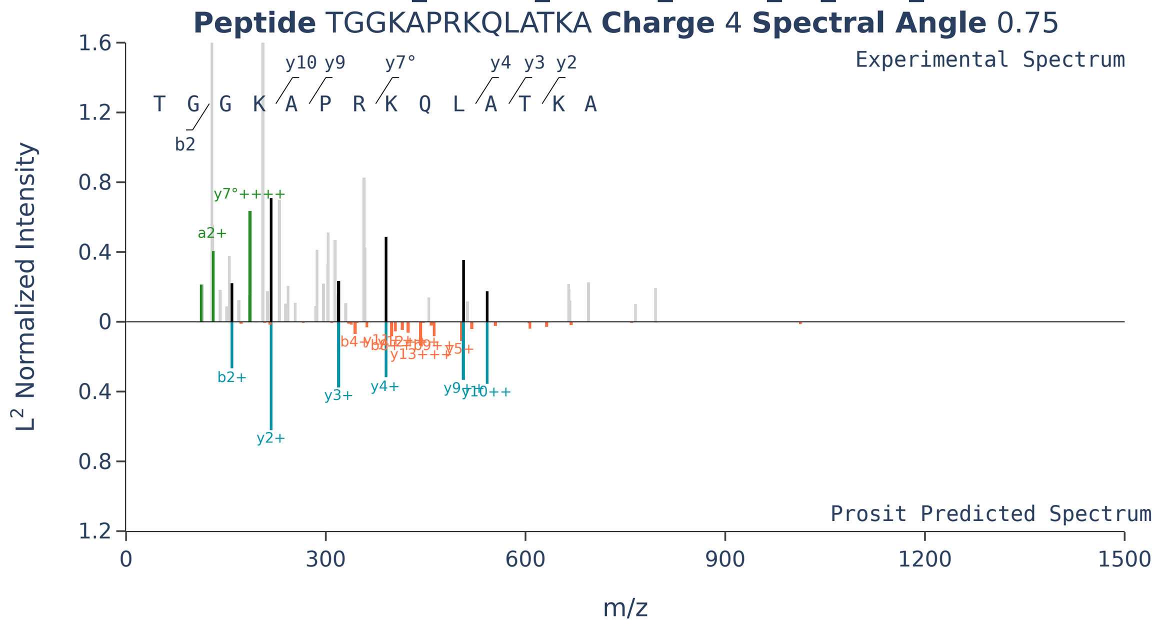


**Source** HRoetschke_271222_120123_HFGoe_H3_b2R2_4h **Scan** 5187
**Peptide** APATGGVKKPHRY **Charge** 2 **Spectral Angle** 0.57

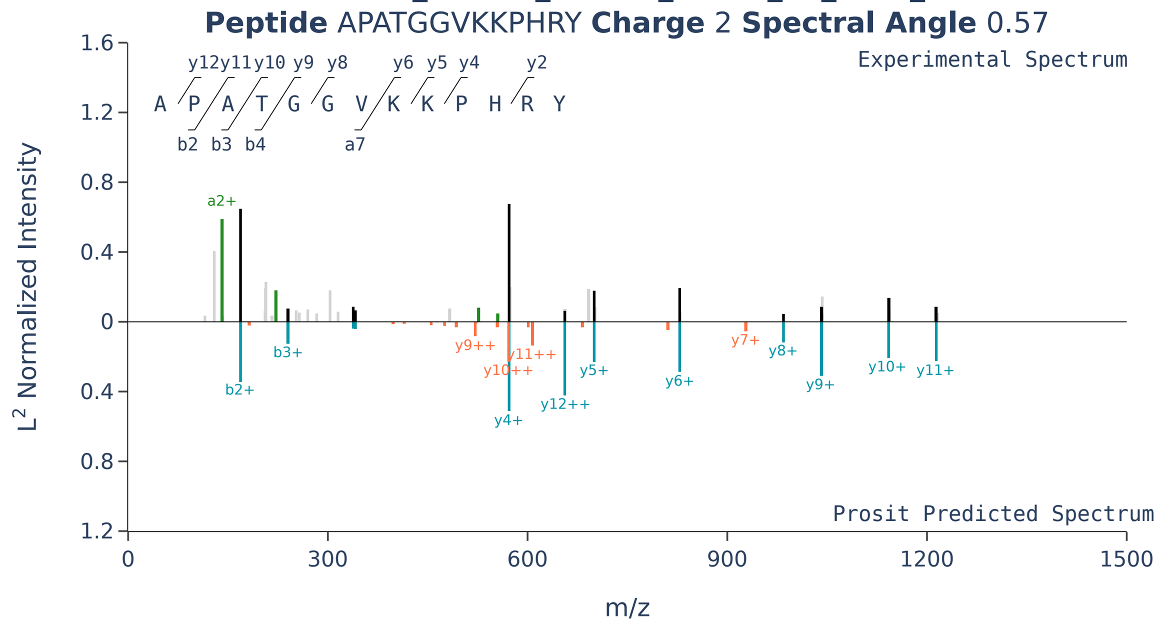


**Source** HRoetschke_271222_120123_HFGoe_H3_b1R1_4h **Scan** 14953
**Peptide** LVREIAQDFKTD **Charge** 3 **Spectral Angle** 0.72

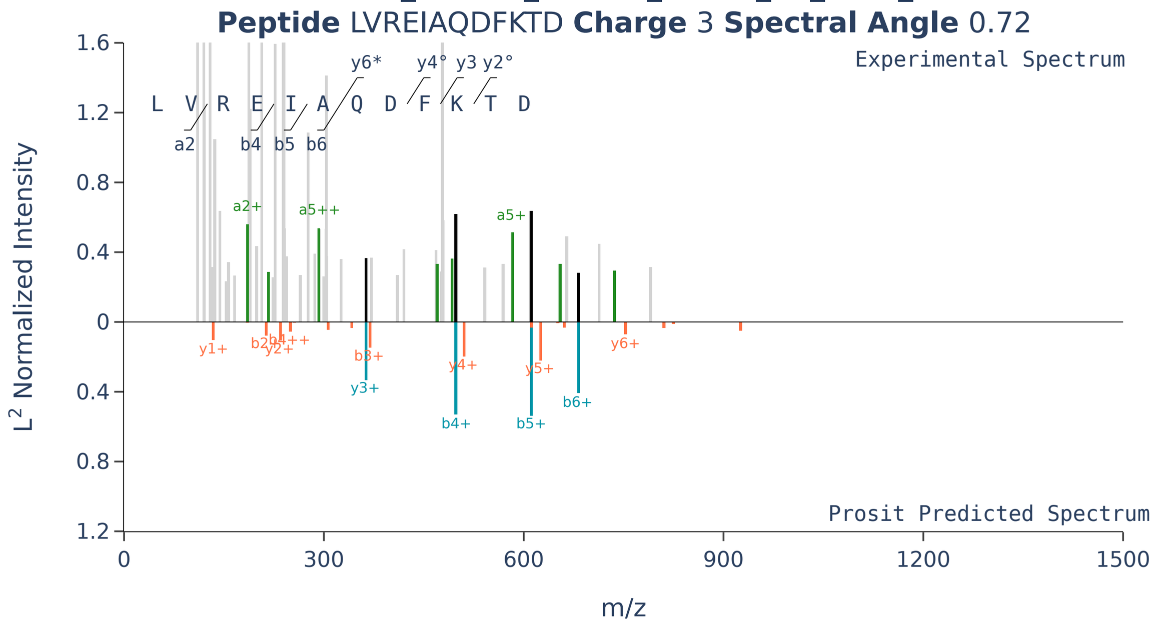


**Source** HRoetschke_271222_120123_HFGoe_H3_b2R2_2h **Scan** 12040
**Peptide** EIRRYQKSTELLIR **Charge** 4 **Spectral Angle** 0.79

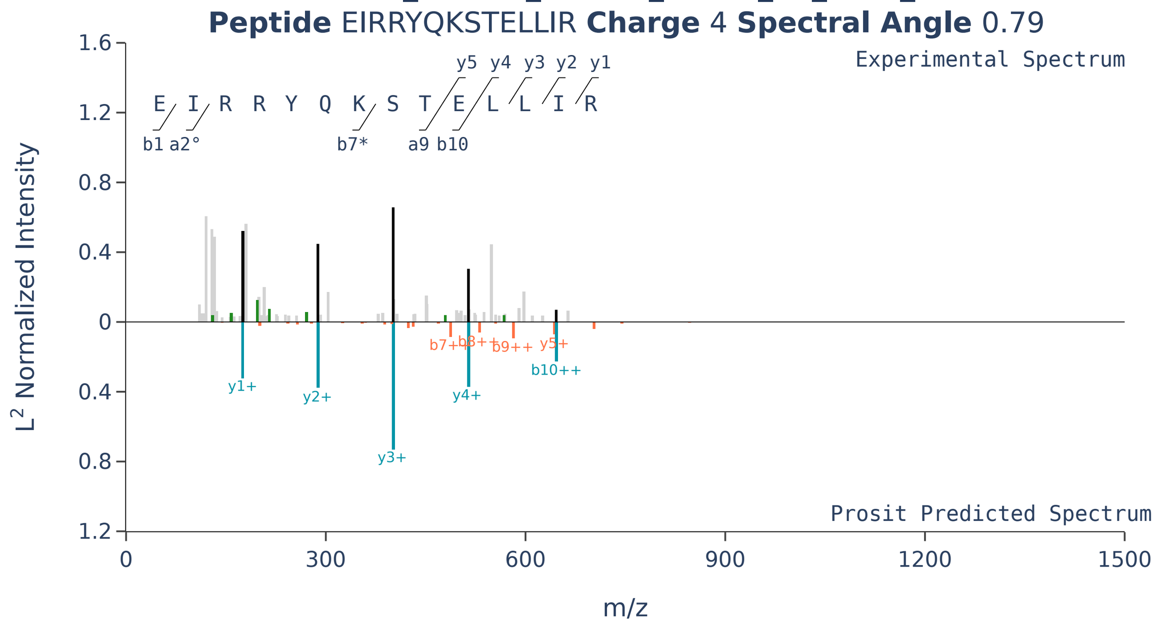


**Source** HRoetschke_271222_120123_HFGoe_H3_b2R2_4h **Scan** 5845
**Peptide** APATGGVKKPHRYRPGTVA **Charge** 4 **Spectral Angle** 0.57

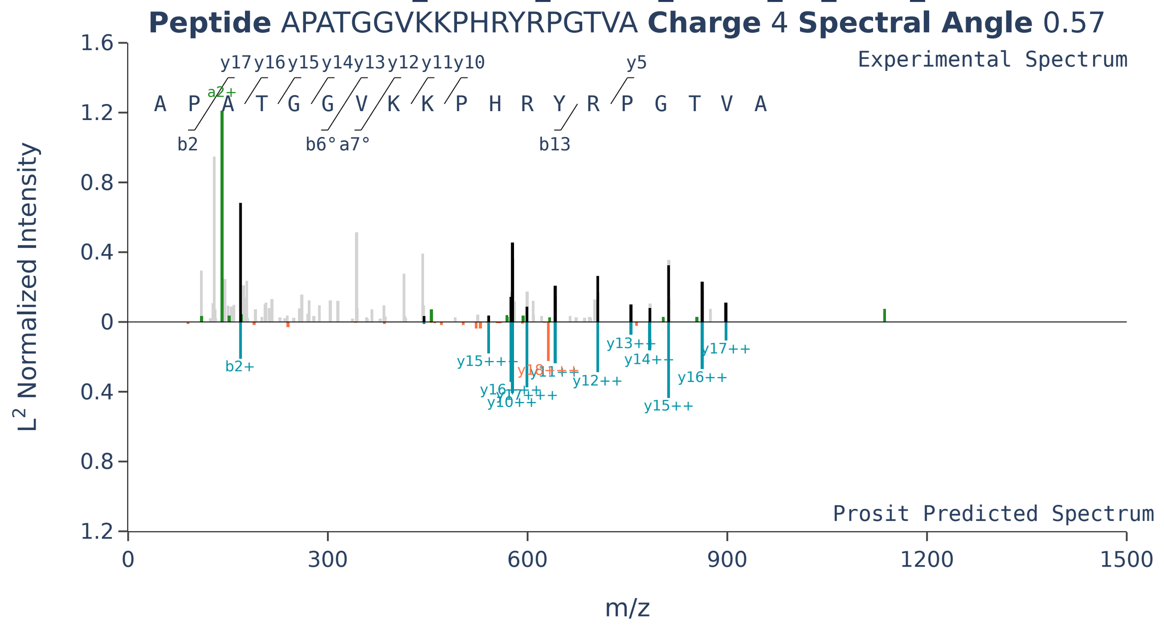

**Source** HRoetschke_271222_120123_HFGoe_H3_b1R1_24h **Scan** 5666
**Peptide** PHRYRPGTVA **Charge** 3 **Spectral Angle** 0.8

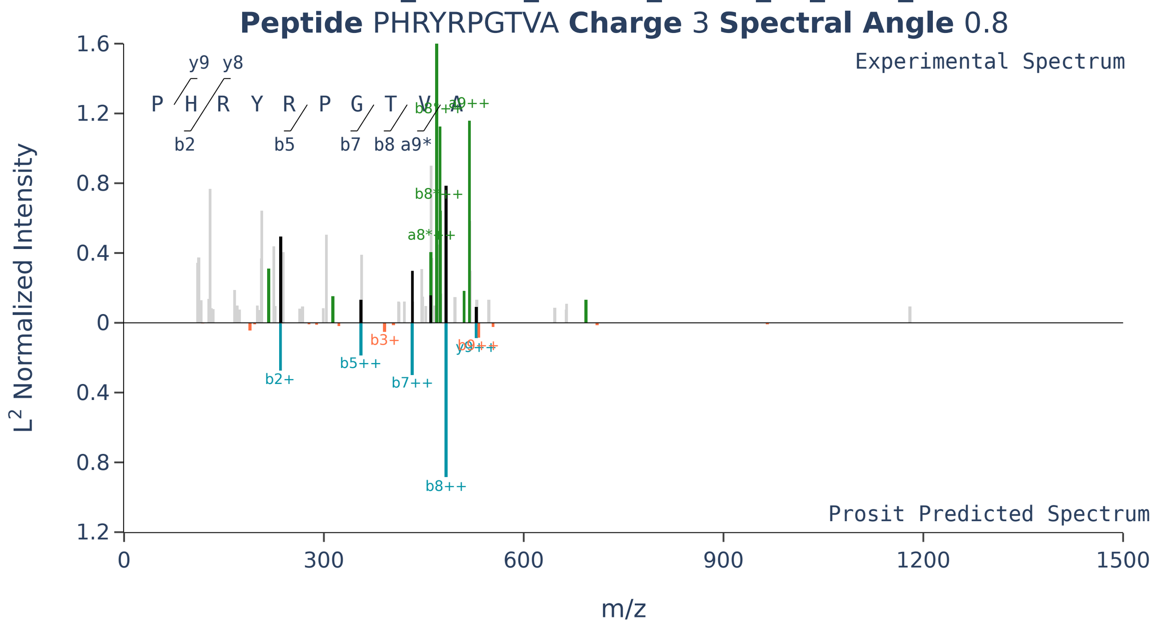


**Source** HRoetschke_271222_120123_HFGoe_H3_b2R2_4h **Scan** 5058
**Peptide** TGGKAPRKQLATKAA **Charge** 4 **Spectral Angle** 0.65

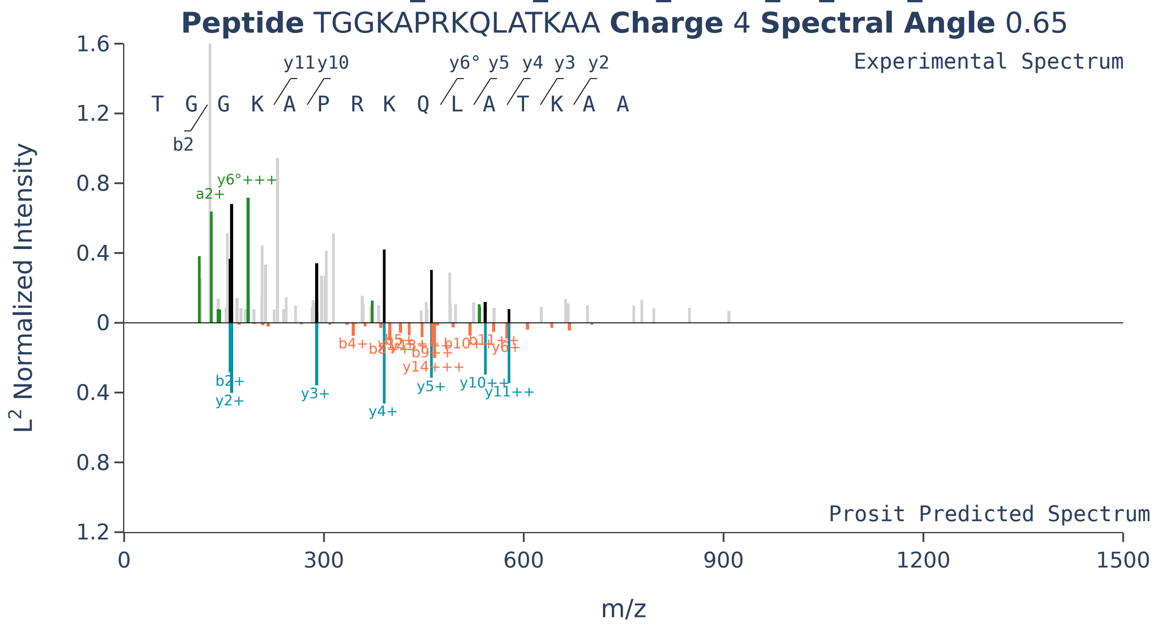


**Source** HRoetschke_271222_120123_HFGoe_H3_b1R1_24h **Scan** 13051
**Peptide** RYRPGTVALREI **Charge** 3 **Spectral Angle** 0.68

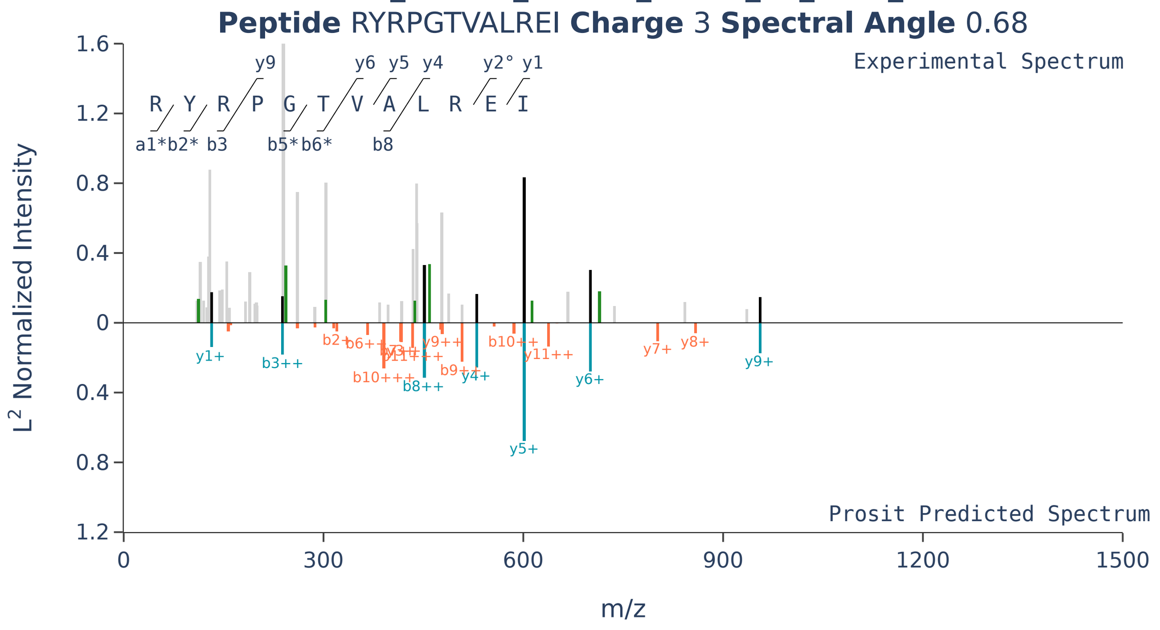


**Source** HRoetschke_271222_120123_HFGoe_H3_b2R2_4h **Scan** 6489
**Peptide** RYRPGTV **Charge** 2 **Spectral Angle** 0.64

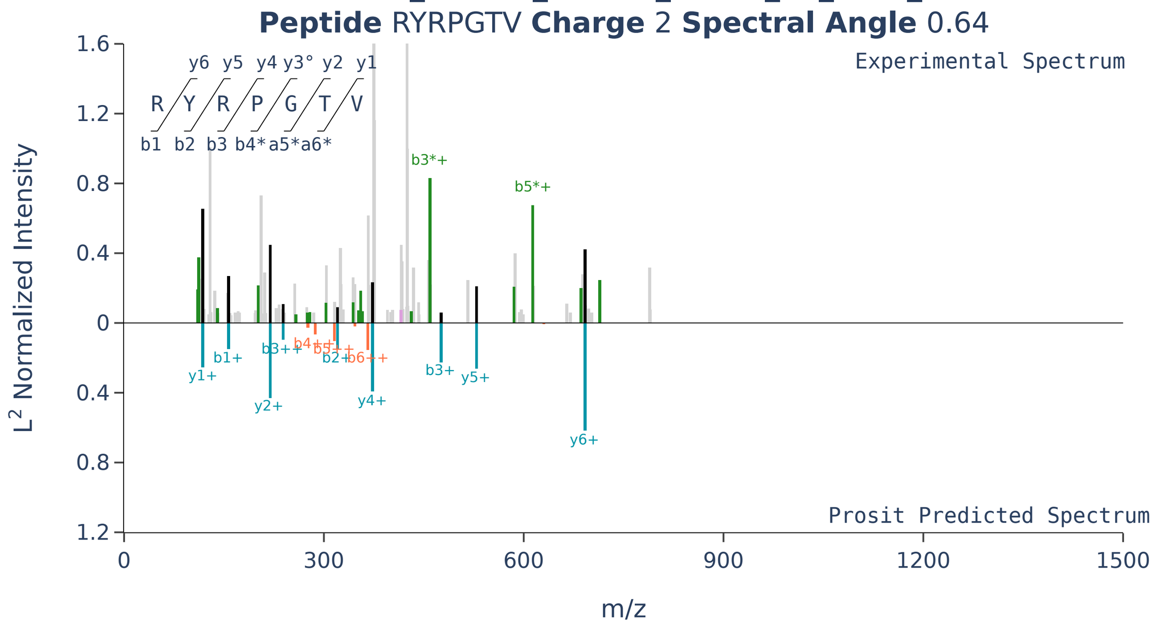


**Source** HRoetschke_271222_120123_HFGoe_H3_b1R2_4h **Scan** 20239
**Peptide** DLRFQSSAVMALQ **Charge** 2 **Spectral Angle** 0.65

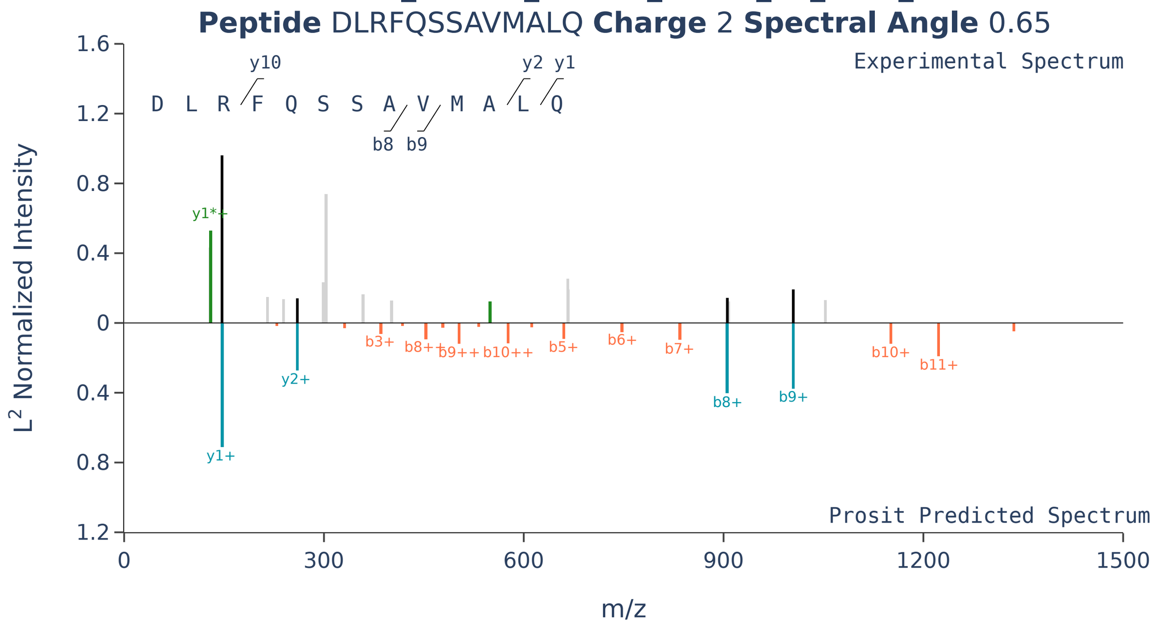


**Source** HRoetschke_271222_120123_HFGoe_H3_b2R1_1h **Scan** 16092
**Peptide** ALFEDTNLCAIHAKRV **Charge** 3 **Spectral Angle** 0.58

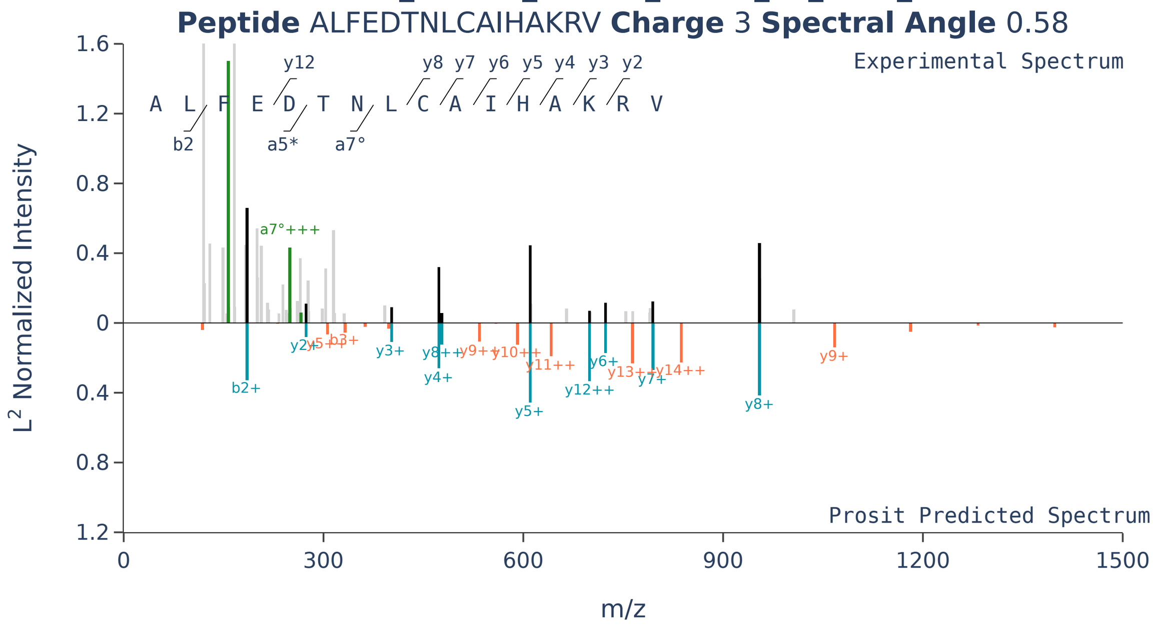


**Source** HRoetschke_271222_120123_HFGoe_H3_b1R1_4h **Scan** 17820
**Peptide** REIAQDFKTDLRFQSSAVM **Charge** 3 **Spectral Angle** 0.78

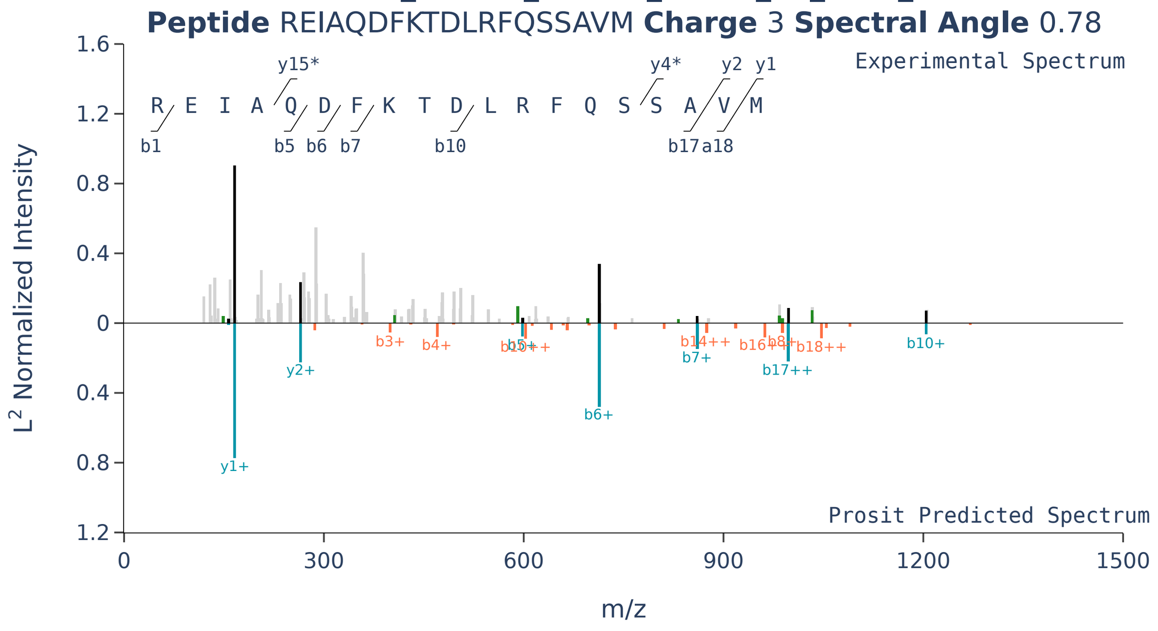


**Source** HRoetschke_271222_120123_HFGoe_H3_b1R1_24h **Scan** 14854
**Peptide** AKRVTIMPKDIQL **Charge** 3 **Spectral Angle** 0.55

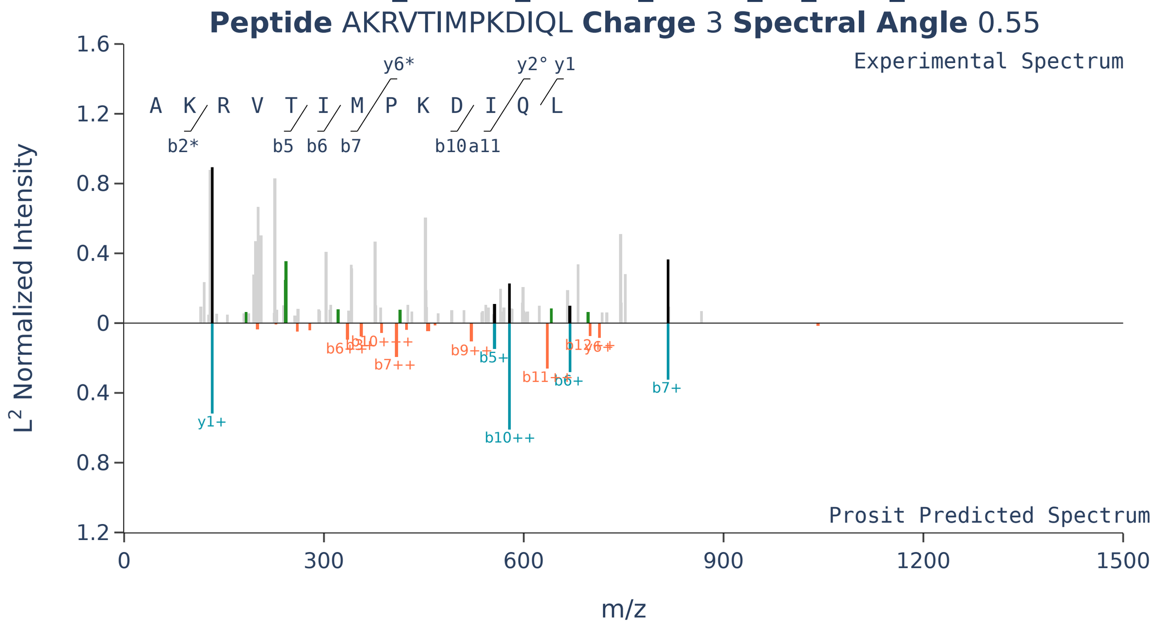


**Source** HRoetschke_271222_120123_HFGoe_H3_b1R1_4h **Scan** 13171
**Peptide** HAKRVTIMPKDIQL **Charge** 4 **Spectral Angle** 0.57

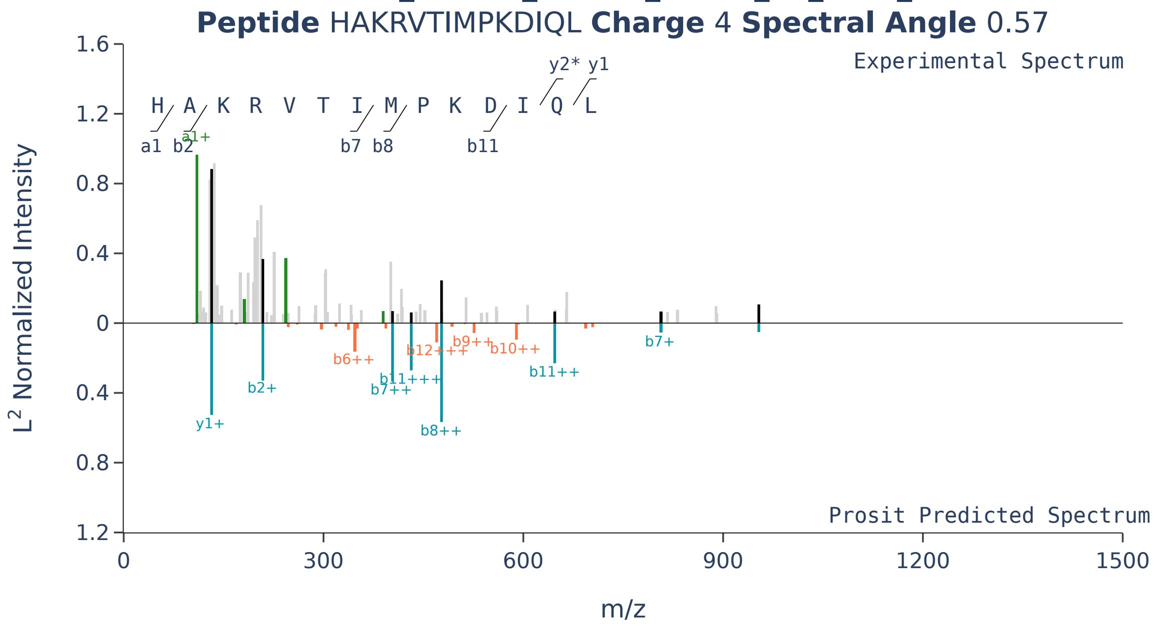


**Source** HRoetschke_271222_120123_HFGoe_H3_b2R1_24h **Scan** 8812
**Peptide** RLVREIAQD **Charge** 3 **Spectral Angle** 0.59

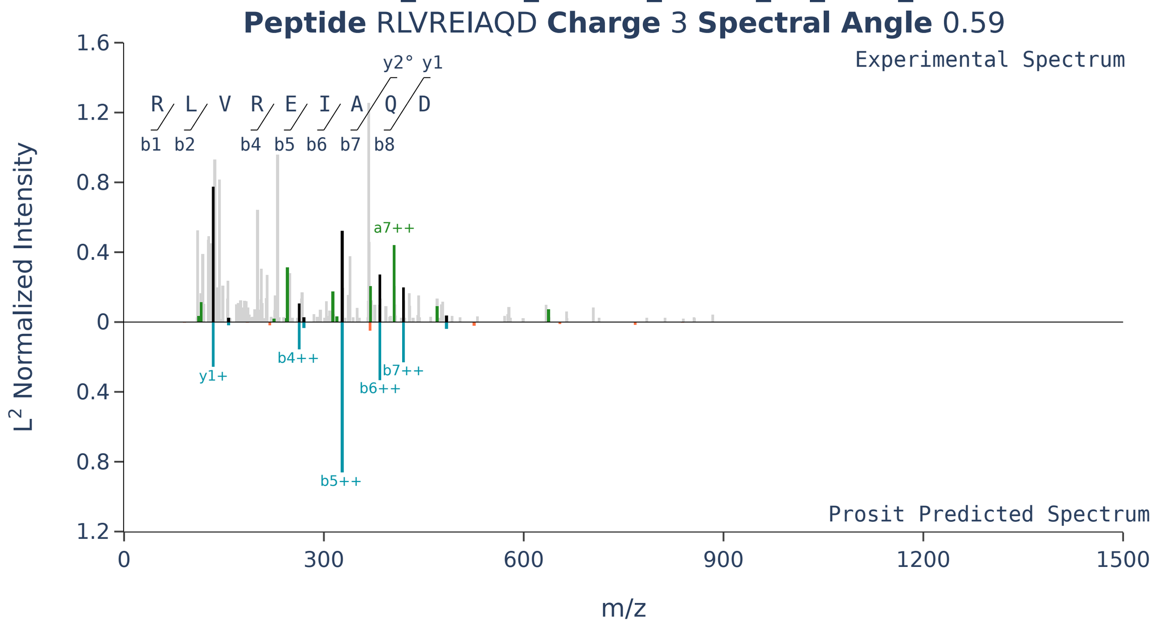


**Source** HRoetschke_271222_120123_HFGoe_H3_b1R1_24h **Scan** 5121
**Peptide** PATGGVKKPHRY **Charge** 3 **Spectral Angle** 0.61

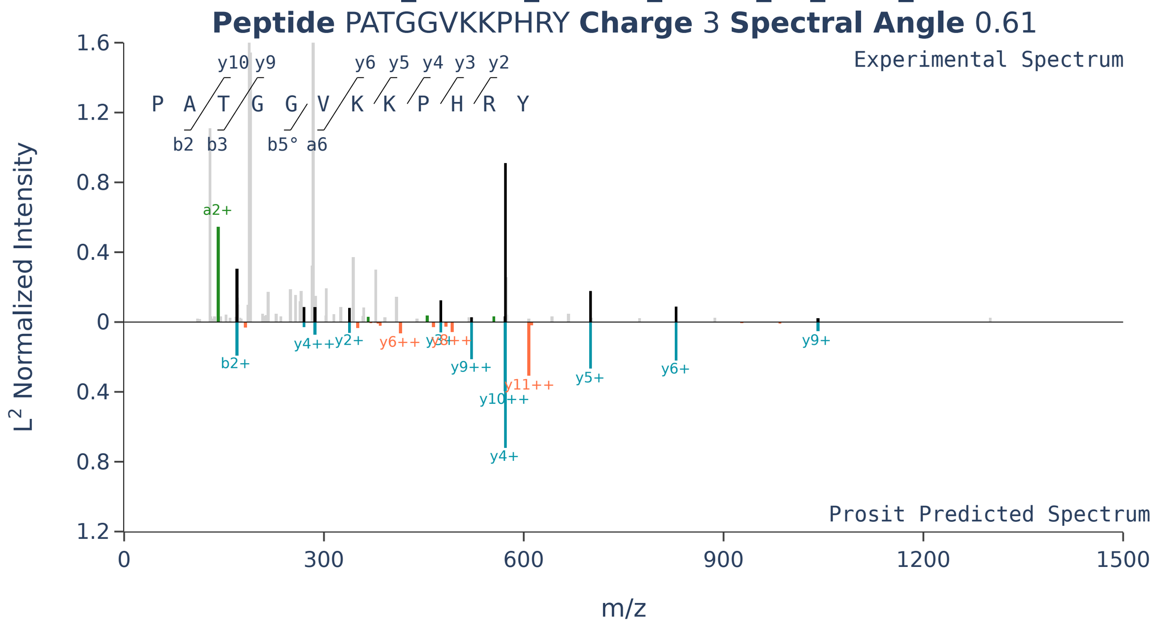


**Source** HRoetschke_271222_120123_HFGoe_H3_b1R1_4h **Scan** 9974
**Peptide** PGTVALREIRRYQKS **Charge** 3 **Spectral Angle** 0.64

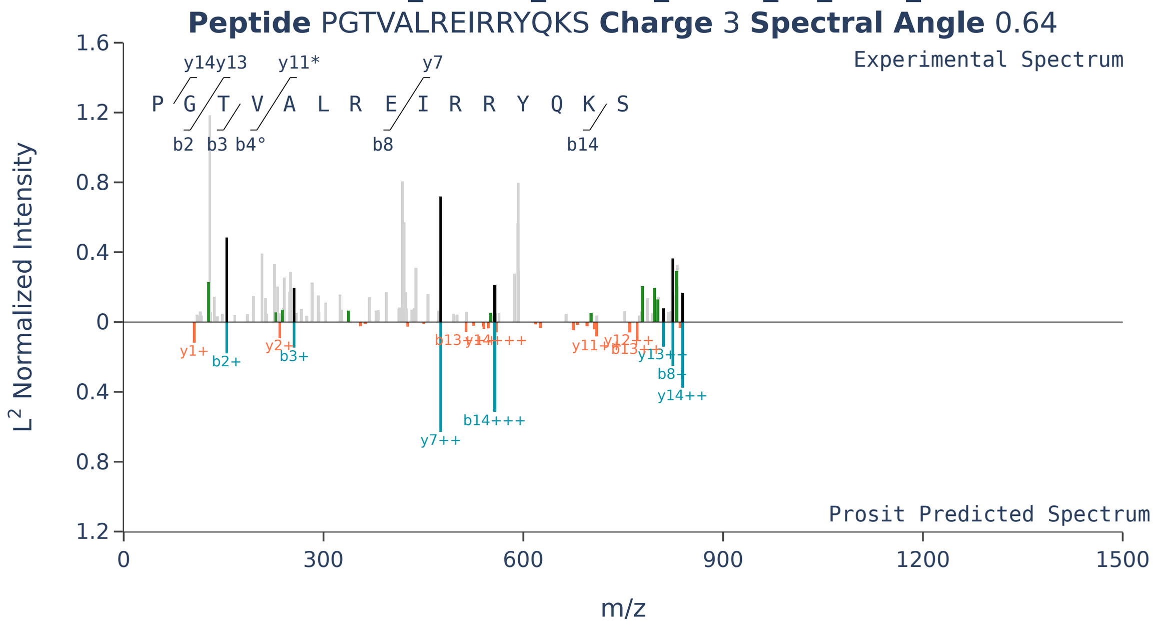


**Source** HRoetschke_271222_120123_HFGoe_H3_b1R1_1h **Scan** 10049
**Peptide** FQRLVRE **Charge** 2 **Spectral Angle** 0.71

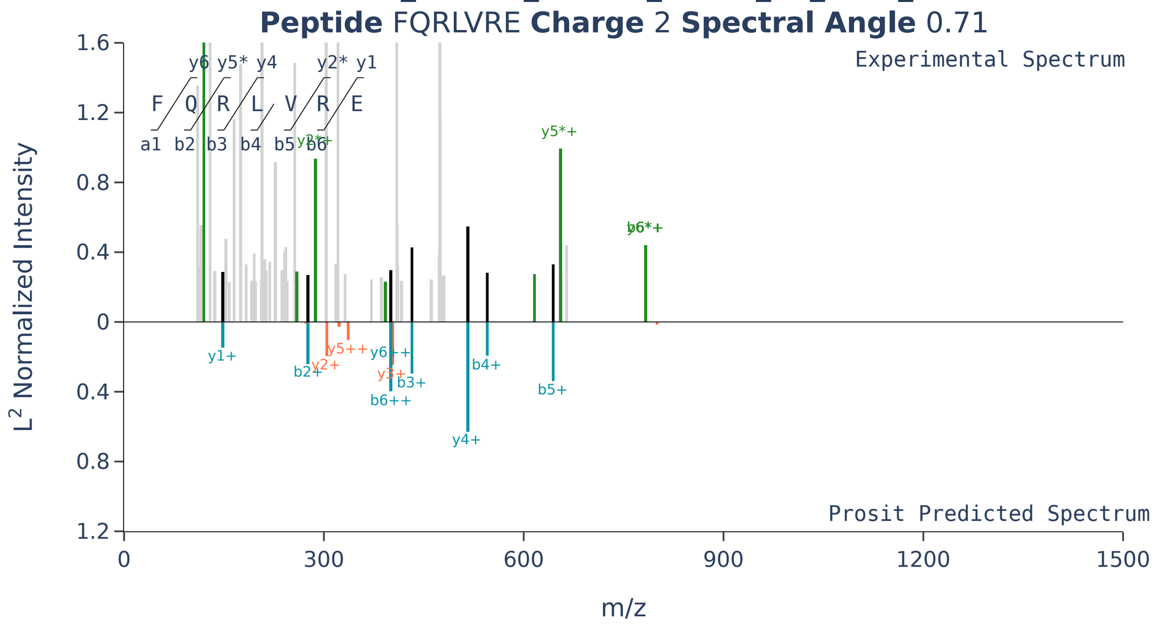


**Source** HRoetschke_271222_120123_HFGoe_H3_b2R2_2h **Scan** 22031
**Peptide** ALFEDTNLCAIH **Charge** 2 **Spectral Angle** 0.69

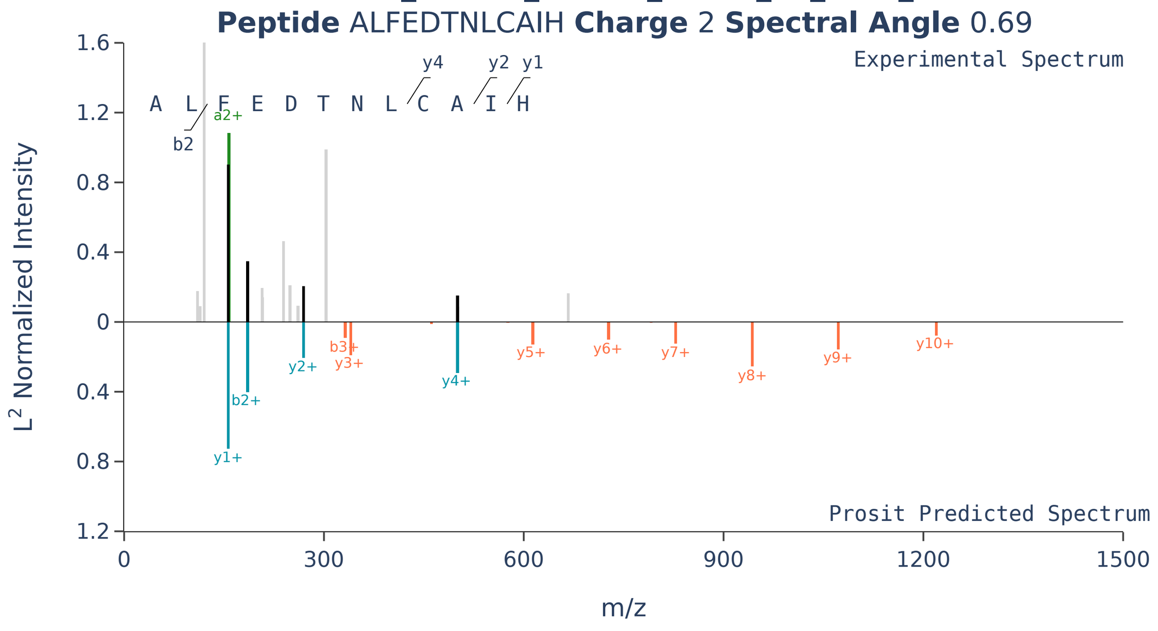


**Source** HRoetschke_271222_120123_HFGoe_H3_b2R2_4h **Scan** 4959
**Peptide** REIRRYQKST **Charge** 3 **Spectral Angle** 0.62

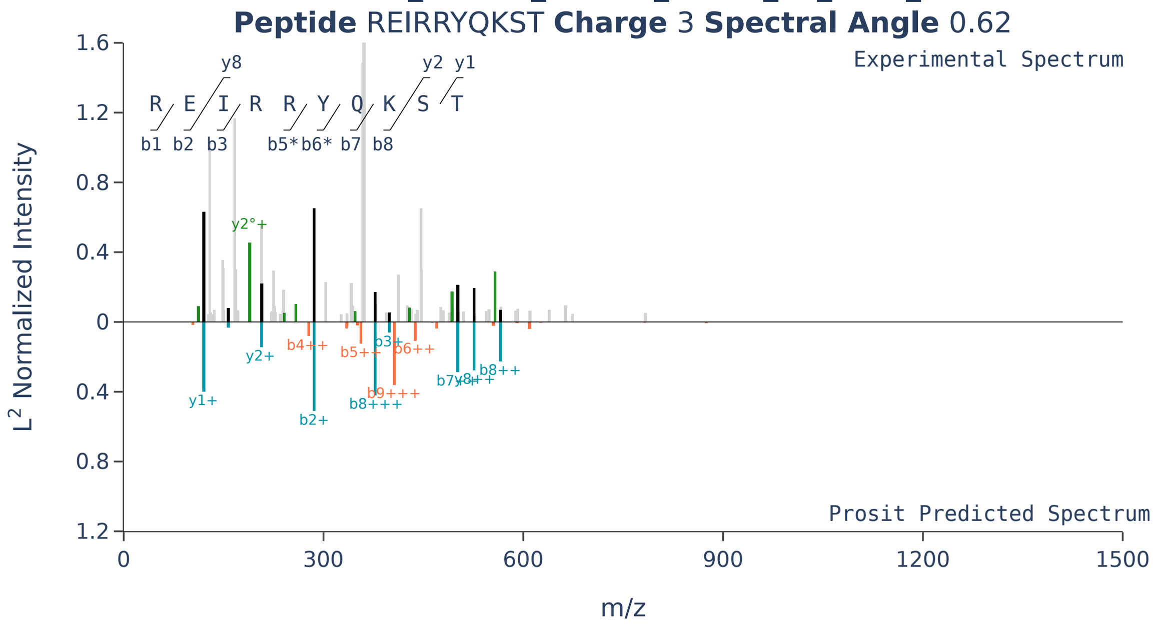

**Source** HRoetschke_271222_120123_HFGoe_H3_b2R1_2h **Scan** 7939
**Peptide** IQLARRIR **Charge** 2 **Spectral Angle** 0.74

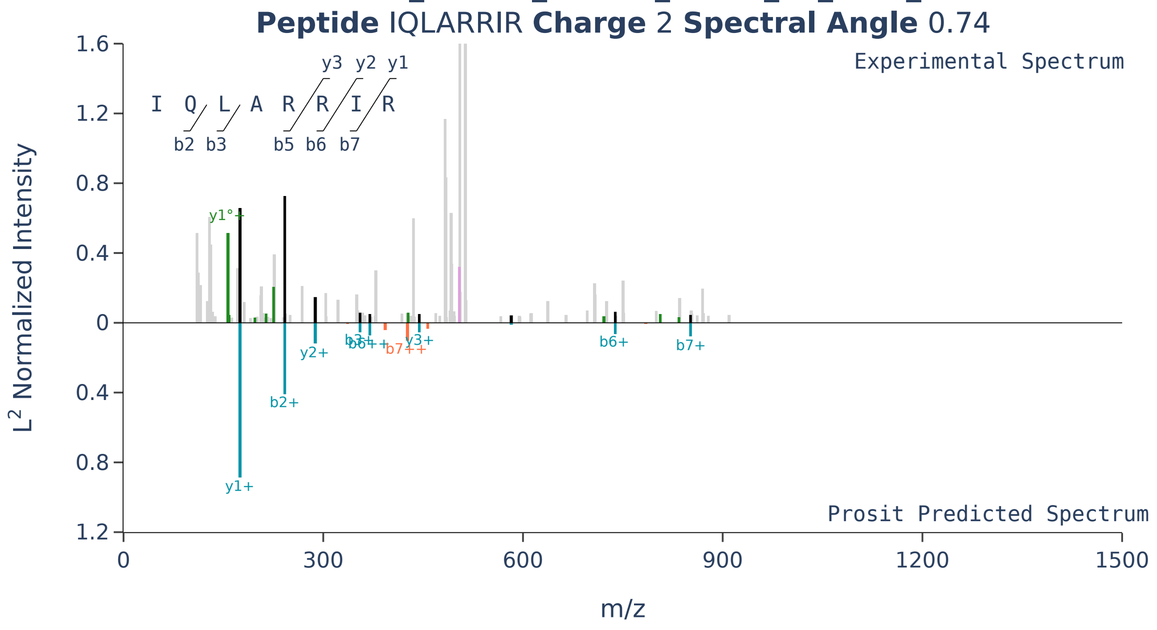


**Source** HRoetschke_271222_120123_HFGoe_H3_b2R1_1h **Scan** 19548
**Peptide** LVREIAQDFKTDLRFQ **Charge** 3 **Spectral Angle** 0.69

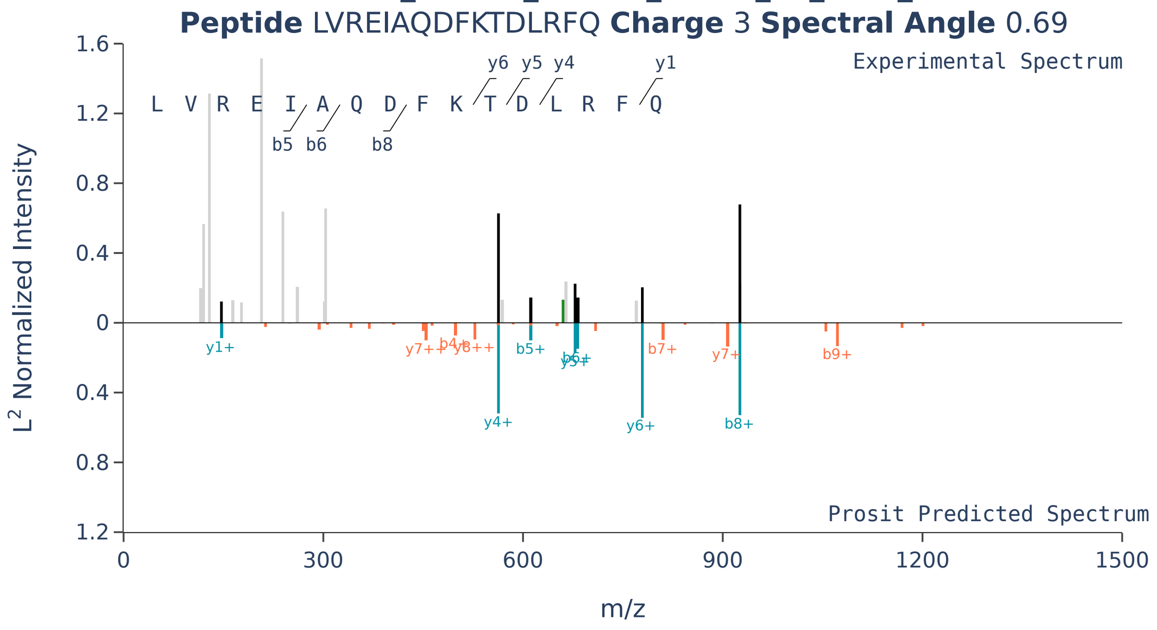


**Source** HRoetschke_271222_120123_HFGoe_H3_b2R1_4h **Scan** 4981
**Peptide** ATGGVKKPHRYR **Charge** 3 **Spectral Angle** 0.76

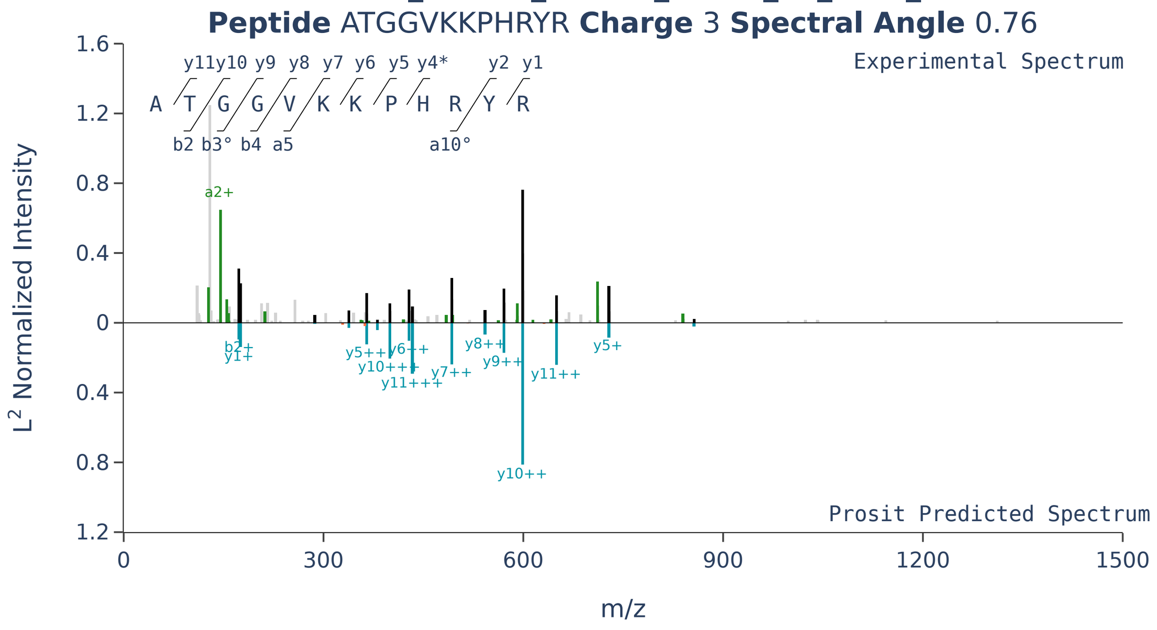


**Source** HRoetschke_271222_120123_HFGoe_H3_b2R2_4h **Scan** 5962
**Peptide** ALREIRRYQKS **Charge** 4 **Spectral Angle** 0.7

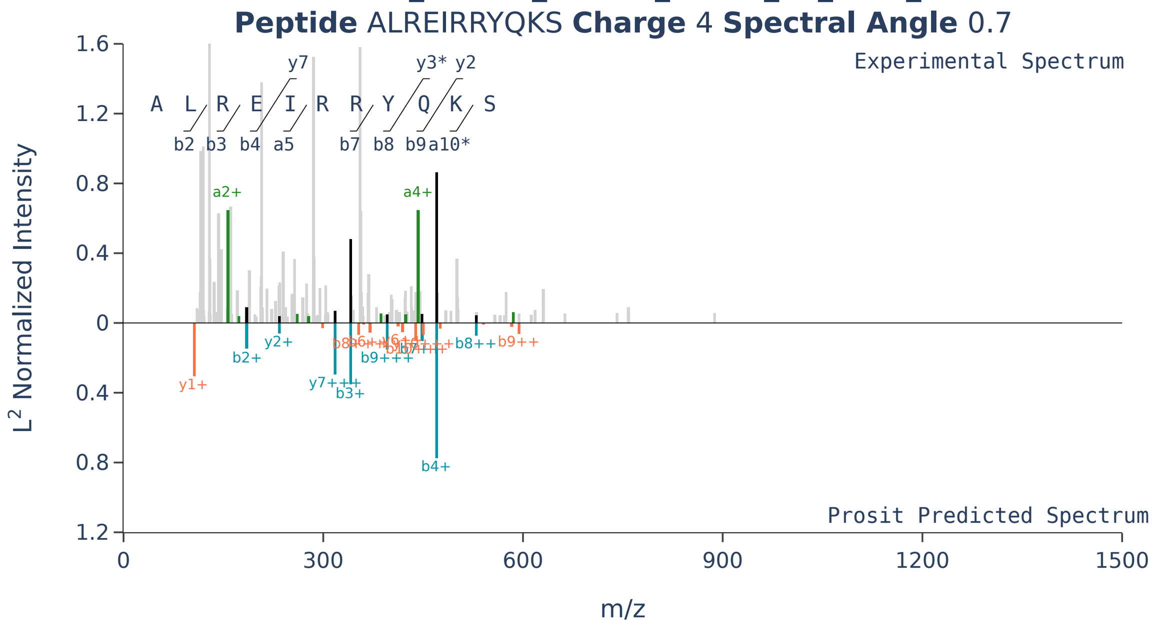


**Source** HRoetschke_271222_120123_HFGoe_H3_b2R2_4h **Scan** 12143
**Peptide** ELAQDFKTD **Charge** 2 **Spectral Angle** 0.61

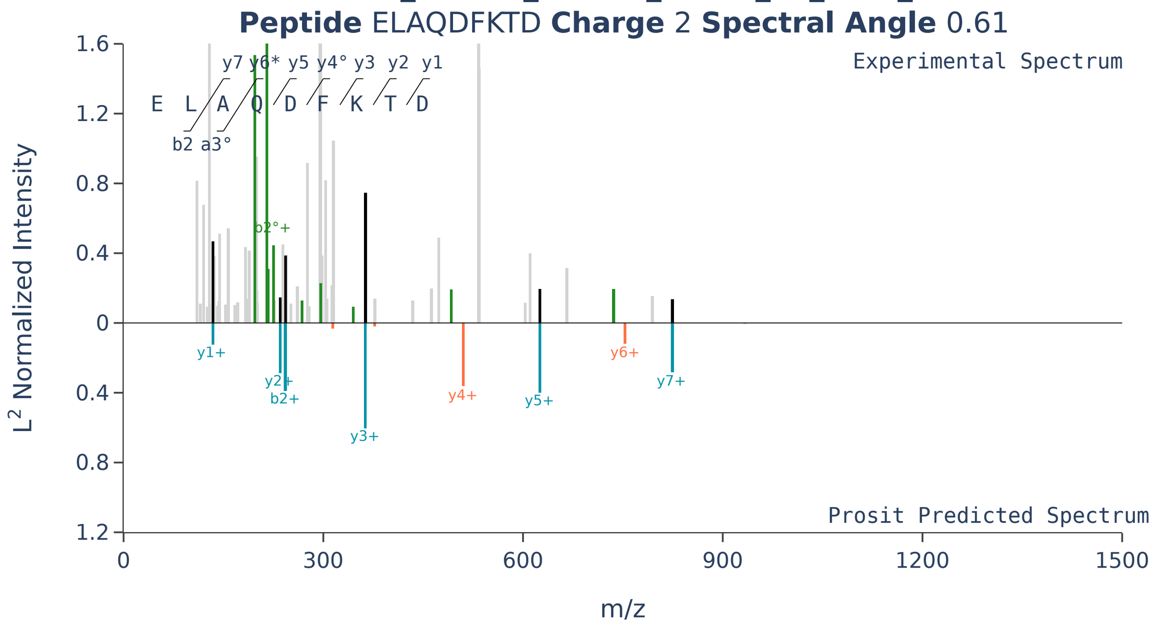


**Source** HRoetschke_271222_120123_HFGoe_H3_b2R1_1h **Scan** 29170
**Peptide** SEAYLVALFE **Charge** 2 **Spectral Angle** 0.69

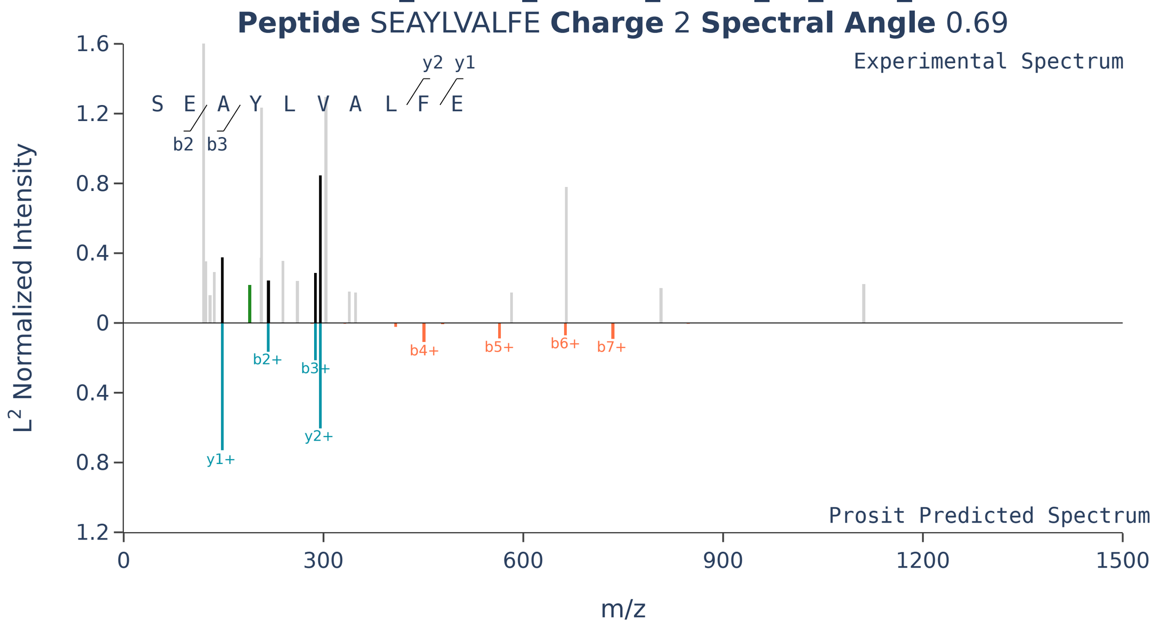


**Source** HRoetschke_271222_120123_HFGoe_H3_b1R2_24h **Scan** 16857
**Peptide** ELRRYQKSTELLI **Charge** 3 **Spectral Angle** 0.52

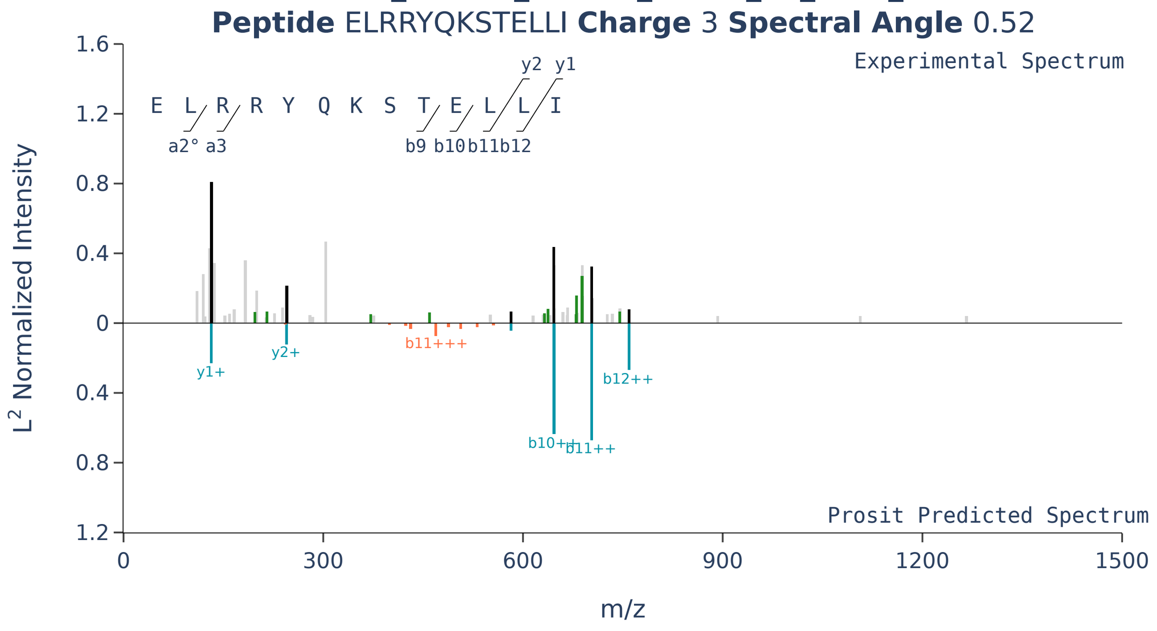


**Source** HRoetschke_271222_120123_HFGoe_H3_b1R1_24h **Scan** 6045
**Peptide** ALREIRRYQKSTE **Charge** 3 **Spectral Angle** 0.68

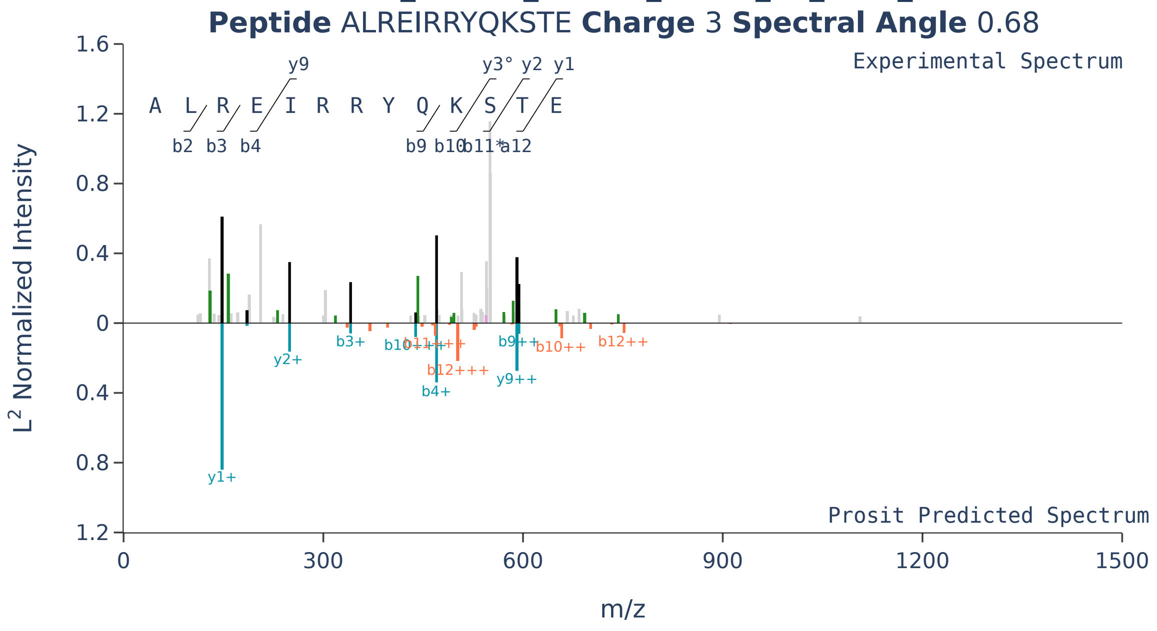


**Source** HRoetschke_271222_120123_HFGoe_H3_b2R1_4h **Scan** 13434
**Peptide** NLCAIHAKRVTIMPKD **Charge** 3 **Spectral Angle** 0.61

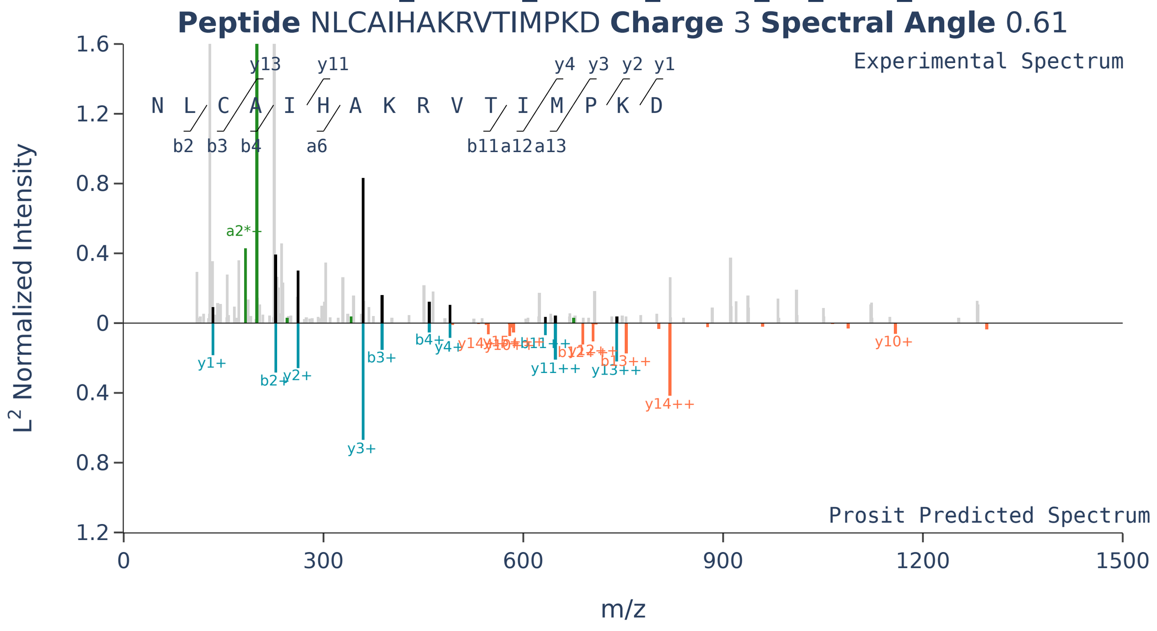


**Source** HRoetschke_271222_120123_HFGoe_H3_b2R1_1h **Scan** 16376
**Peptide** VREIAQDFKTDLRFQSSA **Charge** 3 **Spectral Angle** 0.69

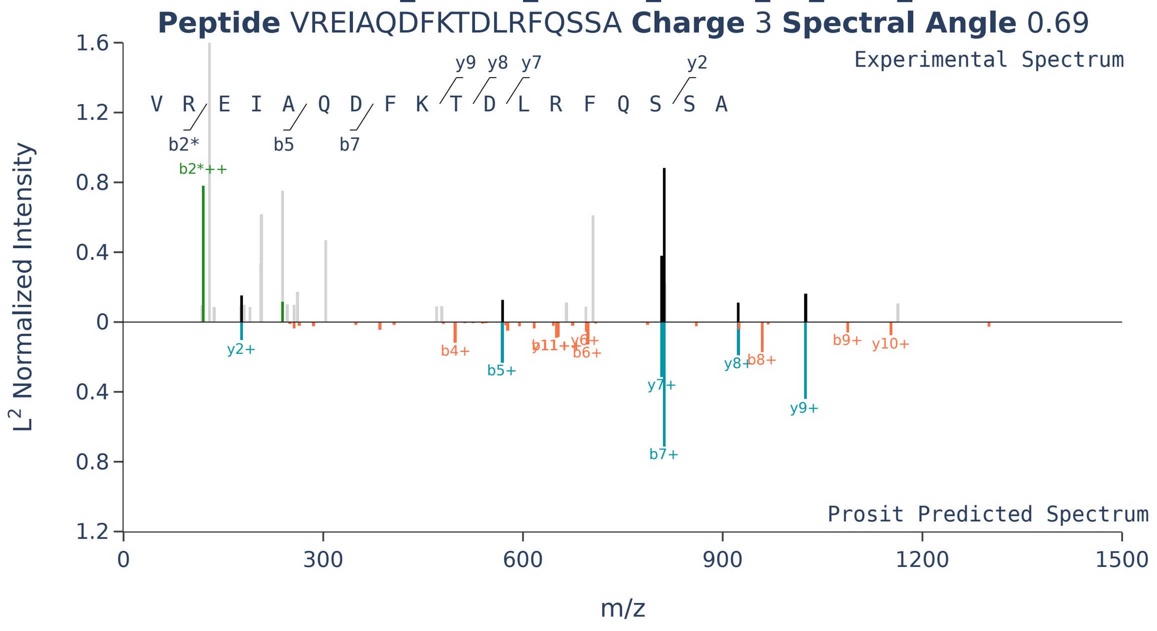


**Source** HRoetschke_271222_120123_HFGoe_H3_b2R1_2h **Scan** 14943
**Peptide** LMPKDIQLA **Charge** 2 **Spectral Angle** 0.58

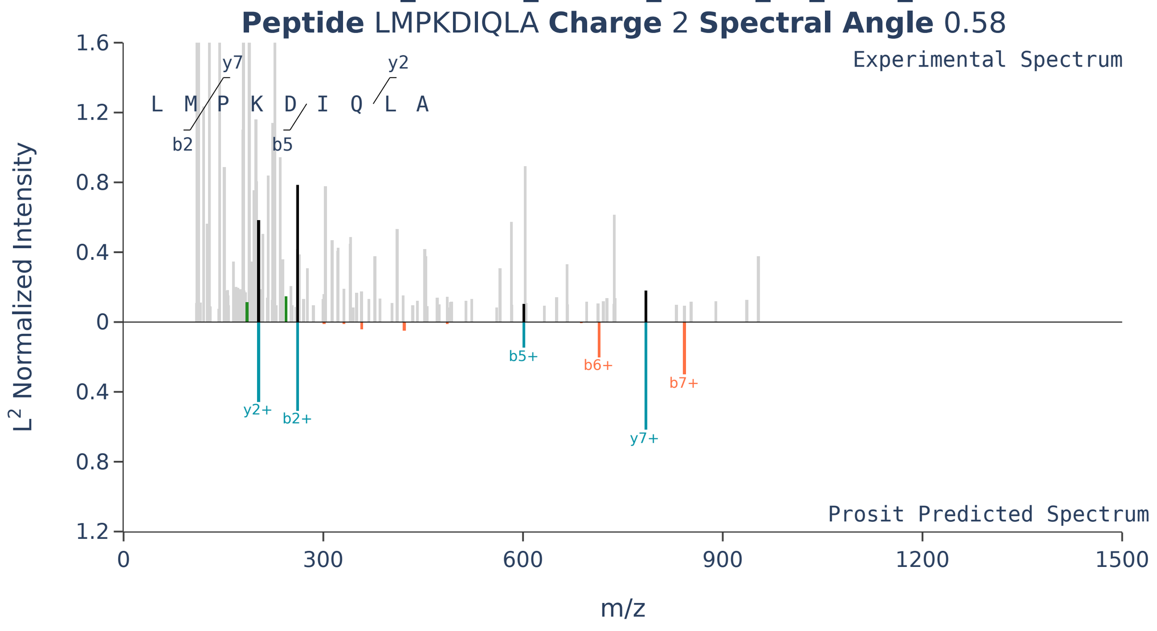


**Source** HRoetschke_271222_120123_HFGoe_H3_b2R2_4h **Scan** 16191
**Peptide** KLPFQRLVRE **Charge** 3 **Spectral Angle** 0.52

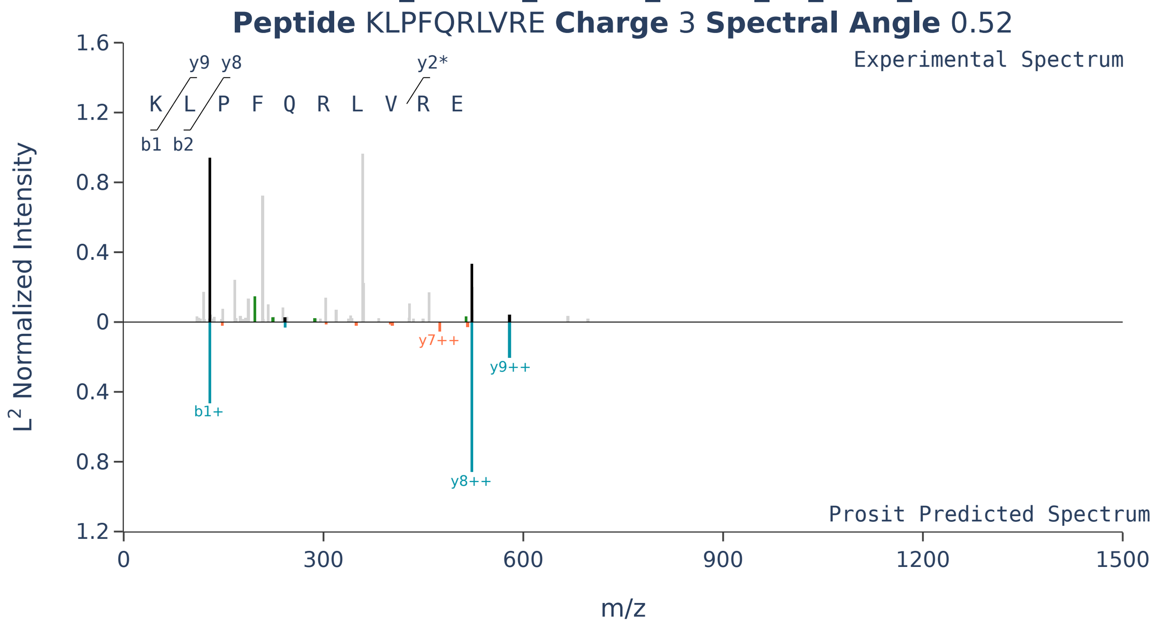


**Source** HRoetschke_271222_120123_HFGoe_H3_b2R2_4h **Scan** 15191
**Peptide** AKRVTIMPKDIQLA **Charge** 3 **Spectral Angle** 0.55

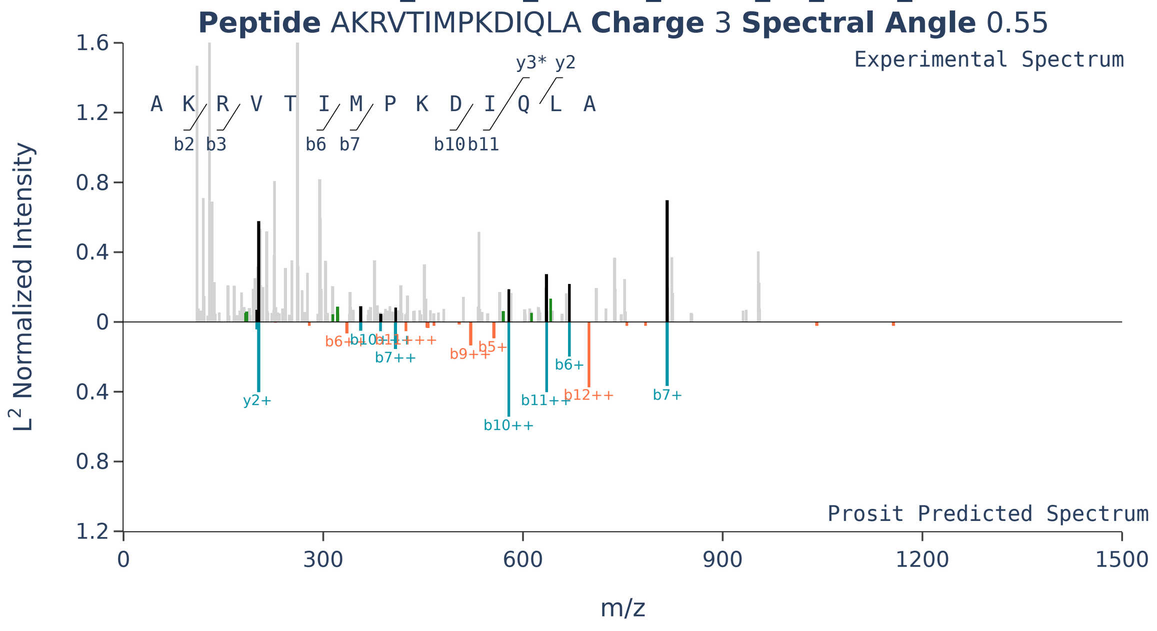


**Source** HRoetschke_271222_120123_HFGoe_H3_b1R2_4h **Scan** 16943
**Peptide** LAQDFKTDLRFQSS **Charge** 2 **Spectral Angle** 0.71

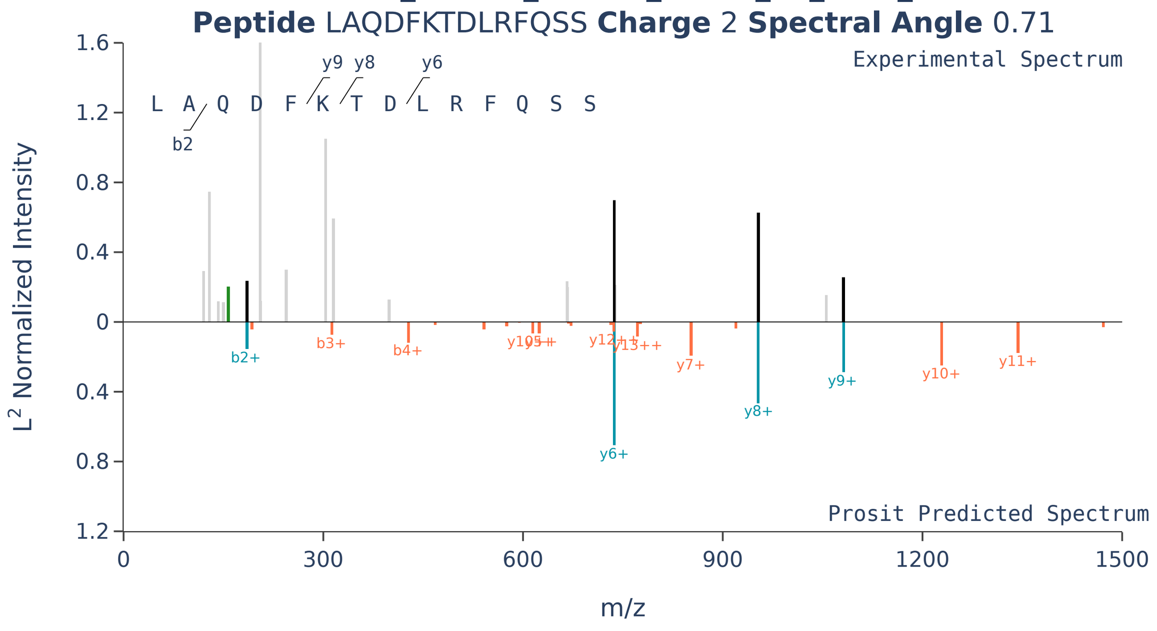


**Source** HRoetschke_271222_120123_HFGoe_H3_b2R2_4h **Scan** 5109
**Peptide** LATKAARKSAPATG **Charge** 2 **Spectral Angle** 0.57

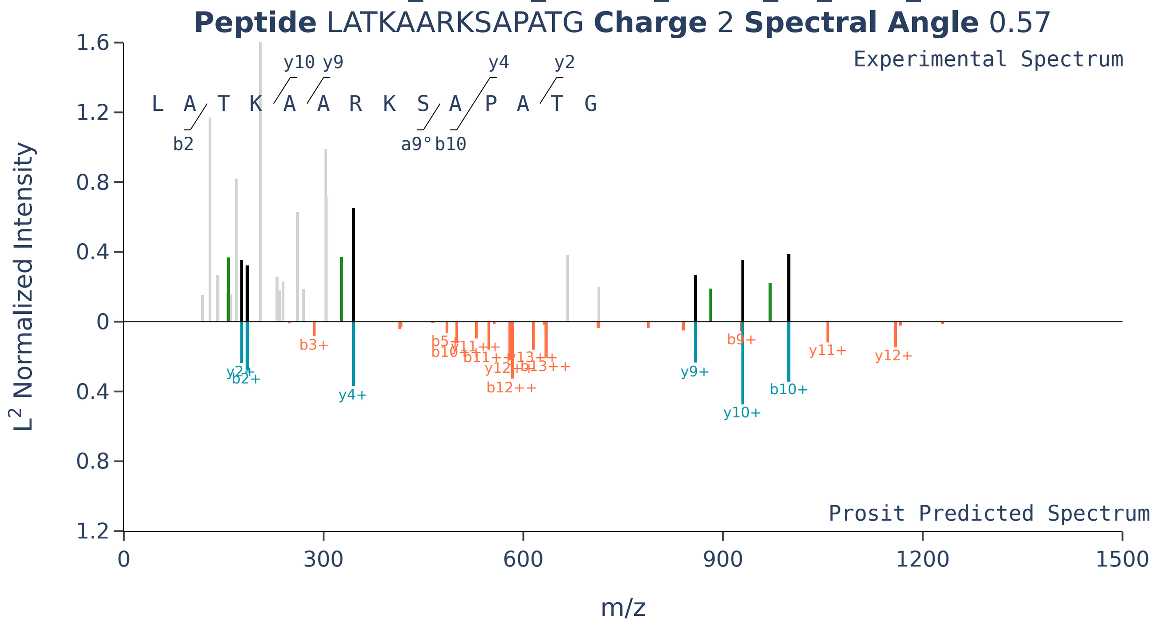

**Source** HRoetschke_271222_120123_HFGoe_H3_b2R1_4h **Scan** 7893
**Peptide** QLARRIRGERA **Charge** 3 **Spectral Angle** 0.64

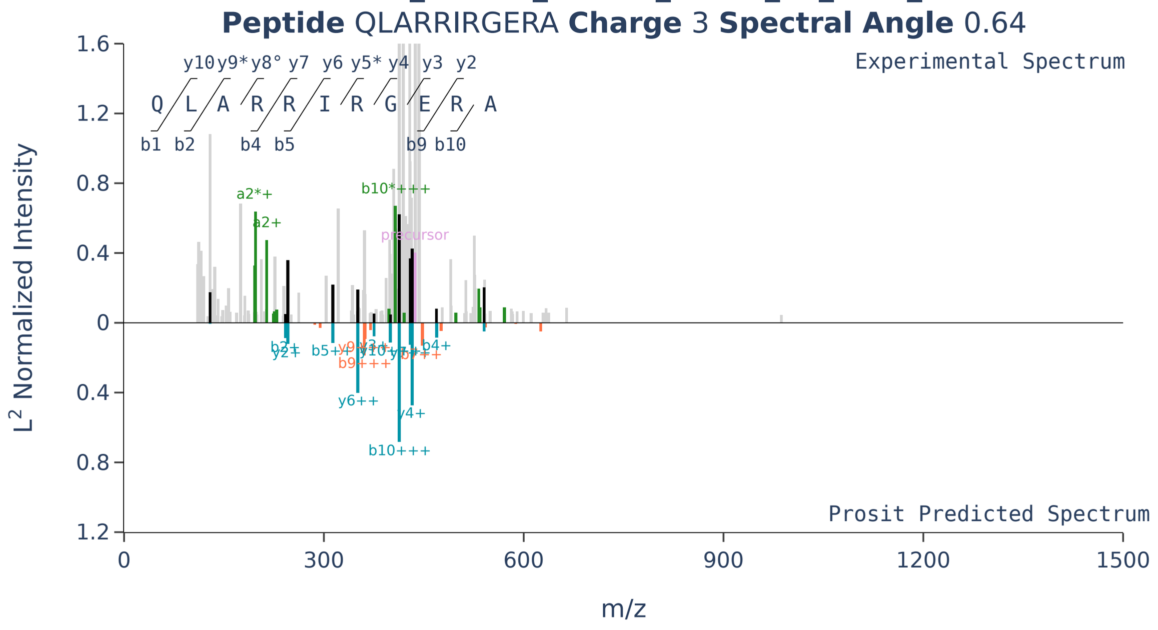


**Source** HRoetschke_271222_120123_HFGoe_H3_b2R1_4h **Scan** 12322
**Peptide** KRVTIMPKDIQLARRIR **Charge** 4 **Spectral Angle** 0.6

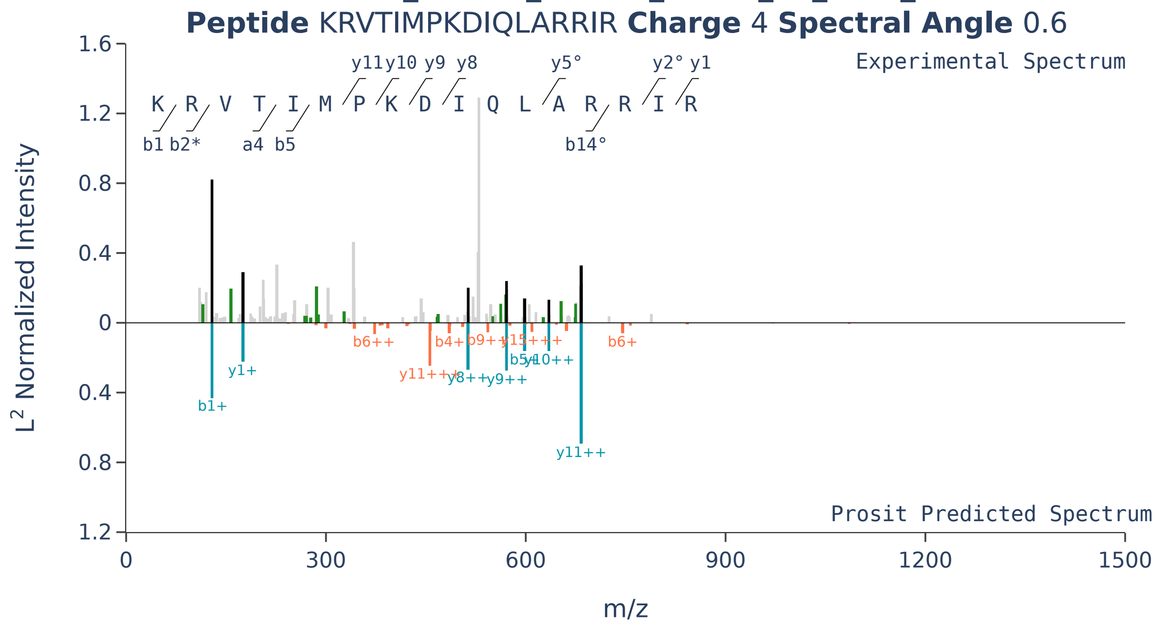


**Source** HRoetschke_271222_120123_HFGoe_H3_b2R2_2h **Scan** 9737
**Peptide** EDTNLCAIHAK **Charge** 3 **Spectral Angle** 0.7

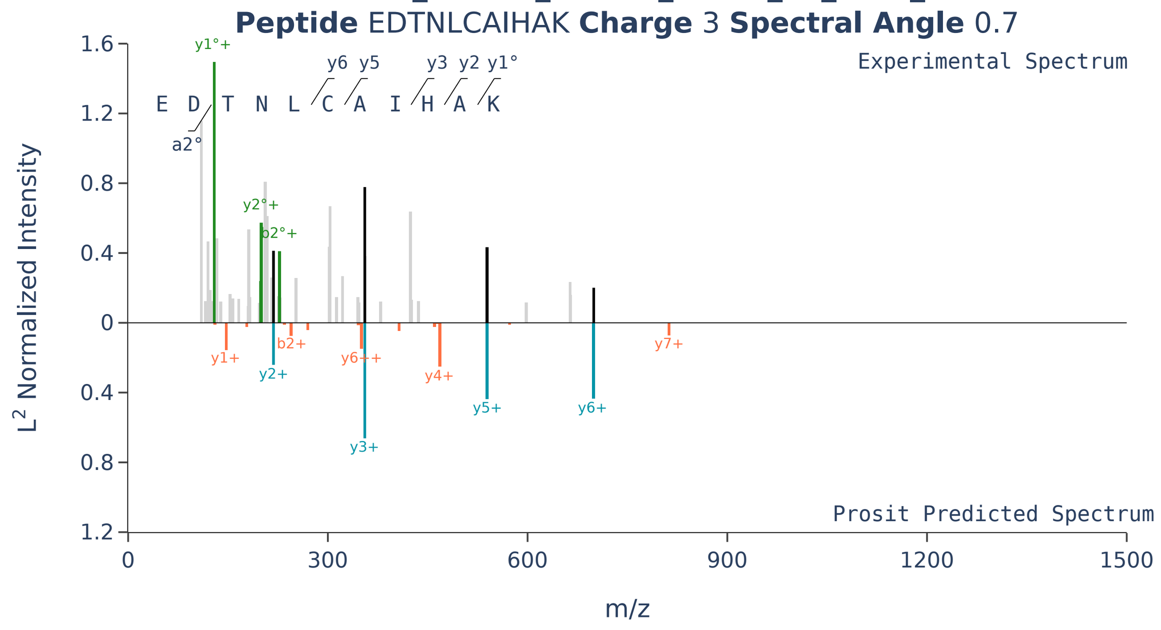


**Source** HRoetschke_271222_120123_HFGoe_H3_b2R1_1h **Scan** 18853
**Peptide** DLRFQSSAVMALQE **Charge** 2 **Spectral Angle** 0.61

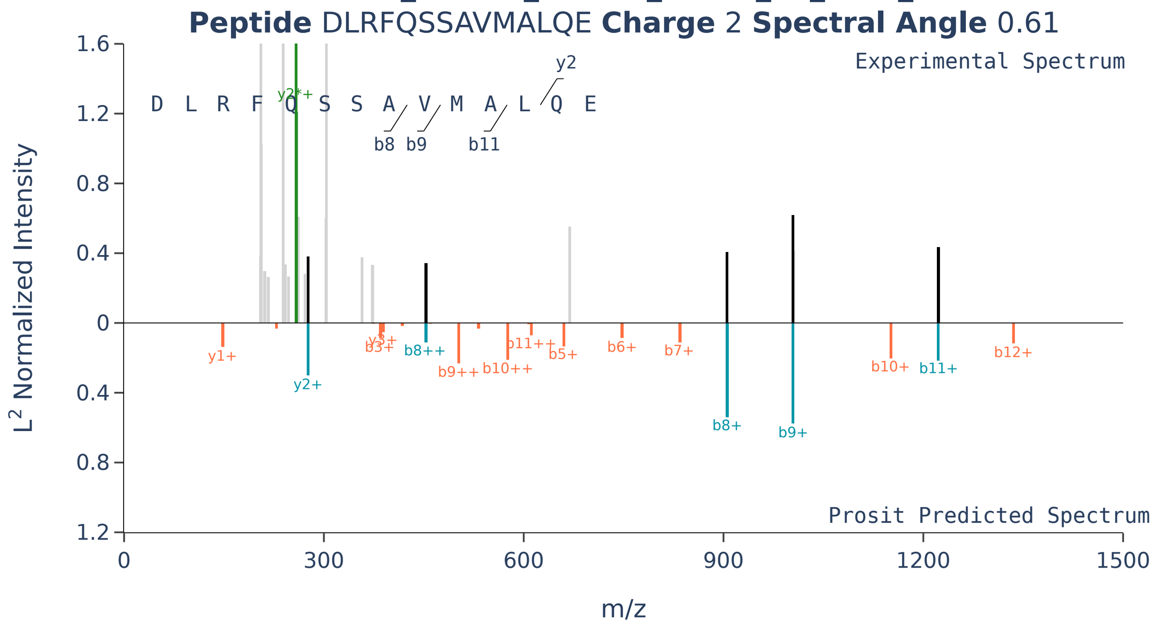


**Source** HRoetschke_271222_120123_HFGoe_H3_b2R1_4h **Scan** 14284
**Peptide** TNLCAIHAKRVTI **Charge** 3 **Spectral Angle** 0.55

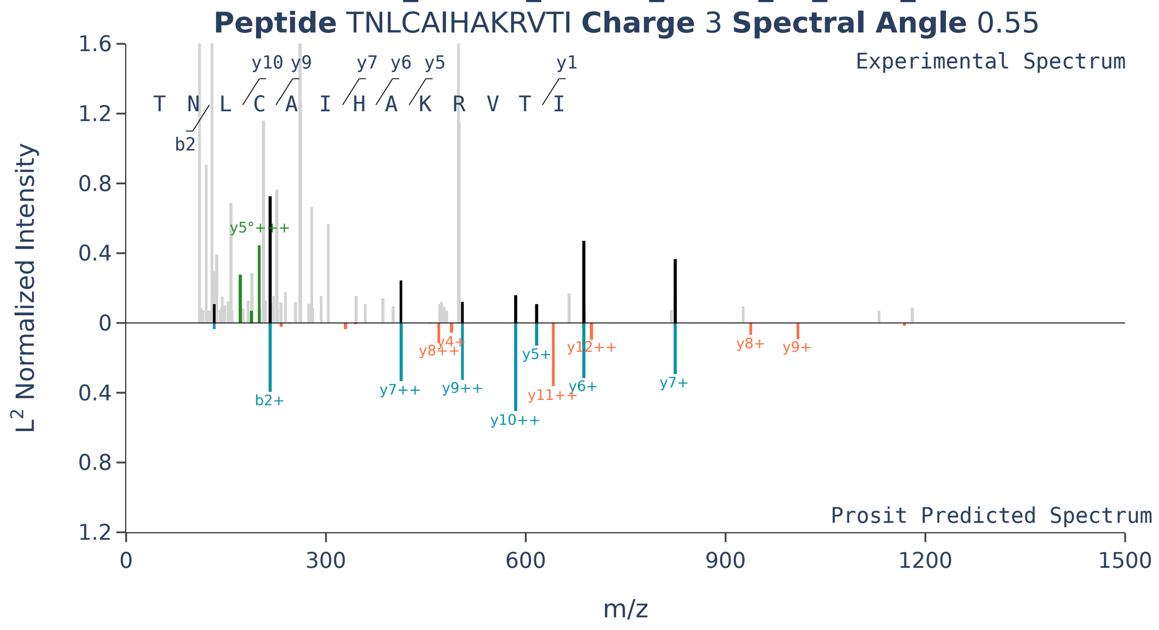


**Source** HRoetschke_271222_120123_HFGoe_H3_b2R1_1h **Scan** 17590
**Peptide** VALFEDTNLCAIHAKRV **Charge** 4 **Spectral Angle** 0.52

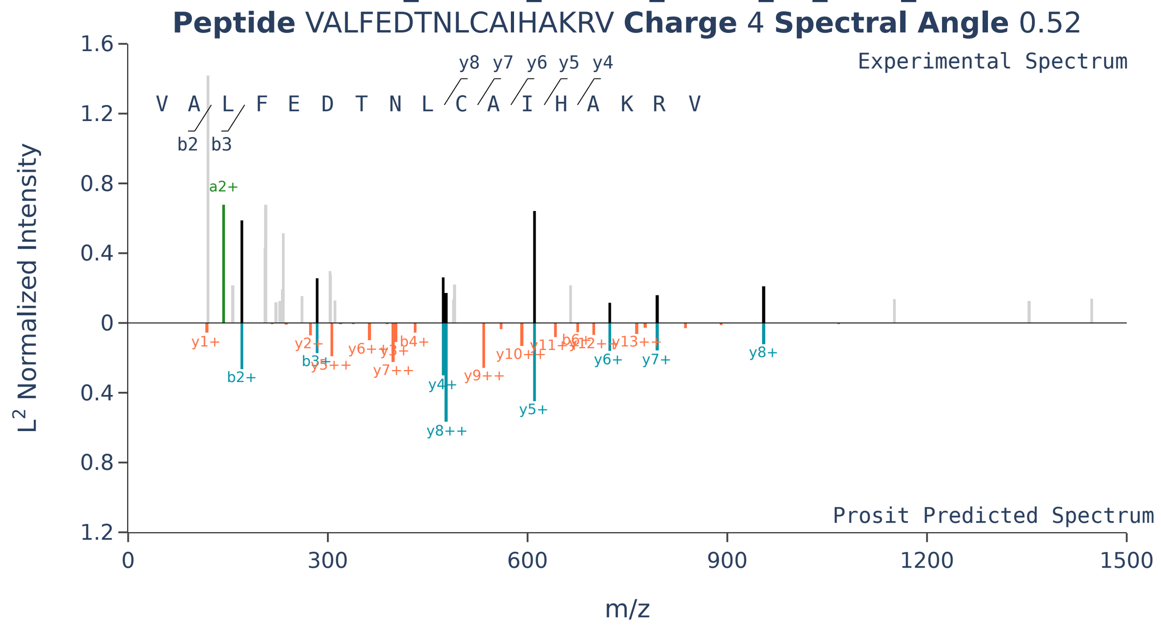


**Source** HRoetschke_271222_120123_HFGoe_H3_b2R1_4h **Scan** 5019
**Peptide** SAPATGGVKKPHRYR **Charge** 3 **Spectral Angle** 0.62

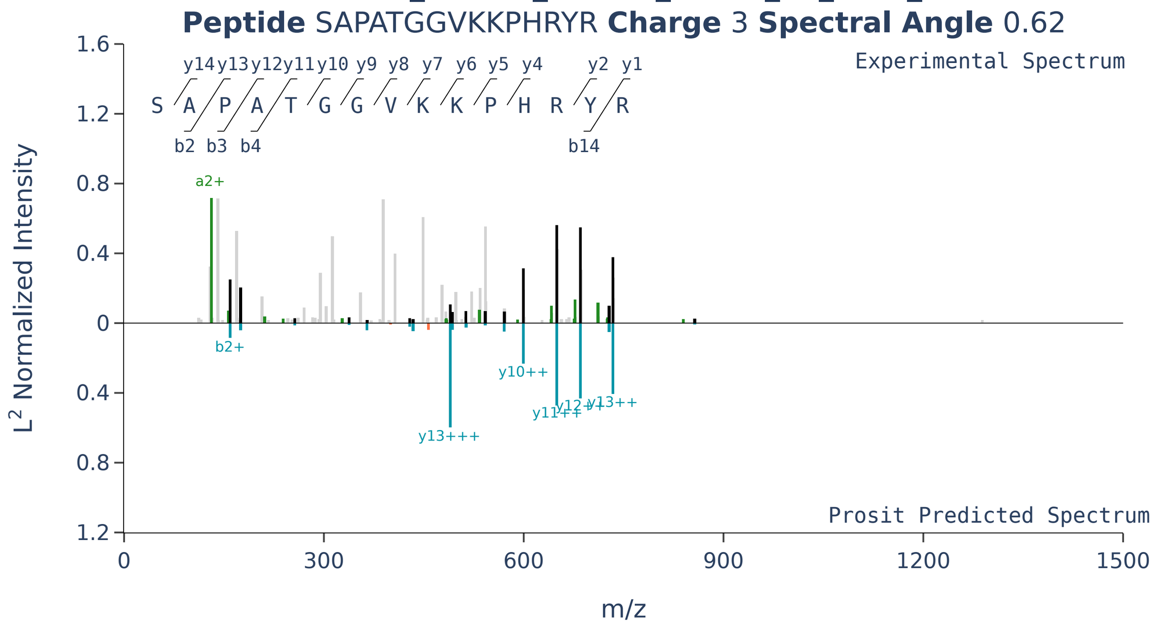


**Source** HRoetschke_271222_120123_HFGoe_H3_b2R2_4h **Scan** 20076
**Peptide** ALFEDTNLCAIHAK **Charge** 3 **Spectral Angle** 0.69

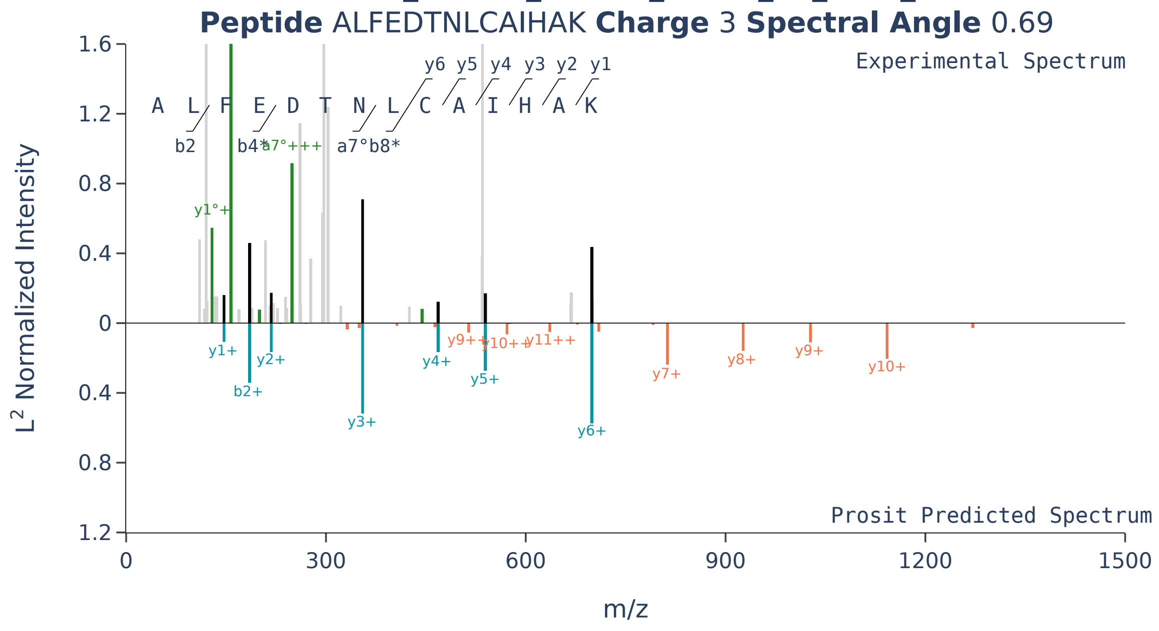


**Source** HRoetschke_271222_120123_HFGoe_H3_b1R1_24h **Scan** 8747
**Peptide** LQLARRIRGE **Charge** 3 **Spectral Angle** 0.68

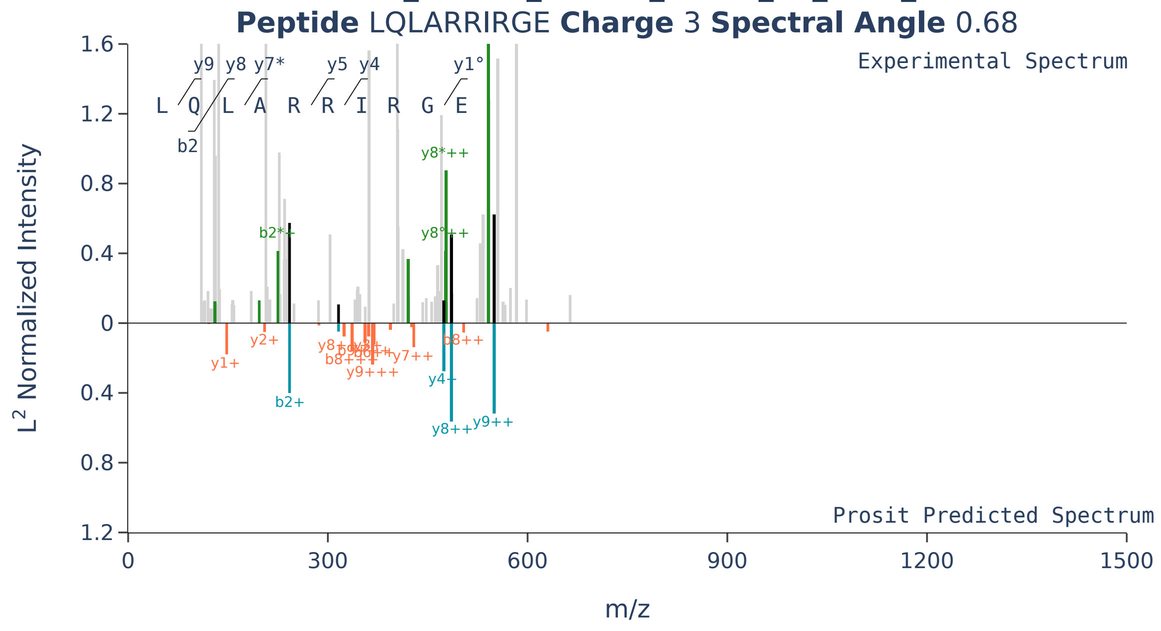


**Source** HRoetschke_271222_120123_HFGoe_H3_b2R2_24h **Scan** 12405
**Peptide** EIRRYQKSTELL **Charge** 3 **Spectral Angle** 0.45

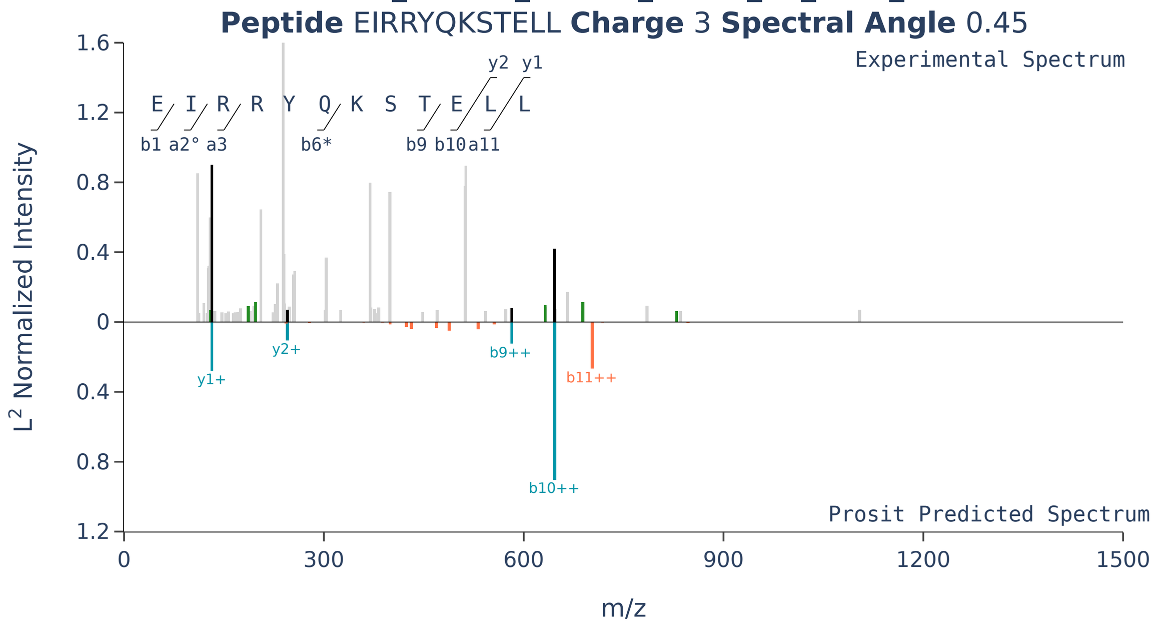


**Source** HRoetschke_271222_120123_HFGoe_H3_b2R2_4h **Scan** 7420
**Peptide** EIRRYQKSTEL **Charge** 2 **Spectral Angle** 0.69

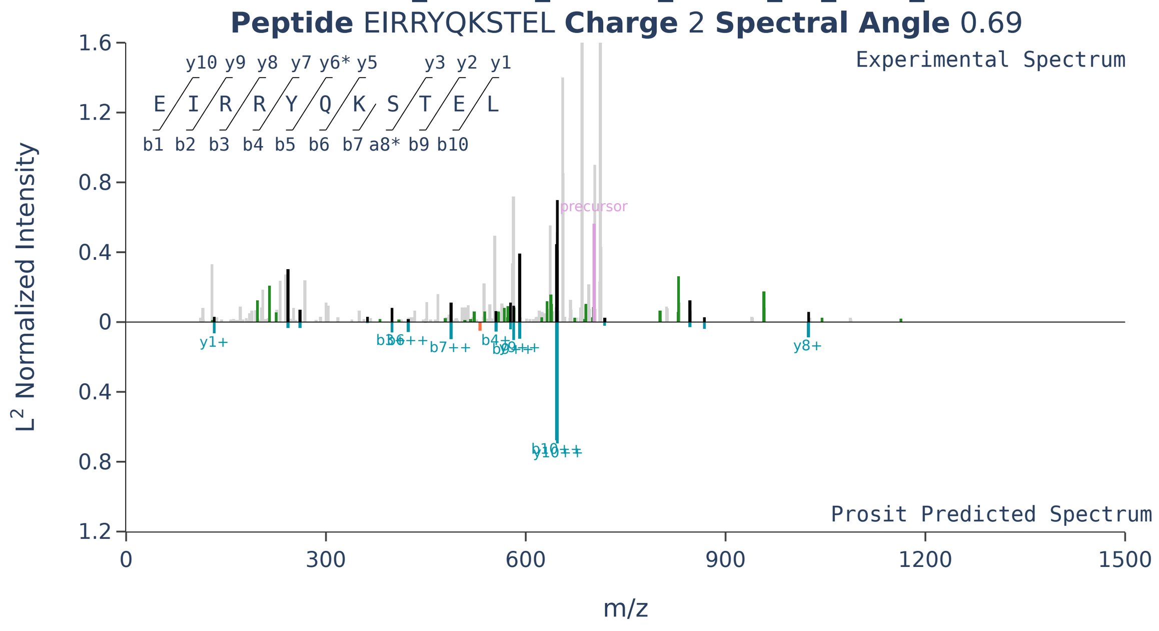


**Source** HRoetschke_271222_120123_HFGoe_H3_b2R2_4h **Scan** 5509
**Peptide** LREIRRYQKST **Charge** 3 **Spectral Angle** 0.57

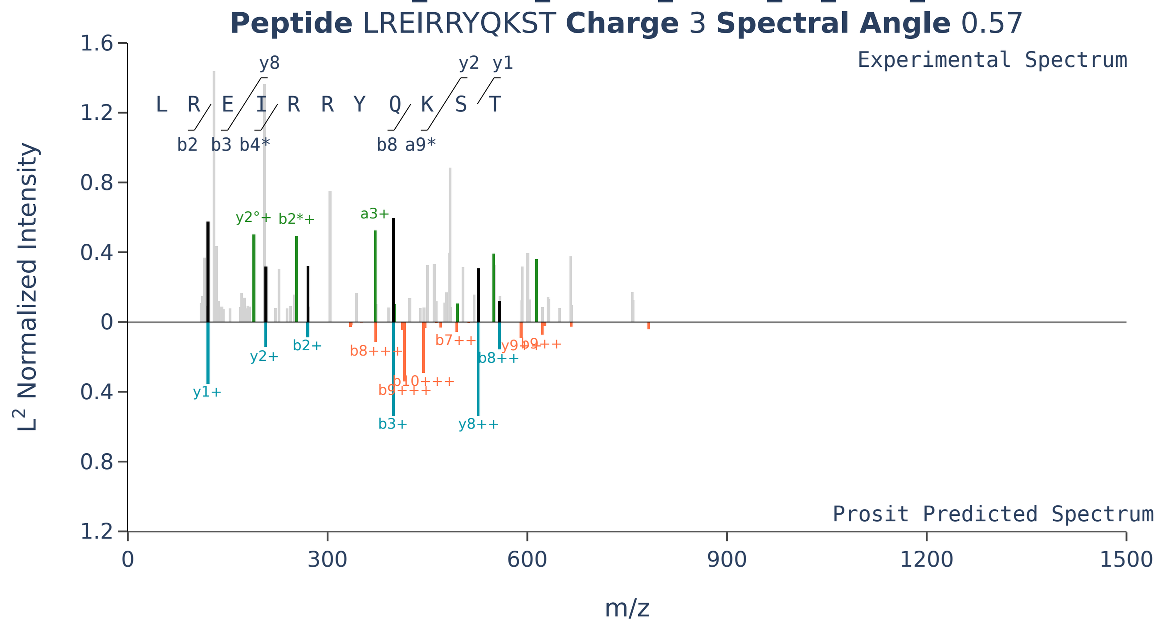


**Source** HRoetschke_271222_120123_HFGoe_H3_b2R1_4h **Scan** 11038
**Peptide** PGTVALREIRRYQK **Charge** 3 **Spectral Angle** 0.61

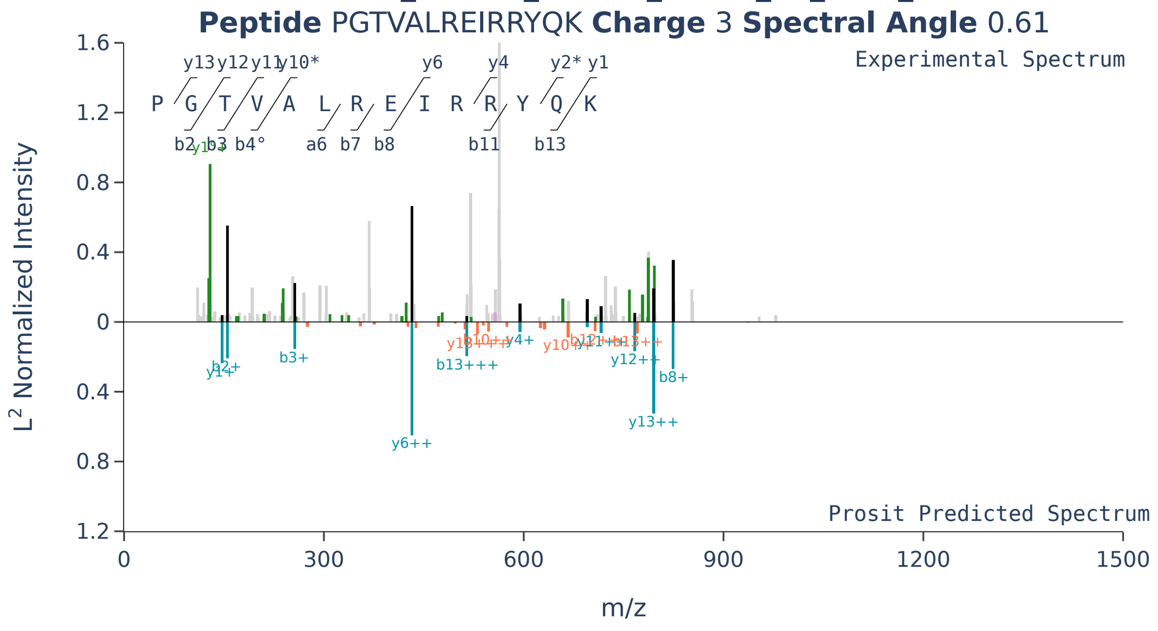


**Source** HRoetschke_271222_120123_HFGoe_H3_b2R1_4h **Scan** 15526
**Peptide** ALHAKRVTIMPKDIQLA **Charge** 4 **Spectral Angle** 0.65

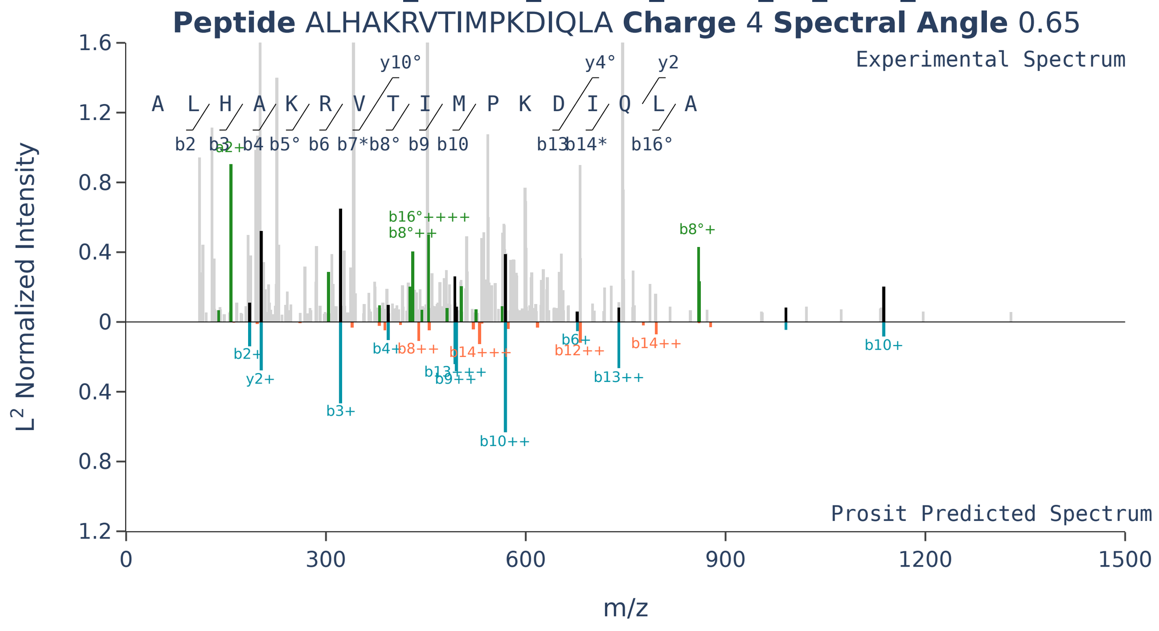


**Source** HRoetschke_271222_120123_HFGoe_H3_b2R1_4h **Scan** 11049
**Peptide** APATGGVKKPHRYRPGTVALREI **Charge** 5 **Spectral Angle** 0.5

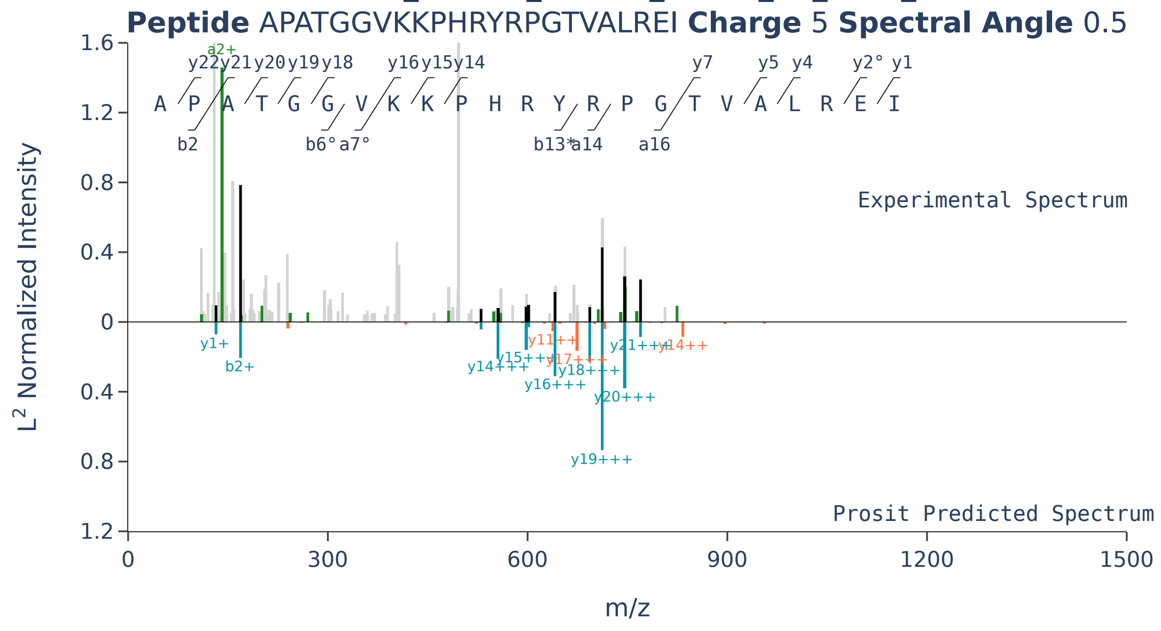

**Source** HRoetschke_271222_120123_HFGoe_H3_b1R1_4h **Scan** 20262
**Peptide** QKSTELLIRKLPFQRL **Charge** 4 **Spectral Angle** 0.57

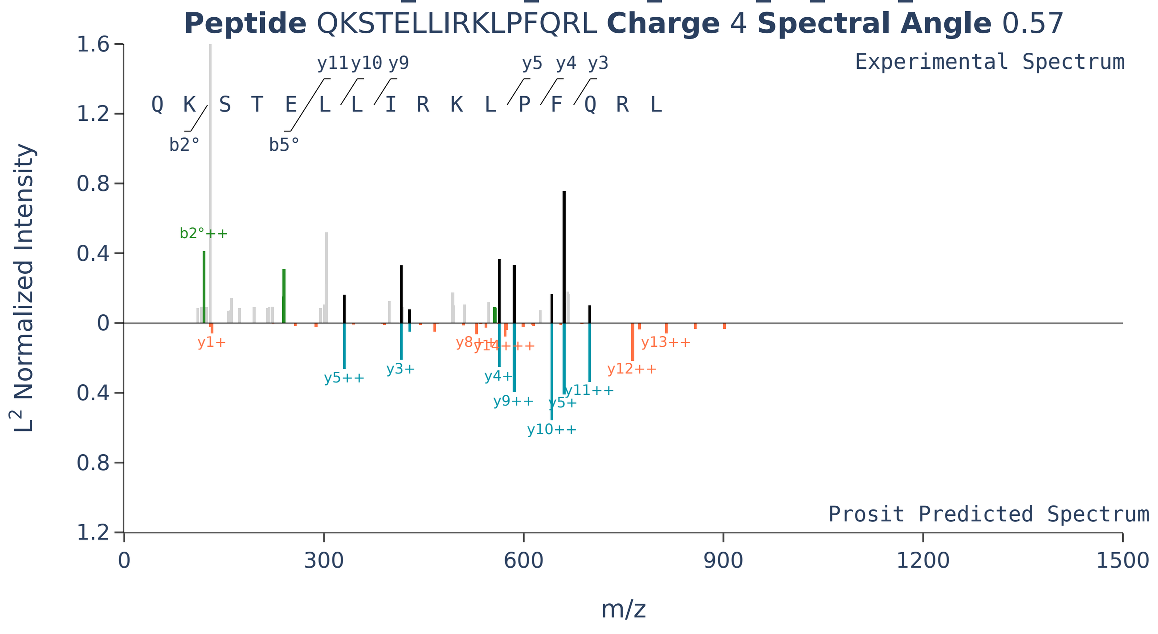


**Source** HRoetschke_271222_120123_HFGoe_H3_b1R1_24h **Scan** 7712
**Peptide** LREIRRYQKSTEL **Charge** 4 **Spectral Angle** 0.66

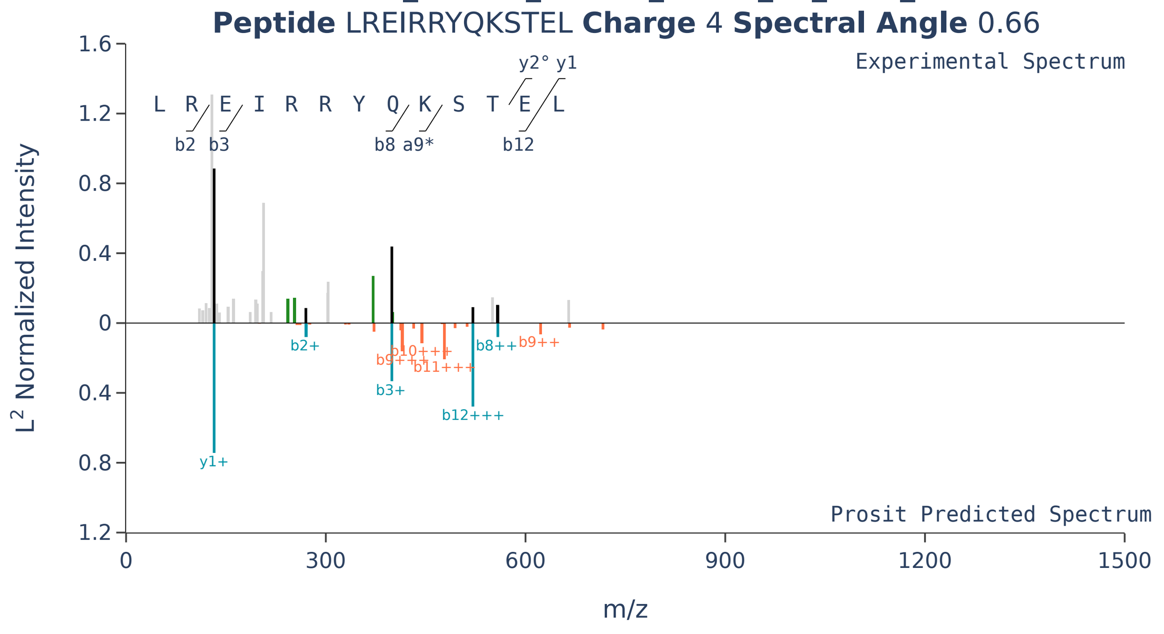


**Source** HRoetschke_271222_120123_HFGoe_H3_b1R2_24h **Scan** 19983
**Peptide** KSTELLI **Charge** 1 **Spectral Angle** 0.56

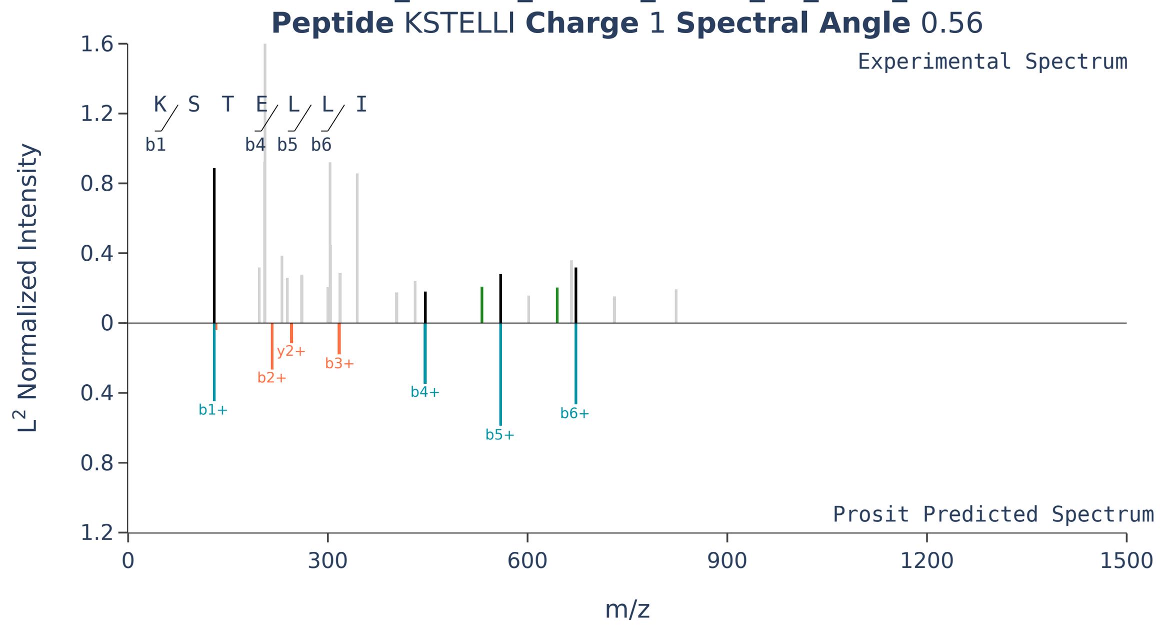


**Source** HRoetschke_271222_120123_HFGoe_H3_b1R1_4h **Scan** 19741
**Peptide** ELLIRKLPFQR **Charge** 3 **Spectral Angle** 0.73

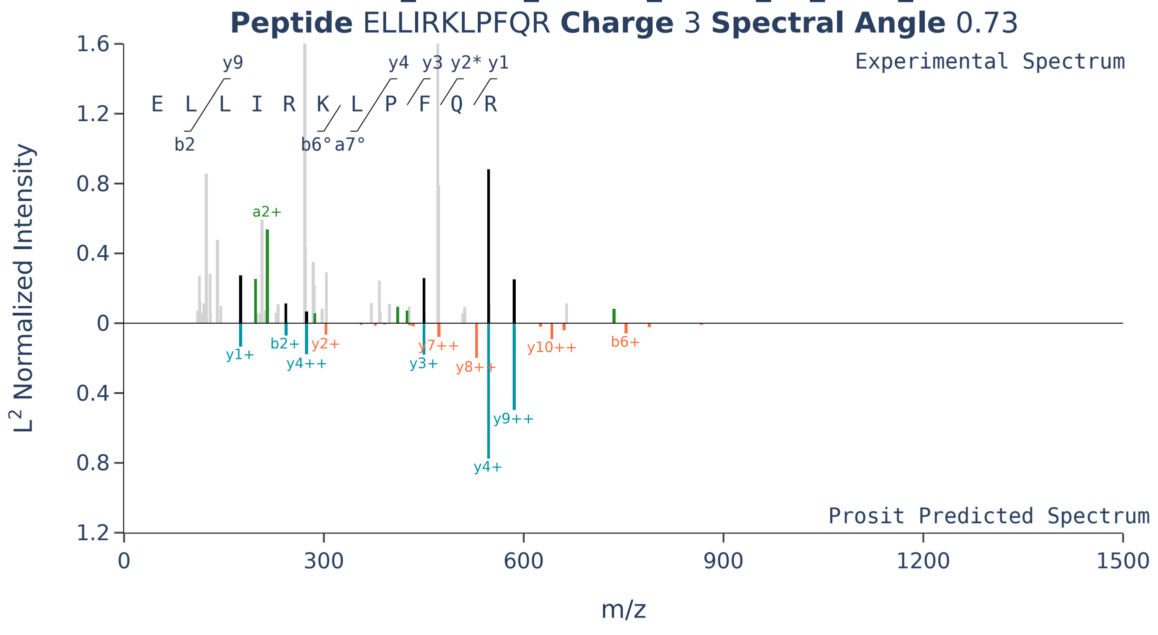


**Source** HRoetschke_271222_120123_HFGoe_H3_b2R1_4h **Scan** 11201
**Peptide** RPGTVALREIRRY **Charge** 4 **Spectral Angle** 0.66

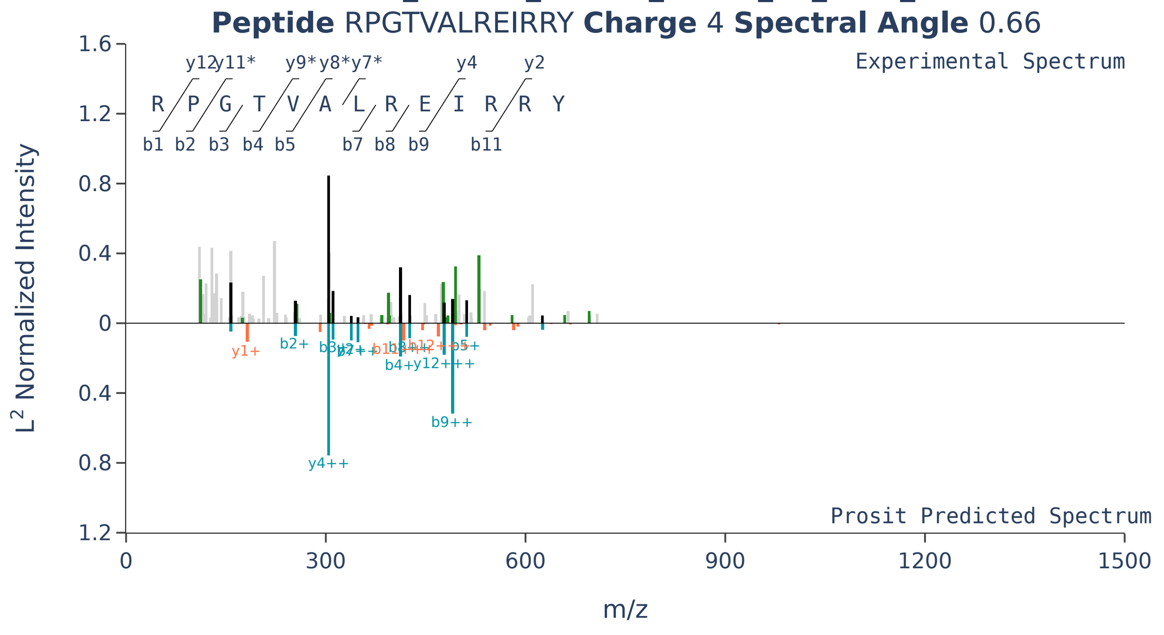


**Source** HRoetschke_271222_120123_HFGoe_H3_b2R1_4h **Scan** 7586
**Peptide** IQLARRIRGER **Charge** 3 **Spectral Angle** 0.87

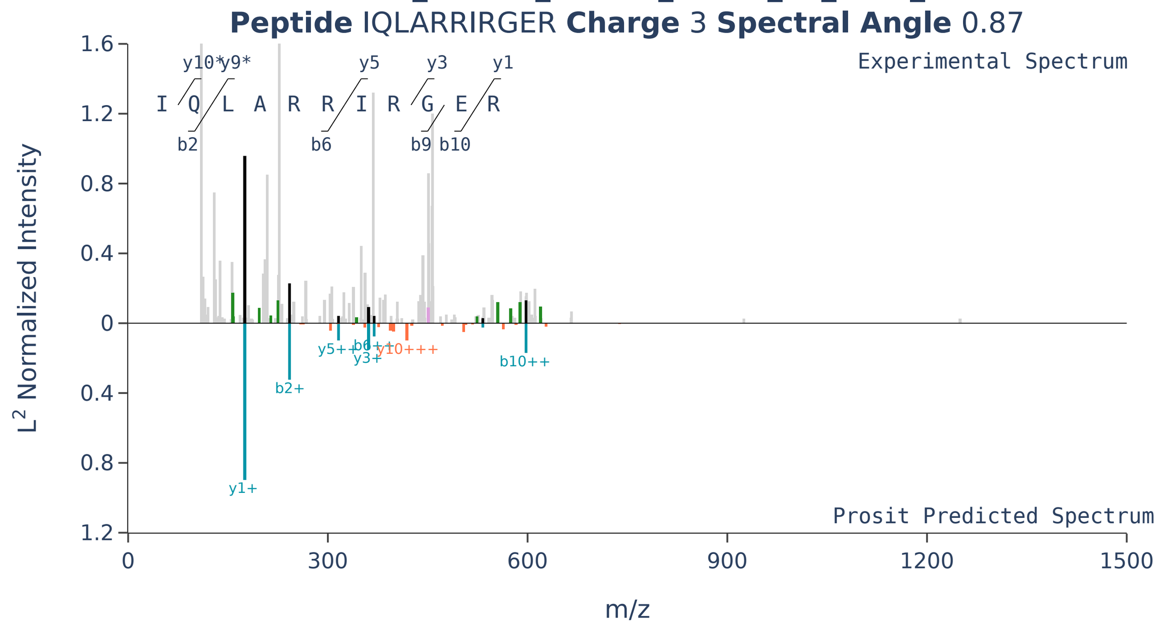


**Source** HRoetschke_271222_120123_HFGoe_H3_b1R2_24h **Scan** 5171
**Peptide** TKAARKSAPAT **Charge** 2 **Spectral Angle** 0.66

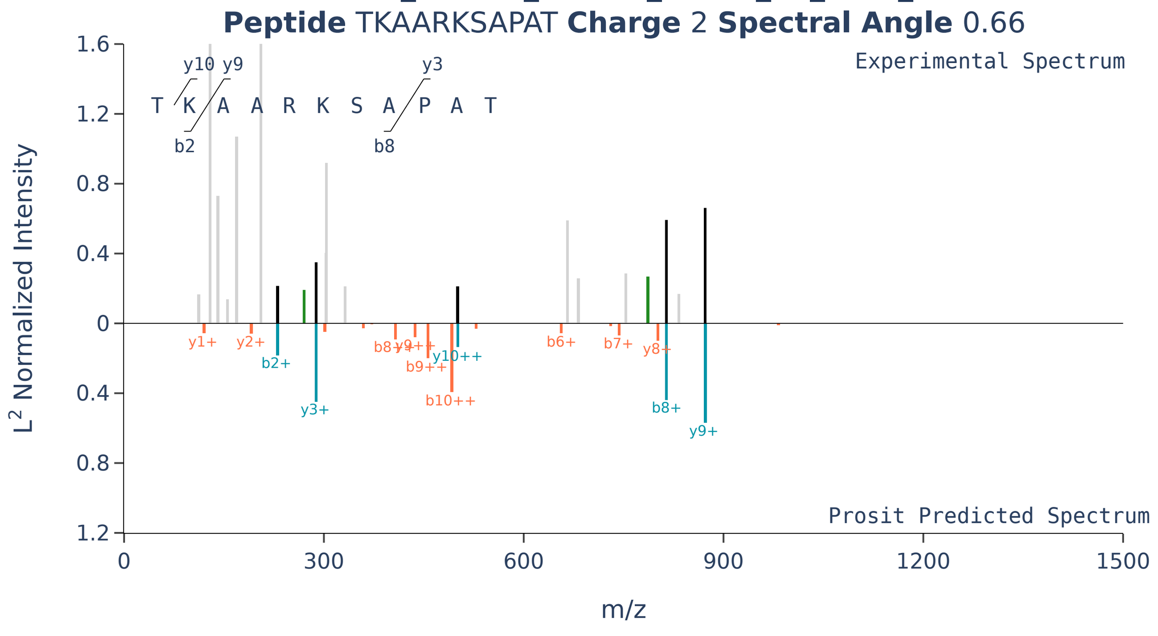


**Source** HRoetschke_271222_120123_HFGoe_H3_b2R2_2h **Scan** 18553
**Peptide** LAQDFKTDLRFQ **Charge** 2 **Spectral Angle** 0.63

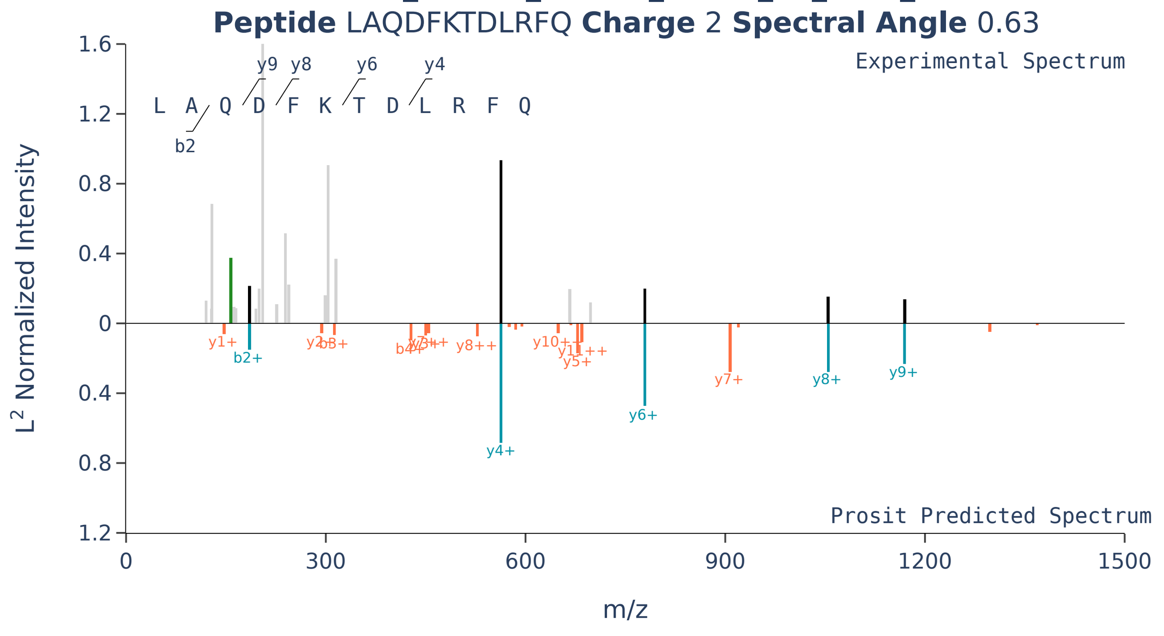


**Source** HRoetschke_271222_120123_HFGoe_H3_b2R1_4h **Scan** 5393
**Peptide** GVKKPHRYRPGTV **Charge** 3 **Spectral Angle** 0.85

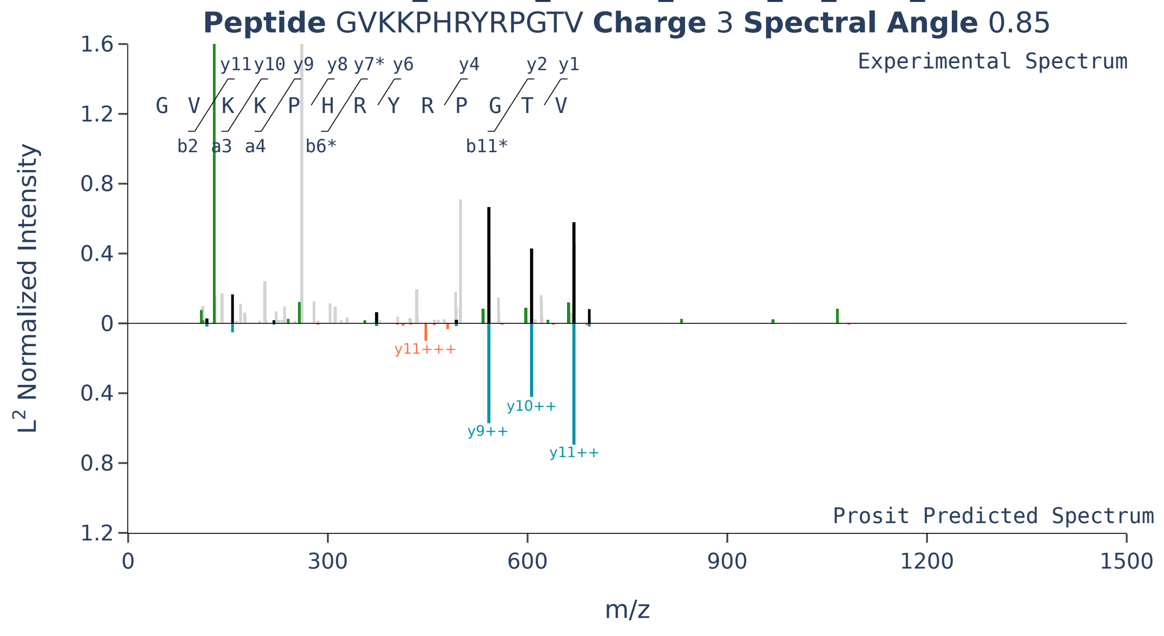


**Source** HRoetschke_271222_120123_HFGoe_H3_b2R1_4h **Scan** 4925
**Peptide** TGGKAPRKQLATKAARKS **Charge** 3 **Spectral Angle** 0.73

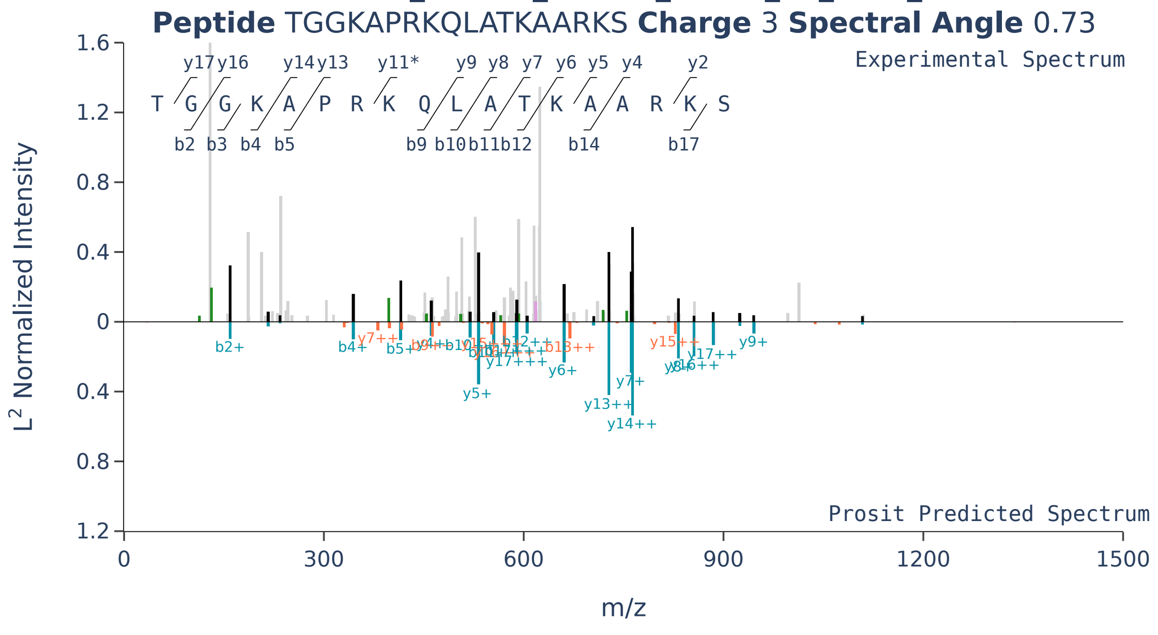


**Source** HRoetschke_271222_120123_HFGoe_H3_b2R1_1h **Scan** 17676
**Peptide** VREIAQDFKTDLRFQSSAV **Charge** 3 **Spectral Angle** 0.65

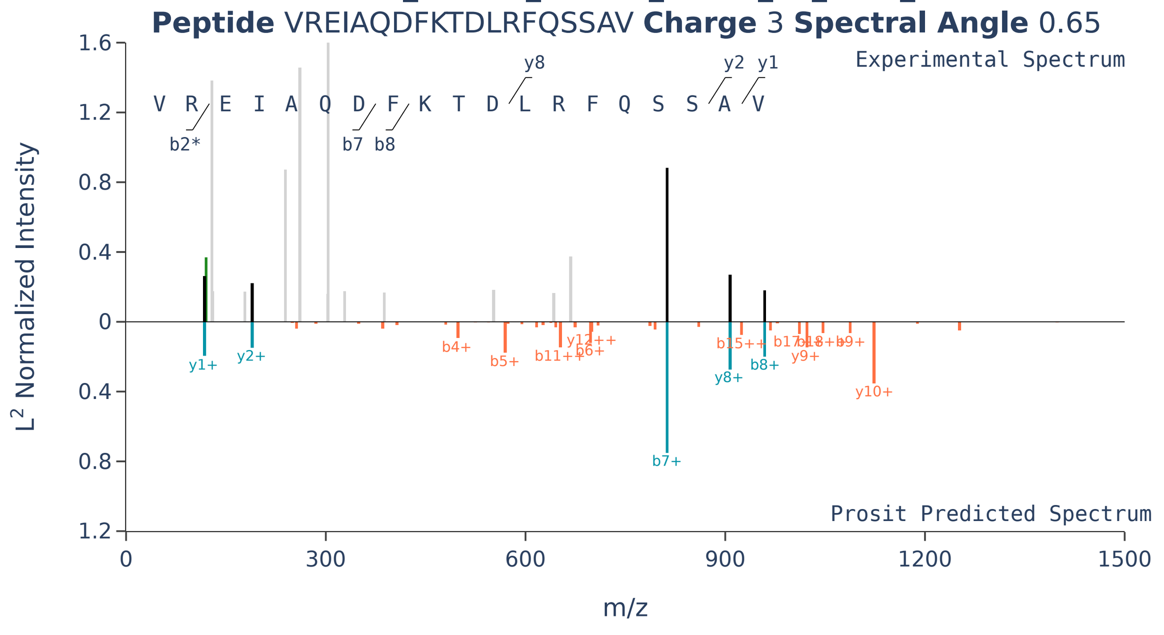


**Source** HRoetschke_271222_120123_HFGoe_H3_b2R1_1h **Scan** 16962
**Peptide** REIAQDFKTDLRFQSSAV **Charge** 4 **Spectral Angle** 0.72

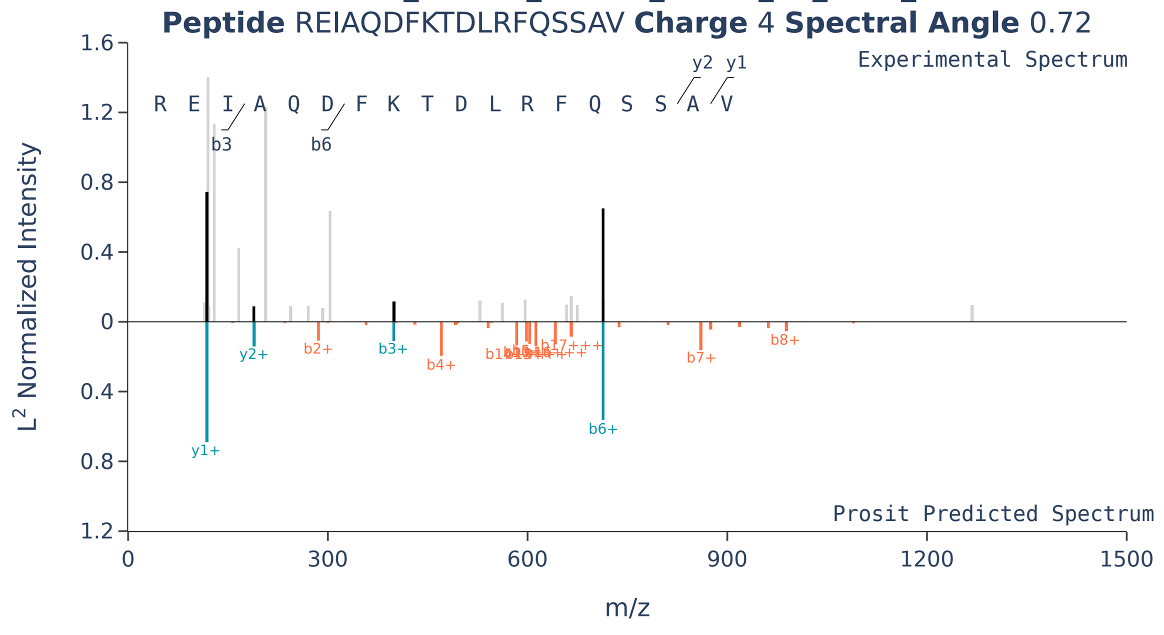


**Source** HRoetschke_271222_120123_HFGoe_H3_b2R1_4h **Scan** 12484
**Peptide** LREIRRYQKSTELL **Charge** 3 **Spectral Angle** 0.38

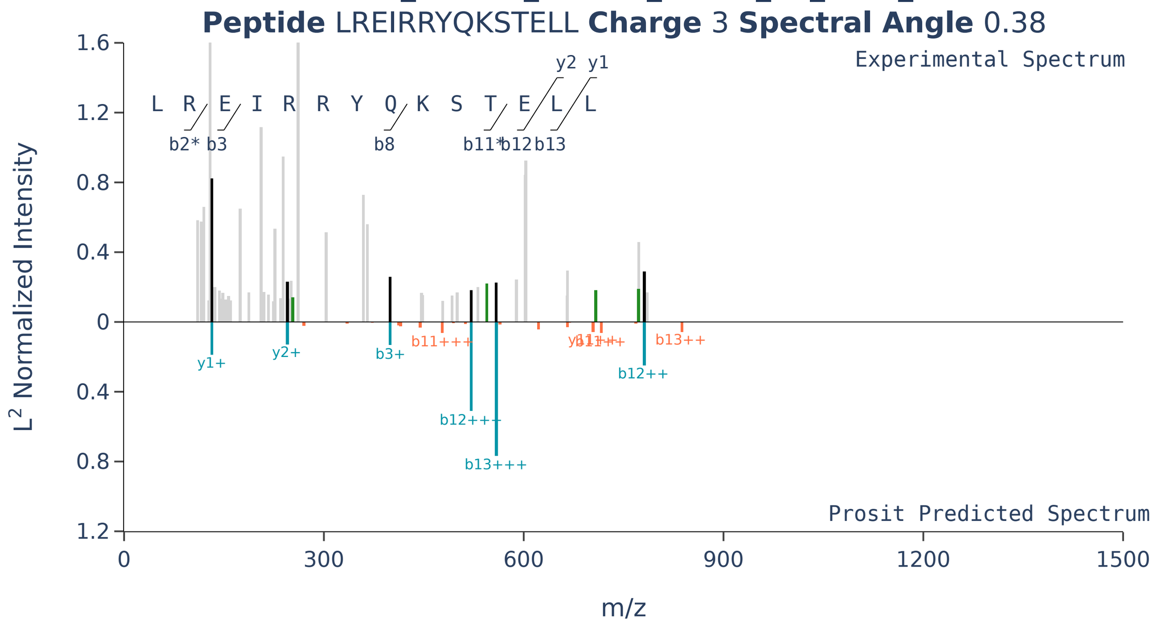


**Source** HRoetschke_271222_120123_HFGoe_H3_b2R2_4h **Scan** 8487
**Peptide** VALREIRRYQKSTE **Charge** 3 **Spectral Angle** 0.65

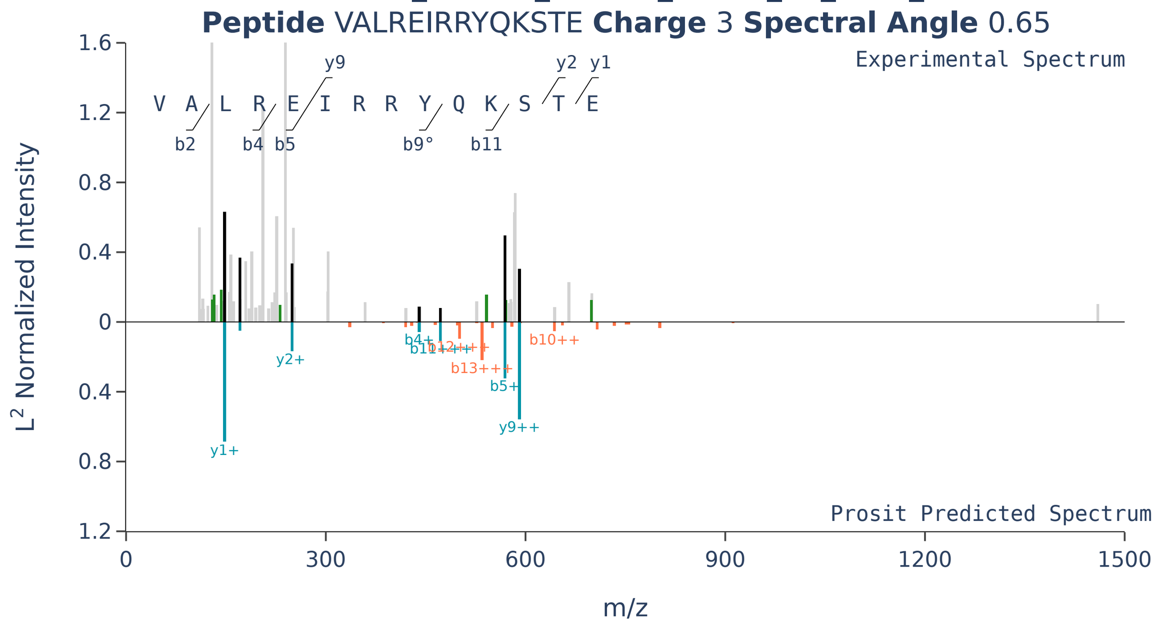


**Source** HRoetschke_271222_120123_HFGoe_H3_b1R2_24h **Scan** 18938
**Peptide** RVTIMPKDIQL **Charge** 3 **Spectral Angle** 0.7

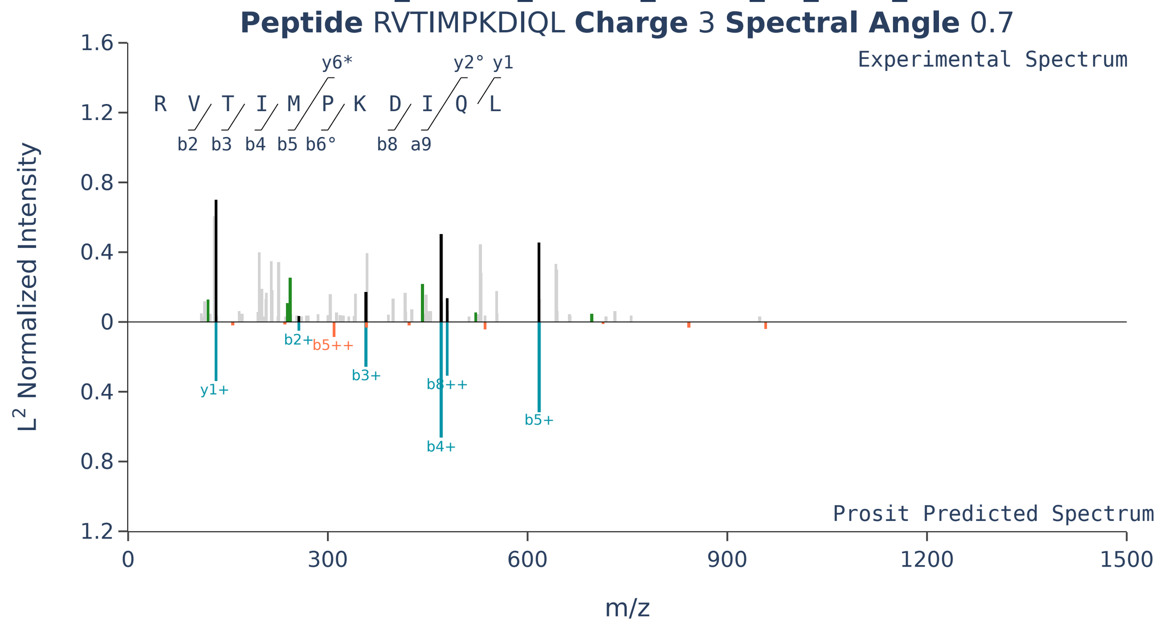

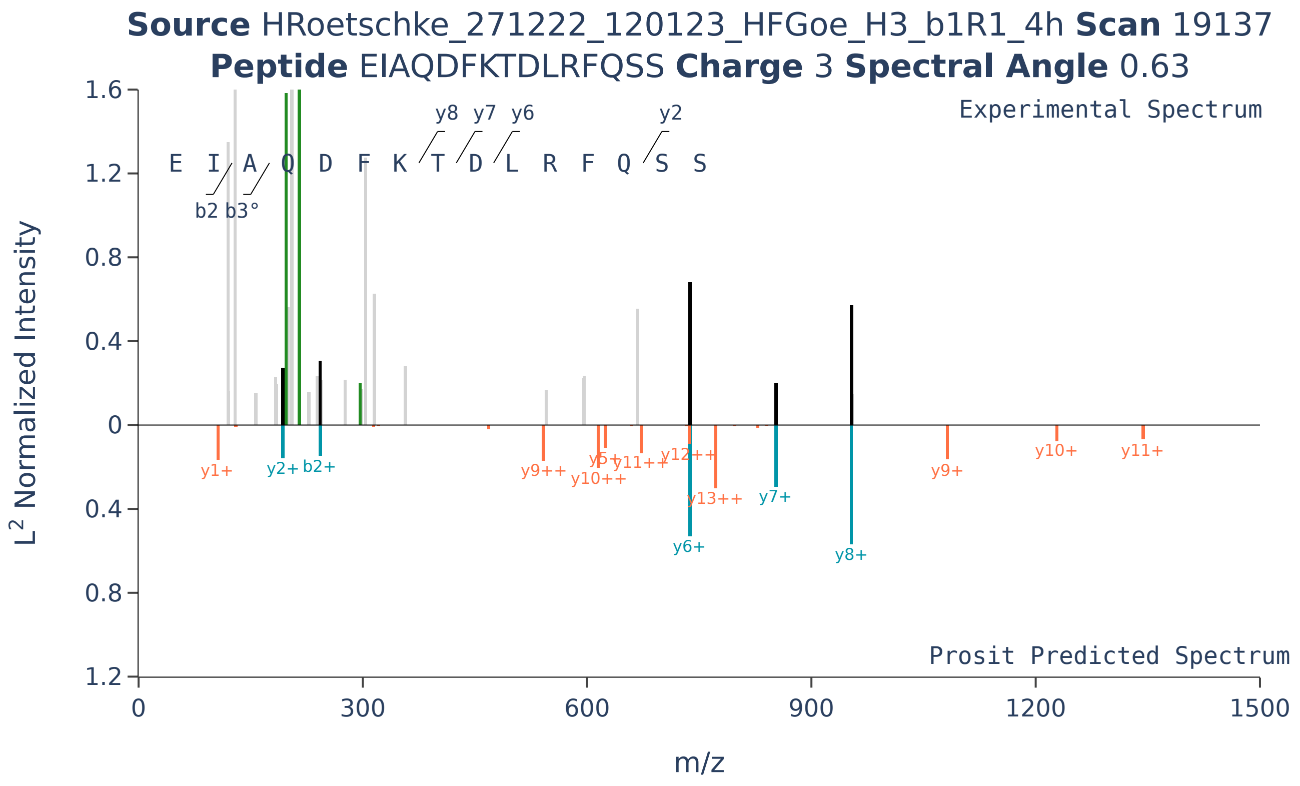
Source HRoetschke_271222_120123_HFGoe_H3_b1R1_4h Scan 19137
Peptide EIAQDFKTDLRFQSS Charge 3 Spectral Angle 0.63
Experimental Spectrum
Prosit Predicted Spectrum
$L^2$ Normalized Intensity
m/z

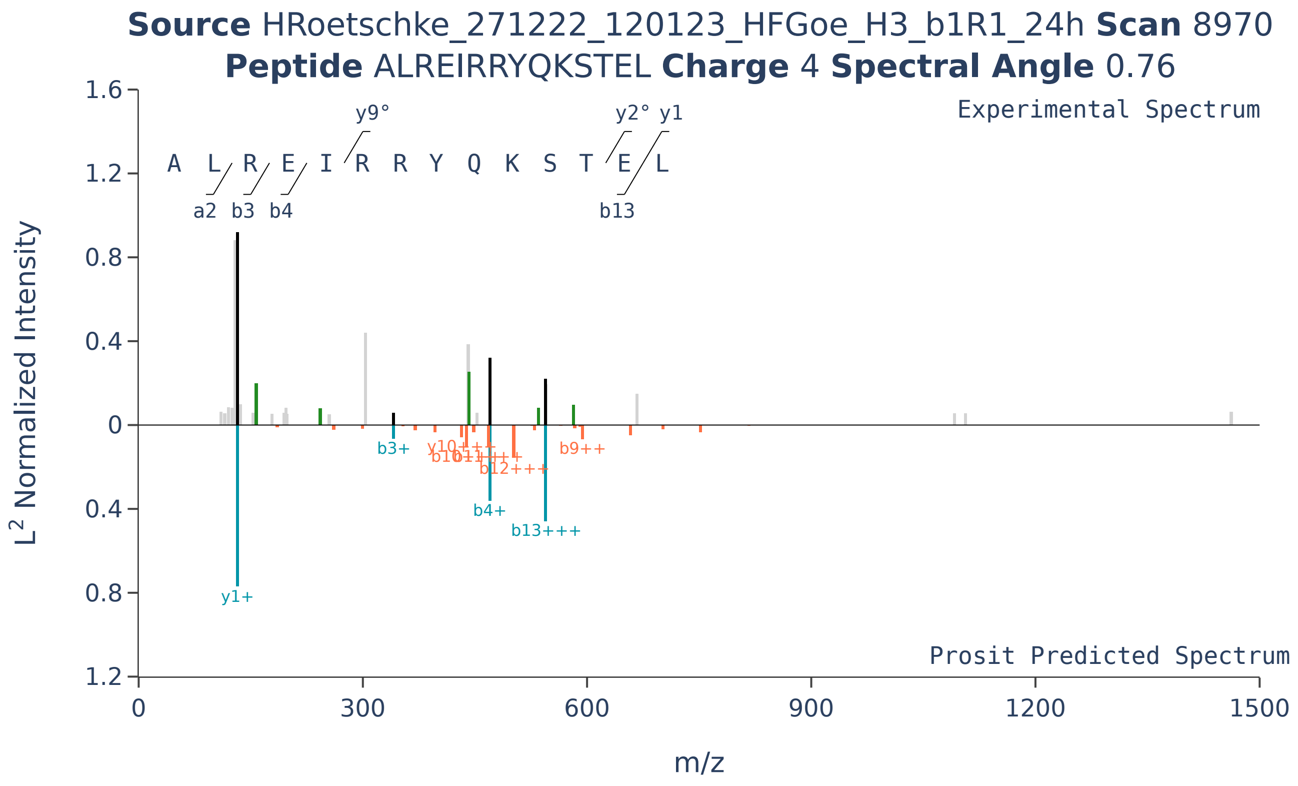
Source HRoetschke_271222_120123_HFGoe_H3_b1R1_24h Scan 8970
Peptide ALREIRRYQKSTEL Charge 4 Spectral Angle 0.76
Experimental Spectrum
Prosit Predicted Spectrum
$L^2$ Normalized Intensity
m/z

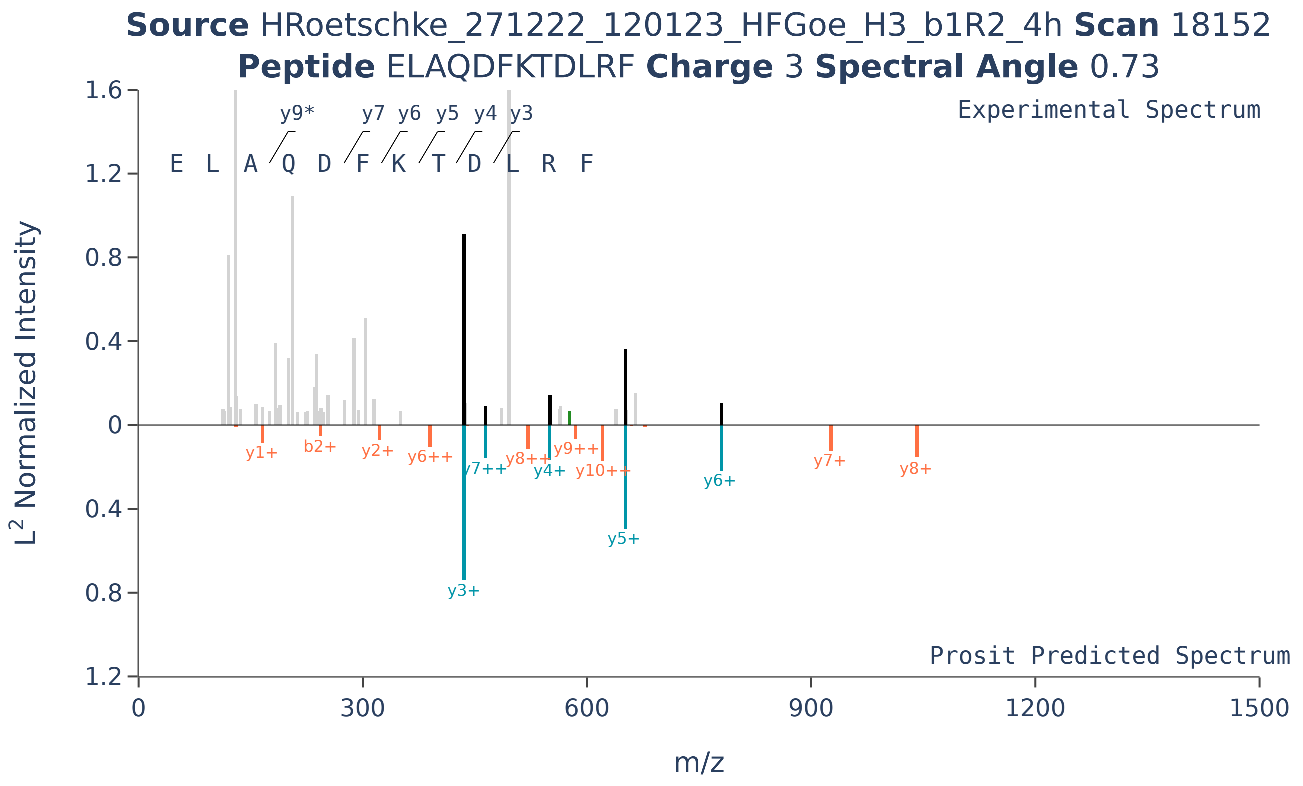
Source HRoetschke_271222_120123_HFGoe_H3_b1R2_4h Scan 18152
Peptide ELAQDFKTDLRF Charge 3 Spectral Angle 0.73
Experimental Spectrum
Prosit Predicted Spectrum
$L^2$ Normalized Intensity
m/z

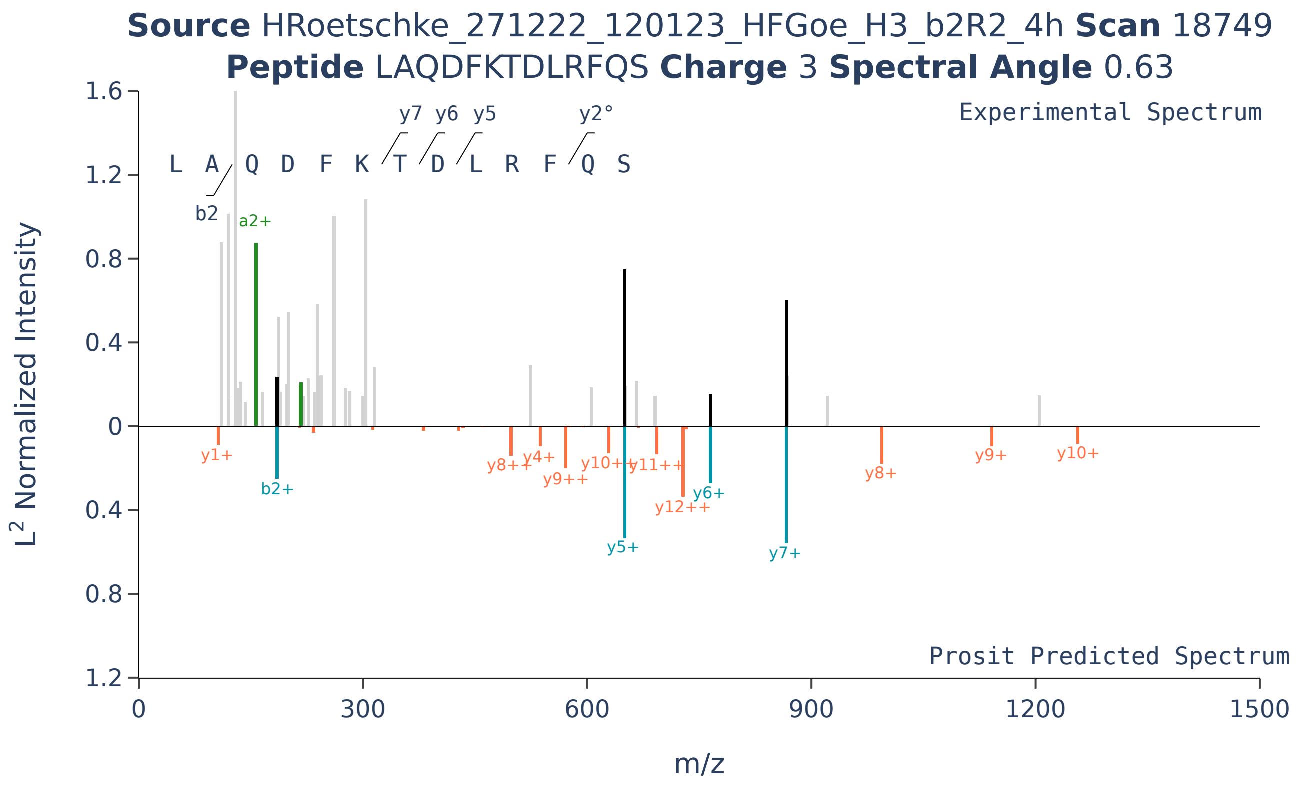
Source HRoetschke_271222_120123_HFGoe_H3_b2R2_4h Scan 18749
Peptide LAQDFKTDLRFQS Charge 3 Spectral Angle 0.63
Experimental Spectrum
Prosit Predicted Spectrum
$L^2$ Normalized Intensity
m/z

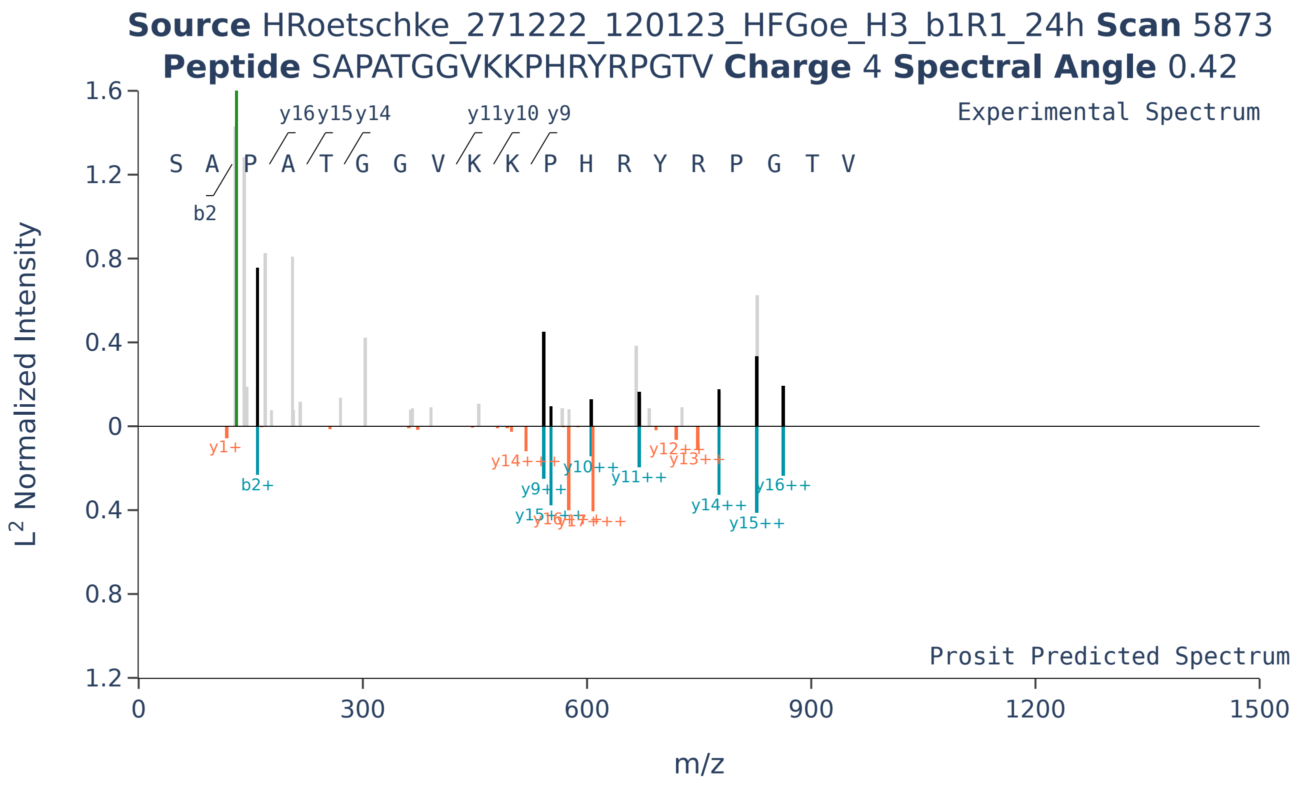
Source HRoetschke_271222_120123_HFGoe_H3_b1R1_24h Scan 5873
Peptide SAPATGGVKKPHRYRPGTV Charge 4 Spectral Angle 0.42
Experimental Spectrum
Prosit Predicted Spectrum
$L^2$ Normalized Intensity
m/z

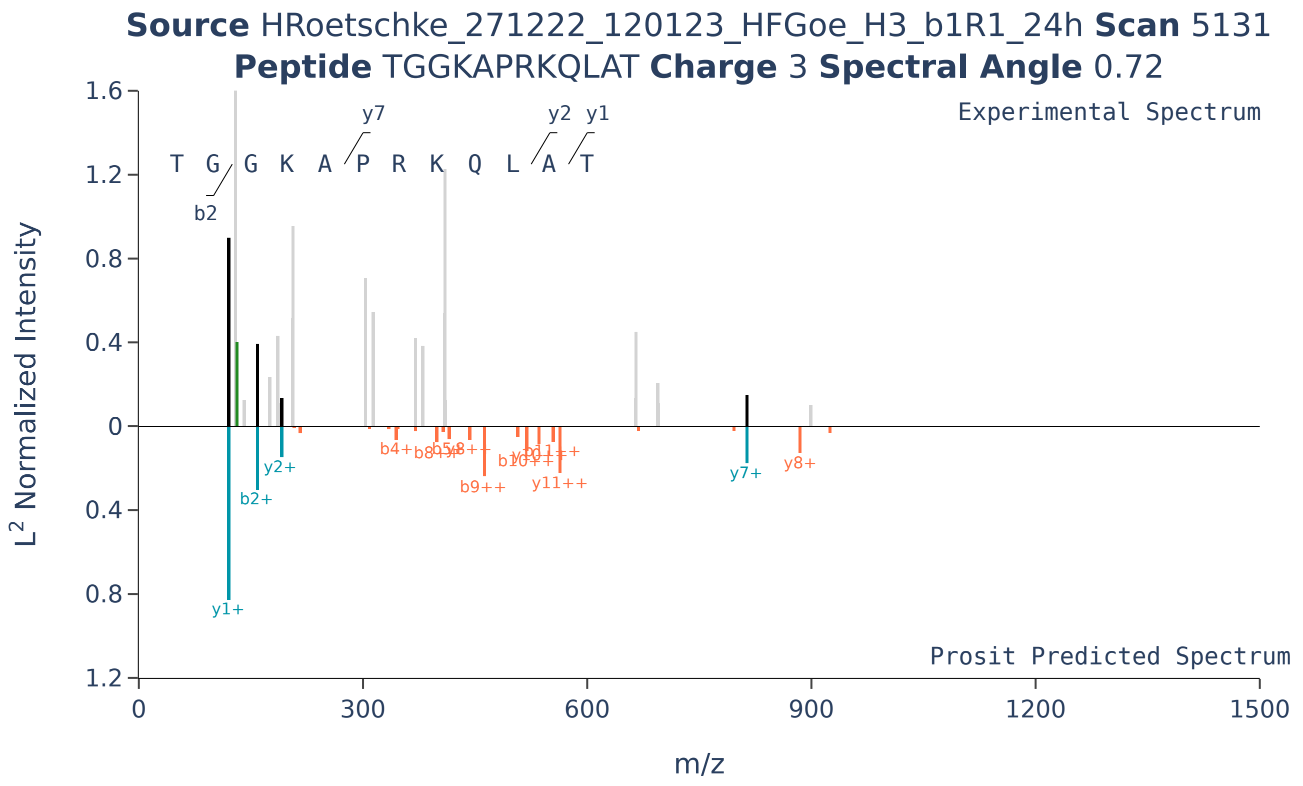
Source HRoetschke_271222_120123_HFGoe_H3_b1R1_24h Scan 5131
Peptide TGGKAPRKQLAT Charge 3 Spectral Angle 0.72
Experimental Spectrum
Prosit Predicted Spectrum
$L^2$ Normalized Intensity
m/z

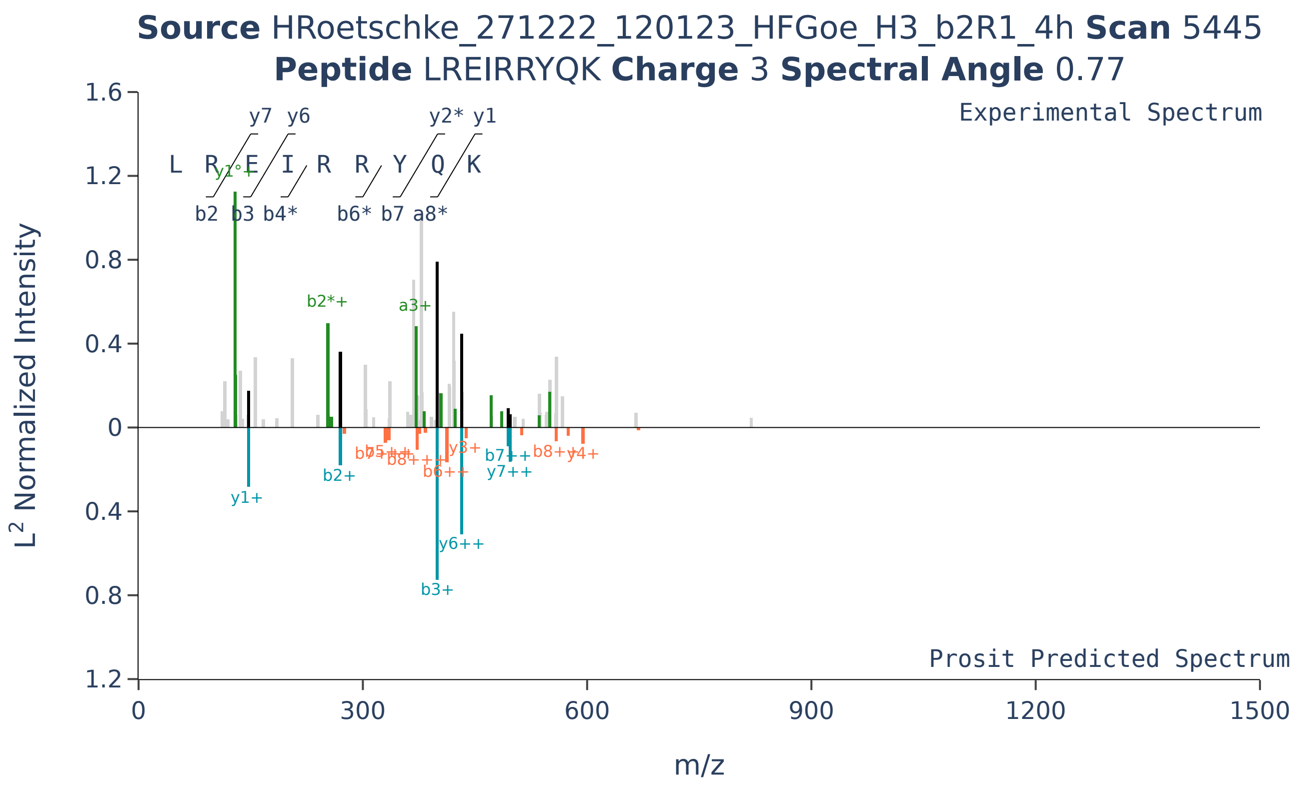
Source HRoetschke_271222_120123_HFGoe_H3_b2R1_4h Scan 5445
Peptide LREIRRYQK Charge 3 Spectral Angle 0.77
Experimental Spectrum
Prosit Predicted Spectrum
$L^2$ Normalized Intensity
m/z

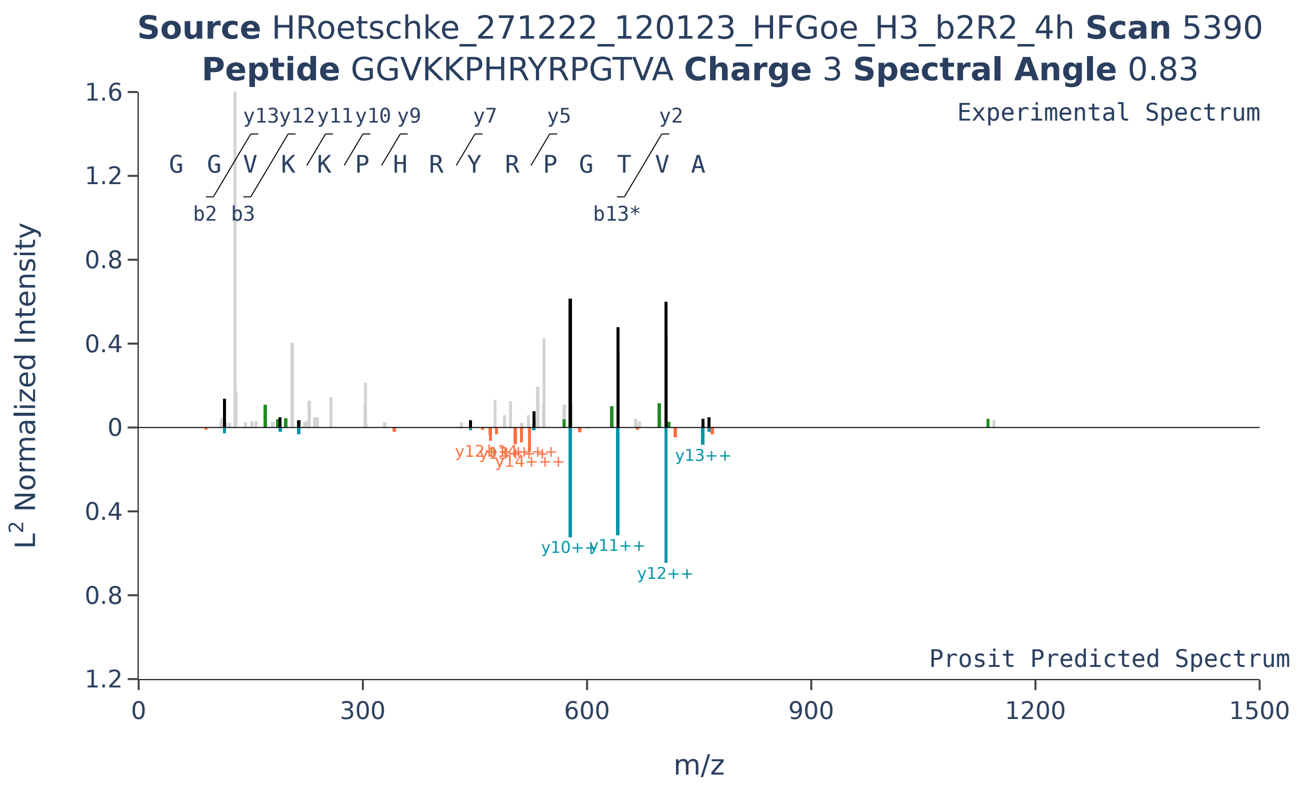
Source HRoetschke_271222_120123_HFGoe_H3_b2R2_4h Scan 5390
Peptide GGVKKPHRYRPGTVA Charge 3 Spectral Angle 0.83
Experimental Spectrum
Prosit Predicted Spectrum
$L^2$ Normalized Intensity
m/z

**Source** HRoetschke_291222_140123_HFGoe_H4_b2R1_24h **Scan** 12849
**Peptide** AMDVVYA **Charge** 1 **Spectral Angle** 0.9

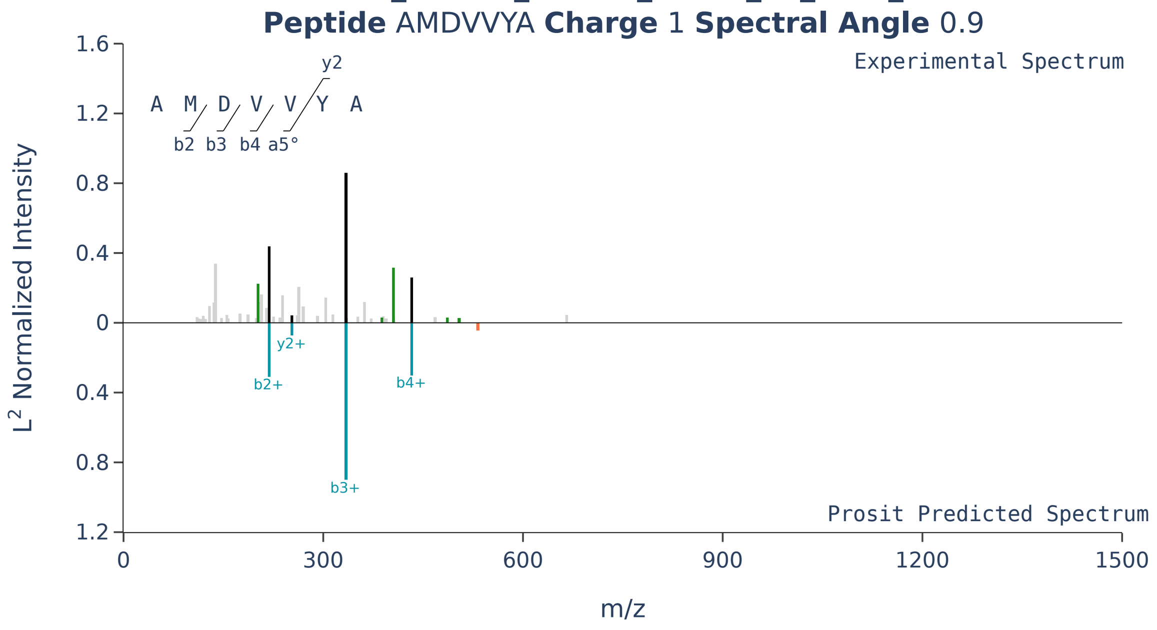


**Source** HRoetschke_291222_140123_HFGoe_H4_b1R1_2h **Scan** 15297
**Peptide** MDVVYALKRQGR **Charge** 4 **Spectral Angle** 0.94

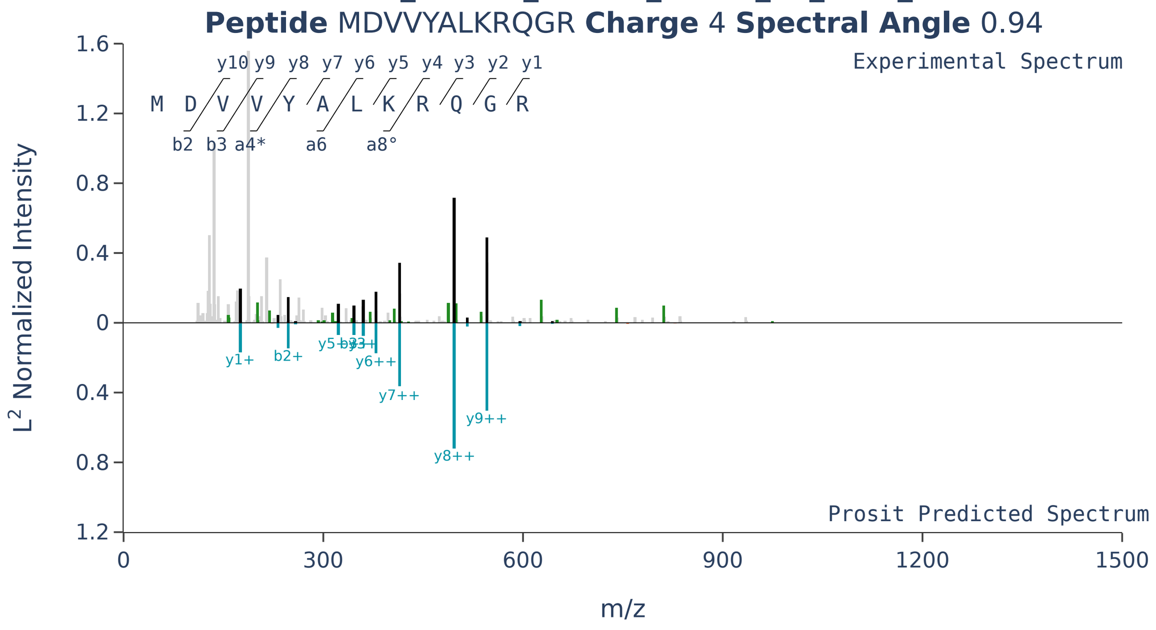


**Source** HRoetschke_291222_140123_HFGoe_H4_b2R1_24h **Scan** 10874
**Peptide** TRGVLKV **Charge** 2 **Spectral Angle** 0.87

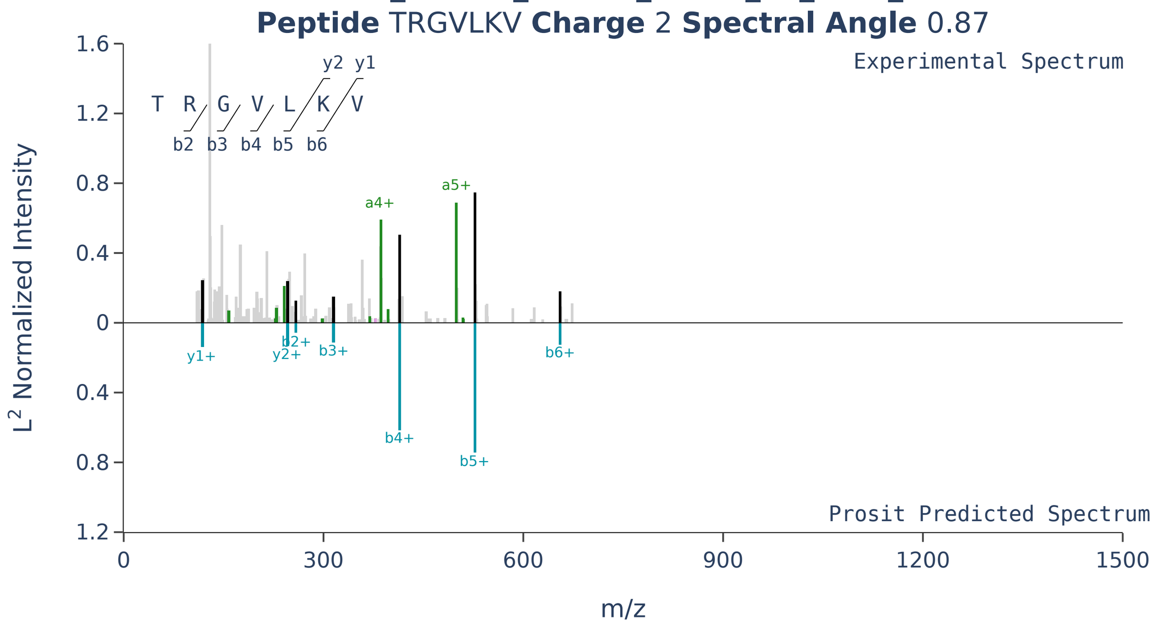


**Source** HRoetschke_291222_140123_HFGoe_H4_b1R1_2h **Scan** 17154
**Peptide** VTAMDVVY **Charge** 1 **Spectral Angle** 0.93

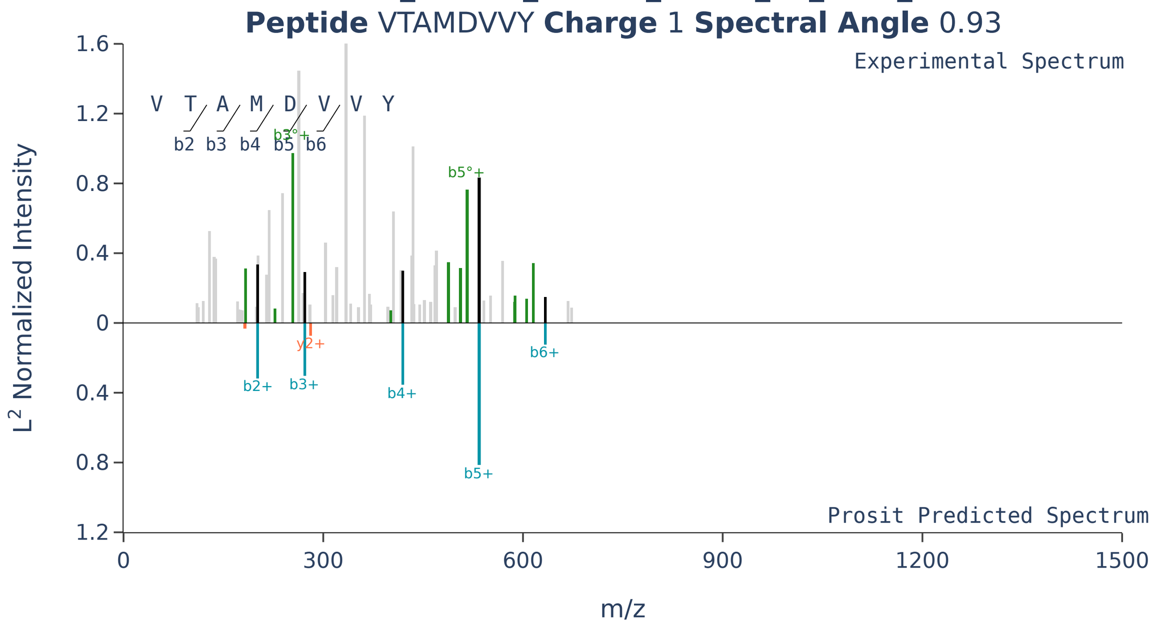


**Source** HRoetschke_291222_140123_HFGoe_H4_b1R1_24h **Scan** 15198
**Peptide** NVIRDAVTY **Charge** 1 **Spectral Angle** 0.93

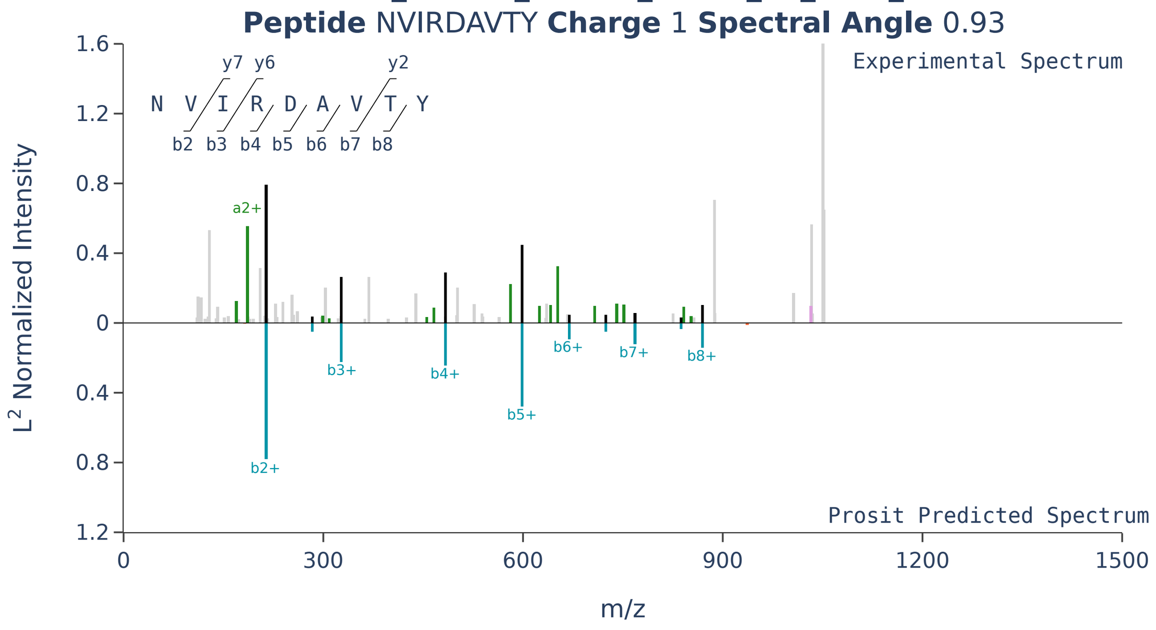


**Source** HRoetschke_291222_140123_HFGoe_H4_b2R1_1h **Scan** 15731
**Peptide** MDVVYALKR **Charge** 3 **Spectral Angle** 0.93

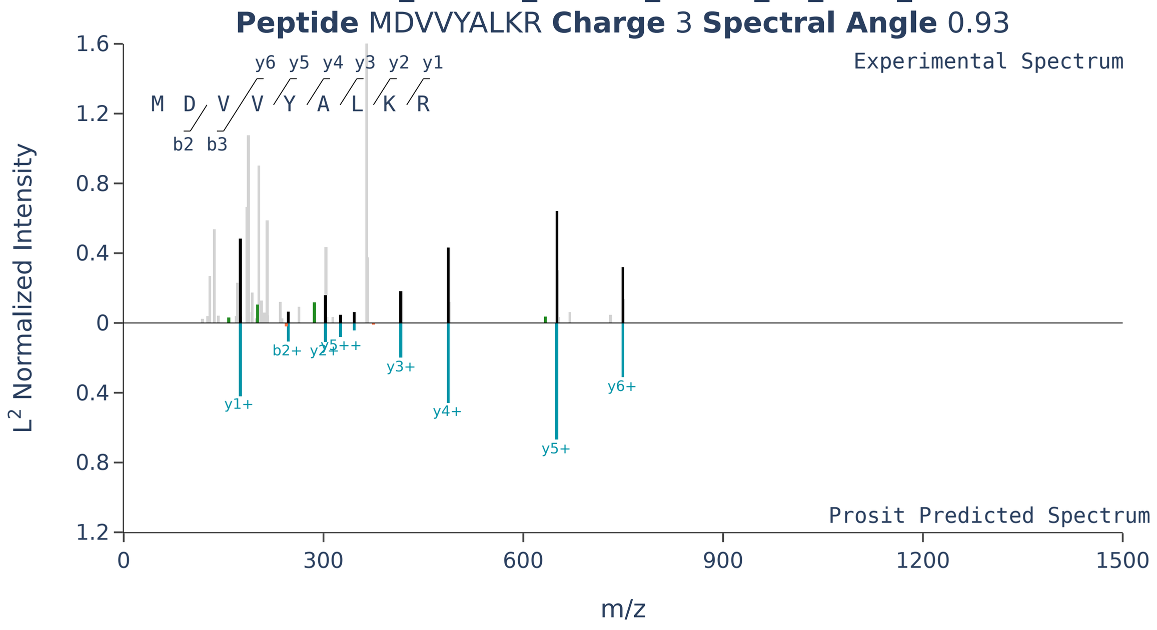


**Source** HRoetschke_291222_140123_HFGoe_H4_b2R1_1h **Scan** 17042
**Peptide** SGLIYEET **Charge** 1 **Spectral Angle** 0.94

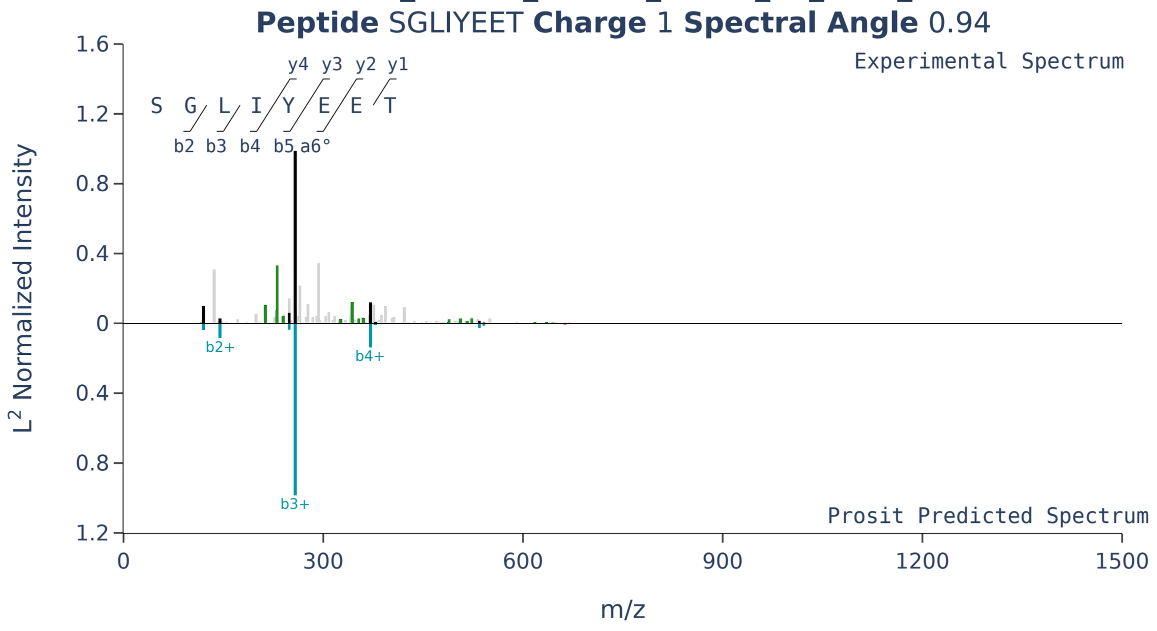


**Source** HRoetschke_291222_140123_HFGoe_H4_b2R1_24h **Scan** 12818
**Peptide** QGITKPAI **Charge** 2 **Spectral Angle** 0.95

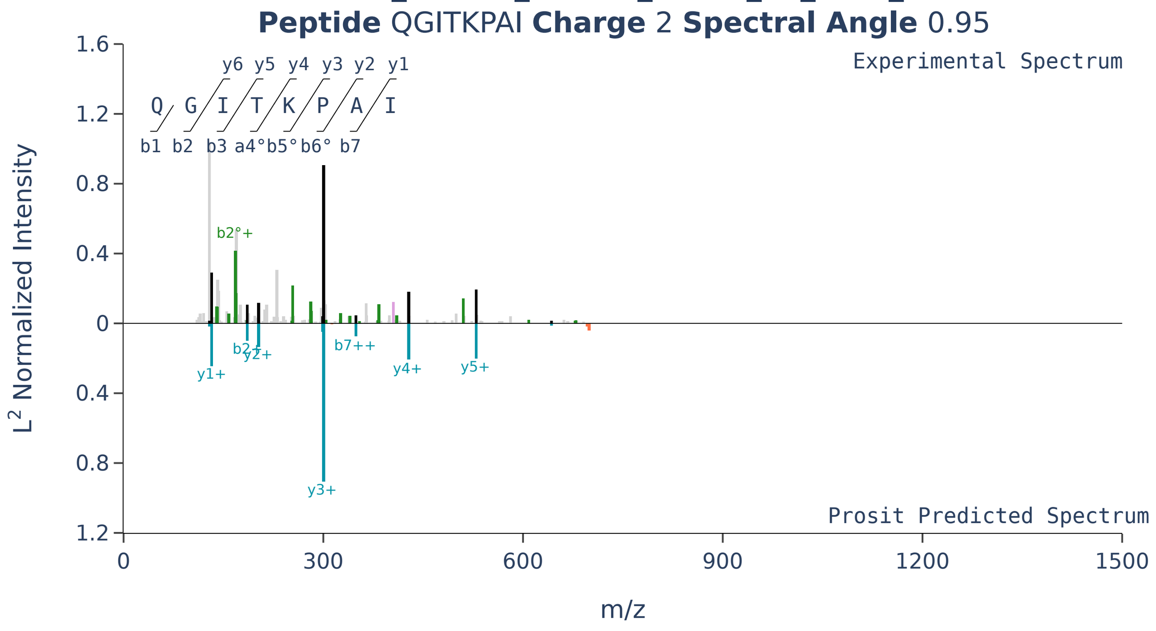


**Source** HRoetschke_291222_140123_HFGoe_H4_b2R2_24h **Scan** 12565
**Peptide** VIRDAVTY **Charge** 1 **Spectral Angle** 0.91

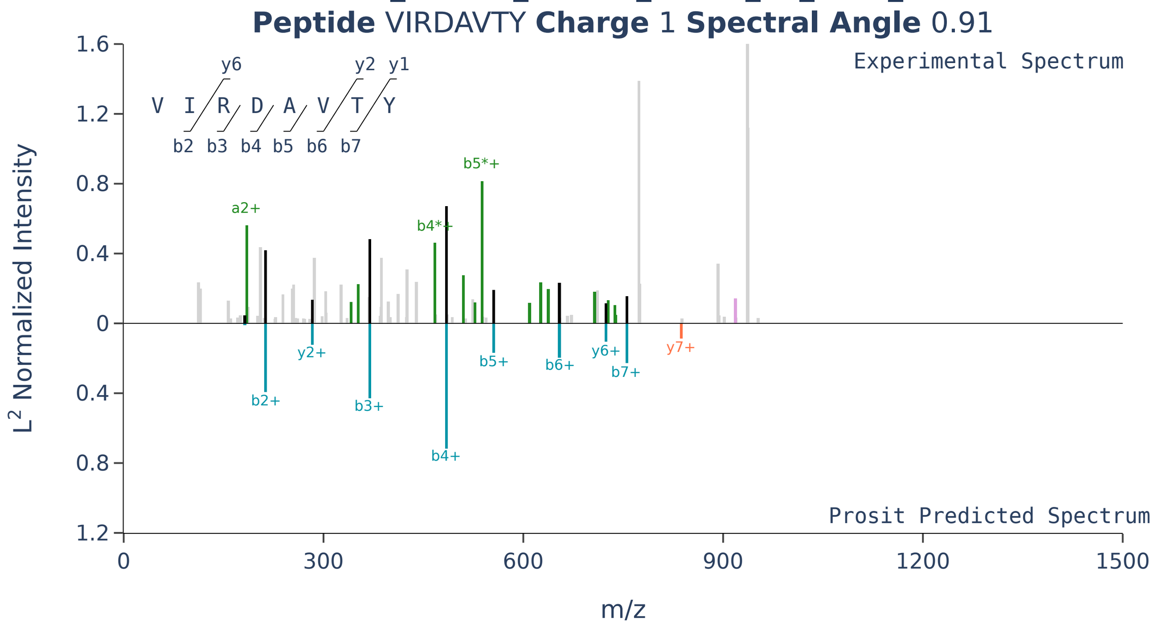


**Source** HRoetschke_291222_140123_HFGoe_H4_b1R2_4h **Scan** 18835
**Peptide** QGRTLYGF **Charge** 2 **Spectral Angle** 0.92

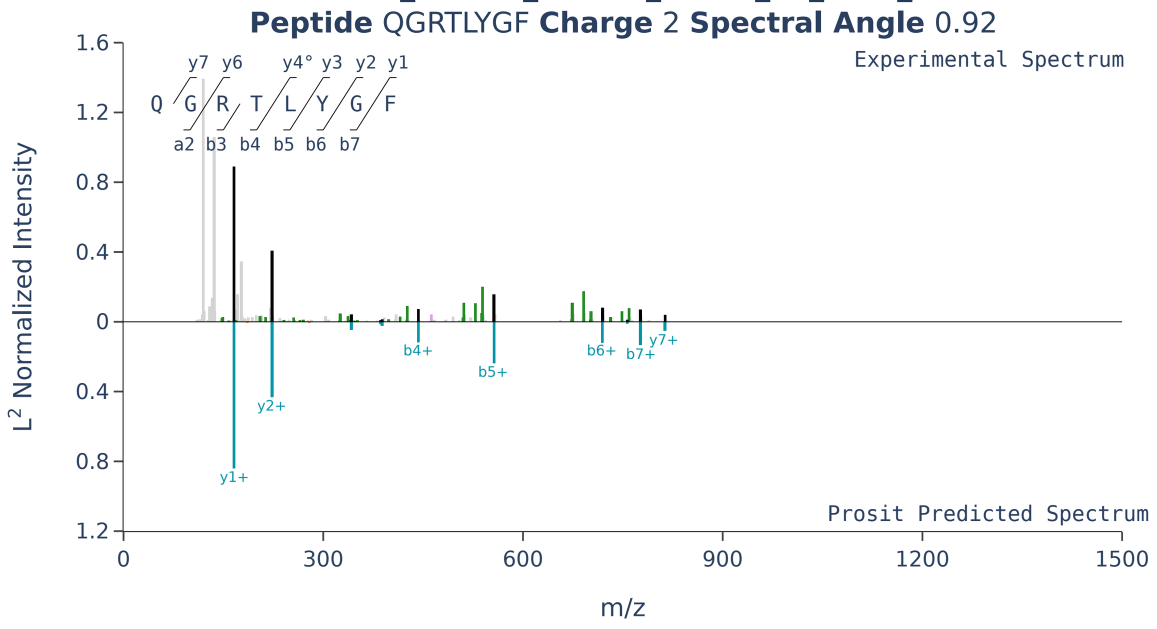


**Source** HRoetschke_291222_140123_HFGoe_H4_b2R1_24h **Scan** 13204
**Peptide** TAMDVVY **Charge** 1 **Spectral Angle** 0.92

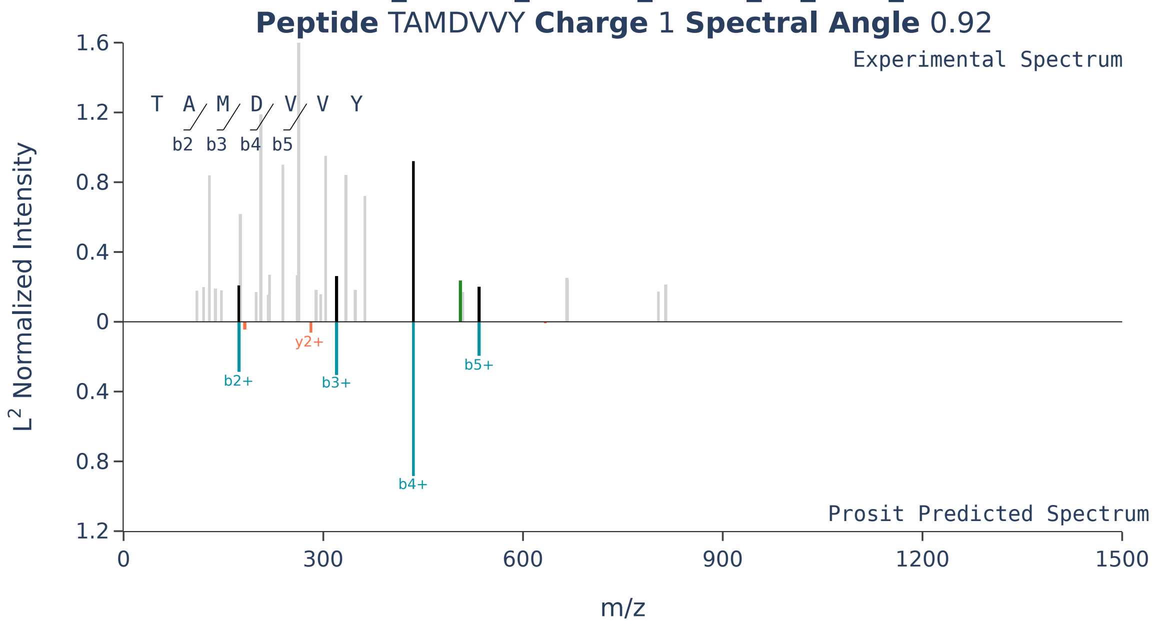


**Source** HRoetschke_291222_140123_HFGoe_H4_b2R1_1h **Scan** 16897
**Peptide** GLIYEET **Charge** 1 **Spectral Angle** 0.92

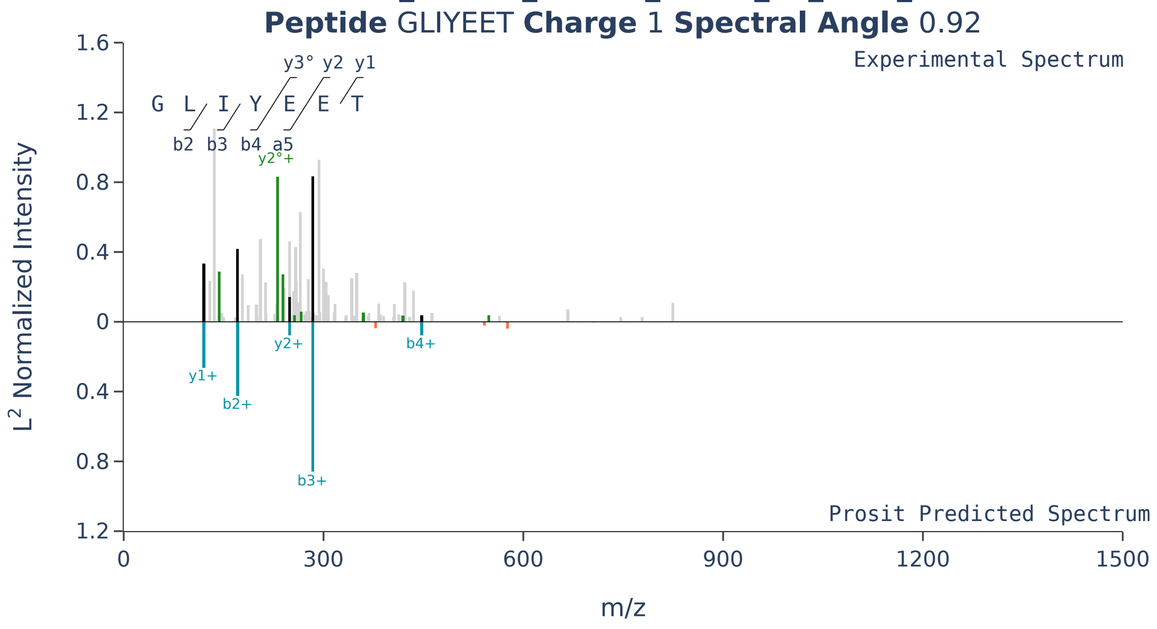


**Source** HRoetschke_291222_140123_HFGoe_H4_b2R1_1h **Scan** 27285
**Peptide** AMDVVYAL **Charge** 1 **Spectral Angle** 0.95

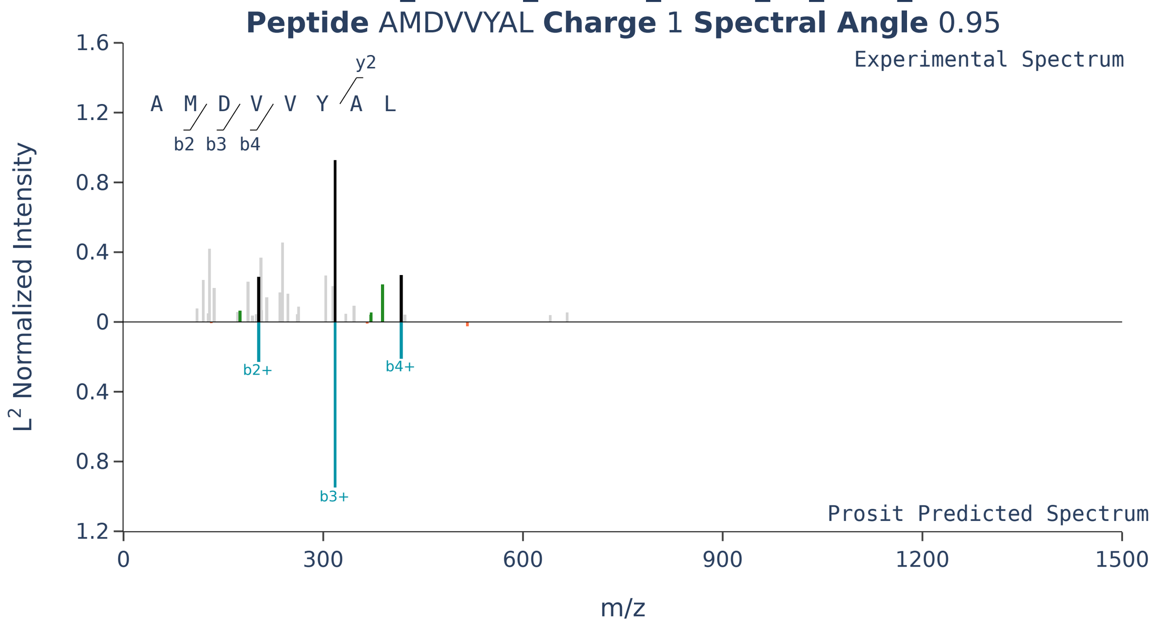


**Source** HRoetschke_291222_140123_HFGoe_H4_b2R2_24h **Scan** 10127
**Peptide** LRDNIQGITK **Charge** 3 **Spectral Angle** 0.93

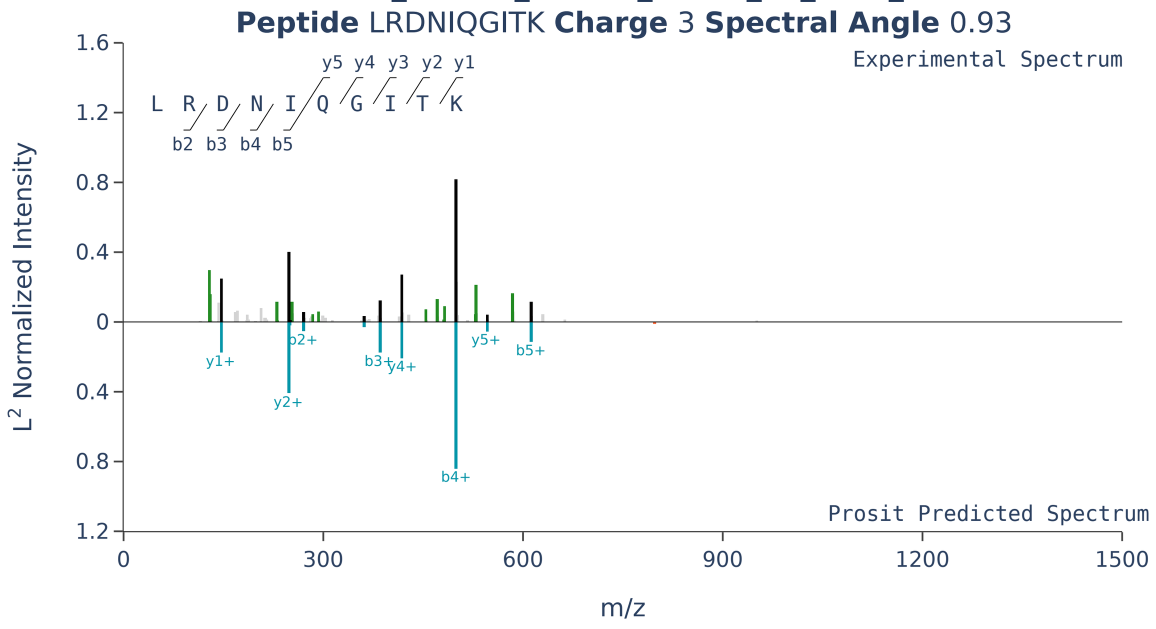


**Source** HRoetschke_291222_140123_HFGoe_H4_b2R2_2h **Scan** 13734
**Peptide** SGLIYEETRG **Charge** 2 **Spectral Angle** 0.86

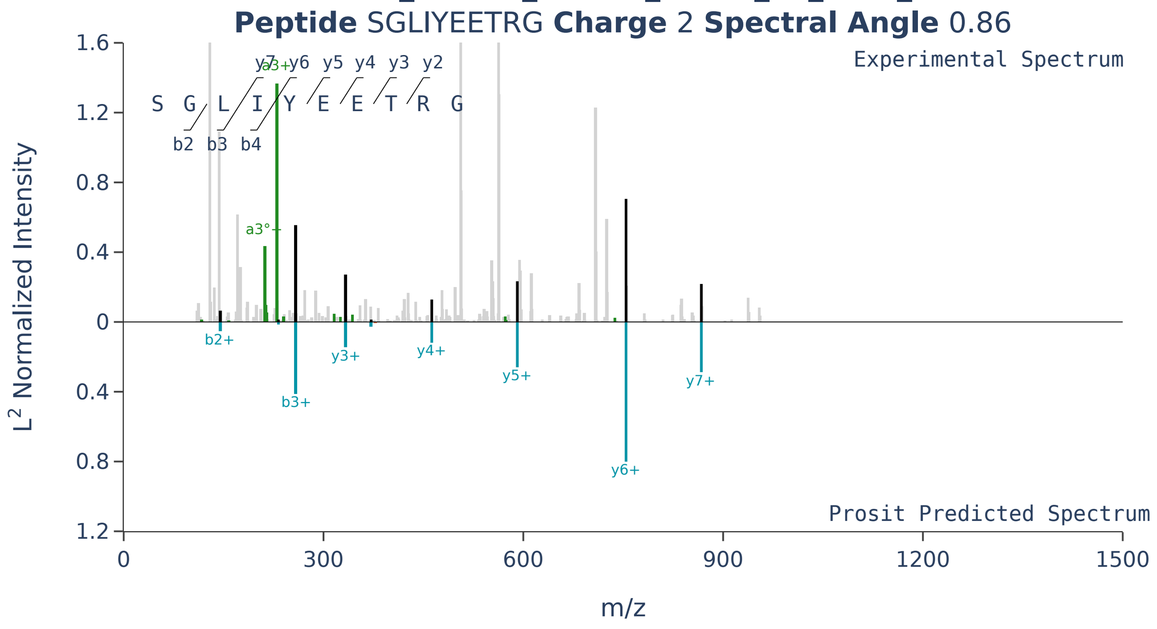

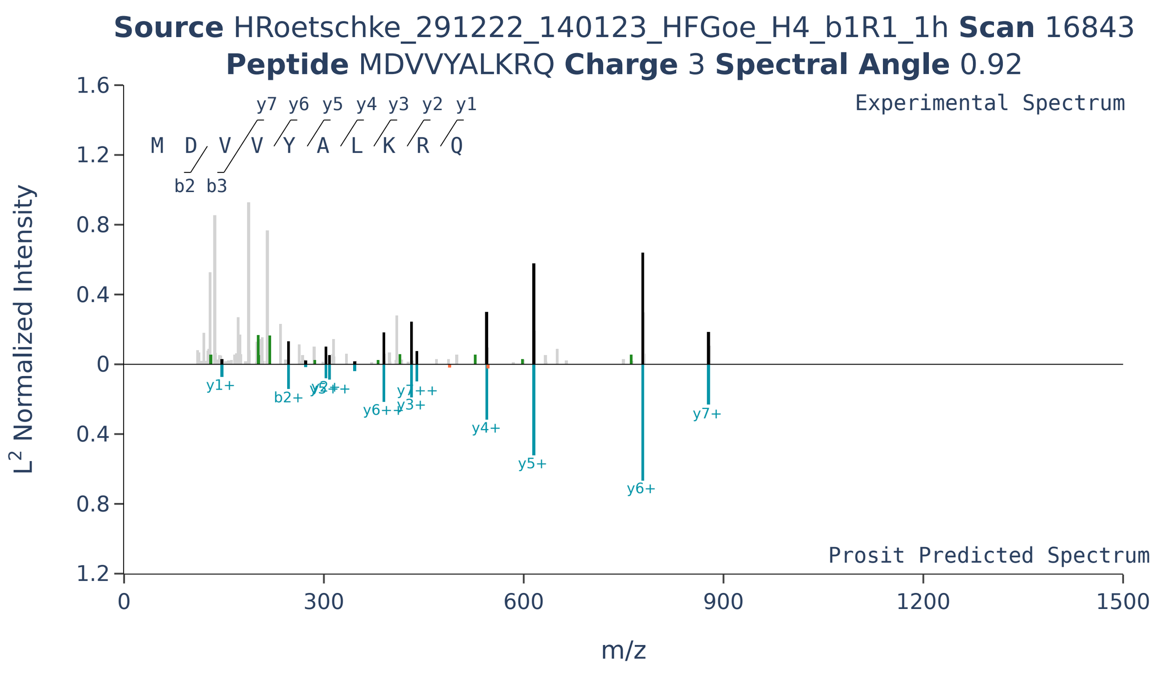
**Source** HRoetschke_291222_140123_HFGoe_H4_b1R1_1h **Scan** 16843
**Peptide** MDVVYALKRQ **Charge** 3 **Spectral Angle** 0.92
Experimental Spectrum
Prosit Predicted Spectrum
L² Normalized Intensity
m/z

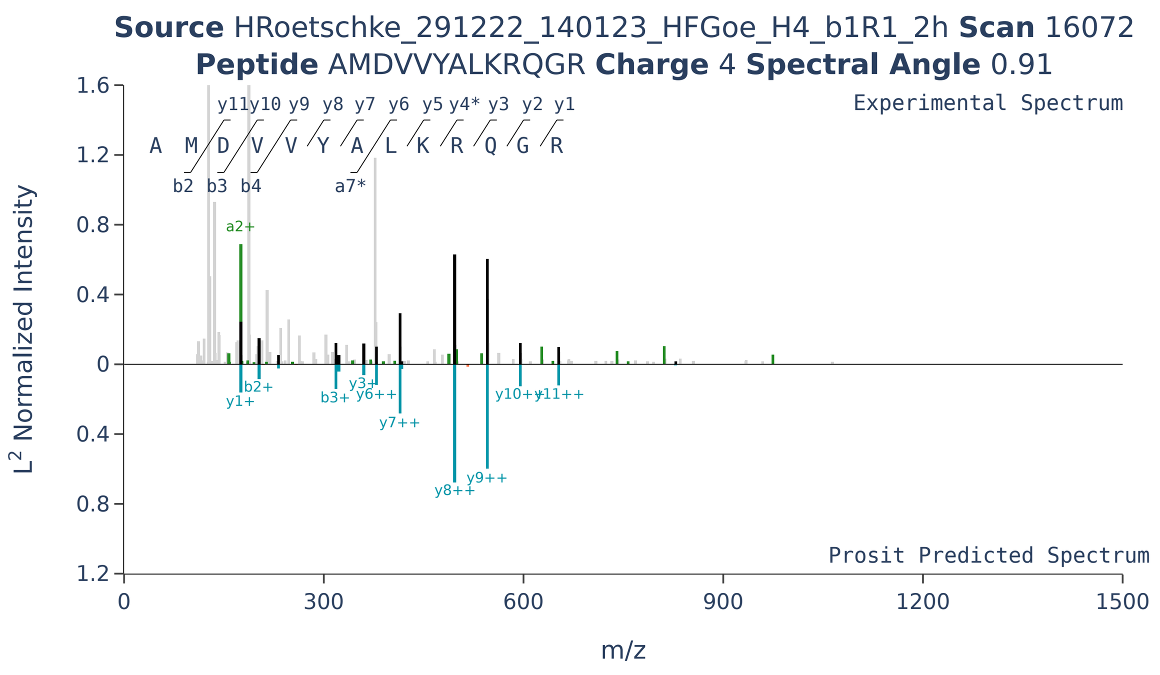
**Source** HRoetschke_291222_140123_HFGoe_H4_b1R1_2h **Scan** 16072
**Peptide** AMDVVYALKRQGR **Charge** 4 **Spectral Angle** 0.91
Experimental Spectrum
Prosit Predicted Spectrum
L² Normalized Intensity
m/z

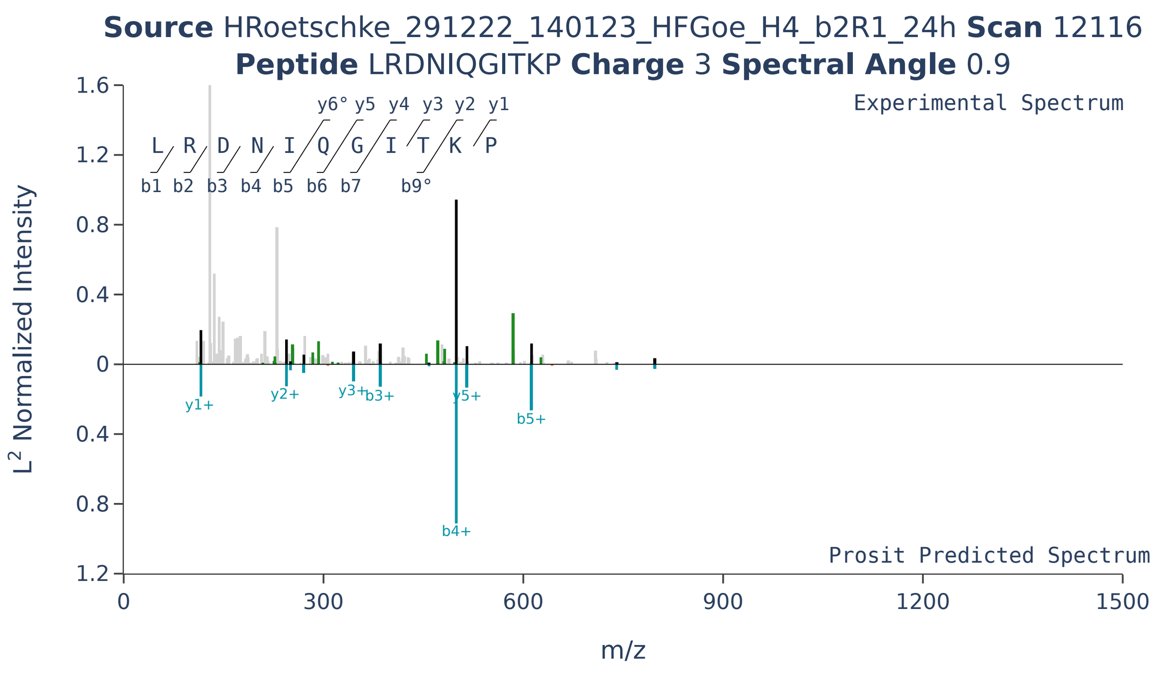
**Source** HRoetschke_291222_140123_HFGoe_H4_b2R1_24h **Scan** 12116
**Peptide** LRDNIQGITKP **Charge** 3 **Spectral Angle** 0.9
Experimental Spectrum
Prosit Predicted Spectrum
L² Normalized Intensity
m/z

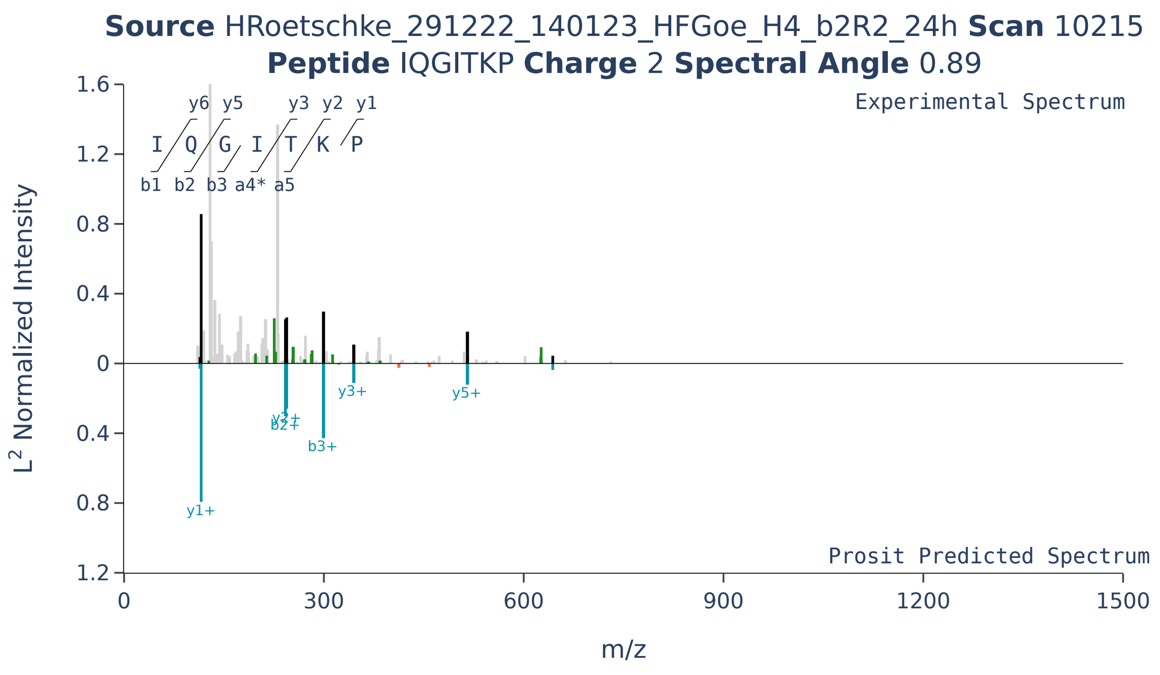
**Source** HRoetschke_291222_140123_HFGoe_H4_b2R2_24h **Scan** 10215
**Peptide** IQGITKP **Charge** 2 **Spectral Angle** 0.89
Experimental Spectrum
Prosit Predicted Spectrum
L² Normalized Intensity
m/z

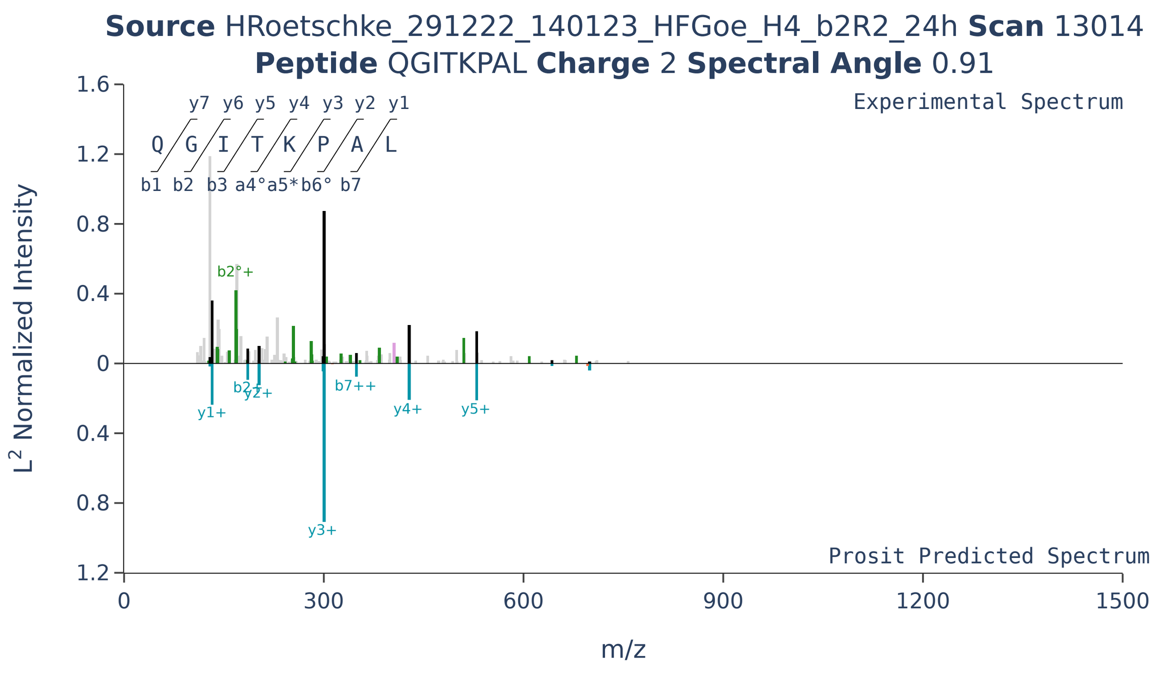
**Source** HRoetschke_291222_140123_HFGoe_H4_b2R2_24h **Scan** 13014
**Peptide** QGITKPAL **Charge** 2 **Spectral Angle** 0.91
Experimental Spectrum
Prosit Predicted Spectrum
L² Normalized Intensity
m/z

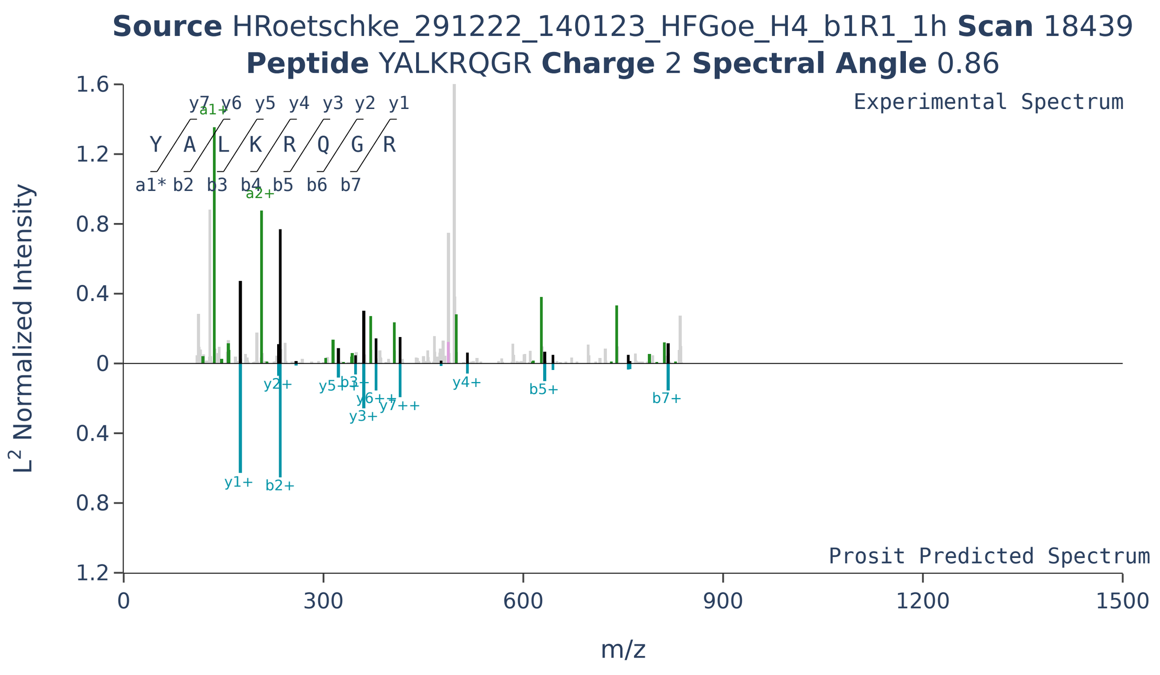
**Source** HRoetschke_291222_140123_HFGoe_H4_b1R1_1h **Scan** 18439
**Peptide** YALKRQGR **Charge** 2 **Spectral Angle** 0.86
Experimental Spectrum
Prosit Predicted Spectrum
L² Normalized Intensity
m/z

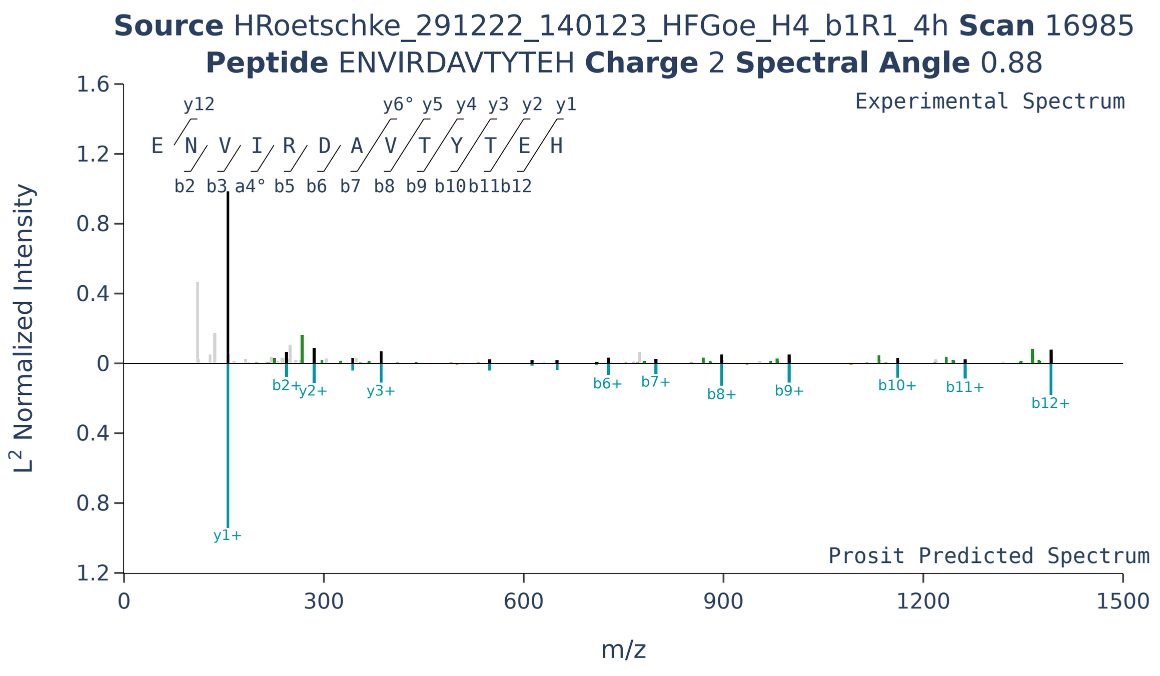
**Source** HRoetschke_291222_140123_HFGoe_H4_b1R1_4h **Scan** 16985
**Peptide** ENVIRDAVTYTEH **Charge** 2 **Spectral Angle** 0.88
Experimental Spectrum
Prosit Predicted Spectrum
L² Normalized Intensity
m/z

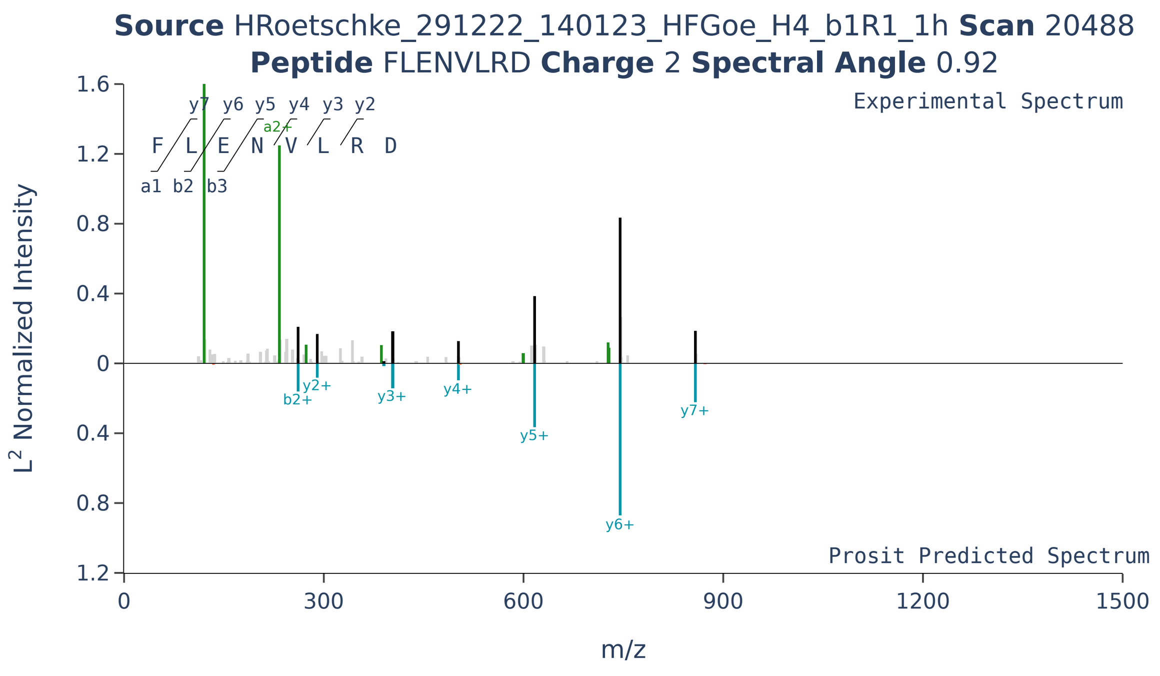
**Source** HRoetschke_291222_140123_HFGoe_H4_b1R1_1h **Scan** 20488
**Peptide** FLENVLRD **Charge** 2 **Spectral Angle** 0.92
Experimental Spectrum
Prosit Predicted Spectrum
L² Normalized Intensity
m/z

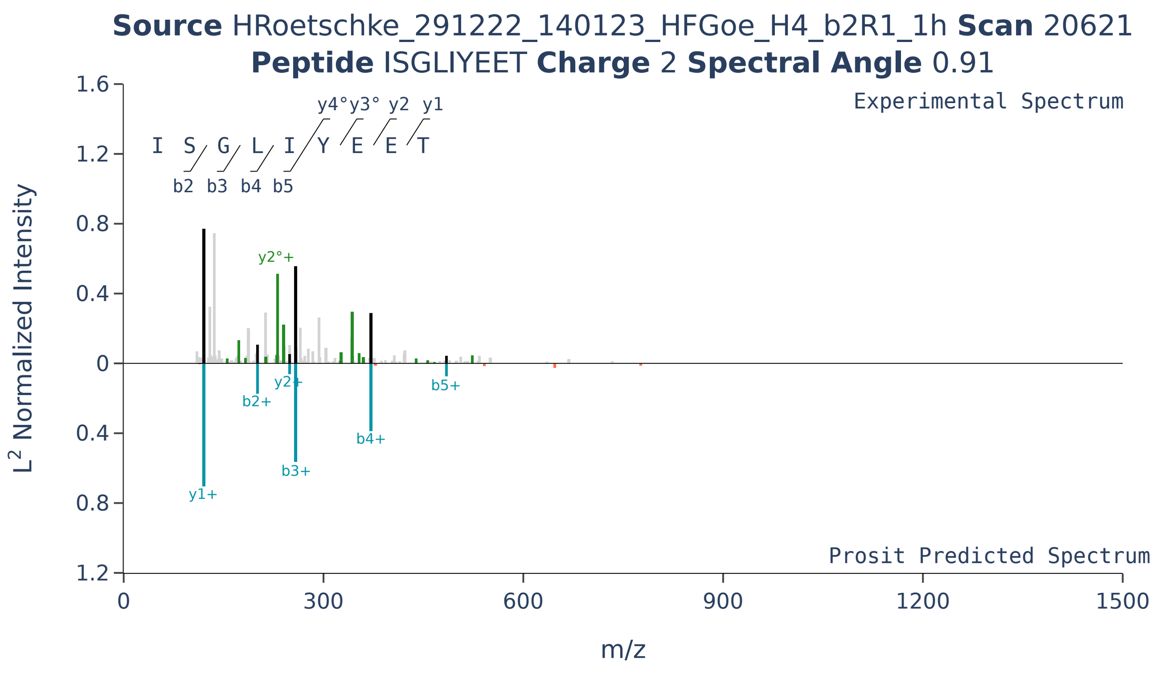
**Source** HRoetschke_291222_140123_HFGoe_H4_b2R1_1h **Scan** 20621
**Peptide** ISGLIYEET **Charge** 2 **Spectral Angle** 0.91
Experimental Spectrum
Prosit Predicted Spectrum
L² Normalized Intensity
m/z

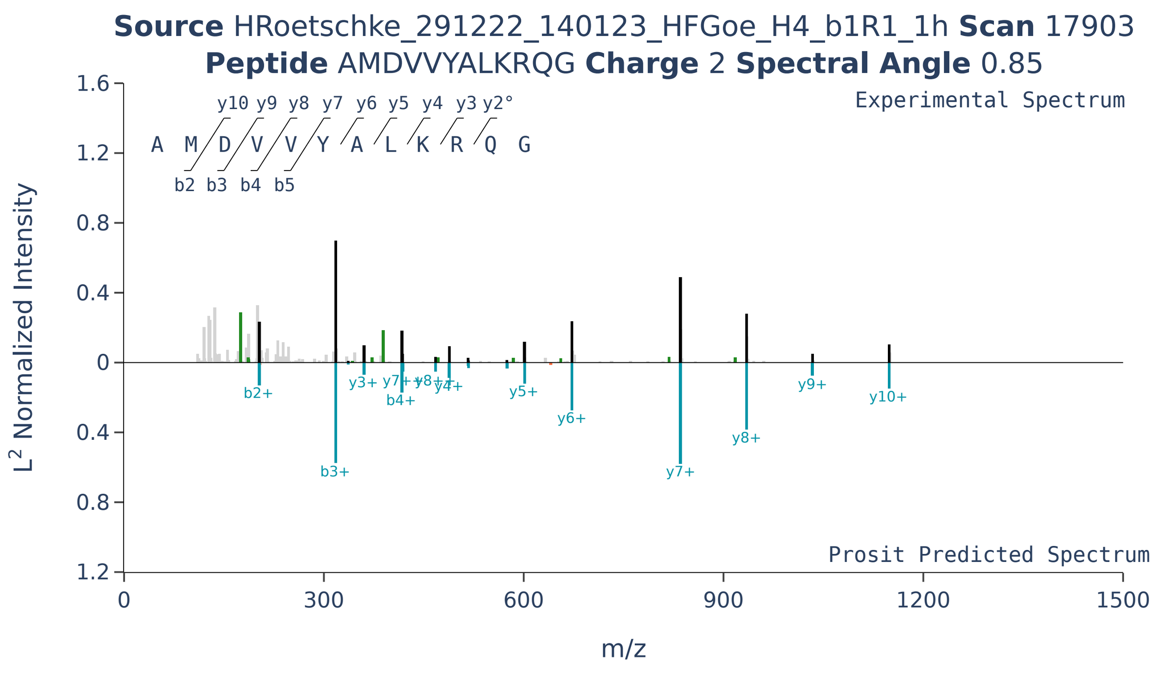
**Source** HRoetschke_291222_140123_HFGoe_H4_b1R1_1h **Scan** 17903
**Peptide** AMDVVYALKRQG **Charge** 2 **Spectral Angle** 0.85
Experimental Spectrum
Prosit Predicted Spectrum
L² Normalized Intensity
m/z

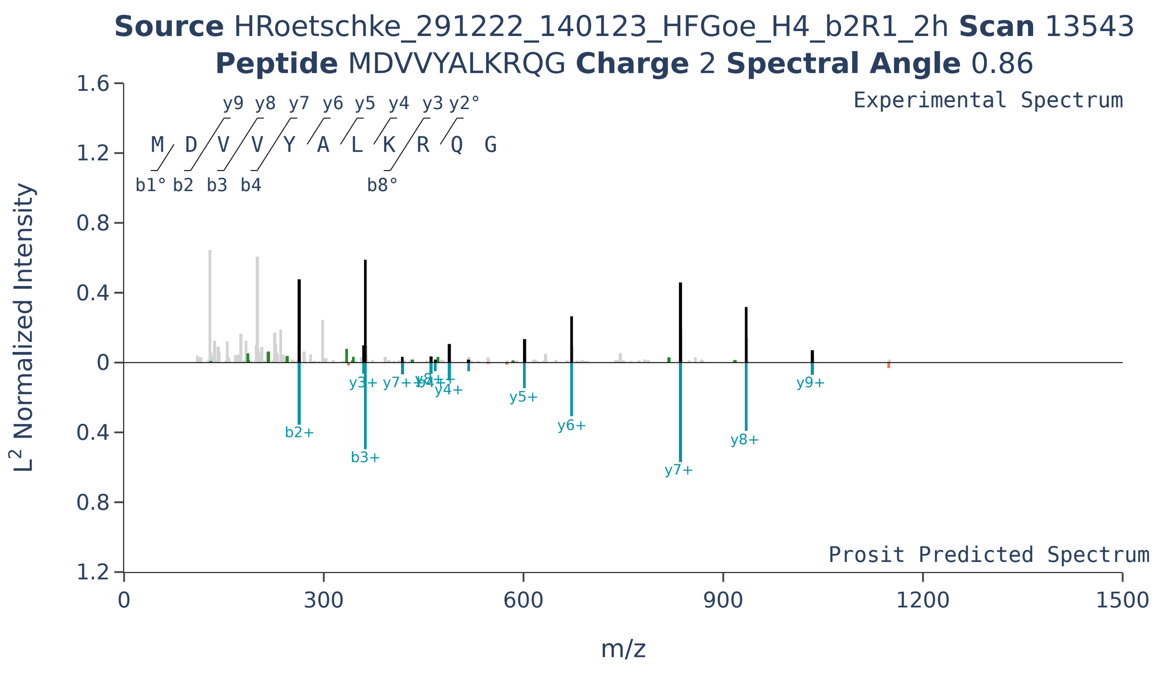
**Source** HRoetschke_291222_140123_HFGoe_H4_b2R1_2h **Scan** 13543
**Peptide** MDVVYALKRQG **Charge** 2 **Spectral Angle** 0.86
Experimental Spectrum
Prosit Predicted Spectrum
L² Normalized Intensity
m/z

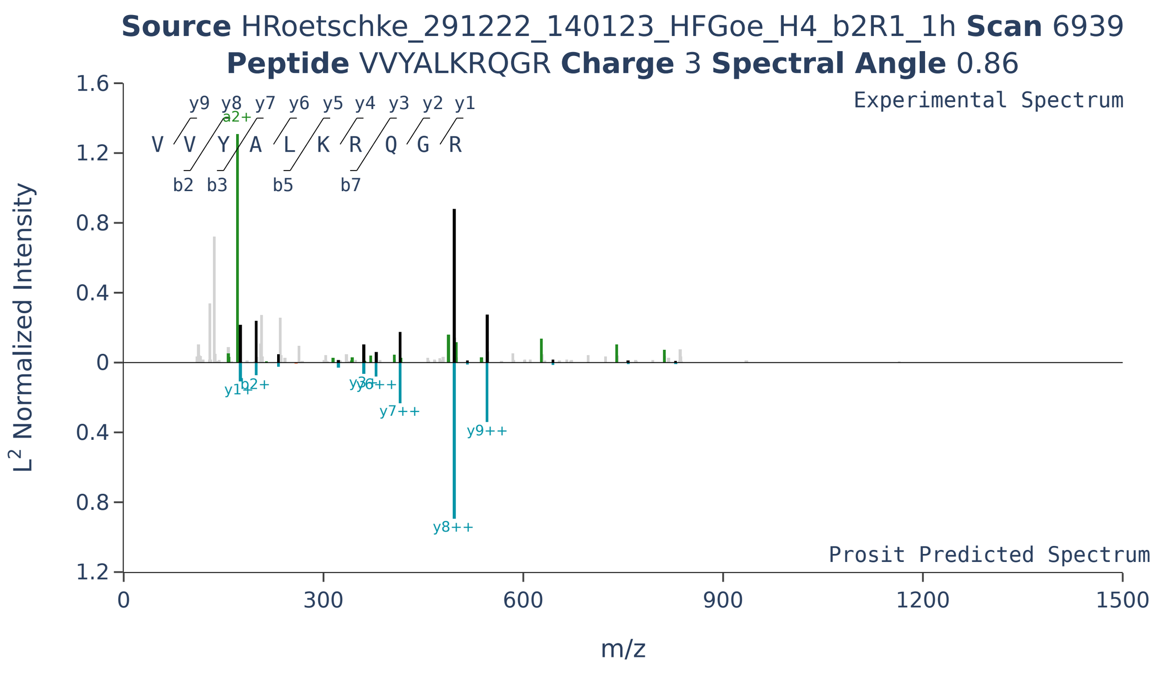
**Source** HRoetschke_291222_140123_HFGoe_H4_b2R1_1h **Scan** 6939
**Peptide** VVYALKRQGR **Charge** 3 **Spectral Angle** 0.86
Experimental Spectrum
Prosit Predicted Spectrum
L² Normalized Intensity
m/z

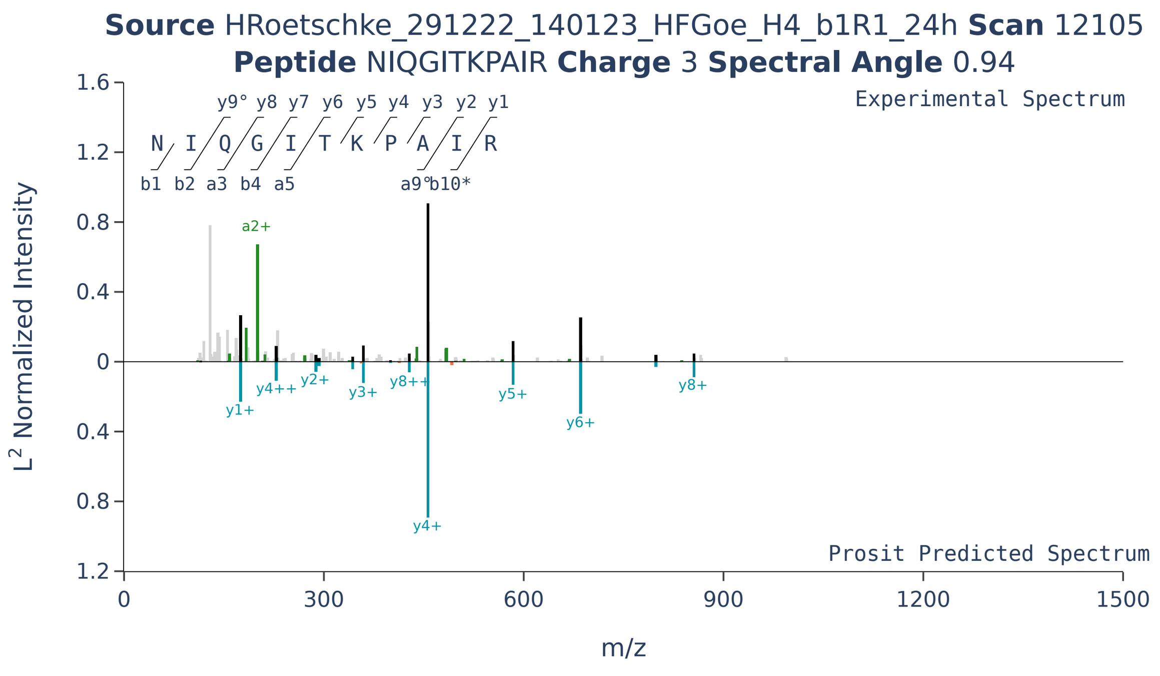
**Source** HRoetschke_291222_140123_HFGoe_H4_b1R1_24h **Scan** 12105
**Peptide** NIQGITKPAIR **Charge** 3 **Spectral Angle** 0.94
Experimental Spectrum
Prosit Predicted Spectrum
L² Normalized Intensity
m/z

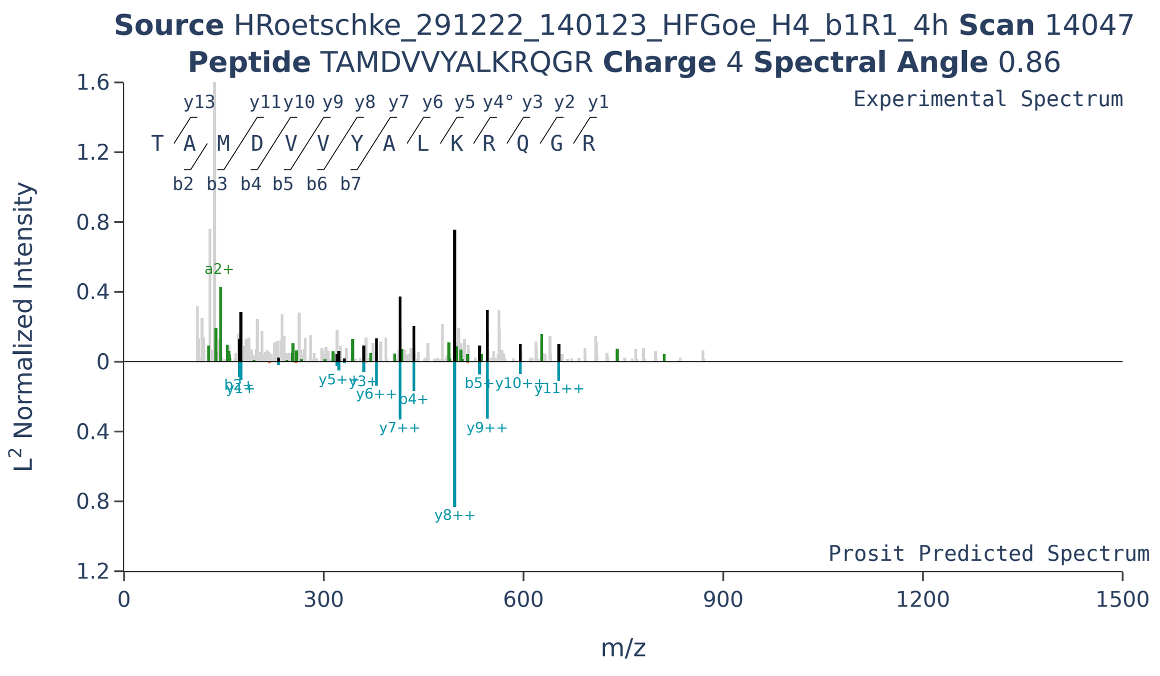
**Source** HRoetschke_291222_140123_HFGoe_H4_b1R1_4h **Scan** 14047
**Peptide** TAMDVVYALKRQGR **Charge** 4 **Spectral Angle** 0.86
Experimental Spectrum
Prosit Predicted Spectrum
L² Normalized Intensity
m/z

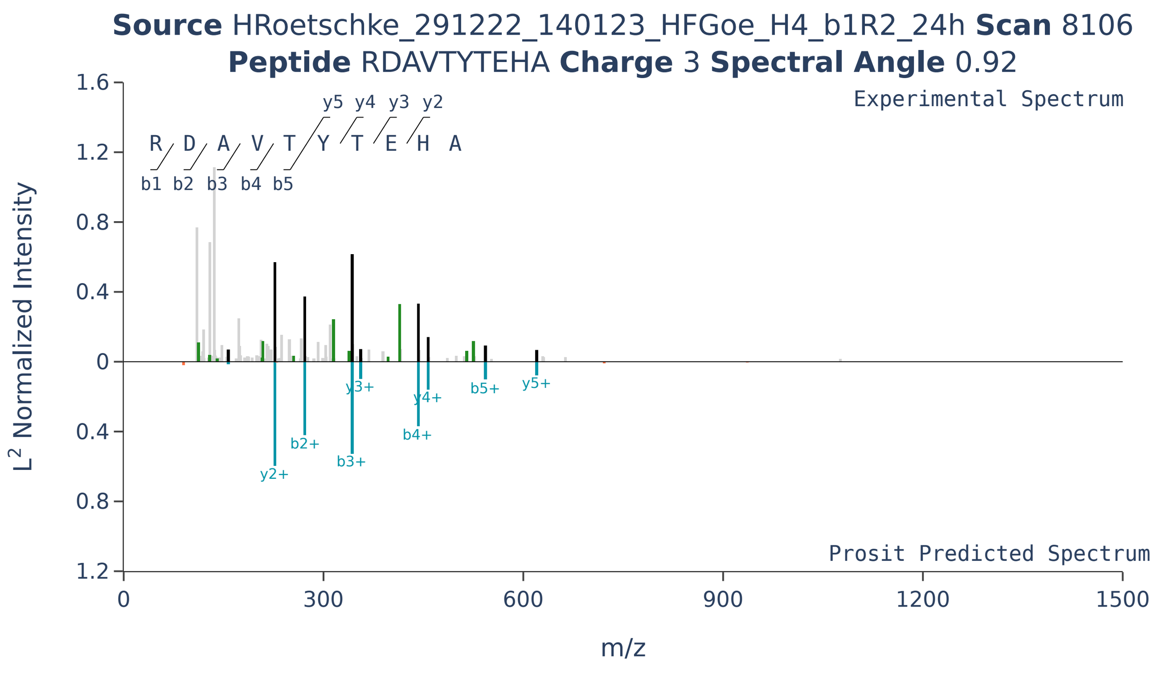
**Source** HRoetschke_291222_140123_HFGoe_H4_b1R2_24h **Scan** 8106
**Peptide** RDAVTYTEHA **Charge** 3 **Spectral Angle** 0.92
Experimental Spectrum
Prosit Predicted Spectrum
L² Normalized Intensity
m/z

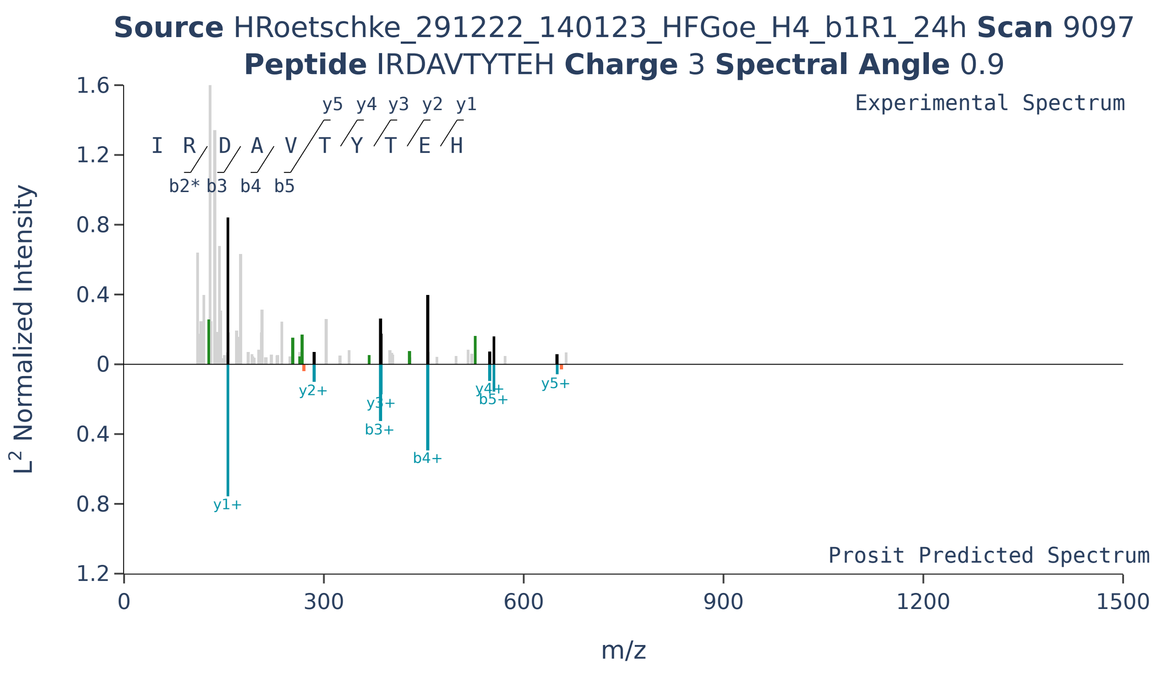
**Source** HRoetschke_291222_140123_HFGoe_H4_b1R1_24h **Scan** 9097
**Peptide** IRDAVTYTEH **Charge** 3 **Spectral Angle** 0.9
Experimental Spectrum
Prosit Predicted Spectrum
L² Normalized Intensity
m/z

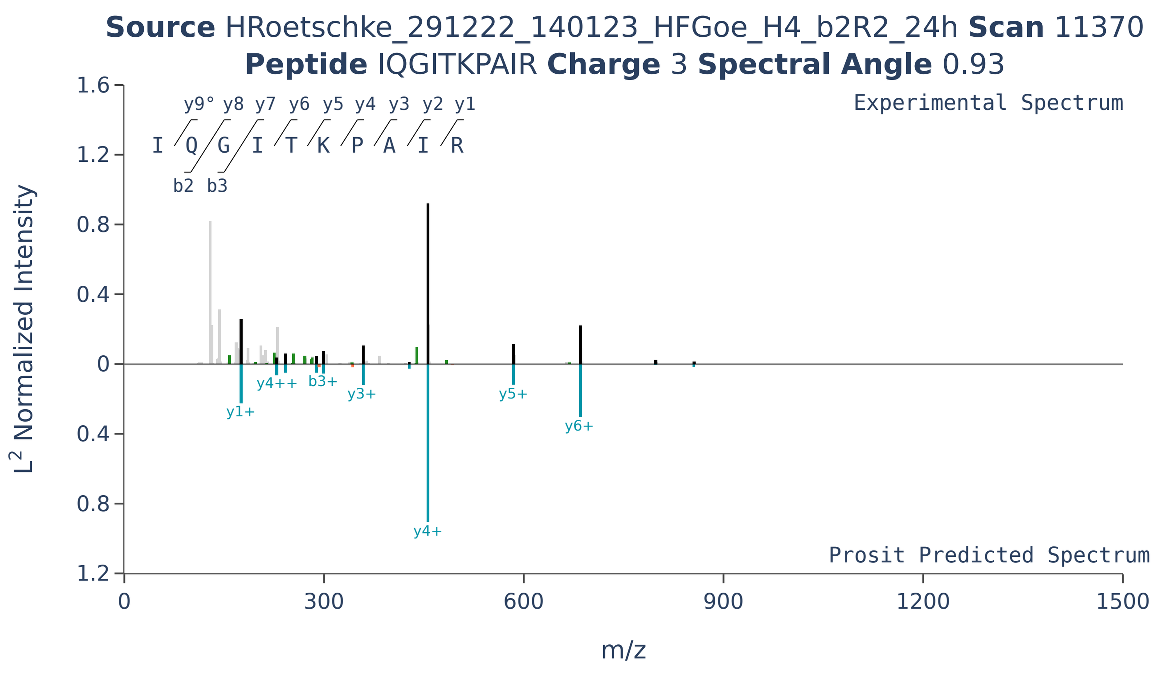
**Source** HRoetschke_291222_140123_HFGoe_H4_b2R2_24h **Scan** 11370
**Peptide** IQGITKPAIR **Charge** 3 **Spectral Angle** 0.93
Experimental Spectrum
Prosit Predicted Spectrum
L² Normalized Intensity
m/z

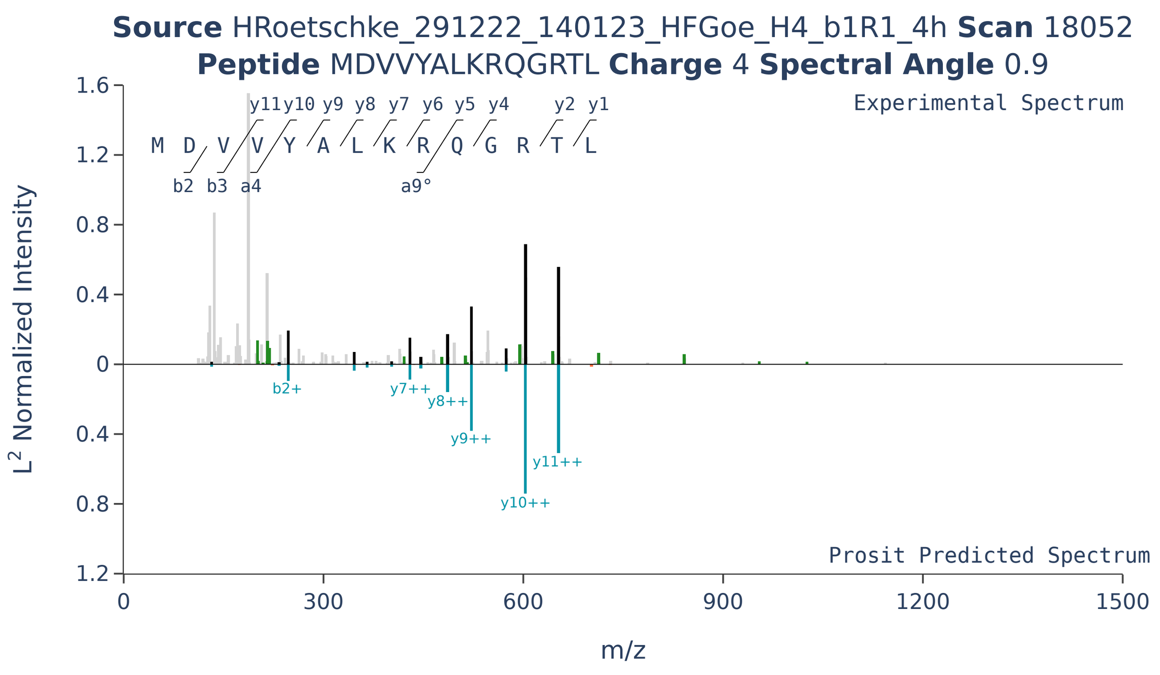
**Source** HRoetschke_291222_140123_HFGoe_H4_b1R1_4h **Scan** 18052
**Peptide** MDVVYALKRQGRTL **Charge** 4 **Spectral Angle** 0.9
Experimental Spectrum
Prosit Predicted Spectrum
L² Normalized Intensity
m/z

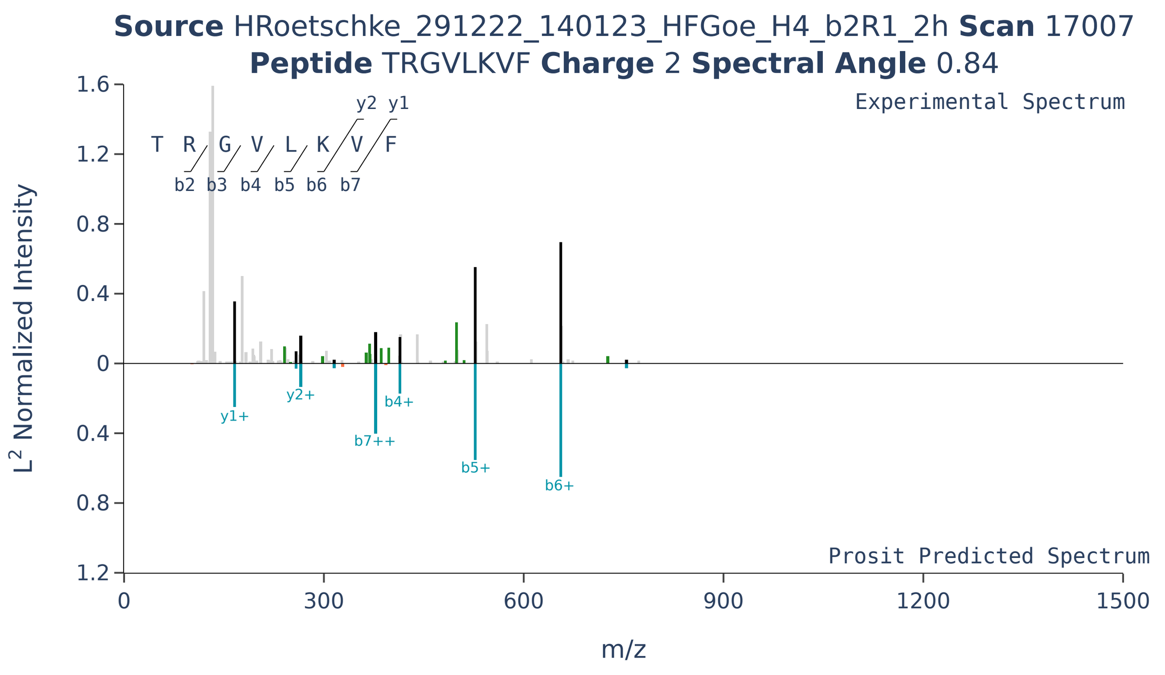
**Source** HRoetschke_291222_140123_HFGoe_H4_b2R1_2h **Scan** 17007
**Peptide** TRGVLKVF **Charge** 2 **Spectral Angle** 0.84
Experimental Spectrum
Prosit Predicted Spectrum
L² Normalized Intensity
m/z

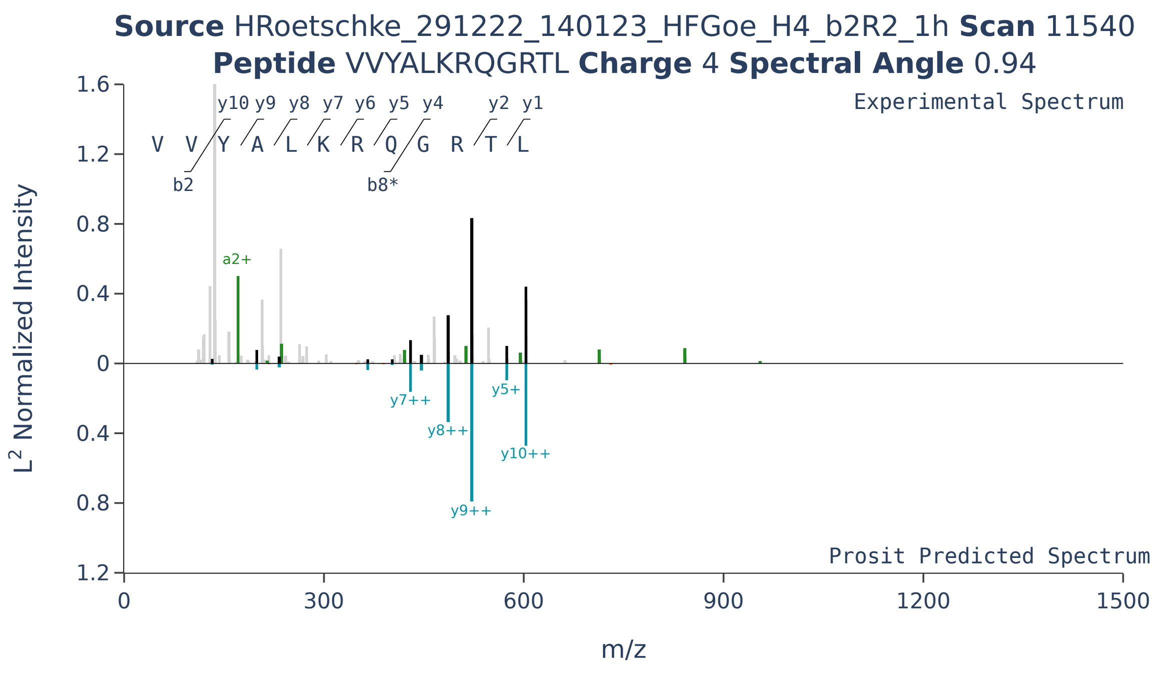
**Source** HRoetschke_291222_140123_HFGoe_H4_b2R2_1h **Scan** 11540
**Peptide** VVYALKRQGRTL **Charge** 4 **Spectral Angle** 0.94
Experimental Spectrum
Prosit Predicted Spectrum
L² Normalized Intensity
m/z

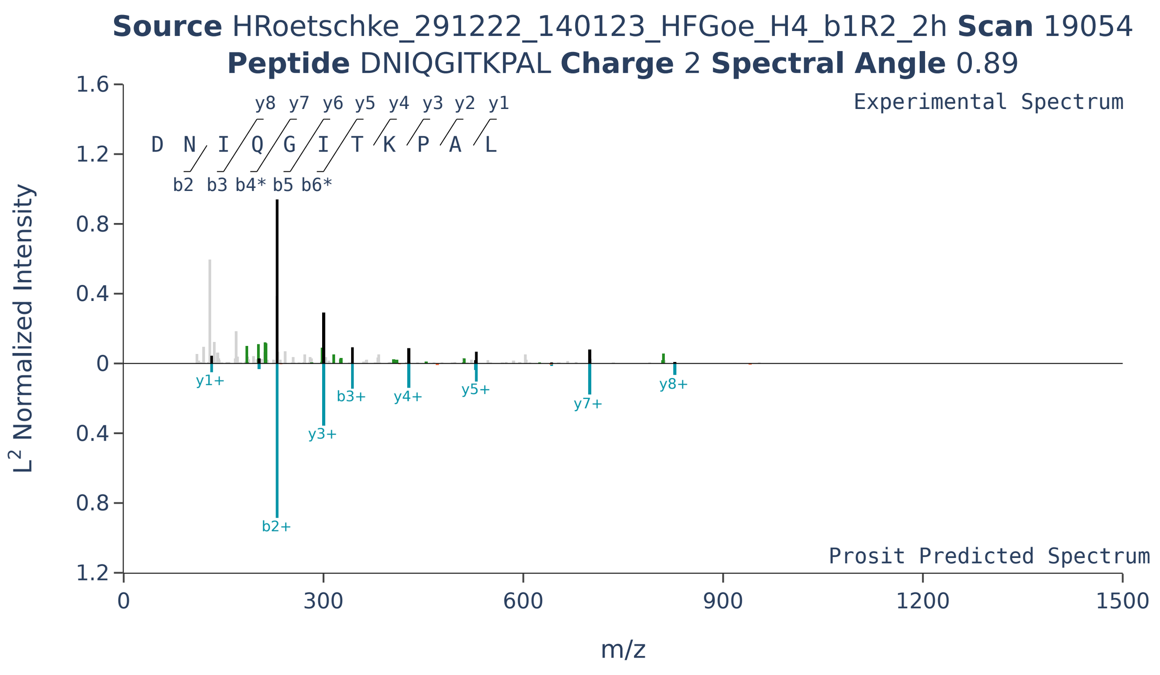
**Source** HRoetschke_291222_140123_HFGoe_H4_b1R2_2h **Scan** 19054
**Peptide** DNIQGITKPAL **Charge** 2 **Spectral Angle** 0.89
Experimental Spectrum
Prosit Predicted Spectrum
L² Normalized Intensity
m/z

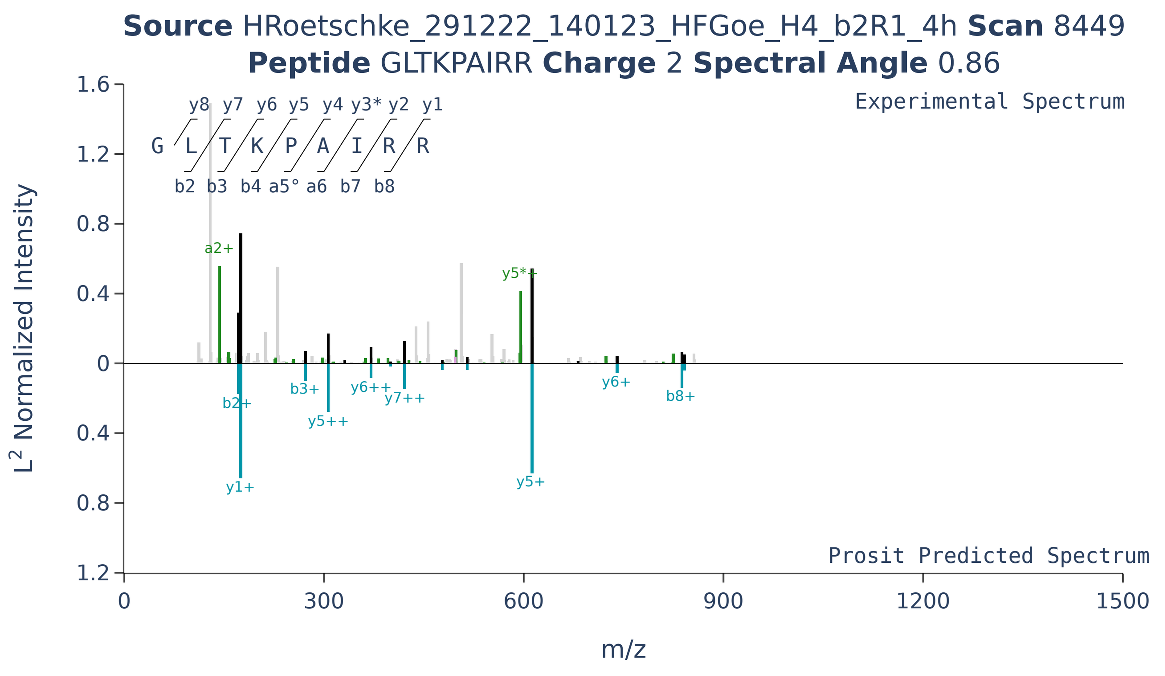
**Source** HRoetschke_291222_140123_HFGoe_H4_b2R1_4h **Scan** 8449
**Peptide** GLTKPAIRR **Charge** 2 **Spectral Angle** 0.86
Experimental Spectrum
Prosit Predicted Spectrum
L² Normalized Intensity
m/z

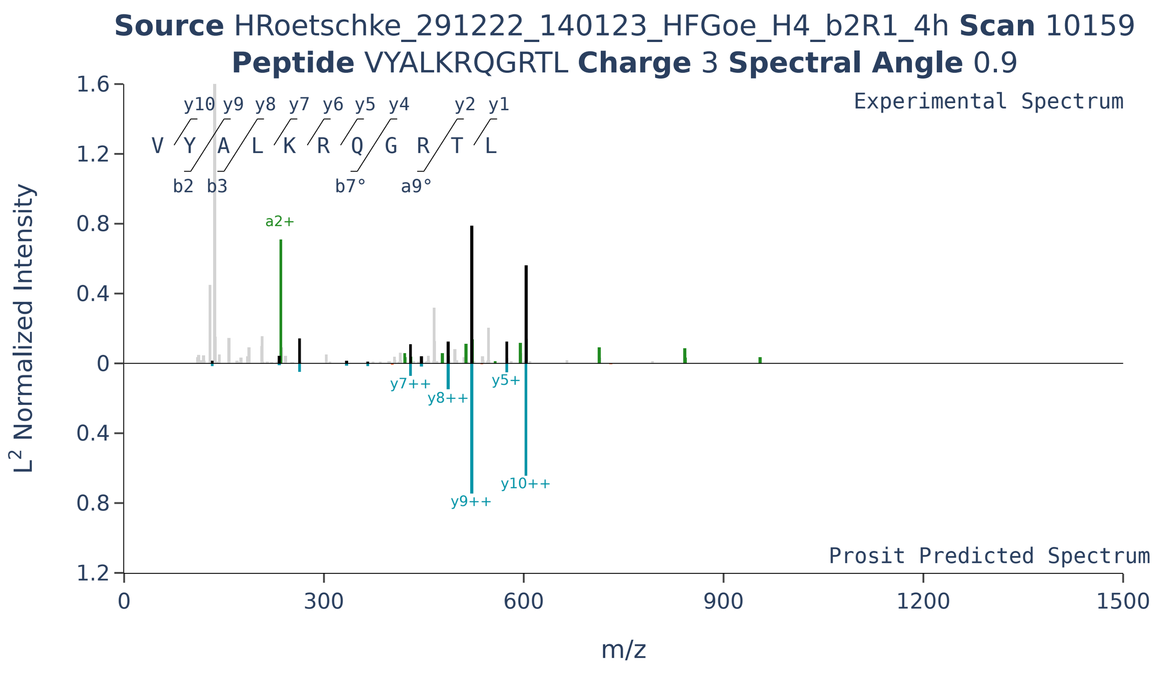
**Source** HRoetschke_291222_140123_HFGoe_H4_b2R1_4h **Scan** 10159
**Peptide** VYALKRQGRTL **Charge** 3 **Spectral Angle** 0.9
Experimental Spectrum
Prosit Predicted Spectrum
L² Normalized Intensity
m/z

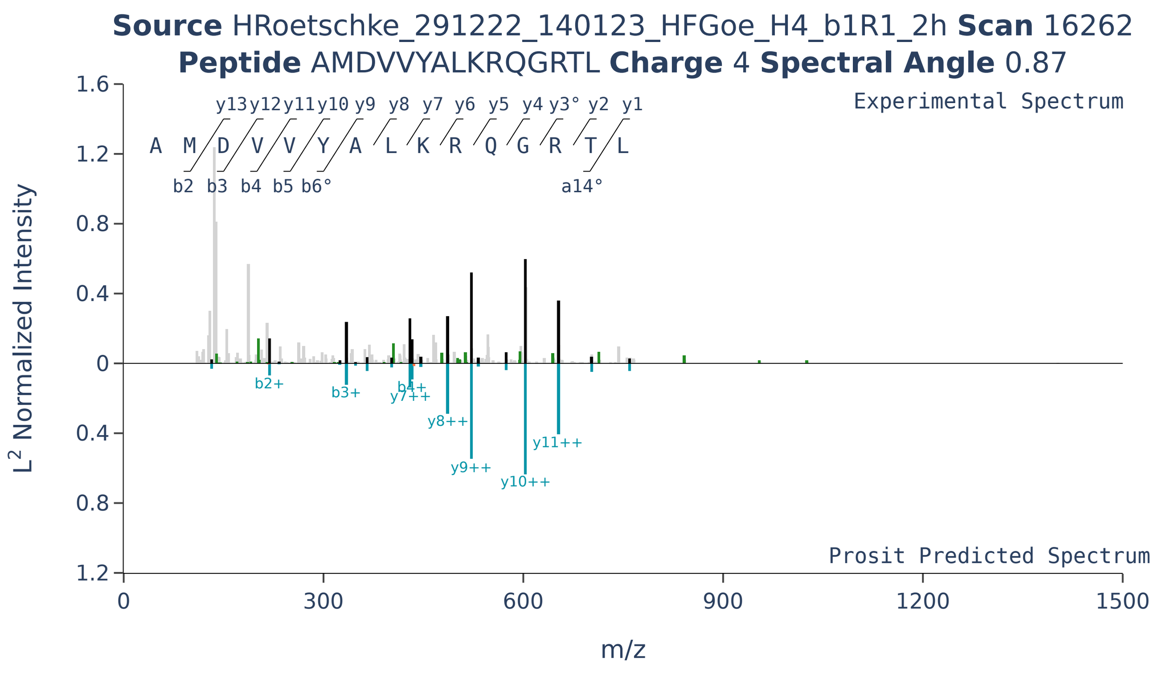
**Source** HRoetschke_291222_140123_HFGoe_H4_b1R1_2h **Scan** 16262
**Peptide** AMDVVYALKRQGRTL **Charge** 4 **Spectral Angle** 0.87
Experimental Spectrum
Prosit Predicted Spectrum
L² Normalized Intensity
m/z

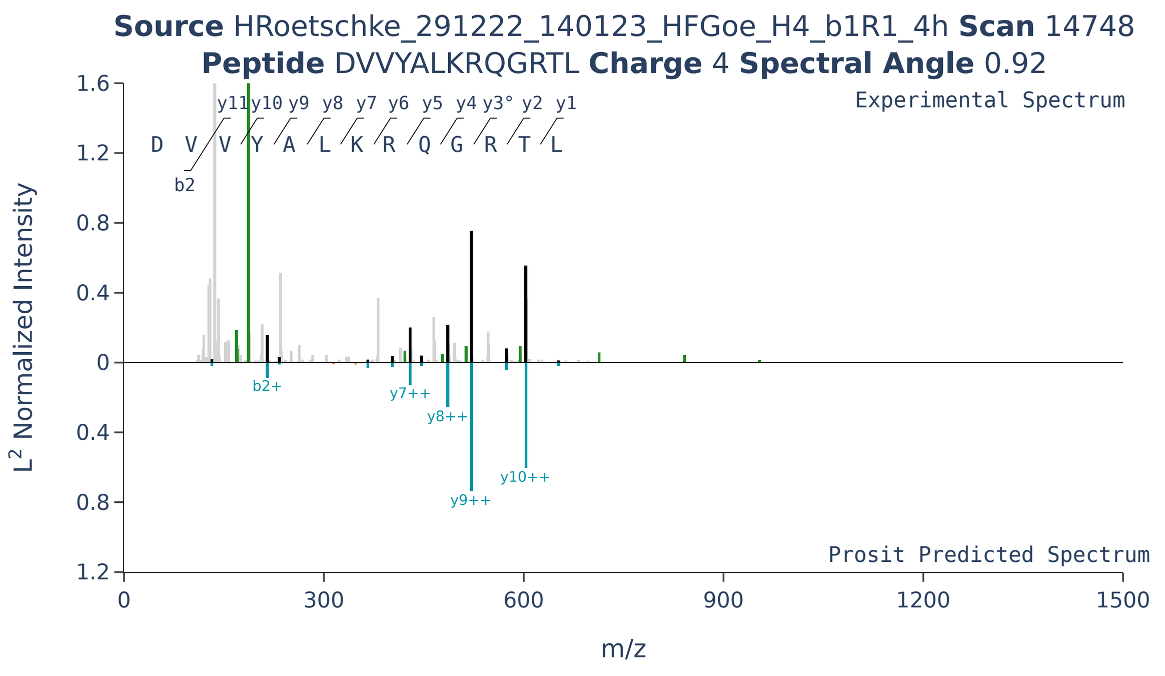
**Source** HRoetschke_291222_140123_HFGoe_H4_b1R1_4h **Scan** 14748
**Peptide** DVVYALKRQGRTL **Charge** 4 **Spectral Angle** 0.92
Experimental Spectrum
Prosit Predicted Spectrum
L² Normalized Intensity
m/z

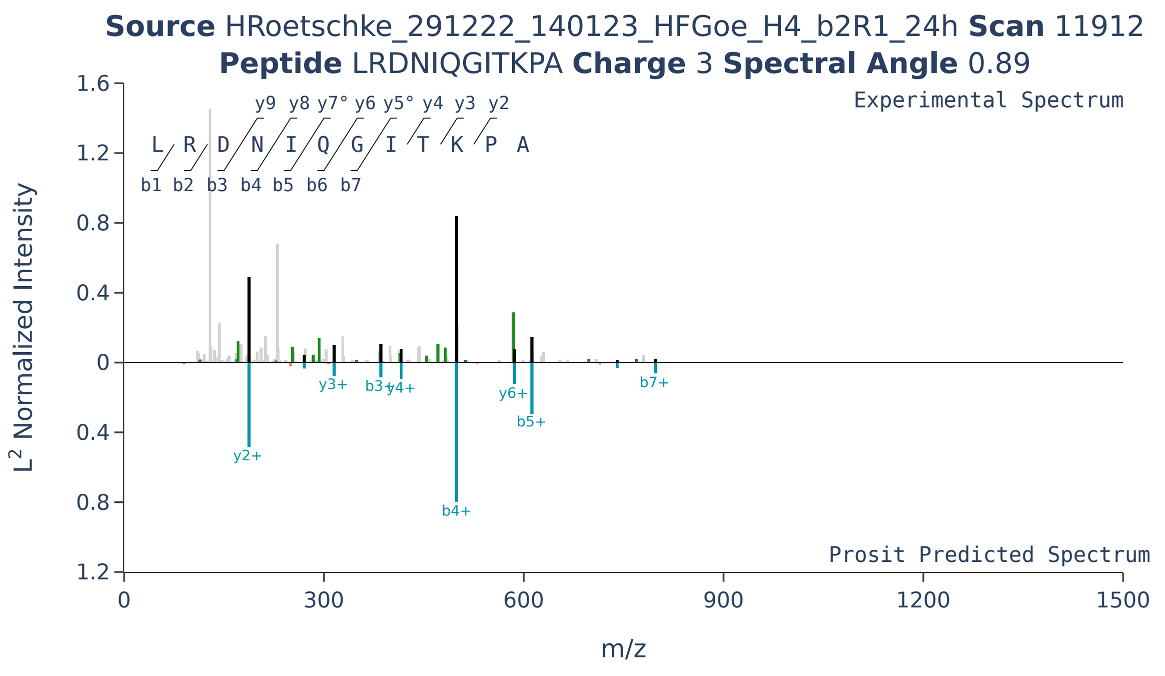
**Source** HRoetschke_291222_140123_HFGoe_H4_b2R1_24h **Scan** 11912
**Peptide** LRDNIQGITKPA **Charge** 3 **Spectral Angle** 0.89
Experimental Spectrum
Prosit Predicted Spectrum
L² Normalized Intensity
m/z

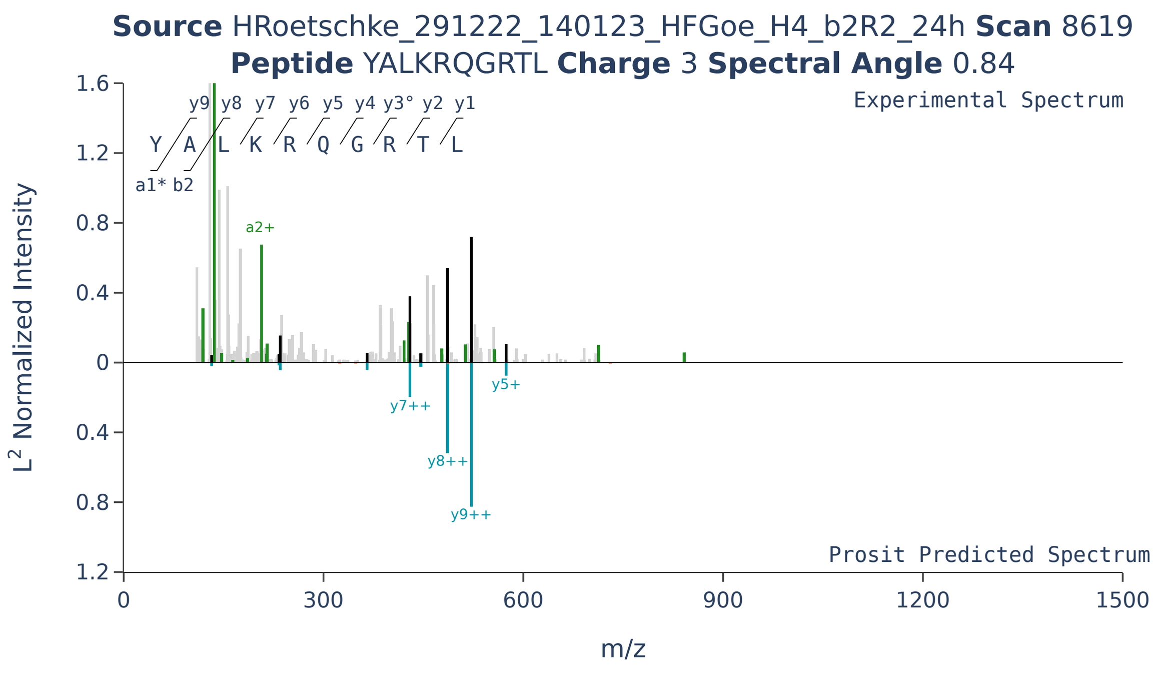
**Source** HRoetschke_291222_140123_HFGoe_H4_b2R2_24h **Scan** 8619
**Peptide** YALKRQGRTL **Charge** 3 **Spectral Angle** 0.84
Experimental Spectrum
Prosit Predicted Spectrum
L² Normalized Intensity
m/z

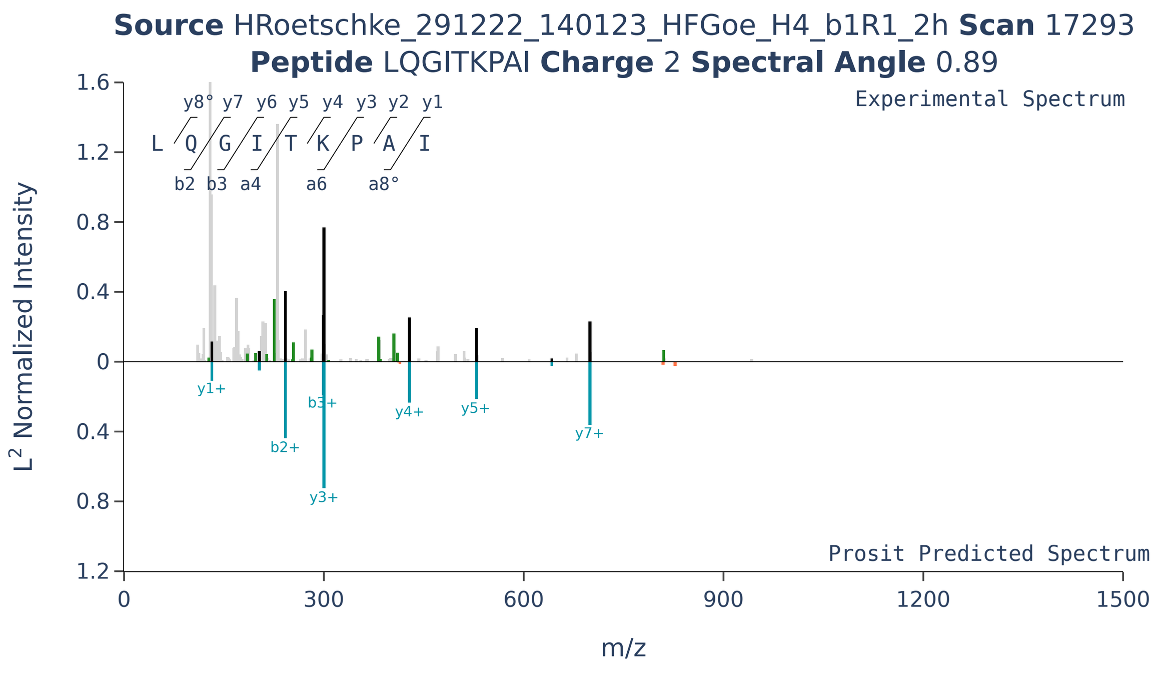
**Source** HRoetschke_291222_140123_HFGoe_H4_b1R1_2h **Scan** 17293
**Peptide** LQGITKPAI **Charge** 2 **Spectral Angle** 0.89
Experimental Spectrum
Prosit Predicted Spectrum
L² Normalized Intensity
m/z

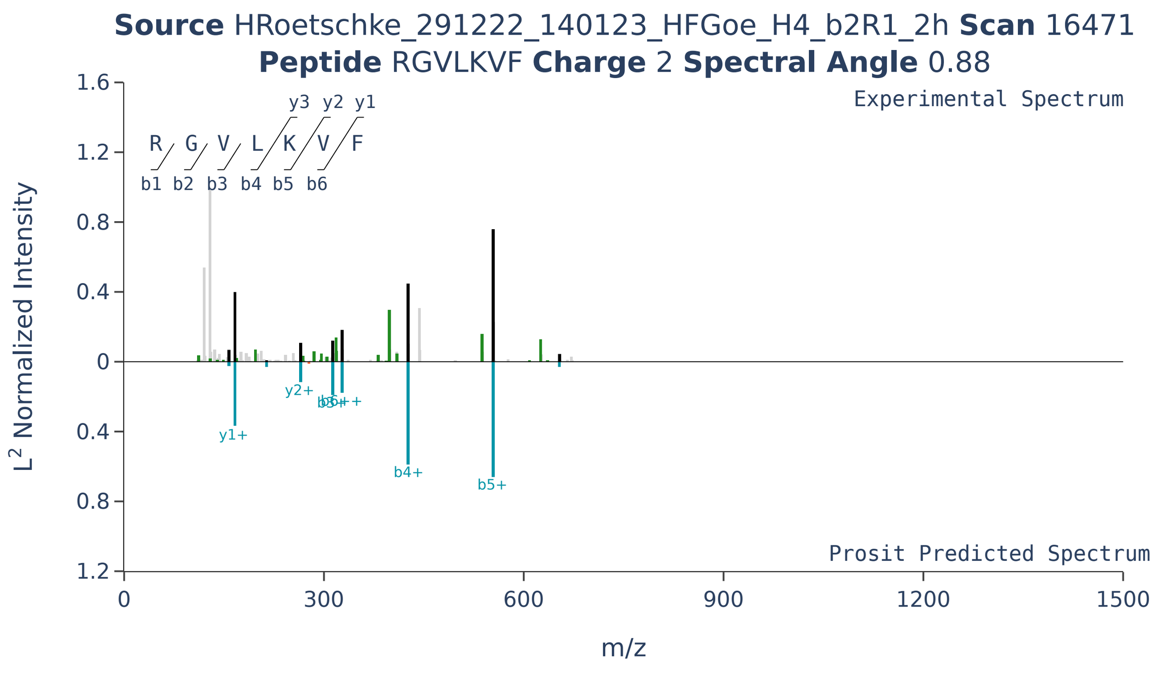
**Source** HRoetschke_291222_140123_HFGoe_H4_b2R1_2h **Scan** 16471
**Peptide** RGVLKVF **Charge** 2 **Spectral Angle** 0.88
Experimental Spectrum
Prosit Predicted Spectrum
L² Normalized Intensity
m/z

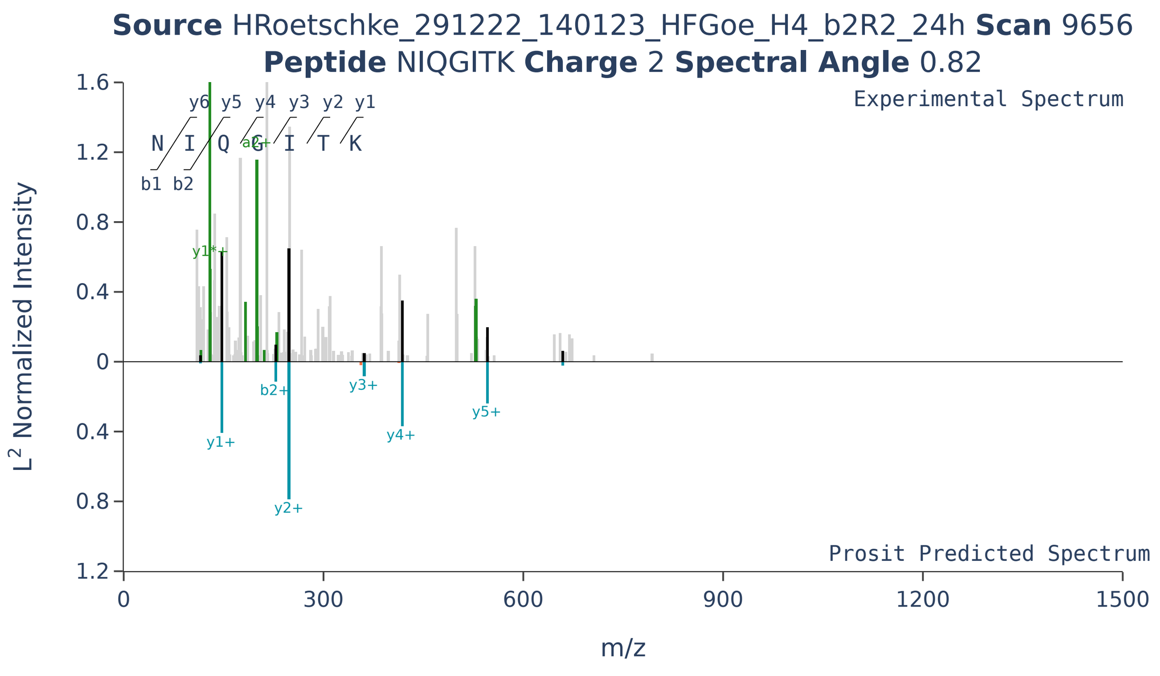
**Source** HRoetschke_291222_140123_HFGoe_H4_b2R2_24h **Scan** 9656
**Peptide** NIQGITK **Charge** 2 **Spectral Angle** 0.82
Experimental Spectrum
Prosit Predicted Spectrum
L² Normalized Intensity
m/z

**Source** HRoetschke_291222_140123_HFGoe_H4_b2R2_1h **Scan** 10047
**Peptide** IRDAVTYTEHA **Charge** 3 **Spectral Angle** 0.89

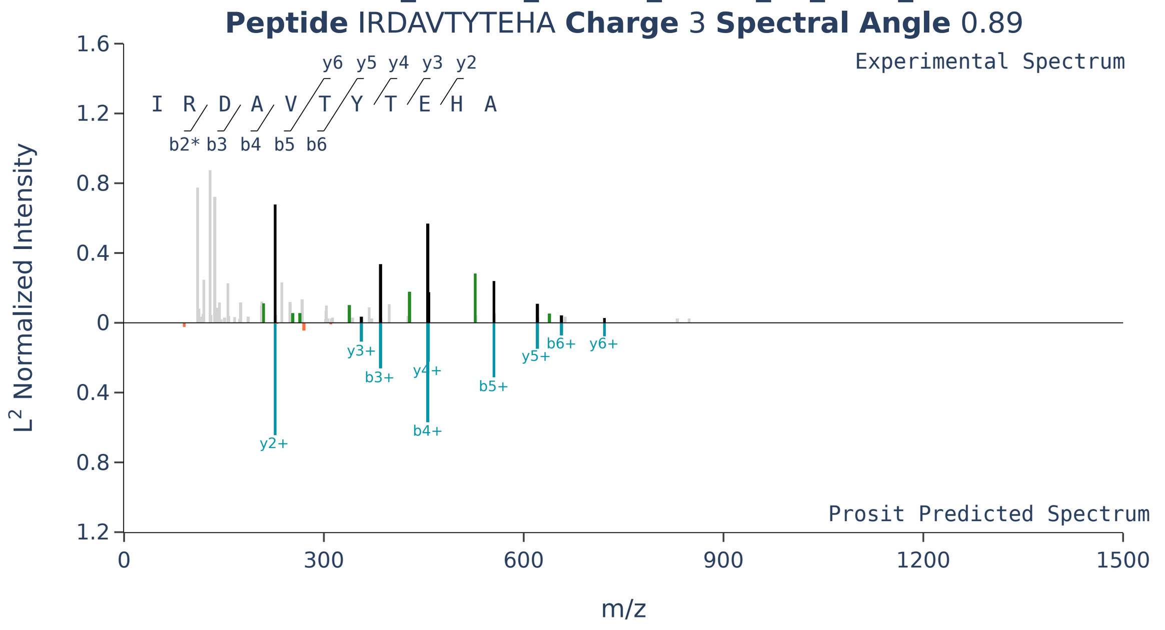


**Source** HRoetschke_291222_140123_HFGoe_H4_b2R1_24h **Scan** 12676
**Peptide** NIQGITKPALR **Charge** 3 **Spectral Angle** 0.88

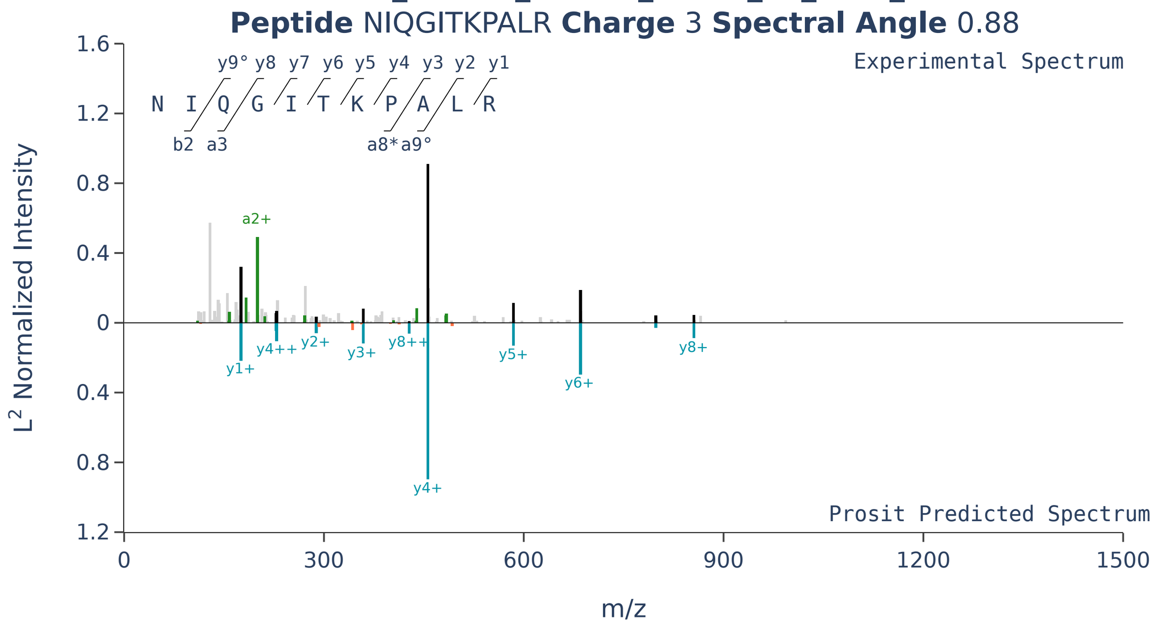


**Source** HRoetschke_291222_140123_HFGoe_H4_b2R2_24h **Scan** 11132
**Peptide** ETRGVLKV **Charge** 2 **Spectral Angle** 0.88

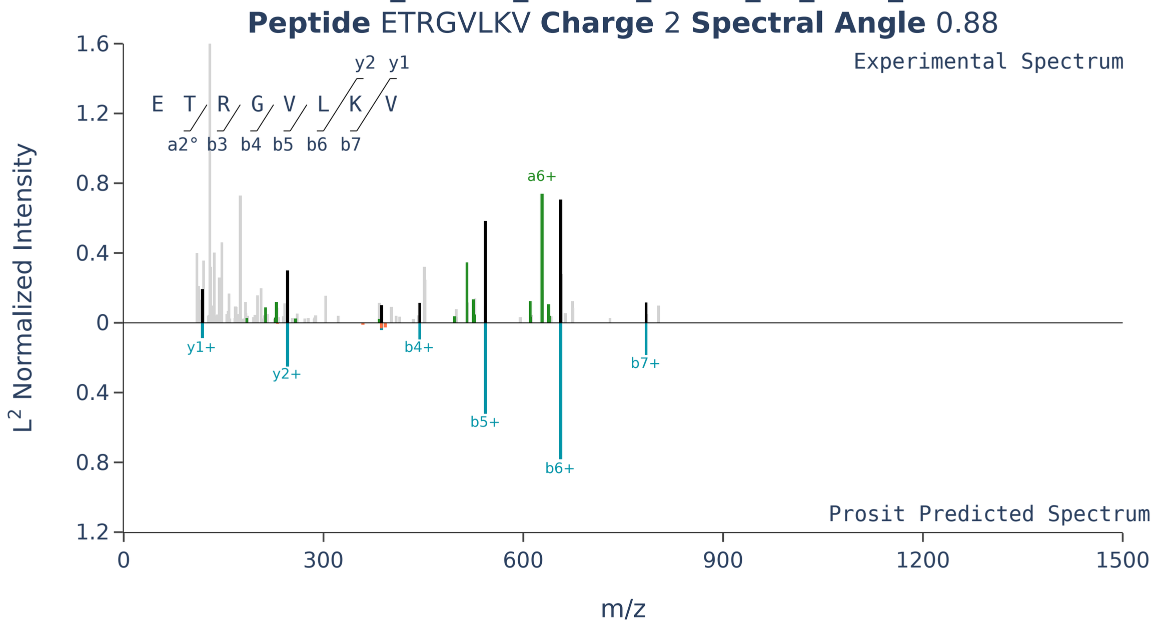


**Source** HRoetschke_291222_140123_HFGoe_H4_b1R1_4h **Scan** 16016
**Peptide** IYEETRGVL **Charge** 2 **Spectral Angle** 0.89

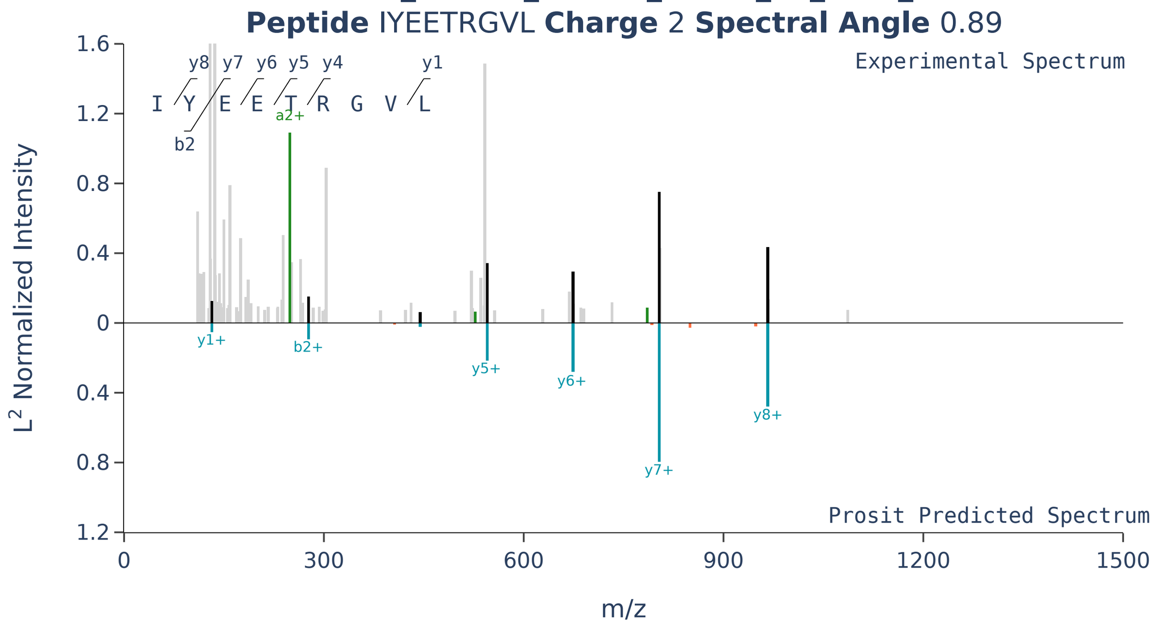


**Source** HRoetschke_291222_140123_HFGoe_H4_b2R2_2h **Scan** 13405
**Peptide** SGLIYEETR **Charge** 2 **Spectral Angle** 0.8

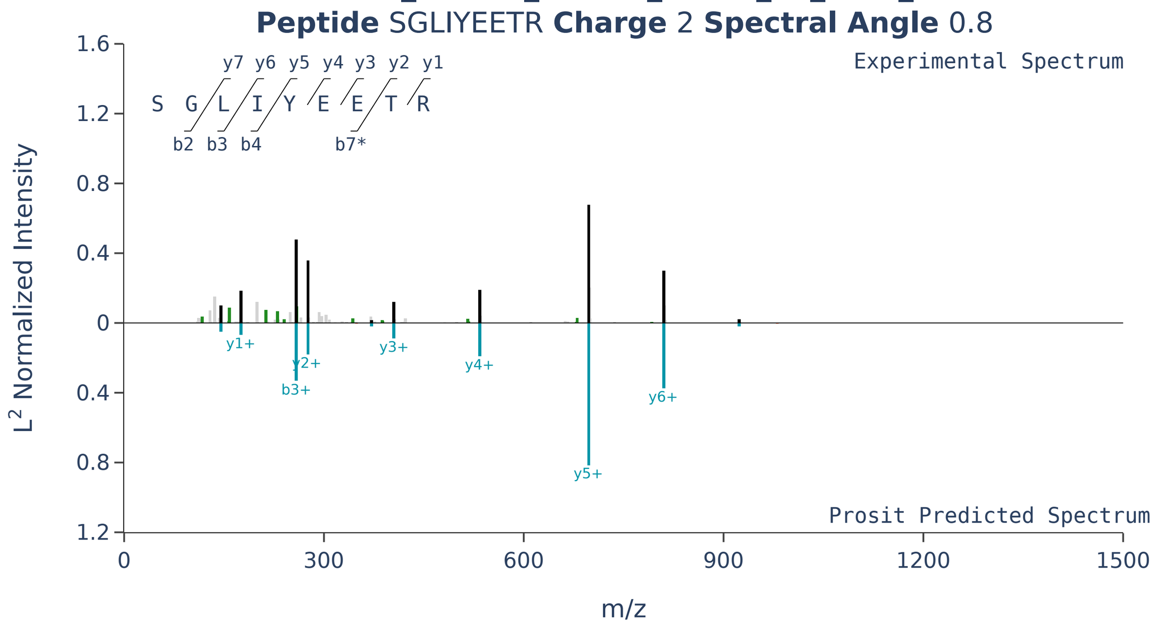


**Source** HRoetschke_291222_140123_HFGoe_H4_b2R2_24h **Scan** 11477
**Peptide** NIQGITKP **Charge** 2 **Spectral Angle** 0.87

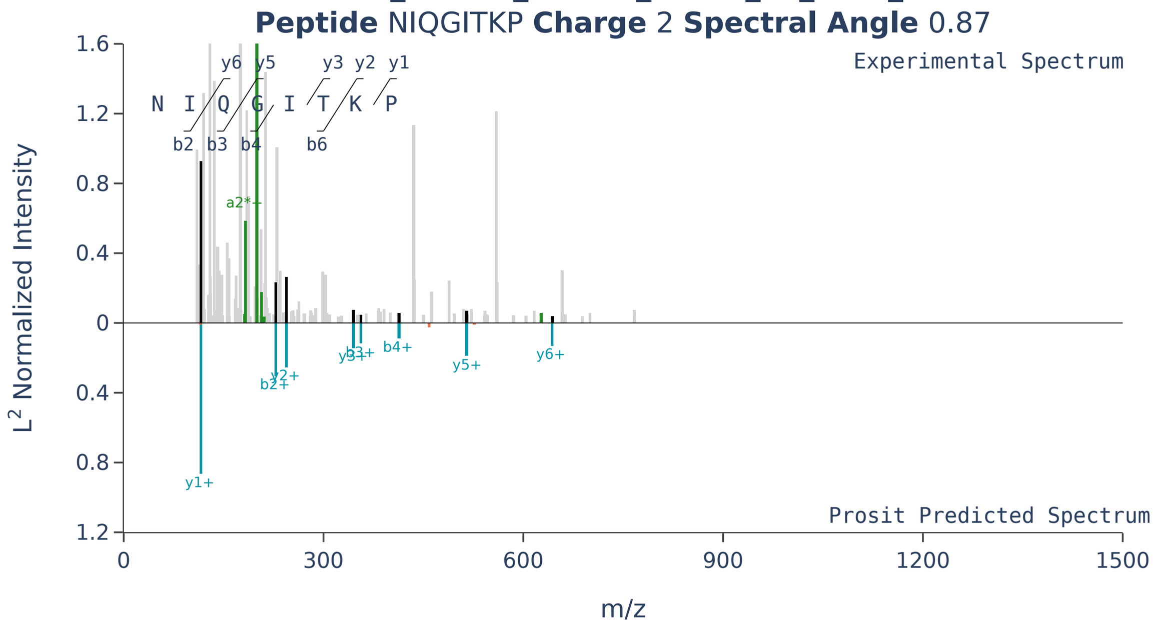


**Source** HRoetschke_291222_140123_HFGoe_H4_b1R2_1h **Scan** 21438
**Peptide** RGVLKVFL **Charge** 2 **Spectral Angle** 0.84

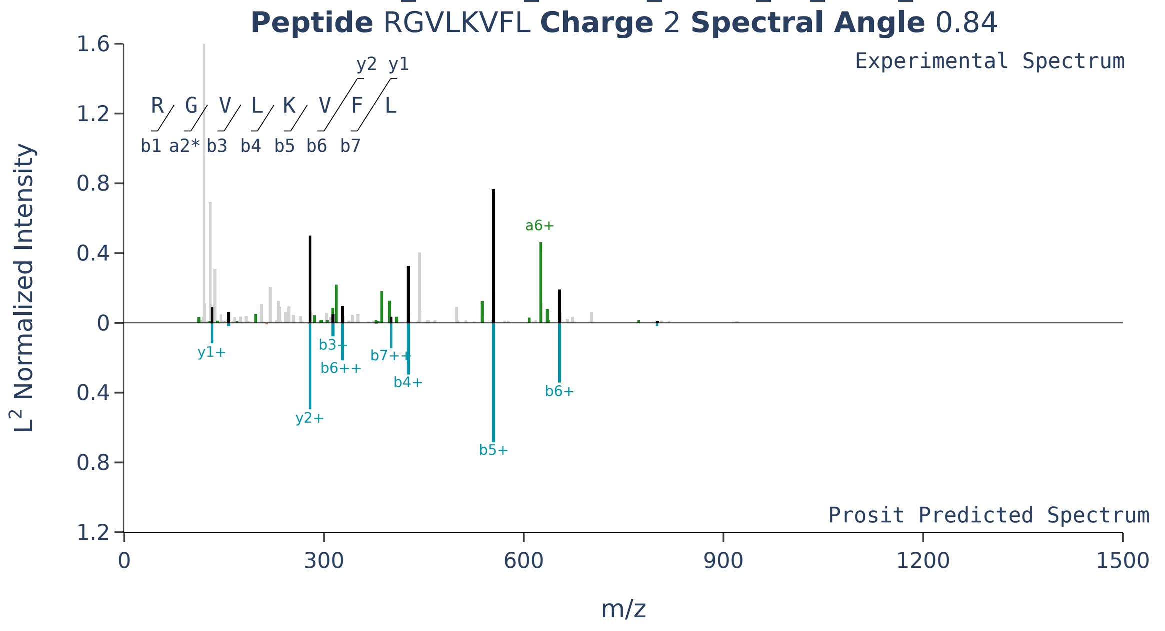


**Source** HRoetschke_291222_140123_HFGoe_H4_b1R1_1h **Scan** 23768
**Peptide** MDVVYAL **Charge** 1 **Spectral Angle** 0.95

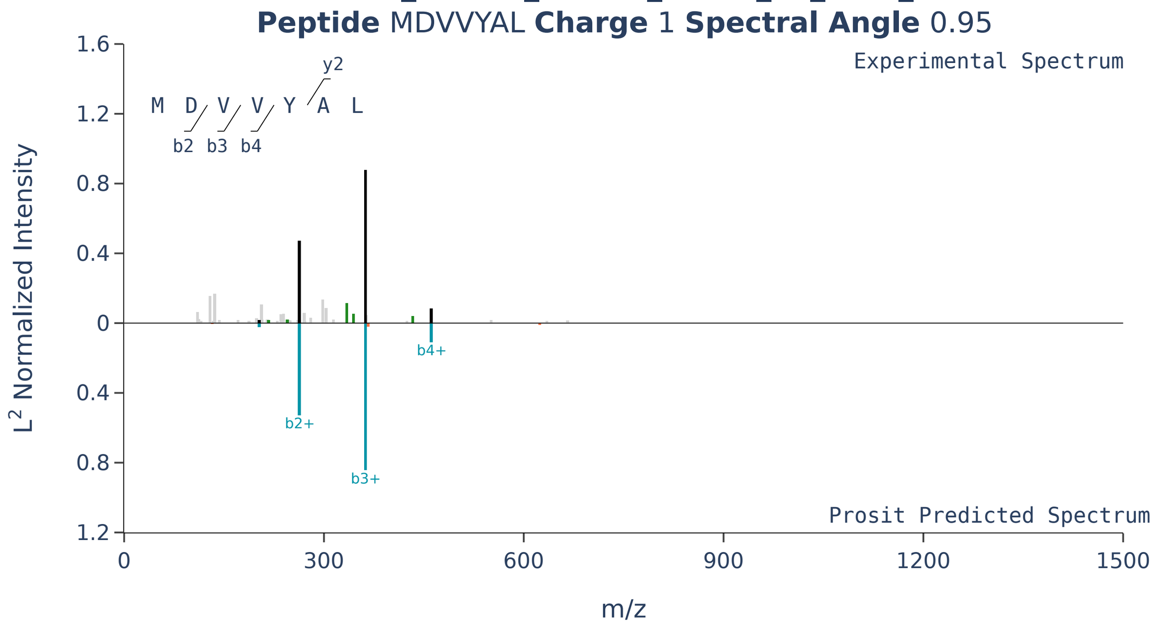


**Source** HRoetschke_291222_140123_HFGoe_H4_b1R2_1h **Scan** 10403
**Peptide** GITKPAIR **Charge** 2 **Spectral Angle** 0.92

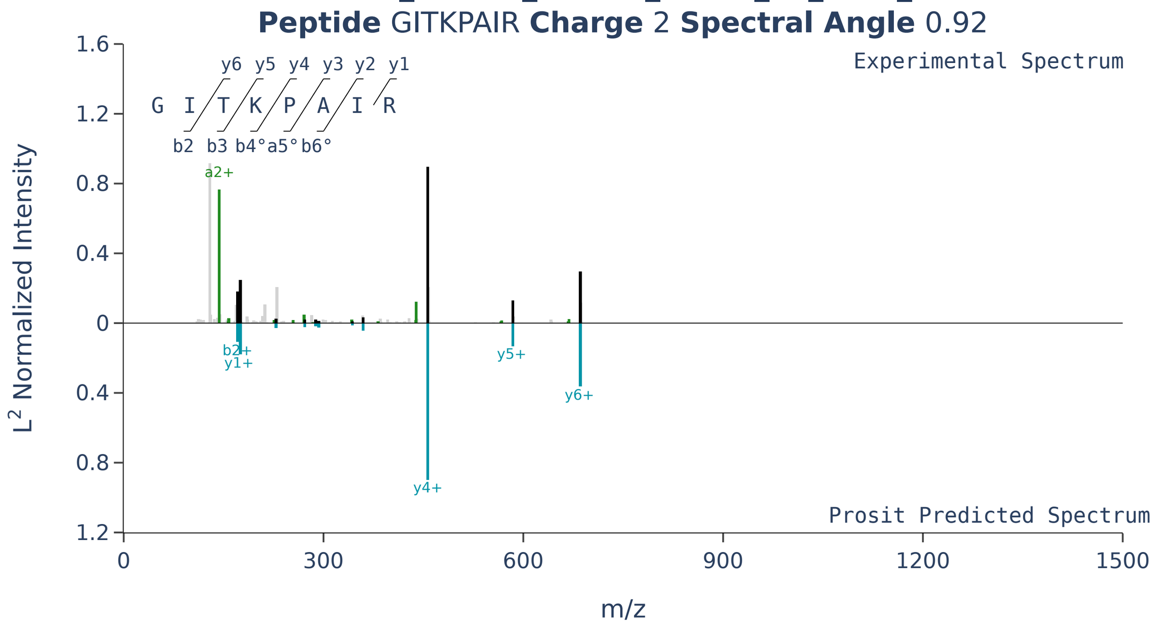


**Source** HRoetschke_291222_140123_HFGoe_H4_b1R1_1h **Scan** 14364
**Peptide** AKRKTVTAMDVVYA **Charge** 2 **Spectral Angle** 0.78

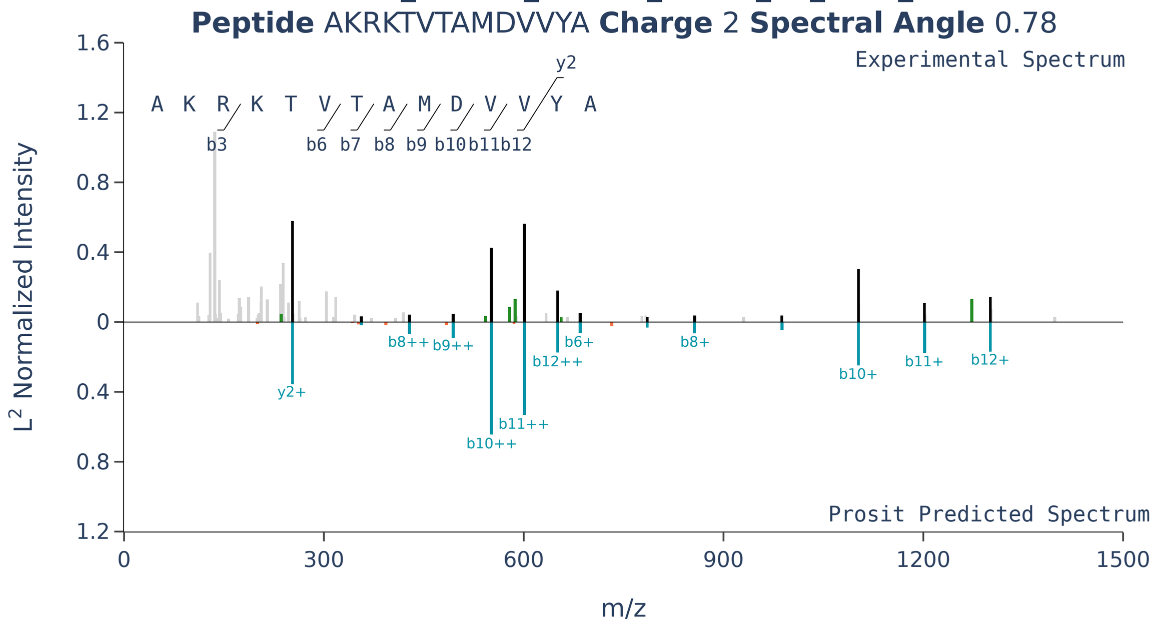


**Source** HRoetschke_291222_140123_HFGoe_H4_b1R2_1h **Scan** 17253
**Peptide** ISGLIYEETRG **Charge** 2 **Spectral Angle** 0.82

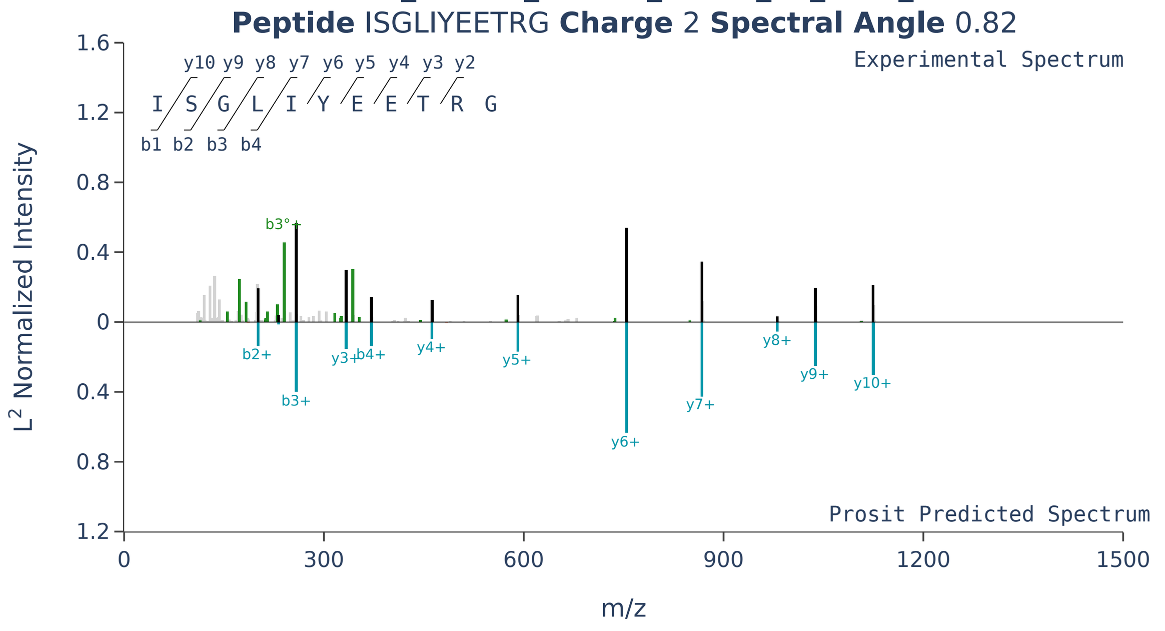


**Source** HRoetschke_291222_140123_HFGoe_H4_b1R2_1h **Scan** 19395
**Peptide** FLENVIRD **Charge** 1 **Spectral Angle** 0.89

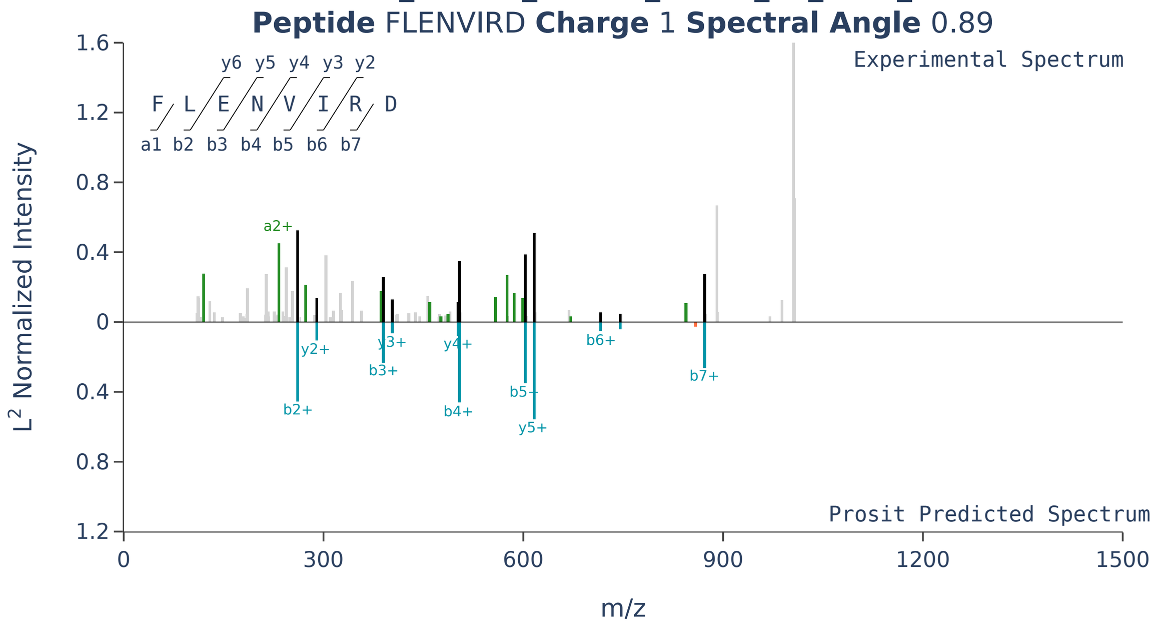


**Source** HRoetschke_291222_140123_HFGoe_H4_b2R1_4h **Scan** 15338
**Peptide** NVIRDAVTYT **Charge** 1 **Spectral Angle** 0.87

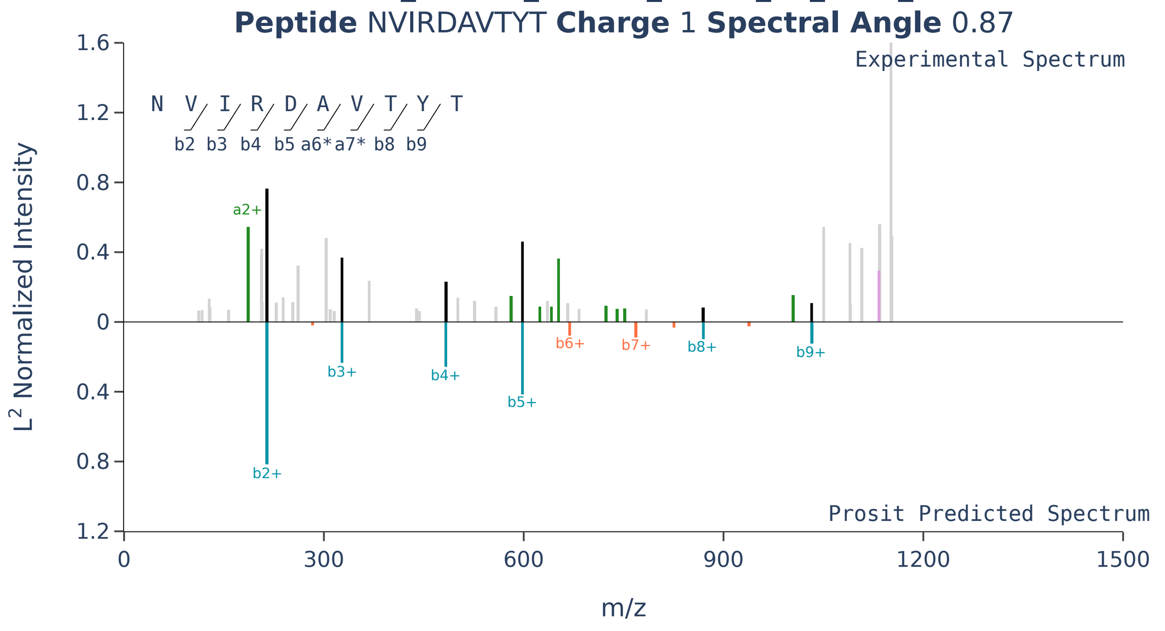


**Source** HRoetschke_291222_140123_HFGoe_H4_b2R1_1h **Scan** 23039
**Peptide** TRGVLKVFL **Charge** 2 **Spectral Angle** 0.87

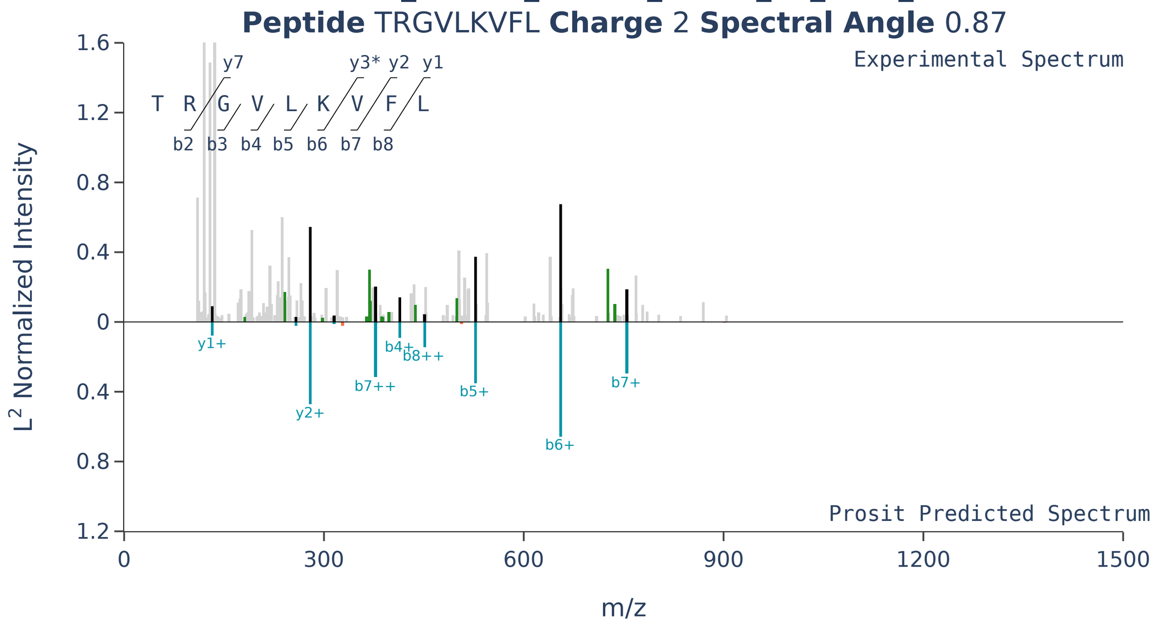


**Source** HRoetschke_291222_140123_HFGoe_H4_b1R2_24h **Scan** 6744
**Peptide** VTYTEHA **Charge** 1 **Spectral Angle** 0.97

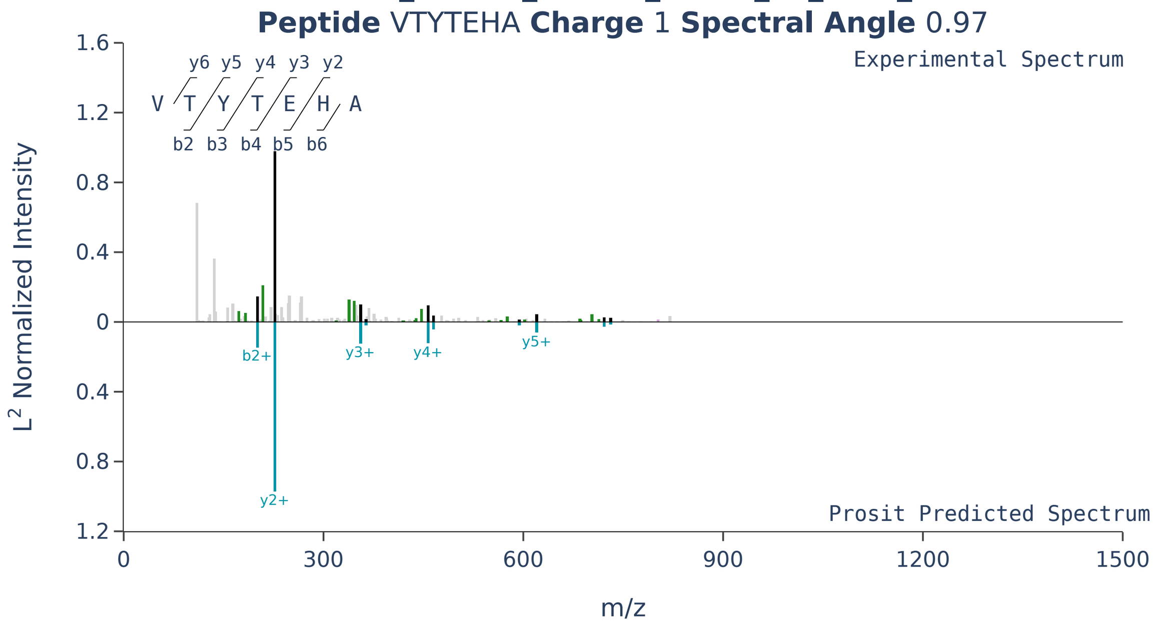

**Source** HRoetschke_291222_140123_HFGoe_H4_b1R2_24h **Scan** 8172
**Peptide** ITKPAIR **Charge** 2 **Spectral Angle** 0.94

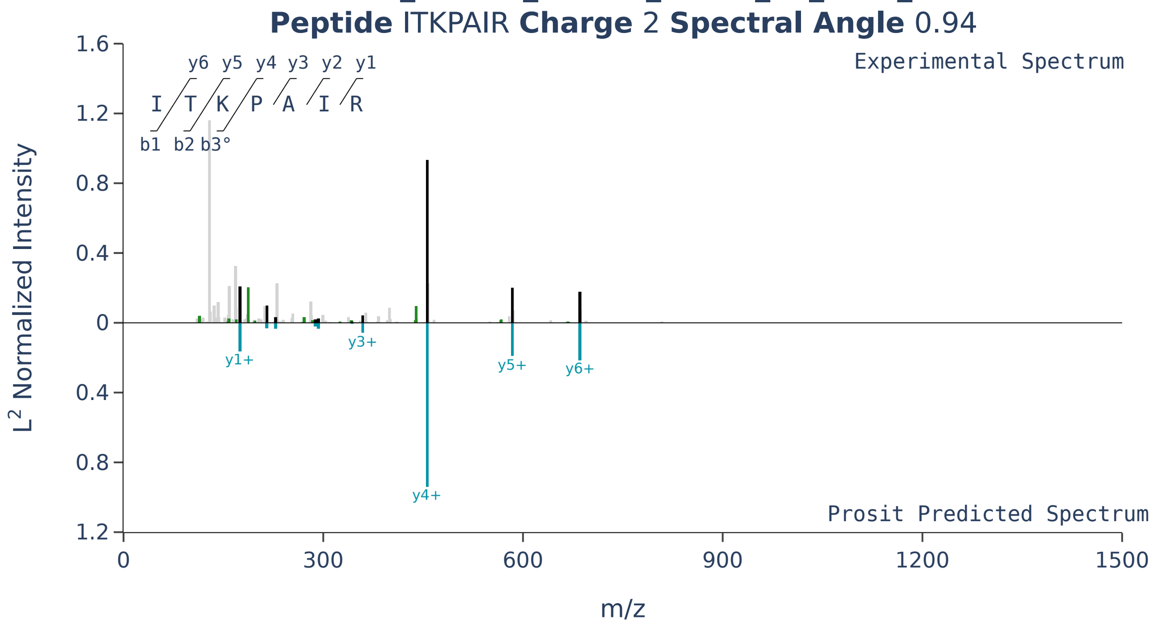


**Source** HRoetschke_291222_140123_HFGoe_H4_b2R2_24h **Scan** 11253
**Peptide** LENVIRD **Charge** 1 **Spectral Angle** 0.88

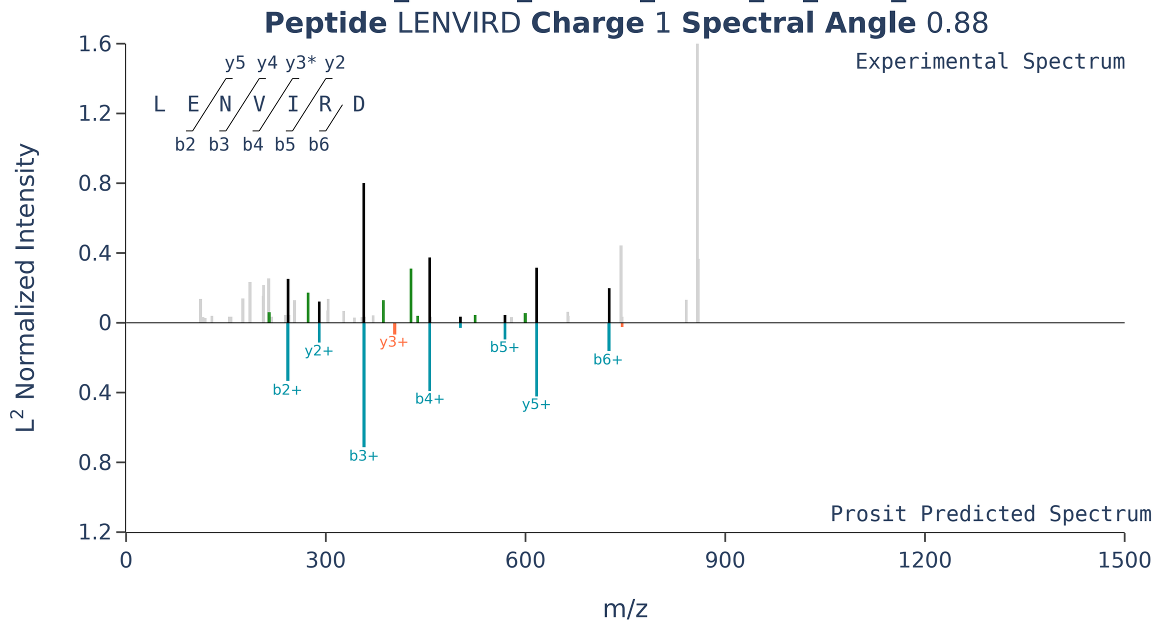


**Source** HRoetschke_291222_140123_HFGoe_H4_b2R2_24h **Scan** 10764
**Peptide** IRDAVTY **Charge** 2 **Spectral Angle** 0.89

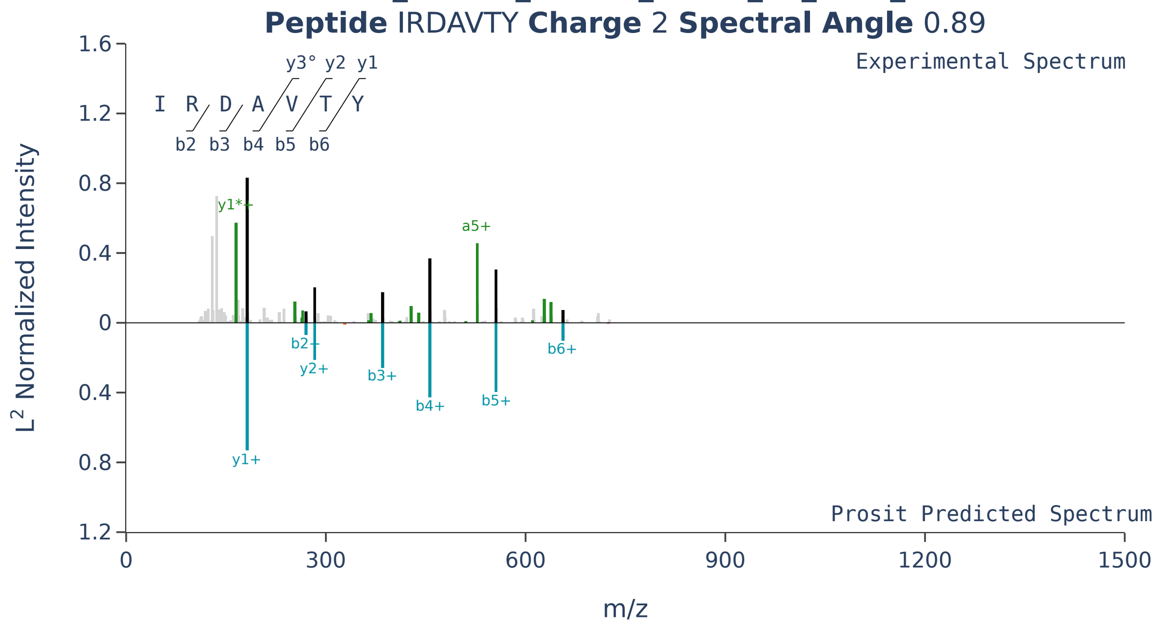


**Source** HRoetschke_291222_140123_HFGoe_H4_b2R1_1h **Scan** 25482
**Peptide** ISGLIYEETRGVL **Charge** 2 **Spectral Angle** 0.85

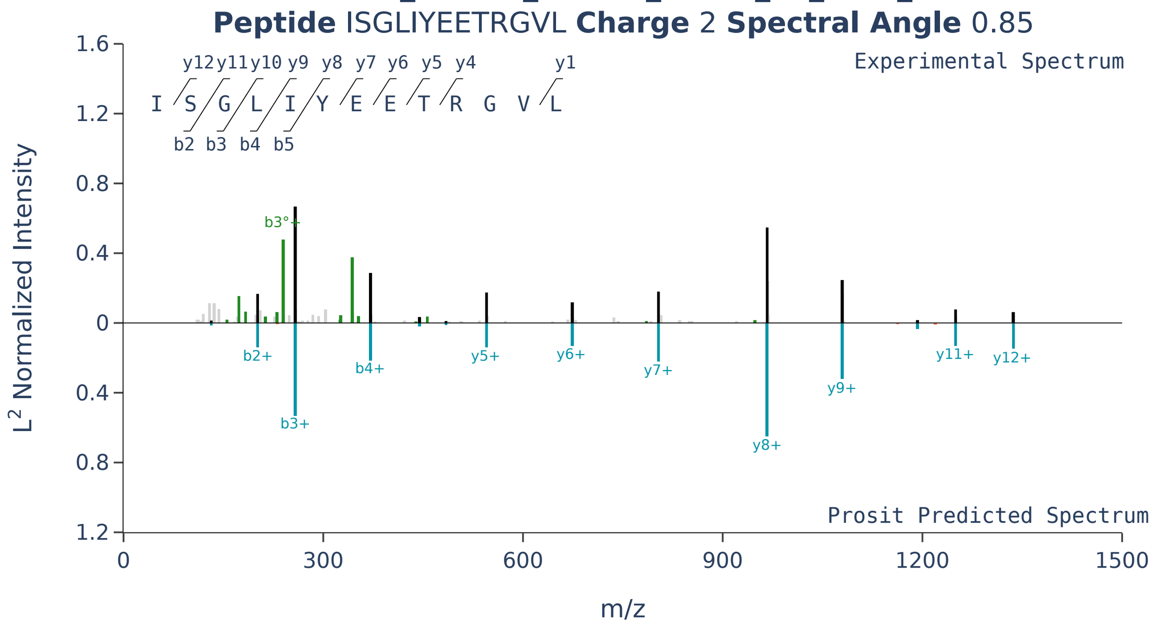


**Source** HRoetschke_291222_140123_HFGoe_H4_b2R2_1h **Scan** 17452
**Peptide** ENVIRDAVTY **Charge** 2 **Spectral Angle** 0.78

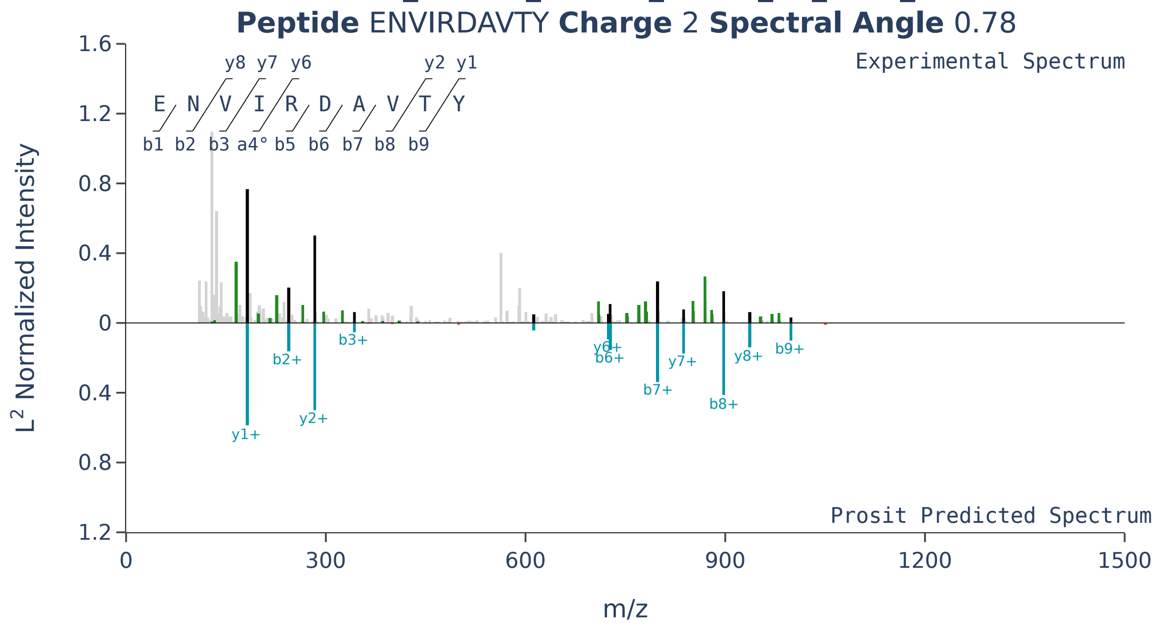


**Source** HRoetschke_291222_140123_HFGoe_H4_b1R1_1h **Scan** 13265
**Peptide** VVYALKRQGRTLY **Charge** 4 **Spectral Angle** 0.92

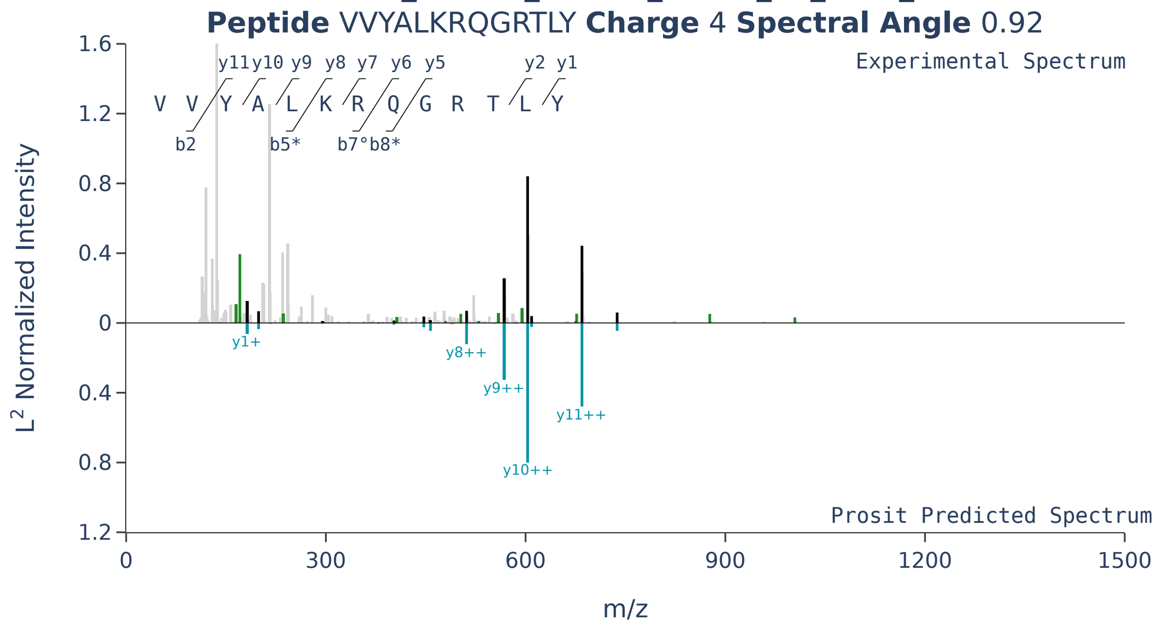


**Source** HRoetschke_291222_140123_HFGoe_H4_b1R2_24h **Scan** 7651
**Peptide** AVTYTEHA **Charge** 2 **Spectral Angle** 0.87

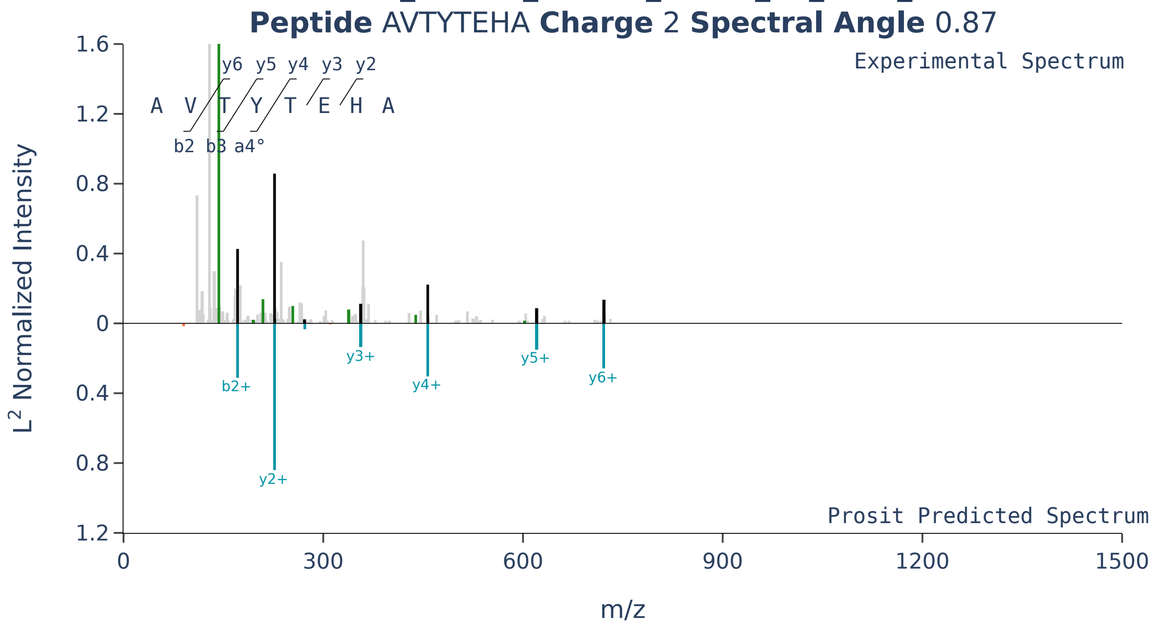


**Source** HRoetschke_291222_140123_HFGoe_H4_b1R1_24h **Scan** 12334
**Peptide** ENVIRDAVT **Charge** 1 **Spectral Angle** 0.92

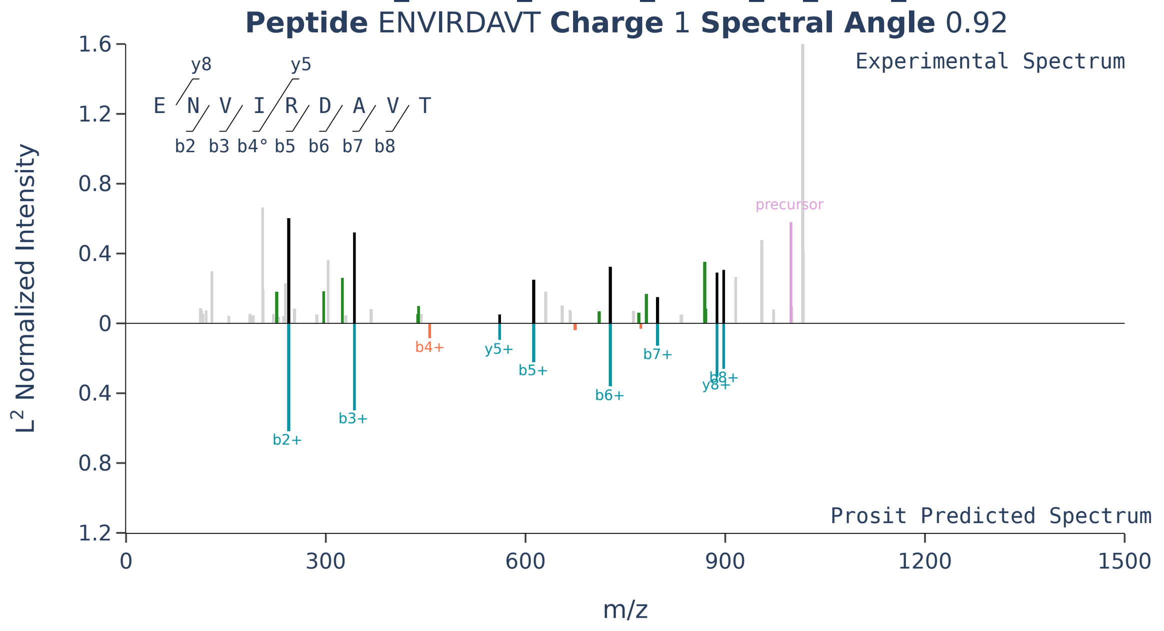


**Source** HRoetschke_291222_140123_HFGoe_H4_b2R1_1h **Scan** 25020
**Peptide** VLKVFLENVIRD **Charge** 3 **Spectral Angle** 0.9

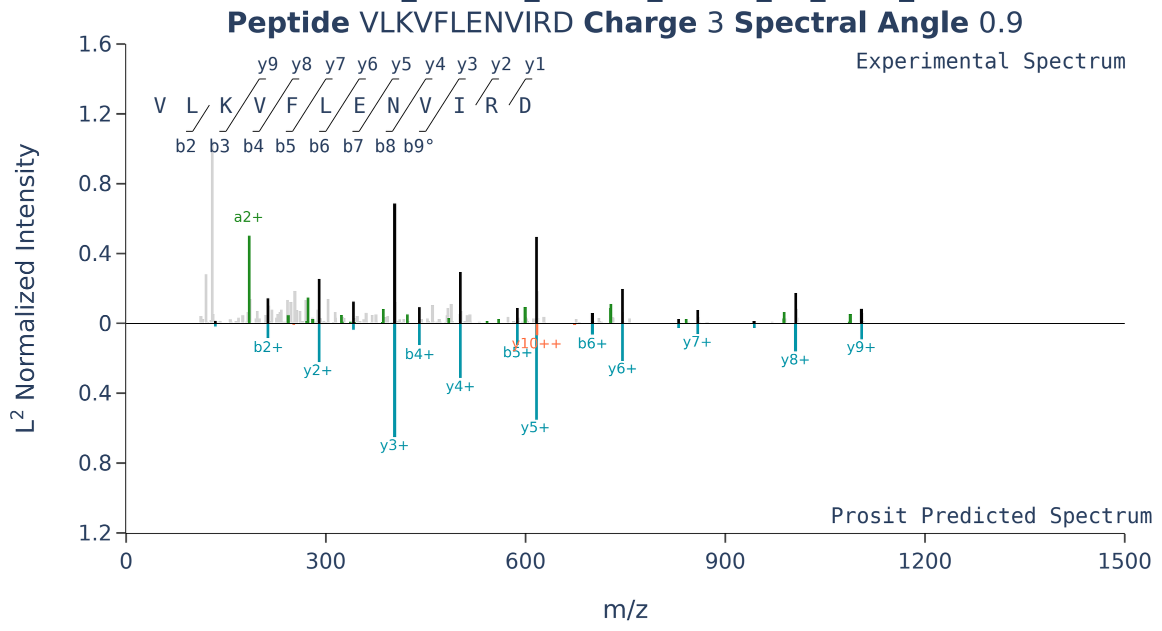


**Source** HRoetschke_291222_140123_HFGoe_H4_b2R1_24h **Scan** 8486
**Peptide** QGITKPAIRR **Charge** 3 **Spectral Angle** 0.87

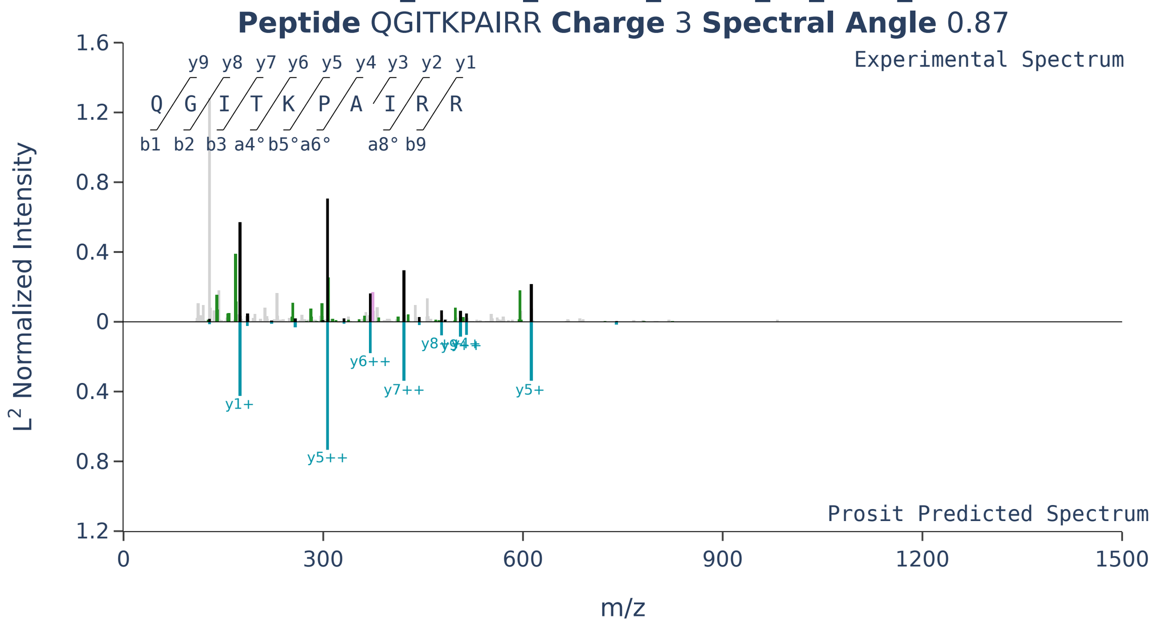


**Source** HRoetschke_291222_140123_HFGoe_H4_b2R2_4h **Scan** 15108
**Peptide** NVIRDAVTYTEH **Charge** 2 **Spectral Angle** 0.86

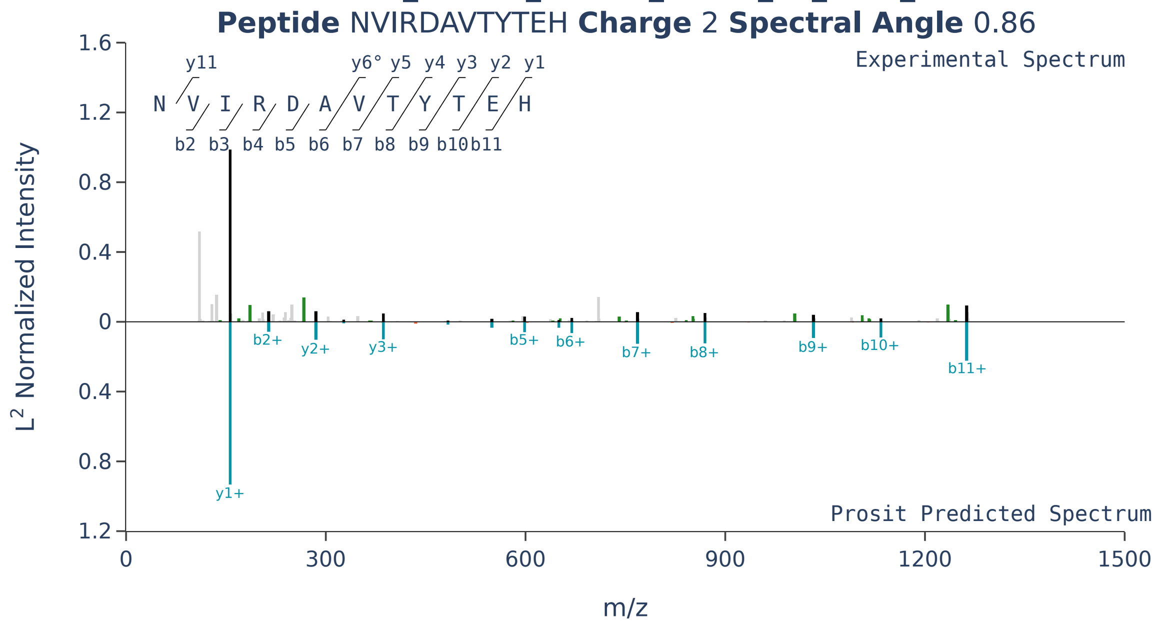


**Source** HRoetschke_291222_140123_HFGoe_H4_b2R1_2h **Scan** 19567
**Peptide** KVFLENVLRD **Charge** 2 **Spectral Angle** 0.8

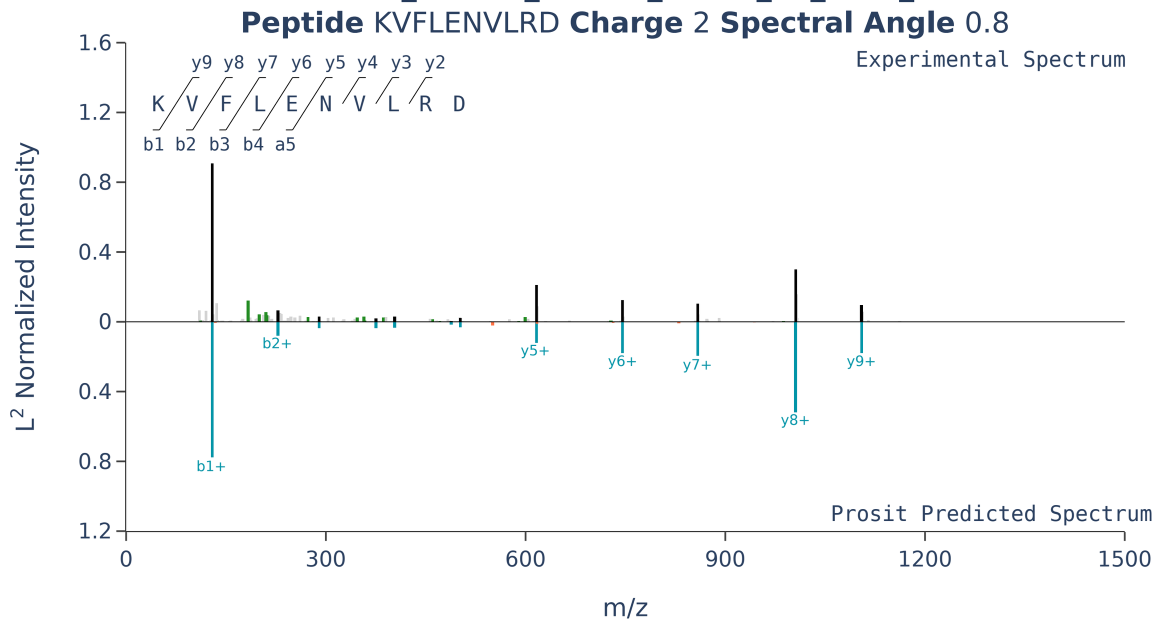


**Source** HRoetschke_291222_140123_HFGoe_H4_b1R1_2h **Scan** 16489
**Peptide** RKTVTAMDVVY **Charge** 2 **Spectral Angle** 0.89

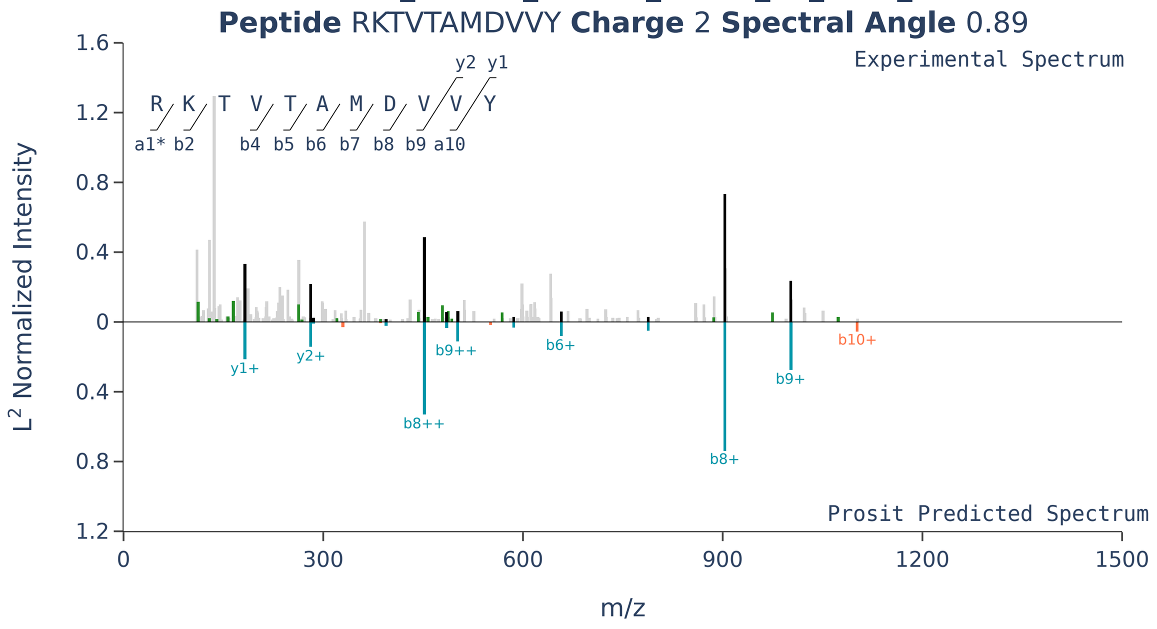


**Source** HRoetschke_291222_140123_HFGoe_H4_b2R2_4h **Scan** 9221
**Peptide** VYALKRQG **Charge** 2 **Spectral Angle** 0.85

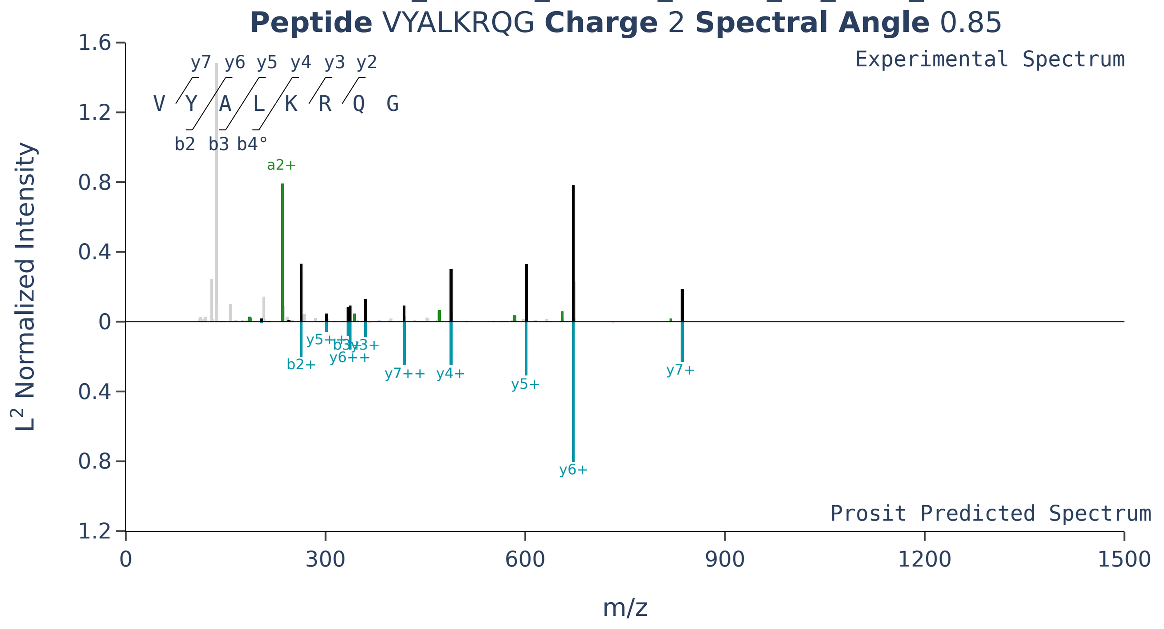


**Source** HRoetschke_291222_140123_HFGoe_H4_b1R1_1h **Scan** 18166
**Peptide** ENVIRDAVTYT **Charge** 1 **Spectral Angle** 0.78

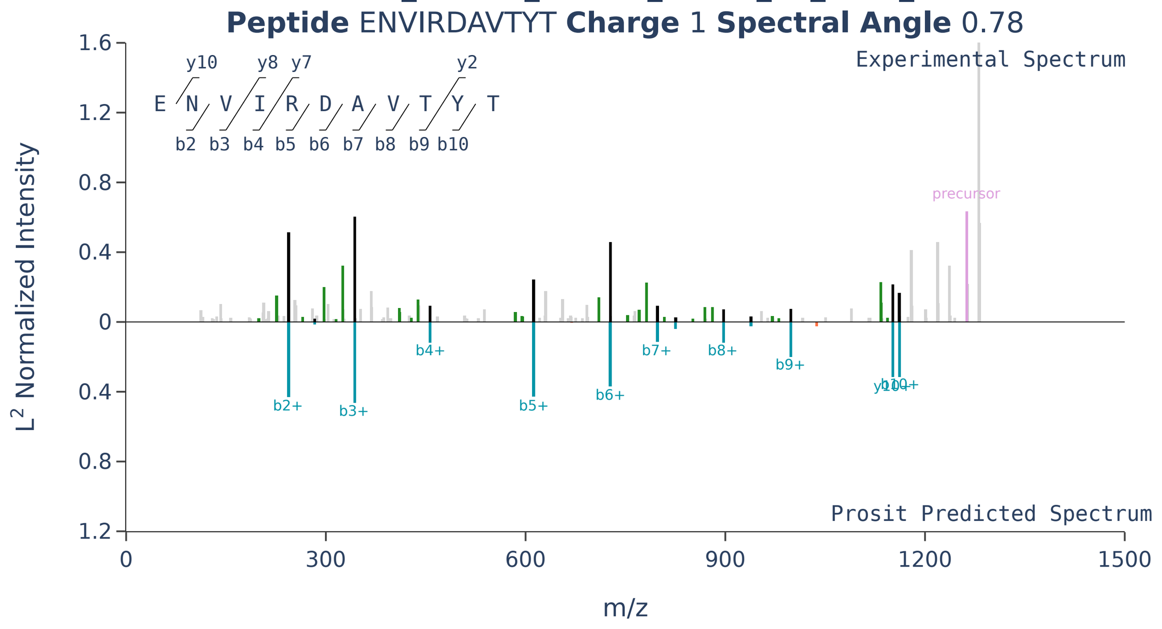

**Source** HRoetschke_291222_140123_HFGoe_H4_b1R1_2h **Scan** 13821
**Peptide** AKRKTVTAMDVVY **Charge** 2 **Spectral Angle** 0.84

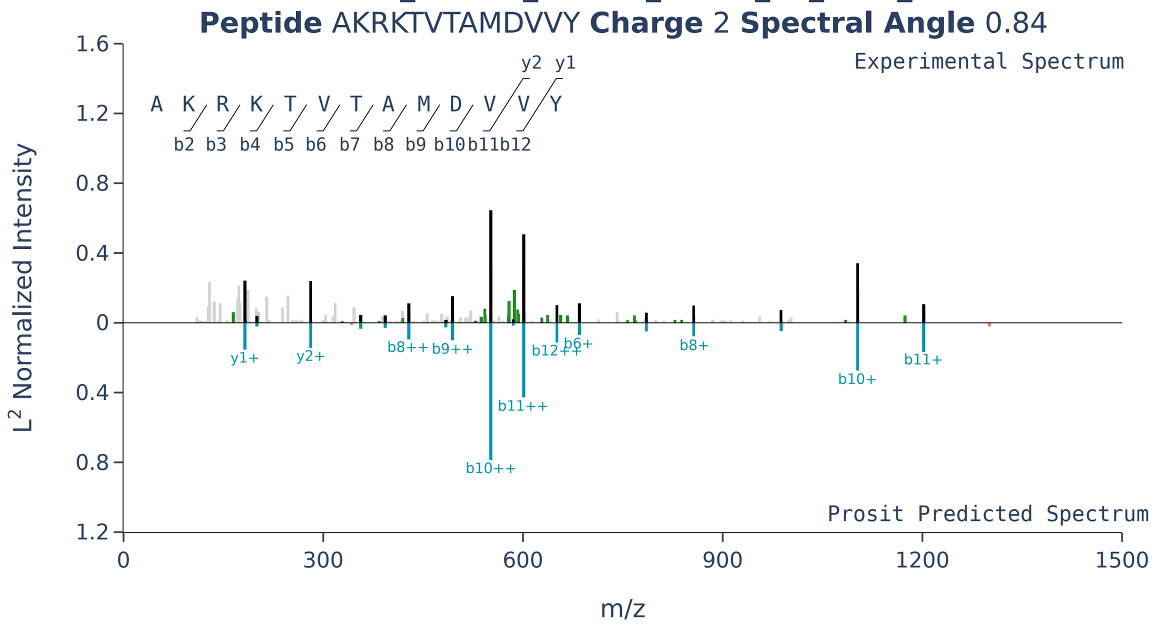

**Source** HRoetschke_291222_140123_HFGoe_H4_b2R1_1h **Scan** 23215
**Peptide** AMDVVYALKRQGRTLYGFGG **Charge** 3 **Spectral Angle** 0.81

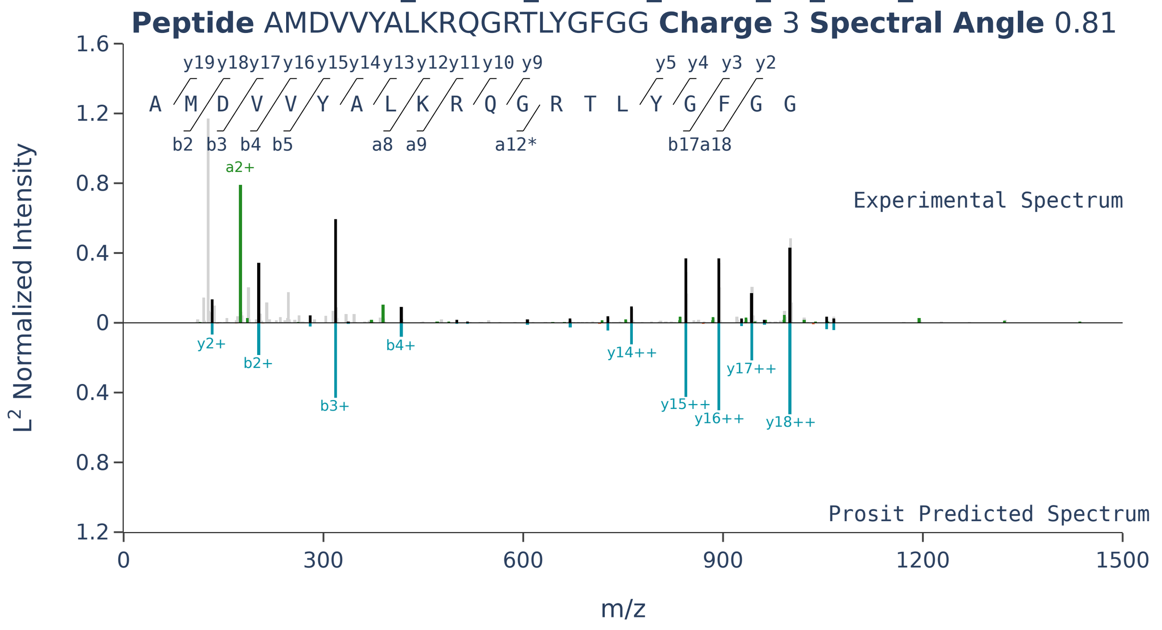

**Source** HRoetschke_291222_140123_HFGoe_H4_b2R2_1h **Scan** 19487
**Peptide** KVFLENVIRD **Charge** 3 **Spectral Angle** 0.91

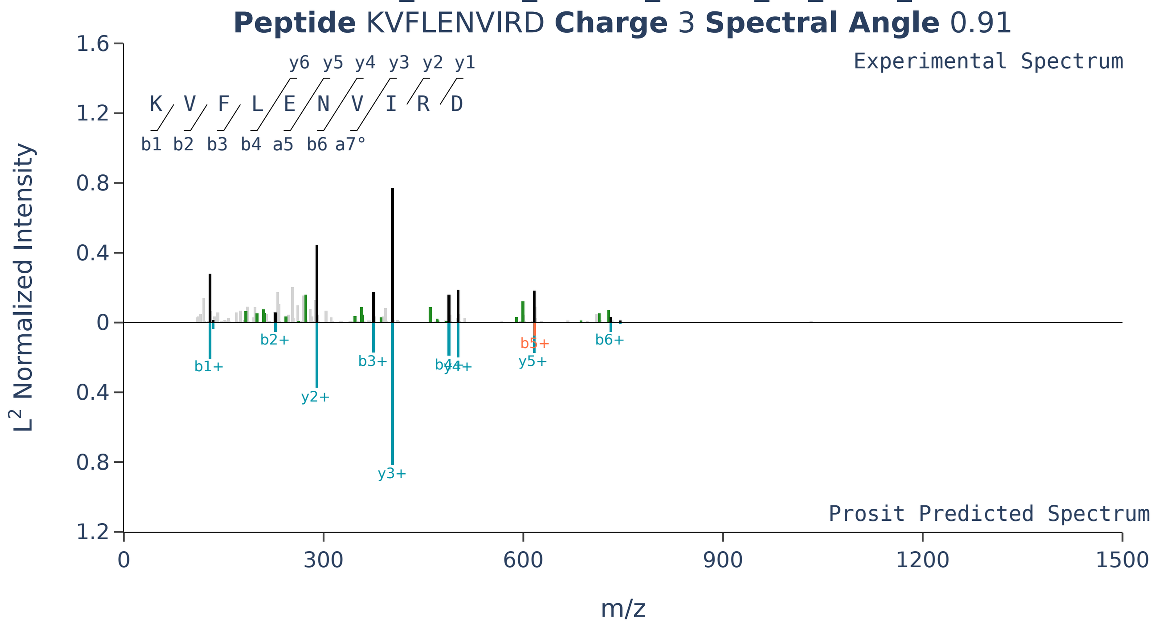

**Source** HRoetschke_291222_140123_HFGoe_H4_b2R1_1h **Scan** 18887
**Peptide** FLENVIR **Charge** 2 **Spectral Angle** 0.85

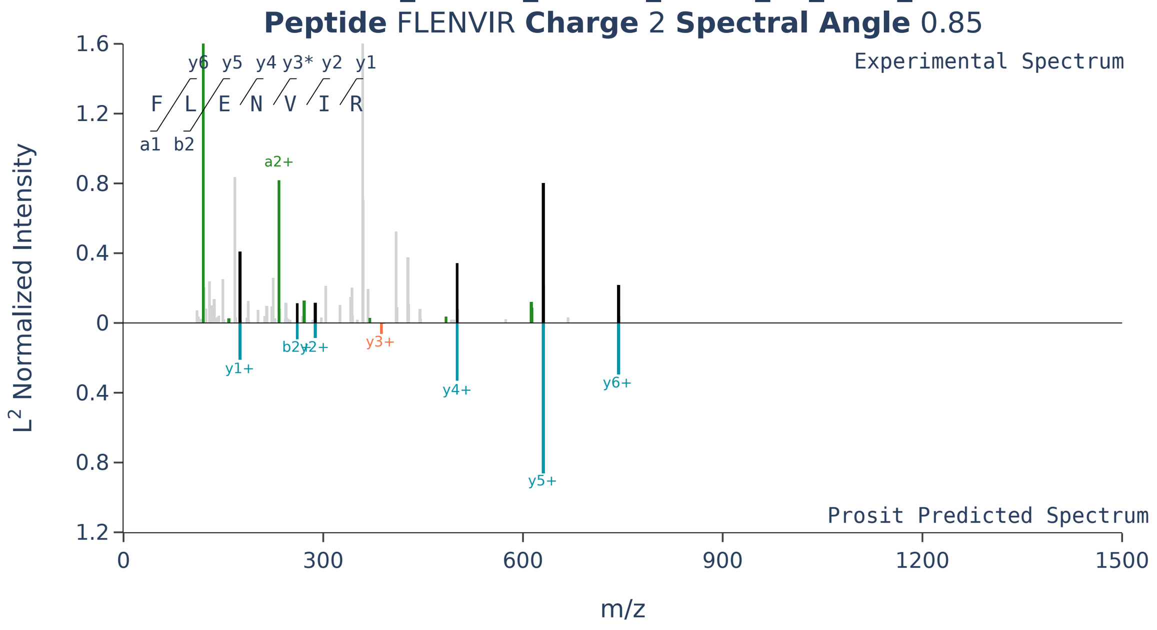

**Source** HRoetschke_291222_140123_HFGoe_H4_b1R1_2h **Scan** 18977
**Peptide** ENVIRDAVTYTE **Charge** 2 **Spectral Angle** 0.82

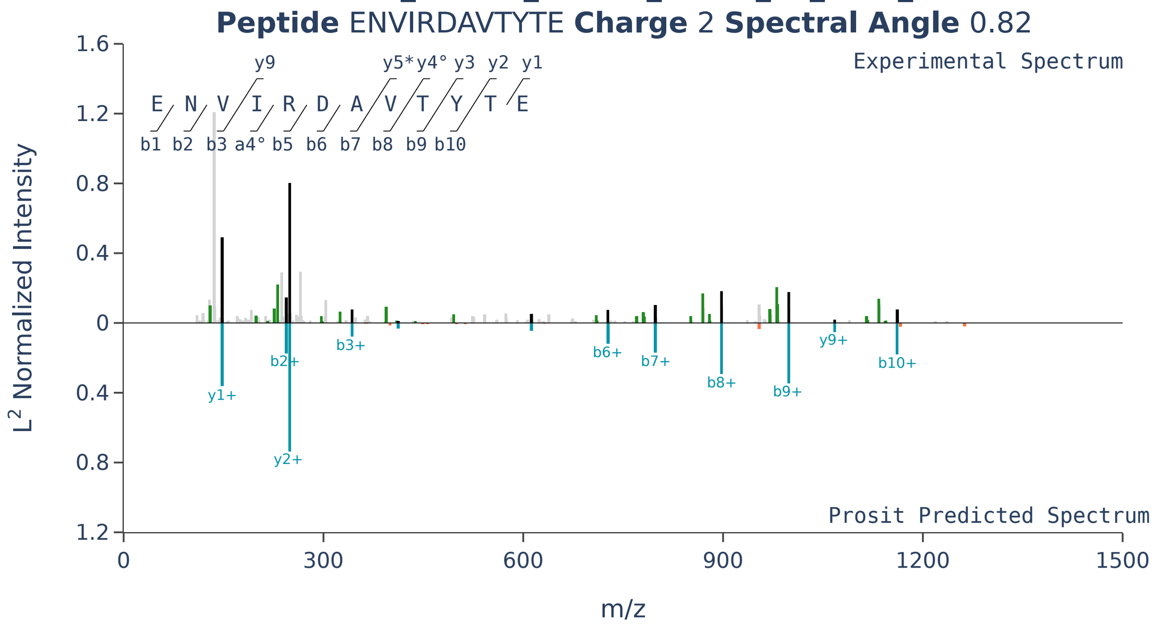

**Source** HRoetschke_291222_140123_HFGoe_H4_b2R1_1h **Scan** 26860
**Peptide** FLENVIRDAVTYTEH **Charge** 2 **Spectral Angle** 0.89

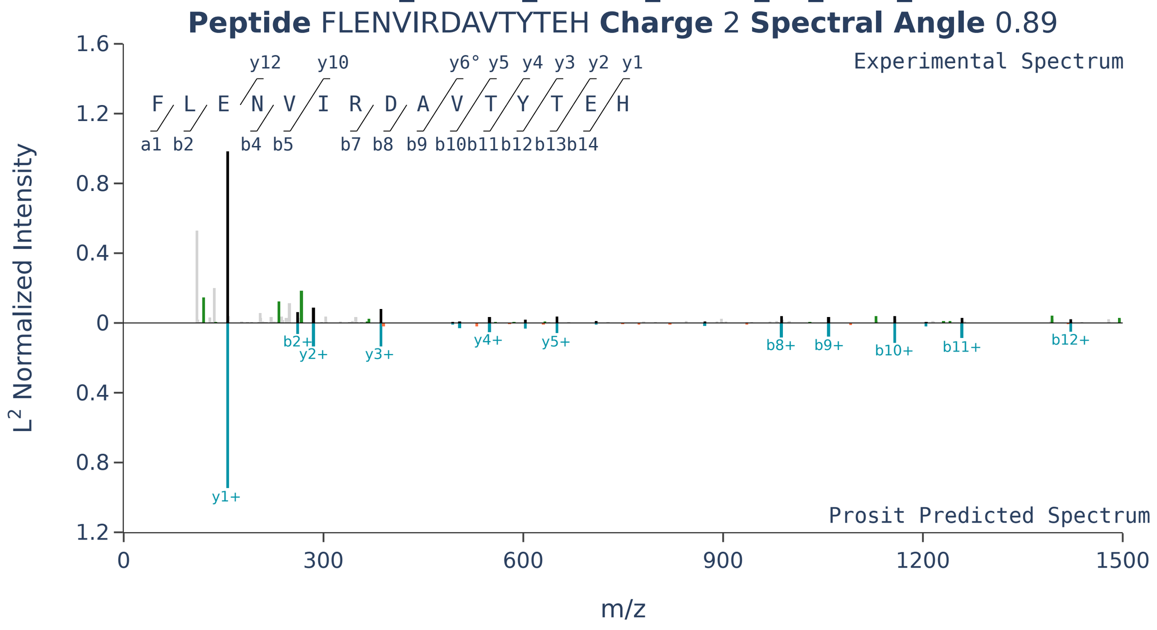

**Source** HRoetschke_291222_140123_HFGoe_H4_b2R1_1h **Scan** 21512
**Peptide** KRKTVTAMDVVYAL **Charge** 2 **Spectral Angle** 0.78

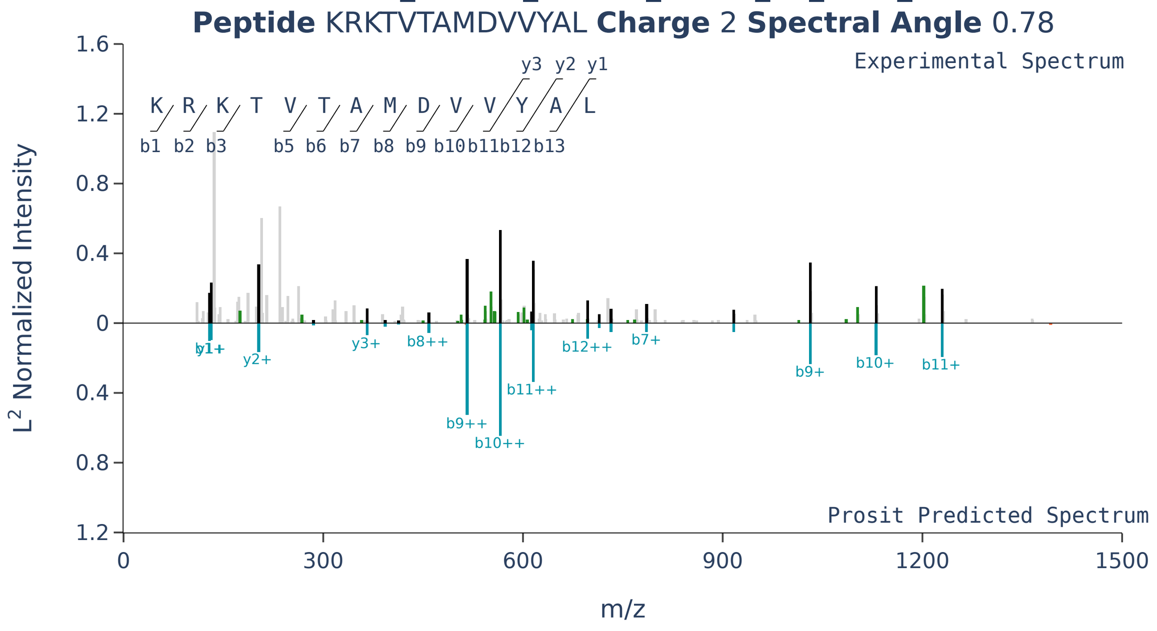

**Source** HRoetschke_291222_140123_HFGoe_H4_b1R1_1h **Scan** 18070
**Peptide** SGLIYEE **Charge** 1 **Spectral Angle** 0.91

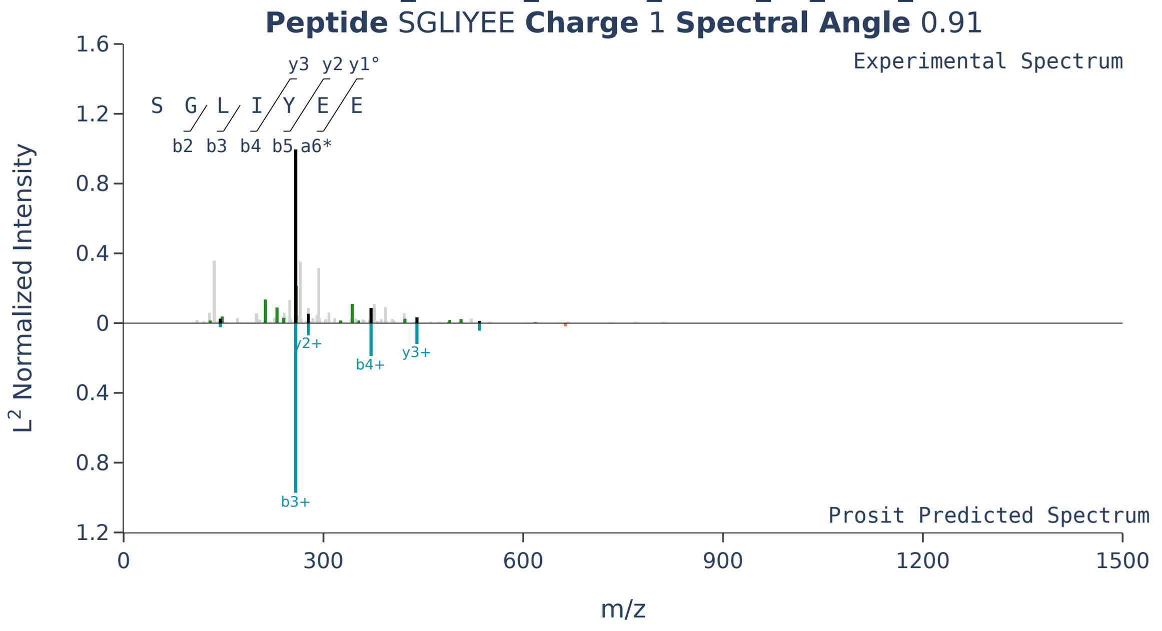

**Source** HRoetschke_291222_140123_HFGoe_H4_b1R2_1h **Scan** 21247
**Peptide** ISGLIYEE **Charge** 1 **Spectral Angle** 0.94

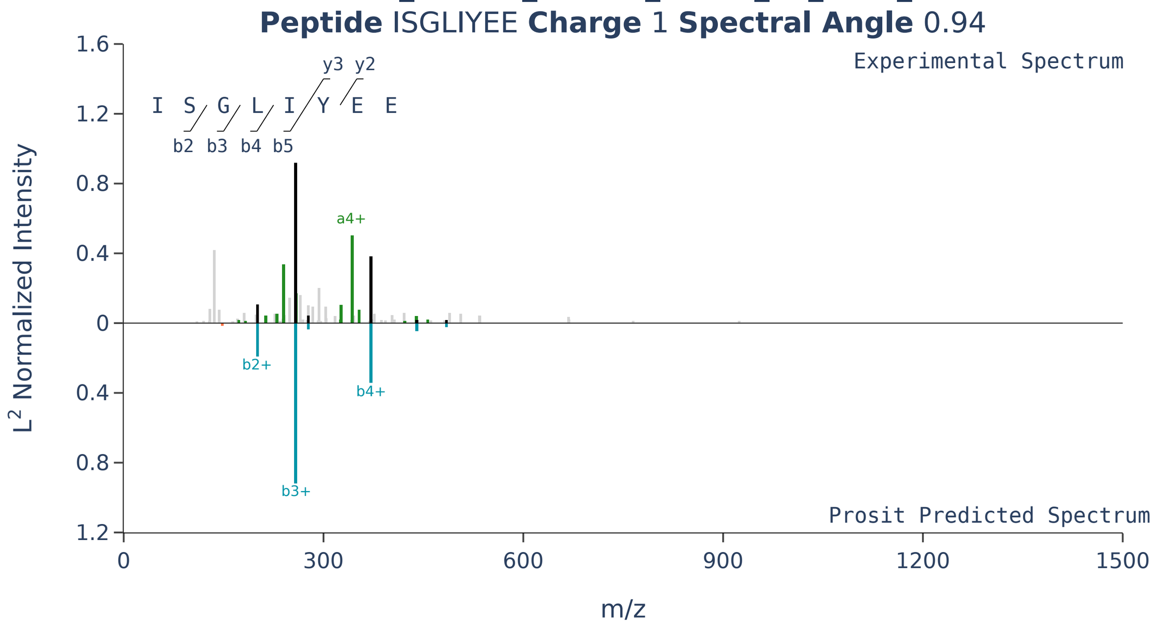

**Source** HRoetschke_291222_140123_HFGoe_H4_b1R1_4h **Scan** 8214
**Peptide** RDAVTYTEH **Charge** 2 **Spectral Angle** 0.83

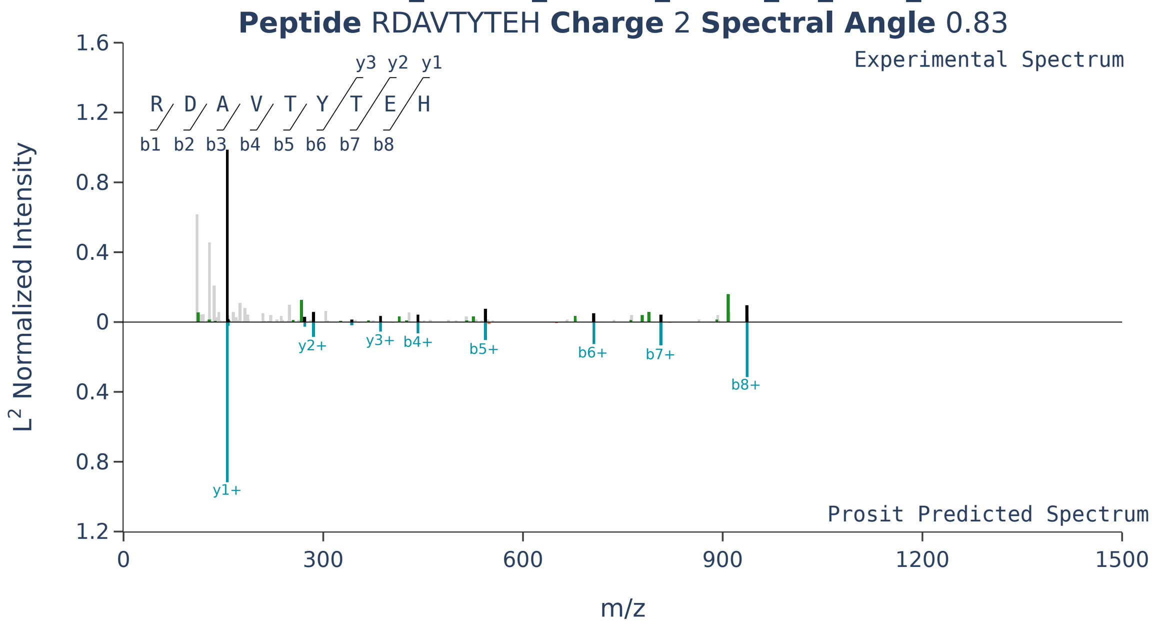

**Source** HRoetschke_291222_140123_HFGoe_H4_b2R2_24h **Scan** 11105
**Peptide** VIRDAVTYTEHA **Charge** 3 **Spectral Angle** 0.88

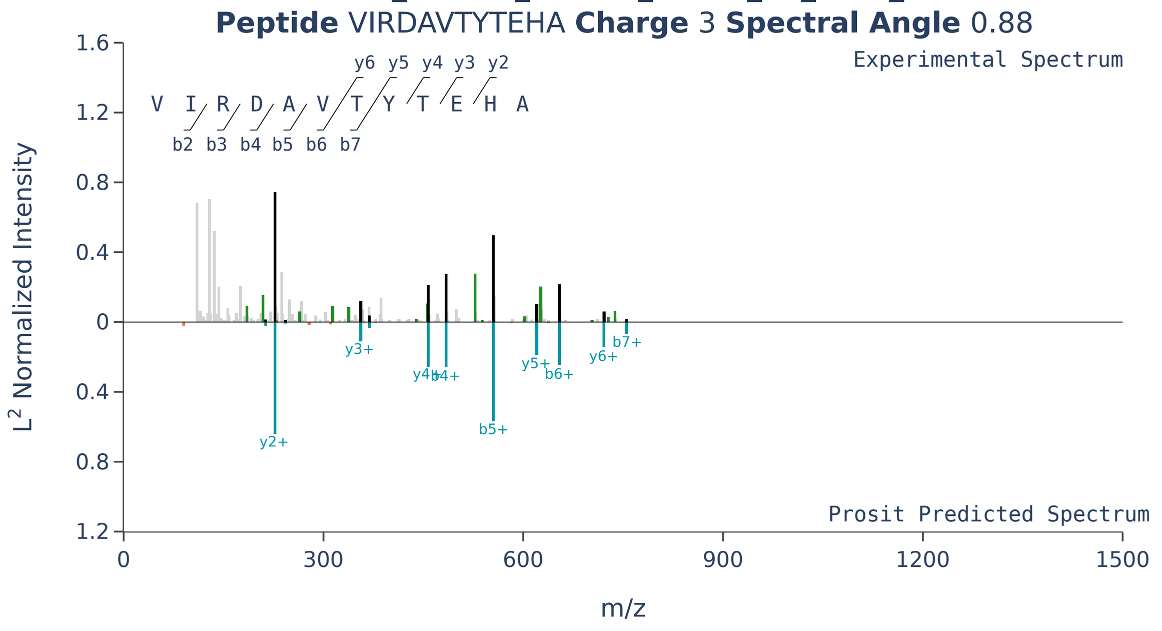

**Source** HRoetschke_291222_140123_HFGoe_H4_b2R2_1h **Scan** 8848
**Peptide** LRDAVTYTEH **Charge** 3 **Spectral Angle** 0.88

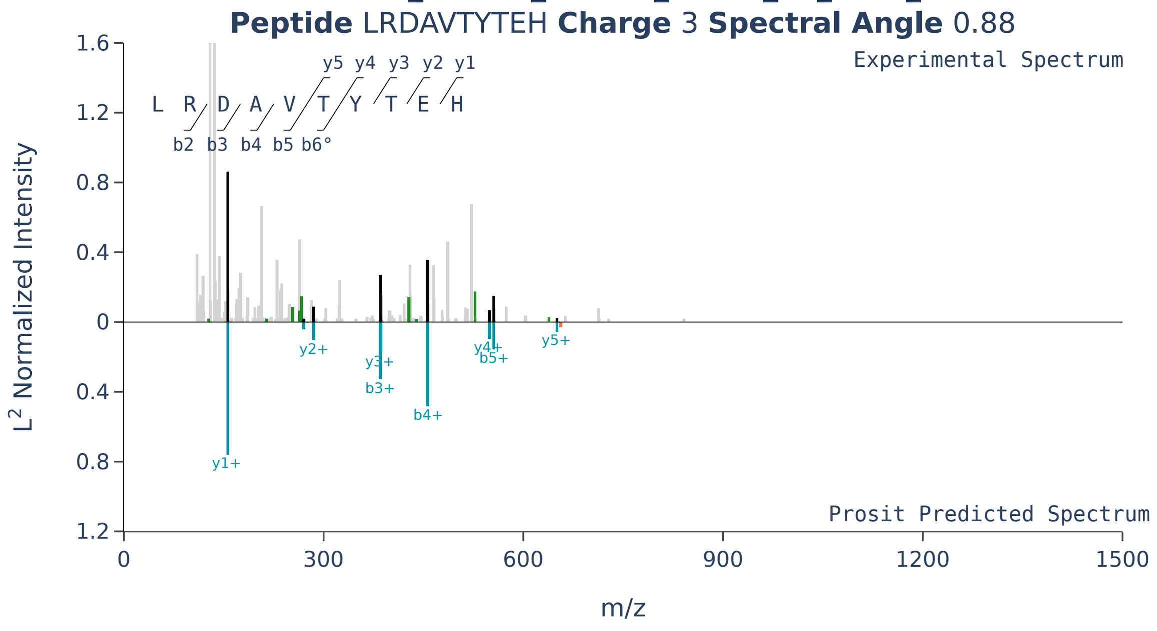

**Source** HRoetschke_291222_140123_HFGoe_H4_b1R2_24h **Scan** 14379
**Peptide** LRDNIQGI **Charge** 2 **Spectral Angle** 0.83

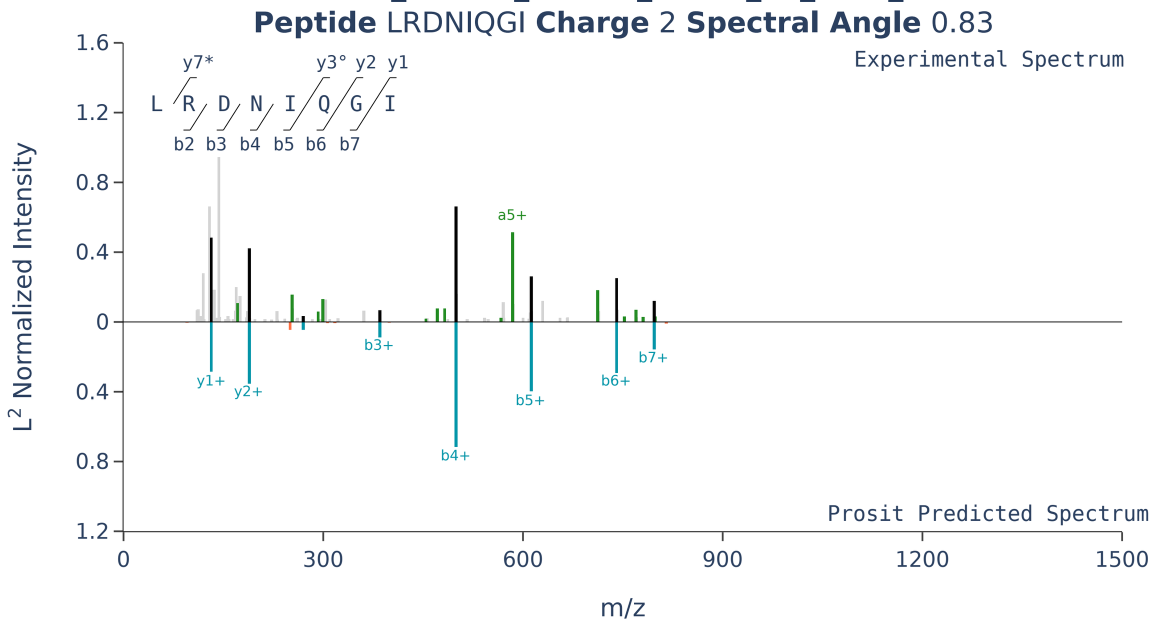

**Source** HRoetschke_291222_140123_HFGoe_H4_b1R1_2h **Scan** 21517
**Peptide** RGVLKVFLE **Charge** 3 **Spectral Angle** 0.78

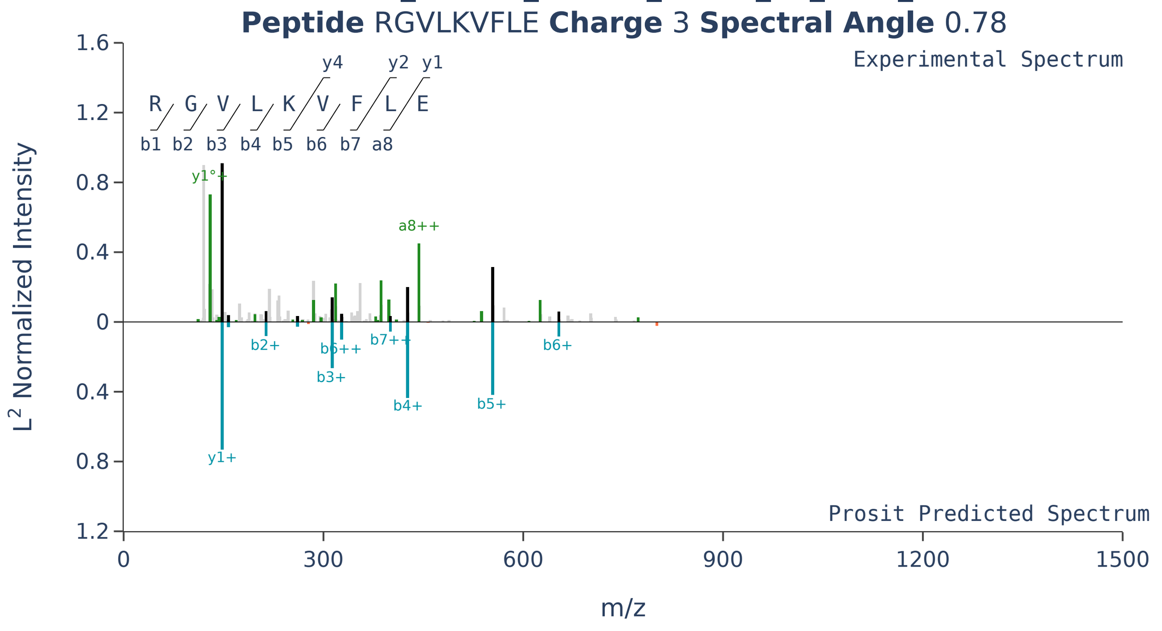

**Source** HRoetschke_291222_140123_HFGoe_H4_b1R1_24h **Scan** 13280
**Peptide** DNIQGITKPAIR **Charge** 3 **Spectral Angle** 0.87

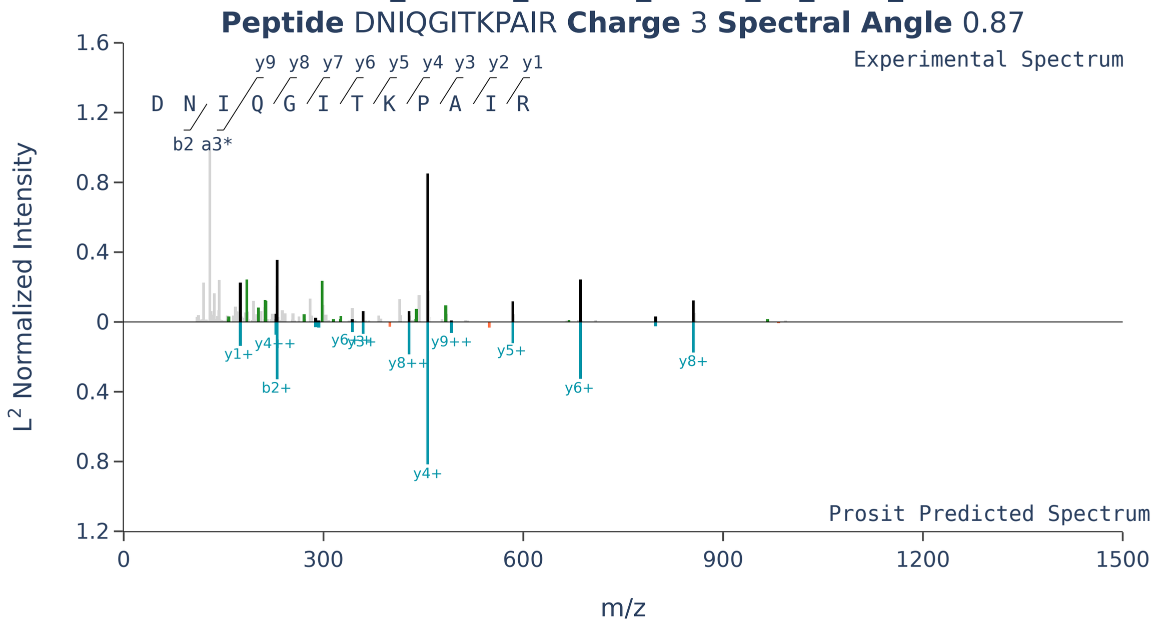

**Source** HRoetschke_291222_140123_HFGoe_H4_b1R2_24h **Scan** 8151
**Peptide** GLTKPAIR **Charge** 2 **Spectral Angle** 0.92

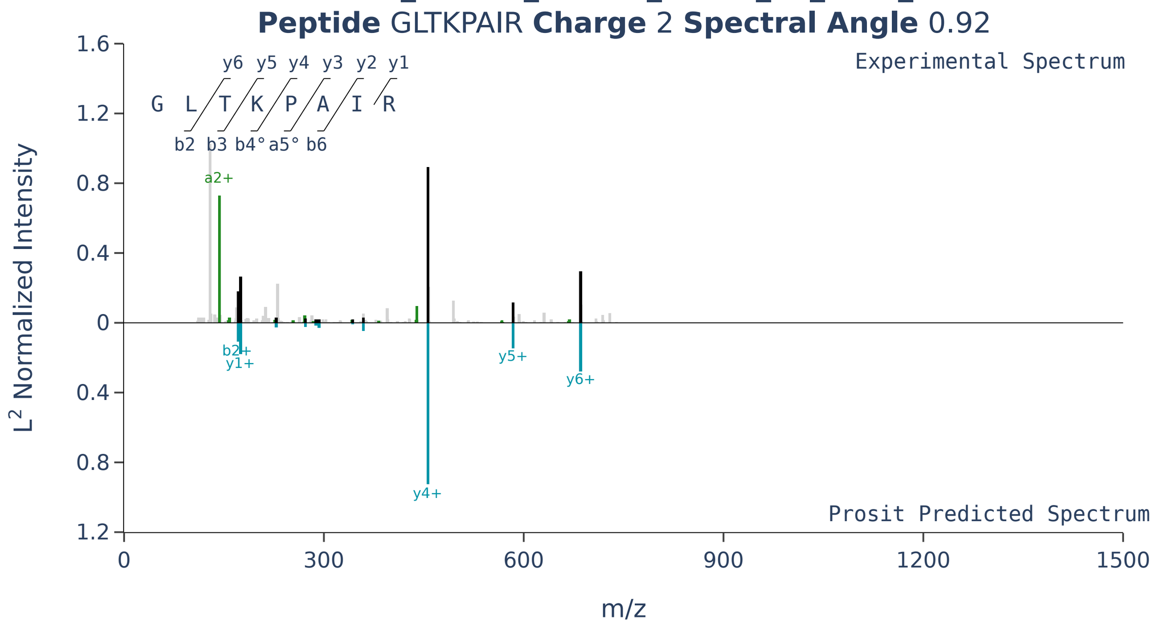


**Source** HRoetschke_291222_140123_HFGoe_H4_b2R1_2h **Scan** 17511
**Peptide** NIQGITKPAI **Charge** 1 **Spectral Angle** 0.88

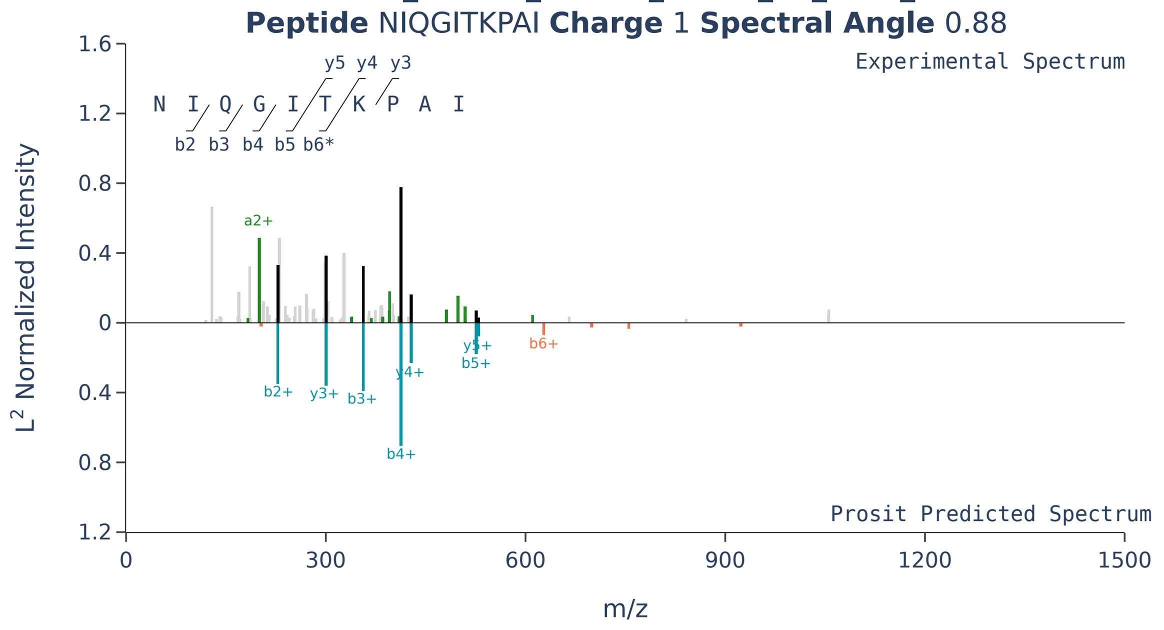


**Source** HRoetschke_291222_140123_HFGoe_H4_b1R1_1h **Scan** 17636
**Peptide** QGRTLYGFGG **Charge** 2 **Spectral Angle** 0.81

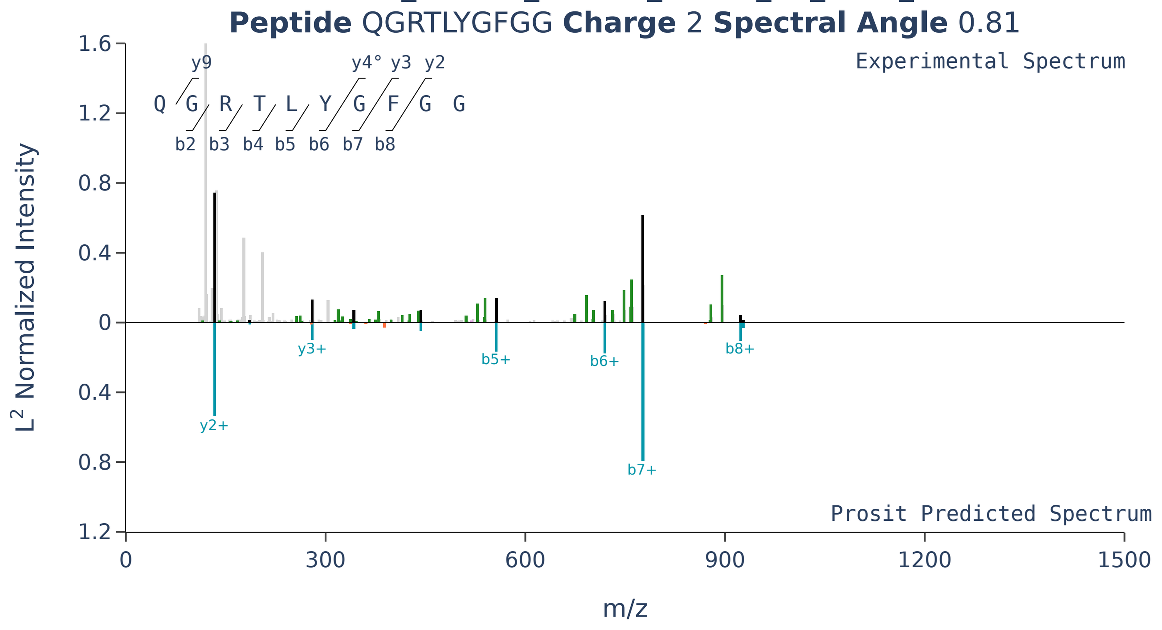


**Source** HRoetschke_291222_140123_HFGoe_H4_b2R2_4h **Scan** 13445
**Peptide** ENVIRDAV **Charge** 2 **Spectral Angle** 0.86

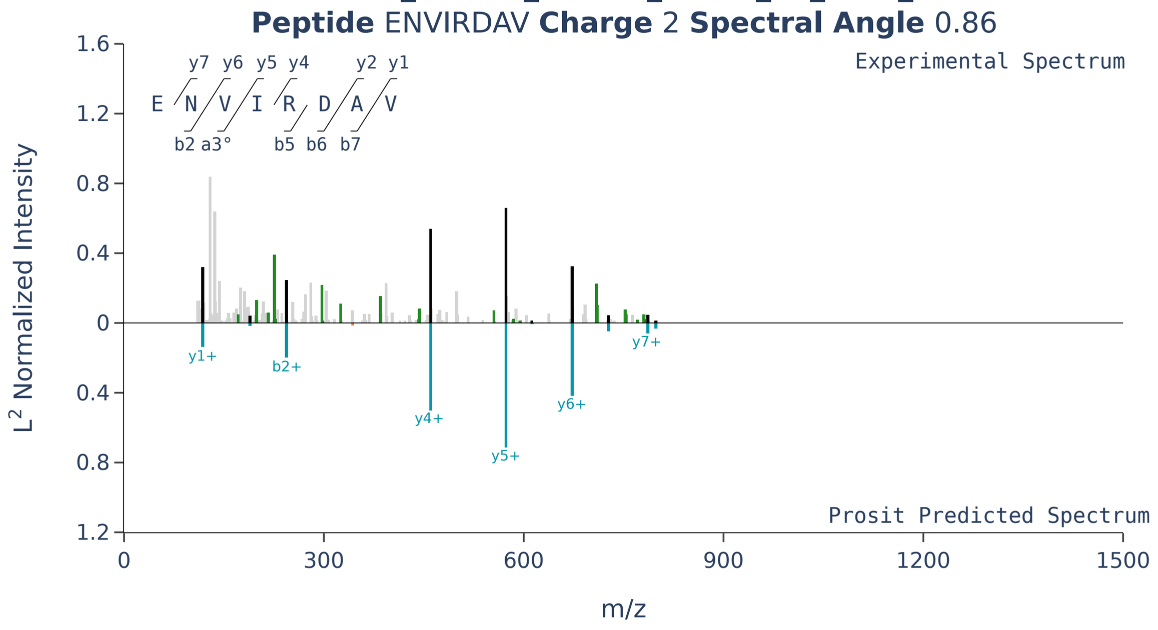


**Source** HRoetschke_291222_140123_HFGoe_H4_b1R1_24h **Scan** 11094
**Peptide** QGITKPAIRRL **Charge** 3 **Spectral Angle** 0.88

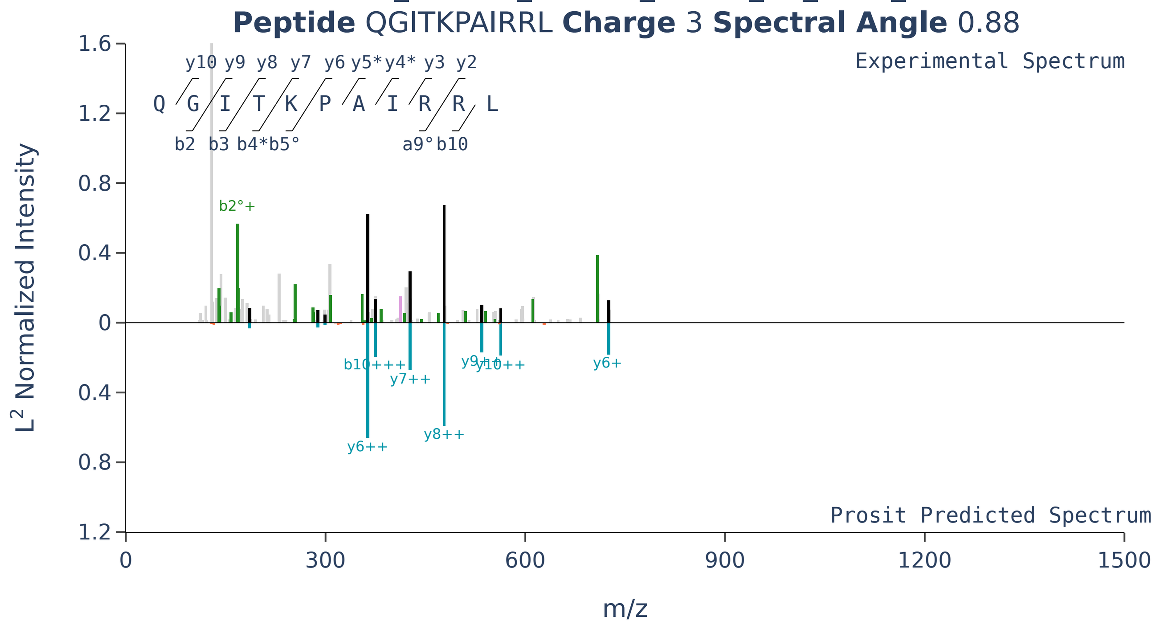


**Source** HRoetschke_291222_140123_HFGoe_H4_b1R2_24h **Scan** 11043
**Peptide** LQGITKPAIR **Charge** 3 **Spectral Angle** 0.9

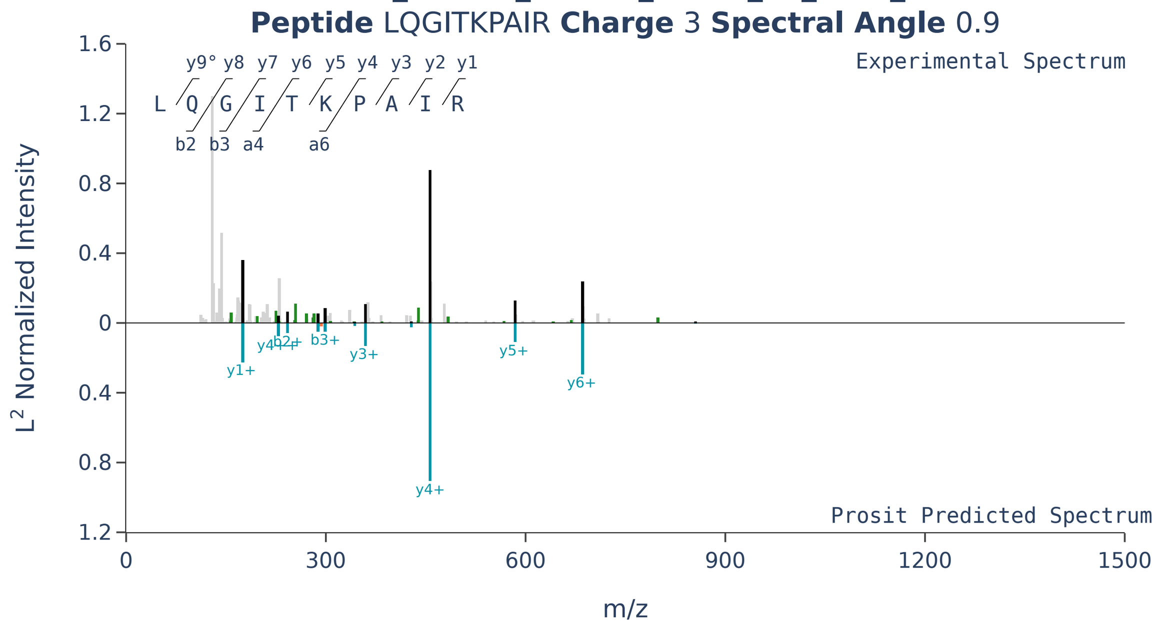


**Source** HRoetschke_291222_140123_HFGoe_H4_b1R2_4h **Scan** 19844
**Peptide** FLENVLR **Charge** 2 **Spectral Angle** 0.84

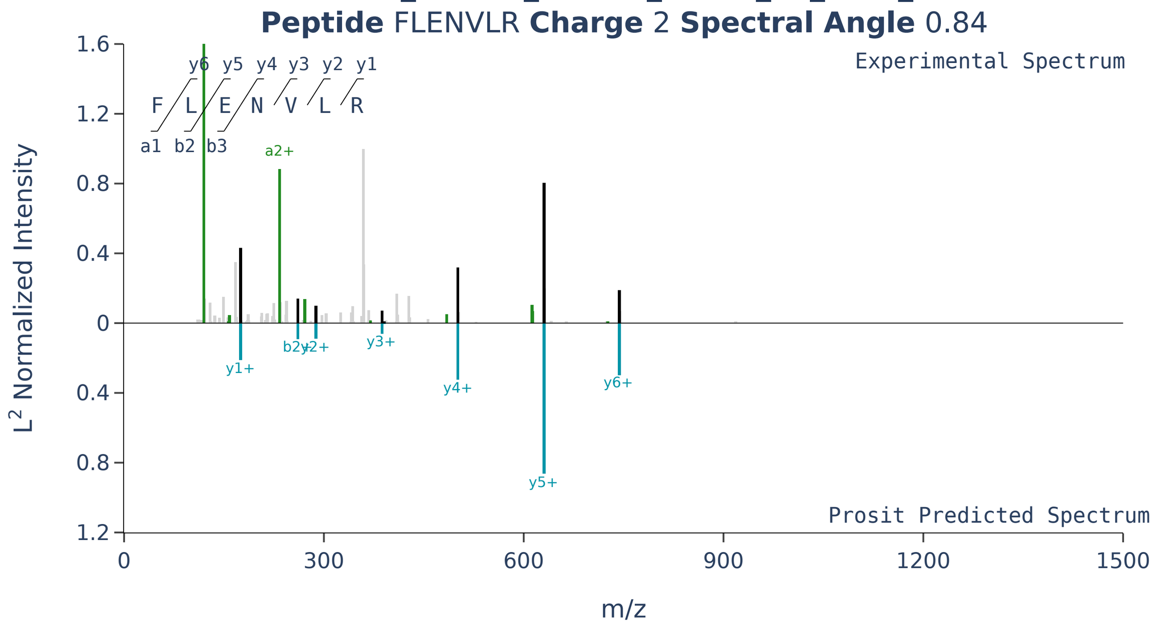


**Source** HRoetschke_291222_140123_HFGoe_H4_b2R1_24h **Scan** 13487
**Peptide** DNIQGITKPALR **Charge** 2 **Spectral Angle** 0.78

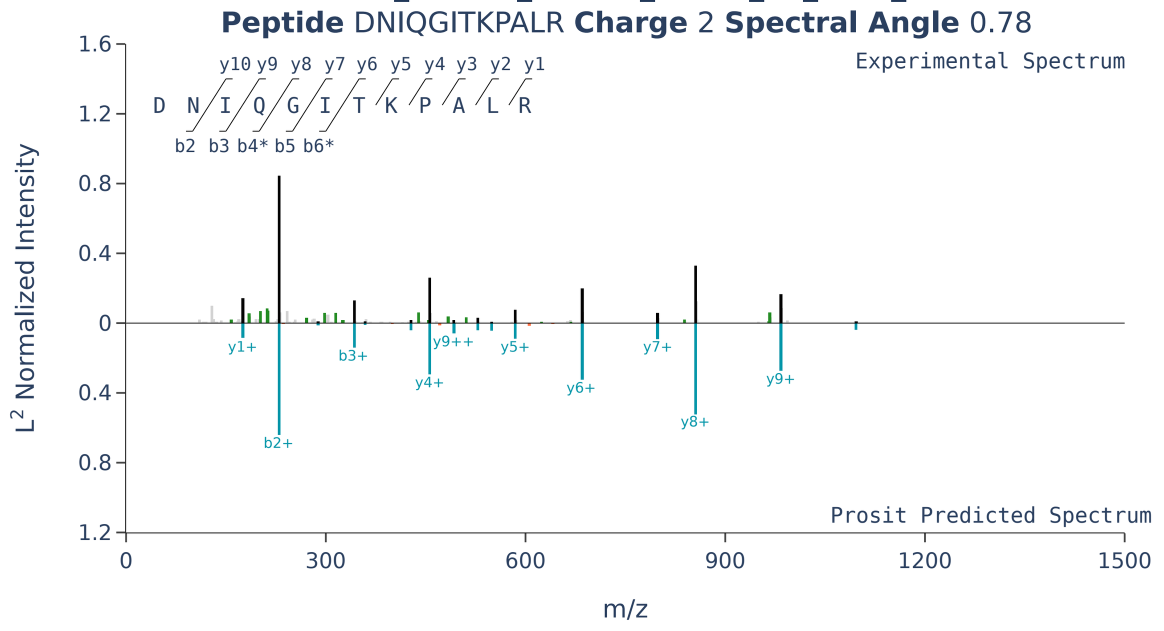


**Source** HRoetschke_291222_140123_HFGoe_H4_b2R1_4h **Scan** 17073
**Peptide** LRDNIQGITKPAL **Charge** 3 **Spectral Angle** 0.85

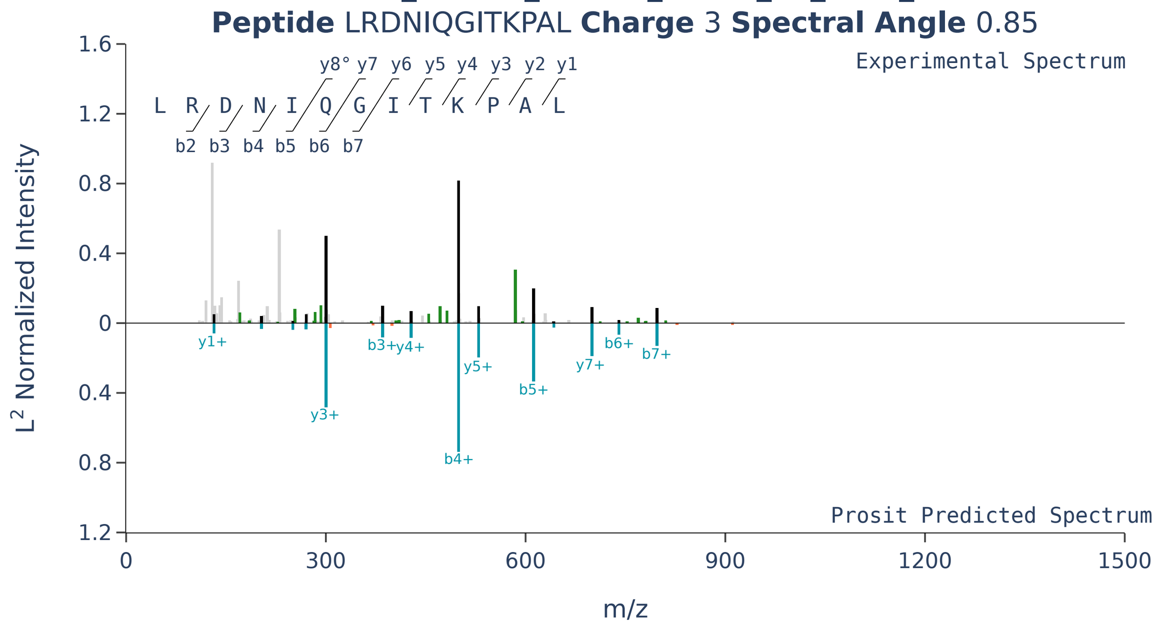


**Source** HRoetschke_291222_140123_HFGoe_H4_b2R1_2h **Scan** 17545
**Peptide** LSGLIYEETRG **Charge** 2 **Spectral Angle** 0.83

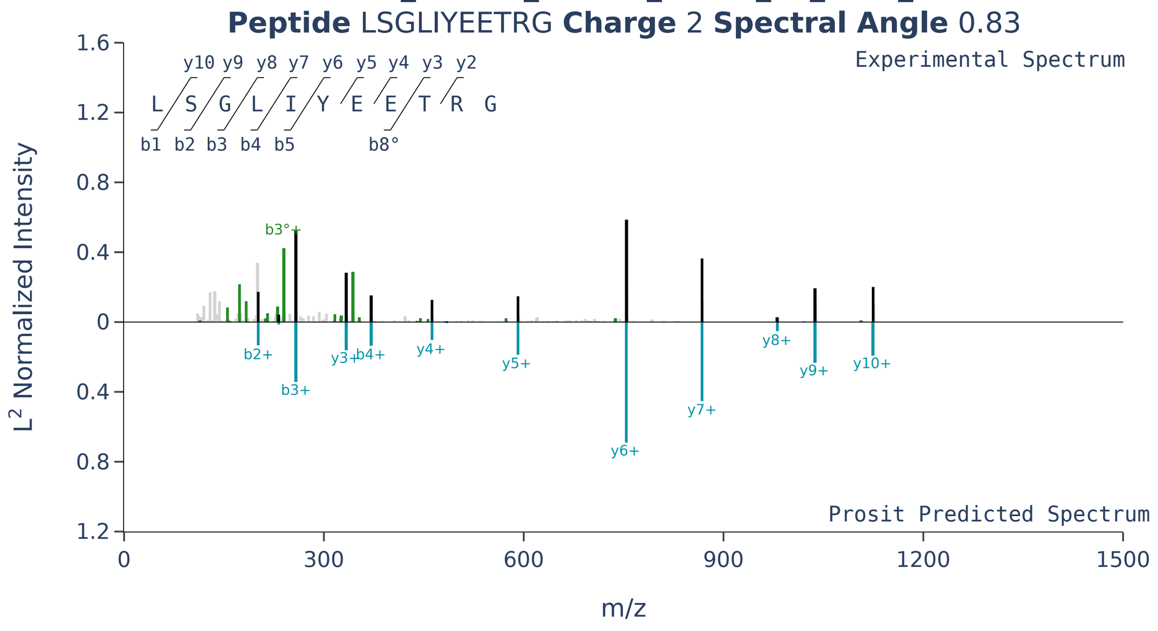


**Source** HRoetschke_291222_140123_HFGoe_H4_b2R1_2h **Scan** 16279
**Peptide** GVKRISGLIYE **Charge** 3 **Spectral Angle** 0.81

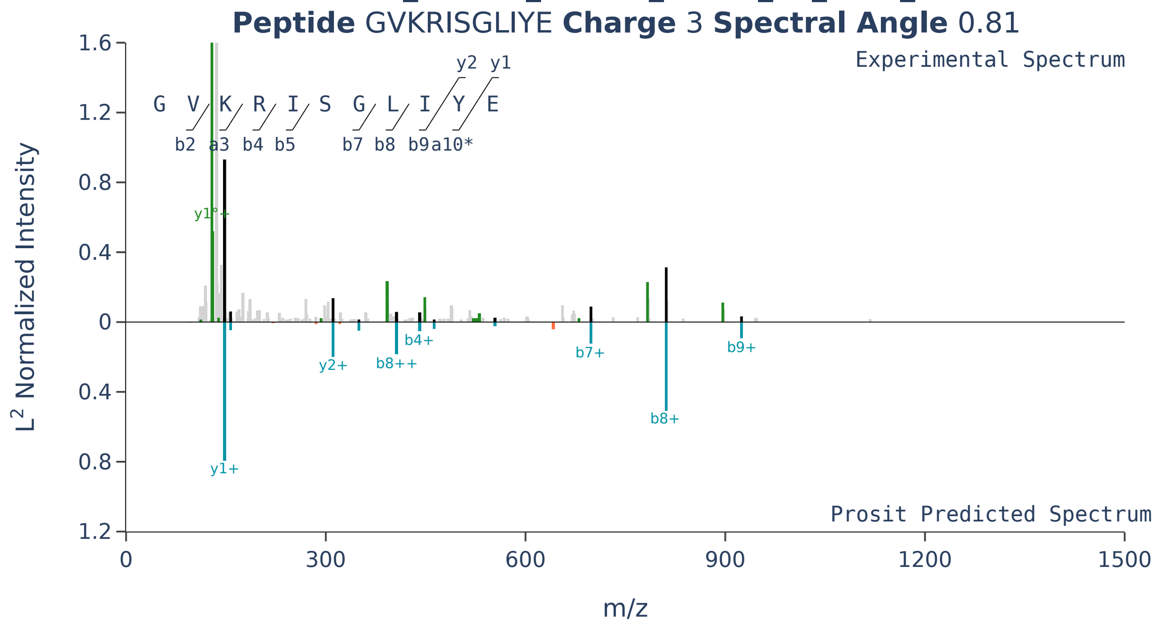


**Source** HRoetschke_291222_140123_HFGoe_H4_b1R2_4h **Scan** 9975
**Peptide** IRDAVTYT **Charge** 1 **Spectral Angle** 0.94

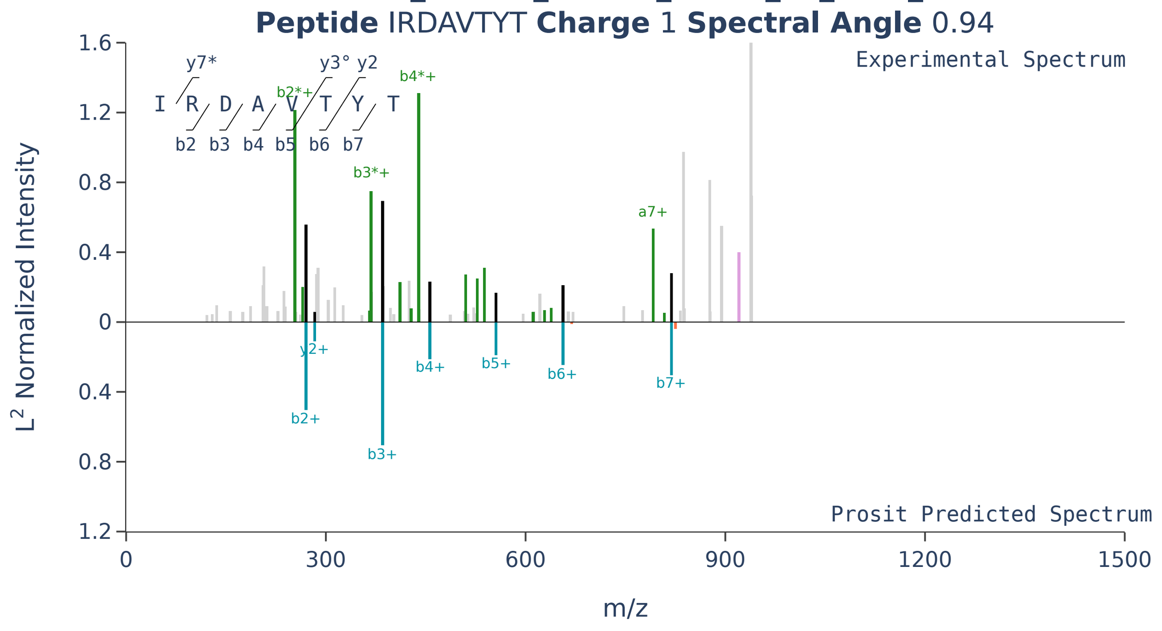


**Source** HRoetschke_291222_140123_HFGoe_H4_b1R2_4h **Scan** 14789
**Peptide** RDNIQGITKPAI **Charge** 3 **Spectral Angle** 0.83

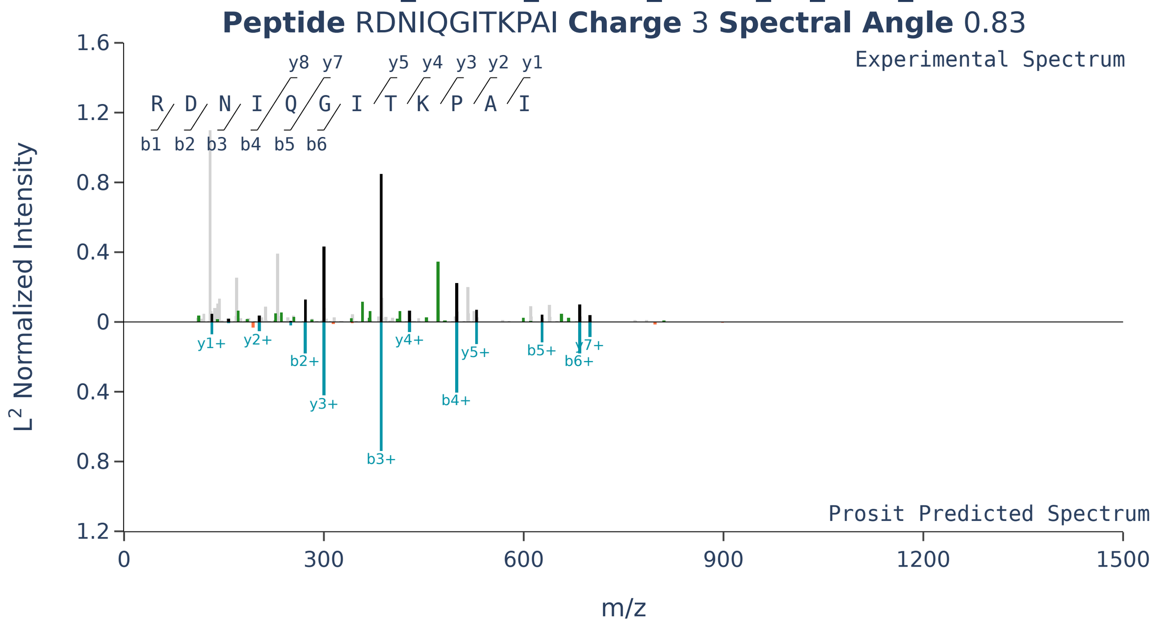


**Source** HRoetschke_291222_140123_HFGoe_H4_b2R1_24h **Scan** 11398
**Peptide** DNIQGITKPA **Charge** 2 **Spectral Angle** 0.91

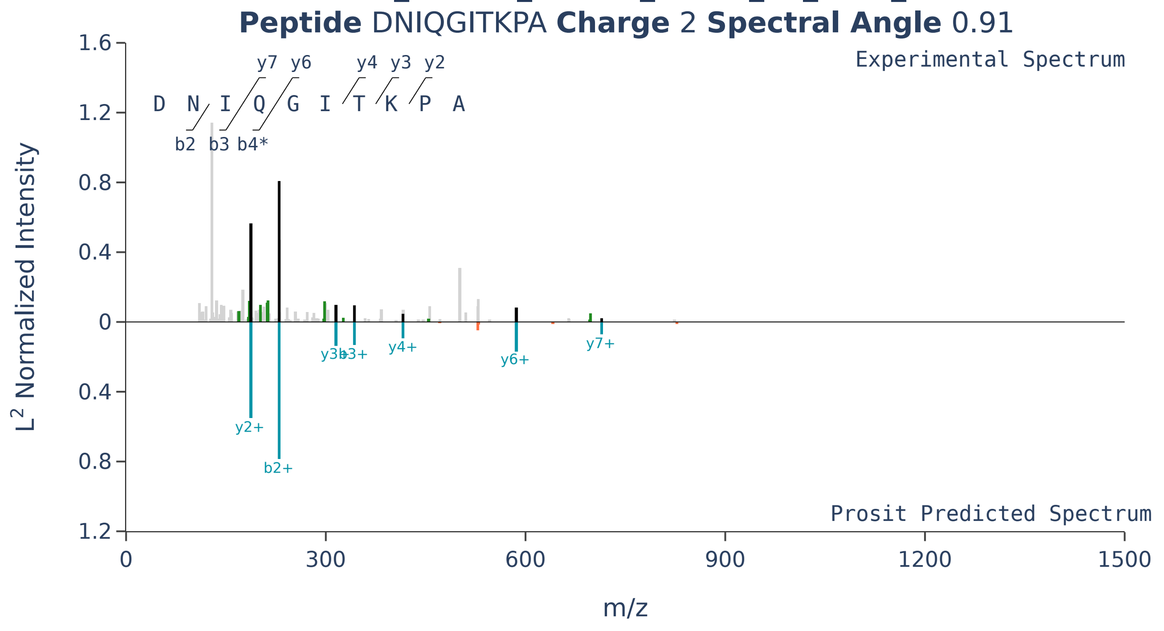


**Source** HRoetschke_291222_140123_HFGoe_H4_b1R1_1h **Scan** 25252
**Peptide** RGVLKVFLENVLR **Charge** 3 **Spectral Angle** 0.84

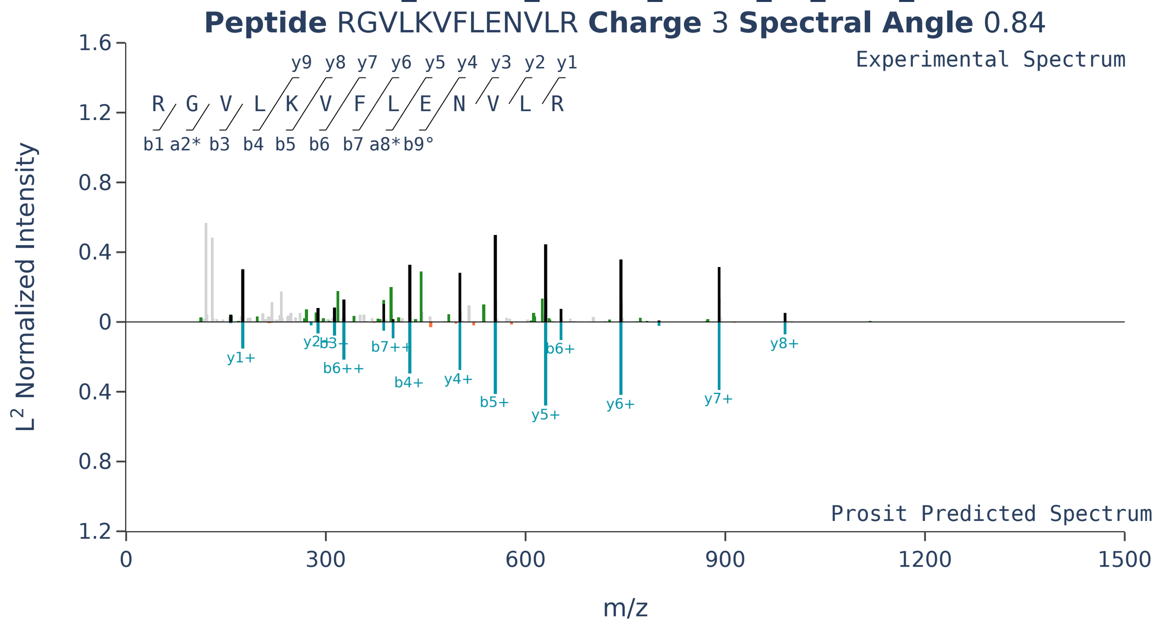

**Source** HRoetschke_291222_140123_HFGoe_H4_b1R1_4h **Scan** 15340
**Peptide** TAMDVVYALKRQG **Charge** 3 **Spectral Angle** 0.83

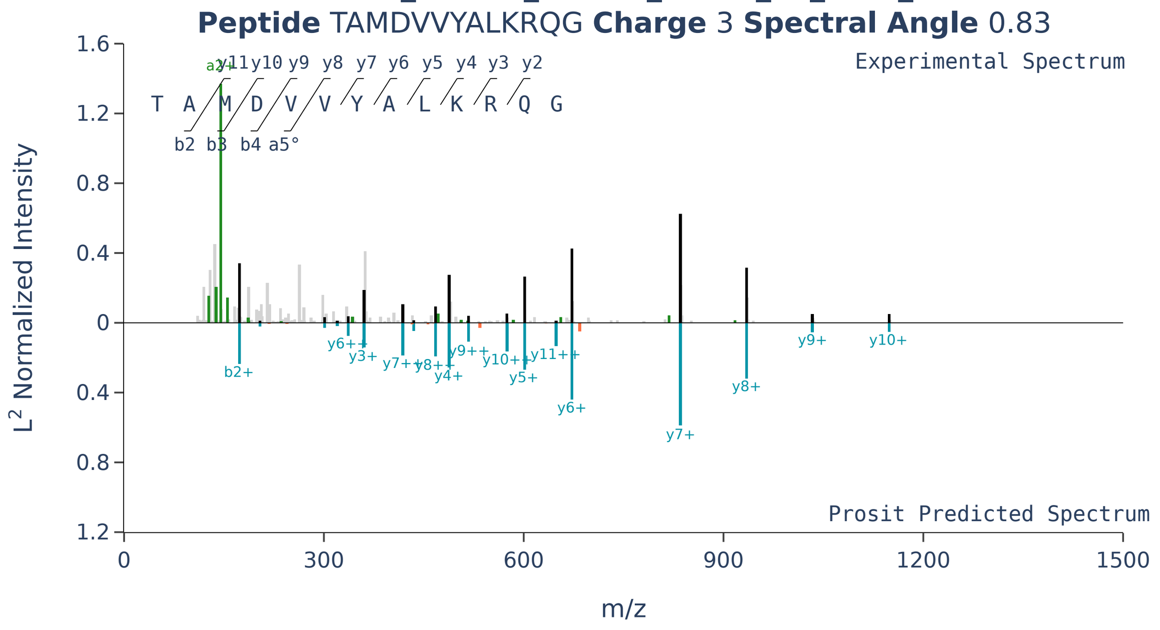


**Source** HRoetschke_291222_140123_HFGoe_H4_b2R2_24h **Scan** 14295
**Peptide** TEHAKRKT **Charge** 2 **Spectral Angle** 0.84

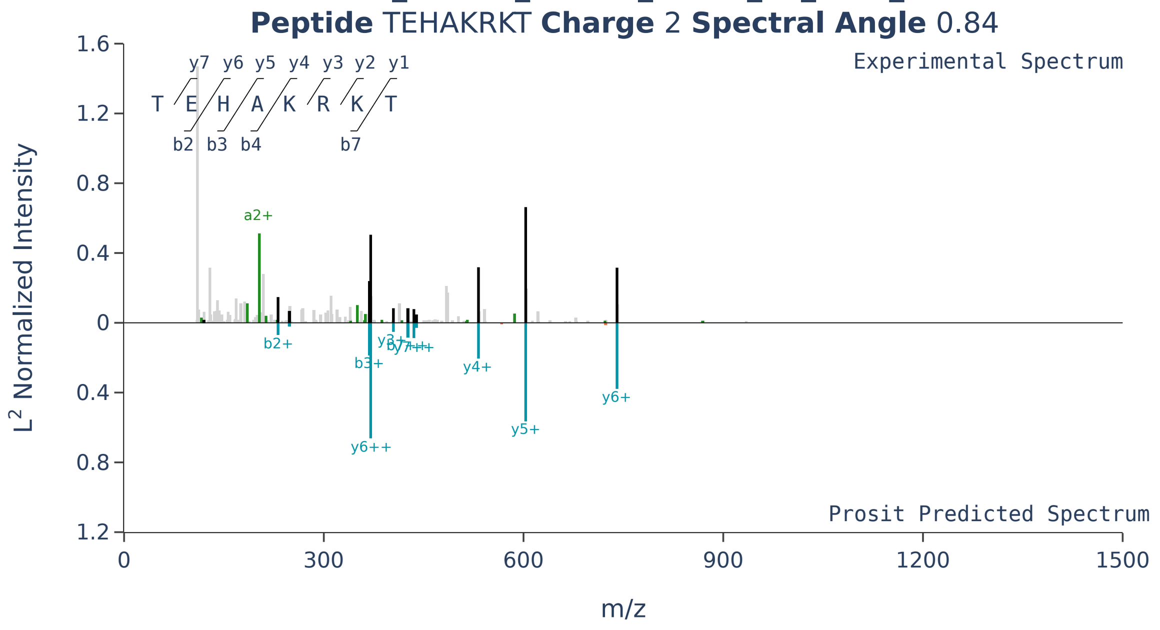


**Source** HRoetschke_291222_140123_HFGoe_H4_b2R1_4h **Scan** 15438
**Peptide** VKRISGLIYE **Charge** 3 **Spectral Angle** 0.85

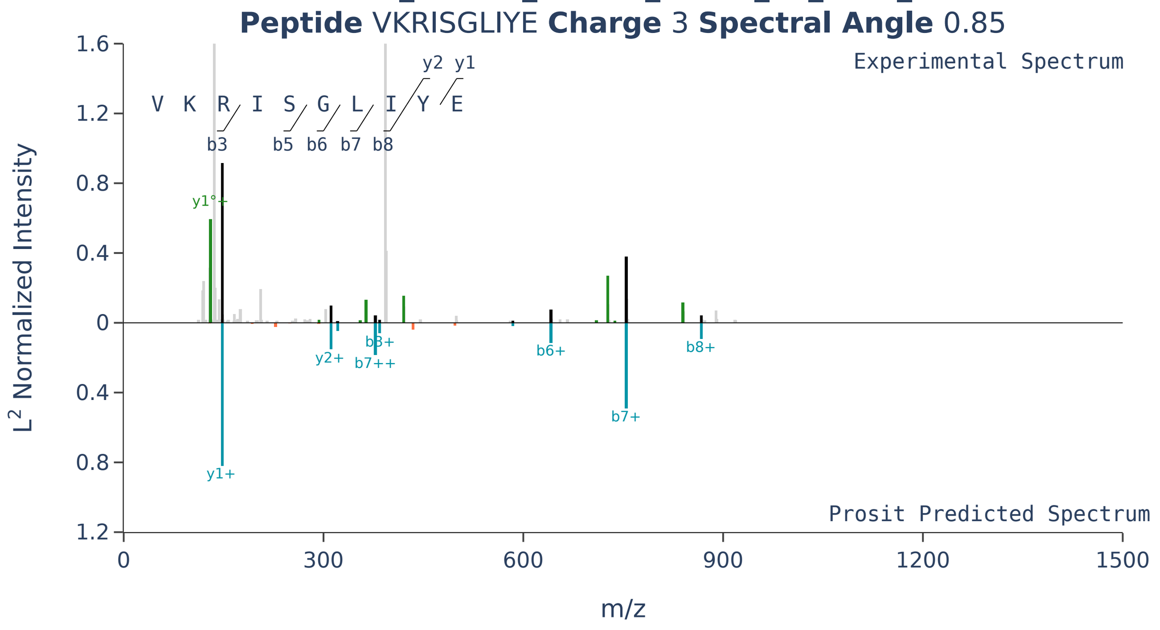


**Source** HRoetschke_291222_140123_HFGoe_H4_b1R1_2h **Scan** 19745
**Peptide** EETRGVLKVF **Charge** 2 **Spectral Angle** 0.84

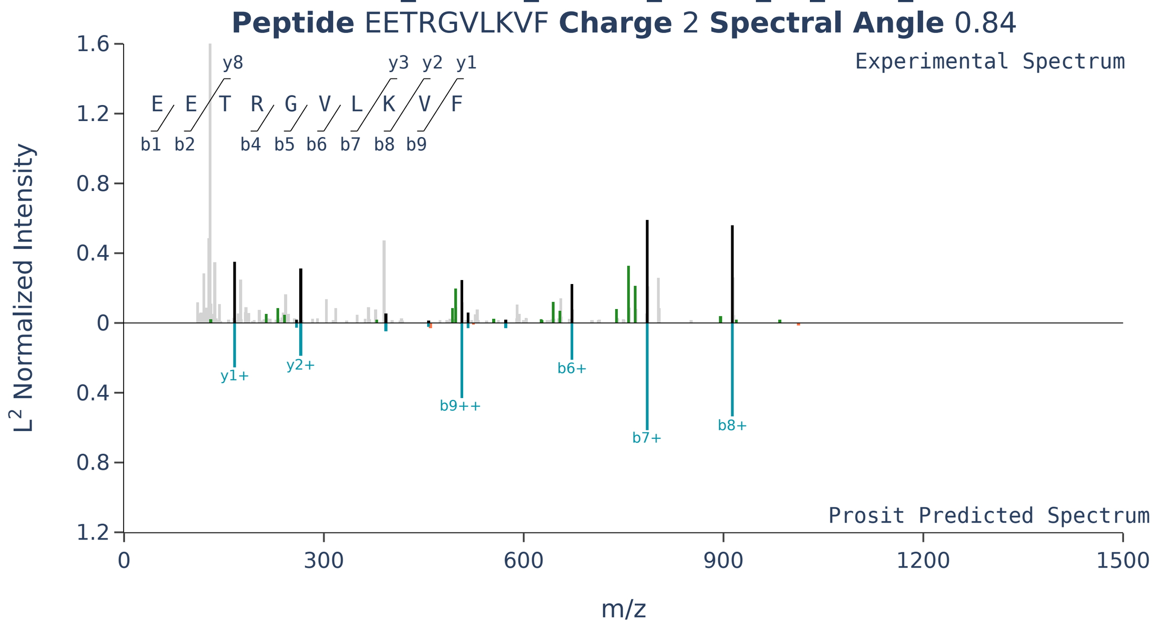


**Source** HRoetschke_291222_140123_HFGoe_H4_b1R1_1h **Scan** 16250
**Peptide** ENVIRDAVTYTEHAKR **Charge** 3 **Spectral Angle** 0.81

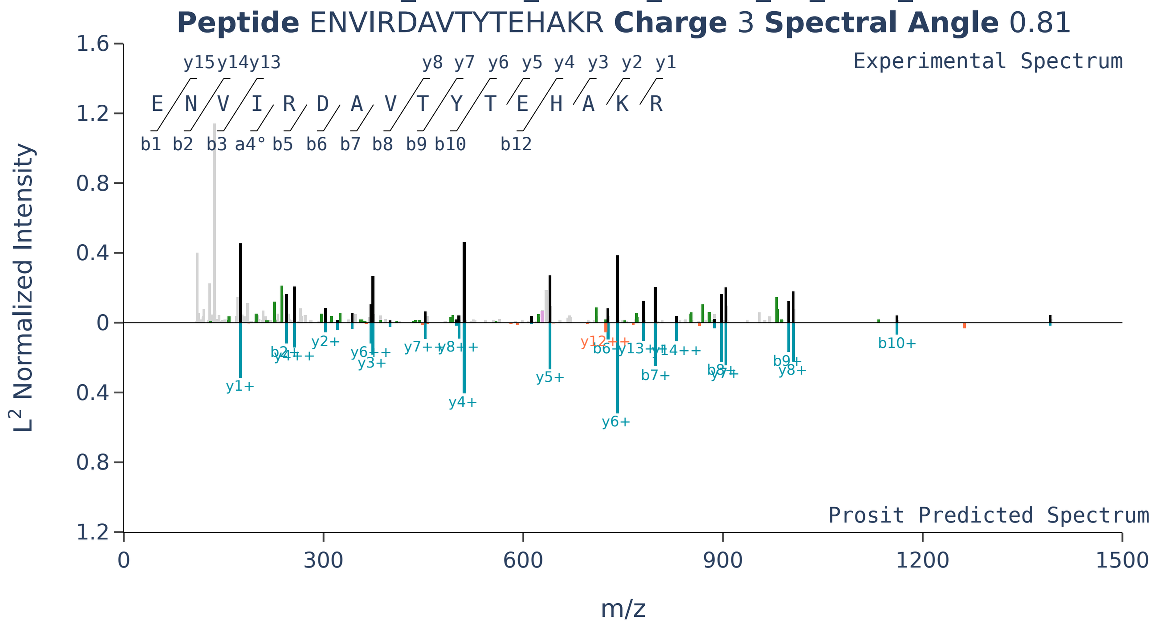


**Source** HRoetschke_291222_140123_HFGoe_H4_b2R1_4h **Scan** 13785
**Peptide** LQGITKPAIRRL **Charge** 3 **Spectral Angle** 0.79

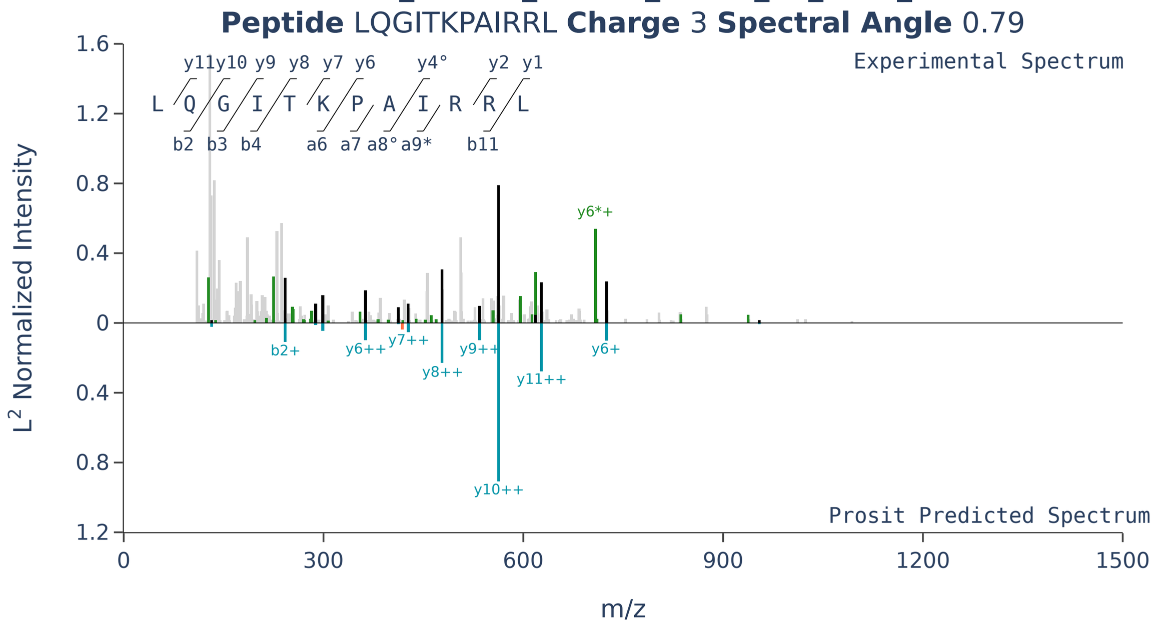


**Source** HRoetschke_291222_140123_HFGoe_H4_b1R2_24h **Scan** 10658
**Peptide** IRDAVTYTE **Charge** 2 **Spectral Angle** 0.8

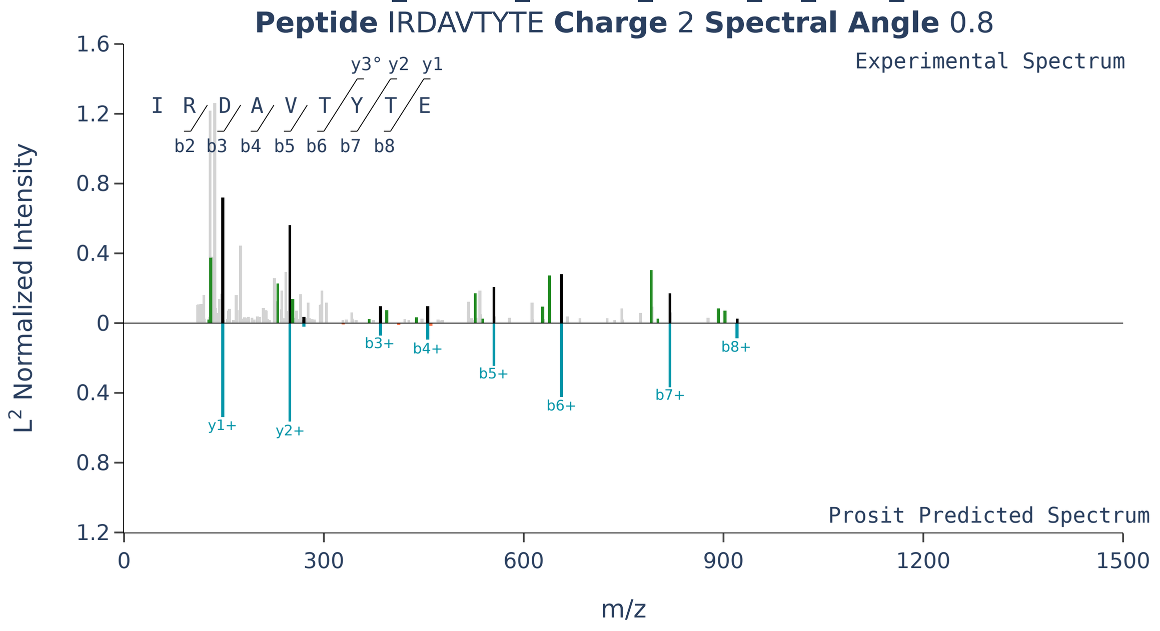


**Source** HRoetschke_291222_140123_HFGoe_H4_b2R1_2h **Scan** 18679
**Peptide** KVFLENV **Charge** 2 **Spectral Angle** 0.74

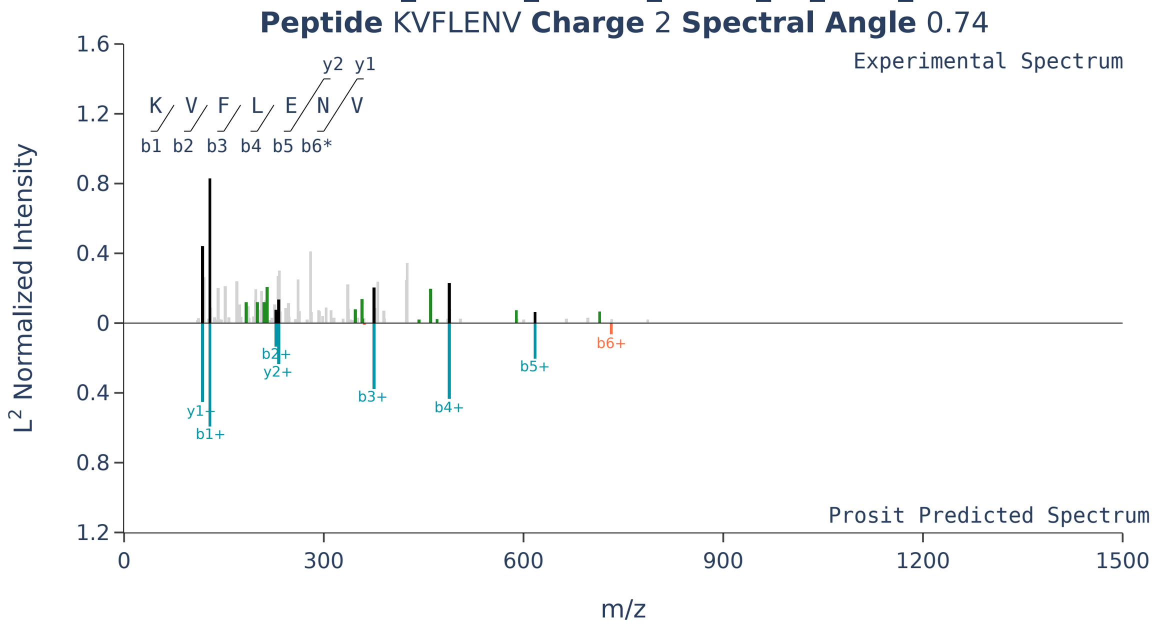


**Source** HRoetschke_291222_140123_HFGoe_H4_b1R2_24h **Scan** 9313
**Peptide** RDNIQGITKP **Charge** 3 **Spectral Angle** 0.89

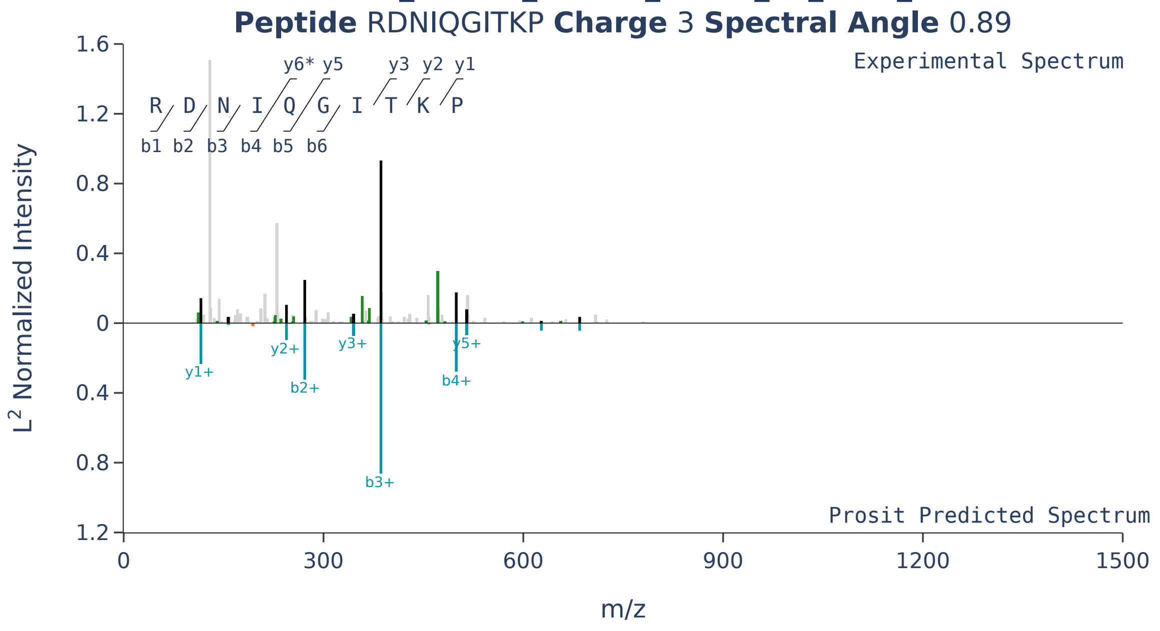


**Source** HRoetschke_291222_140123_HFGoe_H4_b2R2_4h **Scan** 18722
**Peptide** LKRQGRTL **Charge** 2 **Spectral Angle** 0.95

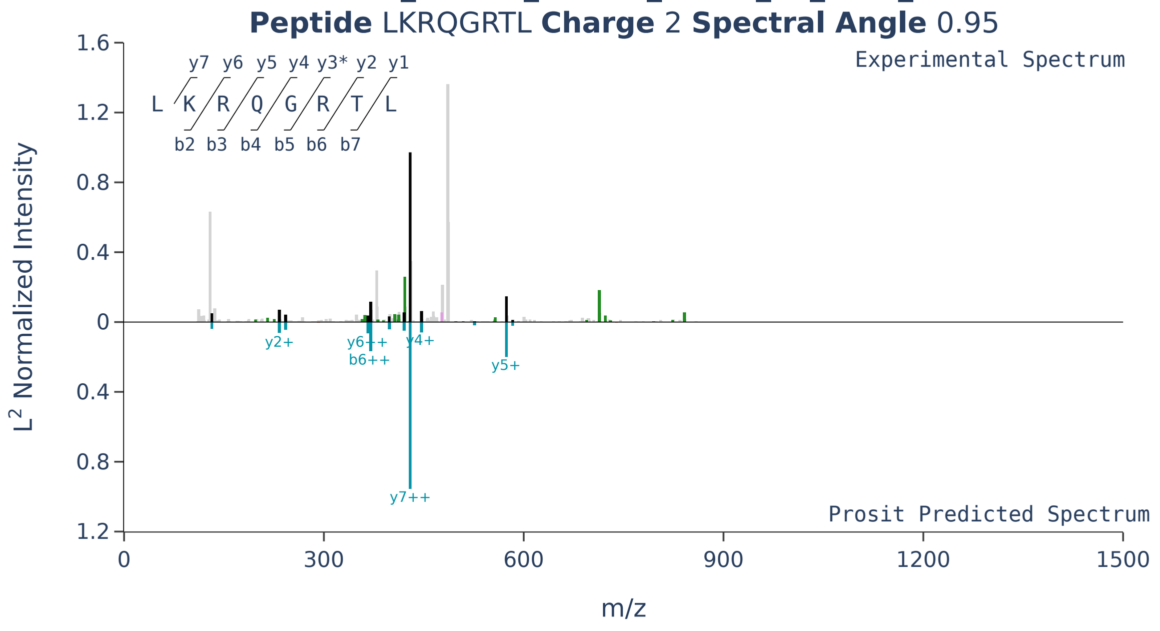


**Source** HRoetschke_291222_140123_HFGoe_H4_b1R2_4h **Scan** 19861
**Peptide** LKVFLEN **Charge** 2 **Spectral Angle** 0.94

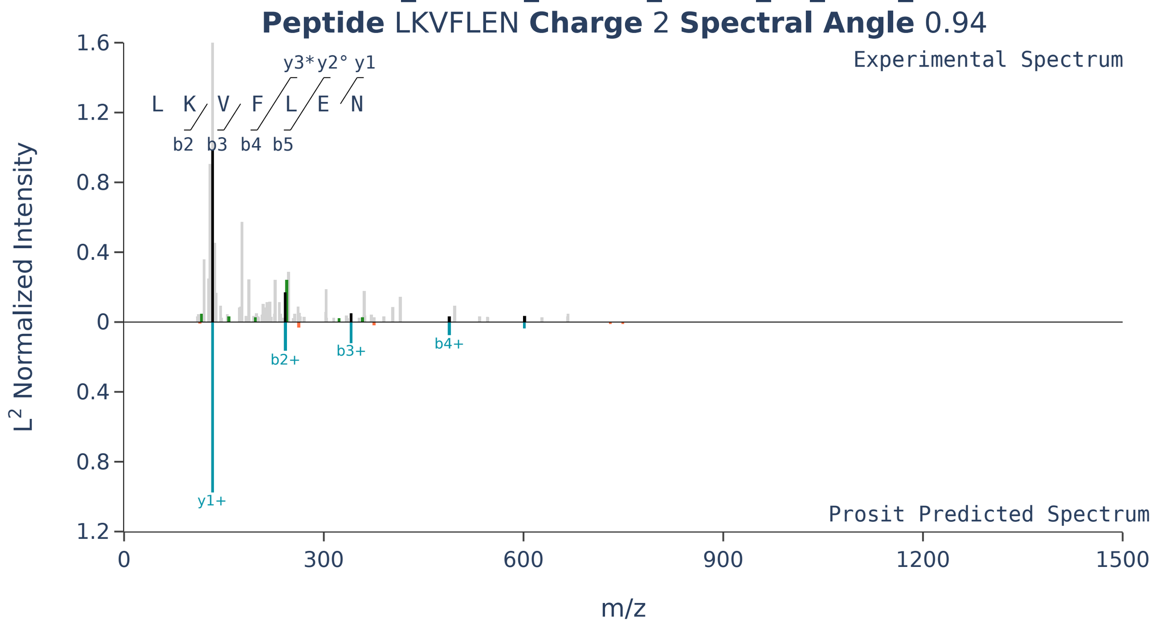


**Source** HRoetschke_291222_140123_HFGoe_H4_b2R1_2h **Scan** 16472
**Peptide** IQGITKPAI **Charge** 2 **Spectral Angle** 0.88

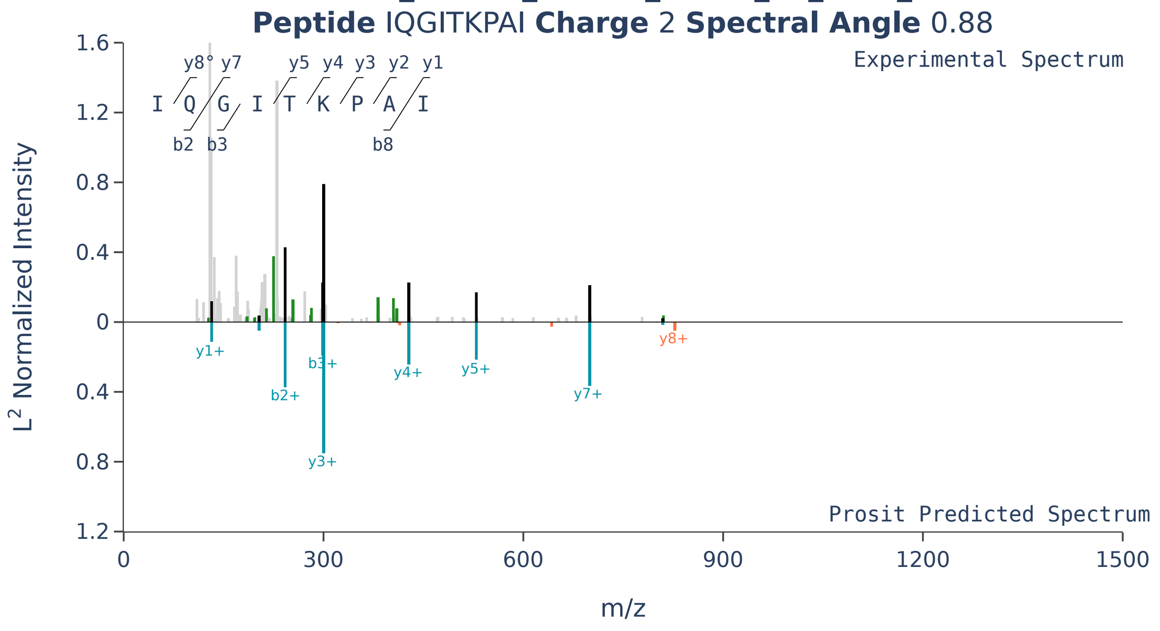


**Source** HRoetschke_291222_140123_HFGoe_H4_b1R1_4h **Scan** 16636
**Peptide** GGVKRISGLIYE **Charge** 2 **Spectral Angle** 0.77

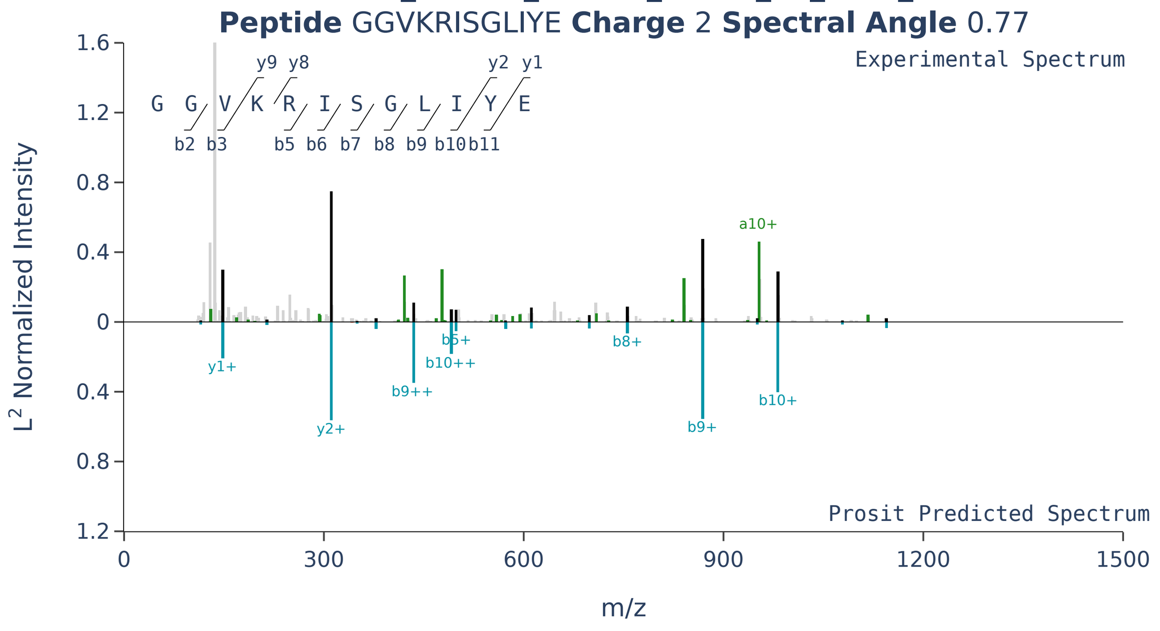


**Source** HRoetschke_291222_140123_HFGoe_H4_b2R1_1h **Scan** 5644
**Peptide** RDAVTYTEHAK **Charge** 3 **Spectral Angle** 0.89

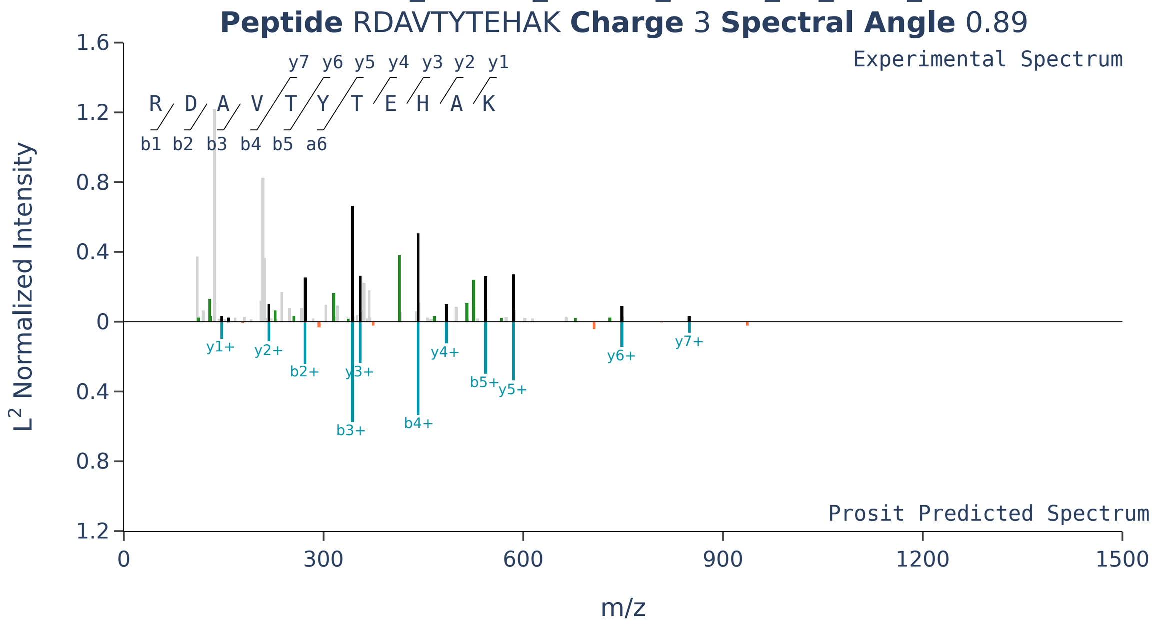


**Source** HRoetschke_291222_140123_HFGoe_H4_b1R1_2h **Scan** 18555
**Peptide** SGLIYEETRGV **Charge** 2 **Spectral Angle** 0.81

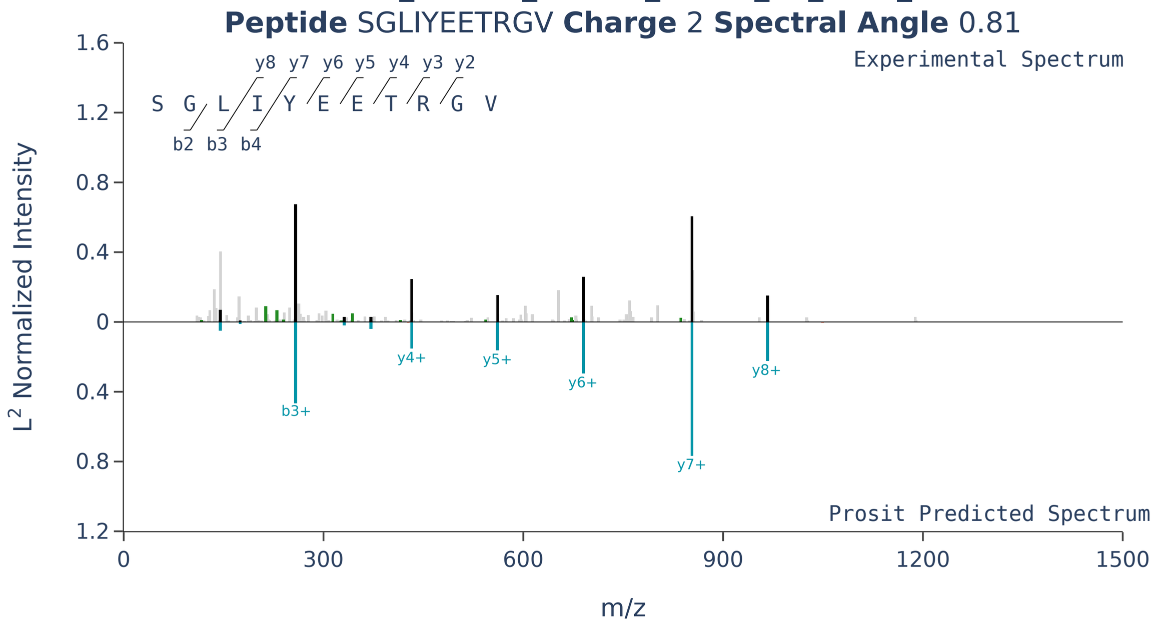

**Source** HRoetschke_291222_140123_HFGoe_H4_b2R1_1h **Scan** 29081
**Peptide** LIYEETRGVLKVFL **Charge** 2 **Spectral Angle** 0.8

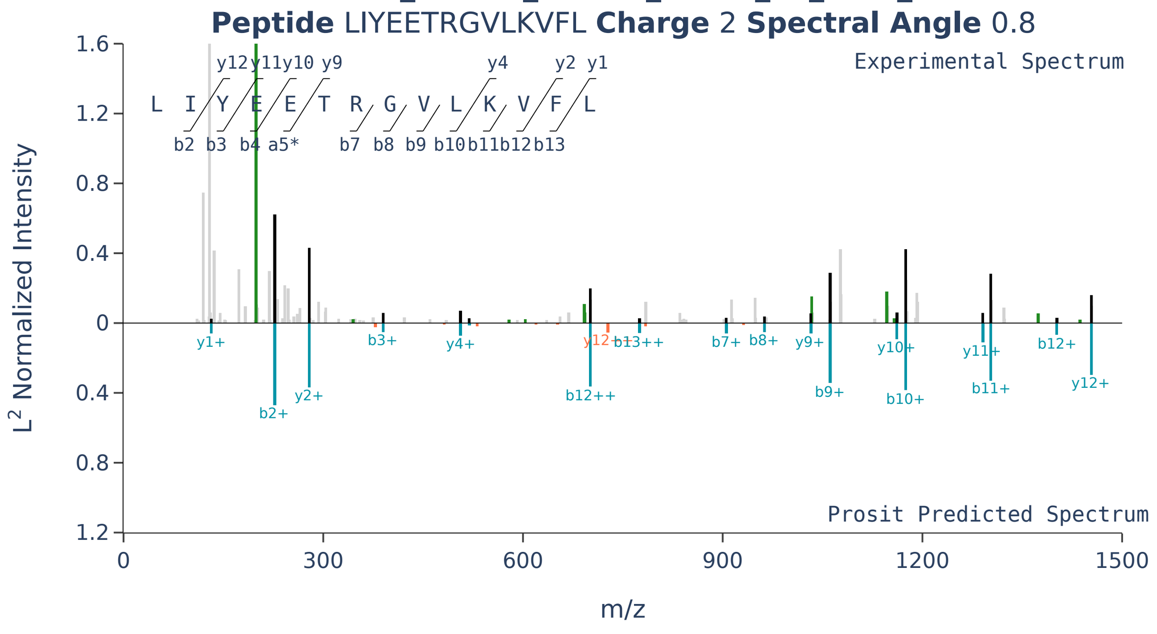


**Source** HRoetschke_291222_140123_HFGoe_H4_b2R1_1h **Scan** 31904
**Peptide** SGLIYEETRGVLKVFLE **Charge** 2 **Spectral Angle** 0.78

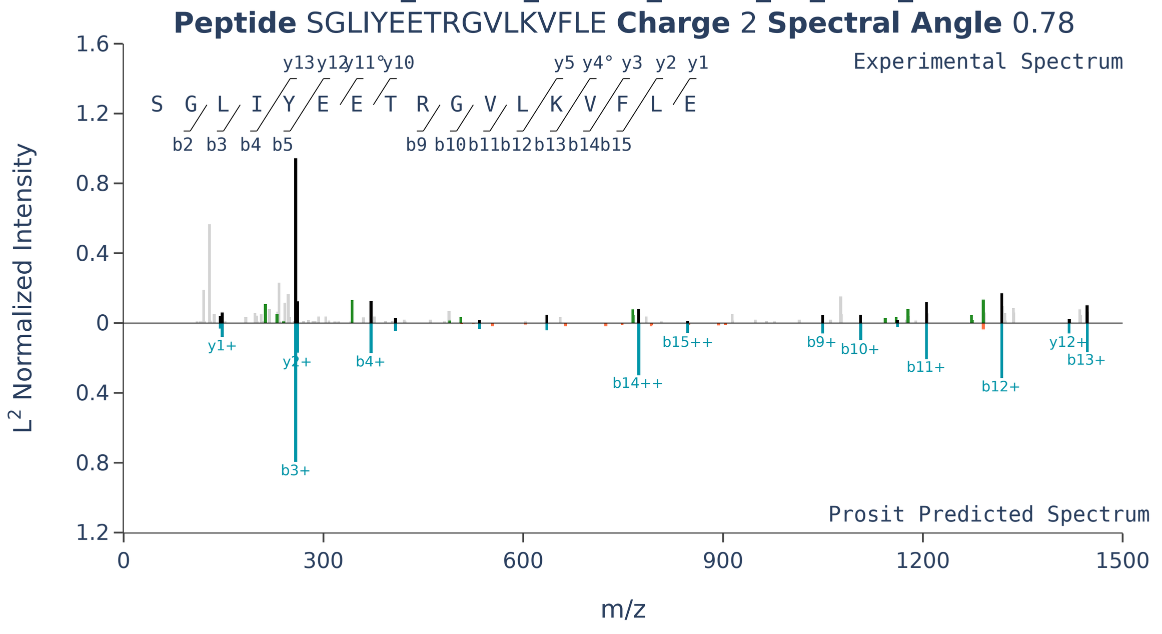


**Source** HRoetschke_291222_140123_HFGoe_H4_b2R1_1h **Scan** 24251
**Peptide** LSGLIYEETRGVLKV **Charge** 2 **Spectral Angle** 0.85

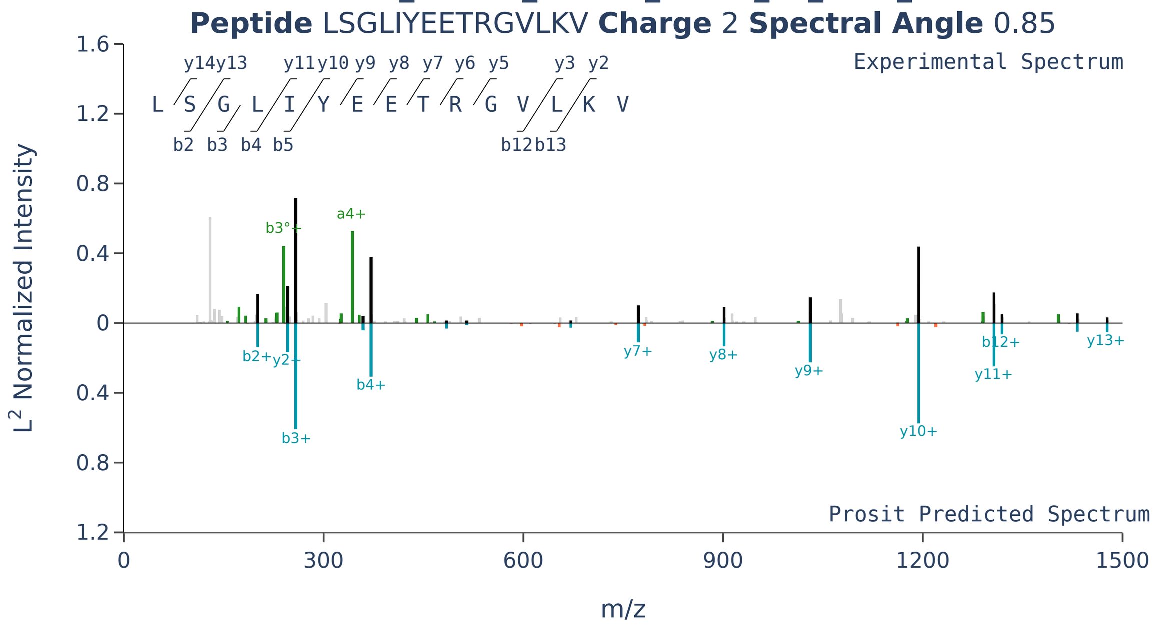


**Source** HRoetschke_291222_140123_HFGoe_H4_b1R1_1h **Scan** 17844
**Peptide** GRTLYGFGG **Charge** 2 **Spectral Angle** 0.73

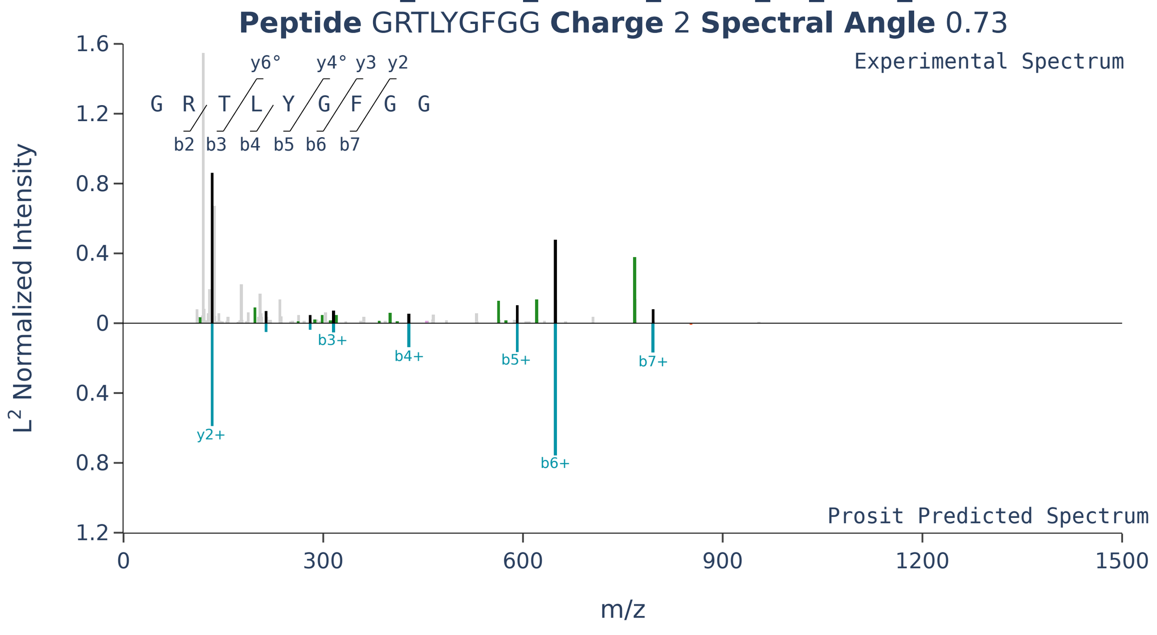


**Source** HRoetschke_291222_140123_HFGoe_H4_b1R1_2h **Scan** 15557
**Peptide** RDNIQGITKPAL **Charge** 2 **Spectral Angle** 0.82

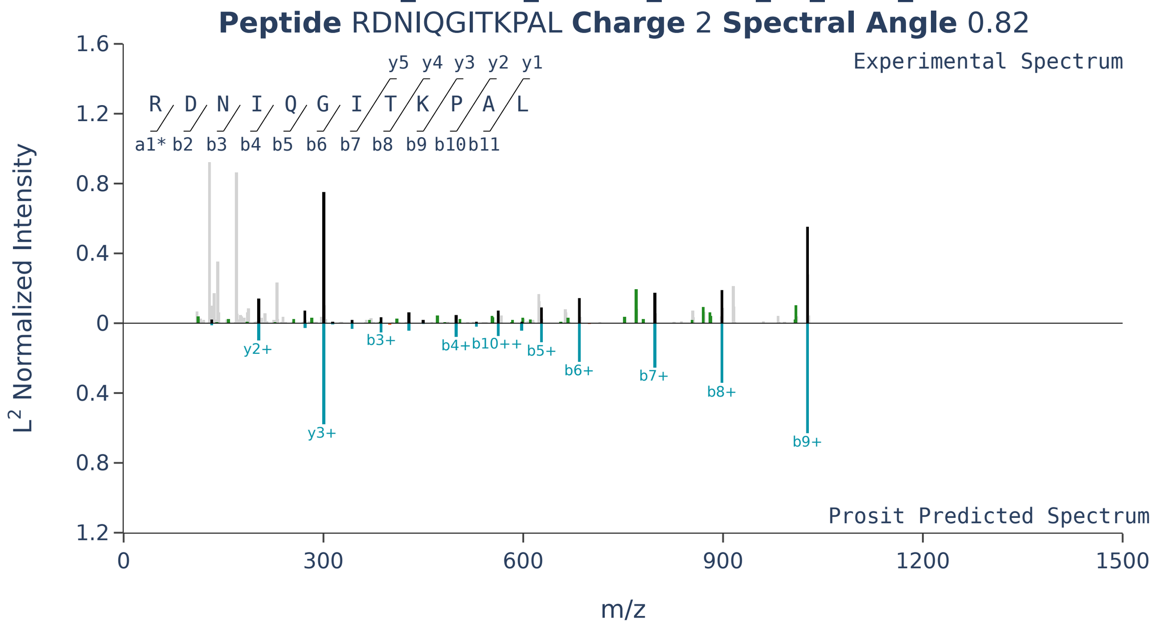


**Source** HRoetschke_291222_140123_HFGoe_H4_b2R1_24h **Scan** 12091
**Peptide** VIRDAVTYTE **Charge** 2 **Spectral Angle** 0.8

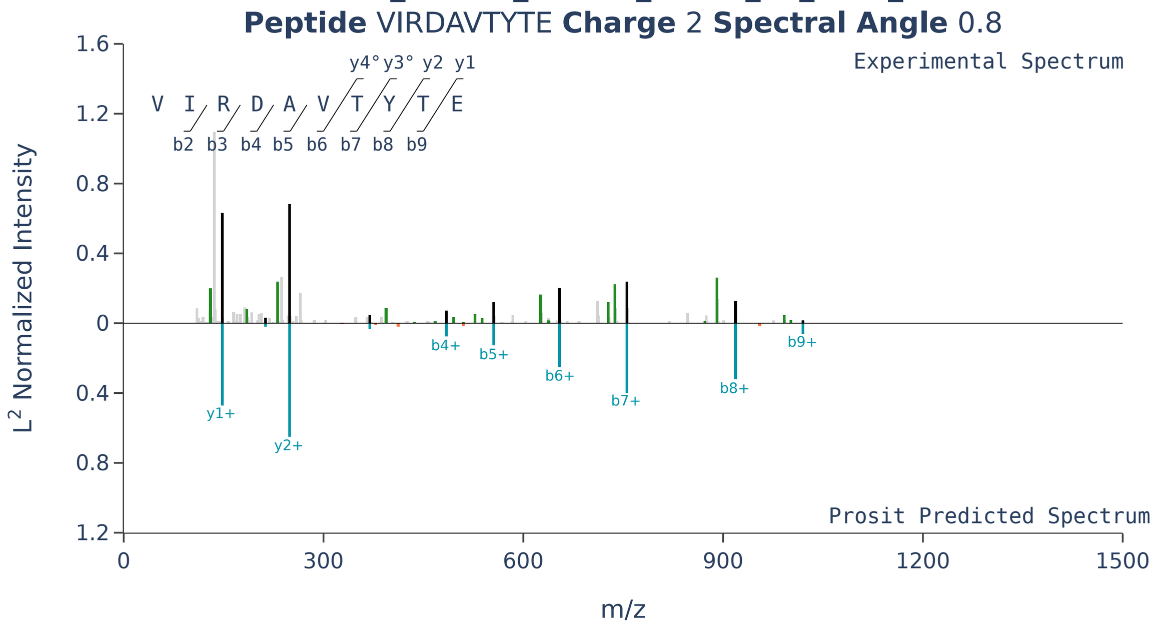


**Source** HRoetschke_291222_140123_HFGoe_H4_b1R1_4h **Scan** 6748
**Peptide** ALKRQGRTLYG **Charge** 3 **Spectral Angle** 0.88

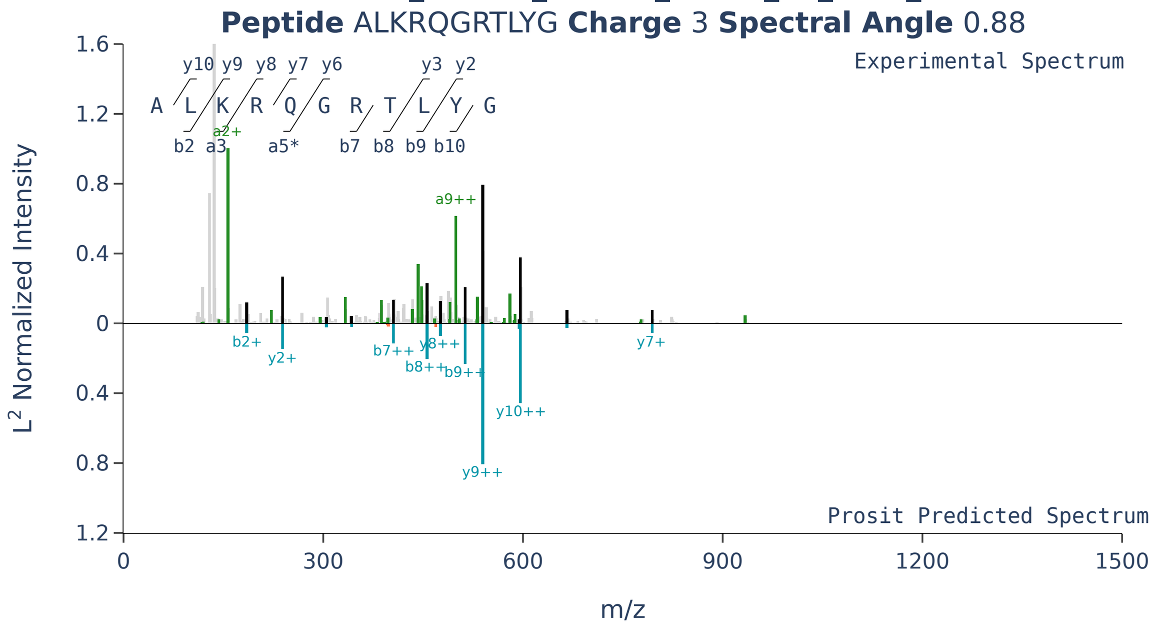


**Source** HRoetschke_291222_140123_HFGoe_H4_b2R1_1h **Scan** 31569
**Peptide** SGLIYEETRGVLKVFL **Charge** 2 **Spectral Angle** 0.79

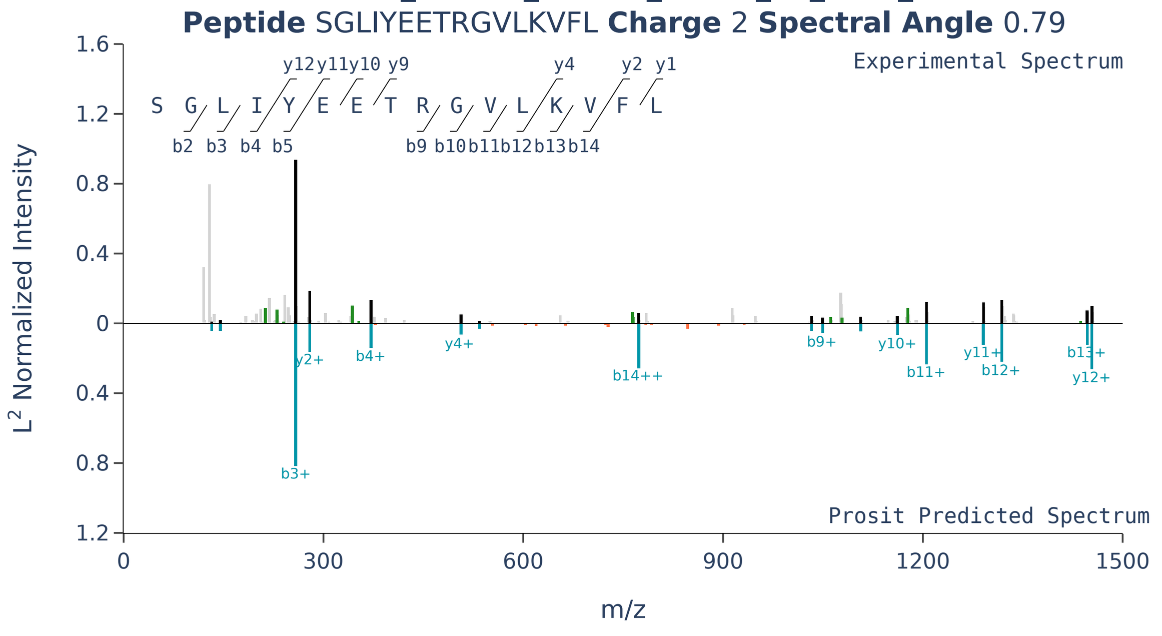


**Source** HRoetschke_291222_140123_HFGoe_H4_b2R1_1h **Scan** 26429
**Peptide** KVFLENVIRDAVTY **Charge** 2 **Spectral Angle** 0.84

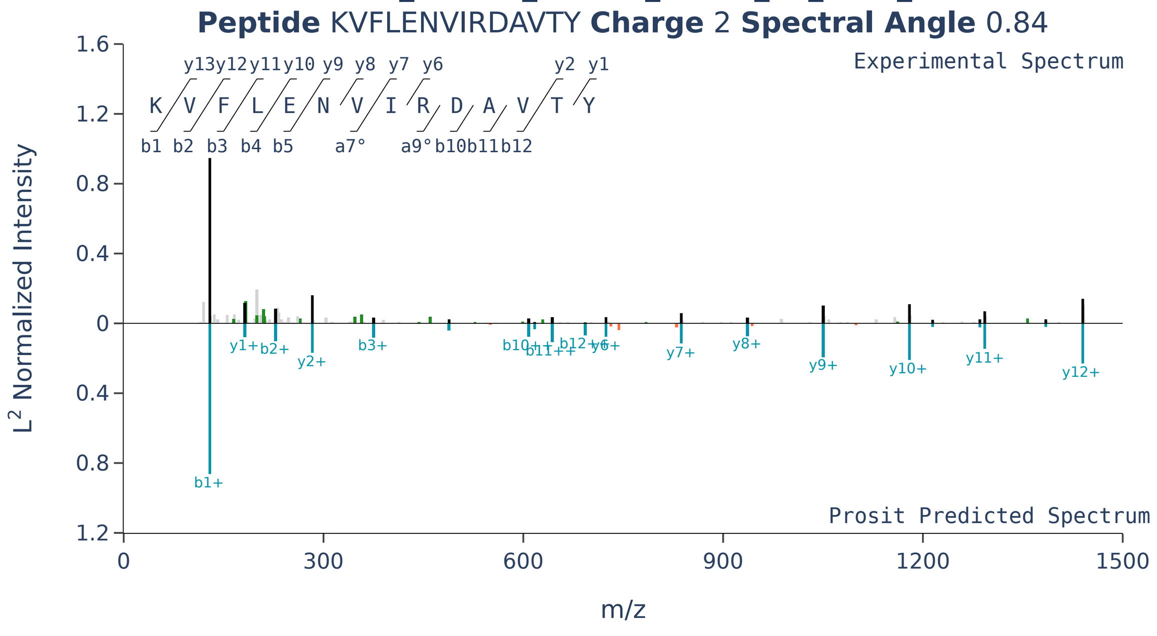


**Source** HRoetschke_291222_140123_HFGoe_H4_b1R2_1h **Scan** 20210
**Peptide** LENVIRDAVTYTEH **Charge** 2 **Spectral Angle** 0.88

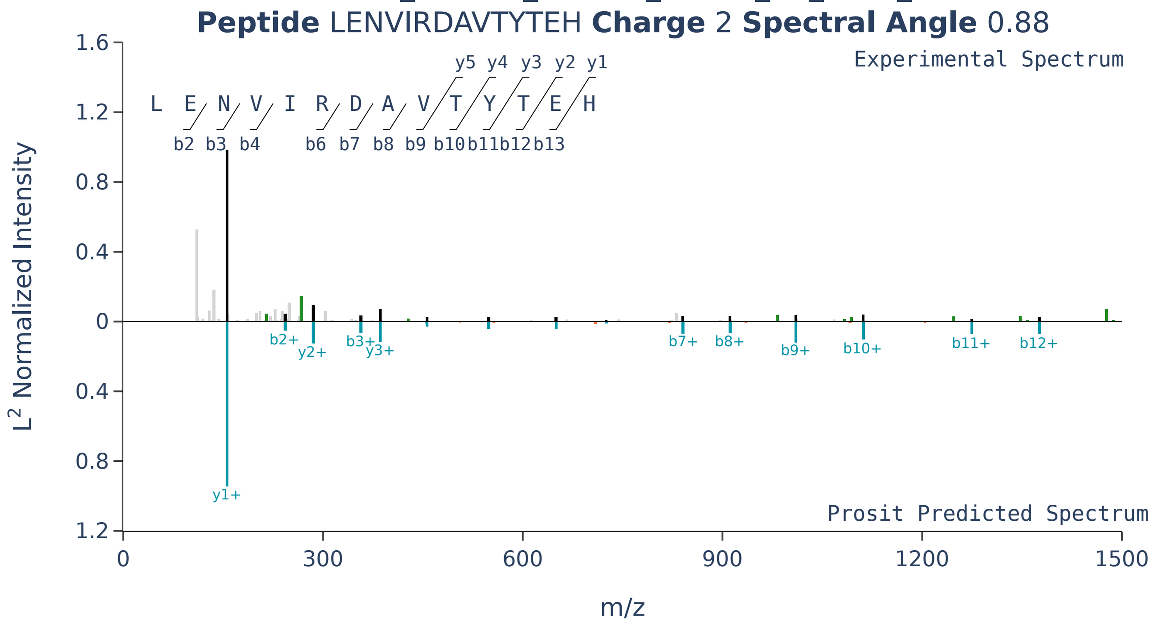


**Source** HRoetschke_291222_140123_HFGoe_H4_b1R2_24h **Scan** 5310
**Peptide** VTYTEHAKRKTV **Charge** 4 **Spectral Angle** 0.85

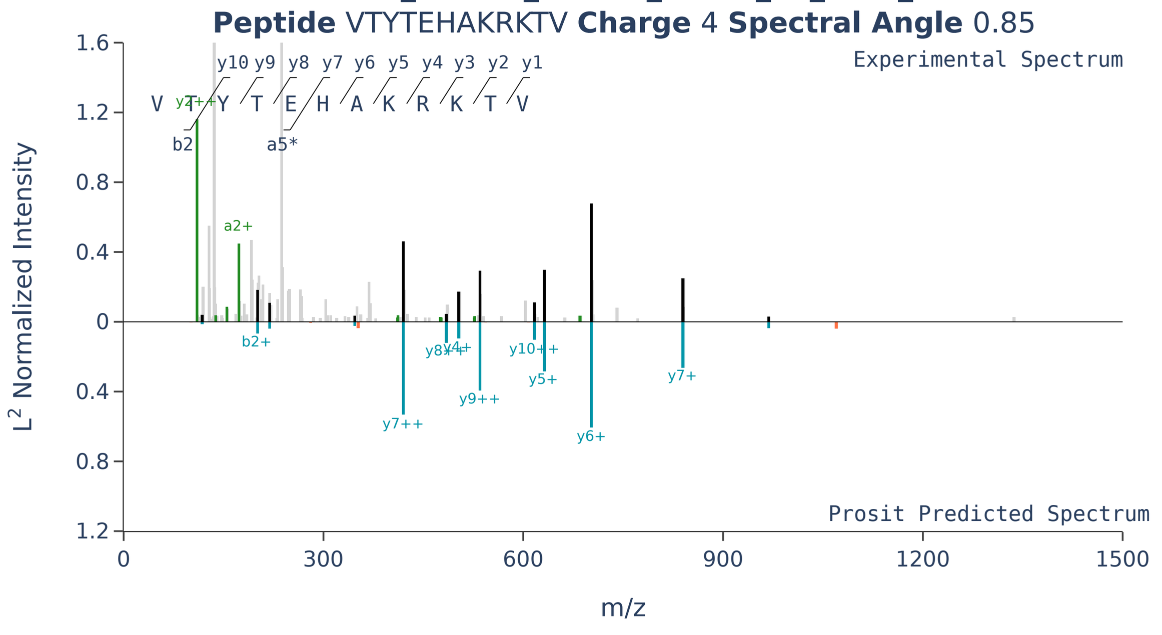


**Source** HRoetschke_291222_140123_HFGoe_H4_b1R1_1h **Scan** 19247
**Peptide** RLSGLIYEET **Charge** 2 **Spectral Angle** 0.8

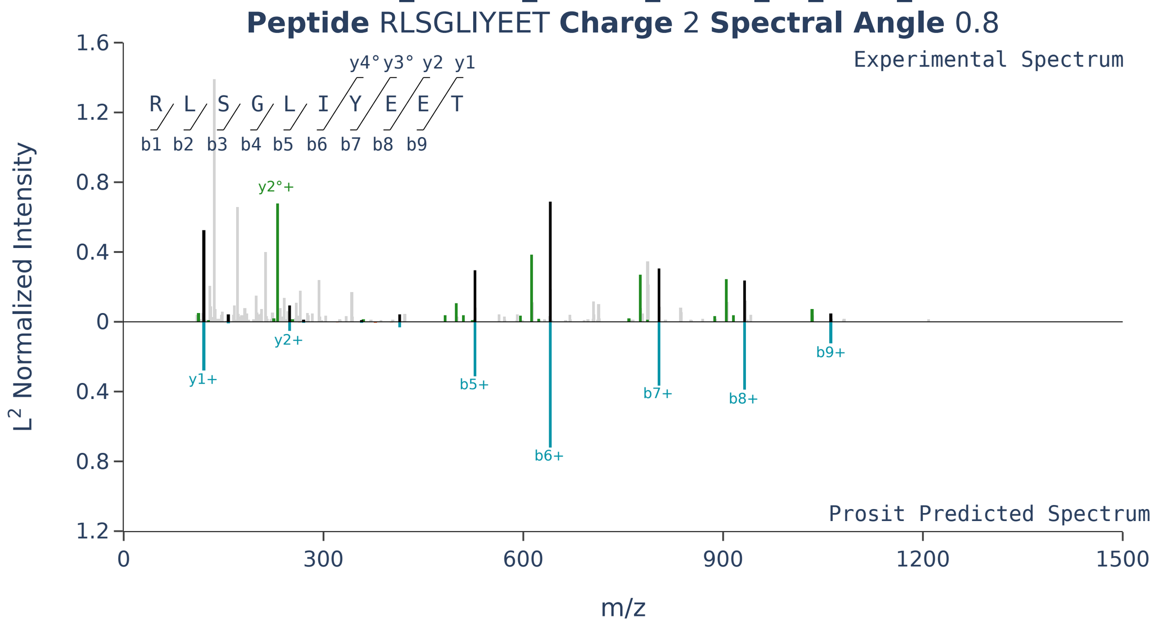


**Source** HRoetschke_291222_140123_HFGoe_H4_b2R2_1h **Scan** 24560
**Peptide** RGVLKVFLENVIR **Charge** 3 **Spectral Angle** 0.83

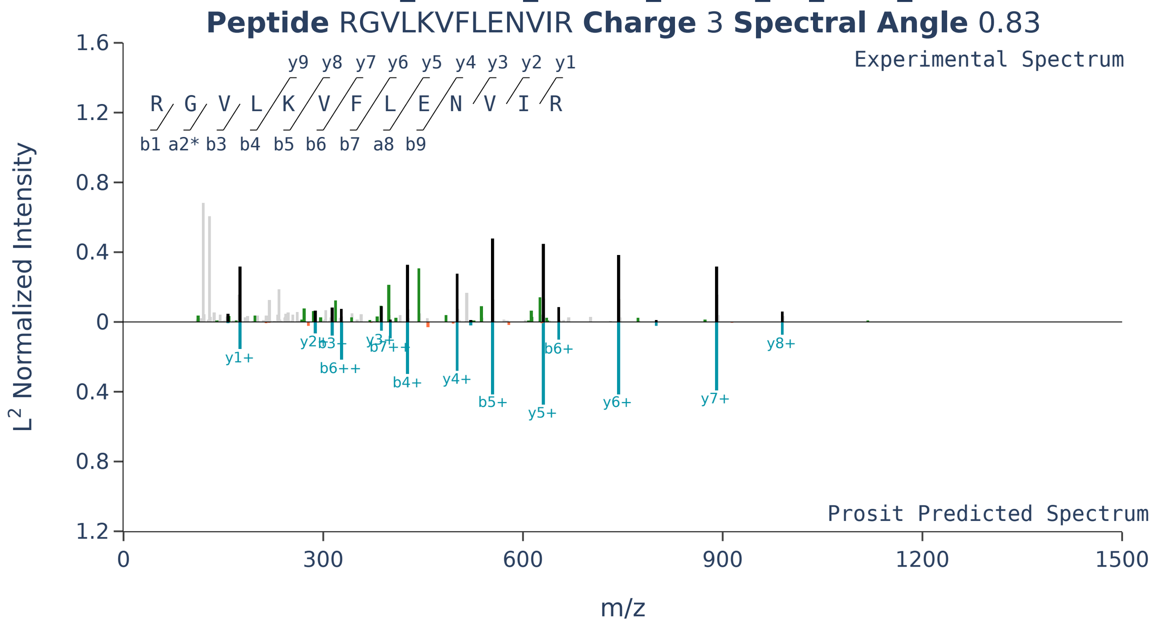


**Source** HRoetschke_291222_140123_HFGoe_H4_b1R2_24h **Scan** 8879
**Peptide** DNIQGITK **Charge** 2 **Spectral Angle** 0.88

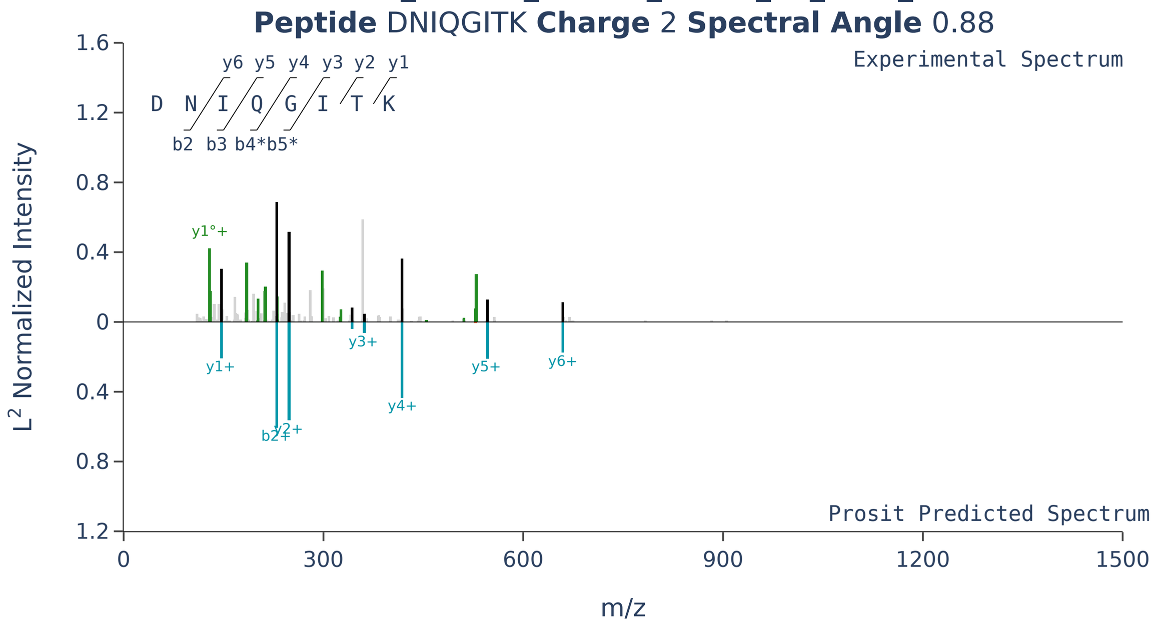


**Source** HRoetschke_291222_140123_HFGoe_H4_b1R1_4h **Scan** 19623
**Peptide** TAMDVVYA **Charge** 1 **Spectral Angle** 0.92

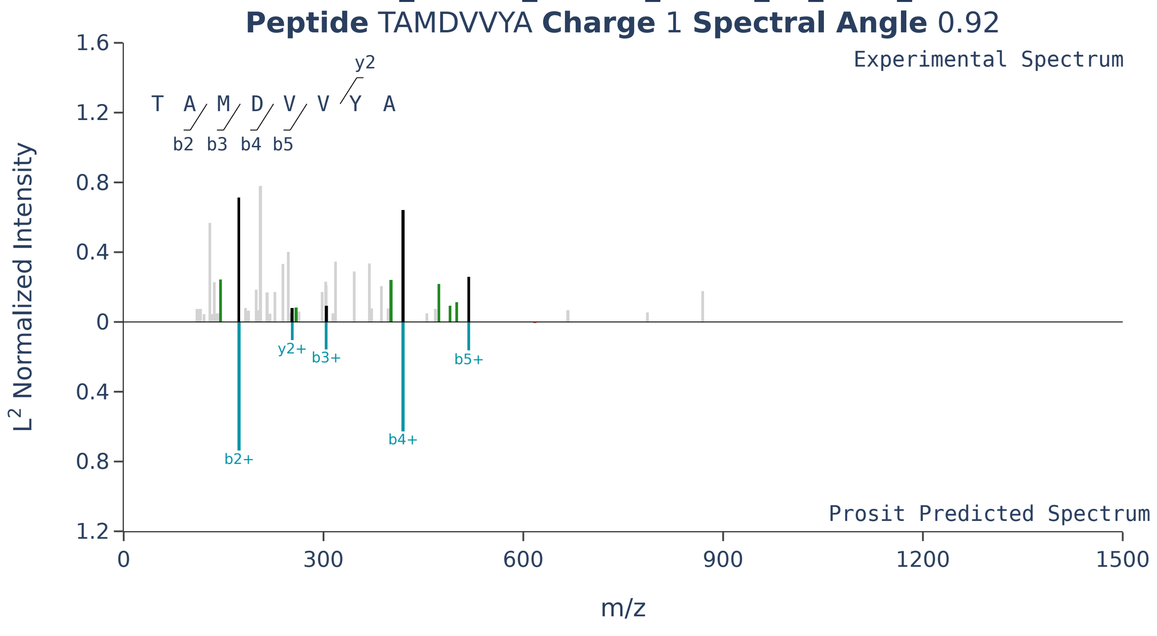

**Source** HRoetschke_291222_140123_HFGoe_H4_b2R2_1h **Scan** 23528
**Peptide** LKVFLENVIRD **Charge** 3 **Spectral Angle** 0.89

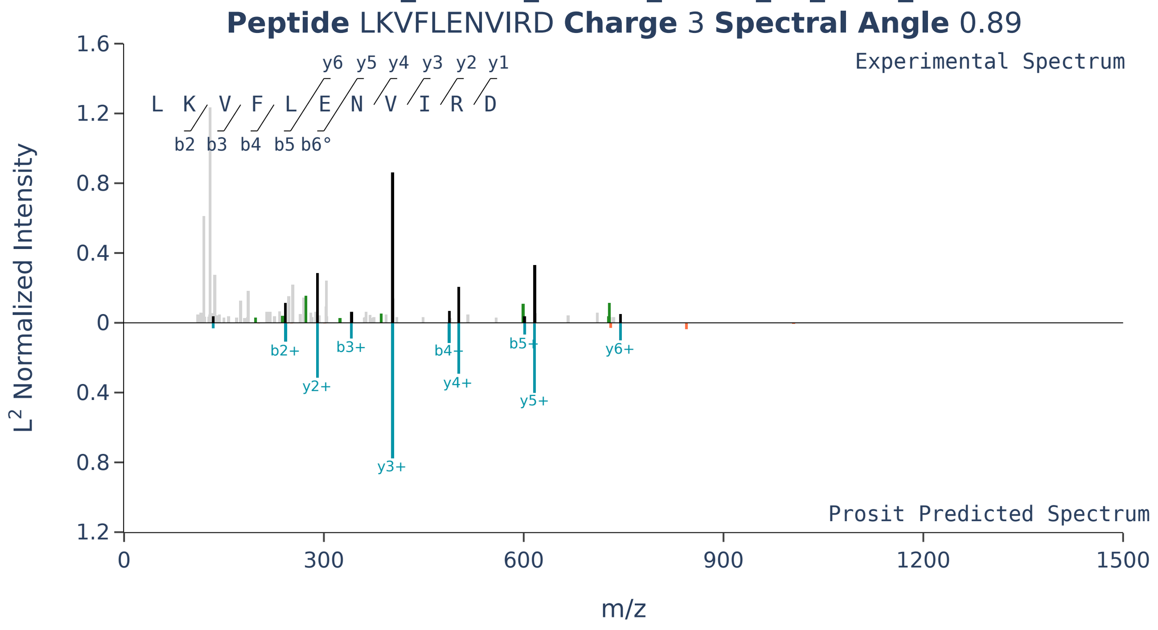


**Source** HRoetschke_291222_140123_HFGoe_H4_b1R1_2h **Scan** 11507
**Peptide** KRKTVTAMDVVY **Charge** 2 **Spectral Angle** 0.83

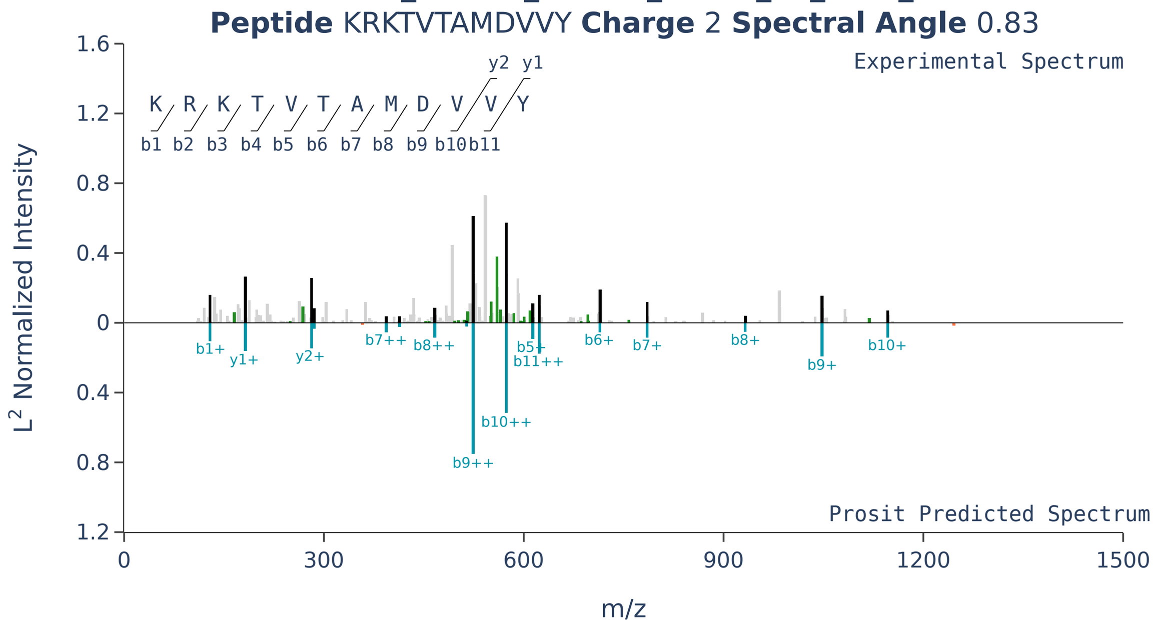


**Source** HRoetschke_291222_140123_HFGoe_H4_b2R2_4h **Scan** 18676
**Peptide** ALKRQGRTL **Charge** 2 **Spectral Angle** 0.84

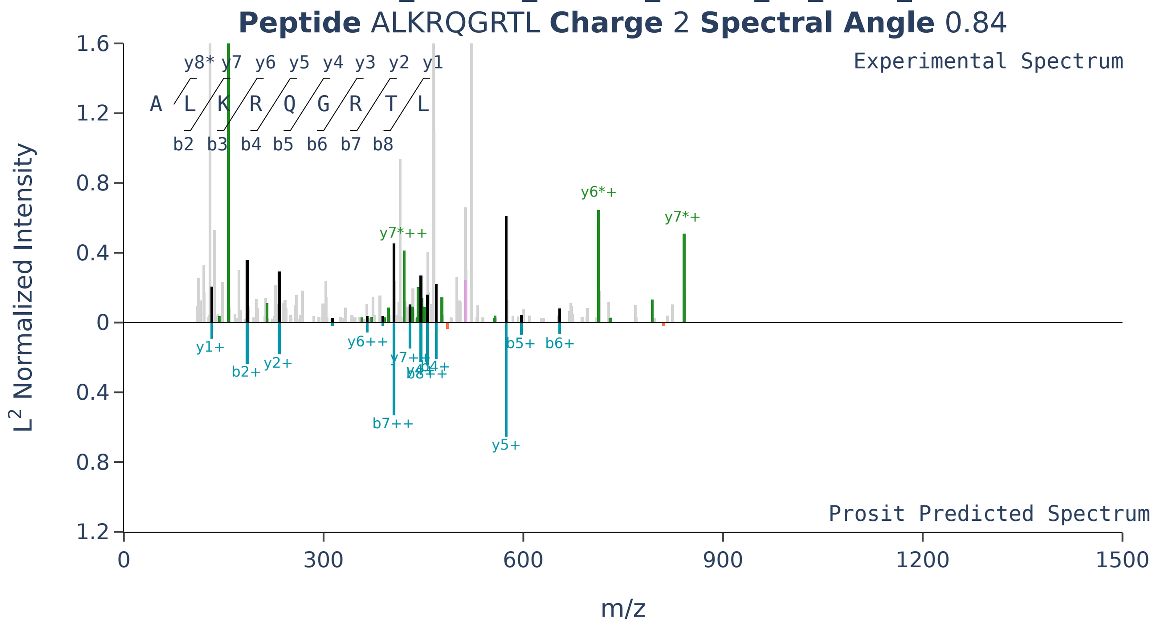


**Source** HRoetschke_291222_140123_HFGoe_H4_b1R2_4h **Scan** 17626
**Peptide** TVTAMDVVY **Charge** 2 **Spectral Angle** 0.92

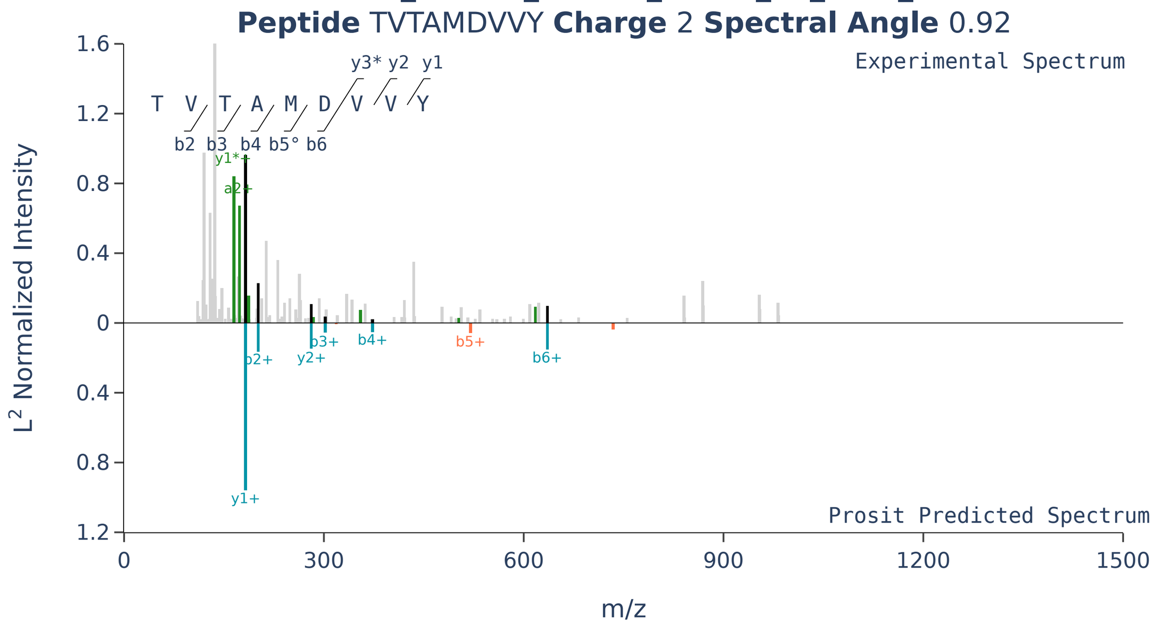


**Source** HRoetschke_291222_140123_HFGoe_H4_b2R1_1h **Scan** 7019
**Peptide** RDNIQGITK **Charge** 2 **Spectral Angle** 0.86

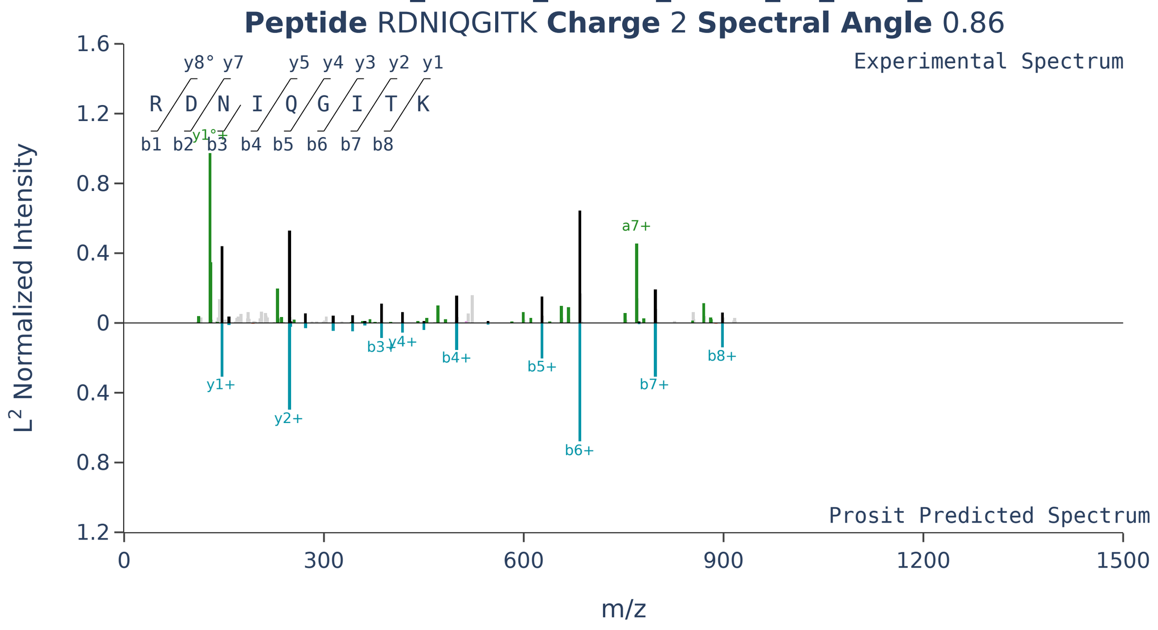


**Source** HRoetschke_291222_140123_HFGoe_H4_b1R1_2h **Scan** 6046
**Peptide** RDAVTYTEHAKRK **Charge** 4 **Spectral Angle** 0.77

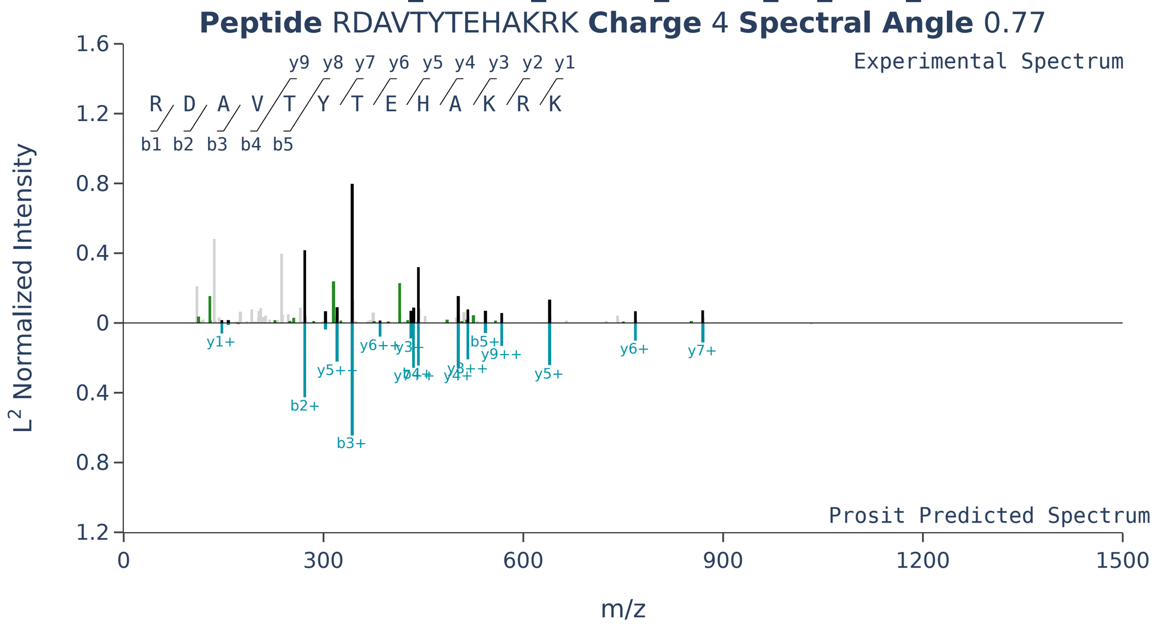


**Source** HRoetschke_291222_140123_HFGoe_H4_b2R2_2h **Scan** 12769
**Peptide** YEETRGVLKV **Charge** 2 **Spectral Angle** 0.82

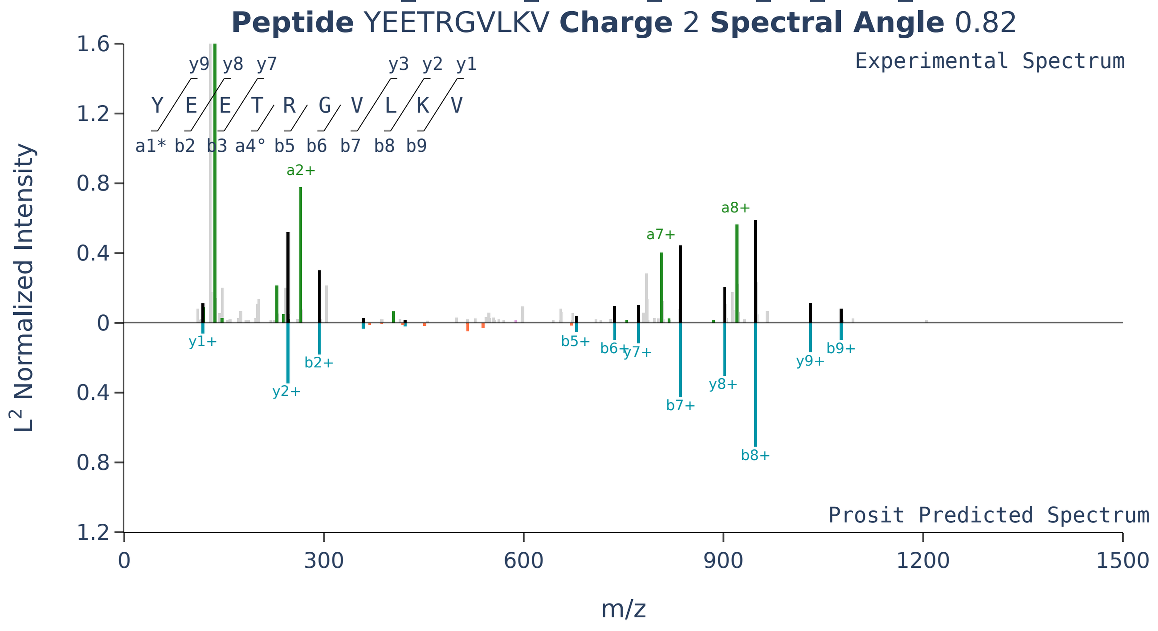


**Source** HRoetschke_291222_140123_HFGoe_H4_b2R1_2h **Scan** 17029
**Peptide** LRDNIQGITKPAI **Charge** 3 **Spectral Angle** 0.85

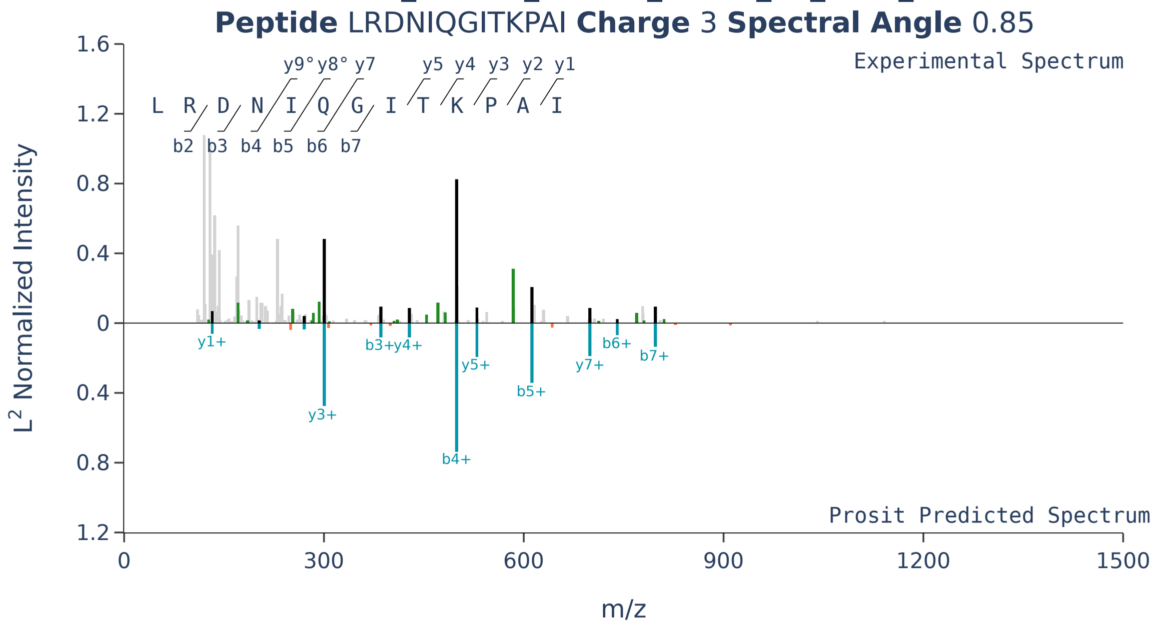


**Source** HRoetschke_291222_140123_HFGoe_H4_b2R1_1h **Scan** 27125
**Peptide** SGLIYEETRGVLKVF **Charge** 2 **Spectral Angle** 0.79

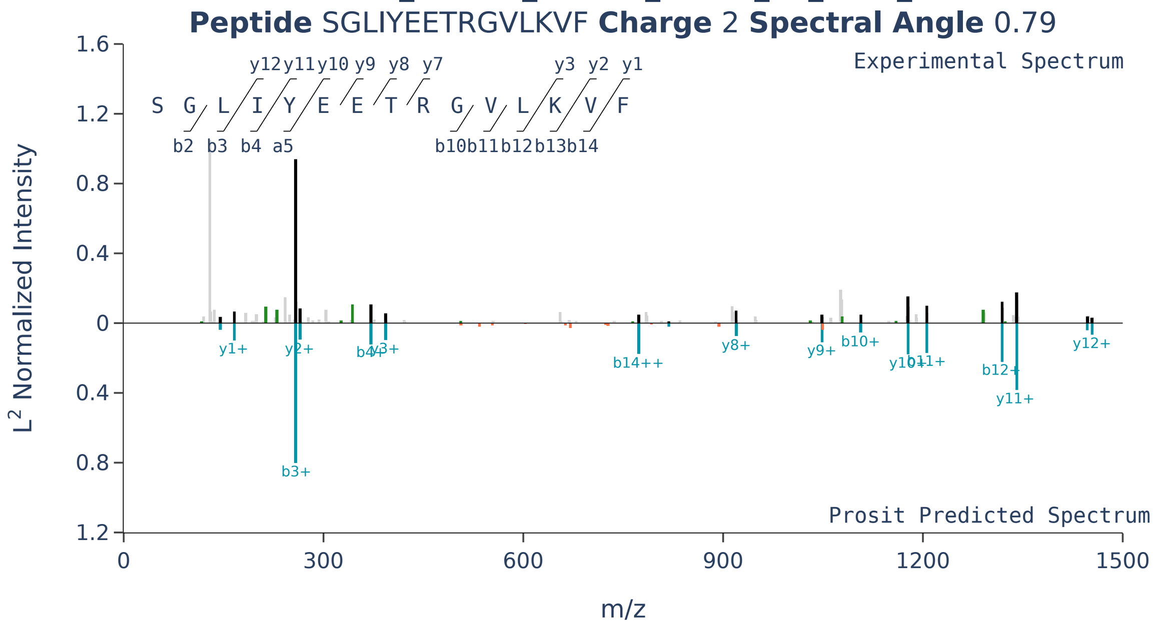


**Source** HRoetschke_291222_140123_HFGoe_H4_b1R1_24h **Scan** 12135
**Peptide** ALKRQGRTLYGFGG **Charge** 4 **Spectral Angle** 0.82

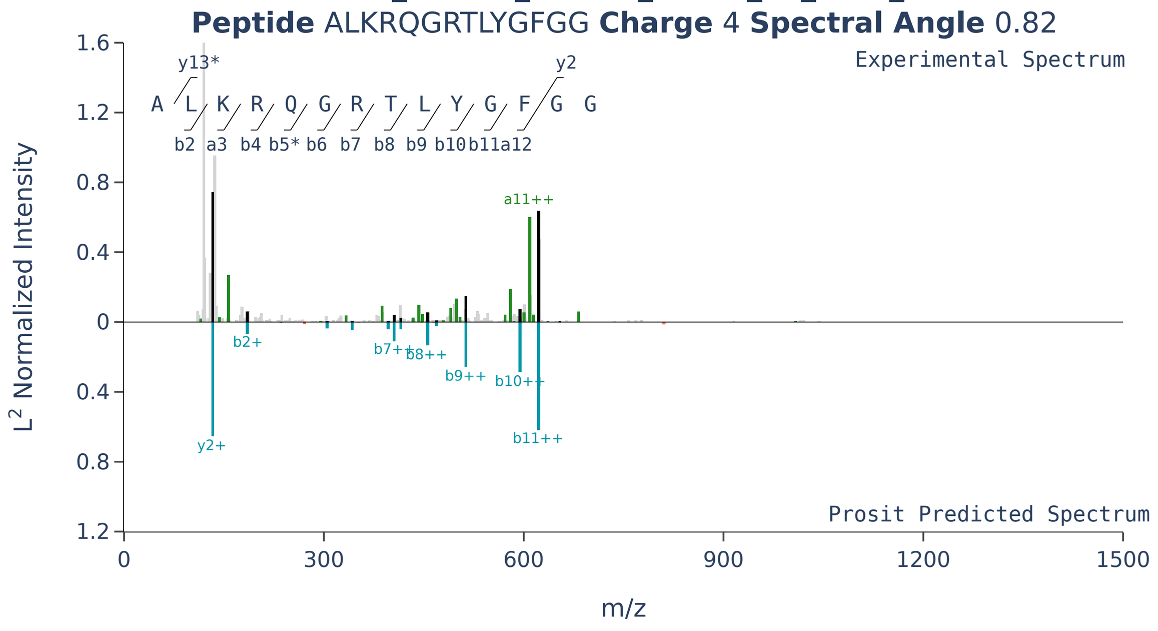


**Source** HRoetschke_291222_140123_HFGoe_H4_b1R2_4h **Scan** 18994
**Peptide** GRTLYGF **Charge** 2 **Spectral Angle** 0.89

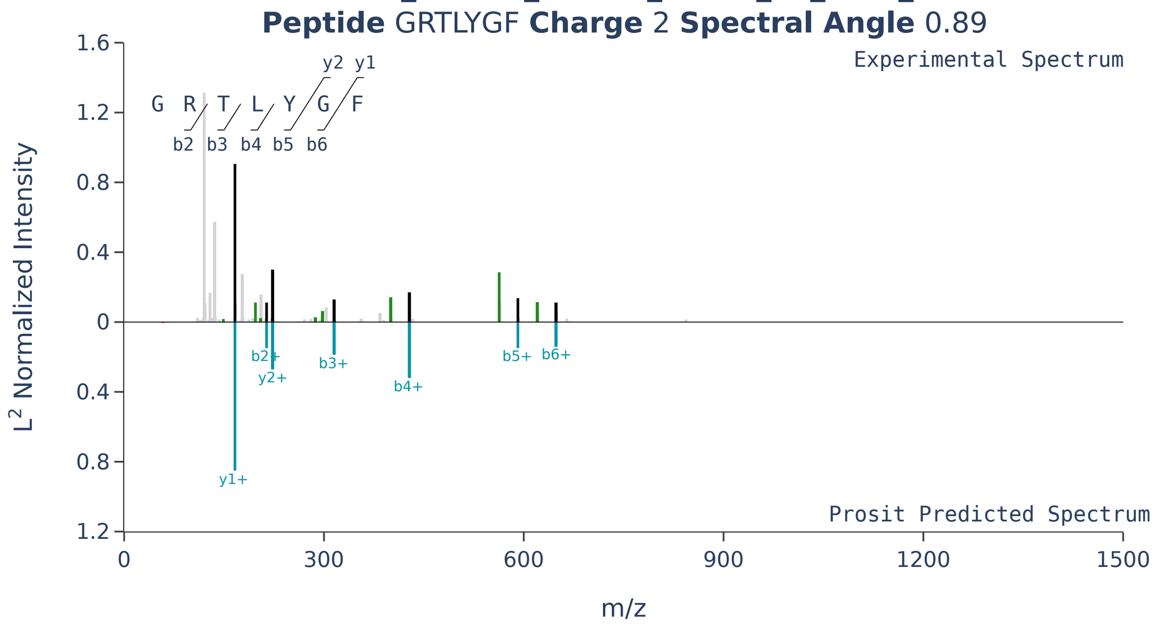


**Source** HRoetschke_291222_140123_HFGoe_H4_b1R2_2h **Scan** 25895
**Peptide** RGVLKVFLENVIRD **Charge** 3 **Spectral Angle** 0.83

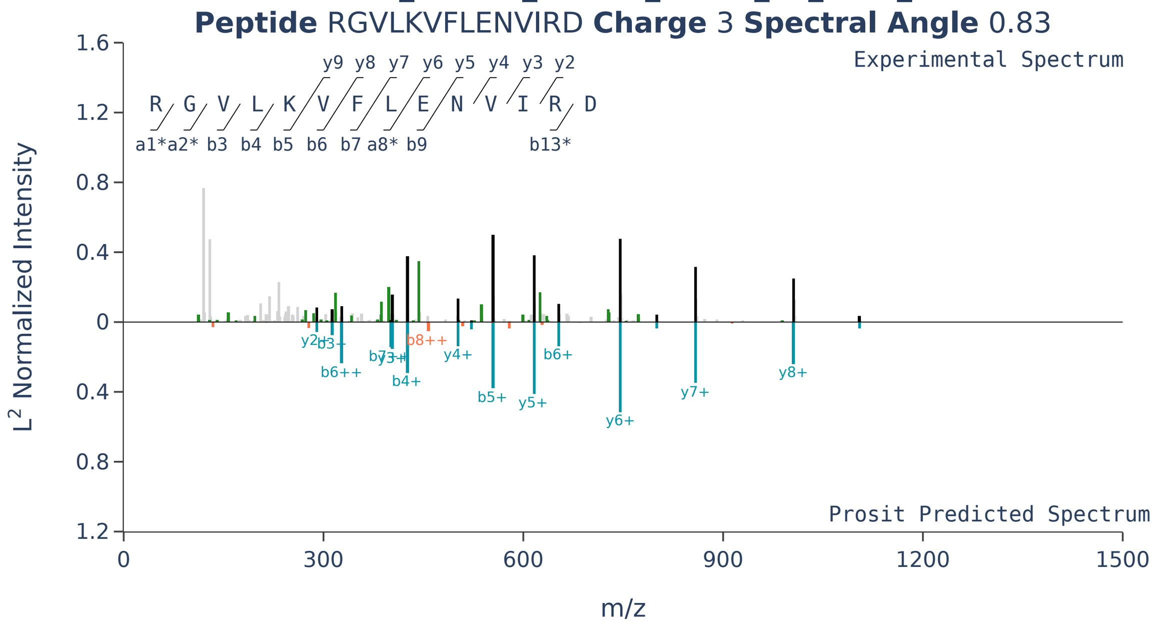


**Source** HRoetschke_291222_140123_HFGoe_H4_b2R2_1h **Scan** 21099
**Peptide** TLYGFGG **Charge** 1 **Spectral Angle** 0.87

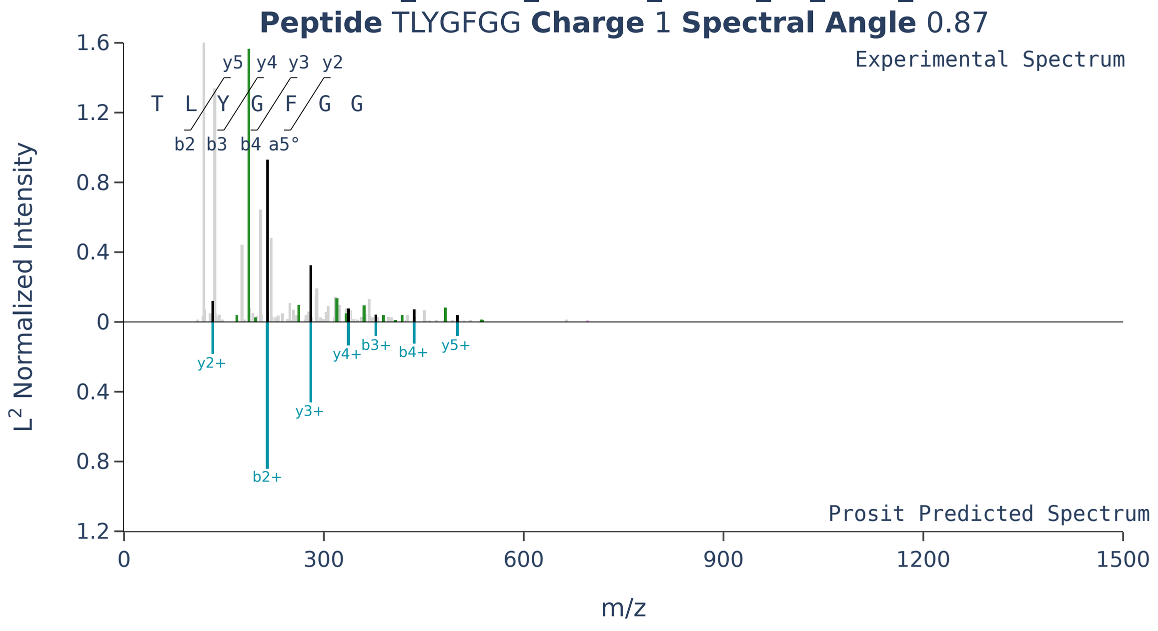


**Source** HRoetschke_291222_140123_HFGoe_H4_b2R1_1h **Scan** 5305
**Peptide** RDAVTYTEHAKR **Charge** 4 **Spectral Angle** 0.77

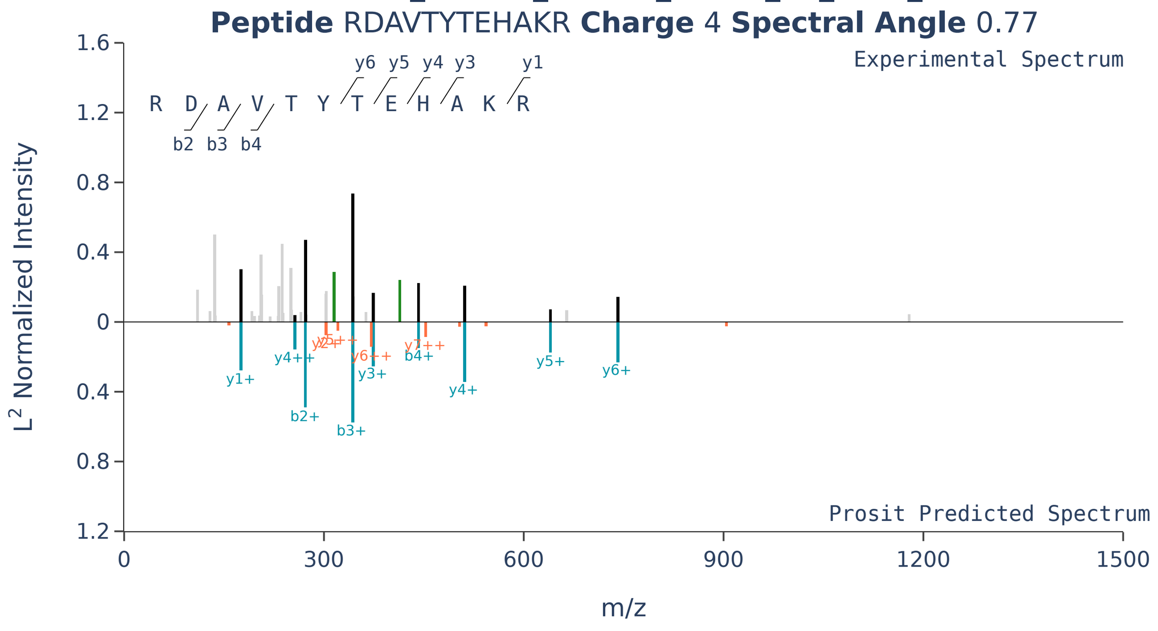


**Source** HRoetschke_291222_140123_HFGoe_H4_b1R1_1h **Scan** 18107
**Peptide** LSGLIYEETR **Charge** 2 **Spectral Angle** 0.77

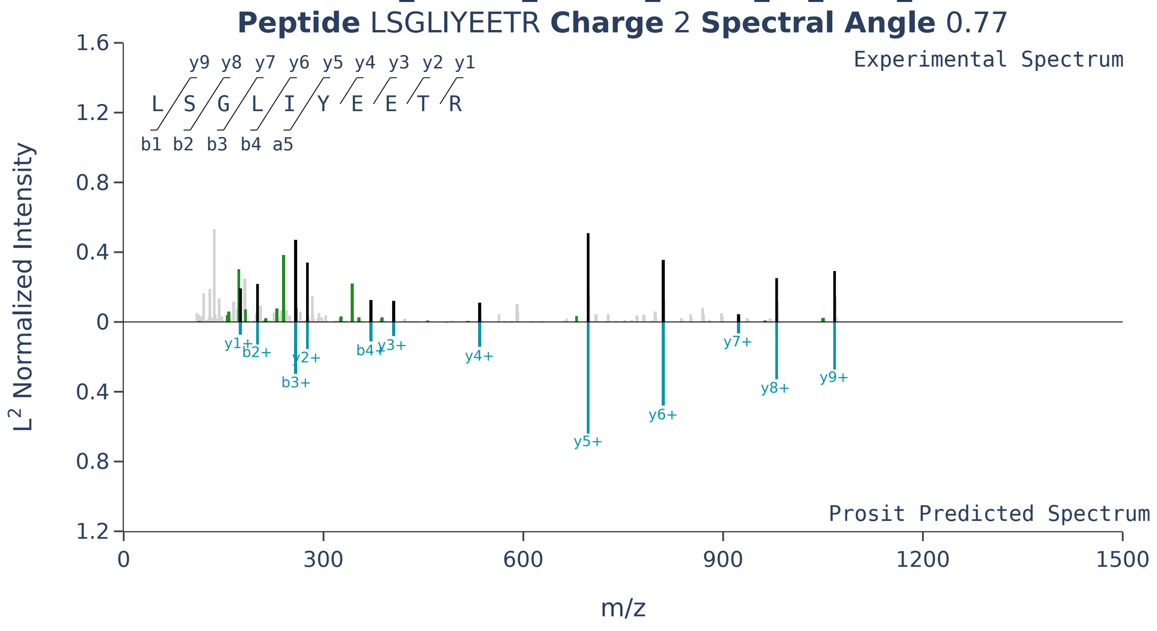

**Source** HRoetschke_291222_140123_HFGoe_H4_b1R2_2h **Scan** 12986
**Peptide** VVYALKRQGRTLYG **Charge** 4 **Spectral Angle** 0.83

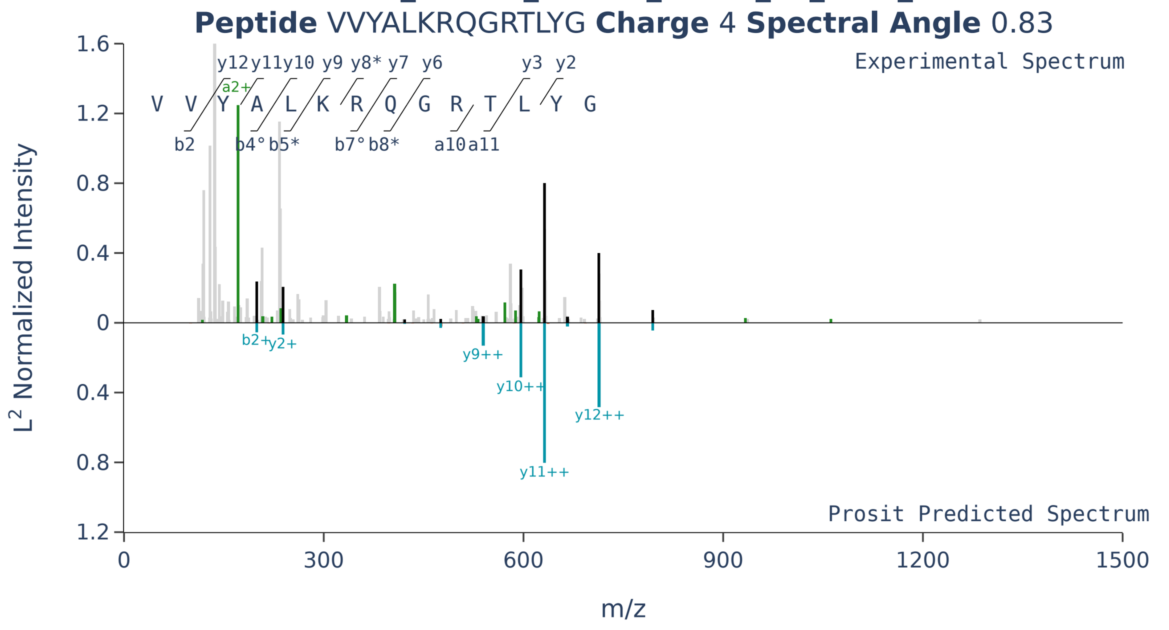


**Source** HRoetschke_291222_140123_HFGoe_H4_b1R1_2h **Scan** 14261
**Peptide** KRKTVTAMDVVYA **Charge** 2 **Spectral Angle** 0.74

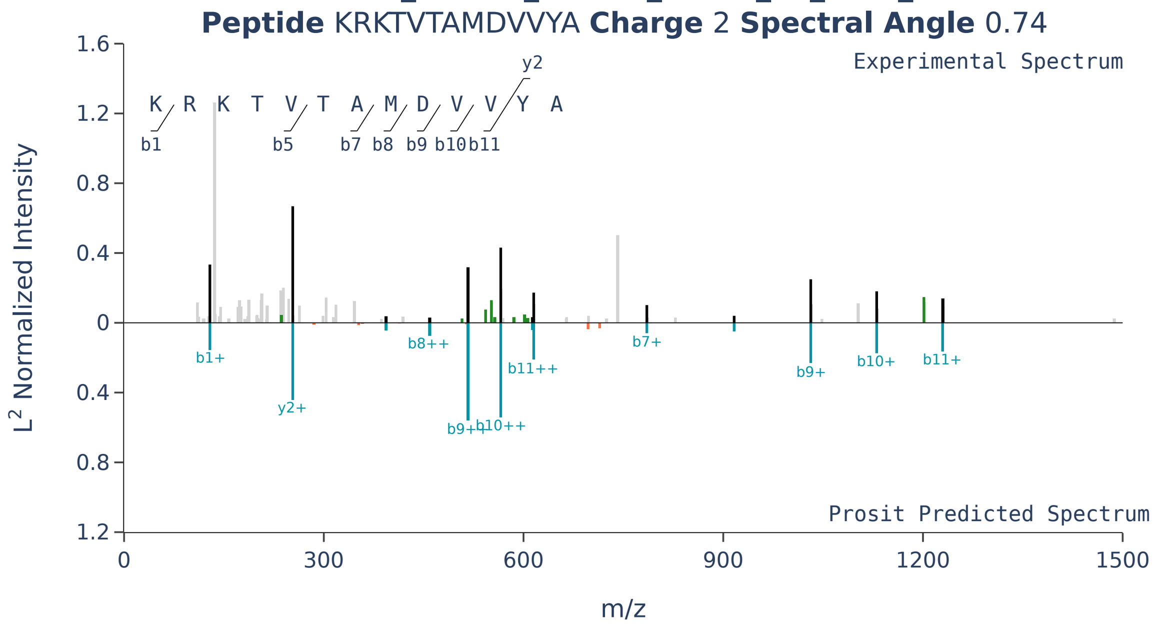


**Source** HRoetschke_291222_140123_HFGoe_H4_b2R1_4h **Scan** 14955
**Peptide** AMDVVYALKRQGRT **Charge** 4 **Spectral Angle** 0.9

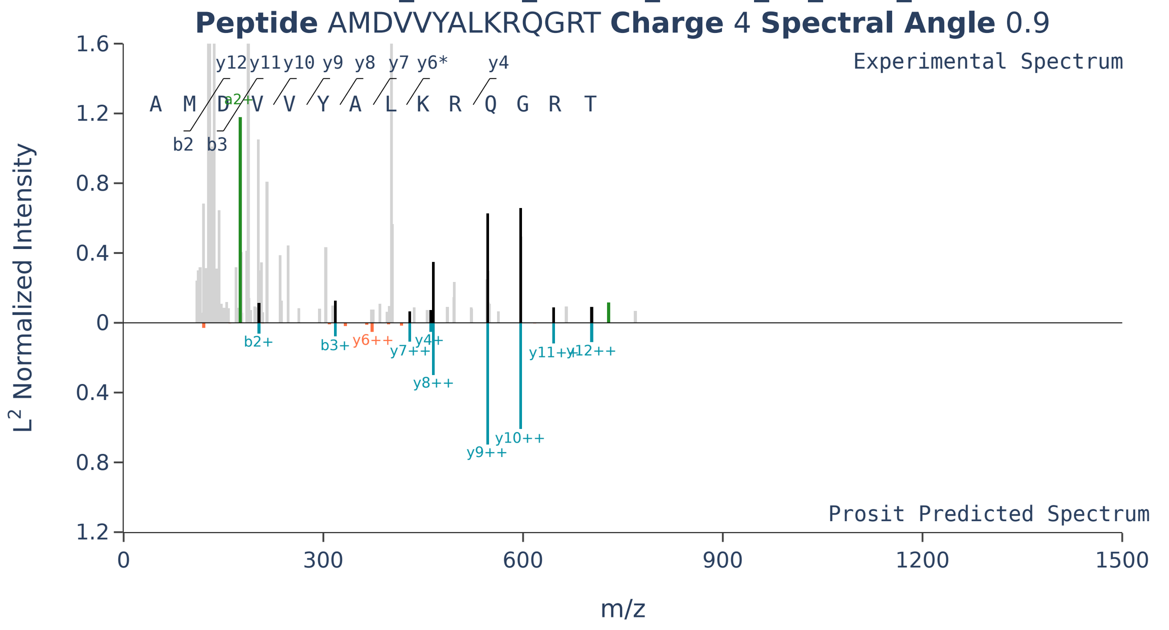


**Source** HRoetschke_291222_140123_HFGoe_H4_b2R1_24h **Scan** 11898
**Peptide** DNIQGITKP **Charge** 2 **Spectral Angle** 0.82

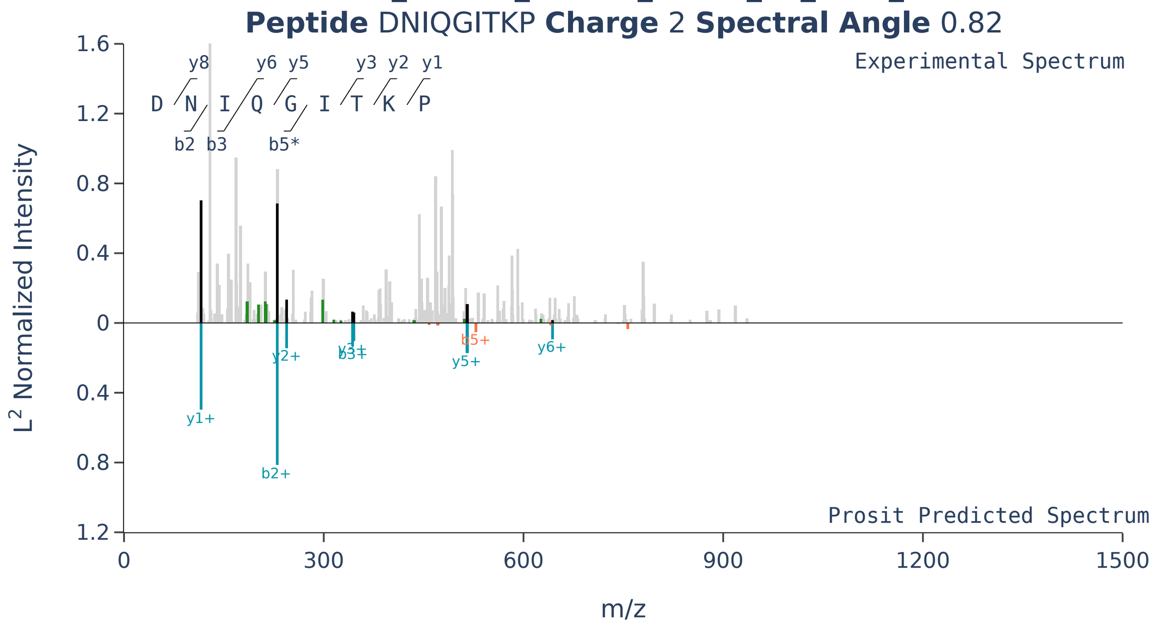


**Source** HRoetschke_291222_140123_HFGoe_H4_b2R1_1h **Scan** 27404
**Peptide** RGVLKVFLENVIRDA **Charge** 3 **Spectral Angle** 0.84

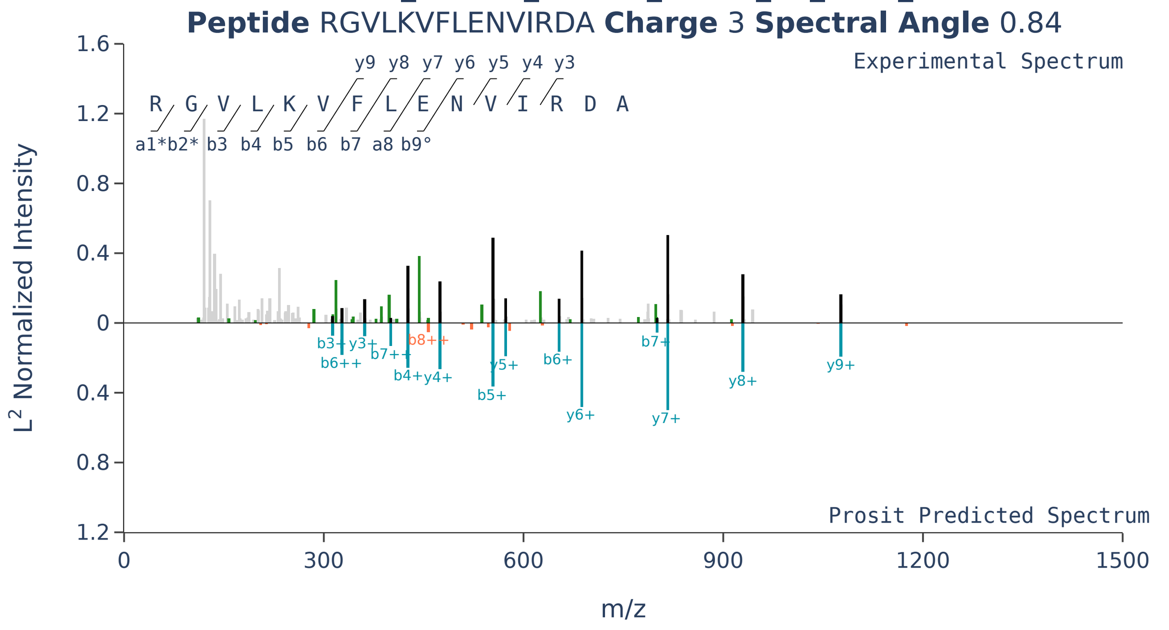


**Source** HRoetschke_291222_140123_HFGoe_H4_b2R2_24h **Scan** 12755
**Peptide** YEETRGVL **Charge** 2 **Spectral Angle** 0.9

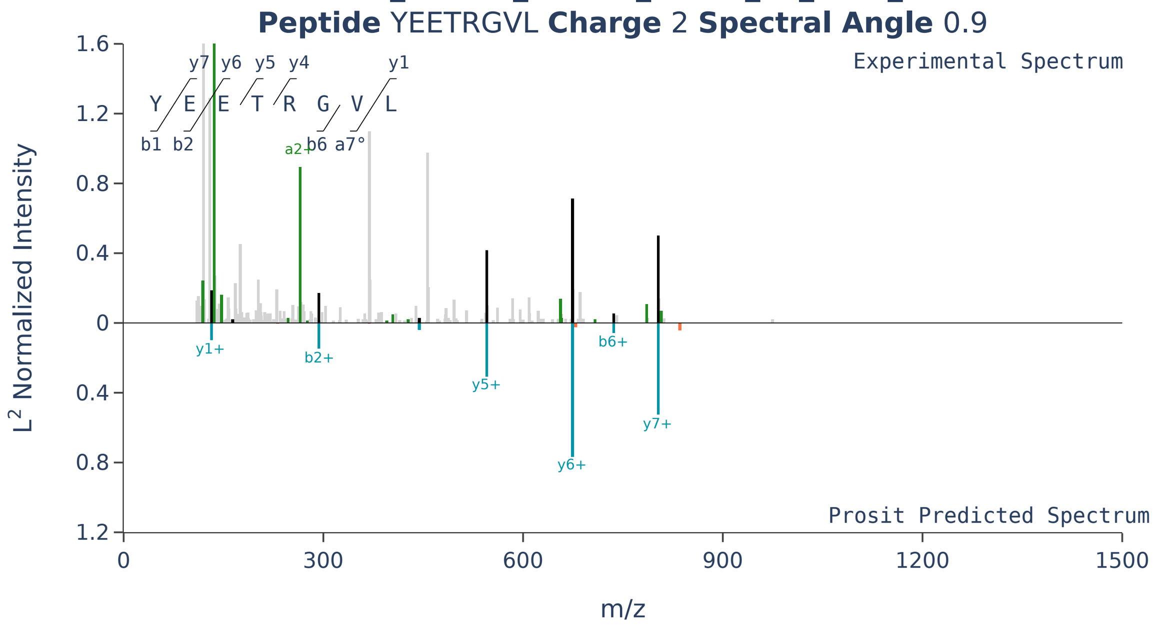


**Source** HRoetschke_291222_140123_HFGoe_H4_b2R1_2h **Scan** 17025
**Peptide** RTLYGFGG **Charge** 2 **Spectral Angle** 0.79

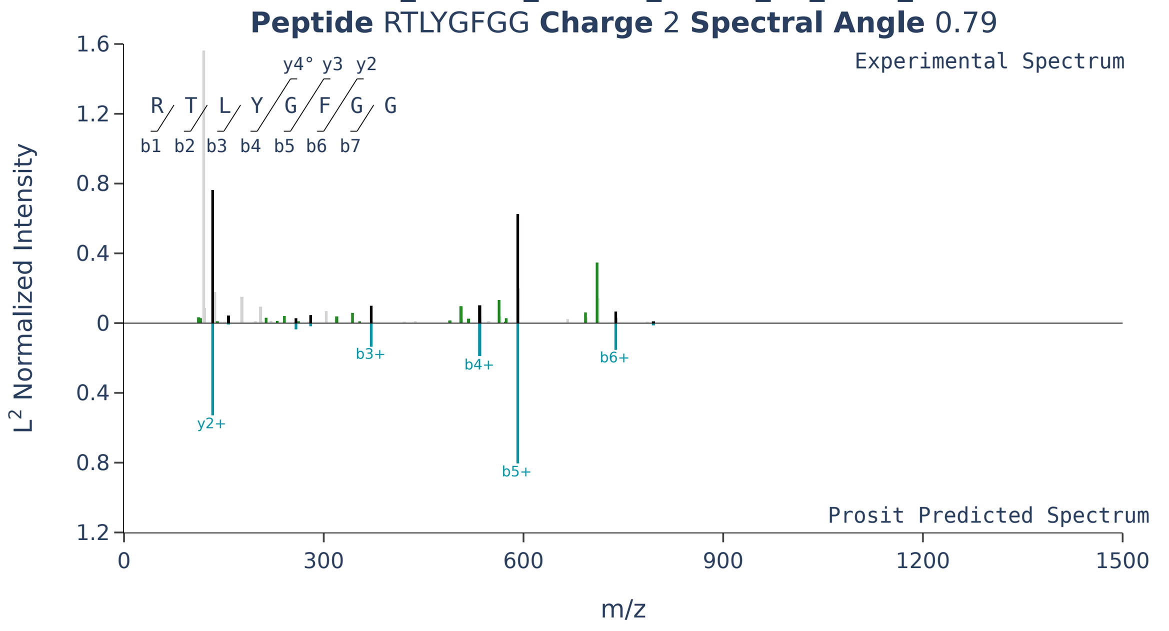


**Source** HRoetschke_291222_140123_HFGoe_H4_b2R2_1h **Scan** 9442
**Peptide** LTKPAIRRL **Charge** 2 **Spectral Angle** 0.91

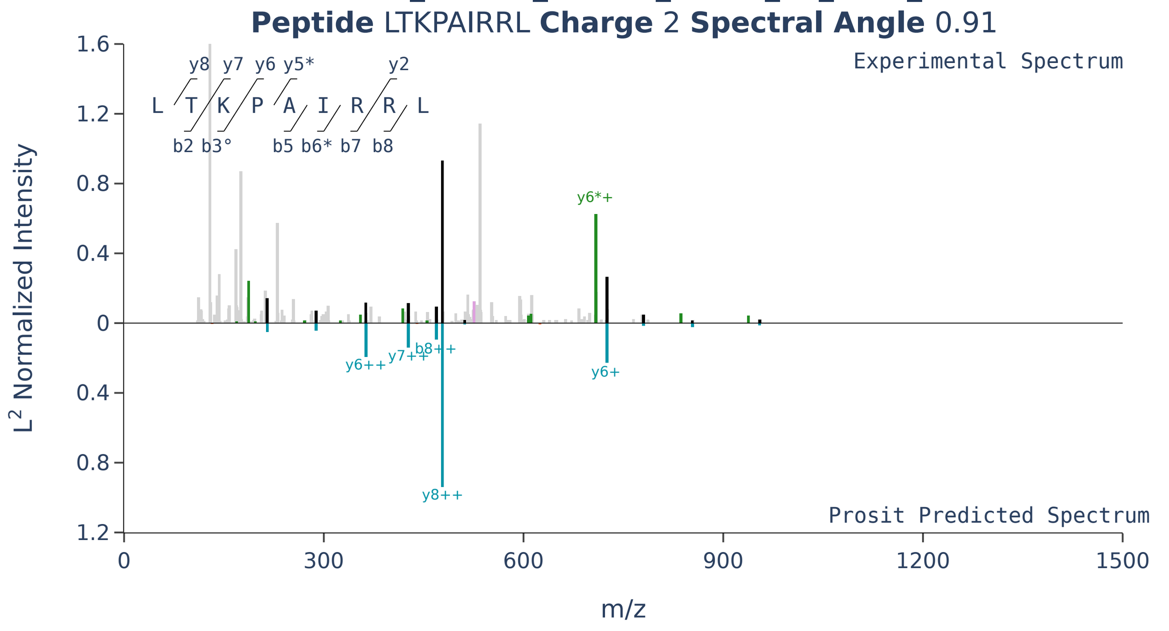


**Source** HRoetschke_291222_140123_HFGoe_H4_b1R1_24h **Scan** 5404
**Peptide** TYTEHAKRKTV **Charge** 2 **Spectral Angle** 0.76

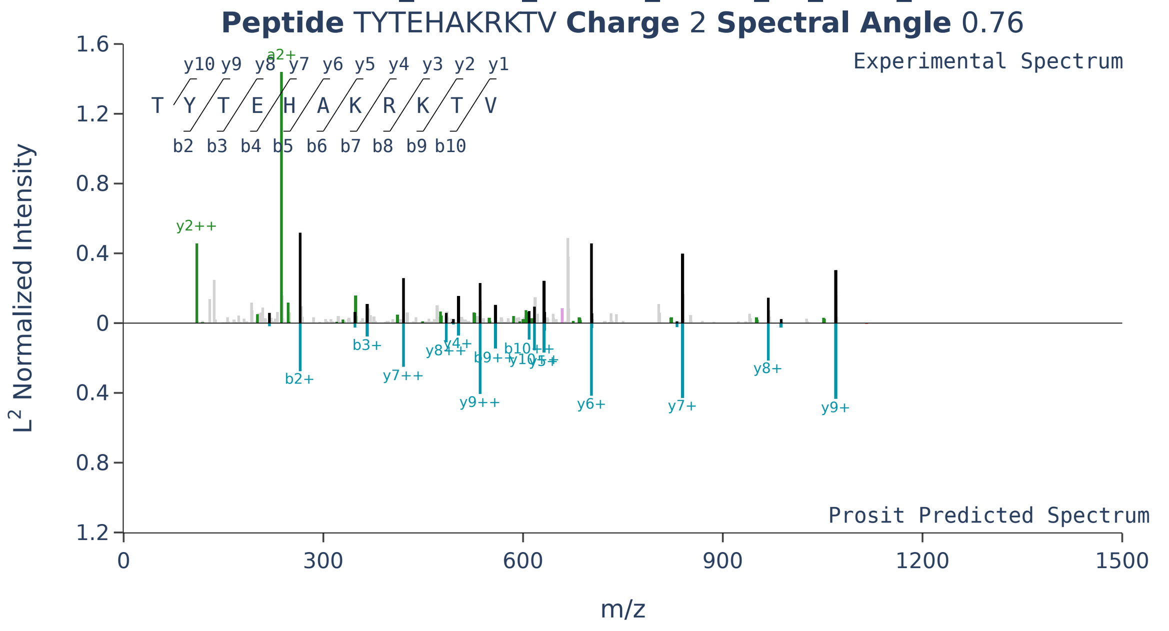


**Source** HRoetschke_291222_140123_HFGoe_H4_b1R2_2h **Scan** 16586
**Peptide** LYEETRGVL **Charge** 2 **Spectral Angle** 0.89

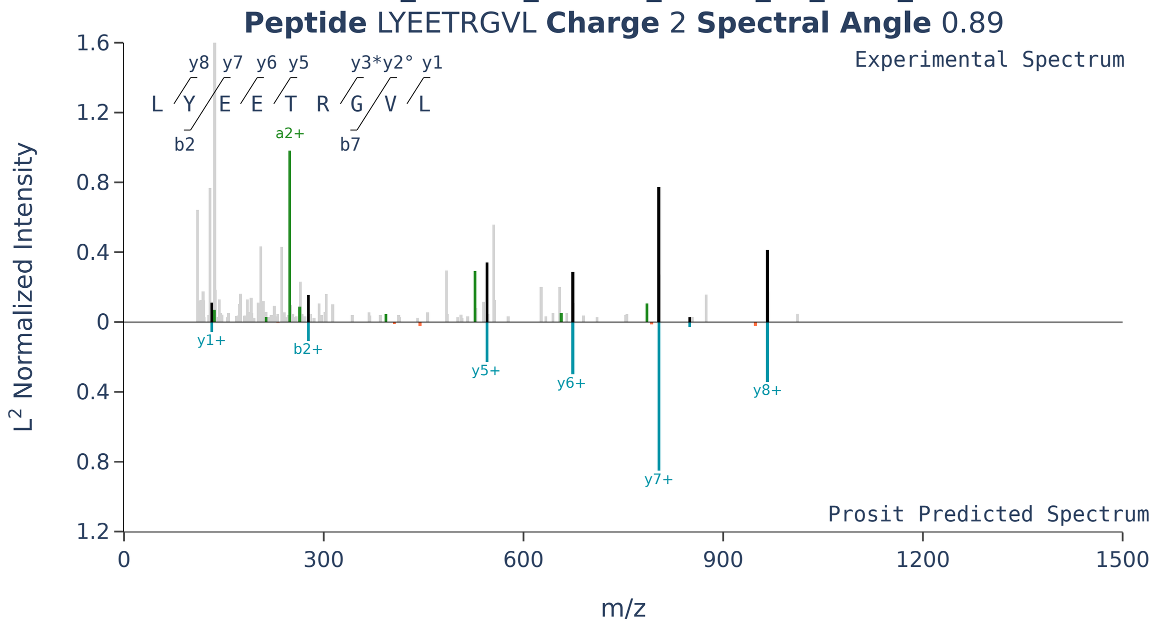


**Source** HRoetschke_291222_140123_HFGoe_H4_b2R1_1h **Scan** 22484
**Peptide** VFLENVIR **Charge** 2 **Spectral Angle** 0.82

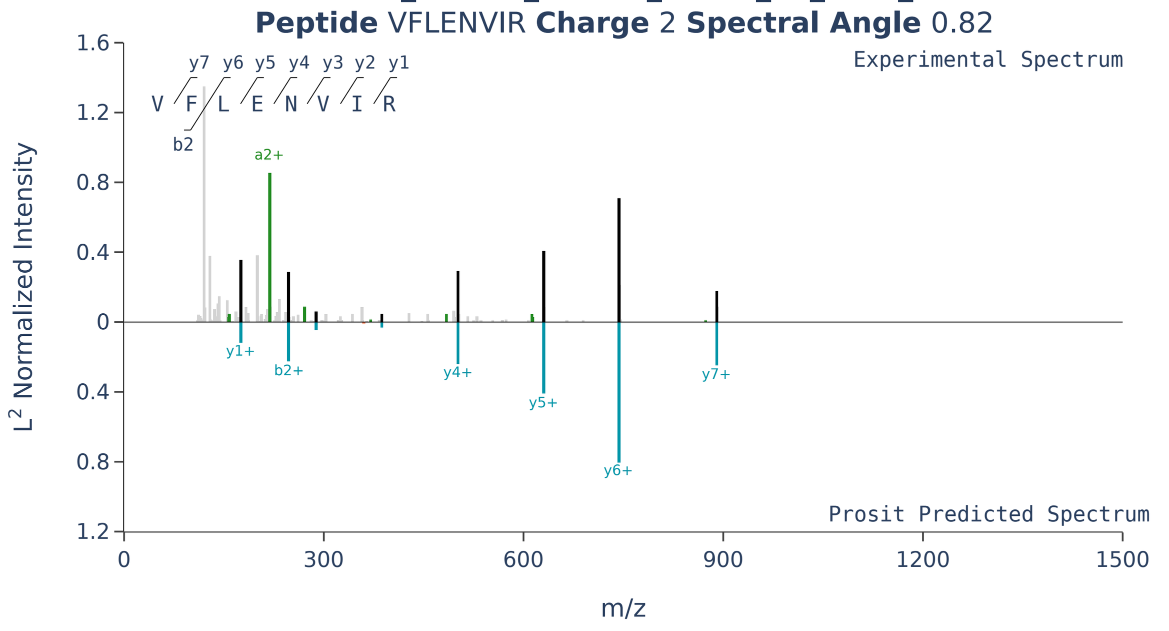


**Source** HRoetschke_291222_140123_HFGoe_H4_b2R2_0h **Scan** 19804
**Peptide** SGLIYEETRGVL **Charge** 2 **Spectral Angle** 0.73

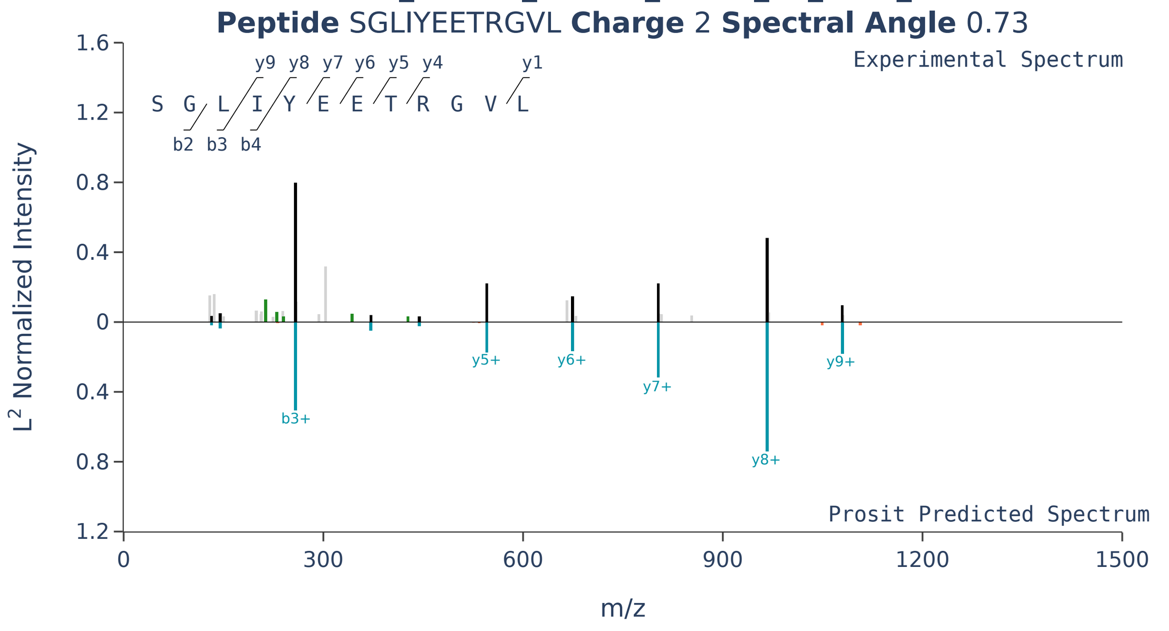


**Source** HRoetschke_291222_140123_HFGoe_H4_b2R2_1h **Scan** 29534
**Peptide** GVLKVFLENVLRD **Charge** 3 **Spectral Angle** 0.83

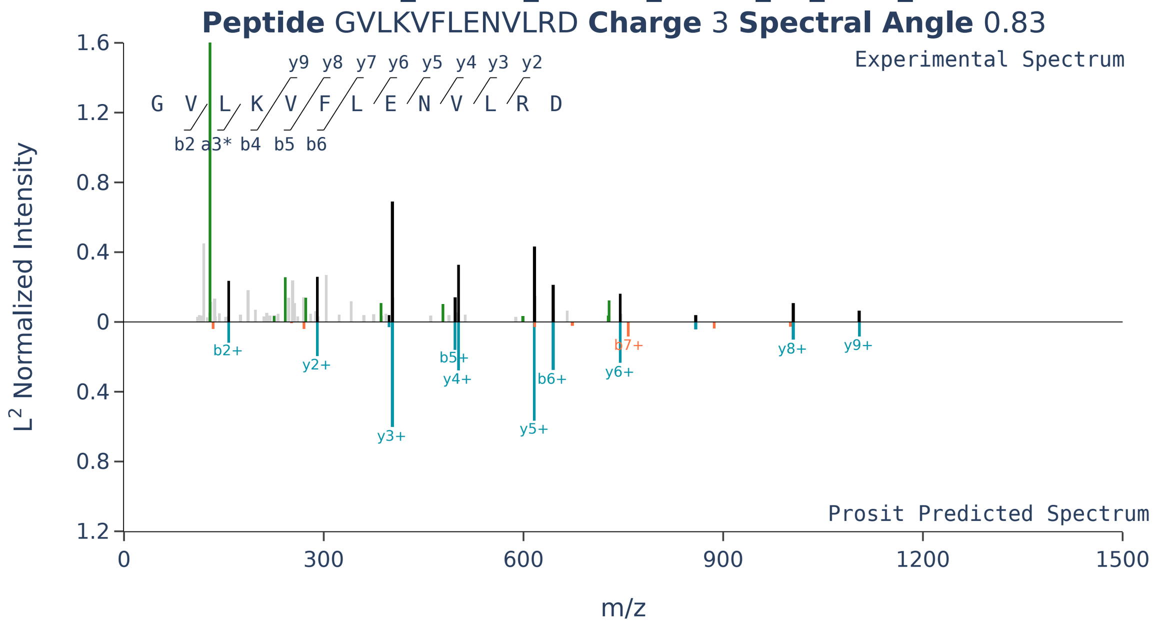


**Source** HRoetschke_291222_140123_HFGoe_H4_b1R1_1h **Scan** 9450
**Peptide** VVYALKRQ **Charge** 2 **Spectral Angle** 0.86

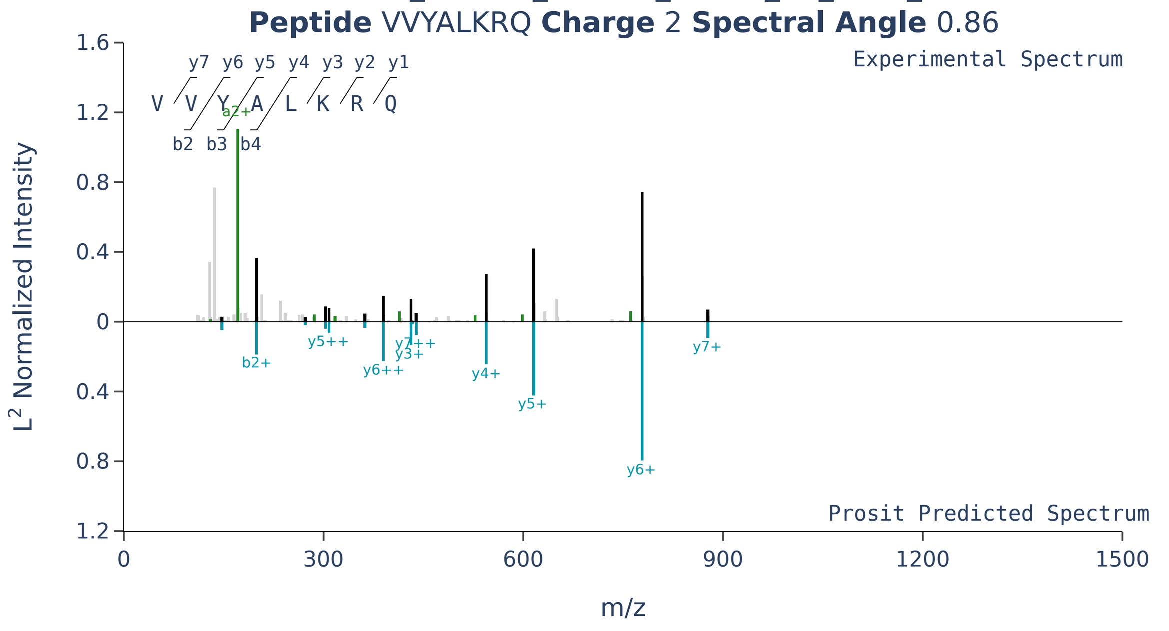


**Source** HRoetschke_291222_140123_HFGoe_H4_b1R1_4h **Scan** 13221
**Peptide** ALKRQGRTLYGF **Charge** 4 **Spectral Angle** 0.83

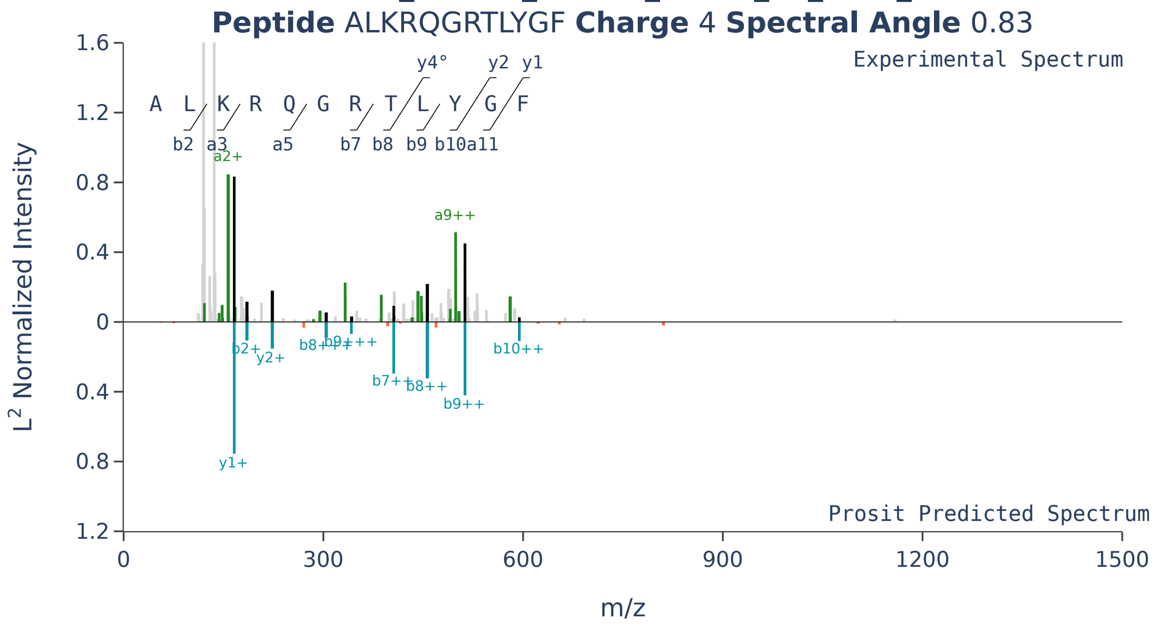

**Source** HRoetschke_291222_140123_HFGoe_H4_b2R1_1h **Scan** 21849
**Peptide** SGLIYEETRGVLKV **Charge** 2 **Spectral Angle** 0.75

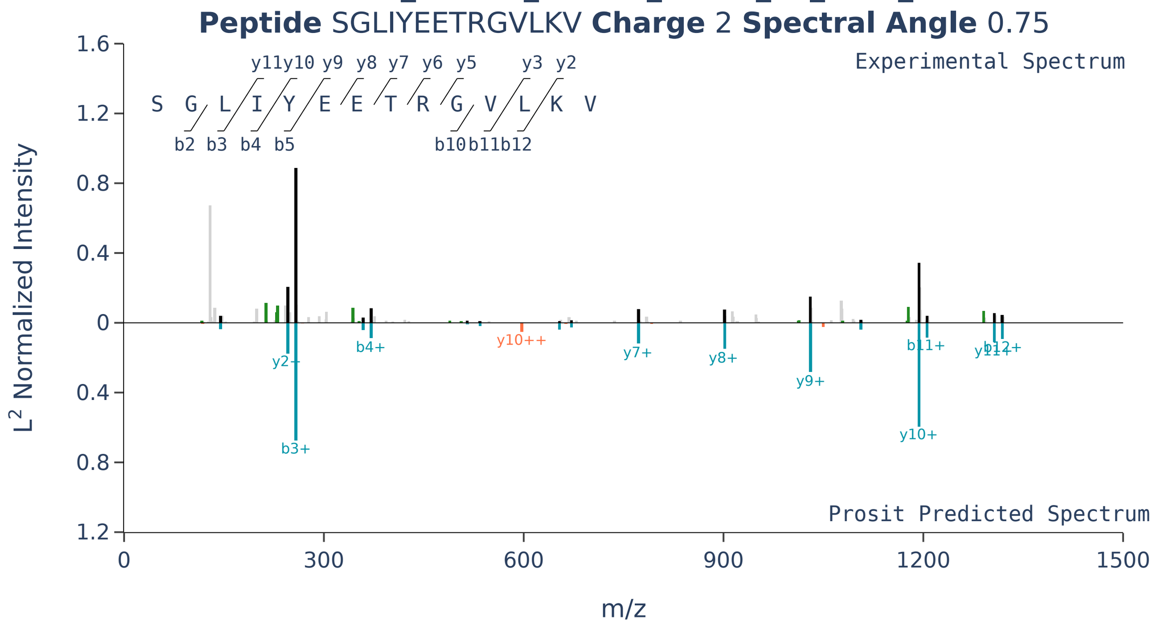


**Source** HRoetschke_291222_140123_HFGoe_H4_b1R1_2h **Scan** 14224
**Peptide** NVIRDAVTYTEHAKR **Charge** 4 **Spectral Angle** 0.79

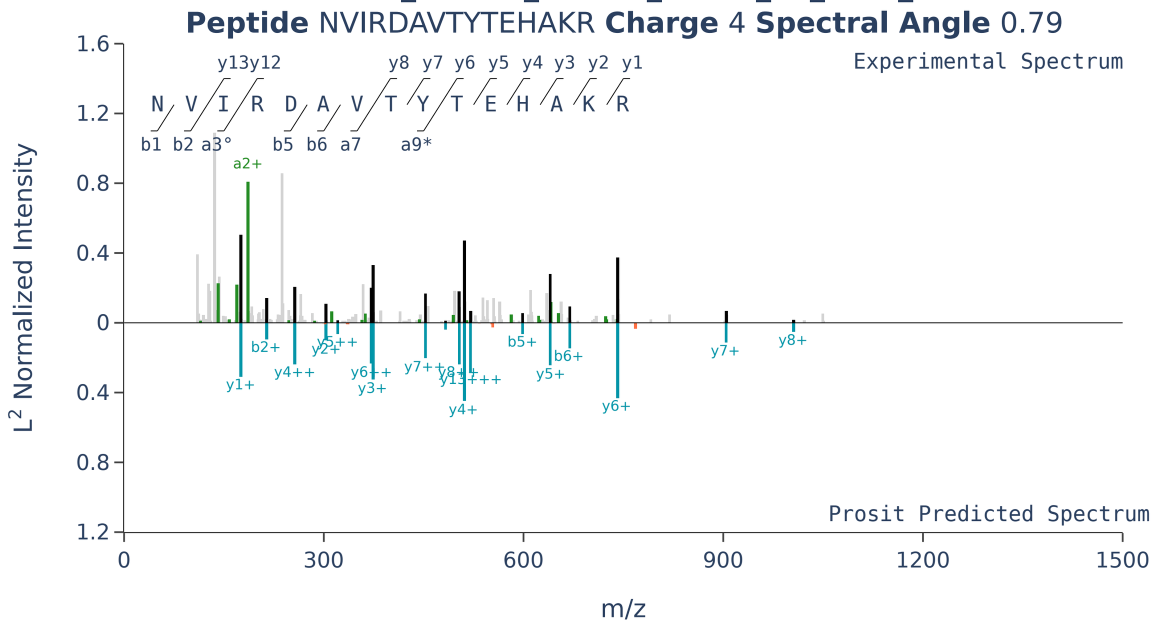


**Source** HRoetschke_291222_140123_HFGoe_H4_b2R1_2h **Scan** 25582
**Peptide** VLKVFLENVLRD **Charge** 3 **Spectral Angle** 0.89

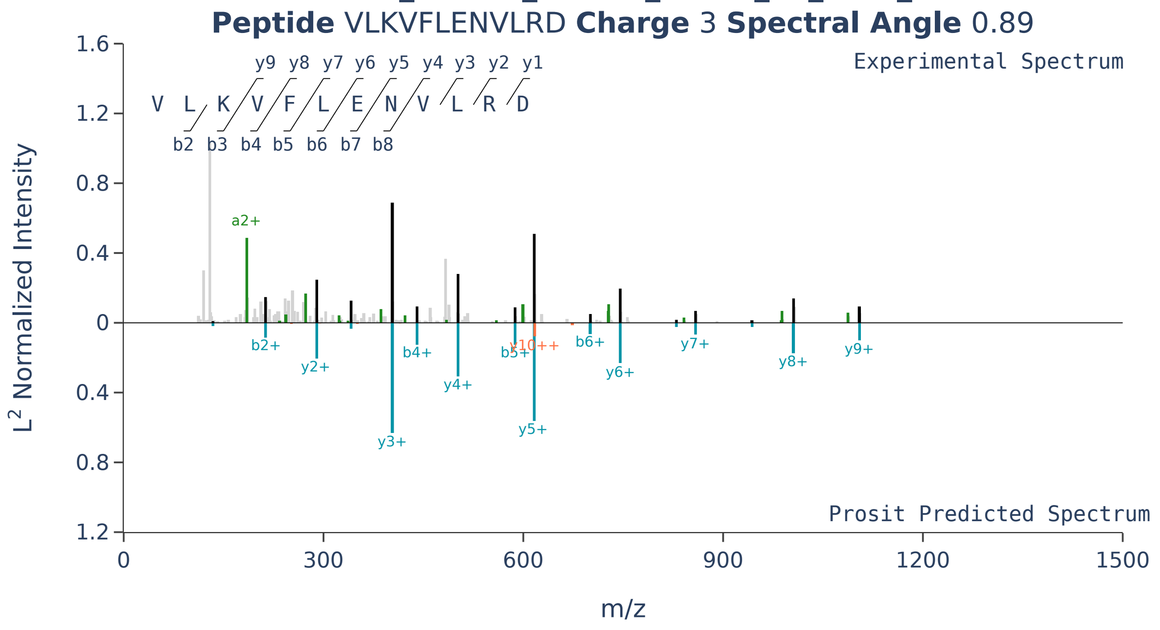


**Source** HRoetschke_291222_140123_HFGoe_H4_b2R1_1h **Scan** 22400
**Peptide** VFLENVIRD **Charge** 2 **Spectral Angle** 0.81

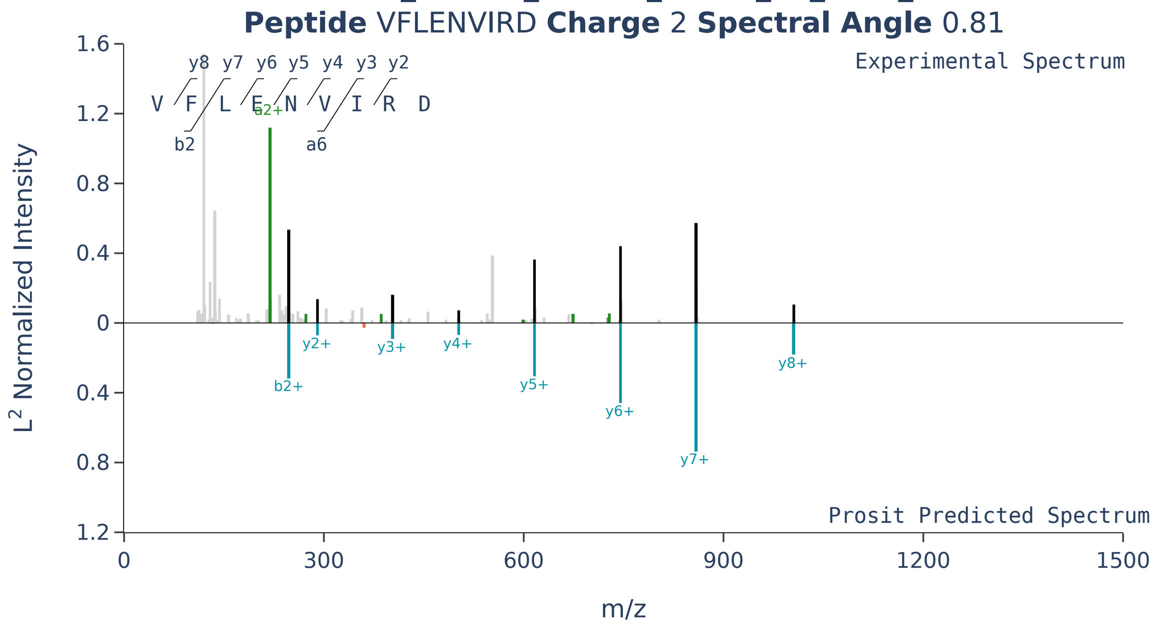


**Source** HRoetschke_291222_140123_HFGoe_H4_b2R1_1h **Scan** 8708
**Peptide** VVYALKR **Charge** 2 **Spectral Angle** 0.8

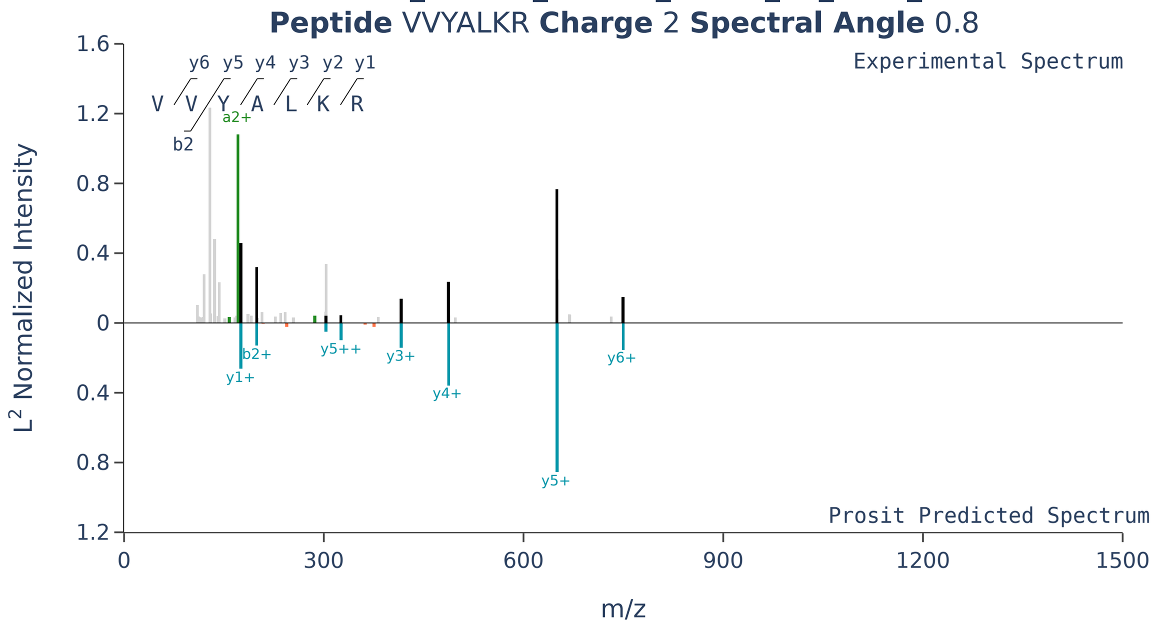


**Source** HRoetschke_291222_140123_HFGoe_H4_b1R1_24h **Scan** 10042
**Peptide** ITKPAIRRL **Charge** 3 **Spectral Angle** 0.81

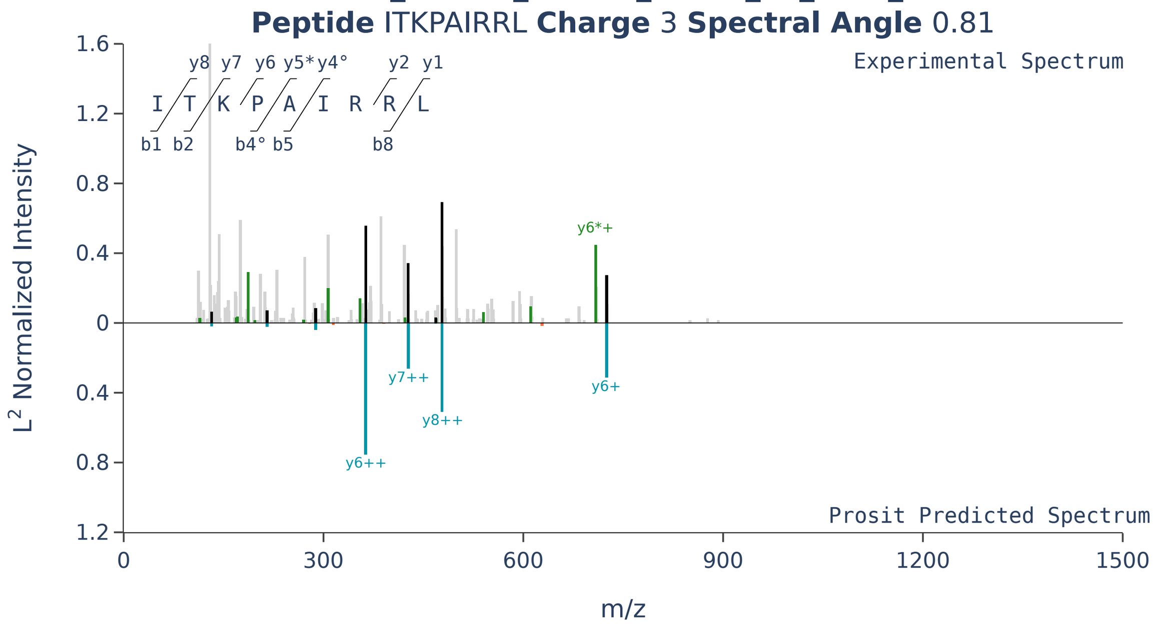


**Source** HRoetschke_291222_140123_HFGoe_H4_b1R2_24h **Scan** 6548
**Peptide** AVTYTEH **Charge** 2 **Spectral Angle** 0.92

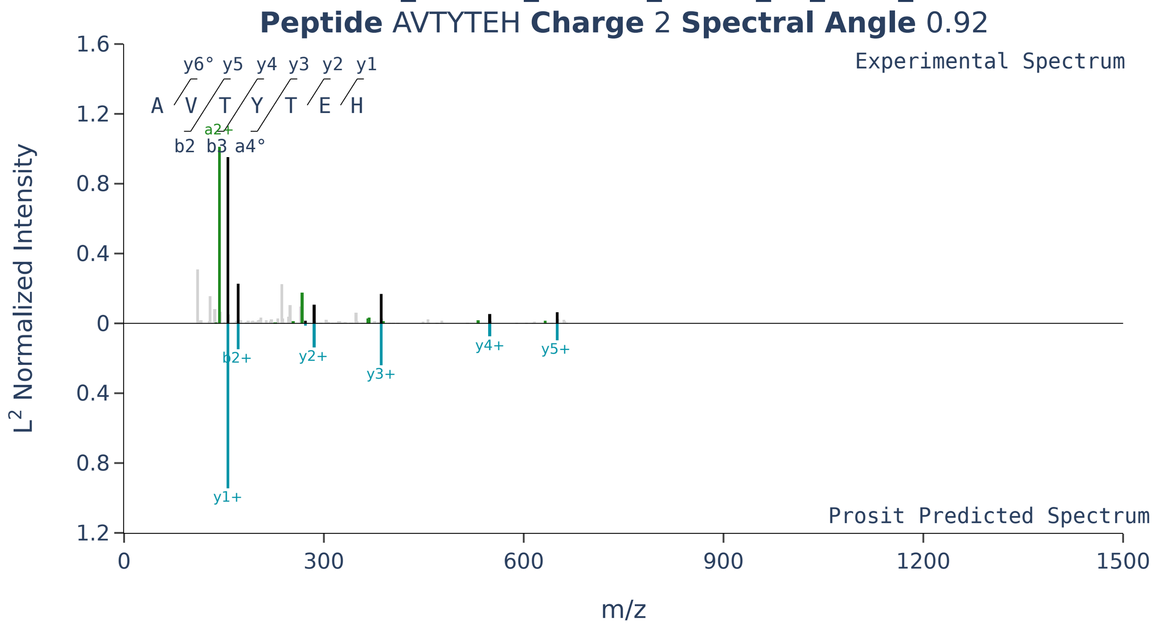


**Source** HRoetschke_291222_140123_HFGoe_H4_b2R2_1h **Scan** 26136
**Peptide** TRGVLKVFLENVIRD **Charge** 3 **Spectral Angle** 0.84

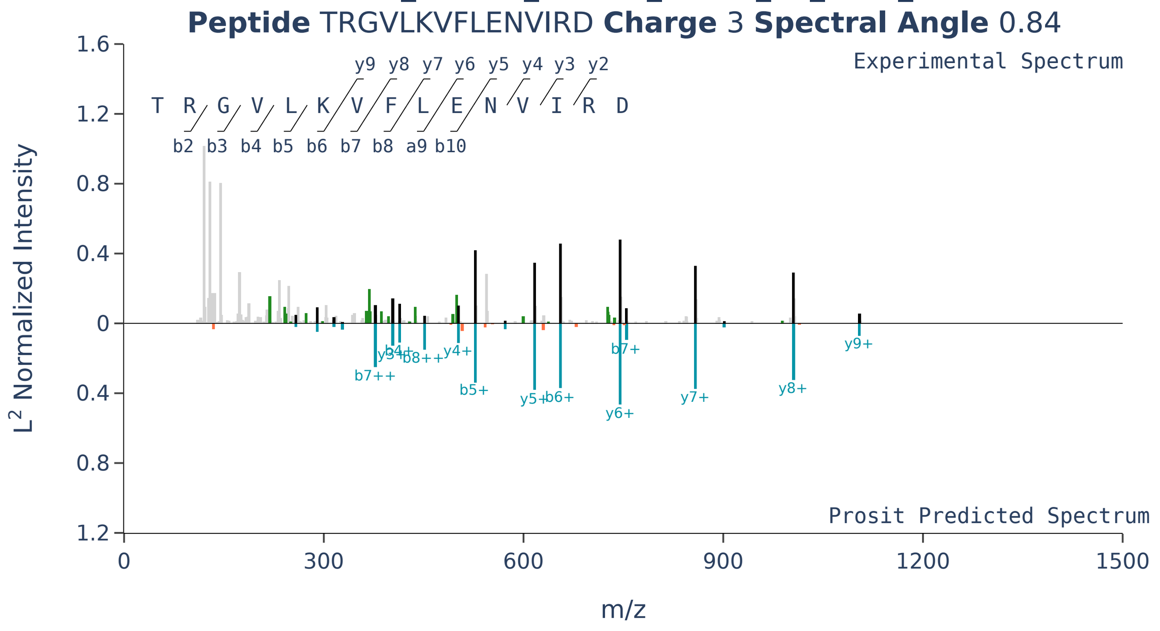


**Source** HRoetschke_291222_140123_HFGoe_H4_b2R1_24h **Scan** 10439
**Peptide** QGITKPAIR **Charge** 2 **Spectral Angle** 0.89

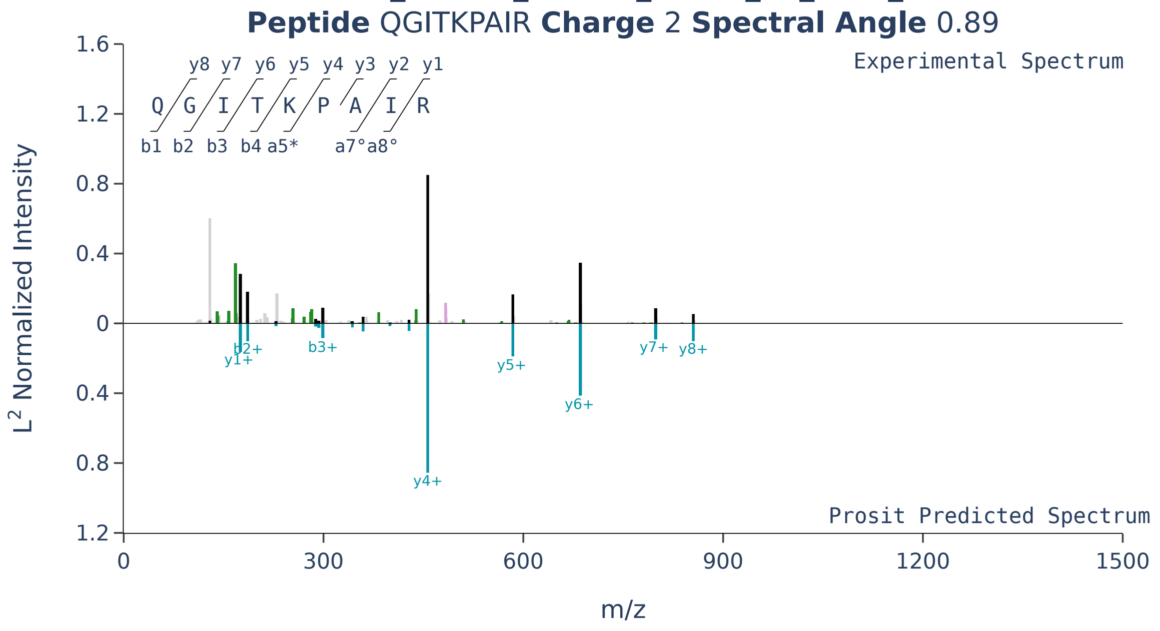


**Source** HRoetschke_291222_140123_HFGoe_H4_b2R2_24h **Scan** 10939
**Peptide** LIYEETRG **Charge** 2 **Spectral Angle** 0.82

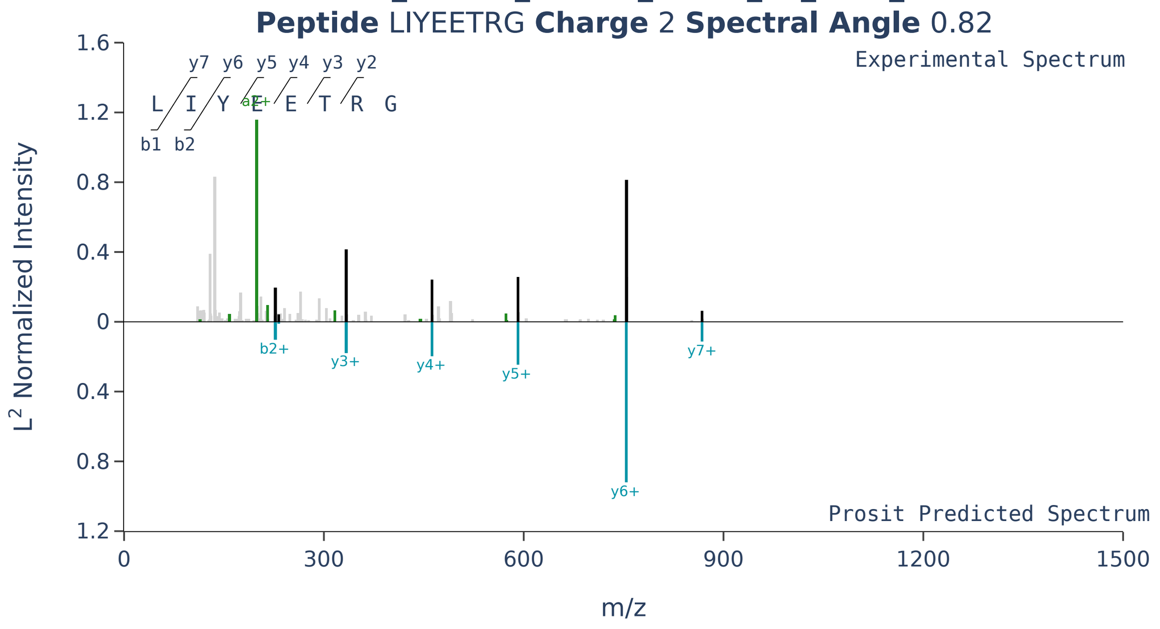


**Source** HRoetschke_291222_140123_HFGoe_H4_b2R1_1h **Scan** 20245
**Peptide** VTAMDVVYALKRQG **Charge** 2 **Spectral Angle** 0.89

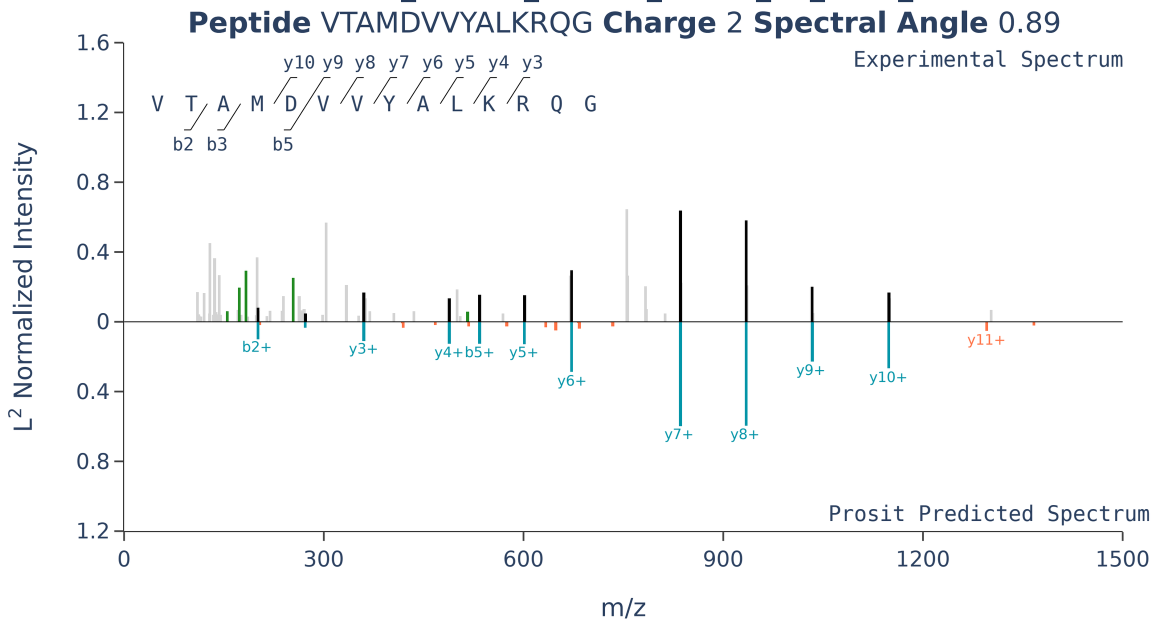


**Source** HRoetschke_291222_140123_HFGoe_H4_b2R2_4h **Scan** 11177
**Peptide** LRDAVTY **Charge** 2 **Spectral Angle** 0.91

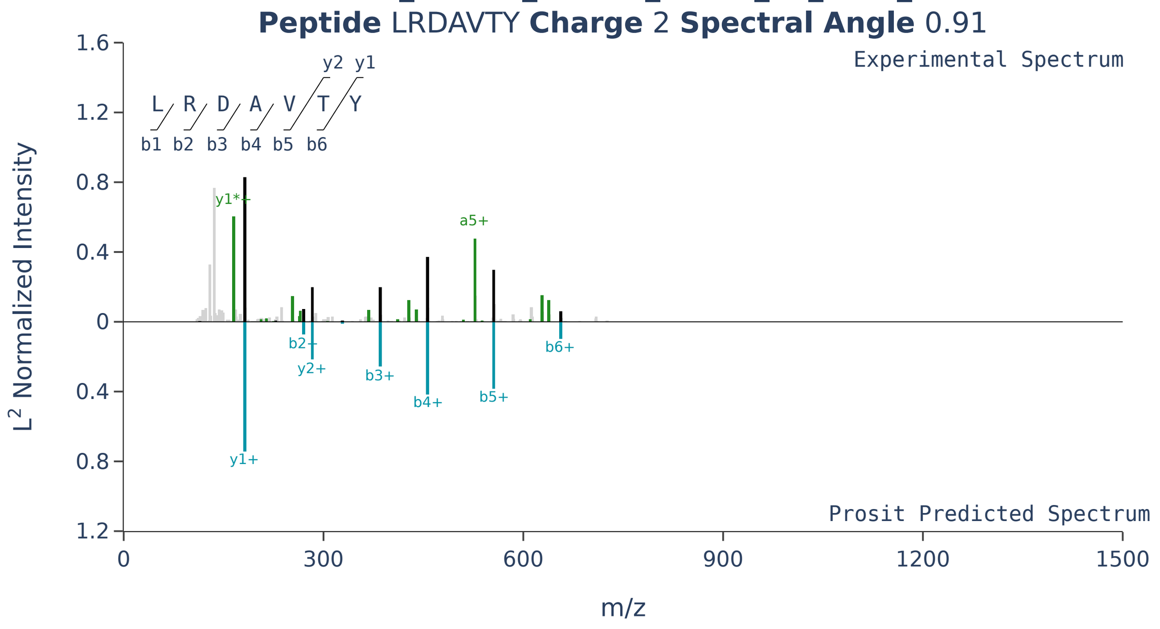


**Source** HRoetschke_291222_140123_HFGoe_H4_b2R1_1h **Scan** 21013
**Peptide** ISGLIYE **Charge** 1 **Spectral Angle** 0.87

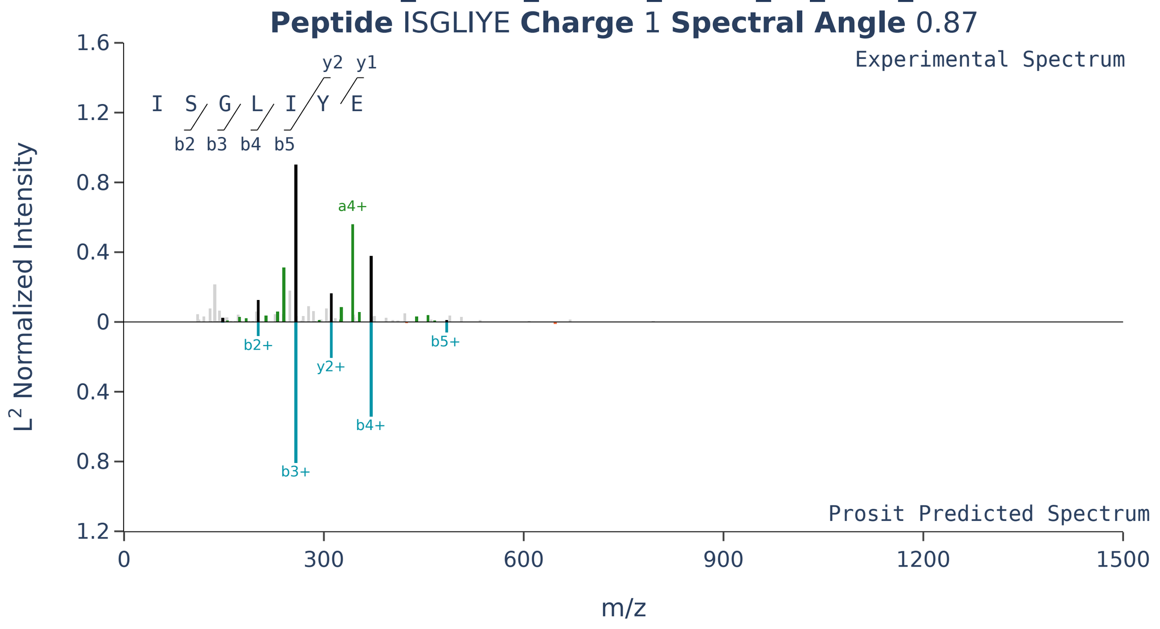


**Source** HRoetschke_291222_140123_HFGoe_H4_b1R1_2h **Scan** 19181
**Peptide** DNIQGITKPAI **Charge** 1 **Spectral Angle** 0.88

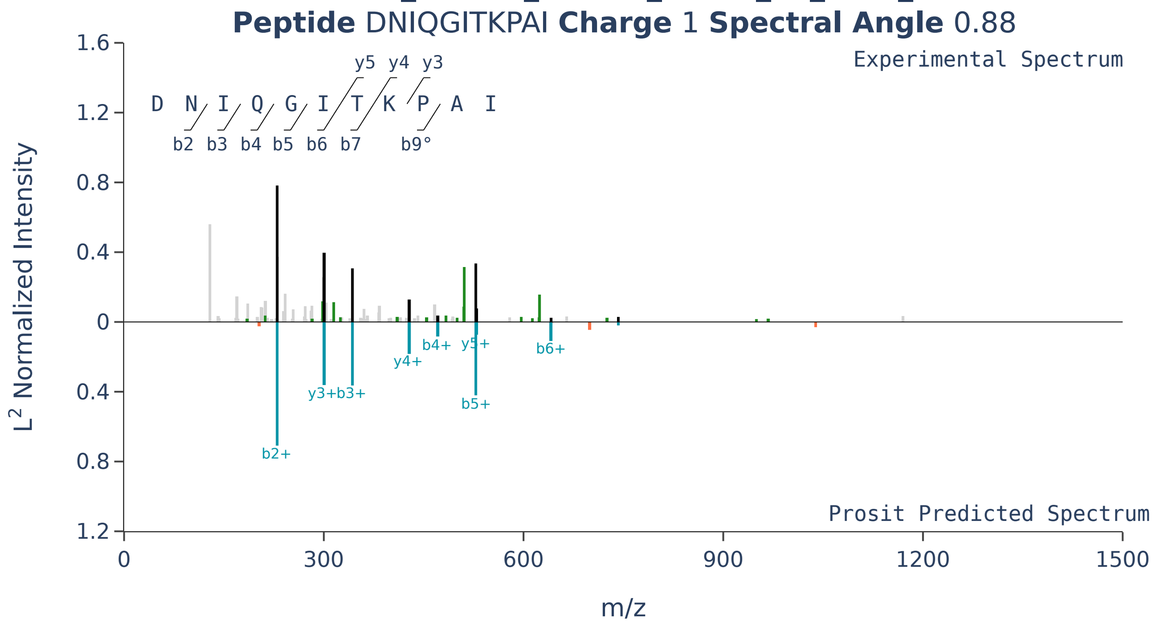


**Source** HRoetschke_291222_140123_HFGoe_H4_b1R2_4h **Scan** 14781
**Peptide** GVKRISGLIYEETRG **Charge** 3 **Spectral Angle** 0.88

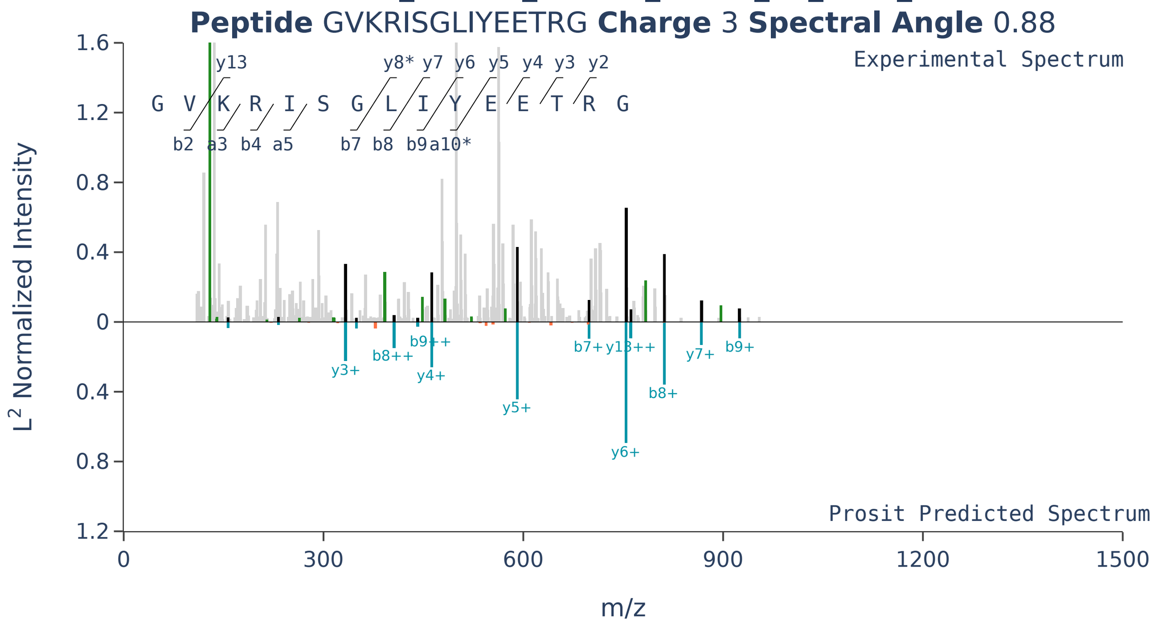

**Source** HRoetschke_291222_140123_HFGoe_H4_b2R2_4h **Scan** 12712
**Peptide** DVVYALKRQ **Charge** 2 **Spectral Angle** 0.79

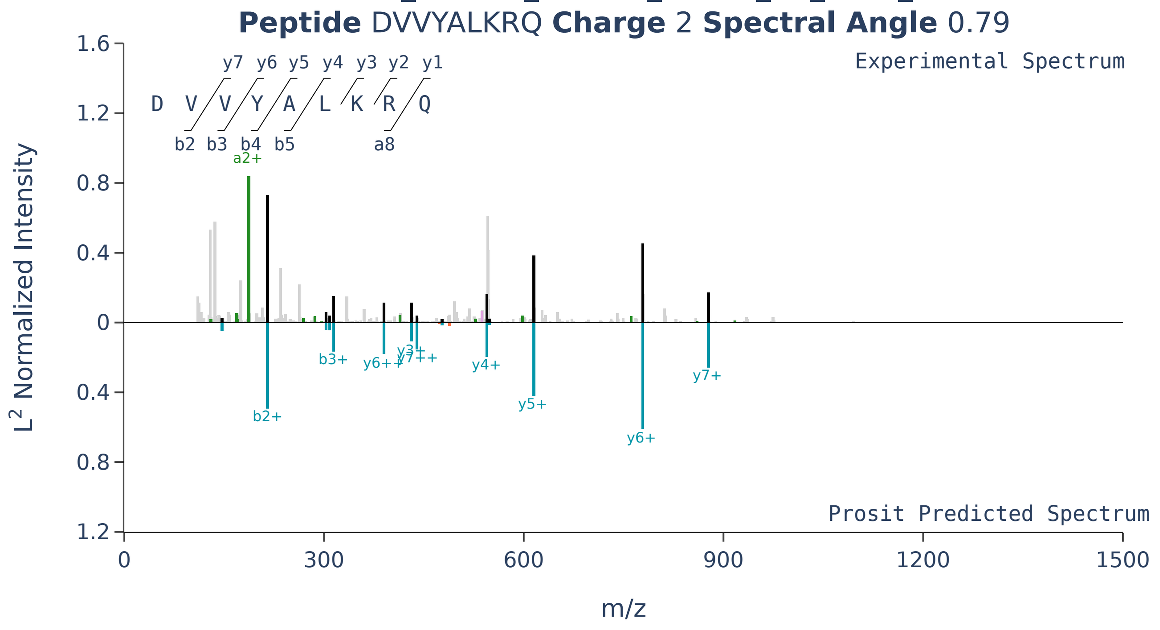


**Source** HRoetschke_291222_140123_HFGoe_H4_b2R2_24h **Scan** 12254
**Peptide** DVVYALKRQG **Charge** 2 **Spectral Angle** 0.76

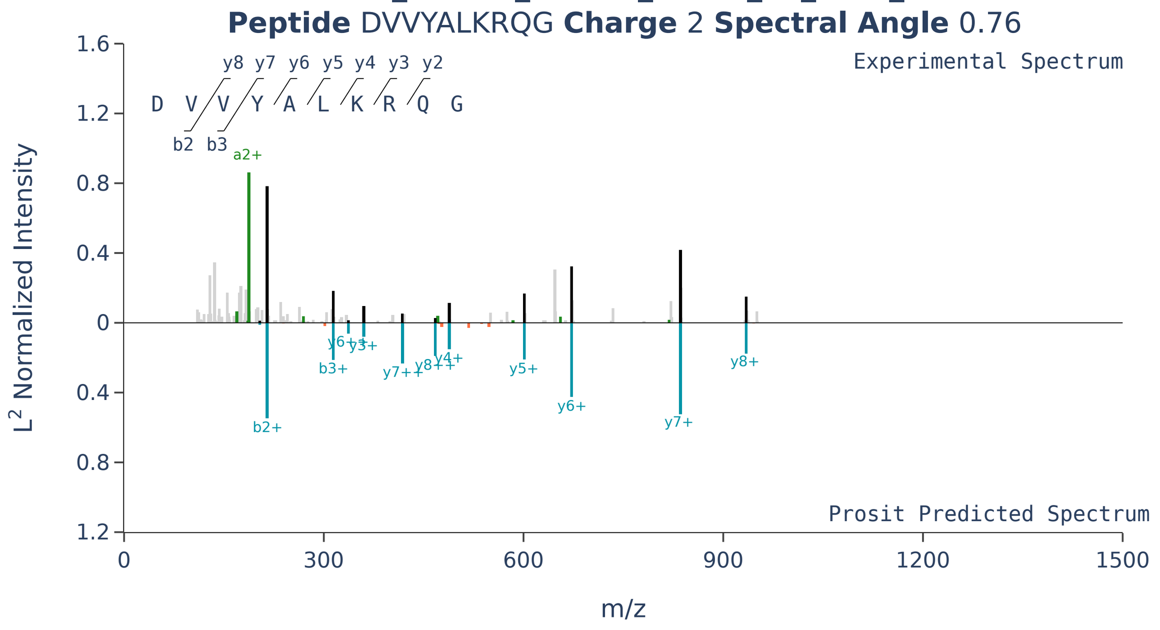


**Source** HRoetschke_291222_140123_HFGoe_H4_b1R1_1h **Scan** 20614
**Peptide** RGVLKVFLEN **Charge** 3 **Spectral Angle** 0.75

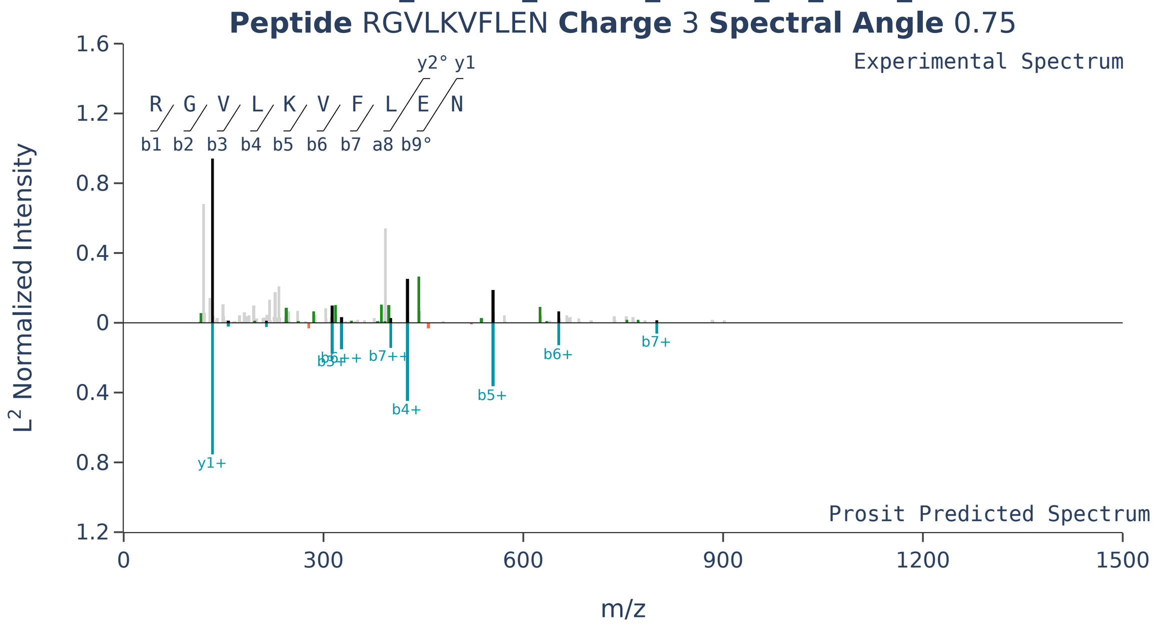


**Source** HRoetschke_291222_140123_HFGoe_H4_b2R1_1h **Scan** 18958
**Peptide** TAMDVVYALKR **Charge** 3 **Spectral Angle** 0.79

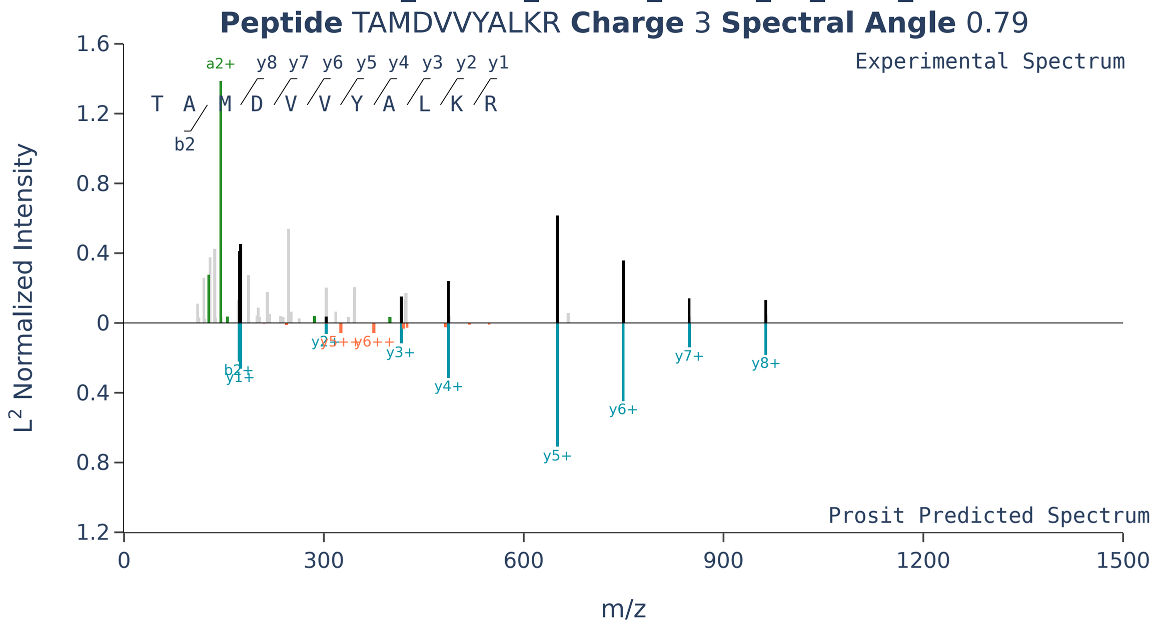


**Source** HRoetschke_291222_140123_HFGoe_H4_b2R1_1h **Scan** 21500
**Peptide** GLIYEETRGVLKV **Charge** 2 **Spectral Angle** 0.69

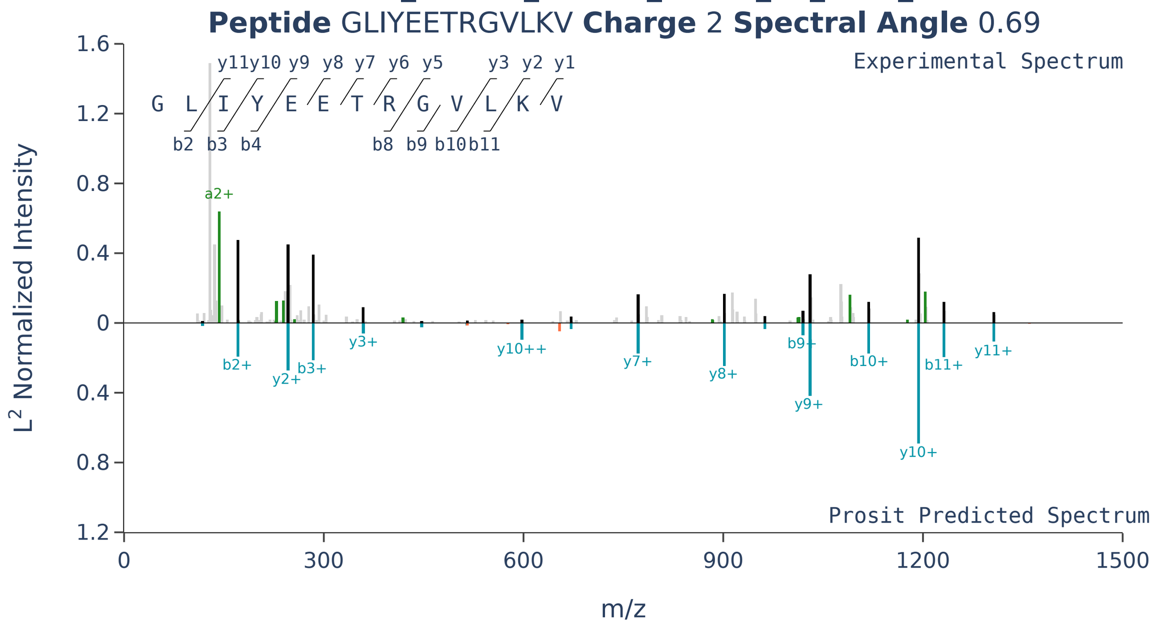


**Source** HRoetschke_291222_140123_HFGoe_H4_b2R1_1h **Scan** 11608
**Peptide** TEHAKRKTVTAMDVVYALKRQGR **Charge** 6 **Spectral Angle** 0.88

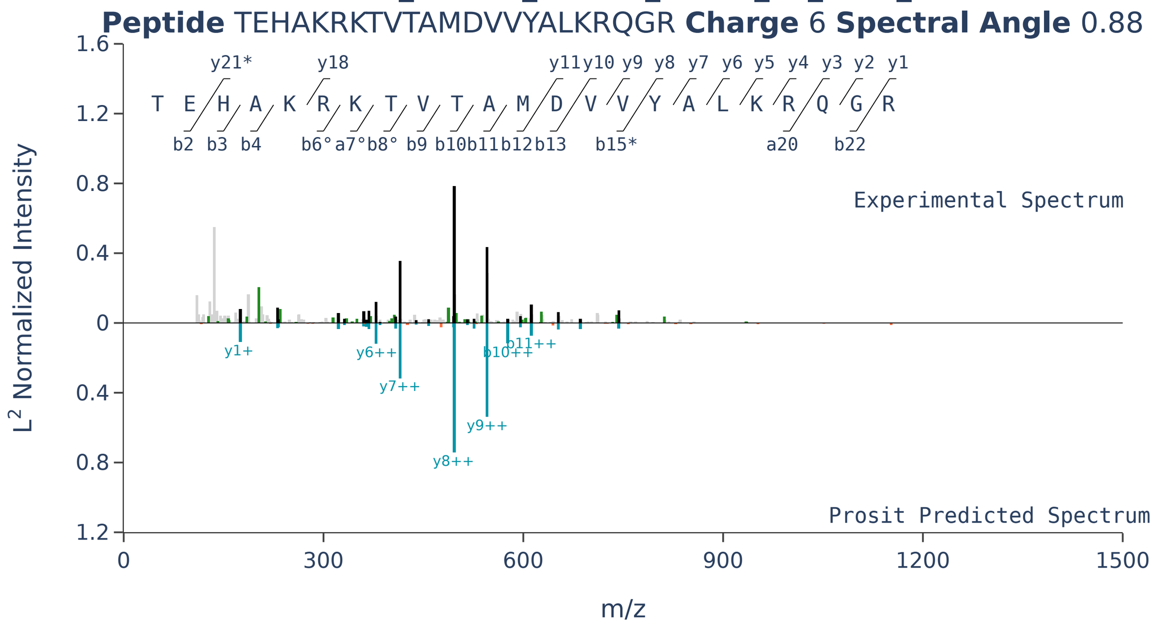


**Source** HRoetschke_291222_140123_HFGoe_H4_b2R1_4h **Scan** 14607
**Peptide** RQGRTLYGF **Charge** 3 **Spectral Angle** 0.72

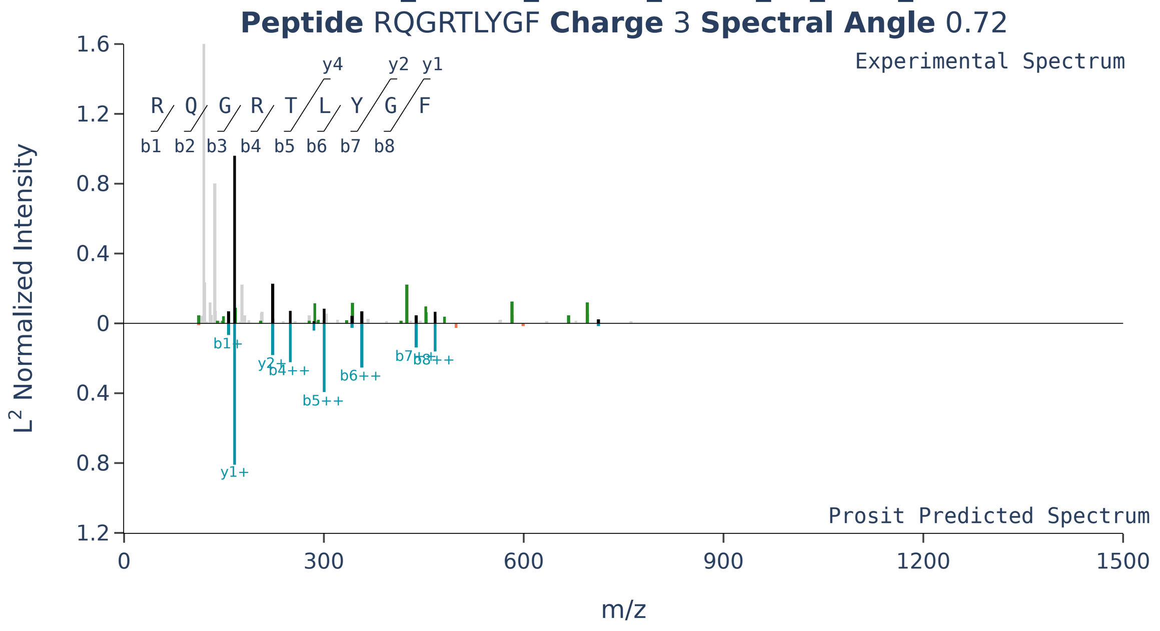


**Source** HRoetschke_291222_140123_HFGoe_H4_b1R1_2h **Scan** 10285
**Peptide** LQGITKPAIRR **Charge** 3 **Spectral Angle** 0.75

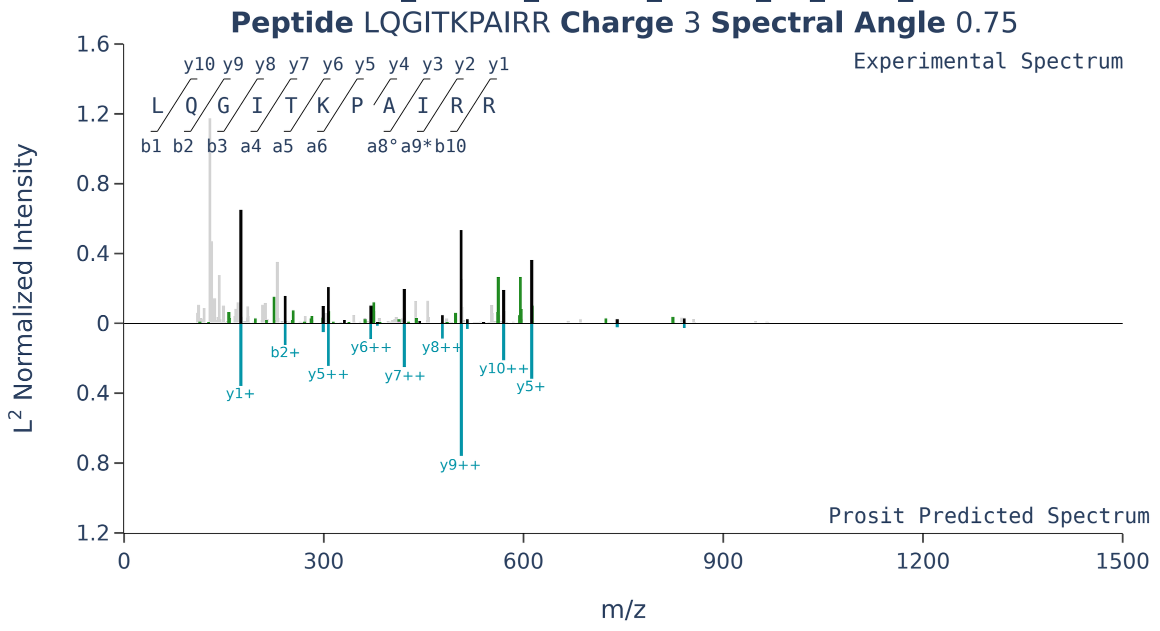


**Source** HRoetschke_291222_140123_HFGoe_H4_b2R2_24h **Scan** 8819
**Peptide** VVYALKRQG **Charge** 2 **Spectral Angle** 0.84

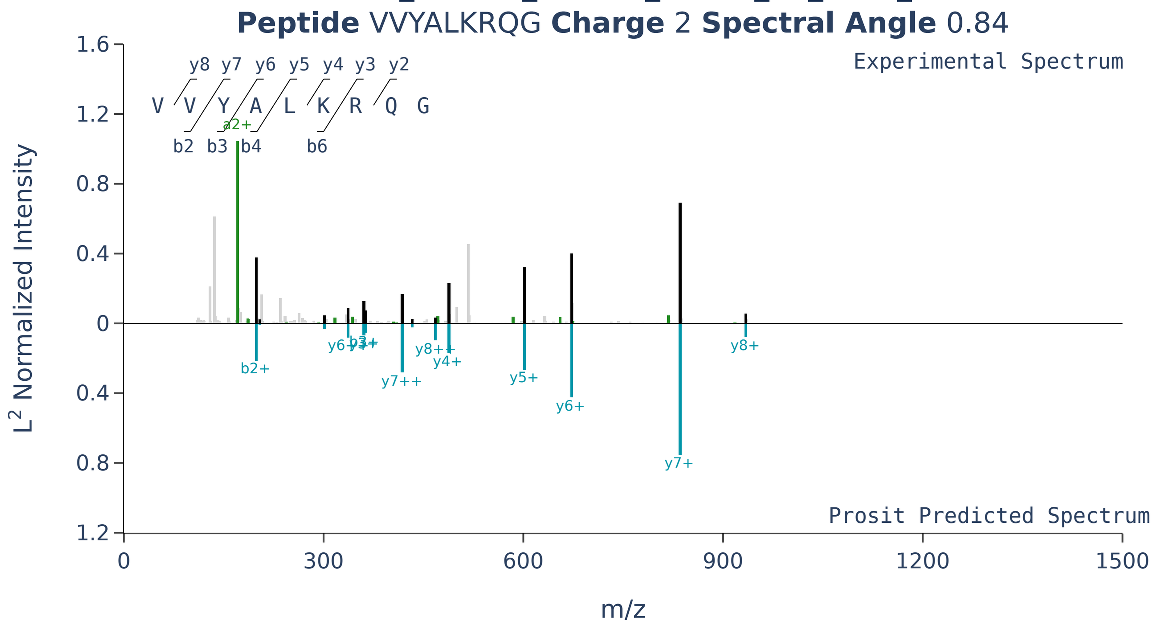


**Source** HRoetschke_291222_140123_HFGoe_H4_b1R1_4h **Scan** 12081
**Peptide** VLRDAVTY **Charge** 1 **Spectral Angle** 0.92

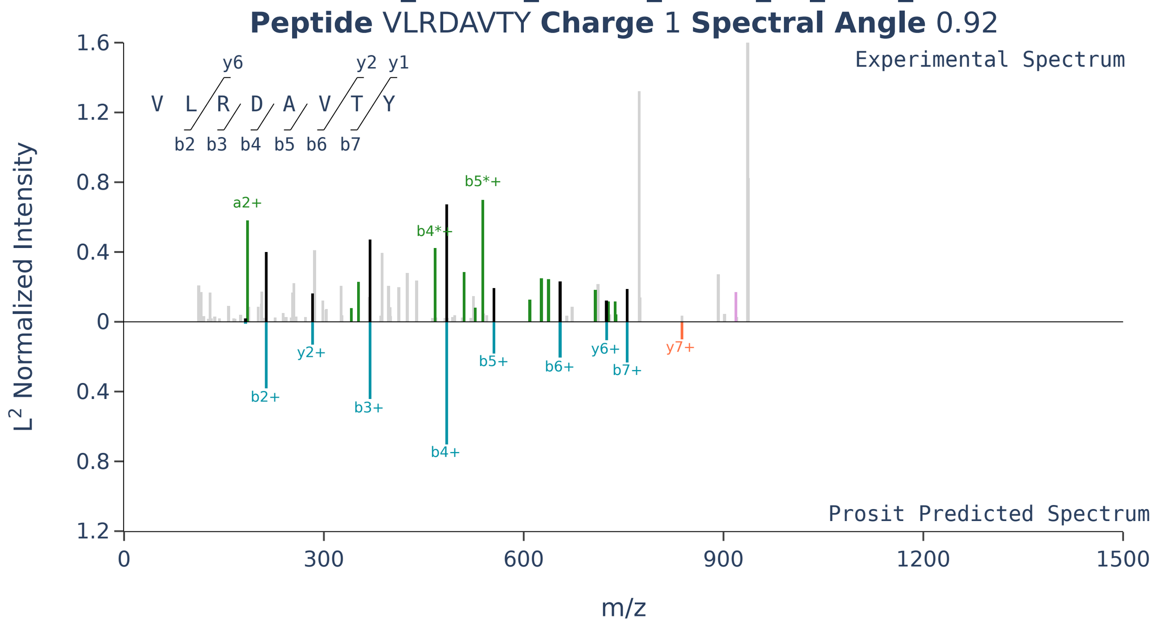


**Source** HRoetschke_291222_140123_HFGoe_H4_b1R2_4h **Scan** 8899
**Peptide** DAVTYTEHA **Charge** 1 **Spectral Angle** 0.89

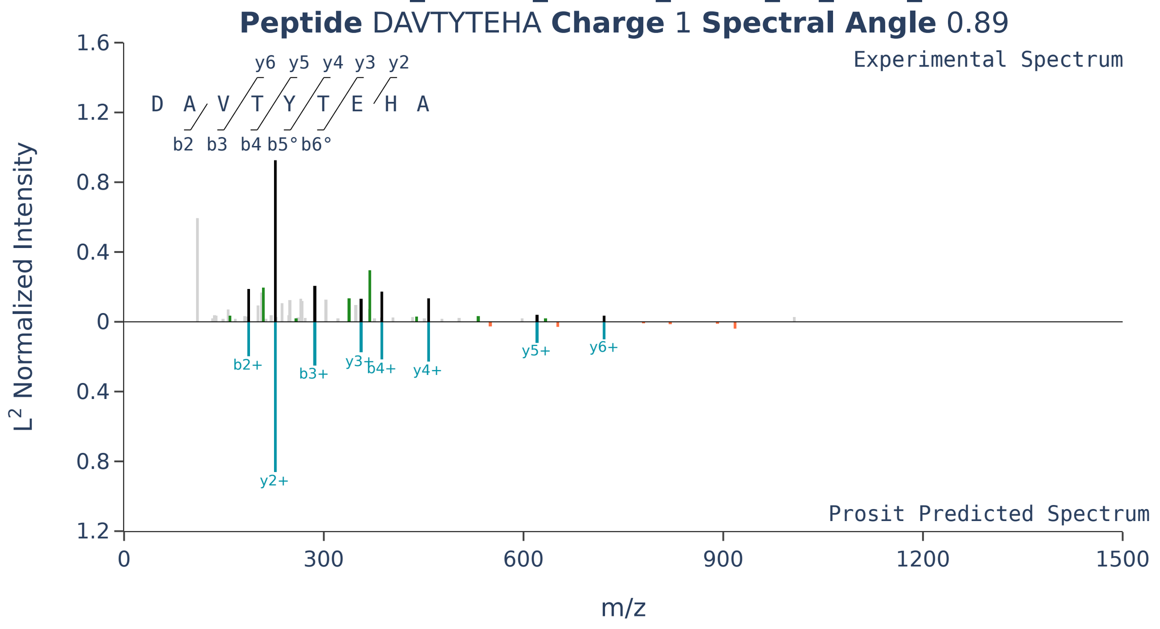


**Source** HRoetschke_291222_140123_HFGoe_H4_b1R2_24h **Scan** 5634
**Peptide** YTEHAKRKTV **Charge** 2 **Spectral Angle** 0.79

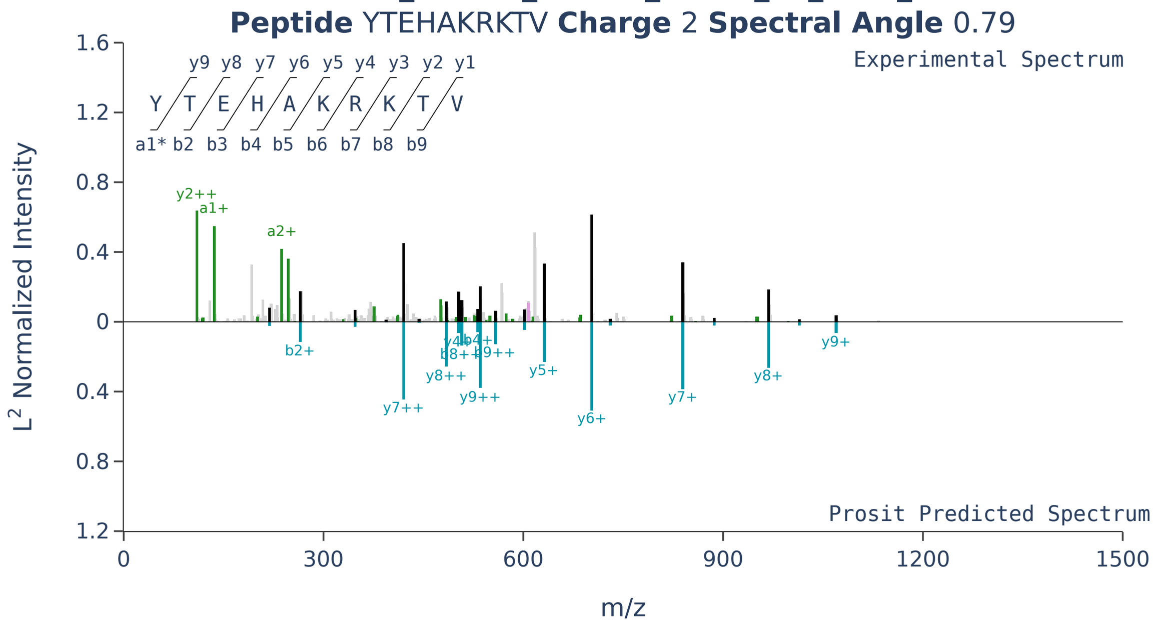


**Source** HRoetschke_291222_140123_HFGoe_H4_b1R2_1h **Scan** 13795
**Peptide** IQGITKPAIRRL **Charge** 3 **Spectral Angle** 0.79

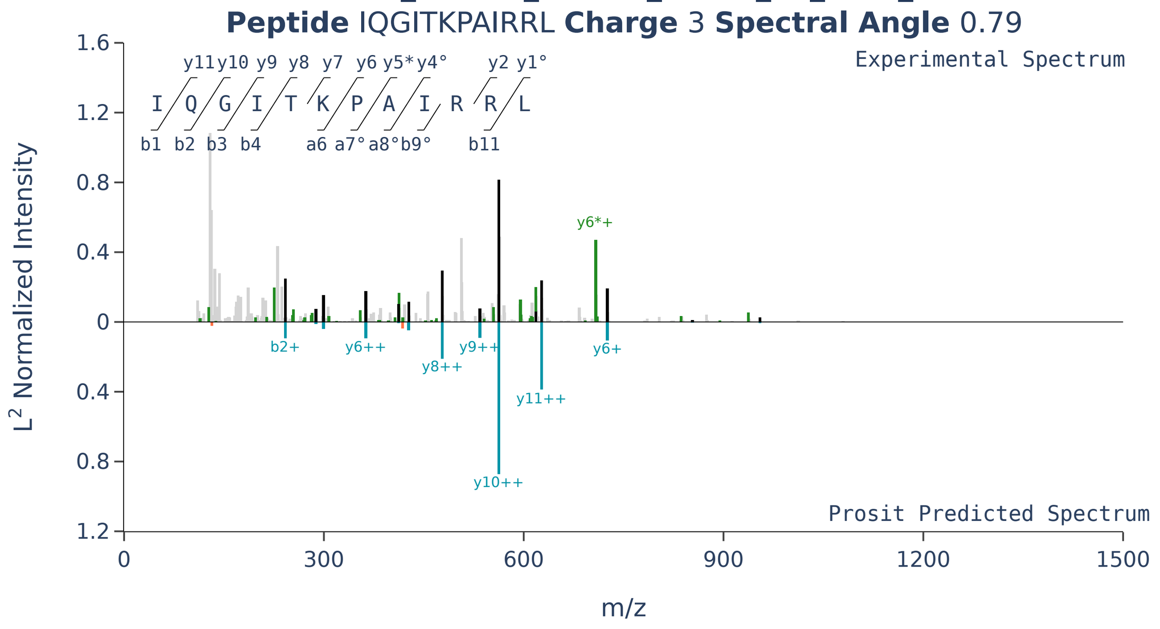


**Source** HRoetschke_291222_140123_HFGoe_H4_b1R1_1h **Scan** 18960
**Peptide** TAMDVVYALKRQGRTL **Charge** 4 **Spectral Angle** 0.86

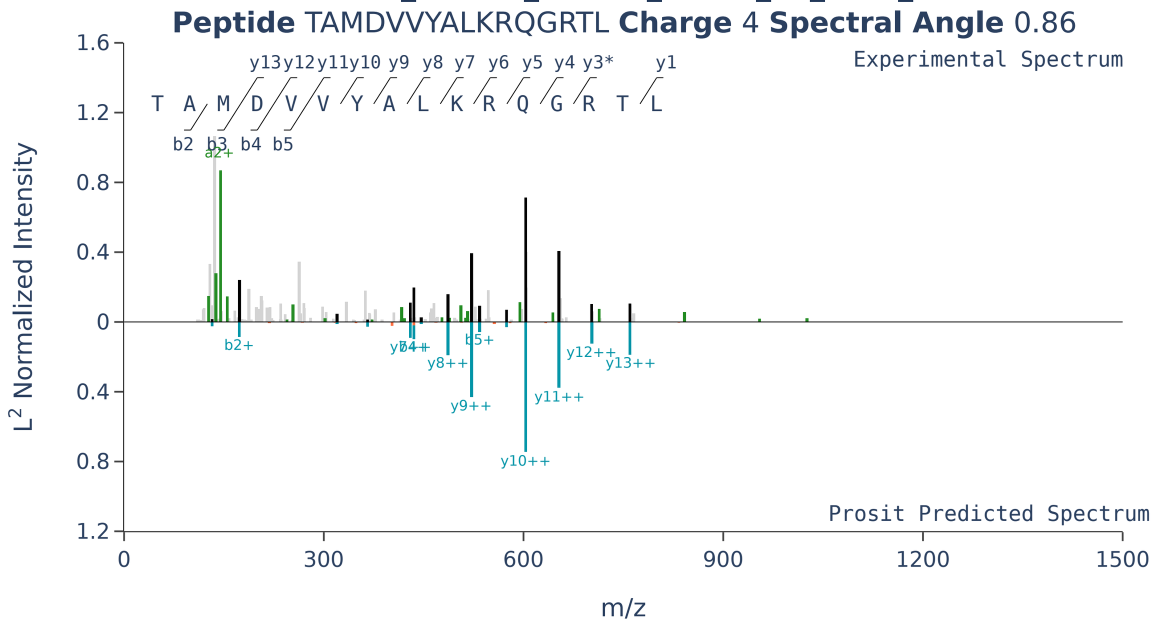


**Source** HRoetschke_291222_140123_HFGoe_H4_b1R1_24h **Scan** 6829
**Peptide** ALKRQGRTLY **Charge** 3 **Spectral Angle** 0.82

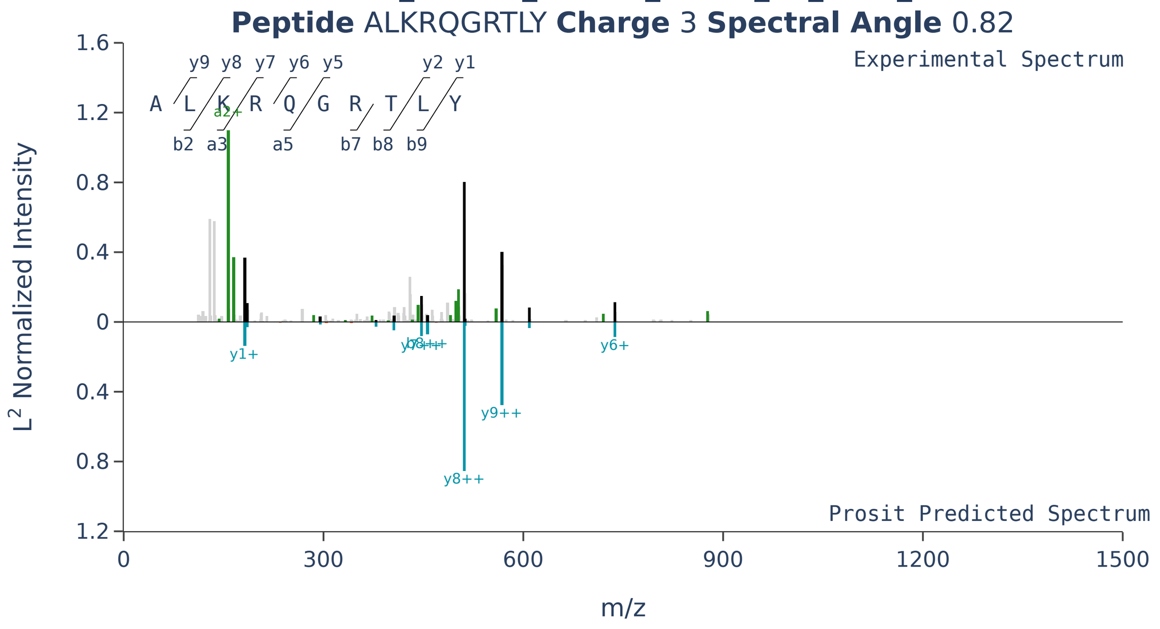

**Source** HRoetschke_291222_140123_HFGoe_H4_b2R1_1h **Scan** 24558
**Peptide** TVTAMDVVYALKRQG **Charge** 2 **Spectral Angle** 0.81

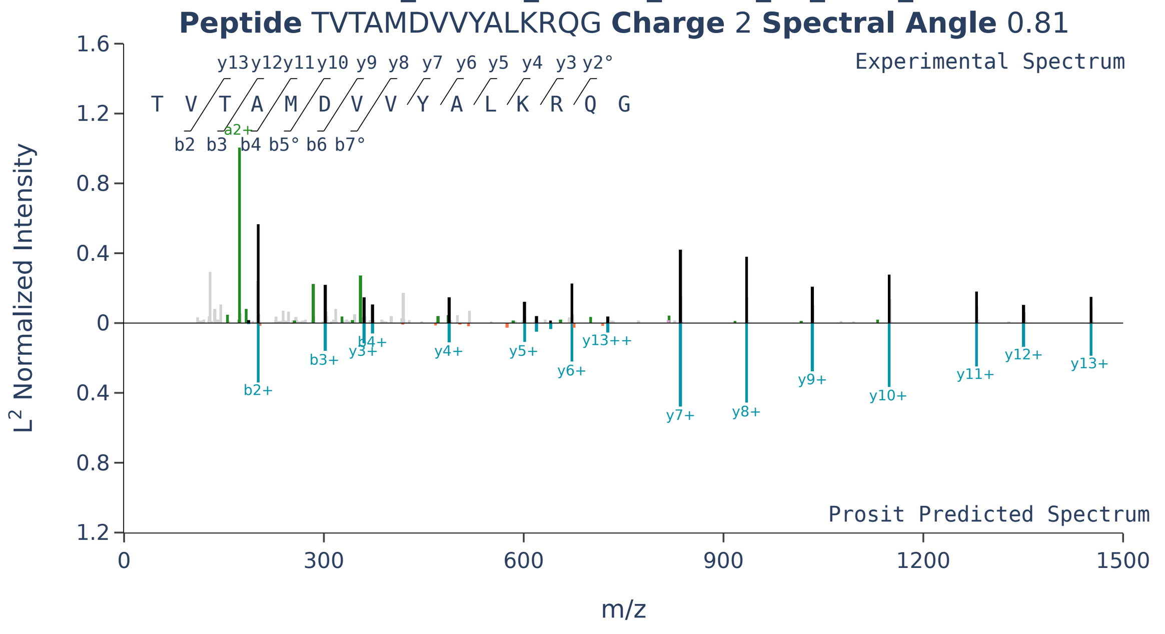


**Source** HRoetschke_291222_140123_HFGoe_H4_b1R1_2h **Scan** 13452
**Peptide** AKRKTVTAMDVVYALKRQGR **Charge** 5 **Spectral Angle** 0.91

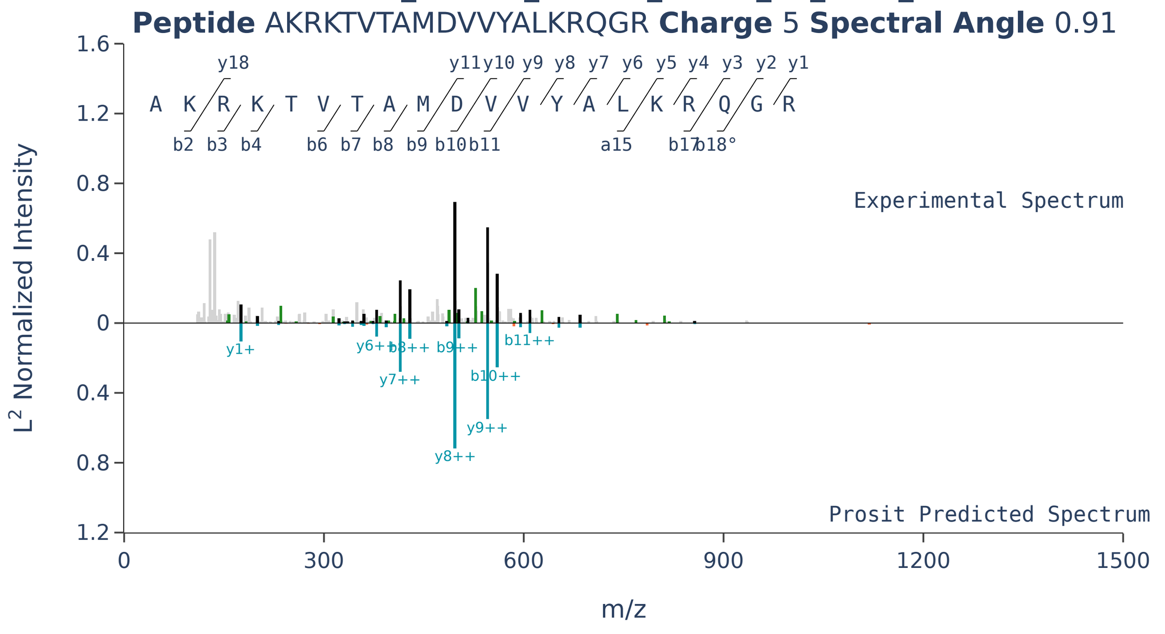


**Source** HRoetschke_291222_140123_HFGoe_H4_b2R1_2h **Scan** 17248
**Peptide** AVTYTEHAKRKTVTAMDVVYALKRQG **Charge** 5 **Spectral Angle** 0.85

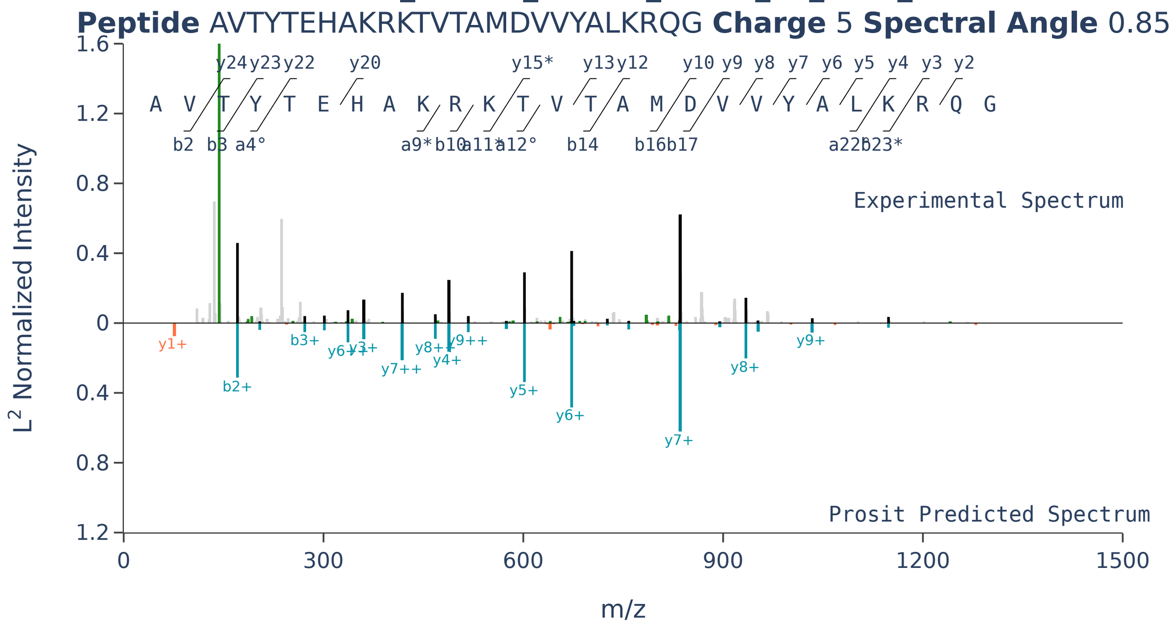


**Source** HRoetschke_291222_140123_HFGoe_H4_b1R2_2h **Scan** 13892
**Peptide** LRDNIQGITKPAIR **Charge** 4 **Spectral Angle** 0.88

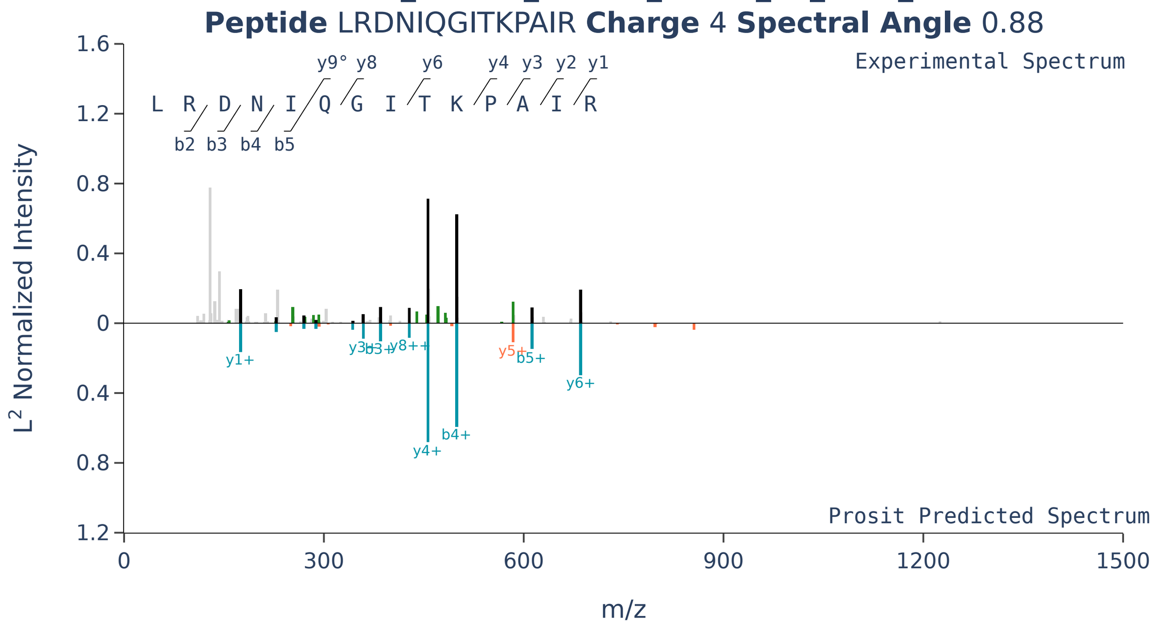


**Source** HRoetschke_291222_140123_HFGoe_H4_b1R1_1h **Scan** 22305
**Peptide** LSGLIYEET **Charge** 1 **Spectral Angle** 0.88

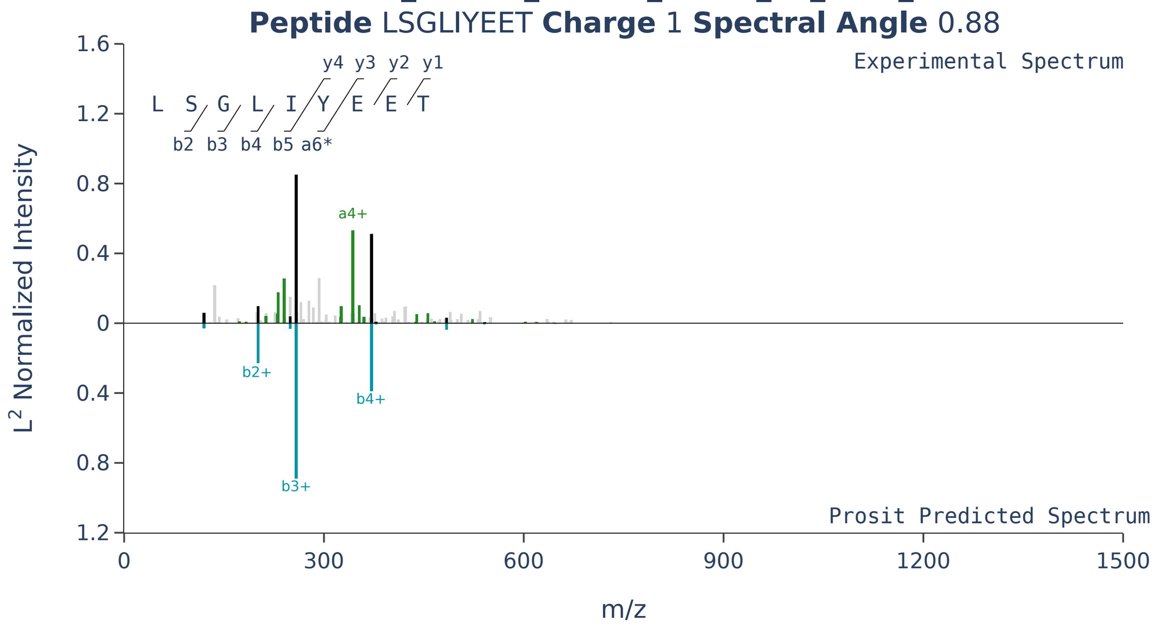


**Source** HRoetschke_291222_140123_HFGoe_H4_b2R1_24h **Scan** 11273
**Peptide** RDNIQGITKPAIR **Charge** 4 **Spectral Angle** 0.88

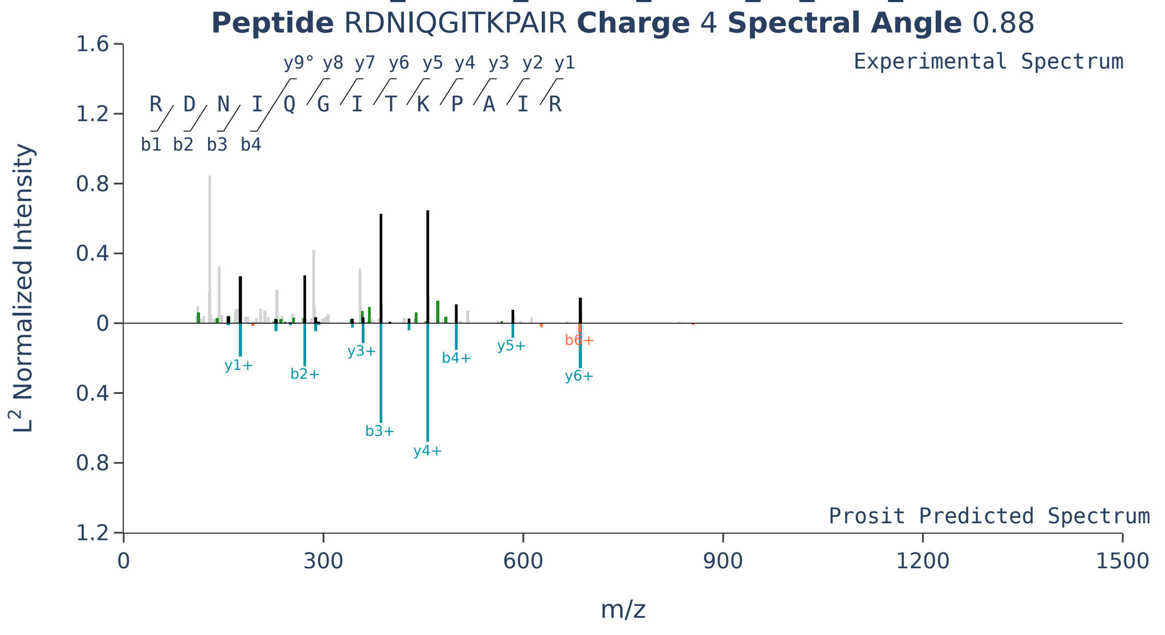


**Source** HRoetschke_291222_140123_HFGoe_H4_b2R1_1h **Scan** 12761
**Peptide** TEHAKRKTVTAMDVVYALKRQG **Charge** 5 **Spectral Angle** 0.81

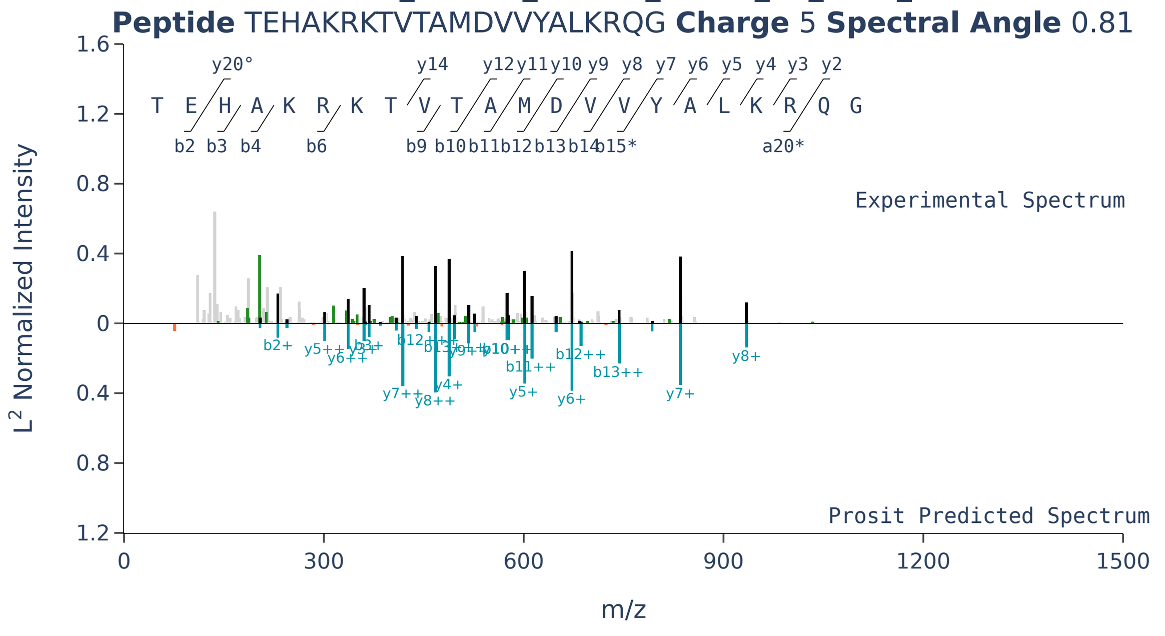


**Source** HRoetschke_291222_140123_HFGoe_H4_b1R2_24h **Scan** 9662
**Peptide** VLRDAVTYTEH **Charge** 3 **Spectral Angle** 0.87

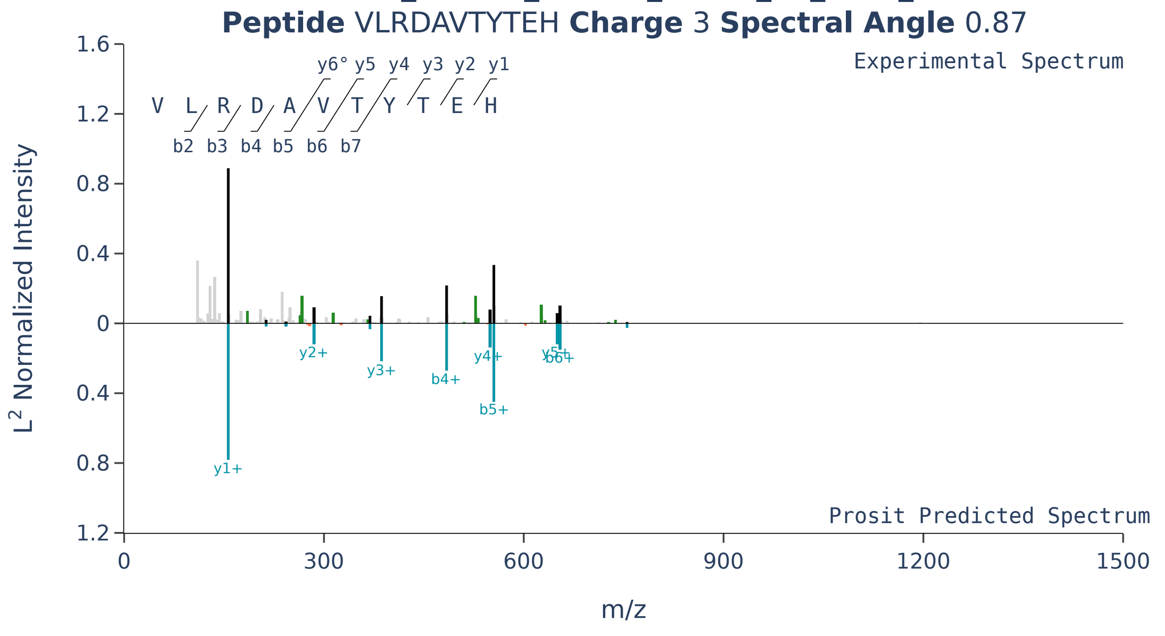


**Source** HRoetschke_291222_140123_HFGoe_H4_b2R2_24h **Scan** 11943
**Peptide** VLRDNIQGITK **Charge** 3 **Spectral Angle** 0.83

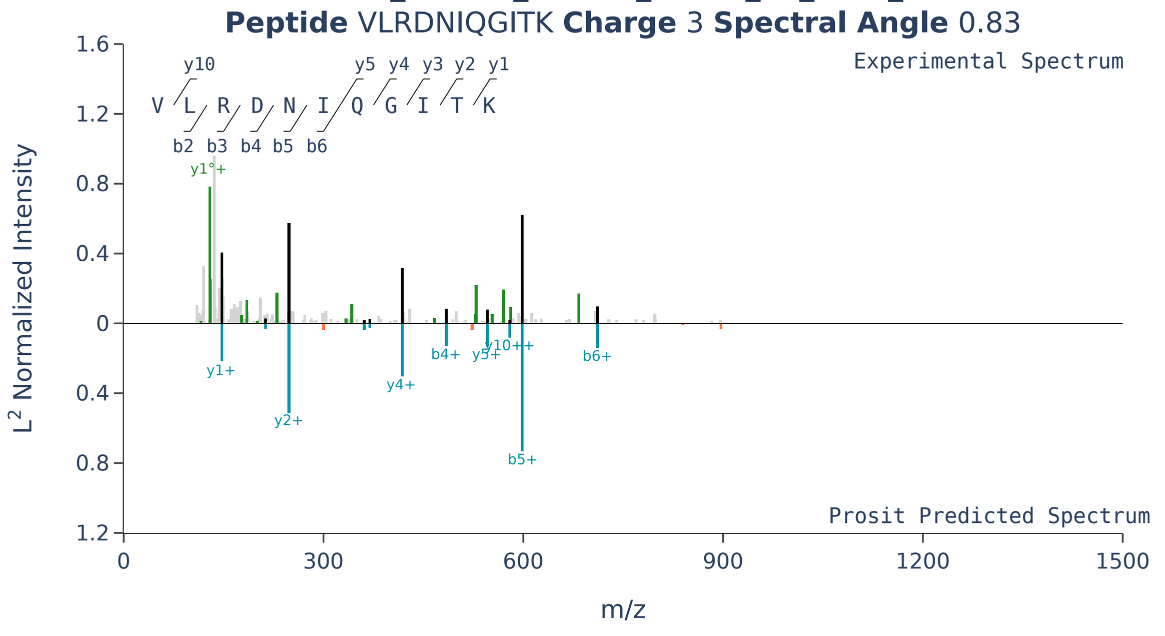


**Source** HRoetschke_291222_140123_HFGoe_H4_b1R1_2h **Scan** 14131
**Peptide** RDNIQGITKPAIRRL **Charge** 4 **Spectral Angle** 0.82

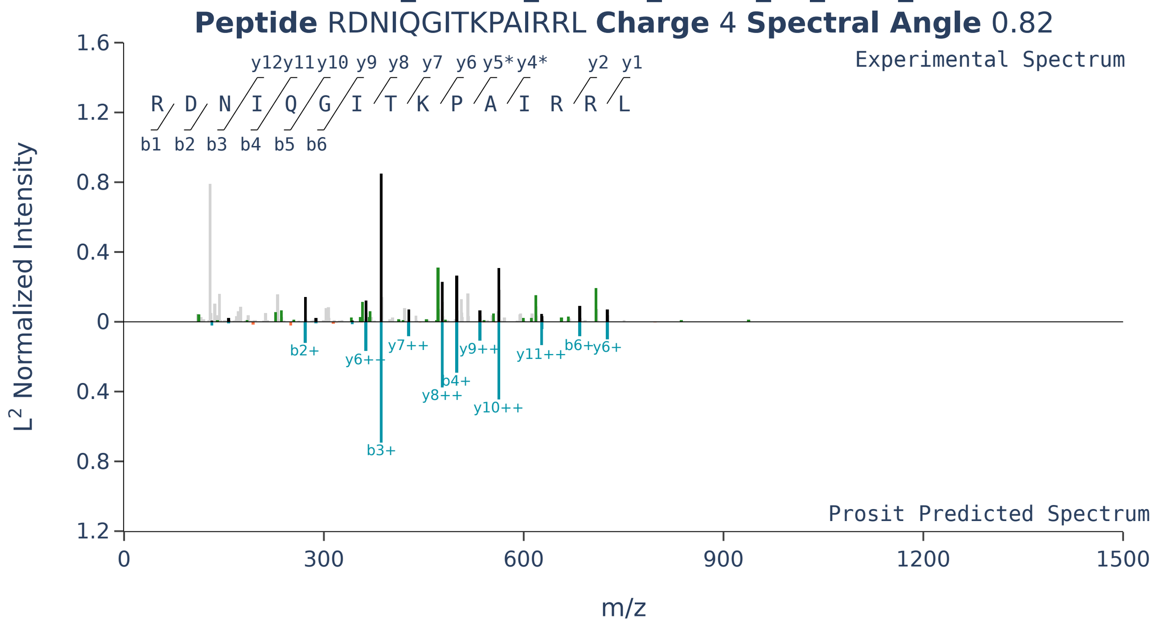


**Source** HRoetschke_291222_140123_HFGoe_H4_b1R1_4h **Scan** 12840
**Peptide** KVLRDNIQGI **Charge** 3 **Spectral Angle** 0.83

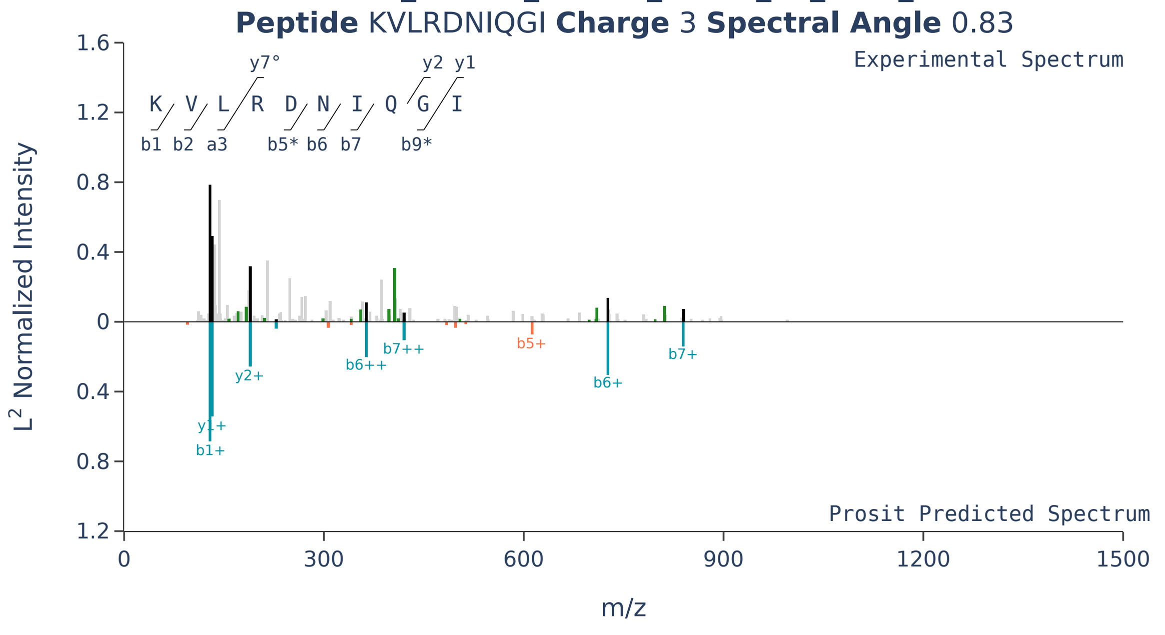


**Source** HRoetschke_291222_140123_HFGoe_H4_b1R1_4h **Scan** 15442
**Peptide** DNIQGITKPAIRRL **Charge** 4 **Spectral Angle** 0.84

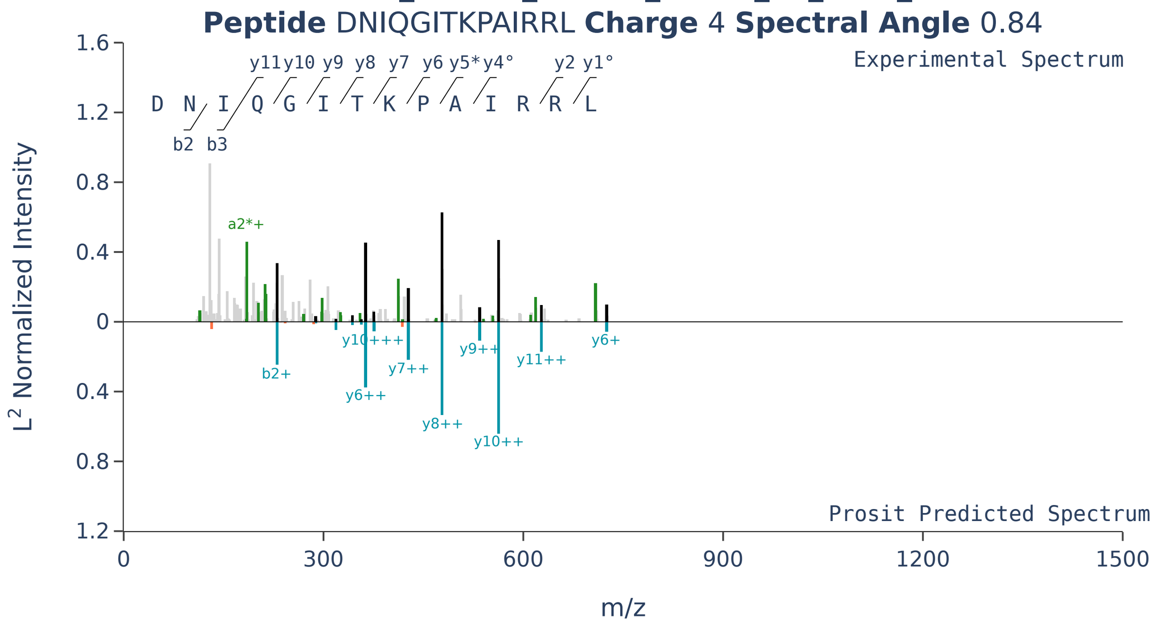


**Source** HRoetschke_291222_140123_HFGoe_H4_b2R1_1h **Scan** 22025
**Peptide** VTAMDVVYALKRQGR **Charge** 4 **Spectral Angle** 0.83

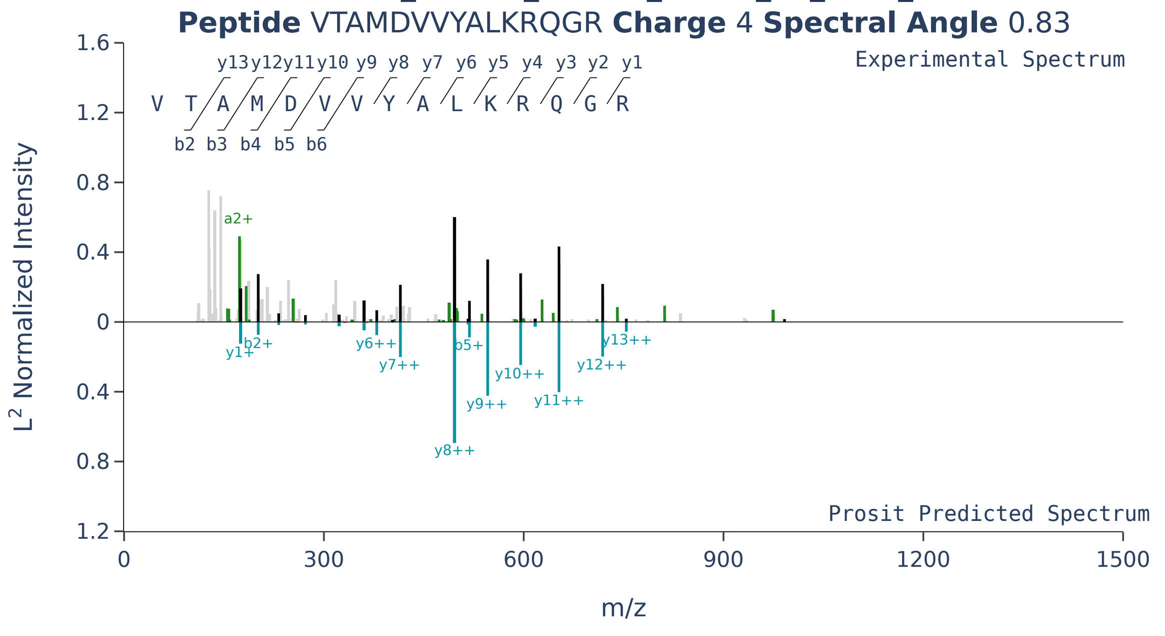


**Source** HRoetschke_291222_140123_HFGoe_H4_b1R1_1h **Scan** 17263
**Peptide** GGVKRISGLIYEE **Charge** 2 **Spectral Angle** 0.73

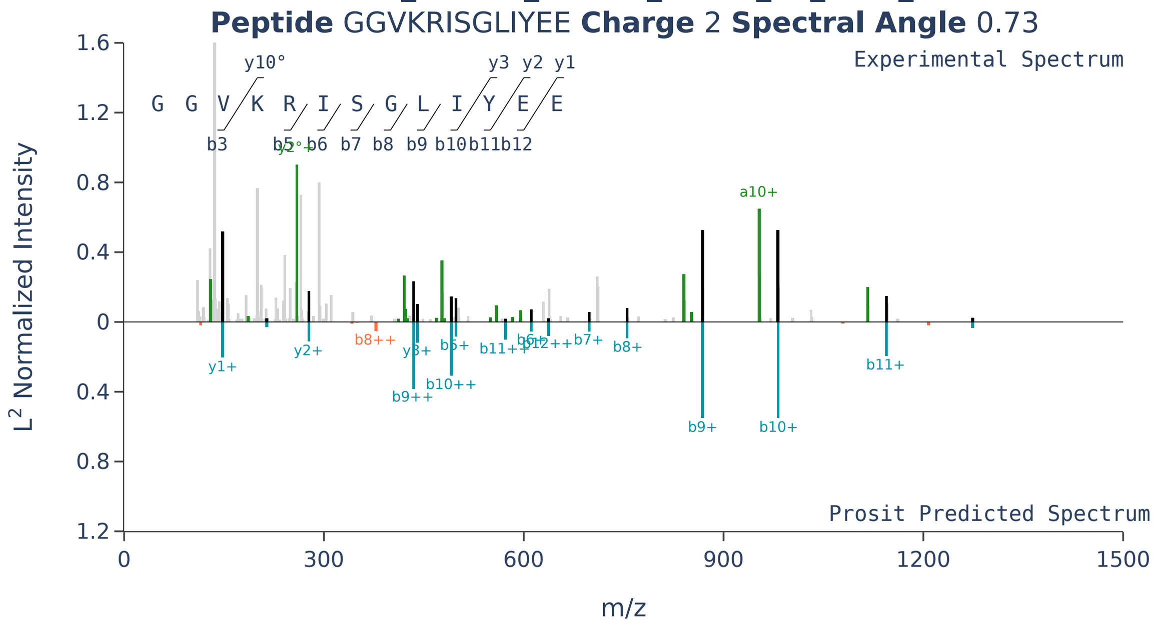


**Source** HRoetschke_291222_140123_HFGoe_H4_b2R1_1h **Scan** 15845
**Peptide** MDVVYALKRQGRTLYG **Charge** 3 **Spectral Angle** 0.71

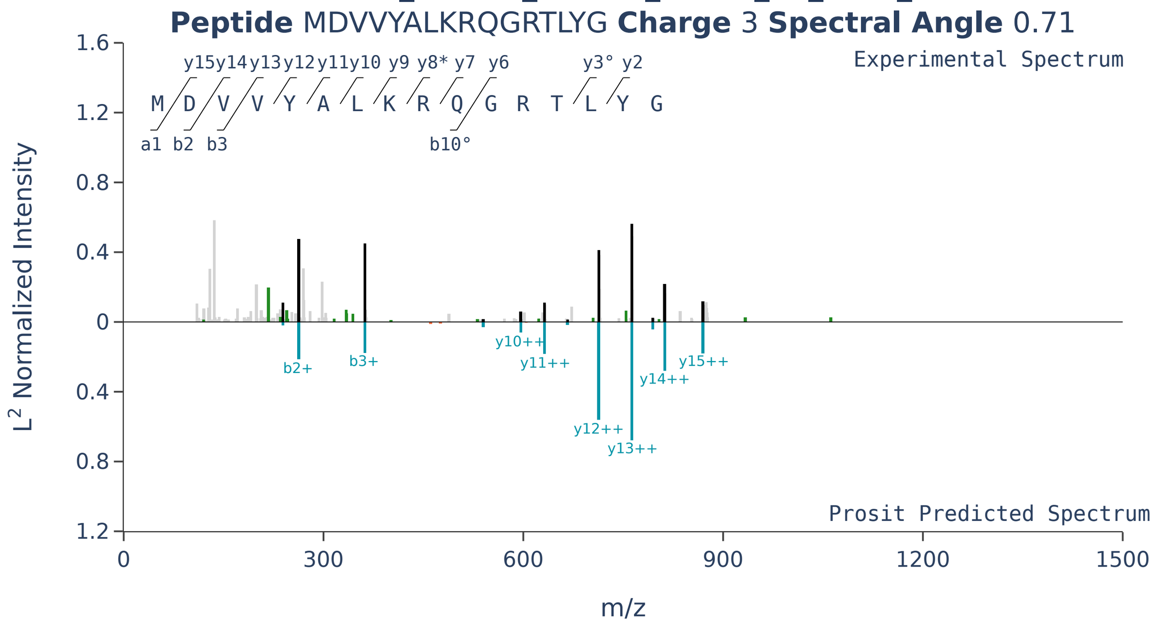

**Source** HRoetschke_291222_140123_HFGoe_H4_b2R1_1h **Scan** 28063
**Peptide** ETRGVLKVFLENVIRD **Charge** 3 **Spectral Angle** 0.85

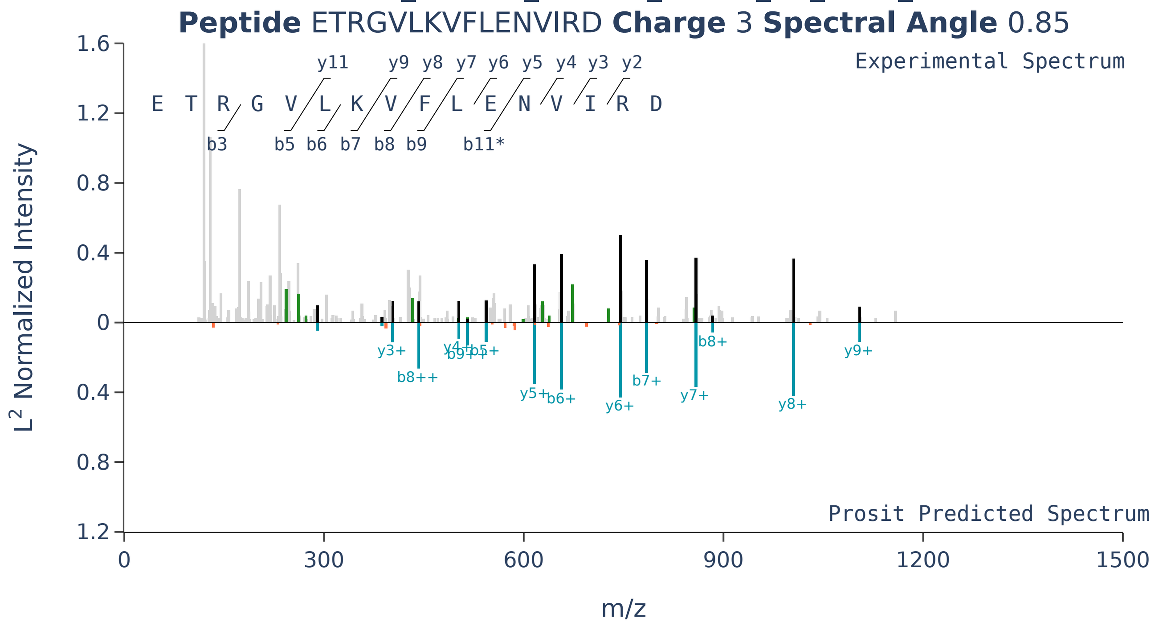


**Source** HRoetschke_291222_140123_HFGoe_H4_b1R2_1h **Scan** 16339
**Peptide** AVTYTEHAKRKTVTAMDVVYALKR **Charge** 6 **Spectral Angle** 0.83

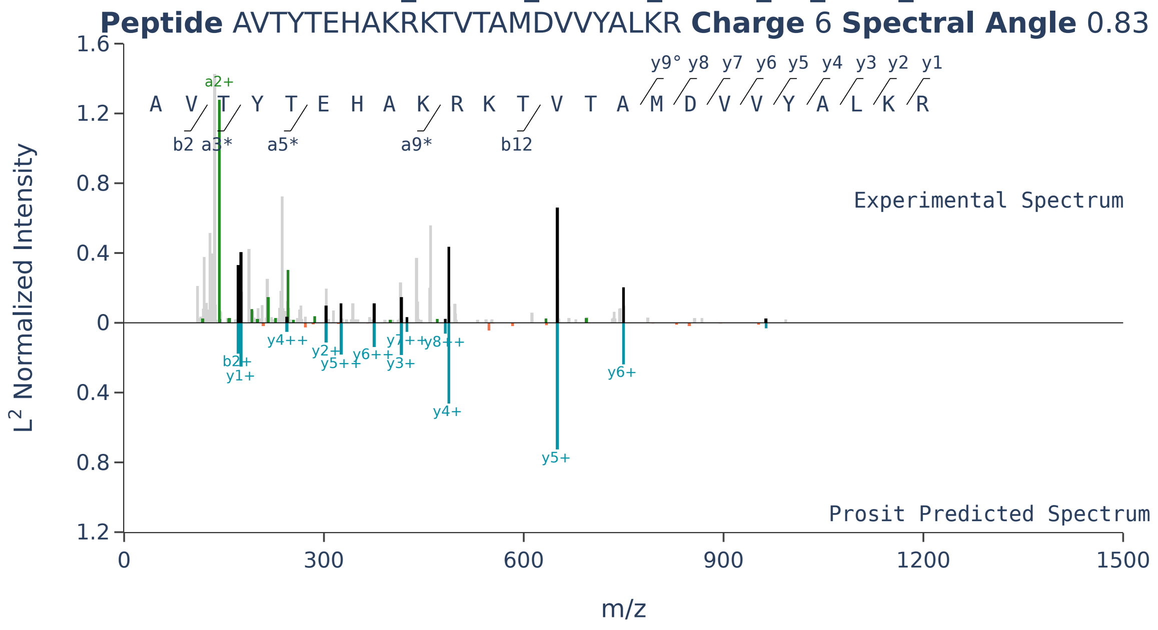


**Source** HRoetschke_291222_140123_HFGoe_H4_b2R2_1h **Scan** 24180
**Peptide** EETRGVLKVFL **Charge** 2 **Spectral Angle** 0.76

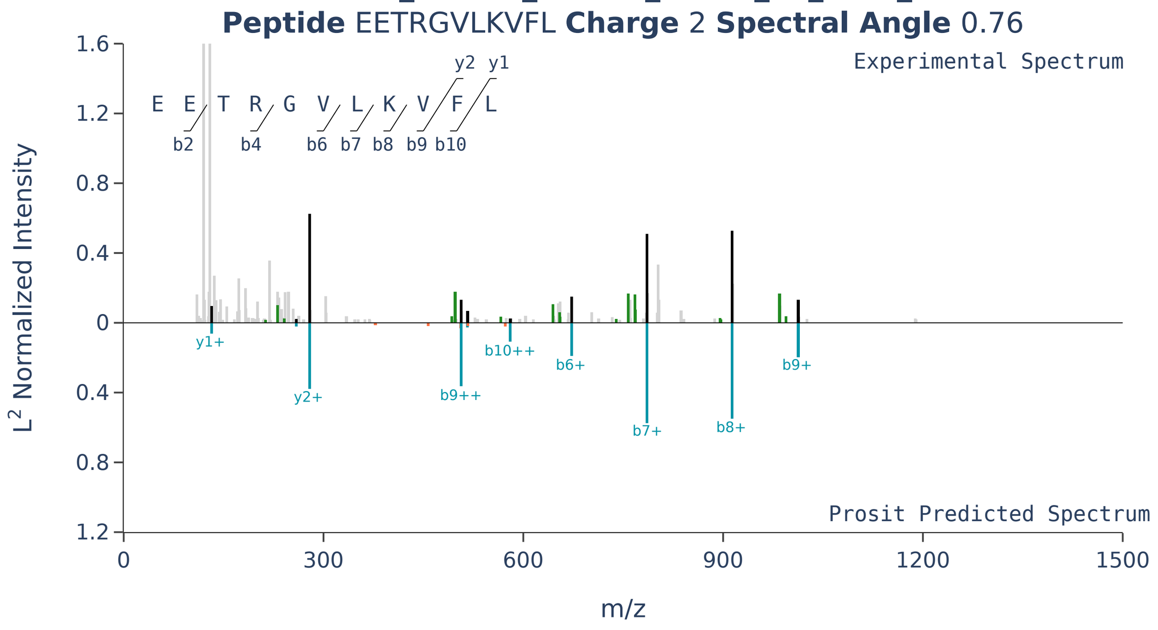


**Source** HRoetschke_291222_140123_HFGoe_H4_b2R1_24h **Scan** 9611
**Peptide** DAVTYTE **Charge** 1 **Spectral Angle** 0.85

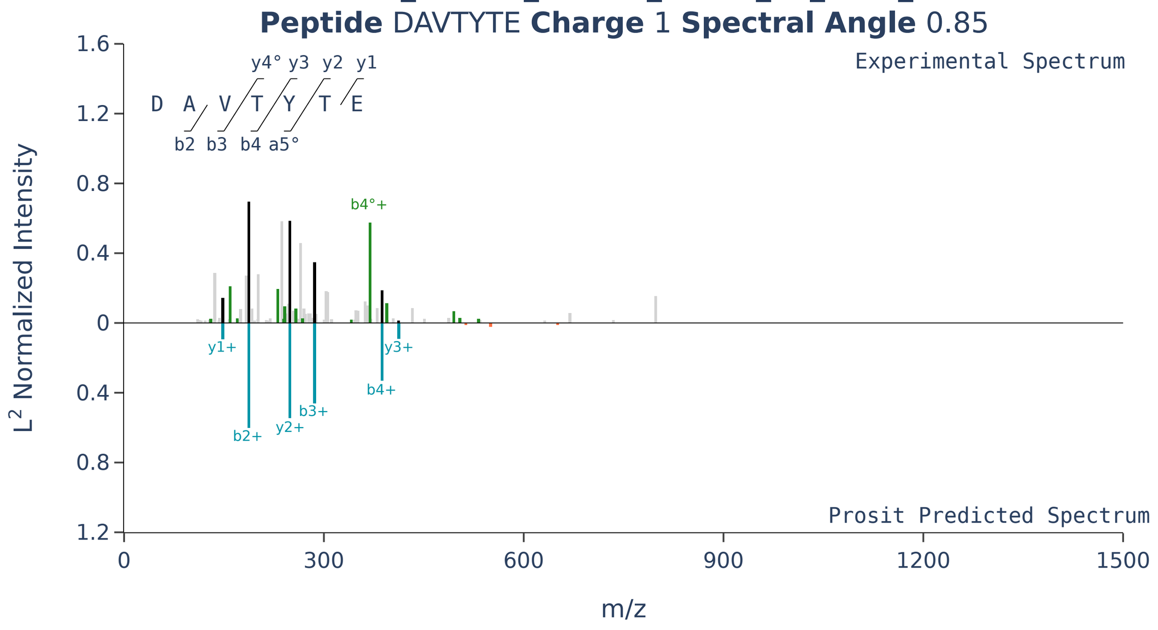


**Source** HRoetschke_291222_140123_HFGoe_H4_b1R2_1h **Scan** 17248
**Peptide** ISGLIYEETR **Charge** 2 **Spectral Angle** 0.77

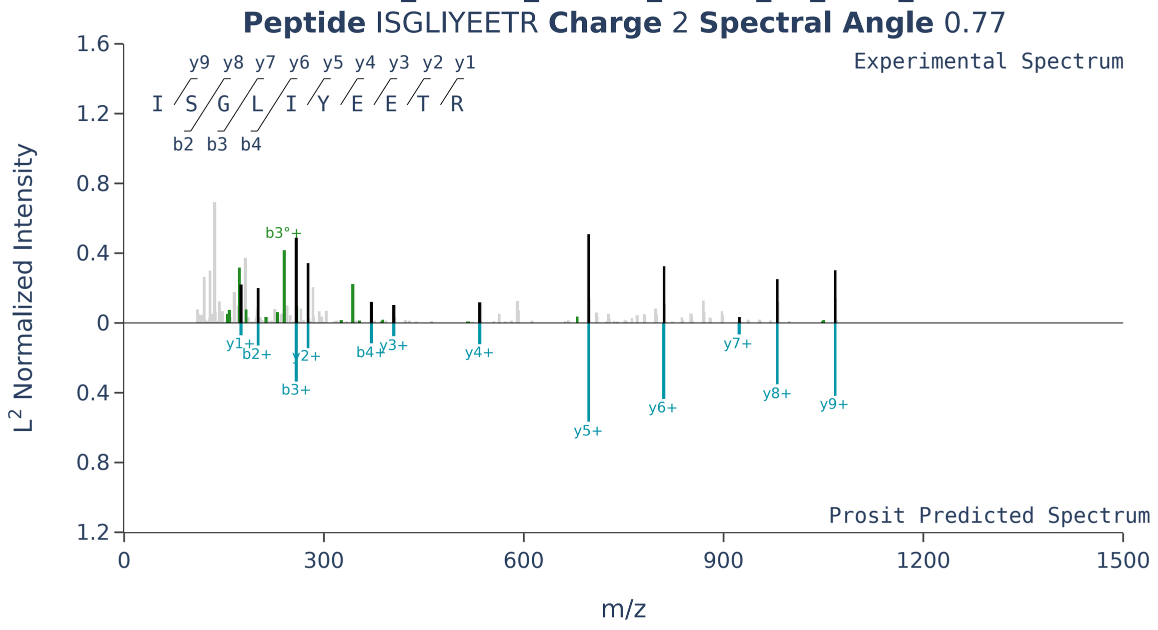


**Source** HRoetschke_291222_140123_HFGoe_H4_b1R1_1h **Scan** 22142
**Peptide** TRGVLKVFLE **Charge** 3 **Spectral Angle** 0.79

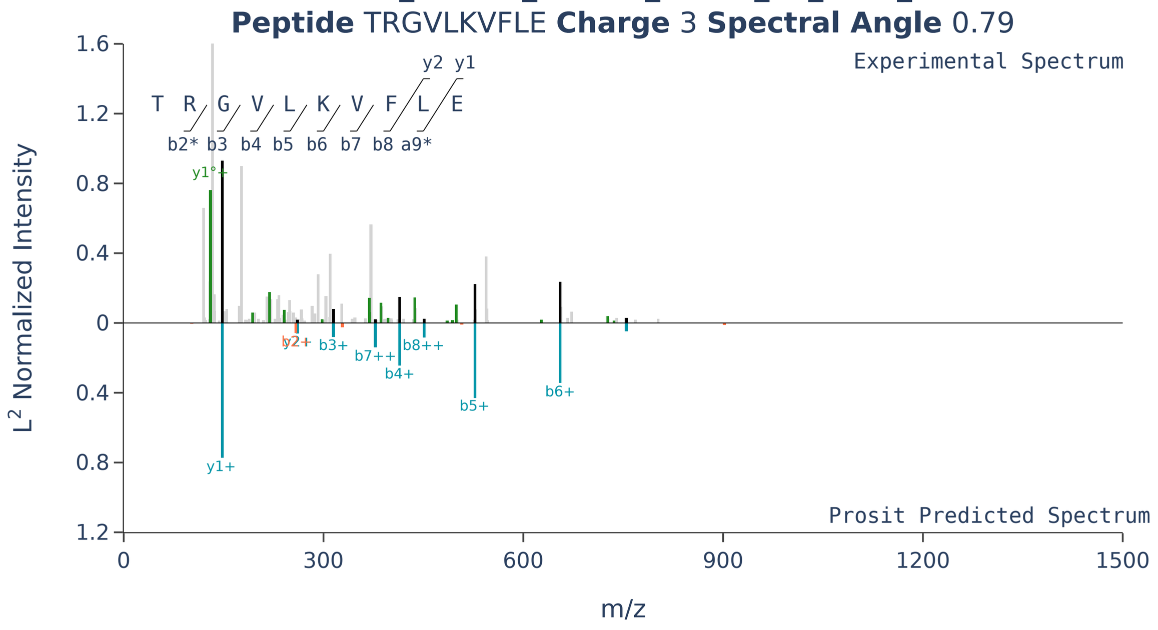


**Source** HRoetschke_291222_140123_HFGoe_H4_b2R2_4h **Scan** 14148
**Peptide** GLIYEETRG **Charge** 1 **Spectral Angle** 0.85

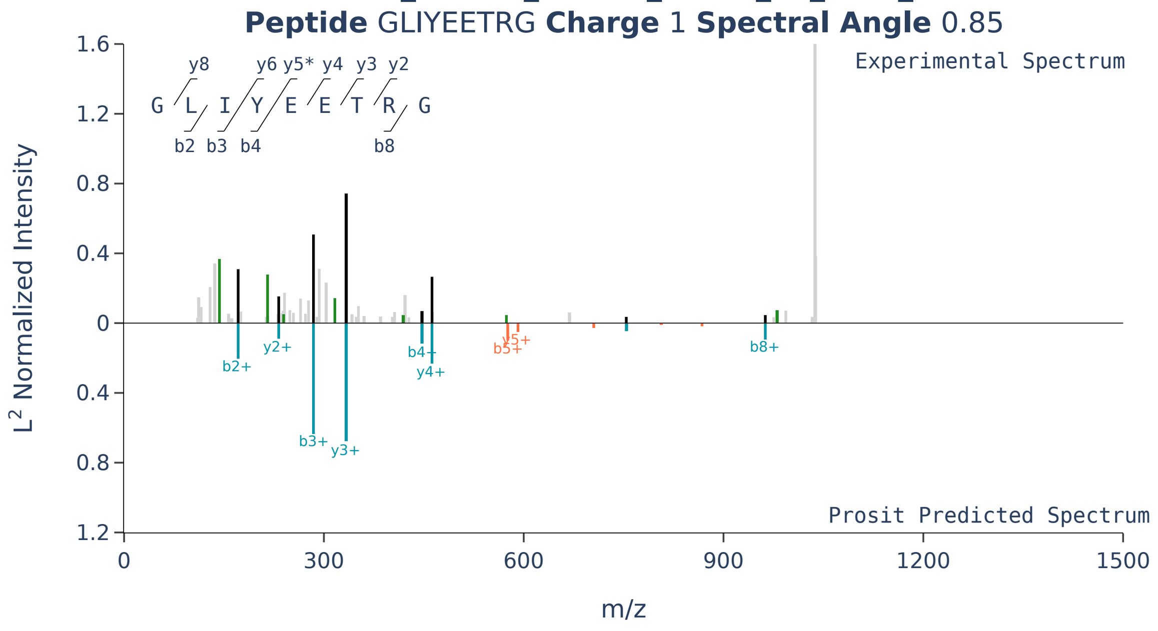


**Source** HRoetschke_291222_140123_HFGoe_H4_b2R1_4h **Scan** 13214
**Peptide** GLIYEETR **Charge** 2 **Spectral Angle** 0.79

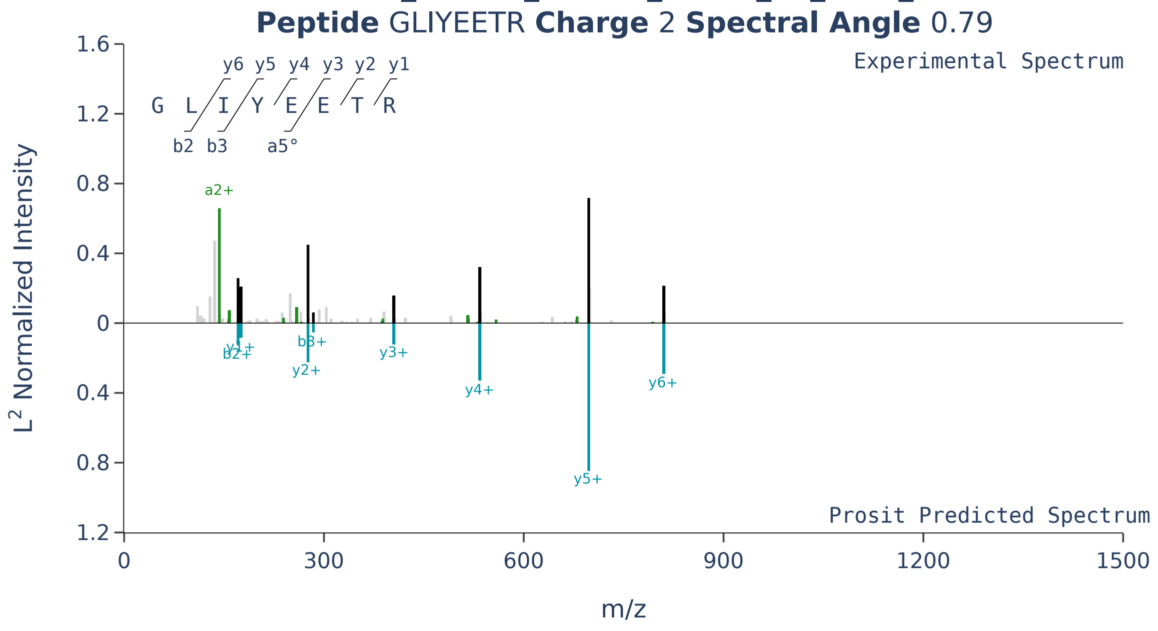


**Source** HRoetschke_291222_140123_HFGoe_H4_b2R1_1h **Scan** 7952
**Peptide** ENVIRDA **Charge** 2 **Spectral Angle** 0.84

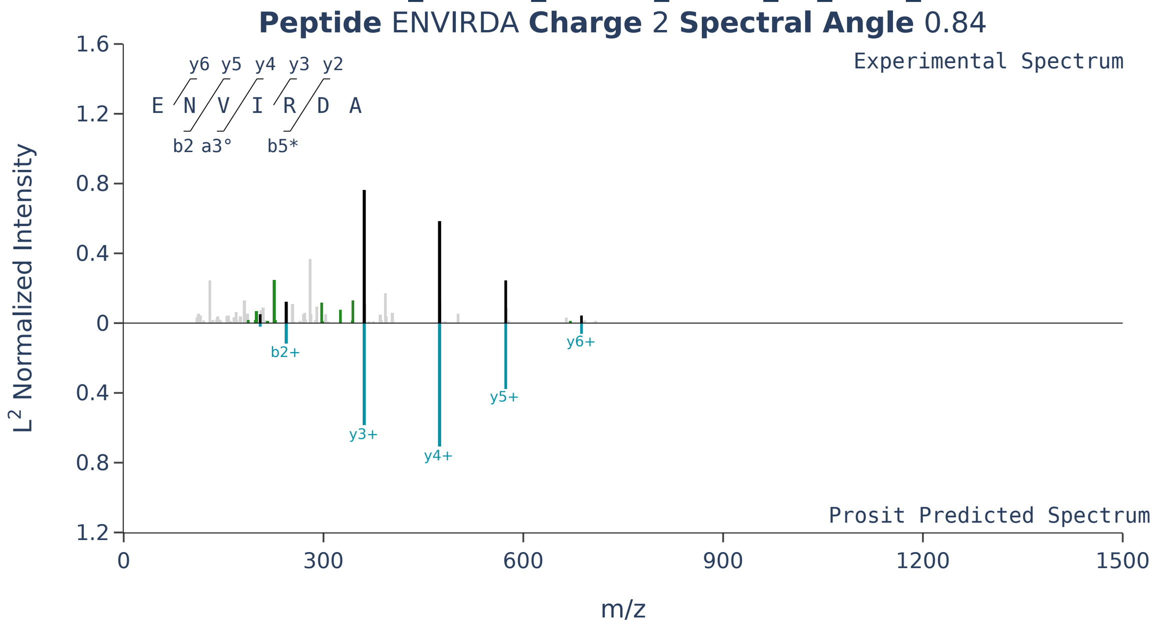


**Source** HRoetschke_291222_140123_HFGoe_H4_b2R2_1h **Scan** 10892
**Peptide** VYALKRQGRTLYG **Charge** 3 **Spectral Angle** 0.88

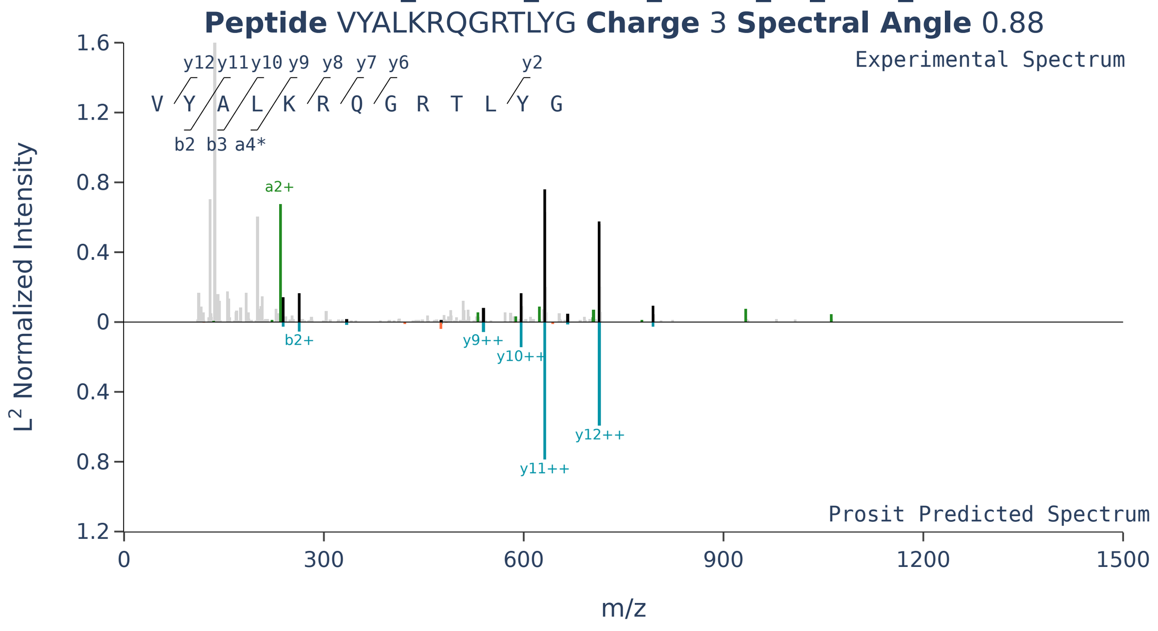


**Source** HRoetschke_291222_140123_HFGoe_H4_b1R1_1h **Scan** 15730
**Peptide** NIQGITKPAIRRL **Charge** 3 **Spectral Angle** 0.8

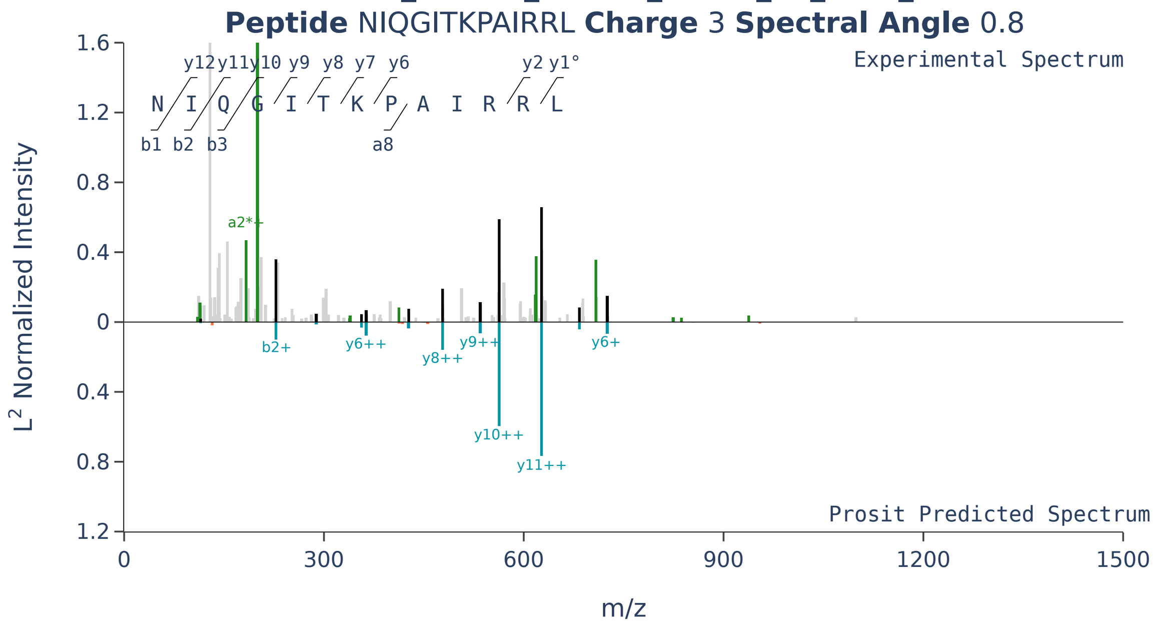


**Source** HRoetschke_291222_140123_HFGoe_H4_b1R2_24h **Scan** 11672
**Peptide** TYTEHAKRK **Charge** 2 **Spectral Angle** 0.76

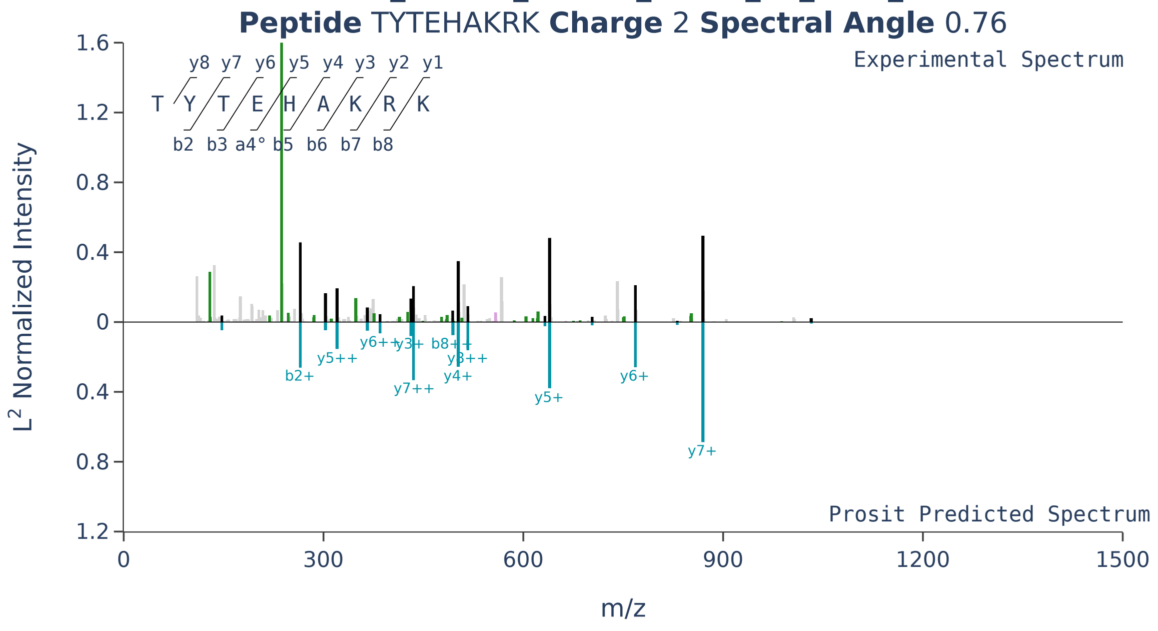


**Source** HRoetschke_291222_140123_HFGoe_H4_b2R2_24h **Scan** 6866
**Peptide** GITKPAIRR **Charge** 2 **Spectral Angle** 0.82

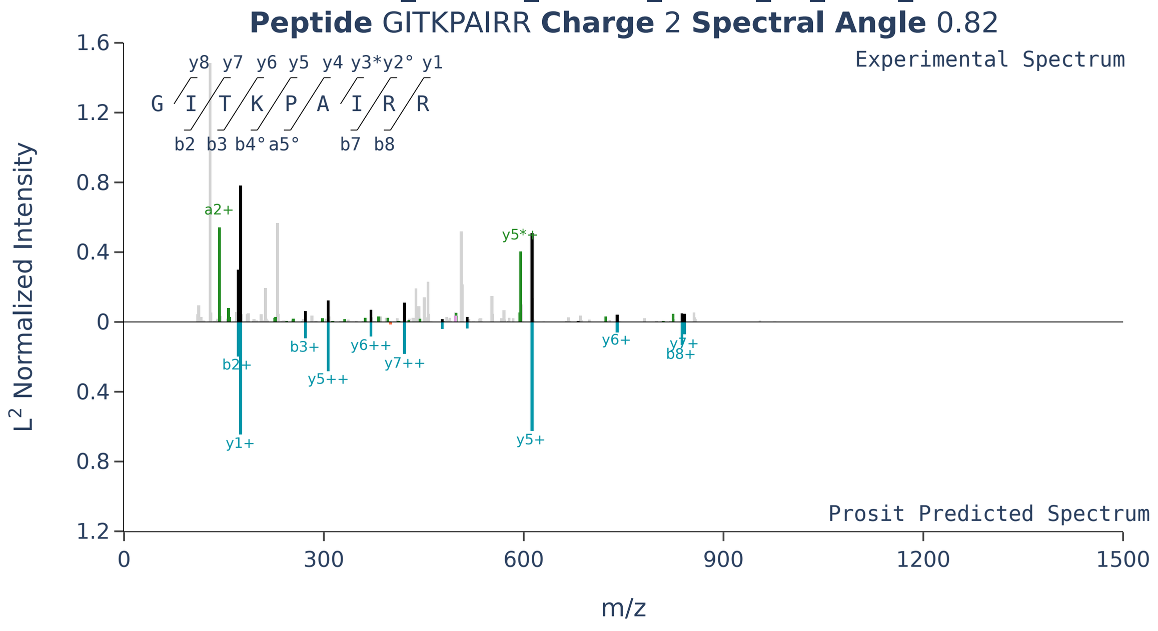


**Source** HRoetschke_291222_140123_HFGoe_H4_b2R2_4h **Scan** 16496
**Peptide** YTEHAKR **Charge** 2 **Spectral Angle** 0.79

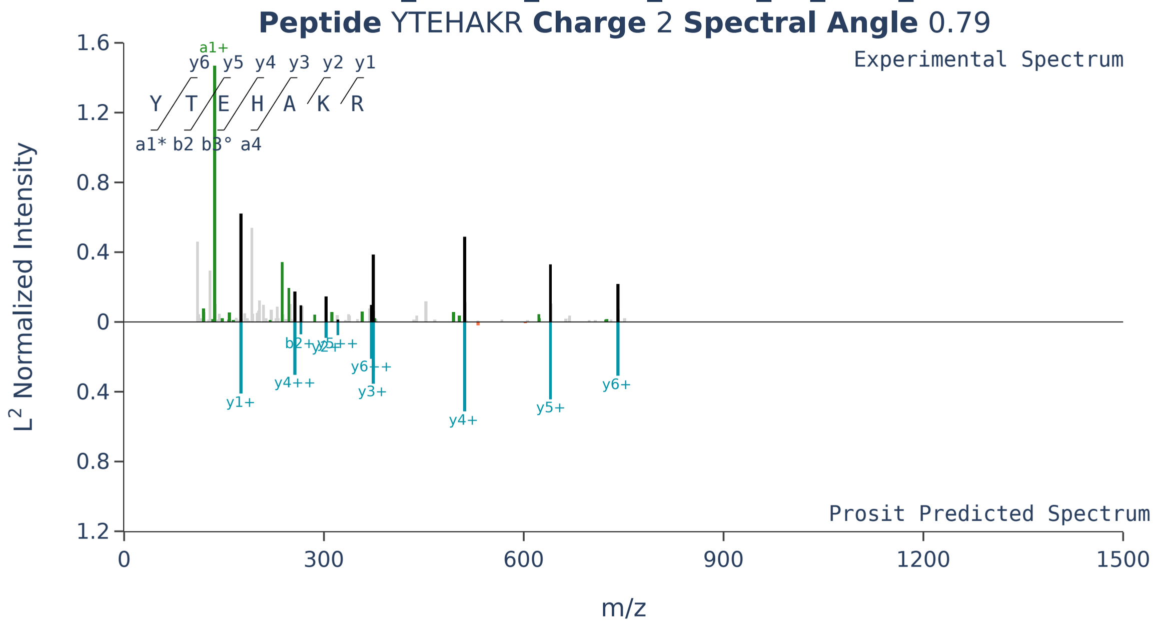


**Source** HRoetschke_291222_140123_HFGoe_H4_b1R2_24h **Scan** 6367
**Peptide** IRDAVTYTEHAKR **Charge** 4 **Spectral Angle** 0.86

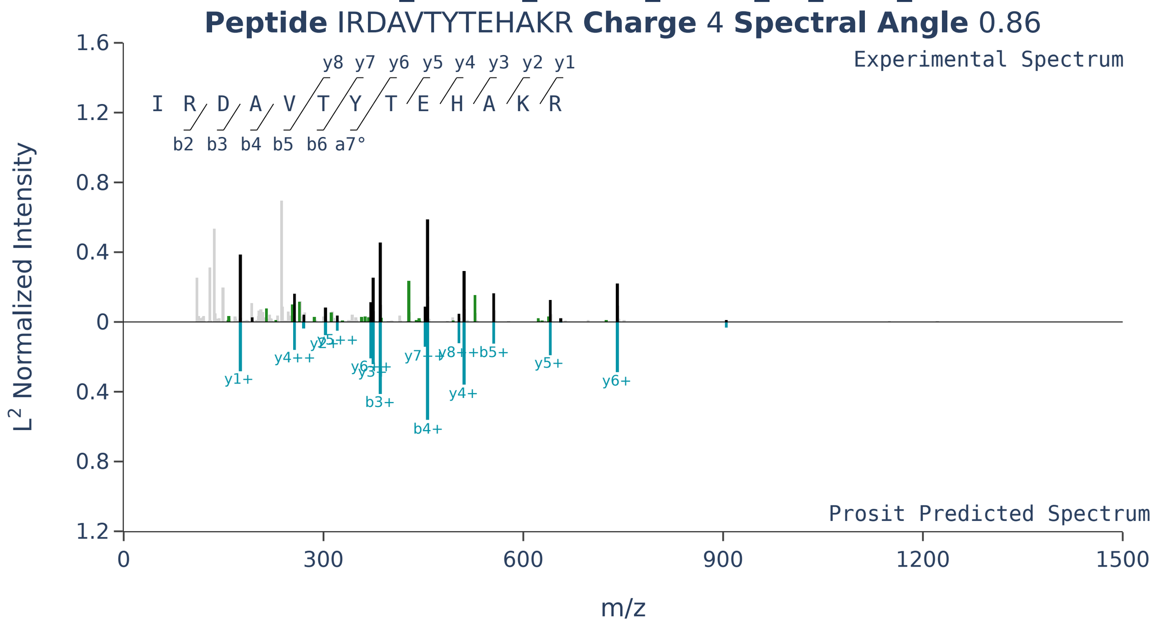

**Source** HRoetschke_291222_140123_HFGoe_H4_b2R1_1h **Scan** 26601
**Peptide** GVLKVFL **Charge** 2 **Spectral Angle** 0.76

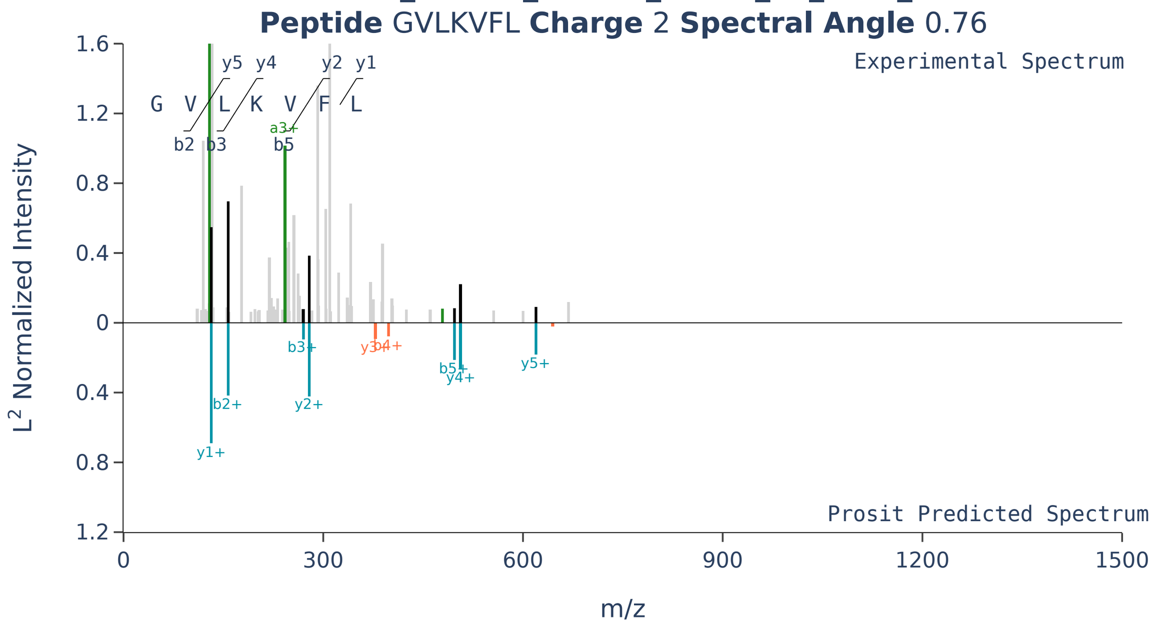


**Source** HRoetschke_291222_140123_HFGoe_H4_b1R2_2h **Scan** 12869
**Peptide** KVLRDNIQGITKPAIR **Charge** 3 **Spectral Angle** 0.85

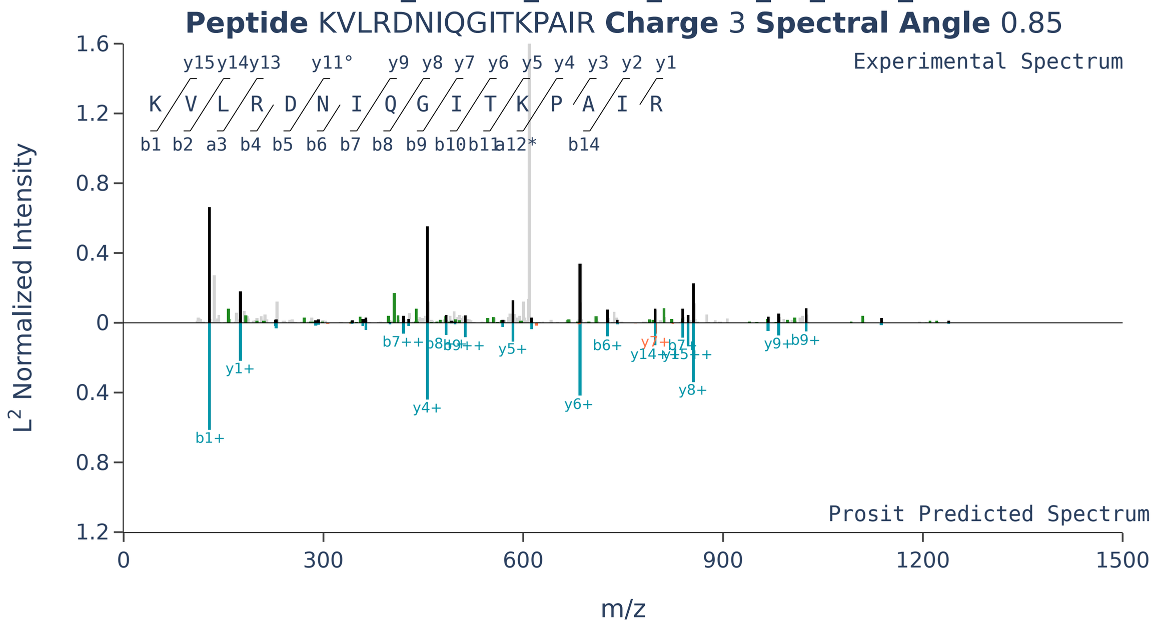


**Source** HRoetschke_291222_140123_HFGoe_H4_b1R1_4h **Scan** 5830
**Peptide** TYTEHAKRKT **Charge** 3 **Spectral Angle** 0.87

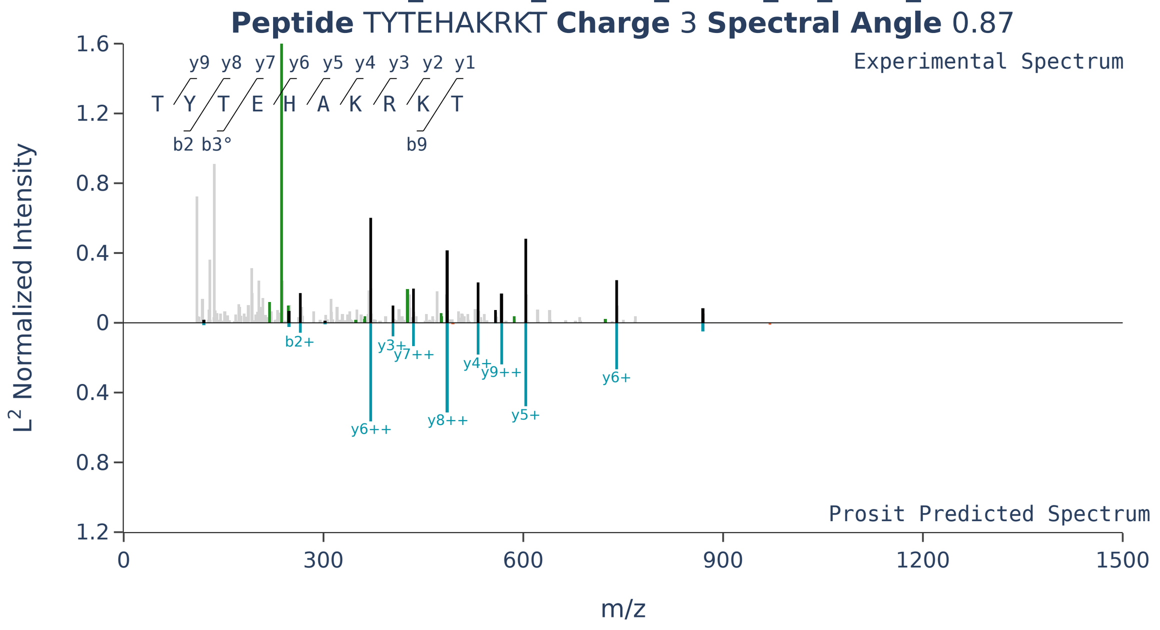


**Source** HRoetschke_291222_140123_HFGoe_H4_b1R2_1h **Scan** 12523
**Peptide** KRKTVTAMDVVYALKRQGR **Charge** 5 **Spectral Angle** 0.88

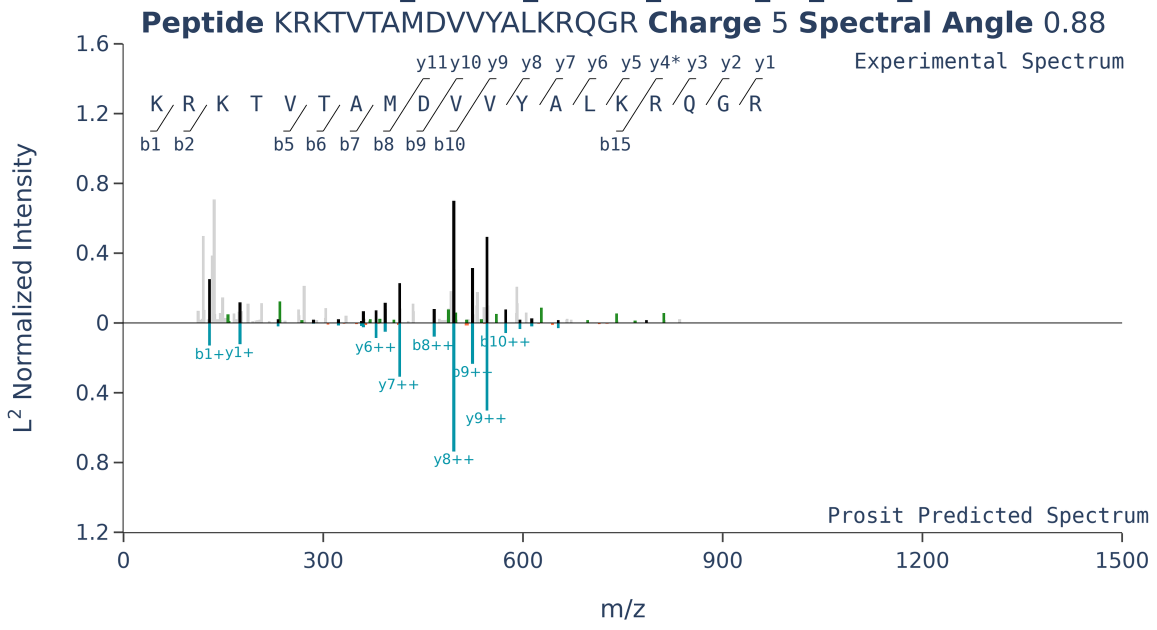


**Source** HRoetschke_291222_140123_HFGoe_H4_b1R2_24h **Scan** 11964
**Peptide** ARRGGVKRISGLIYE **Charge** 3 **Spectral Angle** 0.77

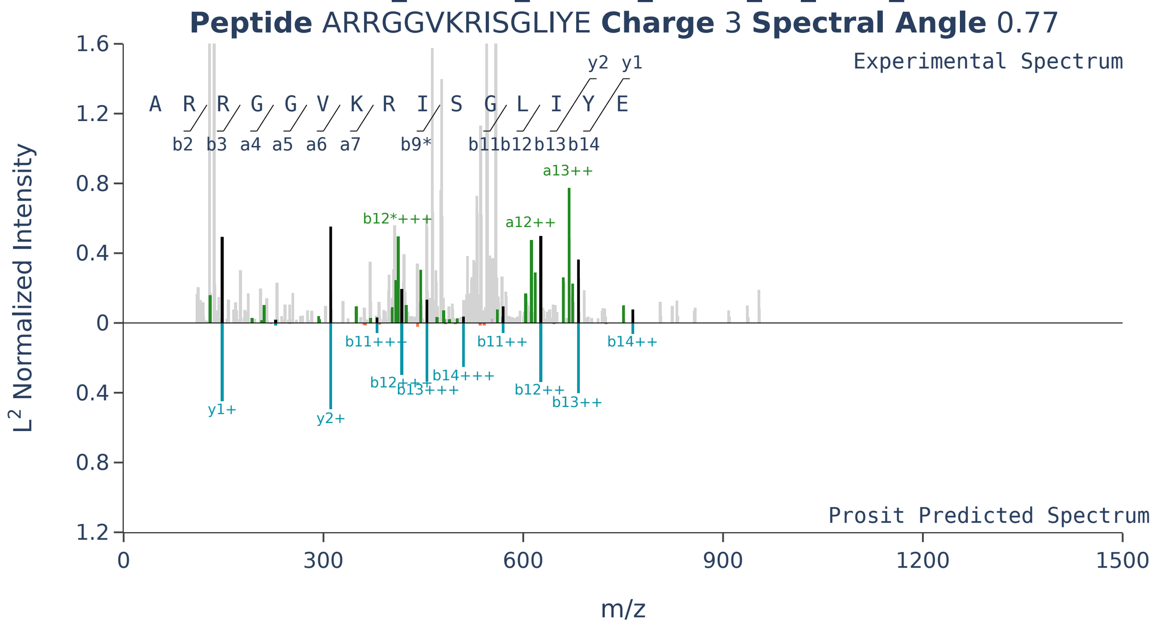


**Source** HRoetschke_291222_140123_HFGoe_H4_b1R2_4h **Scan** 17197
**Peptide** NVIRDAVTYTEHA **Charge** 2 **Spectral Angle** 0.78

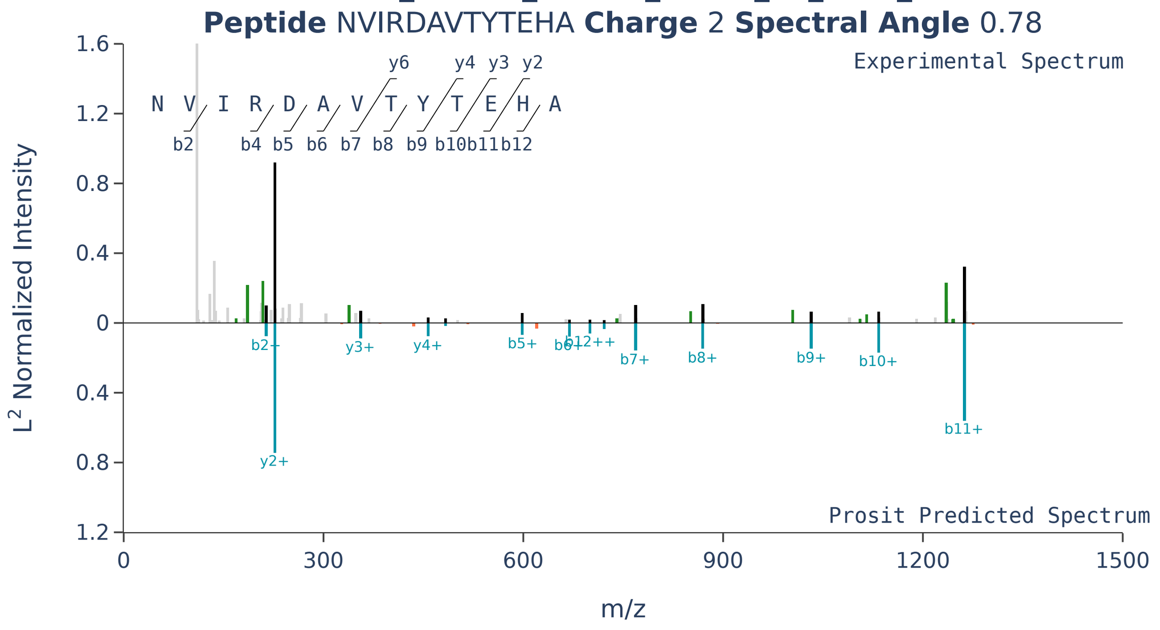


**Source** HRoetschke_291222_140123_HFGoe_H4_b2R1_1h **Scan** 19760
**Peptide** AMDVVYALKRQGRTLY **Charge** 4 **Spectral Angle** 0.87

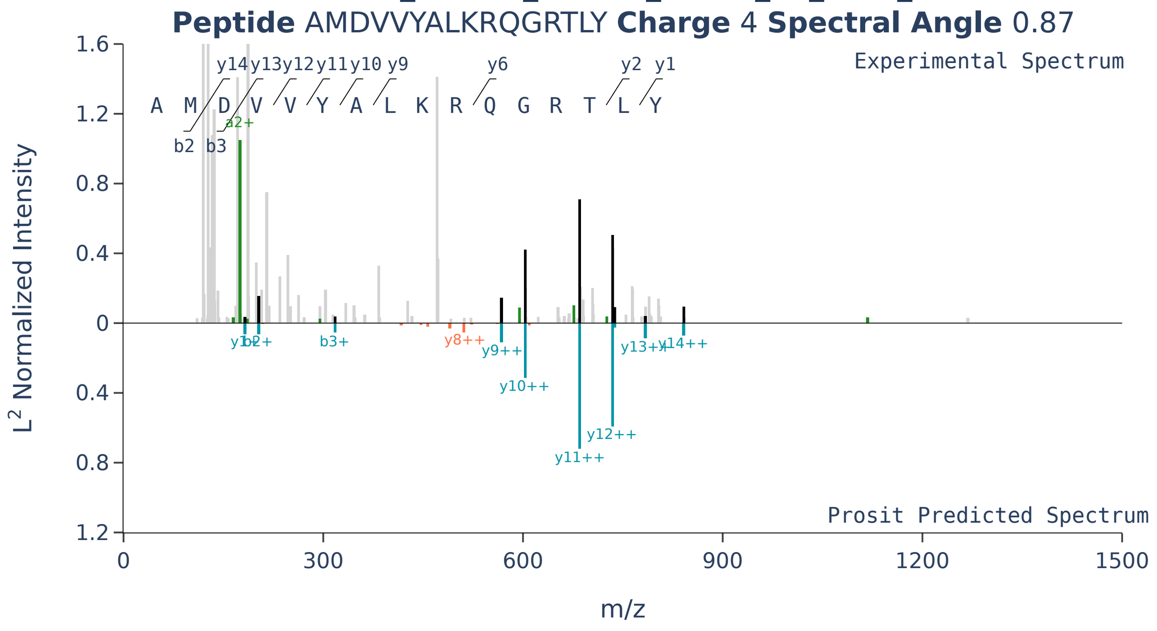


**Source** HRoetschke_291222_140123_HFGoe_H4_b2R1_1h **Scan** 17794
**Peptide** SGLIYEETRGVLK **Charge** 2 **Spectral Angle** 0.72

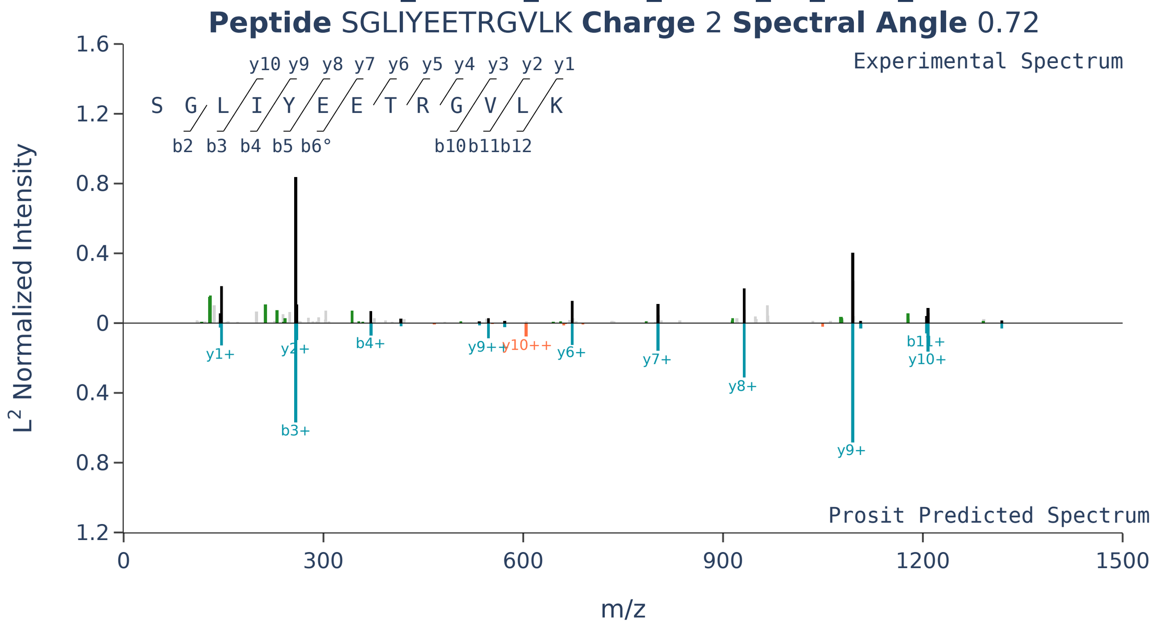


**Source** HRoetschke_291222_140123_HFGoe_H4_b2R2_24h **Scan** 12160
**Peptide** NVIRDAVTYTEHAKRKT **Charge** 5 **Spectral Angle** 0.82

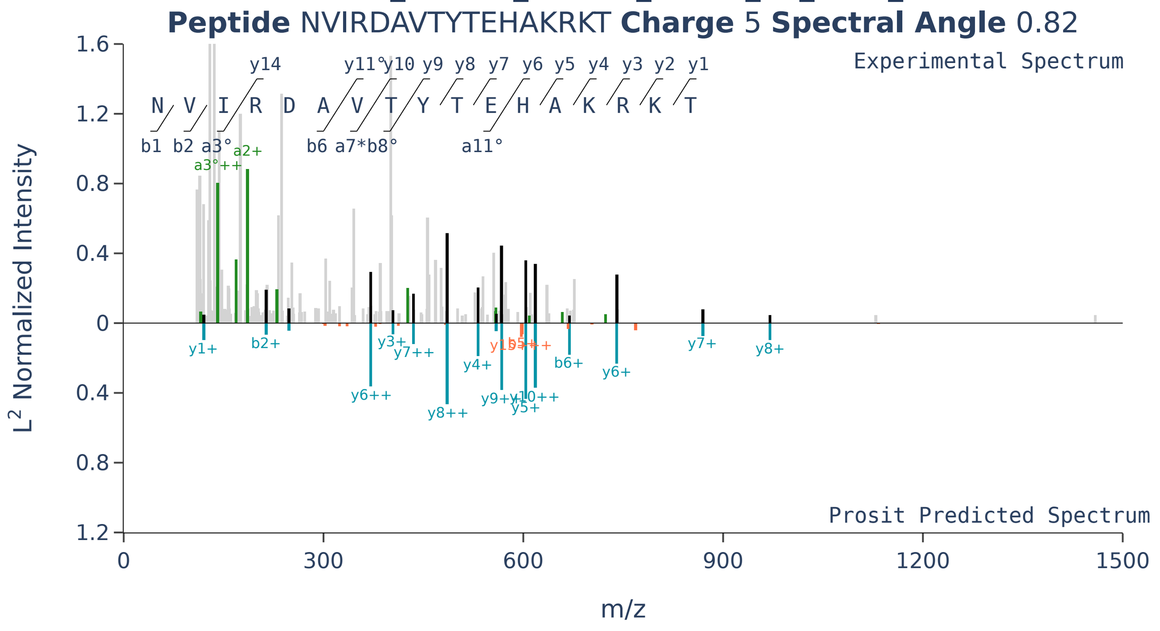


**Source** HRoetschke_291222_140123_HFGoe_H4_b1R2_24h **Scan** 5965
**Peptide** IRDAVTYTEHAKRK **Charge** 4 **Spectral Angle** 0.79

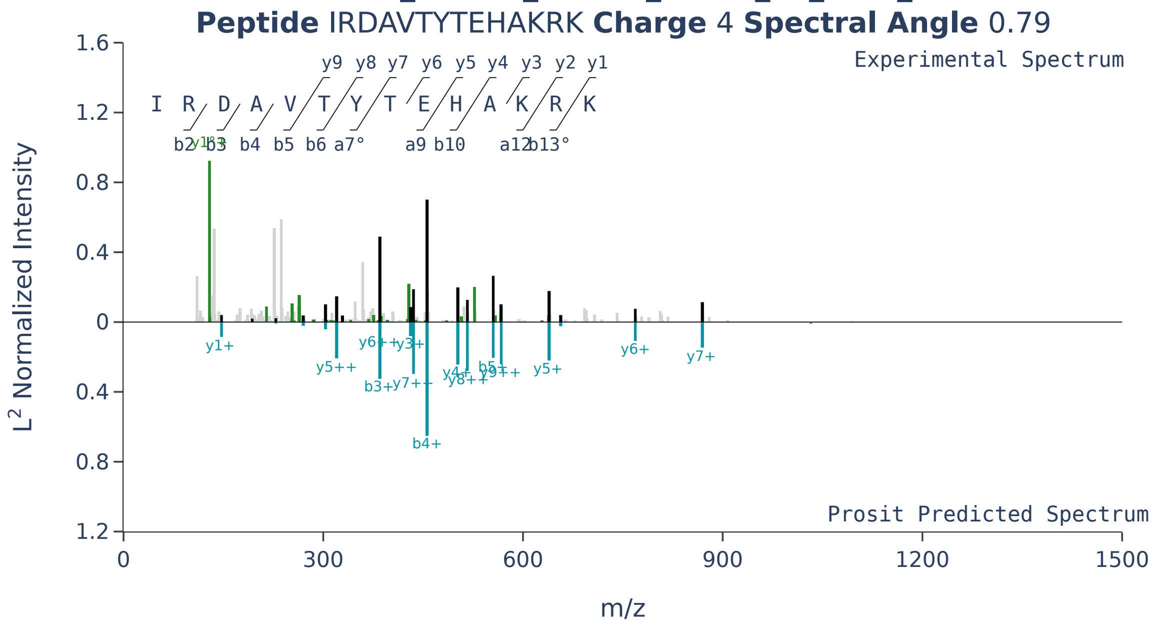


**Source** HRoetschke_291222_140123_HFGoe_H4_b1R1_24h **Scan** 9363
**Peptide** LRDAVTYTEHA **Charge** 2 **Spectral Angle** 0.82

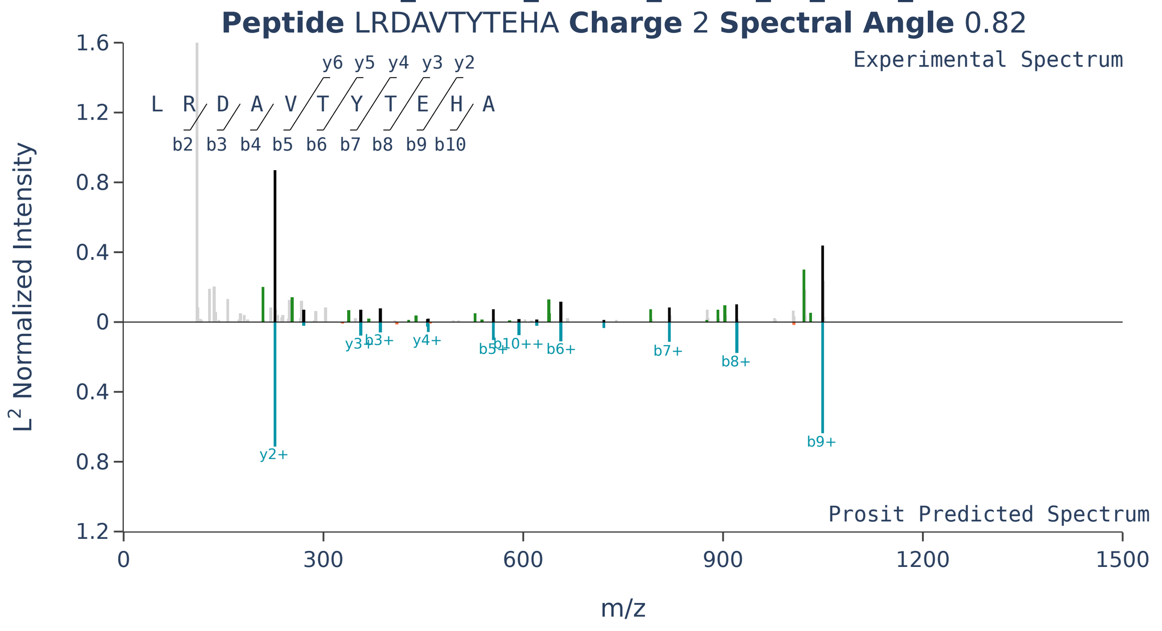


**Source** HRoetschke_291222_140123_HFGoe_H4_b1R2_1h **Scan** 17090
**Peptide** KRKTVTAMDVVYALKRQG **Charge** 3 **Spectral Angle** 0.9

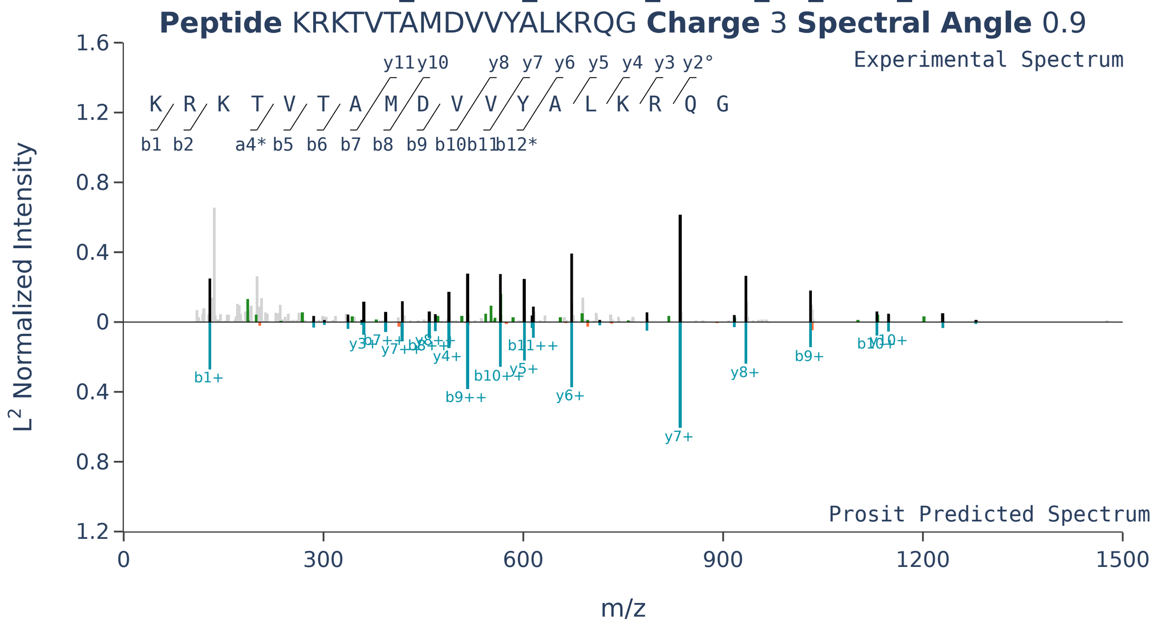


**Source** HRoetschke_291222_140123_HFGoe_H4_b1R2_2h **Scan** 16635
**Peptide** DNIQGITKPAIRRLA **Charge** 4 **Spectral Angle** 0.75

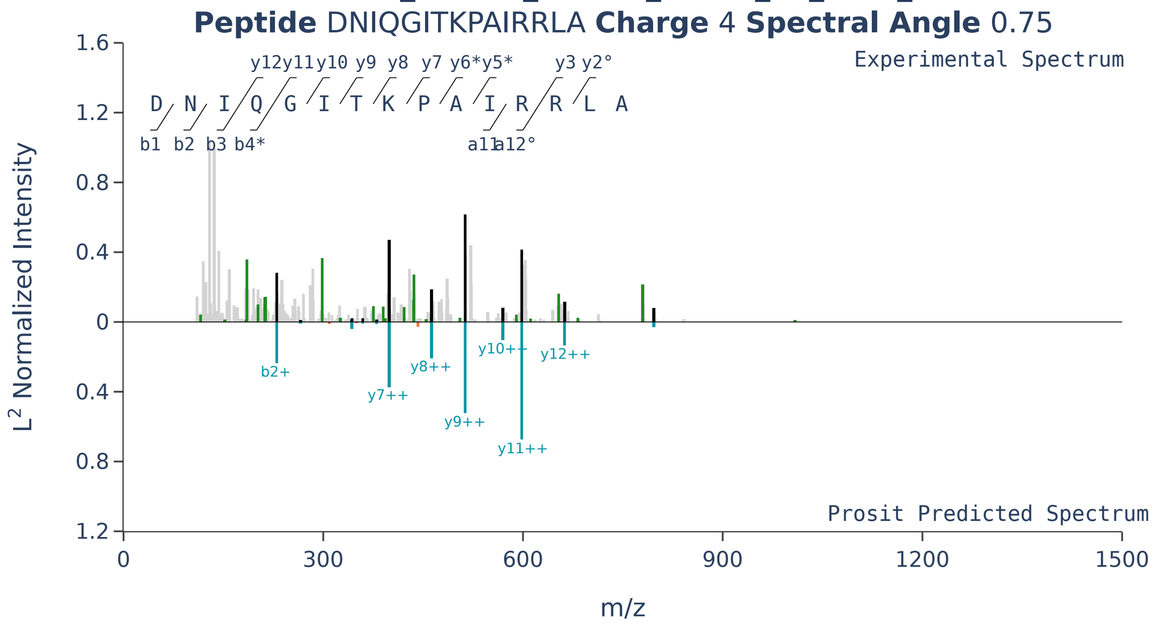


**Source** HRoetschke_291222_140123_HFGoe_H4_b1R1_2h **Scan** 14799
**Peptide** ENVIRDAVTYTEHAKRK **Charge** 3 **Spectral Angle** 0.8

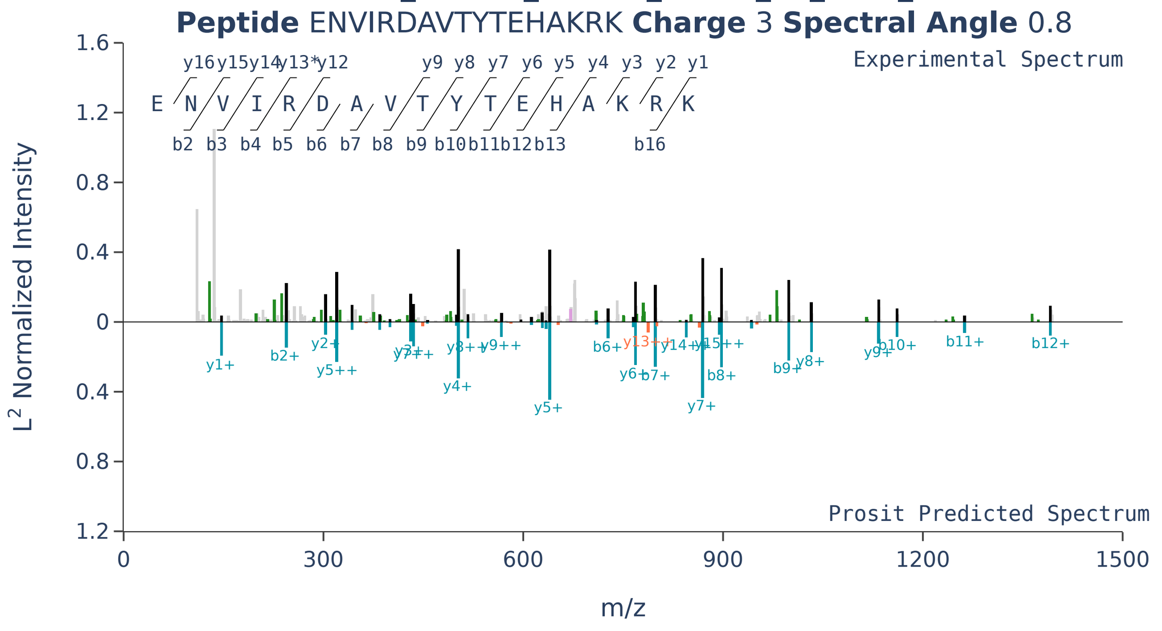


**Source** HRoetschke_291222_140123_HFGoe_H4_b1R1_4h **Scan** 14489
**Peptide** VLRDNIQGITKPALR **Charge** 4 **Spectral Angle** 0.89

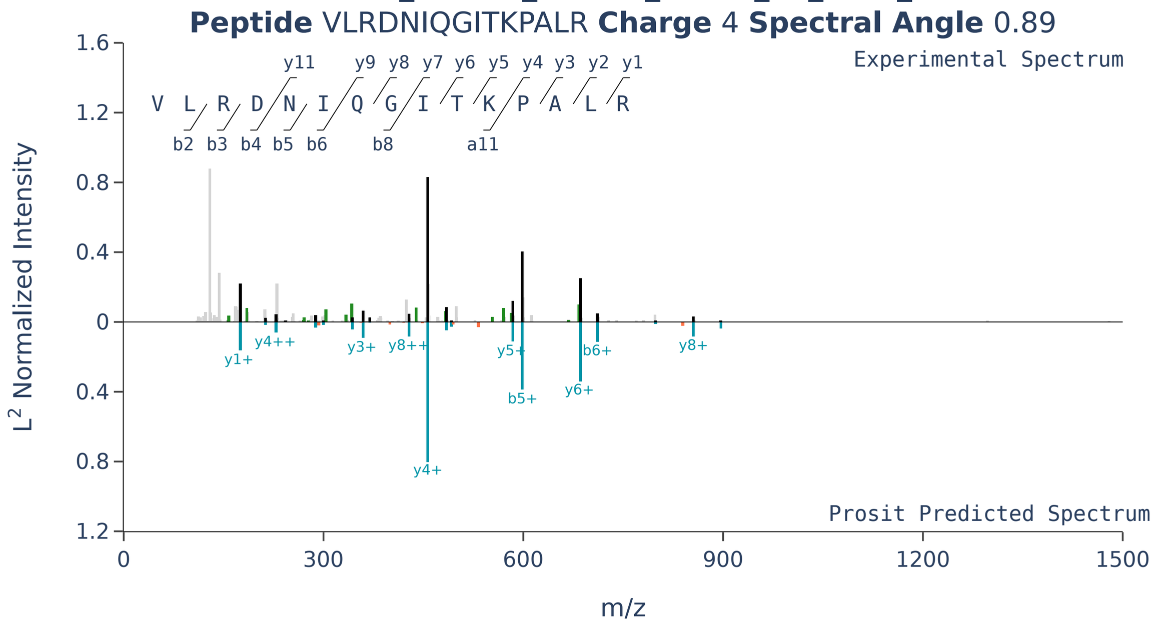

**Source** HRoetschke_291222_140123_HFGoe_H4_b2R1_1h **Scan** 19602
**Peptide** AMDVVYALKRQGRTLYG **Charge** 4 **Spectral Angle** 0.86

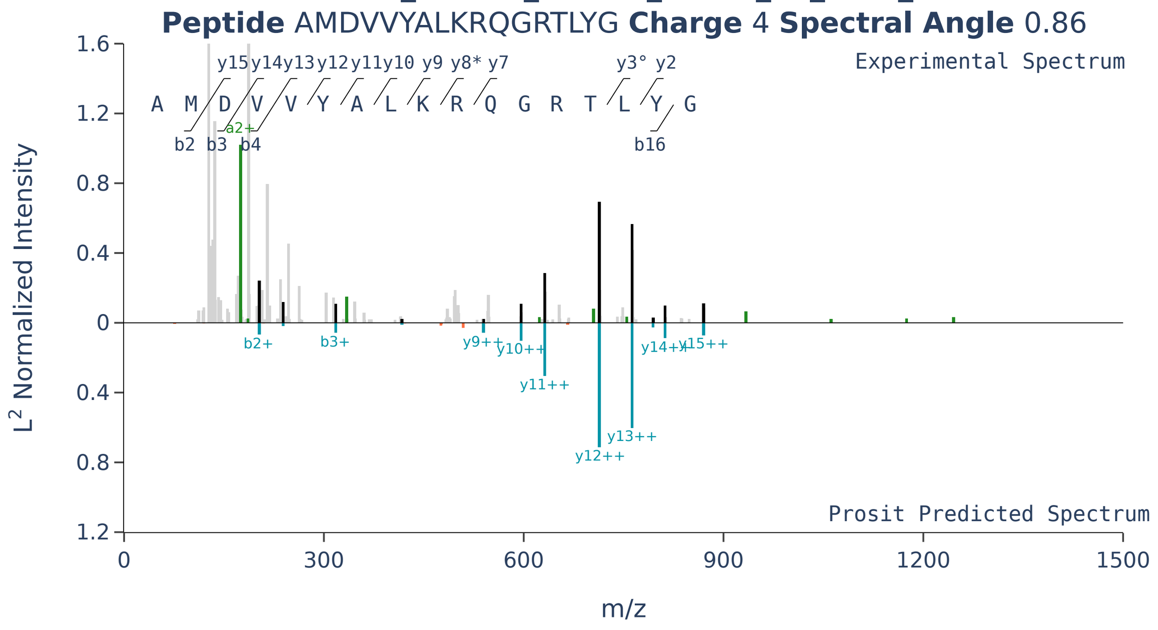


**Source** HRoetschke_291222_140123_HFGoe_H4_b1R2_2h **Scan** 16339
**Peptide** IYEETRGVLKV **Charge** 2 **Spectral Angle** 0.77

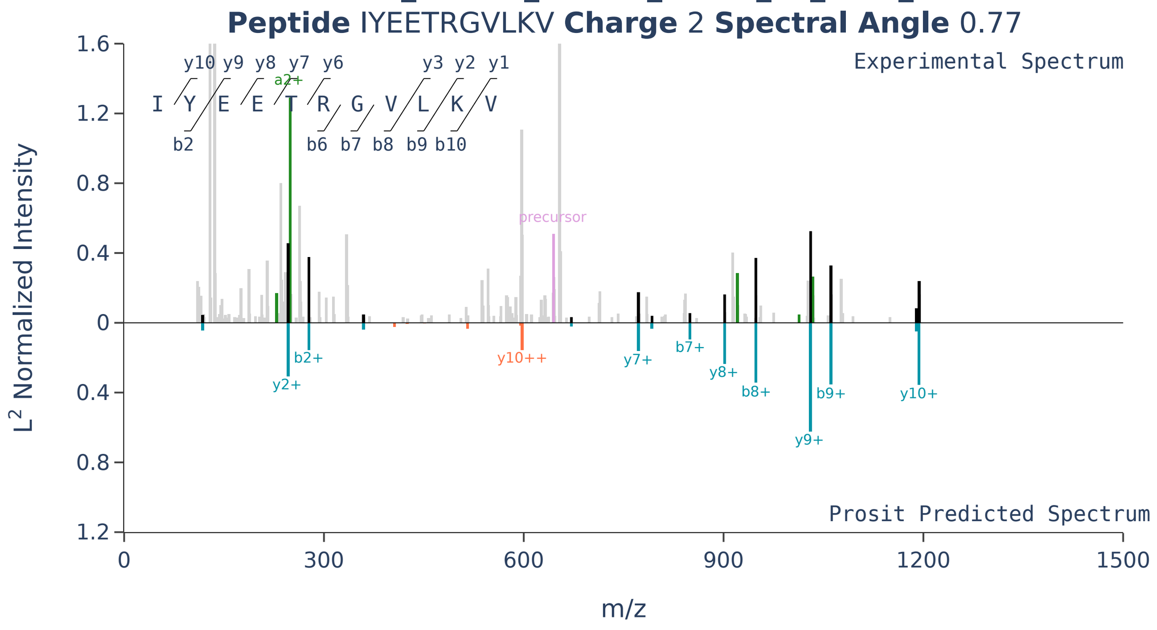


**Source** HRoetschke_291222_140123_HFGoe_H4_b2R2_24h **Scan** 11193
**Peptide** LENVLRD **Charge** 2 **Spectral Angle** 0.89

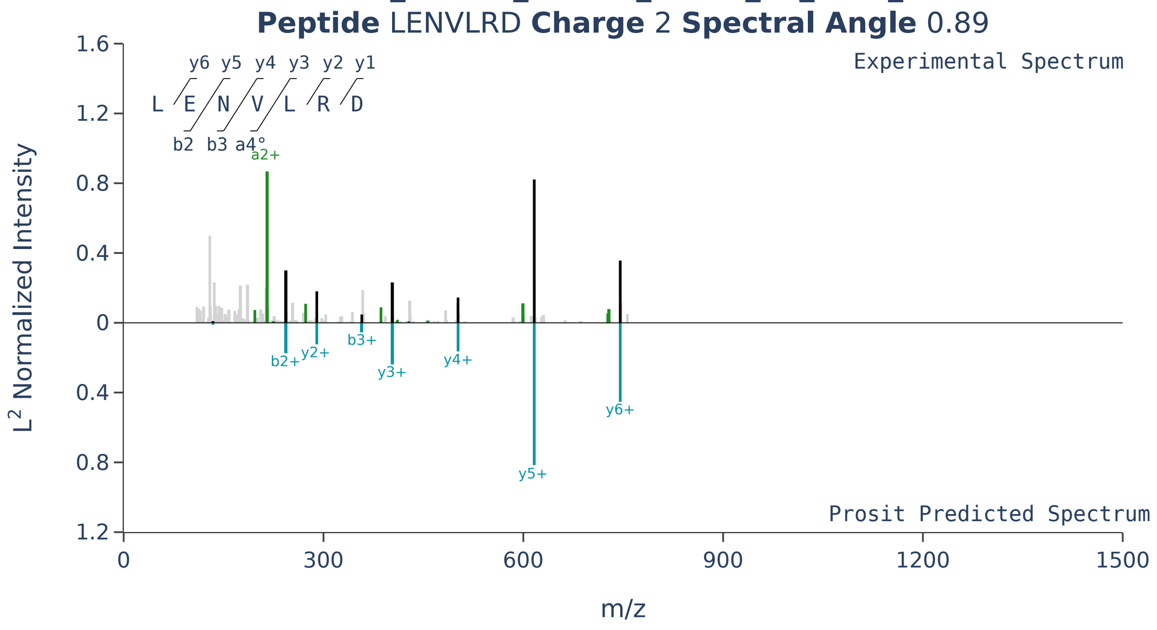


**Source** HRoetschke_291222_140123_HFGoe_H4_b1R2_2h **Scan** 5520
**Peptide** TYTEHAKRKTVT **Charge** 2 **Spectral Angle** 0.74

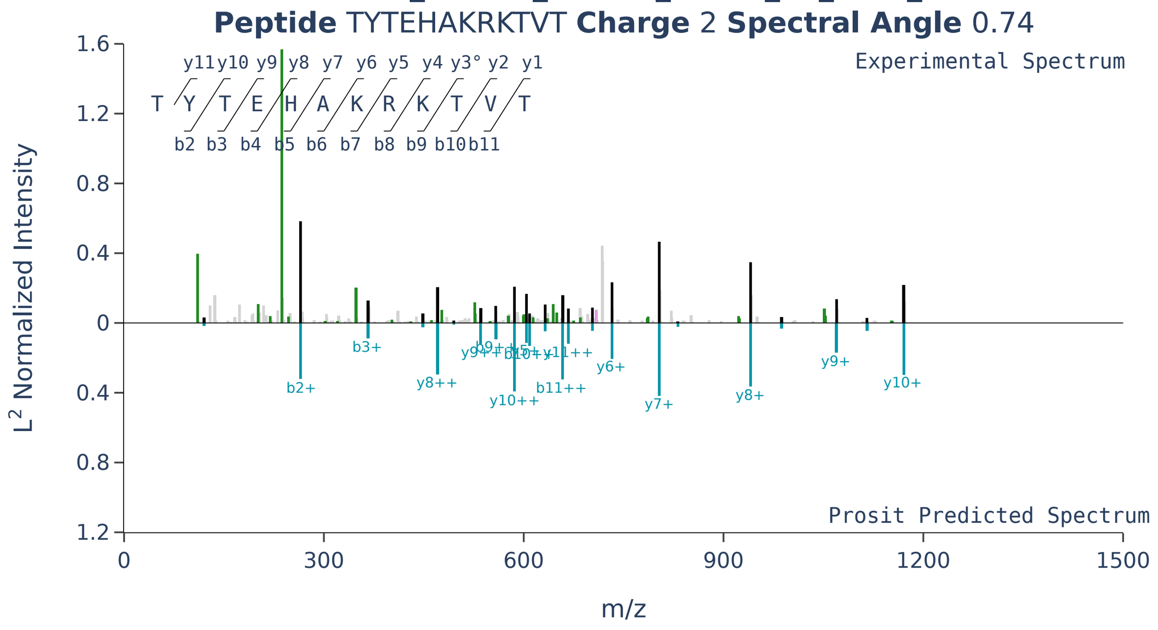


**Source** HRoetschke_291222_140123_HFGoe_H4_b1R1_4h **Scan** 14539
**Peptide** NIQGITKPAIRRLA **Charge** 3 **Spectral Angle** 0.77

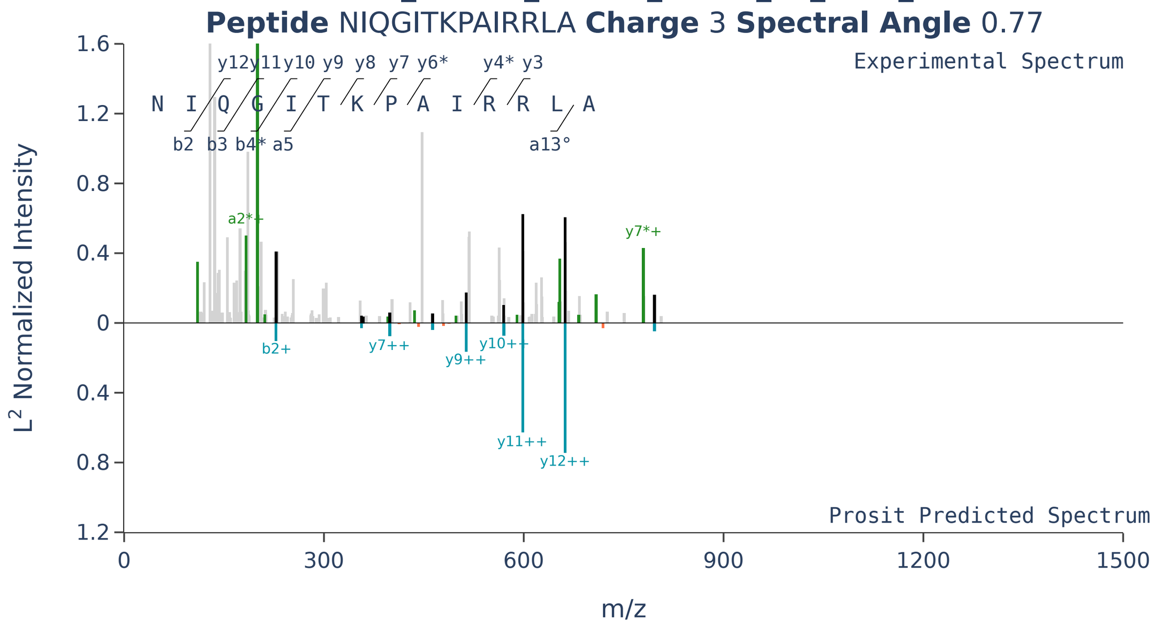


**Source** HRoetschke_291222_140123_HFGoe_H4_b2R2_2h **Scan** 15585
**Peptide** NVIRDAVTYTE **Charge** 2 **Spectral Angle** 0.76

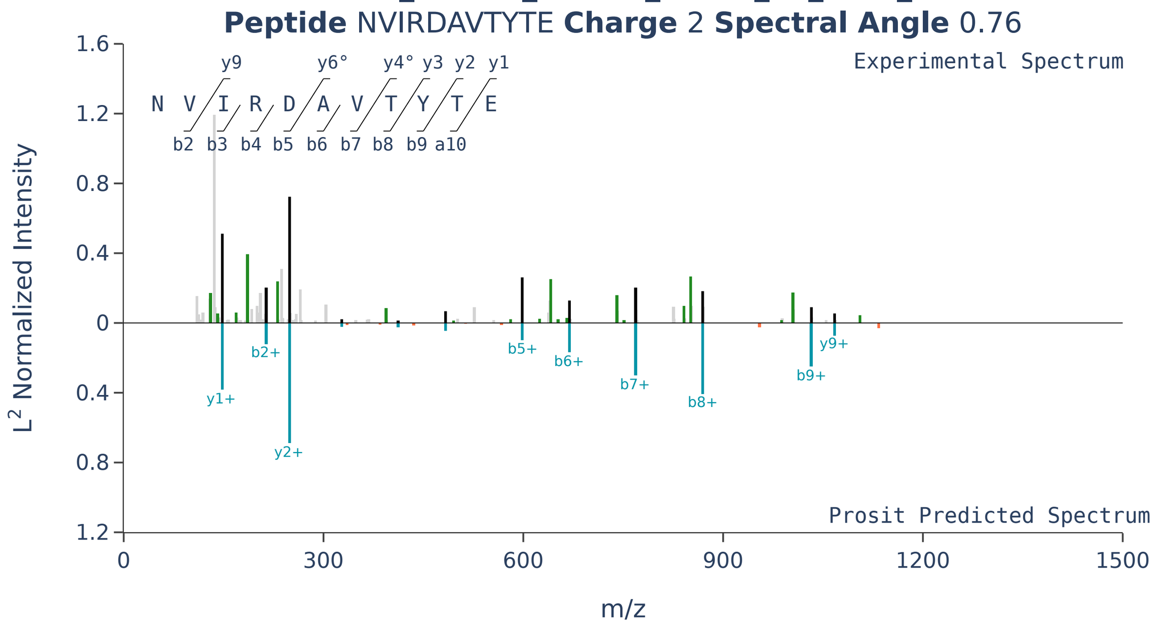


**Source** HRoetschke_291222_140123_HFGoe_H4_b2R1_1h **Scan** 23047
**Peptide** ETRGVLKVFL **Charge** 2 **Spectral Angle** 0.82

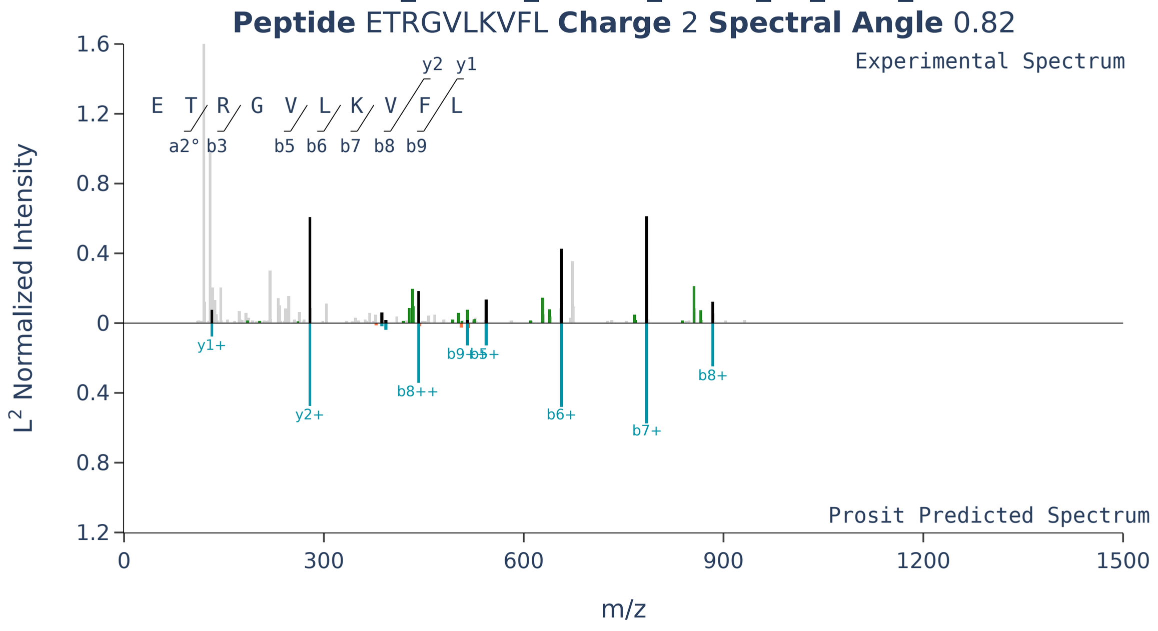


**Source** HRoetschke_291222_140123_HFGoe_H4_b2R1_24h **Scan** 9900
**Peptide** LQGITKP **Charge** 2 **Spectral Angle** 0.92

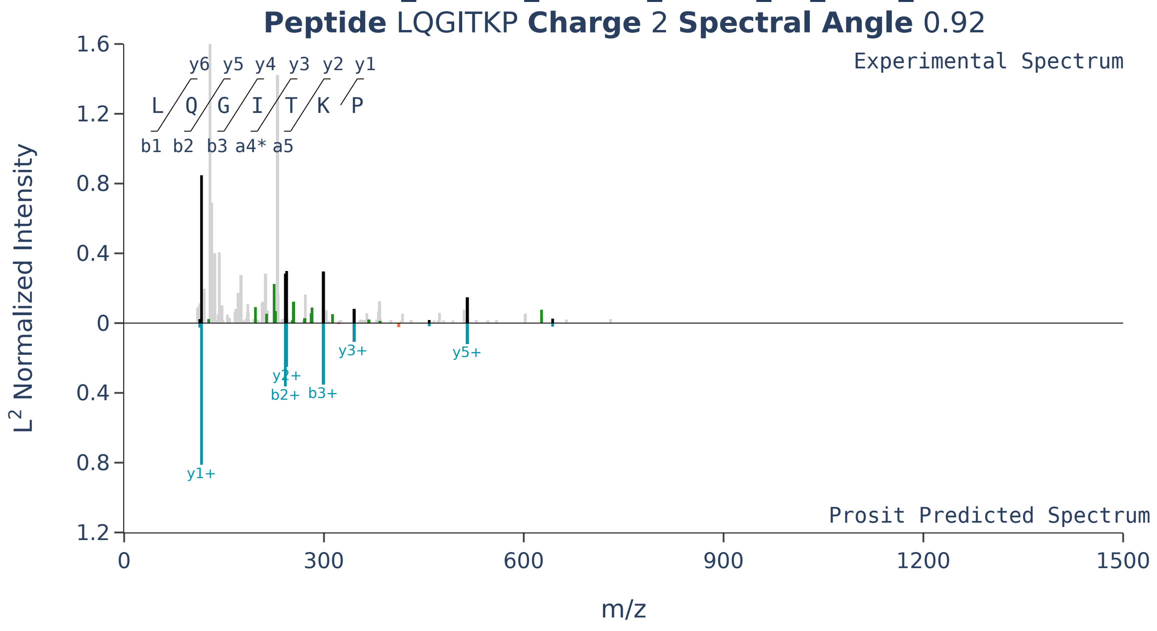


**Source** HRoetschke_291222_140123_HFGoe_H4_b2R2_2h **Scan** 15160
**Peptide** ENVIRDAVTYTEHAKRKTV **Charge** 3 **Spectral Angle** 0.79

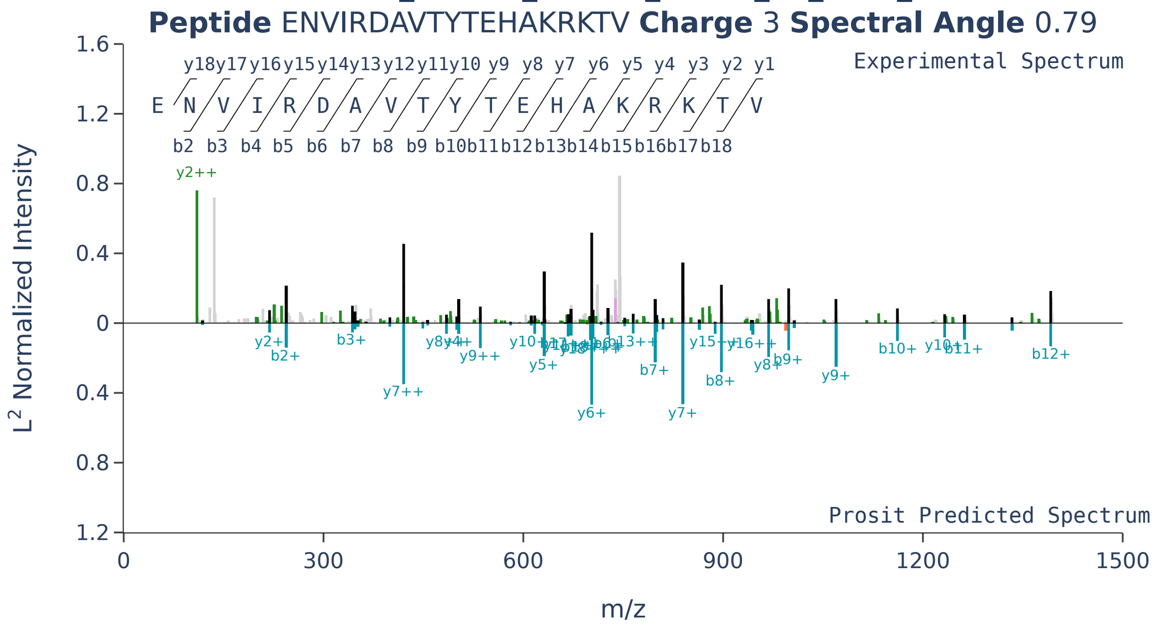


**Source** HRoetschke_291222_140123_HFGoe_H4_b1R2_1h **Scan** 19601
**Peptide** ENVIRDAVTYTEHA **Charge** 2 **Spectral Angle** 0.84

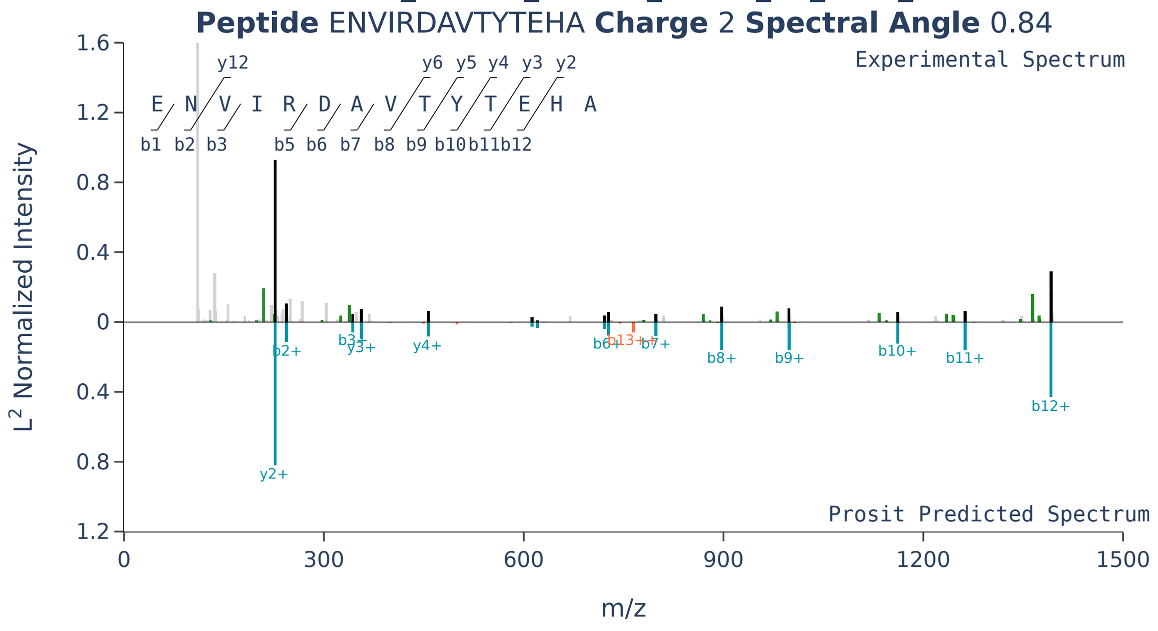


**Source** HRoetschke_291222_140123_HFGoe_H4_b2R1_24h **Scan** 9527
**Peptide** VIRDAVTYTEH **Charge** 3 **Spectral Angle** 0.87

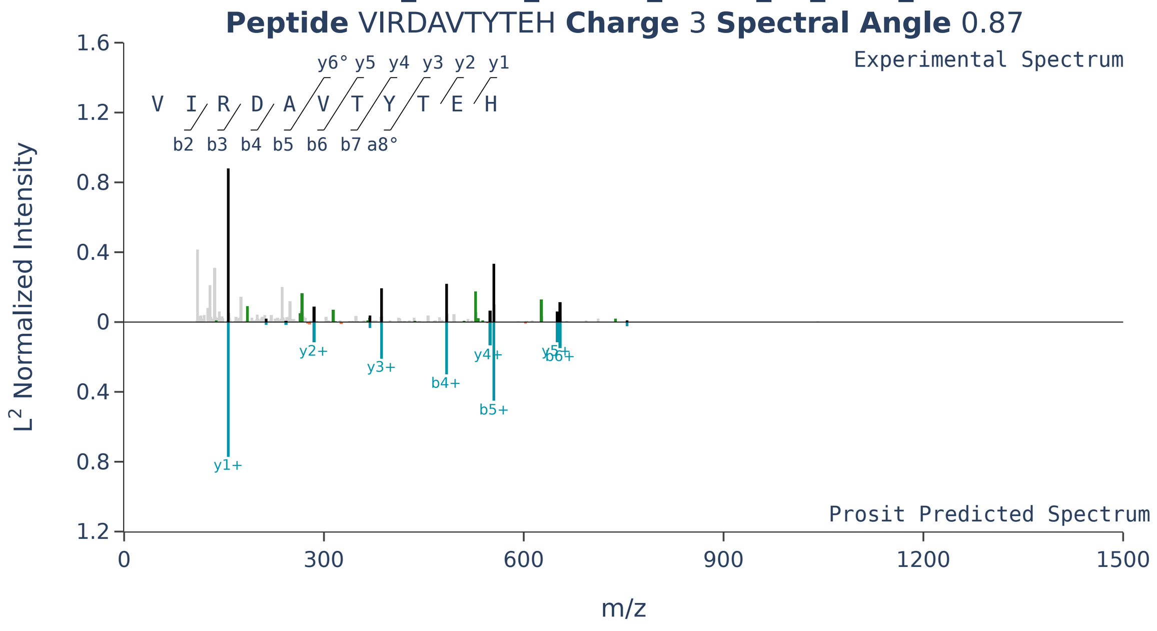


**Source** HRoetschke_291222_140123_HFGoe_H4_b1R1_4h **Scan** 17432
**Peptide** GGVKRISGLIYEET **Charge** 2 **Spectral Angle** 0.75

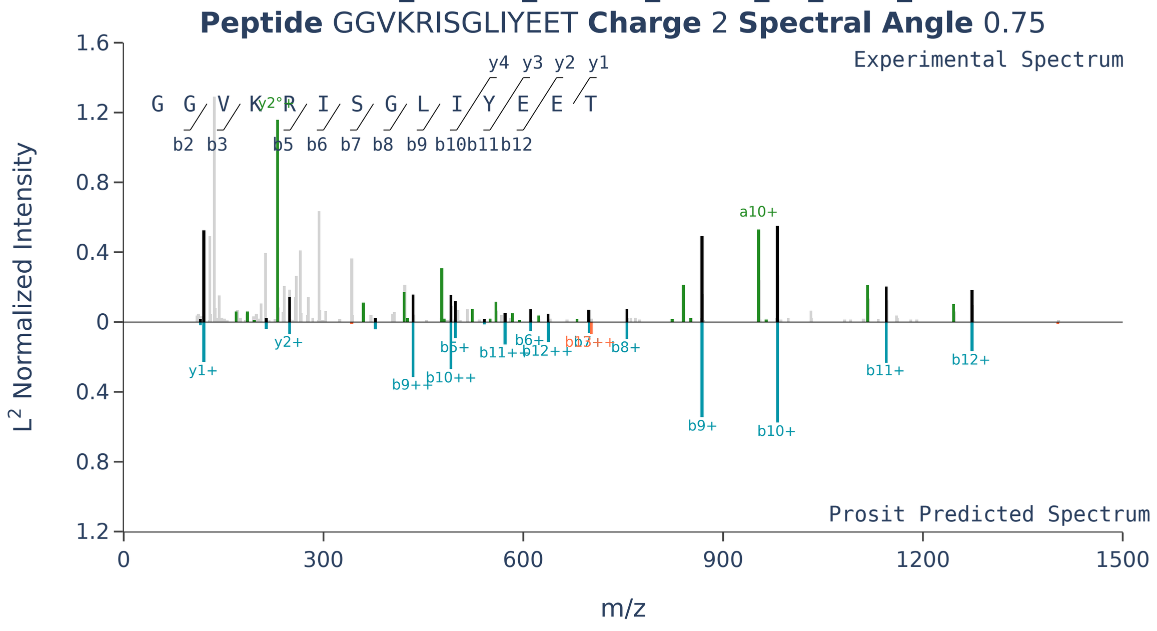


**Source** HRoetschke_291222_140123_HFGoe_H4_b1R1_24h **Scan** 10880
**Peptide** LRDNIQGITKPAIRR **Charge** 5 **Spectral Angle** 0.85

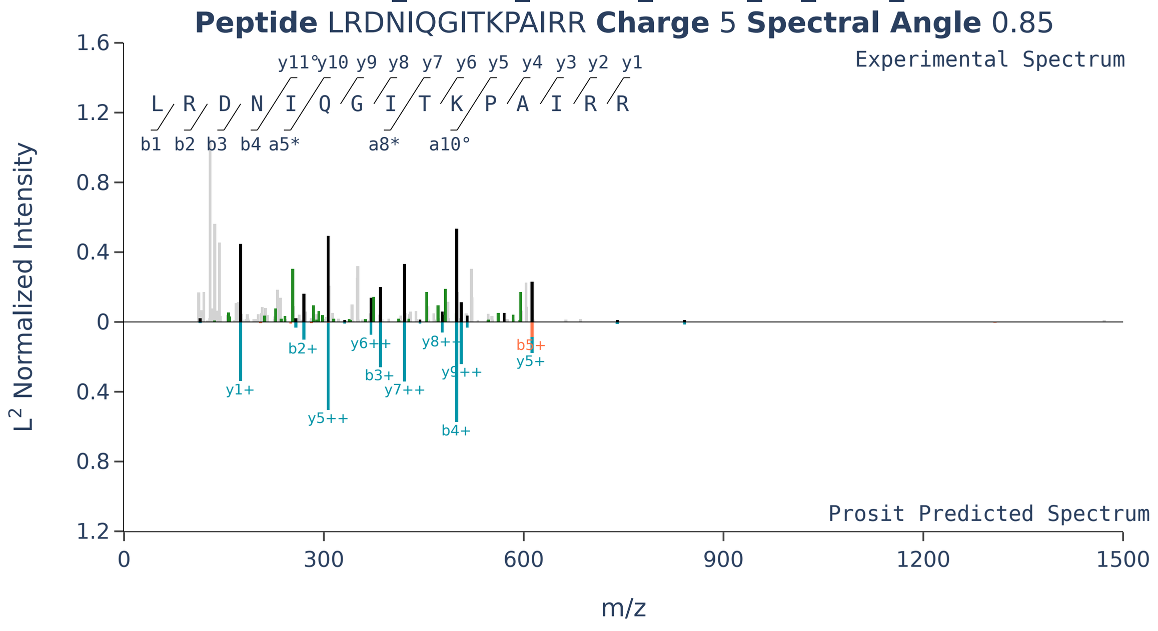


**Source** HRoetschke_291222_140123_HFGoe_H4_b2R1_1h **Scan** 21832
**Peptide** GVKRISGLIYEETRGVLKV **Charge** 3 **Spectral Angle** 0.8

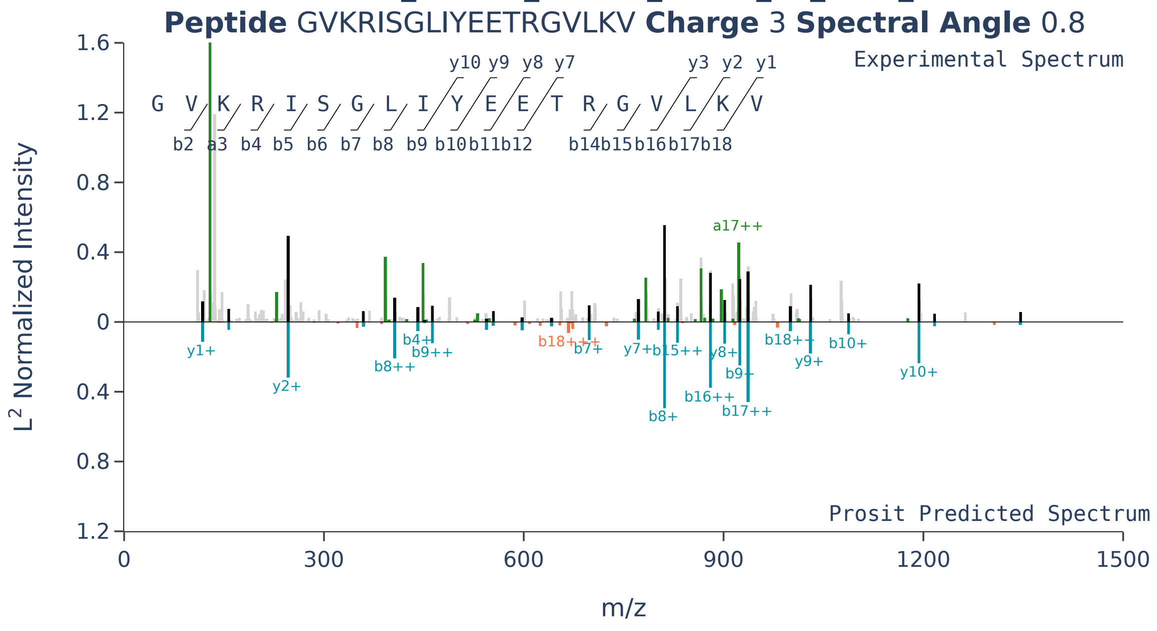


**Source** HRoetschke_291222_140123_HFGoe_H4_b1R1_1h **Scan** 24396
**Peptide** LKVFLENVLRD **Charge** 2 **Spectral Angle** 0.84

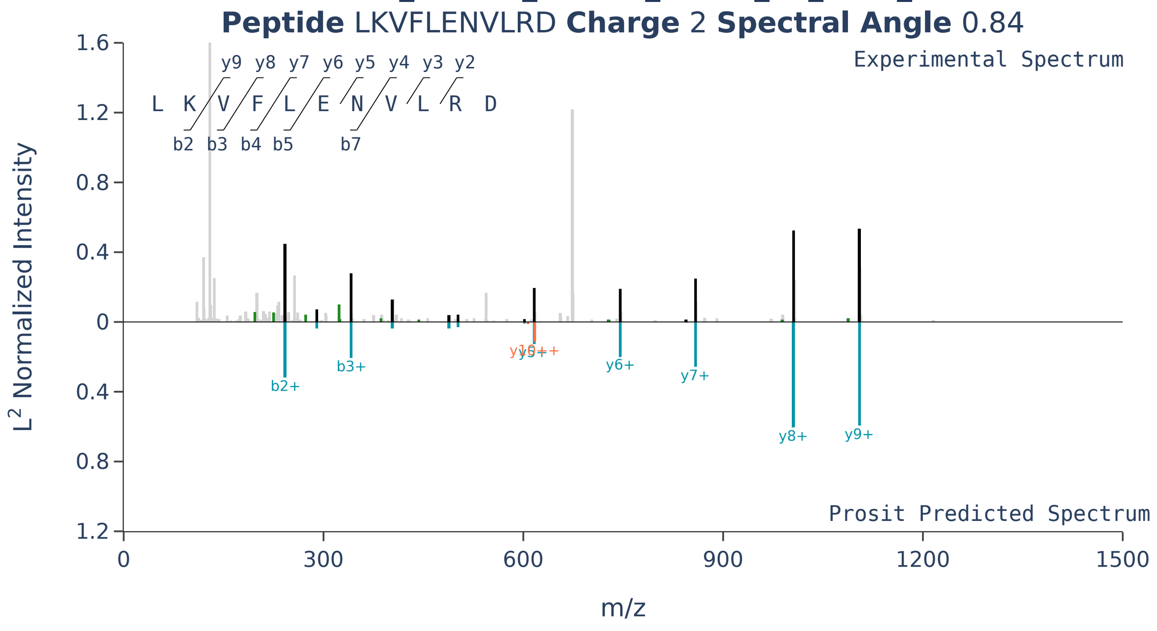

**Source** HRoetschke_291222_140123_HFGoe_H4_b2R1_1h **Scan** 27375
**Peptide** FLENVIRDAVTY **Charge** 2 **Spectral Angle** 0.7

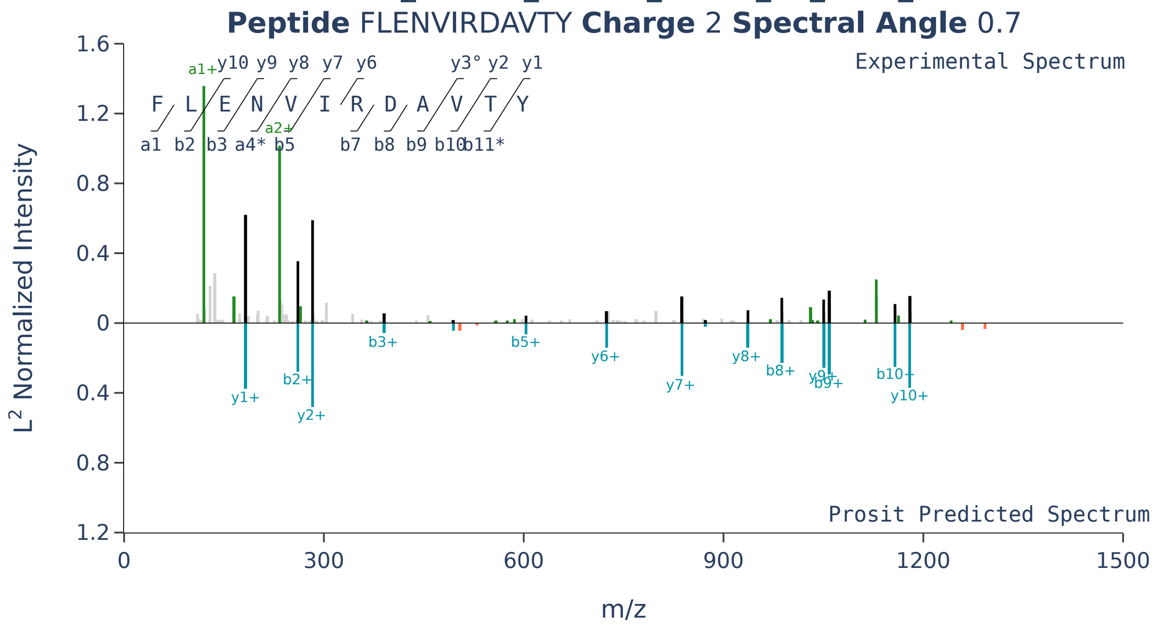


**Source** HRoetschke_291222_140123_HFGoe_H4_b2R2_2h **Scan** 12872
**Peptide** LRDNIQGITKPALR **Charge** 4 **Spectral Angle** 0.87

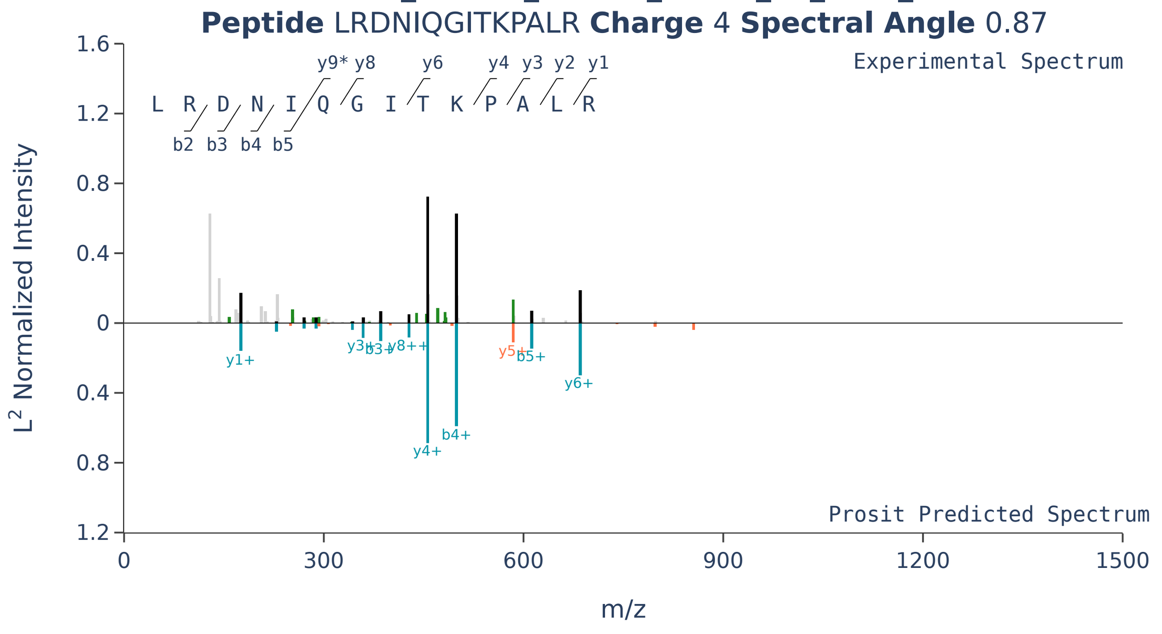


**Source** HRoetschke_291222_140123_HFGoe_H4_b1R1_24h **Scan** 14117
**Peptide** ENVIRDAVTYTEHAKRKT **Charge** 3 **Spectral Angle** 0.81

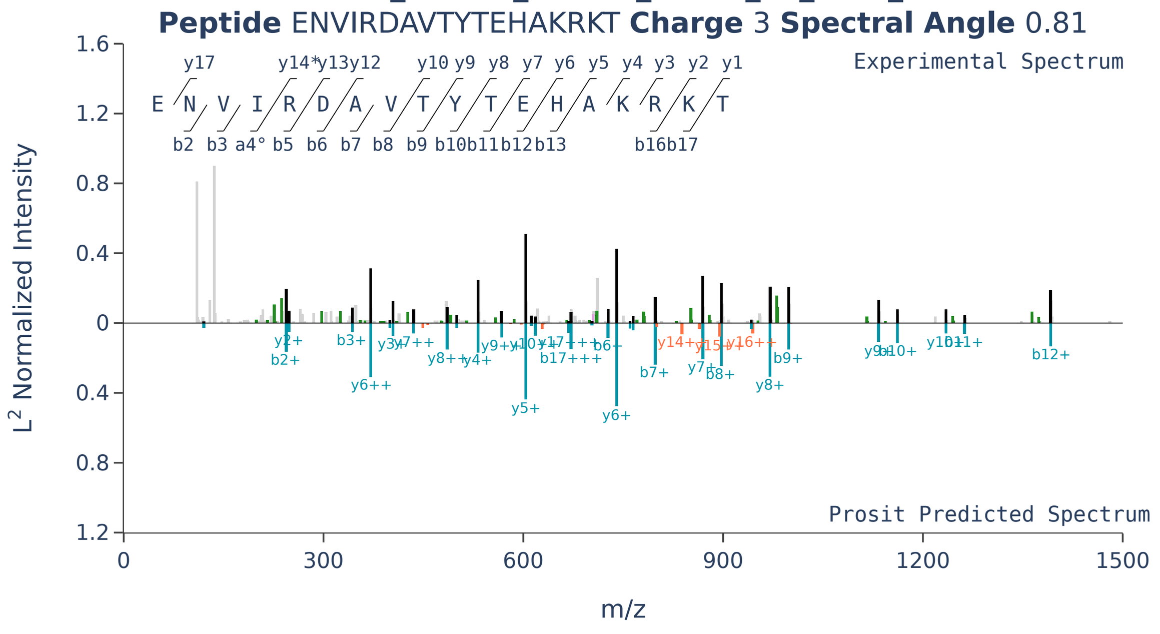


**Source** HRoetschke_291222_140123_HFGoe_H4_b1R1_4h **Scan** 14747
**Peptide** LRDNIQGITKPAIRRLA **Charge** 4 **Spectral Angle** 0.75

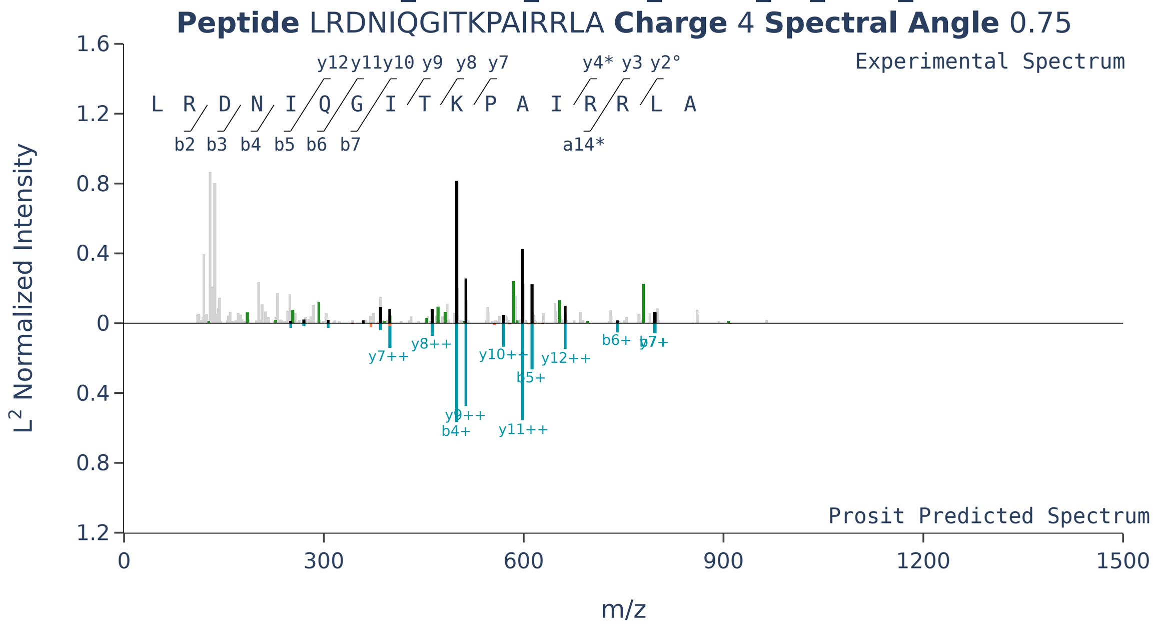


**Source** HRoetschke_291222_140123_HFGoe_H4_b1R1_1h **Scan** 17313
**Peptide** MDVVYALKRQGRTLY **Charge** 3 **Spectral Angle** 0.73

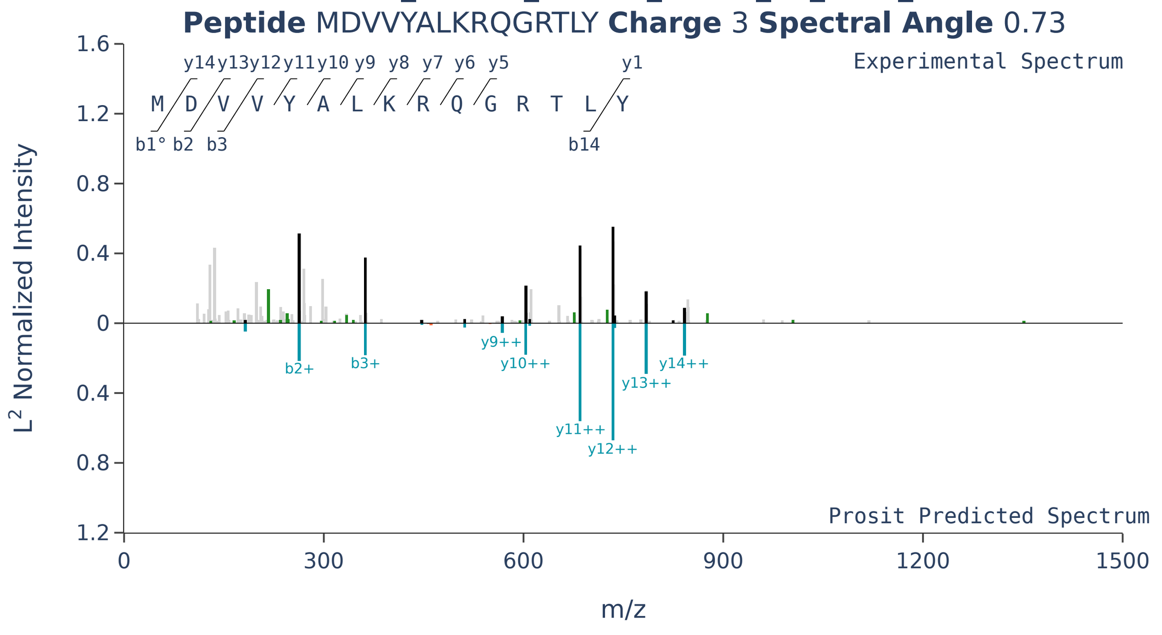


**Source** HRoetschke_291222_140123_HFGoe_H4_b1R2_4h **Scan** 14485
**Peptide** VLRDNIQGITKPAIR **Charge** 4 **Spectral Angle** 0.87

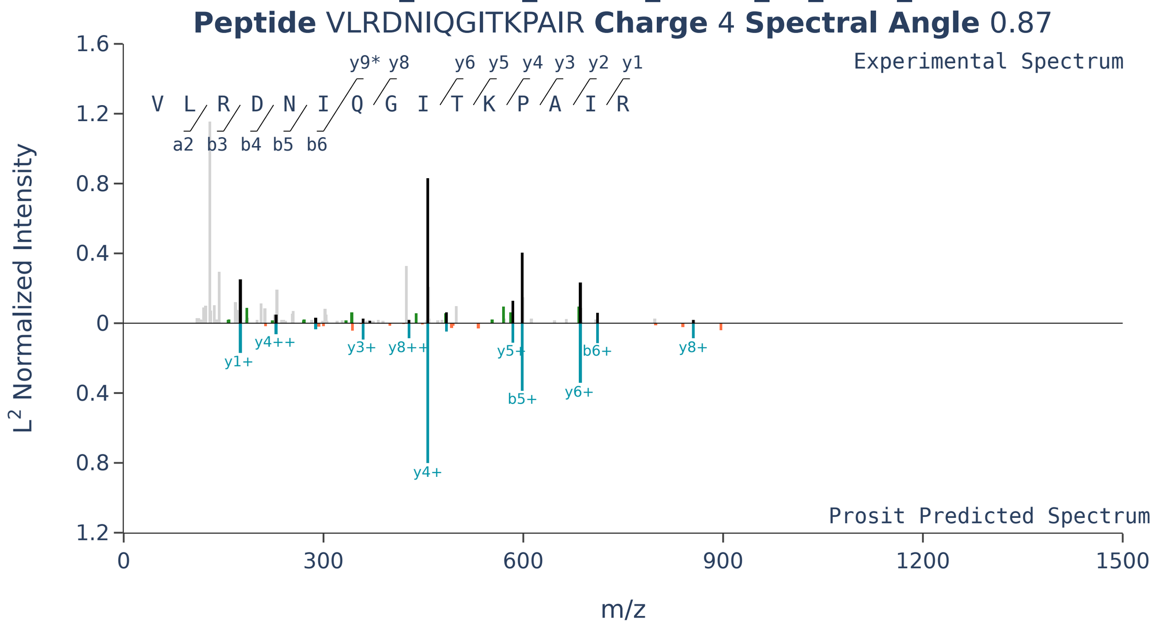


**Source** HRoetschke_291222_140123_HFGoe_H4_b2R1_1h **Scan** 22506
**Peptide** GLIYEETRGVL **Charge** 2 **Spectral Angle** 0.74

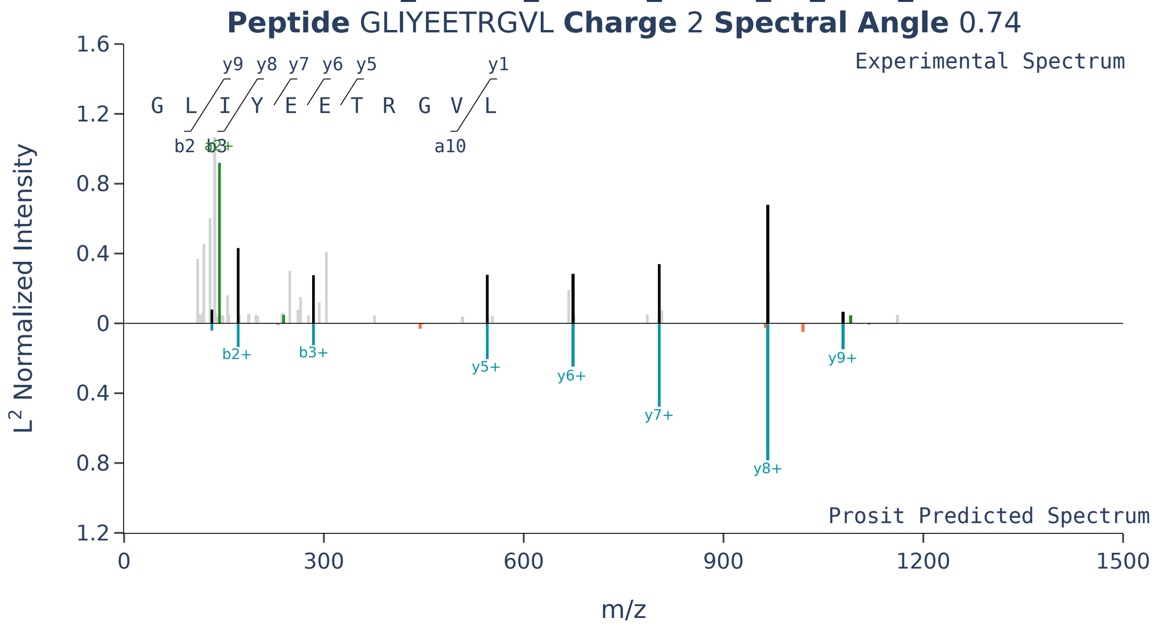


**Source** HRoetschke_291222_140123_HFGoe_H4_b2R1_1h **Scan** 15425
**Peptide** TYTEHAKRKTVTAMDVVYALKRQGR **Charge** 5 **Spectral Angle** 0.89

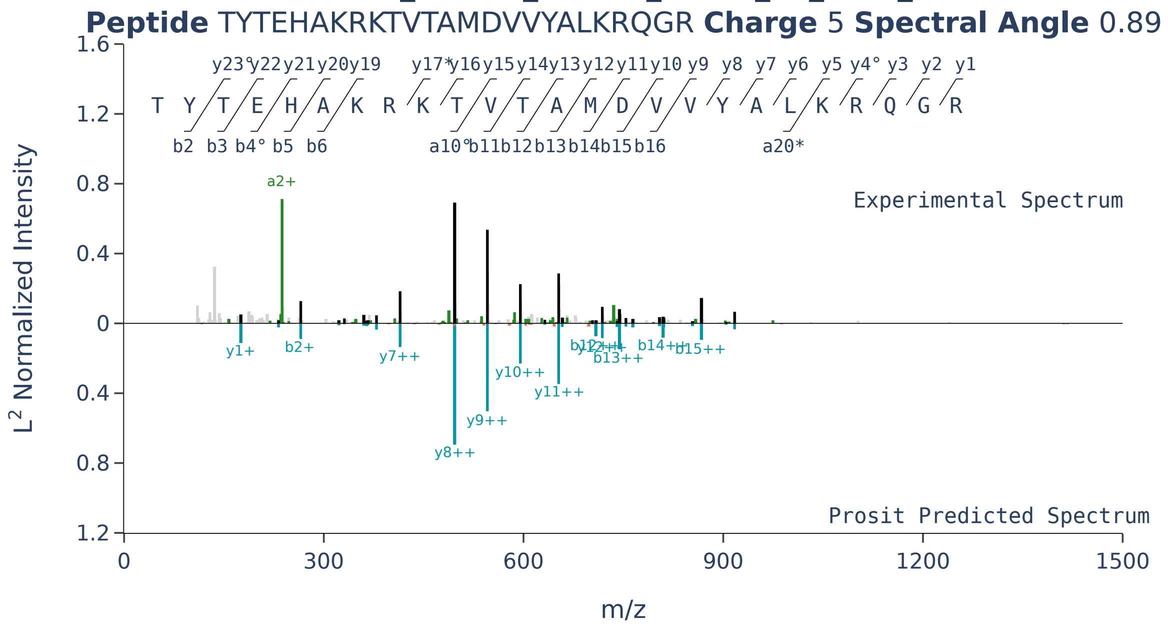


**Source** HRoetschke_291222_140123_HFGoe_H4_b1R1_24h **Scan** 10330
**Peptide** LRDAVTYTE **Charge** 2 **Spectral Angle** 0.78

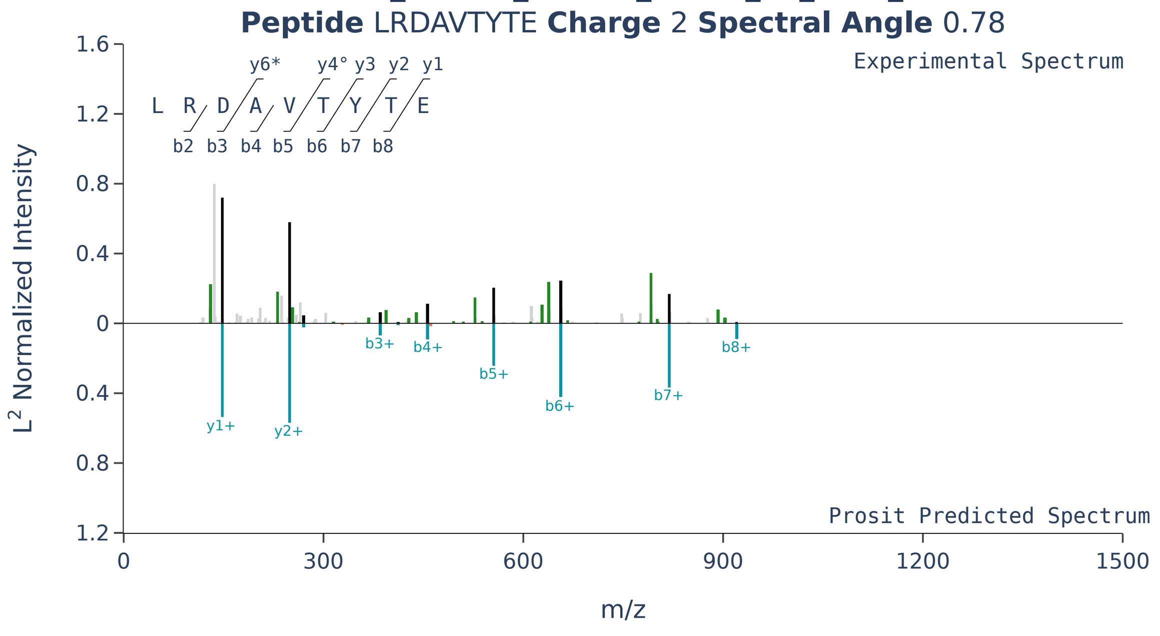


**Source** HRoetschke_291222_140123_HFGoe_H4_b2R1_1h **Scan** 6226
**Peptide** LRDAVTYTEHAKR **Charge** 4 **Spectral Angle** 0.81

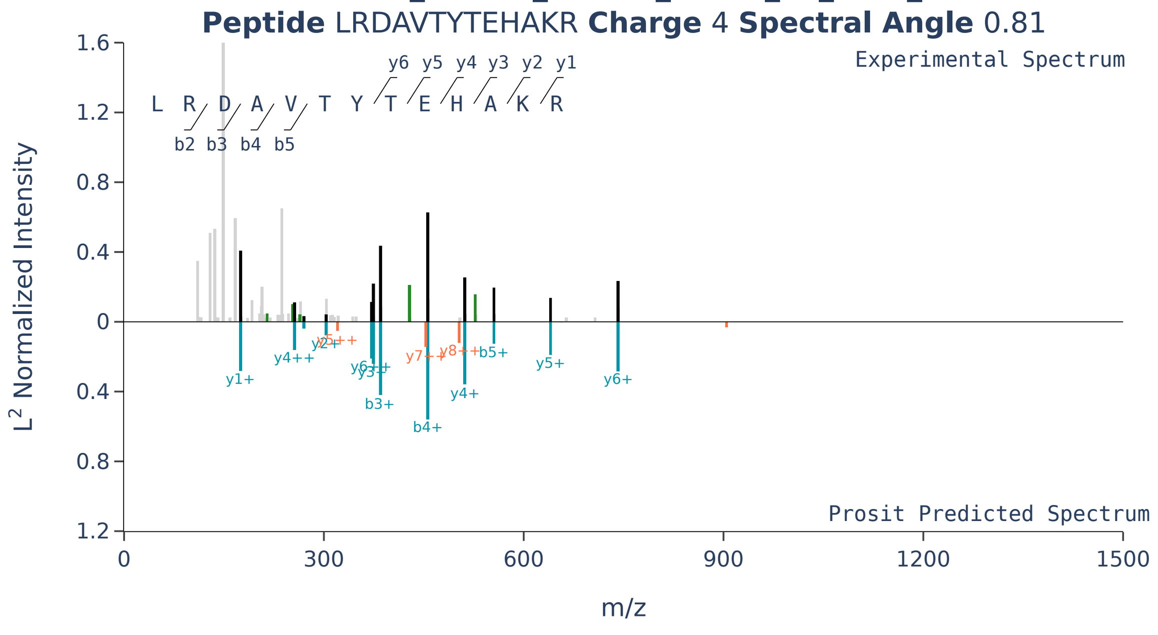


**Source** HRoetschke_291222_140123_HFGoe_H4_b2R1_1h **Scan** 10448
**Peptide** KRQGRTLYGFGG **Charge** 3 **Spectral Angle** 0.65

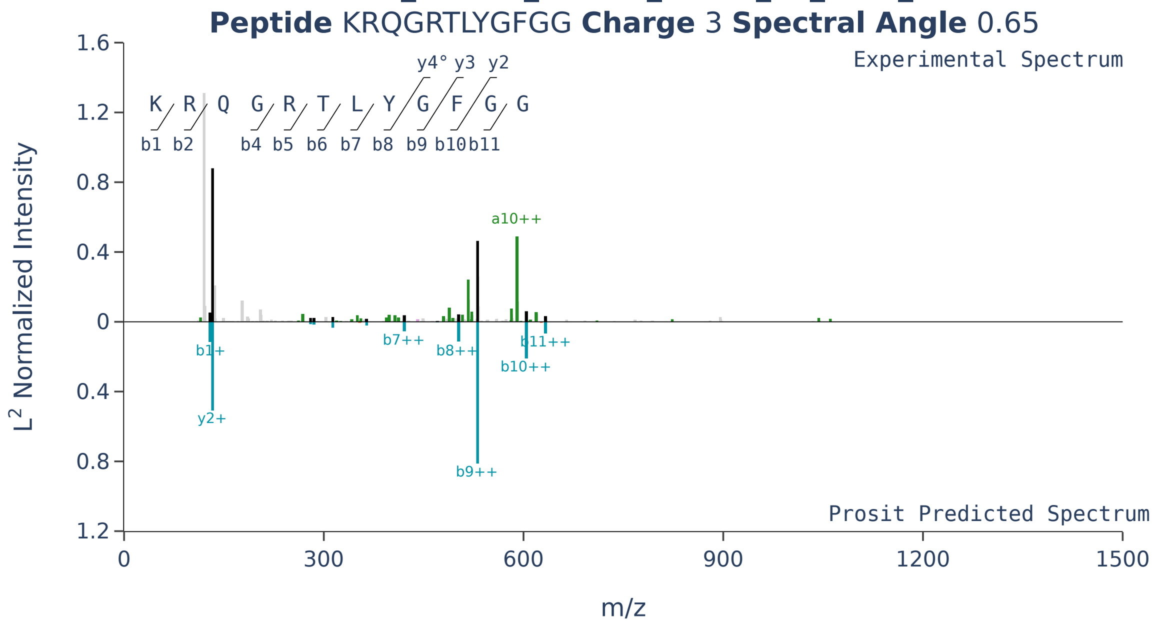


**Source** HRoetschke_291222_140123_HFGoe_H4_b1R1_4h **Scan** 14040
**Peptide** MDVVYALKRQGRT **Charge** 4 **Spectral Angle** 0.89

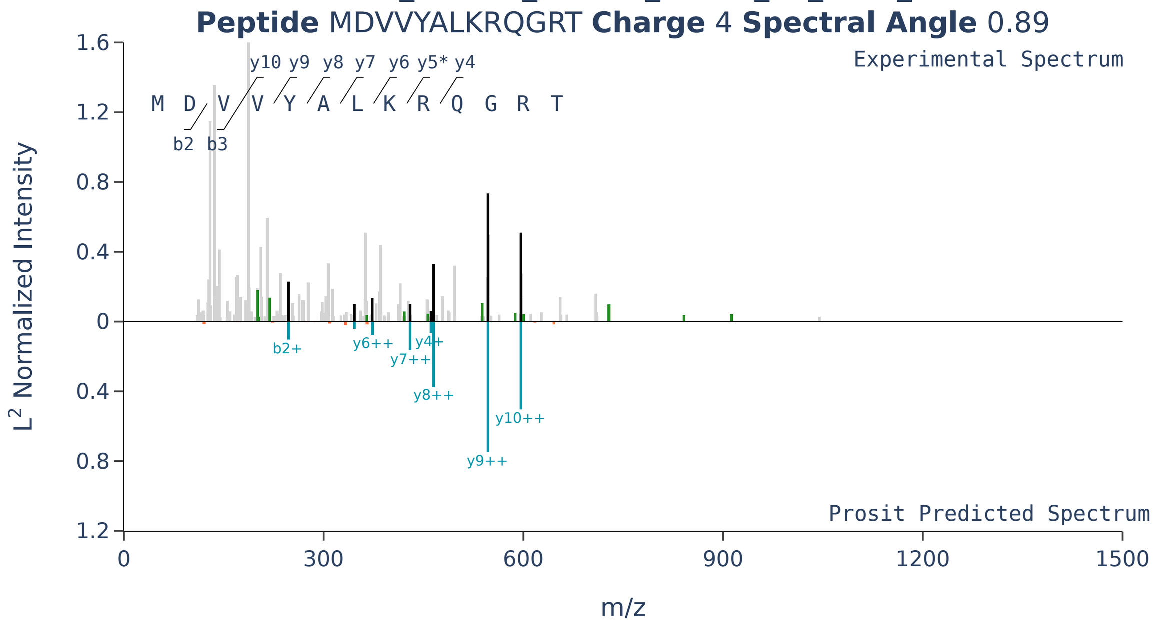


**Source** HRoetschke_291222_140123_HFGoe_H4_b1R2_2h **Scan** 17911
**Peptide** ENVIRDAVTYTEHAK **Charge** 2 **Spectral Angle** 0.78

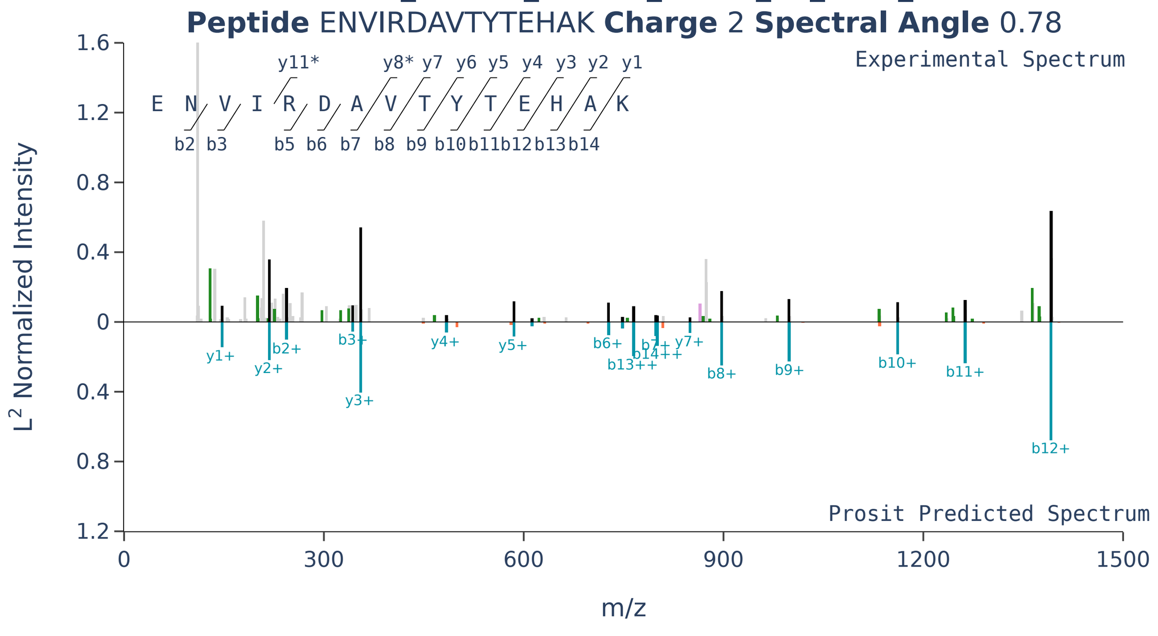


**Source** HRoetschke_291222_140123_HFGoe_H4_b2R1_1h **Scan** 18426
**Peptide** KRKTVTAMDVVYALKRQGRTL **Charge** 4 **Spectral Angle** 0.85

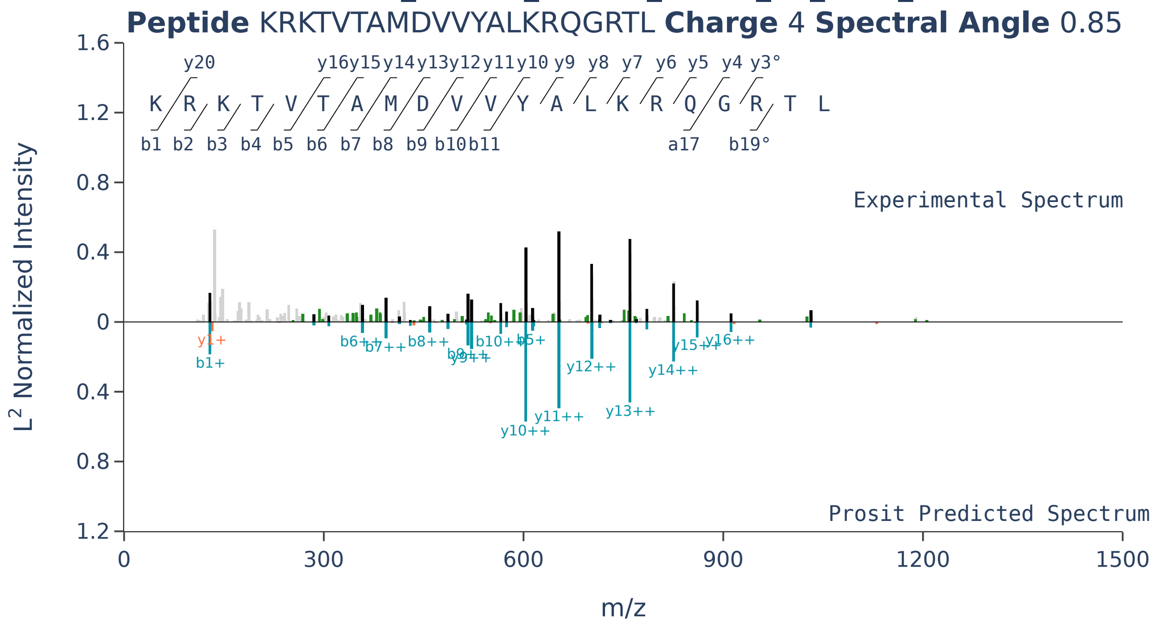


**Source** HRoetschke_291222_140123_HFGoe_H4_b1R1_2h **Scan** 16406
**Peptide** LYEETRGVLKV **Charge** 2 **Spectral Angle** 0.79

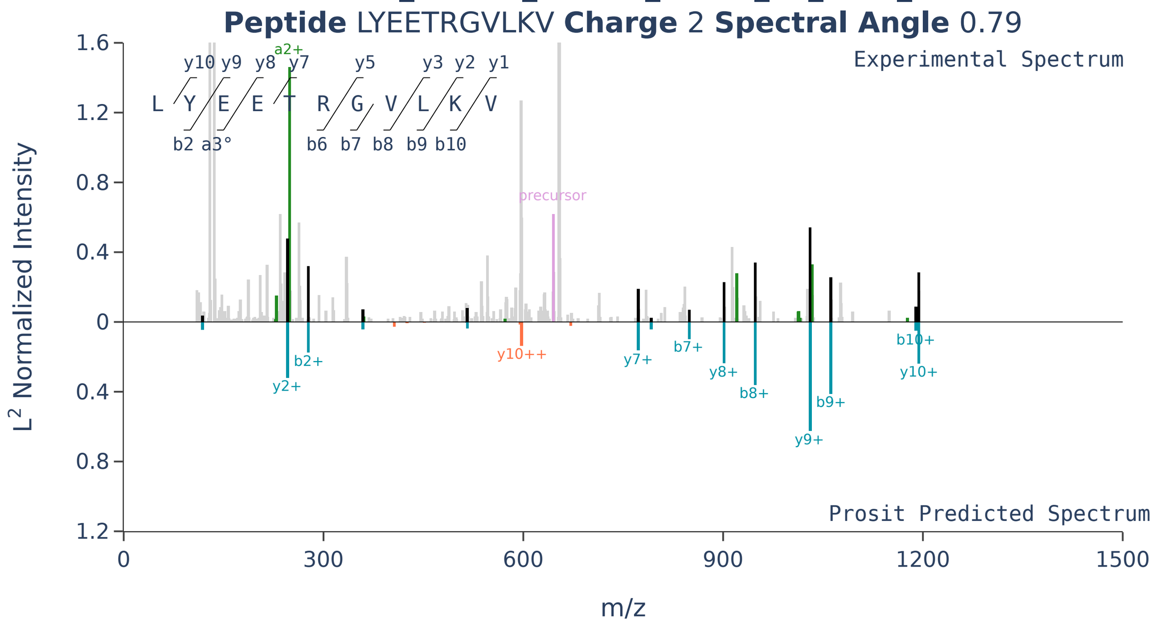

**Source** HRoetschke_291222_140123_HFGoe_H4_b2R1_24h **Scan** 11504
**Peptide** KRQGRTLYGF **Charge** 2 **Spectral Angle** 0.76

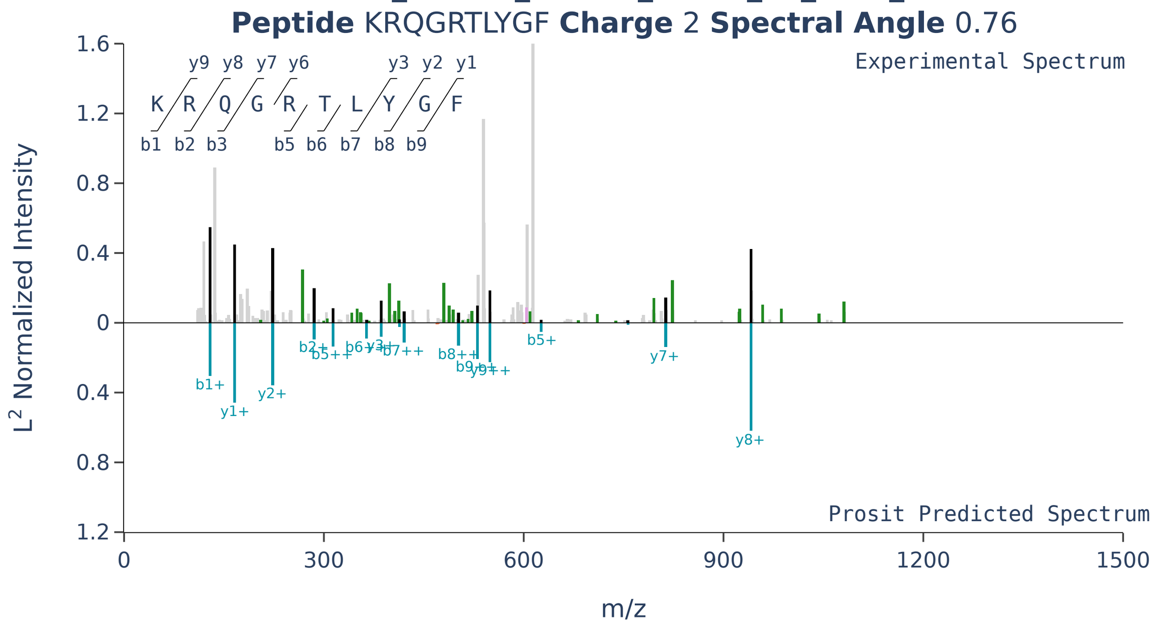


**Source** HRoetschke_291222_140123_HFGoe_H4_b1R1_4h **Scan** 18849
**Peptide** RISGLIYEET **Charge** 2 **Spectral Angle** 0.78

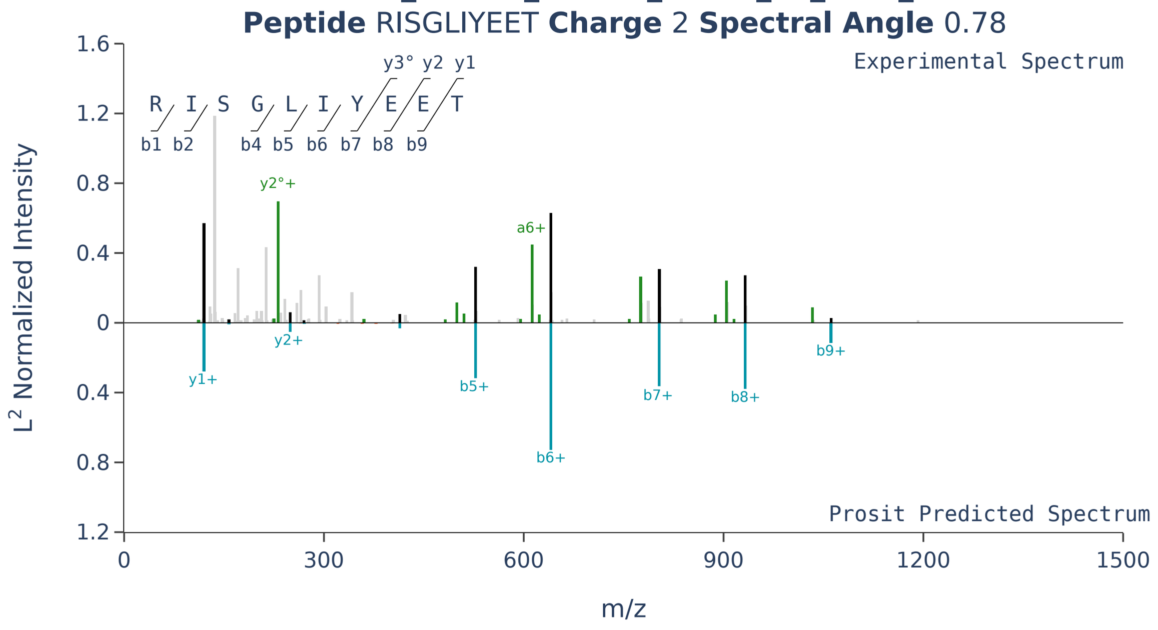


**Source** HRoetschke_291222_140123_HFGoe_H4_b2R1_24h **Scan** 12208
**Peptide** VLRDNIQGITKPAIRR **Charge** 3 **Spectral Angle** 0.82

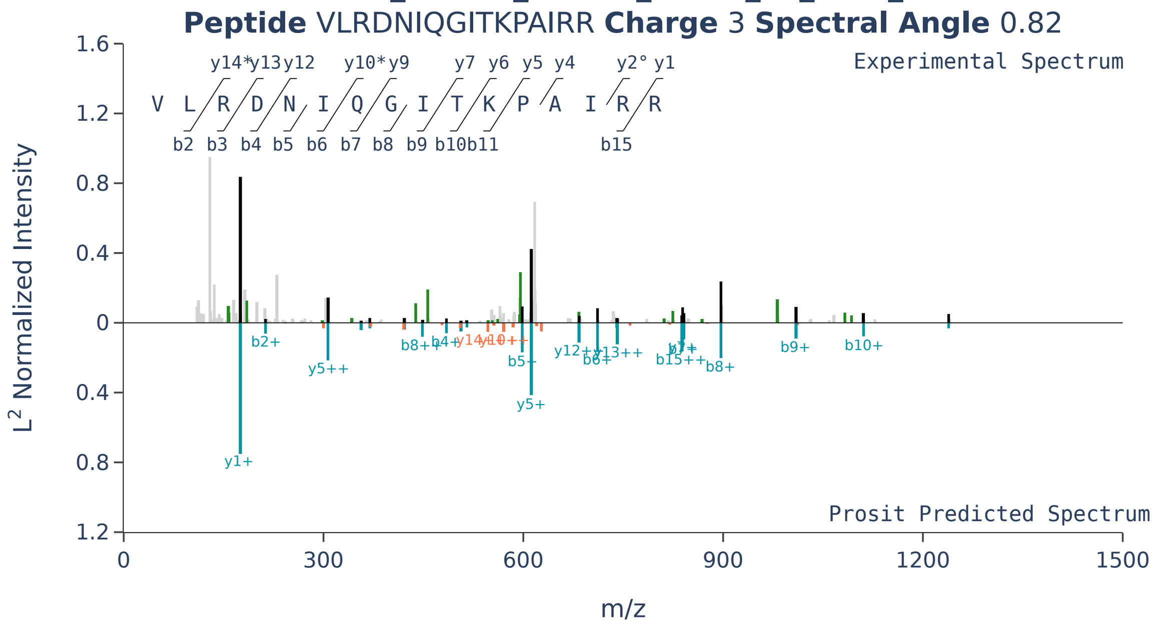


**Source** HRoetschke_291222_140123_HFGoe_H4_b1R1_24h **Scan** 9638
**Peptide** GGVKRISGL **Charge** 2 **Spectral Angle** 0.85

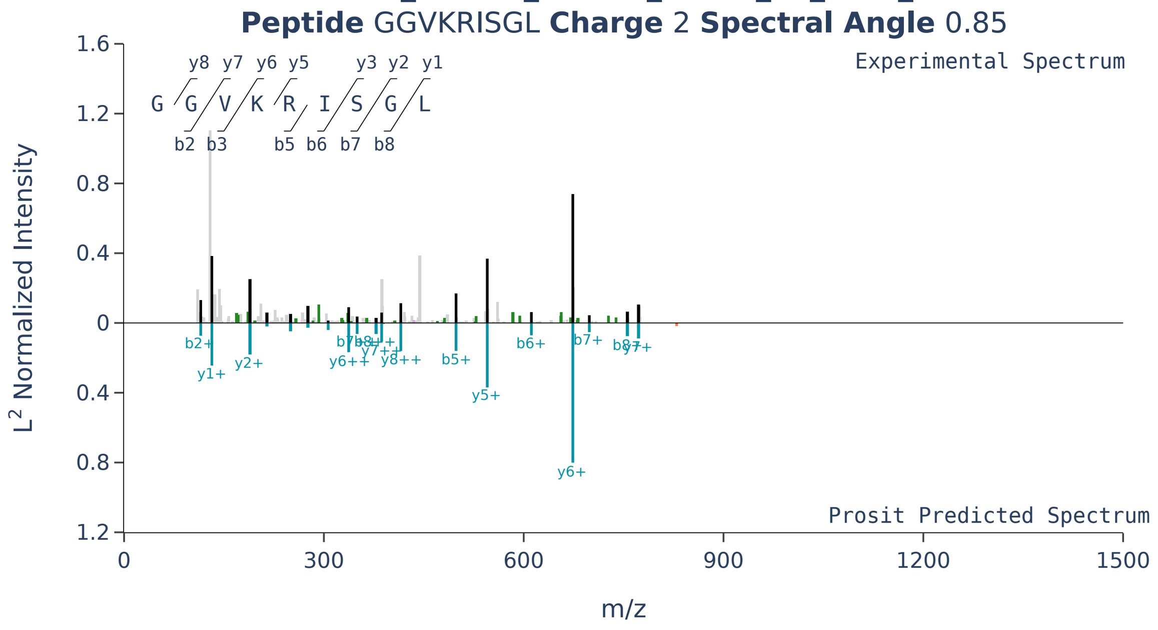


**Source** HRoetschke_291222_140123_HFGoe_H4_b2R2_24h **Scan** 11983
**Peptide** VLRDAVTYT **Charge** 2 **Spectral Angle** 0.67

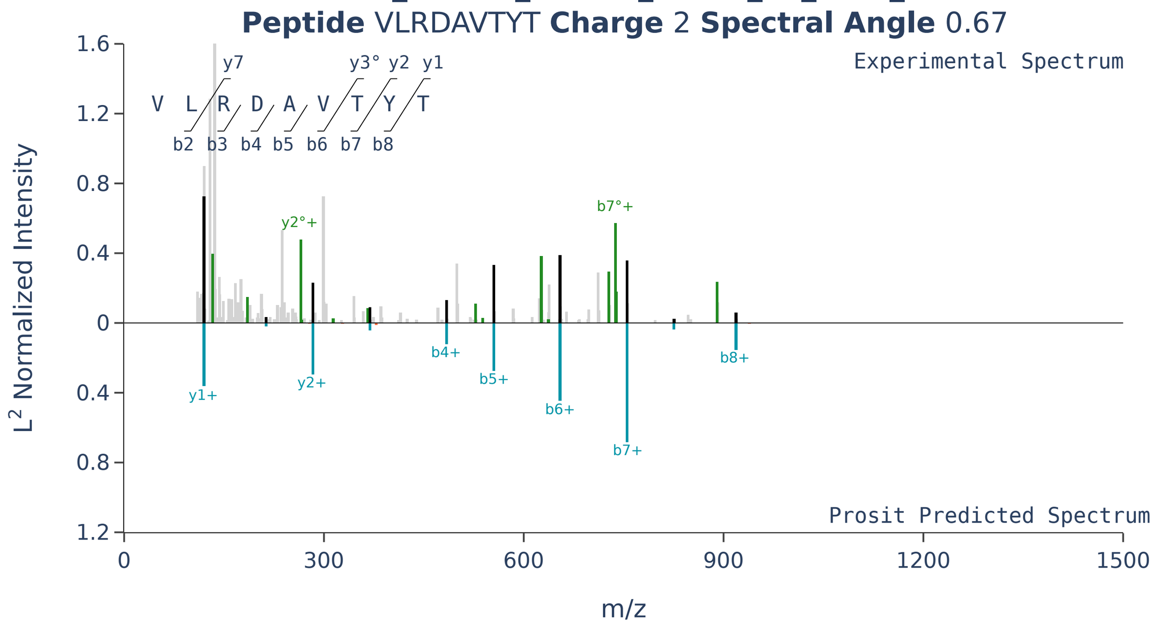


**Source** HRoetschke_291222_140123_HFGoe_H4_b1R2_1h **Scan** 16536
**Peptide** GVKRISGLIYEET **Charge** 2 **Spectral Angle** 0.74

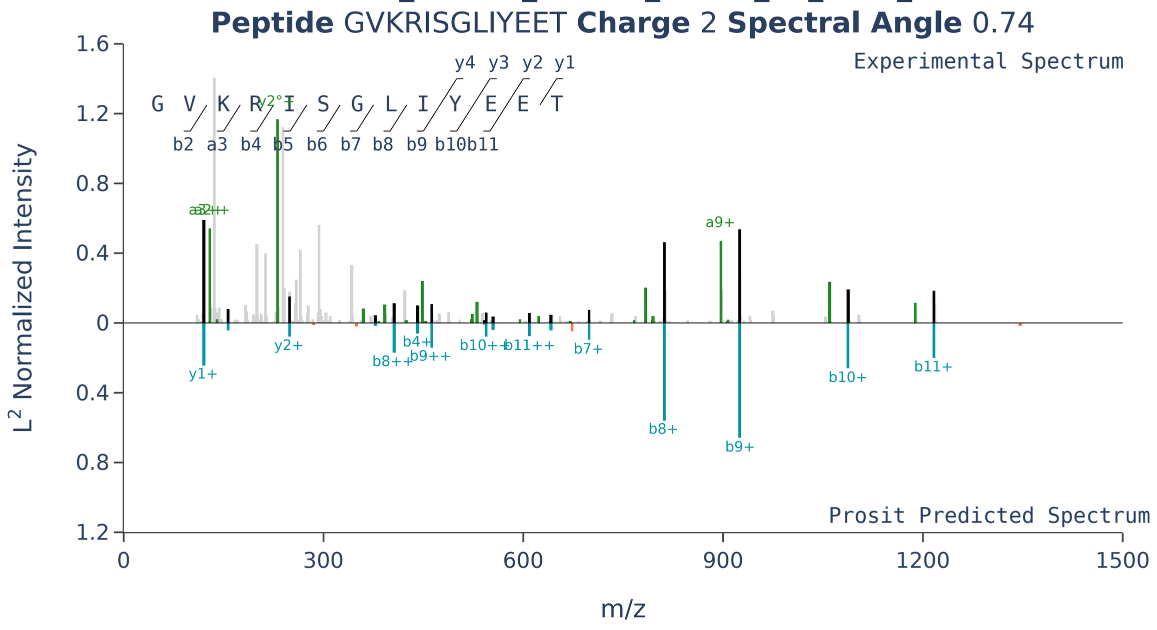


**Source** HRoetschke_291222_140123_HFGoe_H4_b2R1_1h **Scan** 19098
**Peptide** TVTAMDVVYALKRQGR **Charge** 4 **Spectral Angle** 0.8

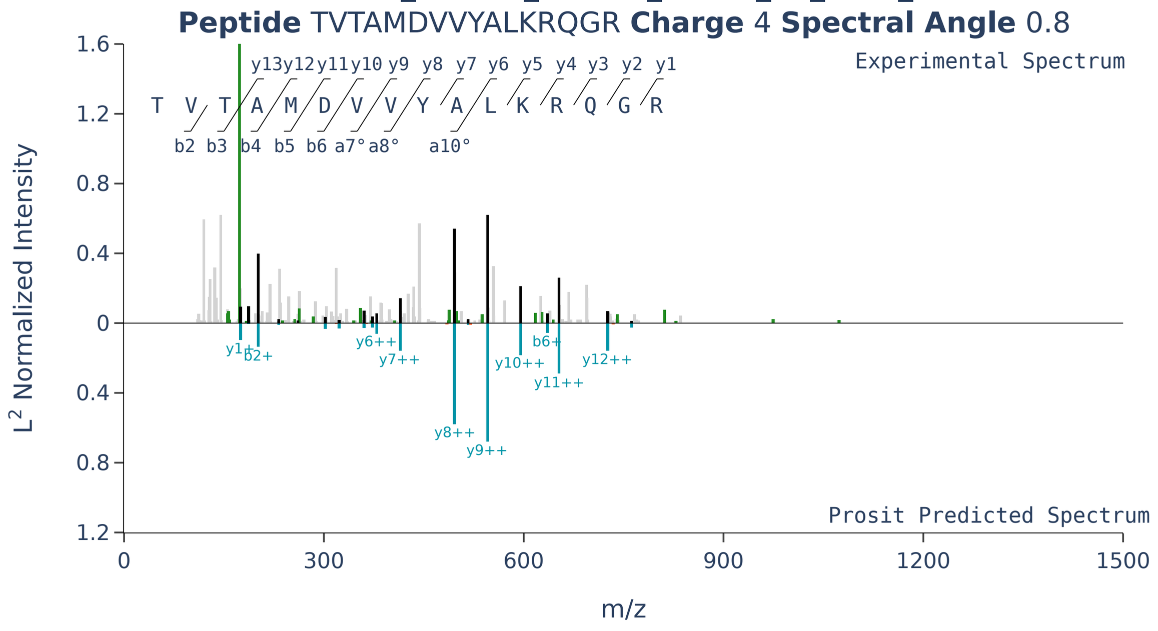


**Source** HRoetschke_291222_140123_HFGoe_H4_b2R1_1h **Scan** 25239
**Peptide** VTAMDVVYALKRQGRTL **Charge** 4 **Spectral Angle** 0.83

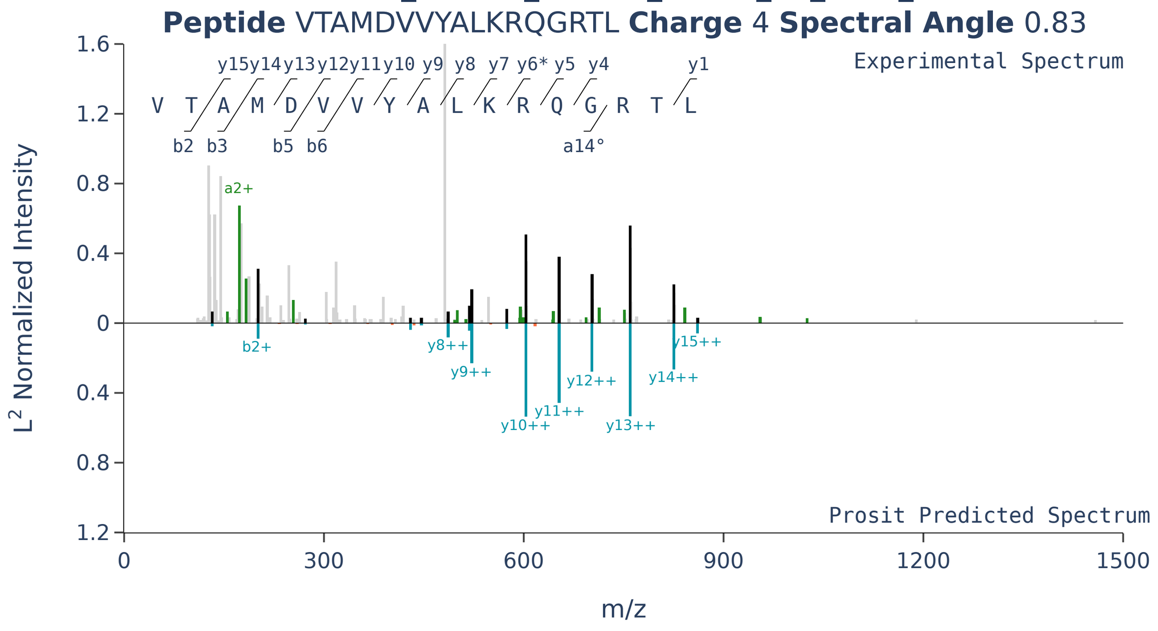


**Source** HRoetschke_291222_140123_HFGoe_H4_b1R2_1h **Scan** 13378
**Peptide** DNIQGITKPAIRRLAR **Charge** 5 **Spectral Angle** 0.83

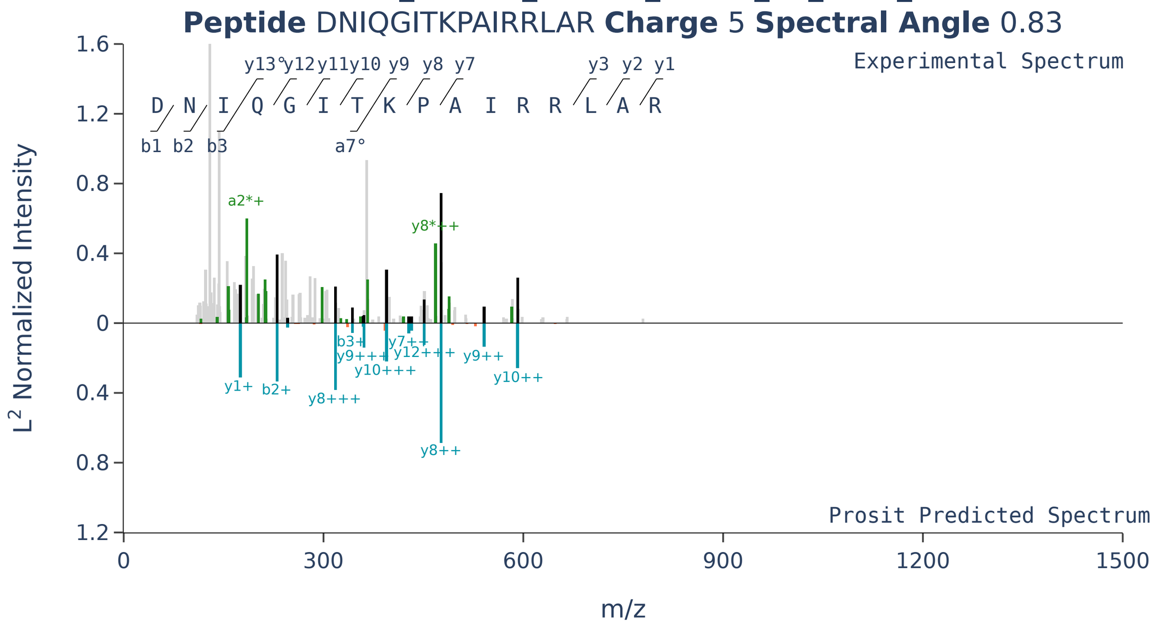


**Source** HRoetschke_291222_140123_HFGoe_H4_b2R2_4h **Scan** 13620
**Peptide** KVLRDNIQGL **Charge** 3 **Spectral Angle** 0.73

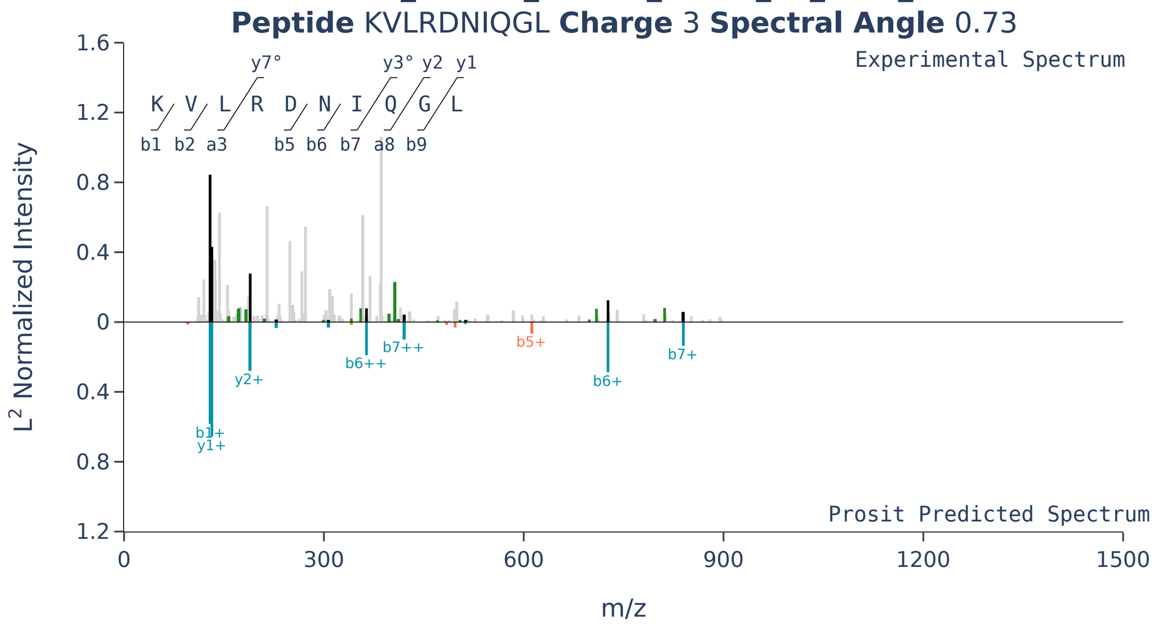


**Source** HRoetschke_291222_140123_HFGoe_H4_b1R2_4h **Scan** 12275
**Peptide** DNIQGITKPAIRRLARR **Charge** 3 **Spectral Angle** 0.86

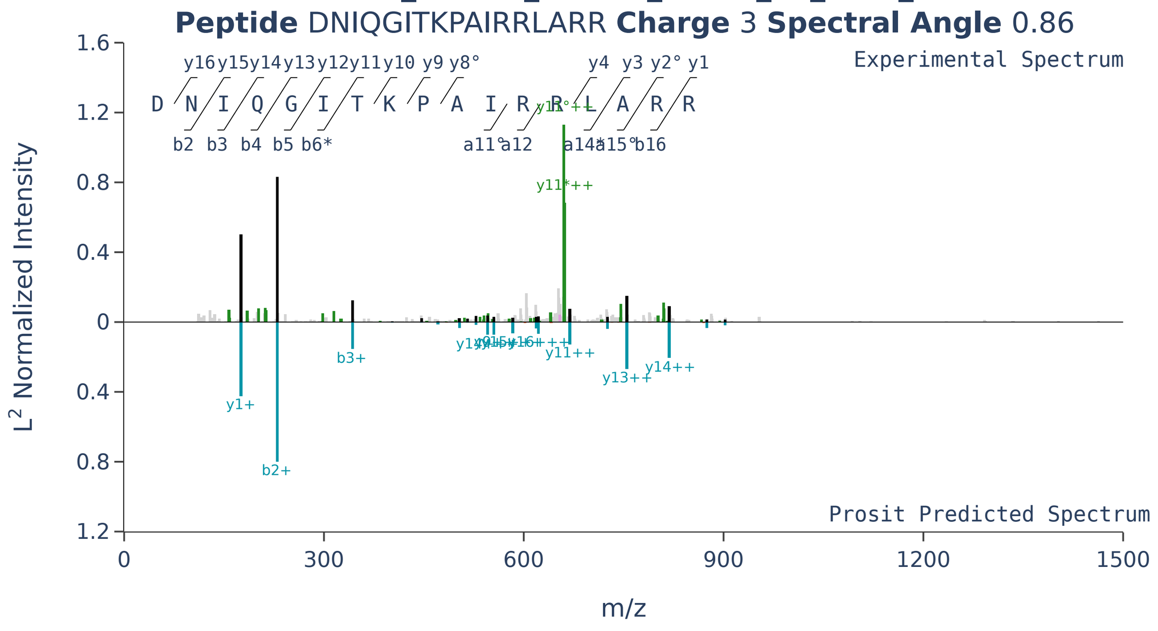


**Source** HRoetschke_291222_140123_HFGoe_H4_b2R1_2h **Scan** 14841
**Peptide** NVIRDAVTYTEHAK **Charge** 3 **Spectral Angle** 0.74

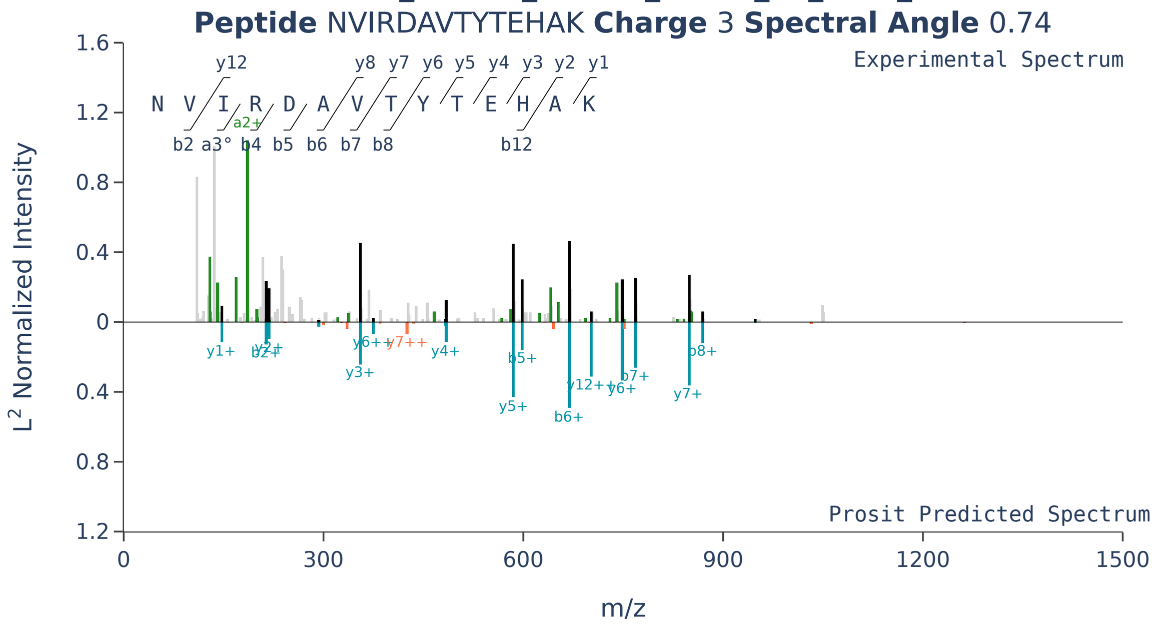


**Source** HRoetschke_291222_140123_HFGoe_H4_b1R2_1h **Scan** 21993
**Peptide** AKRKTVTAMDVVYAL **Charge** 2 **Spectral Angle** 0.84

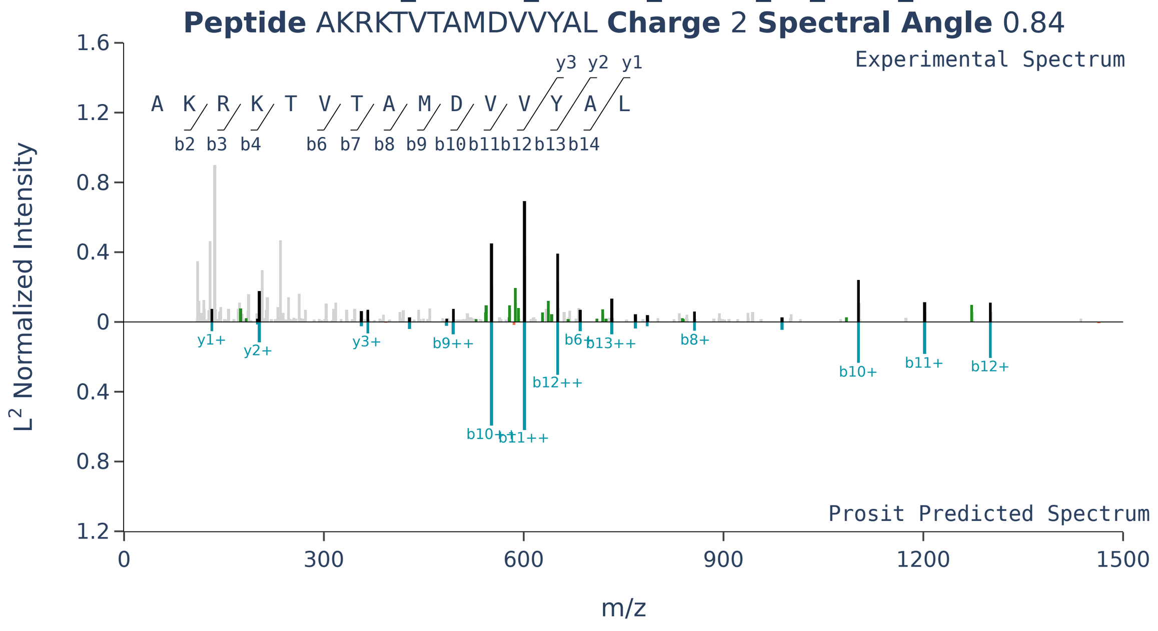


**Source** HRoetschke_291222_140123_HFGoe_H4_b1R1_1h **Scan** 23808
**Peptide** VFLENVLR **Charge** 2 **Spectral Angle** 0.84

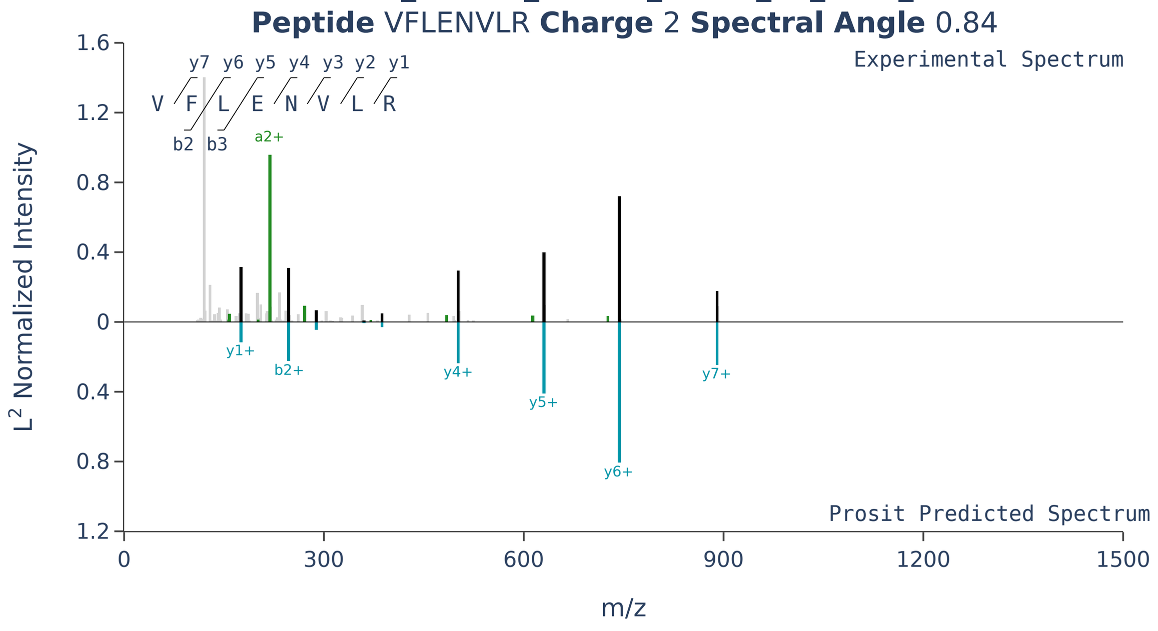


**Source** HRoetschke_291222_140123_HFGoe_H4_b2R1_1h **Scan** 20312
**Peptide** ISGLIYEETRGV **Charge** 2 **Spectral Angle** 0.78

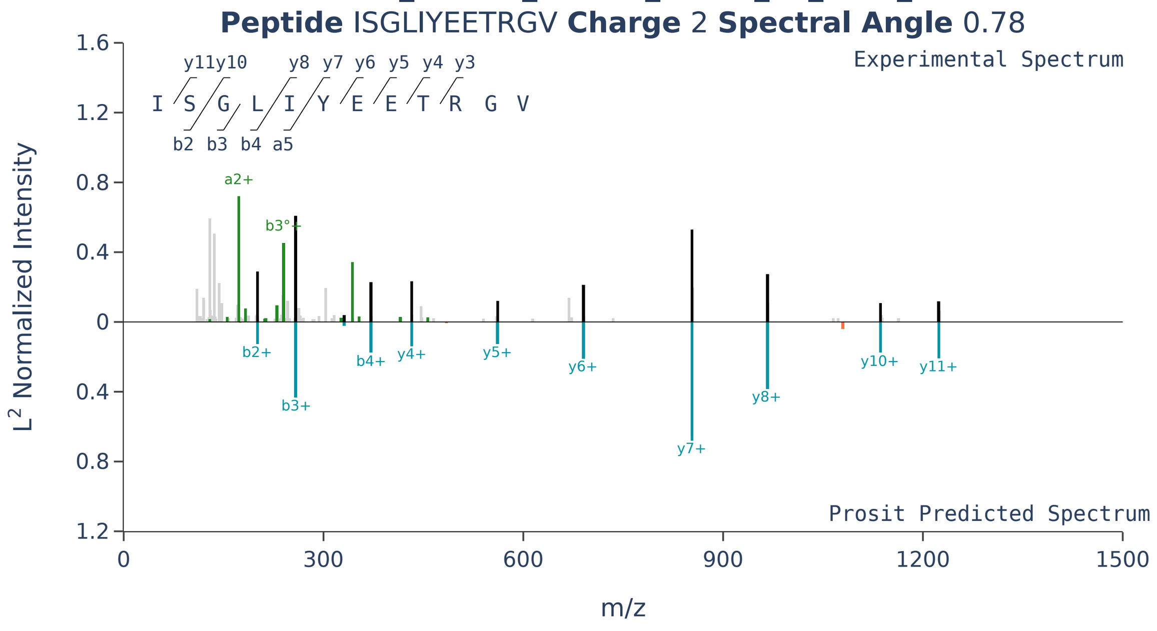

**Source** HRoetschke_291222_140123_HFGoe_H4_b2R1_1h **Scan** 31368
**Peptide** GLIYEETRGVLKVFL **Charge** 2 **Spectral Angle** 0.67

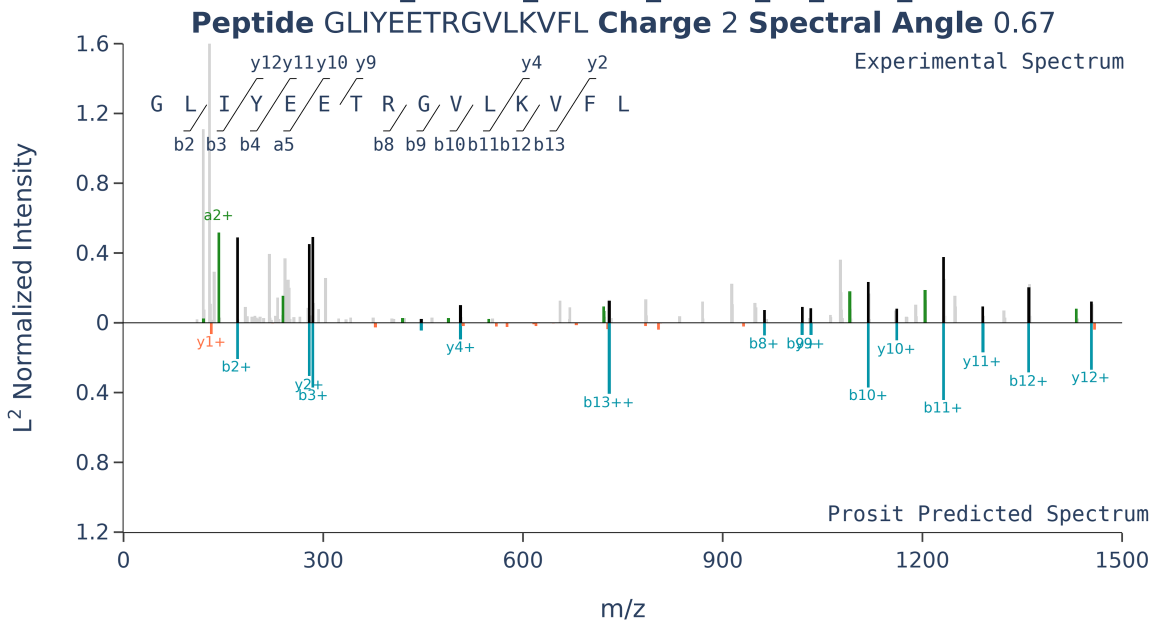


**Source** HRoetschke_291222_140123_HFGoe_H4_b1R2_4h **Scan** 6619
**Peptide** LKRQGRTLY **Charge** 3 **Spectral Angle** 0.91

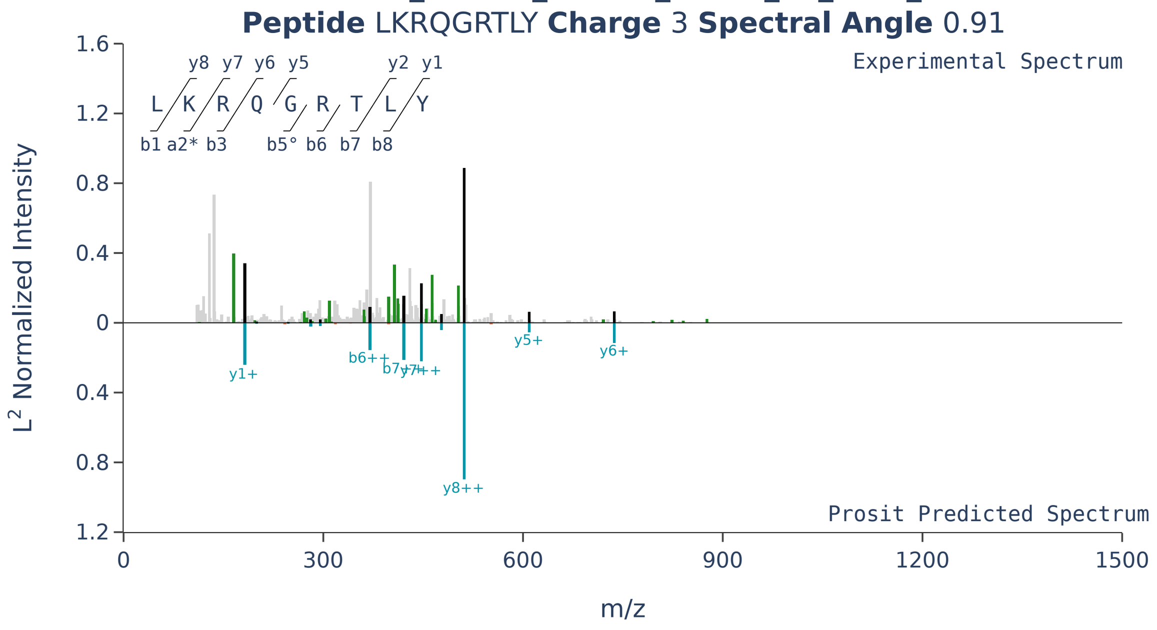


**Source** HRoetschke_291222_140123_HFGoe_H4_b1R1_2h **Scan** 17259
**Peptide** GVKRISGLIYEE **Charge** 3 **Spectral Angle** 0.82

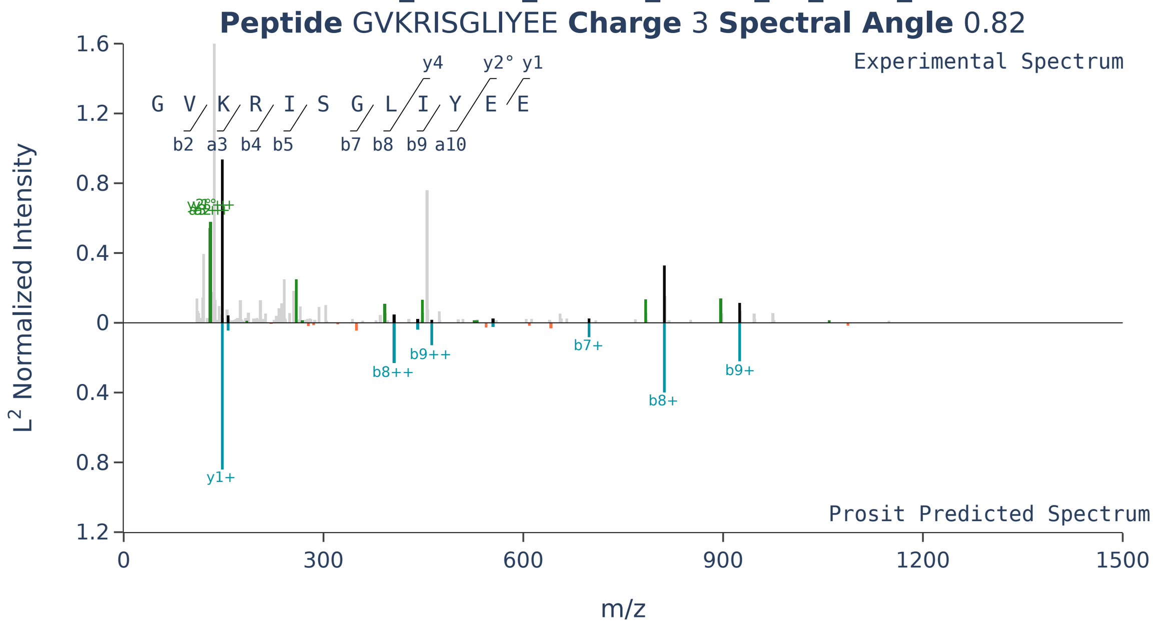


**Source** HRoetschke_291222_140123_HFGoe_H4_b1R1_2h **Scan** 9832
**Peptide** GVKRISGL **Charge** 2 **Spectral Angle** 0.89

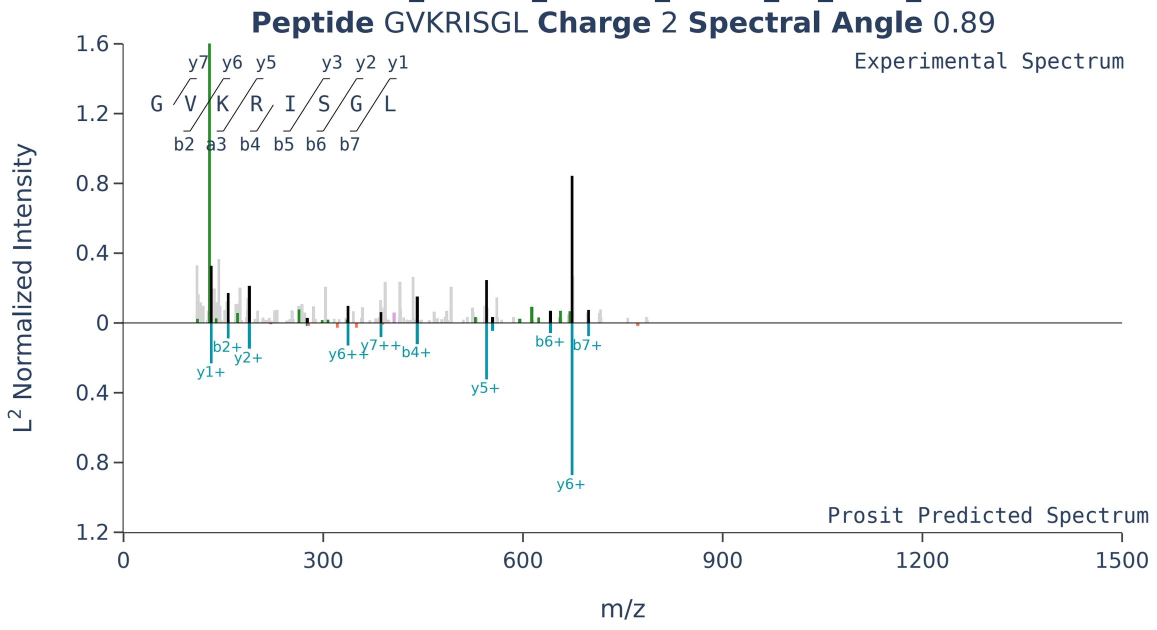


**Source** HRoetschke_291222_140123_HFGoe_H4_b1R1_1h **Scan** 18017
**Peptide** NIQGITKPAL **Charge** 2 **Spectral Angle** 0.76

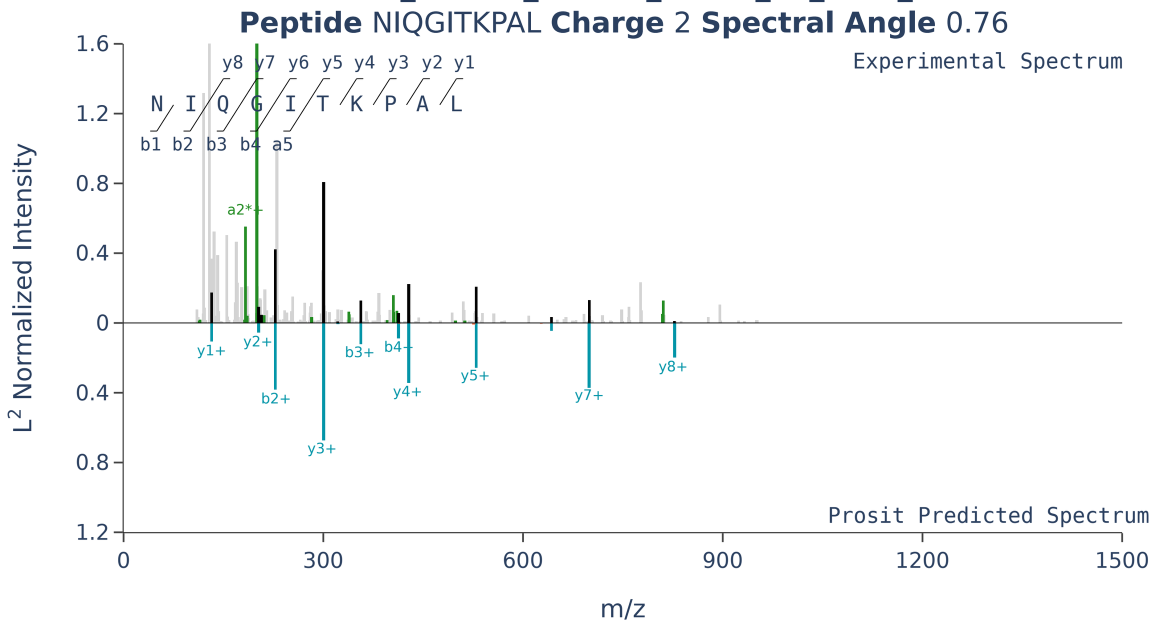


**Source** HRoetschke_291222_140123_HFGoe_H4_b1R1_24h **Scan** 8764
**Peptide** IQGITKPAIRR **Charge** 2 **Spectral Angle** 0.77

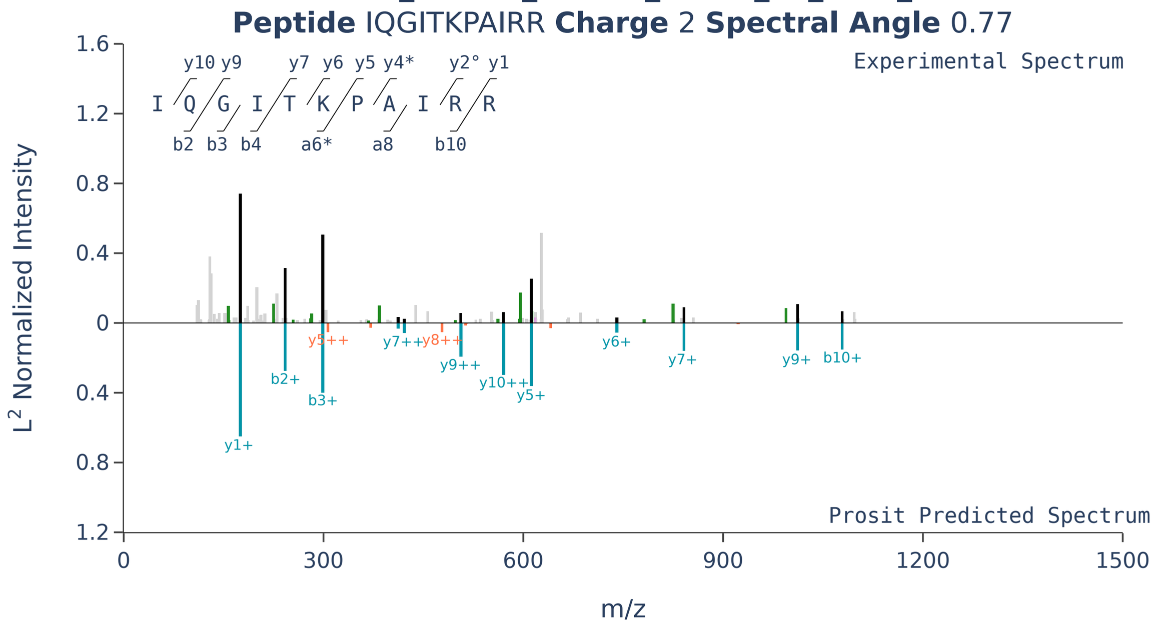


**Source** HRoetschke_291222_140123_HFGoe_H4_b2R1_4h **Scan** 15543
**Peptide** ENVIRDAVTYTEHAKRKTVTA **Charge** 3 **Spectral Angle** 0.75

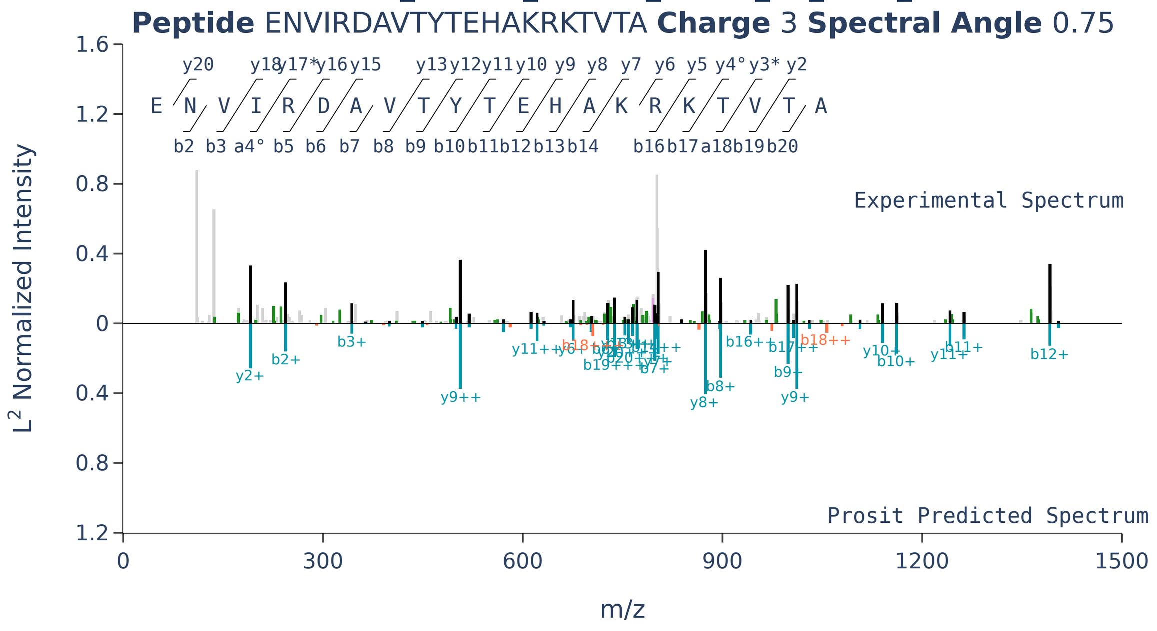


**Source** HRoetschke_291222_140123_HFGoe_H4_b2R1_1h **Scan** 22587
**Peptide** GGVKRISGLIYEETRGVL **Charge** 3 **Spectral Angle** 0.85

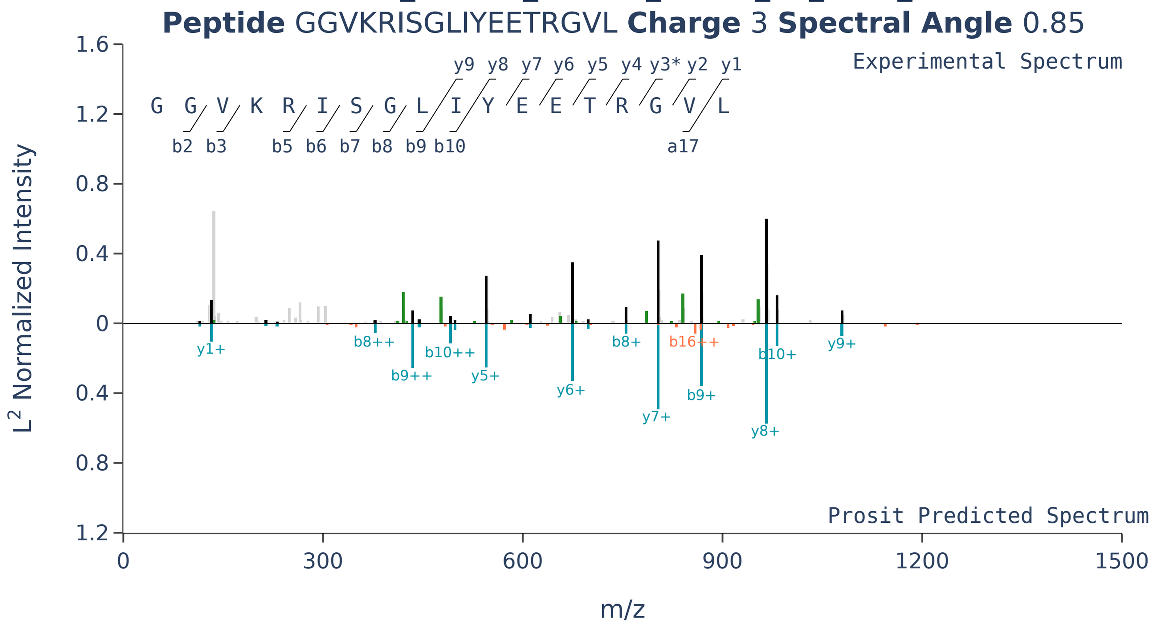


**Source** HRoetschke_291222_140123_HFGoe_H4_b1R1_2h **Scan** 12305
**Peptide** TEHAKRKTVTAMDVVY **Charge** 2 **Spectral Angle** 0.78

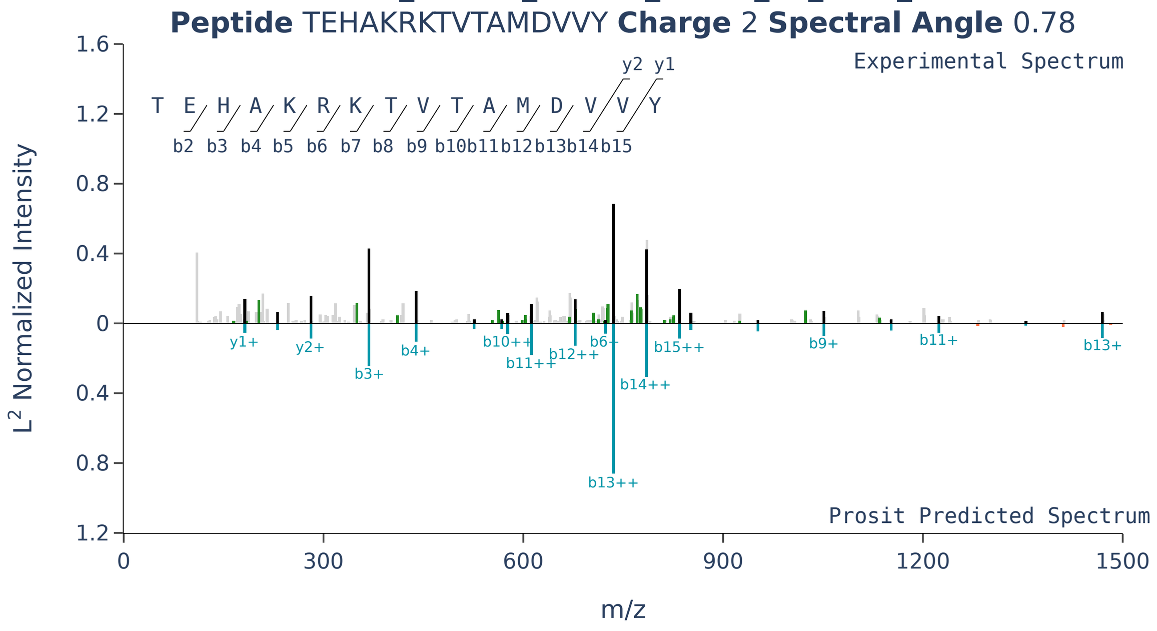


**Source** HRoetschke_291222_140123_HFGoe_H4_b2R1_2h **Scan** 20921
**Peptide** TRGVLKVFLEN **Charge** 3 **Spectral Angle** 0.72

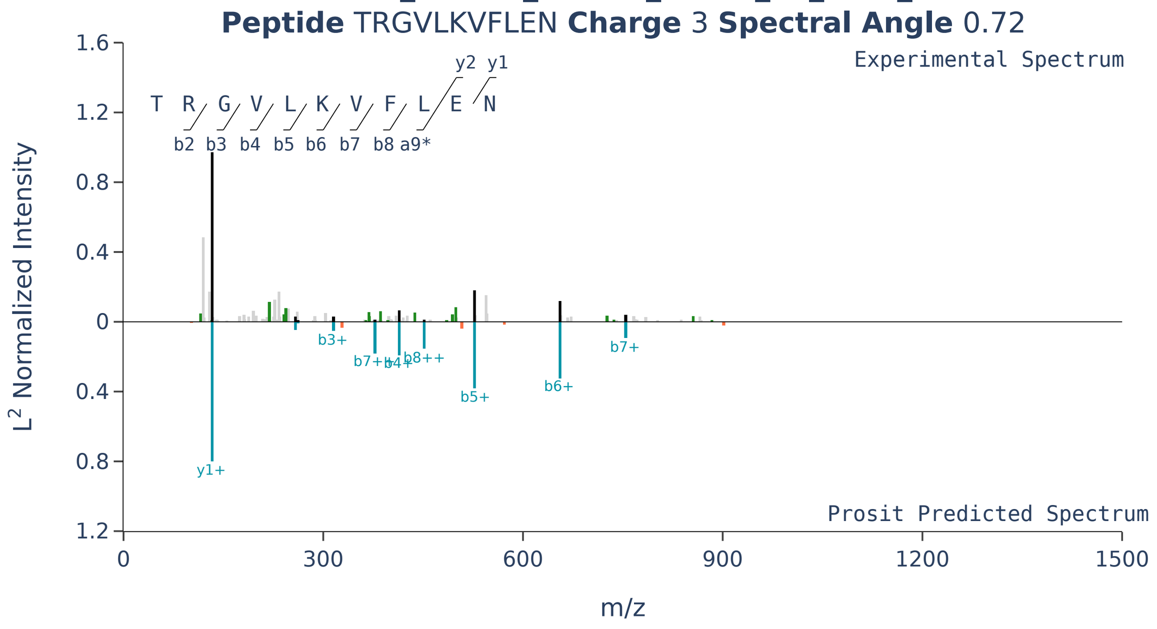


**Source** HRoetschke_291222_140123_HFGoe_H4_b2R1_1h **Scan** 26899
**Peptide** GLIYEETRGVLKVF **Charge** 2 **Spectral Angle** 0.68

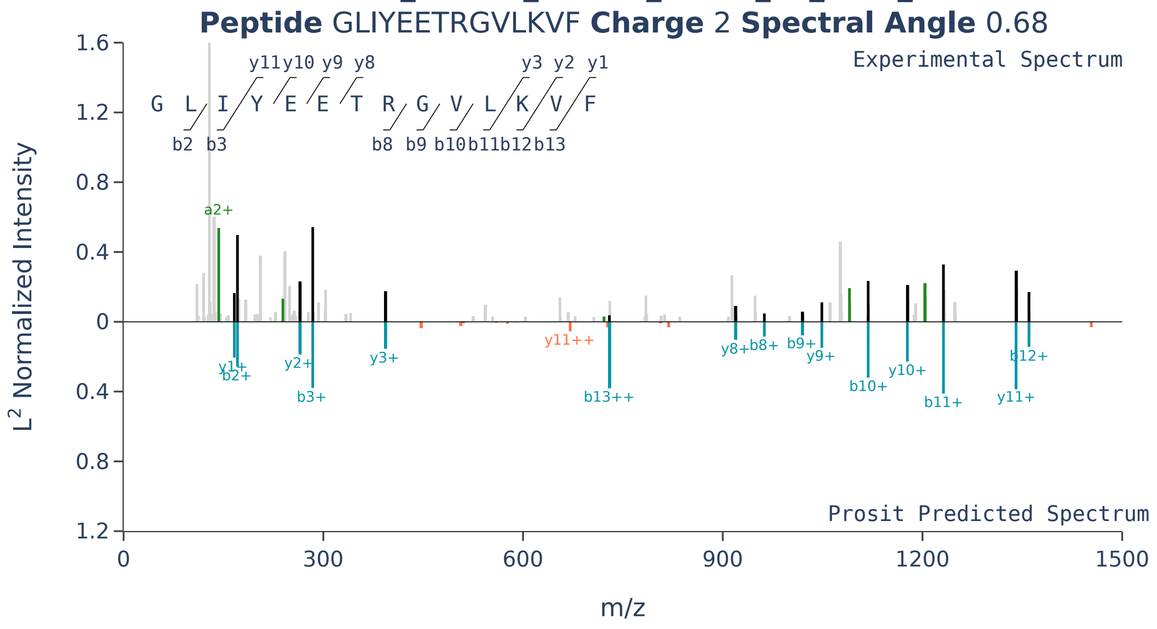


**Source** HRoetschke_291222_140123_HFGoe_H4_b2R1_24h **Scan** 12220
**Peptide** KVLRDNIQGITKPALR **Charge** 3 **Spectral Angle** 0.83

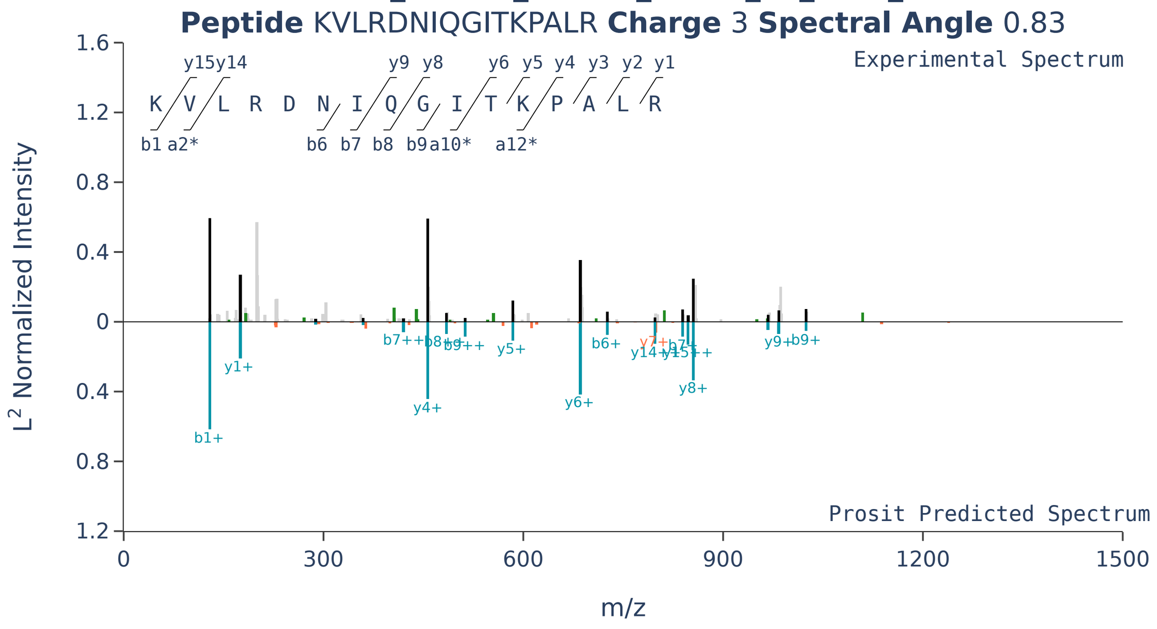


**Source** HRoetschke_291222_140123_HFGoe_H4_b1R1_24h **Scan** 7399
**Peptide** DAVTYTEH **Charge** 2 **Spectral Angle** 0.85

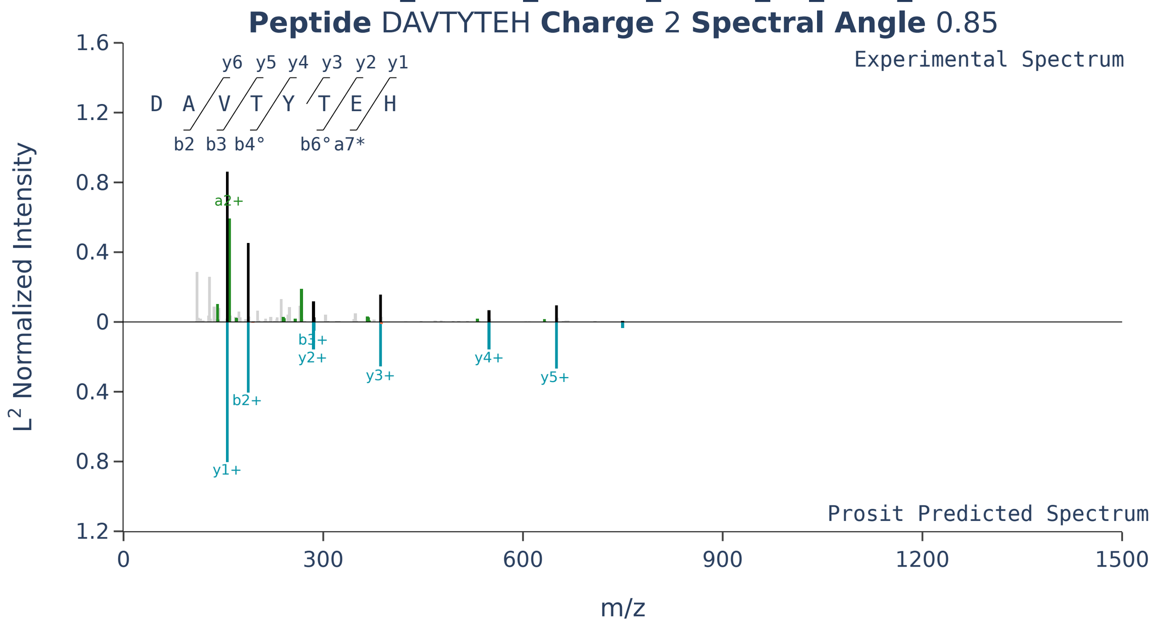


**Source** HRoetschke_291222_140123_HFGoe_H4_b2R2_1h **Scan** 25974
**Peptide** LSGLIYEETRGVL **Charge** 2 **Spectral Angle** 0.79

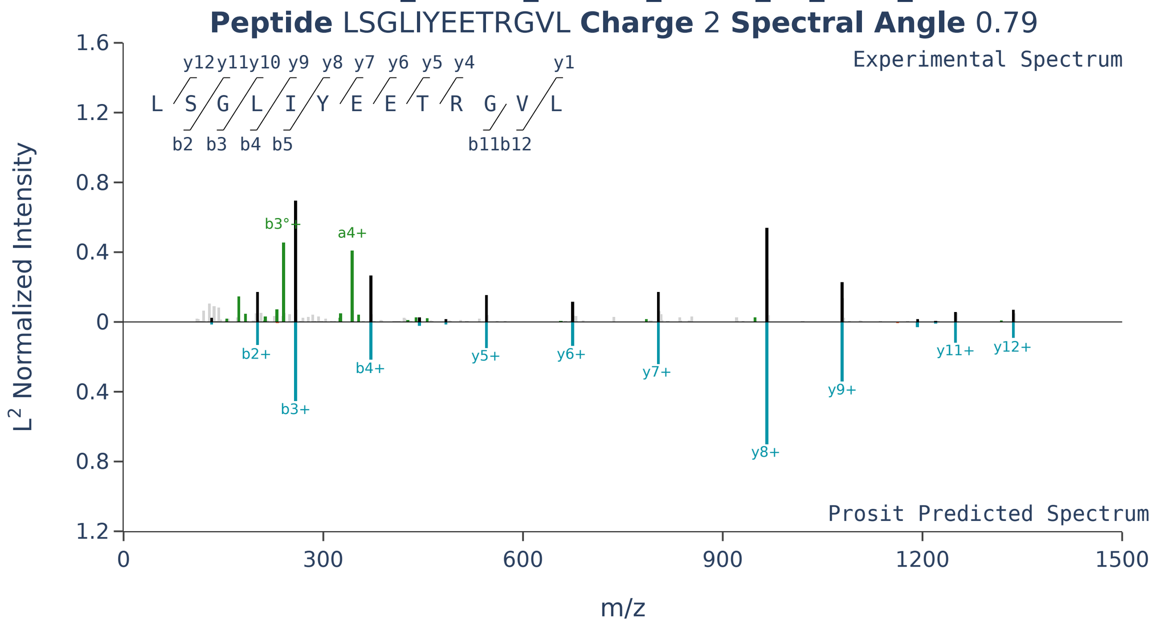


**Source** HRoetschke_291222_140123_HFGoe_H4_b2R1_1h **Scan** 30517
**Peptide** VLKVFLENVI **Charge** 2 **Spectral Angle** 0.7

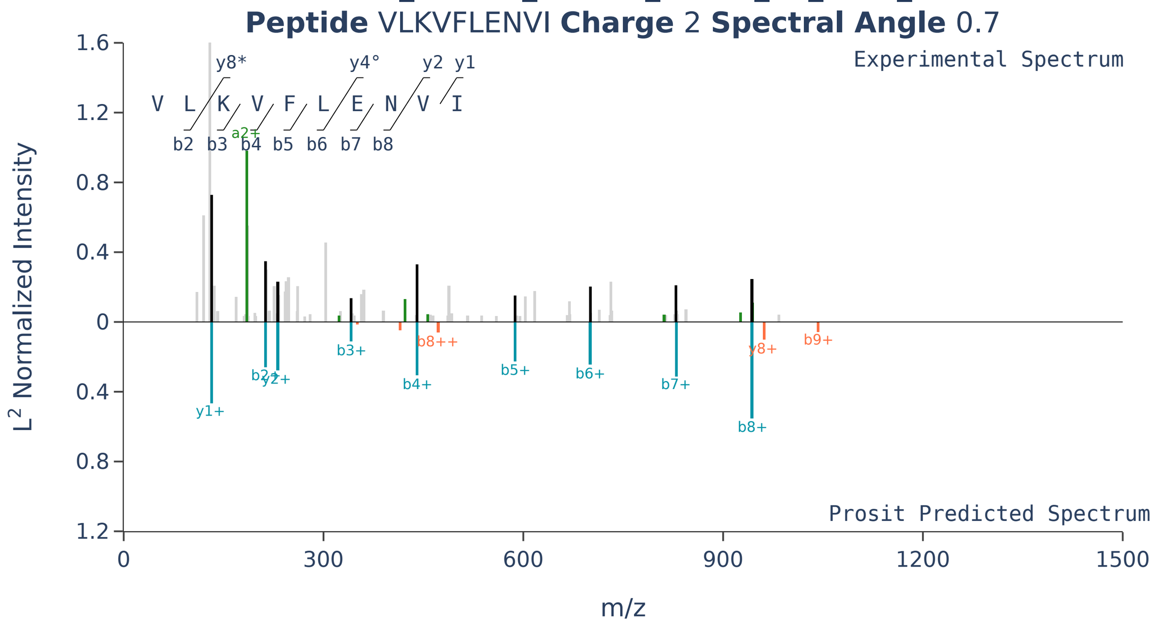

**Source** HRoetschke_291222_140123_HFGoe_H4_b2R2_4h **Scan** 14502
**Peptide** TEHAKRK **Charge** 2 **Spectral Angle** 0.81

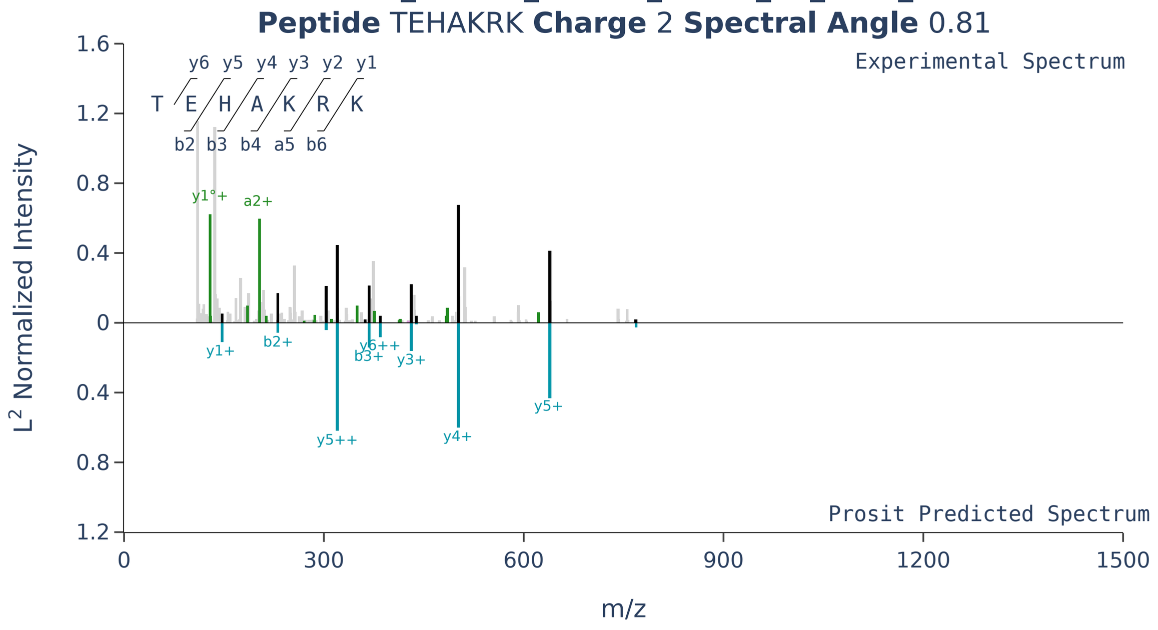


**Source** HRoetschke_291222_140123_HFGoe_H4_b1R1_4h **Scan** 10664
**Peptide** EETRGVLKV **Charge** 2 **Spectral Angle** 0.86

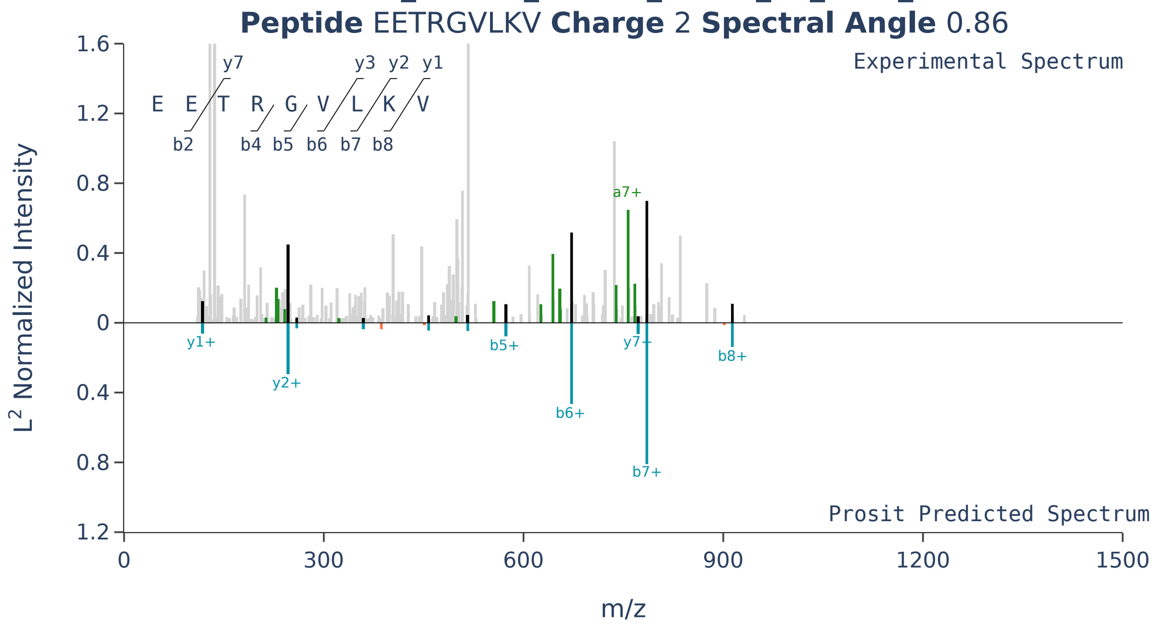


**Source** HRoetschke_291222_140123_HFGoe_H4_b1R1_4h **Scan** 7438
**Peptide** VIRDAVTYTEHAKR **Charge** 4 **Spectral Angle** 0.86

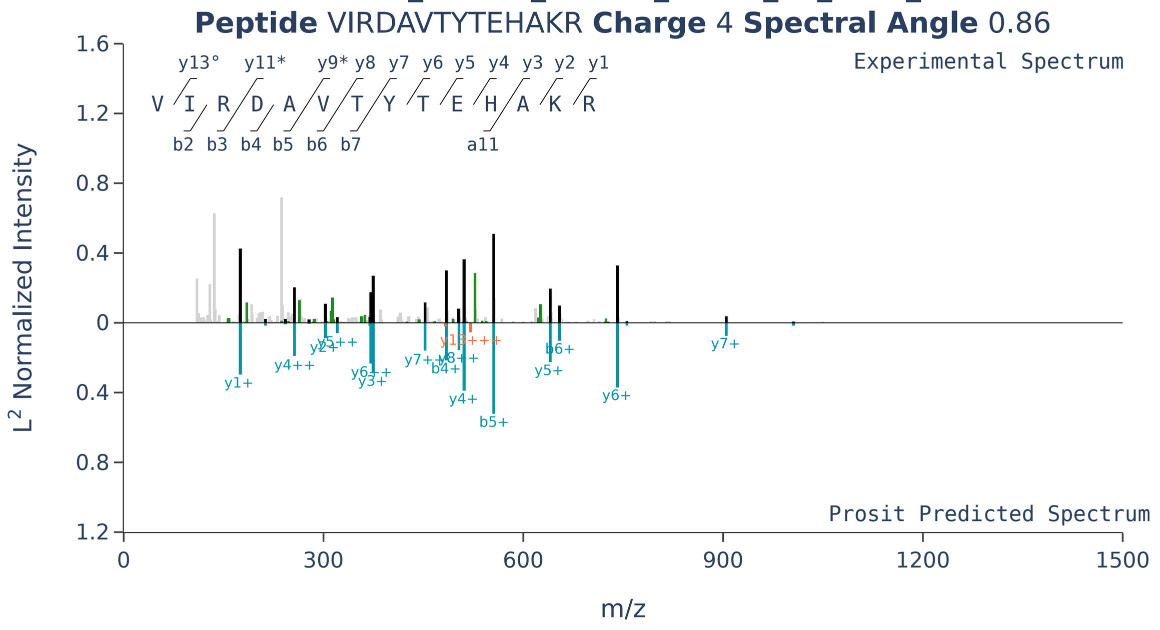


**Source** HRoetschke_291222_140123_HFGoe_H4_b1R2_4h **Scan** 14873
**Peptide** GLTKPAI **Charge** 1 **Spectral Angle** 0.77

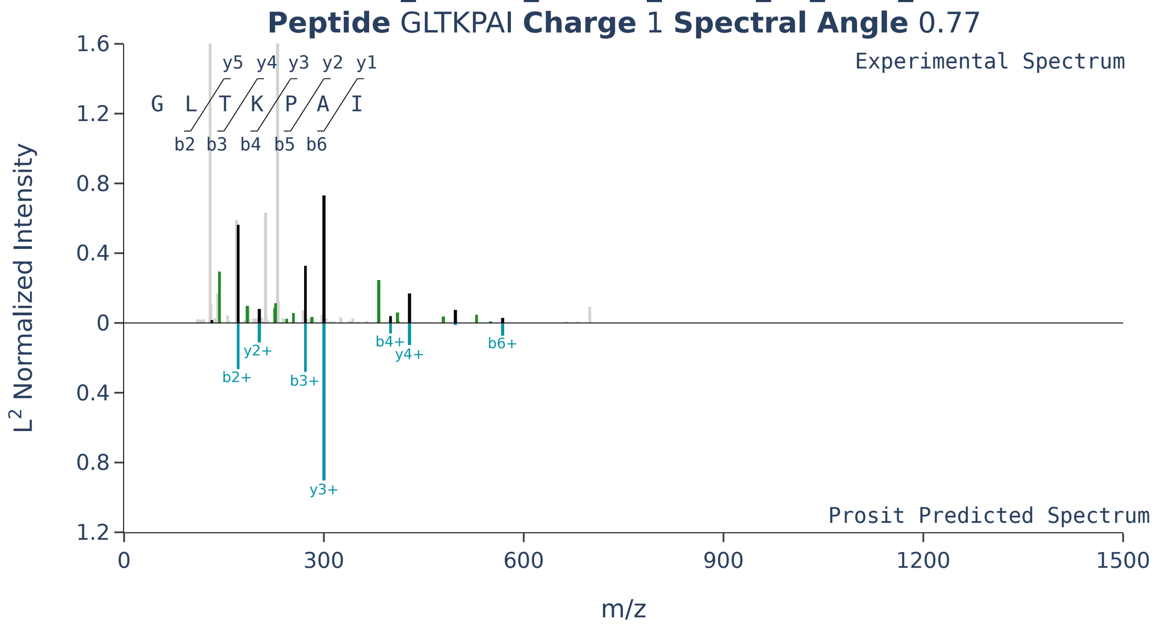


**Source** HRoetschke_291222_140123_HFGoe_H4_b2R1_1h **Scan** 5285
**Peptide** AVTYTEHAKR **Charge** 3 **Spectral Angle** 0.75

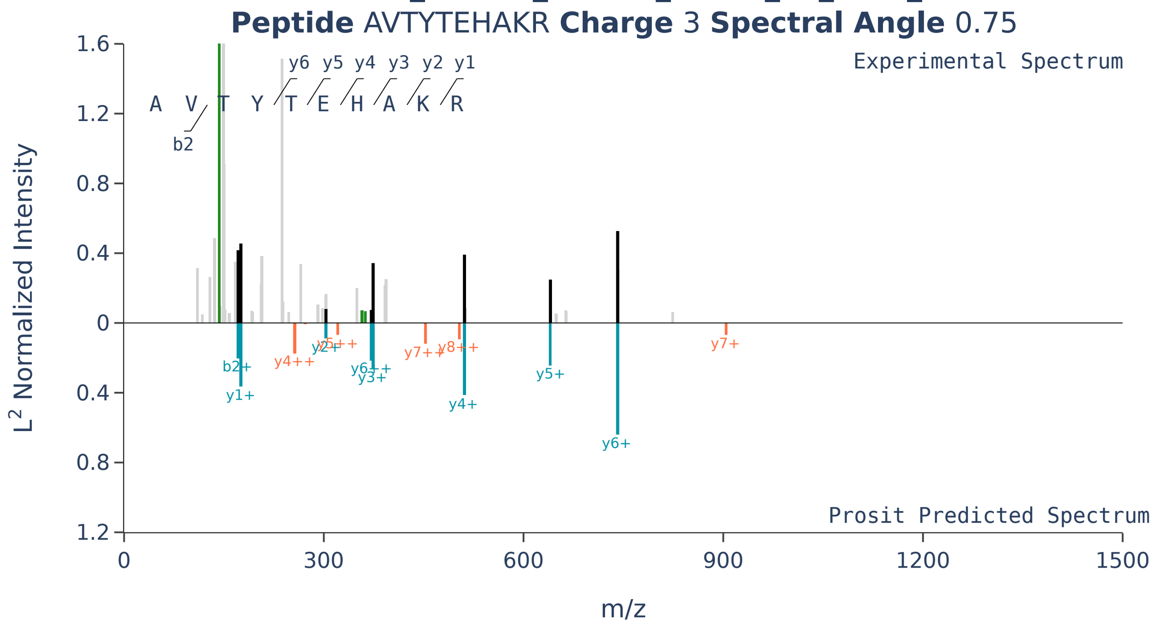


**Source** HRoetschke_291222_140123_HFGoe_H4_b1R1_4h **Scan** 12464
**Peptide** GLTKPAIRRL **Charge** 2 **Spectral Angle** 0.76

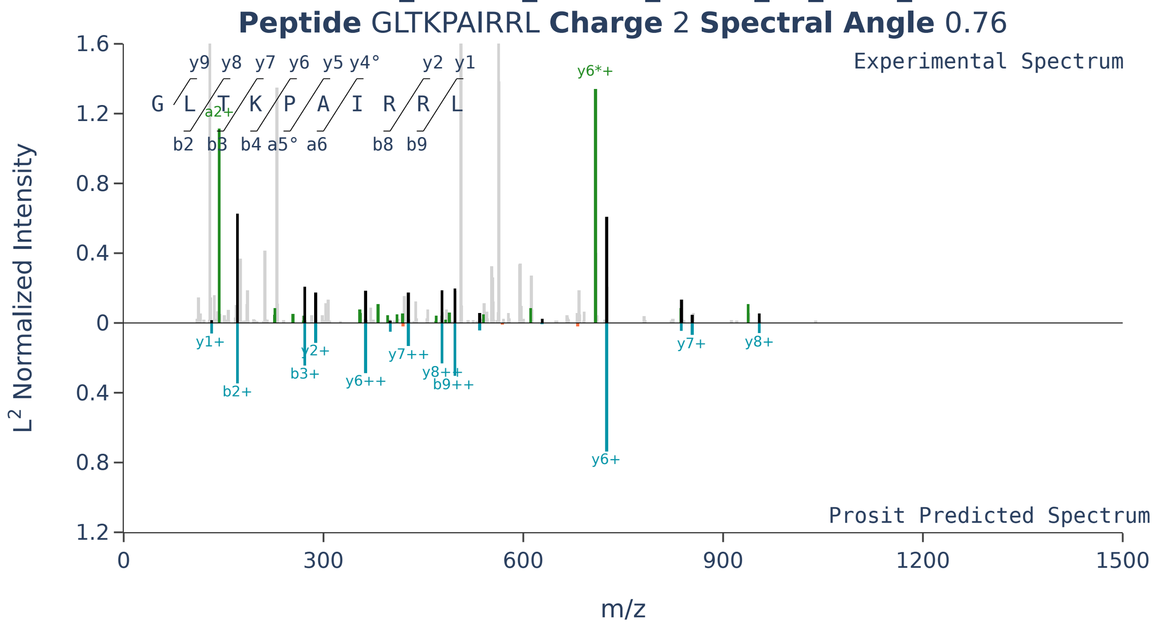


**Source** HRoetschke_291222_140123_HFGoe_H4_b2R2_1h **Scan** 5066
**Peptide** RDAVTYTEHAKRKT **Charge** 4 **Spectral Angle** 0.73

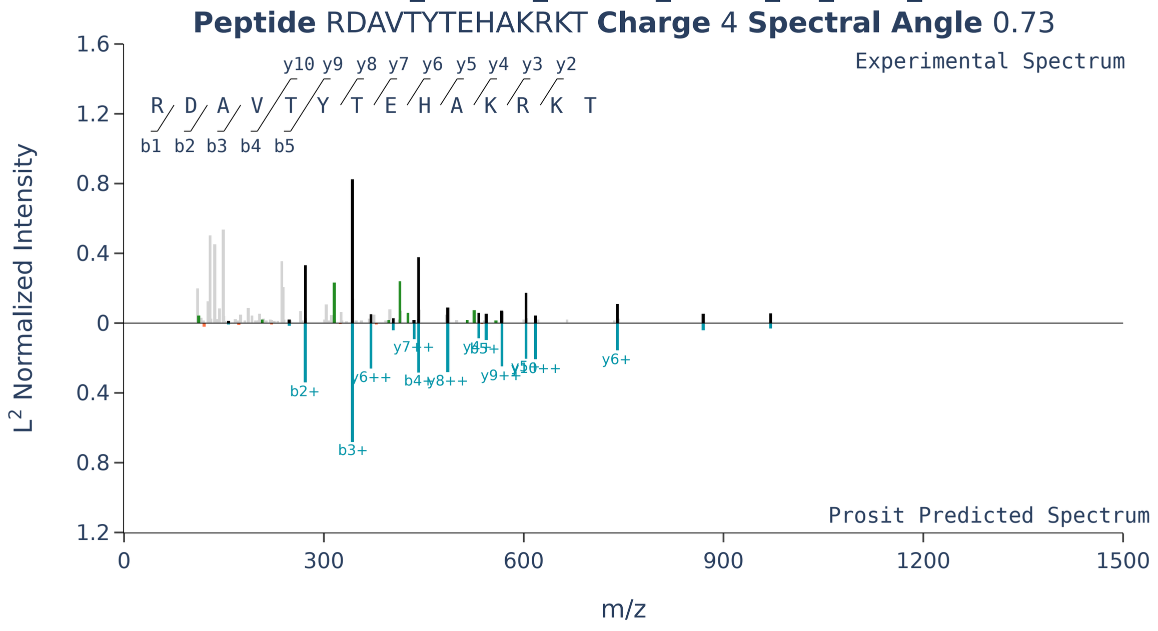


**Source** HRoetschke_291222_140123_HFGoe_H4_b1R1_1h **Scan** 12081
**Peptide** GITKPAIRRL **Charge** 3 **Spectral Angle** 0.81

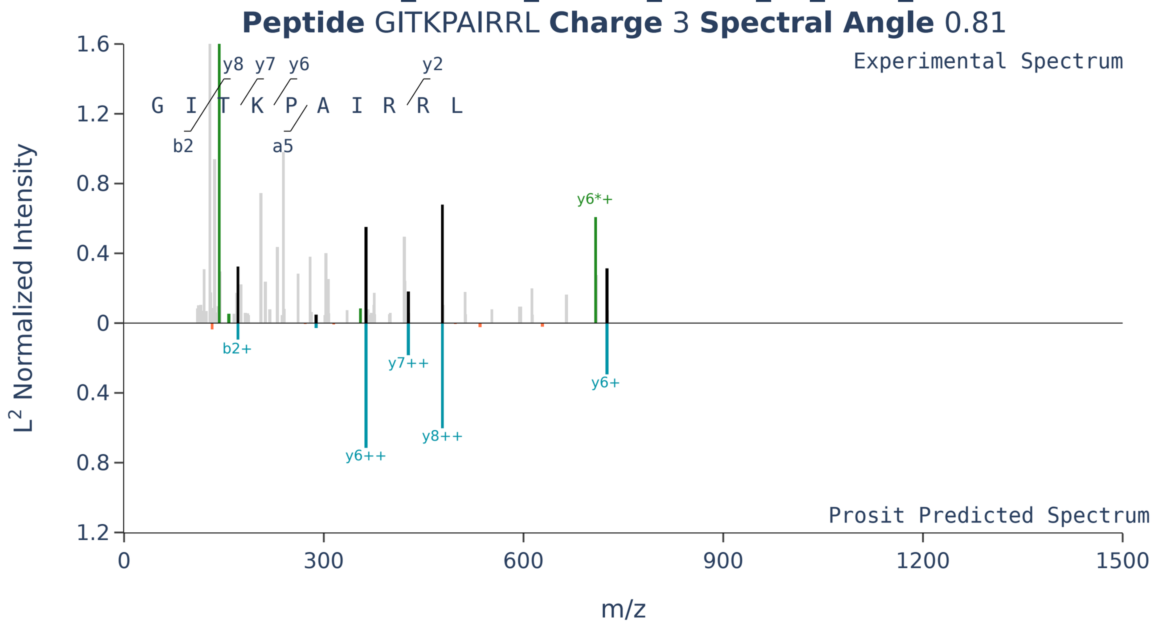


**Source** HRoetschke_291222_140123_HFGoe_H4_b1R1_2h **Scan** 7681
**Peptide** VVYALKRQGRT **Charge** 3 **Spectral Angle** 0.86

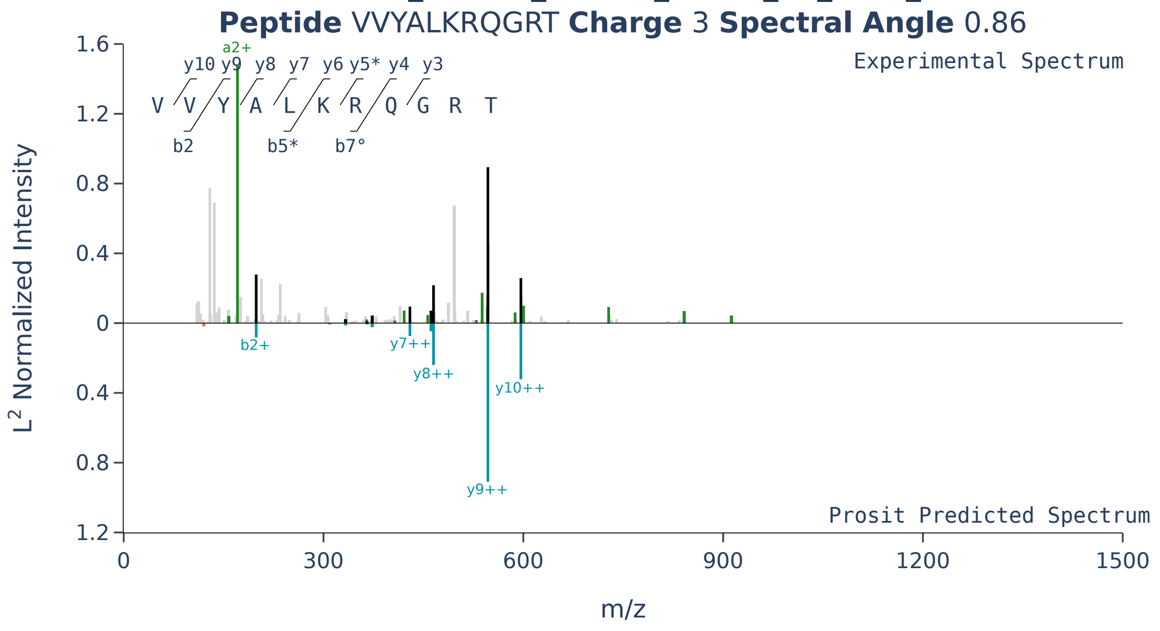


**Source** HRoetschke_291222_140123_HFGoe_H4_b1R1_24h **Scan** 10474
**Peptide** RDNIQGITKPALR **Charge** 3 **Spectral Angle** 0.81

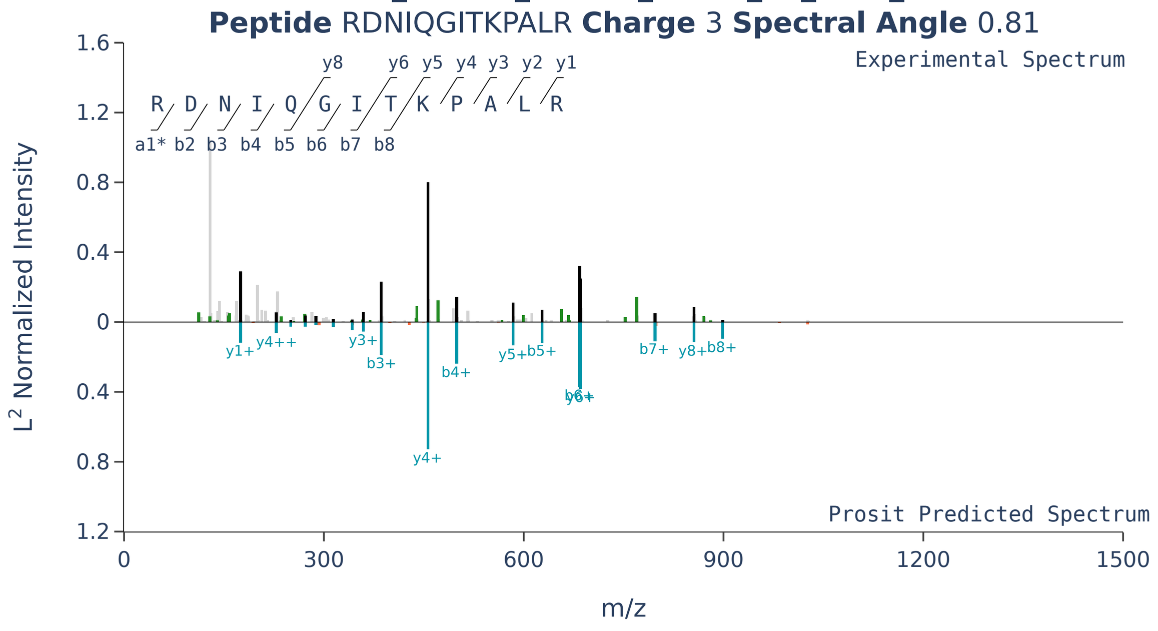


**Source** HRoetschke_291222_140123_HFGoe_H4_b1R2_1h **Scan** 16731
**Peptide** VYALKRQGRTLYGF **Charge** 3 **Spectral Angle** 0.83

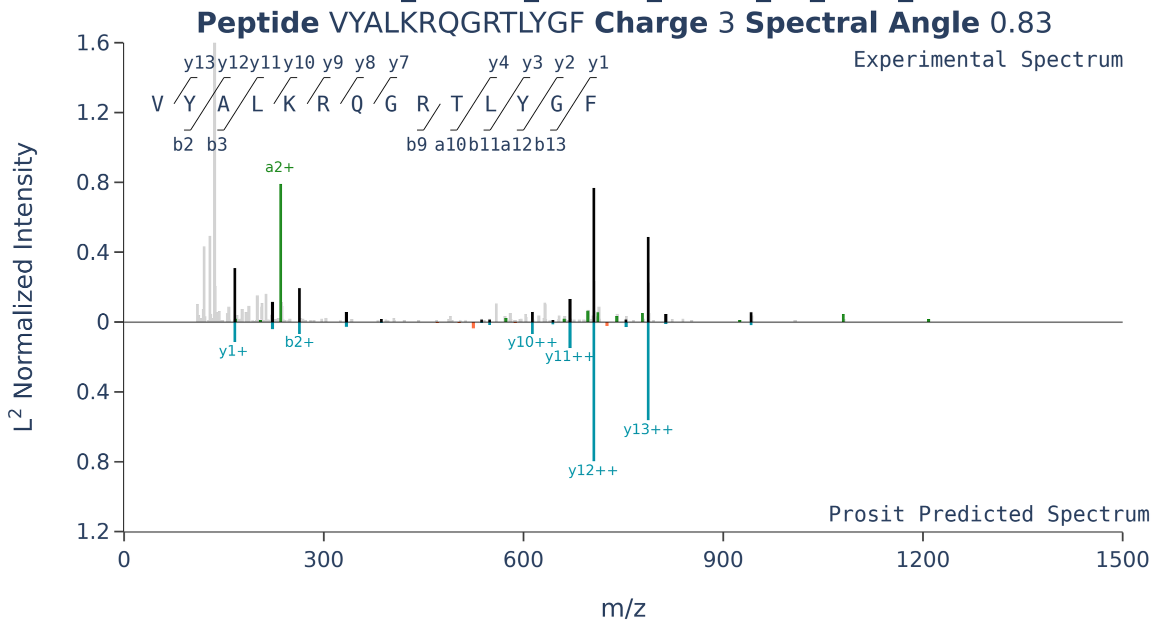


**Source** HRoetschke_291222_140123_HFGoe_H4_b1R2_4h **Scan** 22024
**Peptide** FLENVIRDA **Charge** 2 **Spectral Angle** 0.87

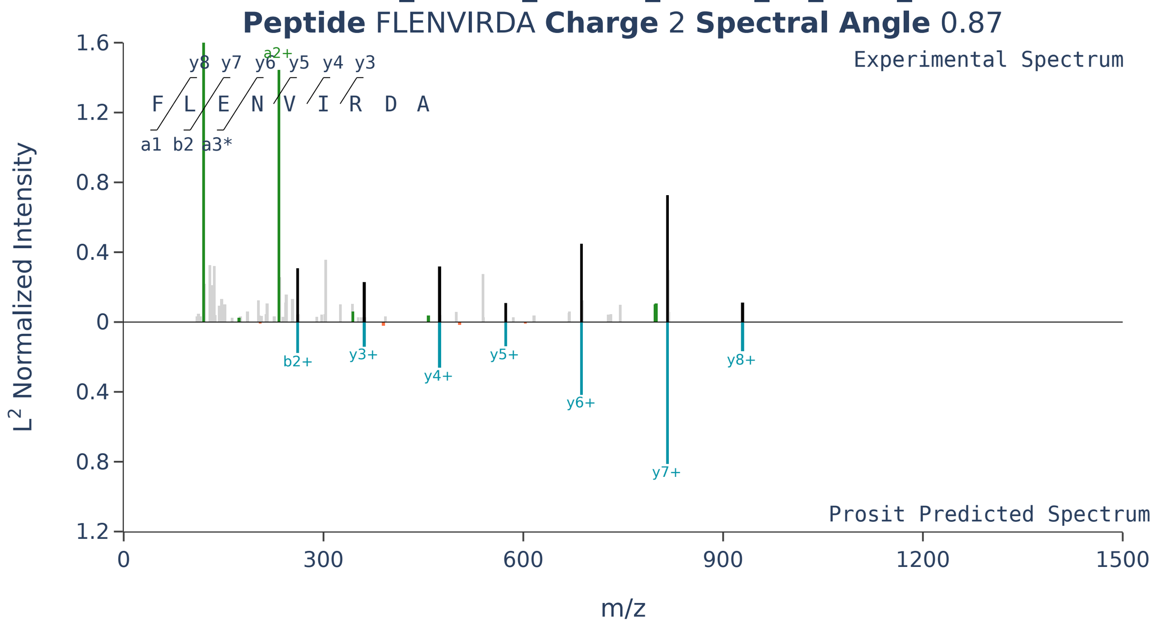


**Source** HRoetschke_291222_140123_HFGoe_H4_b1R2_2h **Scan** 15973
**Peptide** ENVIRDAVTYTEHAKRKTVT **Charge** 3 **Spectral Angle** 0.78

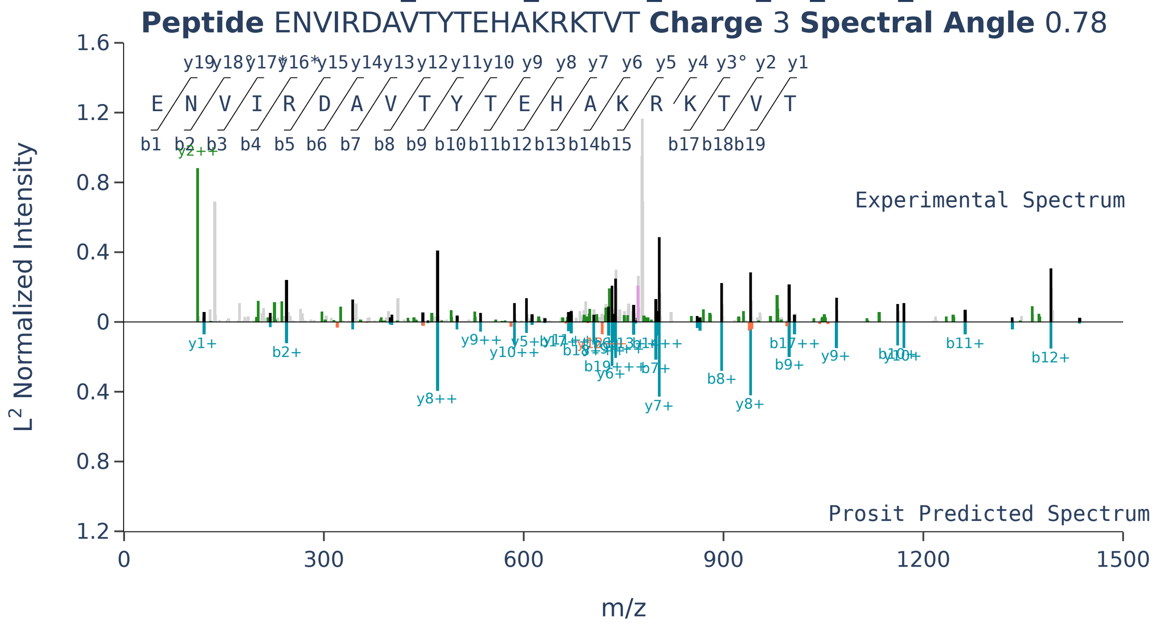


**Source** HRoetschke_291222_140123_HFGoe_H4_b1R1_24h **Scan** 13037
**Peptide** RDAVTYTEHAKRKTVTAMDVVY **Charge** 5 **Spectral Angle** 0.83

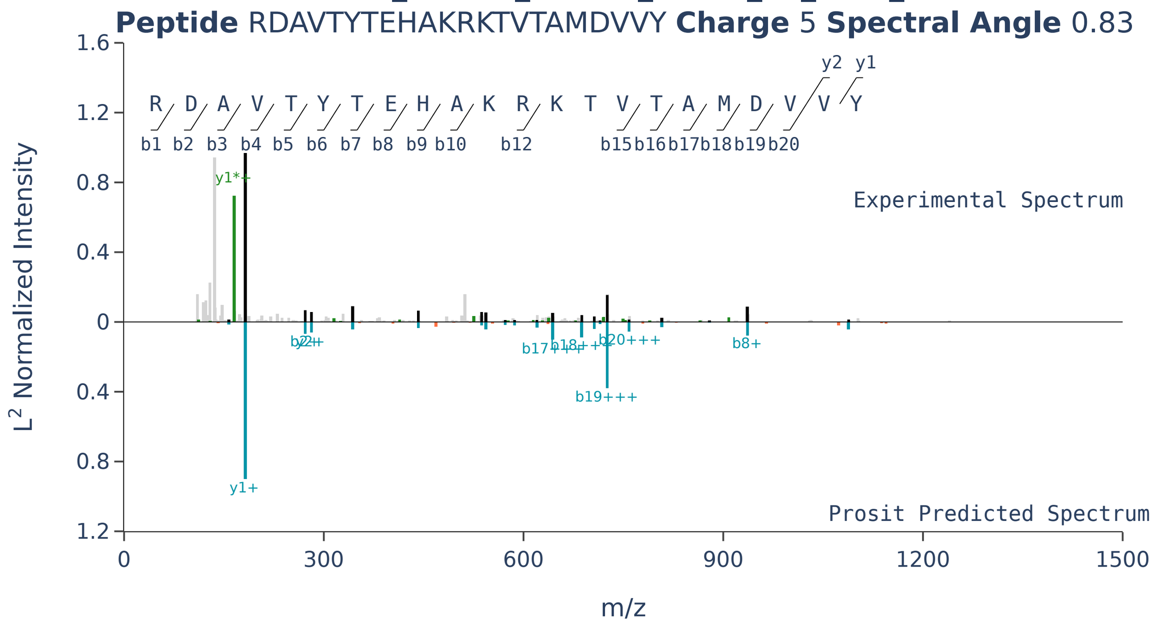


**Source** HRoetschke_291222_140123_HFGoe_H4_b1R1_24h **Scan** 13959
**Peptide** NVIRDAVTYTEHAKRKTVTA **Charge** 3 **Spectral Angle** 0.69

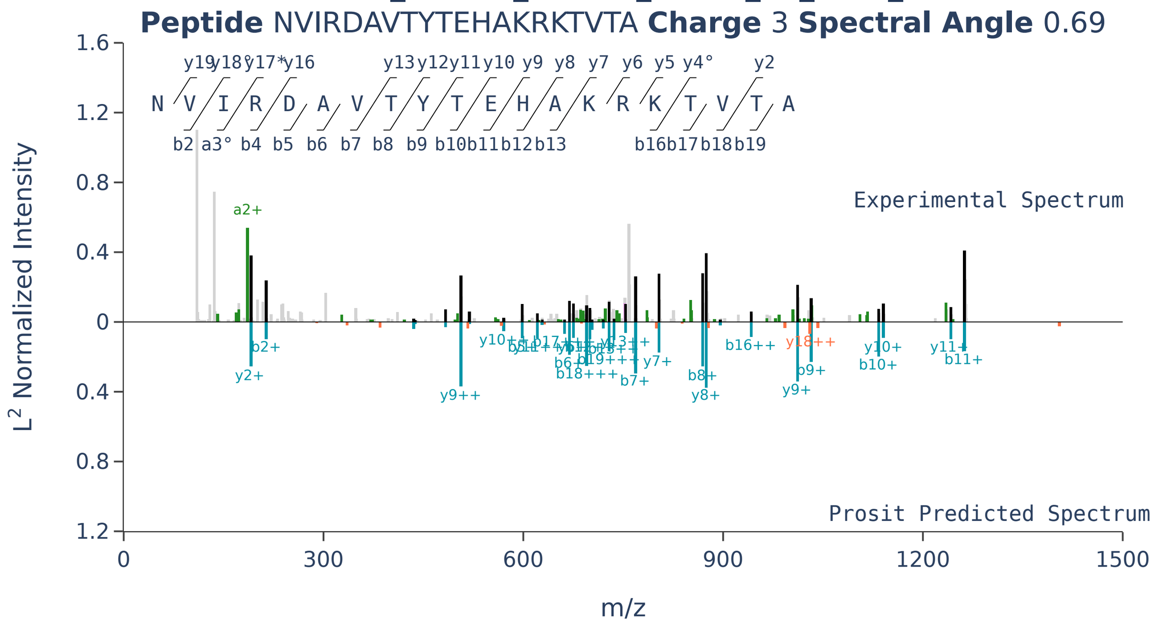

**Source** HRoetschke_291222_140123_HFGoe_H4_b2R1_2h **Scan** 15716
**Peptide** KVLRDNIQGITKPAI **Charge** 2 **Spectral Angle** 0.79

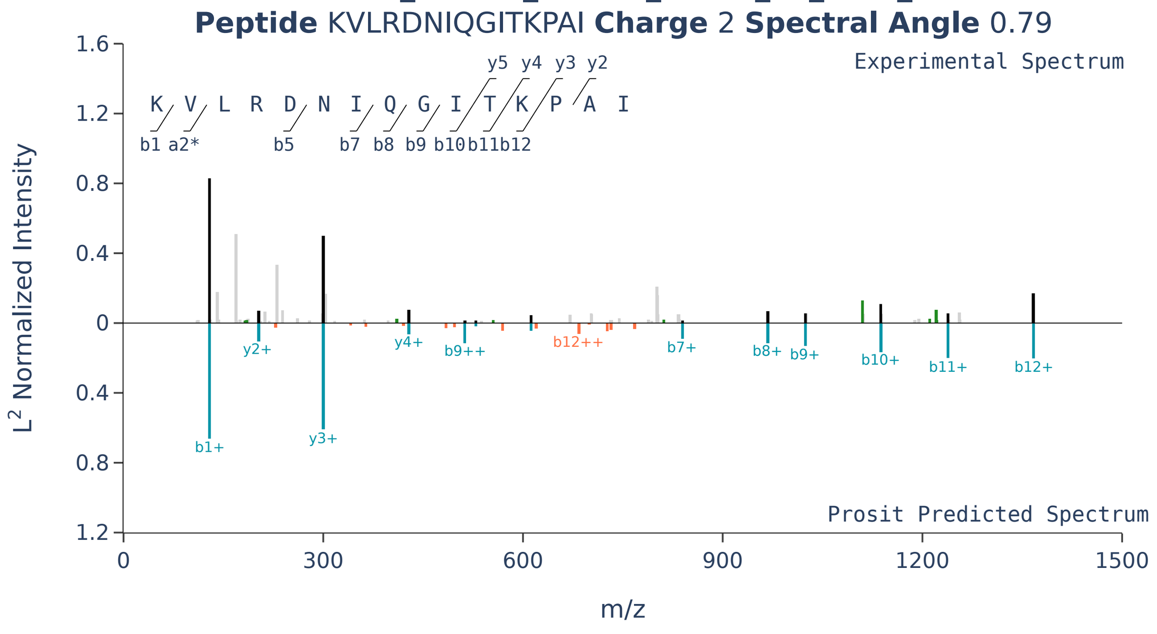


**Source** HRoetschke_291222_140123_HFGoe_H4_b2R1_1h **Scan** 22883
**Peptide** FLENVIRDAVTYTEHAKRK **Charge** 3 **Spectral Angle** 0.73

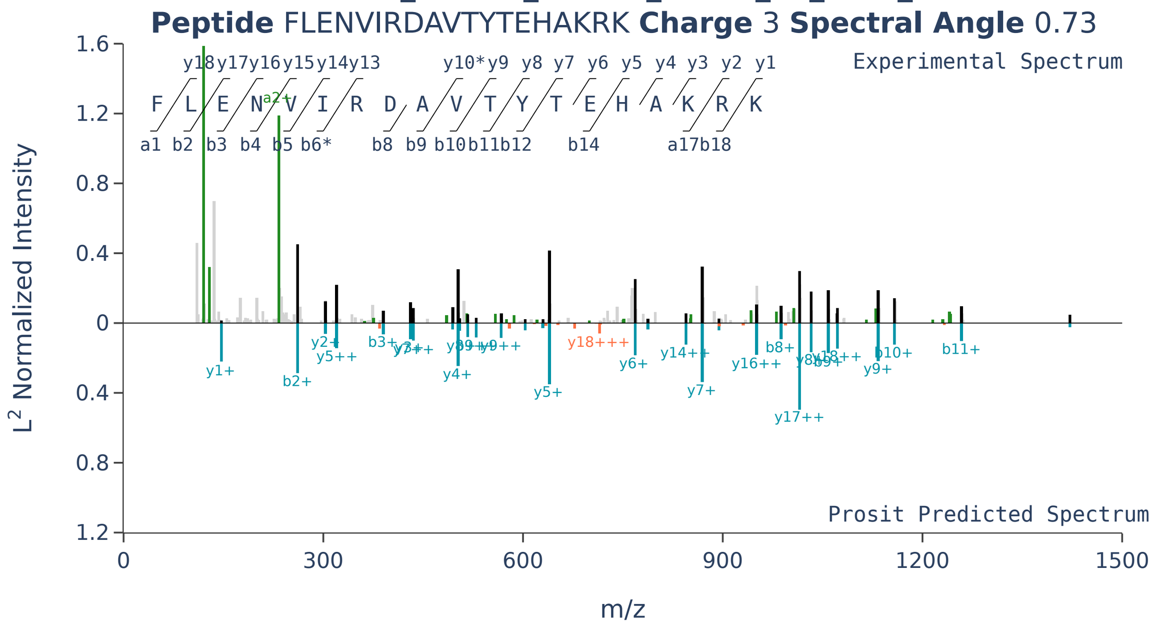


**Source** HRoetschke_291222_140123_HFGoe_H4_b1R2_4h **Scan** 13790
**Peptide** YTEHAKRK **Charge** 2 **Spectral Angle** 0.79

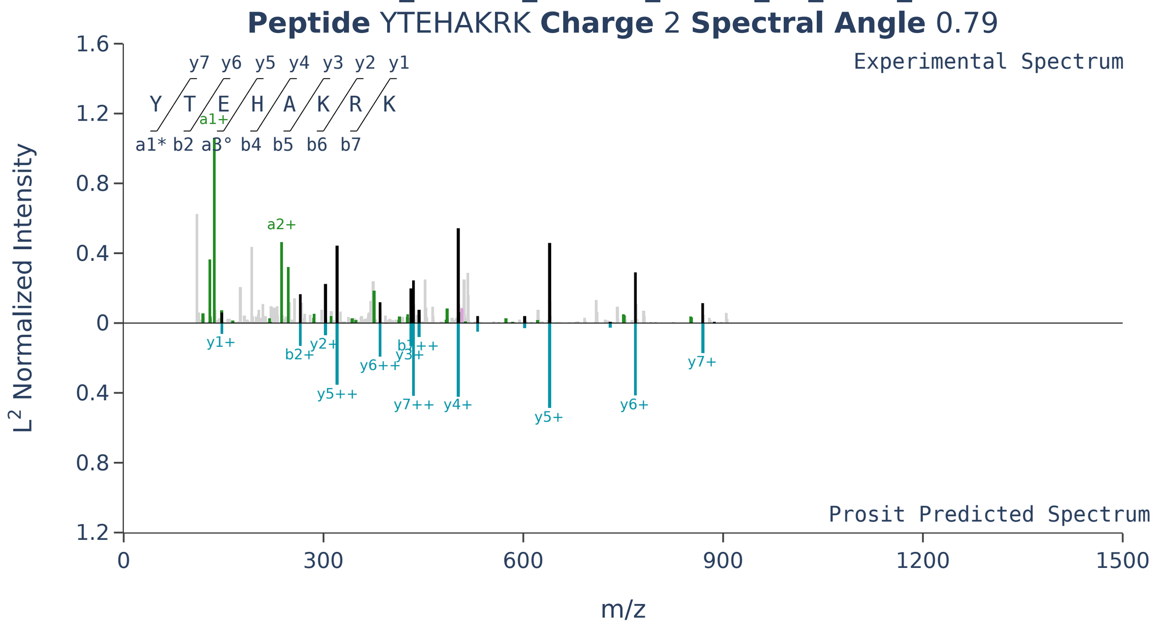


**Source** HRoetschke_291222_140123_HFGoe_H4_b2R1_1h **Scan** 32134
**Peptide** RGVLKVFLENVIRDAVTY **Charge** 3 **Spectral Angle** 0.76

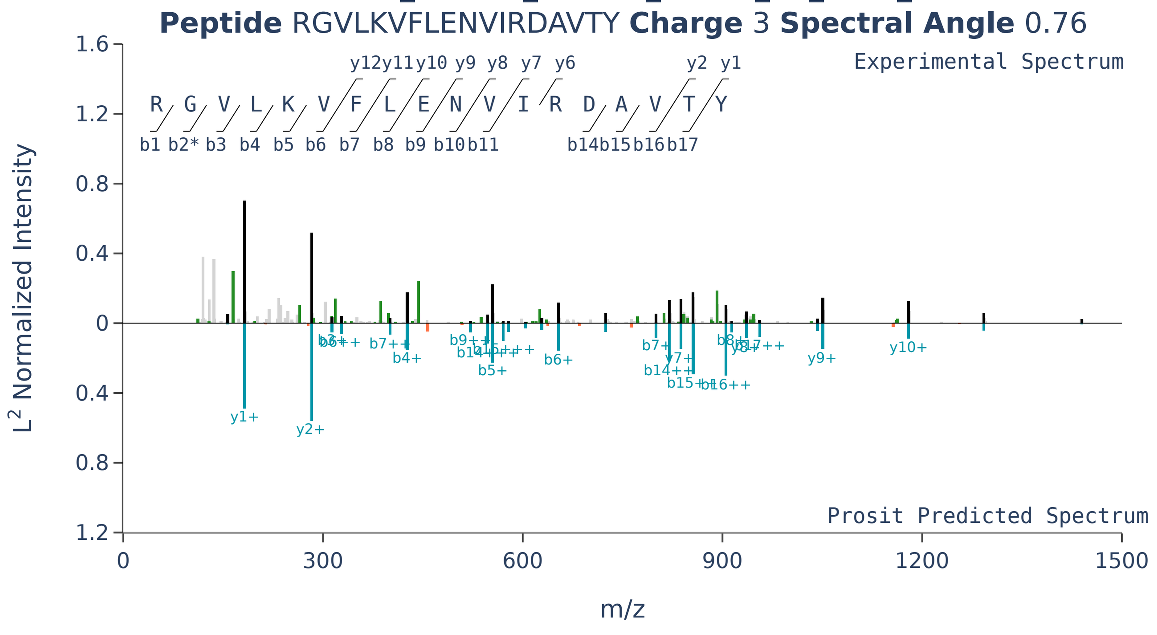


**Source** HRoetschke_291222_140123_HFGoe_H4_b2R1_1h **Scan** 30939
**Peptide** GGVKRISGLIYEETRGVLKVFLE **Charge** 3 **Spectral Angle** 0.76

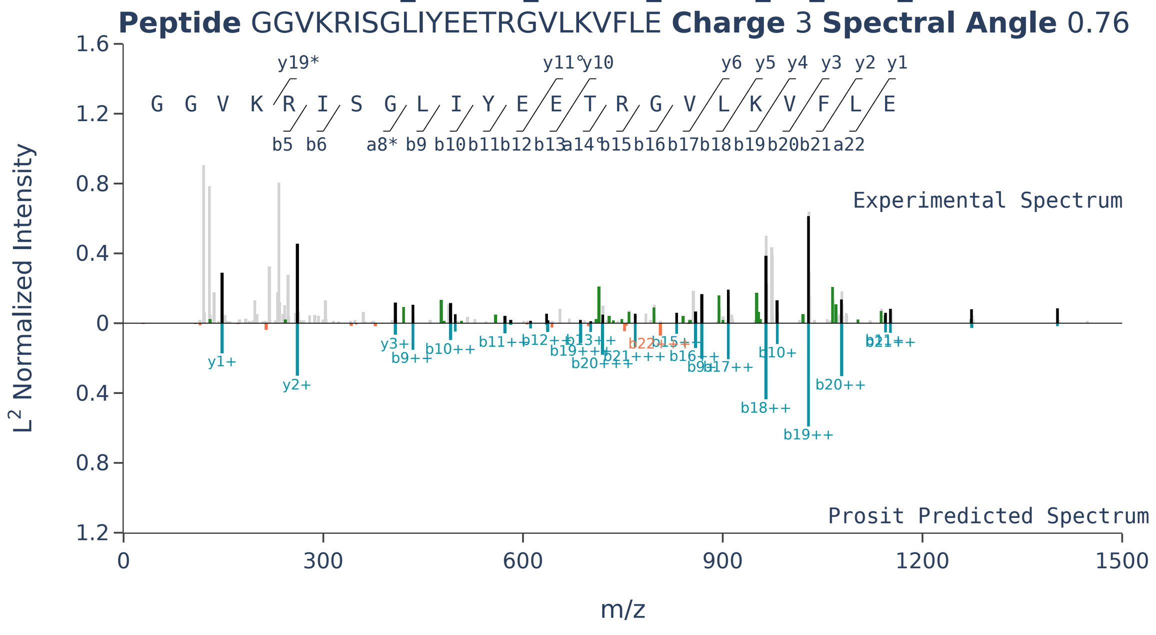


**Source** HRoetschke_291222_140123_HFGoe_H4_b1R1_1h **Scan** 23691
**Peptide** ETRGVLKVFLE **Charge** 2 **Spectral Angle** 0.79

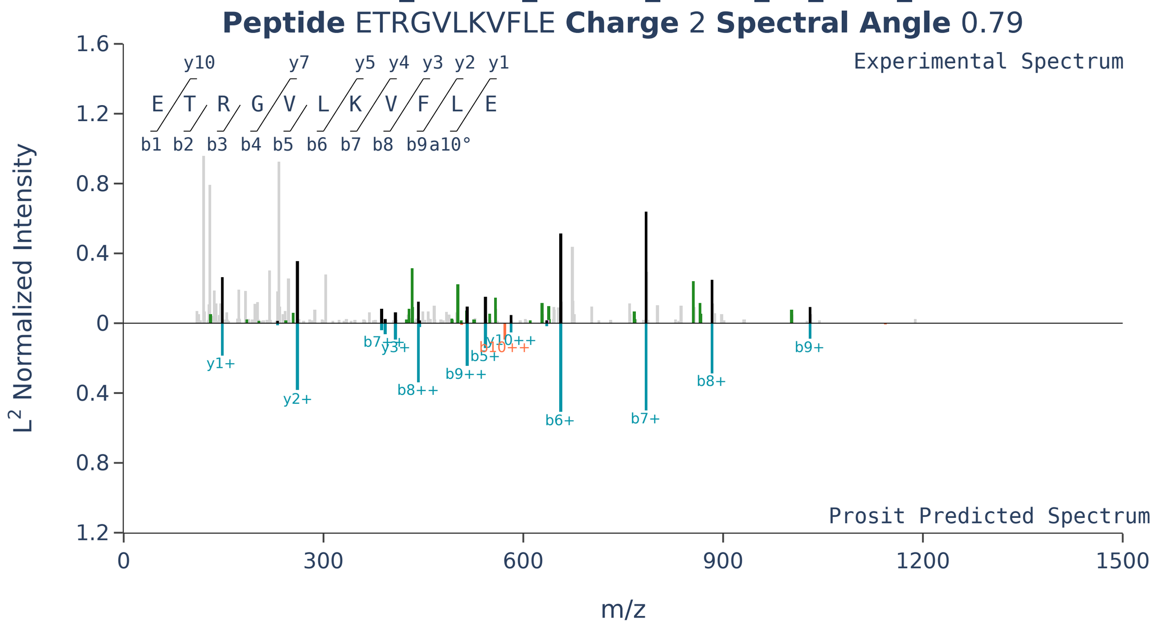


**Source** HRoetschke_291222_140123_HFGoe_H4_b1R1_2h **Scan** 5971
**Peptide** TYTEHAKRKTVTA **Charge** 2 **Spectral Angle** 0.7

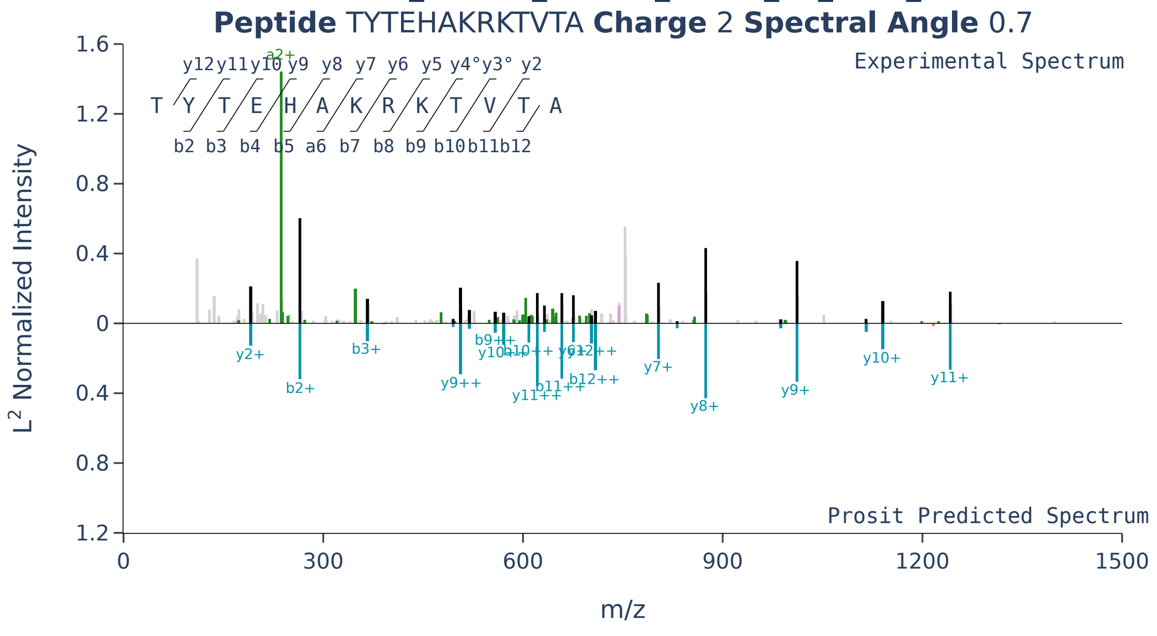


**Source** HRoetschke_291222_140123_HFGoe_H4_b1R1_4h **Scan** 15516
**Peptide** GGVKRISGLI **Charge** 2 **Spectral Angle** 0.69

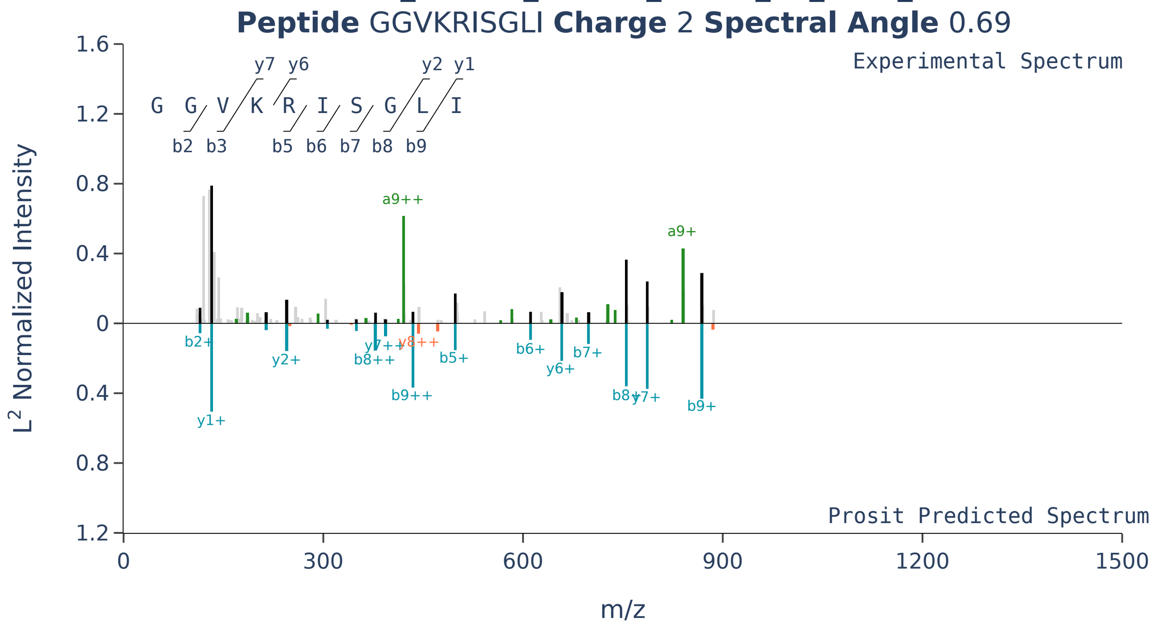


**Source** HRoetschke_291222_140123_HFGoe_H4_b1R2_24h **Scan** 9249
**Peptide** YALKRQGRTLY **Charge** 3 **Spectral Angle** 0.79

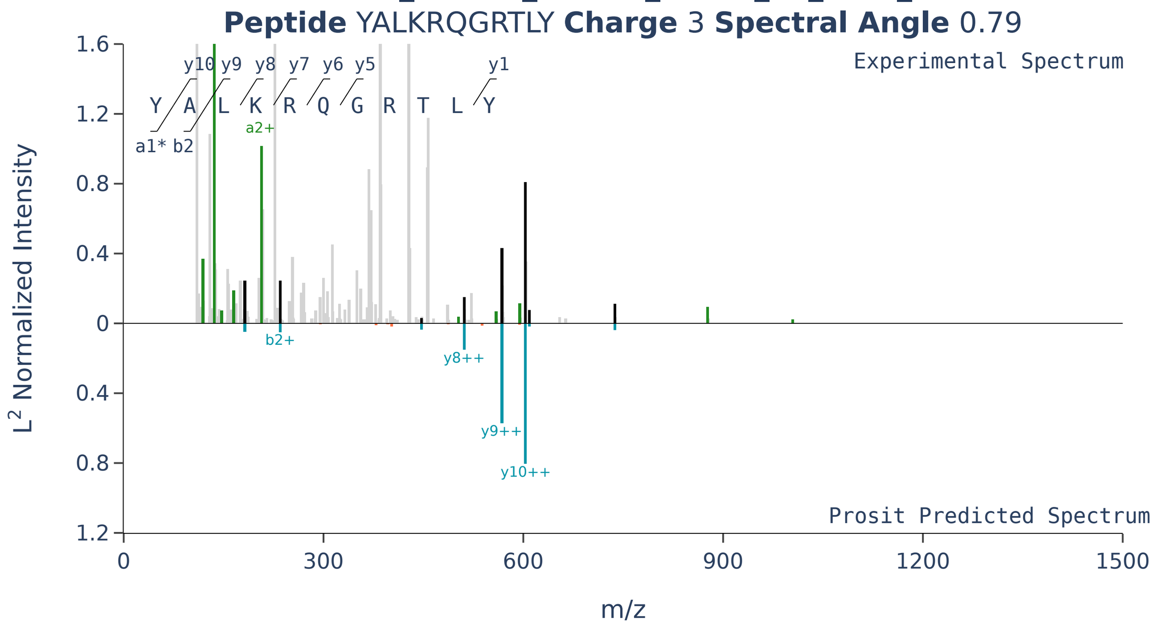


**Source** HRoetschke_291222_140123_HFGoe_H4_b1R1_2h **Scan** 11047
**Peptide** KRKTVTAMDVV **Charge** 2 **Spectral Angle** 0.83

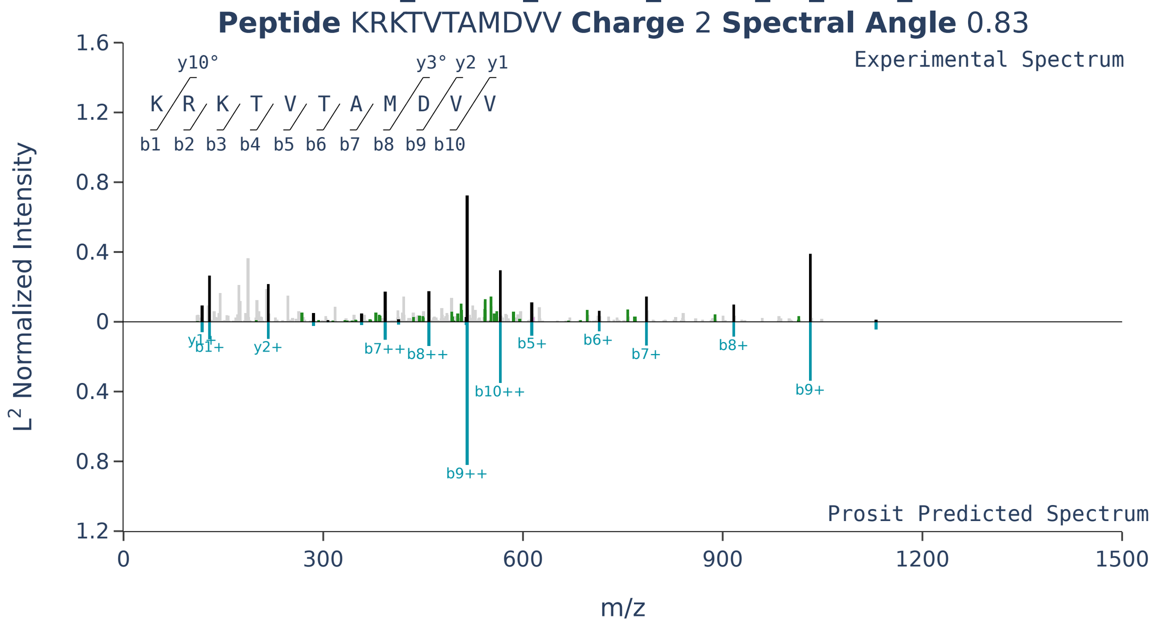


**Source** HRoetschke_291222_140123_HFGoe_H4_b2R1_1h **Scan** 16708
**Peptide** VVYALKRQGRTLYGFGG **Charge** 3 **Spectral Angle** 0.68

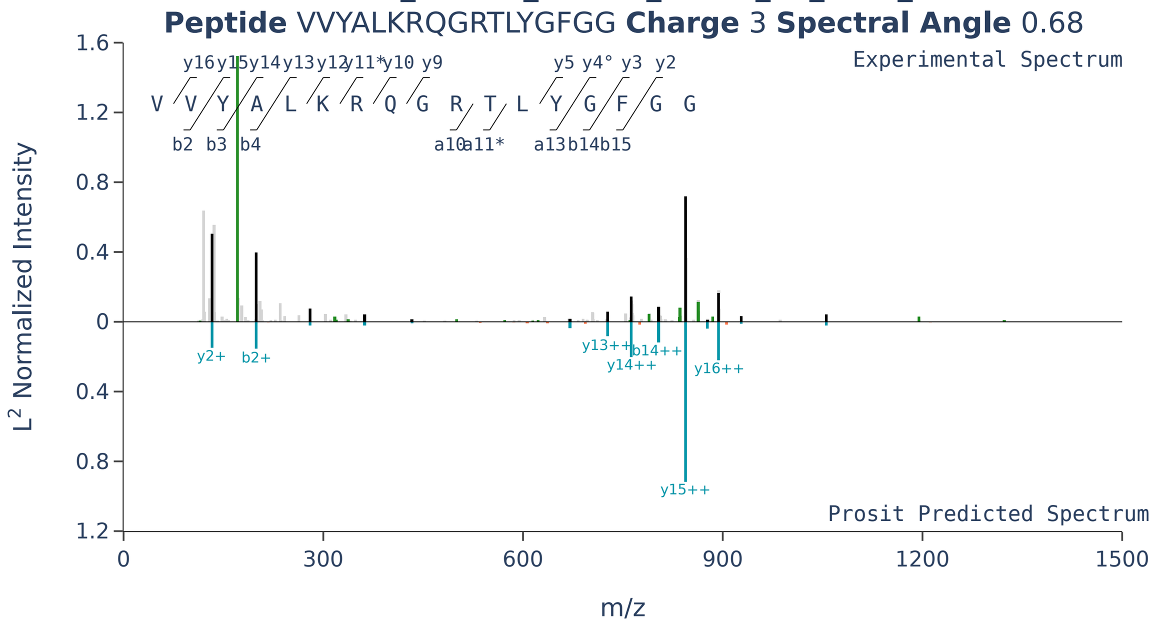


**Source** HRoetschke_291222_140123_HFGoe_H4_b1R2_24h **Scan** 6607
**Peptide** VIRDAVTYTEHAKRK **Charge** 5 **Spectral Angle** 0.78

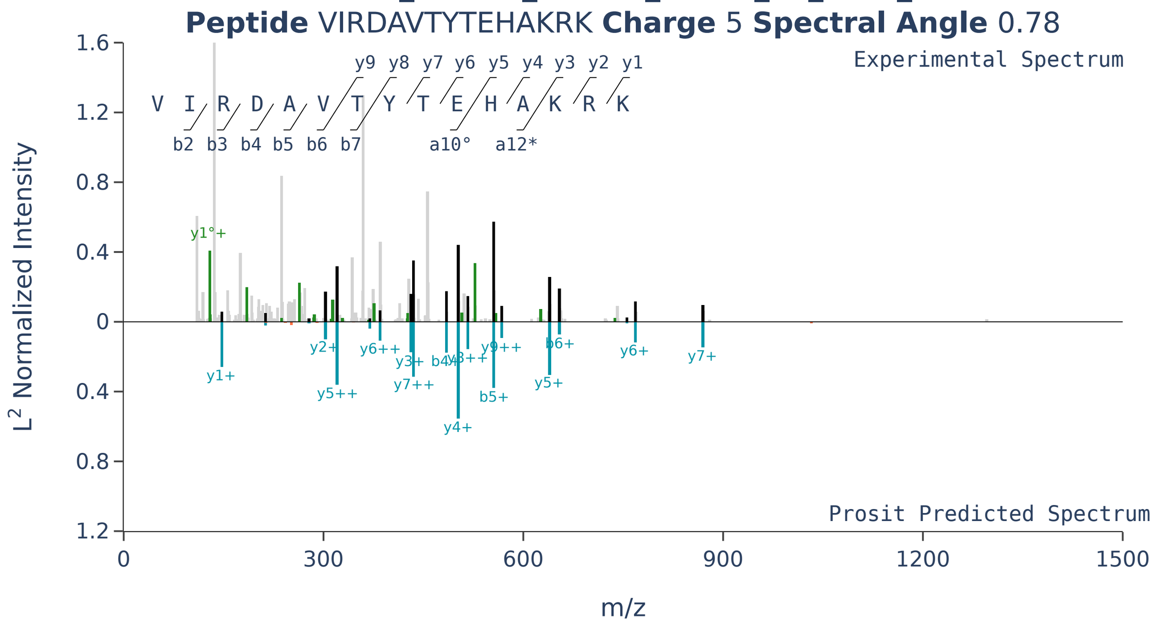


**Source** HRoetschke_291222_140123_HFGoe_H4_b1R1_24h **Scan** 5438
**Peptide** TKPAIRRLARR **Charge** 3 **Spectral Angle** 0.82

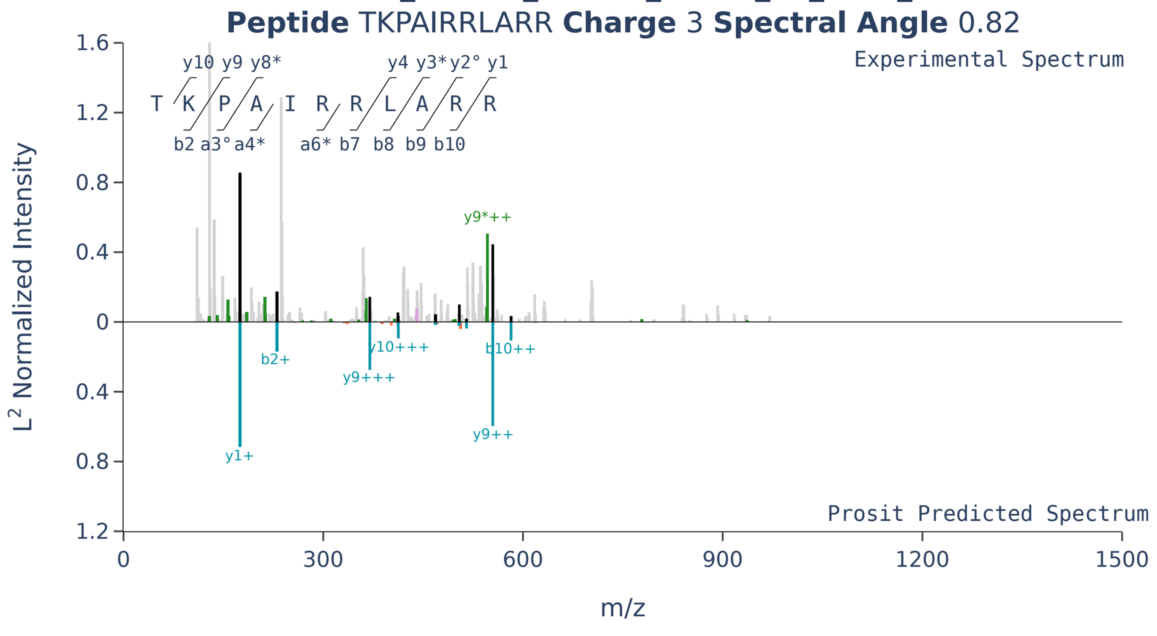


**Source** HRoetschke_291222_140123_HFGoe_H4_b2R2_4h **Scan** 12422
**Peptide** HRKVLRDNIQGITKPAIRRLA **Charge** 5 **Spectral Angle** 0.85

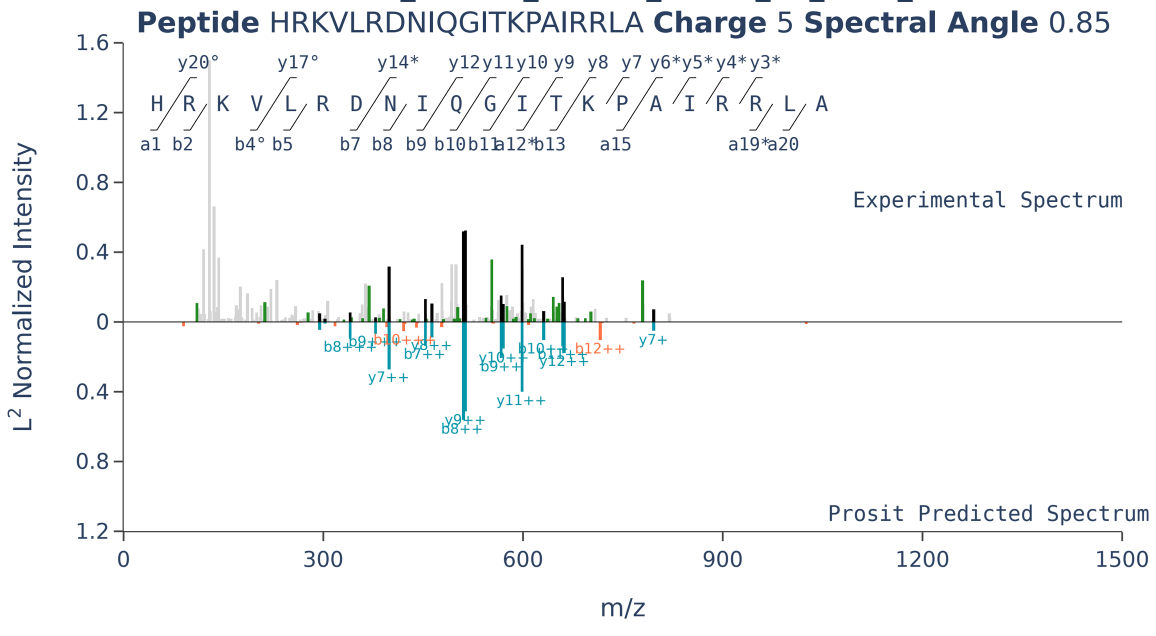


**Source** HRoetschke_291222_140123_HFGoe_H4_b1R1_1h **Scan** 7439
**Peptide** TKPAIRRLA **Charge** 2 **Spectral Angle** 0.86

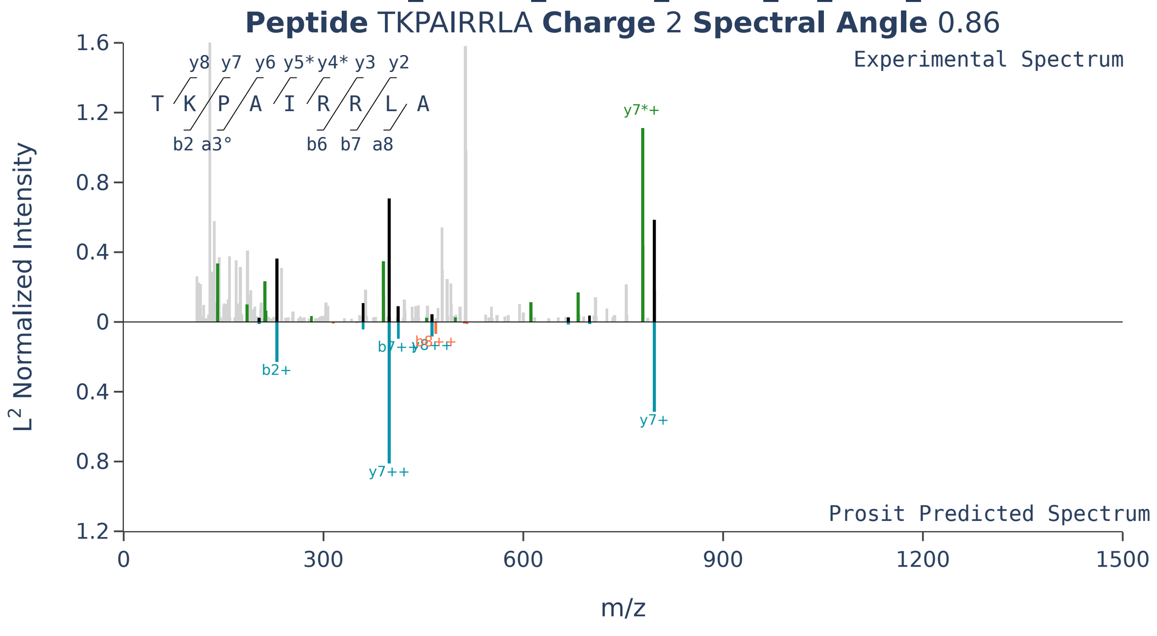

**Source** HRoetschke_291222_140123_HFGoe_H4_b2R2_24h **Scan** 14625
**Peptide** TKPAIRRL **Charge** 2 **Spectral Angle** 0.86

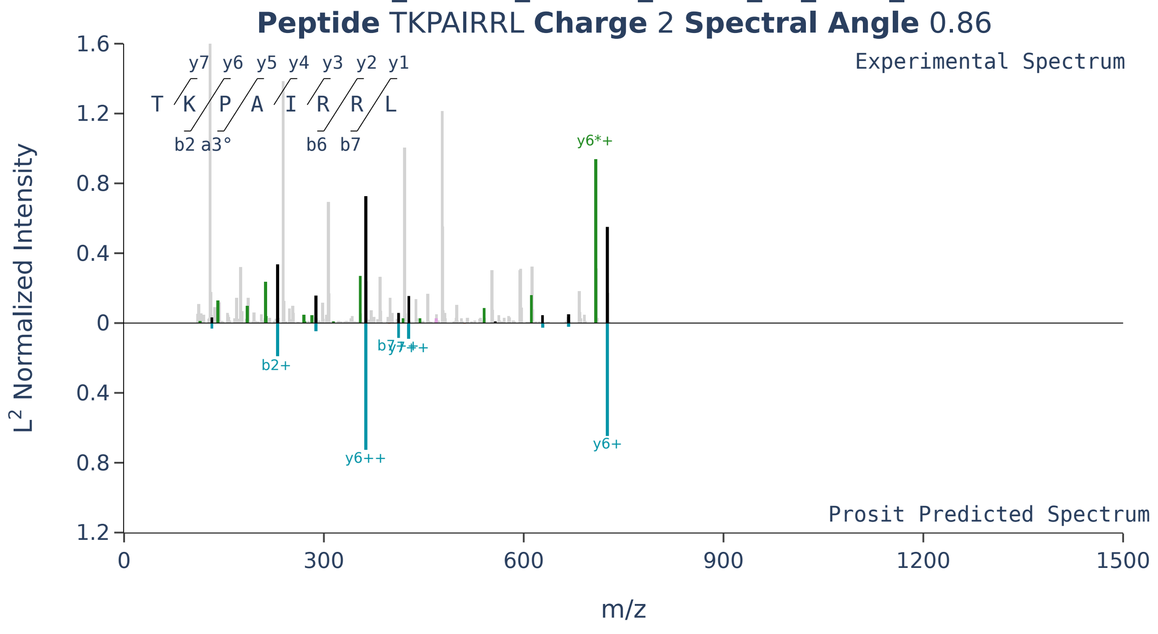


**Source** HRoetschke_291222_140123_HFGoe_H4_b1R2_24h **Scan** 5788
**Peptide** KRKTVTAMDV **Charge** 3 **Spectral Angle** 0.81

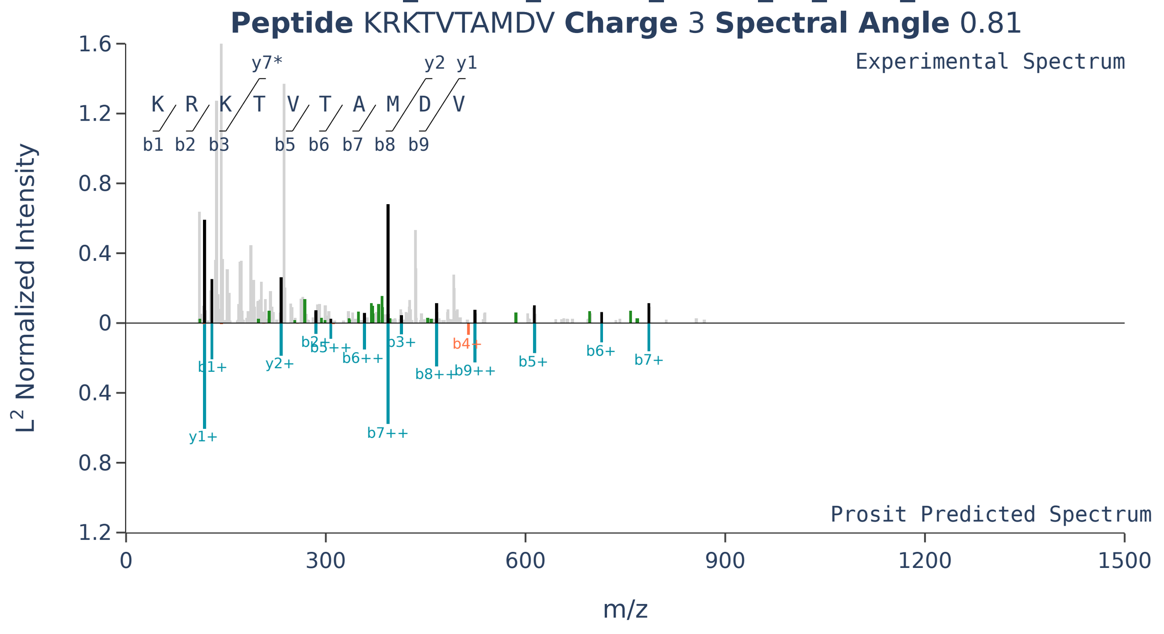


**Source** HRoetschke_291222_140123_HFGoe_H4_b2R1_1h **Scan** 22125
**Peptide** TAMDVVYALKRQGRTLYG **Charge** 3 **Spectral Angle** 0.67

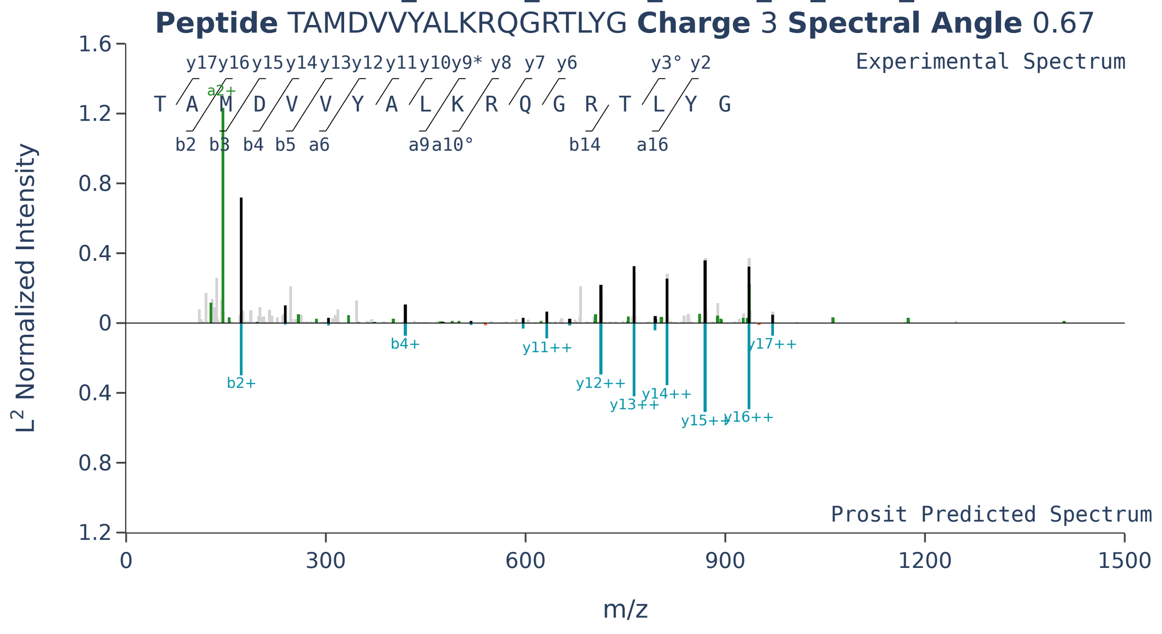


**Source** HRoetschke_291222_140123_HFGoe_H4_b2R2_4h **Scan** 12358
**Peptide** NVIRDAVTYTEHAKRK **Charge** 5 **Spectral Angle** 0.73

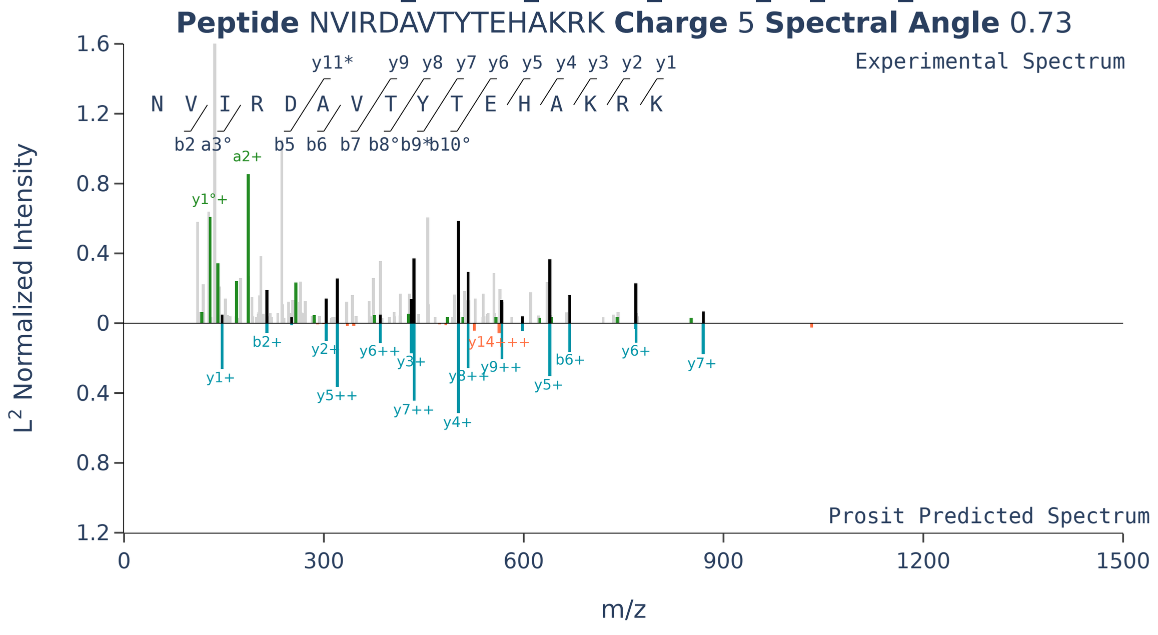


**Source** HRoetschke_291222_140123_HFGoe_H4_b1R1_4h **Scan** 5910
**Peptide** LRDAVTYTEHAKRKT **Charge** 4 **Spectral Angle** 0.8

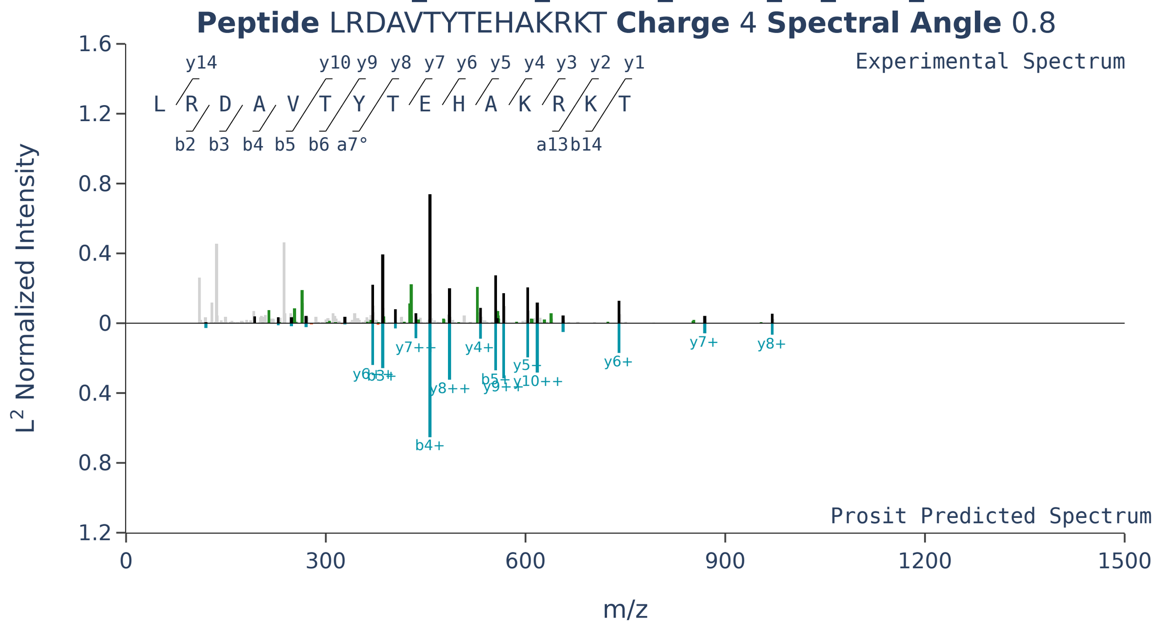


**Source** HRoetschke_291222_140123_HFGoe_H4_b1R1_2h **Scan** 12628
**Peptide** TEHAKRKTVTAMDVVYALKR **Charge** 4 **Spectral Angle** 0.73

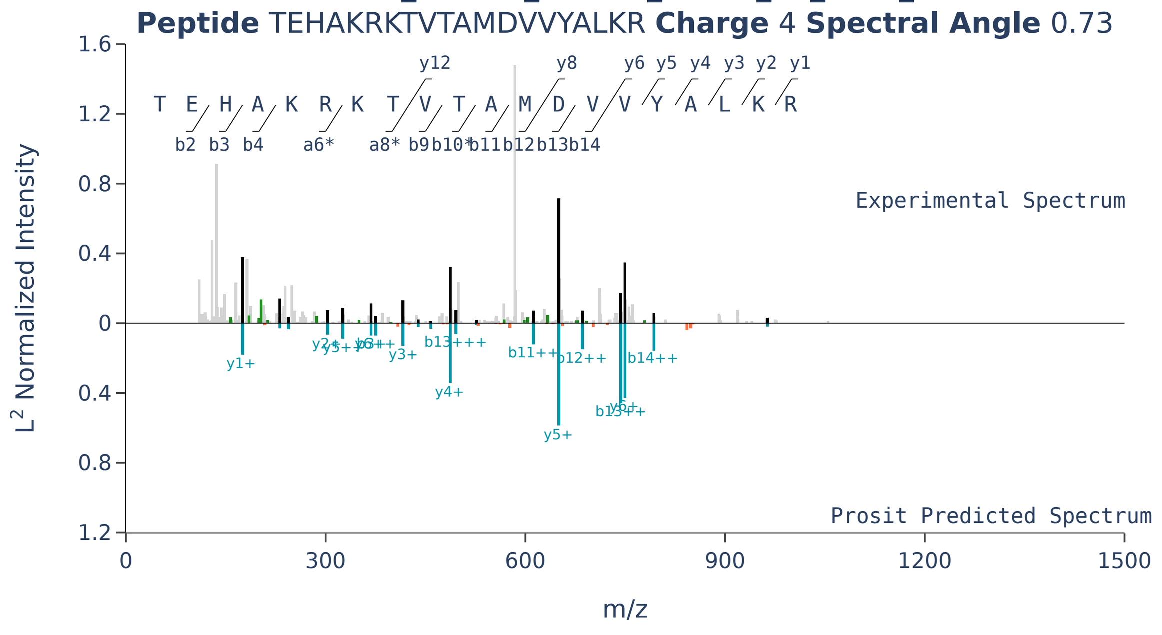


**Source** HRoetschke_291222_140123_HFGoe_H4_b1R2_24h **Scan** 12123
**Peptide** RGGVKRISGLI **Charge** 3 **Spectral Angle** 0.69

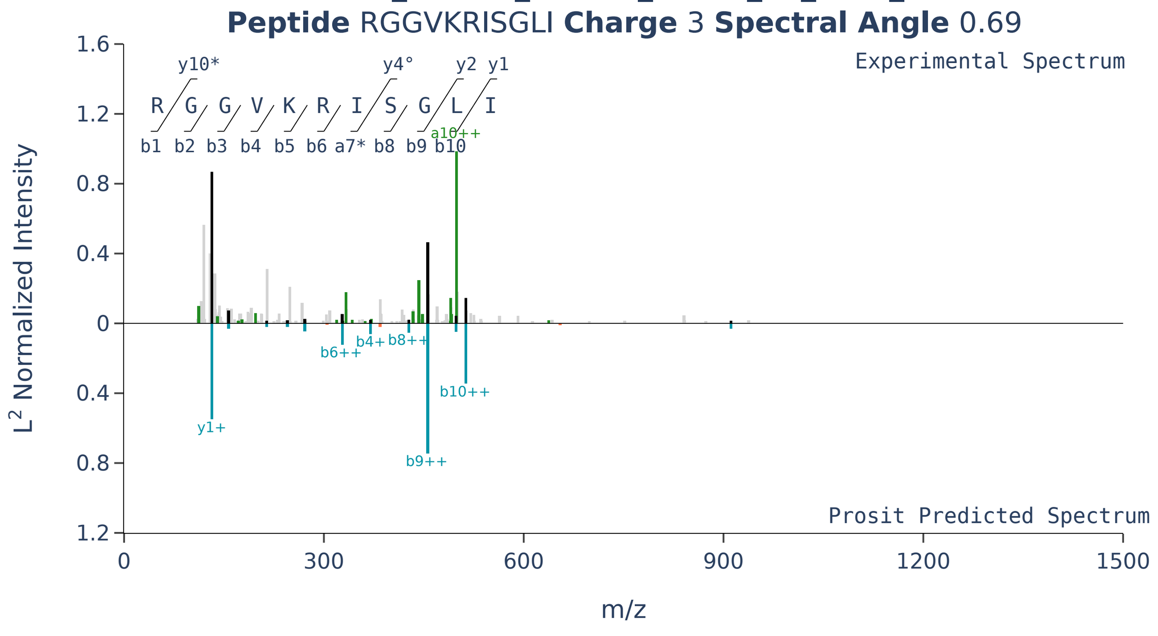


**Source** HRoetschke_291222_140123_HFGoe_H4_b1R1_24h **Scan** 7461
**Peptide** VLRDAVTYTEHAKR **Charge** 4 **Spectral Angle** 0.86

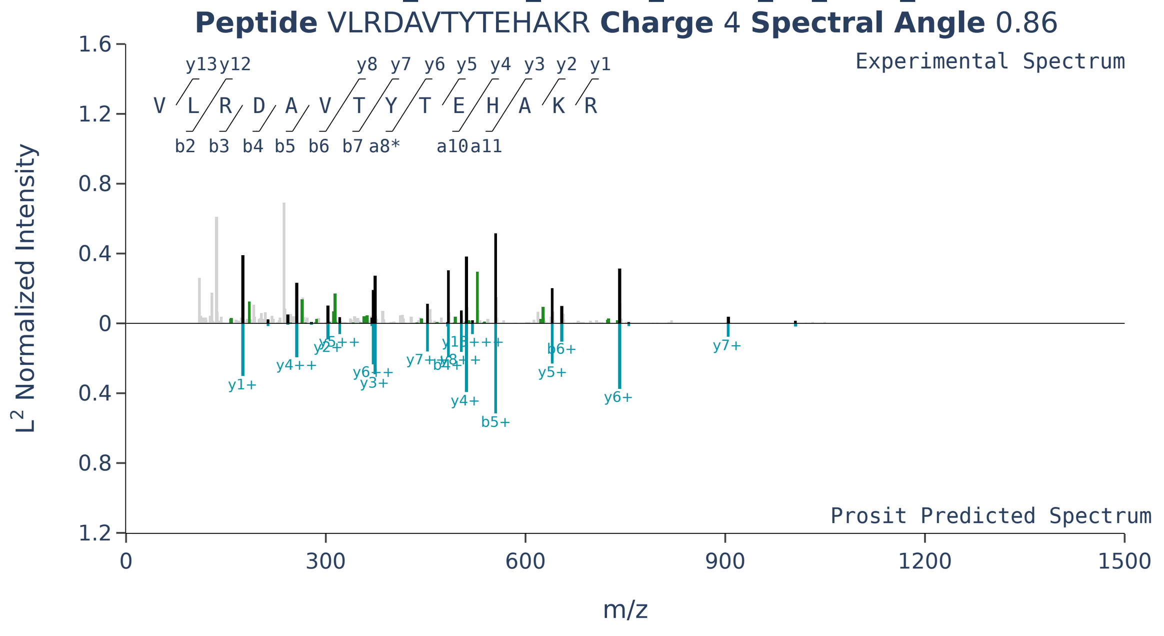


**Source** HRoetschke_291222_140123_HFGoe_H4_b1R1_1h **Scan** 17255
**Peptide** ARRGGVKRISGLIYEETRGVLK **Charge** 4 **Spectral Angle** 0.76

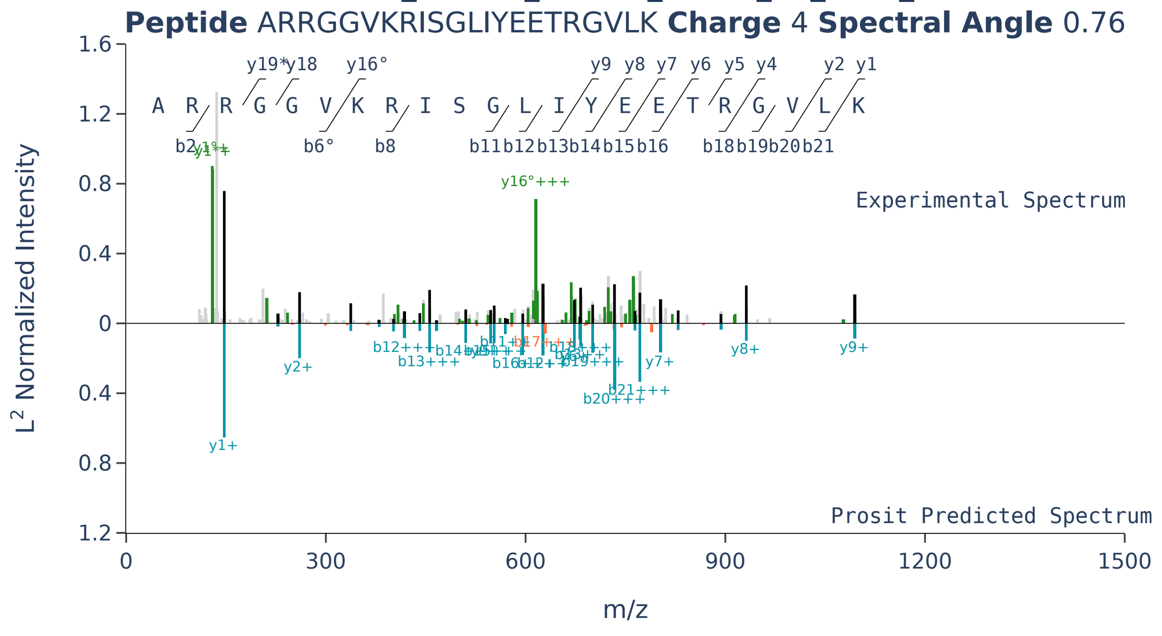


**Source** HRoetschke_291222_140123_HFGoe_H4_b1R1_2h **Scan** 16828
**Peptide** VKRISGLIYEET **Charge** 3 **Spectral Angle** 0.79

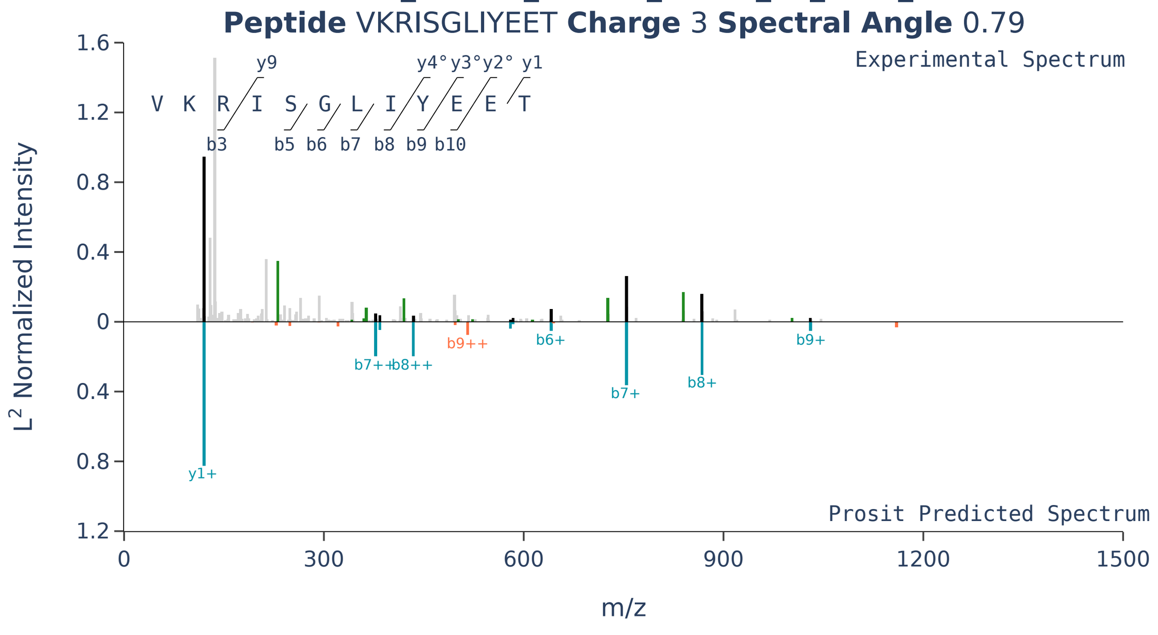


**Source** HRoetschke_291222_140123_HFGoe_H4_b2R1_1h **Scan** 15401
**Peptide** TEHAKRKTVTAMDVVYALKRQGRTL **Charge** 6 **Spectral Angle** 0.89

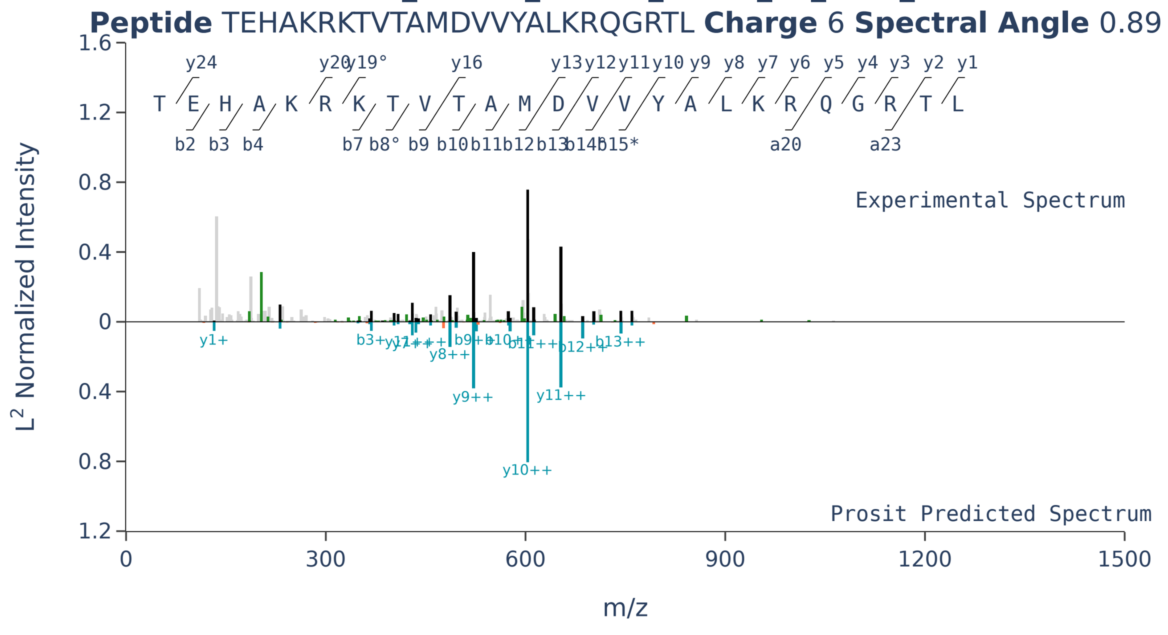


**Source** HRoetschke_291222_140123_HFGoe_H4_b2R1_24h **Scan** 12603
**Peptide** LKRQGRTLYGF **Charge** 3 **Spectral Angle** 0.65

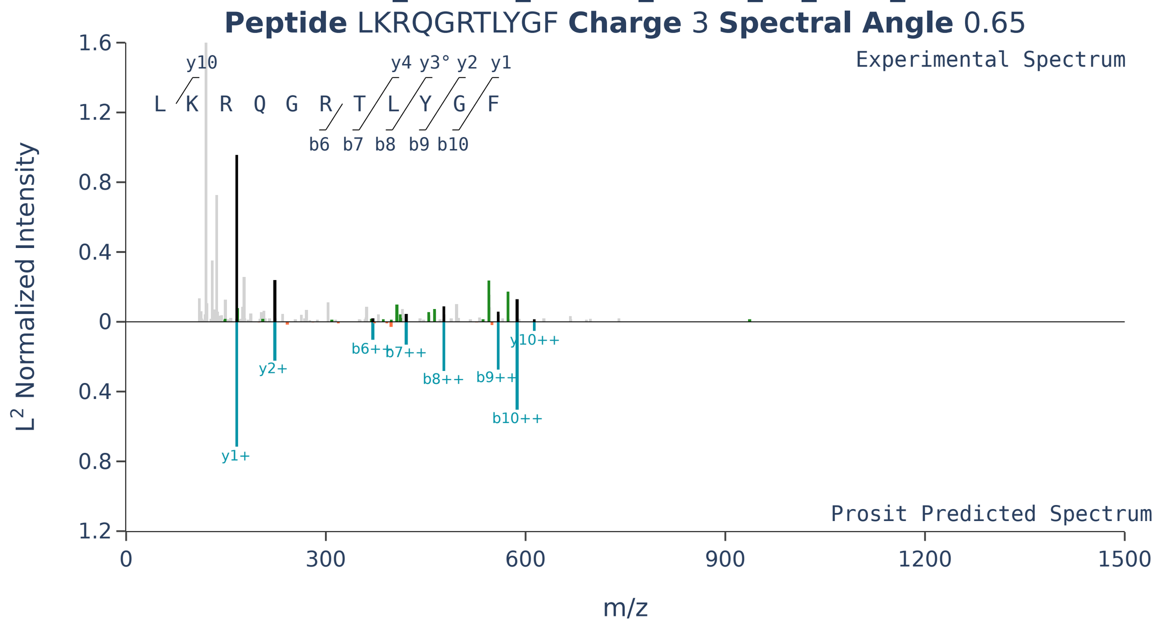


**Source** HRoetschke_291222_140123_HFGoe_H4_b2R1_2h **Scan** 12737
**Peptide** VYALKRQGRTLY **Charge** 3 **Spectral Angle** 0.88

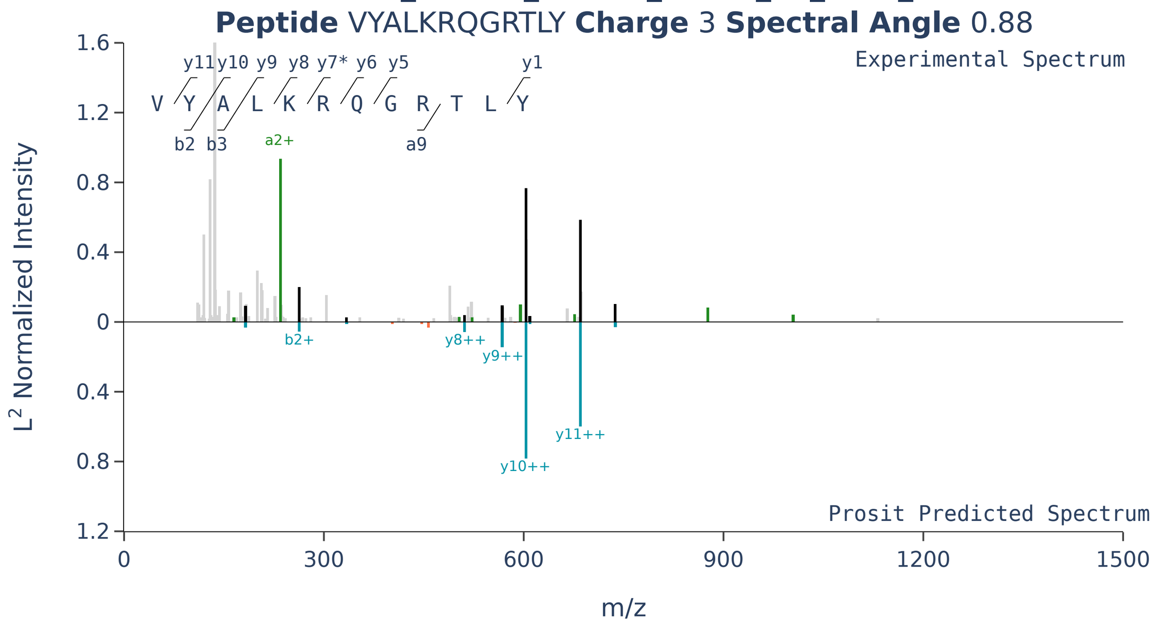


**Source** HRoetschke_291222_140123_HFGoe_H4_b1R1_24h **Scan** 5243
**Peptide** VTYTEHAKRKTVT **Charge** 2 **Spectral Angle** 0.69

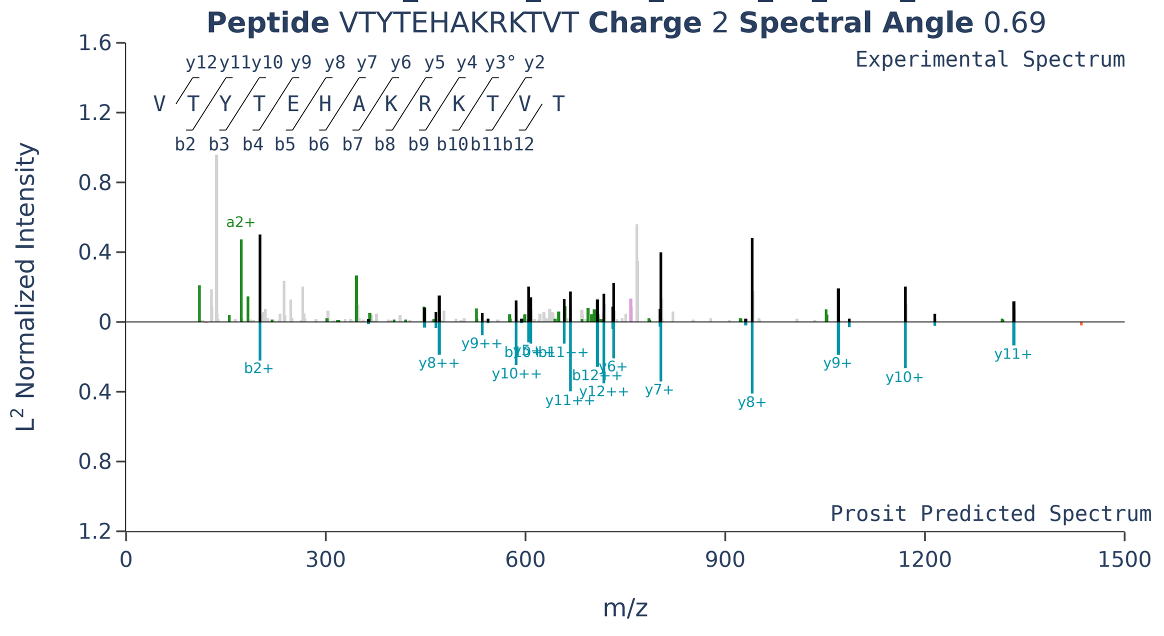


**Source** HRoetschke_291222_140123_HFGoe_H4_b1R1_24h **Scan** 10525
**Peptide** RDNIQGI **Charge** 2 **Spectral Angle** 0.82

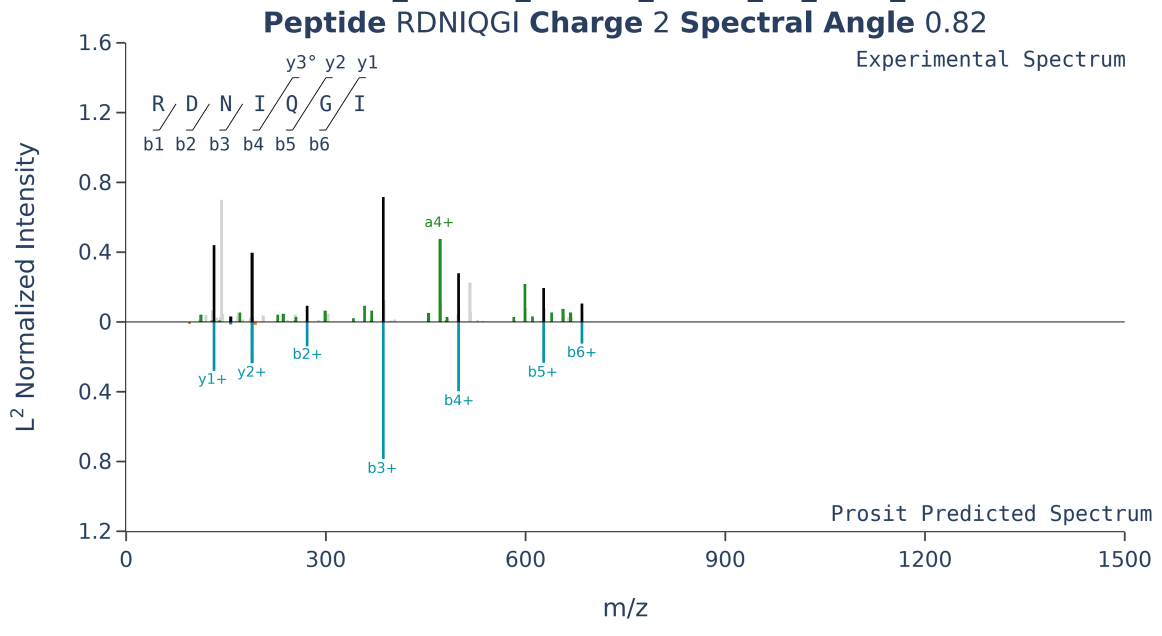

**Source** HRoetschke_291222_140123_HFGoe_H4_b2R1_4h **Scan** 12031
**Peptide** KVLRDNIQGIT **Charge** 3 **Spectral Angle** 0.85

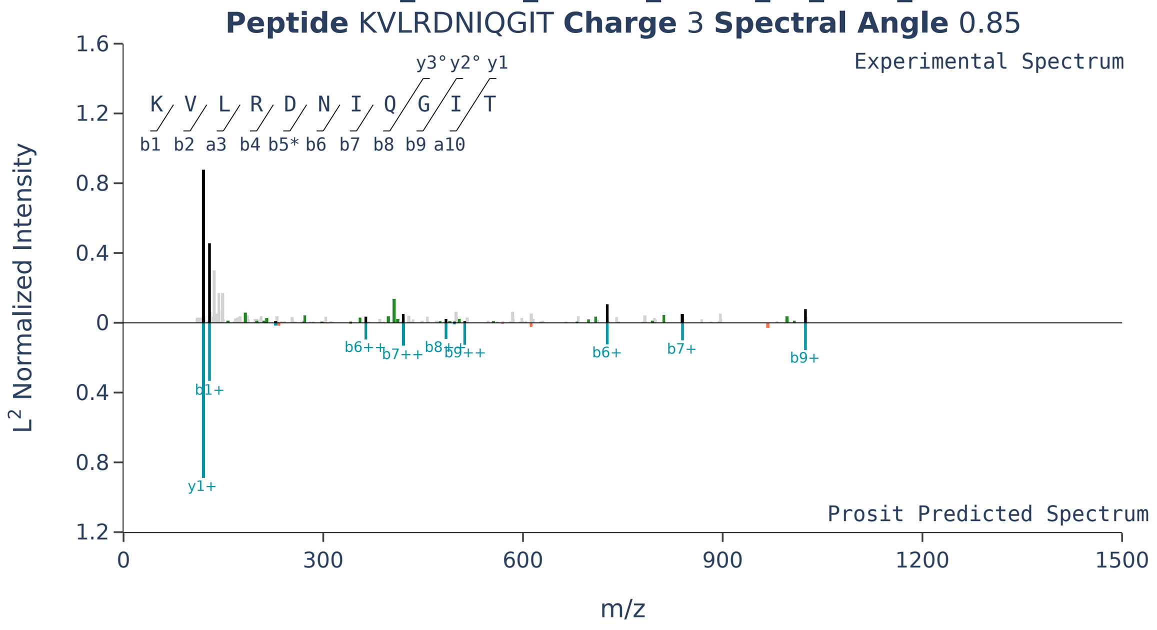


**Source** HRoetschke_291222_140123_HFGoe_H4_b2R1_1h **Scan** 22141
**Peptide** TVTAMDVVYALK **Charge** 2 **Spectral Angle** 0.75

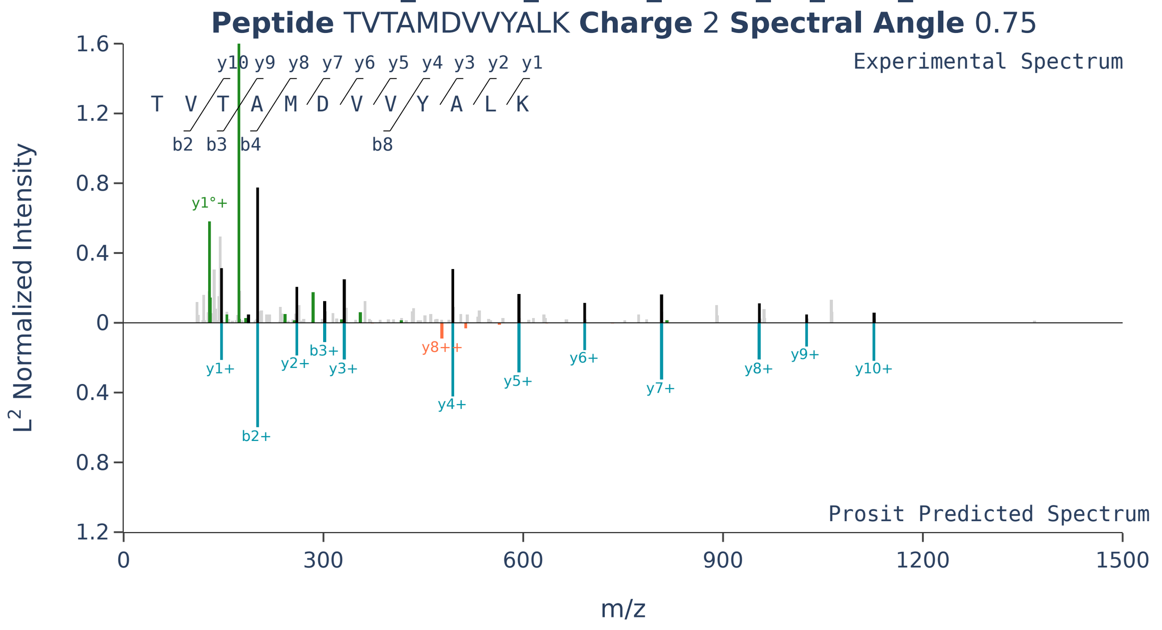


**Source** HRoetschke_291222_140123_HFGoe_H4_b2R1_24h **Scan** 4967
**Peptide** SGRGKGGKGLGKGGAKRHRKVL **Charge** 6 **Spectral Angle** 0.85

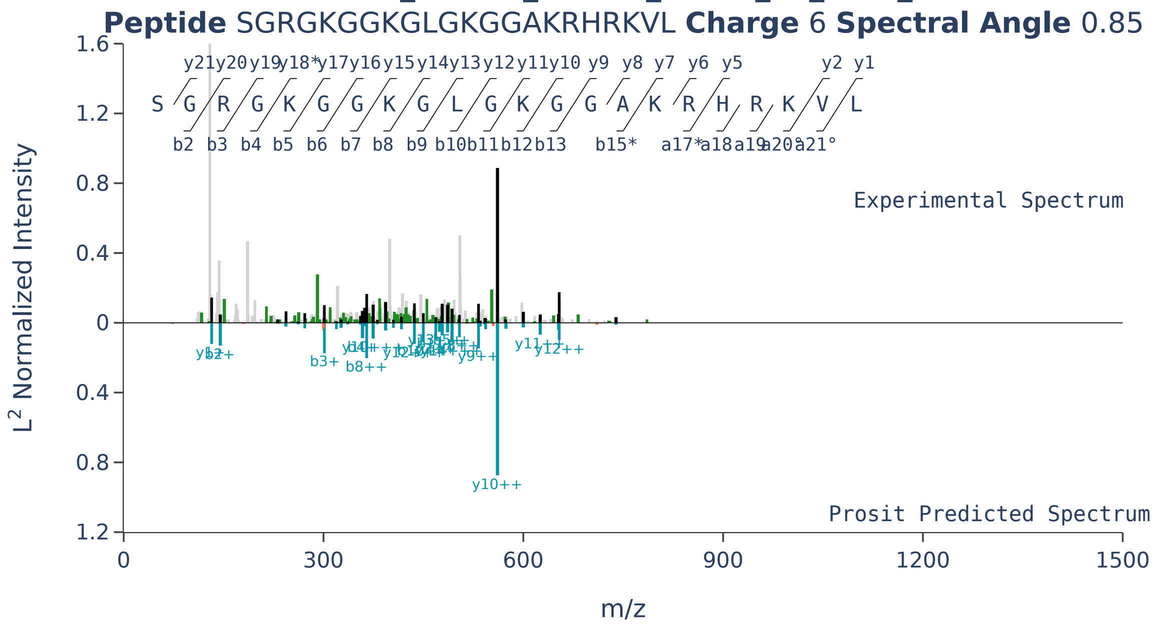


**Source** HRoetschke_291222_140123_HFGoe_H4_b2R2_4h **Scan** 12406
**Peptide** ARRGGVKRISGLIYEE **Charge** 3 **Spectral Angle** 0.75

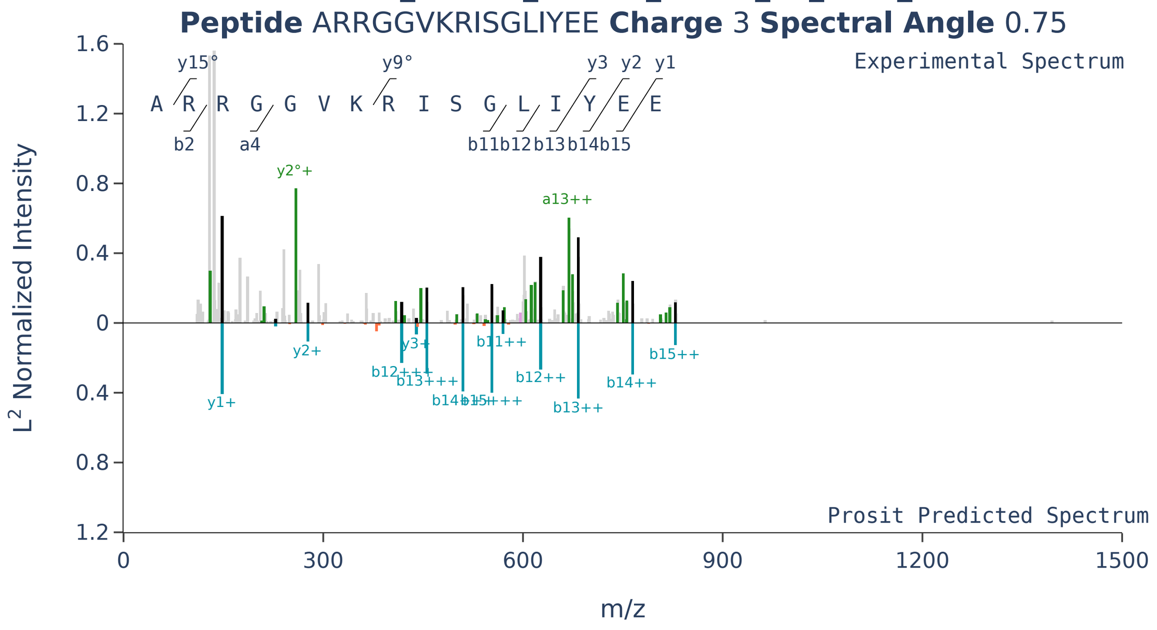


**Source** HRoetschke_291222_140123_HFGoe_H4_b2R1_1h **Scan** 20847
**Peptide** VKRISGLIYEETRGVL **Charge** 3 **Spectral Angle** 0.85

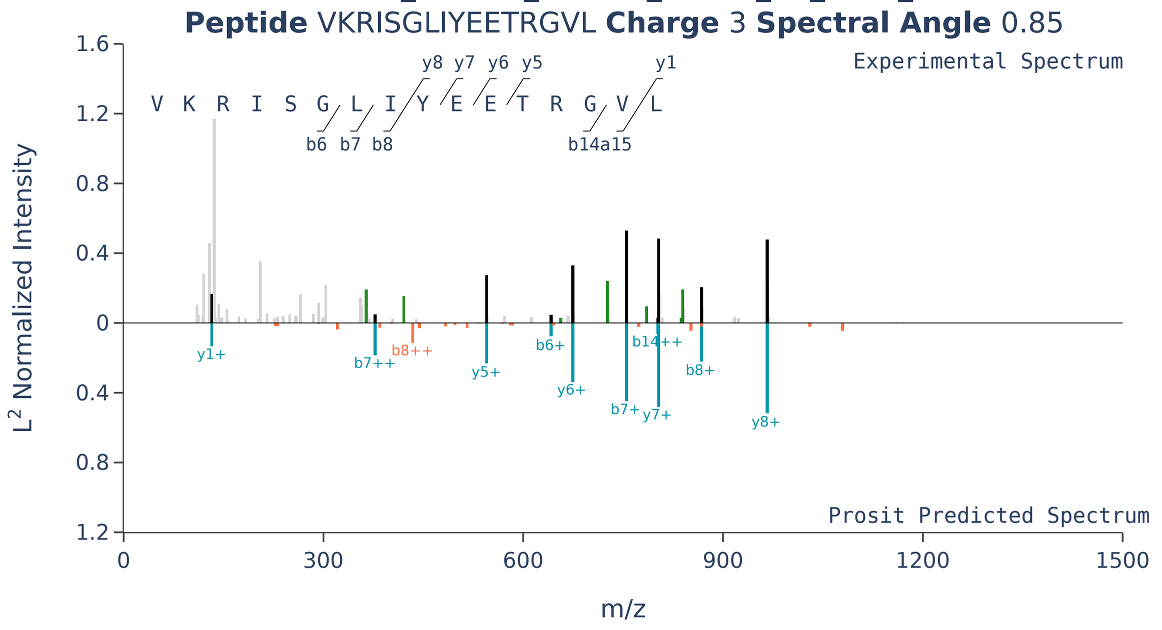


**Source** HRoetschke_291222_140123_HFGoe_H4_b1R1_2h **Scan** 15124
**Peptide** AKRKTVTAMDVVYALKRQG **Charge** 4 **Spectral Angle** 0.76

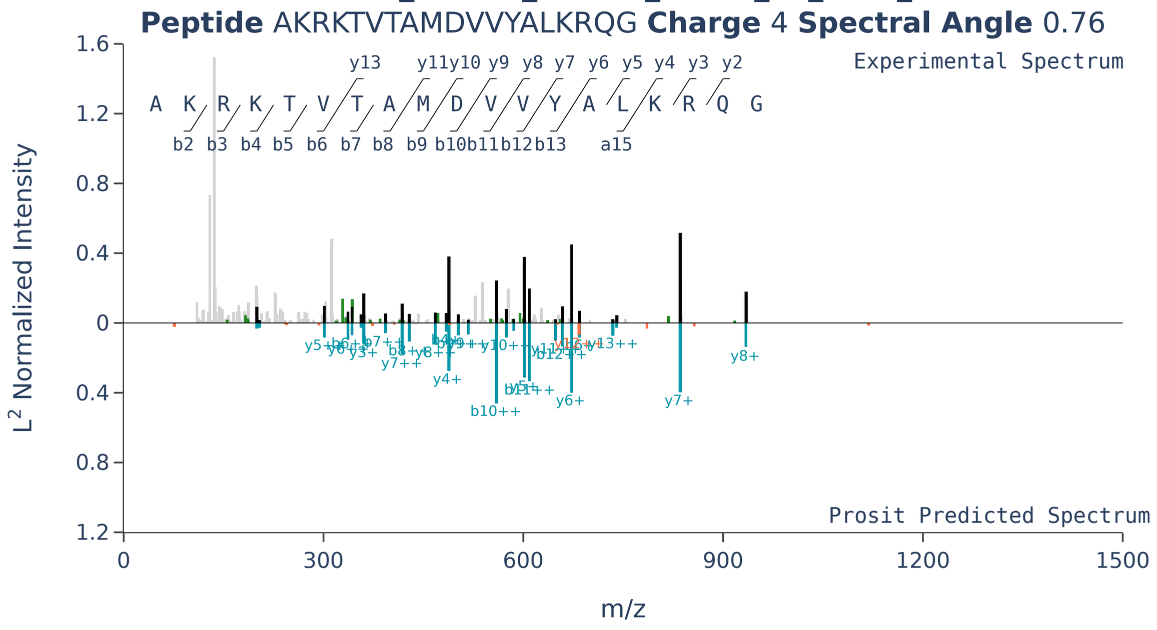


**Source** HRoetschke_291222_140123_HFGoe_H4_b1R1_2h **Scan** 16922
**Peptide** RDAVTYTEHAKRKTVTAMDVVYALKR **Charge** 5 **Spectral Angle** 0.85

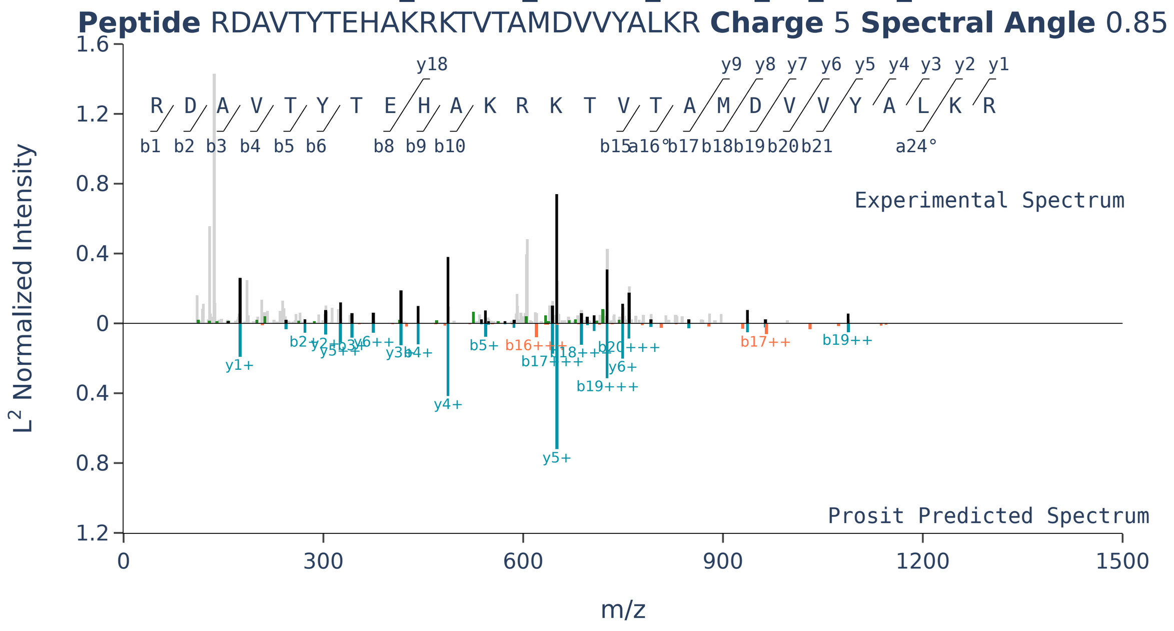


**Source** HRoetschke_291222_140123_HFGoe_H4_b2R1_1h **Scan** 18411
**Peptide** AKRKTVTAMDVVYALKRQGRTL **Charge** 5 **Spectral Angle** 0.89

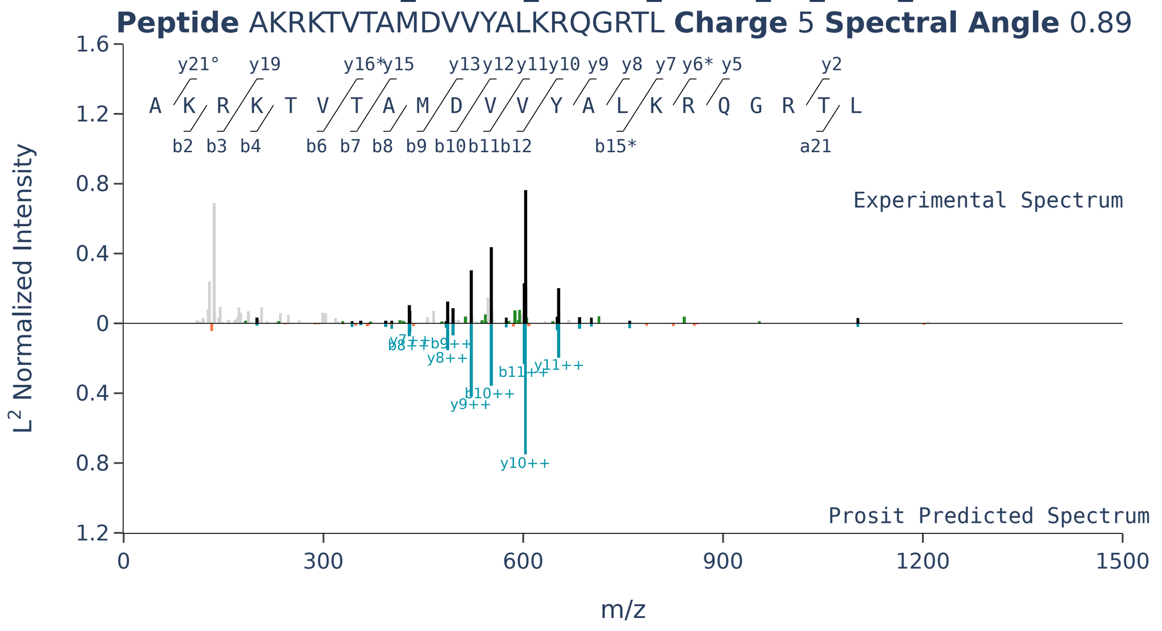


**Source** HRoetschke_291222_140123_HFGoe_H4_b1R1_24h **Scan** 7016
**Peptide** LRDAVTYTEHAK **Charge** 3 **Spectral Angle** 0.81

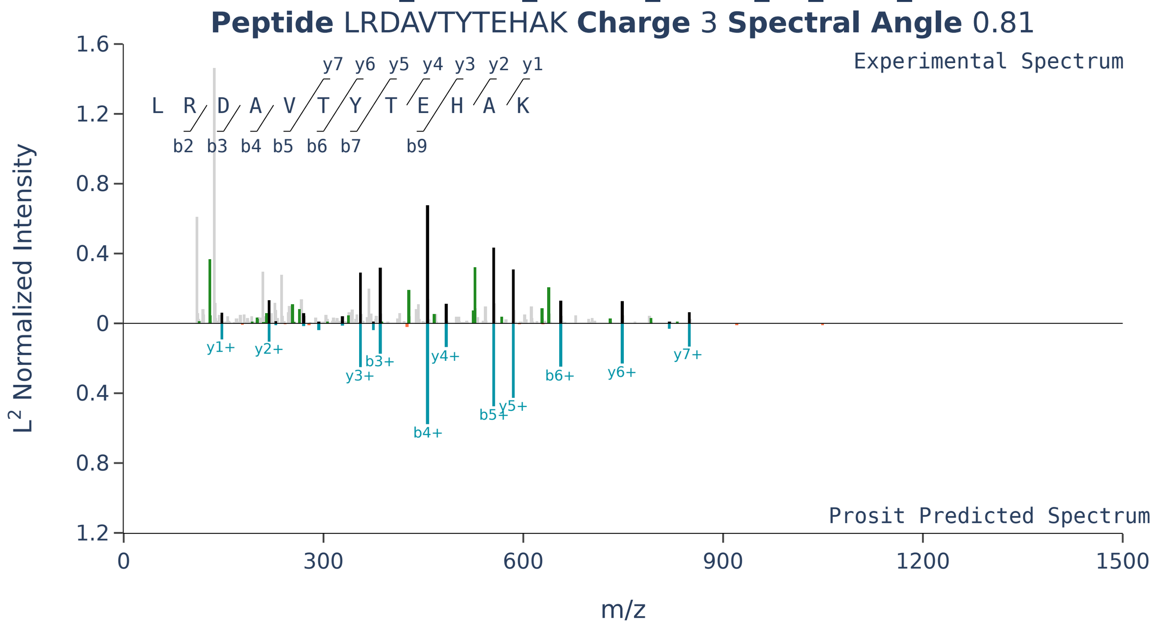


**Source** HRoetschke_291222_140123_HFGoe_H4_b1R1_24h **Scan** 14426
**Peptide** LRDNIQGITKPAIRRL **Charge** 5 **Spectral Angle** 0.81

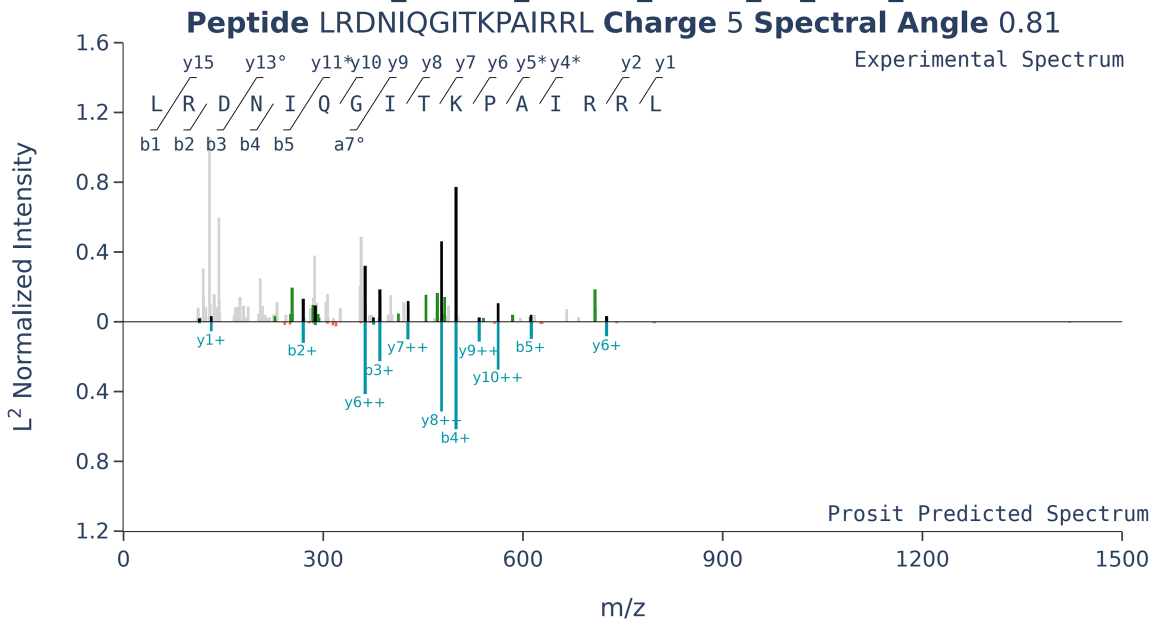


**Source** HRoetschke_291222_140123_HFGoe_H4_b2R2_1h **Scan** 29610
**Peptide** ISGLIYEETRGVLKVF **Charge** 2 **Spectral Angle** 0.9

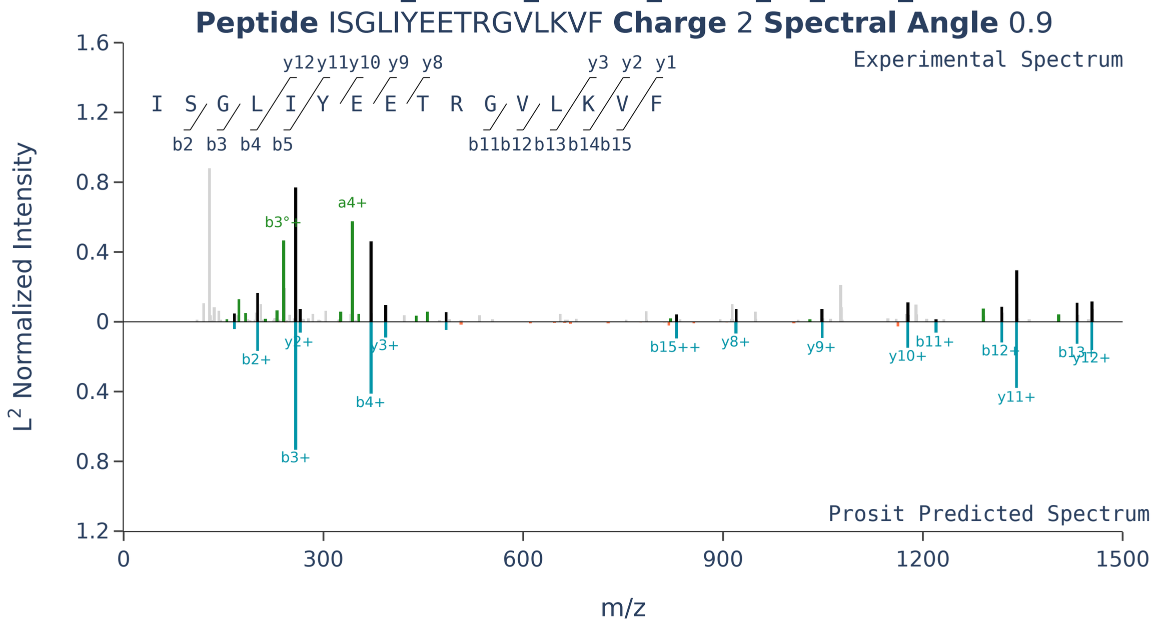


**Source** HRoetschke_291222_140123_HFGoe_H4_b2R2_1h **Scan** 13874
**Peptide** LRDNIQGIT **Charge** 2 **Spectral Angle** 0.83

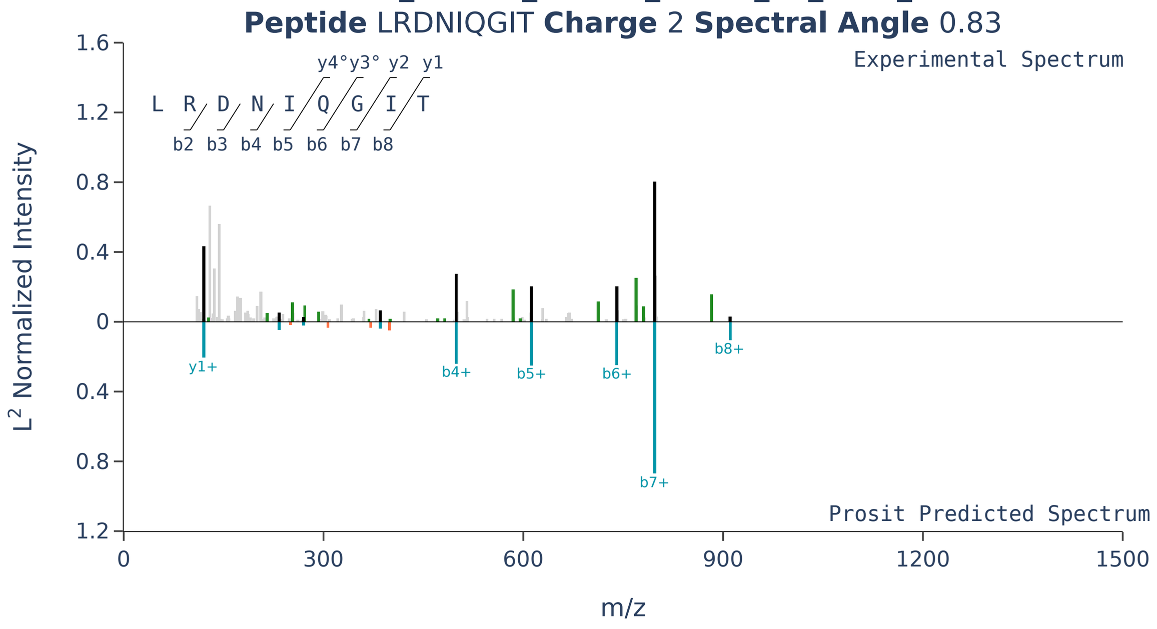


**Source** HRoetschke_291222_140123_HFGoe_H4_b2R1_24h **Scan** 10690
**Peptide** DNIQGITKPAIRR **Charge** 2 **Spectral Angle** 0.72

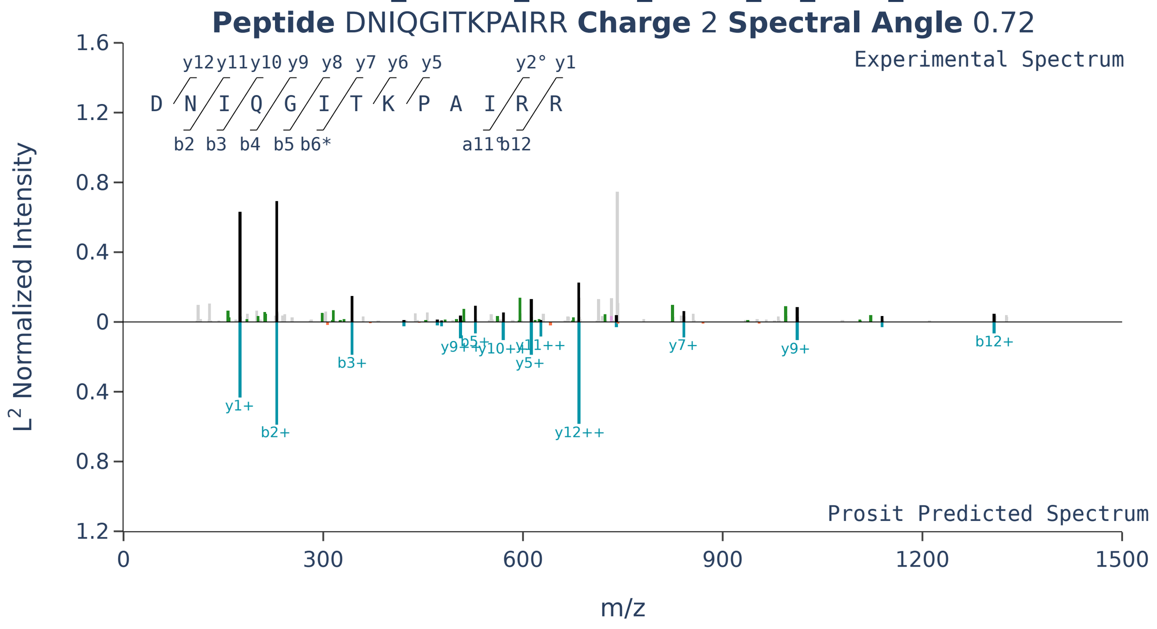


**Source** HRoetschke_291222_140123_HFGoe_H4_b2R1_1h **Scan** 19874
**Peptide** LENVIRDAVTY **Charge** 2 **Spectral Angle** 0.7

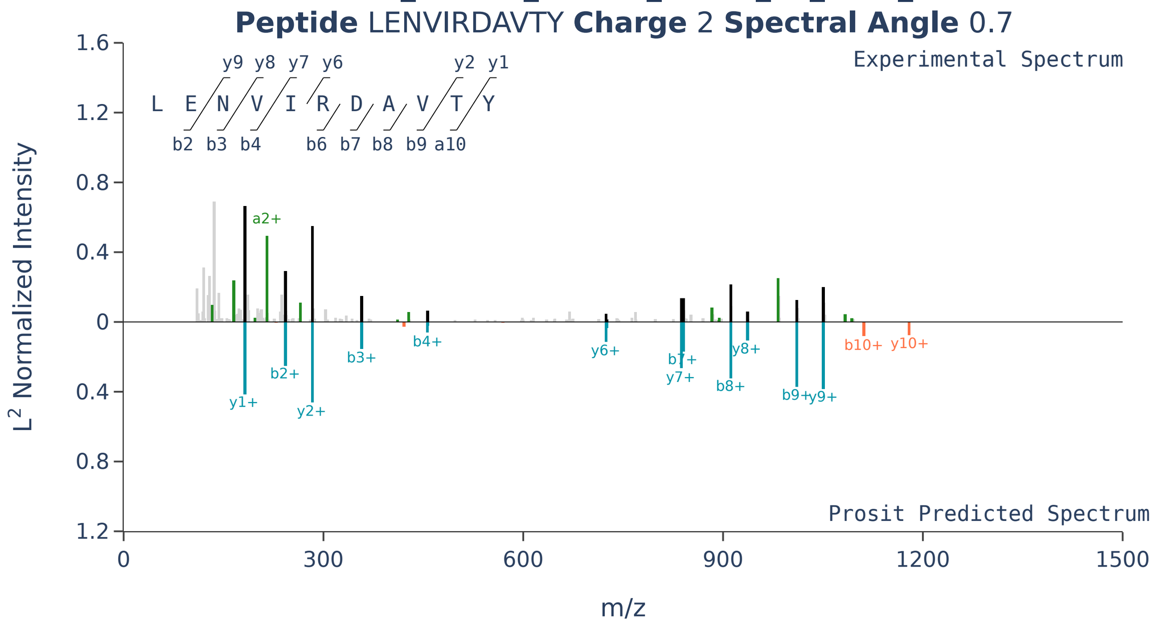


**Source** HRoetschke_291222_140123_HFGoe_H4_b1R2_24h **Scan** 11885
**Peptide** VIRDAVTYT **Charge** 2 **Spectral Angle** 0.66

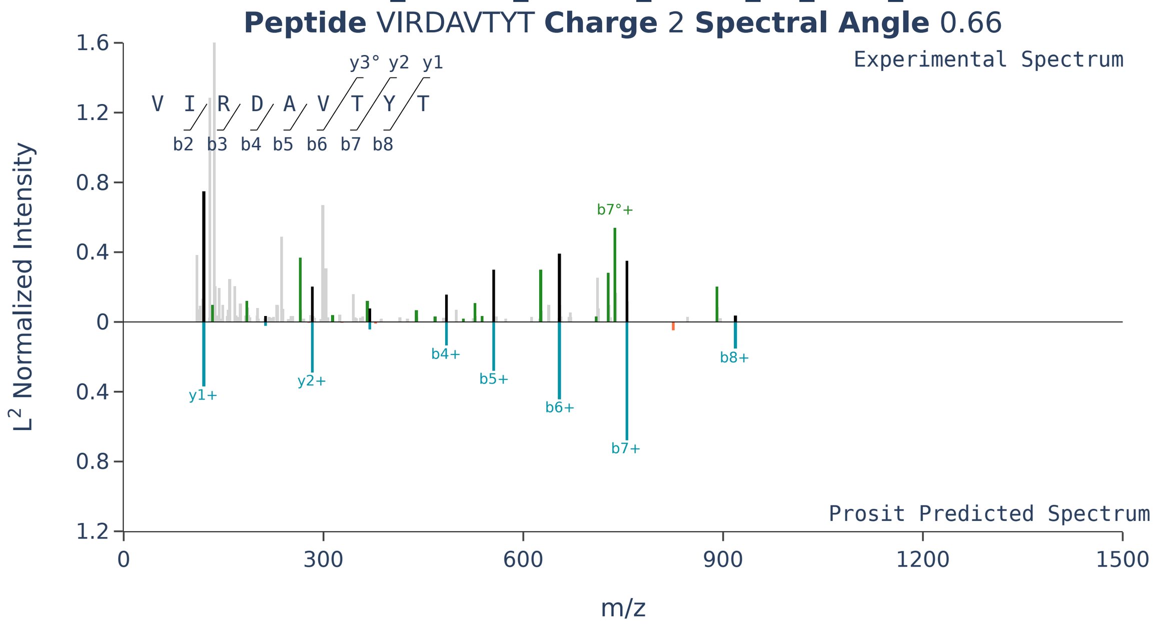

**Source** HRoetschke_291222_140123_HFGoe_H4_b1R2_24h **Scan** 6751
**Peptide** RKVLRDNL **Charge** 2 **Spectral Angle** 0.77

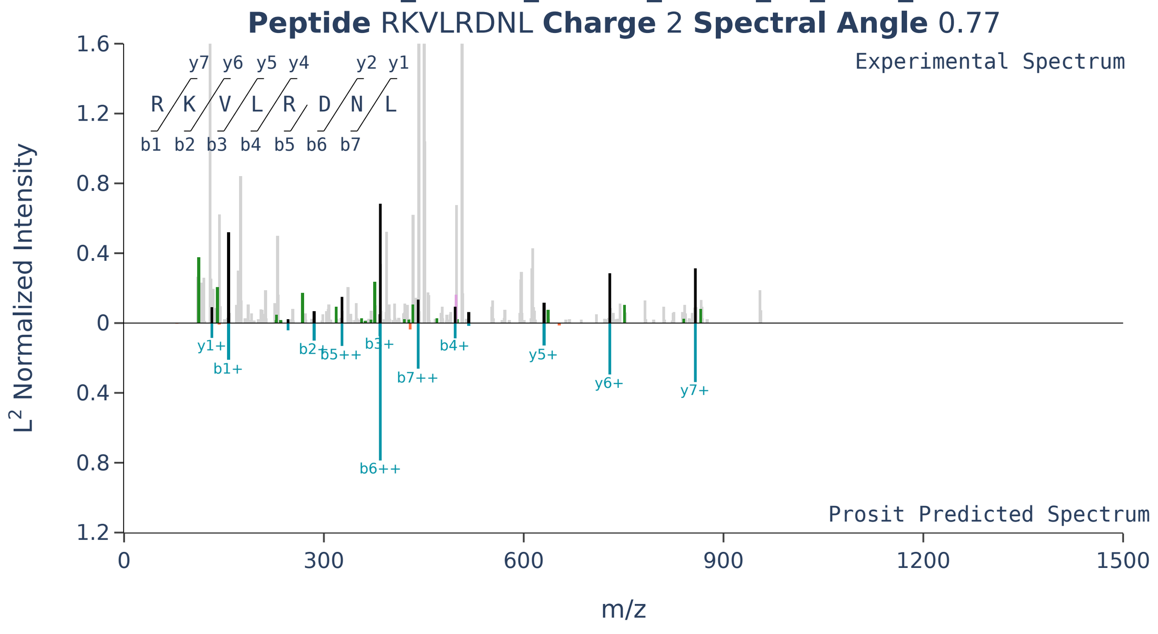


**Source** HRoetschke_291222_140123_HFGoe_H4_b1R2_1h **Scan** 16941
**Peptide** LENVIRDAVTYTEHAKRKT **Charge** 5 **Spectral Angle** 0.73

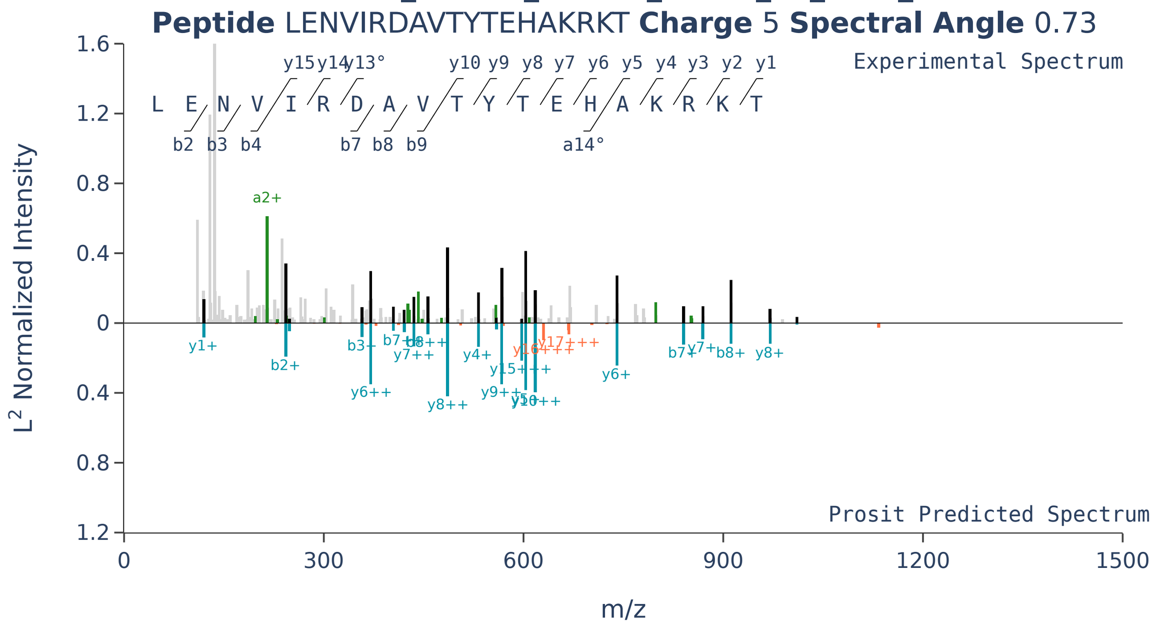


**Source** HRoetschke_291222_140123_HFGoe_H4_b2R2_1h **Scan** 28166
**Peptide** ETRGVLKVFLENVLRD **Charge** 3 **Spectral Angle** 0.84

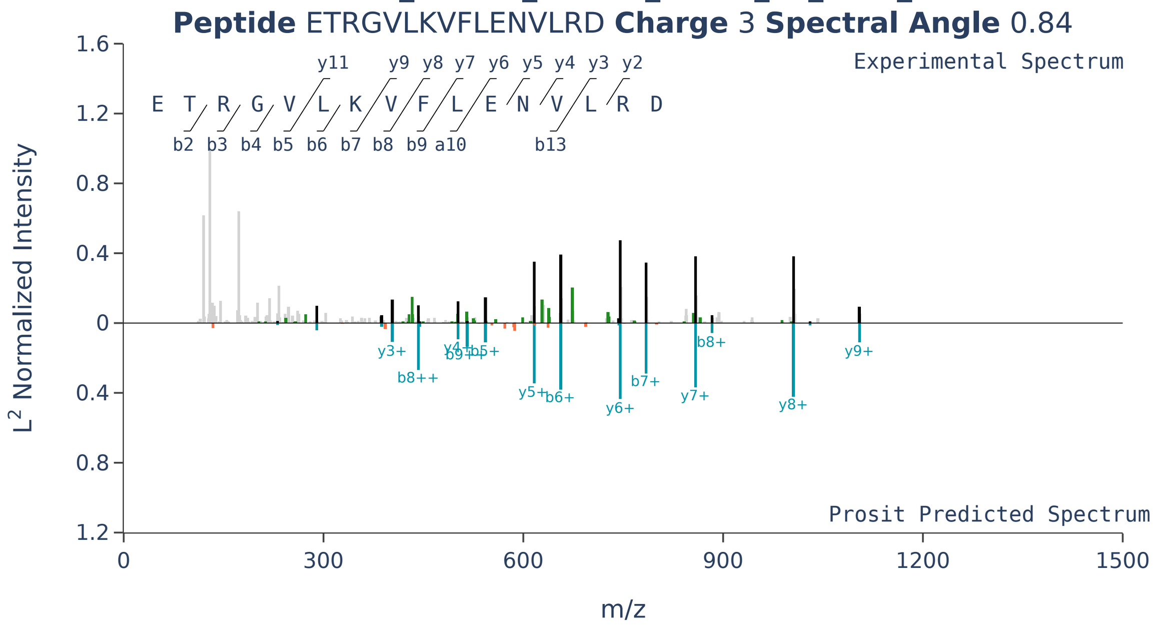


**Source** HRoetschke_291222_140123_HFGoe_H4_b1R2_4h **Scan** 7412
**Peptide** RDAVTYT **Charge** 2 **Spectral Angle** 0.81

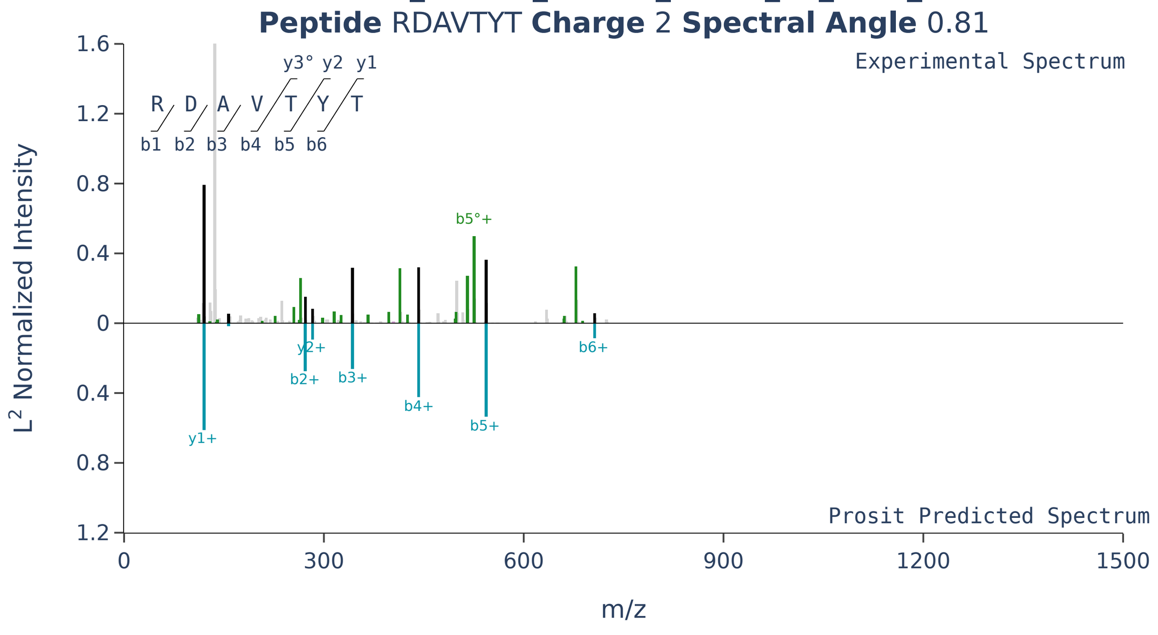


**Source** HRoetschke_291222_140123_HFGoe_H4_b2R1_1h **Scan** 23476
**Peptide** ARRGGVKRISGLIYEETRGVLKVF **Charge** 4 **Spectral Angle** 0.83

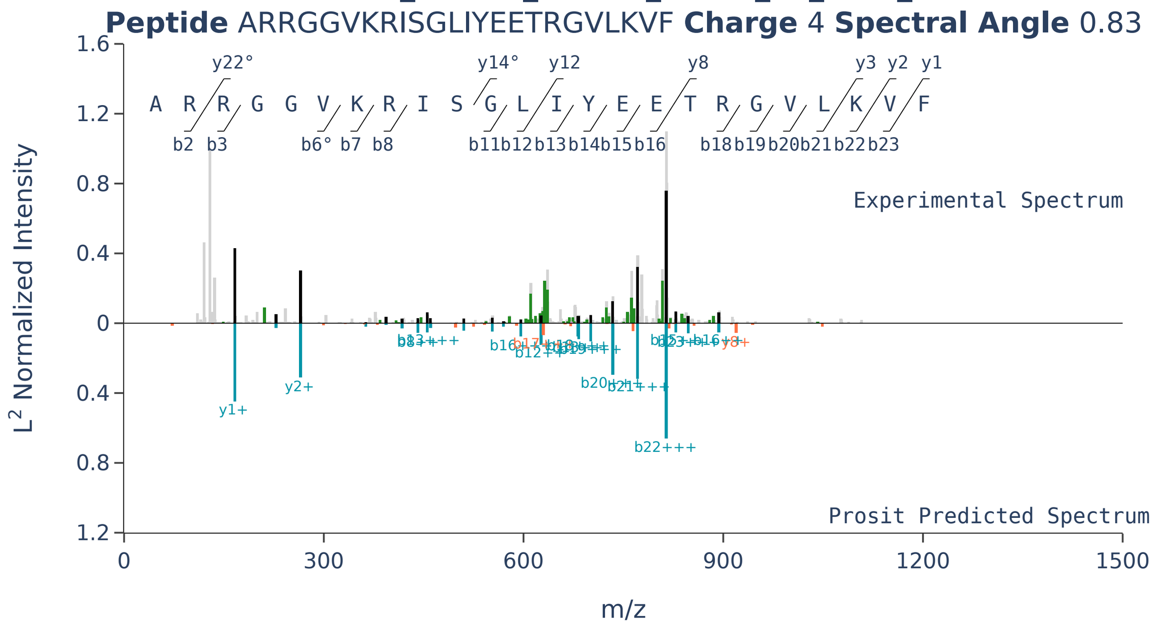


**Source** HRoetschke_291222_140123_HFGoe_H4_b2R1_24h **Scan** 10590
**Peptide** RDNIQGITKPAIRR **Charge** 4 **Spectral Angle** 0.74

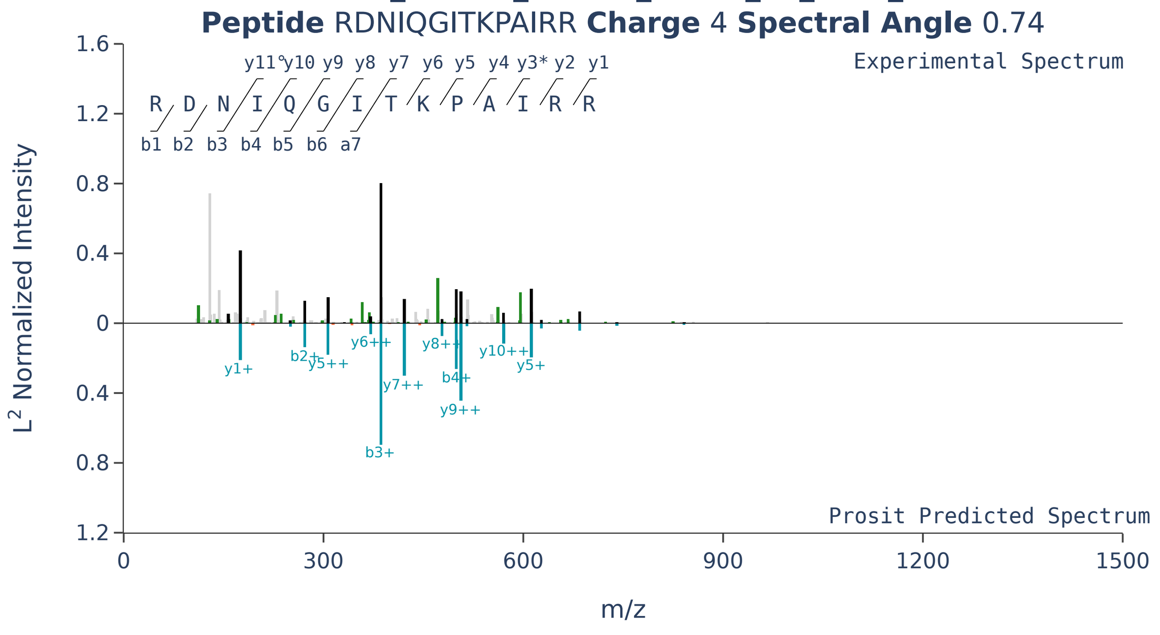


**Source** HRoetschke_291222_140123_HFGoe_H4_b1R1_24h **Scan** 11412
**Peptide** HRKVLRDNIQGITKPAIRRL **Charge** 5 **Spectral Angle** 0.79

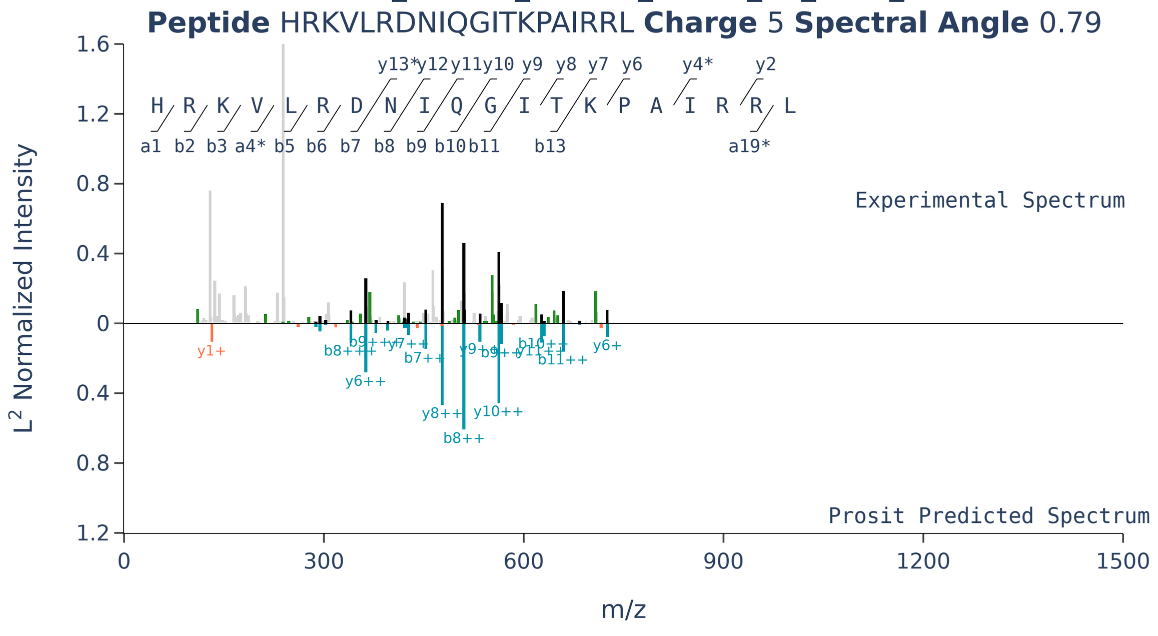


**Source** HRoetschke_291222_140123_HFGoe_H4_b1R1_1h **Scan** 17862
**Peptide** DNIQGITKPAIRRLARRGGVKRISGLI **Charge** 5 **Spectral Angle** 0.79

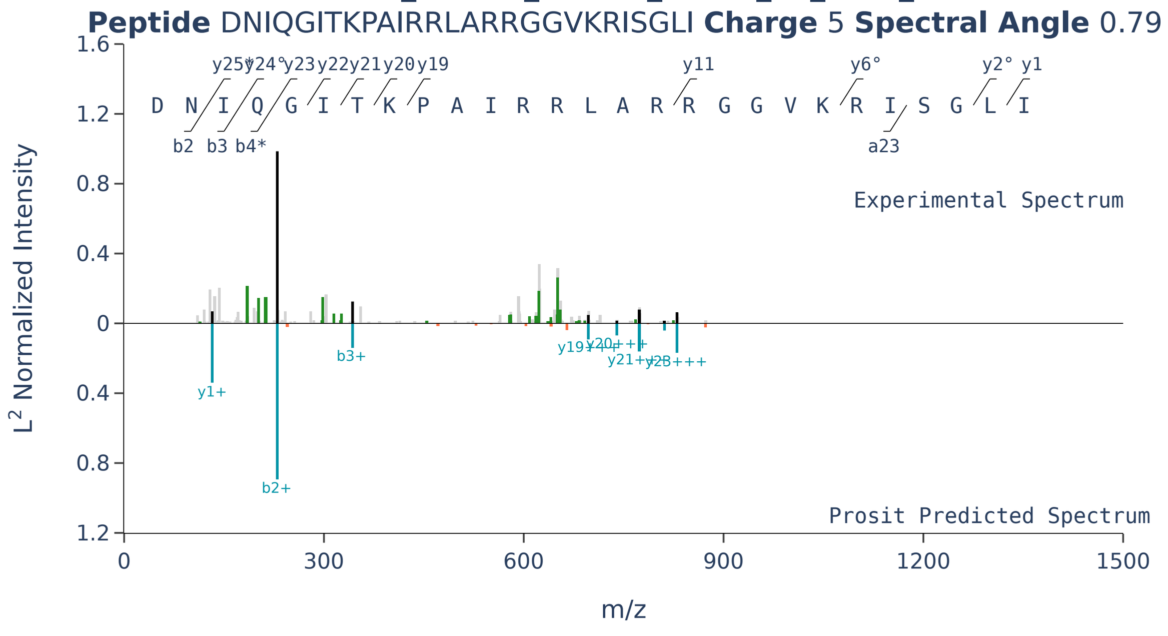


**Source** HRoetschke_291222_140123_HFGoe_H4_b1R1_1h **Scan** 15528
**Peptide** GGVKRISGLIYEETR **Charge** 4 **Spectral Angle** 0.68

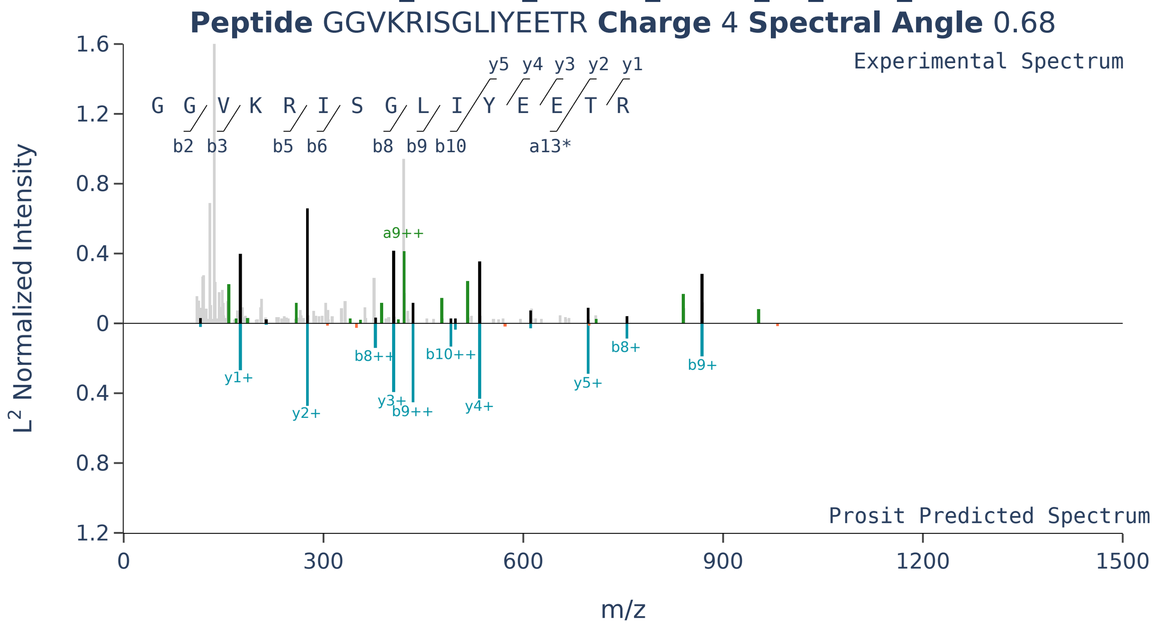


**Source** HRoetschke_291222_140123_HFGoe_H4_b1R1_24h **Scan** 8442
**Peptide** RDAVTYTE **Charge** 2 **Spectral Angle** 0.81

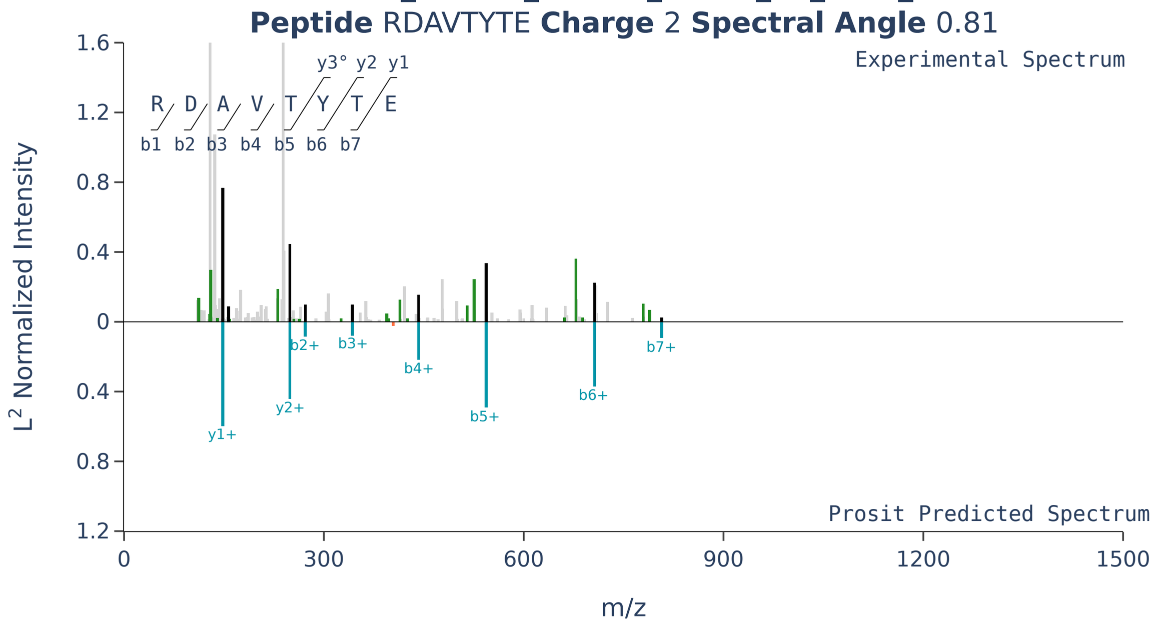


**Source** HRoetschke_291222_140123_HFGoe_H4_b2R2_1h **Scan** 20966
**Peptide** LIYEETRGVL **Charge** 2 **Spectral Angle** 0.82

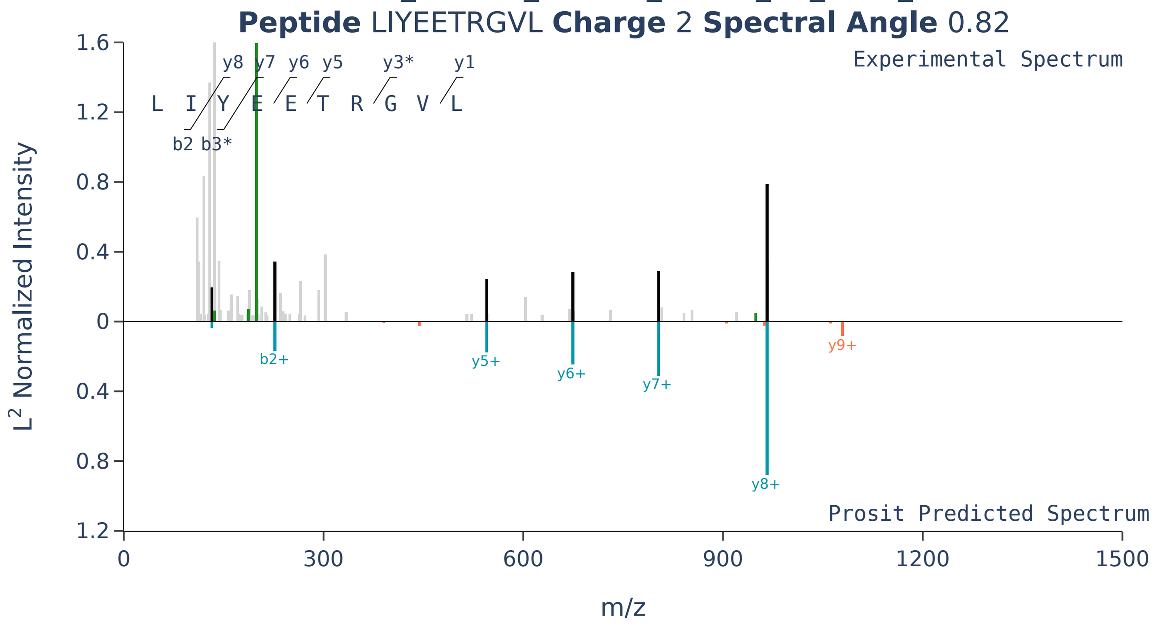


**Source** HRoetschke_291222_140123_HFGoe_H4_b1R2_2h **Scan** 10470
**Peptide** DVVYALKRQGR **Charge** 2 **Spectral Angle** 0.73

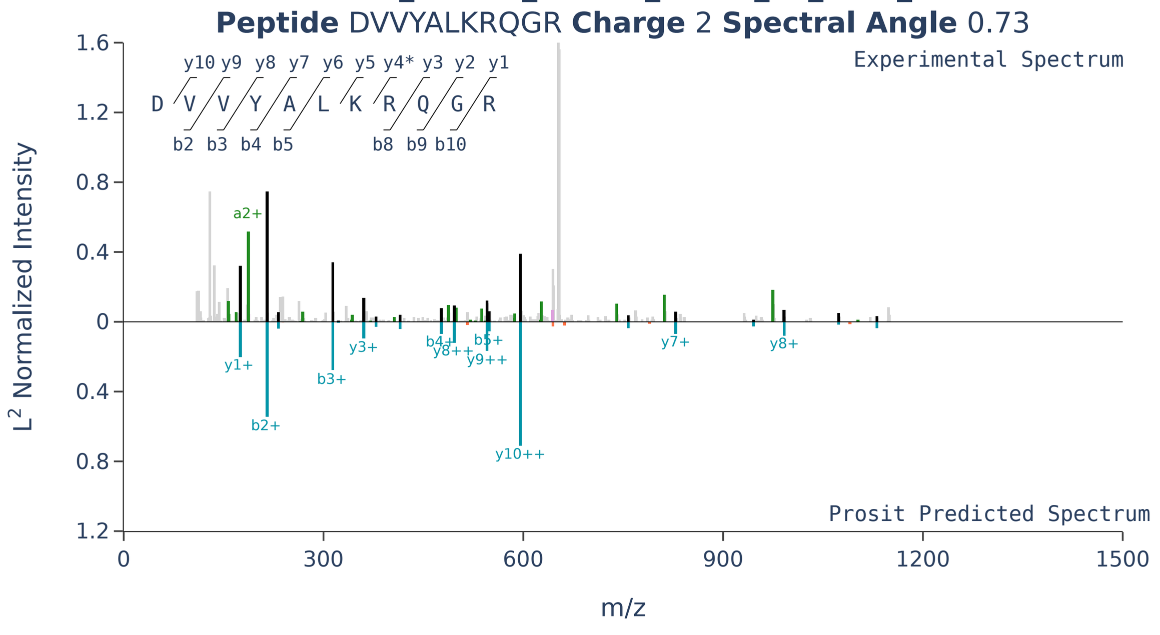


**Source** HRoetschke_291222_140123_HFGoe_H4_b1R2_24h **Scan** 10828
**Peptide** VLRDAVTYTEHA **Charge** 2 **Spectral Angle** 0.73

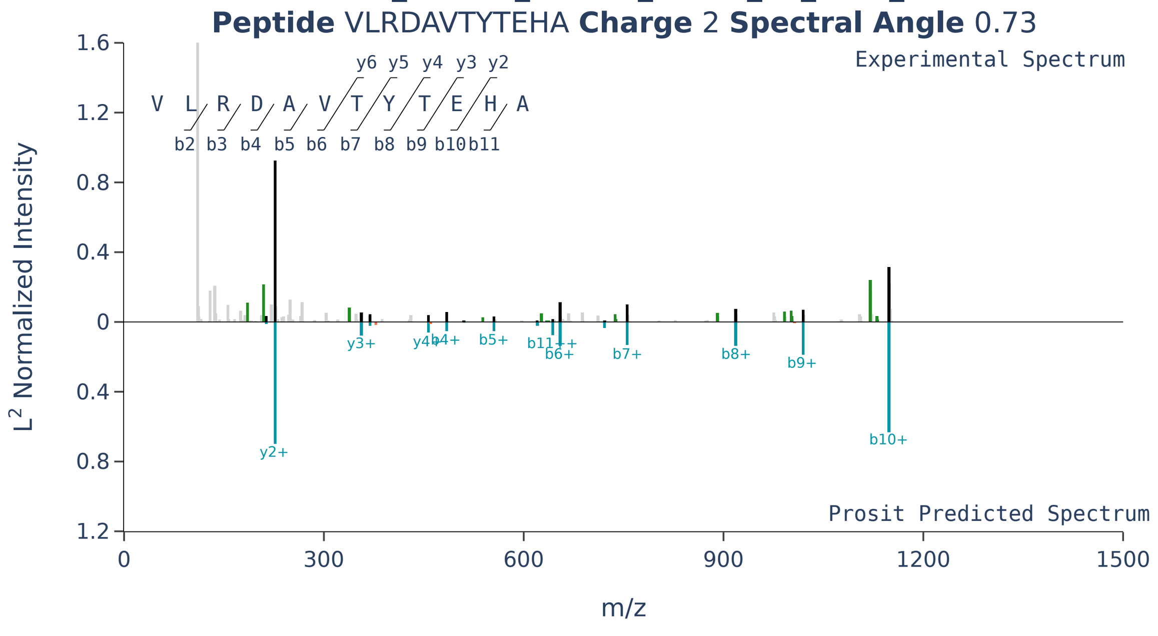


**Source** HRoetschke_291222_140123_HFGoe_H4_b1R1_4h **Scan** 11668
**Peptide** EHAKRKTVTAMDVVY **Charge** 2 **Spectral Angle** 0.66

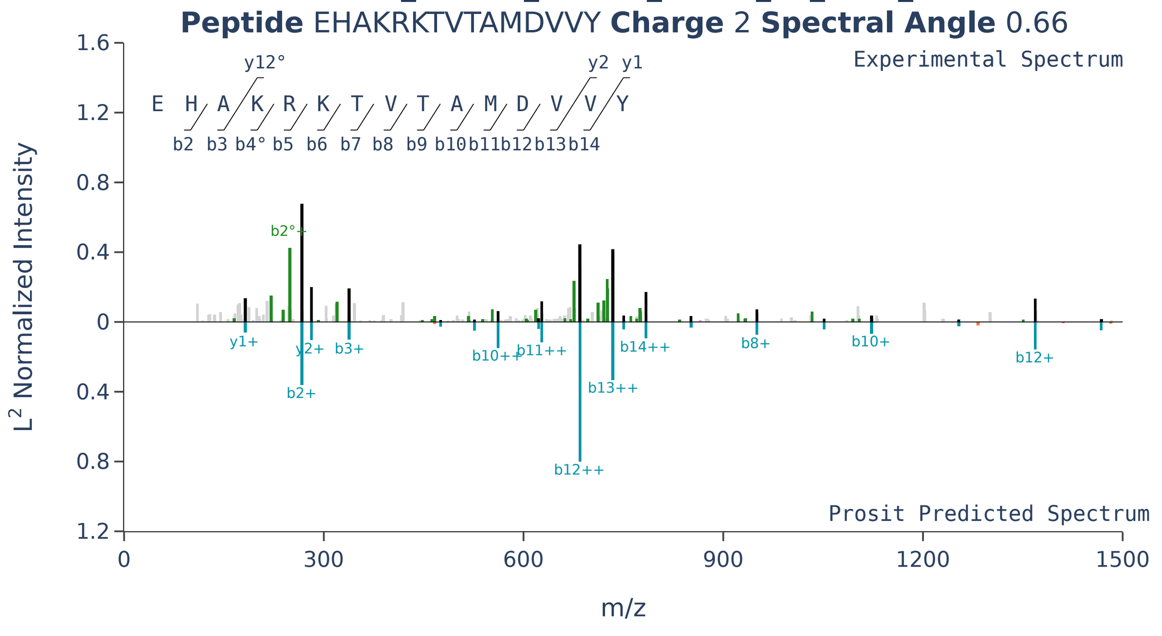


**Source** HRoetschke_291222_140123_HFGoe_H4_b1R2_24h **Scan** 10503
**Peptide** LTKPAIR **Charge** 2 **Spectral Angle** 0.95

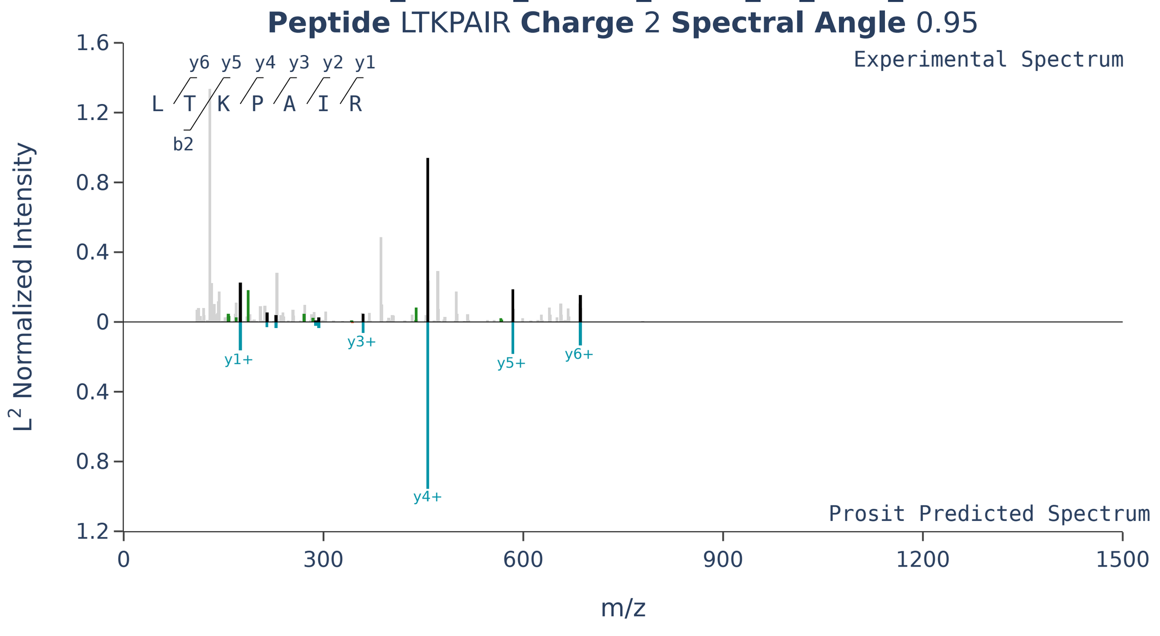

**Source** HRoetschke_291222_140123_HFGoe_H4_b1R2_24h **Scan** 12266
**Peptide** RKVLRDNIQGITKPAIRRL **Charge** 5 **Spectral Angle** 0.82

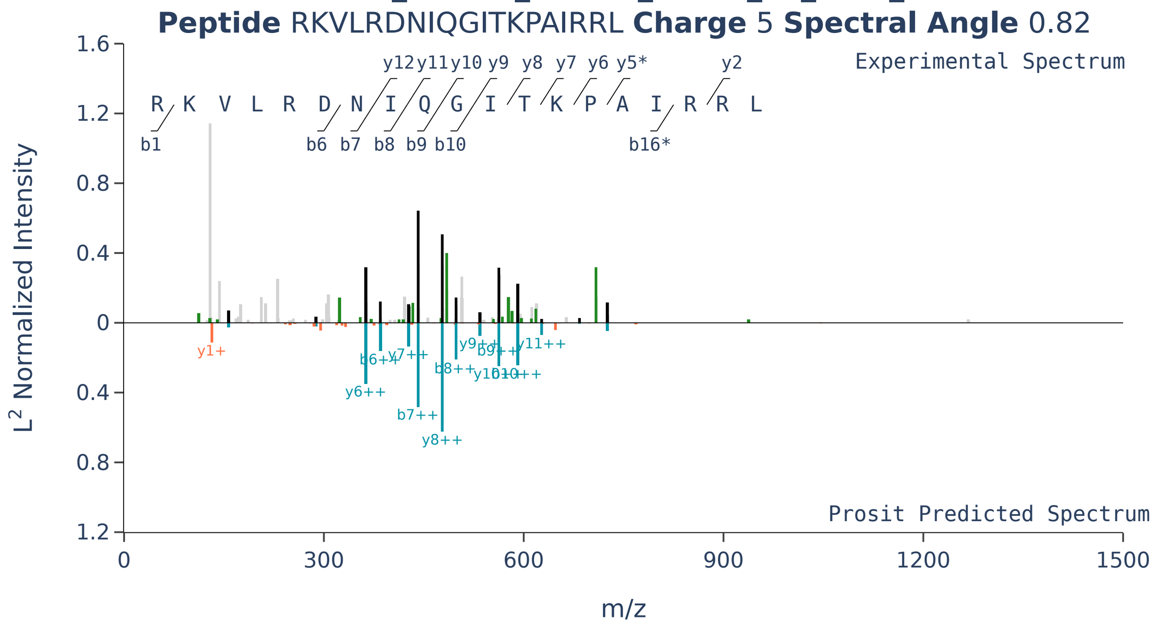


**Source** HRoetschke_291222_140123_HFGoe_H4_b1R2_24h **Scan** 5743
**Peptide** LRDAVTYTEHAKRK **Charge** 3 **Spectral Angle** 0.81

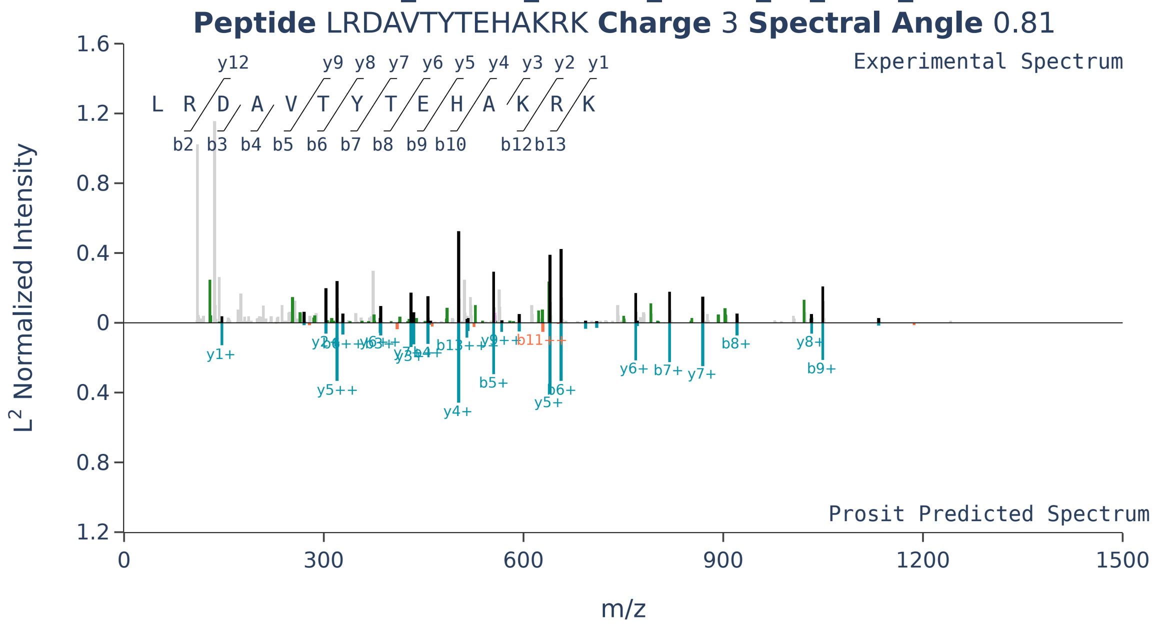


**Source** HRoetschke_291222_140123_HFGoe_H4_b2R1_2h **Scan** 19898
**Peptide** TAMDVVYALKRQ **Charge** 2 **Spectral Angle** 0.73

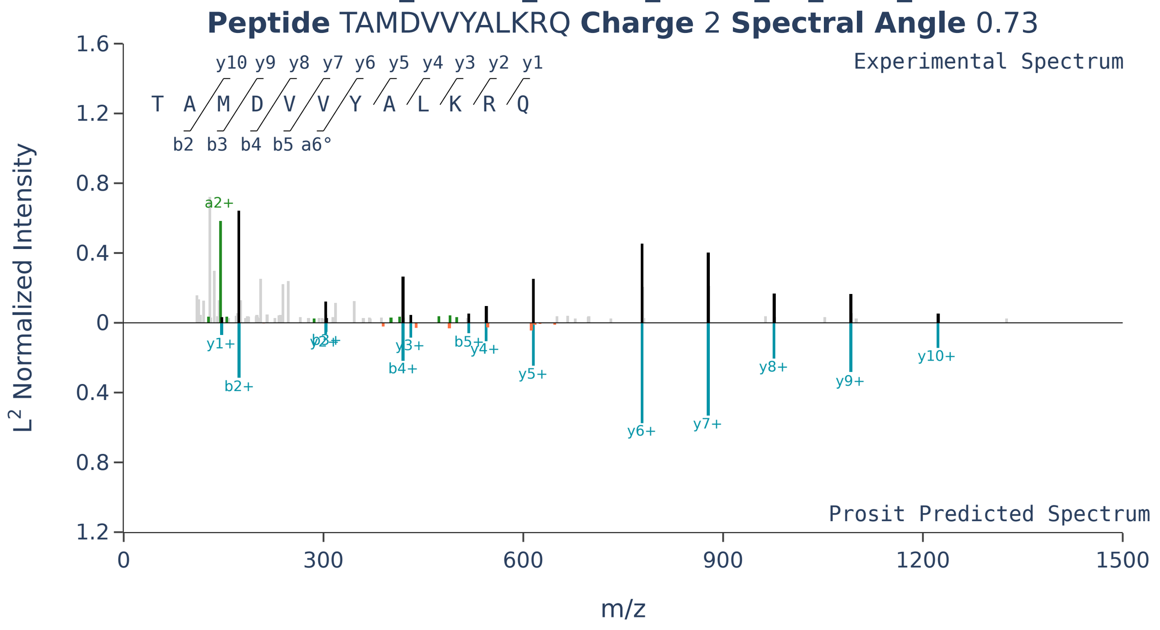


**Source** HRoetschke_291222_140123_HFGoe_H4_b2R2_1h **Scan** 18486
**Peptide** ETRGVLKVF **Charge** 2 **Spectral Angle** 0.78

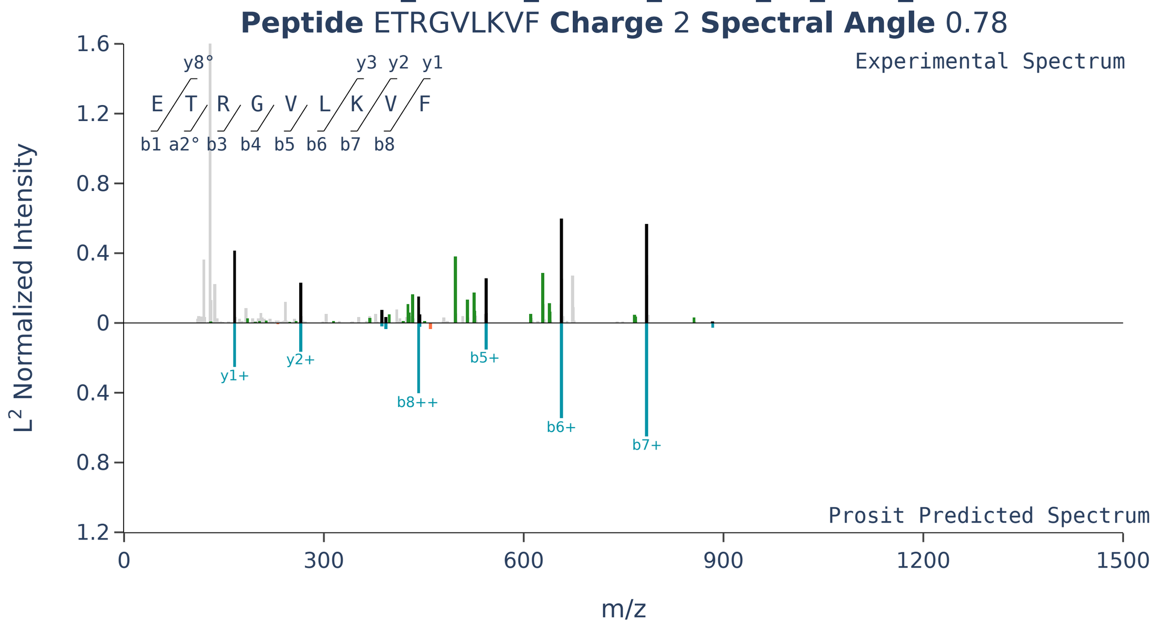


**Source** HRoetschke_291222_140123_HFGoe_H4_b1R1_2h **Scan** 19262
**Peptide** GGVKRISGLIYEETRGV **Charge** 3 **Spectral Angle** 0.89

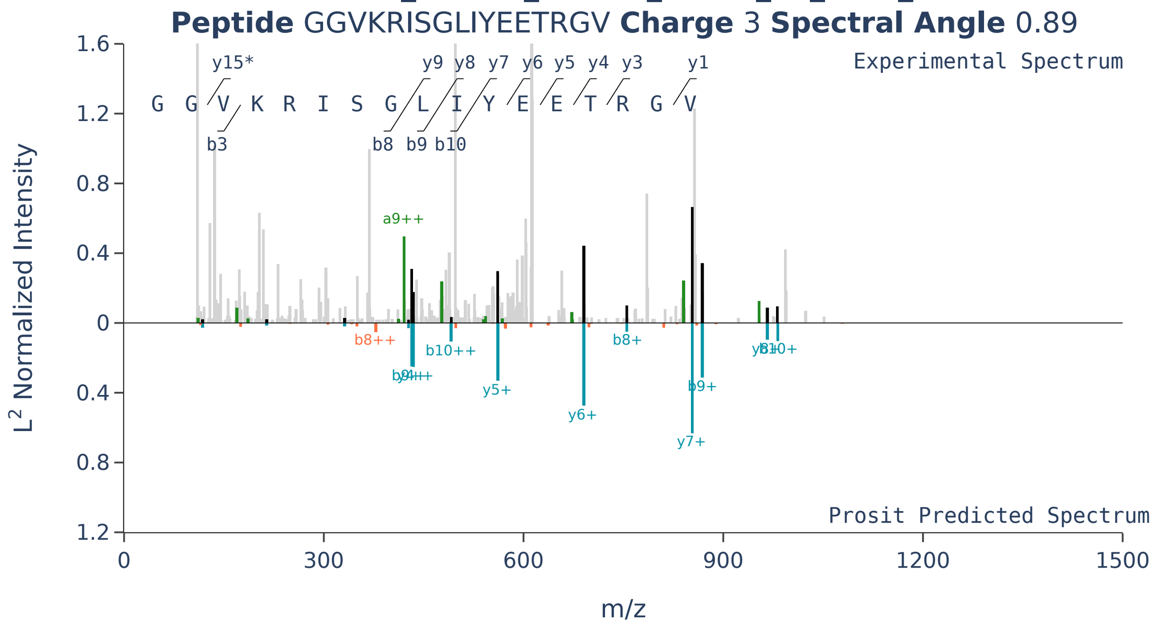


**Source** HRoetschke_291222_140123_HFGoe_H4_b2R1_1h **Scan** 6343
**Peptide** LKRQGRTLYG **Charge** 3 **Spectral Angle** 0.75

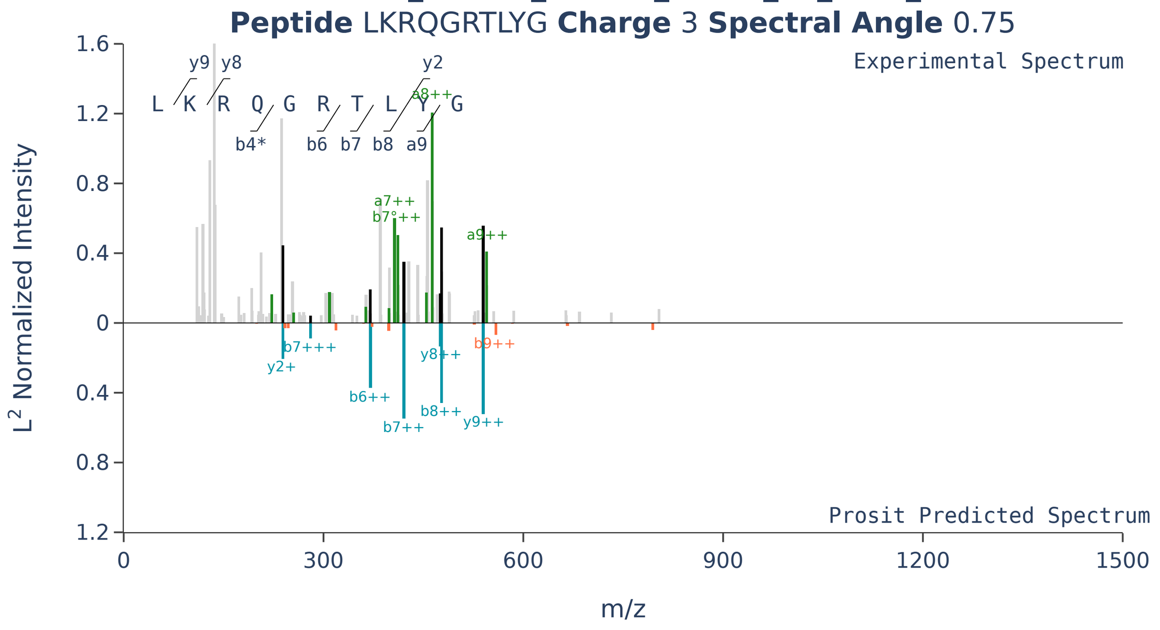


**Source** HRoetschke_291222_140123_HFGoe_H4_b2R2_1h **Scan** 5900
**Peptide** IRDAVTYTEHAKRKT **Charge** 4 **Spectral Angle** 0.72

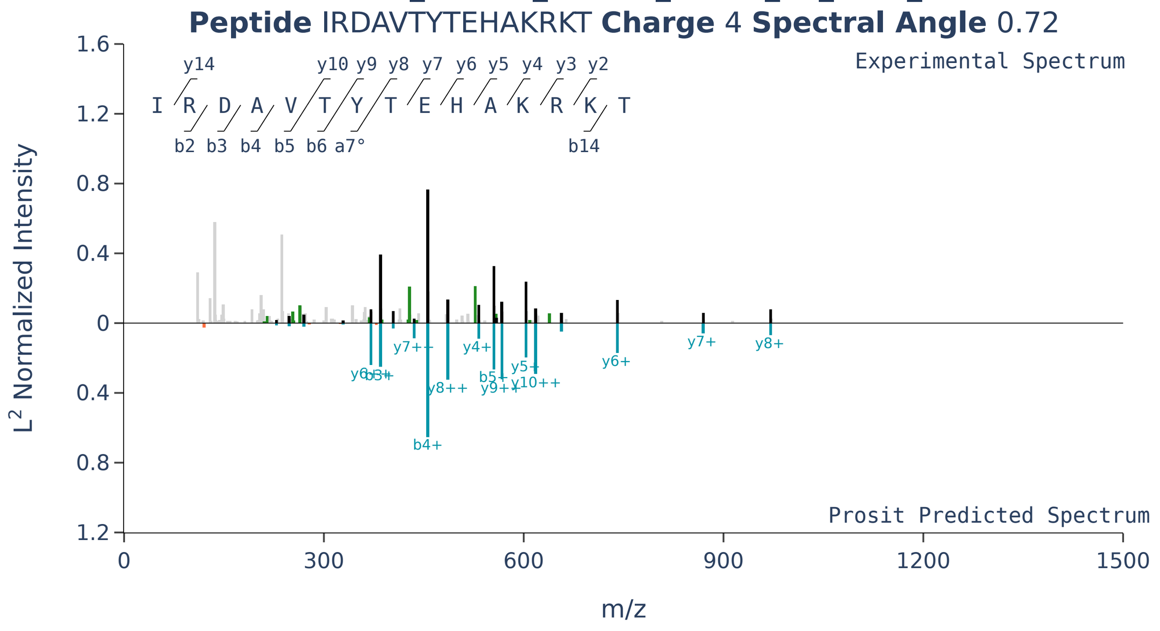


**Source** HRoetschke_291222_140123_HFGoe_H4_b2R1_1h **Scan** 33996
**Peptide** ISGLIYEETRGVLKVFL **Charge** 2 **Spectral Angle** 0.85

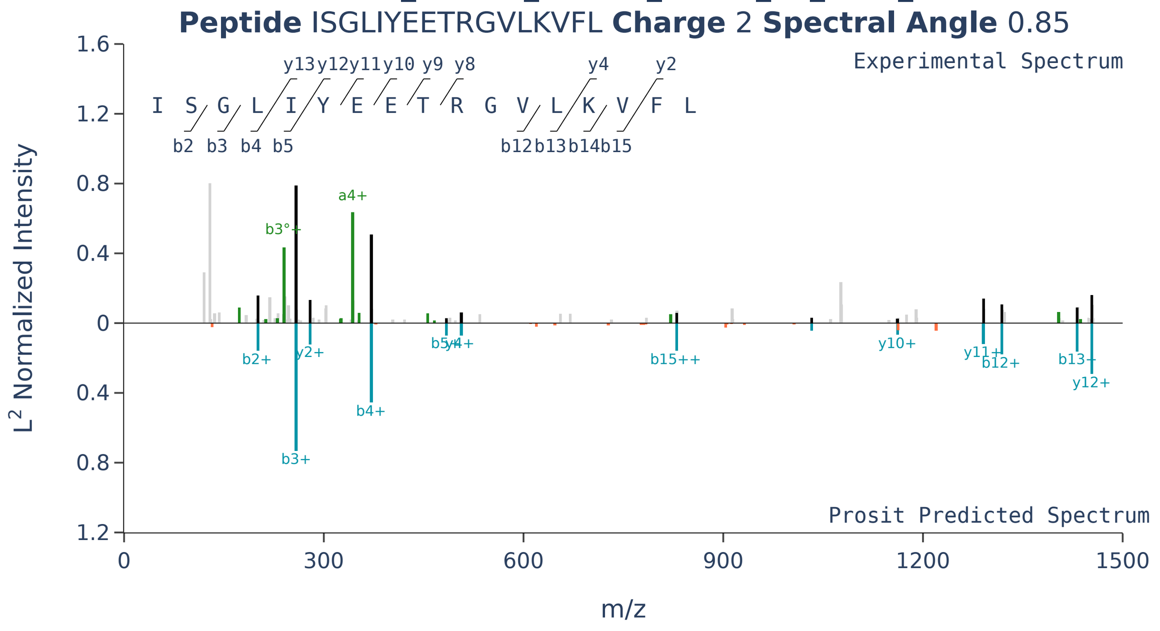


**Source** HRoetschke_291222_140123_HFGoe_H4_b2R1_1h **Scan** 19939
**Peptide** NVIRDAVTYTEHAKRKTVTAMDVVY **Charge** 5 **Spectral Angle** 0.85

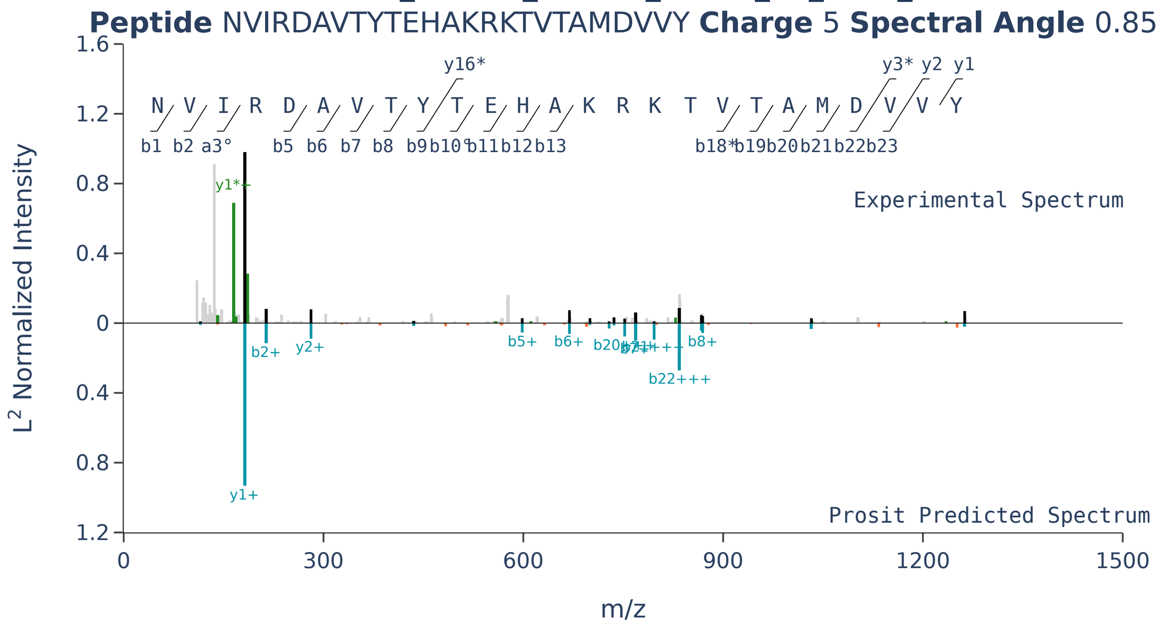


**Source** HRoetschke_291222_140123_HFGoe_H4_b2R1_4h **Scan** 14492
**Peptide** YALKRQGRTLYGFGG **Charge** 3 **Spectral Angle** 0.67

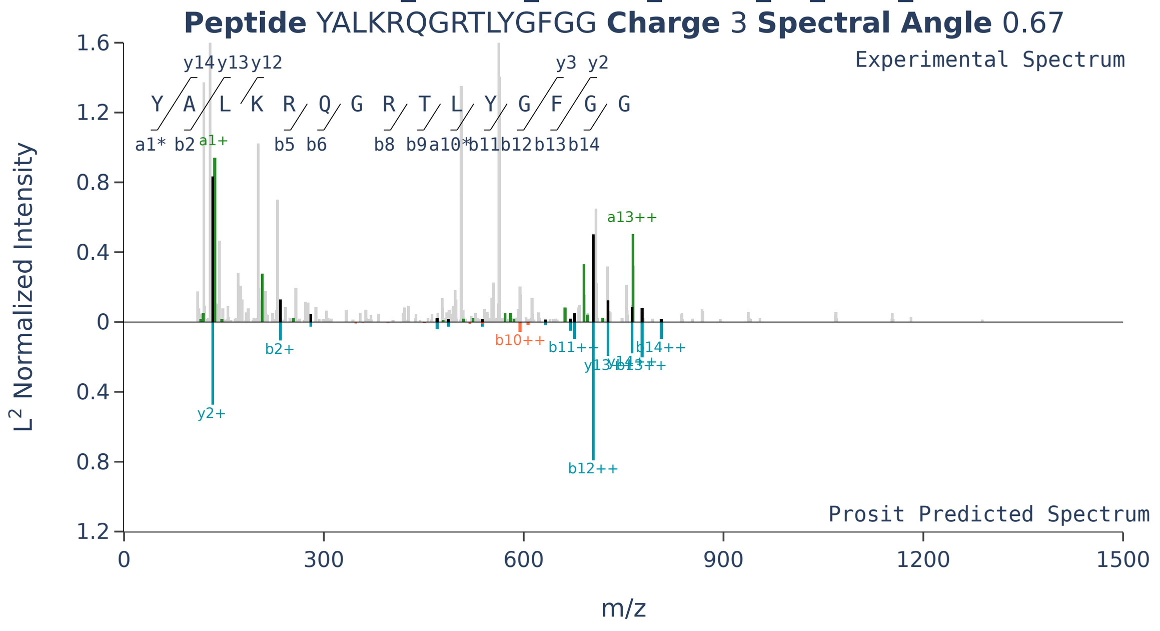


**Source** HRoetschke_291222_140123_HFGoe_H4_b1R1_1h **Scan** 15848
**Peptide** TEHAKRKTVTAMDVVYALKRQGRT **Charge** 5 **Spectral Angle** 0.88

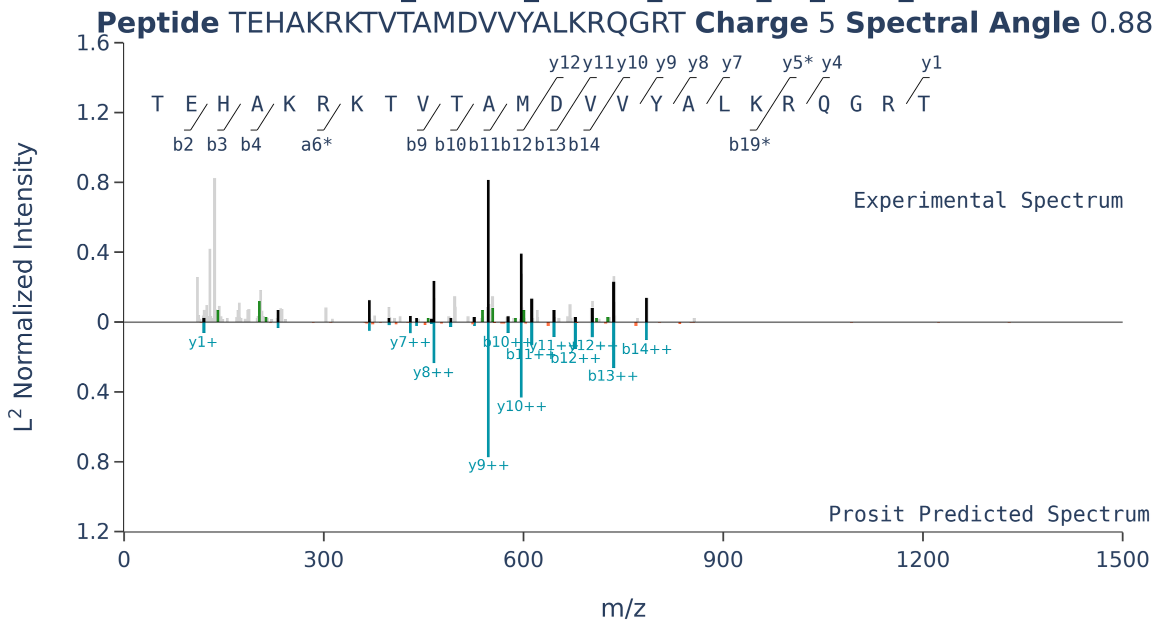


**Source** HRoetschke_291222_140123_HFGoe_H4_b2R2_4h **Scan** 13488
**Peptide** LRDNIQGITKPAIRRLAR **Charge** 5 **Spectral Angle** 0.73

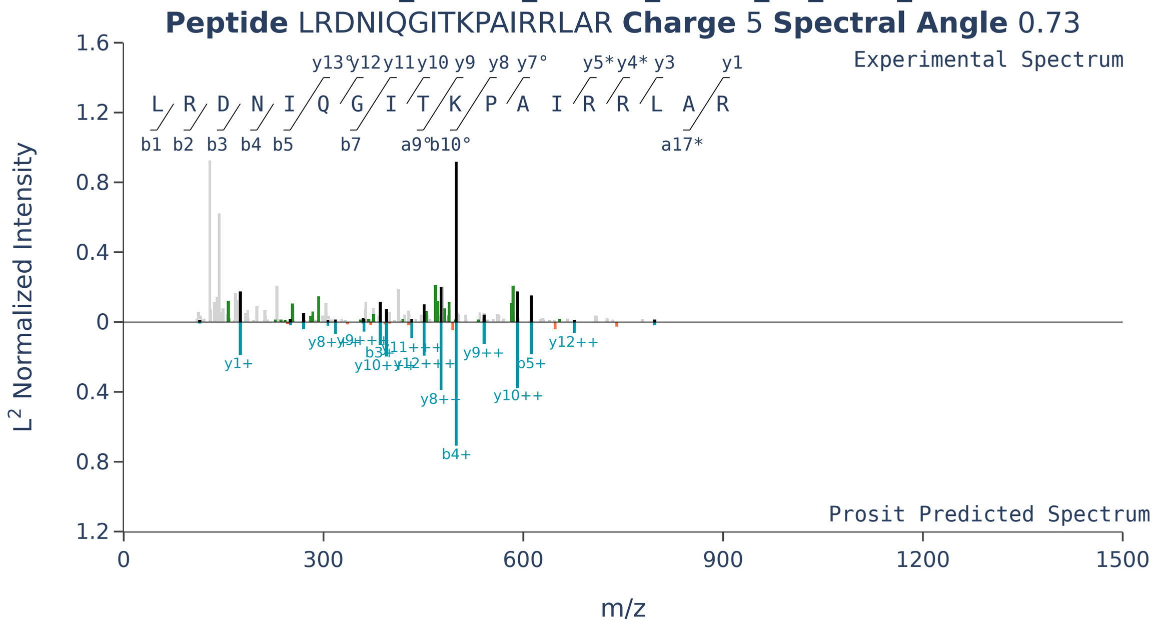


**Source** HRoetschke_291222_140123_HFGoe_H4_b1R2_24h **Scan** 15822
**Peptide** TYTEHAKR **Charge** 2 **Spectral Angle** 0.78

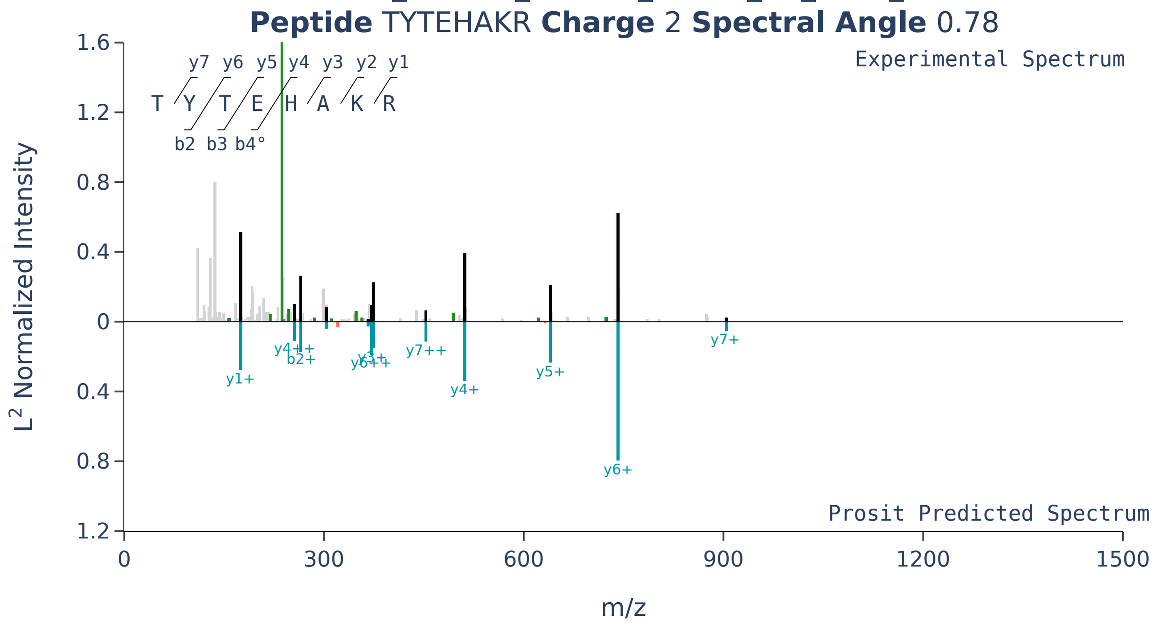


**Source** HRoetschke_291222_140123_HFGoe_H4_b2R1_1h **Scan** 18894
**Peptide** ARRGGVKRISGLIYEETRGVL **Charge** 5 **Spectral Angle** 0.64

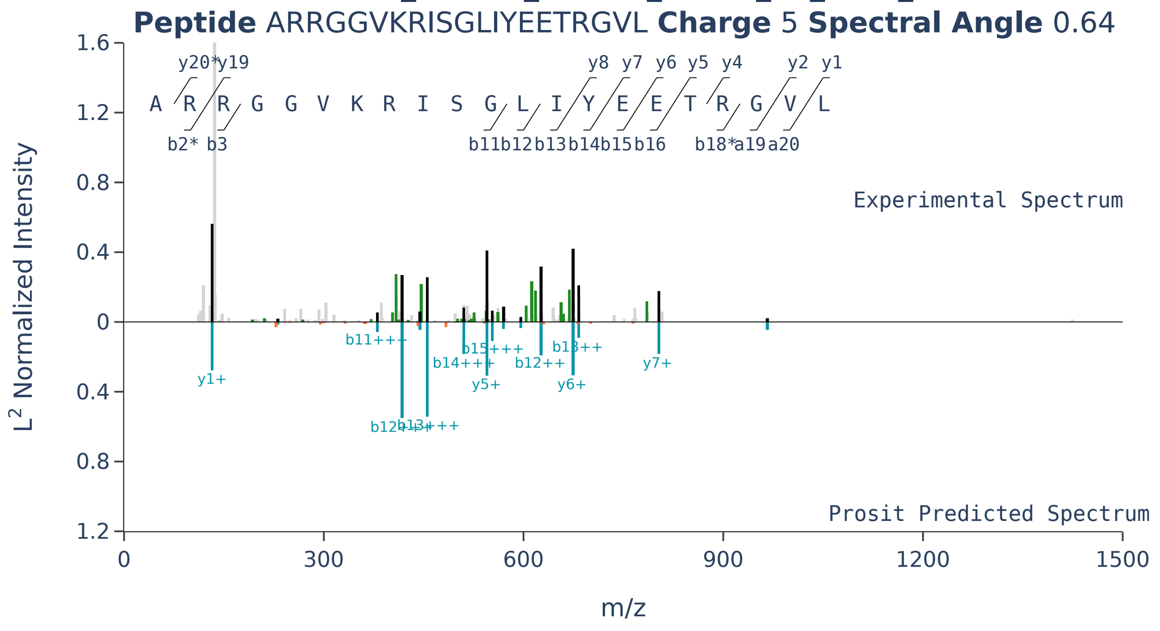


**Source** HRoetschke_291222_140123_HFGoe_H4_b2R2_4h **Scan** 11029
**Peptide** NIQGITKPAIRRLARRGGVKR **Charge** 4 **Spectral Angle** 0.66

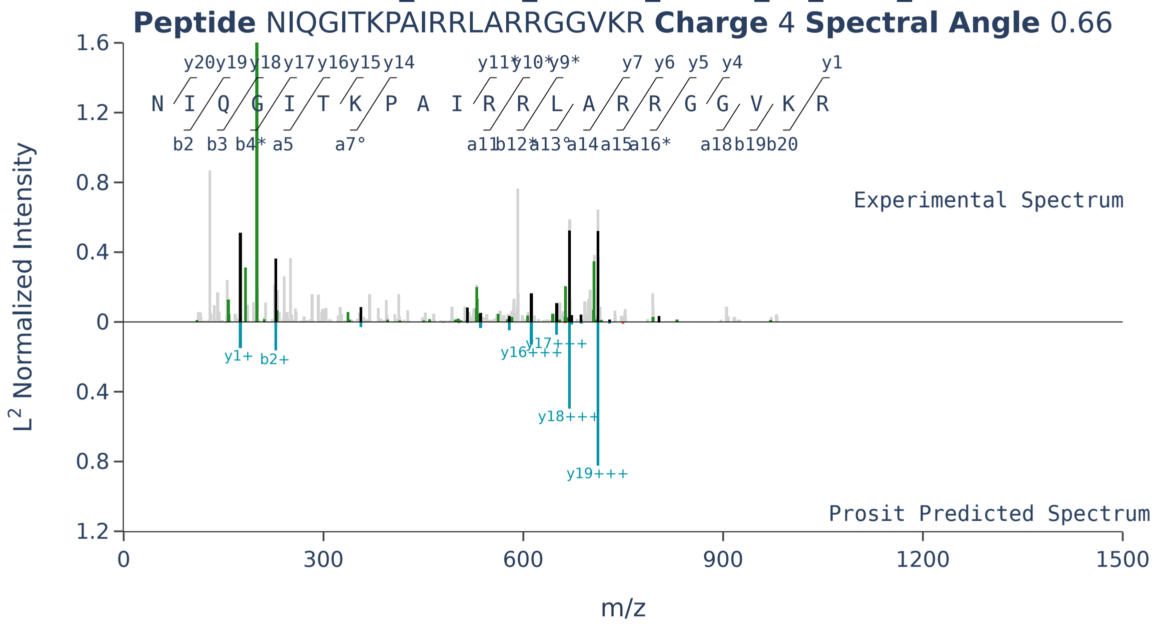

**Source** HRoetschke_291222_140123_HFGoe_H4_b1R1_2h **Scan** 24897
**Peptide** TAMDVVYAL **Charge** 1 **Spectral Angle** 0.82

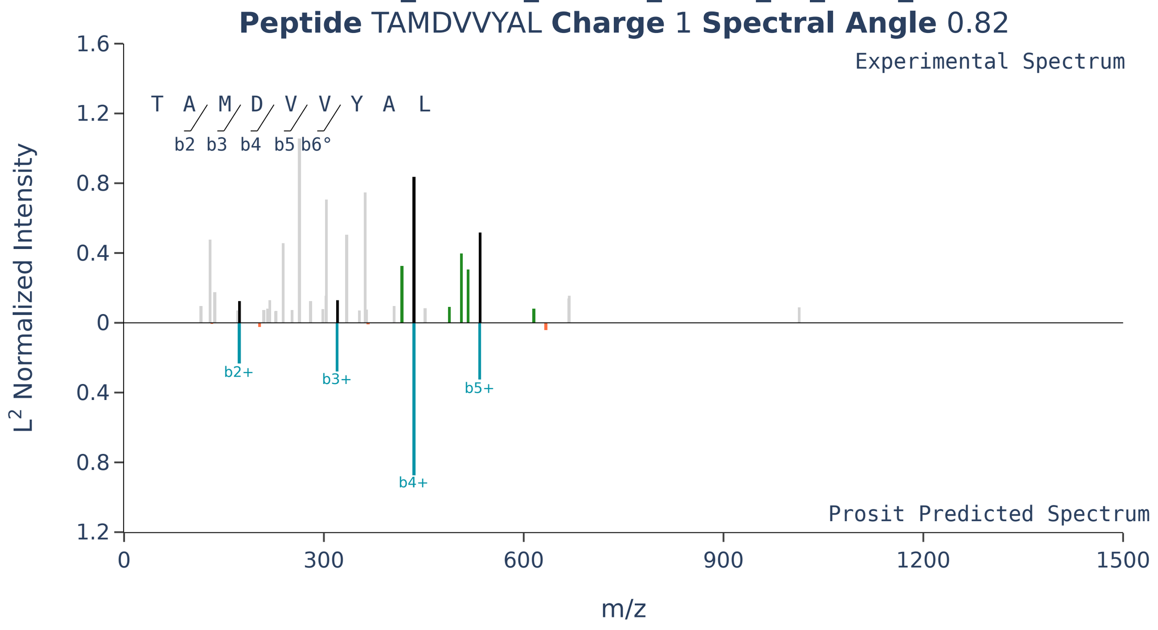


**Source** HRoetschke_291222_140123_HFGoe_H4_b2R1_1h **Scan** 22483
**Peptide** VLKVFLE **Charge** 2 **Spectral Angle** 0.74

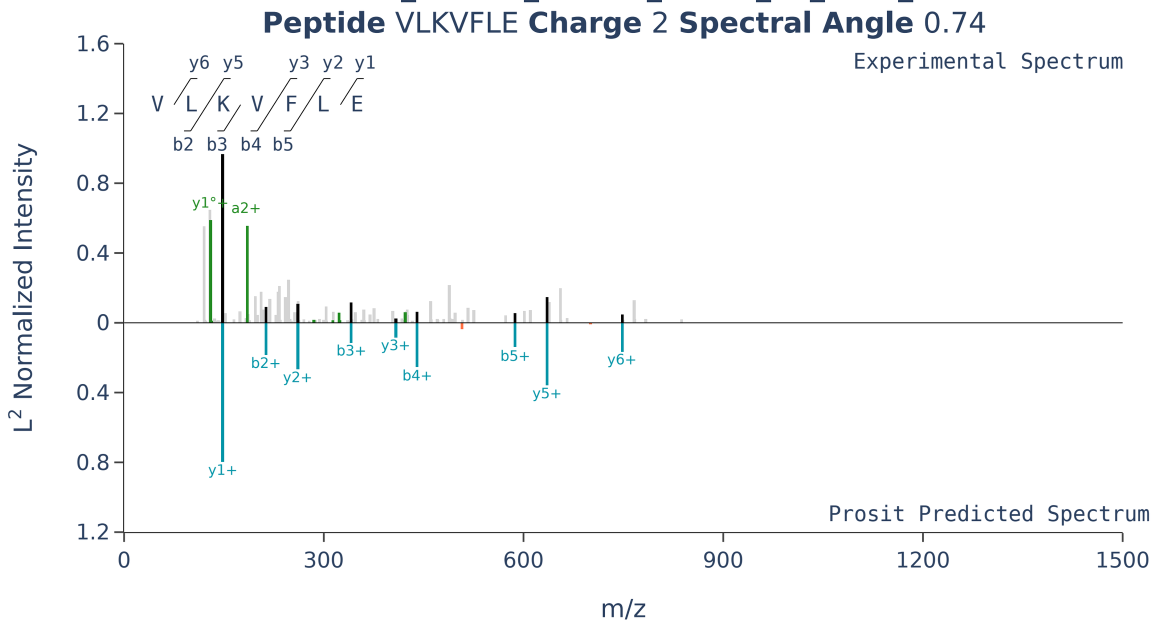


**Source** HRoetschke_291222_140123_HFGoe_H4_b2R2_1h **Scan** 24202
**Peptide** RGVLKVFLENV **Charge** 3 **Spectral Angle** 0.71

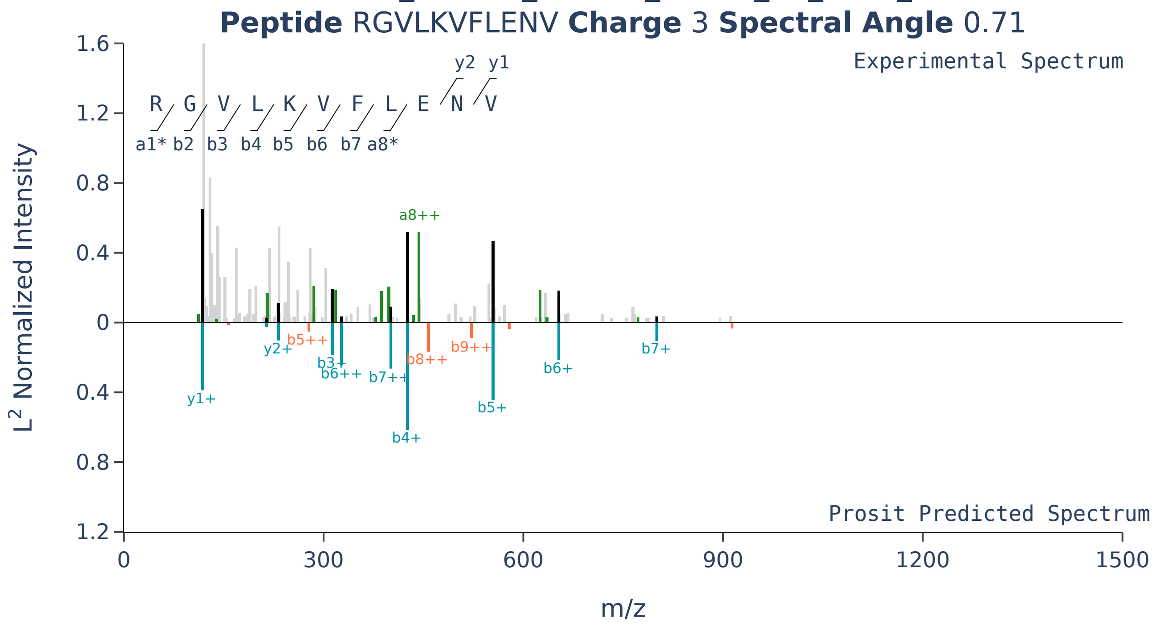


**Source** HRoetschke_291222_140123_HFGoe_H4_b1R2_4h **Scan** 17304
**Peptide** LENVIRDAVTYTEHAKRK **Charge** 3 **Spectral Angle** 0.76

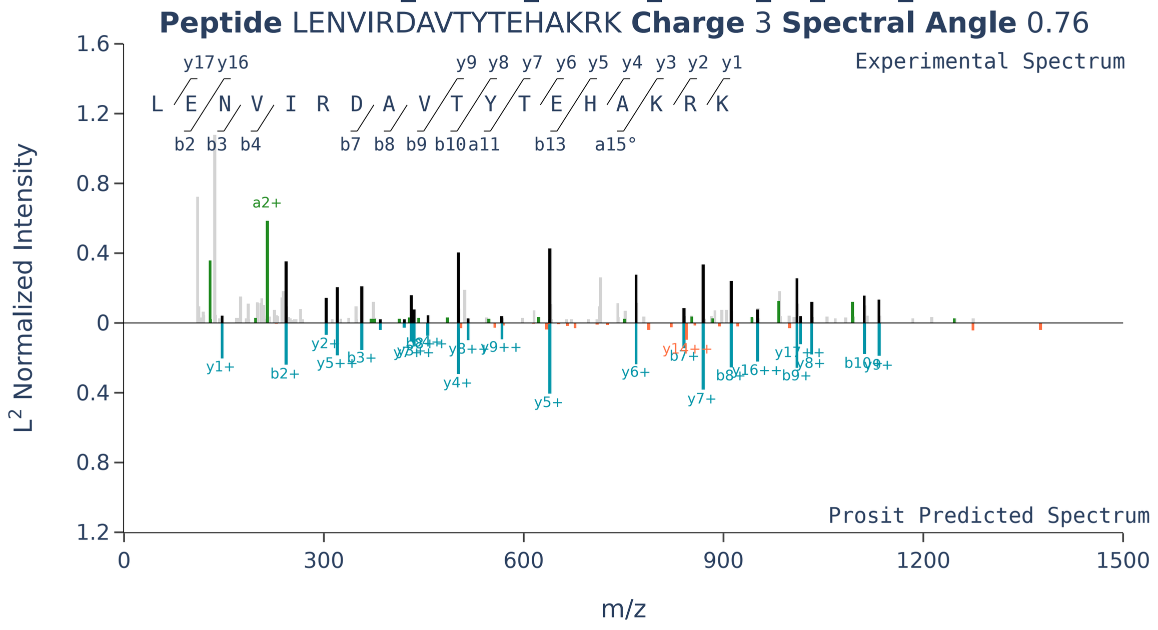


**Source** HRoetschke_291222_140123_HFGoe_H4_b2R1_1h **Scan** 33655
**Peptide** TRGVLKVFLENVIRDAVTY **Charge** 3 **Spectral Angle** 0.79

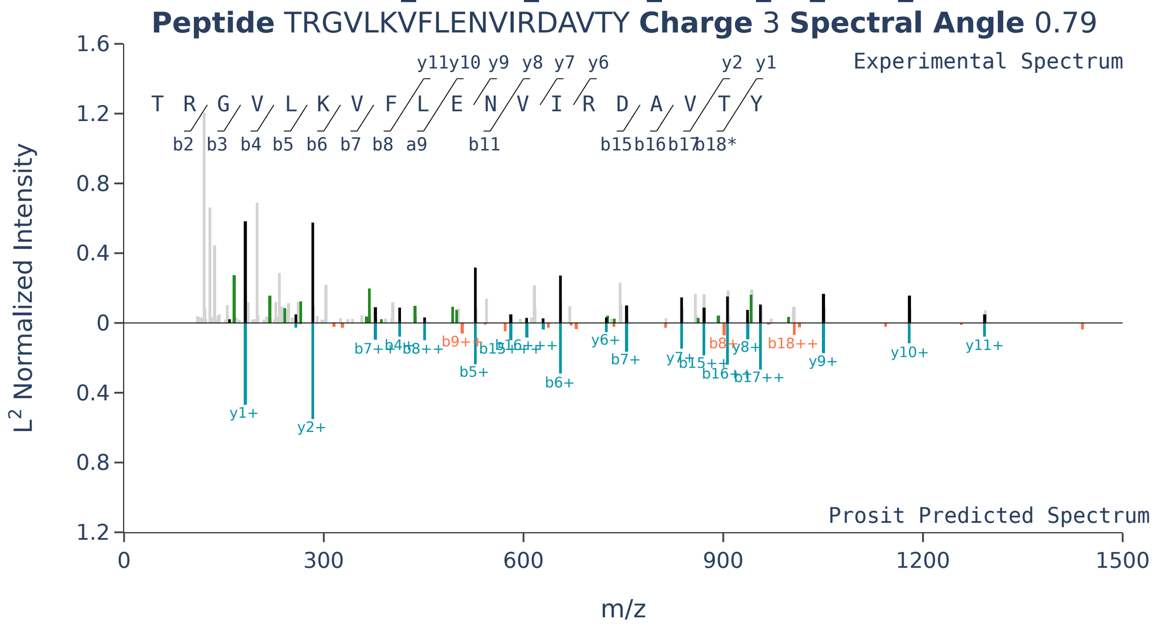


**Source** HRoetschke_291222_140123_HFGoe_H4_b2R1_1h **Scan** 21341
**Peptide** LYEETRGVLKVF **Charge** 2 **Spectral Angle** 0.76

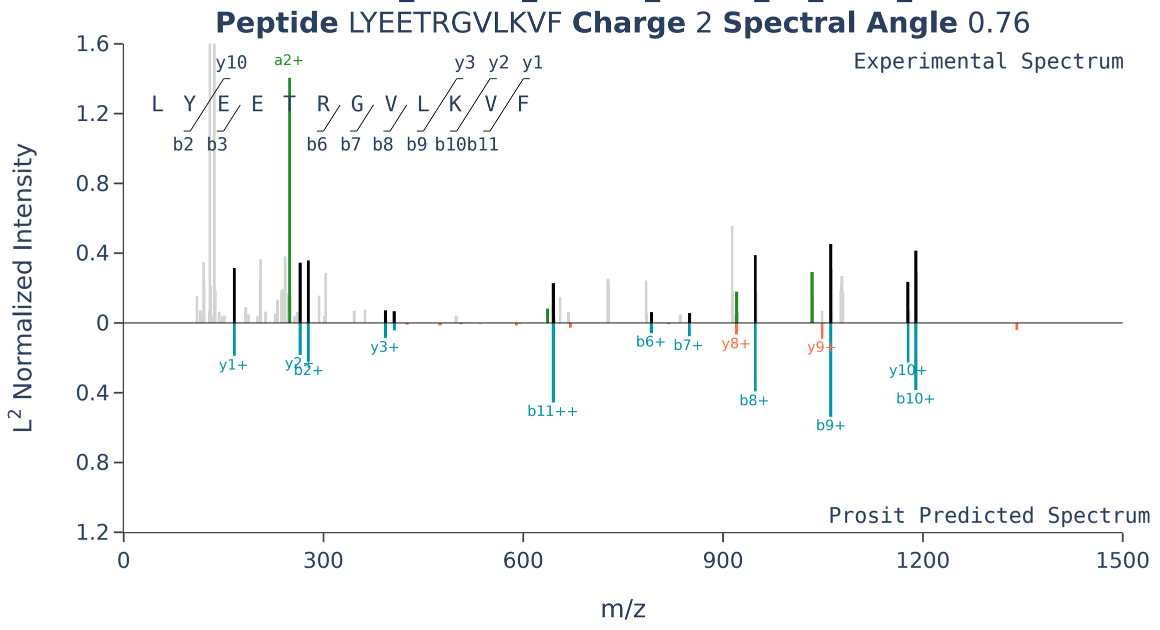


**Source** HRoetschke_291222_140123_HFGoe_H4_b2R1_1h **Scan** 16297
**Peptide** AVTYTEHAKRKTVTAMDVVYALKRQ **Charge** 5 **Spectral Angle** 0.8

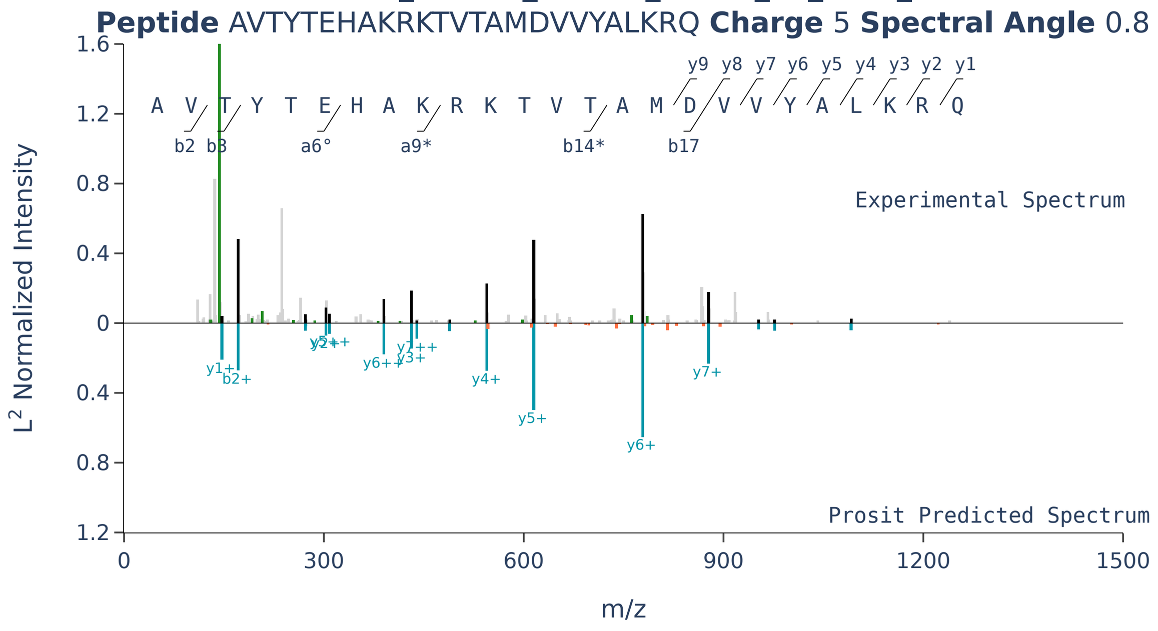


**Source** HRoetschke_291222_140123_HFGoe_H4_b2R1_24h **Scan** 10881
**Peptide** NIQGITKPAIRR **Charge** 3 **Spectral Angle** 0.51

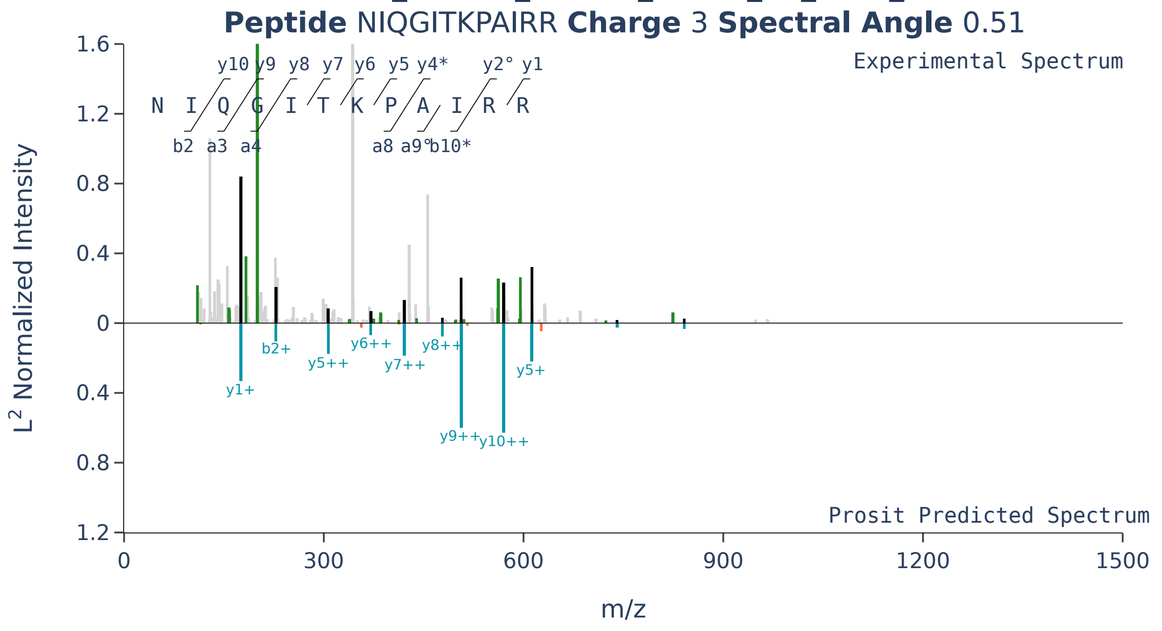


**Source** HRoetschke_291222_140123_HFGoe_H4_b1R1_1h **Scan** 12591
**Peptide** VLRDAVTYTE **Charge** 2 **Spectral Angle** 0.81

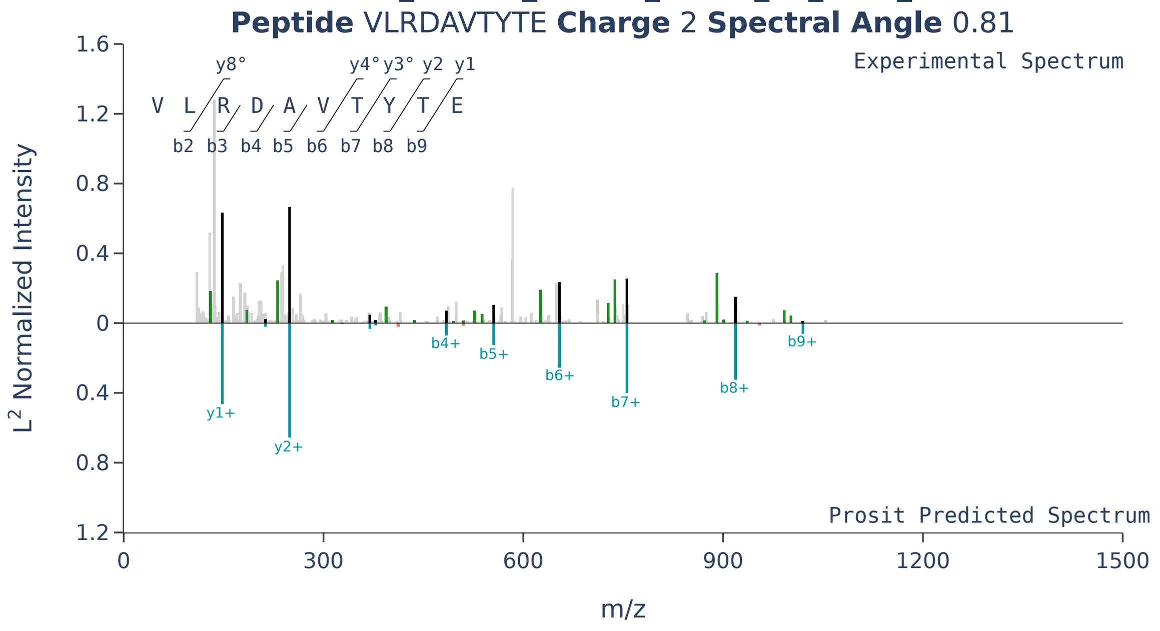


**Source** HRoetschke_291222_140123_HFGoe_H4_b1R1_24h **Scan** 6405
**Peptide** LRDAVTYTEHAKRKTVT **Charge** 3 **Spectral Angle** 0.82

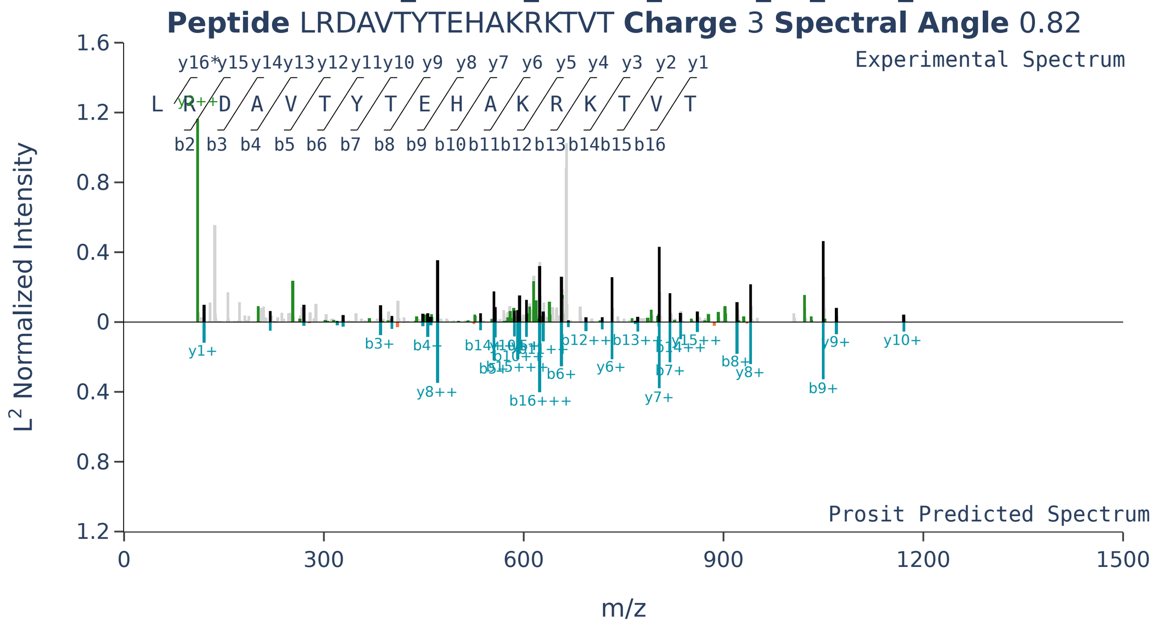


**Source** HRoetschke_291222_140123_HFGoe_H4_b1R1_24h **Scan** 12011
**Peptide** LRDNIQGITKPAIRRLARR **Charge** 5 **Spectral Angle** 0.65

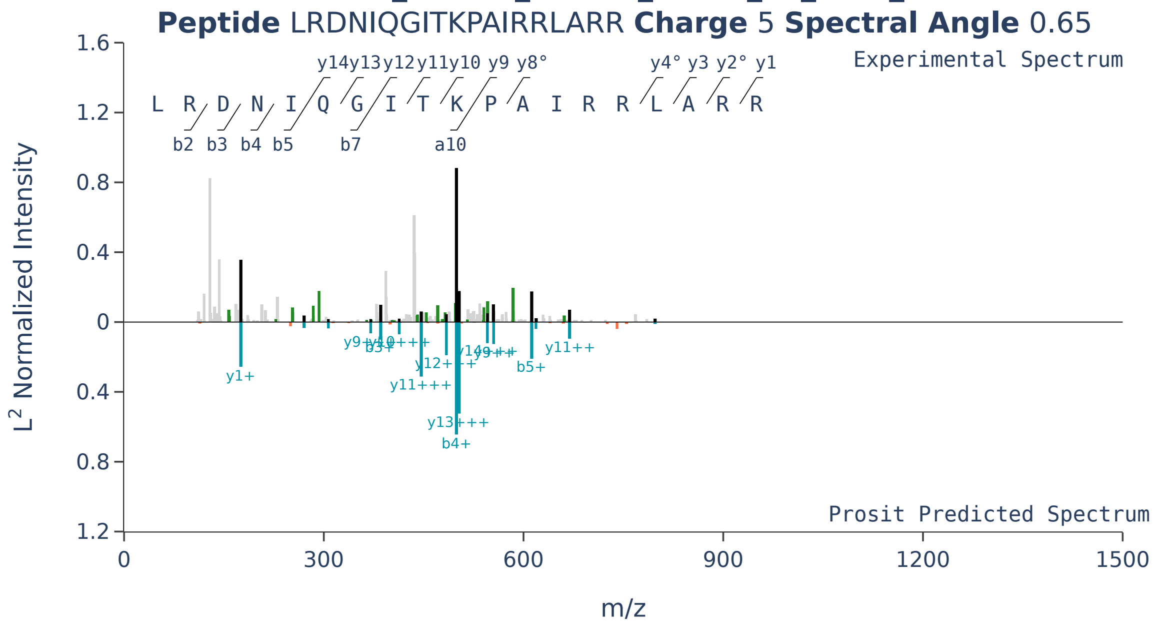


**Source** HRoetschke_291222_140123_HFGoe_H4_b2R1_1h **Scan** 20295
**Peptide** MDVVYALKRQGRTLYGFGG **Charge** 3 **Spectral Angle** 0.75

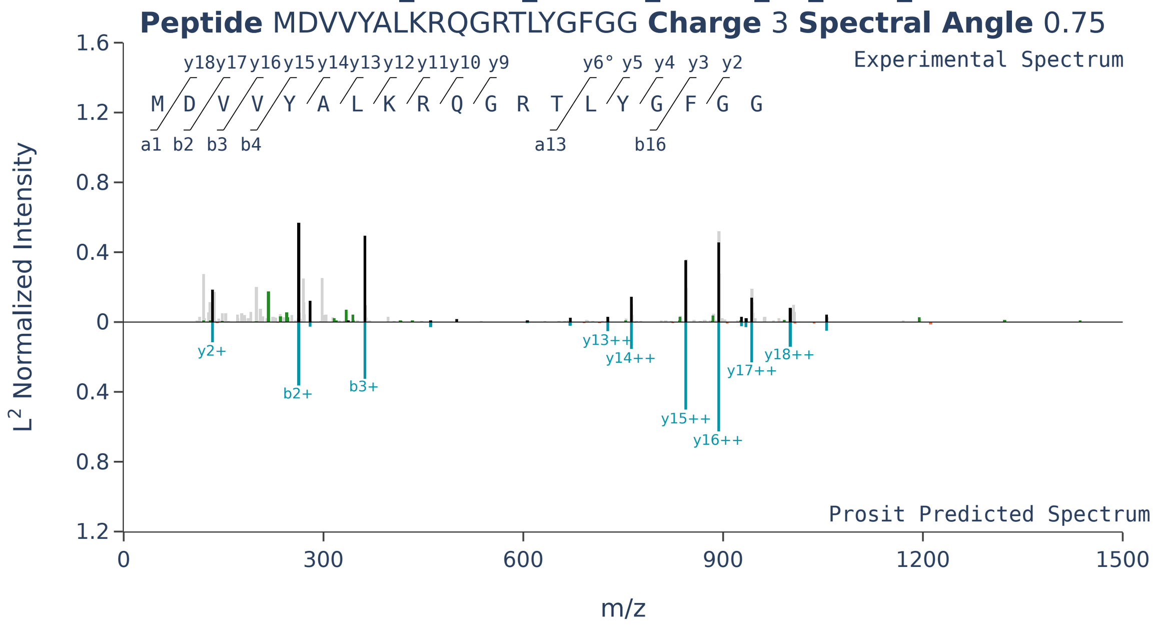


**Source** HRoetschke_291222_140123_HFGoe_H4_b2R2_1h **Scan** 24080
**Peptide** KVFLENVIRDAVTYTEHAKRKTV **Charge** 4 **Spectral Angle** 0.86

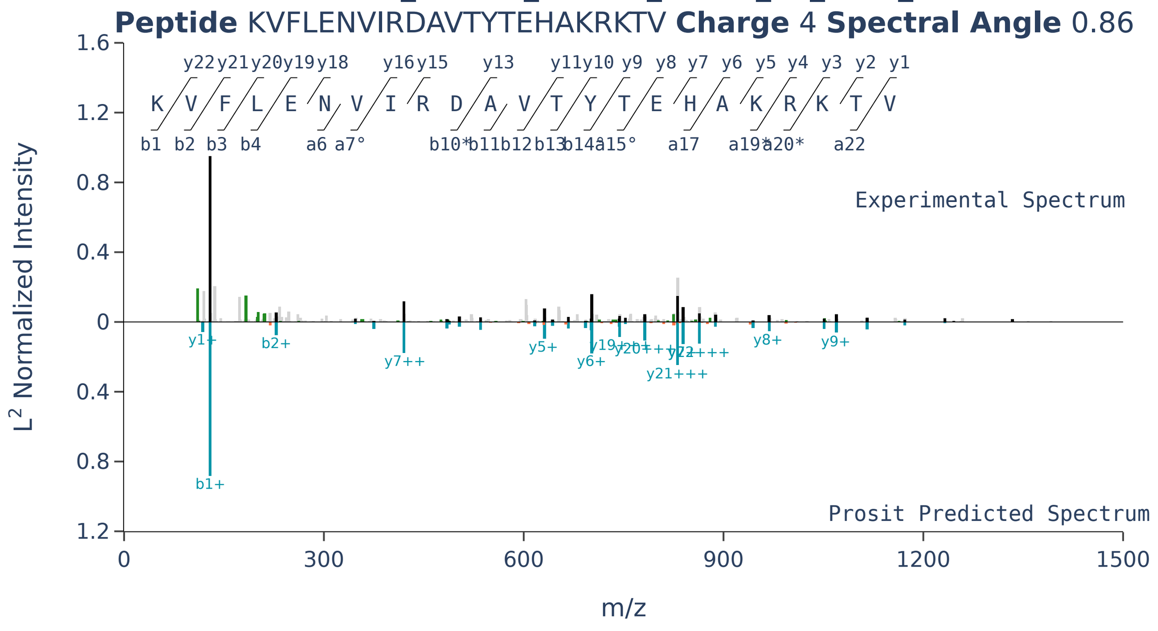


**Source** HRoetschke_291222_140123_HFGoe_H4_b2R2_2h **Scan** 17047
**Peptide** AMDVVYALKRQ **Charge** 2 **Spectral Angle** 0.8

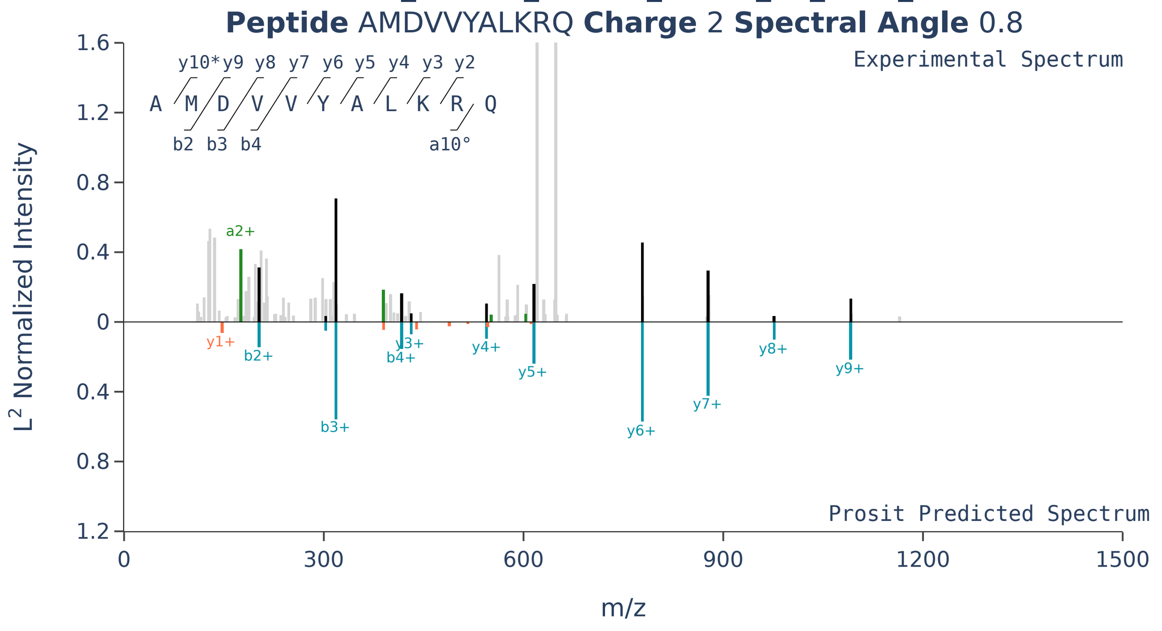


**Source** HRoetschke_291222_140123_HFGoe_H4_b1R1_1h **Scan** 22210
**Peptide** MDVVYALKRQGRTLYGF **Charge** 3 **Spectral Angle** 0.75

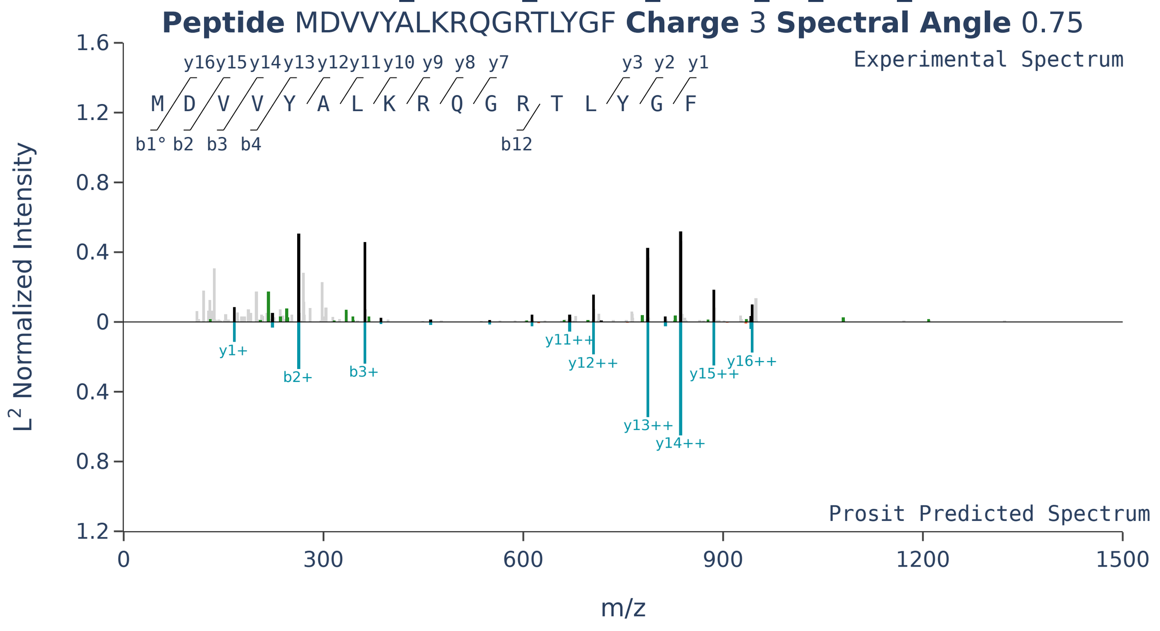

**Source** HRoetschke_291222_140123_HFGoe_H4_b1R1_24h **Scan** 11846
**Peptide** LKRQGRTLYGFGG **Charge** 3 **Spectral Angle** 0.65

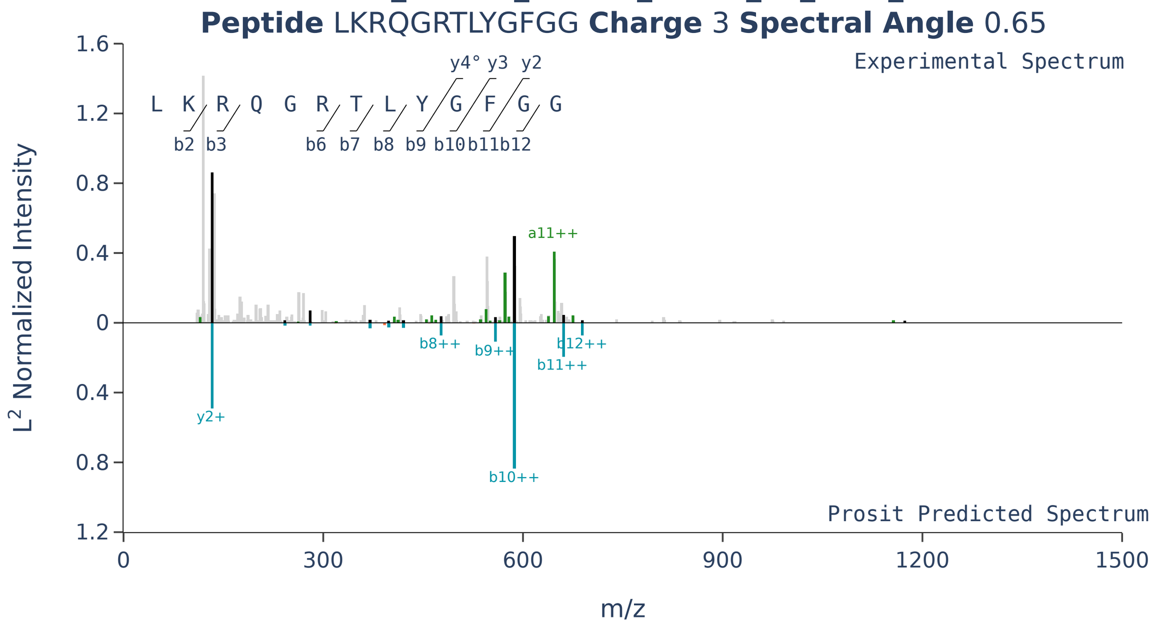


**Source** HRoetschke_291222_140123_HFGoe_H4_b2R2_1h **Scan** 24765
**Peptide** ISGLIYEETRGVLKV **Charge** 2 **Spectral Angle** 0.85

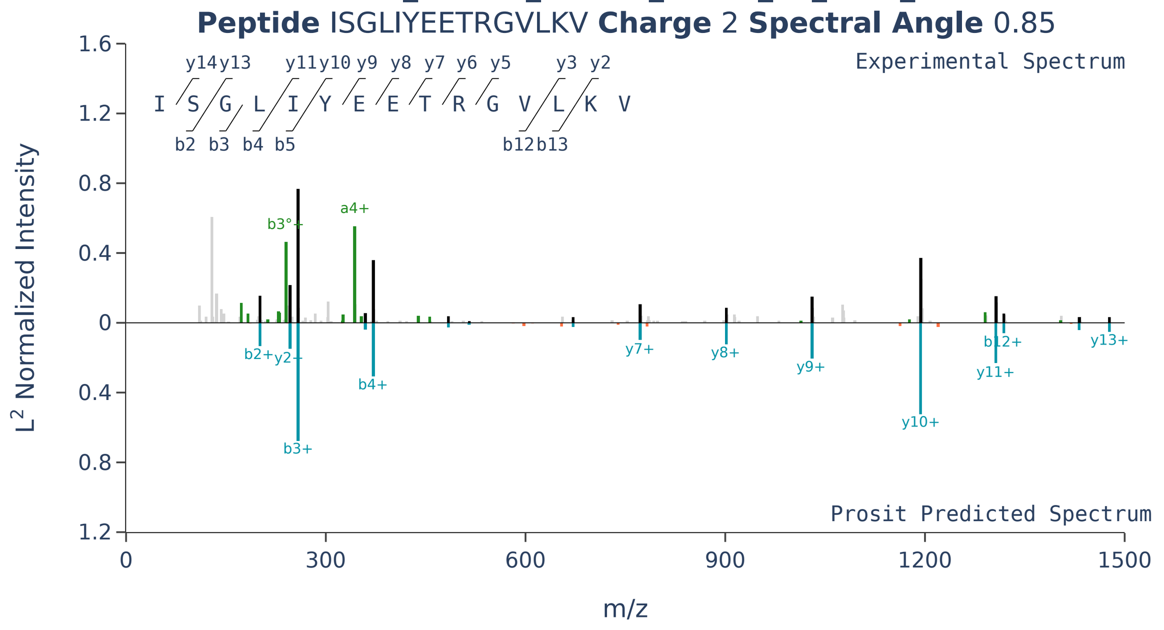


**Source** HRoetschke_291222_140123_HFGoe_H4_b2R1_2h **Scan** 16241
**Peptide** AVTYTEHAKRKTVTAMDVVYALKRQGR **Charge** 5 **Spectral Angle** 0.72

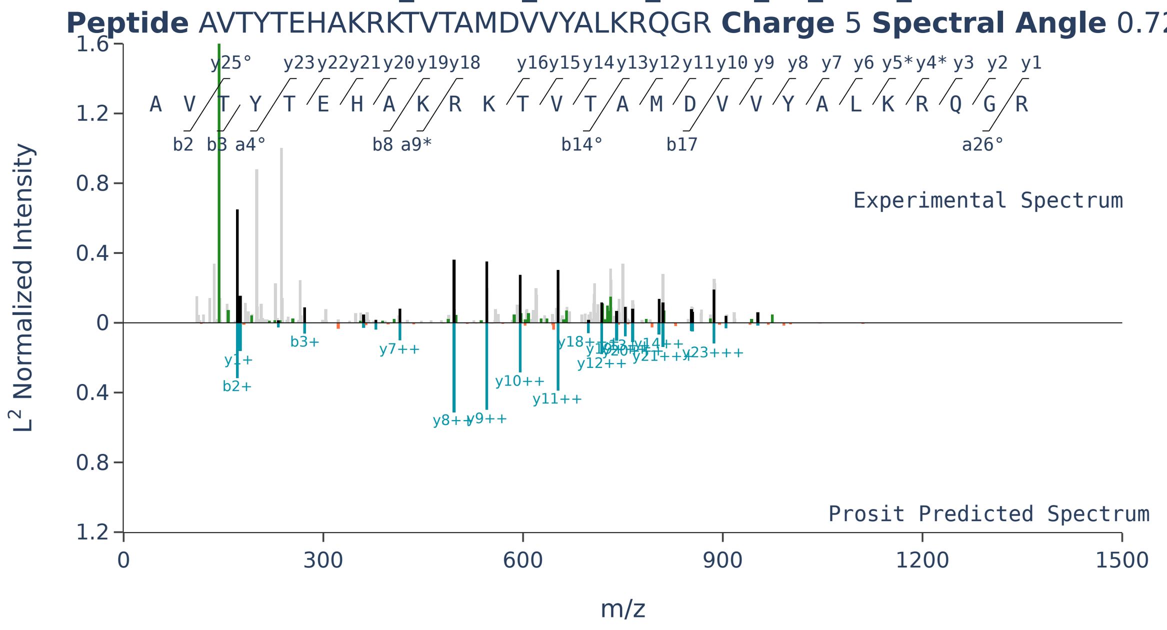


**Source** HRoetschke_291222_140123_HFGoe_H4_b1R1_2h **Scan** 16505
**Peptide** KVLRDNIQGITKPAL **Charge** 4 **Spectral Angle** 0.82

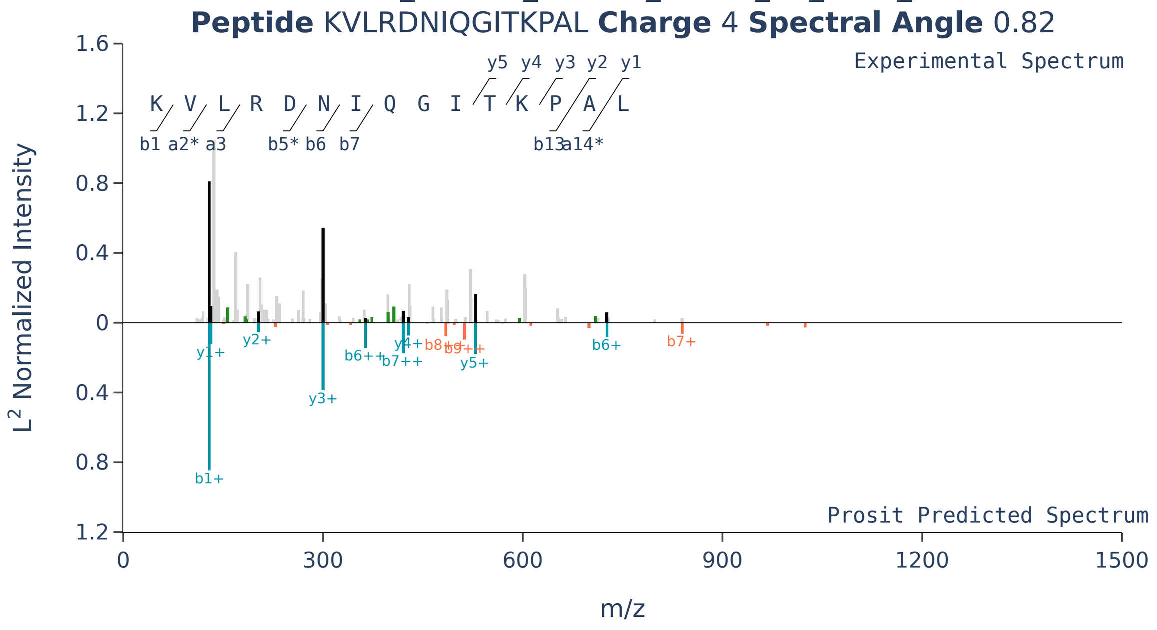


**Source** HRoetschke_291222_140123_HFGoe_H4_b2R1_1h **Scan** 22885
**Peptide** KVFLENVIRDAVTYTEHAKRK **Charge** 5 **Spectral Angle** 0.76

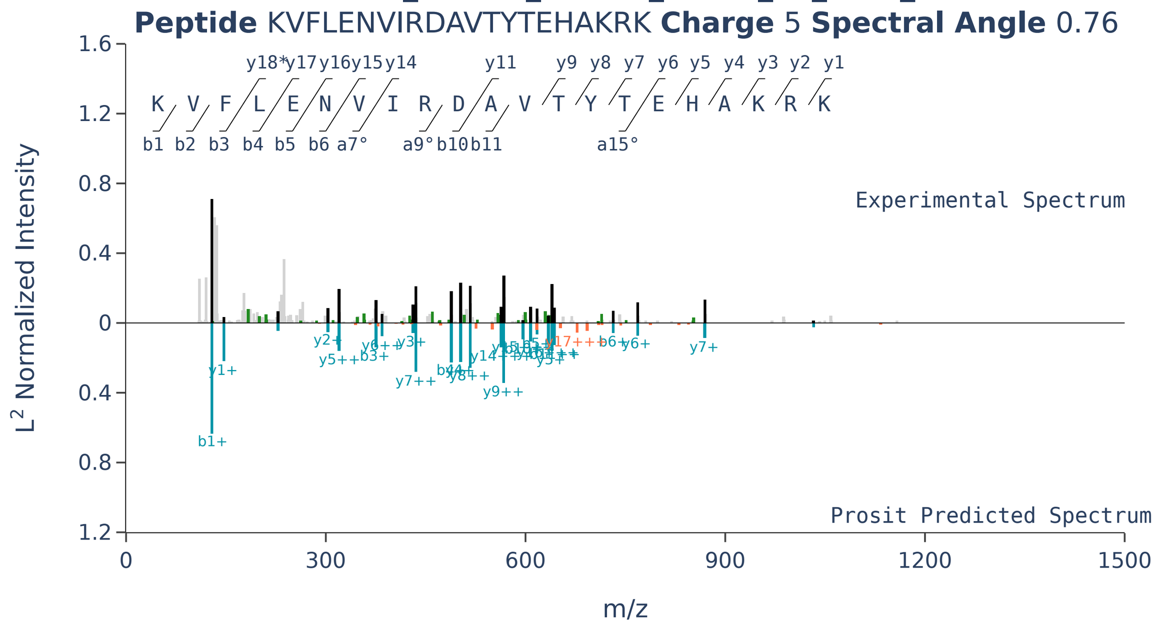


**Source** HRoetschke_291222_140123_HFGoe_H4_b2R2_1h **Scan** 24079
**Peptide** KVFLENVIRDAVTYTEHAKRKTVTA **Charge** 5 **Spectral Angle** 0.84

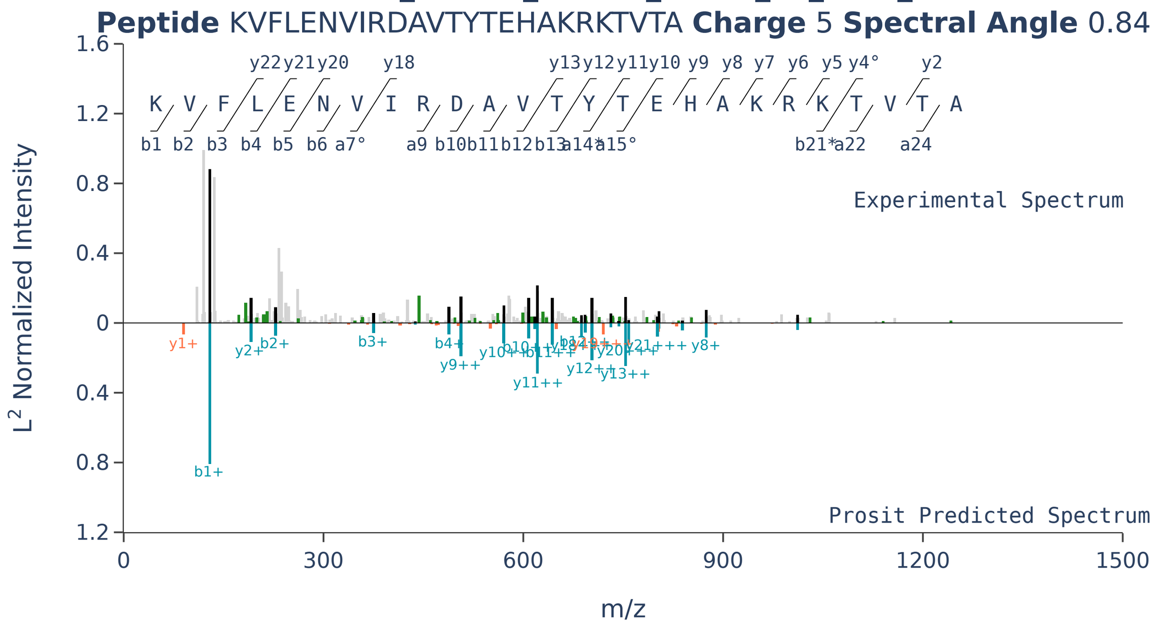


**Source** HRoetschke_291222_140123_HFGoe_H4_b1R1_24h **Scan** 13410
**Peptide** ARRGGVKRISGLIYEET **Charge** 3 **Spectral Angle** 0.57

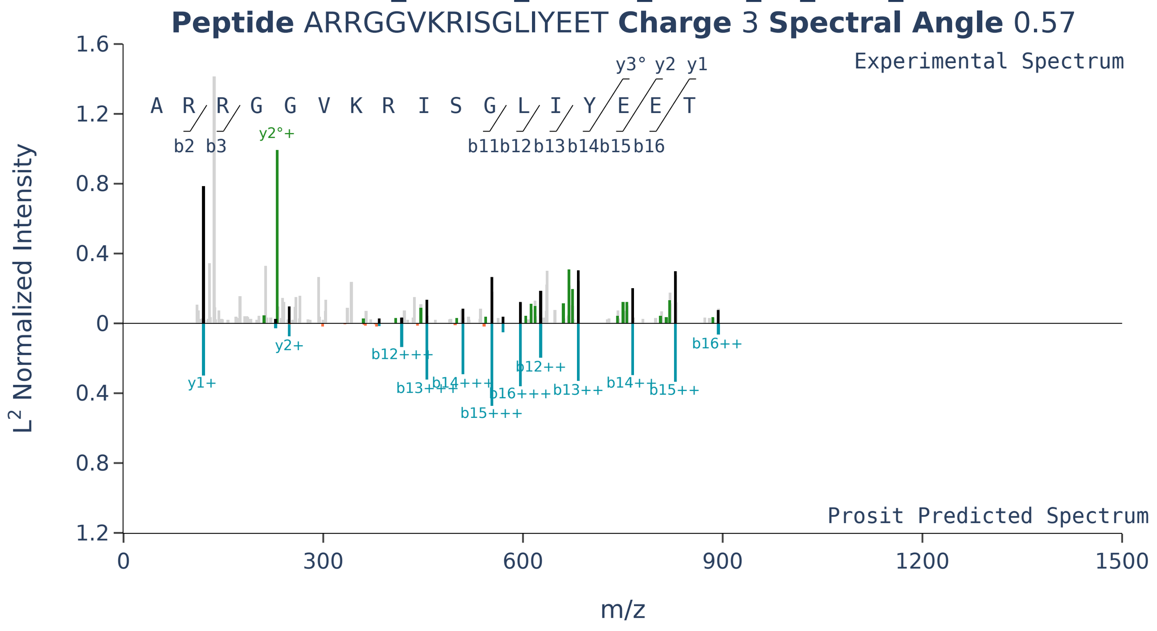


**Source** HRoetschke_291222_140123_HFGoe_H4_b1R1_2h **Scan** 5917
**Peptide** AVTYTEHAKRKTVTA **Charge** 2 **Spectral Angle** 0.74

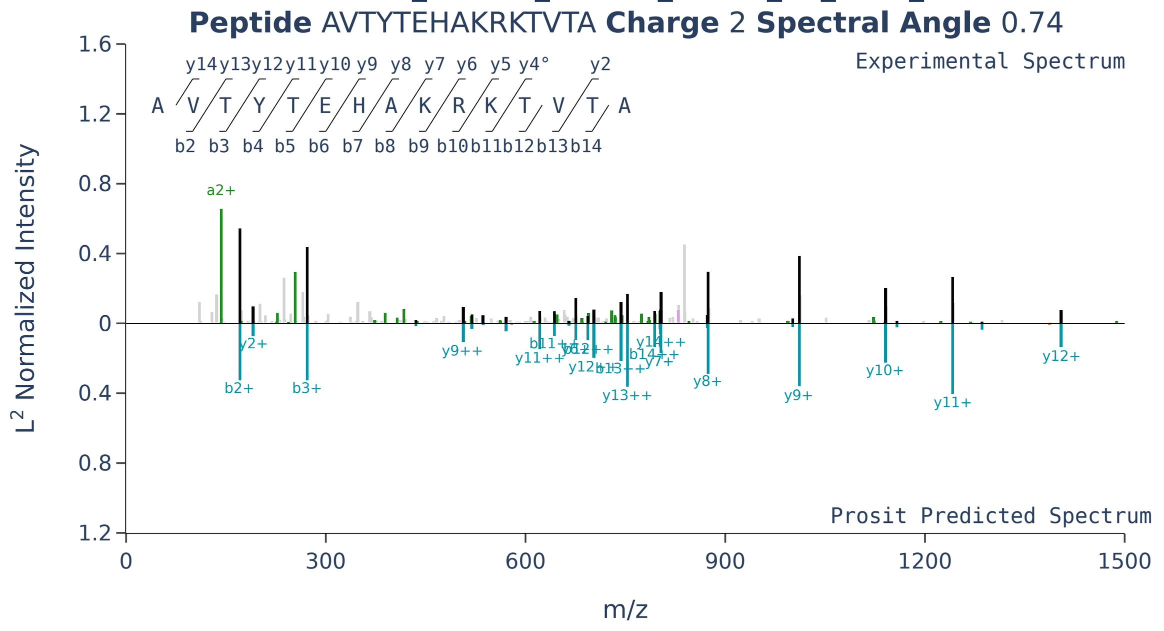


**Source** HRoetschke_291222_140123_HFGoe_H4_b1R1_2h **Scan** 13363
**Peptide** LARRGGVKRISGLIYEE **Charge** 3 **Spectral Angle** 0.73

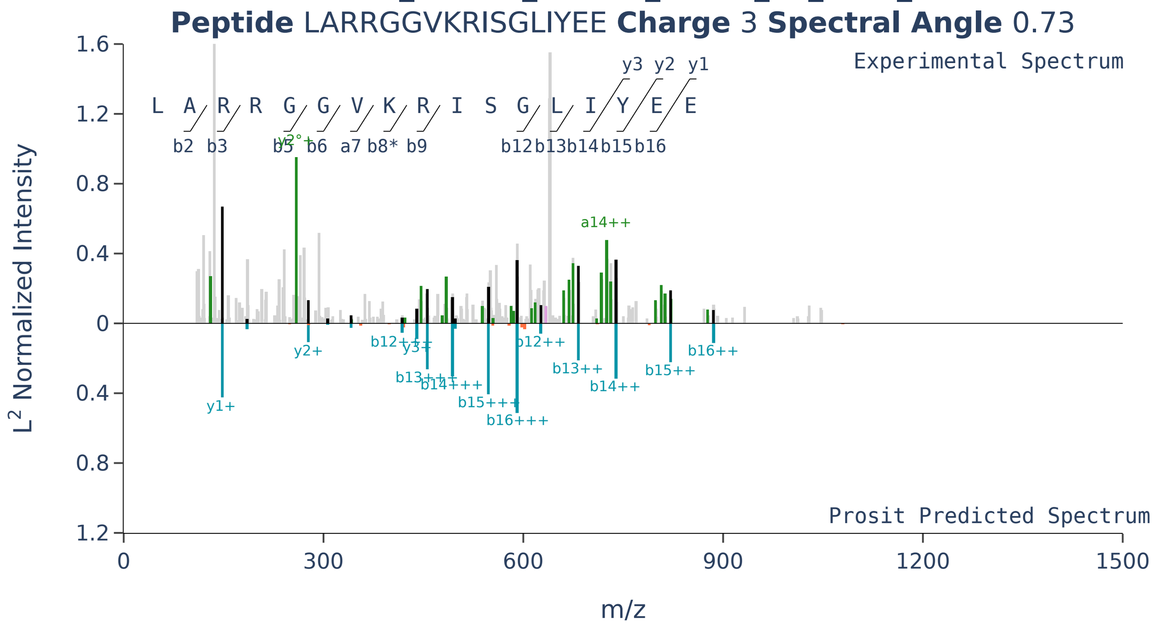


**Source** HRoetschke_291222_140123_HFGoe_H4_b2R1_1h **Scan** 17441
**Peptide** HAKRKTVTAMDVVYALKRQGRTL **Charge** 5 **Spectral Angle** 0.86

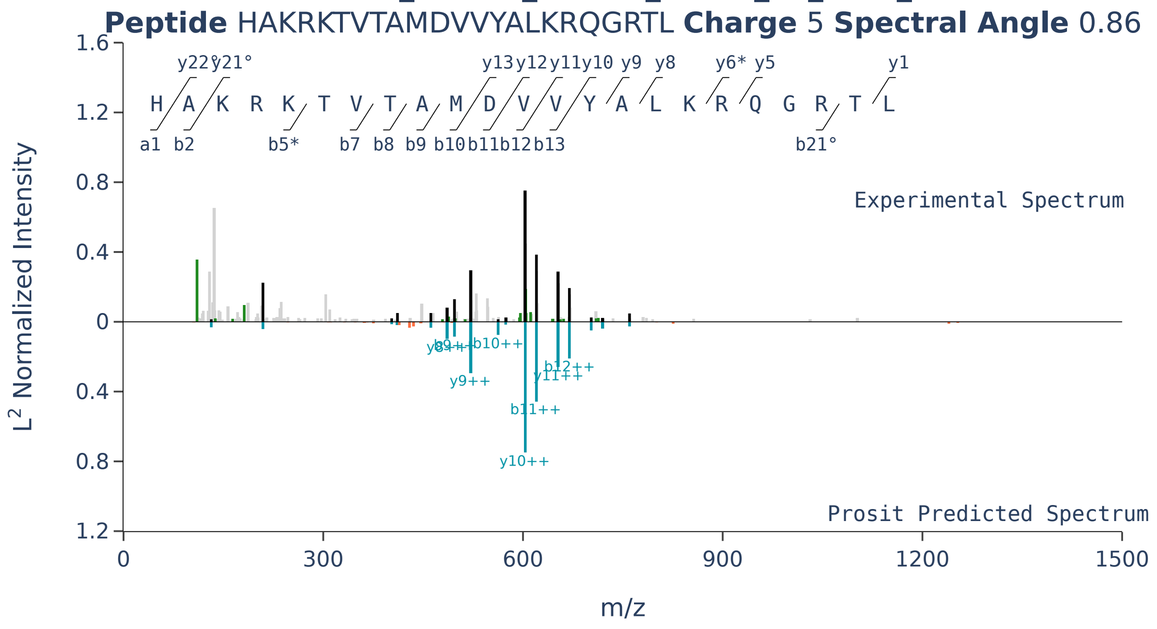


**Source** HRoetschke_291222_140123_HFGoe_H4_b1R1_1h **Scan** 14893
**Peptide** ARRGGVKRISGLIYEETRGV **Charge** 5 **Spectral Angle** 0.67

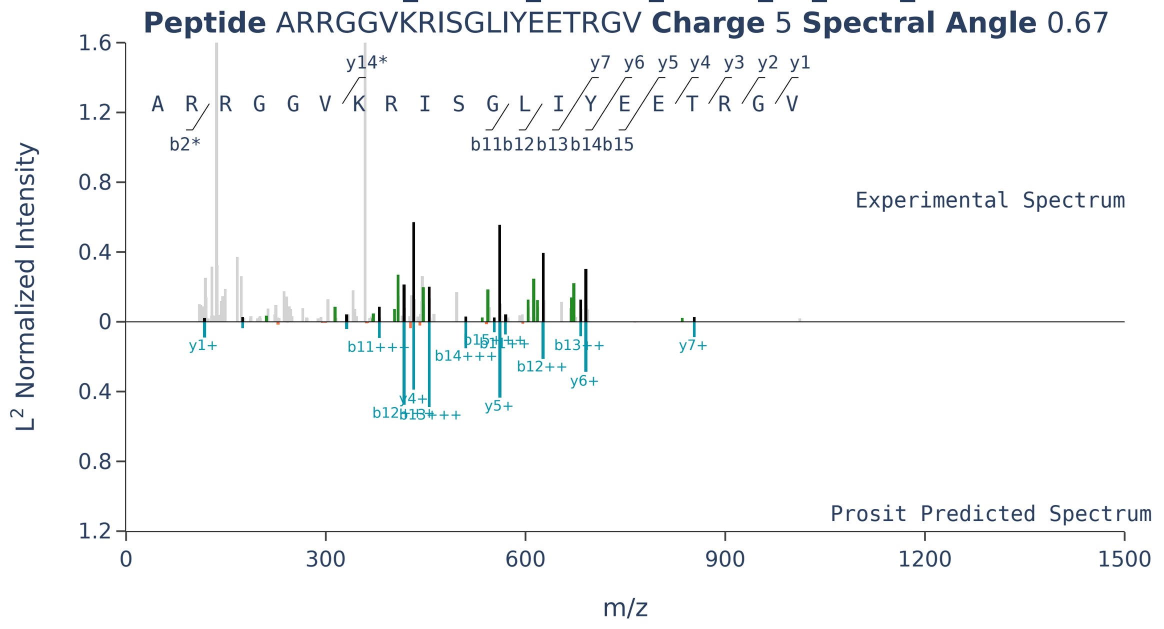


**Source** HRoetschke_291222_140123_HFGoe_H4_b2R1_1h **Scan** 26165
**Peptide** GVKRISGLIYEETRGVLKVF **Charge** 3 **Spectral Angle** 0.73

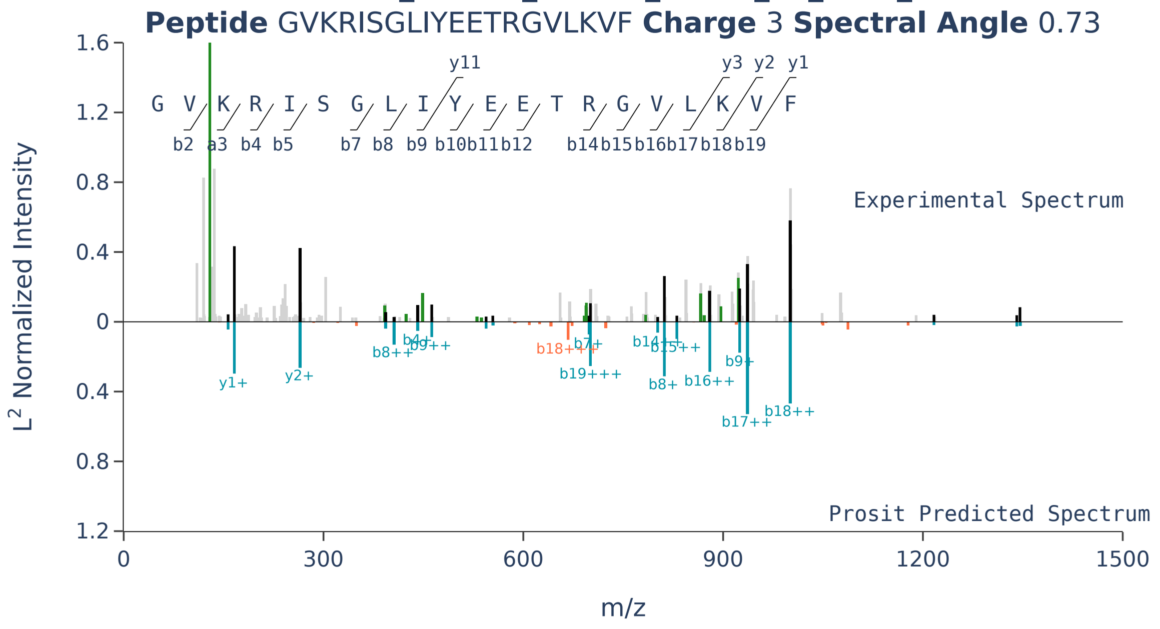


**Source** HRoetschke_291222_140123_HFGoe_H4_b1R2_2h **Scan** 10984
**Peptide** RDNIQGL **Charge** 2 **Spectral Angle** 0.88

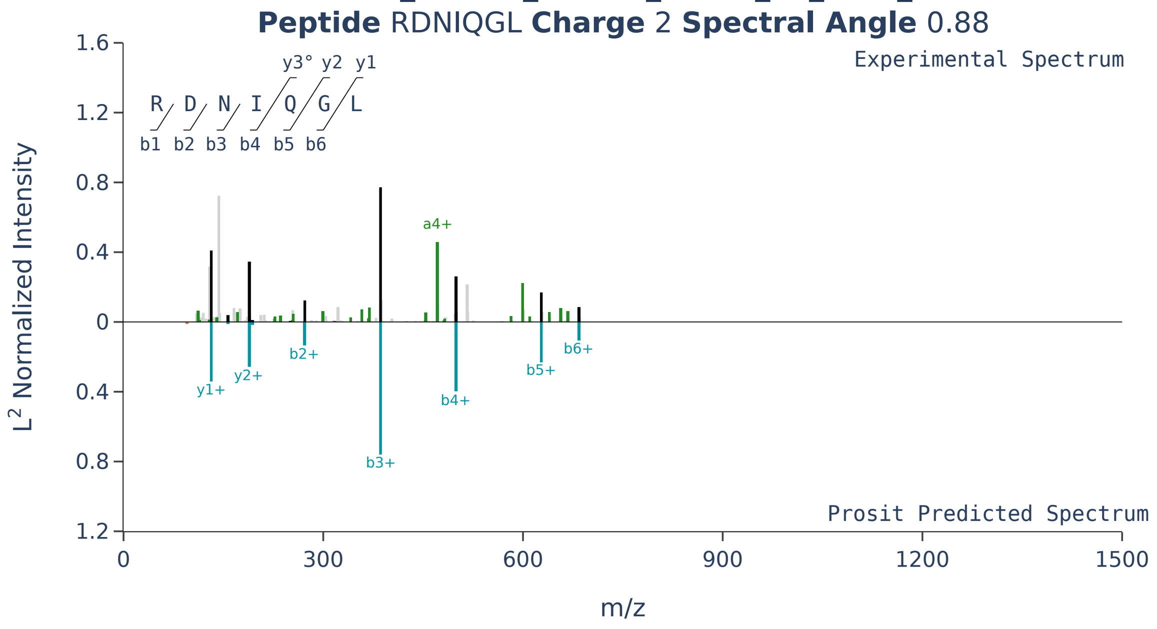


**Source** HRoetschke_291222_140123_HFGoe_H4_b1R2_1h **Scan** 25837
**Peptide** RGVLKVFLENVLRD **Charge** 4 **Spectral Angle** 0.79

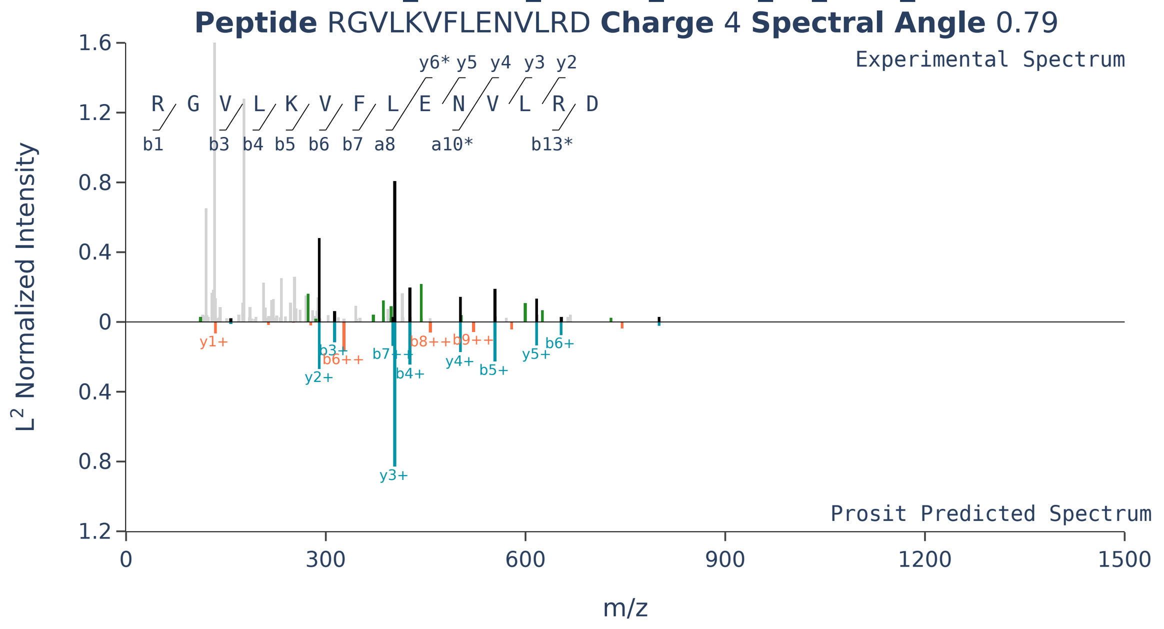


**Source** HRoetschke_291222_140123_HFGoe_H4_b2R2_1h **Scan** 28949
**Peptide** EETRGVLKVFLENVIRD **Charge** 3 **Spectral Angle** 0.85

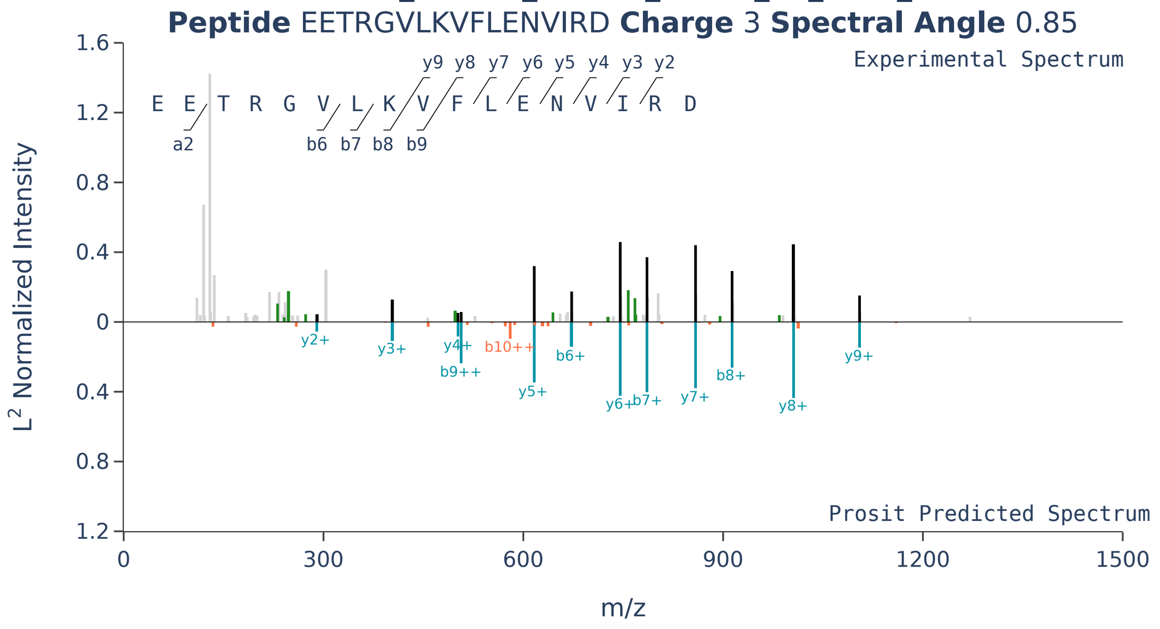

**Source** HRoetschke_291222_140123_HFGoe_H4_b2R2_0h **Scan** 17223
**Peptide** KRISGLIYEETRGVL **Charge** 3 **Spectral Angle** 0.84

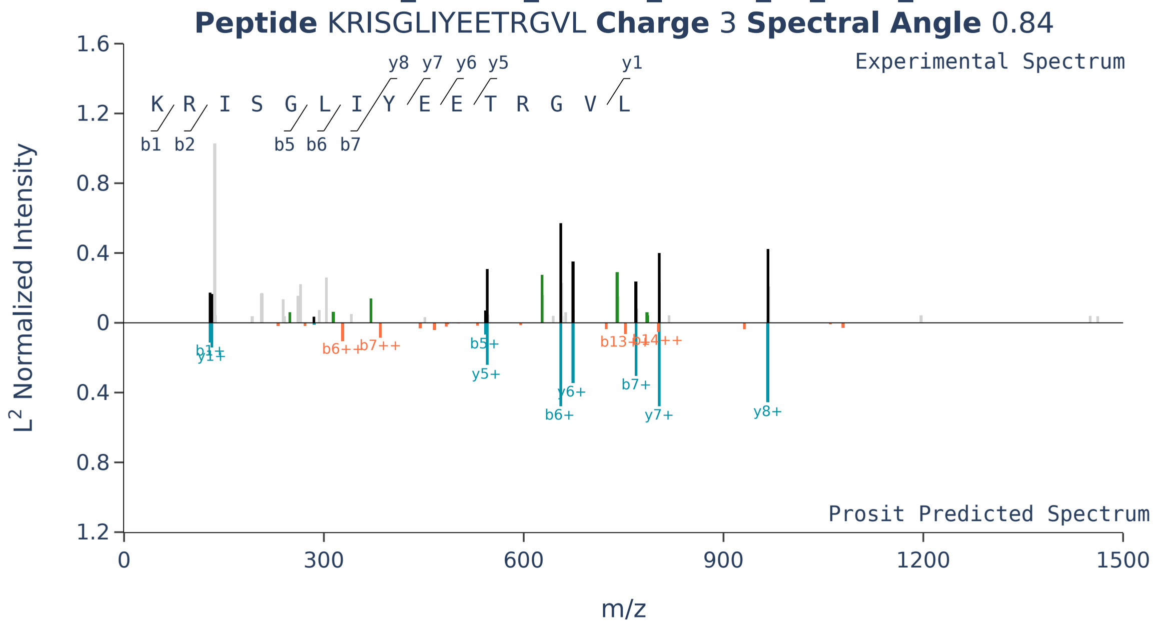


**Source** HRoetschke_291222_140123_HFGoe_H4_b1R1_24h **Scan** 6324
**Peptide** KGLGKGGAKRHRKVLRDNIQGL **Charge** 4 **Spectral Angle** 0.84

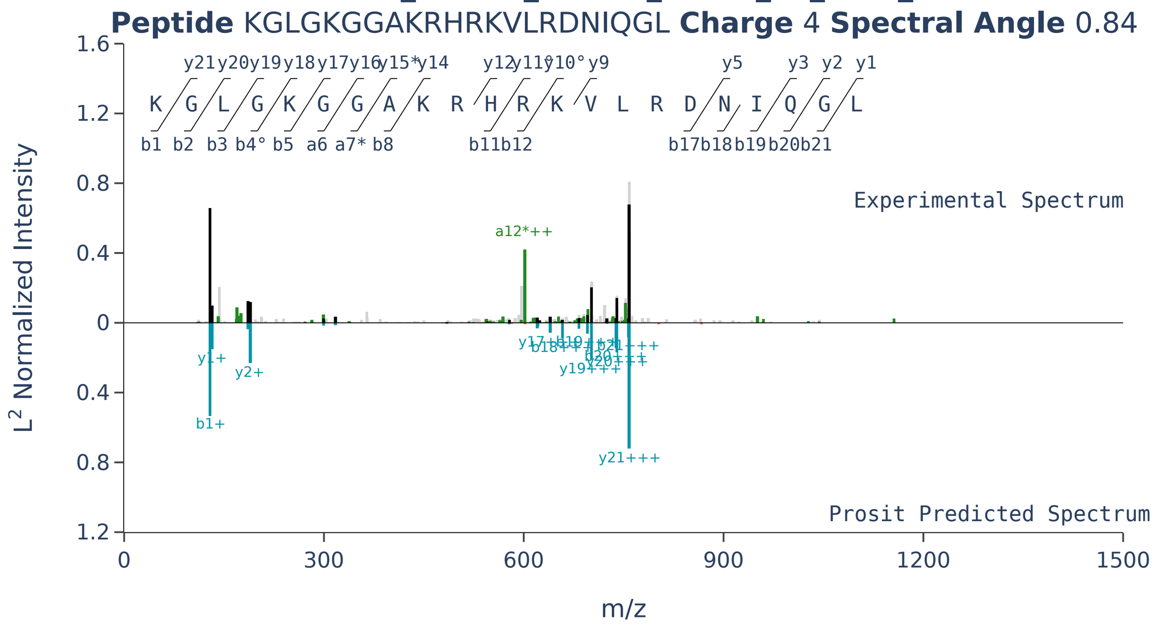


**Source** HRoetschke_291222_140123_HFGoe_H4_b1R1_1h **Scan** 22600
**Peptide** IYEETRGVLKVF **Charge** 2 **Spectral Angle** 0.78

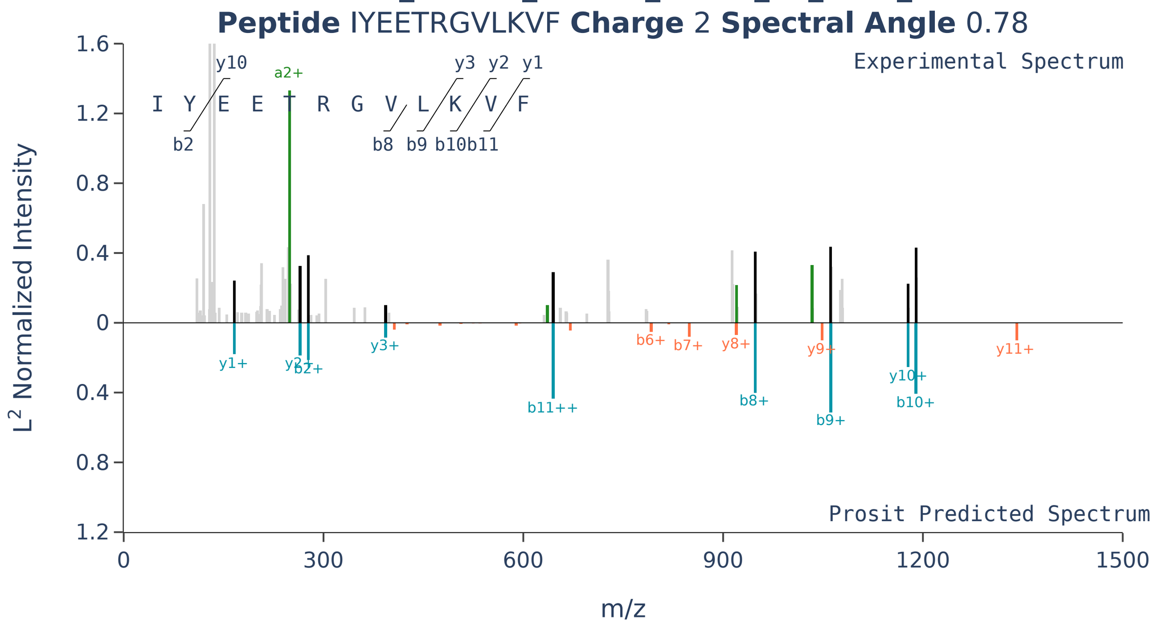


**Source** HRoetschke_291222_140123_HFGoe_H4_b1R1_24h **Scan** 13265
**Peptide** RDNIQGITKPAIRRLA **Charge** 4 **Spectral Angle** 0.76

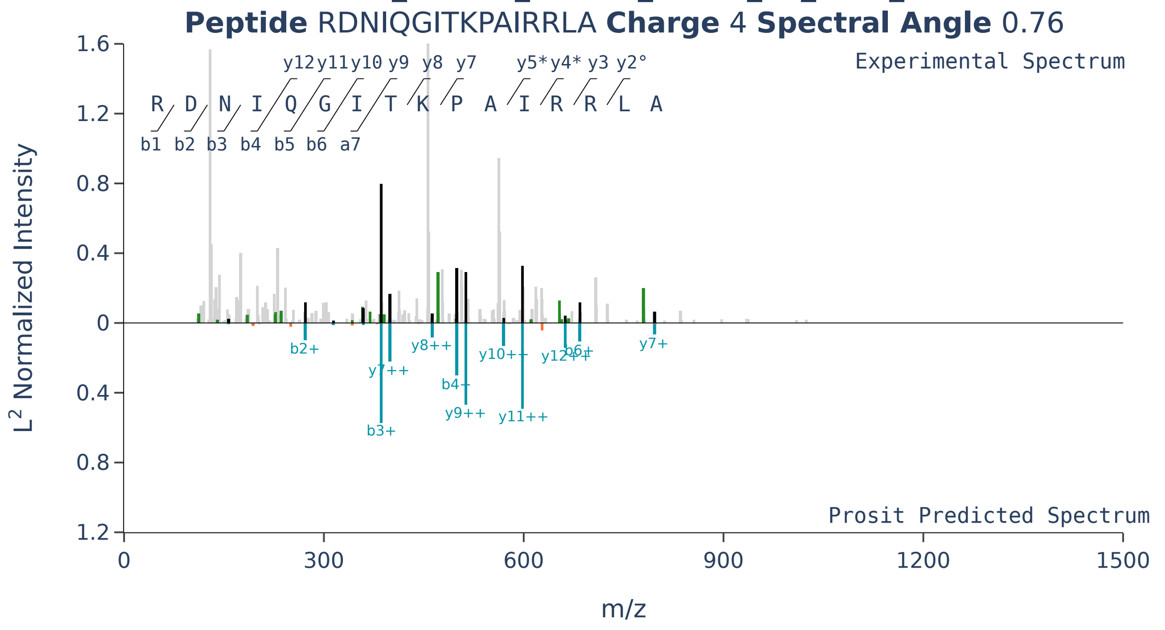


**Source** HRoetschke_291222_140123_HFGoe_H4_b1R1_4h **Scan** 11666
**Peptide** KVLRDNIQGITKPAIRRLARR **Charge** 4 **Spectral Angle** 0.83

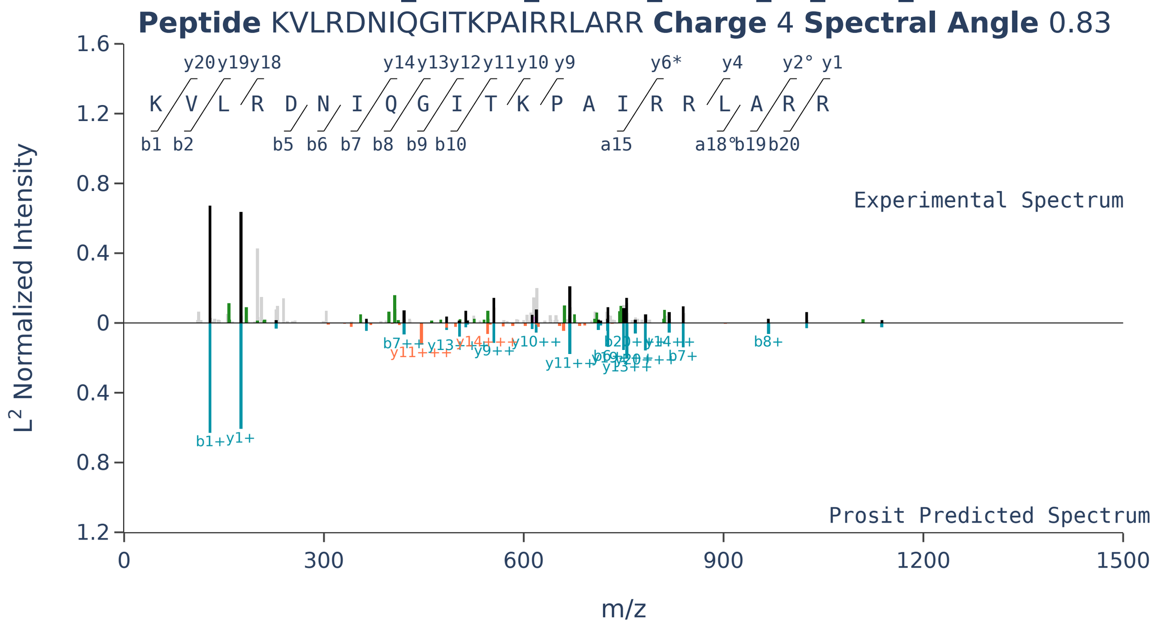


**Source** HRoetschke_291222_140123_HFGoe_H4_b1R1_1h **Scan** 16105
**Peptide** YALKRQGRTLYGF **Charge** 3 **Spectral Angle** 0.62

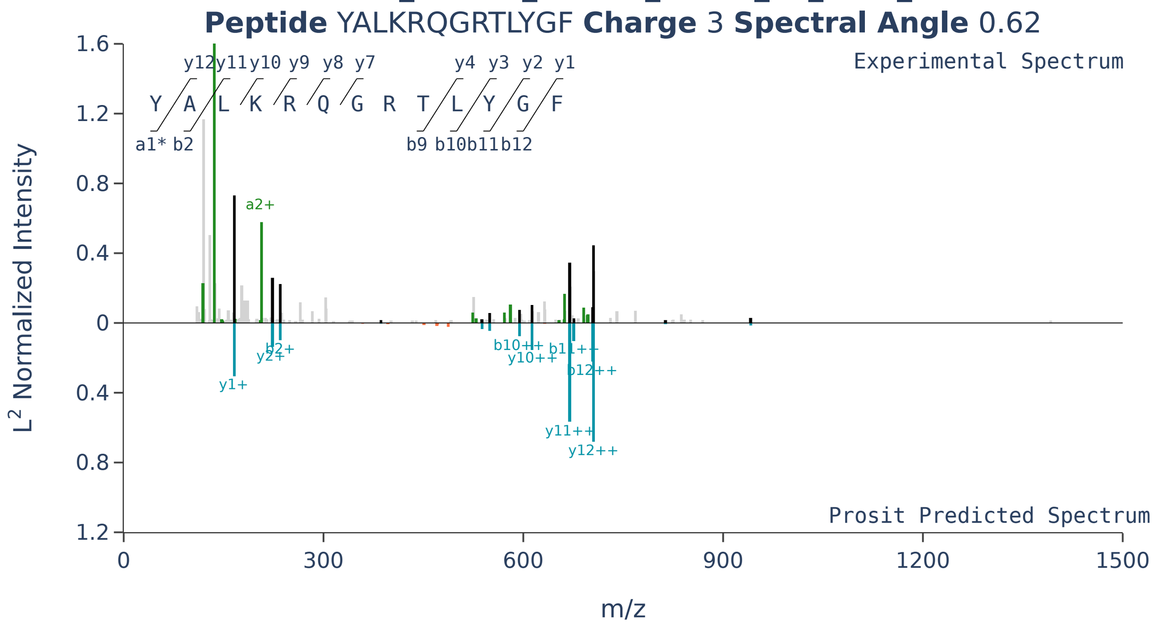


**Source** HRoetschke_291222_140123_HFGoe_H4_b1R1_4h **Scan** 15954
**Peptide** VLRDNIQGITKPAIRRL **Charge** 4 **Spectral Angle** 0.67

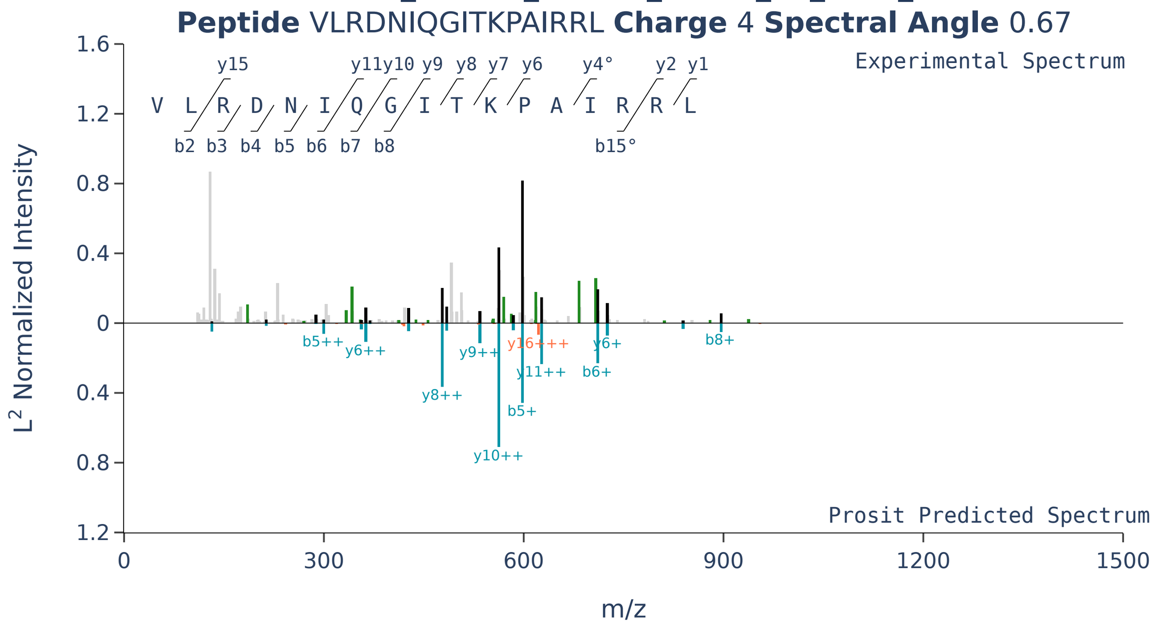


**Source** HRoetschke_291222_140123_HFGoe_H4_b2R1_24h **Scan** 8254
**Peptide** HRKVLRDNIQGITKPAIRR **Charge** 5 **Spectral Angle** 0.74

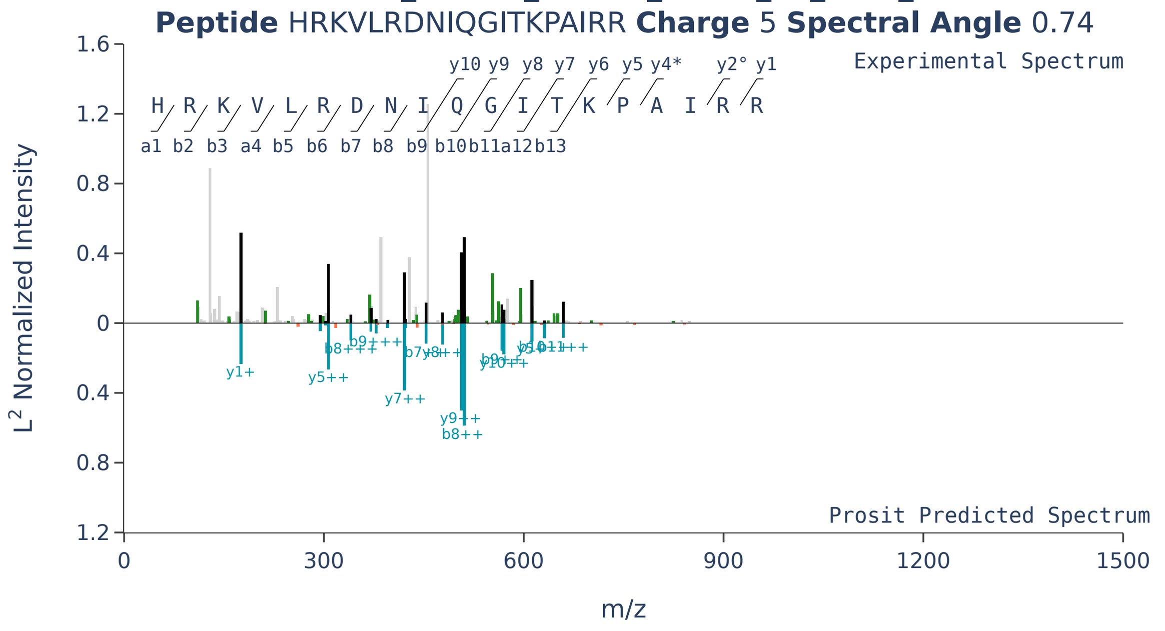


**Source** HRoetschke_291222_140123_HFGoe_H4_b1R1_24h **Scan** 11353
**Peptide** RRGGVKRISGLIYE **Charge** 3 **Spectral Angle** 0.69

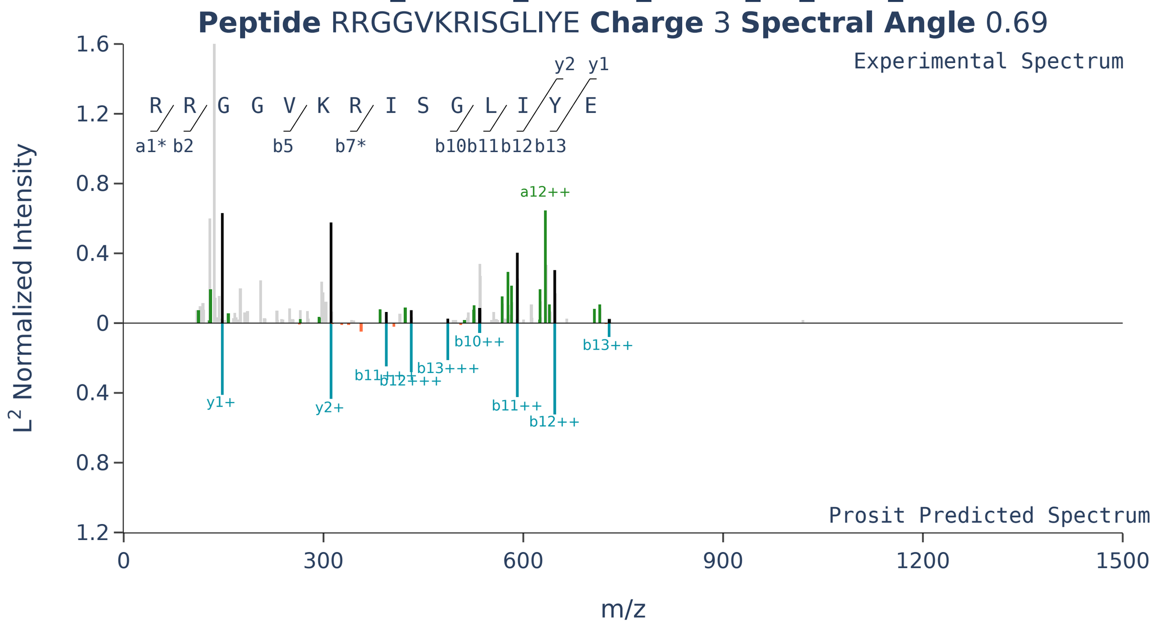


**Source** HRoetschke_291222_140123_HFGoe_H4_b1R2_4h **Scan** 17184
**Peptide** GGVKRISGLIY **Charge** 2 **Spectral Angle** 0.71

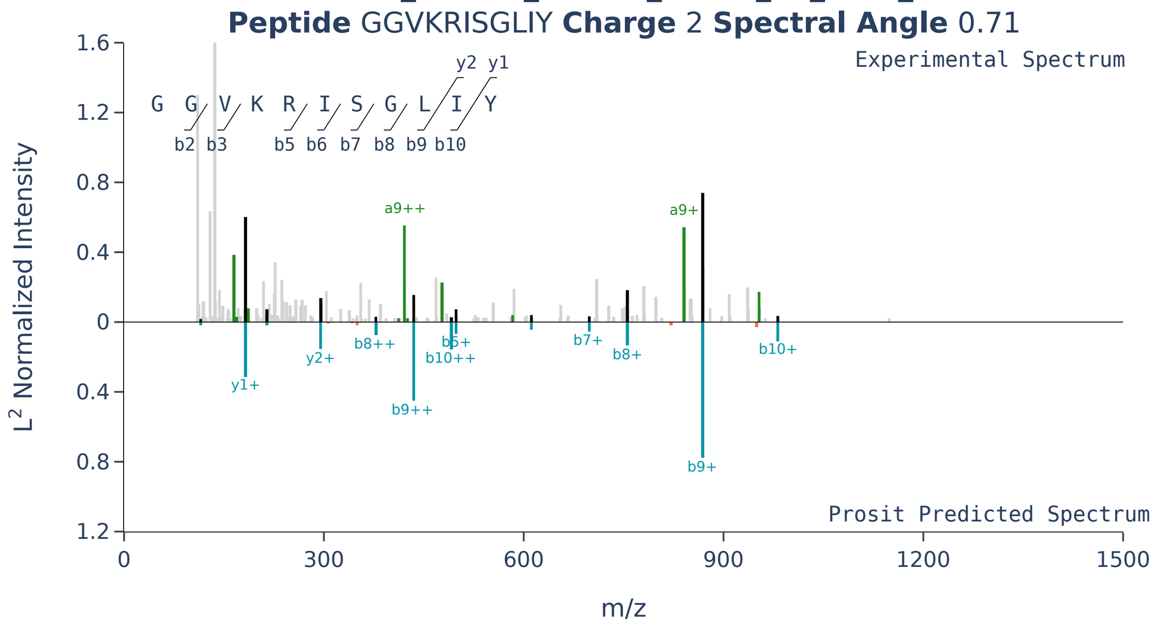


**Source** HRoetschke_291222_140123_HFGoe_H4_b1R2_24h **Scan** 14558
**Peptide** RGGVKRISGLIYEET **Charge** 3 **Spectral Angle** 0.61

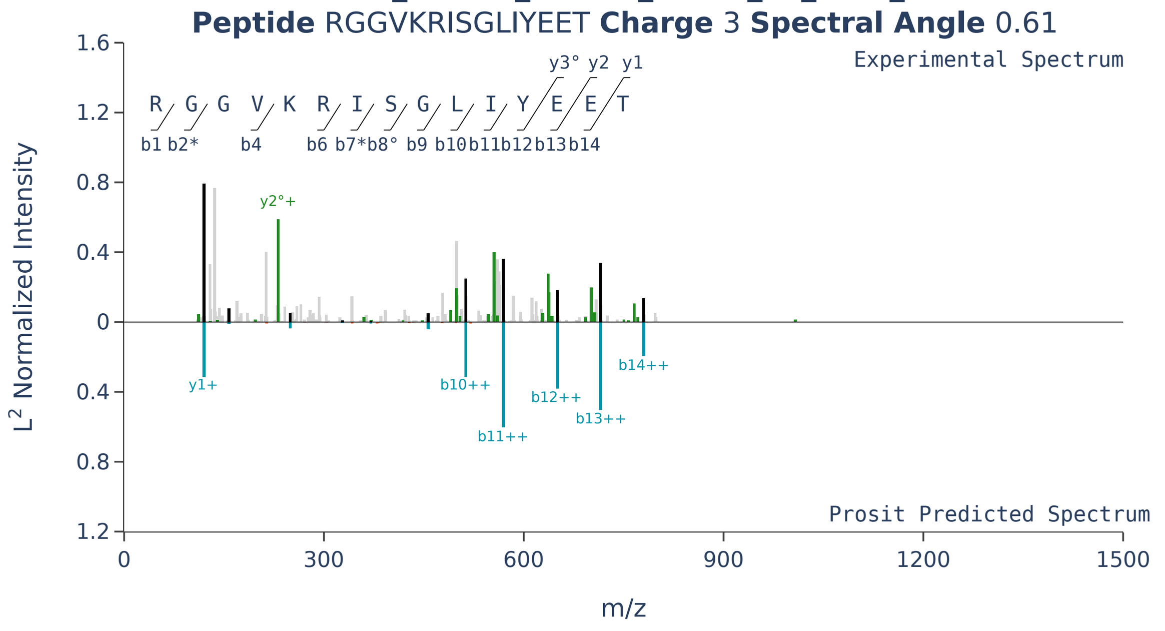


**Source** HRoetschke_291222_140123_HFGoe_H4_b2R1_1h **Scan** 32019
**Peptide** YEETRGVLKVFLENVI **Charge** 3 **Spectral Angle** 0.7

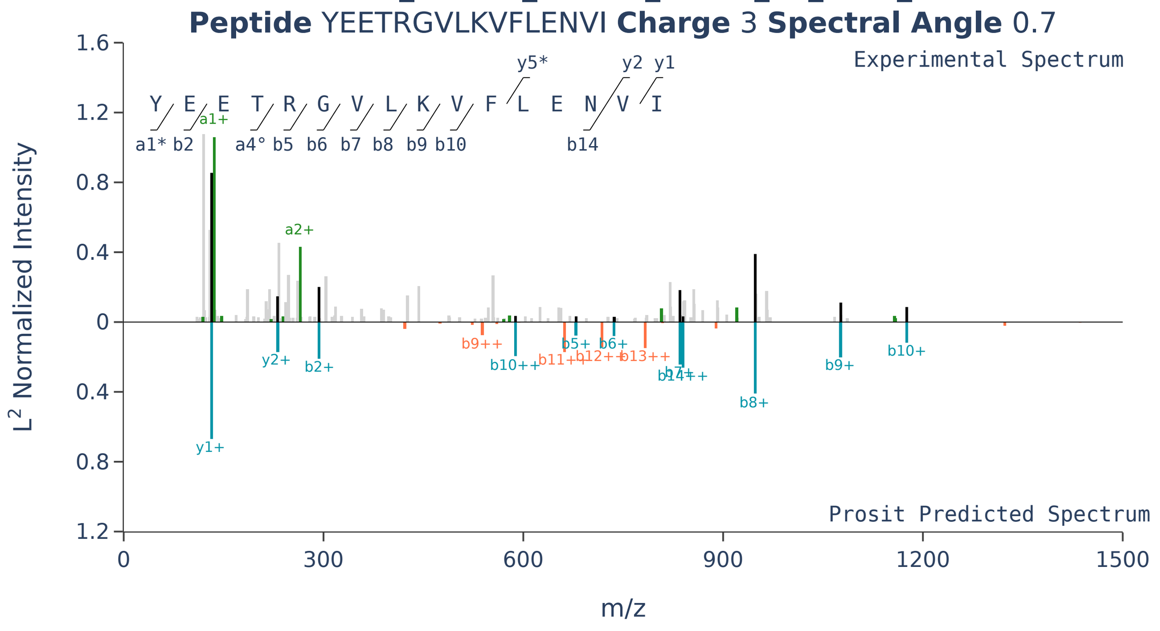


**Source** HRoetschke_291222_140123_HFGoe_H4_b1R1_4h **Scan** 6166
**Peptide** DAVTYTEHAKR **Charge** 3 **Spectral Angle** 0.71

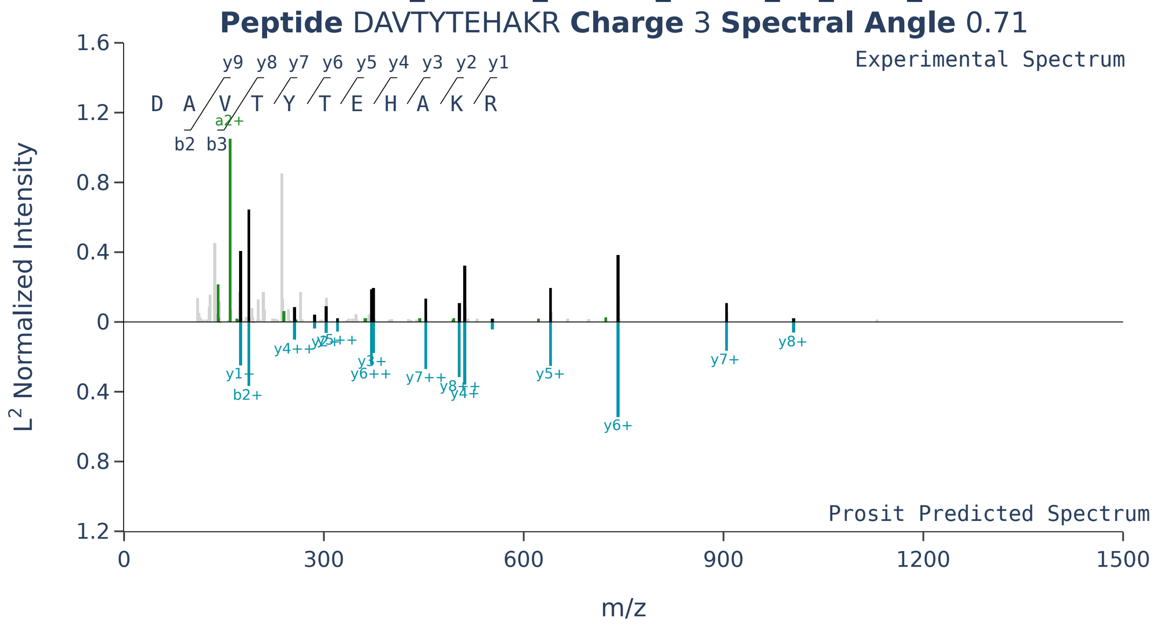


**Source** HRoetschke_291222_140123_HFGoe_H4_b2R2_24h **Scan** 12902
**Peptide** DNIQGIT **Charge** 1 **Spectral Angle** 0.87

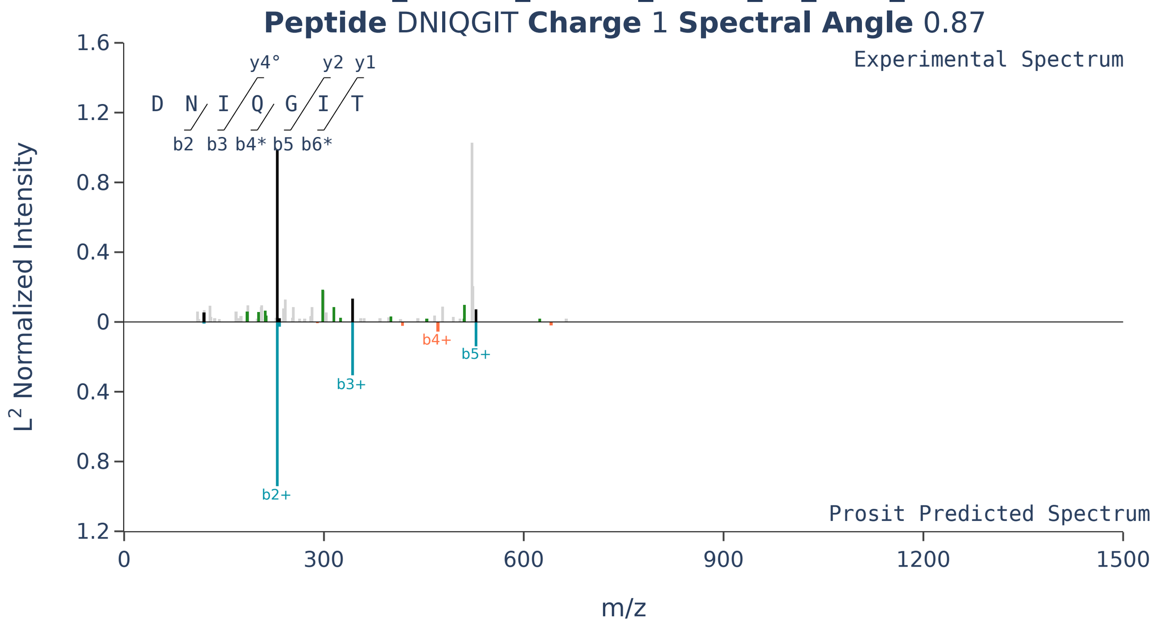


**Source** HRoetschke_291222_140123_HFGoe_H4_b2R1_1h **Scan** 22179
**Peptide** TAMDVVYALKRQGRTLYGFGG **Charge** 3 **Spectral Angle** 0.75

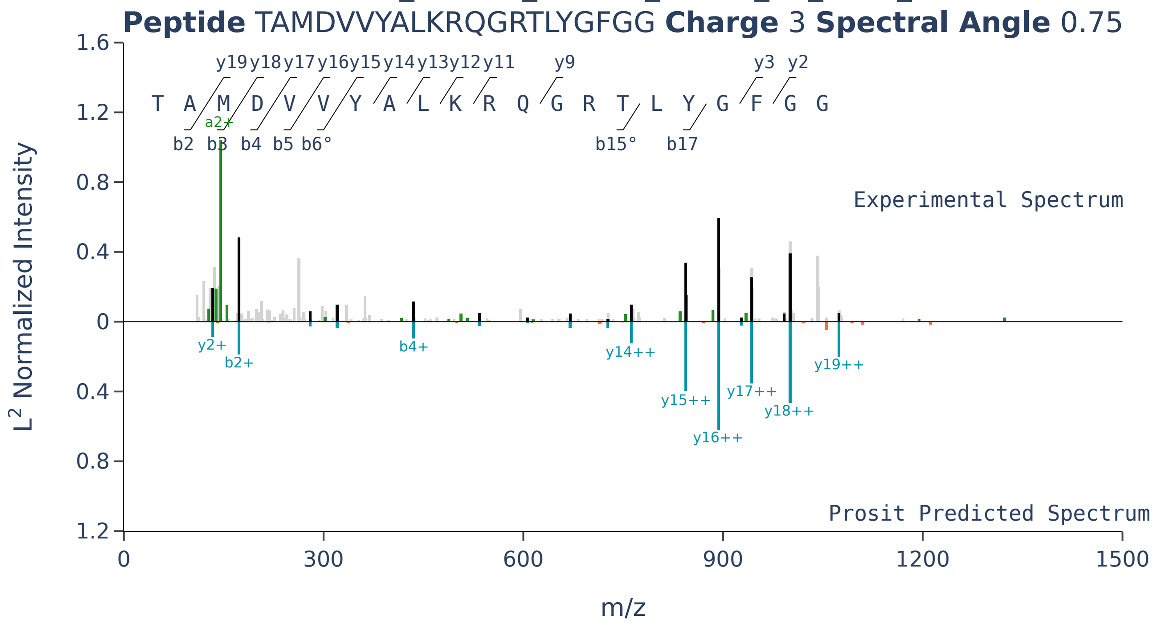

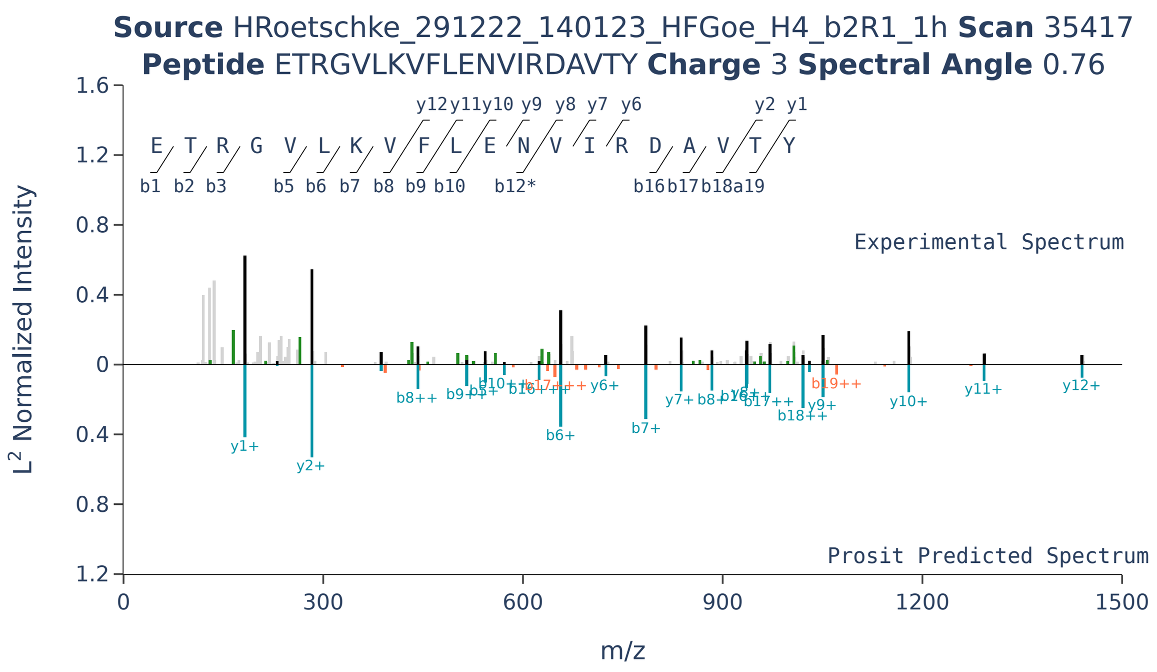
**Source** HRoetschke_291222_140123_HFGoe_H4_b2R1_1h **Scan** 35417
**Peptide** ETRGVLKVFLENVIRDAVTY **Charge** 3 **Spectral Angle** 0.76
Experimental Spectrum
Prosit Predicted Spectrum

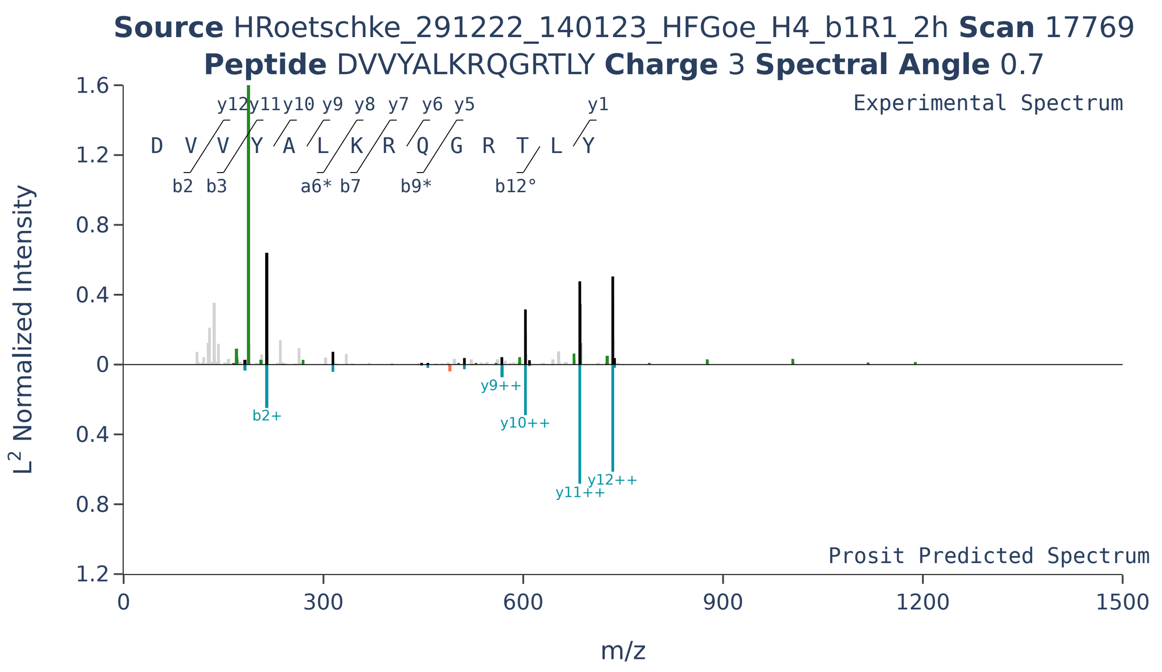
**Source** HRoetschke_291222_140123_HFGoe_H4_b1R1_2h **Scan** 17769
**Peptide** DVVYALKRQGRTLY **Charge** 3 **Spectral Angle** 0.7
Experimental Spectrum
Prosit Predicted Spectrum

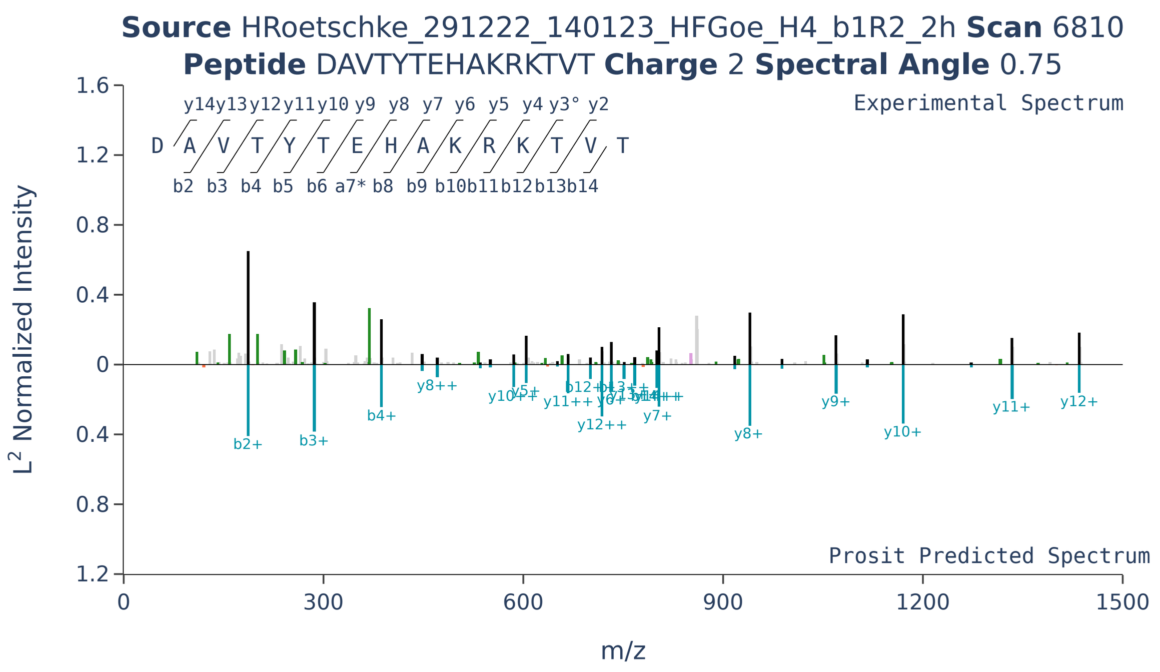
**Source** HRoetschke_291222_140123_HFGoe_H4_b1R2_2h **Scan** 6810
**Peptide** DAVTYTEHAKRKTVT **Charge** 2 **Spectral Angle** 0.75
Experimental Spectrum
Prosit Predicted Spectrum

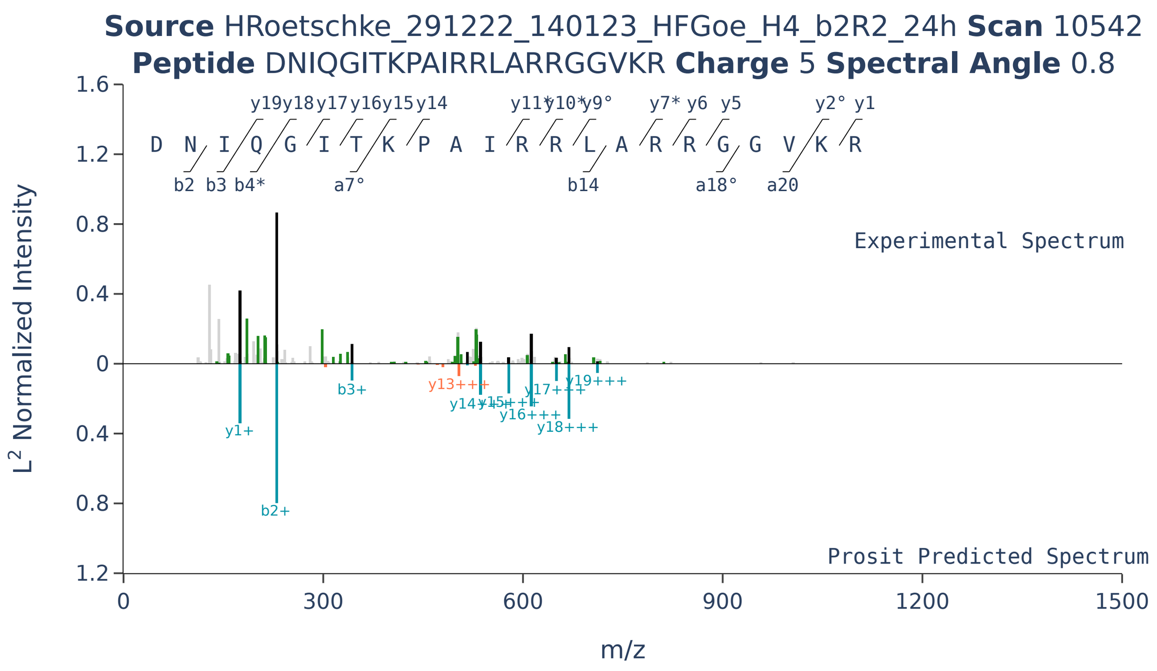
**Source** HRoetschke_291222_140123_HFGoe_H4_b2R2_24h **Scan** 10542
**Peptide** DNIQGITKPAIRRLARRGGVKR **Charge** 5 **Spectral Angle** 0.8
Experimental Spectrum
Prosit Predicted Spectrum

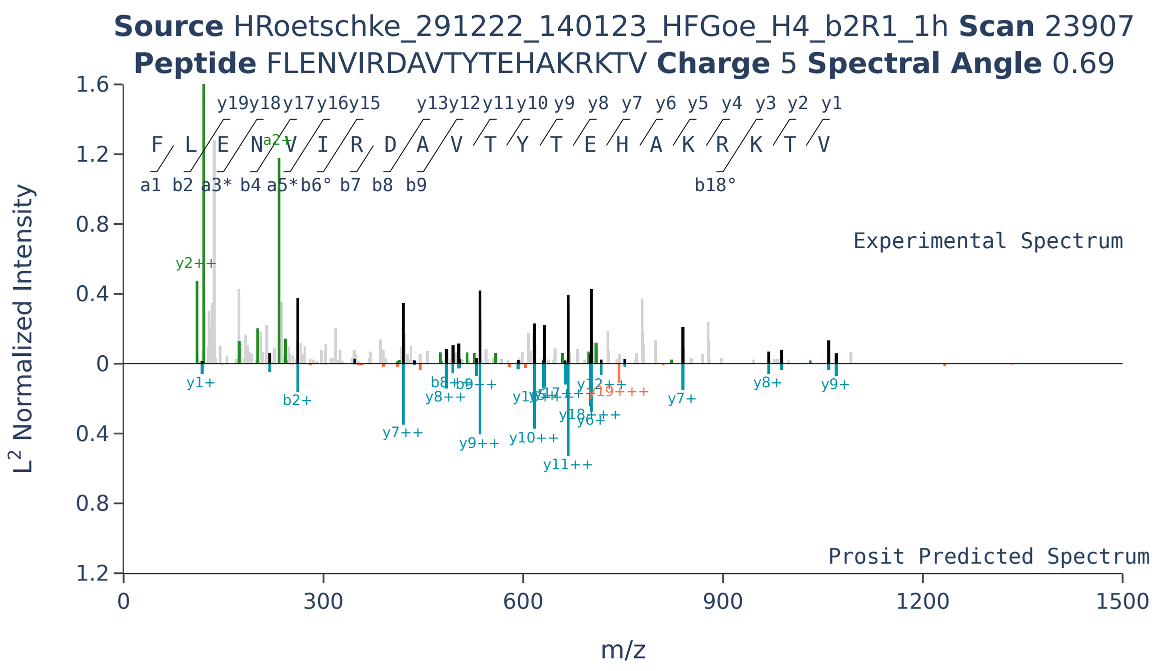
**Source** HRoetschke_291222_140123_HFGoe_H4_b2R1_1h **Scan** 23907
**Peptide** FLENVIRDAVTYTEHAKRKTV **Charge** 5 **Spectral Angle** 0.69
Experimental Spectrum
Prosit Predicted Spectrum

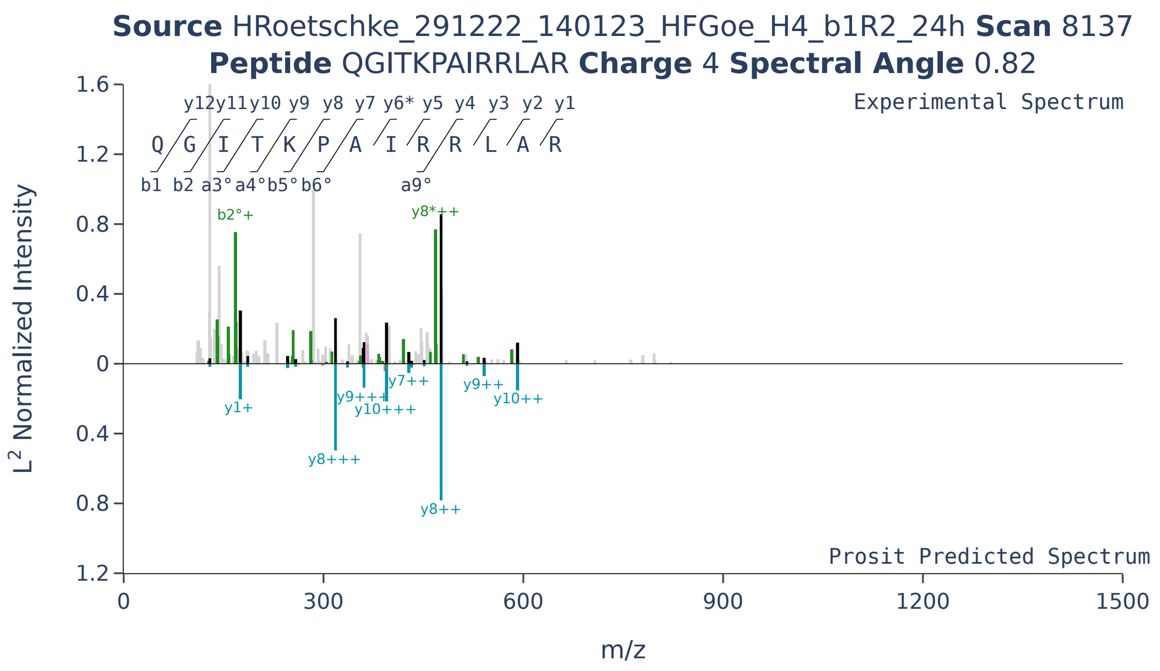
**Source** HRoetschke_291222_140123_HFGoe_H4_b1R2_24h **Scan** 8137
**Peptide** QGITKPAIRRLAR **Charge** 4 **Spectral Angle** 0.82
Experimental Spectrum
Prosit Predicted Spectrum

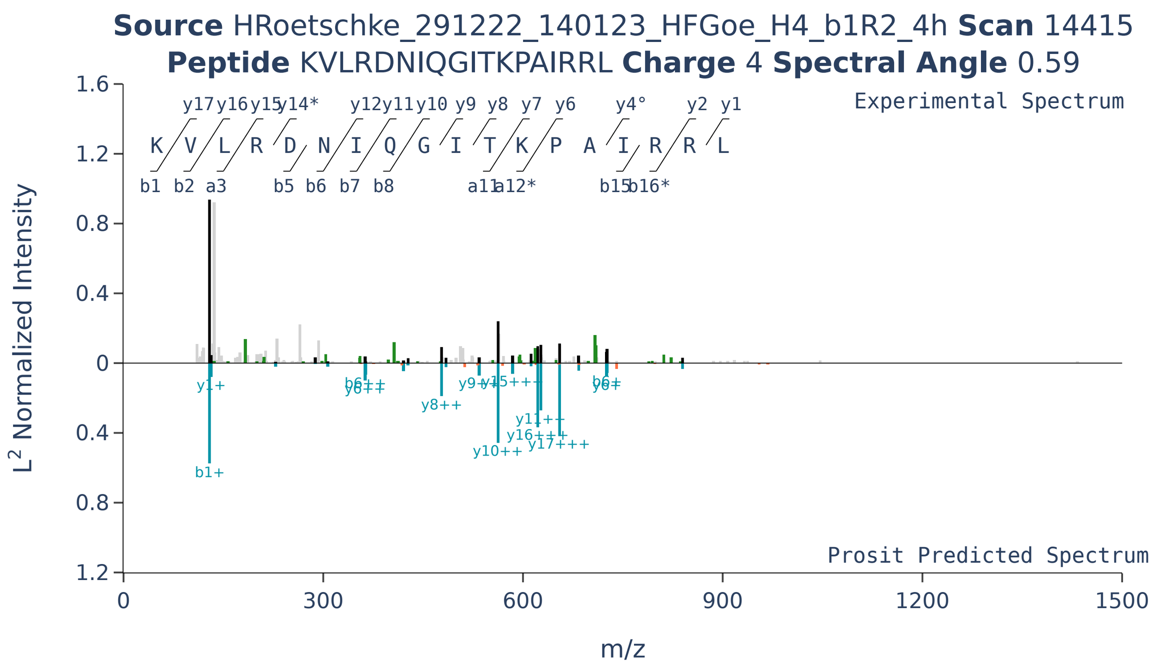
**Source** HRoetschke_291222_140123_HFGoe_H4_b1R2_4h **Scan** 14415
**Peptide** KVLRDNIQGITKPAIRRL **Charge** 4 **Spectral Angle** 0.59
Experimental Spectrum
Prosit Predicted Spectrum

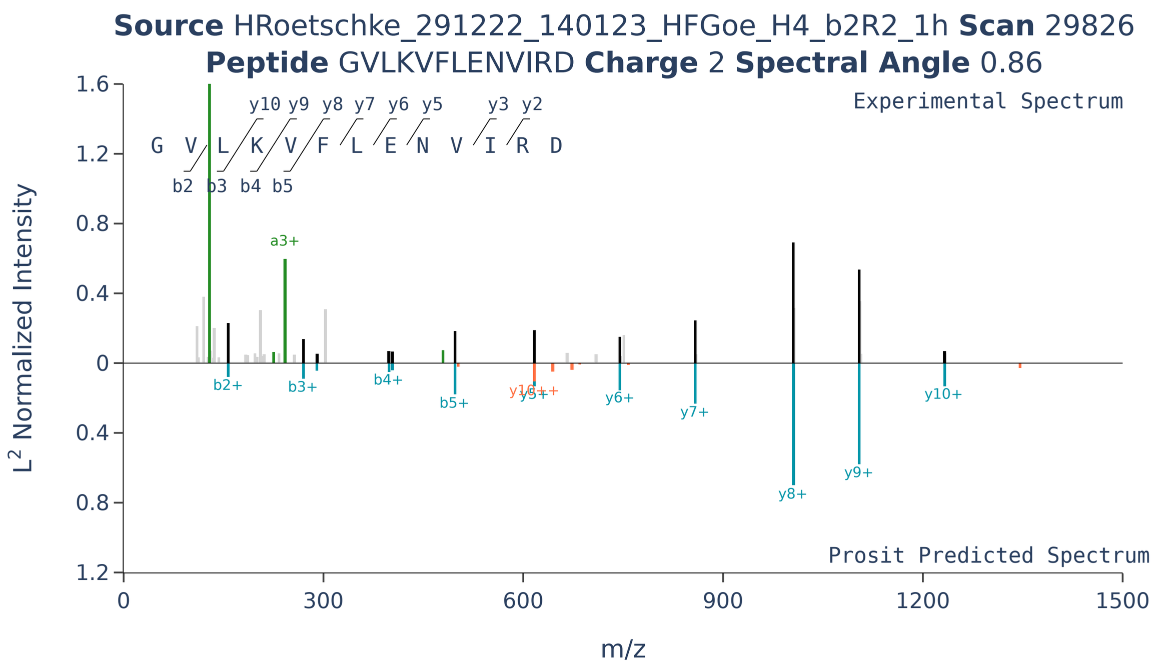
**Source** HRoetschke_291222_140123_HFGoe_H4_b2R2_1h **Scan** 29826
**Peptide** GVLKVFLENVIRD **Charge** 2 **Spectral Angle** 0.86
Experimental Spectrum
Prosit Predicted Spectrum

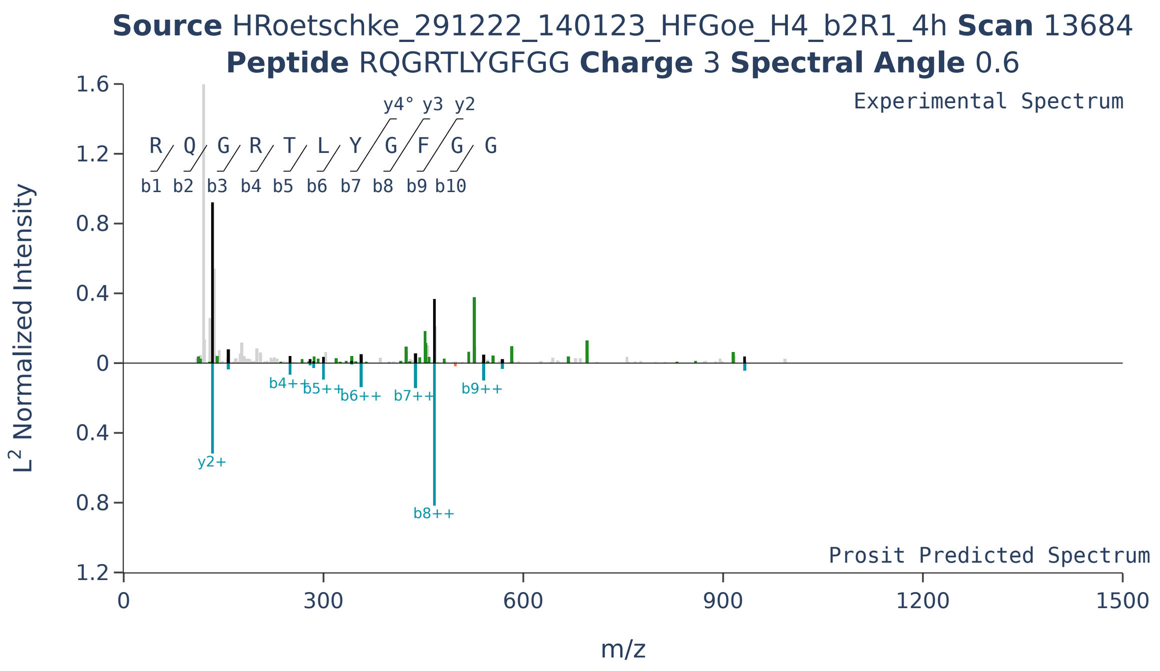
**Source** HRoetschke_291222_140123_HFGoe_H4_b2R1_4h **Scan** 13684
**Peptide** RQGRTLYGFGG **Charge** 3 **Spectral Angle** 0.6
Experimental Spectrum
Prosit Predicted Spectrum

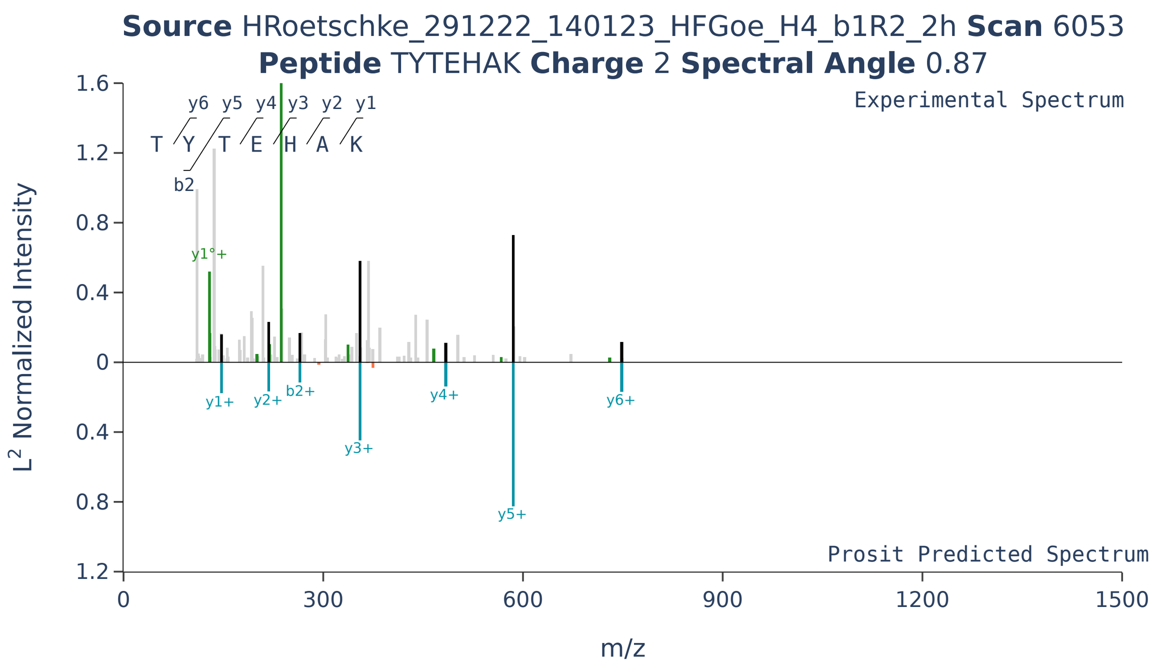
**Source** HRoetschke_291222_140123_HFGoe_H4_b1R2_2h **Scan** 6053
**Peptide** TYTEHAK **Charge** 2 **Spectral Angle** 0.87
Experimental Spectrum
Prosit Predicted Spectrum

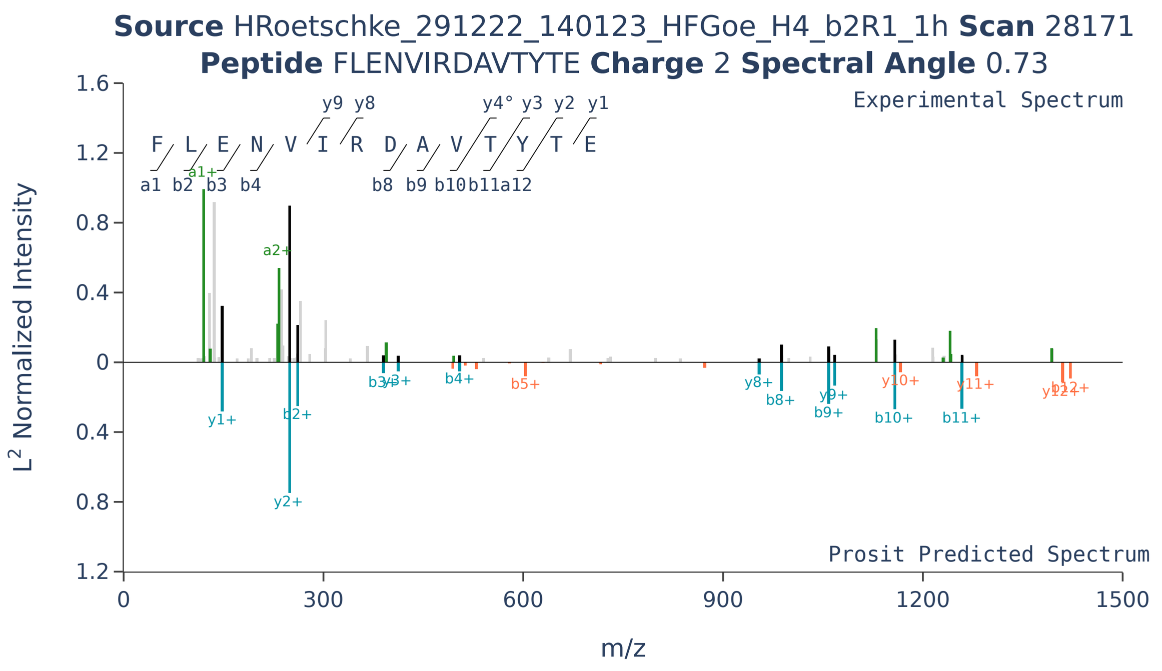
**Source** HRoetschke_291222_140123_HFGoe_H4_b2R1_1h **Scan** 28171
**Peptide** FLENVIRDAVTYTE **Charge** 2 **Spectral Angle** 0.73
Experimental Spectrum
Prosit Predicted Spectrum

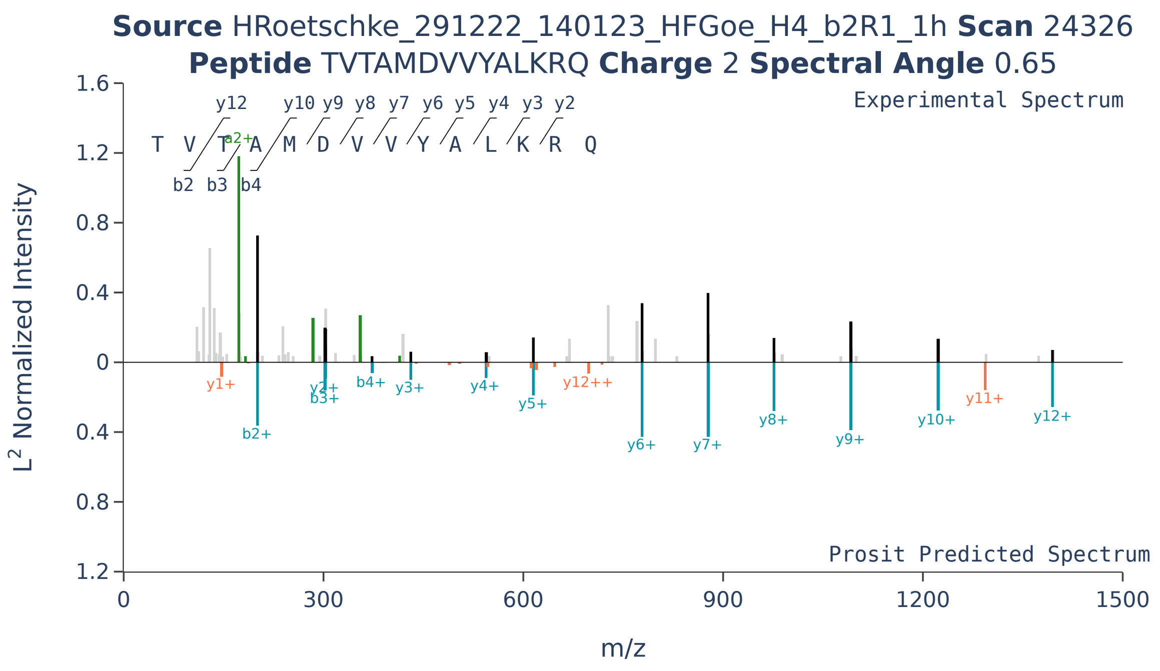
**Source** HRoetschke_291222_140123_HFGoe_H4_b2R1_1h **Scan** 24326
**Peptide** TVTAMDVVYALKRQ **Charge** 2 **Spectral Angle** 0.65
Experimental Spectrum
Prosit Predicted Spectrum

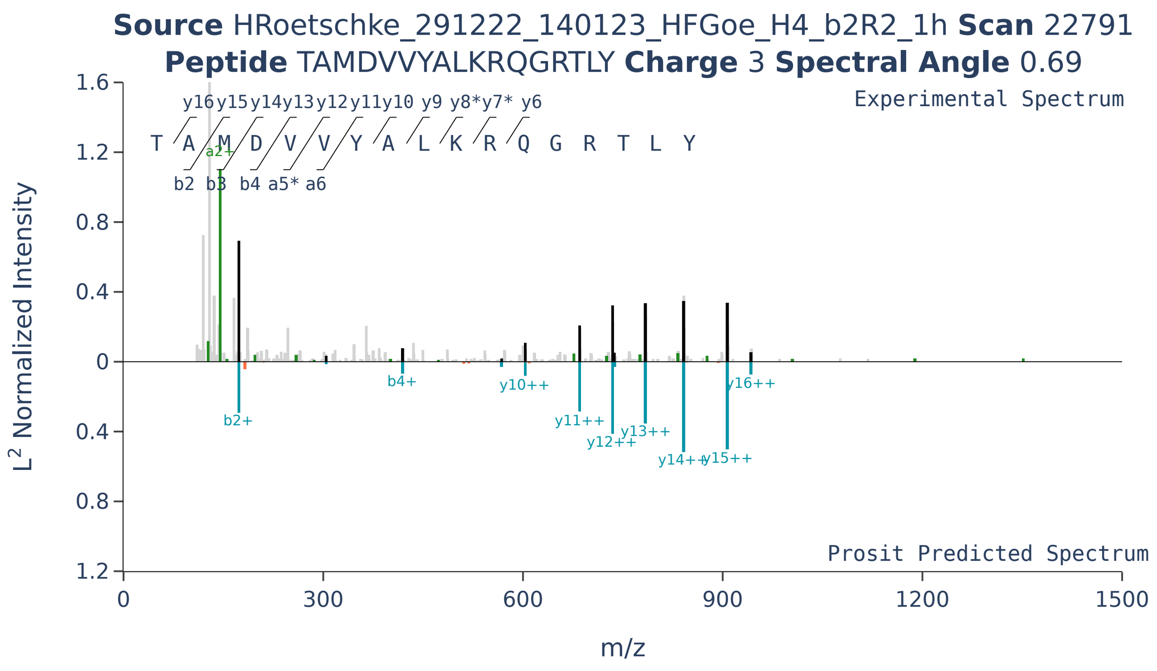
**Source** HRoetschke_291222_140123_HFGoe_H4_b2R2_1h **Scan** 22791
**Peptide** TAMDVVYALKRQGRTLY **Charge** 3 **Spectral Angle** 0.69
Experimental Spectrum
Prosit Predicted Spectrum

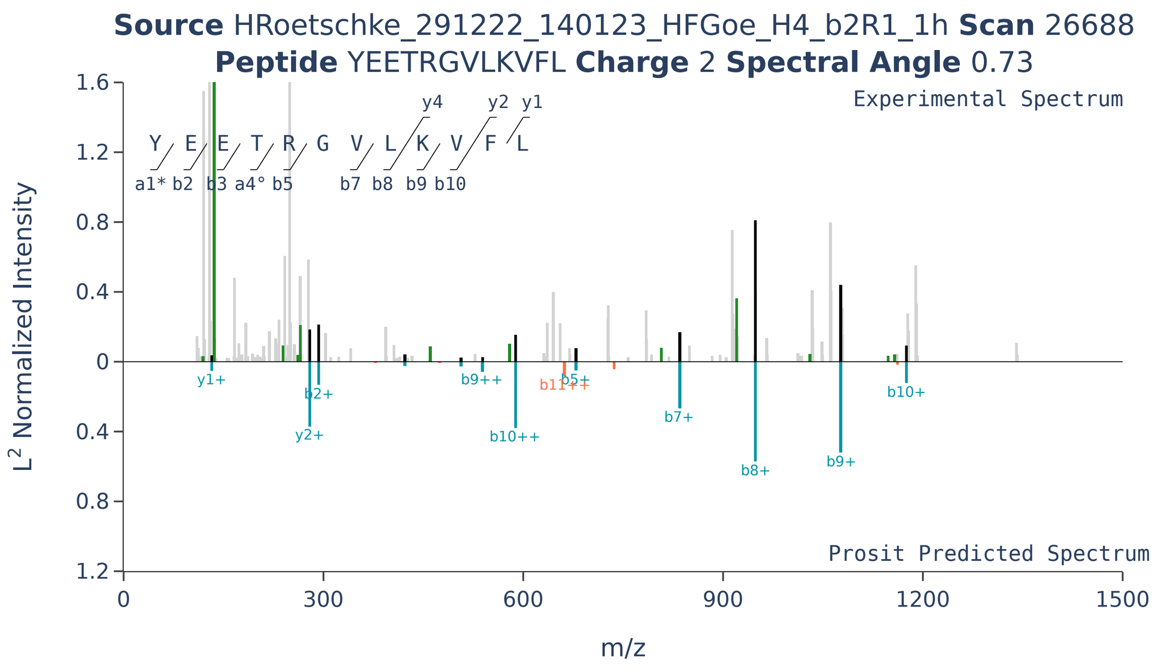
**Source** HRoetschke_291222_140123_HFGoe_H4_b2R1_1h **Scan** 26688
**Peptide** YEETRGVLKVFL **Charge** 2 **Spectral Angle** 0.73
Experimental Spectrum
Prosit Predicted Spectrum

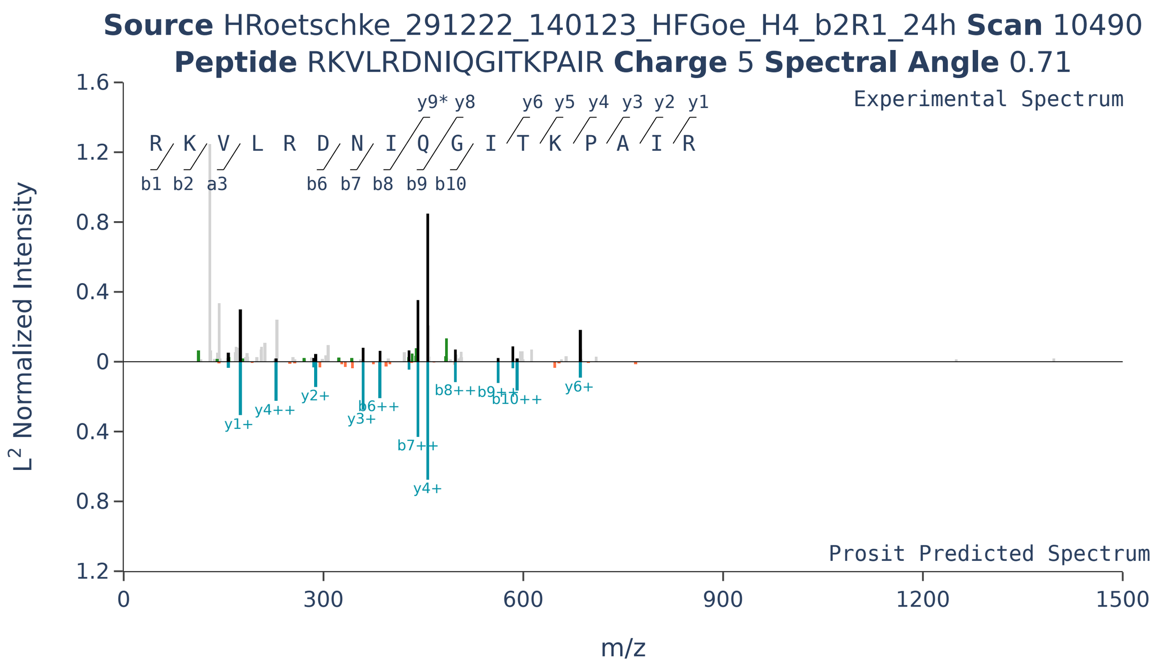
**Source** HRoetschke_291222_140123_HFGoe_H4_b2R1_24h **Scan** 10490
**Peptide** RKVLRDNIQGITKPAIR **Charge** 5 **Spectral Angle** 0.71
Experimental Spectrum
Prosit Predicted Spectrum

**Source** HRoetschke_291222_140123_HFGoe_H4_b1R2_24h **Scan** 5508
**Peptide** DAVTYTEHAKRK **Charge** 4 **Spectral Angle** 0.7

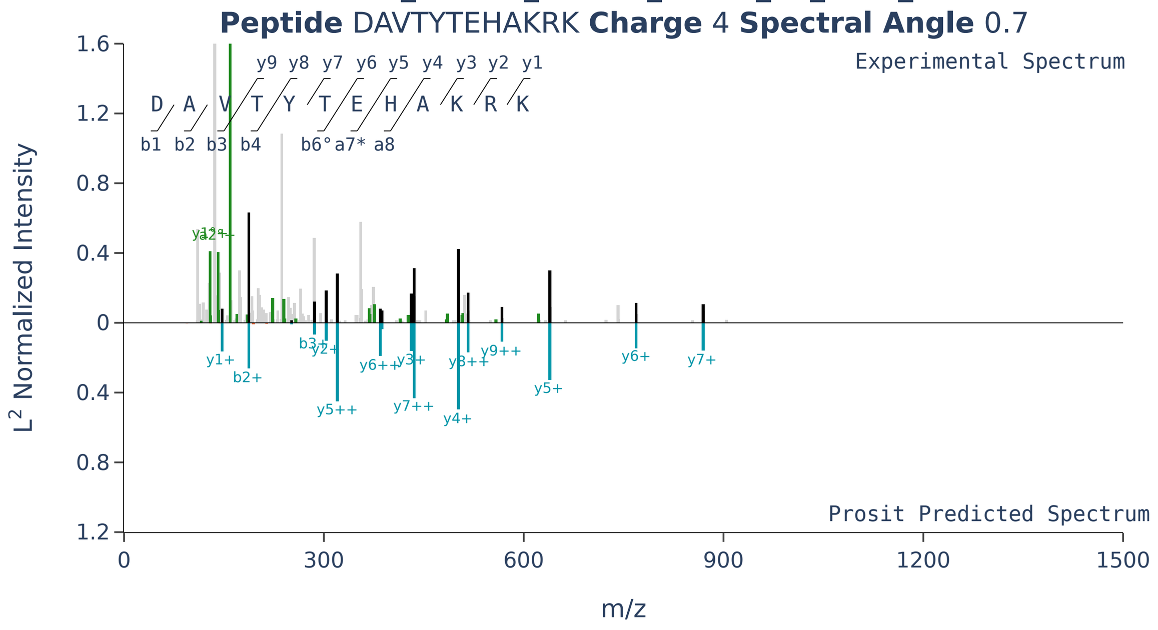


**Source** HRoetschke_291222_140123_HFGoe_H4_b1R2_1h **Scan** 15945
**Peptide** NIQGITKPAIRRLARRGGVKRISGLLY **Charge** 5 **Spectral Angle** 0.9

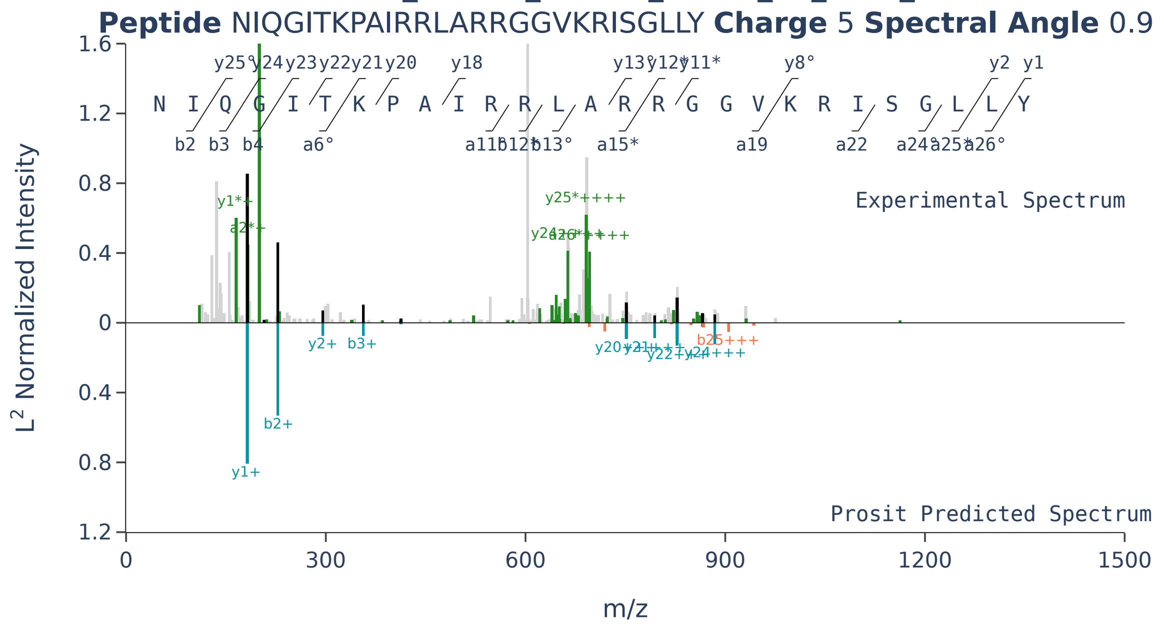


**Source** HRoetschke_291222_140123_HFGoe_H4_b1R1_1h **Scan** 19050
**Peptide** VVYALKRQGRTLYGF **Charge** 3 **Spectral Angle** 0.82

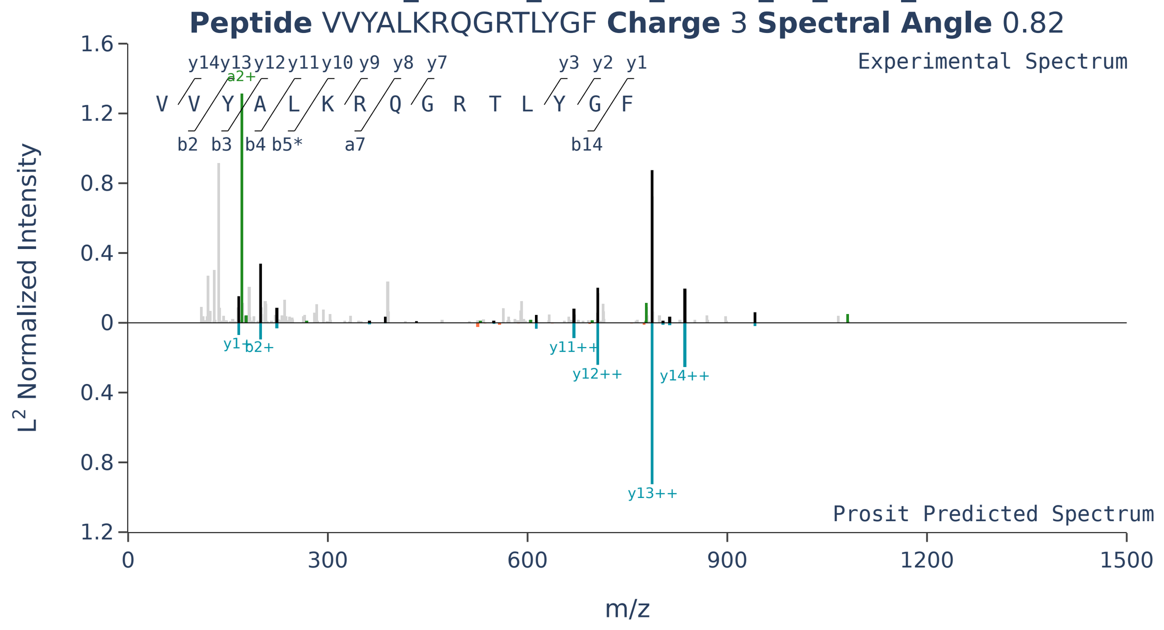


**Source** HRoetschke_291222_140123_HFGoe_H4_b1R1_1h **Scan** 17323
**Peptide** DVVYALKRQGRTLYG **Charge** 3 **Spectral Angle** 0.64

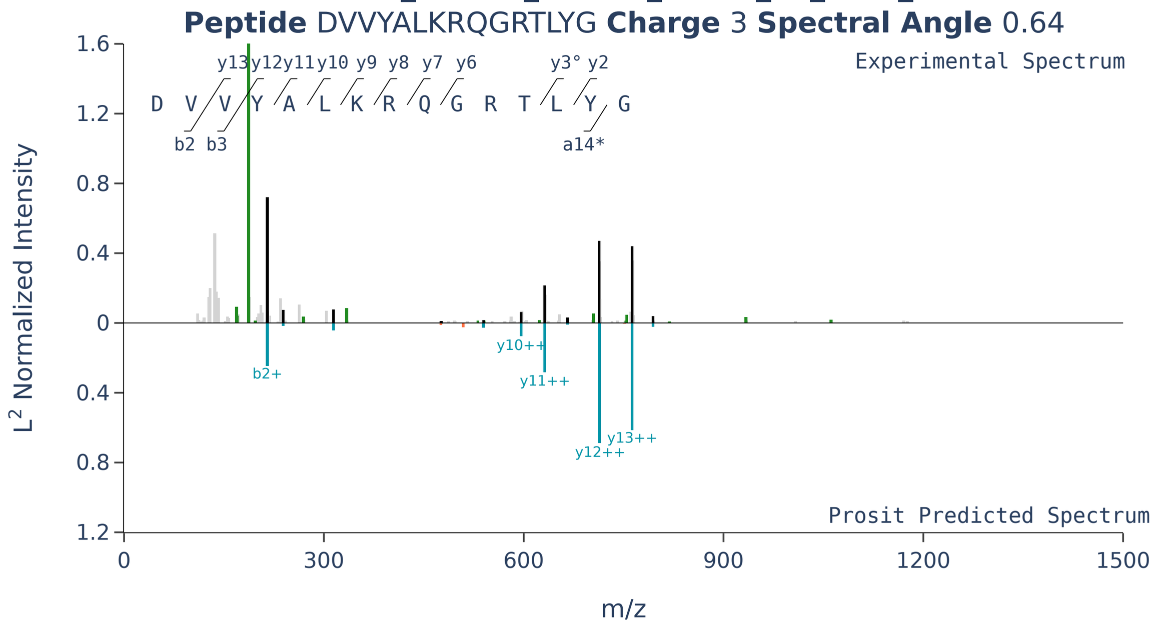


**Source** HRoetschke_291222_140123_HFGoe_H4_b1R1_4h **Scan** 5371
**Peptide** AVTYTEHAKRKTVT **Charge** 4 **Spectral Angle** 0.69

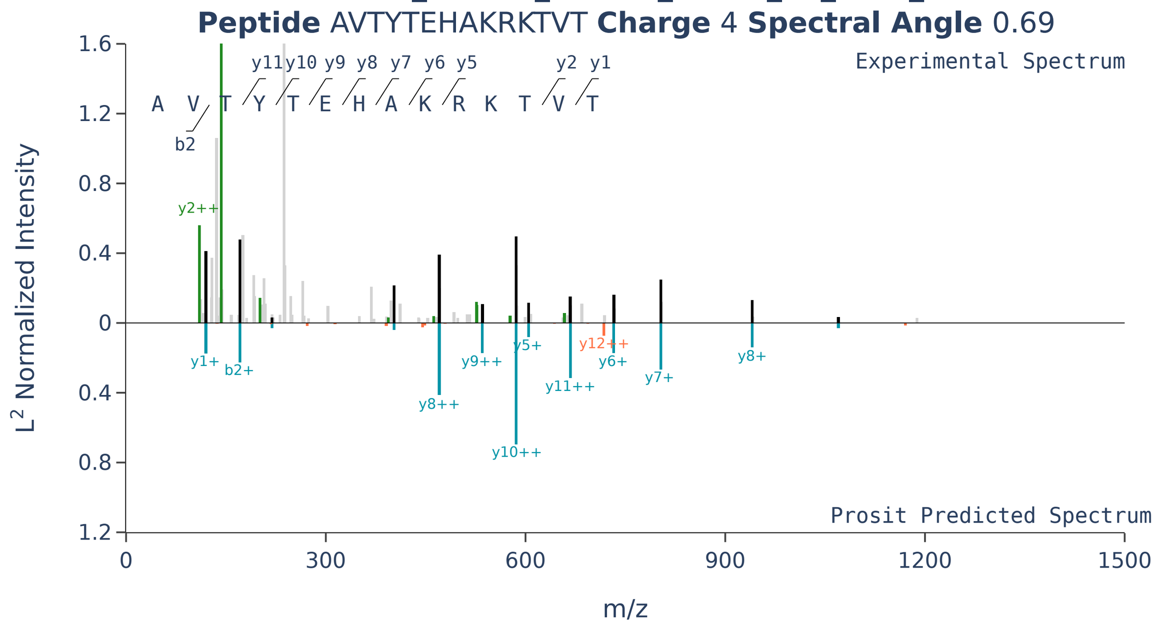


**Source** HRoetschke_291222_140123_HFGoe_H4_b2R1_1h **Scan** 30395
**Peptide** GGVKRISGLIYEETRGVLKVFL **Charge** 4 **Spectral Angle** 0.69

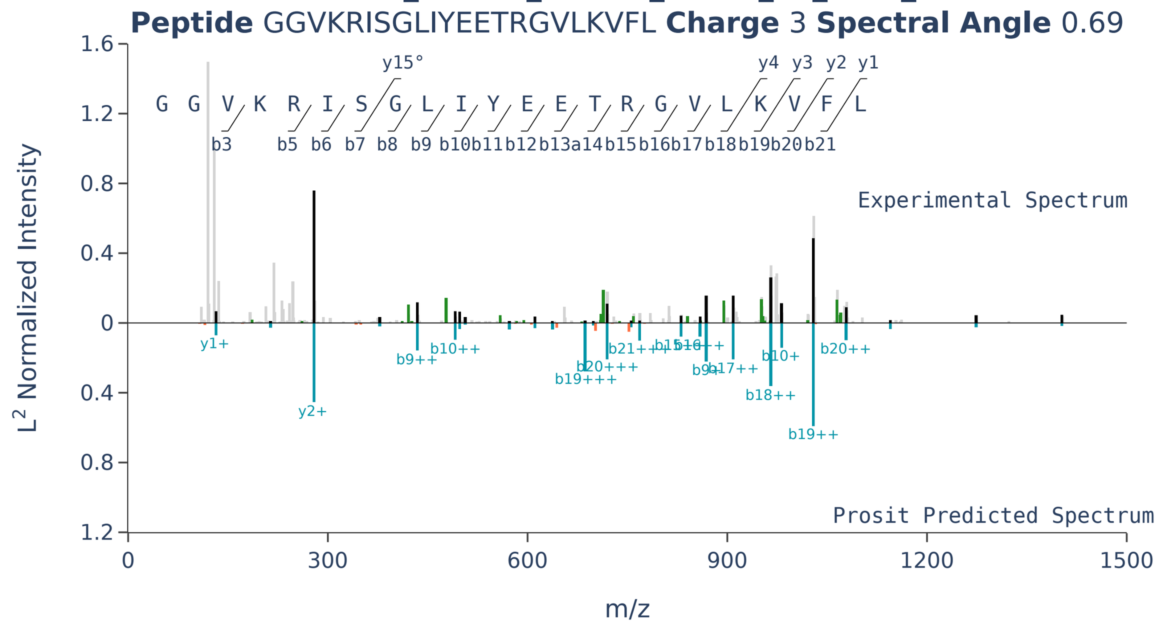


**Source** HRoetschke_291222_140123_HFGoe_H4_b1R1_2h **Scan** 14153
**Peptide** AKRKTVTAMDVVYALKR **Charge** 5 **Spectral Angle** 0.81

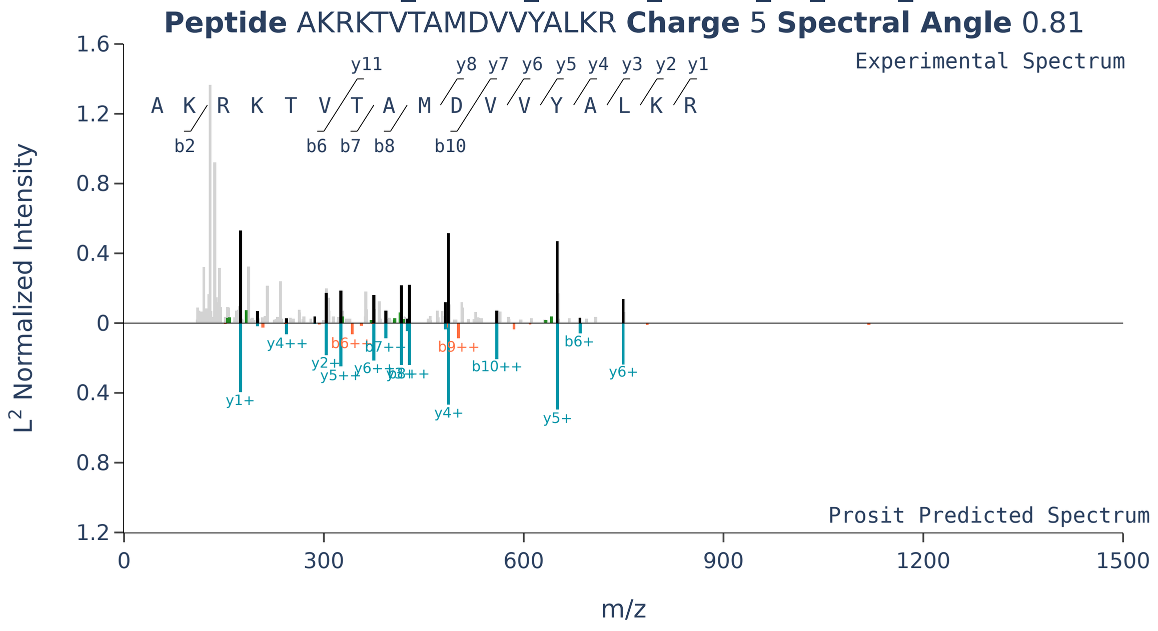


**Source** HRoetschke_291222_140123_HFGoe_H4_b1R1_24h **Scan** 12641
**Peptide** KVLRDNIQGITKPAIRRLAR **Charge** 4 **Spectral Angle** 0.69

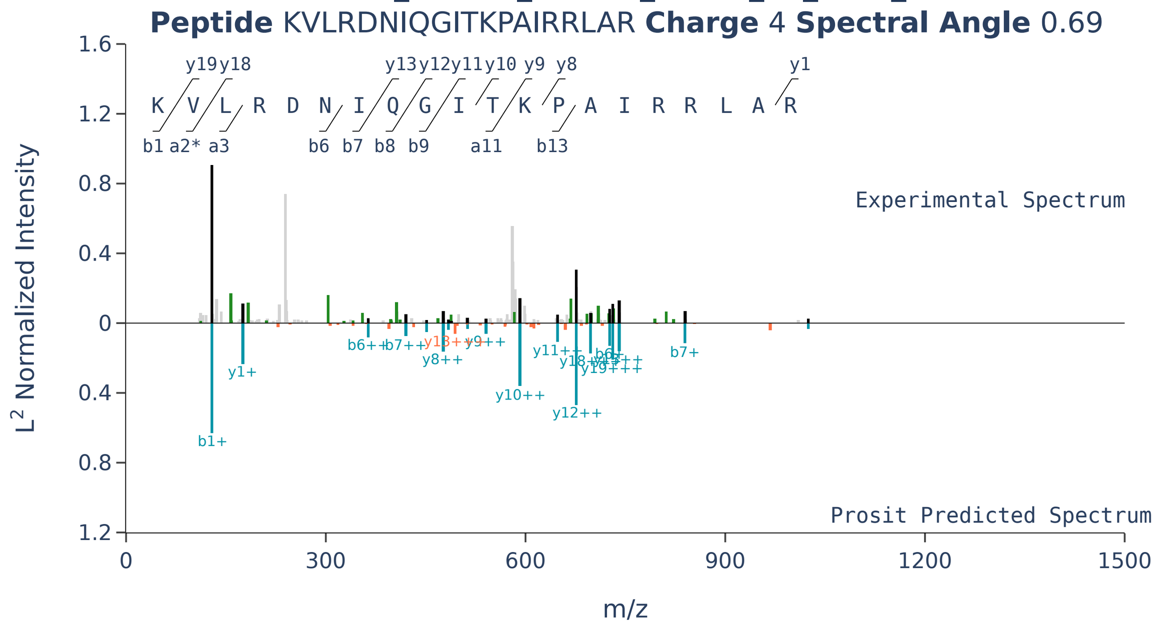


**Source** HRoetschke_291222_140123_HFGoe_H4_b1R1_4h **Scan** 6423
**Peptide** GGKGLGKGGAKRHRKVLRDNIQGL **Charge** 4 **Spectral Angle** 0.81

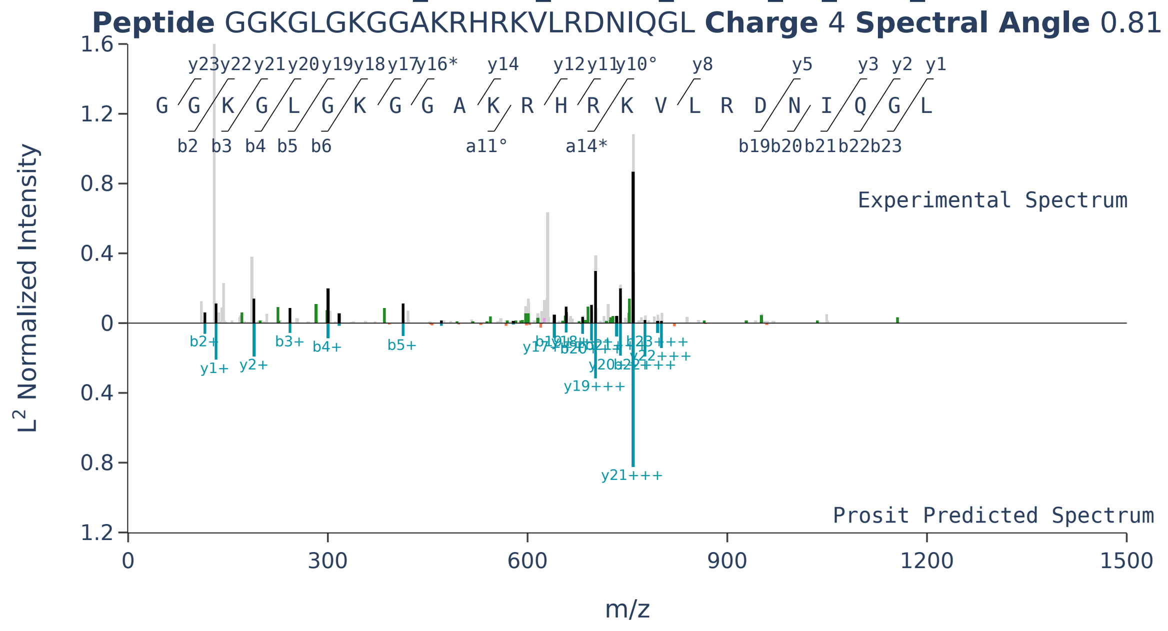


**Source** HRoetschke_291222_140123_HFGoe_H4_b2R1_1h **Scan** 17824
**Peptide** EHAKRKTVTAMDVVYALKRQGRTL **Charge** 5 **Spectral Angle** 0.87

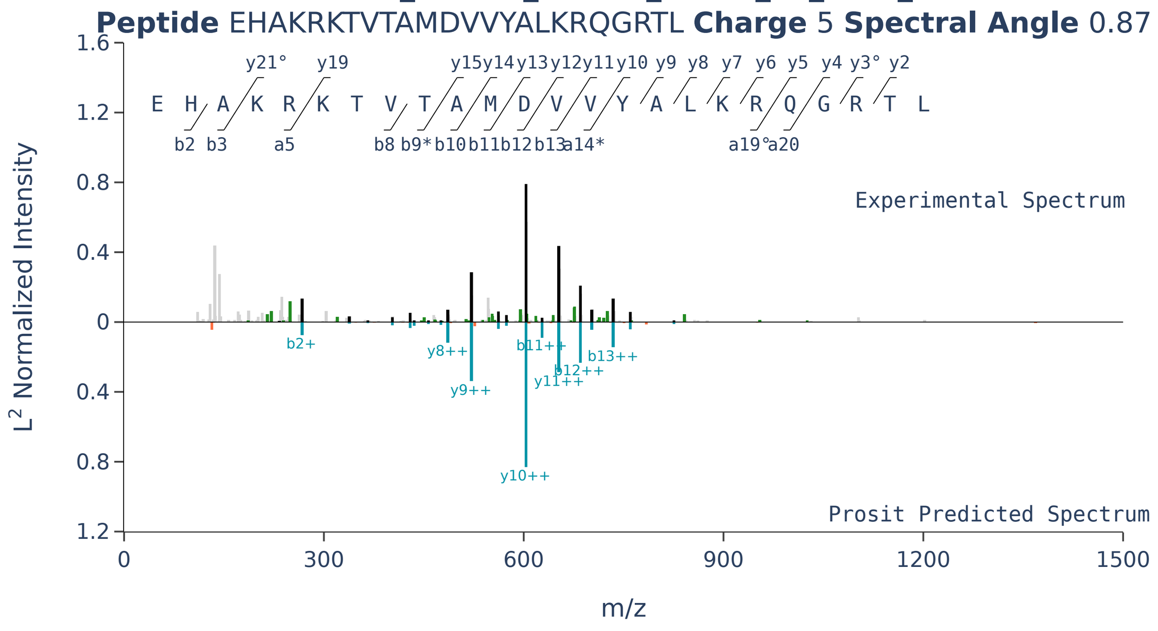


**Source** HRoetschke_291222_140123_HFGoe_H4_b2R2_1h **Scan** 23921
**Peptide** KVFLENVIRDAVTYTEHAKRKTVT **Charge** 4 **Spectral Angle** 0.81

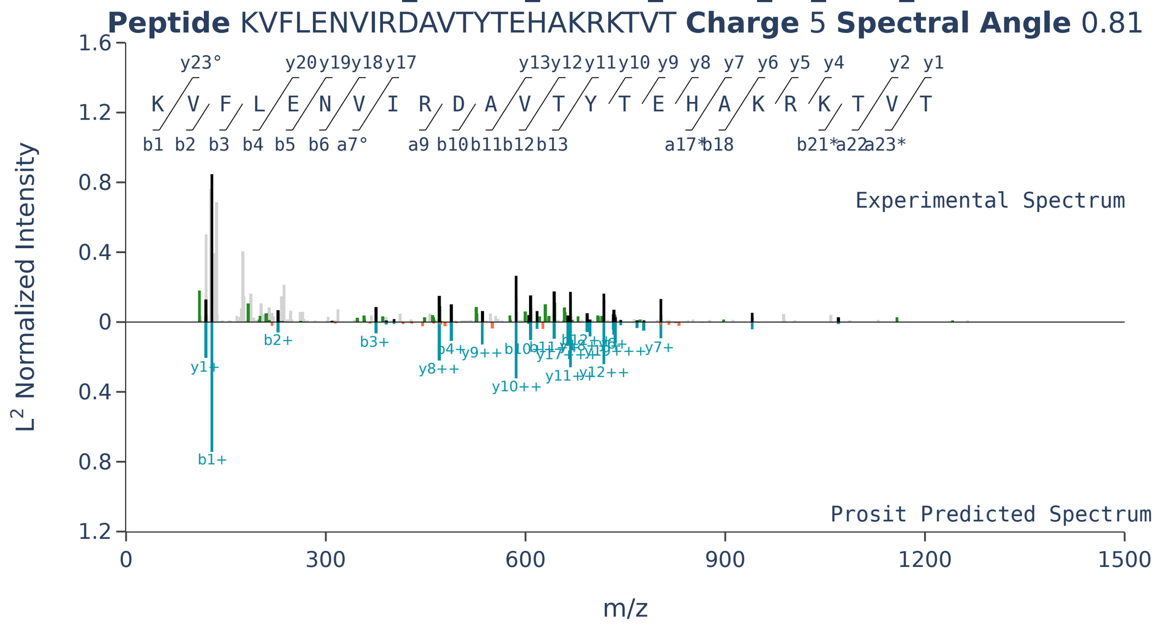


**Source** HRoetschke_291222_140123_HFGoe_H4_b1R1_1h **Scan** 18979
**Peptide** LENVIRDAVTYTEHAKRKTV **Charge** 3 **Spectral Angle** 0.78

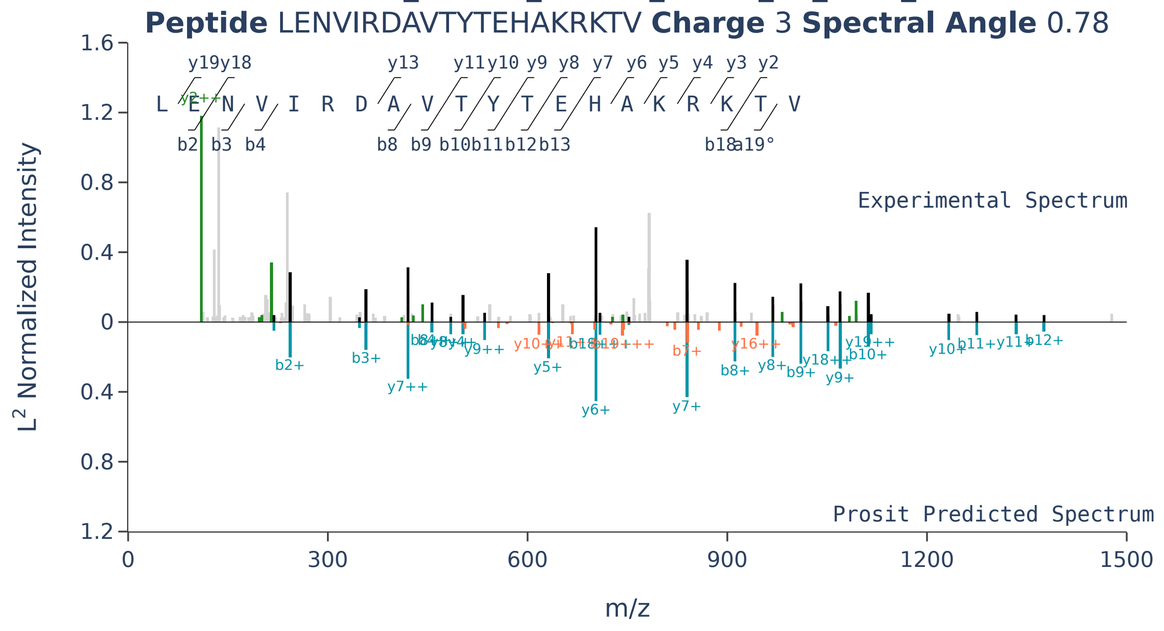


**Source** HRoetschke_291222_140123_HFGoe_H4_b1R2_1h **Scan** 10354
**Peptide** QGITKPAIRRLARR **Charge** 3 **Spectral Angle** 0.73

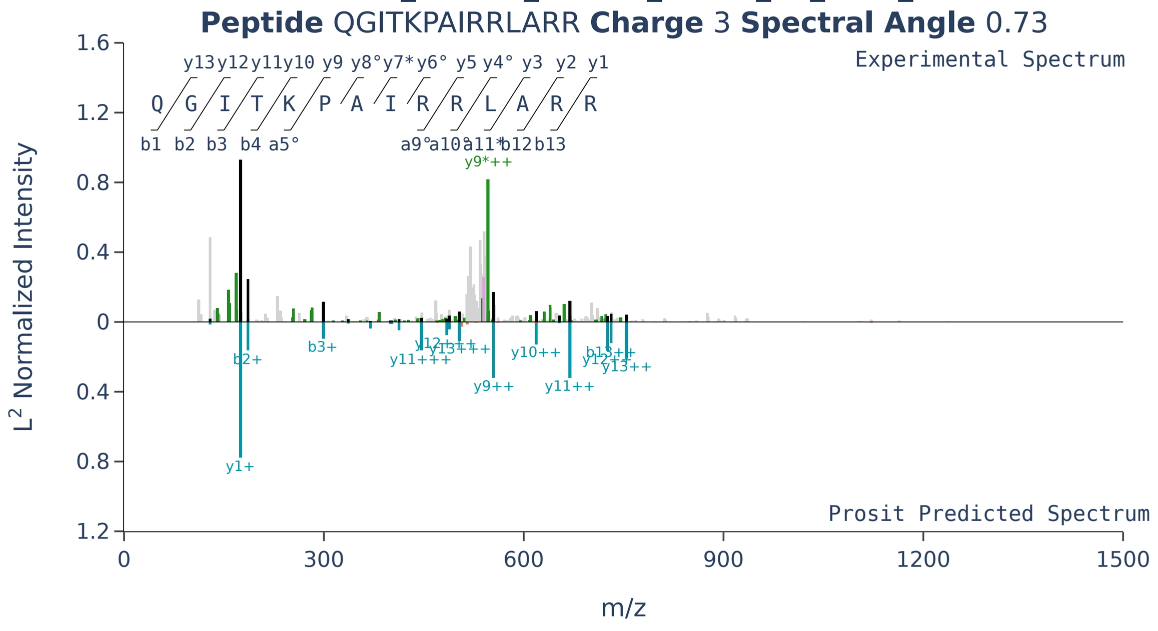


**Source** HRoetschke_291222_140123_HFGoe_H4_b1R2_4h **Scan** 13499
**Peptide** NVIRDAVTYTEHAKRKTV **Charge** 3 **Spectral Angle** 0.78

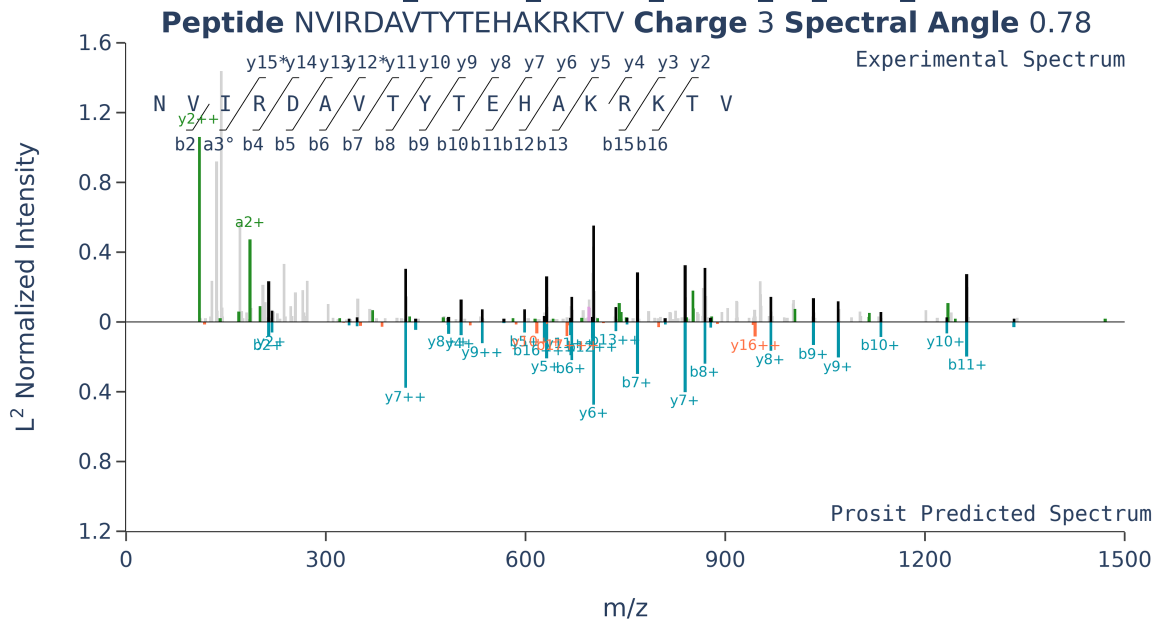


**Source** HRoetschke_291222_140123_HFGoe_H4_b1R1_4h **Scan** 6718
**Peptide** VIRDAVTYTEHAKRKT **Charge** 4 **Spectral Angle** 0.79

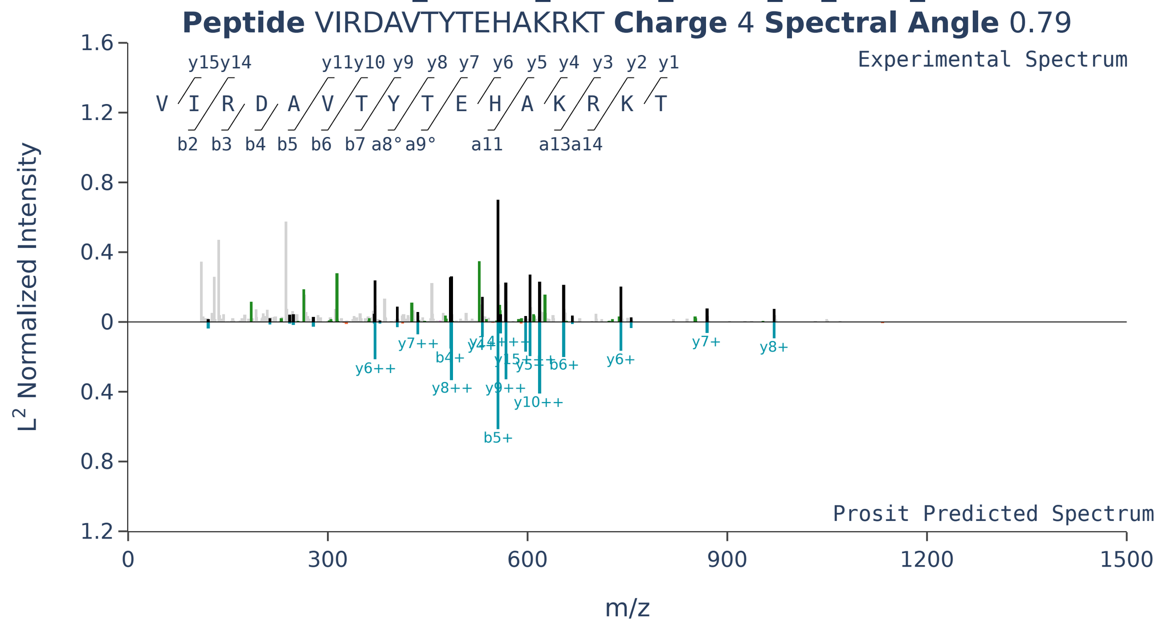

**Source** HRoetschke_291222_140123_HFGoe_H4_b1R2_1h **Scan** 15710
**Peptide** TEHAKRKTVTAMDVVYAL **Charge** 3 **Spectral Angle** 0.45

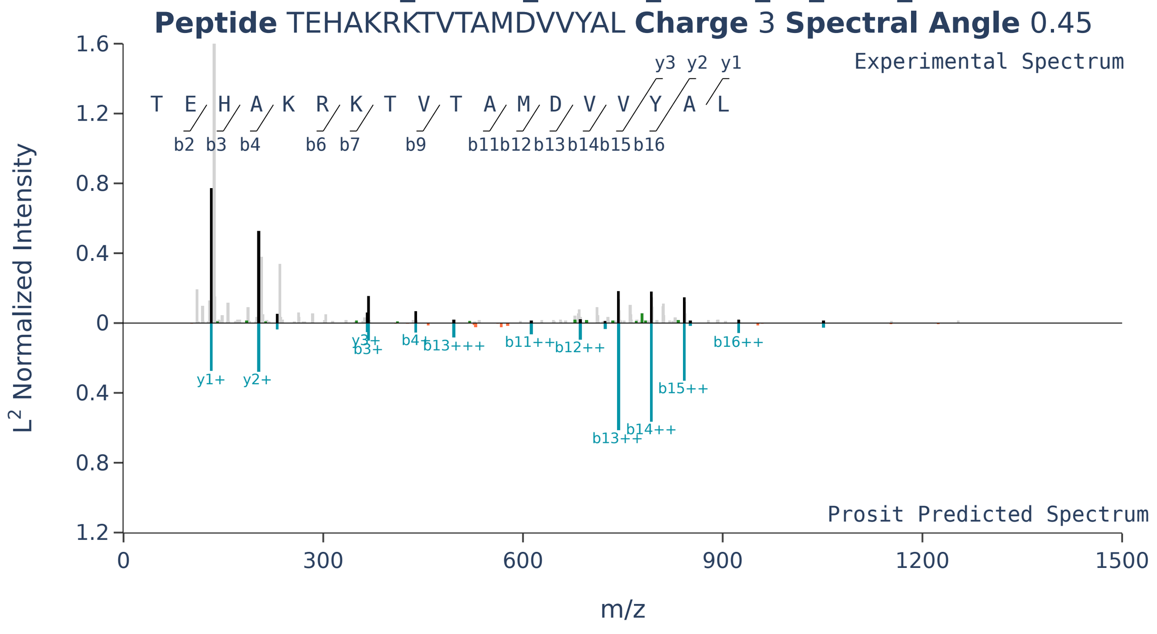


**Source** HRoetschke_291222_140123_HFGoe_H4_b2R1_1h **Scan** 19140
**Peptide** ENVIRDAVTYTEHAKRKTVTAMDVVY **Charge** 5 **Spectral Angle** 0.89

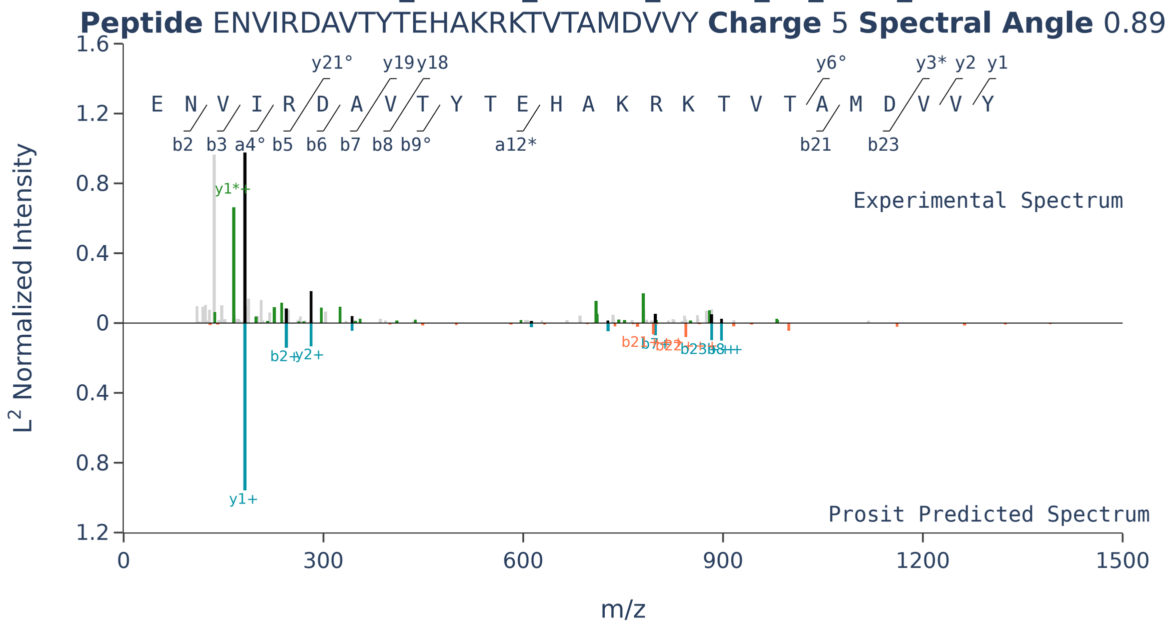


**Source** HRoetschke_291222_140123_HFGoe_H4_b2R2_2h **Scan** 13774
**Peptide** RDAVTYTEHAKRKTVTAMDVVYALKRQG **Charge** 5 **Spectral Angle** 0.79

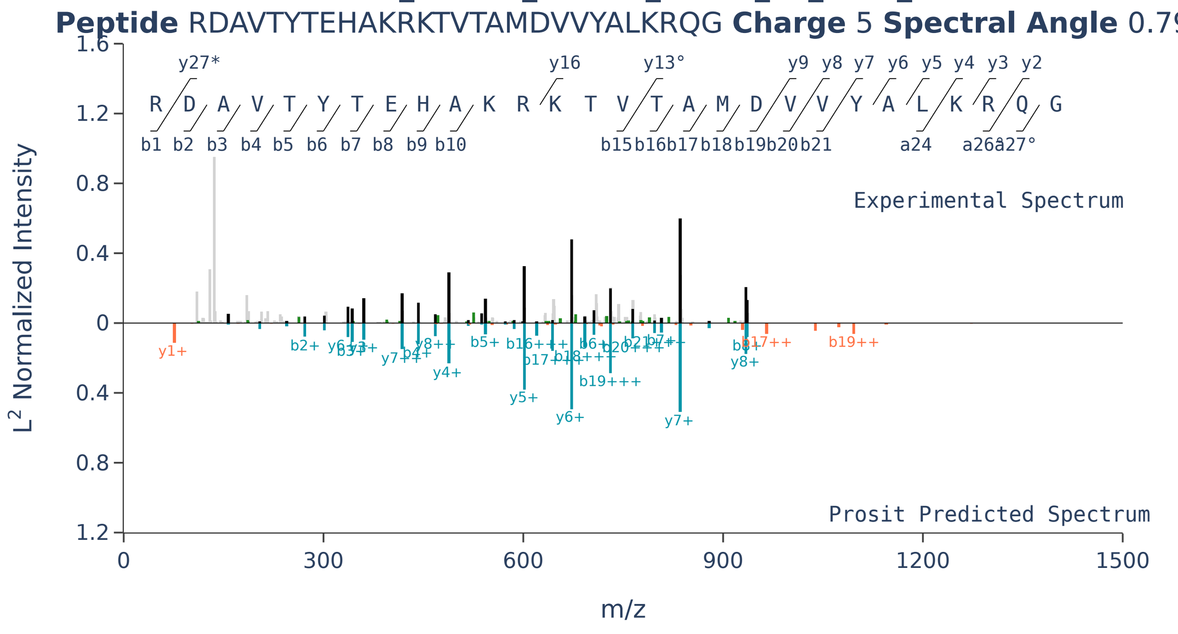


**Source** HRoetschke_291222_140123_HFGoe_H4_b2R1_1h **Scan** 24114
**Peptide** AMDVVYALKRQGRTLYGF **Charge** 4 **Spectral Angle** 0.77

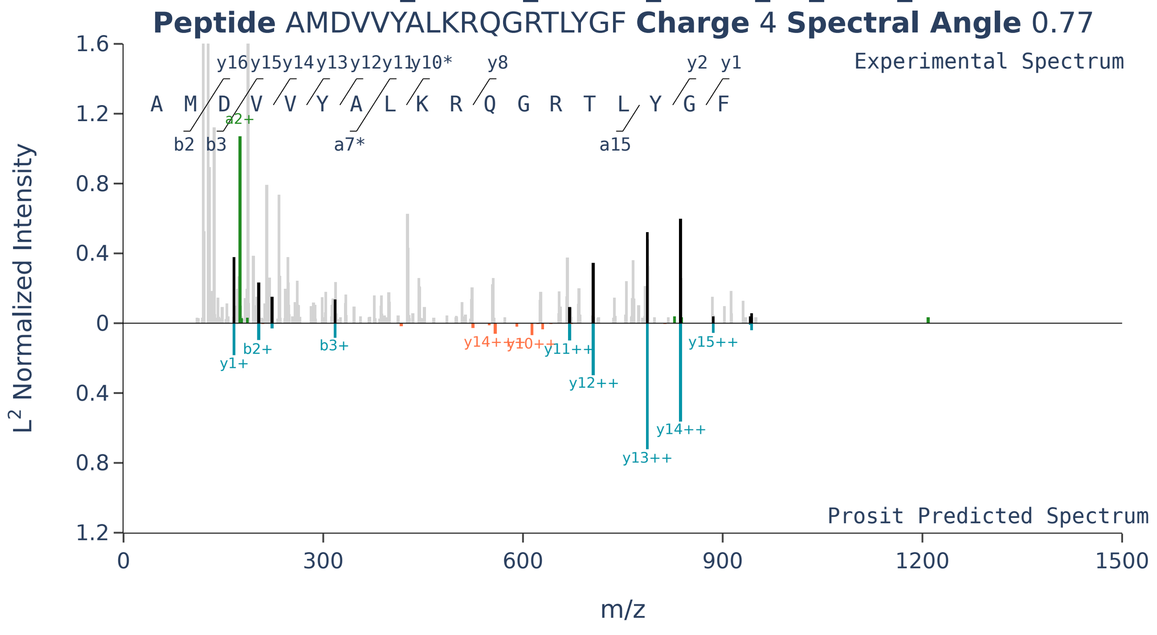


**Source** HRoetschke_291222_140123_HFGoe_H4_b1R2_1h **Scan** 25431
**Peptide** GVLKVFLE **Charge** 2 **Spectral Angle** 0.78

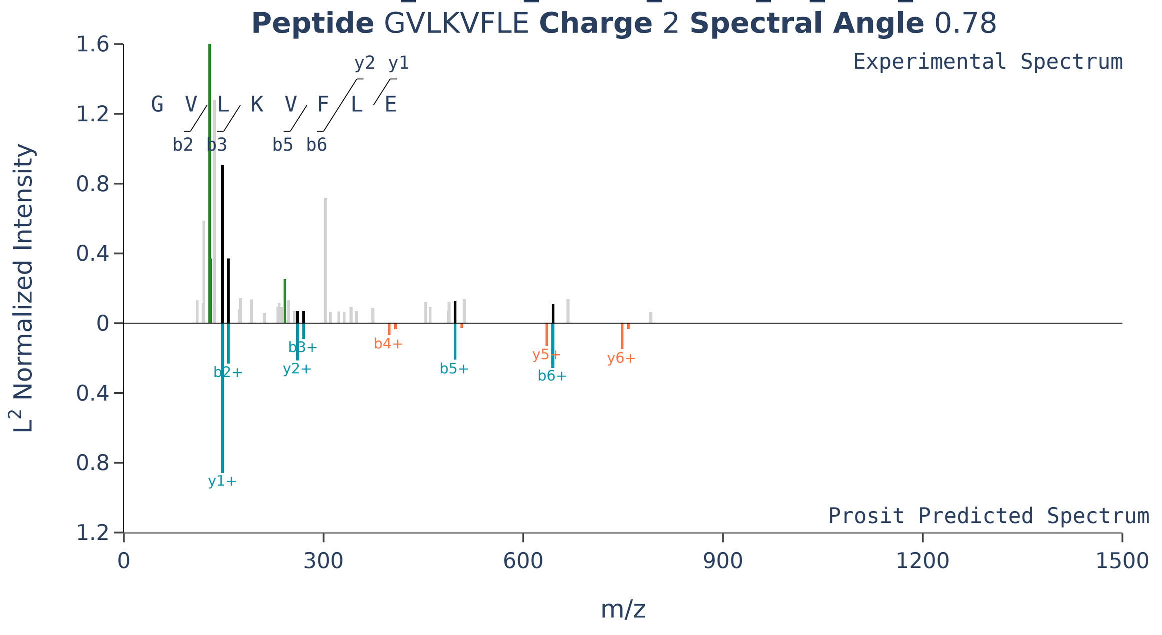


**Source** HRoetschke_291222_140123_HFGoe_H4_b1R2_2h **Scan** 22409
**Peptide** LENVIRDAVTYTE **Charge** 2 **Spectral Angle** 0.79

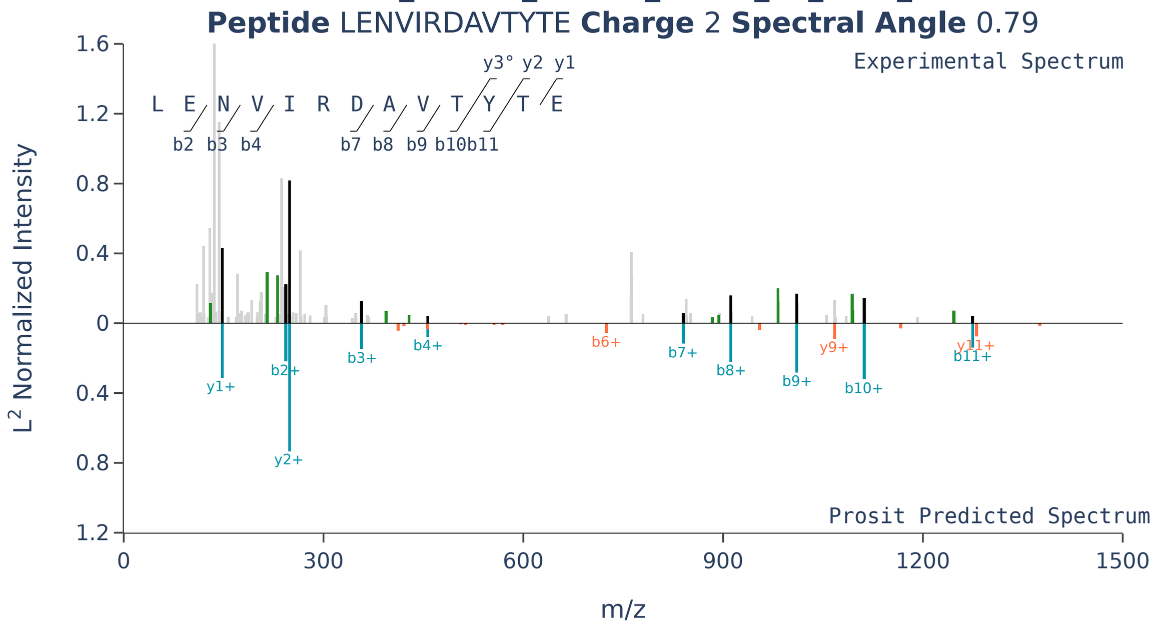


**Source** HRoetschke_291222_140123_HFGoe_H4_b1R2_2h **Scan** 10937
**Peptide** NVIRDAVT **Charge** 2 **Spectral Angle** 0.67

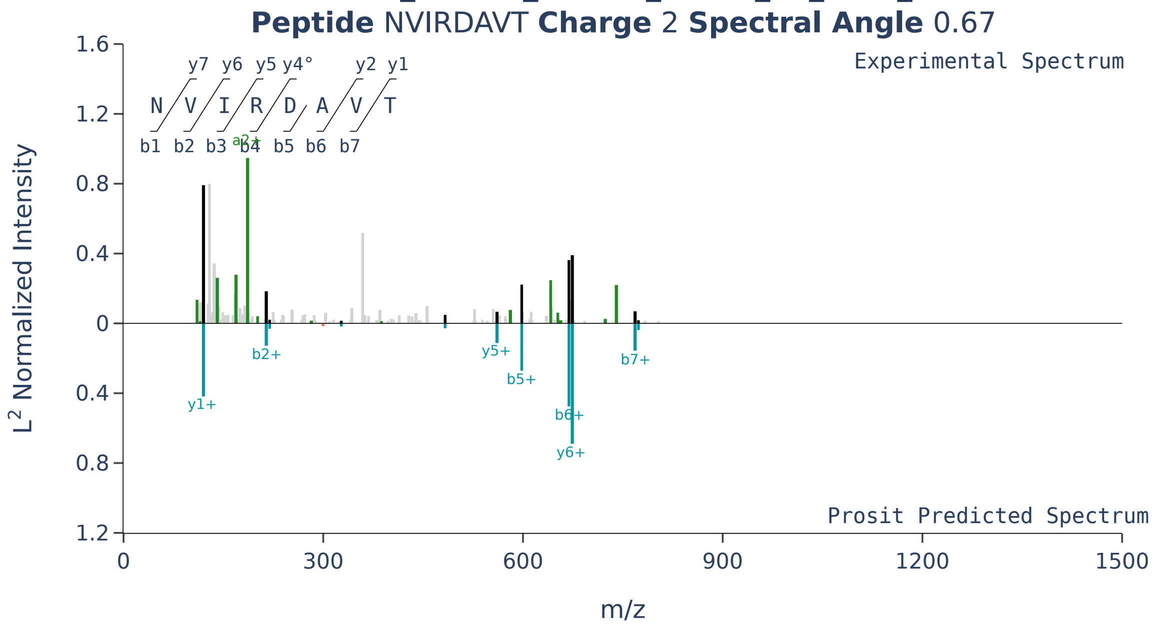


**Source** HRoetschke_291222_140123_HFGoe_H4_b2R1_1h **Scan** 28457
**Peptide** RGVLKVFLENVI **Charge** 2 **Spectral Angle** 0.72

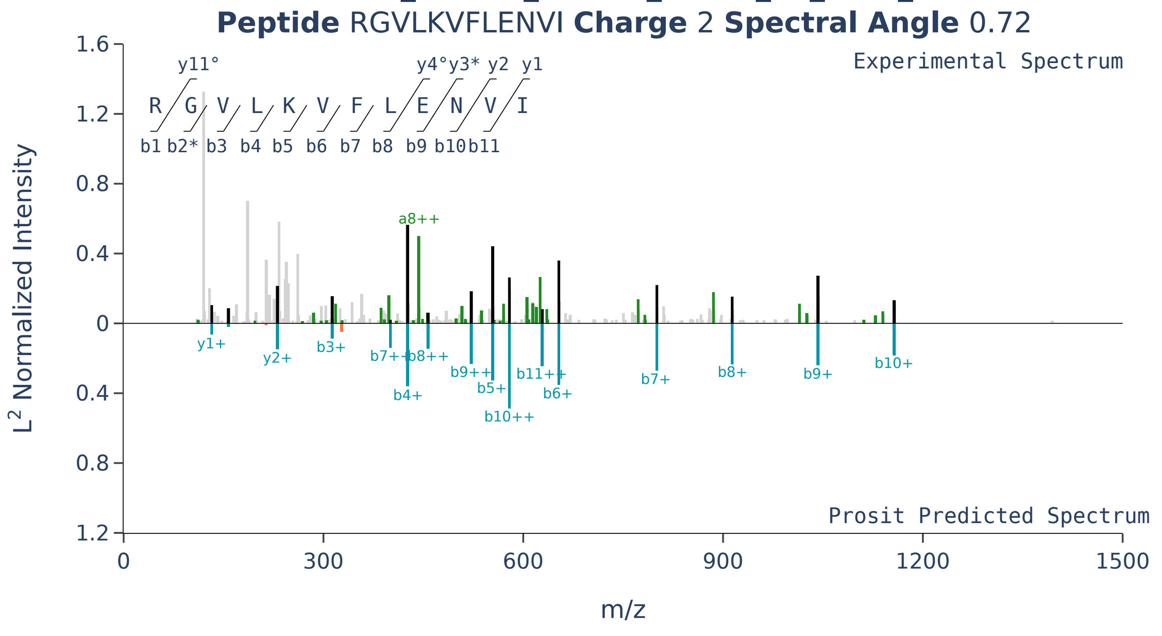


**Source** HRoetschke_291222_140123_HFGoe_H4_b2R2_1h **Scan** 10158
**Peptide** RDNIQGIT **Charge** 2 **Spectral Angle** 0.81

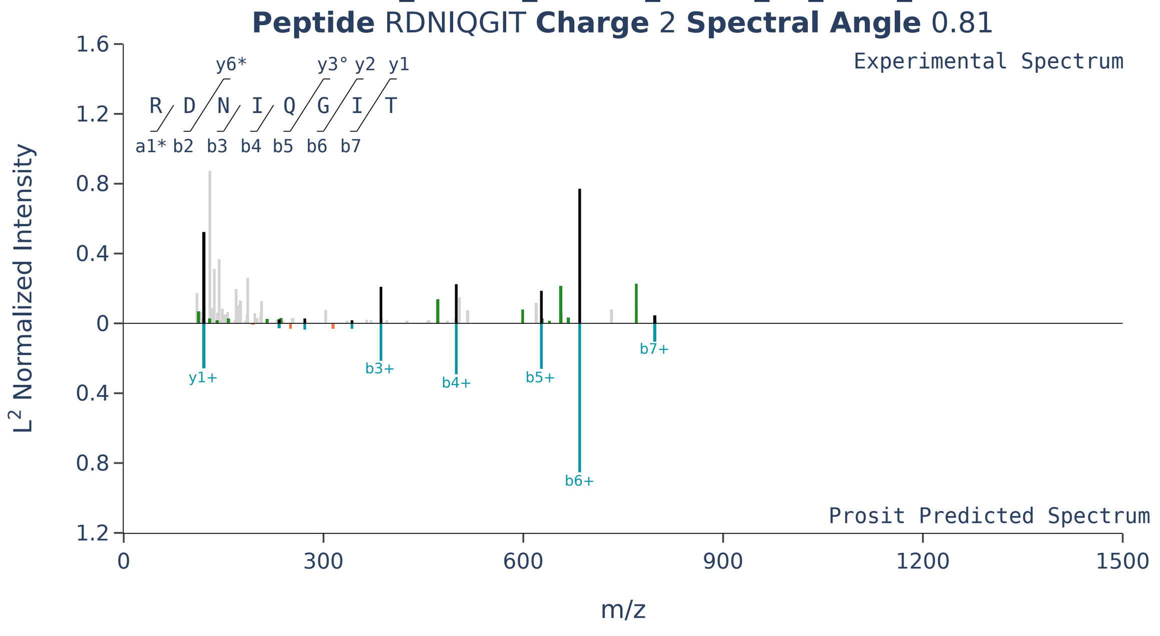


**Source** HRoetschke_291222_140123_HFGoe_H4_b1R2_24h **Scan** 5897
**Peptide** AKRKTVTAMDV **Charge** 3 **Spectral Angle** 0.66

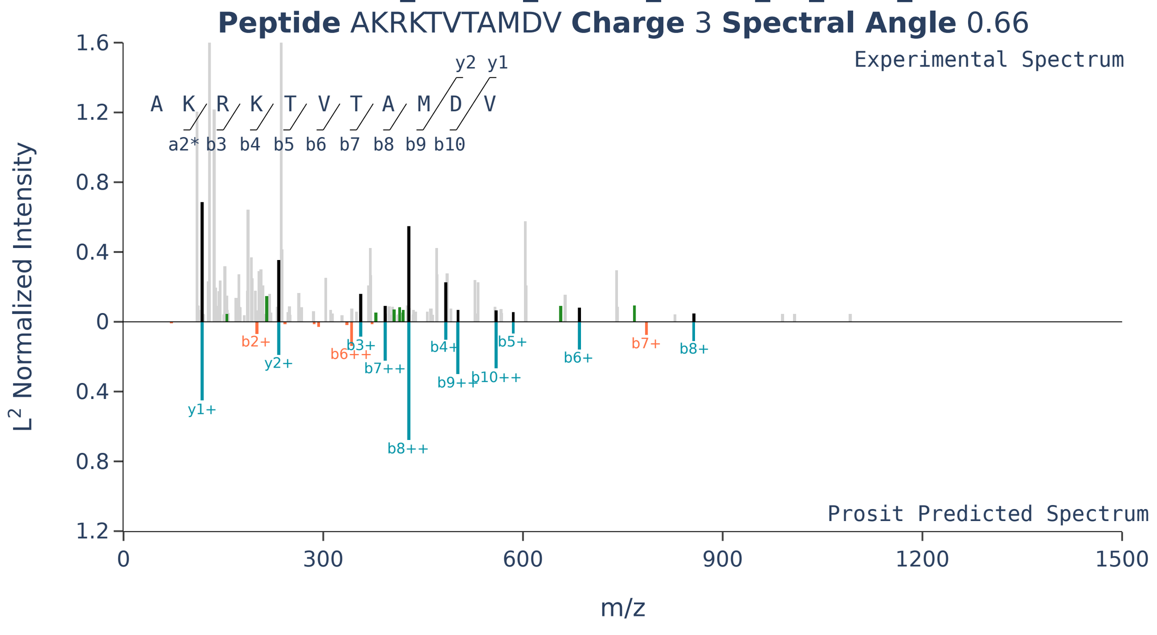


**Source** HRoetschke_291222_140123_HFGoe_H4_b2R1_24h **Scan** 8823
**Peptide** RDNIQGITKPA **Charge** 3 **Spectral Angle** 0.86

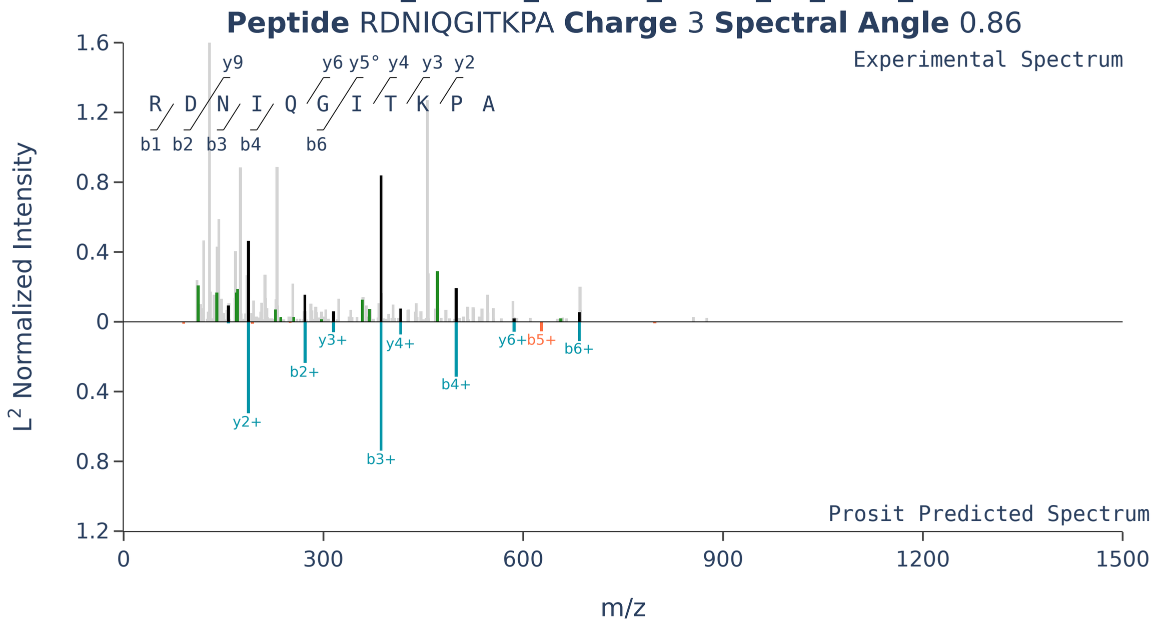


**Source** HRoetschke_291222_140123_HFGoe_H4_b1R2_1h **Scan** 15713
**Peptide** VYALKRQGRTLYGFGG **Charge** 3 **Spectral Angle** 0.68

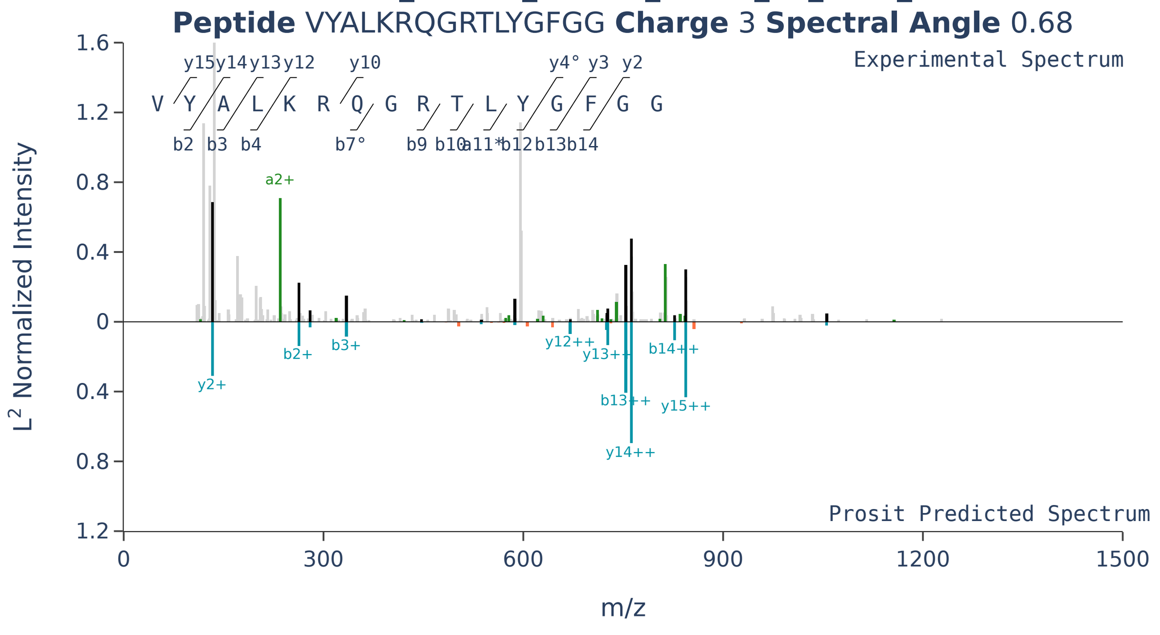


**Source** HRoetschke_291222_140123_HFGoe_H4_b1R1_24h **Scan** 13929
**Peptide** KTVTAMDVVY **Charge** 2 **Spectral Angle** 0.68

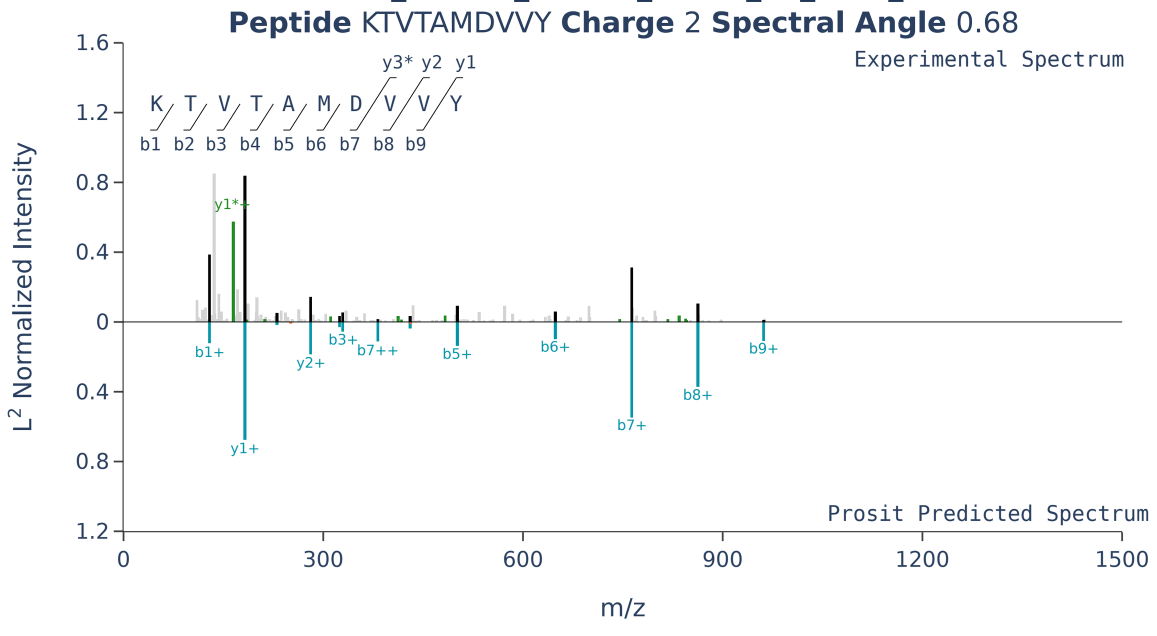


**Source** HRoetschke_291222_140123_HFGoe_H4_b2R2_1h **Scan** 16296
**Peptide** TEHAKRKTVTAMDVVYALKRQ **Charge** 5 **Spectral Angle** 0.7

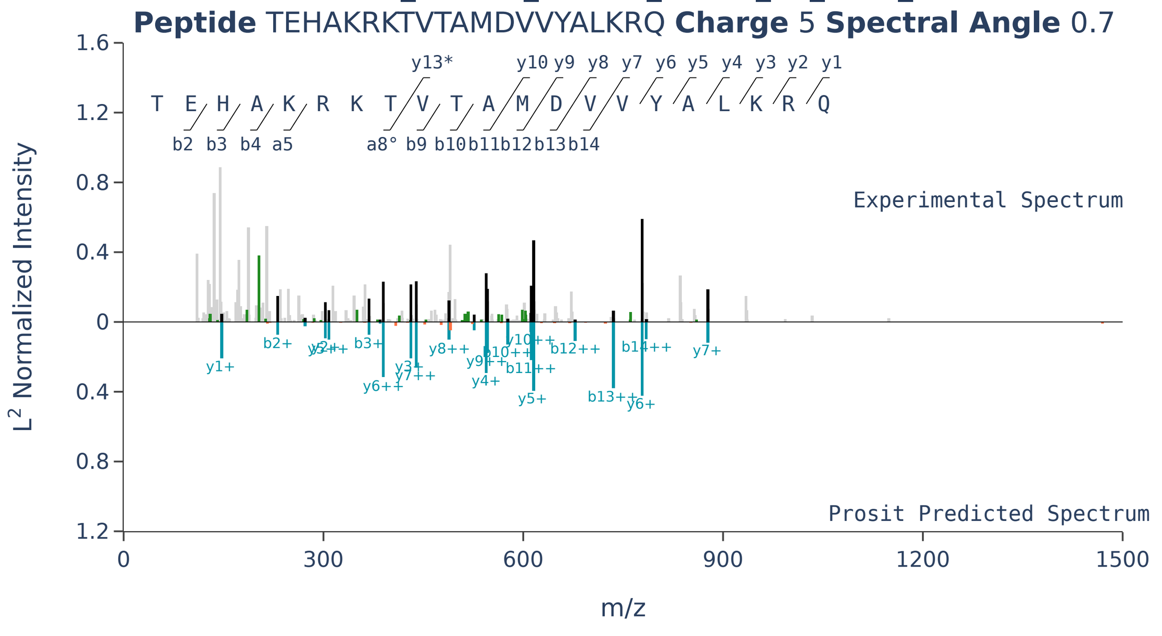


**Source** HRoetschke_291222_140123_HFGoe_H4_b1R1_24h **Scan** 5257
**Peptide** TKPAIRRLARRGGVKR **Charge** 3 **Spectral Angle** 0.83

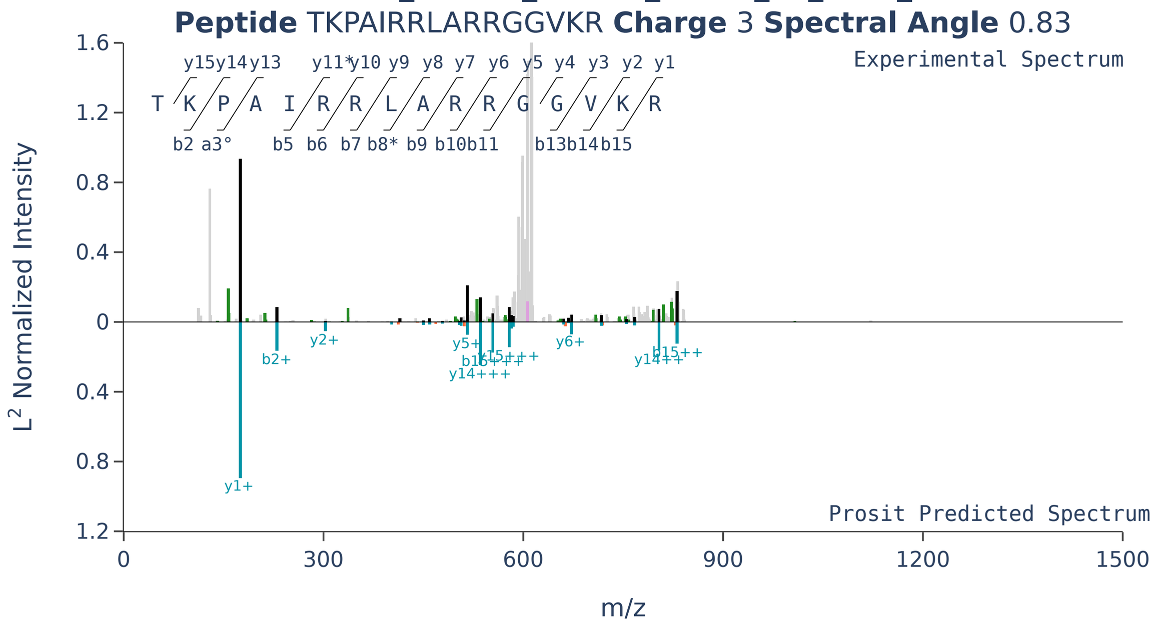

**Source** HRoetschke_291222_140123_HFGoe_H4_b1R1_2h **Scan** 15953
**Peptide** EHAKRKTVTAMDVVYALKRQGR **Charge** 5 **Spectral Angle** 0.9

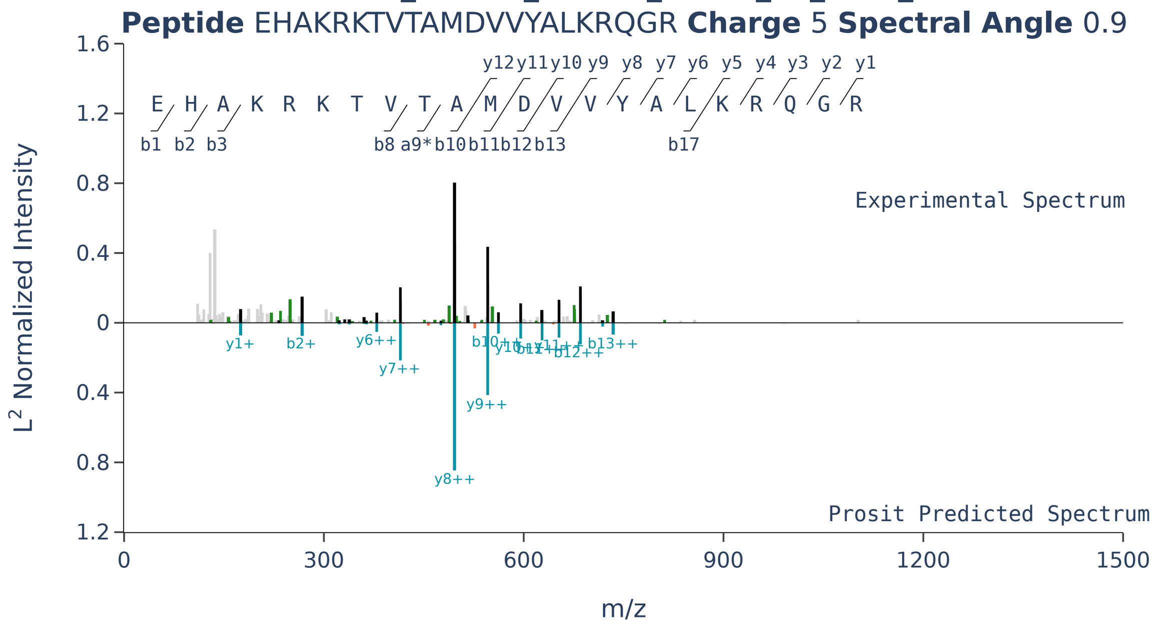


**Source** HRoetschke_291222_140123_HFGoe_H4_b1R2_24h **Scan** 12090
**Peptide** HRKVLRDNIQGITKPAI **Charge** 4 **Spectral Angle** 0.67

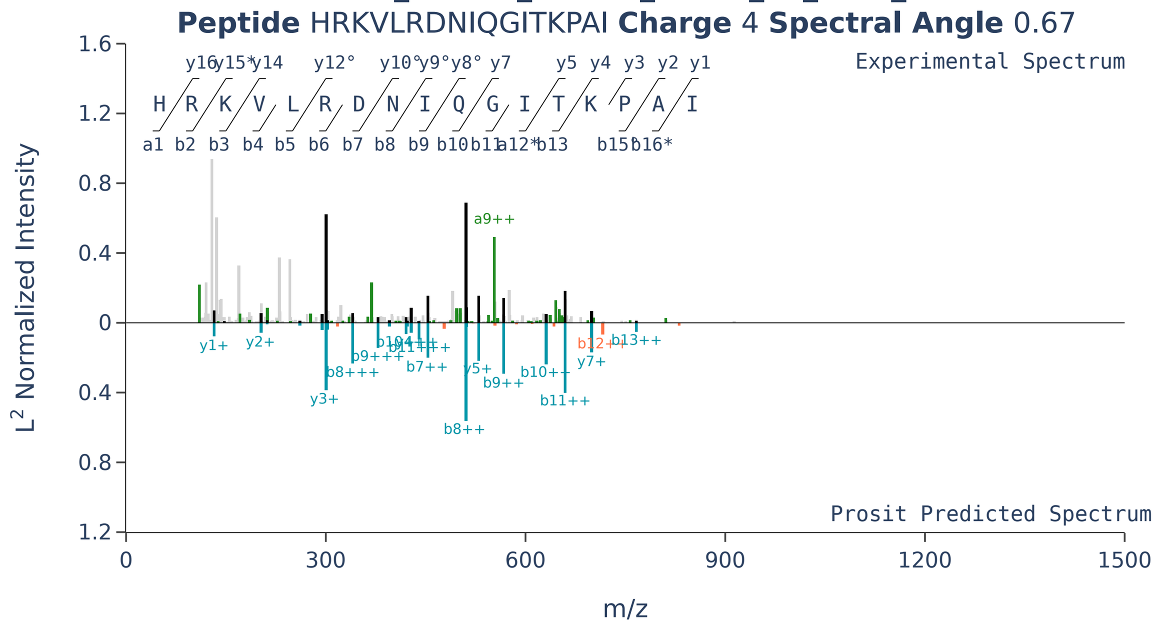


**Source** HRoetschke_291222_140123_HFGoe_H4_b1R1_2h **Scan** 7094
**Peptide** VLRDAVTYTEHAKRK **Charge** 3 **Spectral Angle** 0.82

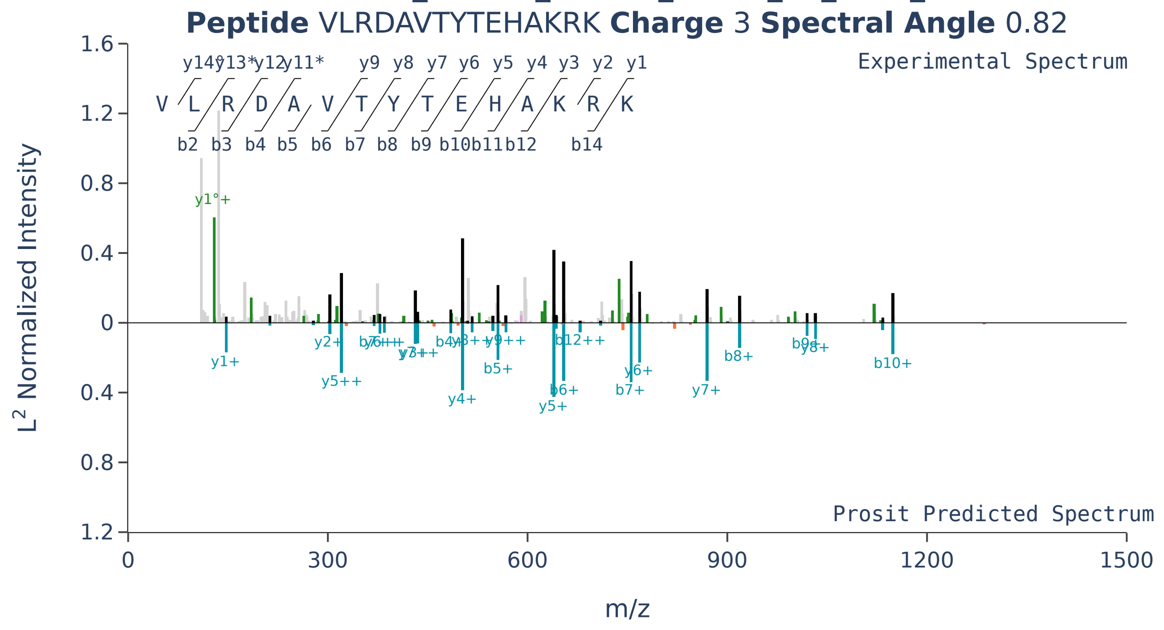


**Source** HRoetschke_291222_140123_HFGoe_H4_b2R1_1h **Scan** 21991
**Peptide** TVTAMDVVYALKRQGRTL **Charge** 3 **Spectral Angle** 0.58

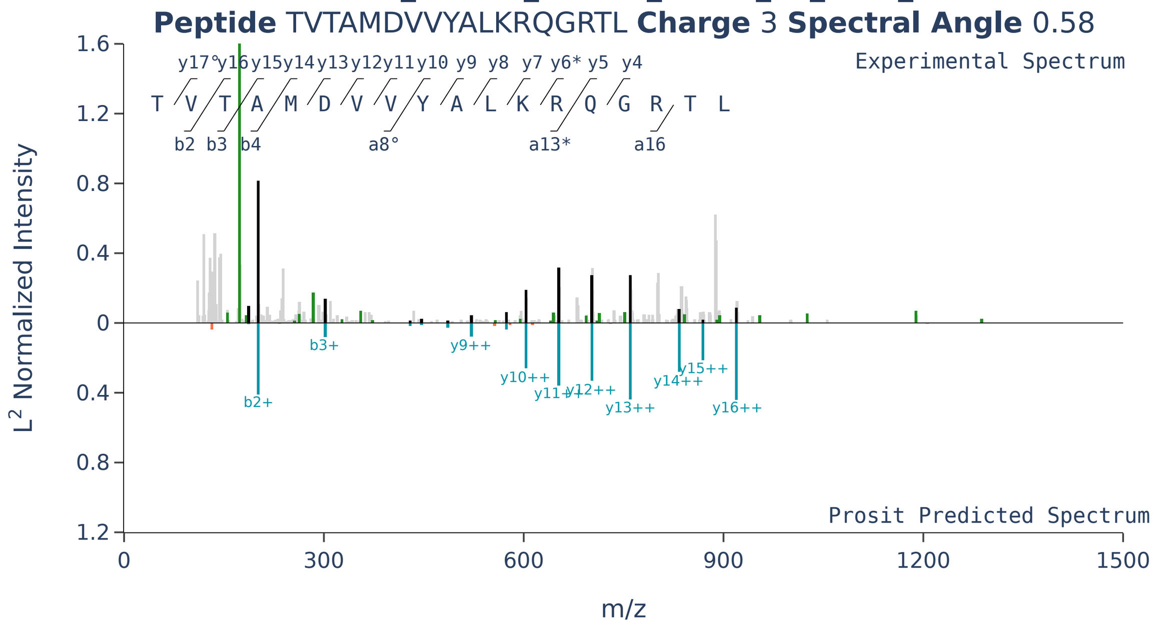


**Source** HRoetschke_291222_140123_HFGoe_H4_b2R2_1h **Scan** 17083
**Peptide** VTYTEHAKRKTVTAMDVVYALKRQG **Charge** 5 **Spectral Angle** 0.85

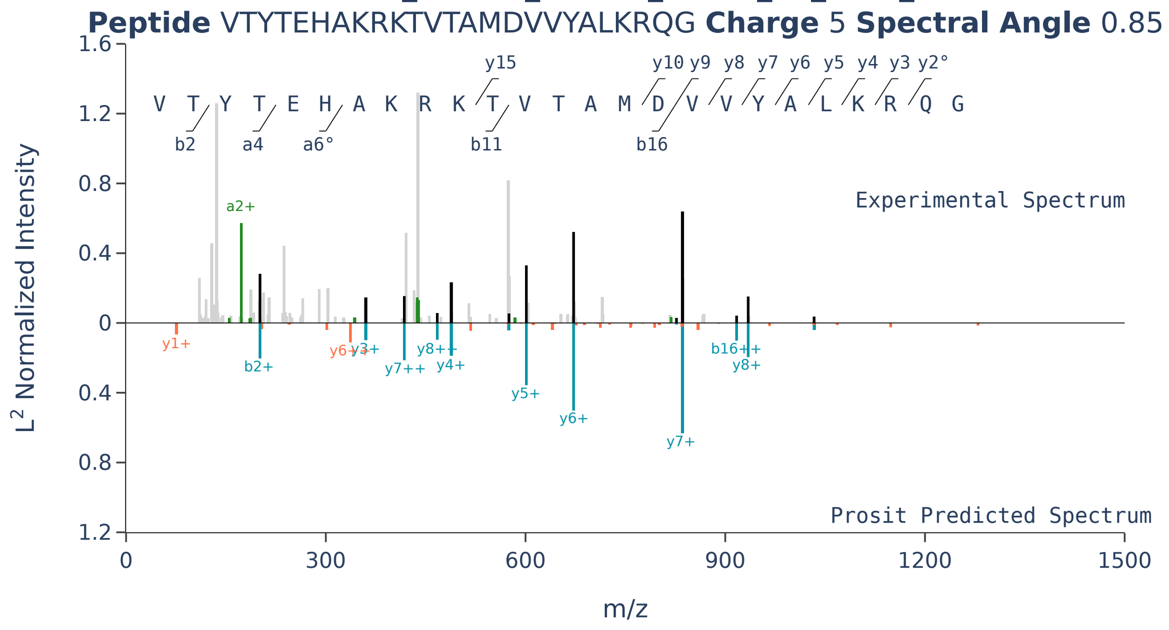


**Source** HRoetschke_291222_140123_HFGoe_H4_b1R2_24h **Scan** 7220
**Peptide** GKGGAKRHRKVLRDNIQGITKPALR **Charge** 5 **Spectral Angle** 0.84

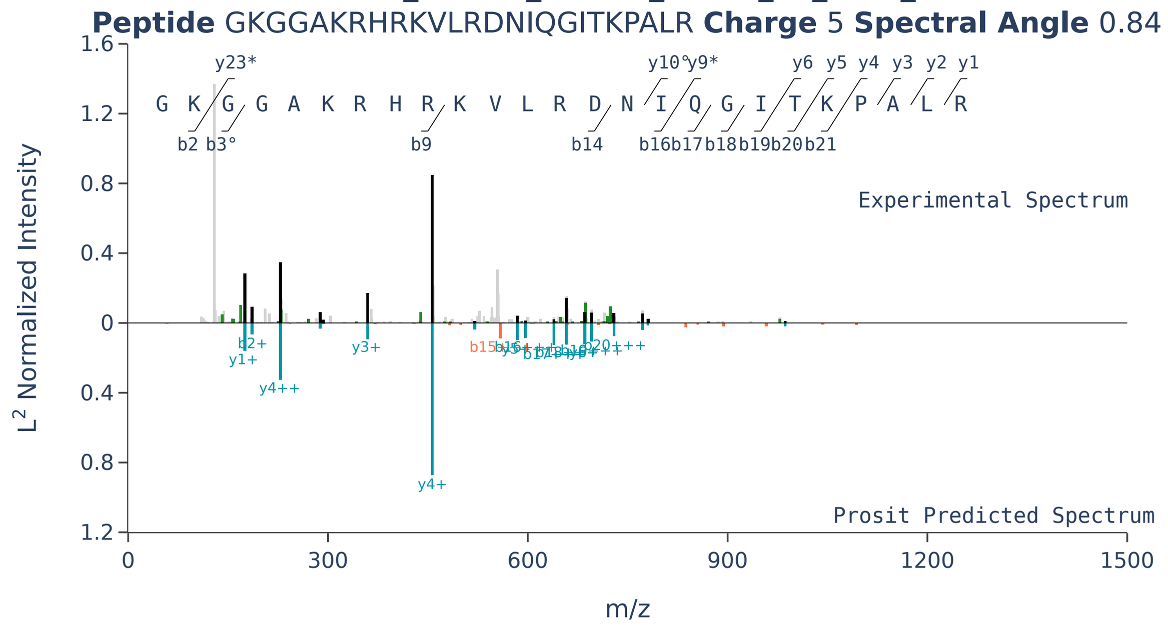


**Source** HRoetschke_291222_140123_HFGoe_H4_b2R1_24h **Scan** 13004
**Peptide** IQGITKPAIRRLA **Charge** 3 **Spectral Angle** 0.75

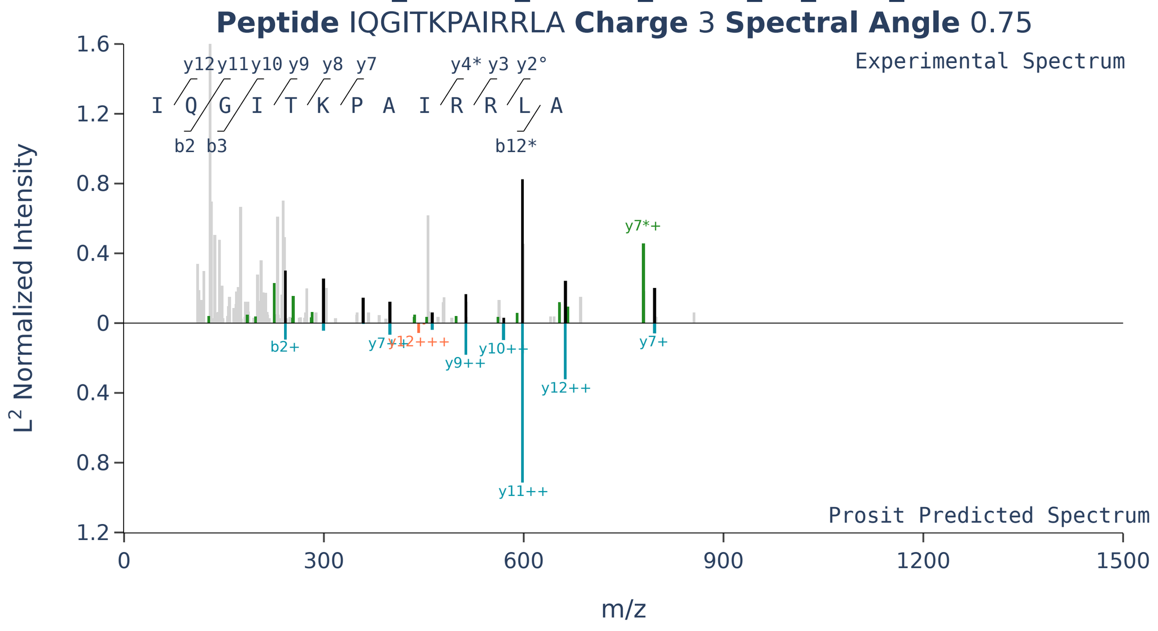


**Source** HRoetschke_291222_140123_HFGoe_H4_b1R2_24h **Scan** 10280
**Peptide** KVLRDNIQGITKPAIRRLARRGGVKR **Charge** 5 **Spectral Angle** 0.77

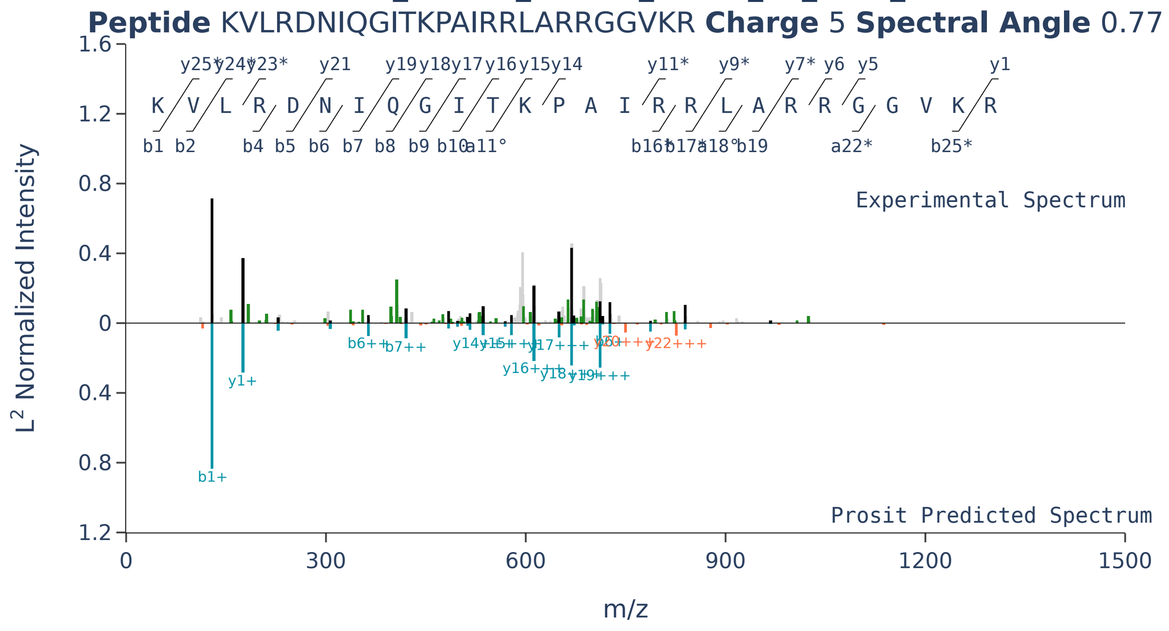


**Source** HRoetschke_291222_140123_HFGoe_H4_b2R1_1h **Scan** 22650
**Peptide** AKRKTVTAMDVVYALKRQGRTLYGFGG **Charge** 4 **Spectral Angle** 0.82

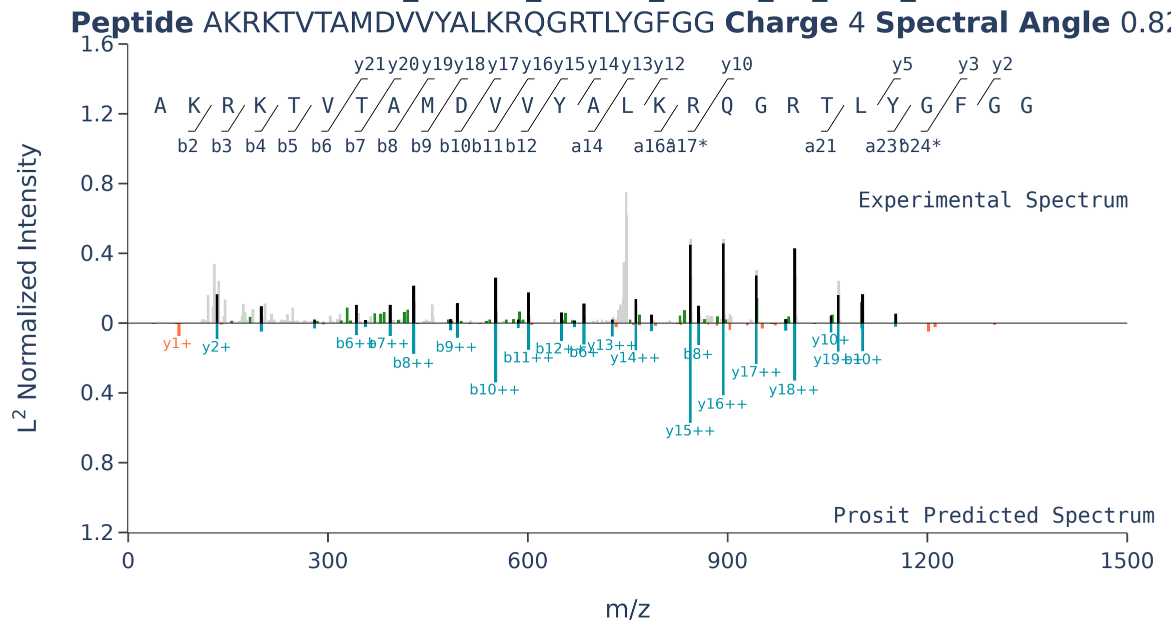


**Source** HRoetschke_291222_140123_HFGoe_H4_b2R1_1h **Scan** 20241
**Peptide** RKTVTAMDVVYALKRQGRTL **Charge** 4 **Spectral Angle** 0.81

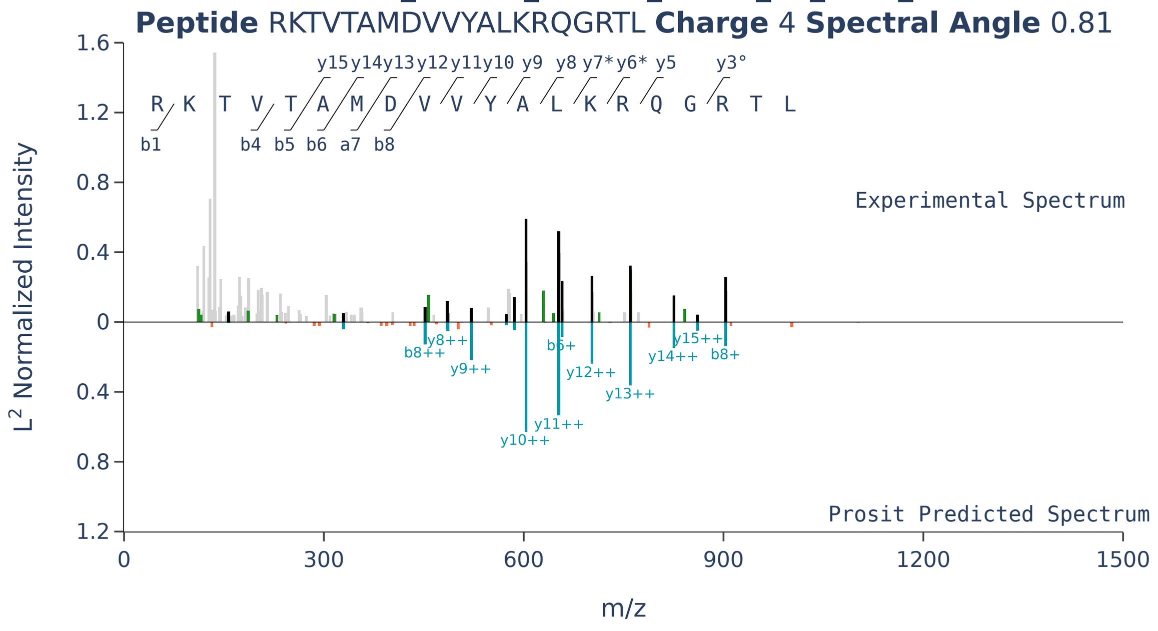


**Source** HRoetschke_291222_140123_HFGoe_H4_b1R1_2h **Scan** 17902
**Peptide** AVTYTEHAKRKTVTAMDVVYAL **Charge** 3 **Spectral Angle** 0.6

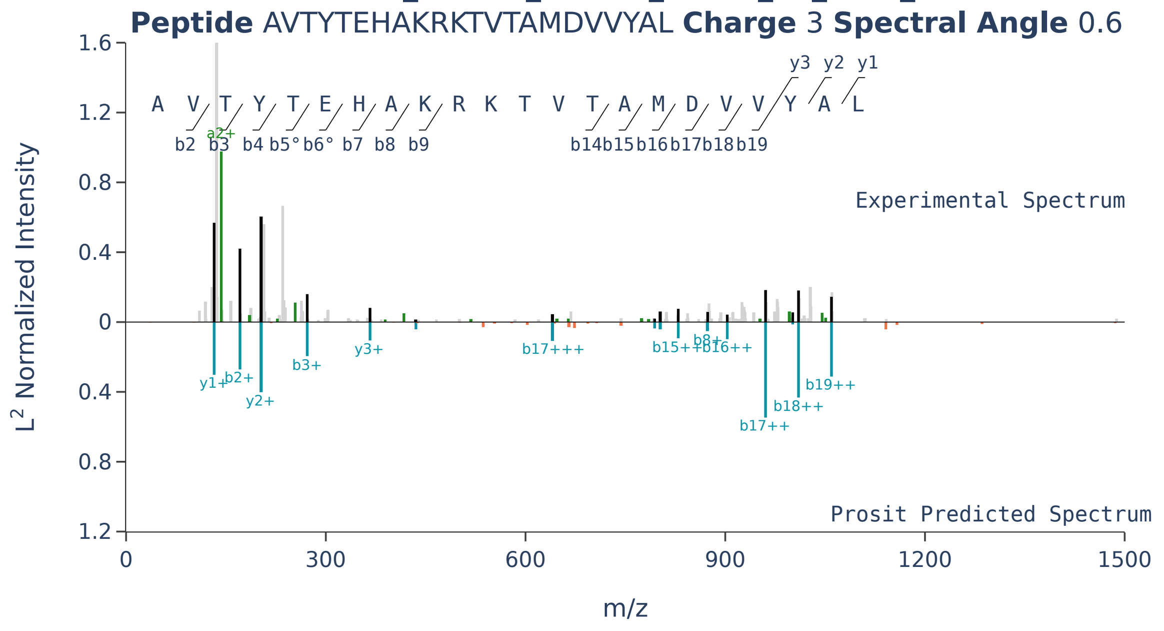


**Source** HRoetschke_291222_140123_HFGoe_H4_b1R1_24h **Scan** 10010
**Peptide** GLTKPAIRRLA **Charge** 3 **Spectral Angle** 0.9

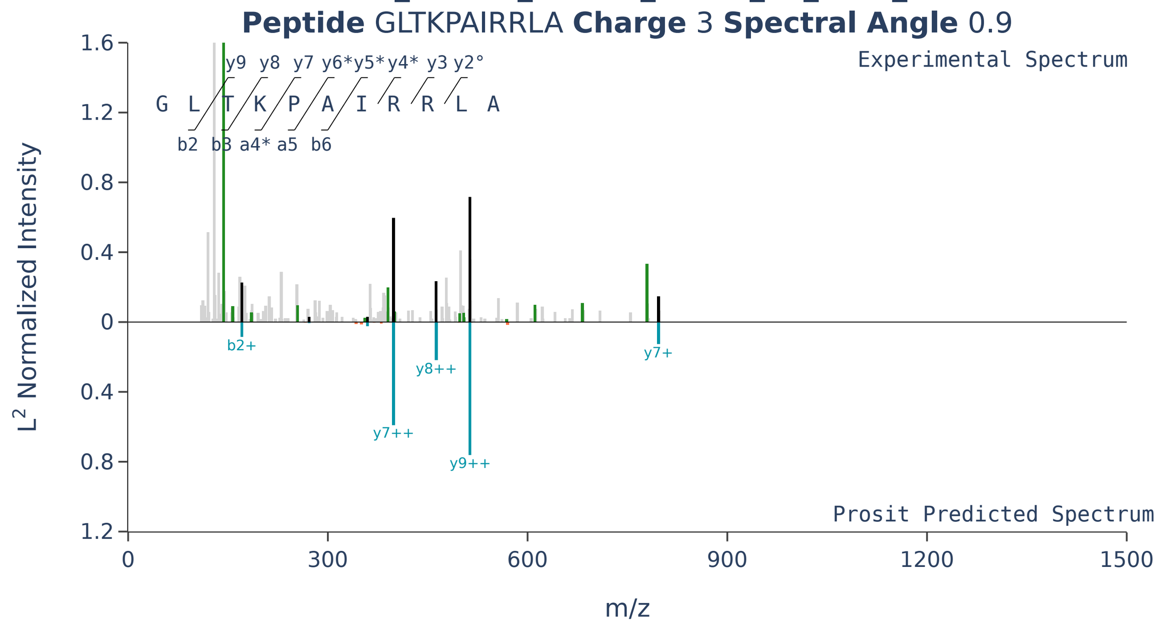


**Source** HRoetschke_291222_140123_HFGoe_H4_b1R2_1h **Scan** 15433
**Peptide** VTYTEHAKRKTVTAMDVVYALKRQGR **Charge** 5 **Spectral Angle** 0.8

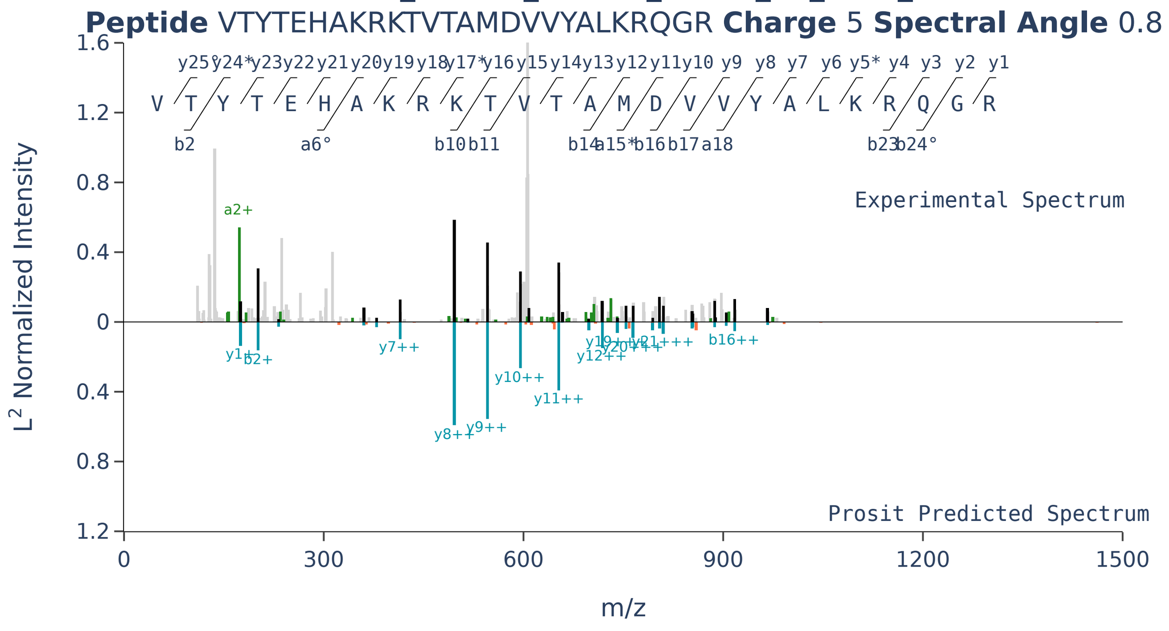


**Source** HRoetschke_291222_140123_HFGoe_H4_b1R1_1h **Scan** 17122
**Peptide** NIQGITKPAIRRLARRGGVKRISGLIYE **Charge** 4 **Spectral Angle** 0.74

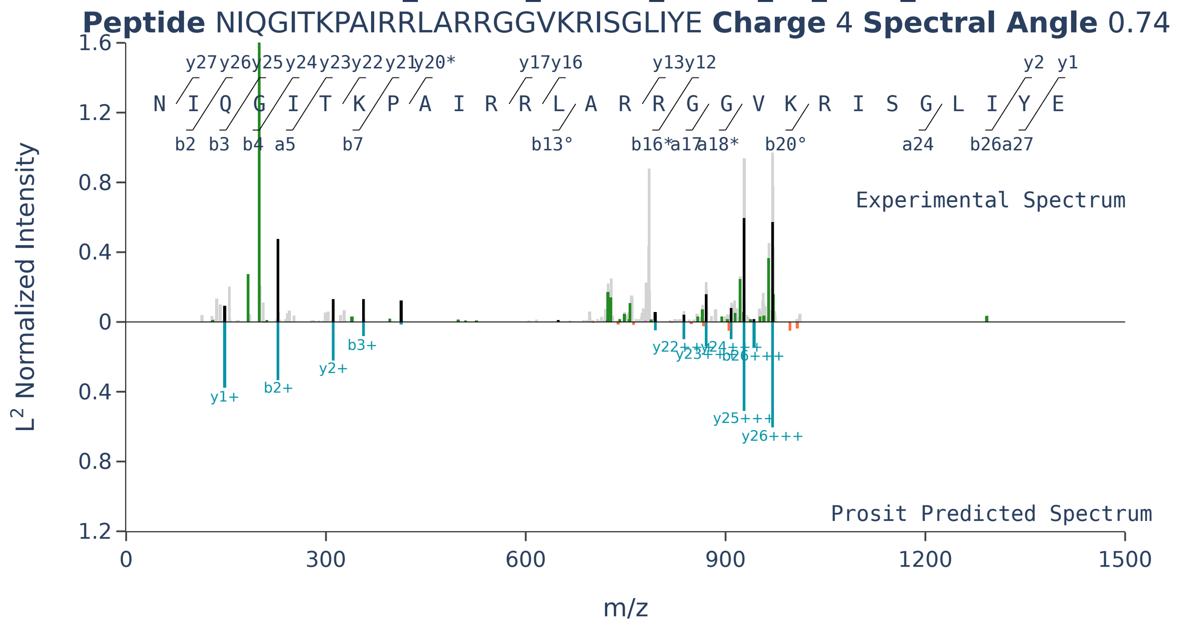


**Source** HRoetschke_291222_140123_HFGoe_H4_b2R1_1h **Scan** 26780
**Peptide** ARRGGVKRISGLIYEETRGVLKVFL **Charge** 5 **Spectral Angle** 0.65

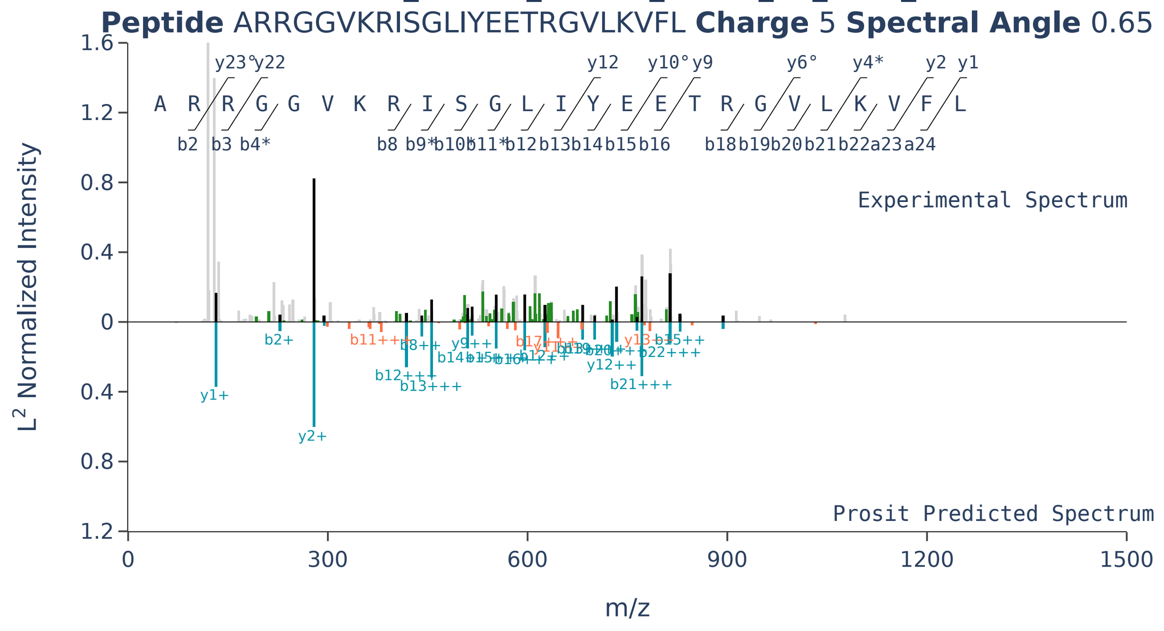

**Source** HRoetschke_291222_140123_HFGoe_H4_b2R2_1h **Scan** 25168
**Peptide** TRGVLKVFLENV **Charge** 3 **Spectral Angle** 0.69

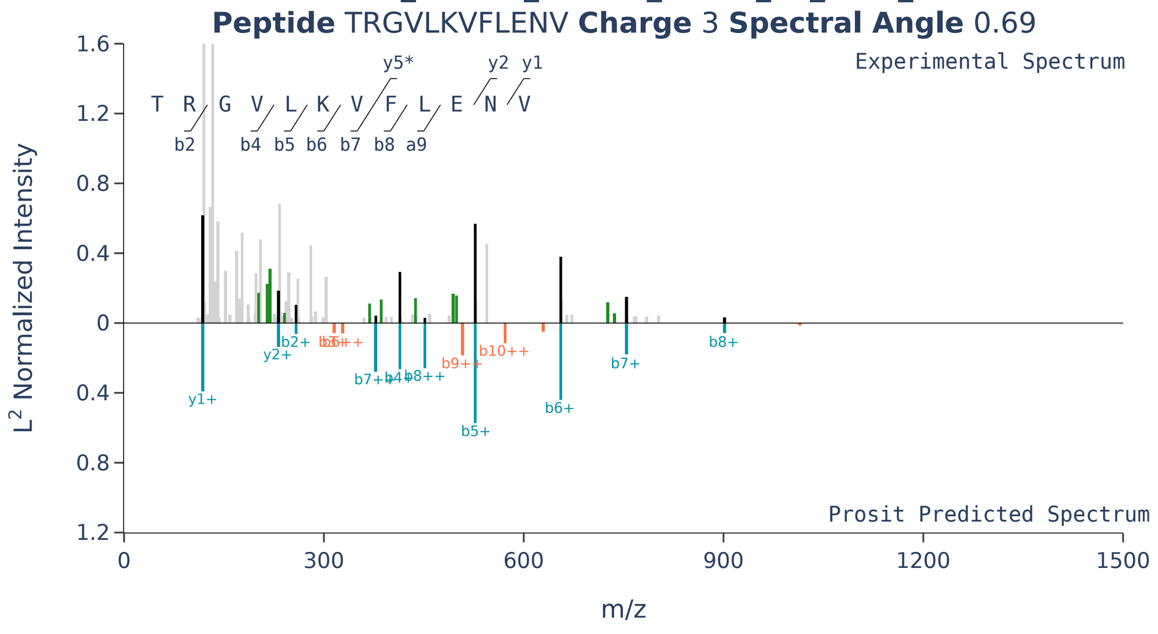


**Source** HRoetschke_291222_140123_HFGoe_H4_b1R2_1h **Scan** 16233
**Peptide** RDAVTYTEHAKRKTVTAMDVVYAL **Charge** 5 **Spectral Angle** 0.62

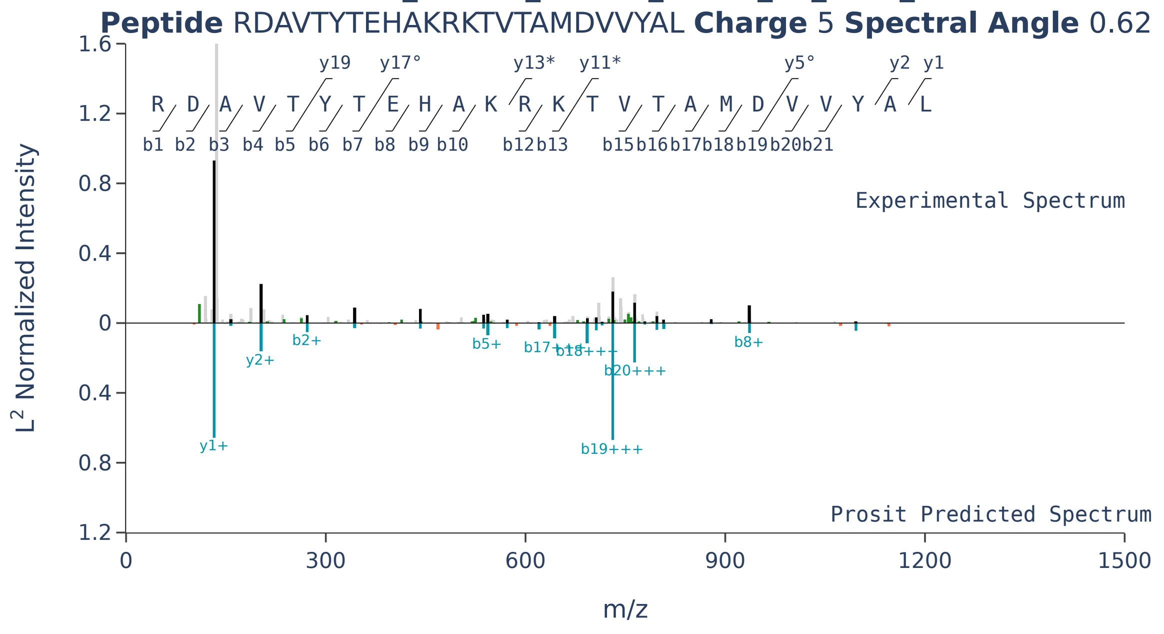


**Source** HRoetschke_291222_140123_HFGoe_H4_b2R2_4h **Scan** 14692
**Peptide** ALKRQGR **Charge** 2 **Spectral Angle** 0.91

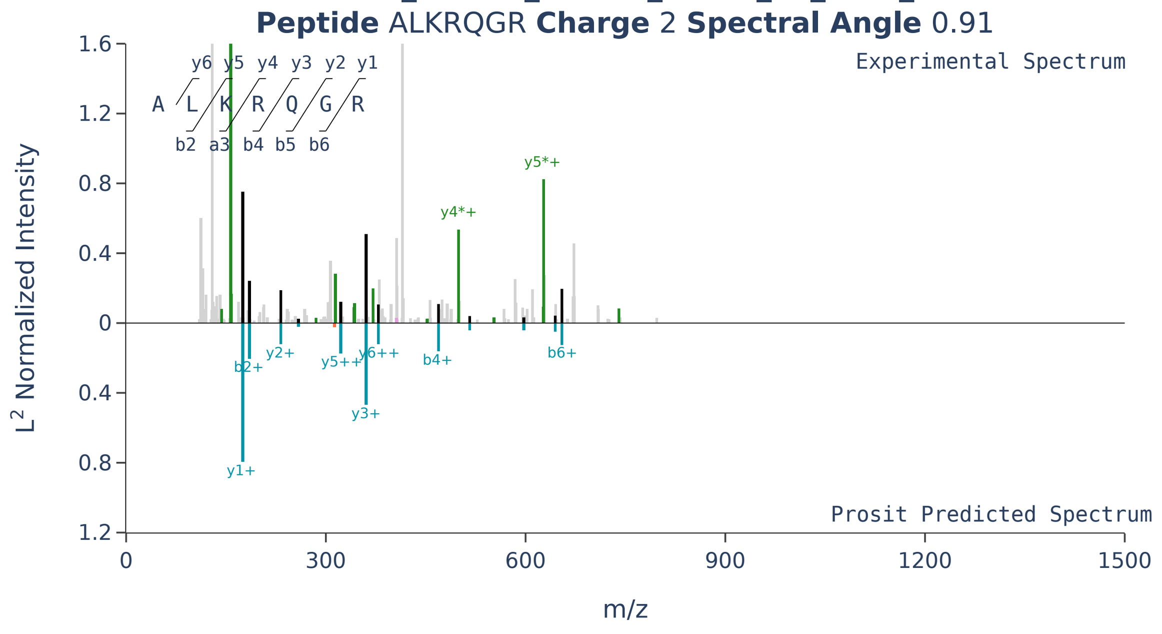


**Source** HRoetschke_291222_140123_HFGoe_H4_b1R1_24h **Scan** 9575
**Peptide** HRKVLRDNIQGITKPAIR **Charge** 5 **Spectral Angle** 0.71

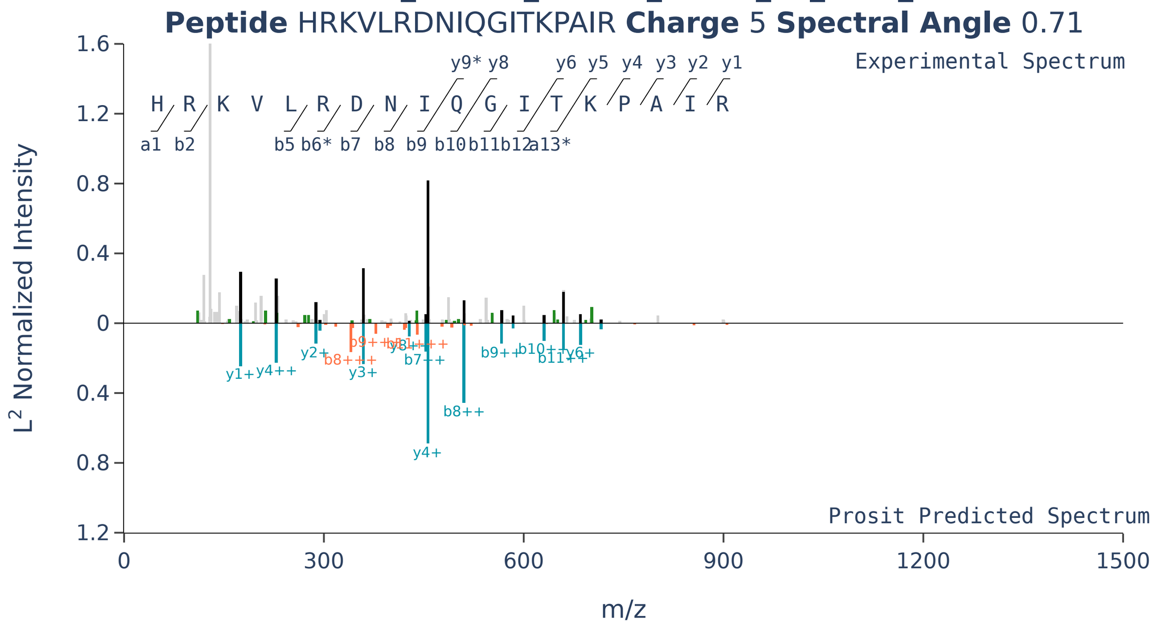


**Source** HRoetschke_291222_140123_HFGoe_H4_b1R1_2h **Scan** 22464
**Peptide** KVFLENVIRDA **Charge** 3 **Spectral Angle** 0.72

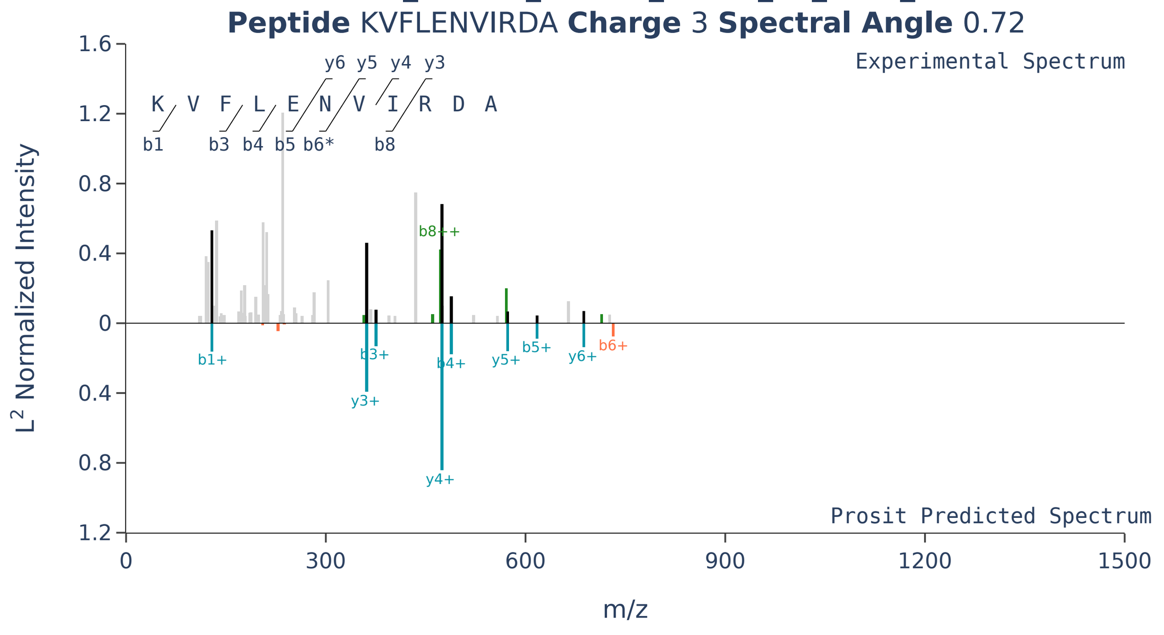


**Source** HRoetschke_291222_140123_HFGoe_H4_b2R2_2h **Scan** 8791
**Peptide** QGITKPAIRRLARRGGVKR **Charge** 4 **Spectral Angle** 0.62

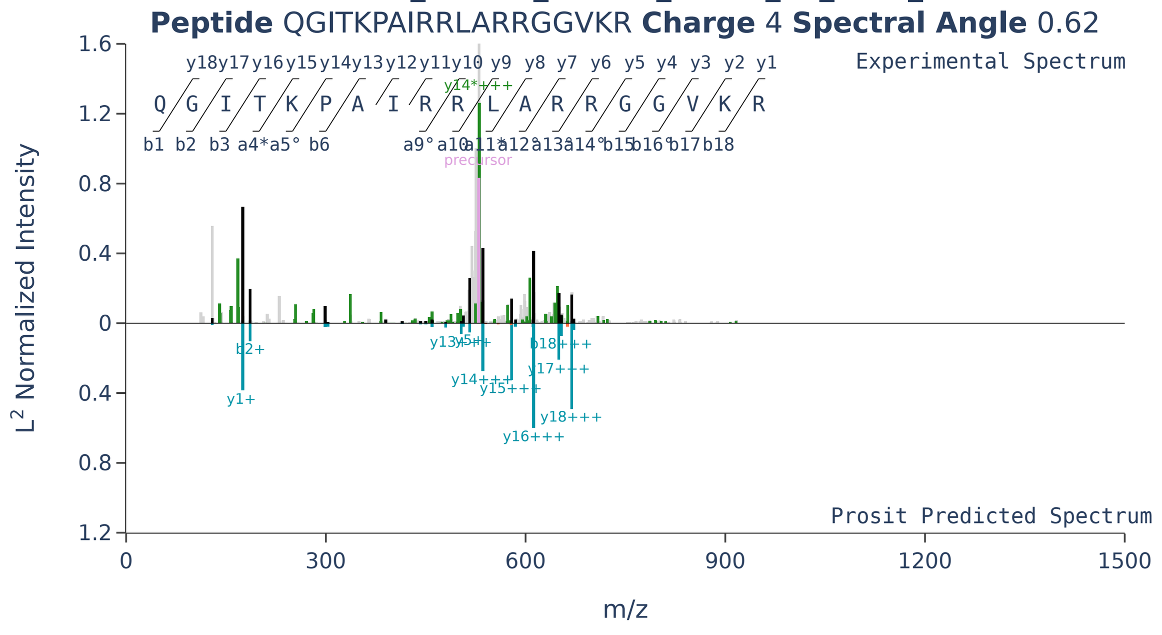


**Source** HRoetschke_291222_140123_HFGoe_H4_b1R1_1h **Scan** 17409
**Peptide** KRKTVTAMDVVYALKR **Charge** 4 **Spectral Angle** 0.8

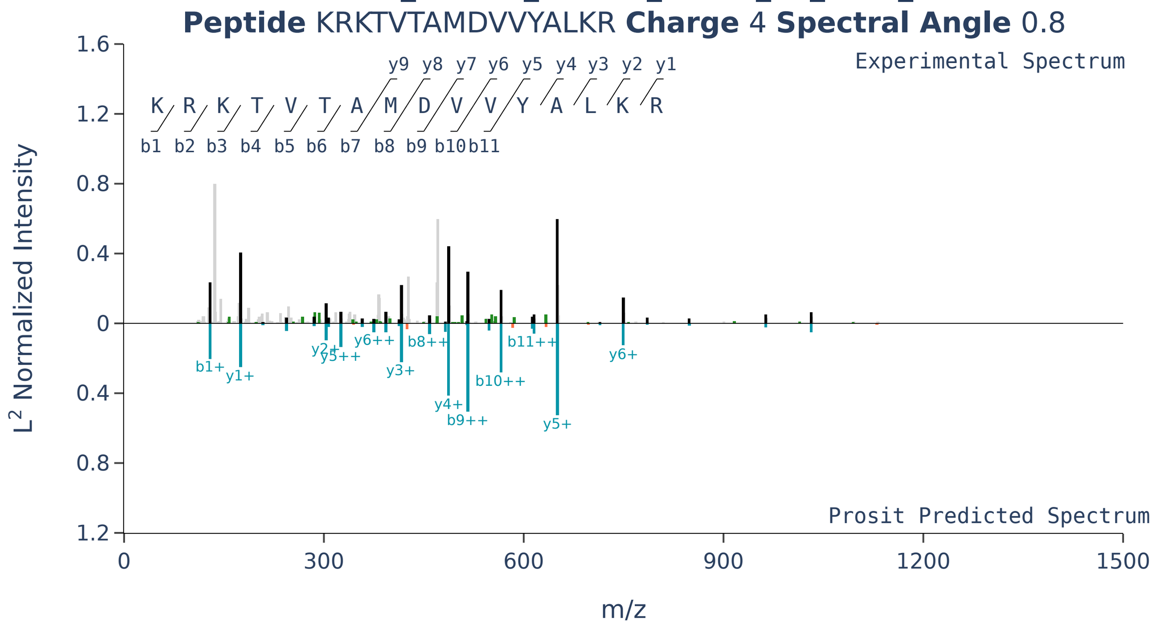


**Source** HRoetschke_291222_140123_HFGoe_H4_b1R1_4h **Scan** 5386
**Peptide** AVTYTEHAKRKTV **Charge** 4 **Spectral Angle** 0.71

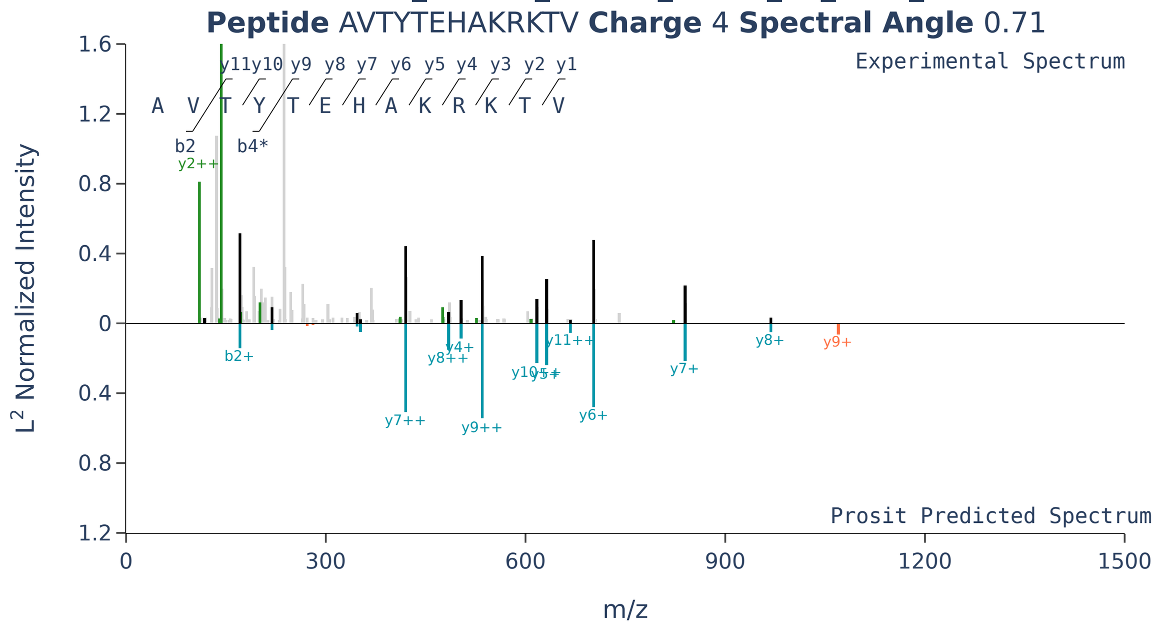


**Source** HRoetschke_291222_140123_HFGoe_H4_b1R1_24h **Scan** 9679
**Peptide** KVLRDNIQGITK **Charge** 3 **Spectral Angle** 0.7

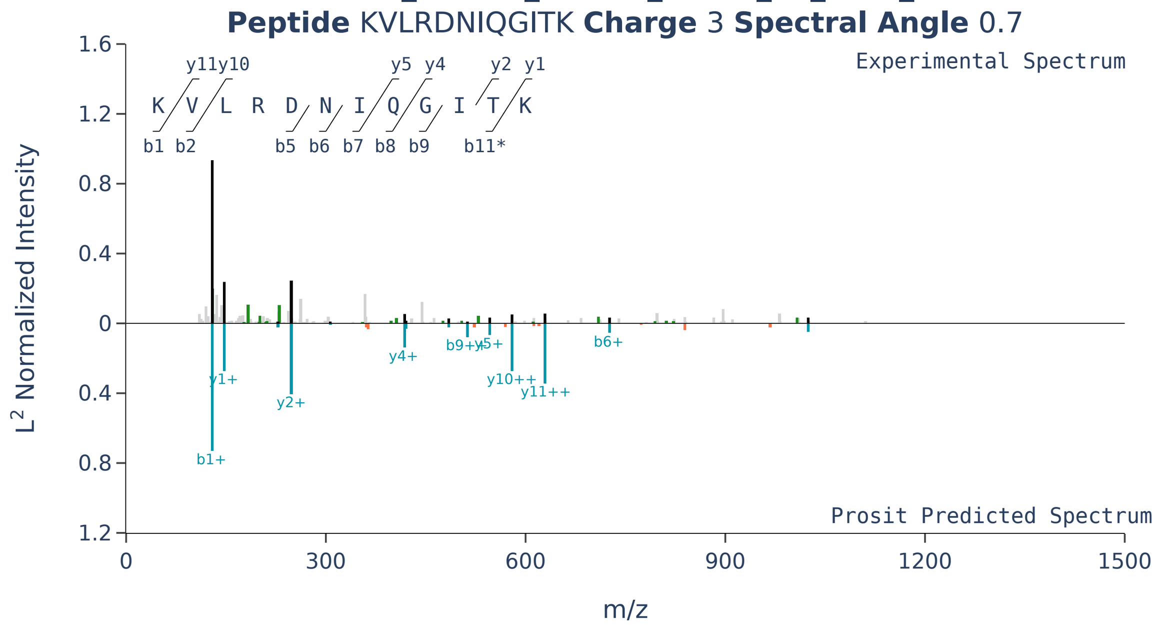


**Source** HRoetschke_291222_140123_HFGoe_H4_b1R1_1h **Scan** 16832
**Peptide** NIQGITKPAIRRLARRGGVKRISGLL **Charge** 5 **Spectral Angle** 0.73

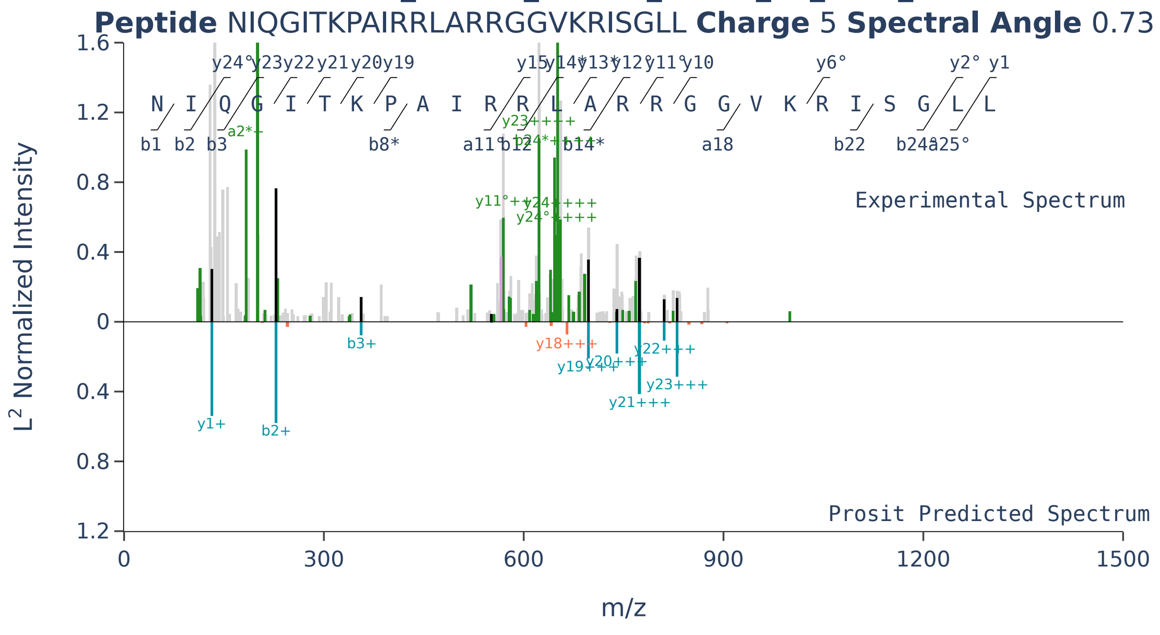


**Source** HRoetschke_291222_140123_HFGoe_H4_b1R1_4h **Scan** 18051
**Peptide** ENVIRDAVTYTEHAKRKTVTAMD **Charge** 4 **Spectral Angle** 0.58

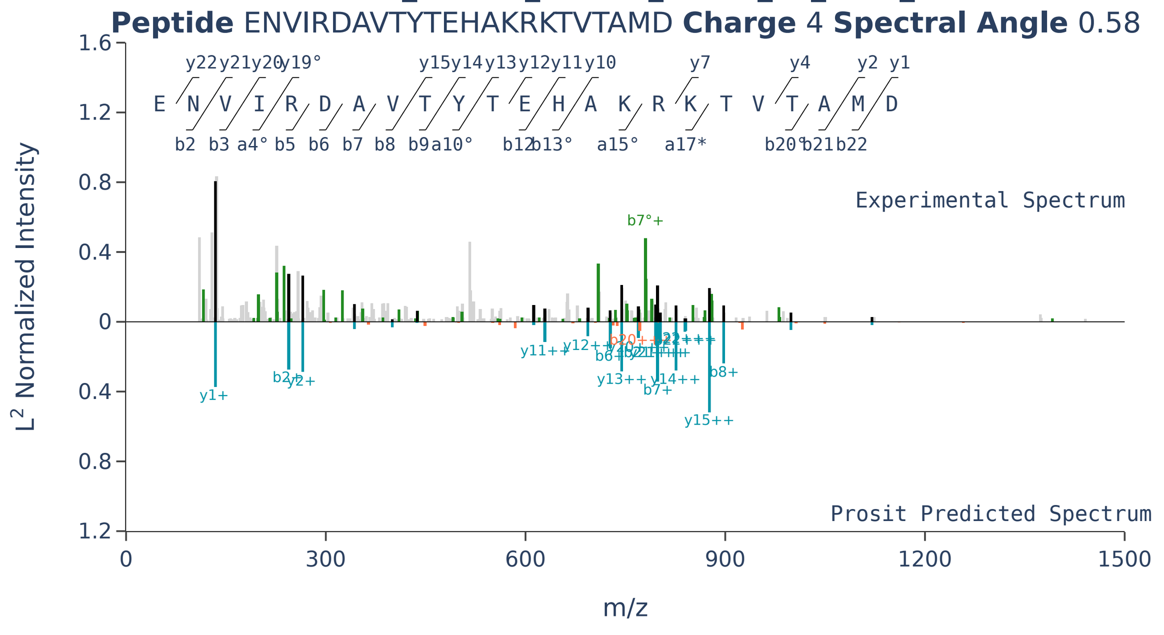


**Source** HRoetschke_291222_140123_HFGoe_H4_b1R2_2h **Scan** 23599
**Peptide** LENVIRDAVTYTEHA **Charge** 2 **Spectral Angle** 0.86

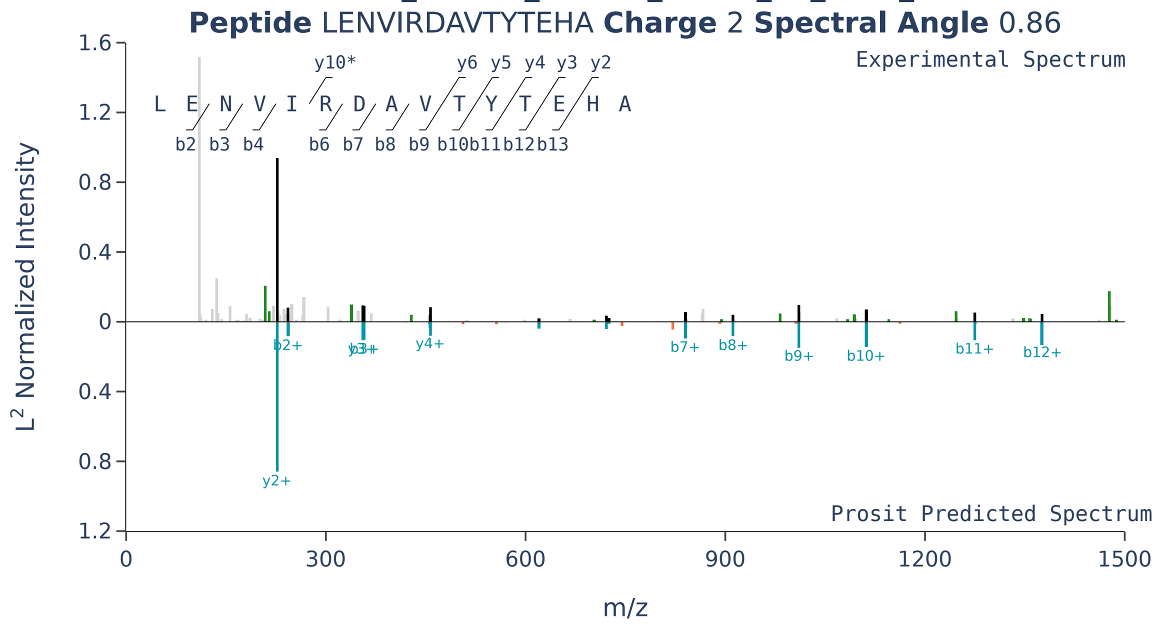


**Source** HRoetschke_291222_140123_HFGoe_H4_b1R2_24h **Scan** 6939
**Peptide** QGITKPAIRRLARRGGVK **Charge** 4 **Spectral Angle** 0.8

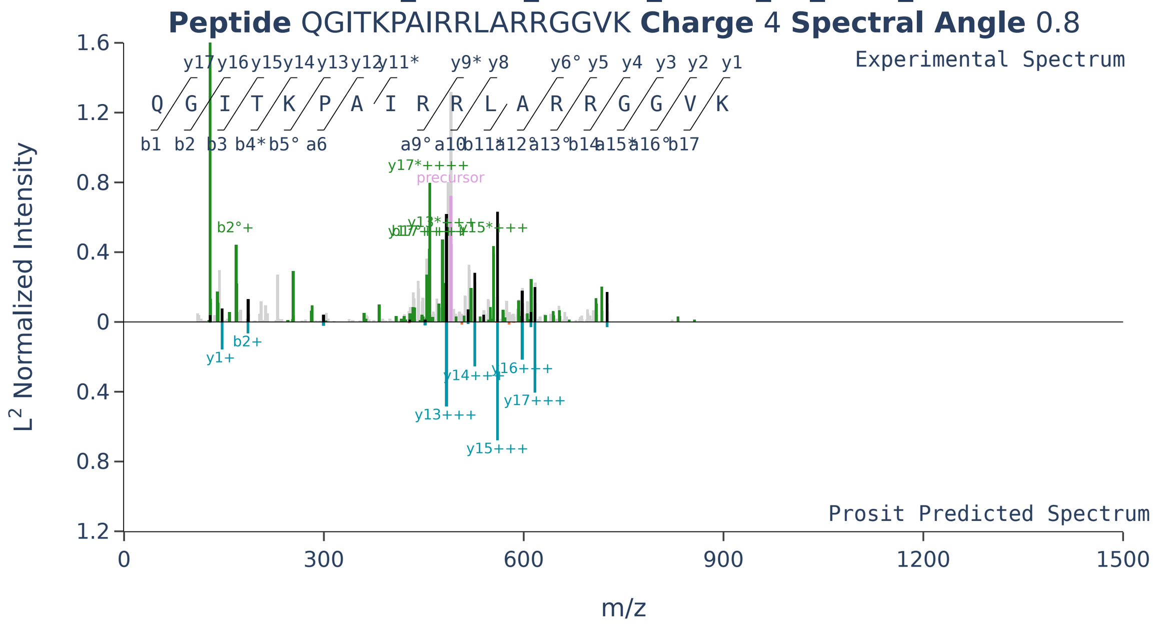


**Source** HRoetschke_291222_140123_HFGoe_H4_b1R1_24h **Scan** 5253
**Peptide** YTEHAKRKTVT **Charge** 3 **Spectral Angle** 0.51

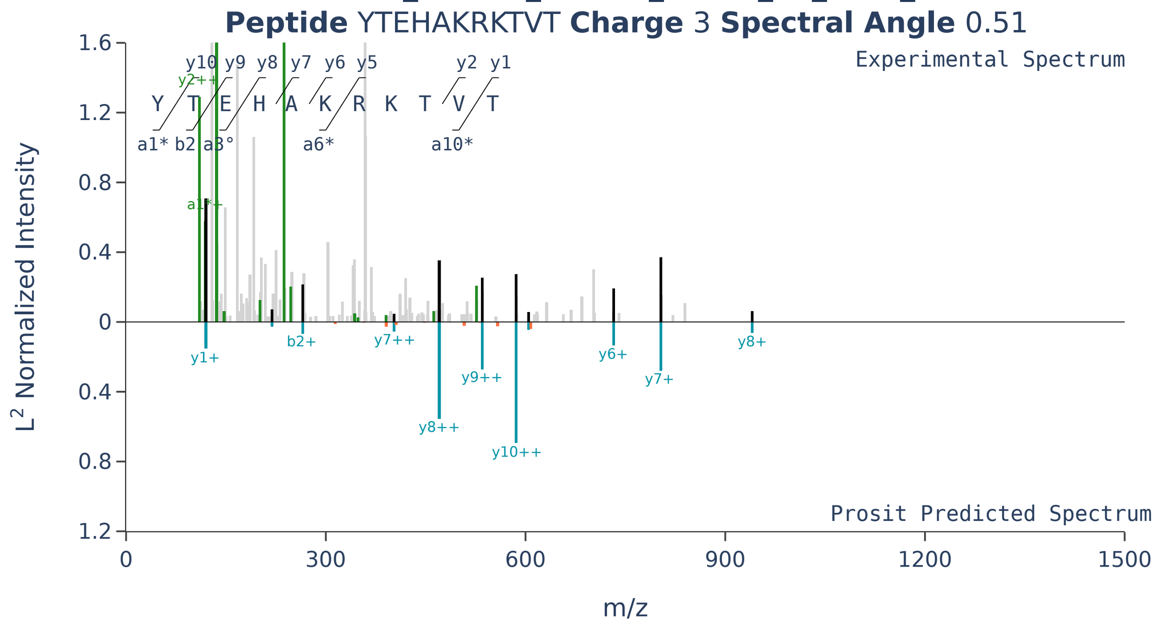


**Source** HRoetschke_291222_140123_HFGoe_H4_b1R1_24h **Scan** 13415
**Peptide** NVIRDAVTYTEHAKRKTVT **Charge** 3 **Spectral Angle** 0.7

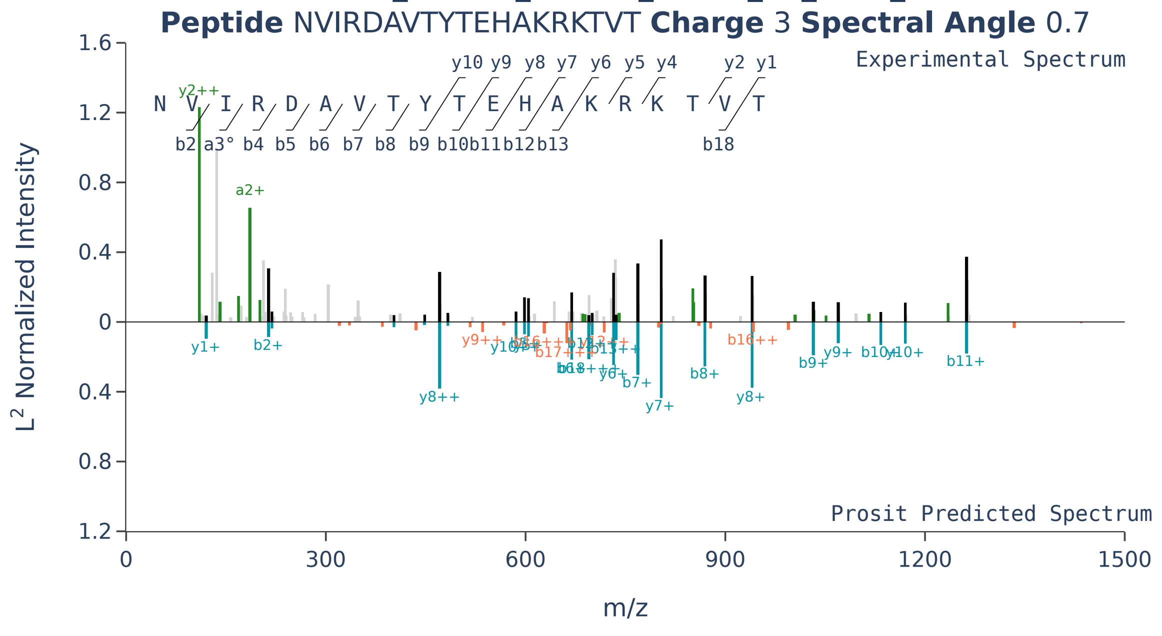

**Source** HRoetschke_291222_140123_HFGoe_H4_b2R2_2h **Scan** 12007
**Peptide** TEHAKRKTVTAMDVVYA **Charge** 3 **Spectral Angle** 0.54

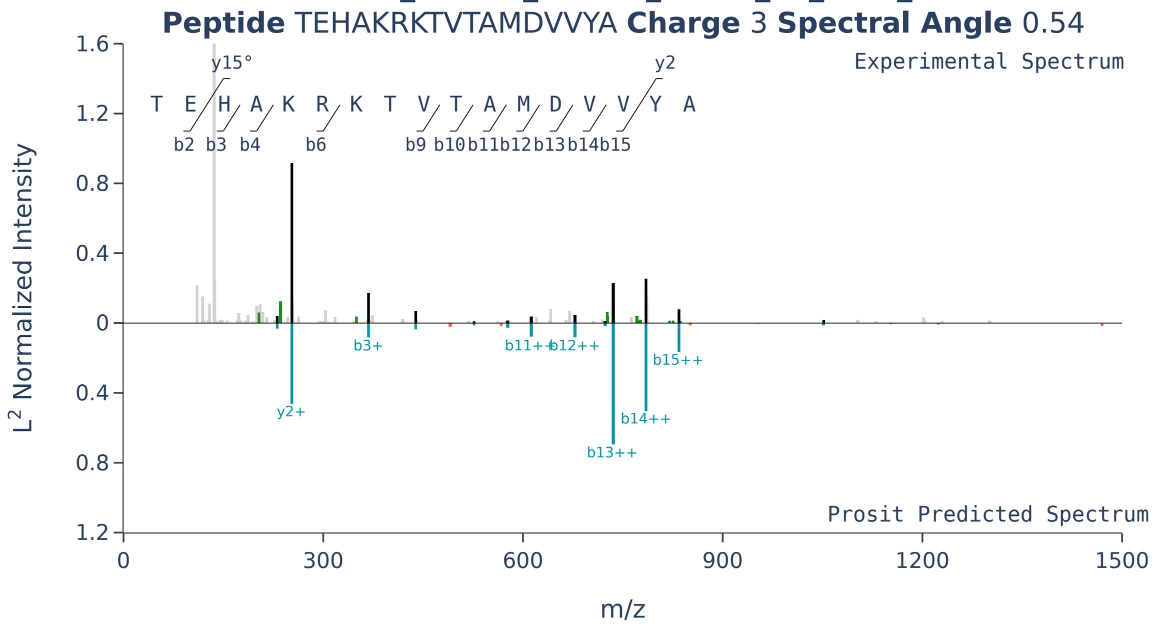

**Source** HRoetschke_291222_140123_HFGoe_H4_b1R1_1h **Scan** 20618
**Peptide** LIYEETRGVLKV **Charge** 2 **Spectral Angle** 0.75

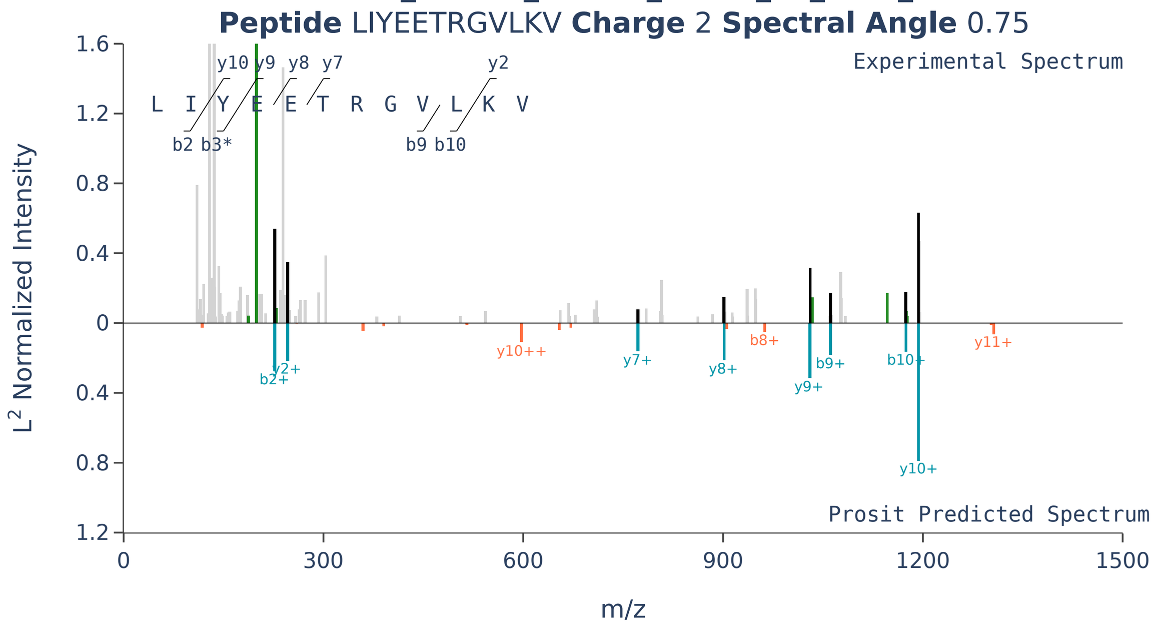

**Source** HRoetschke_291222_140123_HFGoe_H4_b2R1_1h **Scan** 15472
**Peptide** QGITKPAIRRLARRGGVKRISGLIYEET **Charge** 5 **Spectral Angle** 0.81

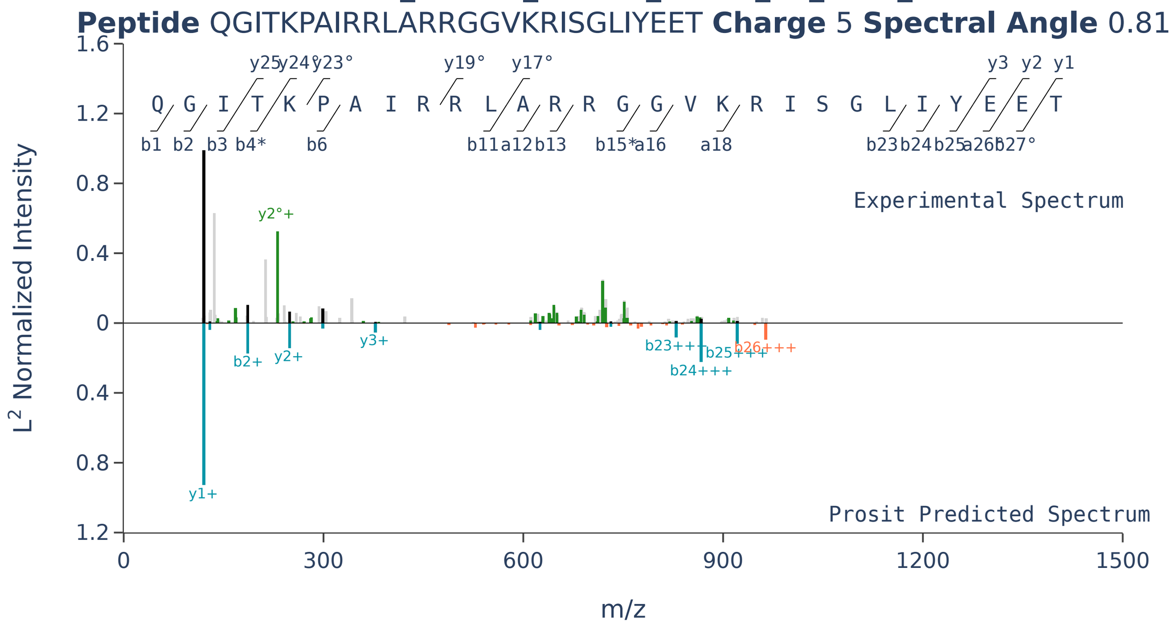

**Source** HRoetschke_291222_140123_HFGoe_H4_b2R1_1h **Scan** 36188
**Peptide** GLIYEETRGVLKVFLENVIRD **Charge** 3 **Spectral Angle** 0.74

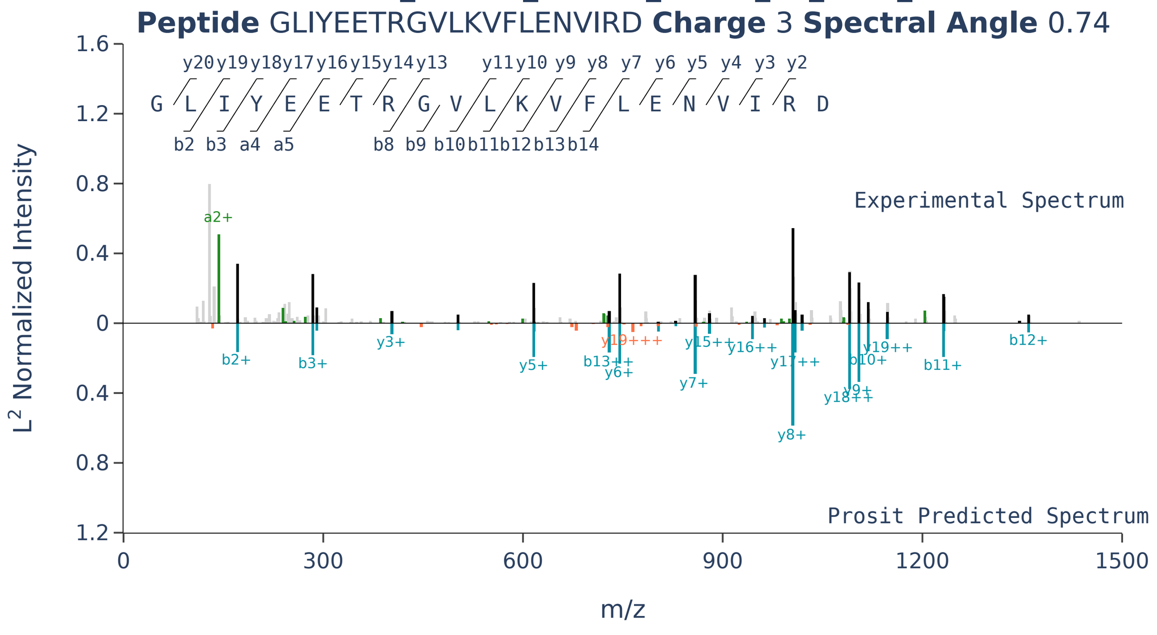

**Source** HRoetschke_291222_140123_HFGoe_H4_b1R1_2h **Scan** 16942
**Peptide** KRKTVTAMDVVYALKRQGRT **Charge** 4 **Spectral Angle** 0.8

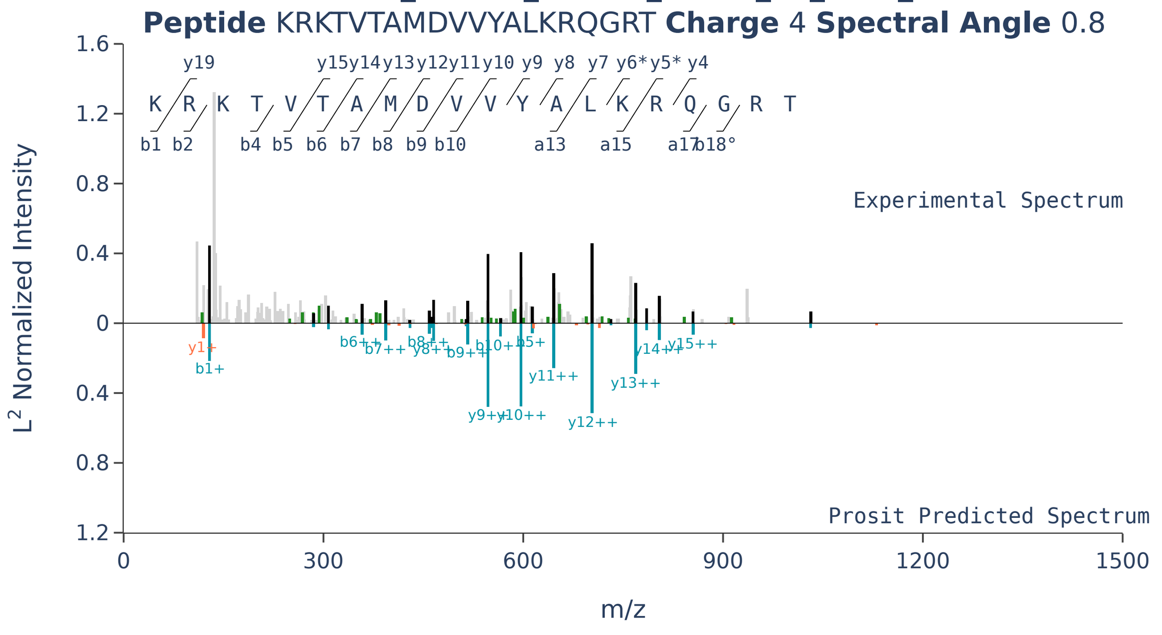

**Source** HRoetschke_291222_140123_HFGoe_H4_b2R2_24h **Scan** 10711
**Peptide** PAIRRLA **Charge** 2 **Spectral Angle** 0.88

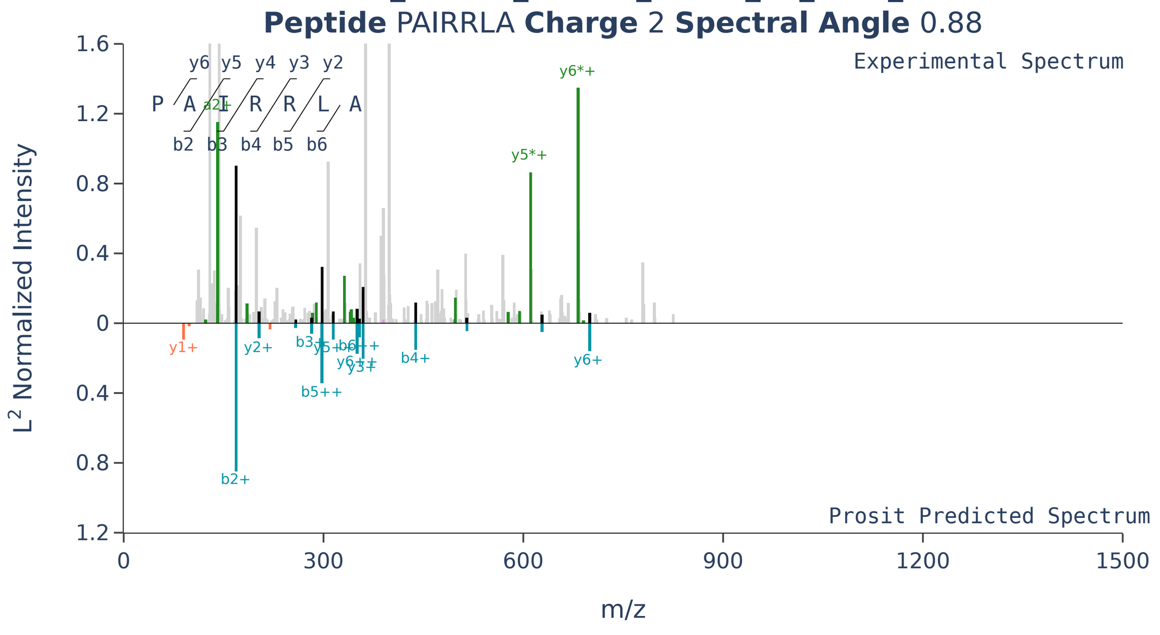

**Source** HRoetschke_291222_140123_HFGoe_H4_b2R2_4h **Scan** 12861
**Peptide** QGITKPALR **Charge** 2 **Spectral Angle** 0.88

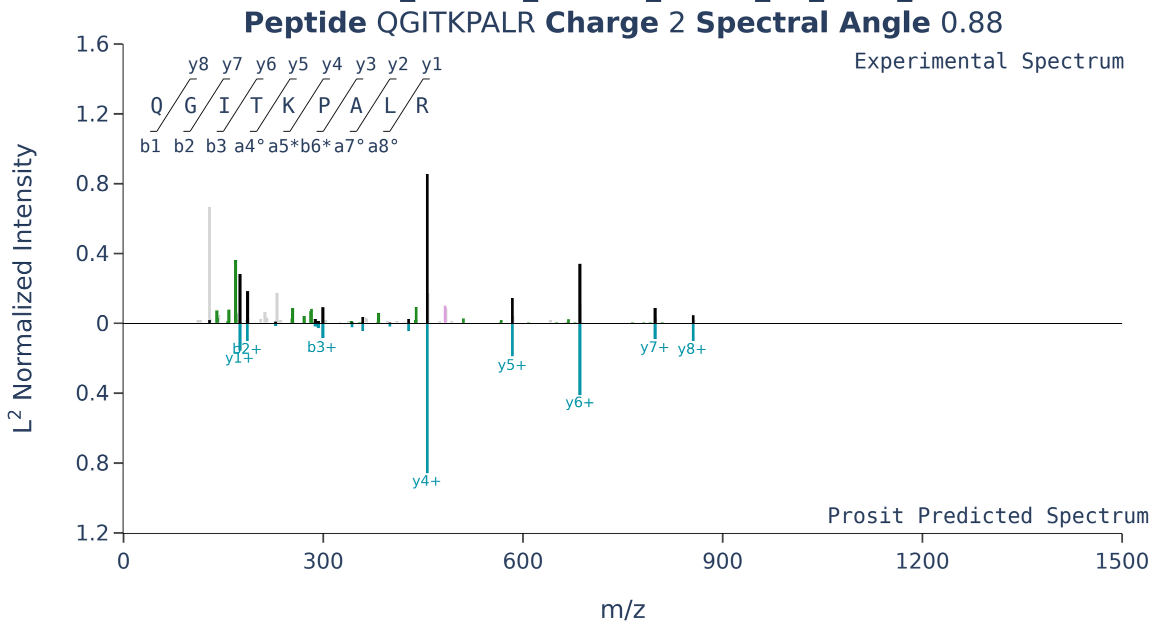

**Source** HRoetschke_291222_140123_HFGoe_H4_b2R2_1h **Scan** 16613
**Peptide** RDAVTYTEHAKRKTVTAMDVVYALKRQ **Charge** 5 **Spectral Angle** 0.78

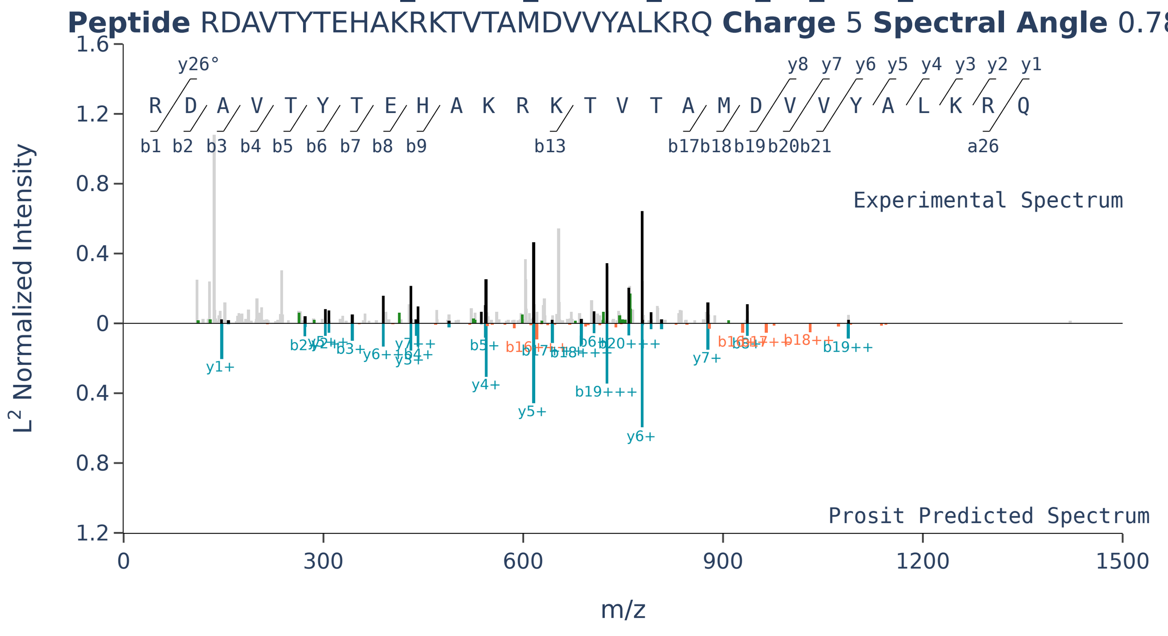

**Source** HRoetschke_291222_140123_HFGoe_H4_b2R1_4h **Scan** 15742
**Peptide** RDAVTYTEHAKRKTVTAMDVVYALKRQGR **Charge** 5 **Spectral Angle** 0.66

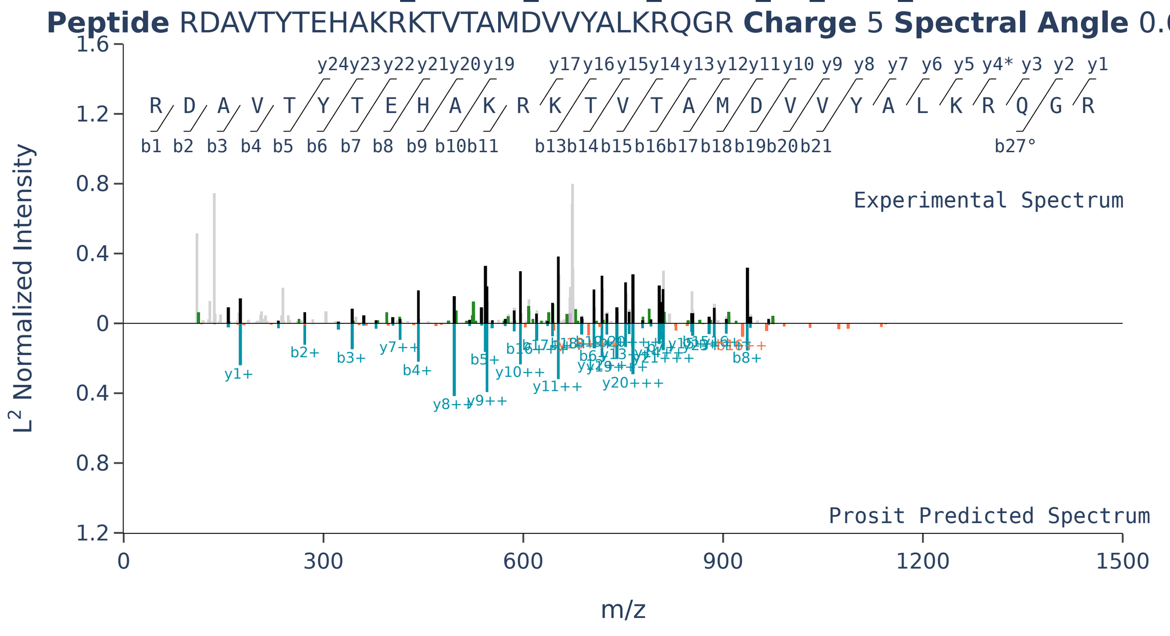

**Source** HRoetschke_291222_140123_HFGoe_H4_b1R1_1h **Scan** 22895
**Peptide** ETRGVLKVFLEN **Charge** 2 **Spectral Angle** 0.72

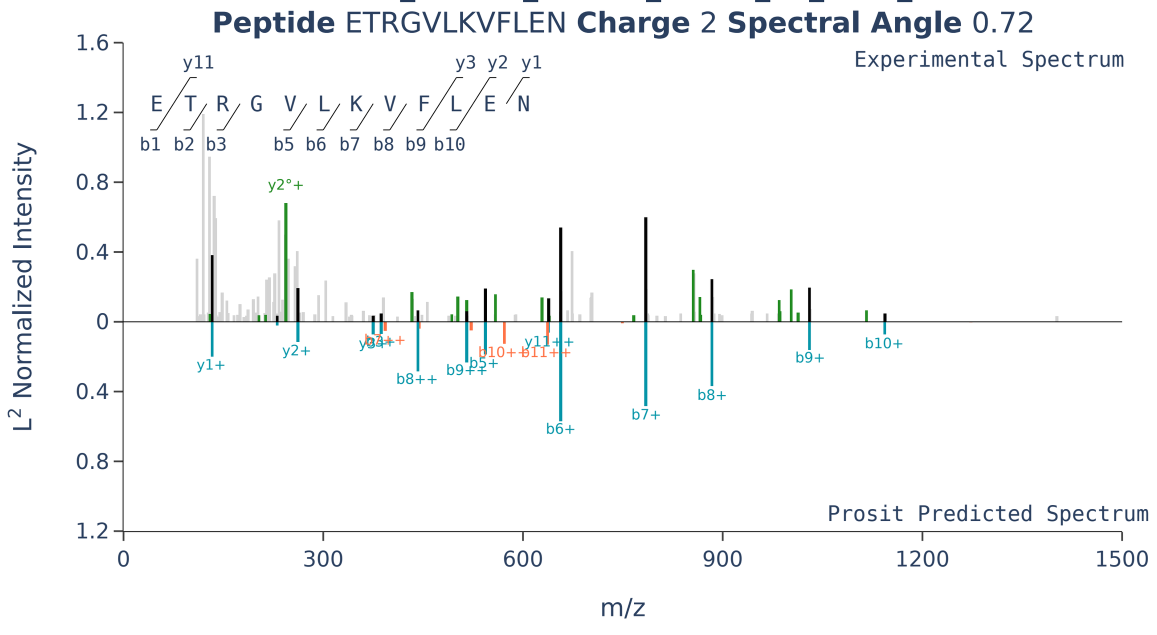

**Source** HRoetschke_291222_140123_HFGoe_H4_b1R1_24h **Scan** 14076
**Peptide** RGGVKRISGLIYEE **Charge** 3 **Spectral Angle** 0.64

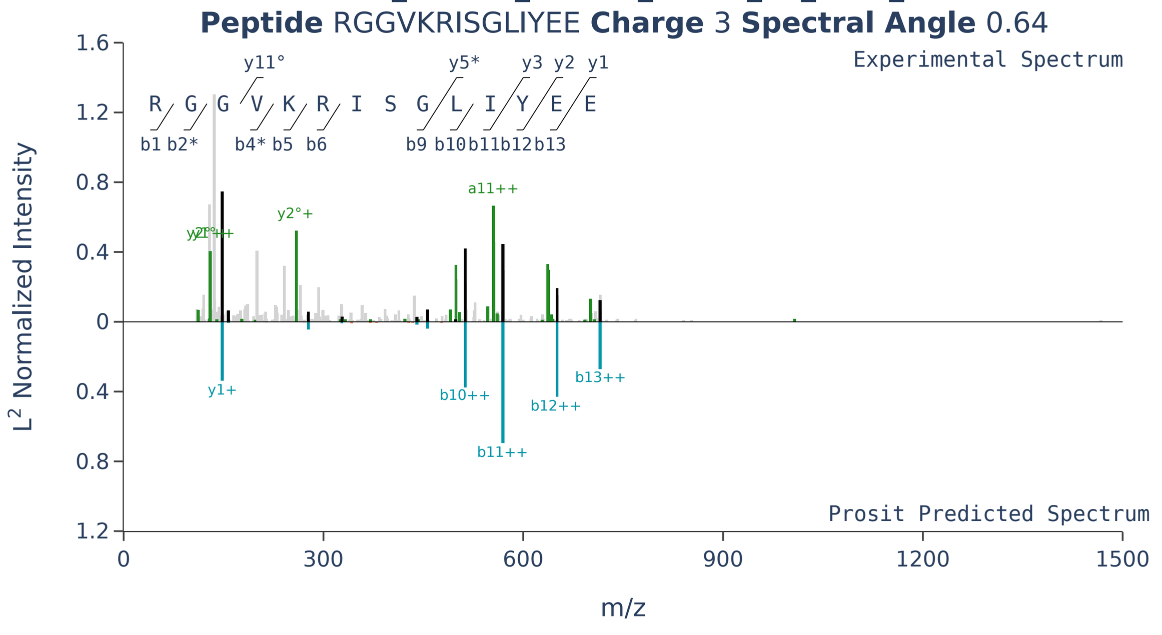

**Source** HRoetschke_291222_140123_HFGoe_H4_b2R1_1h **Scan** 5431
**Peptide** RDAVTYTEHAKRKTV **Charge** 4 **Spectral Angle** 0.7

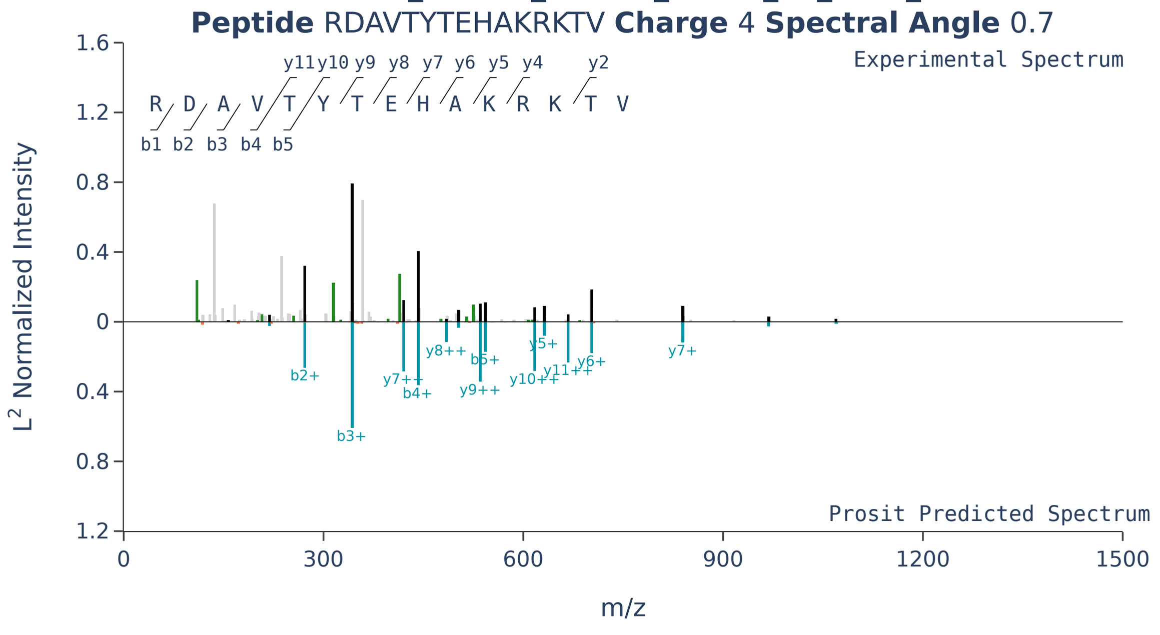

**Source** HRoetschke_291222_140123_HFGoe_H4_b1R2_24h **Scan** 7838
**Peptide** RKVLRDNIQGITK **Charge** 4 **Spectral Angle** 0.74

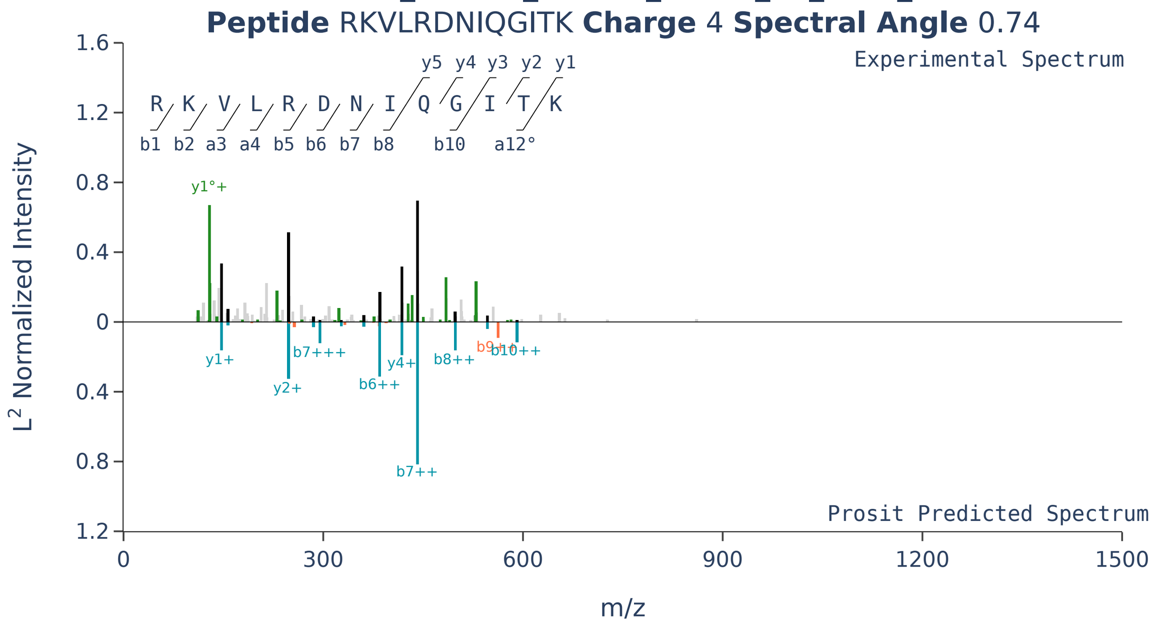

**Source** HRoetschke_291222_140123_HFGoe_H4_b2R1_1h **Scan** 22561
**Peptide** KTVTAMDVVYALKRQGRTL **Charge** 3 **Spectral Angle** 0.66

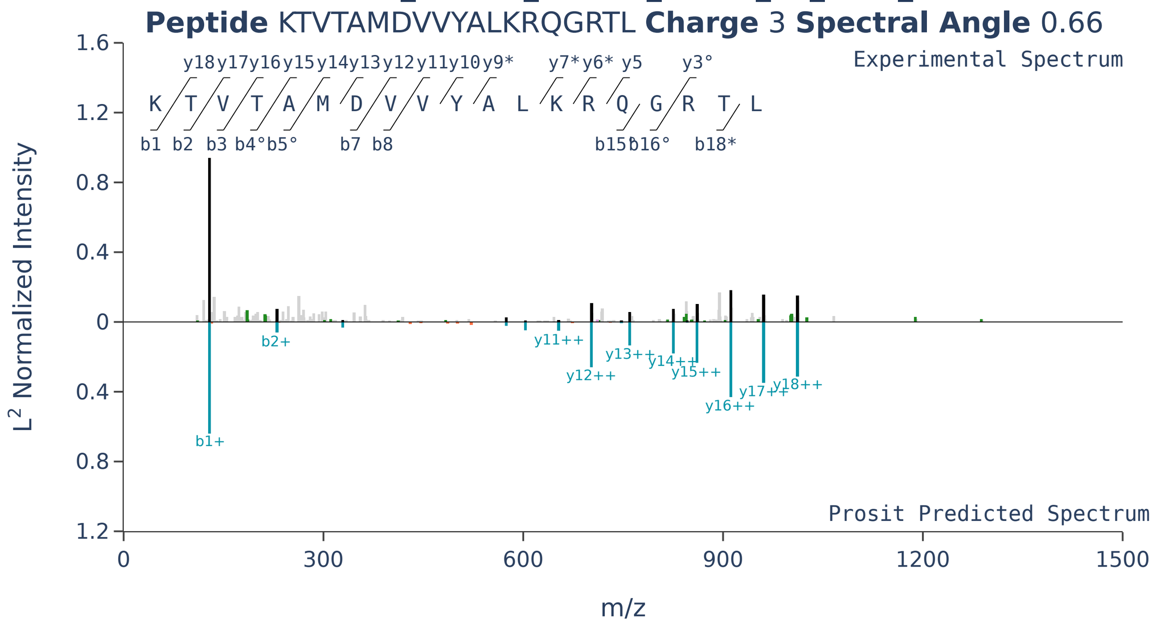

**Source** HRoetschke_291222_140123_HFGoe_H4_b1R1_4h **Scan** 6456
**Peptide** IRDAVTYTEHAKRKTVT **Charge** 3 **Spectral Angle** 0.8

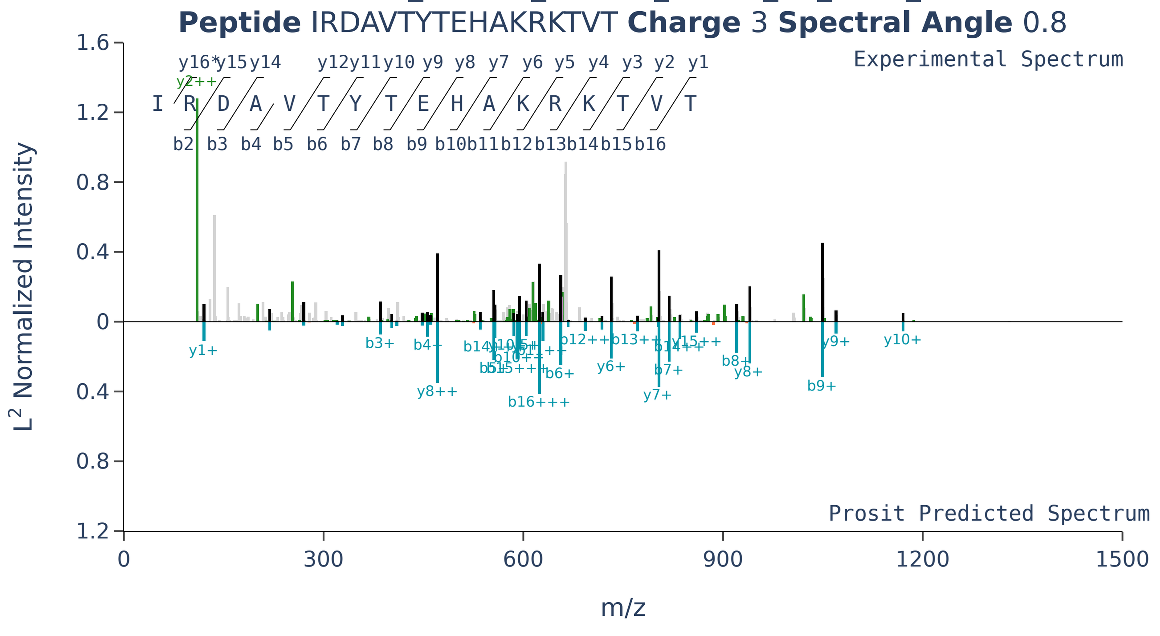

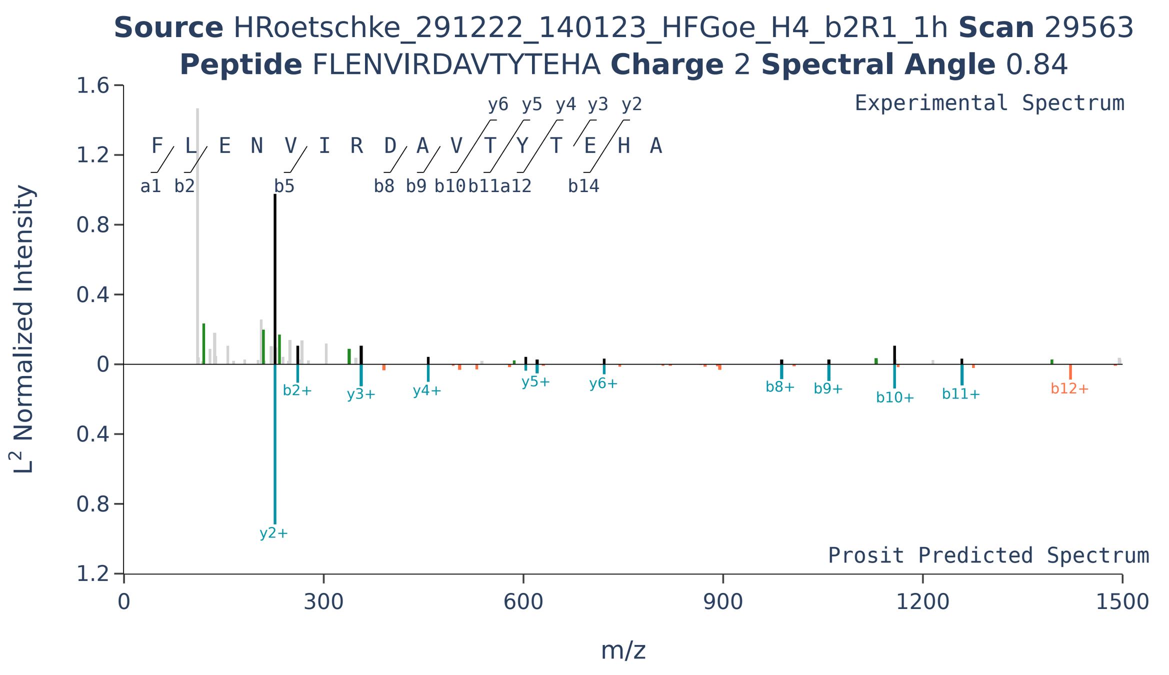
Source HRoetschke_291222_140123_HFGoe_H4_b2R1_1h Scan 29563
Peptide FLENVIRDAVTYTEHA Charge 2 Spectral Angle 0.84
Experimental Spectrum
Prosit Predicted Spectrum
L² Normalized Intensity
m/z

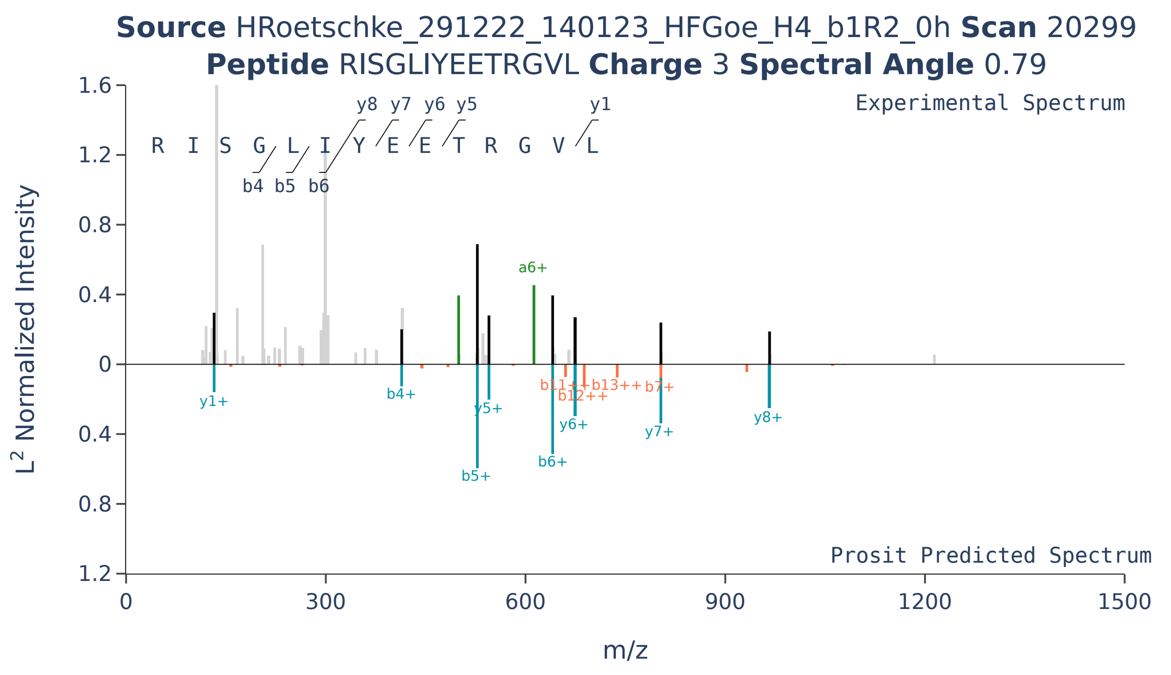
Source HRoetschke_291222_140123_HFGoe_H4_b1R2_0h Scan 20299
Peptide RISGLIYEETRGVL Charge 3 Spectral Angle 0.79
Experimental Spectrum
Prosit Predicted Spectrum
L² Normalized Intensity
m/z

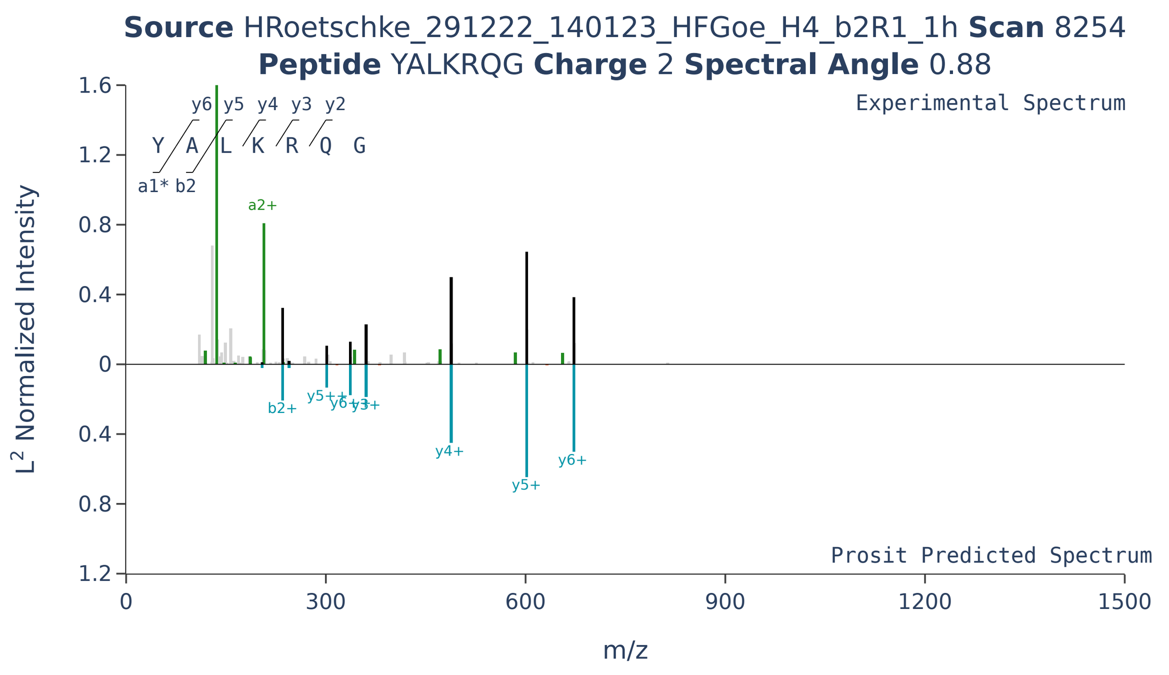
Source HRoetschke_291222_140123_HFGoe_H4_b2R1_1h Scan 8254
Peptide YALKRQG Charge 2 Spectral Angle 0.88
Experimental Spectrum
Prosit Predicted Spectrum
L² Normalized Intensity
m/z

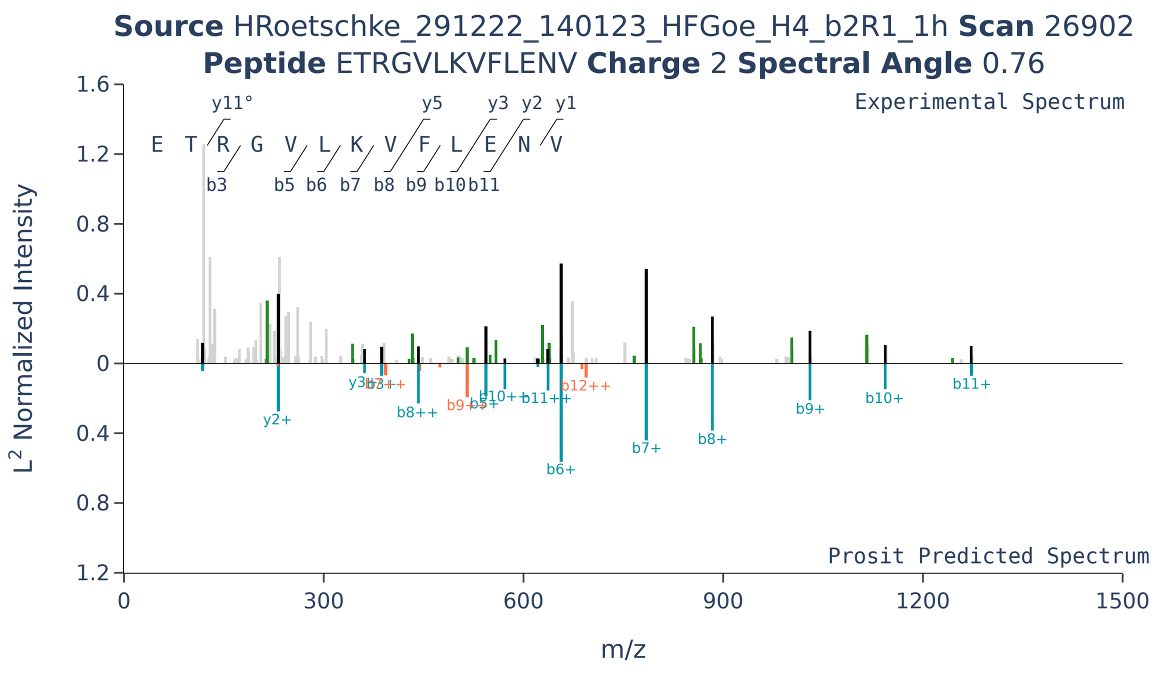
Source HRoetschke_291222_140123_HFGoe_H4_b2R1_1h Scan 26902
Peptide ETRGVLKVFLENV Charge 2 Spectral Angle 0.76
Experimental Spectrum
Prosit Predicted Spectrum
L² Normalized Intensity
m/z

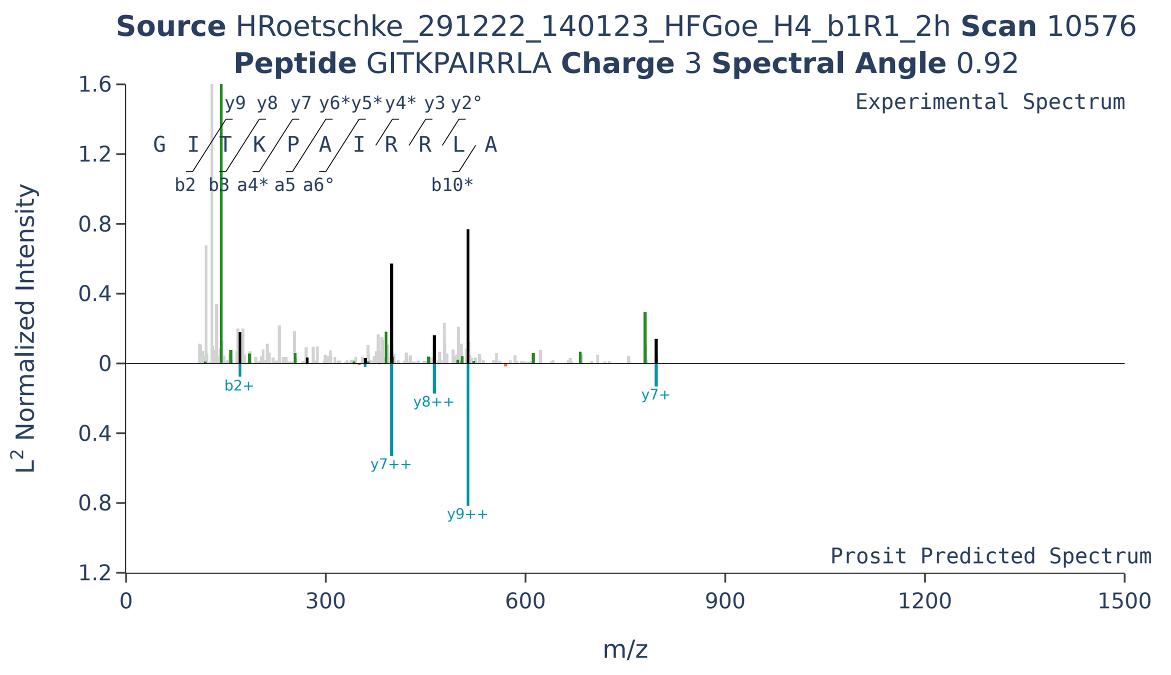
Source HRoetschke_291222_140123_HFGoe_H4_b1R1_2h Scan 10576
Peptide GITKPAIRRLA Charge 3 Spectral Angle 0.92
Experimental Spectrum
Prosit Predicted Spectrum
L² Normalized Intensity
m/z

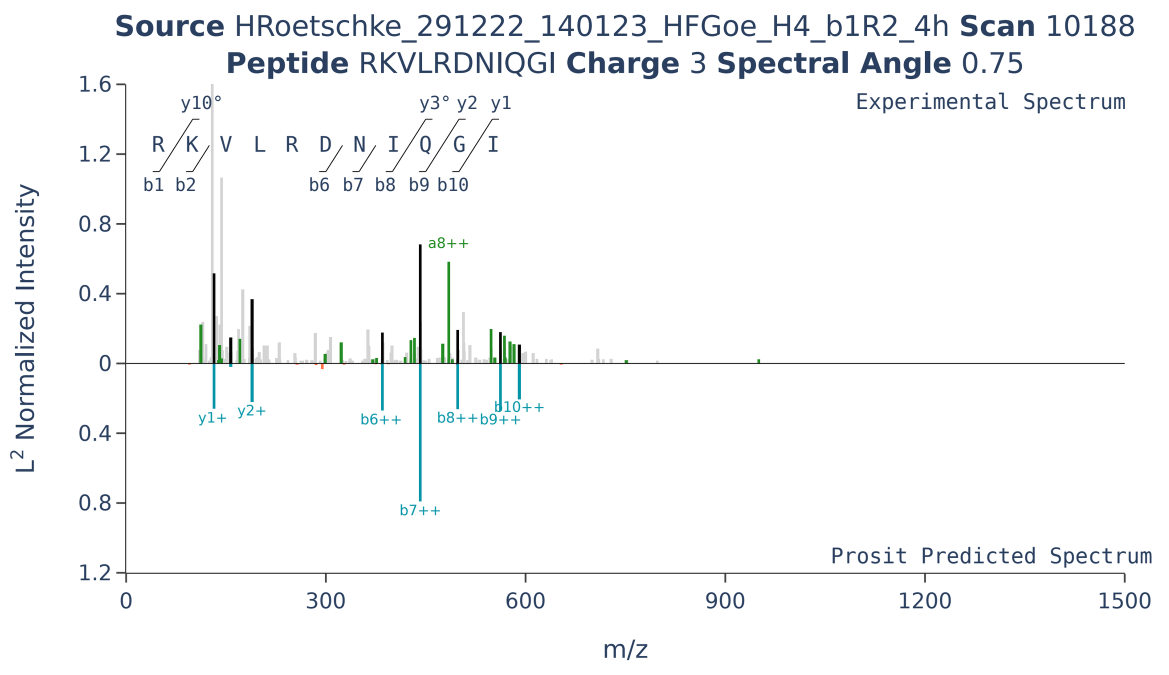
Source HRoetschke_291222_140123_HFGoe_H4_b1R2_4h Scan 10188
Peptide RKVLRDNIQGI Charge 3 Spectral Angle 0.75
Experimental Spectrum
Prosit Predicted Spectrum
L² Normalized Intensity
m/z

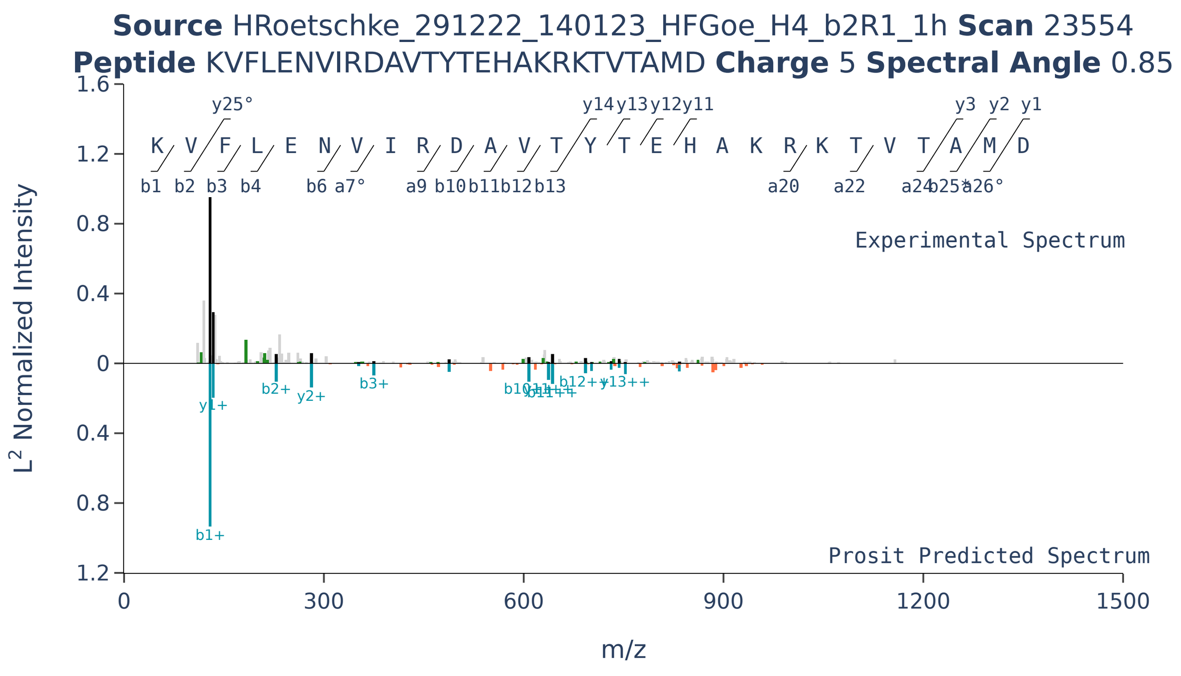
Source HRoetschke_291222_140123_HFGoe_H4_b2R1_1h Scan 23554
Peptide KVFLENVIRDAVTYTEHAKRKTVTAMD Charge 5 Spectral Angle 0.85
Experimental Spectrum
Prosit Predicted Spectrum
L² Normalized Intensity
m/z

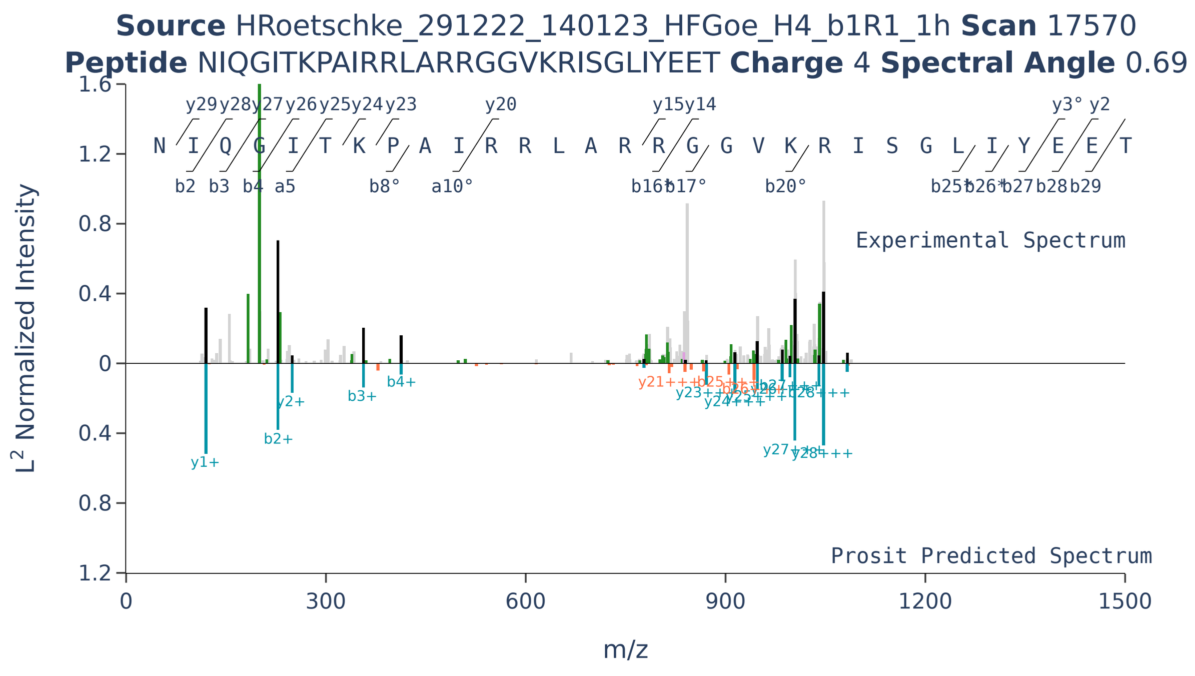
Source HRoetschke_291222_140123_HFGoe_H4_b1R1_1h Scan 17570
Peptide NIQGITKPAIRRLARRGGVKRISGLIYEET Charge 4 Spectral Angle 0.69
Experimental Spectrum
Prosit Predicted Spectrum
L² Normalized Intensity
m/z

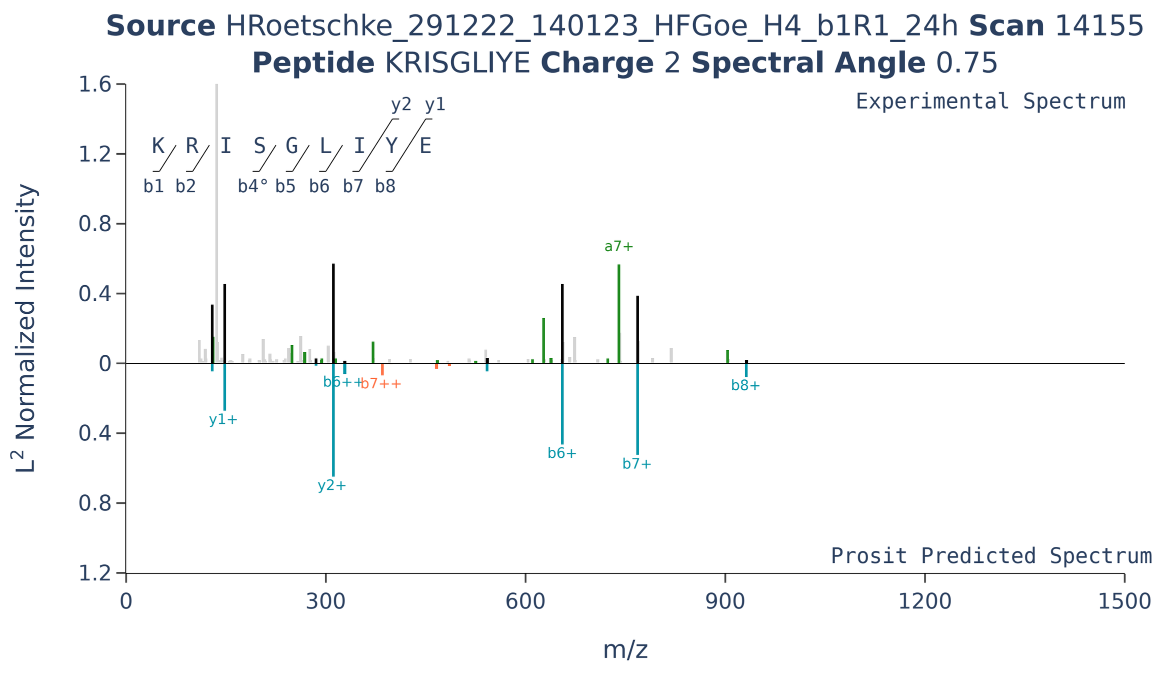
Source HRoetschke_291222_140123_HFGoe_H4_b1R1_24h Scan 14155
Peptide KRISGLIYE Charge 2 Spectral Angle 0.75
Experimental Spectrum
Prosit Predicted Spectrum
L² Normalized Intensity
m/z

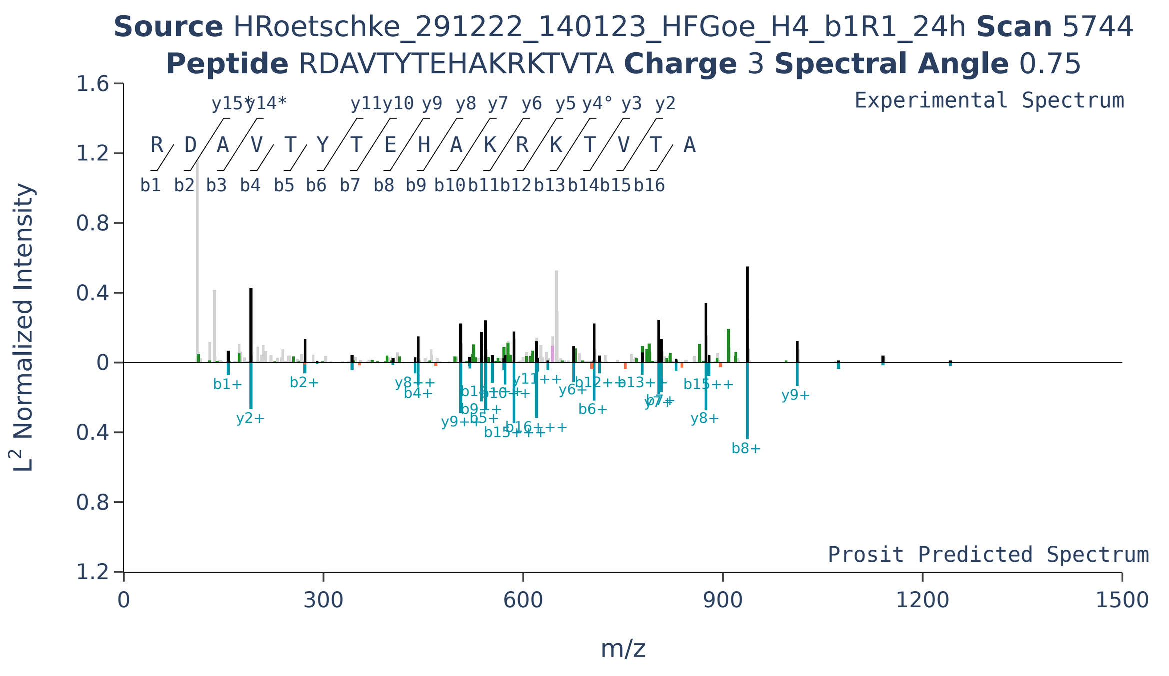
Source HRoetschke_291222_140123_HFGoe_H4_b1R1_24h Scan 5744
Peptide RDAVTYTEHAKRKTVTA Charge 3 Spectral Angle 0.75
Experimental Spectrum
Prosit Predicted Spectrum
L² Normalized Intensity
m/z

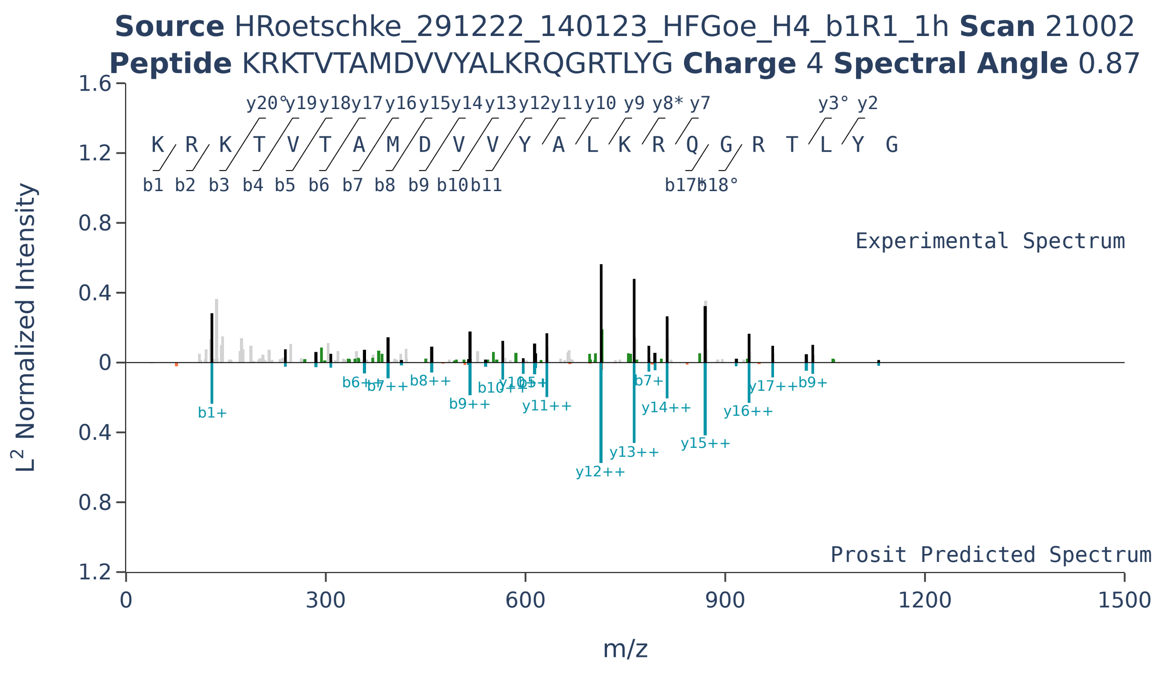
Source HRoetschke_291222_140123_HFGoe_H4_b1R1_1h Scan 21002
Peptide KRKTVTAMDVVYALKRQGRTLYG Charge 4 Spectral Angle 0.87
Experimental Spectrum
Prosit Predicted Spectrum
L² Normalized Intensity
m/z

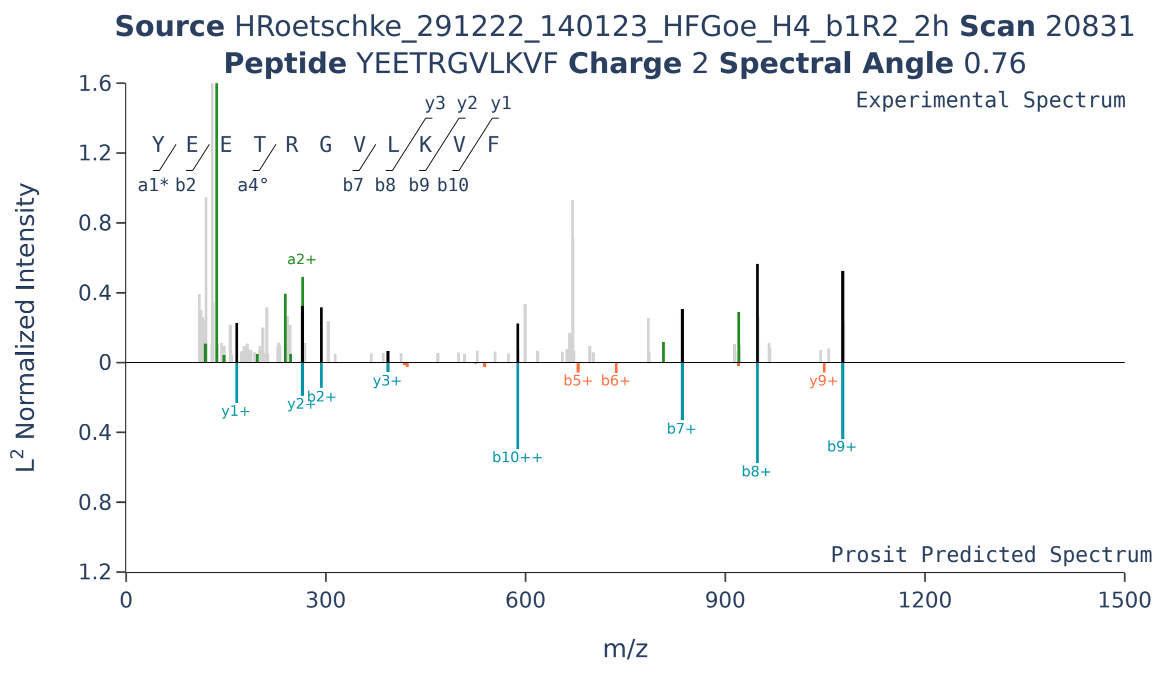
Source HRoetschke_291222_140123_HFGoe_H4_b1R2_2h Scan 20831
Peptide YEETRGVLKVF Charge 2 Spectral Angle 0.76
Experimental Spectrum
Prosit Predicted Spectrum
L² Normalized Intensity
m/z

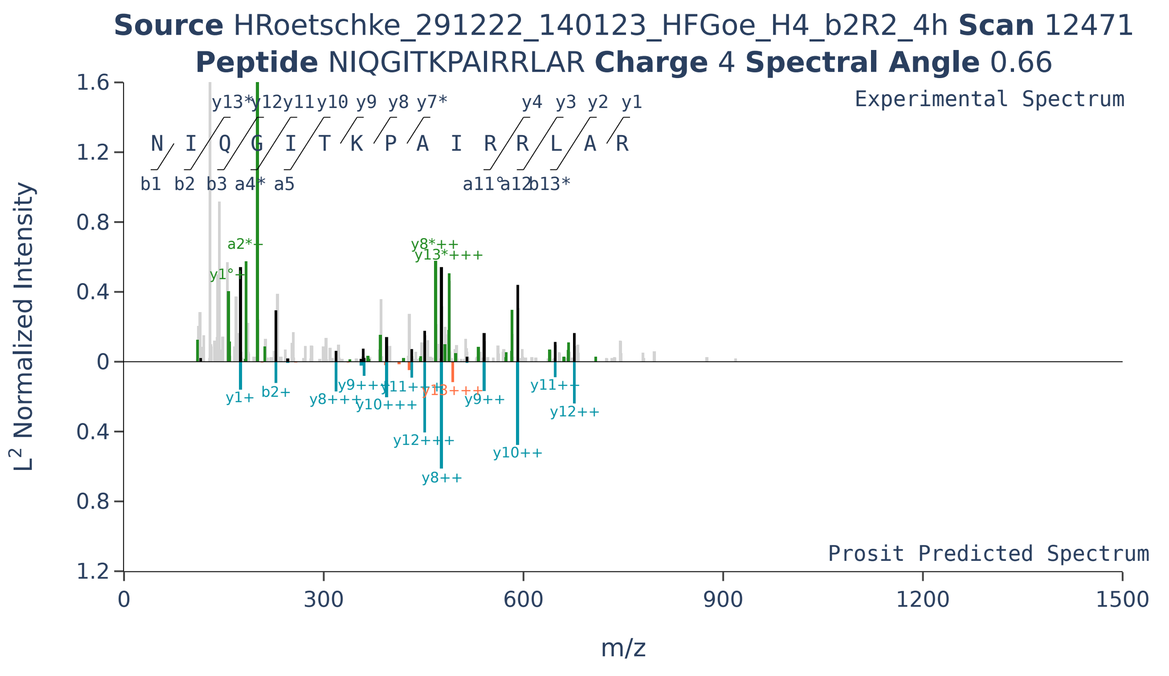
Source HRoetschke_291222_140123_HFGoe_H4_b2R2_4h Scan 12471
Peptide NIQGITKPAIRRLAR Charge 4 Spectral Angle 0.66
Experimental Spectrum
Prosit Predicted Spectrum
L² Normalized Intensity
m/z

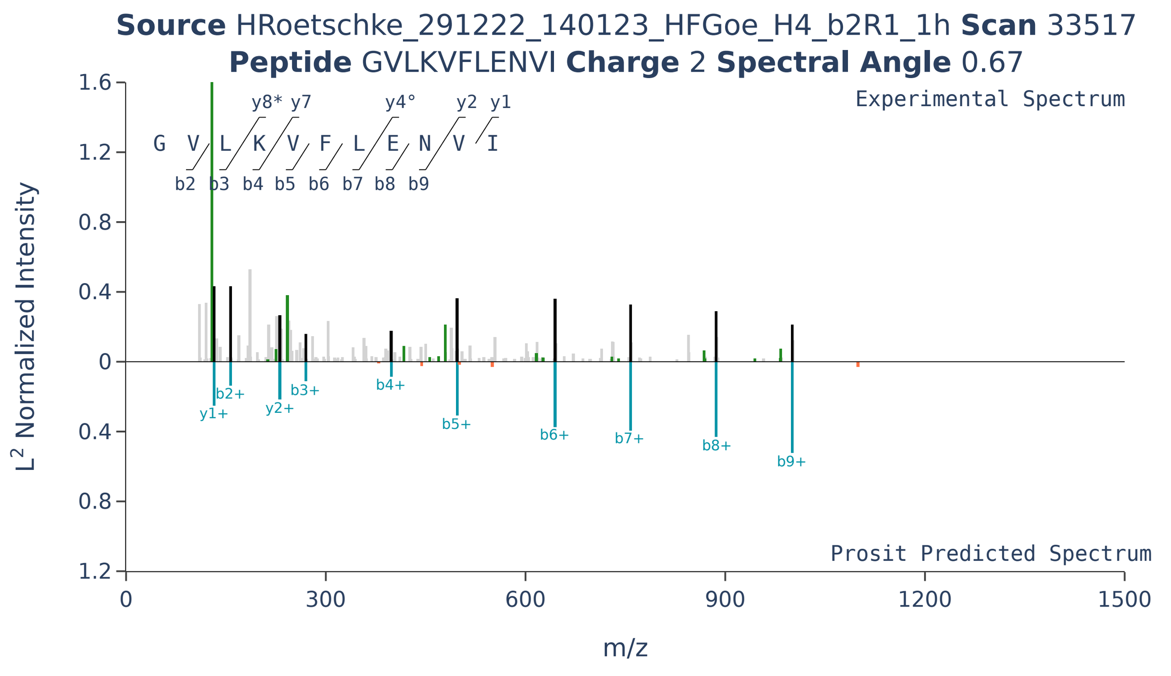
Source HRoetschke_291222_140123_HFGoe_H4_b2R1_1h Scan 33517
Peptide GVLKVFLENVI Charge 2 Spectral Angle 0.67
Experimental Spectrum
Prosit Predicted Spectrum
L² Normalized Intensity
m/z

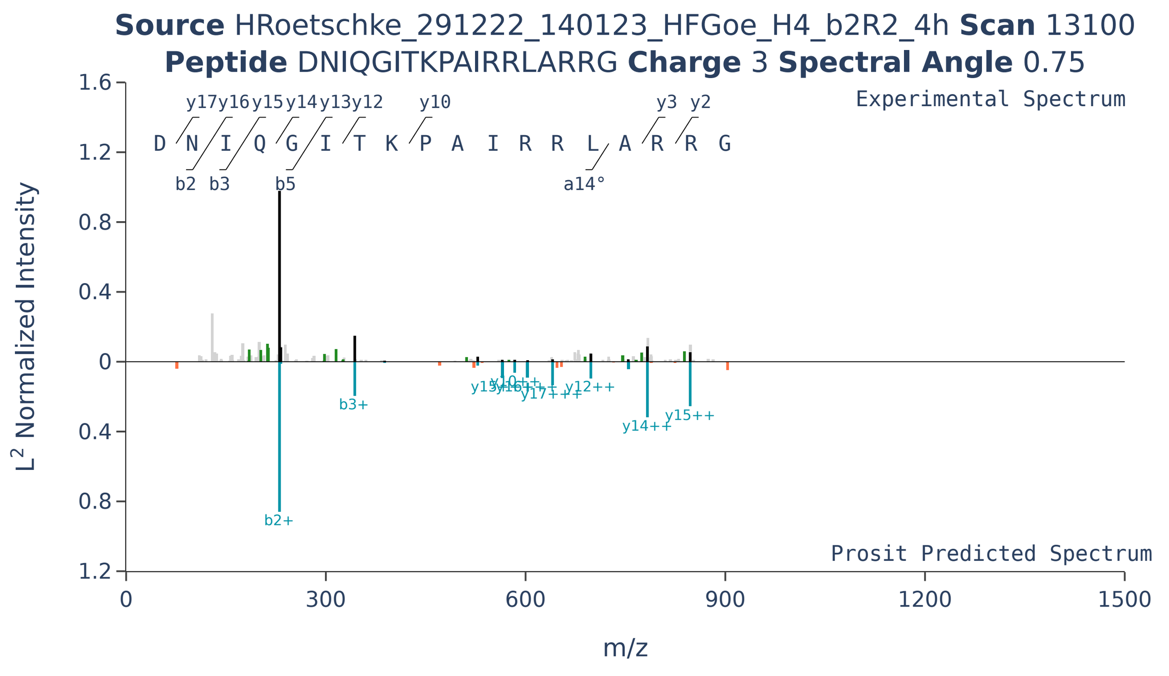
Source HRoetschke_291222_140123_HFGoe_H4_b2R2_4h Scan 13100
Peptide DNIQGITKPAIRRLARRG Charge 3 Spectral Angle 0.75
Experimental Spectrum
Prosit Predicted Spectrum
L² Normalized Intensity
m/z

**Source** HRoetschke_291222_140123_HFGoe_H4_b1R1_2h **Scan** 15856
**Peptide** RISGLIYEETR **Charge** 2 **Spectral Angle** 0.64

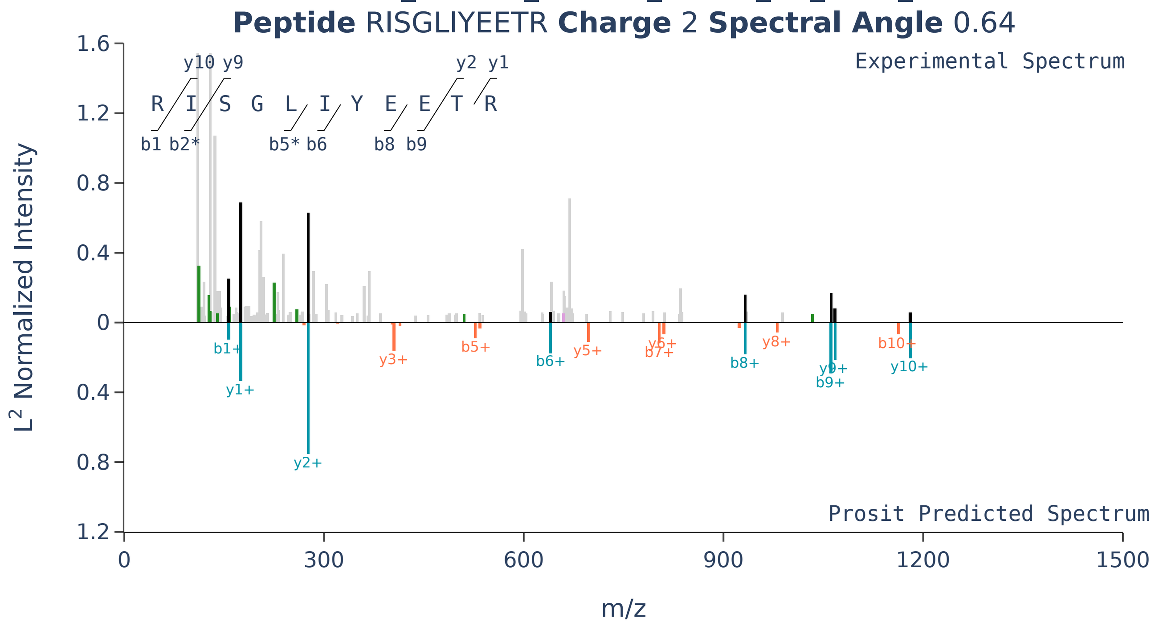


**Source** HRoetschke_291222_140123_HFGoe_H4_b2R1_24h **Scan** 11742
**Peptide** NIQGITKPAIRRLARRGGVKRL **Charge** 4 **Spectral Angle** 0.72

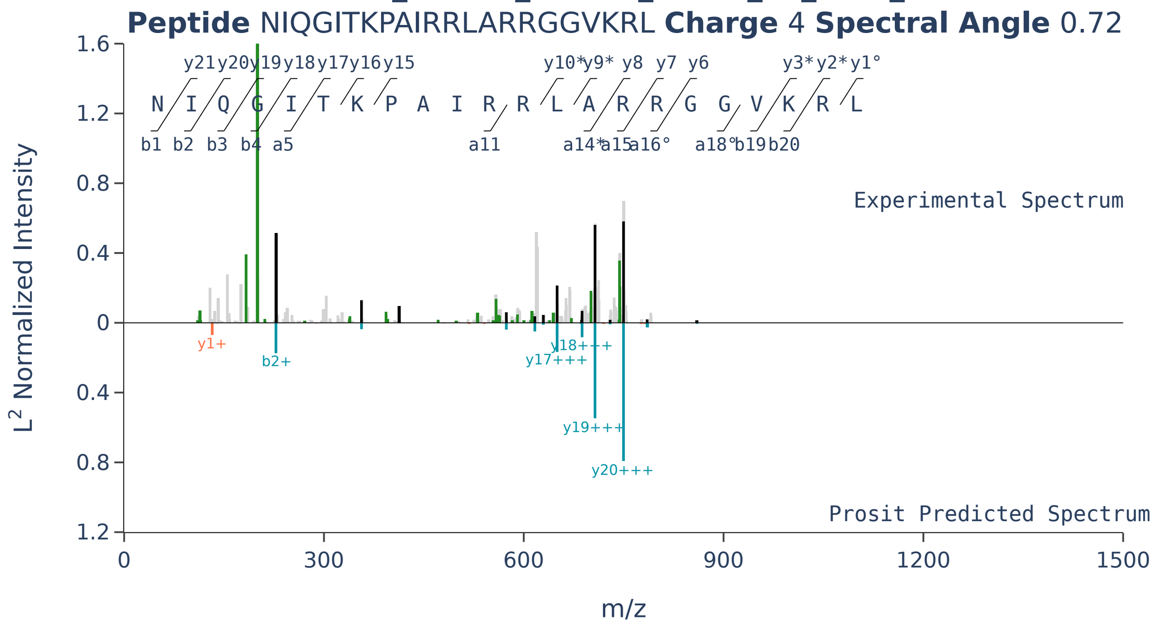


**Source** HRoetschke_291222_140123_HFGoe_H4_b2R1_1h **Scan** 19552
**Peptide** KTVTAMDVVYALKRQGR **Charge** 4 **Spectral Angle** 0.66

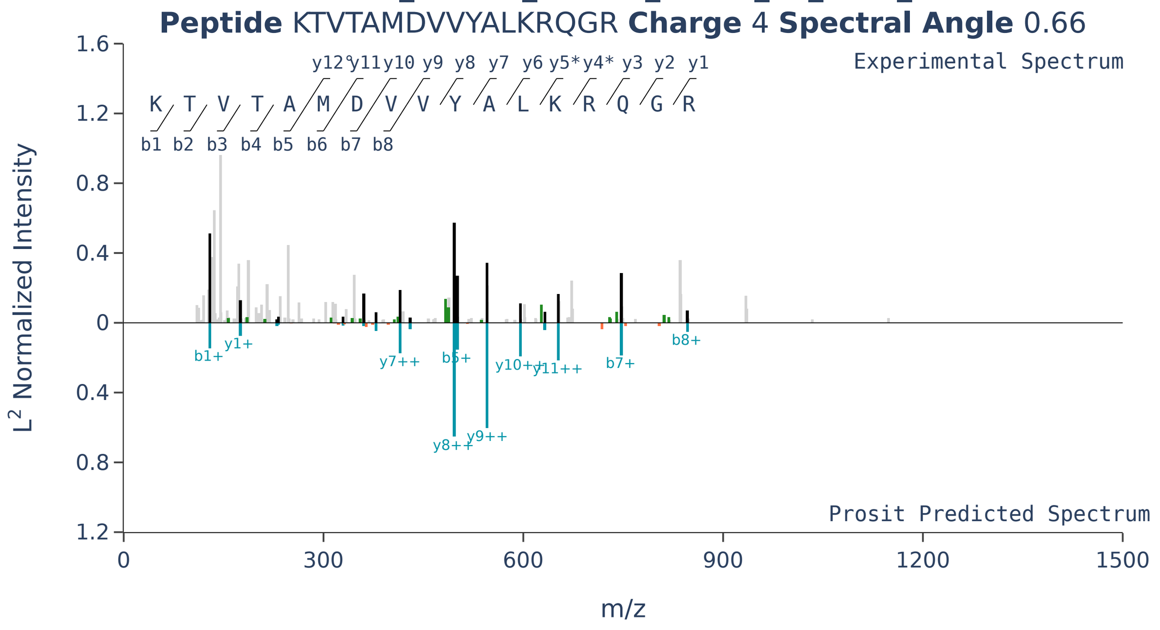


**Source** HRoetschke_291222_140123_HFGoe_H4_b1R1_24h **Scan** 10584
**Peptide** RKVLRDNIQGITKPALR **Charge** 5 **Spectral Angle** 0.74

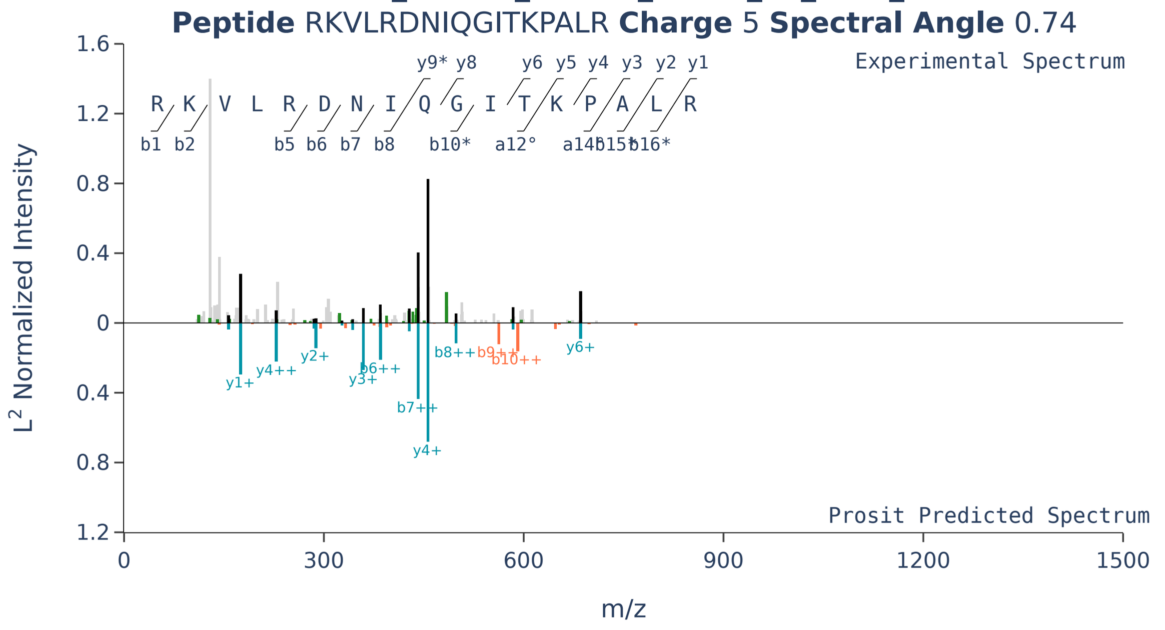


**Source** HRoetschke_291222_140123_HFGoe_H4_b2R1_1h **Scan** 22899
**Peptide** FLENVIRDAVTYTEHAKRKT **Charge** 5 **Spectral Angle** 0.71

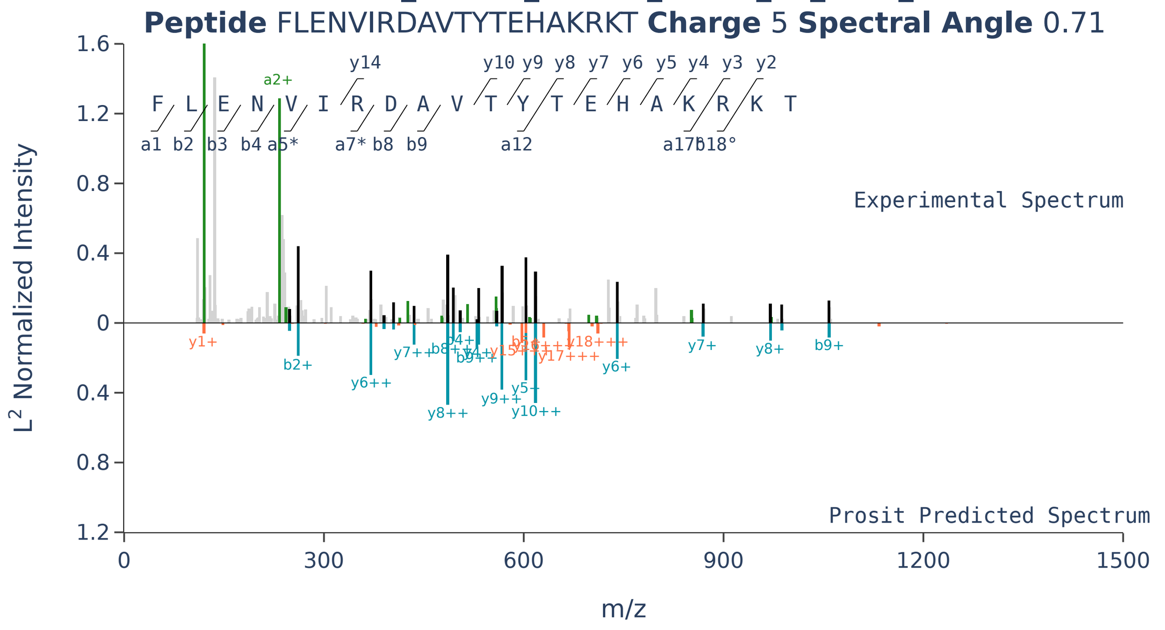


**Source** HRoetschke_291222_140123_HFGoe_H4_b2R1_1h **Scan** 24297
**Peptide** KTVTAMDVVYALKRQGRTLYGFGG **Charge** 4 **Spectral Angle** 0.69

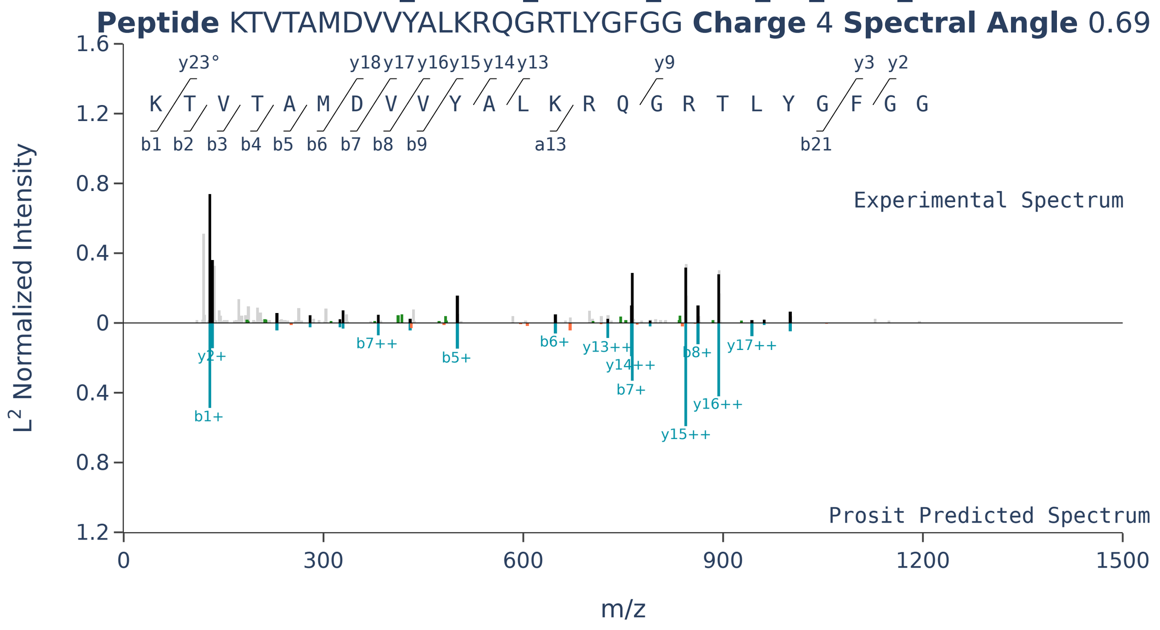


**Source** HRoetschke_291222_140123_HFGoe_H4_b2R1_1h **Scan** 24880
**Peptide** FLENVIRDAVTYTEHAKR **Charge** 5 **Spectral Angle** 0.7

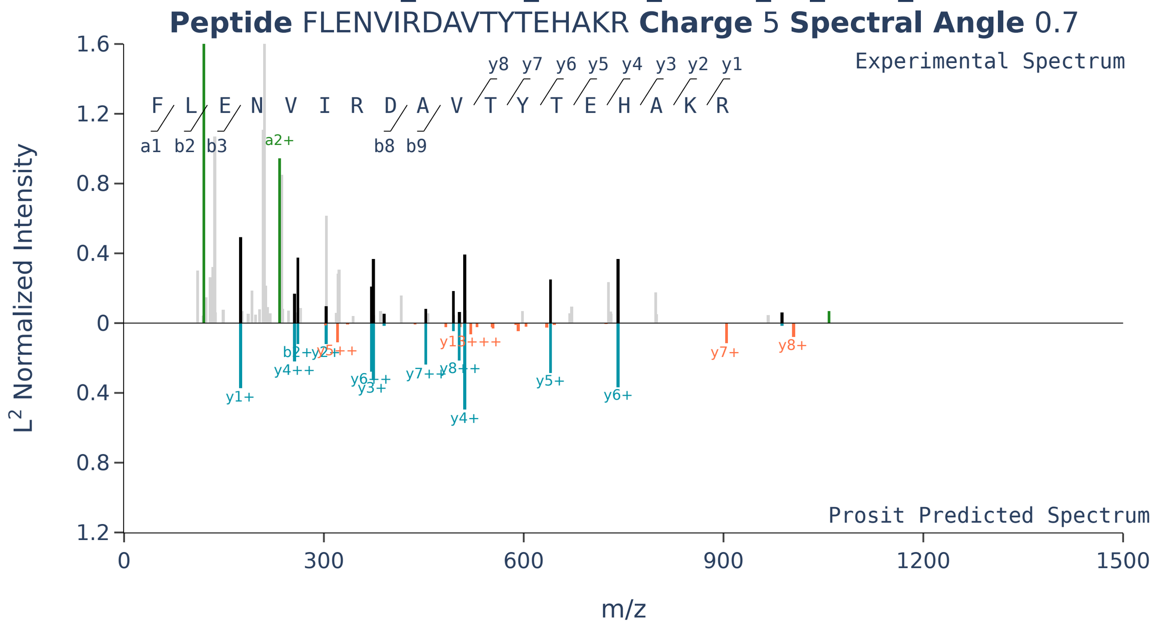


**Source** HRoetschke_291222_140123_HFGoe_H4_b2R1_1h **Scan** 33303
**Peptide** RGVLKVFLENVIRDAVTYTEH **Charge** 4 **Spectral Angle** 0.84

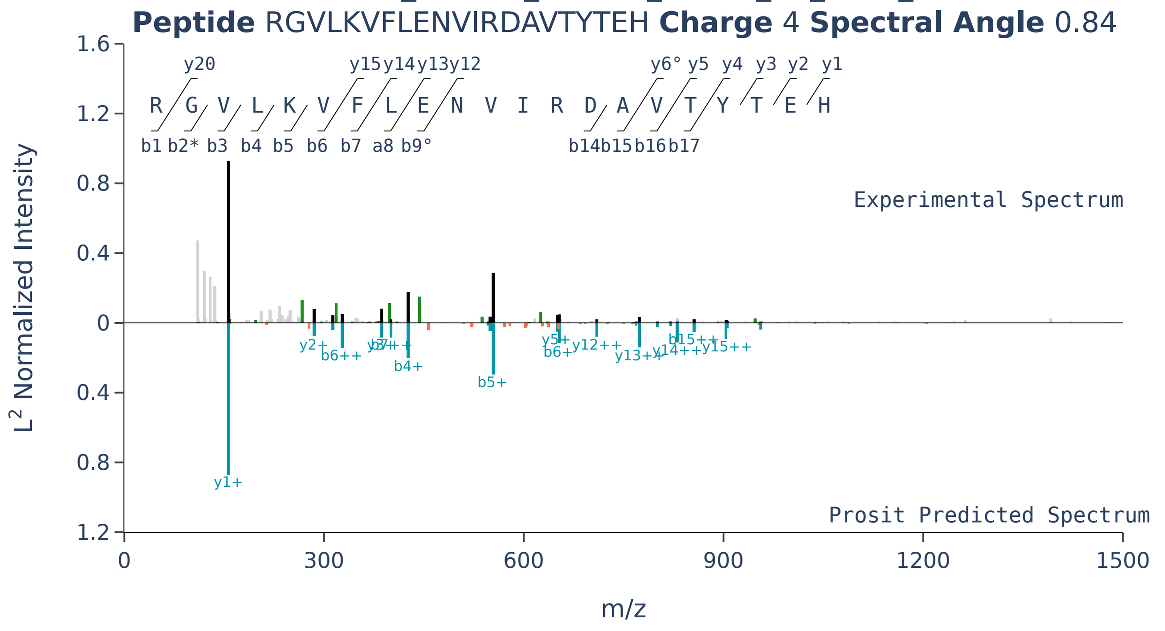


**Source** HRoetschke_291222_140123_HFGoe_H4_b1R1_1h **Scan** 11813
**Peptide** HAKRKTVTAMDVVY **Charge** 3 **Spectral Angle** 0.53

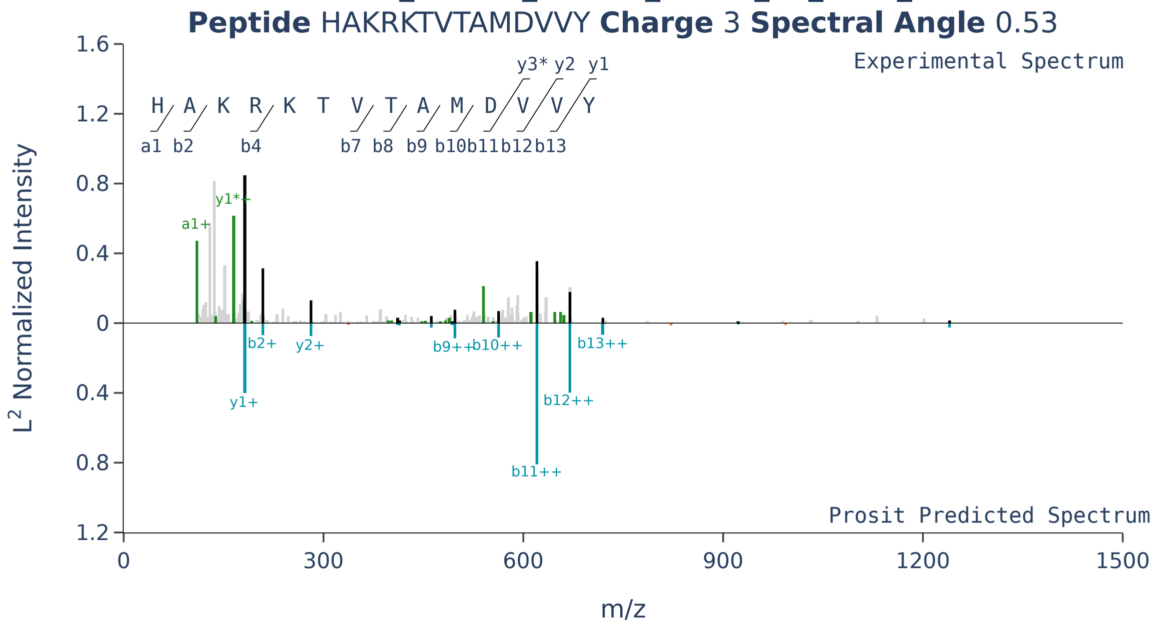


**Source** HRoetschke_291222_140123_HFGoe_H4_b1R2_24h **Scan** 6000
**Peptide** KGLGKGGAKRHRKVLRDNIQGITK **Charge** 6 **Spectral Angle** 0.93

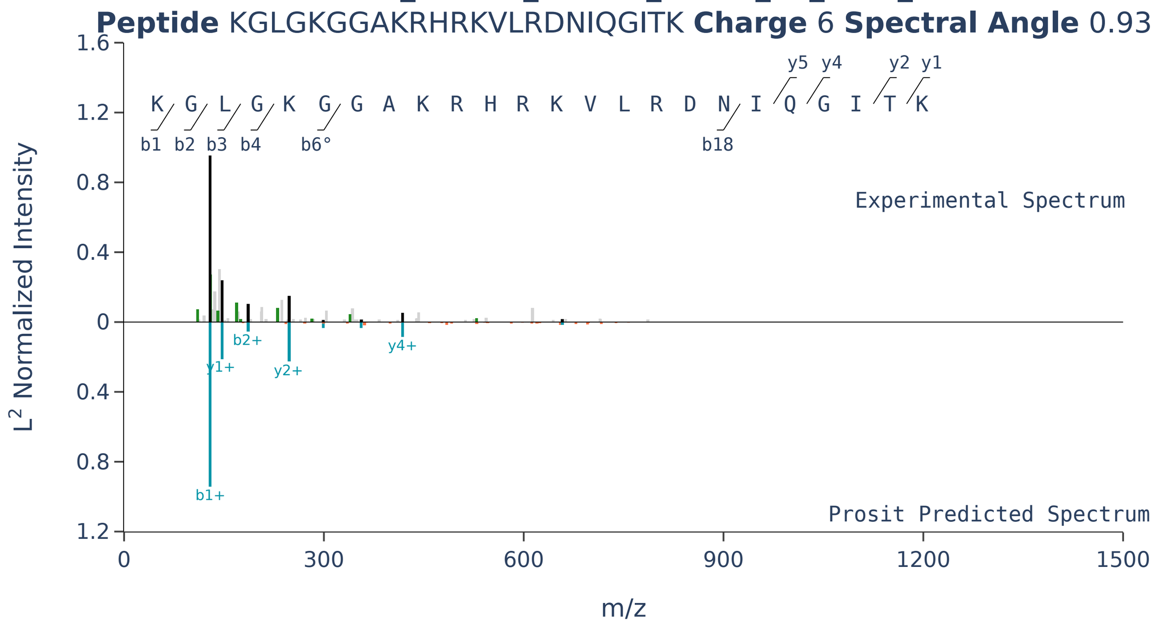


**Source** HRoetschke_291222_140123_HFGoe_H4_b1R1_24h **Scan** 10991
**Peptide** NIQGITKPAIRRLARRG **Charge** 4 **Spectral Angle** 0.59

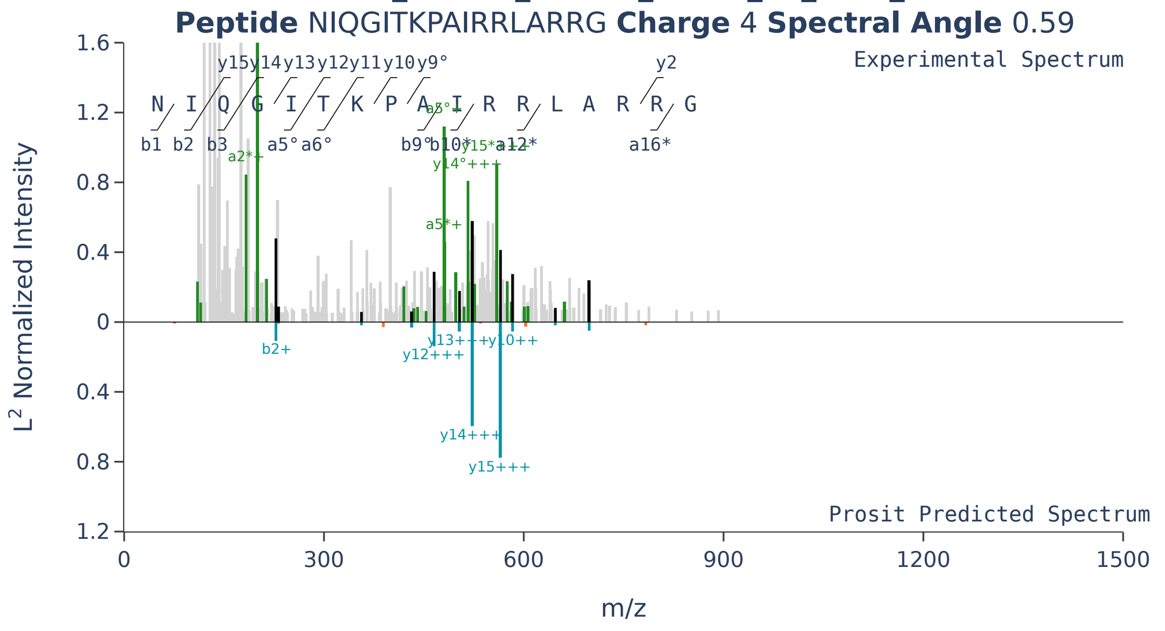


**Source** HRoetschke_291222_140123_HFGoe_H4_b2R1_2h **Scan** 5758
**Peptide** YTEHAKRKTVTA **Charge** 2 **Spectral Angle** 0.64

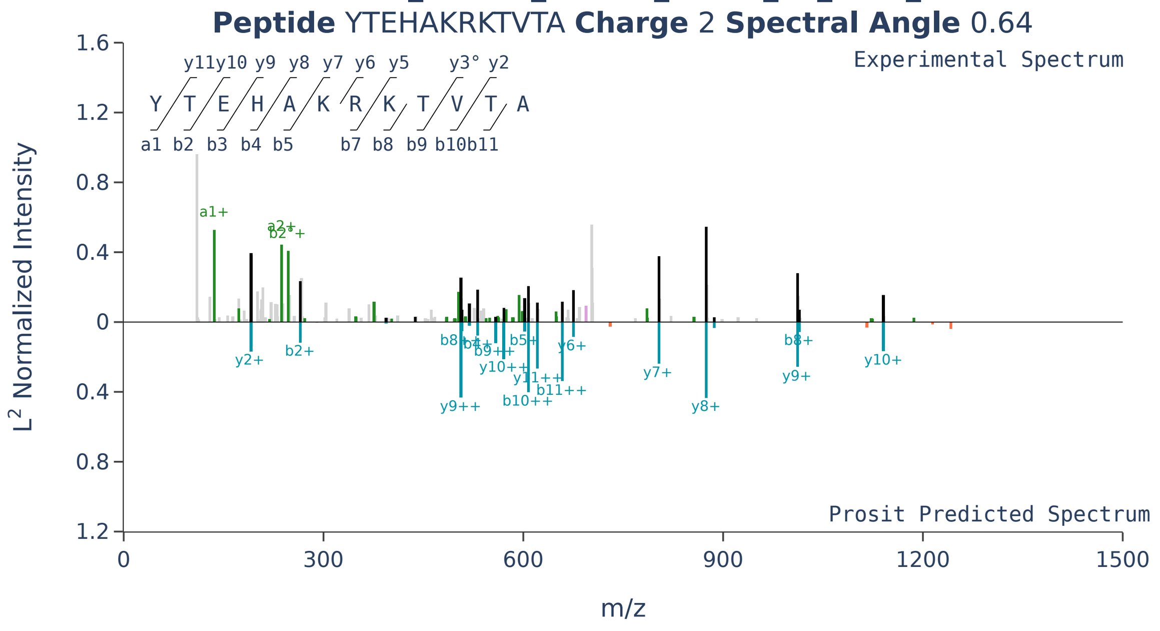


**Source** HRoetschke_291222_140123_HFGoe_H4_b1R2_24h **Scan** 13563
**Peptide** AVTYTEHAKRKTVTAMDVVY **Charge** 4 **Spectral Angle** 0.73

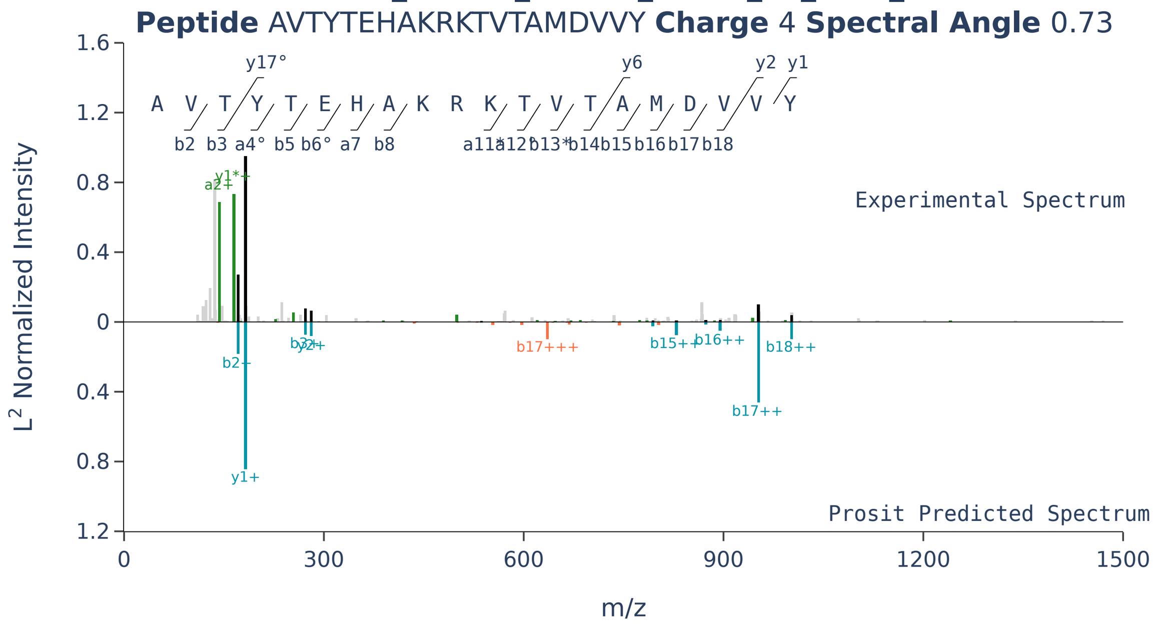


**Source** HRoetschke_291222_140123_HFGoe_H4_b1R2_4h **Scan** 8098
**Peptide** QGRTLYG **Charge** 2 **Spectral Angle** 0.76

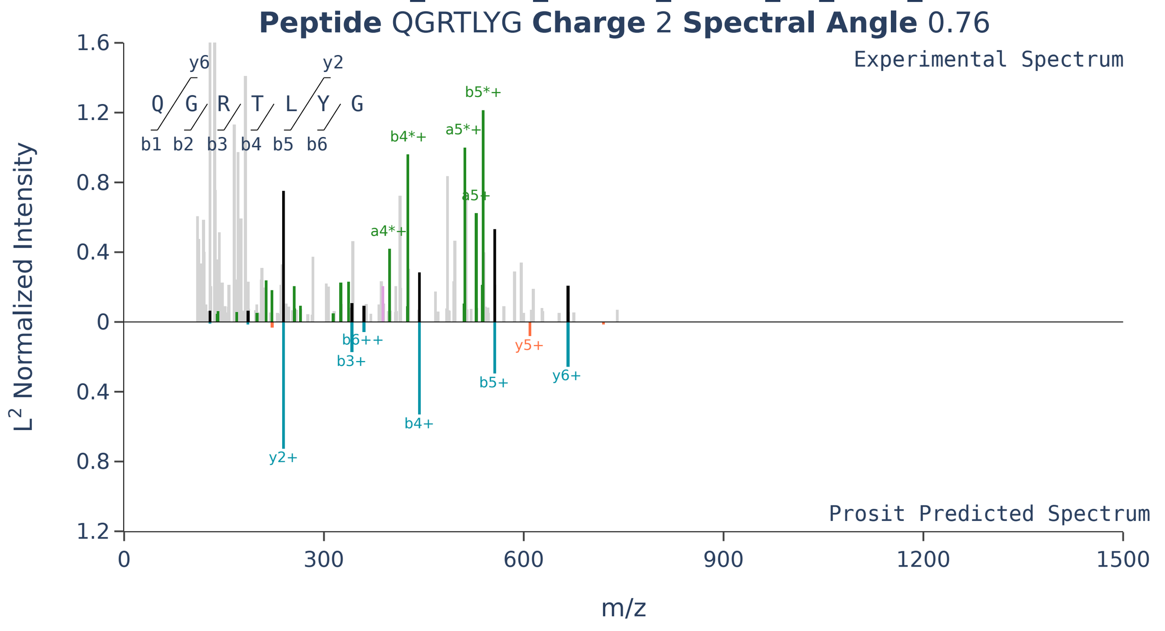


**Source** HRoetschke_291222_140123_HFGoe_H4_b1R2_1h **Scan** 11492
**Peptide** RRGGVKRISGLIYEE **Charge** 3 **Spectral Angle** 0.66

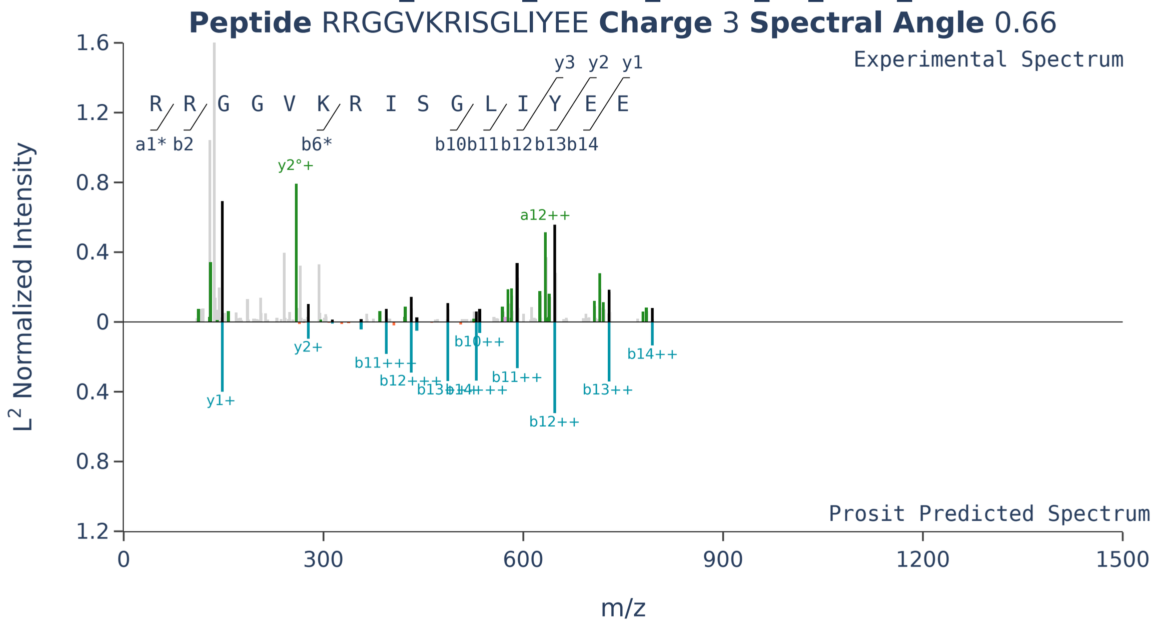

**Source** HRoetschke_291222_140123_HFGoe_H4_b1R1_24h **Scan** 6714
**Peptide** RKVLRDNI **Charge** 2 **Spectral Angle** 0.8

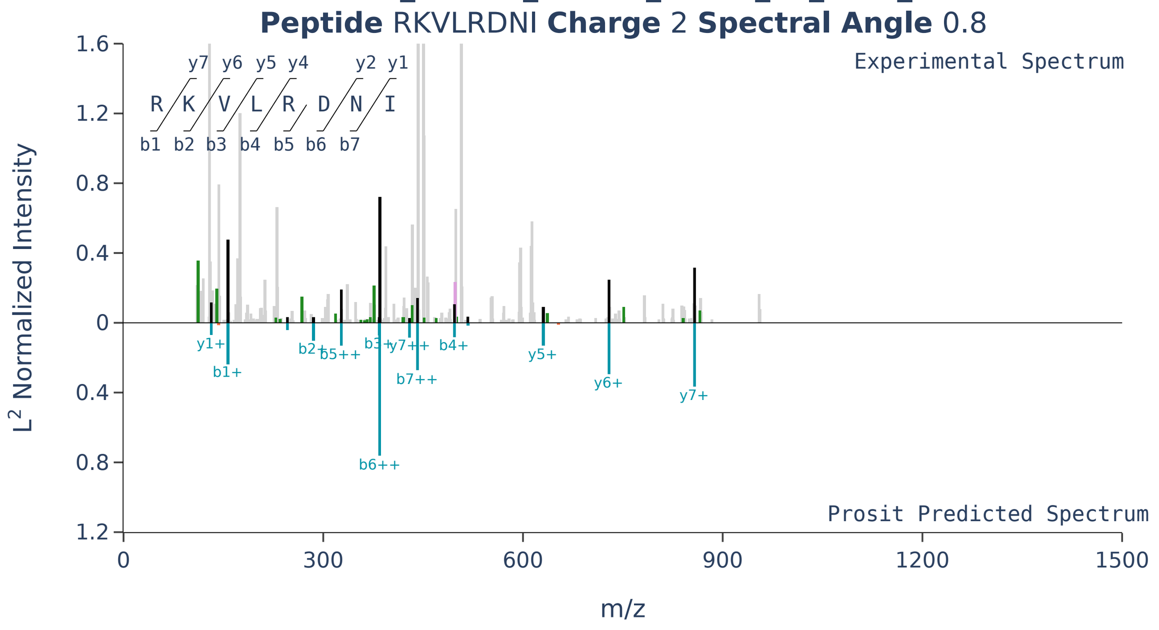


**Source** HRoetschke_291222_140123_HFGoe_H4_b2R1_1h **Scan** 23473
**Peptide** FLENVIRDAVTYTEHAKRKTVT **Charge** 3 **Spectral Angle** 0.74

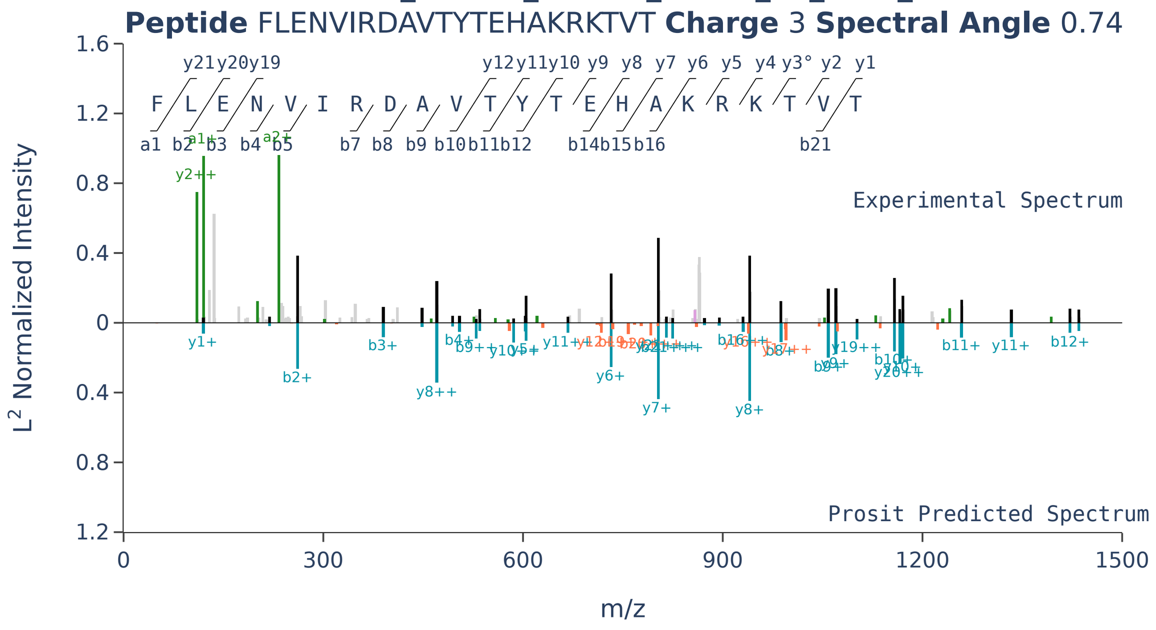


**Source** HRoetschke_291222_140123_HFGoe_H4_b2R2_1h **Scan** 19611
**Peptide** TEHAKRKTVTAMDVVYALKRQGRTLY **Charge** 5 **Spectral Angle** 0.87

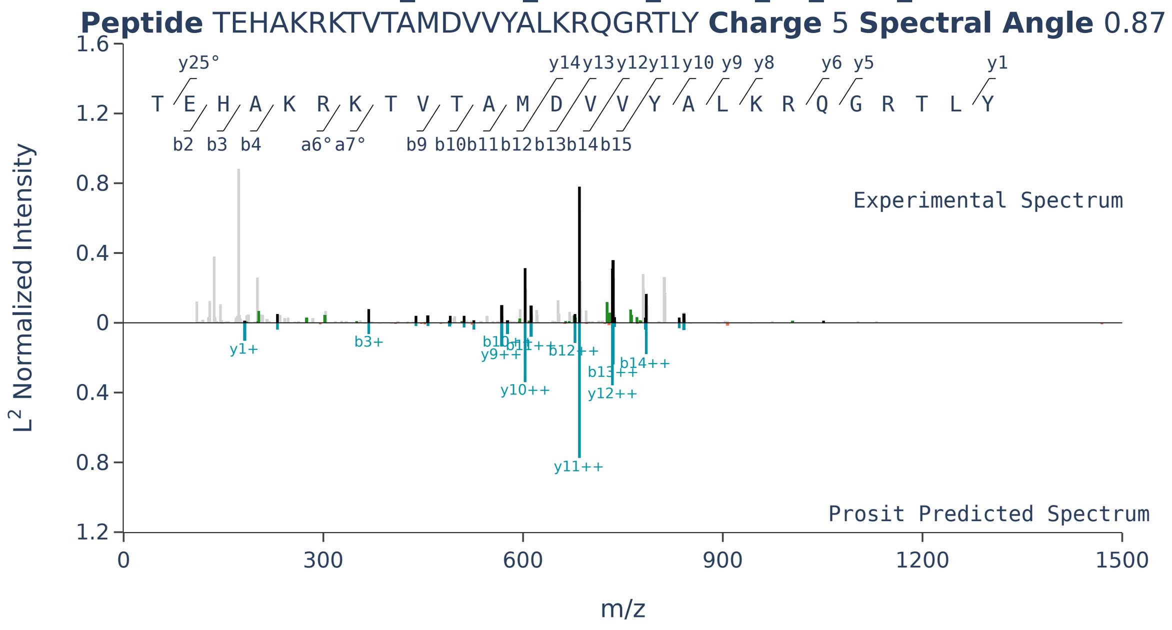


**Source** HRoetschke_291222_140123_HFGoe_H4_b2R2_1h **Scan** 26600
**Peptide** IYEETRGVLKVFL **Charge** 2 **Spectral Angle** 0.74

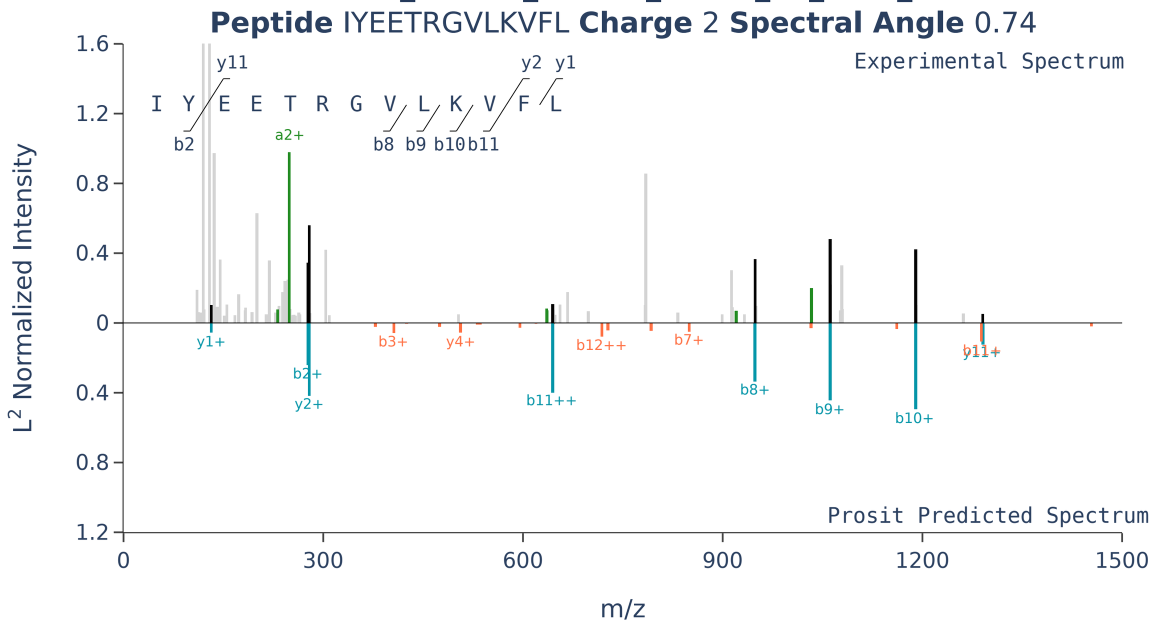


**Source** HRoetschke_291222_140123_HFGoe_H4_b1R2_2h **Scan** 7459
**Peptide** TEHAKRKTVTAMDV **Charge** 2 **Spectral Angle** 0.77

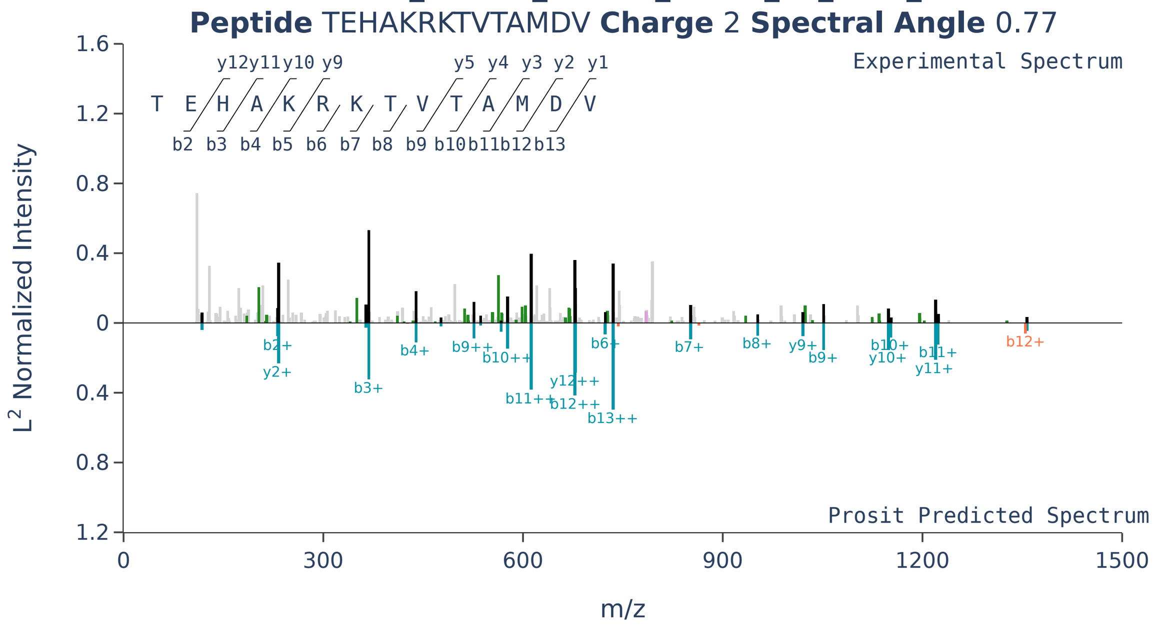


**Source** HRoetschke_291222_140123_HFGoe_H4_b1R1_4h **Scan** 13939
**Peptide** RGGVKRISGLIY **Charge** 3 **Spectral Angle** 0.66

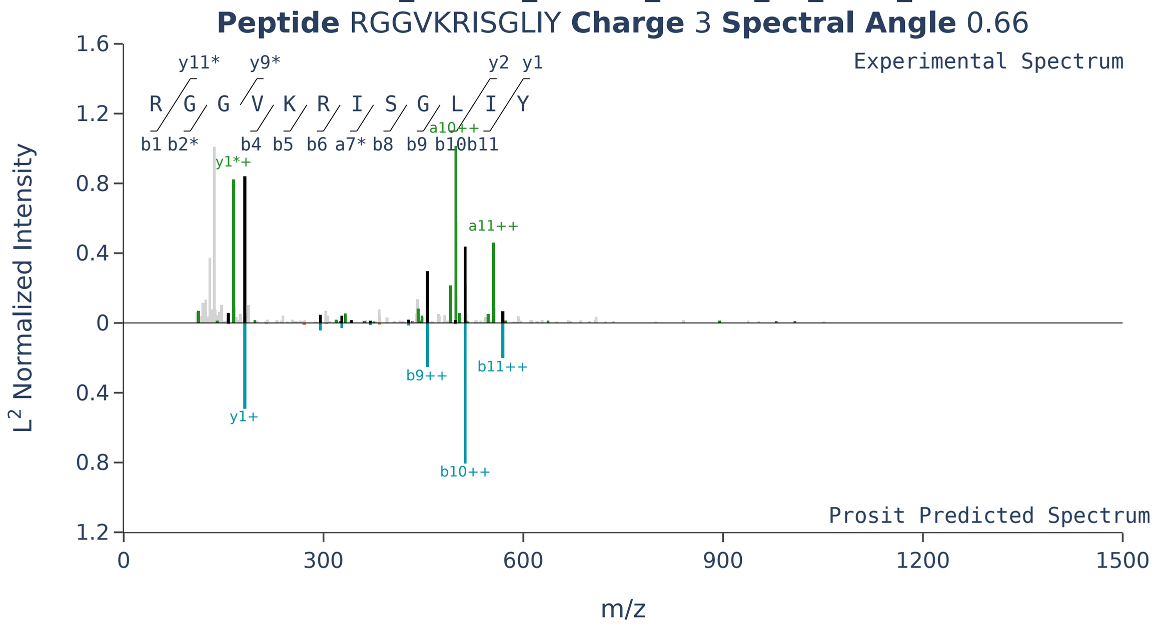


**Source** HRoetschke_291222_140123_HFGoe_H4_b1R1_1h **Scan** 20009
**Peptide** RRGGVKRISGLIYEETRGVLKV **Charge** 4 **Spectral Angle** 0.81

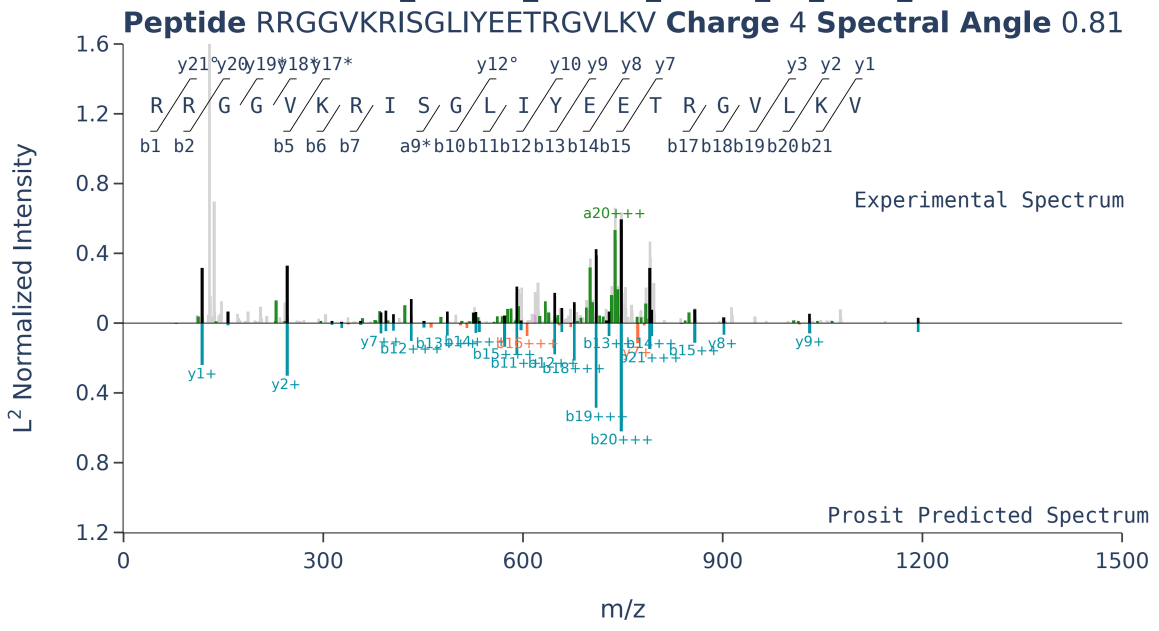


**Source** HRoetschke_291222_140123_HFGoe_H4_b2R2_24h **Scan** 9033
**Peptide** HRKVLRDNIQGI **Charge** 4 **Spectral Angle** 0.75

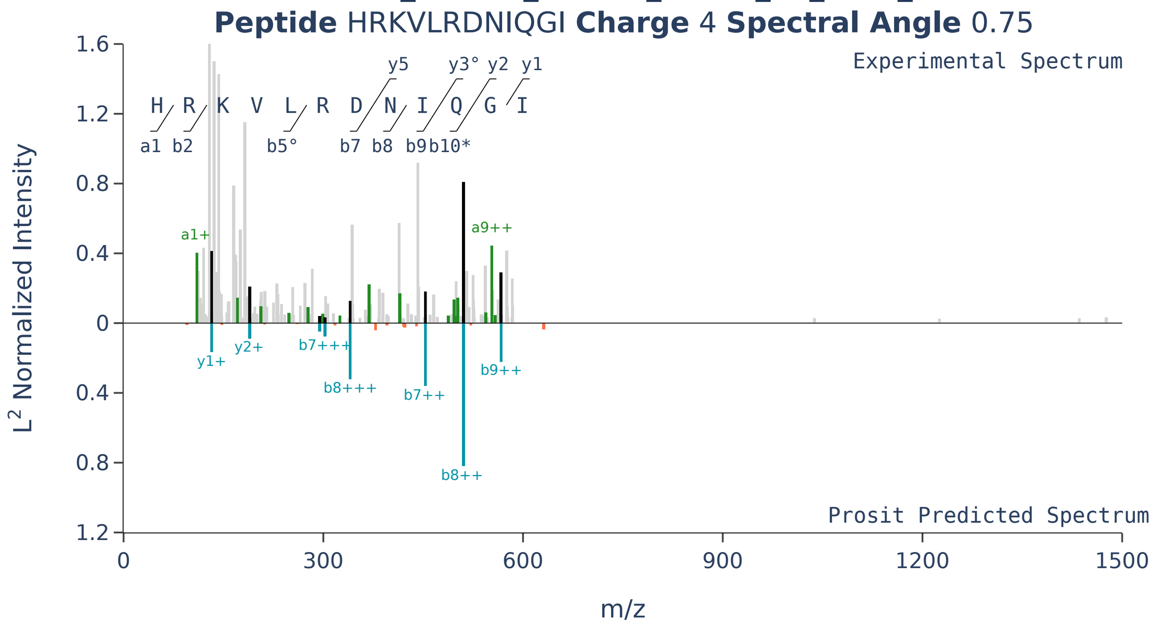


**Source** HRoetschke_291222_140123_HFGoe_H4_b2R1_24h **Scan** 9902
**Peptide** GLTKPAIRRLARR **Charge** 3 **Spectral Angle** 0.73

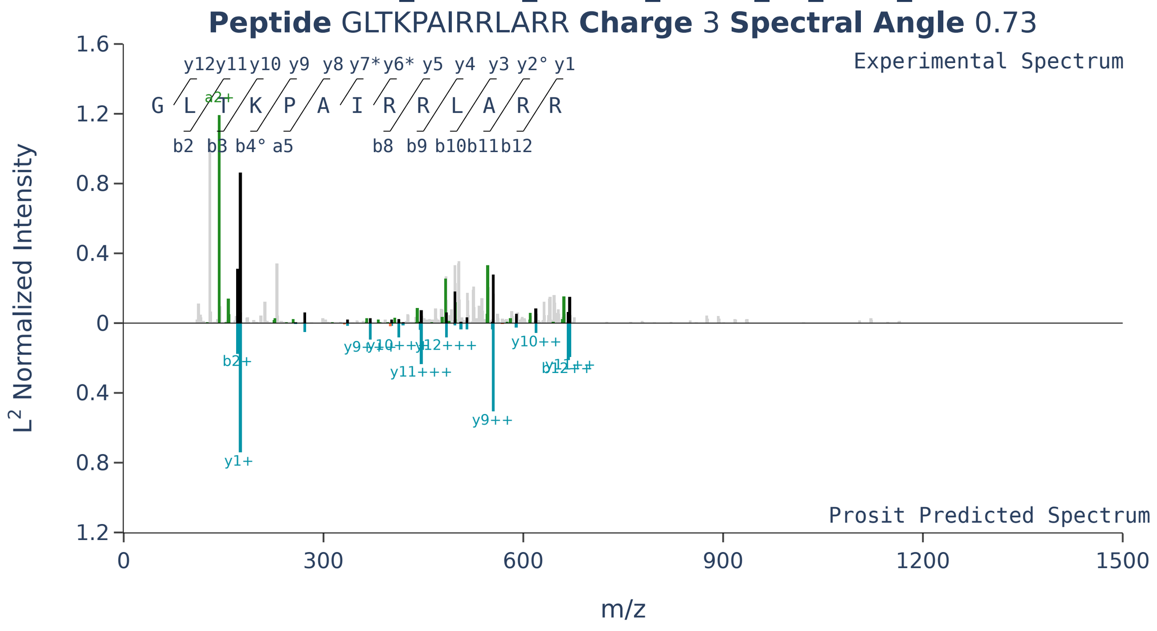


**Source** HRoetschke_291222_140123_HFGoe_H4_b1R2_4h **Scan** 15350
**Peptide** GGVKRISGLIYEETRG **Charge** 2 **Spectral Angle** 0.75

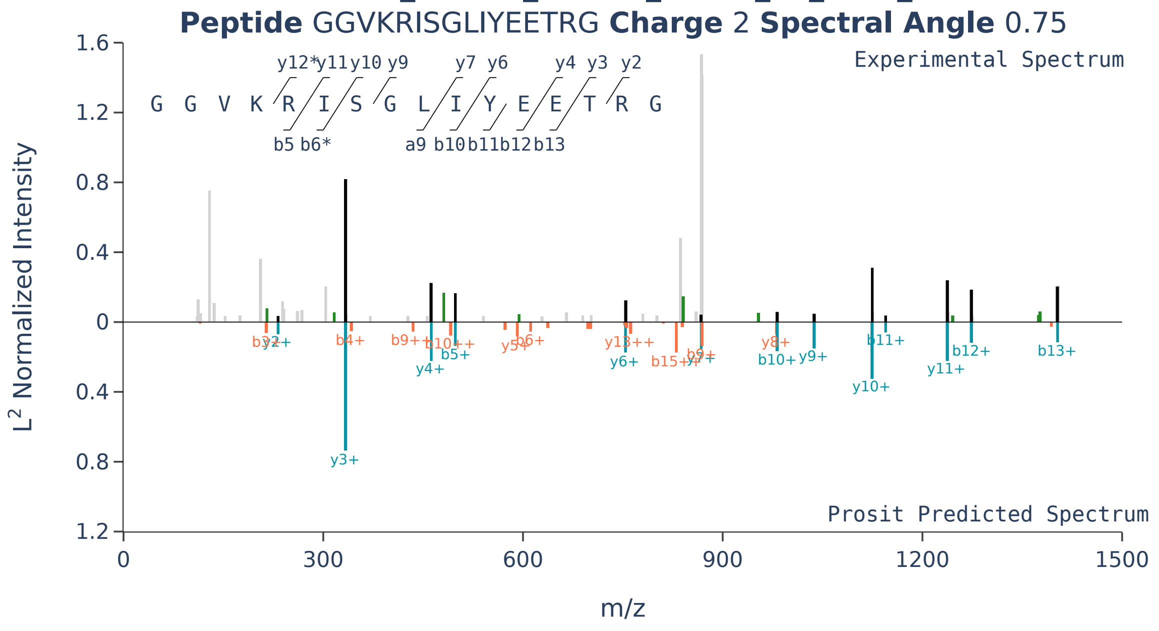


**Source** HRoetschke_291222_140123_HFGoe_H4_b1R1_4h **Scan** 5421
**Peptide** GGAKRHRKVLRDNI **Charge** 4 **Spectral Angle** 0.66

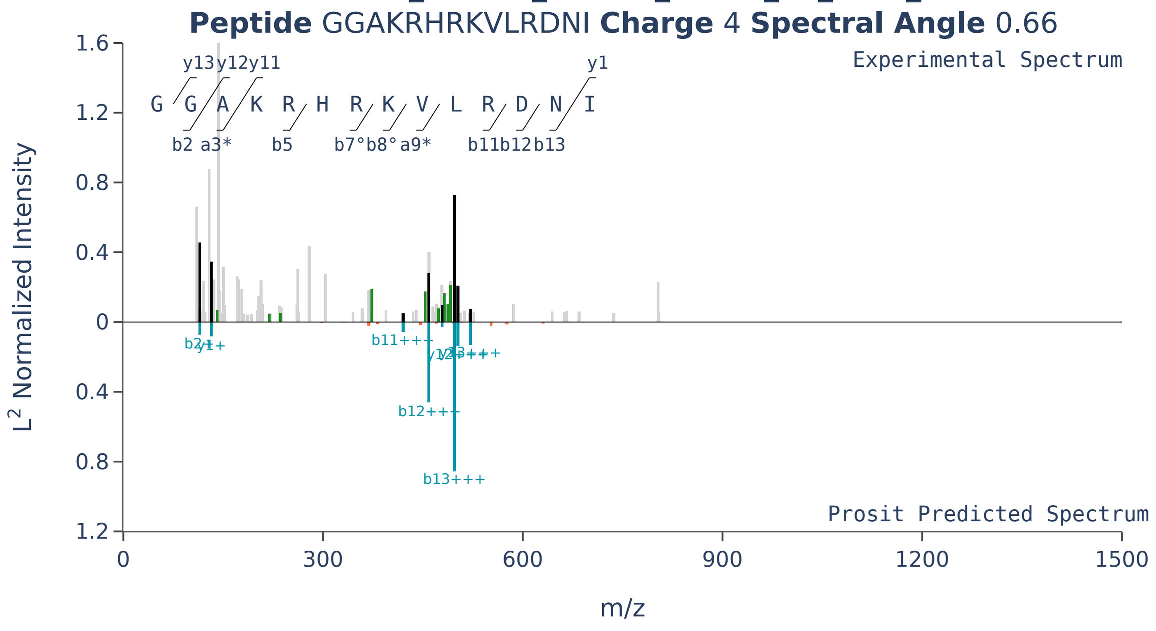


**Source** HRoetschke_291222_140123_HFGoe_H4_b2R1_1h **Scan** 20006
**Peptide** TEHAKRKTVTAMDVVYALKRQGRTLYGF **Charge** 5 **Spectral Angle** 0.85

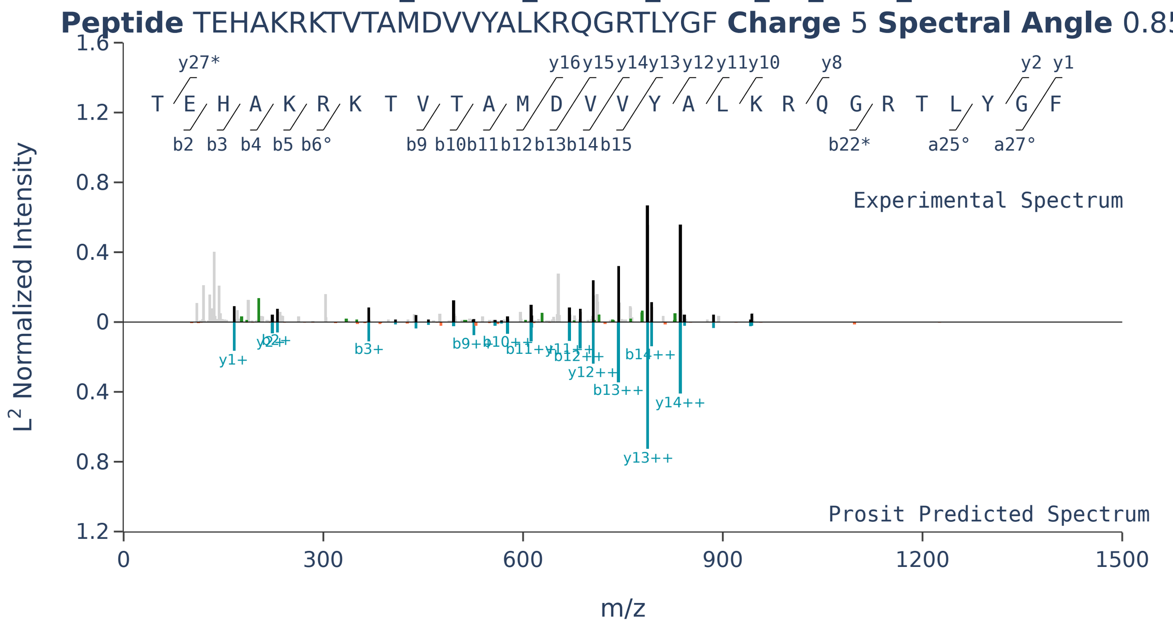


**Source** HRoetschke_291222_140123_HFGoe_H4_b1R2_1h **Scan** 20258
**Peptide** KRKTVTAMDVVYALKRQGRTLY **Charge** 4 **Spectral Angle** 0.85

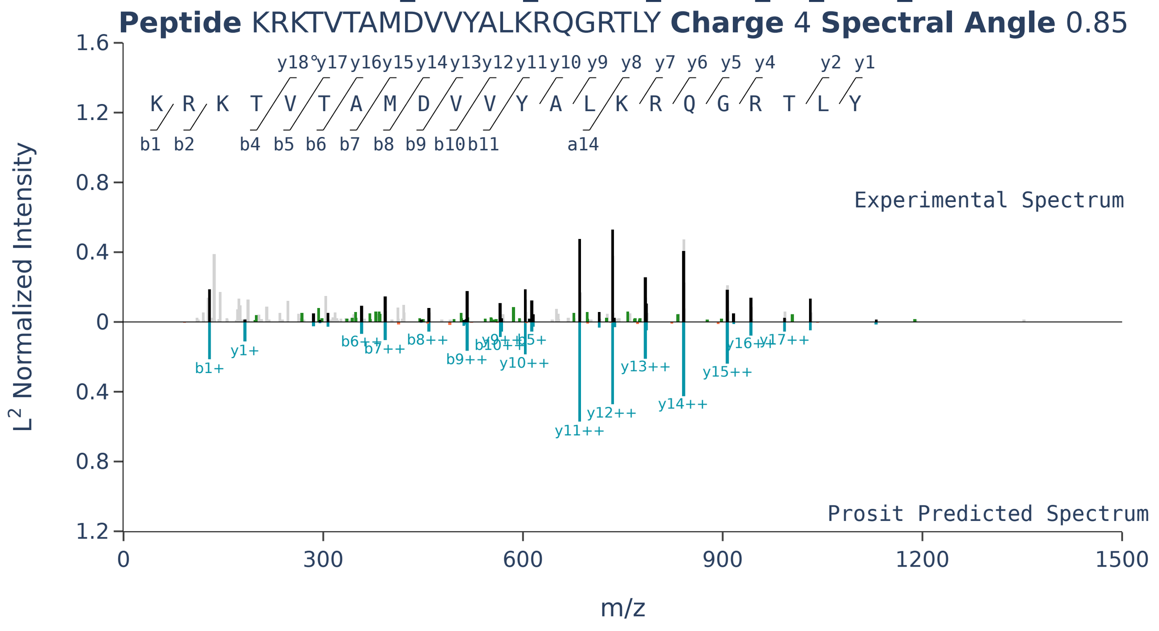


**Source** HRoetschke_291222_140123_HFGoe_H4_b1R1_2h **Scan** 12507
**Peptide** DNIQGITKPAIRRLARRGGVK **Charge** 5 **Spectral Angle** 0.75

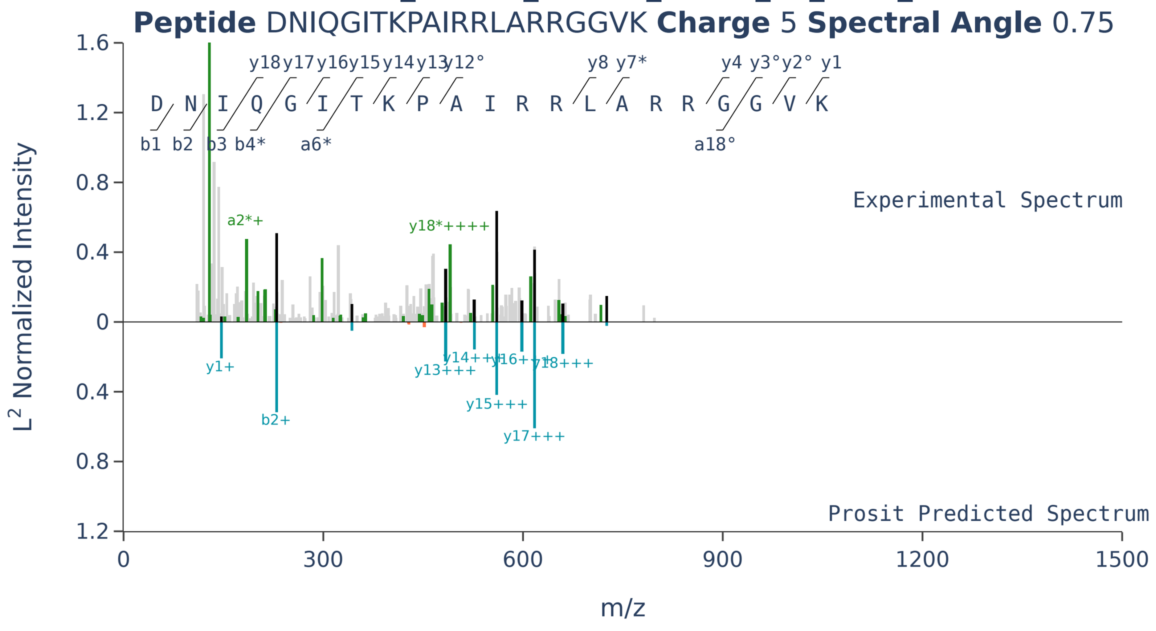


**Source** HRoetschke_291222_140123_HFGoe_H4_b2R1_1h **Scan** 22073
**Peptide** RISGLIYEETRGVLKV **Charge** 3 **Spectral Angle** 0.61

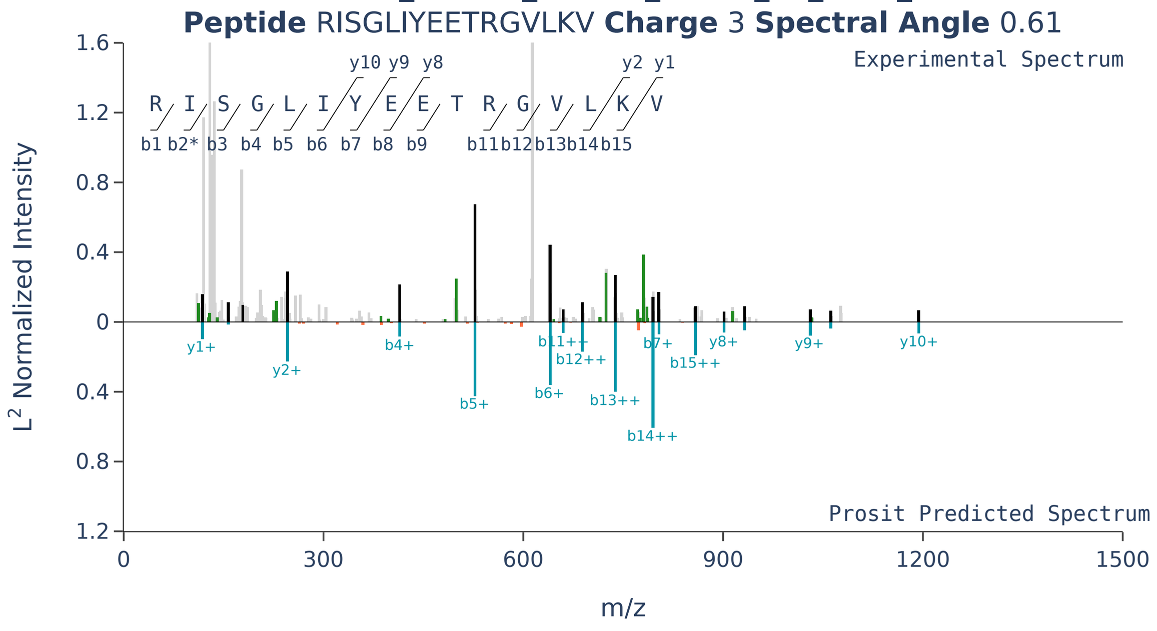

**Source** HRoetschke_291222_140123_HFGoe_H4_b1R1_2h **Scan** 19093
**Peptide** LENVIRDAVTYTEHAKRKTVT **Charge** 4 **Spectral Angle** 0.6

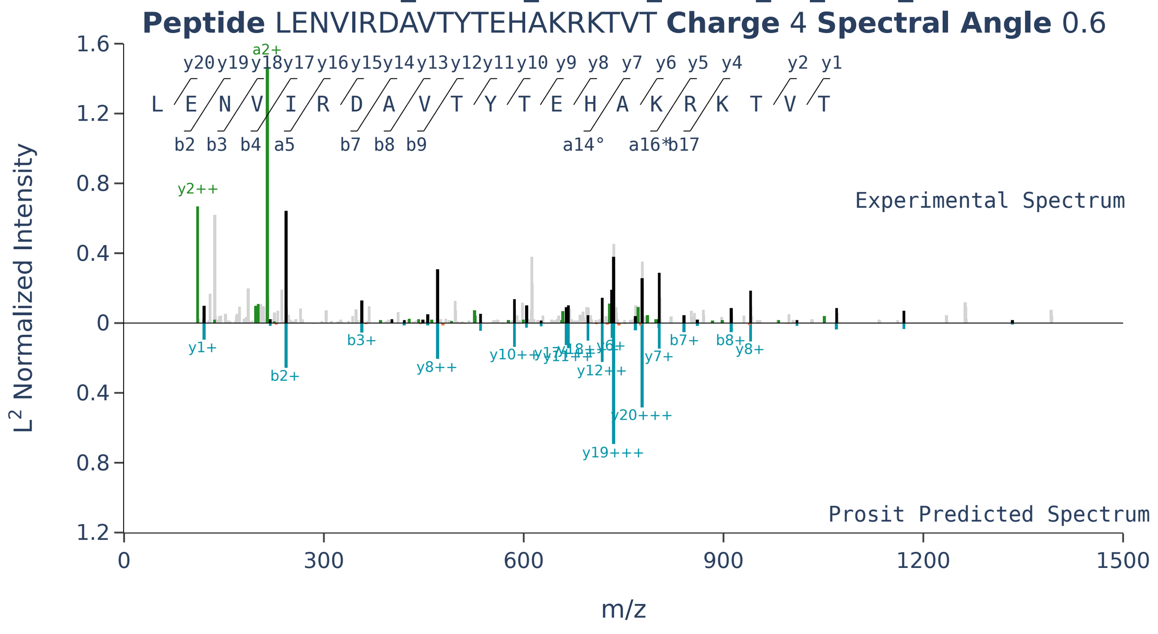

**Source** HRoetschke_291222_140123_HFGoe_H4_b2R2_24h **Scan** 9259
**Peptide** RKVLRDNIQGITKPAIRR **Charge** 5 **Spectral Angle** 0.63

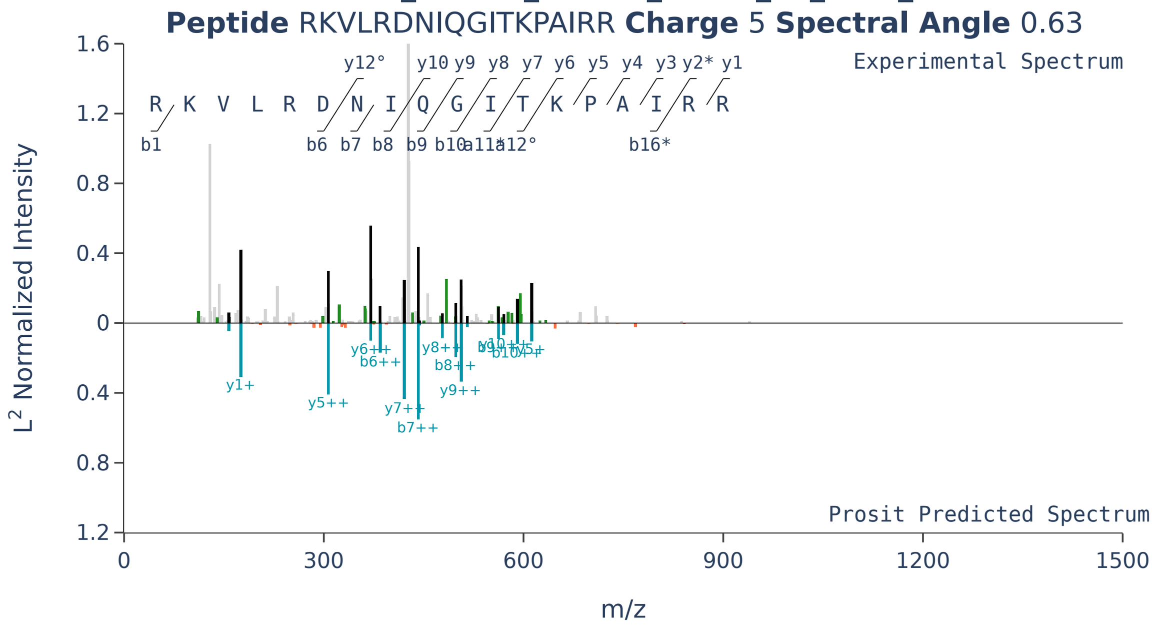

**Source** HRoetschke_291222_140123_HFGoe_H4_b1R1_4h **Scan** 10140
**Peptide** KVLRDNIQGITKPAIRR **Charge** 5 **Spectral Angle** 0.7

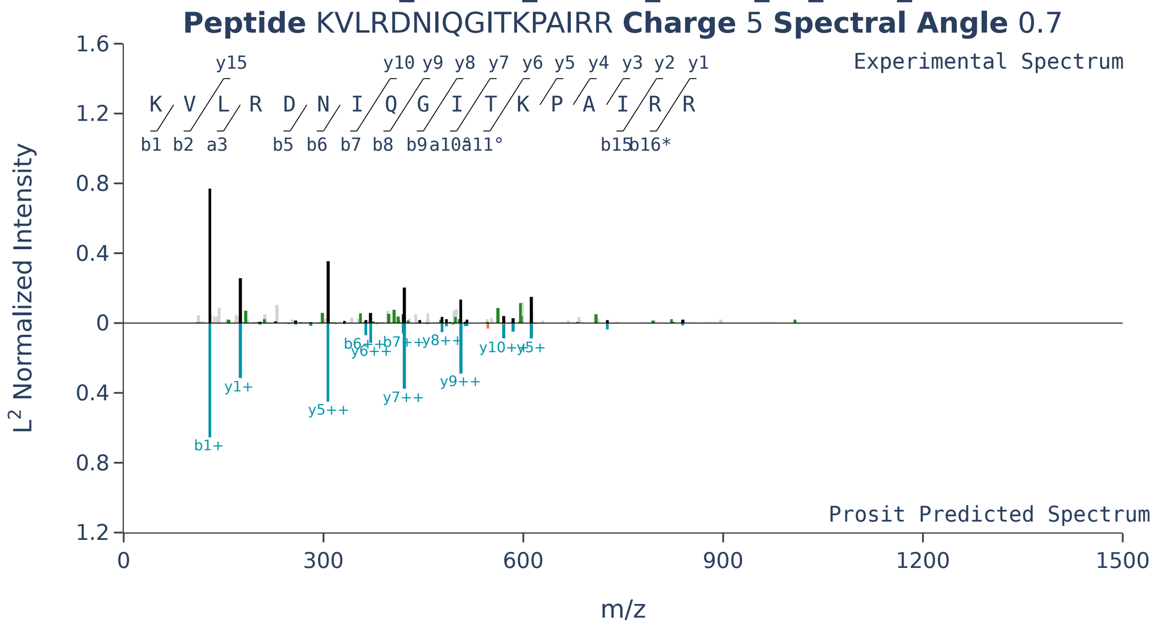

**Source** HRoetschke_291222_140123_HFGoe_H4_b1R1_2h **Scan** 10437
**Peptide** RDNIQGITKPAIRRLARR **Charge** 5 **Spectral Angle** 0.8

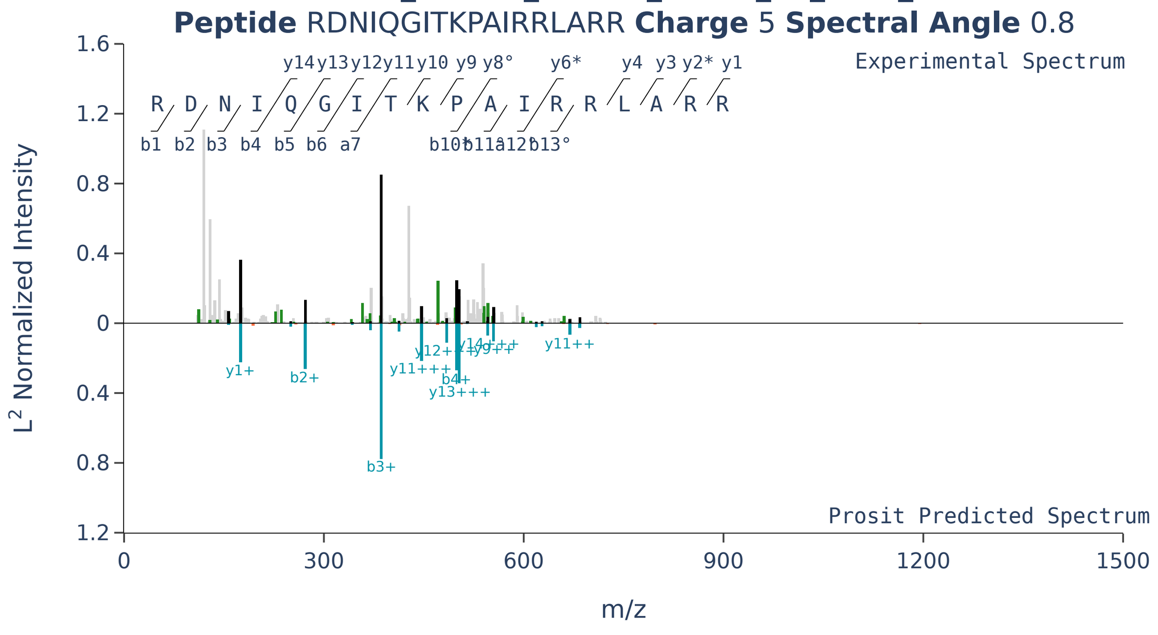

**Source** HRoetschke_291222_140123_HFGoe_H4_b2R1_1h **Scan** 22403
**Peptide** GGVKRISGLIYEETRGVLKV **Charge** 3 **Spectral Angle** 0.68

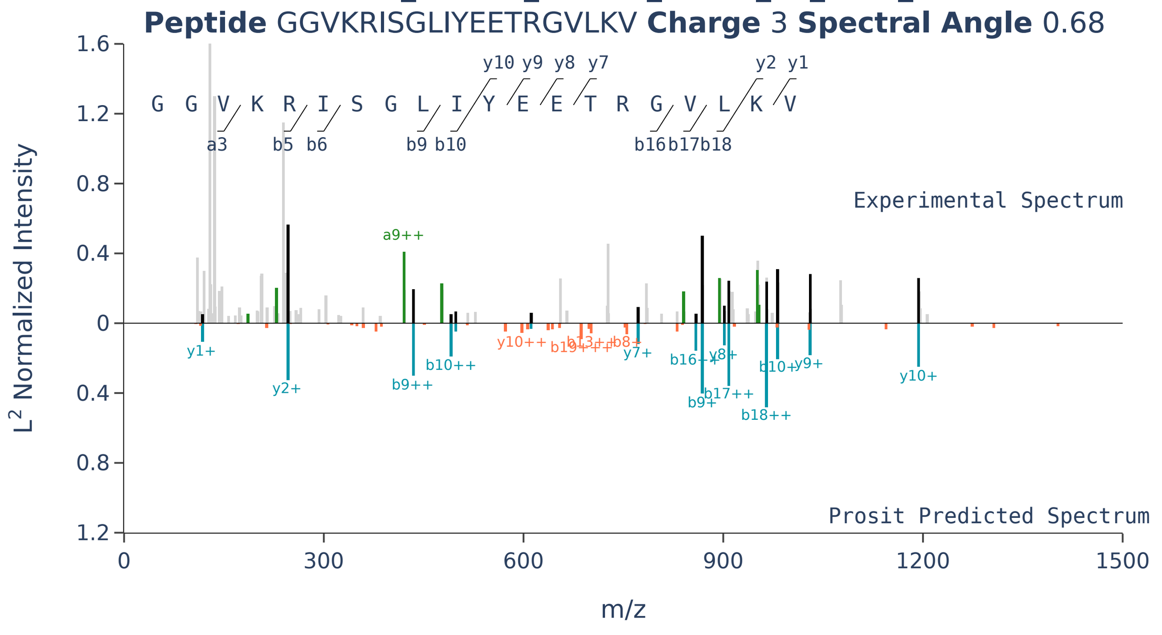

**Source** HRoetschke_291222_140123_HFGoe_H4_b1R1_2h **Scan** 22614
**Peptide** VLKVFLEN **Charge** 2 **Spectral Angle** 0.84

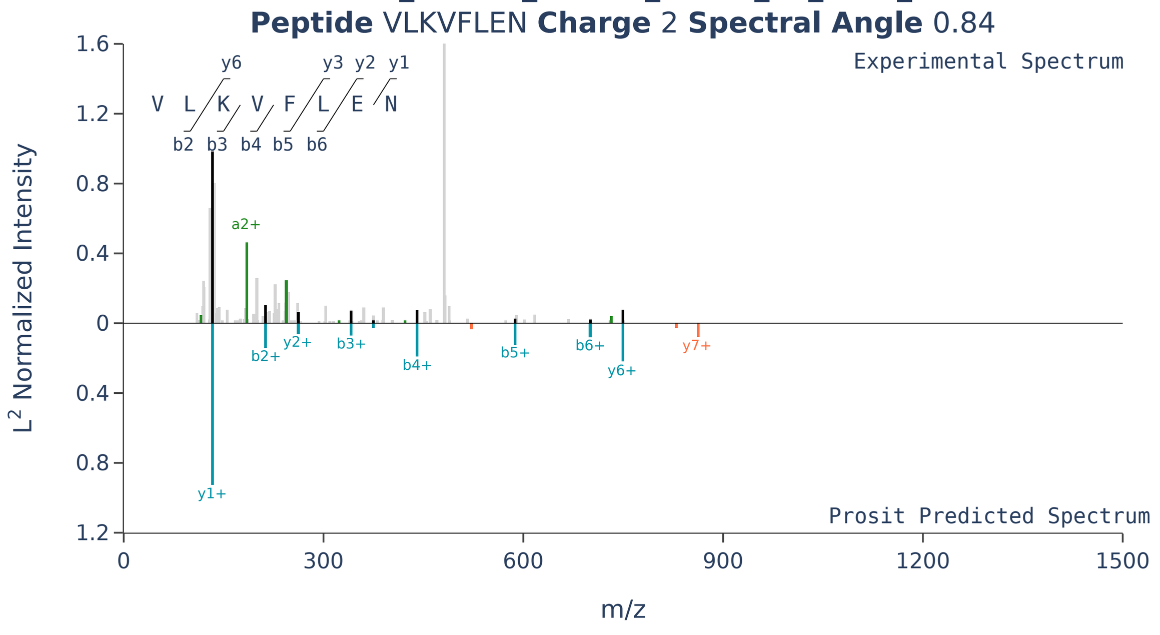

**Source** HRoetschke_291222_140123_HFGoe_H4_b2R1_1h **Scan** 24596
**Peptide** LIYEETRGVLKVF **Charge** 2 **Spectral Angle** 0.62

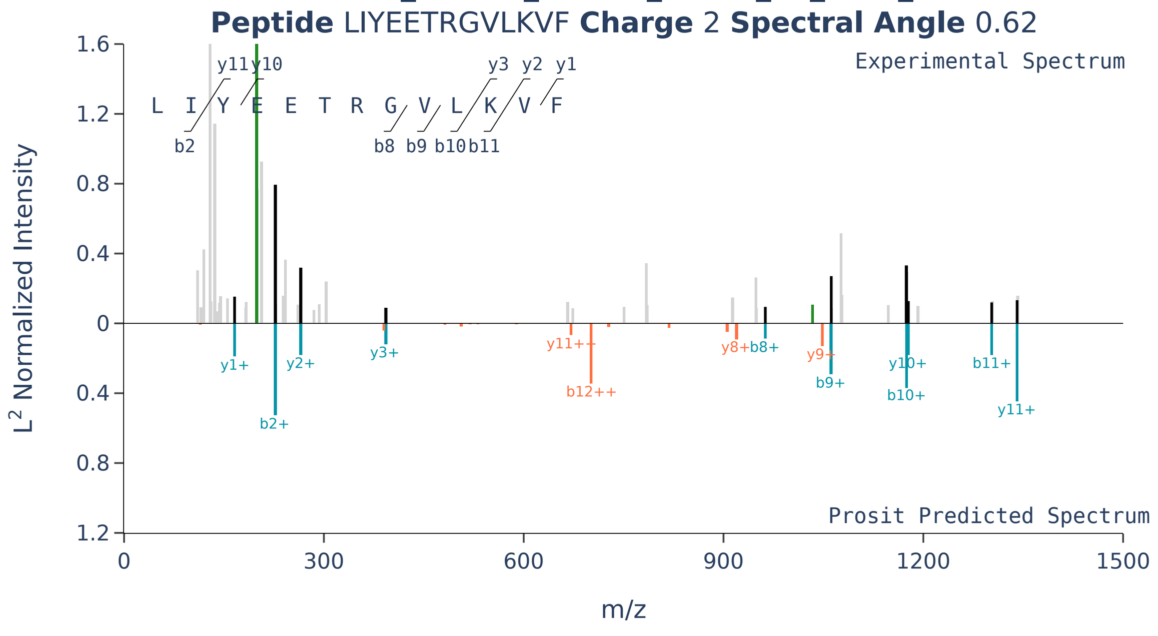

**Source** HRoetschke_291222_140123_HFGoe_H4_b2R2_1h **Scan** 12810
**Peptide** NIQGITKPAIRRLARRGGVKRI **Charge** 4 **Spectral Angle** 0.78

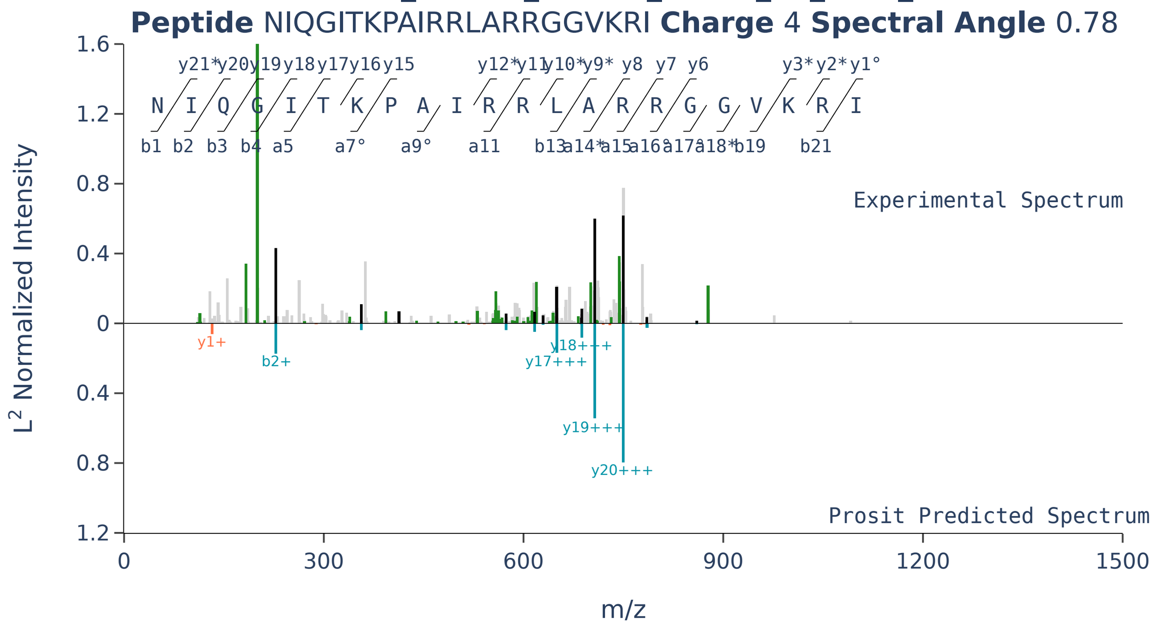

**Source** HRoetschke_291222_140123_HFGoe_H4_b1R2_2h **Scan** 11349
**Peptide** RDNIQGITKPAIRRLAR **Charge** 5 **Spectral Angle** 0.77

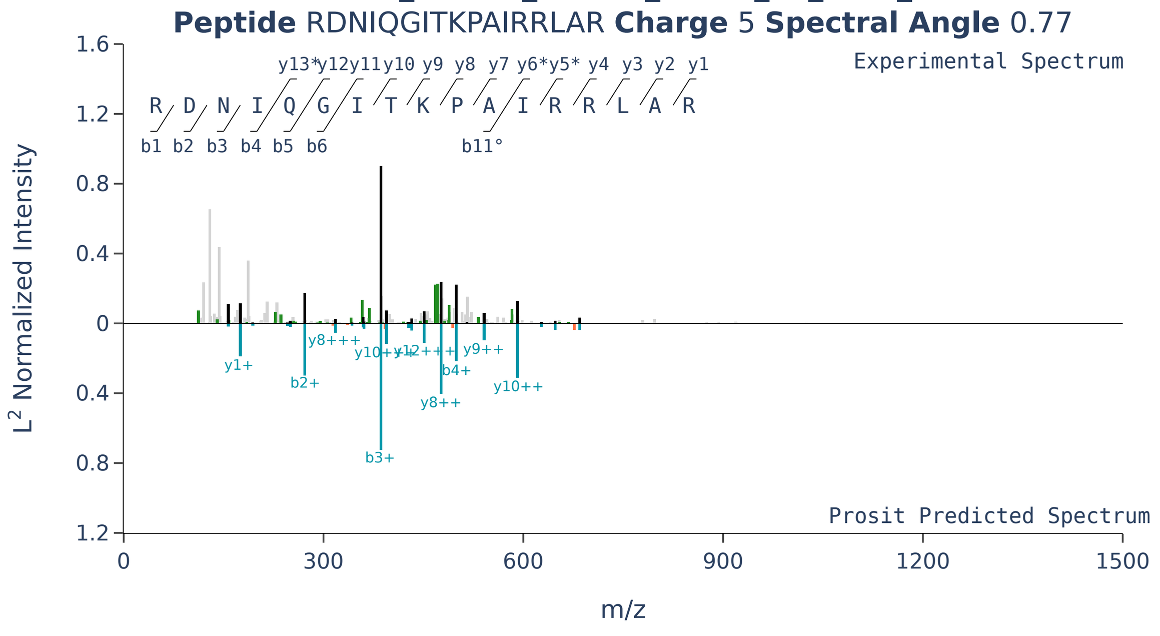

**Source** HRoetschke_291222_140123_HFGoe_H4_b1R2_2h **Scan** 7153
**Peptide** VLRDAVTYTEHAKRKT **Charge** 5 **Spectral Angle** 0.84

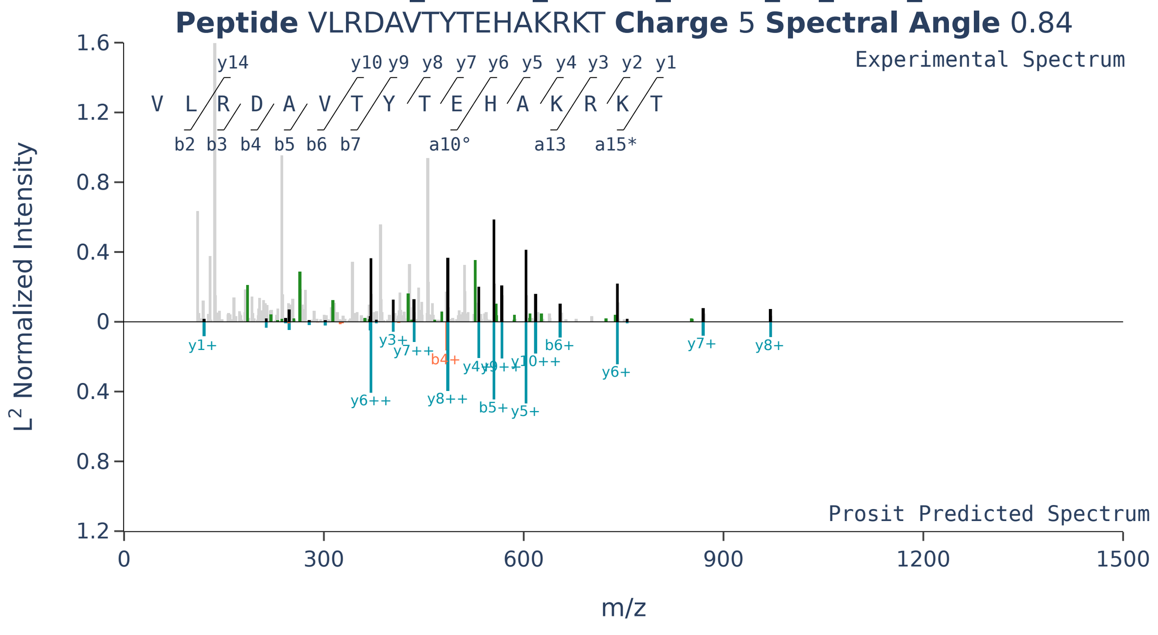

**Source** HRoetschke_291222_140123_HFGoe_H4_b1R1_1h **Scan** 14980
**Peptide** IRDAVTYTEHAKRKTVTAMDVVY **Charge** 5 **Spectral Angle** 0.78

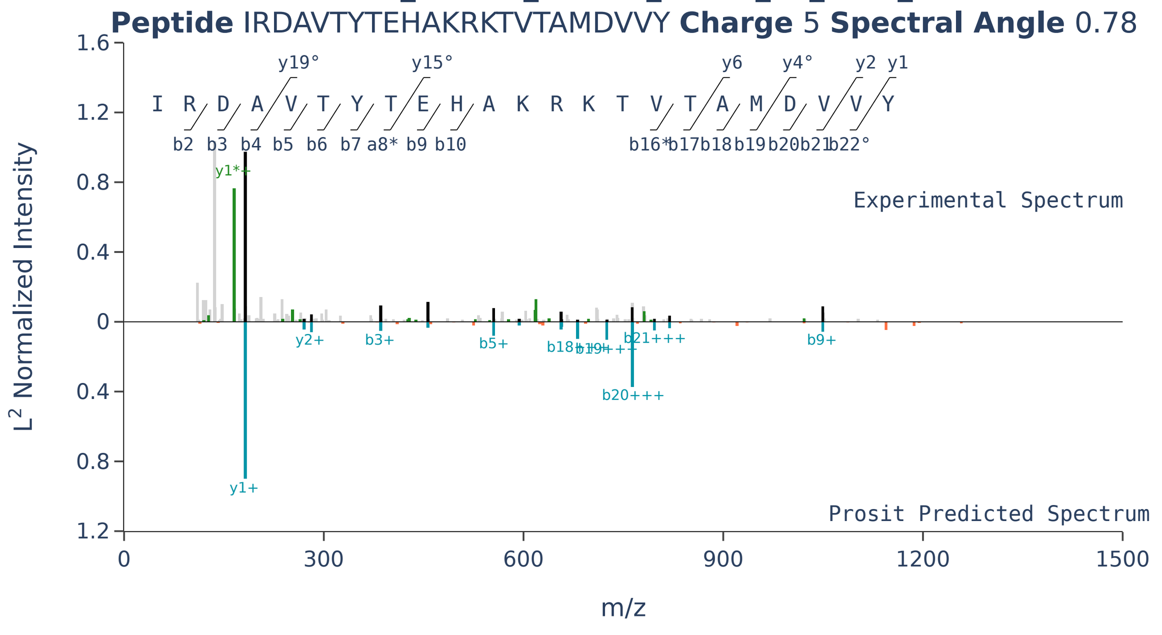

**Source** HRoetschke_291222_140123_HFGoe_H4_b1R1_24h **Scan** 14688
**Peptide** KRISGLIYEET **Charge** 3 **Spectral Angle** 0.49

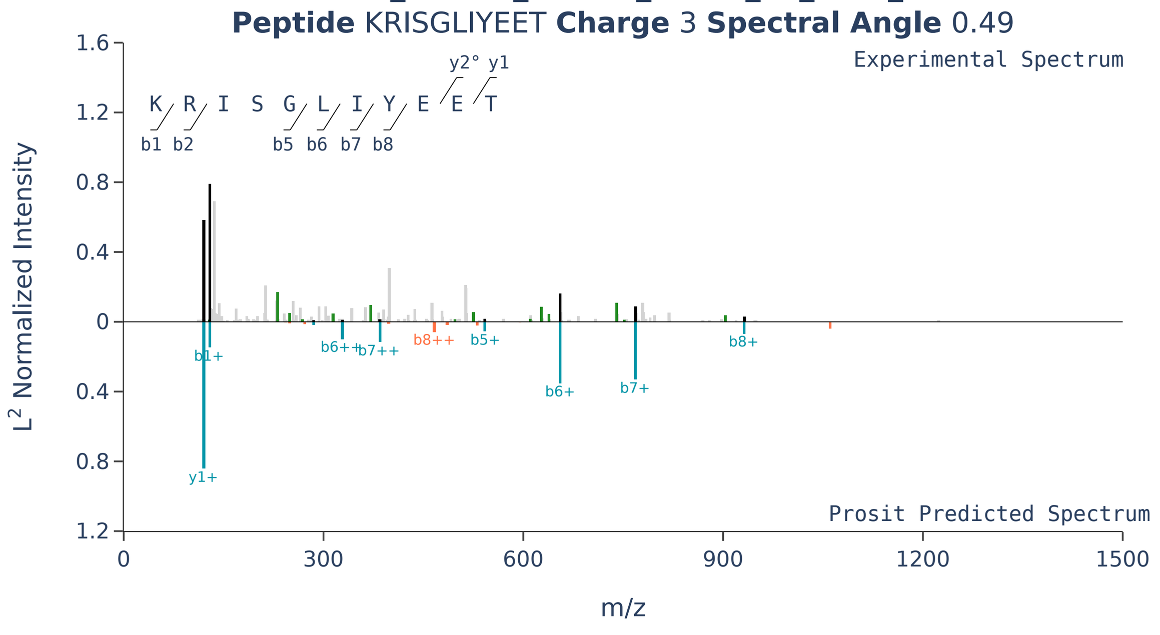

**Source** HRoetschke_291222_140123_HFGoe_H4_b2R1_1h **Scan** 31499
**Peptide** EETRGVLKVFLENVL **Charge** 2 **Spectral Angle** 0.77

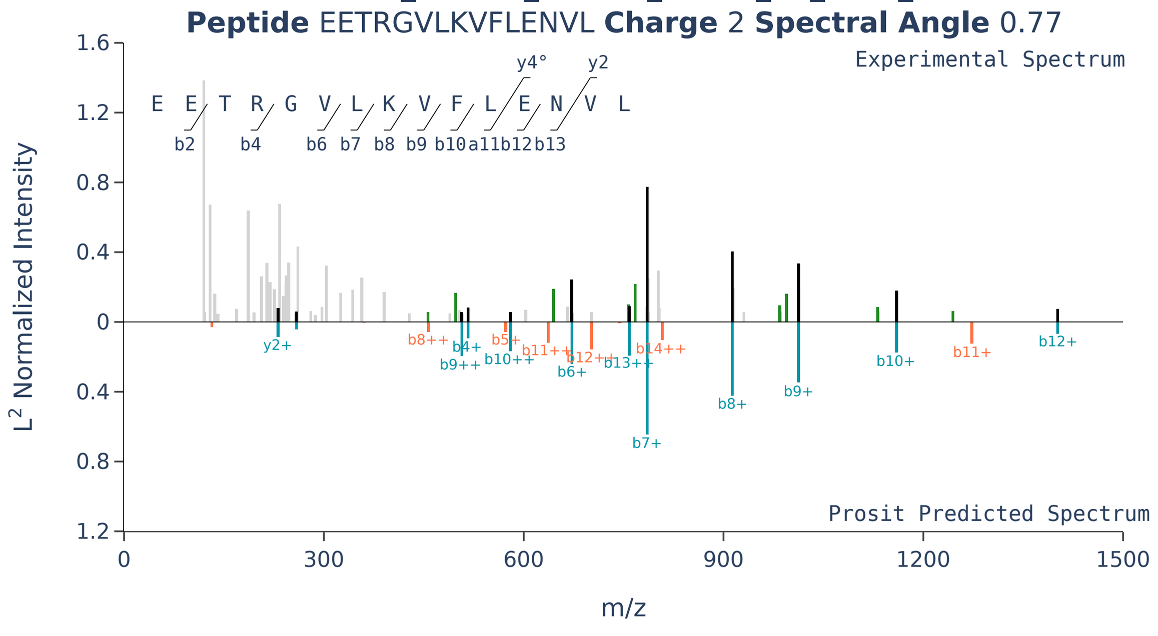

**Source** HRoetschke_291222_140123_HFGoe_H4_b1R1_1h **Scan** 6904
**Peptide** RDAVTYTEHAKRKTVT **Charge** 4 **Spectral Angle** 0.68

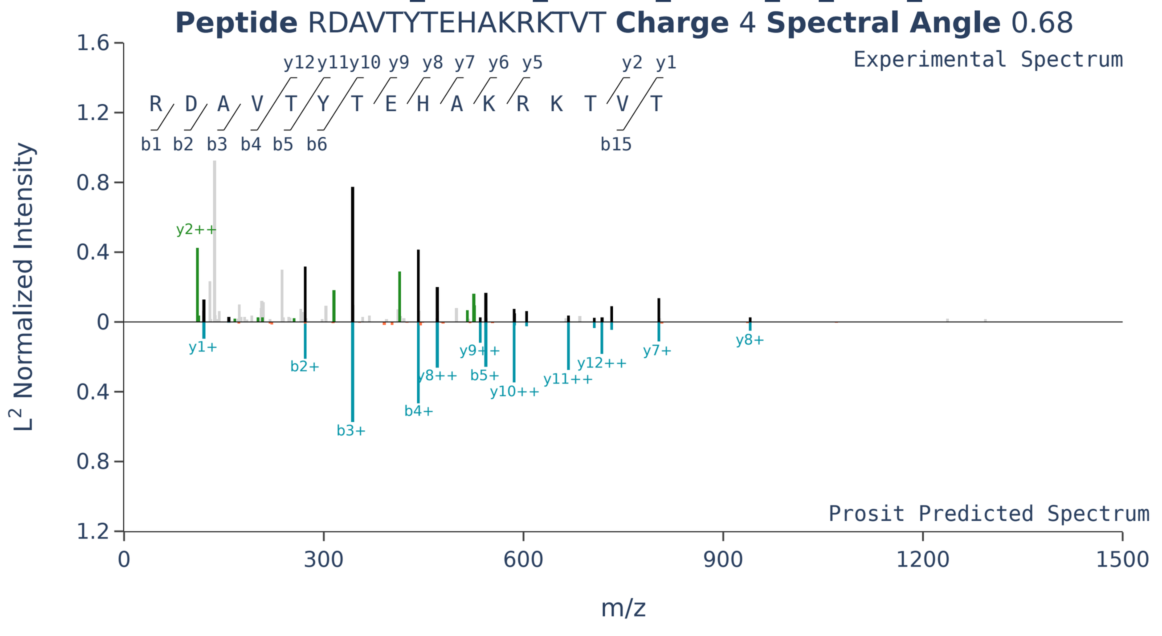

**Source** HRoetschke_291222_140123_HFGoe_H4_b1R1_24h **Scan** 8683
**Peptide** LQGITKPAIRRLARRGGVKR **Charge** 5 **Spectral Angle** 0.76

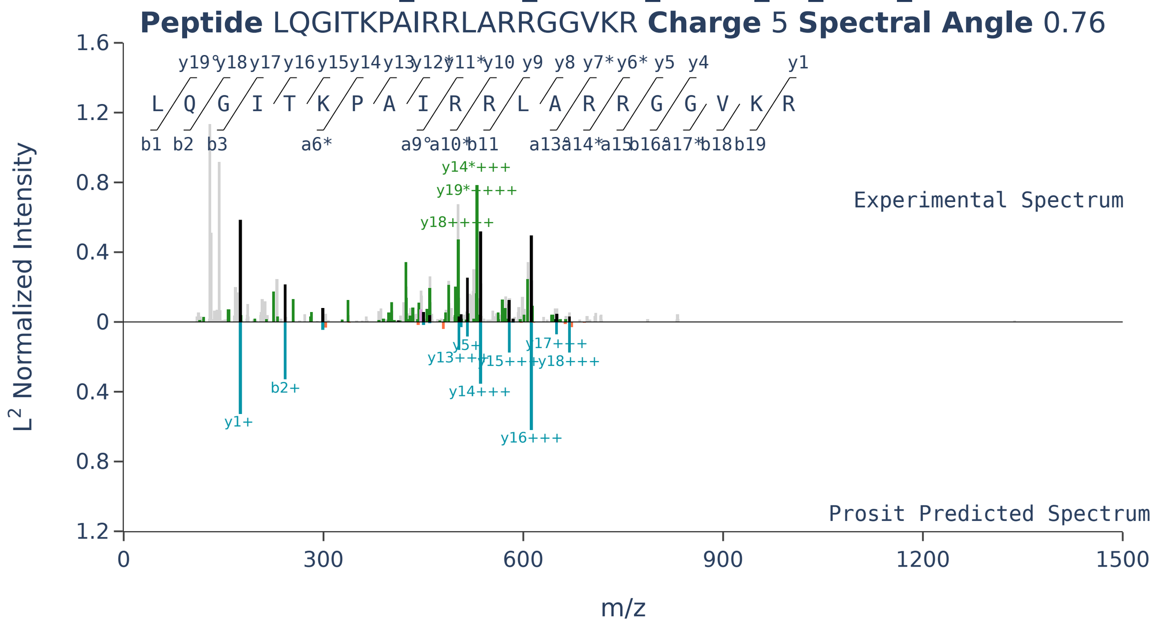

**Source** HRoetschke_291222_140123_HFGoe_H4_b1R1_2h **Scan** 15936
**Peptide** RISGLIYEETRG **Charge** 2 **Spectral Angle** 0.71

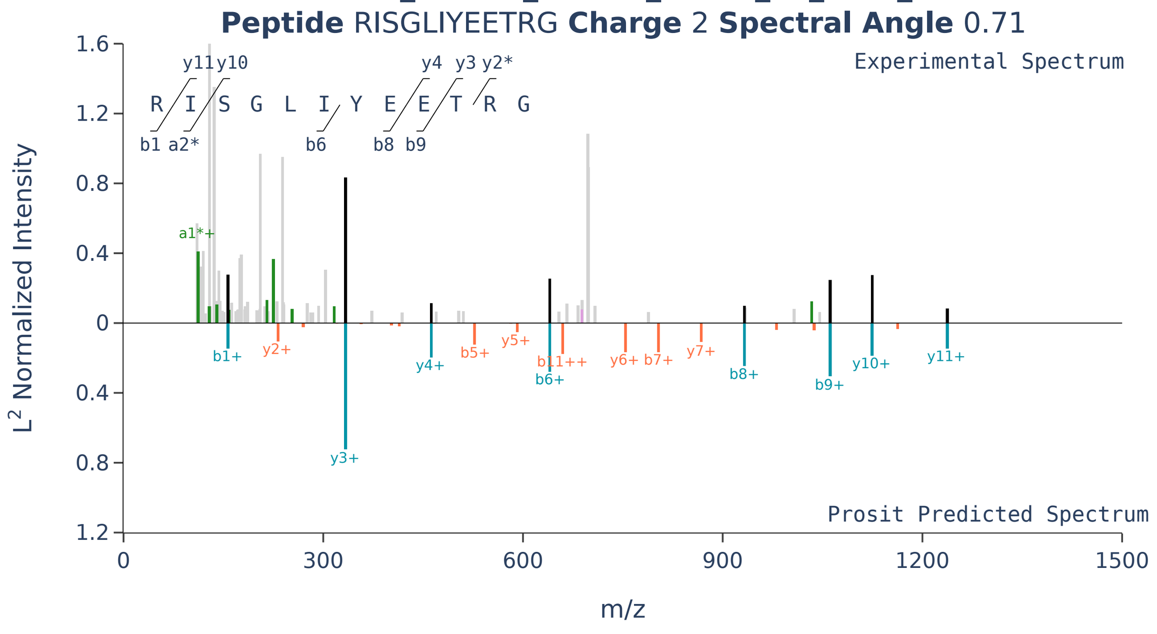


**Source** HRoetschke_291222_140123_HFGoe_H4_b1R2_2h **Scan** 16986
**Peptide** AKRKTVTAMDVVYALKRQGRT **Charge** 5 **Spectral Angle** 0.9

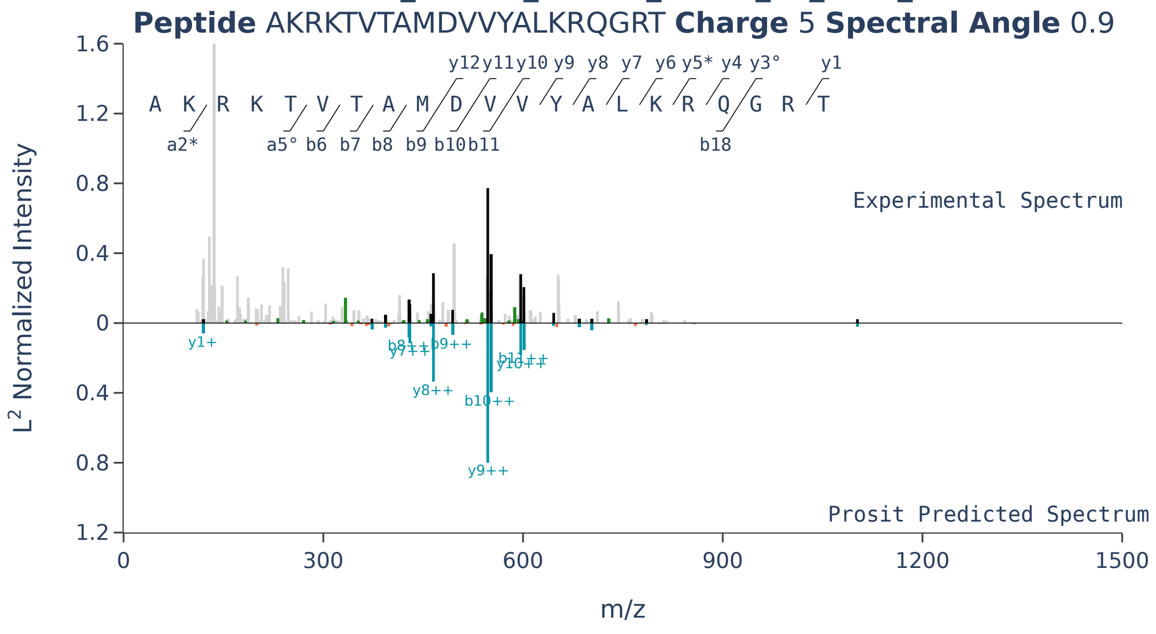


**Source** HRoetschke_291222_140123_HFGoe_H4_b2R1_1h **Scan** 6278
**Peptide** IRDAVTYTEHAKRKTV **Charge** 4 **Spectral Angle** 0.7

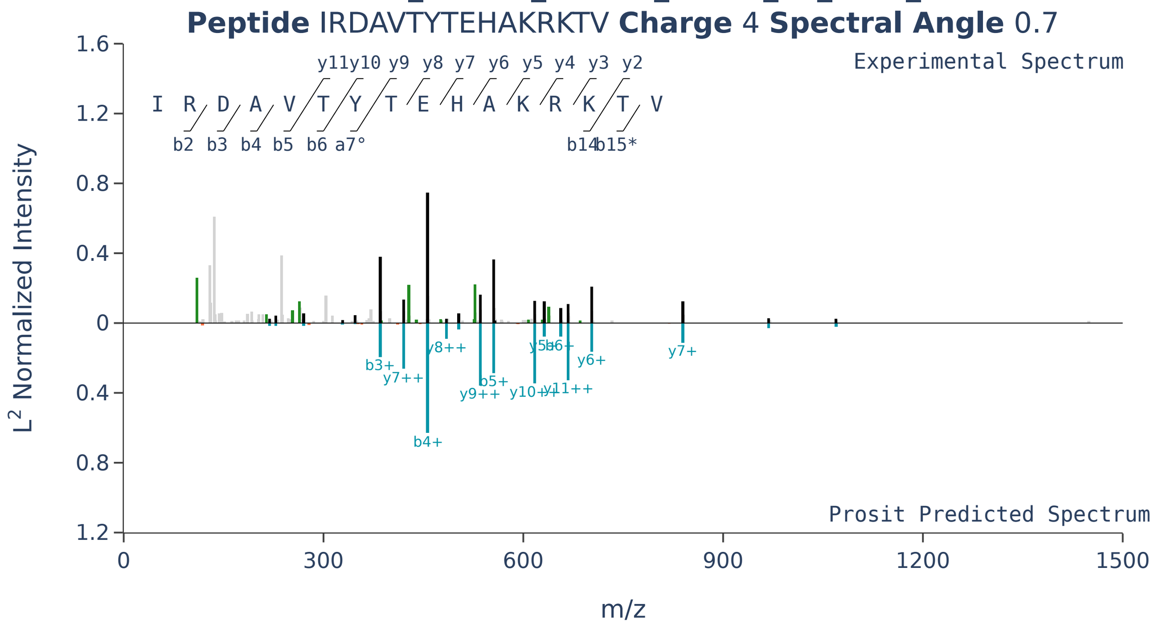


**Source** HRoetschke_291222_140123_HFGoe_H4_b1R1_1h **Scan** 17230
**Peptide** EHAKRKTVTAMDVVYAL **Charge** 3 **Spectral Angle** 0.57

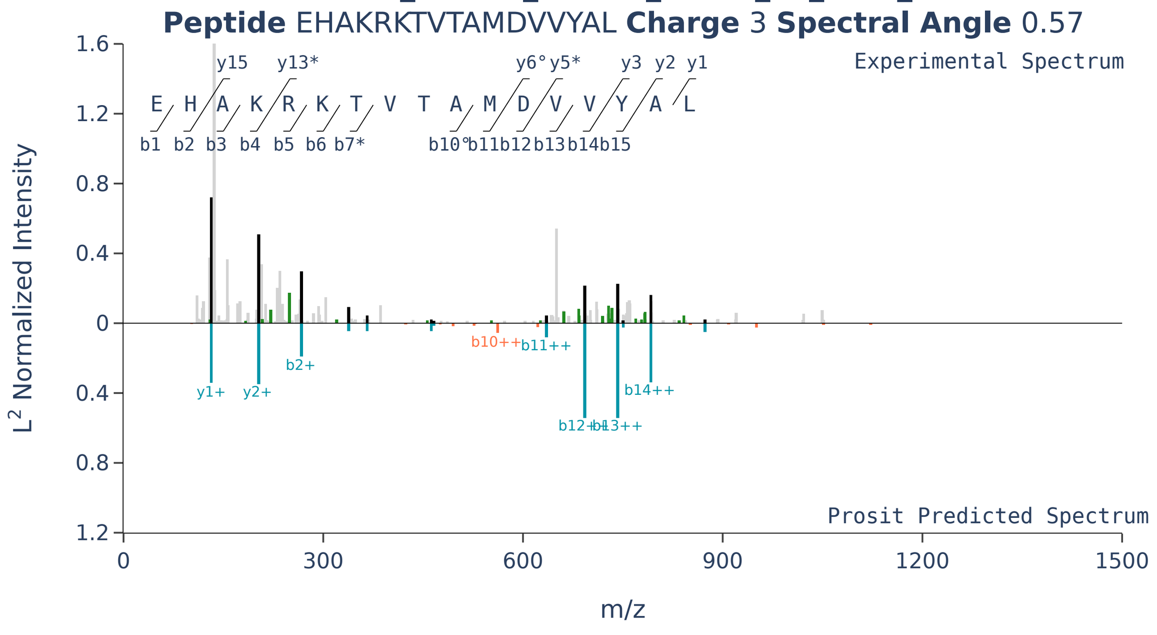


**Source** HRoetschke_291222_140123_HFGoe_H4_b1R1_2h **Scan** 15579
**Peptide** HAKRKTVTAMDVVYALKRQGR **Charge** 5 **Spectral Angle** 0.81

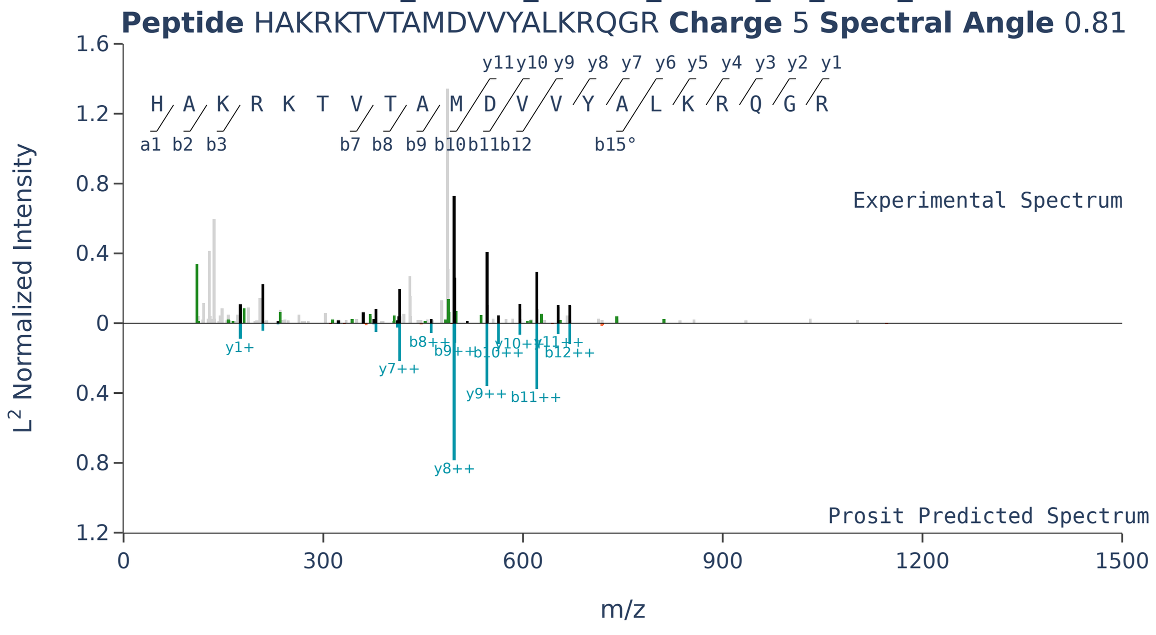


**Source** HRoetschke_291222_140123_HFGoe_H4_b2R1_1h **Scan** 23931
**Peptide** FLENVIRDAVTYTEHAKRKTVTA **Charge** 4 **Spectral Angle** 0.57

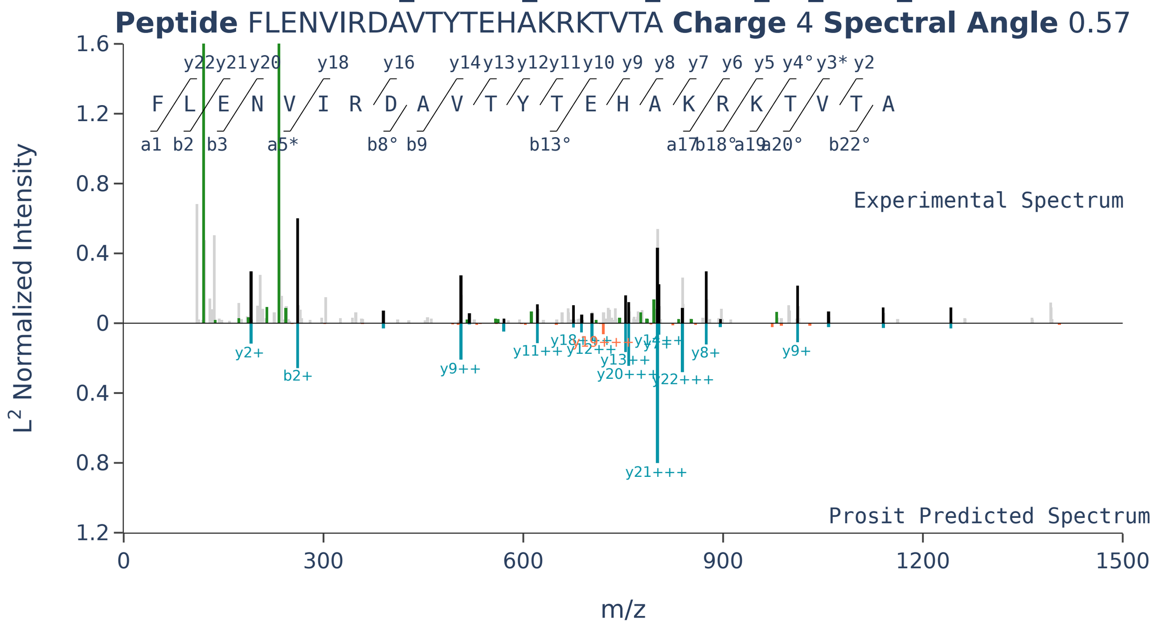


**Source** HRoetschke_291222_140123_HFGoe_H4_b2R2_4h **Scan** 12378
**Peptide** VLRDNIQGITKPAIRRLARRGGVKR **Charge** 5 **Spectral Angle** 0.67

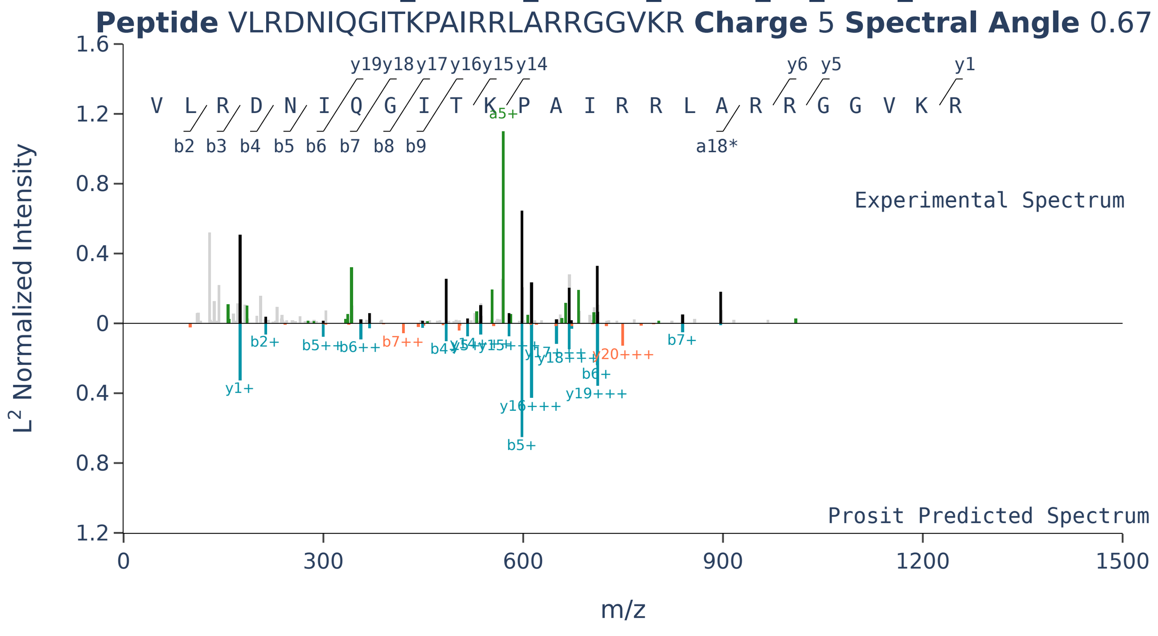


**Source** HRoetschke_291222_140123_HFGoe_H4_b2R2_4h **Scan** 11167
**Peptide** ARRGGVKRISGLIYEETR **Charge** 4 **Spectral Angle** 0.69

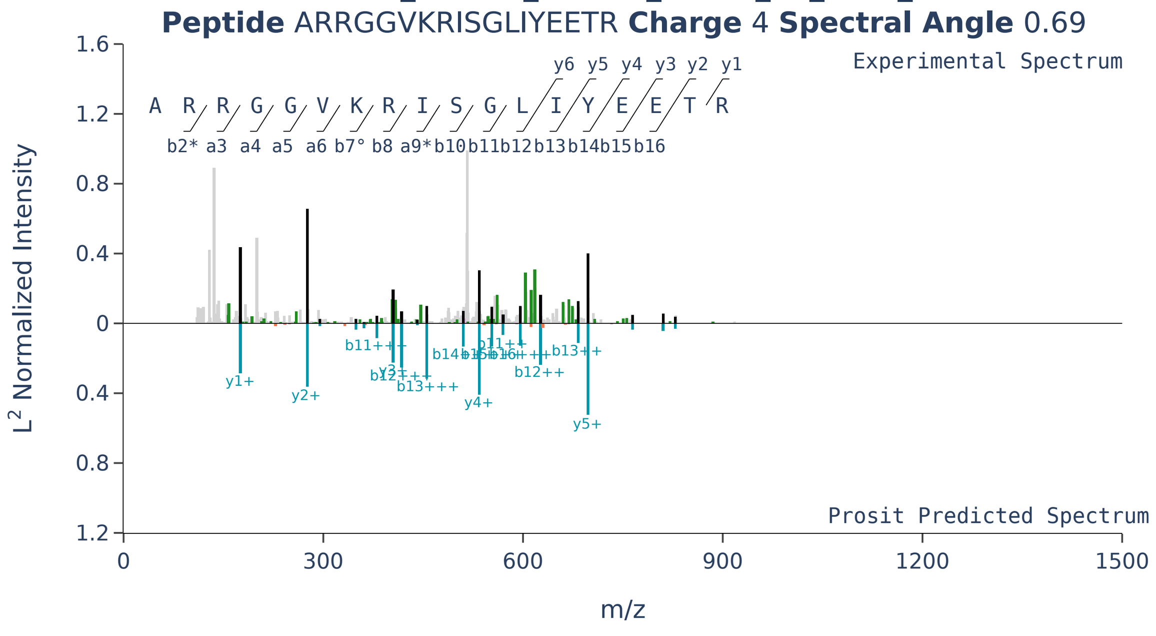


**Source** HRoetschke_291222_140123_HFGoe_H4_b2R1_1h **Scan** 29157
**Peptide** YEETRGVLKVFLENVIRD **Charge** 3 **Spectral Angle** 0.72

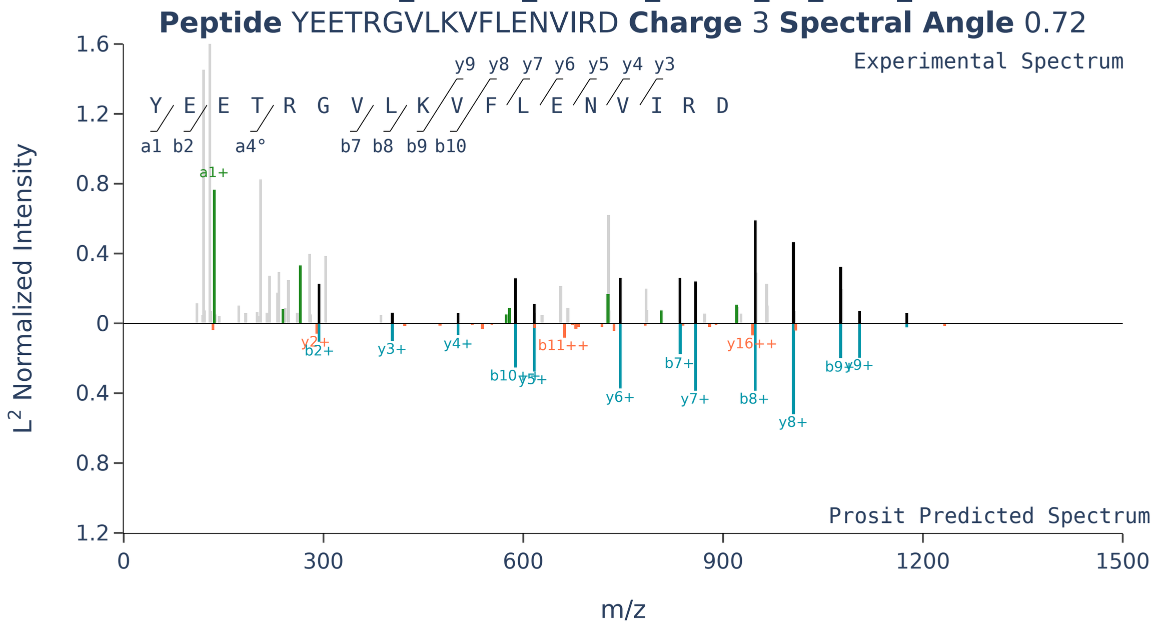


**Source** HRoetschke_291222_140123_HFGoe_H4_b1R1_1h **Scan** 25981
**Peptide** KVFLENVIRDAVTYTEHAKR **Charge** 4 **Spectral Angle** 0.82

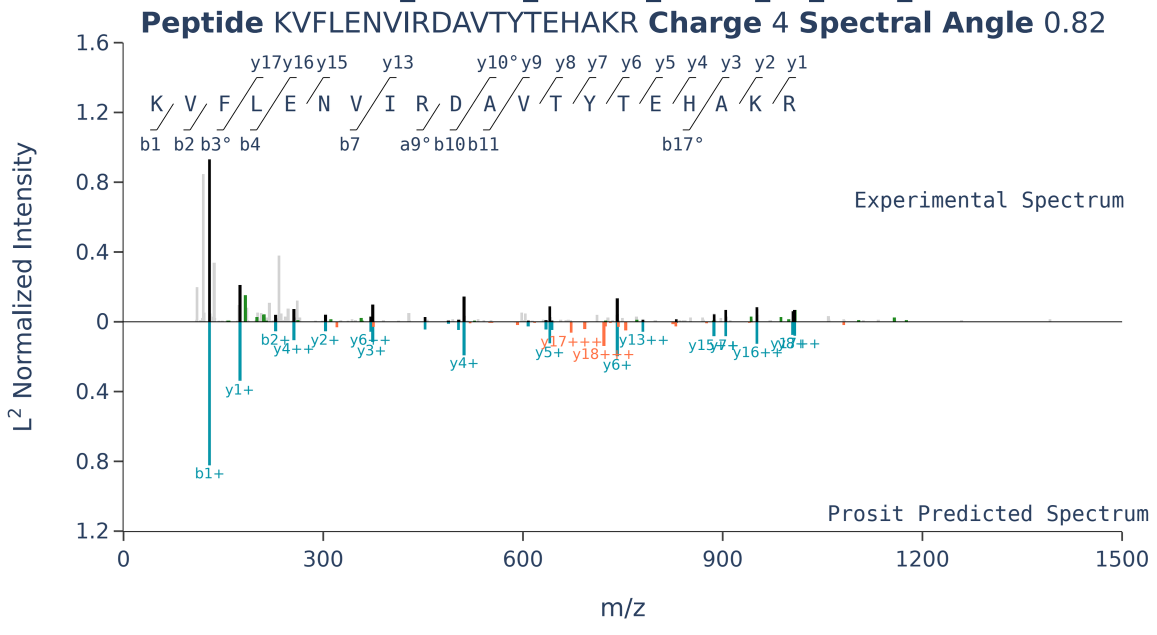


**Source** HRoetschke_291222_140123_HFGoe_H4_b2R1_24h **Scan** 13272
**Peptide** RKVLRDNIQGITKPAIRRLA **Charge** 5 **Spectral Angle** 0.82

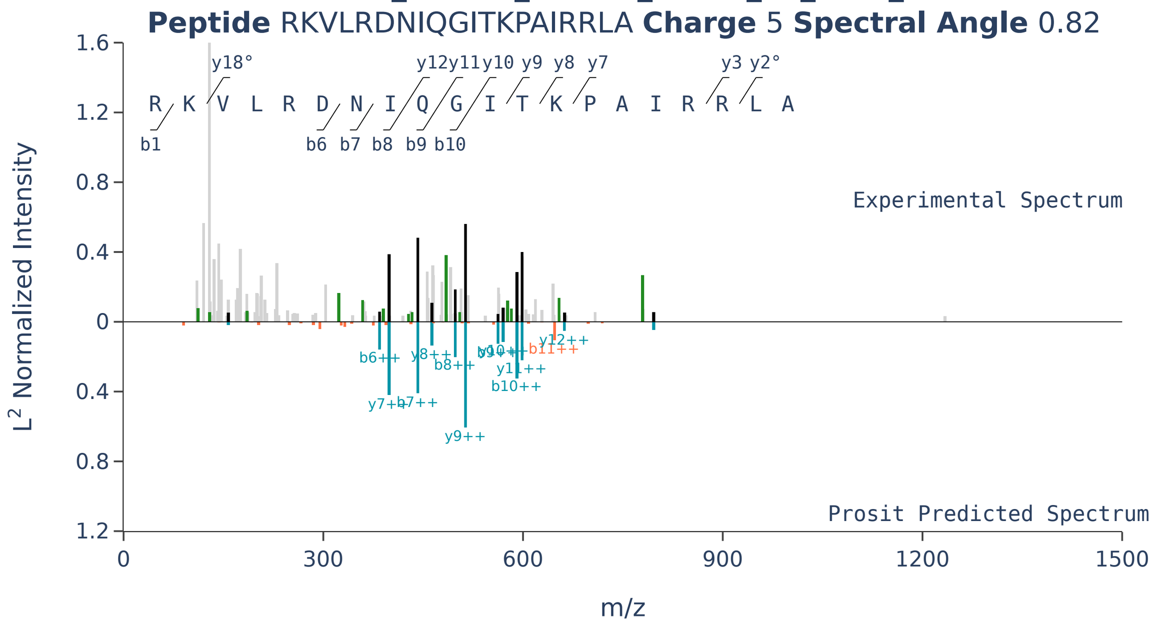


**Source** HRoetschke_291222_140123_HFGoe_H4_b1R1_24h **Scan** 4981
**Peptide** AVTYTEHAKRK **Charge** 3 **Spectral Angle** 0.64

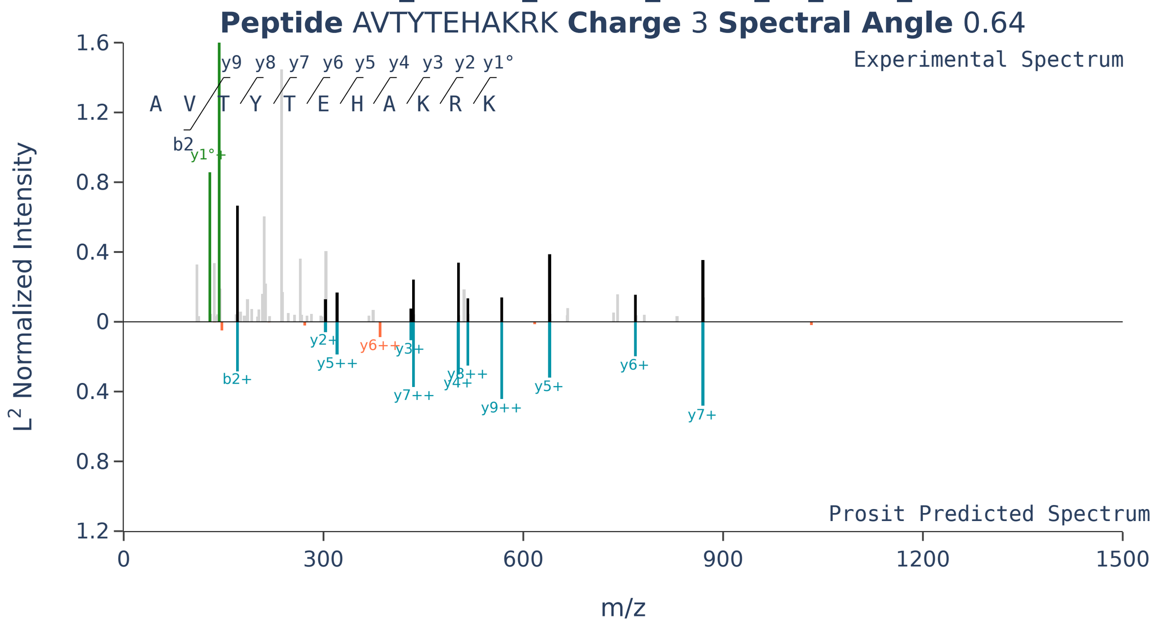


**Source** HRoetschke_291222_140123_HFGoe_H4_b1R1_24h **Scan** 14247
**Peptide** KVLRDNIQGITKPAIRRLA **Charge** 4 **Spectral Angle** 0.71

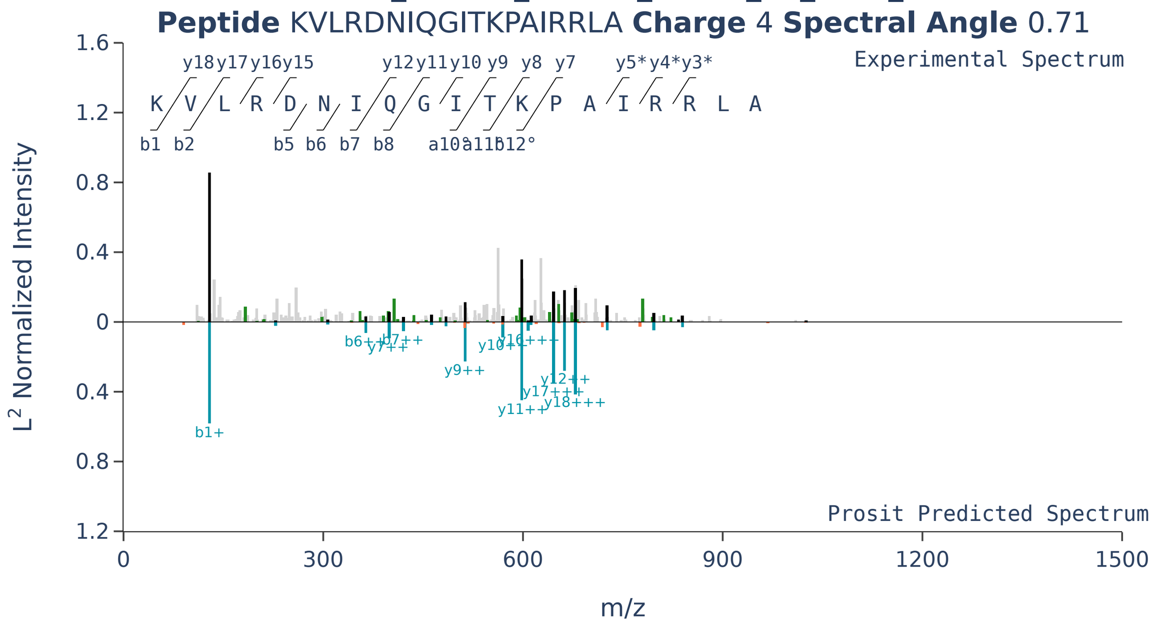


**Source** HRoetschke_291222_140123_HFGoe_H4_b1R1_24h **Scan** 5302
**Peptide** TEHAKRKTVTAMD **Charge** 3 **Spectral Angle** 0.61

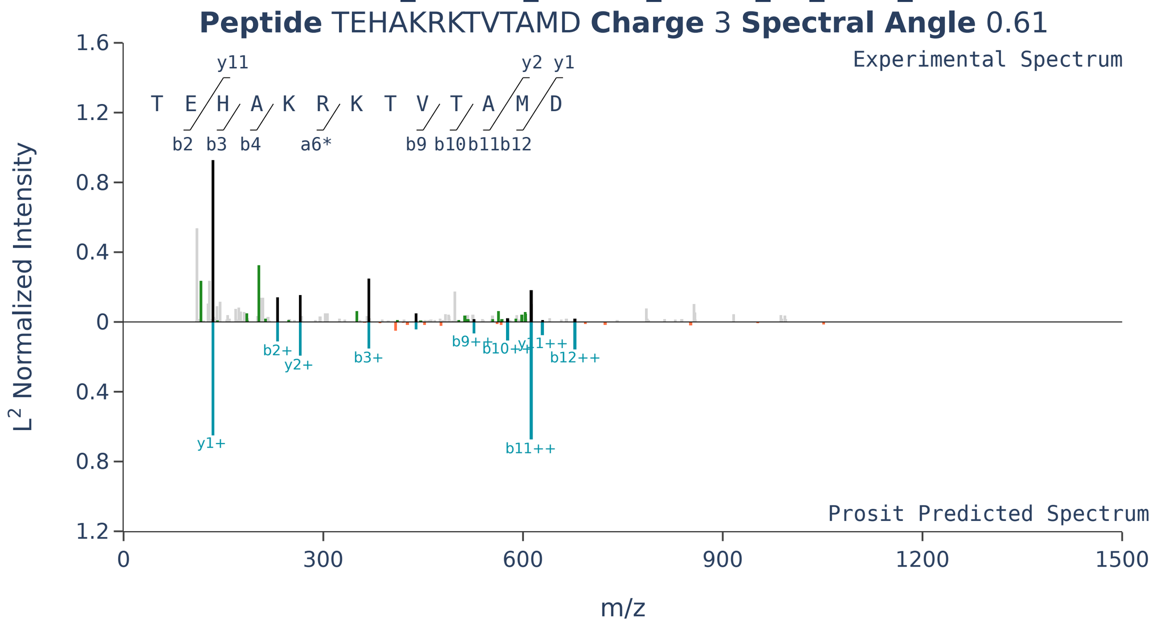


**Source** HRoetschke_291222_140123_HFGoe_H4_b2R1_1h **Scan** 20739
**Peptide** DVVYALKRQGRTLYGFGG **Charge** 3 **Spectral Angle** 0.56

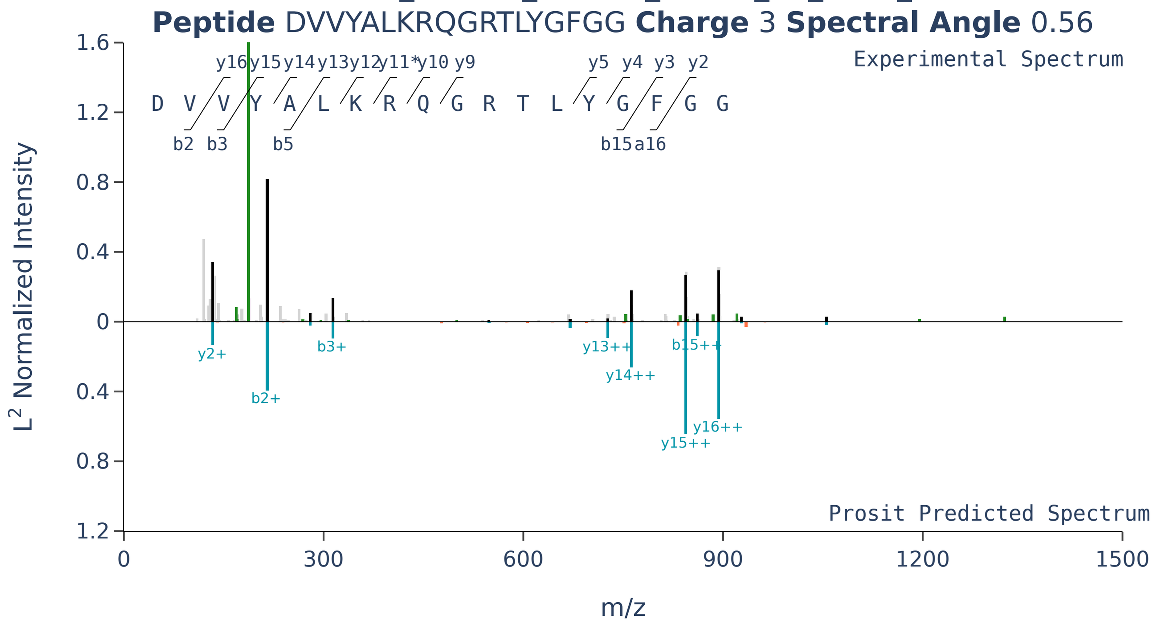

**Source** HRoetschke_291222_140123_HFGoe_H4_b1R1_1h **Scan** 21806
**Peptide** RGGVKRISGLIYEETRGVL **Charge** 4 **Spectral Angle** 0.75

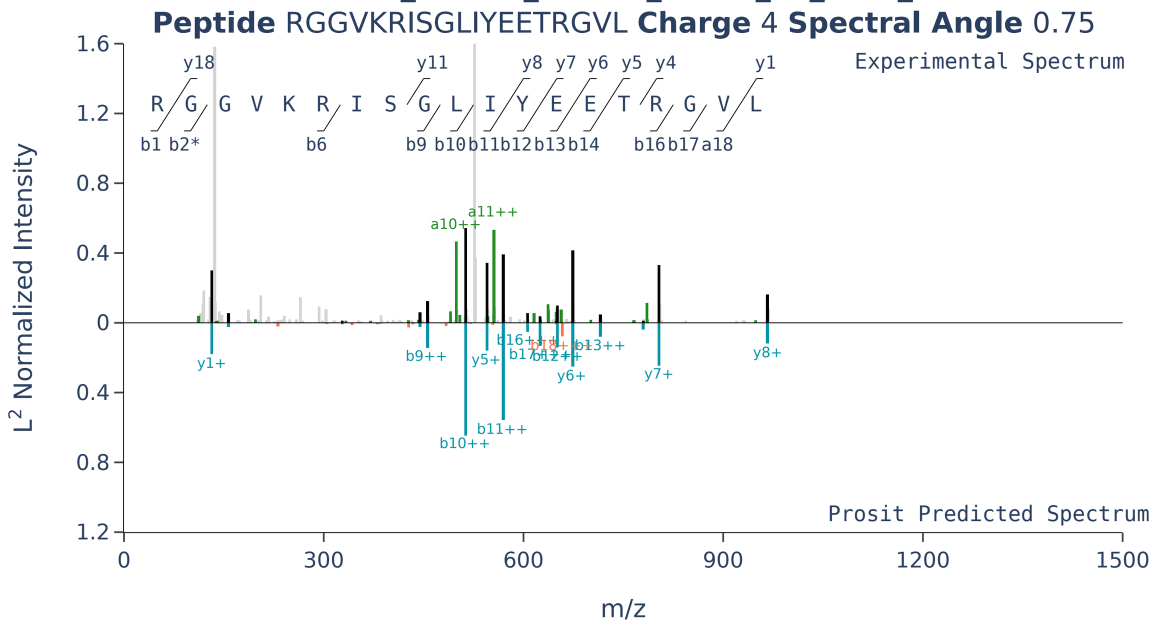


**Source** HRoetschke_291222_140123_HFGoe_H4_b2R2_1h **Scan** 10523
**Peptide** NIQGITKPAIRRLARRGGVKRIS **Charge** 4 **Spectral Angle** 0.84

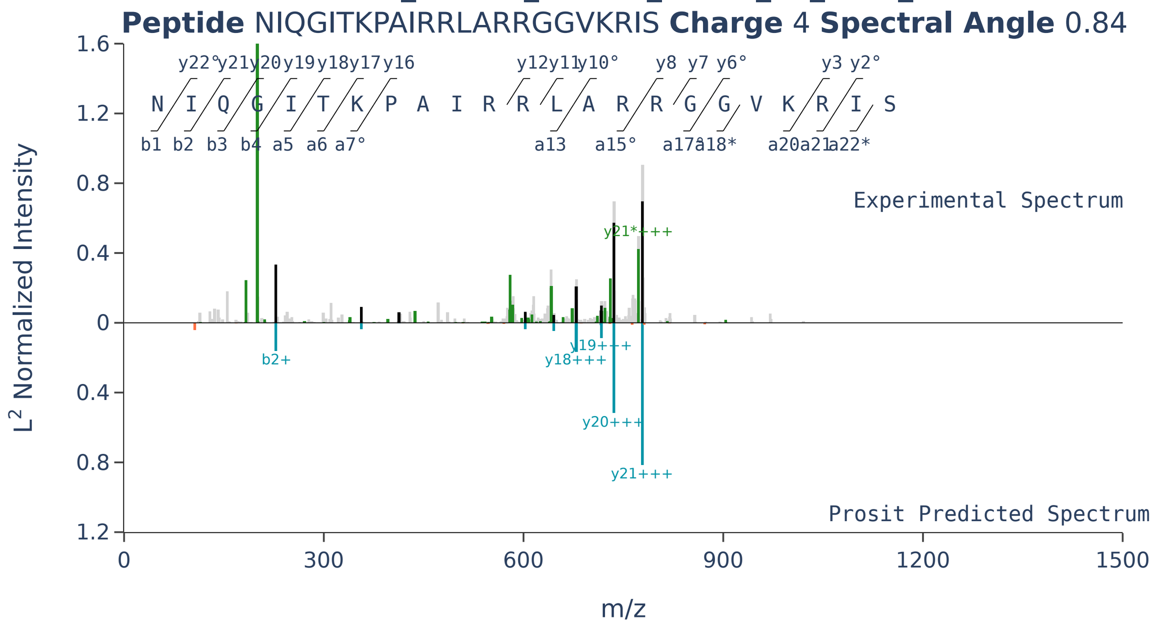


**Source** HRoetschke_291222_140123_HFGoe_H4_b1R1_24h **Scan** 7514
**Peptide** VLRDAVTYTEHAKRKTV **Charge** 5 **Spectral Angle** 0.82

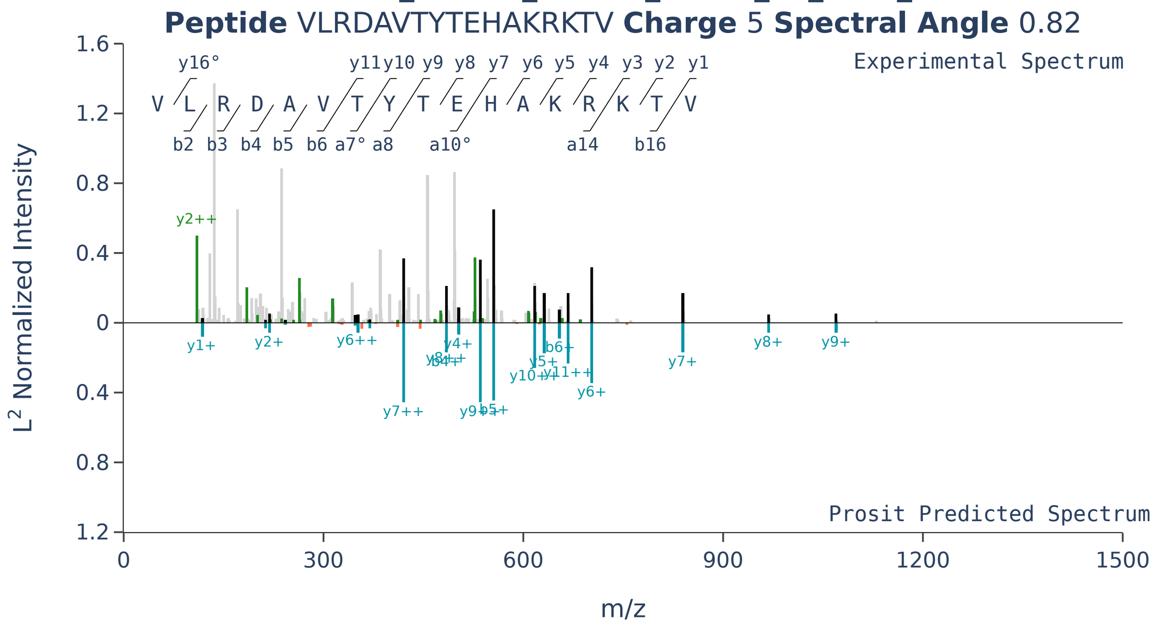


**Source** HRoetschke_291222_140123_HFGoe_H4_b1R2_1h **Scan** 22076
**Peptide** DVVYALKRQGRTLYGF **Charge** 3 **Spectral Angle** 0.7

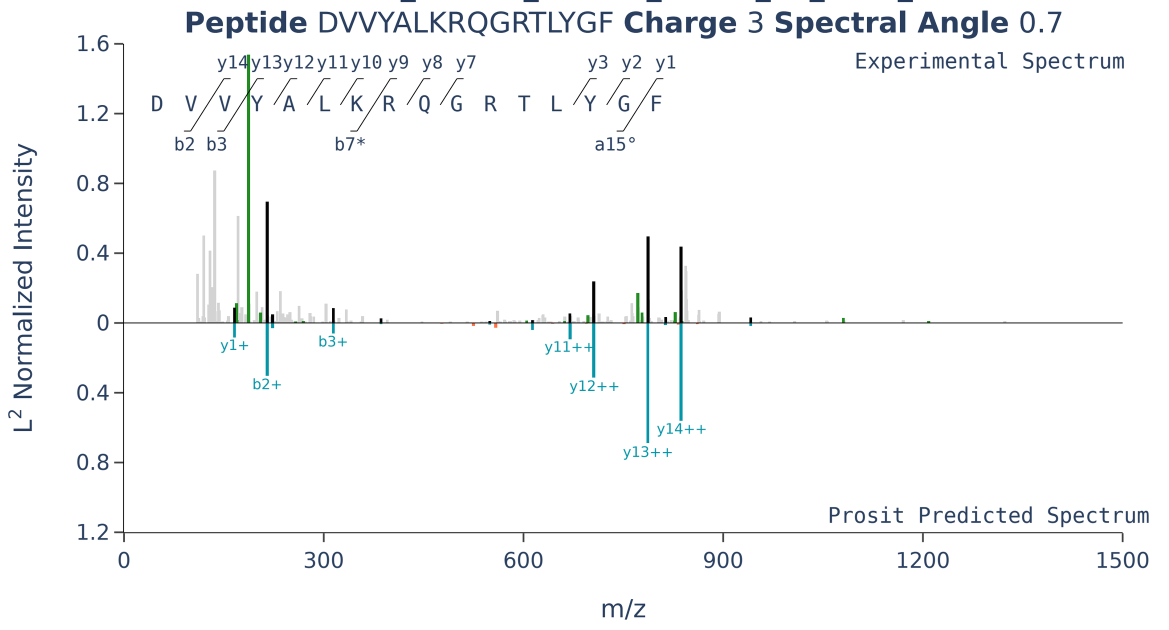


**Source** HRoetschke_291222_140123_HFGoe_H4_b2R2_4h **Scan** 12857
**Peptide** RGGVKRISGLL **Charge** 3 **Spectral Angle** 0.72

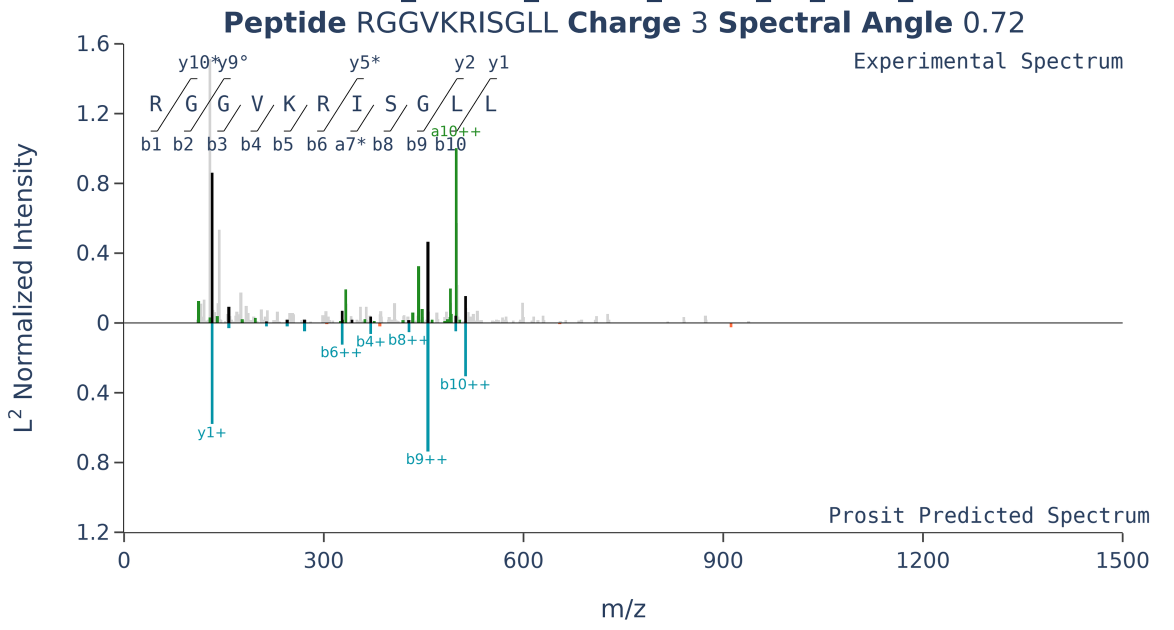


**Source** HRoetschke_291222_140123_HFGoe_H4_b1R1_2h **Scan** 4891
**Peptide** SGRGKGGKGLGKGGAKRHRKV **Charge** 5 **Spectral Angle** 0.81

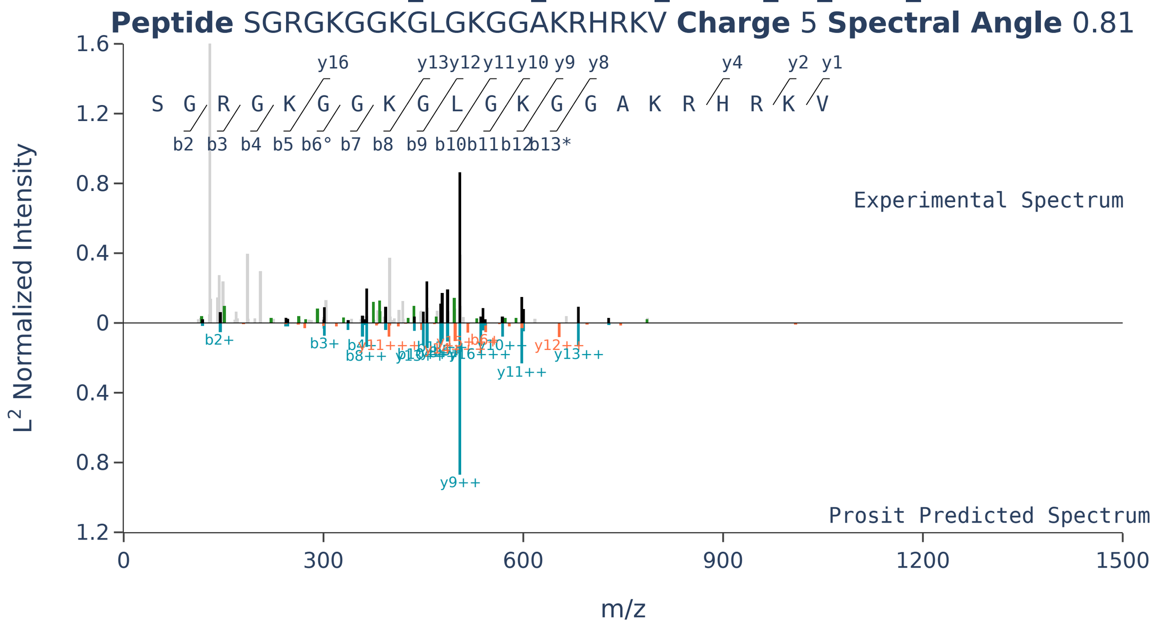


**Source** HRoetschke_291222_140123_HFGoe_H4_b2R1_1h **Scan** 26691
**Peptide** RISGLIYEETRGVLKVF **Charge** 3 **Spectral Angle** 0.59

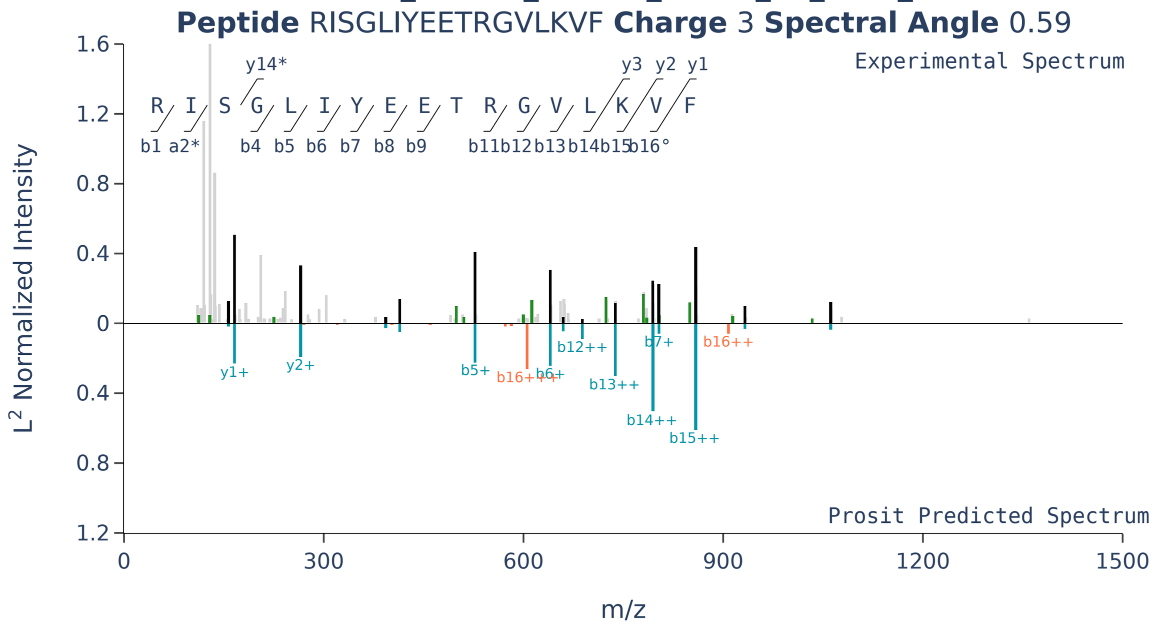


**Source** HRoetschke_291222_140123_HFGoe_H4_b2R1_1h **Scan** 31462
**Peptide** LYEETRGVLKVFL **Charge** 2 **Spectral Angle** 0.83

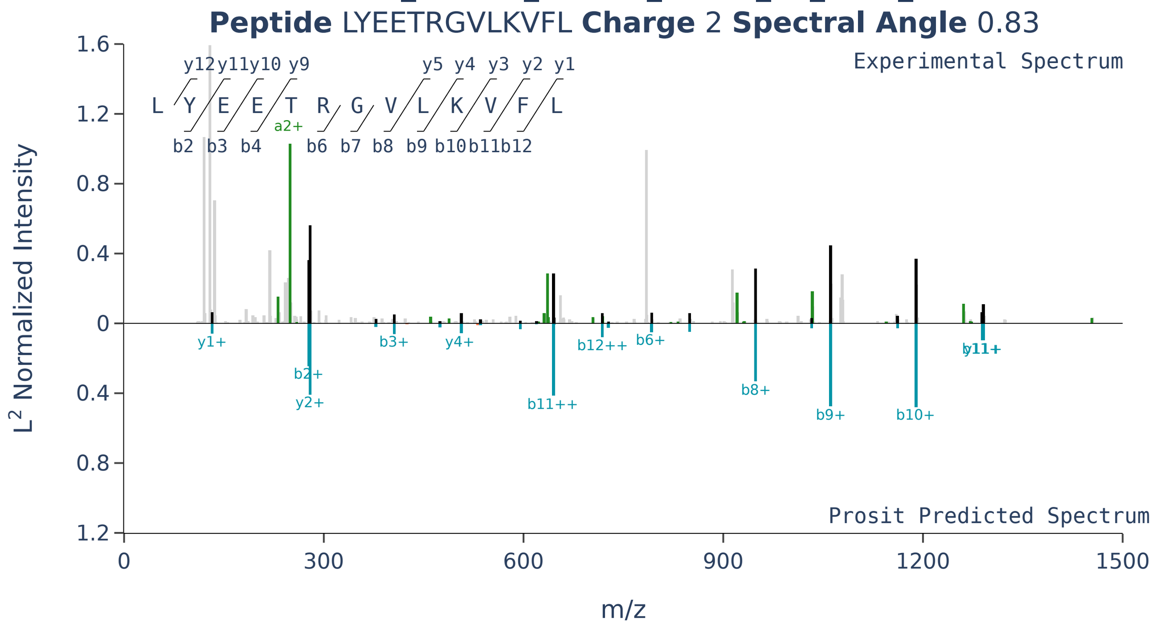


**Source** HRoetschke_291222_140123_HFGoe_H4_b2R1_2h **Scan** 15059
**Peptide** QGITKPAIRRLARRGGVKRISGLIYE **Charge** 5 **Spectral Angle** 0.89

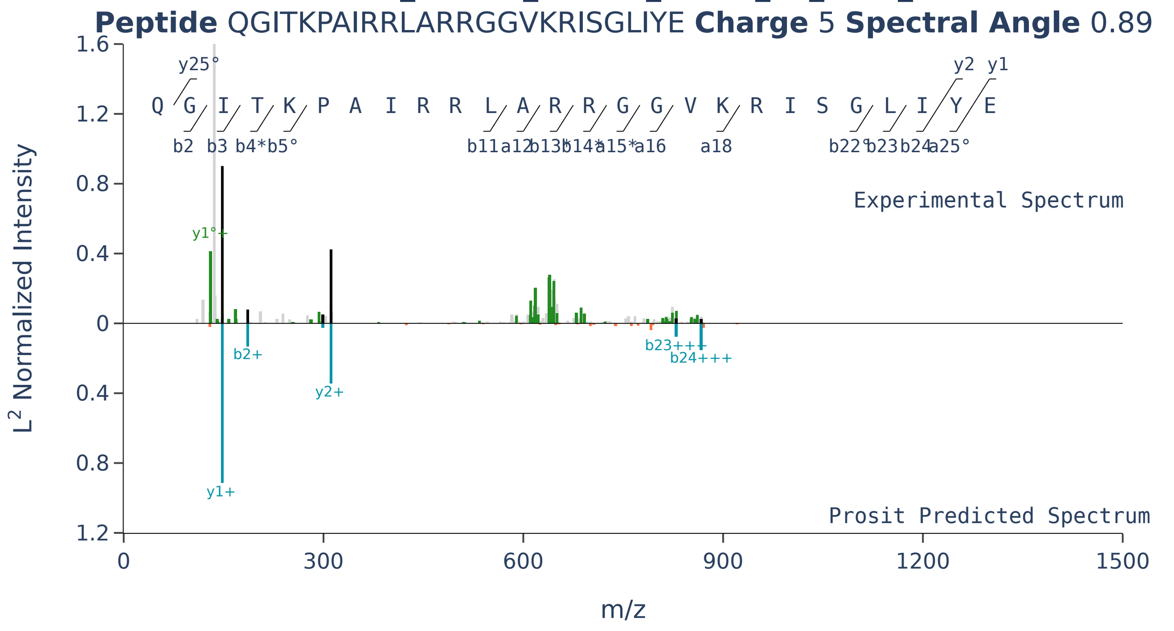


**Source** HRoetschke_291222_140123_HFGoe_H4_b1R2_2h **Scan** 14685
**Peptide** RGGVKRISGLLY **Charge** 3 **Spectral Angle** 0.69

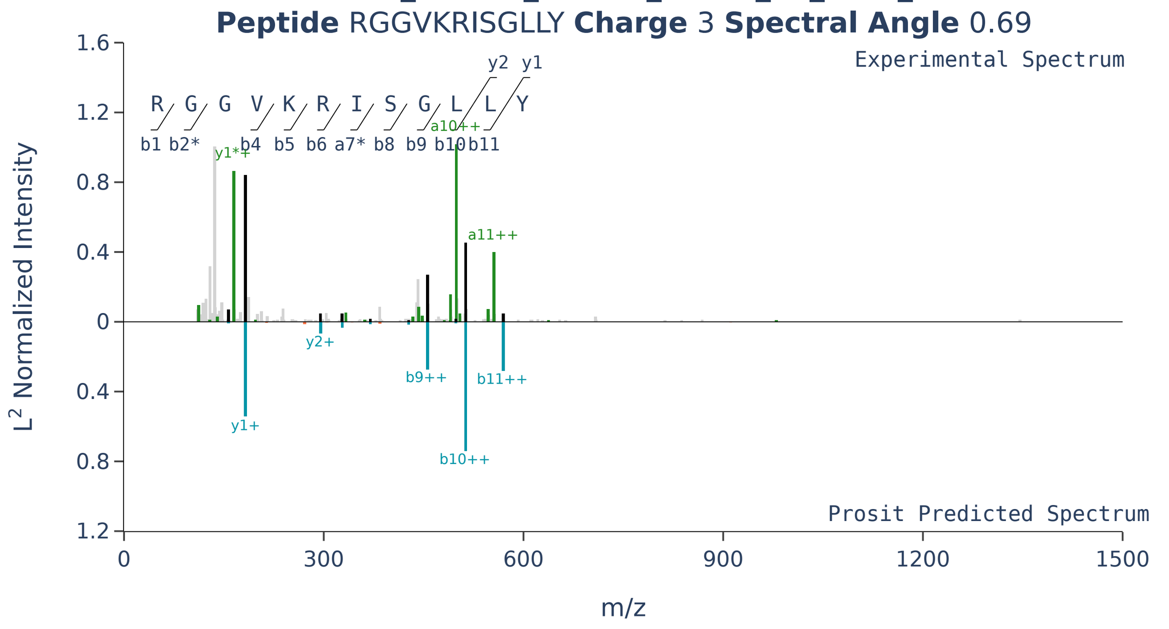


**Source** HRoetschke_291222_140123_HFGoe_H4_b2R2_4h **Scan** 14529
**Peptide** RGGVKRISGLIYE **Charge** 4 **Spectral Angle** 0.6

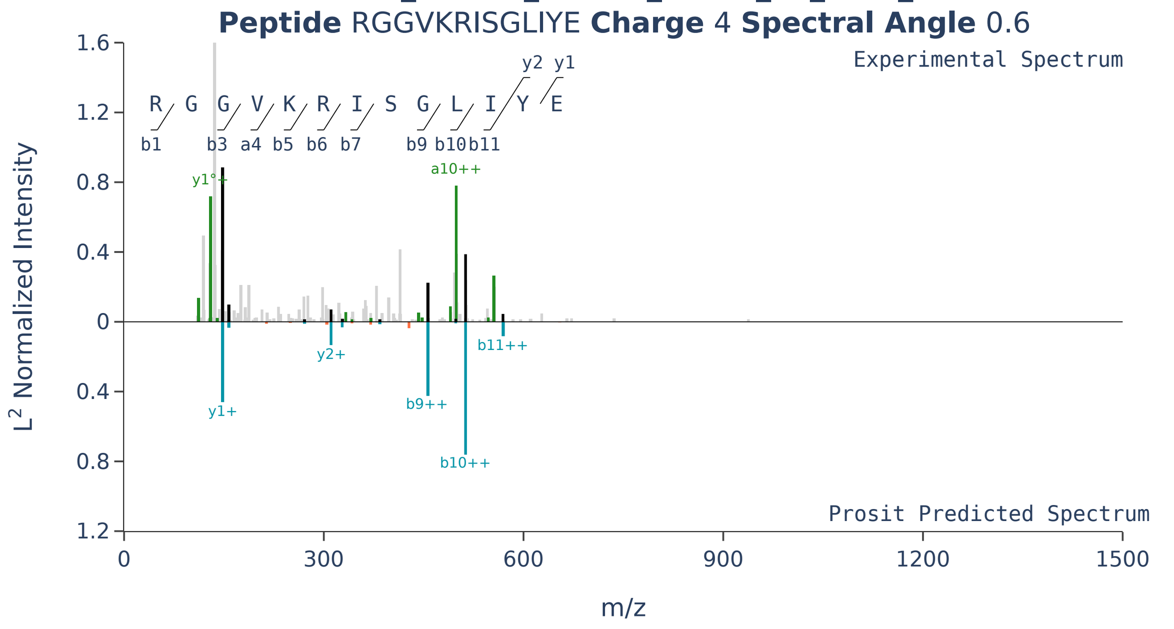


**Source** HRoetschke_291222_140123_HFGoe_H4_b2R1_1h **Scan** 19237
**Peptide** EHAKRKTVTAMDVVYALKRQGRTLYG **Charge** 5 **Spectral Angle** 0.86

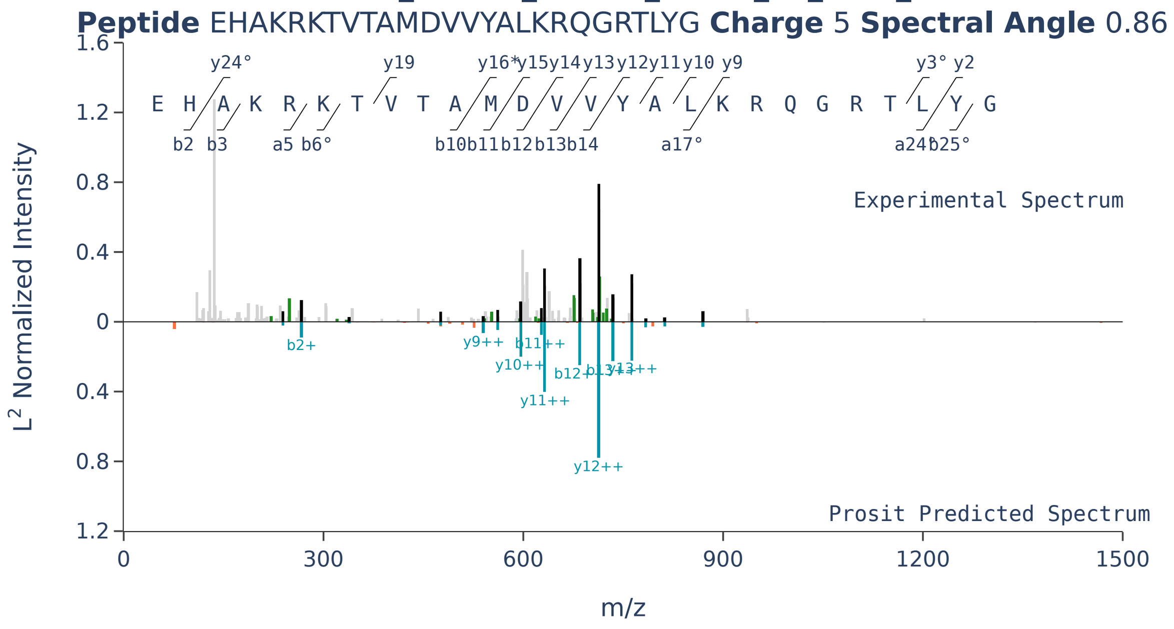


**Source** HRoetschke_291222_140123_HFGoe_H4_b1R2_4h **Scan** 16006
**Peptide** NVIRDAVTYTEHAKRKTVTAMD **Charge** 3 **Spectral Angle** 0.65

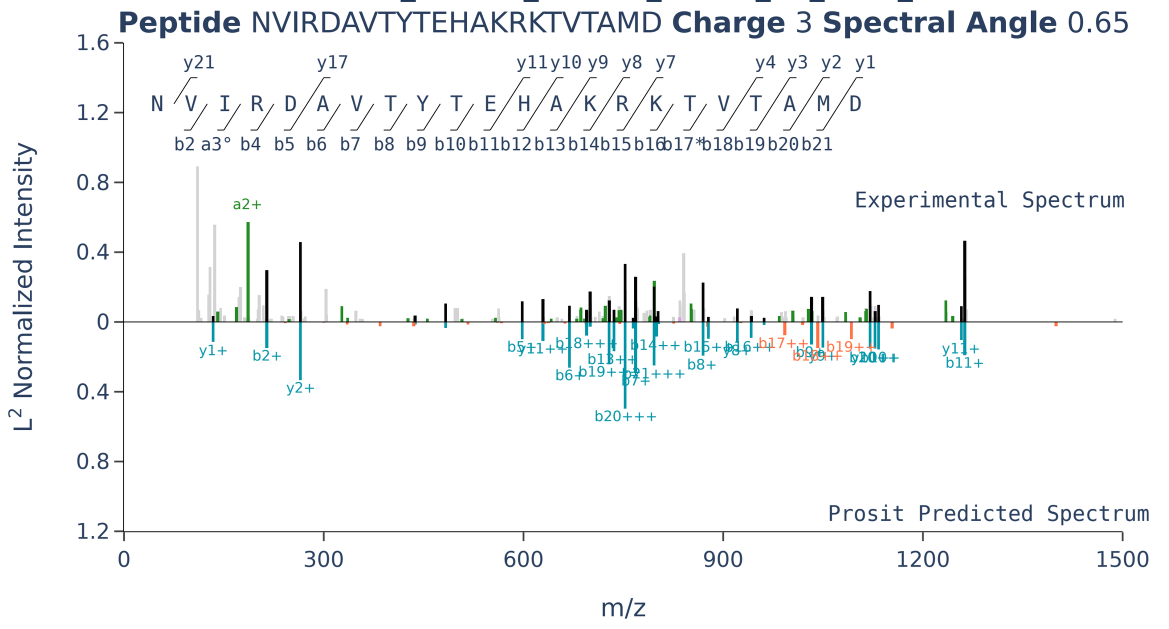


**Source** HRoetschke_291222_140123_HFGoe_H4_b1R1_2h **Scan** 14719
**Peptide** AVTYTEHAKRKTVTAMDVVYA **Charge** 3 **Spectral Angle** 0.63

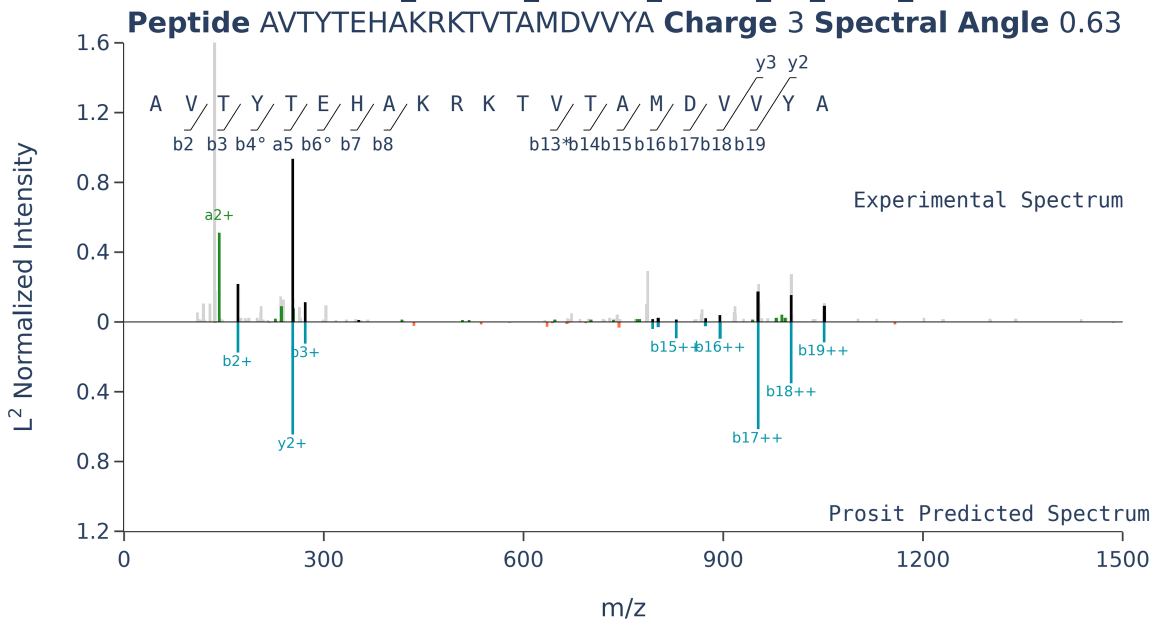


**Source** HRoetschke_291222_140123_HFGoe_H4_b2R1_1h **Scan** 7767
**Peptide** KVLRDNI **Charge** 2 **Spectral Angle** 0.69

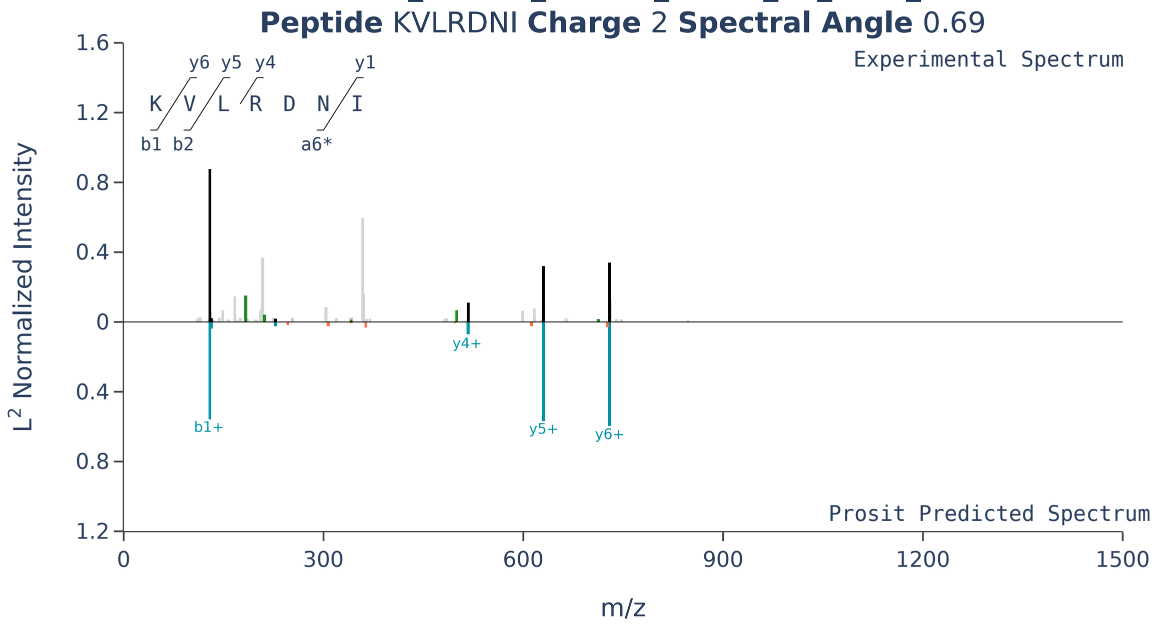

**Source** HRoetschke_291222_140123_HFGoe_H4_b2R1_4h **Scan** 6967
**Peptide** KVLRDNIQG **Charge** 2 **Spectral Angle** 0.67

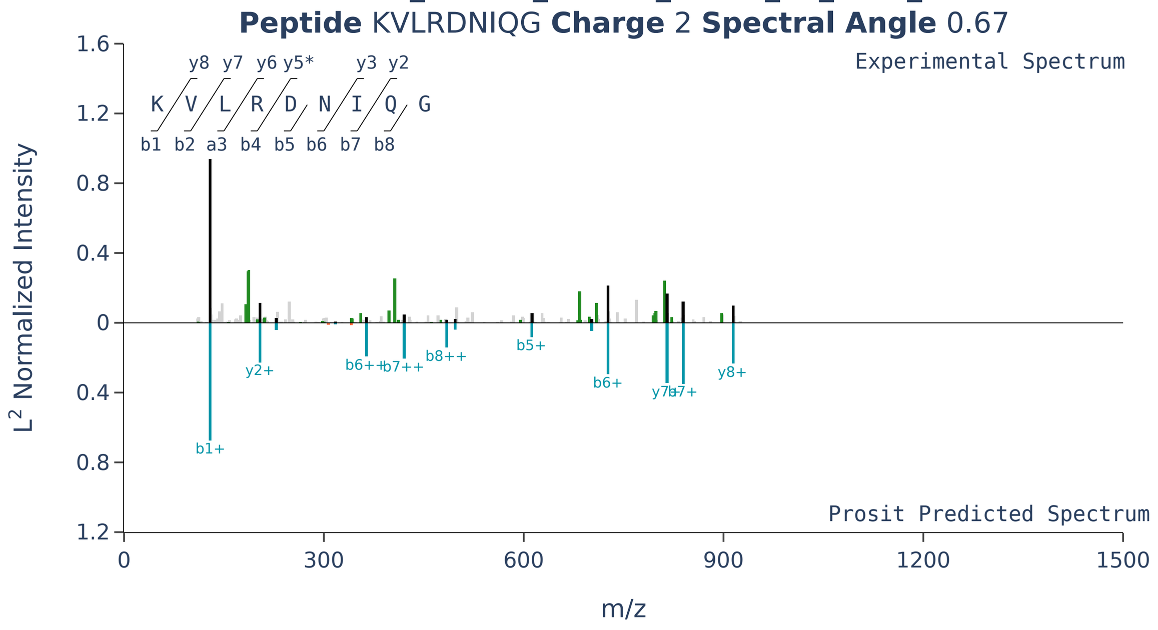


**Source** HRoetschke_291222_140123_HFGoe_H4_b1R2_24h **Scan** 10561
**Peptide** AKRKTVTAMDVV **Charge** 3 **Spectral Angle** 0.57

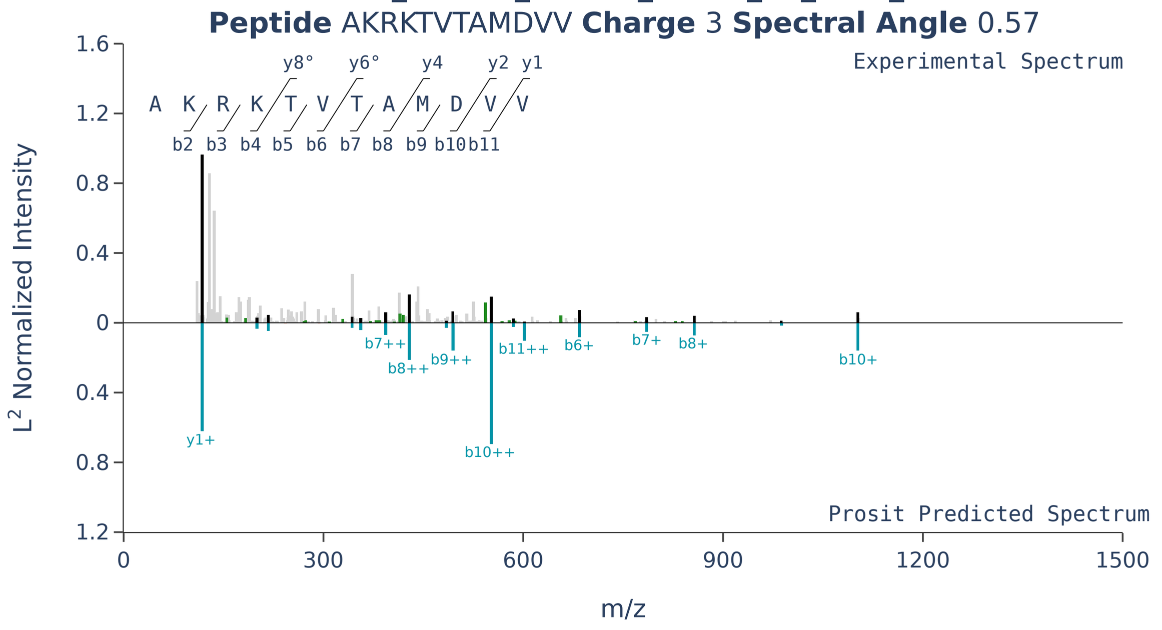


**Source** HRoetschke_291222_140123_HFGoe_H4_b1R1_1h **Scan** 17178
**Peptide** NIQGITKPAIRRLARRGGVKRISGLIY **Charge** 5 **Spectral Angle** 0.89

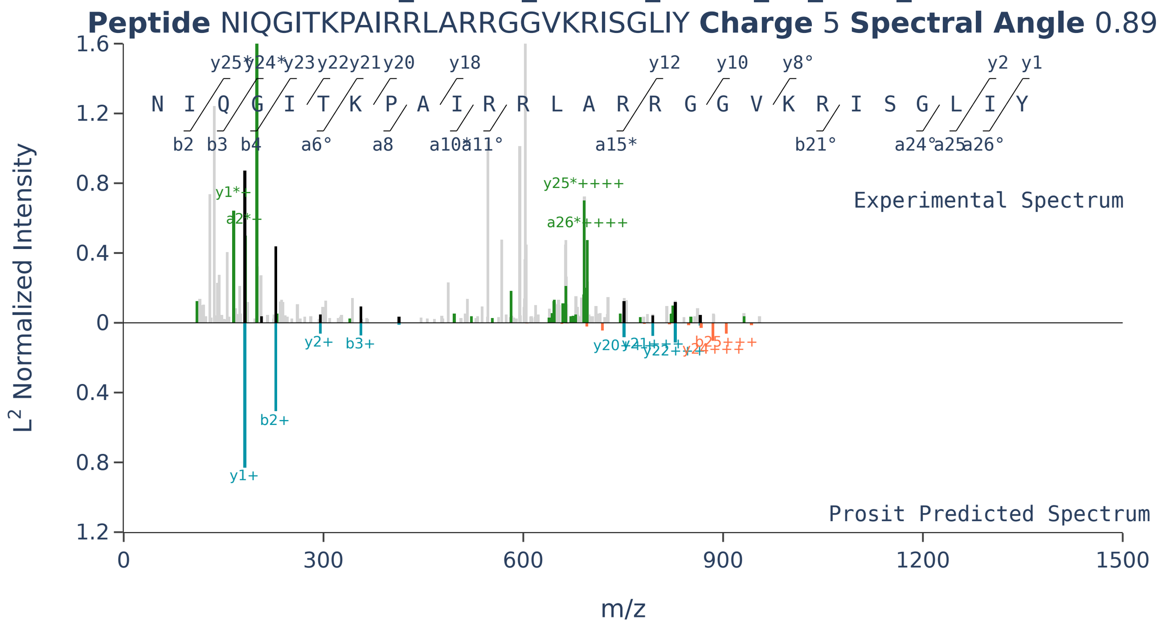


**Source** HRoetschke_291222_140123_HFGoe_H4_b1R1_4h **Scan** 9418
**Peptide** AKRHRKVLRDNIQGITKPAIRRL **Charge** 6 **Spectral Angle** 0.79

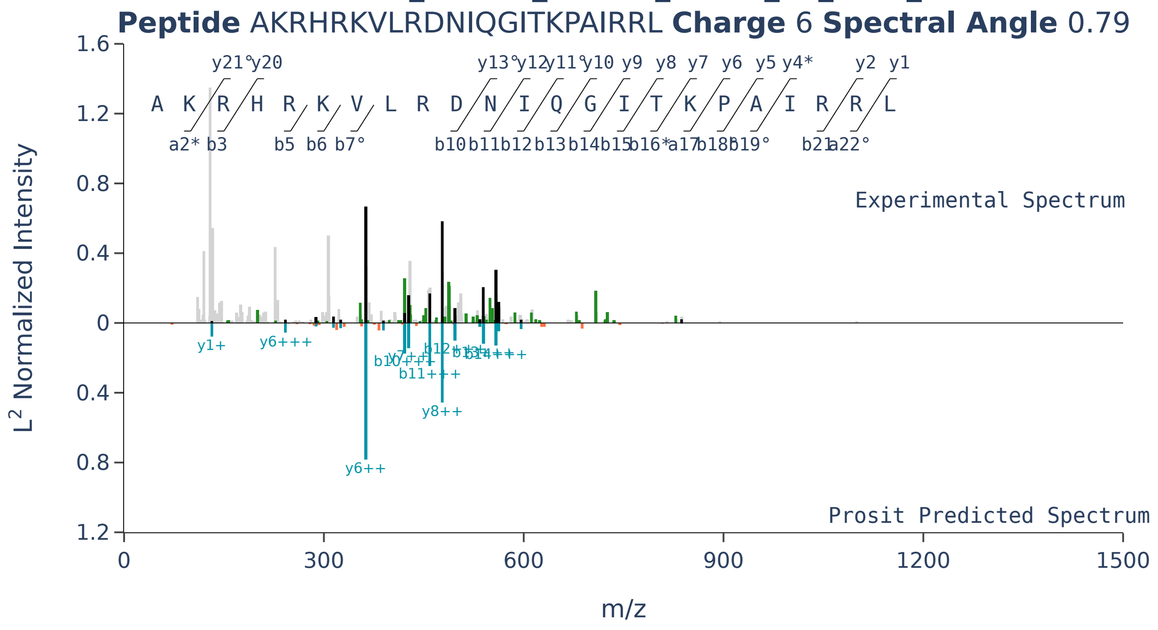


**Source** HRoetschke_291222_140123_HFGoe_H4_b1R1_2h **Scan** 15913
**Peptide** QGITKPAIRRLARRGGVKRISGLIYEE **Charge** 5 **Spectral Angle** 0.88

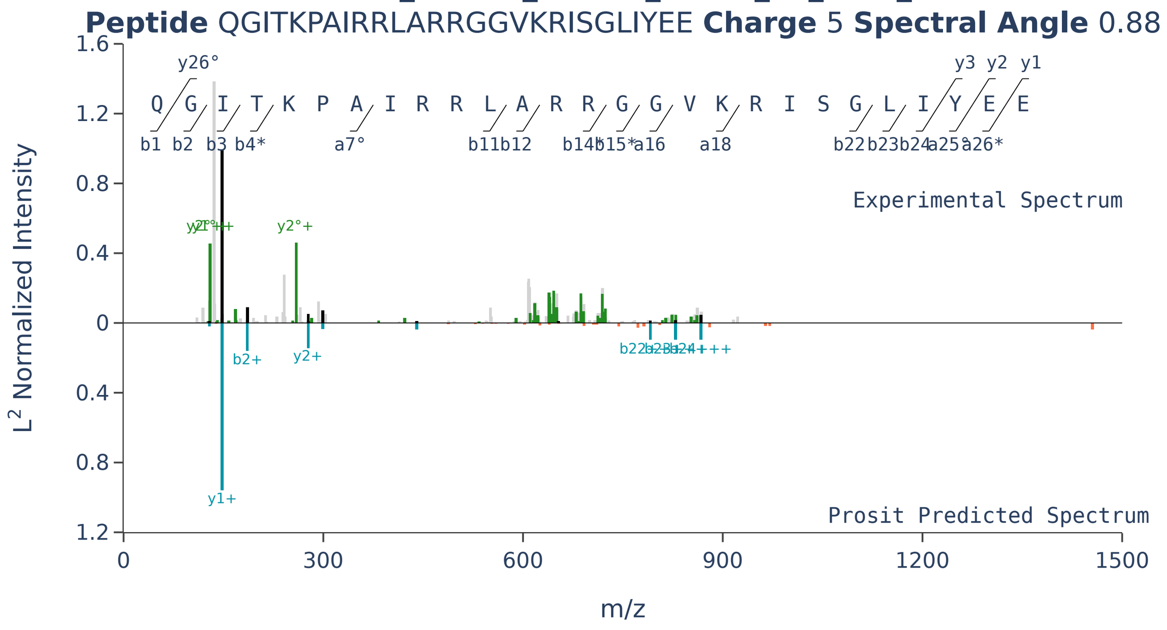


**Source** HRoetschke_291222_140123_HFGoe_H4_b1R1_1h **Scan** 19147
**Peptide** LENVIRDAVTYTEHAKRKTVTA **Charge** 5 **Spectral Angle** 0.76

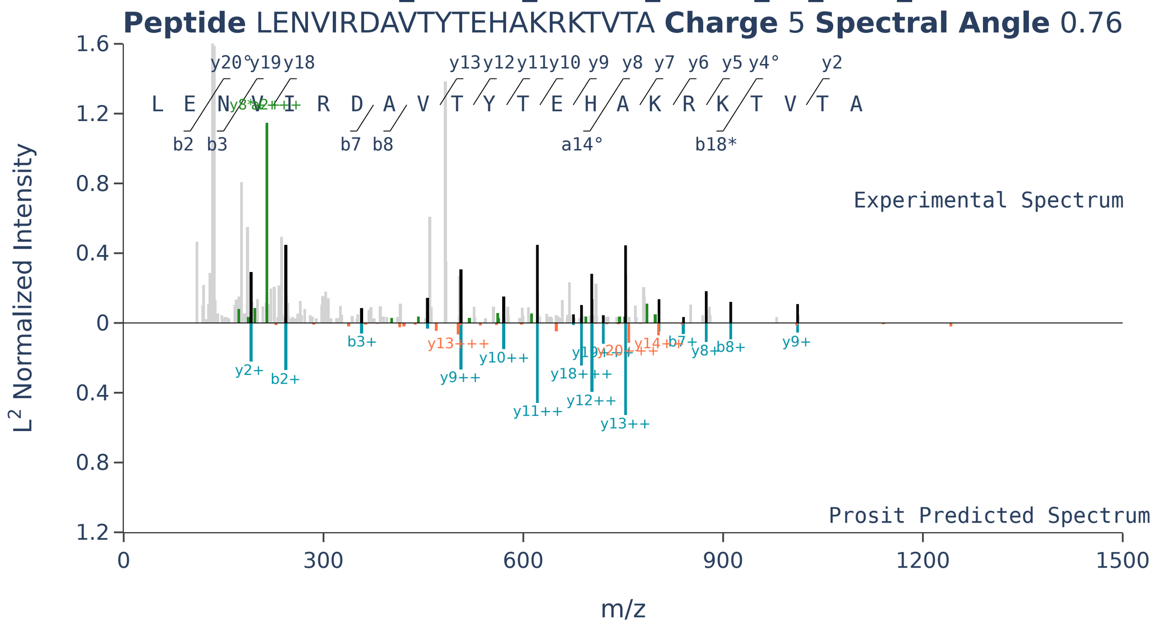


**Source** HRoetschke_291222_140123_HFGoe_H4_b1R1_1h **Scan** 19633
**Peptide** RRGGVKRISGLIYEETRGVL **Charge** 4 **Spectral Angle** 0.75

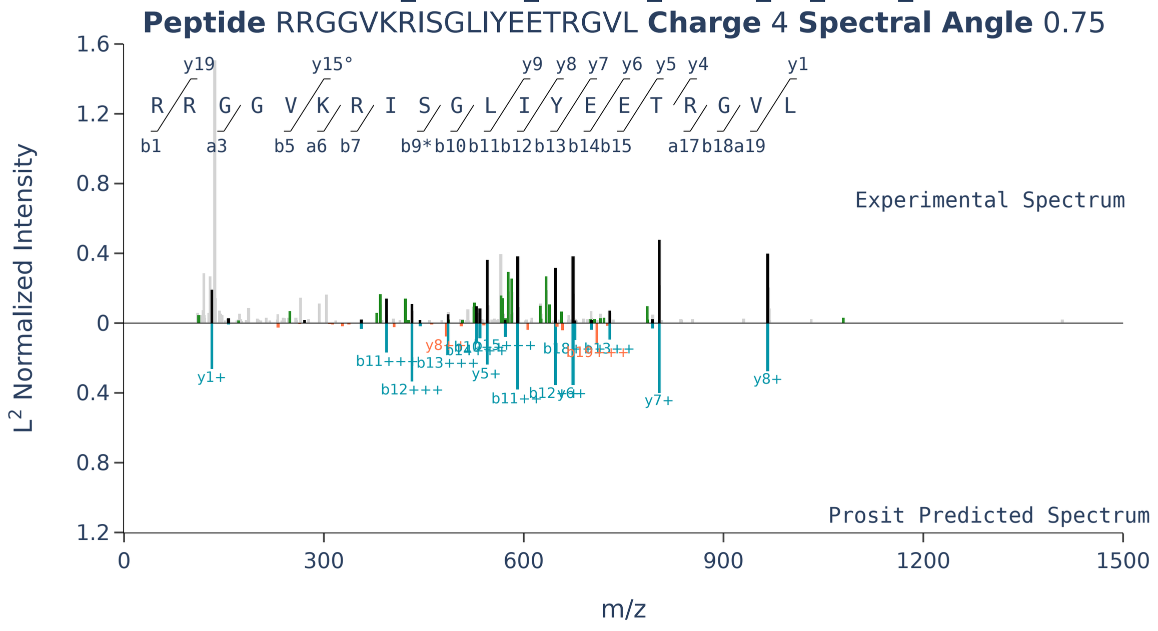


**Source** HRoetschke_291222_140123_HFGoe_H4_b2R1_1h **Scan** 18097
**Peptide** TYTEHAKRKTVTAMDVVYALKRQGRTL **Charge** 6 **Spectral Angle** 0.86

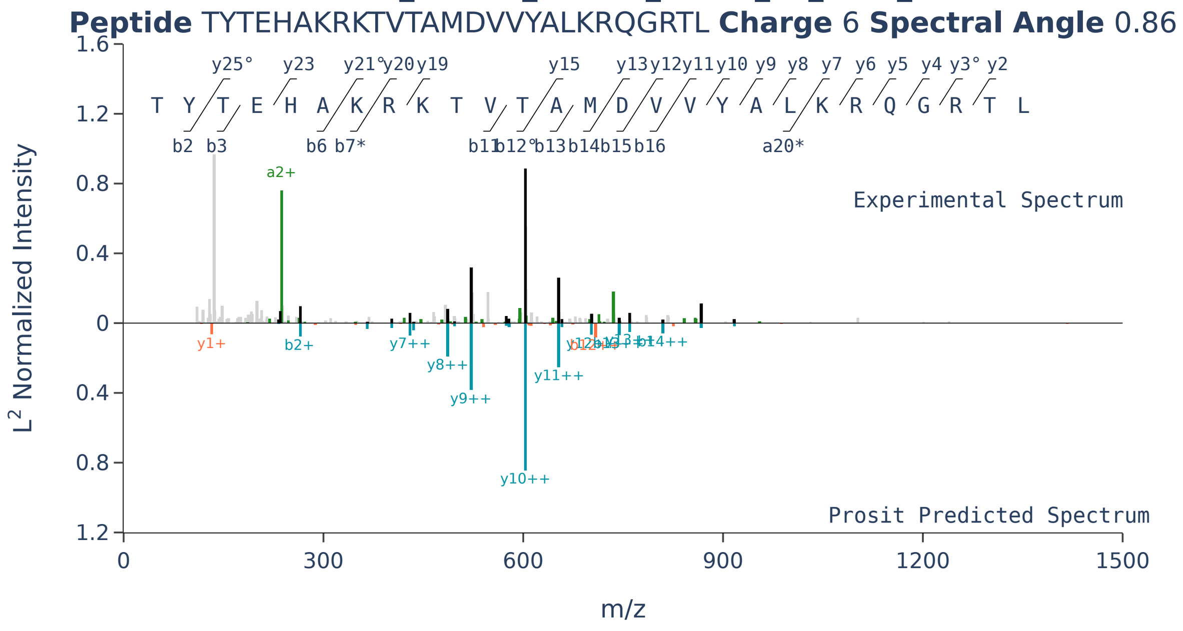


**Source** HRoetschke_291222_140123_HFGoe_H4_b1R1_2h **Scan** 14250
**Peptide** LARRGGVKRISGLIYEET **Charge** 3 **Spectral Angle** 0.59

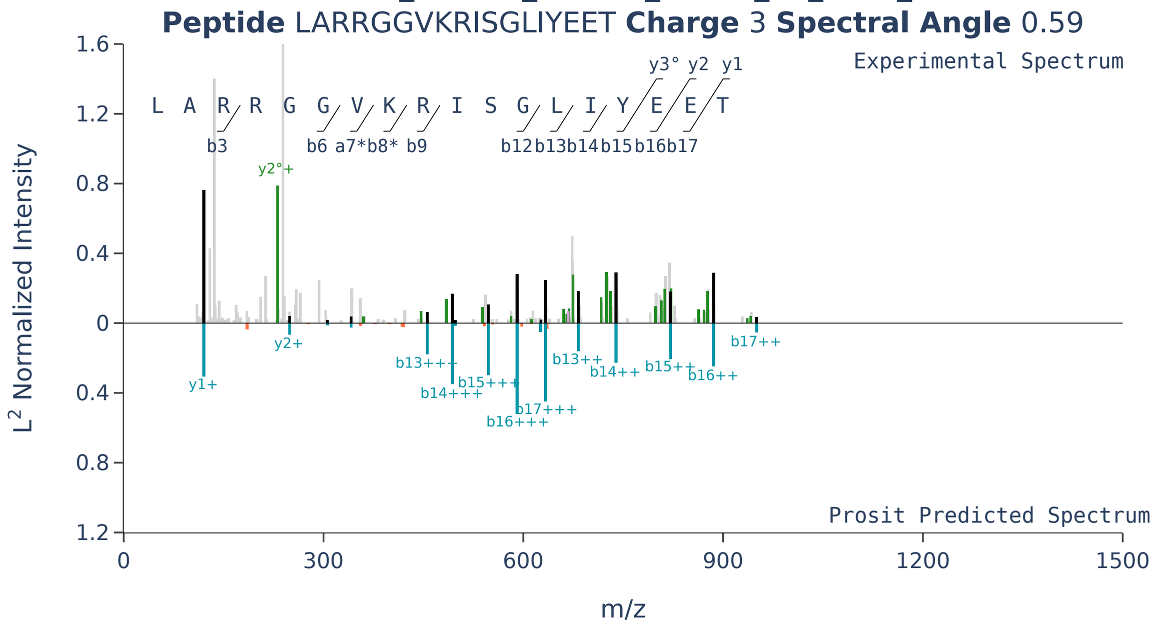


**Source** HRoetschke_291222_140123_HFGoe_H4_b1R1_4h **Scan** 15663
**Peptide** ENVIRDAVTYTEHAKRKTVTAM **Charge** 4 **Spectral Angle** 0.63

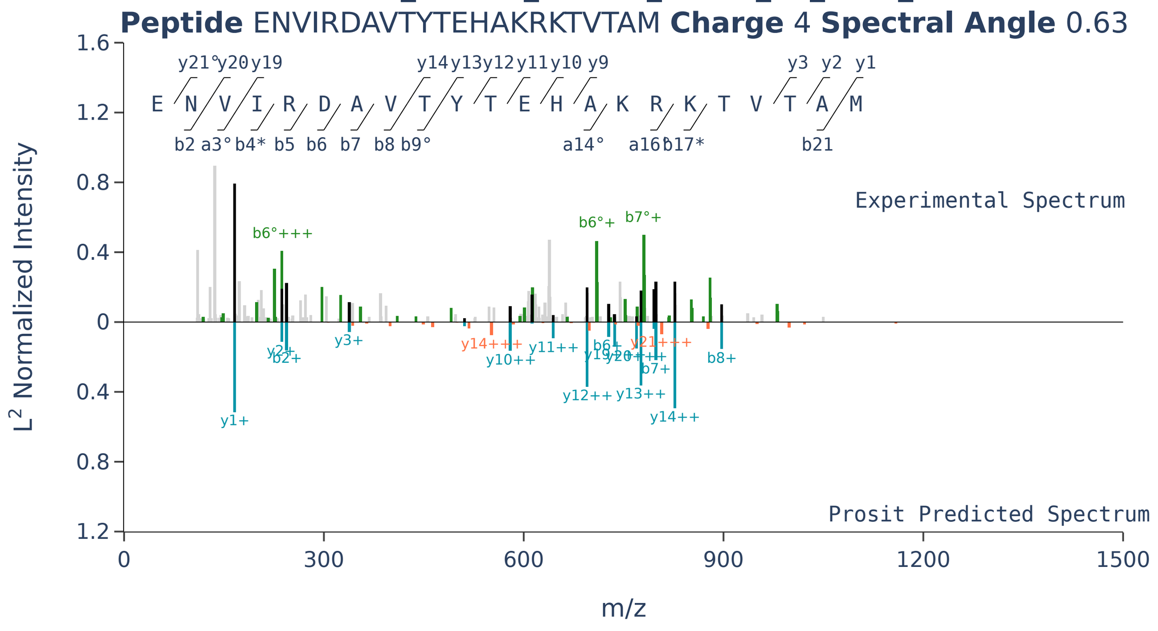


**Source** HRoetschke_291222_140123_HFGoe_H4_b2R1_1h **Scan** 24089
**Peptide** TVTAMDVVYALKR **Charge** 3 **Spectral Angle** 0.75

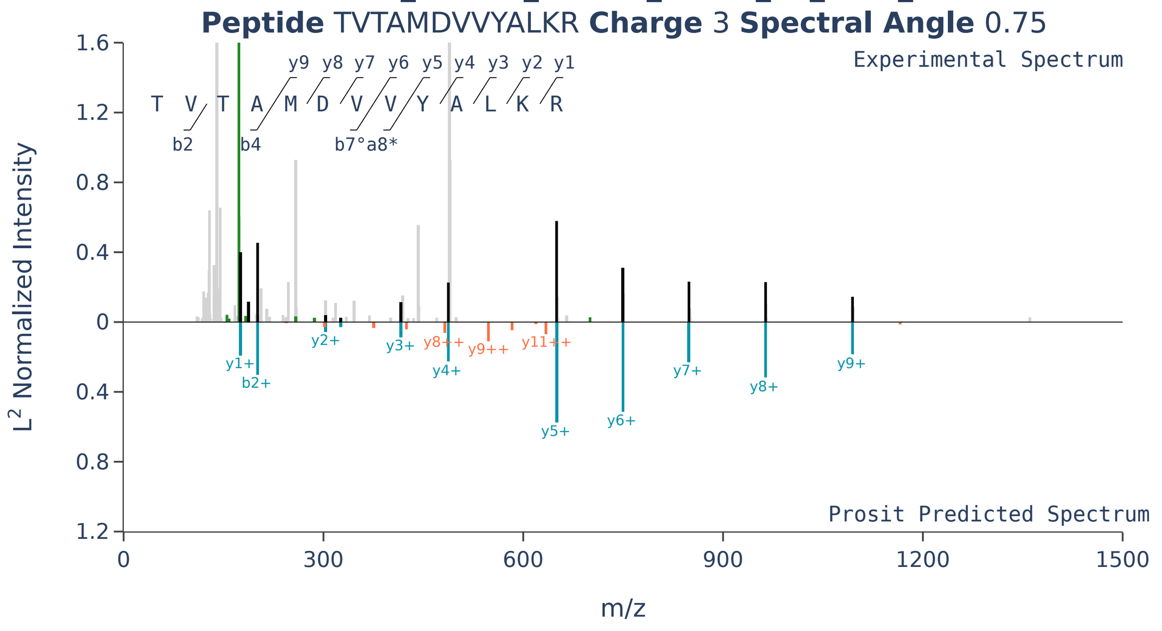


**Source** HRoetschke_291222_140123_HFGoe_H4_b2R1_1h **Scan** 33048
**Peptide** RGVLKVFLENVIRDAVTYTEHAK **Charge** 4 **Spectral Angle** 0.68

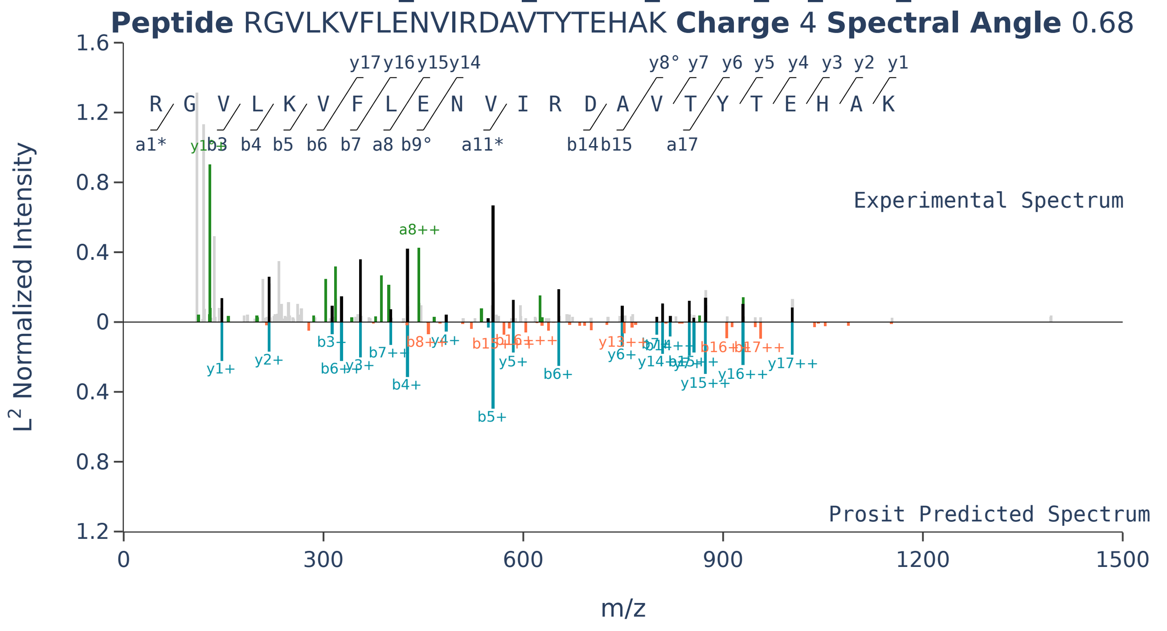


**Source** HRoetschke_291222_140123_HFGoe_H4_b1R2_1h **Scan** 19246
**Peptide** ARRGGVKRISGLIYEETRGVLKV **Charge** 6 **Spectral Angle** 0.59

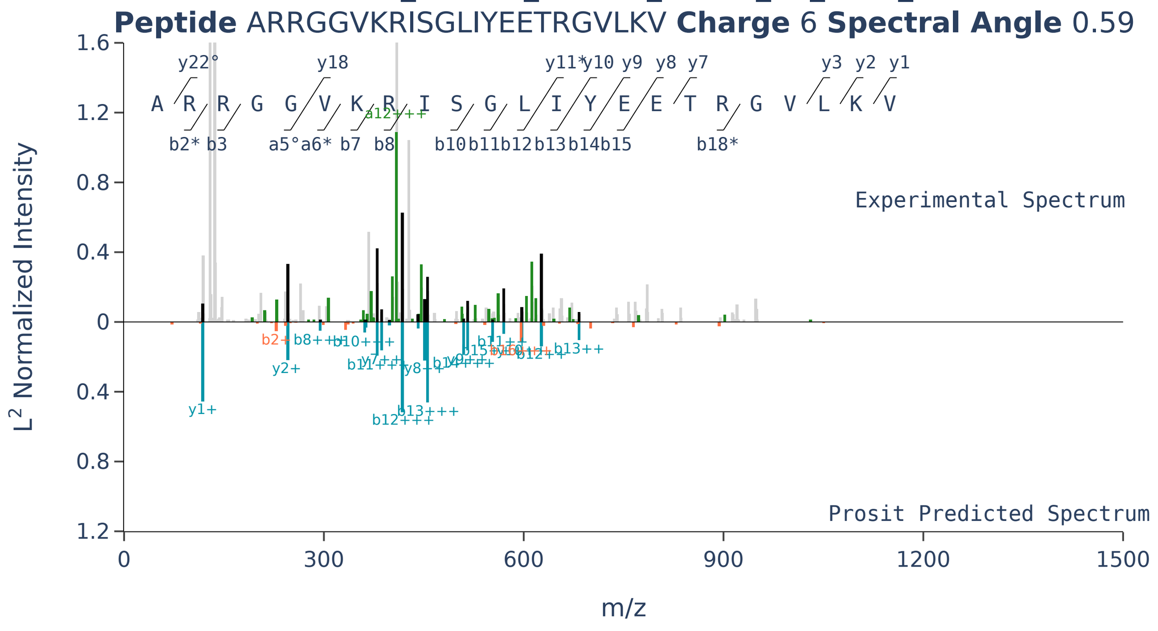


**Source** HRoetschke_291222_140123_HFGoe_H4_b2R1_1h **Scan** 26625
**Peptide** VTAMDVVYALKRQGRTLYGFGG **Charge** 3 **Spectral Angle** 0.71

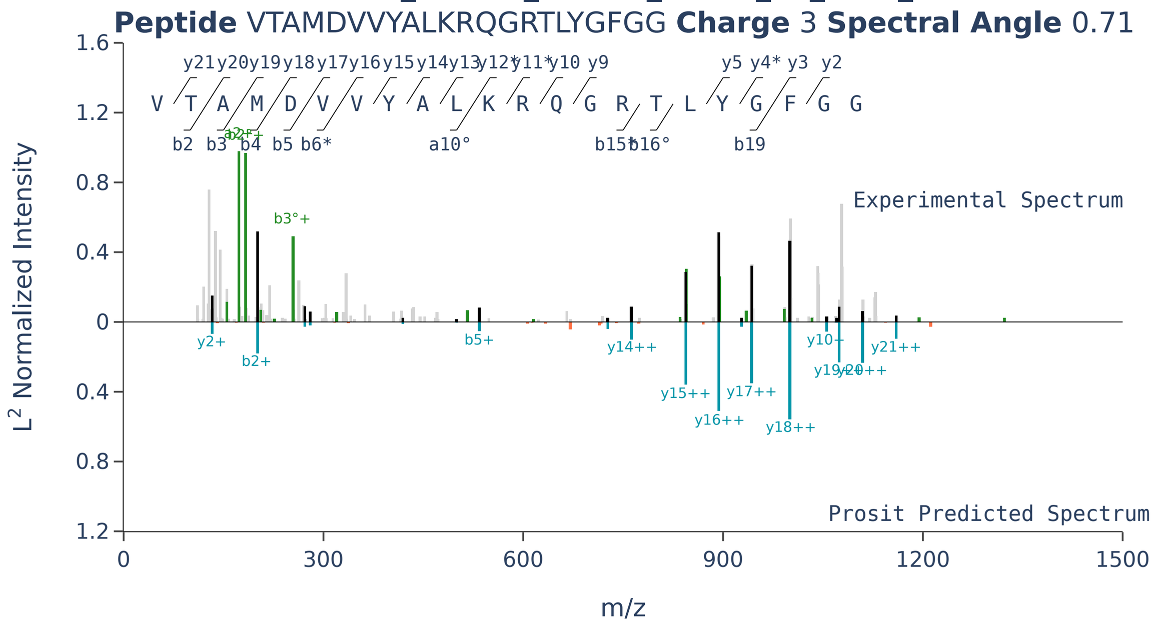


**Source** HRoetschke_291222_140123_HFGoe_H4_b2R1_1h **Scan** 30110
**Peptide** RGVLKVFLENVIRDAVTYTEHAKRKTVTA **Charge** 5 **Spectral Angle** 0.74

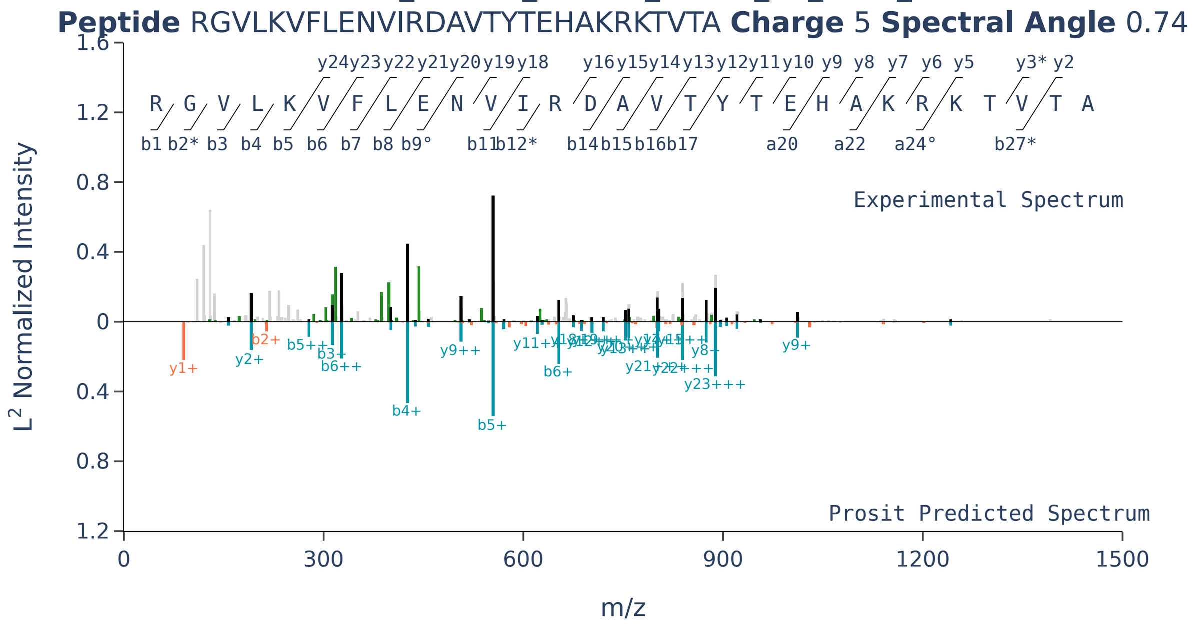

**Source** HRoetschke_291222_140123_HFGoe_H4_b1R2_24h **Scan** 9416
**Peptide** TEHAKRKTVTAMDVV **Charge** 4 **Spectral Angle** 0.67

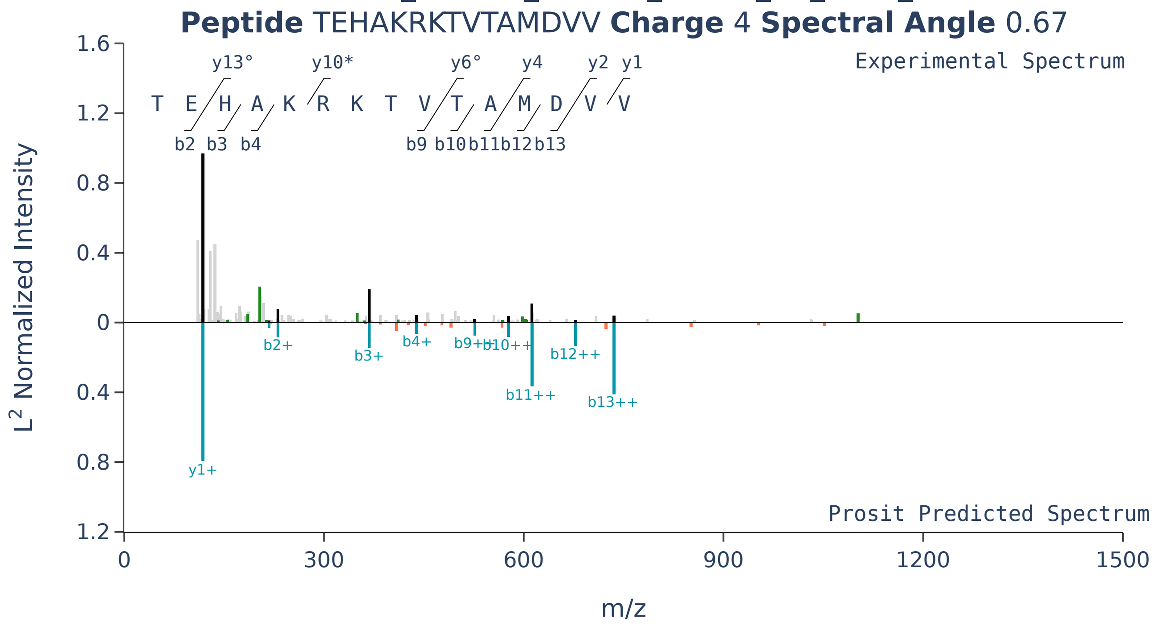


**Source** HRoetschke_291222_140123_HFGoe_H4_b1R1_4h **Scan** 10227
**Peptide** NIQGITKPAIRRLARR **Charge** 3 **Spectral Angle** 0.72

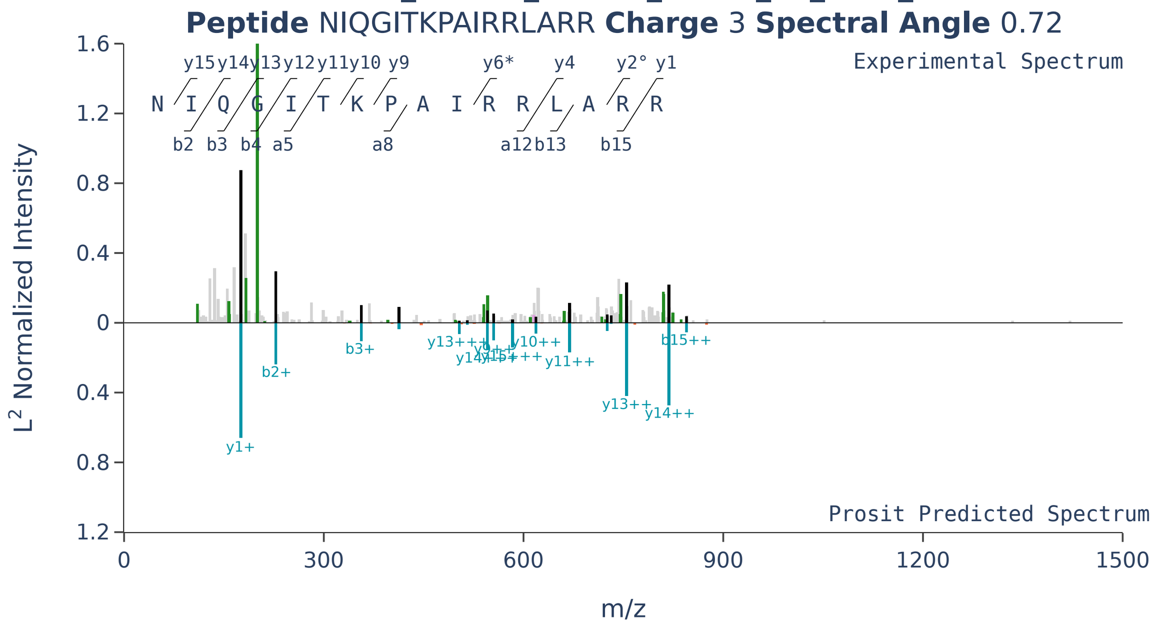


**Source** HRoetschke_291222_140123_HFGoe_H4_b1R1_4h **Scan** 7515
**Peptide** VIRDAVTYTEHAKRKTVT **Charge** 5 **Spectral Angle** 0.78

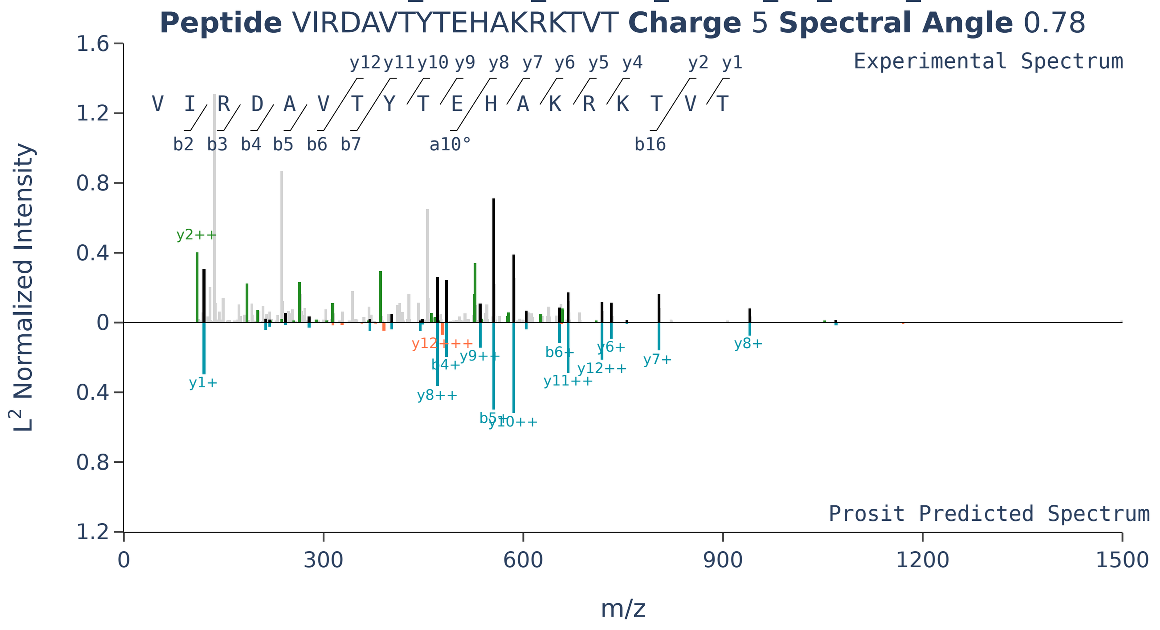


**Source** HRoetschke_291222_140123_HFGoe_H4_b2R1_24h **Scan** 7298
**Peptide** AKRHRKVLRDNIQGITKPAIRR **Charge** 6 **Spectral Angle** 0.82

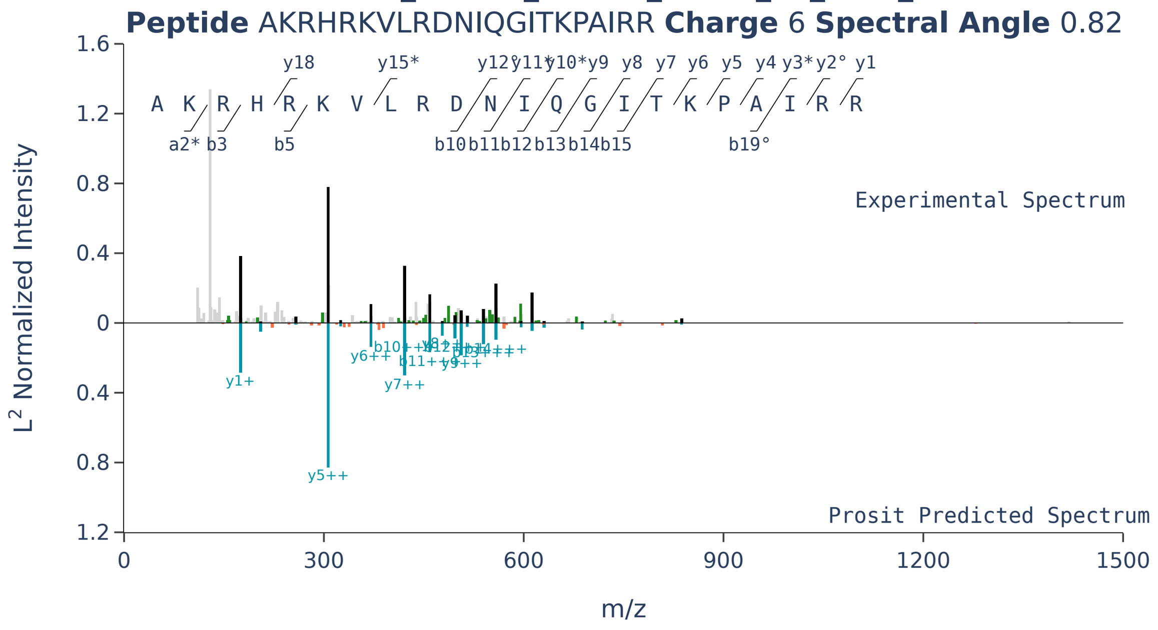


**Source** HRoetschke_291222_140123_HFGoe_H4_b1R1_2h **Scan** 19397
**Peptide** RISGLIYEE **Charge** 2 **Spectral Angle** 0.72

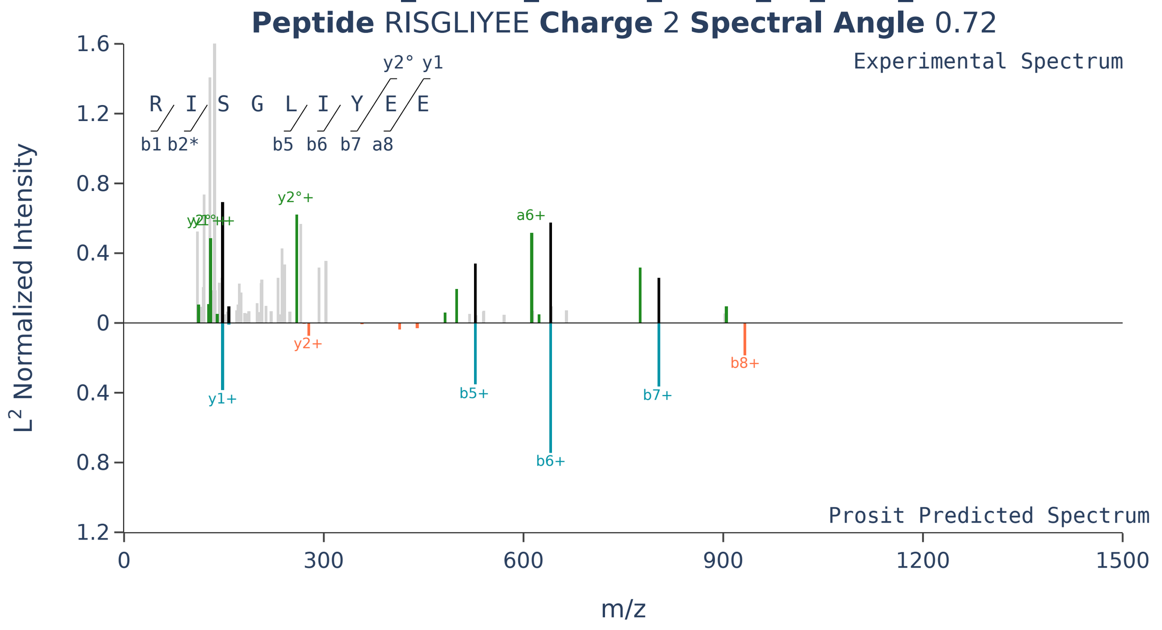


**Source** HRoetschke_291222_140123_HFGoe_H4_b1R1_4h **Scan** 6924
**Peptide** TYTEHAKRKTVTAMD **Charge** 3 **Spectral Angle** 0.57

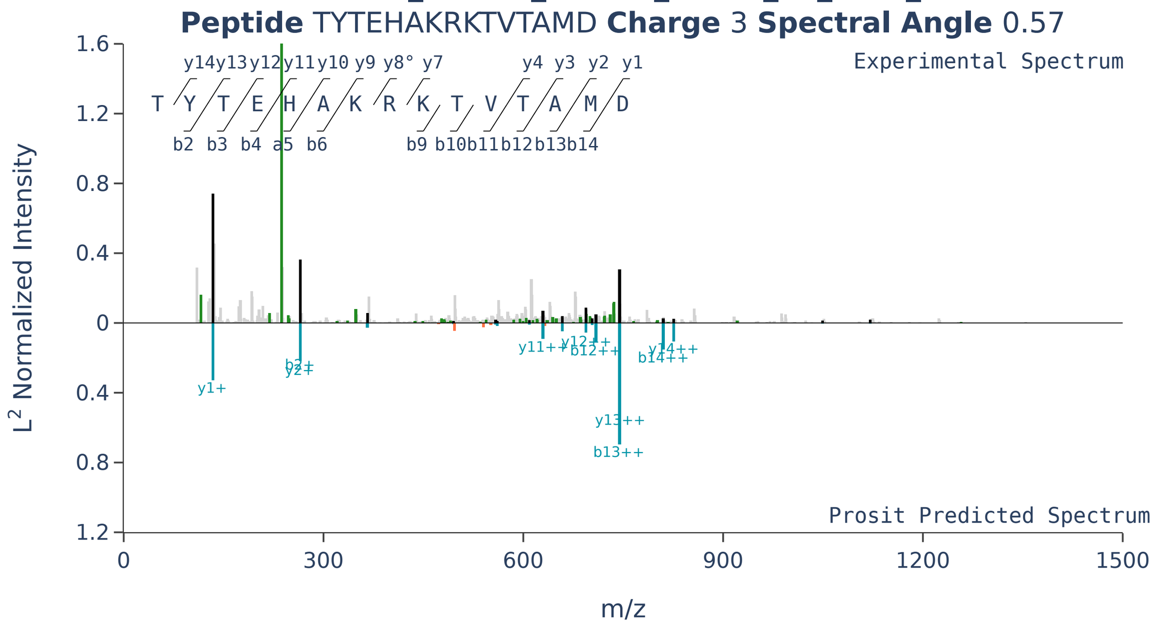


**Source** HRoetschke_291222_140123_HFGoe_H4_b1R1_24h **Scan** 11800
**Peptide** DNIQGITKPAIRRLARRGGVKRIS **Charge** 5 **Spectral Angle** 0.74

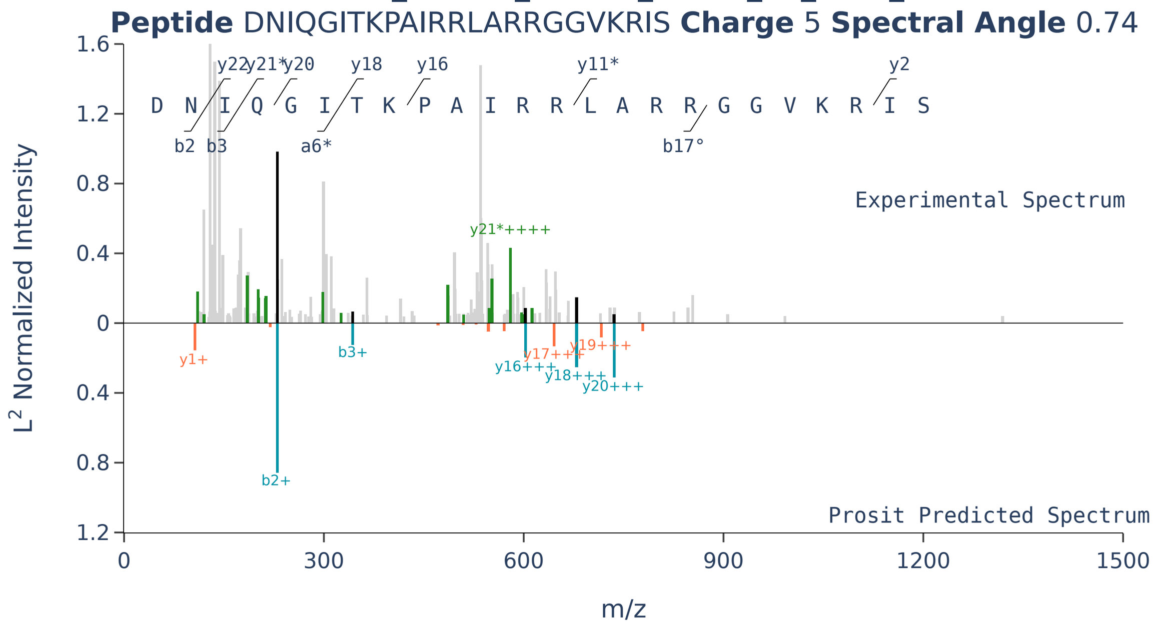


**Source** HRoetschke_291222_140123_HFGoe_H4_b2R2_1h **Scan** 29858
**Peptide** RGVLKVFLENVIRDAV **Charge** 4 **Spectral Angle** 0.8

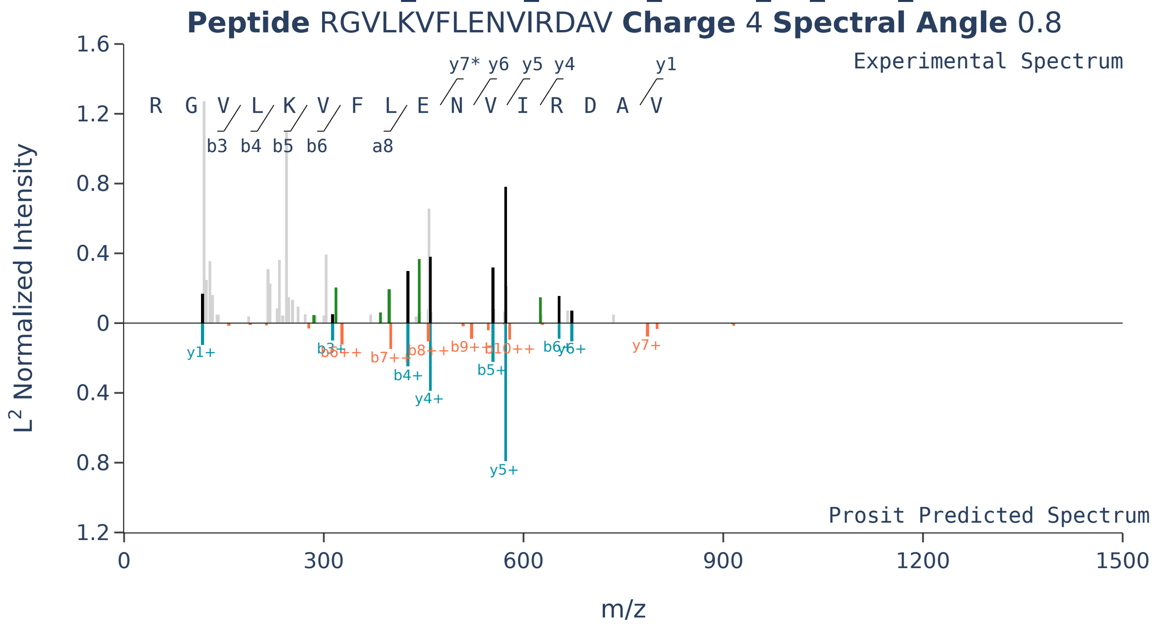


**Source** HRoetschke_291222_140123_HFGoe_H4_b2R2_24h **Scan** 14288
**Peptide** EHAKRKT **Charge** 2 **Spectral Angle** 0.7

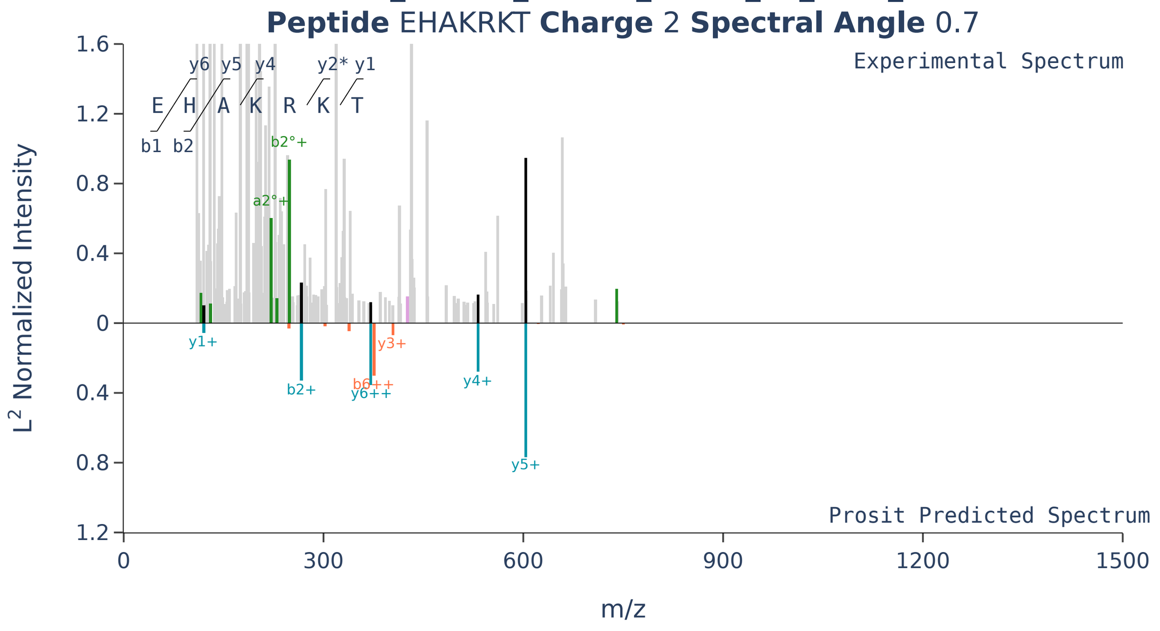


**Source** HRoetschke_291222_140123_HFGoe_H4_b2R1_1h **Scan** 24964
**Peptide** VKRISGLIYEETRGVLKVF **Charge** 3 **Spectral Angle** 0.61

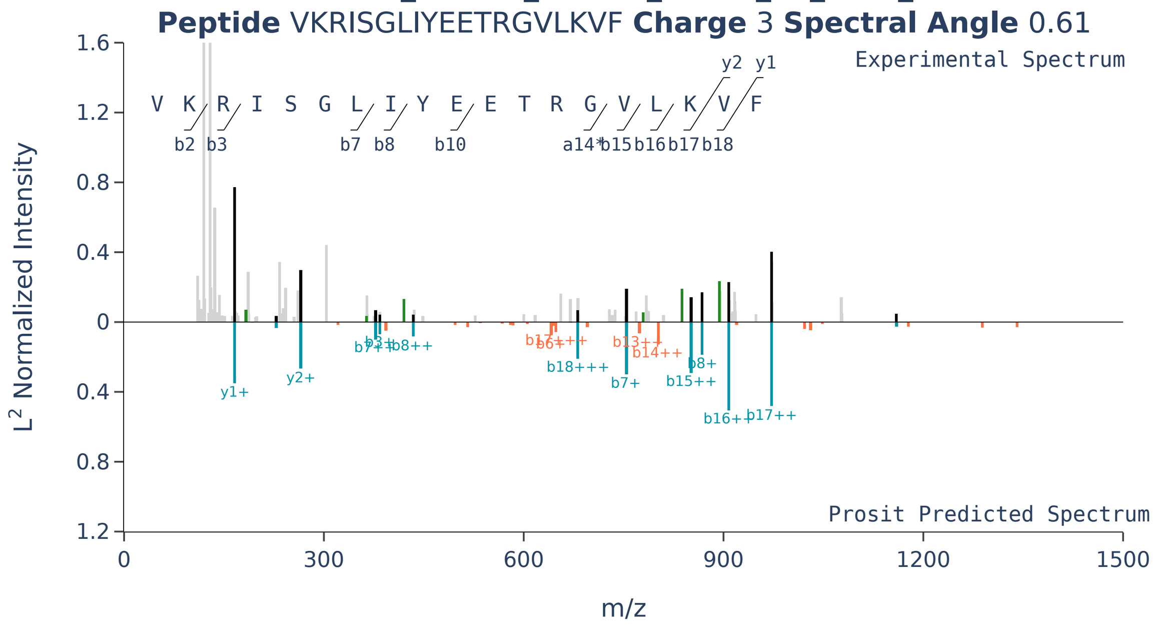


**Source** HRoetschke_291222_140123_HFGoe_H4_b1R2_4h **Scan** 5374
**Peptide** EHAKRKTVTAMD **Charge** 3 **Spectral Angle** 0.76

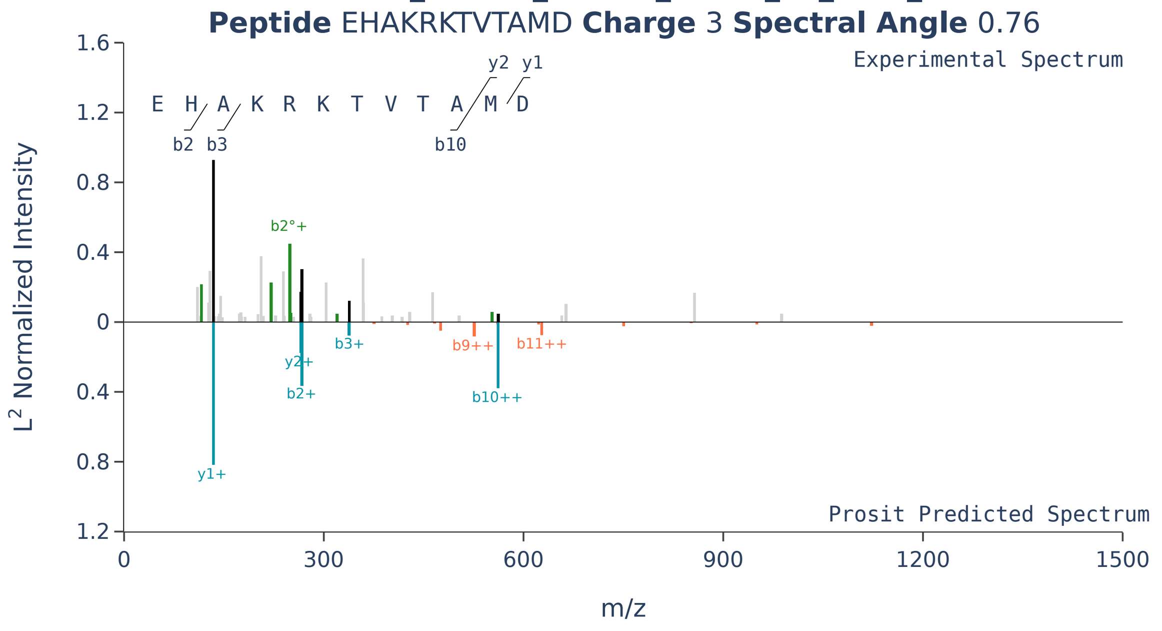


**Source** HRoetschke_291222_140123_HFGoe_H4_b1R2_2h **Scan** 12860
**Peptide** RRGGVKRISGLIYEET **Charge** 3 **Spectral Angle** 0.64

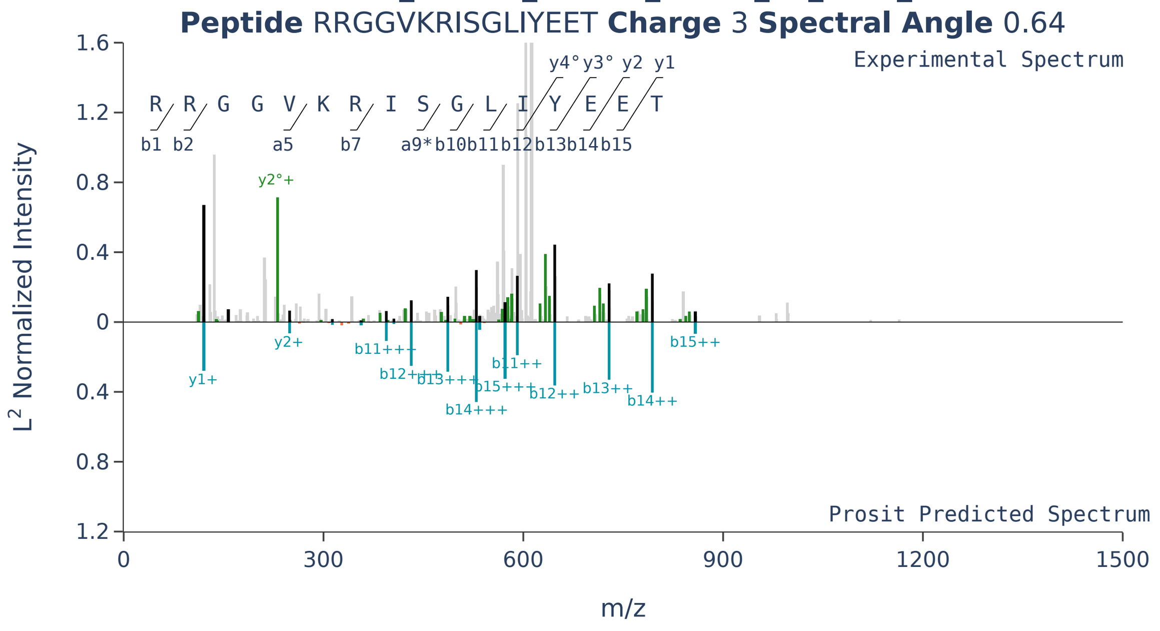


**Source** HRoetschke_291222_140123_HFGoe_H4_b1R2_24h **Scan** 11018
**Peptide** ARRGGVKRISGLIYEETRG **Charge** 5 **Spectral Angle** 0.61

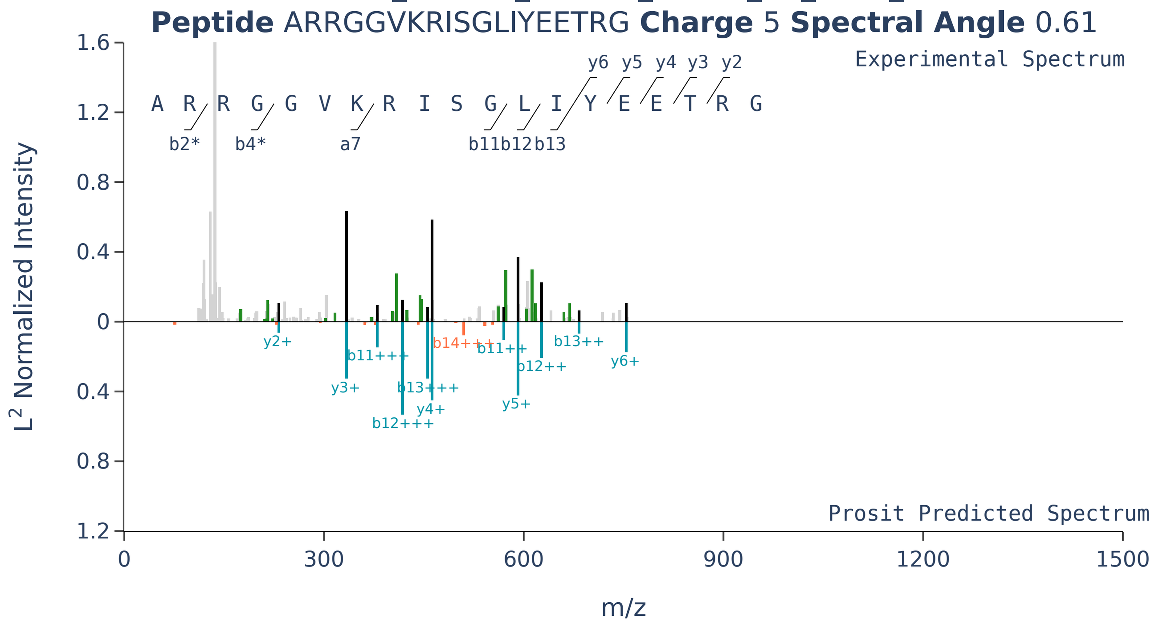


**Source** HRoetschke_291222_140123_HFGoe_H4_b2R2_1h **Scan** 10384
**Peptide** RDNIQGITKPAIRRLARRG **Charge** 5 **Spectral Angle** 0.7

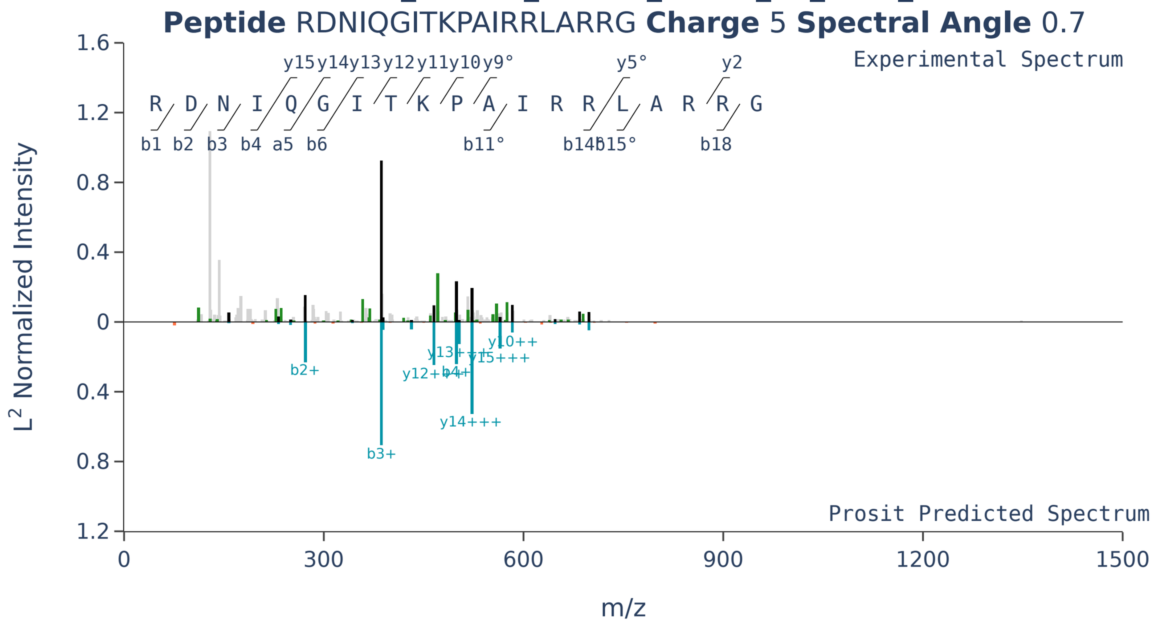


**Source** HRoetschke_291222_140123_HFGoe_H4_b2R1_1h **Scan** 31049
**Peptide** IYEETRGVLKVFLENVIRD **Charge** 3 **Spectral Angle** 0.75

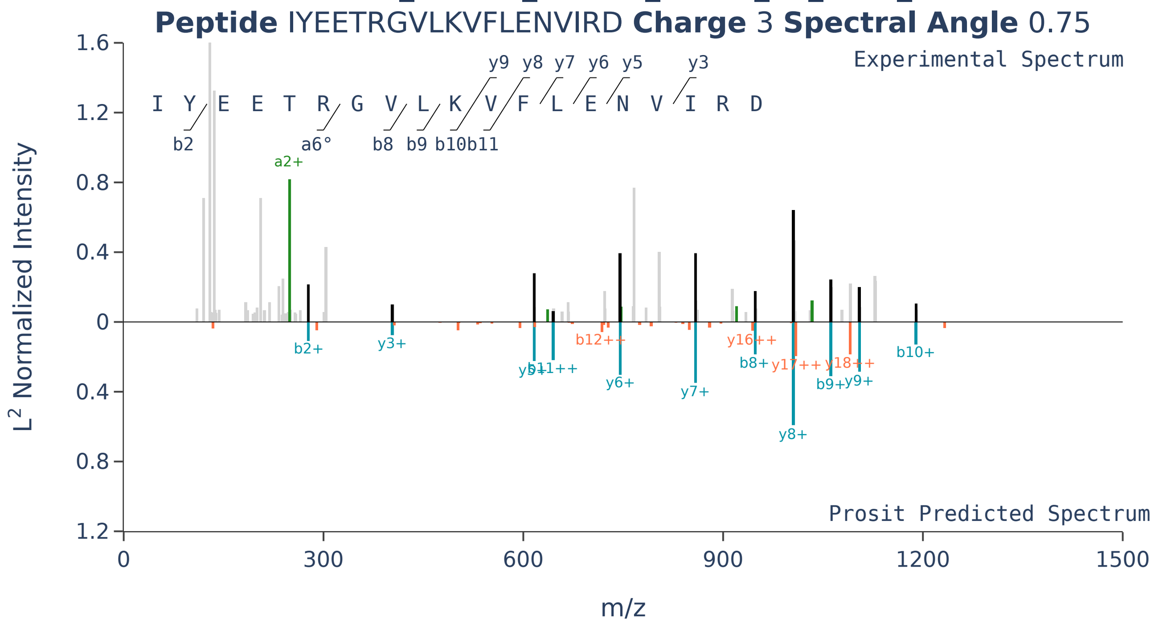

**Source** HRoetschke_291222_140123_HFGoe_H4_b2R2_4h **Scan** 12120
**Peptide** NIQGITKPAIRRLARRGGVK **Charge** 5 **Spectral Angle** 0.68

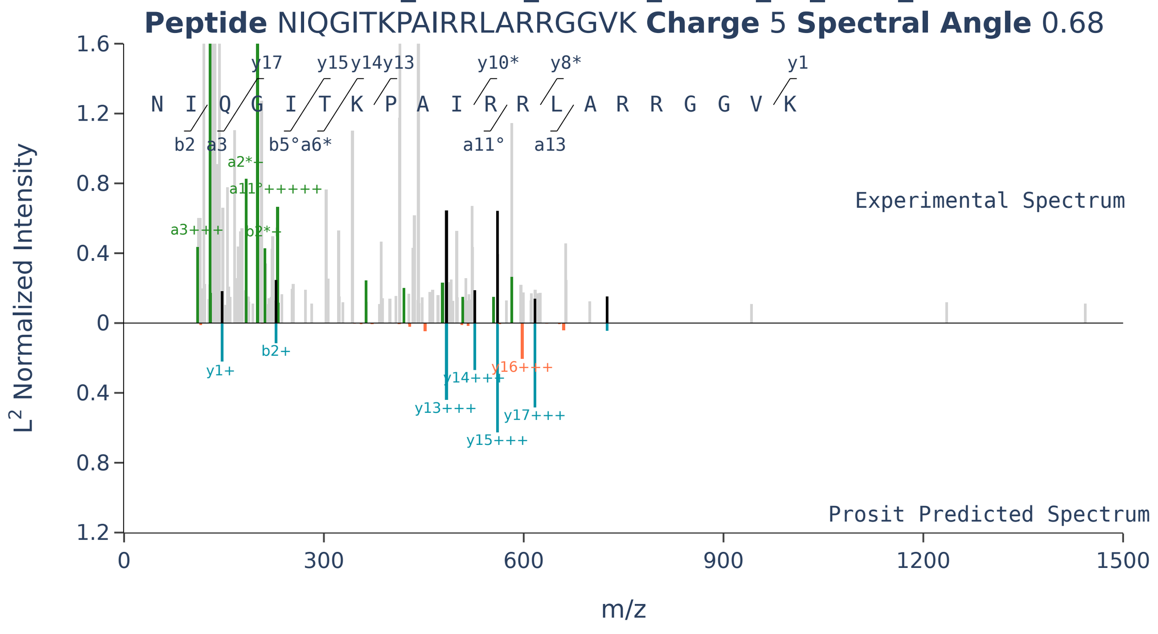


**Source** HRoetschke_291222_140123_HFGoe_H4_b1R1_24h **Scan** 6318
**Peptide** KGLGKGGAKRHRKVLRDNIQGI **Charge** 5 **Spectral Angle** 0.82

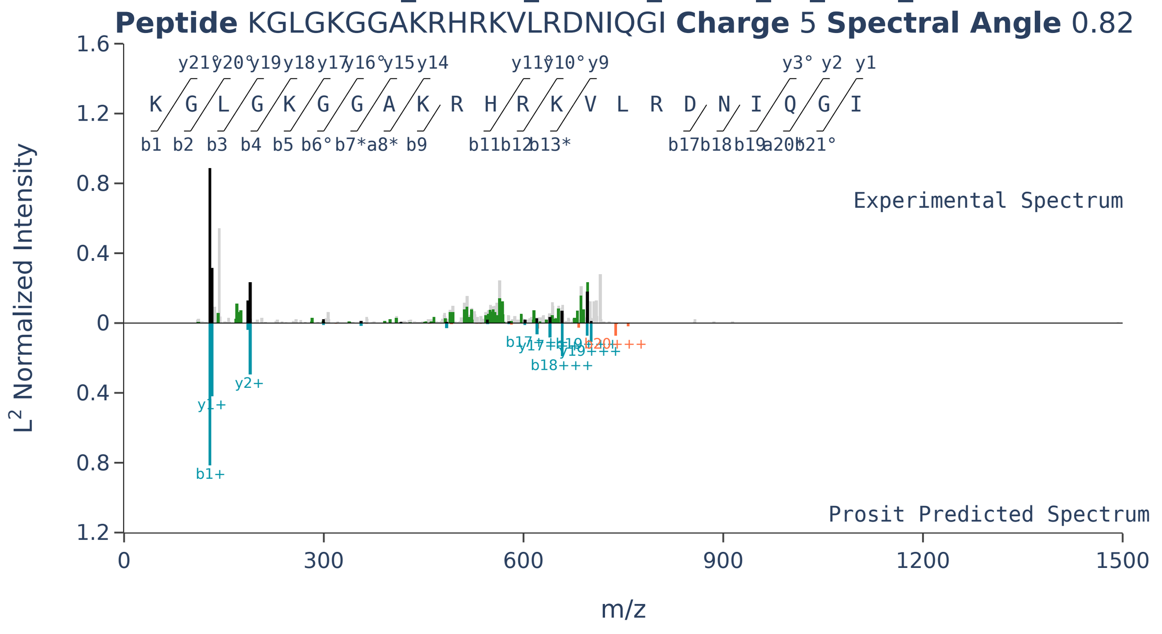


**Source** HRoetschke_291222_140123_HFGoe_H4_b1R1_1h **Scan** 12112
**Peptide** ARRGGVKRISGLIY **Charge** 3 **Spectral Angle** 0.74

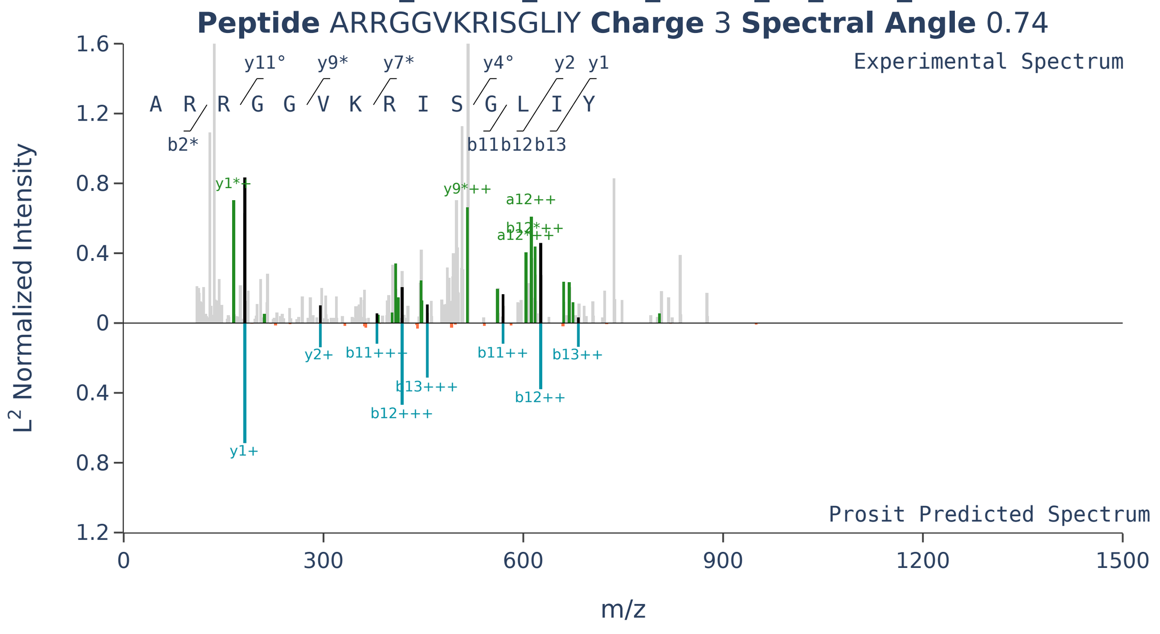


**Source** HRoetschke_291222_140123_HFGoe_H4_b2R1_1h **Scan** 30949
**Peptide** ETRGVLKVFLENVL **Charge** 3 **Spectral Angle** 0.69

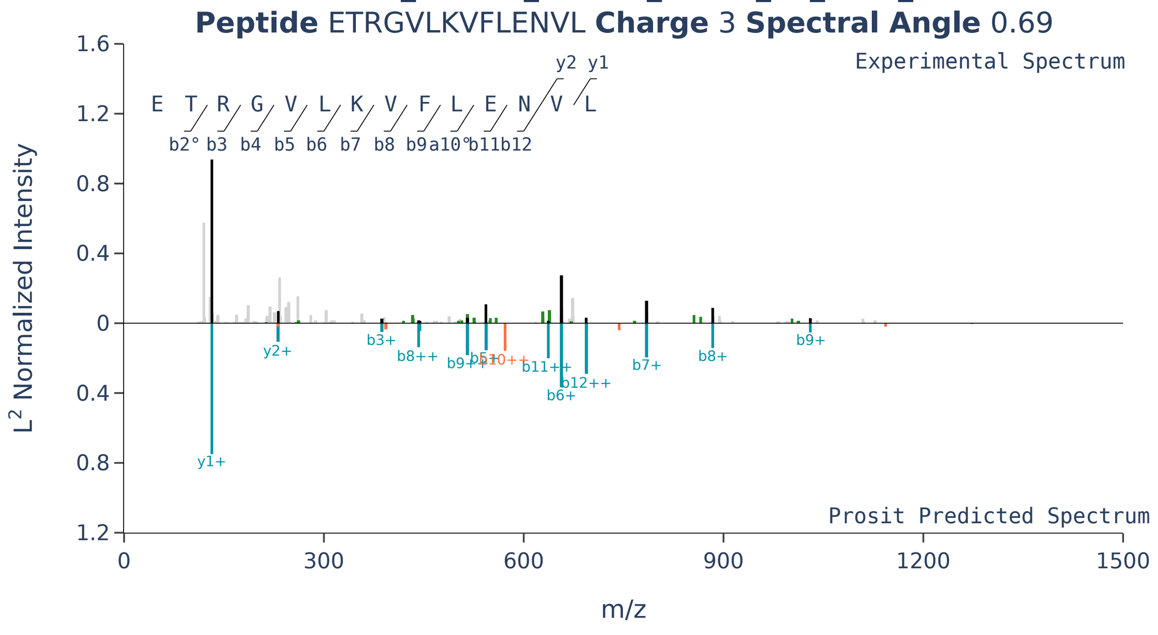


**Source** HRoetschke_291222_140123_HFGoe_H4_b1R2_2h **Scan** 14237
**Peptide** NIQGITKPAIRRLARRGGVKRISGL **Charge** 5 **Spectral Angle** 0.62

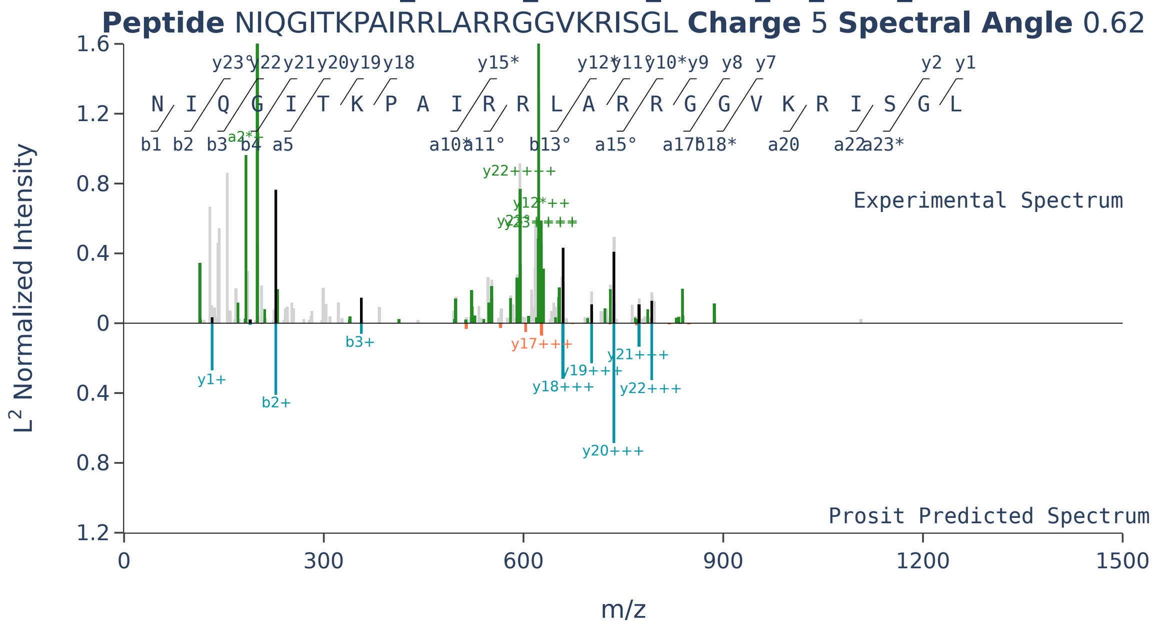


**Source** HRoetschke_291222_140123_HFGoe_H4_b1R2_24h **Scan** 6673
**Peptide** LRDAVTYTEHAKRKTV **Charge** 4 **Spectral Angle** 0.67

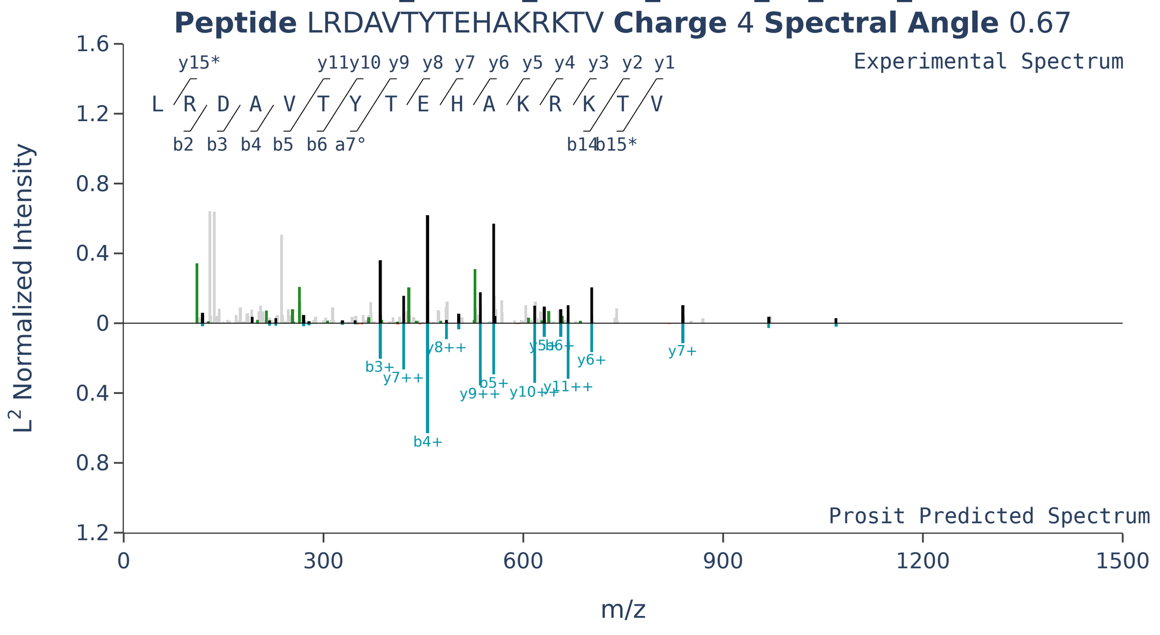


**Source** HRoetschke_291222_140123_HFGoe_H4_b1R2_4h **Scan** 9475
**Peptide** HRKVLRDNIQGITKPALR **Charge** 5 **Spectral Angle** 0.68

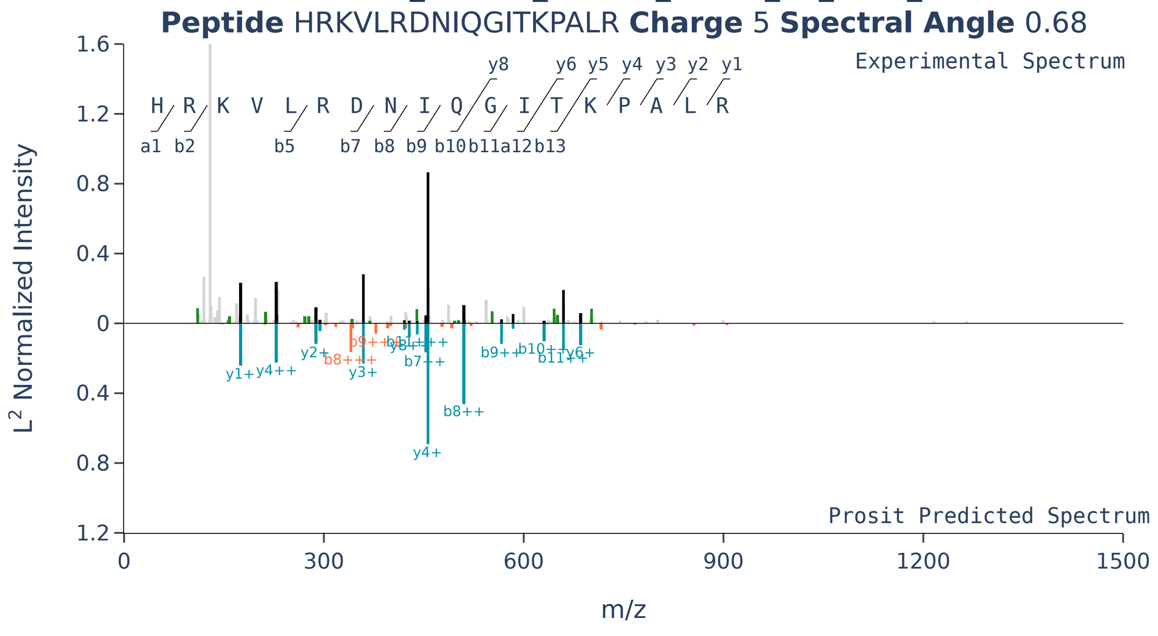


**Source** HRoetschke_291222_140123_HFGoe_H4_b2R2_1h **Scan** 17104
**Peptide** TEHAKRKTVTAMDVVYALKRQGRTLYG **Charge** 5 **Spectral Angle** 0.85

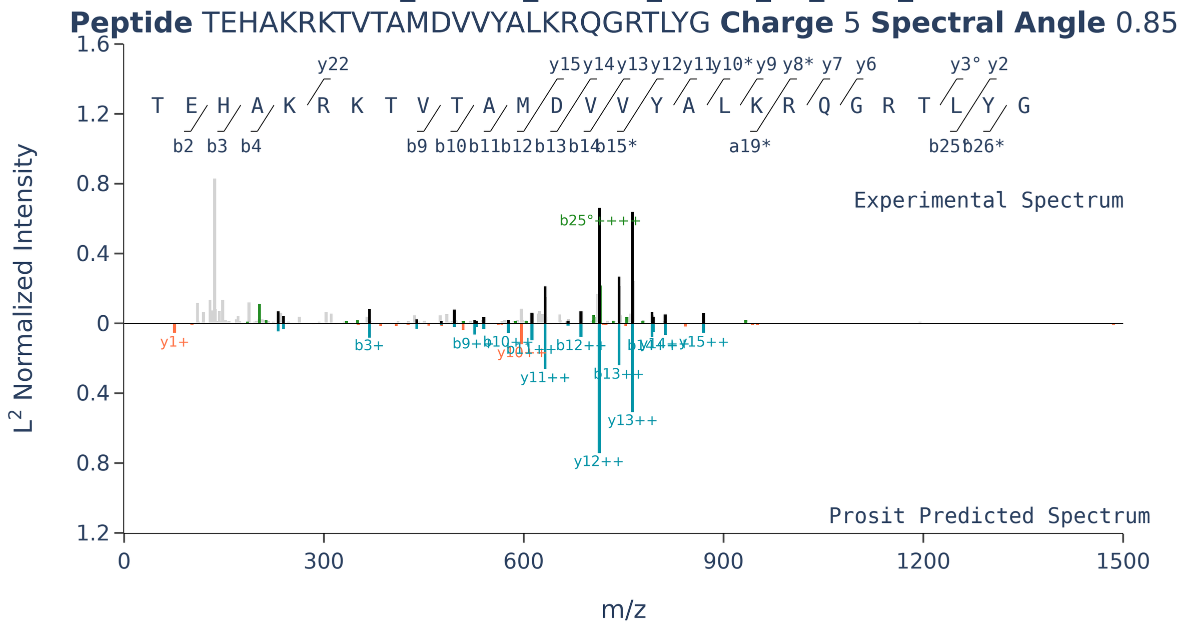


**Source** HRoetschke_291222_140123_HFGoe_H4_b2R1_24h **Scan** 6854
**Peptide** KVLRDNIQ **Charge** 2 **Spectral Angle** 0.77

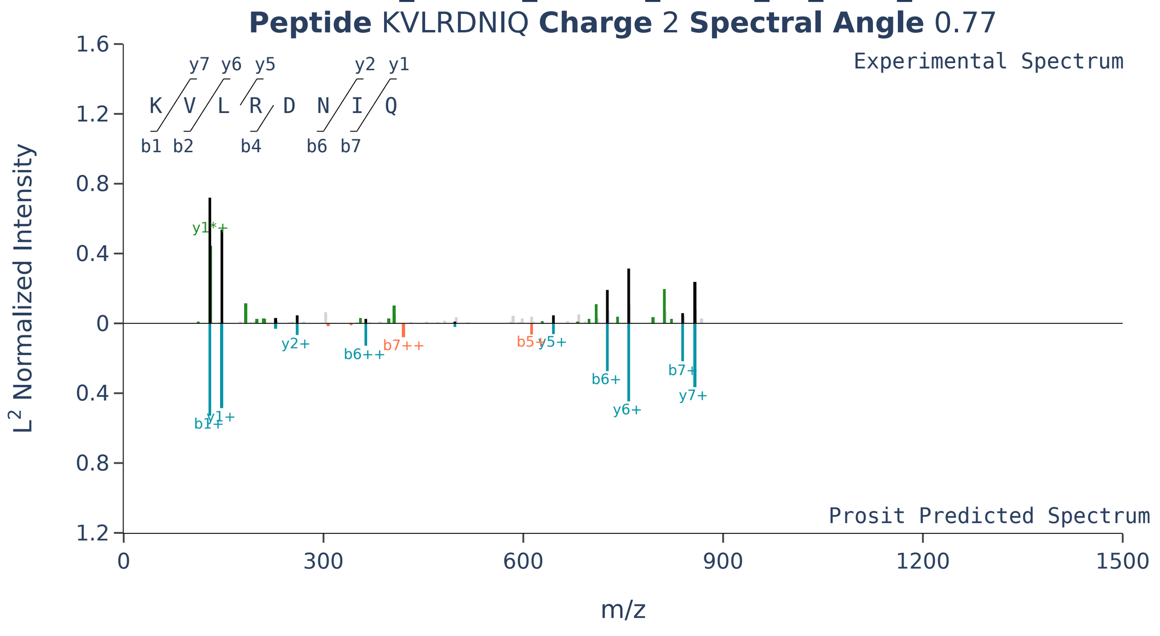


**Source** HRoetschke_291222_140123_HFGoe_H4_b1R2_1h **Scan** 21233
**Peptide** LENVIRDAVTYT **Charge** 2 **Spectral Angle** 0.65

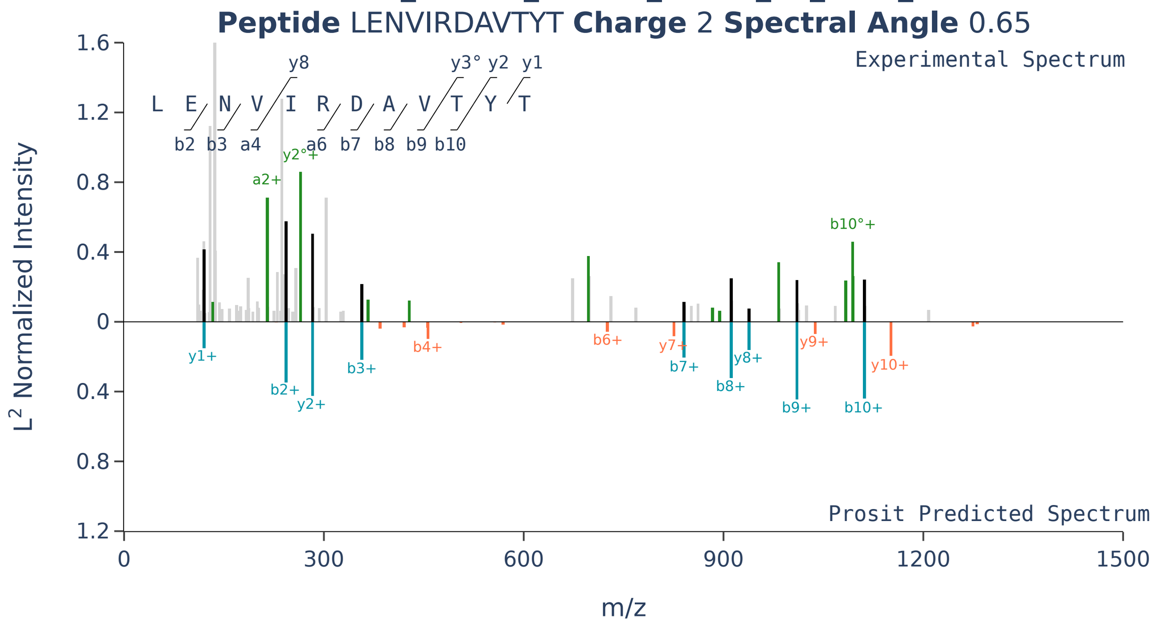


**Source** HRoetschke_291222_140123_HFGoe_H4_b2R1_1h **Scan** 31990
**Peptide** VLKVFLENVIRDAVTY **Charge** 2 **Spectral Angle** 0.64

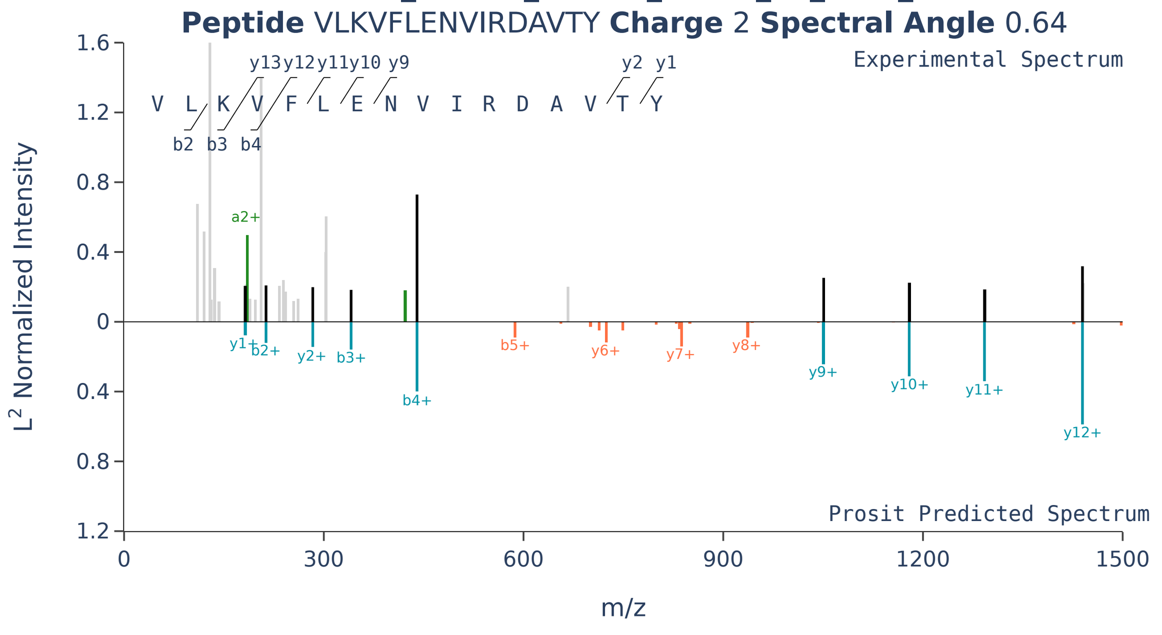


**Source** HRoetschke_291222_140123_HFGoe_H4_b2R1_2h **Scan** 14516
**Peptide** GITKPAIRRLARRGGVKRISGLIYE **Charge** 5 **Spectral Angle** 0.89

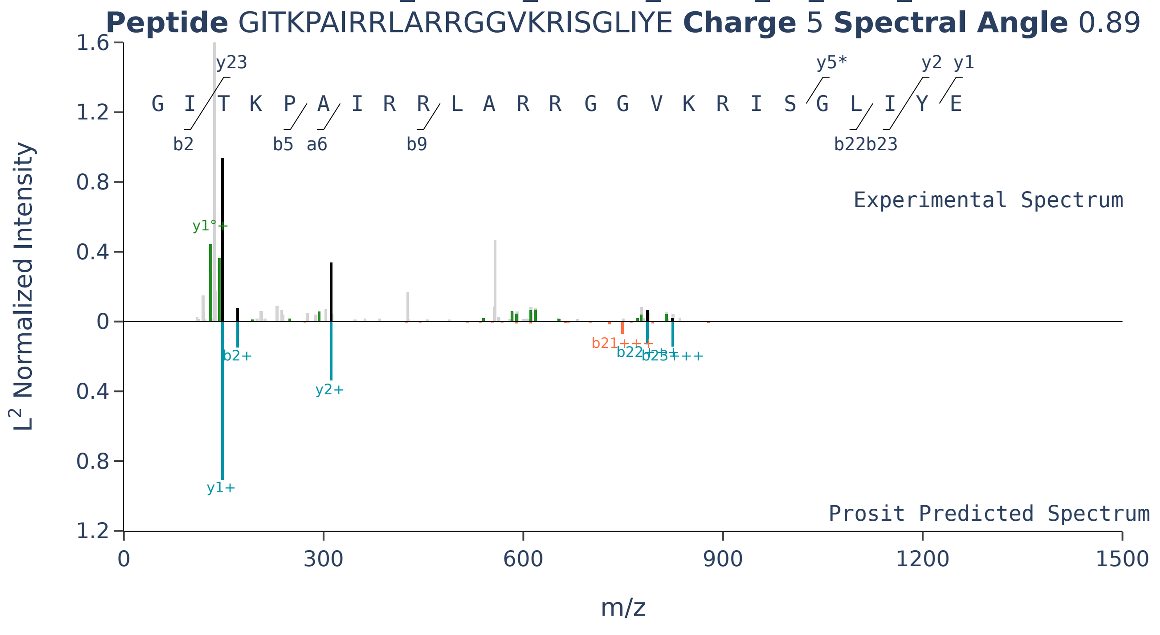


**Source** HRoetschke_291222_140123_HFGoe_H4_b2R1_1h **Scan** 22738
**Peptide** ENVIRDAVTYTEHAKRKTVTAMDVVYAL **Charge** 5 **Spectral Angle** 0.71

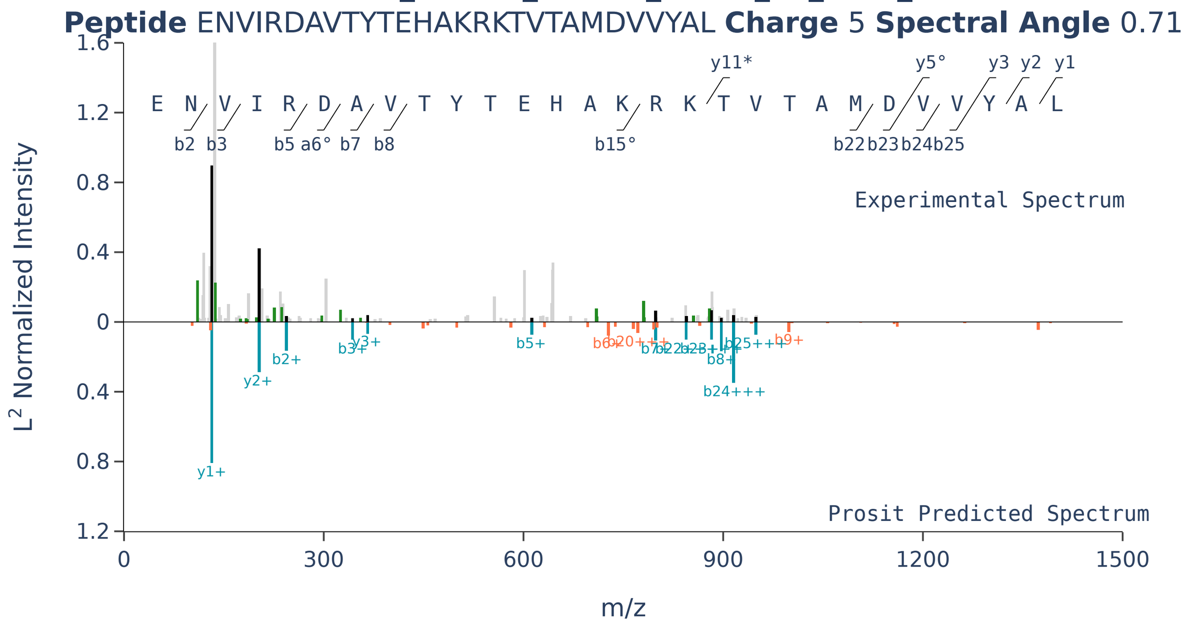


**Source** HRoetschke_291222_140123_HFGoe_H4_b1R2_1h **Scan** 29504
**Peptide** VLKVFLENVIRDAVTYTEHAKRKTV **Charge** 4 **Spectral Angle** 0.78

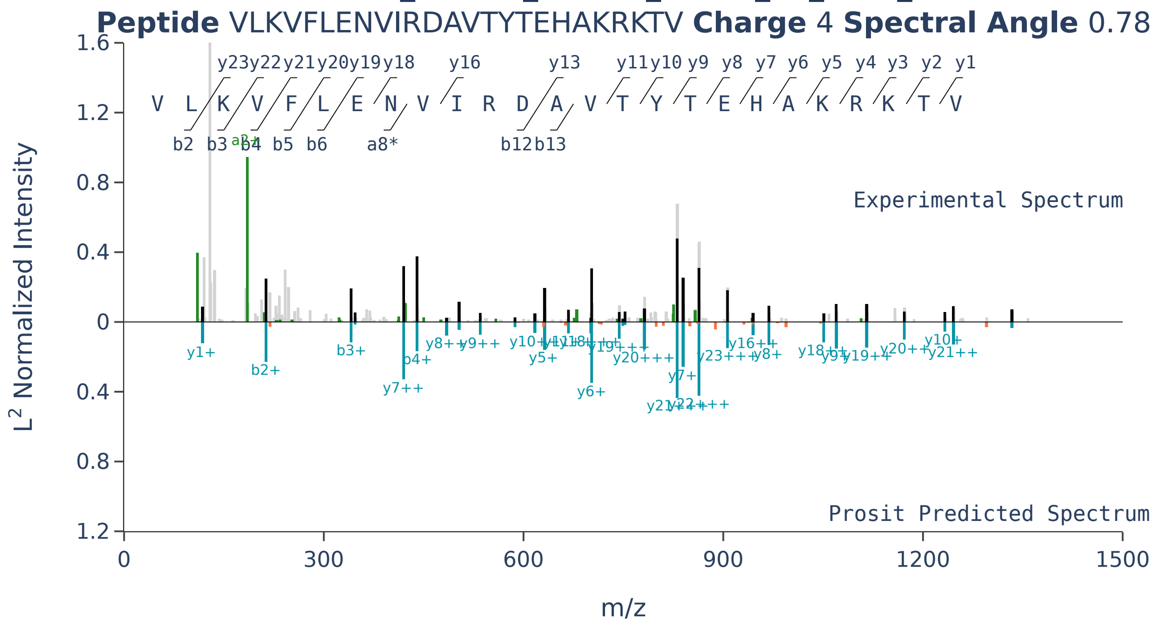


**Source** HRoetschke_291222_140123_HFGoe_H4_b2R2_24h **Scan** 8555
**Peptide** GGKGLGKGGAKRHRKVLRDNIQGITKPALR **Charge** 6 **Spectral Angle** 0.84

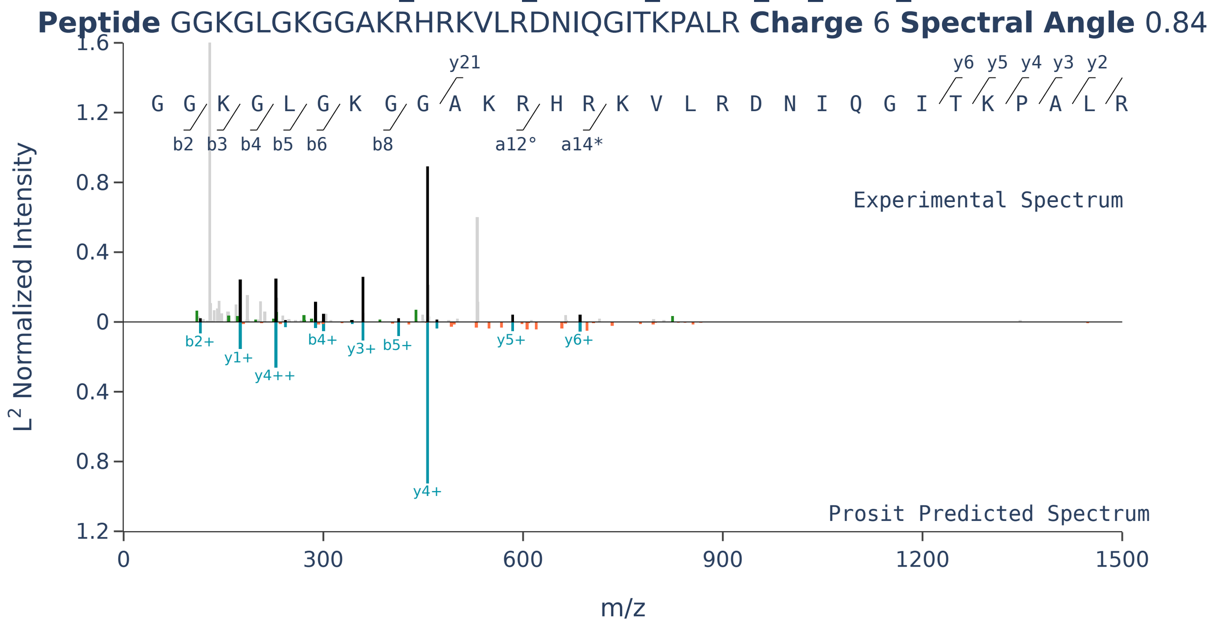

**Source** HRoetschke_291222_140123_HFGoe_H4_b1R2_1h **Scan** 14111
**Peptide** LRDAVTYTEHAKRKTVTAMDVVY **Charge** 5 **Spectral Angle** 0.8

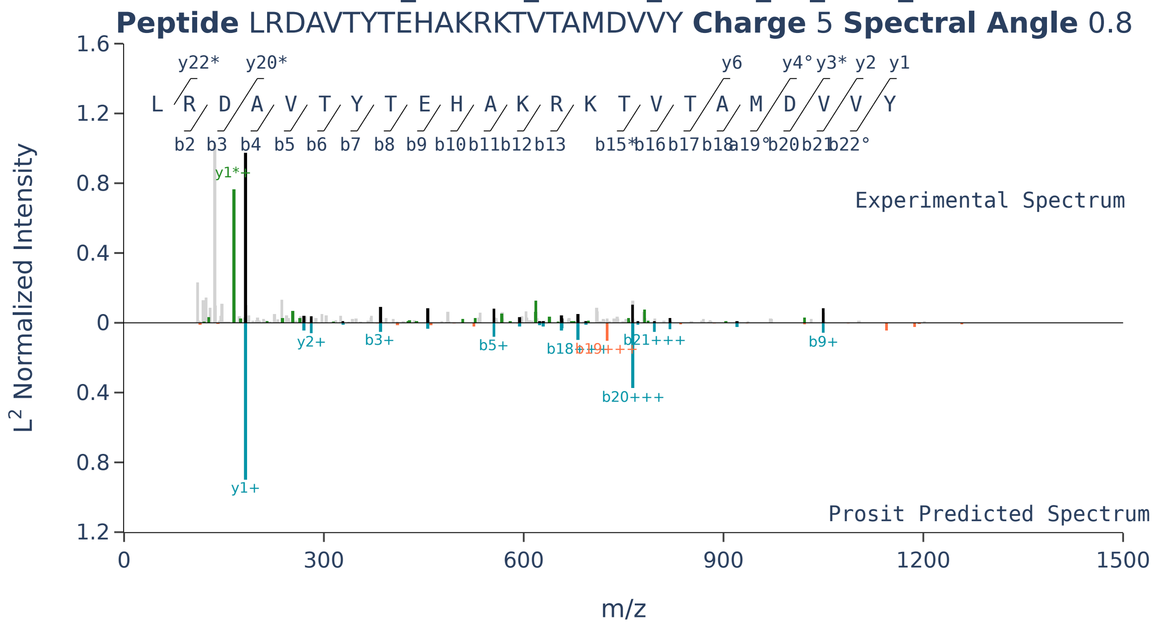


**Source** HRoetschke_291222_140123_HFGoe_H4_b1R2_1h **Scan** 20244
**Peptide** AKRKTVTAMDVVYALKRQGRTLY **Charge** 5 **Spectral Angle** 0.88

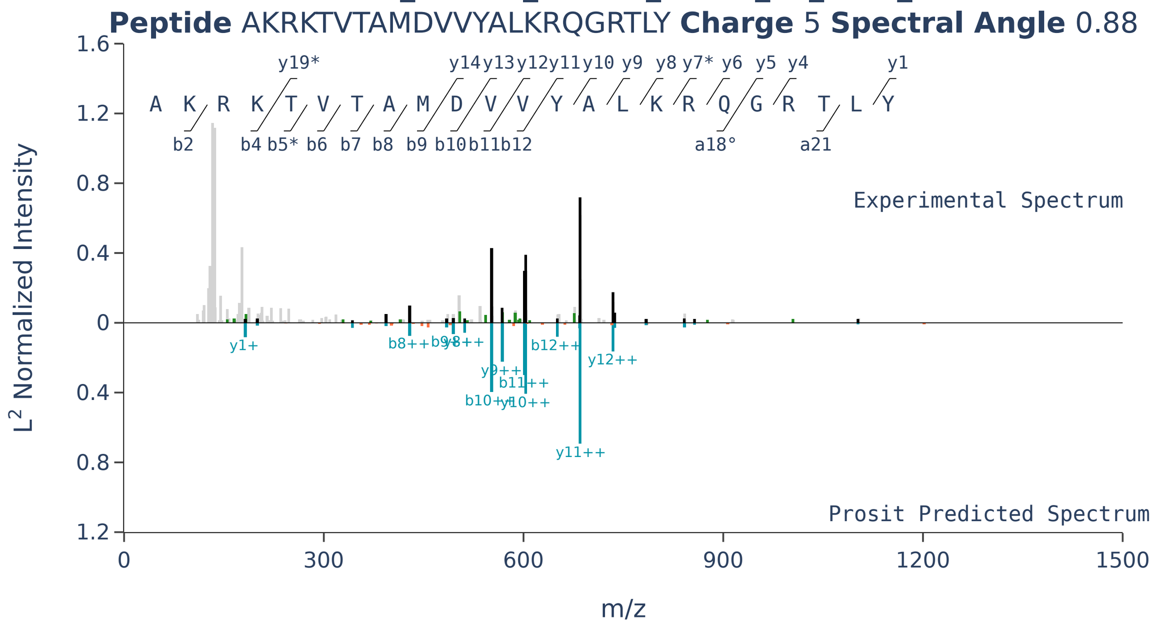


**Source** HRoetschke_291222_140123_HFGoe_H4_b2R1_4h **Scan** 18957
**Peptide** KVFLENVIR **Charge** 3 **Spectral Angle** 0.64

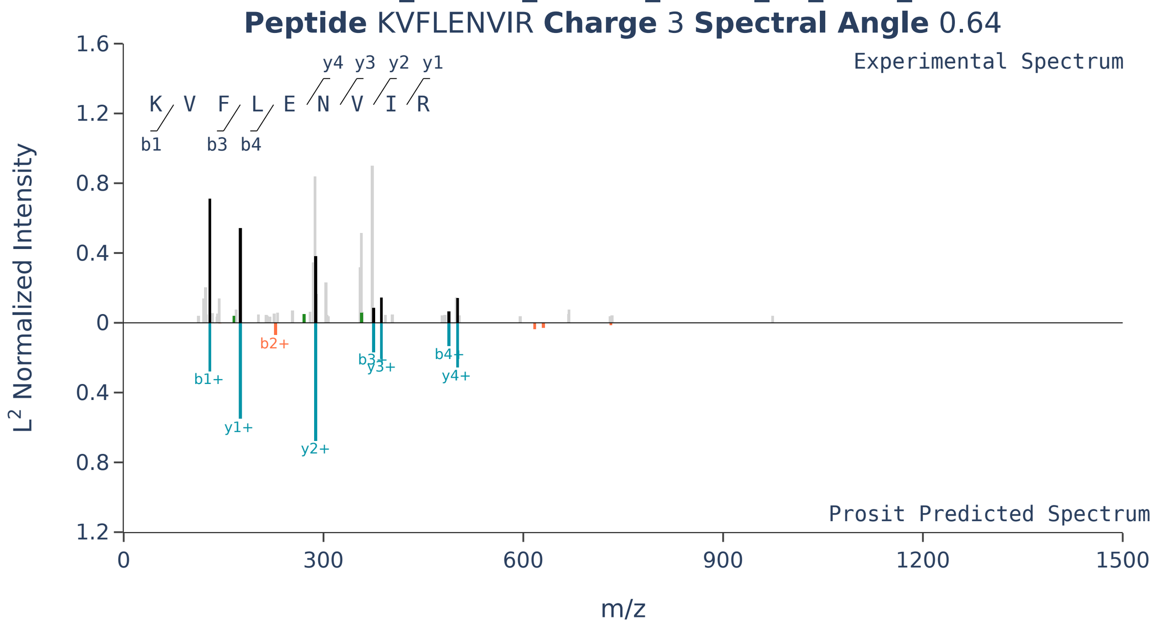


**Source** HRoetschke_291222_140123_HFGoe_H4_b1R2_2h **Scan** 6854
**Peptide** DAVTYTEHAKRKTV **Charge** 2 **Spectral Angle** 0.7

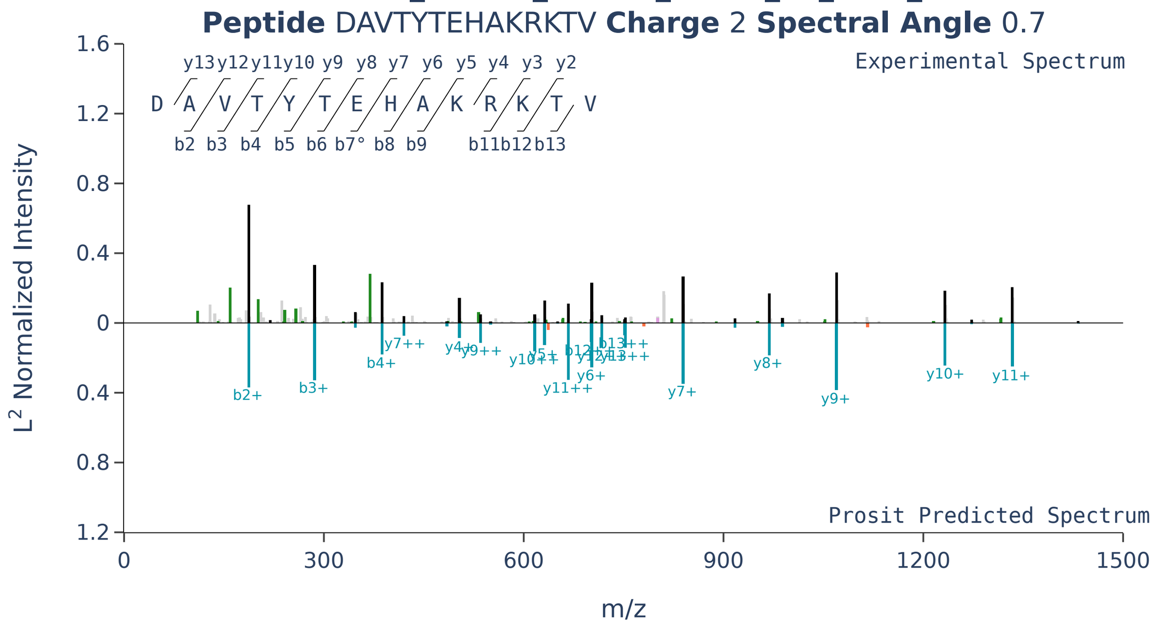


**Source** HRoetschke_291222_140123_HFGoe_H4_b1R2_2h **Scan** 17930
**Peptide** TEHAKRKTVTAMDVVYALK **Charge** 4 **Spectral Angle** 0.56

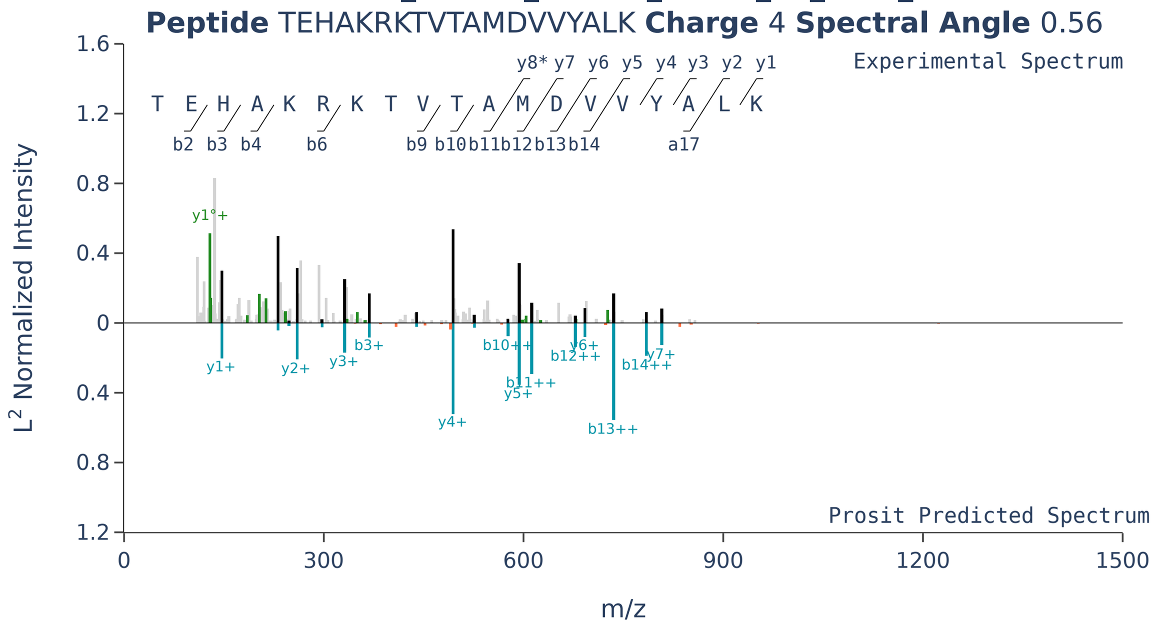


**Source** HRoetschke_291222_140123_HFGoe_H4_b1R1_24h **Scan** 7029
**Peptide** KGLGKGGAKRHRKVLRDNIQGITKPALR **Charge** 5 **Spectral Angle** 0.88

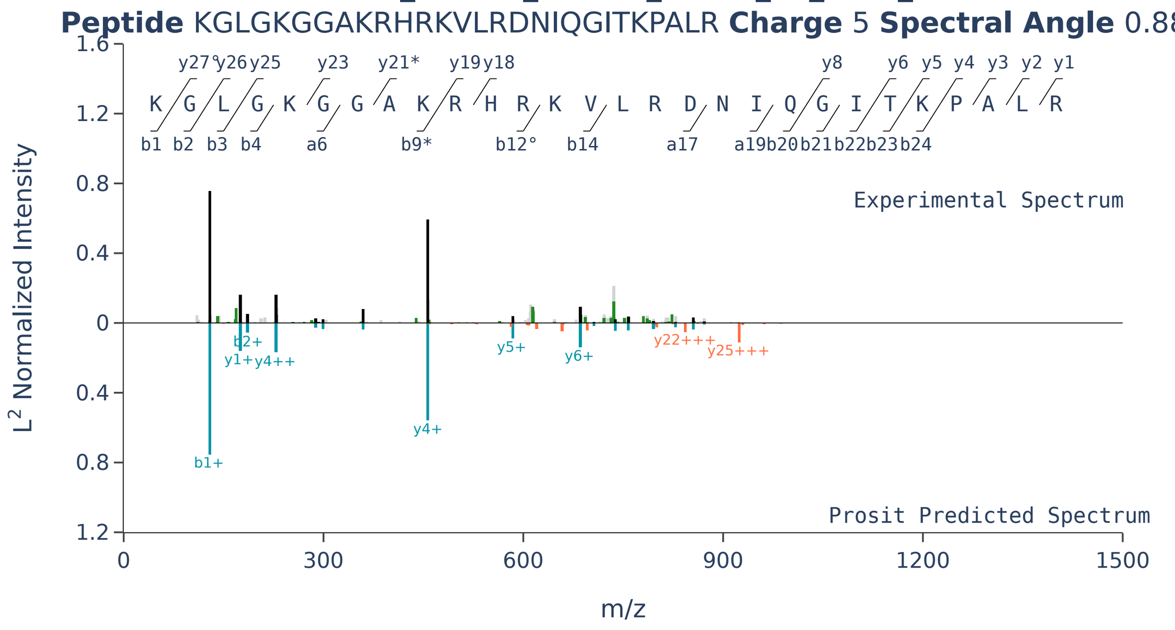


**Source** HRoetschke_291222_140123_HFGoe_H4_b2R1_2h **Scan** 16381
**Peptide** AVTYTEHAKRKTVTAMDVVYALKRQGRTL **Charge** 5 **Spectral Angle** 0.78

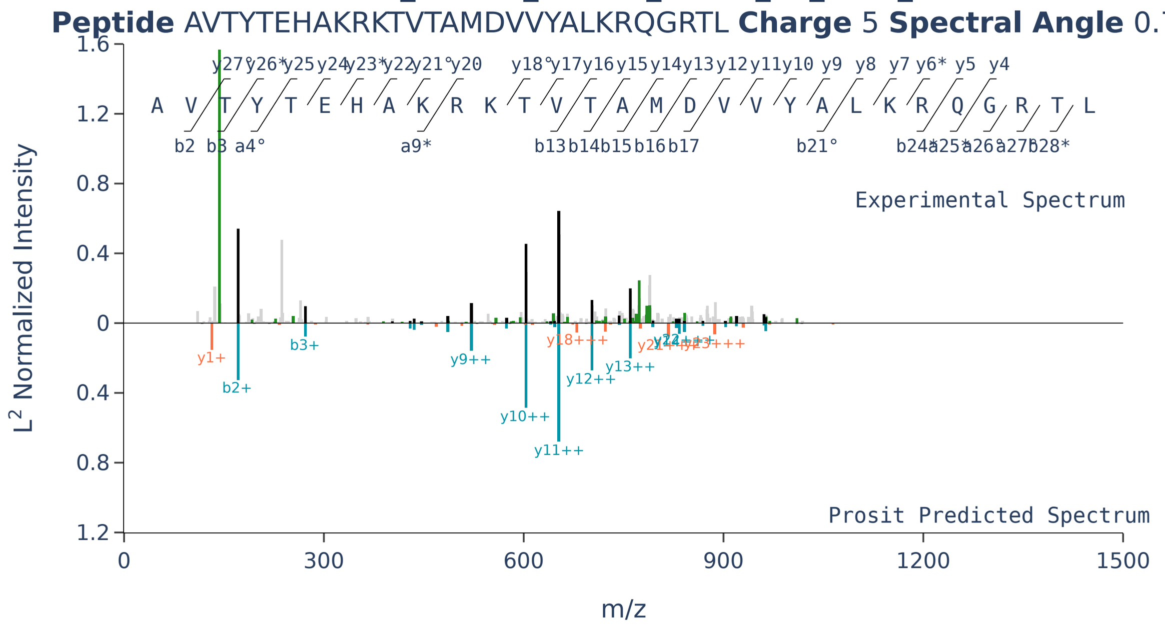


**Source** HRoetschke_291222_140123_HFGoe_H4_b2R1_24h **Scan** 5682
**Peptide** HRKVLRDNIQ **Charge** 3 **Spectral Angle** 0.69

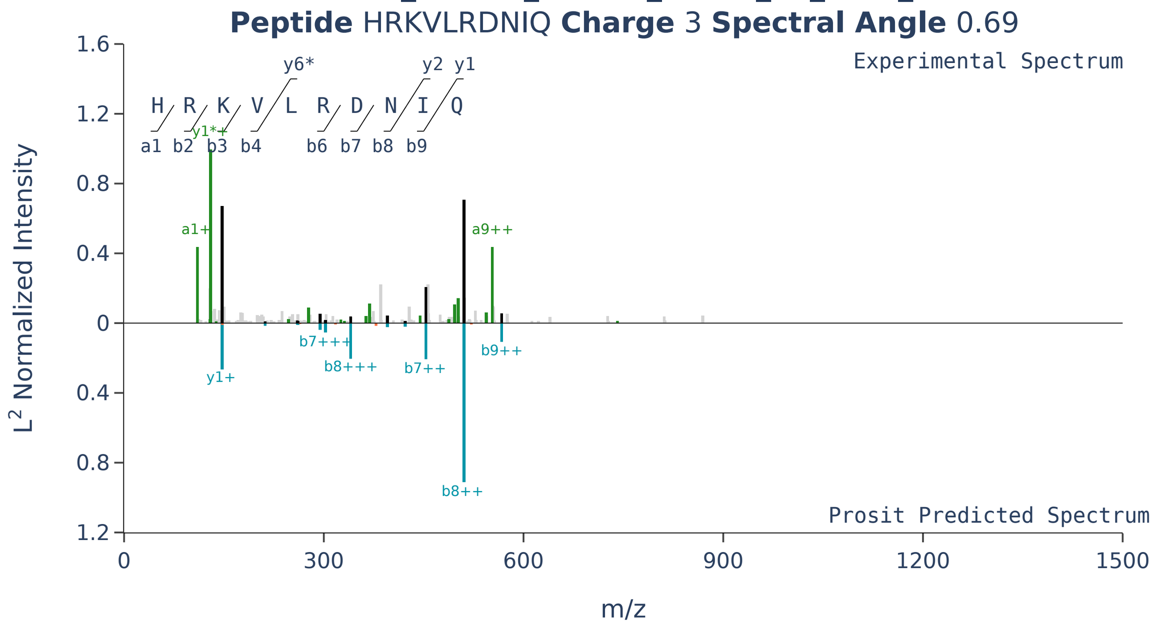


**Source** HRoetschke_291222_140123_HFGoe_H4_b2R2_2h **Scan** 14018
**Peptide** GLTKPAIRRLARRGGVKRISGLIYE **Charge** 5 **Spectral Angle** 0.87

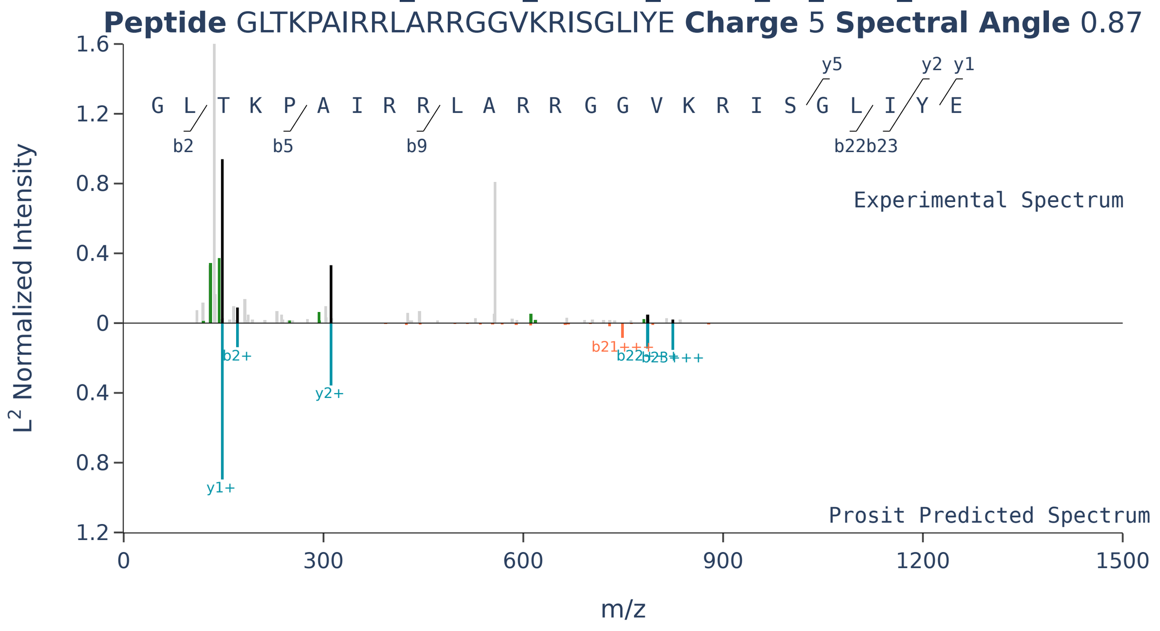


**Source** HRoetschke_291222_140123_HFGoe_H4_b2R1_1h **Scan** 33949
**Peptide** RGVLKVFLENVIRDAVTYT **Charge** 3 **Spectral Angle** 0.68

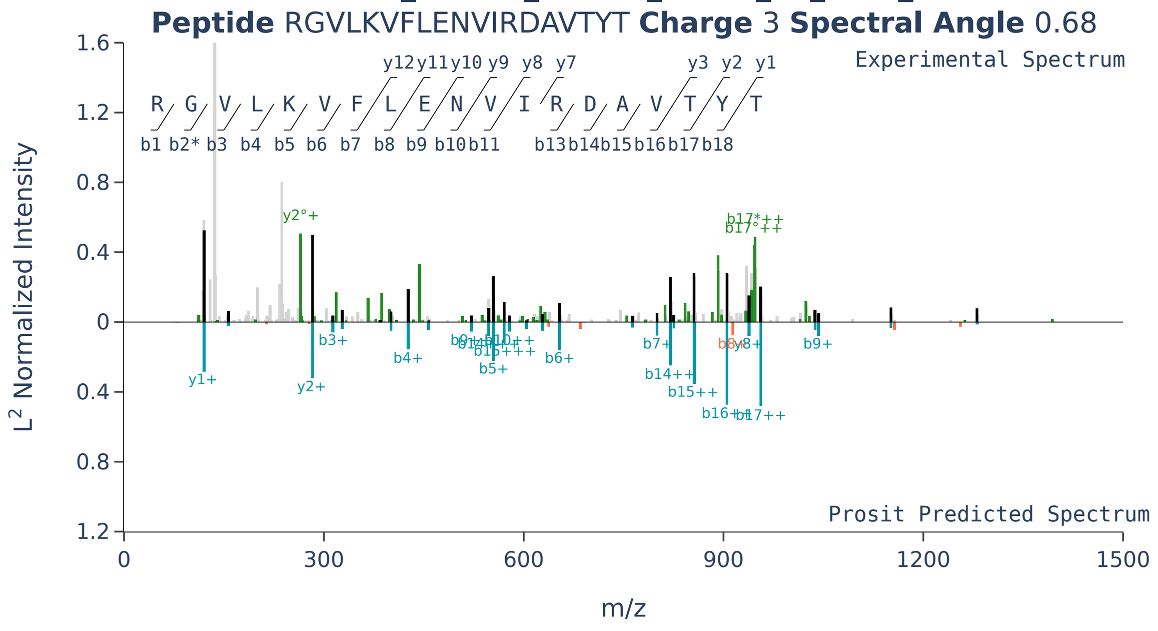


**Source** HRoetschke_291222_140123_HFGoe_H4_b1R2_1h **Scan** 10244
**Peptide** RKVLRDNIQGL **Charge** 3 **Spectral Angle** 0.7

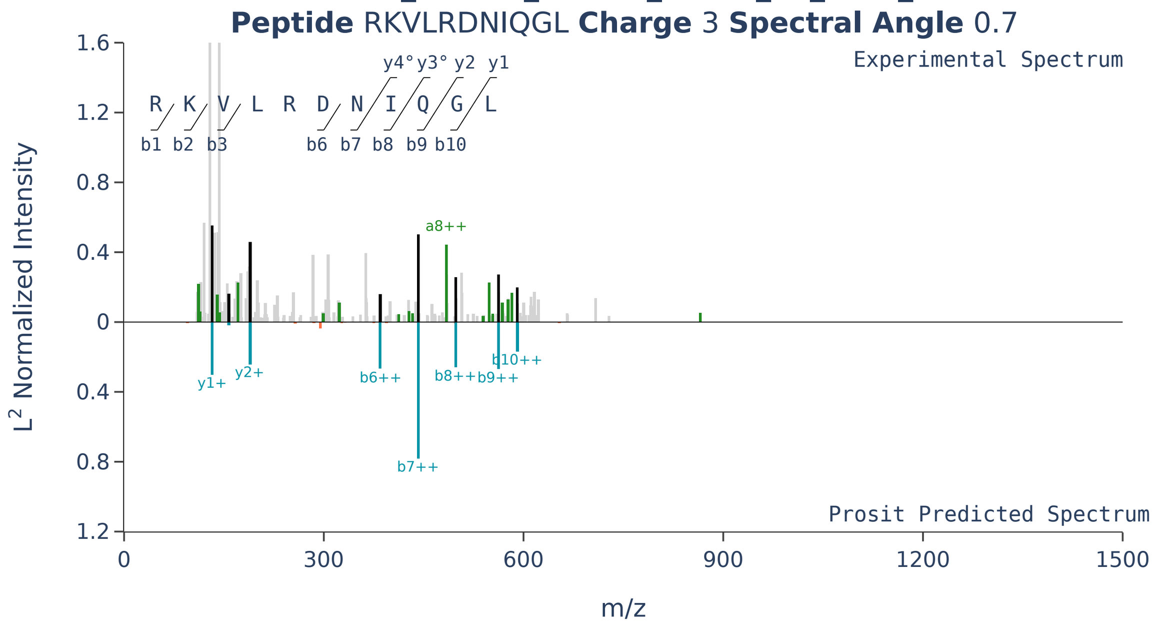


**Source** HRoetschke_291222_140123_HFGoe_H4_b2R2_4h **Scan** 7462
**Peptide** IRDAVTYTEHAK **Charge** 3 **Spectral Angle** 0.81

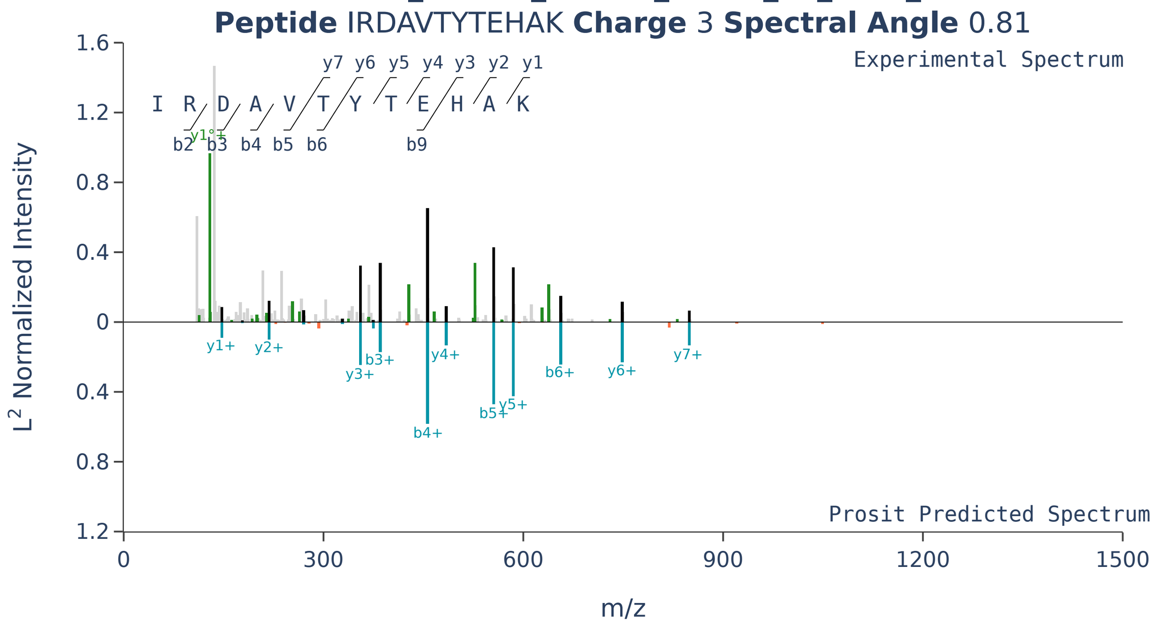


**Source** HRoetschke_291222_140123_HFGoe_H4_b2R1_4h **Scan** 10466
**Peptide** LIYEETR **Charge** 2 **Spectral Angle** 0.75

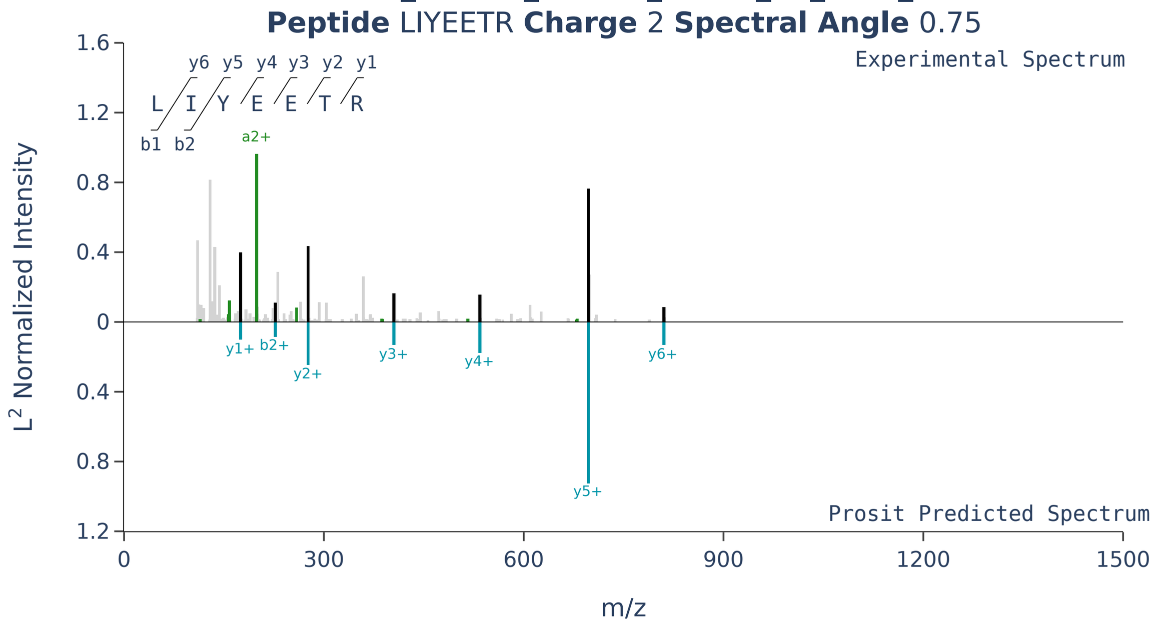


**Source** HRoetschke_291222_140123_HFGoe_H4_b1R1_24h **Scan** 5528
**Peptide** DAVTYTEHAKRKT **Charge** 3 **Spectral Angle** 0.45

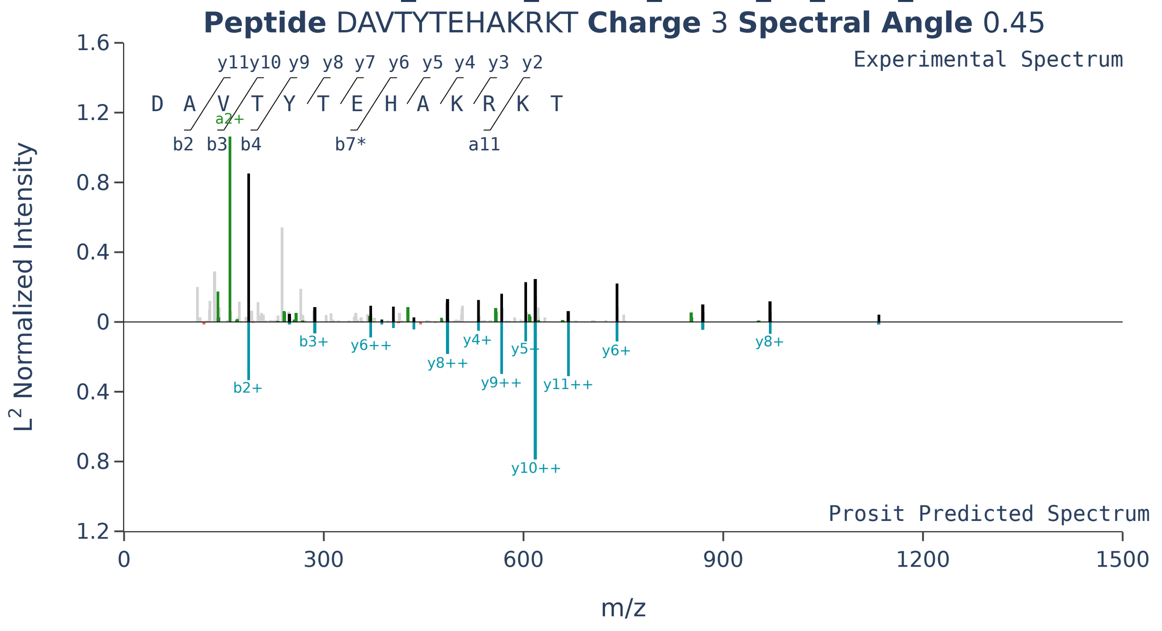


**Source** HRoetschke_291222_140123_HFGoe_H4_b1R1_1h **Scan** 22036
**Peptide** ISGLIYEETRGVLK **Charge** 2 **Spectral Angle** 0.75

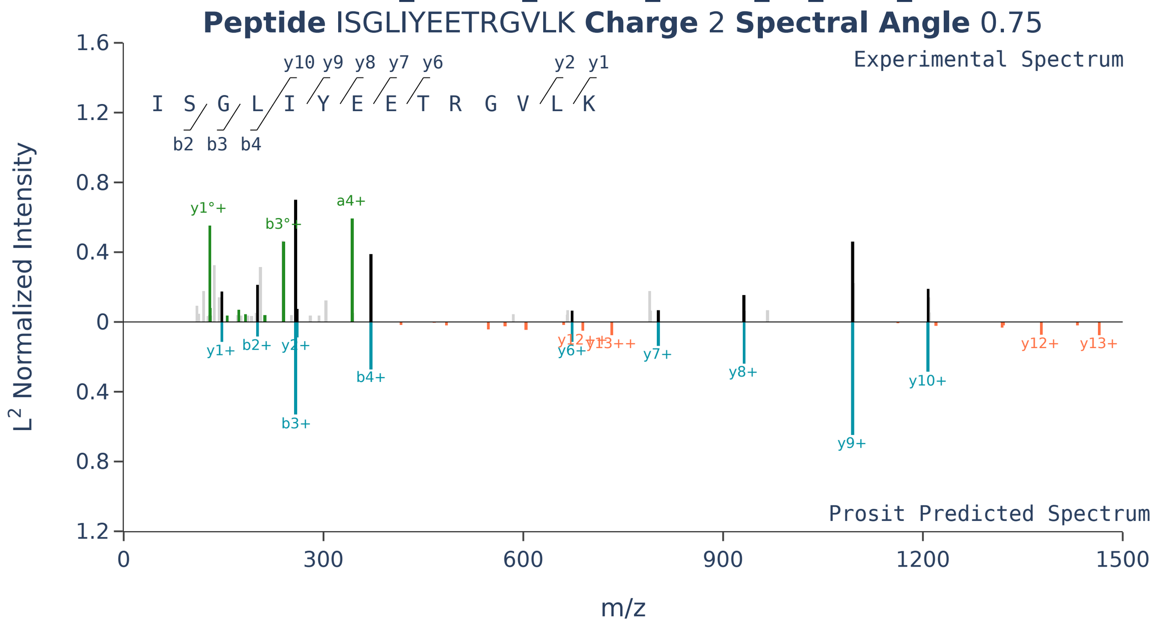

**Source** HRoetschke_291222_140123_HFGoe_H4_b2R2_1h **Scan** 26970
**Peptide** KVFLENVIRDAVTYTEH **Charge** 3 **Spectral Angle** 0.86

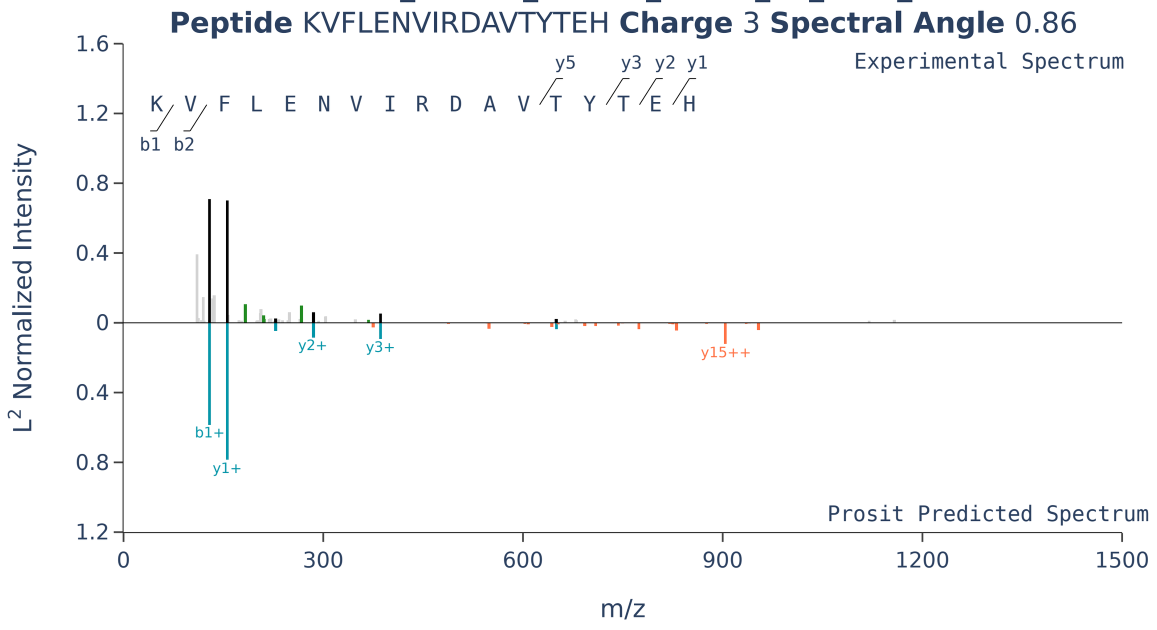


**Source** HRoetschke_291222_140123_HFGoe_H4_b2R2_24h **Scan** 11376
**Peptide** KVLRDNIQGITKPA **Charge** 4 **Spectral Angle** 0.8

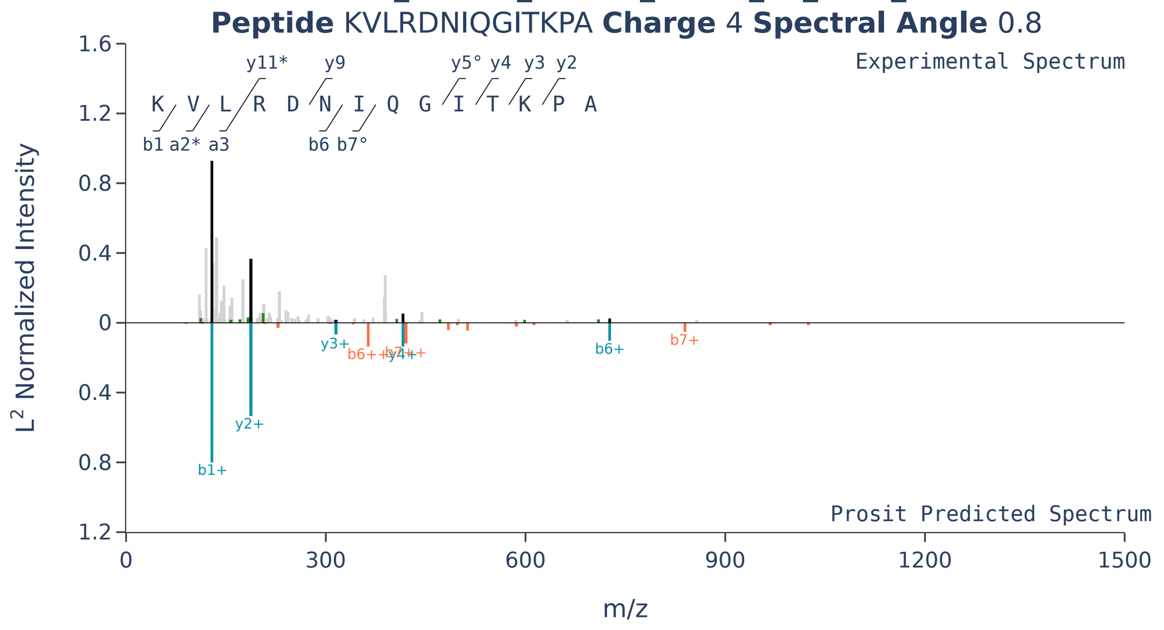


**Source** HRoetschke_291222_140123_HFGoe_H4_b1R1_2h **Scan** 18113
**Peptide** RDAVTYTEHAKRKTVTAMDVVYALK **Charge** 5 **Spectral Angle** 0.7

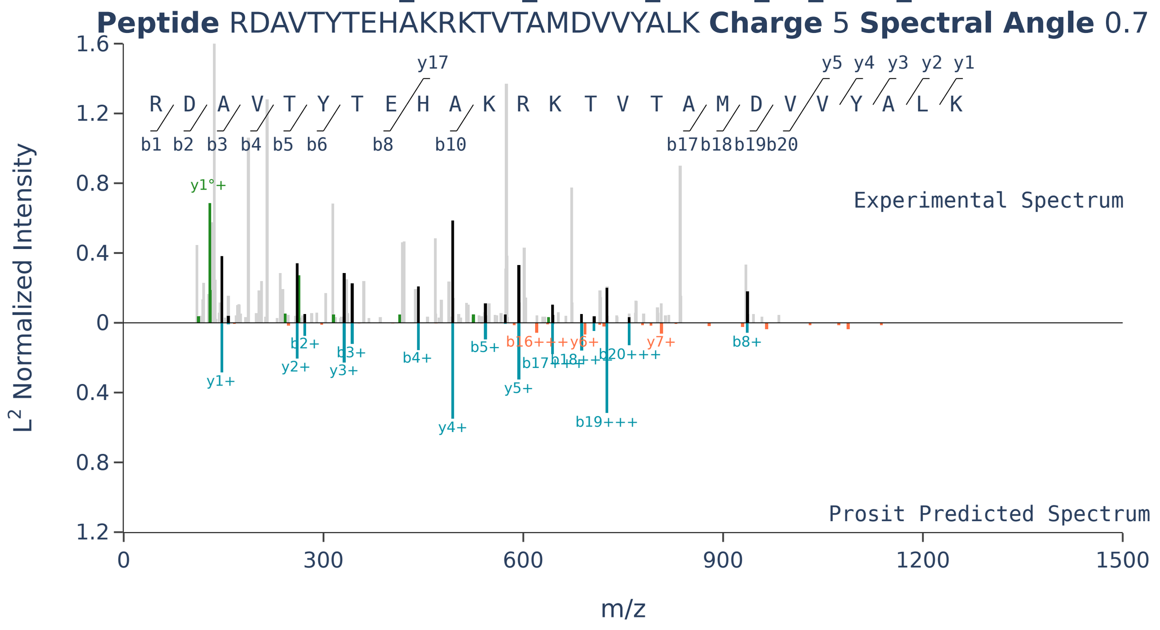


**Source** HRoetschke_291222_140123_HFGoe_H4_b1R2_1h **Scan** 23473
**Peptide** KVFLENVIRDAVTYTEHAKRKT **Charge** 5 **Spectral Angle** 0.7

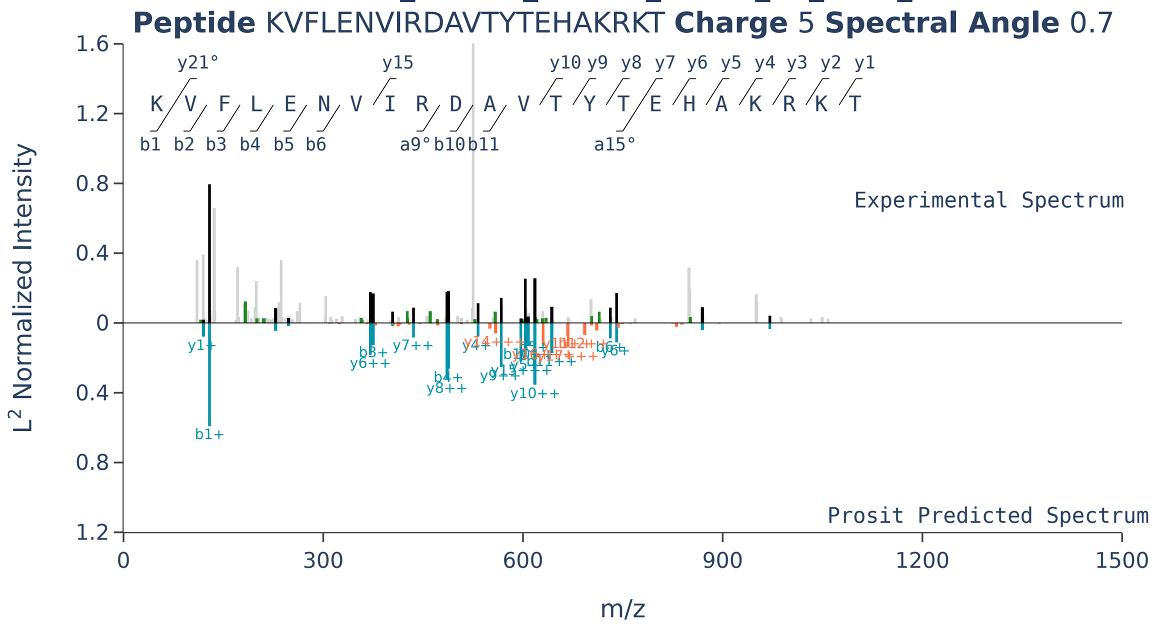


**Source** HRoetschke_291222_140123_HFGoe_H4_b2R1_1h **Scan** 25439
**Peptide** VLKVFLENV **Charge** 2 **Spectral Angle** 0.62

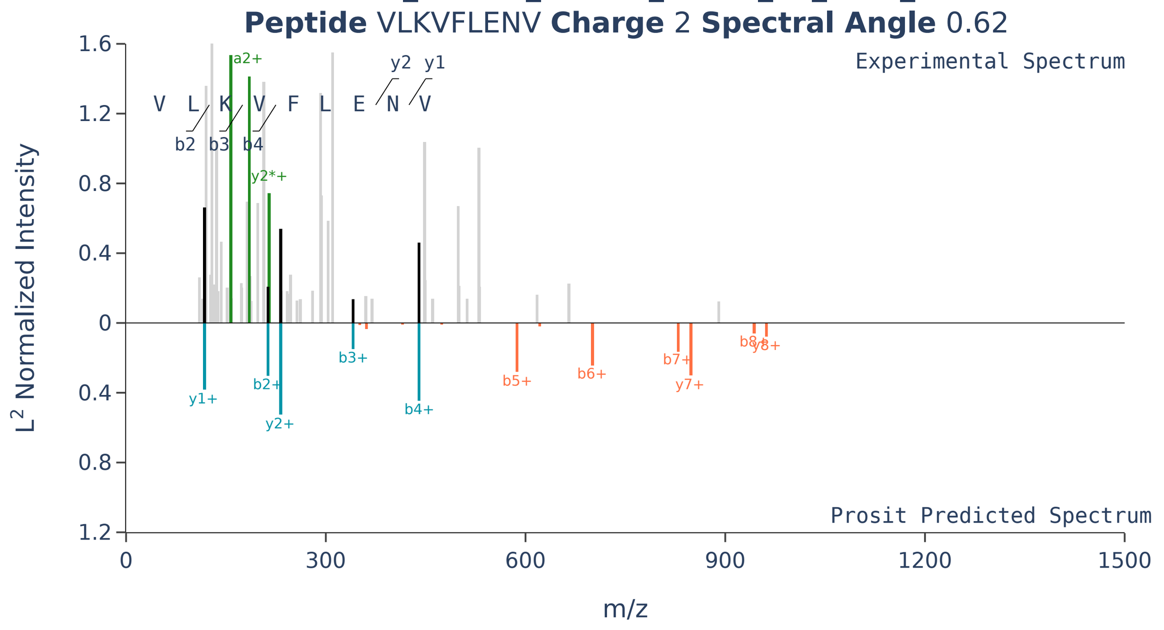


**Source** HRoetschke_291222_140123_HFGoe_H4_b1R1_24h **Scan** 7112
**Peptide** HRKVLRDNIQGITK **Charge** 4 **Spectral Angle** 0.71

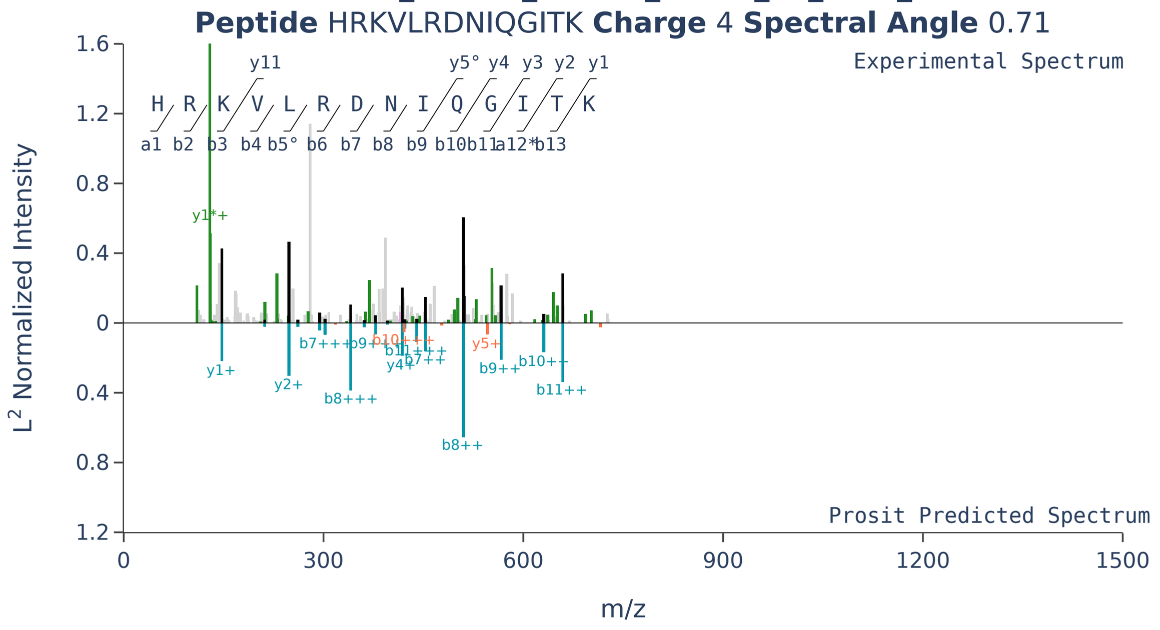


**Source** HRoetschke_291222_140123_HFGoe_H4_b1R1_1h **Scan** 11365
**Peptide** RRGGVKRISGLIYEETRG **Charge** 4 **Spectral Angle** 0.73

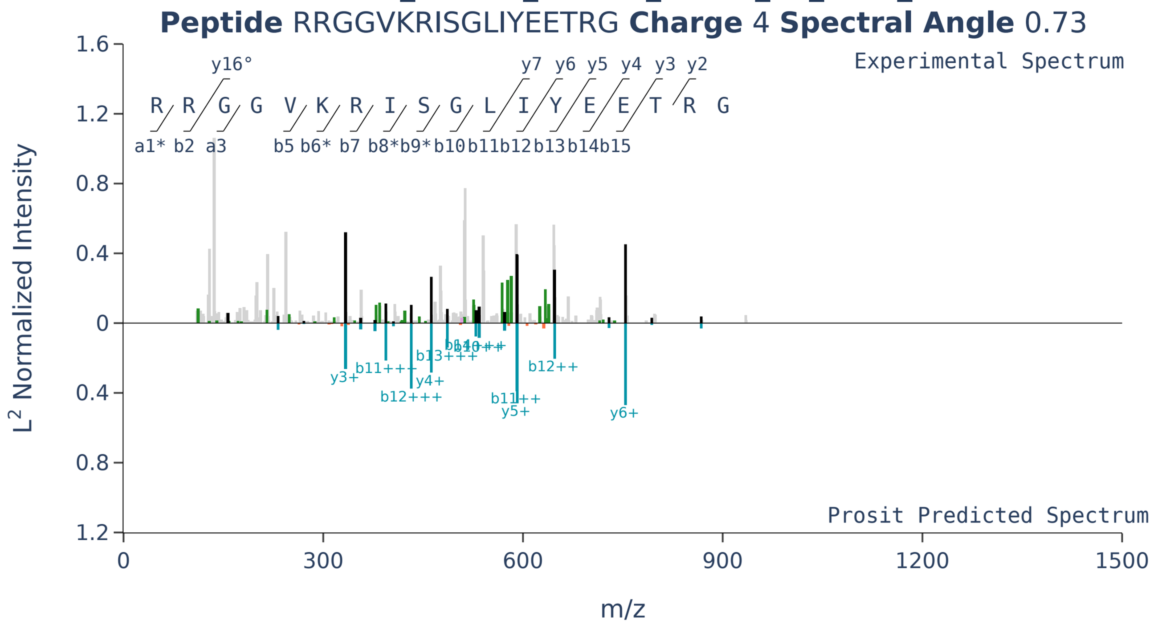


**Source** HRoetschke_291222_140123_HFGoe_H4_b1R2_2h **Scan** 8027
**Peptide** VLRDAVTYTEHAKRKTVT **Charge** 3 **Spectral Angle** 0.82

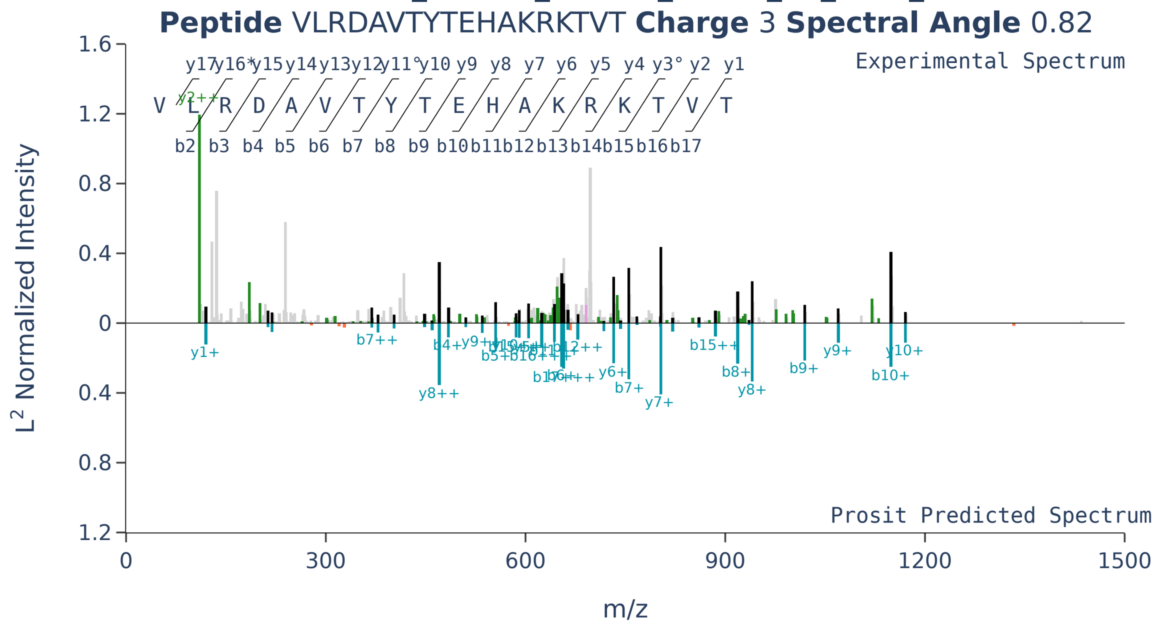


**Source** HRoetschke_291222_140123_HFGoe_H4_b2R1_24h **Scan** 9963
**Peptide** GKGGAKRHRKVLRDNIQGITKPAL **Charge** 5 **Spectral Angle** 0.61

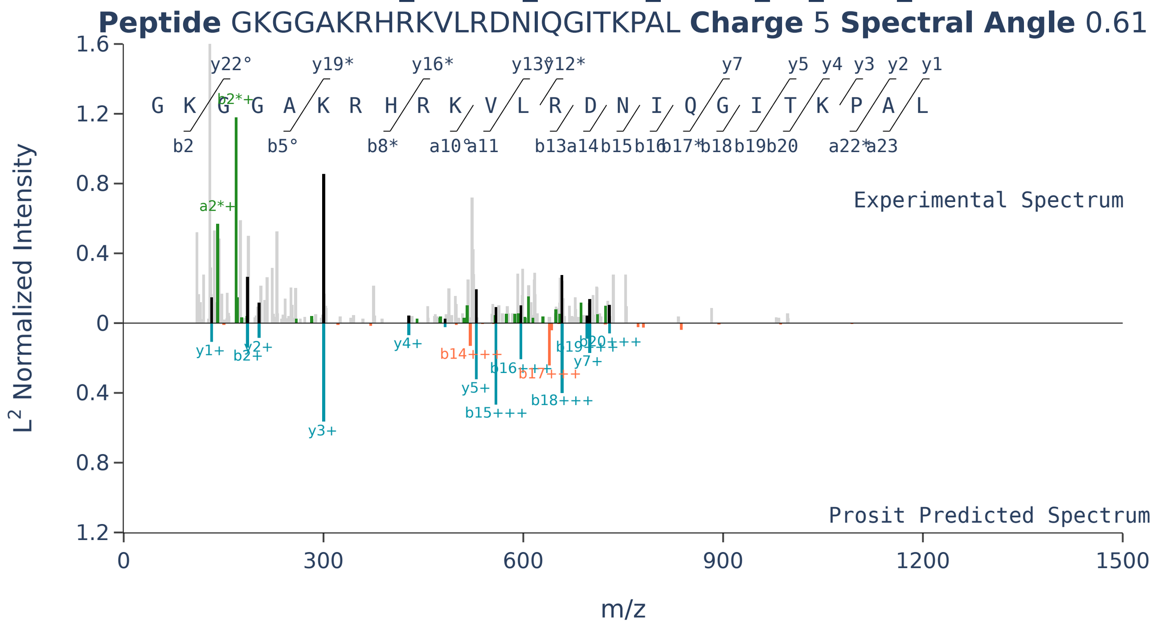


**Source** HRoetschke_291222_140123_HFGoe_H4_b2R1_1h **Scan** 23204
**Peptide** KRKTVTAMDVVYALKRQGRTLYGF **Charge** 5 **Spectral Angle** 0.84

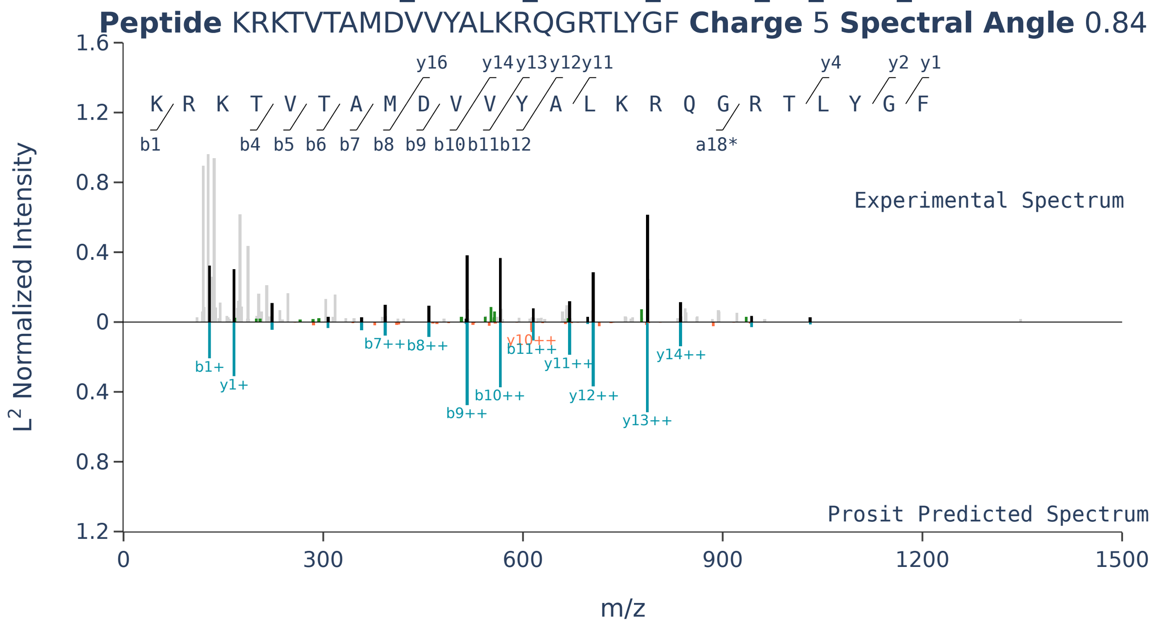


**Source** HRoetschke_291222_140123_HFGoe_H4_b1R1_24h **Scan** 10577
**Peptide** RLARRGGVKRISGLIYE **Charge** 4 **Spectral Angle** 0.71

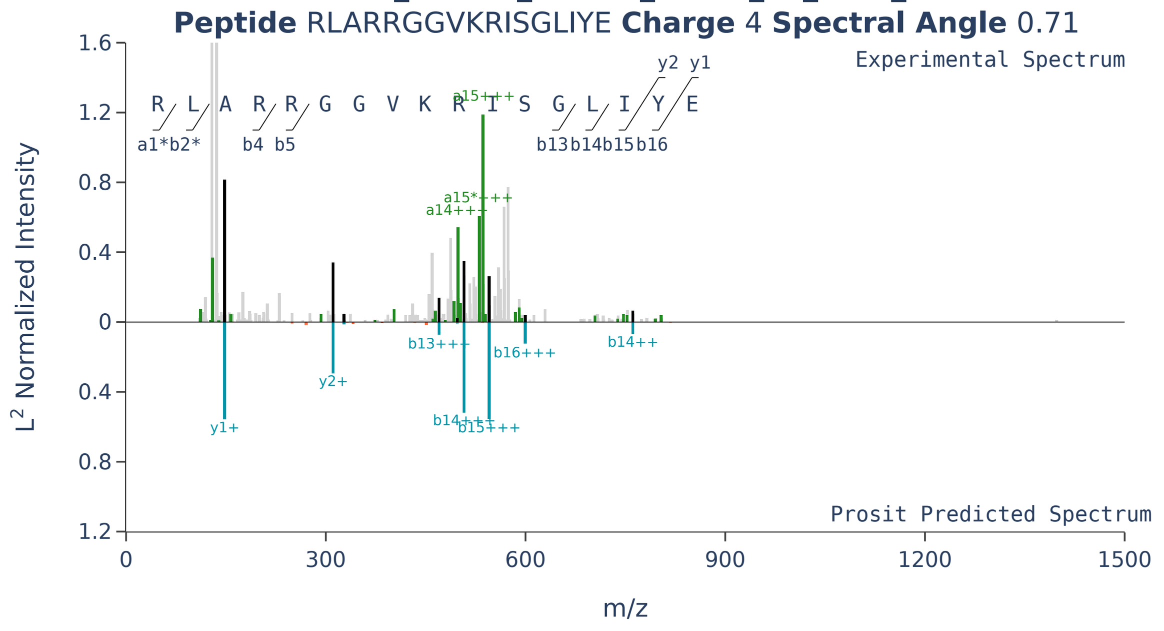


**Source** HRoetschke_291222_140123_HFGoe_H4_b2R1_24h **Scan** 8676
**Peptide** HRKVLRDNIQGL **Charge** 3 **Spectral Angle** 0.7

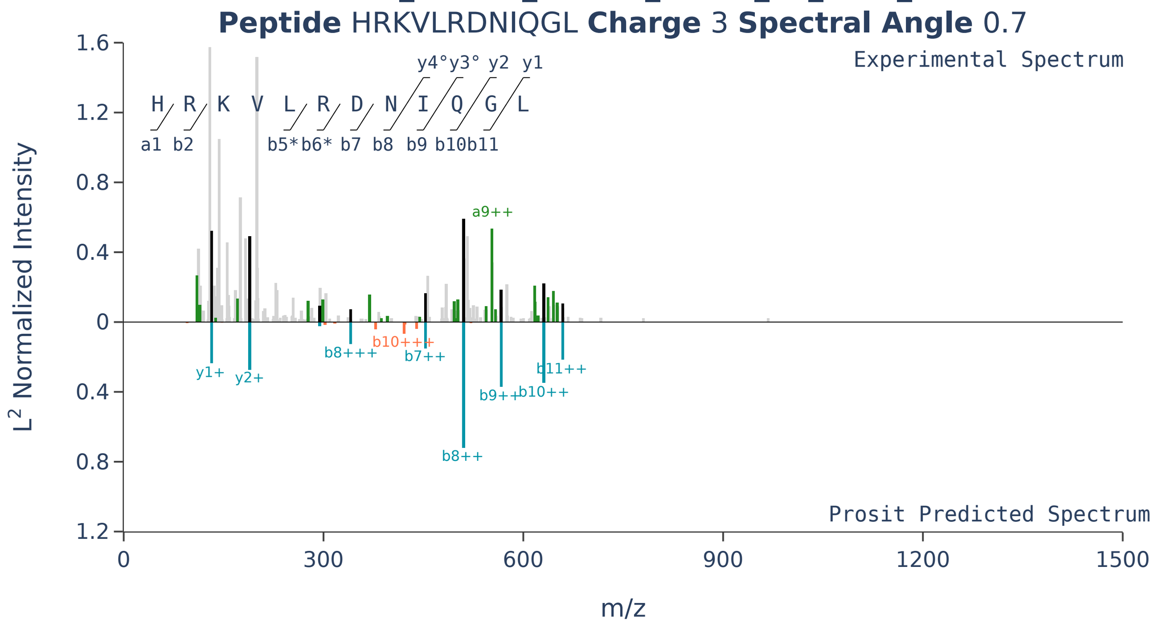


**Source** HRoetschke_291222_140123_HFGoe_H4_b1R1_24h **Scan** 12039
**Peptide** DNIQGITKPAIRRLARRGGVKRISG **Charge** 5 **Spectral Angle** 0.71

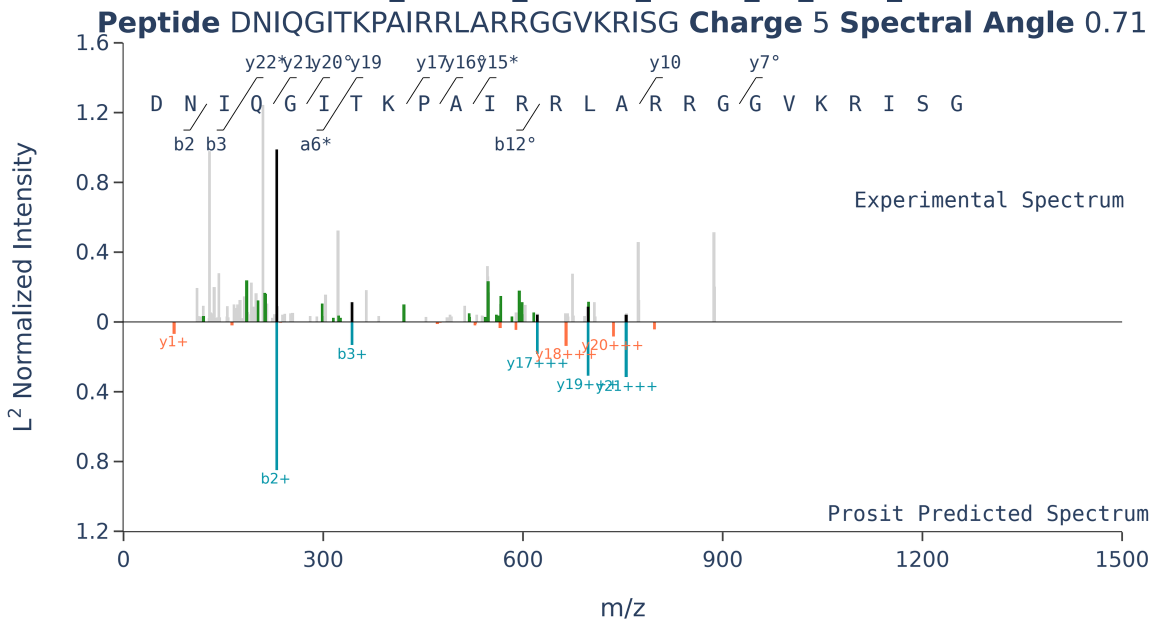


**Source** HRoetschke_291222_140123_HFGoe_H4_b2R1_1h **Scan** 36769
**Peptide** SGLIYEETRGVLKVFLENVIRD **Charge** 3 **Spectral Angle** 0.74

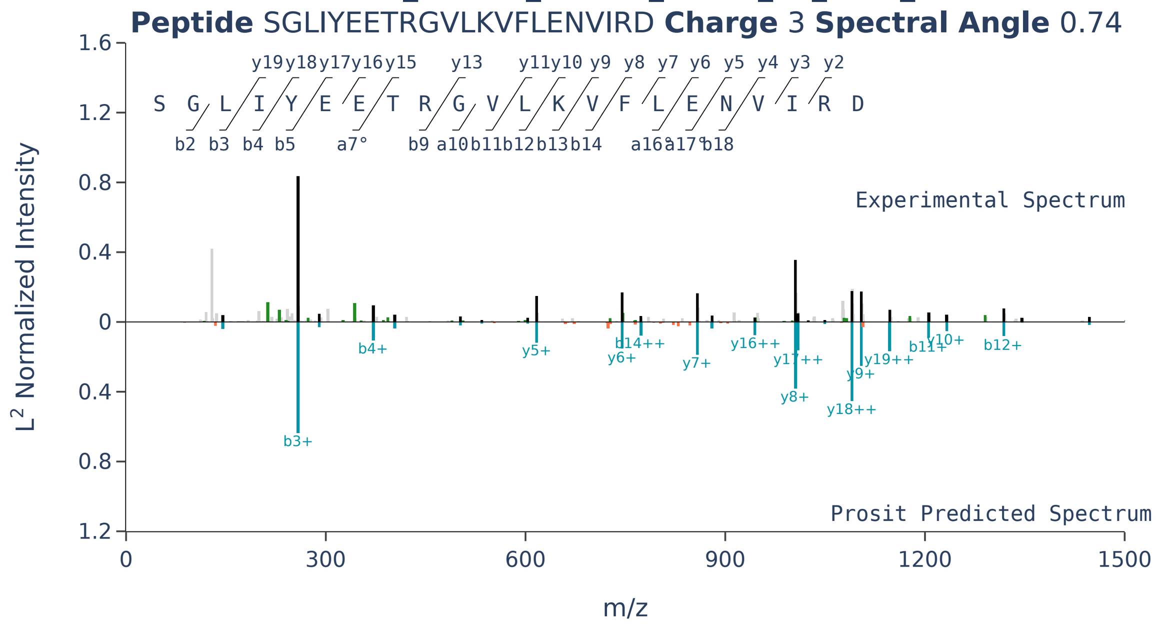


**Source** HRoetschke_291222_140123_HFGoe_H4_b2R1_1h **Scan** 26662
**Peptide** TVTAMDVVYALKRQGRTLYGFGG **Charge** 4 **Spectral Angle** 0.62

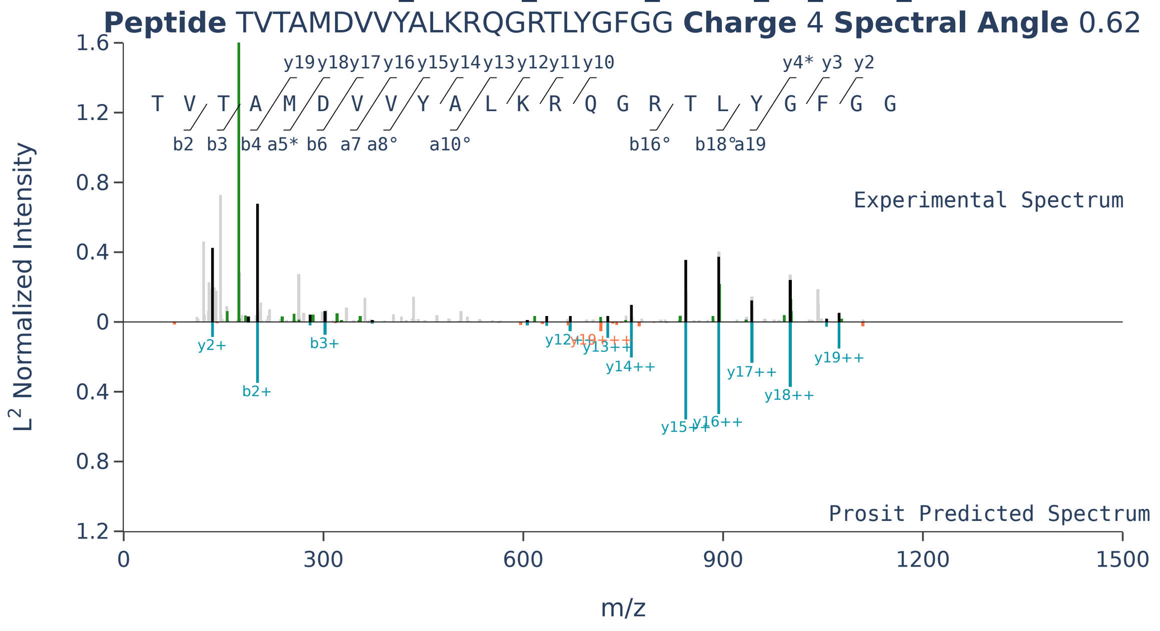

**Source** HRoetschke_291222_140123_HFGoe_H4_b2R2_1h **Scan** 20232
**Peptide** DAVTYTEHAKRKTVTAMDVVYALKRQGRTL **Charge** 5 **Spectral Angle** 0.75

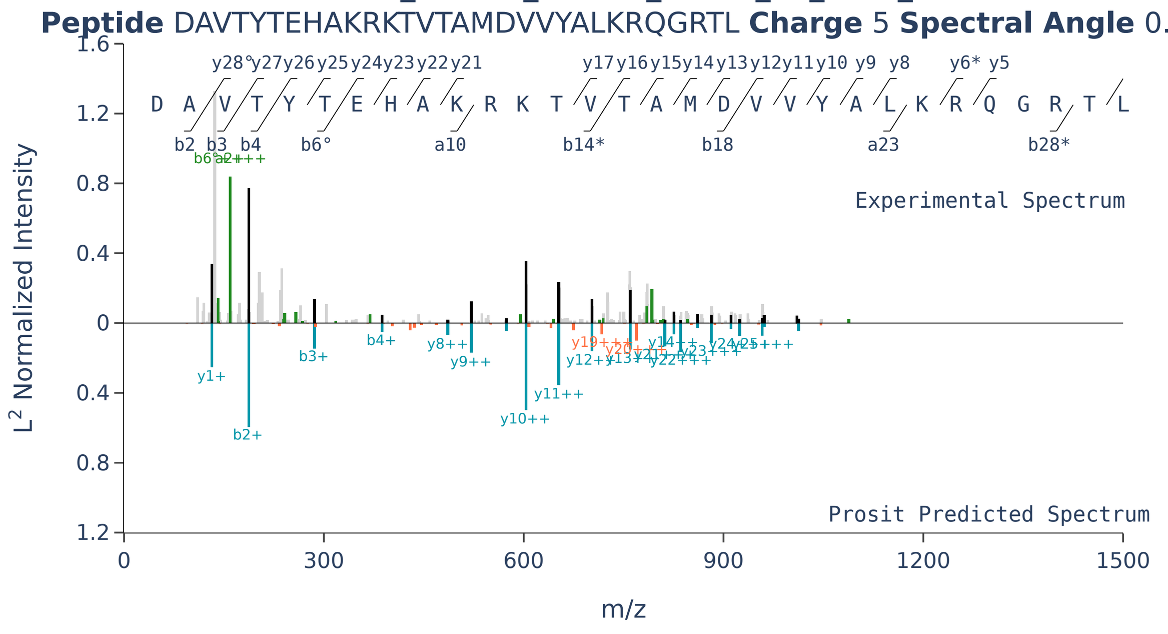


**Source** HRoetschke_291222_140123_HFGoe_H4_b1R1_1h **Scan** 26031
**Peptide** FLENVIRDAV **Charge** 2 **Spectral Angle** 0.79

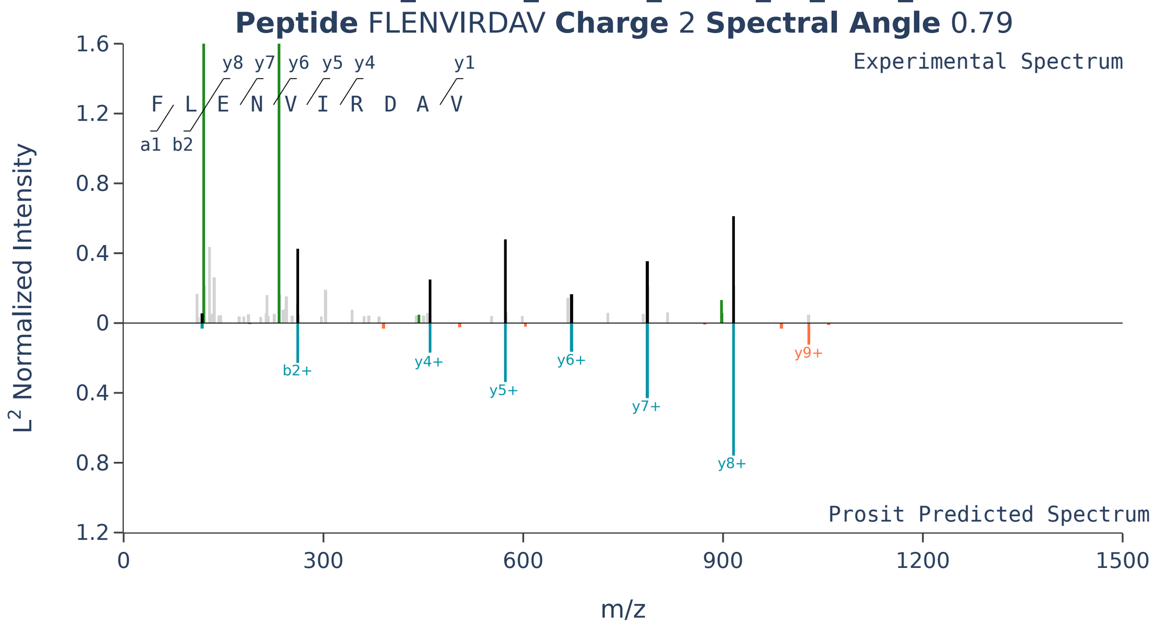


**Source** HRoetschke_291222_140123_HFGoe_H4_b1R2_2h **Scan** 23738
**Peptide** EETRGVLKVFLEN **Charge** 3 **Spectral Angle** 0.91

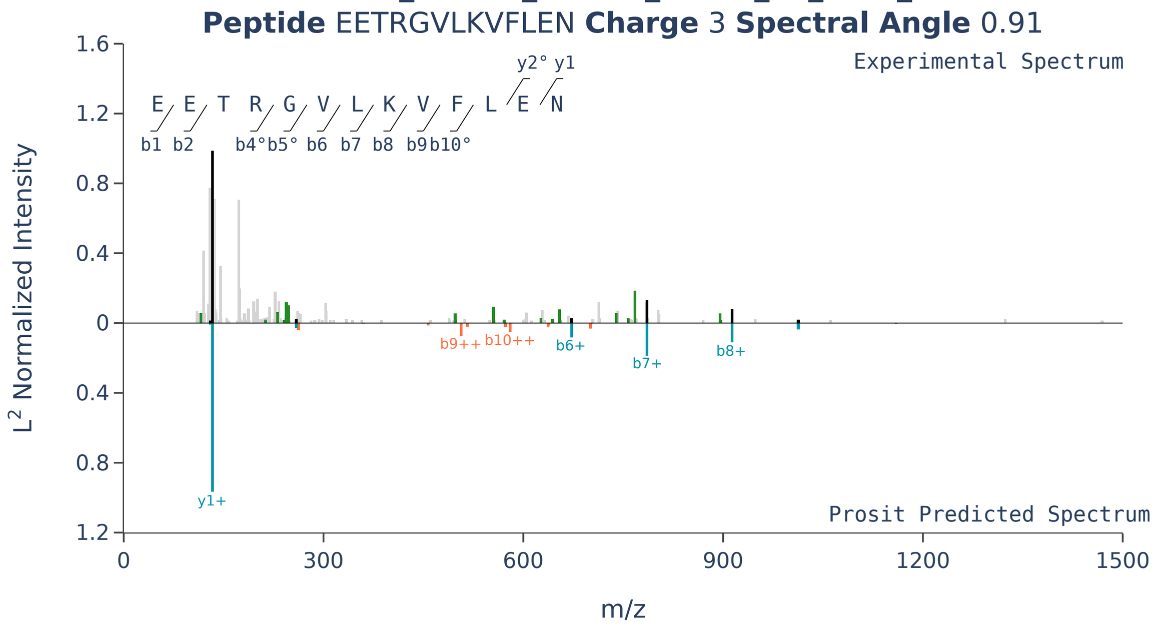


**Source** HRoetschke_291222_140123_HFGoe_H4_b2R1_24h **Scan** 8196
**Peptide** KGGAKRHRKVLRDNIQGITKPAIR **Charge** 5 **Spectral Angle** 0.86

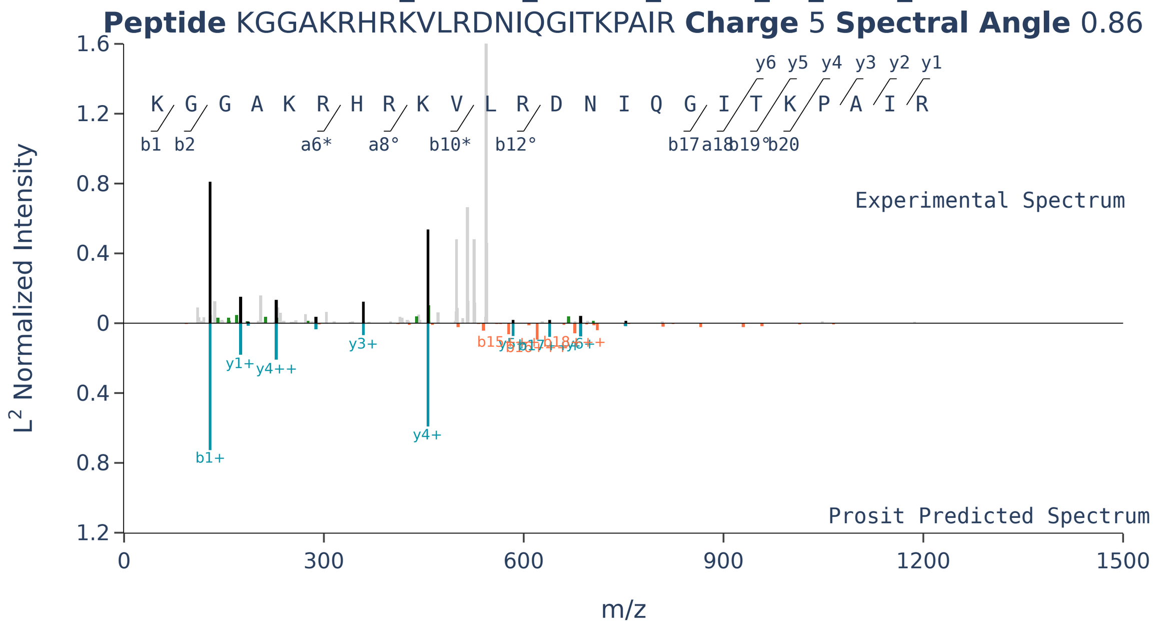


**Source** HRoetschke_291222_140123_HFGoe_H4_b2R2_1h **Scan** 7106
**Peptide** AVTYTEHAKRKTVTAMD **Charge** 2 **Spectral Angle** 0.73

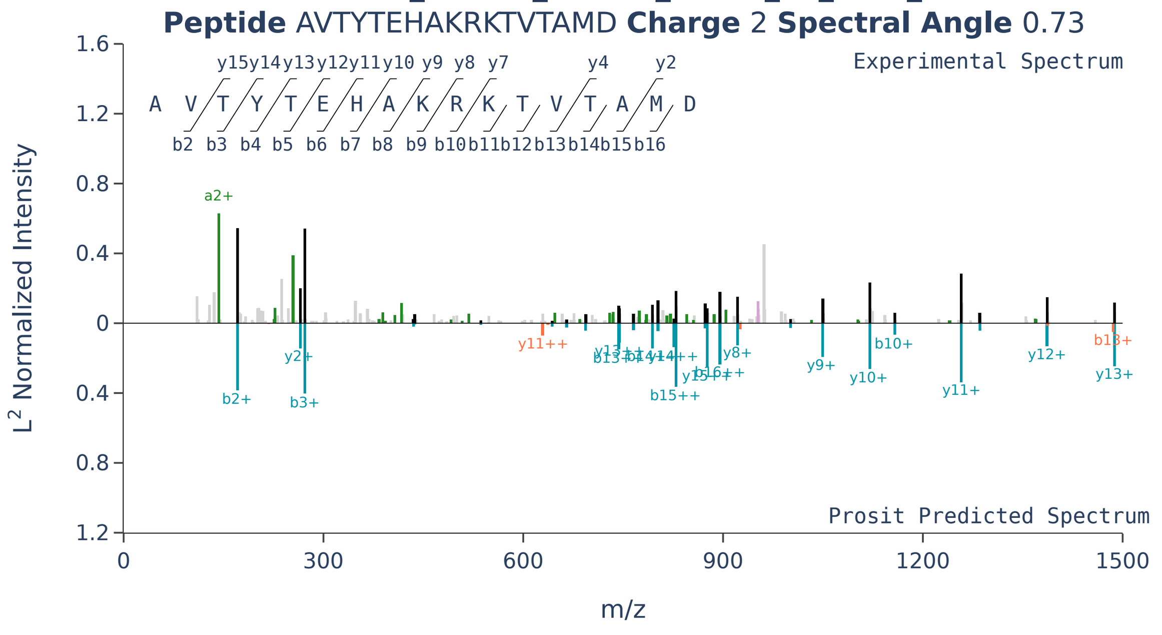


**Source** HRoetschke_291222_140123_HFGoe_H4_b2R1_24h **Scan** 12127
**Peptide** NIQGITKPAIRRLARRGGV **Charge** 4 **Spectral Angle** 0.67

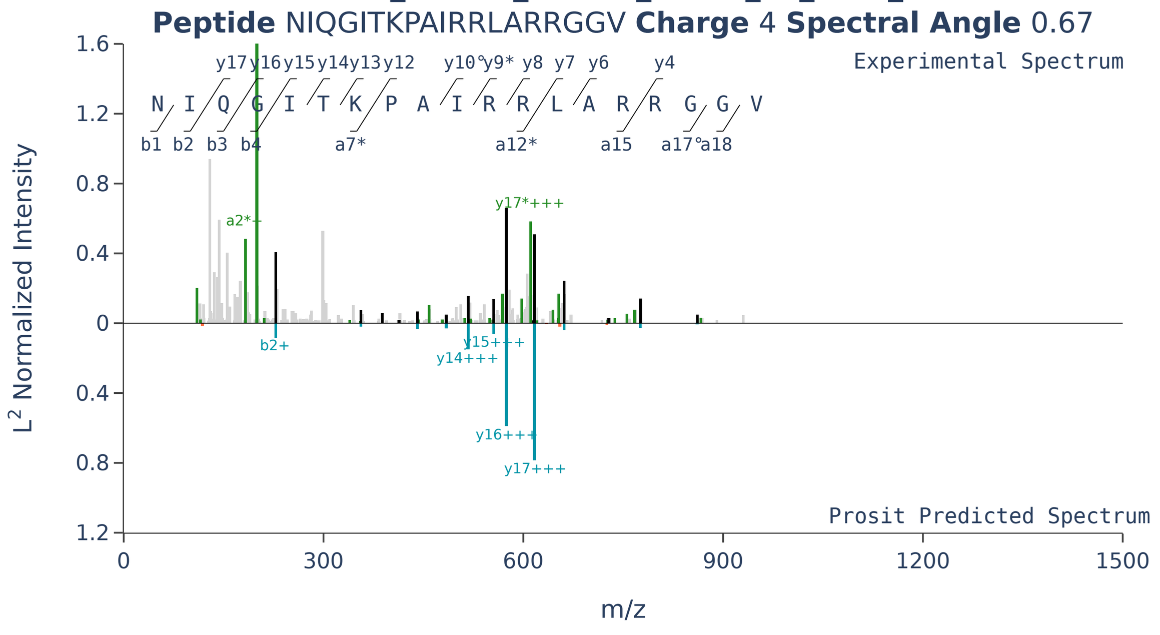


**Source** HRoetschke_291222_140123_HFGoe_H4_b1R1_2h **Scan** 13786
**Peptide** RGGVKRISGLIYEETRG **Charge** 4 **Spectral Angle** 0.79

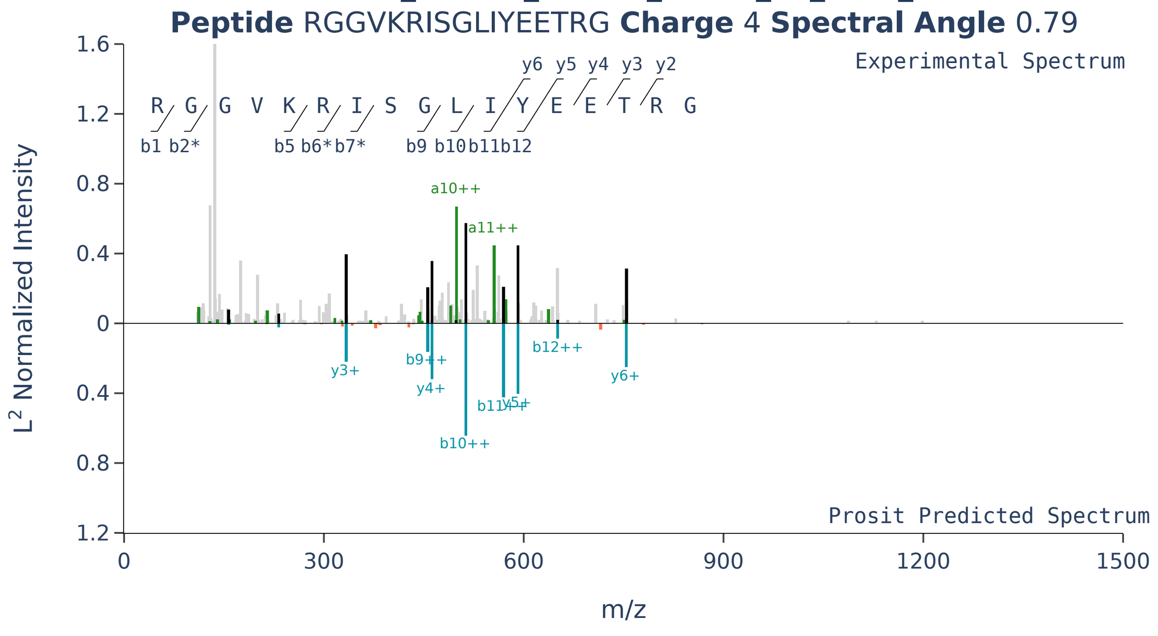


**Source** HRoetschke_291222_140123_HFGoe_H4_b2R1_1h **Scan** 36947
**Peptide** ETRGVLKVFLENVIRDAVTYT **Charge** 3 **Spectral Angle** 0.71

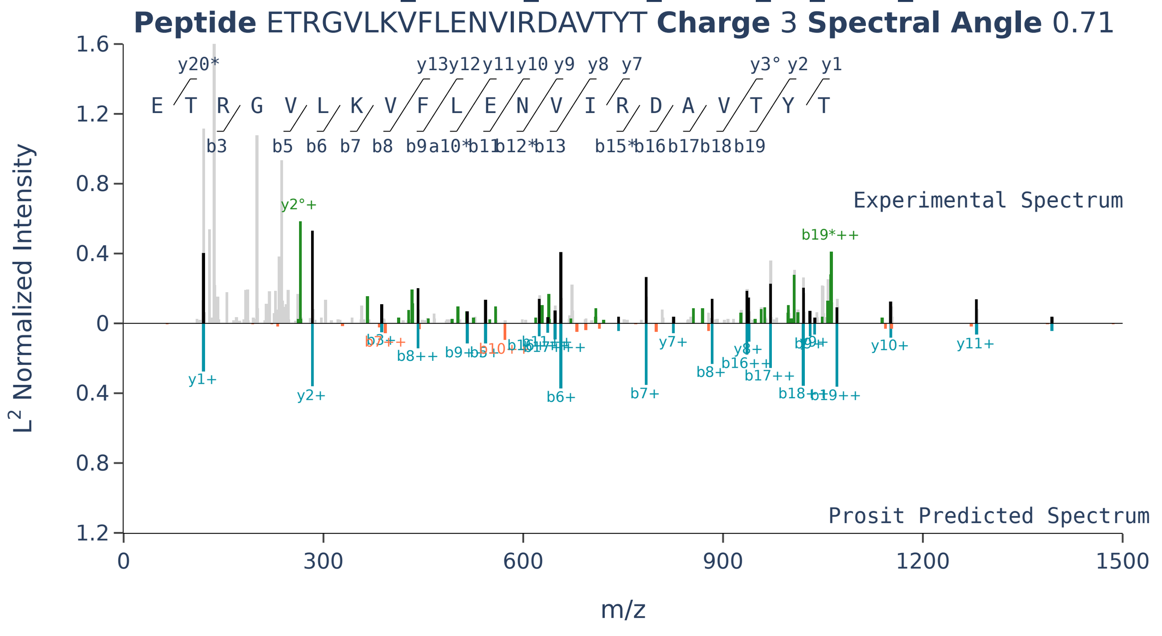


**Source** HRoetschke_291222_140123_HFGoe_H4_b2R1_24h **Scan** 10398
**Peptide** LQGITKPAIRRLARR **Charge** 4 **Spectral Angle** 0.44

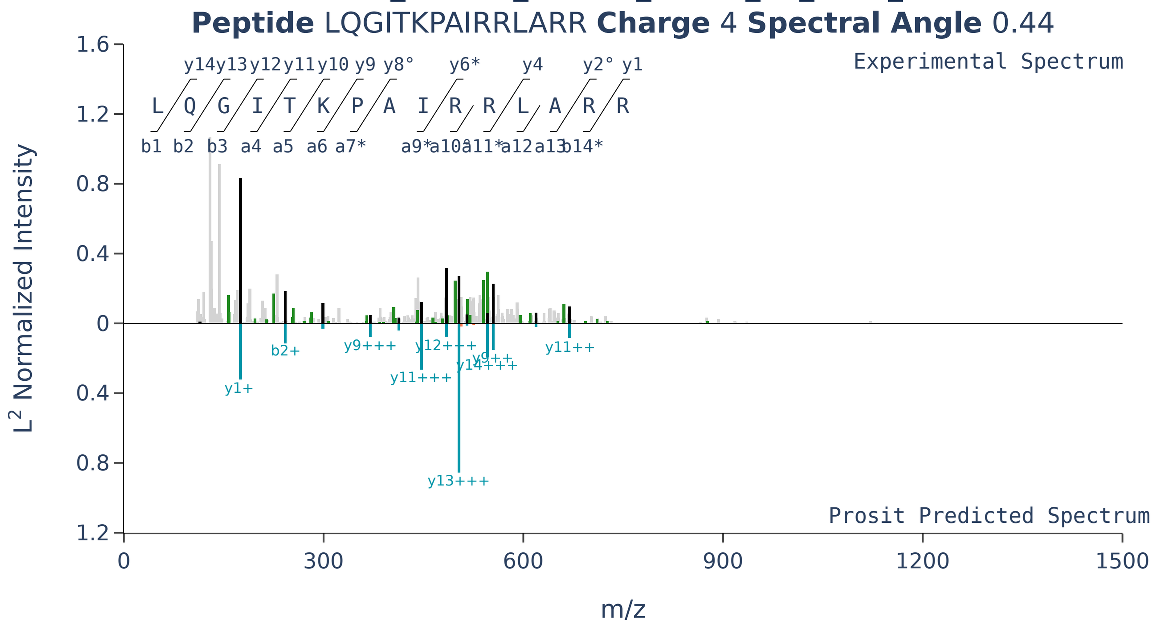


**Source** HRoetschke_291222_140123_HFGoe_H4_b1R1_4h **Scan** 8379
**Peptide** QGITKPAIRRLARRGGV **Charge** 4 **Spectral Angle** 0.79

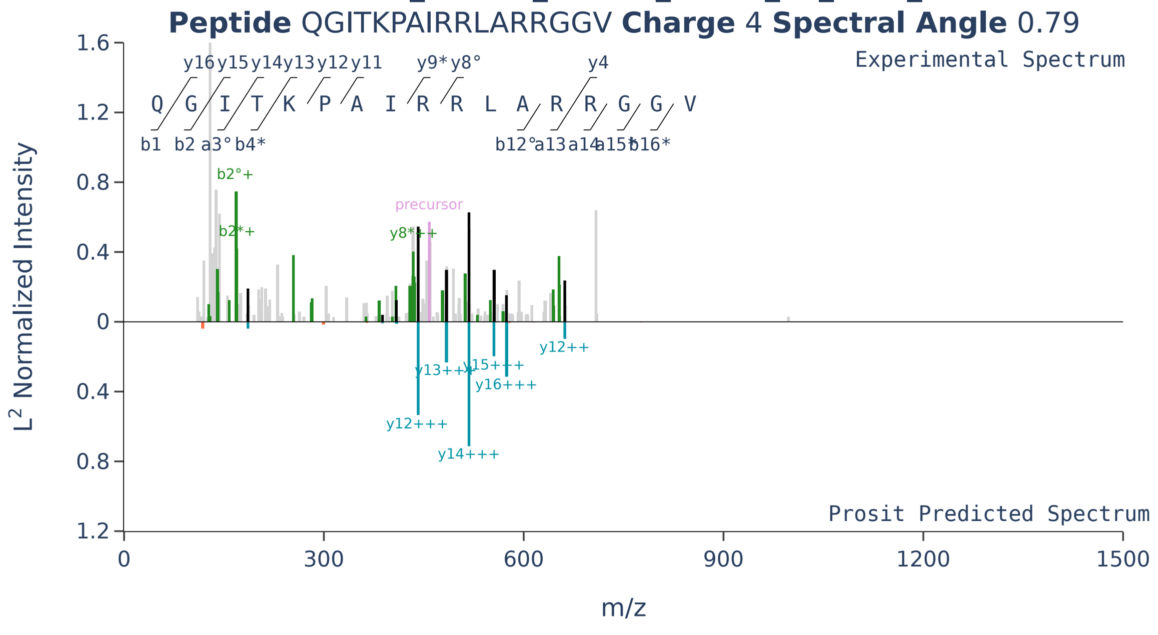


**Source** HRoetschke_291222_140123_HFGoe_H4_b2R1_1h **Scan** 29459
**Peptide** RGVLKVFLENVIRDAVTYTEHAKRKT **Charge** 5 **Spectral Angle** 0.68

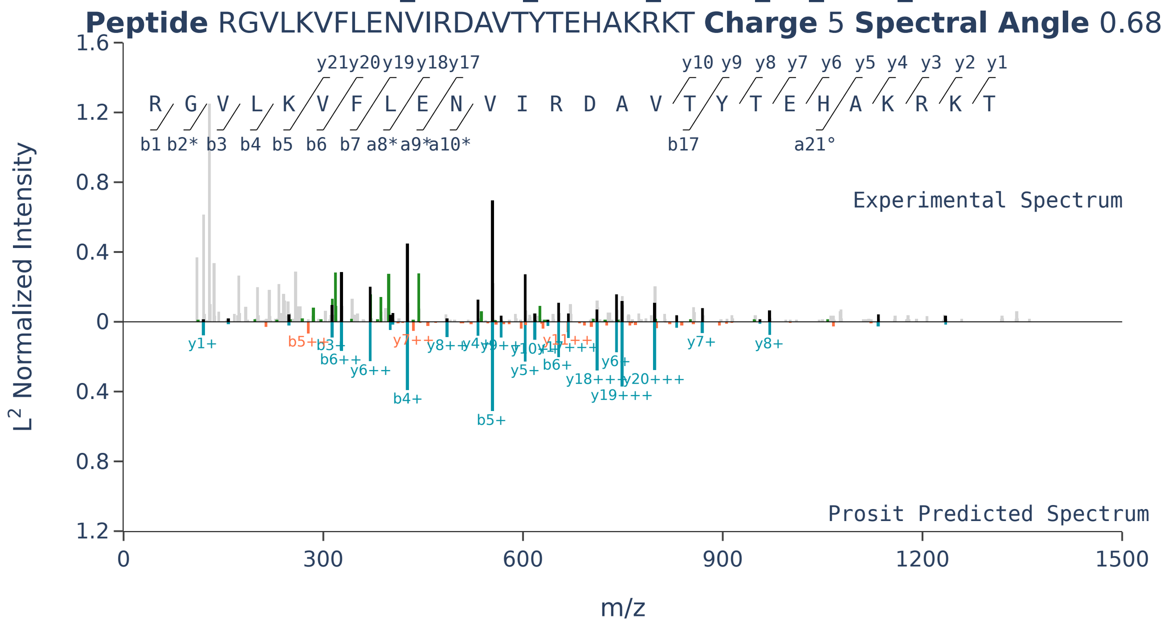


**Source** HRoetschke_291222_140123_HFGoe_H4_b1R1_2h **Scan** 12816
**Peptide** DNIQGITKPAIRRLARRGGVKRL **Charge** 5 **Spectral Angle** 0.78

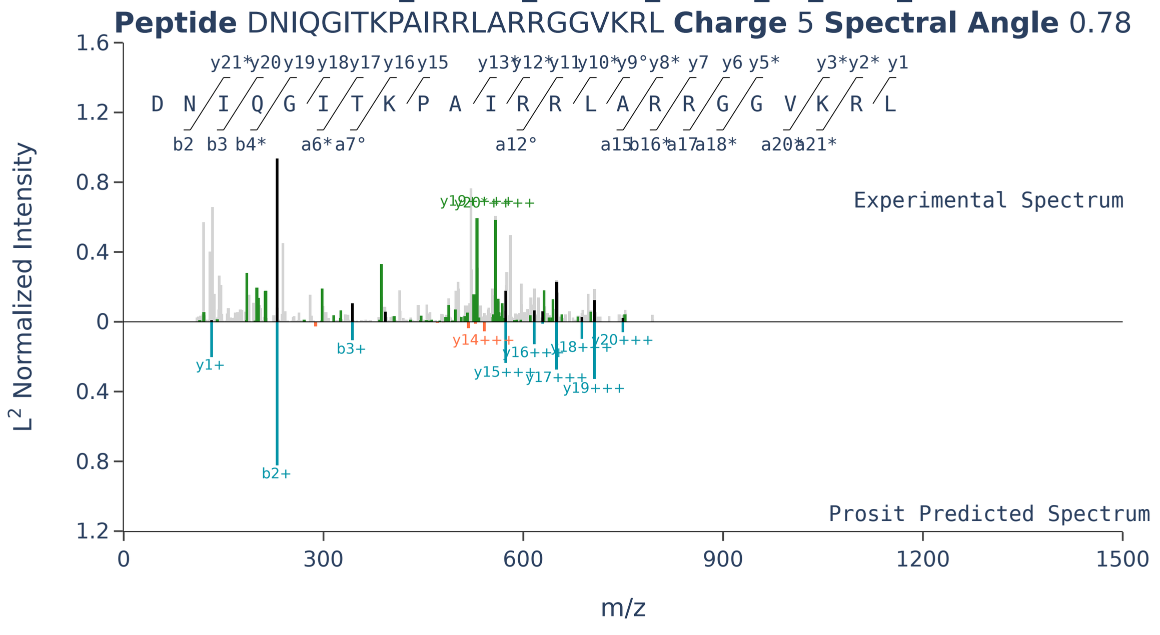


**Source** HRoetschke_291222_140123_HFGoe_H4_b1R1_24h **Scan** 7524
**Peptide** VIRDAVTYTEHAKRKTV **Charge** 3 **Spectral Angle** 0.79

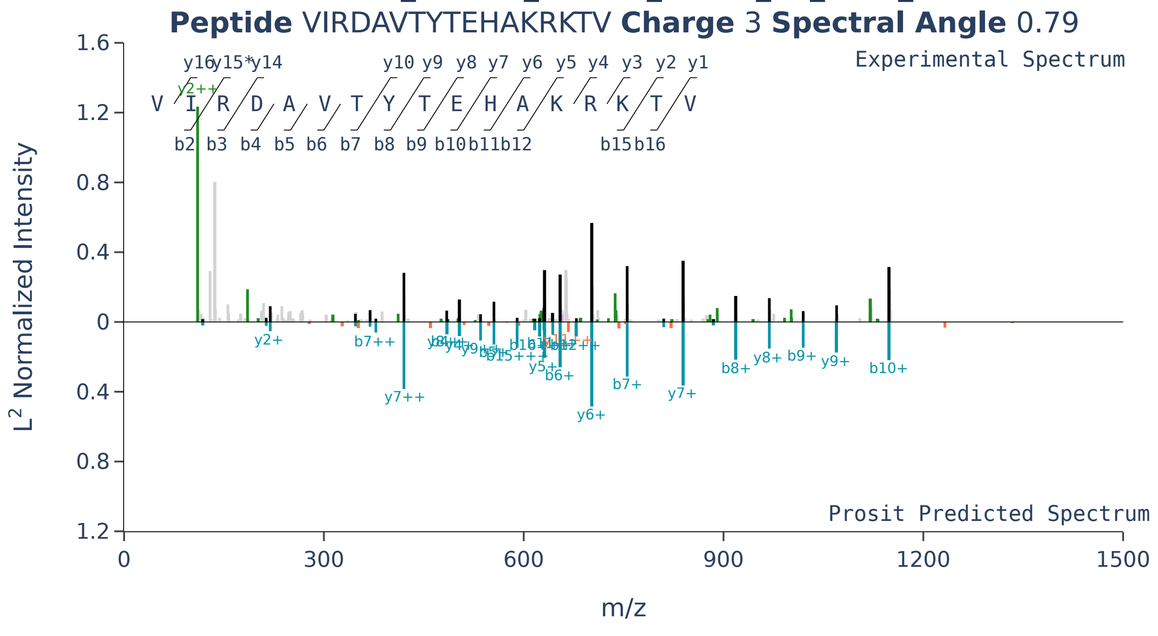


**Source** HRoetschke_291222_140123_HFGoe_H4_b2R2_1h **Scan** 8346
**Peptide** KVLRDNL **Charge** 2 **Spectral Angle** 0.68

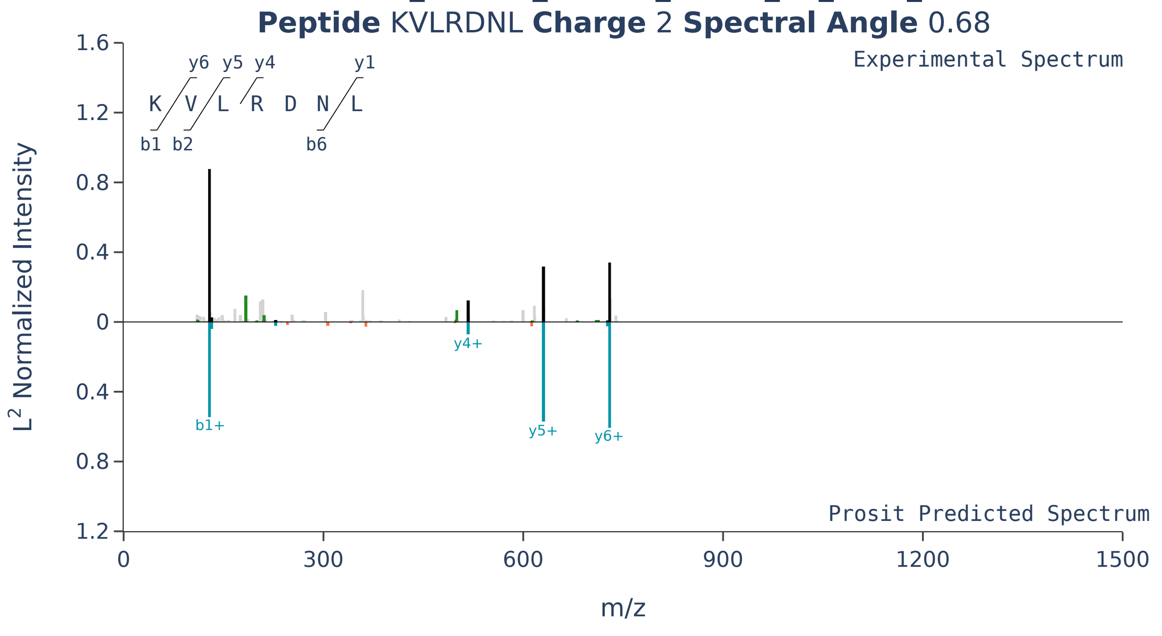


**Source** HRoetschke_291222_140123_HFGoe_H4_b1R1_4h **Scan** 13263
**Peptide** TKPAIRRLARRGGVKRISGLIYEET **Charge** 5 **Spectral Angle** 0.75

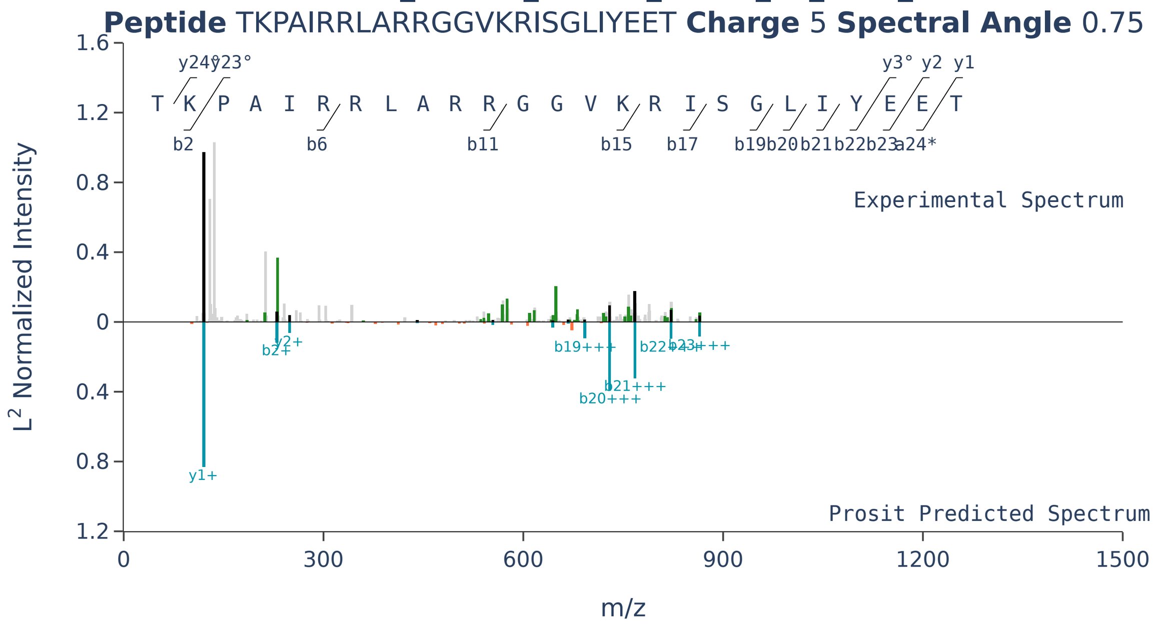

**Source** HRoetschke_291222_140123_HFGoe_H4_b2R1_1h **Scan** 30045
**Peptide** RGVLKVFLENVIRDAVTYTEHAKRKTVT **Charge** 5 **Spectral Angle** 0.67

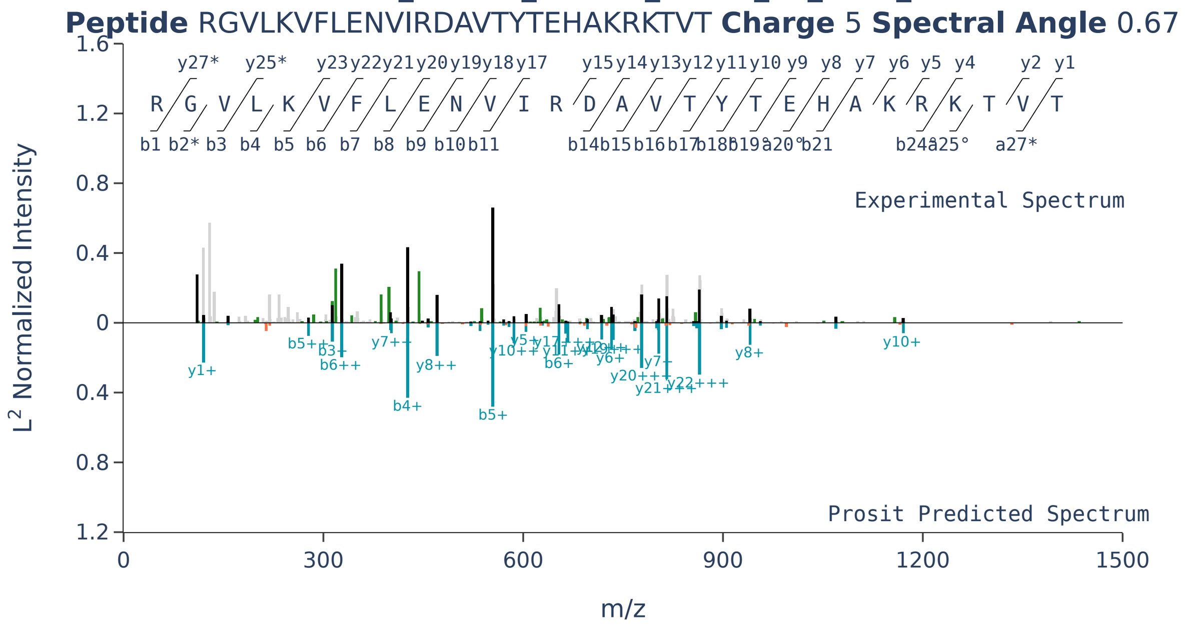


**Source** HRoetschke_291222_140123_HFGoe_H4_b1R2_24h **Scan** 5948
**Peptide** GKGGAKRHRKVLRDNIQGITK **Charge** 5 **Spectral Angle** 0.79

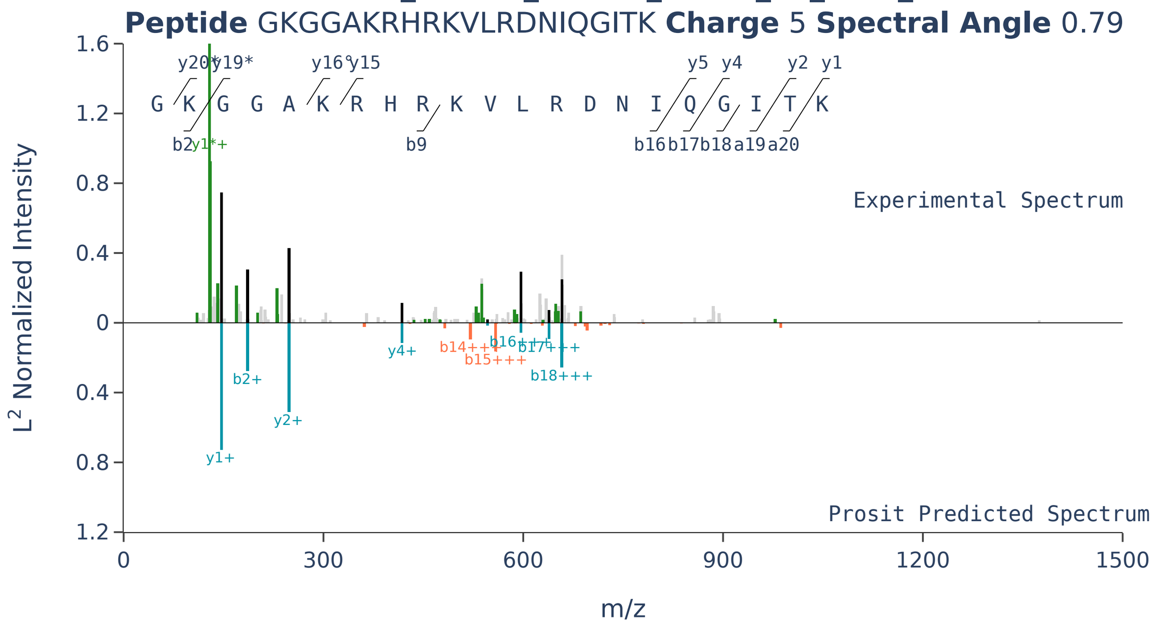


**Source** HRoetschke_291222_140123_HFGoe_H4_b1R1_24h **Scan** 24295
**Peptide** GVLKVFLEN **Charge** 2 **Spectral Angle** 0.83

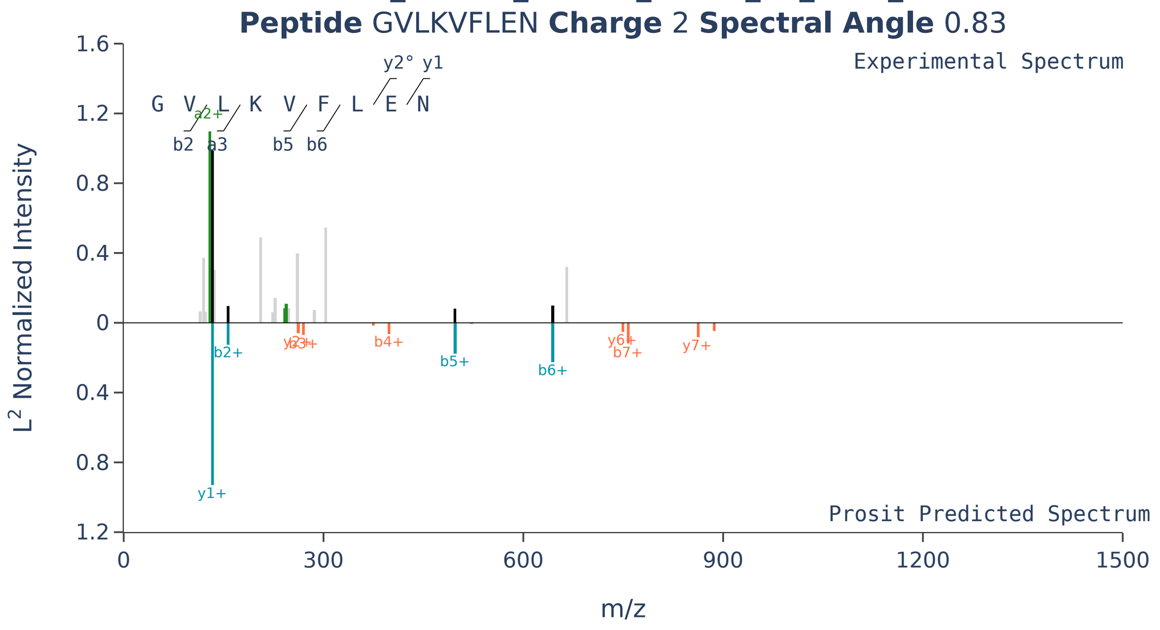


**Source** HRoetschke_291222_140123_HFGoe_H4_b1R2_2h **Scan** 13682
**Peptide** ITKPAIRRLARRGGVKRISGLIYE **Charge** 5 **Spectral Angle** 0.92

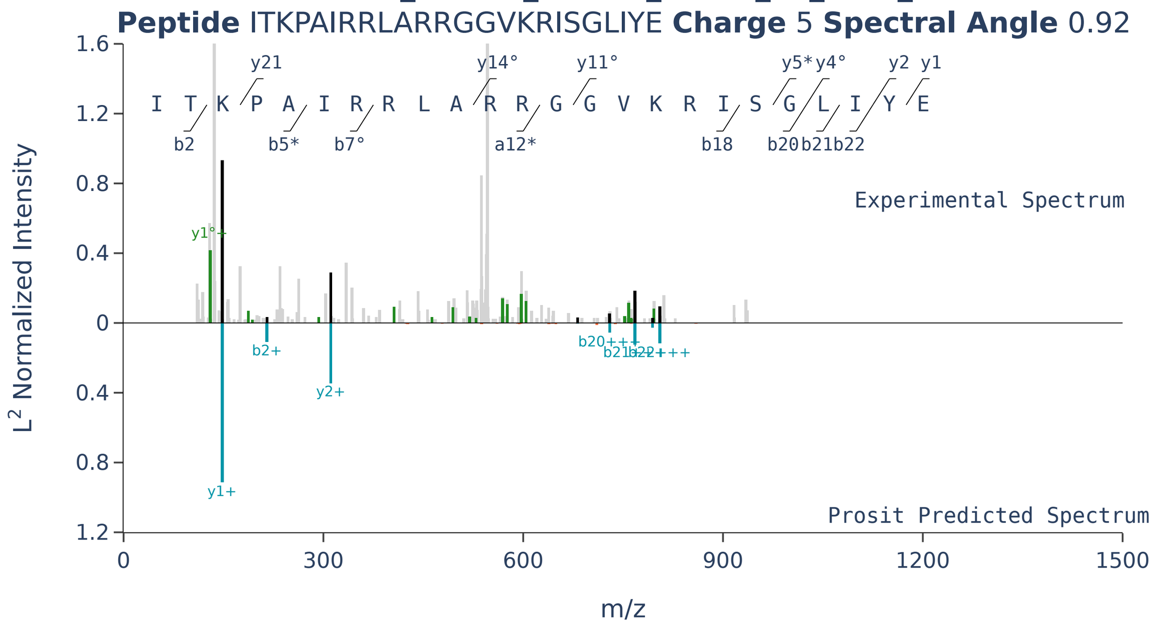


**Source** HRoetschke_291222_140123_HFGoe_H4_b1R2_24h **Scan** 5485
**Peptide** KRHRKVLRDNL **Charge** 4 **Spectral Angle** 0.69

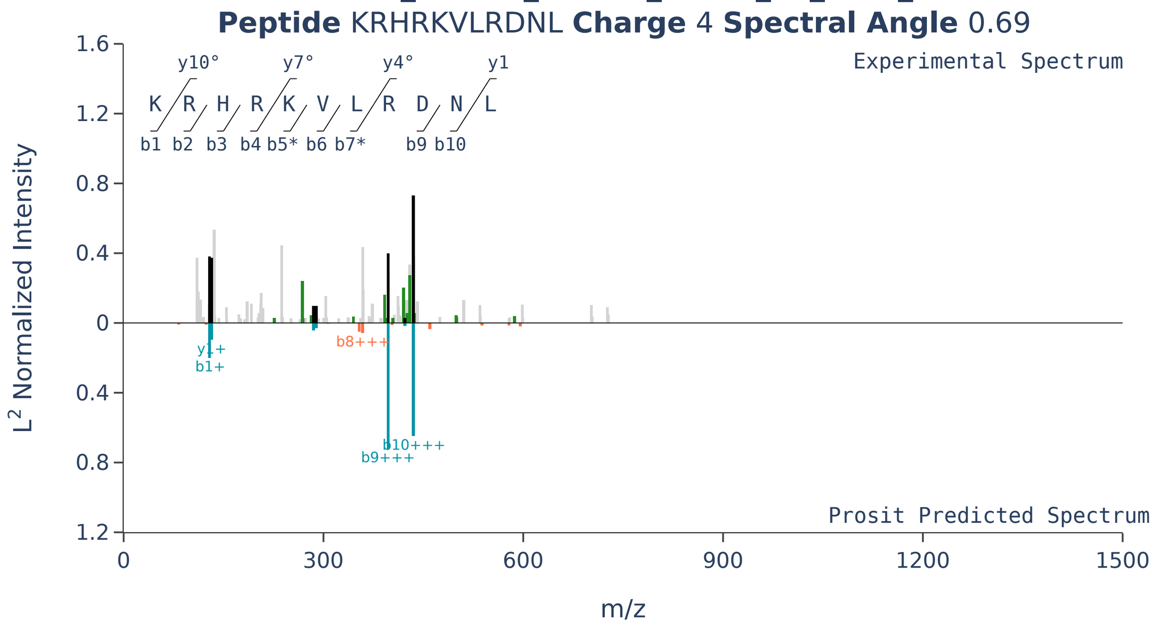


**Source** HRoetschke_291222_140123_HFGoe_H4_b1R1_1h **Scan** 16718
**Peptide** RDNIQGITKPAIRRLARRGGVKRISGLIYE **Charge** 6 **Spectral Angle** 0.75

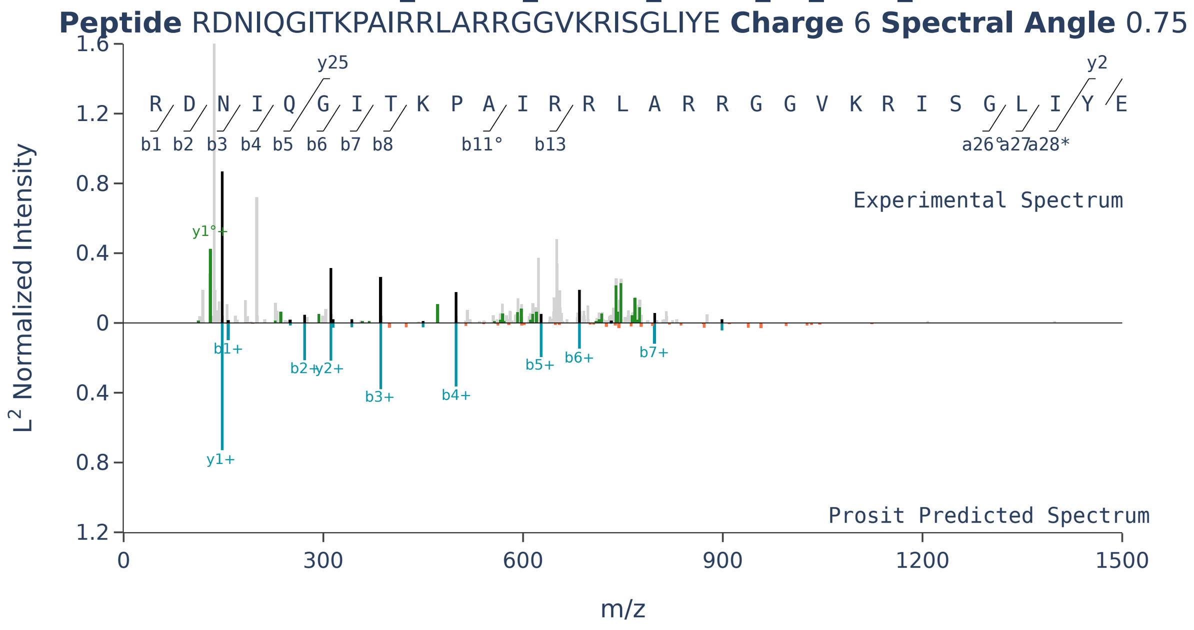


**Source** HRoetschke_291222_140123_HFGoe_H4_b1R1_1h **Scan** 29131
**Peptide** ETRGVLKVFLENVLR **Charge** 3 **Spectral Angle** 0.79

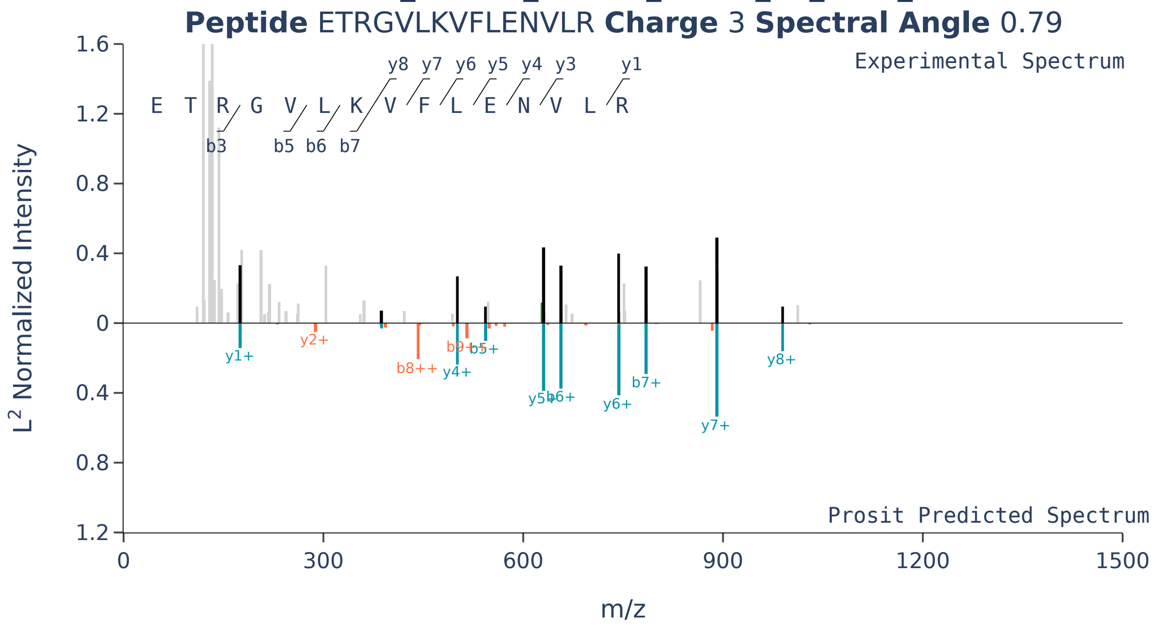


**Source** HRoetschke_291222_140123_HFGoe_H4_b1R1_24h **Scan** 12301
**Peptide** LARRGGVKRISGLIYE **Charge** 4 **Spectral Angle** 0.51

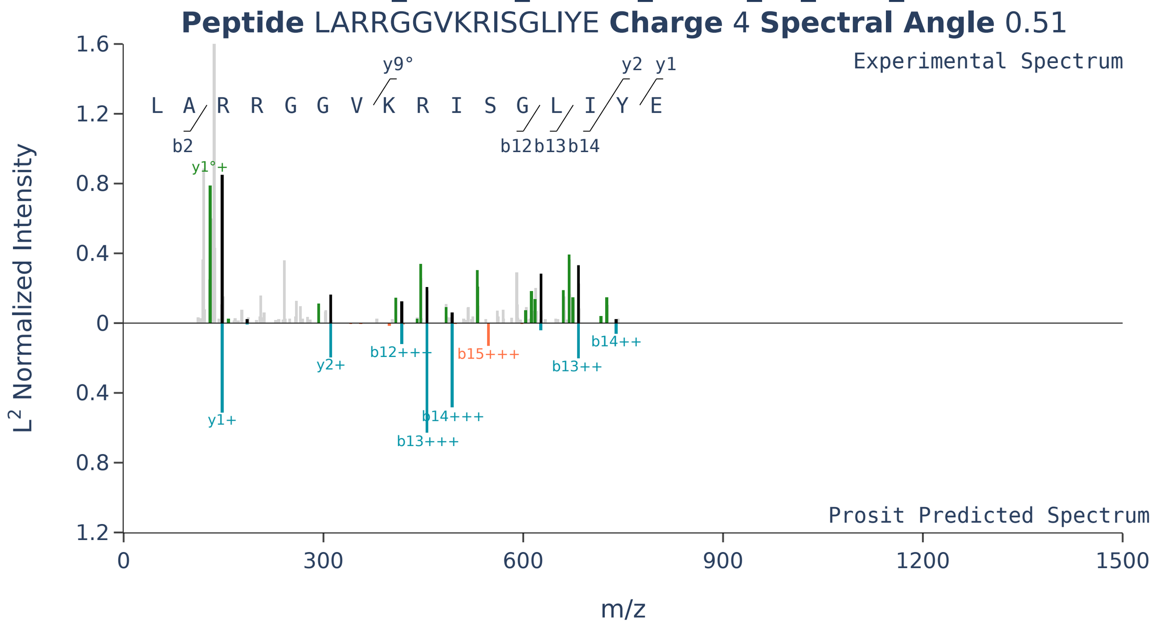


**Source** HRoetschke_291222_140123_HFGoe_H4_b2R1_1h **Scan** 29606
**Peptide** RGVLKVFLENVIRDAVTYTEHAKRK **Charge** 5 **Spectral Angle** 0.67

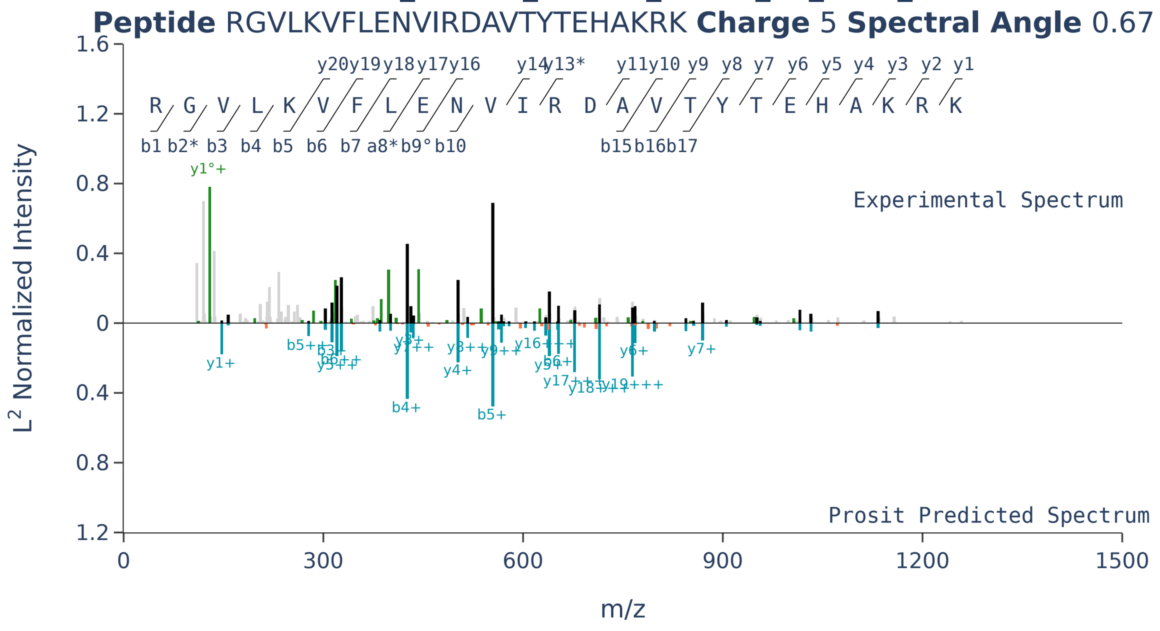


**Source** HRoetschke_291222_140123_HFGoe_H4_b2R1_24h **Scan** 8229
**Peptide** KRHRKVLRDNIQGITKPAIR **Charge** 5 **Spectral Angle** 0.76

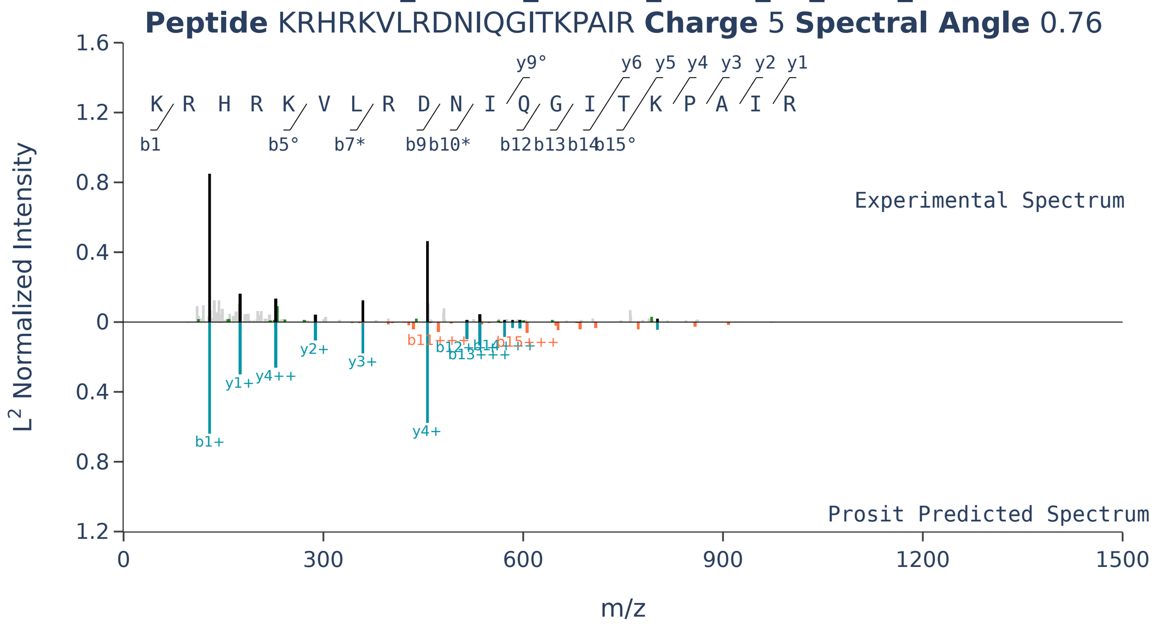


**Source** HRoetschke_291222_140123_HFGoe_H4_b1R1_2h **Scan** 14187
**Peptide** VLRDNIQGITKPAIRRLARR **Charge** 4 **Spectral Angle** 0.75

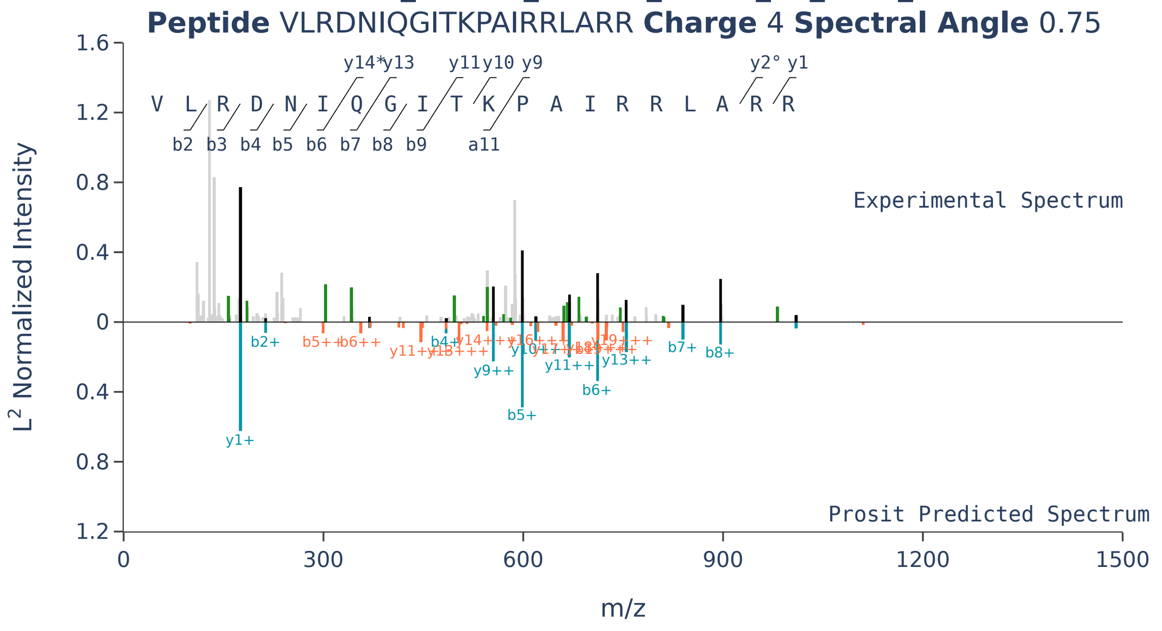


**Source** HRoetschke_291222_140123_HFGoe_H4_b2R2_24h **Scan** 8040
**Peptide** AKRHRKVLRDNIQGITKPALR **Charge** 5 **Spectral Angle** 0.79

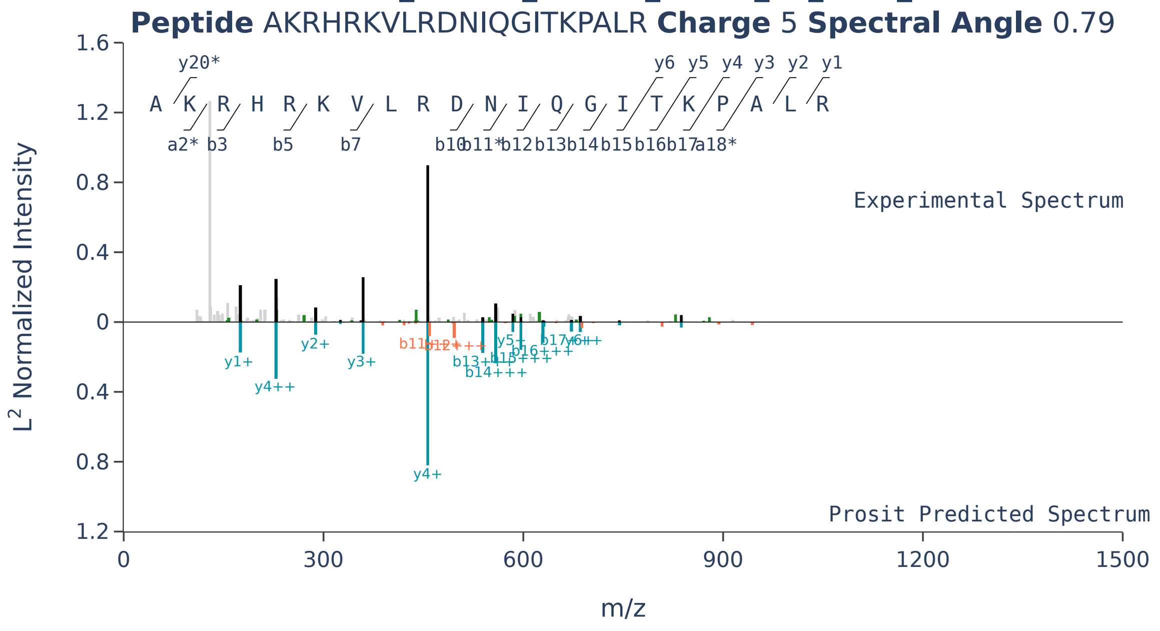


**Source** HRoetschke_291222_140123_HFGoe_H4_b2R2_1h **Scan** 21011
**Peptide** KRKTVTAMDVVYALKRQGRTLYGFGG **Charge** 4 **Spectral Angle** 0.79

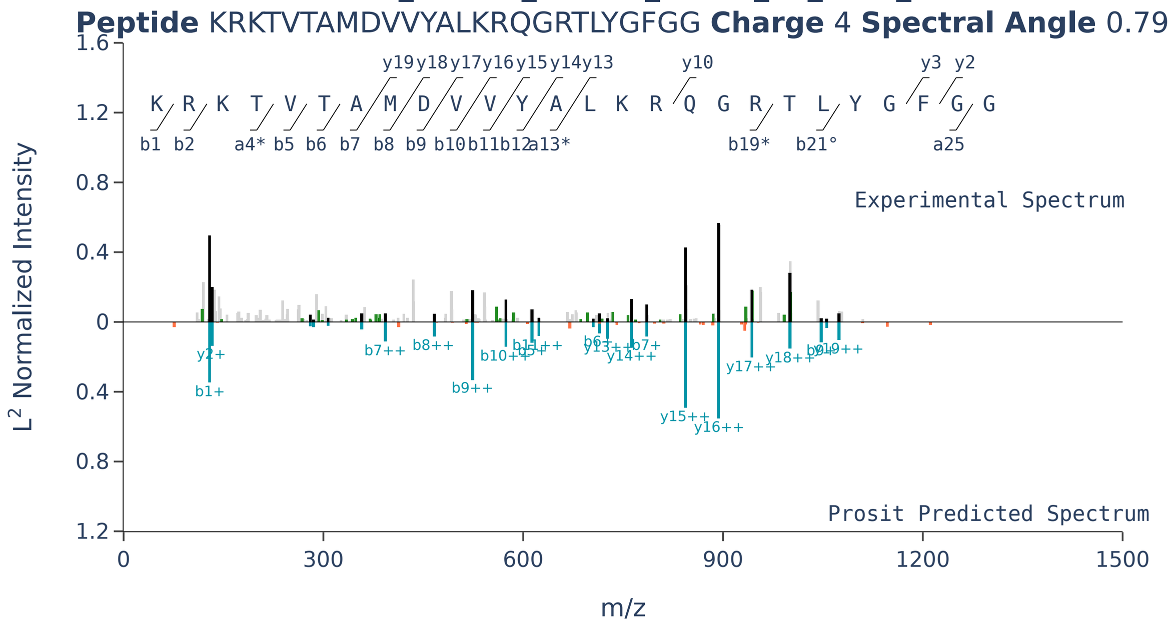


**Source** HRoetschke_291222_140123_HFGoe_H4_b1R1_24h **Scan** 6668
**Peptide** IRDAVTYTEHAKRKTVTA **Charge** 5 **Spectral Angle** 0.85

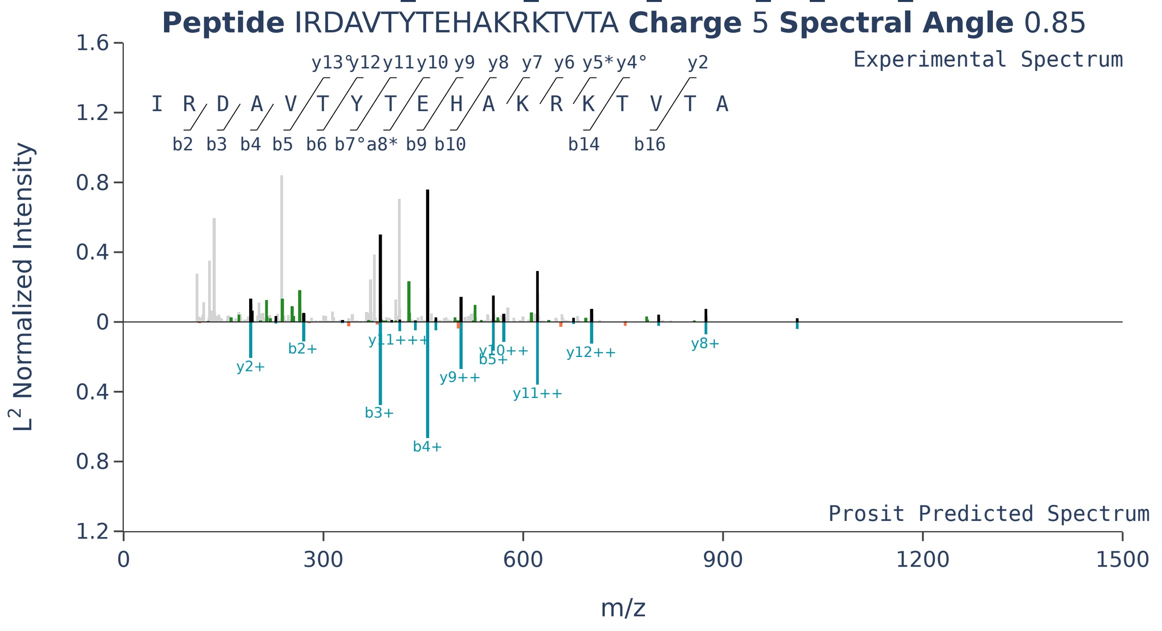


**Source** HRoetschke_291222_140123_HFGoe_H4_b2R1_1h **Scan** 24705
**Peptide** KVFLENVI **Charge** 2 **Spectral Angle** 0.44

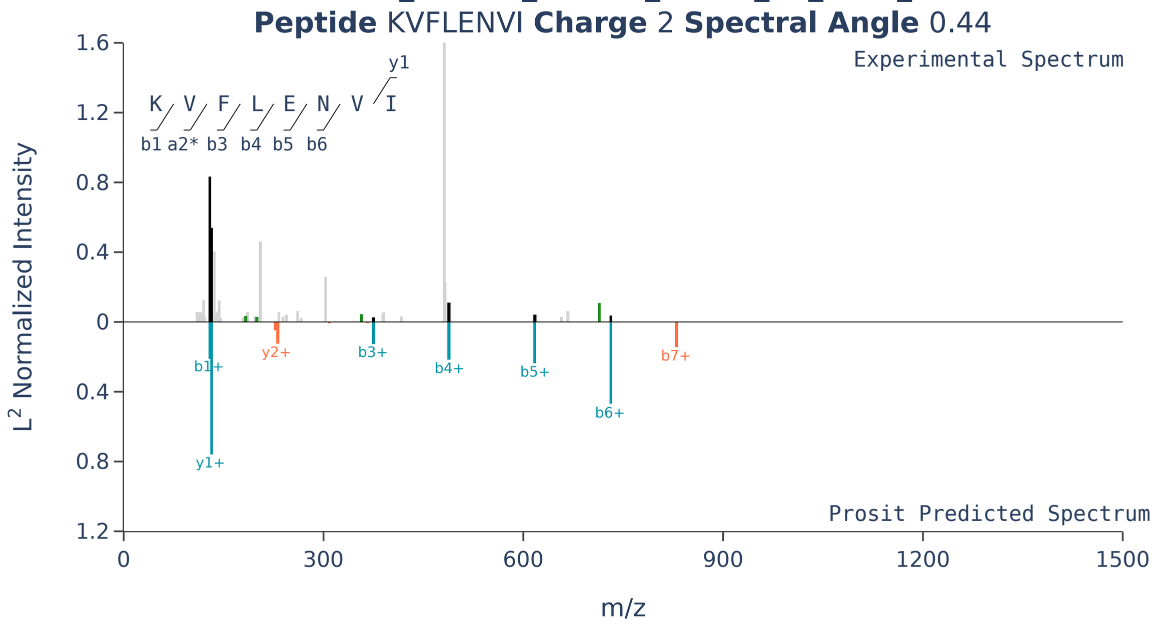

**Source** HRoetschke_291222_140123_HFGoe_H4_b2R1_1h **Scan** 29917
**Peptide** GVKRISGLIYEETRGVLKVFL **Charge** 4 **Spectral Angle** 0.62

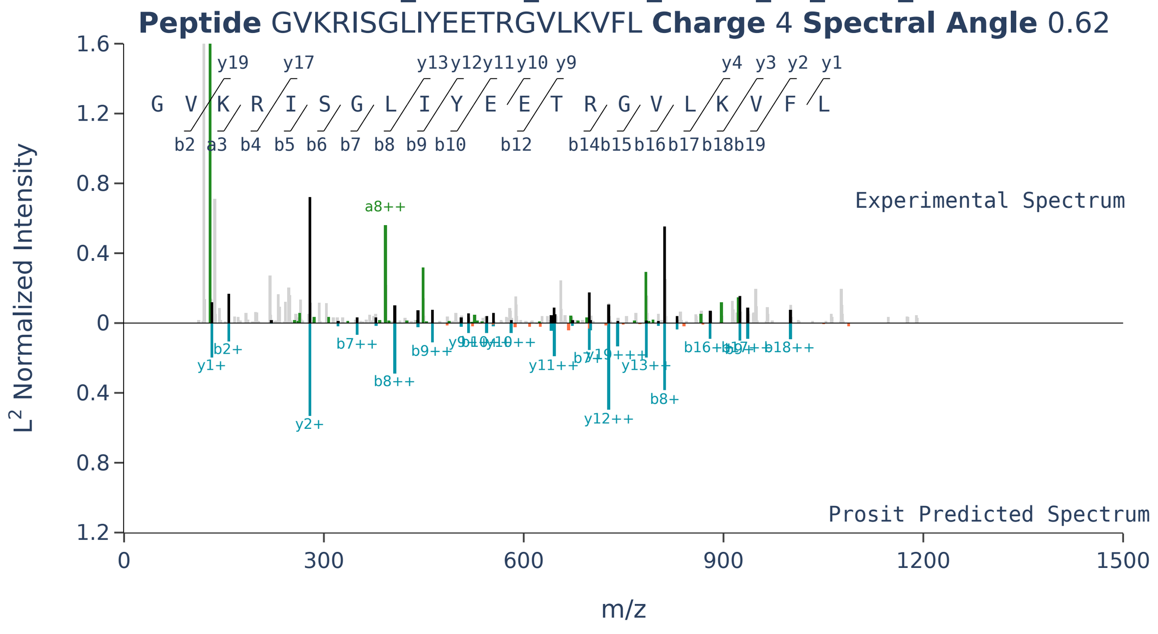


**Source** HRoetschke_291222_140123_HFGoe_H4_b2R1_1h **Scan** 13019
**Peptide** EHAKRKTVTAMDVVYALKRQG **Charge** 4 **Spectral Angle** 0.72

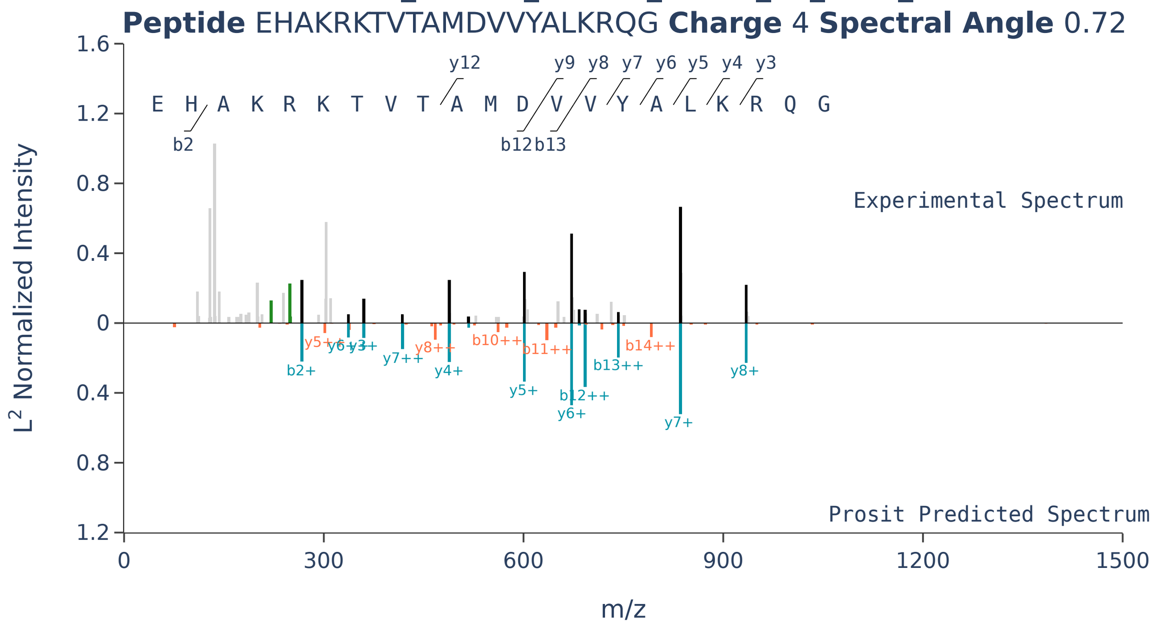


**Source** HRoetschke_291222_140123_HFGoe_H4_b2R1_4h **Scan** 18240
**Peptide** VLRDNIQGITKPAI **Charge** 3 **Spectral Angle** 0.65

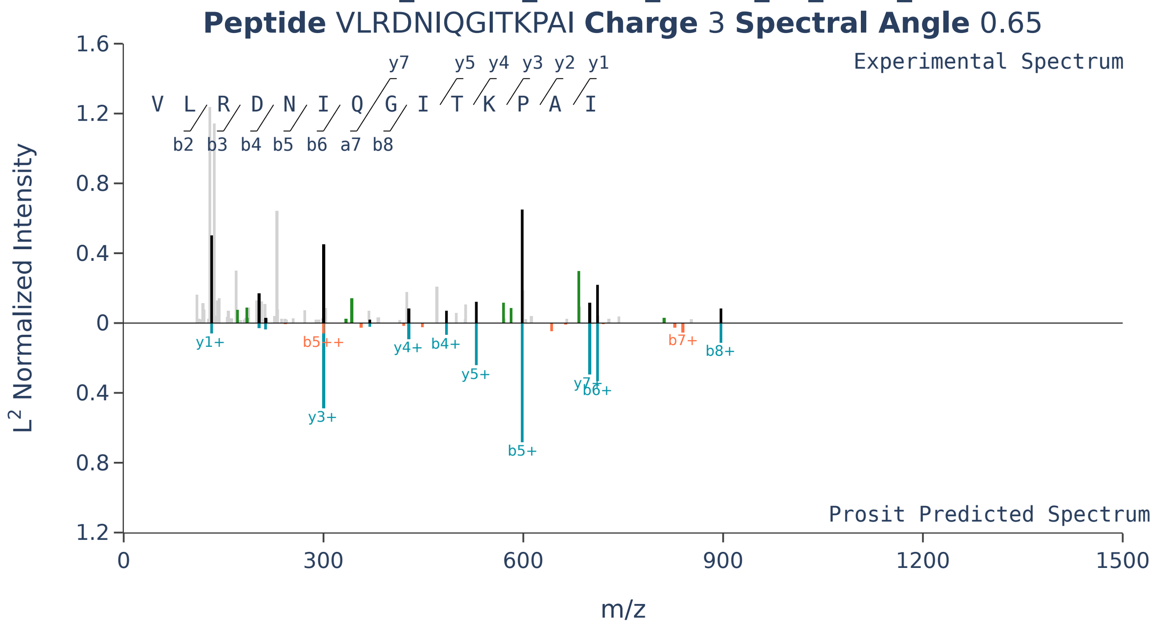


**Source** HRoetschke_291222_140123_HFGoe_H4_b1R1_4h **Scan** 6919
**Peptide** RDAVTYTEHAKRKTVTAM **Charge** 3 **Spectral Angle** 0.78

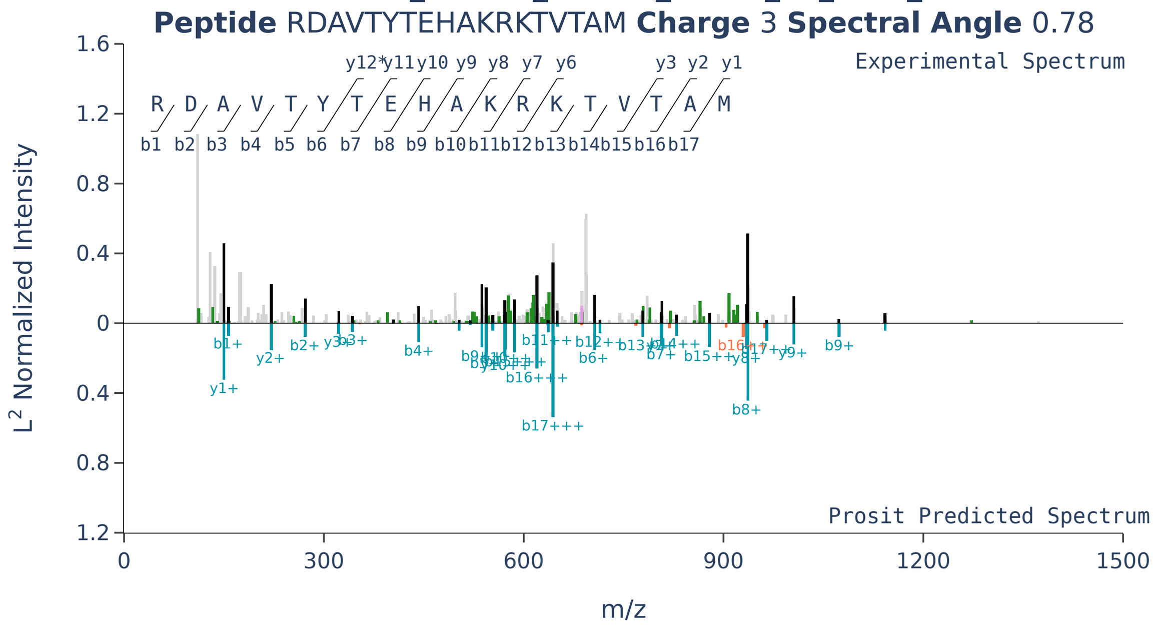


**Source** HRoetschke_291222_140123_HFGoe_H4_b1R1_4h **Scan** 19201
**Peptide** LENVIRDAVTYTEHAKR **Charge** 5 **Spectral Angle** 0.72

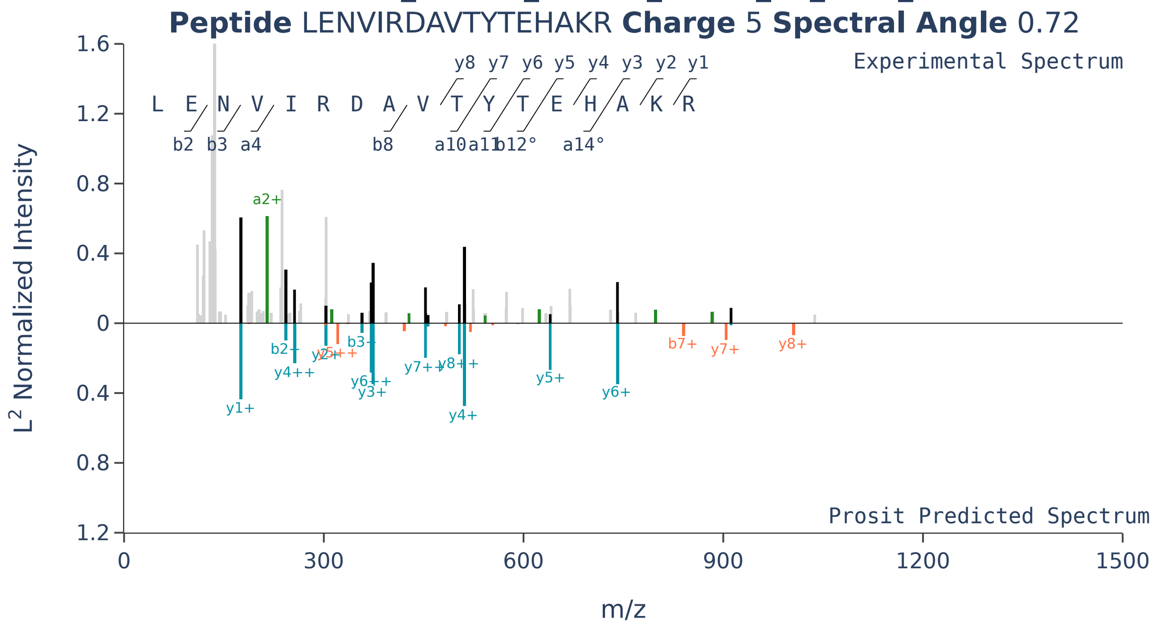


**Source** HRoetschke_291222_140123_HFGoe_H4_b1R1_1h **Scan** 15374
**Peptide** IRDAVTYTEHAKRKTVTAMDVVYA **Charge** 5 **Spectral Angle** 0.66

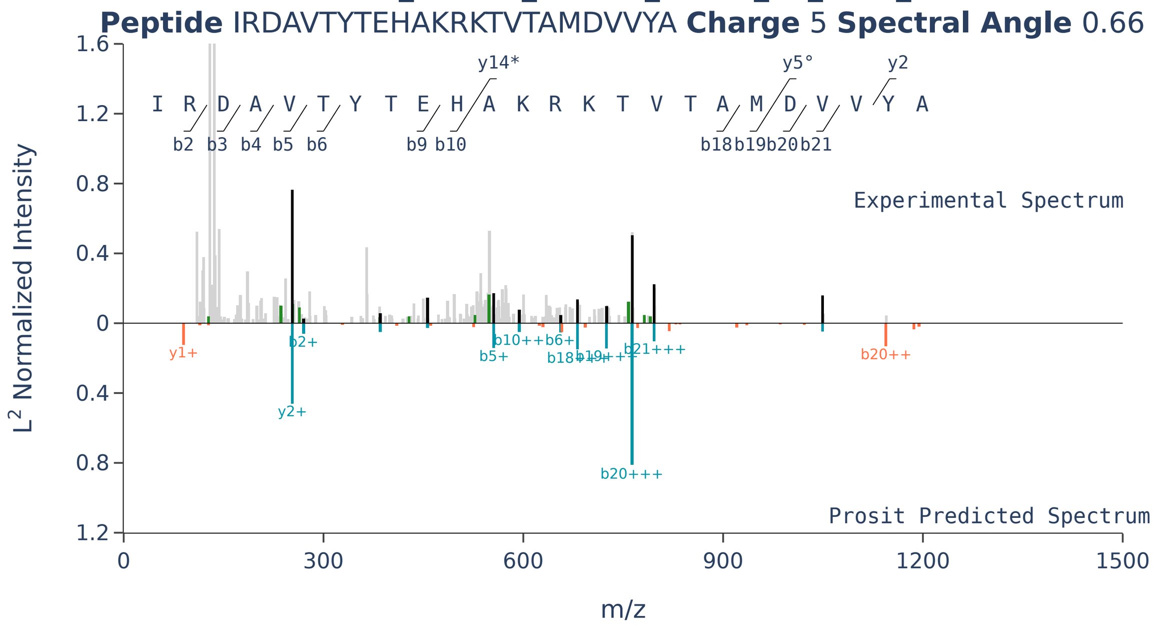


**Source** HRoetschke_291222_140123_HFGoe_H4_b1R1_2h **Scan** 9591
**Peptide** IQGITKPAIRRLARR **Charge** 3 **Spectral Angle** 0.68

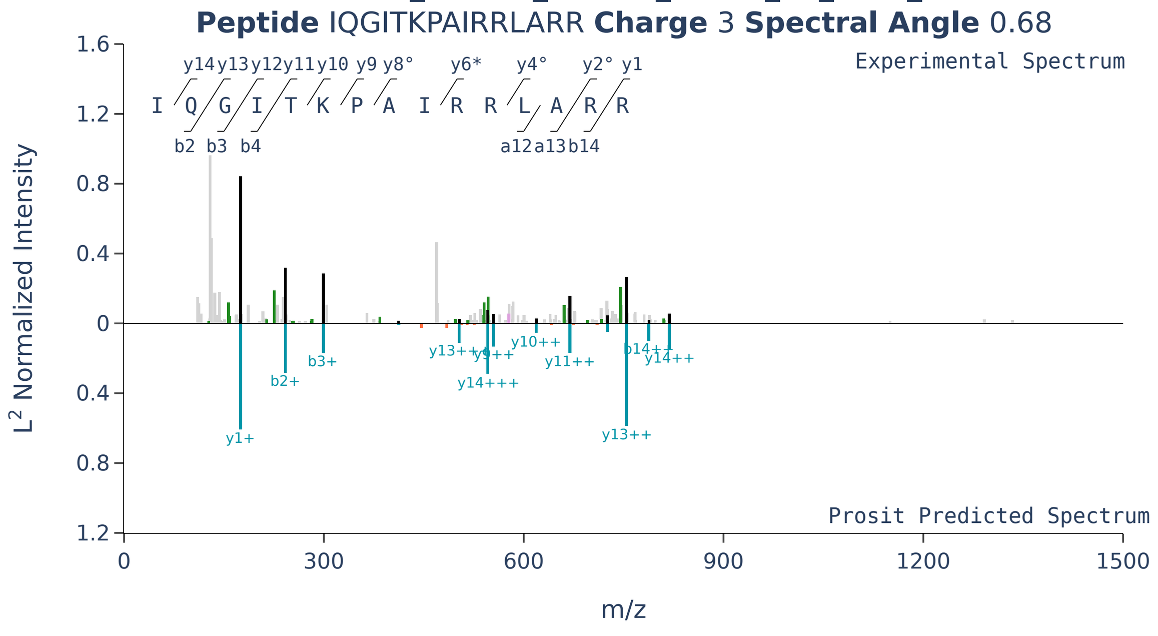


**Source** HRoetschke_291222_140123_HFGoe_H4_b1R1_24h **Scan** 5820
**Peptide** RDAVTYTEHAKRKTVTAMD **Charge** 3 **Spectral Angle** 0.67

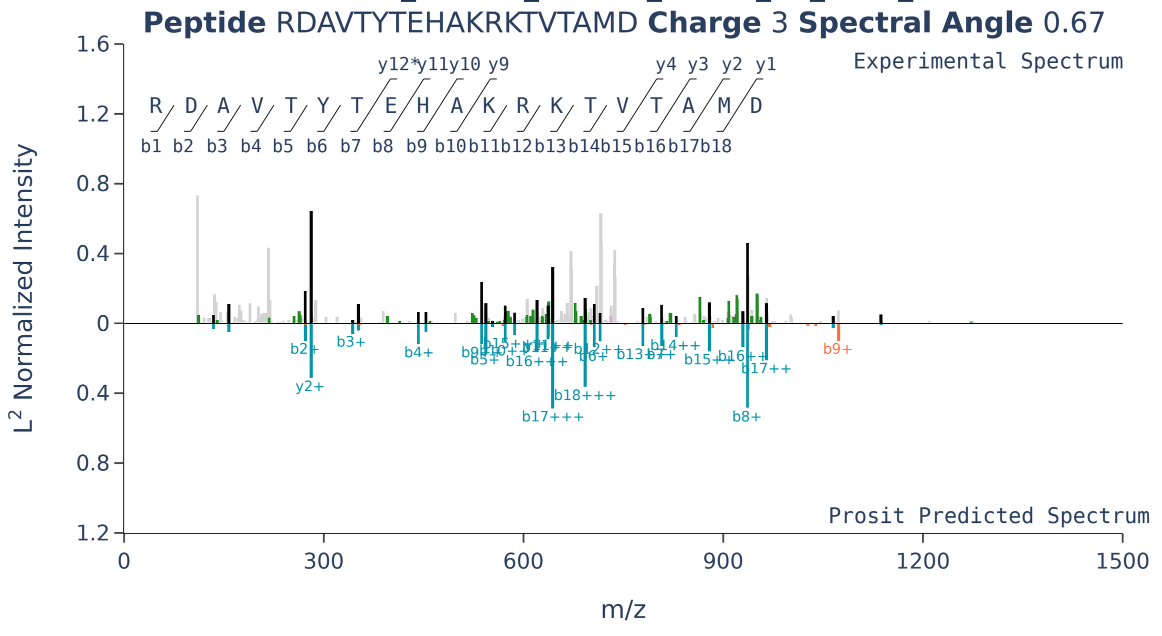


**Source** HRoetschke_291222_140123_HFGoe_H4_b2R1_1h **Scan** 29366
**Peptide** TRGVLKVFLENVI **Charge** 2 **Spectral Angle** 0.68

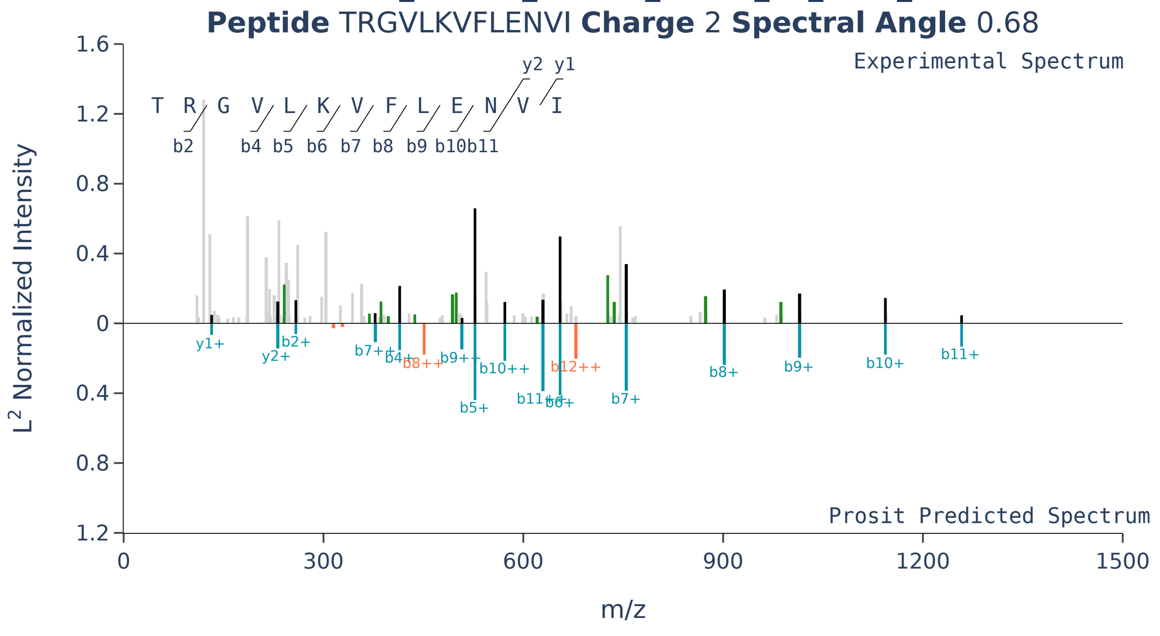


**Source** HRoetschke_291222_140123_HFGoe_H4_b1R2_2h **Scan** 13676
**Peptide** LTKPAIRRLARRGGVKRISGLIYE **Charge** 5 **Spectral Angle** 0.92

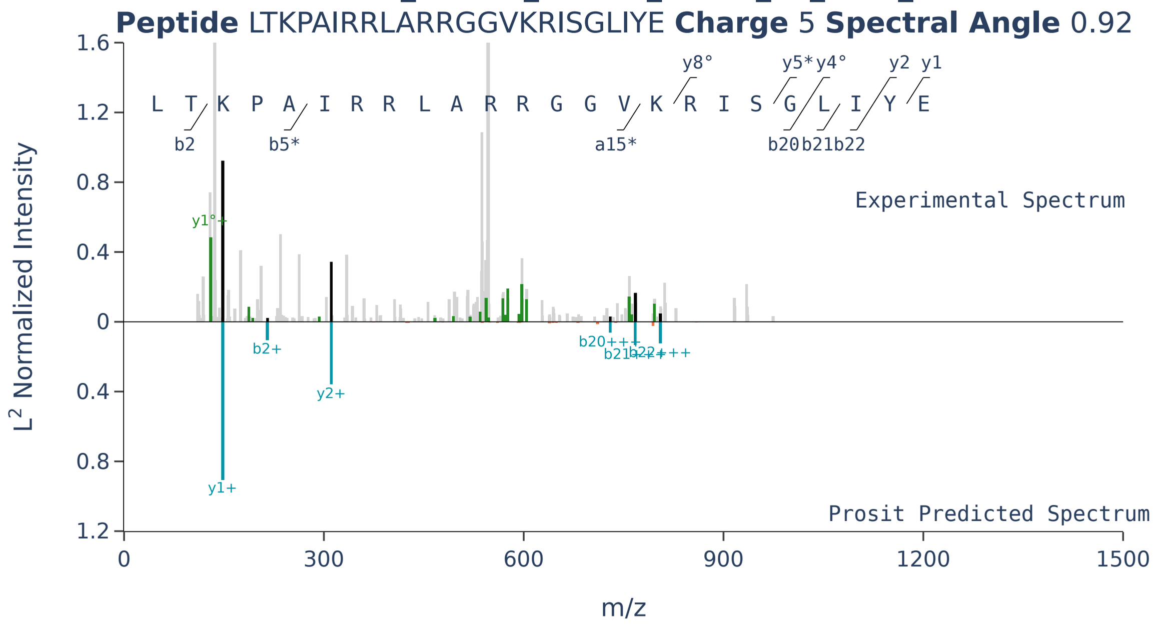


**Source** HRoetschke_291222_140123_HFGoe_H4_b2R1_1h **Scan** 21698
**Peptide** ENVIRDAVTYTEHAKRKTVTAMDVVYA **Charge** 4 **Spectral Angle** 0.58

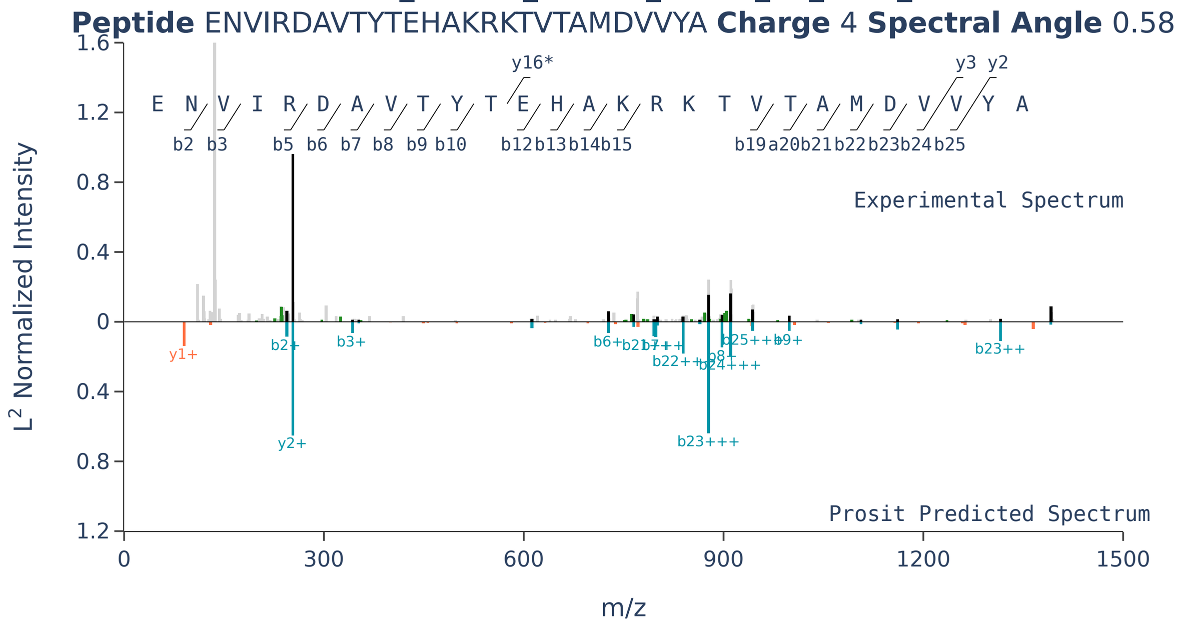


**Source** HRoetschke_291222_140123_HFGoe_H4_b2R1_1h **Scan** 31930
**Peptide** RGVLKVFLENVIRDAVTYTEHAKR **Charge** 5 **Spectral Angle** 0.73

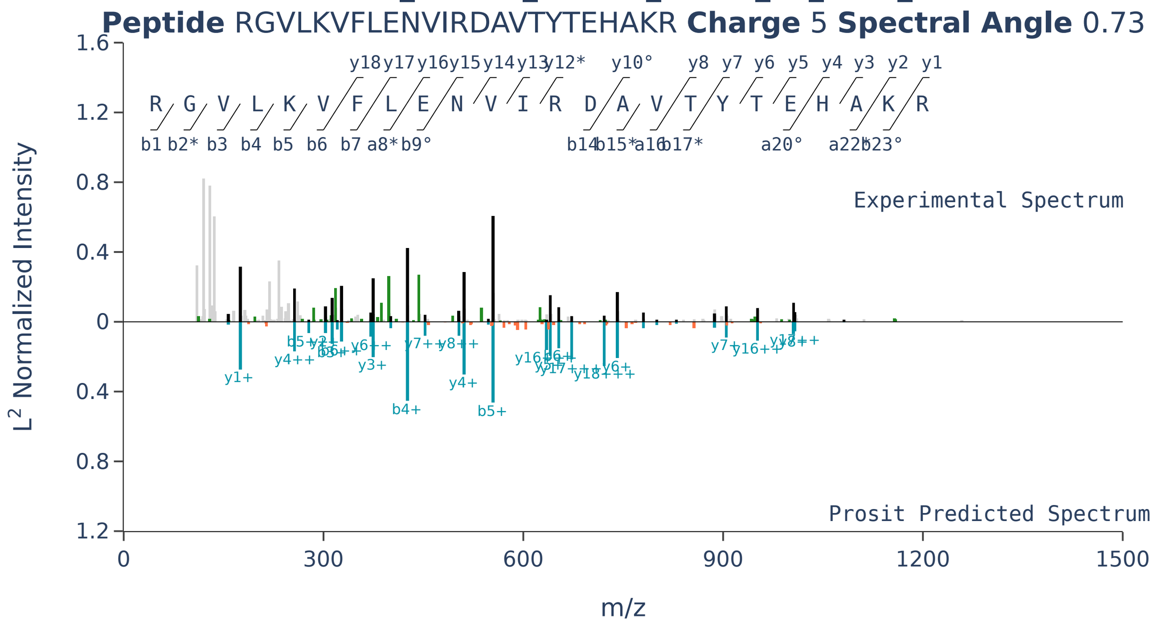


**Source** HRoetschke_291222_140123_HFGoe_H4_b1R1_4h **Scan** 5264
**Peptide** SGRGKGGKGLGKGGAKRHRKVLRDNL **Charge** 6 **Spectral Angle** 0.67

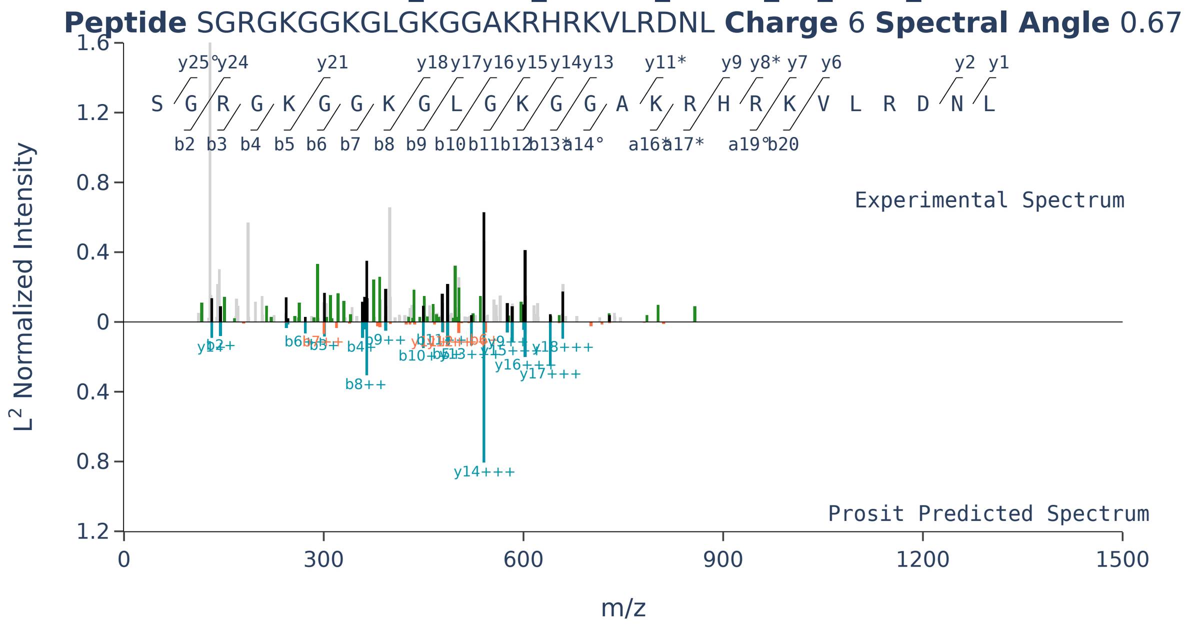


**Source** HRoetschke_291222_140123_HFGoe_H4_b1R1_24h **Scan** 9099
**Peptide** RDNIQGITKPAIRRLARRGGVKR **Charge** 5 **Spectral Angle** 0.7

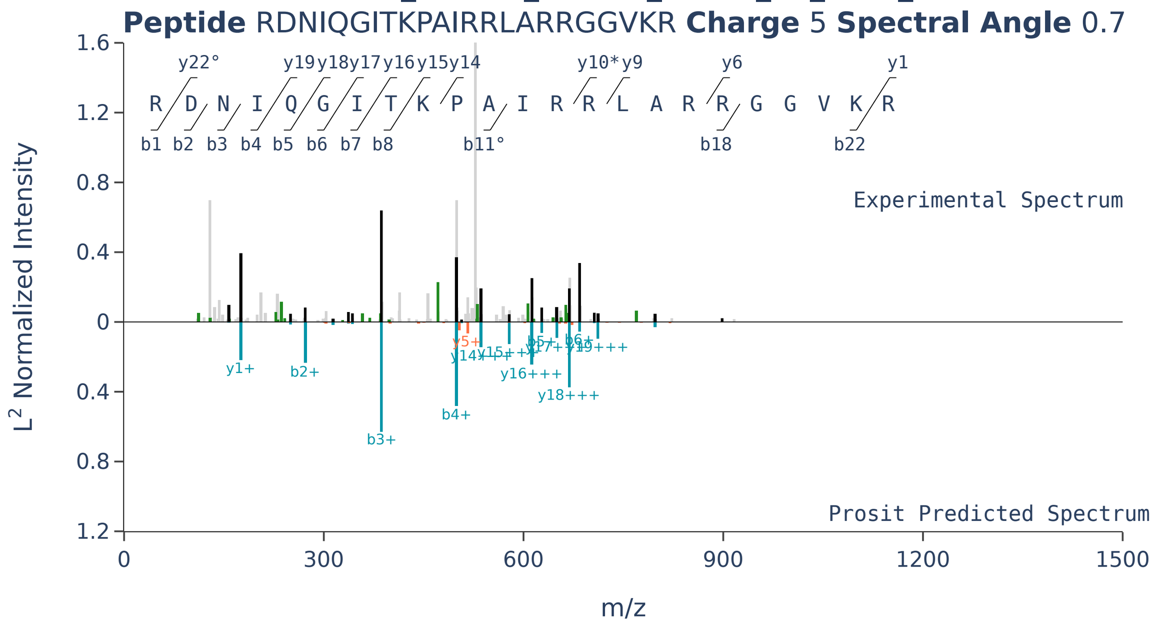


**Source** HRoetschke_291222_140123_HFGoe_H4_b2R1_1h **Scan** 36573
**Peptide** YEETRGVLKVFLENVLRD **Charge** 3 **Spectral Angle** 0.9

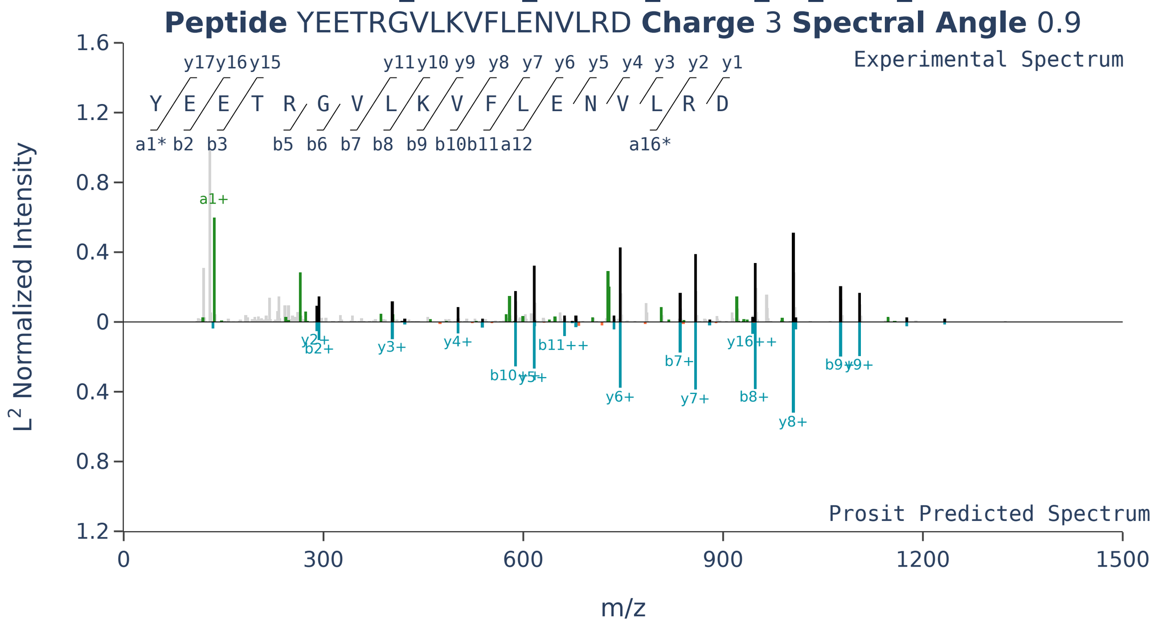

**Source** HRoetschke_291222_140123_HFGoe_H4_b1R2_2h **Scan** 17004
**Peptide** NIQGITKPAIRRLARRGGVKRISGLI **Charge** 5 **Spectral Angle** 0.62

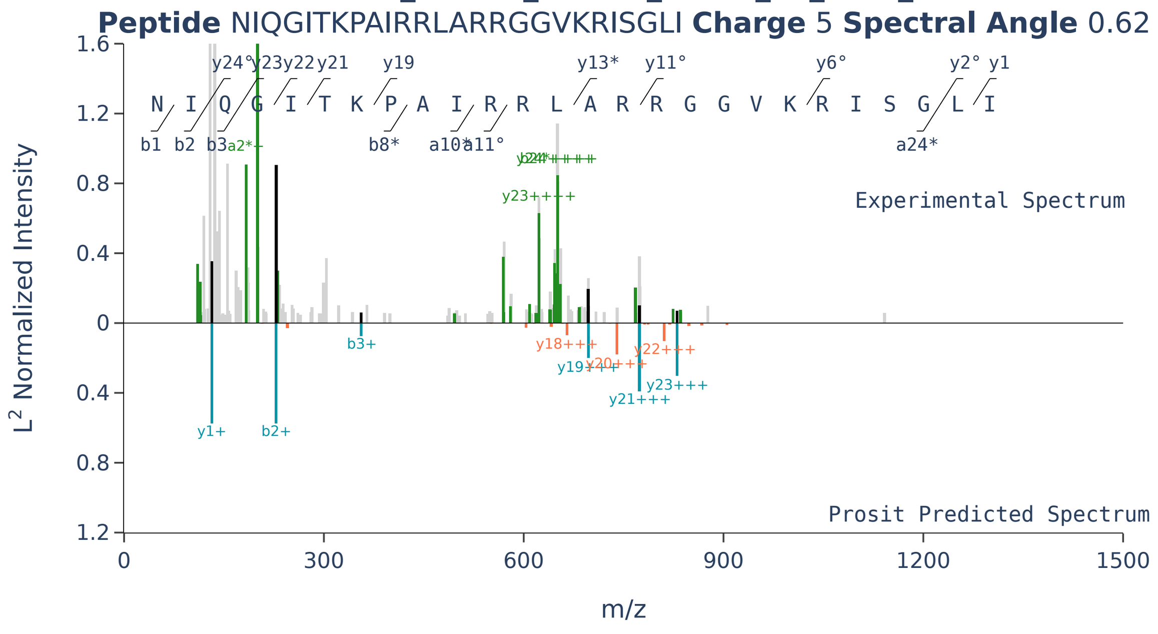


**Source** HRoetschke_291222_140123_HFGoe_H4_b2R1_2h **Scan** 17750
**Peptide** DAVTYTEHAKRKTVTAMDVVYALKRQGR **Charge** 5 **Spectral Angle** 0.61

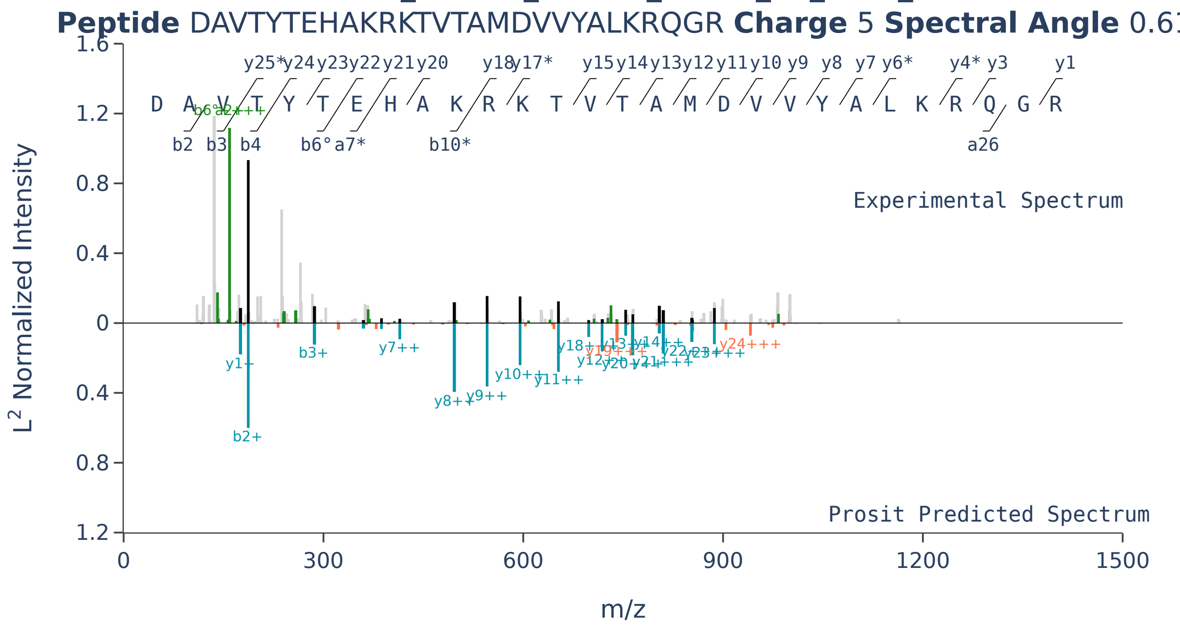


**Source** HRoetschke_291222_140123_HFGoe_H4_b2R2_1h **Scan** 31999
**Peptide** EETRGVLKVFLENVI **Charge** 2 **Spectral Angle** 0.79

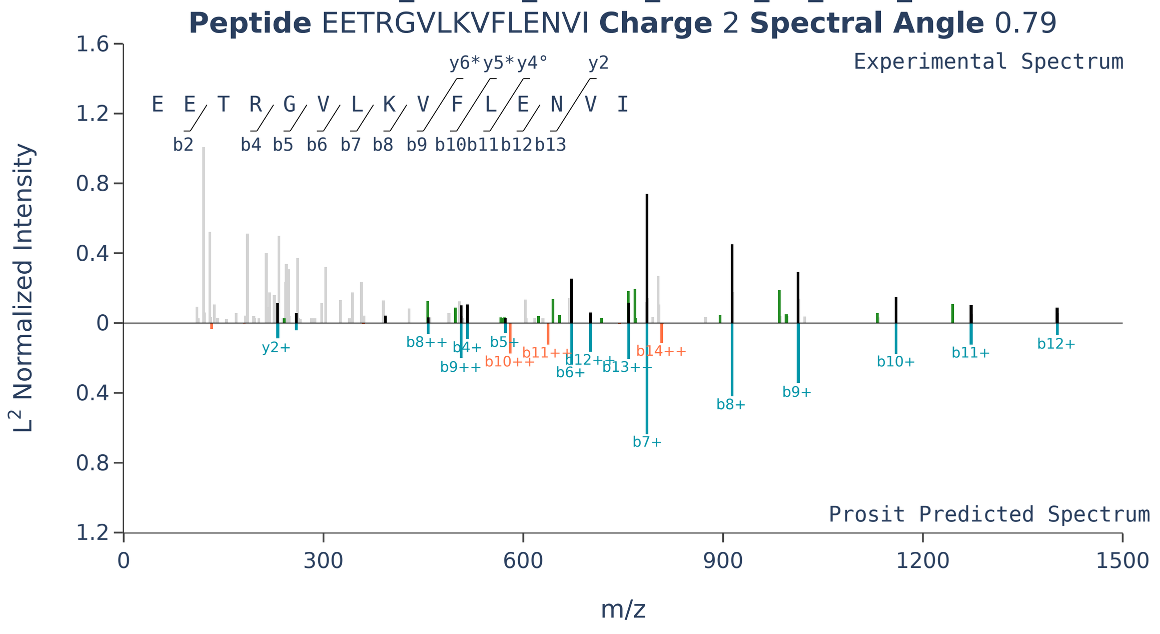


**Source** HRoetschke_291222_140123_HFGoe_H4_b1R1_1h **Scan** 11008
**Peptide** QGITKPAIRRLA **Charge** 2 **Spectral Angle** 0.61

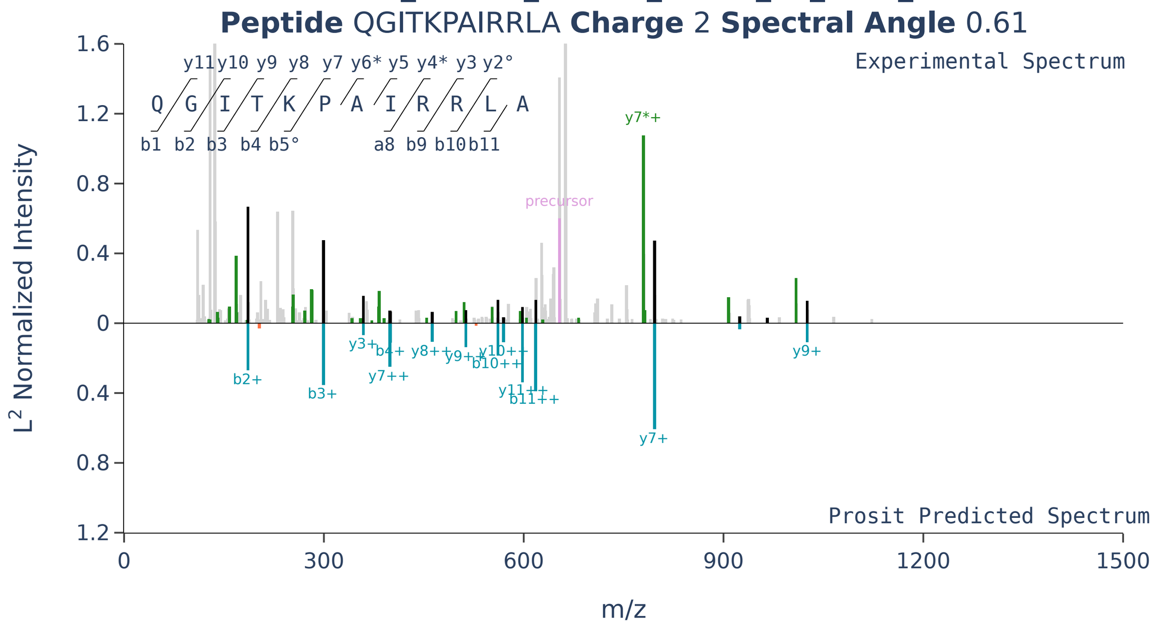


**Source** HRoetschke_291222_140123_HFGoe_H4_b2R1_4h **Scan** 8248
**Peptide** GLTKPAIRRLARRGGVKR **Charge** 4 **Spectral Angle** 0.66

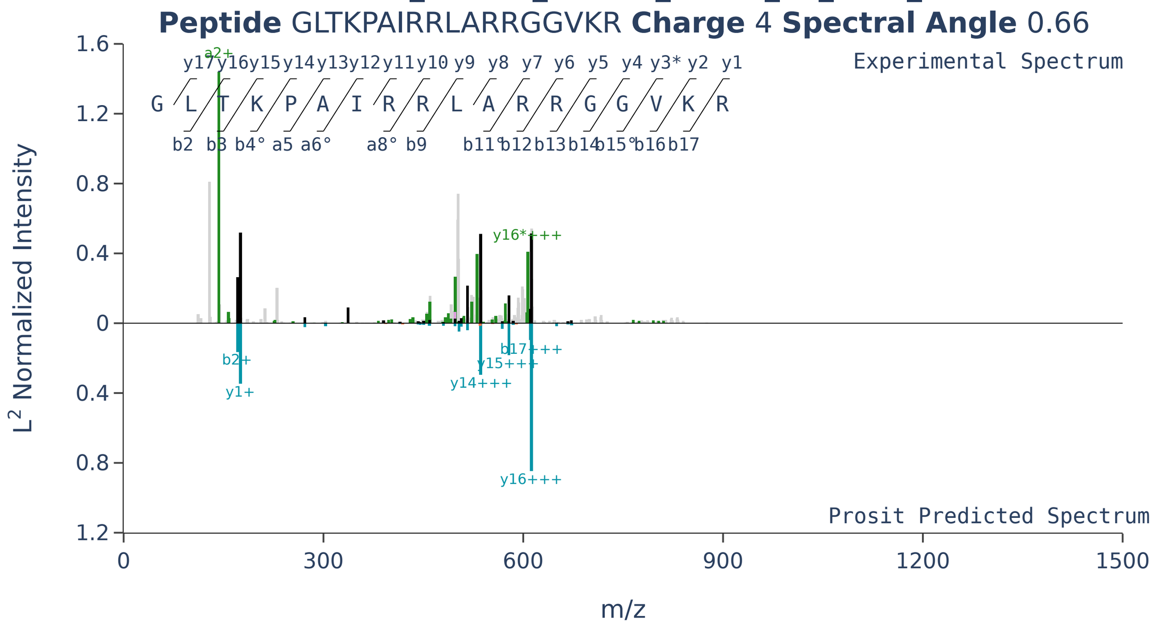


**Source** HRoetschke_291222_140123_HFGoe_H4_b2R1_2h **Scan** 5154
**Peptide** KPAIRRLARRGGVKR **Charge** 3 **Spectral Angle** 0.74

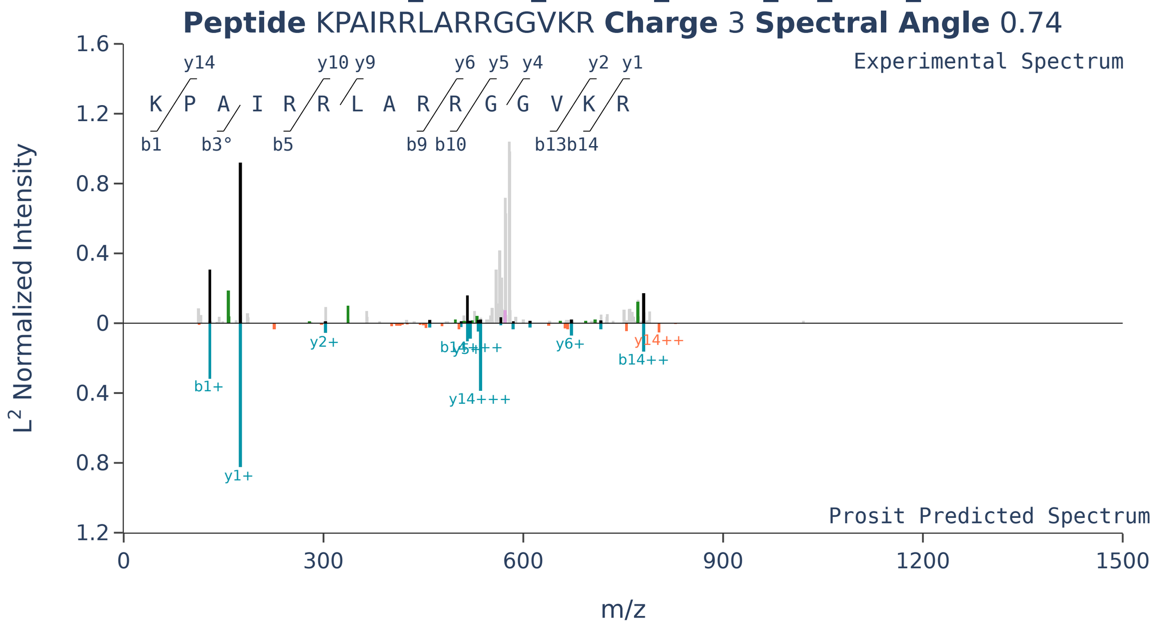


**Source** HRoetschke_291222_140123_HFGoe_H4_b2R2_1h **Scan** 31894
**Peptide** SGLIYEETRGVLKVFLEN **Charge** 3 **Spectral Angle** 0.7

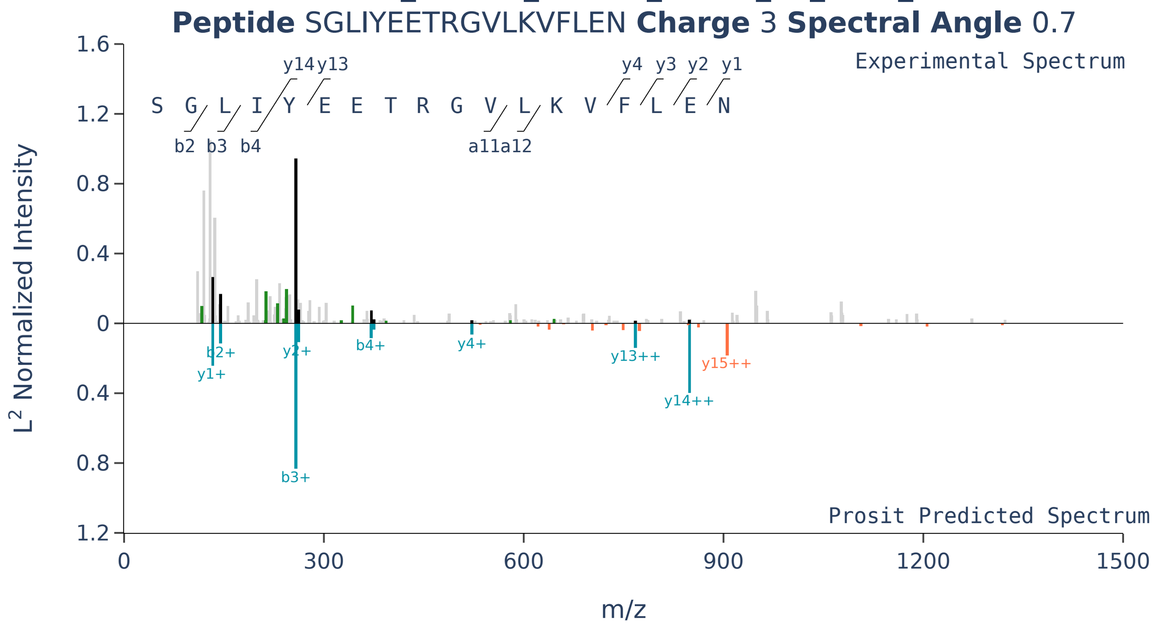


**Source** HRoetschke_291222_140123_HFGoe_H4_b1R2_24h **Scan** 11790
**Peptide** KVLRDNIQGITKPAIRRLARRG **Charge** 4 **Spectral Angle** 0.65

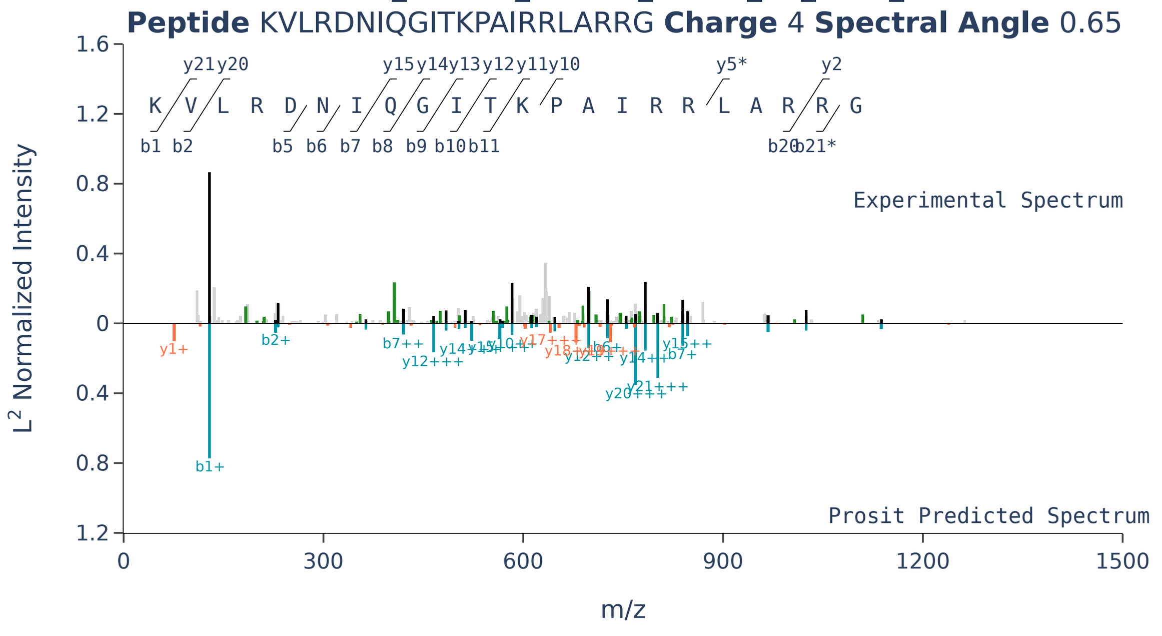


**Source** HRoetschke_291222_140123_HFGoe_H4_b1R1_1h **Scan** 16364
**Peptide** VIRDAVTYTEHAKRKTVTAMDVVY **Charge** 4 **Spectral Angle** 0.6

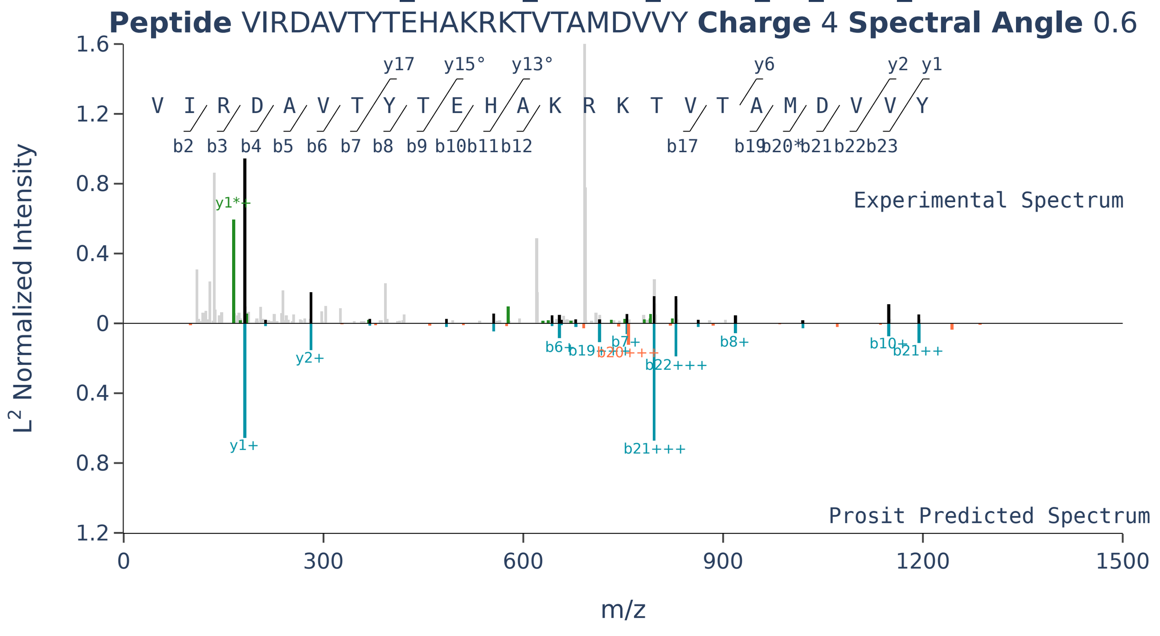


**Source** HRoetschke_291222_140123_HFGoe_H4_b1R1_24h **Scan** 12195
**Peptide** LRDNIQGITKPAIRRLARRGGVKRL **Charge** 5 **Spectral Angle** 0.71

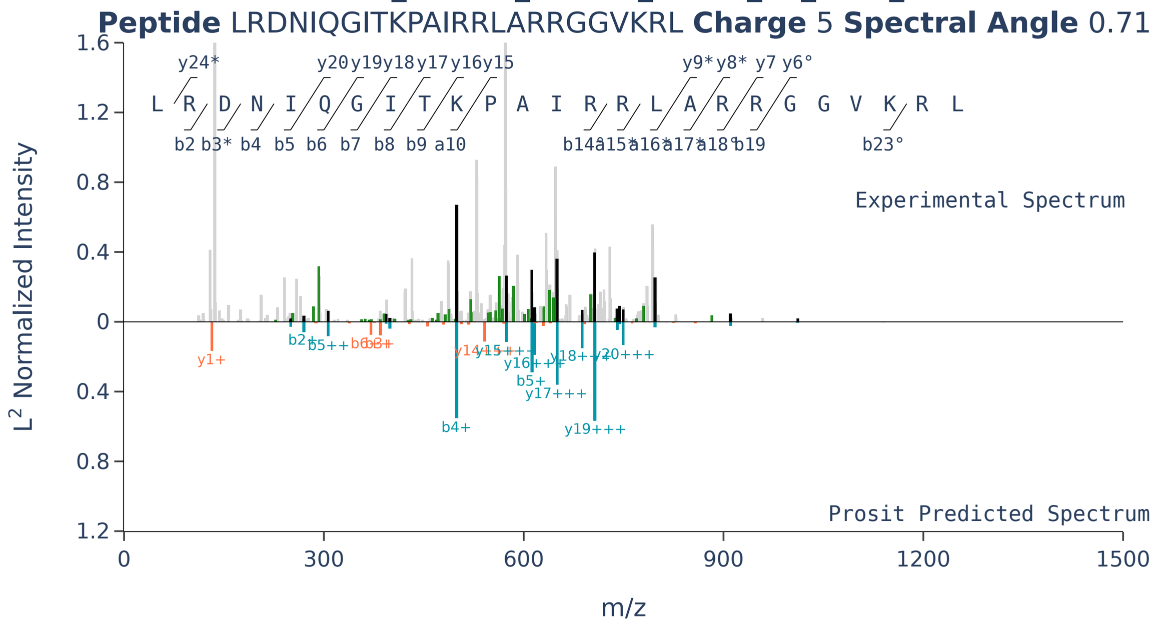


**Source** HRoetschke_291222_140123_HFGoe_H4_b1R2_4h **Scan** 11691
**Peptide** LRDNIQGITKPAIRRLARRG **Charge** 5 **Spectral Angle** 0.59

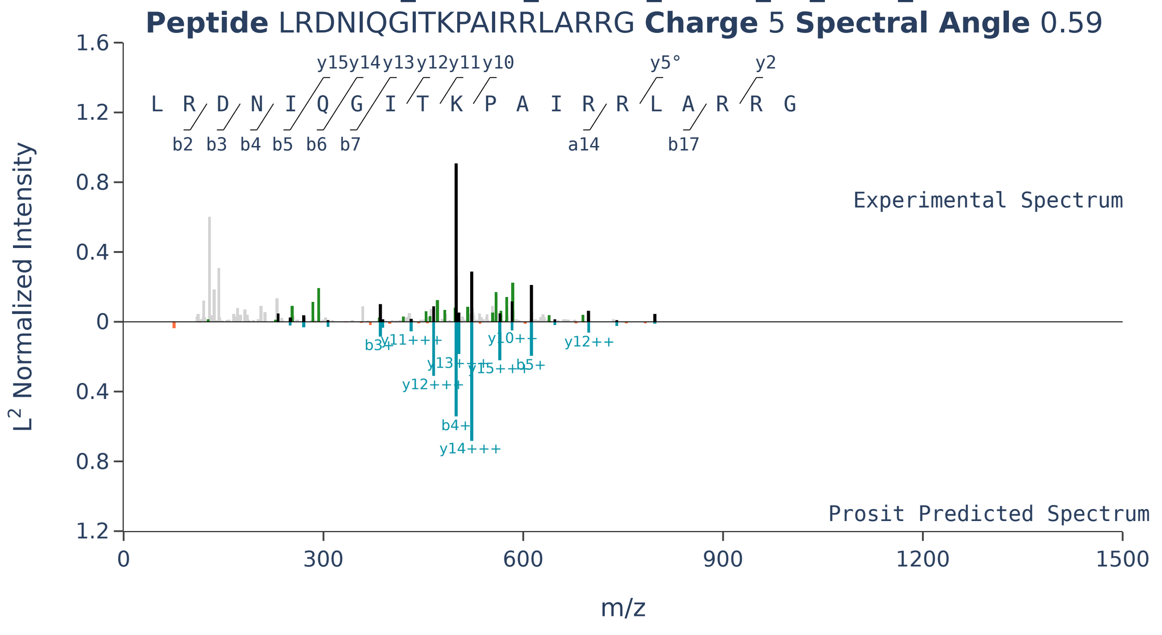


**Source** HRoetschke_291222_140123_HFGoe_H4_b1R1_2h **Scan** 23640
**Peptide** VTAMDVVYA **Charge** 1 **Spectral Angle** 0.87

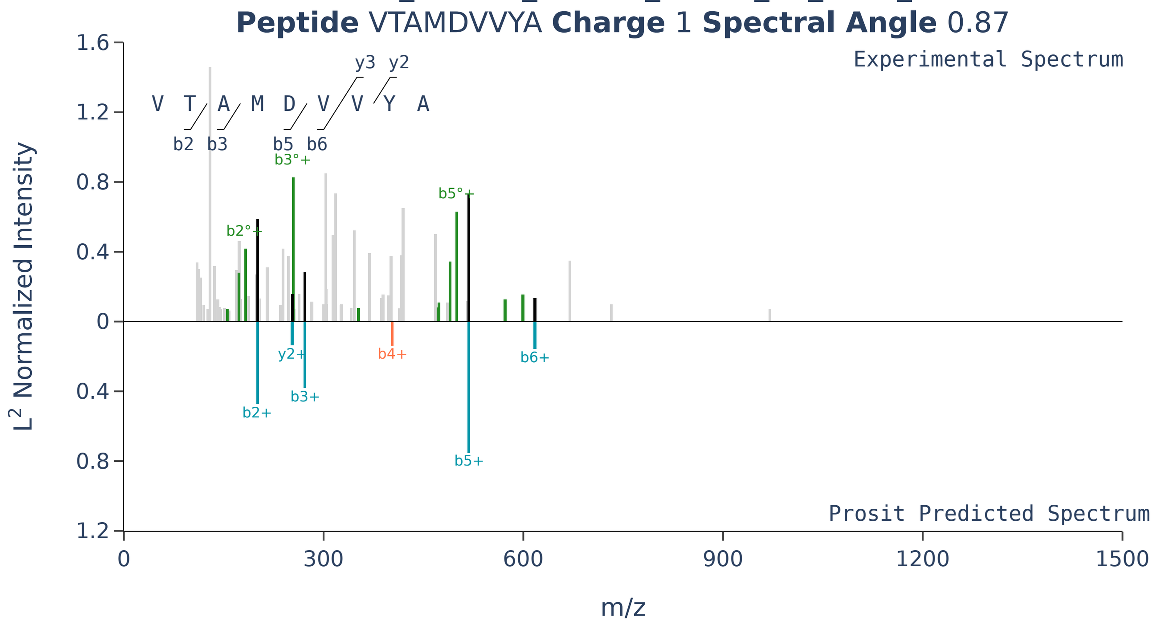


**Source** HRoetschke_291222_140123_HFGoe_H4_b1R1_24h **Scan** 5951
**Peptide** GGKGLGKGGAKRHRKVLRDNIQGITK **Charge** 6 **Spectral Angle** 0.74

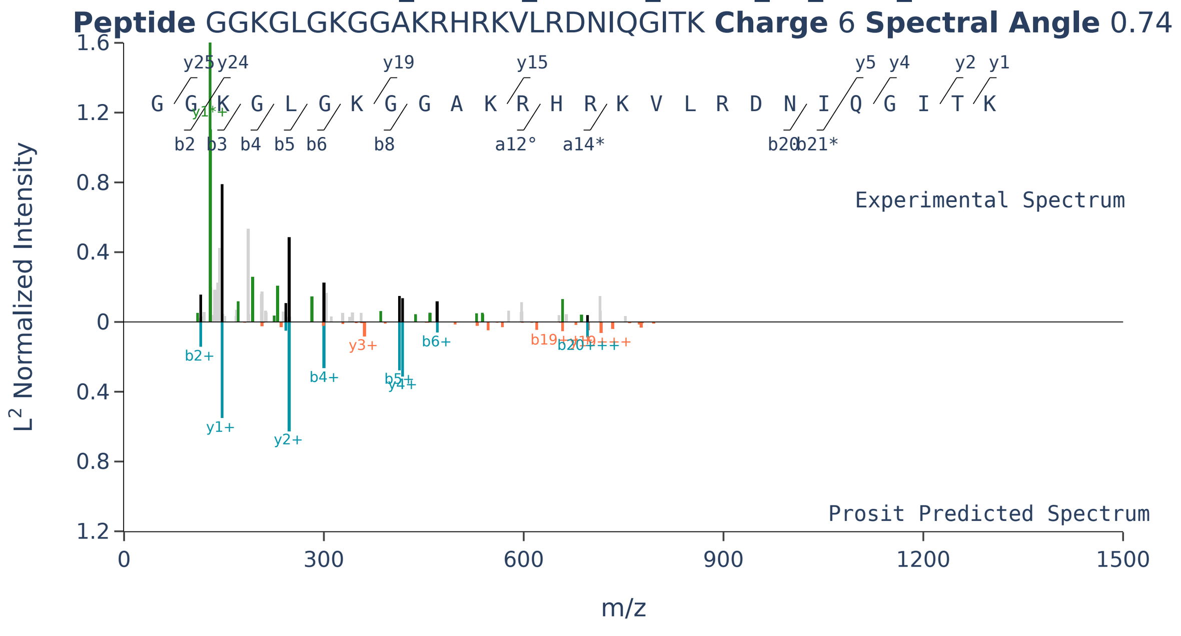


**Source** HRoetschke_291222_140123_HFGoe_H4_b1R1_2h **Scan** 15238
**Peptide** QGITKPAIRRLARRGGVKRISGLIY **Charge** 5 **Spectral Angle** 0.88

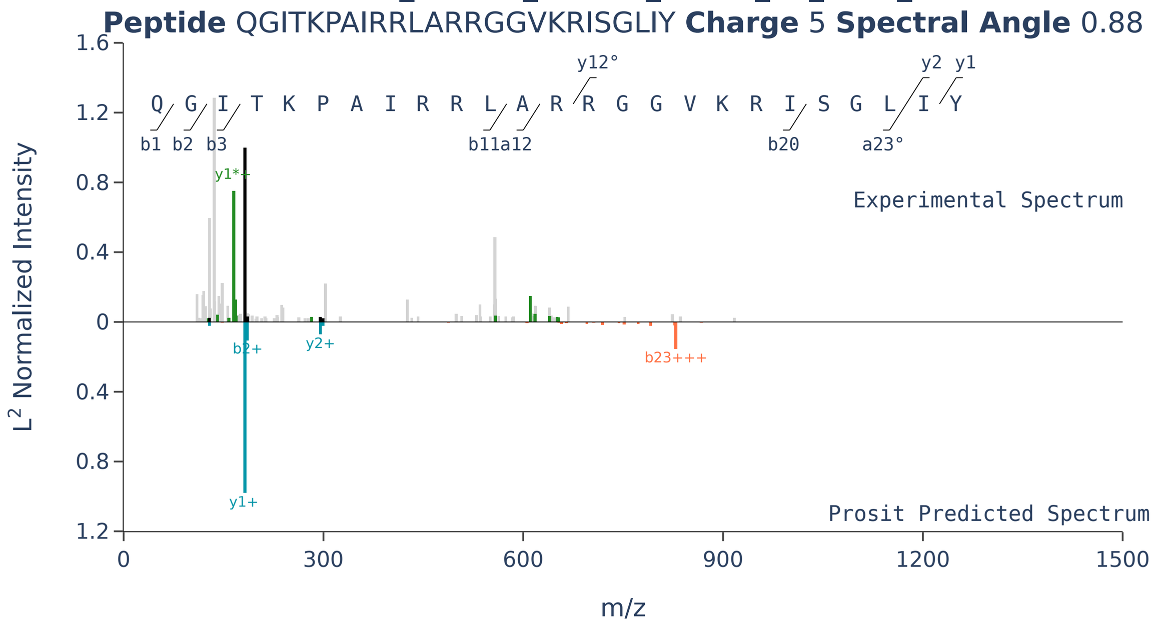


**Source** HRoetschke_291222_140123_HFGoe_H4_b2R1_1h **Scan** 26313
**Peptide** VTAMDVVYALKRQGRTLYG **Charge** 4 **Spectral Angle** 0.74

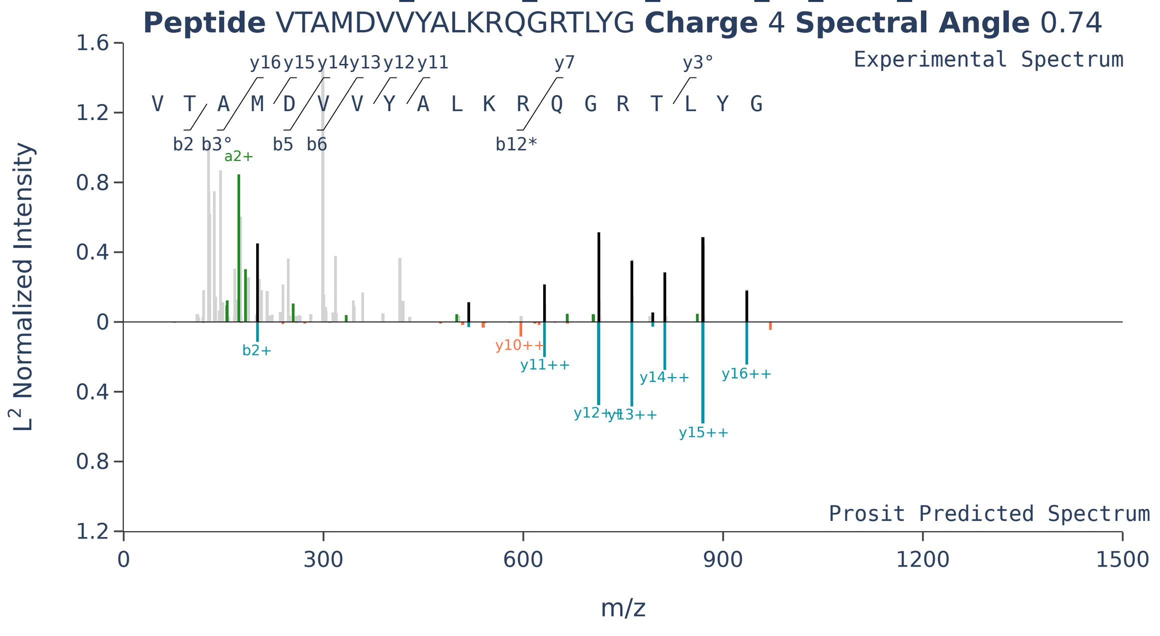

**Source** HRoetschke_291222_140123_HFGoe_H4_b1R1_1h **Scan** 25179
**Peptide** LKVFLENV **Charge** 2 **Spectral Angle** 0.68

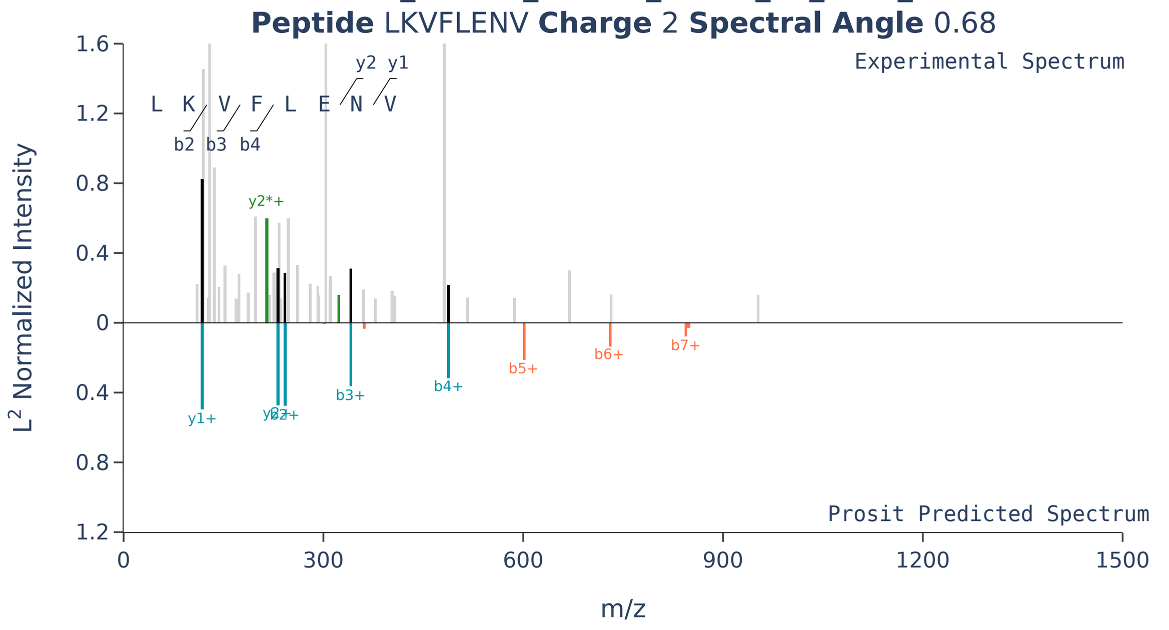


**Source** HRoetschke_291222_140123_HFGoe_H4_b2R1_1h **Scan** 27485
**Peptide** ARRGGVKRISGLIYEETRGVLKVFLE **Charge** 5 **Spectral Angle** 0.68

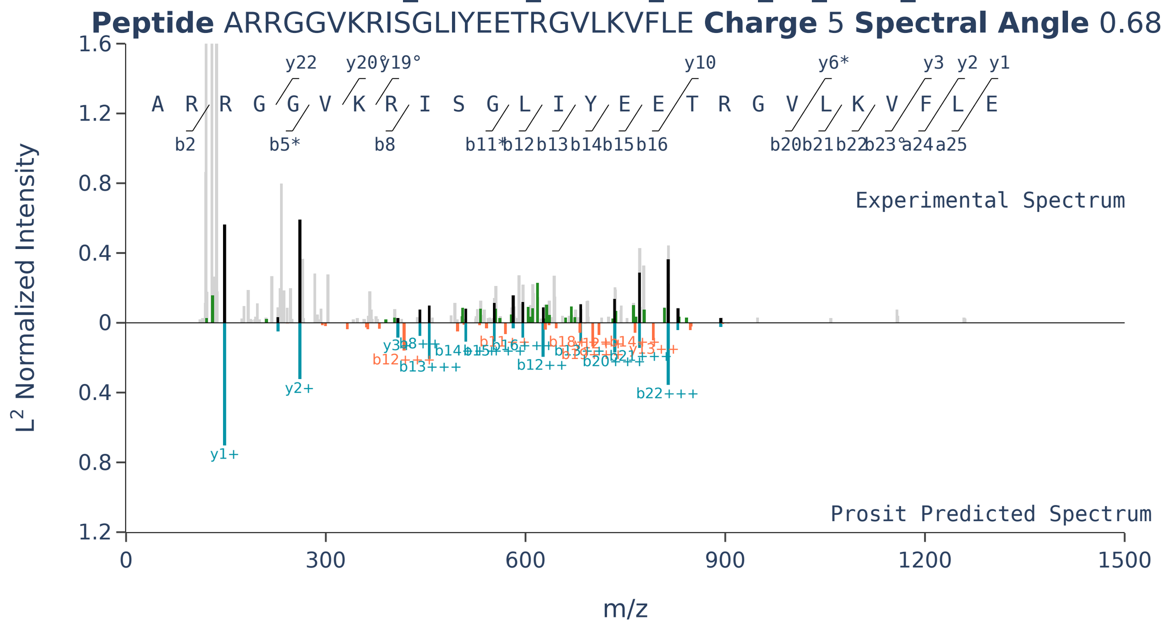


**Source** HRoetschke_291222_140123_HFGoe_H4_b2R1_24h **Scan** 8314
**Peptide** GKGGAKRHRKVLRDNIQGITKPAIR **Charge** 5 **Spectral Angle** 0.8

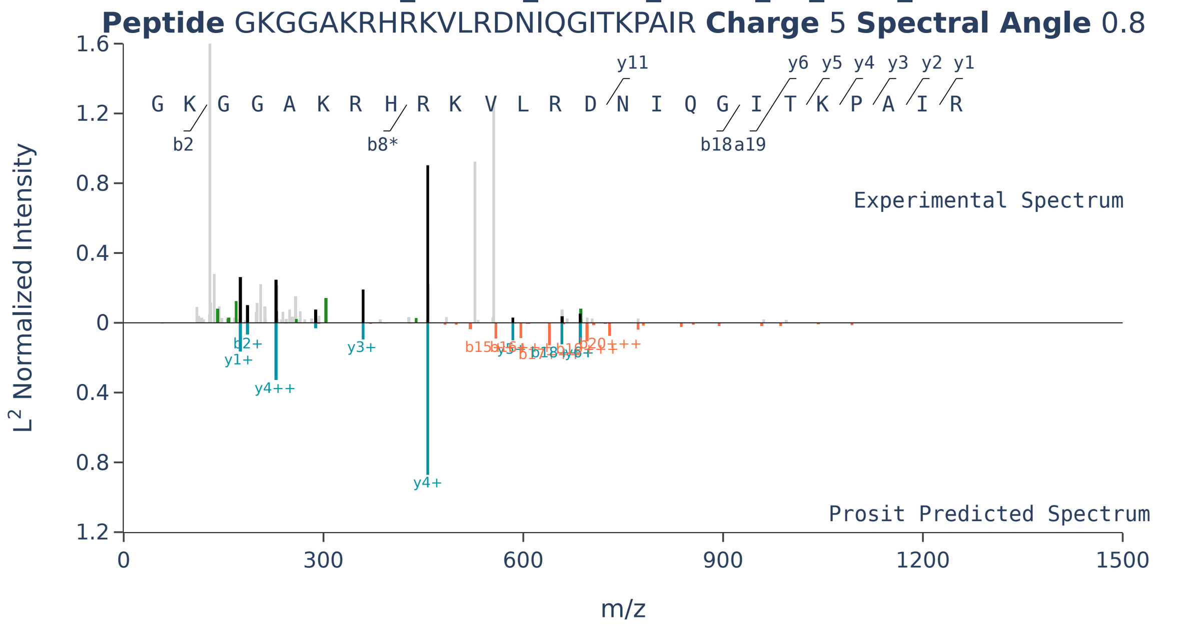


**Source** HRoetschke_291222_140123_HFGoe_H4_b1R1_1h **Scan** 16684
**Peptide** RDNIQGITKPAIRRLARRGGVKRISGLIY **Charge** 6 **Spectral Angle** 0.8

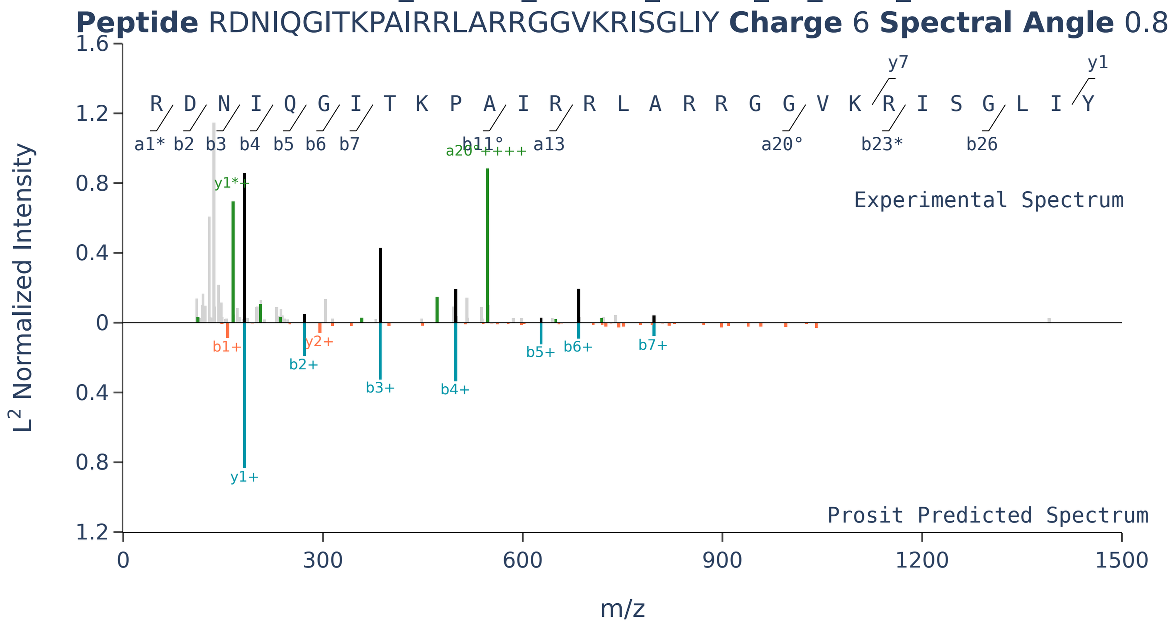


**Source** HRoetschke_291222_140123_HFGoe_H4_b2R1_1h **Scan** 33807
**Peptide** IYEETRGVLKVFLENVI **Charge** 2 **Spectral Angle** 0.68

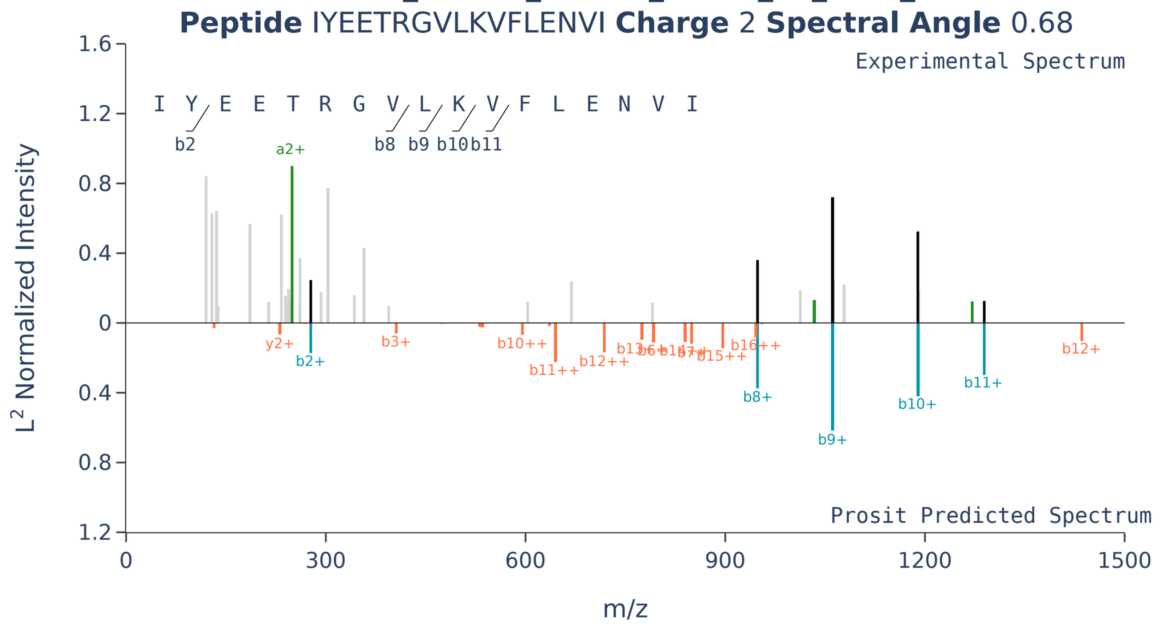


**Source** HRoetschke_291222_140123_HFGoe_H4_b1R1_2h **Scan** 7098
**Peptide** ARRGGVKRISG **Charge** 3 **Spectral Angle** 0.59

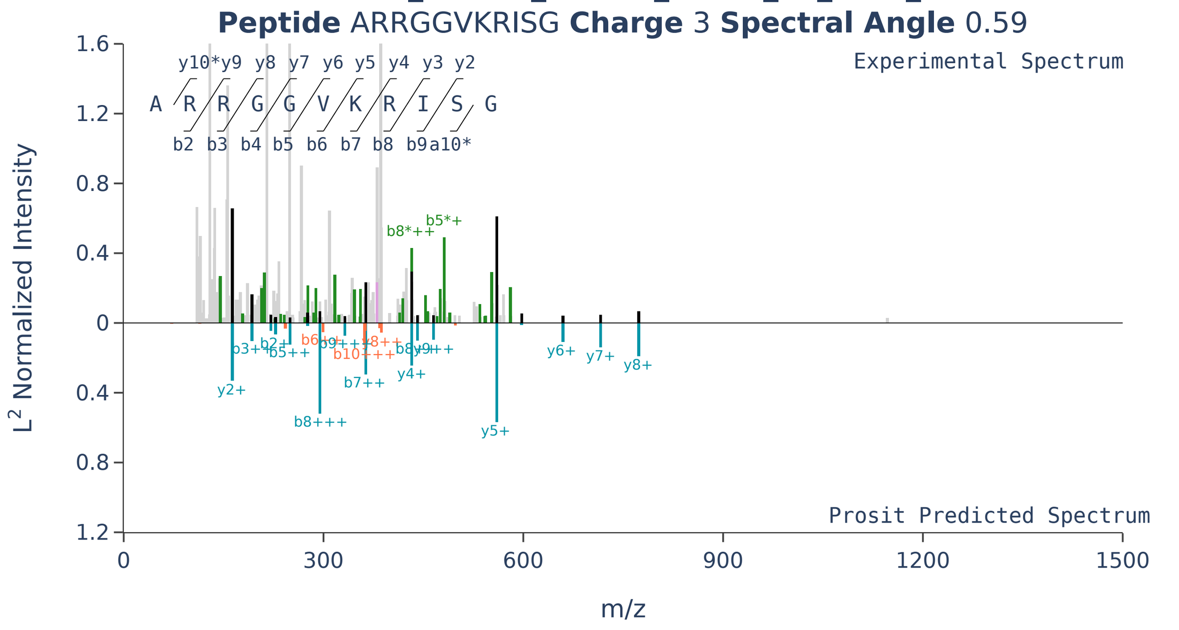


**Source** HRoetschke_291222_140123_HFGoe_H4_b2R1_1h **Scan** 15685
**Peptide** RDNIQGITKPAIRRLARRGGVKRISGLI **Charge** 5 **Spectral Angle** 0.58

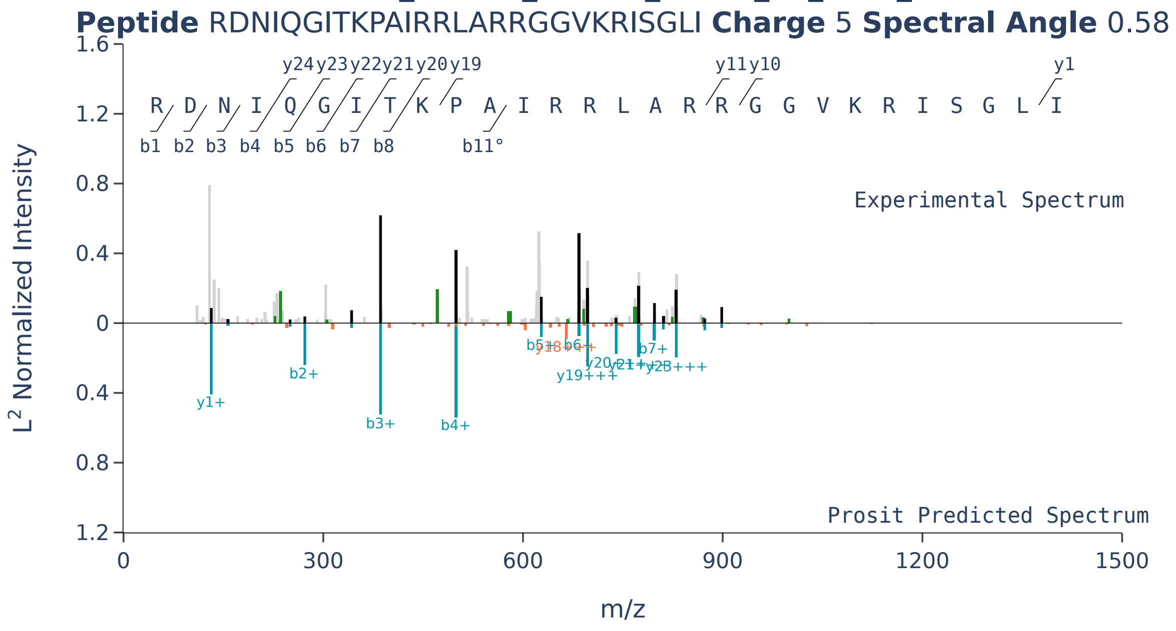


**Source** HRoetschke_291222_140123_HFGoe_H4_b2R1_2h **Scan** 10593
**Peptide** LQGITKPAIRRLARRG **Charge** 4 **Spectral Angle** 0.53

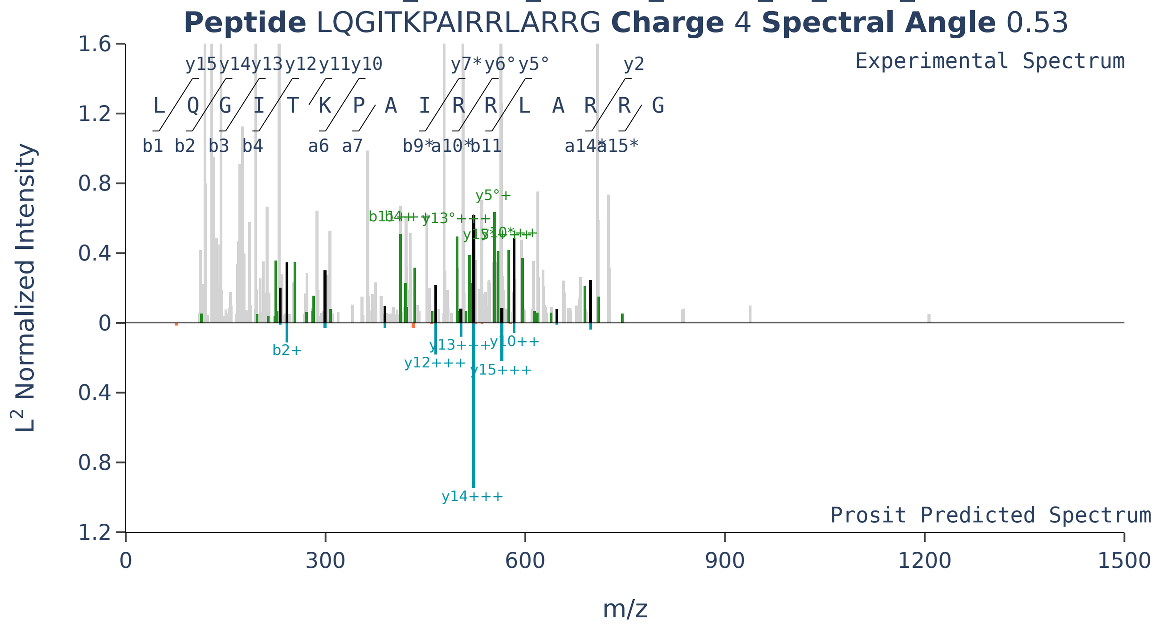


**Source** HRoetschke_291222_140123_HFGoe_H4_b1R1_2h **Scan** 16982
**Peptide** VTYTEHAKRKTVTAMDVVYALKRQGRTL **Charge** 6 **Spectral Angle** 0.82

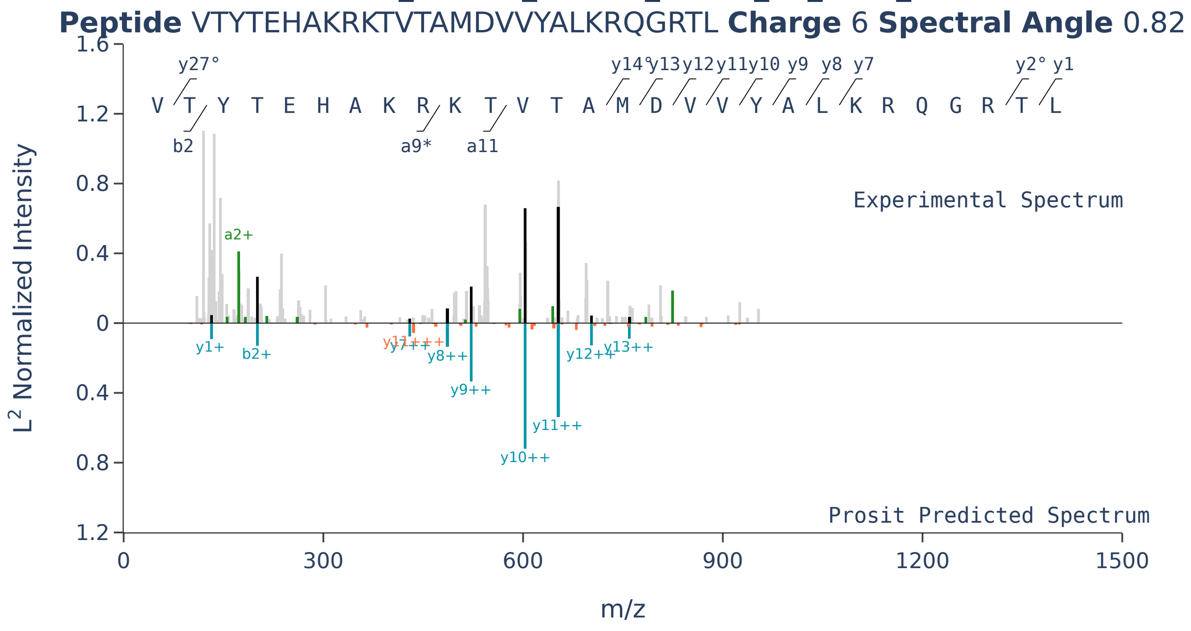


**Source** HRoetschke_291222_140123_HFGoe_H4_b1R1_1h **Scan** 19148
**Peptide** LENVIRDAVTYTEHAKRKTVTAMD **Charge** 4 **Spectral Angle** 0.56

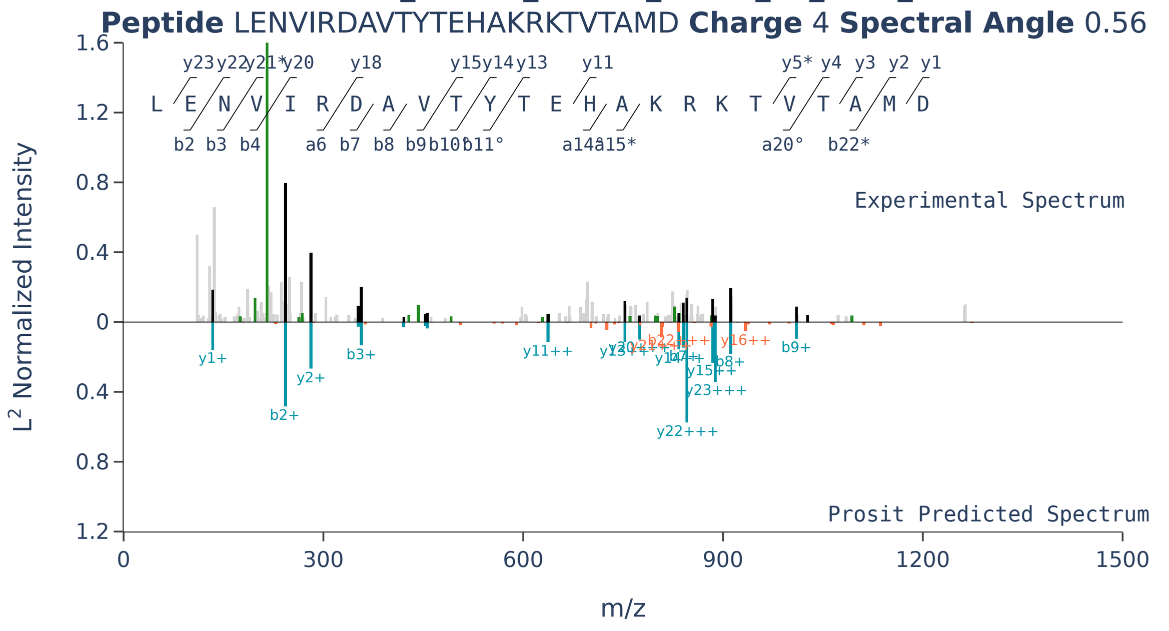


**Source** HRoetschke_291222_140123_HFGoe_H4_b2R1_1h **Scan** 26637
**Peptide** VFLENVIRDAVTYTEHAKRKTVT **Charge** 4 **Spectral Angle** 0.53

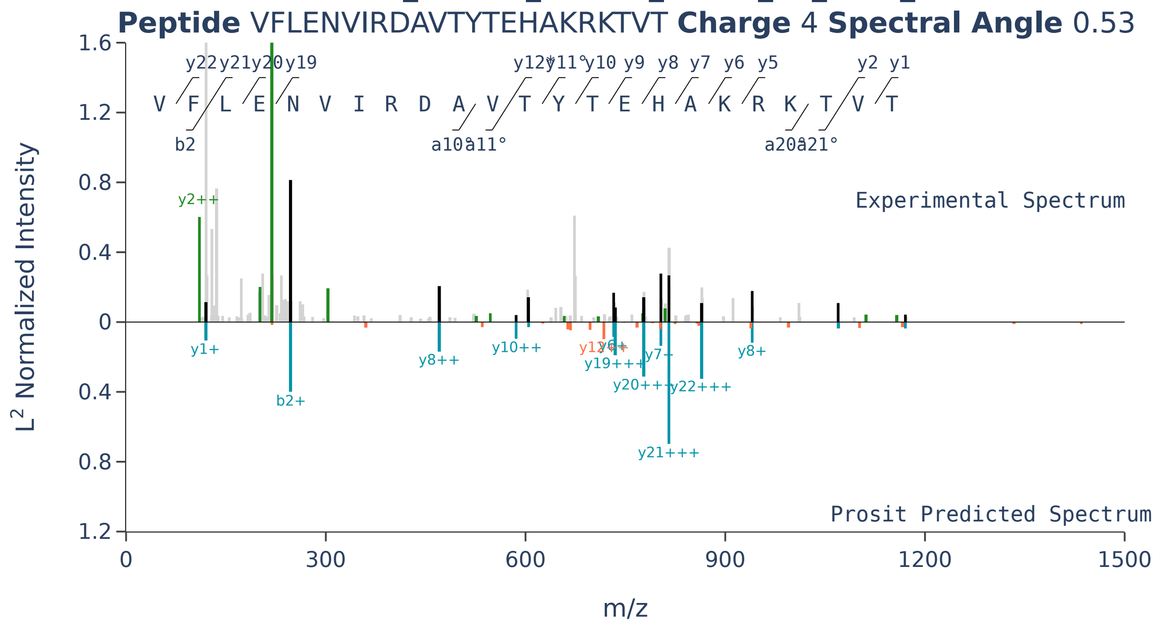


**Source** HRoetschke_291222_140123_HFGoe_H4_b1R1_2h **Scan** 12143
**Peptide** LRDNIQGITKPAIRRLARRGGVKRIS **Charge** 6 **Spectral Angle** 0.85

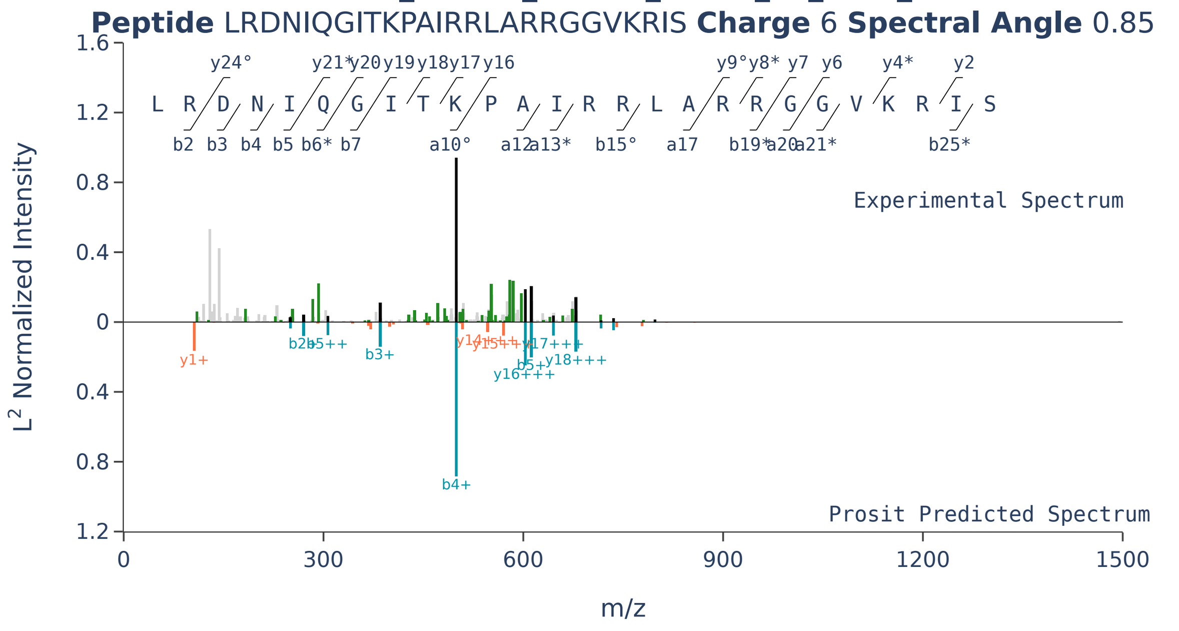


**Source** HRoetschke_291222_140123_HFGoe_H4_b2R1_1h **Scan** 26243
**Peptide** KVFLENVIRDAVTYTEHAK **Charge** 4 **Spectral Angle** 0.67

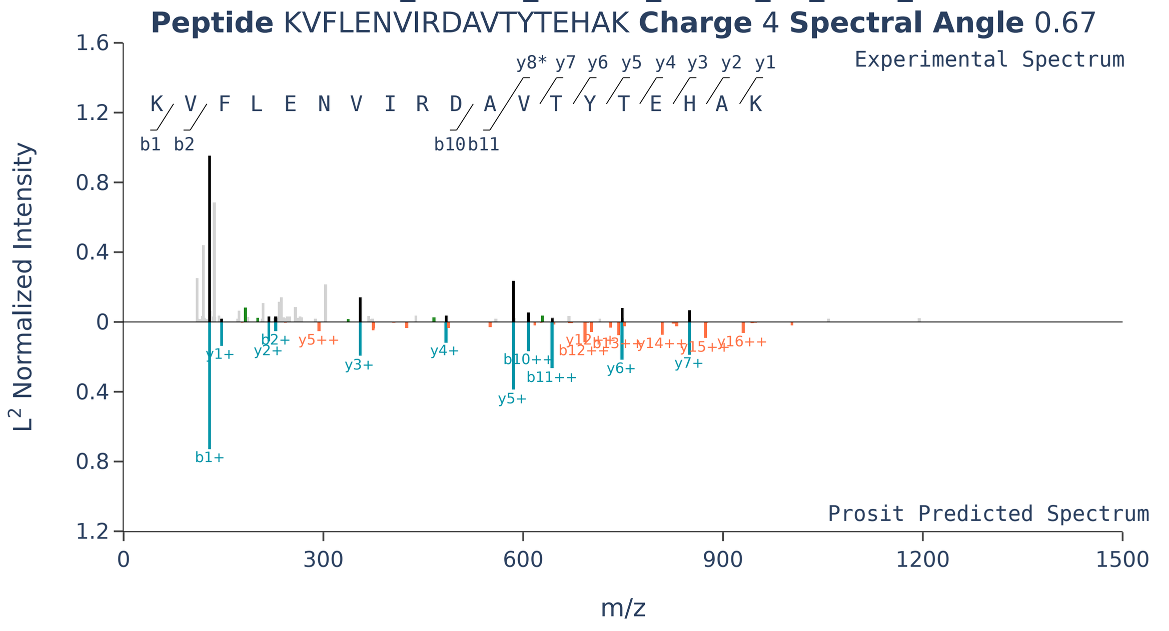


**Source** HRoetschke_291222_140123_HFGoe_H4_b2R1_1h **Scan** 37395
**Peptide** SGLIYEETRGVLKVFLENVLRD **Charge** 3 **Spectral Angle** 0.76

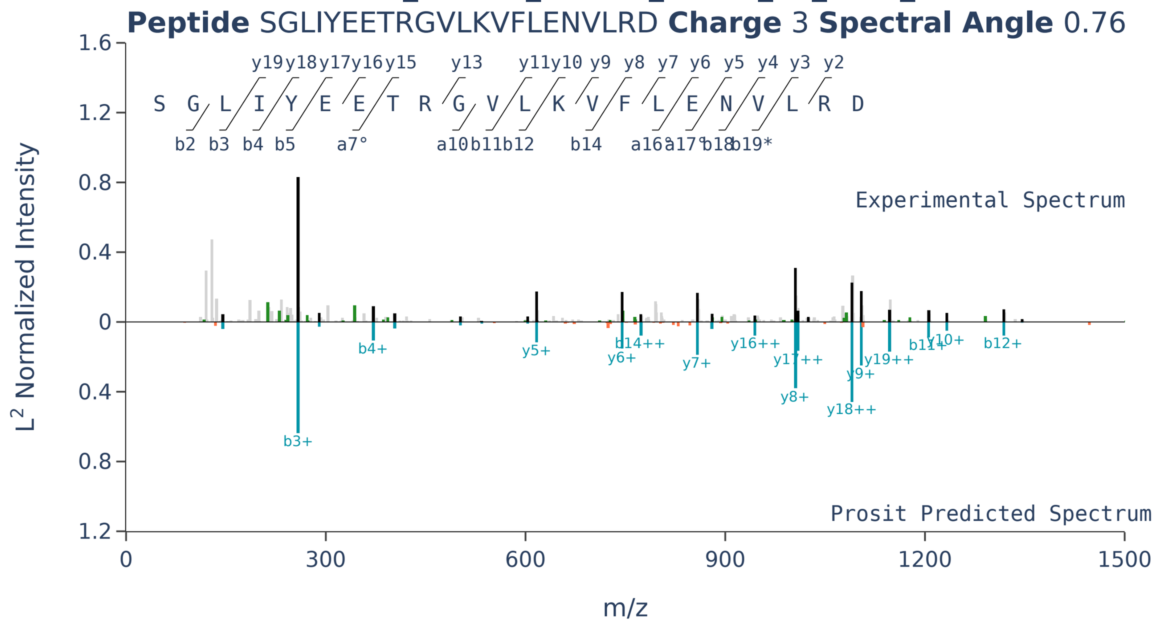


**Source** HRoetschke_291222_140123_HFGoe_H4_b1R2_4h **Scan** 5735
**Peptide** SGRGKGGKGLGKGGAKRHRKVLRDNI **Charge** 6 **Spectral Angle** 0.68

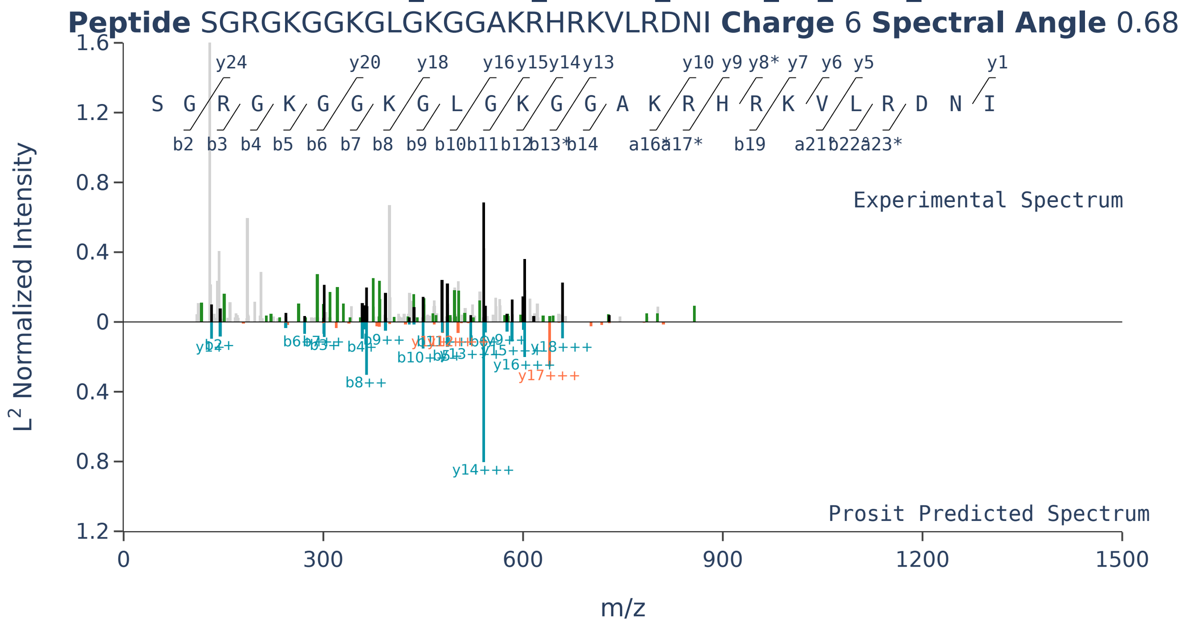

**Source** HRoetschke_291222_140123_HFGoe_H4_b2R2_24h **Scan** 10824
**Peptide** NIQGITKPAIRRLARRGG **Charge** 3 **Spectral Angle** 0.57

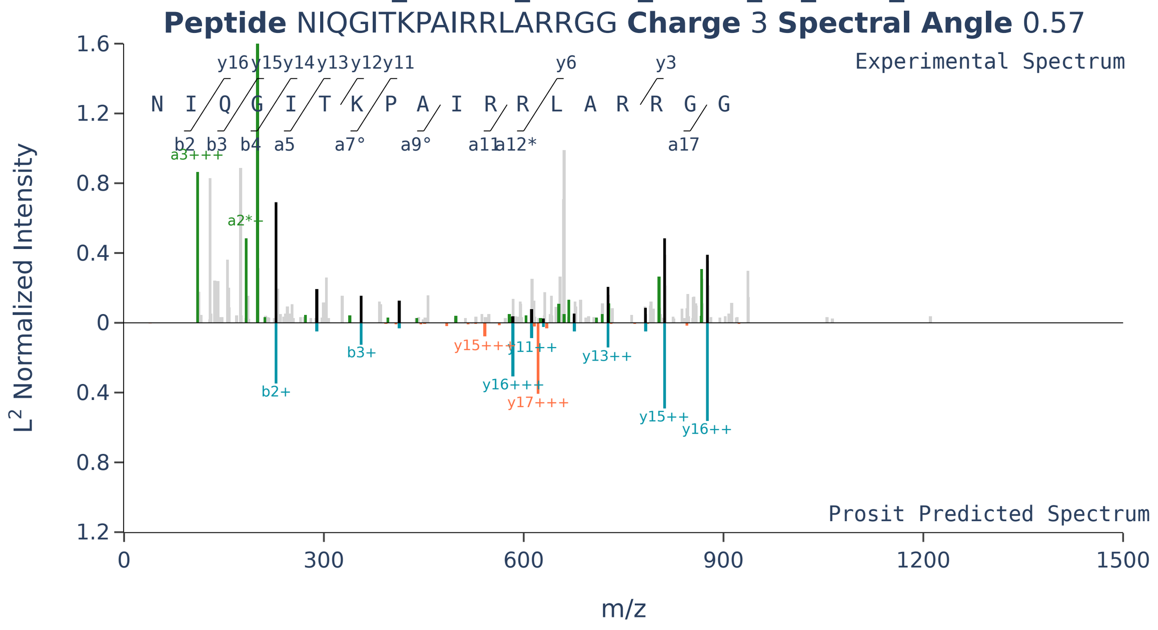


**Source** HRoetschke_291222_140123_HFGoe_H4_b1R1_1h **Scan** 20403
**Peptide** MDVVYALK **Charge** 2 **Spectral Angle** 0.69

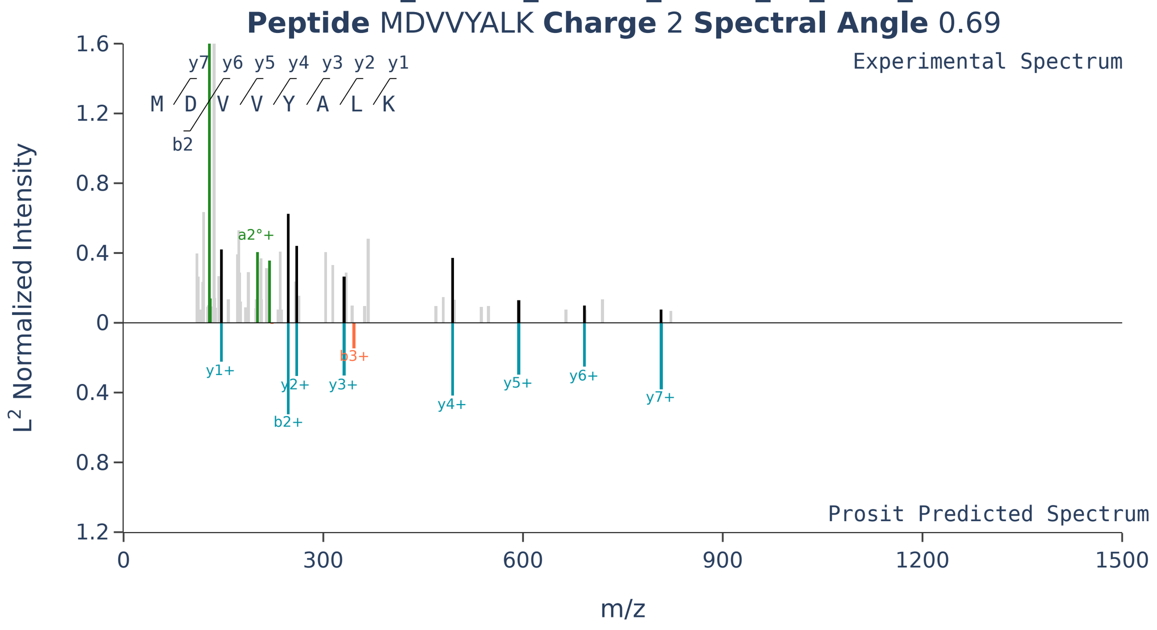


**Source** HRoetschke_291222_140123_HFGoe_H4_b1R2_24h **Scan** 6784
**Peptide** DAVTYTEHAKRKTVTA **Charge** 3 **Spectral Angle** 0.52

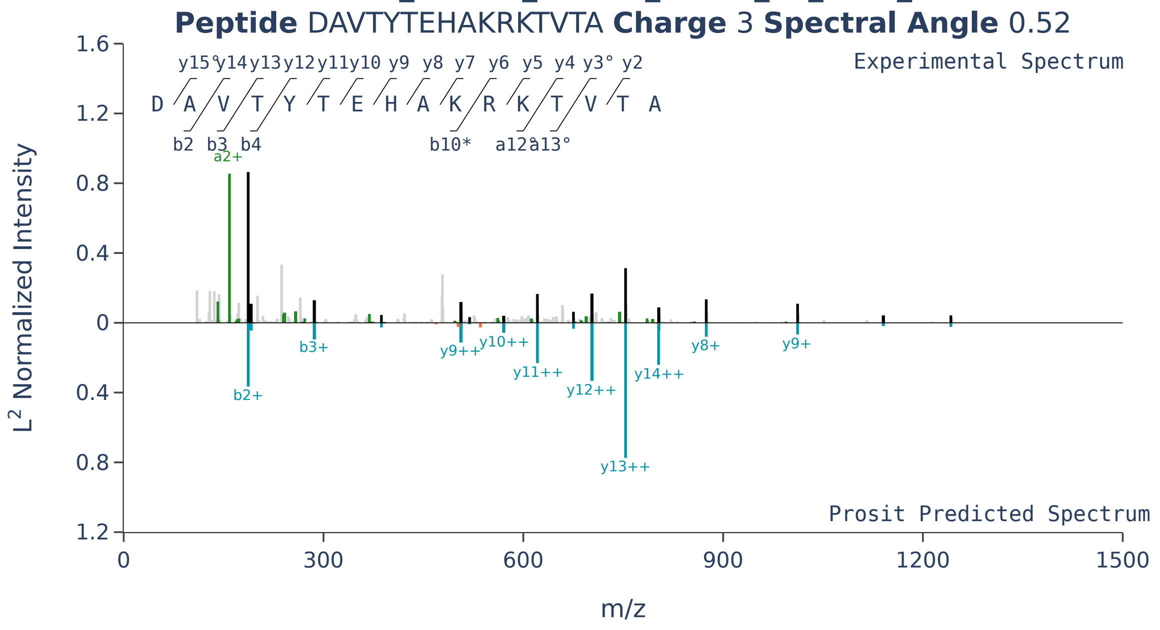


**Source** HRoetschke_291222_140123_HFGoe_H4_b1R2_1h **Scan** 21188
**Peptide** VKRISGLIYEETRGVLKV **Charge** 3 **Spectral Angle** 0.73

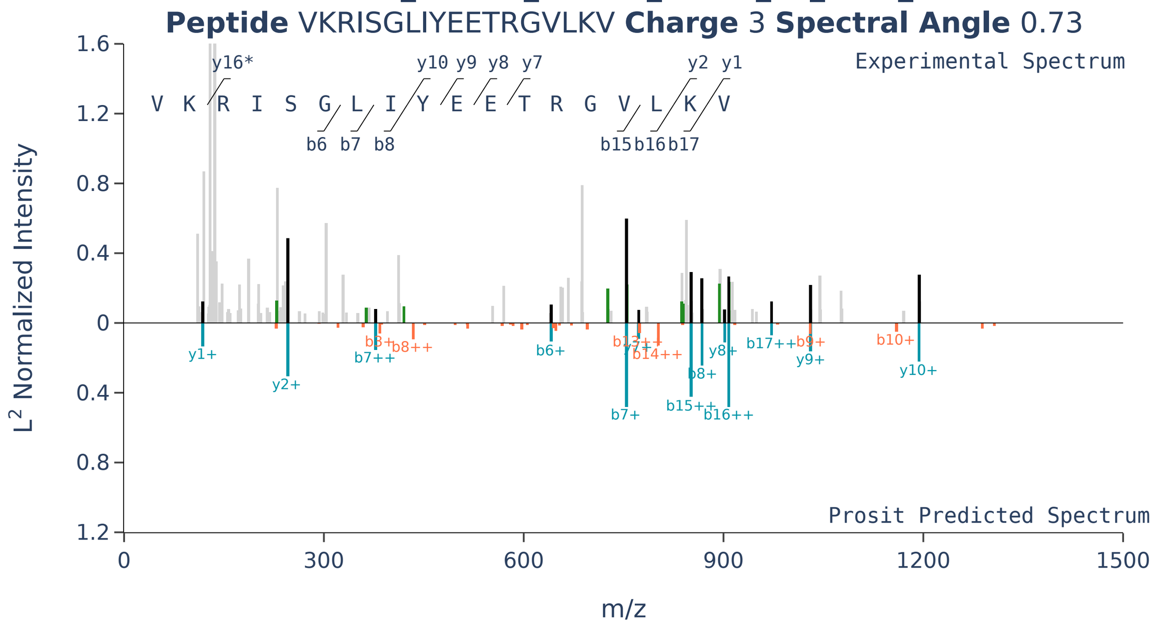


**Source** HRoetschke_291222_140123_HFGoe_H4_b2R2_1h **Scan** 16620
**Peptide** NIQGITKPAIRRLARRGGVKRISGLIYEE **Charge** 4 **Spectral Angle** 0.59

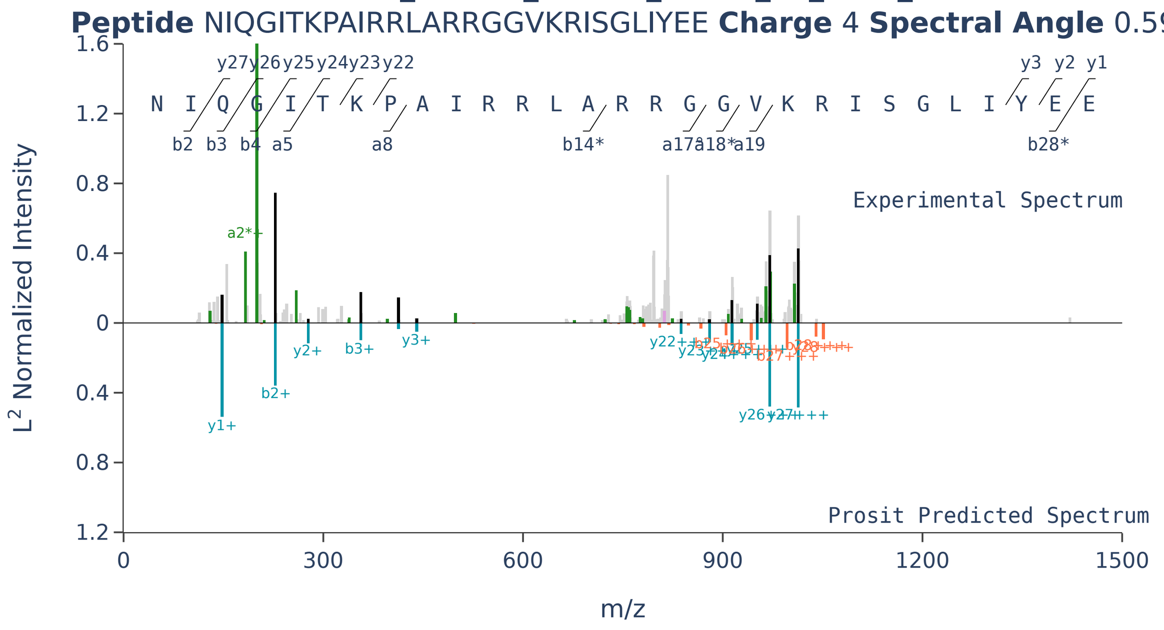


**Source** HRoetschke_291222_140123_HFGoe_H4_b1R1_4h **Scan** 6856
**Peptide** IRDAVTYTEHAKRKTVTAMD **Charge** 3 **Spectral Angle** 0.67

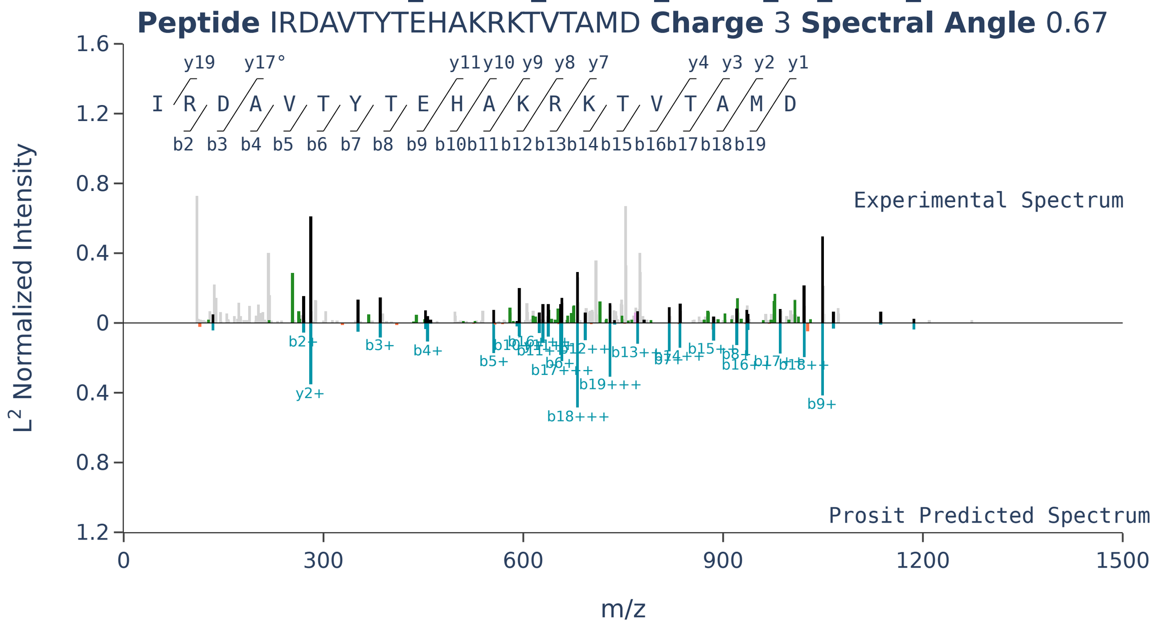


**Source** HRoetschke_291222_140123_HFGoe_H4_b1R2_2h **Scan** 13201
**Peptide** RGGVKRISGLIYEETR **Charge** 4 **Spectral Angle** 0.73

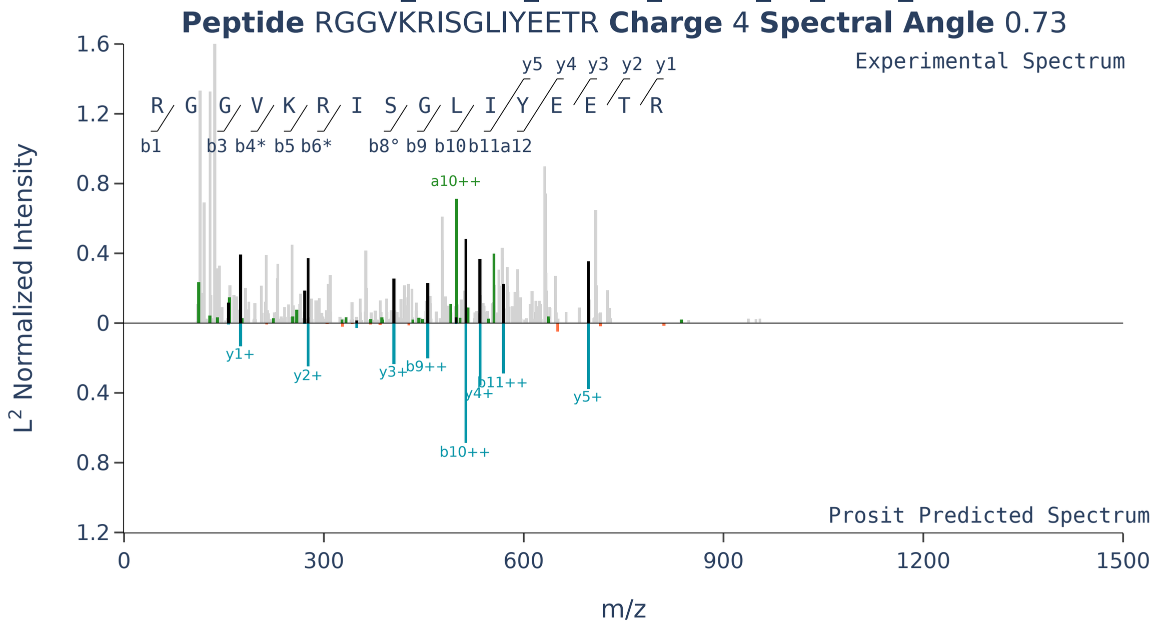


**Source** HRoetschke_291222_140123_HFGoe_H4_b1R2_1h **Scan** 21377
**Peptide** RGGVKRISGLIYEETRGVLKV **Charge** 4 **Spectral Angle** 0.74

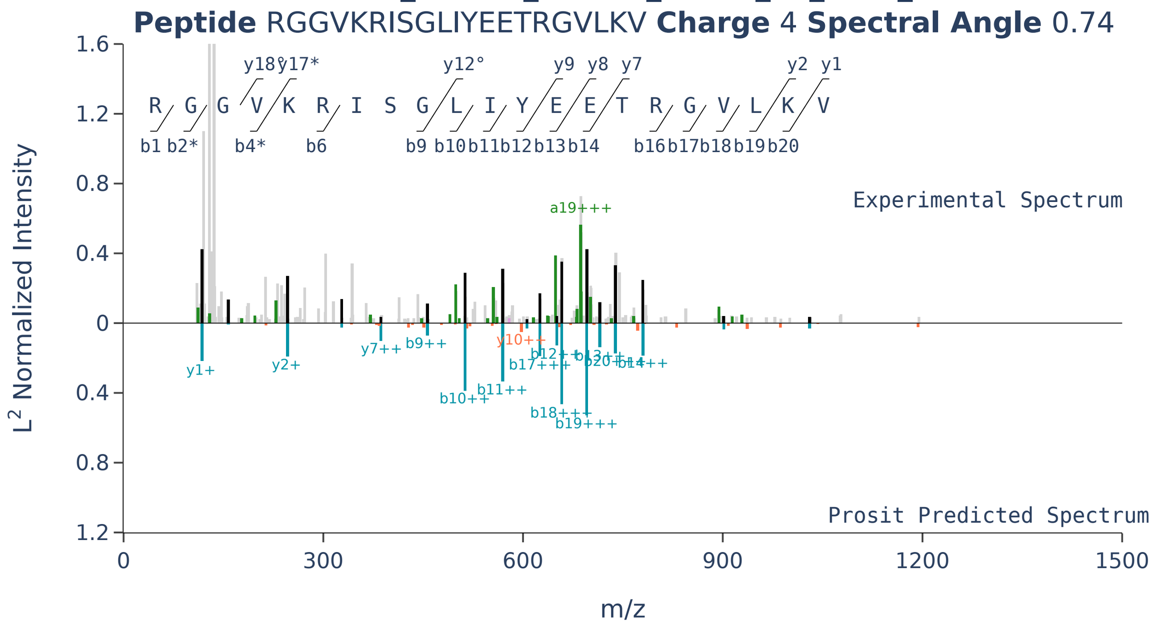


**Source** HRoetschke_291222_140123_HFGoe_H4_b1R2_24h **Scan** 5543
**Peptide** AKRKTVTAMD **Charge** 3 **Spectral Angle** 0.83

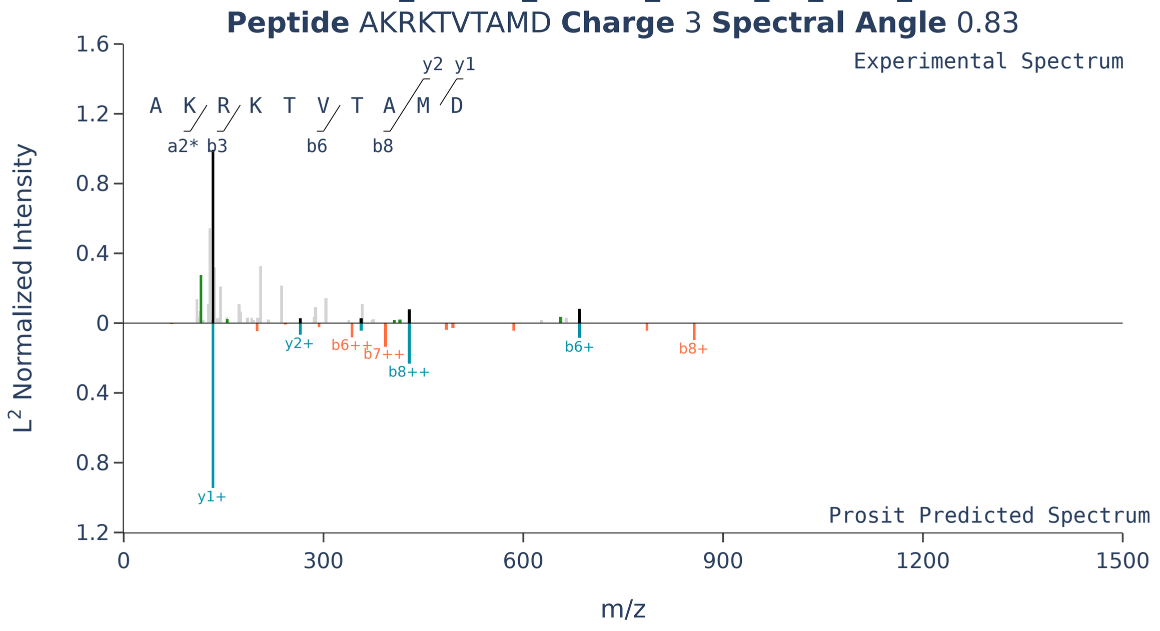


**Source** HRoetschke_291222_140123_HFGoe_H4_b2R1_1h **Scan** 30194
**Peptide** RGVLKVFLENVIRDAVTYTEHAKRKTV **Charge** 5 **Spectral Angle** 0.72

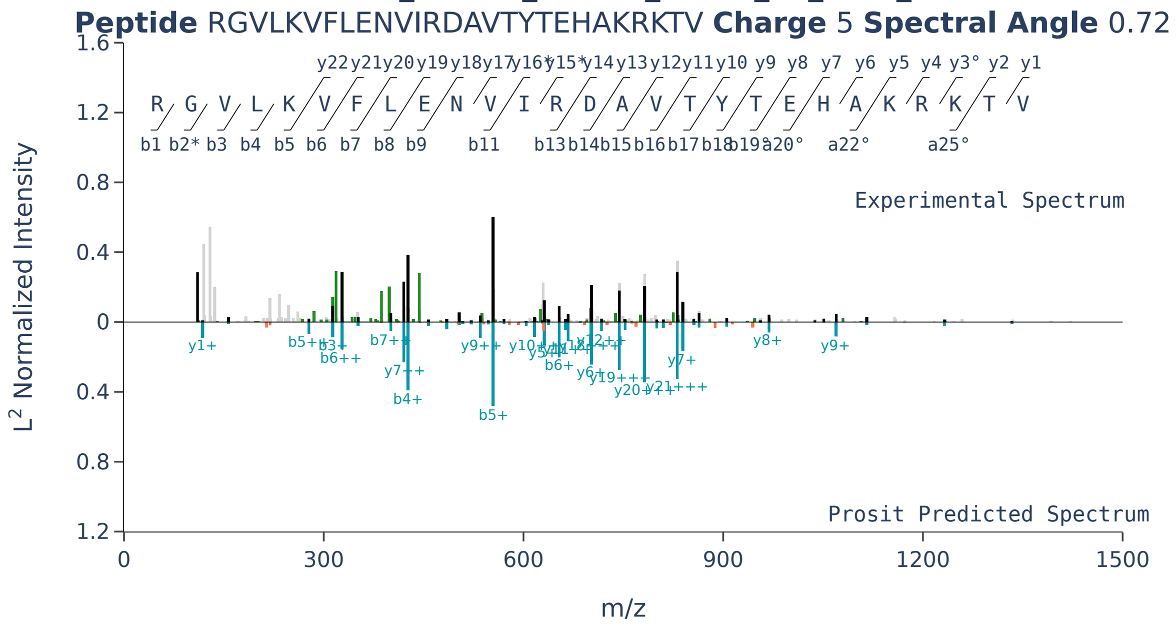


**Source** HRoetschke_291222_140123_HFGoe_H4_b2R2_4h **Scan** 12938
**Peptide** DNIQGITKPAIRRLARRGGVKRI **Charge** 3 **Spectral Angle** 0.74

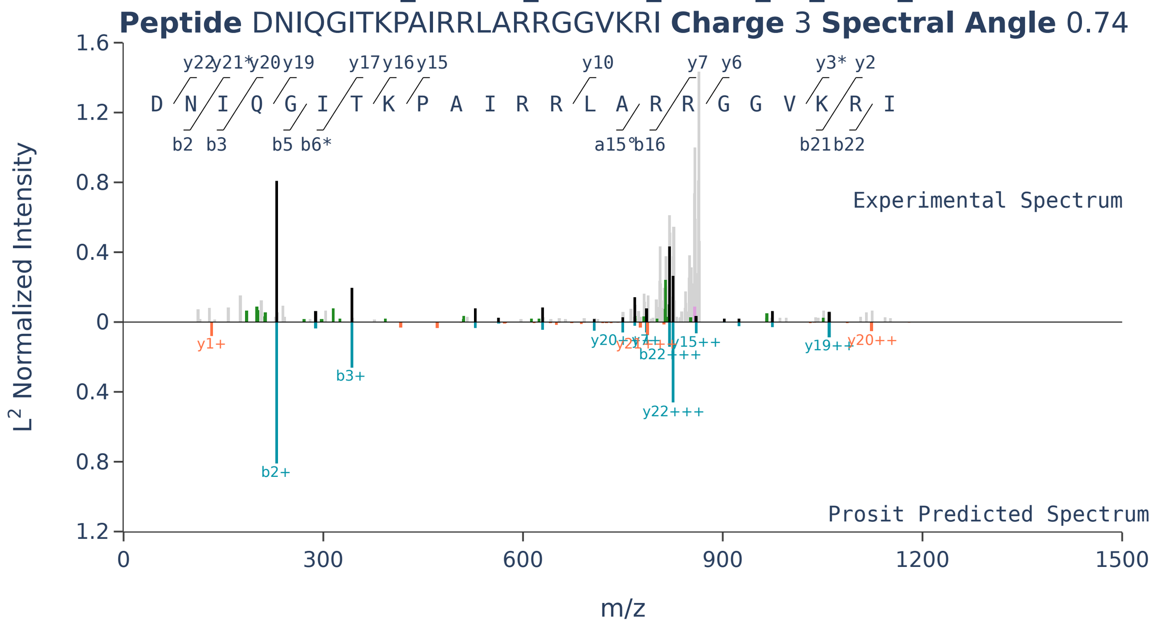


**Source** HRoetschke_291222_140123_HFGoe_H4_b2R1_1h **Scan** 23167
**Peptide** TAMDVVYALKRQGRTLYGF **Charge** 4 **Spectral Angle** 0.56

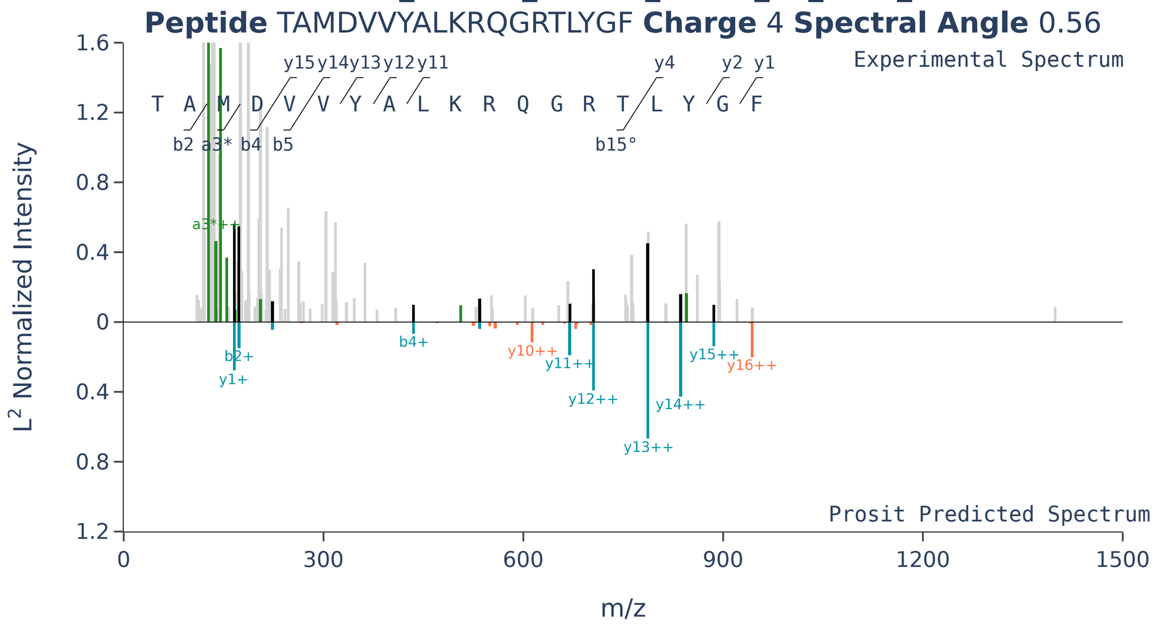


**Source** HRoetschke_291222_140123_HFGoe_H4_b2R1_1h **Scan** 11432
**Peptide** LRDNIQGITKPAIRRLARRGGVKRI **Charge** 5 **Spectral Angle** 0.67

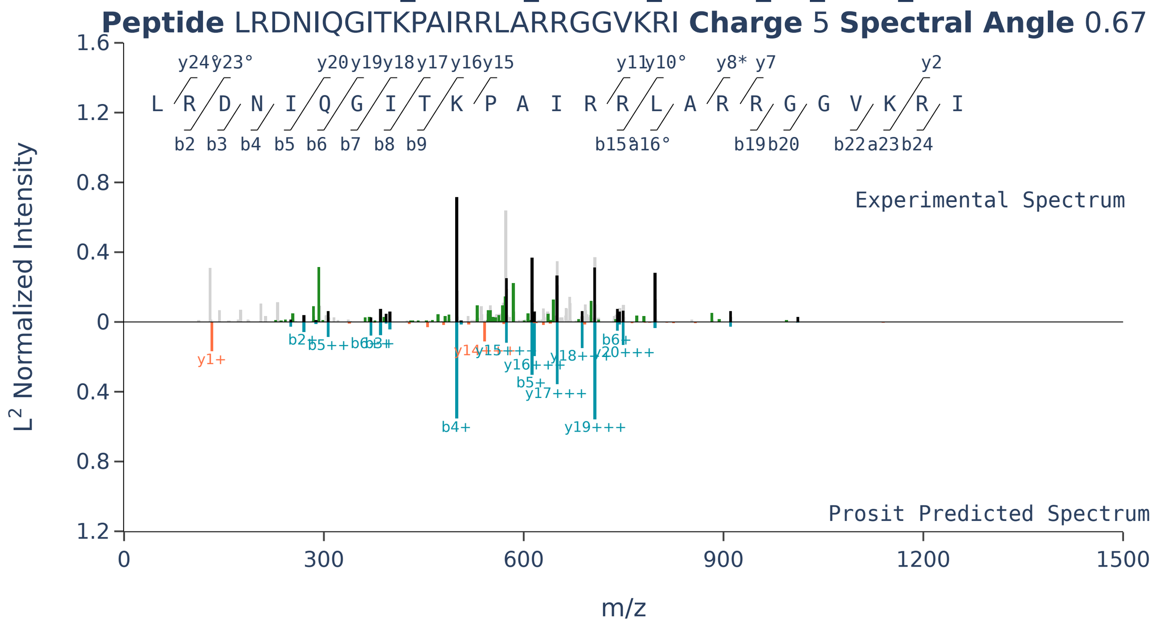


**Source** HRoetschke_291222_140123_HFGoe_H4_b1R1_2h **Scan** 6607
**Peptide** HRKVLRDNI **Charge** 3 **Spectral Angle** 0.71

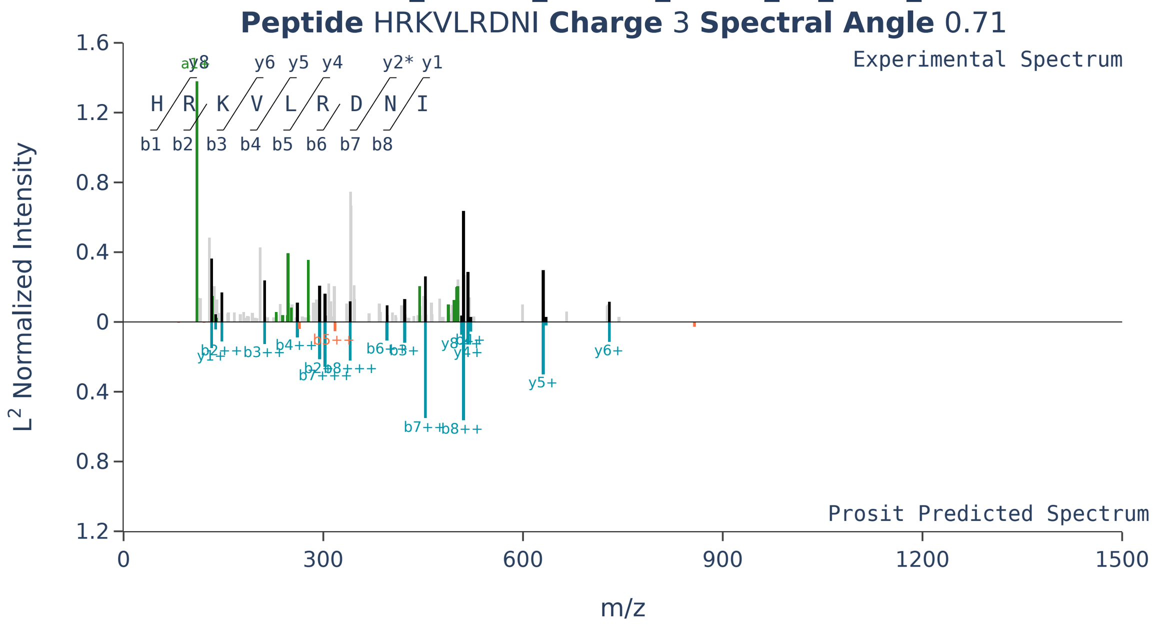


**Source** HRoetschke_291222_140123_HFGoe_H4_b1R1_1h **Scan** 7276
**Peptide** LRDAVTYTEHAKRKTVTAMD **Charge** 3 **Spectral Angle** 0.7

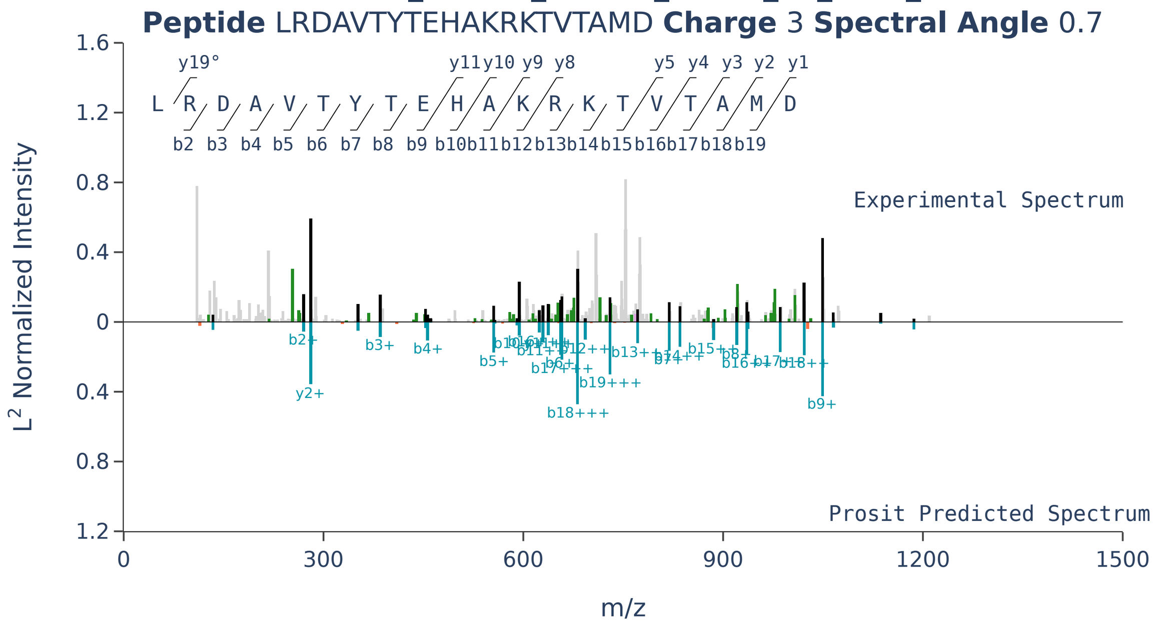

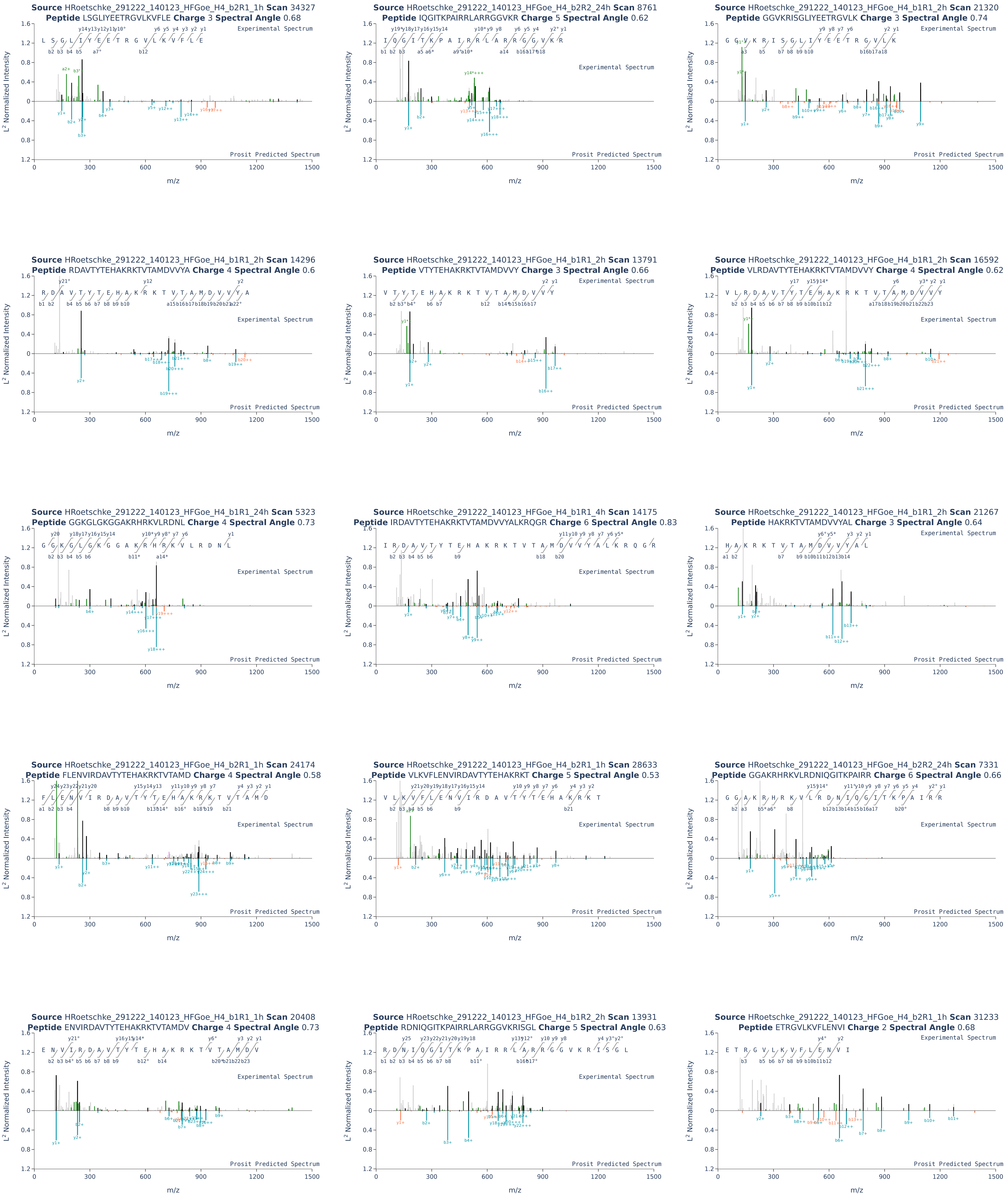
Source HRoetschke_291222_140123_HFGoe_H4_b2R1_1h Scan 34327
Peptide LSGLIYEETRGVLKVFLE Charge 3 Spectral Angle 0.68
Source HRoetschke_291222_140123_HFGoe_H4_b2R2_24h Scan 8761
Peptide IQGITKPAIRRLARRGGVKR Charge 5 Spectral Angle 0.62
Source HRoetschke_291222_140123_HFGoe_H4_b1R1_2h Scan 21320
Peptide GGVKRISGLIYEETRGVLK Charge 3 Spectral Angle 0.74
Source HRoetschke_291222_140123_HFGoe_H4_b1R1_2h Scan 14296
Peptide RDAVTYTEHAKRKTVTAMDVVYA Charge 4 Spectral Angle 0.6
Source HRoetschke_291222_140123_HFGoe_H4_b1R1_2h Scan 13791
Peptide VTYTEHAKRKTVTAMDVVY Charge 3 Spectral Angle 0.66
Source HRoetschke_291222_140123_HFGoe_H4_b1R1_2h Scan 16592
Peptide VLRDAVTYTEHAKRKTVTAMDVVY Charge 4 Spectral Angle 0.62
Source HRoetschke_291222_140123_HFGoe_H4_b1R1_24h Scan 5323
Peptide GGKGLGKGGAKRHRKVLRDNL Charge 4 Spectral Angle 0.73
Source HRoetschke_291222_140123_HFGoe_H4_b1R1_4h Scan 14175
Peptide IRDAVTYTEHAKRKTVTAMDVVYALKRQGR Charge 6 Spectral Angle 0.83
Source HRoetschke_291222_140123_HFGoe_H4_b1R1_2h Scan 21267
Peptide HAKRKTVTAMDVVYAL Charge 3 Spectral Angle 0.64
Source HRoetschke_291222_140123_HFGoe_H4_b2R1_1h Scan 24174
Peptide FLENVIRDAVTYTEHAKRKTVTAMD Charge 4 Spectral Angle 0.58
Source HRoetschke_291222_140123_HFGoe_H4_b2R1_1h Scan 28633
Peptide VLKVFLENVIRDAVTYTEHAKRKT Charge 5 Spectral Angle 0.53
Source HRoetschke_291222_140123_HFGoe_H4_b2R2_24h Scan 7331
Peptide GGAKRHRKVLRDNIQGITKPAIRR Charge 6 Spectral Angle 0.66
Source HRoetschke_291222_140123_HFGoe_H4_b1R1_1h Scan 20408
Peptide ENVIRDAVTYTEHAKRKTVTAMDV Charge 4 Spectral Angle 0.73
Source HRoetschke_291222_140123_HFGoe_H4_b1R2_2h Scan 13931
Peptide RDNIQGITKPAIRRLARRGGVKRISGL Charge 5 Spectral Angle 0.63
Source HRoetschke_291222_140123_HFGoe_H4_b2R1_1h Scan 31233
Peptide ETRGVLKVFLENVI Charge 2 Spectral Angle 0.68
Experimental Spectrum
Prosit Predicted Spectrum
L² Normalized Intensity
m/z

**Source** HRoetschke_291222_140123_HFGoe_H4_b1R2_4h **Scan** 13707
**Peptide** QGITKPAIRRLARRGGVKRISGLI **Charge** 5 **Spectral Angle** 0.88

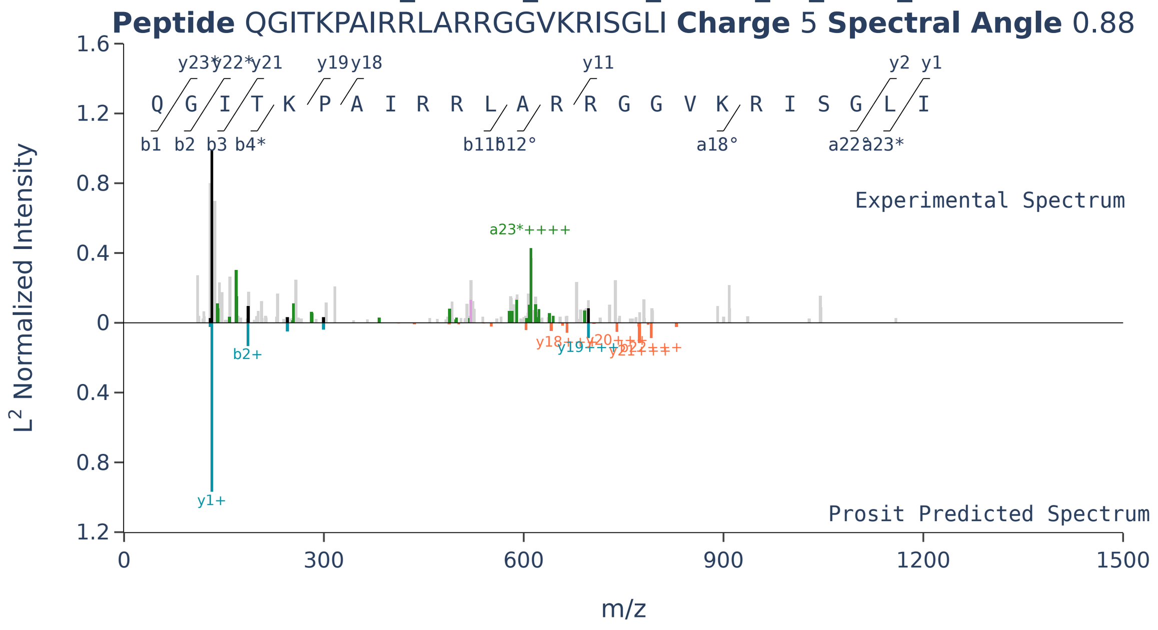

**Source** HRoetschke_291222_140123_HFGoe_H4_b2R2_4h **Scan** 12871
**Peptide** HRKVLRDNIQGITKPAL **Charge** 4 **Spectral Angle** 0.57

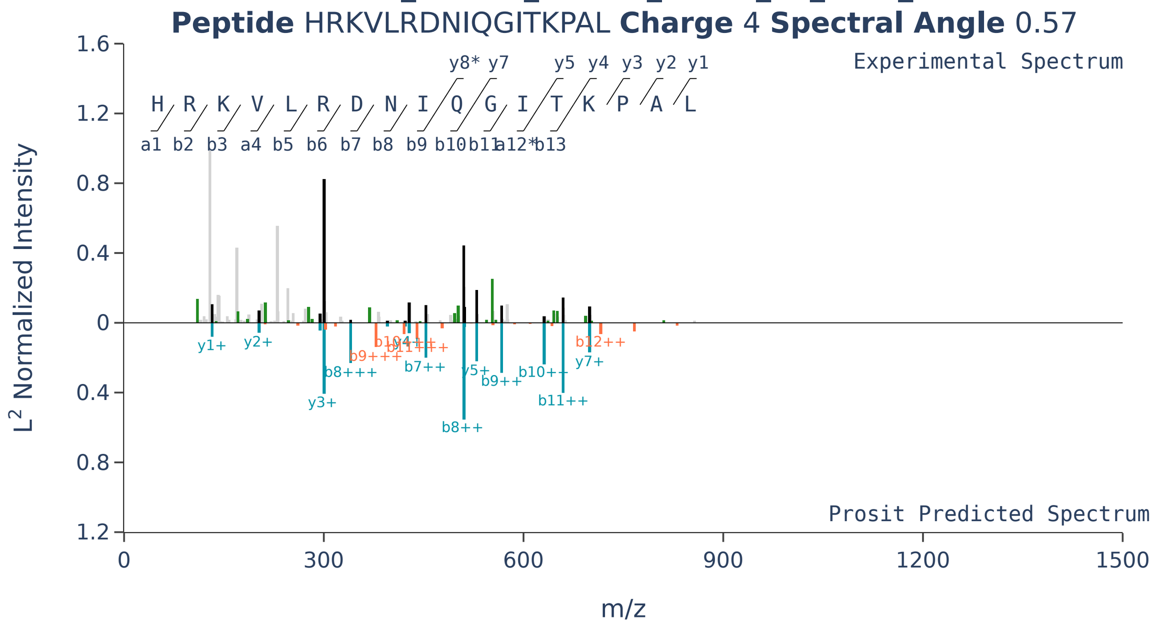

**Source** HRoetschke_291222_140123_HFGoe_H4_b2R1_1h **Scan** 26803
**Peptide** VFLENVIRDAVTYTEHAKRKTVTA **Charge** 5 **Spectral Angle** 0.58

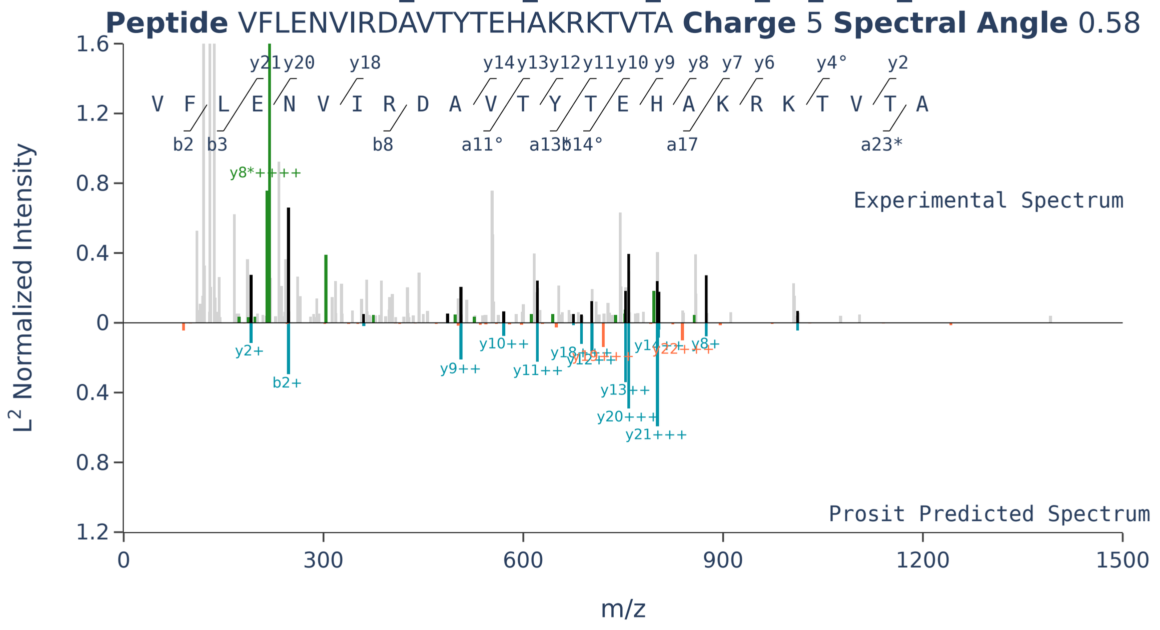

**Source** HRoetschke_291222_140123_HFGoe_H4_b1R1_24h **Scan** 13379
**Peptide** LRDNIQGITKPAIRRLARRGGV **Charge** 6 **Spectral Angle** 0.66

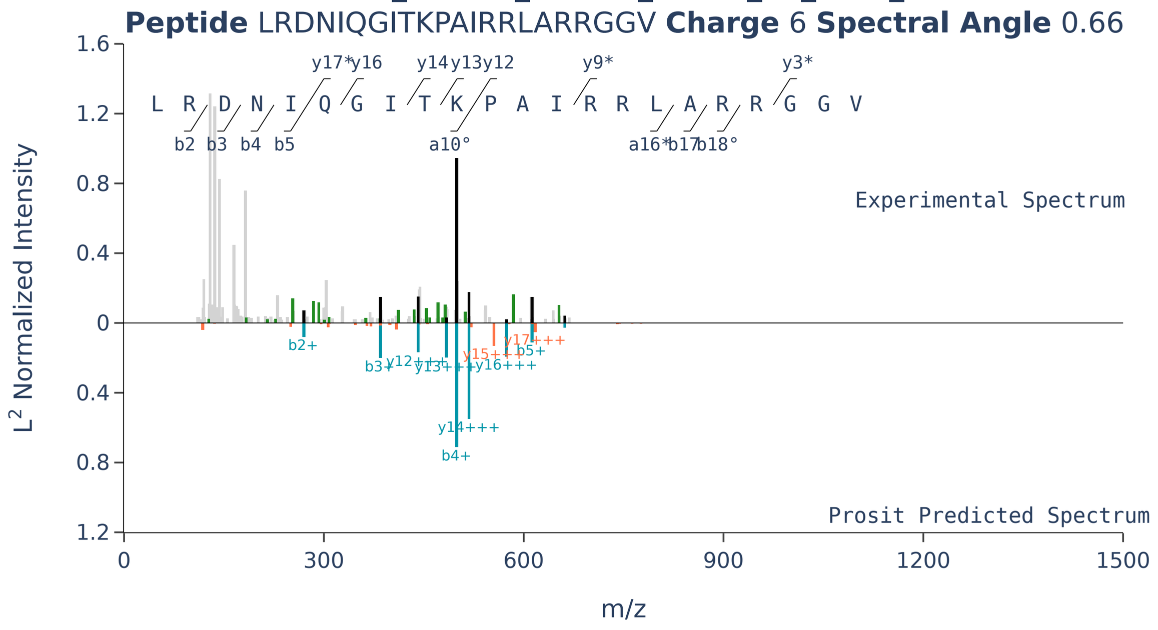

**Source** HRoetschke_291222_140123_HFGoe_H4_b2R1_24h **Scan** 4949
**Peptide** GGKGLGKGGAKRHRKVLRD **Charge** 5 **Spectral Angle** 0.74

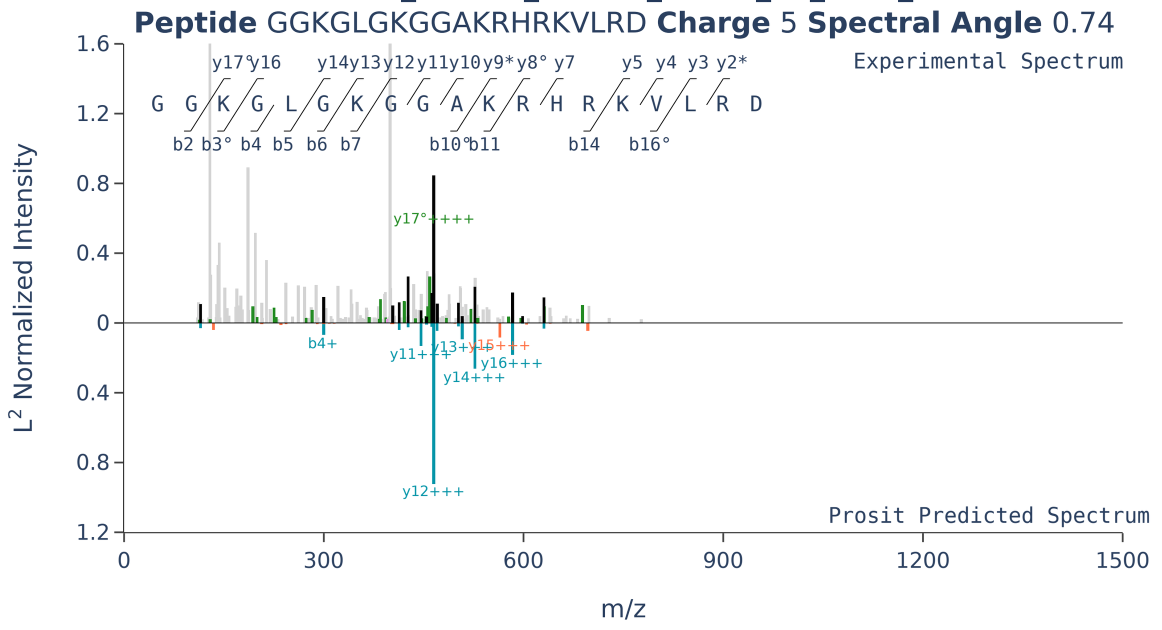

**Source** HRoetschke_291222_140123_HFGoe_H4_b1R1_2h **Scan** 12208
**Peptide** RDAVTYTEHAKRKTVTAMDVV **Charge** 5 **Spectral Angle** 0.68

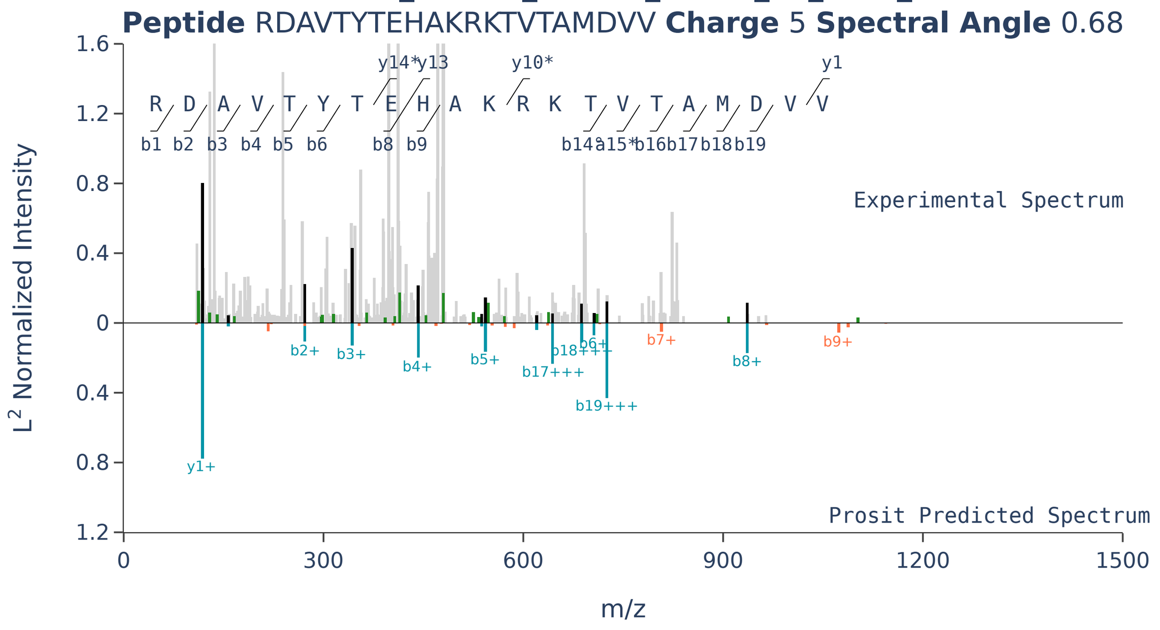

**Source** HRoetschke_291222_140123_HFGoe_H4_b1R2_2h **Scan** 5624
**Peptide** TKPAIRRLARRG **Charge** 3 **Spectral Angle** 0.65

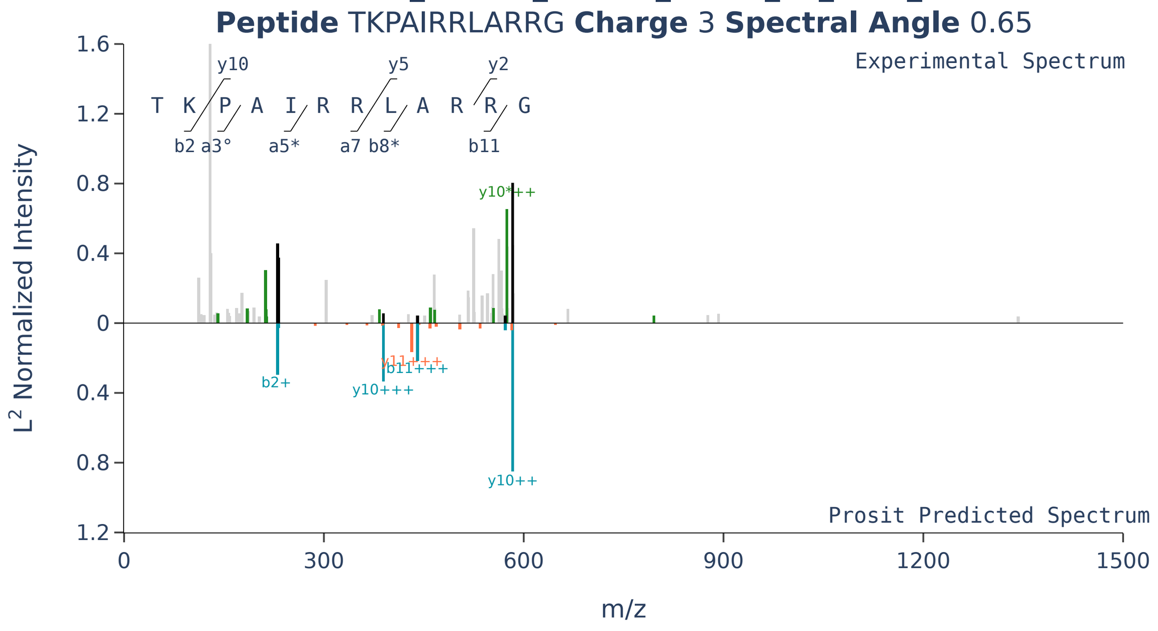

**Source** HRoetschke_291222_140123_HFGoe_H4_b2R1_1h **Scan** 15616
**Peptide** IQGITKPAIRRLARRGGVKRISGLIYE **Charge** 4 **Spectral Angle** 0.58

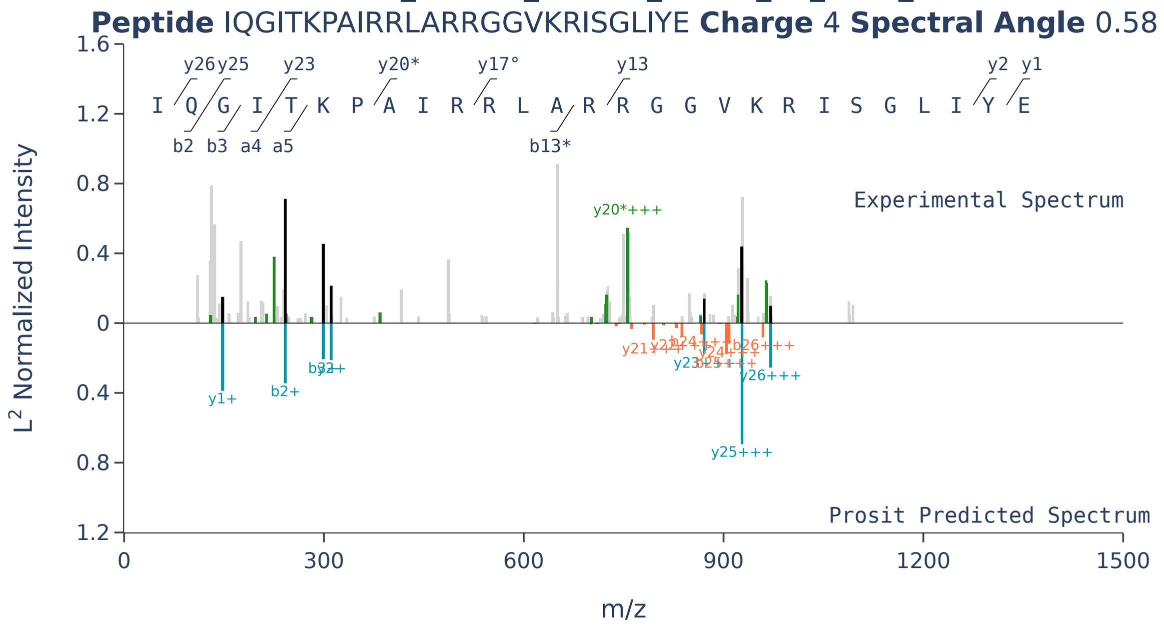

**Source** HRoetschke_291222_140123_HFGoe_H4_b1R2_2h **Scan** 16806
**Peptide** LQGITKPAIRRLARRGGVKRISGLIYE **Charge** 5 **Spectral Angle** 0.84

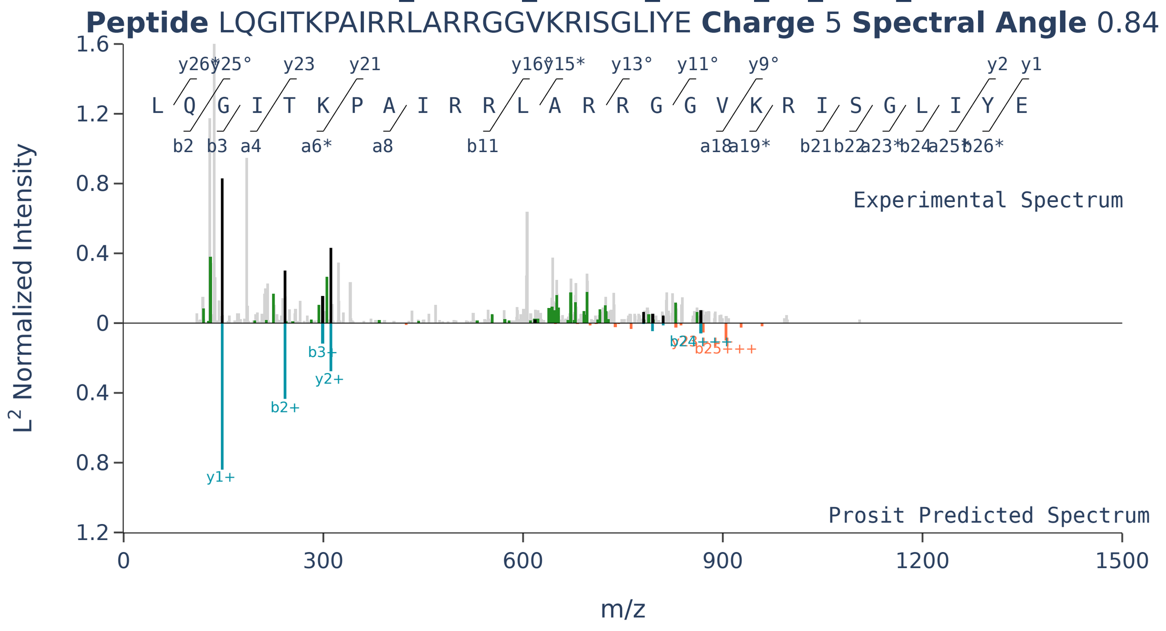

**Source** HRoetschke_291222_140123_HFGoe_H4_b2R1_2h **Scan** 18151
**Peptide** AVTYTEHAKRKTVTAMDVVYALK **Charge** 5 **Spectral Angle** 0.79

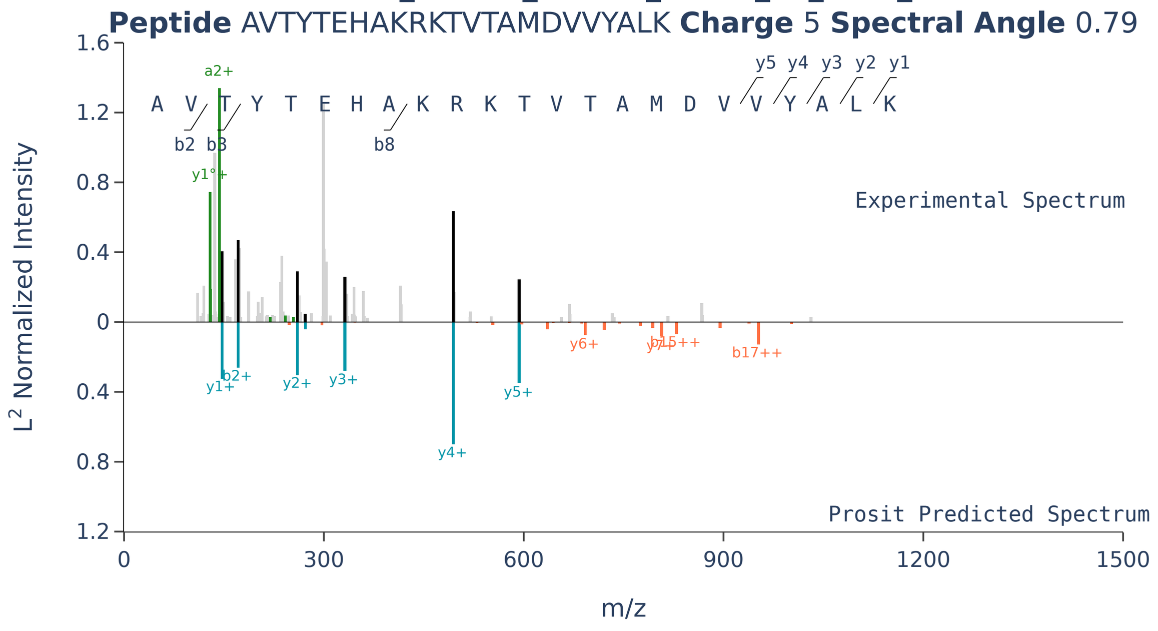

**Source** HRoetschke_291222_140123_HFGoe_H4_b1R2_24h **Scan** 5733
**Peptide** VTYTEHAKRKTVTA **Charge** 3 **Spectral Angle** 0.47

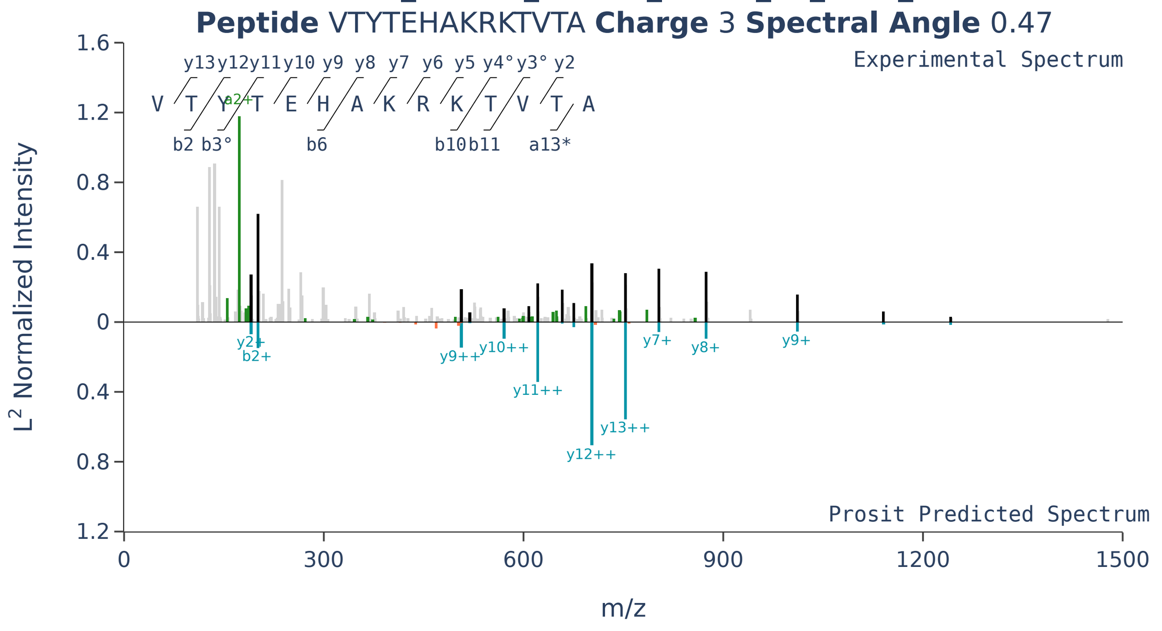

**Source** HRoetschke_291222_140123_HFGoe_H4_b1R1_2h **Scan** 21070
**Peptide** KRISGLIYEETRGVLKV **Charge** 3 **Spectral Angle** 0.83

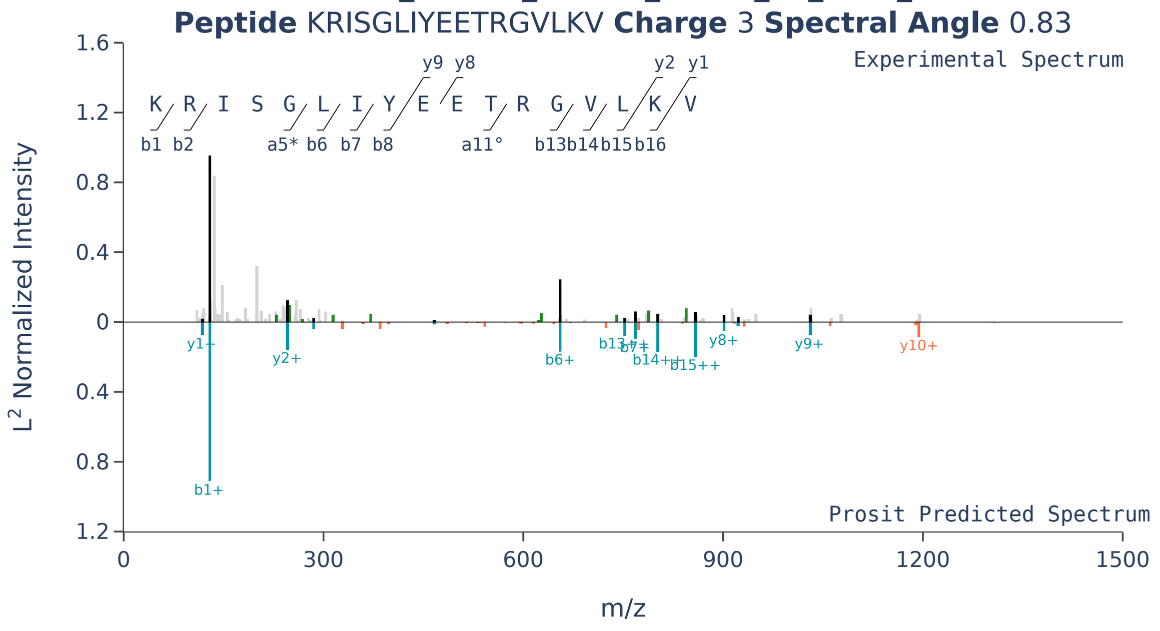

**Source** HRoetschke_291222_140123_HFGoe_H4_b1R1_4h **Scan** 8585
**Peptide** DAVTYTEHAKRKTVTAMD **Charge** 4 **Spectral Angle** 0.75

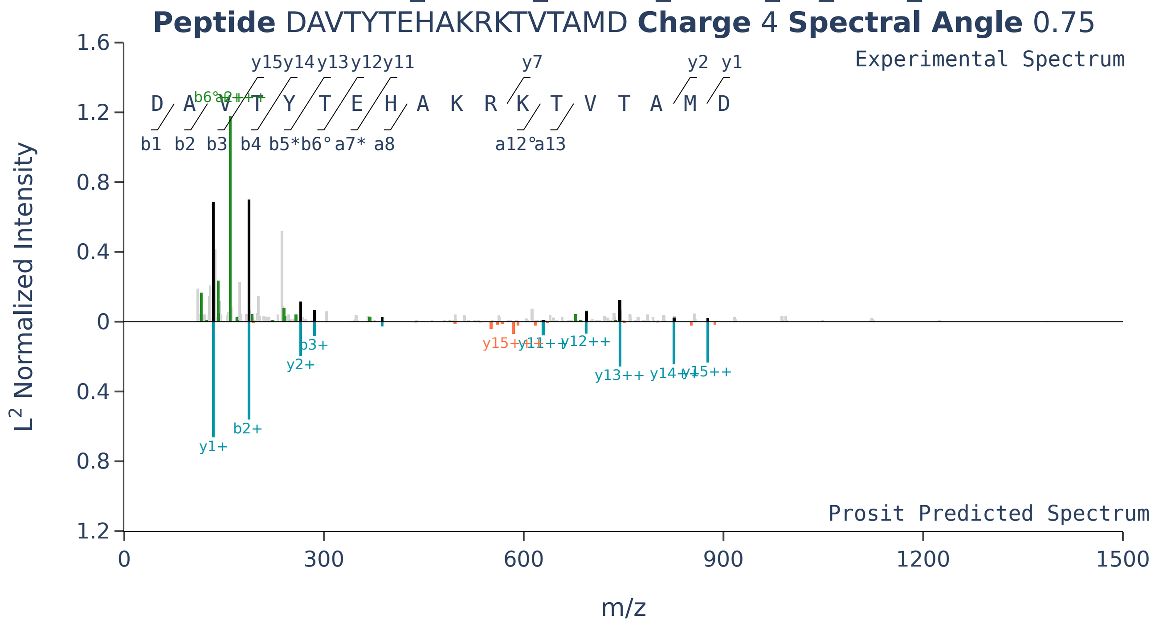

**Source** HRoetschke_291222_140123_HFGoe_H4_b1R1_2h **Scan** 10316
**Peptide** EHAKRKTVTAMDVV **Charge** 3 **Spectral Angle** 0.48

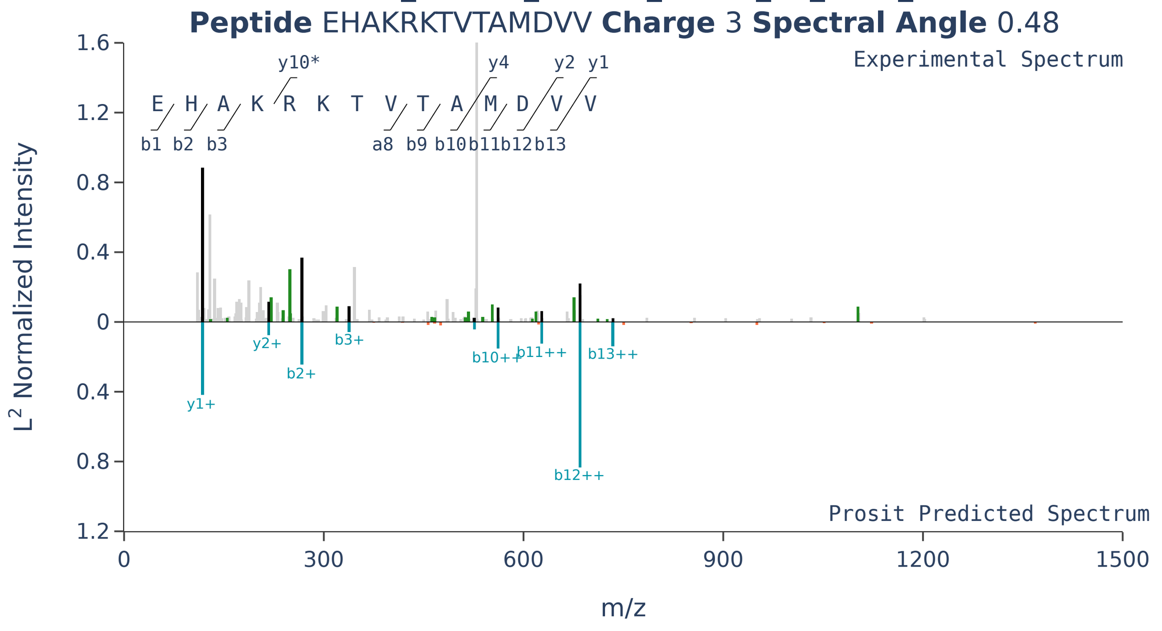

**Source** HRoetschke_291222_140123_HFGoe_H4_b1R1_4h **Scan** 9657
**Peptide** HRKVLRDNIQGITKPAIRRLARR **Charge** 6 **Spectral Angle** 0.72

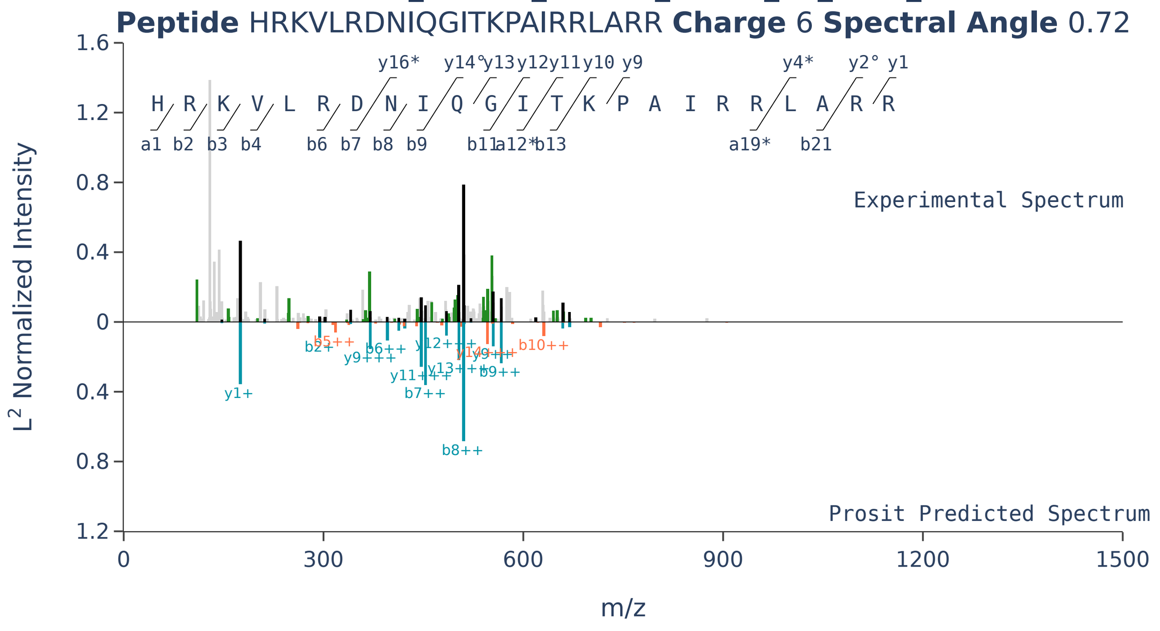

**Source** HRoetschke_291222_140123_HFGoe_H4_b1R2_1h **Scan** 10391
**Peptide** LQGITKPAIRRLARRGGVKRI **Charge** 5 **Spectral Angle** 0.75

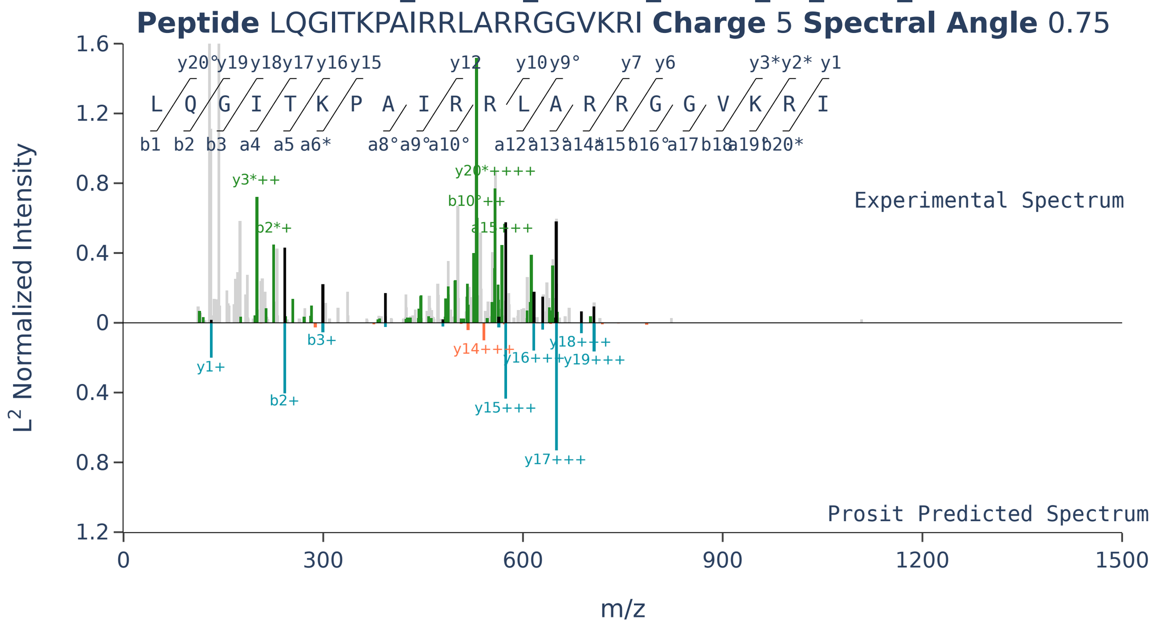


**Source** HRoetschke_291222_140123_HFGoe_H4_b2R2_4h **Scan** 10510
**Peptide** RDNIQGITKPAIRRLARRGGVK **Charge** 5 **Spectral Angle** 0.59

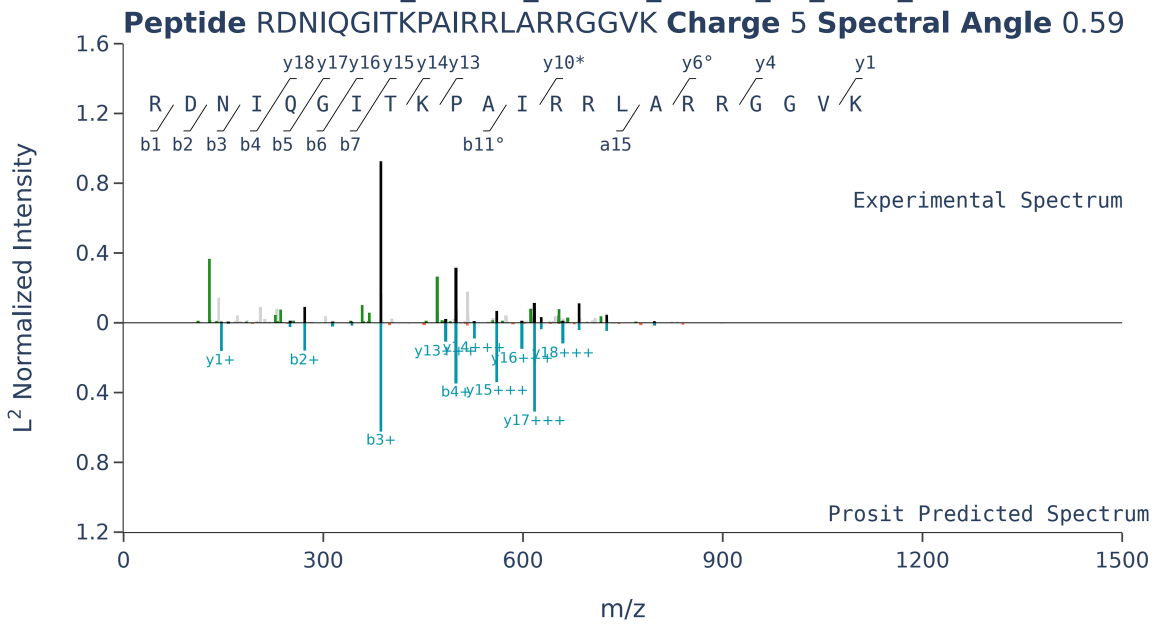


**Source** HRoetschke_291222_140123_HFGoe_H4_b1R2_2h **Scan** 6315
**Peptide** TKPAIRRLAR **Charge** 3 **Spectral Angle** 0.51

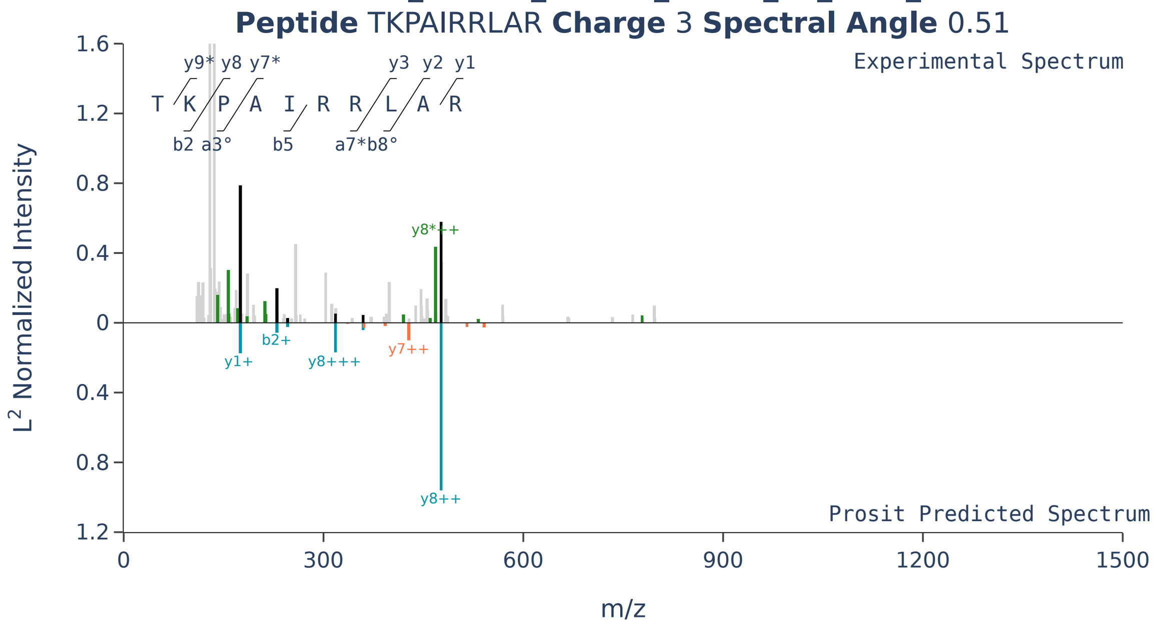


**Source** HRoetschke_291222_140123_HFGoe_H4_b2R1_4h **Scan** 11683
**Peptide** LQGITKPAIRRLAR **Charge** 4 **Spectral Angle** 0.55

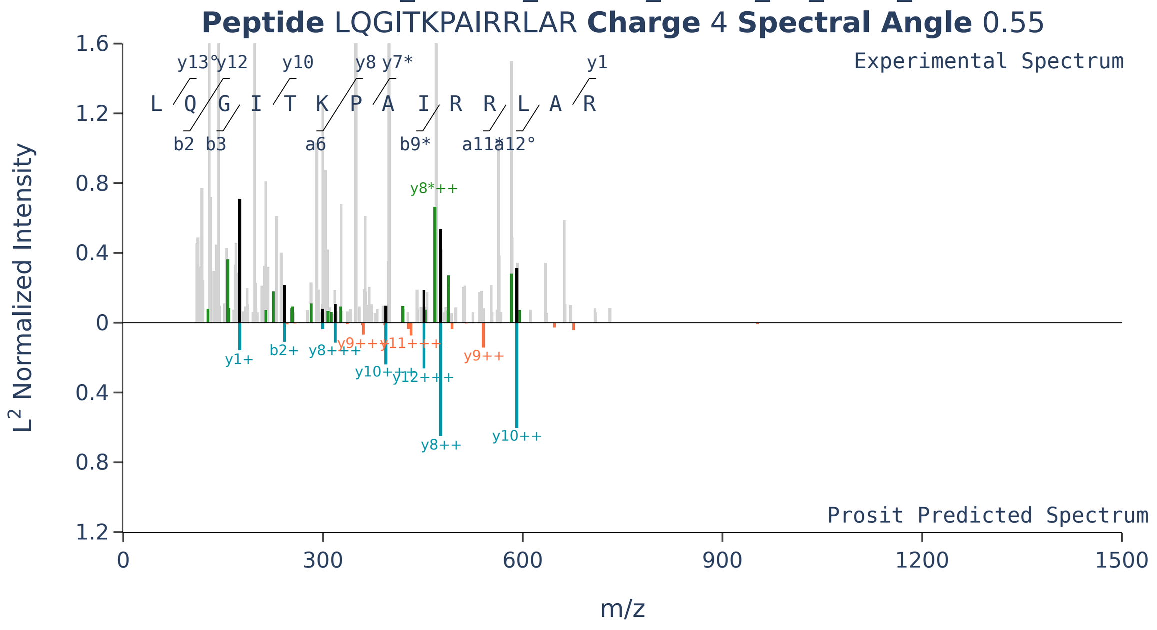


**Source** HRoetschke_291222_140123_HFGoe_H4_b2R1_1h **Scan** 26352
**Peptide** TVTAMDVVYALKRQGRTLY **Charge** 3 **Spectral Angle** 0.47

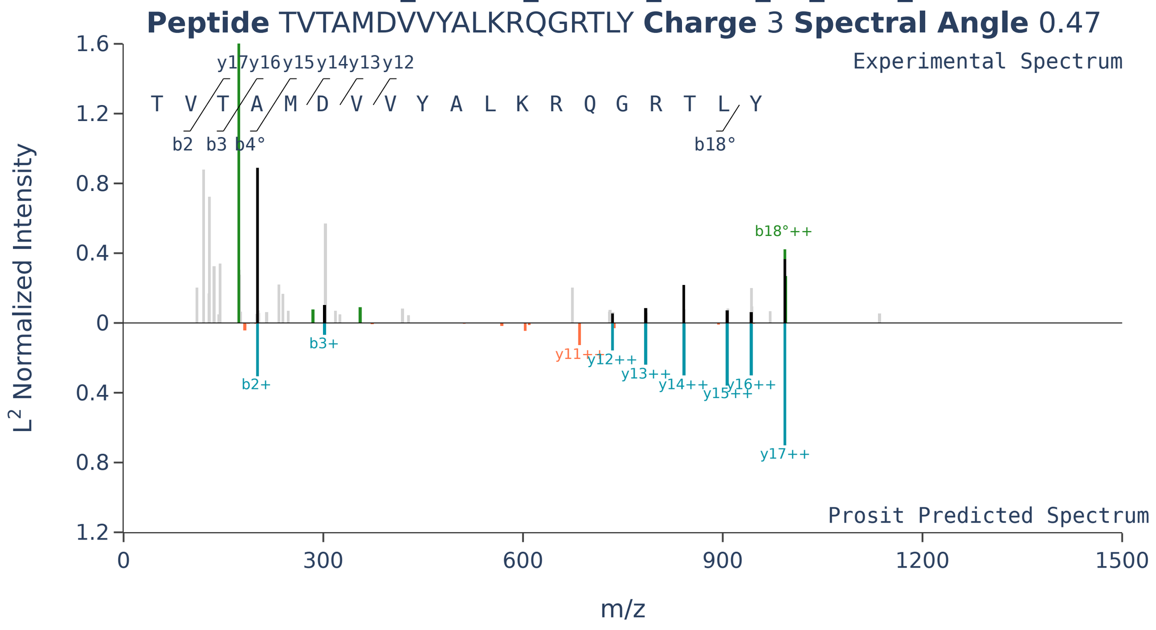


**Source** HRoetschke_291222_140123_HFGoe_H4_b1R1_1h **Scan** 25503
**Peptide** FLENVIRDAVT **Charge** 2 **Spectral Angle** 0.67

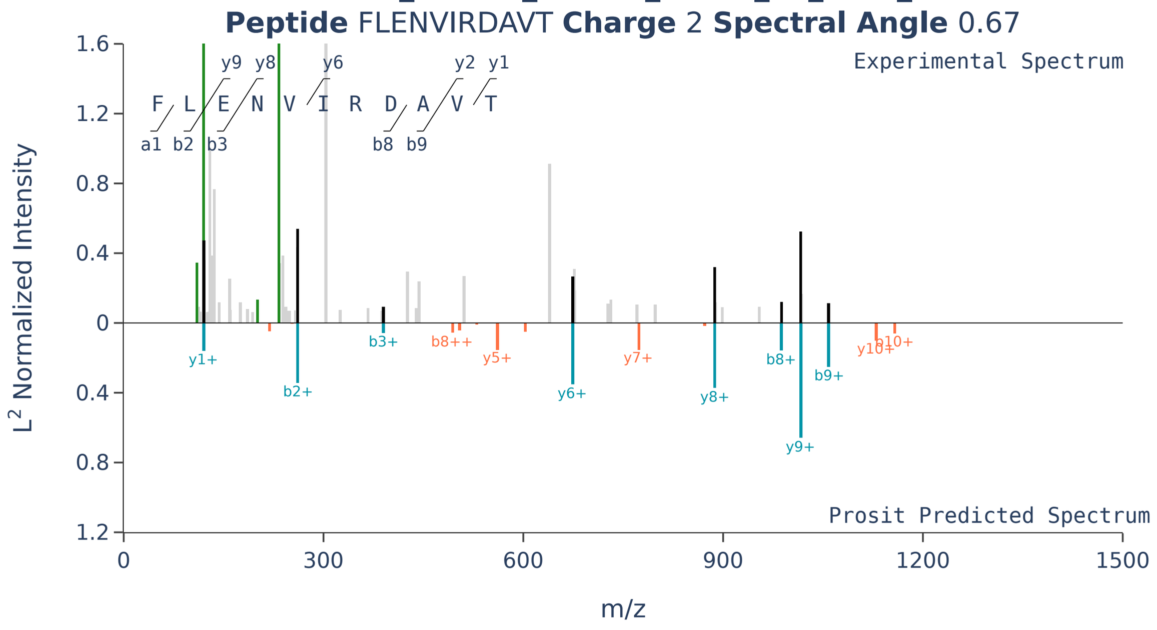


**Source** HRoetschke_291222_140123_HFGoe_H4_b2R1_1h **Scan** 17273
**Peptide** LRDNIQGITKPAIRRLARRGGVKRISGLI **Charge** 5 **Spectral Angle** 0.59

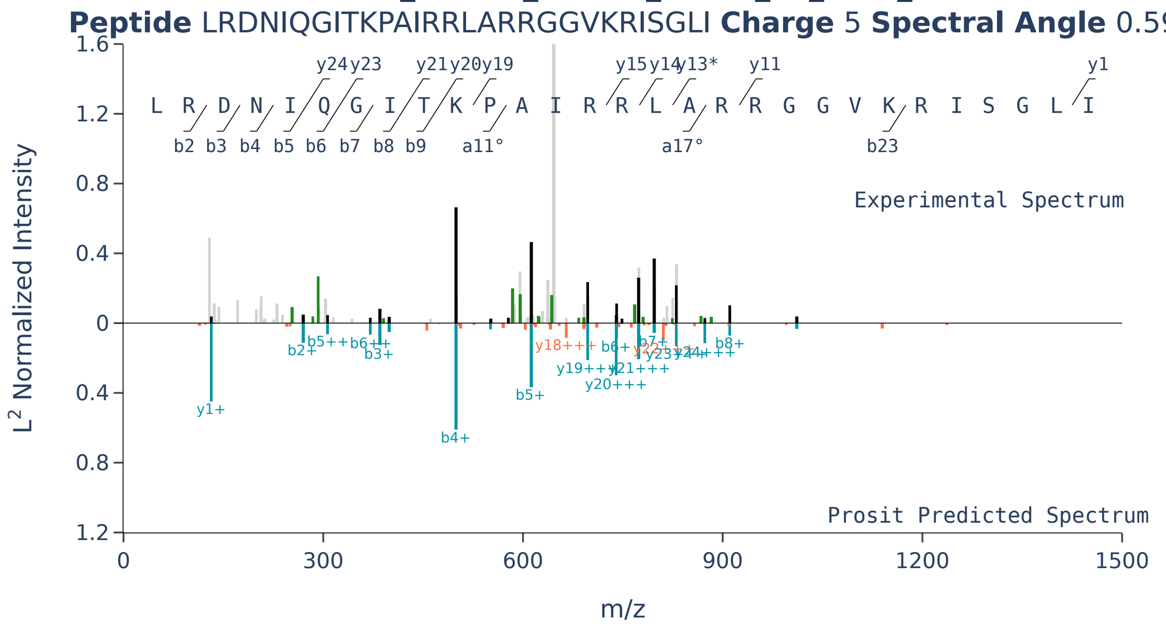


**Source** HRoetschke_291222_140123_HFGoe_H4_b1R2_24h **Scan** 5108
**Peptide** SGRGKGGKGLGKGGAKRHRKVLRDNIQ **Charge** 6 **Spectral Angle** 0.66

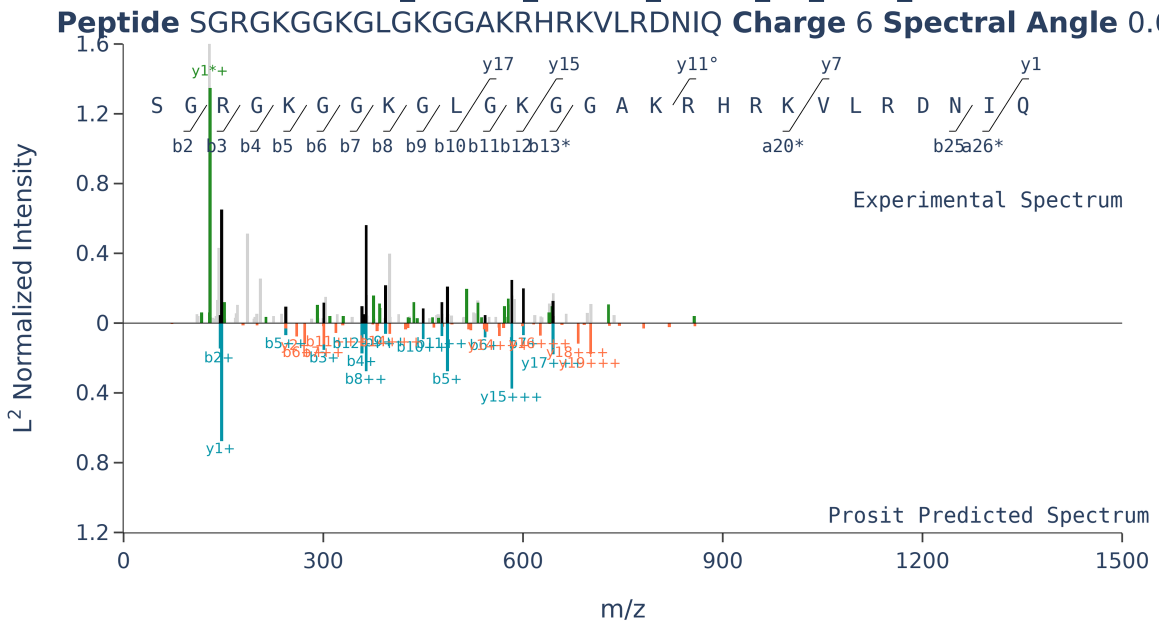


**Source** HRoetschke_291222_140123_HFGoe_H4_b2R1_24h **Scan** 8367
**Peptide** LQGITKPAIRRLARRGGVK **Charge** 5 **Spectral Angle** 0.69

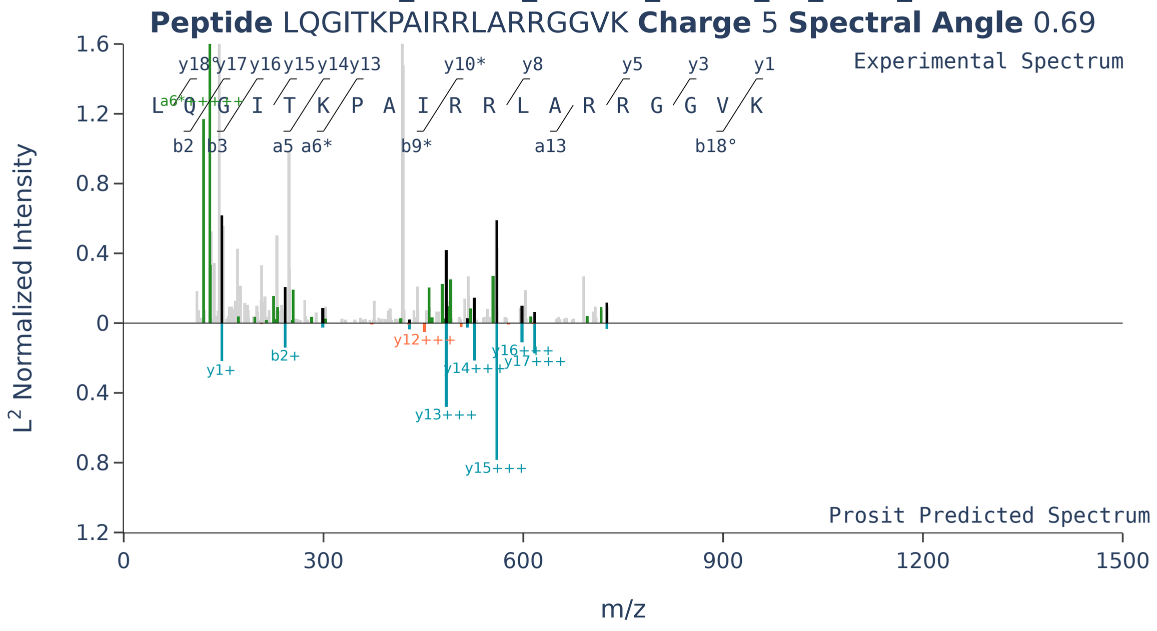


**Source** HRoetschke_291222_140123_HFGoe_H4_b2R1_4h **Scan** 13497
**Peptide** IYEETRG **Charge** 1 **Spectral Angle** 0.93

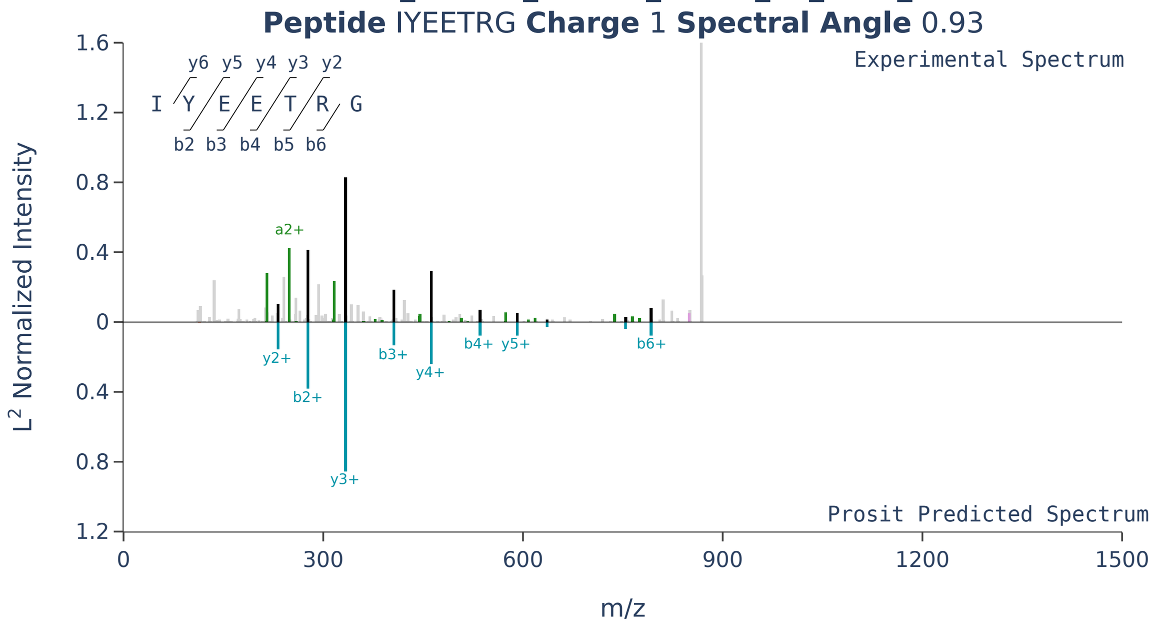


**Source** HRoetschke_291222_140123_HFGoe_H4_b2R2_24h **Scan** 7687
**Peptide** KGGAKRHRKVLRDNIQGITKPALR **Charge** 5 **Spectral Angle** 0.89

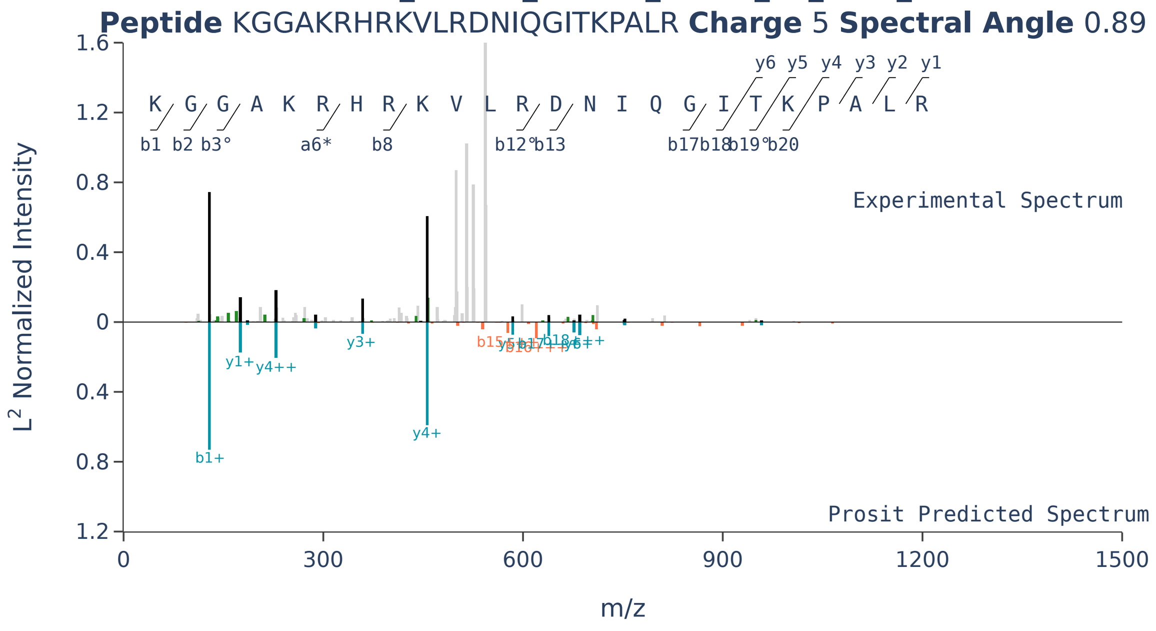


**Source** HRoetschke_291222_140123_HFGoe_H4_b2R1_1h **Scan** 33965
**Peptide** LSGLIYEETRGVLKVFL **Charge** 3 **Spectral Angle** 0.71

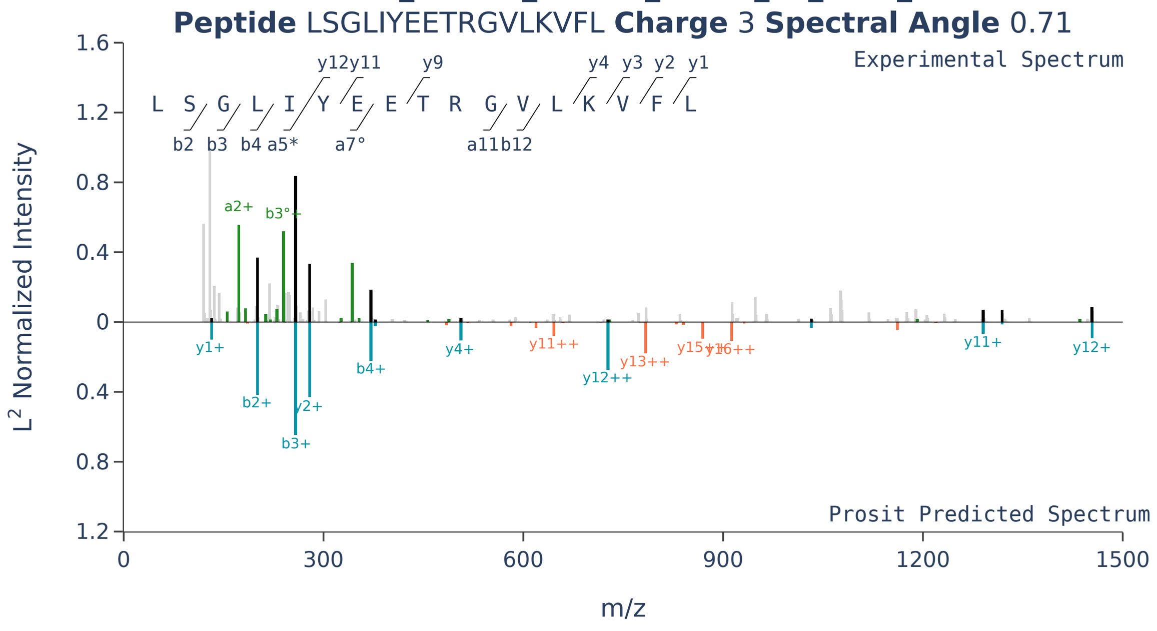


**Source** HRoetschke_291222_140123_HFGoe_H4_b1R1_4h **Scan** 17182
**Peptide** GGVKRISGLLY **Charge** 3 **Spectral Angle** 0.84

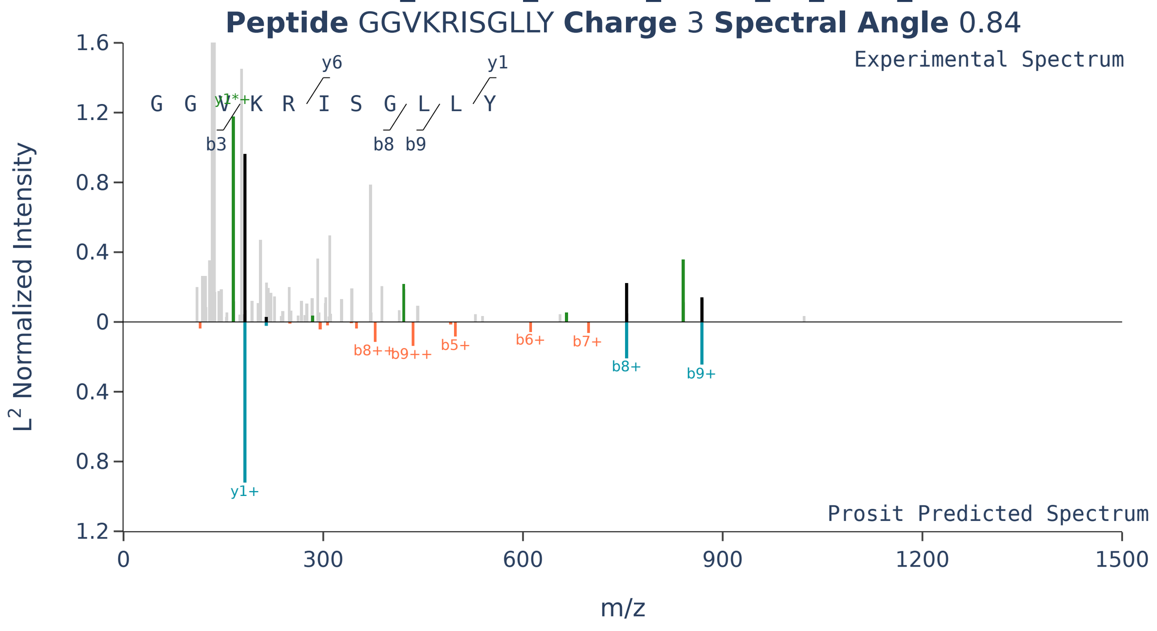


**Source** HRoetschke_291222_140123_HFGoe_H4_b1R1_4h **Scan** 9162
**Peptide** IQGITKPAIRRLARRGGVKRI **Charge** 4 **Spectral Angle** 0.76

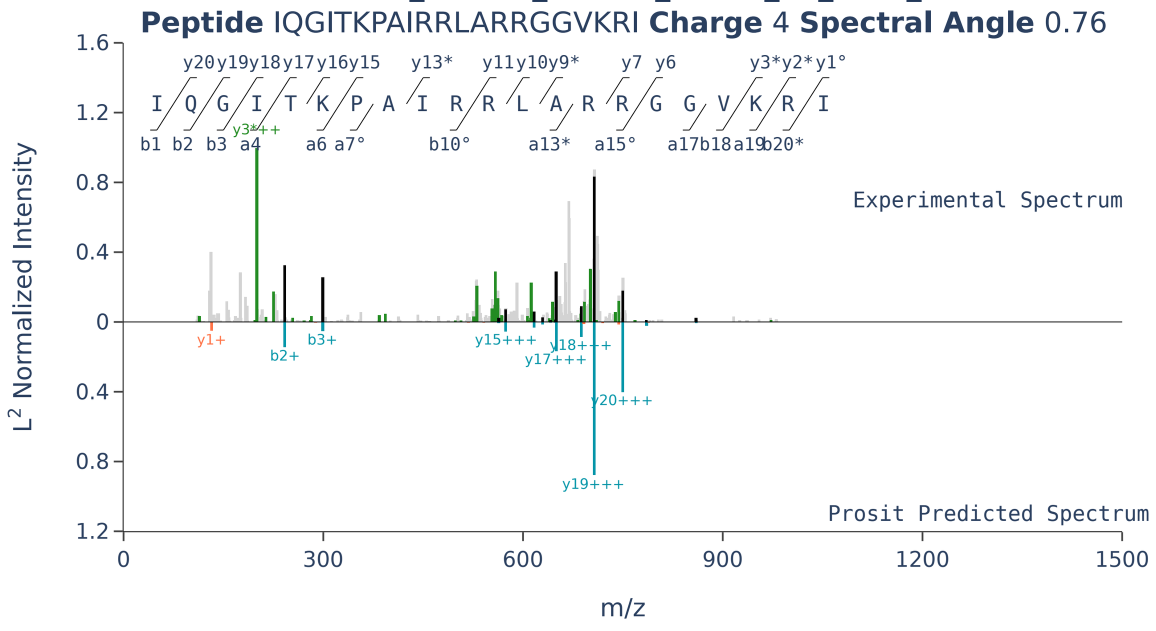


**Source** HRoetschke_291222_140123_HFGoe_H4_b2R1_4h **Scan** 8664
**Peptide** IQGITKPAIRRLARRG **Charge** 4 **Spectral Angle** 0.7

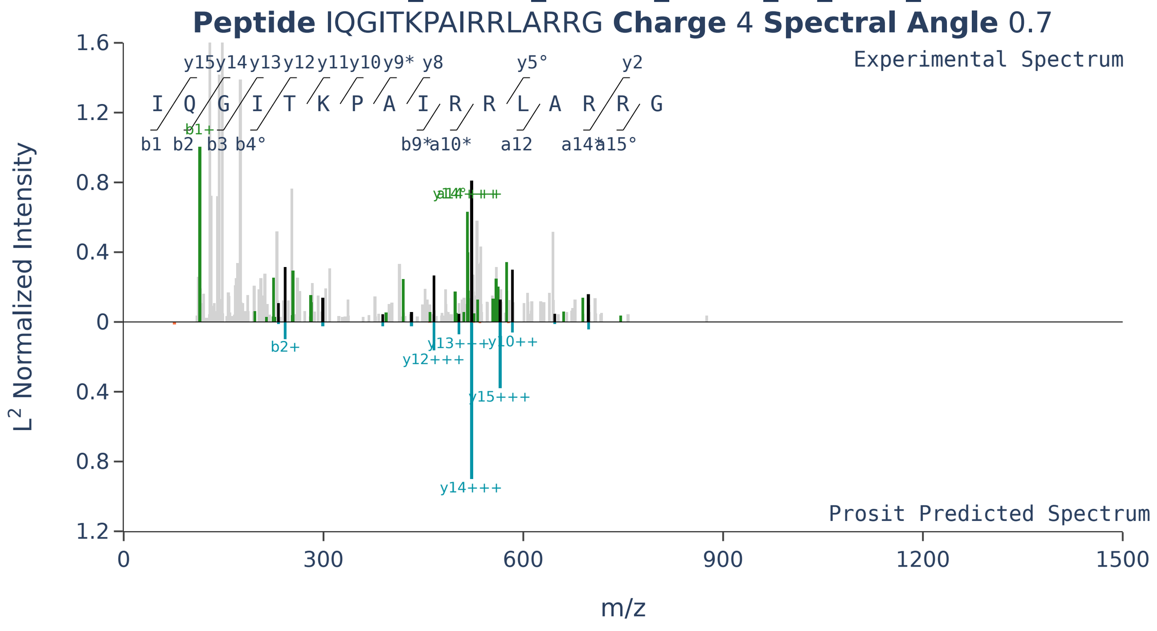

**Source** HRoetschke_291222_140123_HFGoe_H4_b1R1_4h **Scan** 8036
**Peptide** VIRDAVTYTEHAKRKTVTA **Charge** 5 **Spectral Angle** 0.72

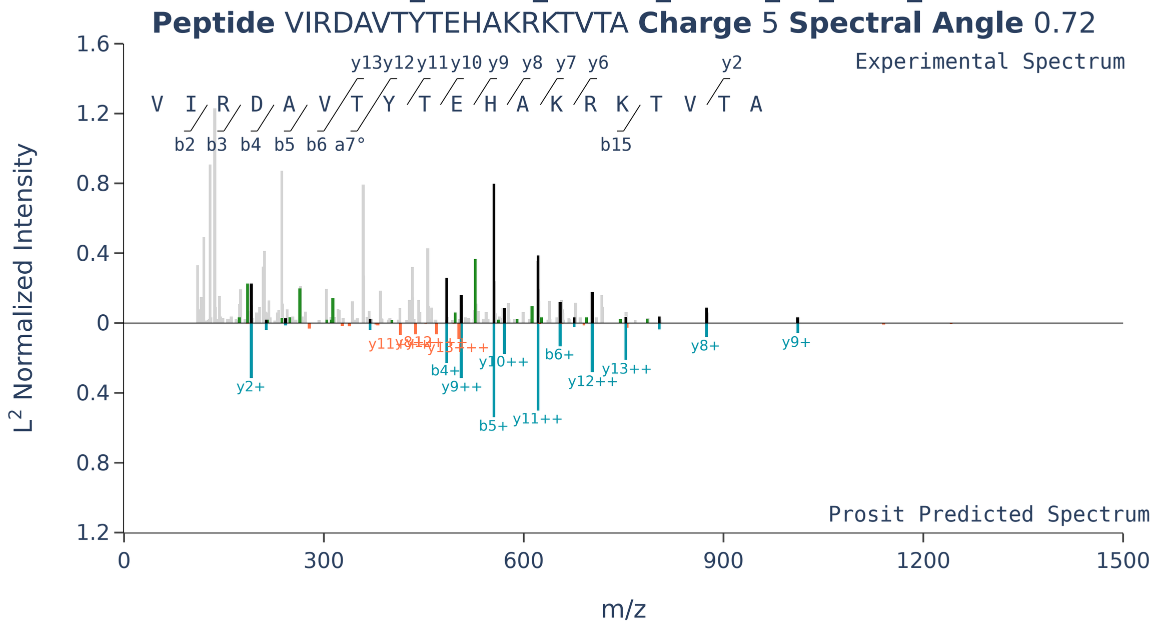


**Source** HRoetschke_291222_140123_HFGoe_H4_b1R2_2h **Scan** 10845
**Peptide** RKVLRDNIQGITKPAIRRLARR **Charge** 6 **Spectral Angle** 0.65

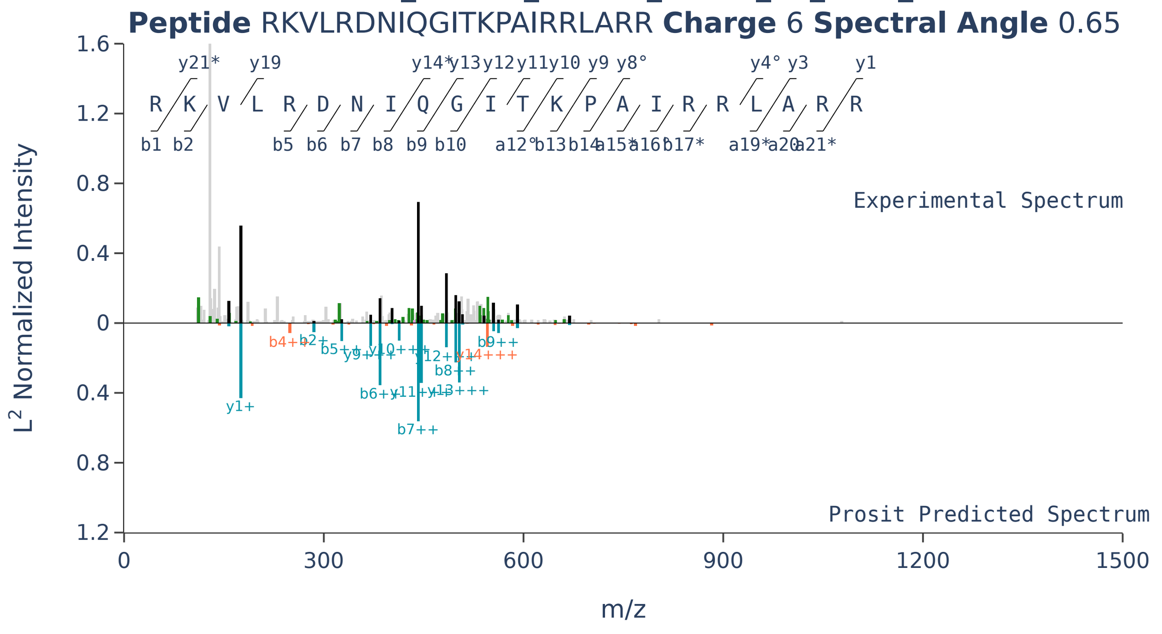


**Source** HRoetschke_291222_140123_HFGoe_H4_b1R1_24h **Scan** 16083
**Peptide** VYALKRQGR **Charge** 2 **Spectral Angle** 0.88

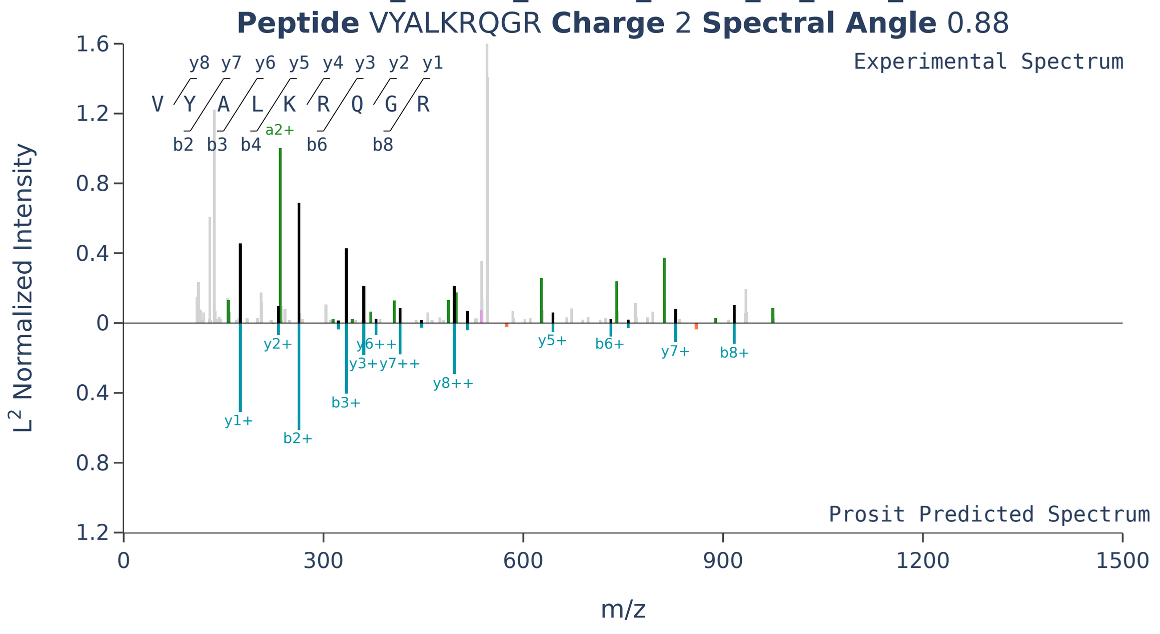


**Source** HRoetschke_291222_140123_HFGoe_H4_b1R1_4h **Scan** 9515
**Peptide** KRHRKVLRDNIQGITKPAIRRL **Charge** 6 **Spectral Angle** 0.66

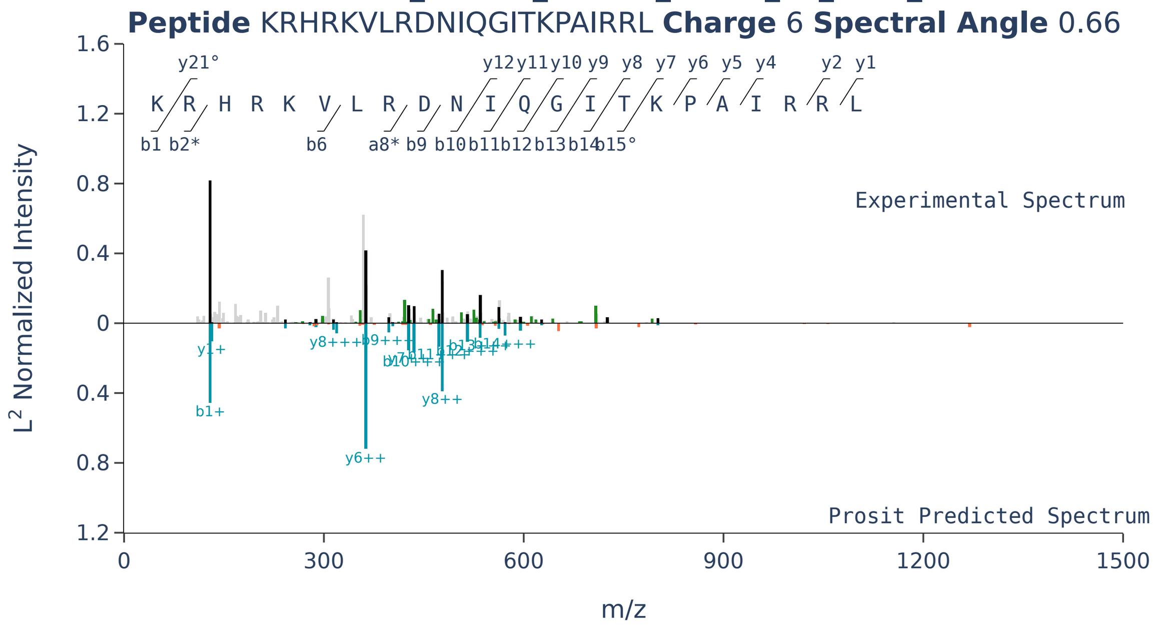


**Source** HRoetschke_291222_140123_HFGoe_H4_b2R1_1h **Scan** 36061
**Peptide** SGLIYEETRGVLKVFLENV **Charge** 3 **Spectral Angle** 0.79

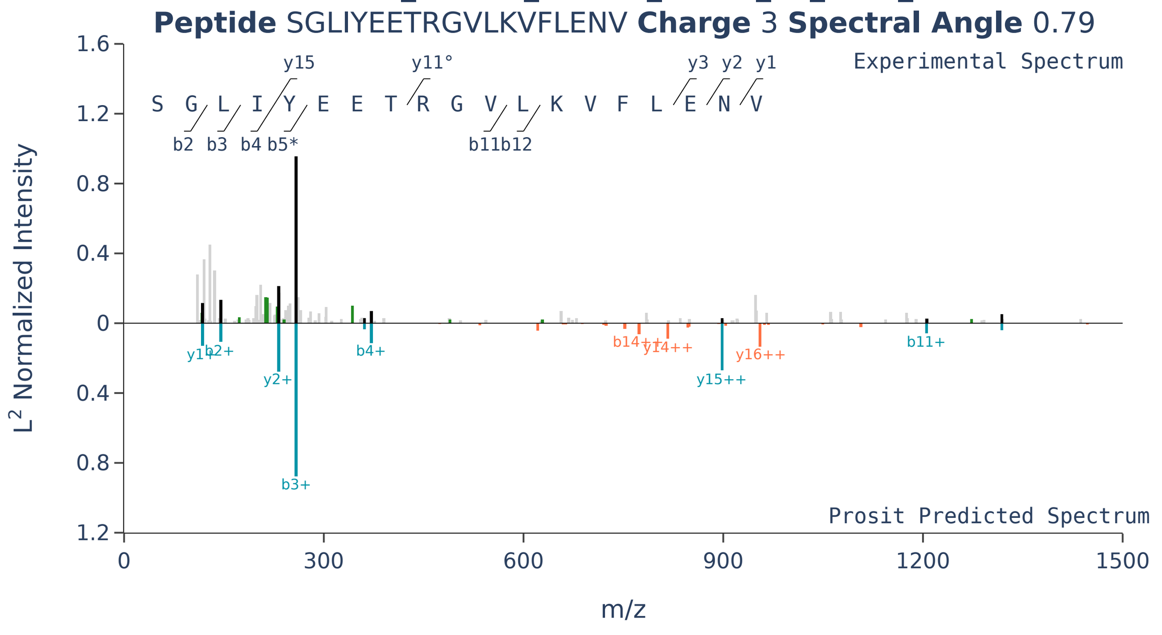


**Source** HRoetschke_291222_140123_HFGoe_H4_b2R1_1h **Scan** 28936
**Peptide** VLKVFLENVIRDAVTYTEHAKRKTVTA **Charge** 6 **Spectral Angle** 0.67

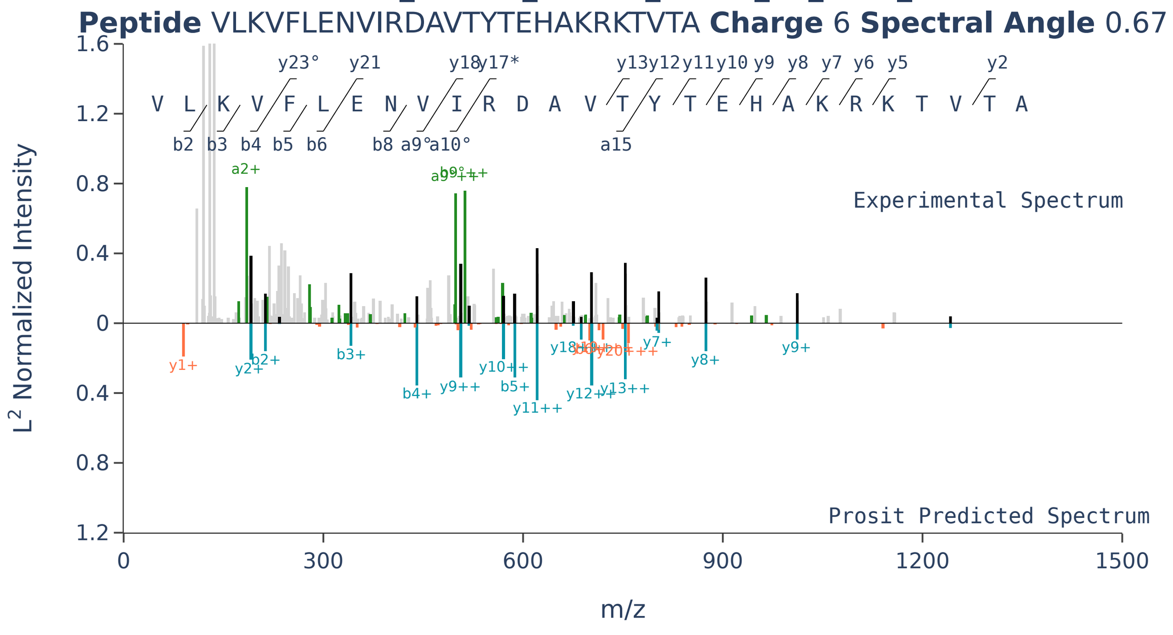


**Source** HRoetschke_291222_140123_HFGoe_H4_b1R1_2h **Scan** 8573
**Peptide** VLRDAVTYTEHAKRKTVTA **Charge** 5 **Spectral Angle** 0.77

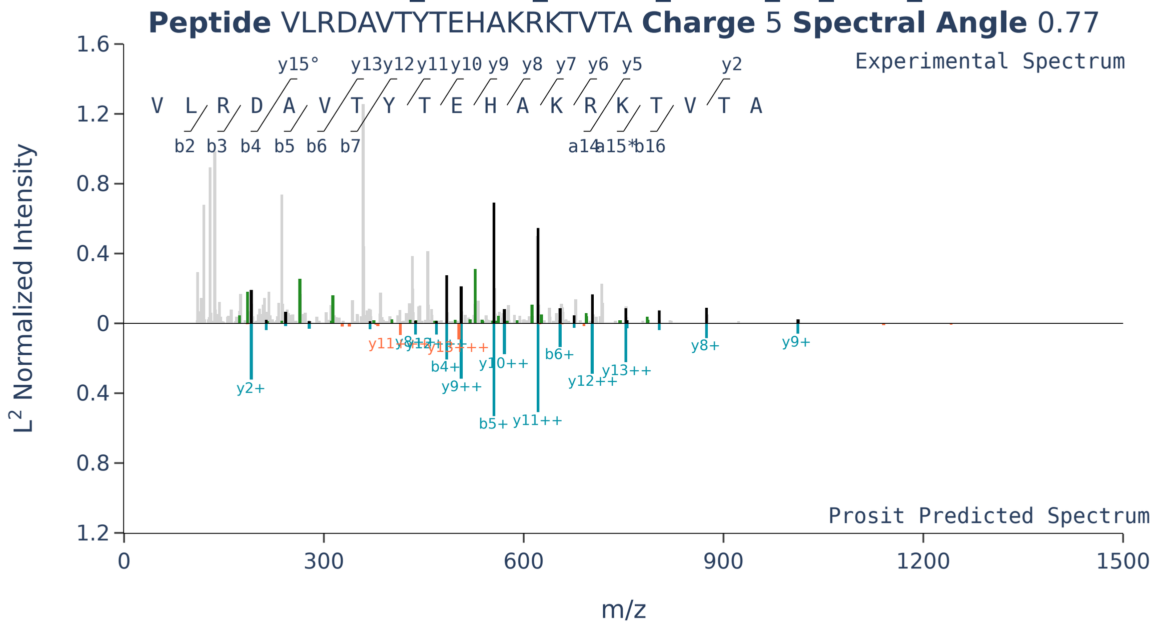


**Source** HRoetschke_291222_140123_HFGoe_H4_b2R1_1h **Scan** 33814
**Peptide** ISGLIYEETRGVLKVFLEN **Charge** 3 **Spectral Angle** 0.75

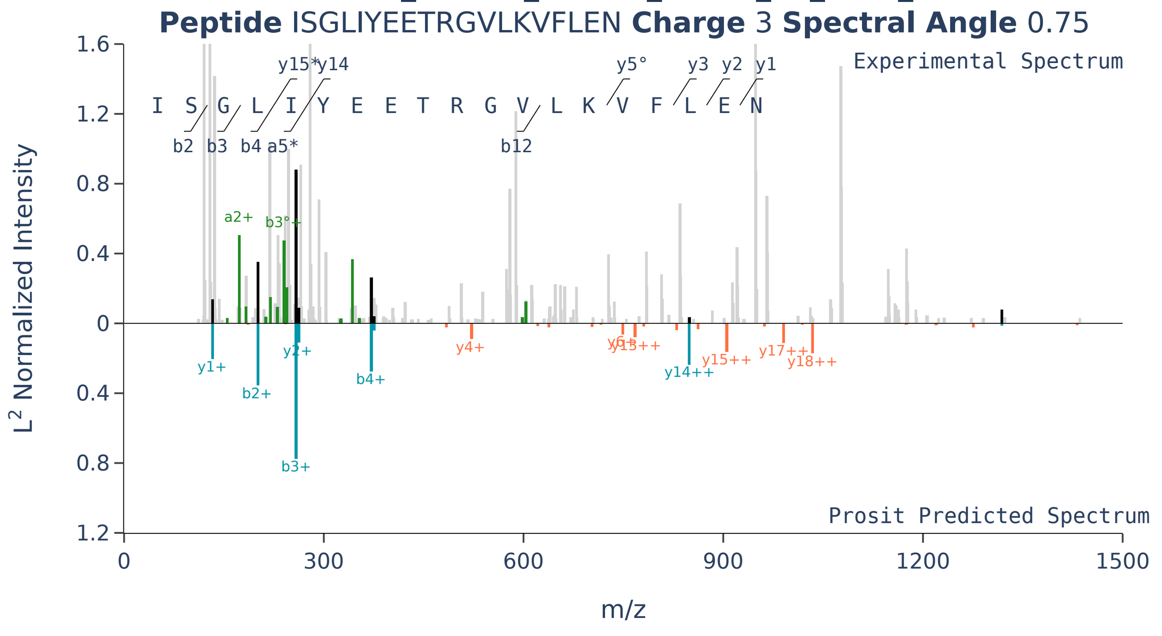


**Source** HRoetschke_291222_140123_HFGoe_H4_b1R1_2h **Scan** 15657
**Peptide** LRDAVTYTEHAKRKTVTAMDVVYA **Charge** 5 **Spectral Angle** 0.66

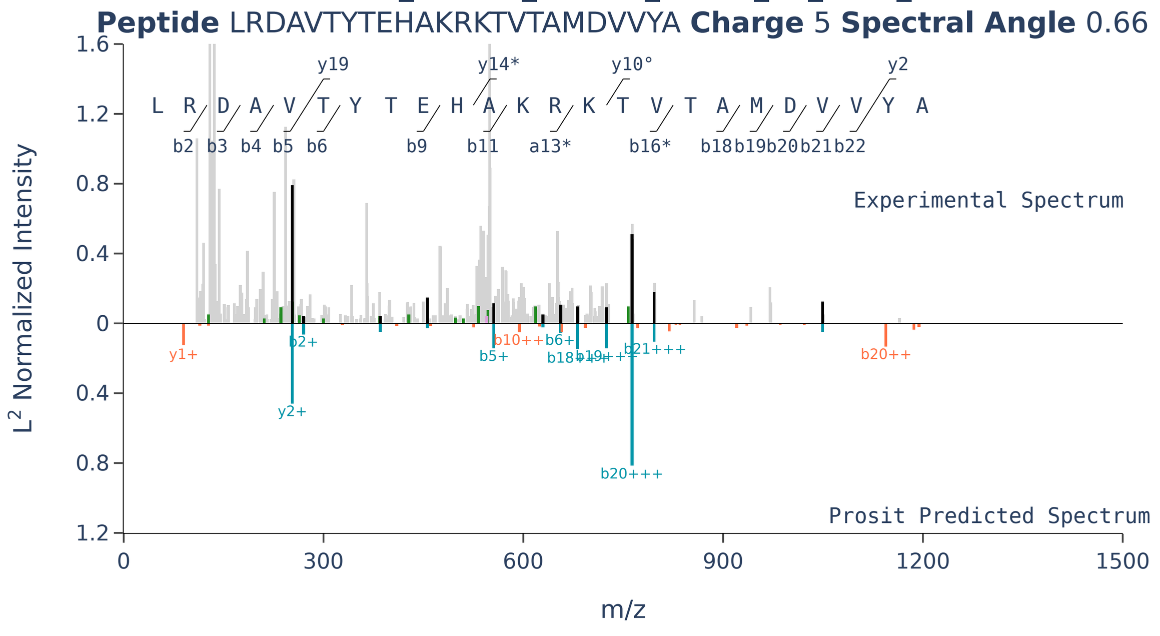


**Source** HRoetschke_291222_140123_HFGoe_H4_b1R1_2h **Scan** 7234
**Peptide** LRDAVTYTEHAKRKTVTA **Charge** 5 **Spectral Angle** 0.81

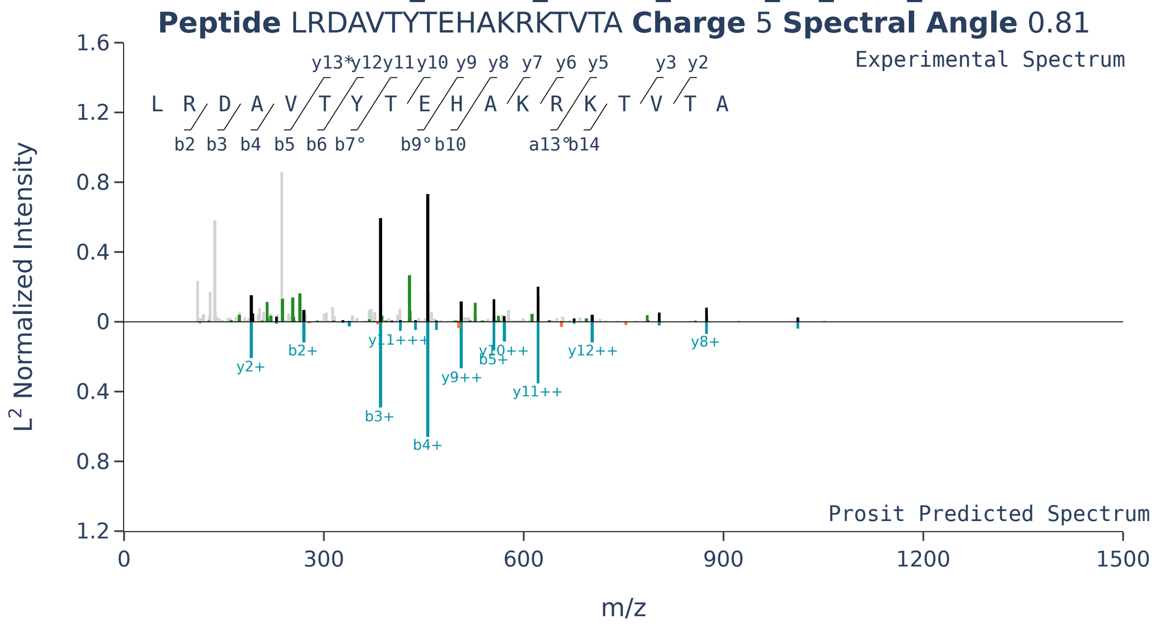


**Source** HRoetschke_291222_140123_HFGoe_H4_b2R1_2h **Scan** 4962
**Peptide** AVTYTEHAKRKT **Charge** 3 **Spectral Angle** 0.5

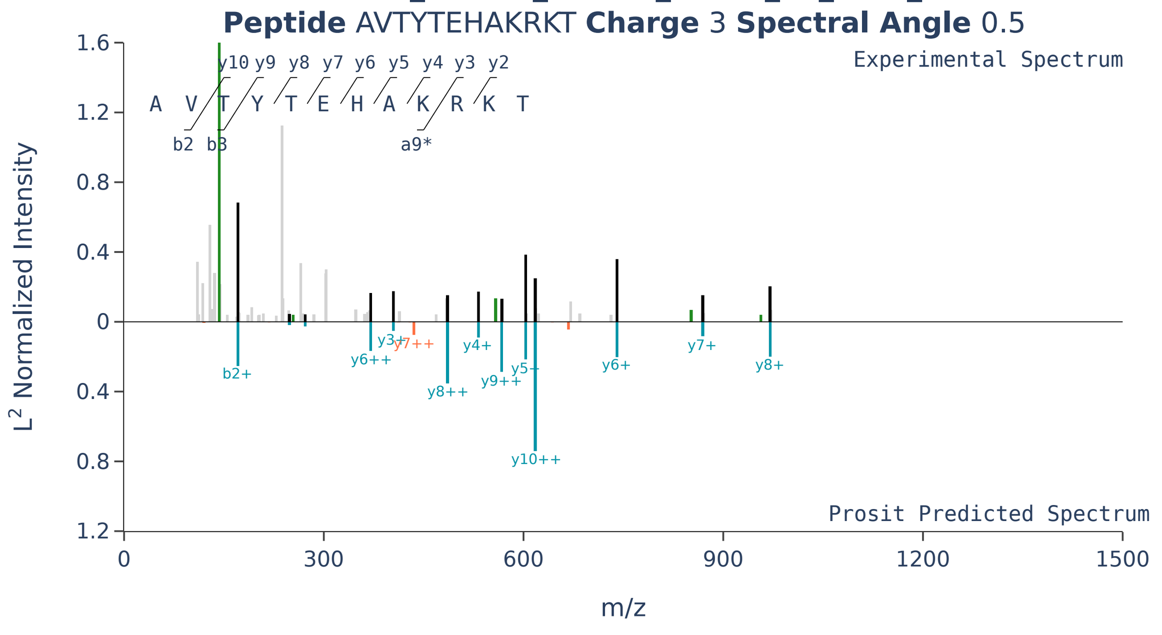


**Source** HRoetschke_291222_140123_HFGoe_H4_b2R1_1h **Scan** 36274
**Peptide** RGVLKVFLENVIRDAVTYTEHA **Charge** 4 **Spectral Angle** 0.72

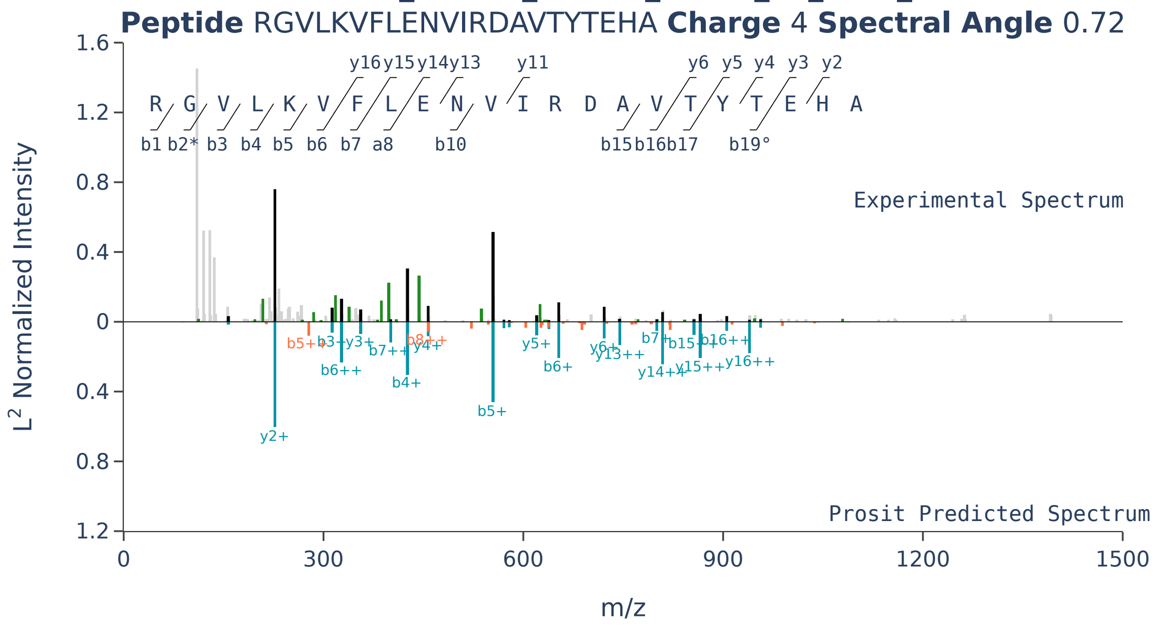


**Source** HRoetschke_291222_140123_HFGoe_H4_b2R2_4h **Scan** 12285
**Peptide** RDNIQGITKPAIRRLARRGGV **Charge** 5 **Spectral Angle** 0.62

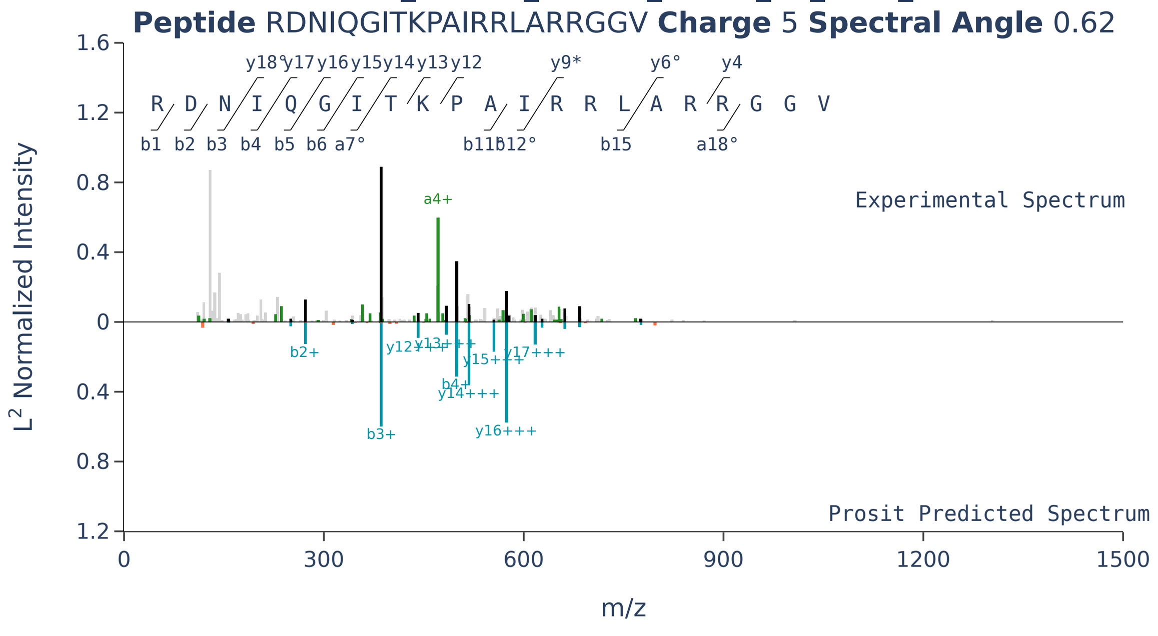


**Source** HRoetschke_291222_140123_HFGoe_H4_b2R2_1h **Scan** 20678
**Peptide** LRDAVTYTEHAKRKTVTAMDVVYAL **Charge** 4 **Spectral Angle** 0.63

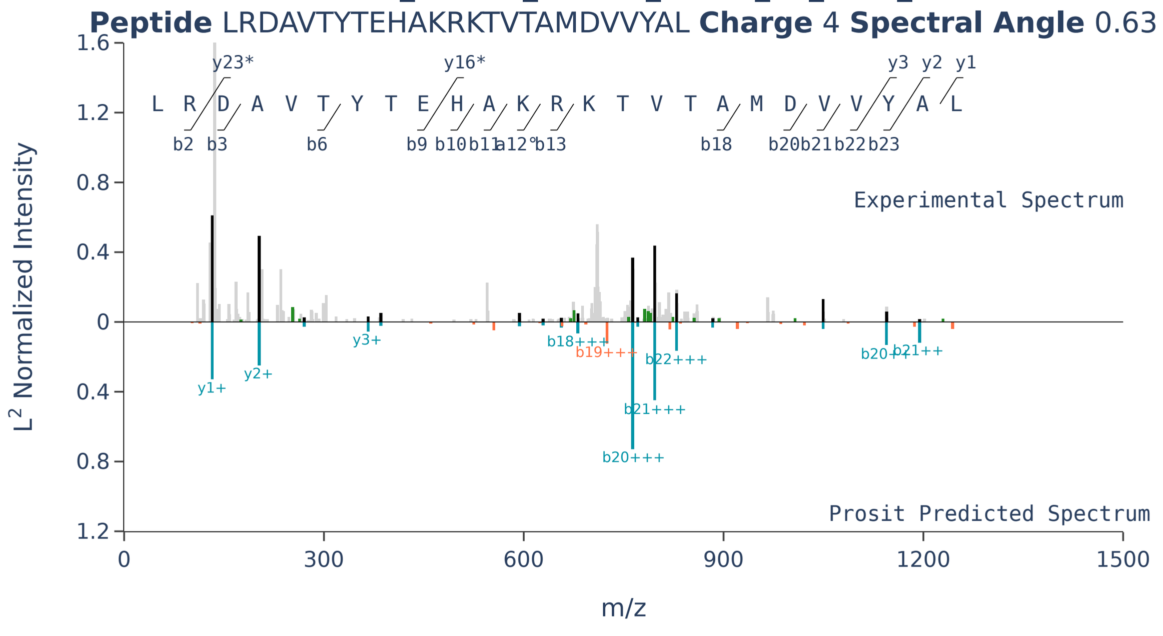


**Source** HRoetschke_291222_140123_HFGoe_H4_b1R1_2h **Scan** 13684
**Peptide** PAIRRLARRGGVKRISGLIYEET **Charge** 5 **Spectral Angle** 0.84

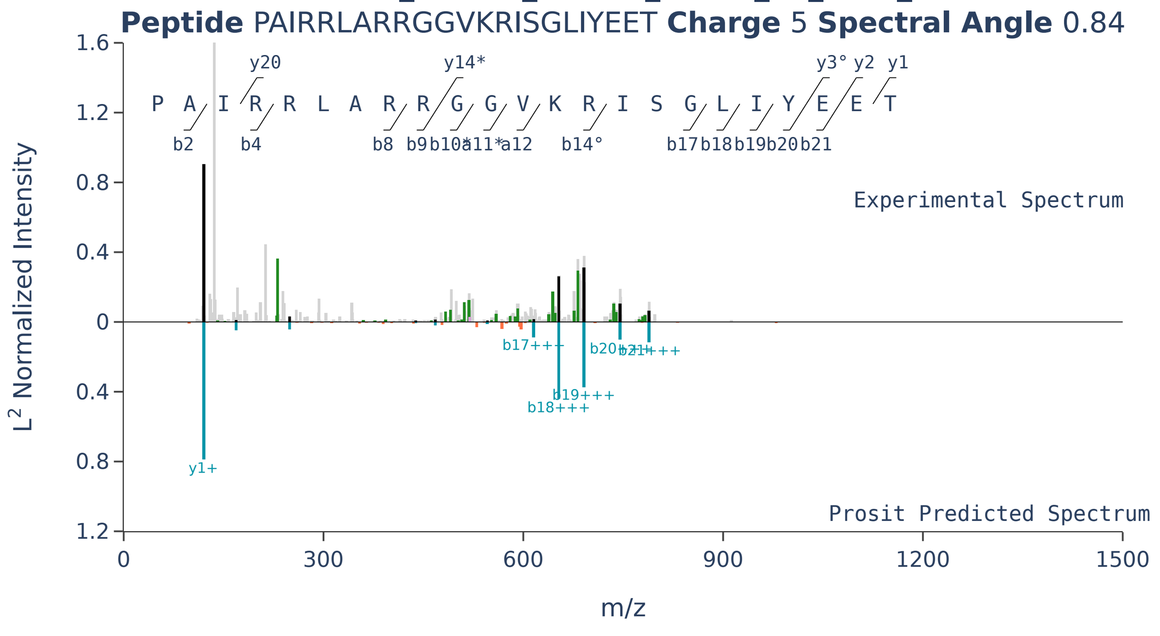

**Source** HRoetschke_291222_140123_HFGoe_H4_b2R2_1h **Scan** 9303
**Peptide** IQGITKPAIRRLARRGGVKRIS **Charge** 4 **Spectral Angle** 0.59

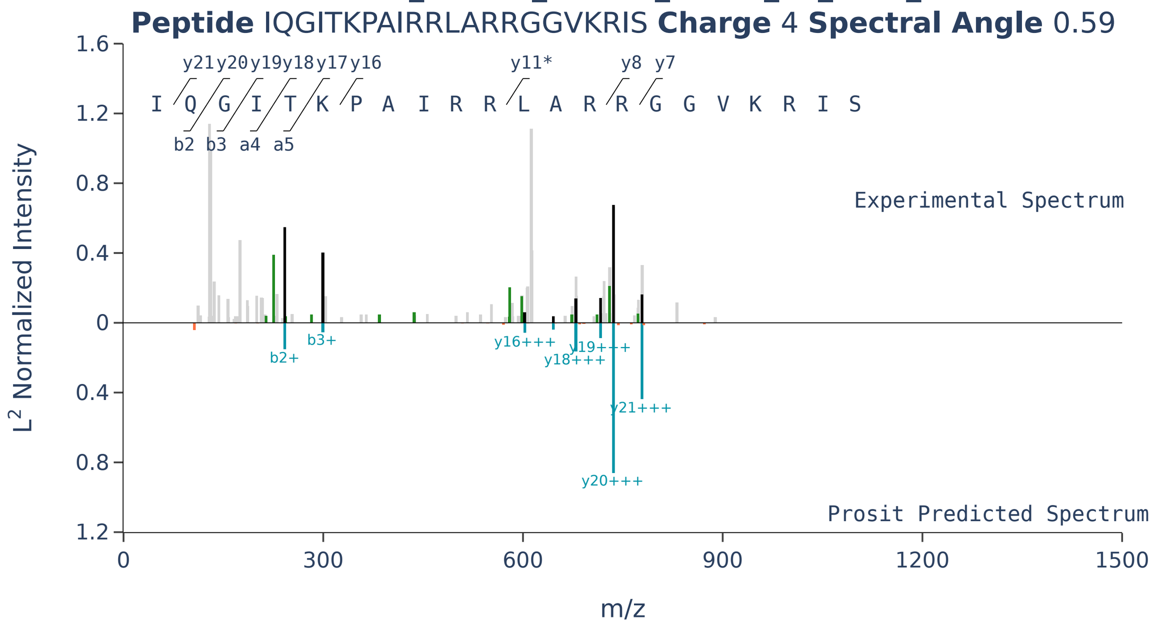


**Source** HRoetschke_291222_140123_HFGoe_H4_b1R2_24h **Scan** 6664
**Peptide** KGLGKGGAKRHRKVLRDNIQGITKPAIRR **Charge** 6 **Spectral Angle** 0.72

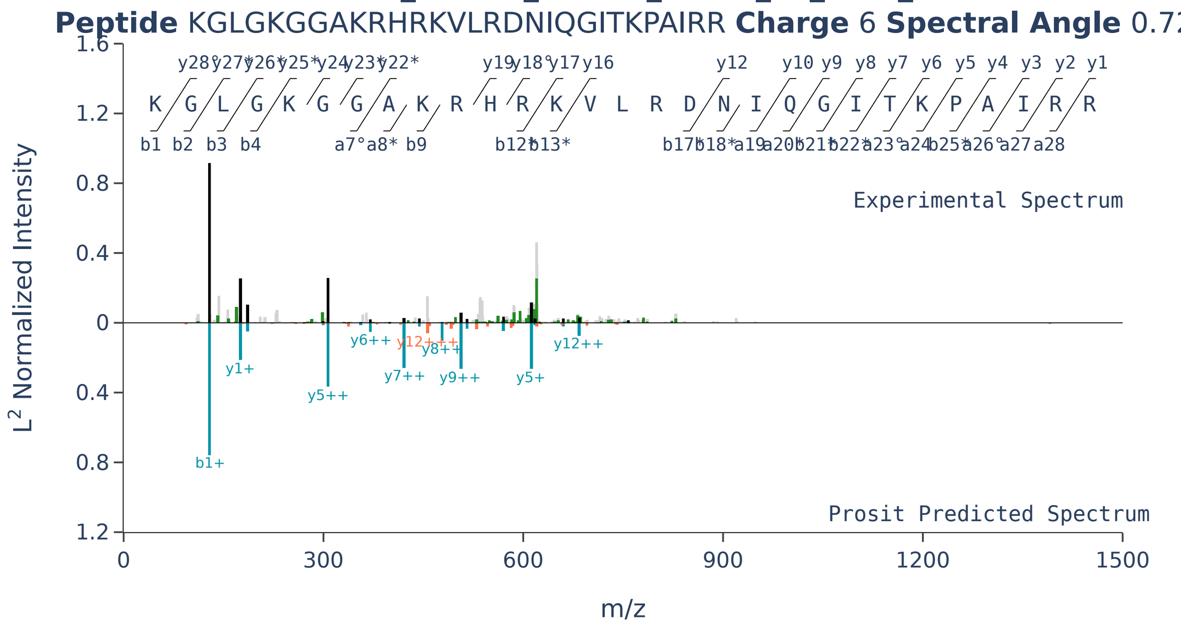


**Source** HRoetschke_291222_140123_HFGoe_H4_b2R2_1h **Scan** 28146
**Peptide** ETRGVLKVFLENVIR **Charge** 3 **Spectral Angle** 0.7

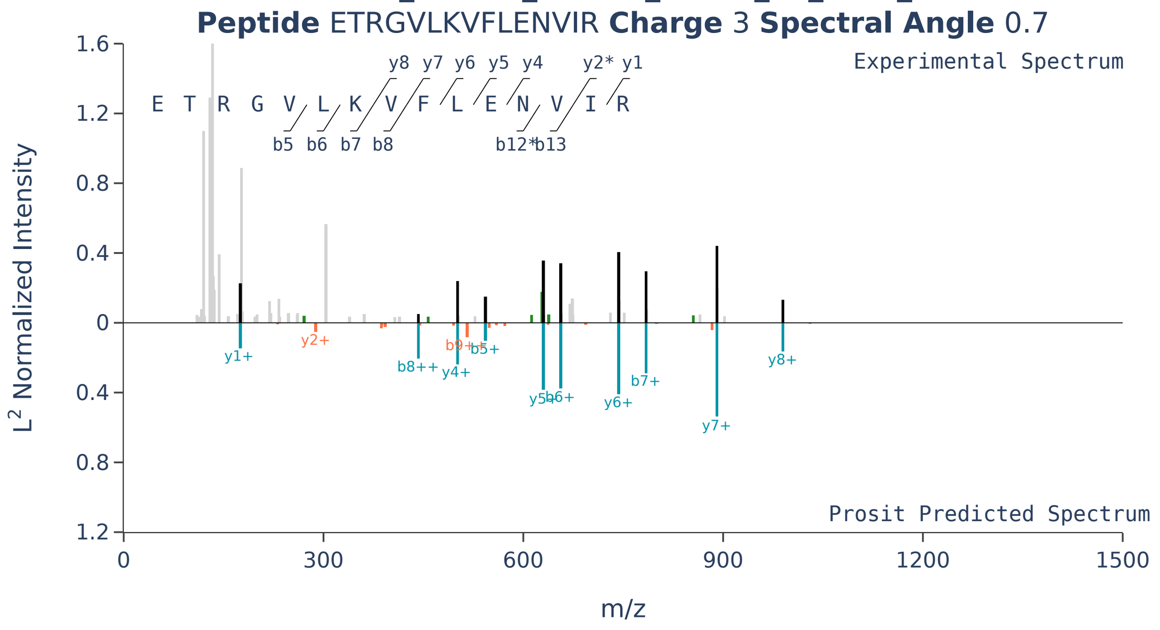


**Source** HRoetschke_291222_140123_HFGoe_H4_b2R1_4h **Scan** 9829
**Peptide** RDNIQGITKPAIRRLARRGG **Charge** 5 **Spectral Angle** 0.71

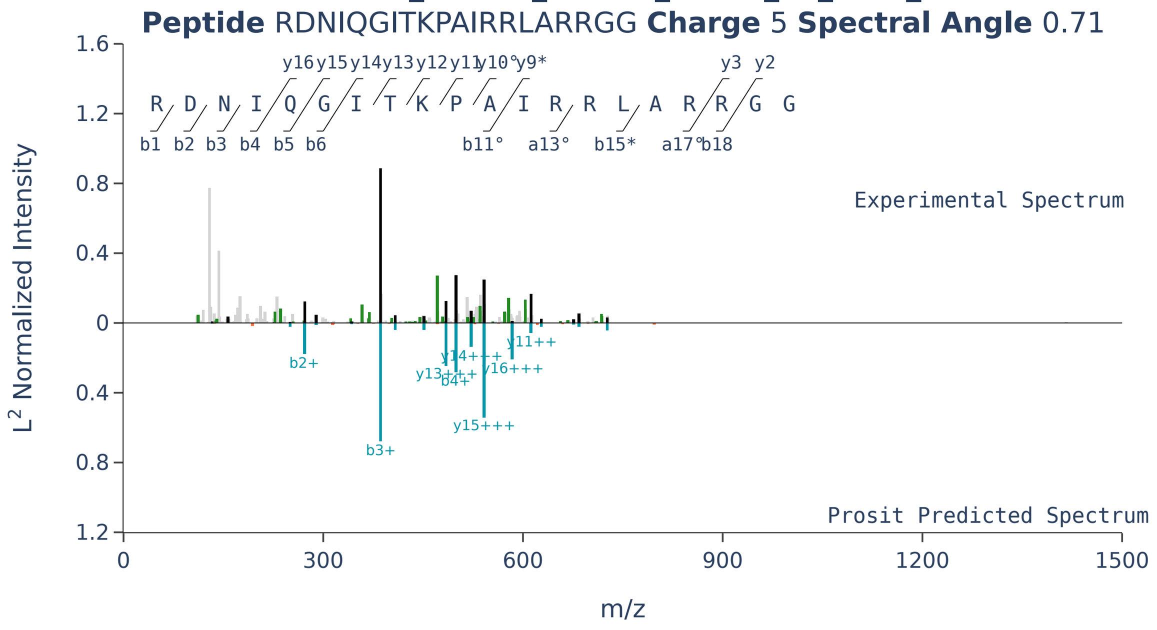


**Source** HRoetschke_291222_140123_HFGoe_H4_b2R1_1h **Scan** 36831
**Peptide** SGLIYEETRGVLKVFLENVIR **Charge** 3 **Spectral Angle** 0.65

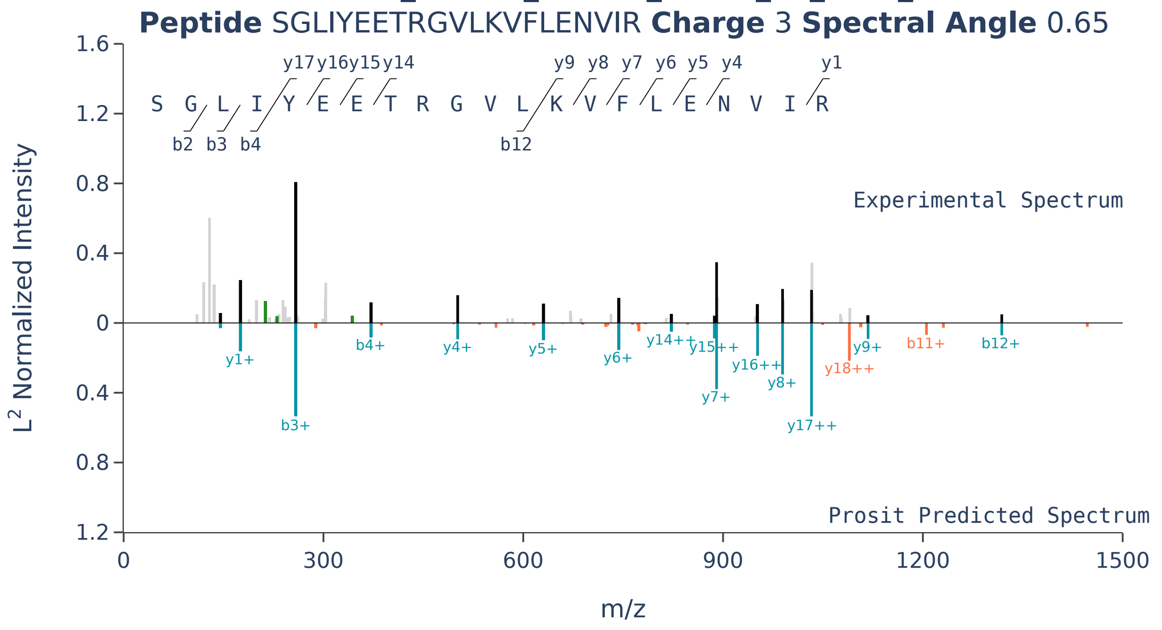


**Source** HRoetschke_291222_140123_HFGoe_H4_b1R2_24h **Scan** 5663
**Peptide** PAIRRLARRGGVK **Charge** 4 **Spectral Angle** 0.64

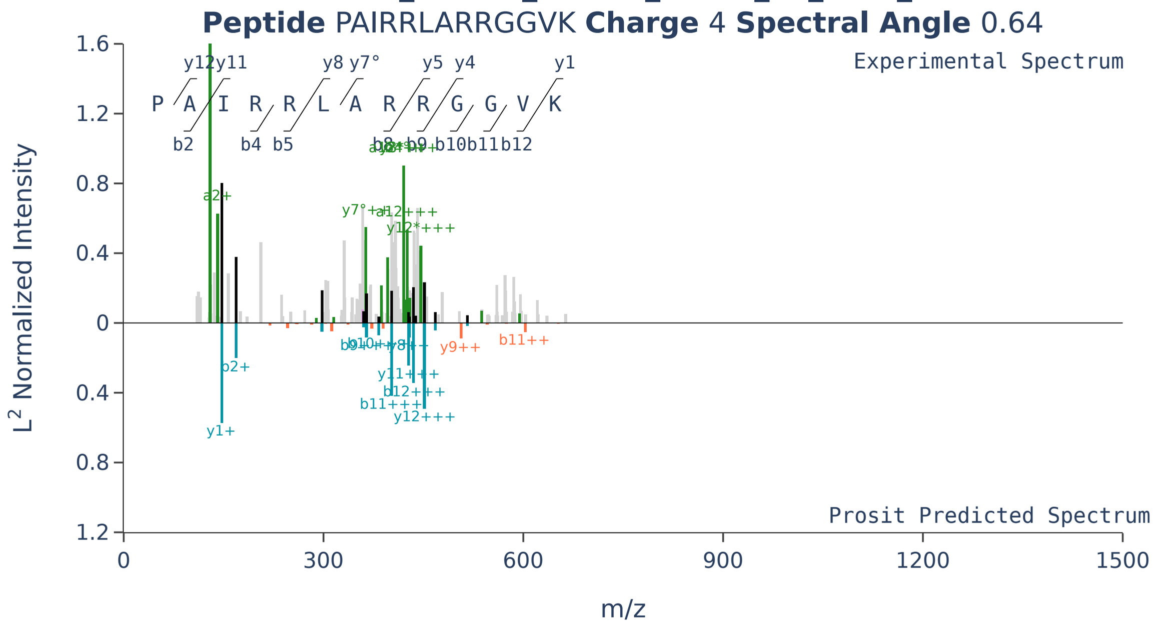


**Source** HRoetschke_291222_140123_HFGoe_H4_b2R1_2h **Scan** 13728
**Peptide** LYEETRG **Charge** 1 **Spectral Angle** 0.91

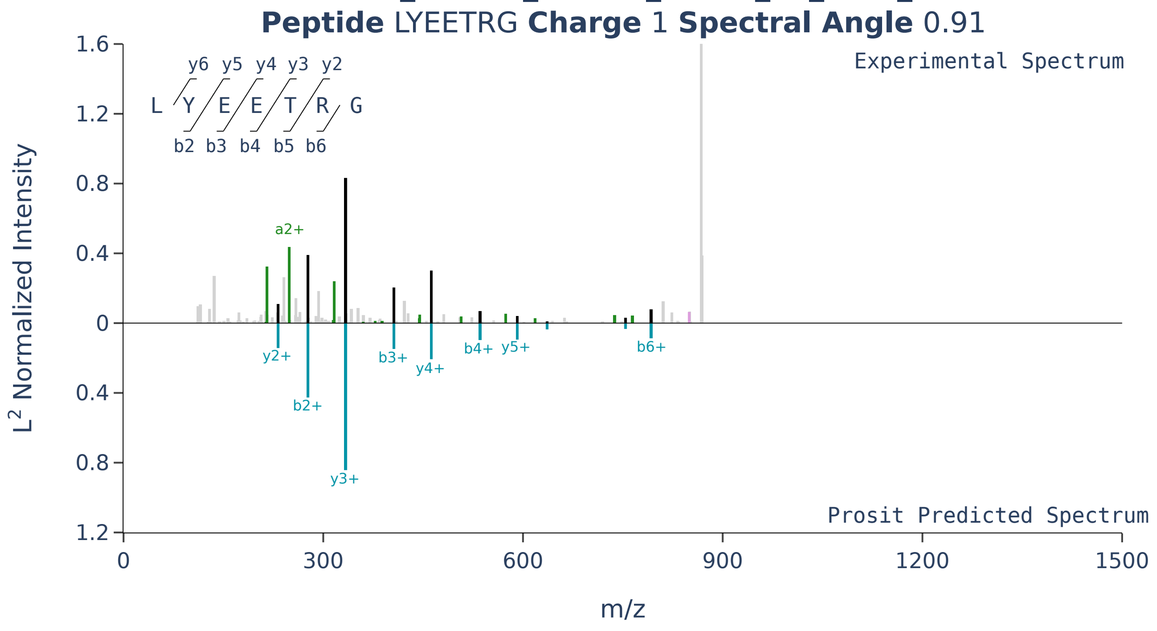


**Source** HRoetschke_291222_140123_HFGoe_H4_b2R1_1h **Scan** 29497
**Peptide** RGVLKVFLENVIRDAVT **Charge** 3 **Spectral Angle** 0.62

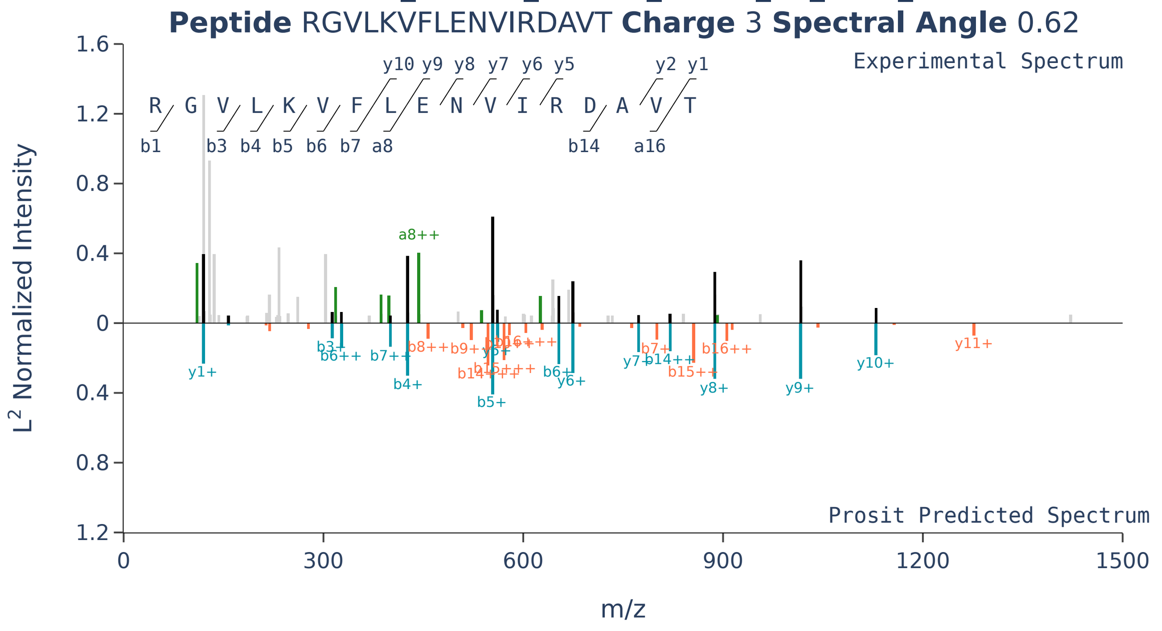


**Source** HRoetschke_291222_140123_HFGoe_H4_b1R2_24h **Scan** 11774
**Peptide** AVTYTEHAKRKTVTAMDVV **Charge** 4 **Spectral Angle** 0.75

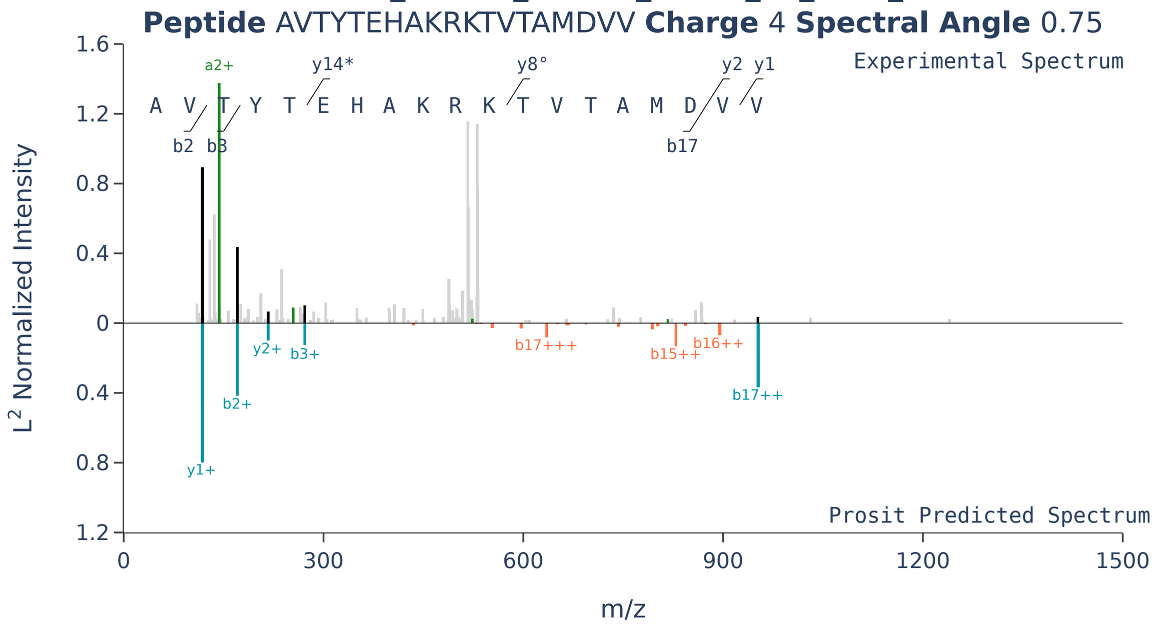


**Source** HRoetschke_291222_140123_HFGoe_H4_b1R2_24h **Scan** 10460
**Peptide** NIQGITKPAIRRLARRGGVKRISG **Charge** 4 **Spectral Angle** 0.77

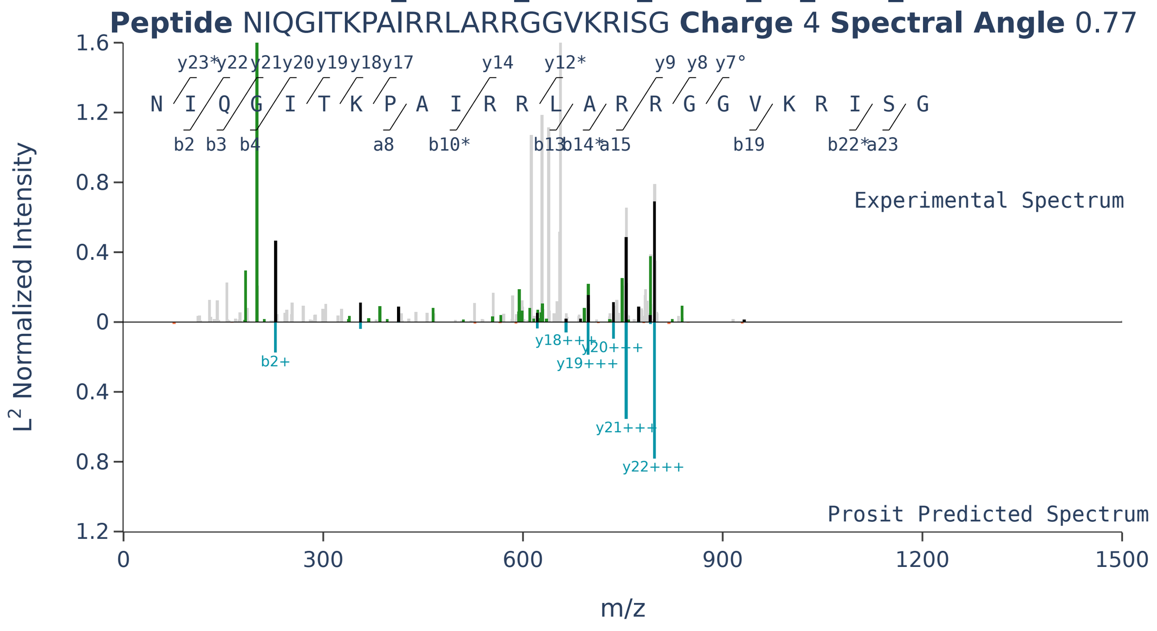


**Source** HRoetschke_291222_140123_HFGoe_H4_b2R1_1h **Scan** 26495
**Peptide** GGVKRISGLIYEETRGVLKVF **Charge** 3 **Spectral Angle** 0.56

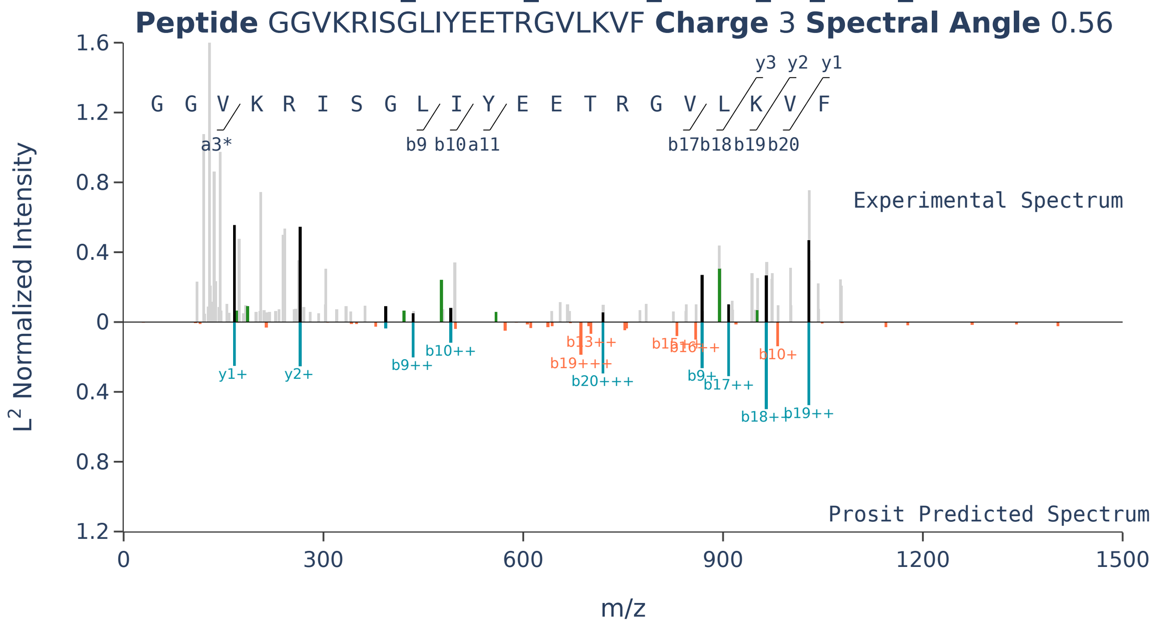


**Source** HRoetschke_291222_140123_HFGoe_H4_b2R1_4h **Scan** 12685
**Peptide** GLTKPAIRRLAR **Charge** 3 **Spectral Angle** 0.67

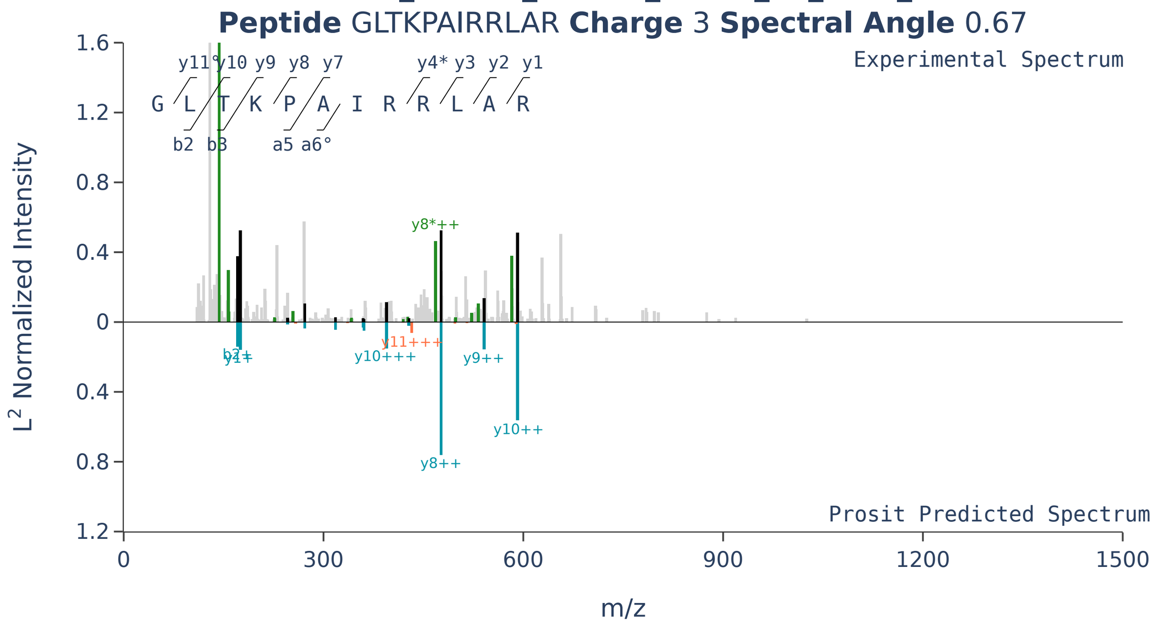


**Source** HRoetschke_291222_140123_HFGoe_H4_b1R1_2h **Scan** 18074
**Peptide** KRKTVTAMDVVYALKRQ **Charge** 5 **Spectral Angle** 0.55

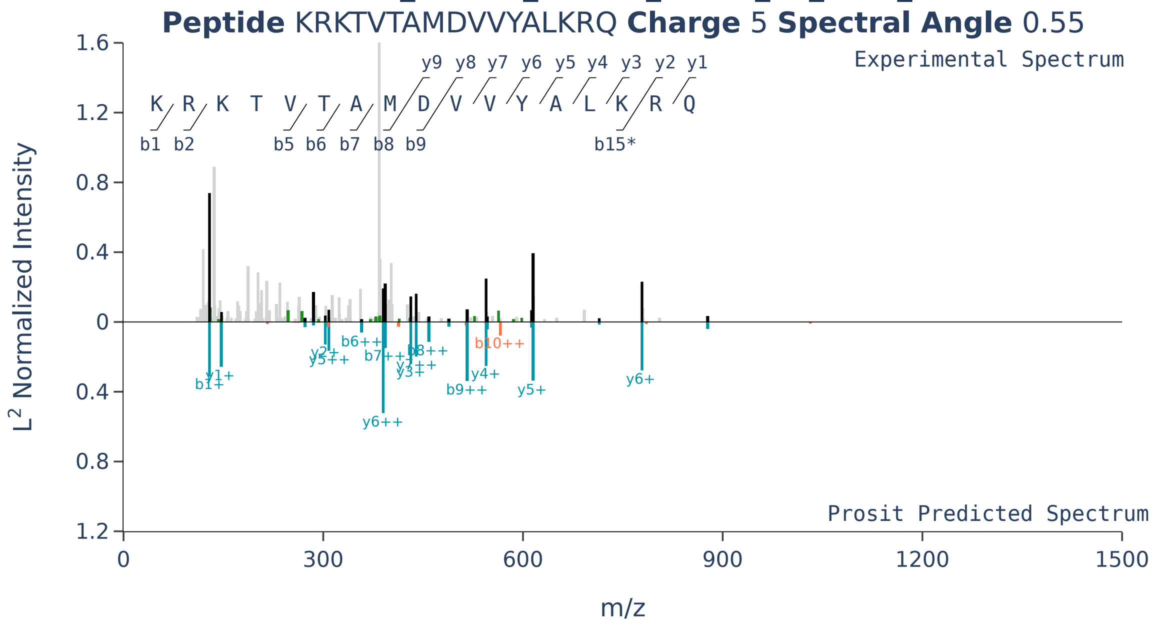


**Source** HRoetschke_291222_140123_HFGoe_H4_b1R1_24h **Scan** 11196
**Peptide** RDNIQGITKPAIRRLARRGGVKRL **Charge** 5 **Spectral Angle** 0.68

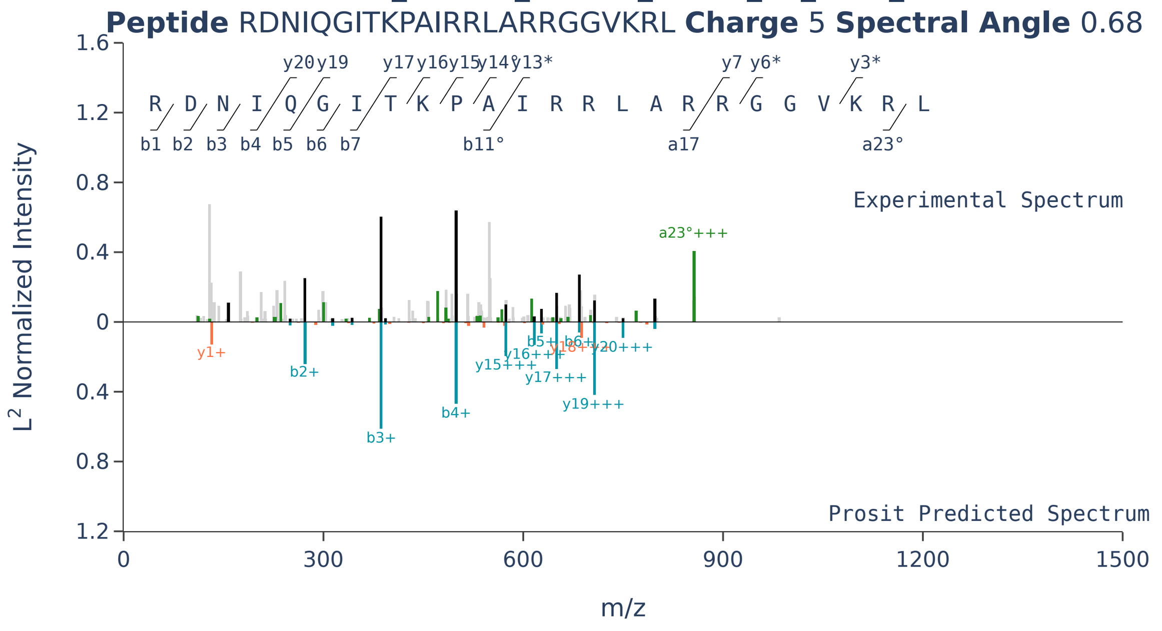


**Source** HRoetschke_291222_140123_HFGoe_H4_b2R2_1h **Scan** 10632
**Peptide** RRLARRGGVKRISGLIYEET **Charge** 4 **Spectral Angle** 0.8

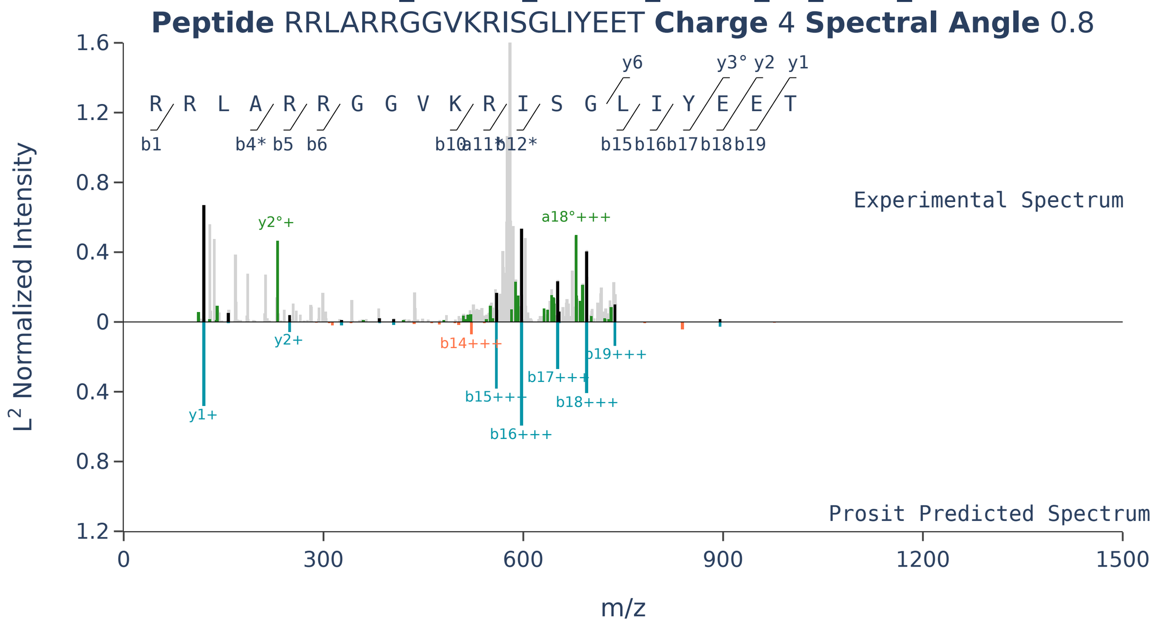

**Source** HRoetschke_291222_140123_HFGoe_H4_b2R1_24h **Scan** 11920
**Peptide** IQGITKPAIRRLAR **Charge** 3 **Spectral Angle** 0.47

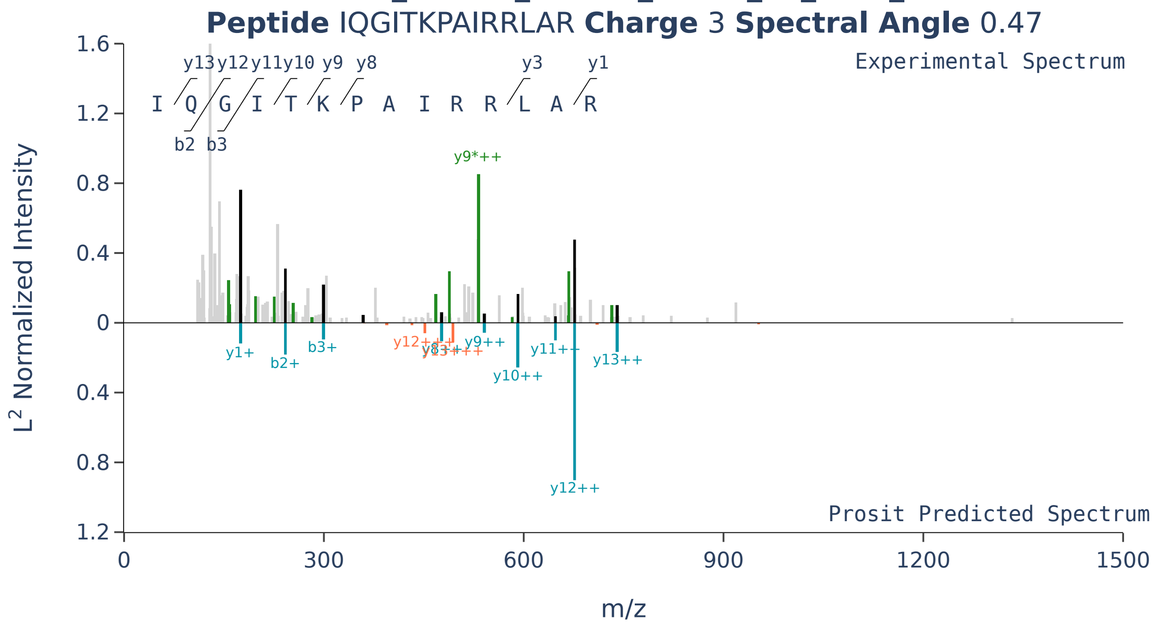


**Source** HRoetschke_291222_140123_HFGoe_H4_b2R2_24h **Scan** 8235
**Peptide** KGLGKGGAKRHRKVLRDNIQGITKPAIR **Charge** 5 **Spectral Angle** 0.78

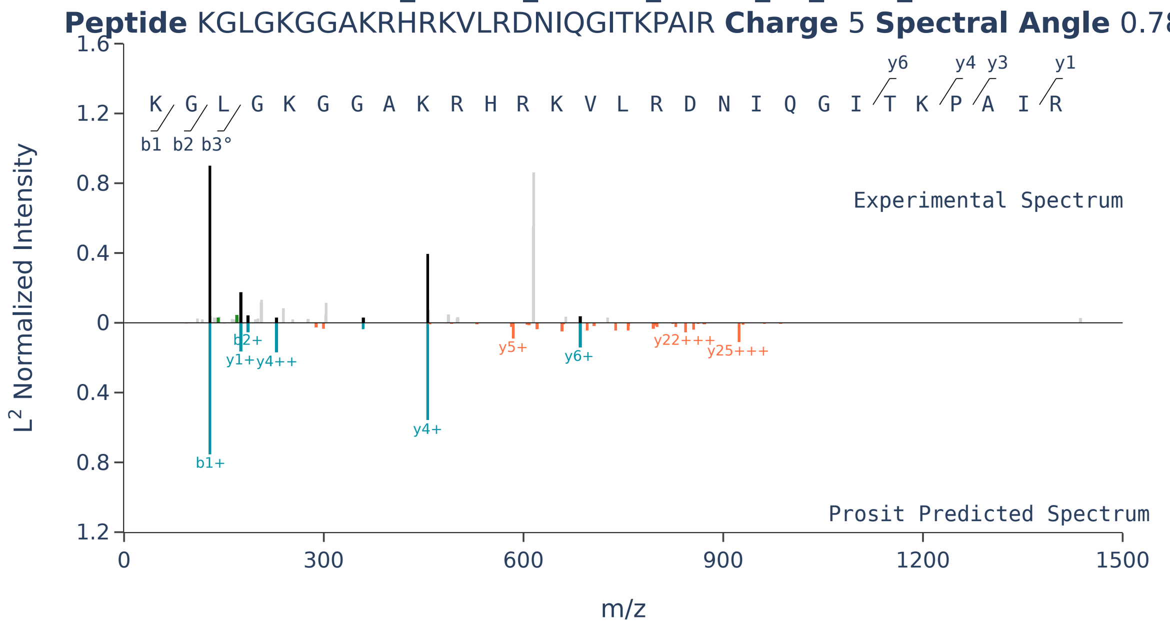


**Source** HRoetschke_291222_140123_HFGoe_H4_b1R1_1h **Scan** 21418
**Peptide** ENVIRDAVTYTEHAKRKTVTAMDVVYALKR **Charge** 5 **Spectral Angle** 0.8

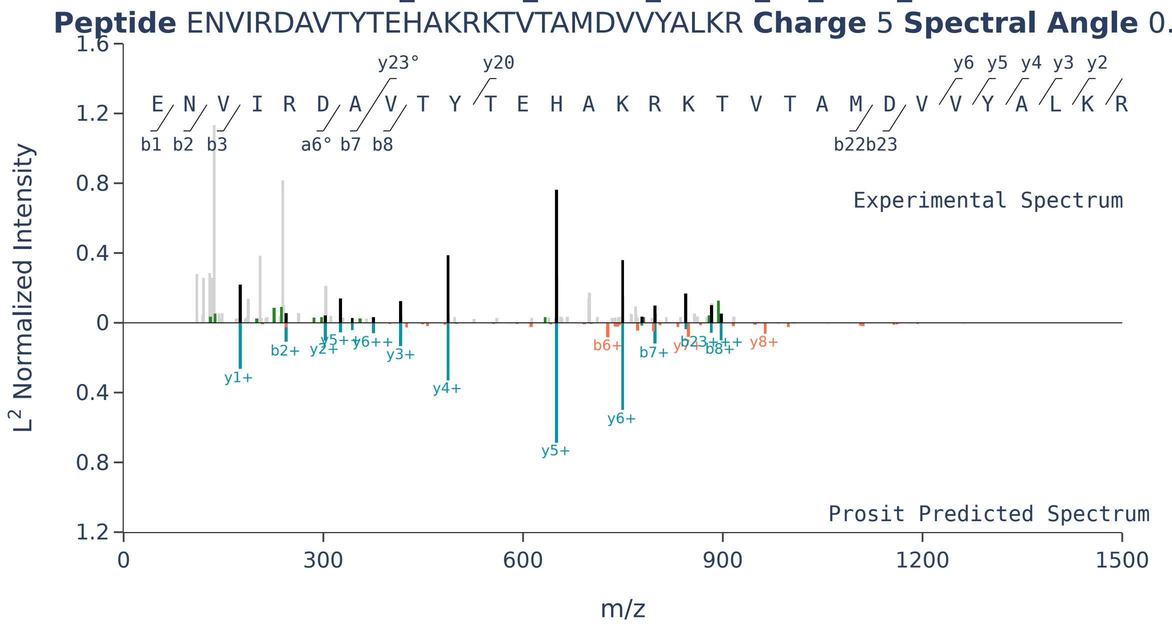


**Source** HRoetschke_291222_140123_HFGoe_H4_b2R2_24h **Scan** 8727
**Peptide** IQGITKPAIRRLARRGGVK **Charge** 4 **Spectral Angle** 0.64

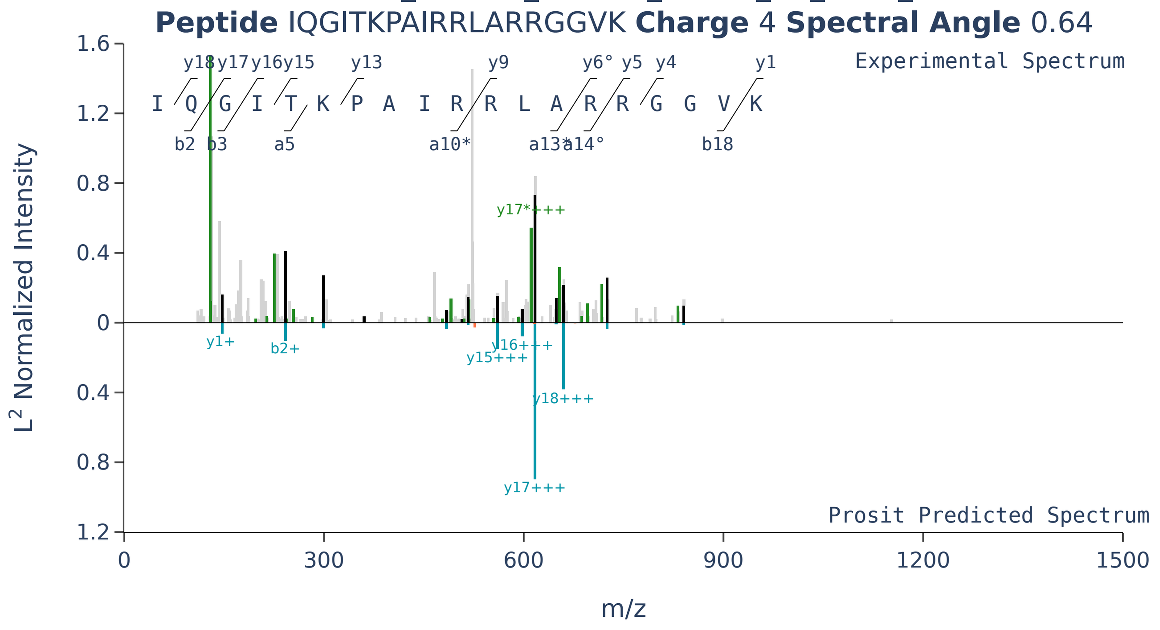


**Source** HRoetschke_291222_140123_HFGoe_H4_b1R1_4h **Scan** 6504
**Peptide** VTYTEHAKRKT **Charge** 3 **Spectral Angle** 0.57

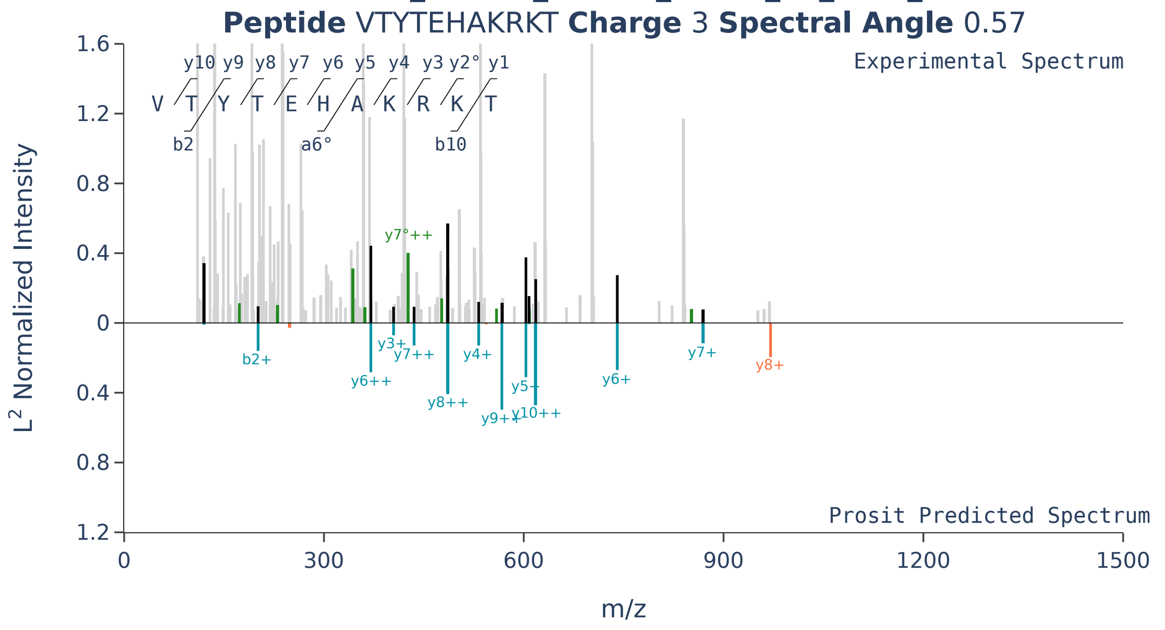


**Source** HRoetschke_291222_140123_HFGoe_H4_b2R1_24h **Scan** 8058
**Peptide** GGAKRHRKVLRDNIQGITKPAIR **Charge** 5 **Spectral Angle** 0.73

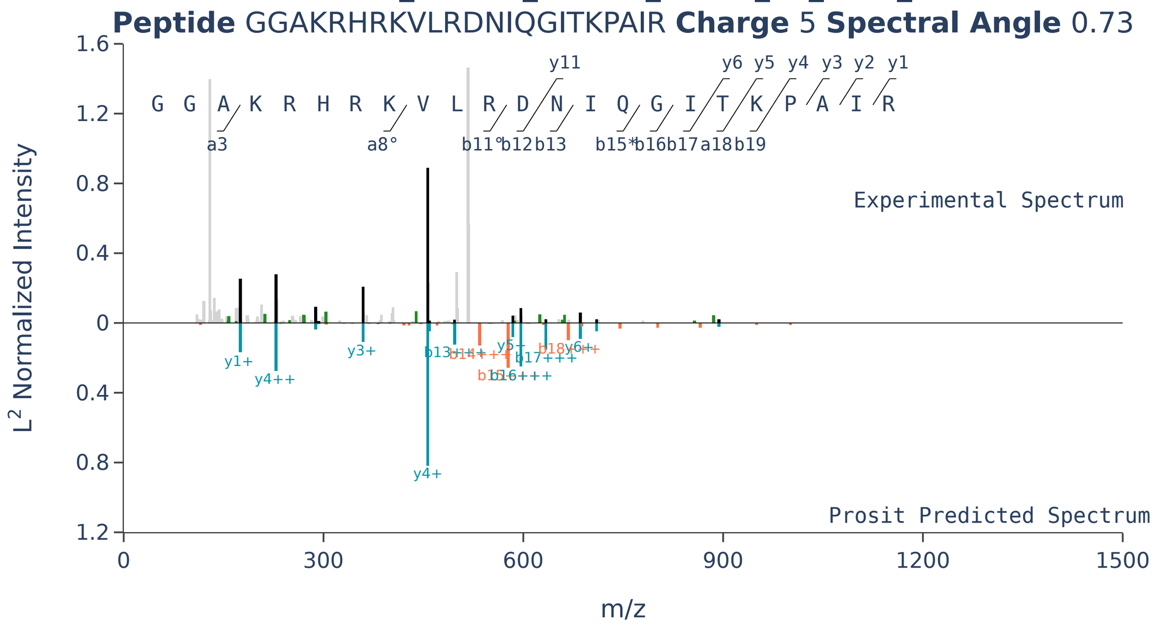


**Source** HRoetschke_291222_140123_HFGoe_H4_b2R1_24h **Scan** 11780
**Peptide** LRDNIQGITKPAIRRLARRGGVK **Charge** 5 **Spectral Angle** 0.42

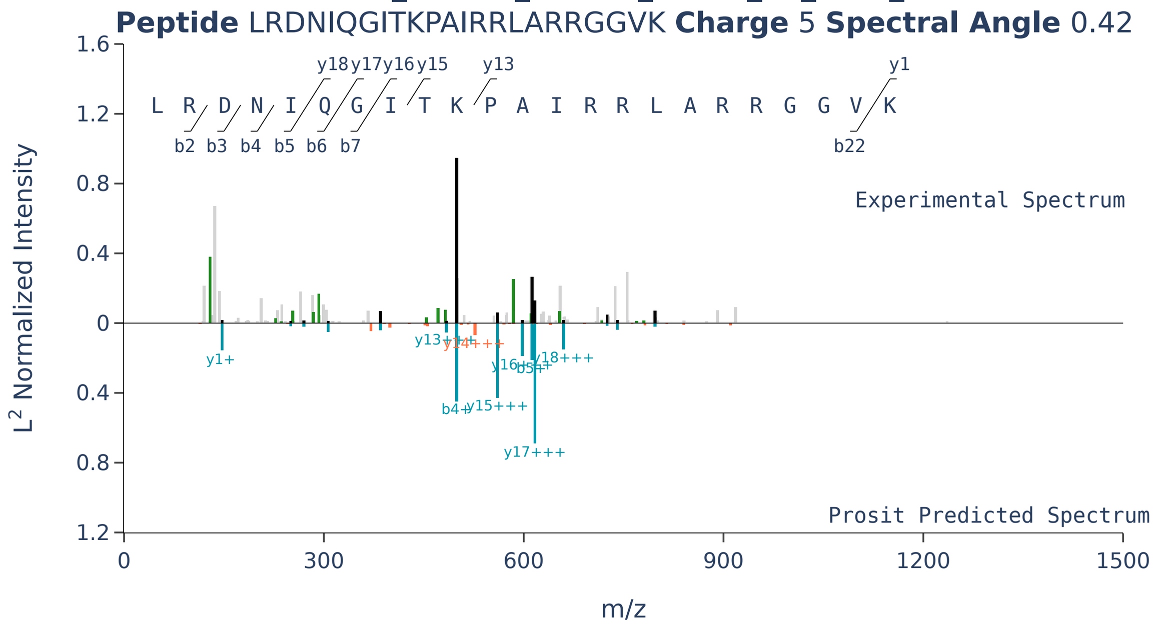


**Source** HRoetschke_291222_140123_HFGoe_H4_b1R1_2h **Scan** 30055
**Peptide** RGVLKVFLENVL **Charge** 3 **Spectral Angle** 0.74

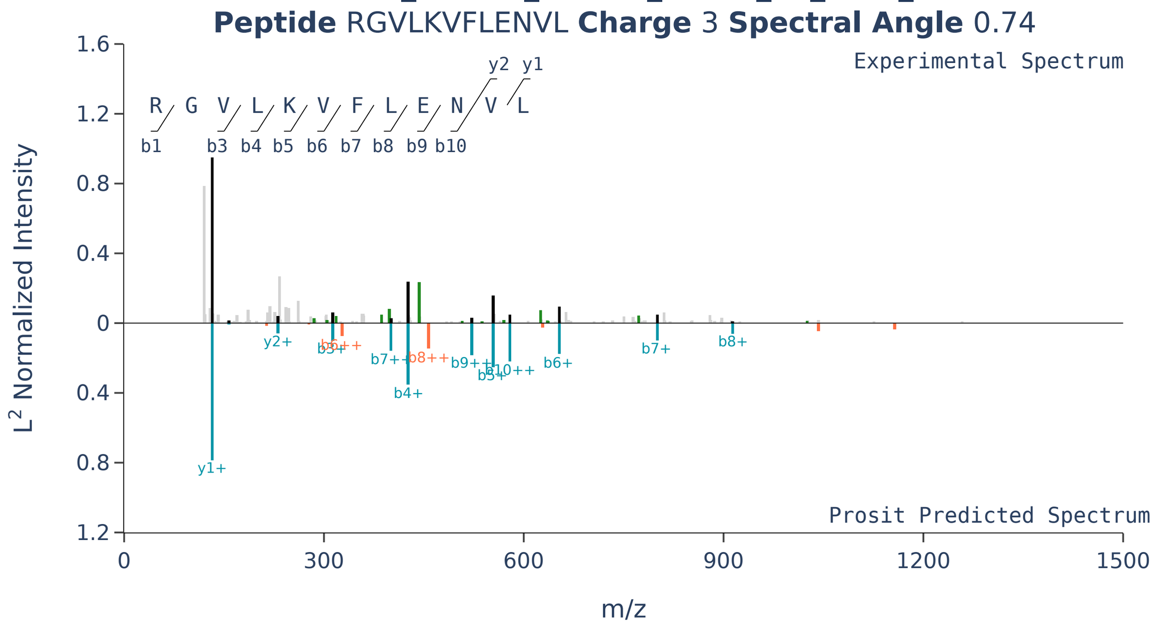


**Source** HRoetschke_291222_140123_HFGoe_H4_b2R2_4h **Scan** 7298
**Peptide** KTVTAMD **Charge** 2 **Spectral Angle** 0.78

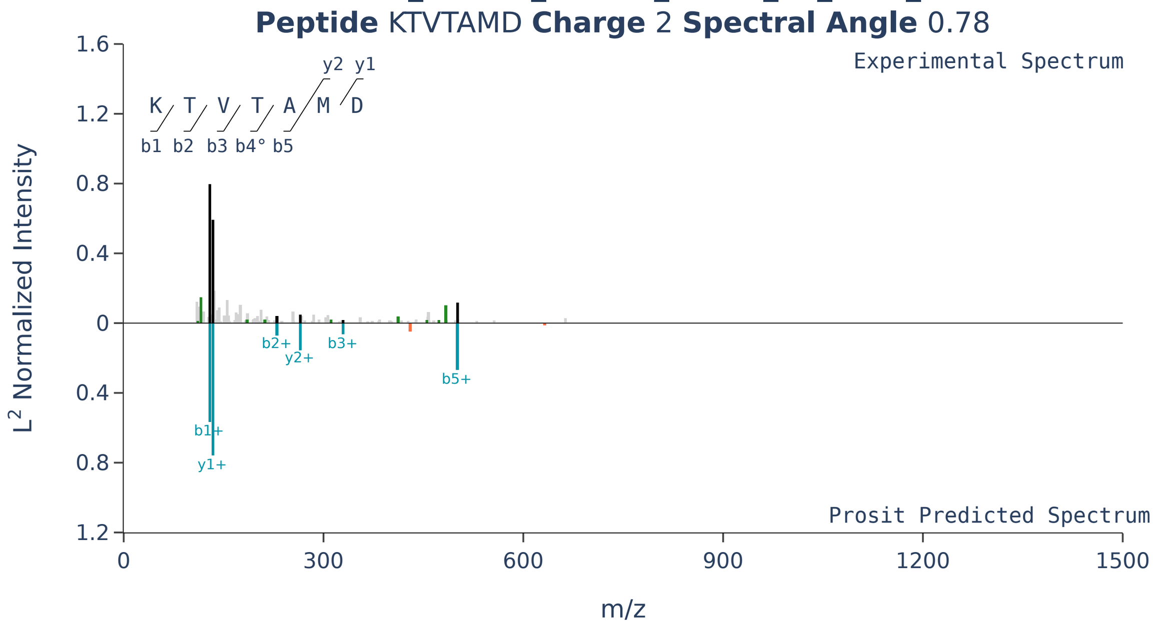


**Source** HRoetschke_291222_140123_HFGoe_H4_b1R1_24h **Scan** 10496
**Peptide** QGITKPAIRRLARRG **Charge** 4 **Spectral Angle** 0.69

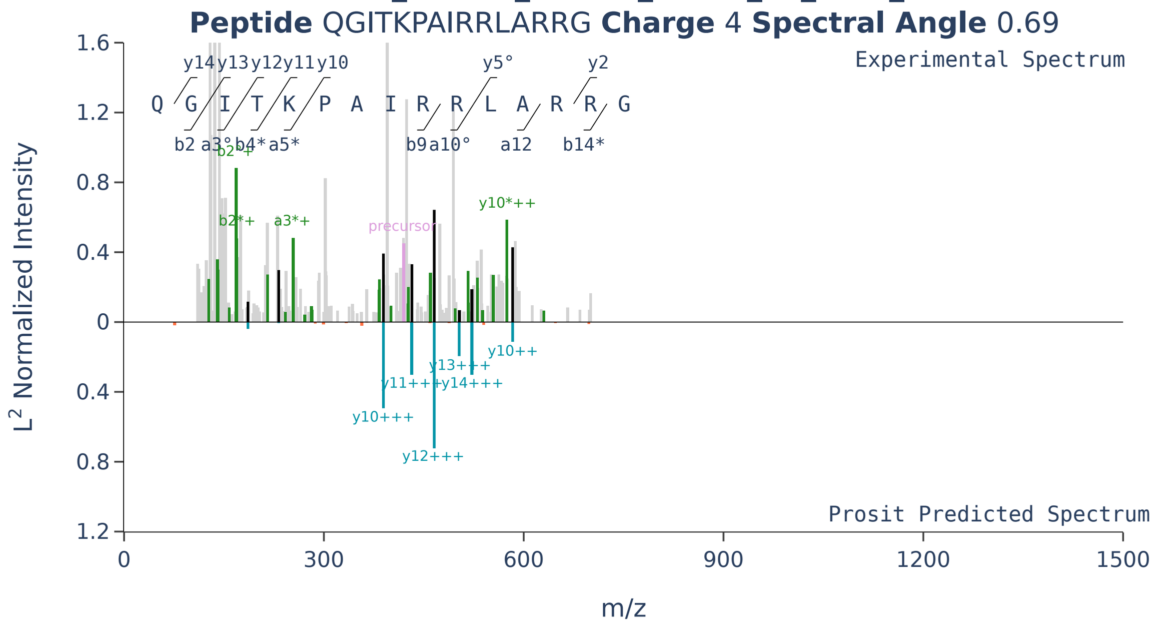


**Source** HRoetschke_291222_140123_HFGoe_H4_b2R1_1h **Scan** 36206
**Peptide** GLIYEETRGVLKVFLENVLRD **Charge** 4 **Spectral Angle** 0.56

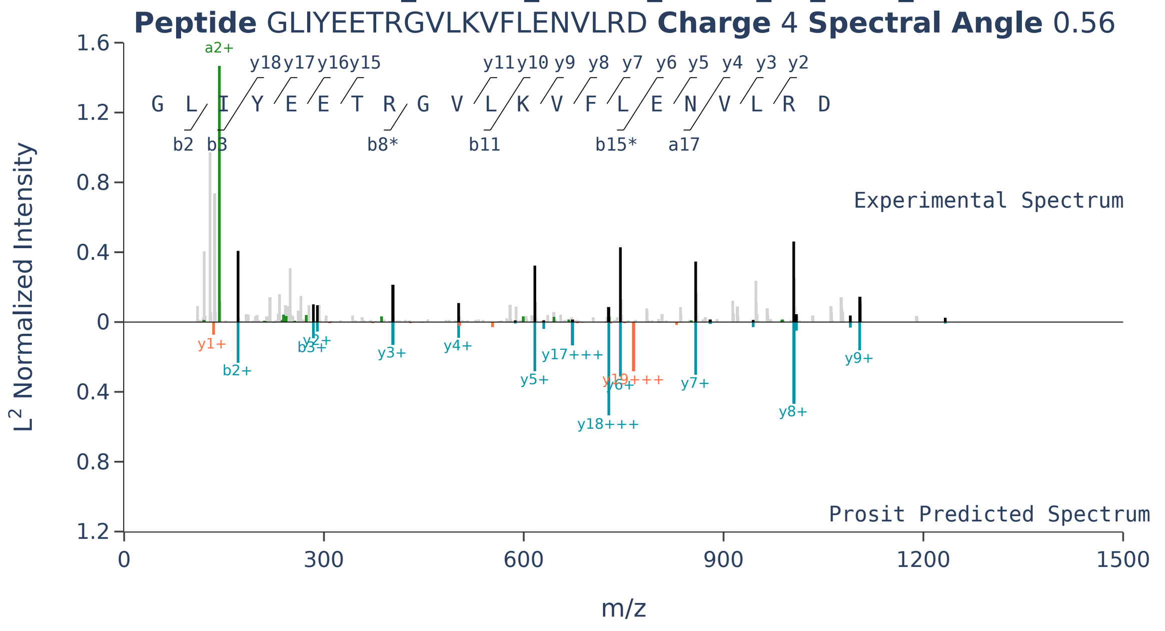


**Source** HRoetschke_291222_140123_HFGoe_H4_b1R1_1h **Scan** 30424
**Peptide** TRGVLKVFLENVL **Charge** 3 **Spectral Angle** 0.72

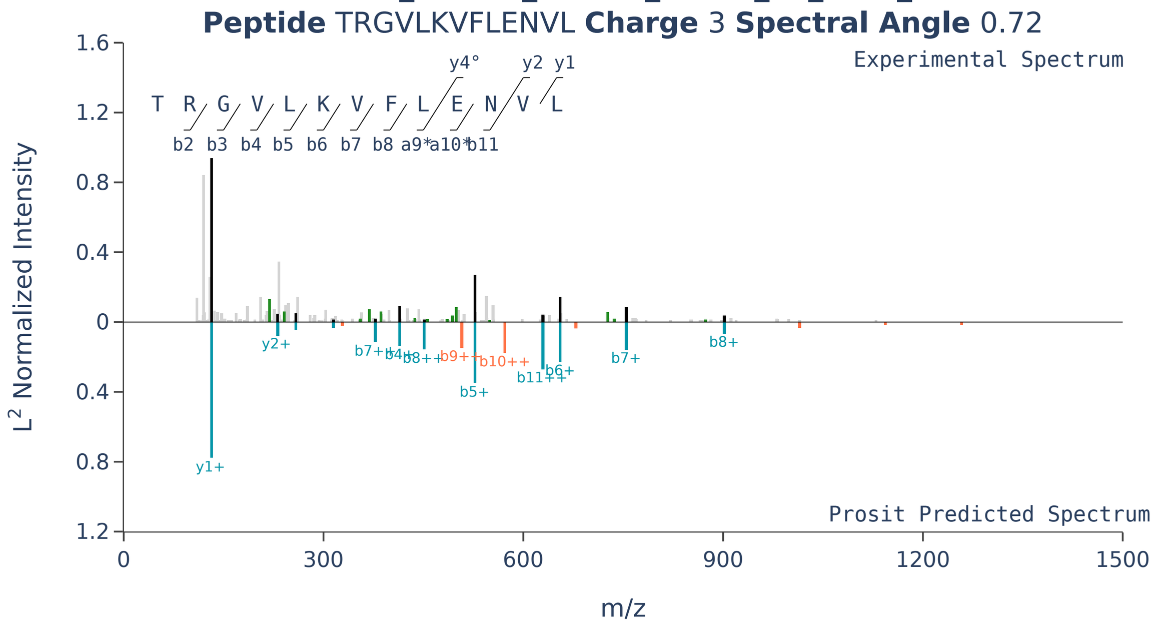


**Source** HRoetschke_291222_140123_HFGoe_H4_b2R2_24h **Scan** 10491
**Peptide** LRDNIQGITKPAIRRLARRGGVKR **Charge** 6 **Spectral Angle** 0.76

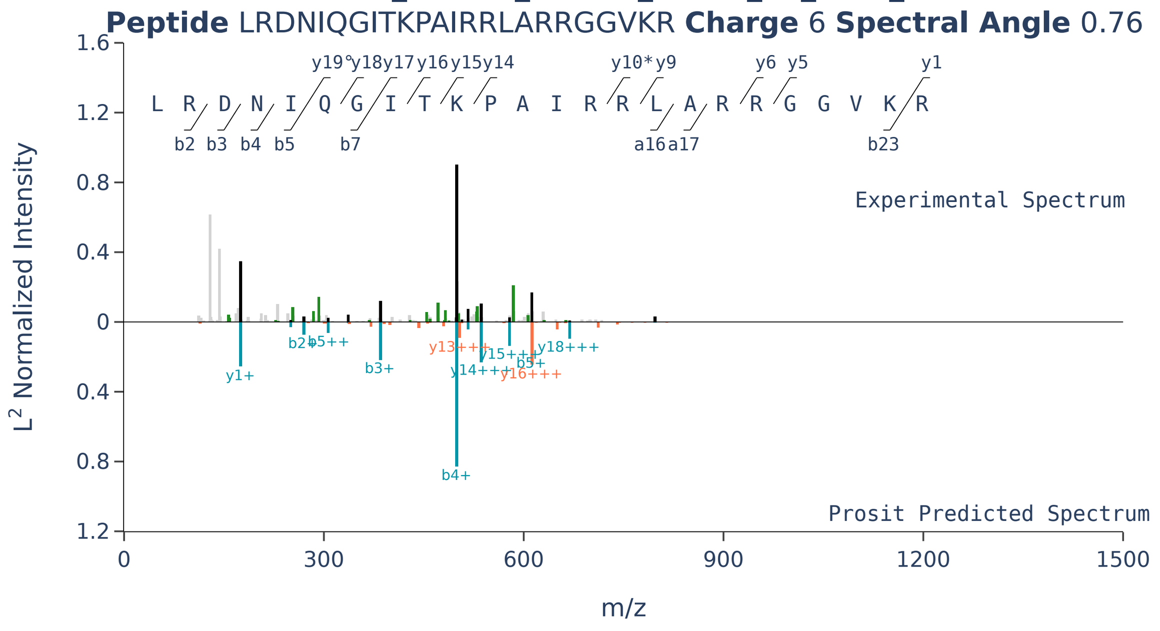


**Source** HRoetschke_291222_140123_HFGoe_H4_b1R1_24h **Scan** 9261
**Peptide** AVTYTEHAKRKTVTAMDV **Charge** 4 **Spectral Angle** 0.85

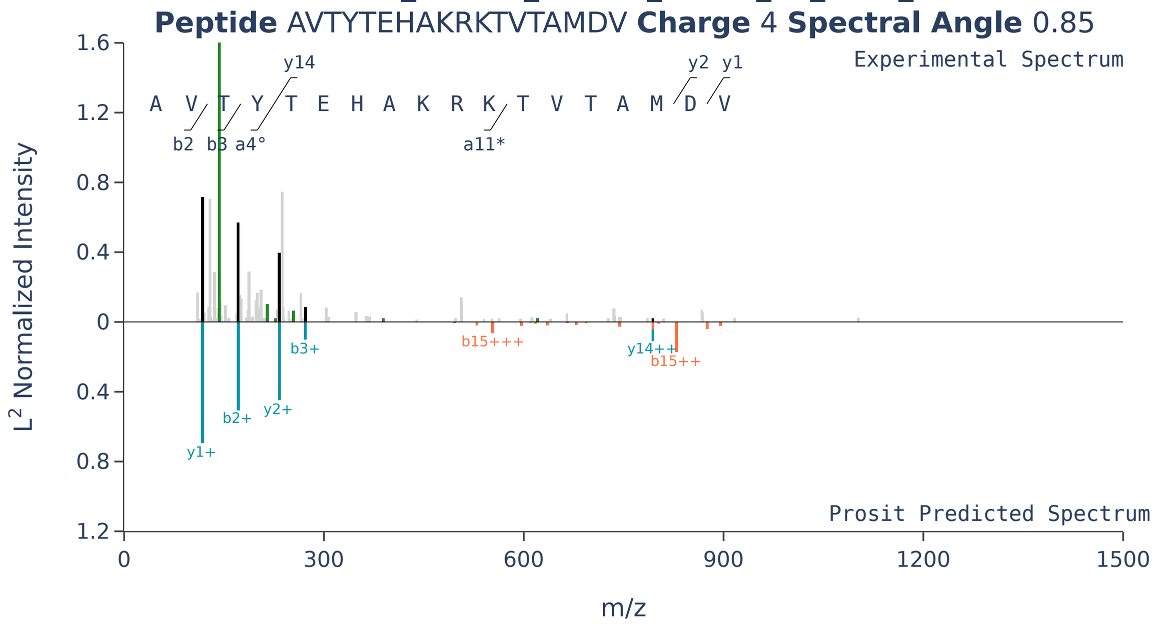


**Source** HRoetschke_291222_140123_HFGoe_H4_b1R1_24h **Scan** 10426
**Peptide** RRGGVKRISGLIYEETR **Charge** 4 **Spectral Angle** 0.64

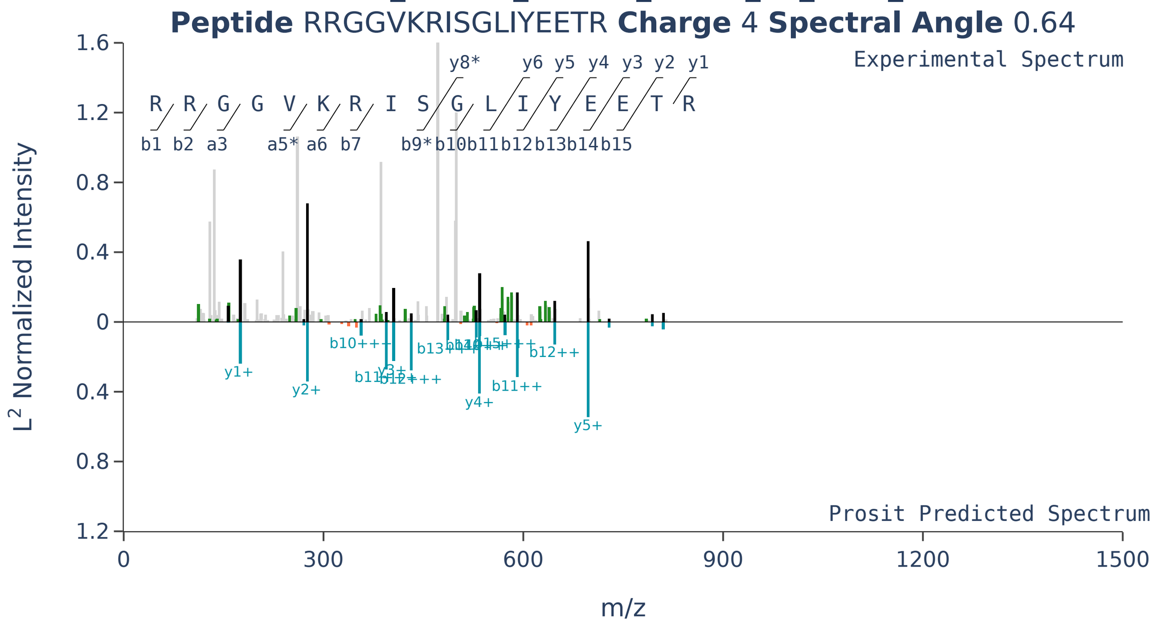

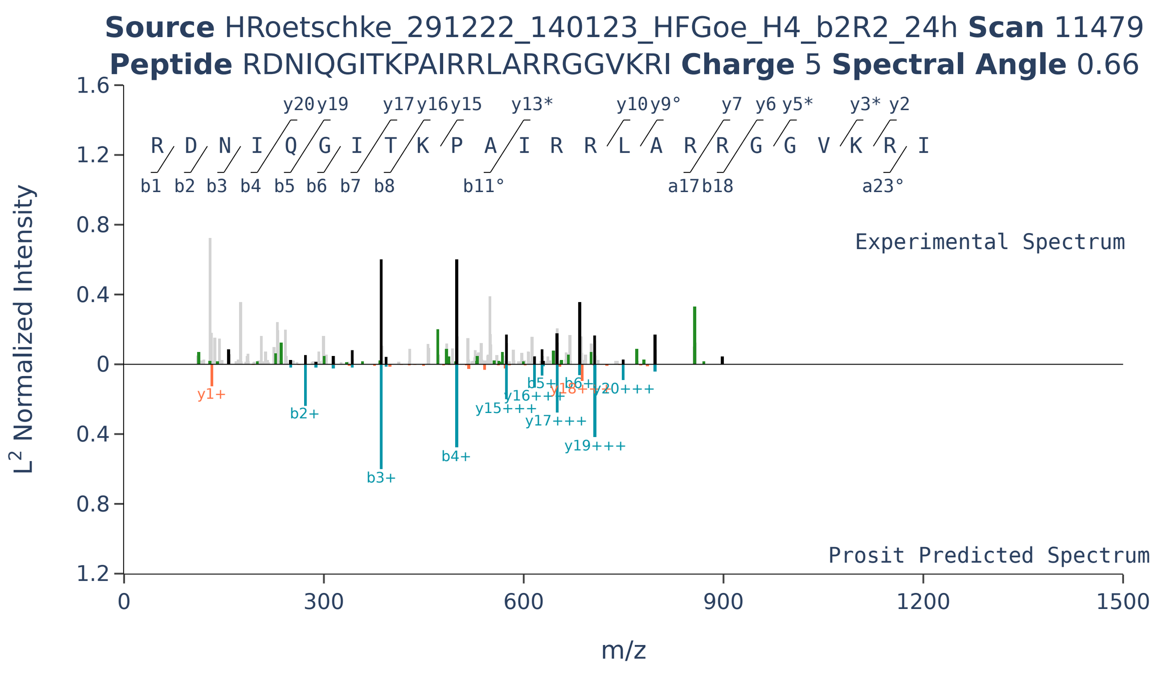
Source HRoetschke_291222_140123_HFGoe_H4_b2R2_24h Scan 11479
Peptide RDNIQGITKPAIRRLARRGGVKRI Charge 5 Spectral Angle 0.66
Experimental Spectrum
Prosit Predicted Spectrum
L² Normalized Intensity
m/z

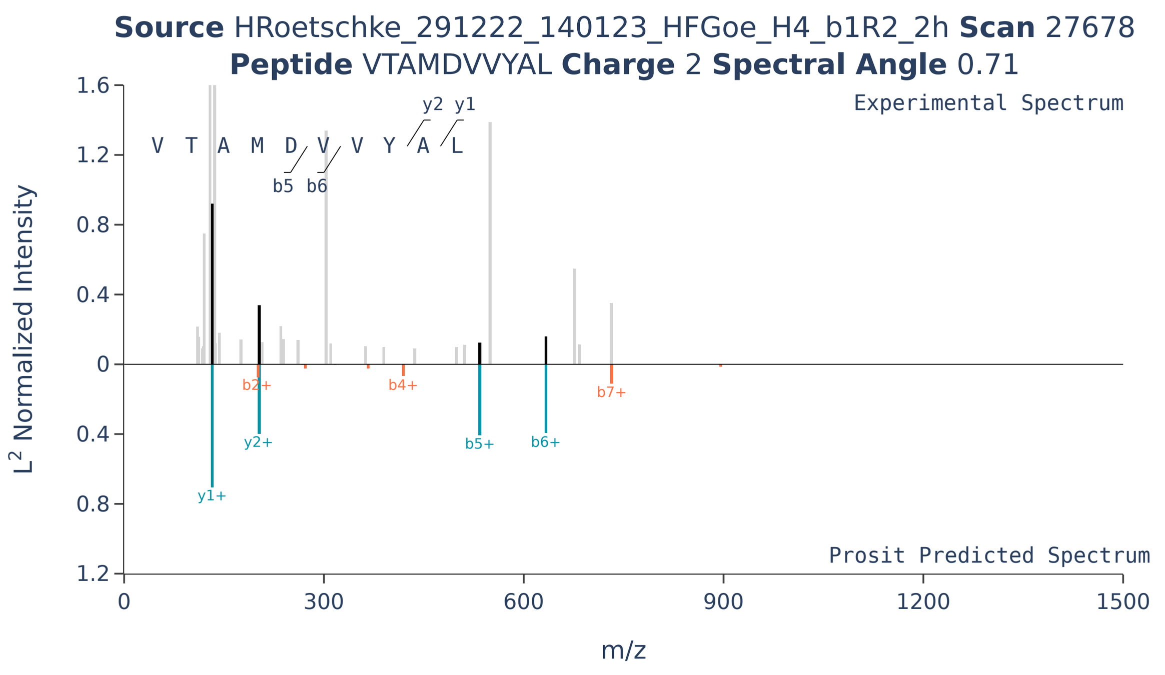
Source HRoetschke_291222_140123_HFGoe_H4_b1R2_2h Scan 27678
Peptide VTAMDVVYAL Charge 2 Spectral Angle 0.71
Experimental Spectrum
Prosit Predicted Spectrum
L² Normalized Intensity
m/z

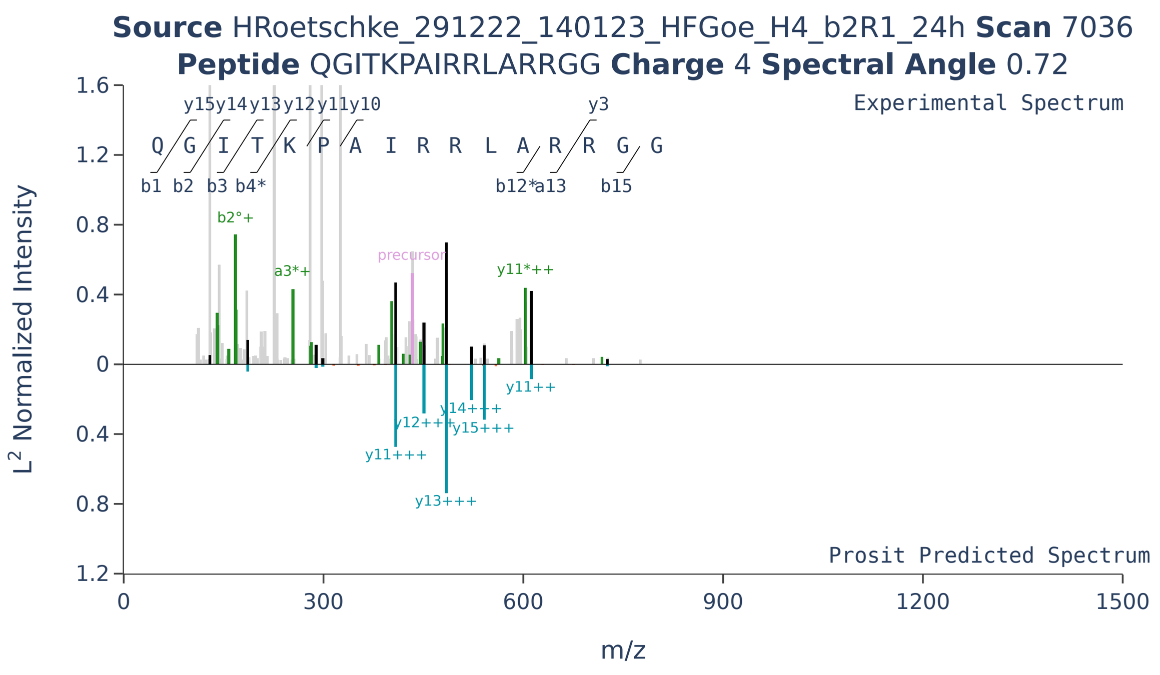
Source HRoetschke_291222_140123_HFGoe_H4_b2R1_24h Scan 7036
Peptide QGITKPAIRRLARRGG Charge 4 Spectral Angle 0.72
Experimental Spectrum
Prosit Predicted Spectrum
L² Normalized Intensity
m/z

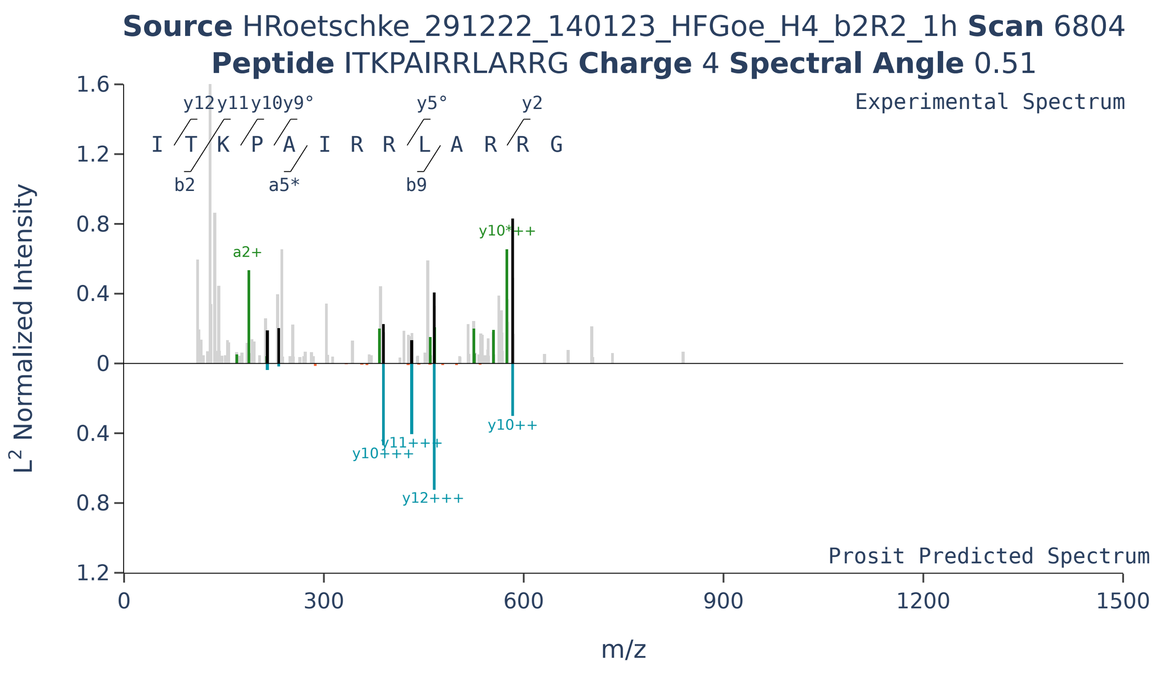
Source HRoetschke_291222_140123_HFGoe_H4_b2R2_1h Scan 6804
Peptide ITKPAIRRLARRG Charge 4 Spectral Angle 0.51
Experimental Spectrum
Prosit Predicted Spectrum
L² Normalized Intensity
m/z

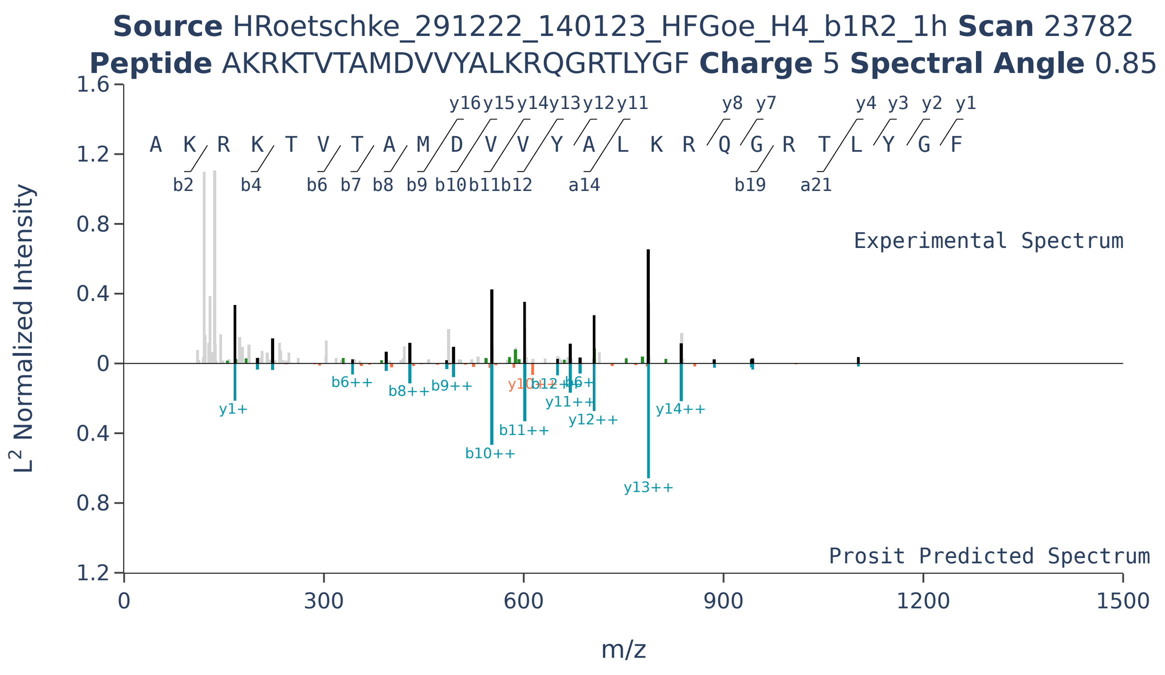
Source HRoetschke_291222_140123_HFGoe_H4_b1R2_1h Scan 23782
Peptide AKRKTVTAMDVVYALKRQGRTLYGF Charge 5 Spectral Angle 0.85
Experimental Spectrum
Prosit Predicted Spectrum
L² Normalized Intensity
m/z

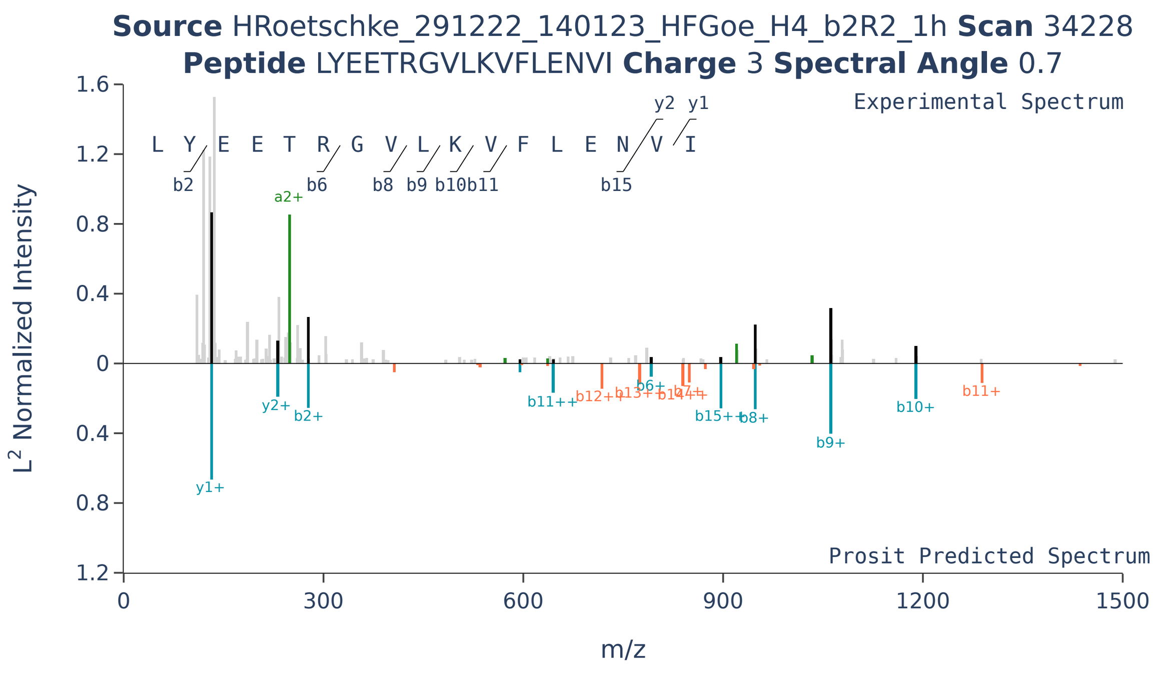
Source HRoetschke_291222_140123_HFGoe_H4_b2R2_1h Scan 34228
Peptide LYEETRGVLKVFLENVI Charge 3 Spectral Angle 0.7
Experimental Spectrum
Prosit Predicted Spectrum
L² Normalized Intensity
m/z

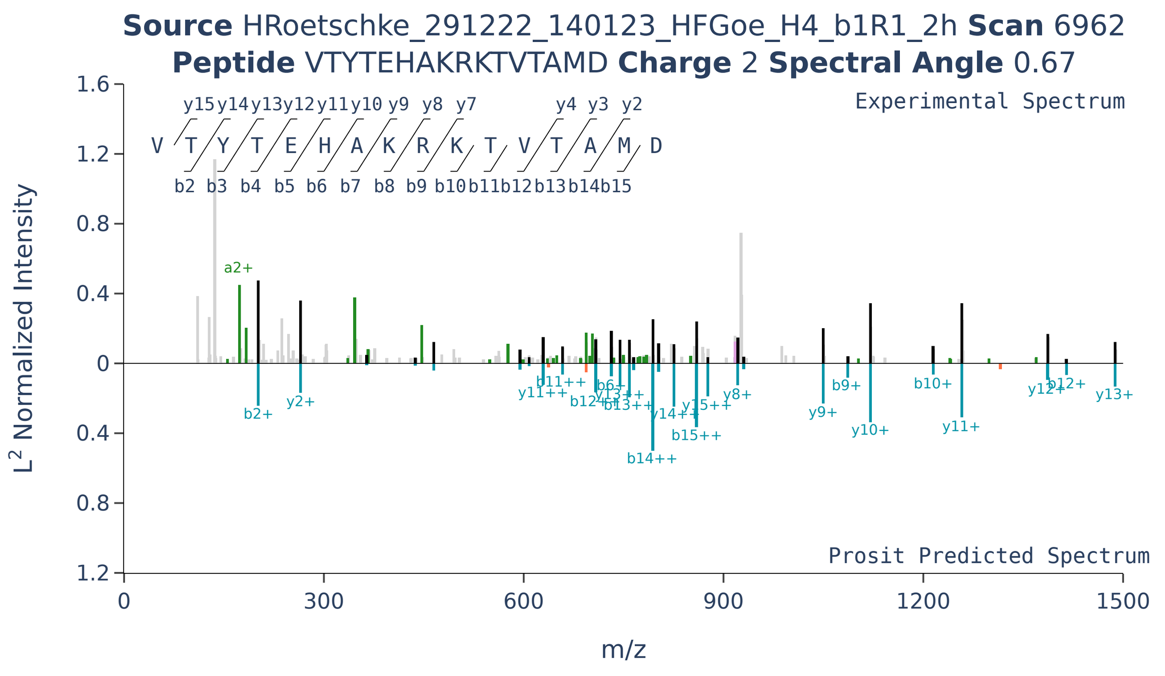
Source HRoetschke_291222_140123_HFGoe_H4_b1R1_2h Scan 6962
Peptide VTYTEHAKRKTVTAMD Charge 2 Spectral Angle 0.67
Experimental Spectrum
Prosit Predicted Spectrum
L² Normalized Intensity
m/z

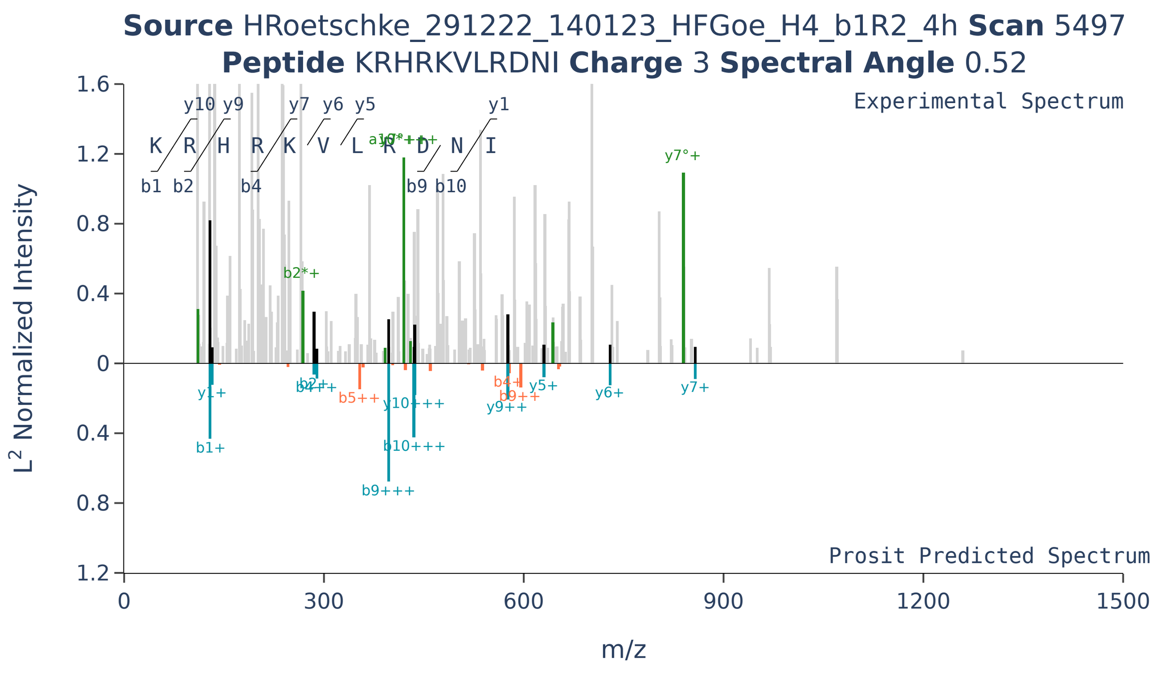
Source HRoetschke_291222_140123_HFGoe_H4_b1R2_4h Scan 5497
Peptide KRHRKVLRDNI Charge 3 Spectral Angle 0.52
Experimental Spectrum
Prosit Predicted Spectrum
L² Normalized Intensity
m/z

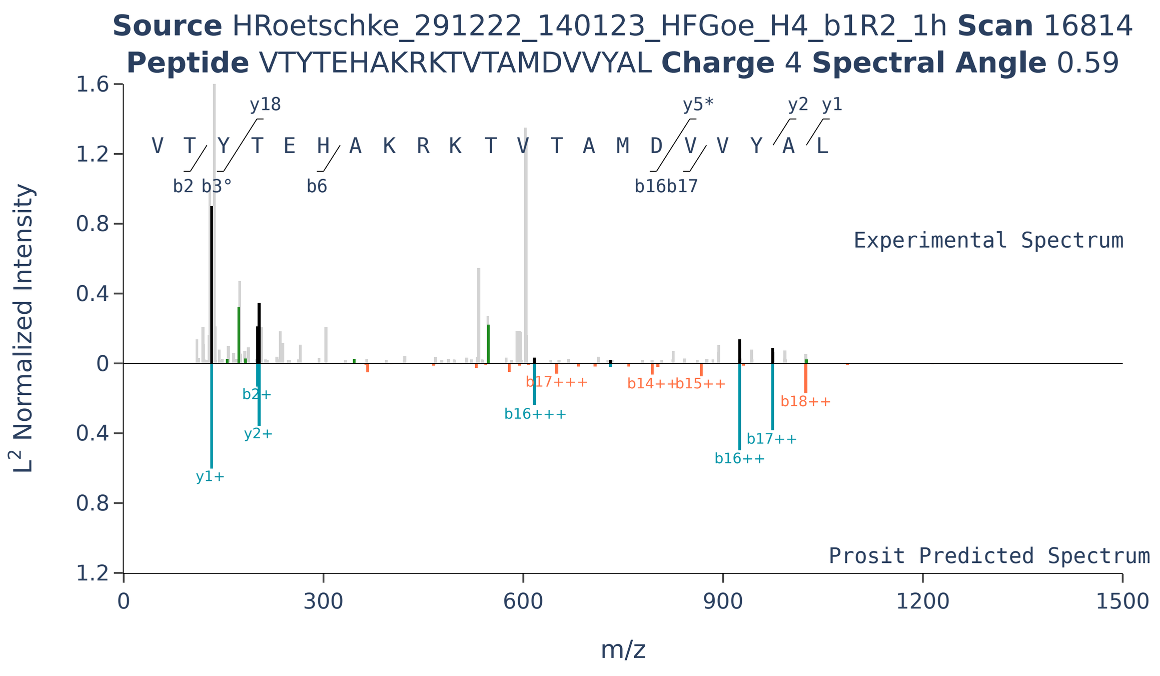
Source HRoetschke_291222_140123_HFGoe_H4_b1R2_1h Scan 16814
Peptide VTYTEHAKRKTVTAMDVVYAL Charge 4 Spectral Angle 0.59
Experimental Spectrum
Prosit Predicted Spectrum
L² Normalized Intensity
m/z

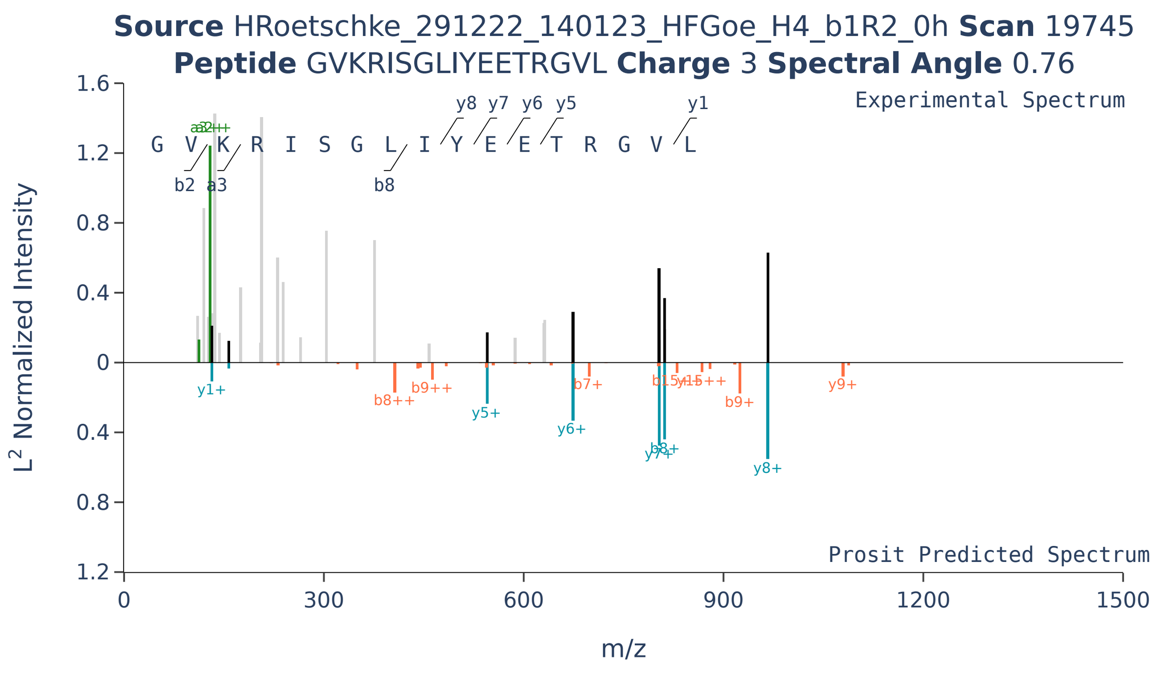
Source HRoetschke_291222_140123_HFGoe_H4_b1R2_0h Scan 19745
Peptide GVKRISGLIYEETRGVL Charge 3 Spectral Angle 0.76
Experimental Spectrum
Prosit Predicted Spectrum
L² Normalized Intensity
m/z

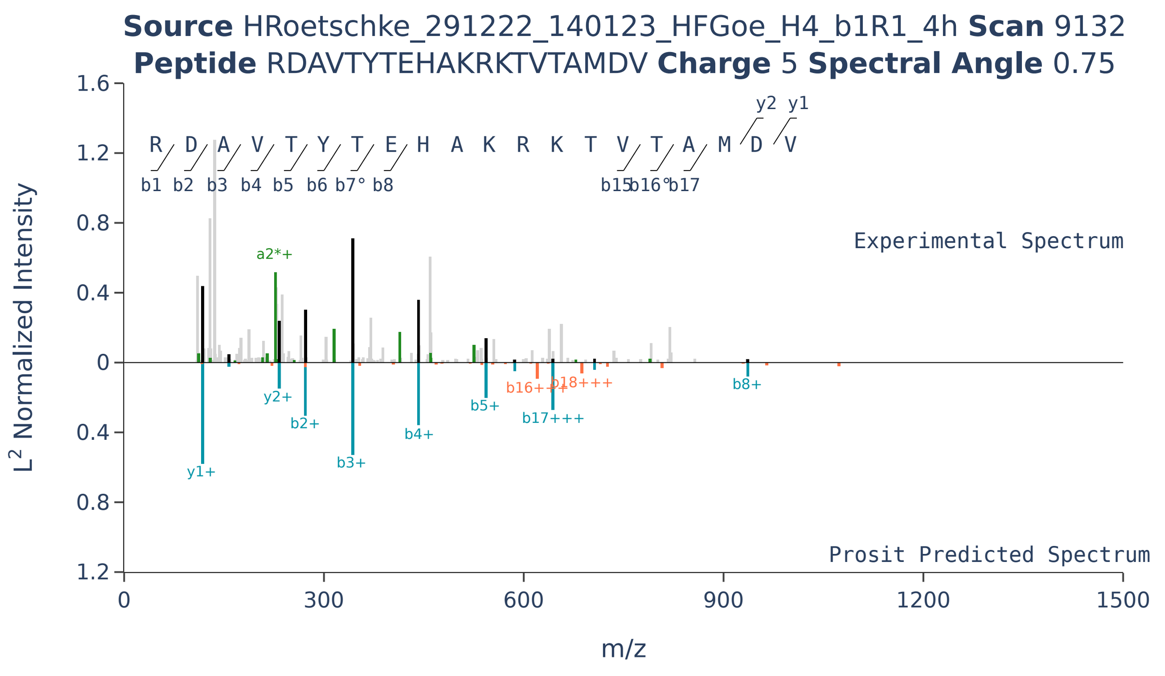
Source HRoetschke_291222_140123_HFGoe_H4_b1R1_4h Scan 9132
Peptide RDAVTYTEHAKRKTVTAMDV Charge 5 Spectral Angle 0.75
Experimental Spectrum
Prosit Predicted Spectrum
L² Normalized Intensity
m/z

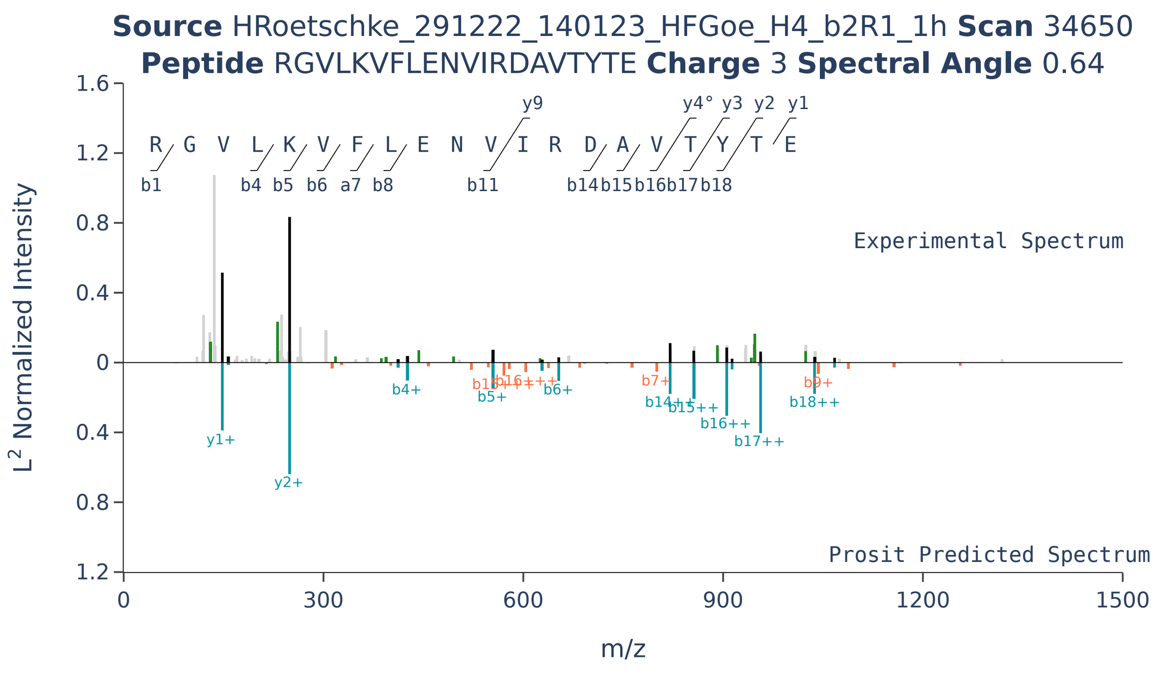
Source HRoetschke_291222_140123_HFGoe_H4_b2R1_1h Scan 34650
Peptide RGVLKVFLENVIRDAVTYTE Charge 3 Spectral Angle 0.64
Experimental Spectrum
Prosit Predicted Spectrum
L² Normalized Intensity
m/z

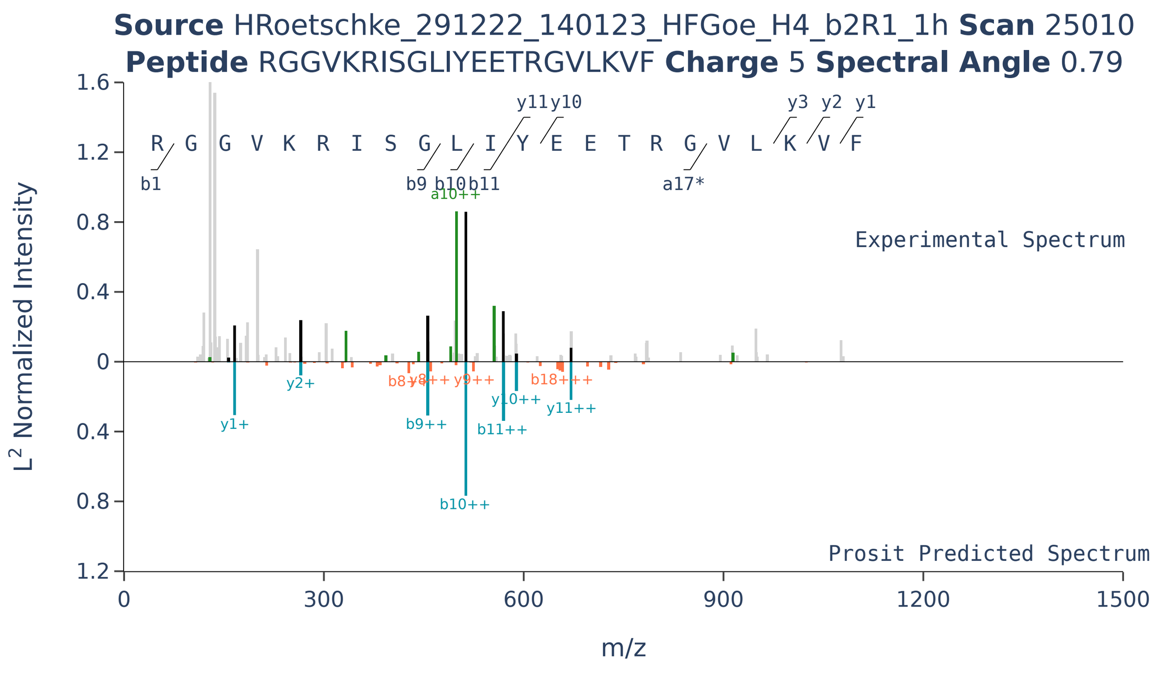
Source HRoetschke_291222_140123_HFGoe_H4_b2R1_1h Scan 25010
Peptide RGGVKRISGLIYEETRGVLKVF Charge 5 Spectral Angle 0.79
Experimental Spectrum
Prosit Predicted Spectrum
L² Normalized Intensity
m/z

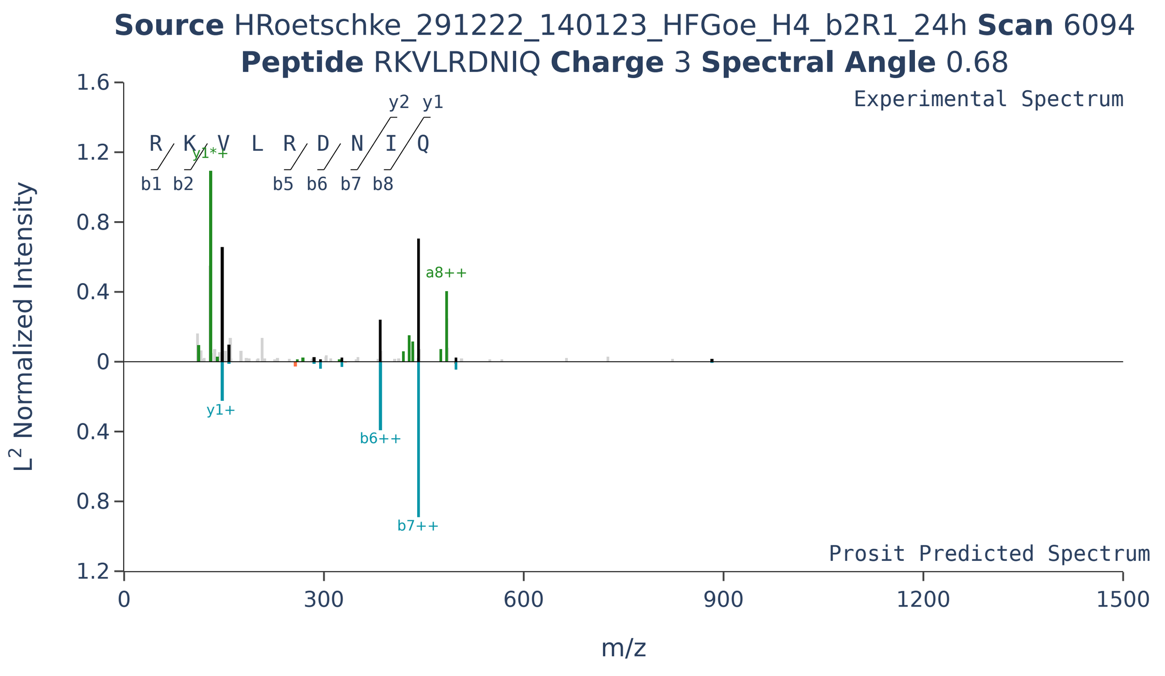
Source HRoetschke_291222_140123_HFGoe_H4_b2R1_24h Scan 6094
Peptide RKVLRDNIQ Charge 3 Spectral Angle 0.68
Experimental Spectrum
Prosit Predicted Spectrum
L² Normalized Intensity
m/z

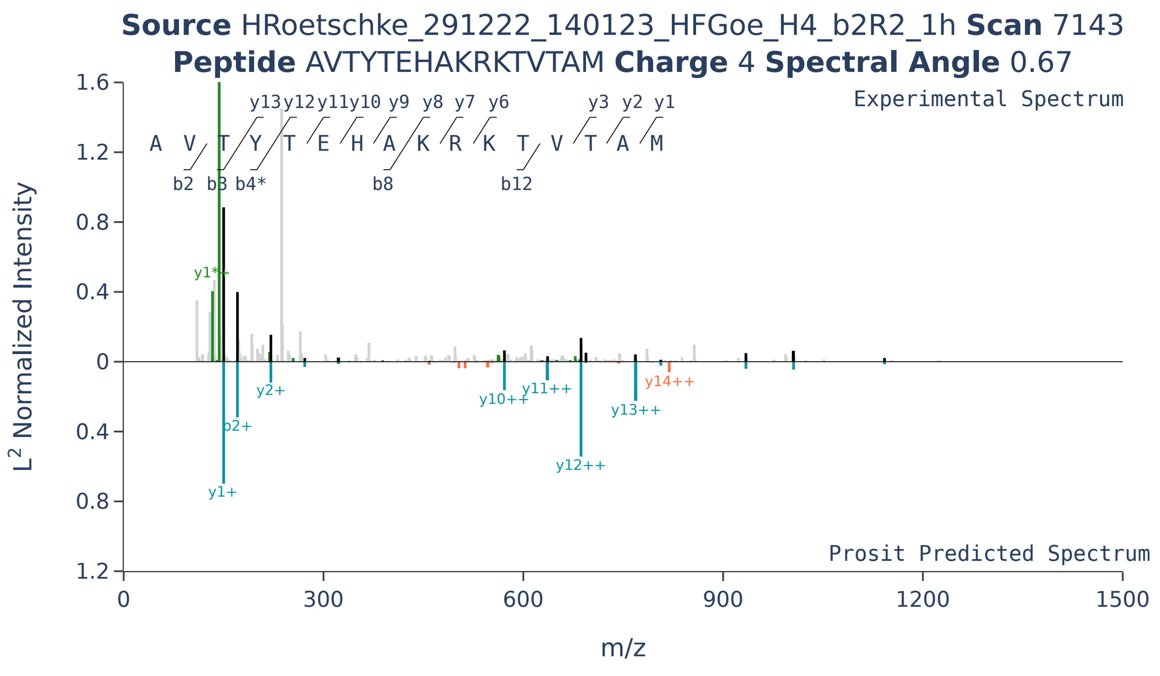
Source HRoetschke_291222_140123_HFGoe_H4_b2R2_1h Scan 7143
Peptide AVTYTEHAKRKTVTAM Charge 4 Spectral Angle 0.67
Experimental Spectrum
Prosit Predicted Spectrum
L² Normalized Intensity
m/z

**Source** HRoetschke_291222_140123_HFGoe_H4_b1R2_1h **Scan** 17374
**Peptide** DNIQGITKPAIRRLARRGGVKRISGLIYE **Charge** 4 **Spectral Angle** 0.64

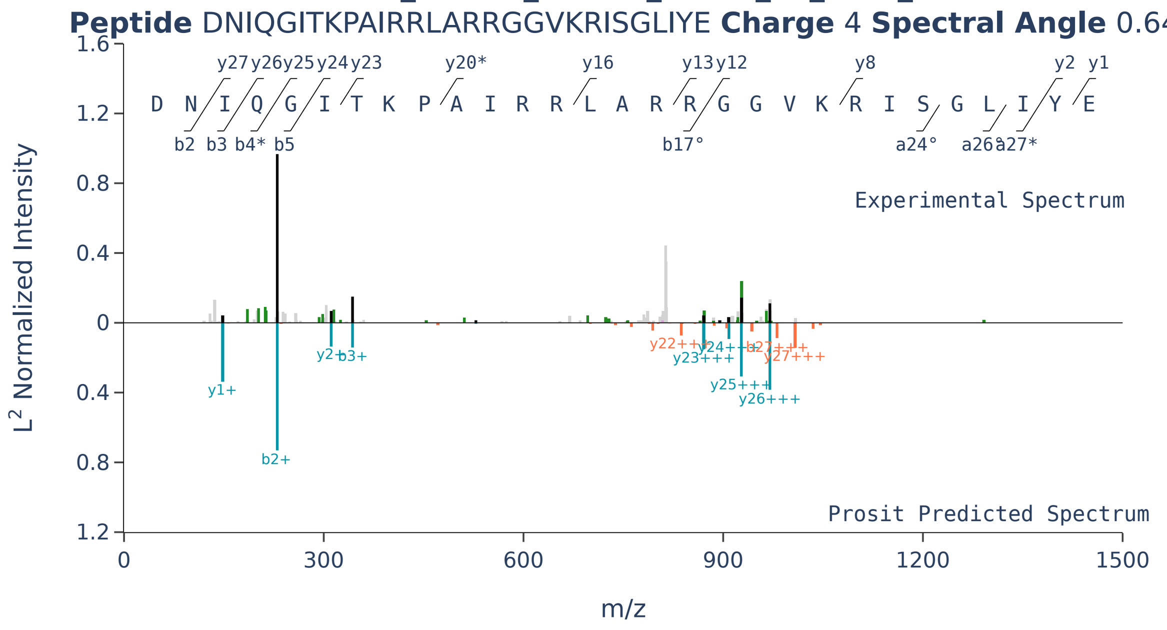


**Source** HRoetschke_291222_140123_HFGoe_H4_b1R1_24h **Scan** 5278
**Peptide** PAIRRLARRGGVKR **Charge** 4 **Spectral Angle** 0.63

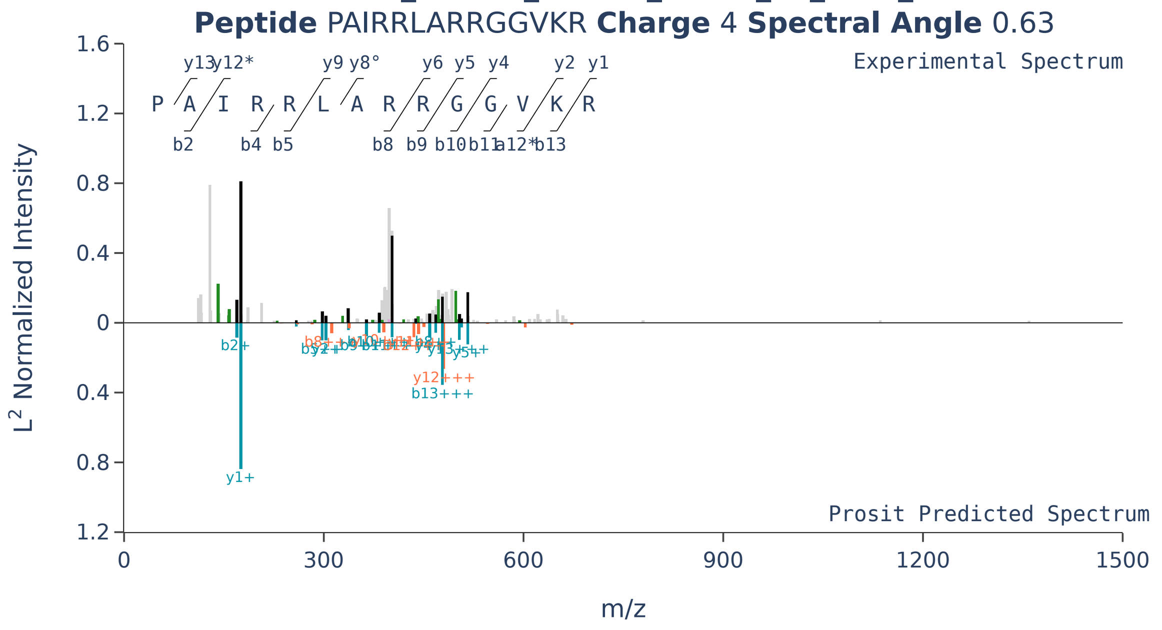


**Source** HRoetschke_291222_140123_HFGoe_H4_b2R1_1h **Scan** 15687
**Peptide** IQGITKPAIRRLARRGGVKRISGLIYEE **Charge** 5 **Spectral Angle** 0.82

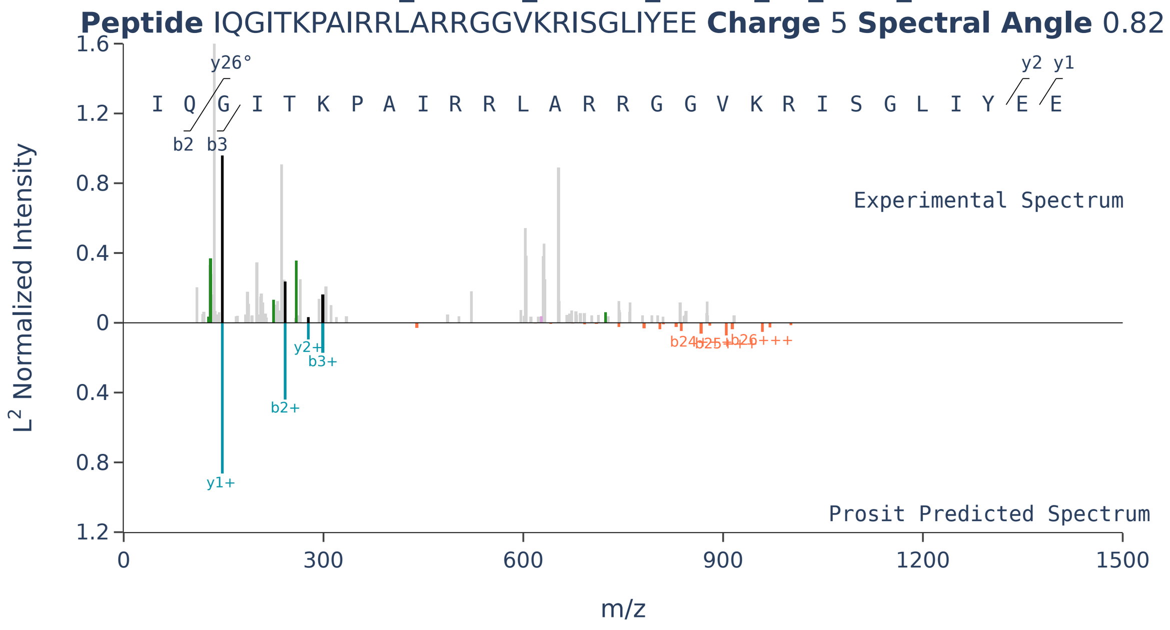


**Source** HRoetschke_291222_140123_HFGoe_H4_b2R1_4h **Scan** 17809
**Peptide** TAMDVVYALKRQGRT **Charge** 3 **Spectral Angle** 0.43

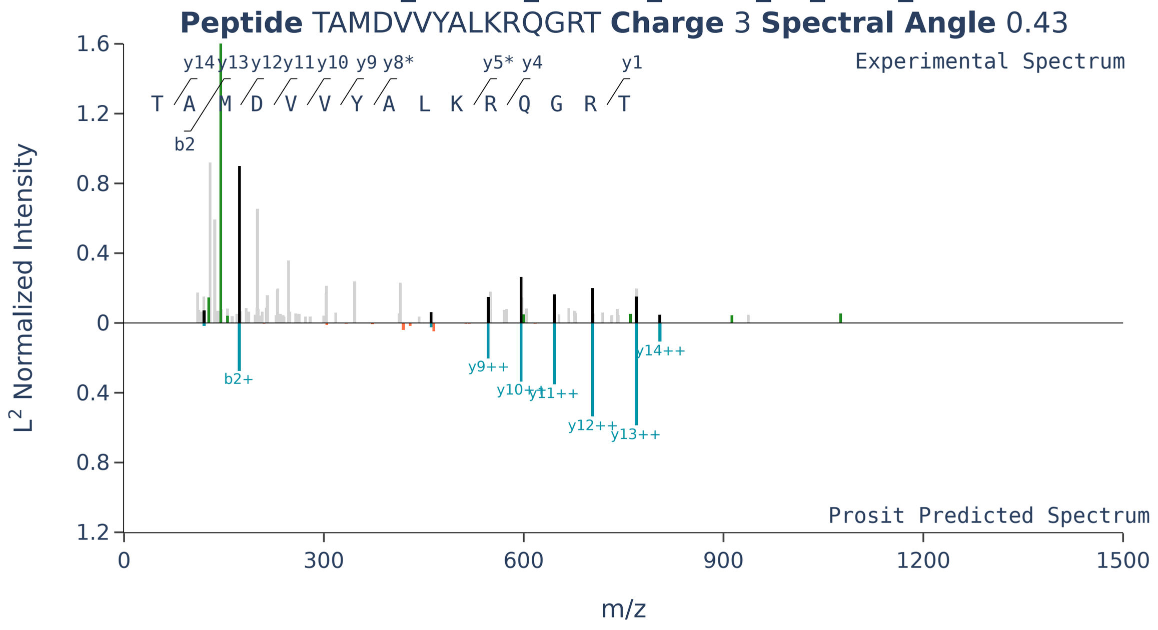


**Source** HRoetschke_291222_140123_HFGoe_H4_b1R1_2h **Scan** 13141
**Peptide** DAVTYTEHAKRKTVTAMDVVY **Charge** 3 **Spectral Angle** 0.68

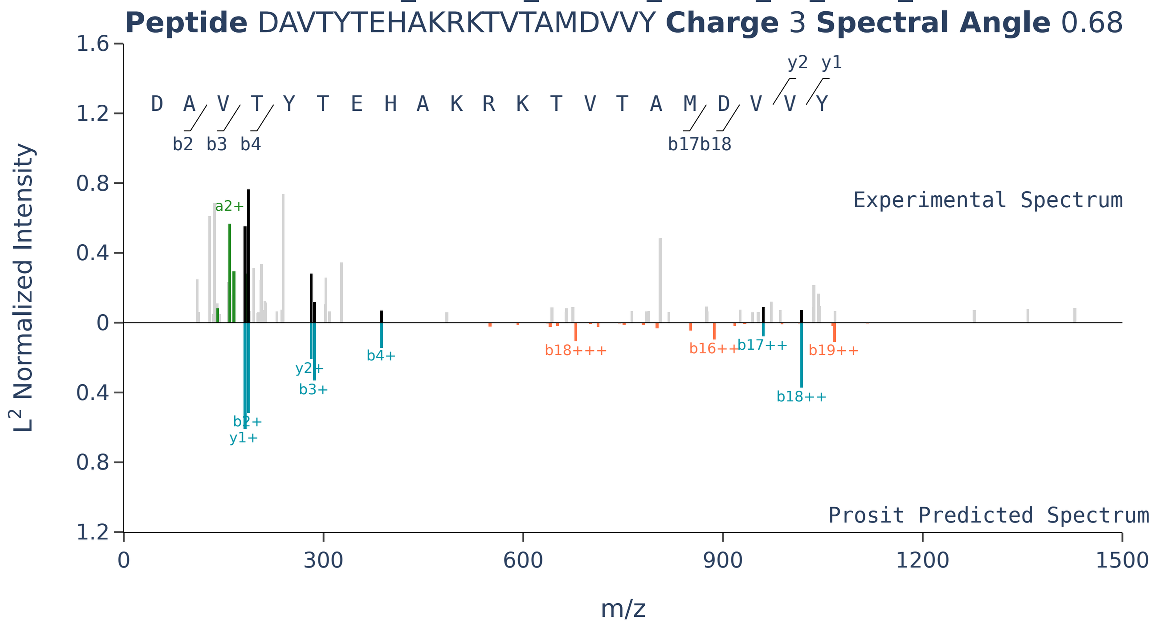


**Source** HRoetschke_291222_140123_HFGoe_H4_b2R1_1h **Scan** 28814
**Peptide** RGGVKRISGLIYEETRGVLKVFL **Charge** 5 **Spectral Angle** 0.74

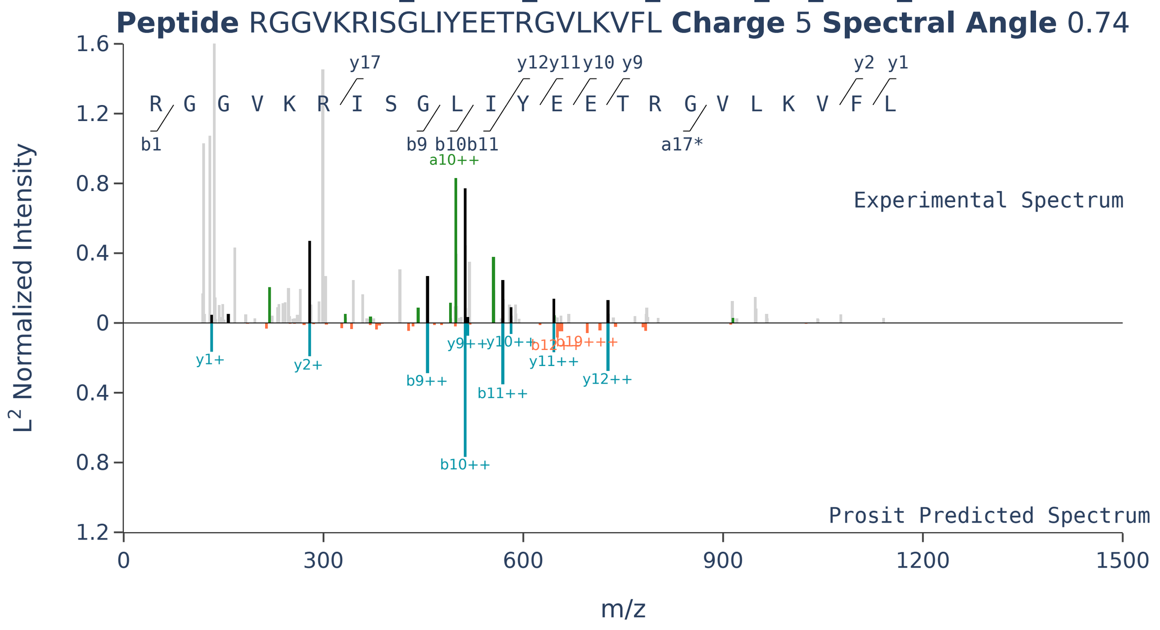


**Source** HRoetschke_291222_140123_HFGoe_H4_b1R1_1h **Scan** 11661
**Peptide** TKPAIRRLARRGGVKRISGLIYE **Charge** 4 **Spectral Angle** 0.72

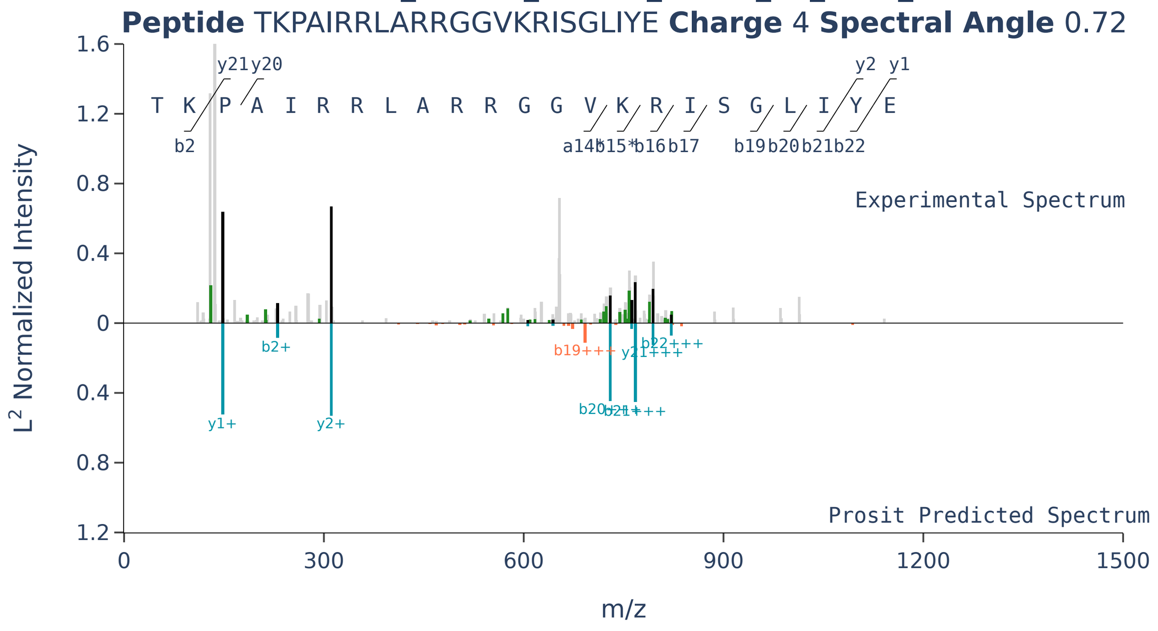


**Source** HRoetschke_291222_140123_HFGoe_H4_b1R2_2h **Scan** 21228
**Peptide** AKRKTVTAMDVVYALKRQGRTLYG **Charge** 5 **Spectral Angle** 0.86

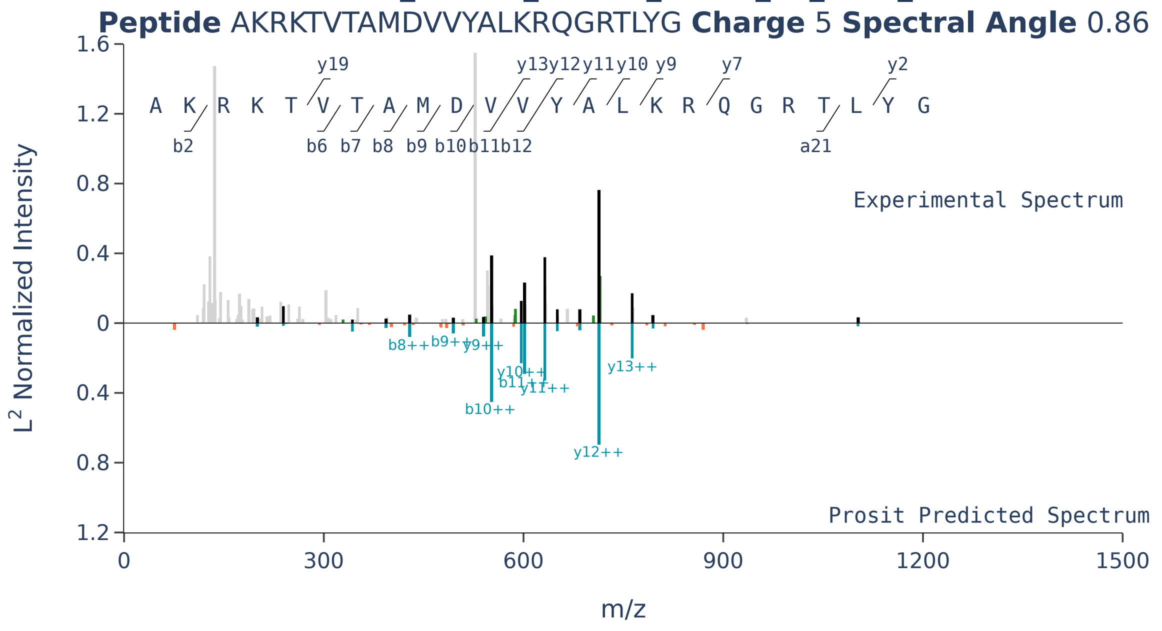


**Source** HRoetschke_291222_140123_HFGoe_H4_b1R2_4h **Scan** 14246
**Peptide** KTVTAMDVVYA **Charge** 2 **Spectral Angle** 0.43

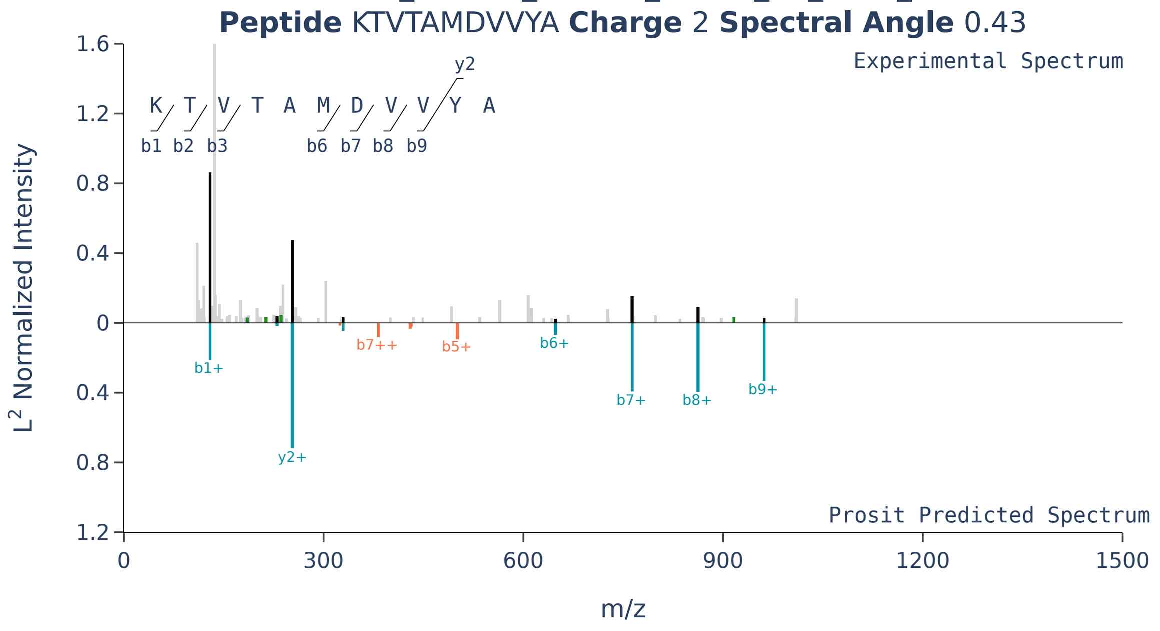


**Source** HRoetschke_291222_140123_HFGoe_H4_b1R2_2h **Scan** 12144
**Peptide** RLARRGGVKRISGLIYEET **Charge** 4 **Spectral Angle** 0.52

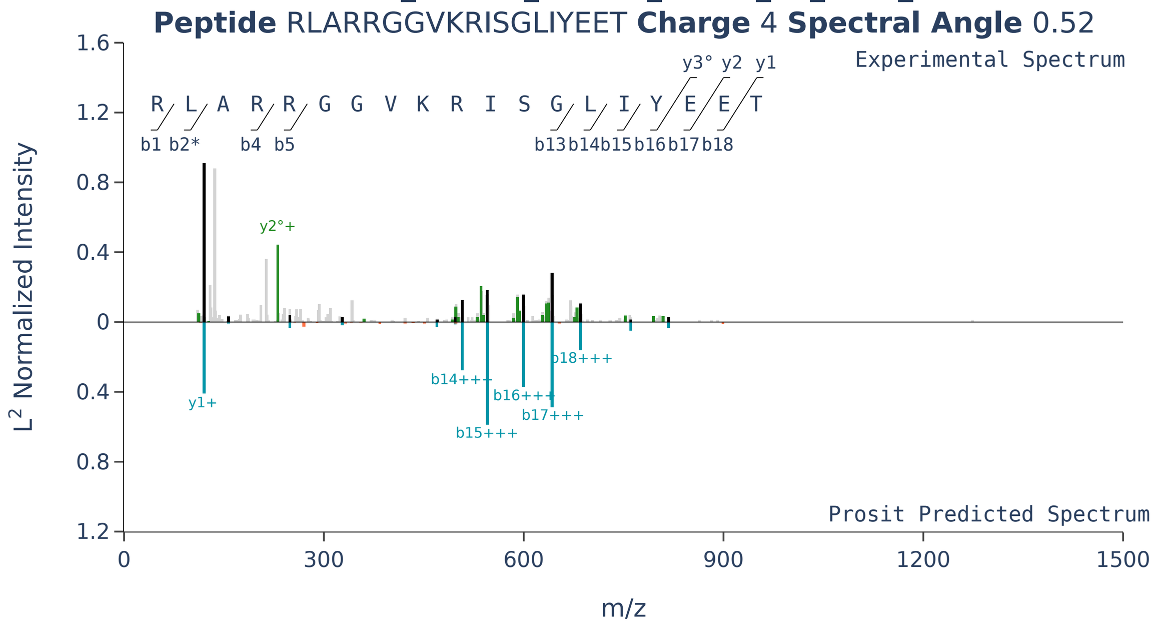


**Source** HRoetschke_291222_140123_HFGoe_H4_b1R2_2h **Scan** 23600
**Peptide** RLSGLIYEETRGVLKV **Charge** 3 **Spectral Angle** 0.62

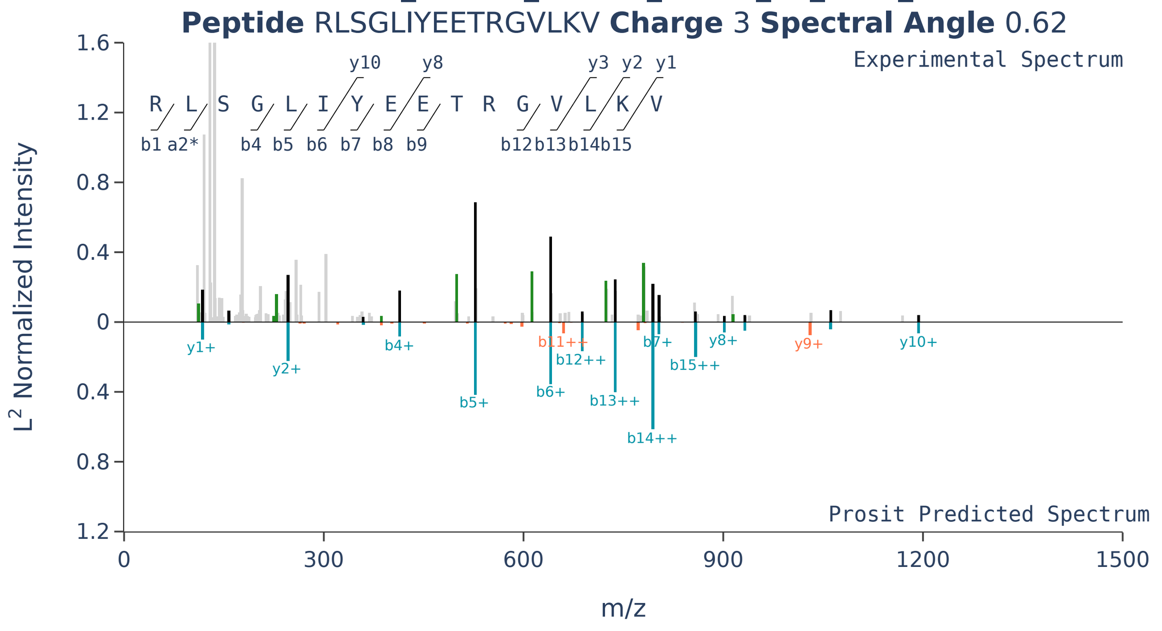


**Source** HRoetschke_291222_140123_HFGoe_H4_b2R1_24h **Scan** 10907
**Peptide** QGITKPAIRRLARRGGVKRL **Charge** 4 **Spectral Angle** 0.68

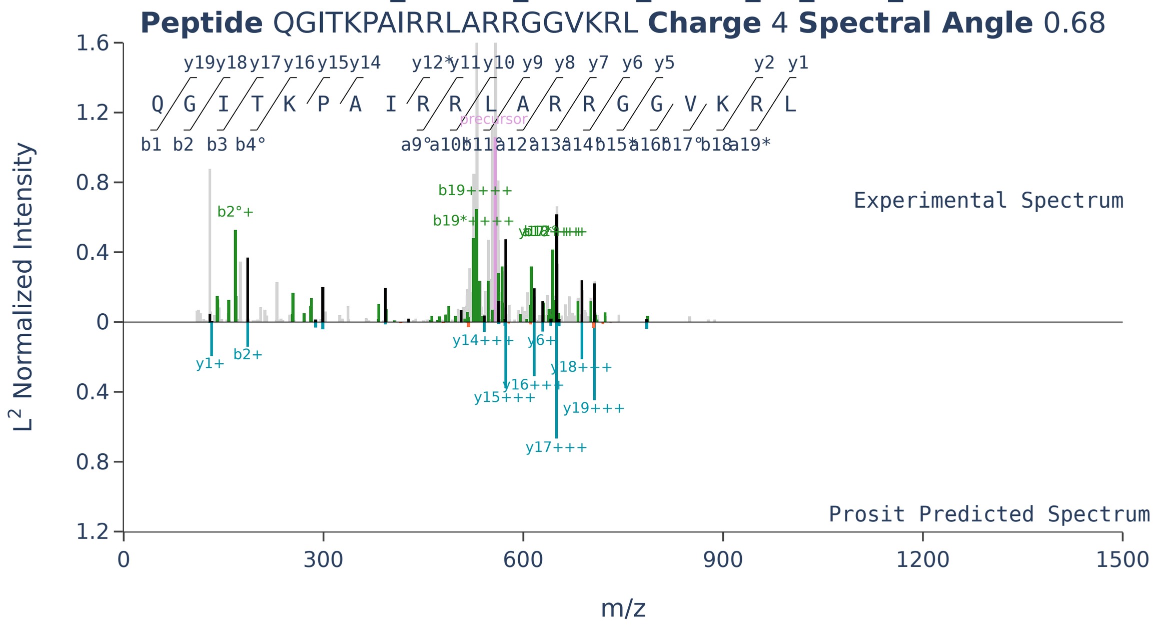


**Source** HRoetschke_291222_140123_HFGoe_H4_b1R2_1h **Scan** 5443
**Peptide** KRKTVTAMD **Charge** 3 **Spectral Angle** 0.79

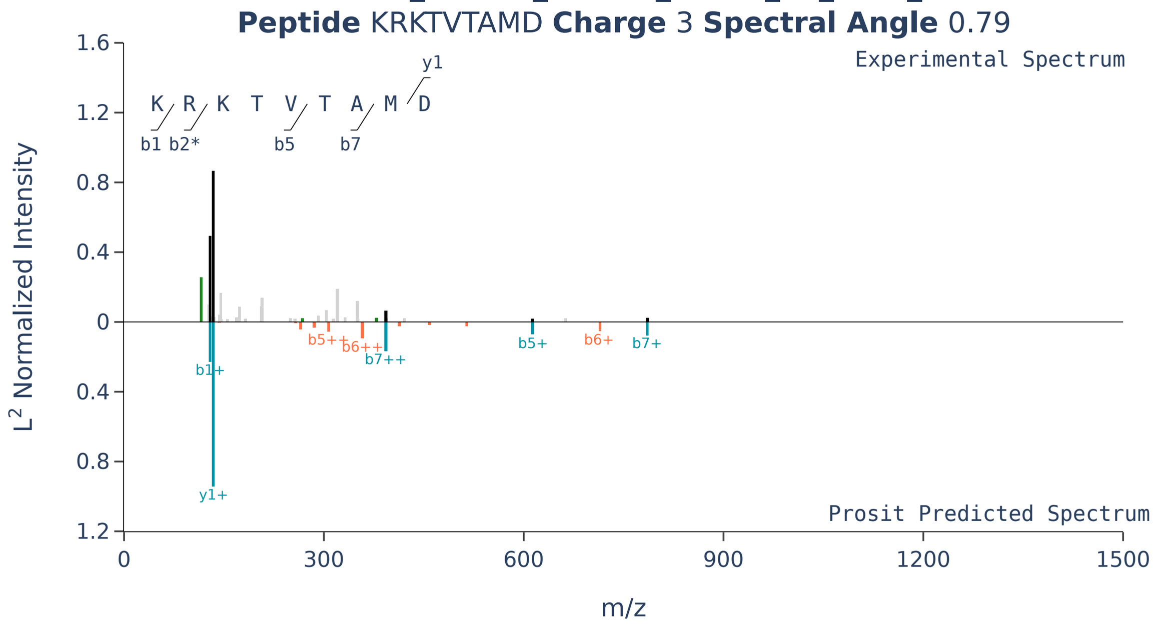


**Source** HRoetschke_291222_140123_HFGoe_H4_b2R2_1h **Scan** 29512
**Peptide** LKVFLENVI **Charge** 2 **Spectral Angle** 0.63

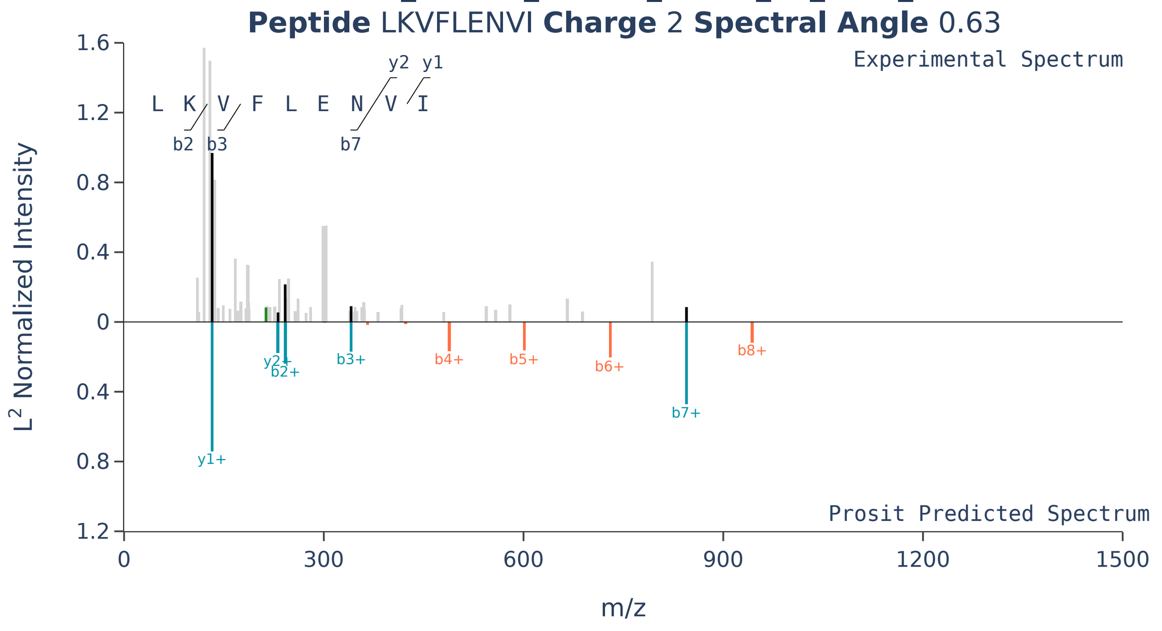


**Source** HRoetschke_291222_140123_HFGoe_H4_b2R1_1h **Scan** 29586
**Peptide** LKVFLENVIRDAVTY **Charge** 3 **Spectral Angle** 0.75

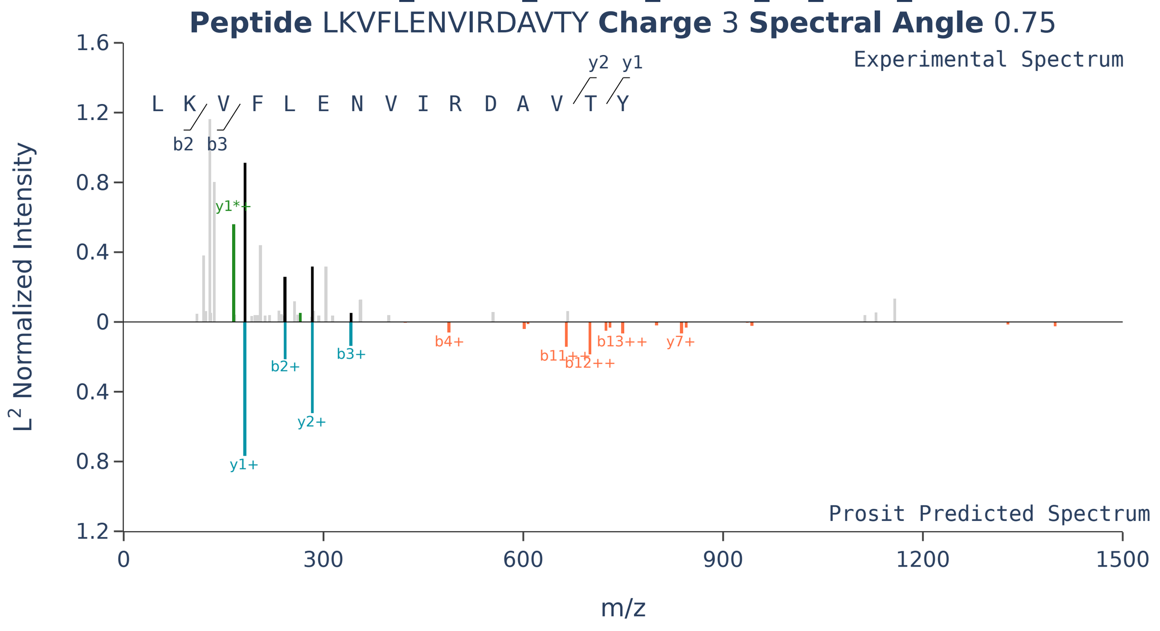

**Source** HRoetschke_291222_140123_HFGoe_H4_b2R2_24h **Scan** 7693
**Peptide** GITKPAIRRLARRGGVKR **Charge** 4 **Spectral Angle** 0.51

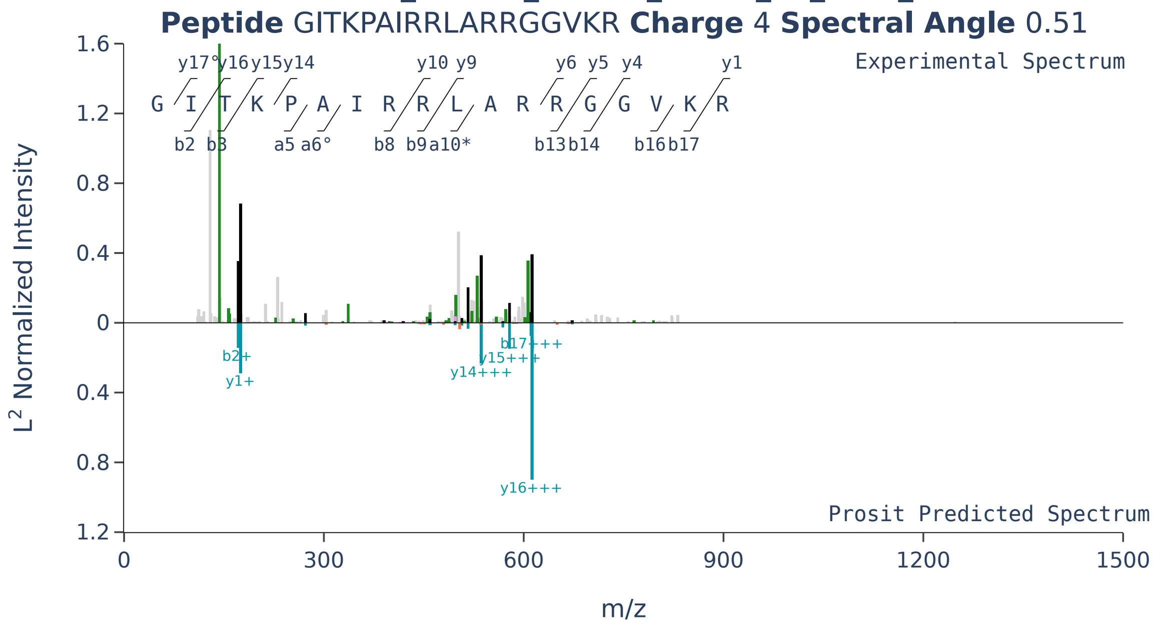


**Source** HRoetschke_291222_140123_HFGoe_H4_b2R1_1h **Scan** 37729
**Peptide** GVLKVFLENVIRDAVTY **Charge** 3 **Spectral Angle** 0.8

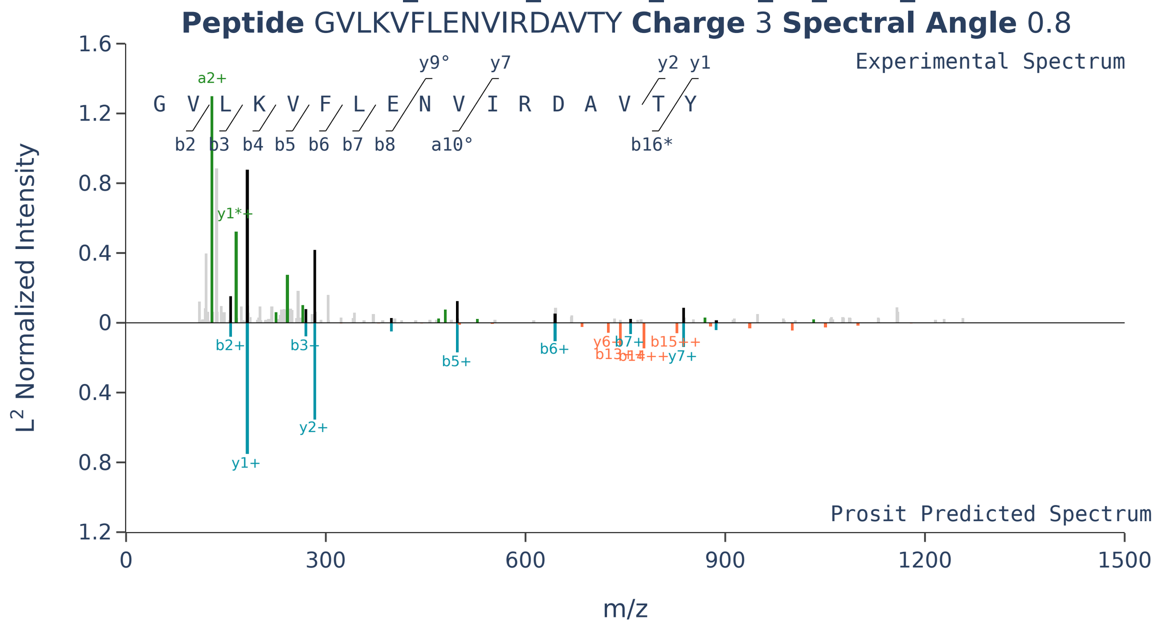


**Source** HRoetschke_291222_140123_HFGoe_H4_b2R2_X **Scan** 6621
**Peptide** DAVTYTEHAK **Charge** 3 **Spectral Angle** 0.55

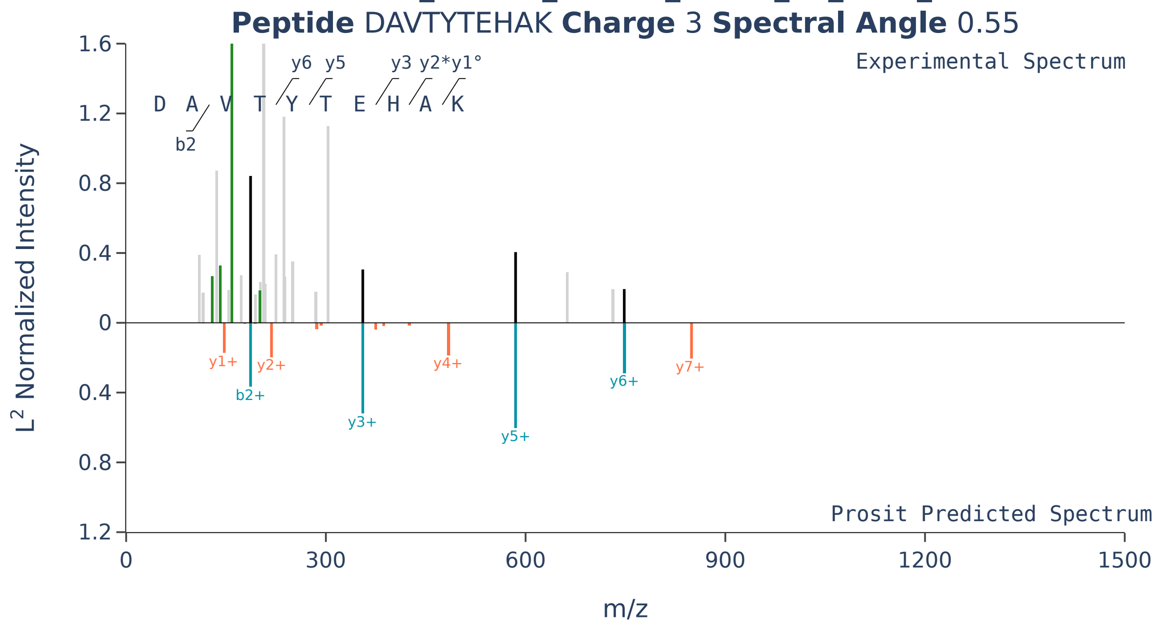


**Source** HRoetschke_291222_140123_HFGoe_H4_b2R1_1h **Scan** 32626
**Peptide** ETRGVLKVFLENVIRDAVTYTEHAKRKTVT **Charge** 5 **Spectral Angle** 0.63

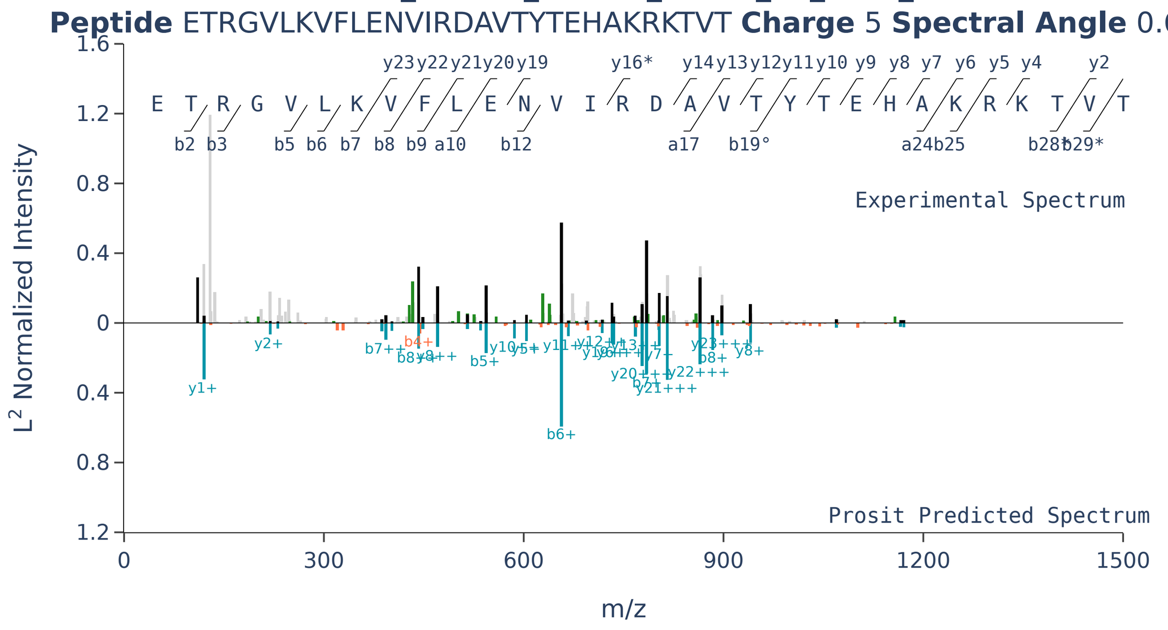


**Source** HRoetschke_291222_140123_HFGoe_H4_b2R1_1h **Scan** 28616
**Peptide** KVFLENVIRDAVTYTEHA **Charge** 4 **Spectral Angle** 0.44

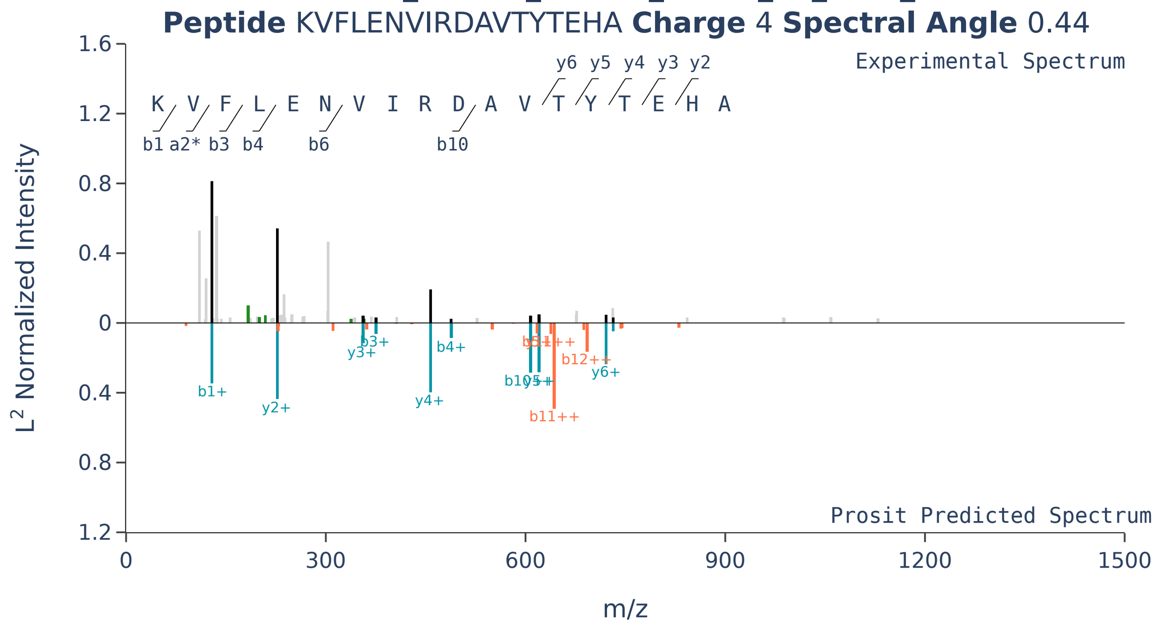


**Source** HRoetschke_291222_140123_HFGoe_H4_b2R1_1h **Scan** 26627
**Peptide** FLENVIRDAVTYTEHAK **Charge** 4 **Spectral Angle** 0.66

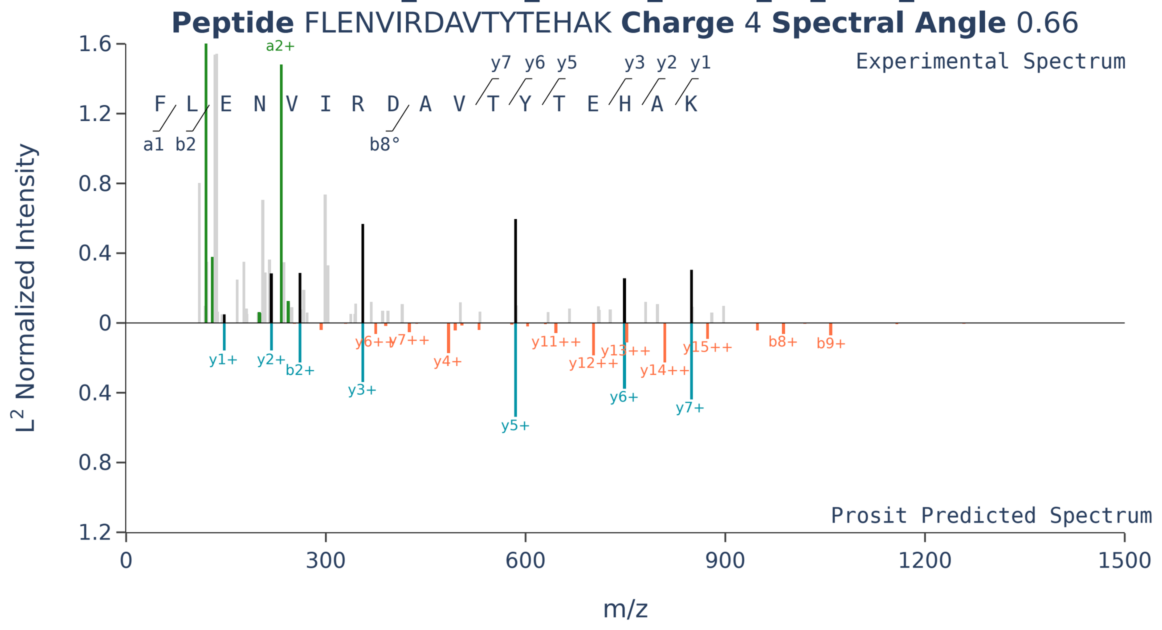


**Source** HRoetschke_291222_140123_HFGoe_H4_b1R1_1h **Scan** 21985
**Peptide** ENVIRDAVTYTEHAKRKTVTAMDVV **Charge** 5 **Spectral Angle** 0.83

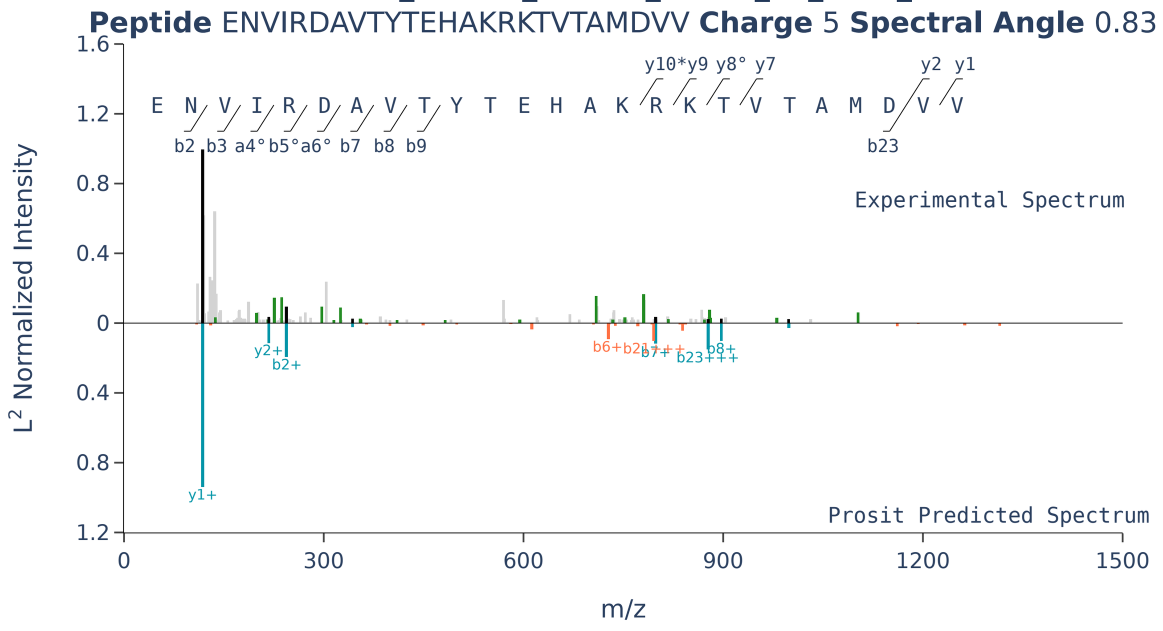


**Source** HRoetschke_291222_140123_HFGoe_H4_b2R1_4h **Scan** 7190
**Peptide** TKPAIRRLARRGGVKRL **Charge** 4 **Spectral Angle** 0.84

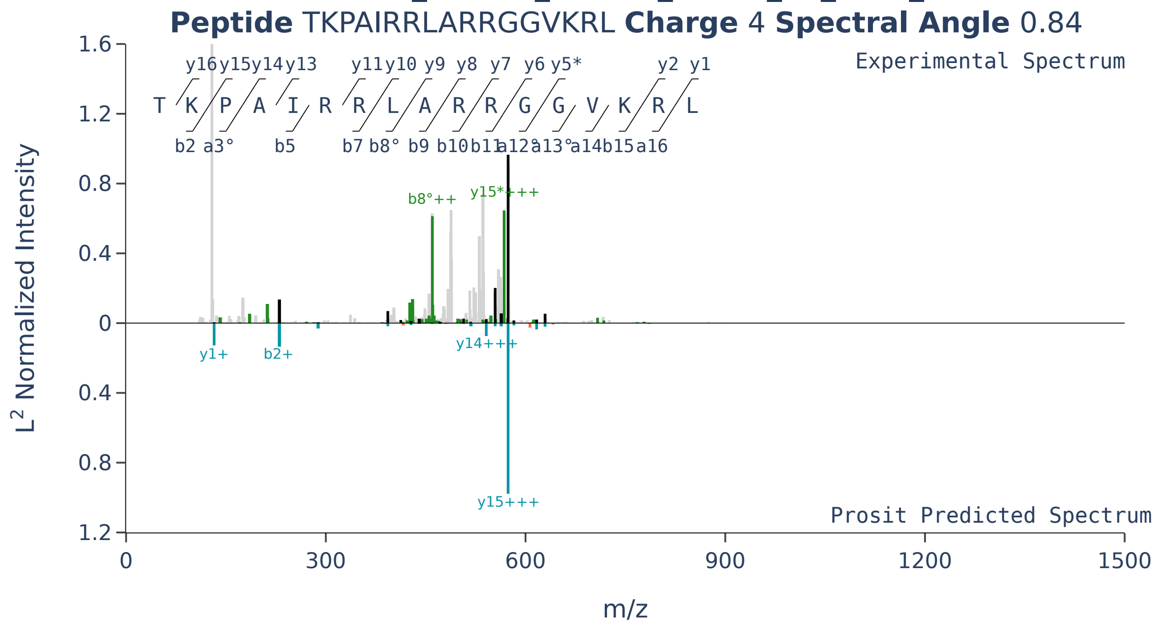


**Source** HRoetschke_291222_140123_HFGoe_H4_b2R2_1h **Scan** 24874
**Peptide** RKTVTAMDVVYALKRQGRTLYGFGG **Charge** 4 **Spectral Angle** 0.67

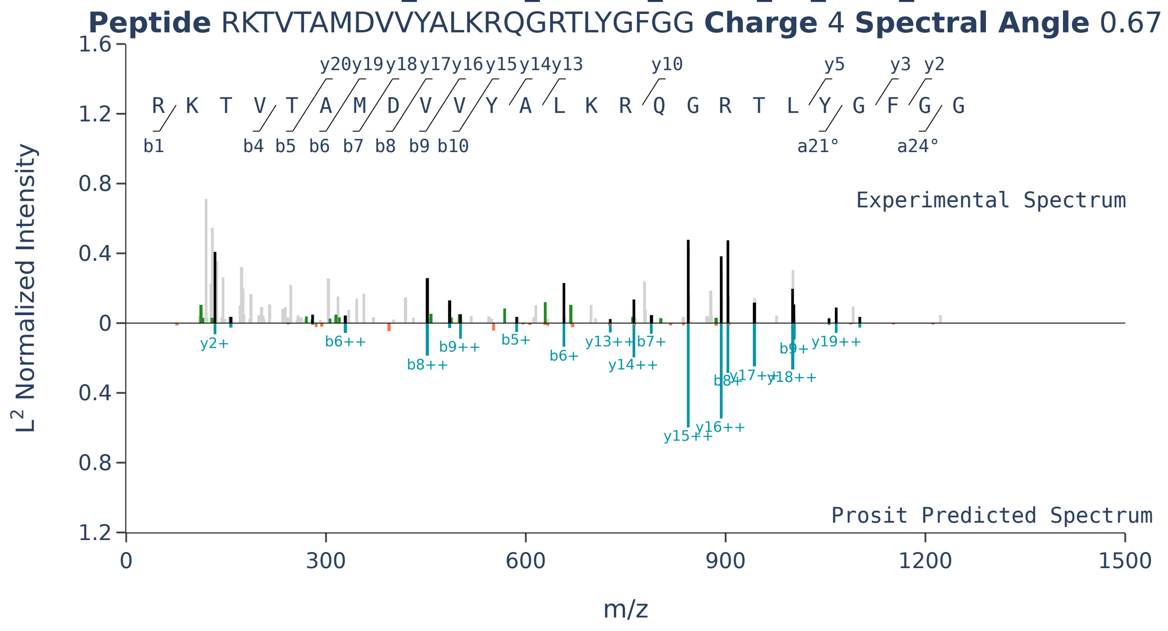


**Source** HRoetschke_291222_140123_HFGoe_H4_b1R1_4h **Scan** 13272
**Peptide** VLRDNIQGITKPAIRRLARRGGVKRL **Charge** 5 **Spectral Angle** 0.67

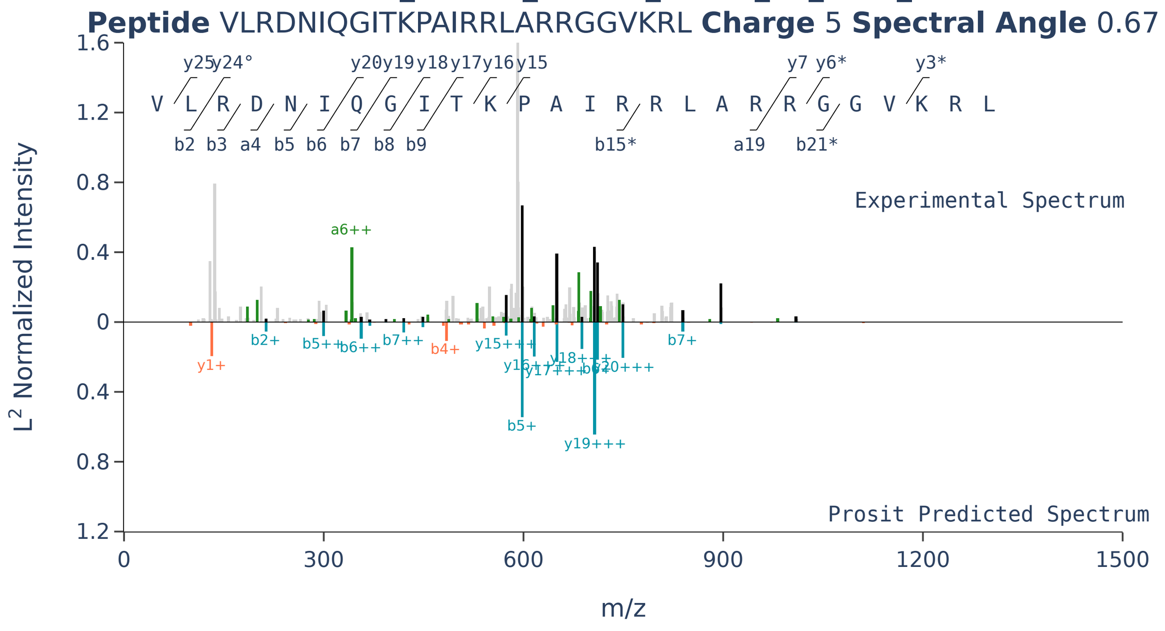


**Source** HRoetschke_291222_140123_HFGoe_H4_b1R2_2h **Scan** 15324
**Peptide** LRDNIQGITKPAIRRLARRGGVKRISGL **Charge** 6 **Spectral Angle** 0.74

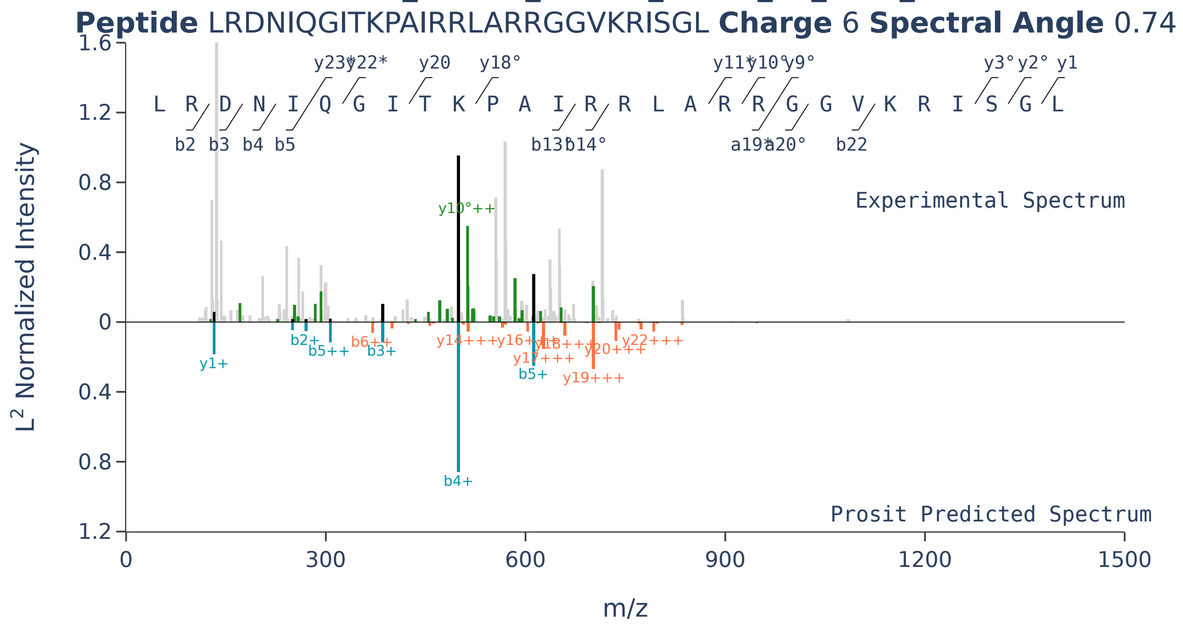


**Source** HRoetschke_291222_140123_HFGoe_H4_b1R1_2h **Scan** 12609
**Peptide** PAIRRLARRGGVKRISGLIYE **Charge** 4 **Spectral Angle** 0.79

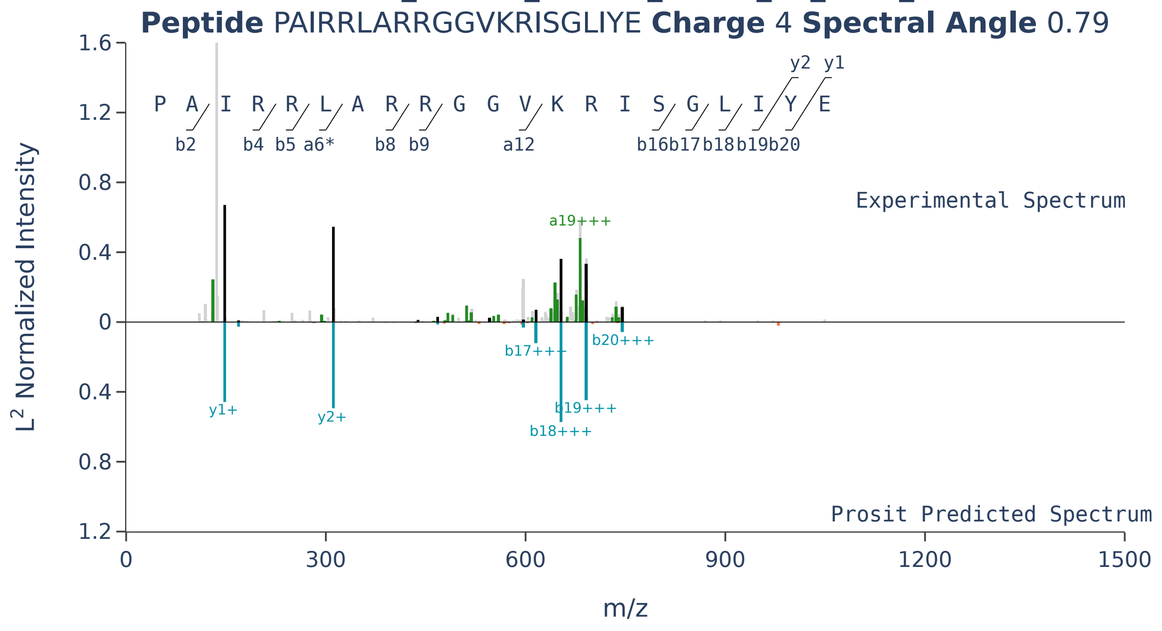


**Source** HRoetschke_291222_140123_HFGoe_H4_b2R1_1h **Scan** 34348
**Peptide** ISGLIYEETRGVLKVFLE **Charge** 2 **Spectral Angle** 0.74

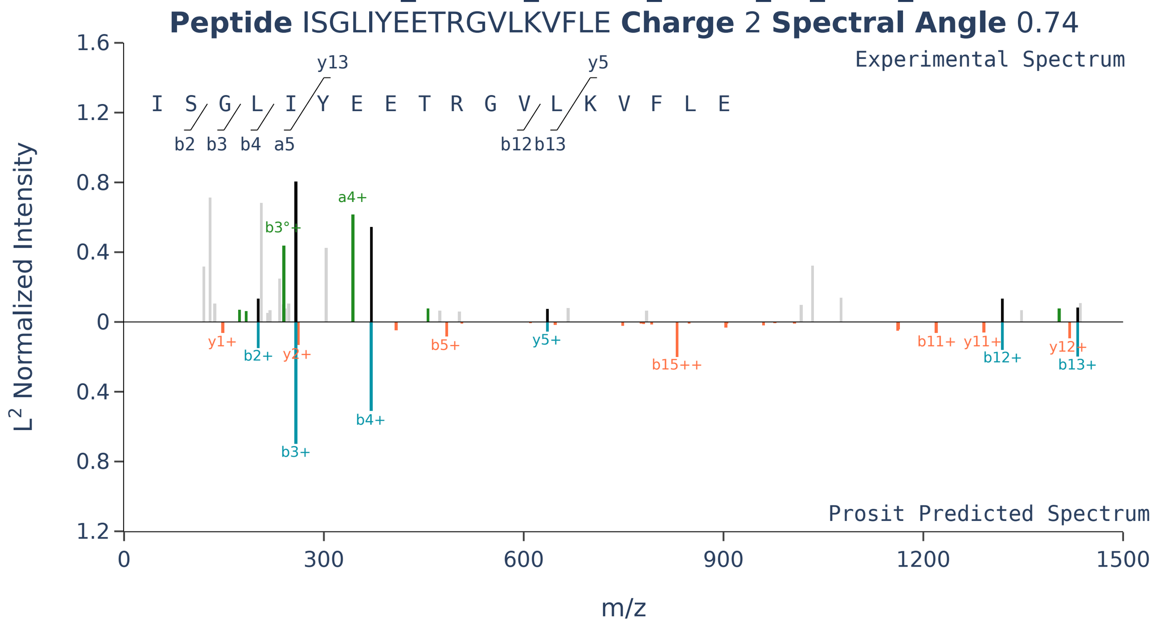


**Source** HRoetschke_291222_140123_HFGoe_H4_b1R1_2h **Scan** 8825
**Peptide** LRDAVTYTEHAKRKTVTAM **Charge** 3 **Spectral Angle** 0.78

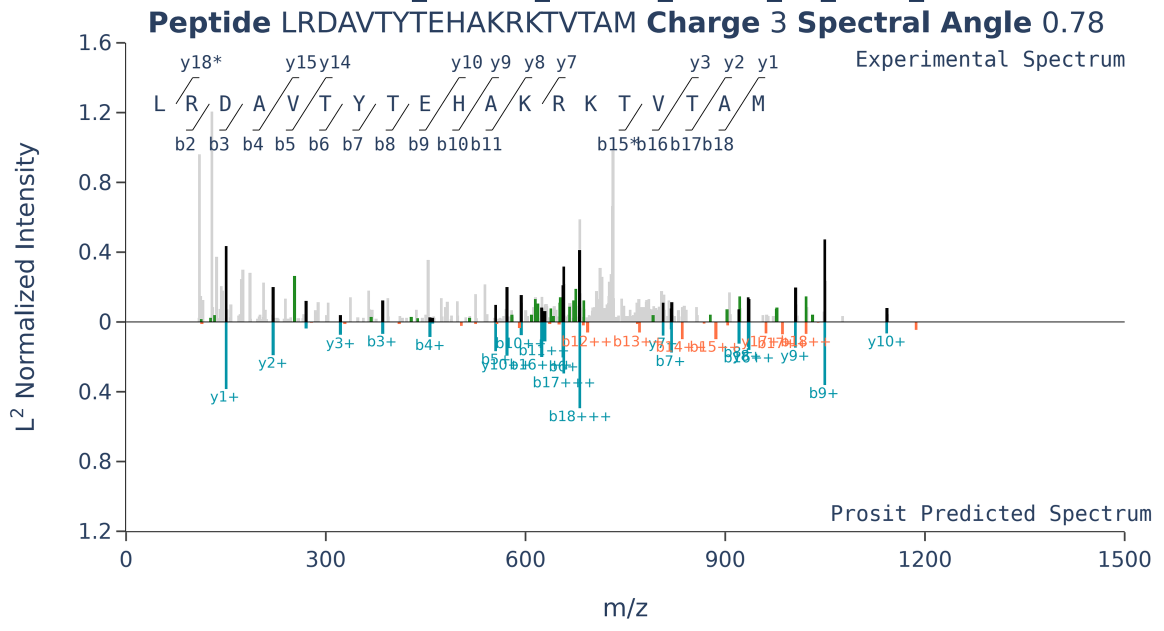


**Source** HRoetschke_291222_140123_HFGoe_H4_b1R1_1h **Scan** 15810
**Peptide** QGITKPAIRRLARRGGVKRISGLIYEETRG **Charge** 6 **Spectral Angle** 0.73

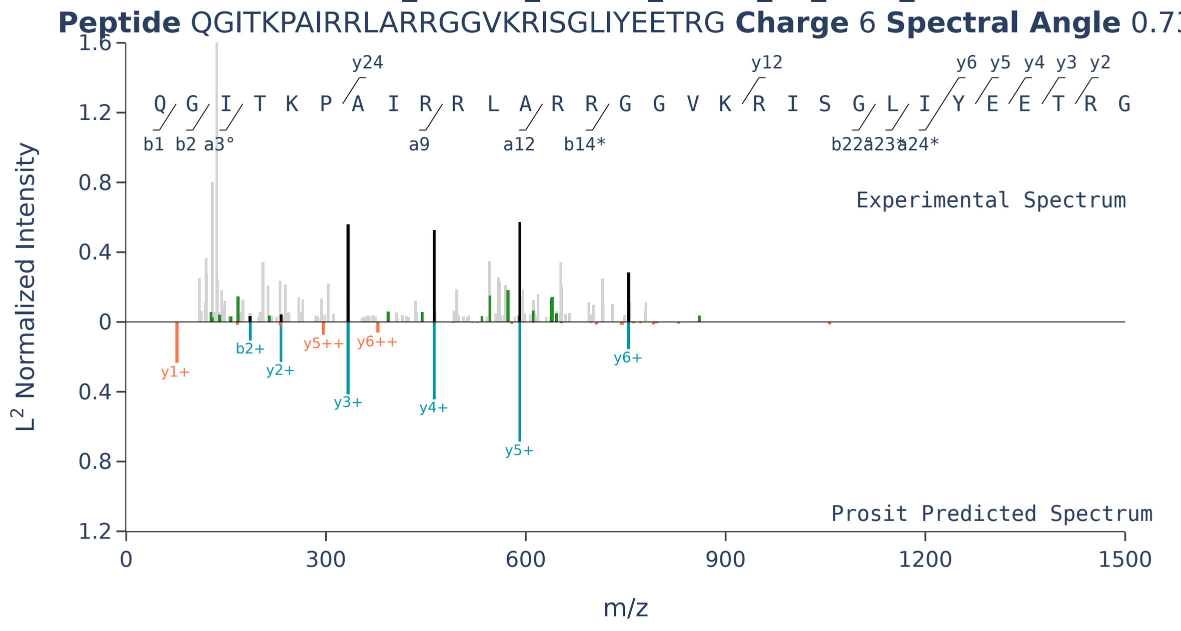

**Source** HRoetschke_291222_140123_HFGoe_H4_b1R1_4h **Scan** 8675
**Peptide** EHAKRKTVTAMDVVYA **Charge** 3 **Spectral Angle** 0.57

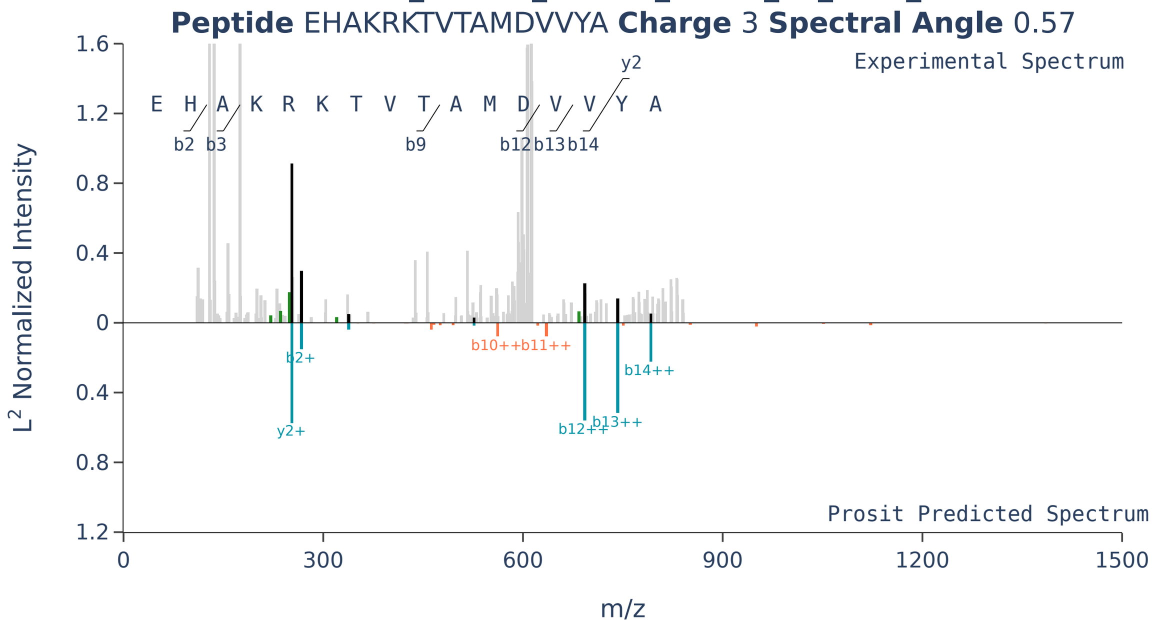


**Source** HRoetschke_291222_140123_HFGoe_H4_b1R1_24h **Scan** 5477
**Peptide** TKPAIRRLARRGGVK **Charge** 4 **Spectral Angle** 0.72

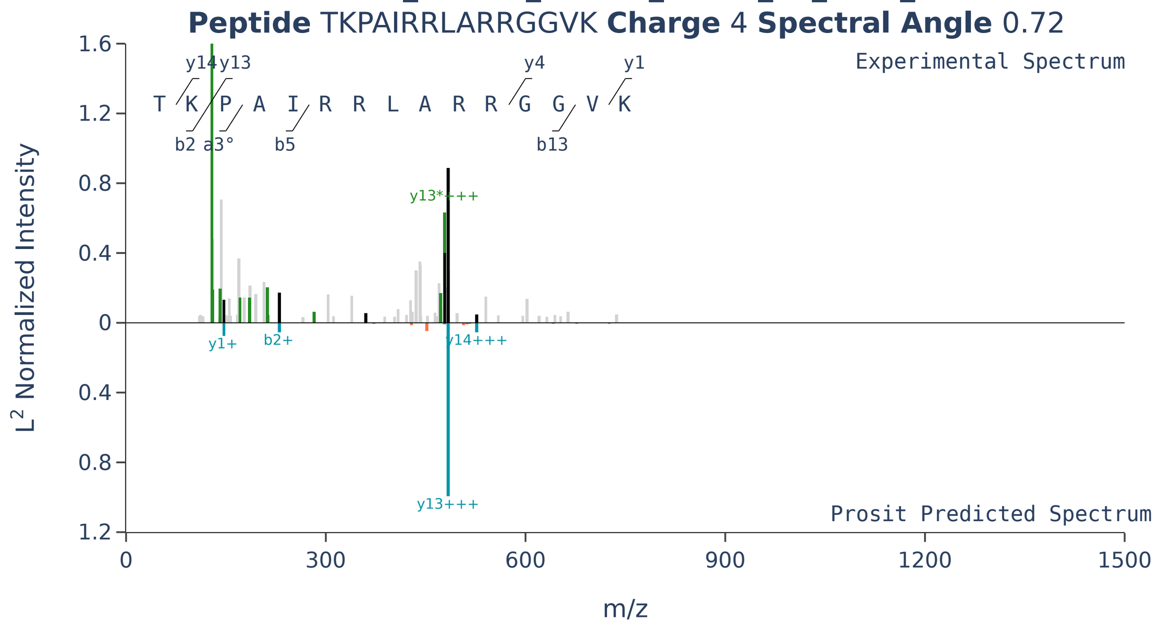


**Source** HRoetschke_291222_140123_HFGoe_H4_b1R1_2h **Scan** 13769
**Peptide** IRDAVTYTEHAKRKTVTAMDVV **Charge** 4 **Spectral Angle** 0.51

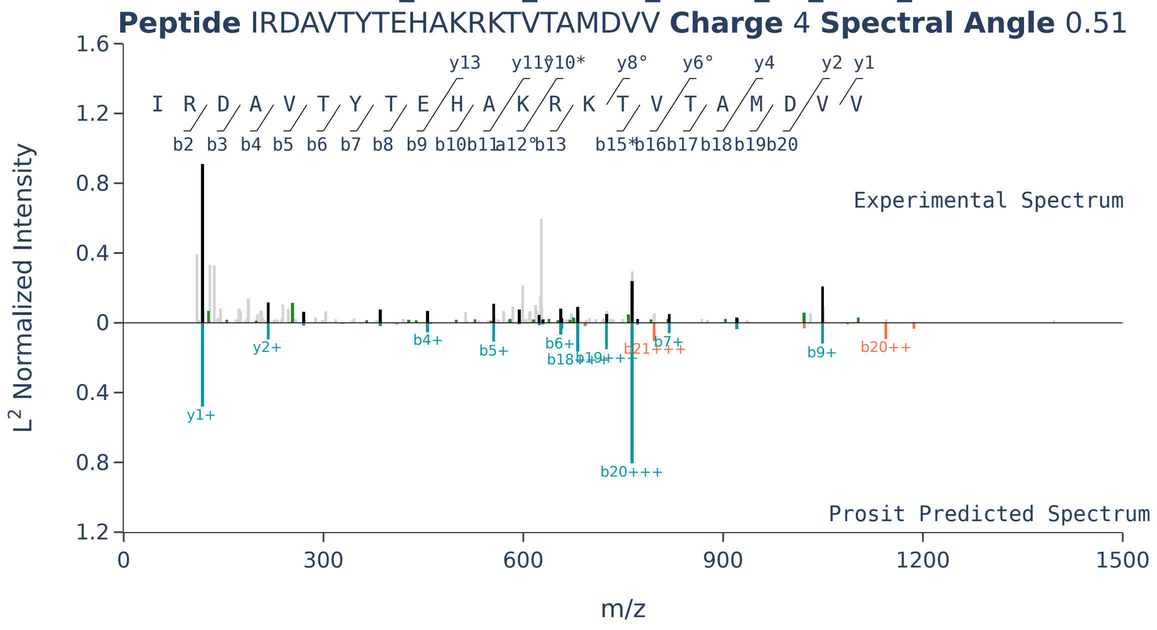


**Source** HRoetschke_291222_140123_HFGoe_H4_b2R2_1h **Scan** 28033
**Peptide** KVFLENVIRDAVTYTE **Charge** 3 **Spectral Angle** 0.68

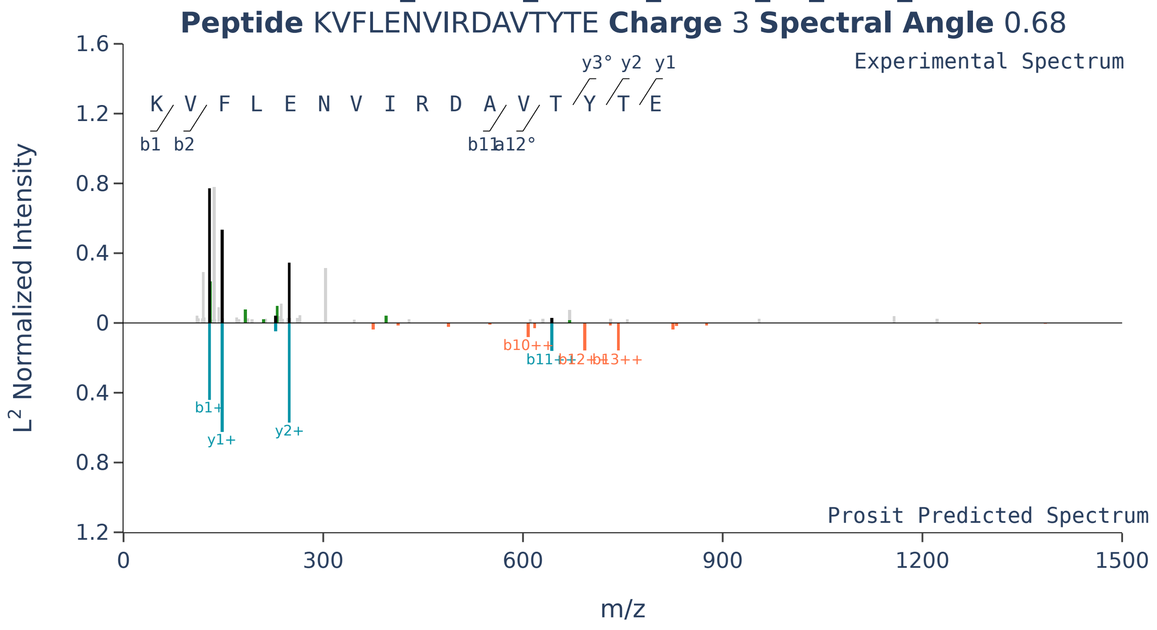


**Source** HRoetschke_291222_140123_HFGoe_H4_b1R1_1h **Scan** 6831
**Peptide** GGKGLGKGGAKRHRKVLRDNIQGI **Charge** 5 **Spectral Angle** 0.74

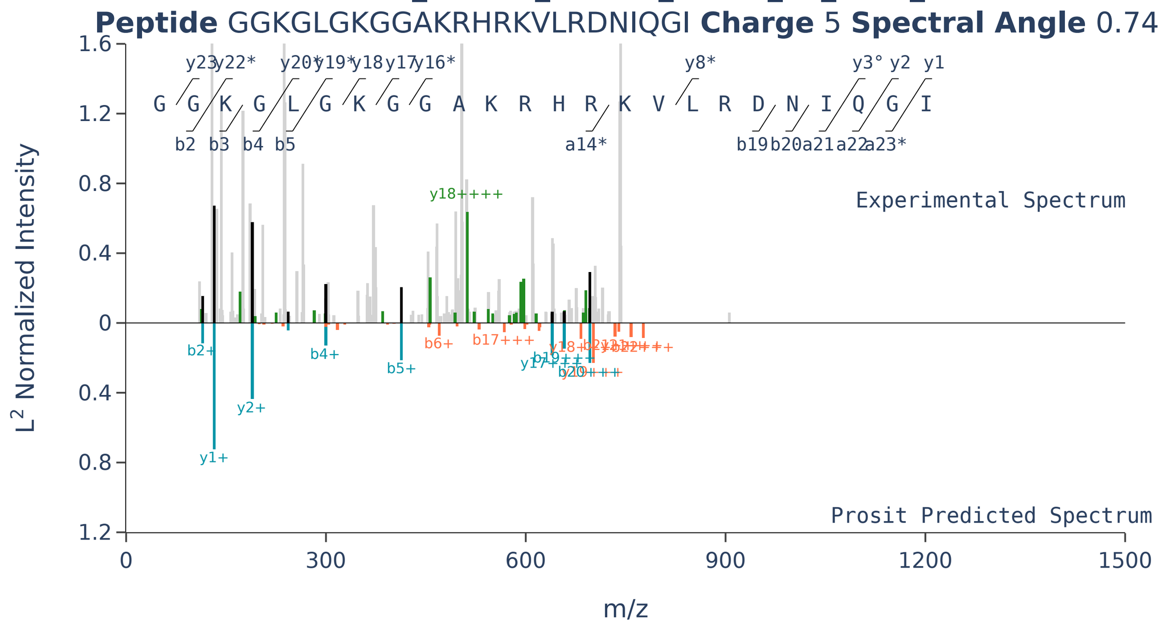


**Source** HRoetschke_291222_140123_HFGoe_H4_b2R2_1h **Scan** 29584
**Peptide** LSGLIYEETRGVLKVF **Charge** 3 **Spectral Angle** 0.71

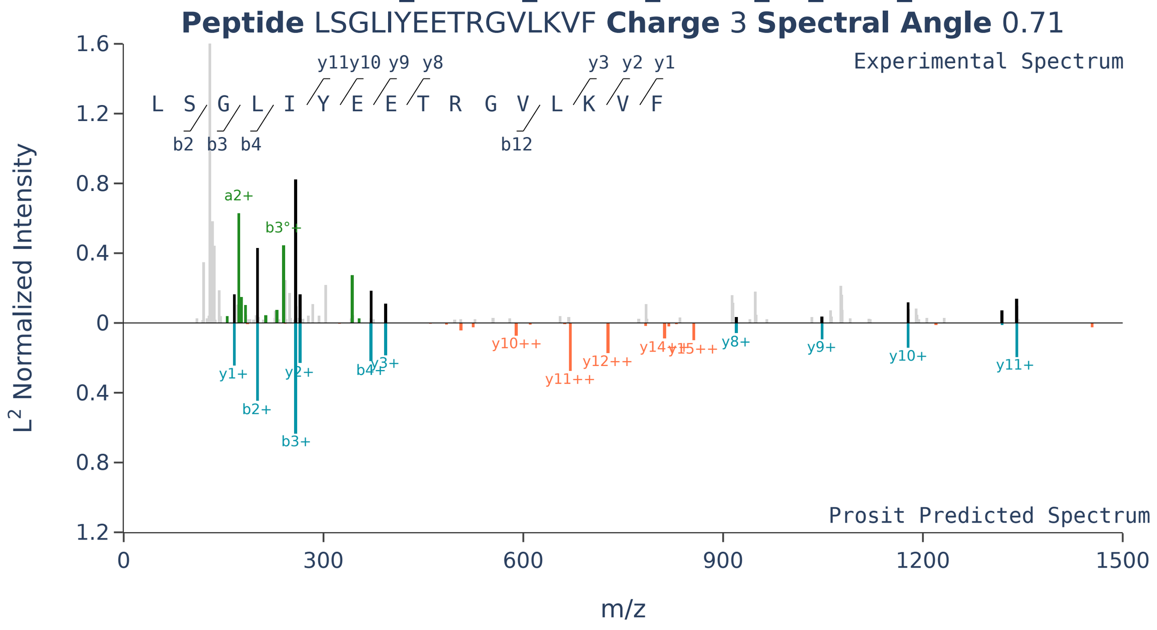


**Source** HRoetschke_291222_140123_HFGoe_H4_b2R1_1h **Scan** 34079
**Peptide** LIYEETRGVLKVFLENVIRD **Charge** 4 **Spectral Angle** 0.56

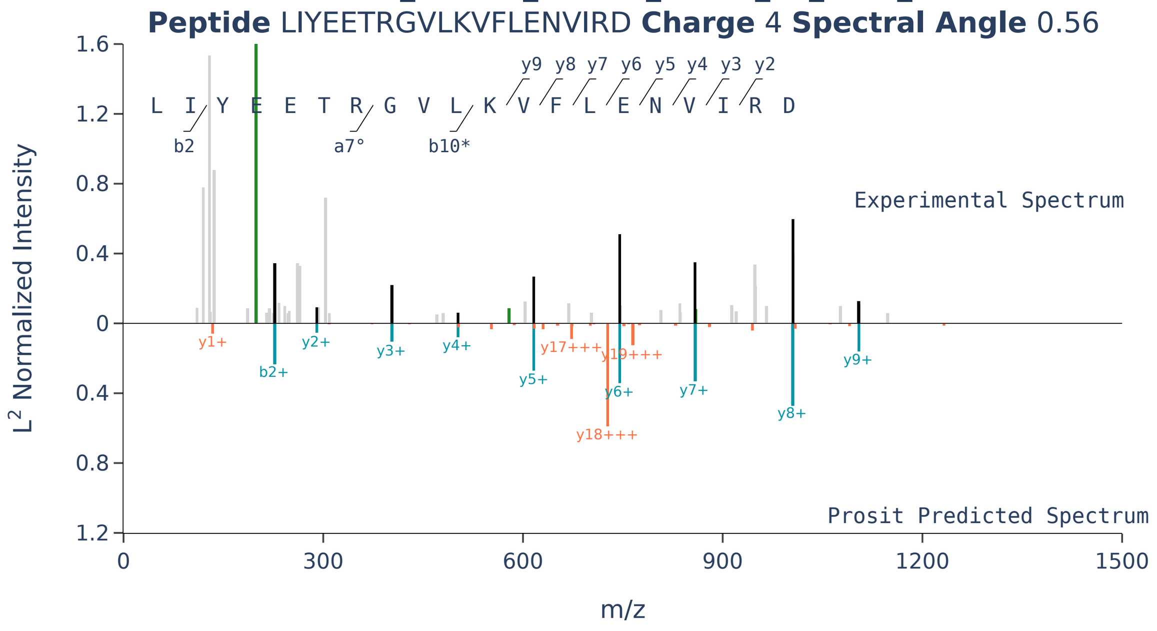


**Source** HRoetschke_291222_140123_HFGoe_H4_b2R2_4h **Scan** 12732
**Peptide** KVLRDNIQGITKPAIRRLARRGGVKRL **Charge** 5 **Spectral Angle** 0.72

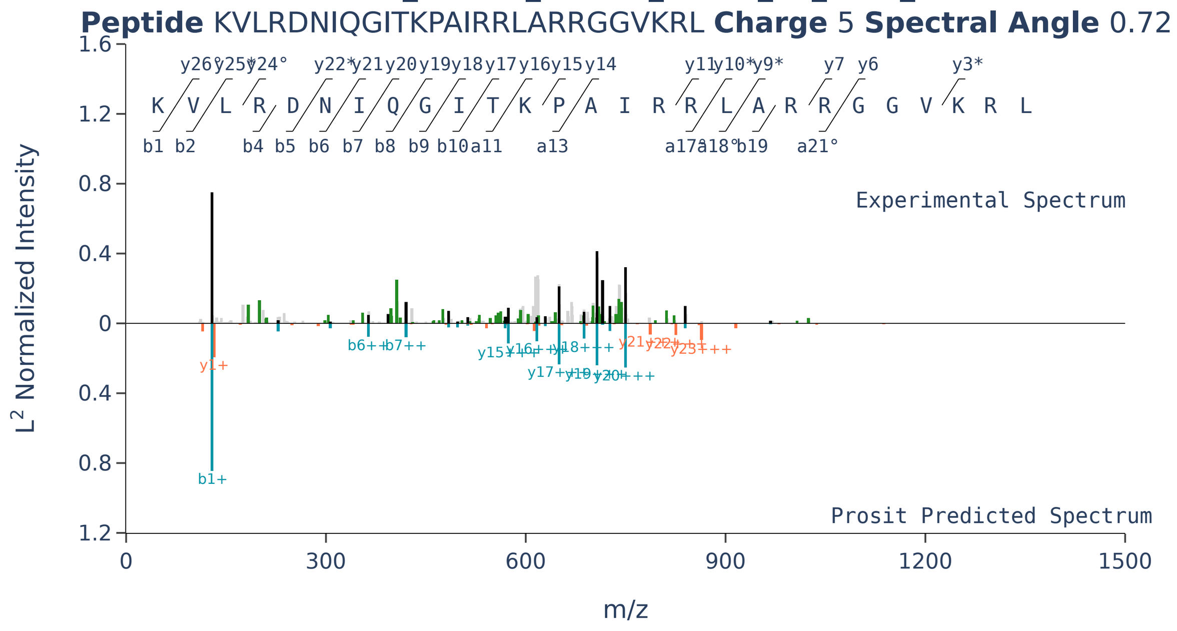


**Source** HRoetschke_291222_140123_HFGoe_H4_b2R2_24h **Scan** 10449
**Peptide** RDNIQGITKPAIRRLARRGGVKRIS **Charge** 5 **Spectral Angle** 0.66

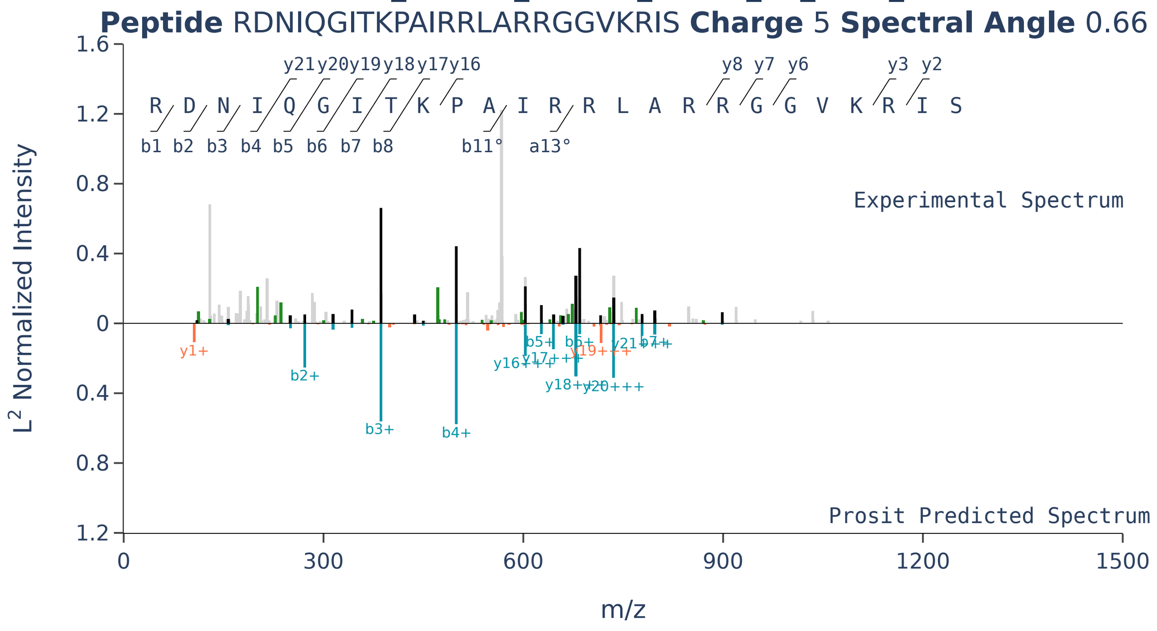


**Source** HRoetschke_291222_140123_HFGoe_H4_b1R2_1h **Scan** 17000
**Peptide** AKRKTVTAMDVVYALKRQ **Charge** 4 **Spectral Angle** 0.68

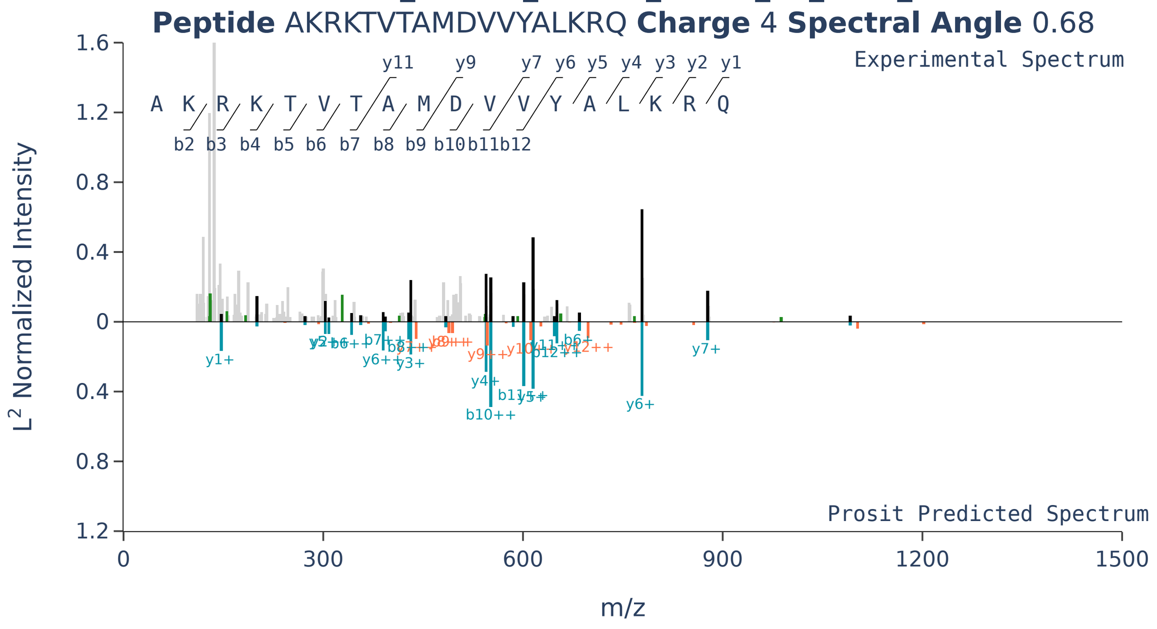


**Source** HRoetschke_291222_140123_HFGoe_H4_b2R1_1h **Scan** 27411
**Peptide** KVFLENVIRDAVTYT **Charge** 2 **Spectral Angle** 0.76

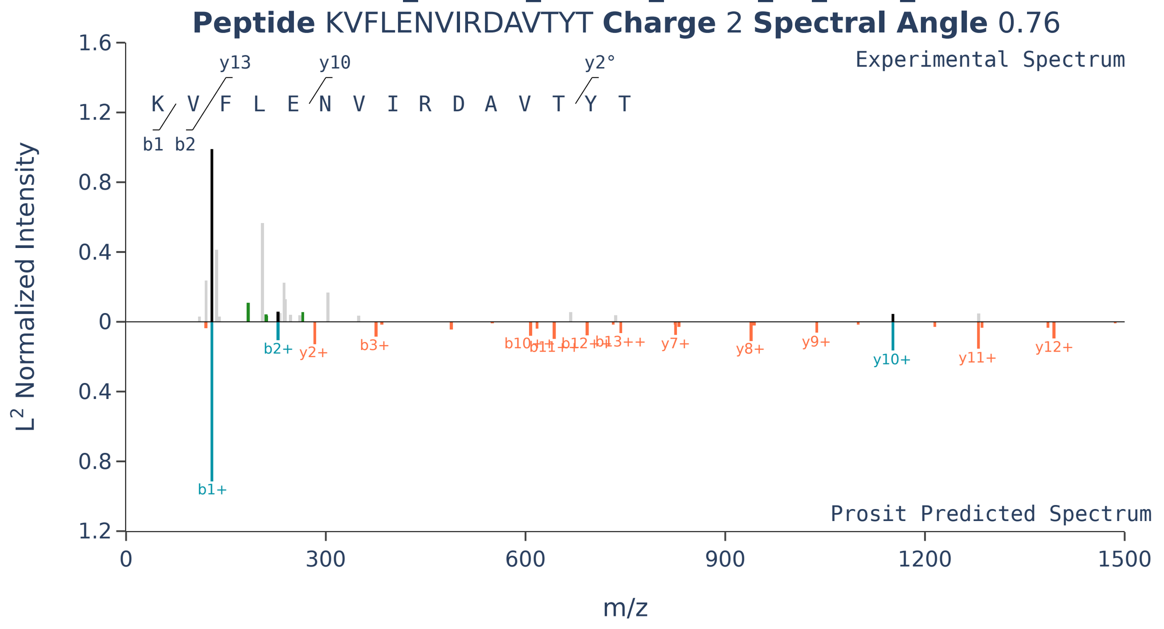


**Source** HRoetschke_291222_140123_HFGoe_H4_b1R1_24h **Scan** 5333
**Peptide** KGLGKGGAKRHRKVLRDNI **Charge** 4 **Spectral Angle** 0.44

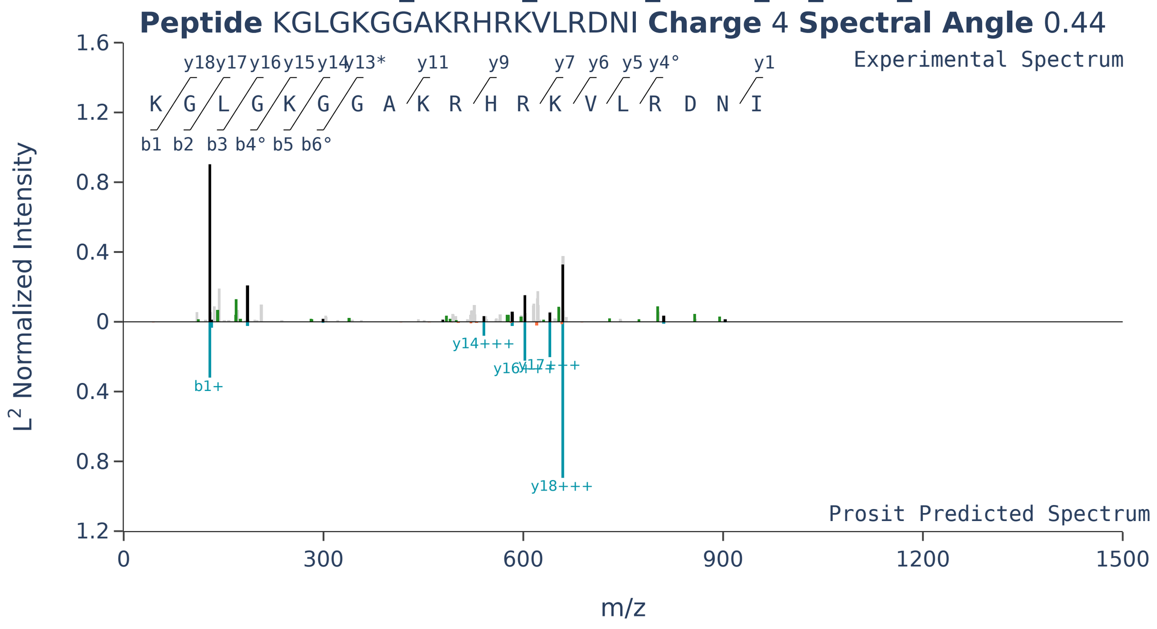


**Source** HRoetschke_291222_140123_HFGoe_H4_b1R1_1h **Scan** 27115
**Peptide** TRGVLKVFLENVIR **Charge** 3 **Spectral Angle** 0.68

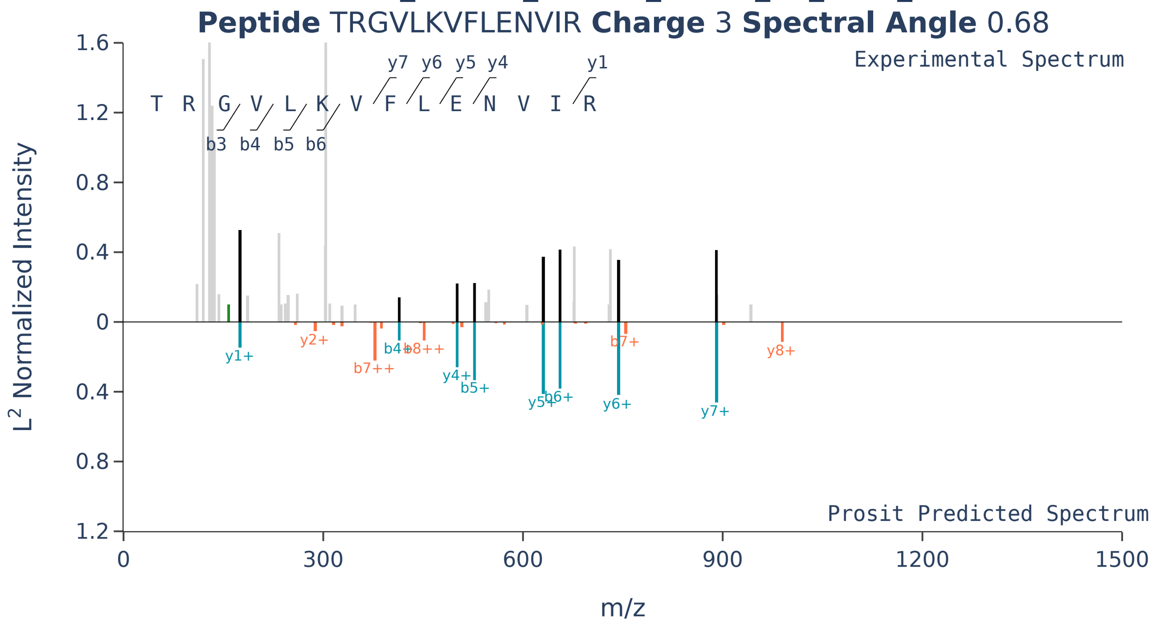


**Source** HRoetschke_291222_140123_HFGoe_H4_b1R2_24h **Scan** 6195
**Peptide** AKRHRKVLRDNIQGITK **Charge** 5 **Spectral Angle** 0.47

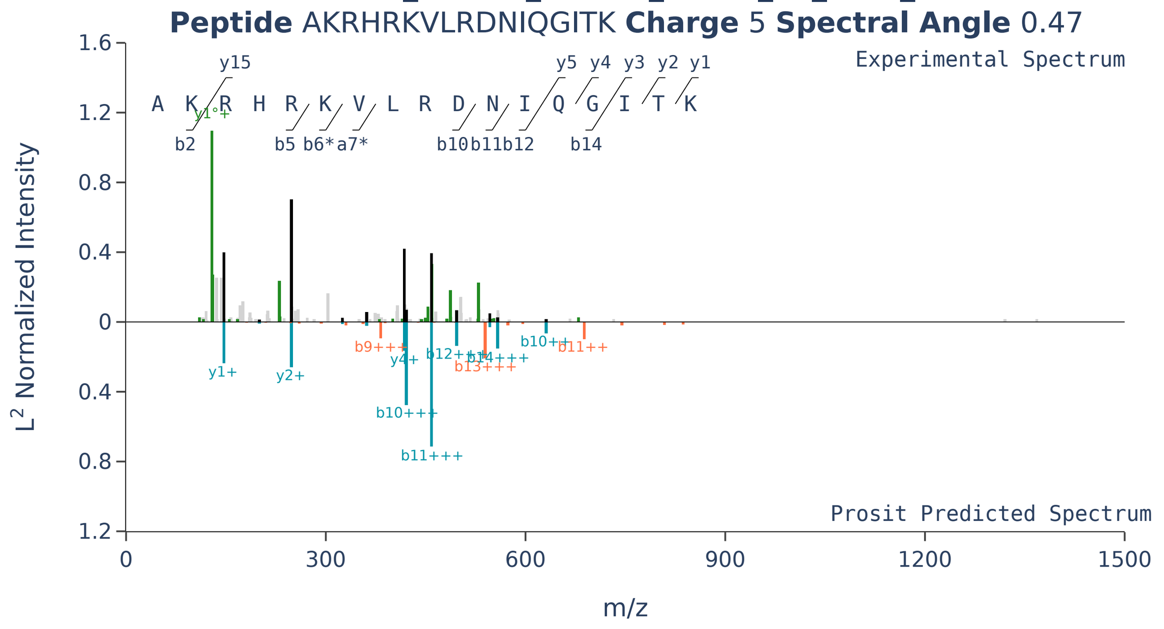


**Source** HRoetschke_291222_140123_HFGoe_H4_b1R2_4h **Scan** 6109
**Peptide** TKPAIRRLARRGGVKRI **Charge** 3 **Spectral Angle** 0.59

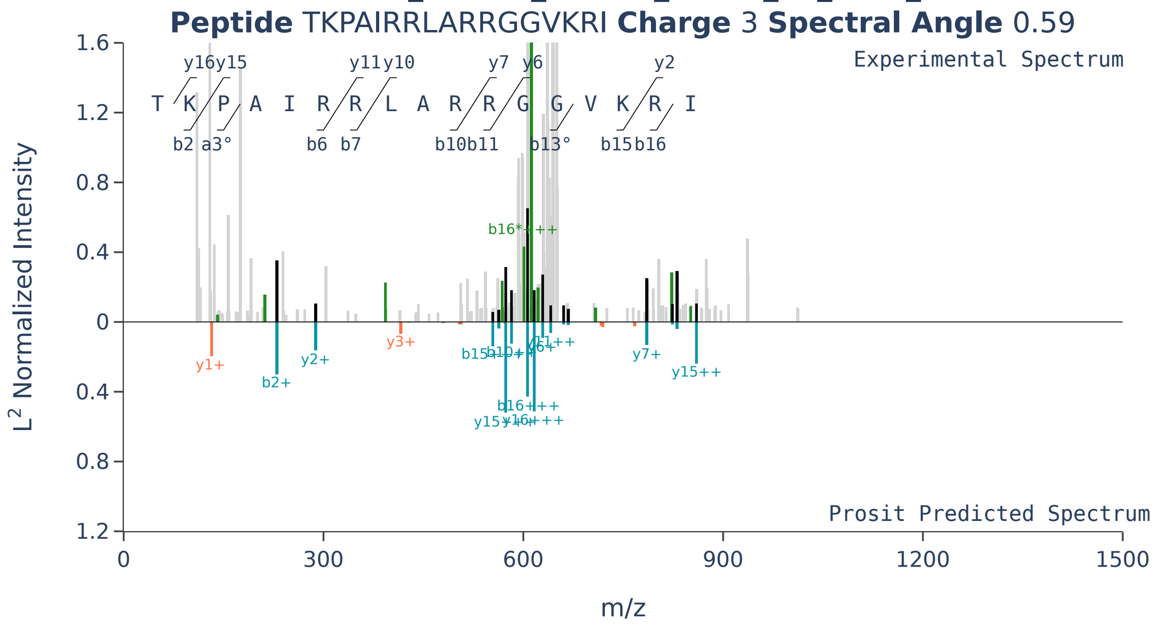

**Source** HRoetschke_291222_140123_HFGoe_H4_b1R2_24h **Scan** 8238
**Peptide** VIRDAVTYTEHAKRKTVTAMD **Charge** 5 **Spectral Angle** 0.84

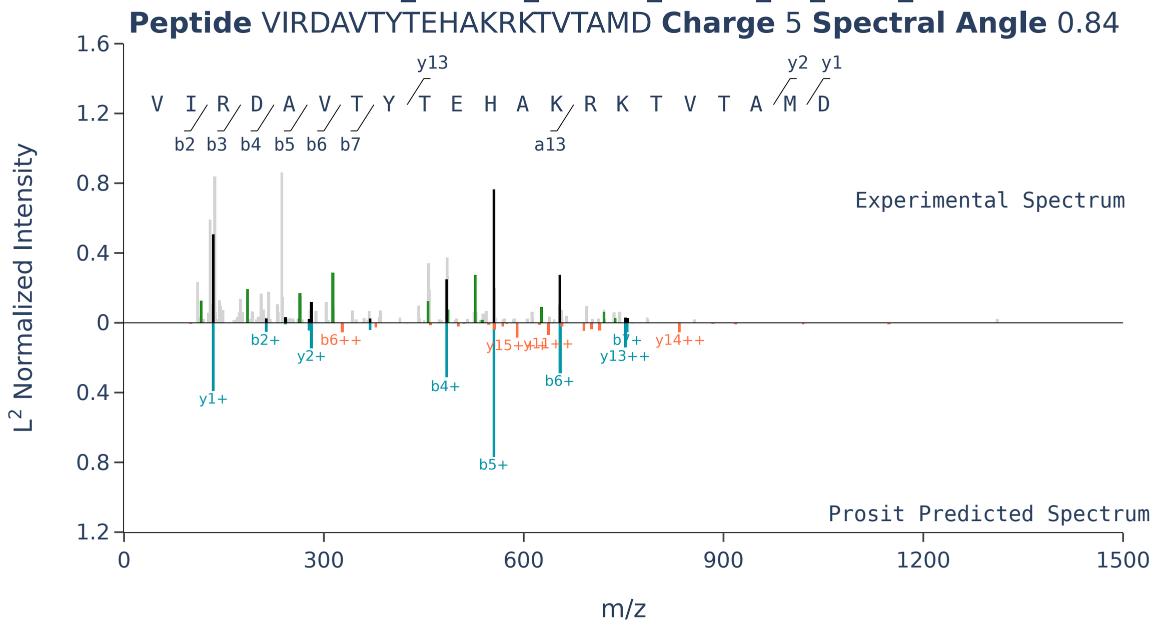


**Source** HRoetschke_291222_140123_HFGoe_H4_b1R2_2h **Scan** 17172
**Peptide** LRDAVTYTEHAKRKTVTAMDVVYALKRQGR **Charge** 5 **Spectral Angle** 0.54

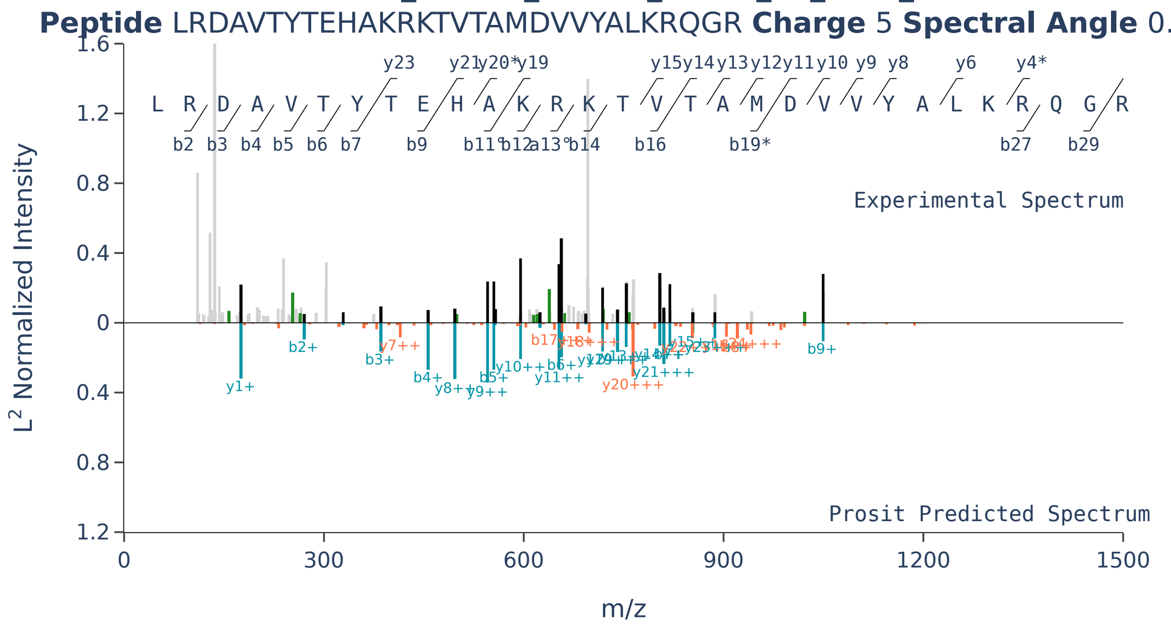


**Source** HRoetschke_291222_140123_HFGoe_H4_b1R2_2h **Scan** 17561
**Peptide** RRGGVKRISGLIYEETRGVLK **Charge** 5 **Spectral Angle** 0.69

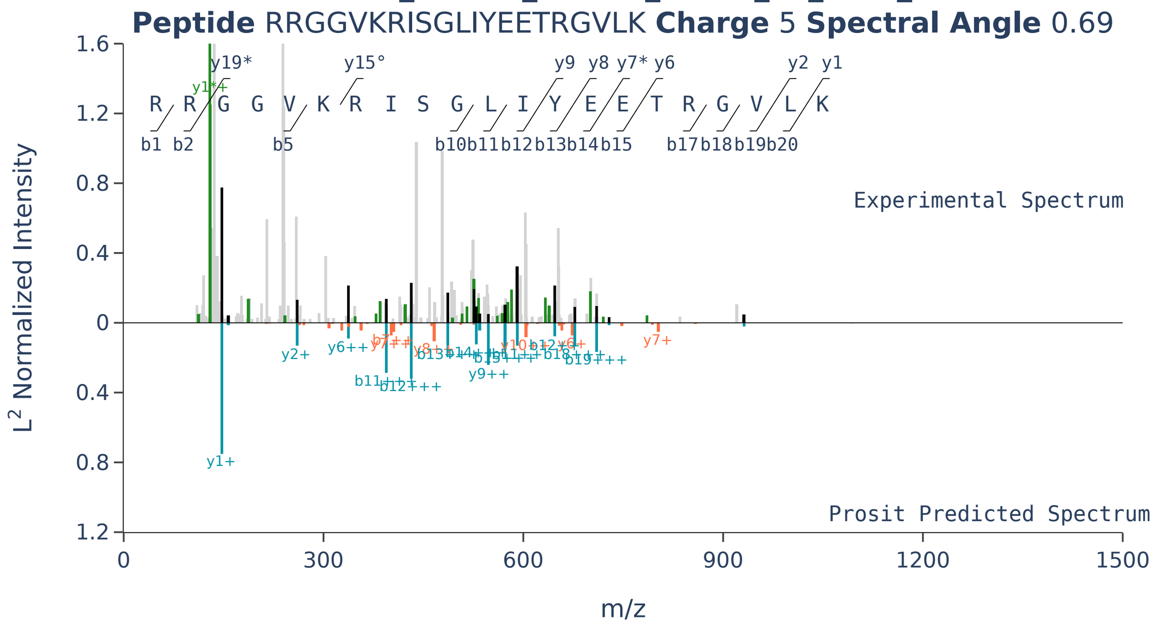


**Source** HRoetschke_291222_140123_HFGoe_H4_b2R2_4h **Scan** 7486
**Peptide** GGKGLGKGGAKRHRKVLRDNIQGITKPAIR **Charge** 6 **Spectral Angle** 0.85

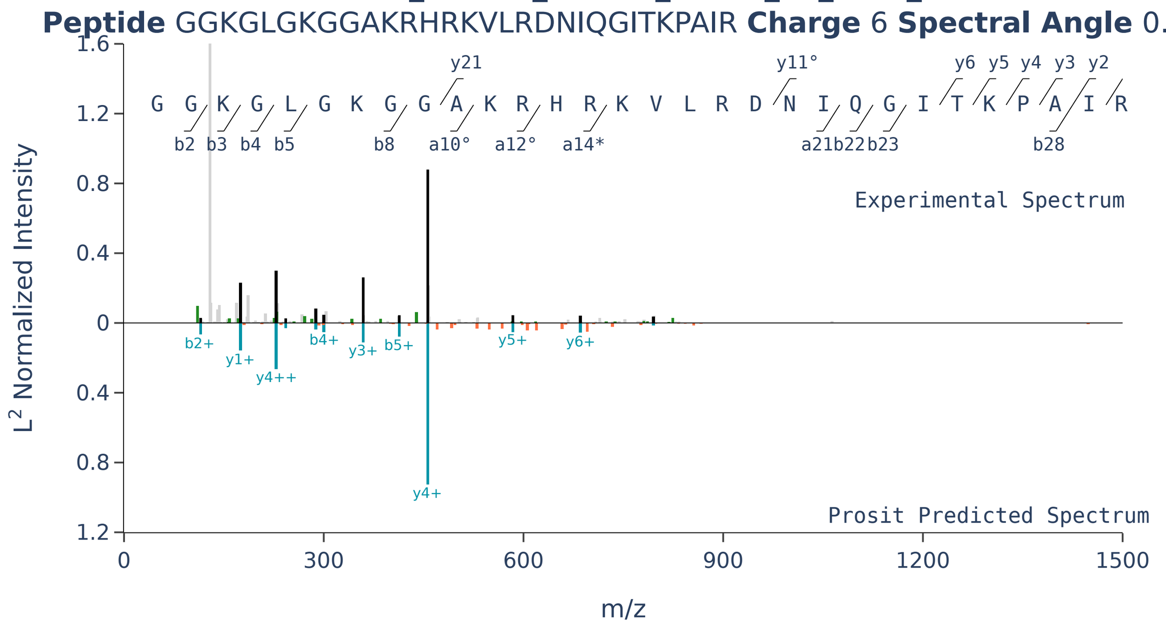


**Source** HRoetschke_291222_140123_HFGoe_H4_b2R1_24h **Scan** 9965
**Peptide** GGAKRHRKVLRDNIQGITKPAI **Charge** 5 **Spectral Angle** 0.5

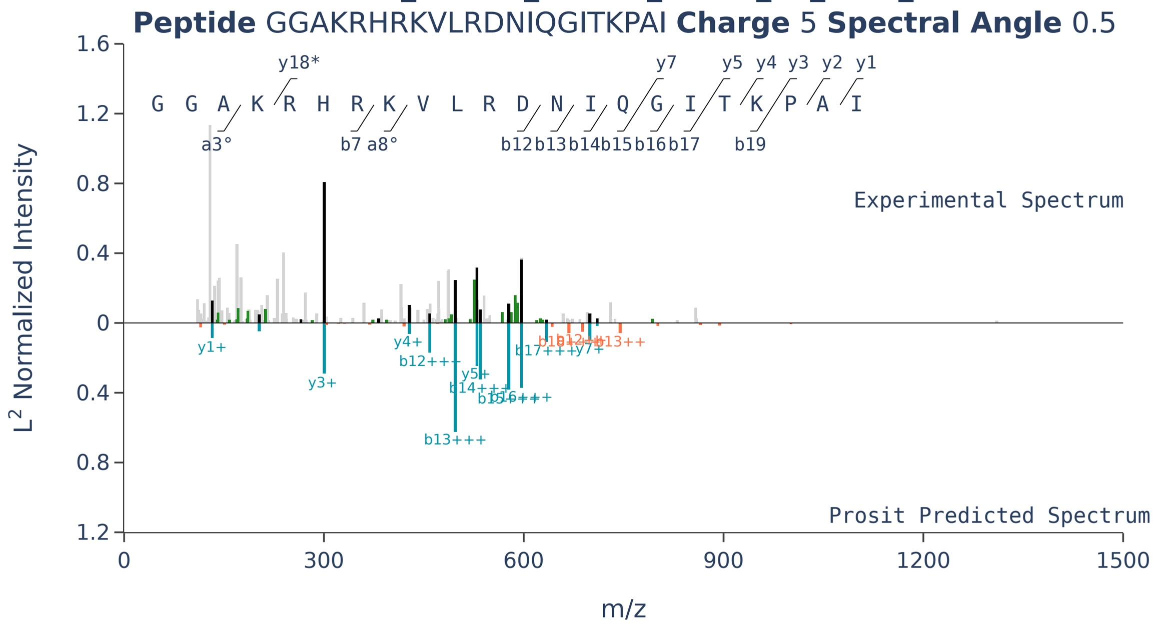


**Source** HRoetschke_291222_140123_HFGoe_H4_b2R2_1h **Scan** 31445
**Peptide** GLIYEETRGVLKVFLE **Charge** 2 **Spectral Angle** 0.64

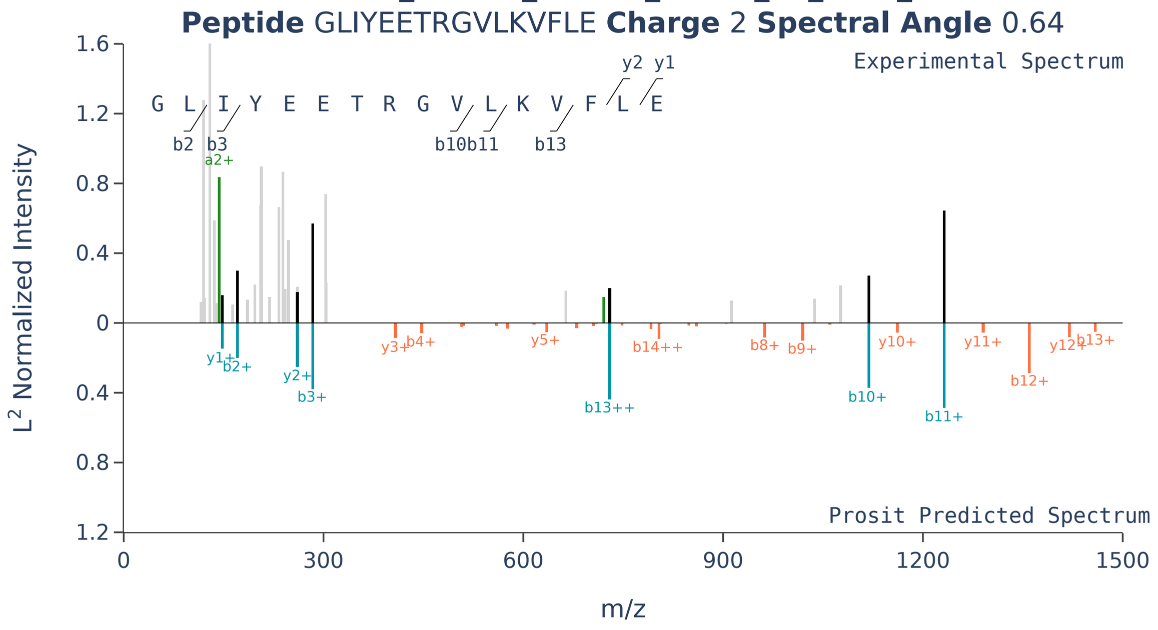


**Source** HRoetschke_291222_140123_HFGoe_H4_b2R2_1h **Scan** 31346
**Peptide** VFLENVIRDAVTY **Charge** 2 **Spectral Angle** 0.63

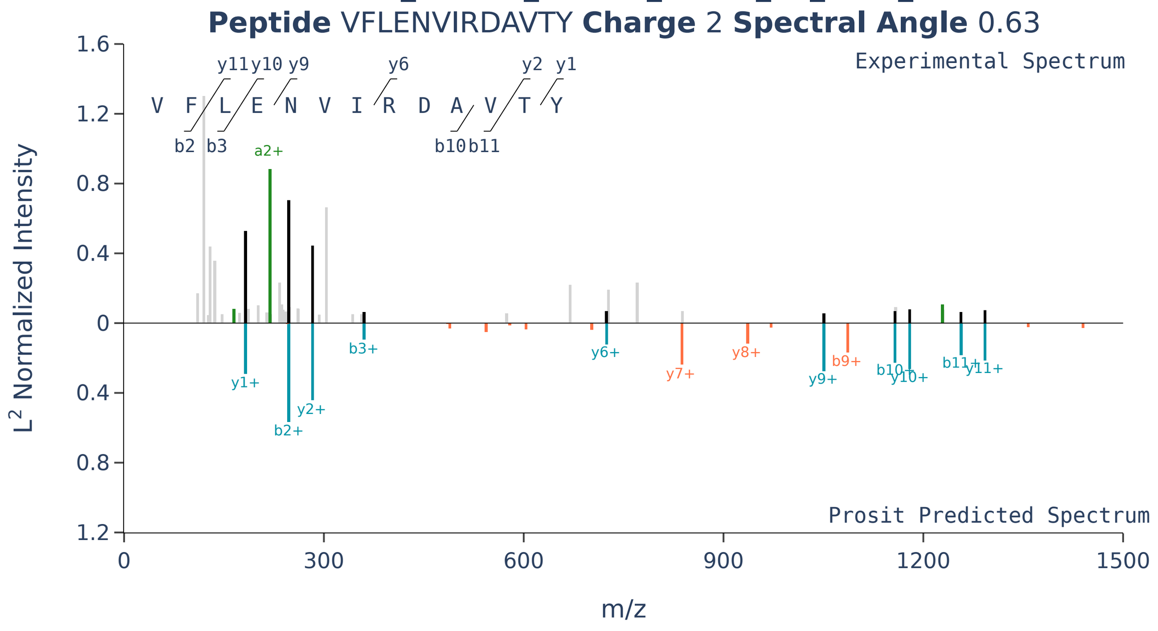


**Source** HRoetschke_291222_140123_HFGoe_H4_b1R2_2h **Scan** 6105
**Peptide** YTEHAKRKT **Charge** 2 **Spectral Angle** 0.66

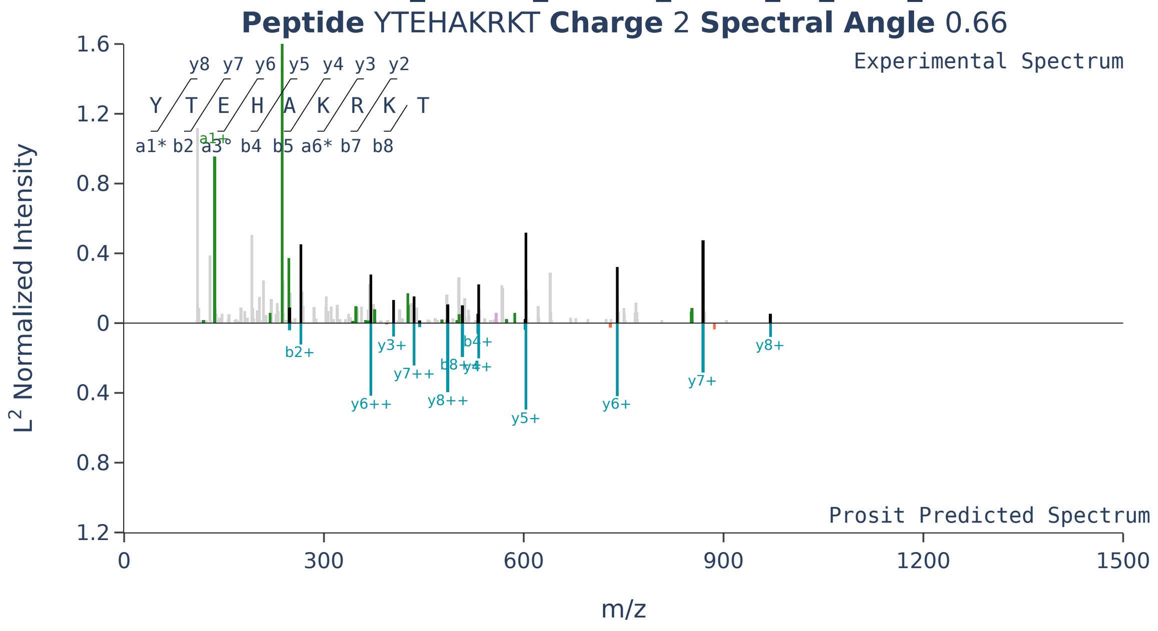


**Source** HRoetschke_291222_140123_HFGoe_H4_b1R1_2h **Scan** 11574
**Peptide** RKVLRDNIQGITKPAIRRLARRGGVKRI **Charge** 6 **Spectral Angle** 0.57

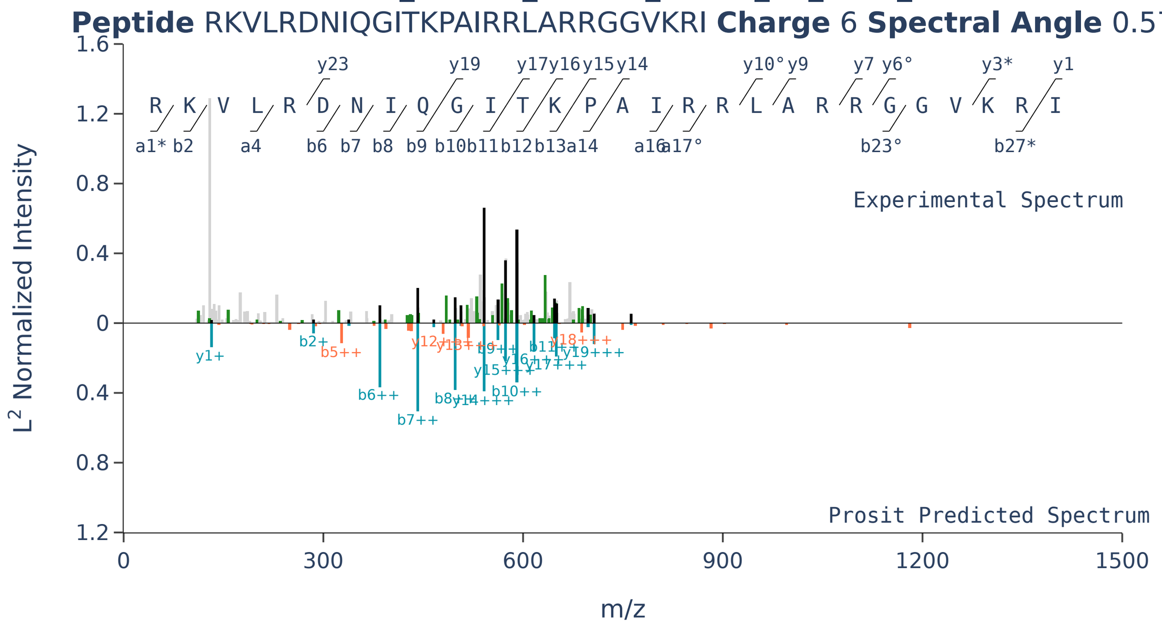


**Source** HRoetschke_291222_140123_HFGoe_H4_b2R1_1h **Scan** 31716
**Peptide** YEETRGVLKVFLE **Charge** 2 **Spectral Angle** 0.82

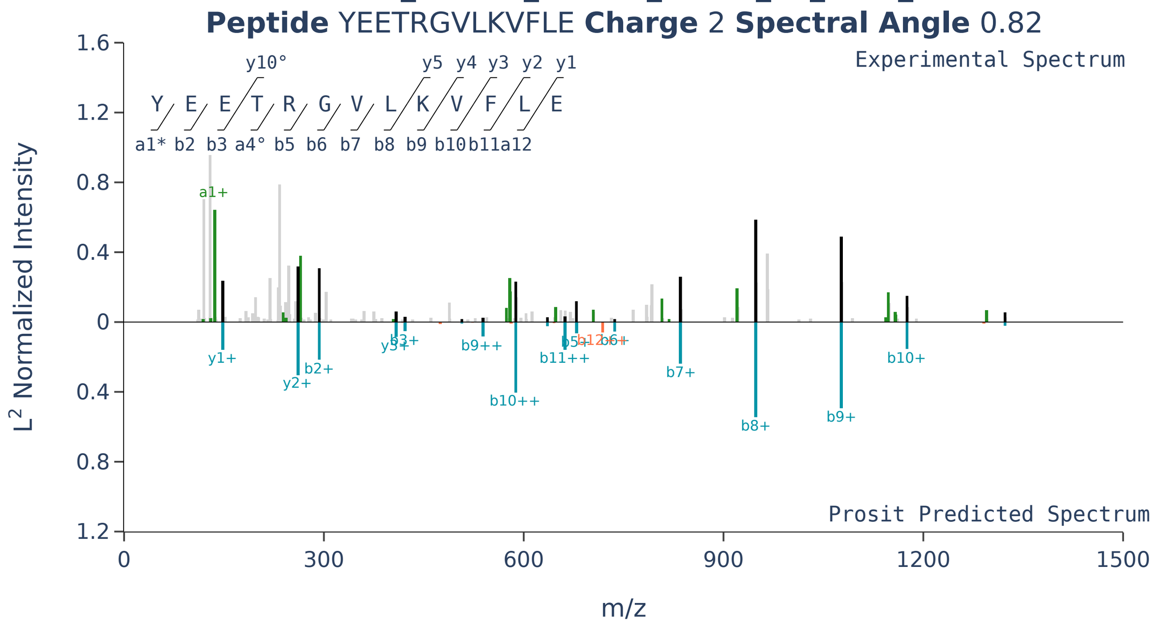


**Source** HRoetschke_291222_140123_HFGoe_H4_b1R1_24h **Scan** 5270
**Peptide** GKGGKGLGKGGAKRHRKVLRDNL **Charge** 5 **Spectral Angle** 0.59

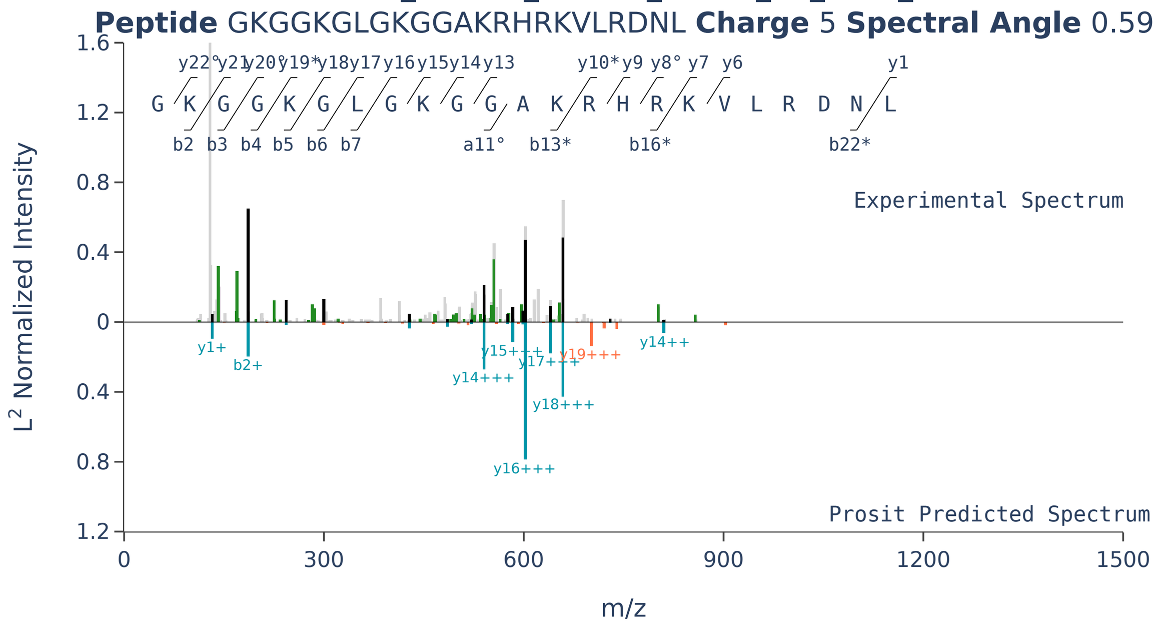


**Source** HRoetschke_291222_140123_HFGoe_H4_b1R1_2h **Scan** 15884
**Peptide** LTKPAIRRLARRGGVKRISGLIYEET **Charge** 5 **Spectral Angle** 0.81

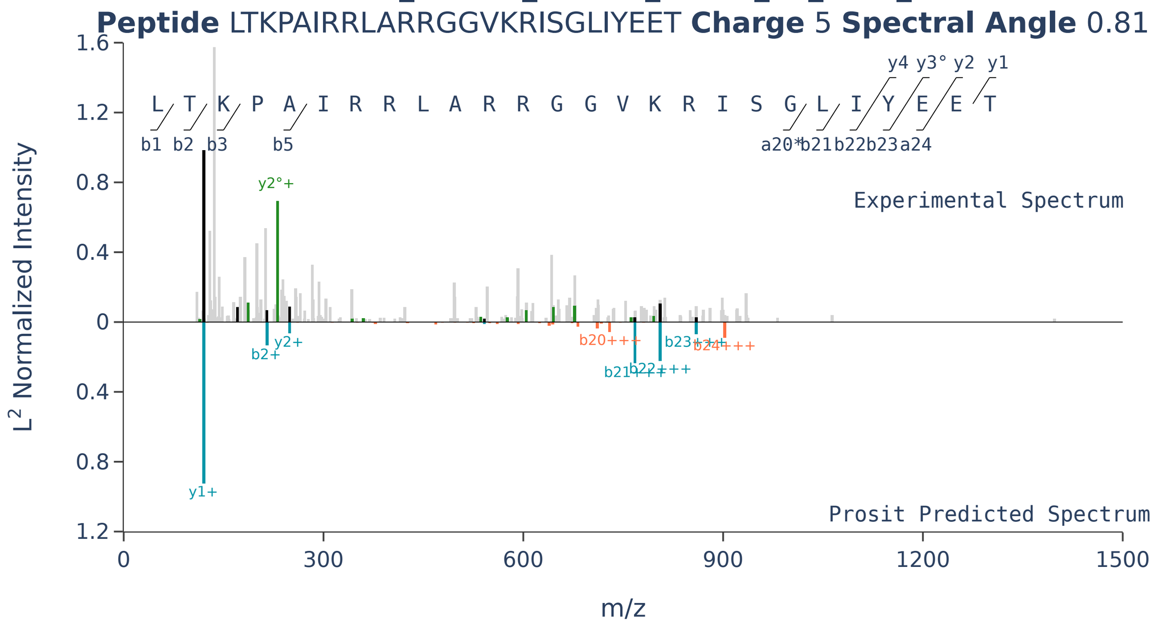


**Source** HRoetschke_291222_140123_HFGoe_H4_b1R1_1h **Scan** 20490
**Peptide** LARRGGVKRISGLIYEETRGVL **Charge** 4 **Spectral Angle** 0.67

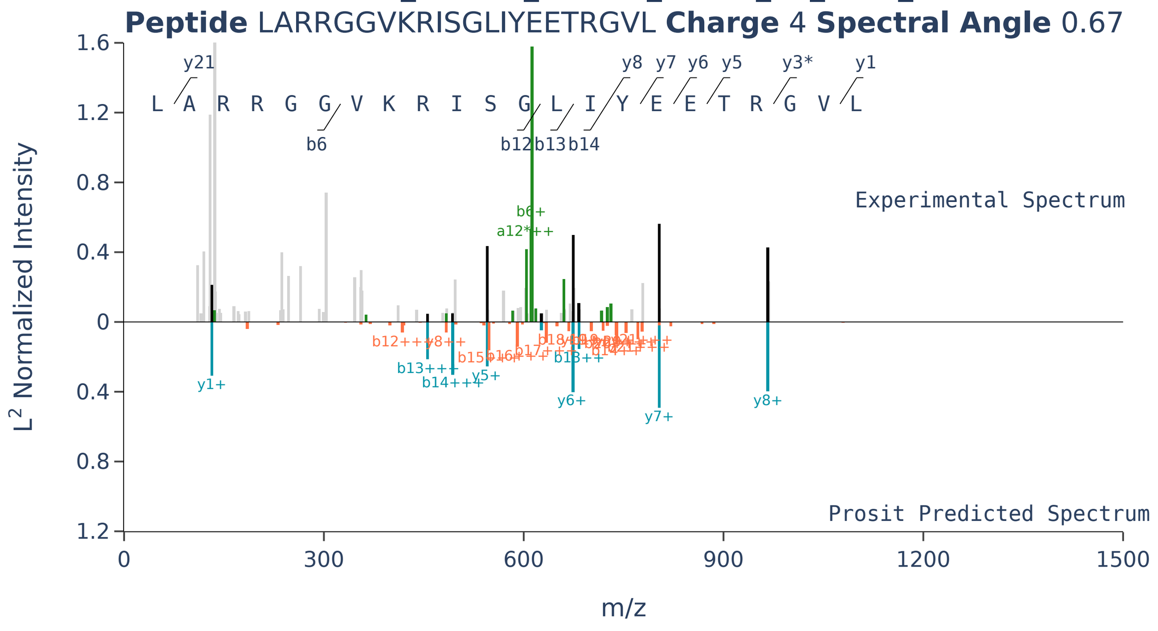


**Source** HRoetschke_291222_140123_HFGoe_H4_b2R1_1h **Scan** 22004
**Peptide** EHAKRKTVTAMDVVYALKRQGRTLYGFGG **Charge** 4 **Spectral Angle** 0.64

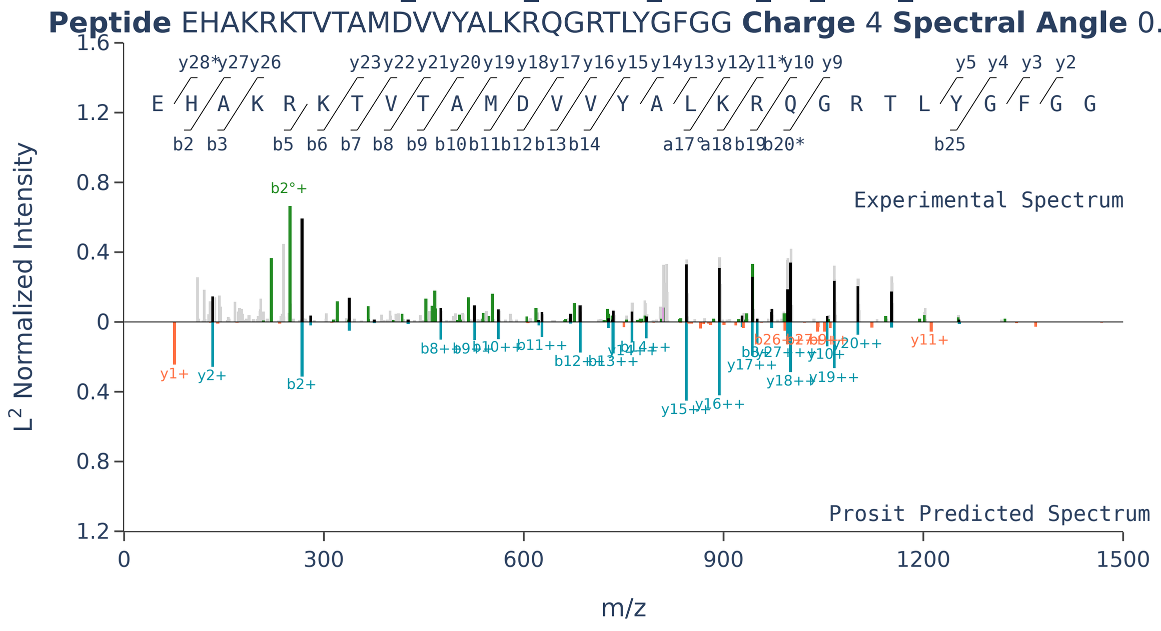


**Source** HRoetschke_291222_140123_HFGoe_H4_b2R1_1h **Scan** 38683
**Peptide** ISGLIYEETRGVLKVFLENVIRD **Charge** 3 **Spectral Angle** 0.67

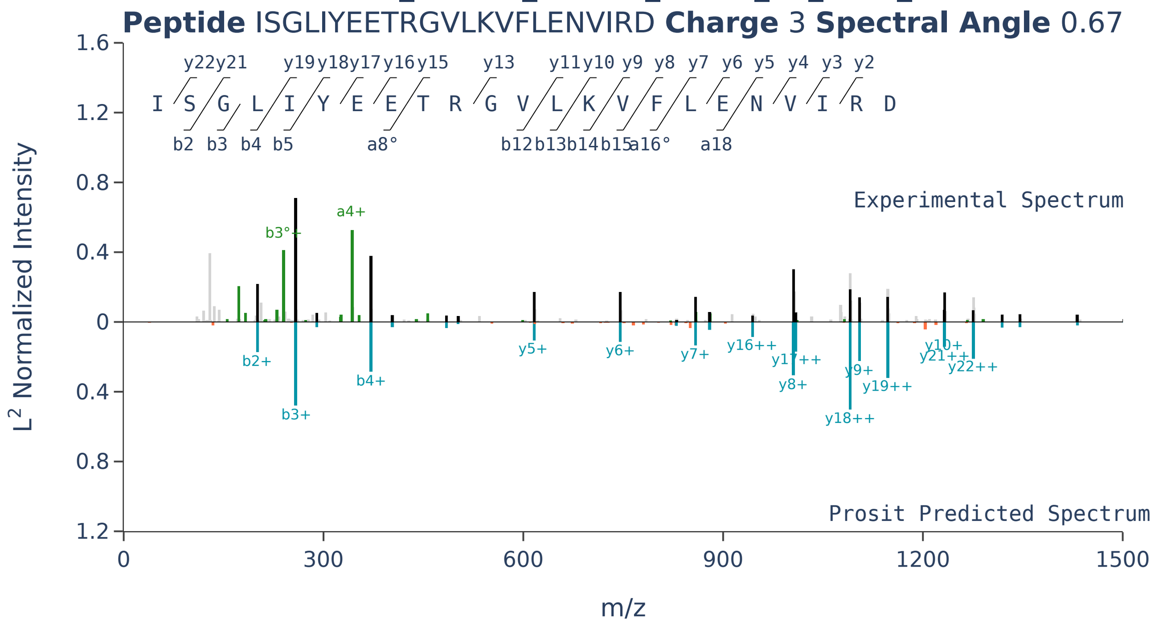

**Source** HRoetschke_291222_140123_HFGoe_H4_b2R1_1h **Scan** 30444
**Peptide** GVKRISGLIYEETRGVLKVFLE **Charge** 3 **Spectral Angle** 0.61

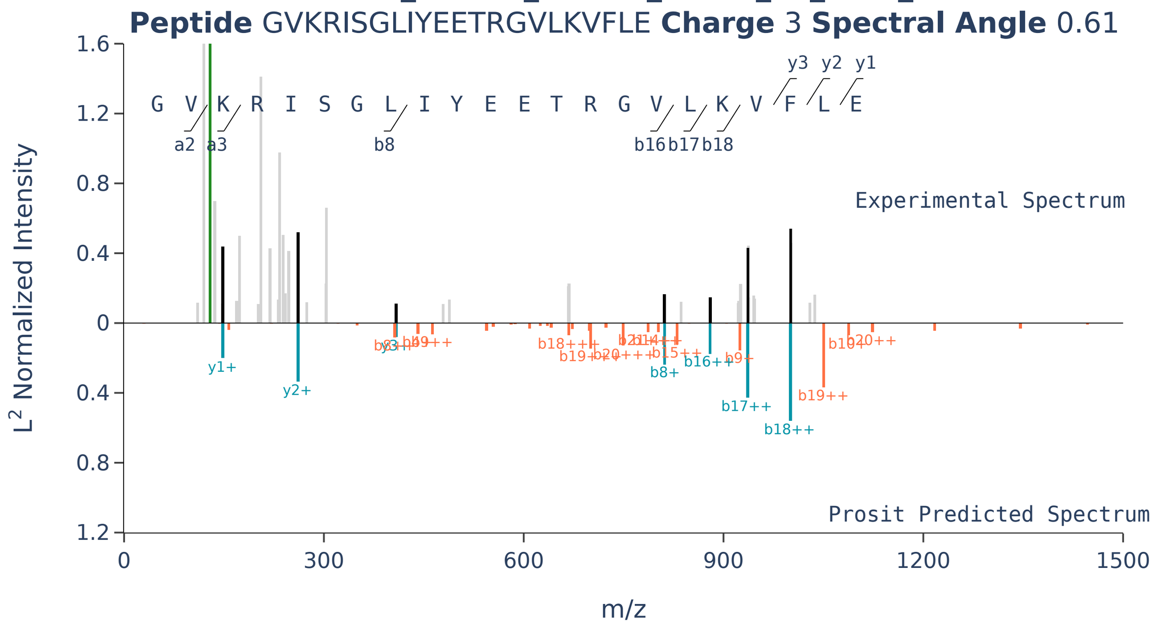


**Source** HRoetschke_291222_140123_HFGoe_H4_b1R1_4h **Scan** 6589
**Peptide** ARRGGVKRISGL **Charge** 3 **Spectral Angle** 0.59

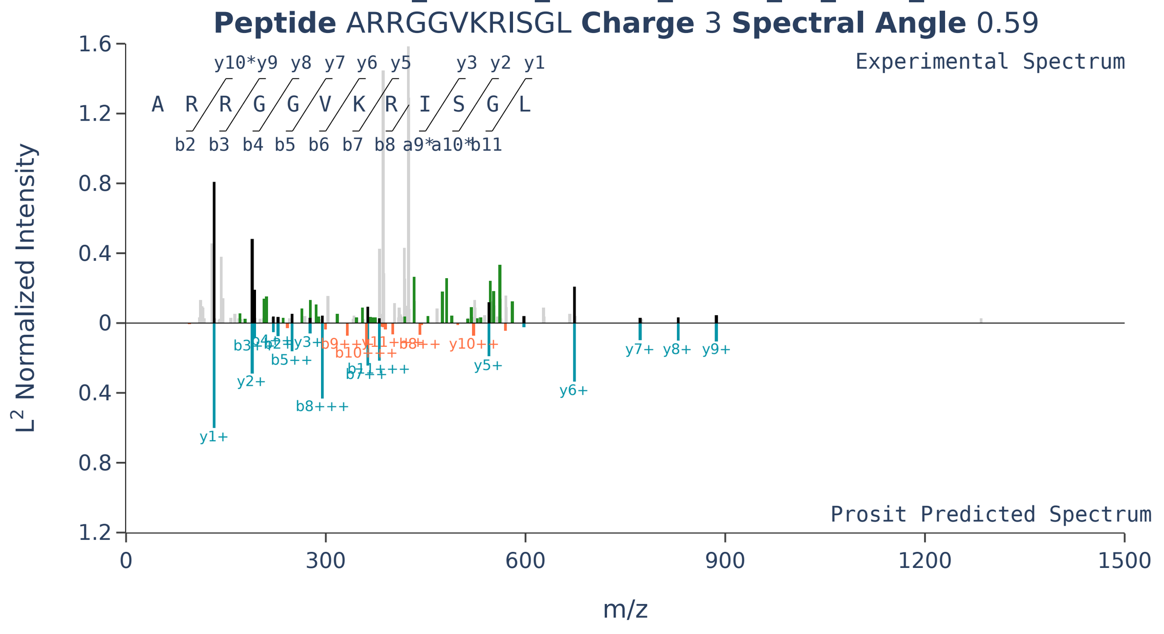


**Source** HRoetschke_291222_140123_HFGoe_H4_b1R1_2h **Scan** 18654
**Peptide** RDNIQGITKPAIRRLARRGGVKRISGLL **Charge** 5 **Spectral Angle** 0.63

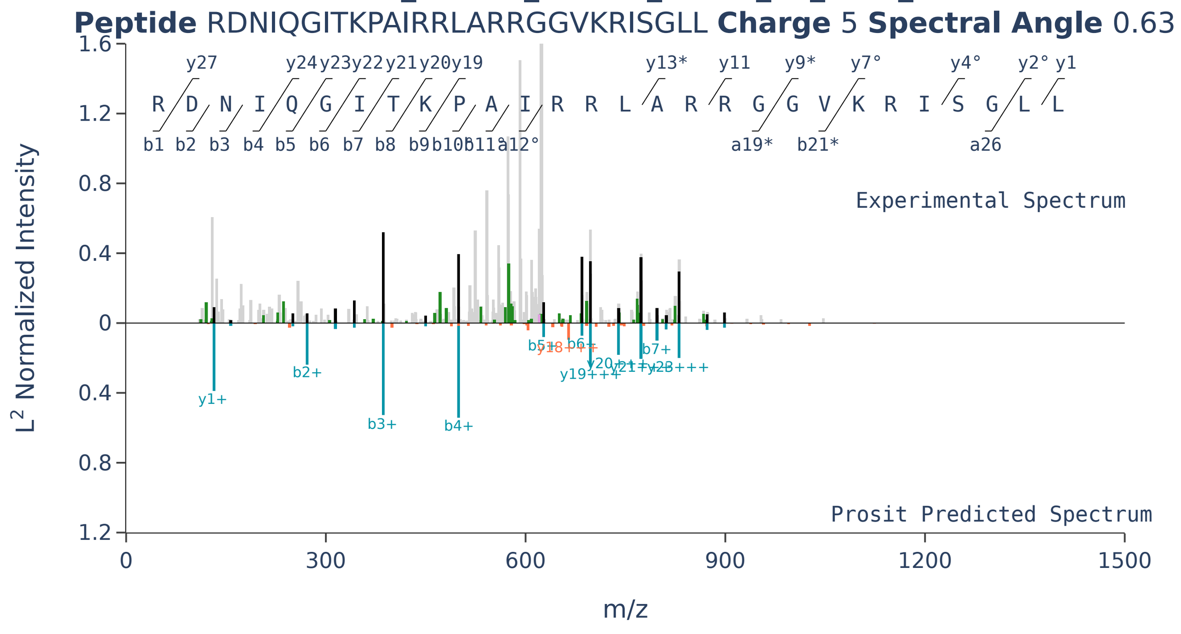


**Source** HRoetschke_291222_140123_HFGoe_H4_b2R2_1h **Scan** 22104
**Peptide** KTVTAMDVVYALKRQG **Charge** 3 **Spectral Angle** 0.71

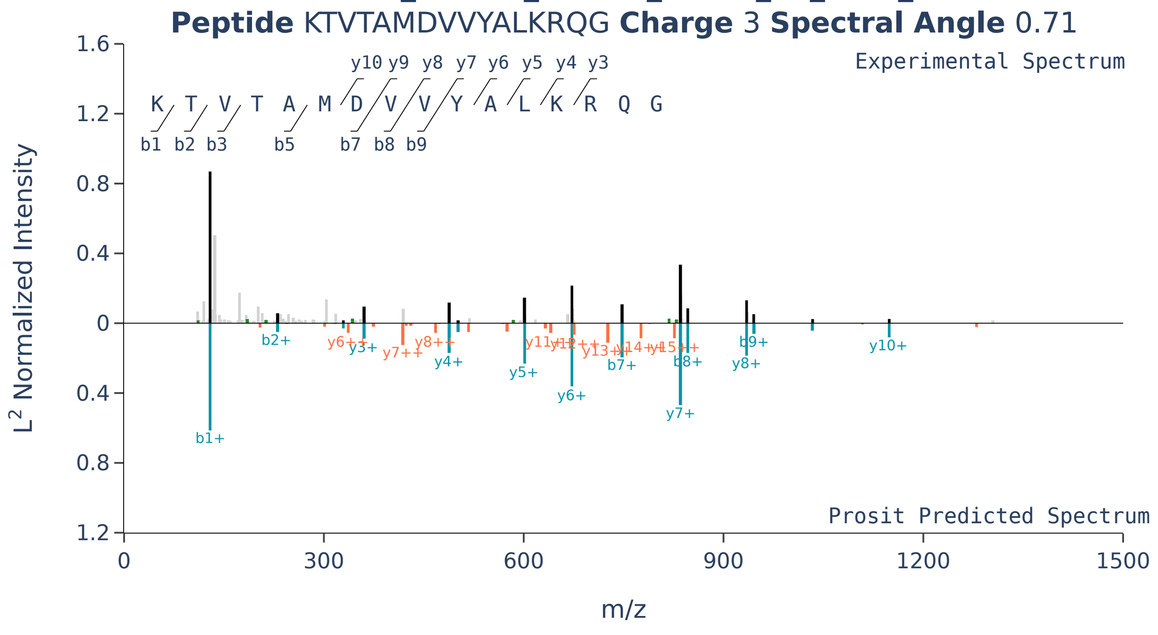


**Source** HRoetschke_291222_140123_HFGoe_H4_b2R1_1h **Scan** 38403
**Peptide** GLIYEETRGVLKVFLENVI **Charge** 3 **Spectral Angle** 0.7

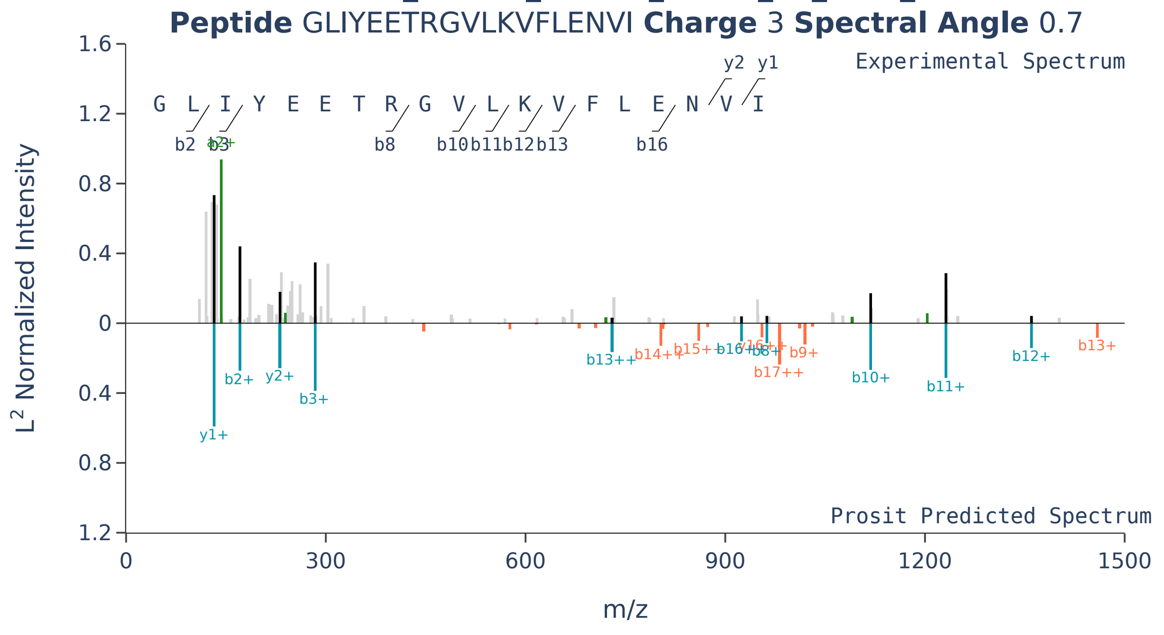


**Source** HRoetschke_291222_140123_HFGoe_H4_b2R2_4h **Scan** 14009
**Peptide** VKRISGLIYEETR **Charge** 3 **Spectral Angle** 0.4

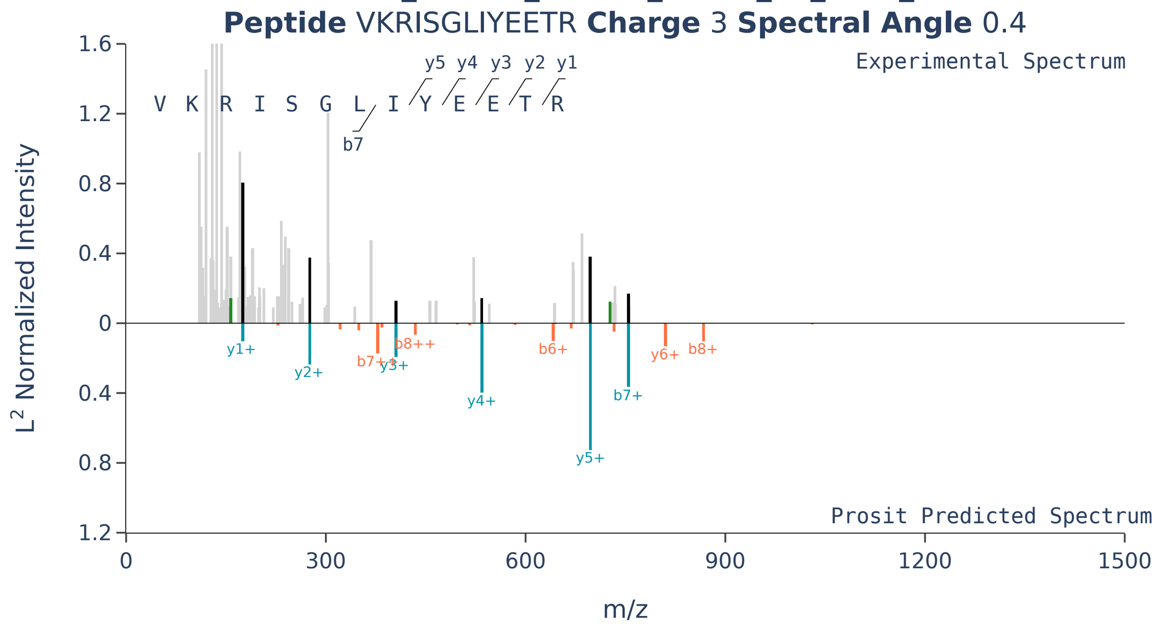


**Source** HRoetschke_291222_140123_HFGoe_H4_b2R1_2h **Scan** 12207
**Peptide** KVLRDNIQGITKPAIRRLARRGGVKRI **Charge** 5 **Spectral Angle** 0.76

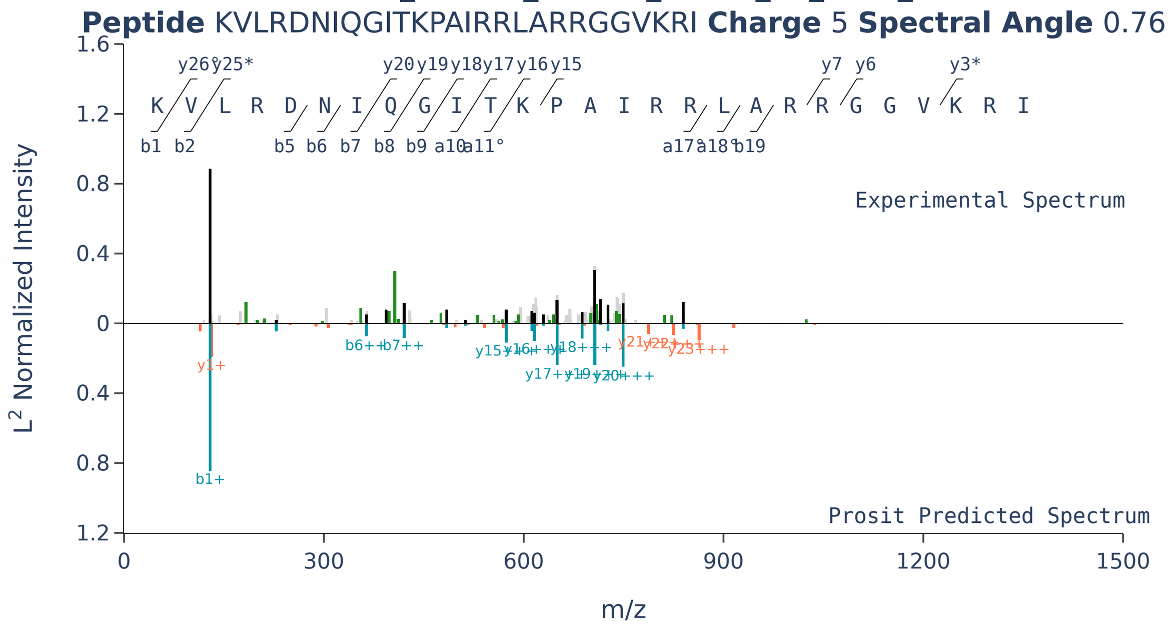


**Source** HRoetschke_291222_140123_HFGoe_H4_b1R1_4h **Scan** 5124
**Peptide** SGRGKGGKGLGKGGAKRHRKVLRDNIQG **Charge** 5 **Spectral Angle** 0.57

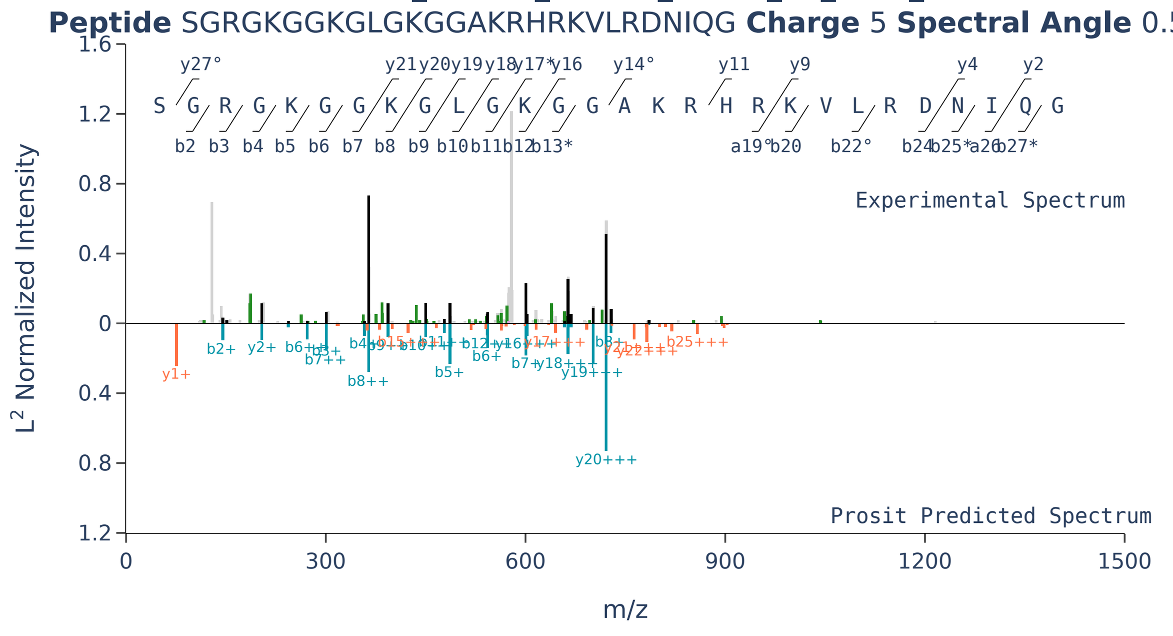


**Source** HRoetschke_291222_140123_HFGoe_H4_b1R1_2h **Scan** 12092
**Peptide** TKPAIRRLARRGGVKRISGLIYEE **Charge** 4 **Spectral Angle** 0.65

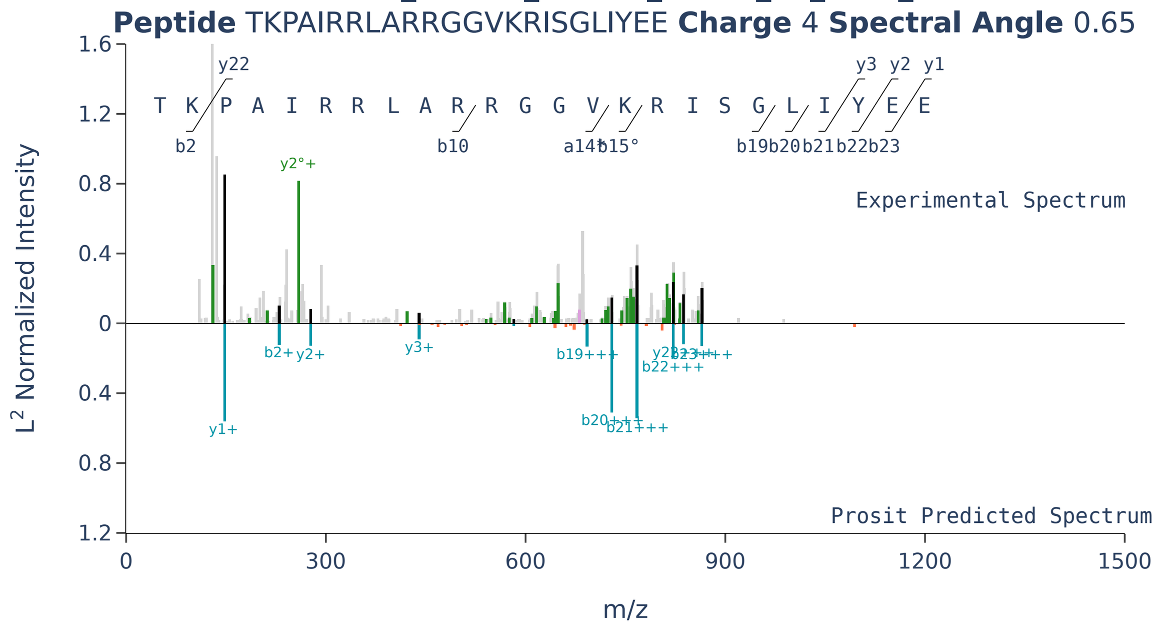


**Source** HRoetschke_291222_140123_HFGoe_H4_b1R1_24h **Scan** 14124
**Peptide** DNIQGITKPAIRRLARRGGV **Charge** 4 **Spectral Angle** 0.38

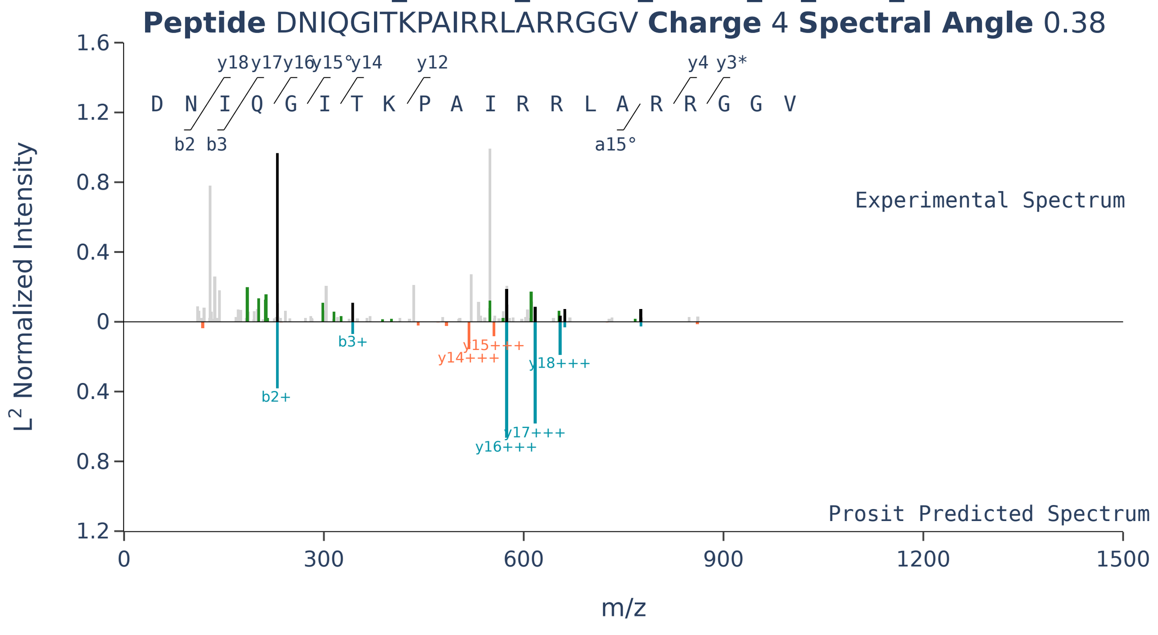


**Source** HRoetschke_291222_140123_HFGoe_H4_b1R1_4h **Scan** 6549
**Peptide** RRGGVKRISGL **Charge** 2 **Spectral Angle** 0.63

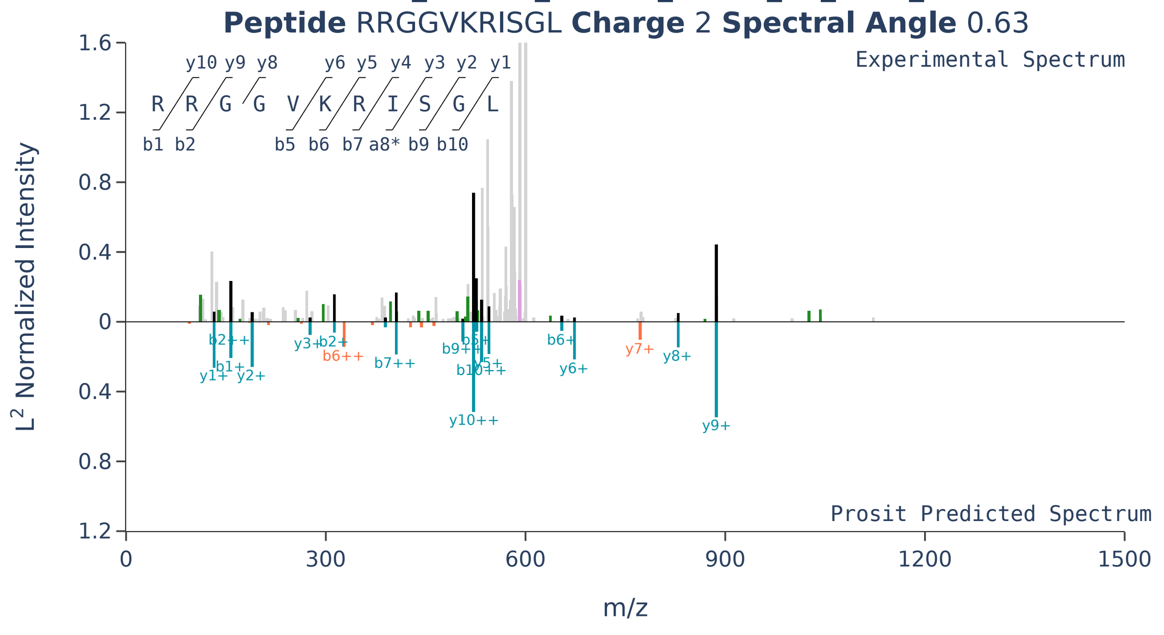


**Source** HRoetschke_291222_140123_HFGoe_H4_b1R1_1h **Scan** 14780
**Peptide** GITKPAIRRLARRGGVKRISGLIYEET **Charge** 5 **Spectral Angle** 0.77

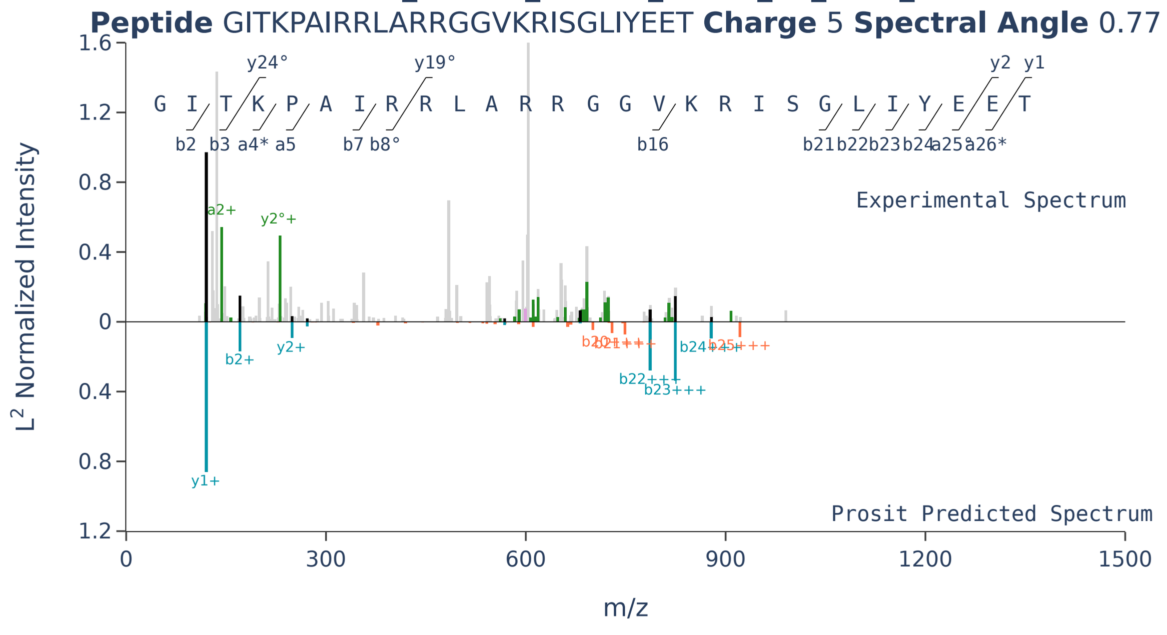


**Source** HRoetschke_291222_140123_HFGoe_H4_b2R1_1h **Scan** 32456
**Peptide** ETRGVLKVFLENVIRDAVTYTEHAKRK **Charge** 6 **Spectral Angle** 0.64

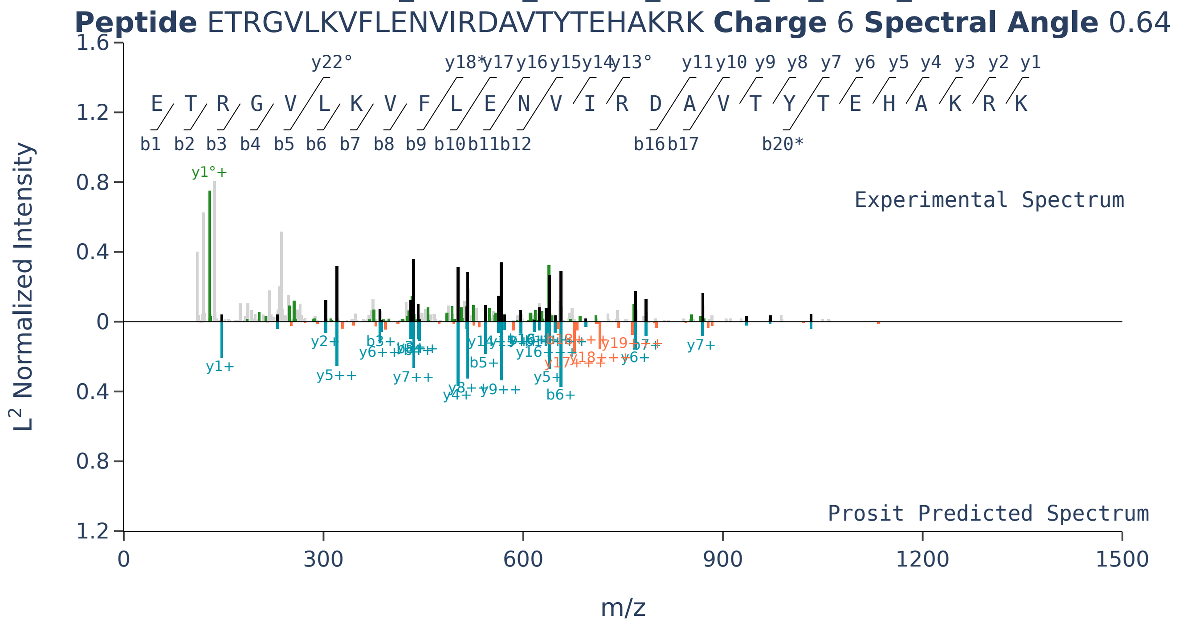


**Source** HRoetschke_291222_140123_HFGoe_H4_b2R1_1h **Scan** 28977
**Peptide** LIYEETRGVLKVFLE **Charge** 3 **Spectral Angle** 0.44

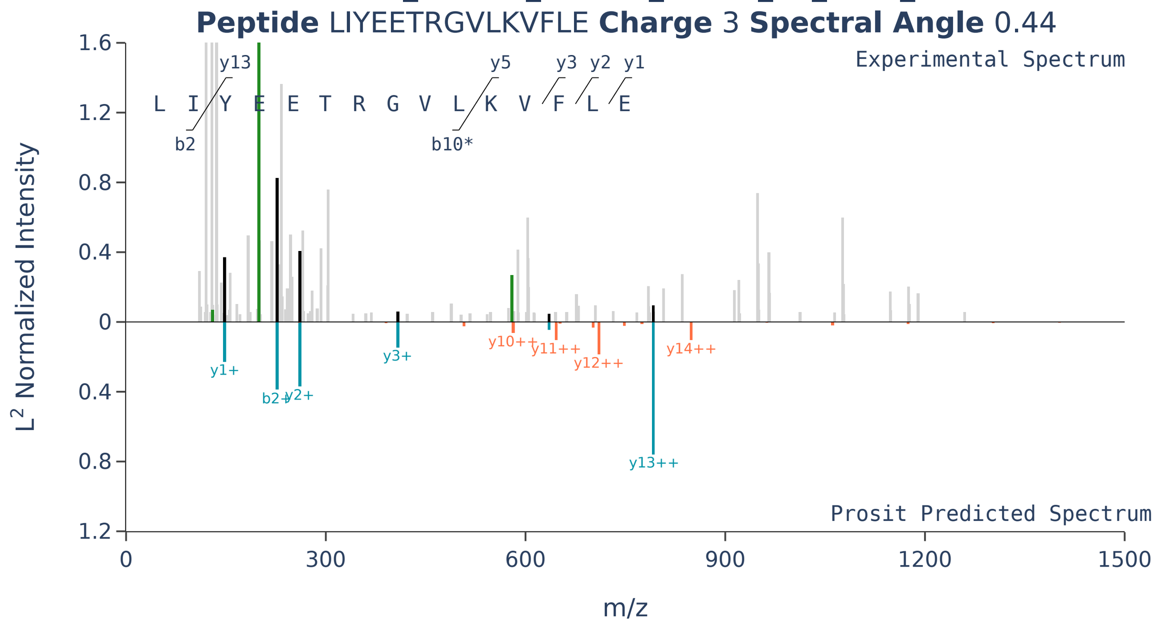


**Source** HRoetschke_291222_140123_HFGoe_H4_b2R1_24h **Scan** 10162
**Peptide** VTYTEHAKRKTVTAMDVVYA **Charge** 3 **Spectral Angle** 0.63

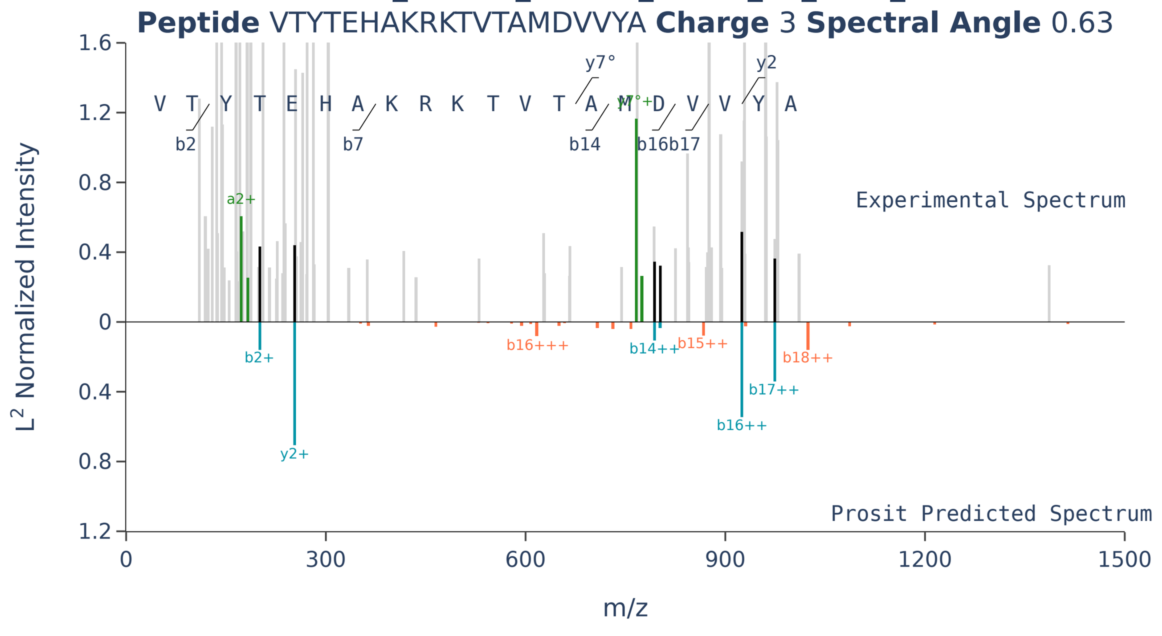

**Source** HRoetschke_291222_140123_HFGoe_H4_b1R2_2h **Scan** 13969
**Peptide** VLRDNIQGITKPAIRRLARRGGVKRI **Charge** 5 **Spectral Angle** 0.65

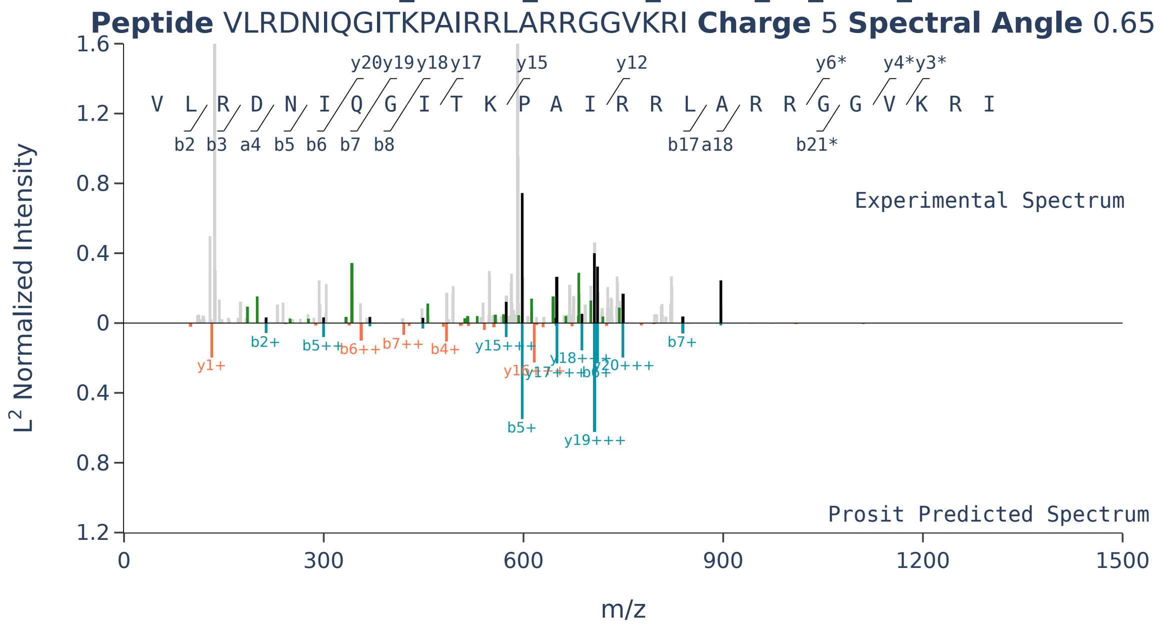


**Source** HRoetschke_291222_140123_HFGoe_H4_b2R2_24h **Scan** 11535
**Peptide** ARRGGVKRISGLLY **Charge** 3 **Spectral Angle** 0.63

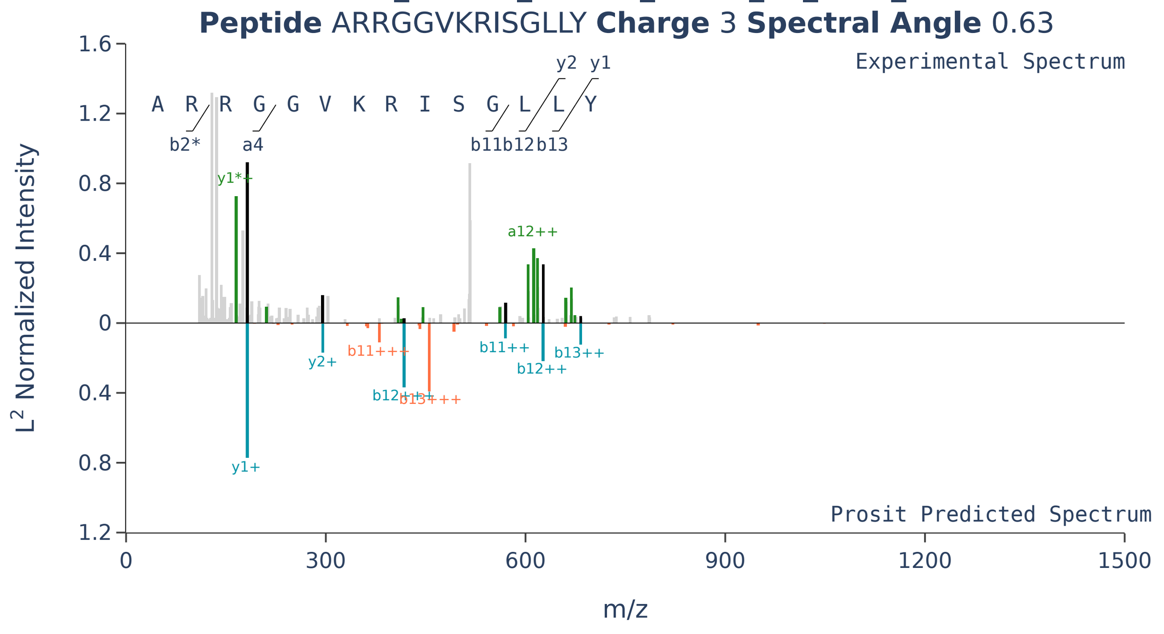


**Source** HRoetschke_291222_140123_HFGoe_H4_b2R2_2h **Scan** 8431
**Peptide** KGLGKGGAKRHRKVLRDNIQGITKPAL **Charge** 6 **Spectral Angle** 0.87

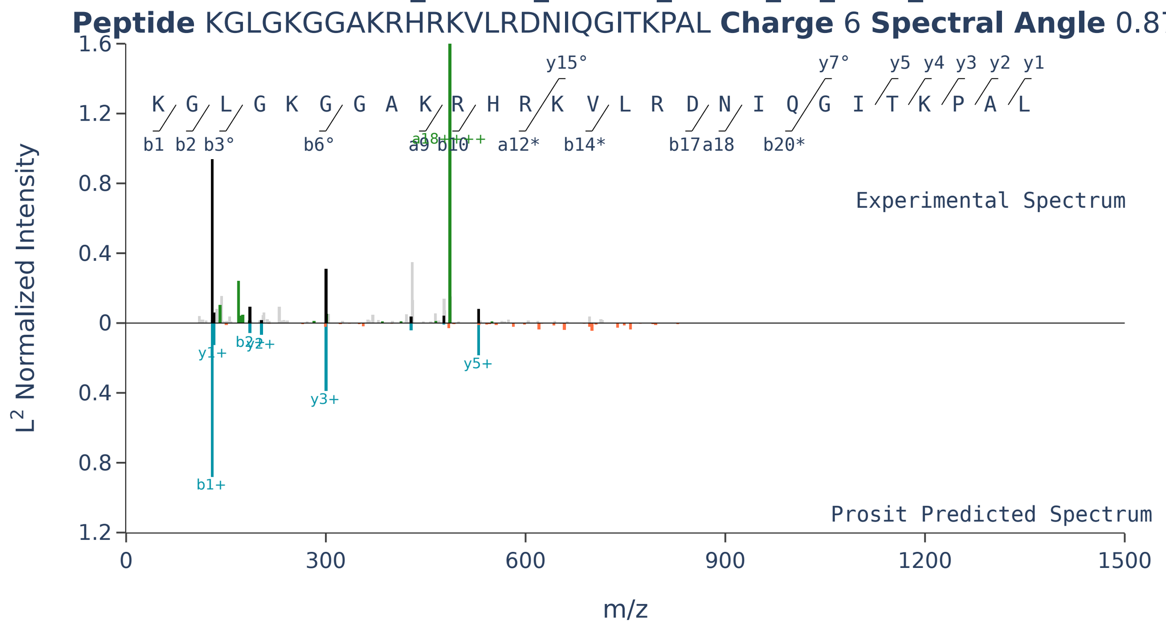


**Source** HRoetschke_291222_140123_HFGoe_H4_b1R1_24h **Scan** 10486
**Peptide** GITKPAIRRLARR **Charge** 3 **Spectral Angle** 0.69

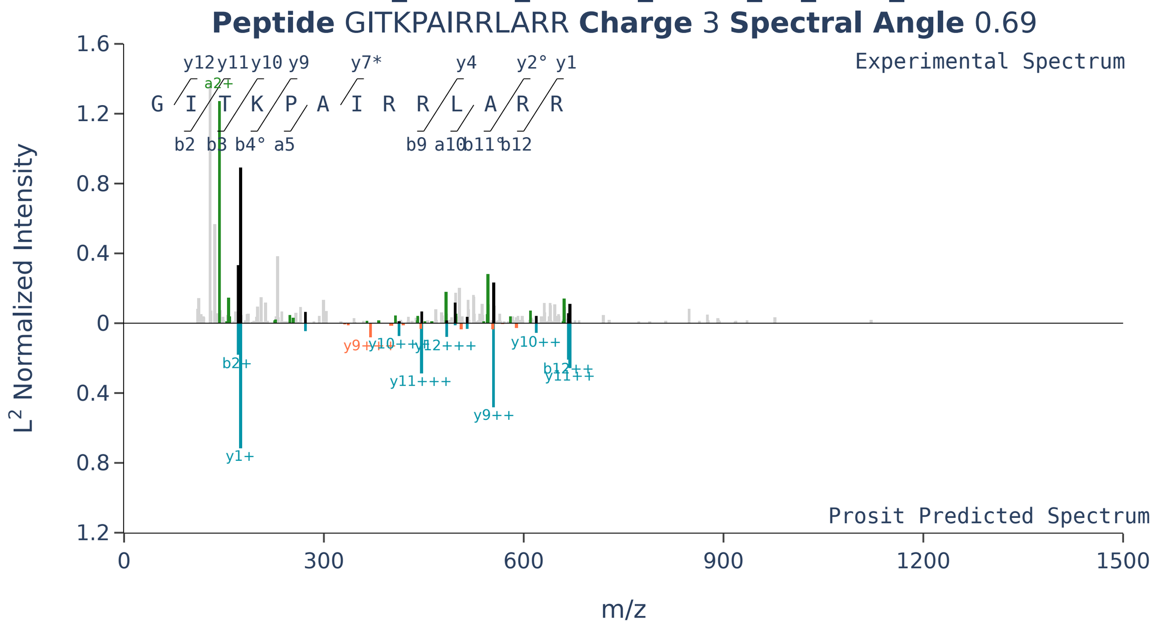


**Source** HRoetschke_291222_140123_HFGoe_H4_b2R1_24h **Scan** 13525
**Peptide** RKVLRDNIQGITKPAI **Charge** 4 **Spectral Angle** 0.48

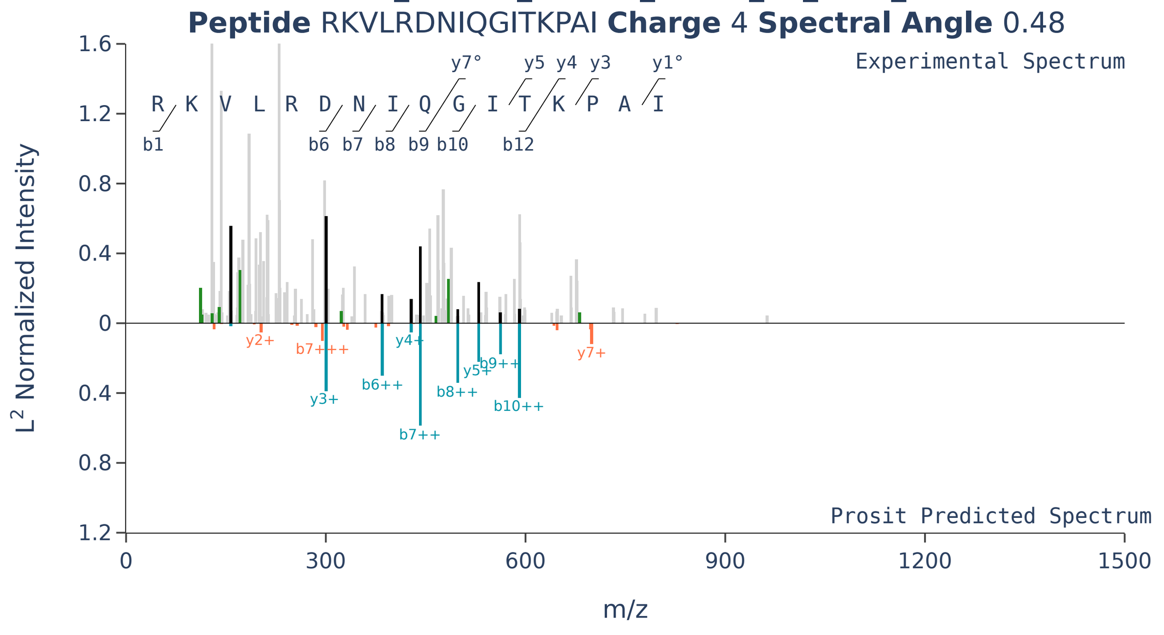


**Source** HRoetschke_291222_140123_HFGoe_H4_b1R1_1h **Scan** 18473
**Peptide** DNIQGITKPAIRRLARRGGVKRISGLIYEE **Charge** 5 **Spectral Angle** 0.74

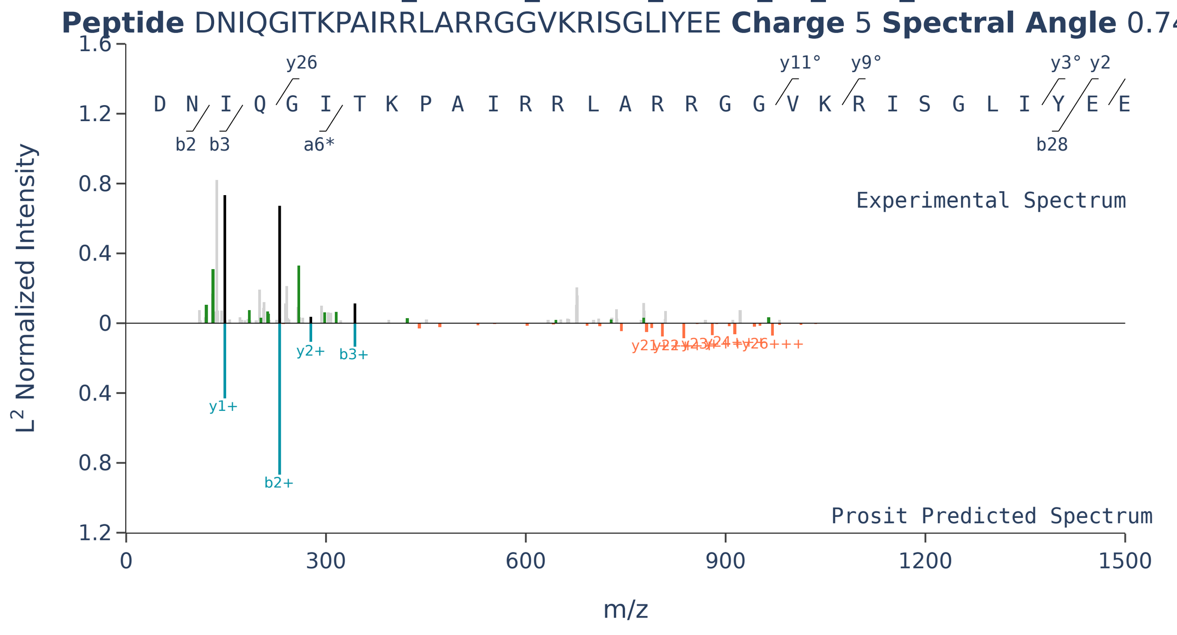


**Source** HRoetschke_291222_140123_HFGoe_H4_b2R1_4h **Scan** 5143
**Peptide** SGRGKGGKGLGKGGAKRHRKVLRD **Charge** 6 **Spectral Angle** 0.57

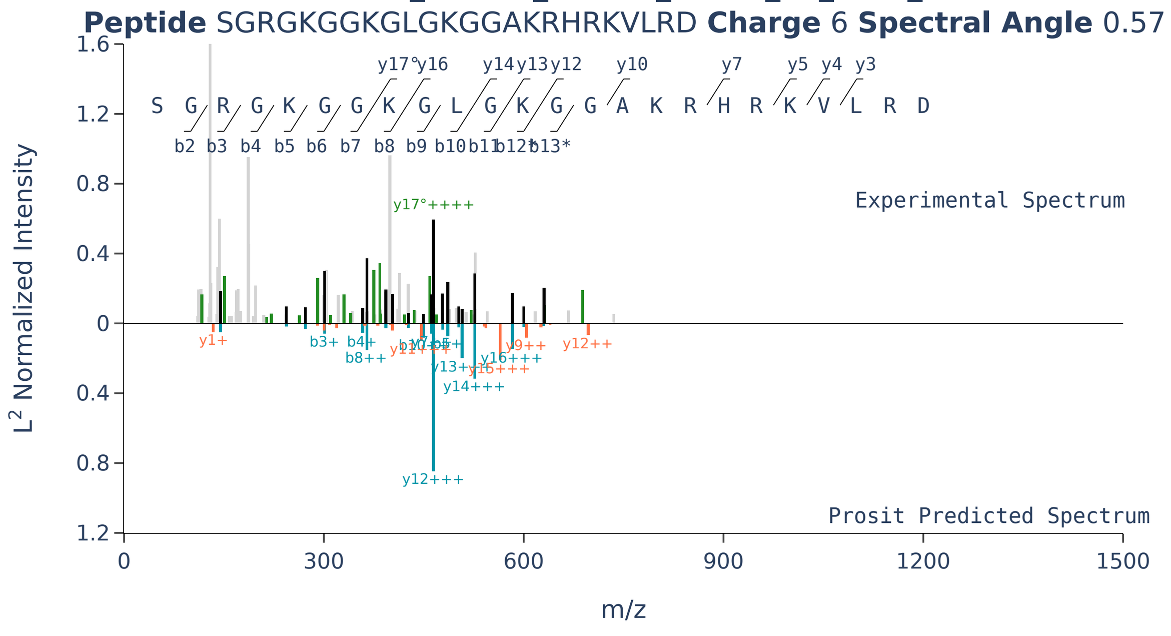


**Source** HRoetschke_291222_140123_HFGoe_H4_b1R2_24h **Scan** 6685
**Peptide** GKGGAKRHRKVLRDNIQGITKPAIRR **Charge** 6 **Spectral Angle** 0.58

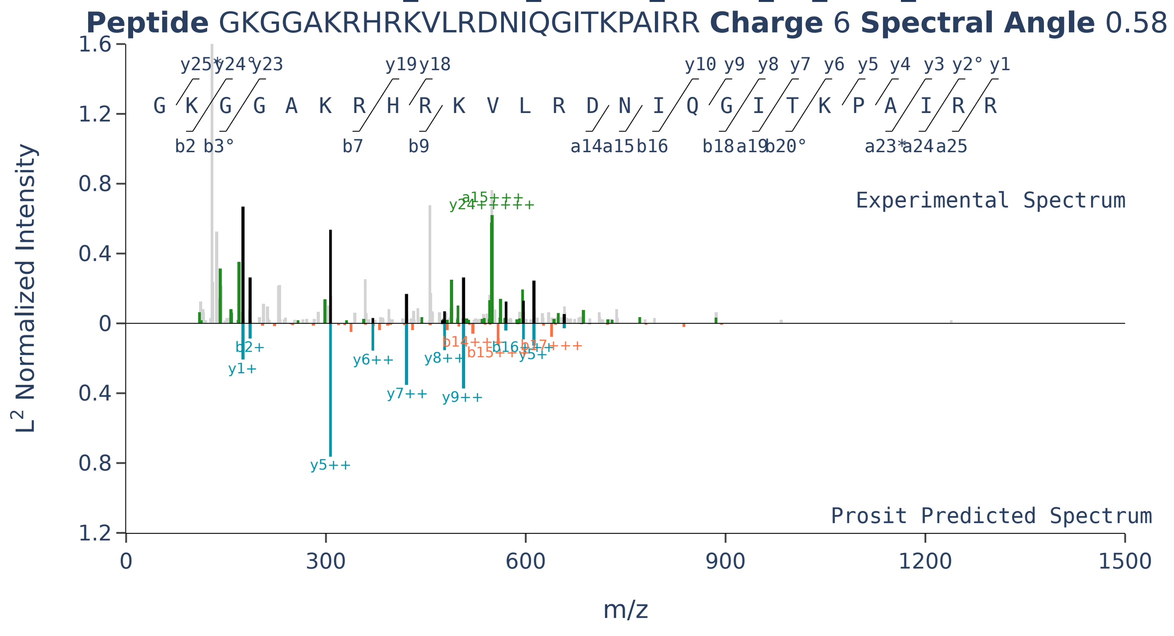


**Source** HRoetschke_291222_140123_HFGoe_H4_b1R2_1h **Scan** 17998
**Peptide** NVIRDAVTYTEHAKRKTVTAMDV **Charge** 4 **Spectral Angle** 0.58

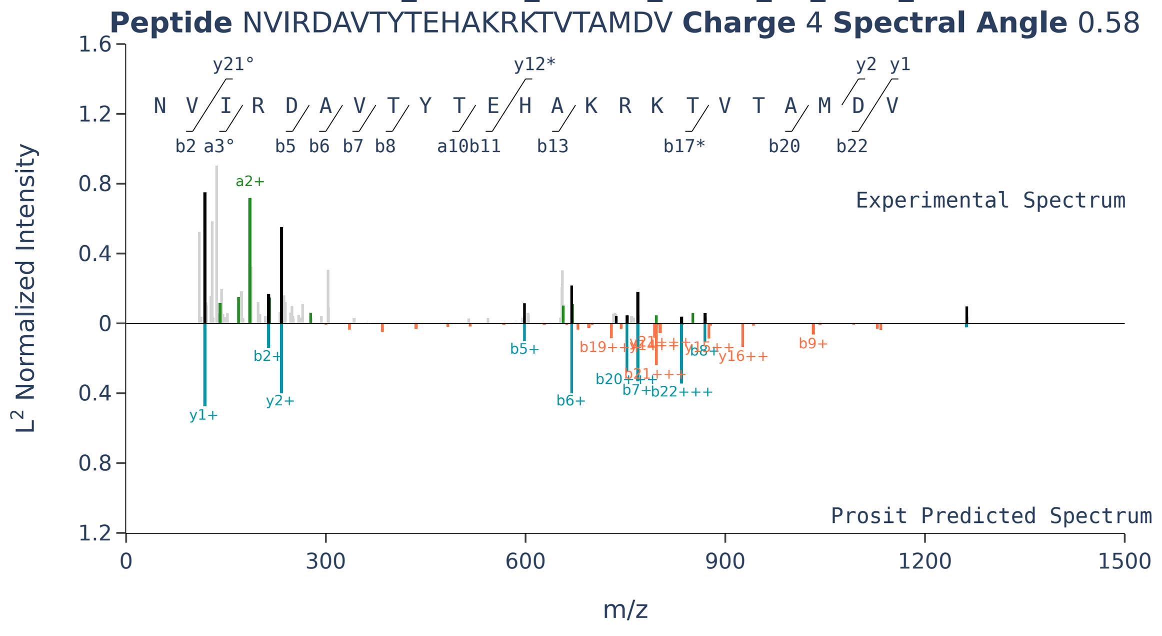


**Source** HRoetschke_291222_140123_HFGoe_H4_b2R2_4h **Scan** 13107
**Peptide** DNIQGITKPAIRRLARRGG **Charge** 4 **Spectral Angle** 0.42

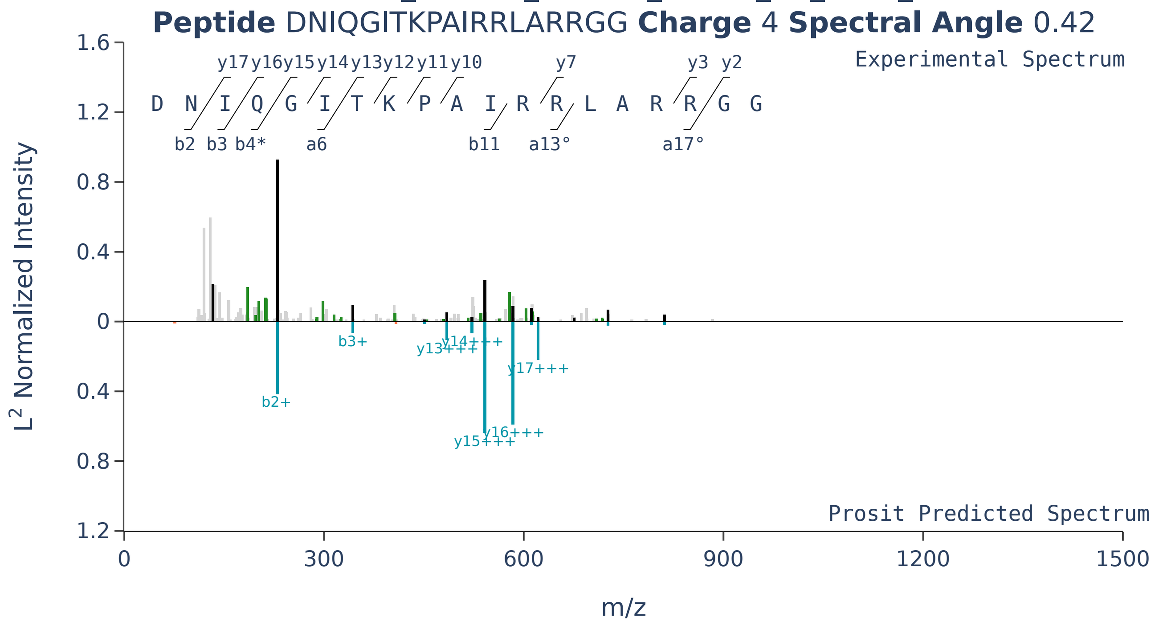


**Source** HRoetschke_291222_140123_HFGoe_H4_b2R1_1h **Scan** 29233
**Peptide** RGGVKRISGLIYEETRGVLKVFLE **Charge** 5 **Spectral Angle** 0.72

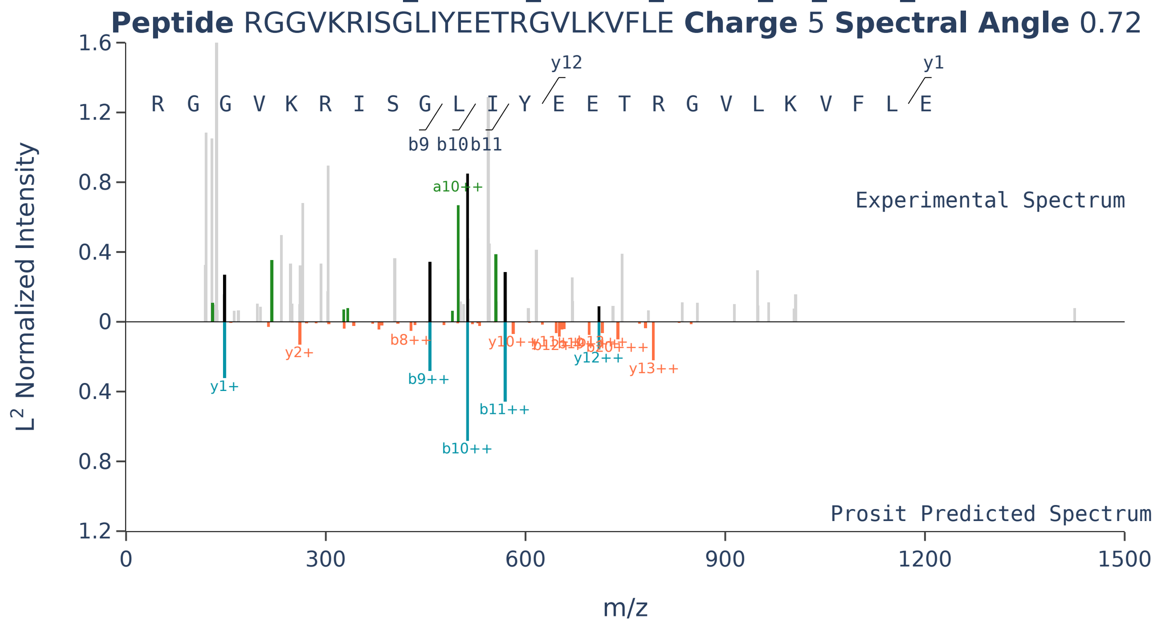


**Source** HRoetschke_291222_140123_HFGoe_H4_b2R2_1h **Scan** 15996
**Peptide** IQGITKPAIRRLARRGGVKRISGLIYEET **Charge** 4 **Spectral Angle** 0.63

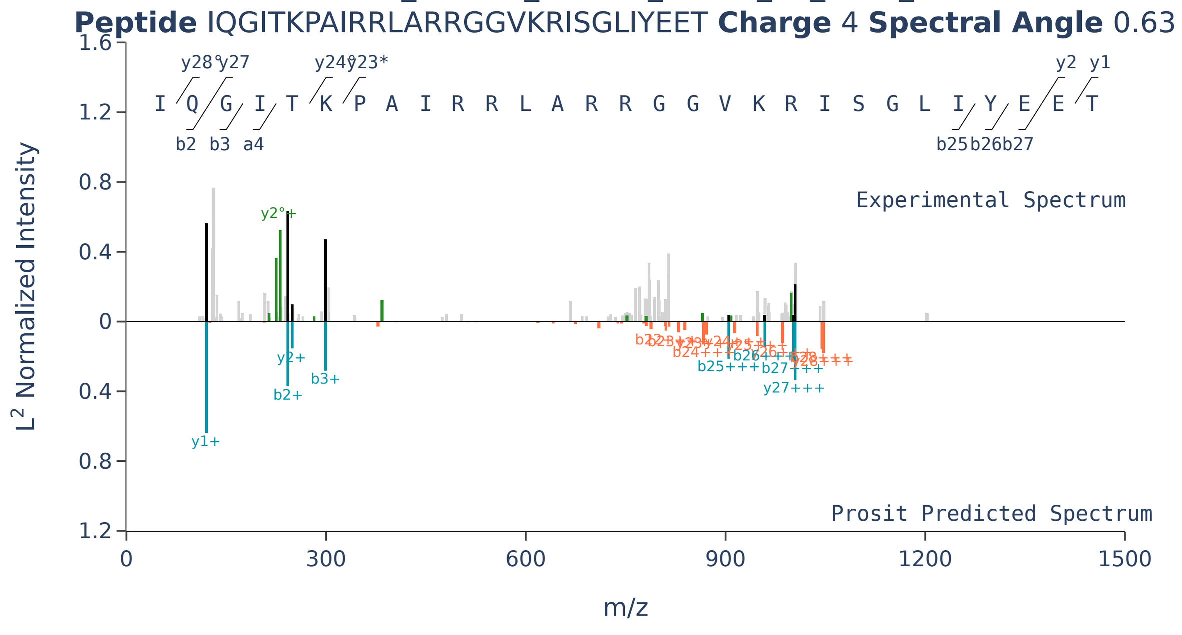


**Source** HRoetschke_291222_140123_HFGoe_H4_b1R1_24h **Scan** 6739
**Peptide** IRDAVTYTEHAKRKTVTAM **Charge** 4 **Spectral Angle** 0.67

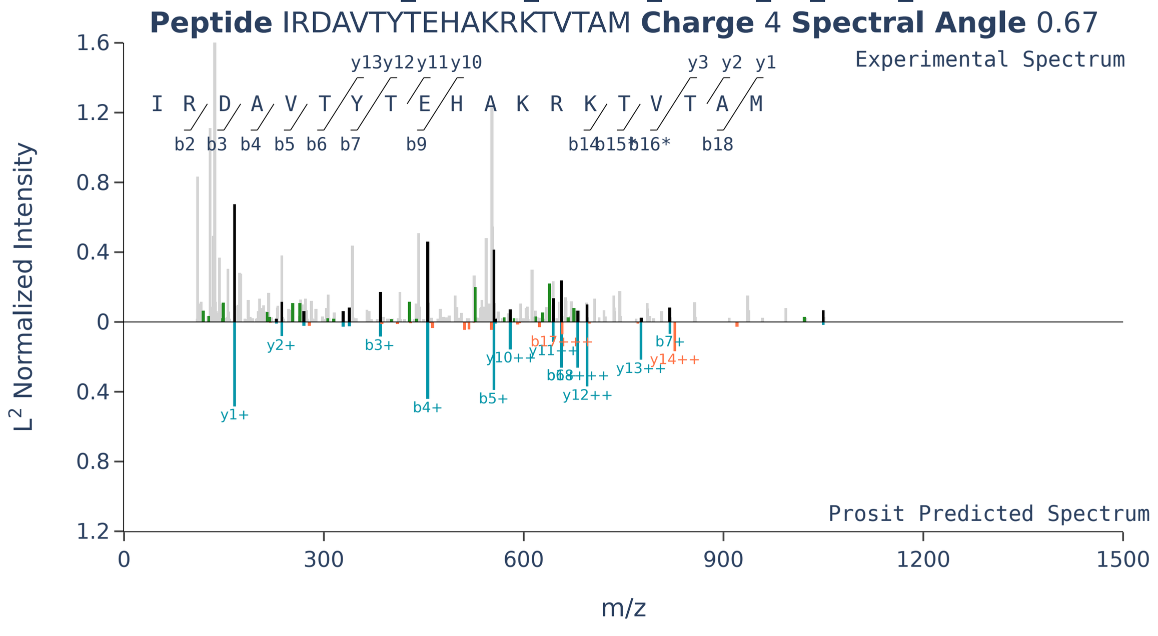


**Source** HRoetschke_291222_140123_HFGoe_H4_b2R2_1h **Scan** 31113
**Peptide** GLIYEETRGVLKVFLEN **Charge** 2 **Spectral Angle** 0.53

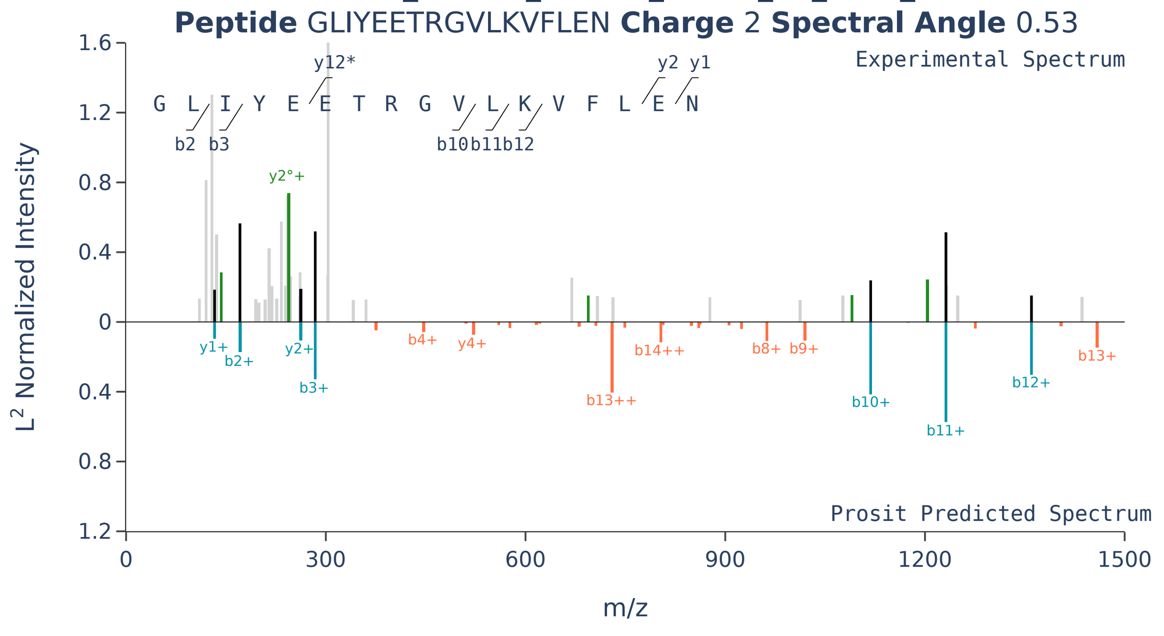


**Source** HRoetschke_291222_140123_HFGoe_H4_b1R2_24h **Scan** 5953
**Peptide** SGRGKGGKGLGKGGAKRHRKVLRDNIQGIT **Charge** 5 **Spectral Angle** 0.55

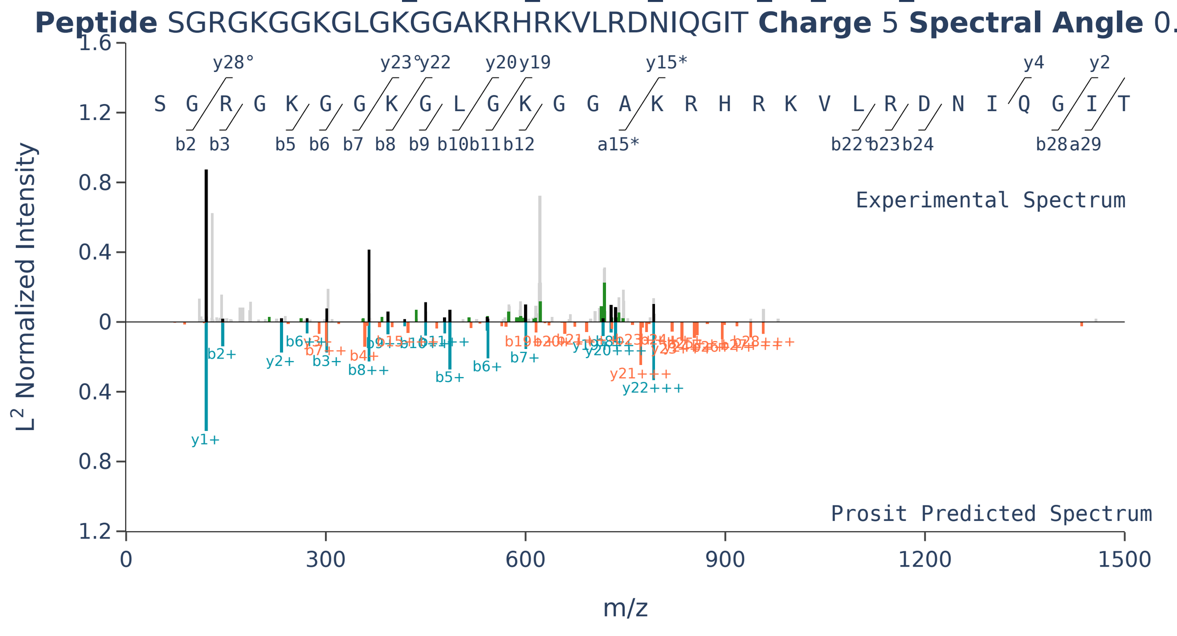

**Source** HRoetschke_291222_140123_HFGoe_H4_b1R2_1h **Scan** 9431
**Peptide** GAKRHRKVLRDNIQGITKPAIRRL **Charge** 6 **Spectral Angle** 0.79

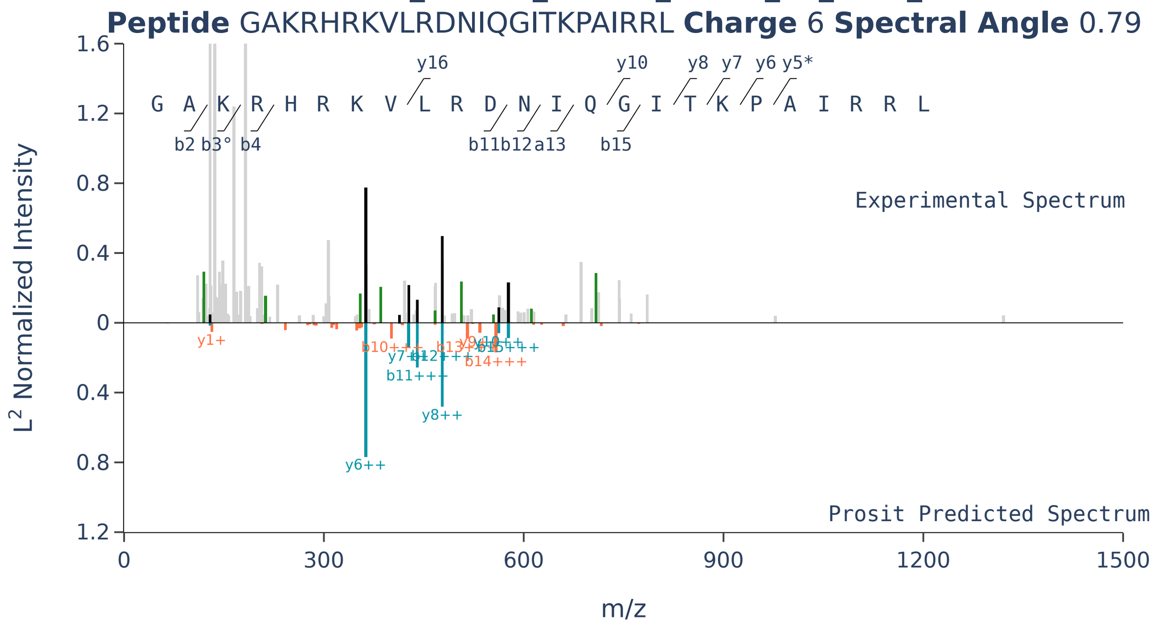


**Source** HRoetschke_291222_140123_HFGoe_H4_b1R2_1h **Scan** 13240
**Peptide** QGITKPAIRRLARRGGVKRISGLLY **Charge** 5 **Spectral Angle** 0.9

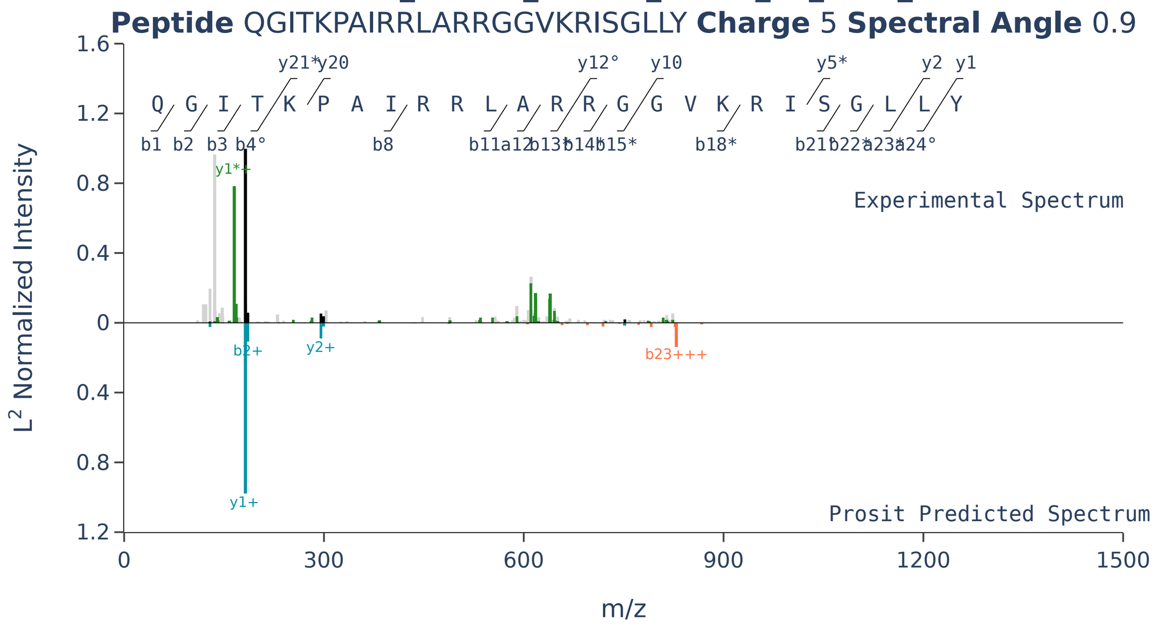


**Source** HRoetschke_291222_140123_HFGoe_H4_b1R1_1h **Scan** 14289
**Peptide** ITKPAIRRLARRGGVKRISGLIYEET **Charge** 4 **Spectral Angle** 0.72

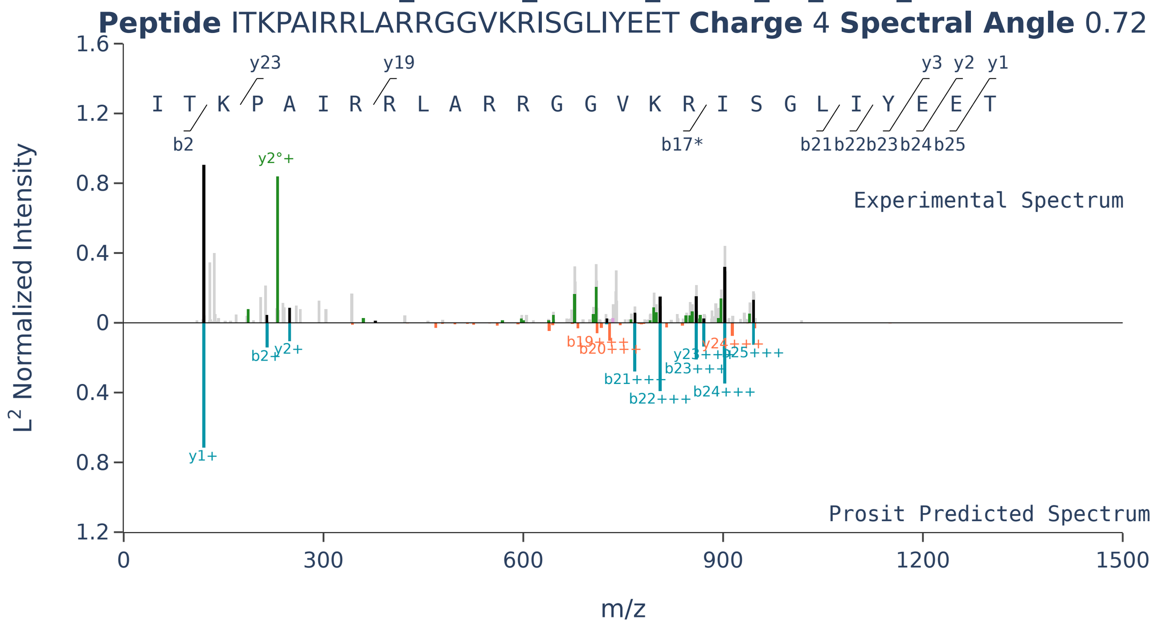


**Source** HRoetschke_291222_140123_HFGoe_H4_b2R2_1h **Scan** 29596
**Peptide** VKRISGLIYEETRGVLKVFLE **Charge** 4 **Spectral Angle** 0.58

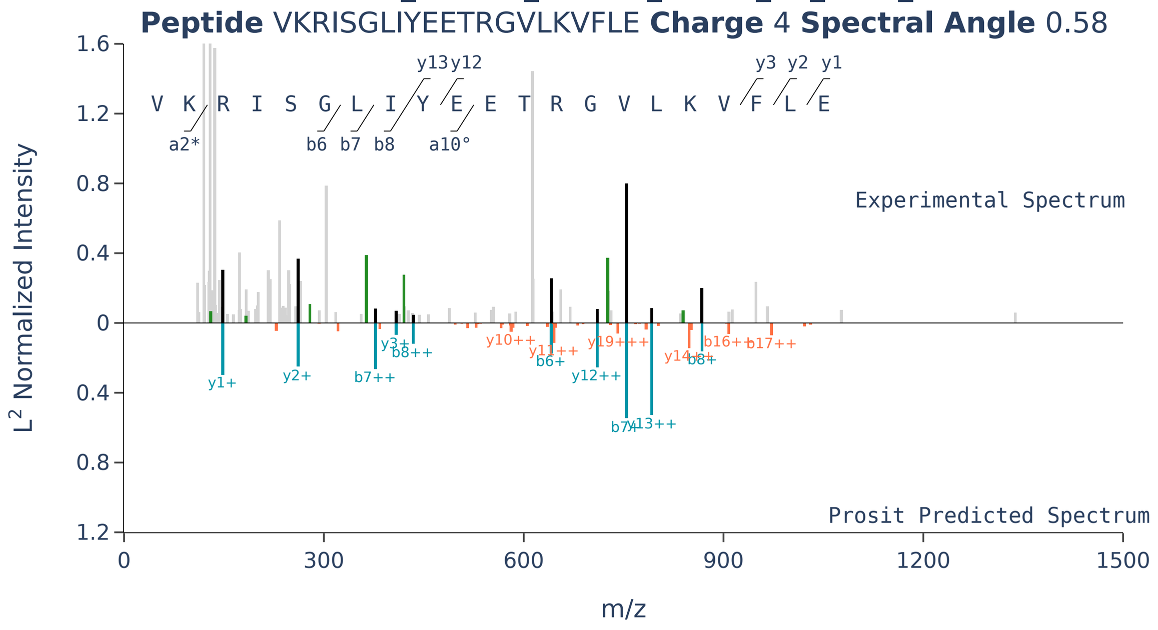


**Source** HRoetschke_291222_140123_HFGoe_H4_b1R2_24h **Scan** 5393
**Peptide** GGKGLGKGGAKRHRKVLRDNI **Charge** 4 **Spectral Angle** 0.68

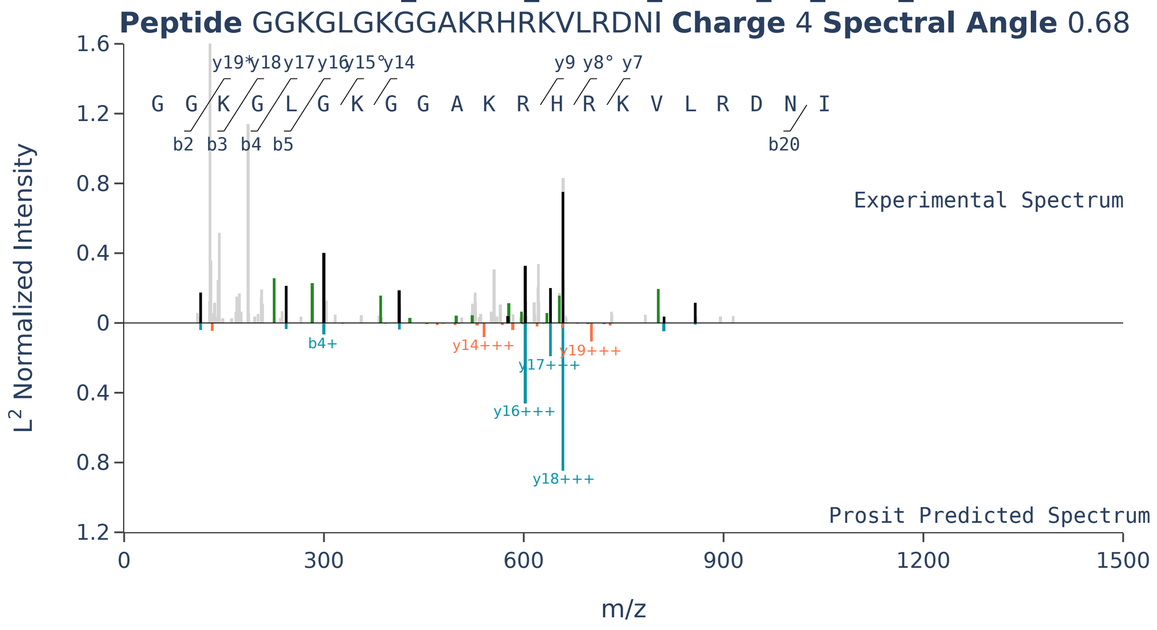


**Source** HRoetschke_291222_140123_HFGoe_H4_b1R2_0h **Scan** 21123
**Peptide** KRISGLIYEETRGVLKVF **Charge** 3 **Spectral Angle** 0.54

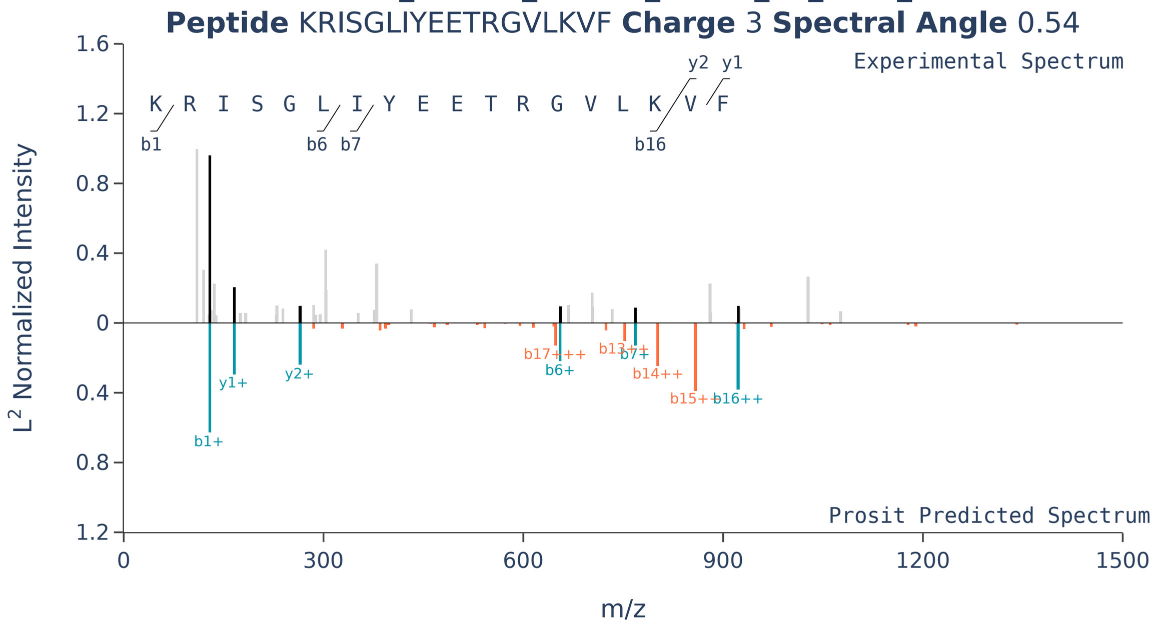


**Source** HRoetschke_291222_140123_HFGoe_H4_b1R2_2h **Scan** 15458
**Peptide** GLTKPAIRRLARRGGVKRISGLIYEET **Charge** 5 **Spectral Angle** 0.73

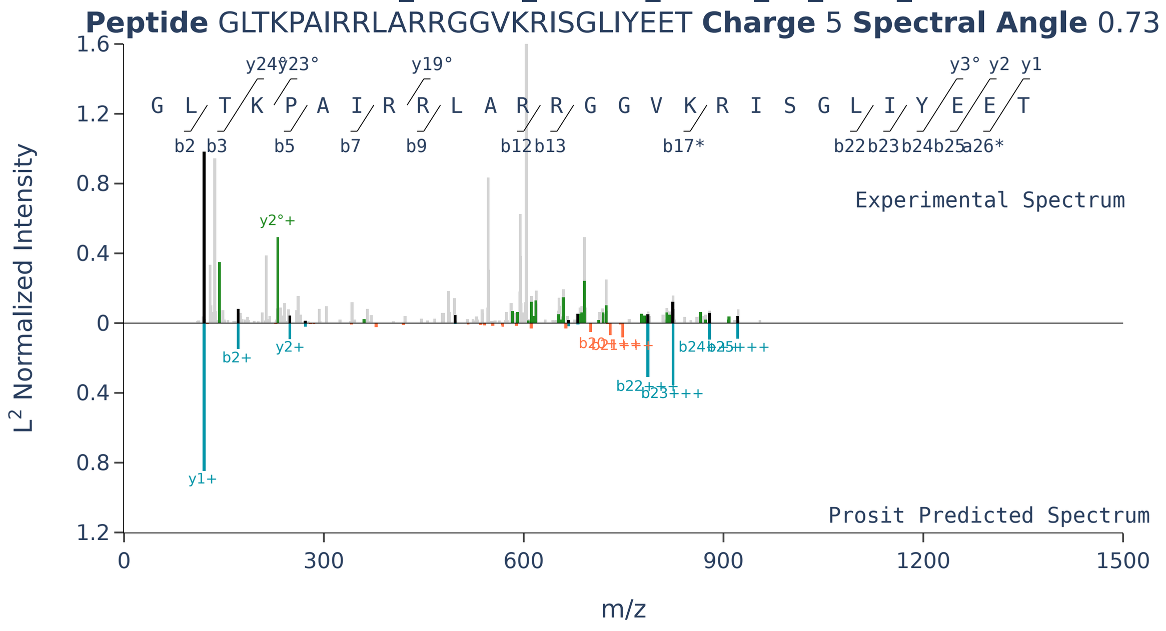


**Source** HRoetschke_291222_140123_HFGoe_H4_b1R1_2h **Scan** 9795
**Peptide** RRLARRGGVKRISGLIYE **Charge** 4 **Spectral Angle** 0.72

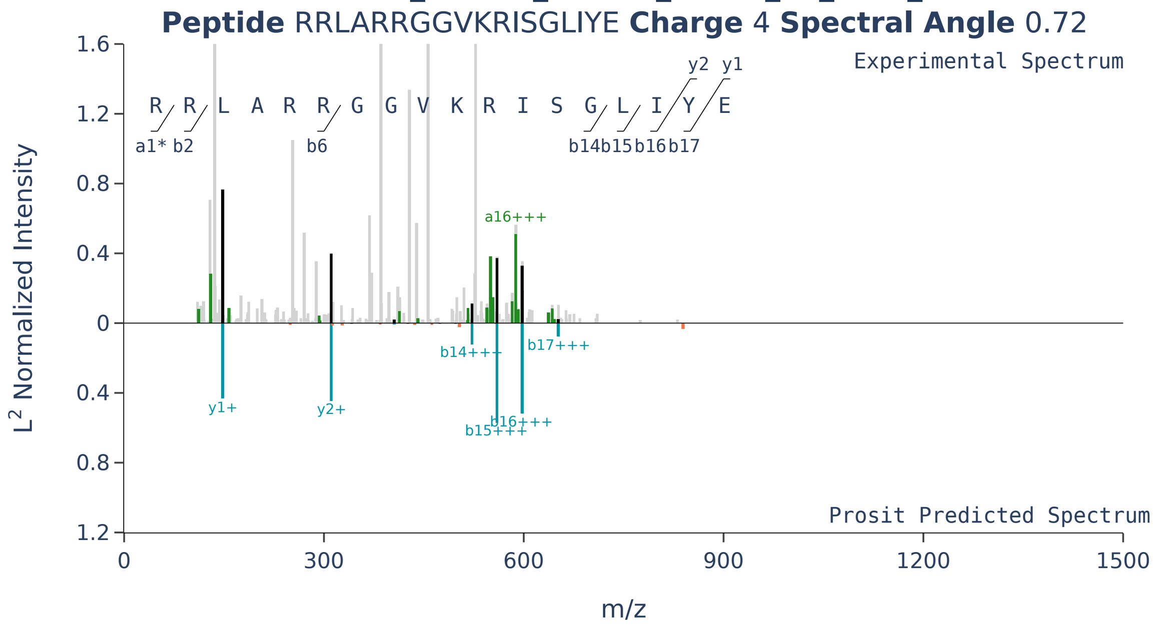


**Source** HRoetschke_291222_140123_HFGoe_H4_b2R1_1h **Scan** 37882
**Peptide** IYEETRGVLKVFLENVIRDAVTY **Charge** 3 **Spectral Angle** 0.67

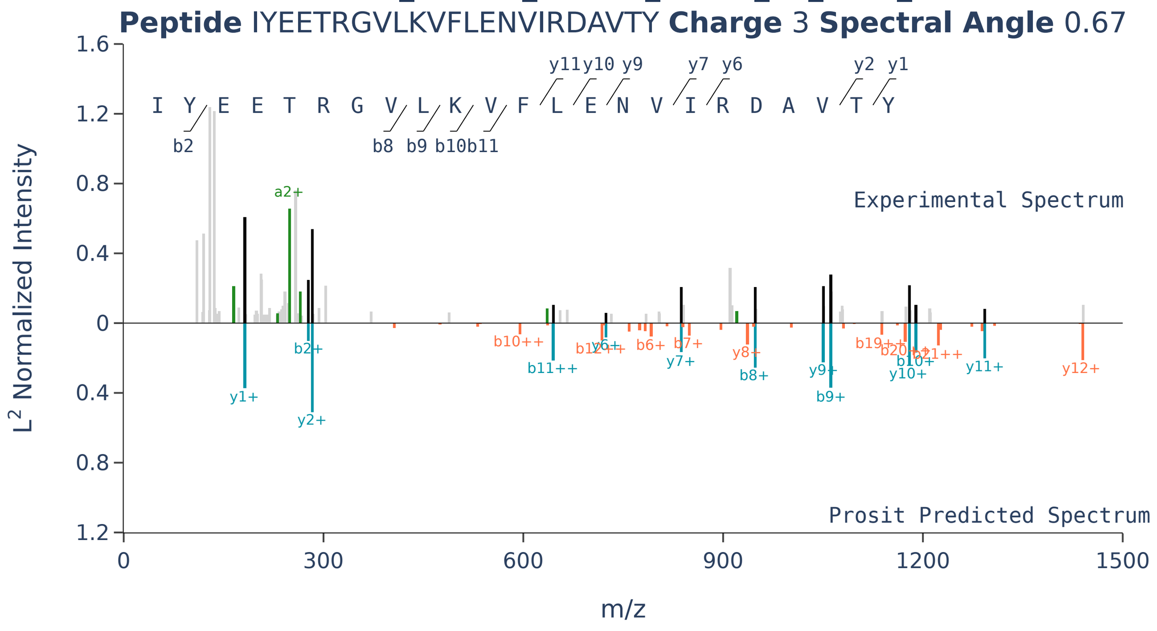


**Source** HRoetschke_291222_140123_HFGoe_H4_b2R1_4h **Scan** 6096
**Peptide** HRKVLRDNL **Charge** 3 **Spectral Angle** 0.77

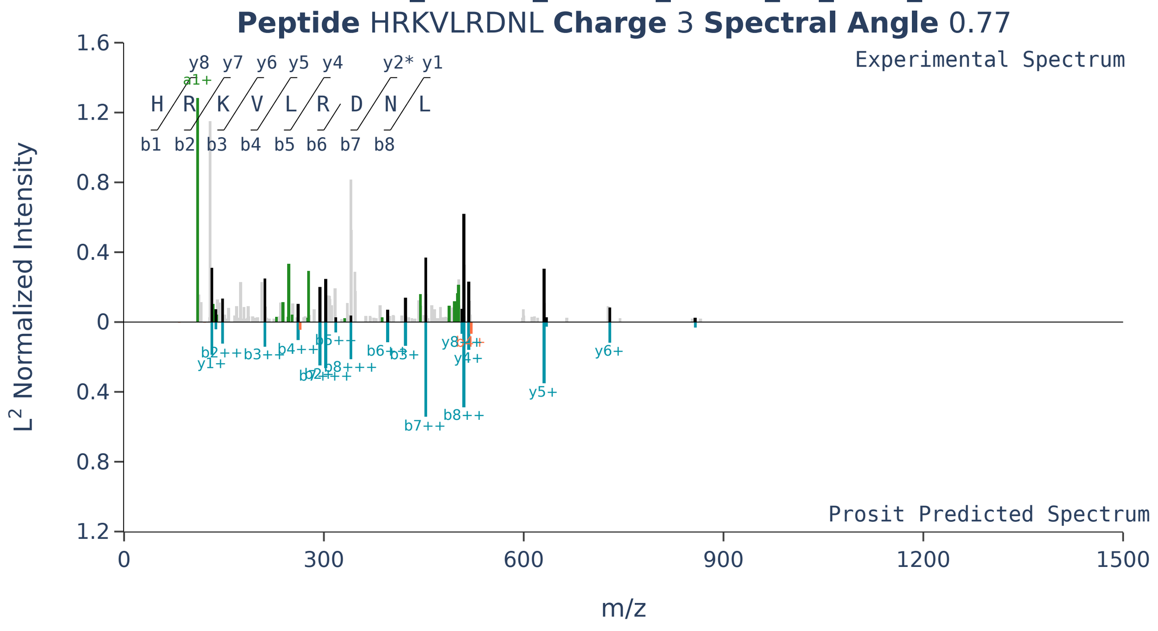


**Source** HRoetschke_291222_140123_HFGoe_H4_b2R1_4h **Scan** 5012
**Peptide** KGLGKGGAKRHRKVL **Charge** 3 **Spectral Angle** 0.48

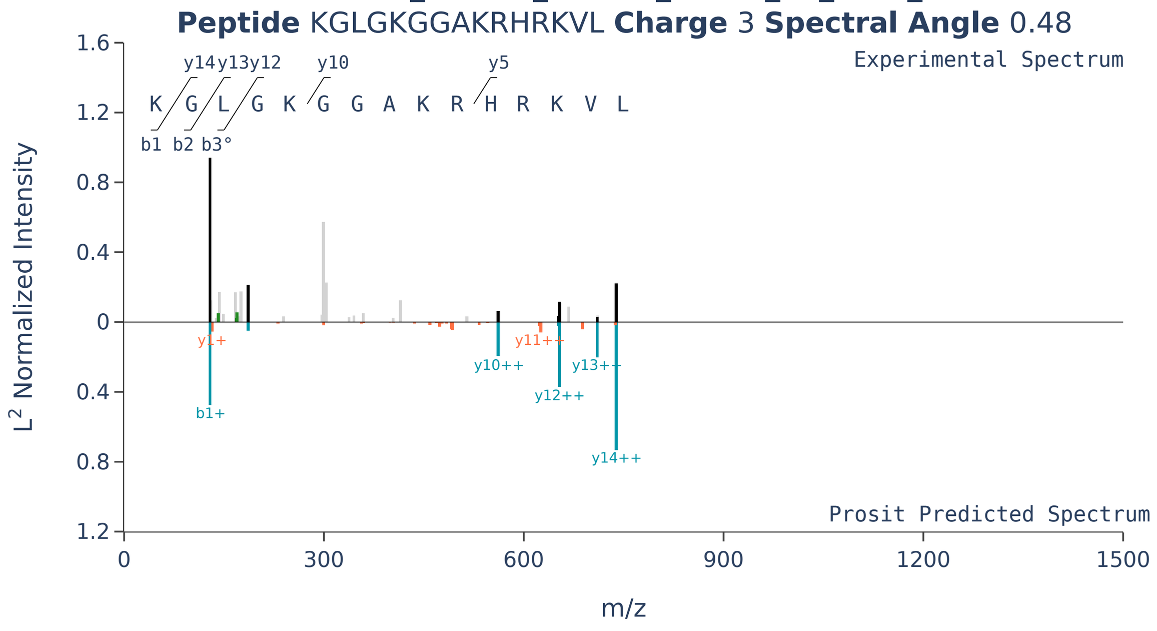


**Source** HRoetschke_291222_140123_HFGoe_H4_b2R2_2h **Scan** 18489
**Peptide** DAVTYTEHAKRKTVTAMDVVYALKRQG **Charge** 5 **Spectral Angle** 0.72

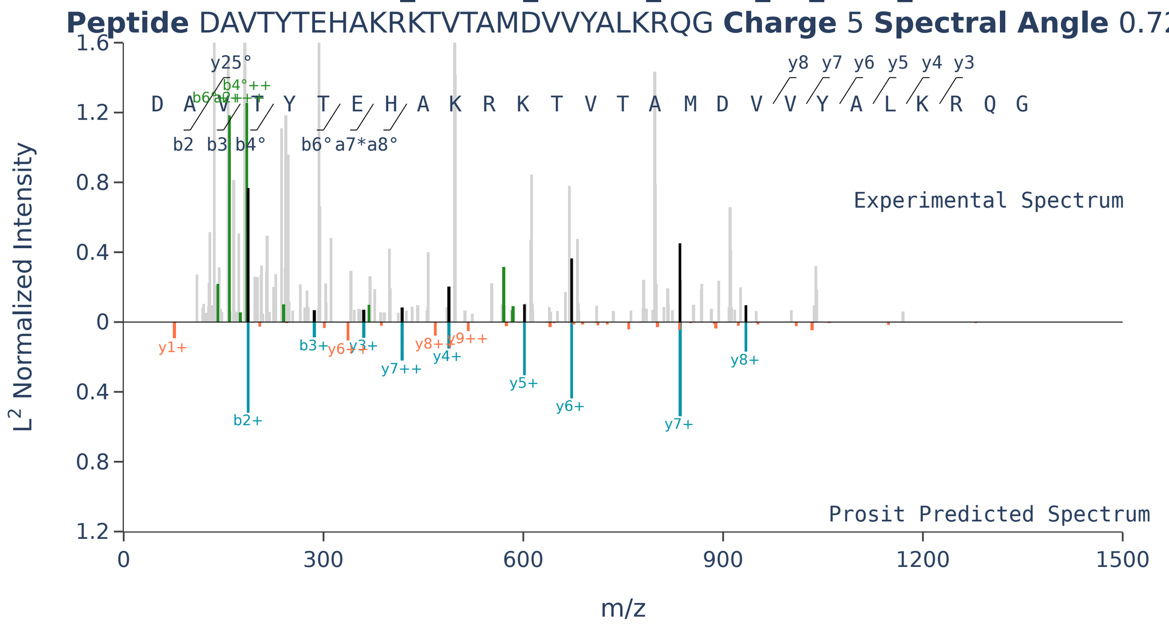


**Source** HRoetschke_291222_140123_HFGoe_H4_b2R1_4h **Scan** 4939
**Peptide** MSGRGKGGKGLGKGGAKRHRKVLRD **Charge** 6 **Spectral Angle** 0.58

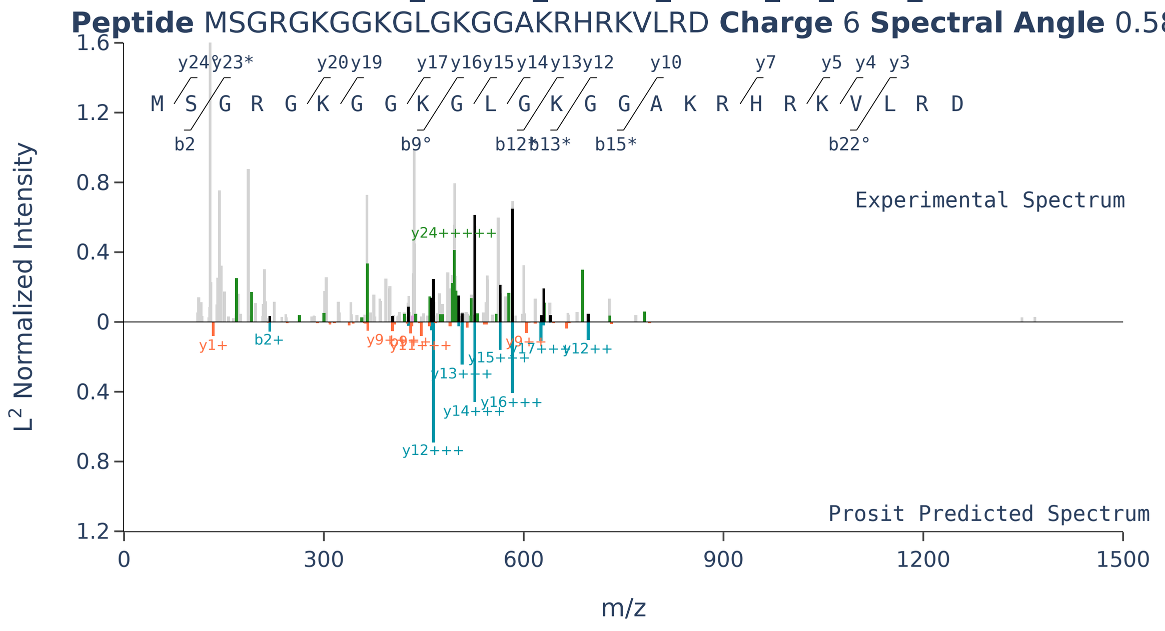


**Source** HRoetschke_291222_140123_HFGoe_H4_b1R1_4h **Scan** 29876
**Peptide** VFLENVI **Charge** 1 **Spectral Angle** 0.91

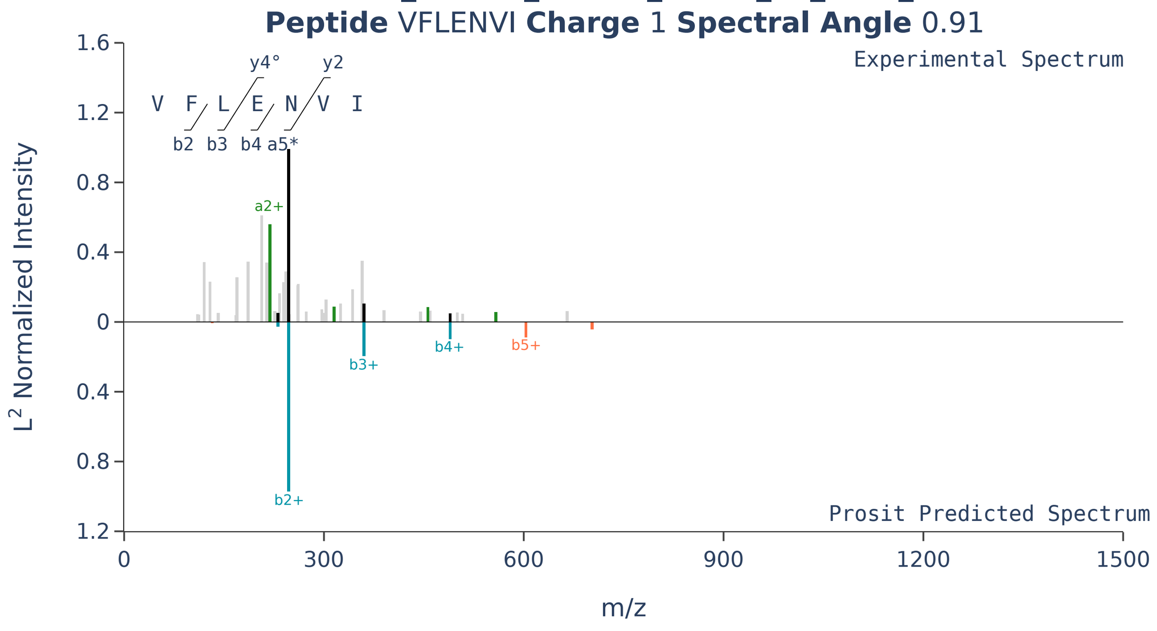


**Source** HRoetschke_291222_140123_HFGoe_H4_b2R2_4h **Scan** 9054
**Peptide** KGLGKGGAKRHRKVLRDNIQGITKPAI **Charge** 5 **Spectral Angle** 0.83

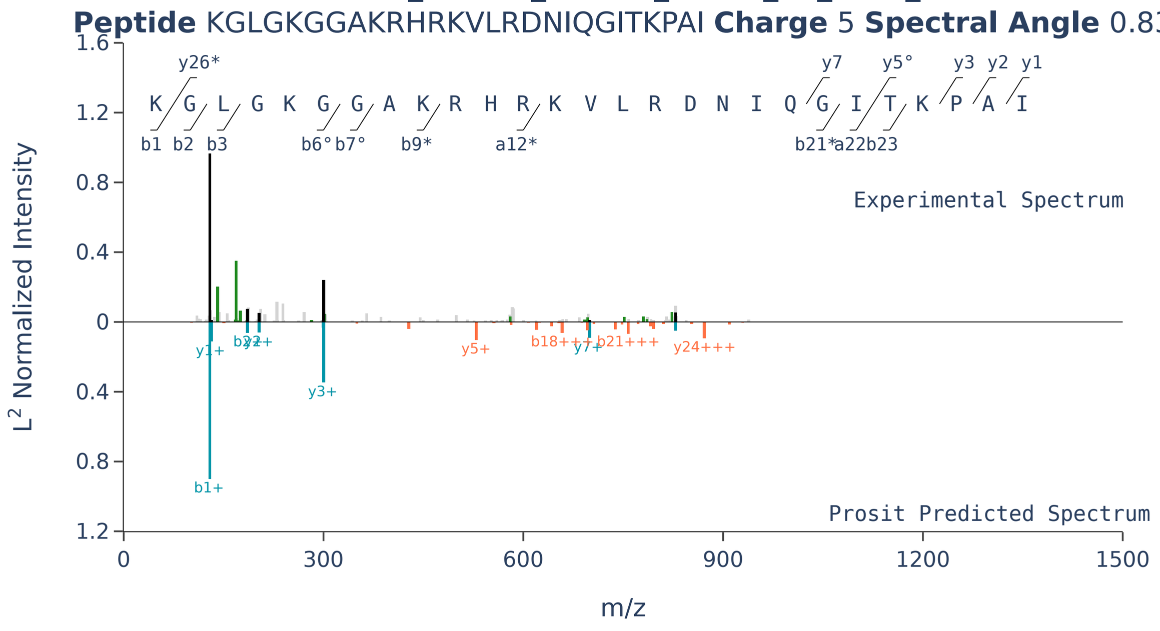

**Source** HRoetschke_291222_140123_HFGoe_H4_b2R1_1h **Scan** 26382
**Peptide** VTAMDVVYALKRQGRTLY **Charge** 4 **Spectral Angle** 0.68

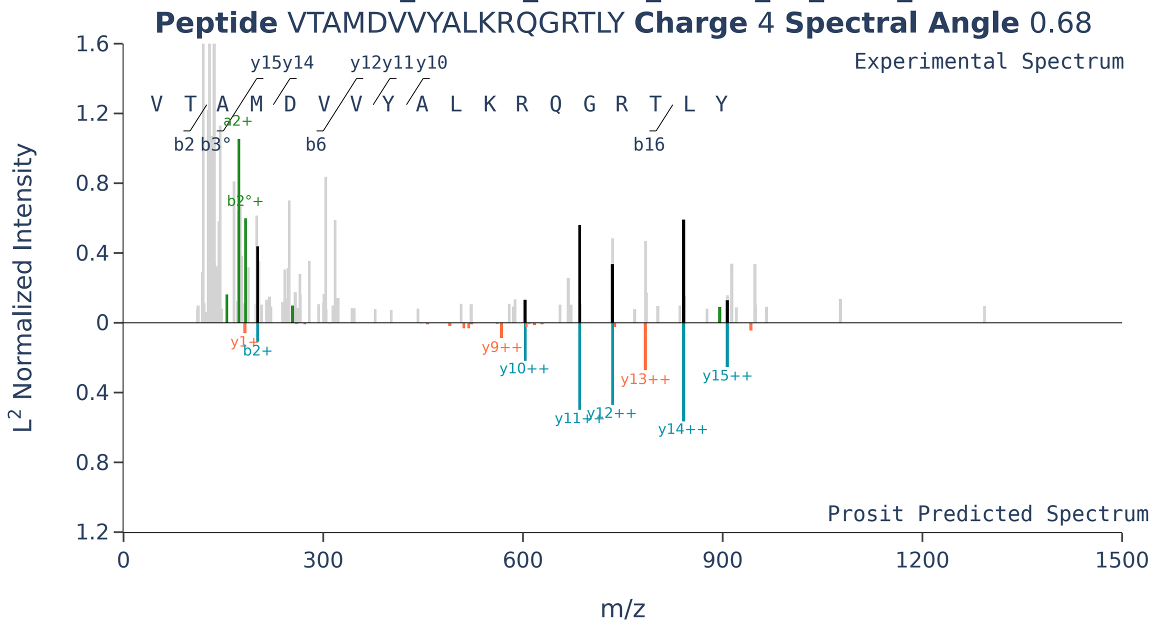


**Source** HRoetschke_291222_140123_HFGoe_H4_b2R1_1h **Scan** 25784
**Peptide** IYEETRGVLKVFLE **Charge** 2 **Spectral Angle** 0.62

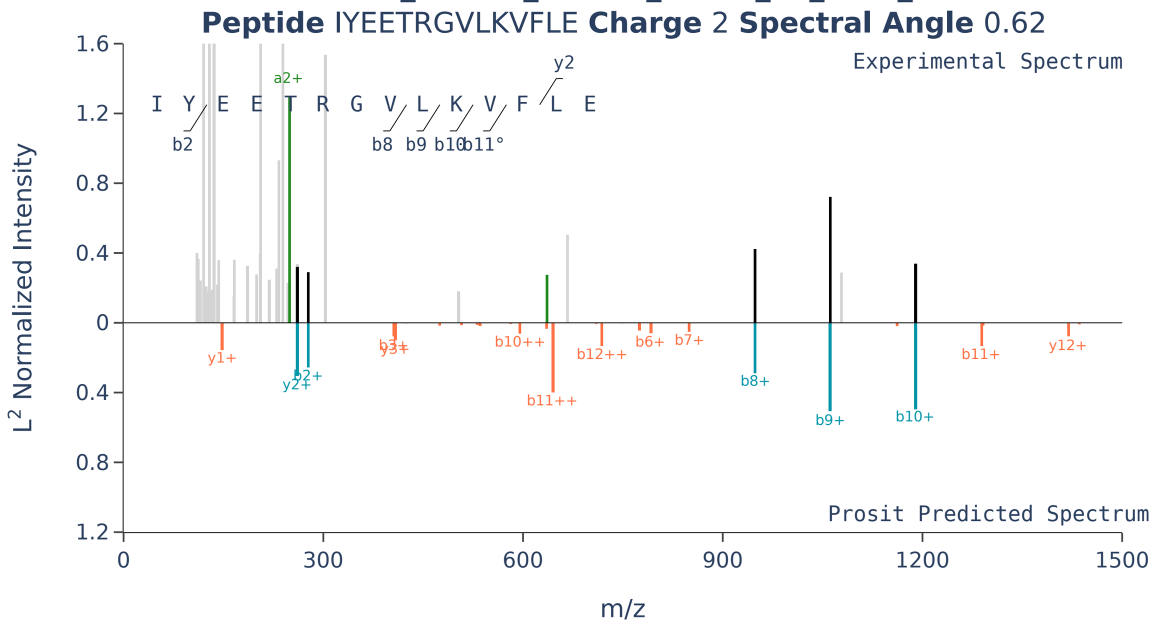


**Source** HRoetschke_291222_140123_HFGoe_H4_b2R1_1h **Scan** 27332
**Peptide** ARRGGVKRISGLIYEETRGVLKVFLEN **Charge** 5 **Spectral Angle** 0.74

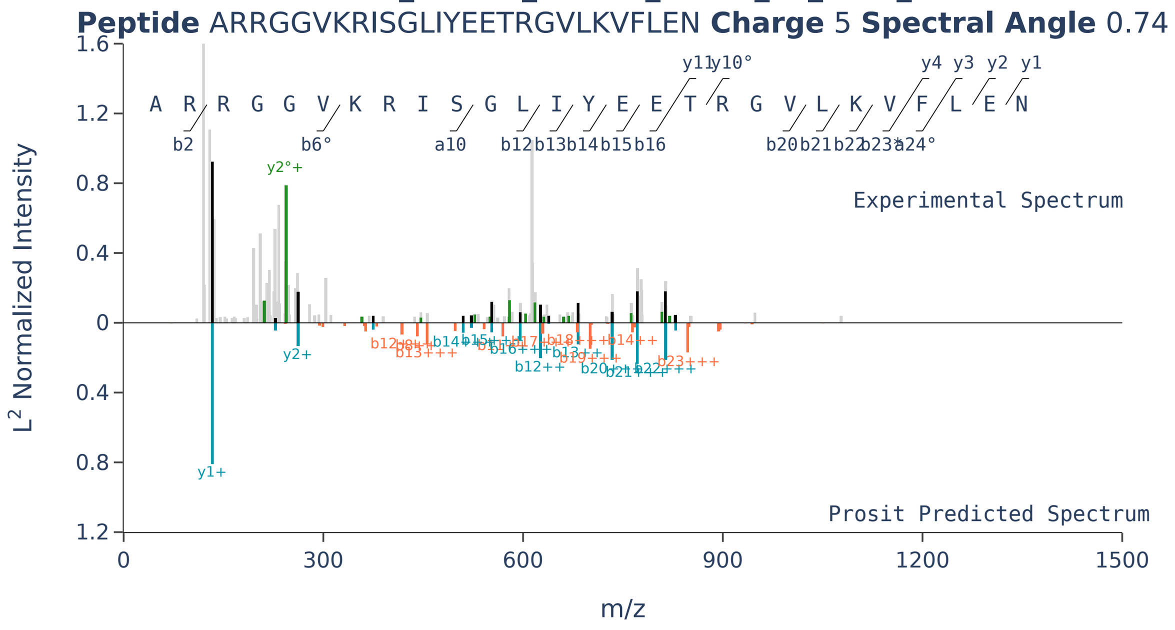


**Source** HRoetschke_291222_140123_HFGoe_H4_b2R1_1h **Scan** 10552
**Peptide** RKVLRDNIQGITKPAIRRLARRGGVKRL **Charge** 6 **Spectral Angle** 0.54

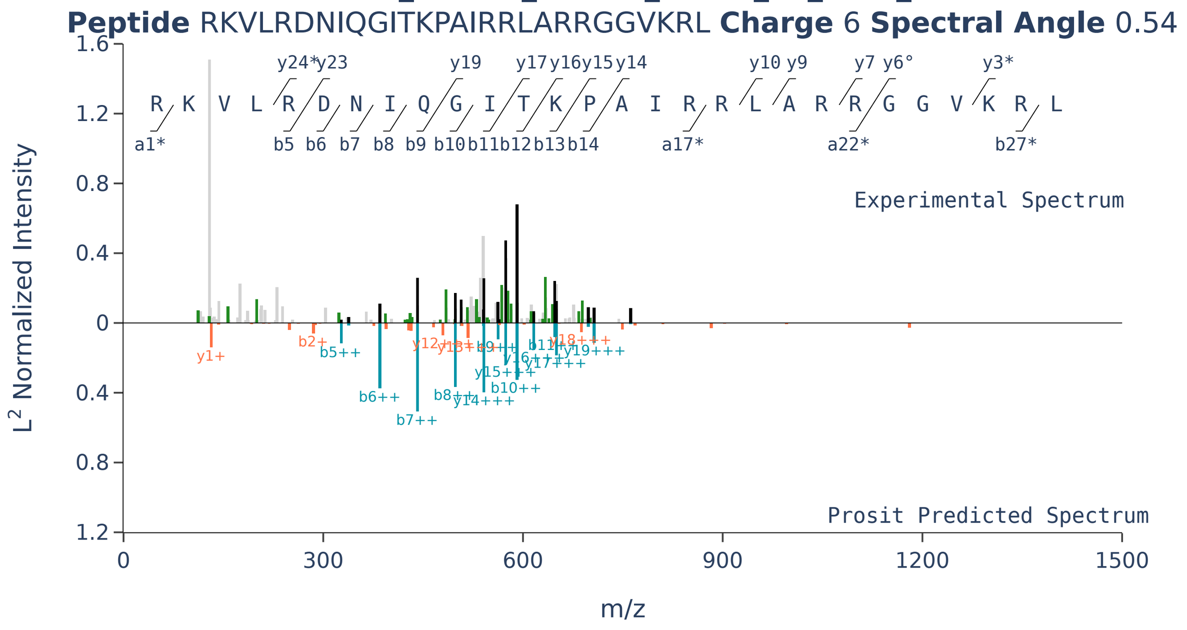


**Source** HRoetschke_291222_140123_HFGoe_H4_b2R2_1h **Scan** 16013
**Peptide** LQGITKPAIRRLARRGGVKRISGLIYEET **Charge** 4 **Spectral Angle** 0.63

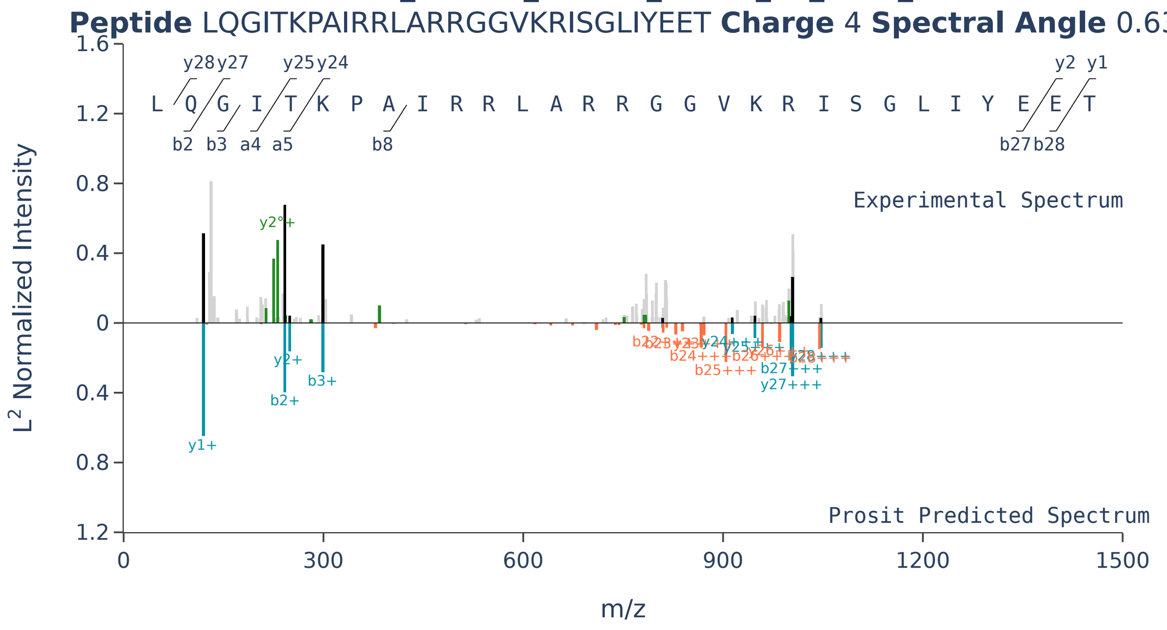


**Source** HRoetschke_291222_140123_HFGoe_H4_b1R2_2h **Scan** 10421
**Peptide** VLRDAVTYTEHAKRKTVTAMD **Charge** 5 **Spectral Angle** 0.85

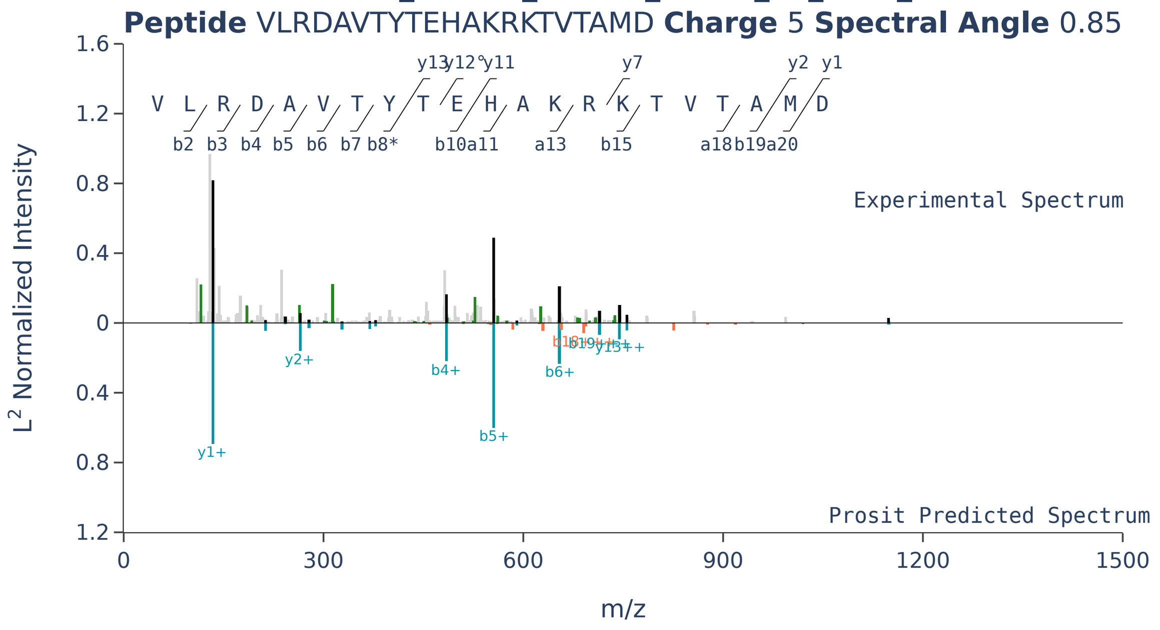


**Source** HRoetschke_291222_140123_HFGoe_H4_b1R1_1h **Scan** 12254
**Peptide** LRDNIQGITKPAIRRLARRGGVKRISG **Charge** 5 **Spectral Angle** 0.6

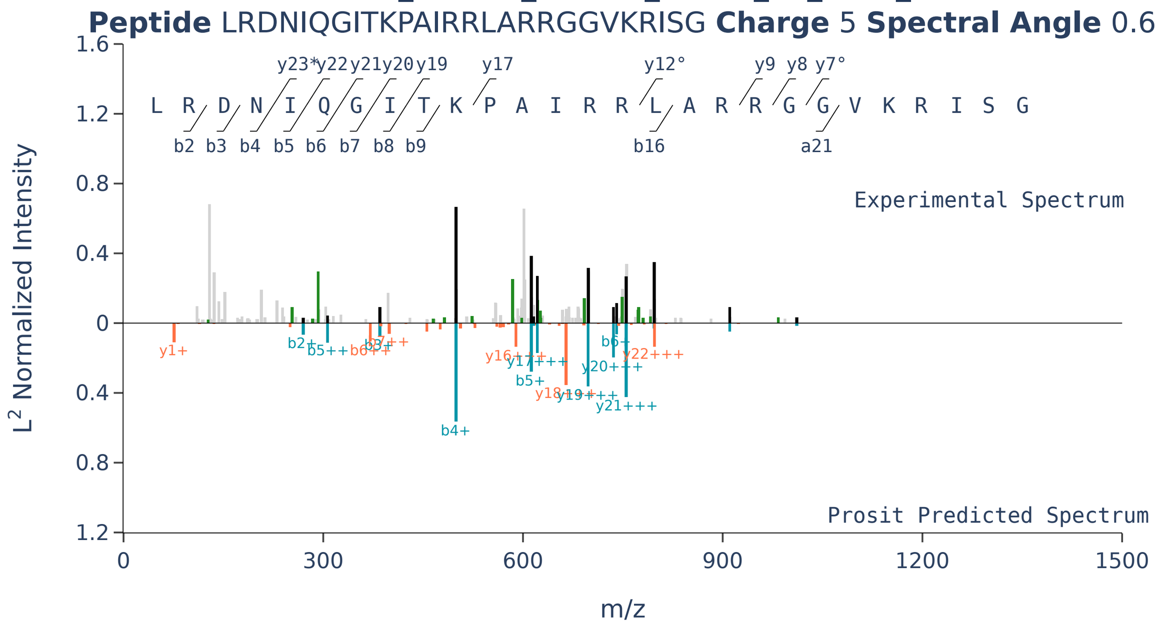


**Source** HRoetschke_291222_140123_HFGoe_H4_b1R2_24h **Scan** 8739
**Peptide** GKGGAKRHRKVLRDNIQGITKPAIRRL **Charge** 6 **Spectral Angle** 0.53

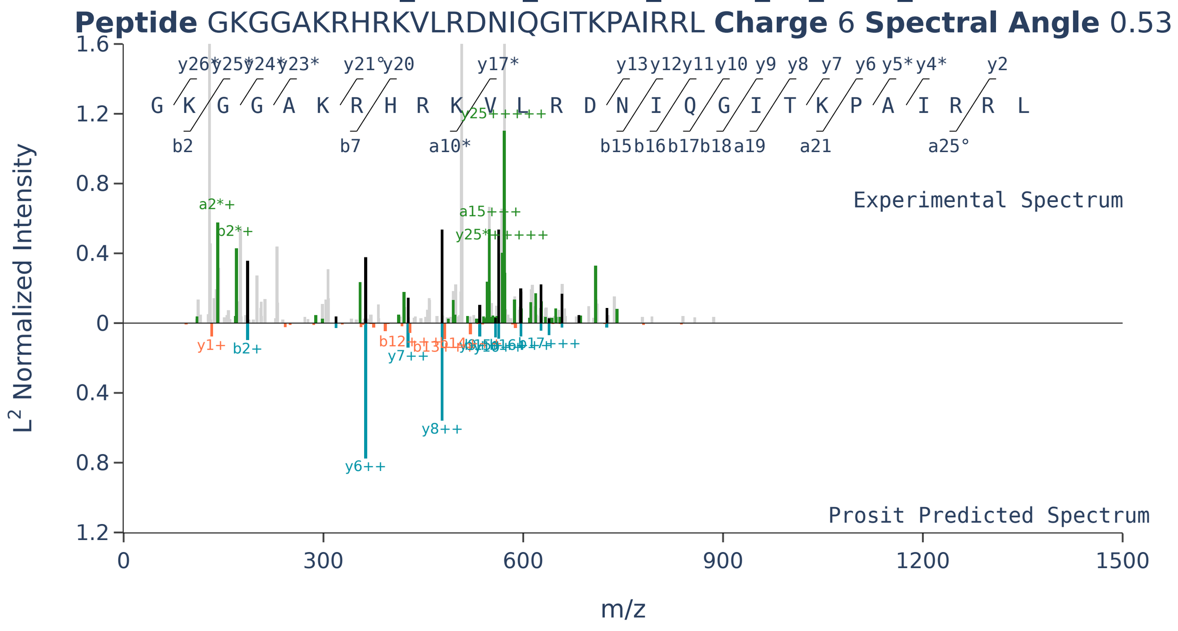


**Source** HRoetschke_291222_140123_HFGoe_H4_b2R1_24h **Scan** 21484
**Peptide** LSGLIYE **Charge** 1 **Spectral Angle** 0.8

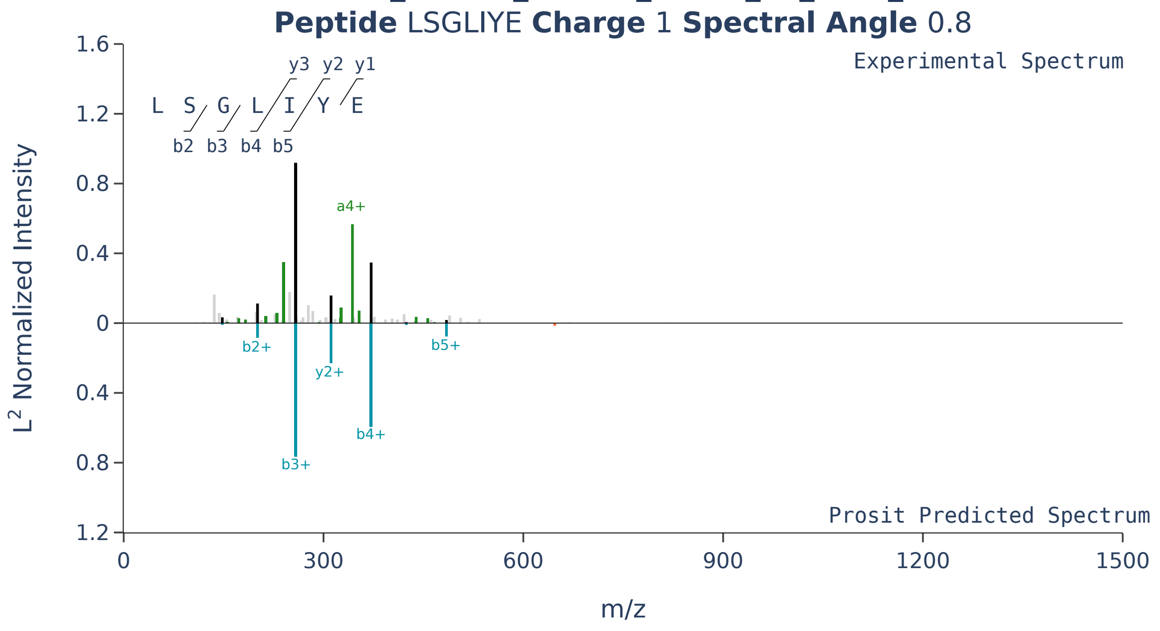


**Source** HRoetschke_291222_140123_HFGoe_H4_b1R2_1h **Scan** 10133
**Peptide** RDNIQGITKPAIRRLARRGGVKRISG **Charge** 6 **Spectral Angle** 0.76

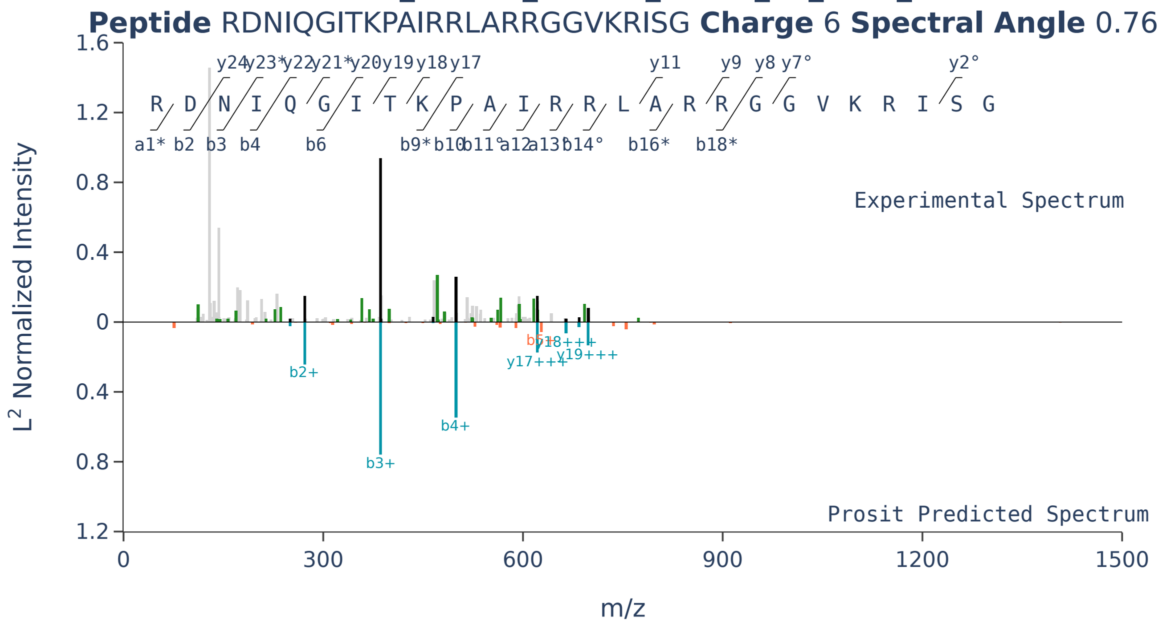


**Source** HRoetschke_291222_140123_HFGoe_H4_b2R2_2h **Scan** 10210
**Peptide** GLTKPAIRRLARRGGVKRI **Charge** 4 **Spectral Angle** 0.59

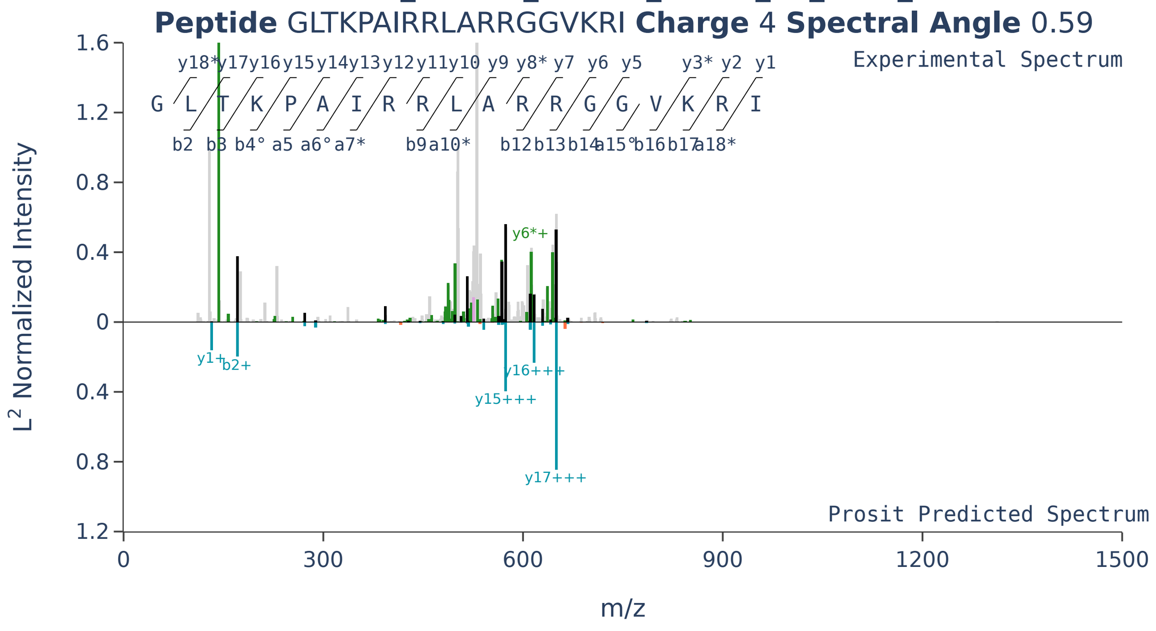


**Source** HRoetschke_291222_140123_HFGoe_H4_b1R1_24h **Scan** 5351
**Peptide** KGLGKGGAKRHRKVLRDNL **Charge** 6 **Spectral Angle** 0.64

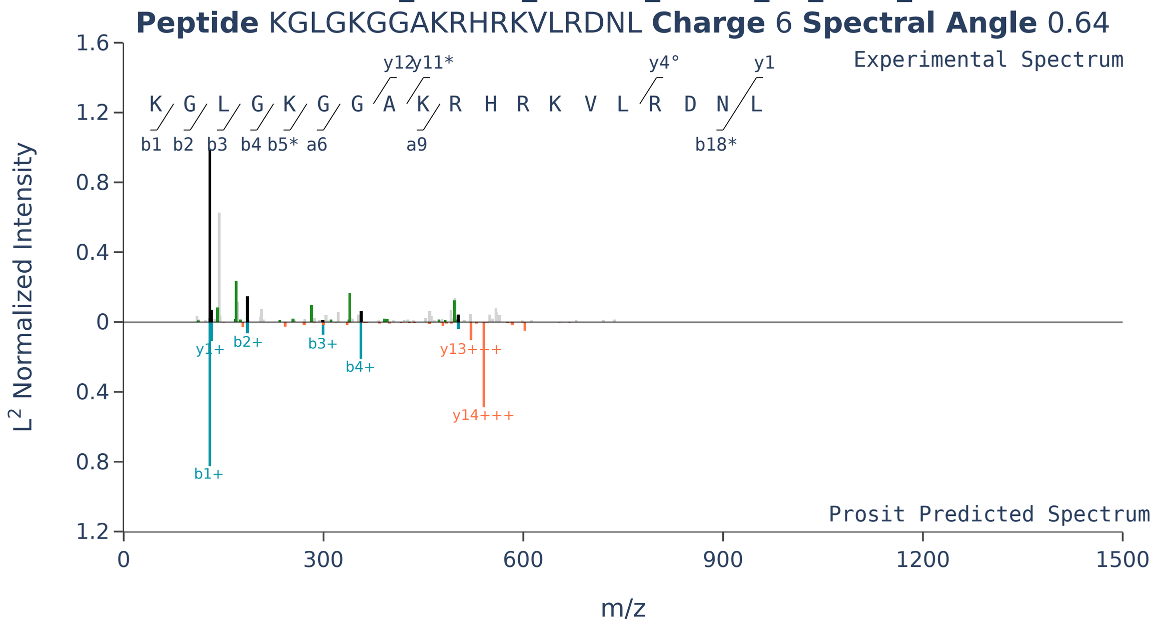


**Source** HRoetschke_291222_140123_HFGoe_H4_b1R1_4h **Scan** 11009
**Peptide** RRGGVKRISGLIY **Charge** 3 **Spectral Angle** 0.69

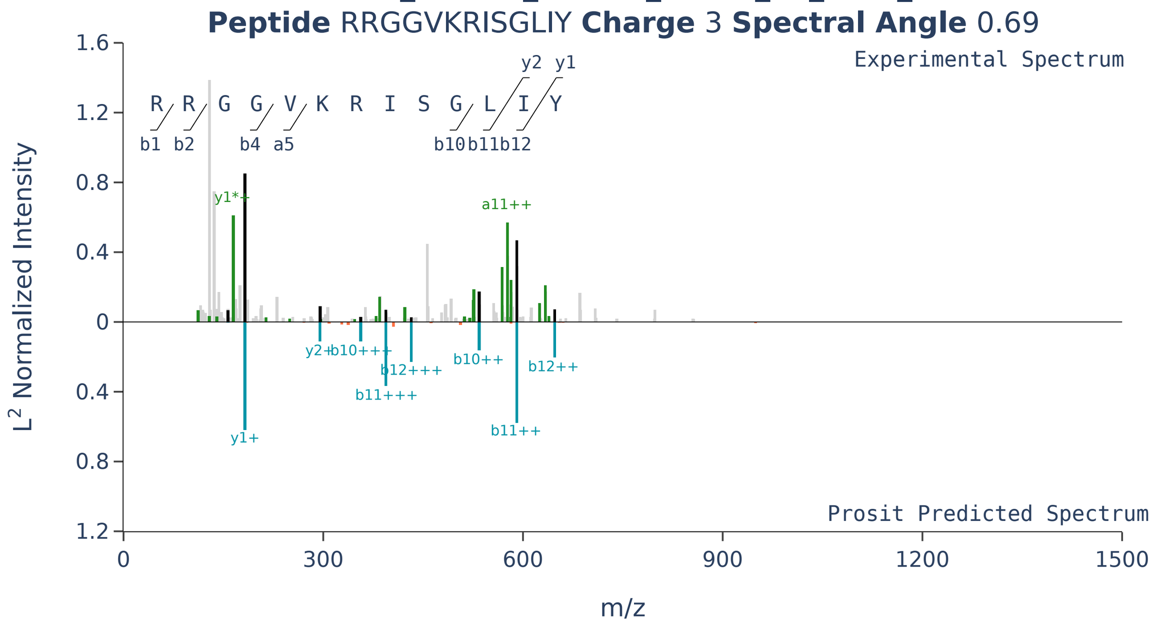


**Source** HRoetschke_291222_140123_HFGoe_H4_b1R2_4h **Scan** 6018
**Peptide** SGRGKGGKGLGKGGAKRHRKVLRDNIQGI **Charge** 5 **Spectral Angle** 0.41

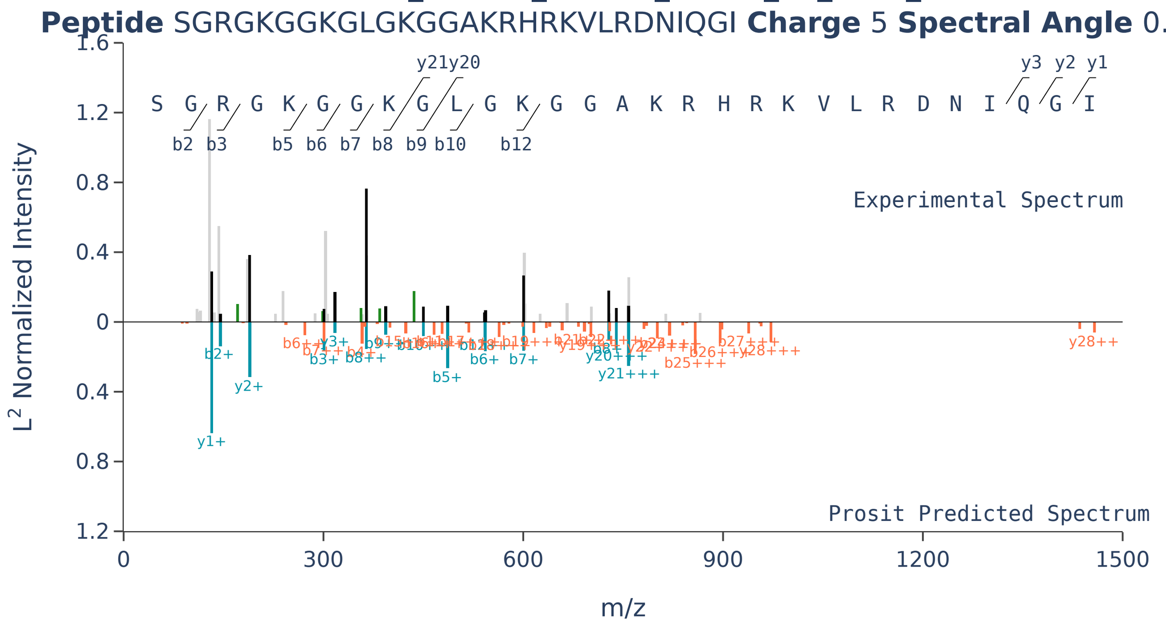


**Source** HRoetschke_291222_140123_HFGoe_H4_b1R1_2h **Scan** 6057
**Peptide** VTYTEHAKRK **Charge** 3 **Spectral Angle** 0.77

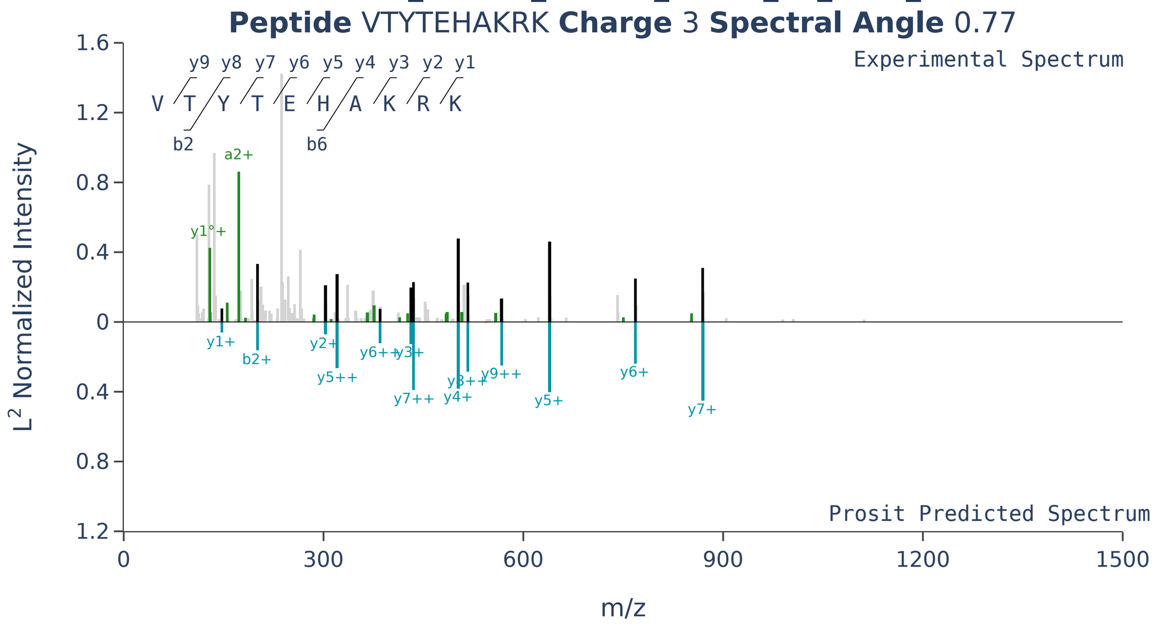

**Source** HRoetschke_291222_140123_HFGoe_H4_b1R2_4h **Scan** 13278
**Peptide** QGITKPAIRRLARRGGVKRISGLL **Charge** 4 **Spectral Angle** 0.6

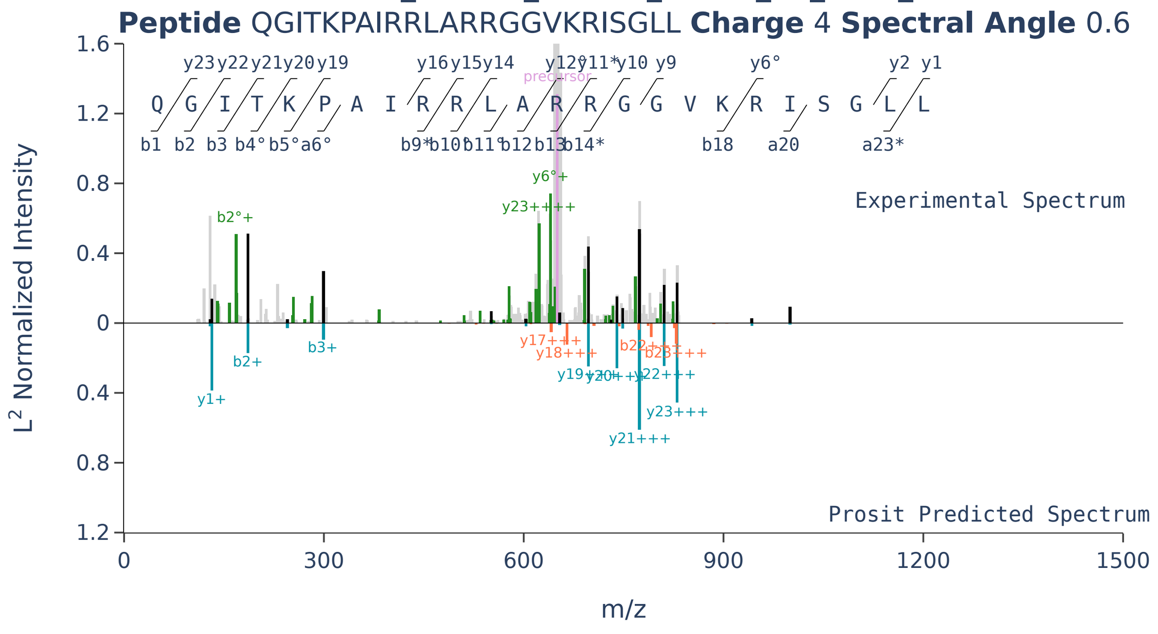


**Source** HRoetschke_291222_140123_HFGoe_H4_b1R1_24h **Scan** 4903
**Peptide** KGLGKGGAKRHRKV **Charge** 3 **Spectral Angle** 0.34

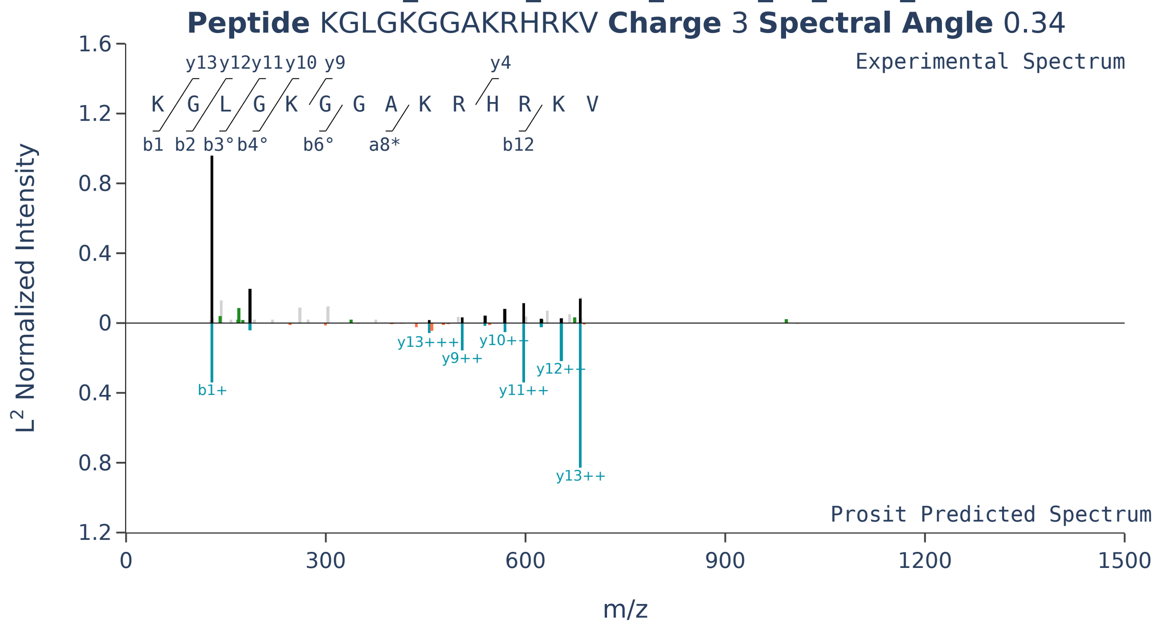


**Source** HRoetschke_291222_140123_HFGoe_H4_b1R1_4h **Scan** 10710
**Peptide** LARRGGVKRISGLI **Charge** 3 **Spectral Angle** 0.71

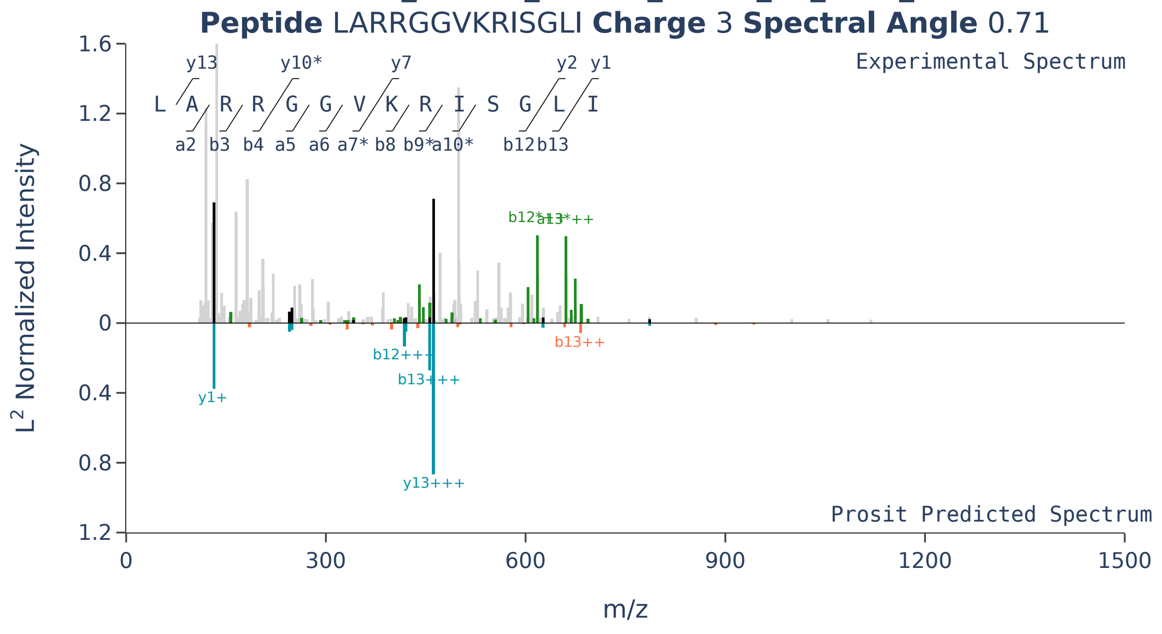


**Source** HRoetschke_291222_140123_HFGoe_H4_b2R1_1h **Scan** 35888
**Peptide** GGVKRISGLIYEETRGVLKVFLENVIRD **Charge** 5 **Spectral Angle** 0.75

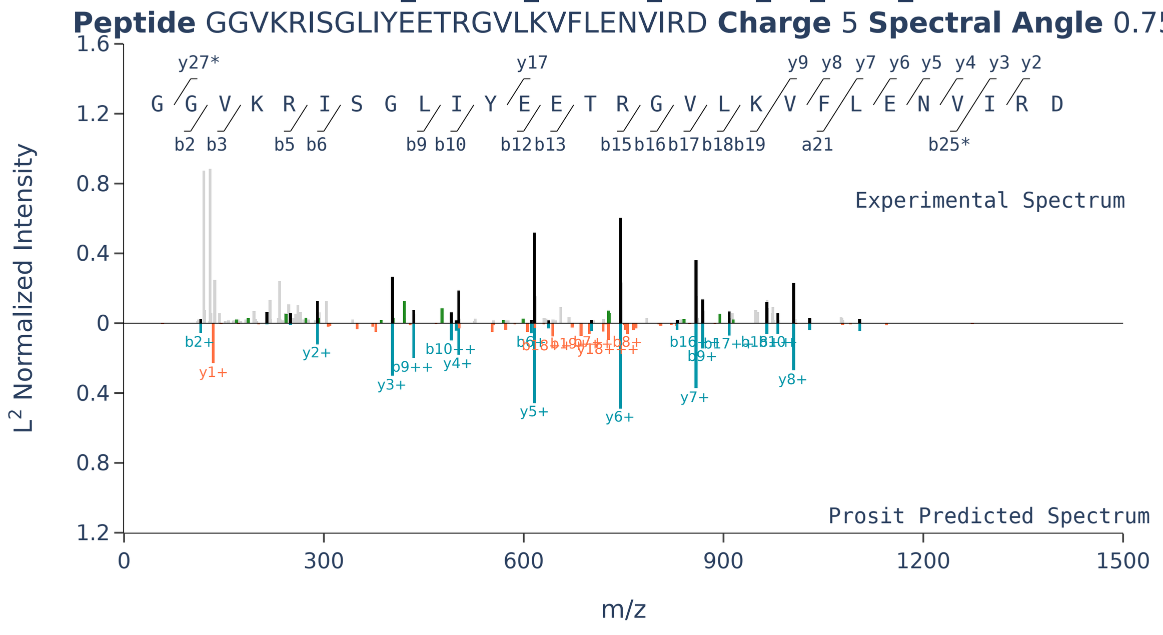


**Source** HRoetschke_291222_140123_HFGoe_H4_b2R1_1h **Scan** 35682
**Peptide** VKRISGLIYEETRGVLKVFLENVI **Charge** 4 **Spectral Angle** 0.75

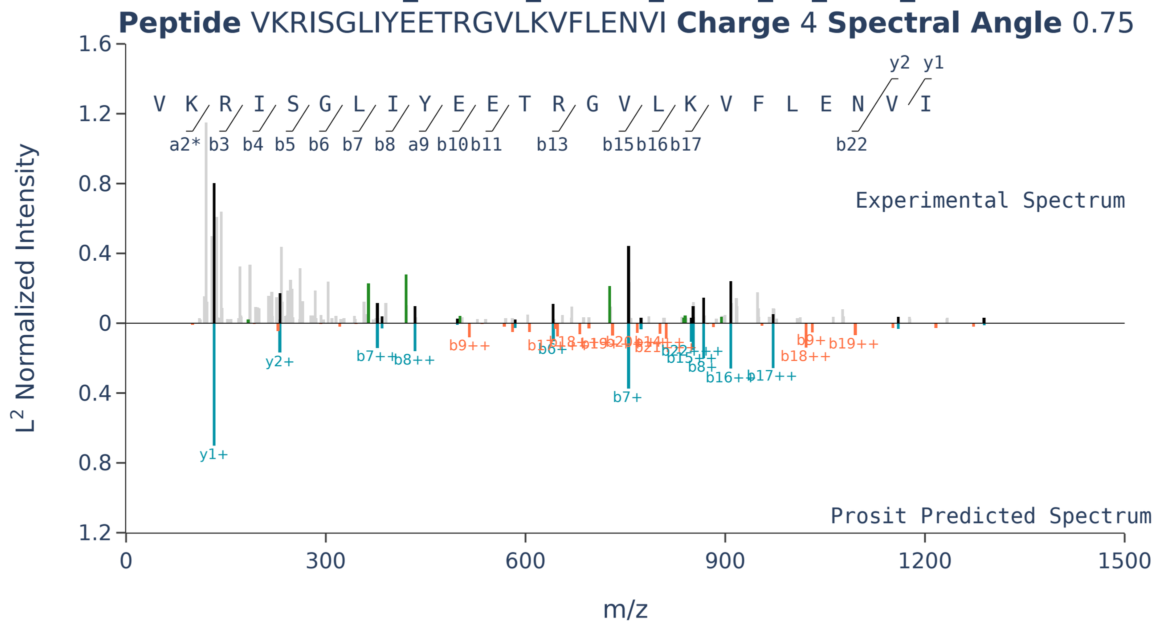


**Source** HRoetschke_291222_140123_HFGoe_H4_b1R2_24h **Scan** 5077
**Peptide** GGKGLGKGGAKRHRKVL **Charge** 3 **Spectral Angle** 0.68

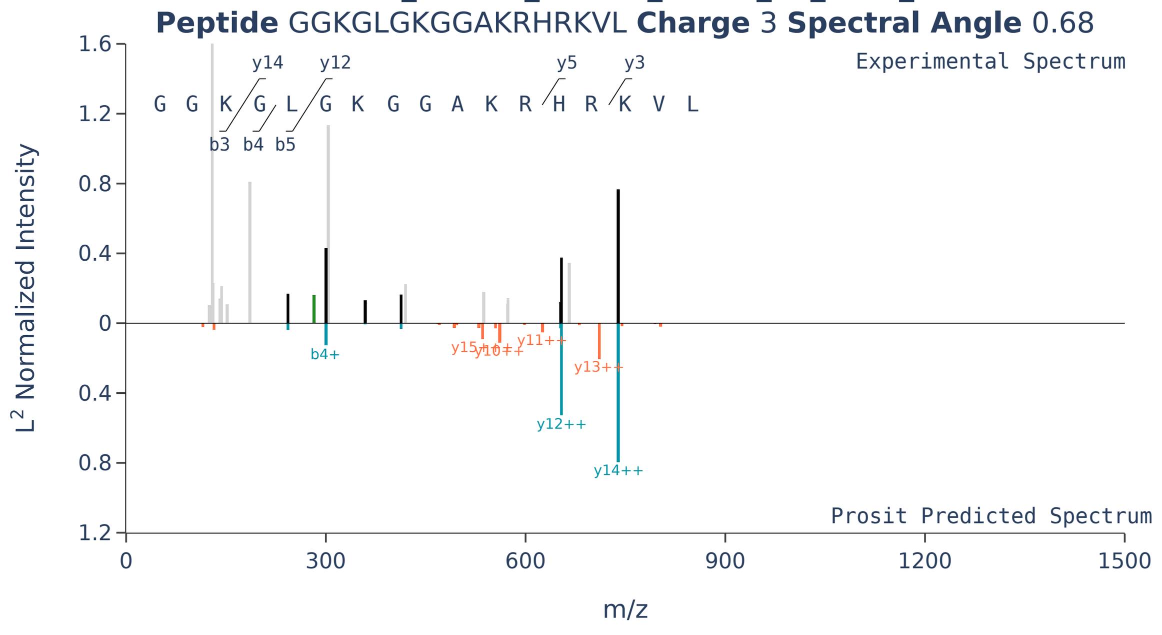


**Source** HRoetschke_291222_140123_HFGoe_H4_b2R1_1h **Scan** 21931
**Peptide** TEHAKRKTVTAMDVVYALKRQGRTLYGFGG **Charge** 4 **Spectral Angle** 0.55

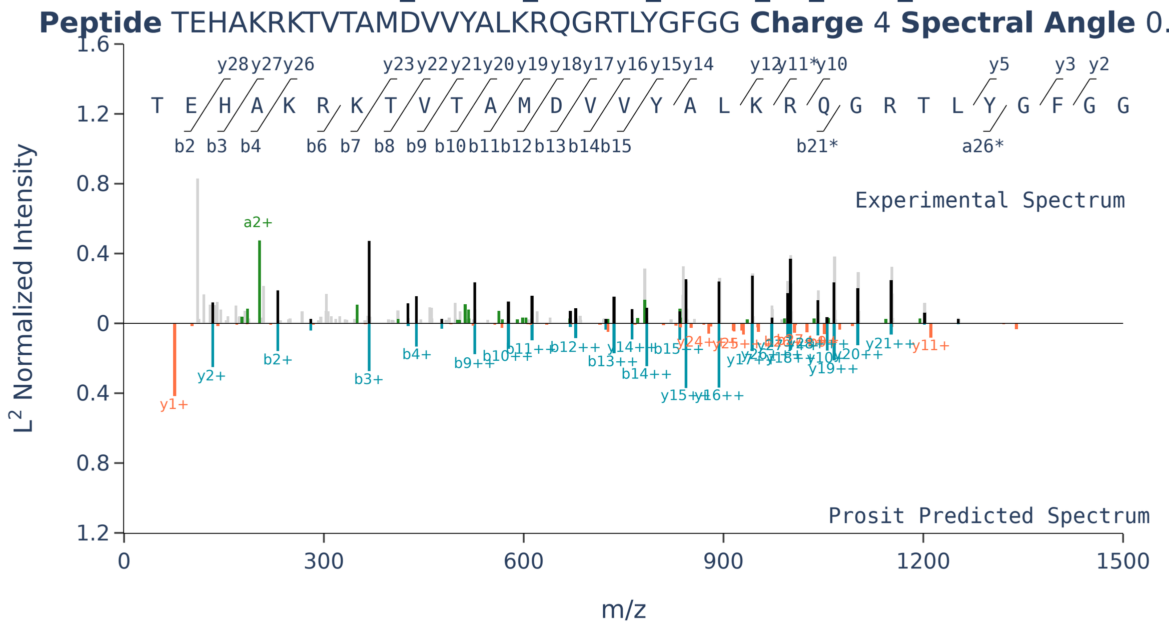


**Source** HRoetschke_291222_140123_HFGoe_H4_b1R1_4h **Scan** 27041
**Peptide** TRGVLKVFLENVLRD **Charge** 3 **Spectral Angle** 0.56

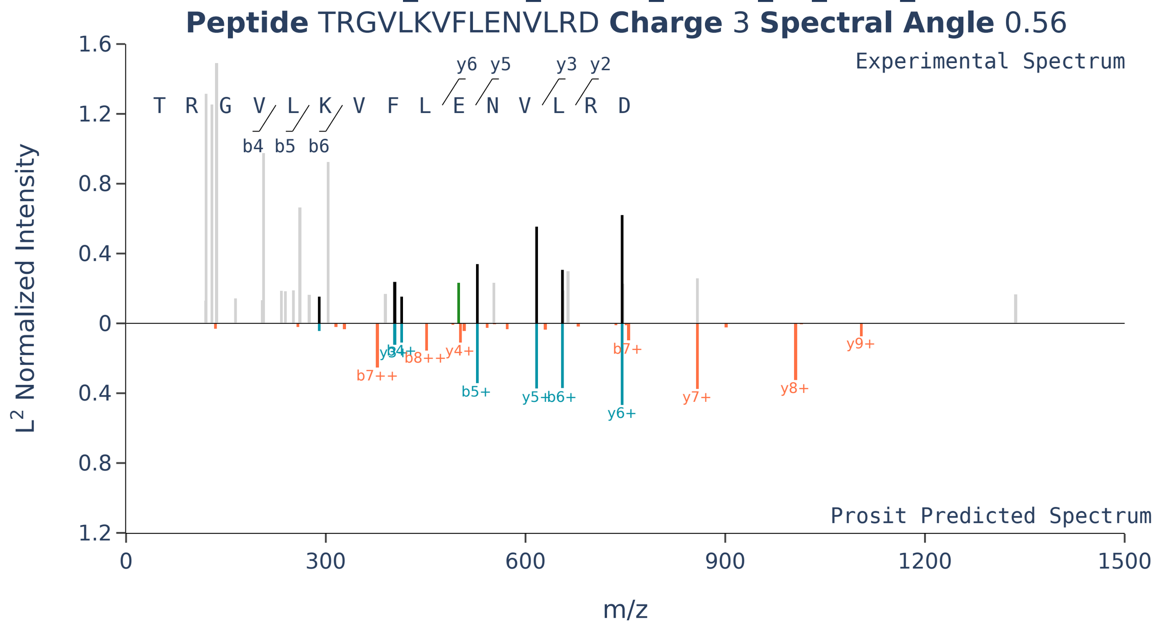


**Source** HRoetschke_291222_140123_HFGoe_H4_b2R1_1h **Scan** 32756
**Peptide** TRGVLKVFLENVIRDAVTYTEHAKR **Charge** 6 **Spectral Angle** 0.64

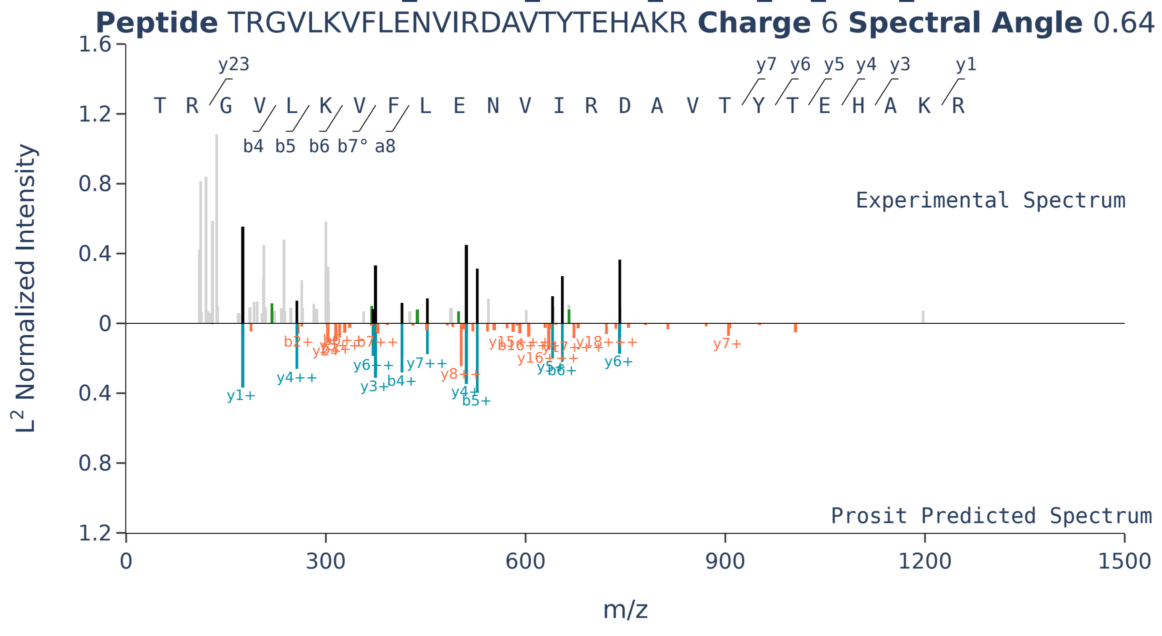


**Source** HRoetschke_291222_140123_HFGoe_H4_b2R1_1h **Scan** 35221
**Peptide** GVKRISGLIYEETRGVLKVFLENVIRD **Charge** 4 **Spectral Angle** 0.74

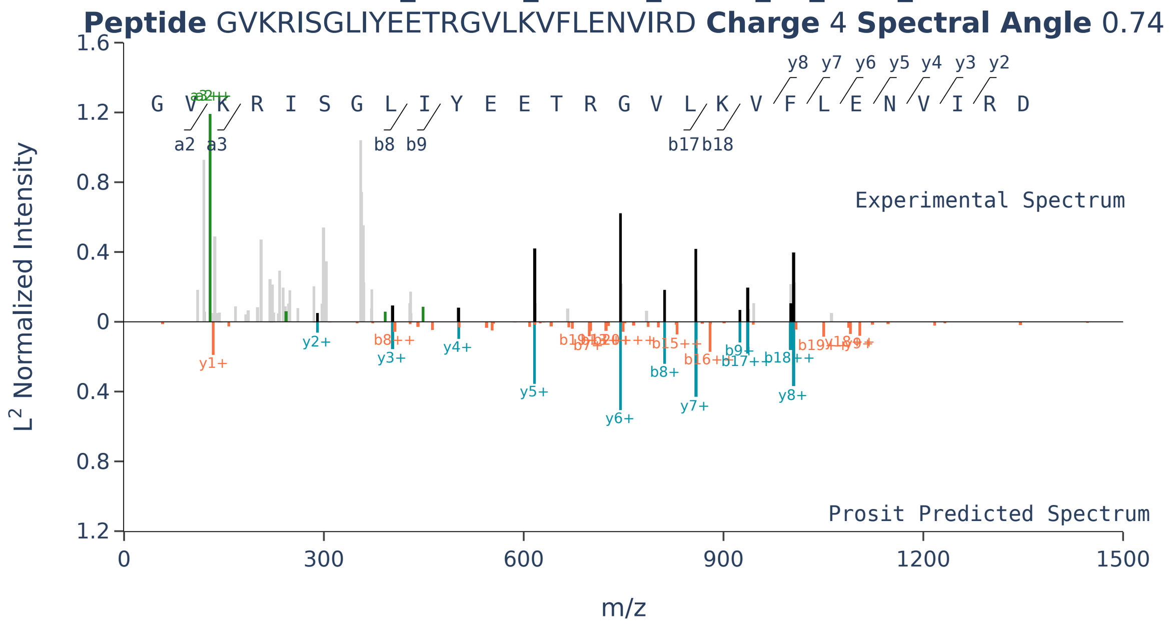


**Source** HRoetschke_291222_140123_HFGoe_H4_b1R1_24h **Scan** 11285
**Peptide** VTYTEHAKRKTVTAMDVV **Charge** 4 **Spectral Angle** 0.68

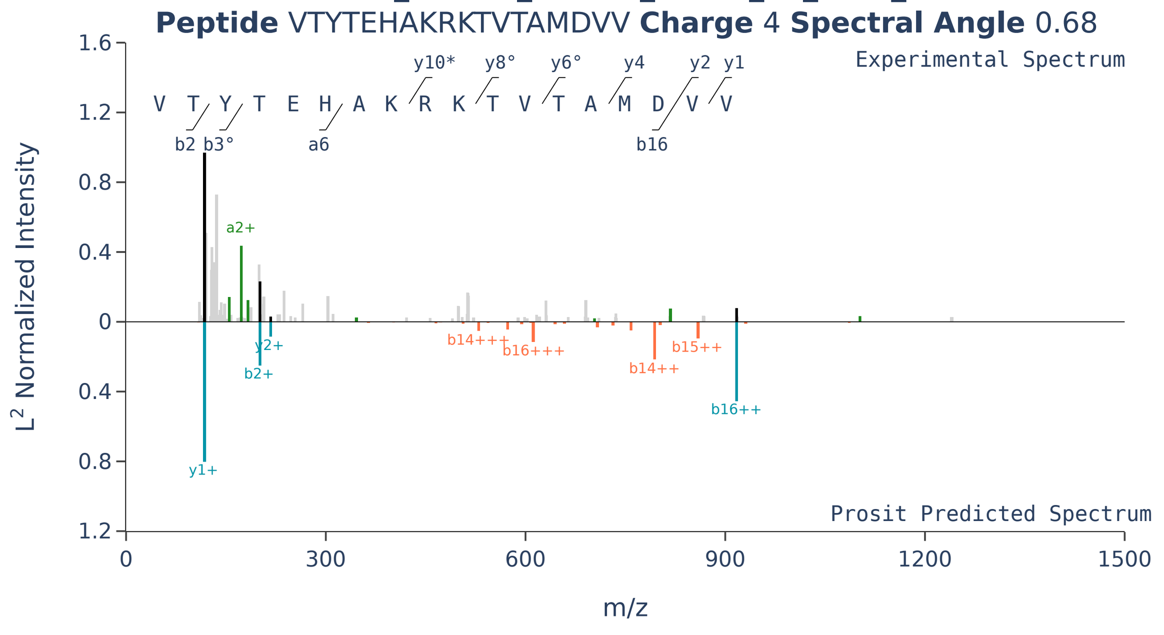


**Source** HRoetschke_291222_140123_HFGoe_H4_b1R1_4h **Scan** 5308
**Peptide** AIRRLARRGGVKR **Charge** 4 **Spectral Angle** 0.76

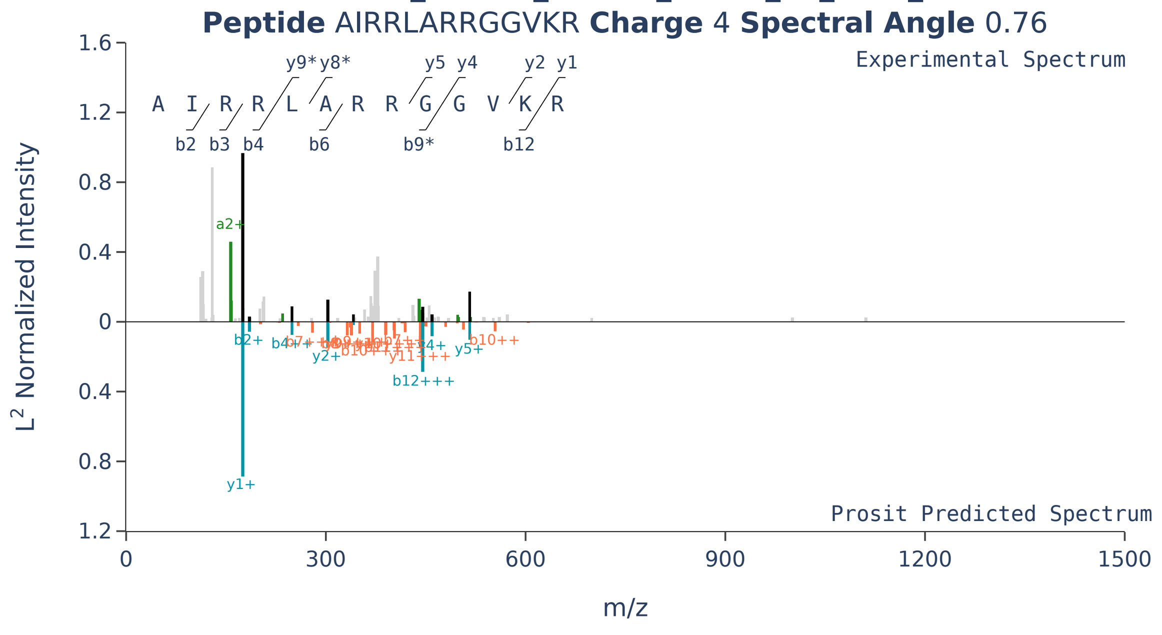


**Source** HRoetschke_291222_140123_HFGoe_H4_b2R1_4h **Scan** 6651
**Peptide** GLTKPAIRRLARRGGVK **Charge** 4 **Spectral Angle** 0.79

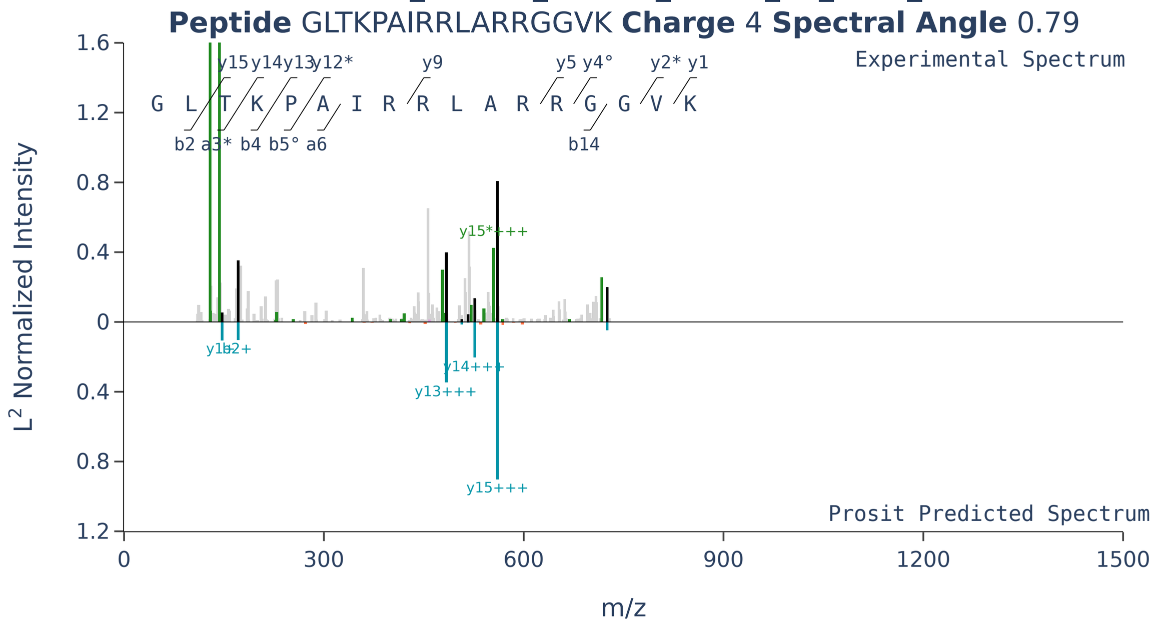


**Source** HRoetschke_291222_140123_HFGoe_H4_b2R1_2h **Scan** 11997
**Peptide** LRDNIQGITKPAIRRLARRGG **Charge** 6 **Spectral Angle** 0.7

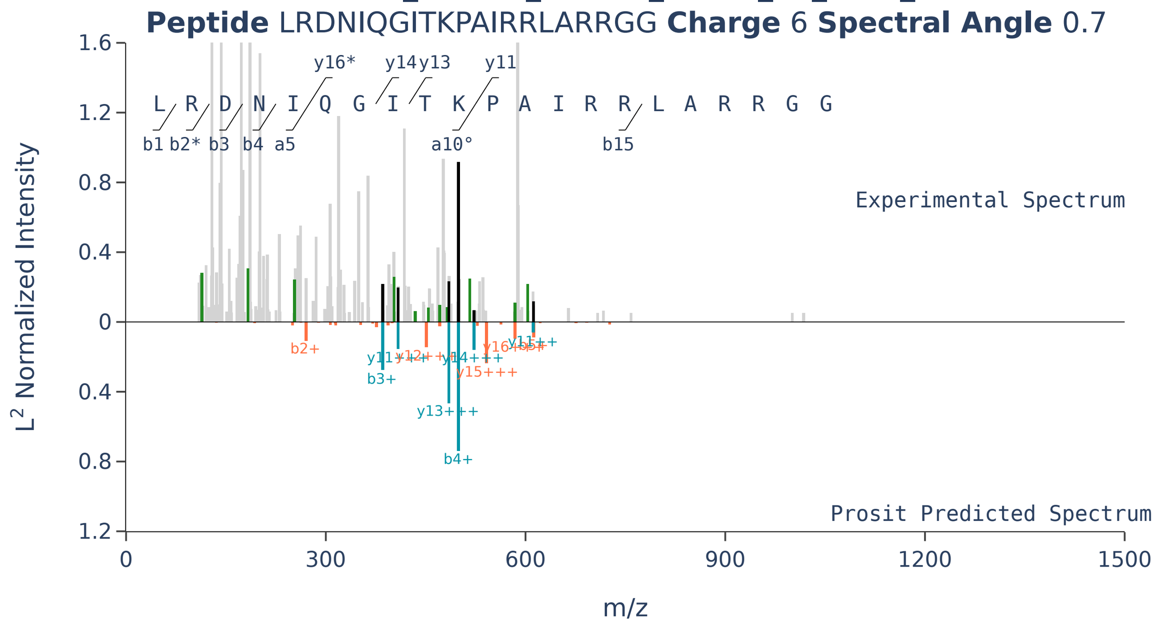


**Source** HRoetschke_291222_140123_HFGoe_H4_b2R1_1h **Scan** 23633
**Peptide** FLENVIRDAVTYTEHAKRKTVTAM **Charge** 4 **Spectral Angle** 0.5

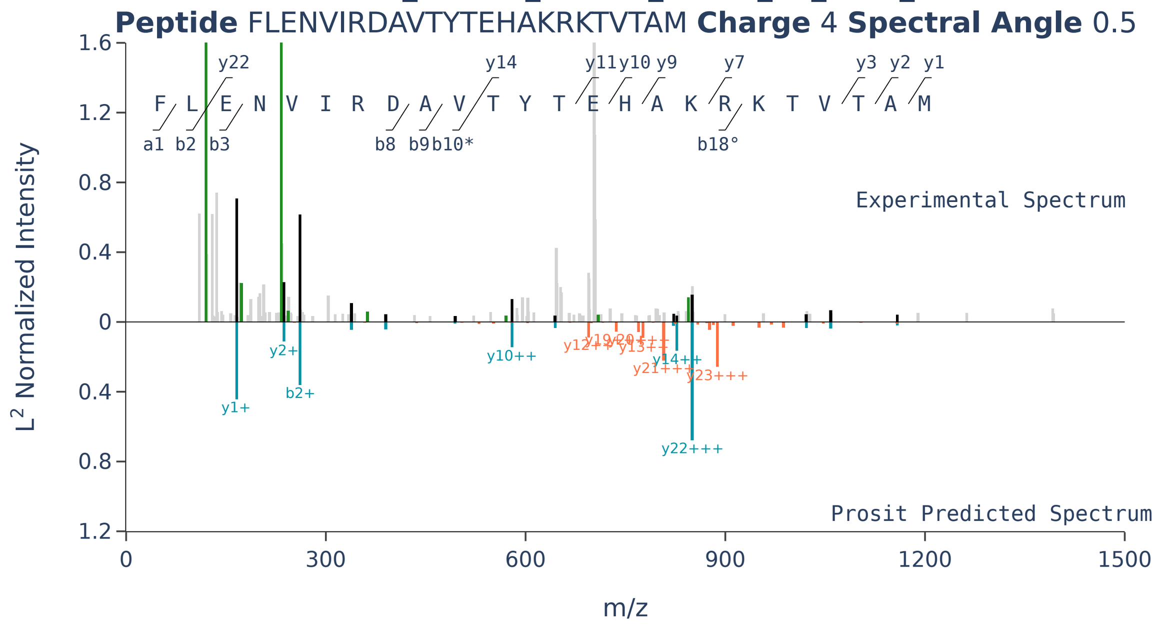

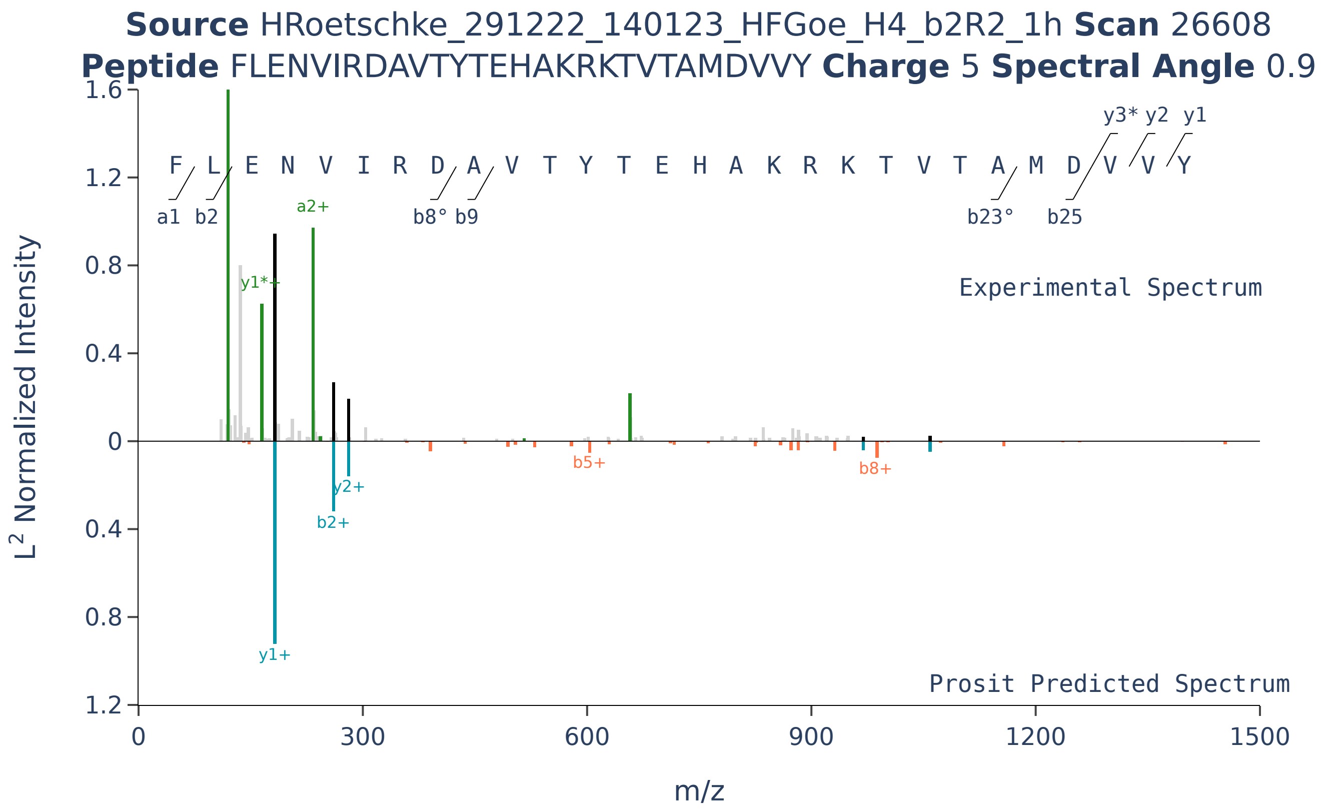
**Source** HRoetschke_291222_140123_HFGoe_H4_b2R2_1h **Scan** 26608
**Peptide** FLENVIRDAVTYTEHAKRKTVTAMDVVY **Charge** 5 **Spectral Angle** 0.9
Experimental Spectrum
Prosit Predicted Spectrum
$L^2$ Normalized Intensity
m/z

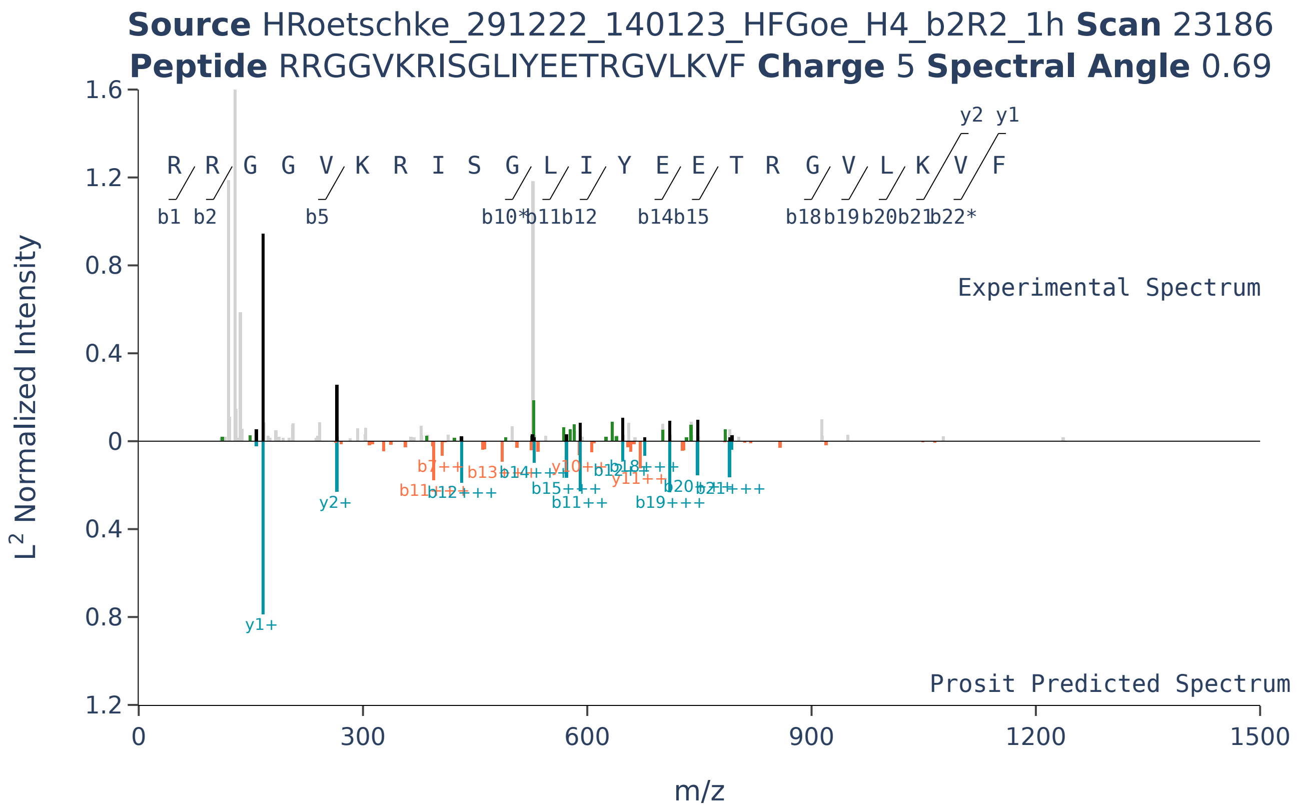
**Source** HRoetschke_291222_140123_HFGoe_H4_b2R2_1h **Scan** 23186
**Peptide** RRGGVKRISGLIYEETRGVLKVF **Charge** 5 **Spectral Angle** 0.69
Experimental Spectrum
Prosit Predicted Spectrum
$L^2$ Normalized Intensity
m/z

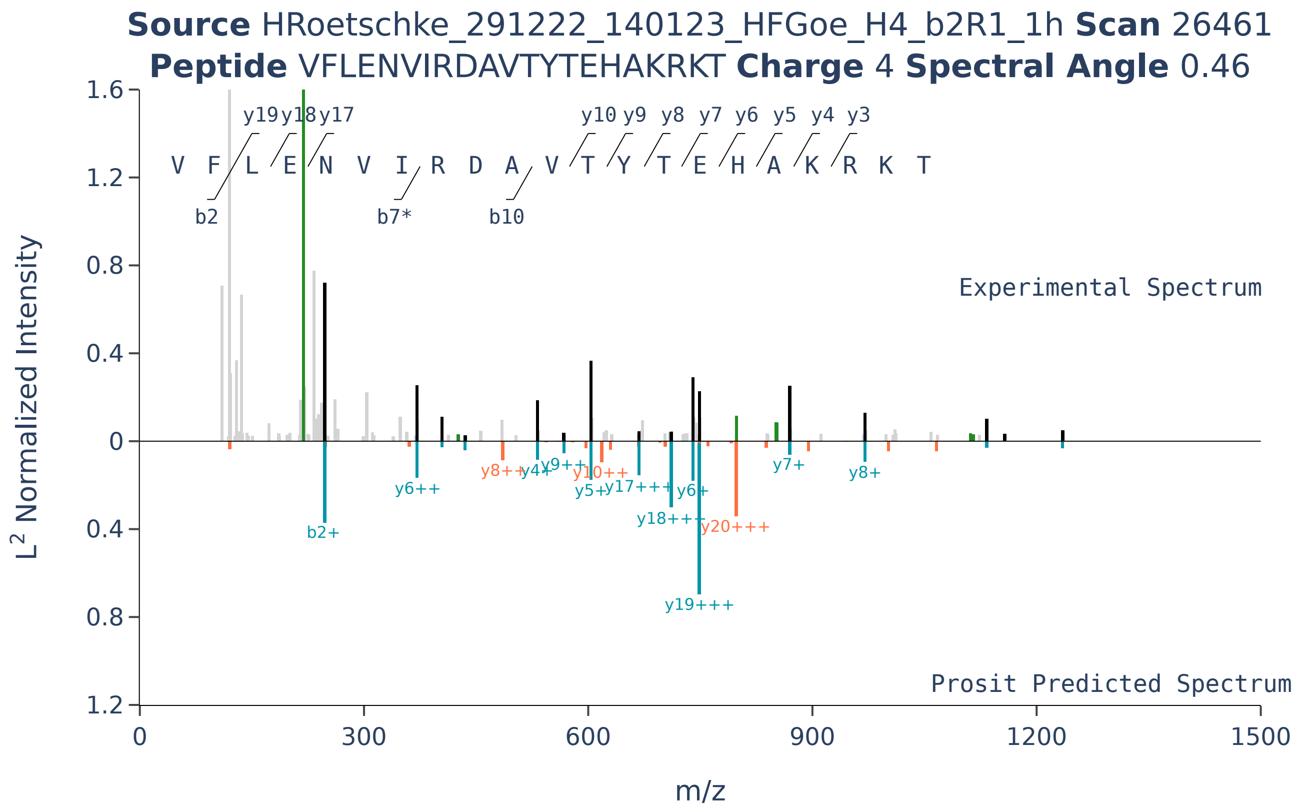
**Source** HRoetschke_291222_140123_HFGoe_H4_b2R1_1h **Scan** 26461
**Peptide** VFLENVIRDAVTYTEHAKRKT **Charge** 4 **Spectral Angle** 0.46
Experimental Spectrum
Prosit Predicted Spectrum
$L^2$ Normalized Intensity
m/z

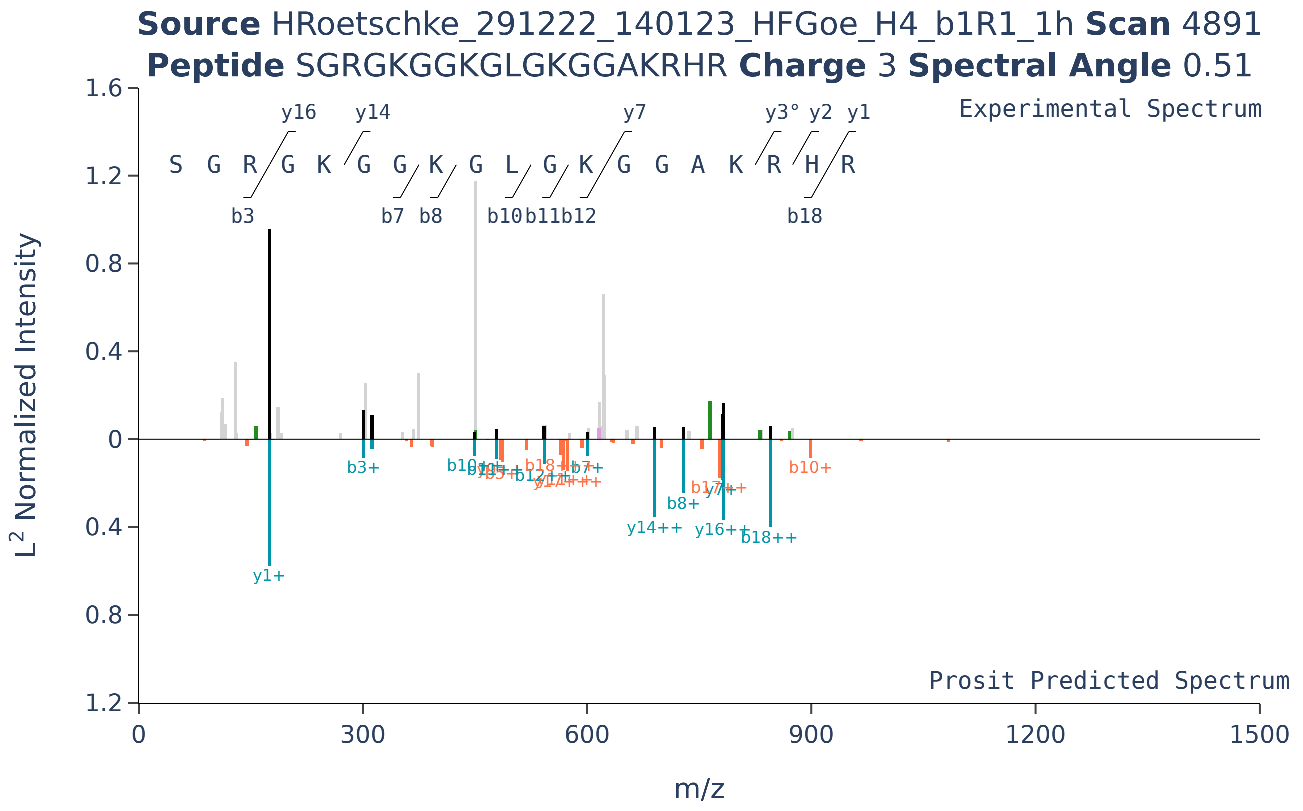
**Source** HRoetschke_291222_140123_HFGoe_H4_b1R1_1h **Scan** 4891
**Peptide** SGRGKGGKGLGKGGAKRHR **Charge** 3 **Spectral Angle** 0.51
Experimental Spectrum
Prosit Predicted Spectrum
$L^2$ Normalized Intensity
m/z

**Source** WSoh_221121_011221_HFGoe_L2_hTau_24h_R2 **Scan** 15518
**Peptide** ATLADEVS **Charge** 1 **Spectral Angle** 0.96

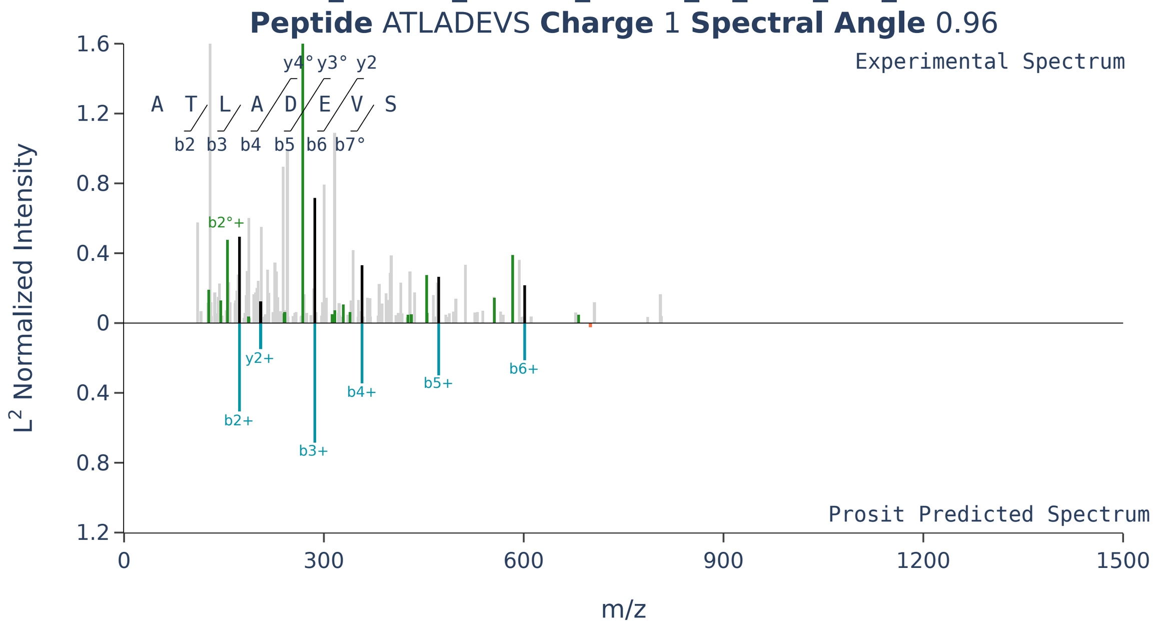


**Source** WSoh_221121_011221_HFGoe_L1_hTau_4h_R1 **Scan** 8551
**Peptide** HGAEIVYKS **Charge** 1 **Spectral Angle** 0.96

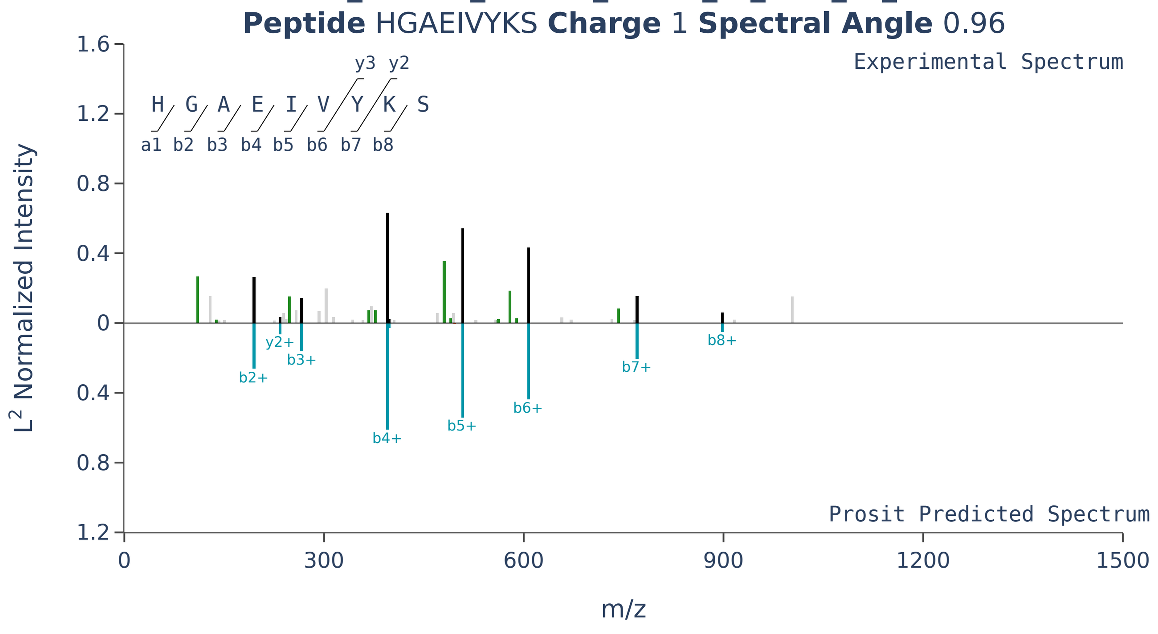


**Source** WSoh_221121_011221_HFGoe_L1_hTau_4h_R1 **Scan** 8731
**Peptide** SGYSSPGSPGTPGSR **Charge** 2 **Spectral Angle** 0.97

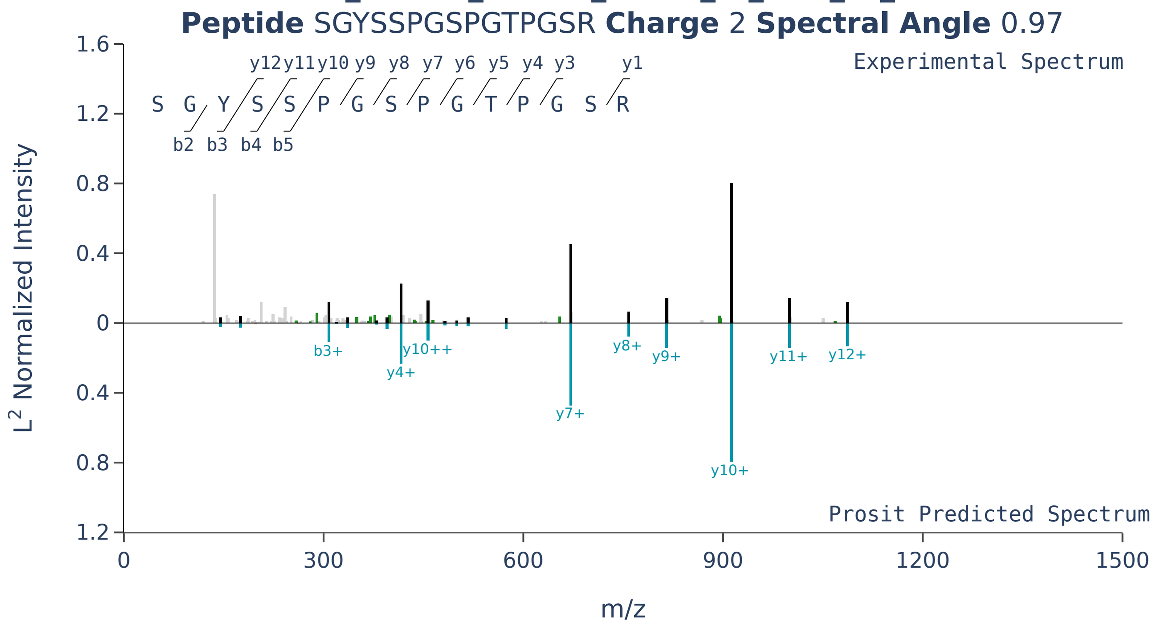


**Source** WSoh_221121_011221_HFGoe_L2_hTau_4h_R1 **Scan** 16168
**Peptide** DHGAEIVY **Charge** 1 **Spectral Angle** 0.94

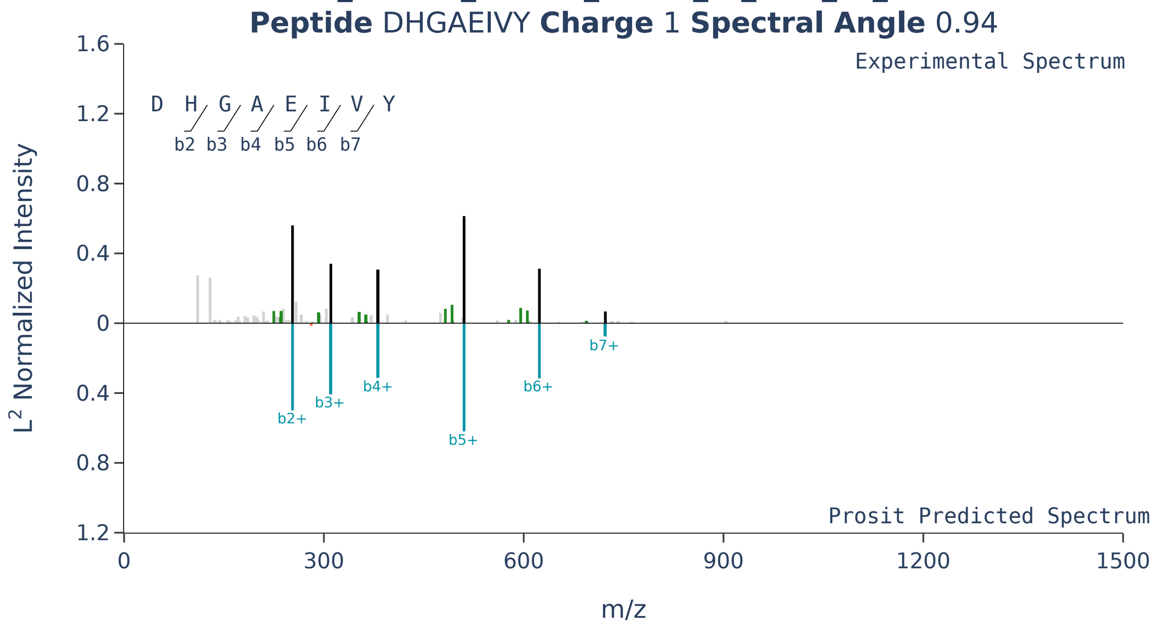


**Source** WSoh_221121_011221_HFGoe_L2_hTau_24h_R1 **Scan** 8366
**Peptide** YSSPGSPGTPGSR **Charge** 2 **Spectral Angle** 0.95

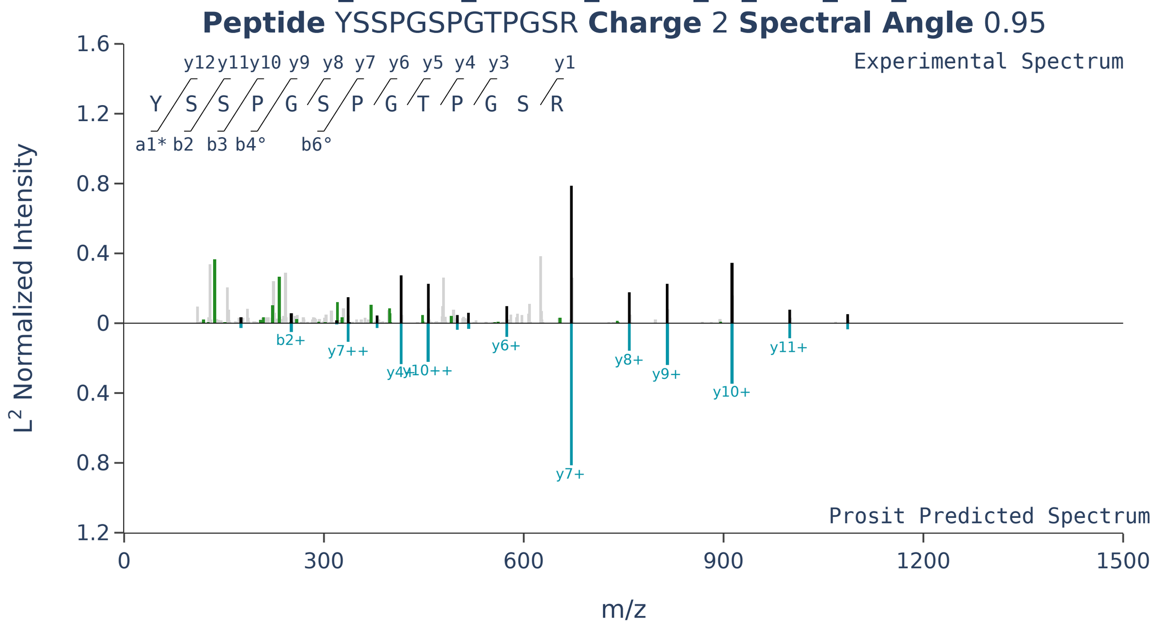


**Source** WSoh_221121_011221_HFGoe_L2_hTau_2h_R1 **Scan** 13671
**Peptide** SPLQTPTED **Charge** 1 **Spectral Angle** 0.92

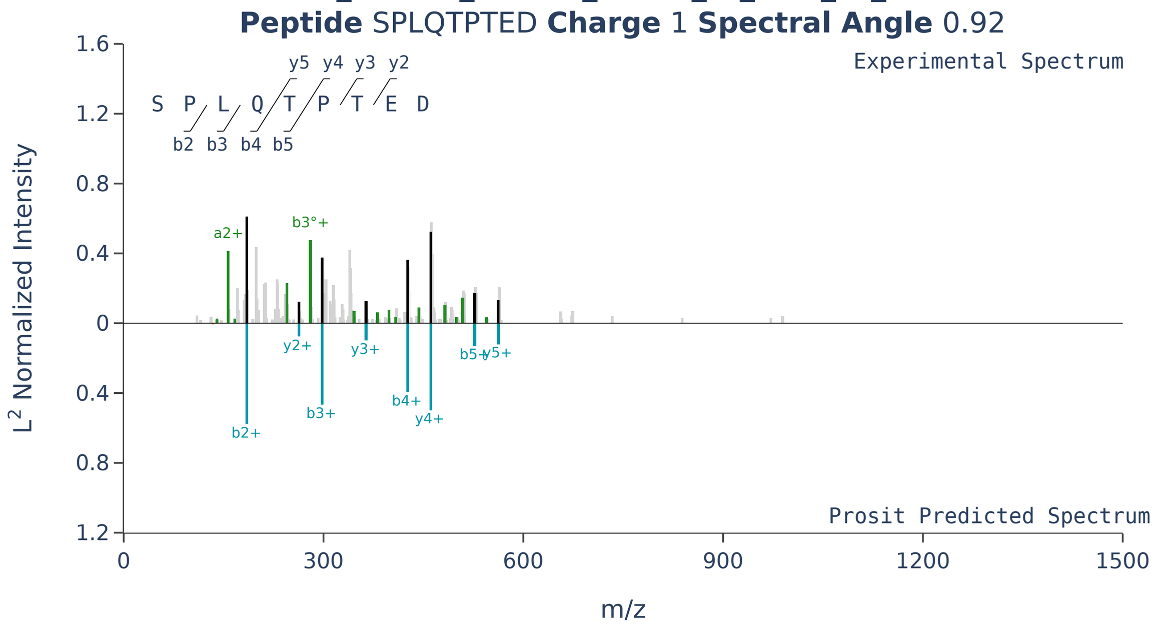


**Source** WSoh_221121_011221_HFGoe_L1_hTau_24h_R1 **Scan** 9658
**Peptide** SNVSSTGSID **Charge** 1 **Spectral Angle** 0.98

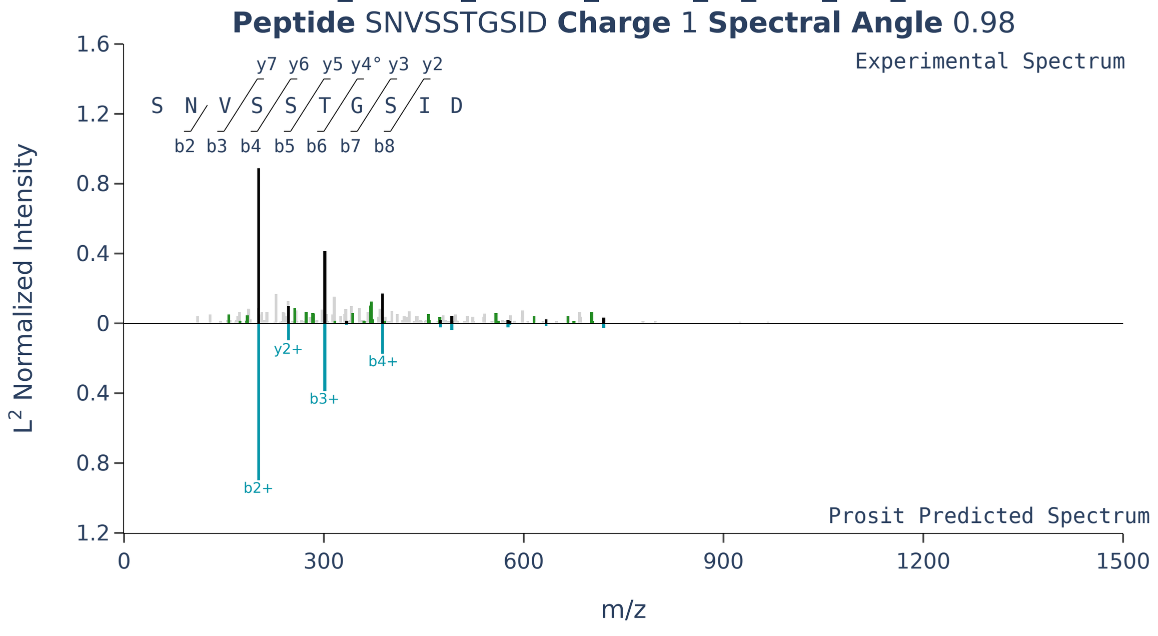


**Source** WSoh_221121_011221_HFGoe_L1_hTau_4h_R2 **Scan** 16066
**Peptide** ADEVSASLA **Charge** 1 **Spectral Angle** 0.9

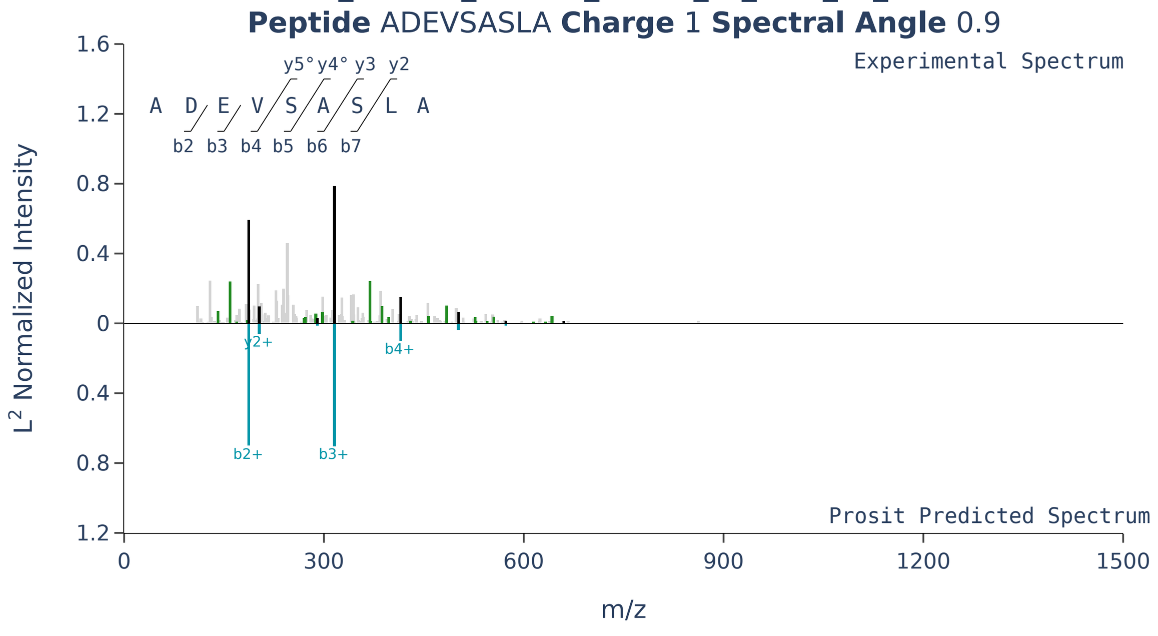


**Source** WSoh_221121_011221_HFGoe_L1_hTau_24h_R1 **Scan** 22041
**Peptide** SPQLATLADE **Charge** 2 **Spectral Angle** 0.96

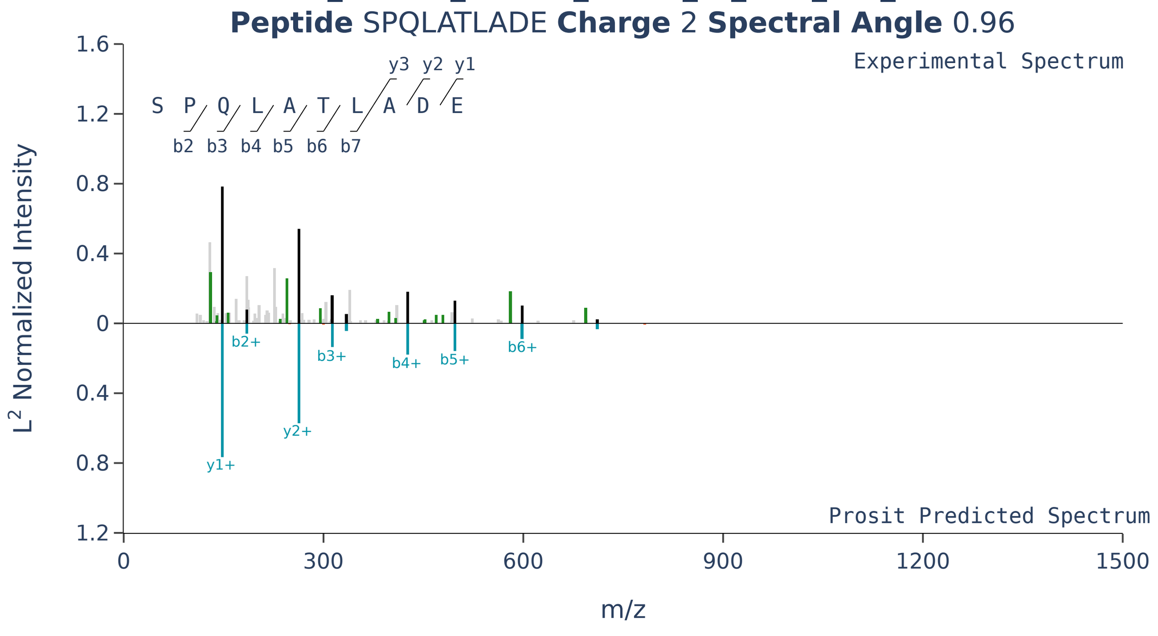


**Source** WSoh_221121_011221_HFGoe_L1_hTau_24h_R2 **Scan** 8194
**Peptide** TYGLGDRKD **Charge** 2 **Spectral Angle** 0.94

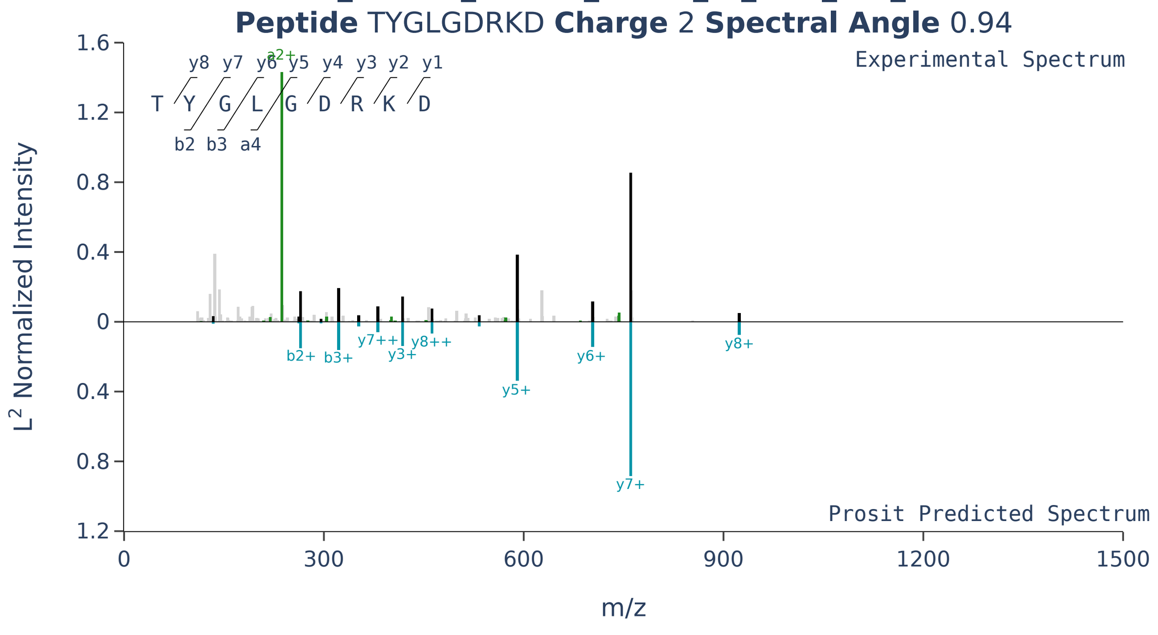


**Source** WSoh_221121_011221_HFGoe_L1_hTau_4h_R2 **Scan** 8692
**Peptide** GYSSPGSPGTPGSR **Charge** 2 **Spectral Angle** 0.95

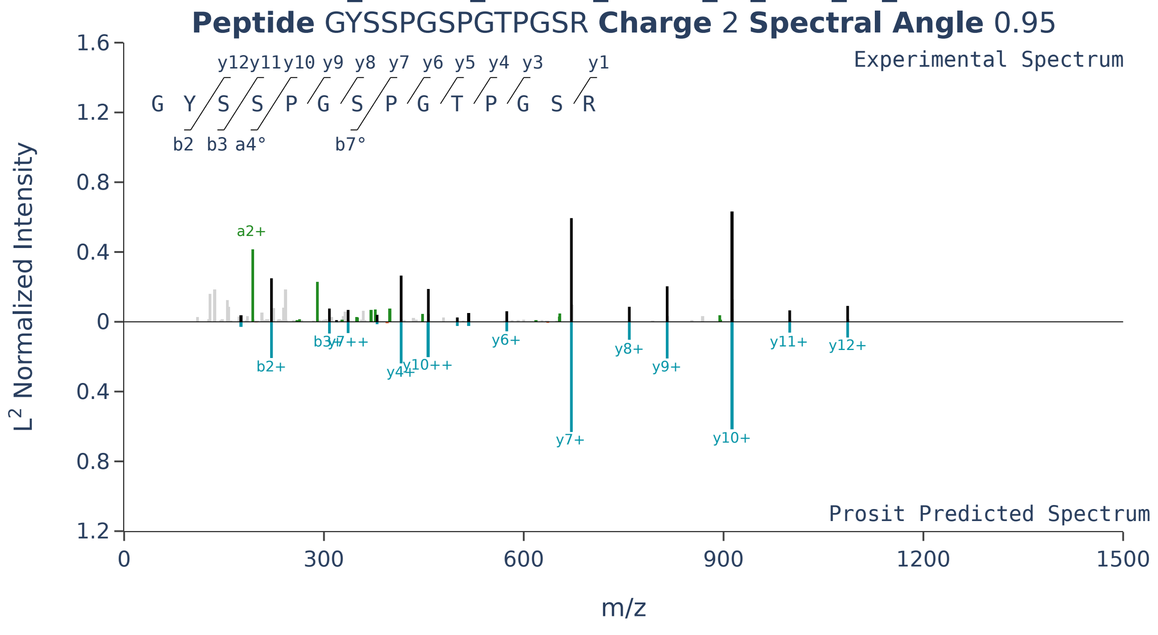


**Source** WSoh_221121_011221_HFGoe_L1_hTau_24h_R2 **Scan** 9607
**Peptide** NVSSTGSID **Charge** 1 **Spectral Angle** 0.96

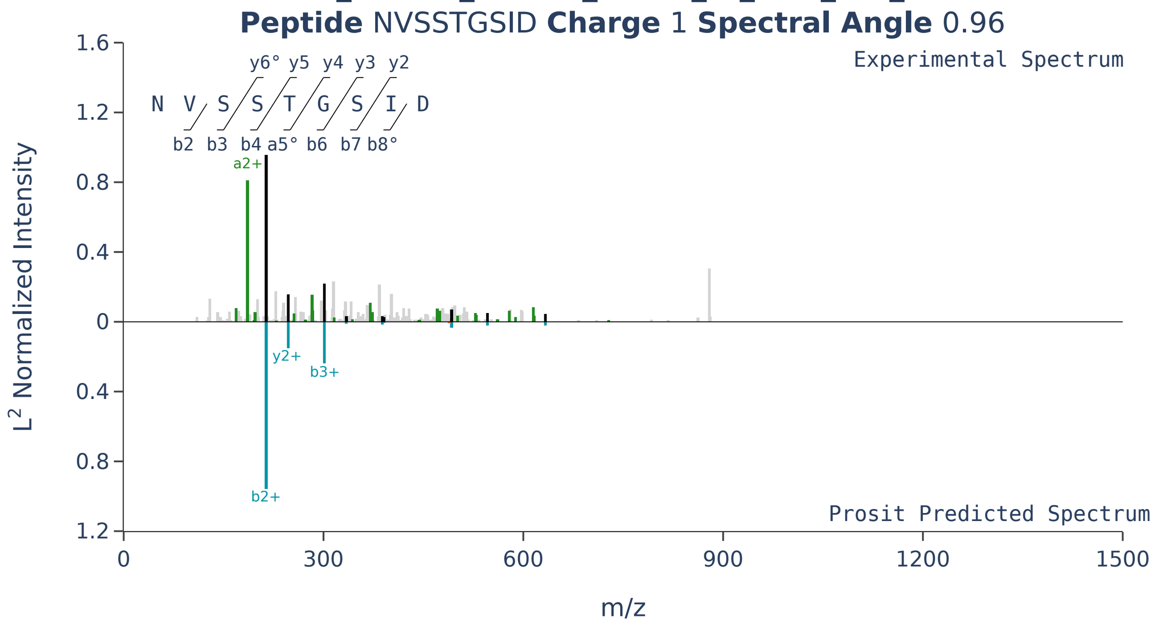


**Source** WSoh_221121_011221_HFGoe_L1_hTau_24h_R2 **Scan** 12945
**Peptide** AAQPHTEIPEGTTA **Charge** 1 **Spectral Angle** 0.94

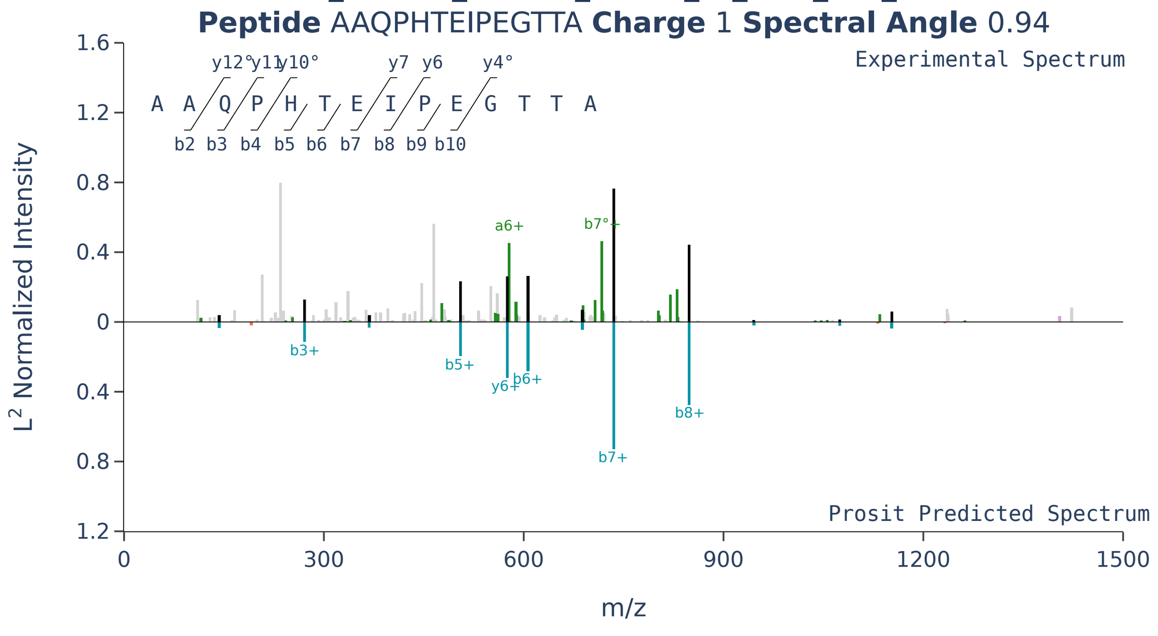


**Source** WSoh_221121_011221_HFGoe_L1_hTau_24h_R1 **Scan** 7252
**Peptide** SGDTSPRHLSNVSS **Charge** 2 **Spectral Angle** 0.93

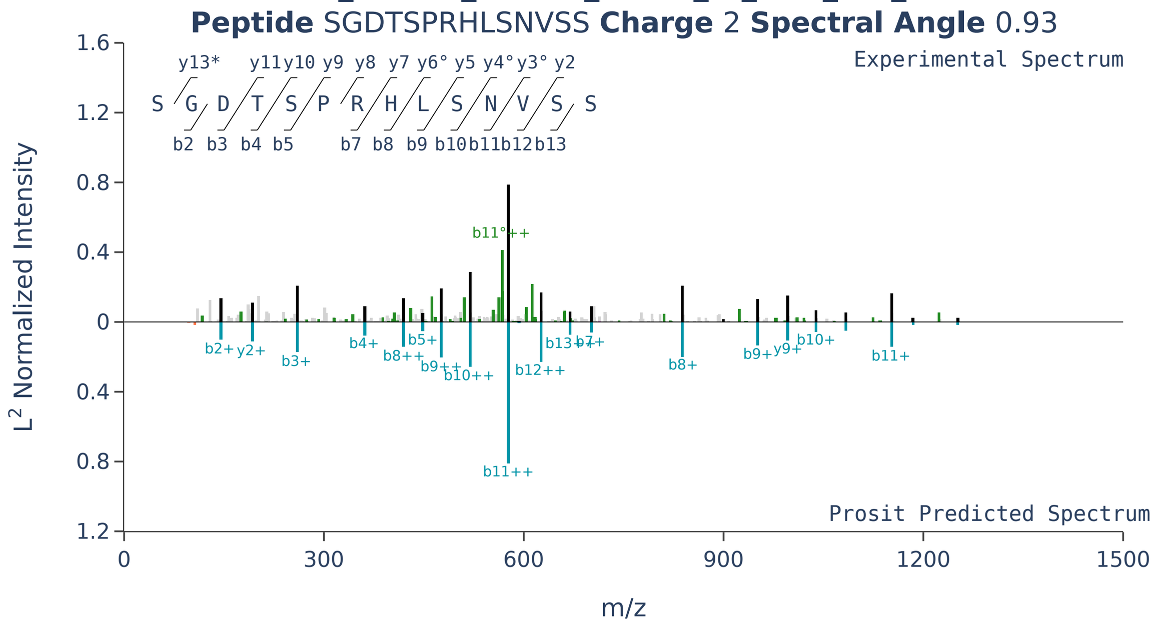


**Source** WSoh_221121_011221_HFGoe_L1_hTau_4h_R2 **Scan** 11732
**Peptide** AAQPHTEIPEGT **Charge** 1 **Spectral Angle** 0.95

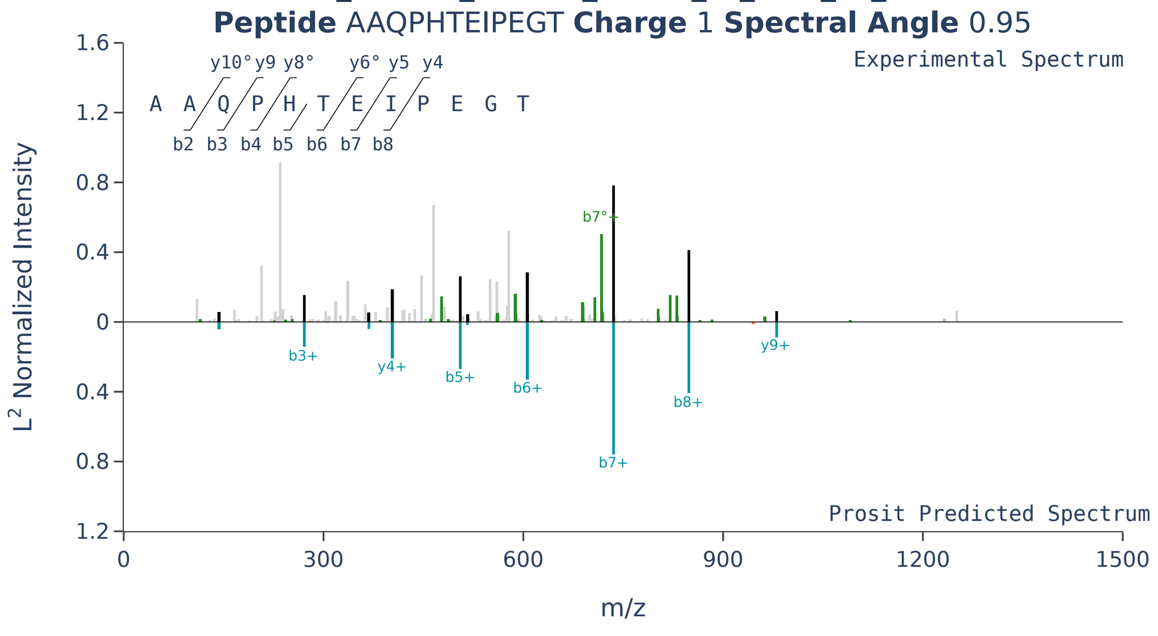

**Source** WSoh_221121_011221_HFGoe_L2_hTau_24h_R1 **Scan** 12780
**Peptide** PVDLSKVTSK **Charge** 3 **Spectral Angle** 0.94

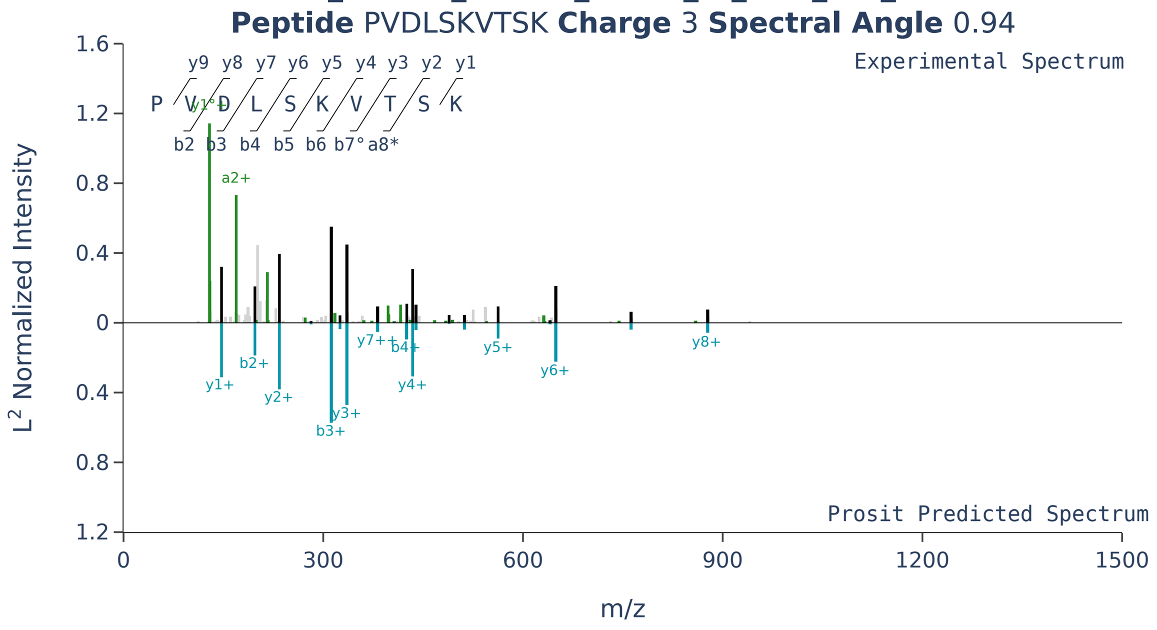


**Source** WSoh_221121_011221_HFGoe_L2_hTau_24h_R1 **Scan** 7634
**Peptide** APPGQKGQANATRL **Charge** 2 **Spectral Angle** 0.96

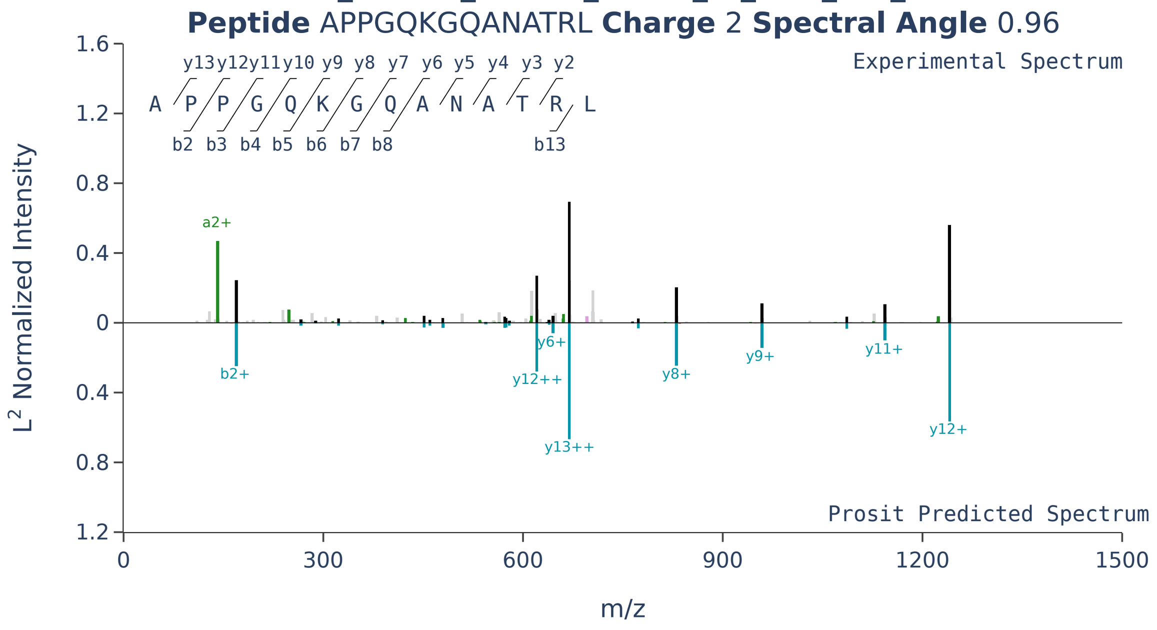


**Source** WSoh_221121_011221_HFGoe_L2_hTau_2h_R1 **Scan** 8403
**Peptide** GYSSPGSPGTPGSRS **Charge** 2 **Spectral Angle** 0.97

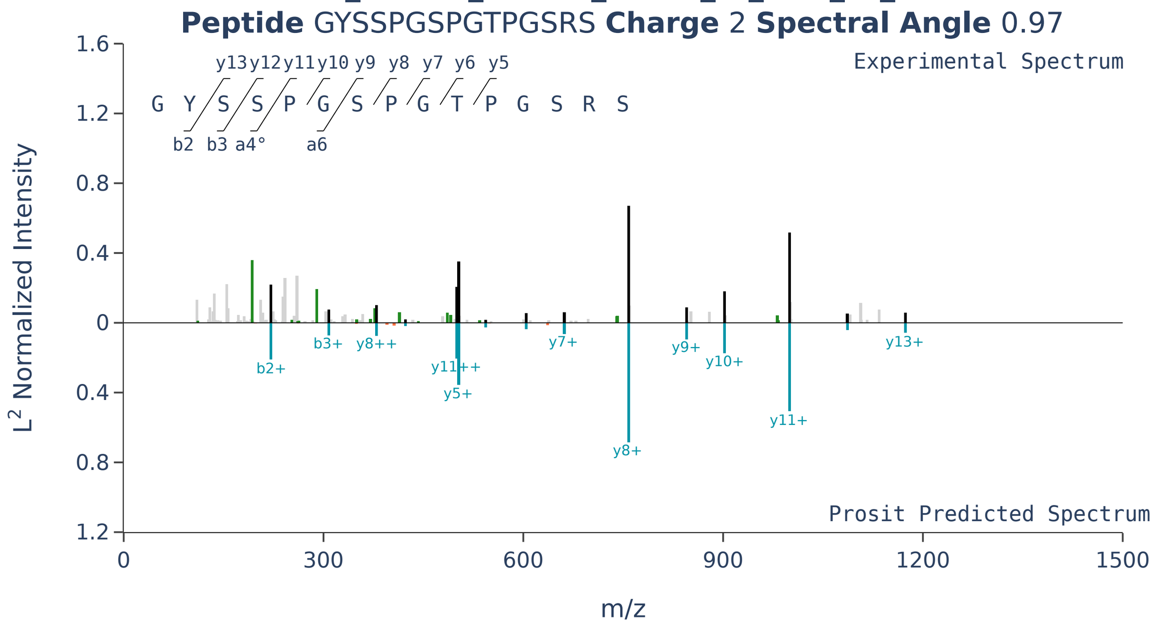


**Source** WSoh_221121_011221_HFGoe_L2_hTau_24h_R2 **Scan** 16038
**Peptide** VYKPVDLSKVTSKC **Charge** 4 **Spectral Angle** 0.91

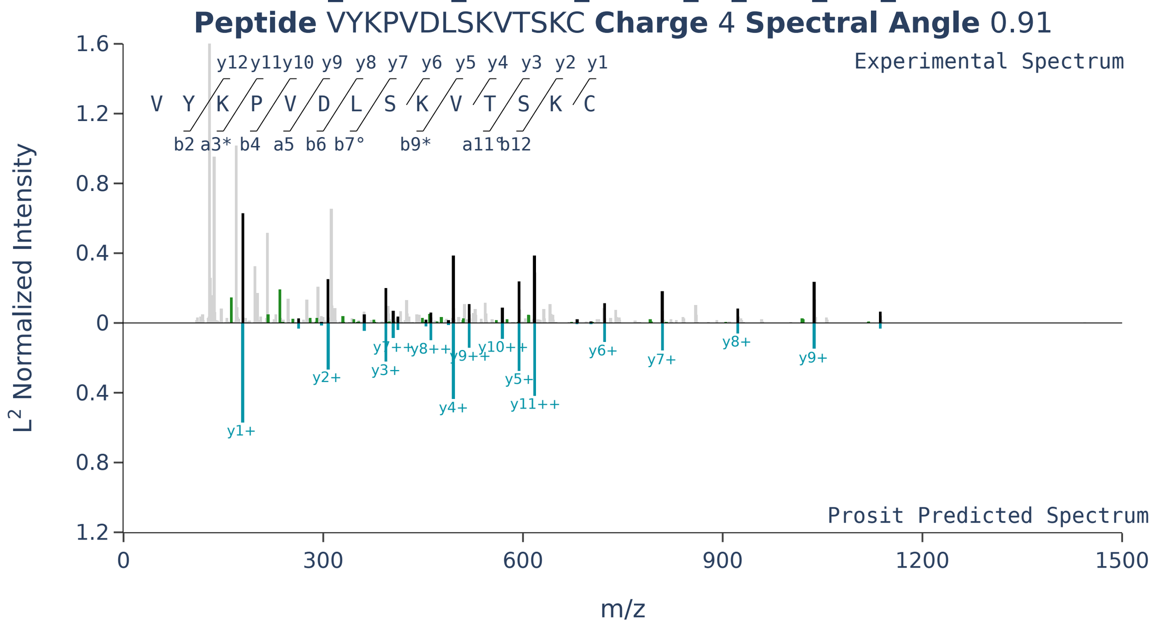


**Source** WSoh_221121_011221_HFGoe_L2_hTau_24h_R2 **Scan** 7649
**Peptide** DHAGTYGLGDRK **Charge** 3 **Spectral Angle** 0.94

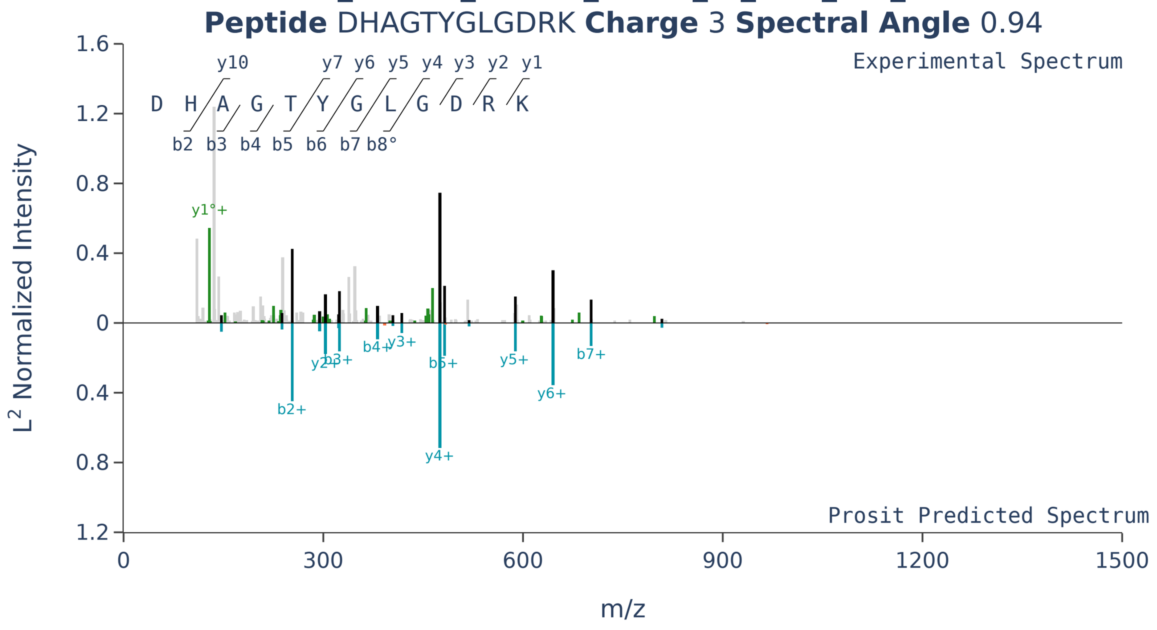


**Source** WSoh_221121_011221_HFGoe_L1_hTau_2h_R2 **Scan** 7319
**Peptide** EAAGHVTQARM **Charge** 2 **Spectral Angle** 0.94

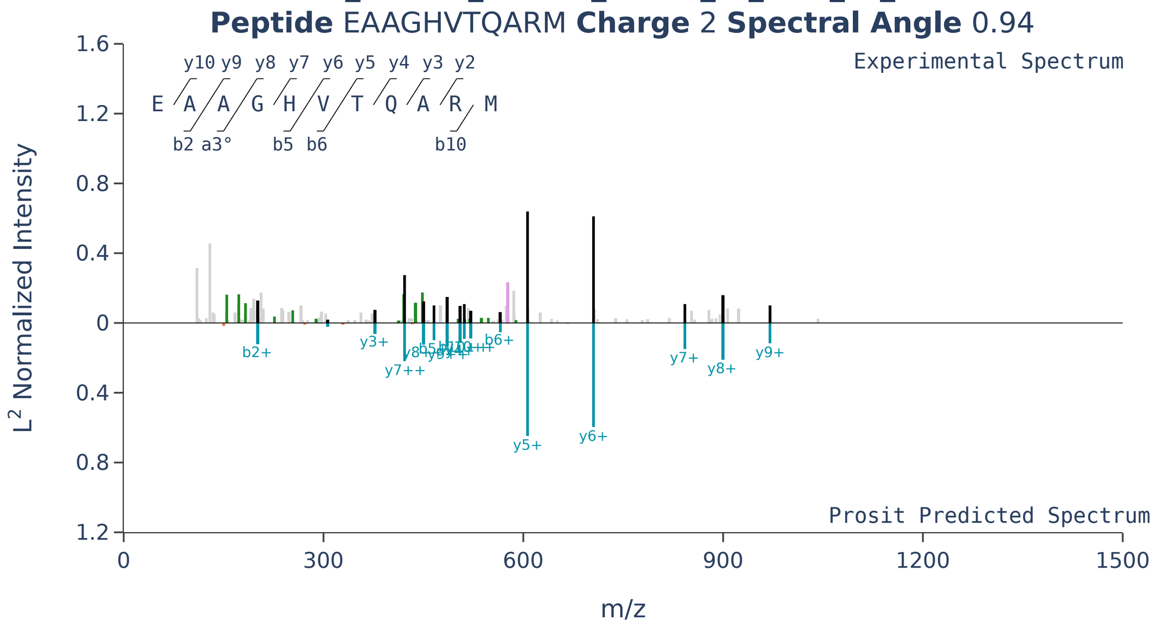


**Source** WSoh_221121_011221_HFGoe_L2_hTau_2h_R2 **Scan** 17166
**Peptide** TGSIDMVD **Charge** 1 **Spectral Angle** 0.92

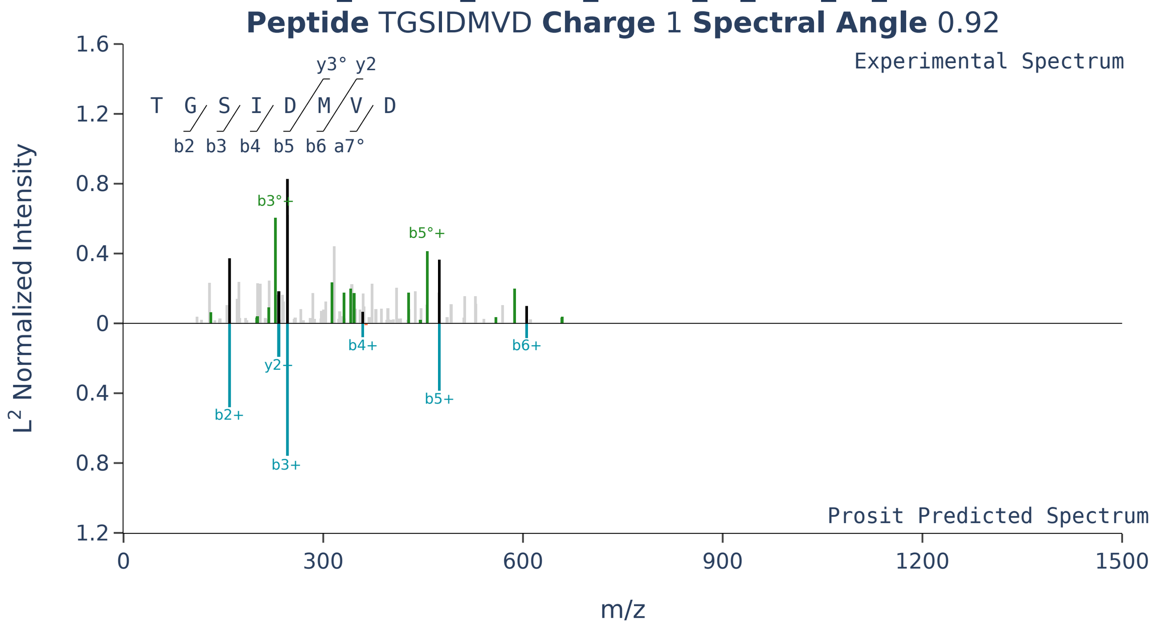


**Source** WSoh_221121_011221_HFGoe_L1_hTau_24h_R2 **Scan** 8482
**Peptide** AGTYGLGDRKD **Charge** 2 **Spectral Angle** 0.96

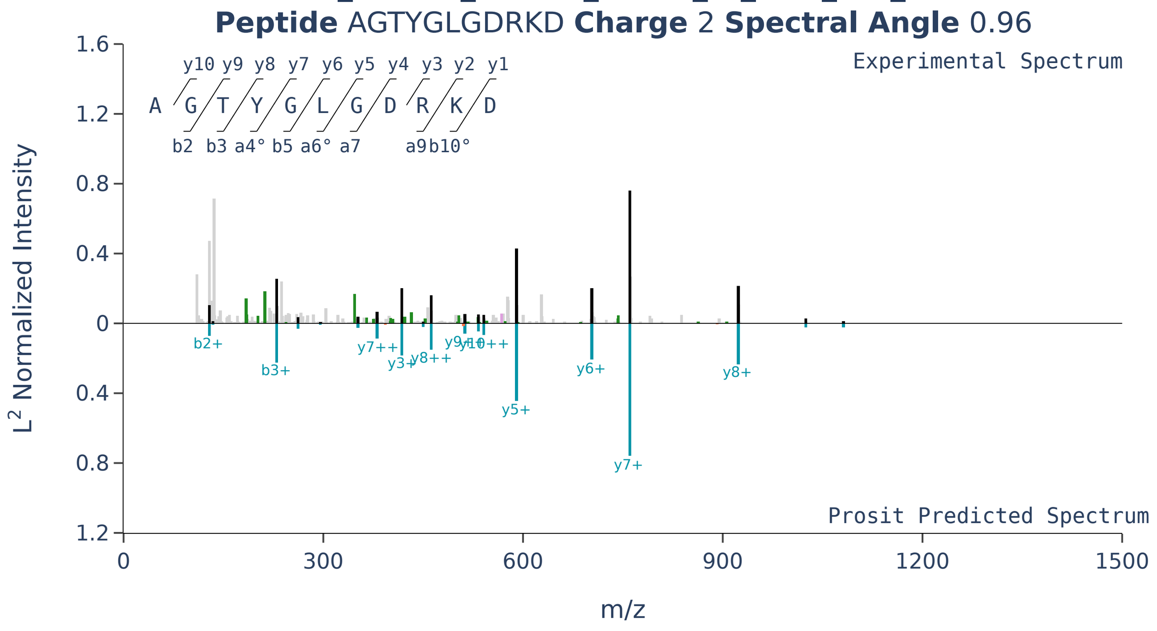


**Source** WSoh_221121_011221_HFGoe_L1_hTau_4h_R2 **Scan** 19197
**Peptide** SKIGSLDNITHV **Charge** 3 **Spectral Angle** 0.94

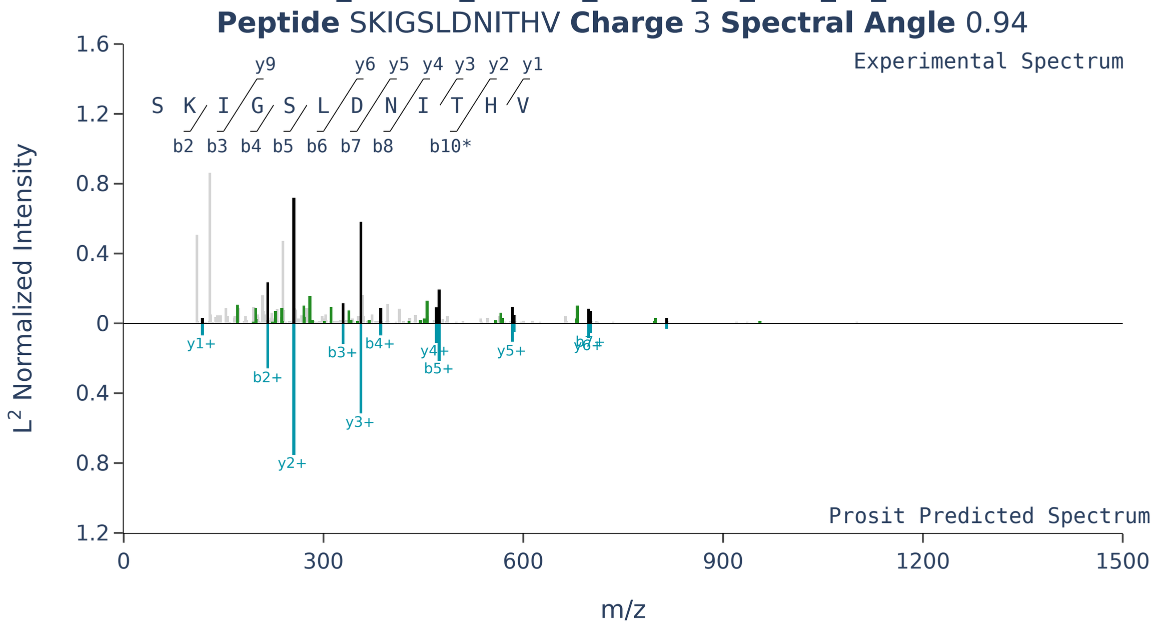


**Source** WSoh_221121_011221_HFGoe_L1_hTau_2h_R1 **Scan** 13739
**Peptide** HGAEIVY **Charge** 2 **Spectral Angle** 0.93

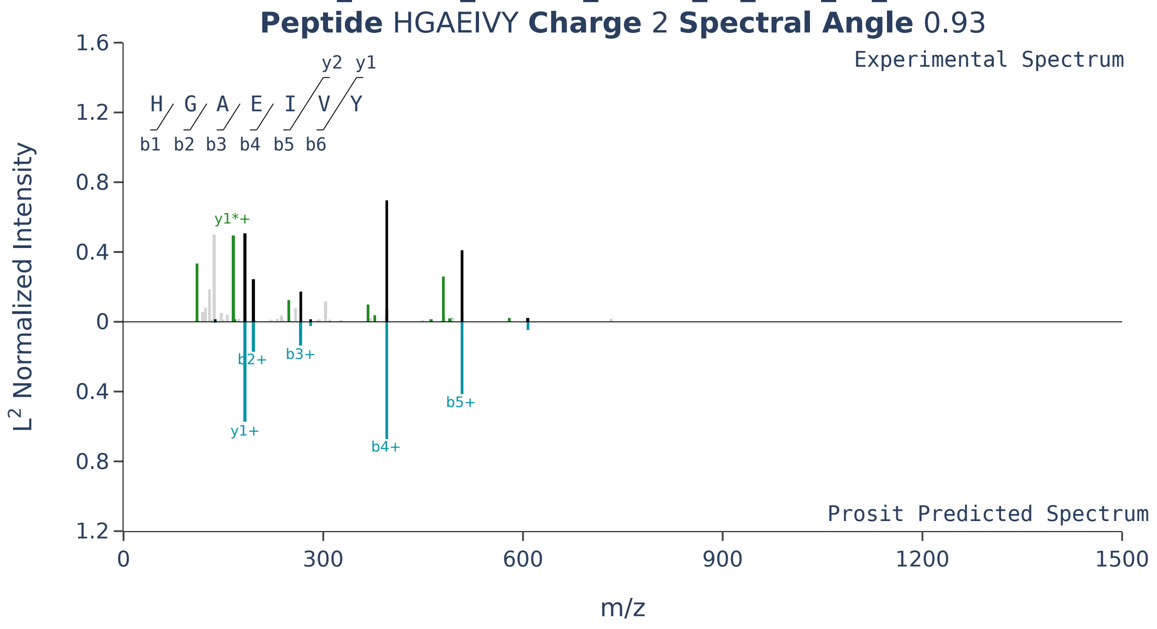


**Source** WSoh_221121_011221_HFGoe_L1_hTau_4h_R1 **Scan** 8662
**Peptide** SGYSSPGSPGTPGSRS **Charge** 2 **Spectral Angle** 0.94

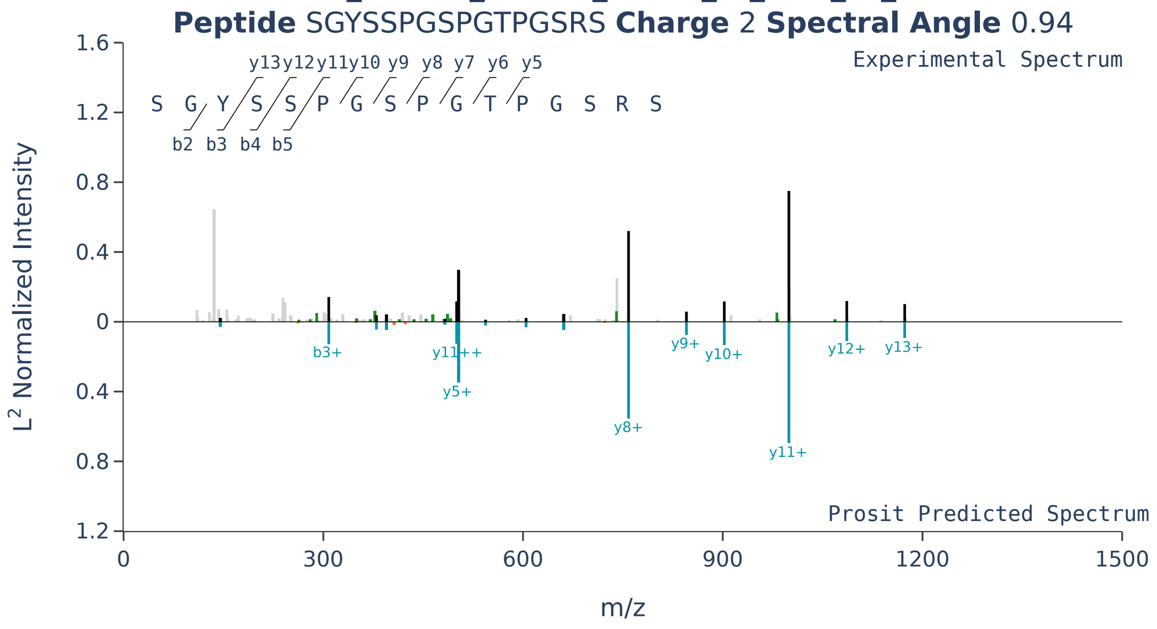


**Source** WSoh_221121_011221_HFGoe_L1_hTau_4h_R1 **Scan** 7158
**Peptide** GYSSPGSPGTPGSRSRT **Charge** 3 **Spectral Angle** 0.96

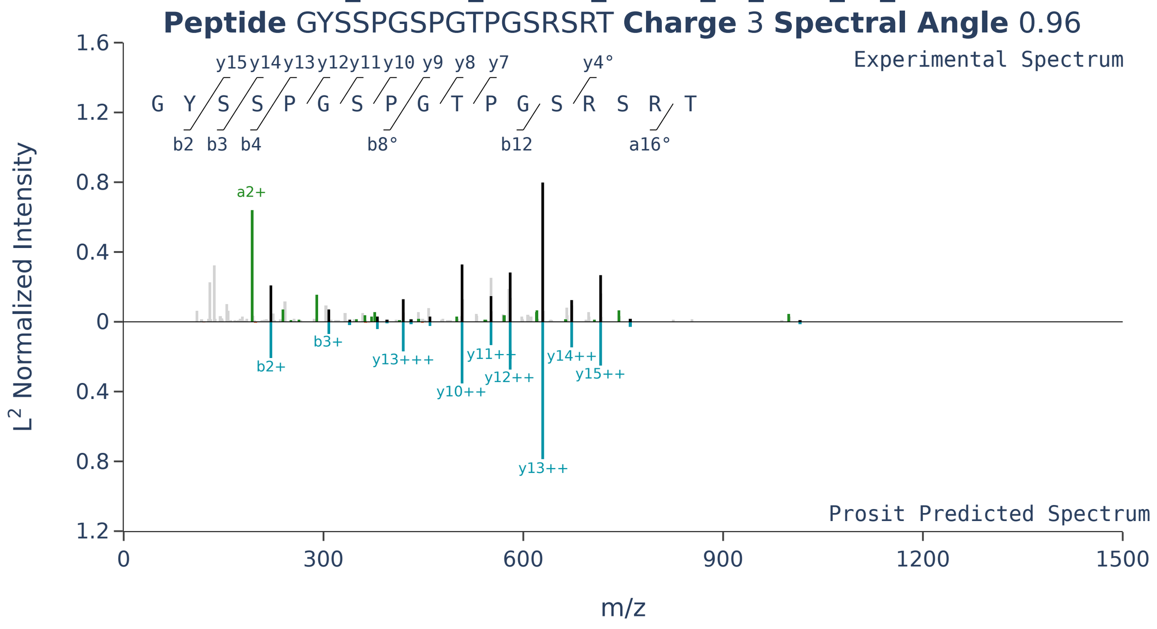


**Source** WSoh_221121_011221_HFGoe_L2_hTau_24h_R1 **Scan** 18310
**Peptide** TDAGLKESPLQTPTED **Charge** 2 **Spectral Angle** 0.91

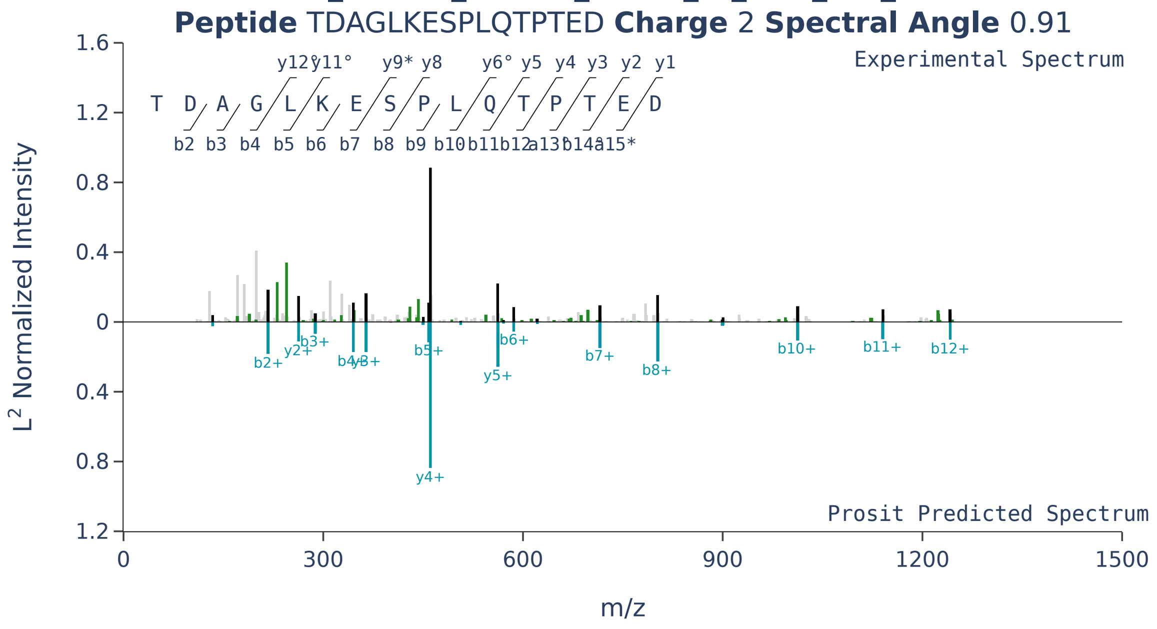


**Source** WSoh_221121_011221_HFGoe_L1_hTau_24h_R1 **Scan** 11836
**Peptide** VYKPVDLSK **Charge** 2 **Spectral Angle** 0.96

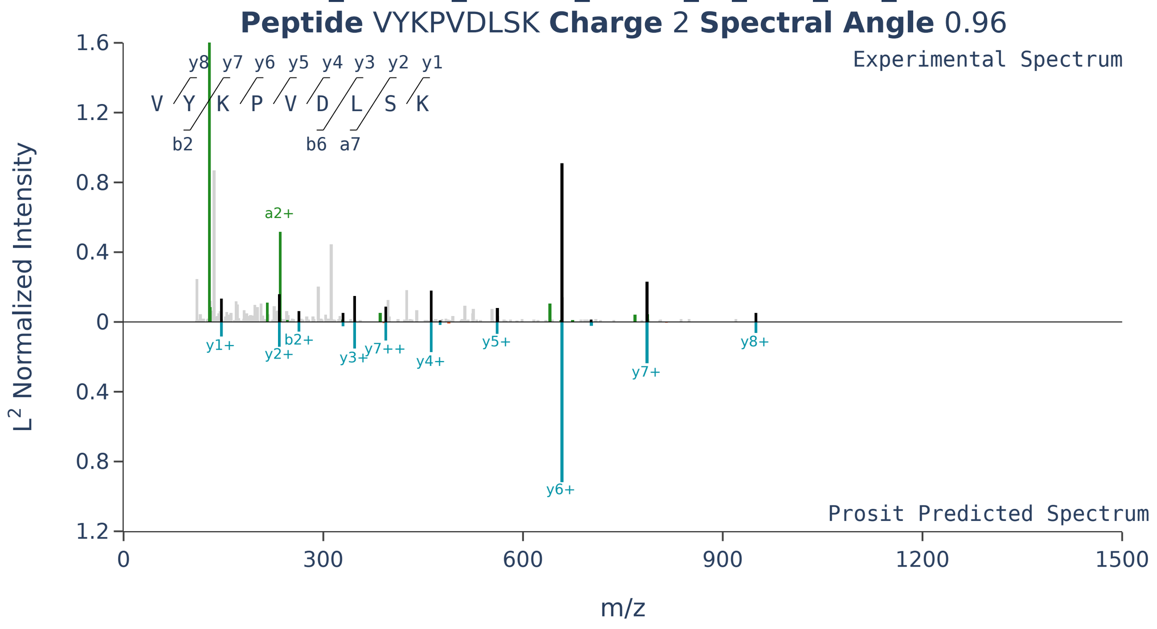


**Source** WSoh_221121_011221_HFGoe_L1_hTau_2h_R1 **Scan** 8201
**Peptide** KLDFKDRVQSK **Charge** 3 **Spectral Angle** 0.91

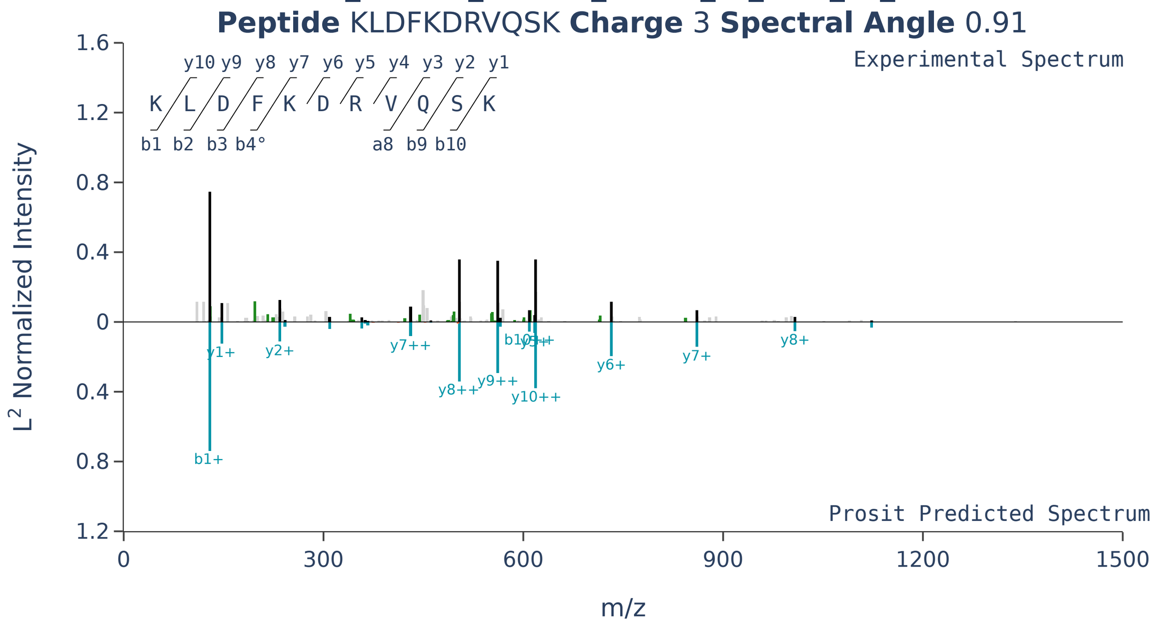

**Source** WSoh_221121_011221_HFGoe_L2_hTau_24h_R2 **Scan** 7472
**Peptide** LGDRKDQGGYTMH **Charge** 2 **Spectral Angle** 0.94

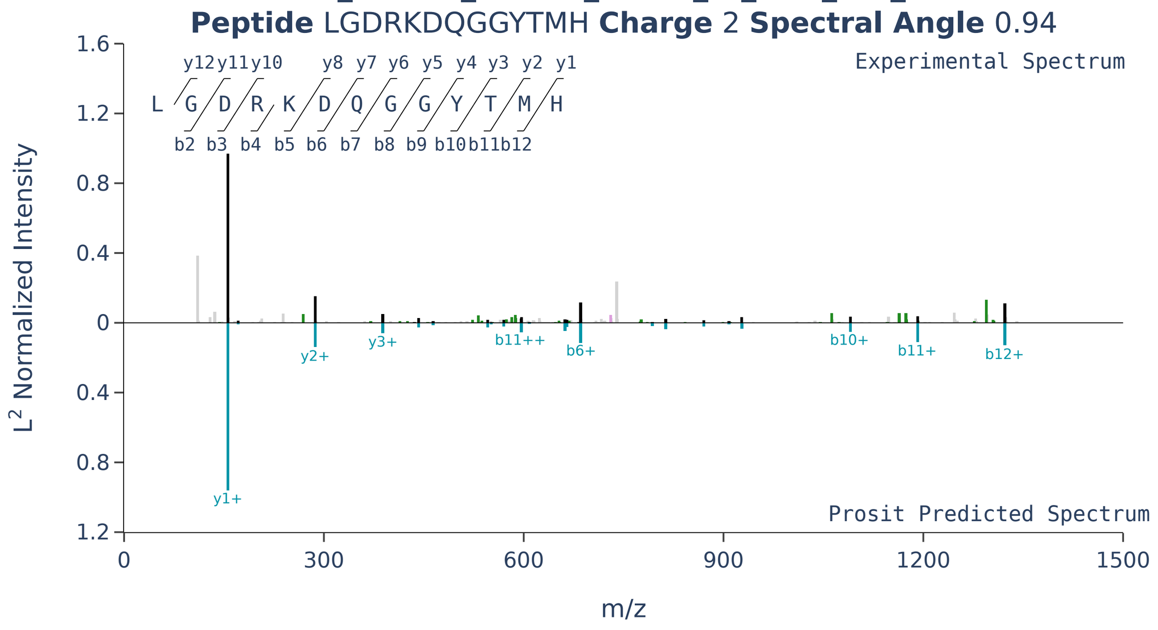


**Source** WSoh_221121_011221_HFGoe_L1_hTau_1h_R1 **Scan** 17420
**Peptide** GSLDNITHV **Charge** 2 **Spectral Angle** 0.93

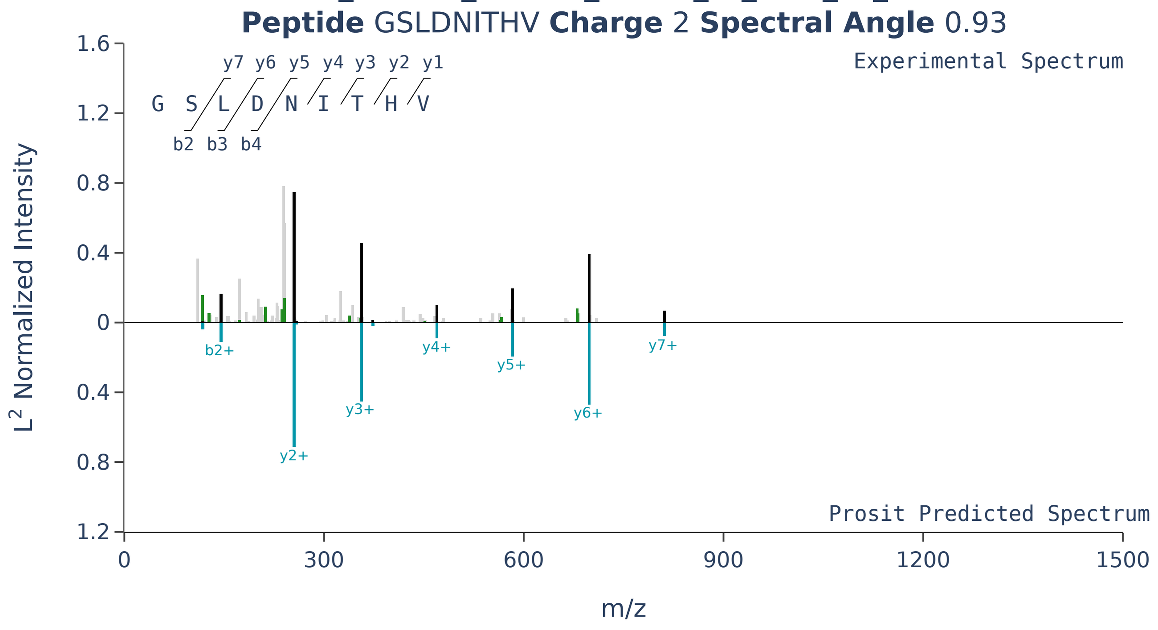


**Source** WSoh_221121_011221_HFGoe_L1_hTau_4h_R2 **Scan** 17032
**Peptide** VTAPLVDEGAPGKQA **Charge** 2 **Spectral Angle** 0.95

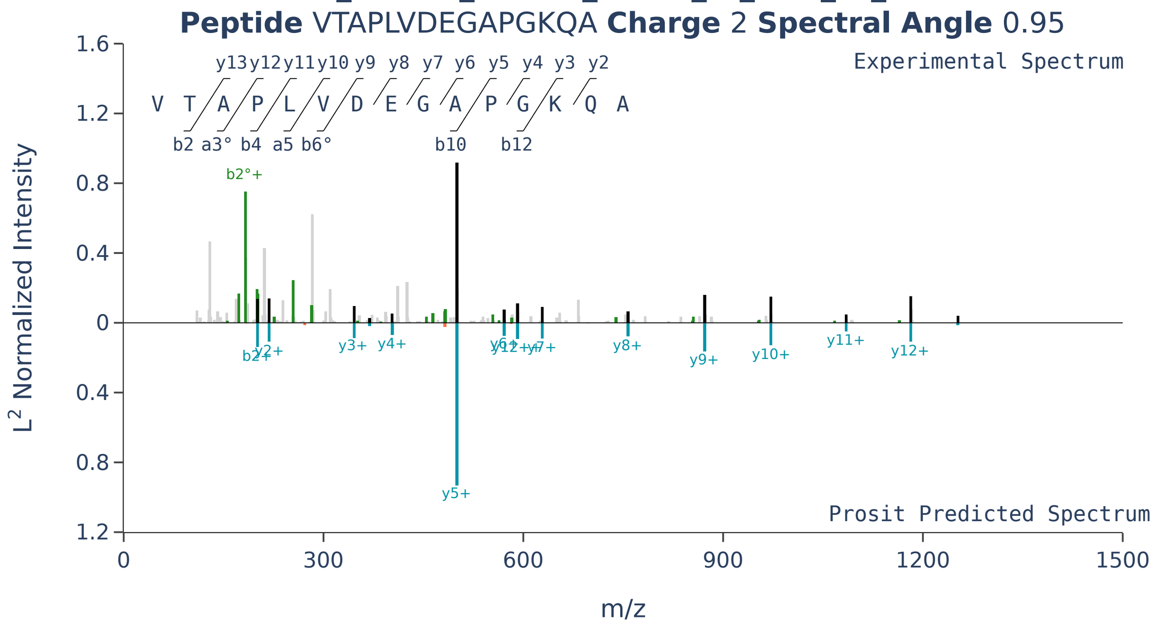


**Source** WSoh_221121_011221_HFGoe_L1_hTau_24h_R2 **Scan** 8748
**Peptide** HGAEIVYK **Charge** 2 **Spectral Angle** 0.94

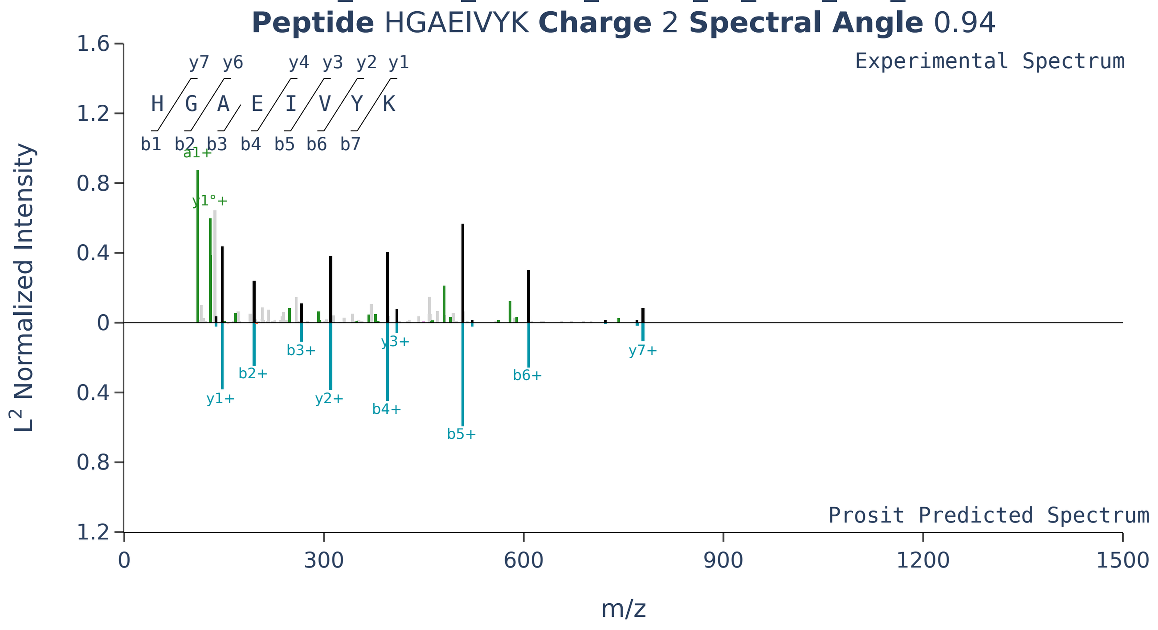


**Source** WSoh_221121_011221_HFGoe_L2_hTau_2h_R1 **Scan** 14832
**Peptide** KLDLSNVQ **Charge** 2 **Spectral Angle** 0.94

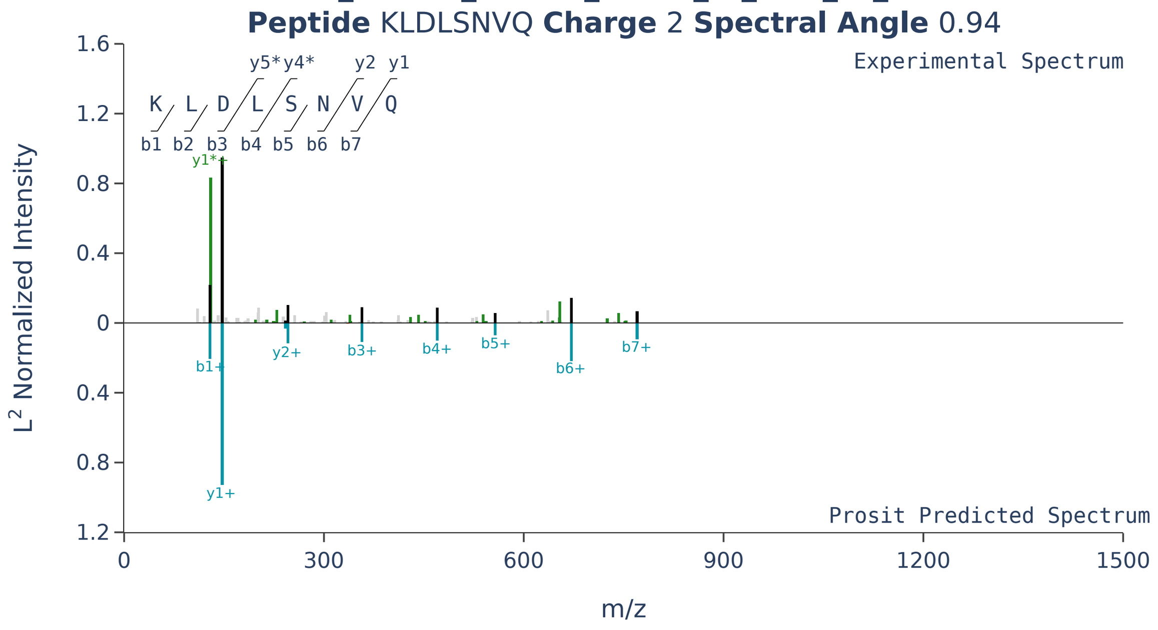


**Source** WSoh_221121_011221_HFGoe_L1_hTau_4h_R2 **Scan** 20478
**Peptide** SPQLATLAD **Charge** 2 **Spectral Angle** 0.89

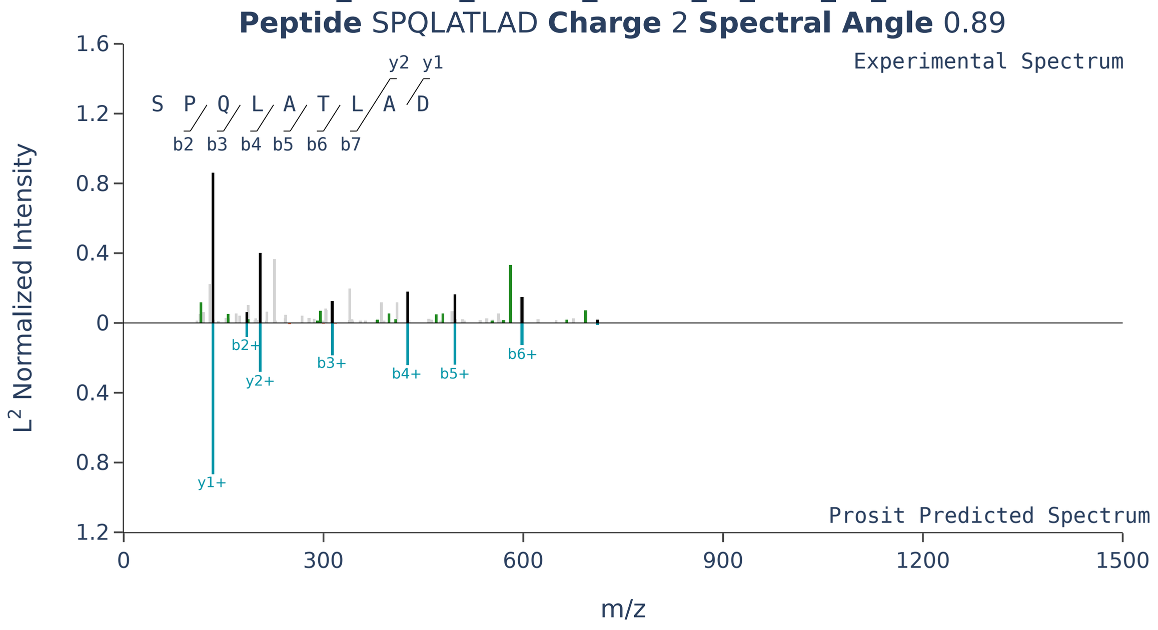


**Source** WSoh_221121_011221_HFGoe_L1_hTau_4h_R1 **Scan** 17336
**Peptide** SLDNITHV **Charge** 2 **Spectral Angle** 0.96

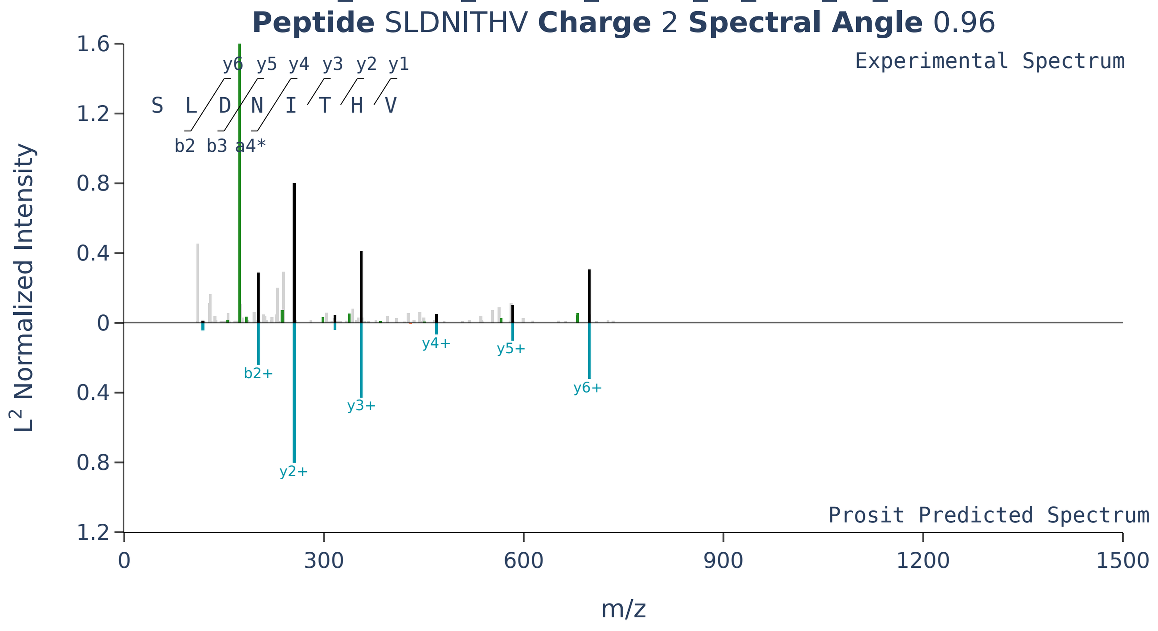


**Source** WSoh_221121_011221_HFGoe_L1_hTau_24h_R2 **Scan** 9171
**Peptide** DEAAGHVTQARM **Charge** 2 **Spectral Angle** 0.93

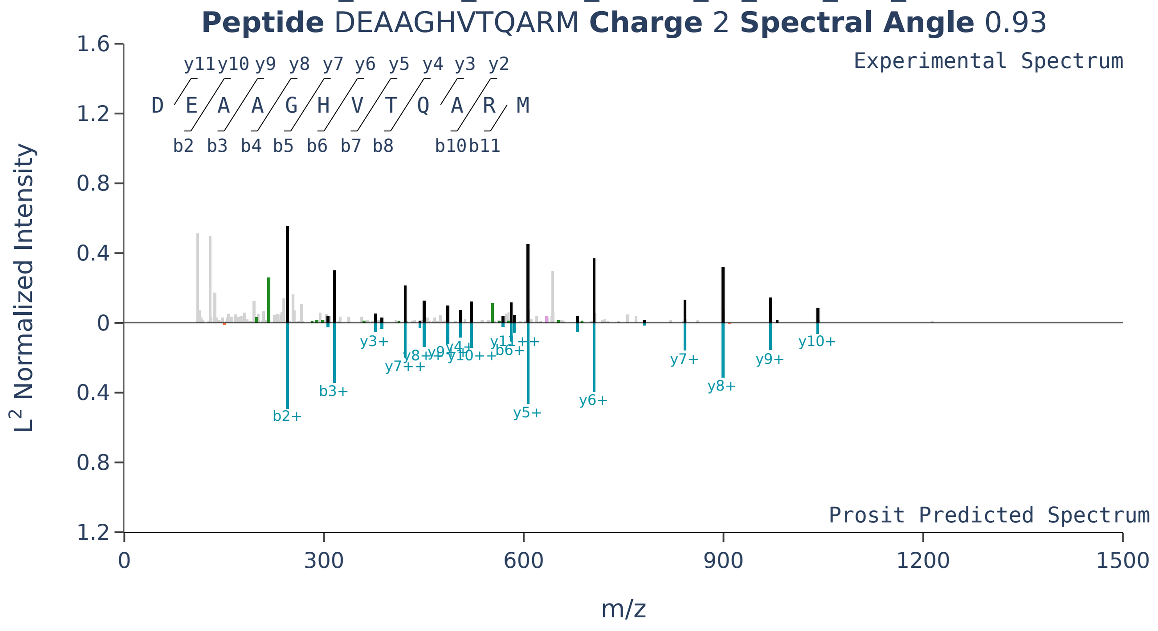


**Source** WSoh_221121_011221_HFGoe_L1_hTau_4h_R1 **Scan** 21770
**Peptide** SPQLATL **Charge** 1 **Spectral Angle** 0.9

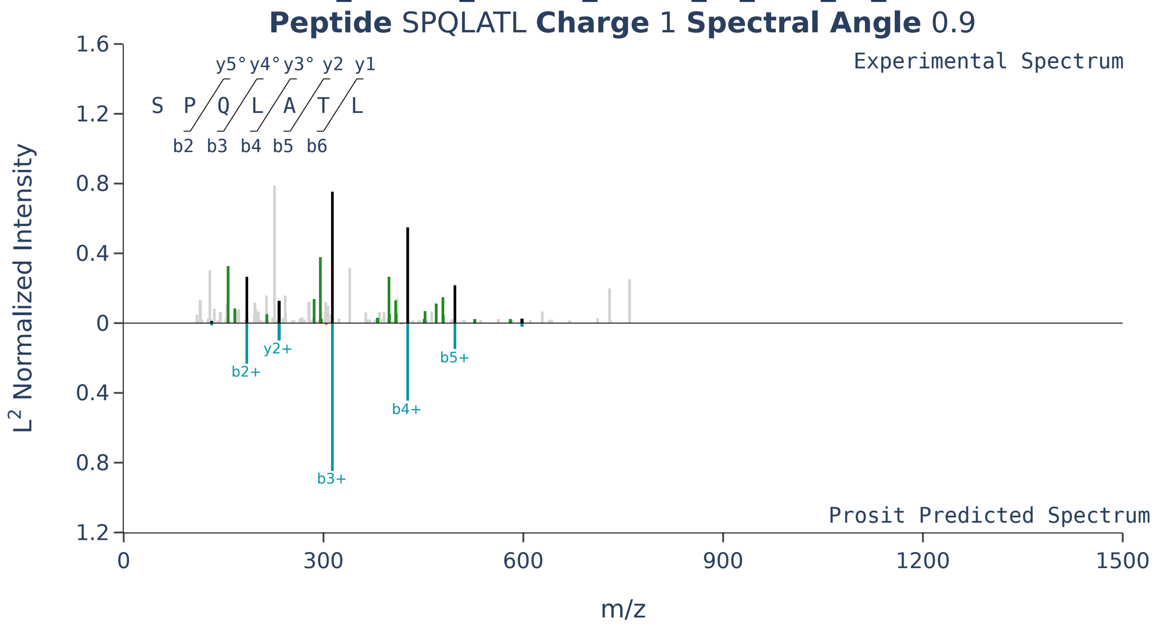


**Source** WSoh_221121_011221_HFGoe_L1_hTau_4h_R2 **Scan** 11478
**Peptide** EKLDFKDRVQS **Charge** 2 **Spectral Angle** 0.91

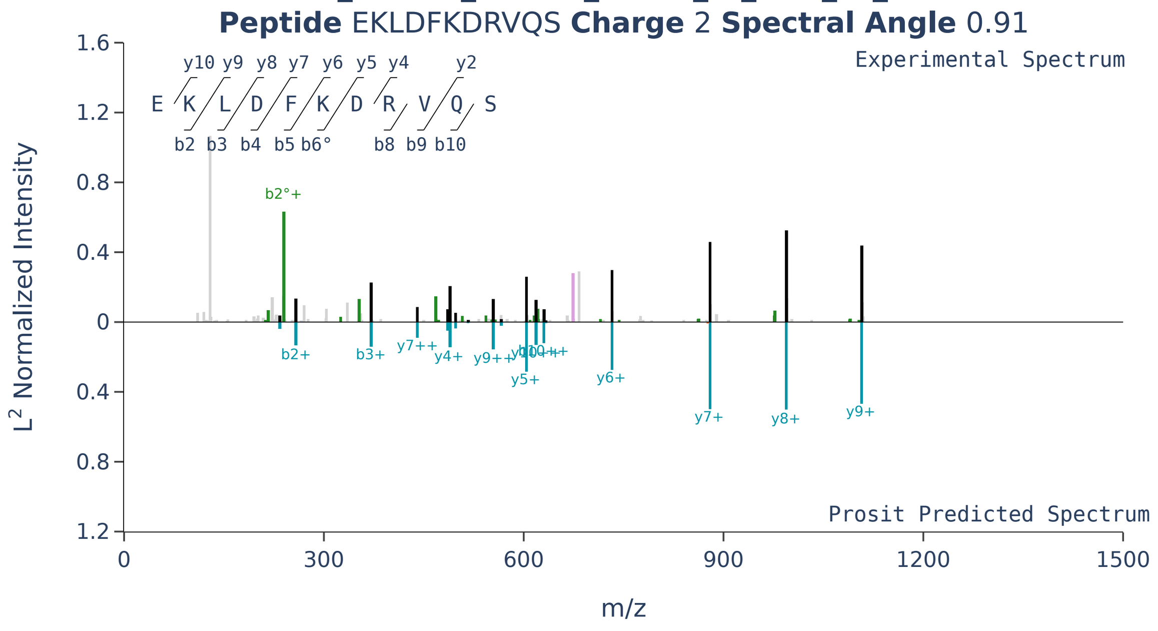


**Source** WSoh_221121_011221_HFGoe_L1_hTau_2h_R1 **Scan** 18769
**Peptide** AEPRQEFEVME **Charge** 2 **Spectral Angle** 0.92

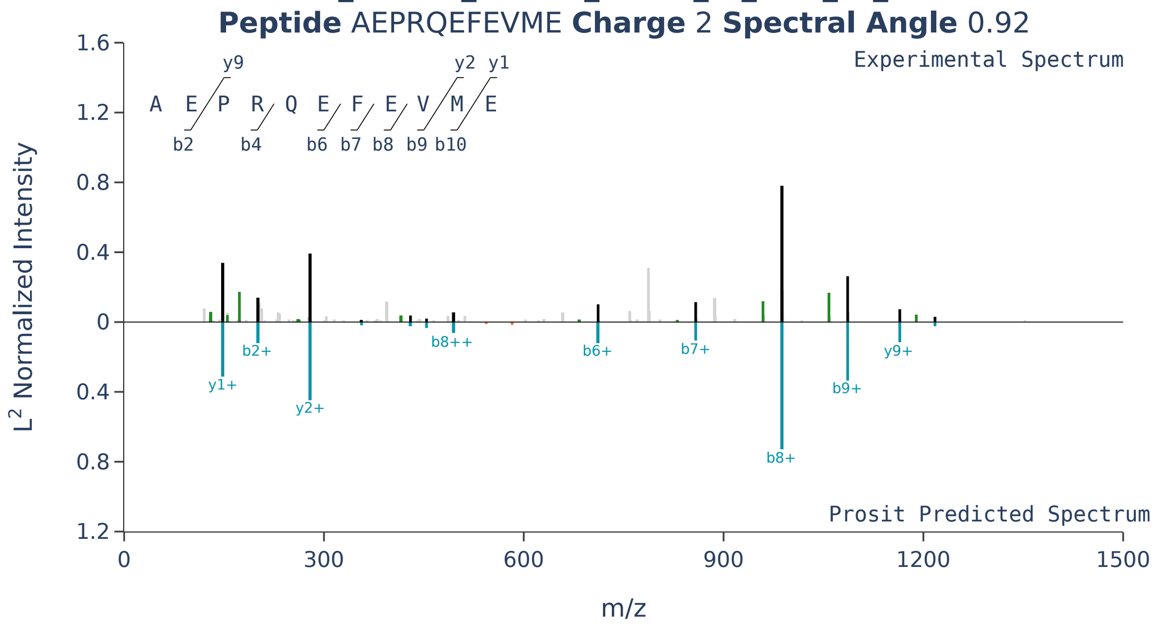


**Source** WSoh_221121_011221_HFGoe_L1_hTau_24h_R2 **Scan** 18821
**Peptide** AGIGDTPSLEDEAAGH **Charge** 2 **Spectral Angle** 0.92

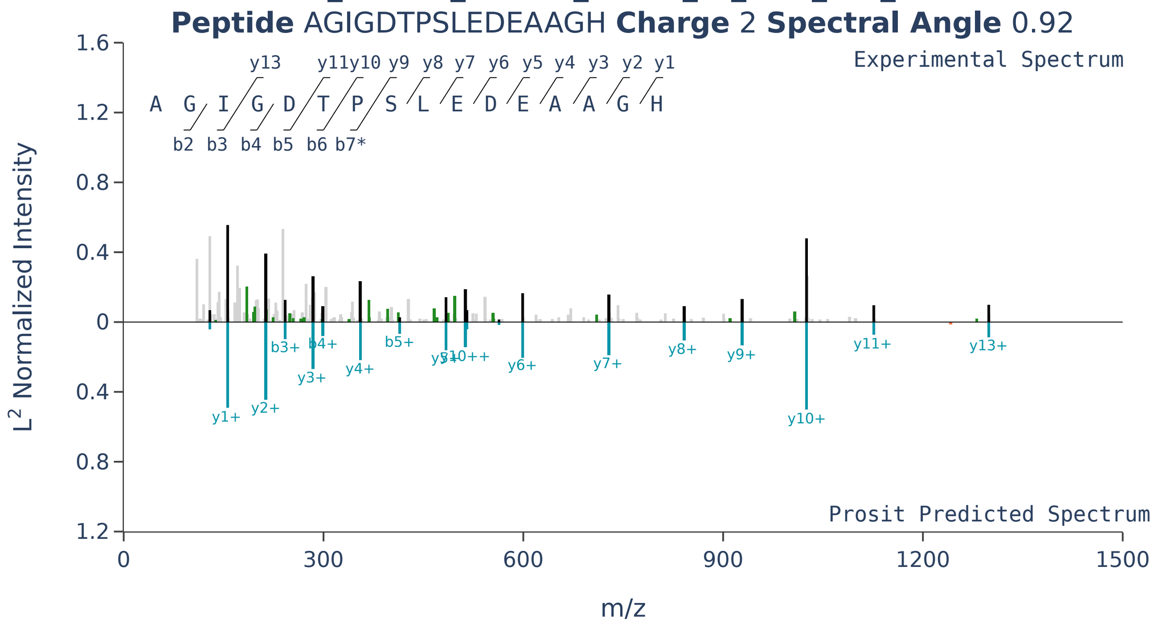


**Source** WSoh_221121_011221_HFGoe_L2_hTau_2h_R1 **Scan** 16487
**Peptide** EVSASLAKQGL **Charge** 1 **Spectral Angle** 0.91

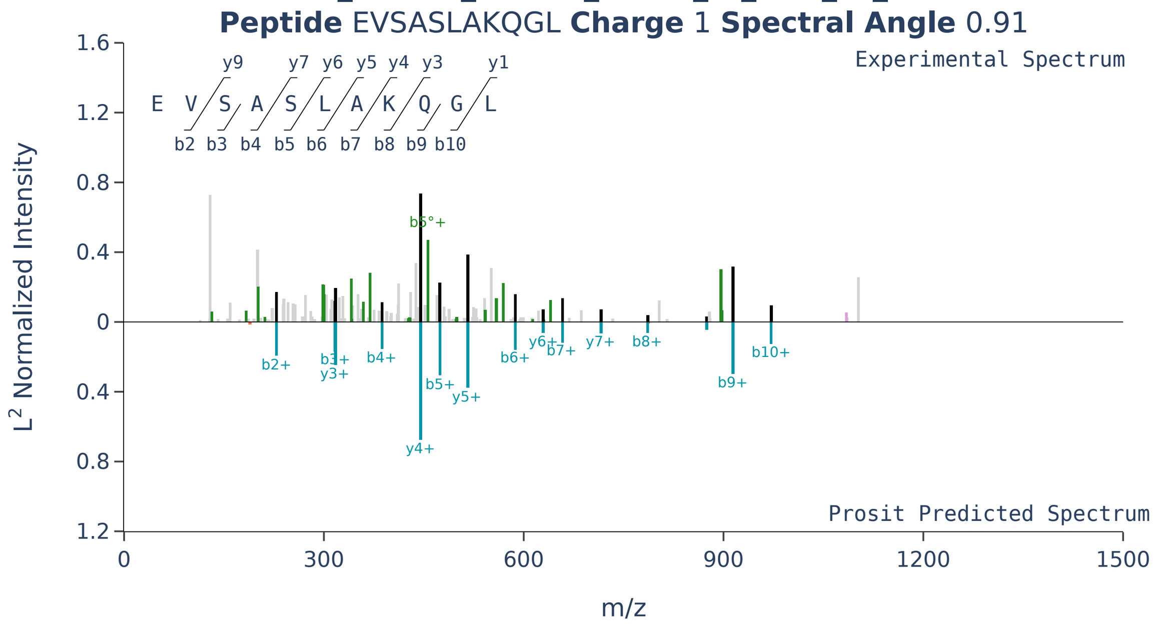


**Source** WSoh_221121_011221_HFGoe_L2_hTau_2h_R1 **Scan** 6218
**Peptide** KCGSLGNIHH **Charge** 2 **Spectral Angle** 0.92

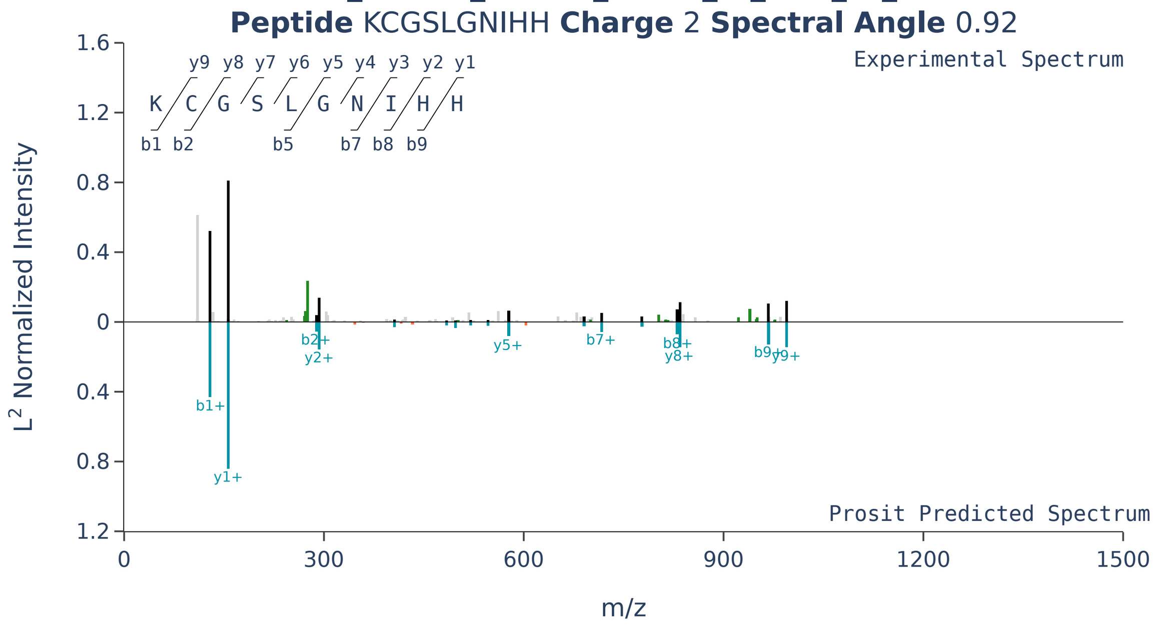


**Source** WSoh_221121_011221_HFGoe_L1_hTau_4h_R1 **Scan** 11911
**Peptide** PLQTPTE **Charge** 1 **Spectral Angle** 0.91

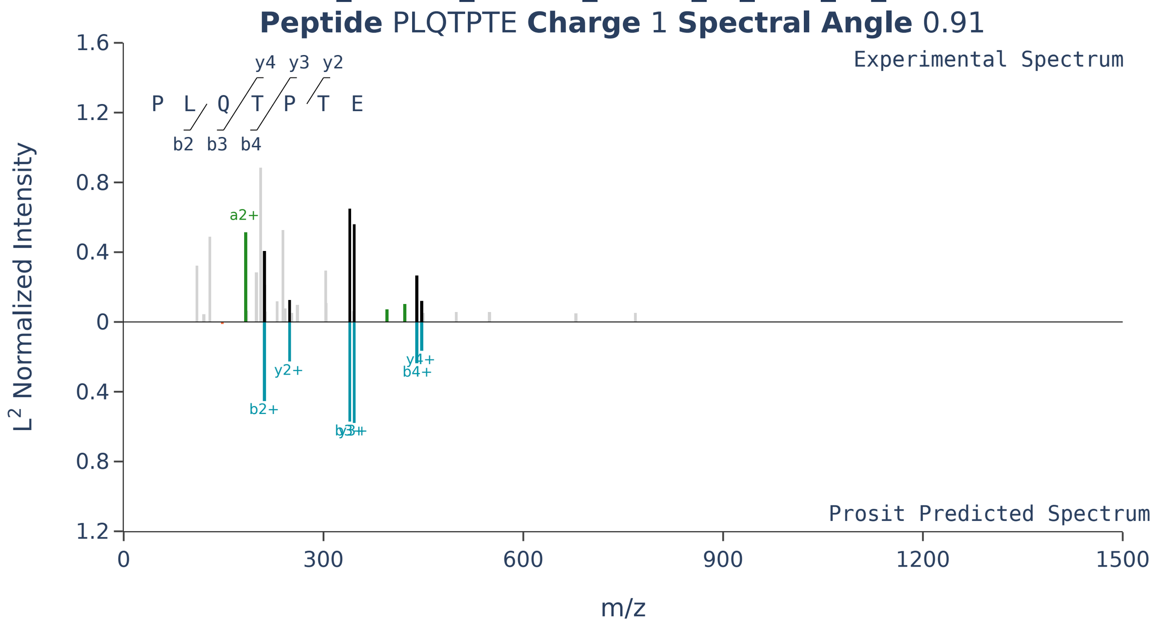

**Source** WSoh_221121_011221_HFGoe_L2_hTau_4h_R1 **Scan** 7305
**Peptide** PGGGQVEVKSE **Charge** 2 **Spectral Angle** 0.92

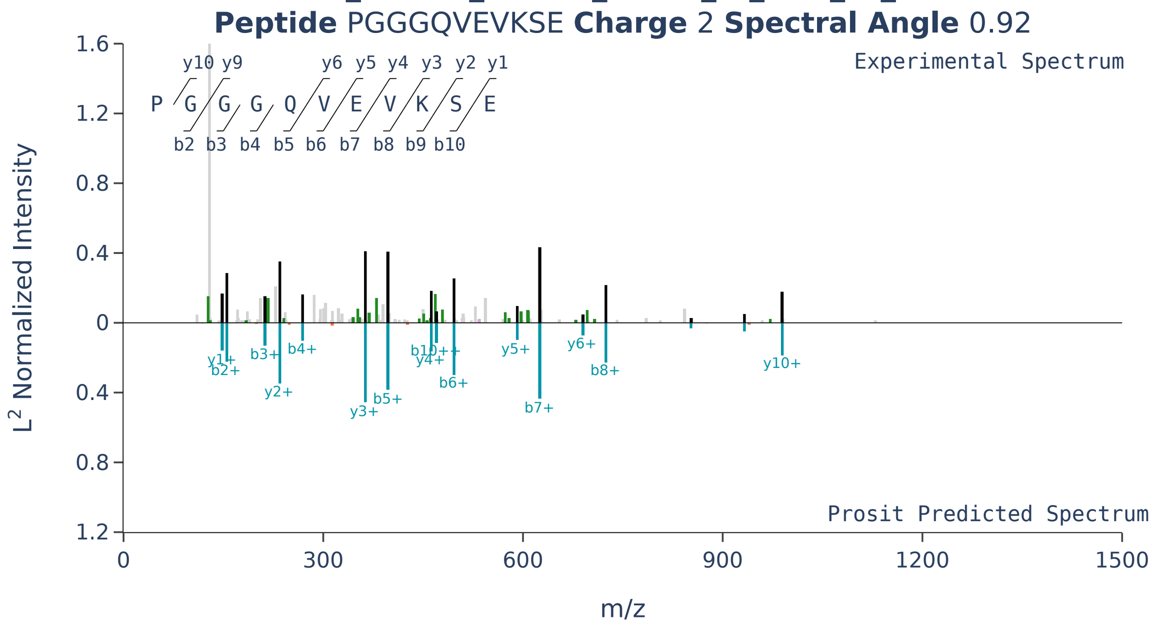


**Source** WSoh_221121_011221_HFGoe_L2_hTau_4h_R2 **Scan** 6820
**Peptide** KAKTDHGAEIVYKS **Charge** 2 **Spectral Angle** 0.93

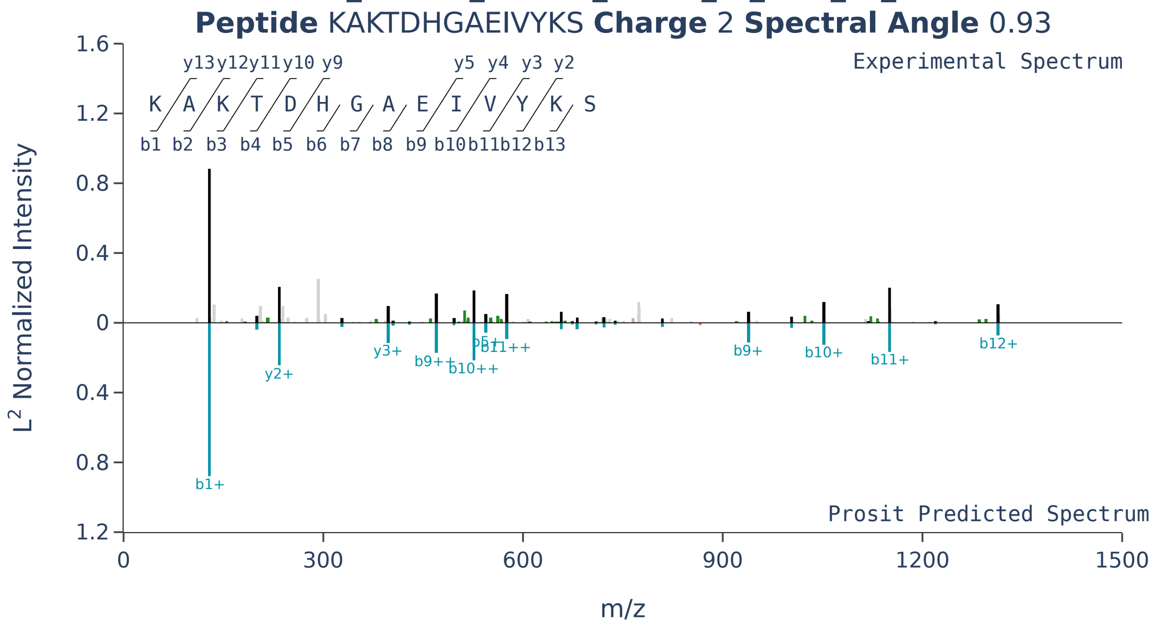


**Source** WSoh_221121_011221_HFGoe_L2_hTau_2h_R1 **Scan** 7847
**Peptide** KLTFRENAKA **Charge** 2 **Spectral Angle** 0.96

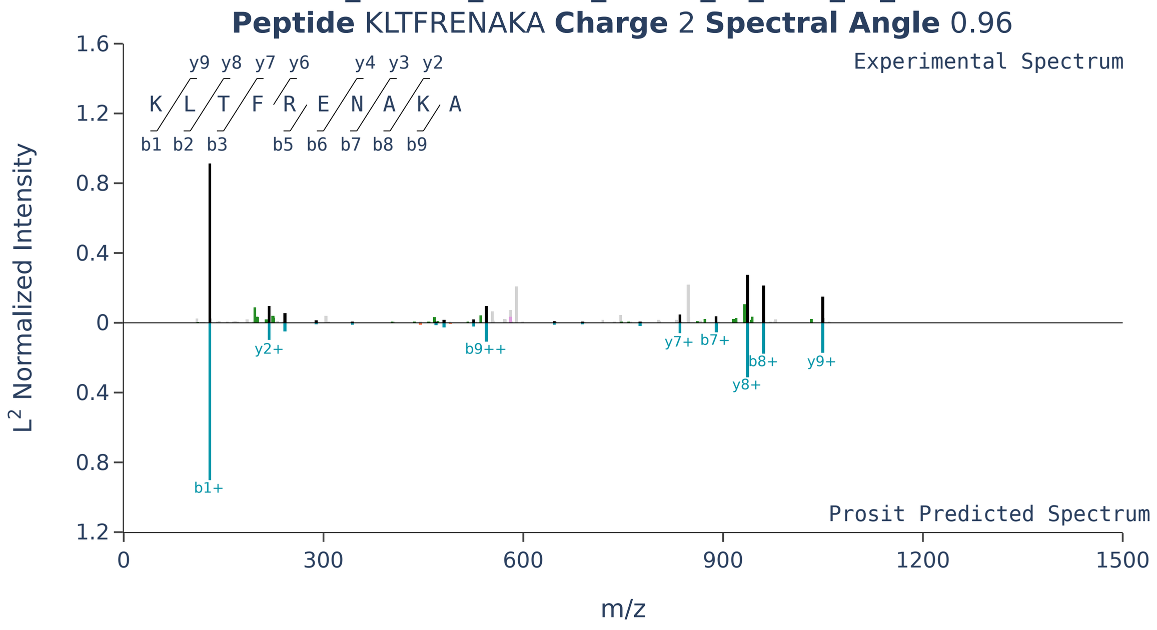


**Source** WSoh_221121_011221_HFGoe_L1_hTau_2h_R1 **Scan** 12455
**Peptide** IVYKPVD **Charge** 2 **Spectral Angle** 0.97

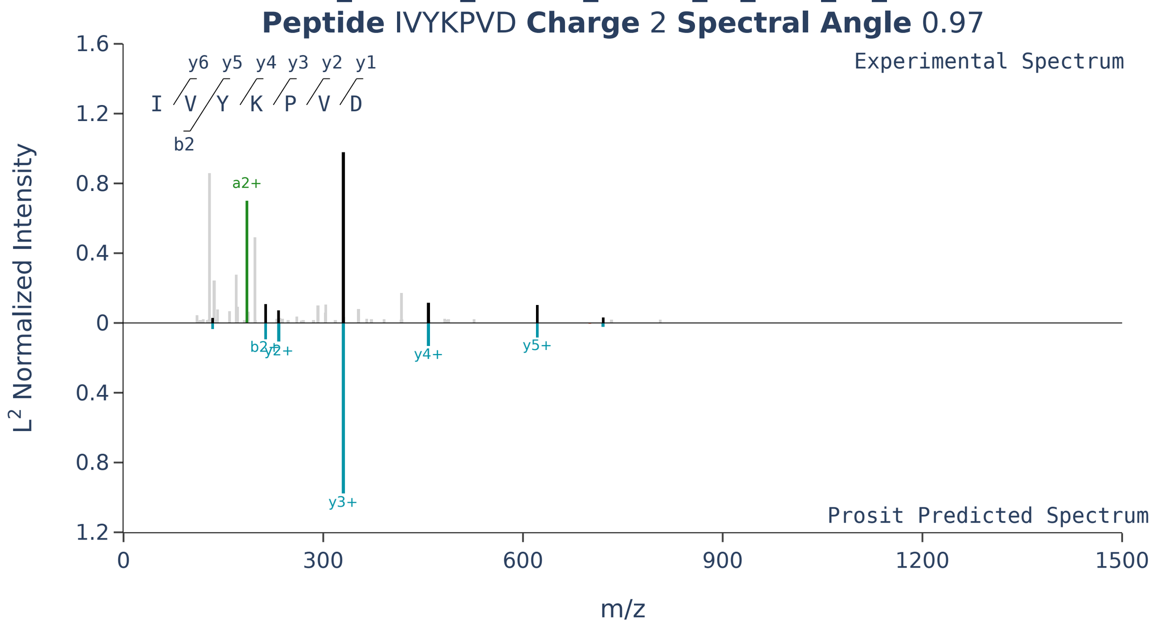


**Source** WSoh_221121_011221_HFGoe_L2_hTau_4h_R1 **Scan** 7444
**Peptide** GSLGNIHH **Charge** 2 **Spectral Angle** 0.94

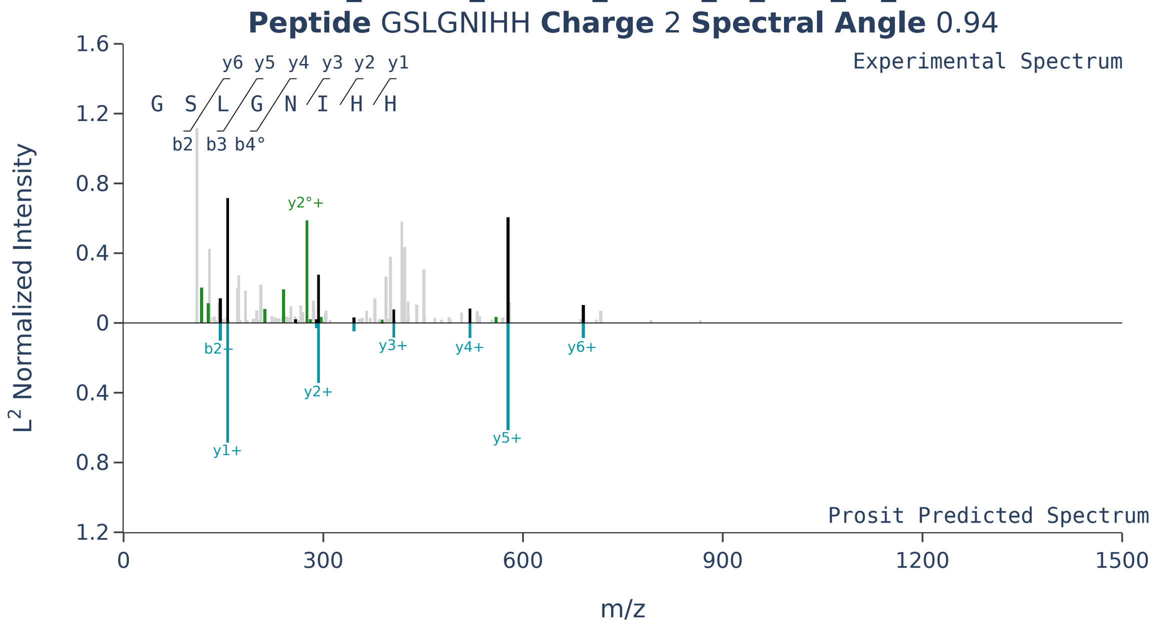


**Source** WSoh_221121_011221_HFGoe_L2_hTau_4h_R1 **Scan** 9946
**Peptide** KLTFRENA **Charge** 2 **Spectral Angle** 0.96

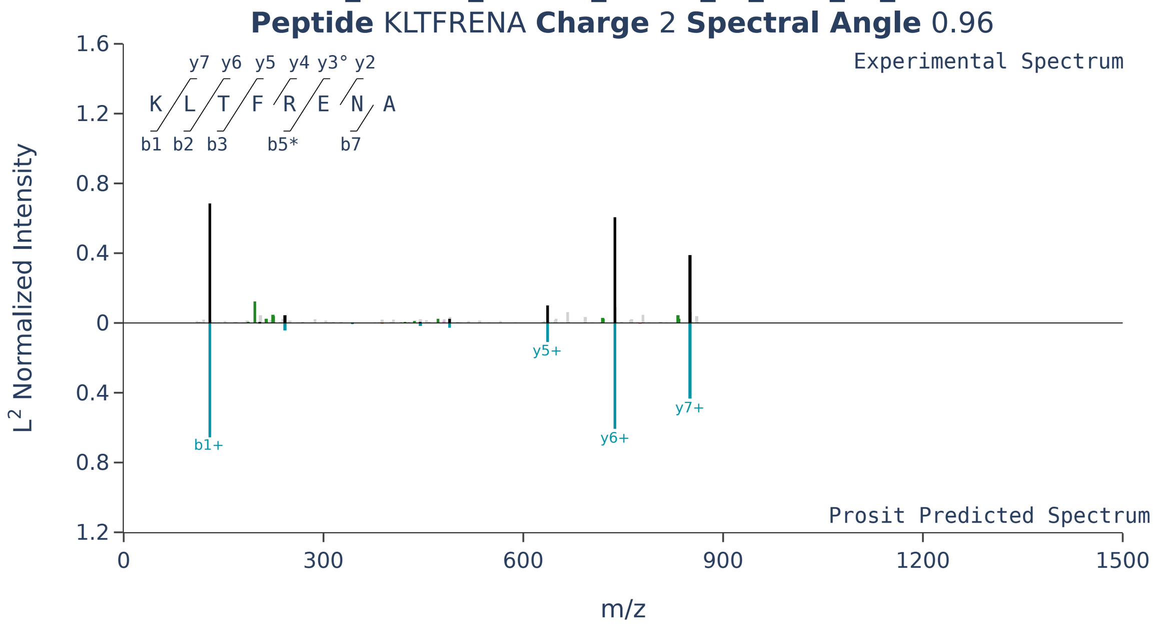


**Source** WSoh_221121_011221_HFGoe_L1_hTau_24h_R1 **Scan** 8082
**Peptide** LEDEAAGHVTQAR **Charge** 3 **Spectral Angle** 0.93

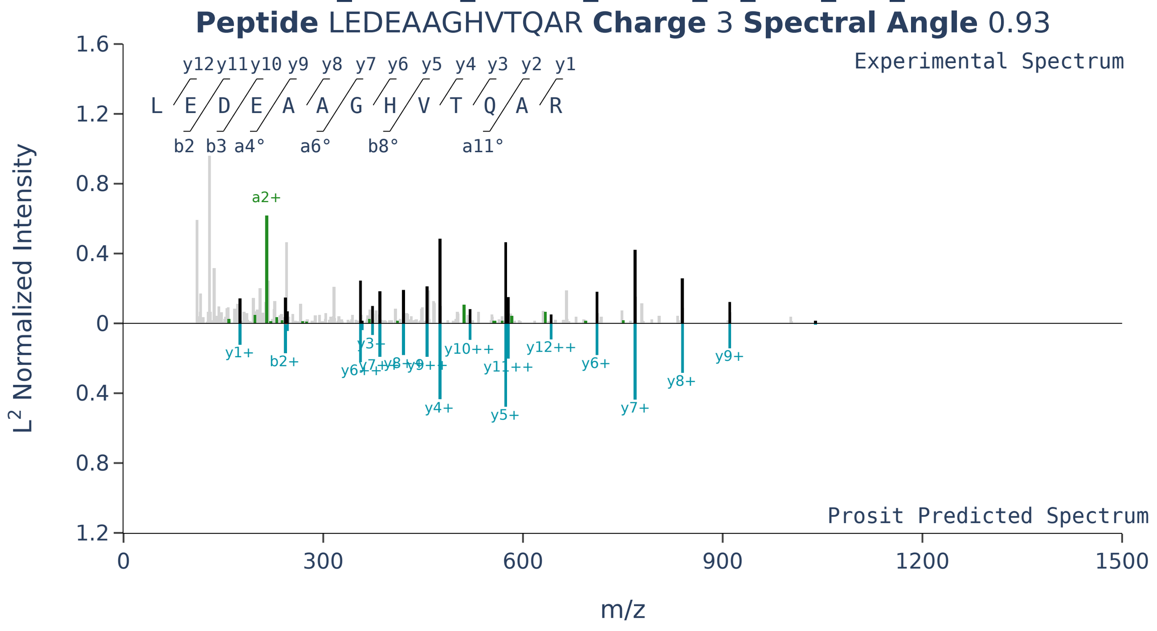


**Source** WSoh_221121_011221_HFGoe_L2_hTau_4h_R1 **Scan** 16897
**Peptide** AGLKESPLQTPTE **Charge** 2 **Spectral Angle** 0.95

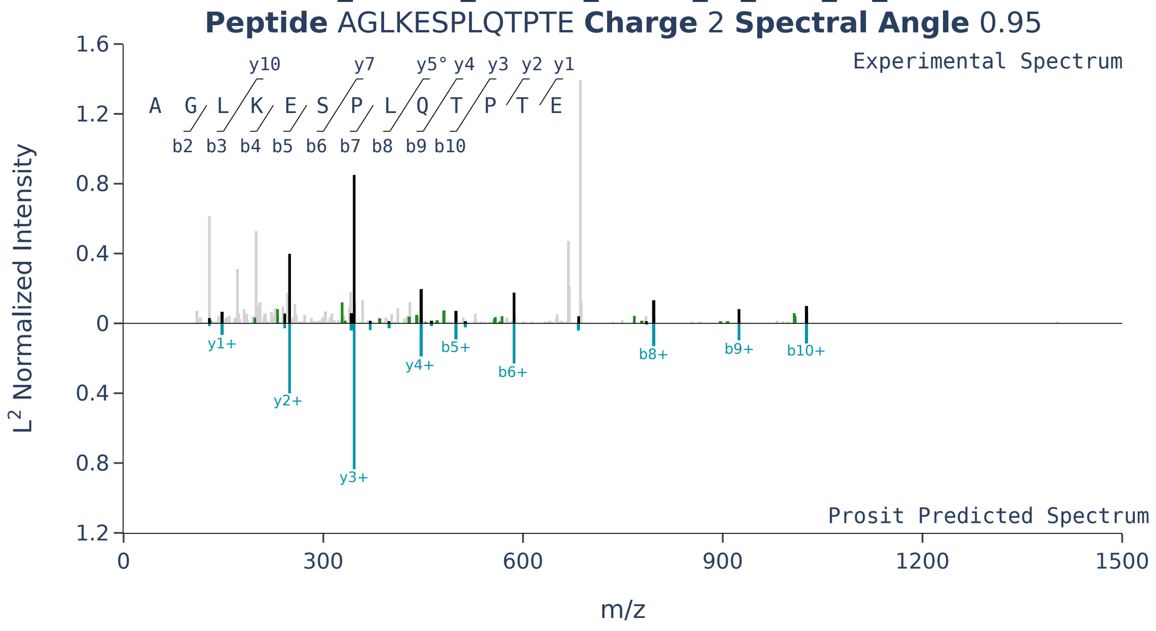


**Source** WSoh_221121_011221_HFGoe_L1_hTau_24h_R2 **Scan** 16773
**Peptide** LADEVSASLAKQ **Charge** 2 **Spectral Angle** 0.9

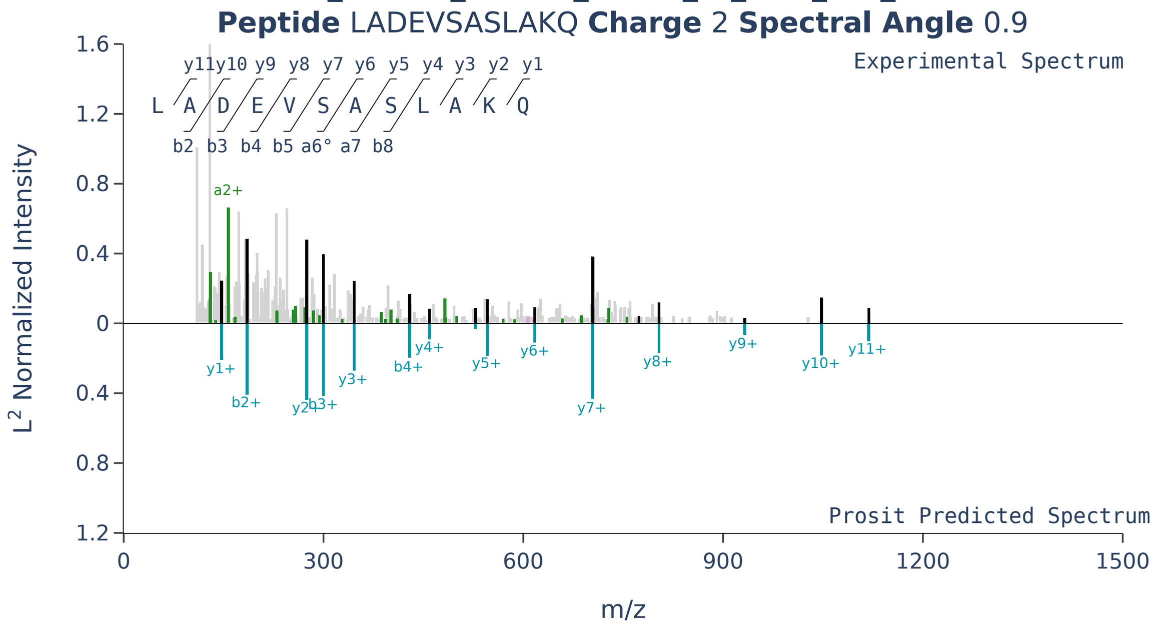


**Source** WSoh_221121_011221_HFGoe_L1_hTau_2h_R2 **Scan** 7920
**Peptide** QEGDTDAGLKE **Charge** 1 **Spectral Angle** 0.92

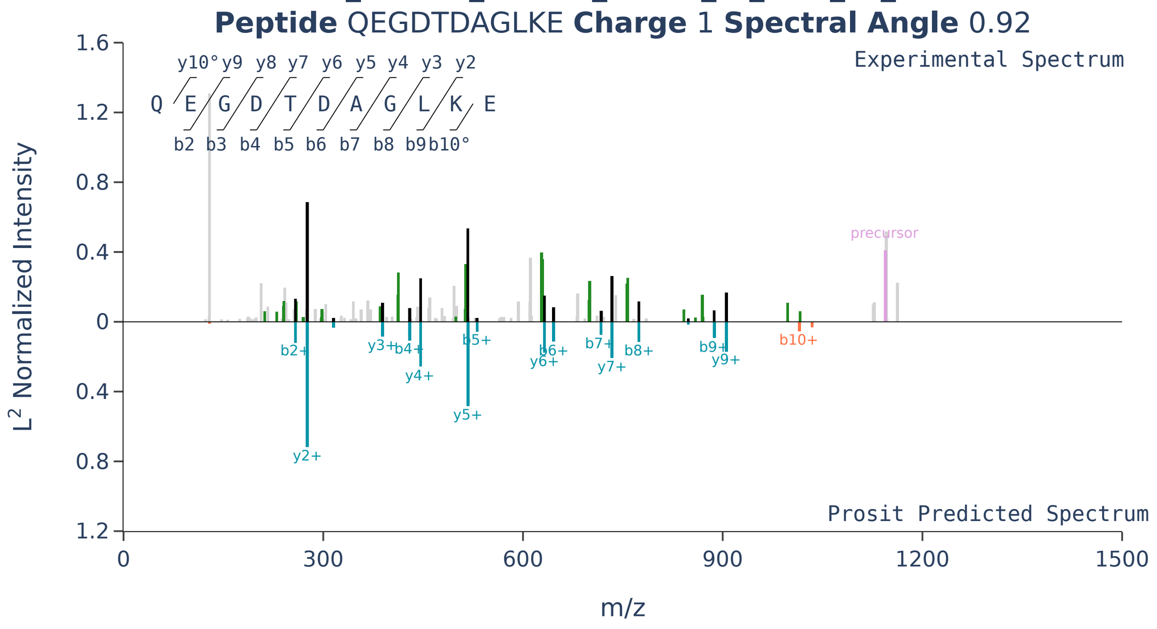


**Source** WSoh_221121_011221_HFGoe_L1_hTau_24h_R1 **Scan** 9106
**Peptide** EDEAAGHVTQARM **Charge** 2 **Spectral Angle** 0.94

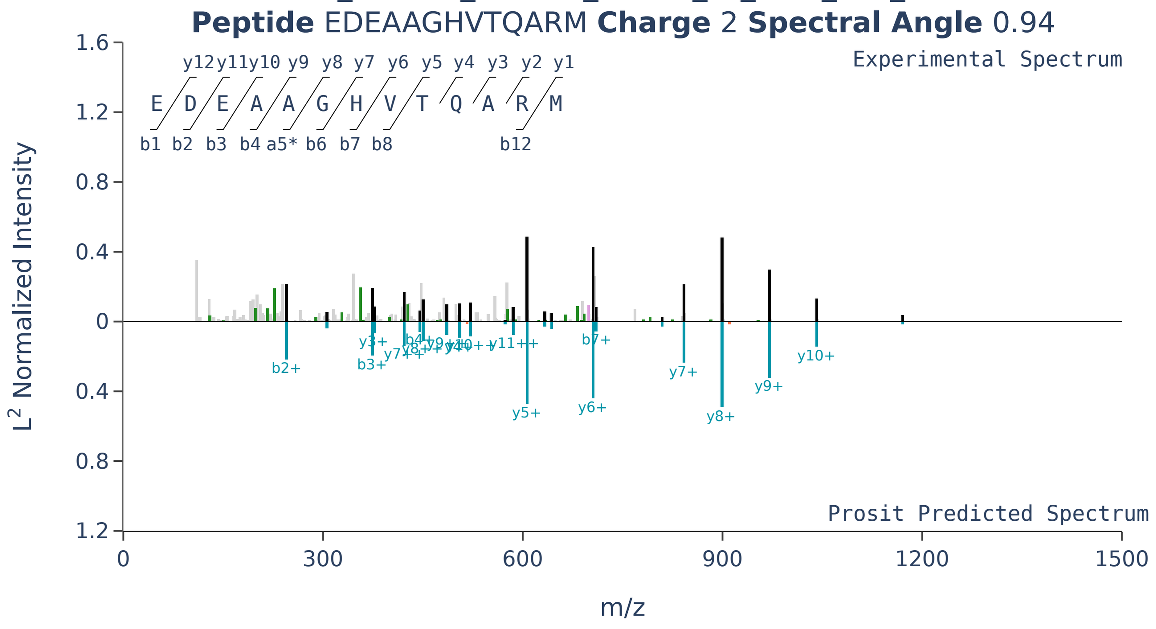


**Source** WSoh_221121_011221_HFGoe_L2_hTau_4h_R1 **Scan** 7180
**Peptide** PGGGQVEVKS **Charge** 1 **Spectral Angle** 0.94

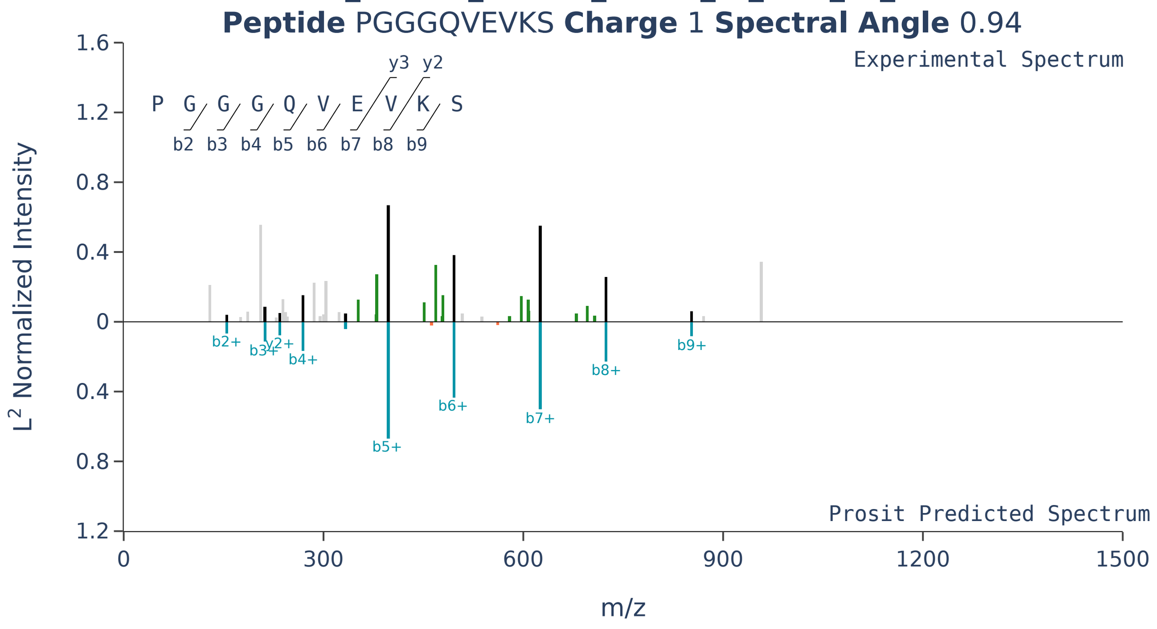


**Source** WSoh_221121_011221_HFGoe_L1_hTau_24h_R2 **Scan** 7846
**Peptide** SGDTSPRHLSNVSST **Charge** 2 **Spectral Angle** 0.94

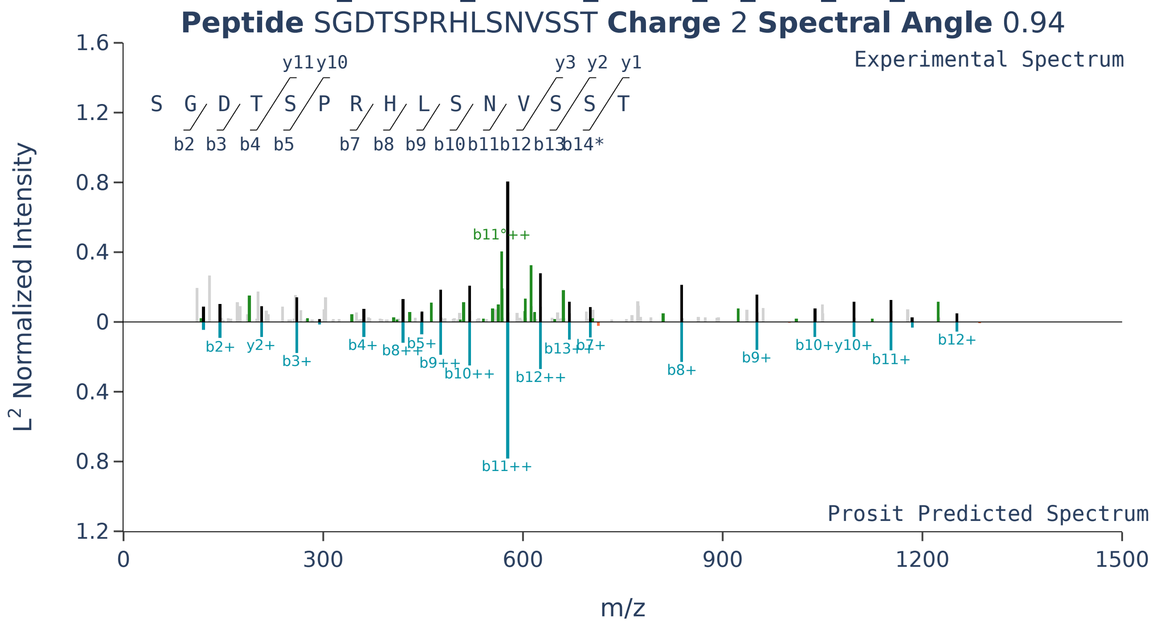


**Source** WSoh_221121_011221_HFGoe_L2_hTau_4h_R2 **Scan** 15204
**Peptide** AEPRQEFEVMED **Charge** 2 **Spectral Angle** 0.91

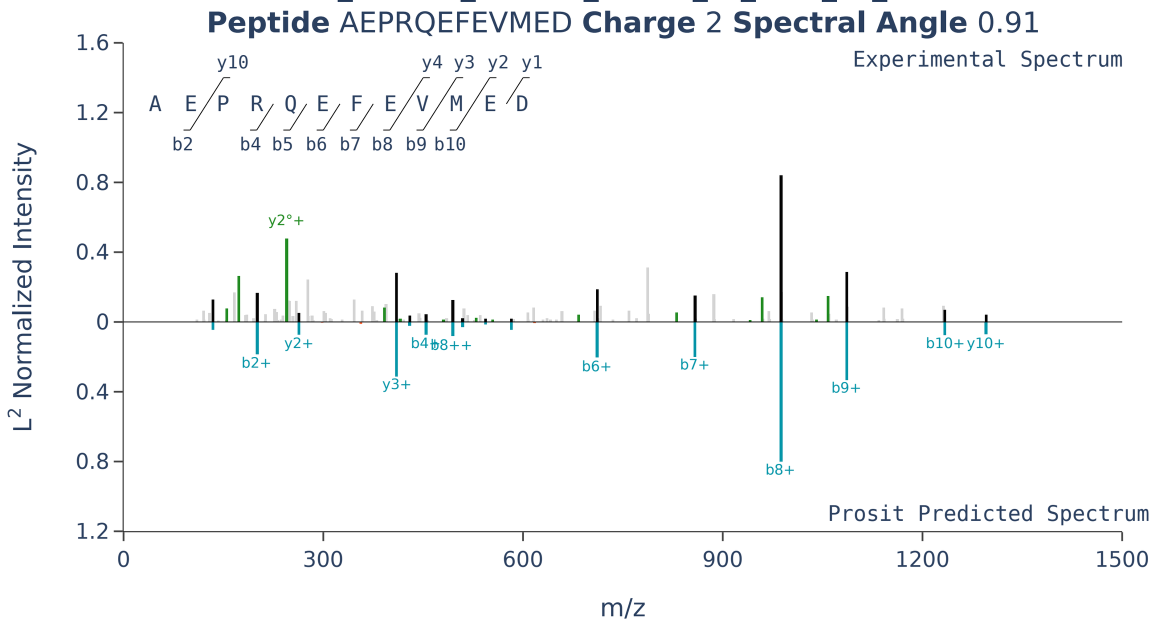


**Source** WSoh_221121_011221_HFGoe_L1_hTau_4h_R1 **Scan** 16427
**Peptide** AEPRQEFEVMEDH **Charge** 2 **Spectral Angle** 0.94

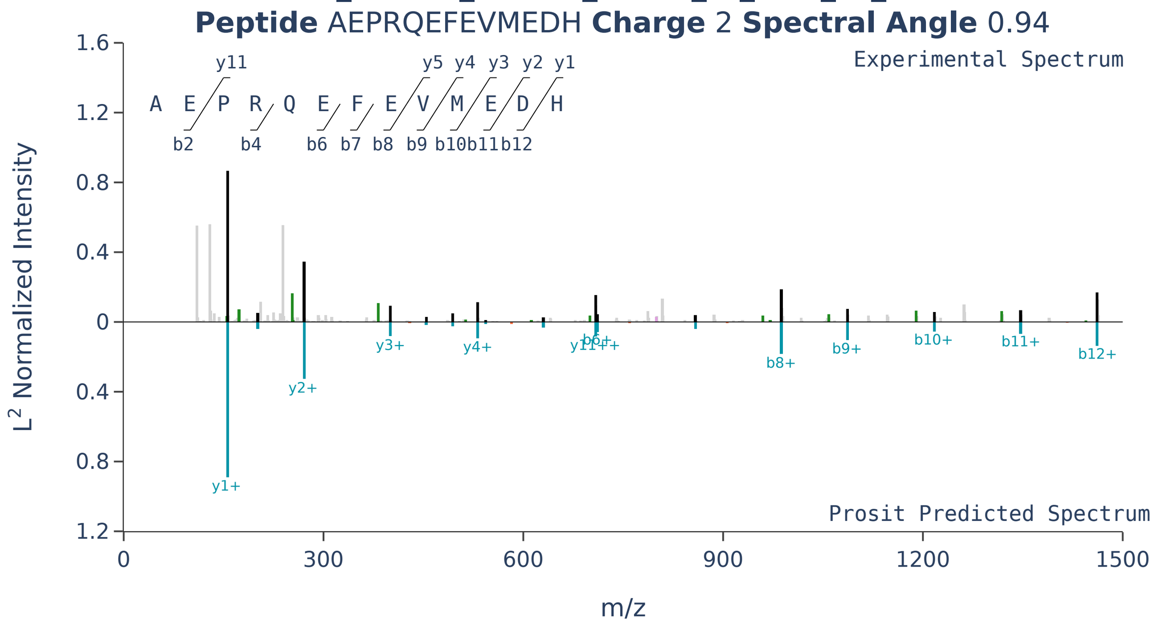

**Source** WSoh_221121_011221_HFGoe_L2_hTau_24h_R2 **Scan** 17876
**Peptide** HGAEIVYKSPVV **Charge** 2 **Spectral Angle** 0.93

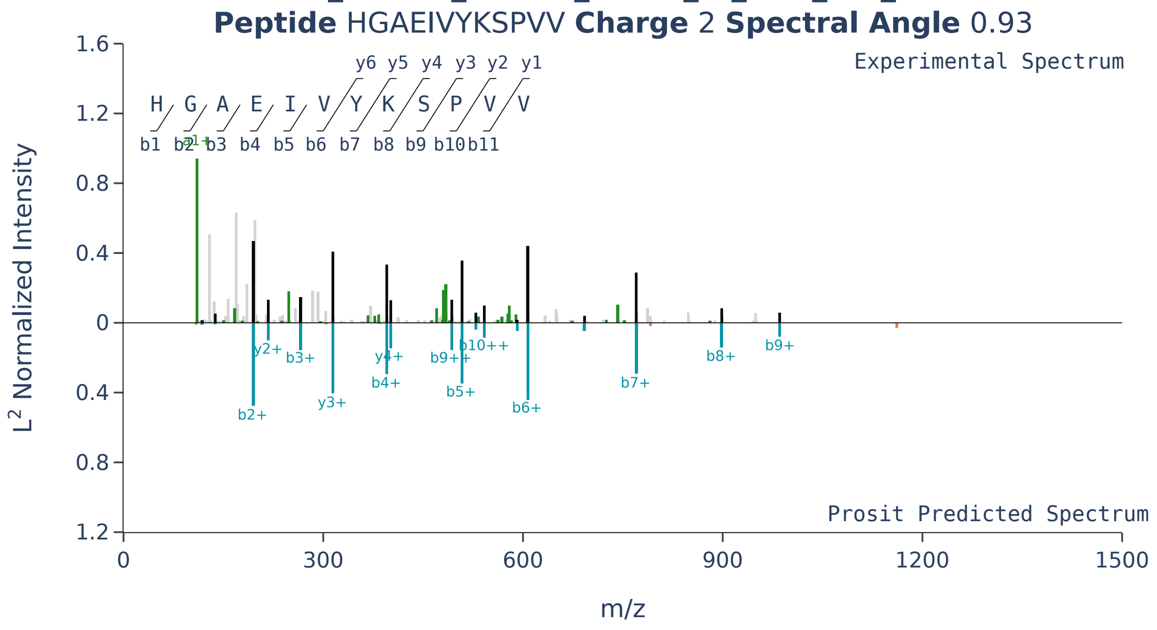


**Source** WSoh_221121_011221_HFGoe_L1_hTau_4h_R2 **Scan** 7035
**Peptide** HQDQEGDTDAGLKE **Charge** 2 **Spectral Angle** 0.93

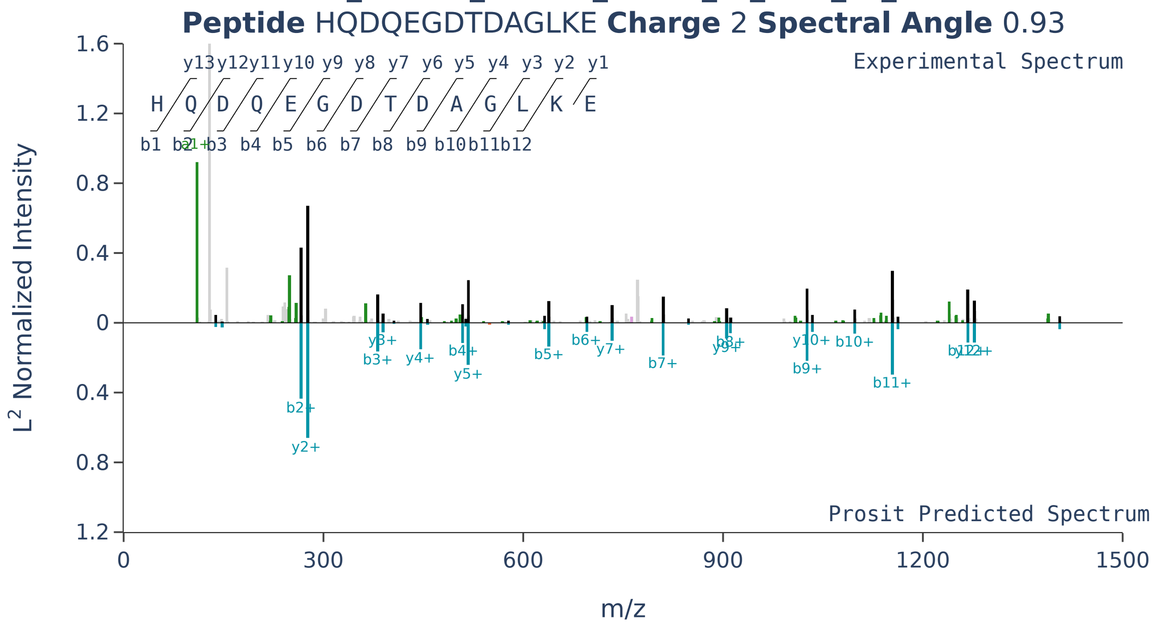


**Source** WSoh_221121_011221_HFGoe_L2_hTau_4h_R2 **Scan** 13811
**Peptide** GYSSPGSPGTPGSRSRTPSL **Charge** 3 **Spectral Angle** 0.96

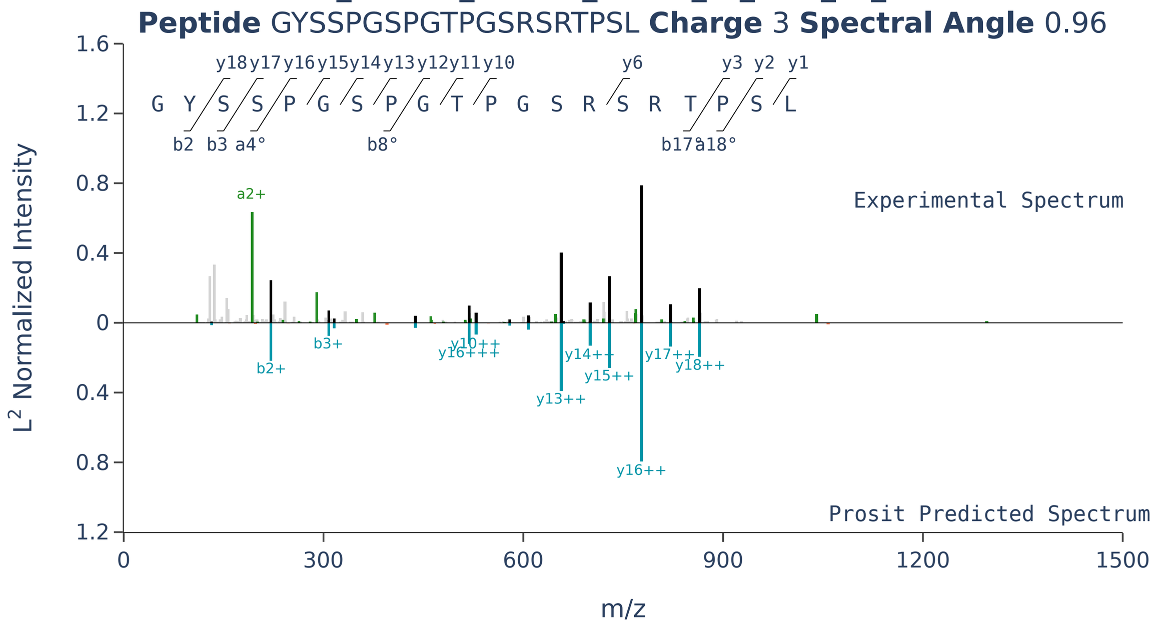


**Source** WSoh_221121_011221_HFGoe_L1_hTau_24h_R1 **Scan** 7388
**Peptide** YGLGDRKD **Charge** 2 **Spectral Angle** 0.92

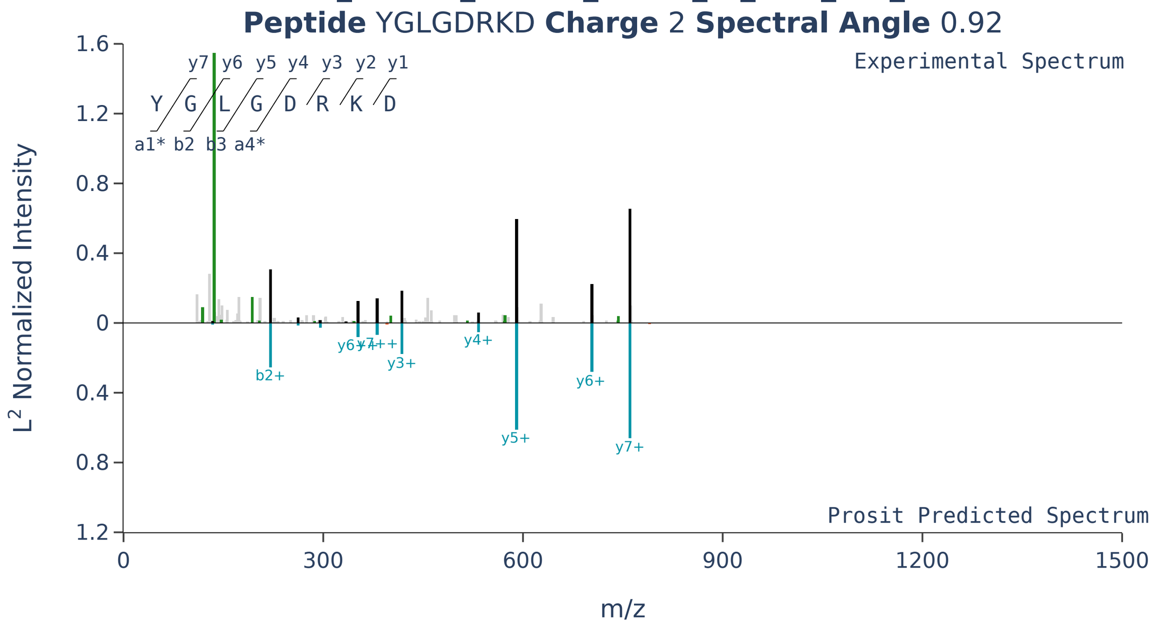


**Source** WSoh_221121_011221_HFGoe_L1_hTau_2h_R2 **Scan** 11700
**Peptide** ADEVSASLAKQ **Charge** 2 **Spectral Angle** 0.93

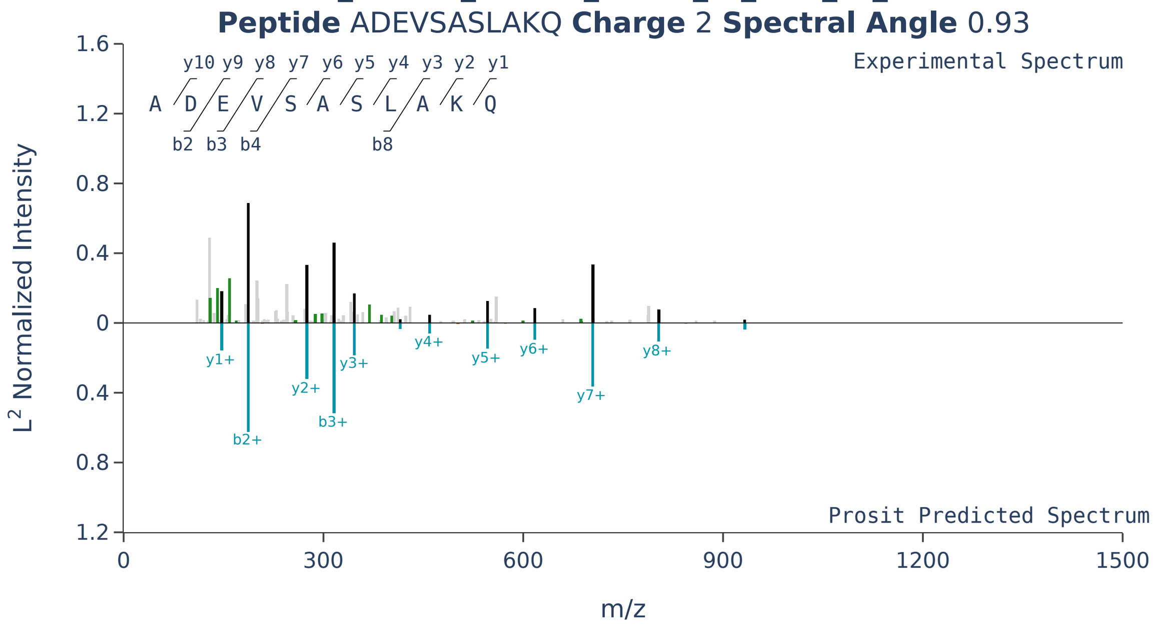


**Source** WSoh_221121_011221_HFGoe_L1_hTau_4h_R2 **Scan** 10585
**Peptide** KLDFKDRVQS **Charge** 2 **Spectral Angle** 0.94

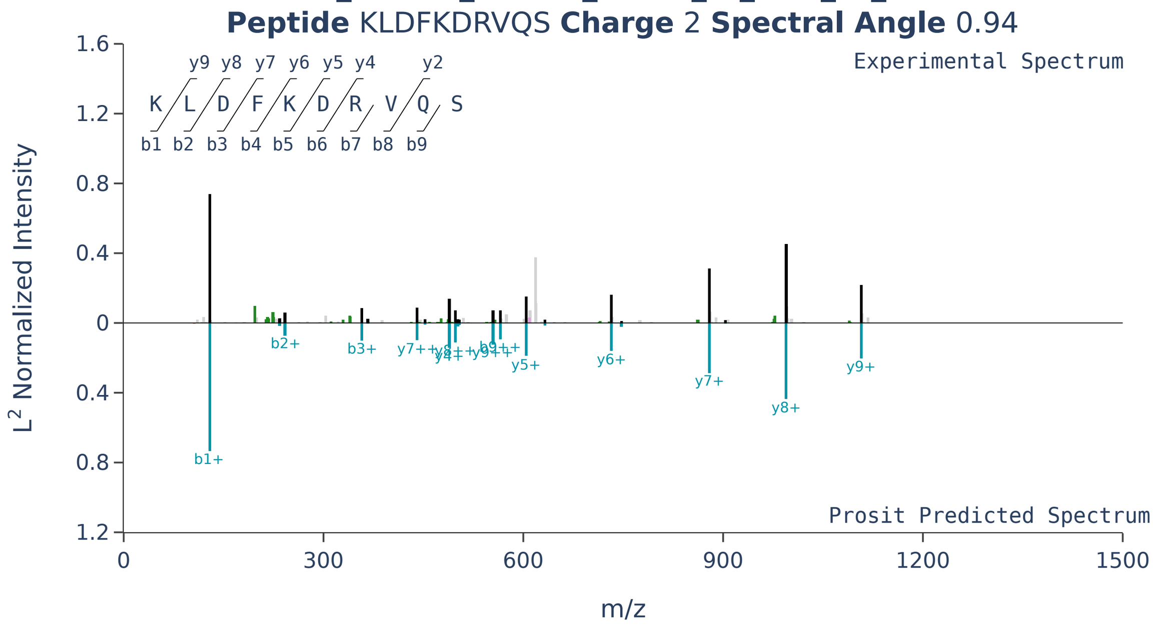


**Source** WSoh_221121_011221_HFGoe_L2_hTau_2h_R2 **Scan** 16747
**Peptide** AGLKESPLQTPTED **Charge** 2 **Spectral Angle** 0.91

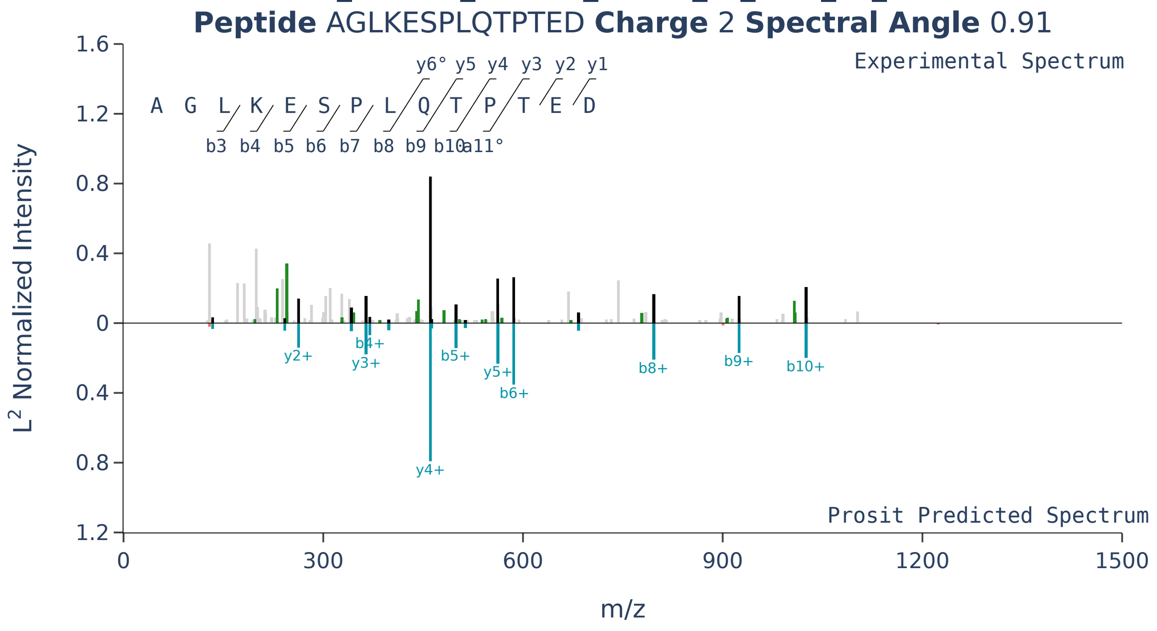


**Source** WSoh_221121_011221_HFGoe_L2_hTau_4h_R1 **Scan** 10537
**Peptide** KLDFKDRVQ **Charge** 2 **Spectral Angle** 0.94

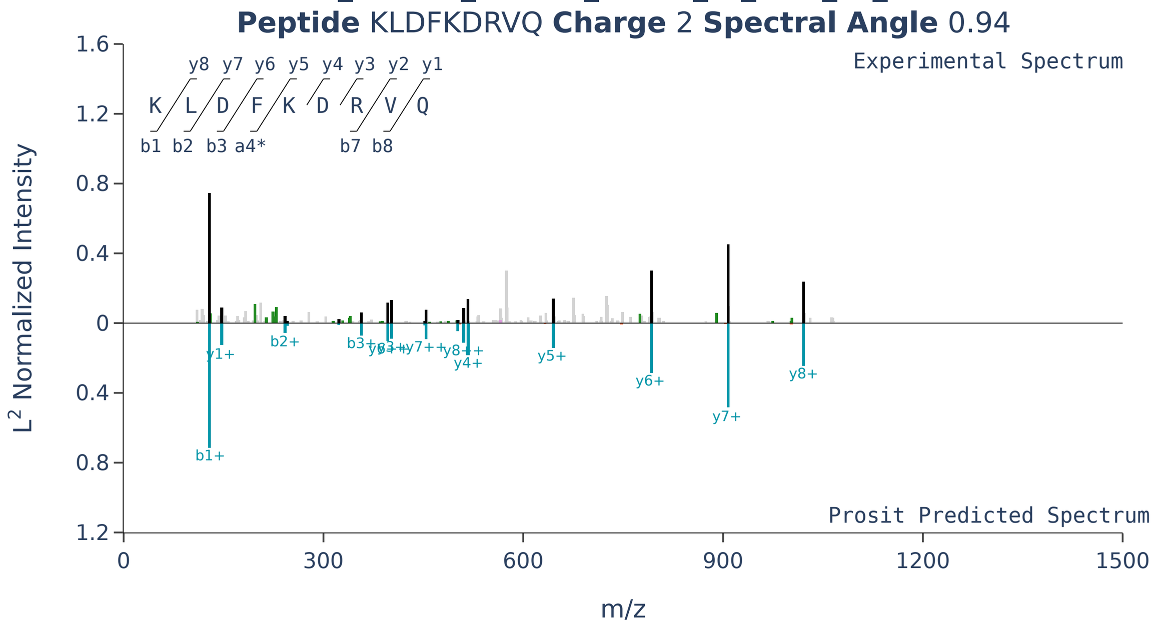


**Source** WSoh_221121_011221_HFGoe_L1_hTau_24h_R2 **Scan** 9340
**Peptide** ETHKLTFRENA **Charge** 2 **Spectral Angle** 0.93

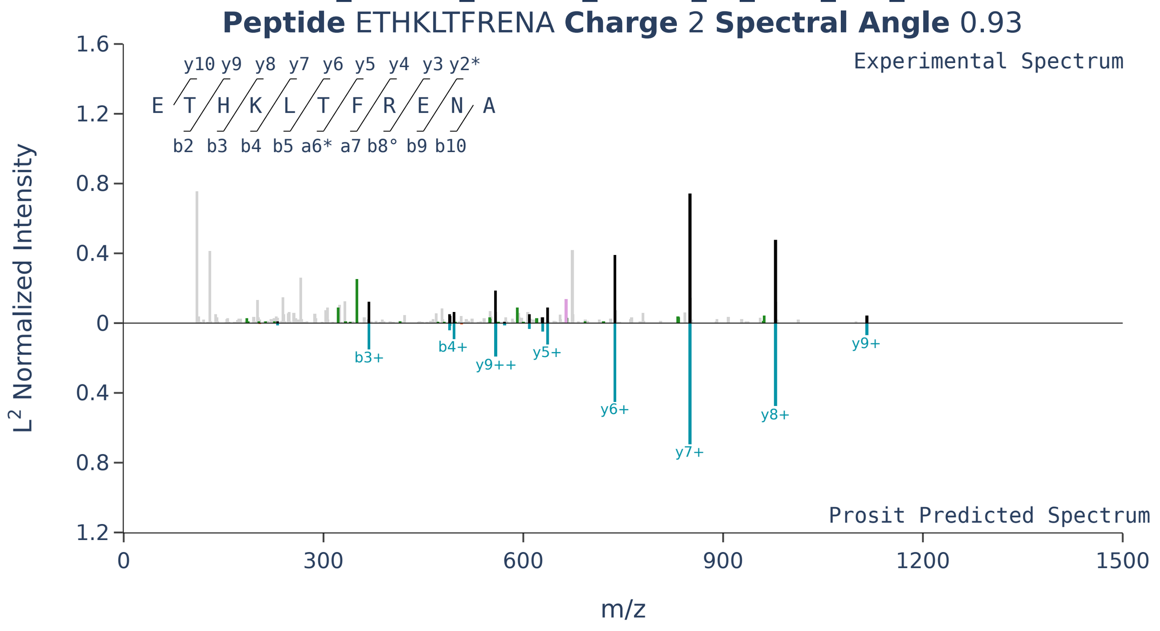


**Source** WSoh_221121_011221_HFGoe_L2_hTau_4h_R1 **Scan** 13738
**Peptide** AAQPHTEIPEGTTAEEA **Charge** 2 **Spectral Angle** 0.93

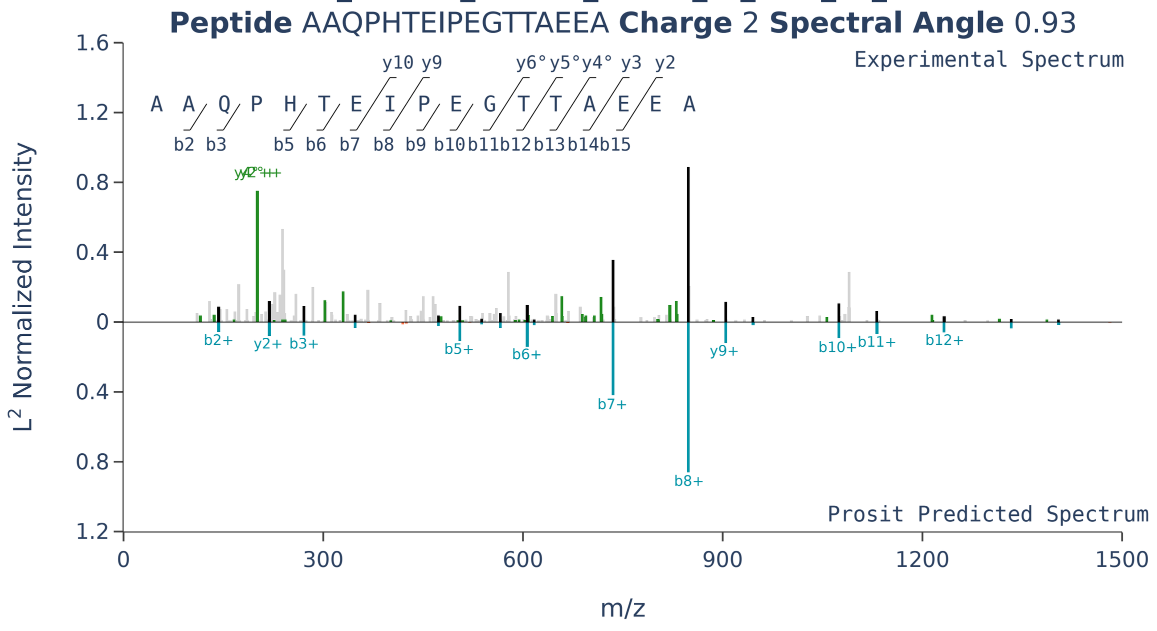


**Source** WSoh_221121_011221_HFGoe_L1_hTau_24h_R1 **Scan** 7745
**Peptide** DHAGTYGLGDRKDQ **Charge** 2 **Spectral Angle** 0.94

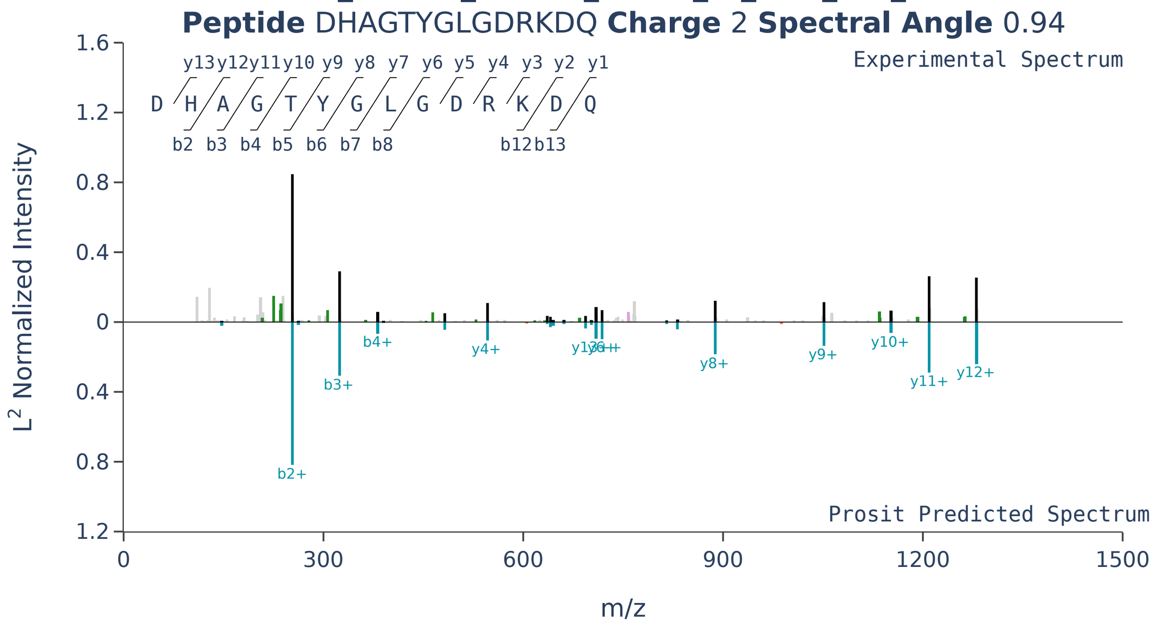


**Source** WSoh_221121_011221_HFGoe_L2_hTau_24h_R1 **Scan** 15812
**Peptide** VYKPVDLSKVTSKCG **Charge** 3 **Spectral Angle** 0.87

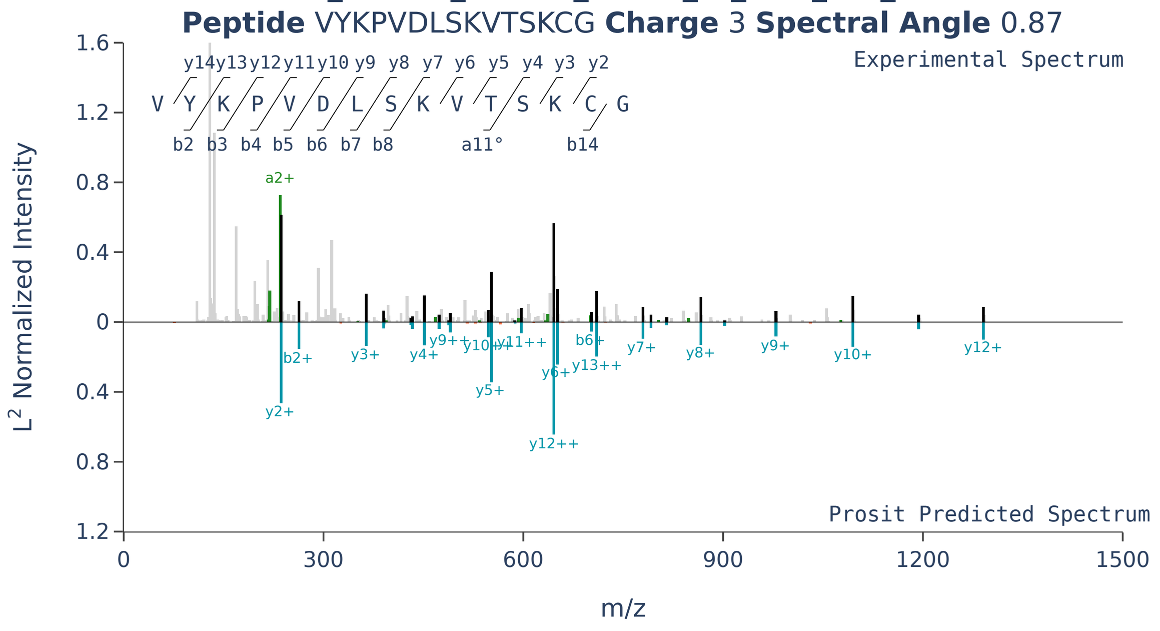


**Source** WSoh_221121_011221_HFGoe_L2_hTau_24h_R2 **Scan** 18332
**Peptide** TDAGLKESPLQTPTE **Charge** 2 **Spectral Angle** 0.95

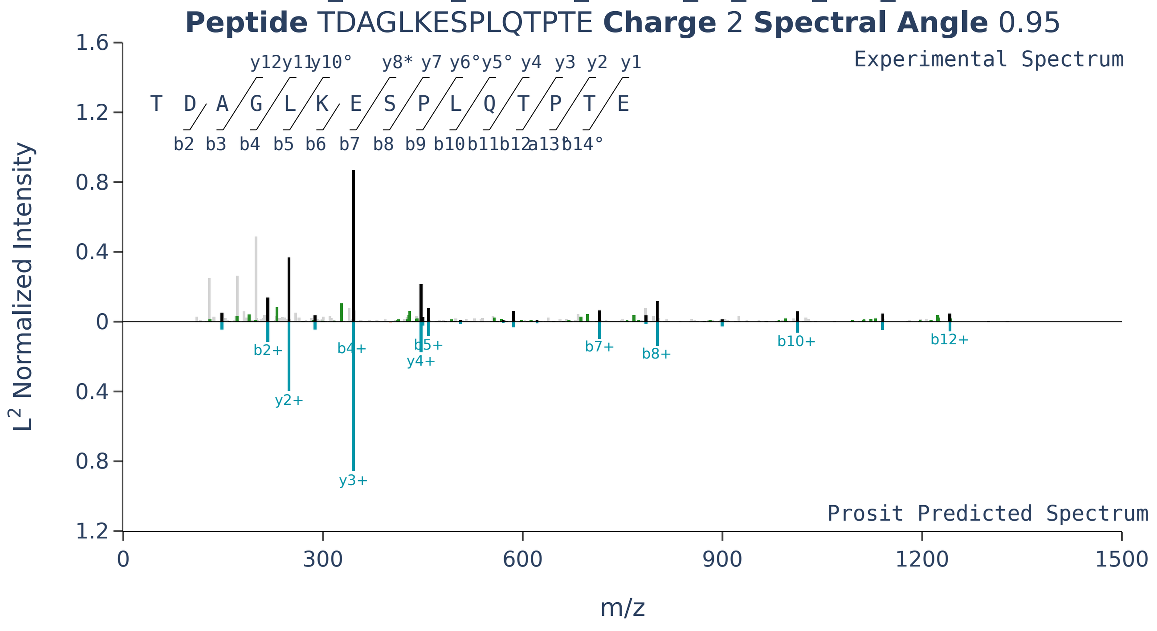


**Source** WSoh_221121_011221_HFGoe_L2_hTau_2h_R2 **Scan** 17873
**Peptide** PRQEFEVME **Charge** 2 **Spectral Angle** 0.92

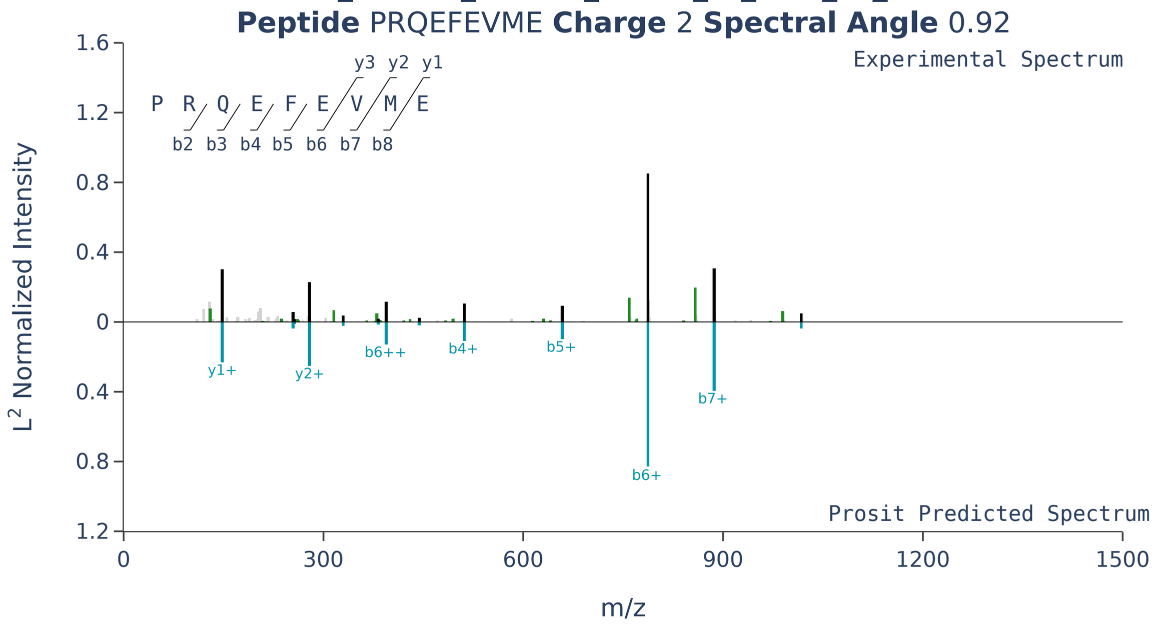


**Source** WSoh_221121_011221_HFGoe_L1_hTau_24h_R1 **Scan** 8381
**Peptide** LVDEGAPGKQA **Charge** 2 **Spectral Angle** 0.91

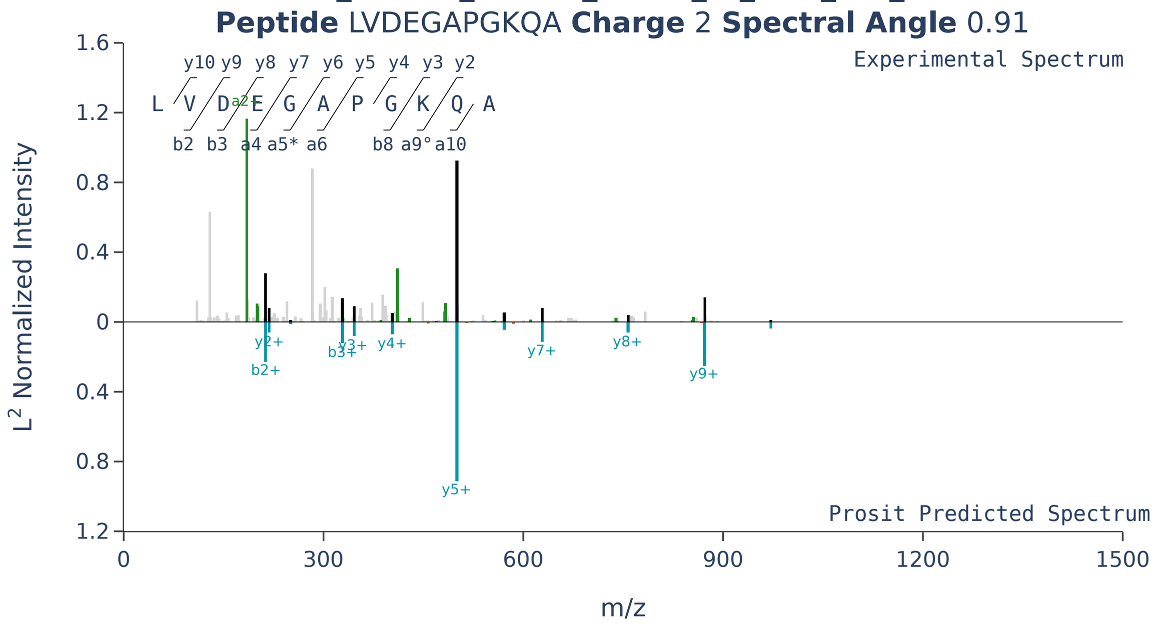

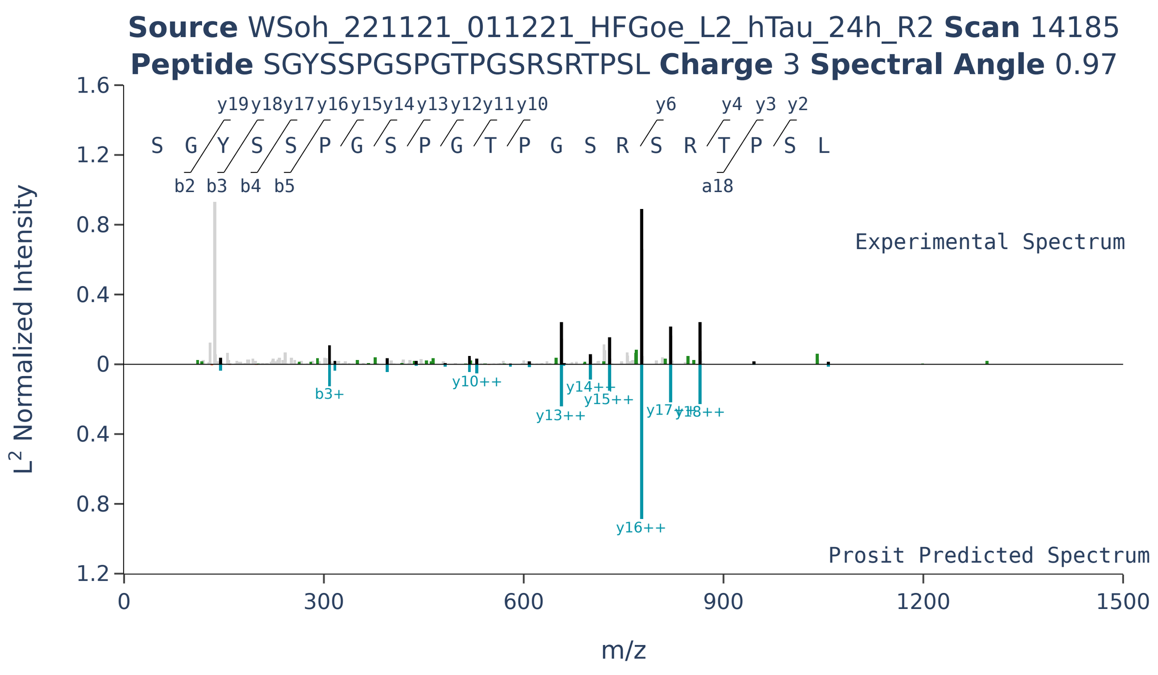
Source WSoh_221121_011221_HFGoe_L2_hTau_24h_R2 Scan 14185
Peptide SGYSSPGSPGTPGSRSRTPSL Charge 3 Spectral Angle 0.97
Experimental Spectrum
Prosit Predicted Spectrum
L² Normalized Intensity
m/z

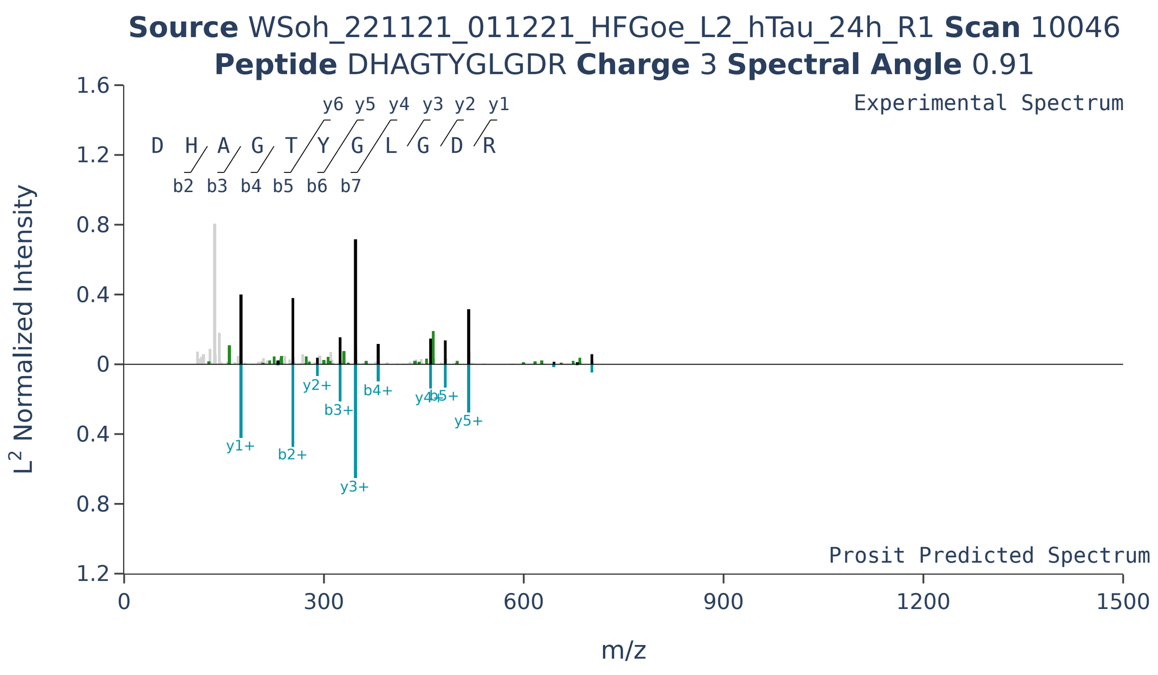
Source WSoh_221121_011221_HFGoe_L2_hTau_24h_R1 Scan 10046
Peptide DHAGTYGLGDR Charge 3 Spectral Angle 0.91
Experimental Spectrum
Prosit Predicted Spectrum
L² Normalized Intensity
m/z

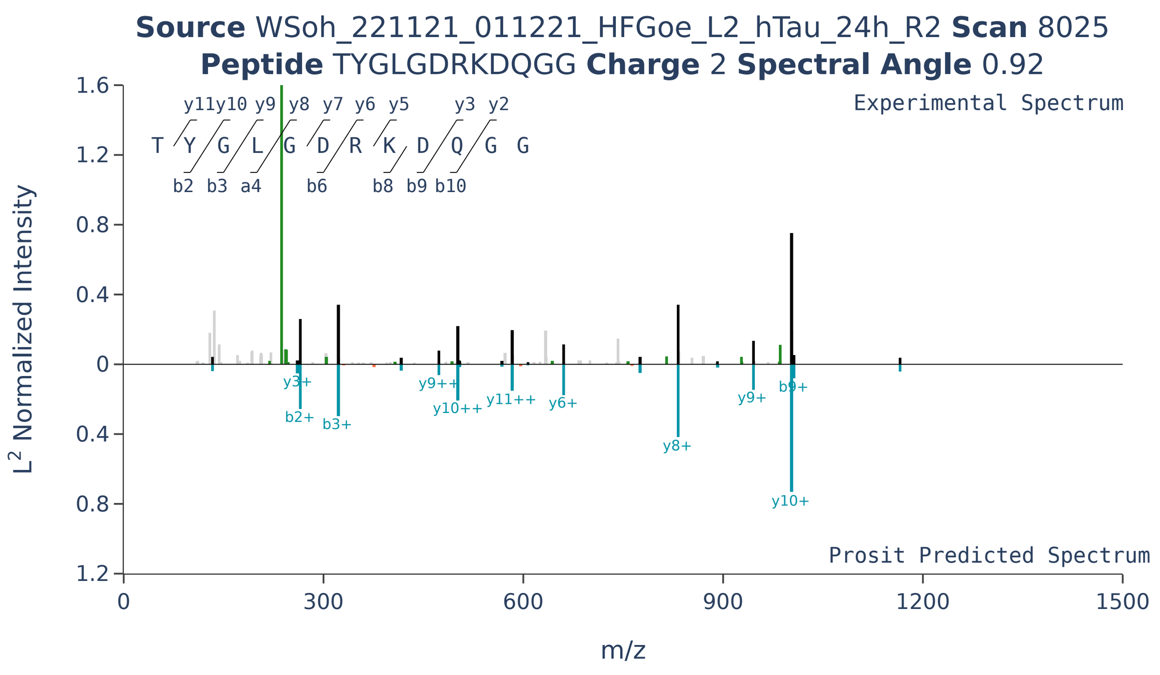
Source WSoh_221121_011221_HFGoe_L2_hTau_24h_R2 Scan 8025
Peptide TYGLGDRKDQGG Charge 2 Spectral Angle 0.92
Experimental Spectrum
Prosit Predicted Spectrum
L² Normalized Intensity
m/z

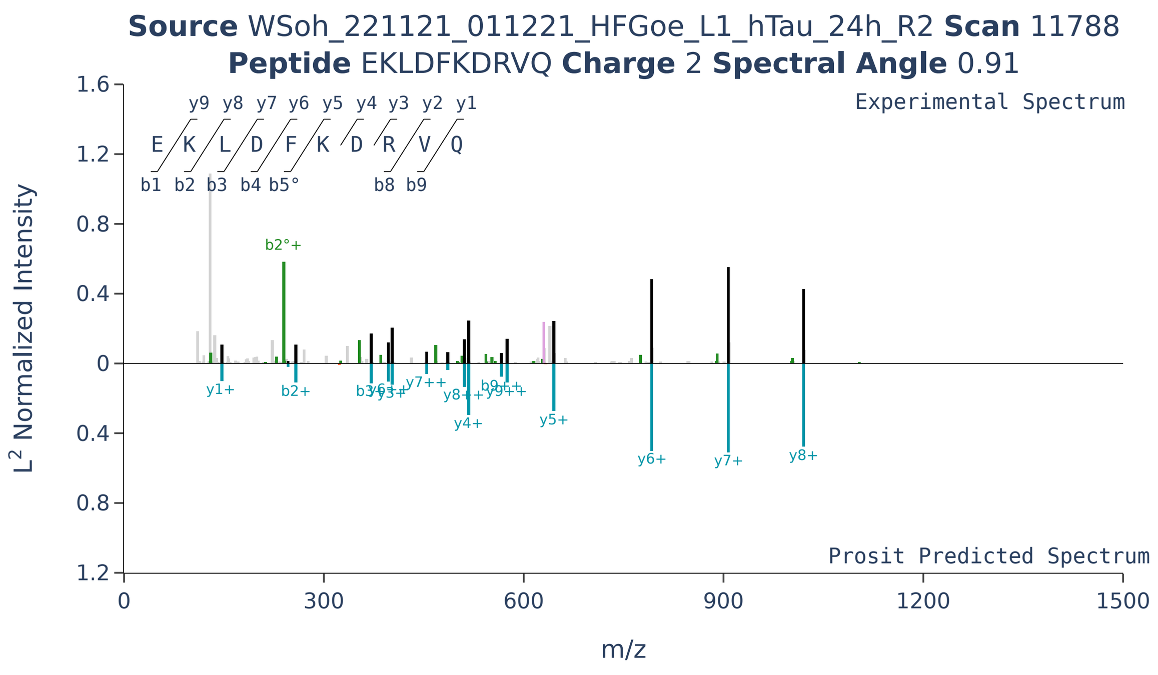
Source WSoh_221121_011221_HFGoe_L1_hTau_24h_R2 Scan 11788
Peptide EKLDFKDRVQ Charge 2 Spectral Angle 0.91
Experimental Spectrum
Prosit Predicted Spectrum
L² Normalized Intensity
m/z

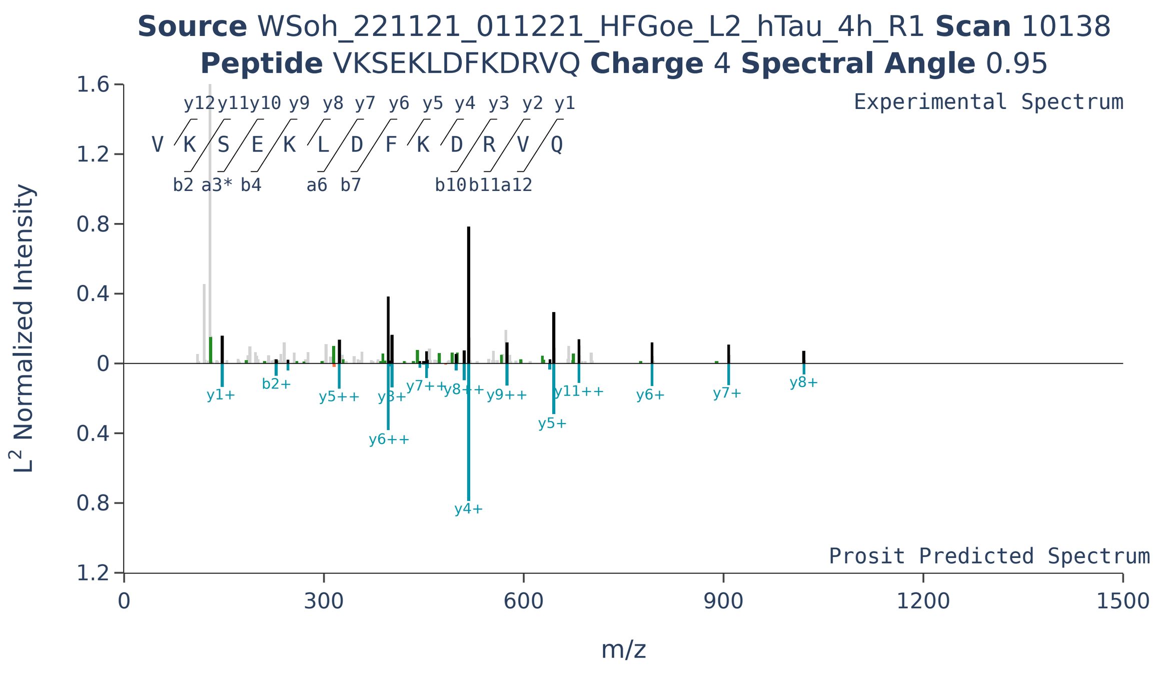
Source WSoh_221121_011221_HFGoe_L2_hTau_4h_R1 Scan 10138
Peptide VKSEKLDFKDRVQ Charge 4 Spectral Angle 0.95
Experimental Spectrum
Prosit Predicted Spectrum
L² Normalized Intensity
m/z

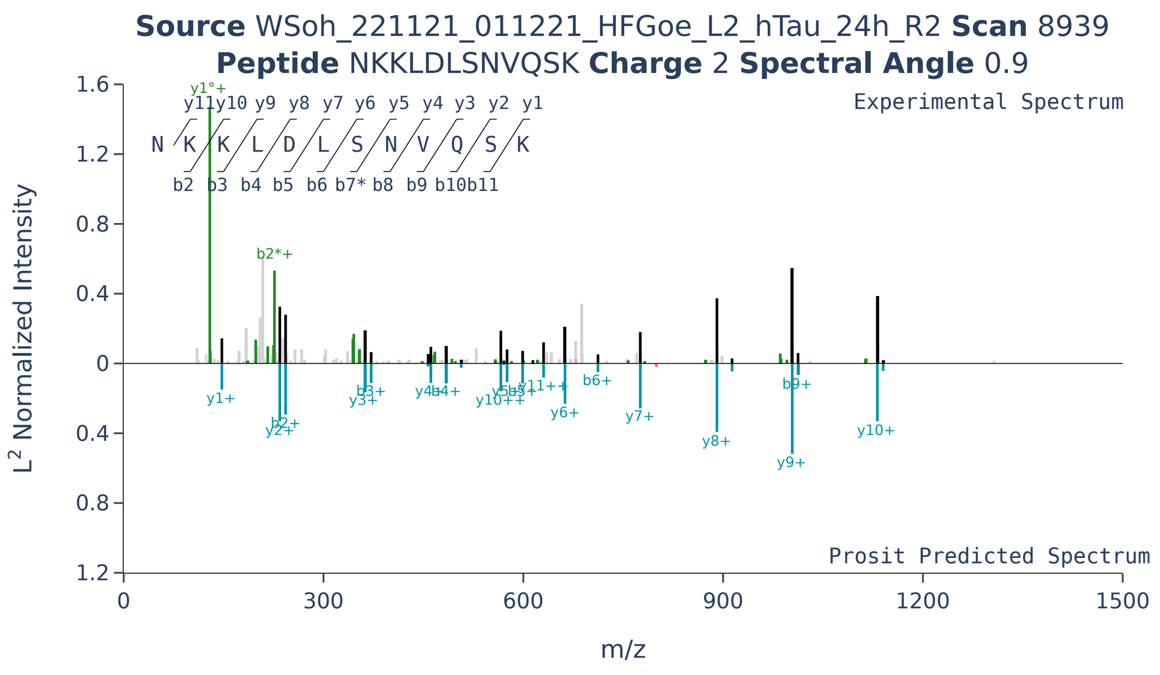
Source WSoh_221121_011221_HFGoe_L2_hTau_24h_R2 Scan 8939
Peptide NKKLDLSNVQSK Charge 2 Spectral Angle 0.9
Experimental Spectrum
Prosit Predicted Spectrum
L² Normalized Intensity
m/z

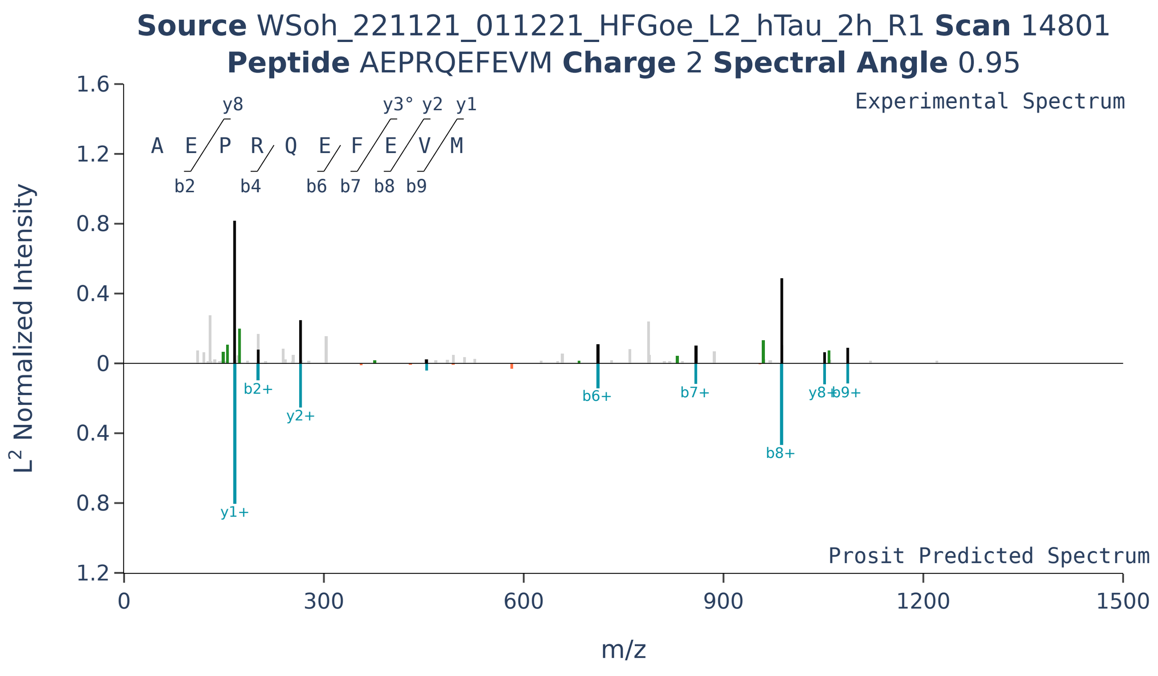
Source WSoh_221121_011221_HFGoe_L2_hTau_2h_R1 Scan 14801
Peptide AEPRQEFEVM Charge 2 Spectral Angle 0.95
Experimental Spectrum
Prosit Predicted Spectrum
L² Normalized Intensity
m/z

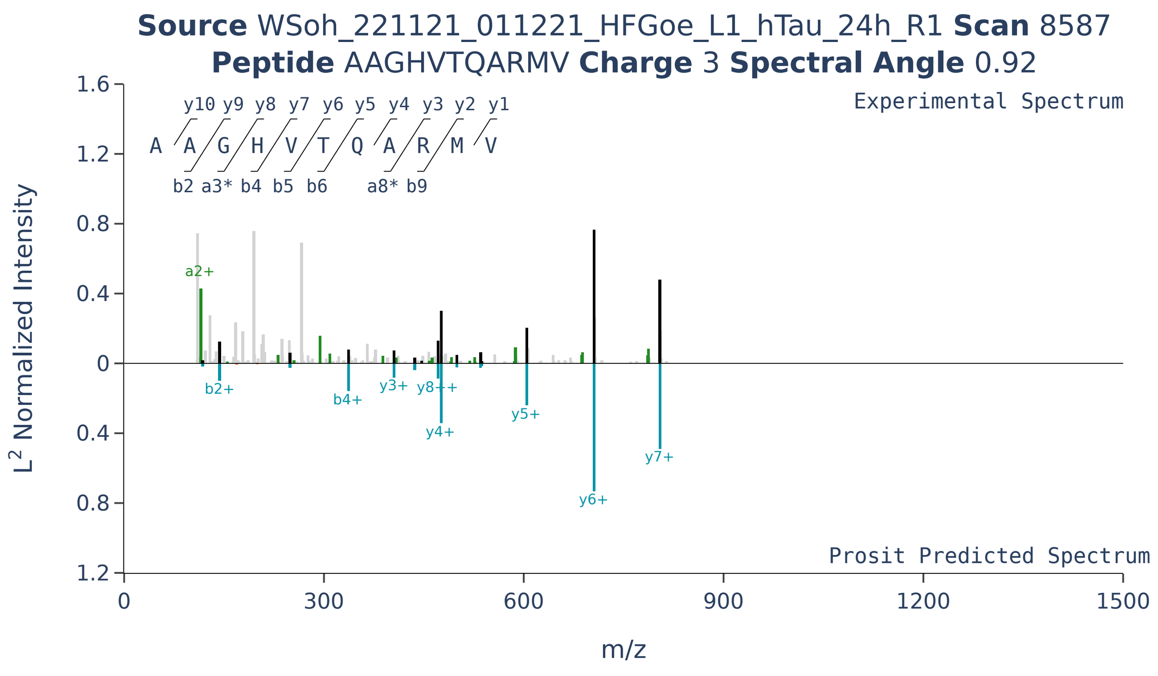
Source WSoh_221121_011221_HFGoe_L1_hTau_24h_R1 Scan 8587
Peptide AAGHVTQARMV Charge 3 Spectral Angle 0.92
Experimental Spectrum
Prosit Predicted Spectrum
L² Normalized Intensity
m/z

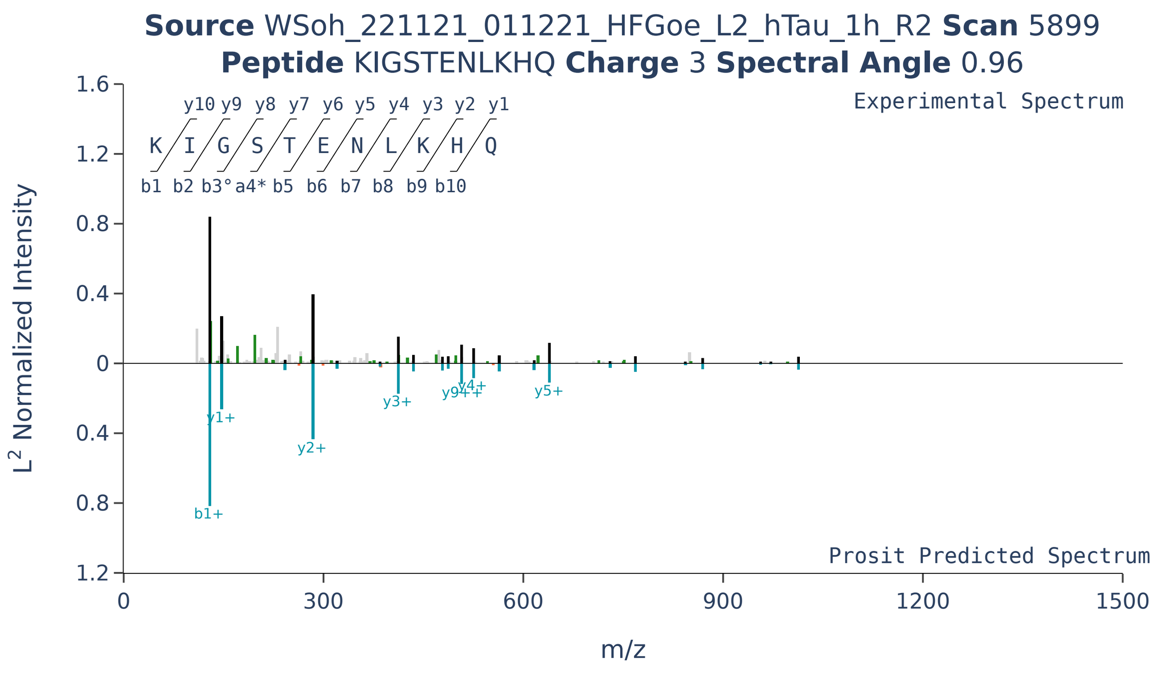
Source WSoh_221121_011221_HFGoe_L2_hTau_1h_R2 Scan 5899
Peptide KIGSTENLKHQ Charge 3 Spectral Angle 0.96
Experimental Spectrum
Prosit Predicted Spectrum
L² Normalized Intensity
m/z

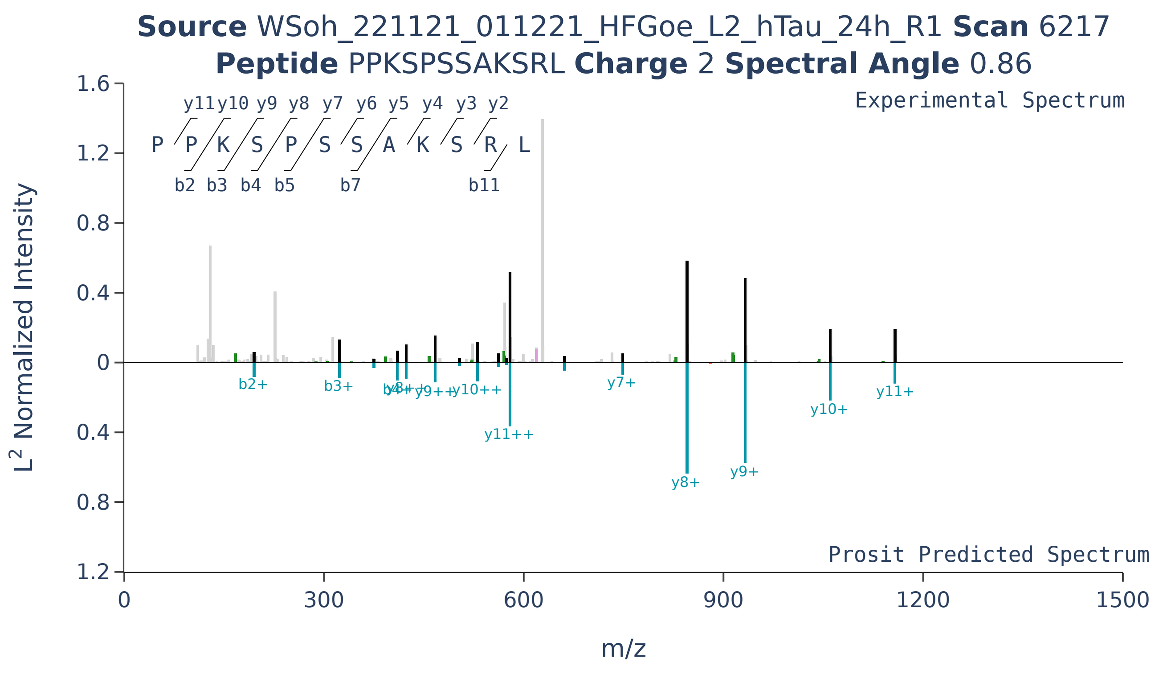
Source WSoh_221121_011221_HFGoe_L2_hTau_24h_R1 Scan 6217
Peptide PPKSPSSAKSRL Charge 2 Spectral Angle 0.86
Experimental Spectrum
Prosit Predicted Spectrum
L² Normalized Intensity
m/z

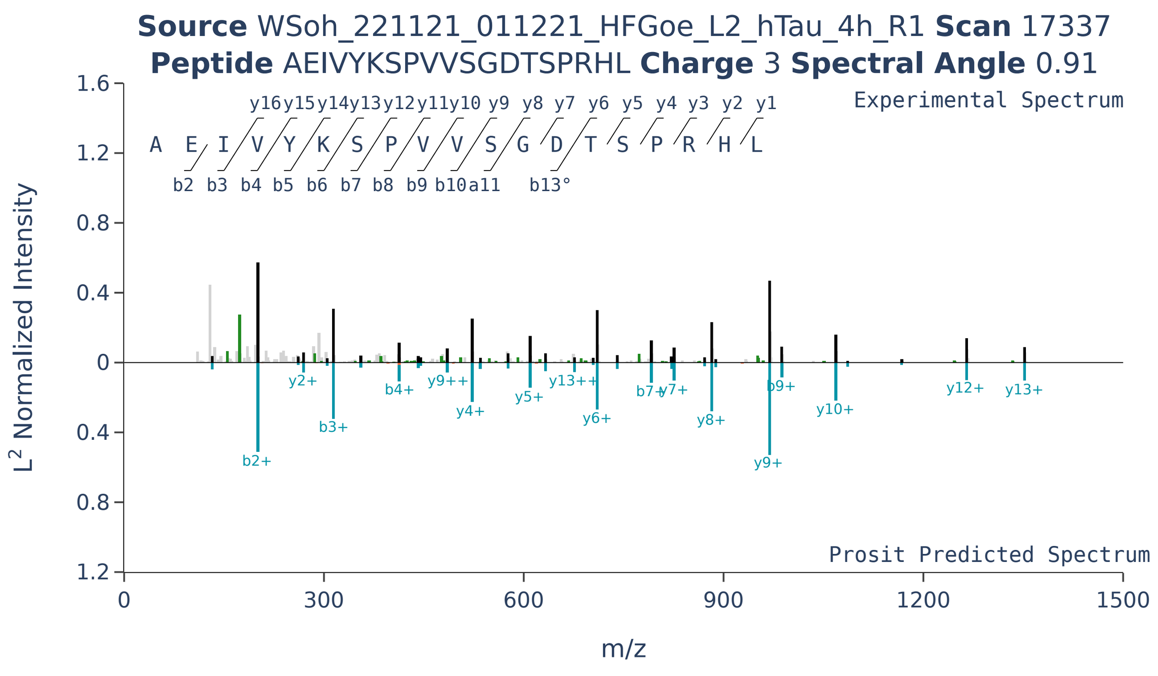
Source WSoh_221121_011221_HFGoe_L2_hTau_4h_R1 Scan 17337
Peptide AEIVYKSPVVSGDTSPRHL Charge 3 Spectral Angle 0.91
Experimental Spectrum
Prosit Predicted Spectrum
L² Normalized Intensity
m/z

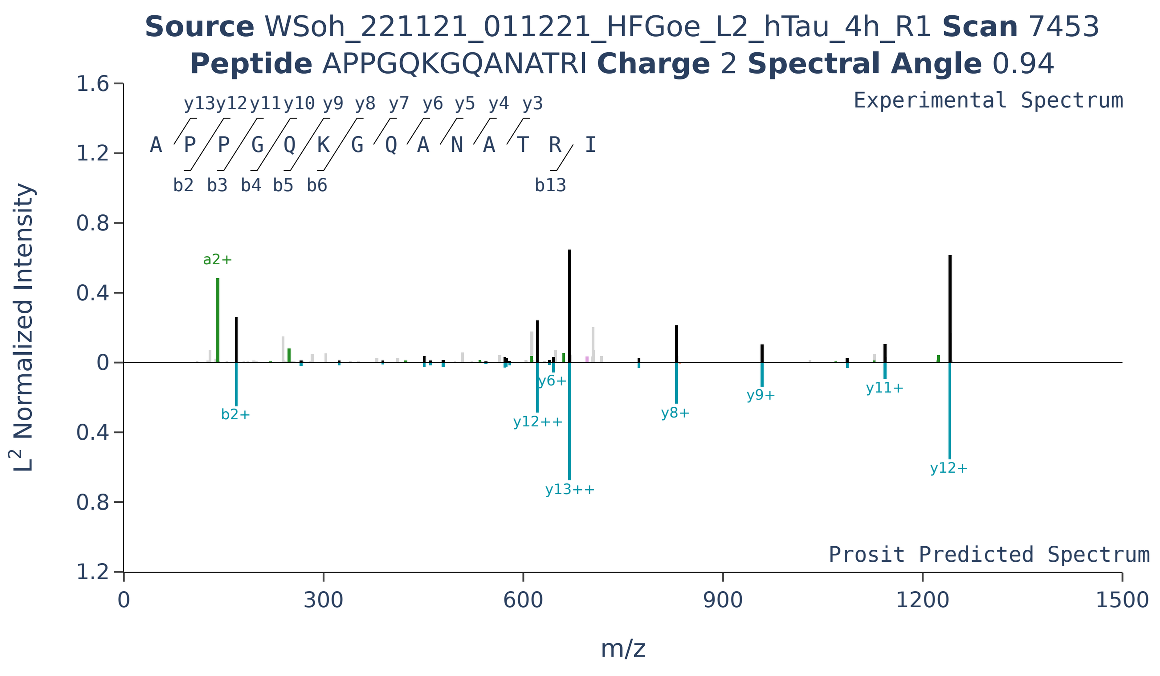
Source WSoh_221121_011221_HFGoe_L2_hTau_4h_R1 Scan 7453
Peptide APPGQKGQANATRI Charge 2 Spectral Angle 0.94
Experimental Spectrum
Prosit Predicted Spectrum
L² Normalized Intensity
m/z

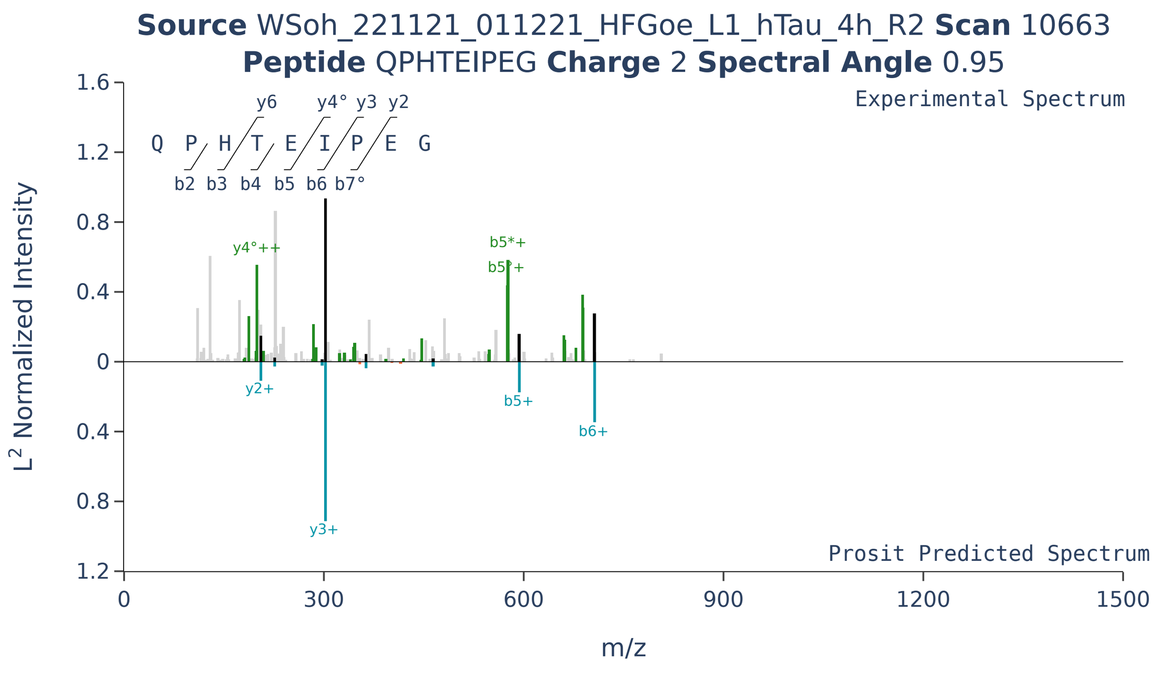
Source WSoh_221121_011221_HFGoe_L1_hTau_4h_R2 Scan 10663
Peptide QPHTEIPEG Charge 2 Spectral Angle 0.95
Experimental Spectrum
Prosit Predicted Spectrum
L² Normalized Intensity
m/z

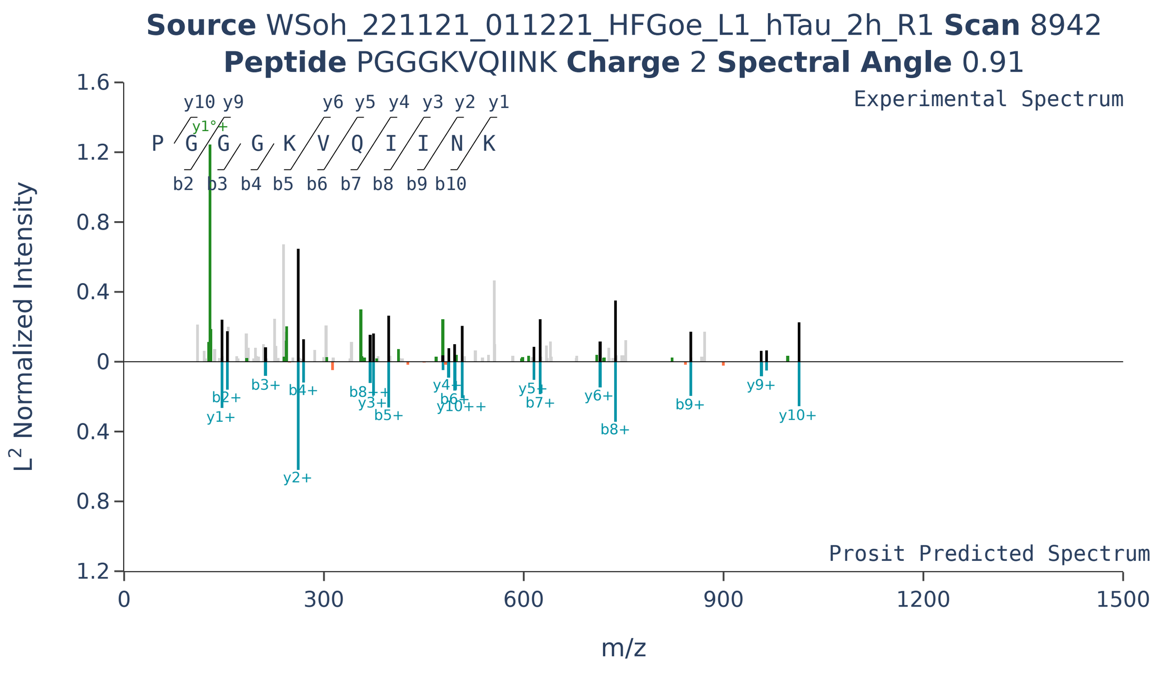
Source WSoh_221121_011221_HFGoe_L1_hTau_2h_R1 Scan 8942
Peptide PGGGKVQIINK Charge 2 Spectral Angle 0.91
Experimental Spectrum
Prosit Predicted Spectrum
L² Normalized Intensity
m/z

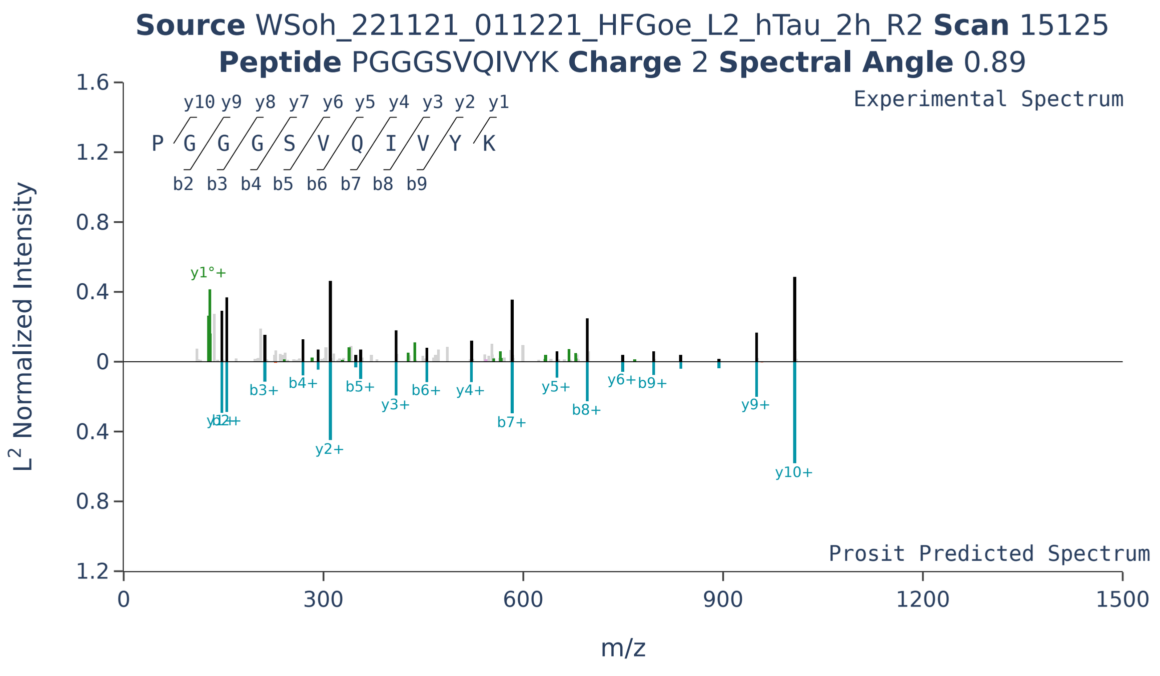
Source WSoh_221121_011221_HFGoe_L2_hTau_2h_R2 Scan 15125
Peptide PGGGSVQIVYK Charge 2 Spectral Angle 0.89
Experimental Spectrum
Prosit Predicted Spectrum
L² Normalized Intensity
m/z

**Source** WSoh_221121_011221_HFGoe_L2_hTau_24h_R2 **Scan** 8767
**Peptide** SPVVSGDTS **Charge** 1 **Spectral Angle** 0.97

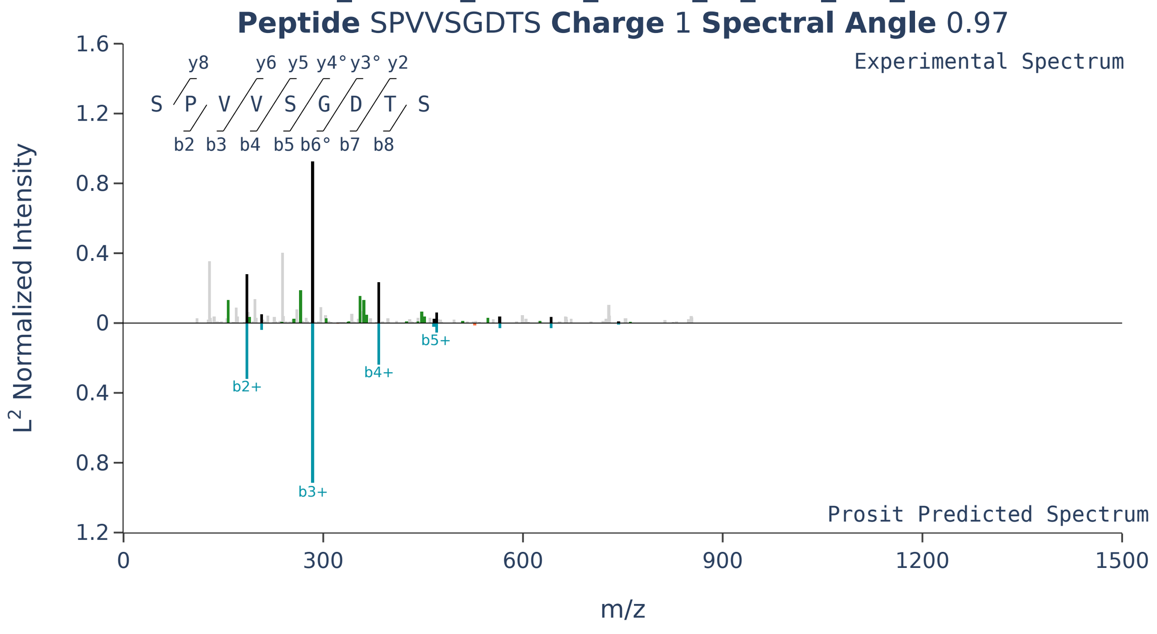


**Source** WSoh_221121_011221_HFGoe_L2_hTau_2h_R2 **Scan** 11116
**Peptide** PVVSGDTSPRHL **Charge** 3 **Spectral Angle** 0.93

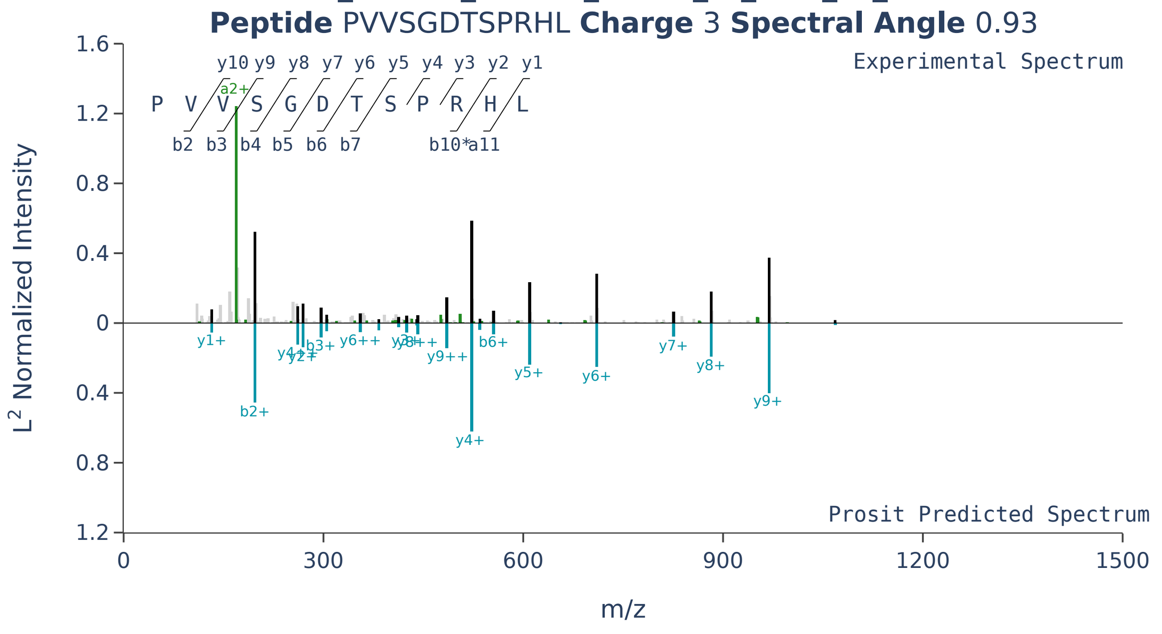


**Source** WSoh_221121_011221_HFGoe_L2_hTau_4h_R1 **Scan** 6340
**Peptide** TPPKSPSSAKSRL **Charge** 2 **Spectral Angle** 0.91

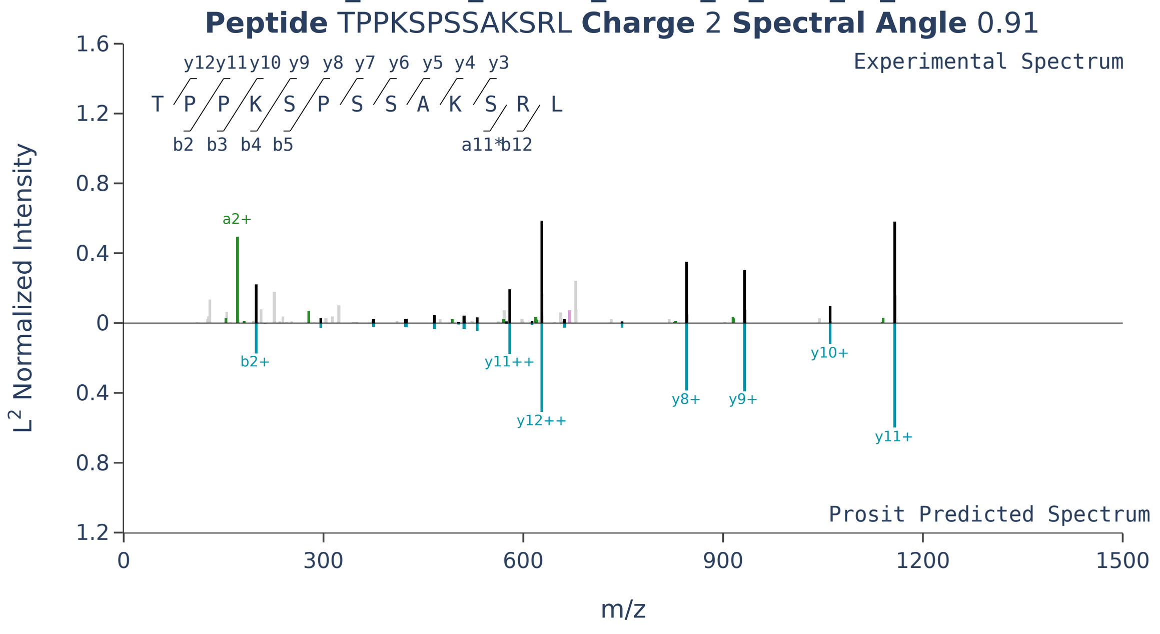


**Source** WSoh_221121_011221_HFGoe_L1_hTau_24h_R2 **Scan** 15153
**Peptide** DHAGTYGL **Charge** 2 **Spectral Angle** 0.92

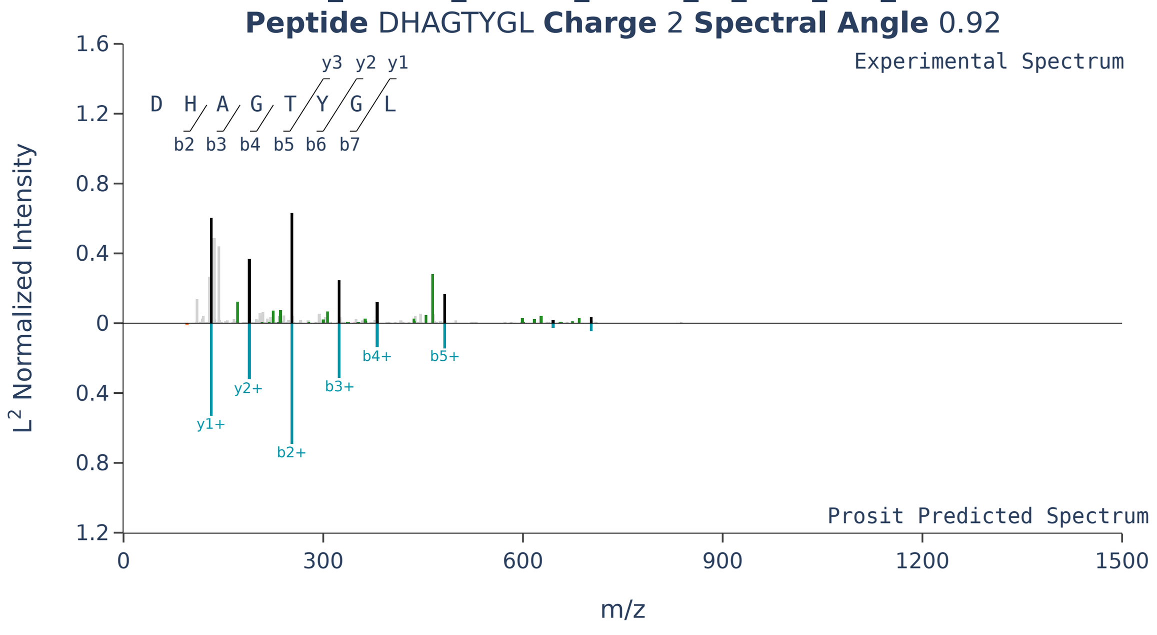


**Source** WSoh_221121_011221_HFGoe_L1_hTau_24h_R1 **Scan** 11087
**Peptide** GAEIVYK **Charge** 2 **Spectral Angle** 0.9

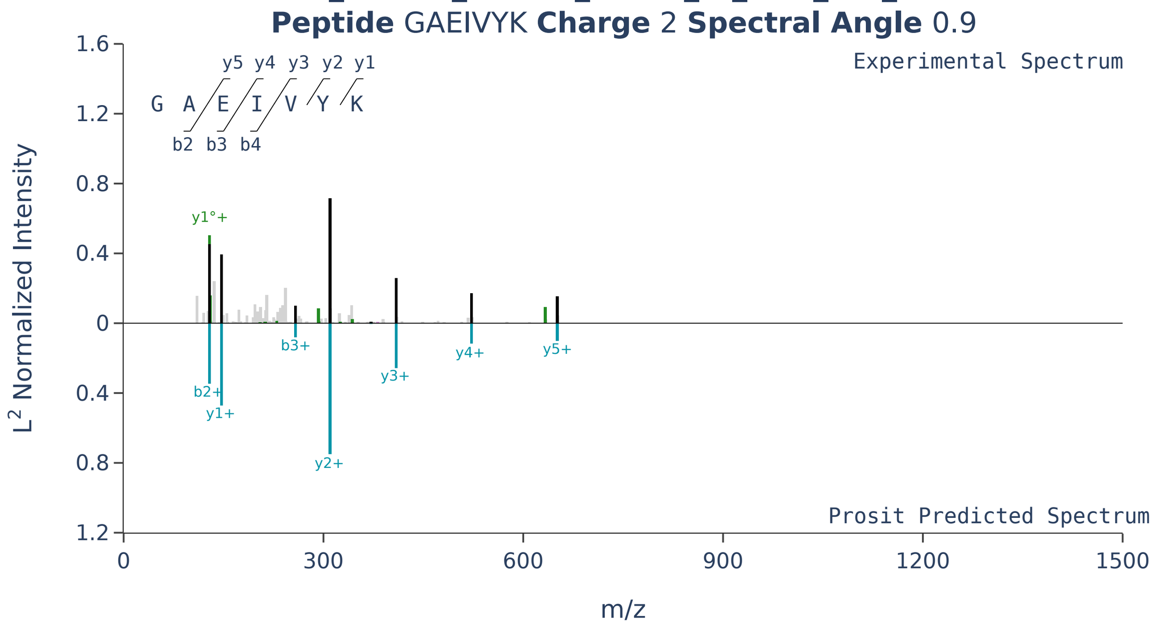


**Source** WSoh_221121_011221_HFGoe_L1_hTau_2h_R1 **Scan** 8267
**Peptide** ETHKLTFREN **Charge** 2 **Spectral Angle** 0.93

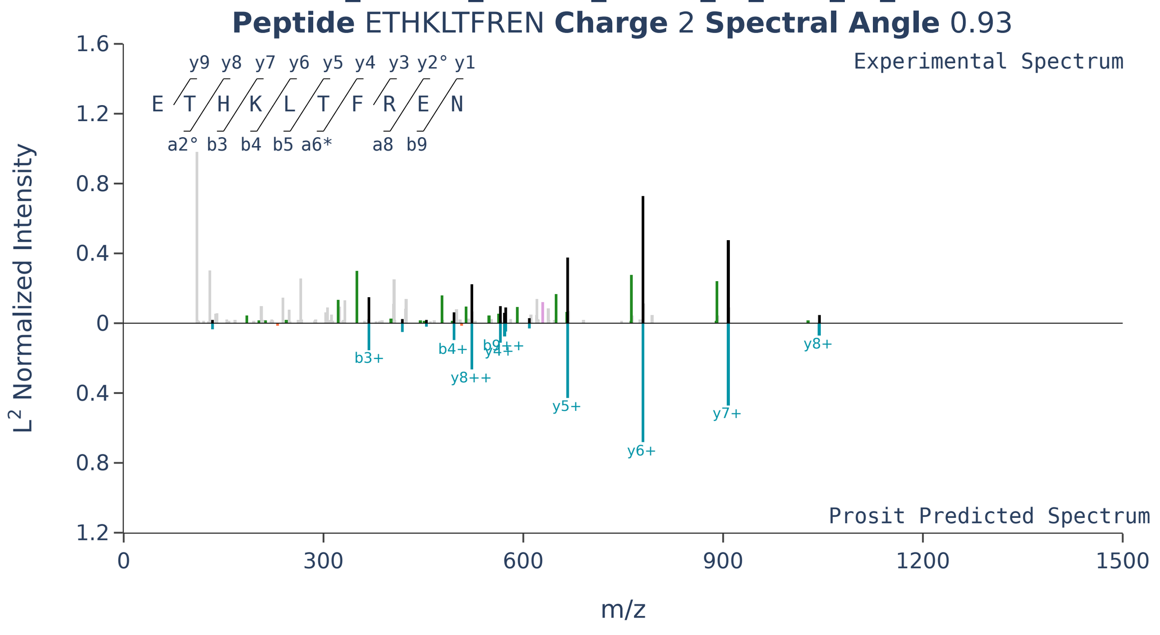


**Source** WSoh_221121_011221_HFGoe_L1_hTau_24h_R1 **Scan** 6429
**Peptide** GDTSPRHL **Charge** 2 **Spectral Angle** 0.94

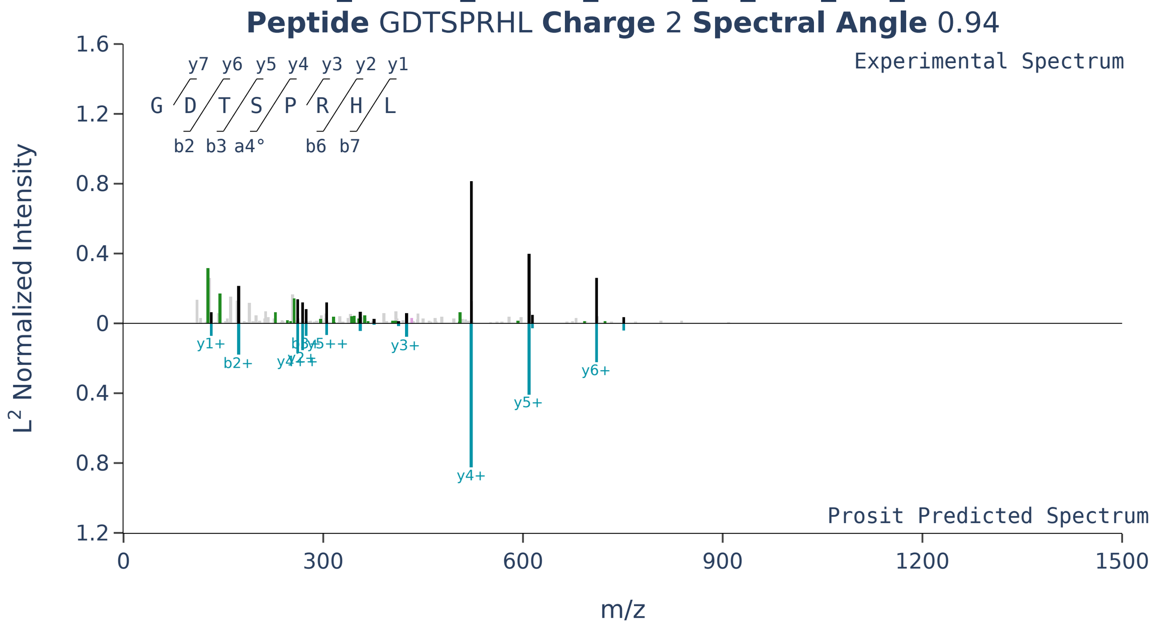


**Source** WSoh_221121_011221_HFGoe_L1_hTau_2h_R1 **Scan** 5949
**Peptide** PRHLSNVSSTG **Charge** 2 **Spectral Angle** 0.91

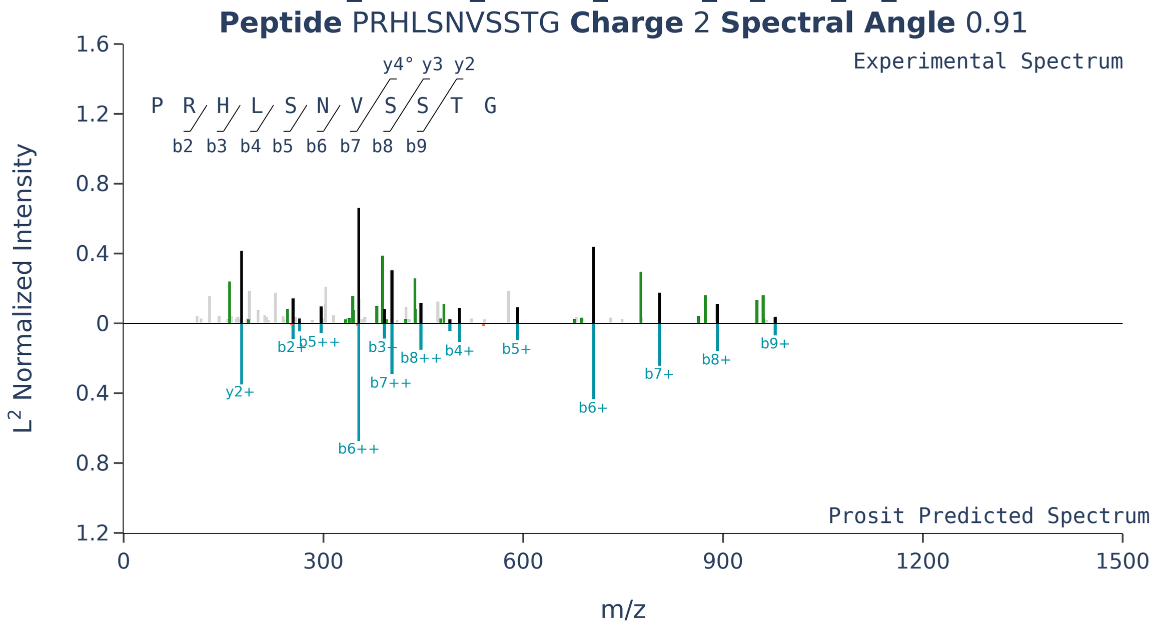


**Source** WSoh_221121_011221_HFGoe_L1_hTau_24h_R1 **Scan** 19507
**Peptide** VTAPLVD **Charge** 1 **Spectral Angle** 0.91

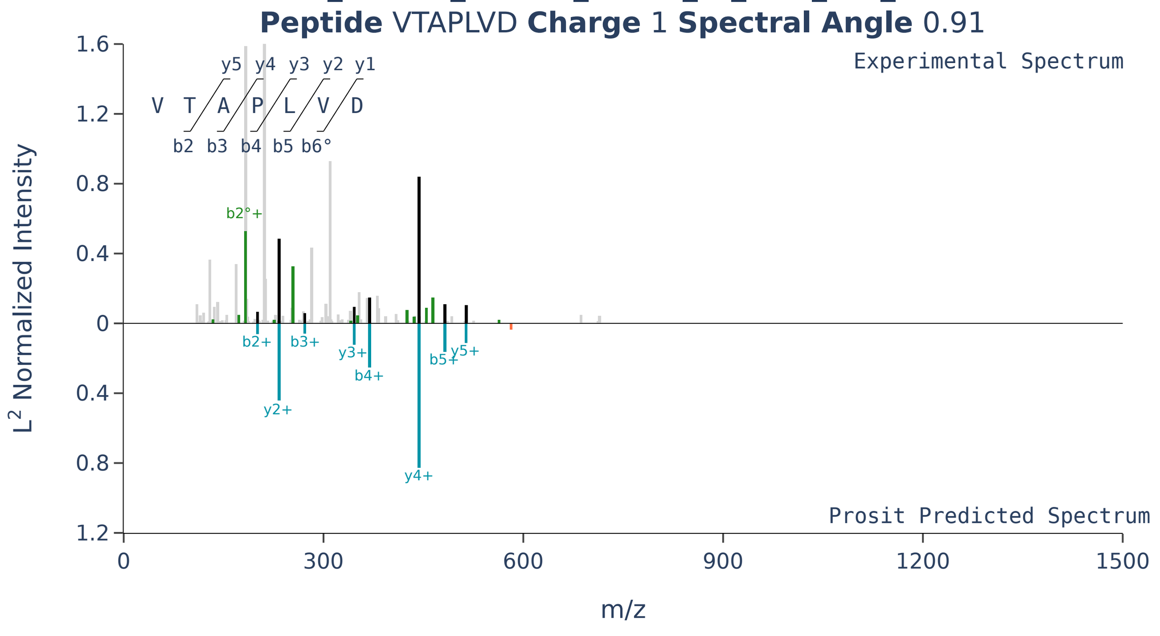


**Source** WSoh_221121_011221_HFGoe_L1_hTau_2h_R1 **Scan** 11564
**Peptide** DEAAGHVTQARMV **Charge** 2 **Spectral Angle** 0.92

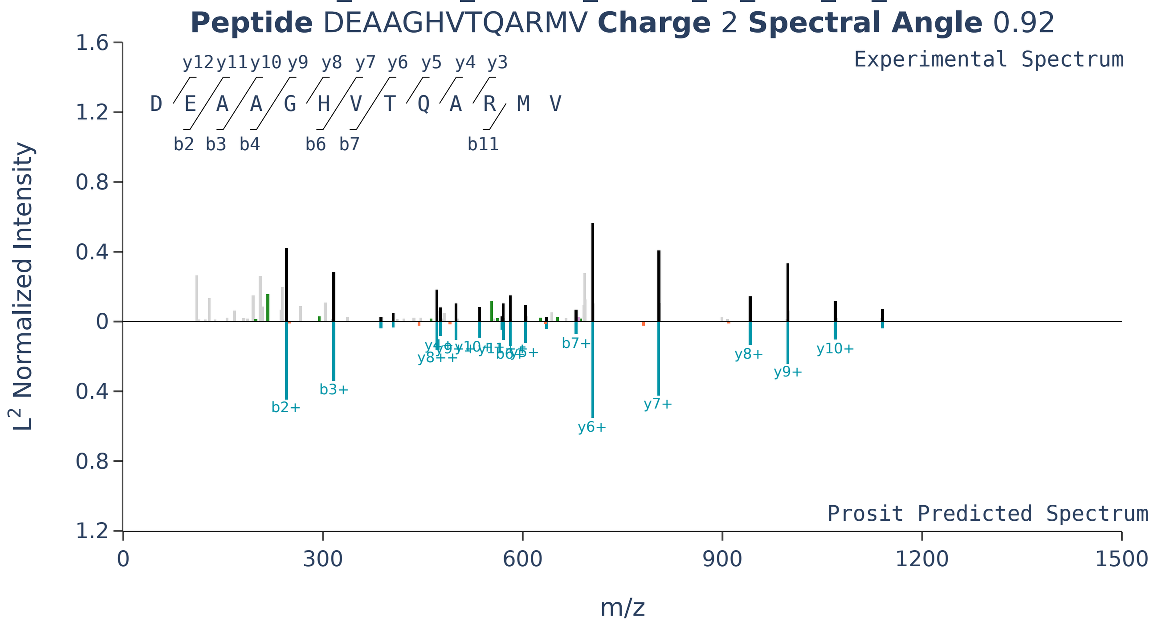


**Source** WSoh_221121_011221_HFGoe_L1_hTau_24h_R1 **Scan** 18637
**Peptide** AEIVYKSPVVSGDTS **Charge** 2 **Spectral Angle** 0.94

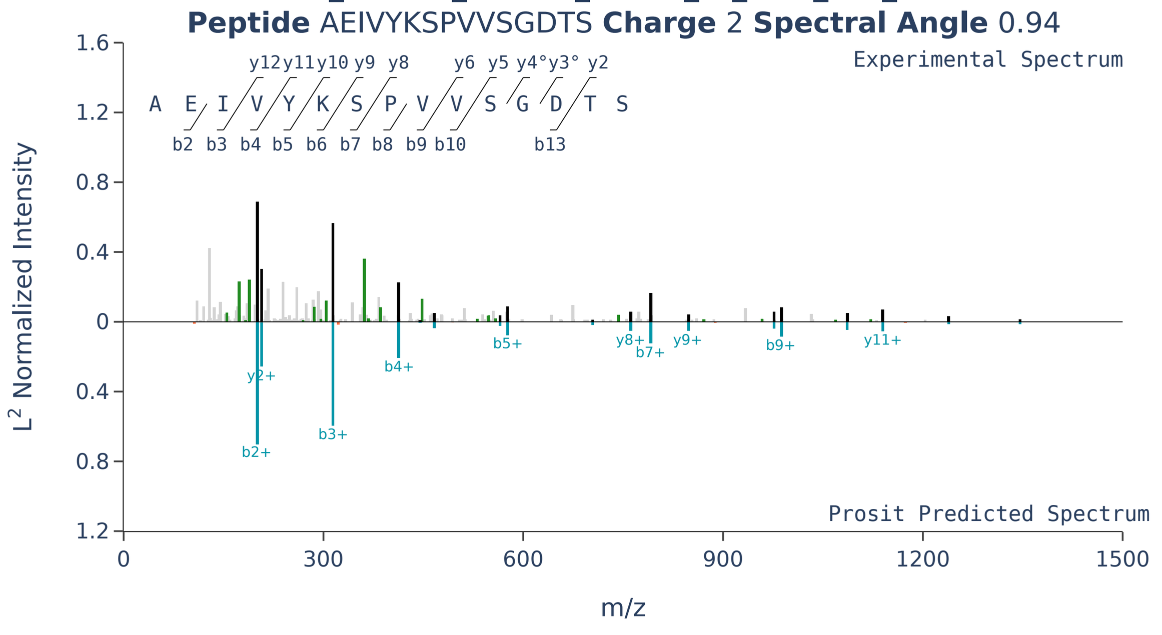


**Source** WSoh_221121_011221_HFGoe_L2_hTau_0h_R1 **Scan** 9013
**Peptide** KIGSTENL **Charge** 2 **Spectral Angle** 0.91

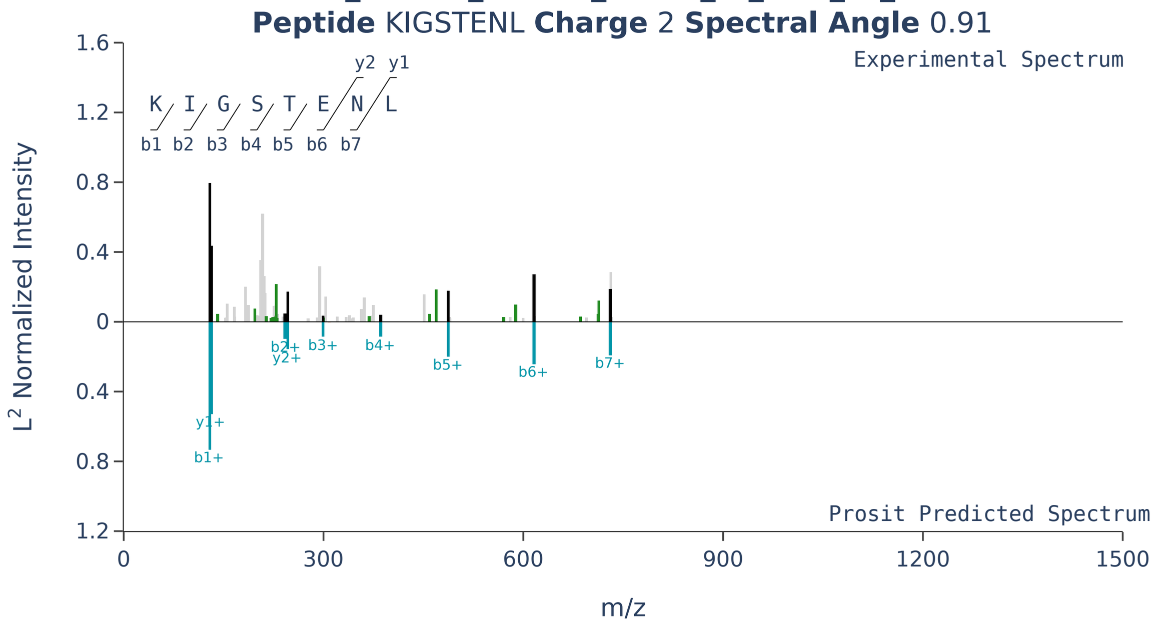


**Source** WSoh_221121_011221_HFGoe_L2_hTau_2h_R1 **Scan** 13109
**Peptide** PVDLSKVTSKC **Charge** 3 **Spectral Angle** 0.92

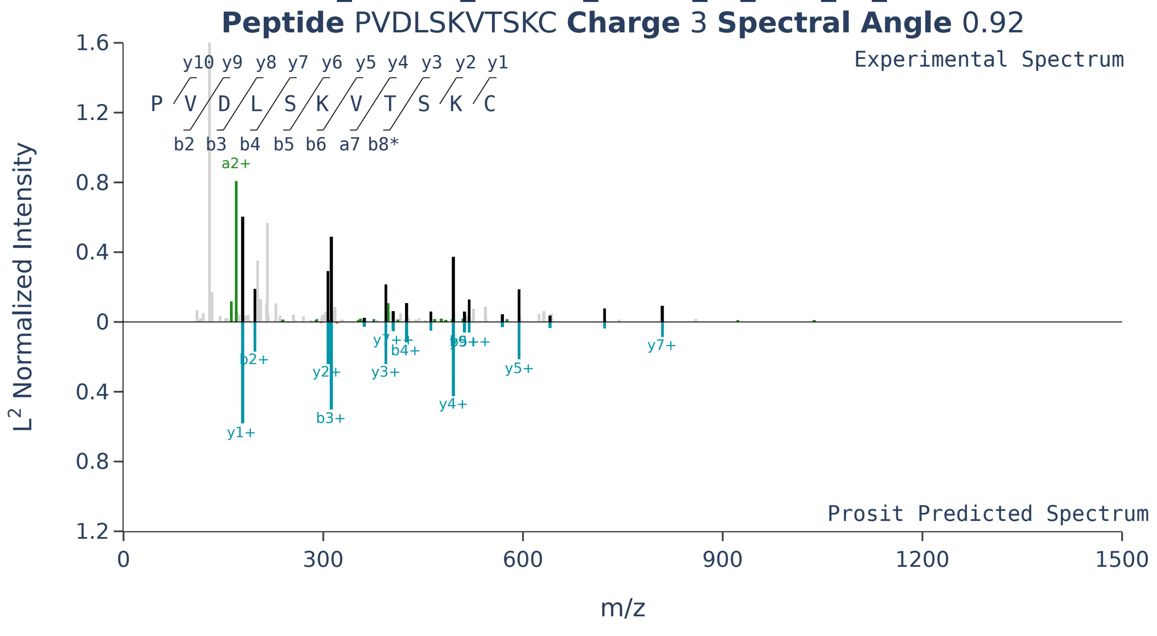


**Source** WSoh_221121_011221_HFGoe_L1_hTau_24h_R1 **Scan** 6268
**Peptide** TRIPAKT **Charge** 2 **Spectral Angle** 0.85

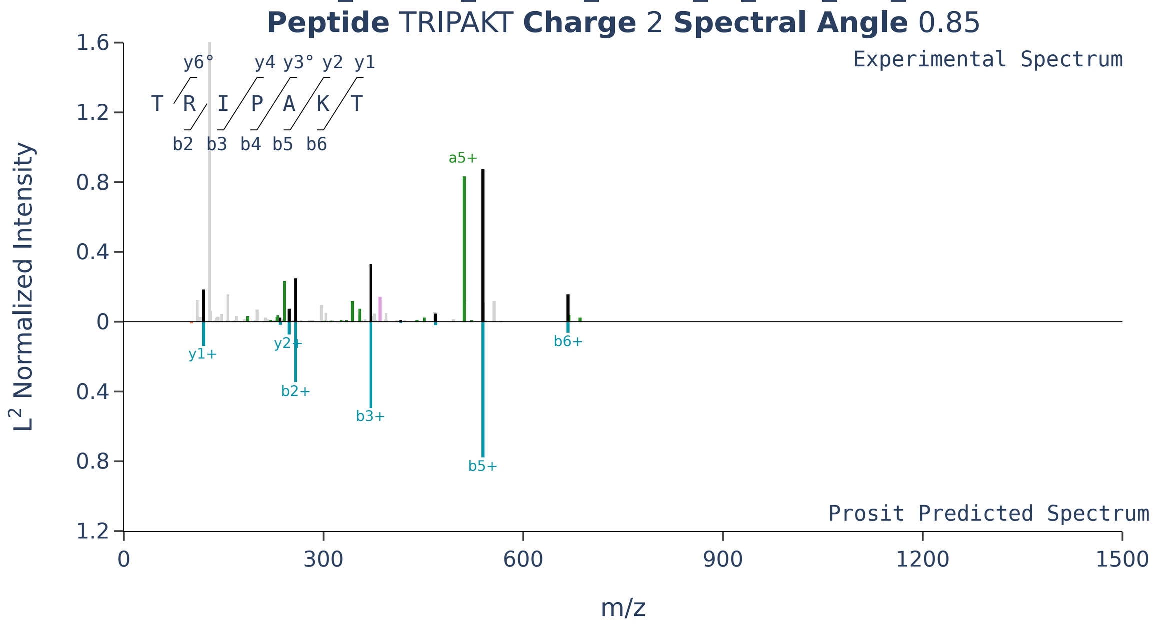


**Source** WSoh_221121_011221_HFGoe_L1_hTau_2h_R1 **Scan** 9842
**Peptide** AEIVYKS **Charge** 2 **Spectral Angle** 0.94

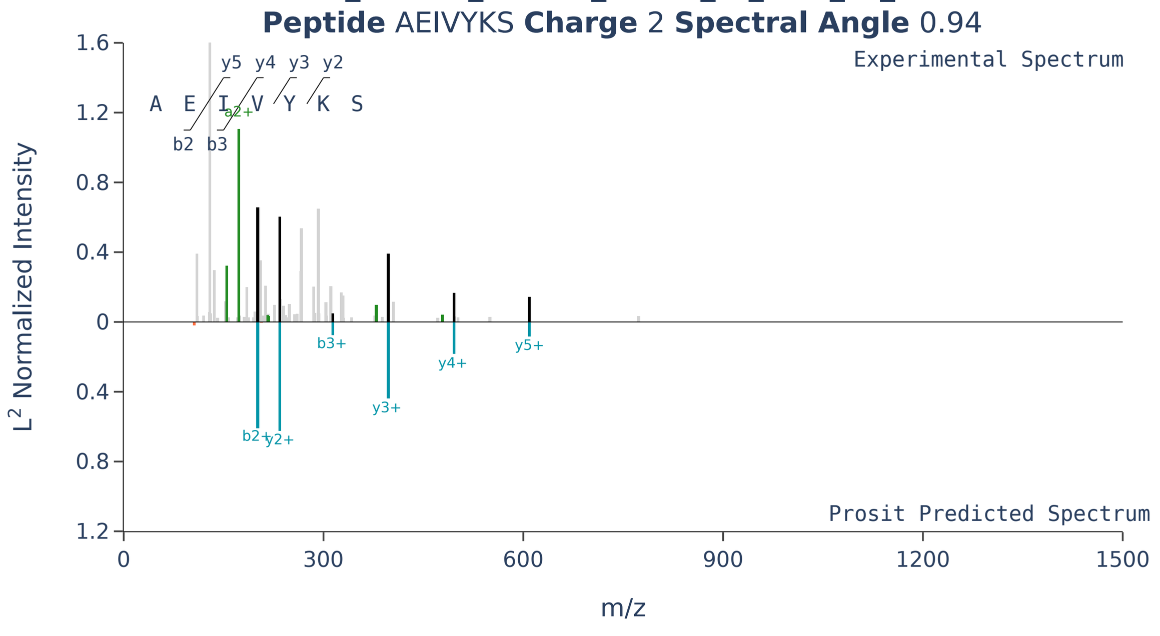

**Source** WSoh_221121_011221_HFGoe_L1_hTau_24h_R2 **Scan** 12250
**Peptide** RTPSLPTPPTREPKKV **Charge** 4 **Spectral Angle** 0.9

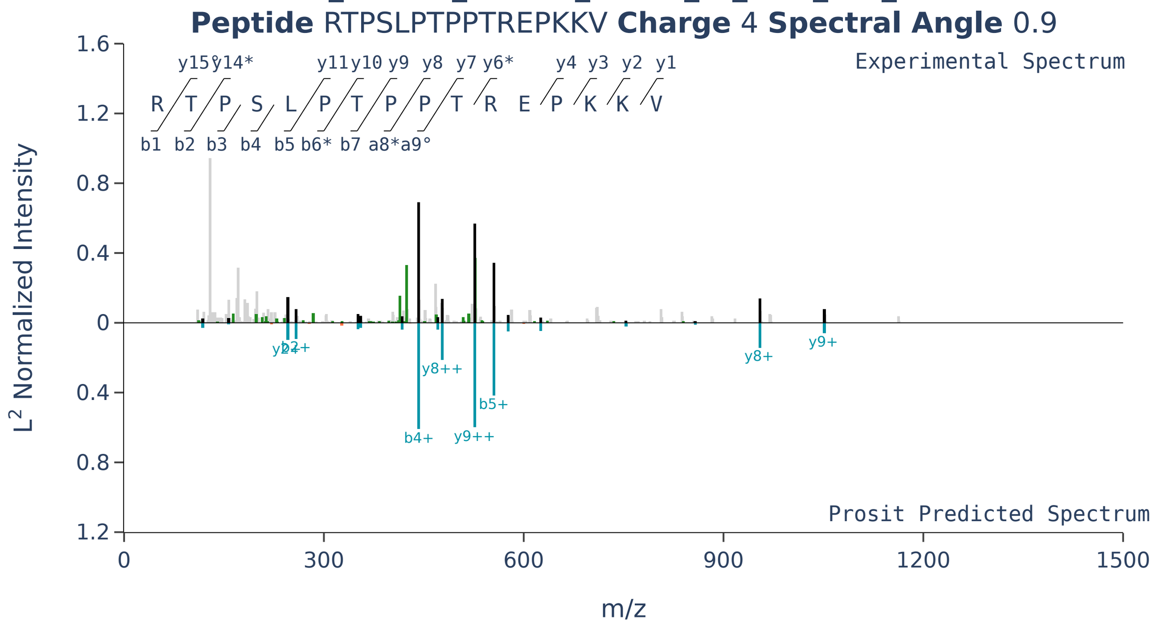


**Source** WSoh_221121_011221_HFGoe_L1_hTau_4h_R1 **Scan** 6630
**Peptide** AKAKTDHGAEIVYK **Charge** 2 **Spectral Angle** 0.87

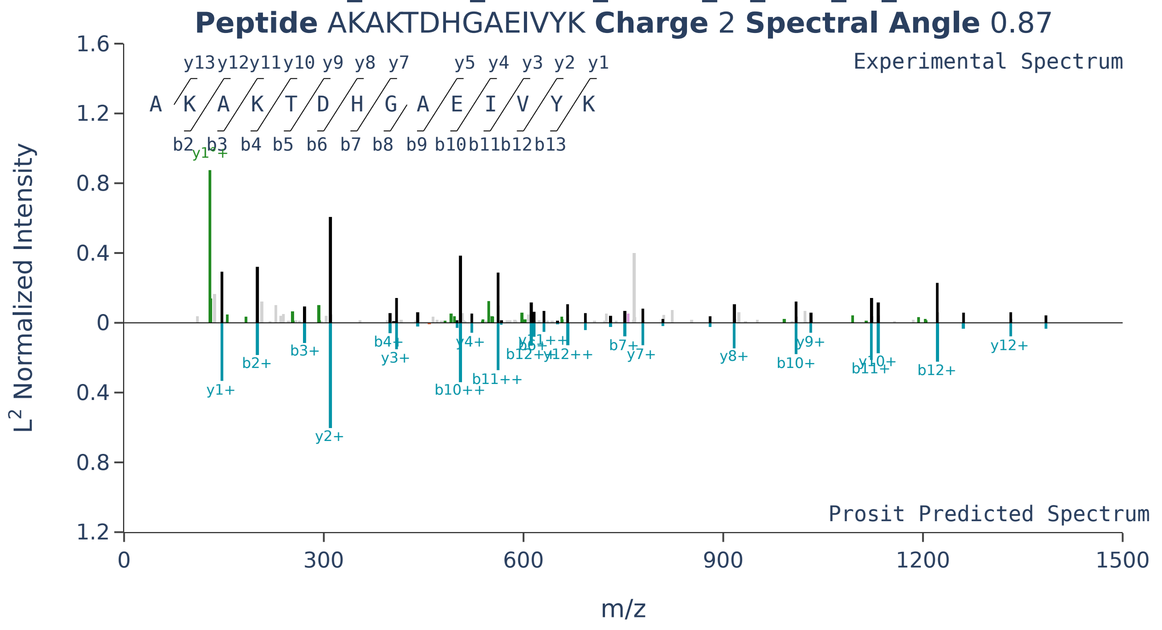


**Source** WSoh_221121_011221_HFGoe_L2_hTau_1h_R2 **Scan** 11268
**Peptide** AAQPHTEIPEG **Charge** 2 **Spectral Angle** 0.95

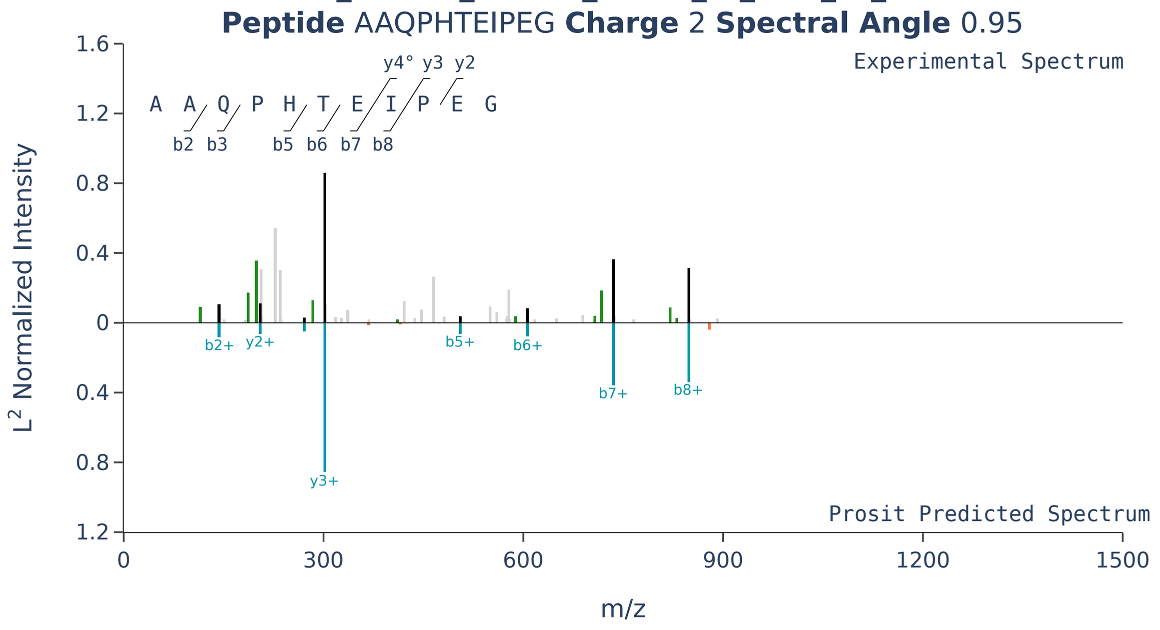


**Source** WSoh_221121_011221_HFGoe_L1_hTau_4h_R2 **Scan** 6878
**Peptide** HAGTYGLGDRKD **Charge** 3 **Spectral Angle** 0.92

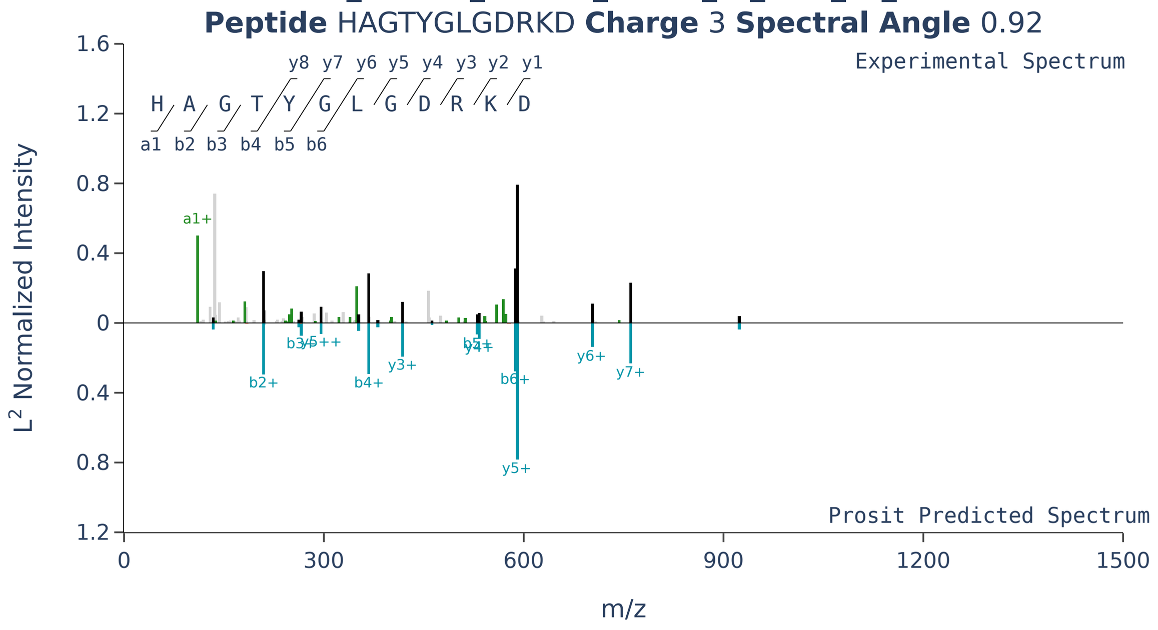


**Source** WSoh_221121_011221_HFGoe_L2_hTau_2h_R1 **Scan** 14946
**Peptide** TDHGAEIVY **Charge** 2 **Spectral Angle** 0.9

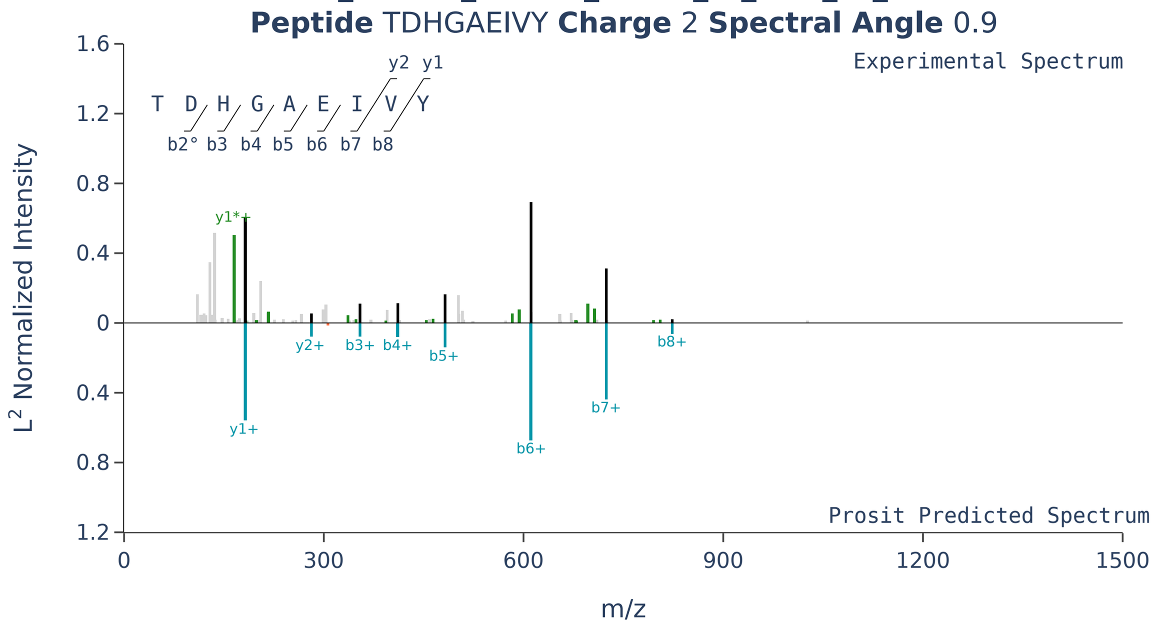


**Source** WSoh_221121_011221_HFGoe_L1_hTau_4h_R2 **Scan** 19549
**Peptide** SNVSSTGSIDMVD **Charge** 2 **Spectral Angle** 0.9

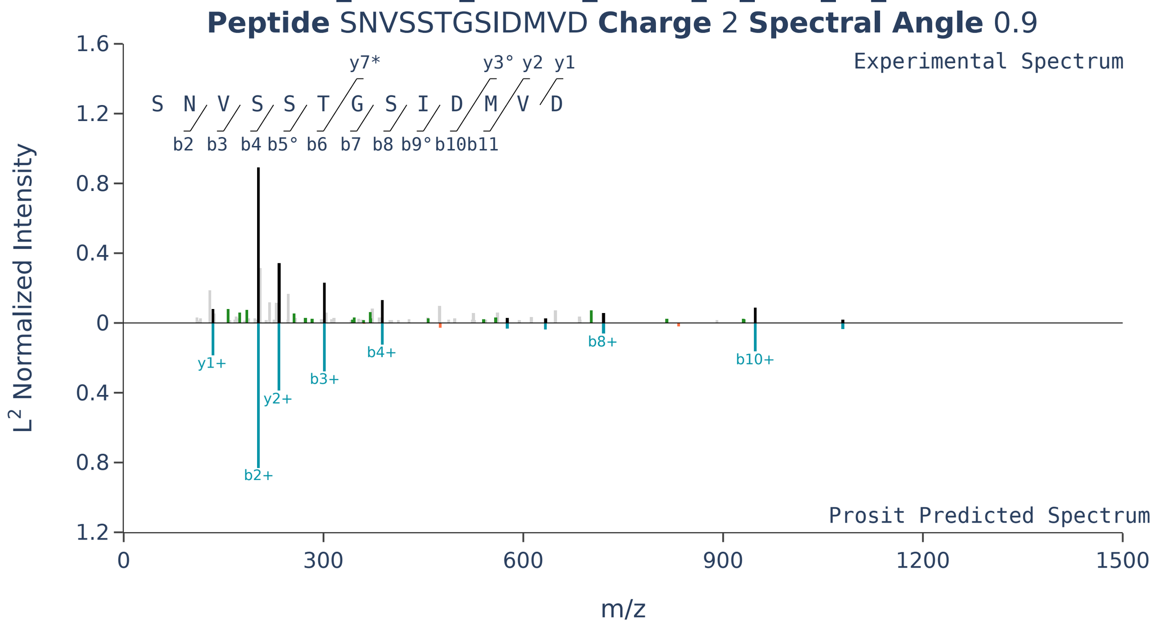


**Source** WSoh_221121_011221_HFGoe_L2_hTau_4h_R2 **Scan** 11945
**Peptide** PLVDEGAPGKQA **Charge** 2 **Spectral Angle** 0.9

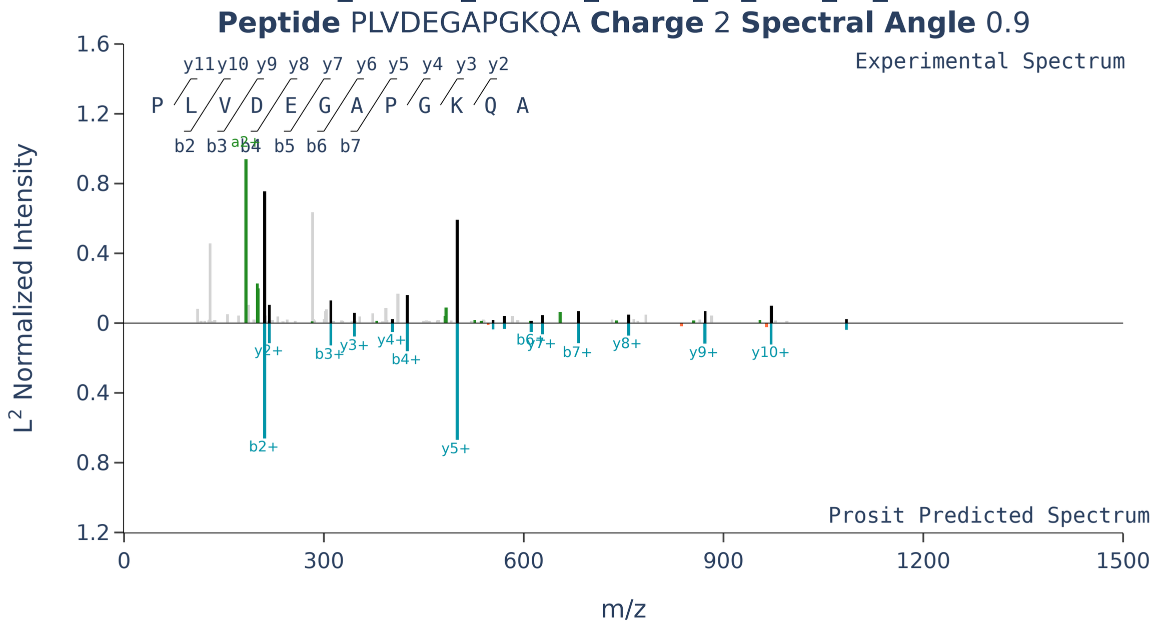


**Source** WSoh_221121_011221_HFGoe_L1_hTau_4h_R1 **Scan** 13078
**Peptide** AAQPHTEIPEGTTAEE **Charge** 2 **Spectral Angle** 0.92

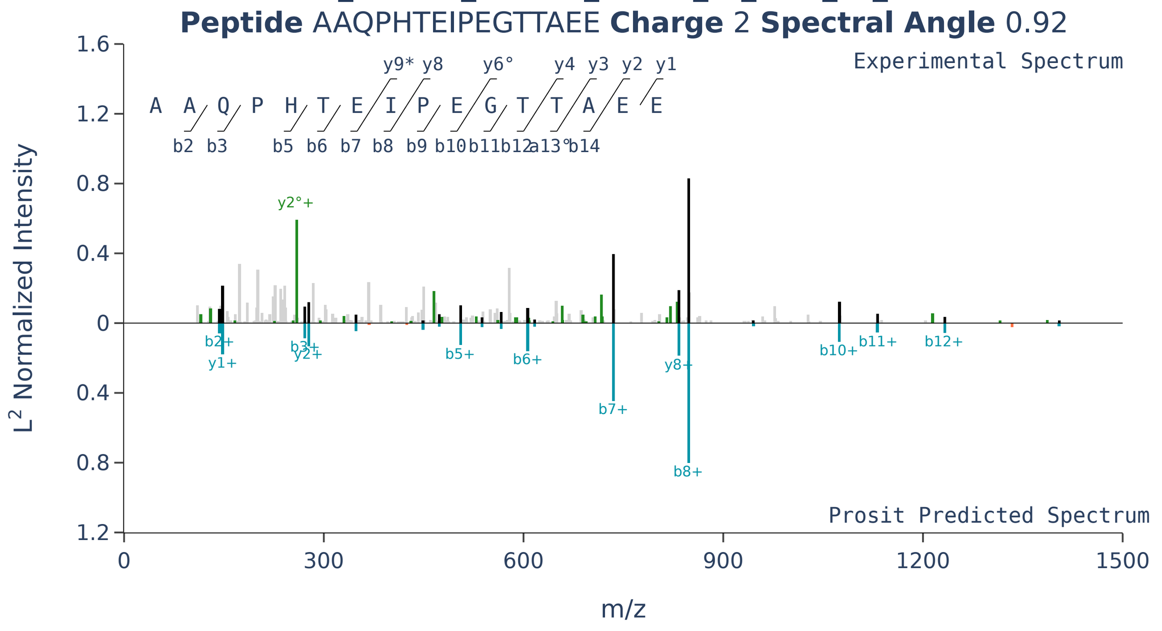


**Source** WSoh_221121_011221_HFGoe_L2_hTau_4h_R1 **Scan** 7713
**Peptide** CGSLGNIHH **Charge** 2 **Spectral Angle** 0.94

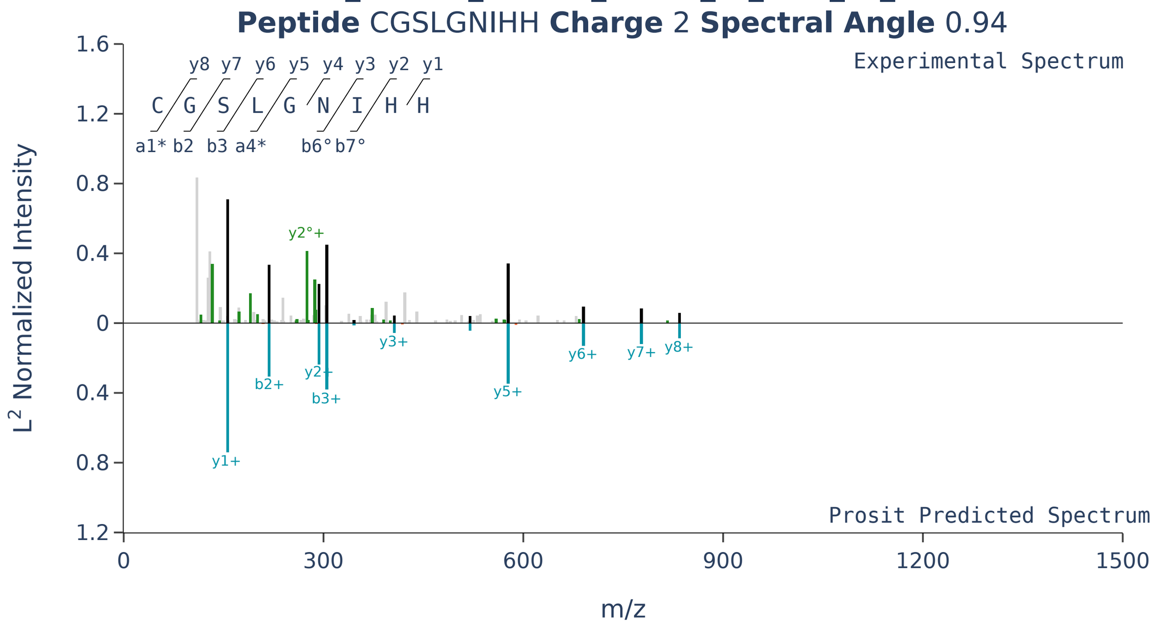


**Source** WSoh_221121_011221_HFGoe_L1_hTau_2h_R1 **Scan** 6363
**Peptide** IGSTENLKHQ **Charge** 3 **Spectral Angle** 0.93

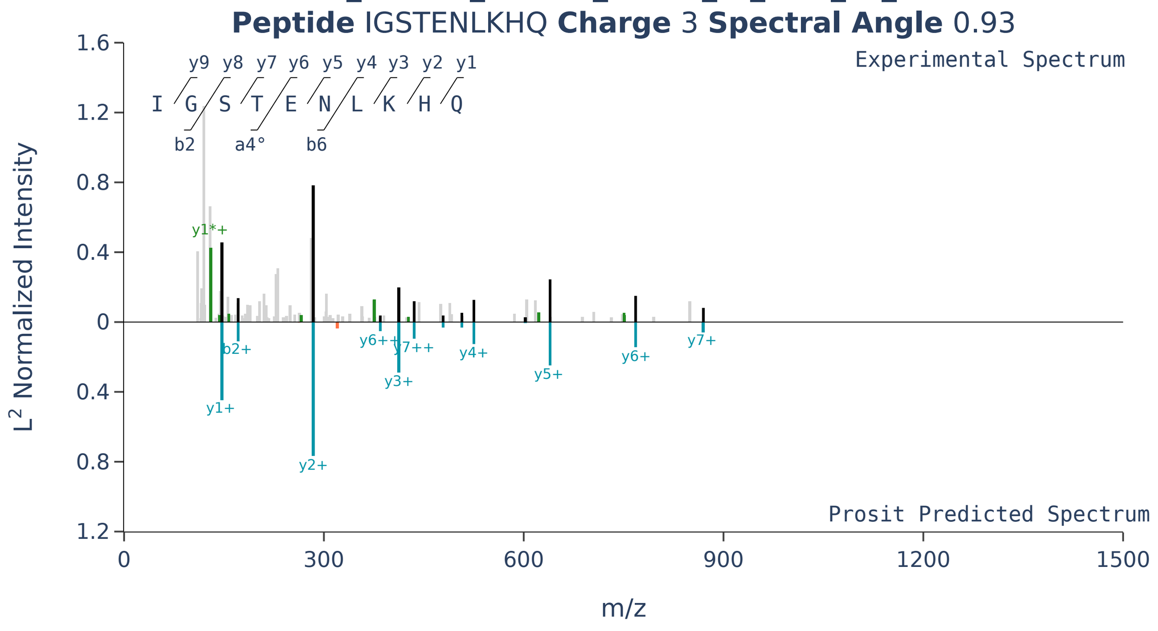


**Source** WSoh_221121_011221_HFGoe_L2_hTau_24h_R2 **Scan** 8071
**Peptide** PKSPSSAKSRL **Charge** 2 **Spectral Angle** 0.87

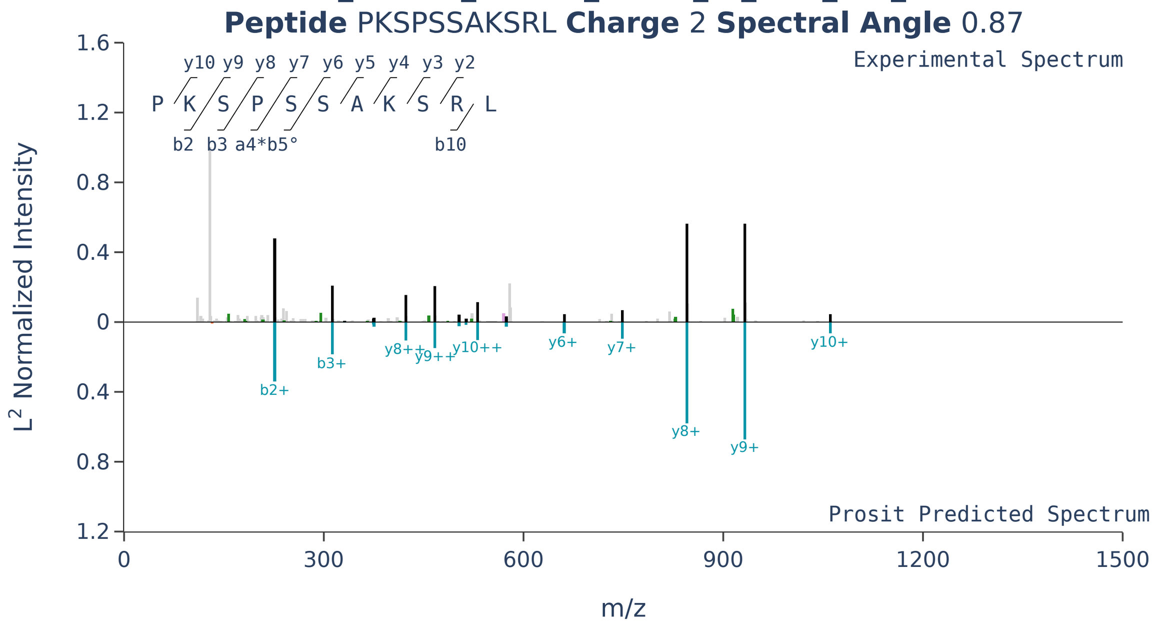


**Source** WSoh_221121_011221_HFGoe_L2_hTau_24h_R2 **Scan** 16615
**Peptide** SPLQTPTEDGSEEPGSE **Charge** 2 **Spectral Angle** 0.92

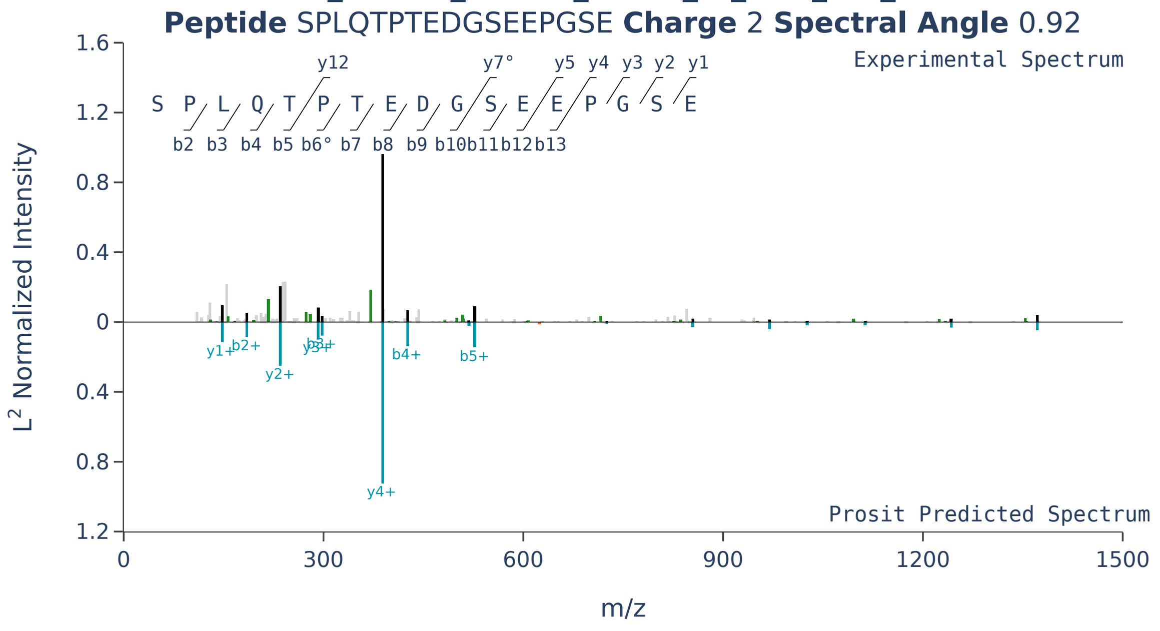


**Source** WSoh_221121_011221_HFGoe_L1_hTau_24h_R2 **Scan** 6827
**Peptide** HAGTYGLGDRK **Charge** 3 **Spectral Angle** 0.91

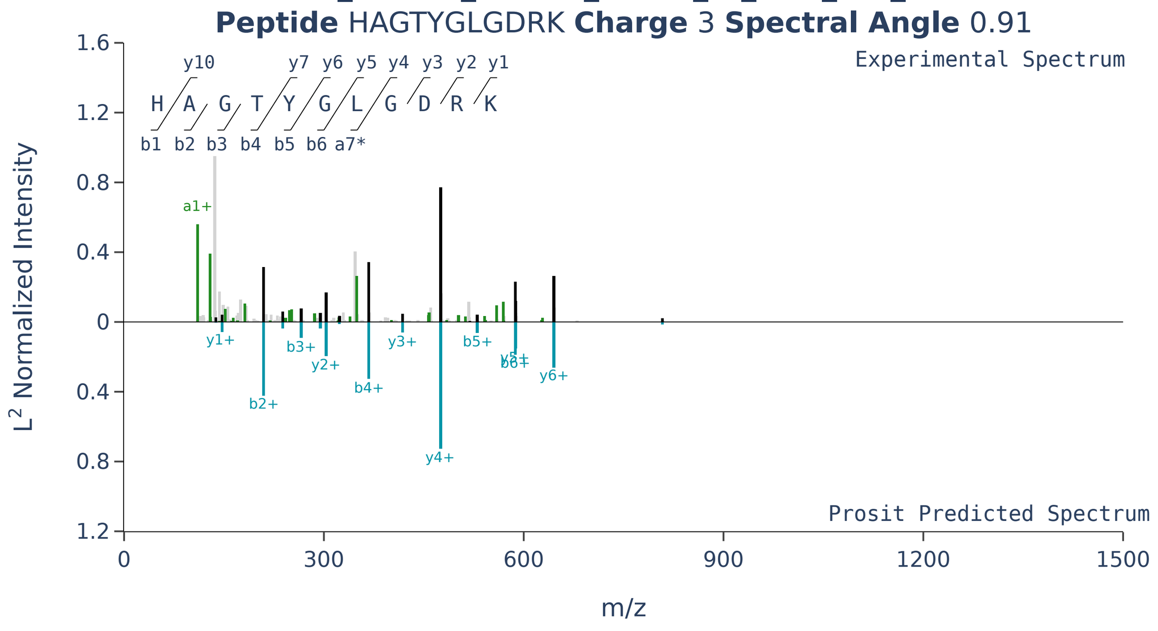


**Source** WSoh_221121_011221_HFGoe_L2_hTau_24h_R1 **Scan** 6762
**Peptide** GSTENLKH **Charge** 2 **Spectral Angle** 0.95

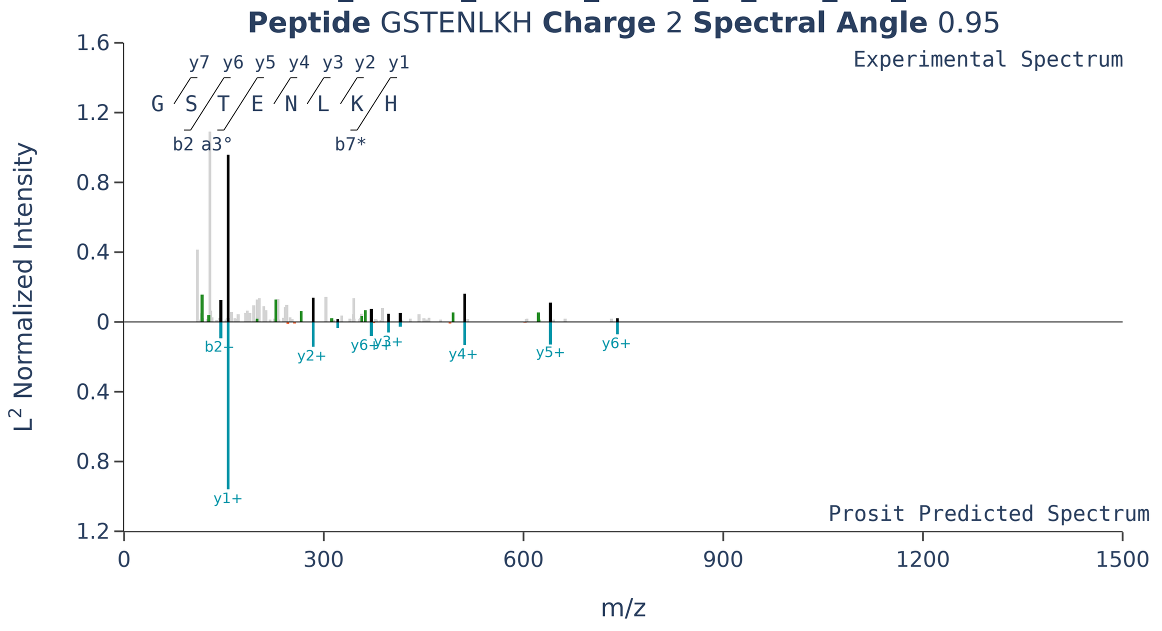


**Source** WSoh_221121_011221_HFGoe_L1_hTau_24h_R1 **Scan** 12907
**Peptide** APLVDEGAPGKQA **Charge** 2 **Spectral Angle** 0.93

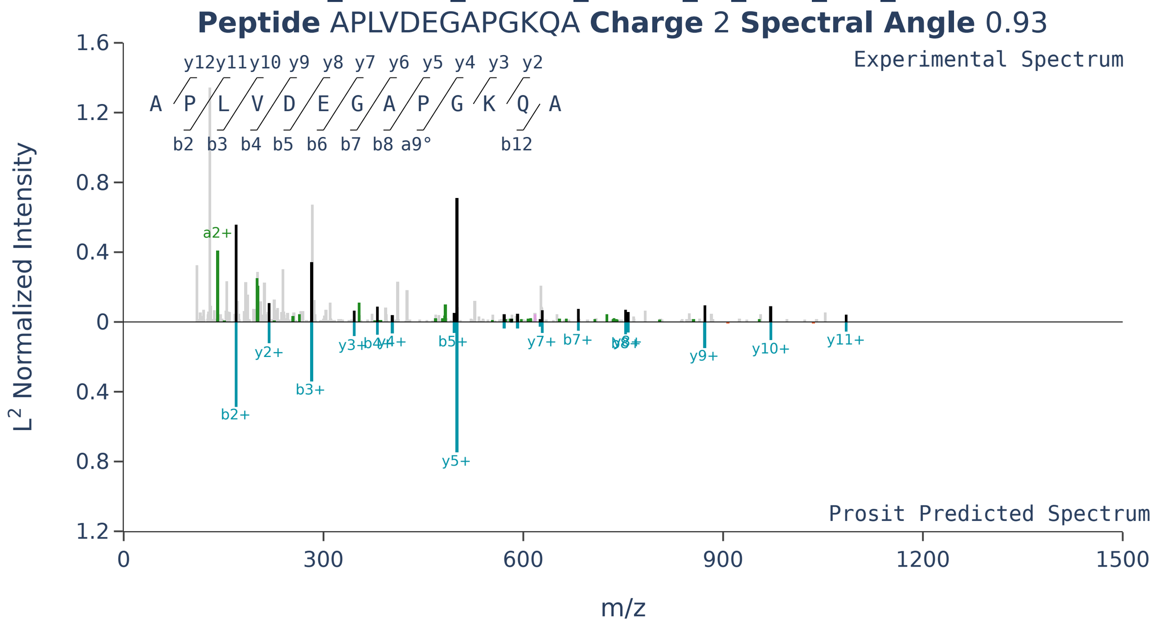

**Source** WSoh_221121_011221_HFGoe_L2_hTau_24h_R1 **Scan** 15249
**Peptide** VYKPVDLSKVTSK **Charge** 3 **Spectral Angle** 0.92

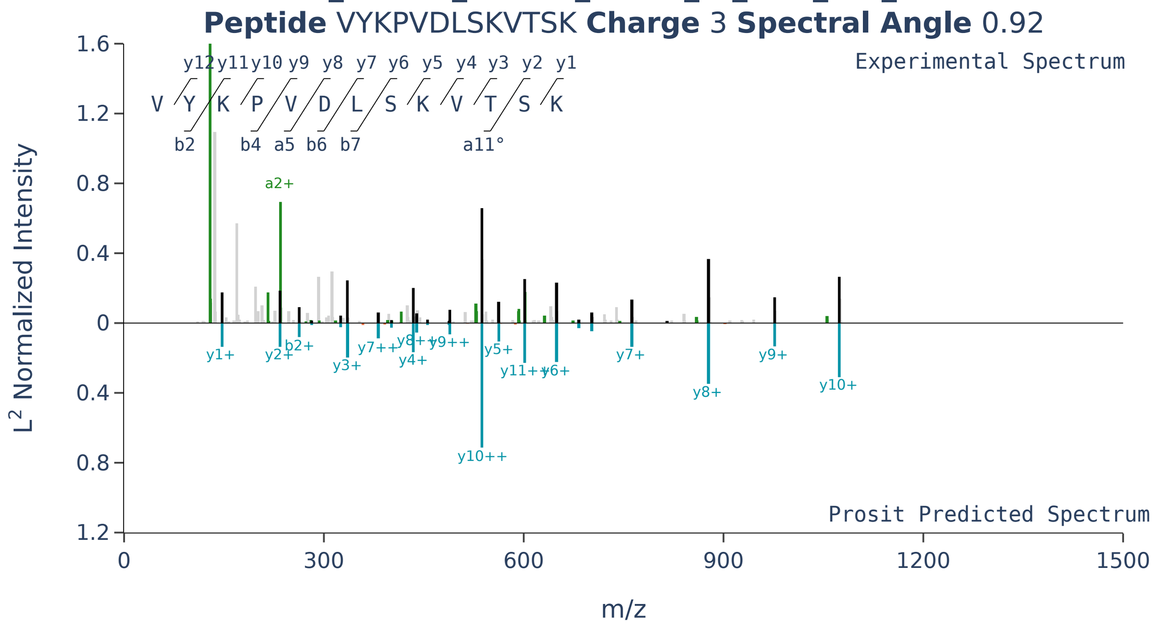


**Source** WSoh_221121_011221_HFGoe_L2_hTau_1h_R2 **Scan** 6083
**Peptide** ADGKTKIATPRG **Charge** 2 **Spectral Angle** 0.91

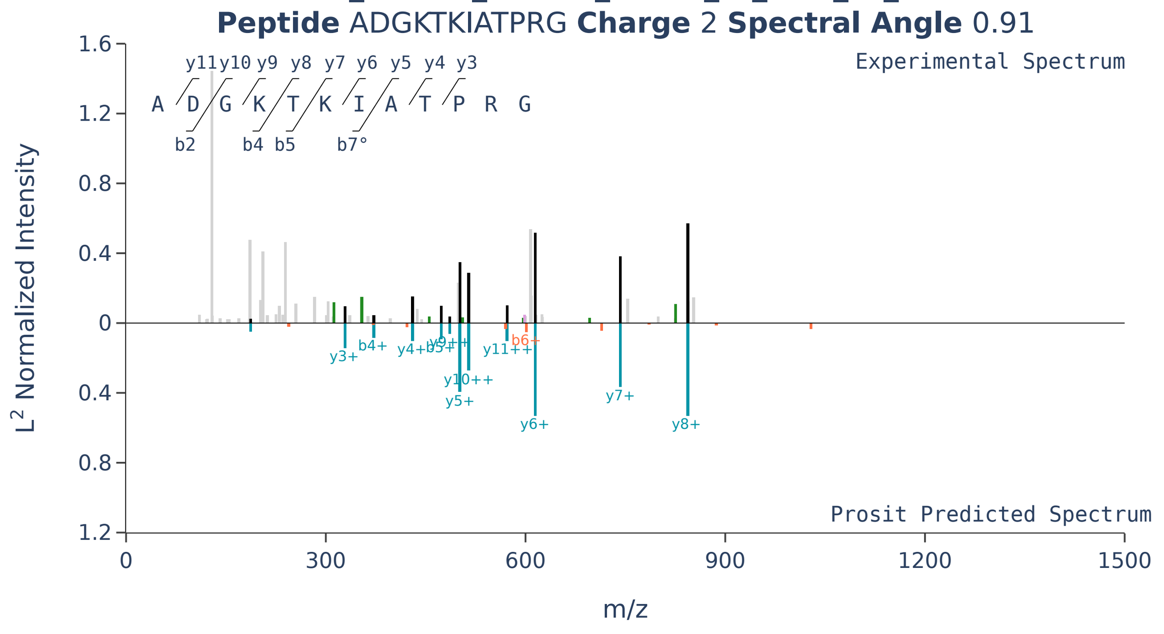


**Source** WSoh_221121_011221_HFGoe_L2_hTau_24h_R2 **Scan** 17245
**Peptide** ADEVSASL **Charge** 1 **Spectral Angle** 0.84

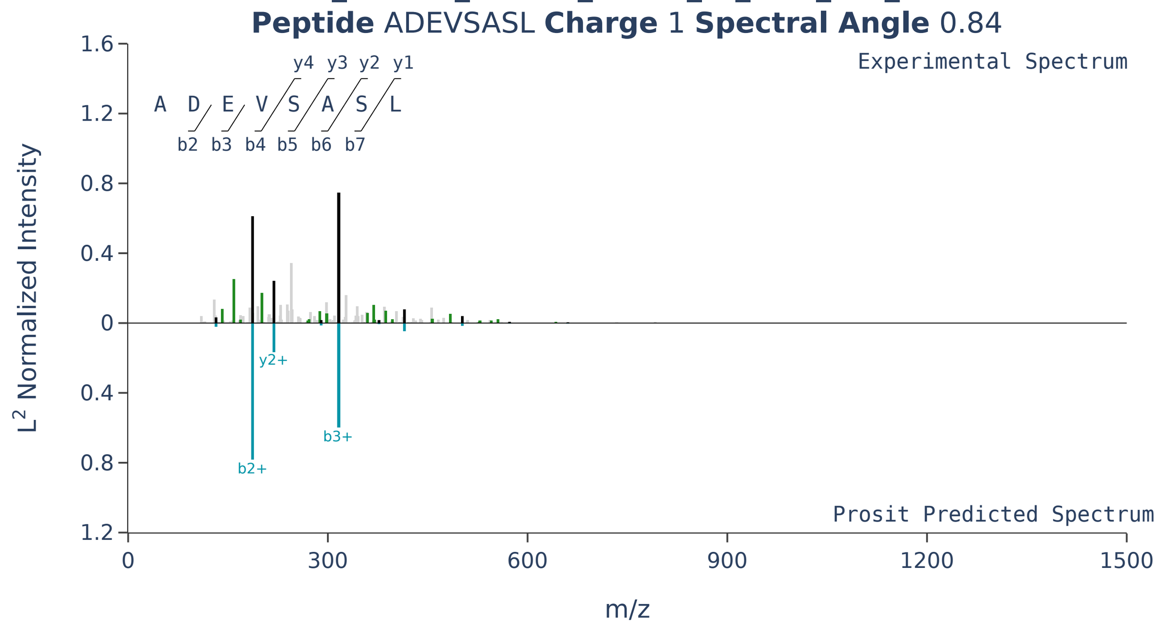


**Source** WSoh_221121_011221_HFGoe_L1_hTau_4h_R1 **Scan** 7626
**Peptide** KTDHGAEIVYK **Charge** 2 **Spectral Angle** 0.93

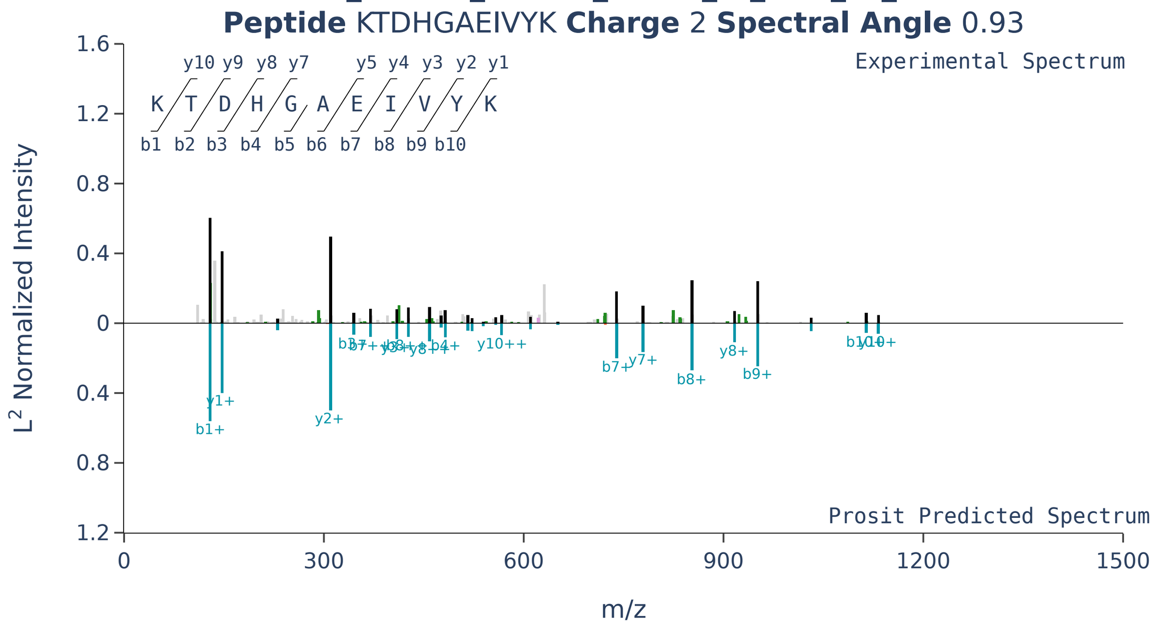


**Source** WSoh_221121_011221_HFGoe_L1_hTau_24h_R2 **Scan** 16458
**Peptide** HGAEIVYKSPVVS **Charge** 2 **Spectral Angle** 0.91

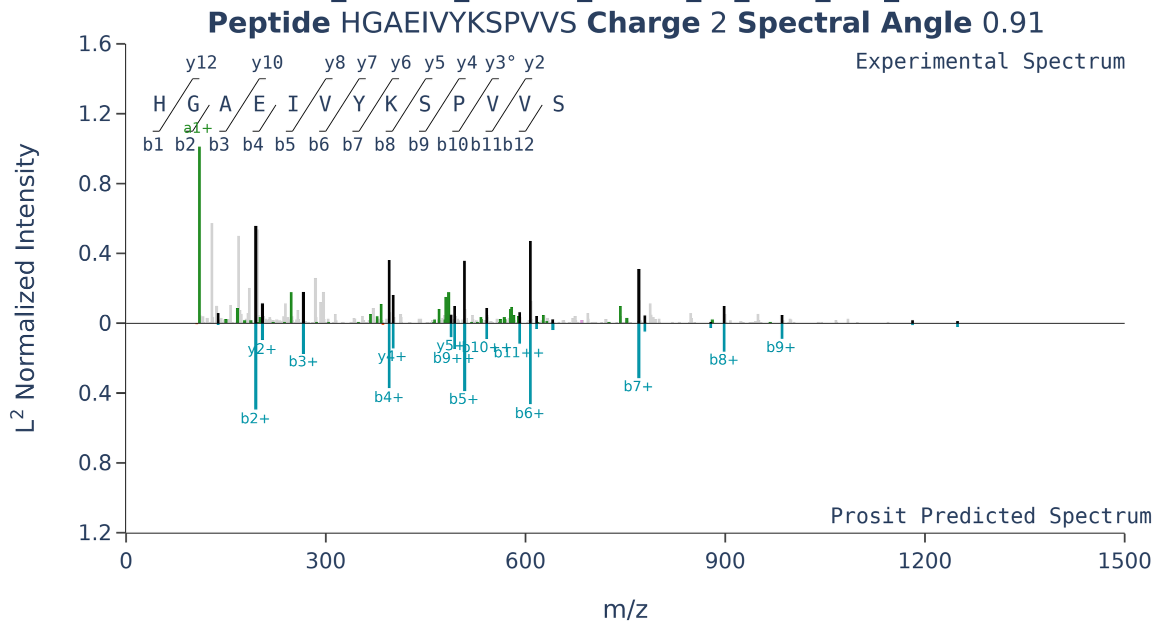


**Source** WSoh_221121_011221_HFGoe_L2_hTau_4h_R2 **Scan** 13057
**Peptide** PHTEIPEGTTAEEA **Charge** 2 **Spectral Angle** 0.89

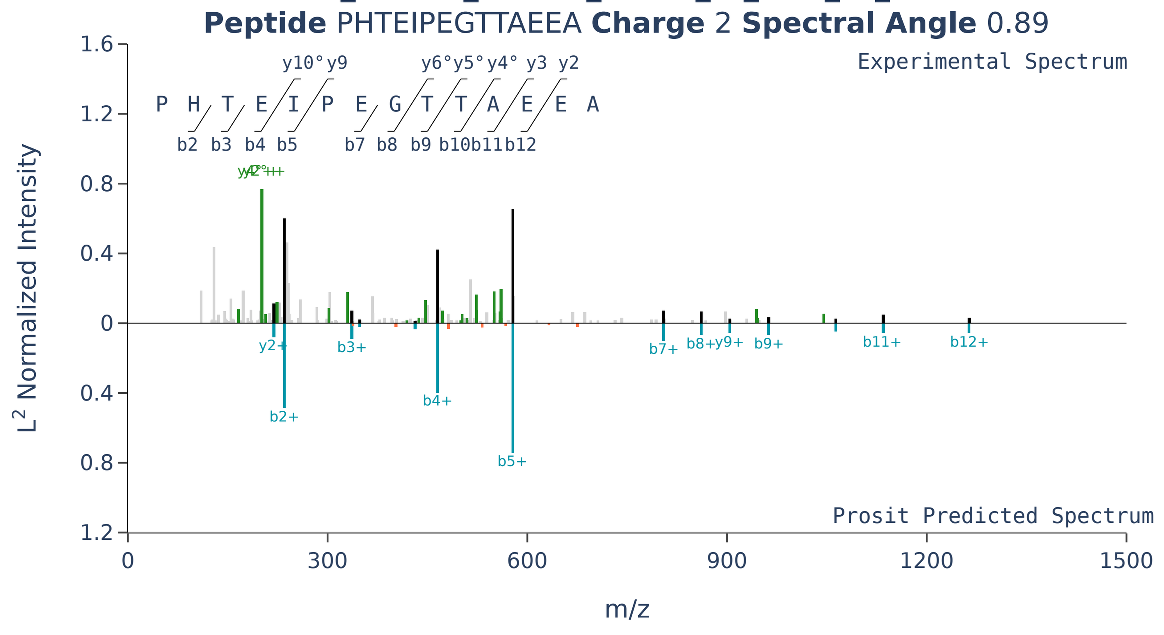


**Source** WSoh_221121_011221_HFGoe_L2_hTau_4h_R1 **Scan** 19272
**Peptide** QSKIGSLDNITHV **Charge** 2 **Spectral Angle** 0.94

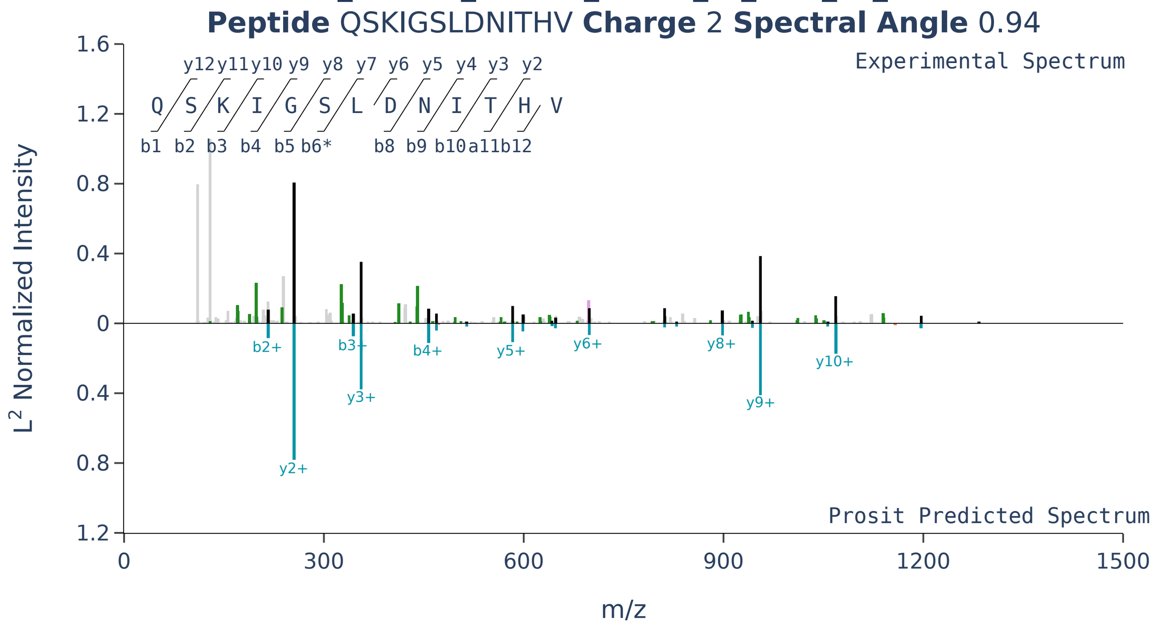


**Source** WSoh_221121_011221_HFGoe_L2_hTau_24h_R2 **Scan** 8909
**Peptide** EAAGHVTQARMVS **Charge** 2 **Spectral Angle** 0.93

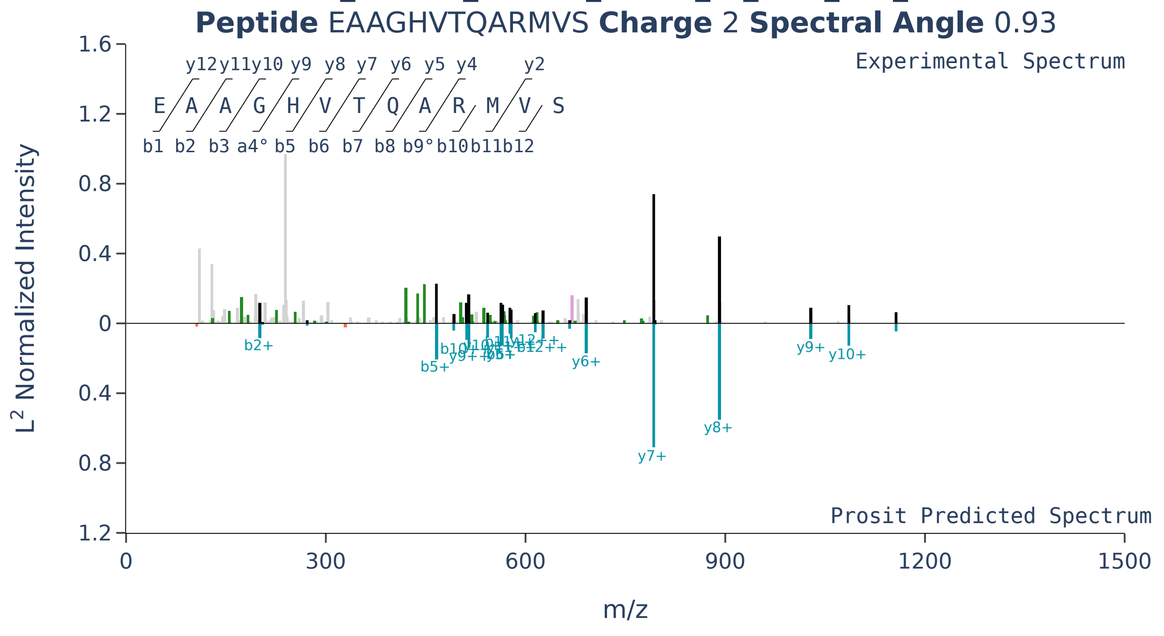


**Source** WSoh_221121_011221_HFGoe_L2_hTau_4h_R1 **Scan** 7746
**Peptide** DHAGTYGLGDRKD **Charge** 2 **Spectral Angle** 0.92

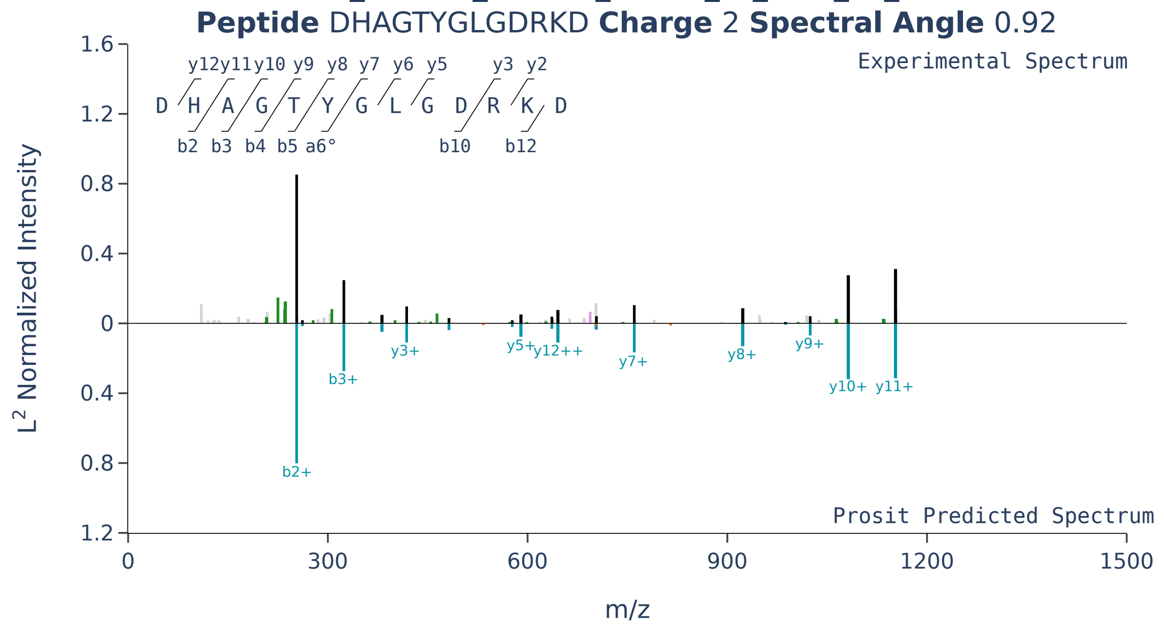


**Source** WSoh_221121_011221_HFGoe_L2_hTau_24h_R2 **Scan** 7135
**Peptide** SPVVSGDTSPRH **Charge** 3 **Spectral Angle** 0.92

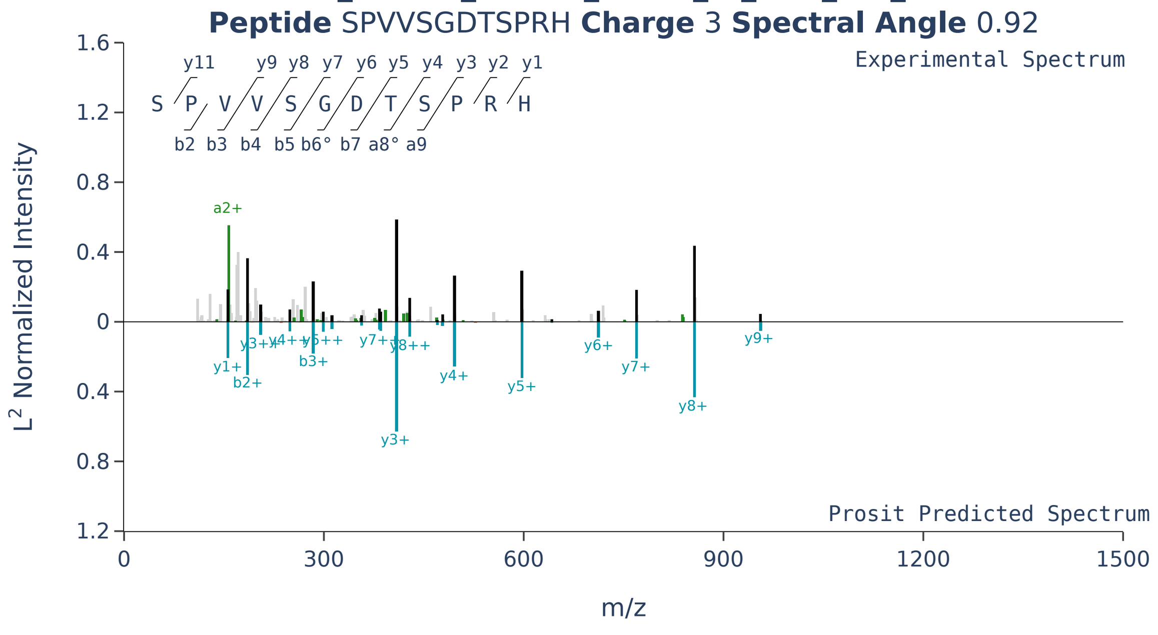


**Source** WSoh_221121_011221_HFGoe_L1_hTau_24h_R1 **Scan** 11908
**Peptide** AAQPHTEIPEGTT **Charge** 1 **Spectral Angle** 0.91

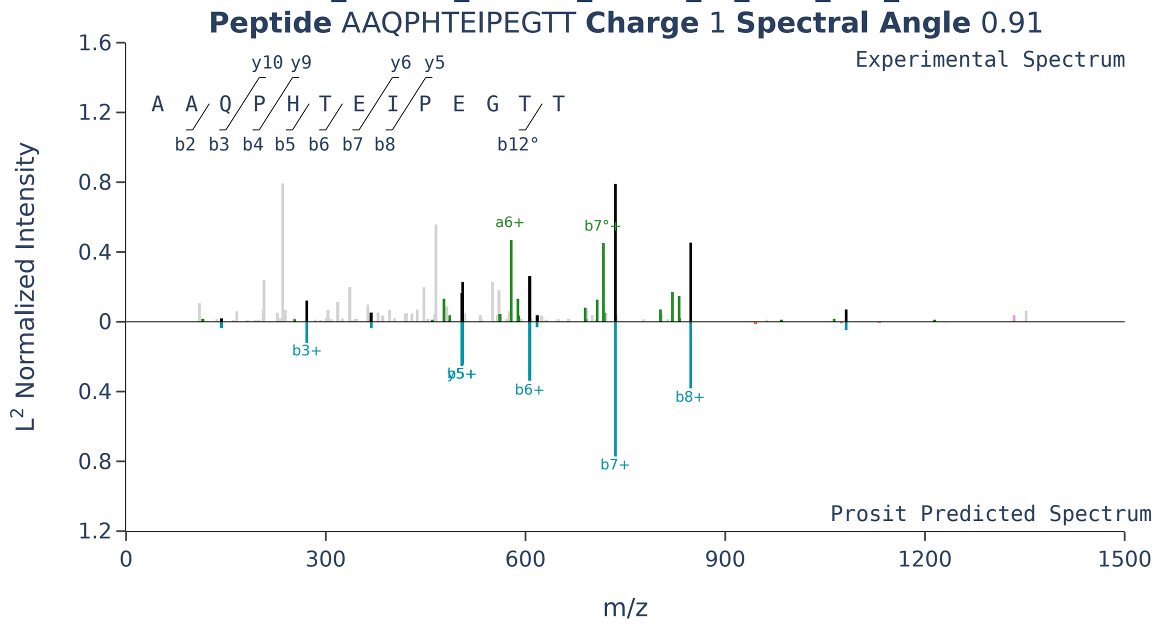


**Source** WSoh_221121_011221_HFGoe_L2_hTau_24h_R1 **Scan** 20173
**Peptide** ADEVSASLAKQGL **Charge** 2 **Spectral Angle** 0.93

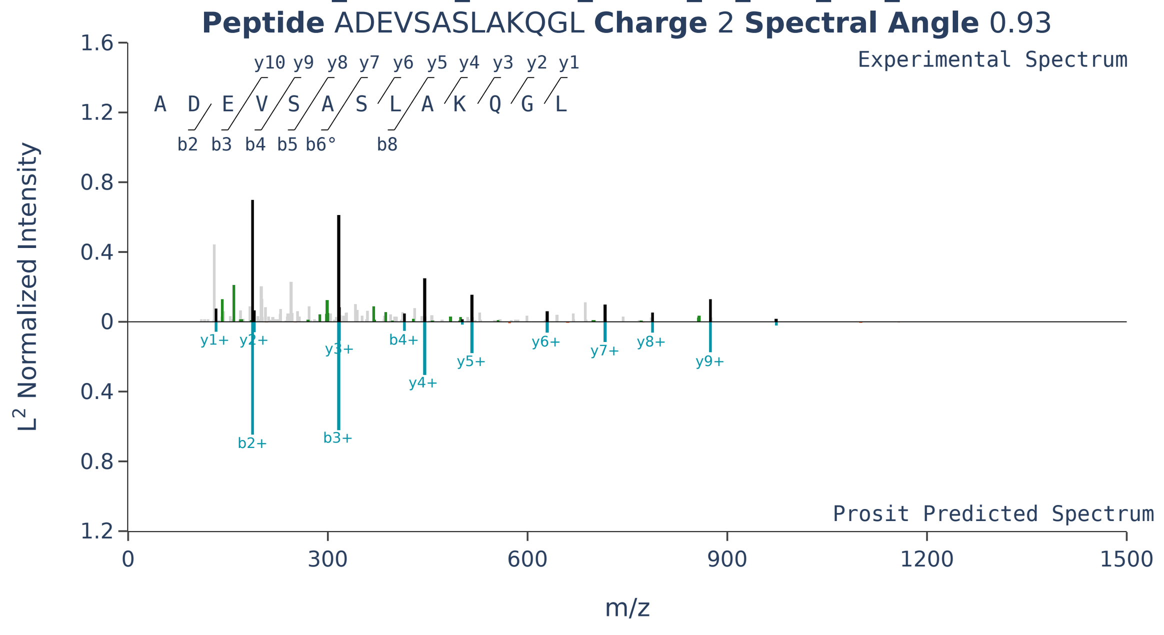


**Source** WSoh_221121_011221_HFGoe_L2_hTau_24h_R2 **Scan** 16509
**Peptide** PVDLSKVT **Charge** 2 **Spectral Angle** 0.9

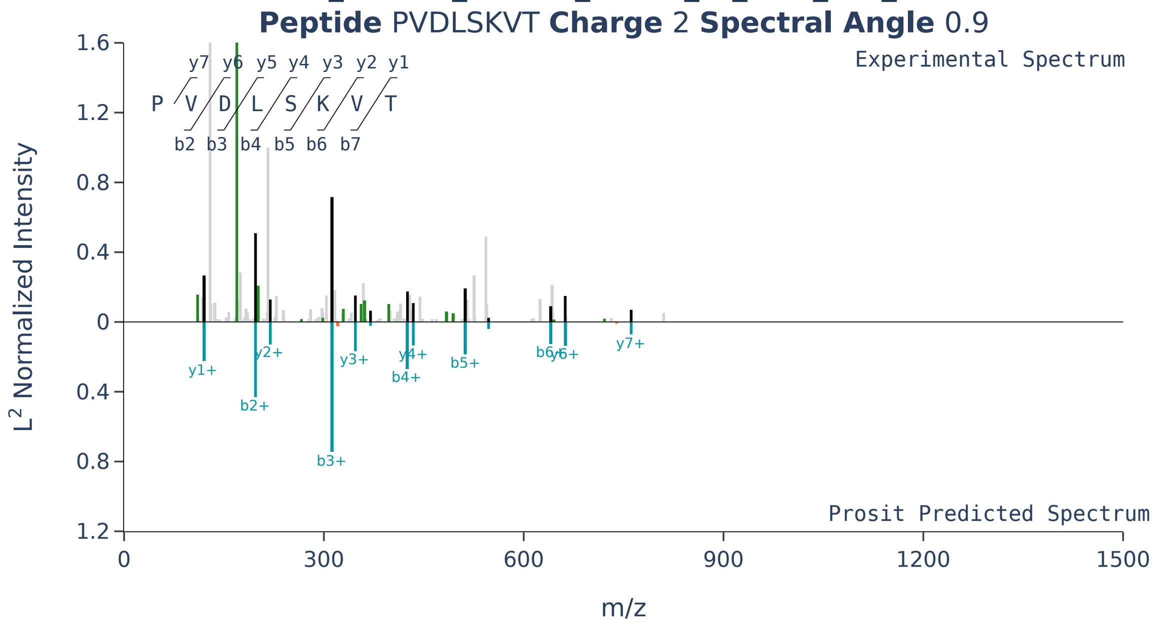


**Source** WSoh_221121_011221_HFGoe_L2_hTau_24h_R2 **Scan** 5807
**Peptide** GEPPKSGDRSGY **Charge** 3 **Spectral Angle** 0.92

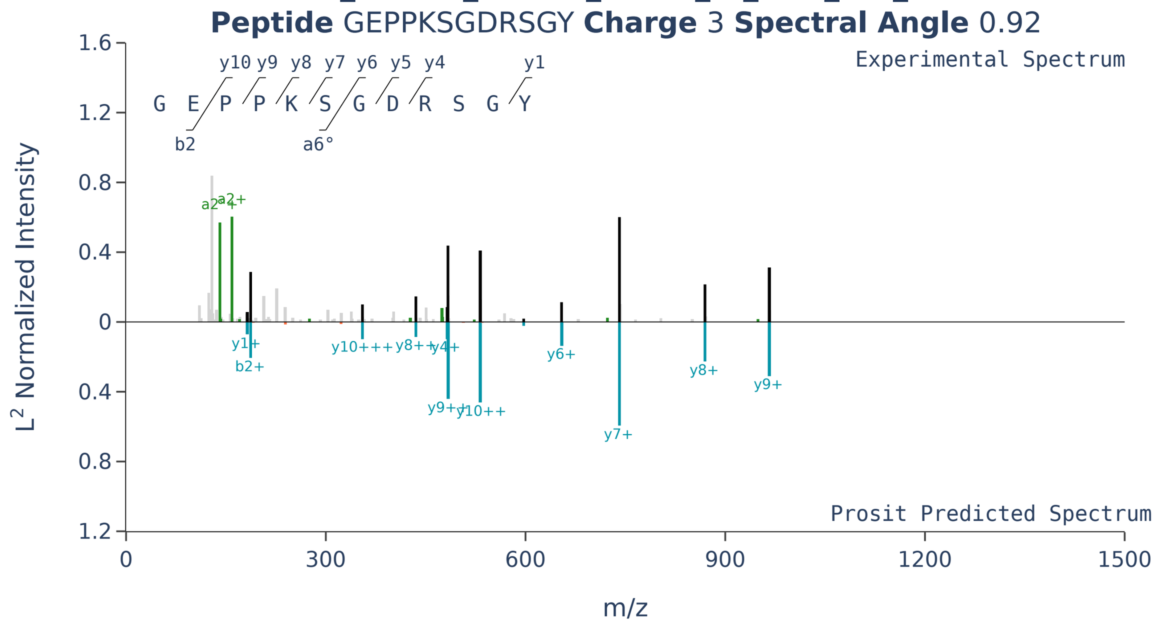


**Source** WSoh_221121_011221_HFGoe_L2_hTau_24h_R2 **Scan** 23454
**Peptide** TLADEVSASLAKQ **Charge** 2 **Spectral Angle** 0.94

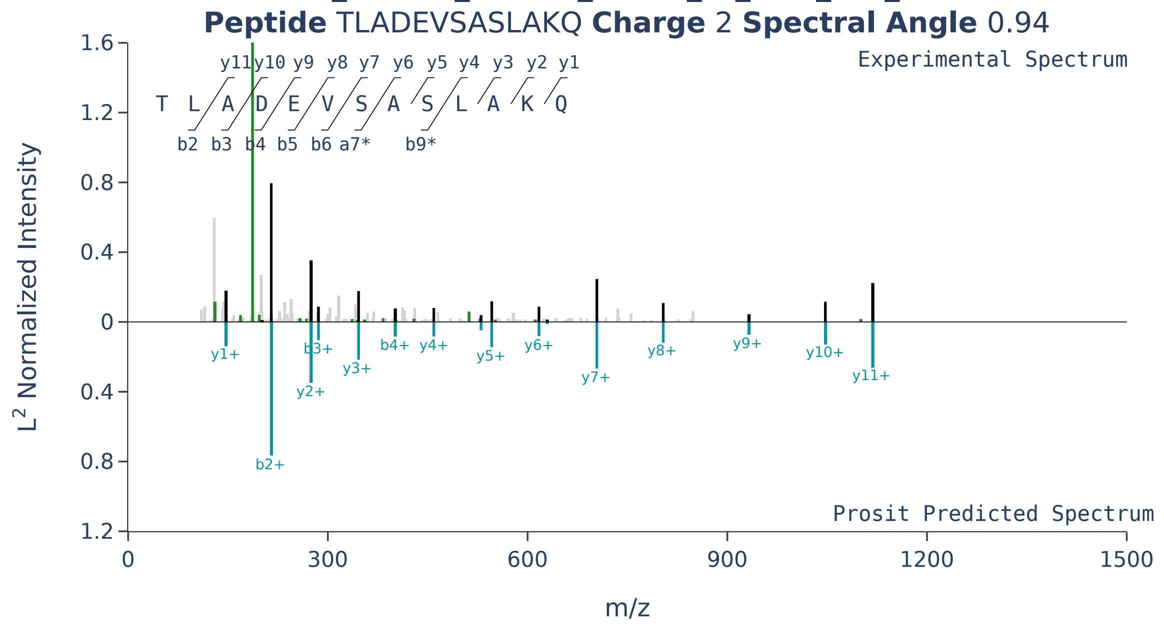

**Source** WSoh_221121_011221_HFGoe_L1_hTau_4h_R2 **Scan** 12786
**Peptide** AQPHTEIPEGTTAEE **Charge** 2 **Spectral Angle** 0.91

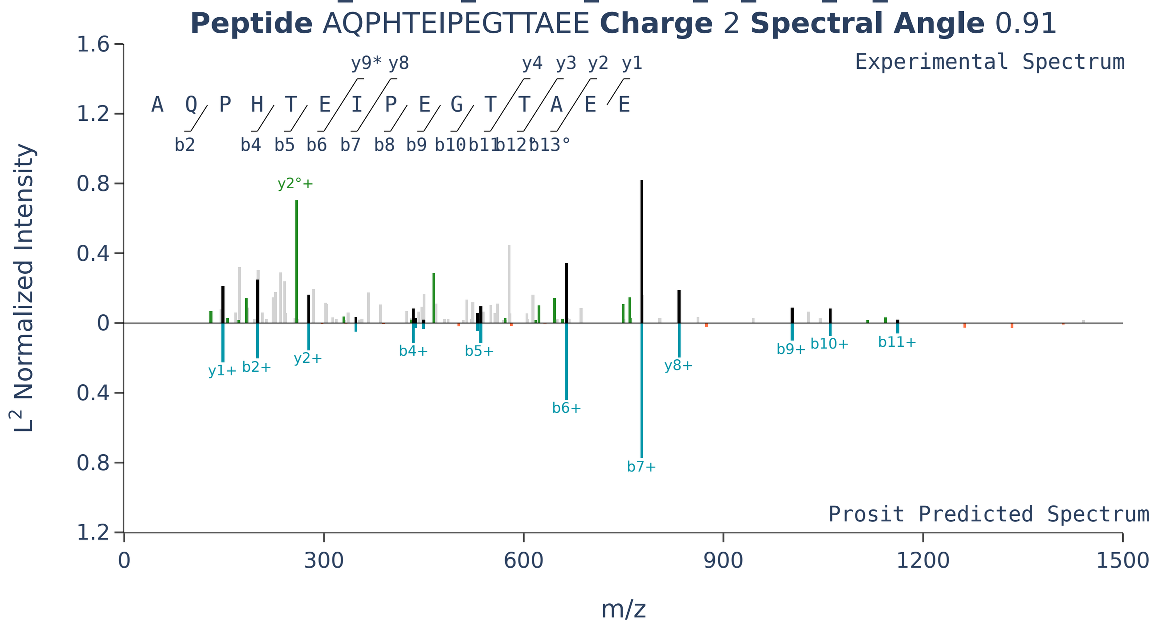


**Source** WSoh_221121_011221_HFGoe_L1_hTau_24h_R2 **Scan** 20395
**Peptide** DEVSASLAKQGL **Charge** 2 **Spectral Angle** 0.94

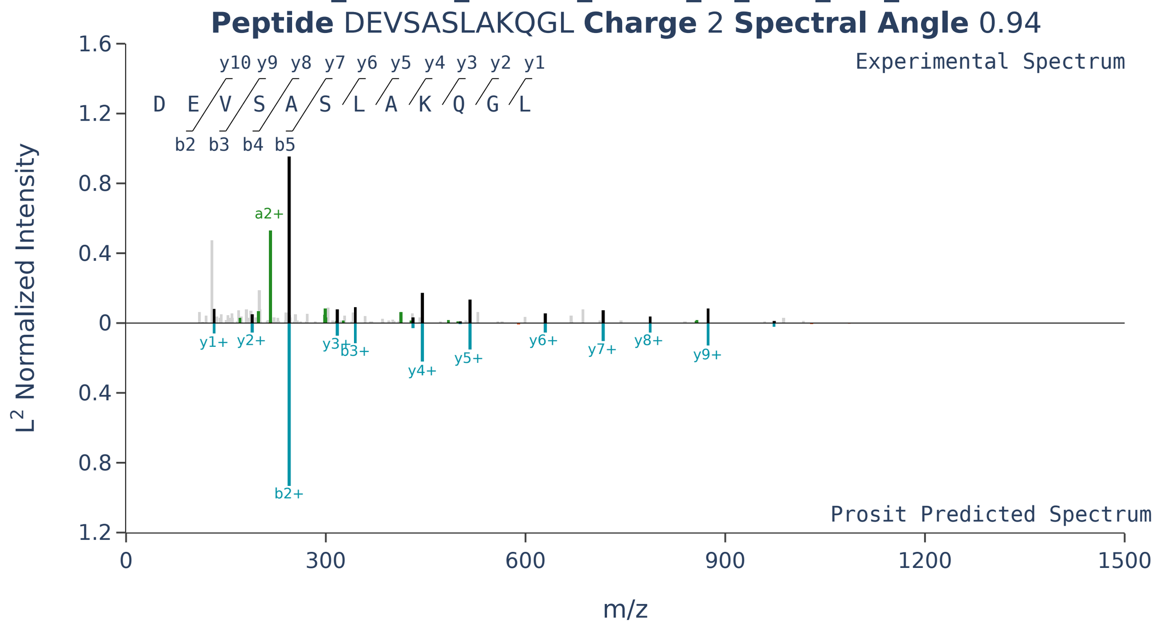


**Source** WSoh_221121_011221_HFGoe_L2_hTau_24h_R1 **Scan** 9091
**Peptide** QDQEGDTDAGLKE **Charge** 2 **Spectral Angle** 0.9

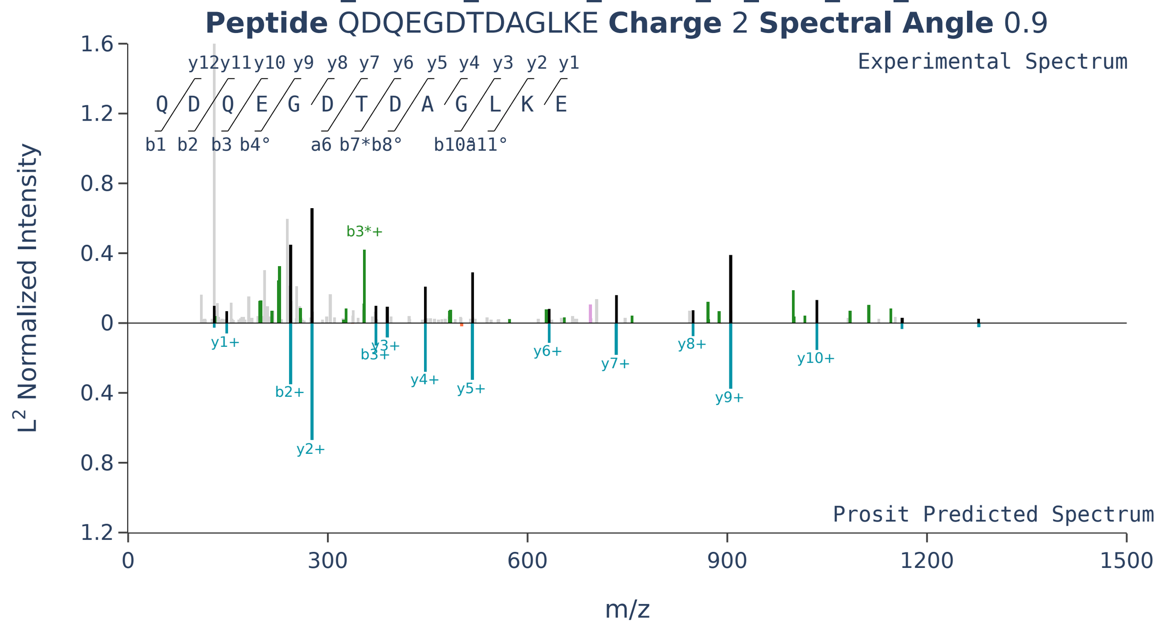


**Source** WSoh_221121_011221_HFGoe_L1_hTau_24h_R1 **Scan** 7807
**Peptide** MHQDQEGDTDAGLKE **Charge** 2 **Spectral Angle** 0.93

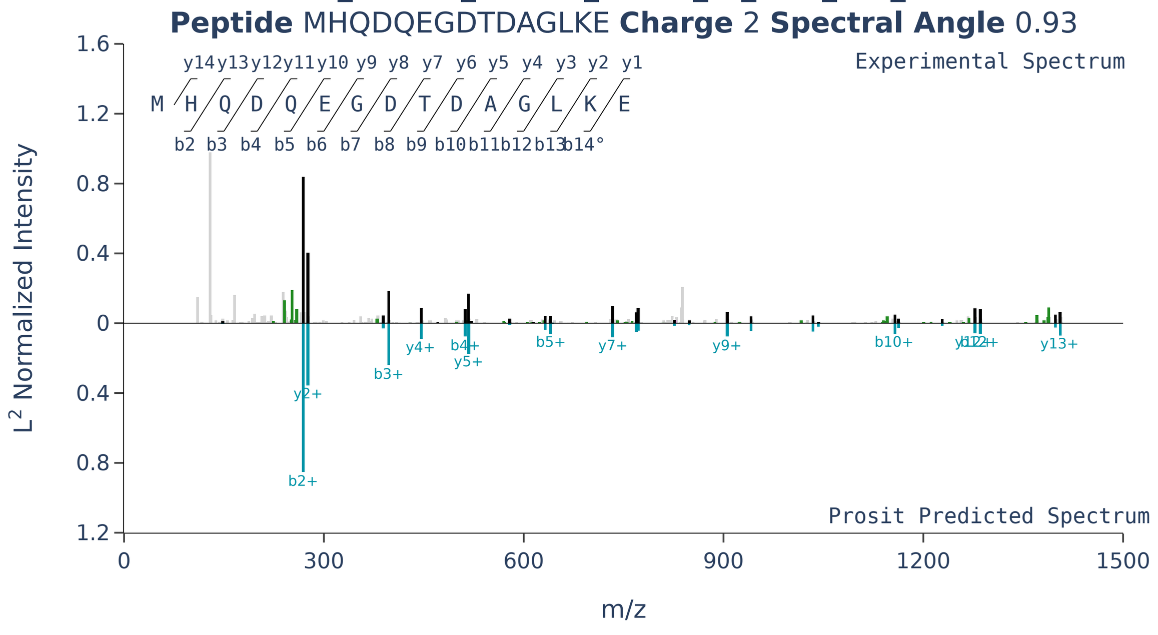


**Source** WSoh_221121_011221_HFGoe_L2_hTau_4h_R1 **Scan** 12829
**Peptide** VYKSPVVSGDTSPRHL **Charge** 2 **Spectral Angle** 0.91

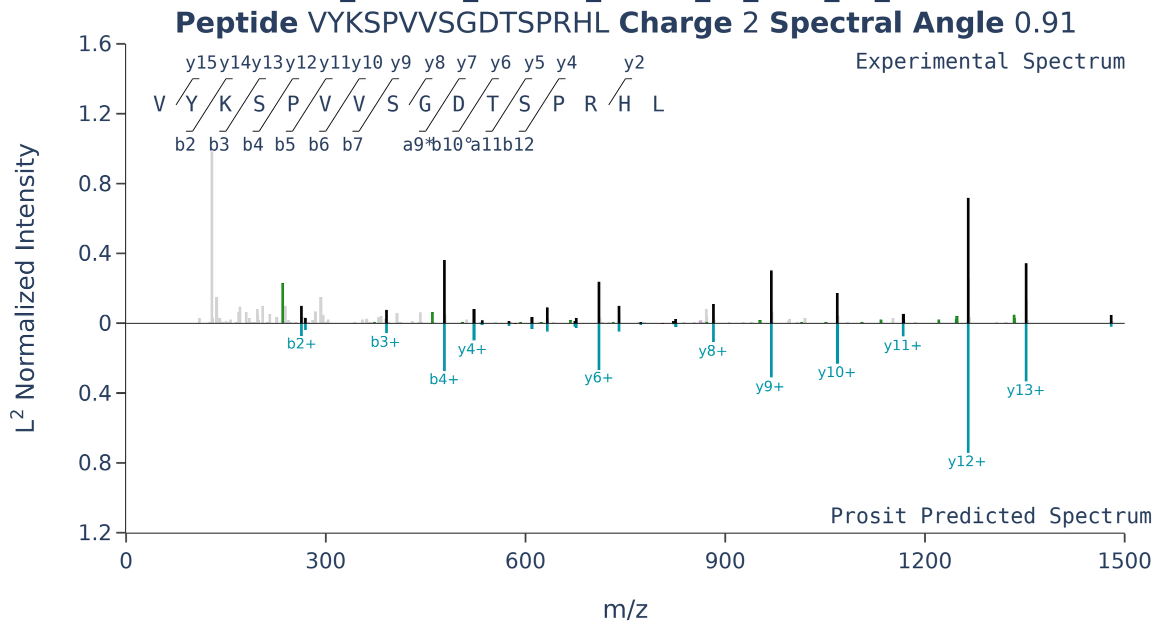


**Source** WSoh_221121_011221_HFGoe_L2_hTau_24h_R2 **Scan** 14107
**Peptide** EVSASLA **Charge** 1 **Spectral Angle** 0.9

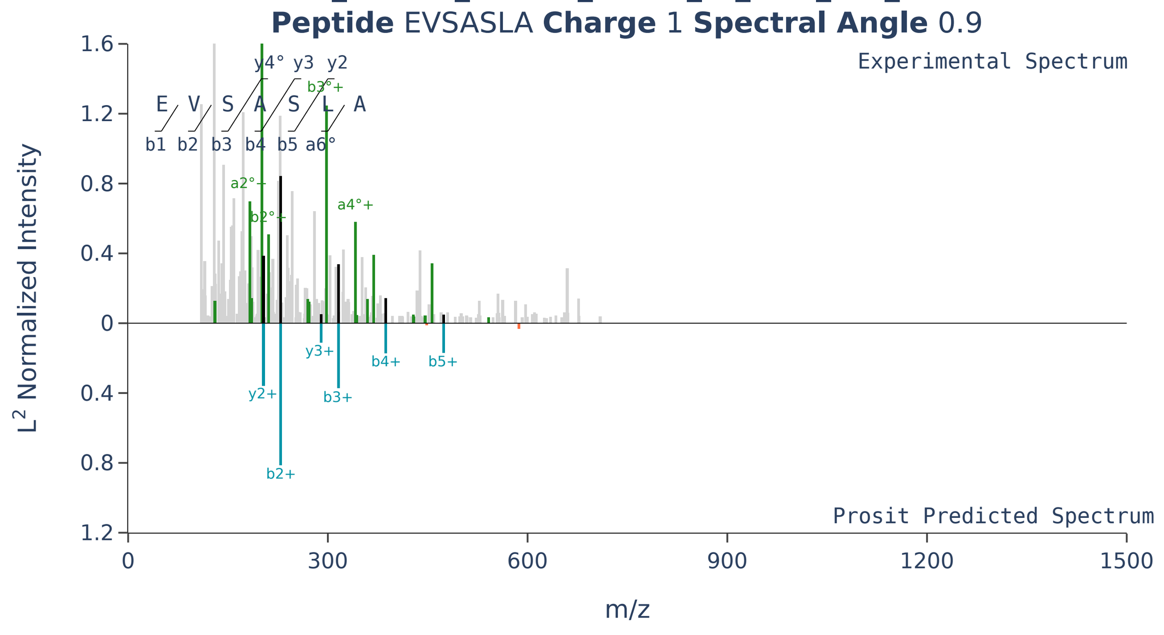


**Source** WSoh_221121_011221_HFGoe_L2_hTau_24h_R2 **Scan** 8454
**Peptide** GLGDRKDQGGYTMH **Charge** 2 **Spectral Angle** 0.96

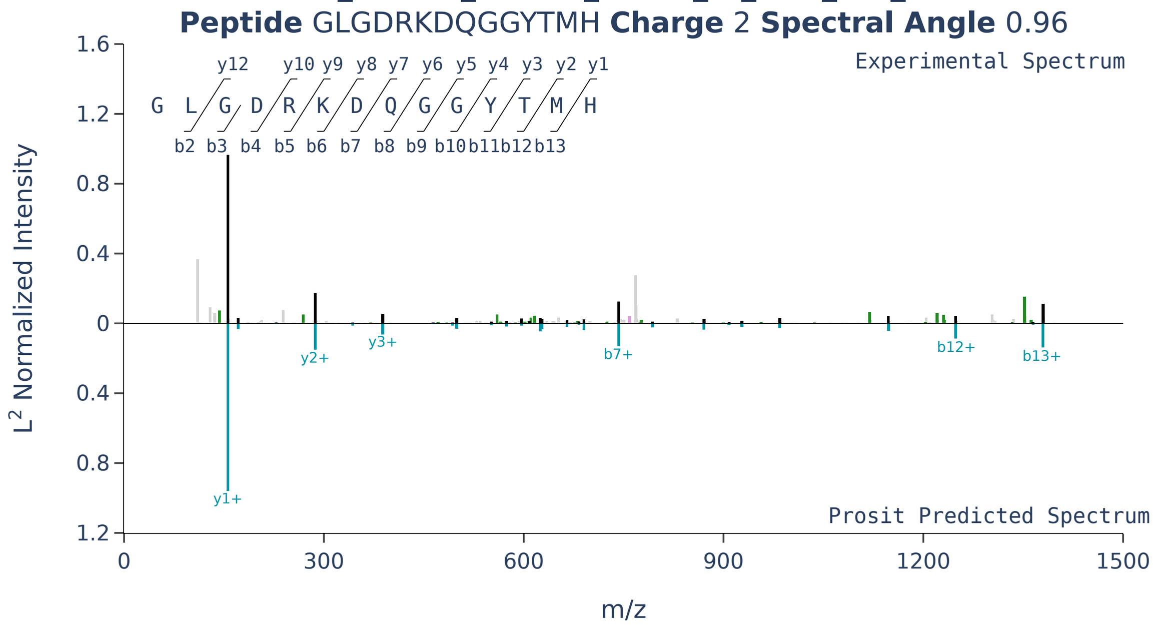


**Source** WSoh_221121_011221_HFGoe_L1_hTau_24h_R2 **Scan** 11816
**Peptide** GLGDRKDQGGYTM **Charge** 2 **Spectral Angle** 0.88

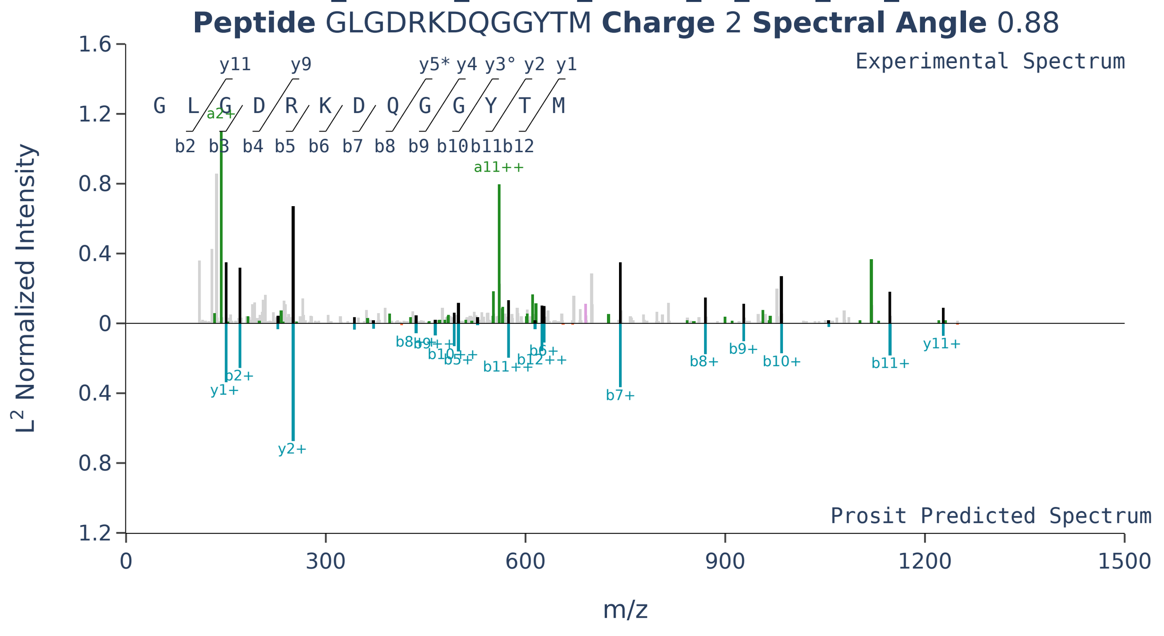


**Source** WSoh_221121_011221_HFGoe_L1_hTau_2h_R1 **Scan** 6727
**Peptide** DGKTKIATPRGA **Charge** 2 **Spectral Angle** 0.91

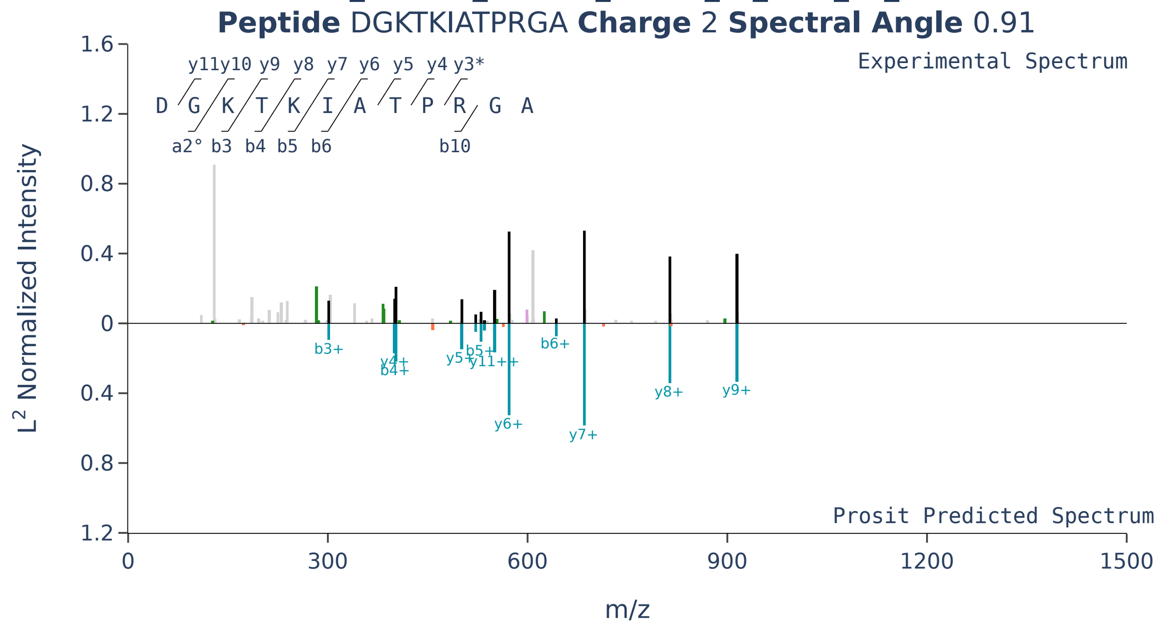


**Source** WSoh_221121_011221_HFGoe_L1_hTau_4h_R2 **Scan** 17079
**Peptide** GSKDNIKHVPGGGSVQIVY **Charge** 2 **Spectral Angle** 0.88

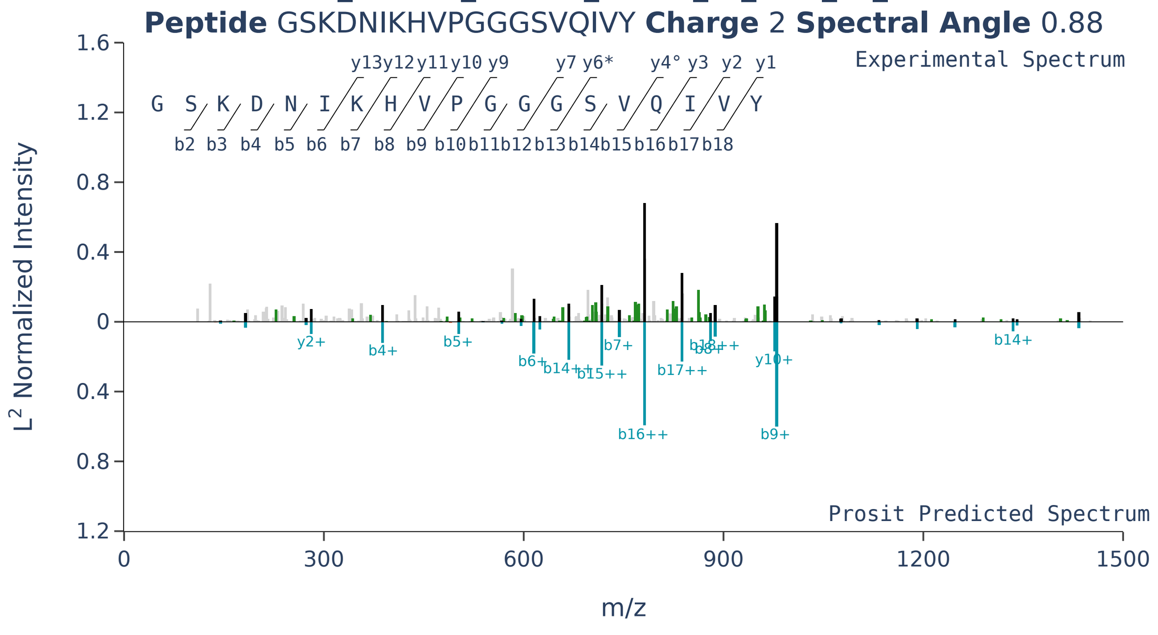


**Source** WSoh_221121_011221_HFGoe_L2_hTau_2h_R1 **Scan** 6681
**Peptide** DEAAGHVTQA **Charge** 2 **Spectral Angle** 0.93

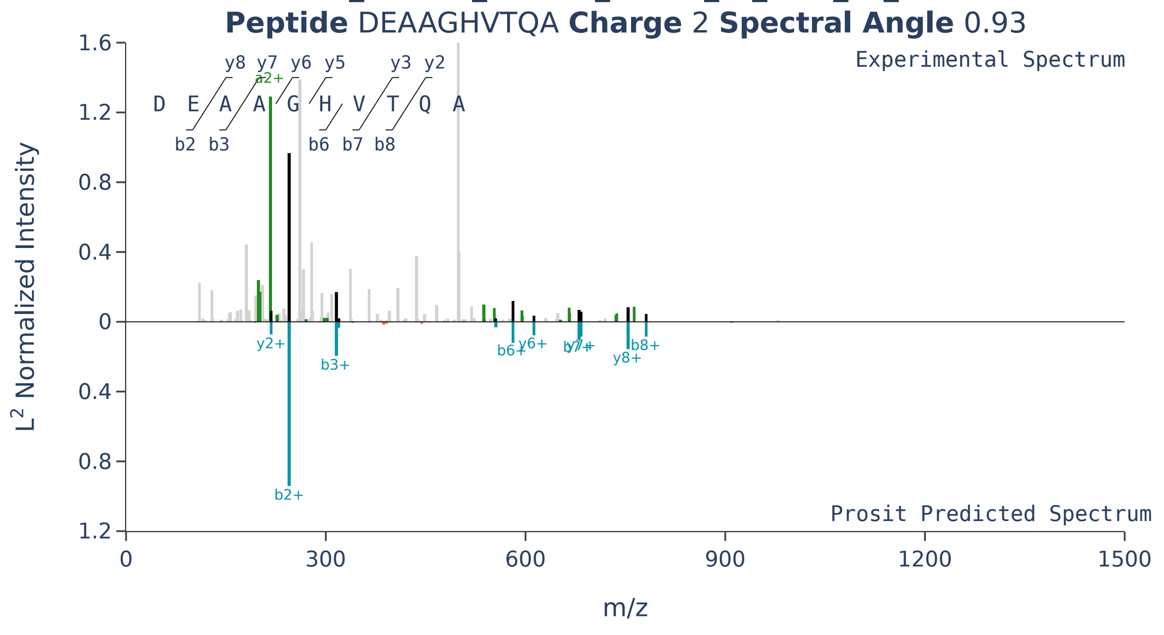


**Source** WSoh_221121_011221_HFGoe_L2_hTau_4h_R2 **Scan** 11154
**Peptide** VDLSKVTSK **Charge** 2 **Spectral Angle** 0.92

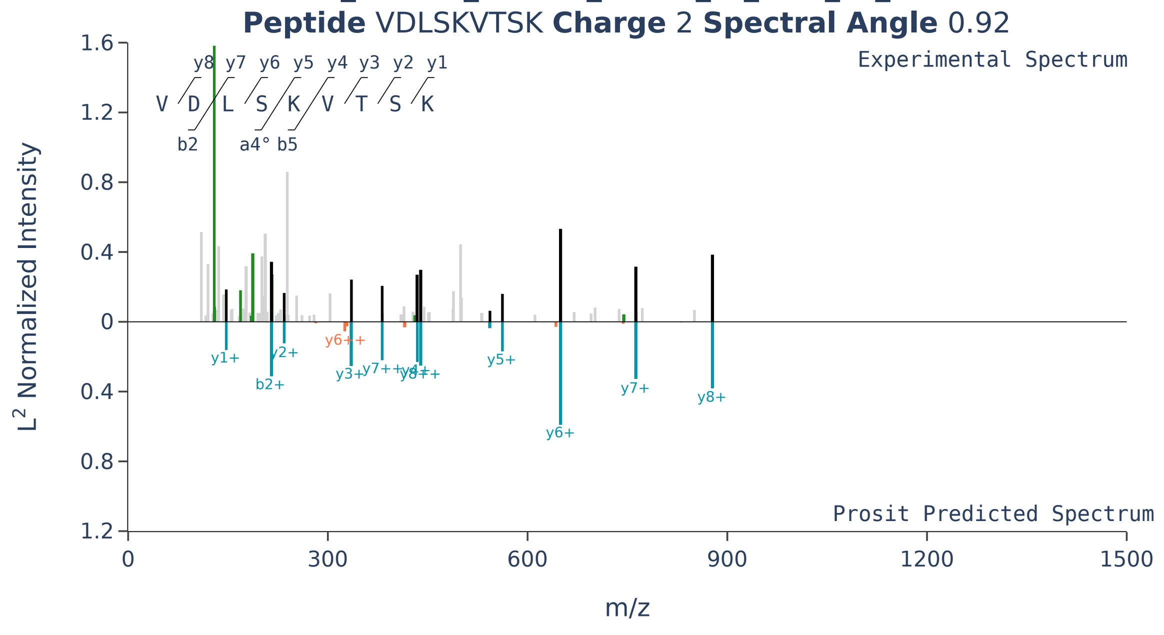


**Source** WSoh_221121_011221_HFGoe_L1_hTau_24h_R1 **Scan** 7919
**Peptide** EDHAGTYGLGDRKD **Charge** 2 **Spectral Angle** 0.9

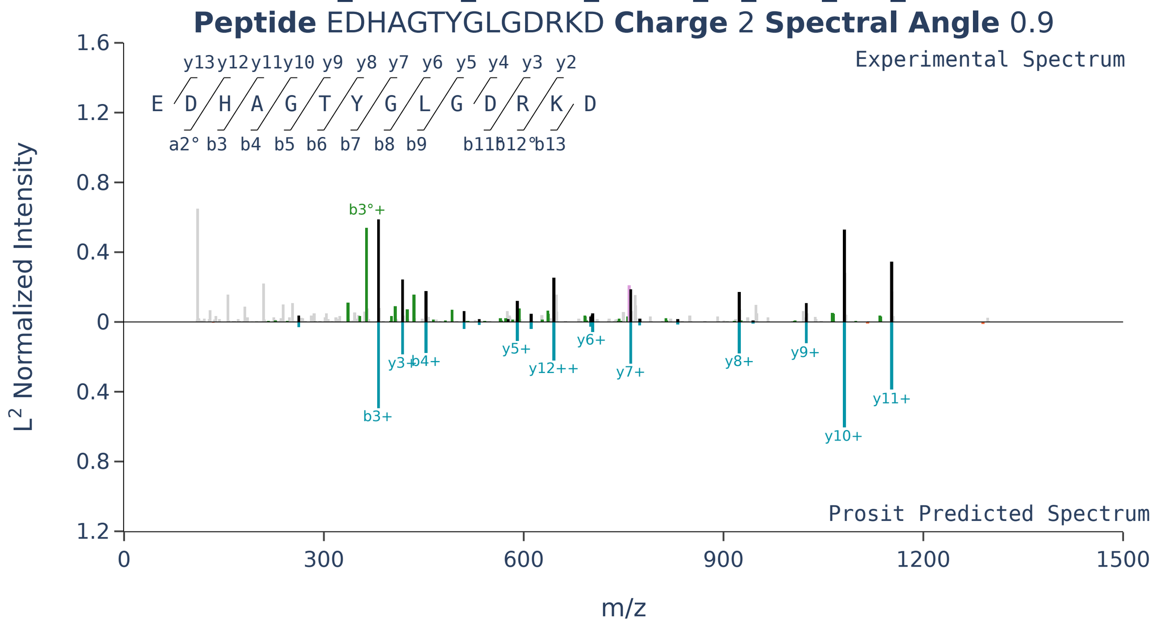


**Source** WSoh_221121_011221_HFGoe_L2_hTau_4h_R1 **Scan** 5993
**Peptide** DGSEEPGSETSDAKST **Charge** 2 **Spectral Angle** 0.88

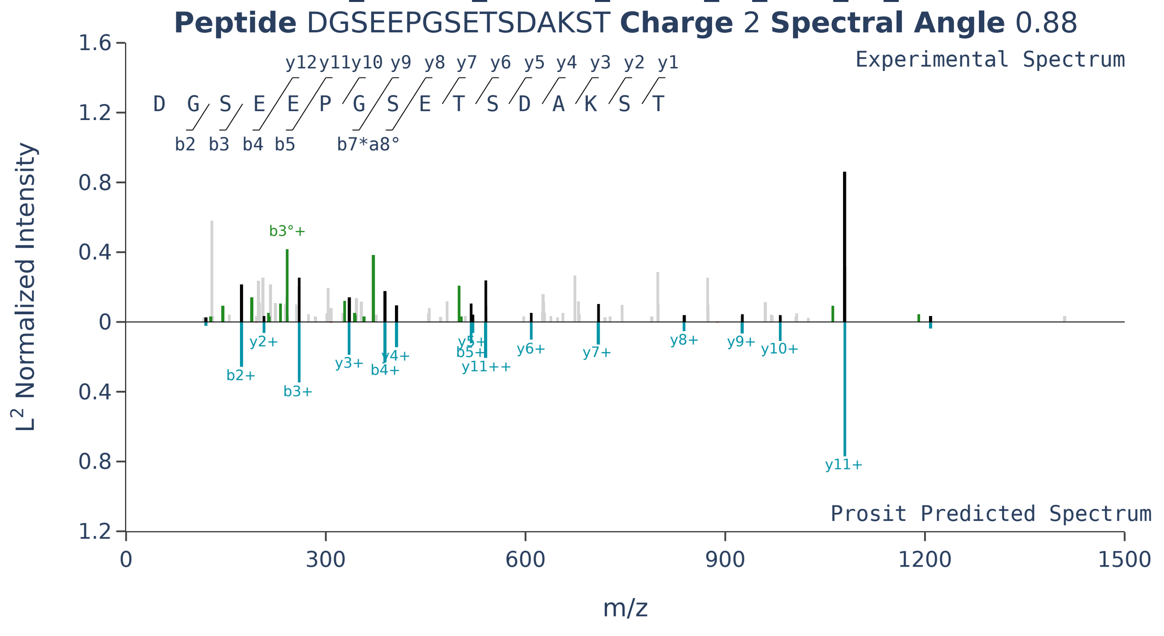


**Source** WSoh_221121_011221_HFGoe_L1_hTau_24h_R1 **Scan** 12999
**Peptide** DHAGTYGLGD **Charge** 1 **Spectral Angle** 0.97

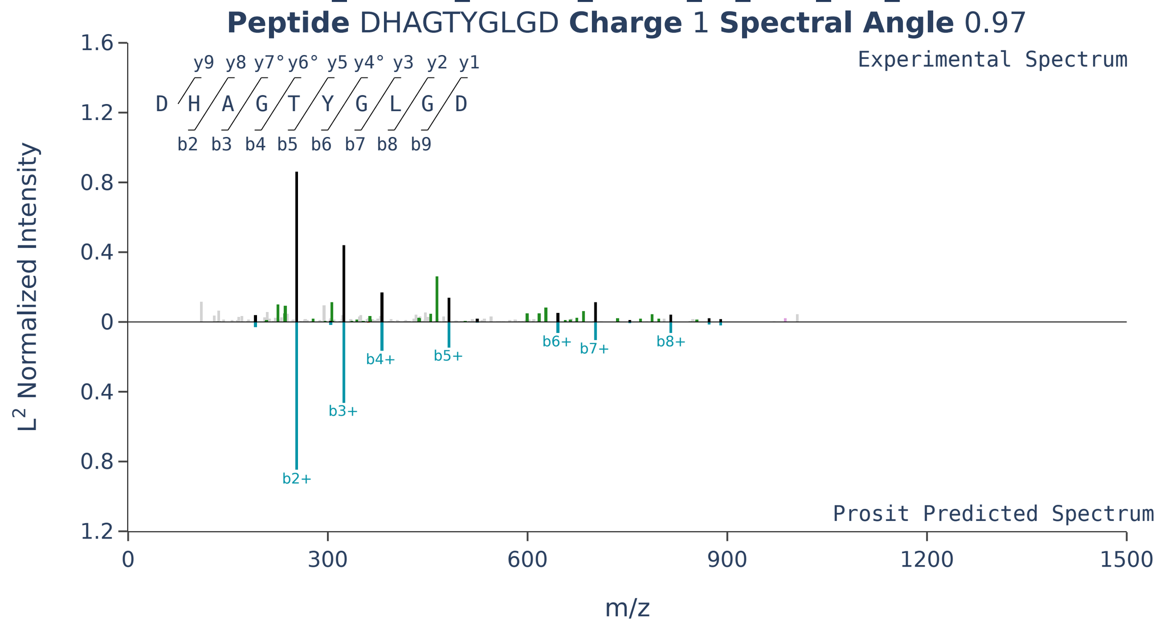

**Source** WSoh_221121_011221_HFGoe_L1_hTau_24h_R2 **Scan** 7263
**Peptide** GSKDNIKHVPGGGSVQ **Charge** 3 **Spectral Angle** 0.91

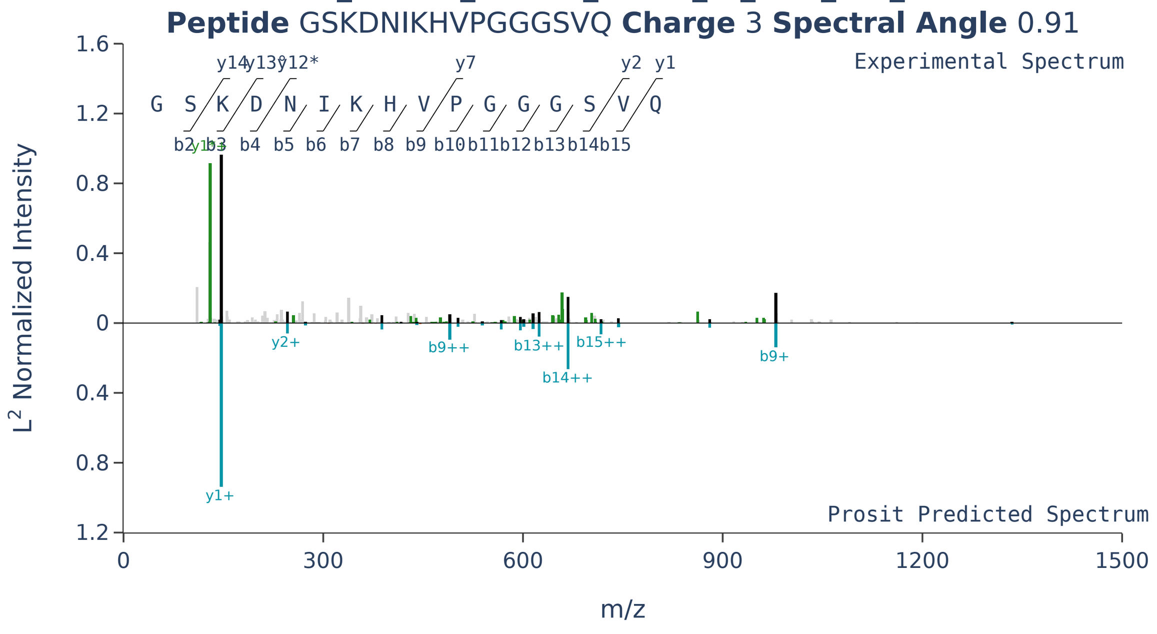


**Source** WSoh_221121_011221_HFGoe_L1_hTau_24h_R2 **Scan** 12605
**Peptide** SPVVSGDTSPRHLSNVS **Charge** 2 **Spectral Angle** 0.93

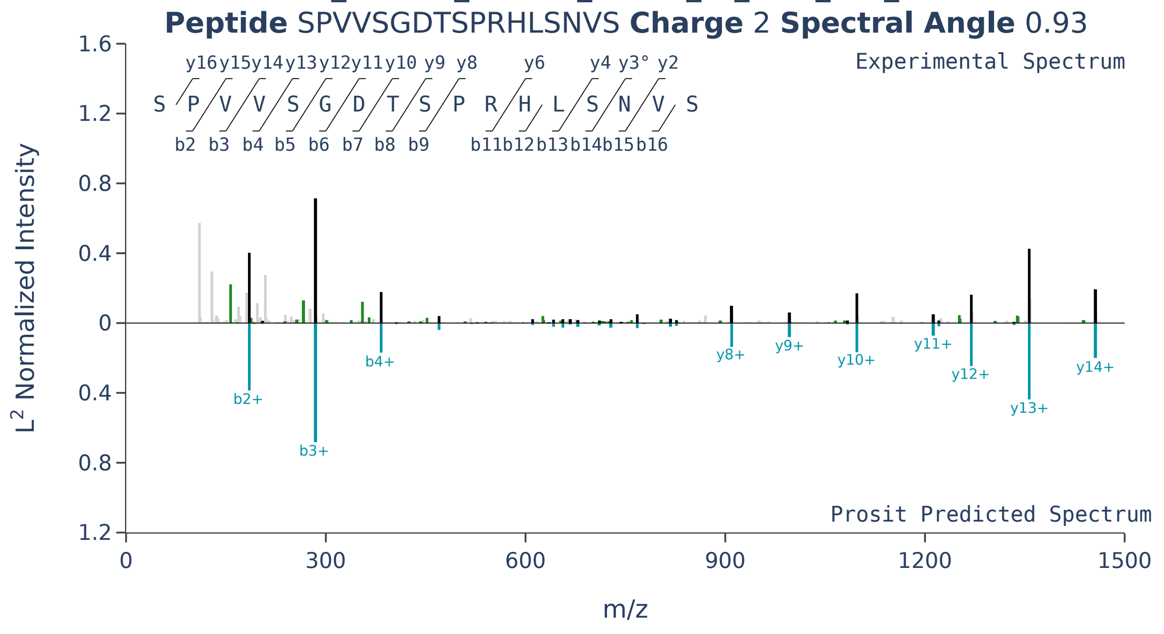


**Source** WSoh_221121_011221_HFGoe_L1_hTau_24h_R1 **Scan** 20007
**Peptide** DVTAPLVDEGAPGKQA **Charge** 2 **Spectral Angle** 0.92

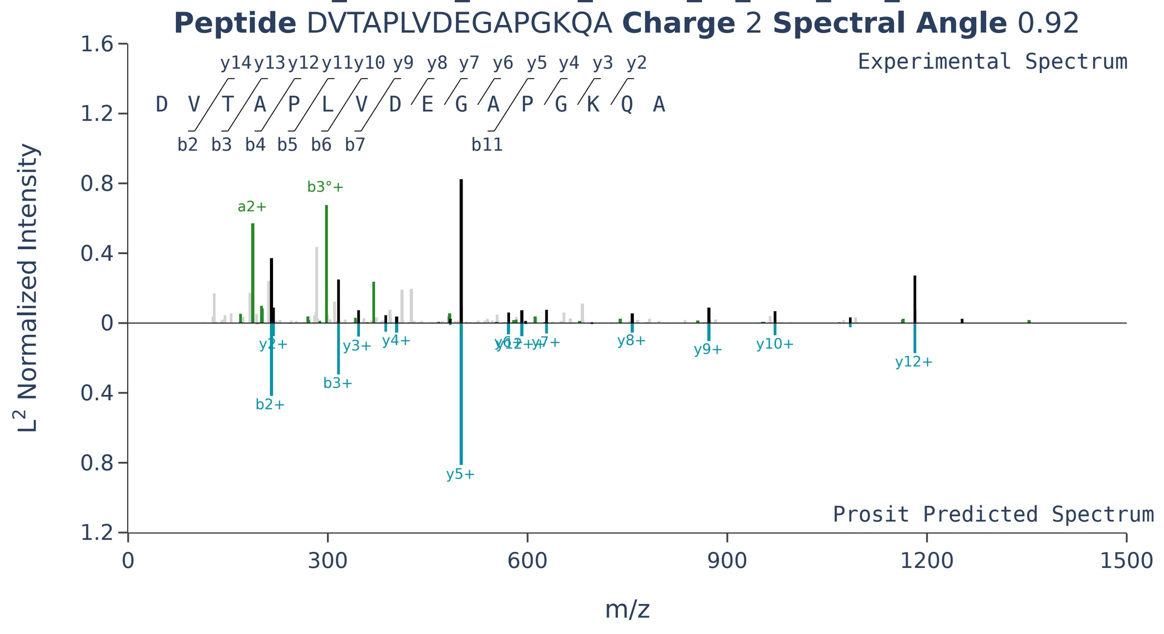


**Source** WSoh_221121_011221_HFGoe_L2_hTau_24h_R2 **Scan** 6279
**Peptide** GKTKIATPRGA **Charge** 2 **Spectral Angle** 0.91

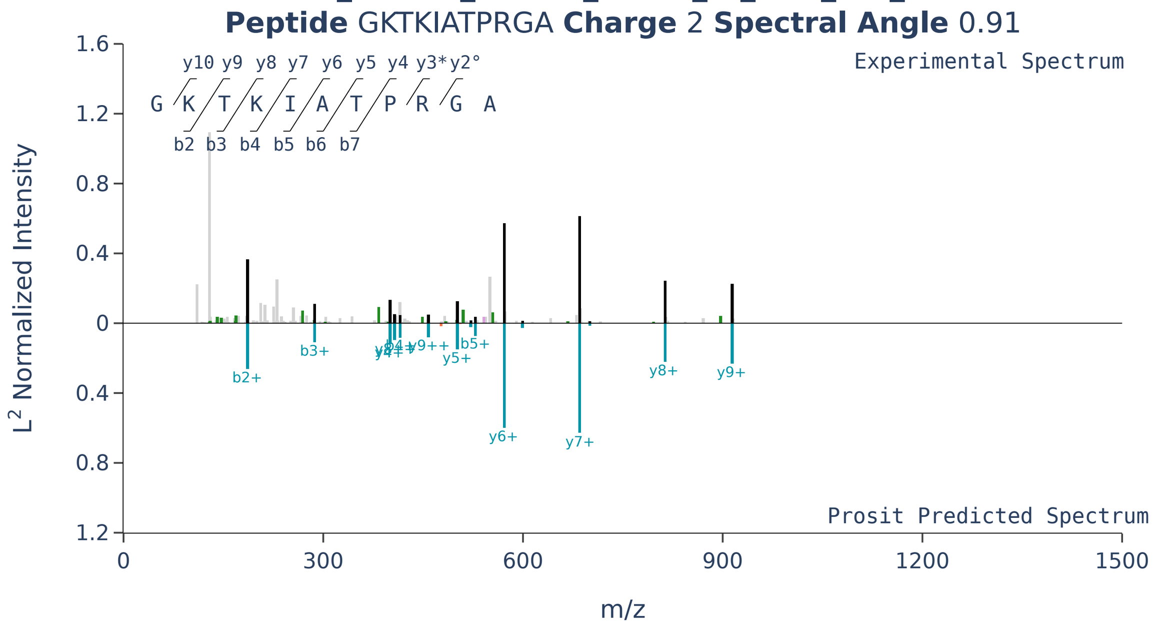


**Source** WSoh_221121_011221_HFGoe_L2_hTau_24h_R2 **Scan** 6556
**Peptide** SGDTSPRHL **Charge** 2 **Spectral Angle** 0.89

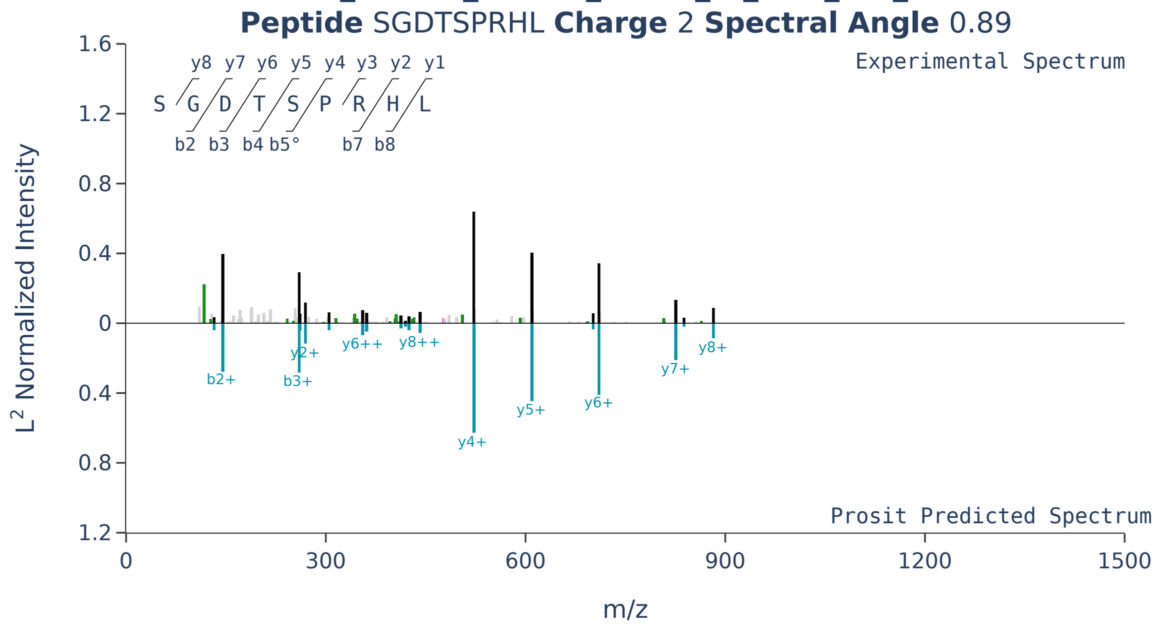


**Source** WSoh_221121_011221_HFGoe_L2_hTau_4h_R2 **Scan** 14430
**Peptide** EDHAGTYGL **Charge** 2 **Spectral Angle** 0.9

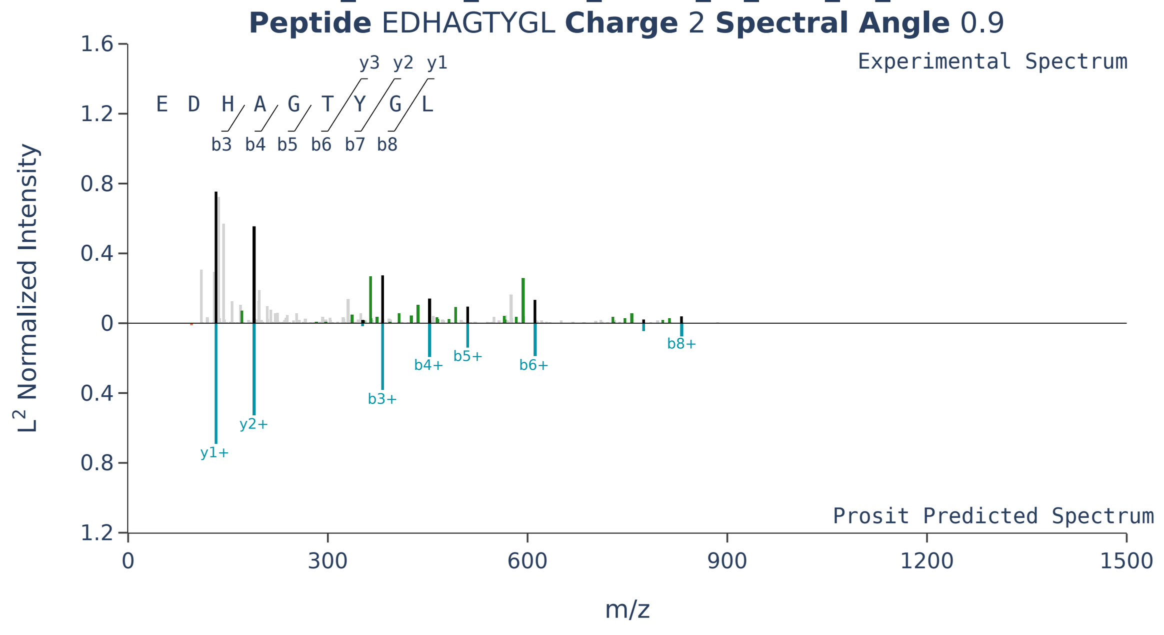


**Source** WSoh_221121_011221_HFGoe_L2_hTau_24h_R2 **Scan** 16163
**Peptide** PVPMPDLKNVKSK **Charge** 3 **Spectral Angle** 0.88

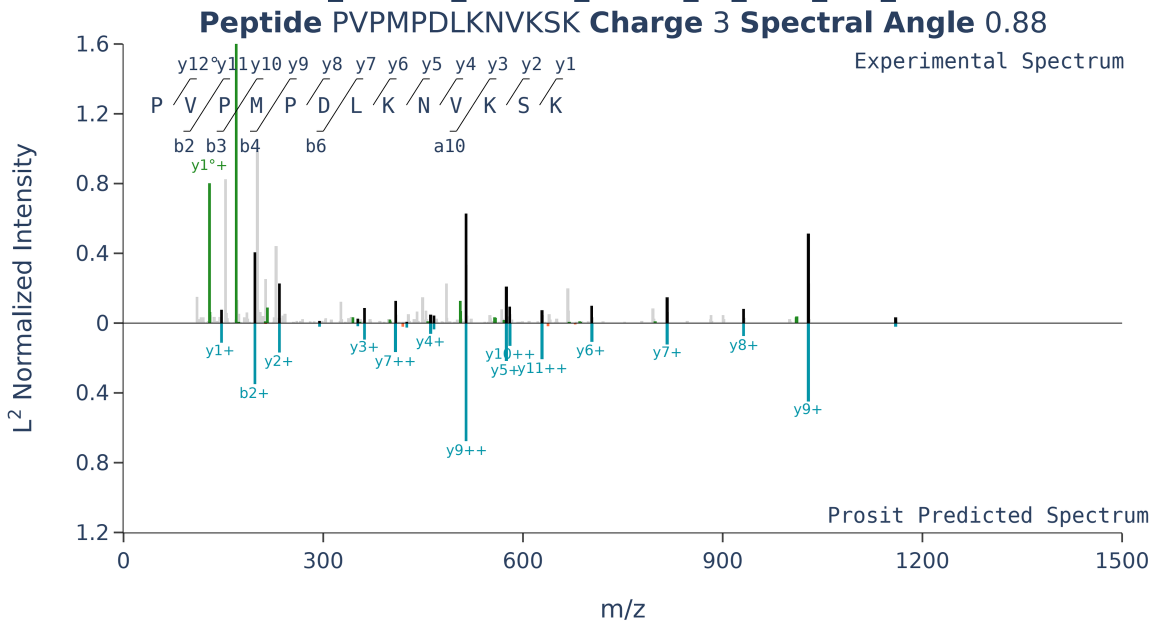


**Source** WSoh_221121_011221_HFGoe_L2_hTau_24h_R2 **Scan** 7840
**Peptide** DHAGTYGLGDRKDQG **Charge** 2 **Spectral Angle** 0.94

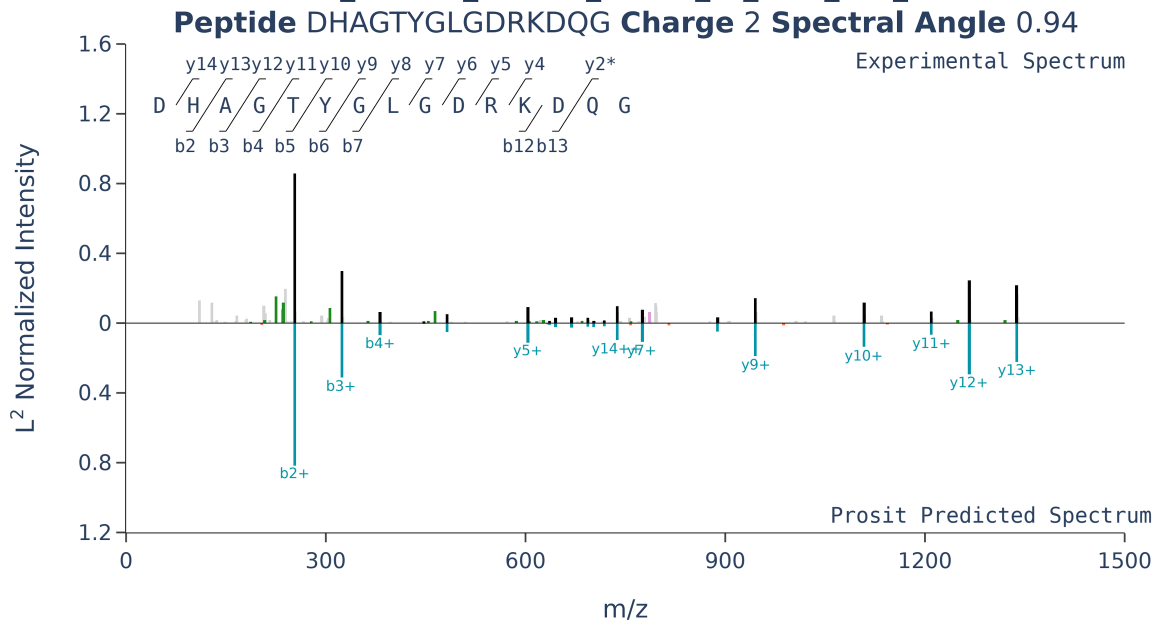


**Source** WSoh_221121_011221_HFGoe_L2_hTau_4h_R2 **Scan** 10514
**Peptide** KPVDLSKVTSKC **Charge** 3 **Spectral Angle** 0.9

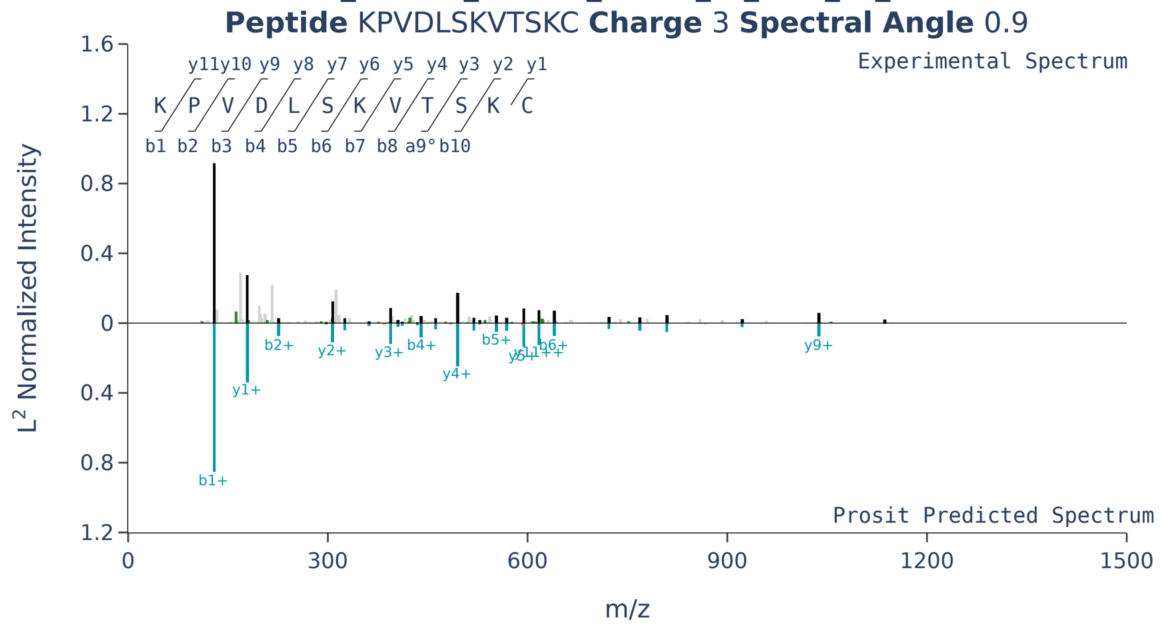


**Source** WSoh_221121_011221_HFGoe_L2_hTau_24h_R2 **Scan** 14508
**Peptide** TPSLPTPPTREPKKV **Charge** 2 **Spectral Angle** 0.87

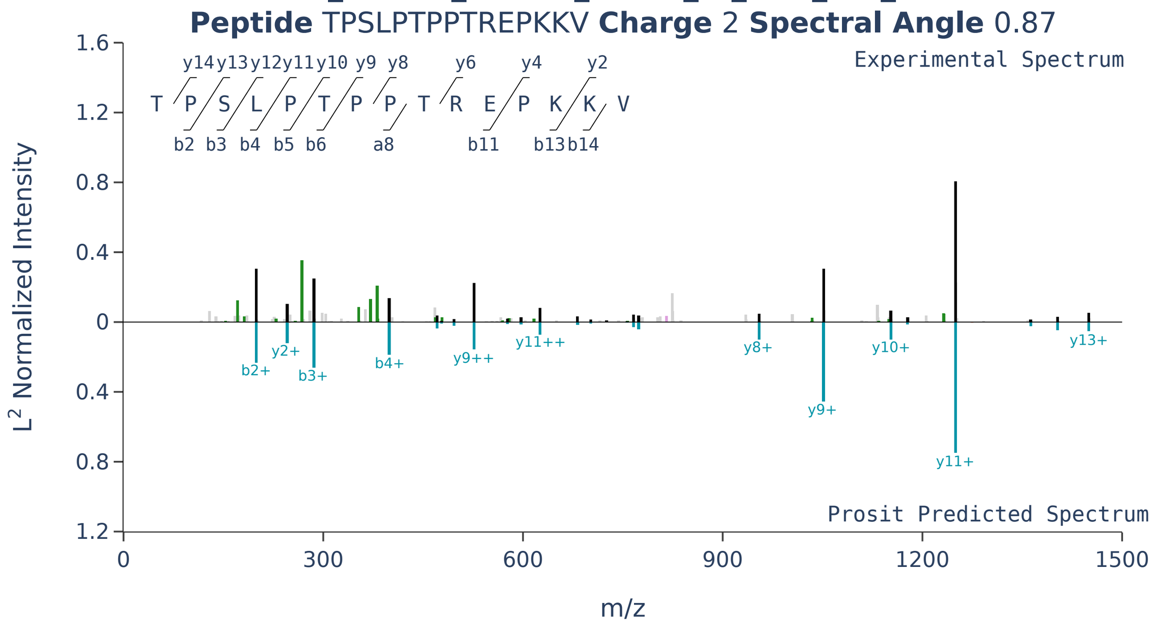


**Source** WSoh_221121_011221_HFGoe_L2_hTau_24h_R1 **Scan** 19842
**Peptide** VYKPVDLSKVTSKCGSL **Charge** 3 **Spectral Angle** 0.91

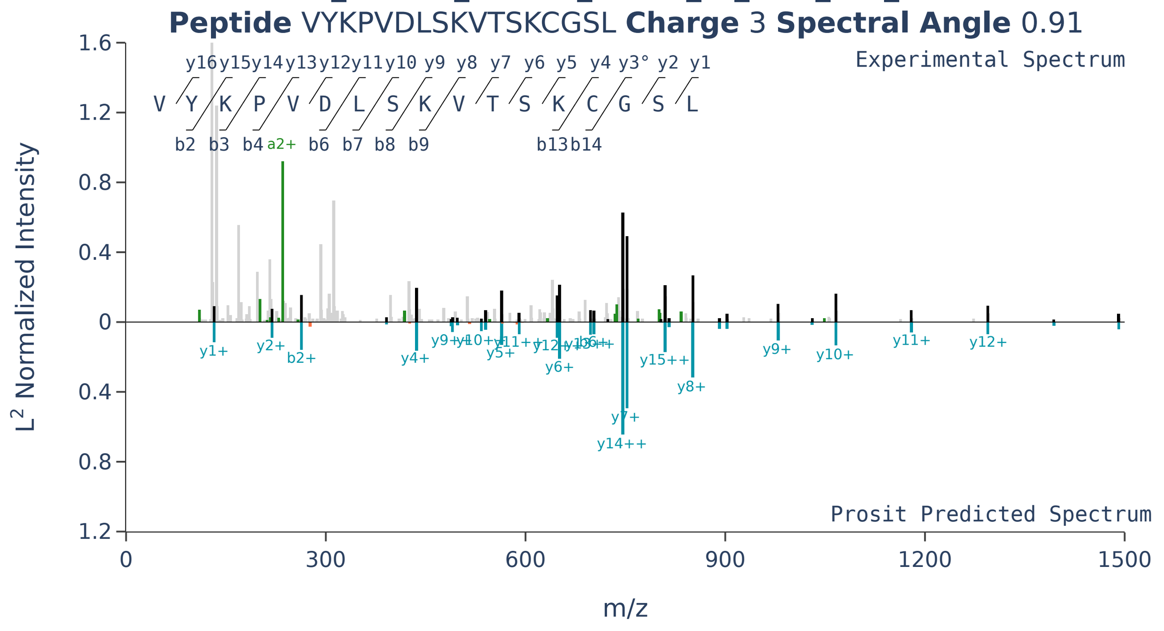


**Source** WSoh_221121_011221_HFGoe_L2_hTau_1h_R2 **Scan** 13814
**Peptide** LDNITHV **Charge** 2 **Spectral Angle** 0.9

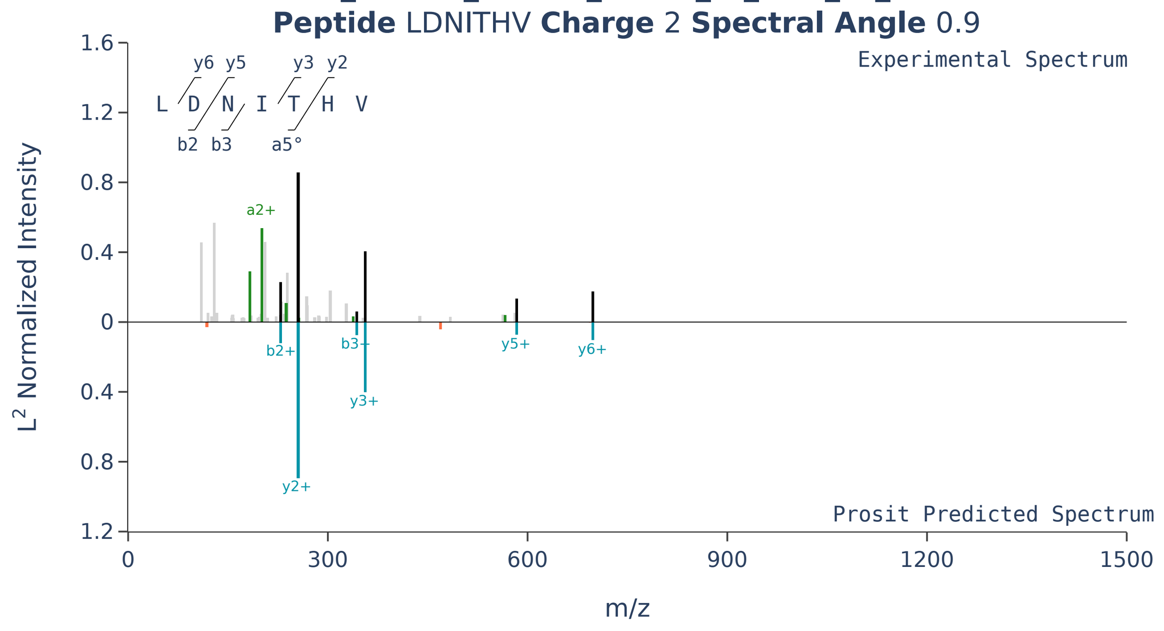


**Source** WSoh_221121_011221_HFGoe_L2_hTau_2h_R1 **Scan** 6556
**Peptide** IHHKPGGGQVEV **Charge** 2 **Spectral Angle** 0.88

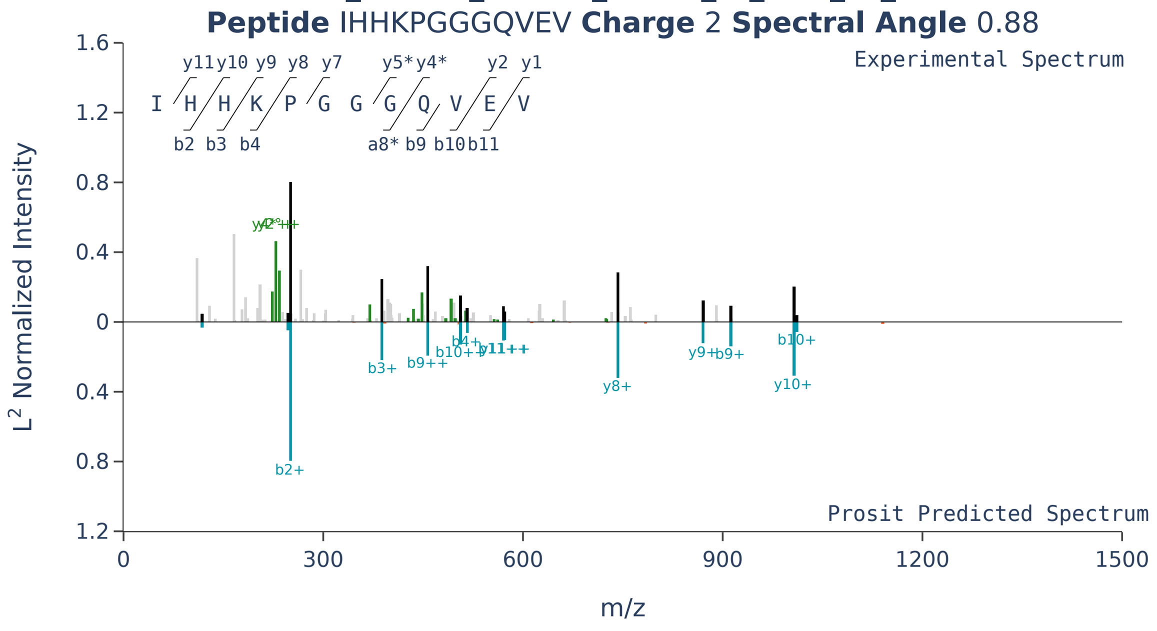


**Source** WSoh_221121_011221_HFGoe_L2_hTau_24h_R2 **Scan** 7765
**Peptide** GDTSPRHLSNVSSTG **Charge** 3 **Spectral Angle** 0.94

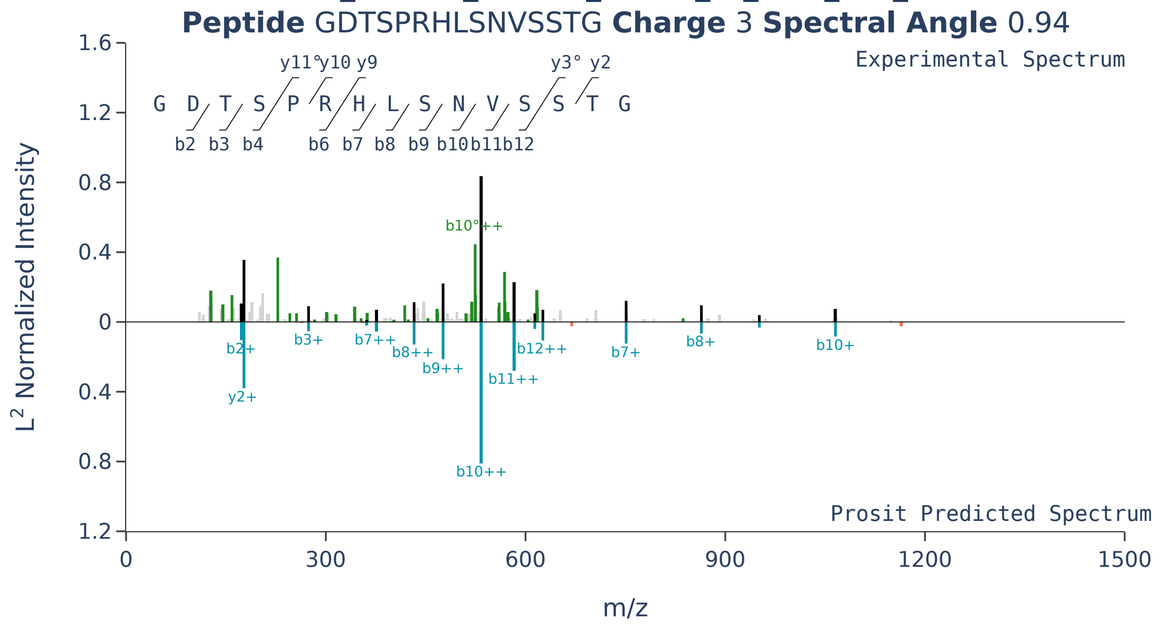


**Source** WSoh_221121_011221_HFGoe_L2_hTau_4h_R2 **Scan** 7144
**Peptide** RSGYSSPGSPGTPGSR **Charge** 3 **Spectral Angle** 0.91

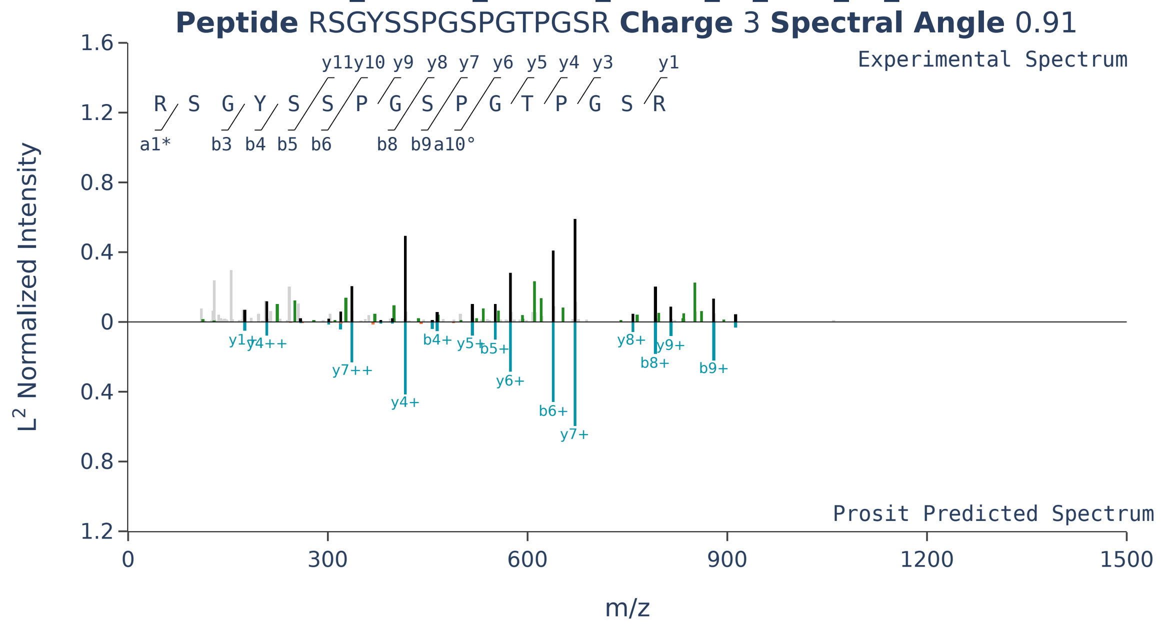

**Source** WSoh_221121_011221_HFGoe_L2_hTau_4h_R1 **Scan** 10438
**Peptide** PHTEIPEGT **Charge** 2 **Spectral Angle** 0.85

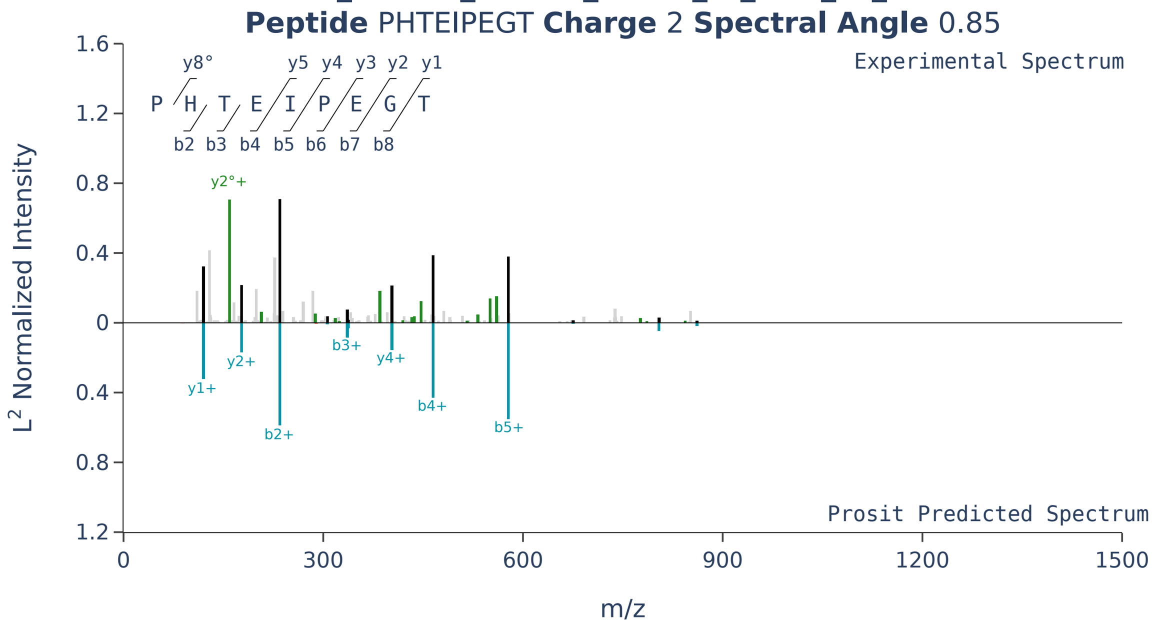


**Source** WSoh_221121_011221_HFGoe_L2_hTau_24h_R2 **Scan** 8648
**Peptide** VSSTGSID **Charge** 1 **Spectral Angle** 0.93

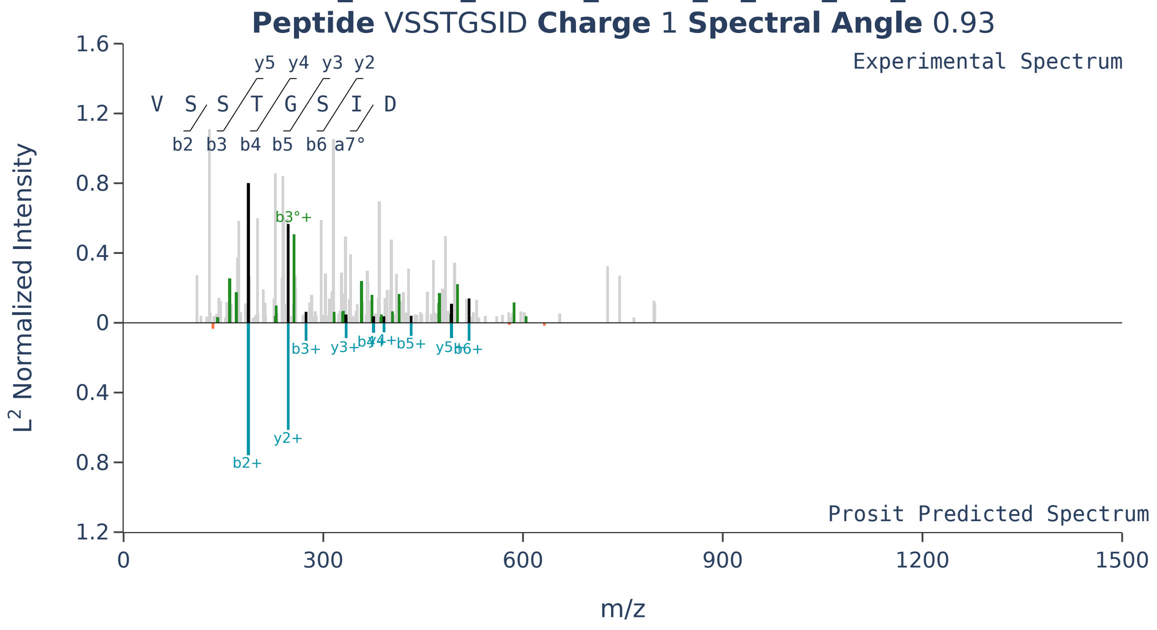


**Source** WSoh_221121_011221_HFGoe_L1_hTau_24h_R1 **Scan** 8066
**Peptide** KVQIINK **Charge** 2 **Spectral Angle** 0.93

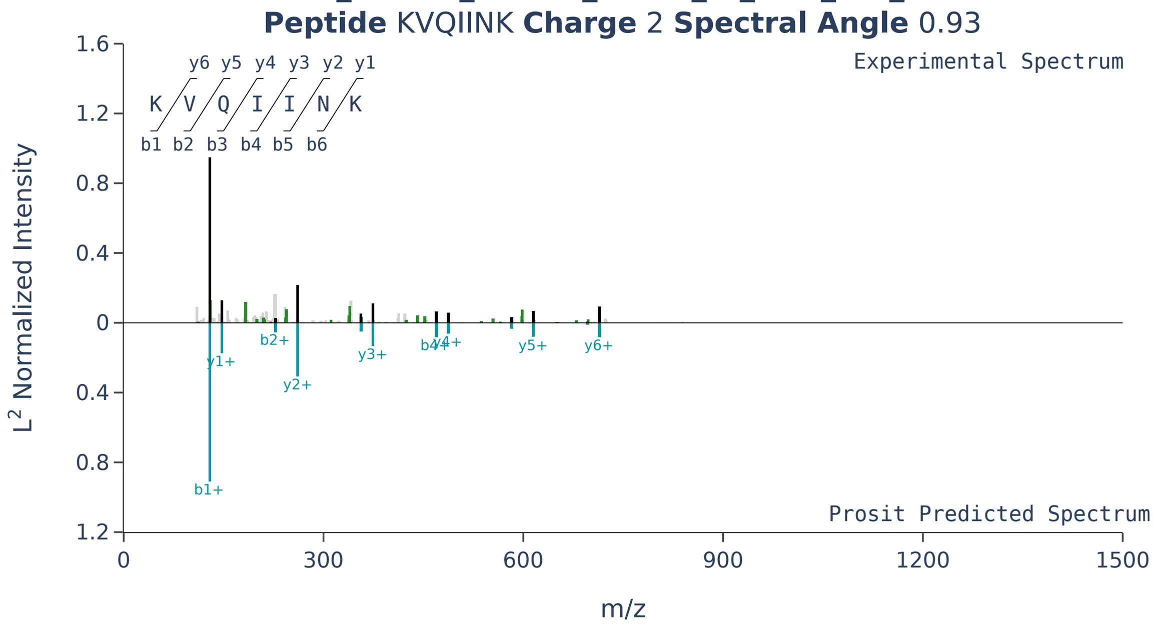


**Source** WSoh_221121_011221_HFGoe_L1_hTau_24h_R2 **Scan** 7783
**Peptide** DTSPRHLSNVSS **Charge** 2 **Spectral Angle** 0.89

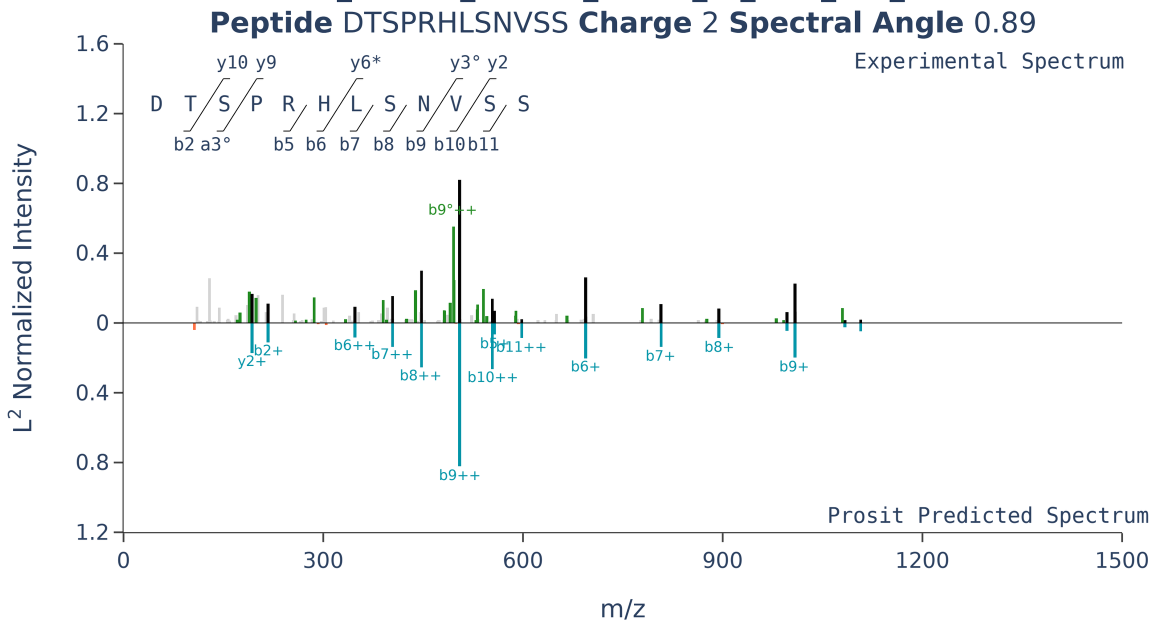


**Source** WSoh_221121_011221_HFGoe_L2_hTau_24h_R2 **Scan** 19194
**Peptide** DAGLKESPLQTPTE **Charge** 2 **Spectral Angle** 0.96

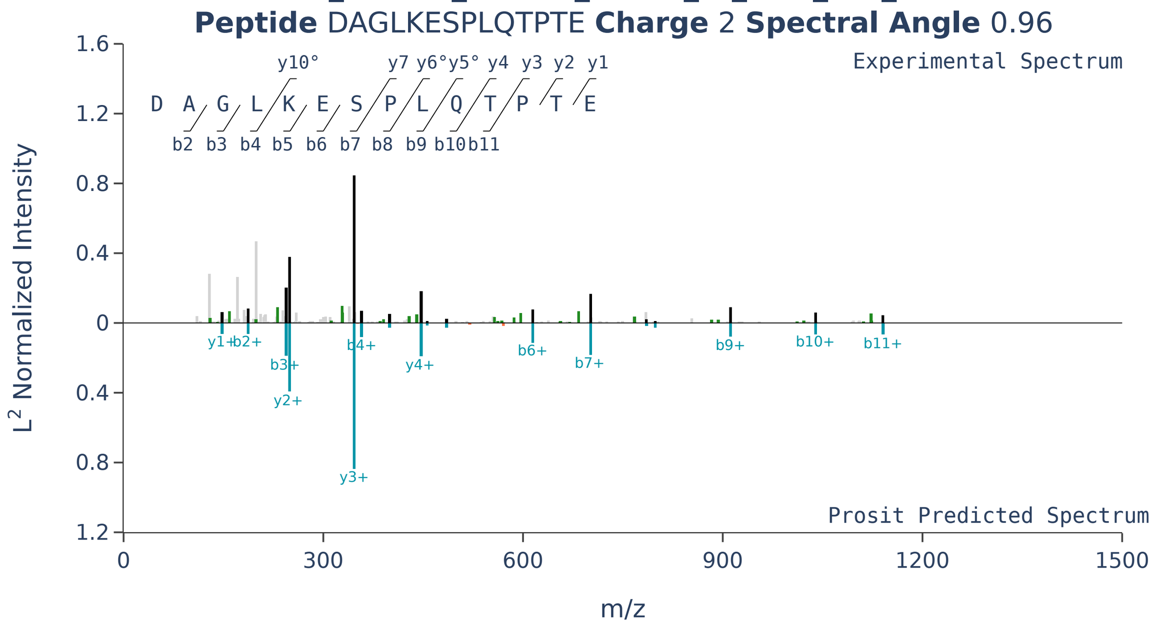


**Source** WSoh_221121_011221_HFGoe_L1_hTau_4h_R2 **Scan** 9327
**Peptide** LGNIHHKPGGGQVEV **Charge** 2 **Spectral Angle** 0.88

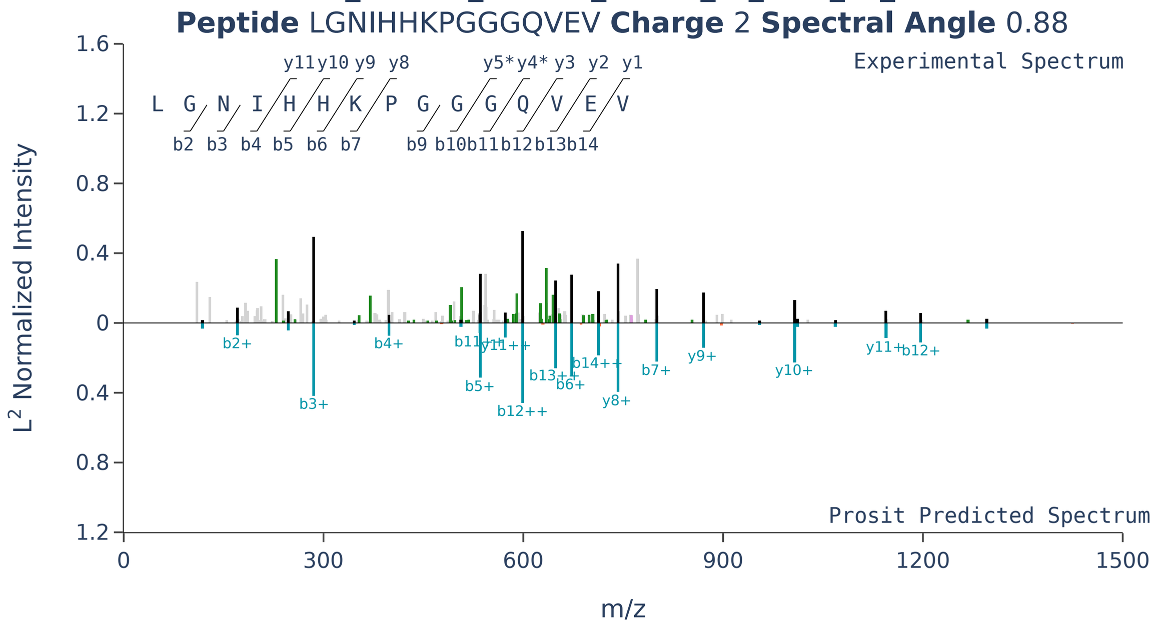


**Source** WSoh_221121_011221_HFGoe_L2_hTau_4h_R2 **Scan** 16068
**Peptide** GDTSPRHLSNVSSTGSIDM **Charge** 3 **Spectral Angle** 0.91

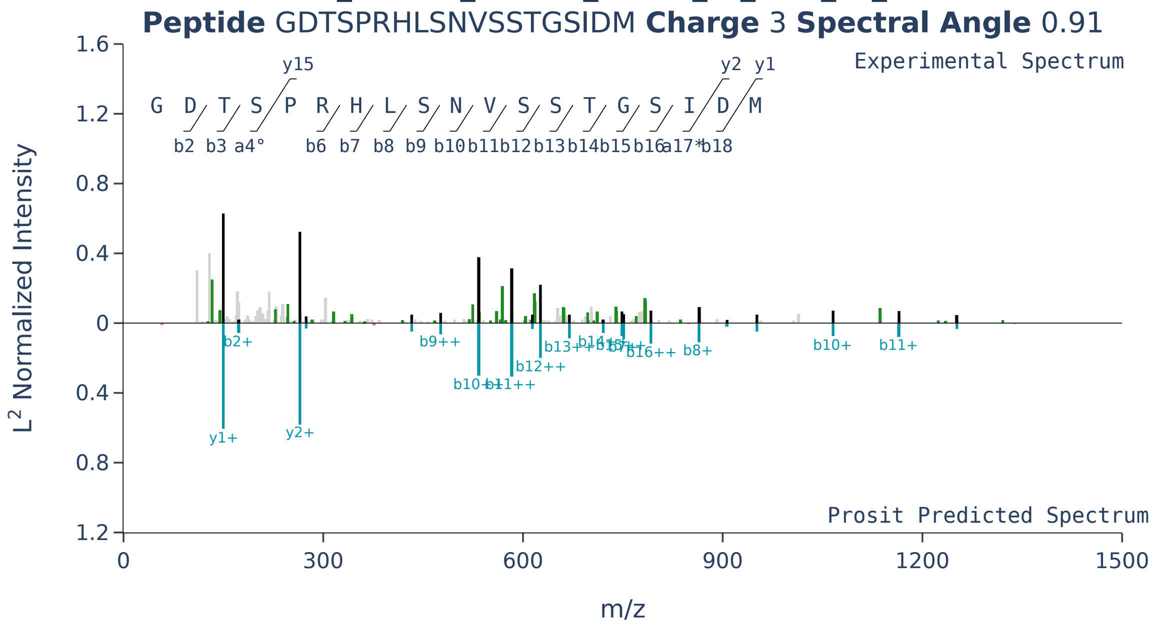


**Source** WSoh_221121_011221_HFGoe_L2_hTau_24h_R2 **Scan** 6090
**Peptide** EDEAAGHVTQAR **Charge** 2 **Spectral Angle** 0.92

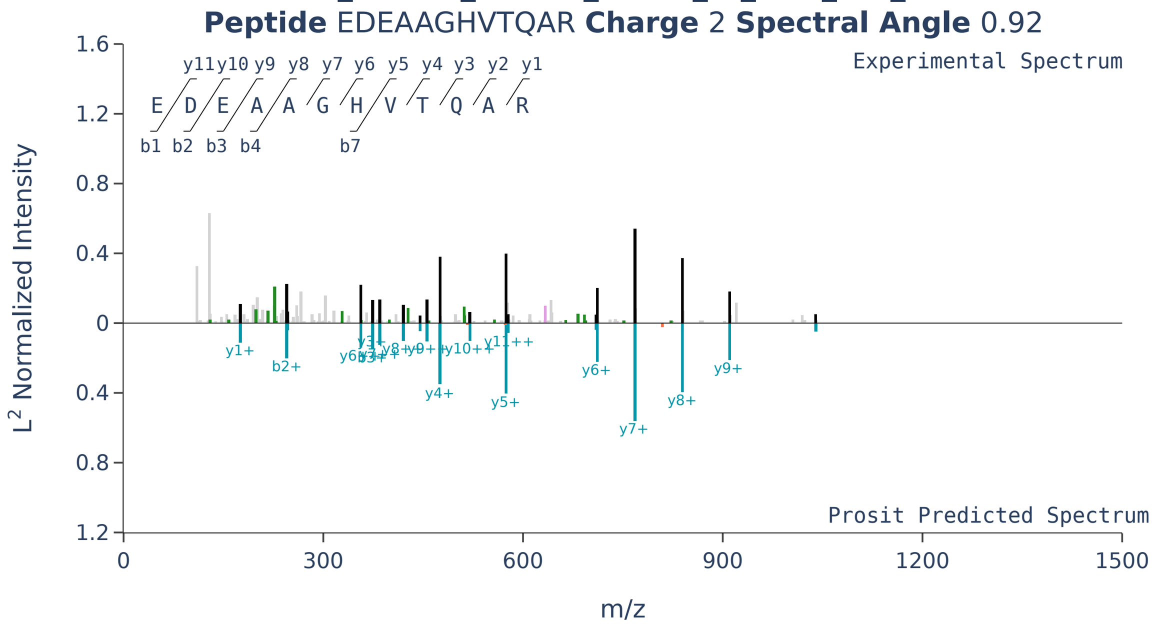


**Source** WSoh_221121_011221_HFGoe_L2_hTau_24h_R1 **Scan** 15961
**Peptide** IINKKLDLSNVQS **Charge** 2 **Spectral Angle** 0.87

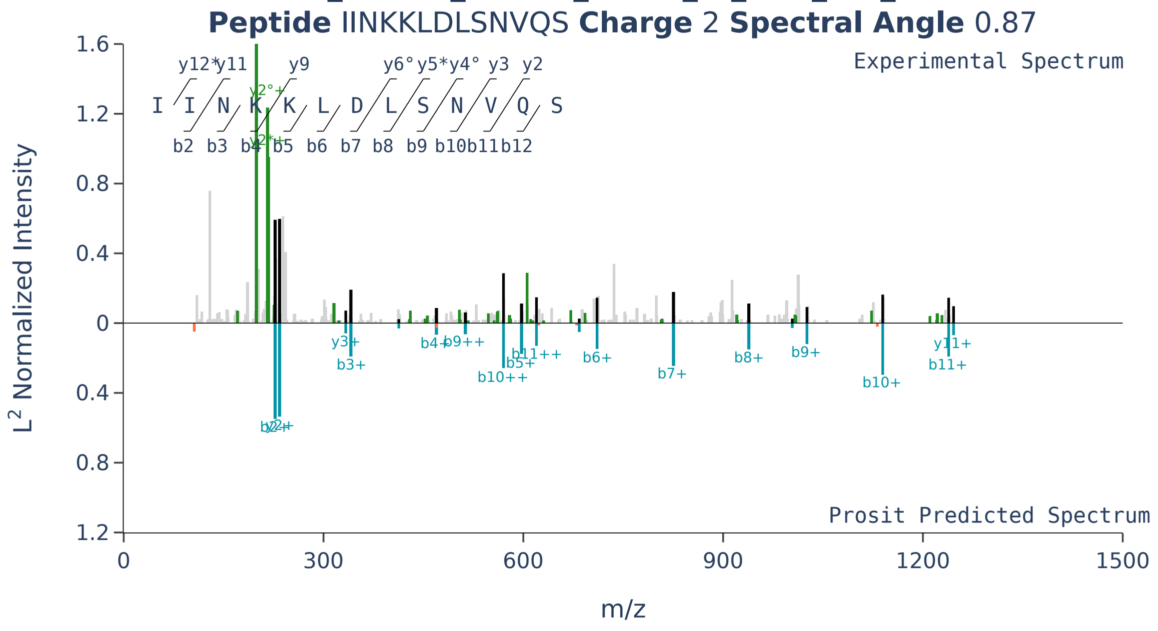


**Source** WSoh_221121_011221_HFGoe_L1_hTau_4h_R2 **Scan** 11333
**Peptide** EVKSEKLDFKDRVQS **Charge** 2 **Spectral Angle** 0.84

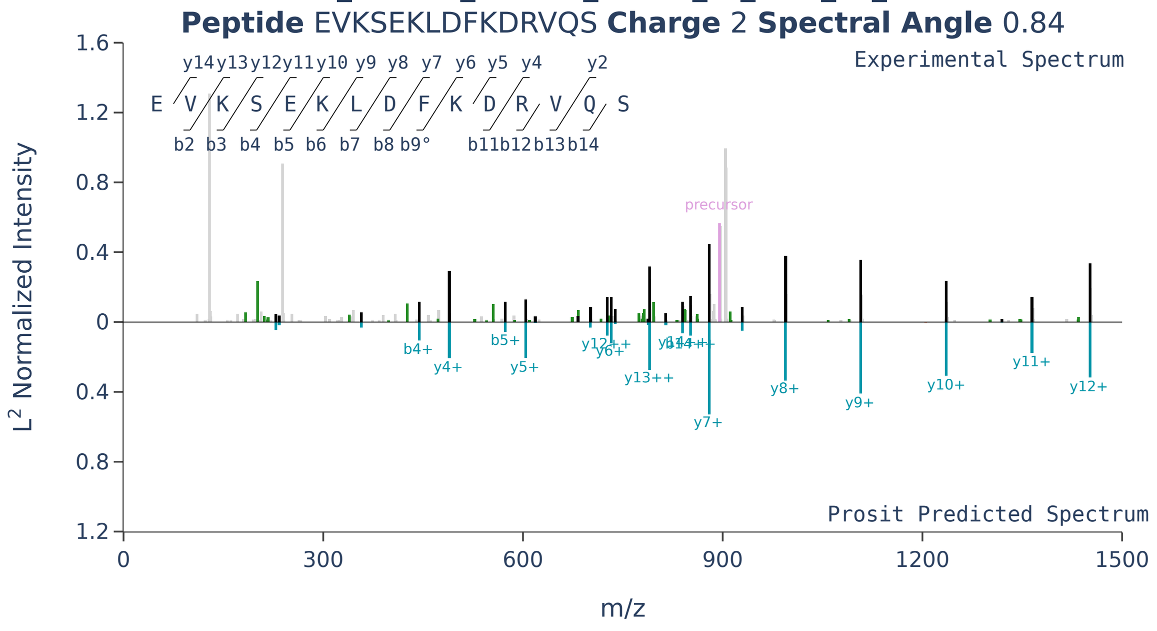


**Source** WSoh_221121_011221_HFGoe_L2_hTau_2h_R1 **Scan** 17665
**Peptide** PRQEFEVMED **Charge** 2 **Spectral Angle** 0.93

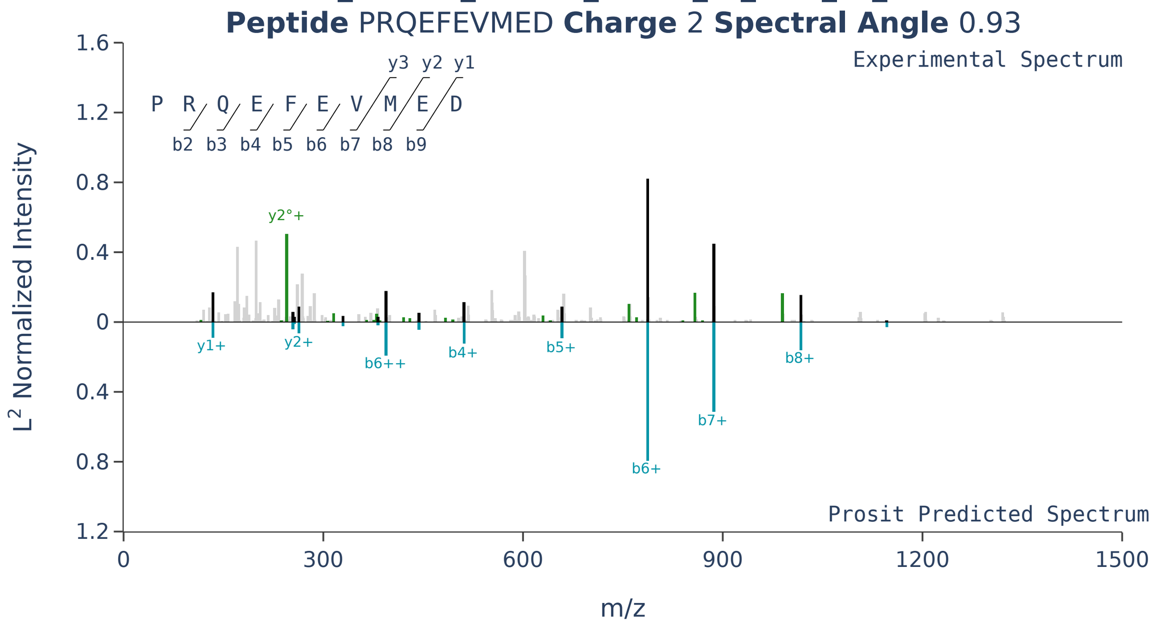


**Source** WSoh_221121_011221_HFGoe_L2_hTau_4h_R2 **Scan** 5538
**Peptide** SNVQSKCGSKDNIKHV **Charge** 4 **Spectral Angle** 0.93

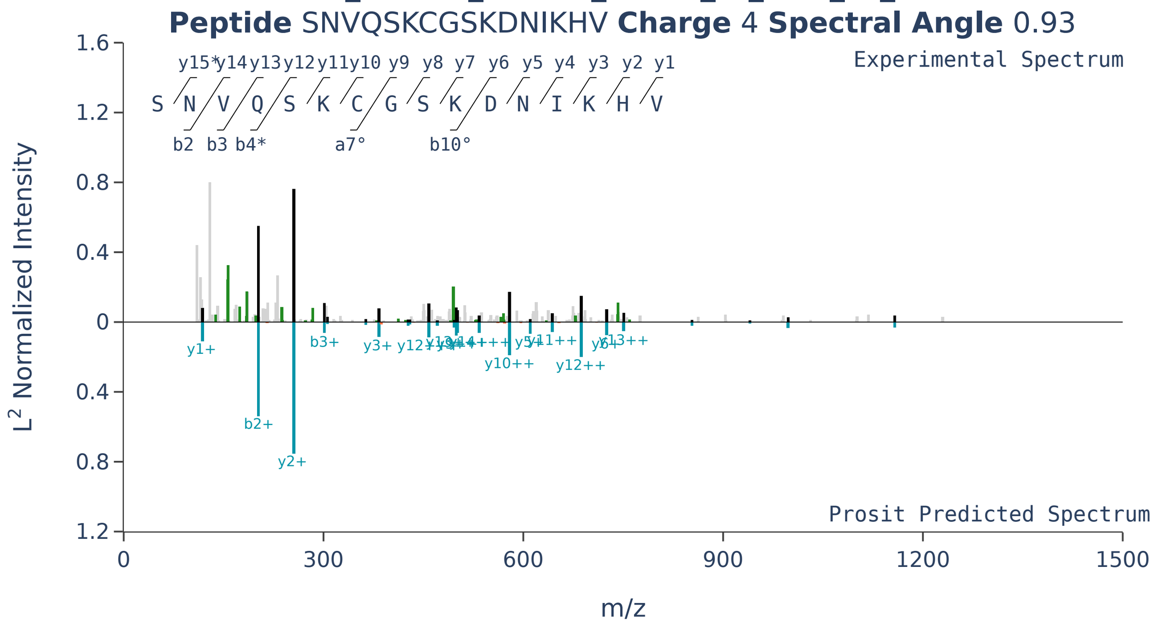


**Source** WSoh_221121_011221_HFGoe_L1_hTau_24h_R1 **Scan** 21851
**Peptide** QTAPVPMPDLKN **Charge** 2 **Spectral Angle** 0.91

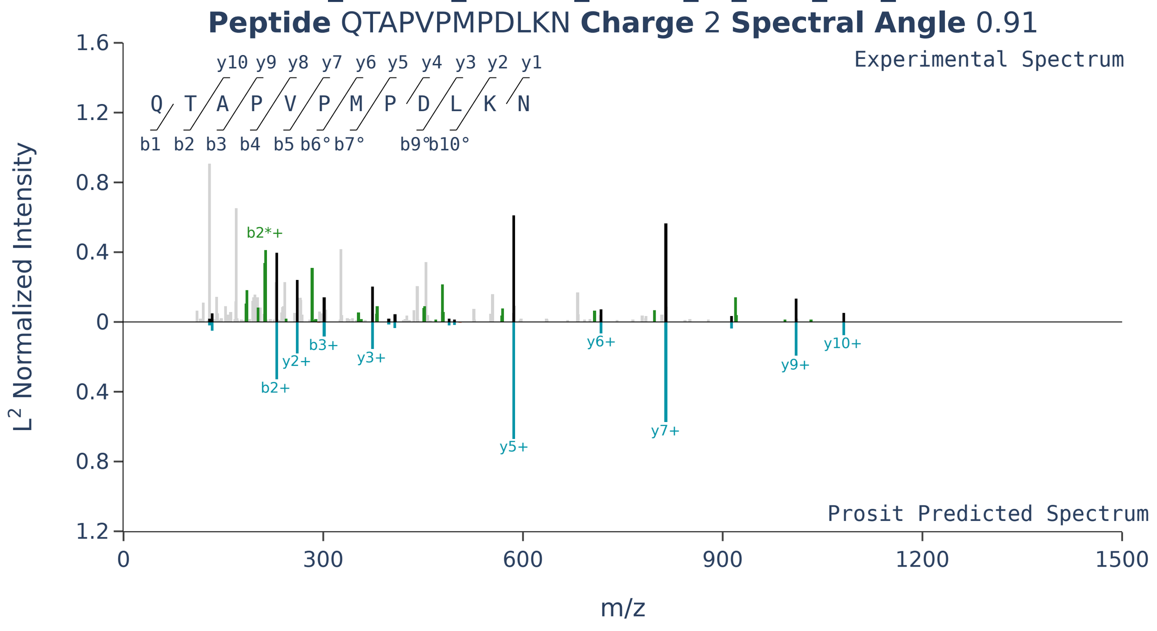


**Source** WSoh_221121_011221_HFGoe_L1_hTau_4h_R1 **Scan** 11272
**Peptide** GSLDNITH **Charge** 2 **Spectral Angle** 0.97

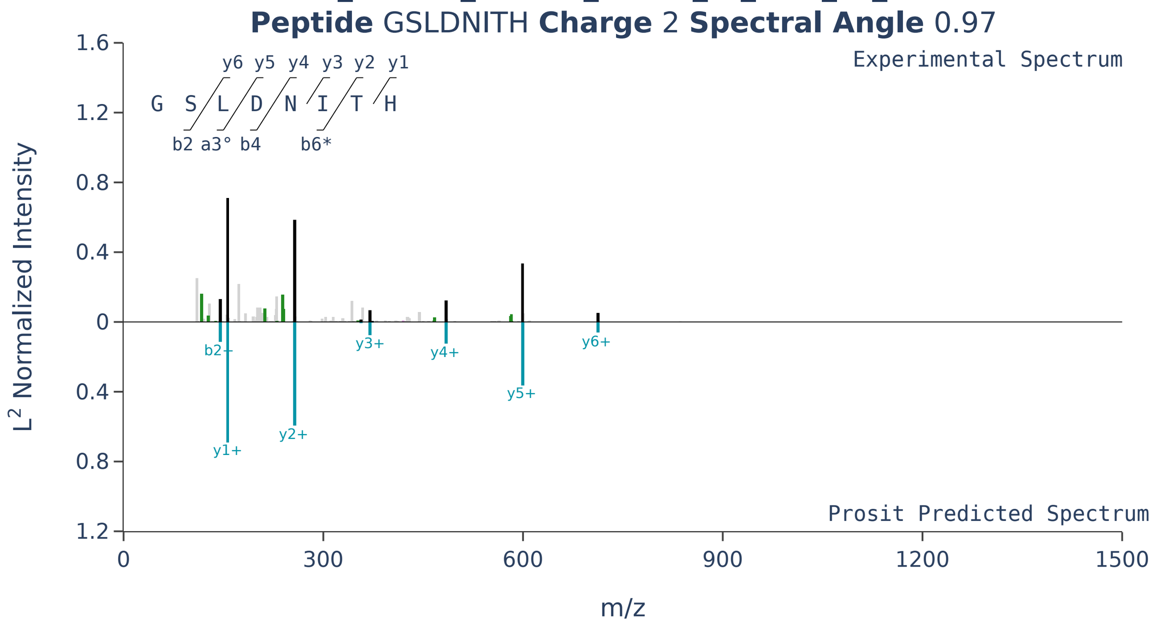


**Source** WSoh_221121_011221_HFGoe_L2_hTau_2h_R2 **Scan** 12223
**Peptide** SRTPSLPTPPTREPKKV **Charge** 4 **Spectral Angle** 0.9

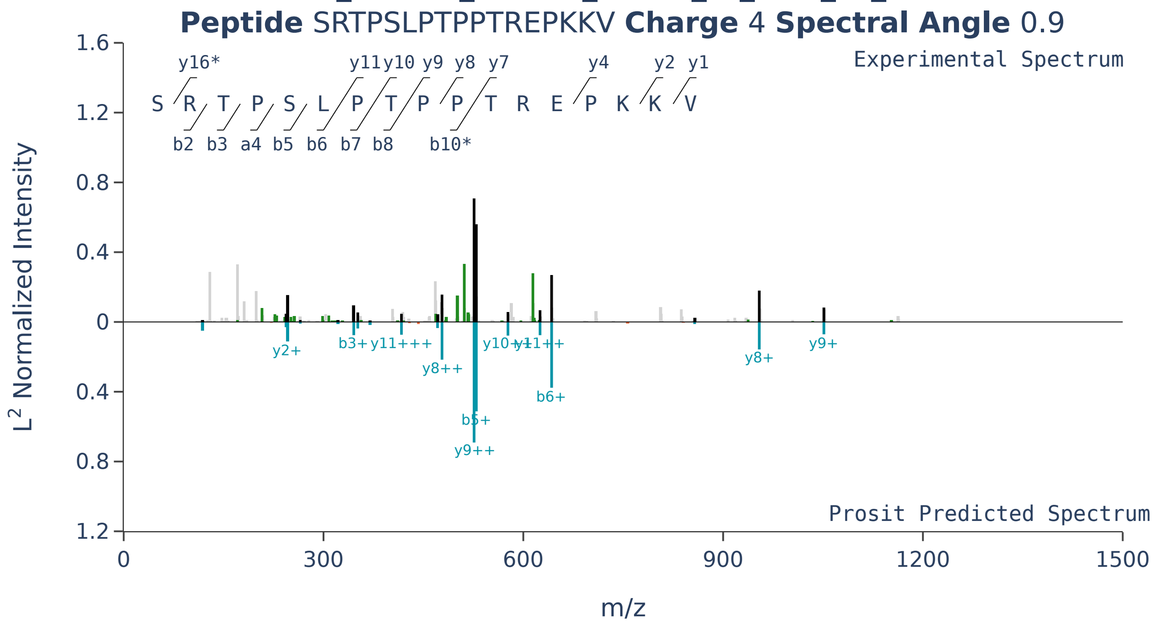

**Source** WSoh_221121_011221_HFGoe_L2_hTau_24h_R2 **Scan** 7101
**Peptide** SKDNIKHVPGGGSVQ **Charge** 2 **Spectral Angle** 0.92

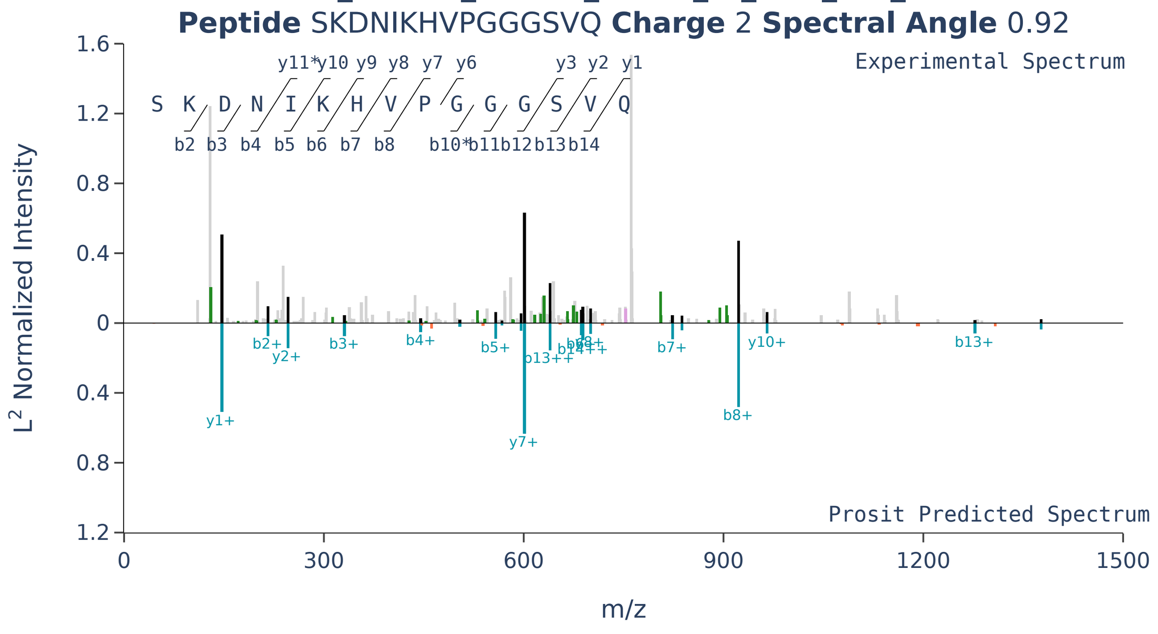


**Source** WSoh_221121_011221_HFGoe_L2_hTau_24h_R1 **Scan** 14138
**Peptide** SPLQTPTEDGSEEPGSETSDAKST **Charge** 2 **Spectral Angle** 0.93

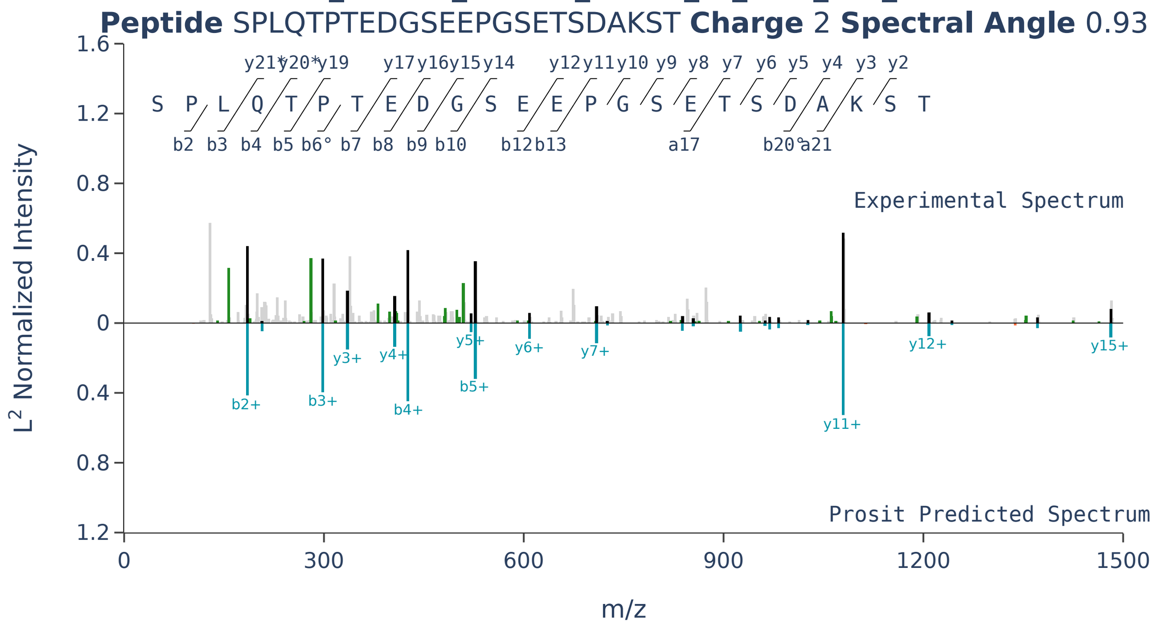


**Source** WSoh_221121_011221_HFGoe_L2_hTau_1h_R2 **Scan** 11728
**Peptide** KTDHGAEIVY **Charge** 2 **Spectral Angle** 0.94

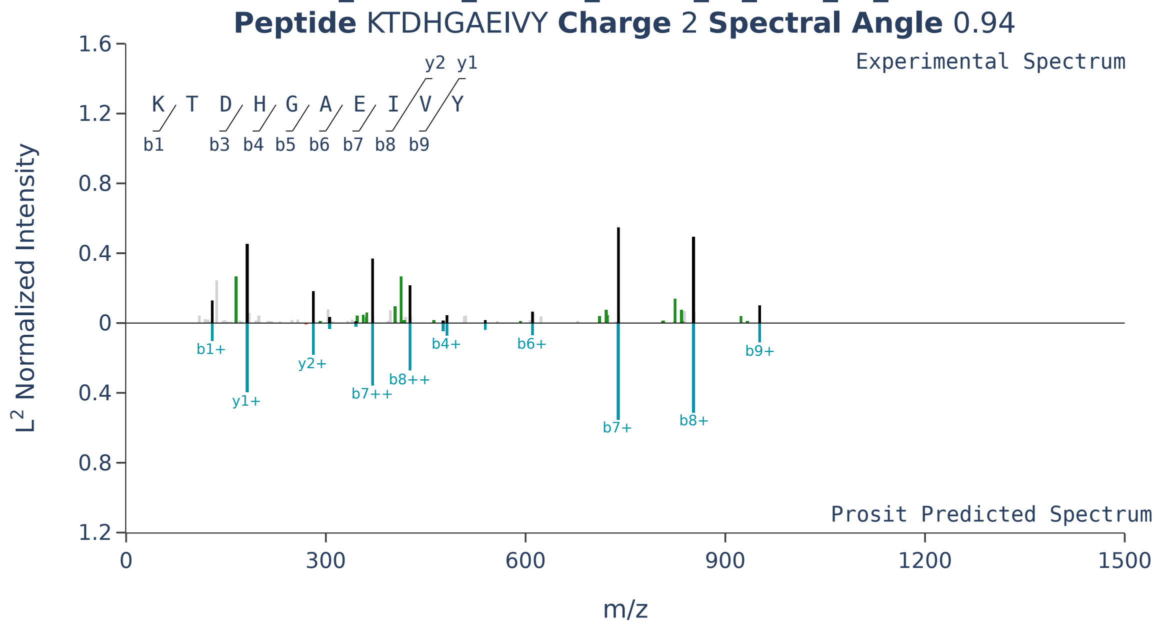


**Source** WSoh_221121_011221_HFGoe_L2_hTau_4h_R1 **Scan** 15386
**Peptide** PGGGSVQI **Charge** 2 **Spectral Angle** 0.86

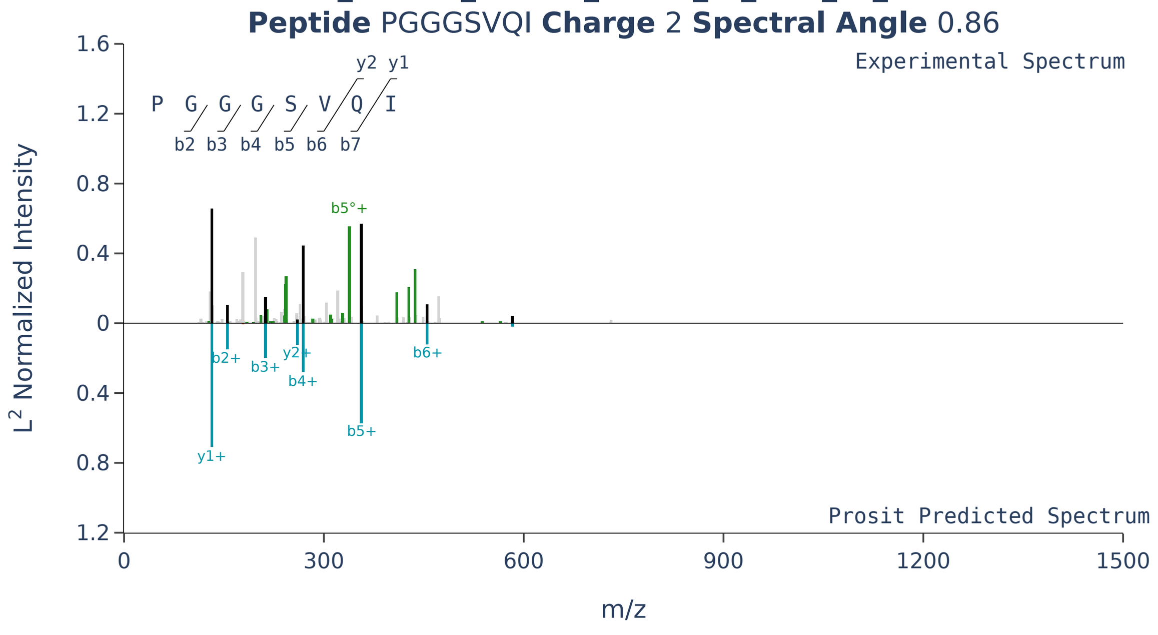


**Source** WSoh_221121_011221_HFGoe_L1_hTau_4h_R2 **Scan** 17706
**Peptide** IVYKSPVV **Charge** 2 **Spectral Angle** 0.85

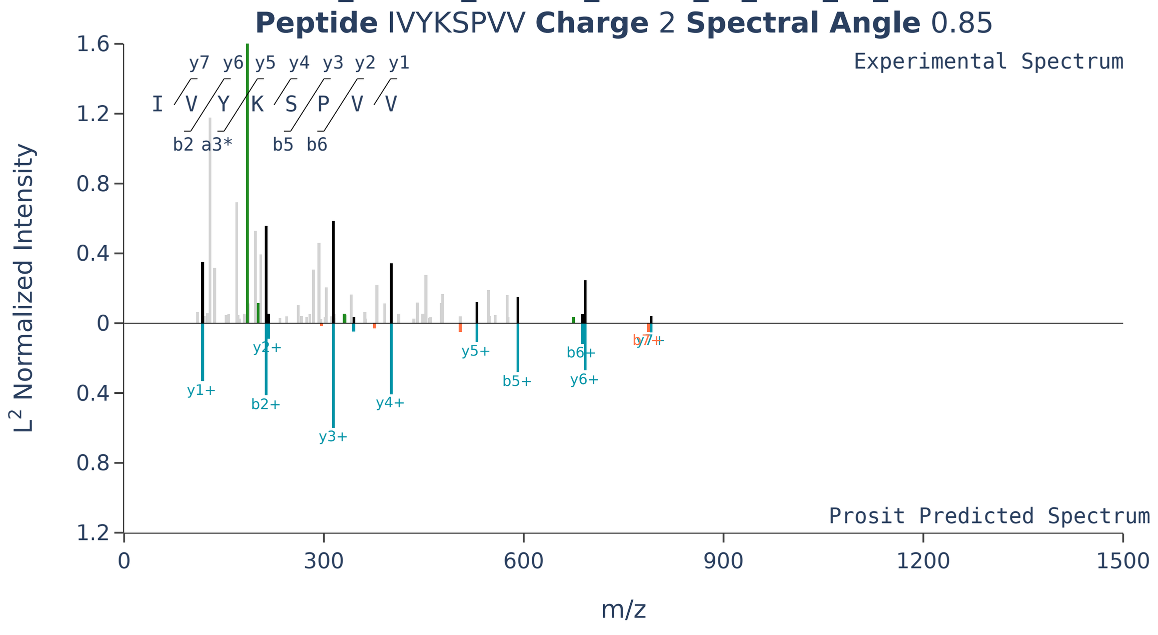


**Source** WSoh_221121_011221_HFGoe_L2_hTau_24h_R1 **Scan** 17678
**Peptide** TDHGAEIVYKSPVVS **Charge** 2 **Spectral Angle** 0.91

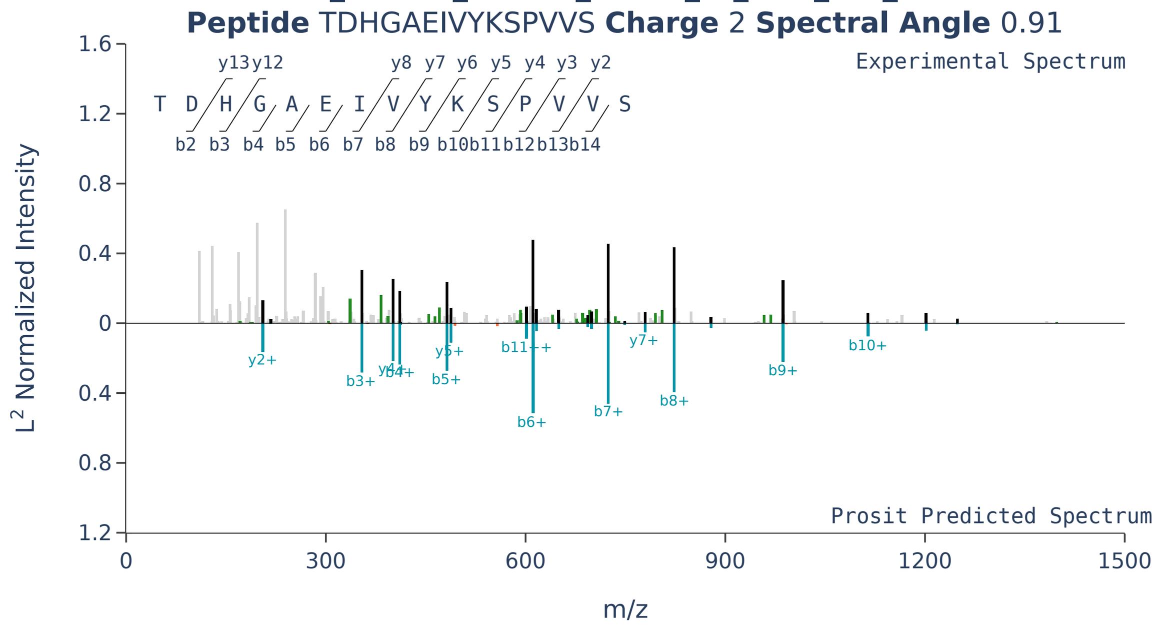


**Source** WSoh_221121_011221_HFGoe_L2_hTau_1h_R2 **Scan** 5586
**Peptide** SSPGSPGTPGSRSR **Charge** 3 **Spectral Angle** 0.94

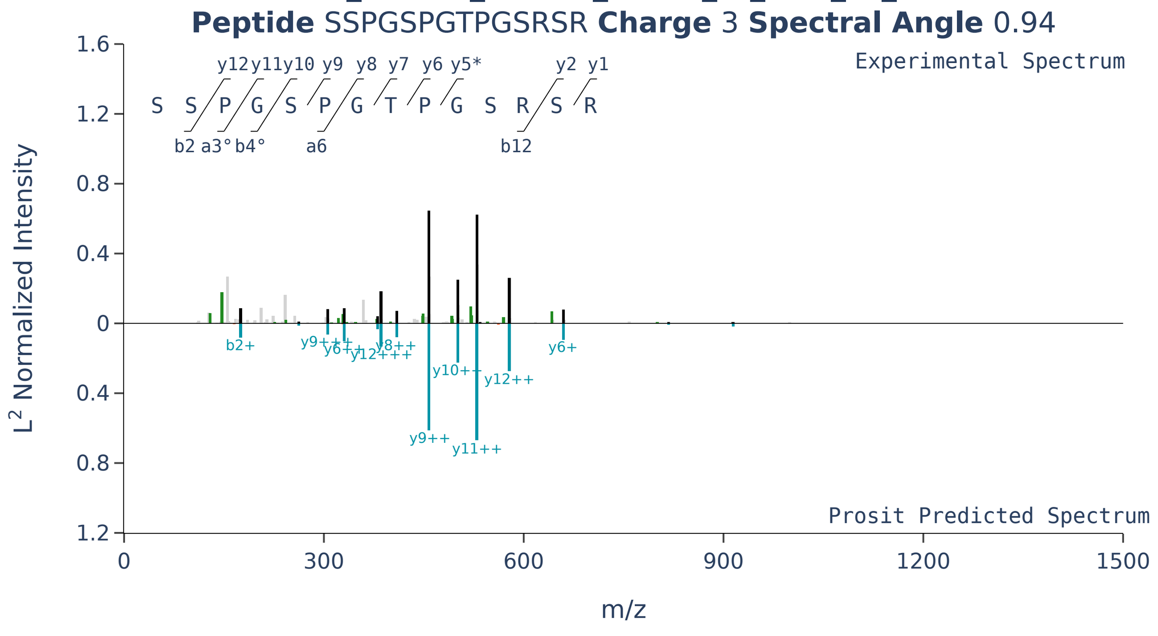


**Source** WSoh_221121_011221_HFGoe_L2_hTau_24h_R1 **Scan** 16518
**Peptide** PVDLSKV **Charge** 2 **Spectral Angle** 0.87

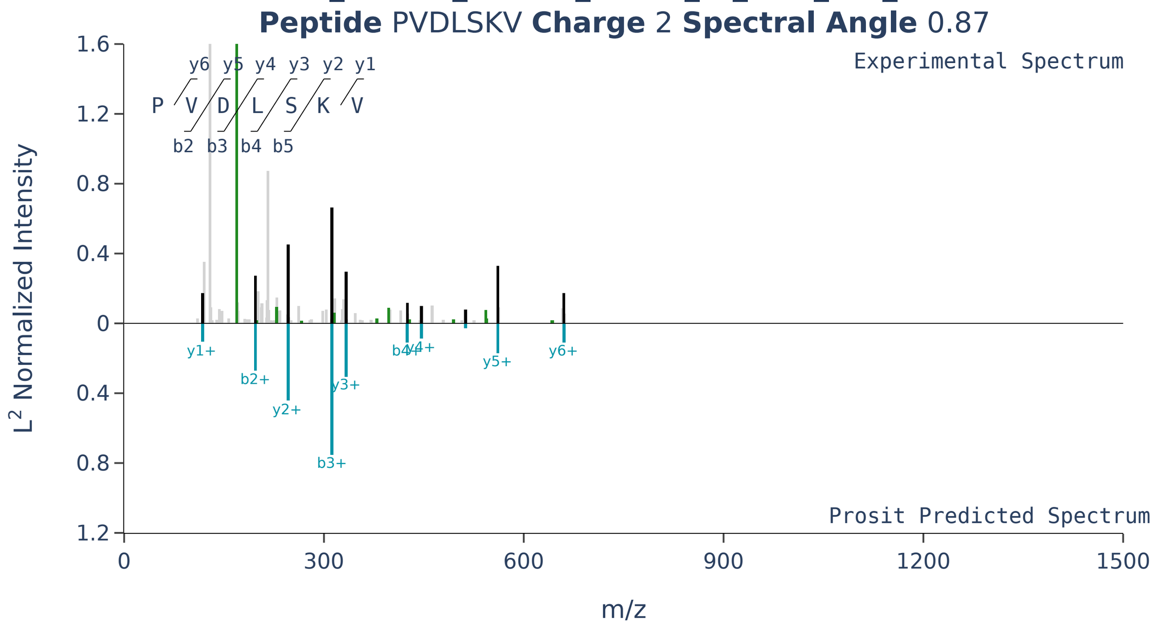


**Source** WSoh_221121_011221_HFGoe_L1_hTau_24h_R1 **Scan** 7764
**Peptide** THKLTFREN **Charge** 2 **Spectral Angle** 0.87

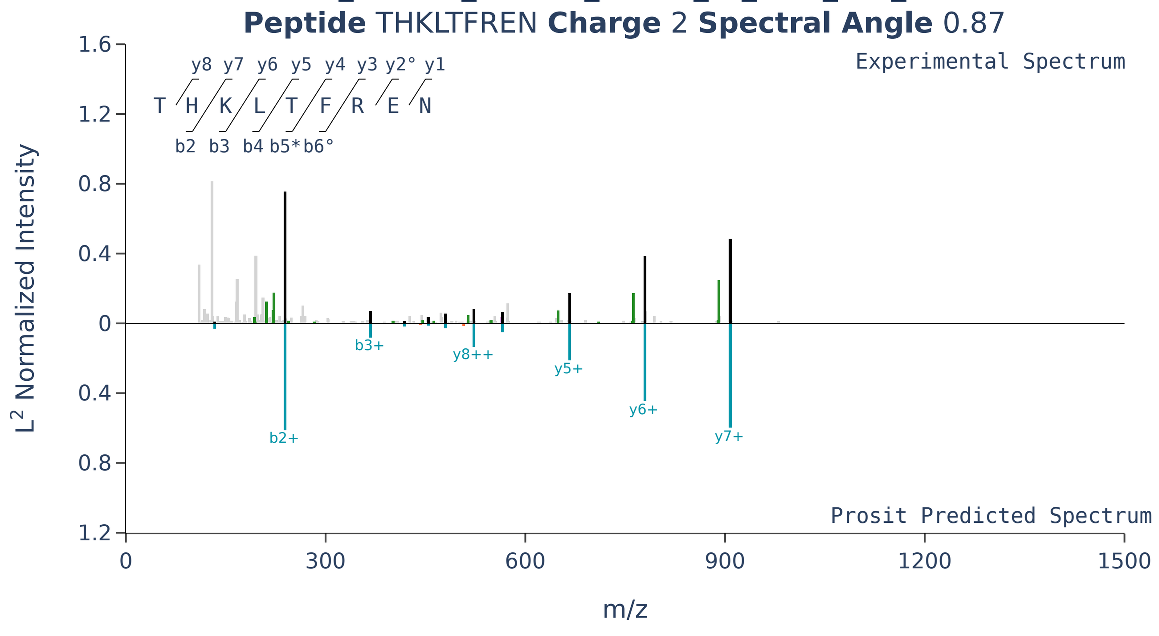


**Source** WSoh_221121_011221_HFGoe_L2_hTau_4h_R1 **Scan** 14986
**Peptide** VSASLAKQGL **Charge** 1 **Spectral Angle** 0.89

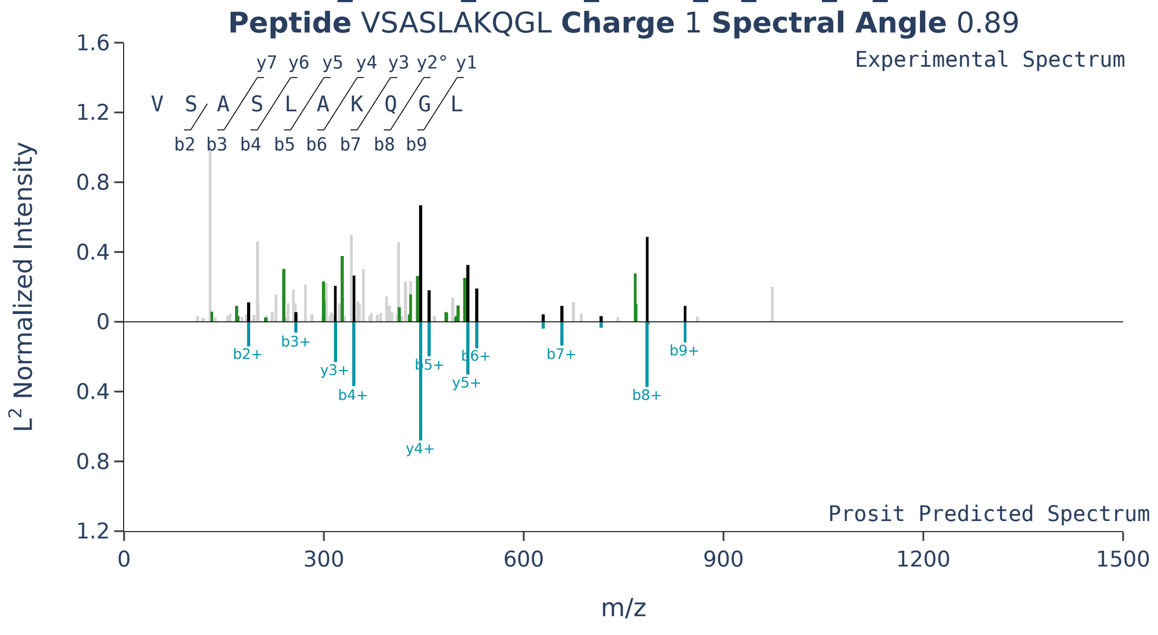


**Source** WSoh_221121_011221_HFGoe_L2_hTau_4h_R1 **Scan** 11487
**Peptide** KLDLSNVQSK **Charge** 2 **Spectral Angle** 0.87

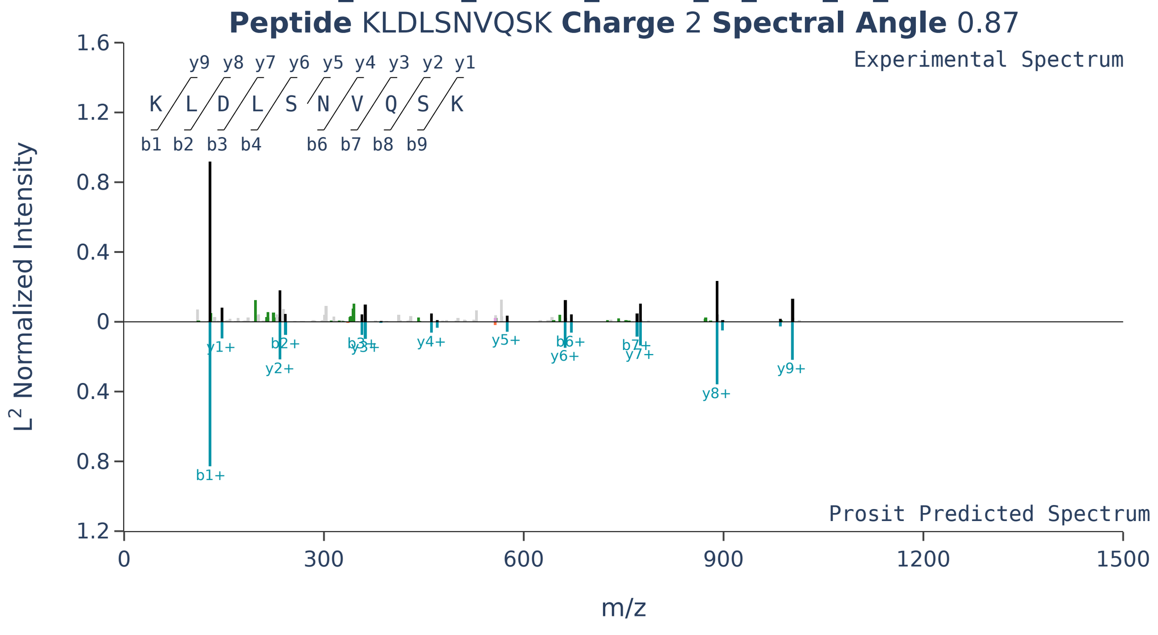


**Source** WSoh_221121_011221_HFGoe_L1_hTau_24h_R1 **Scan** 16822
**Peptide** APVPMPDLKNVKSK **Charge** 2 **Spectral Angle** 0.9

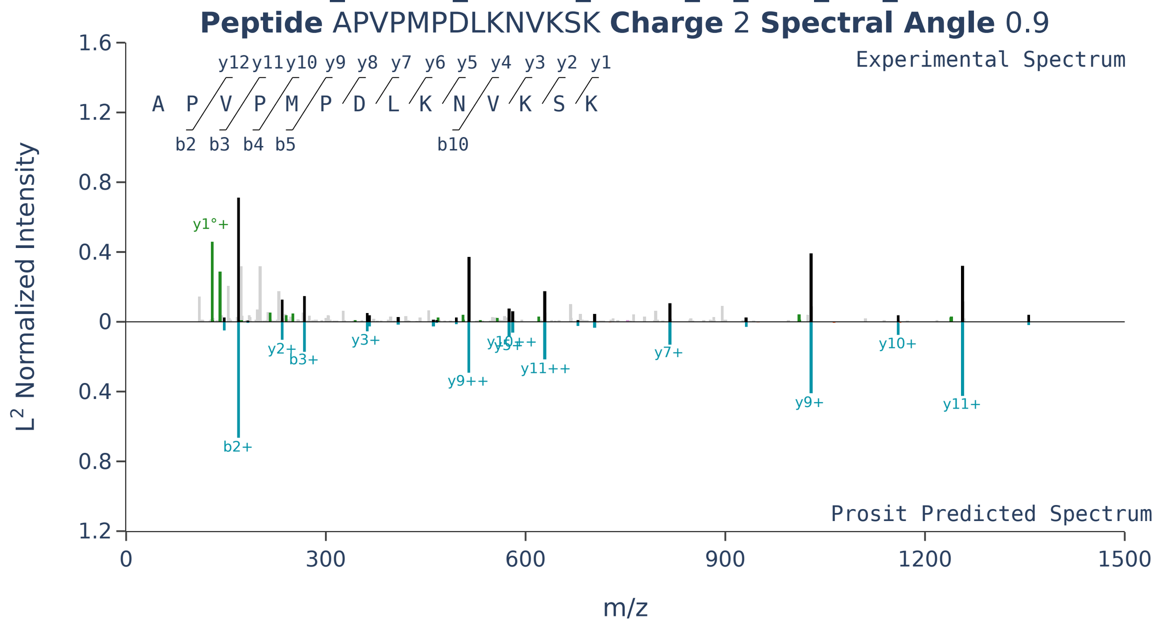


**Source** WSoh_221121_011221_HFGoe_L1_hTau_2h_R1 **Scan** 13805
**Peptide** LDLSNVQSK **Charge** 2 **Spectral Angle** 0.92

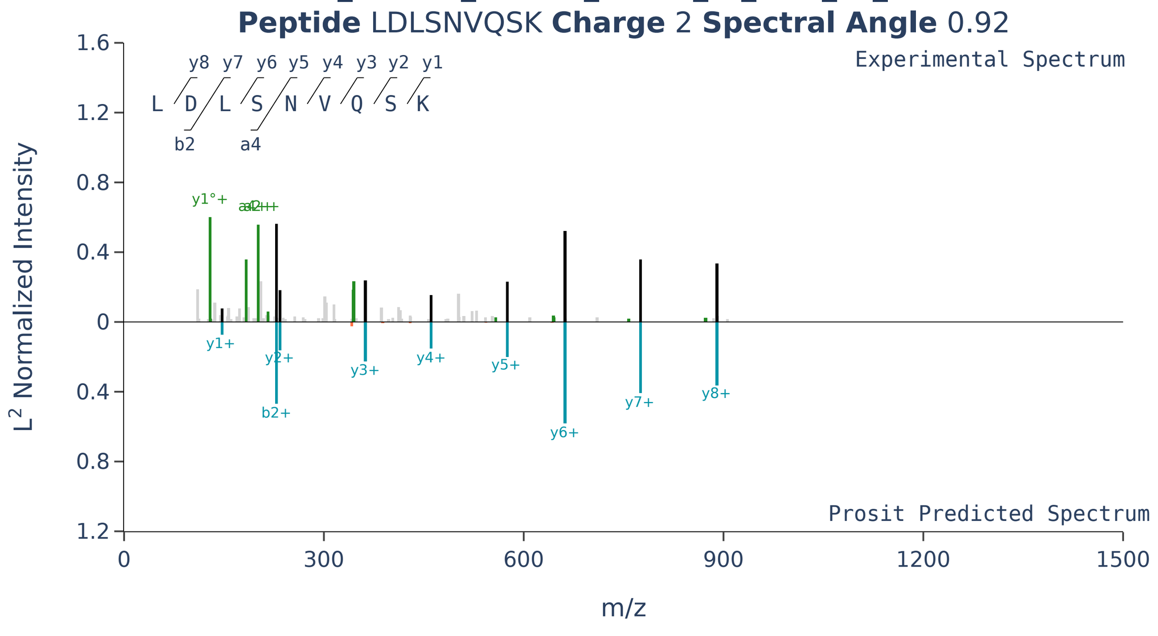


**Source** WSoh_221121_011221_HFGoe_L1_hTau_24h_R1 **Scan** 21551
**Peptide** GIGDTPSLEDEAAGHVTQARM **Charge** 3 **Spectral Angle** 0.88

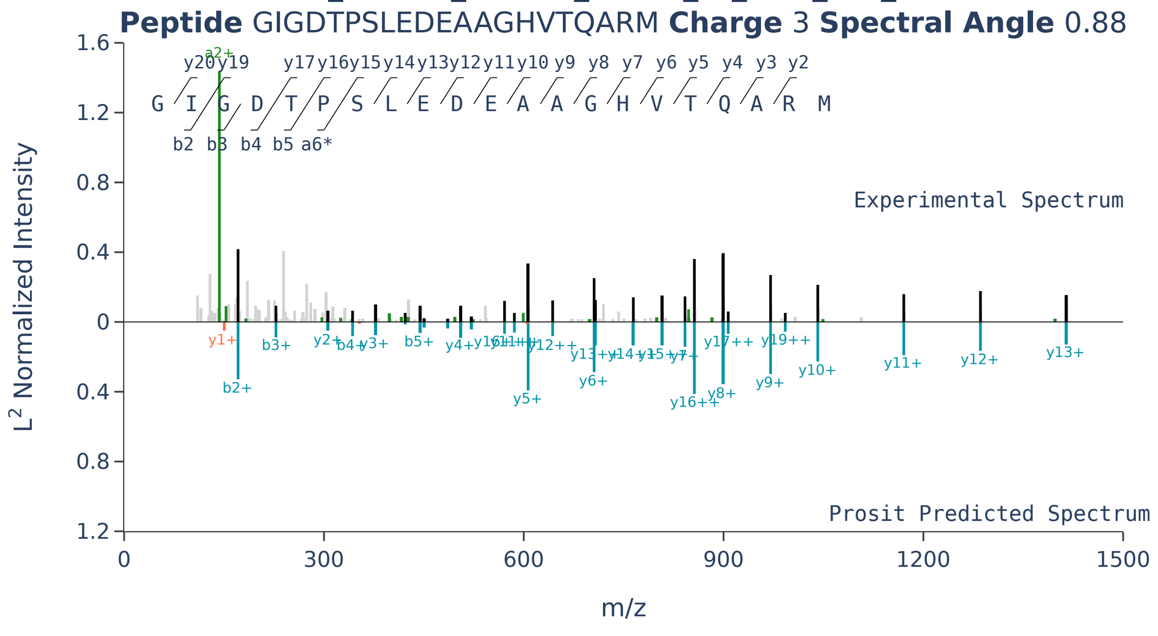


**Source** WSoh_221121_011221_HFGoe_L2_hTau_4h_R2 **Scan** 6737
**Peptide** KAKTDHGAEIVYK **Charge** 2 **Spectral Angle** 0.87

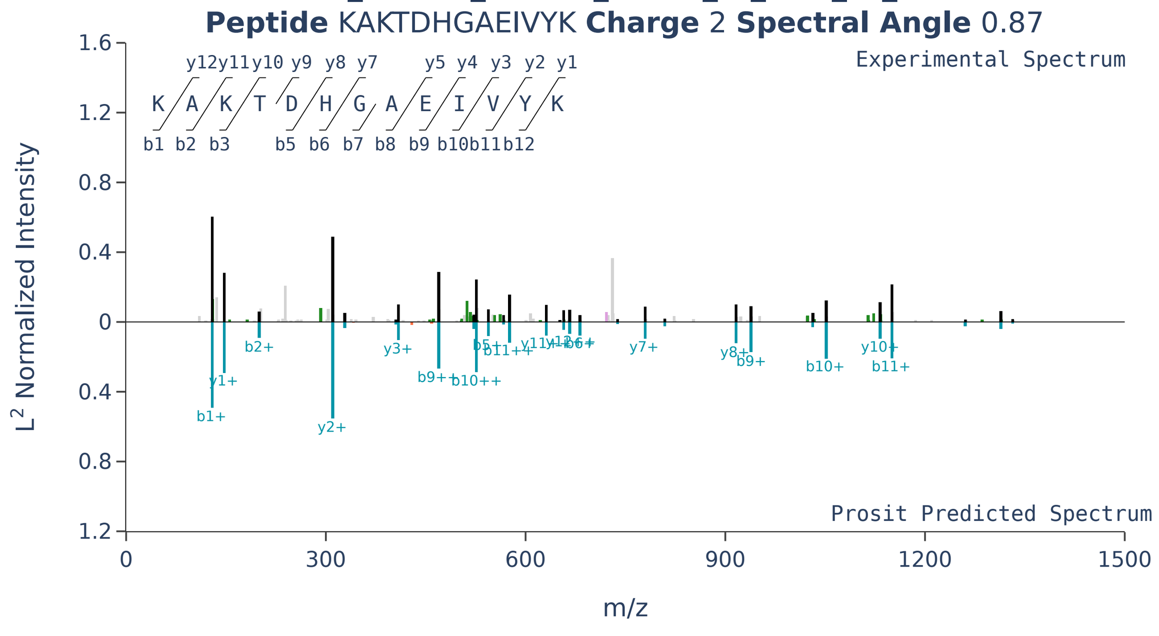

**Source** WSoh_221121_011221_HFGoe_L1_hTau_24h_R2 **Scan** 16878
**Peptide** AEIVYKSPVVSGDTSPRHLS **Charge** 3 **Spectral Angle** 0.93

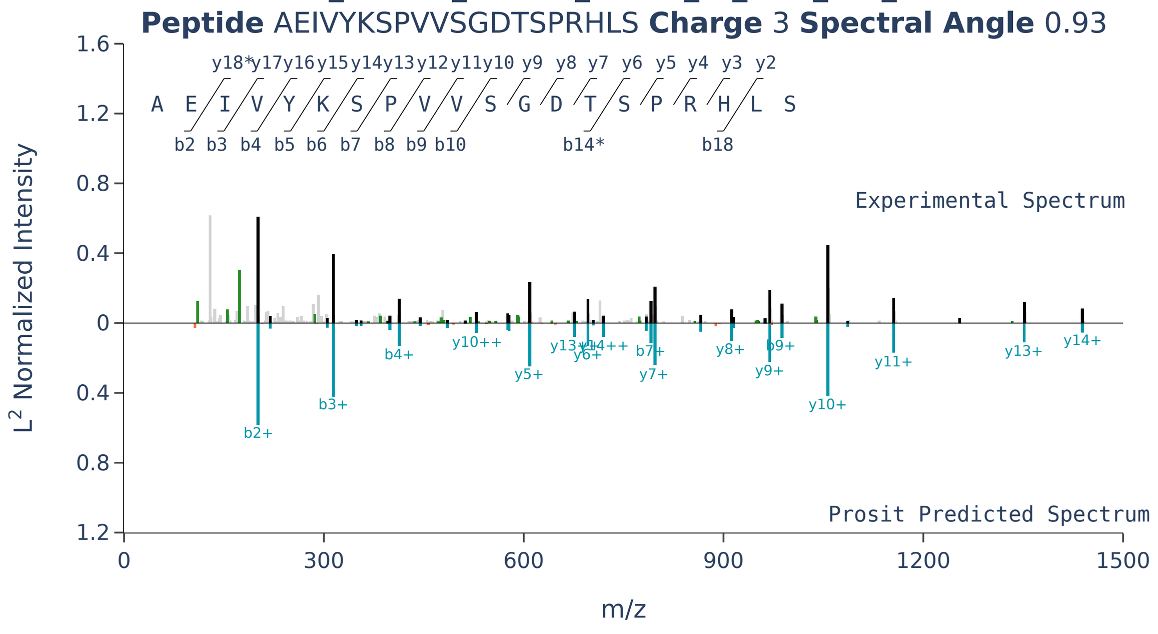


**Source** WSoh_221121_011221_HFGoe_L2_hTau_2h_R2 **Scan** 6369
**Peptide** KLTFRENAKAK **Charge** 2 **Spectral Angle** 0.88

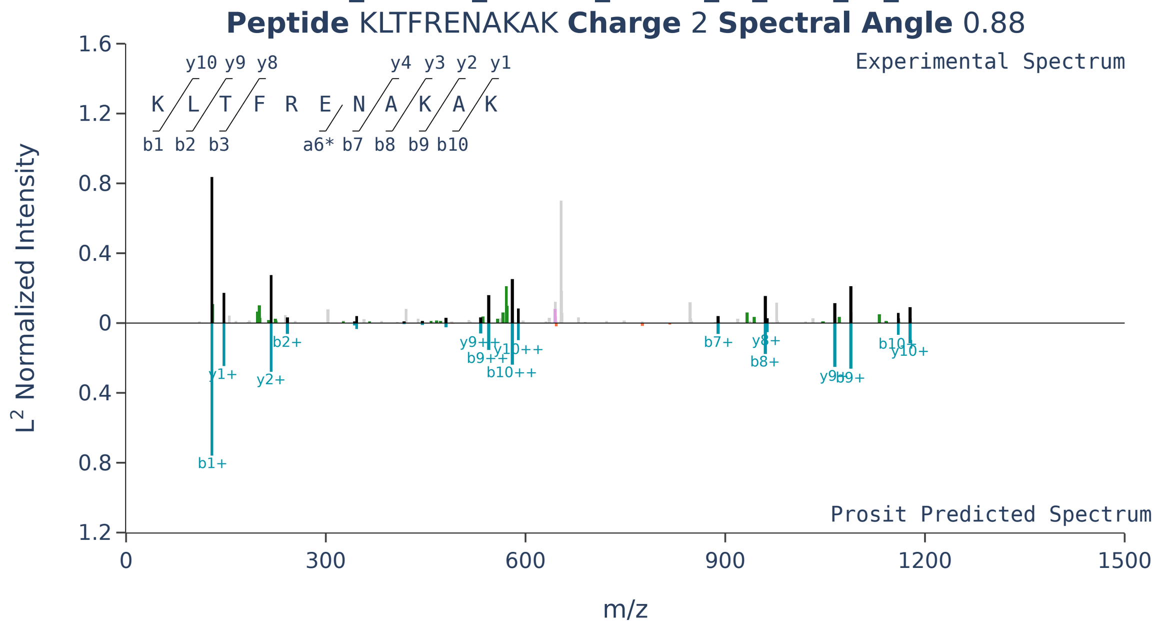


**Source** WSoh_221121_011221_HFGoe_L1_hTau_24h_R1 **Scan** 19022
**Peptide** TDHGAEIVYKSPVV **Charge** 2 **Spectral Angle** 0.92

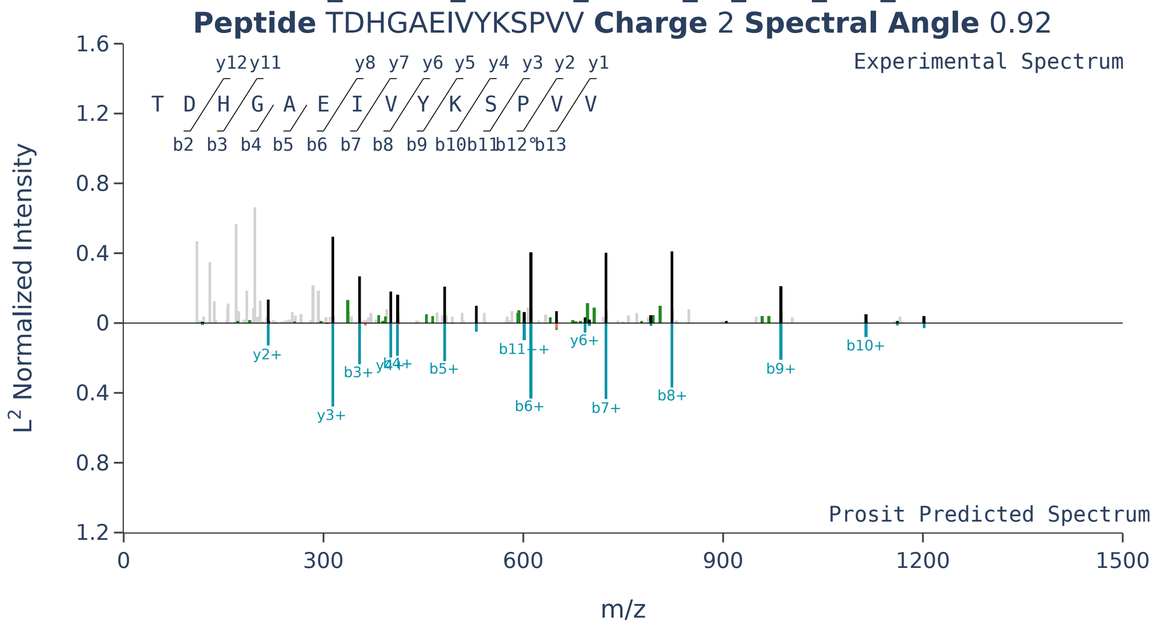


**Source** WSoh_221121_011221_HFGoe_L2_hTau_24h_R1 **Scan** 18319
**Peptide** DHGAEIVYKSPVVS **Charge** 2 **Spectral Angle** 0.94

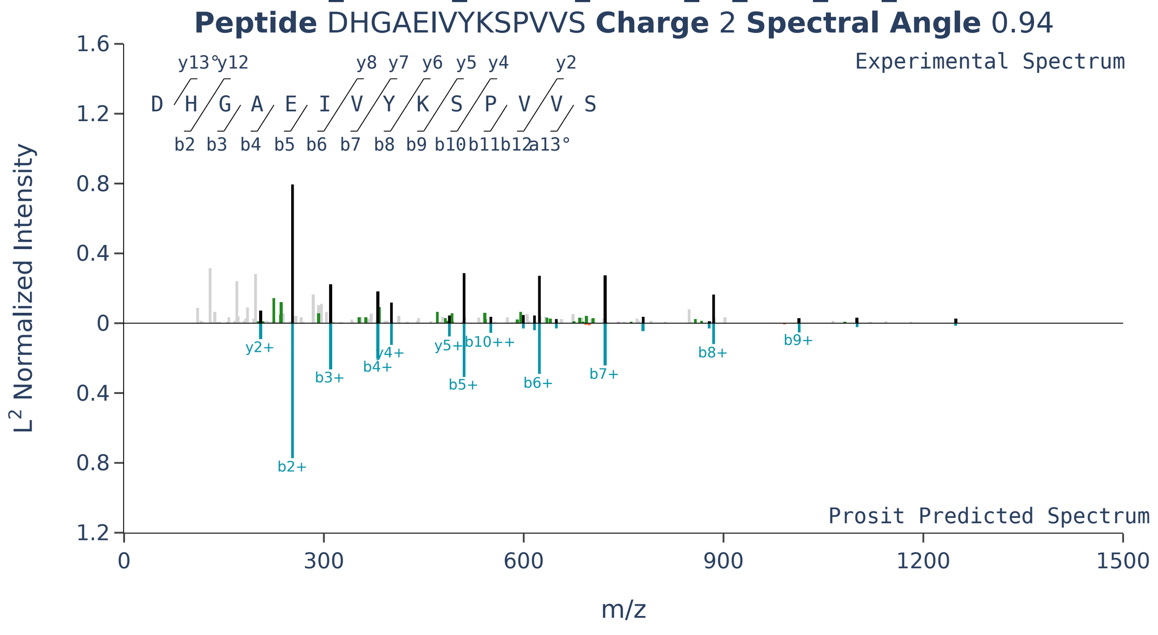


**Source** WSoh_221121_011221_HFGoe_L1_hTau_4h_R2 **Scan** 12638
**Peptide** AAQPHTEIPEGTTAE **Charge** 2 **Spectral Angle** 0.92

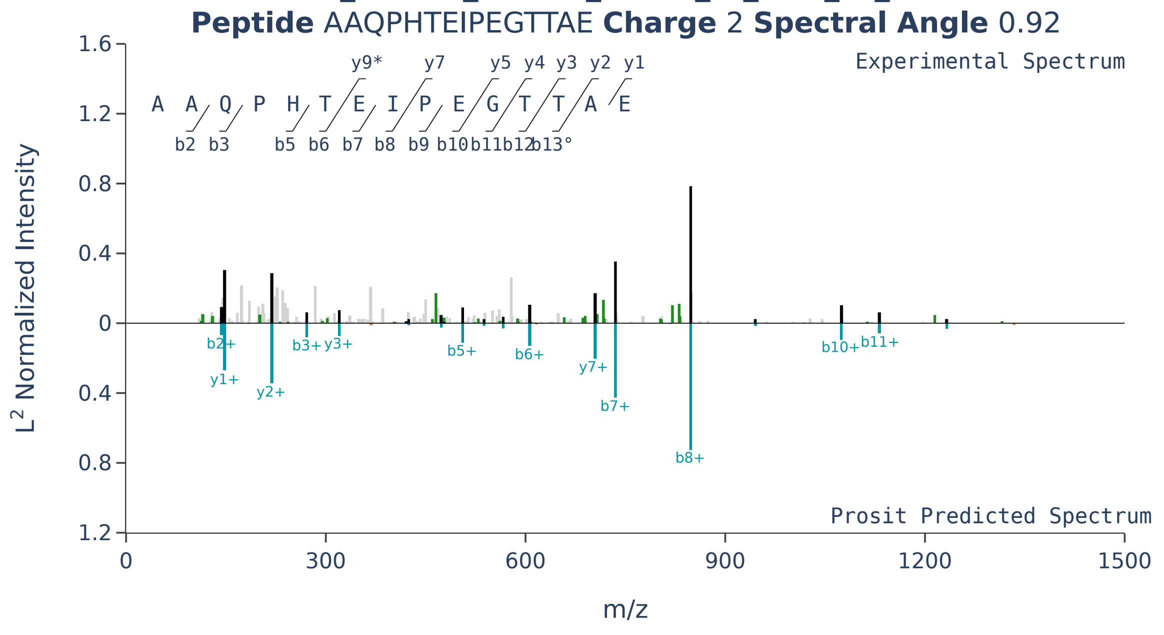


**Source** WSoh_221121_011221_HFGoe_L2_hTau_1h_R1 **Scan** 6481
**Peptide** ATPRGAAPPGQ **Charge** 2 **Spectral Angle** 0.93

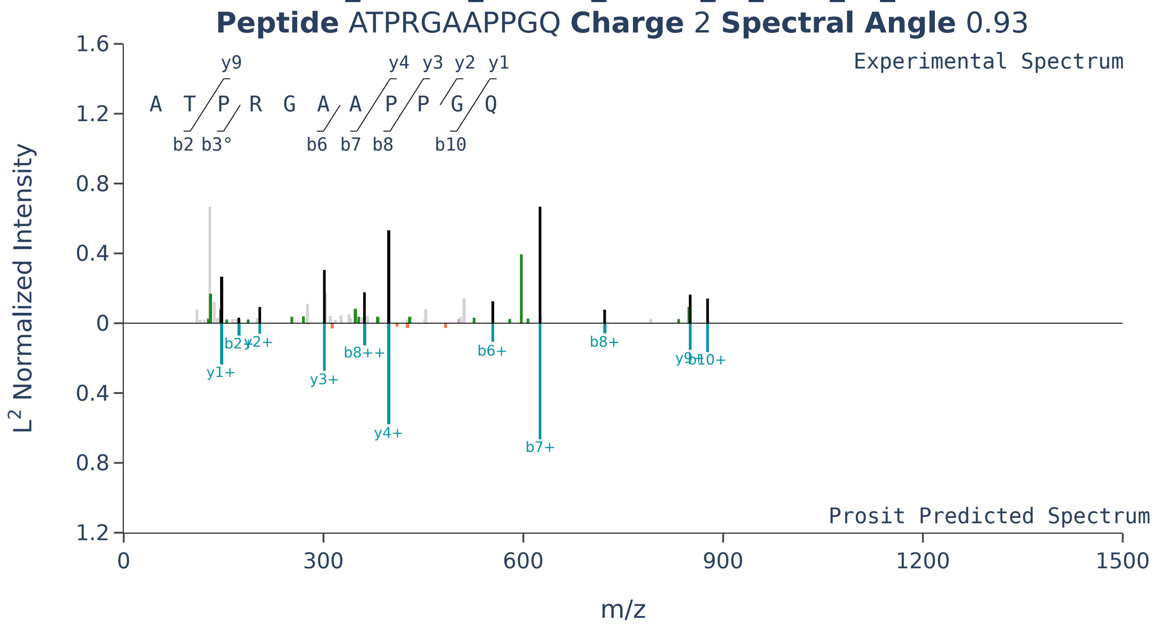


**Source** WSoh_221121_011221_HFGoe_L1_hTau_4h_R2 **Scan** 11637
**Peptide** SPVVSGDTSPRHL **Charge** 3 **Spectral Angle** 0.93

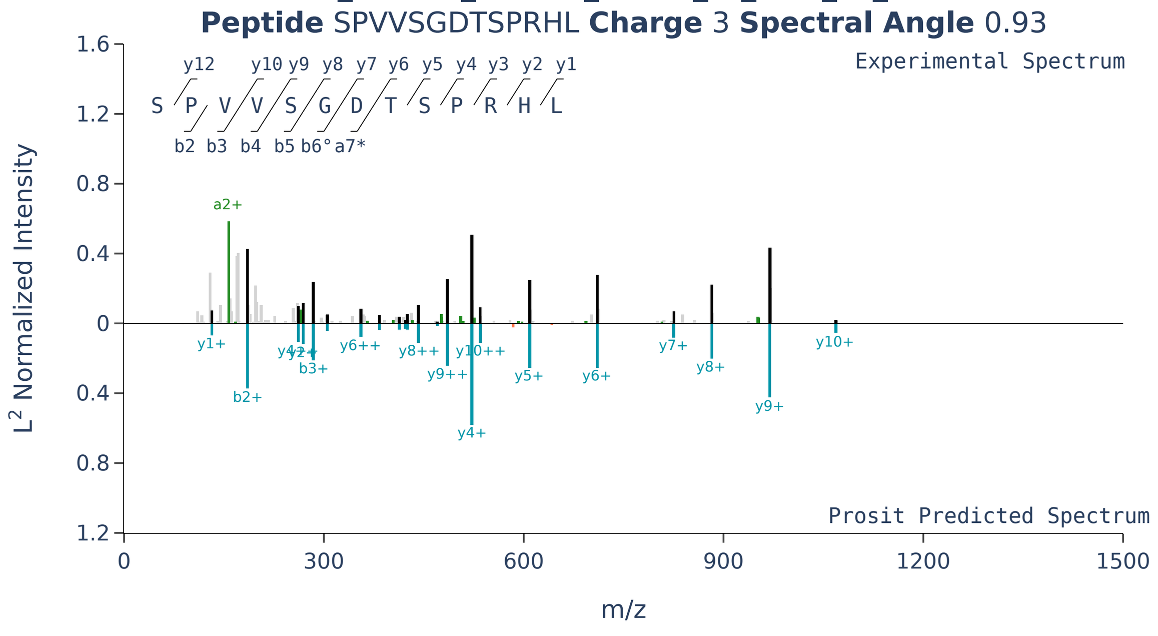


**Source** WSoh_221121_011221_HFGoe_L1_hTau_24h_R1 **Scan** 9421
**Peptide** KLTFREN **Charge** 2 **Spectral Angle** 0.94

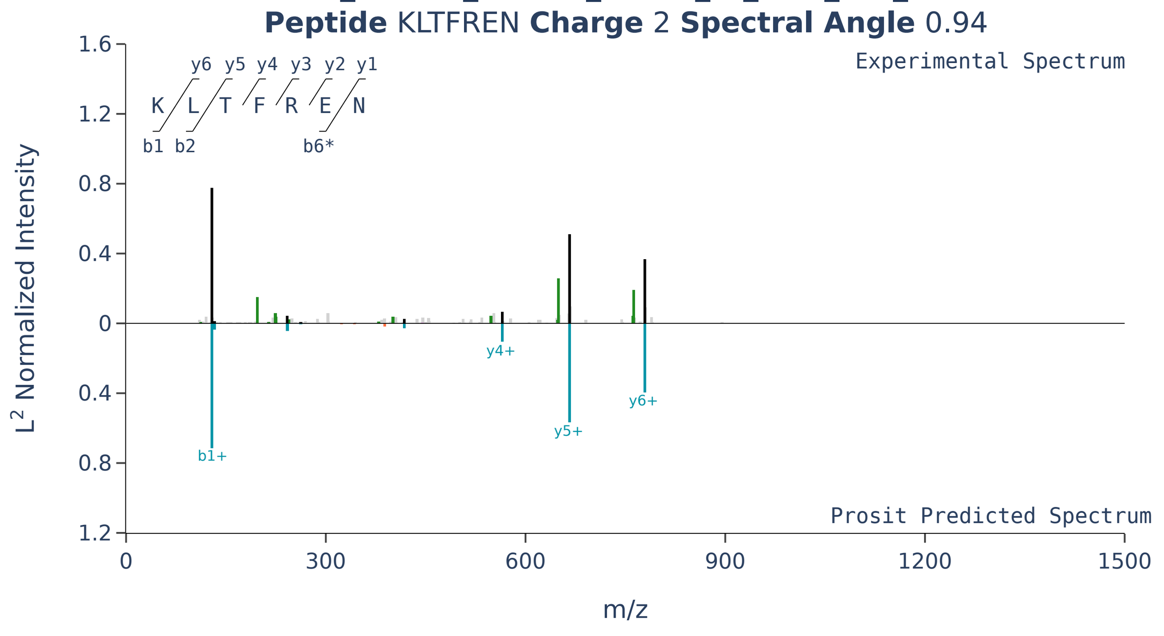


**Source** WSoh_221121_011221_HFGoe_L2_hTau_24h_R1 **Scan** 13413
**Peptide** GDTPSLED **Charge** 1 **Spectral Angle** 0.91

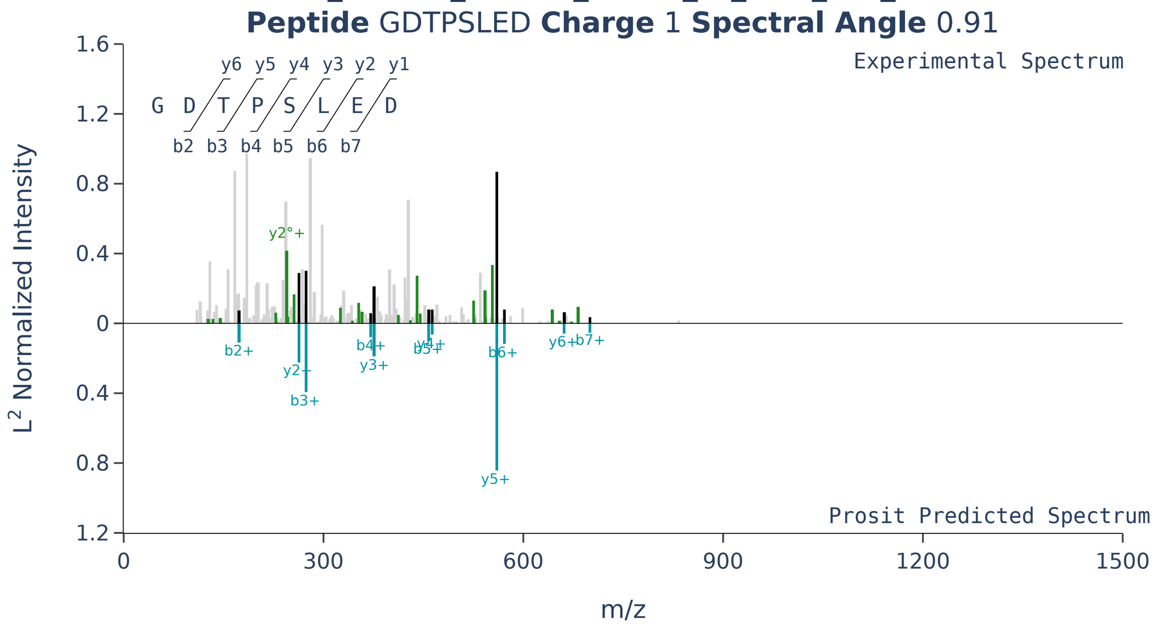


**Source** WSoh_221121_011221_HFGoe_L2_hTau_2h_R2 **Scan** 6306
**Peptide** TSPRHLSNVS **Charge** 2 **Spectral Angle** 0.88

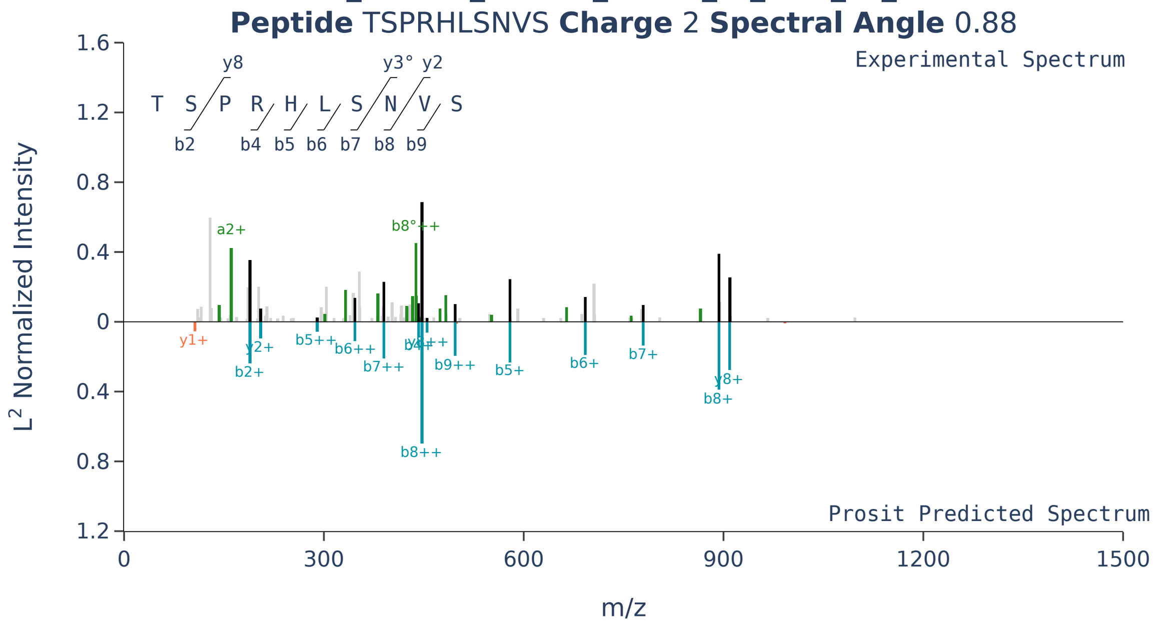


**Source** WSoh_221121_011221_HFGoe_L1_hTau_24h_R1 **Scan** 10532
**Peptide** LADEVSAS **Charge** 1 **Spectral Angle** 0.85

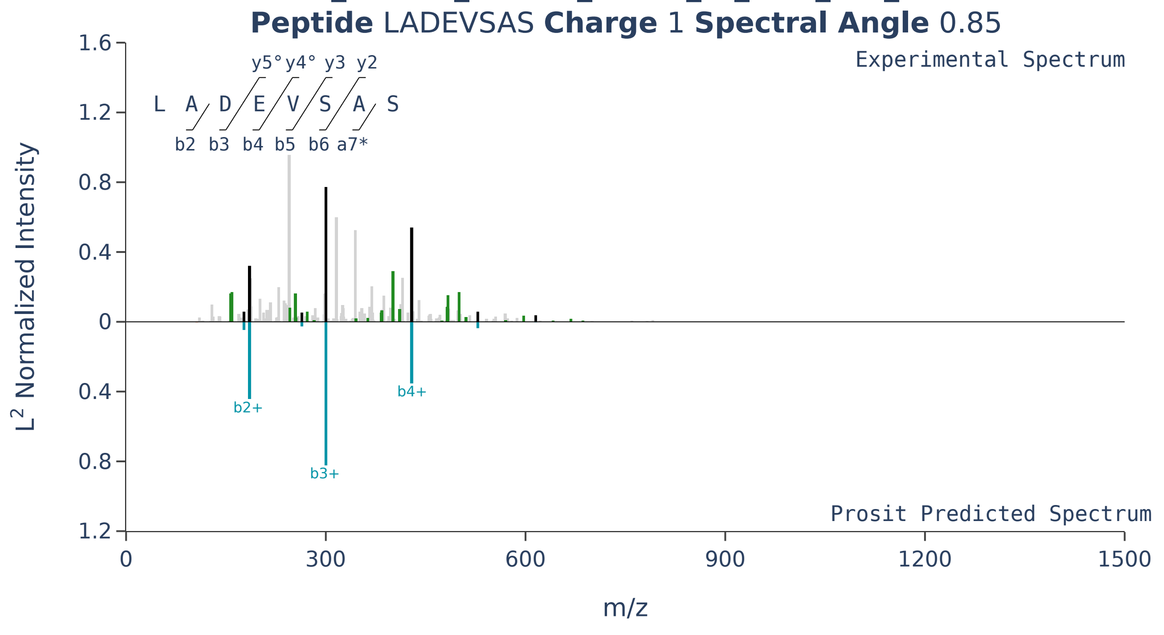


**Source** WSoh_221121_011221_HFGoe_L2_hTau_4h_R2 **Scan** 16018
**Peptide** SGDTSPRHLSNVSSTGSIDM **Charge** 3 **Spectral Angle** 0.91

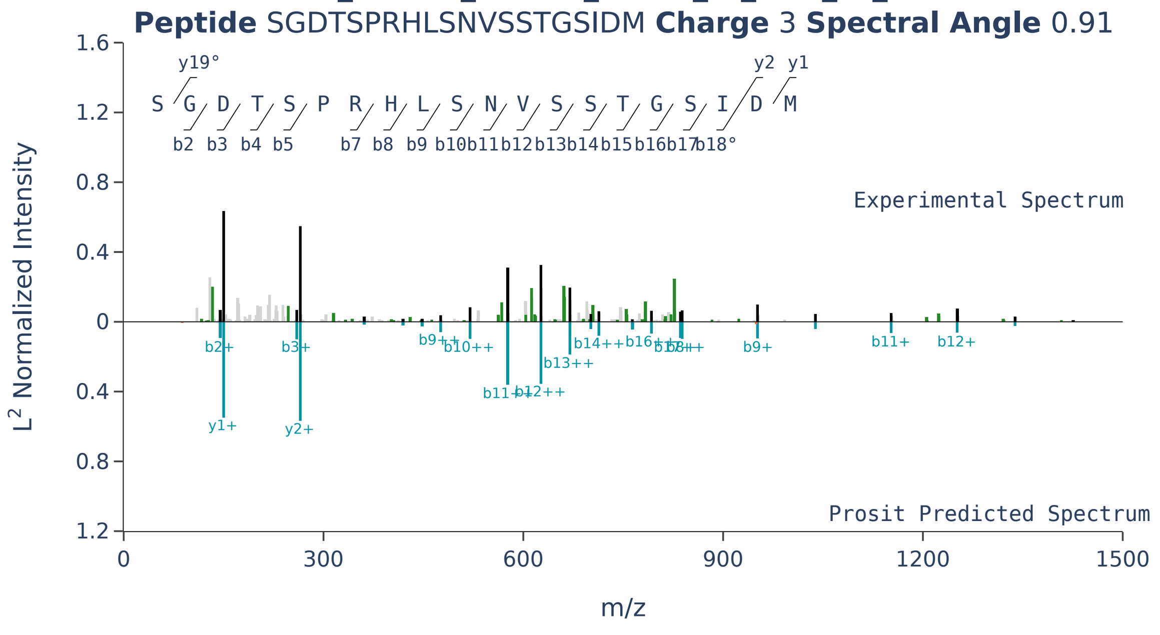


**Source** WSoh_221121_011221_HFGoe_L2_hTau_4h_R2 **Scan** 13429
**Peptide** PVDLSKVTSKCG **Charge** 2 **Spectral Angle** 0.92

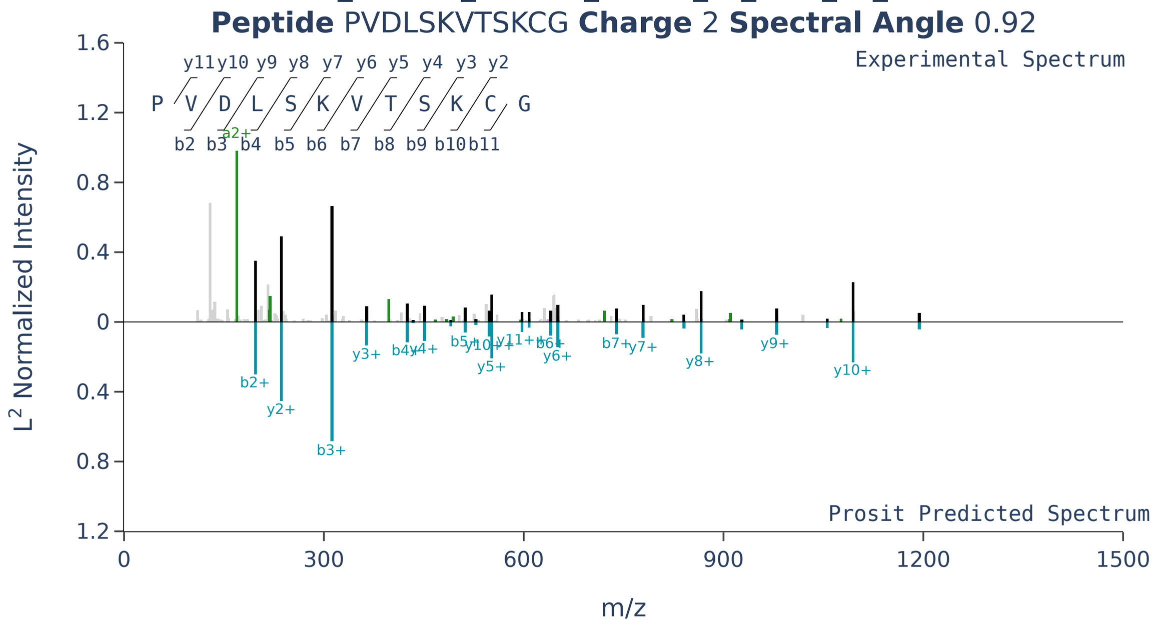


**Source** WSoh_221121_011221_HFGoe_L2_hTau_4h_R1 **Scan** 9738
**Peptide** TDHGAEIVYK **Charge** 3 **Spectral Angle** 0.92

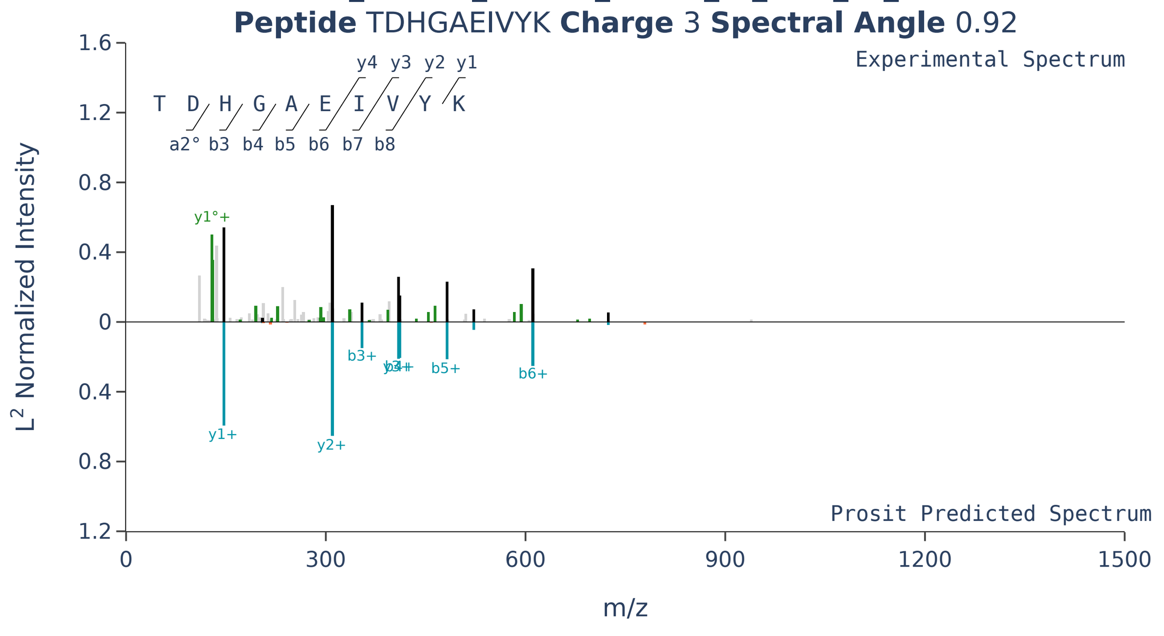


**Source** WSoh_221121_011221_HFGoe_L2_hTau_4h_R2 **Scan** 13538
**Peptide** SPVVSGDTSPRHLSNV **Charge** 2 **Spectral Angle** 0.92

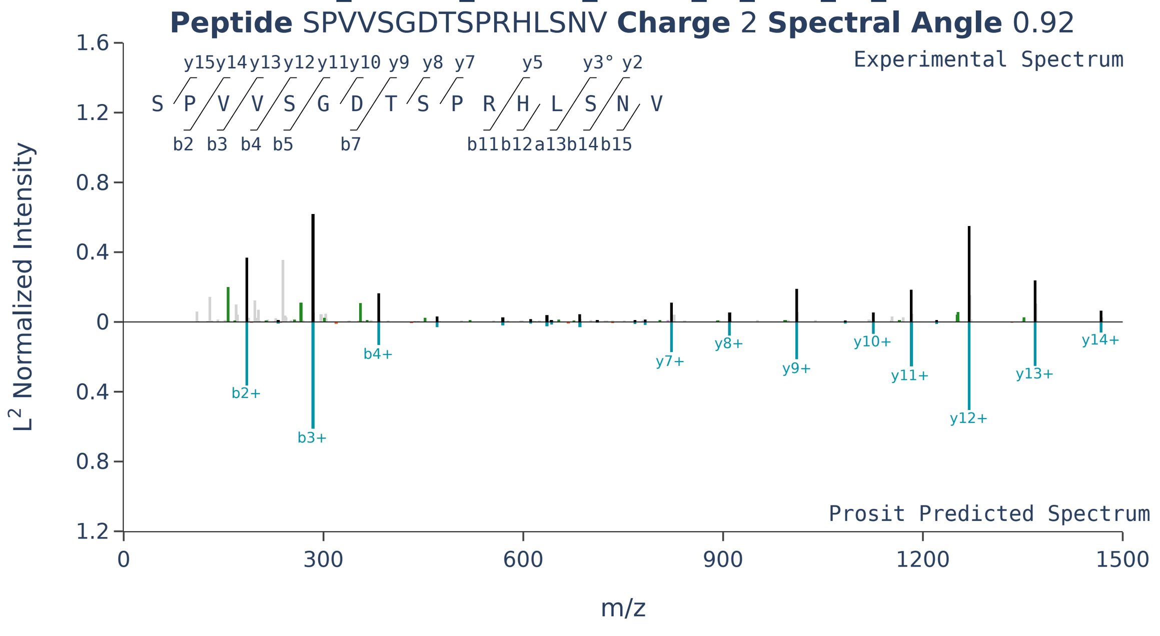

**Source** WSoh_221121_011221_HFGoe_L1_hTau_24h_R1 **Scan** 10163
**Peptide** KVQIINKKL **Charge** 3 **Spectral Angle** 0.94

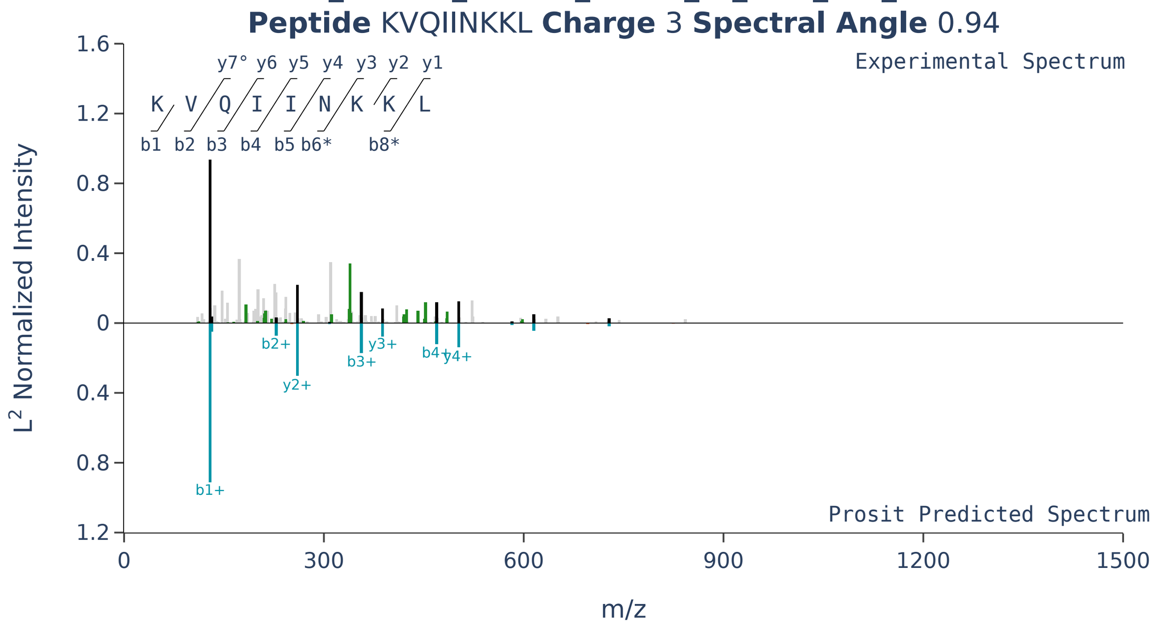


**Source** WSoh_221121_011221_HFGoe_L1_hTau_1h_R2 **Scan** 6100
**Peptide** PTPPTREPKKV **Charge** 2 **Spectral Angle** 0.9

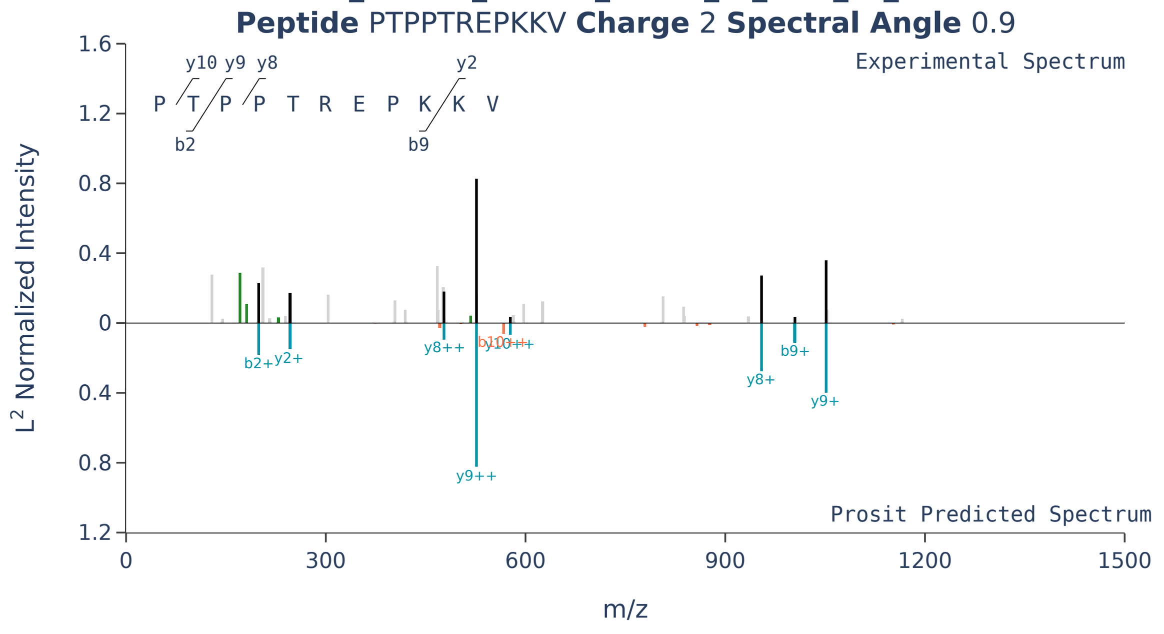


**Source** WSoh_221121_011221_HFGoe_L1_hTau_24h_R1 **Scan** 8152
**Peptide** DEAAGHVTQARMVSK **Charge** 2 **Spectral Angle** 0.85

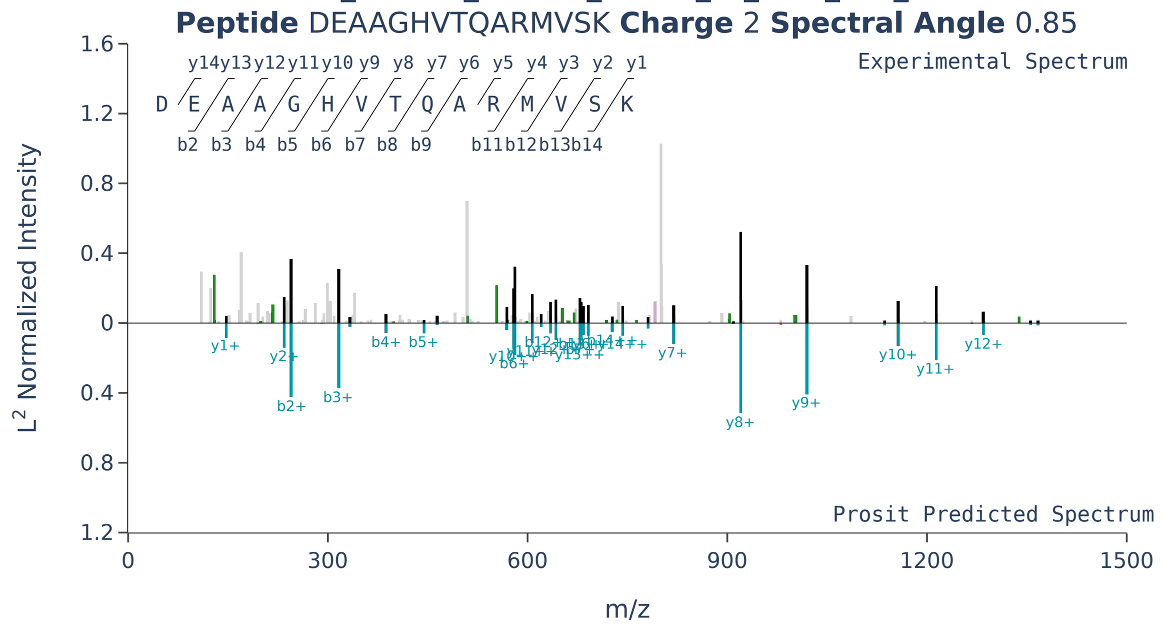


**Source** WSoh_221121_011221_HFGoe_L2_hTau_24h_R1 **Scan** 6514
**Peptide** LGNIHHKPGGGQVE **Charge** 2 **Spectral Angle** 0.9

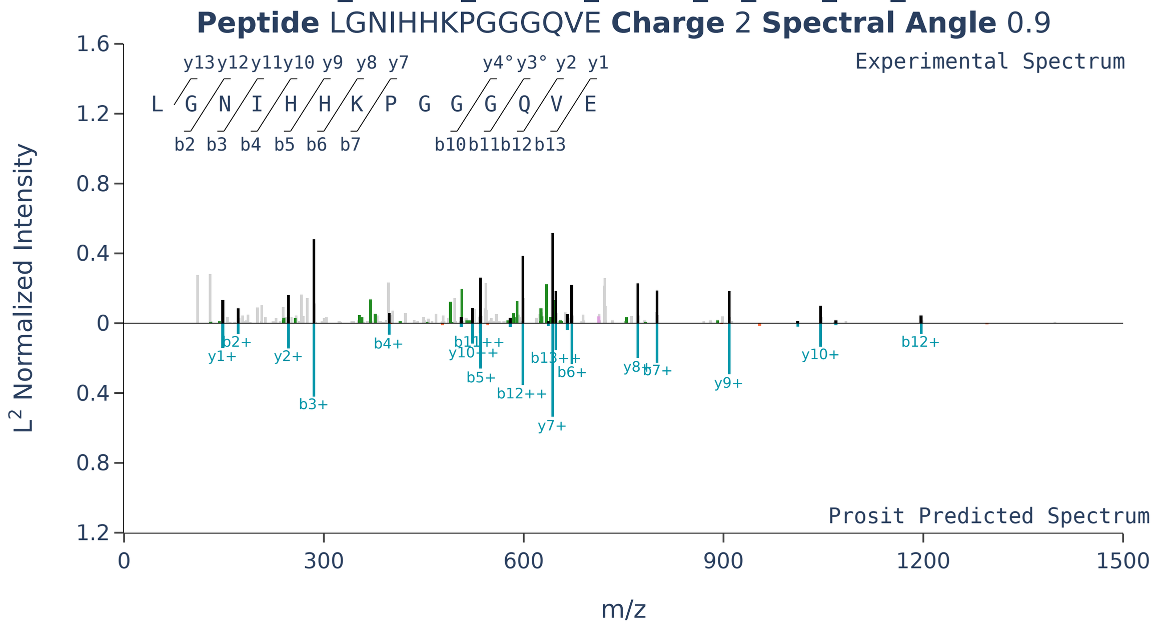


**Source** WSoh_221121_011221_HFGoe_L2_hTau_2h_R1 **Scan** 11674
**Peptide** SASLAKQGL **Charge** 1 **Spectral Angle** 0.91

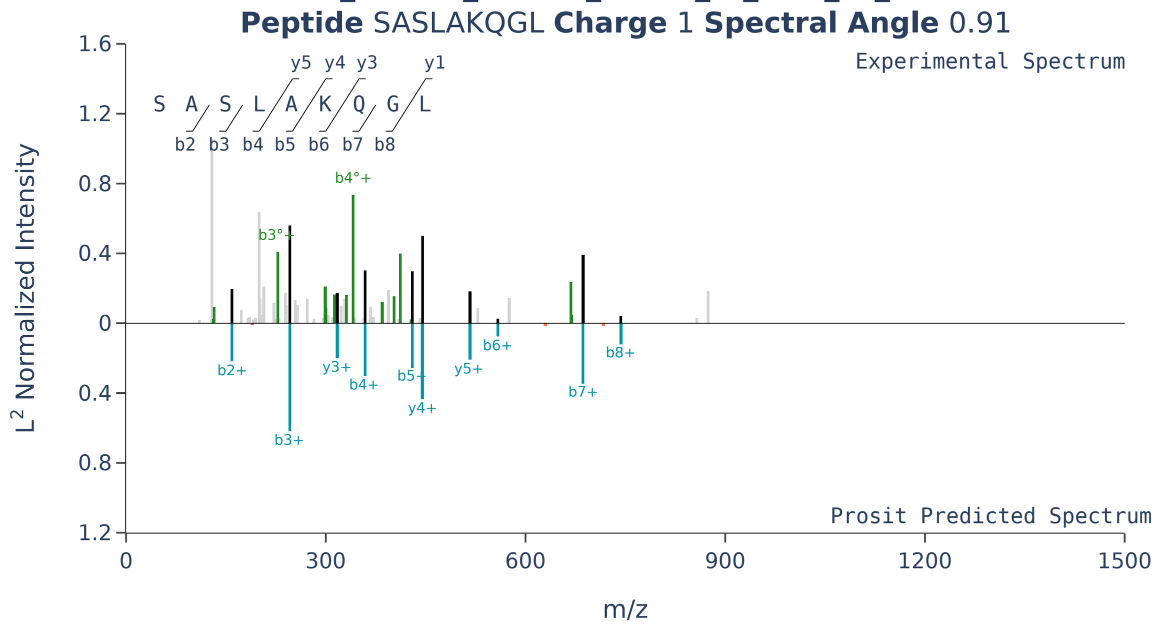


**Source** WSoh_221121_011221_HFGoe_L2_hTau_1h_R2 **Scan** 13520
**Peptide** KIGSLDNITH **Charge** 3 **Spectral Angle** 0.92

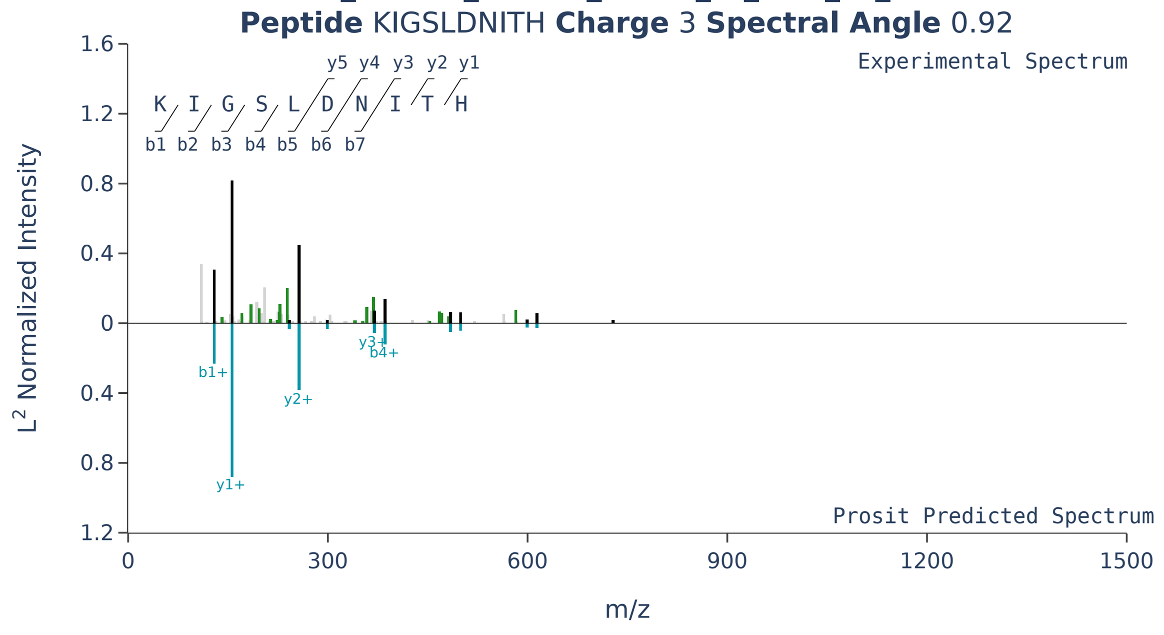


**Source** WSoh_221121_011221_HFGoe_L2_hTau_24h_R1 **Scan** 9074
**Peptide** DQEGDTDAGLKE **Charge** 2 **Spectral Angle** 0.93

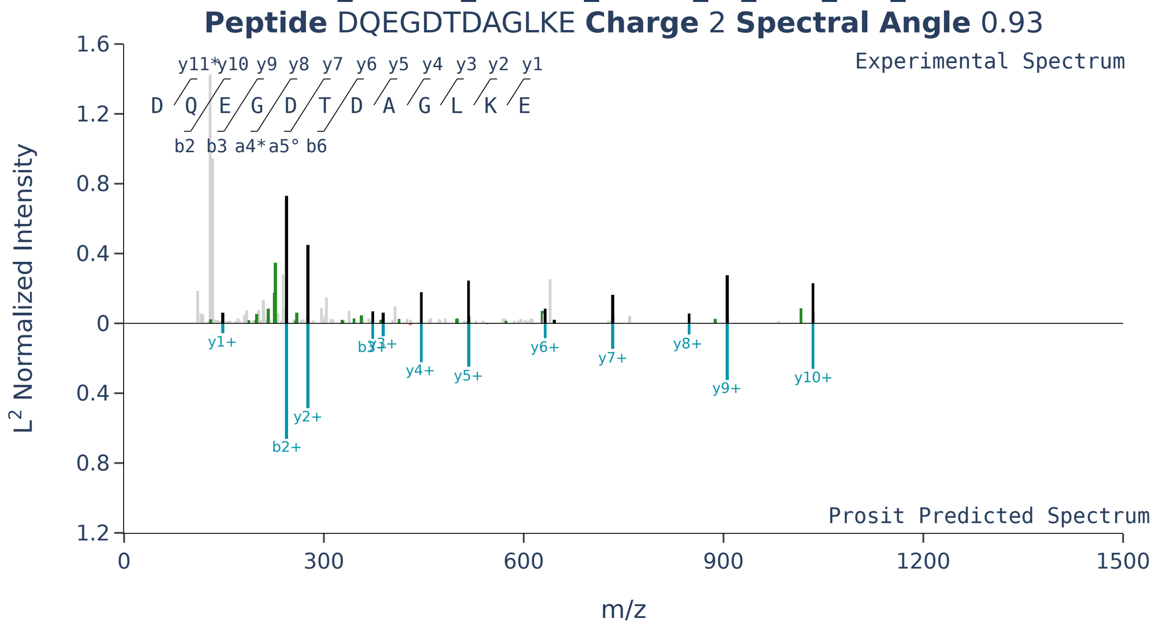


**Source** WSoh_221121_011221_HFGoe_L2_hTau_4h_R1 **Scan** 11086
**Peptide** NKKLDLSNVQ **Charge** 2 **Spectral Angle** 0.92

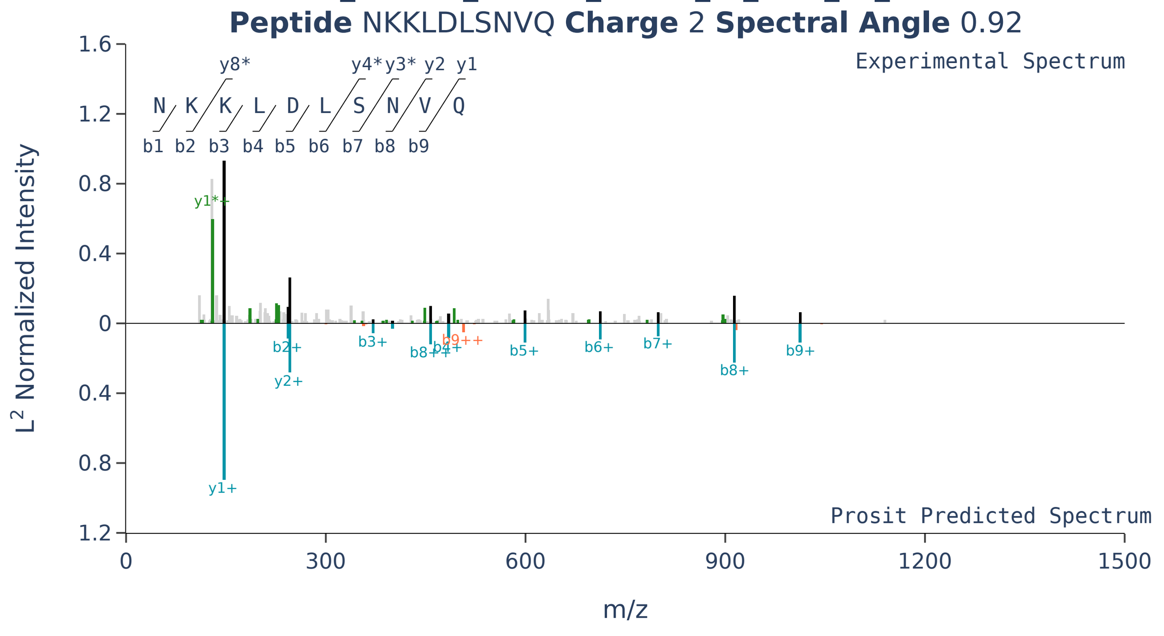


**Source** WSoh_221121_011221_HFGoe_L1_hTau_4h_R1 **Scan** 19707
**Peptide** GIGDTPSLEDEAAGHVTQ **Charge** 2 **Spectral Angle** 0.89

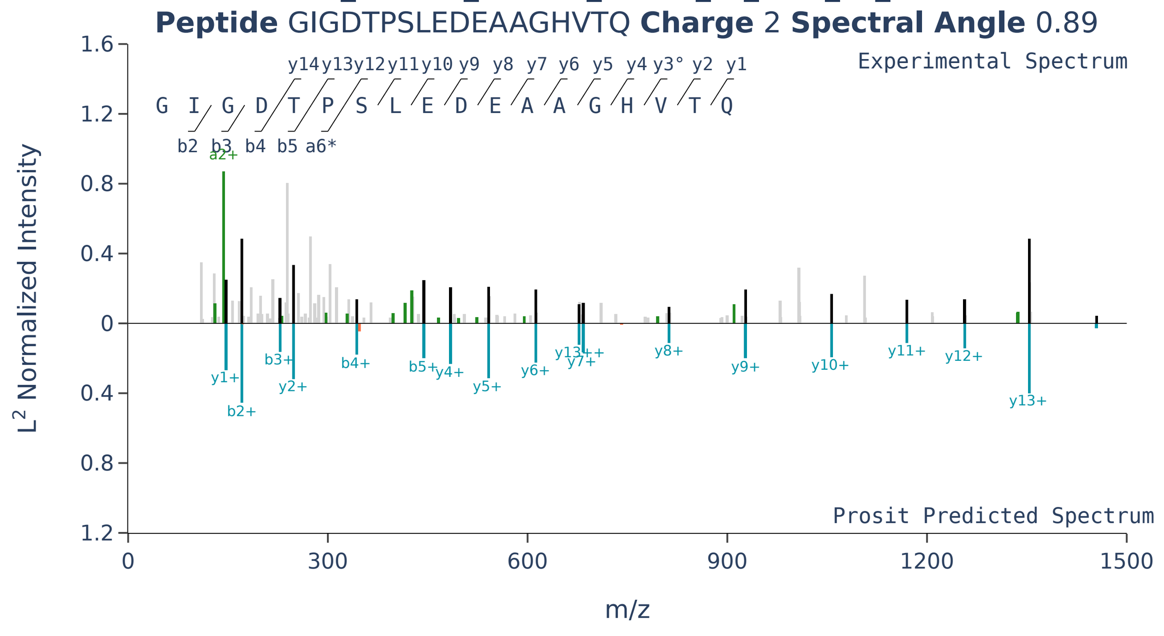


**Source** WSoh_221121_011221_HFGoe_L1_hTau_4h_R2 **Scan** 13038
**Peptide** LDFKDRVQ **Charge** 2 **Spectral Angle** 0.92

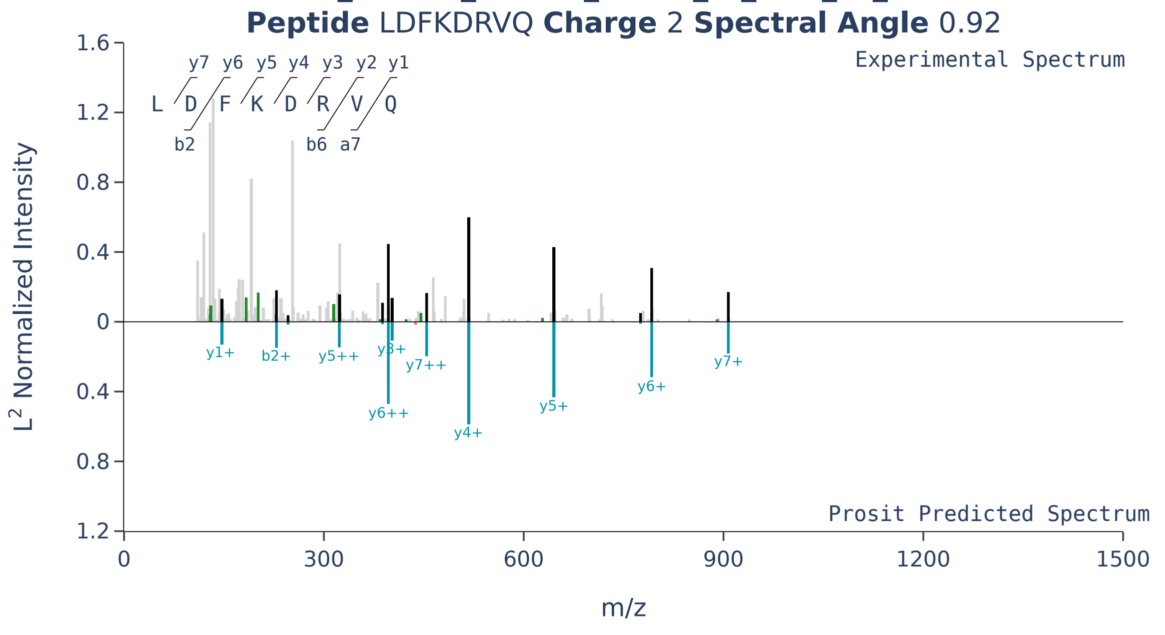


**Source** WSoh_221121_011221_HFGoe_L2_hTau_4h_R1 **Scan** 7262
**Peptide** AGHVTQARMVS **Charge** 2 **Spectral Angle** 0.91

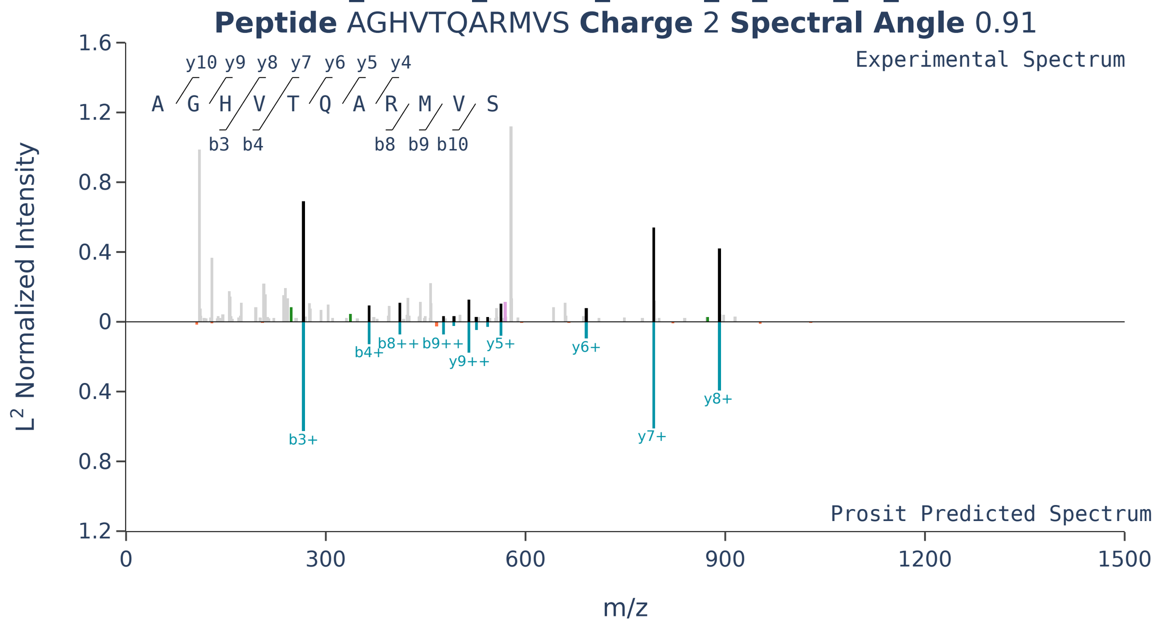


**Source** WSoh_221121_011221_HFGoe_L2_hTau_24h_R2 **Scan** 16426
**Peptide** HGAEIVYKSPVVSGDTSPRHL **Charge** 2 **Spectral Angle** 0.86

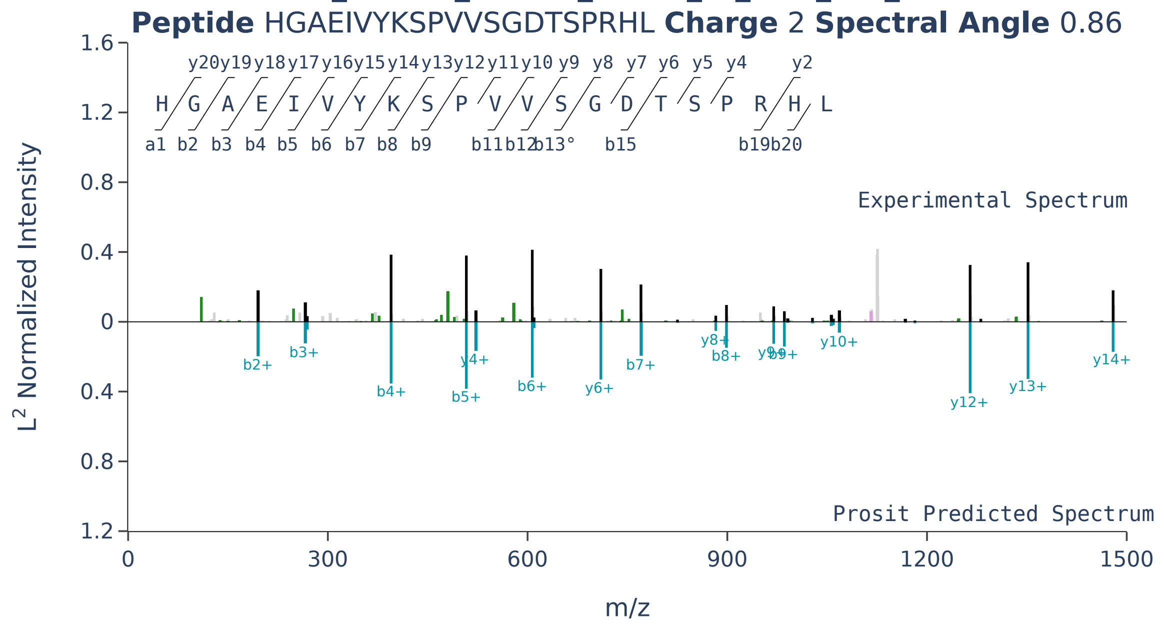


**Source** WSoh_221121_011221_HFGoe_L1_hTau_2h_R1 **Scan** 17674
**Peptide** KIGSLDNI **Charge** 2 **Spectral Angle** 0.92

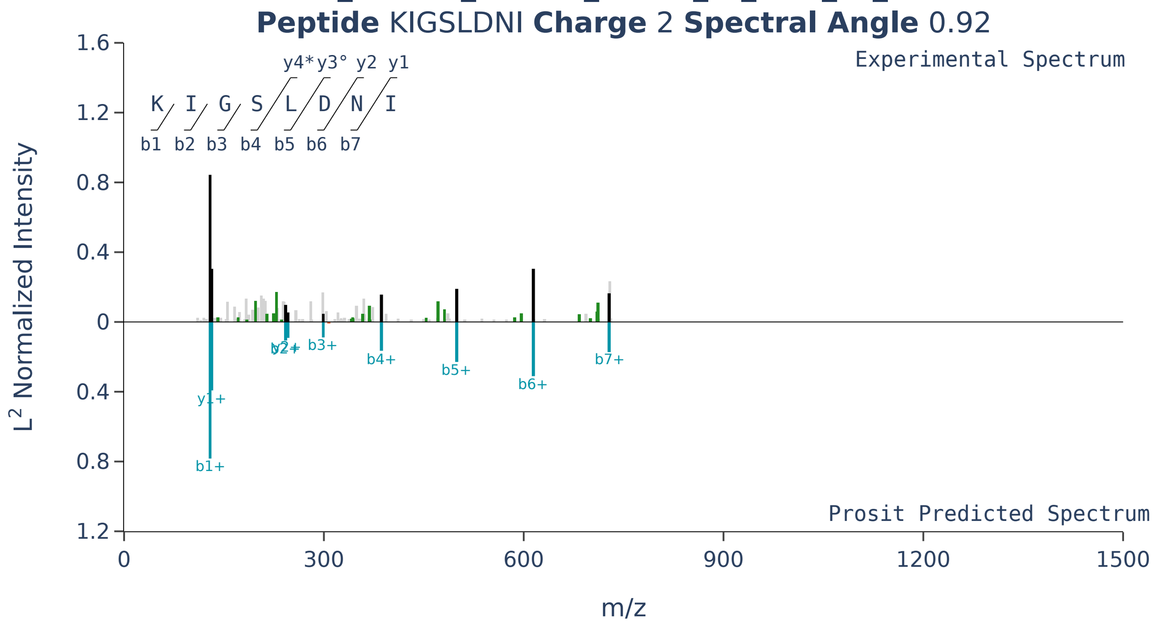


**Source** WSoh_221121_011221_HFGoe_L1_hTau_4h_R1 **Scan** 8487
**Peptide** HAGTYGLGDR **Charge** 2 **Spectral Angle** 0.92

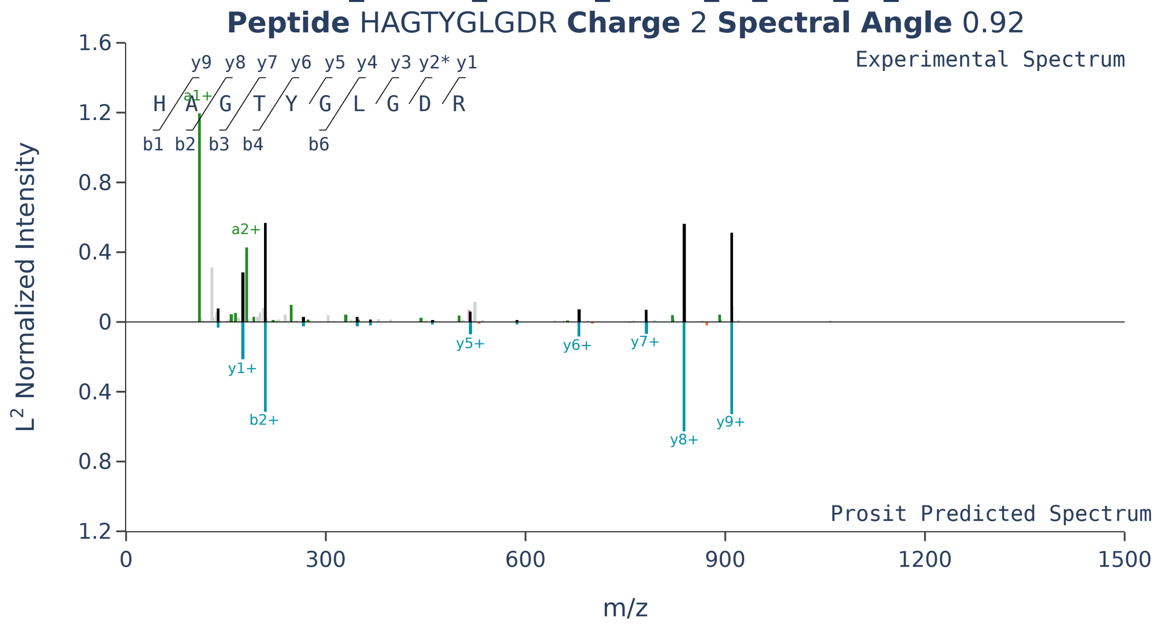


**Source** WSoh_221121_011221_HFGoe_L2_hTau_1h_R1 **Scan** 5784
**Peptide** ATPRGAAPPGQKGQA **Charge** 3 **Spectral Angle** 0.93

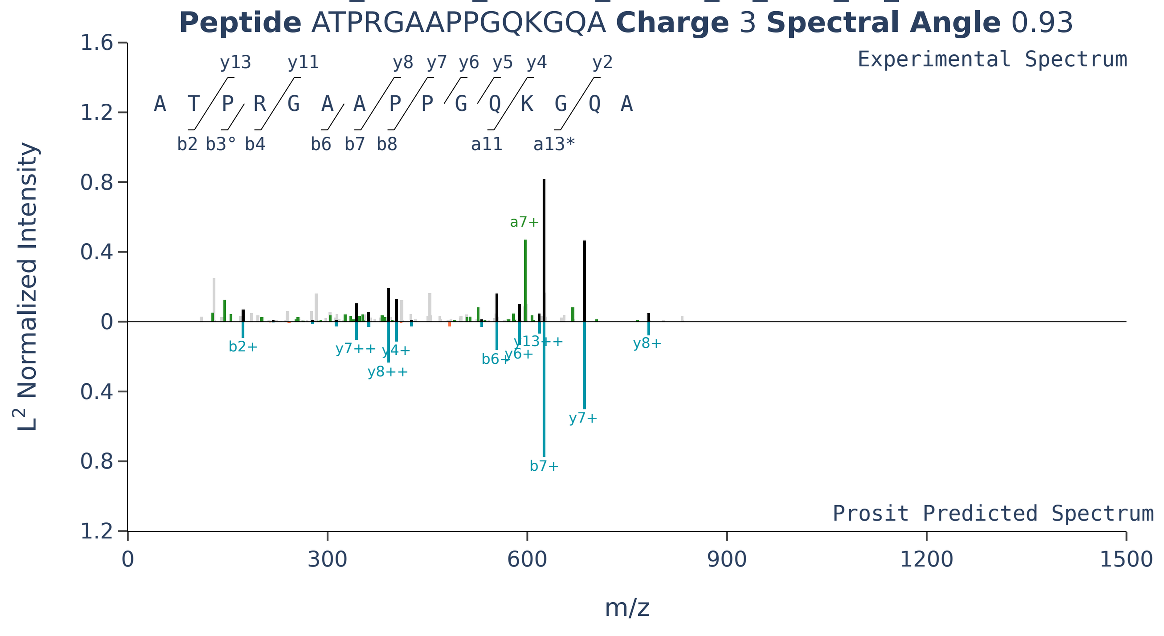

**Source** WSoh_221121_011221_HFGoe_L2_hTau_4h_R2 **Scan** 12951
**Peptide** SKDNIKHVPGGGSVQI **Charge** 2 **Spectral Angle** 0.91

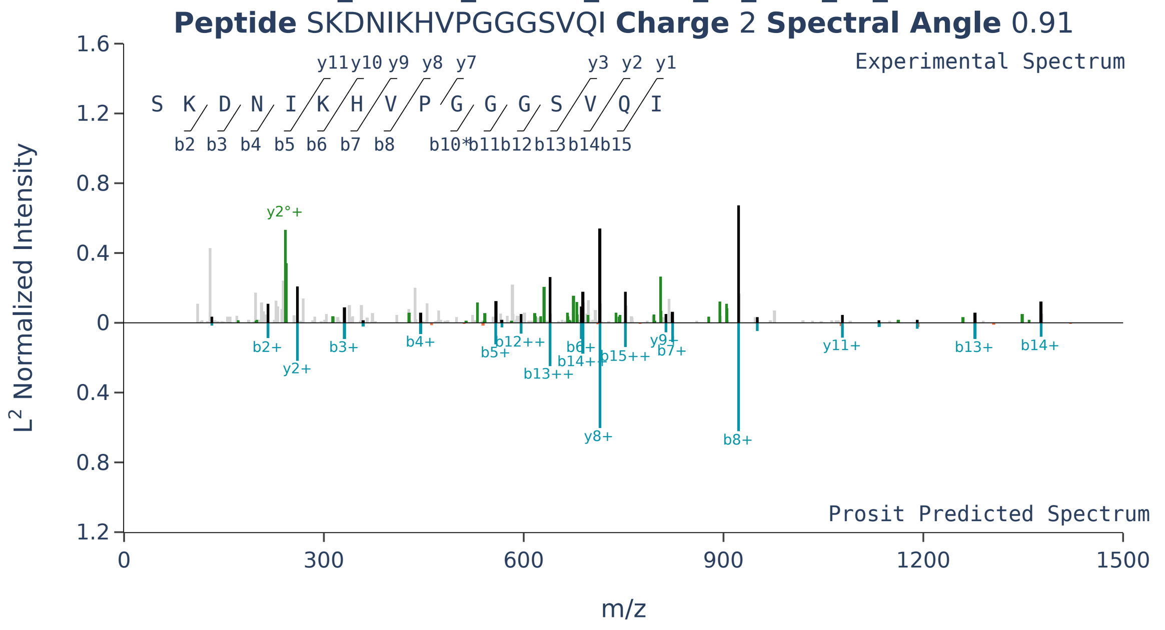


**Source** WSoh_221121_011221_HFGoe_L2_hTau_24h_R2 **Scan** 22475
**Peptide** DVTAPLVD **Charge** 1 **Spectral Angle** 0.9

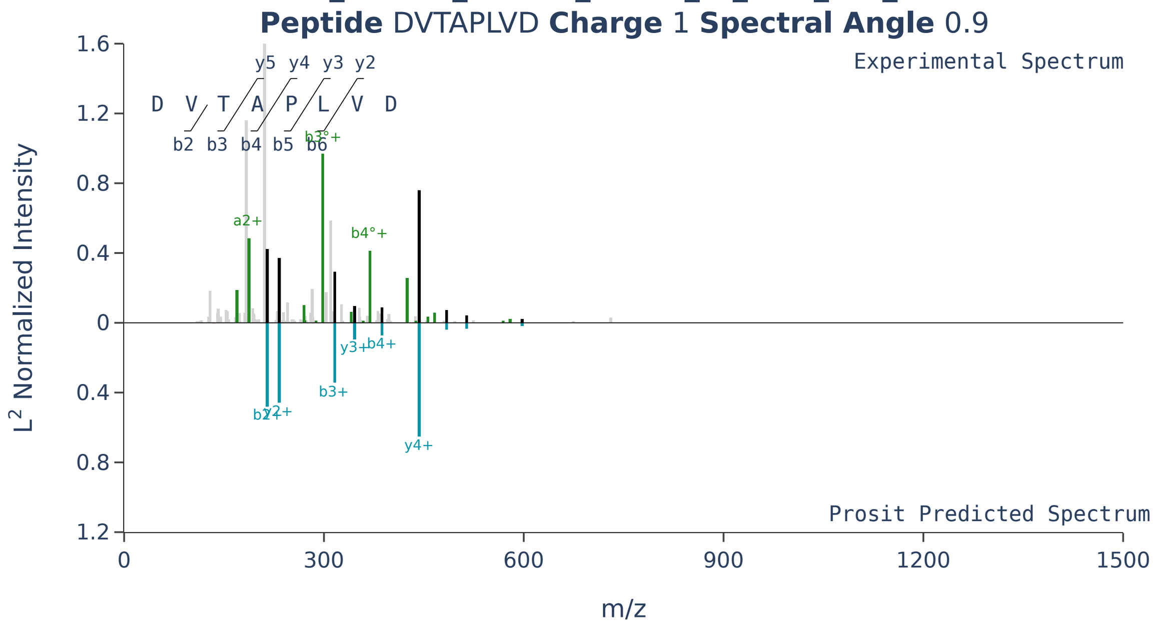


**Source** WSoh_221121_011221_HFGoe_L2_hTau_4h_R2 **Scan** 16893
**Peptide** SKDNIKHVPGGGSVQIVY **Charge** 3 **Spectral Angle** 0.91

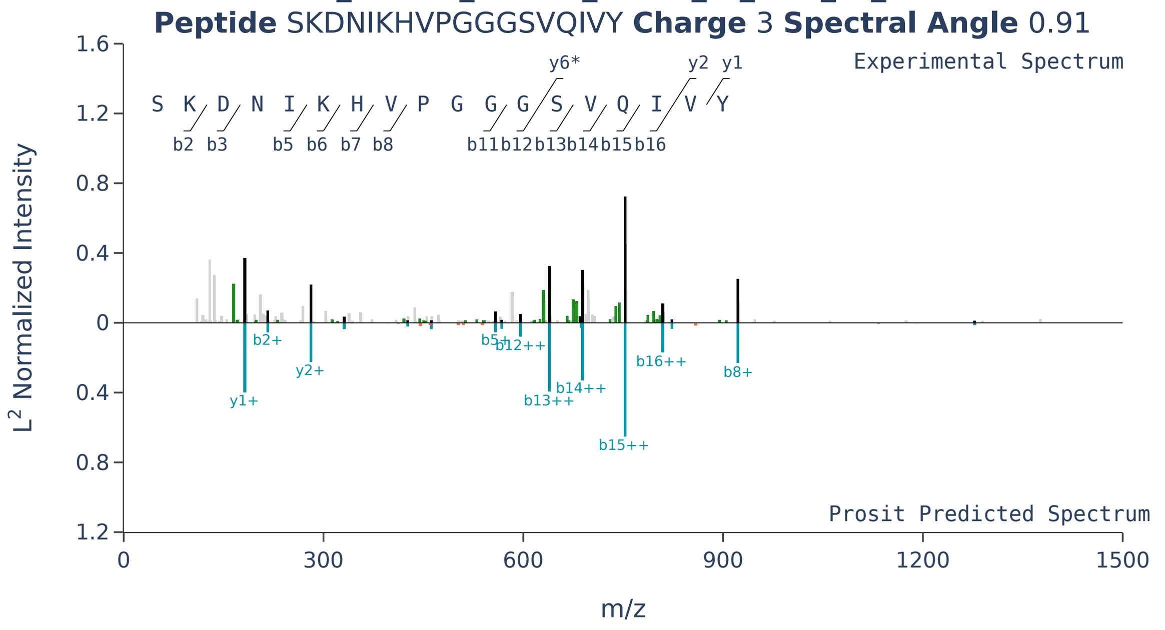


**Source** WSoh_221121_011221_HFGoe_L1_hTau_4h_R2 **Scan** 7163
**Peptide** GDTSPRHLSNVSS **Charge** 2 **Spectral Angle** 0.89

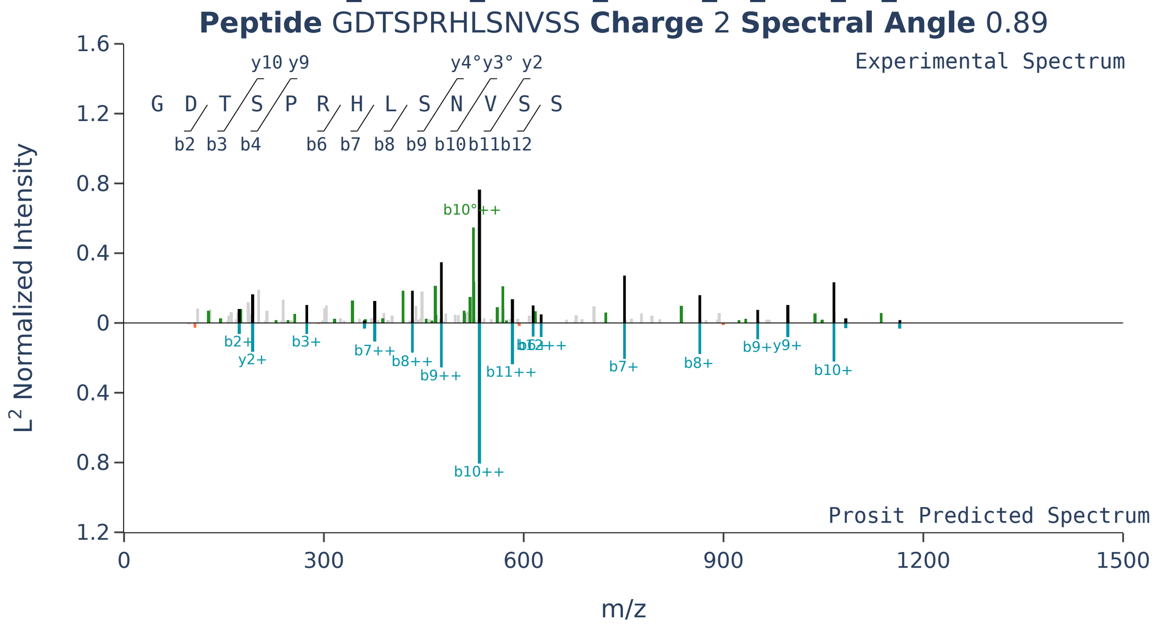


**Source** WSoh_221121_011221_HFGoe_L1_hTau_24h_R1 **Scan** 19679
**Peptide** MVDSPQL **Charge** 1 **Spectral Angle** 0.93

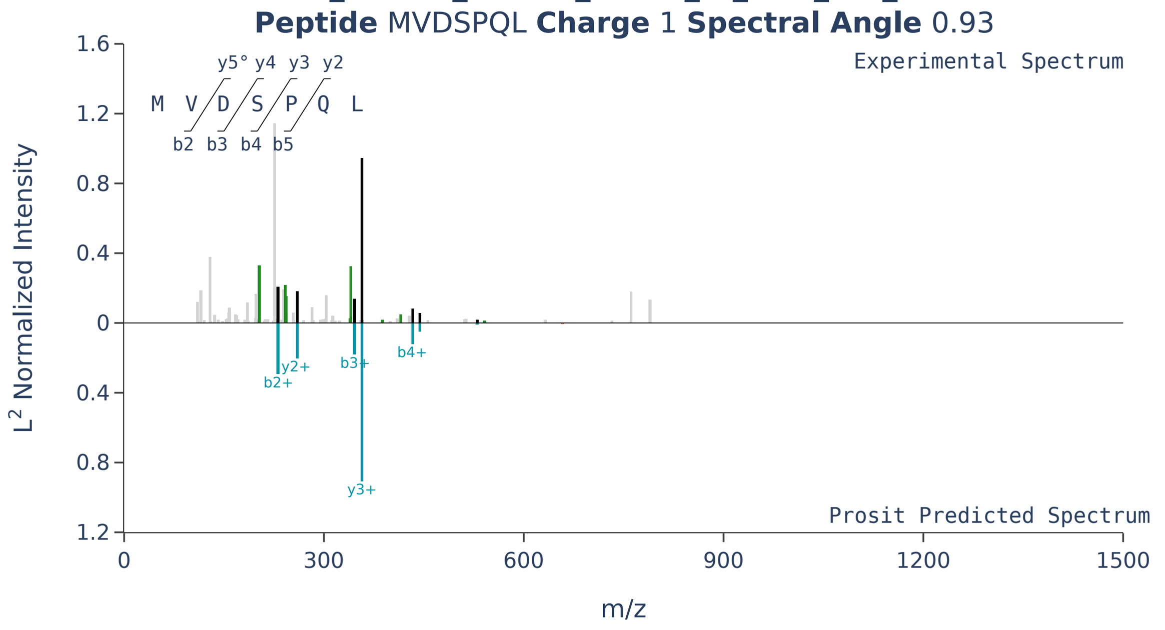


**Source** WSoh_221121_011221_HFGoe_L2_hTau_1h_R2 **Scan** 12946
**Peptide** NKKLDLSNV **Charge** 2 **Spectral Angle** 0.84

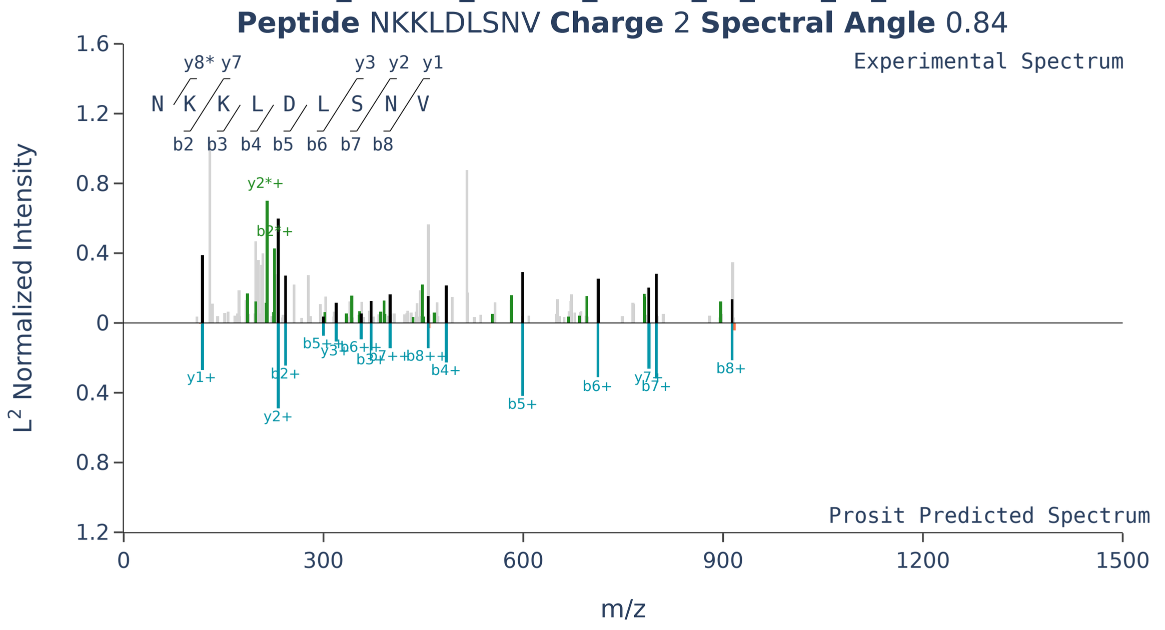


**Source** WSoh_221121_011221_HFGoe_L2_hTau_24h_R1 **Scan** 10299
**Peptide** LEDEAAGHVTQA **Charge** 2 **Spectral Angle** 0.88

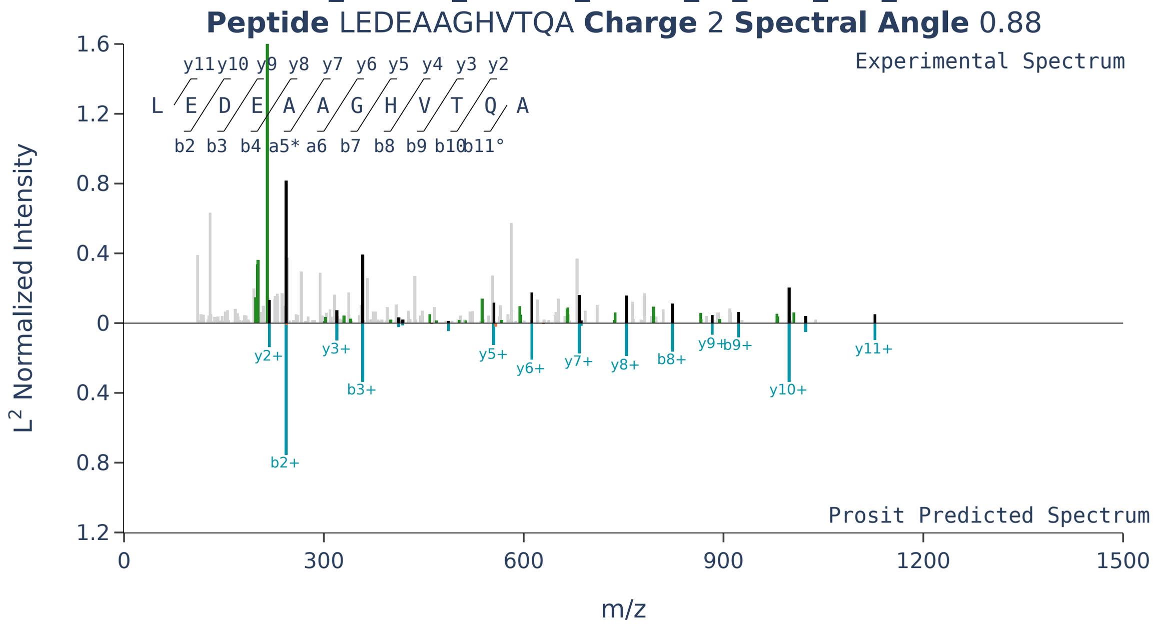


**Source** WSoh_221121_011221_HFGoe_L2_hTau_24h_R2 **Scan** 21685
**Peptide** TTAEEAGIGDTPSLE **Charge** 2 **Spectral Angle** 0.94

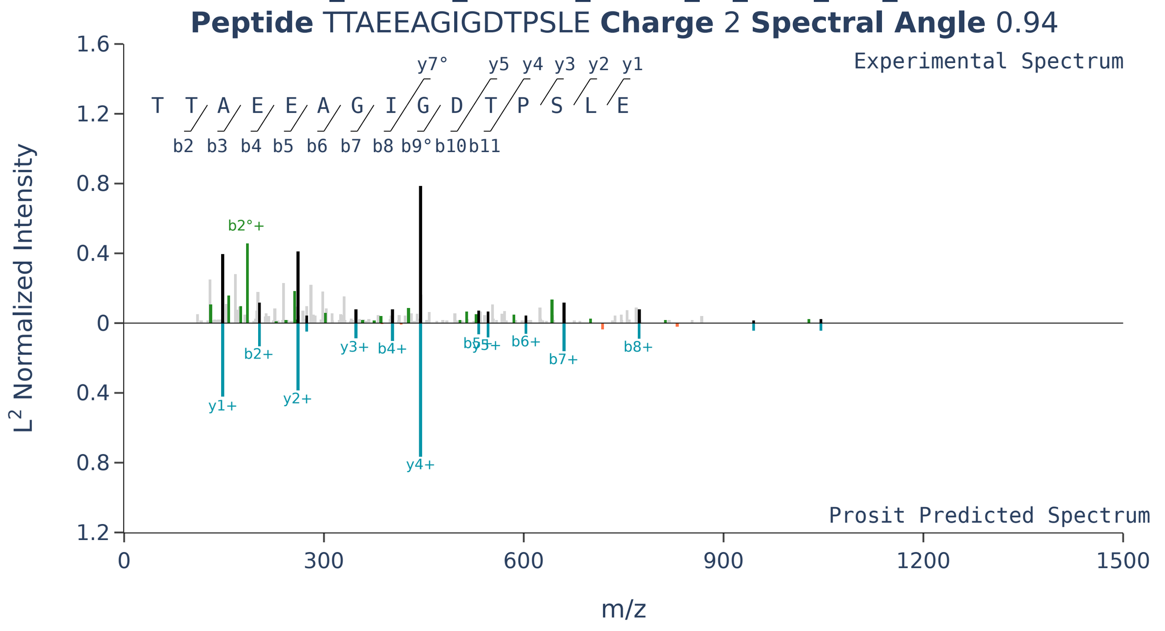


**Source** WSoh_221121_011221_HFGoe_L1_hTau_24h_R2 **Scan** 11226
**Peptide** SLPTPPTREPKKV **Charge** 3 **Spectral Angle** 0.96

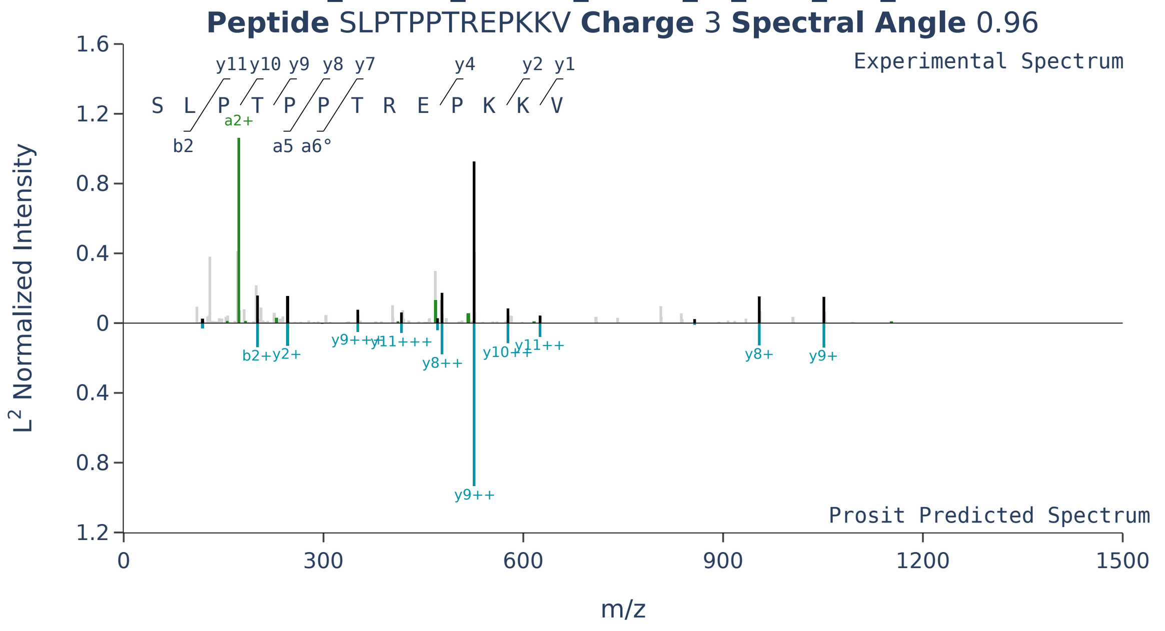


**Source** WSoh_221121_011221_HFGoe_L1_hTau_24h_R1 **Scan** 18607
**Peptide** AEIVYKSPVVSGDTSPRHLSNV **Charge** 3 **Spectral Angle** 0.91

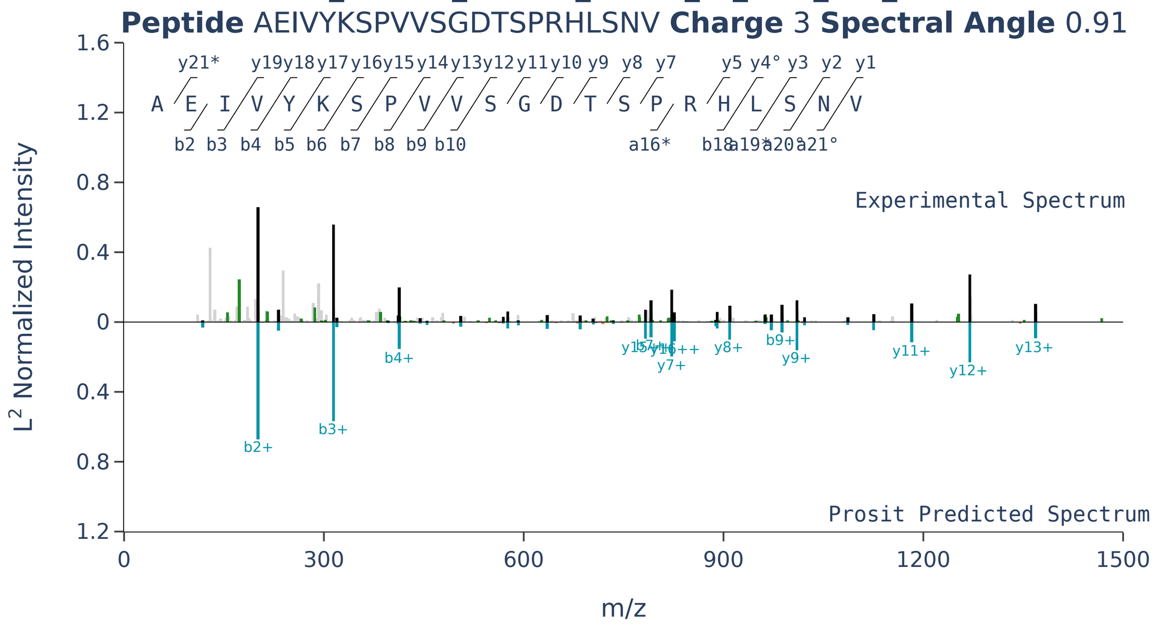


**Source** WSoh_221121_011221_HFGoe_L1_hTau_24h_R1 **Scan** 7581
**Peptide** SGDTSPRHLSNVS **Charge** 2 **Spectral Angle** 0.87

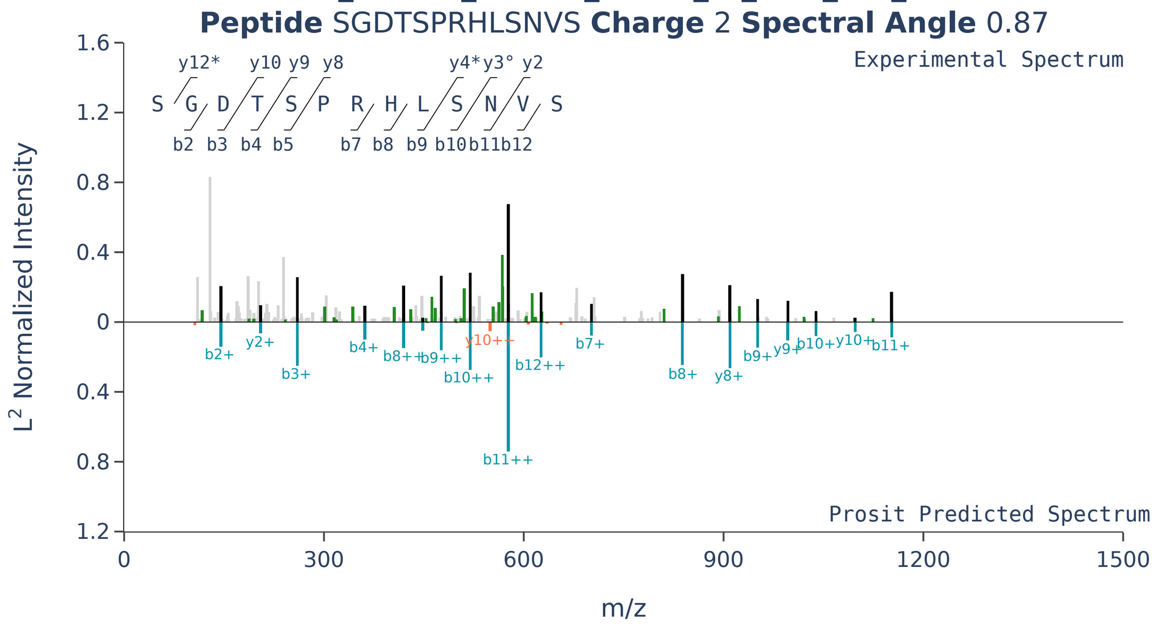


**Source** WSoh_221121_011221_HFGoe_L1_hTau_24h_R1 **Scan** 16166
**Peptide** AKTDHGAEIVYKSPVV **Charge** 3 **Spectral Angle** 0.86

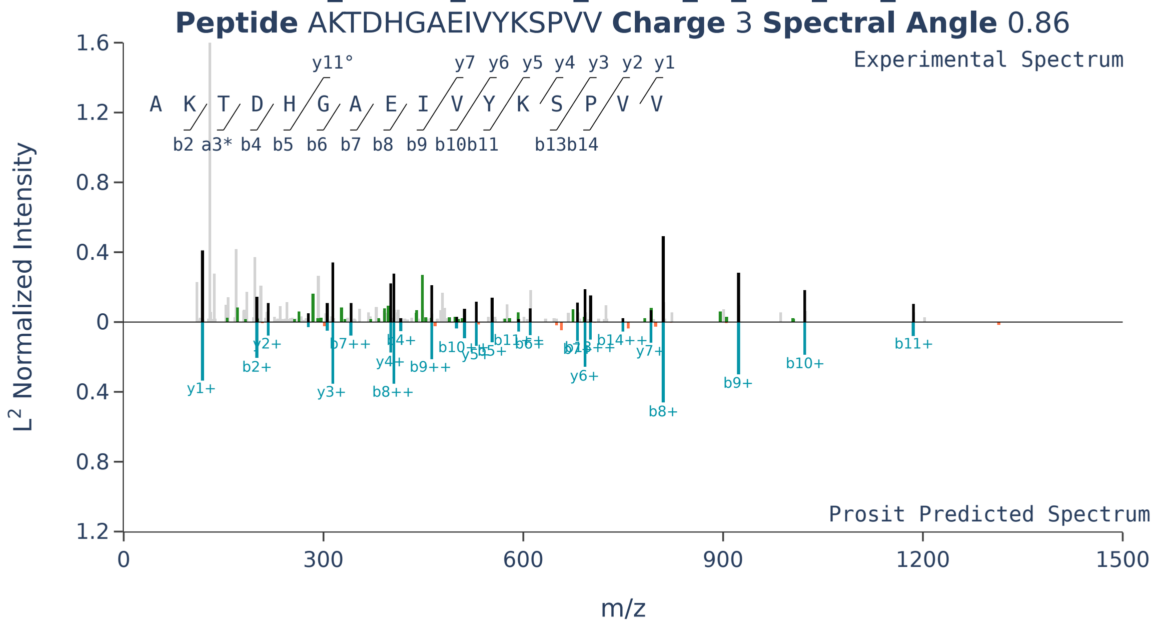


**Source** WSoh_221121_011221_HFGoe_L2_hTau_1h_R2 **Scan** 7343
**Peptide** SGYSSPGSPGTPGSRSR **Charge** 3 **Spectral Angle** 0.96

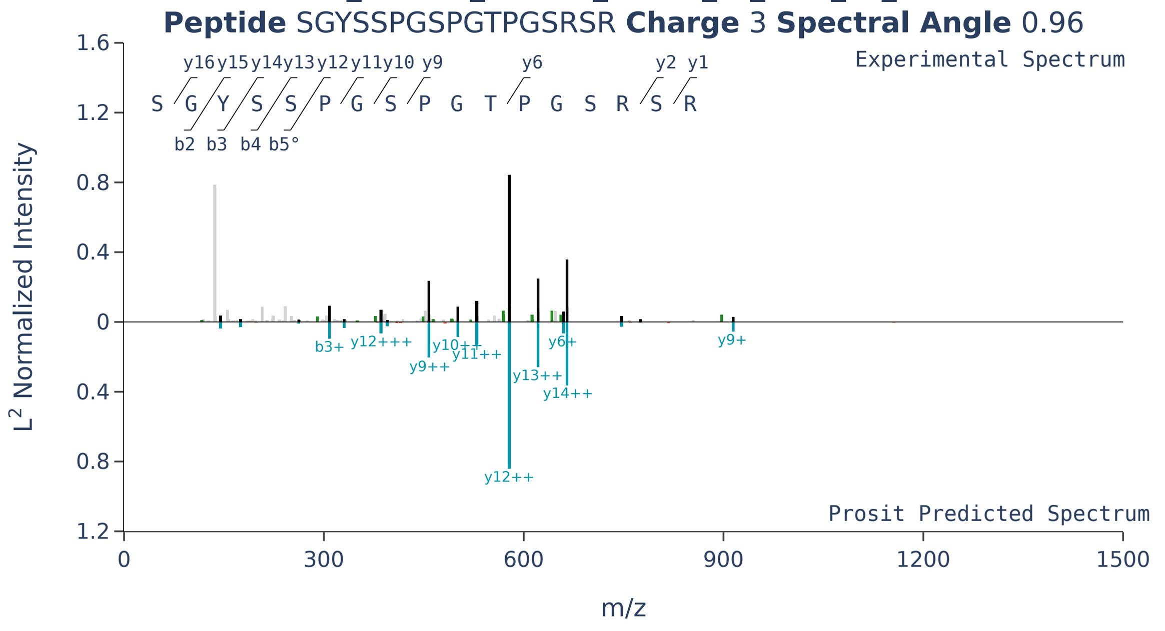


**Source** WSoh_221121_011221_HFGoe_L2_hTau_4h_R1 **Scan** 15814
**Peptide** KTDHGAEIVYKSPVV **Charge** 2 **Spectral Angle** 0.9

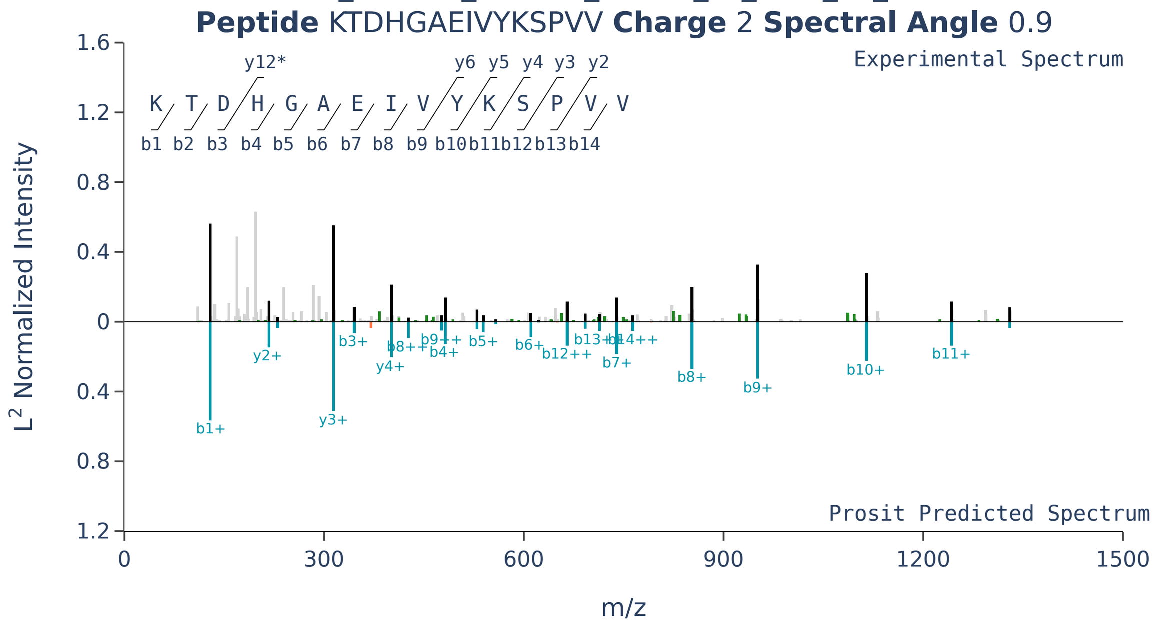


**Source** WSoh_221121_011221_HFGoe_L2_hTau_4h_R2 **Scan** 12038
**Peptide** EDEAAGHVTQARMV **Charge** 2 **Spectral Angle** 0.9

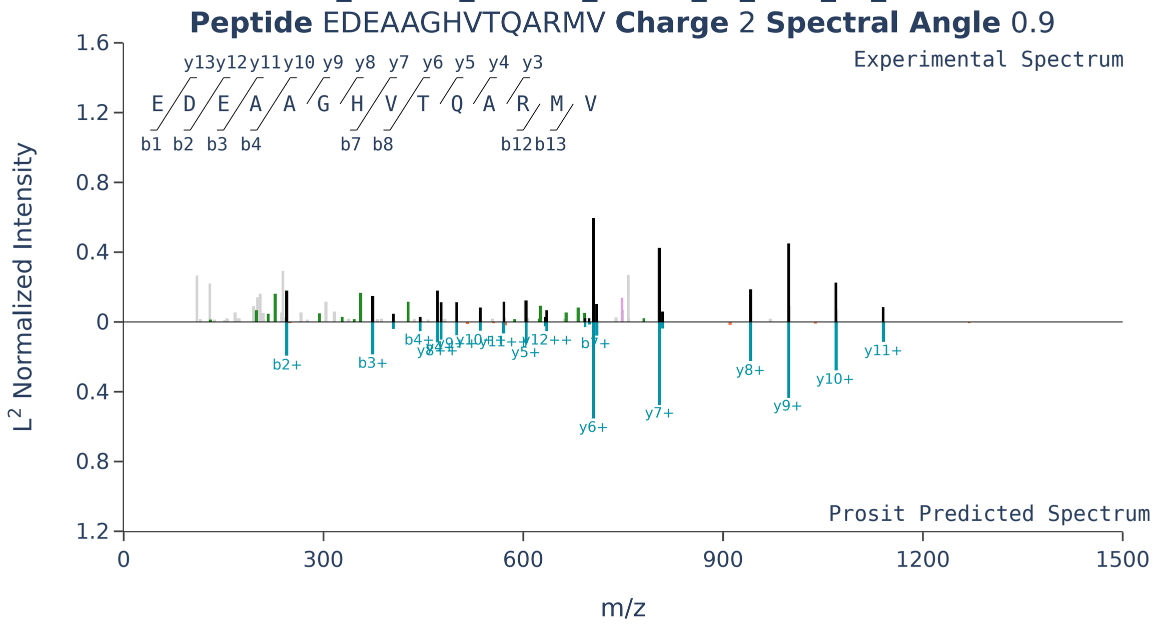

**Source** WSoh_221121_011221_HFGoe_L2_hTau_24h_R1 **Scan** 20296
**Peptide** PGGGKVQIINKKLDLSNV **Charge** 2 **Spectral Angle** 0.9

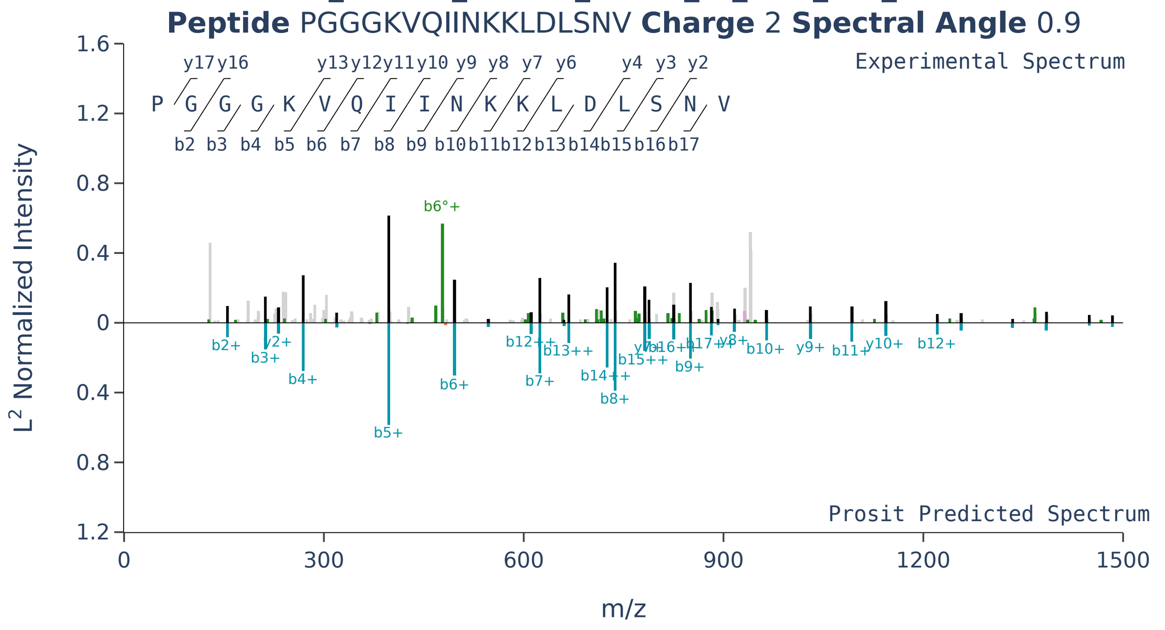


**Source** WSoh_221121_011221_HFGoe_L2_hTau_4h_R2 **Scan** 12214
**Peptide** AQPHTEIPEGTTA **Charge** 2 **Spectral Angle** 0.91

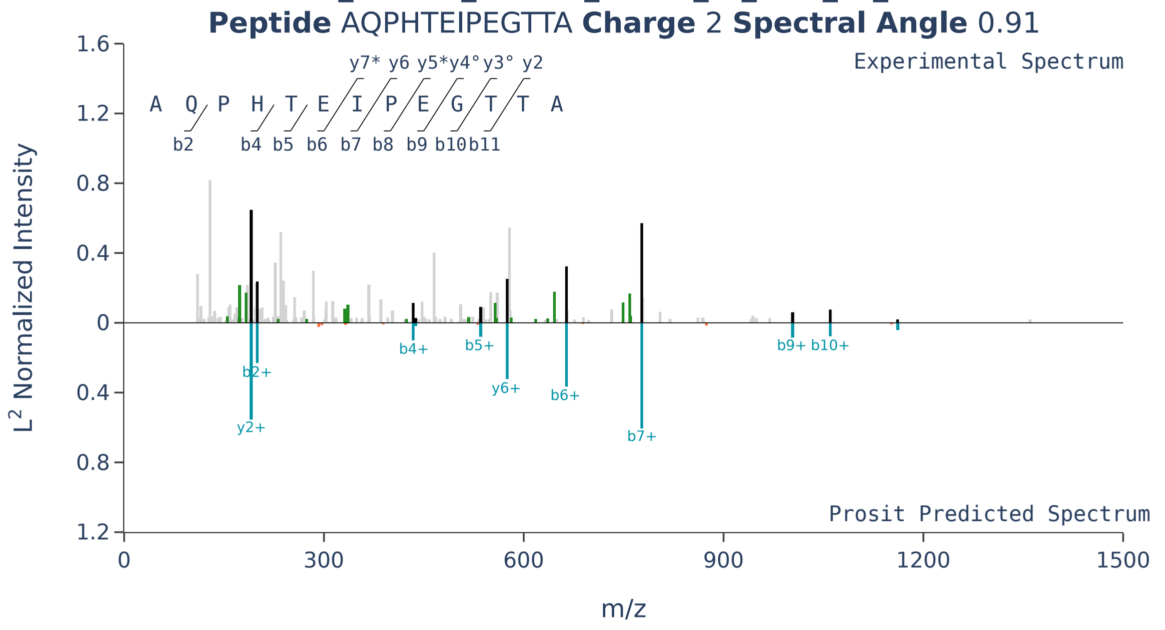


**Source** WSoh_221121_011221_HFGoe_L1_hTau_24h_R1 **Scan** 8149
**Peptide** AGTYGLGDRKDQGG **Charge** 2 **Spectral Angle** 0.91

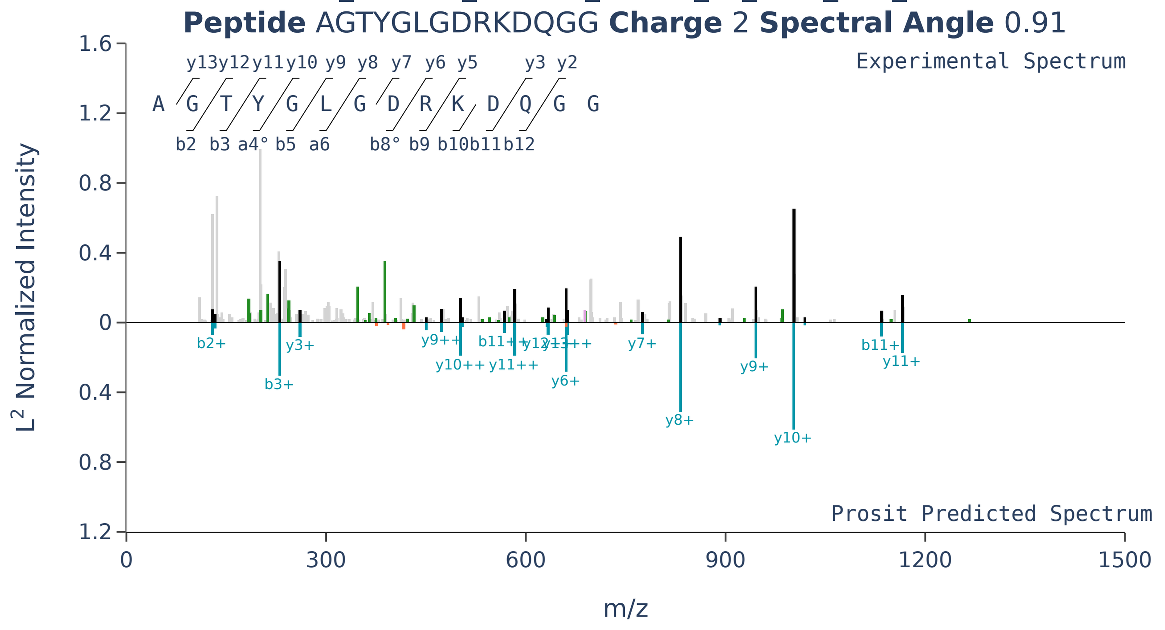


**Source** WSoh_221121_011221_HFGoe_L1_hTau_24h_R1 **Scan** 10333
**Peptide** KPVDLSKVTSKCG **Charge** 3 **Spectral Angle** 0.9

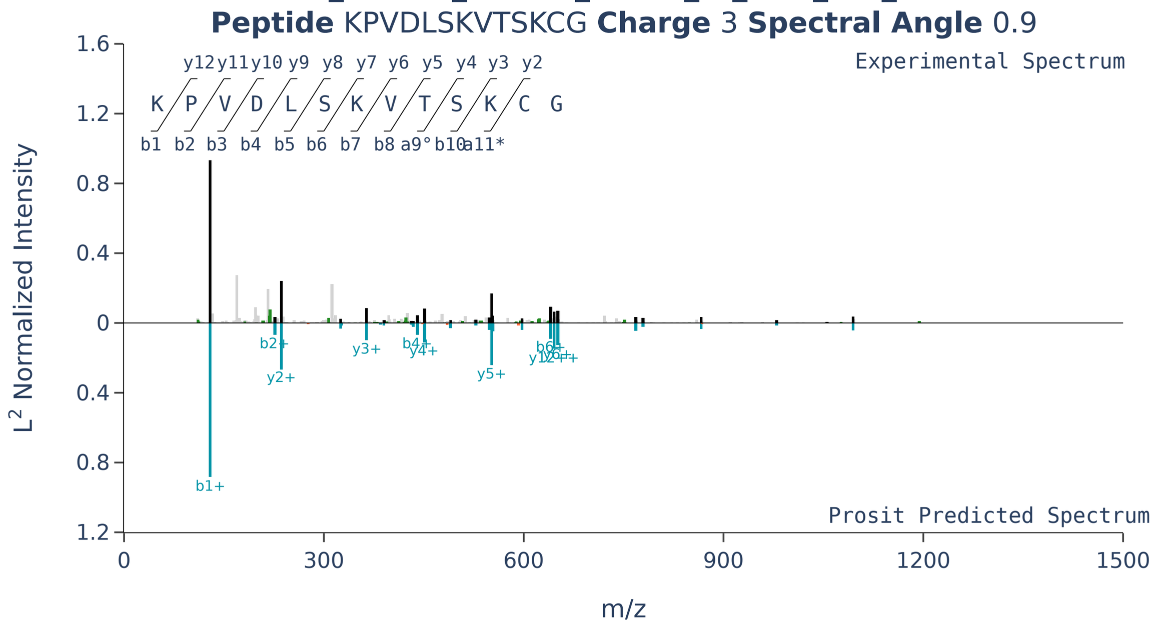


**Source** WSoh_221121_011221_HFGoe_L1_hTau_24h_R2 **Scan** 18409
**Peptide** TDAGLKESPLQTPTEDG **Charge** 2 **Spectral Angle** 0.87

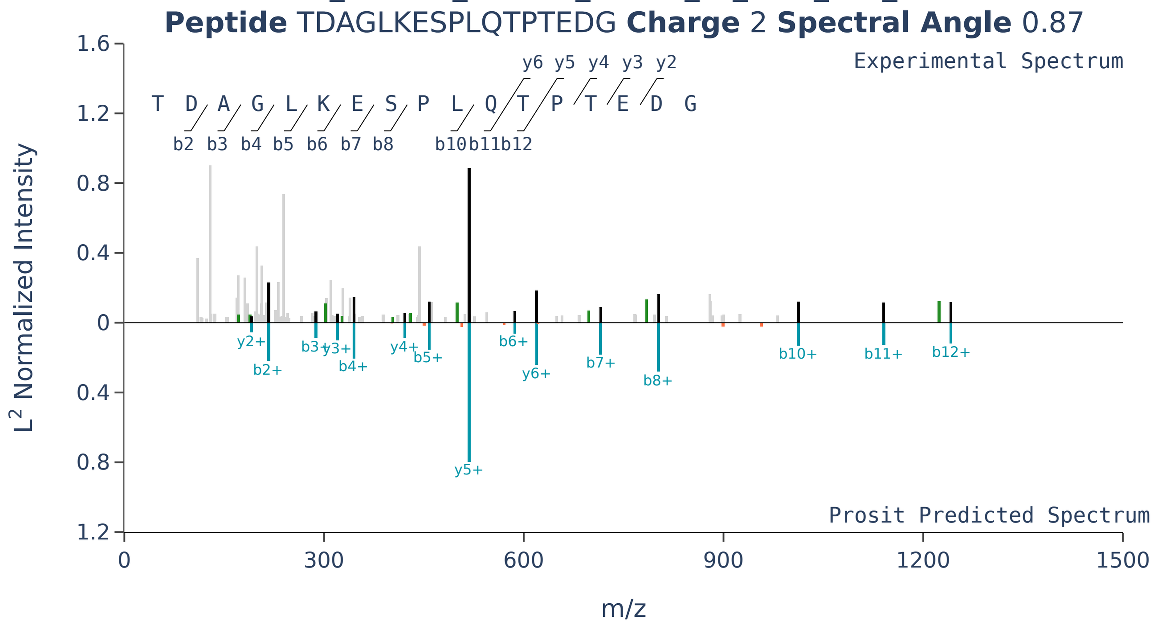


**Source** WSoh_221121_011221_HFGoe_L2_hTau_2h_R2 **Scan** 7911
**Peptide** HKLTFRENA **Charge** 2 **Spectral Angle** 0.93

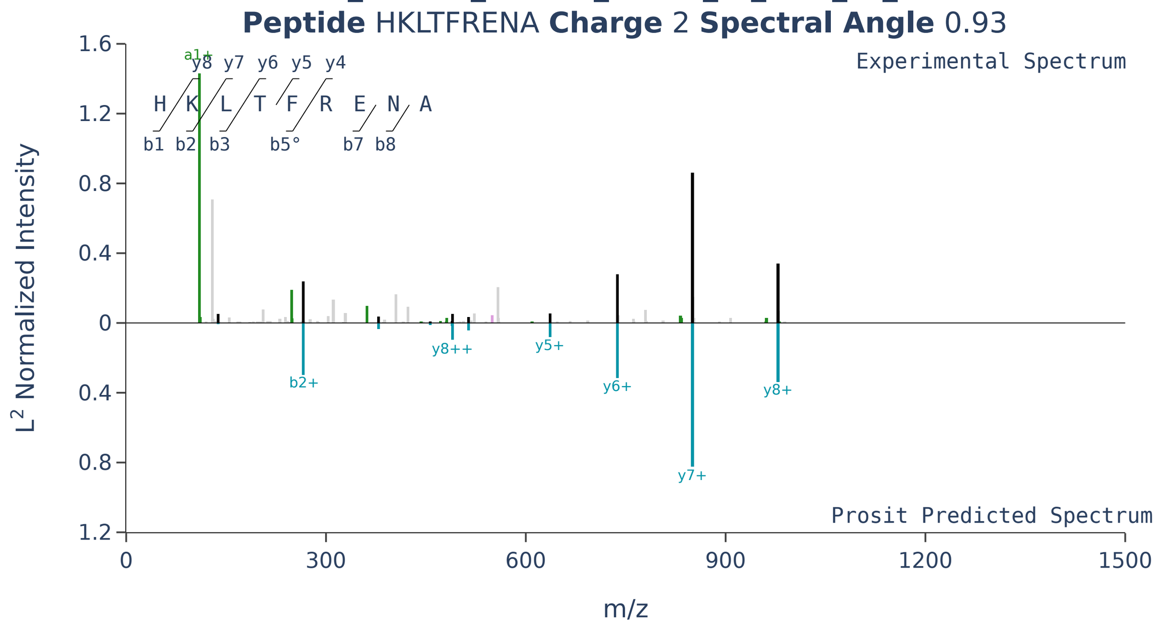


**Source** WSoh_221121_011221_HFGoe_L2_hTau_24h_R1 **Scan** 6795
**Peptide** DNITHVPGGGNKK **Charge** 2 **Spectral Angle** 0.93

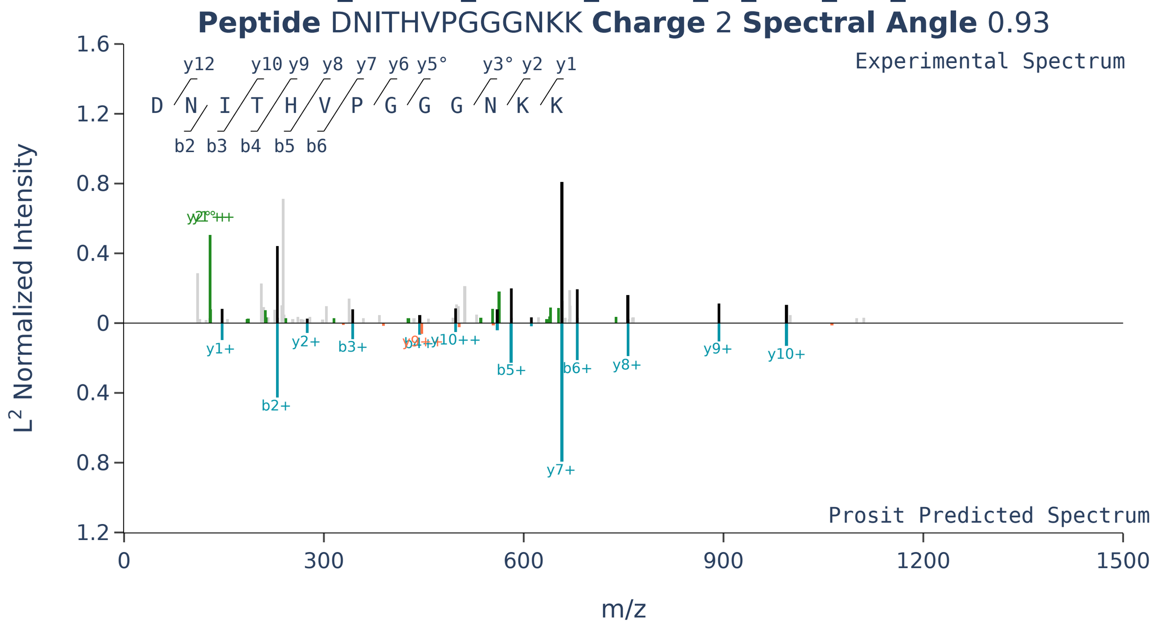


**Source** WSoh_221121_011221_HFGoe_L1_hTau_24h_R1 **Scan** 21913
**Peptide** EFEVMED **Charge** 2 **Spectral Angle** 0.79

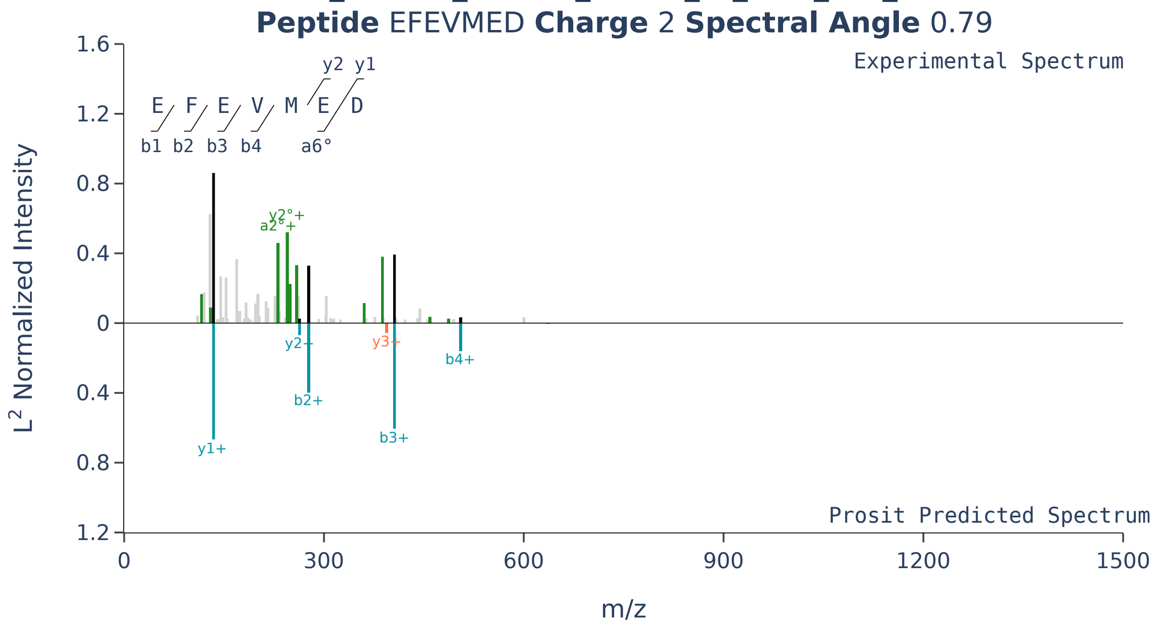


**Source** WSoh_221121_011221_HFGoe_L1_hTau_24h_R1 **Scan** 8883
**Peptide** VYKSPVVSGDTSPRH **Charge** 4 **Spectral Angle** 0.88

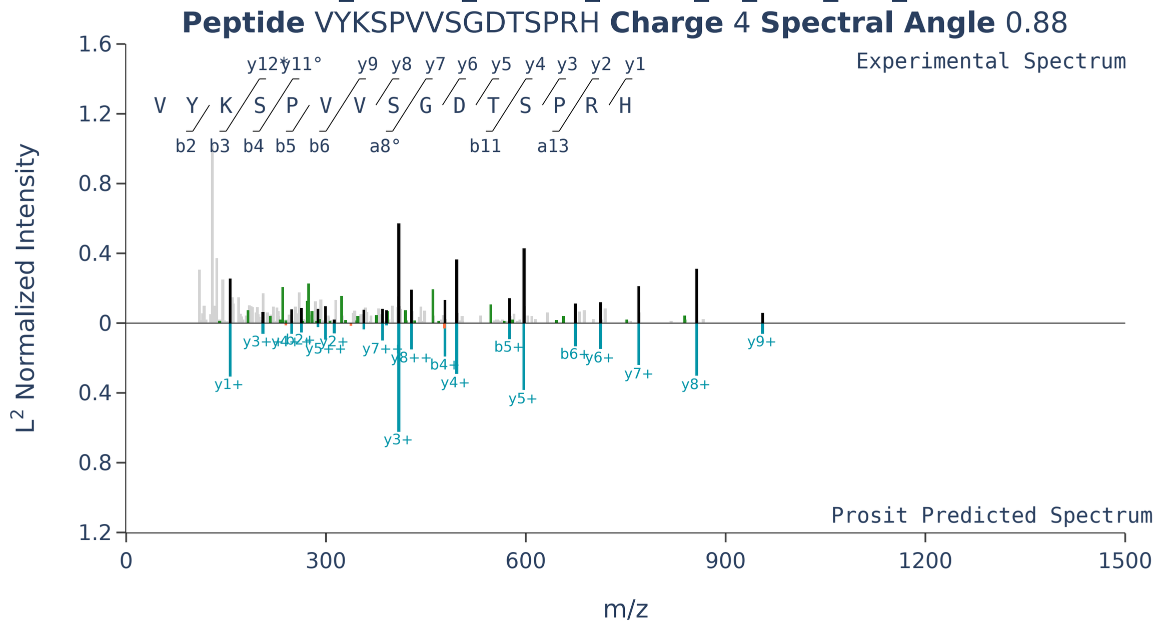


**Source** WSoh_221121_011221_HFGoe_L1_hTau_24h_R2 **Scan** 15781
**Peptide** PGGGQVEVKSEKLDFKDRV **Charge** 3 **Spectral Angle** 0.84

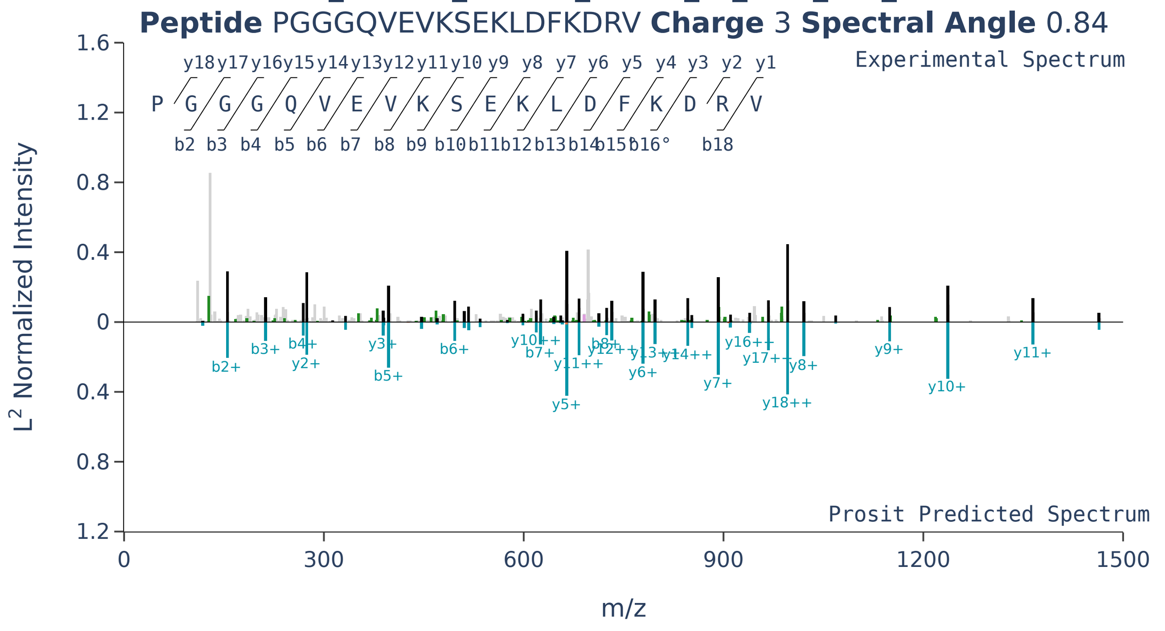


**Source** WSoh_221121_011221_HFGoe_L1_hTau_24h_R1 **Scan** 6369
**Peptide** DGKTKIATPRG **Charge** 2 **Spectral Angle** 0.93

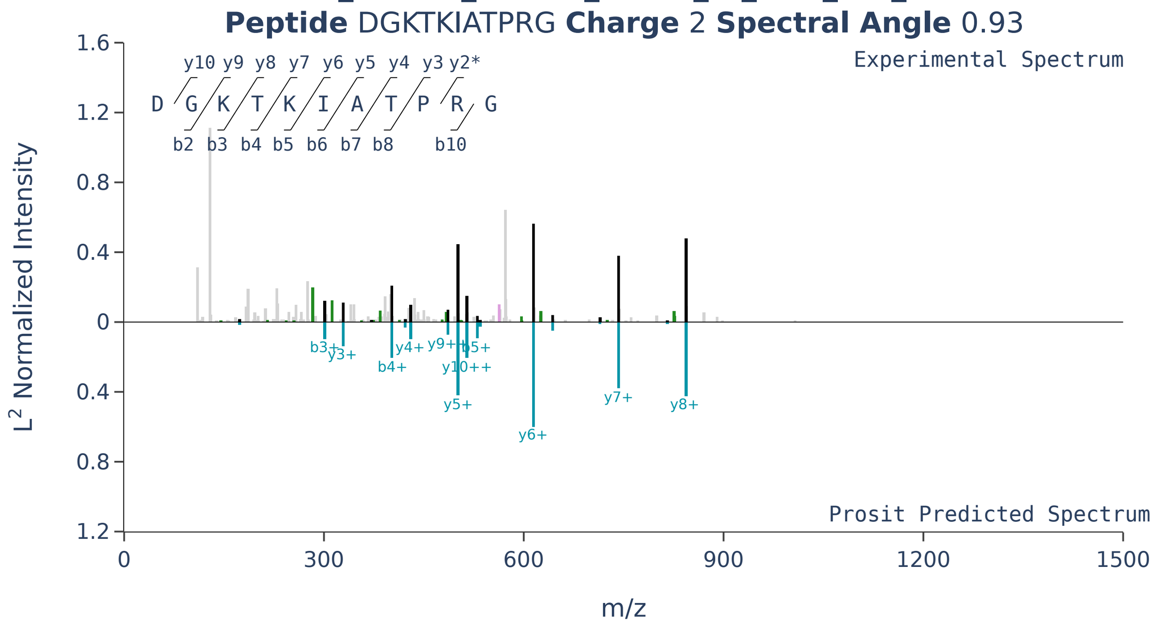


**Source** WSoh_221121_011221_HFGoe_L1_hTau_4h_R1 **Scan** 17657
**Peptide** AEPRQEFEVMEDHA **Charge** 2 **Spectral Angle** 0.88

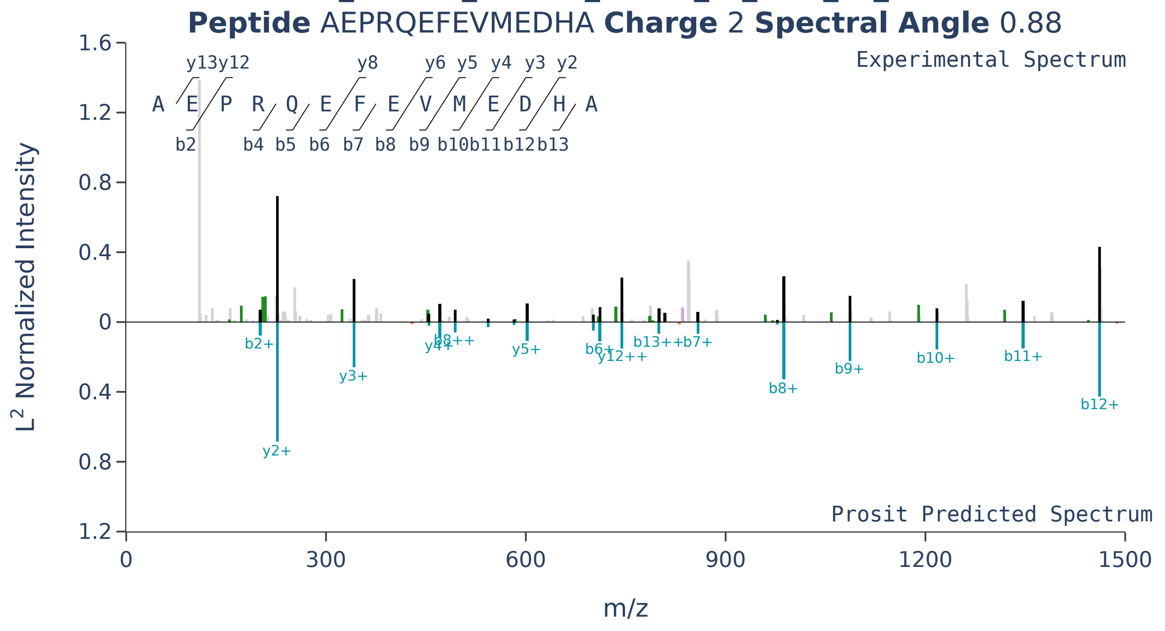


**Source** WSoh_221121_011221_HFGoe_L2_hTau_4h_R1 **Scan** 11178
**Peptide** SKIGSTENL **Charge** 2 **Spectral Angle** 0.95

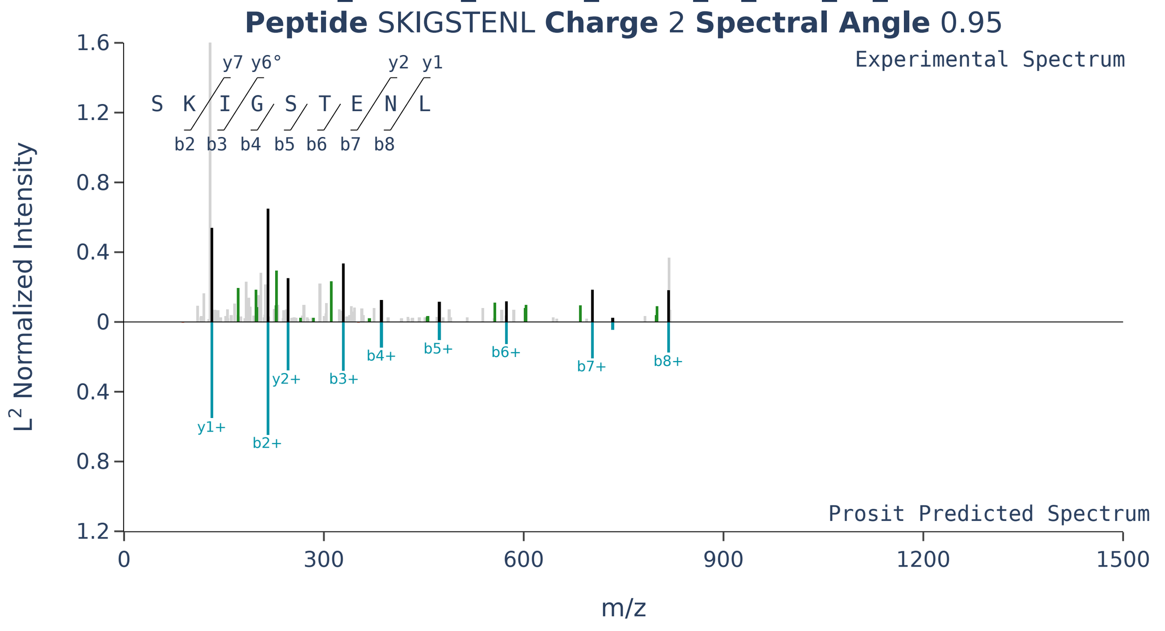


**Source** WSoh_221121_011221_HFGoe_L1_hTau_2h_R2 **Scan** 13475
**Peptide** AQPHTEIPEGTTAEEA **Charge** 2 **Spectral Angle** 0.92

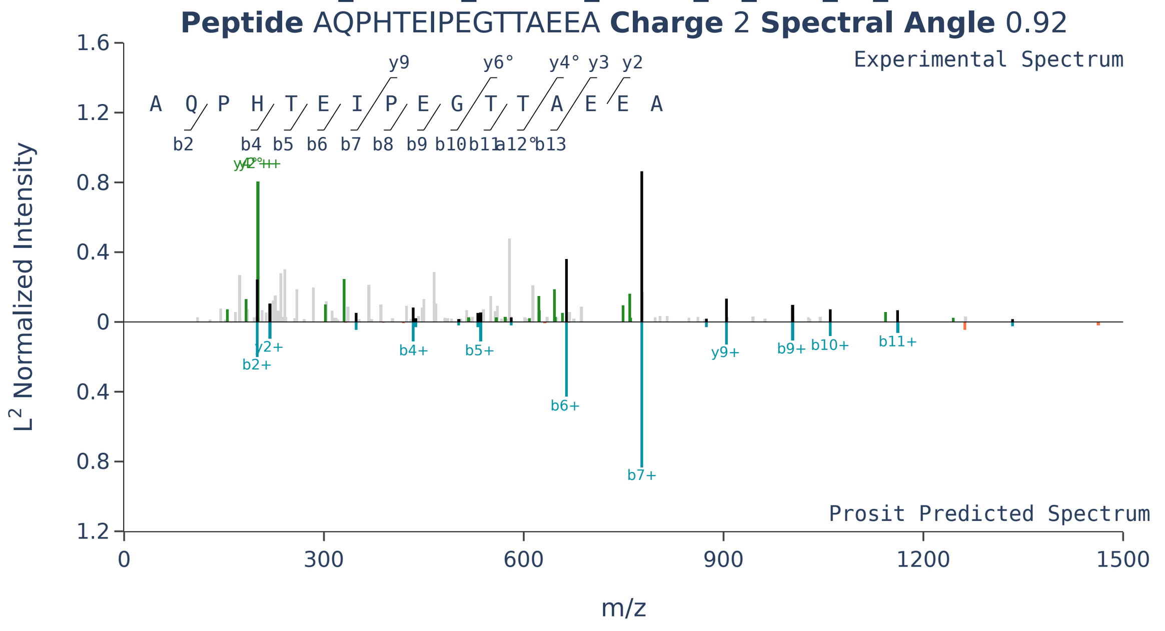


**Source** WSoh_221121_011221_HFGoe_L1_hTau_2h_R2 **Scan** 12032
**Peptide** AAAQPHTEIPEGT **Charge** 2 **Spectral Angle** 0.89

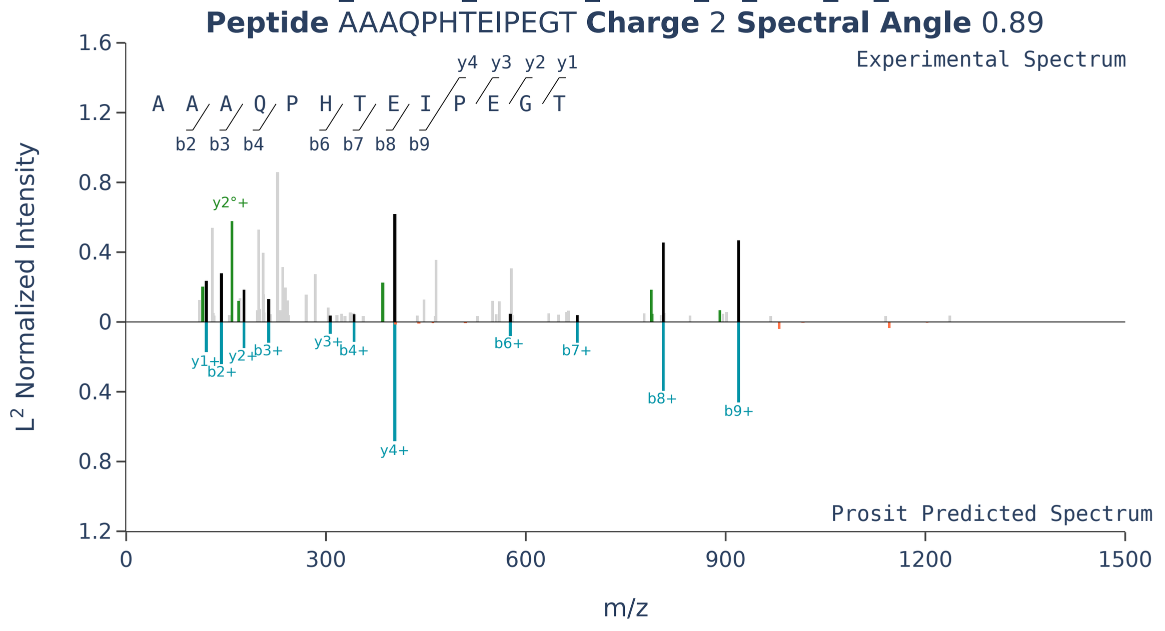

**Source** WSoh_221121_011221_HFGoe_L1_hTau_24h_R1 **Scan** 11304
**Peptide** AQPHTEIPEGT **Charge** 1 **Spectral Angle** 0.91

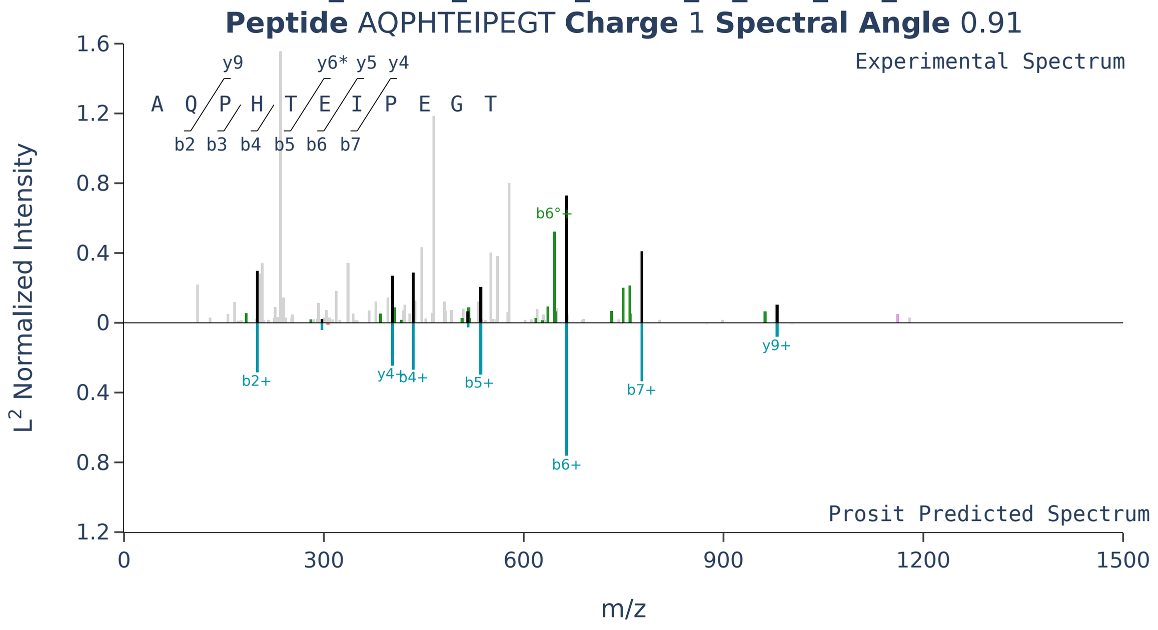


**Source** WSoh_221121_011221_HFGoe_L1_hTau_24h_R2 **Scan** 12731
**Peptide** PGGGQVEV **Charge** 2 **Spectral Angle** 0.92

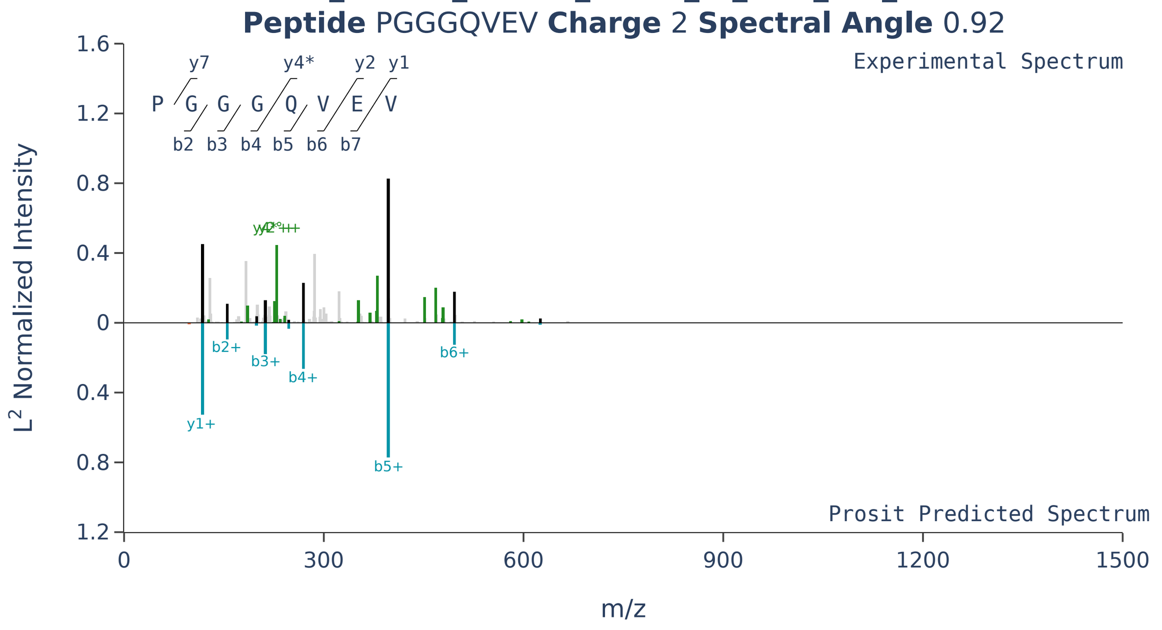


**Source** WSoh_221121_011221_HFGoe_L1_hTau_24h_R1 **Scan** 8329
**Peptide** LATPRGAAPPGQKGQ **Charge** 3 **Spectral Angle** 0.9

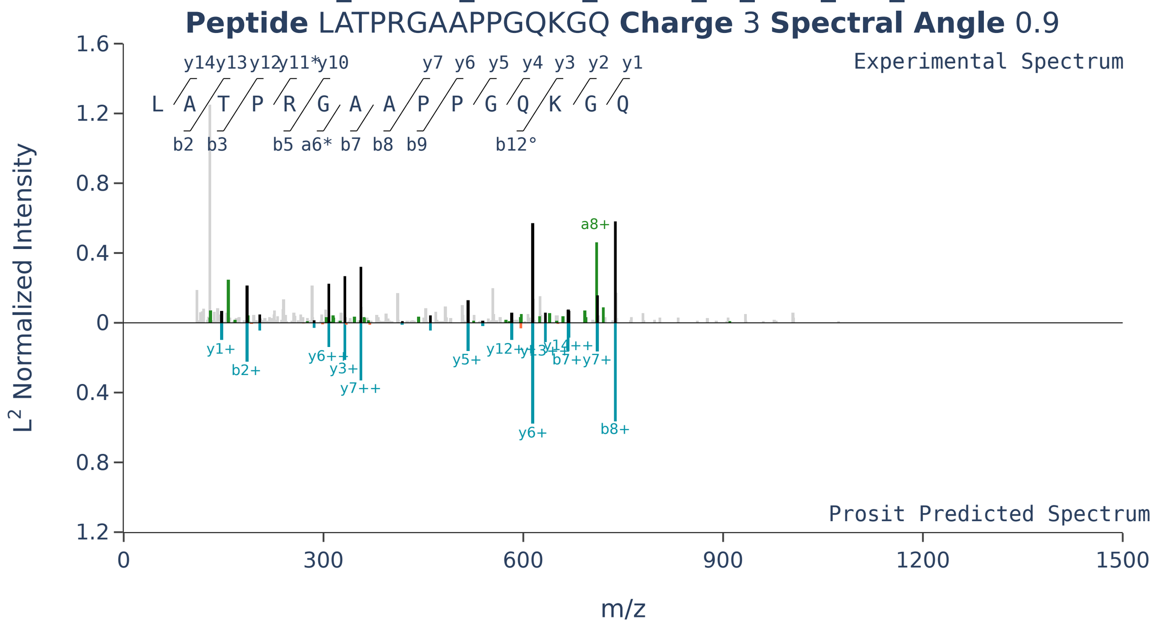


**Source** WSoh_221121_011221_HFGoe_L1_hTau_24h_R1 **Scan** 17163
**Peptide** HQDQEGDTDAGLKESPLQTPTED **Charge** 2 **Spectral Angle** 0.9

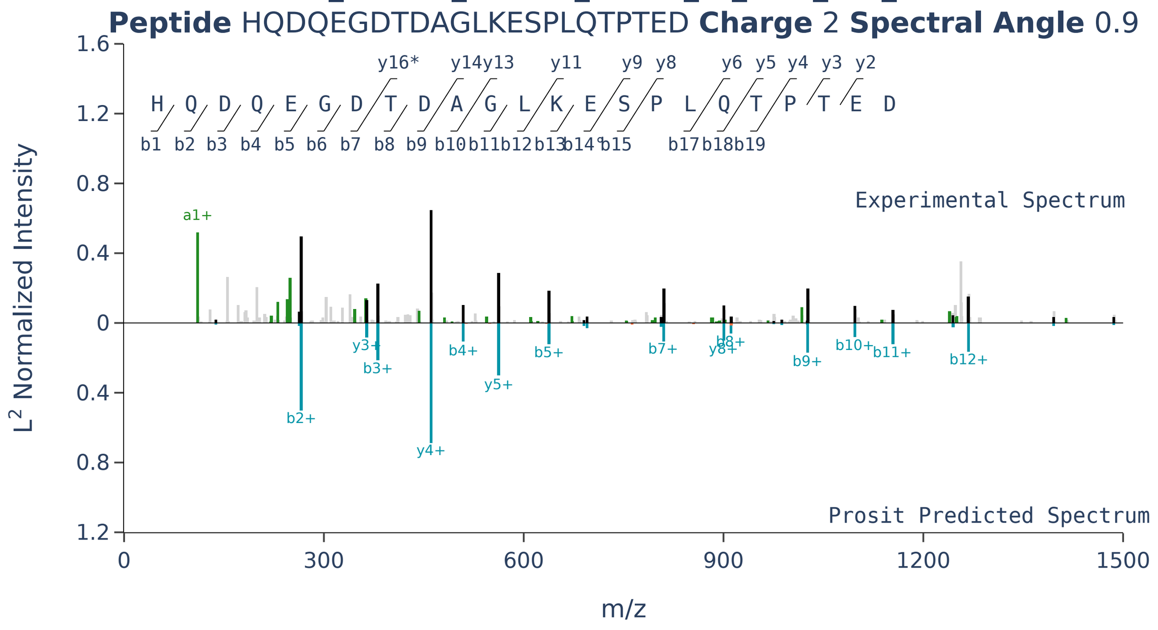


**Source** WSoh_221121_011221_HFGoe_L1_hTau_24h_R1 **Scan** 21183
**Peptide** QTAPVPMPDLKNVKS **Charge** 2 **Spectral Angle** 0.91

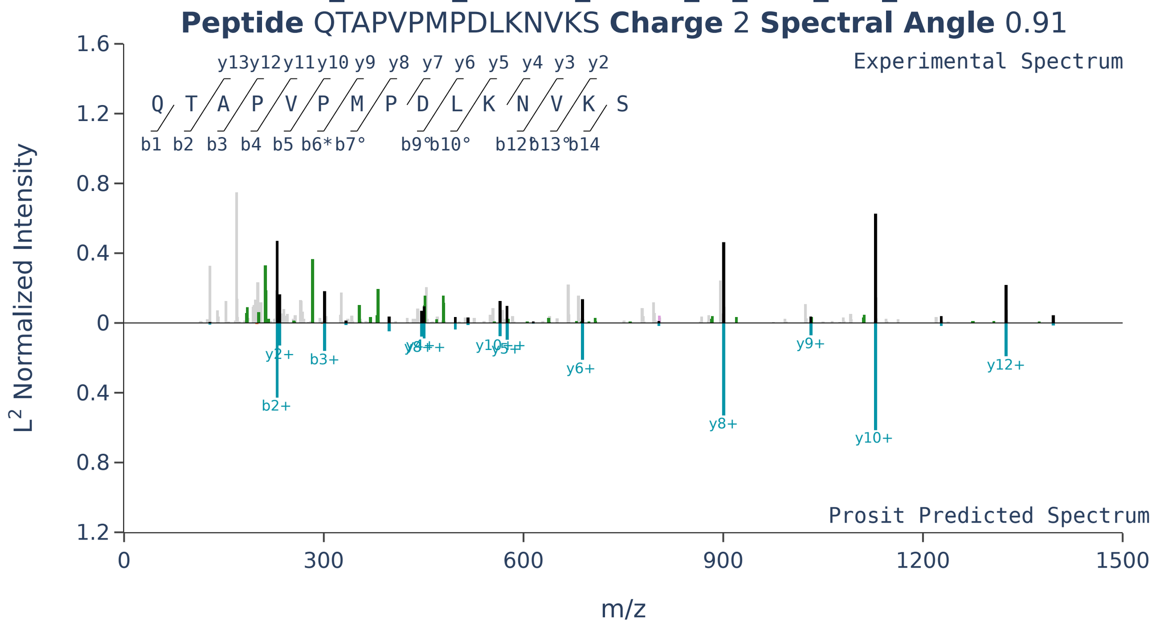


**Source** WSoh_221121_011221_HFGoe_L2_hTau_4h_R2 **Scan** 14936
**Peptide** GSLDNITHVPGGGNK **Charge** 3 **Spectral Angle** 0.92

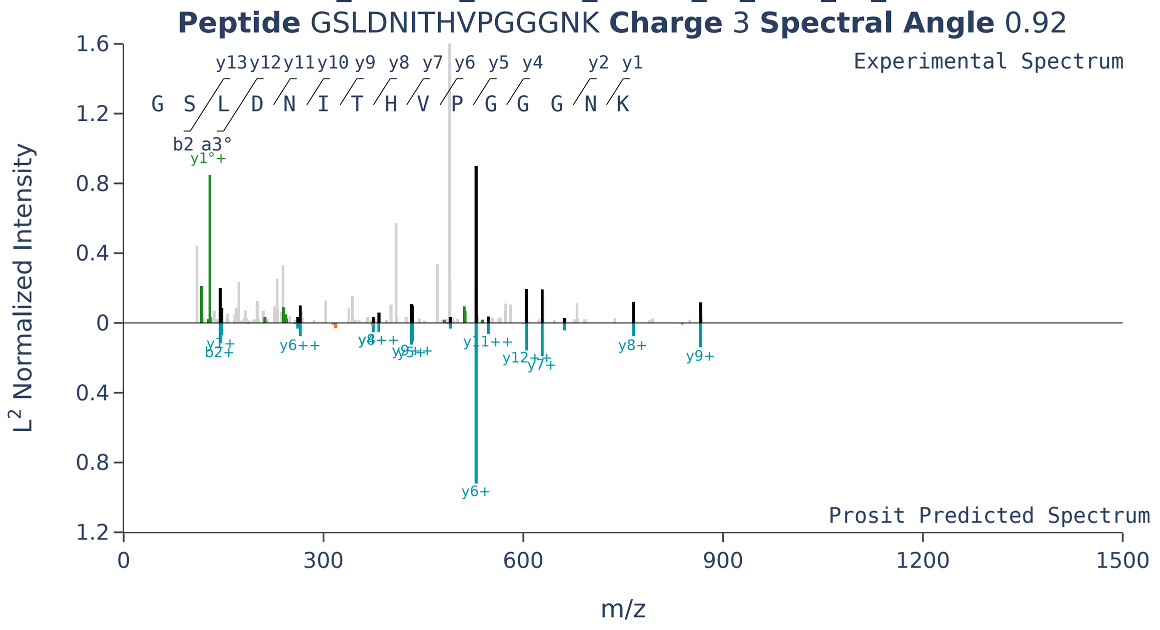


**Source** WSoh_221121_011221_HFGoe_L1_hTau_4h_R1 **Scan** 17432
**Peptide** STGSIDMVD **Charge** 2 **Spectral Angle** 0.9

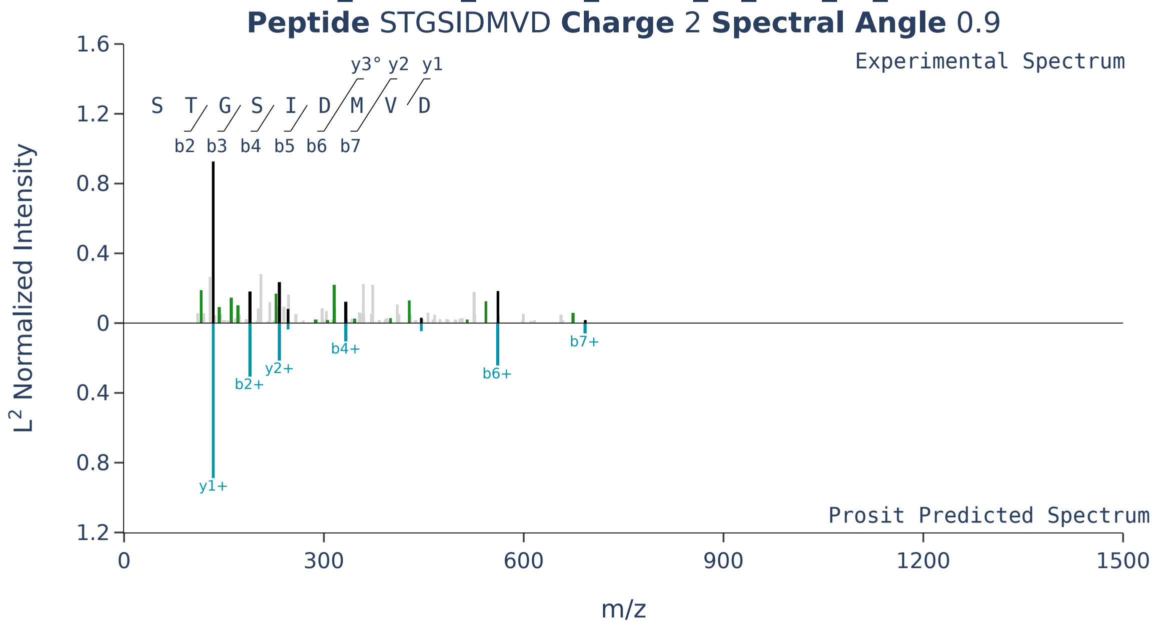


**Source** WSoh_221121_011221_HFGoe_L2_hTau_4h_R2 **Scan** 5827
**Peptide** GSEEPGSETSDAKST **Charge** 2 **Spectral Angle** 0.91

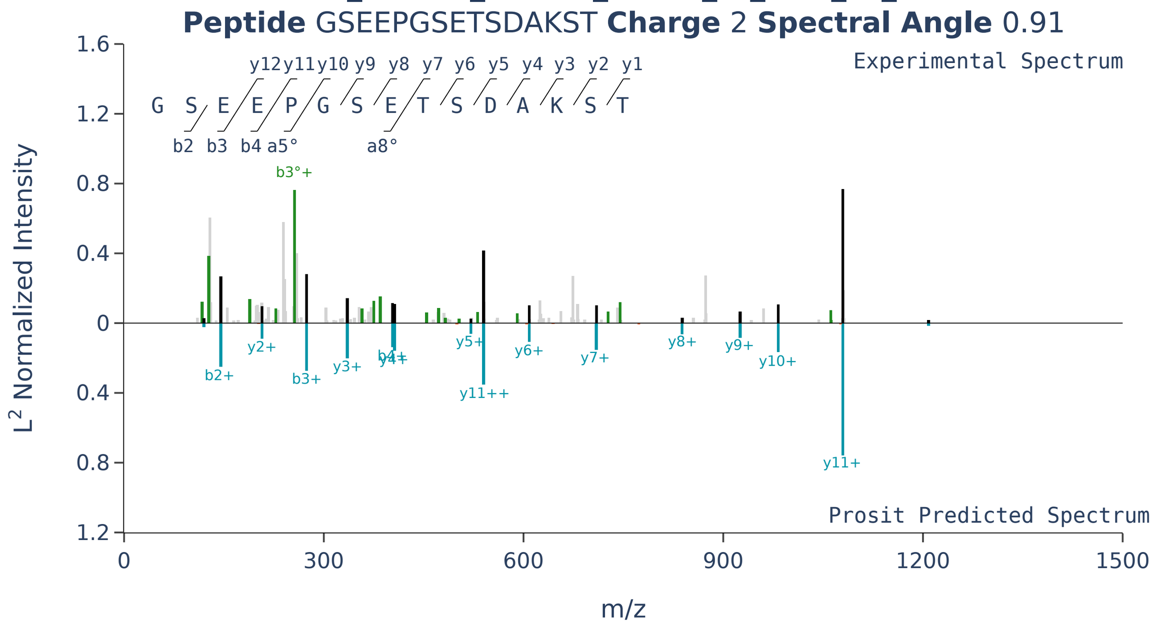


**Source** WSoh_221121_011221_HFGoe_L1_hTau_24h_R2 **Scan** 10549
**Peptide** LQTPTED **Charge** 2 **Spectral Angle** 0.83

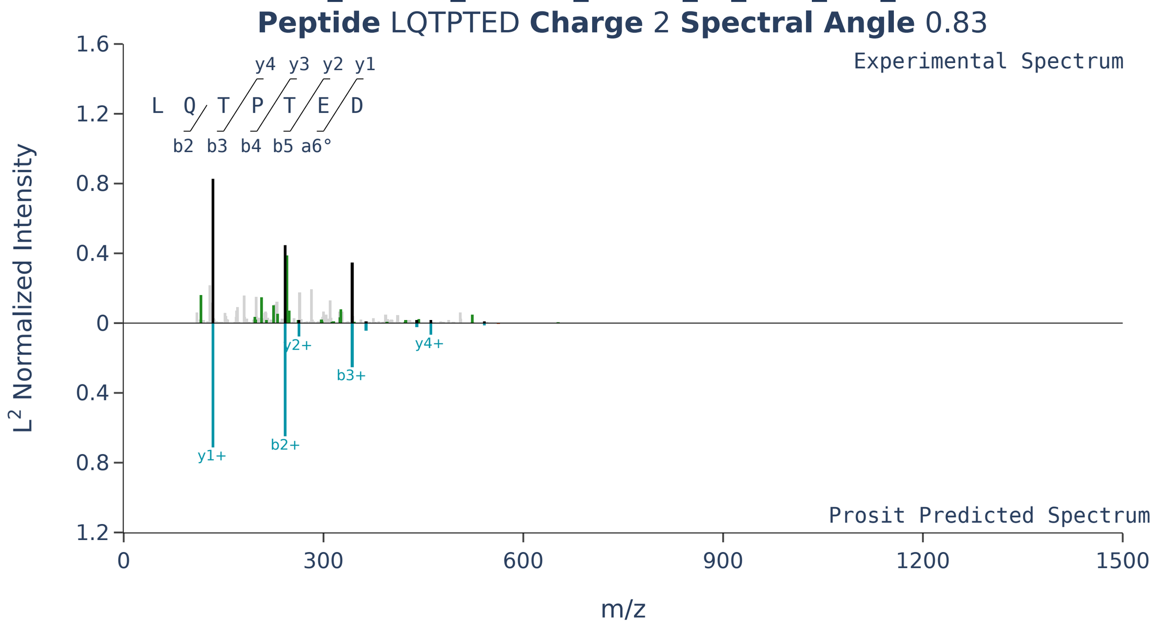


**Source** WSoh_221121_011221_HFGoe_L2_hTau_24h_R2 **Scan** 14172
**Peptide** GAEIVYKSP **Charge** 2 **Spectral Angle** 0.89

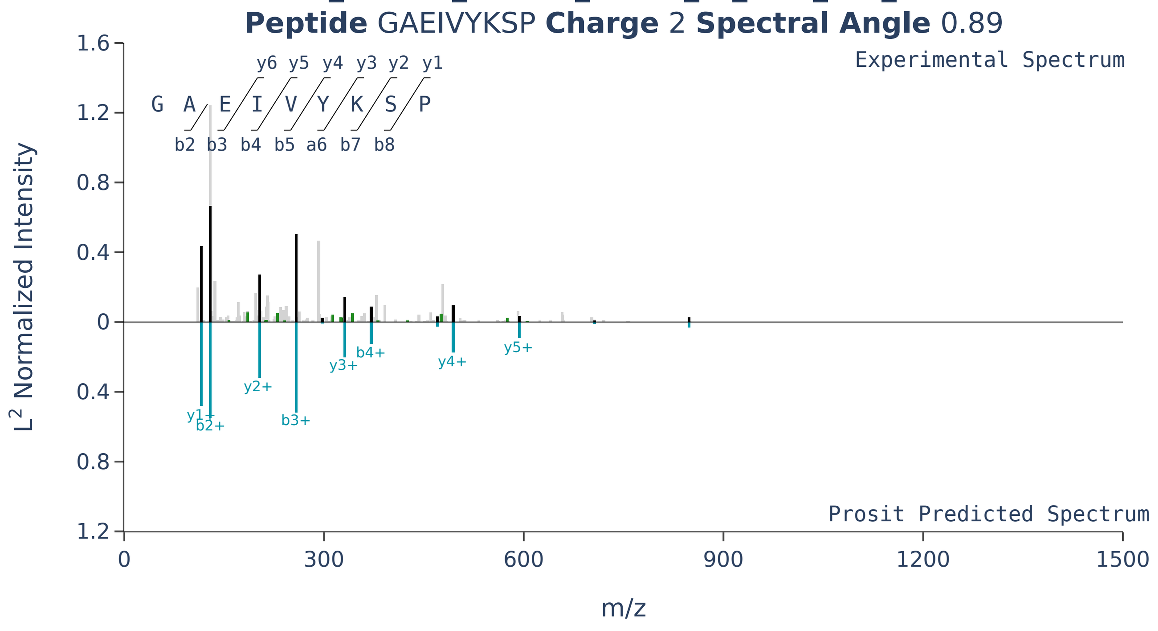


**Source** WSoh_221121_011221_HFGoe_L1_hTau_4h_R1 **Scan** 18630
**Peptide** KIGSLDNITHV **Charge** 2 **Spectral Angle** 0.84

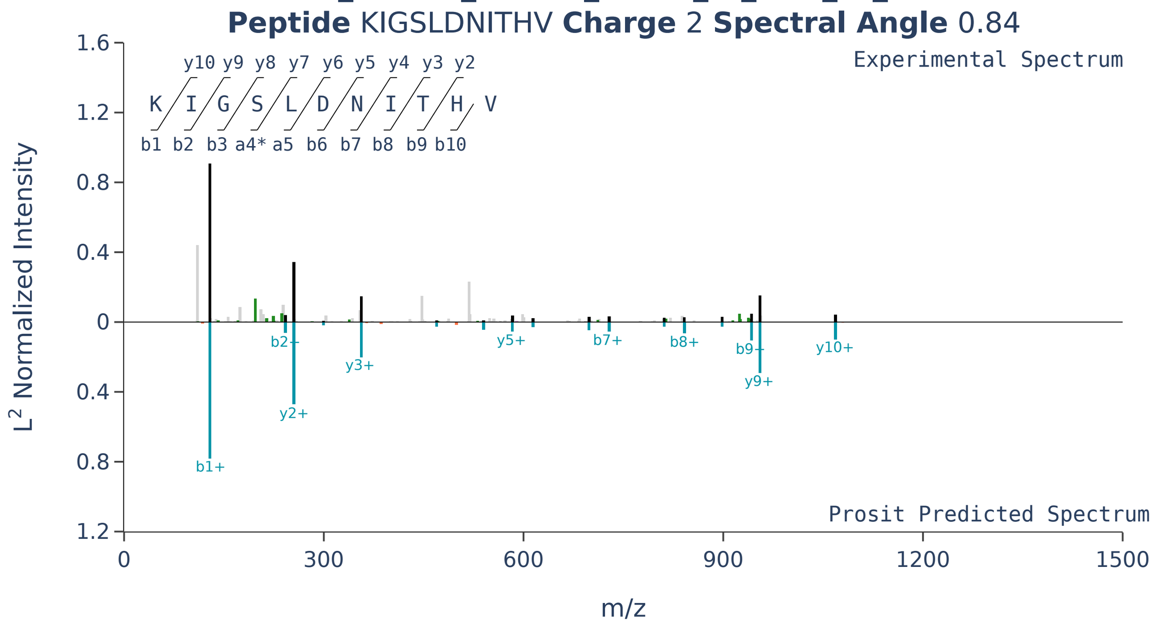


**Source** WSoh_221121_011221_HFGoe_L1_hTau_2h_R1 **Scan** 9451
**Peptide** EAAGHVTQARMV **Charge** 2 **Spectral Angle** 0.92

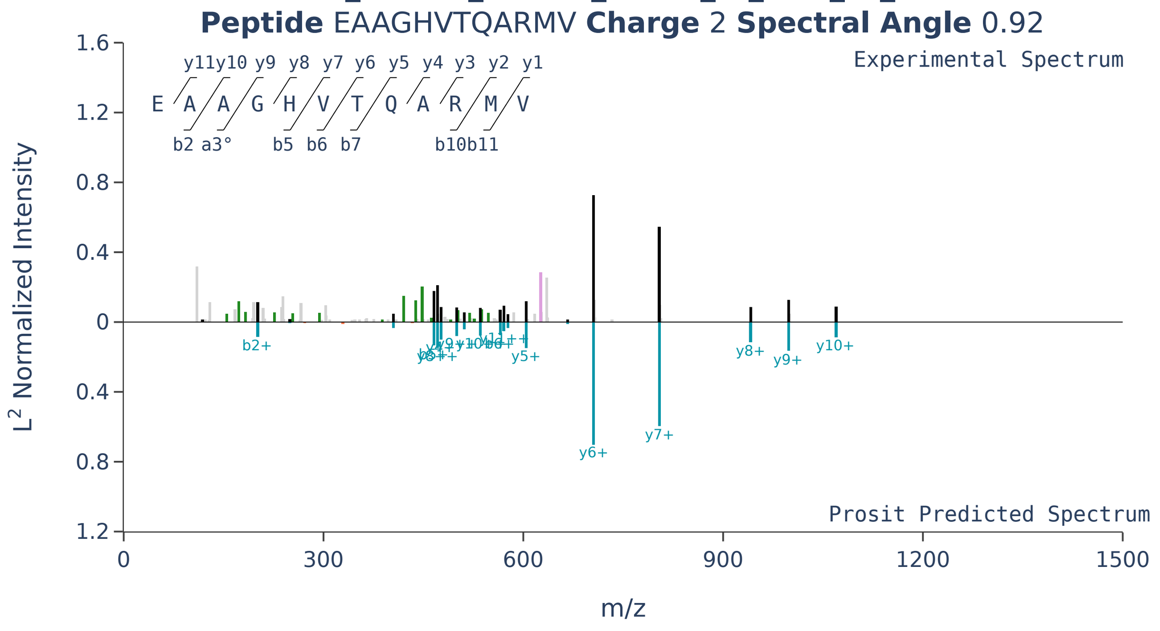


**Source** WSoh_221121_011221_HFGoe_L1_hTau_4h_R2 **Scan** 17353
**Peptide** VTAPLVDEGAPGKQAA **Charge** 2 **Spectral Angle** 0.94

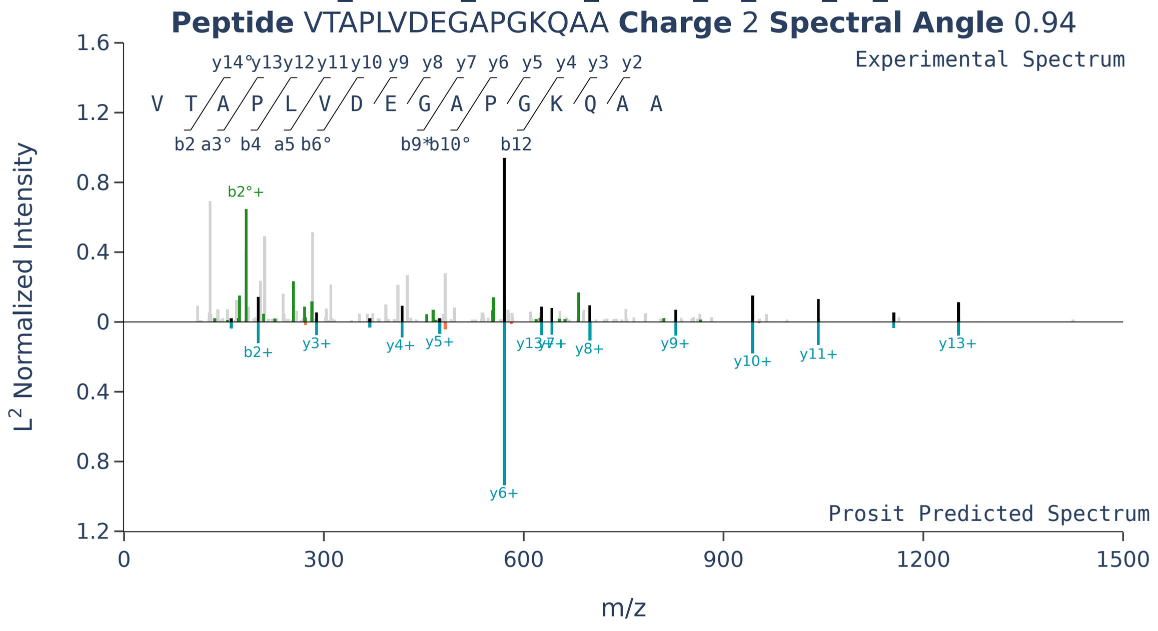


**Source** WSoh_221121_011221_HFGoe_L1_hTau_4h_R2 **Scan** 15038
**Peptide** DLSKVTSK **Charge** 2 **Spectral Angle** 0.93

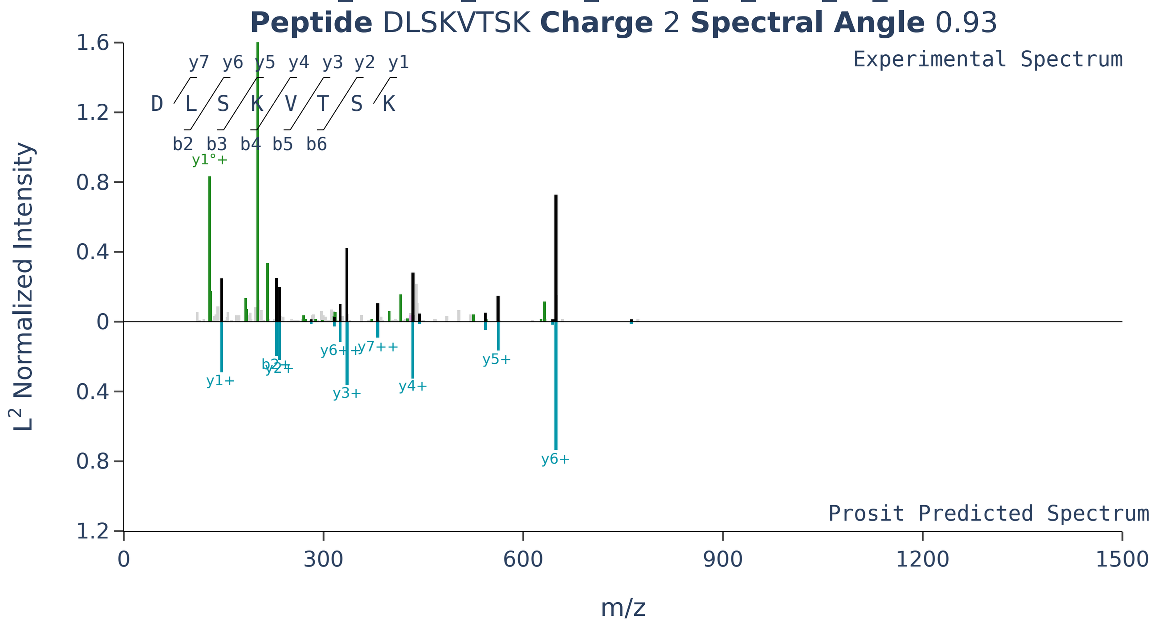


**Source** WSoh_221121_011221_HFGoe_L1_hTau_24h_R2 **Scan** 19335
**Peptide** AGIGDTPSLEDEAAGHVTQAR **Charge** 3 **Spectral Angle** 0.87

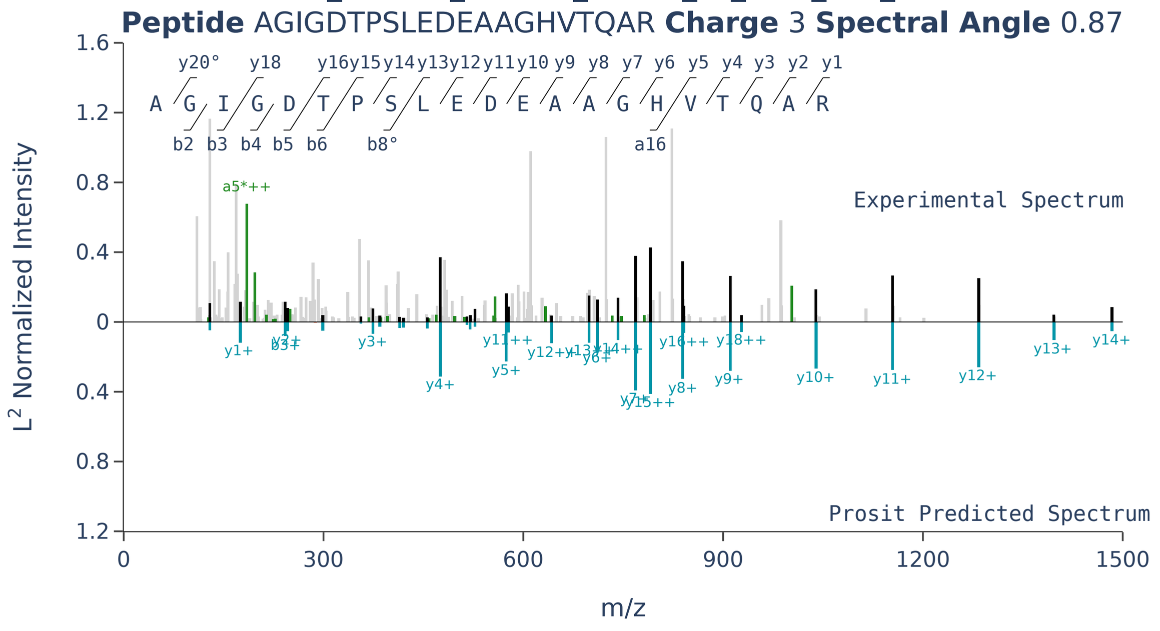

**Source** WSoh_221121_011221_HFGoe_L1_hTau_24h_R1 **Scan** 21118
**Peptide** GDTPSLEDEAAGHVTQARMV **Charge** 2 **Spectral Angle** 0.88

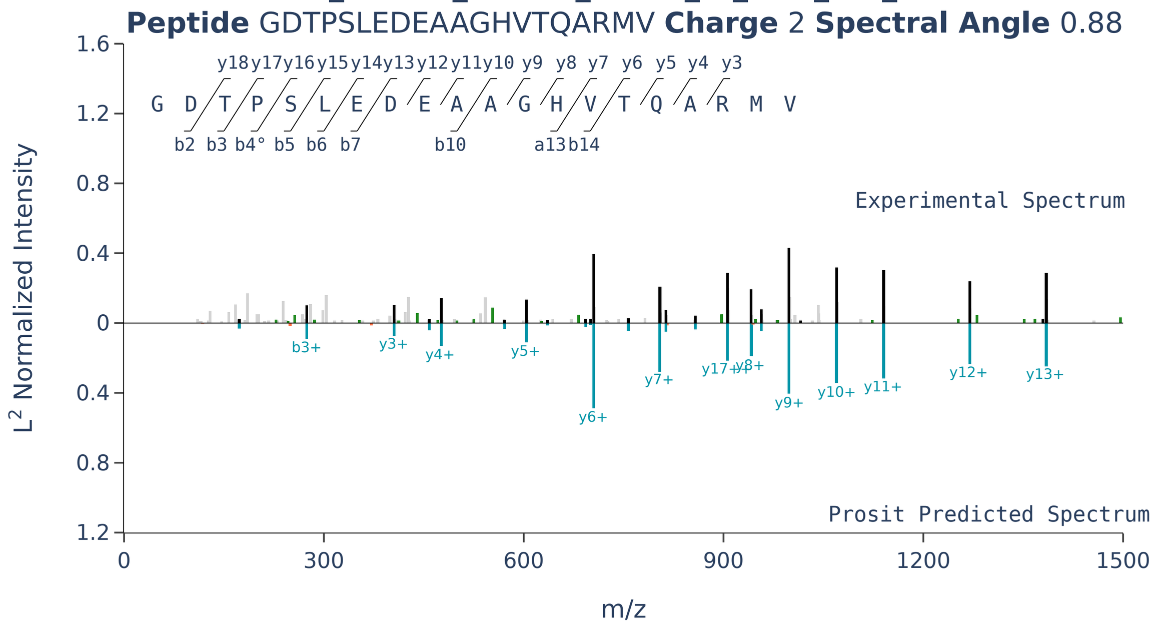


**Source** WSoh_221121_011221_HFGoe_L1_hTau_2h_R1 **Scan** 18060
**Peptide** GDTSPRHLSNVSSTGSIDMV **Charge** 3 **Spectral Angle** 0.86

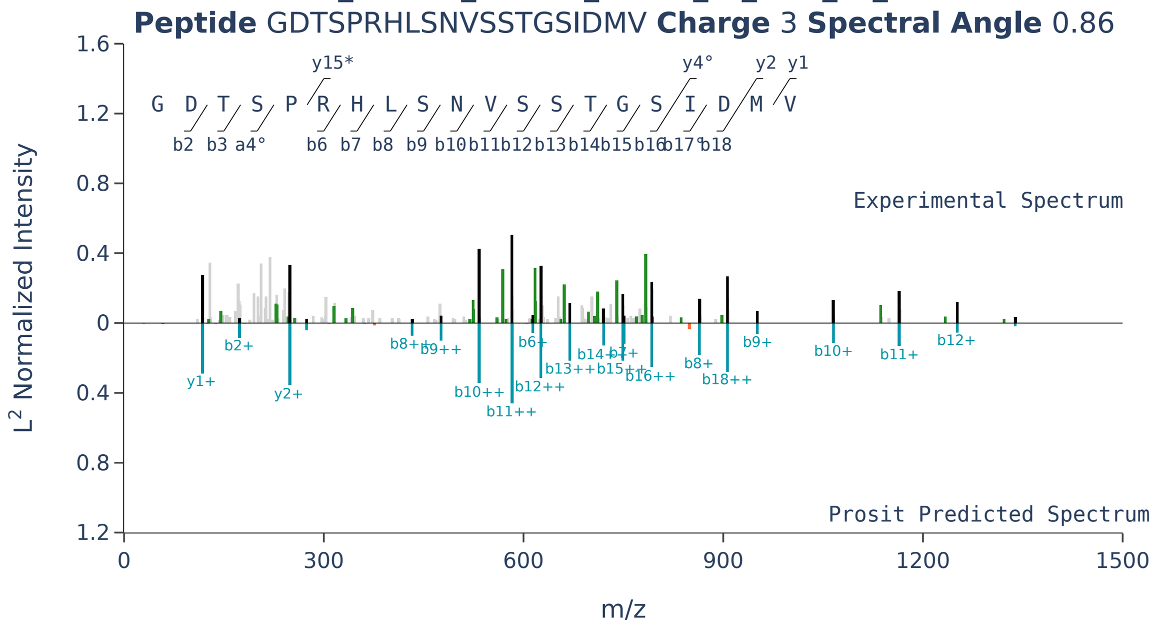


**Source** WSoh_221121_011221_HFGoe_L2_hTau_24h_R2 **Scan** 22567
**Peptide** IGSLDNITHV **Charge** 2 **Spectral Angle** 0.85

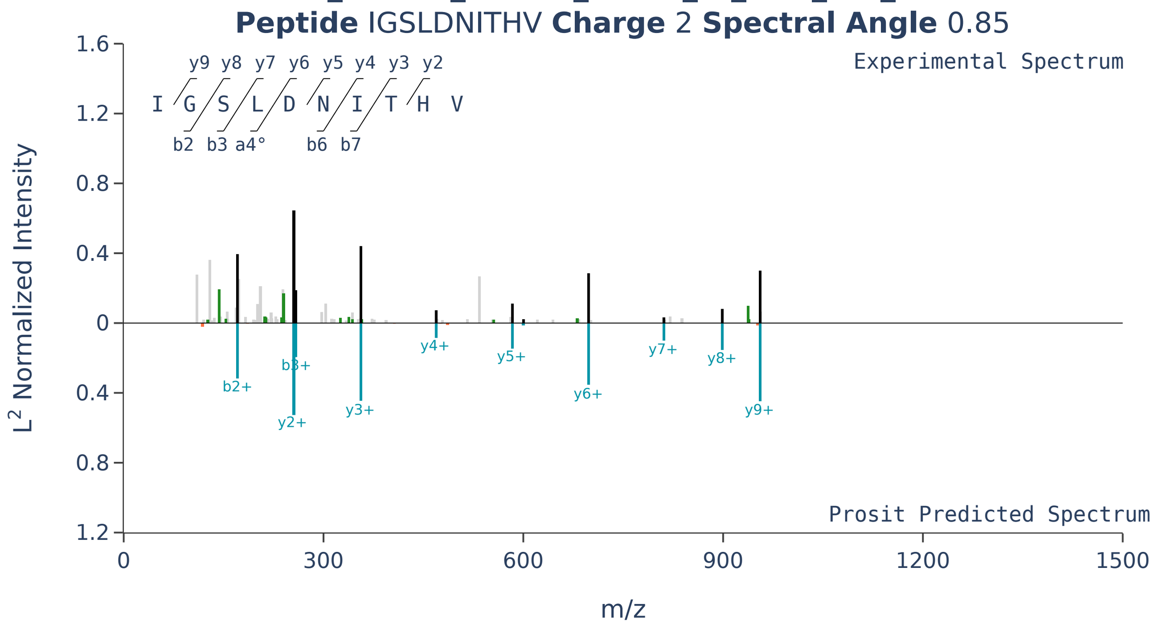


**Source** WSoh_221121_011221_HFGoe_L1_hTau_24h_R1 **Scan** 19009
**Peptide** DAGLKESPLQTPTED **Charge** 2 **Spectral Angle** 0.91

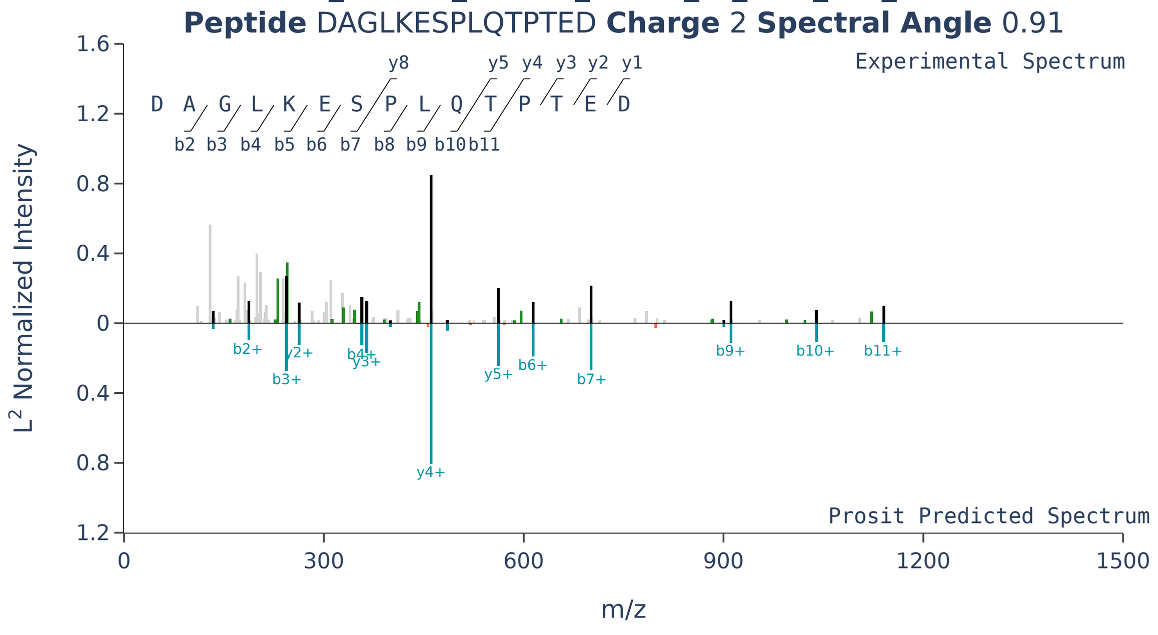


**Source** WSoh_221121_011221_HFGoe_L2_hTau_24h_R2 **Scan** 20903
**Peptide** VQIINKKLDL **Charge** 2 **Spectral Angle** 0.91

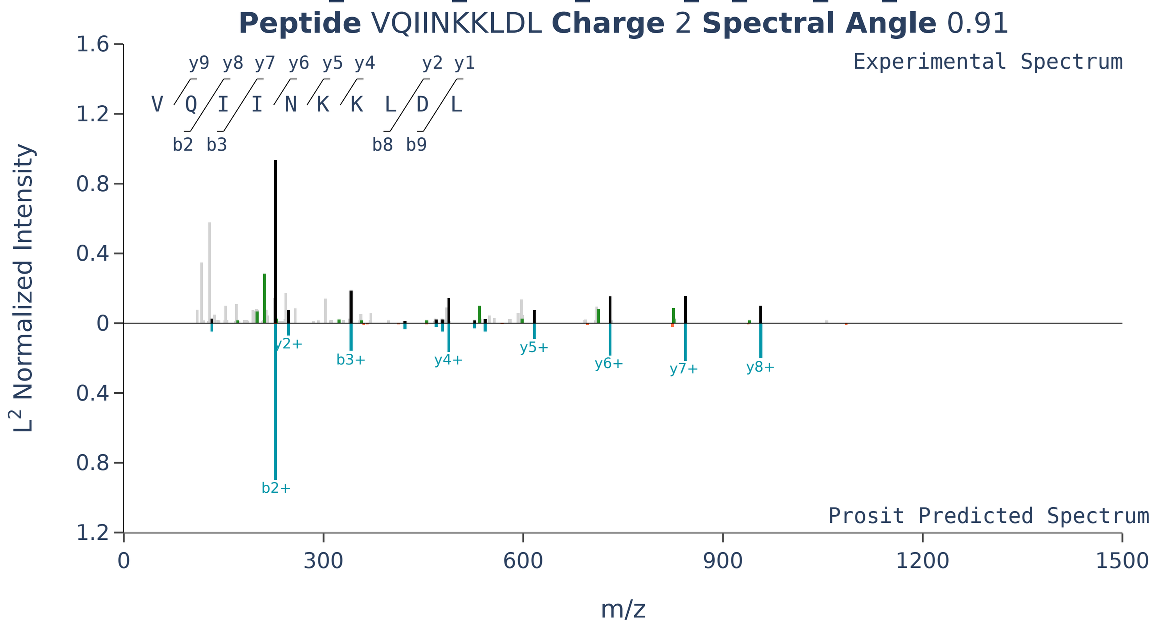


**Source** WSoh_221121_011221_HFGoe_L2_hTau_4h_R1 **Scan** 13127
**Peptide** DFKDRVQSKL **Charge** 2 **Spectral Angle** 0.85

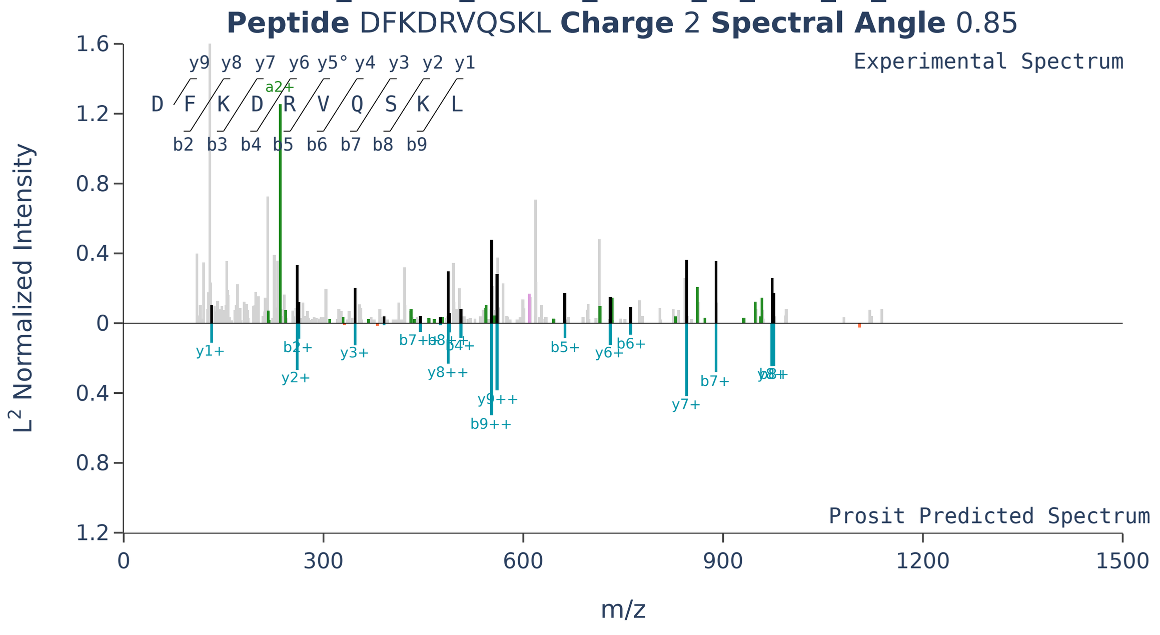


**Source** WSoh_221121_011221_HFGoe_L1_hTau_4h_R1 **Scan** 5944
**Peptide** GDRKDQGGYTMHQD **Charge** 2 **Spectral Angle** 0.85

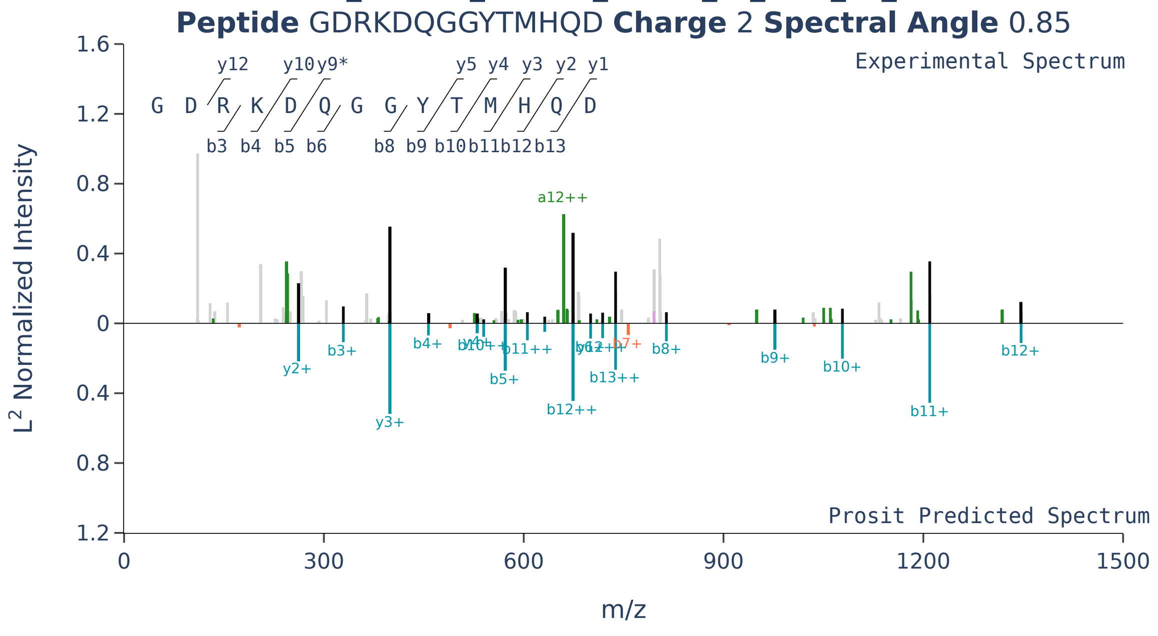


**Source** WSoh_221121_011221_HFGoe_L1_hTau_24h_R2 **Scan** 11582
**Peptide** VYKSPVVSGDTSPRHLSN **Charge** 2 **Spectral Angle** 0.91

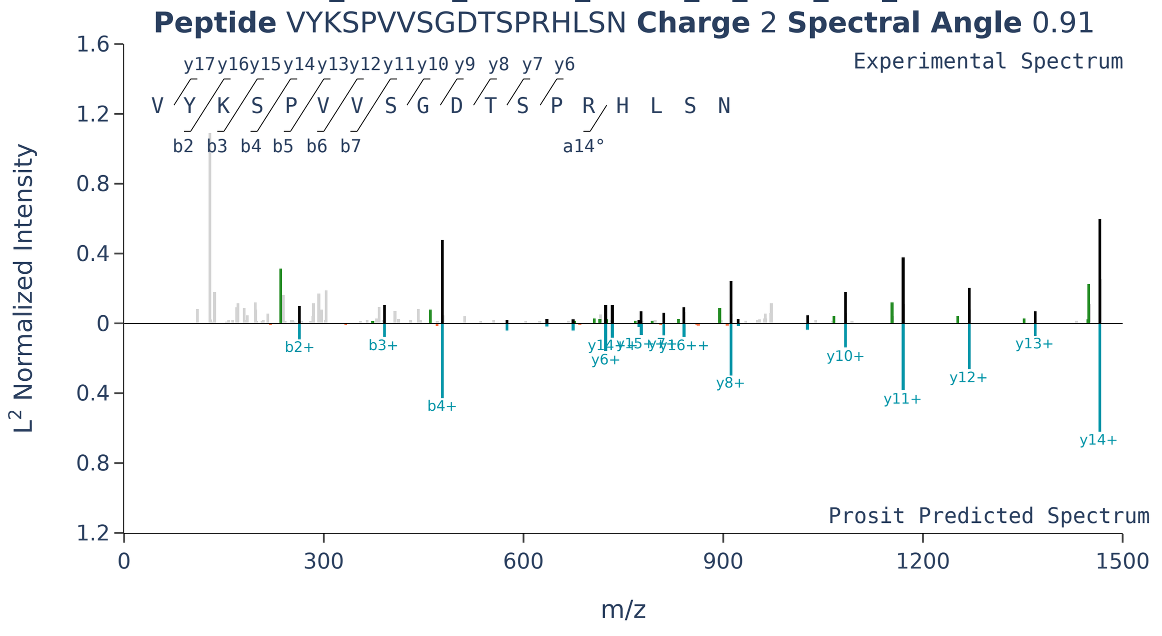


**Source** WSoh_221121_011221_HFGoe_L2_hTau_24h_R1 **Scan** 21076
**Peptide** EEAGIGDTPSLE **Charge** 2 **Spectral Angle** 0.94

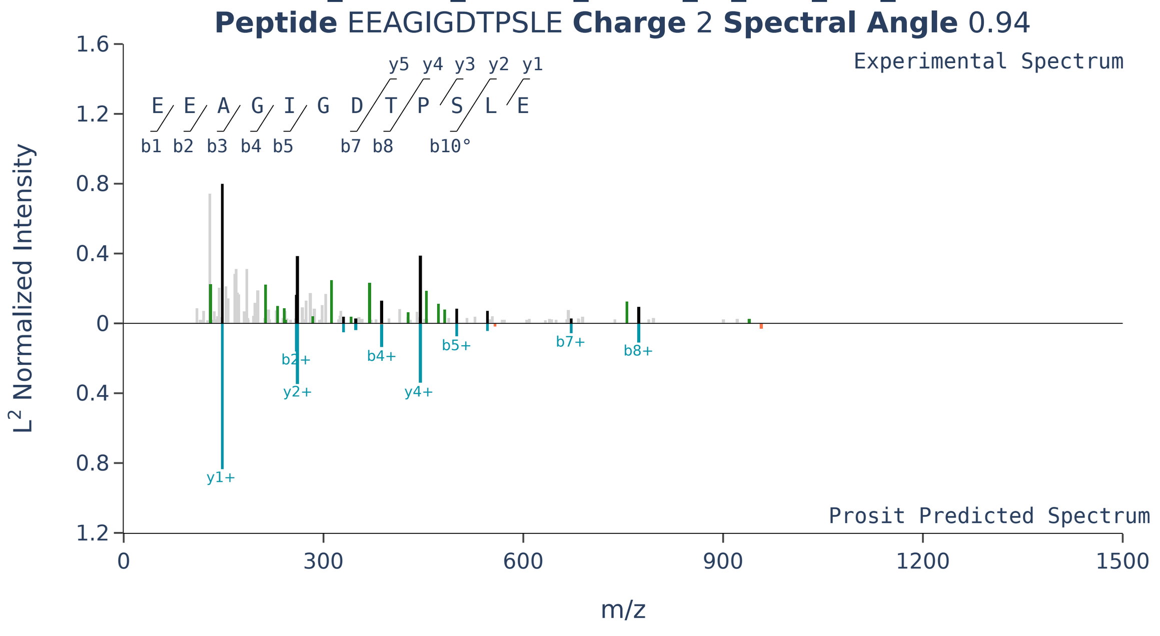


**Source** WSoh_221121_011221_HFGoe_L2_hTau_24h_R2 **Scan** 23861
**Peptide** DSPQLATL **Charge** 2 **Spectral Angle** 0.79

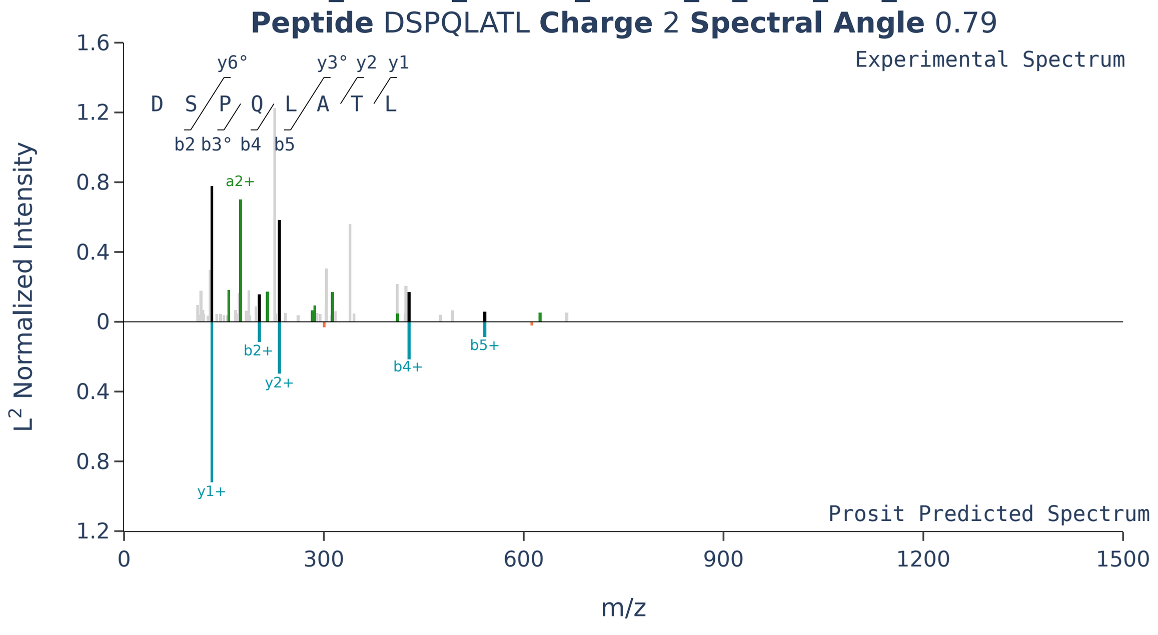


**Source** WSoh_221121_011221_HFGoe_L2_hTau_2h_R1 **Scan** 11416
**Peptide** SGDTSPRHLSNVSSTGSID **Charge** 3 **Spectral Angle** 0.92

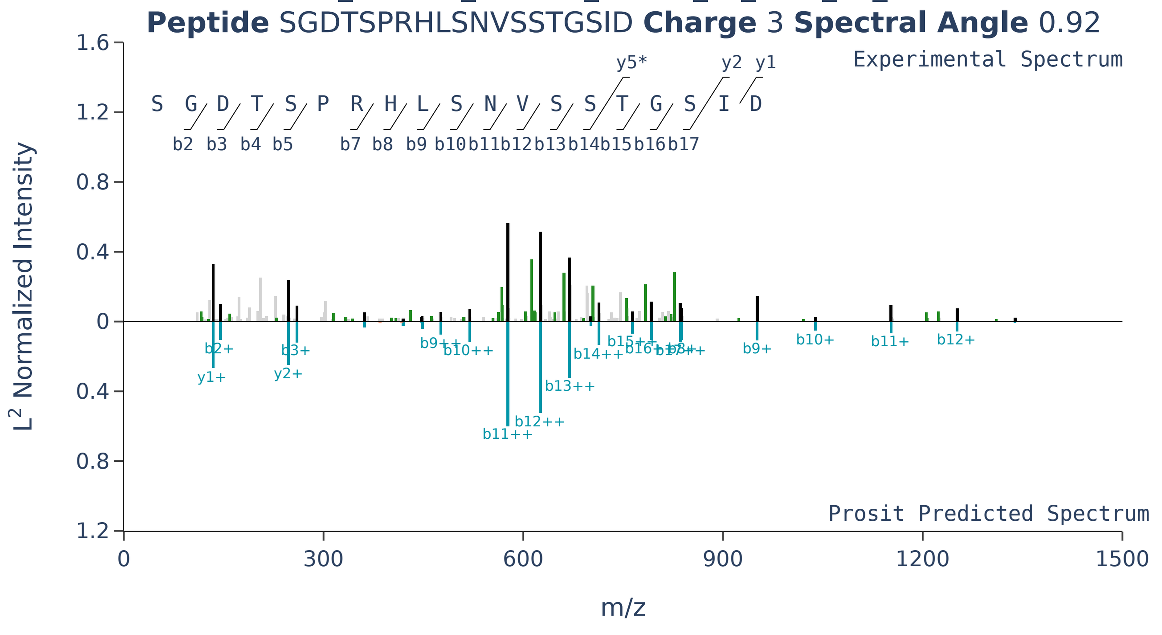


**Source** WSoh_221121_011221_HFGoe_L2_hTau_4h_R2 **Scan** 12024
**Peptide** DFKDRVQSKIG **Charge** 2 **Spectral Angle** 0.89

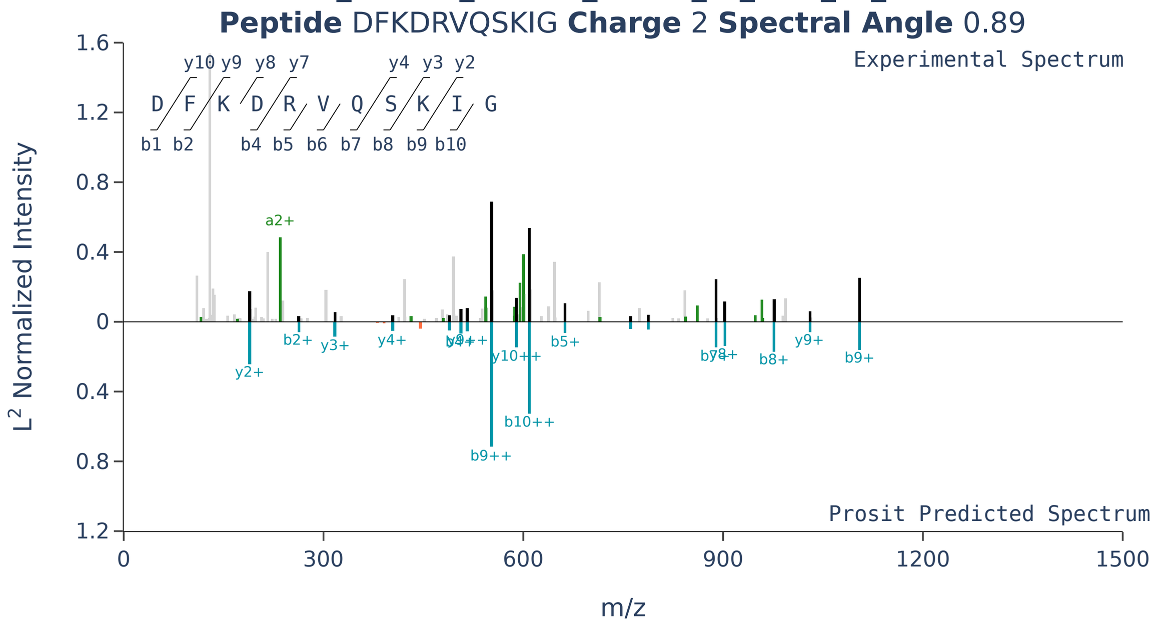


**Source** WSoh_221121_011221_HFGoe_L1_hTau_4h_R2 **Scan** 10066
**Peptide** AEPRQEFE **Charge** 2 **Spectral Angle** 0.9

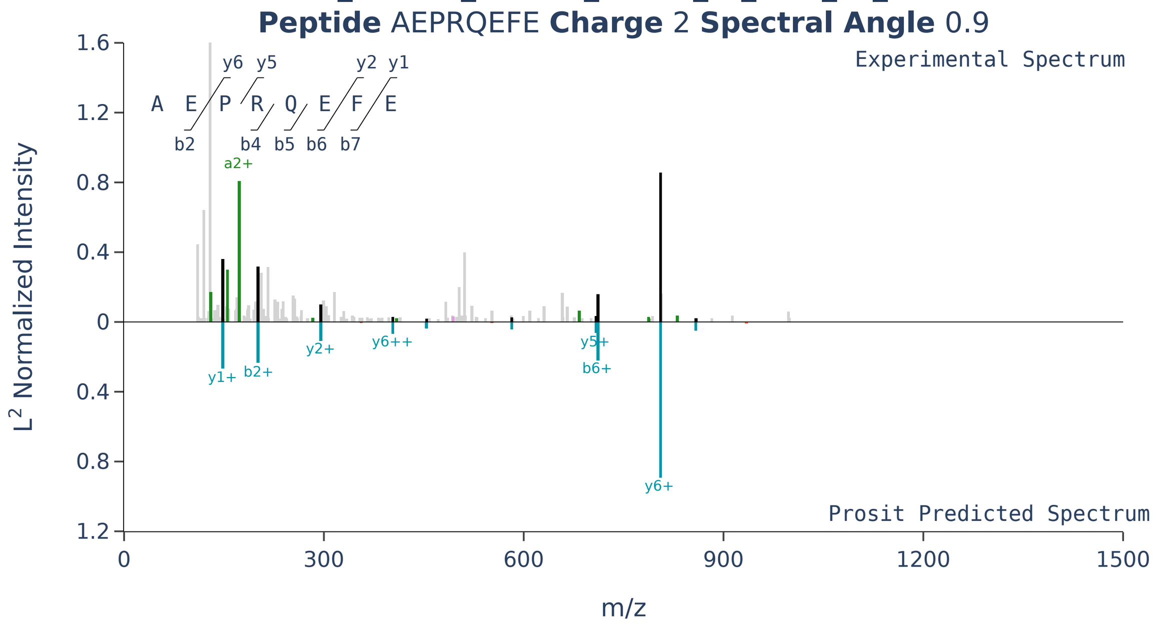


**Source** WSoh_221121_011221_HFGoe_L1_hTau_24h_R1 **Scan** 6683
**Peptide** DLSNVQSKCGSKD **Charge** 3 **Spectral Angle** 0.91

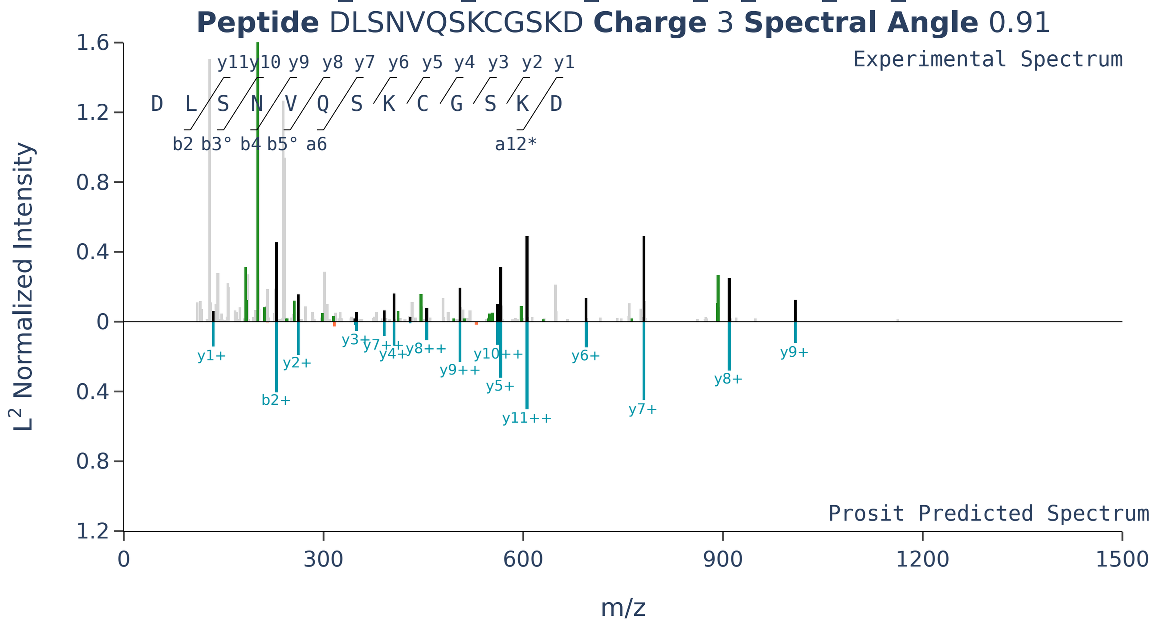


**Source** WSoh_221121_011221_HFGoe_L2_hTau_24h_R1 **Scan** 6728
**Peptide** PVVSGDTSPRH **Charge** 3 **Spectral Angle** 0.93

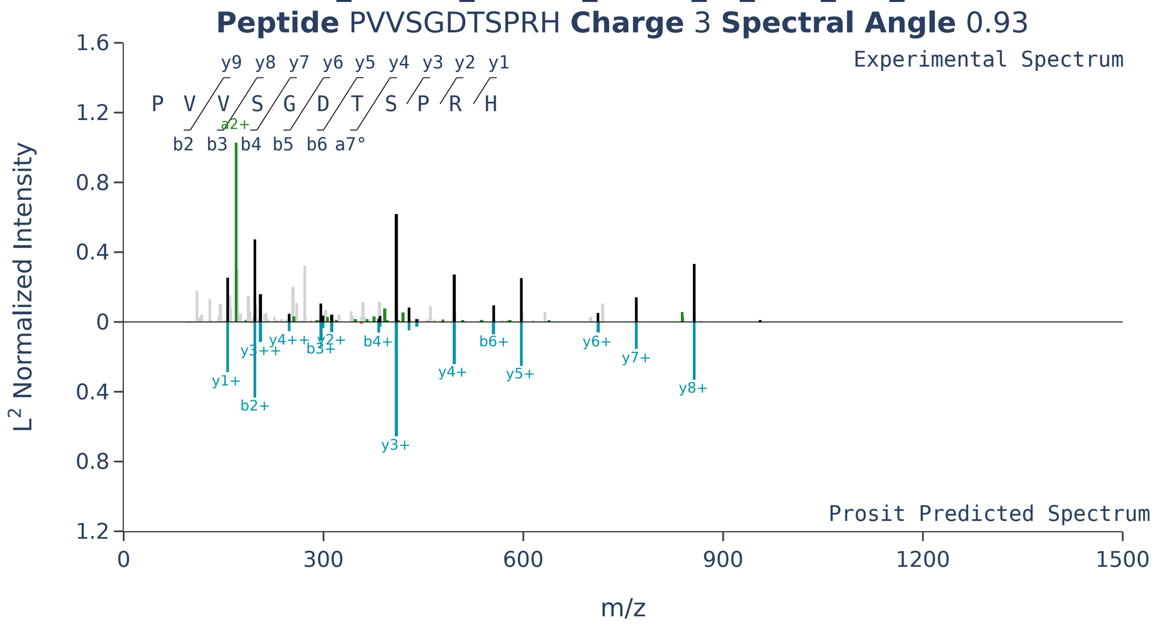

**Source** WSoh_221121_011221_HFGoe_L2_hTau_4h_R1 **Scan** 6964
**Peptide** EDEAAGHVTQA **Charge** 1 **Spectral Angle** 0.84

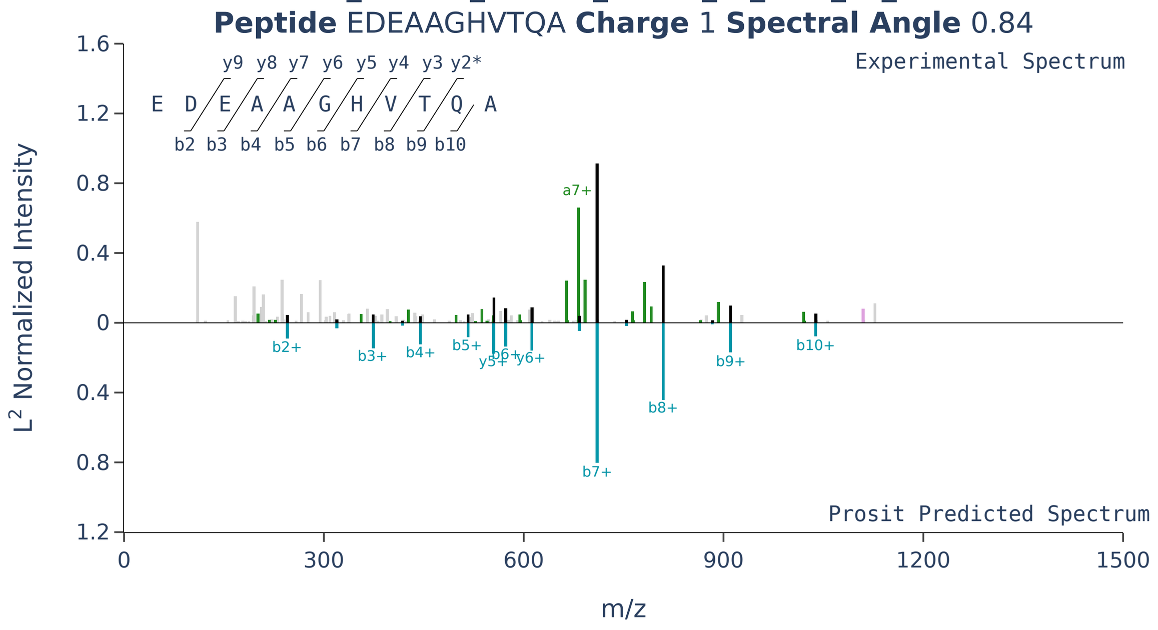


**Source** WSoh_221121_011221_HFGoe_L1_hTau_2h_R2 **Scan** 8631
**Peptide** KPGGGQVEVKSEKL **Charge** 2 **Spectral Angle** 0.9

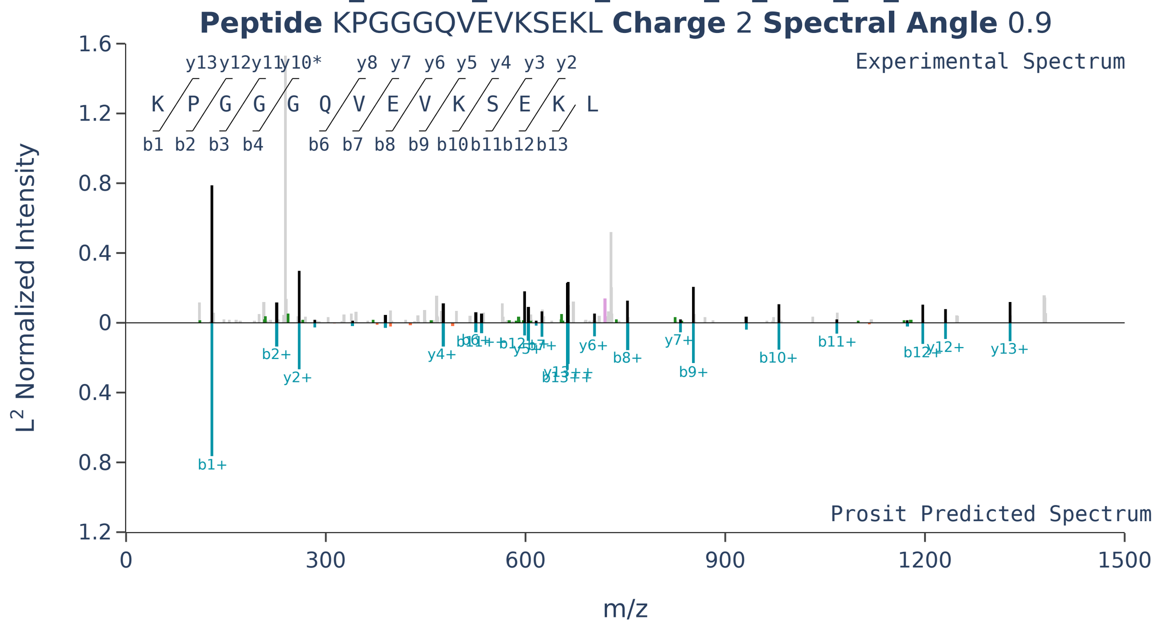


**Source** WSoh_221121_011221_HFGoe_L2_hTau_24h_R2 **Scan** 13132
**Peptide** PSLPTPPTREPKKV **Charge** 2 **Spectral Angle** 0.83

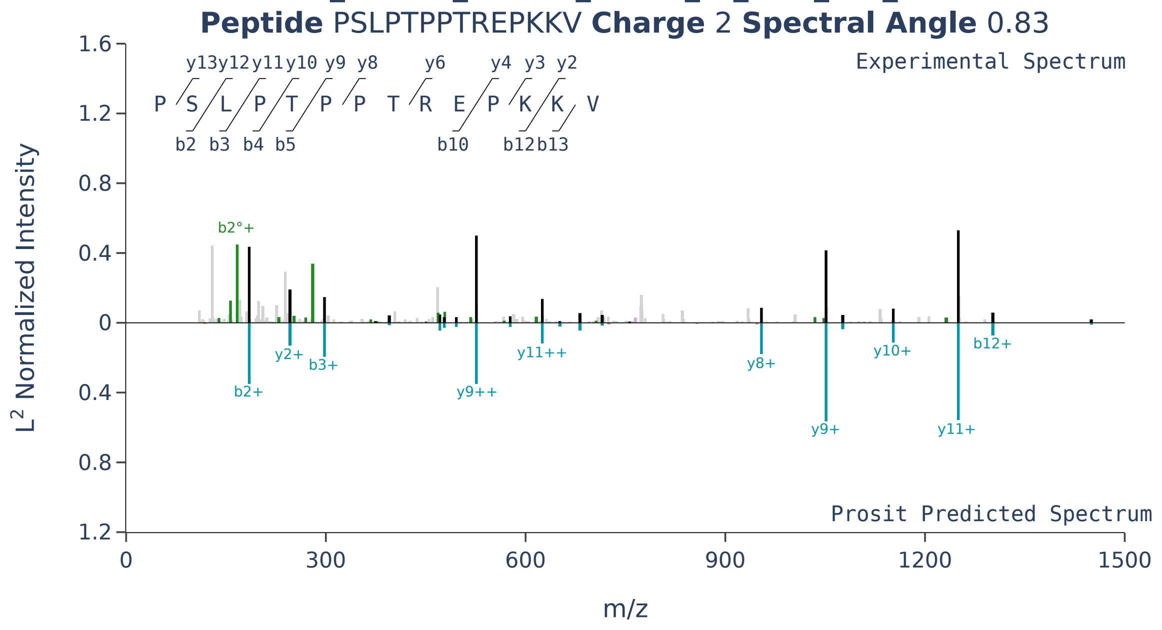


**Source** WSoh_221121_011221_HFGoe_L2_hTau_24h_R1 **Scan** 11254
**Peptide** NKKLDLSNVQS **Charge** 1 **Spectral Angle** 0.87

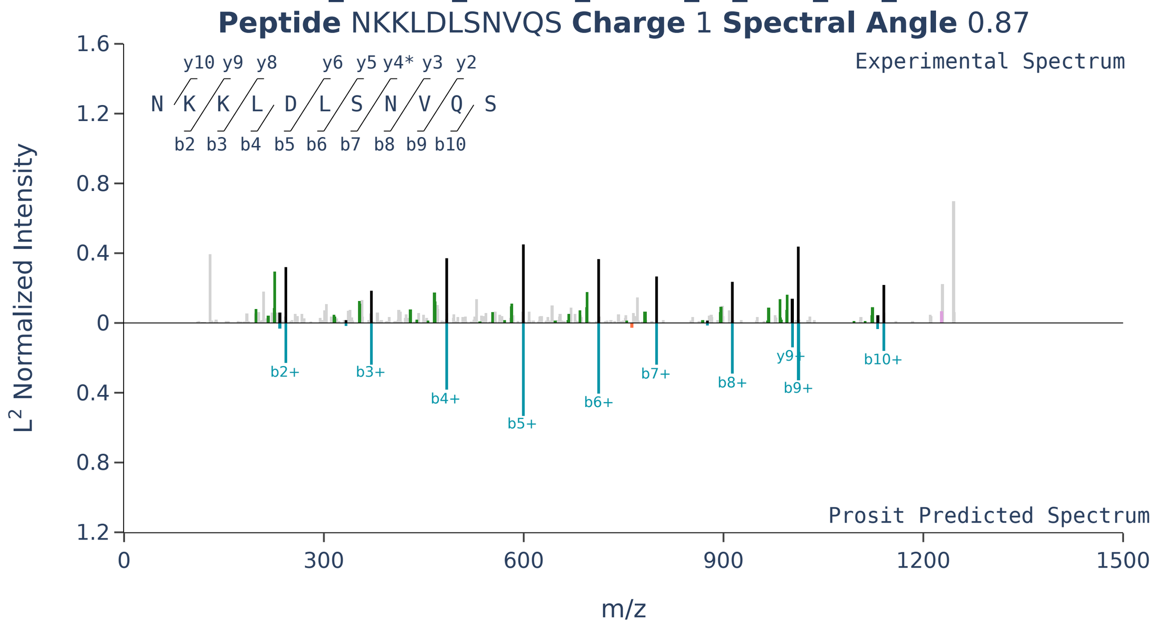


**Source** WSoh_221121_011221_HFGoe_L1_hTau_24h_R1 **Scan** 18003
**Peptide** FEVMEDHAGTYGLGDRKD **Charge** 3 **Spectral Angle** 0.87

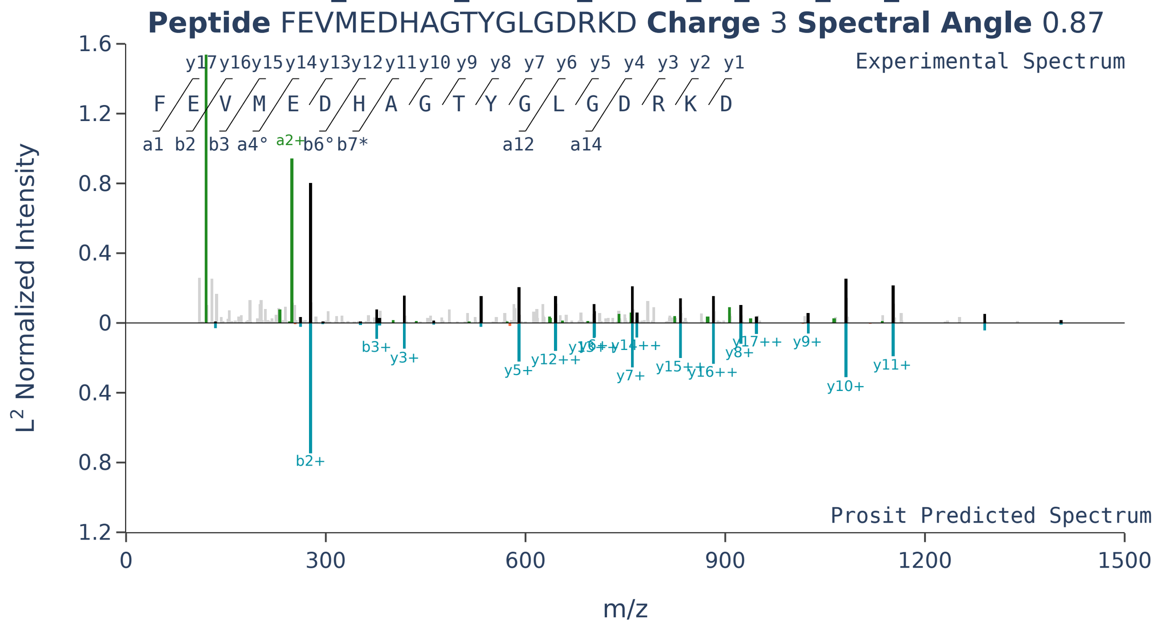


**Source** WSoh_221121_011221_HFGoe_L1_hTau_4h_R2 **Scan** 13352
**Peptide** AAAQPHTEIPEGTTAEE **Charge** 2 **Spectral Angle** 0.91

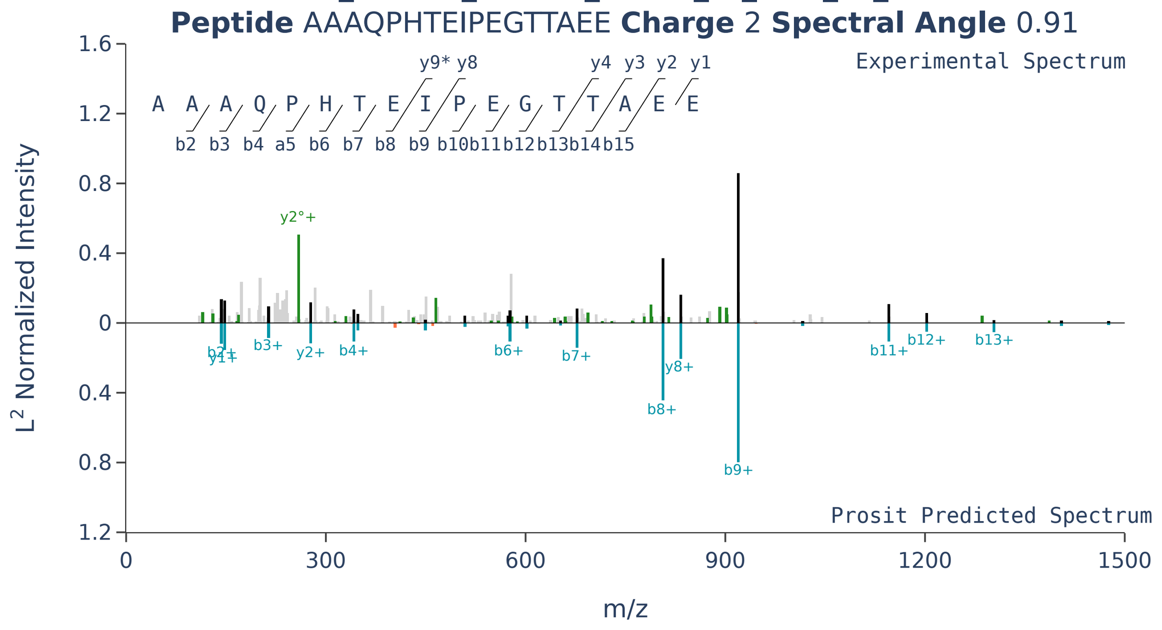


**Source** WSoh_221121_011221_HFGoe_L2_hTau_24h_R1 **Scan** 9751
**Peptide** KPVDLSKVTSK **Charge** 3 **Spectral Angle** 0.91

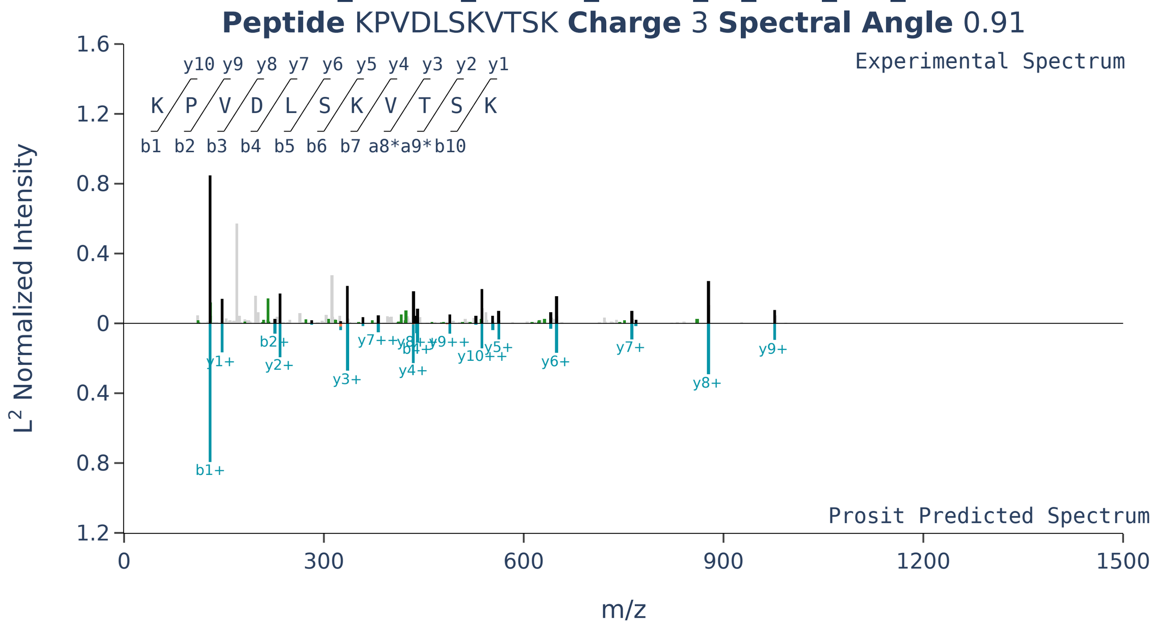


**Source** WSoh_221121_011221_HFGoe_L2_hTau_1h_R1 **Scan** 6051
**Peptide** PTPPTREPKKVA **Charge** 3 **Spectral Angle** 0.87

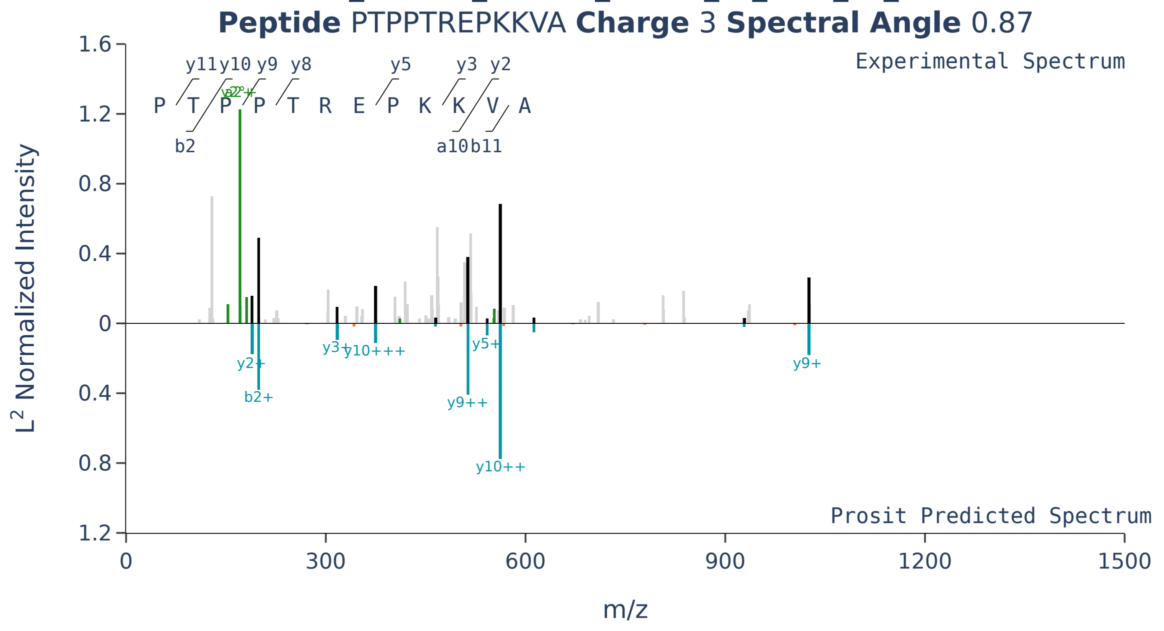


**Source** WSoh_221121_011221_HFGoe_L1_hTau_24h_R1 **Scan** 7464
**Peptide** QEGDTDAGLKES **Charge** 2 **Spectral Angle** 0.88

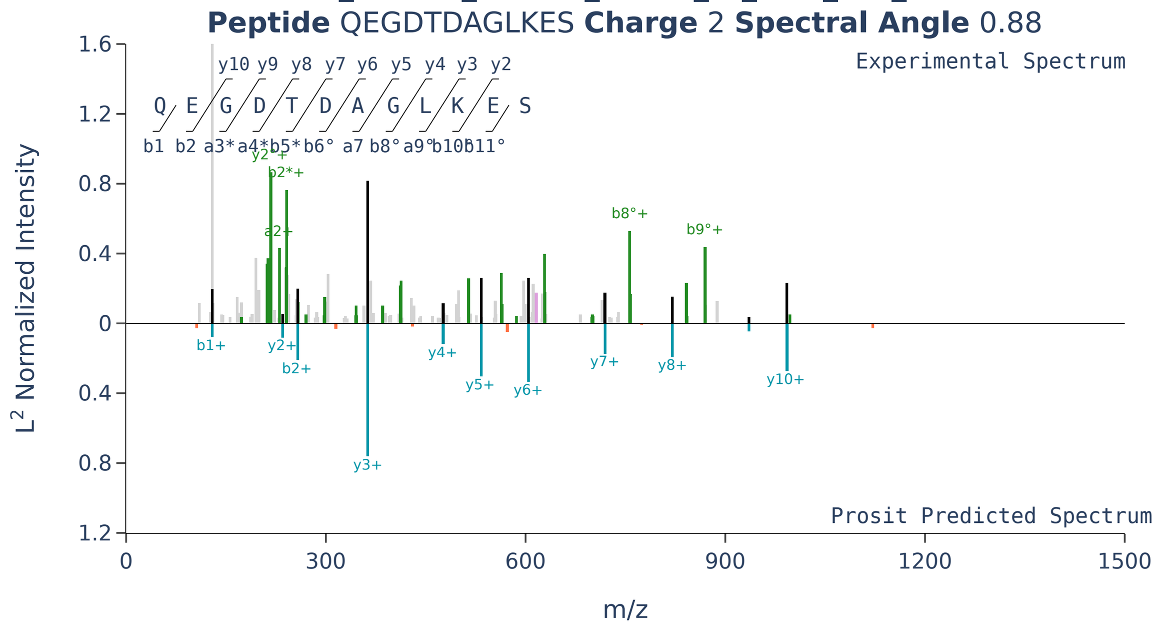


**Source** WSoh_221121_011221_HFGoe_L1_hTau_2h_R2 **Scan** 18504
**Peptide** PVPMPDLKNVKS **Charge** 2 **Spectral Angle** 0.91

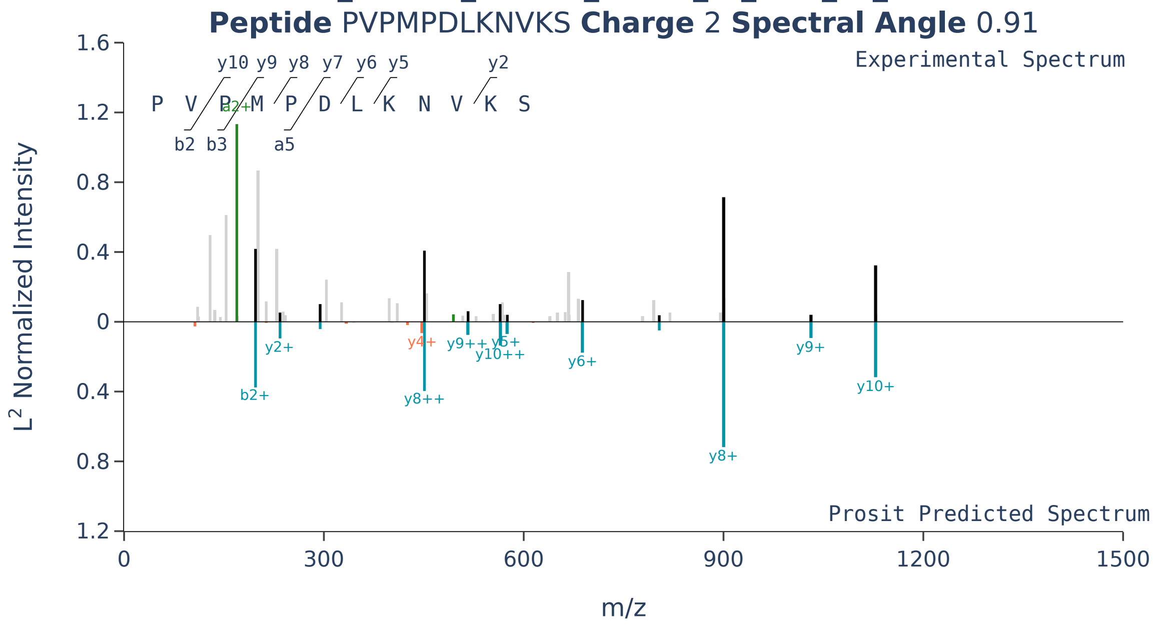


**Source** WSoh_221121_011221_HFGoe_L2_hTau_24h_R1 **Scan** 6239
**Peptide** SSPGSPGTPGSR **Charge** 2 **Spectral Angle** 0.93

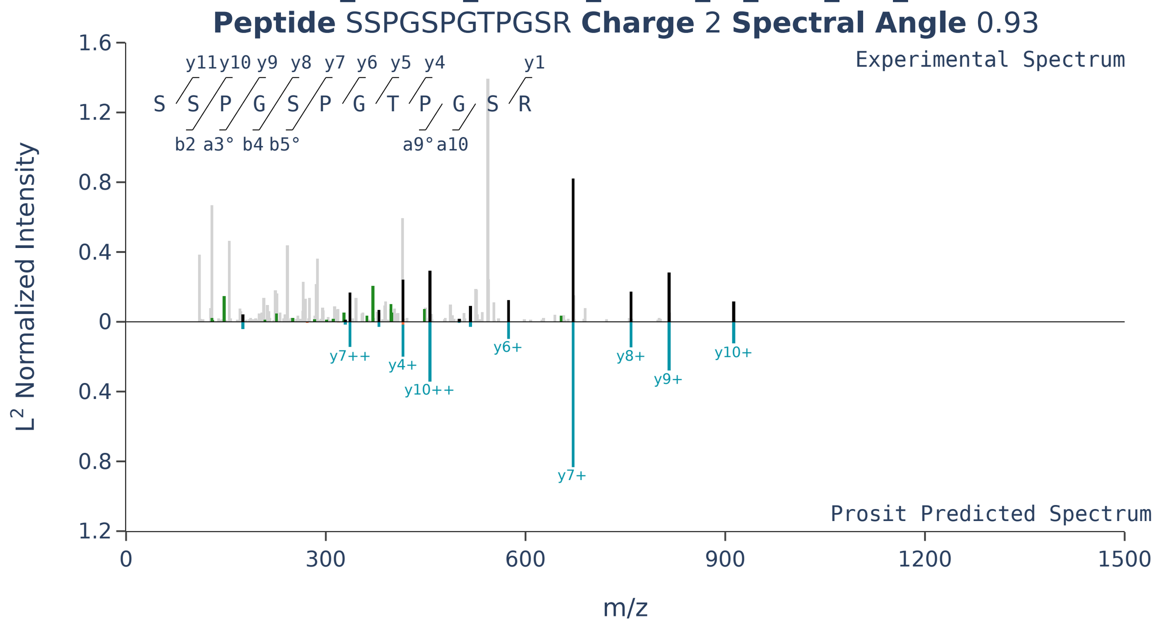


**Source** WSoh_221121_011221_HFGoe_L2_hTau_1h_R1 **Scan** 5818
**Peptide** KIGSTENLKH **Charge** 3 **Spectral Angle** 0.9

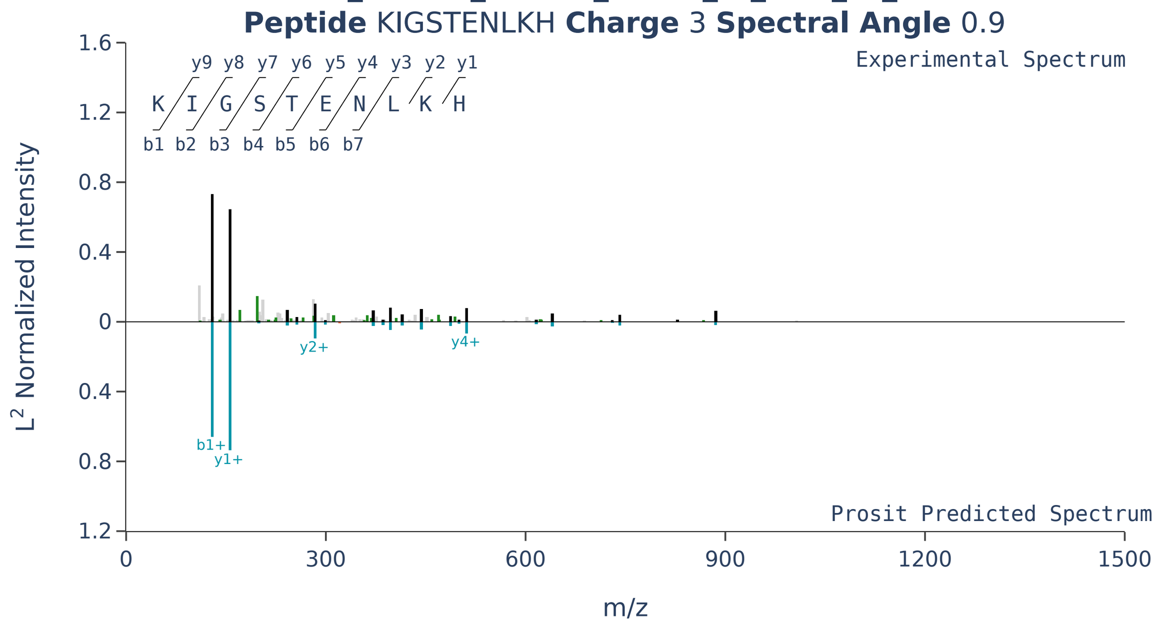


**Source** WSoh_221121_011221_HFGoe_L1_hTau_4h_R1 **Scan** 8544
**Peptide** TRIPAKTPPAPKT **Charge** 3 **Spectral Angle** 0.87

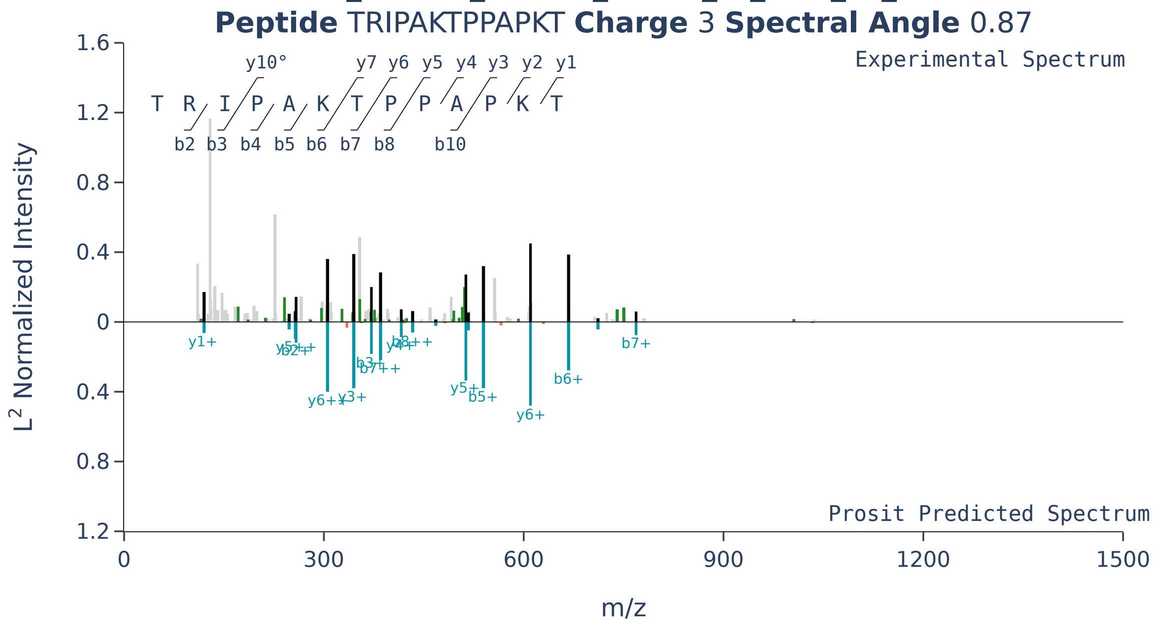


**Source** WSoh_221121_011221_HFGoe_L1_hTau_4h_R1 **Scan** 14685
**Peptide** GDTSPRHLSNVSSTGSIDMVD **Charge** 3 **Spectral Angle** 0.85

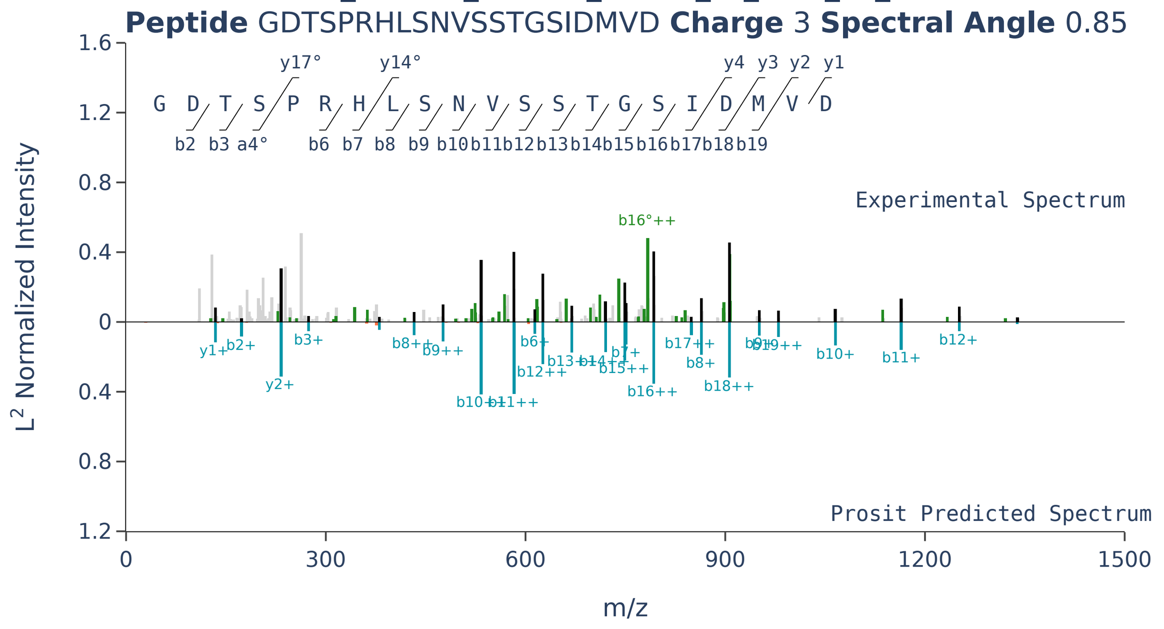


**Source** WSoh_221121_011221_HFGoe_L2_hTau_24h_R1 **Scan** 17247
**Peptide** SNVSSTGSIDM **Charge** 1 **Spectral Angle** 0.94

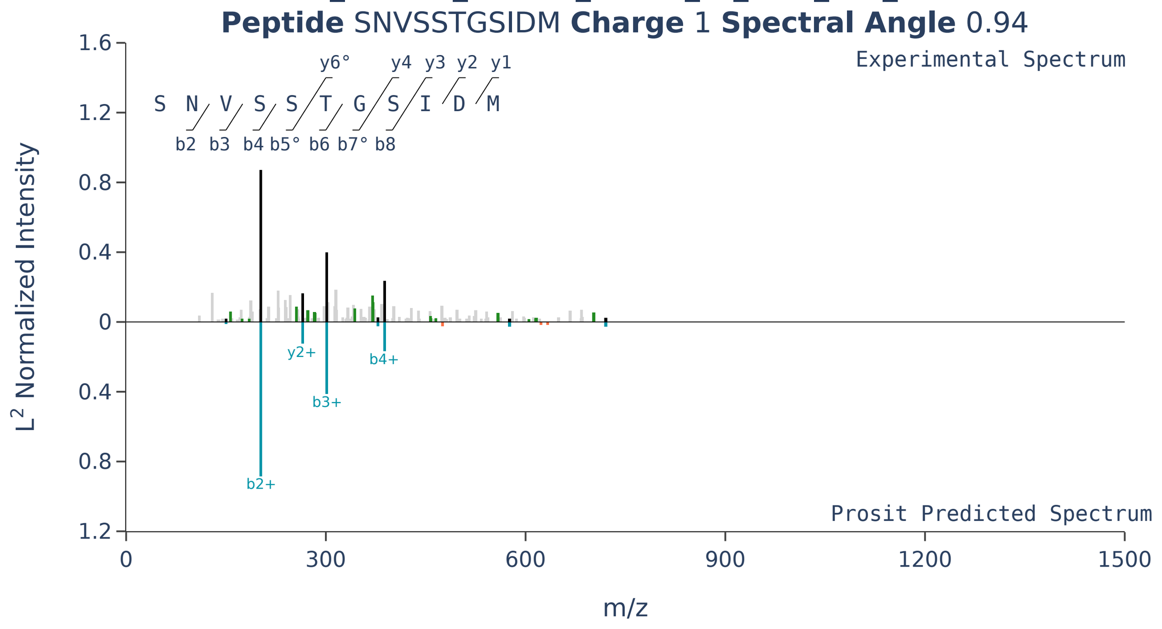

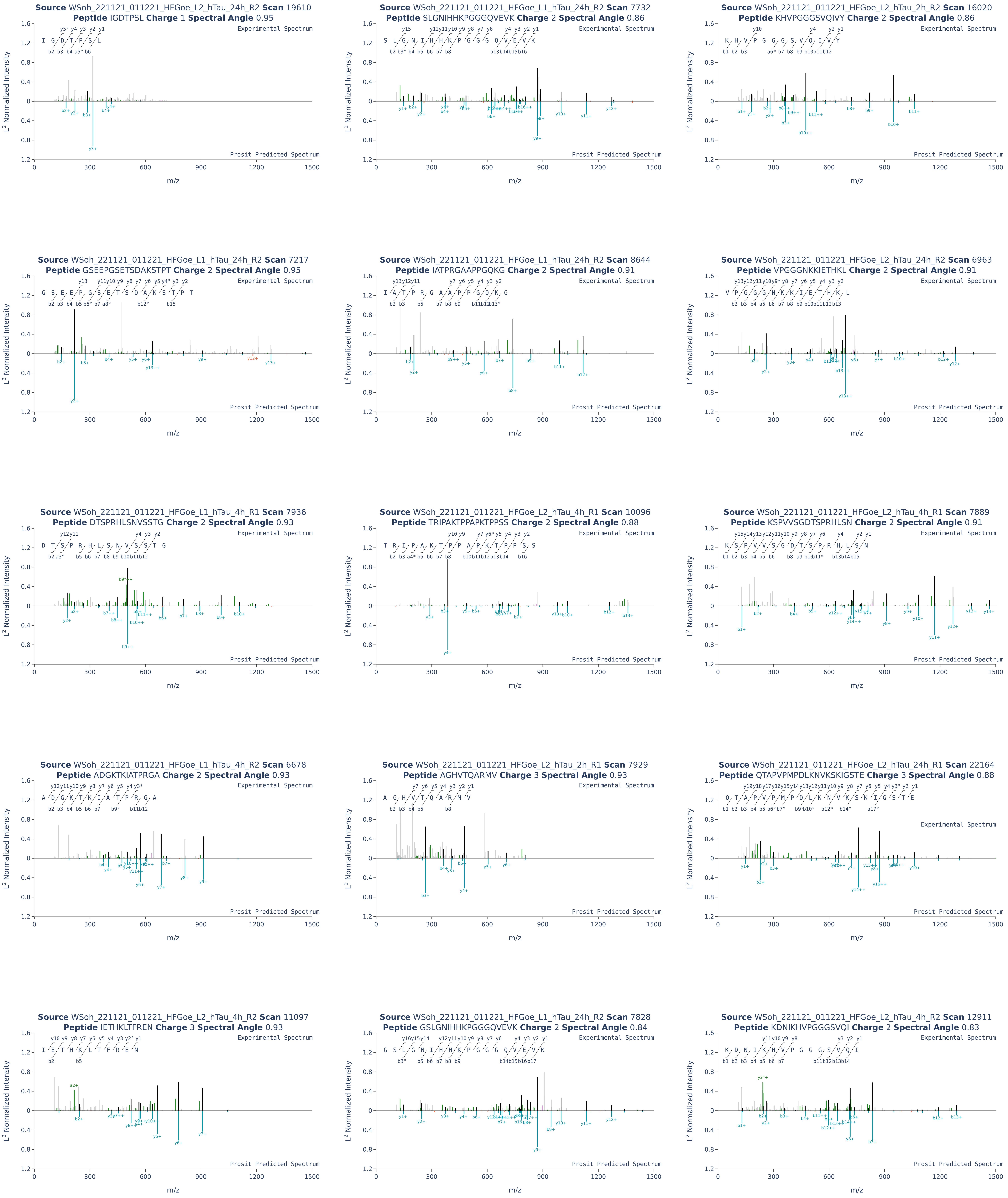
Source WSoh_221121_011221_HFGoe_L2_hTau_24h_R2 Scan 19610
Peptide IGDTPSL Charge 1 Spectral Angle 0.95
Source WSoh_221121_011221_HFGoe_L1_hTau_24h_R2 Scan 7732
Peptide SLGNIHHKPGGGQVEVK Charge 2 Spectral Angle 0.86
Source WSoh_221121_011221_HFGoe_L2_hTau_2h_R2 Scan 16020
Peptide KHVPGGGSVQIVY Charge 2 Spectral Angle 0.86
Source WSoh_221121_011221_HFGoe_L1_hTau_24h_R2 Scan 7217
Peptide GSEEPGSETSDAKSTPT Charge 2 Spectral Angle 0.95
Source WSoh_221121_011221_HFGoe_L1_hTau_24h_R2 Scan 8644
Peptide IATPRGAAPPGQKG Charge 2 Spectral Angle 0.91
Source WSoh_221121_011221_HFGoe_L2_hTau_24h_R2 Scan 6963
Peptide VPGGGNKKIETHKL Charge 2 Spectral Angle 0.91
Source WSoh_221121_011221_HFGoe_L1_hTau_4h_R1 Scan 7936
Peptide DTSPRHLSNVSSTG Charge 2 Spectral Angle 0.93
Source WSoh_221121_011221_HFGoe_L2_hTau_4h_R1 Scan 10096
Peptide TRIPAKTPPAPKTPPSS Charge 2 Spectral Angle 0.88
Source WSoh_221121_011221_HFGoe_L2_hTau_4h_R1 Scan 7889
Peptide KSPVVSGDTSPRHLSN Charge 2 Spectral Angle 0.91
Source WSoh_221121_011221_HFGoe_L1_hTau_4h_R2 Scan 6678
Peptide ADGKTKIATPRGA Charge 2 Spectral Angle 0.93
Source WSoh_221121_011221_HFGoe_L2_hTau_2h_R1 Scan 7929
Peptide AGHVTQARMV Charge 3 Spectral Angle 0.93
Source WSoh_221121_011221_HFGoe_L2_hTau_24h_R1 Scan 22164
Peptide QTAPVPMPDLKNVKSKIGSTE Charge 3 Spectral Angle 0.88
Source WSoh_221121_011221_HFGoe_L2_hTau_4h_R2 Scan 11097
Peptide IETHKLTFREN Charge 3 Spectral Angle 0.93
Source WSoh_221121_011221_HFGoe_L1_hTau_24h_R2 Scan 7828
Peptide GSLGNIHHKPGGGQVEVK Charge 2 Spectral Angle 0.84
Source WSoh_221121_011221_HFGoe_L2_hTau_4h_R2 Scan 12911
Peptide KDNIKHVPGGGSVQI Charge 2 Spectral Angle 0.83
Experimental Spectrum
Prosit Predicted Spectrum
L² Normalized Intensity
m/z

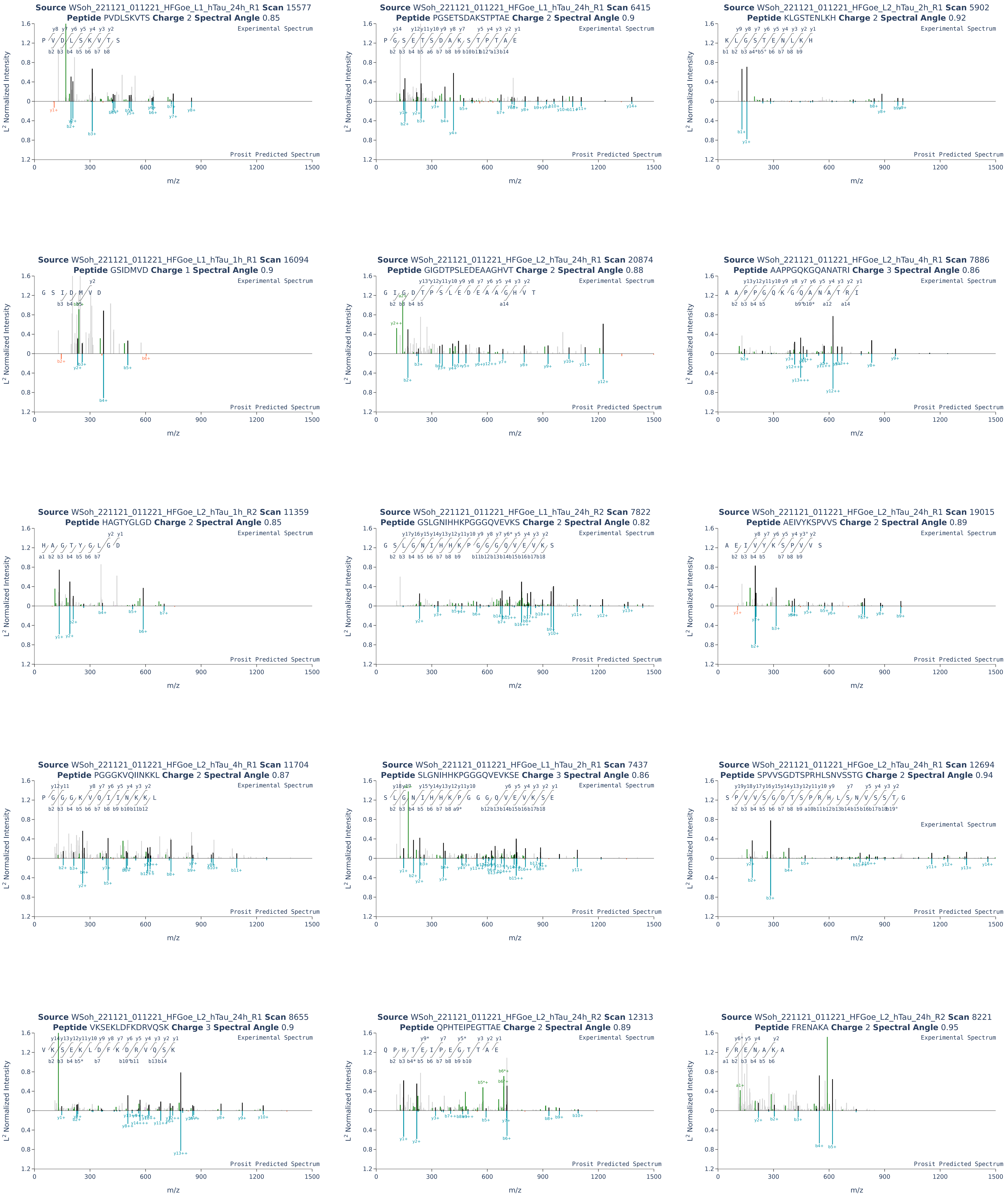
Source WSoh_221121_011221_HFGoe_L1_hTau_24h_R1 Scan 15577
Peptide PVDLSKVTS Charge 2 Spectral Angle 0.85
Source WSoh_221121_011221_HFGoe_L1_hTau_24h_R1 Scan 6415
Peptide PGSETSDAKSTPTAE Charge 2 Spectral Angle 0.9
Source WSoh_221121_011221_HFGoe_L2_hTau_2h_R1 Scan 5902
Peptide KLGSTENLKH Charge 2 Spectral Angle 0.92
Source WSoh_221121_011221_HFGoe_L1_hTau_1h_R1 Scan 16094
Peptide GSIDMVD Charge 1 Spectral Angle 0.9
Source WSoh_221121_011221_HFGoe_L2_hTau_24h_R1 Scan 20874
Peptide GIGDTPSLEDEAAGHVT Charge 2 Spectral Angle 0.88
Source WSoh_221121_011221_HFGoe_L2_hTau_4h_R1 Scan 7886
Peptide AAPPGQKGQANATRI Charge 3 Spectral Angle 0.86
Source WSoh_221121_011221_HFGoe_L2_hTau_1h_R2 Scan 11359
Peptide HAGTYGLGD Charge 2 Spectral Angle 0.85
Source WSoh_221121_011221_HFGoe_L1_hTau_24h_R2 Scan 7822
Peptide GSLGNIHHKPGGGQVEVKS Charge 2 Spectral Angle 0.82
Source WSoh_221121_011221_HFGoe_L2_hTau_24h_R1 Scan 19015
Peptide AEIVYKSPVVS Charge 2 Spectral Angle 0.89
Source WSoh_221121_011221_HFGoe_L2_hTau_4h_R1 Scan 11704
Peptide PGGGKVQIINKKL Charge 2 Spectral Angle 0.87
Source WSoh_221121_011221_HFGoe_L1_hTau_2h_R1 Scan 7437
Peptide SLGNIHHKPGGGQVEVKSE Charge 3 Spectral Angle 0.86
Source WSoh_221121_011221_HFGoe_L2_hTau_24h_R1 Scan 12694
Peptide SPVVSGDTSPRHLSNVSSTG Charge 2 Spectral Angle 0.94
Source WSoh_221121_011221_HFGoe_L2_hTau_24h_R1 Scan 8655
Peptide VKSEKLDFKDRVQSK Charge 3 Spectral Angle 0.9
Source WSoh_221121_011221_HFGoe_L2_hTau_24h_R2 Scan 12313
Peptide QPHTEIPEGTTAE Charge 2 Spectral Angle 0.89
Source WSoh_221121_011221_HFGoe_L2_hTau_24h_R2 Scan 8221
Peptide FRENAKA Charge 2 Spectral Angle 0.95
Experimental Spectrum
Prosit Predicted Spectrum
L² Normalized Intensity
m/z

**Source** WSoh_221121_011221_HFGoe_L1_hTau_24h_R2 **Scan** 20086
**Peptide** DHGAEIVYKSPVV **Charge** 2 **Spectral Angle** 0.94

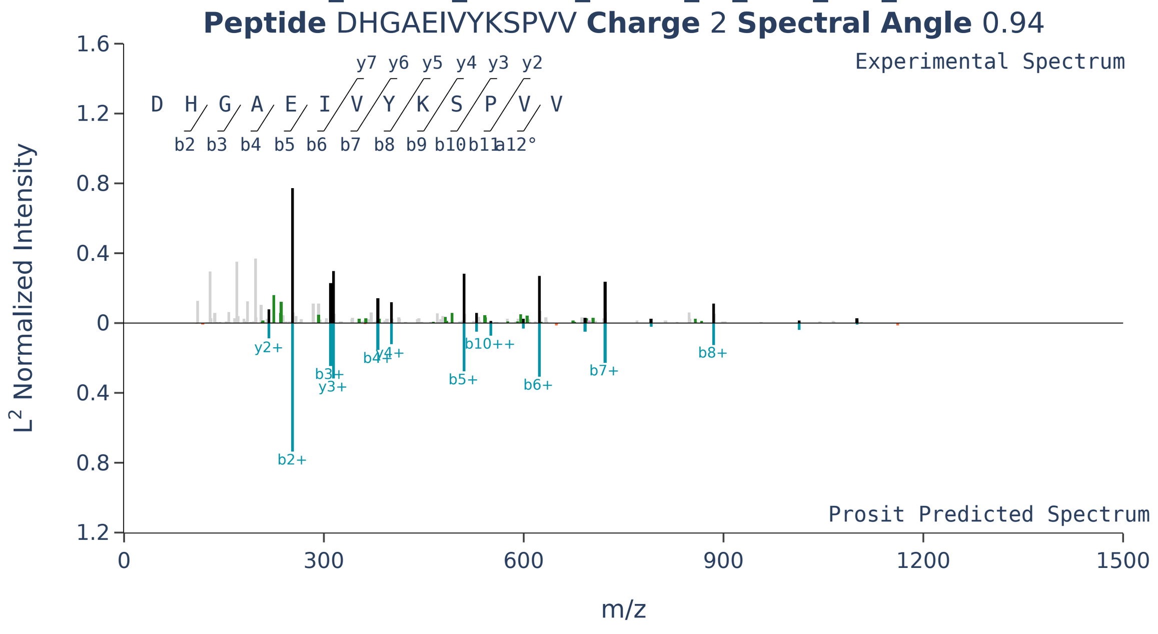


**Source** WSoh_221121_011221_HFGoe_L1_hTau_24h_R1 **Scan** 19707
**Peptide** FEVMEDHAGTY **Charge** 2 **Spectral Angle** 0.89

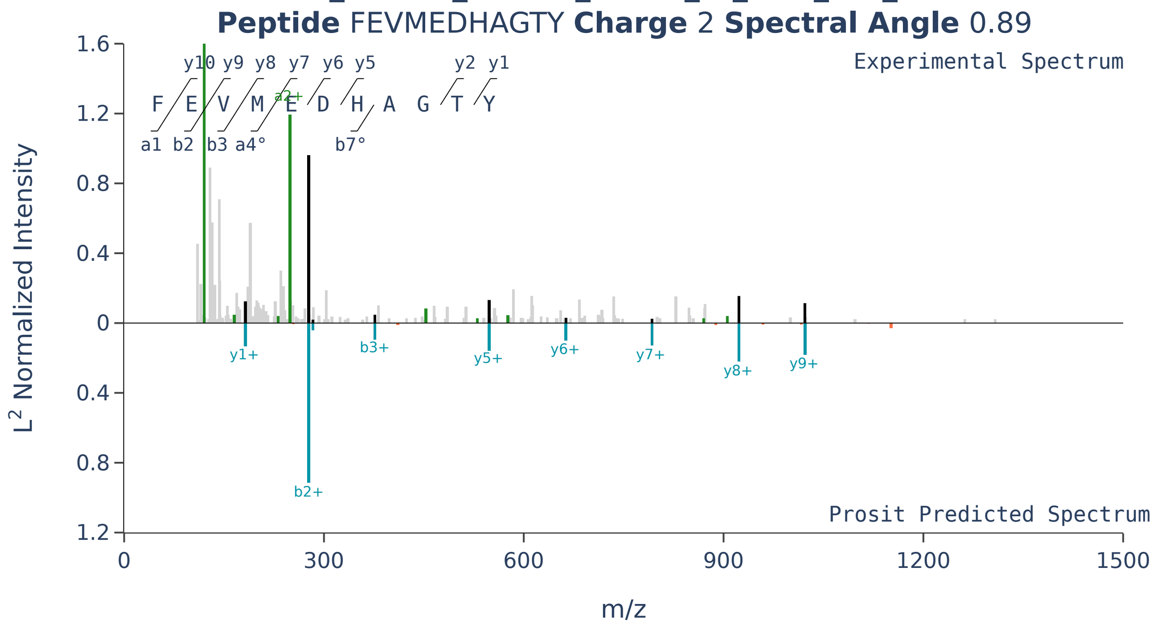


**Source** WSoh_221121_011221_HFGoe_L1_hTau_24h_R2 **Scan** 10932
**Peptide** SLDNITH **Charge** 2 **Spectral Angle** 0.93

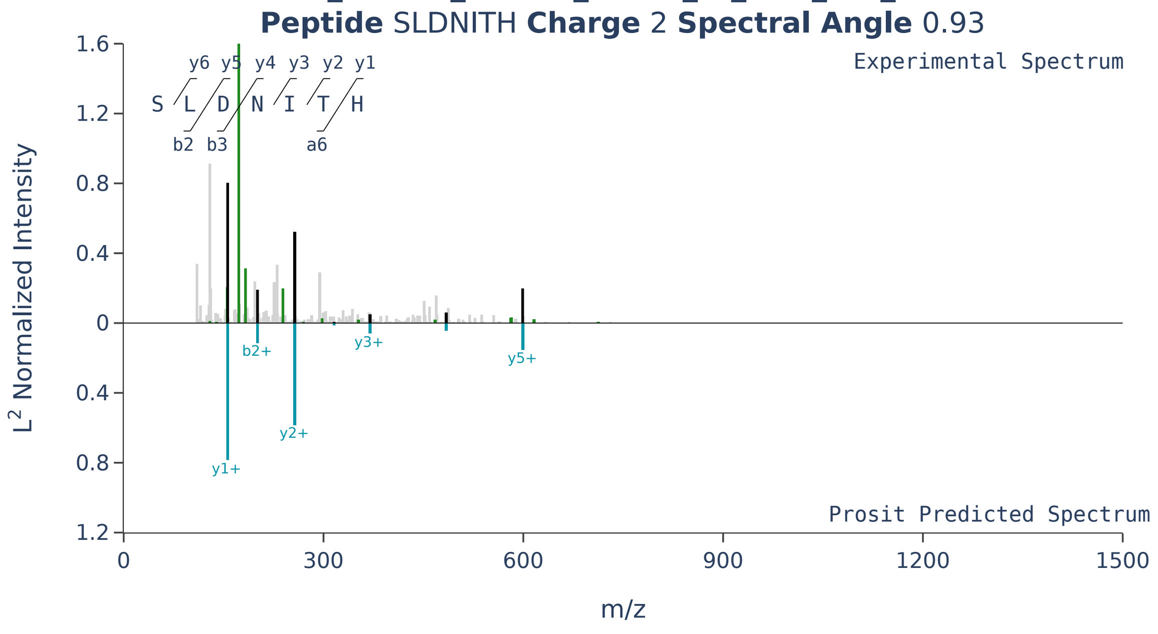


**Source** WSoh_221121_011221_HFGoe_L1_hTau_4h_R1 **Scan** 13049
**Peptide** SKDNIKHVPGGGSVQIVYK **Charge** 3 **Spectral Angle** 0.86

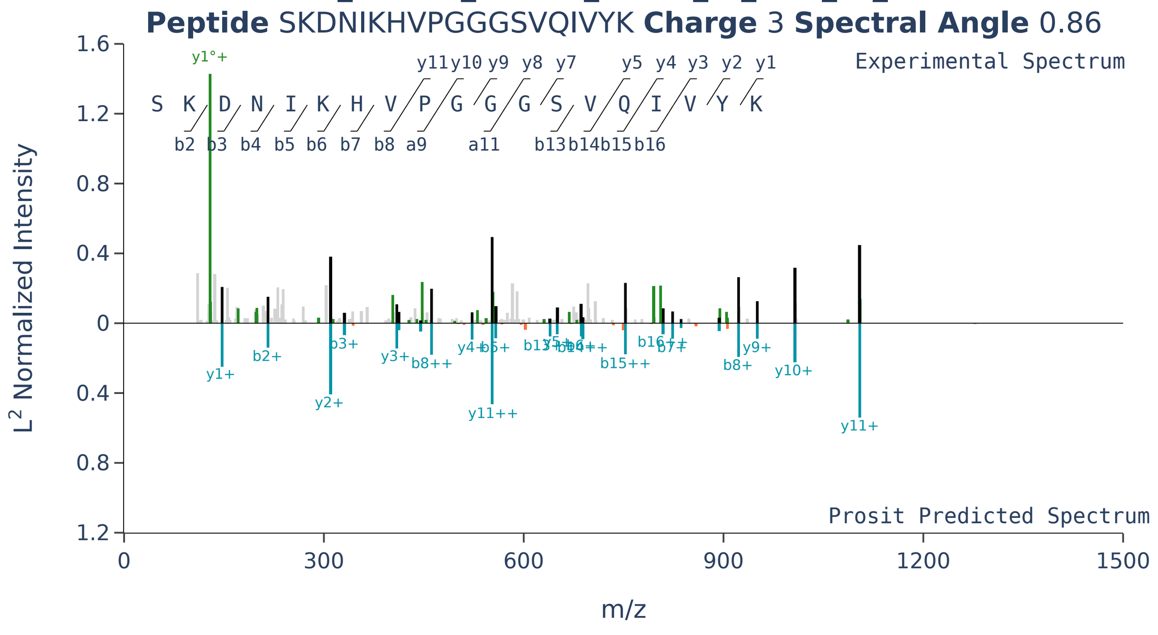


**Source** WSoh_221121_011221_HFGoe_L2_hTau_2h_R2 **Scan** 8540
**Peptide** PTPPTREPKKVAV **Charge** 3 **Spectral Angle** 0.89

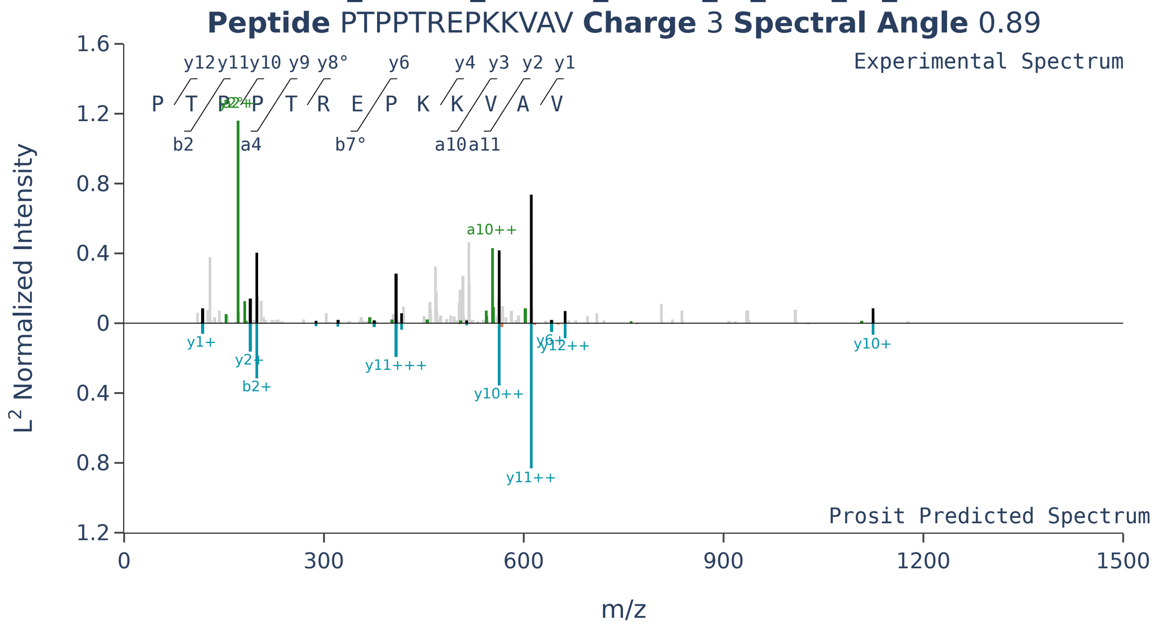


**Source** WSoh_221121_011221_HFGoe_L1_hTau_4h_R2 **Scan** 5480
**Peptide** GDRKDQGGYTM **Charge** 2 **Spectral Angle** 0.9

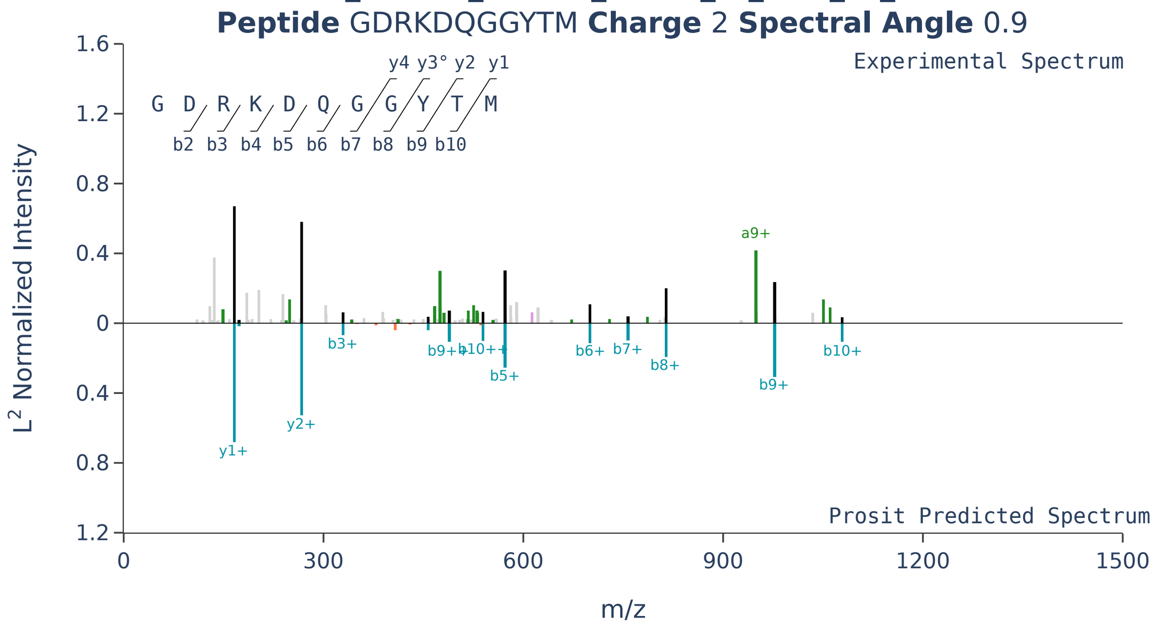


**Source** WSoh_221121_011221_HFGoe_L2_hTau_24h_R1 **Scan** 6718
**Peptide** SKVTSKCGSLGNIHH **Charge** 2 **Spectral Angle** 0.85

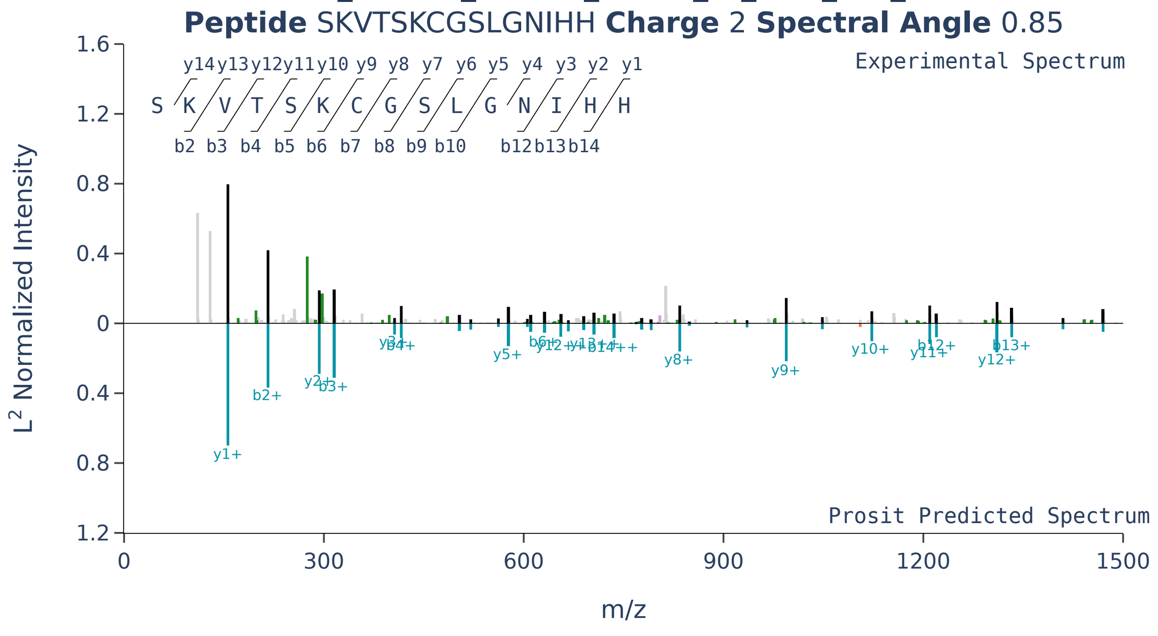


**Source** WSoh_221121_011221_HFGoe_L1_hTau_24h_R1 **Scan** 18242
**Peptide** KLDLSNV **Charge** 2 **Spectral Angle** 0.91

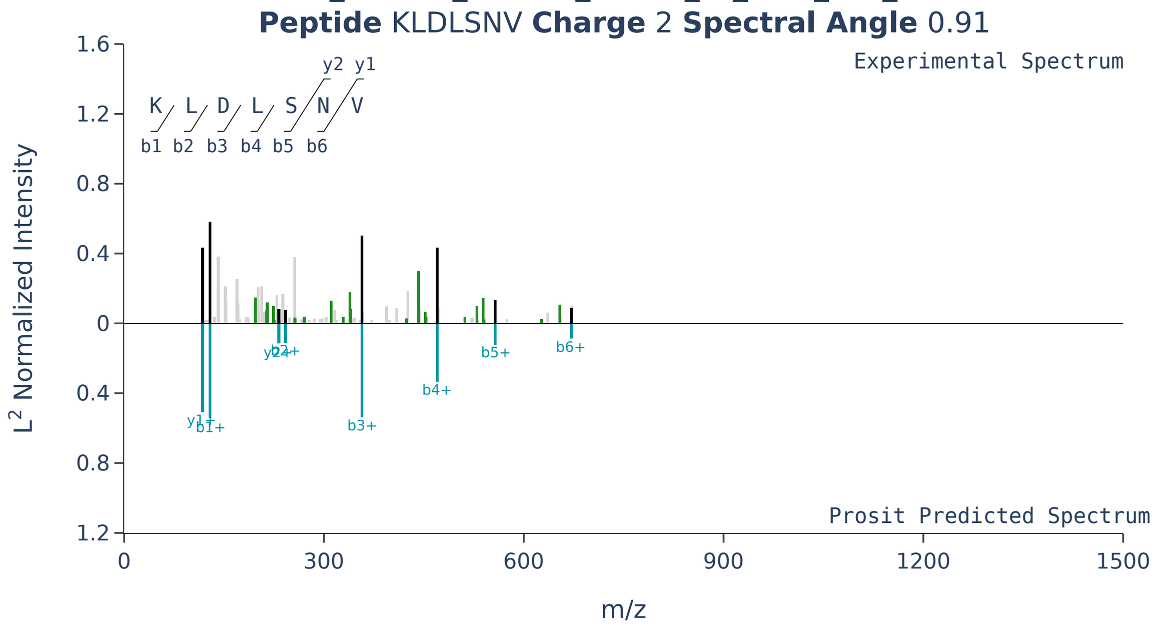


**Source** WSoh_221121_011221_HFGoe_L1_hTau_4h_R1 **Scan** 5898
**Peptide** TPPKSPSSAKSRLQ **Charge** 3 **Spectral Angle** 0.88

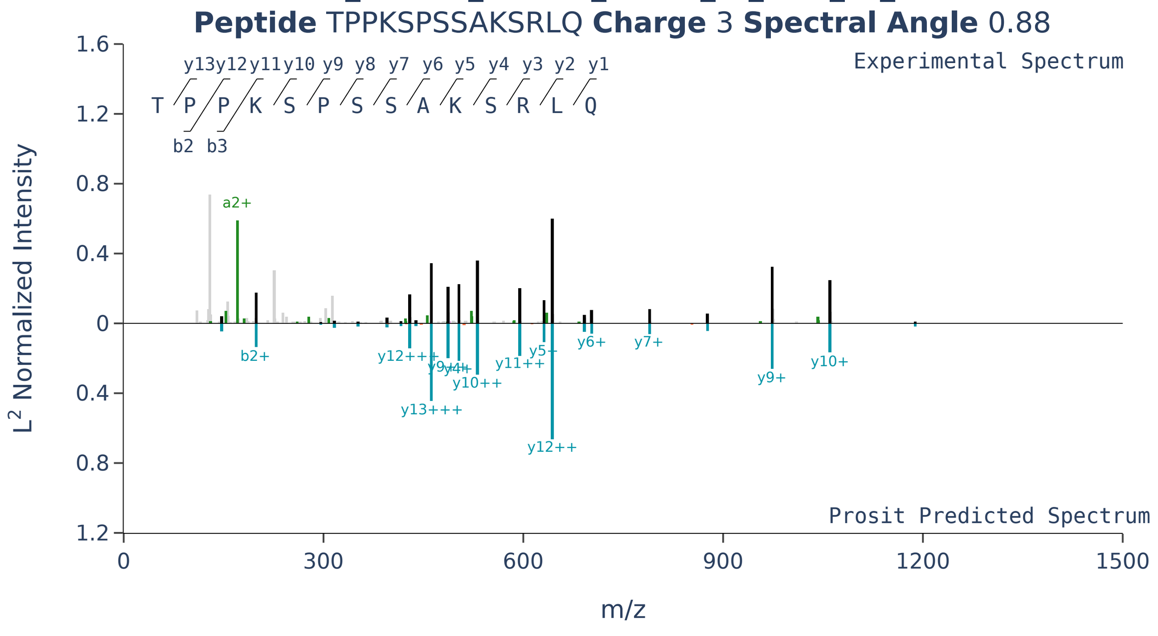


**Source** WSoh_221121_011221_HFGoe_L1_hTau_24h_R2 **Scan** 20440
**Peptide** LQTAPVPMPDLKNVKSK **Charge** 4 **Spectral Angle** 0.89

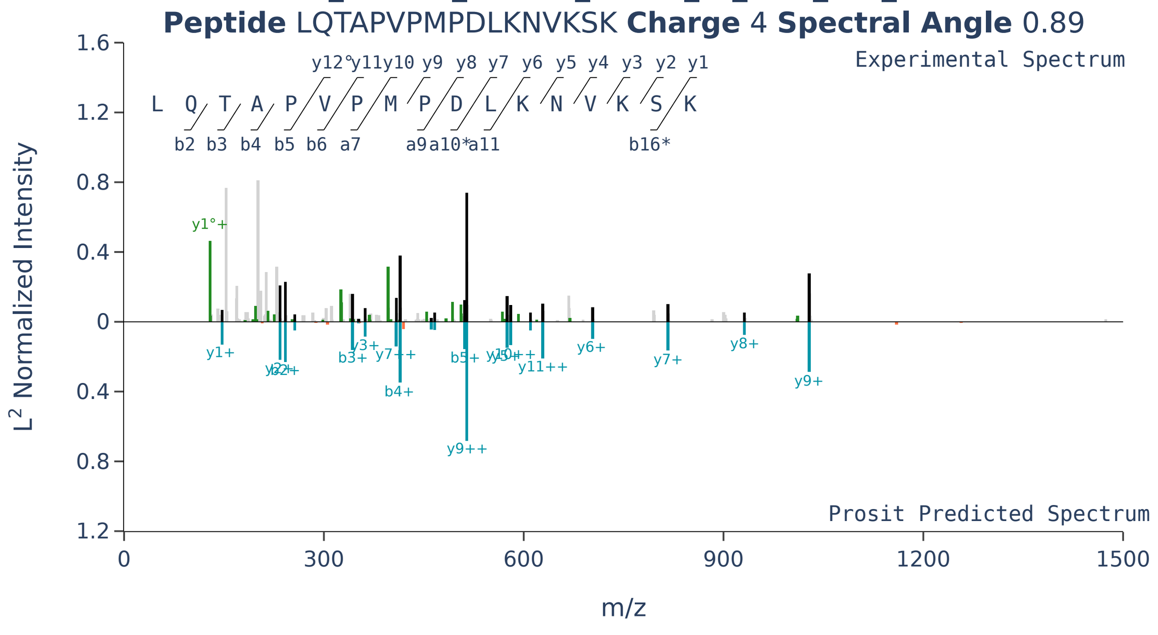


**Source** WSoh_221121_011221_HFGoe_L2_hTau_24h_R1 **Scan** 10309
**Peptide** SPVVSGDTSPRHLS **Charge** 2 **Spectral Angle** 0.94

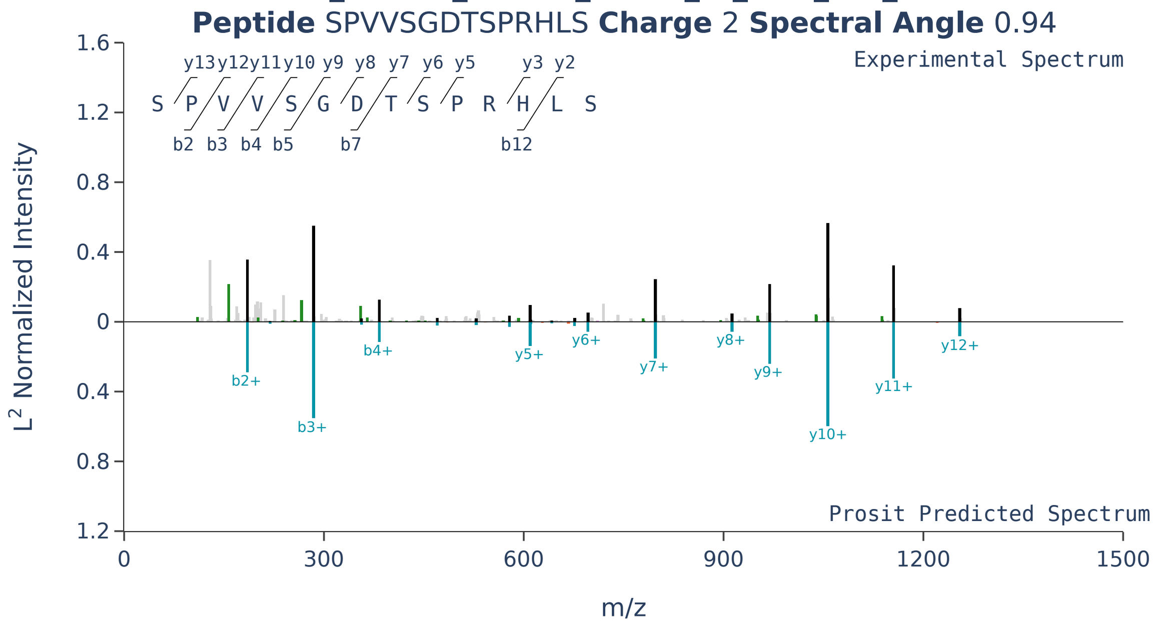


**Source** WSoh_221121_011221_HFGoe_L1_hTau_2h_R2 **Scan** 11583
**Peptide** PHTEIPEGTTAE **Charge** 2 **Spectral Angle** 0.86

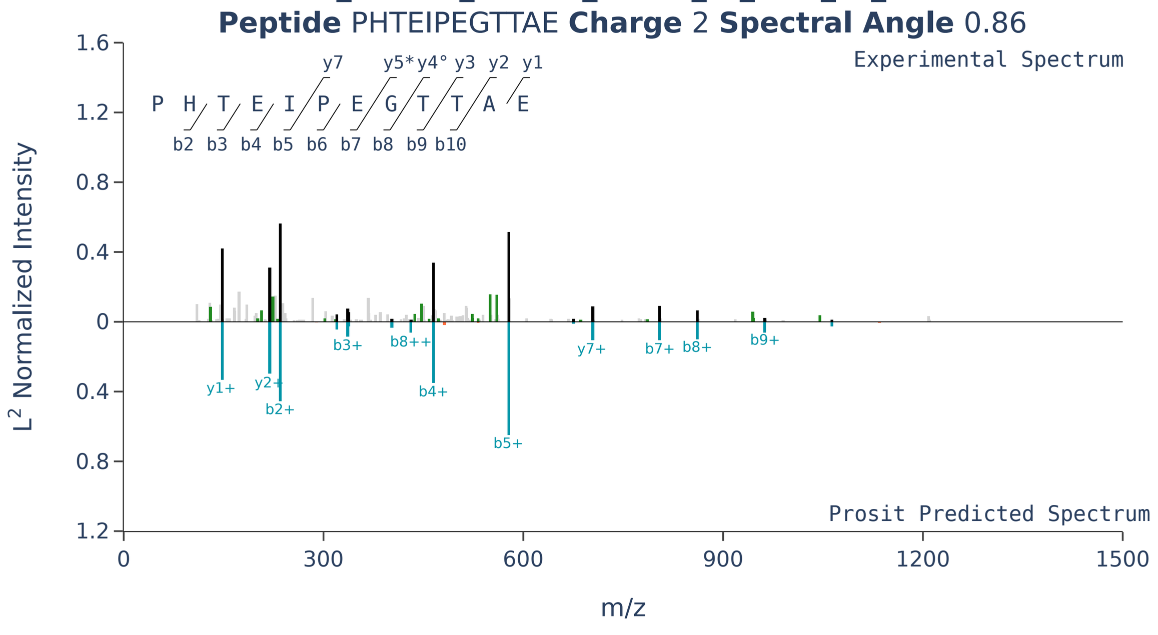


**Source** WSoh_221121_011221_HFGoe_L1_hTau_24h_R1 **Scan** 20235
**Peptide** AGIGDTPSLE **Charge** 2 **Spectral Angle** 0.95

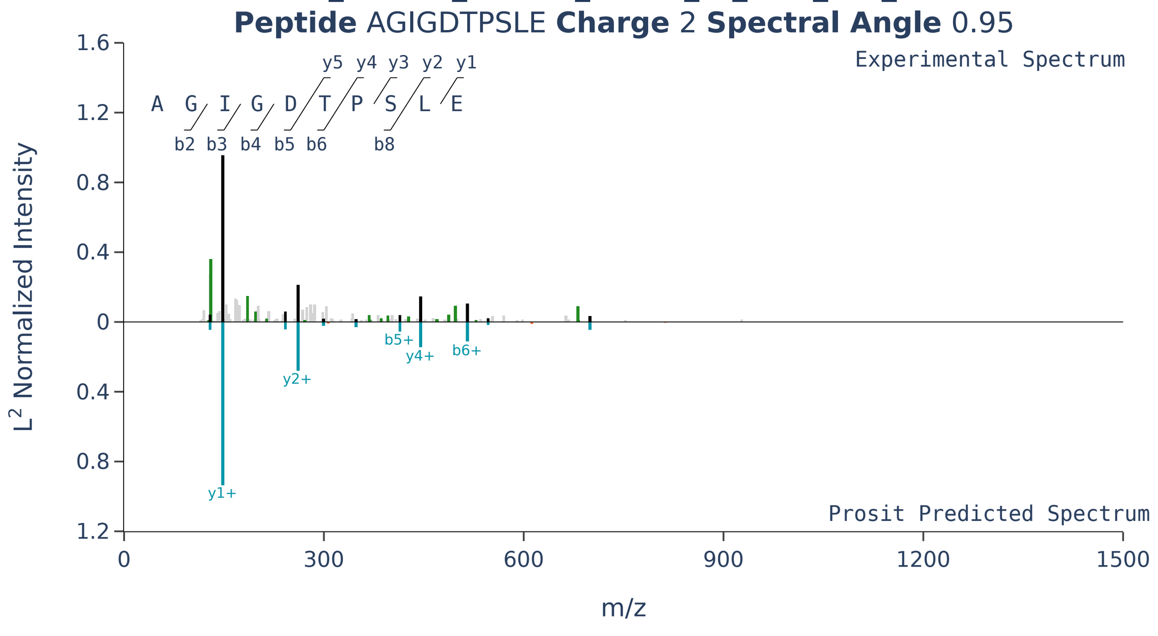


**Source** WSoh_221121_011221_HFGoe_L1_hTau_24h_R1 **Scan** 11950
**Peptide** AKTDHGAEIVY **Charge** 2 **Spectral Angle** 0.88

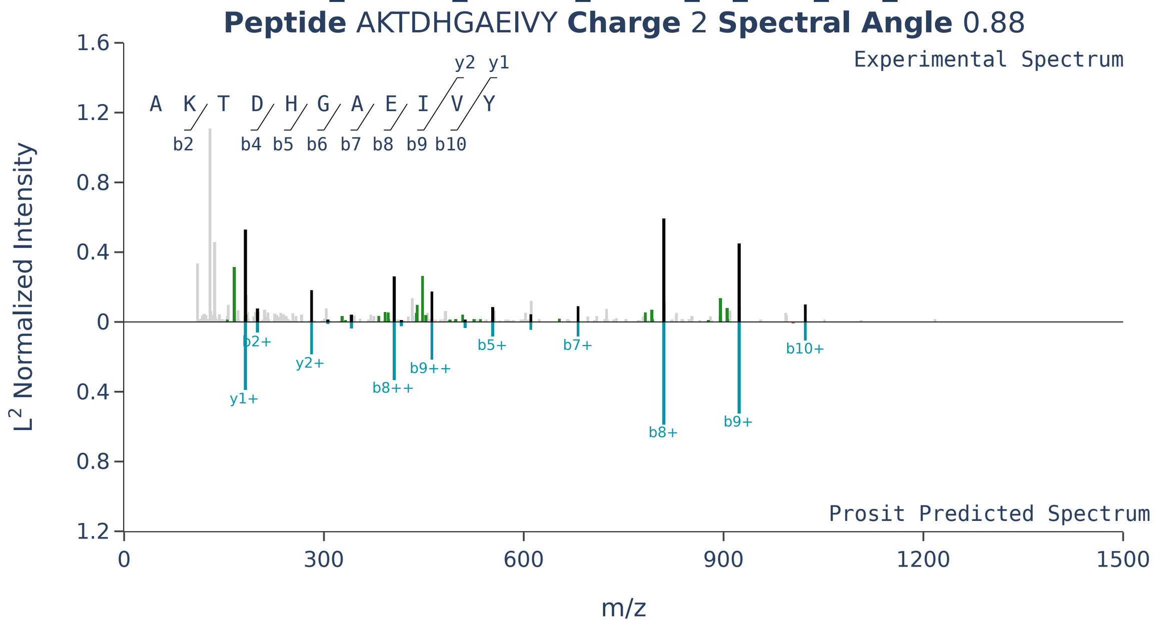


**Source** WSoh_221121_011221_HFGoe_L2_hTau_1h_R1 **Scan** 5946
**Peptide** SSPGSPGTPGSRS **Charge** 2 **Spectral Angle** 0.87

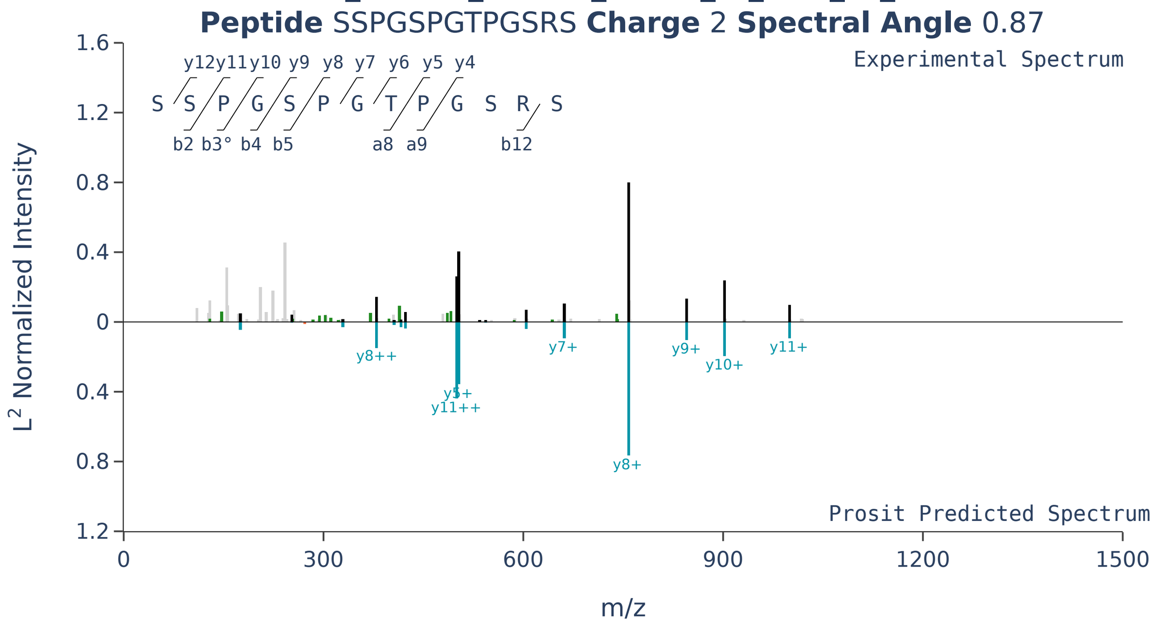

**Source** WSoh_221121_011221_HFGoe_L2_hTau_24h_R1 **Scan** 20691
**Peptide** TAPVPMPDLKNVKS **Charge** 2 **Spectral Angle** 0.91

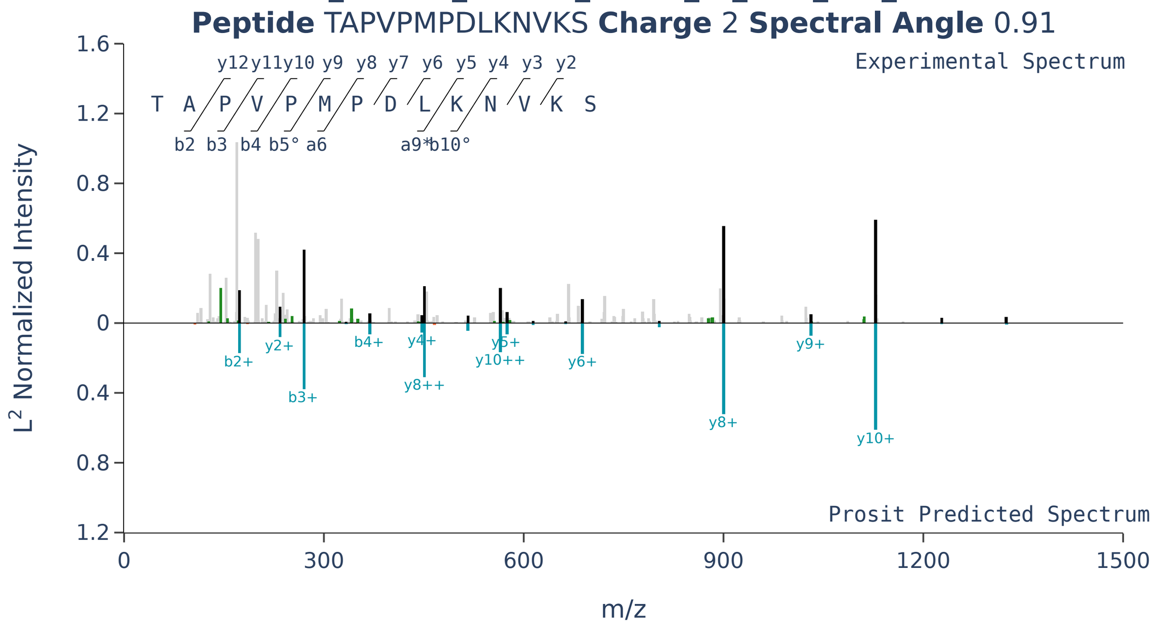


**Source** WSoh_221121_011221_HFGoe_L2_hTau_1h_R2 **Scan** 12454
**Peptide** DFKDRVQSKI **Charge** 3 **Spectral Angle** 0.9

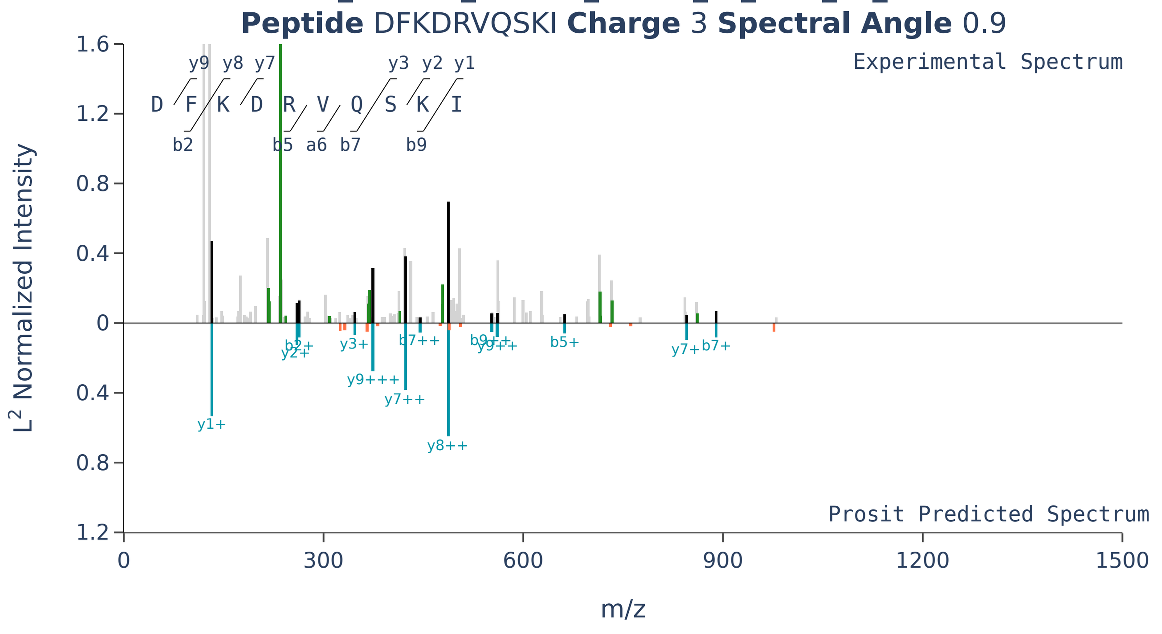


**Source** WSoh_221121_011221_HFGoe_L1_hTau_24h_R2 **Scan** 8489
**Peptide** GLGDRKDQGGYTMHQD **Charge** 2 **Spectral Angle** 0.9

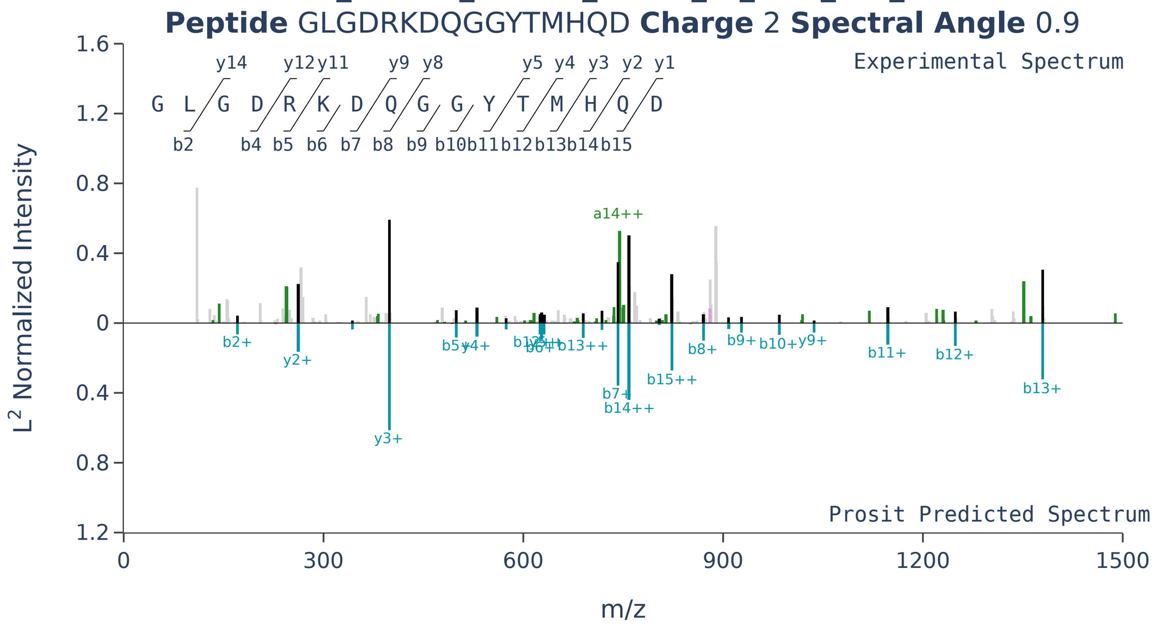


**Source** WSoh_221121_011221_HFGoe_L2_hTau_4h_R2 **Scan** 19816
**Peptide** AGLGDTPSLE **Charge** 2 **Spectral Angle** 0.95

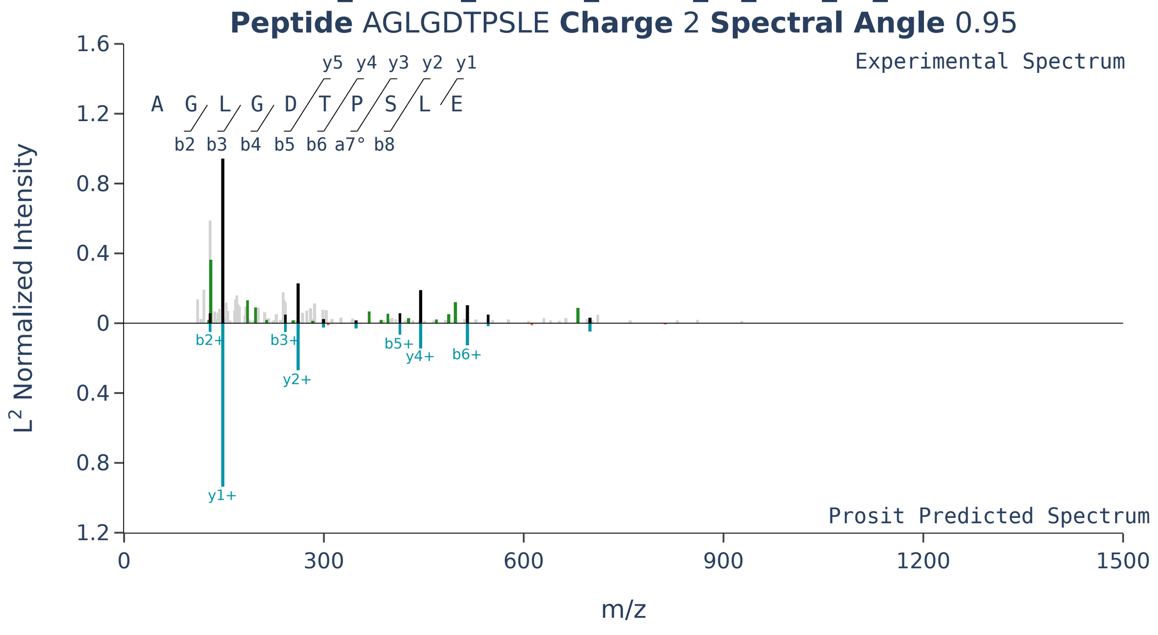


**Source** WSoh_221121_011221_HFGoe_L1_hTau_24h_R1 **Scan** 8160
**Peptide** SGDTSPRHLSNV **Charge** 2 **Spectral Angle** 0.9

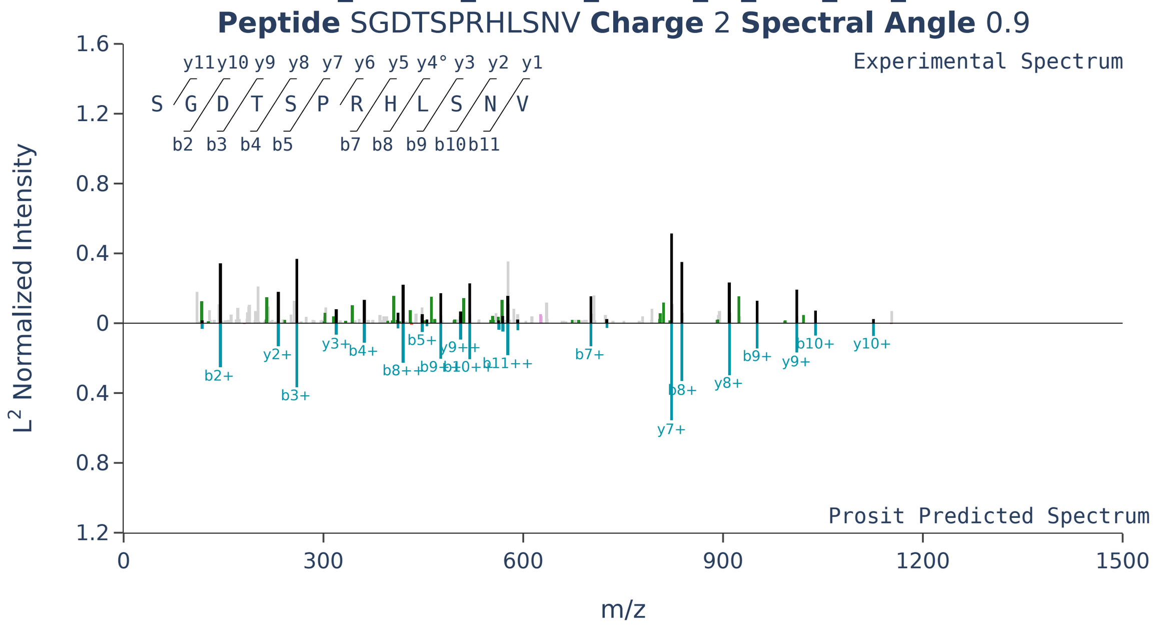


**Source** WSoh_221121_011221_HFGoe_L1_hTau_4h_R2 **Scan** 11809
**Peptide** PVVSGDTSPRHLSNVSS **Charge** 2 **Spectral Angle** 0.9

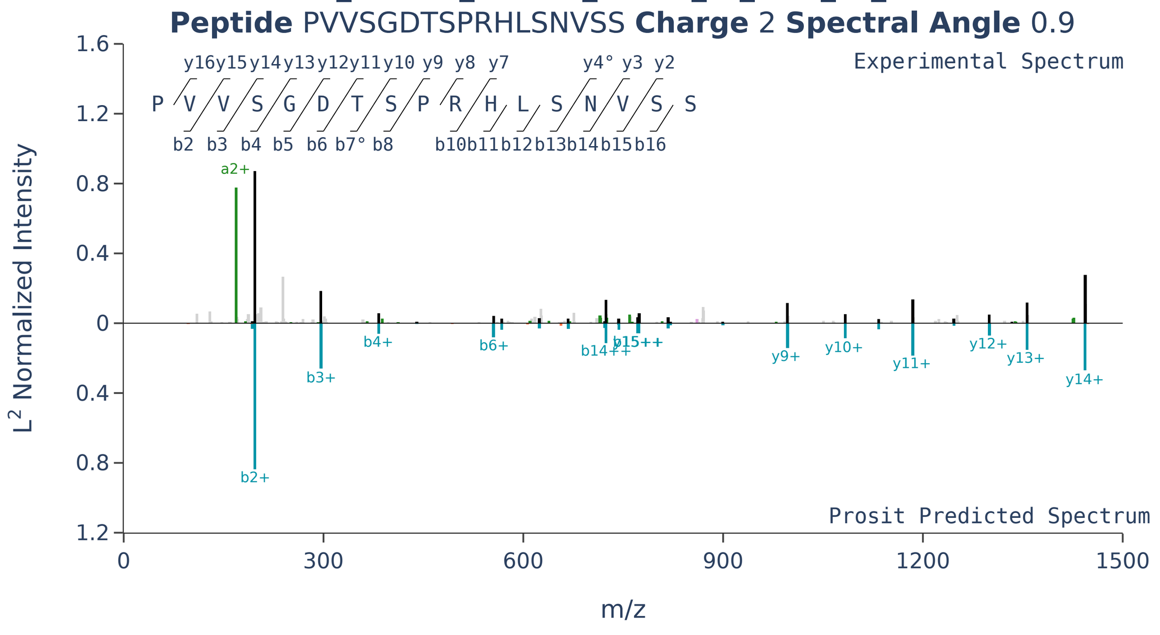


**Source** WSoh_221121_011221_HFGoe_L1_hTau_24h_R1 **Scan** 12487
**Peptide** TPSLPTPPTREPKK **Charge** 2 **Spectral Angle** 0.86

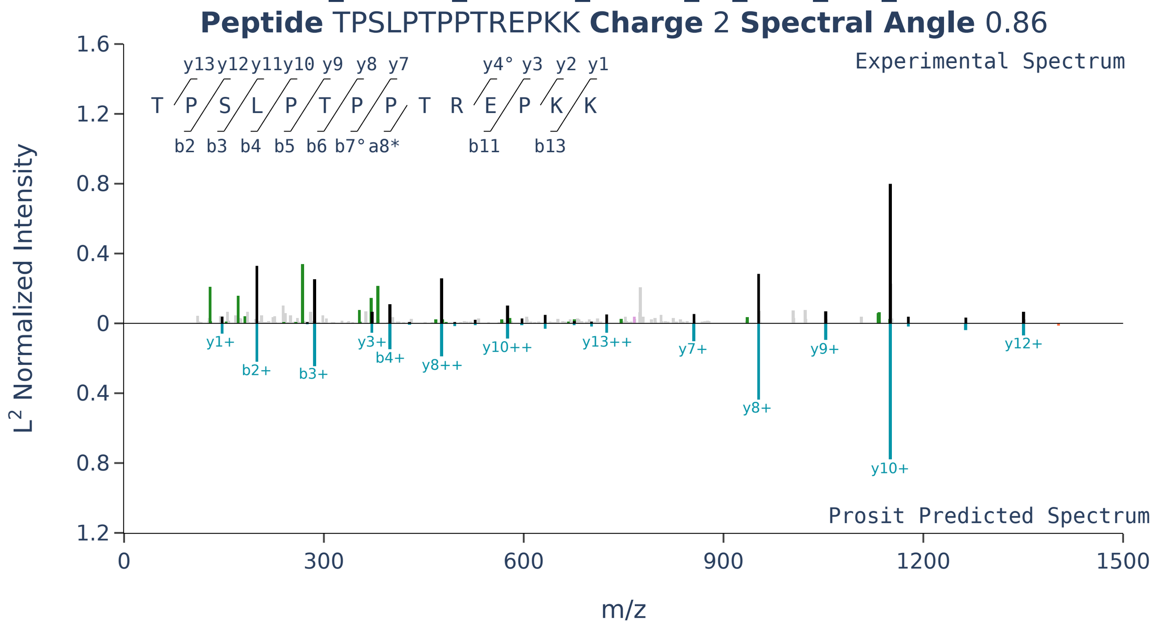


**Source** WSoh_221121_011221_HFGoe_L2_hTau_4h_R2 **Scan** 9513
**Peptide** NVSSTGSLD **Charge** 2 **Spectral Angle** 0.89

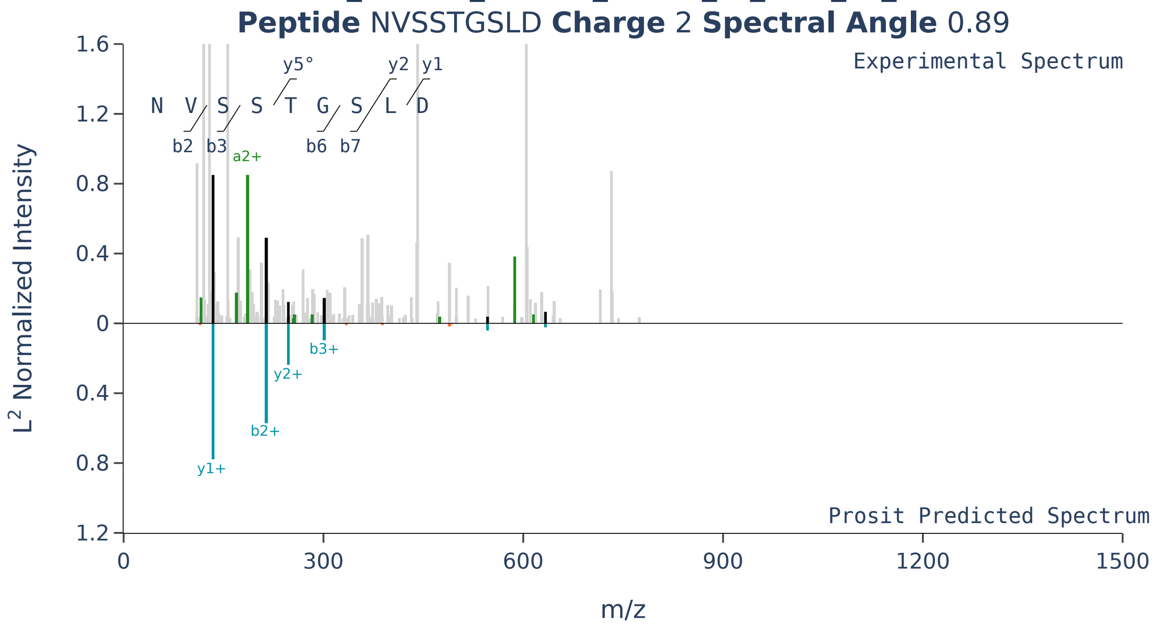


**Source** WSoh_221121_011221_HFGoe_L1_hTau_24h_R2 **Scan** 8699
**Peptide** PTPPTREPKKVAVVRT **Charge** 3 **Spectral Angle** 0.95

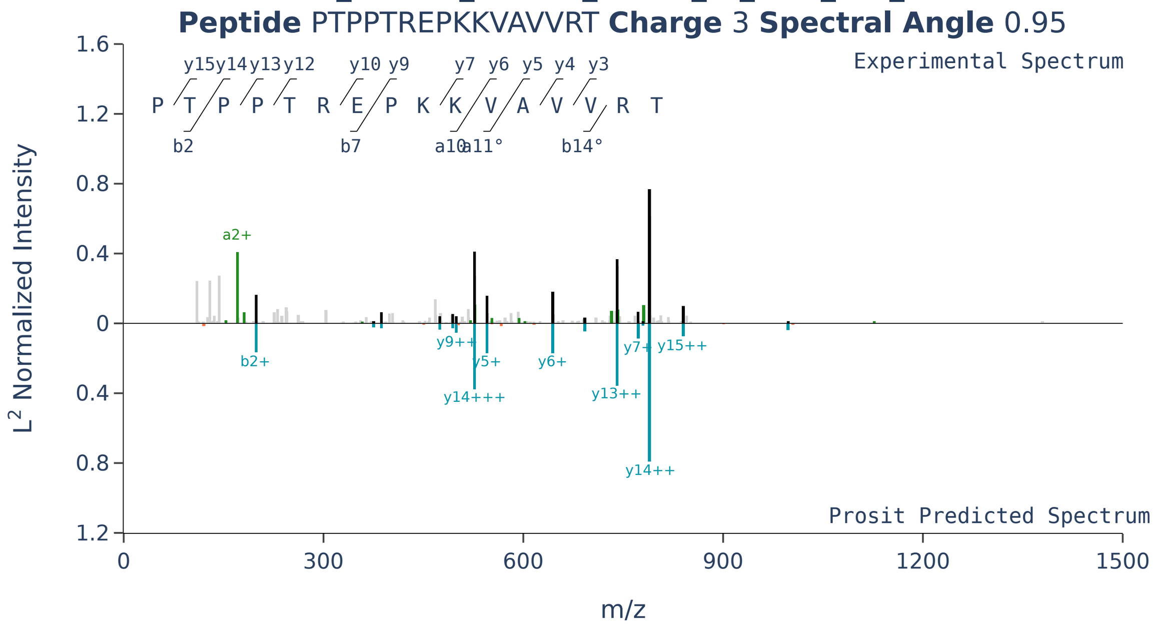


**Source** WSoh_221121_011221_HFGoe_L1_hTau_4h_R2 **Scan** 18614
**Peptide** SGDTSPRHLSNVSSTGSIDMV **Charge** 3 **Spectral Angle** 0.87

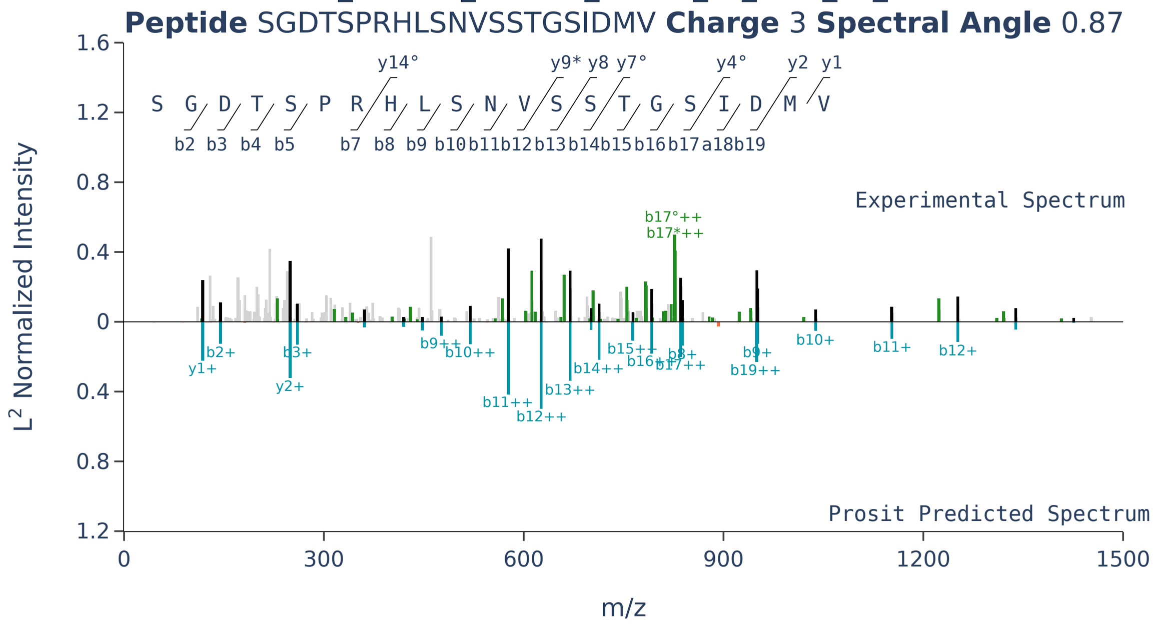


**Source** WSoh_221121_011221_HFGoe_L1_hTau_24h_R2 **Scan** 14725
**Peptide** SSTGSIDM **Charge** 1 **Spectral Angle** 0.88

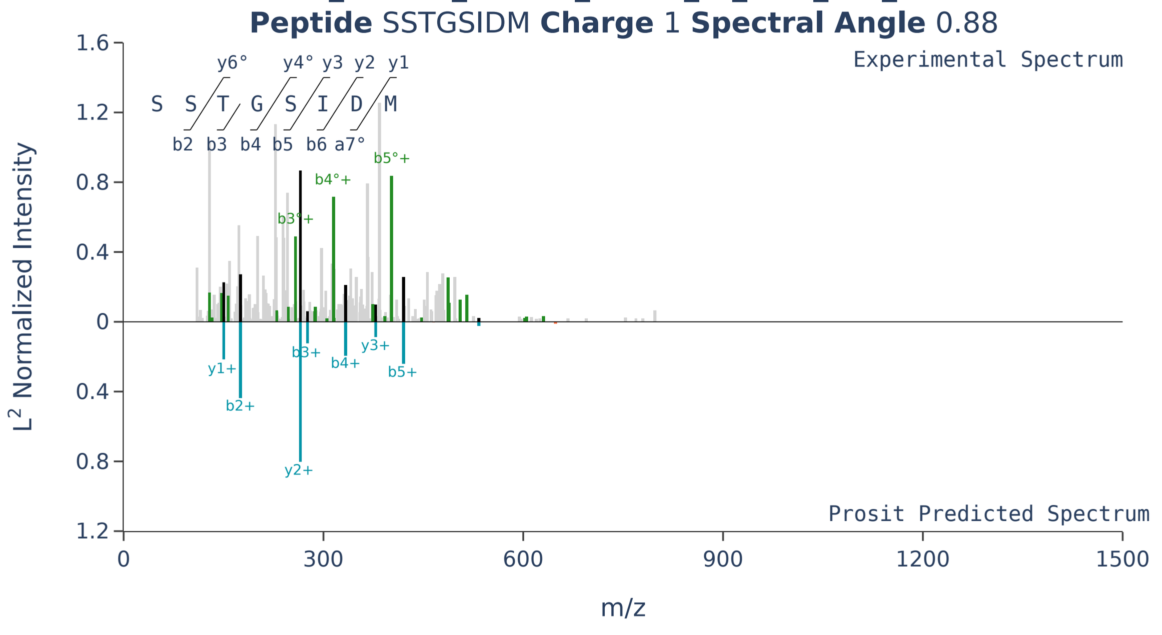


**Source** WSoh_221121_011221_HFGoe_L2_hTau_24h_R2 **Scan** 22971
**Peptide** TAEEAGIGDTPSL **Charge** 2 **Spectral Angle** 0.96

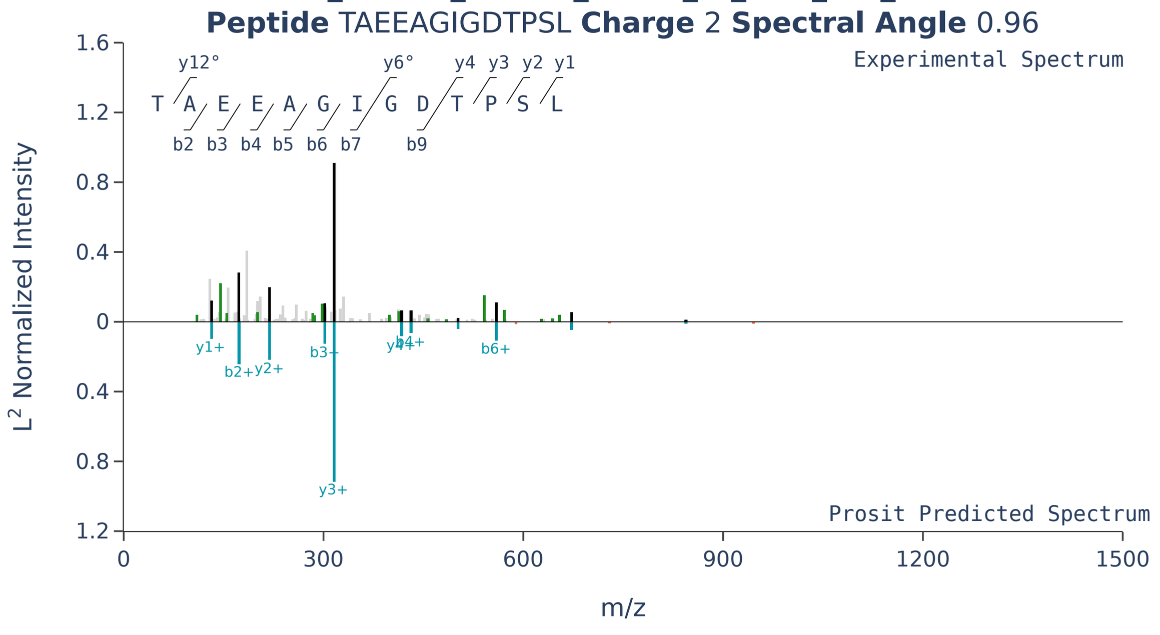


**Source** WSoh_221121_011221_HFGoe_L1_hTau_4h_R2 **Scan** 11894
**Peptide** GTYGLGDRKDQGGYTMH **Charge** 4 **Spectral Angle** 0.95

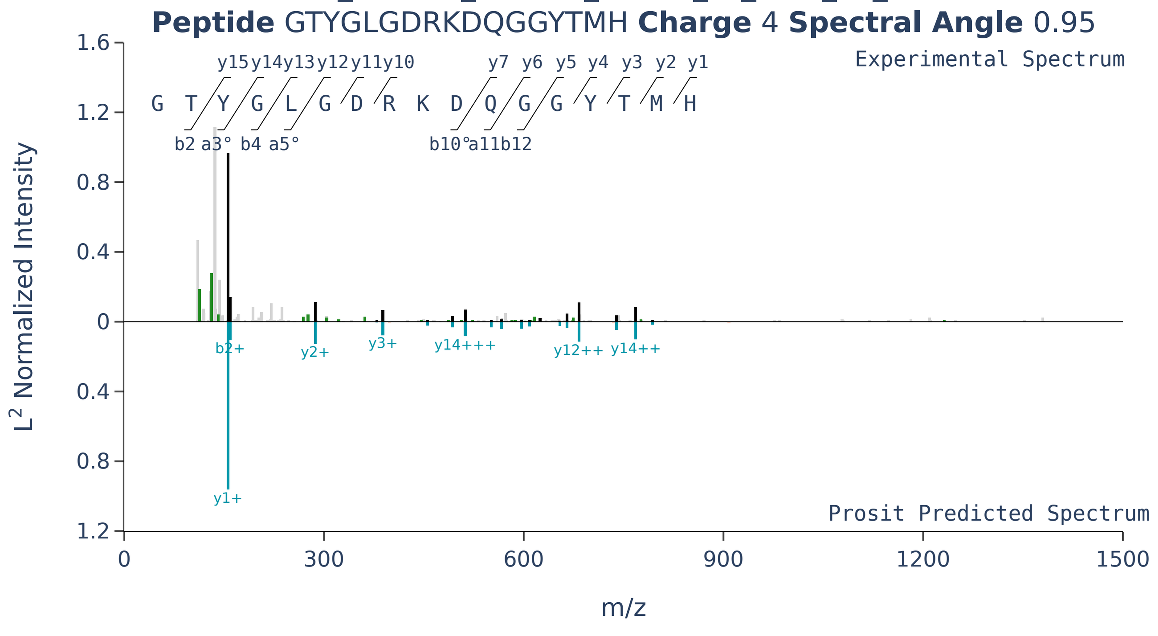


**Source** WSoh_221121_011221_HFGoe_L1_hTau_24h_R1 **Scan** 20236
**Peptide** PGGGSVQIVYKPVD **Charge** 2 **Spectral Angle** 0.9

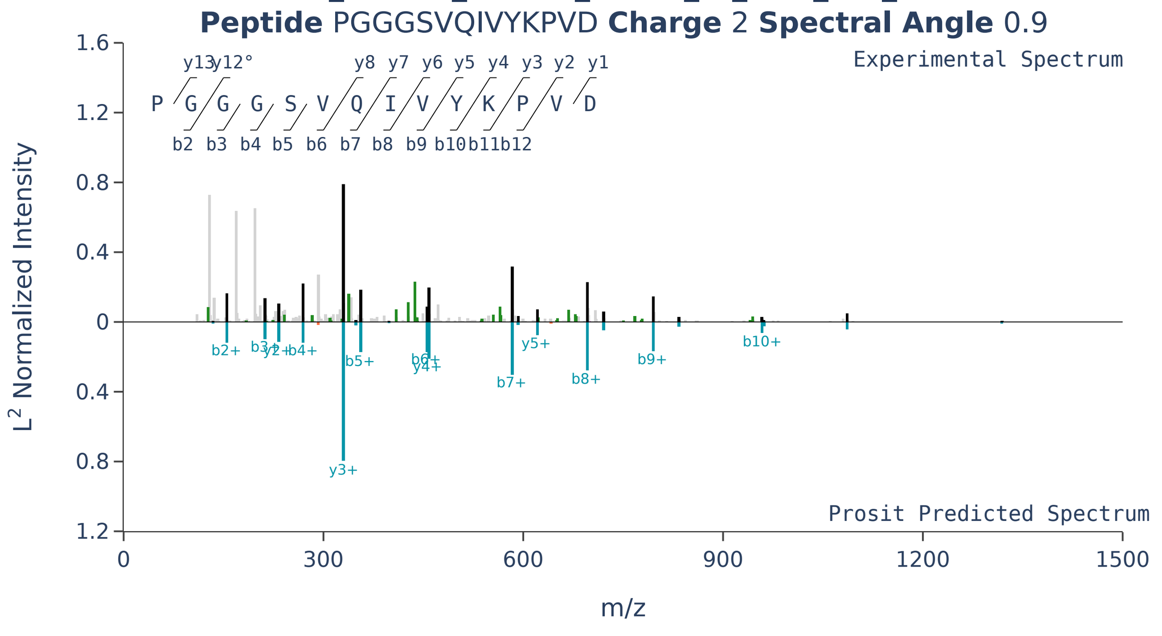


**Source** WSoh_221121_011221_HFGoe_L1_hTau_24h_R1 **Scan** 17829
**Peptide** QTAPVPMPDLKNVKSK **Charge** 3 **Spectral Angle** 0.88

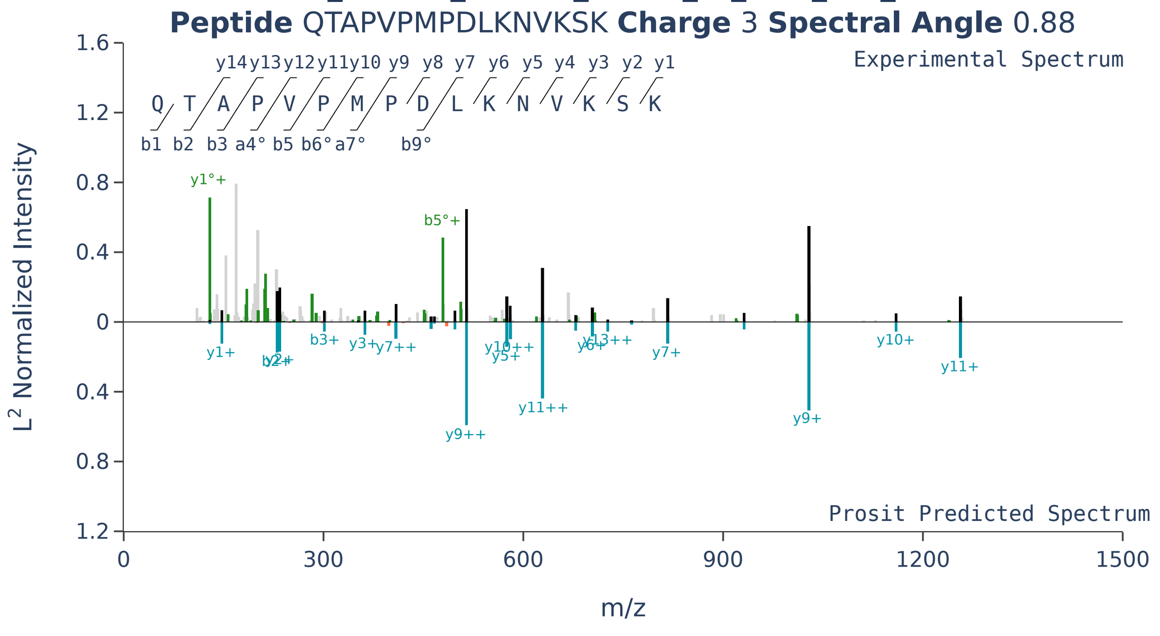

**Source** WSoh_221121_011221_HFGoe_L2_hTau_24h_R2 **Scan** 9649
**Peptide** PRHLSNVSSTGSID **Charge** 3 **Spectral Angle** 0.85

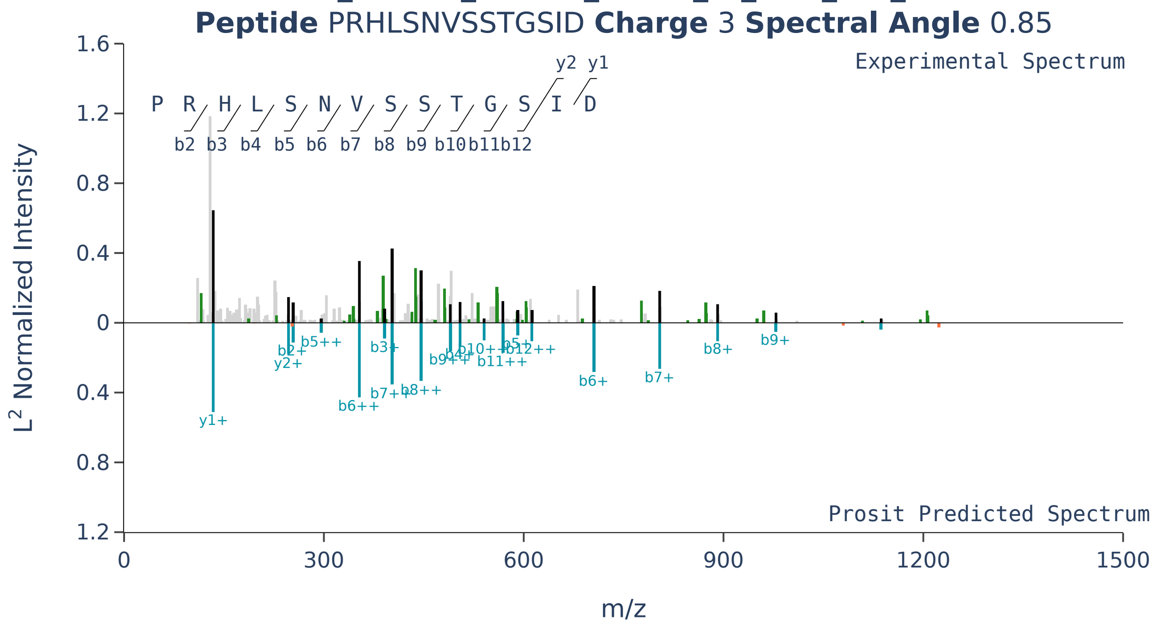


**Source** WSoh_221121_011221_HFGoe_L1_hTau_4h_R2 **Scan** 14529
**Peptide** PLQTPTEDGSEEPGSE **Charge** 2 **Spectral Angle** 0.89

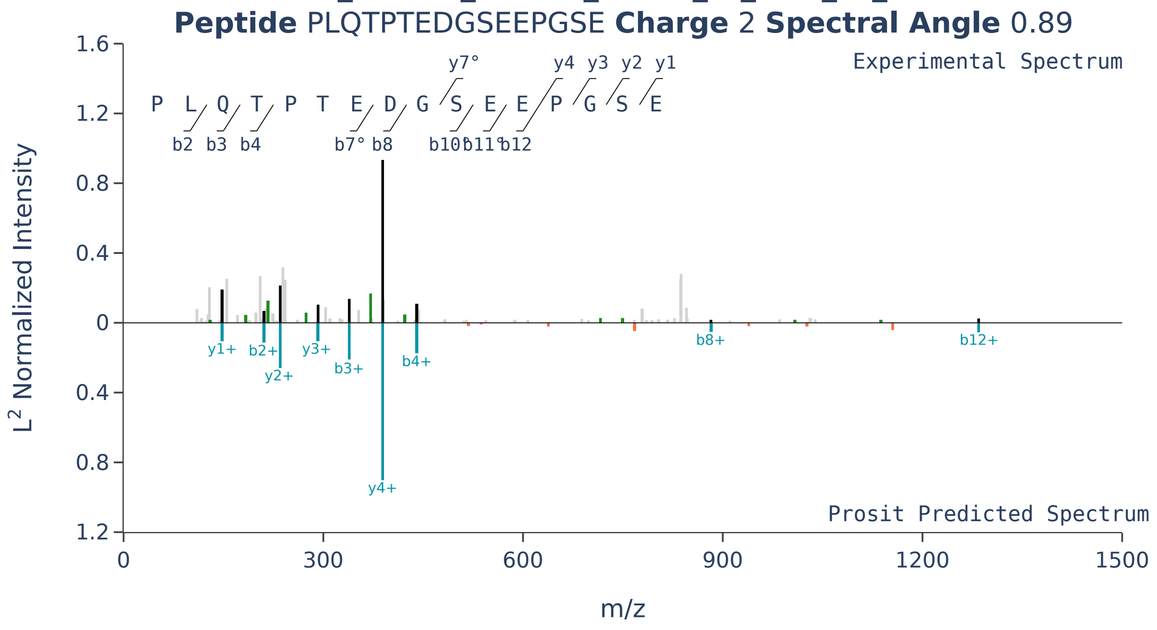


**Source** WSoh_221121_011221_HFGoe_L2_hTau_4h_R1 **Scan** 7020
**Peptide** SLGNIHHKPGGGQVE **Charge** 2 **Spectral Angle** 0.89

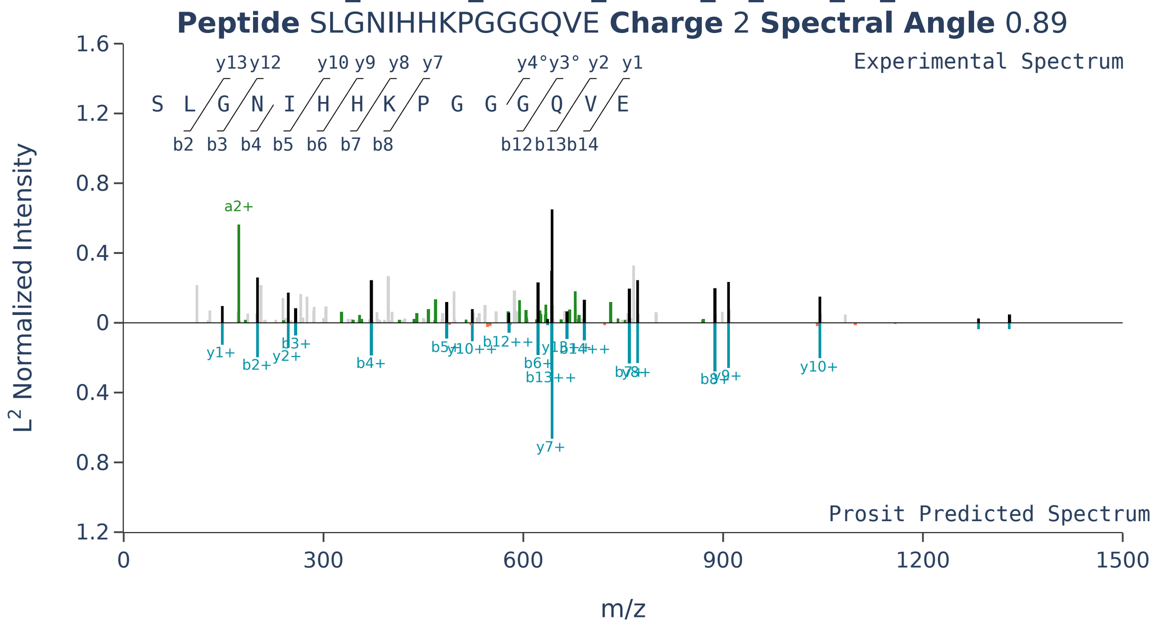


**Source** WSoh_221121_011221_HFGoe_L2_hTau_24h_R2 **Scan** 7803
**Peptide** DHAGTYGLGDRKDQGG **Charge** 2 **Spectral Angle** 0.91

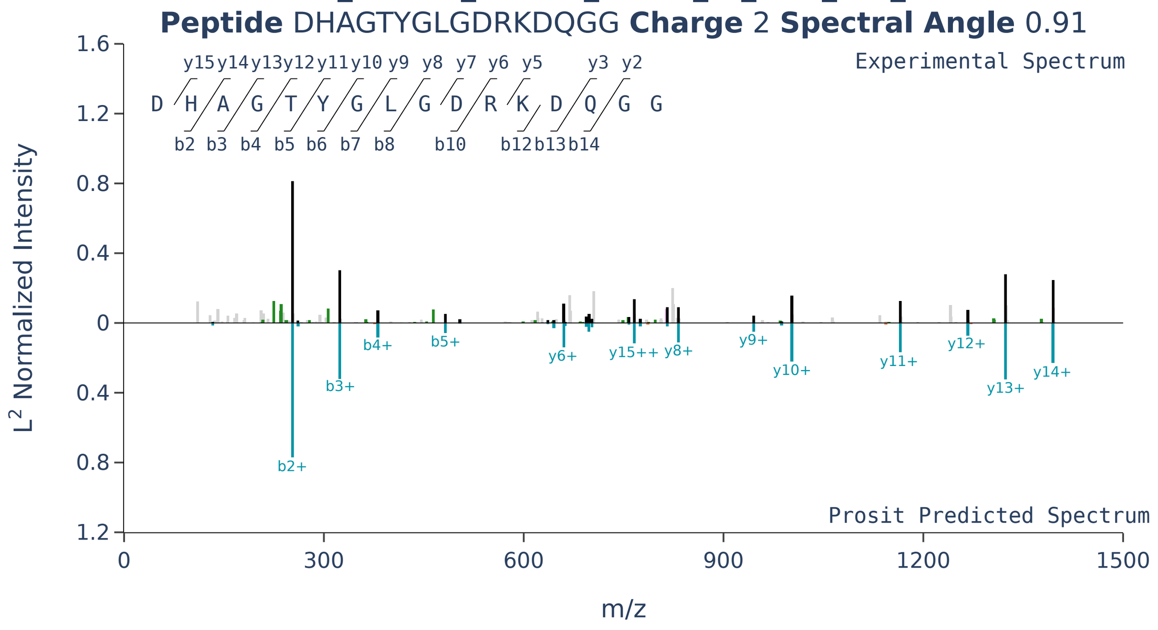


**Source** WSoh_221121_011221_HFGoe_L1_hTau_2h_R1 **Scan** 13245
**Peptide** KAKTDHGAEIVYKSPVV **Charge** 3 **Spectral Angle** 0.9

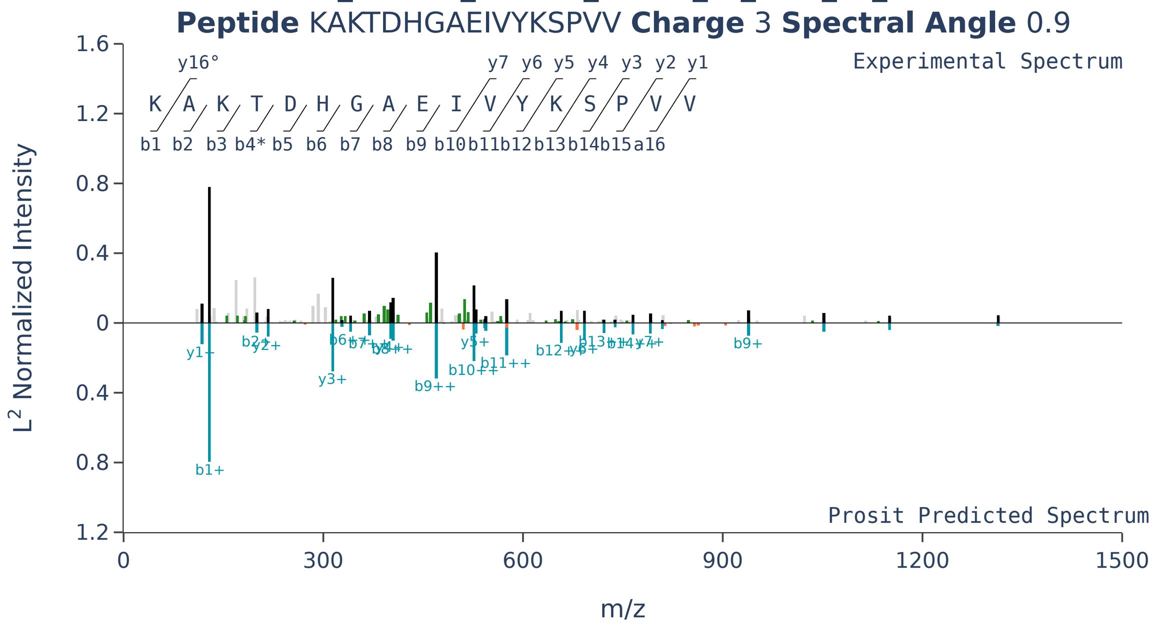


**Source** WSoh_221121_011221_HFGoe_L2_hTau_24h_R2 **Scan** 20255
**Peptide** SNVSSTGSIDMVDSPQ **Charge** 2 **Spectral Angle** 0.94

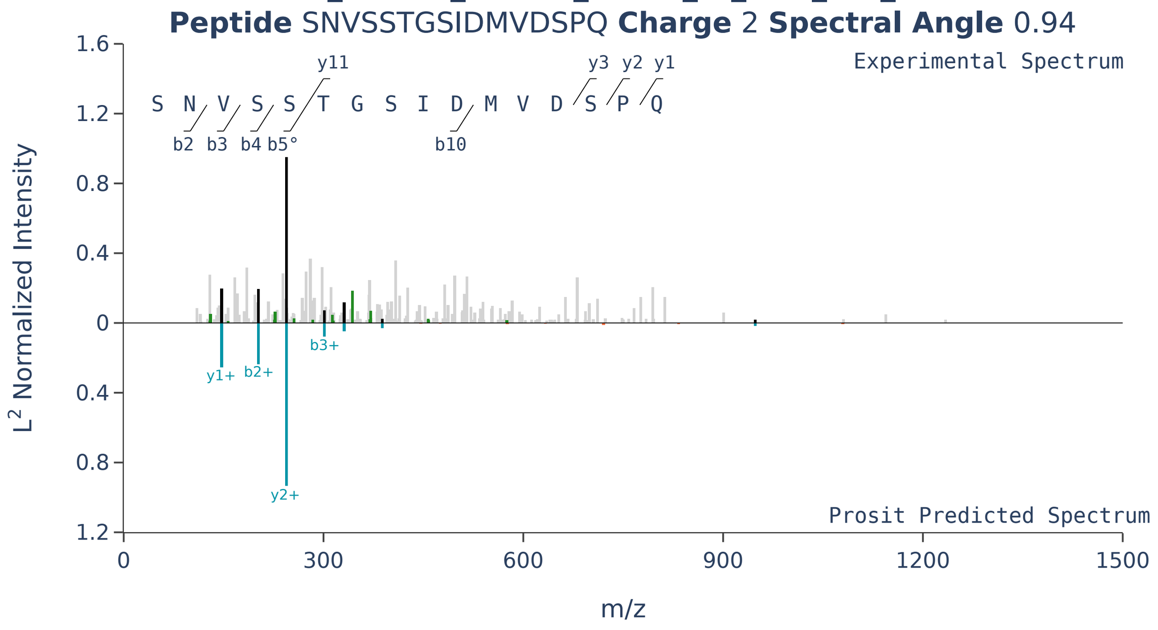


**Source** WSoh_221121_011221_HFGoe_L2_hTau_24h_R1 **Scan** 7216
**Peptide** PGGGKVQIINKK **Charge** 3 **Spectral Angle** 0.87

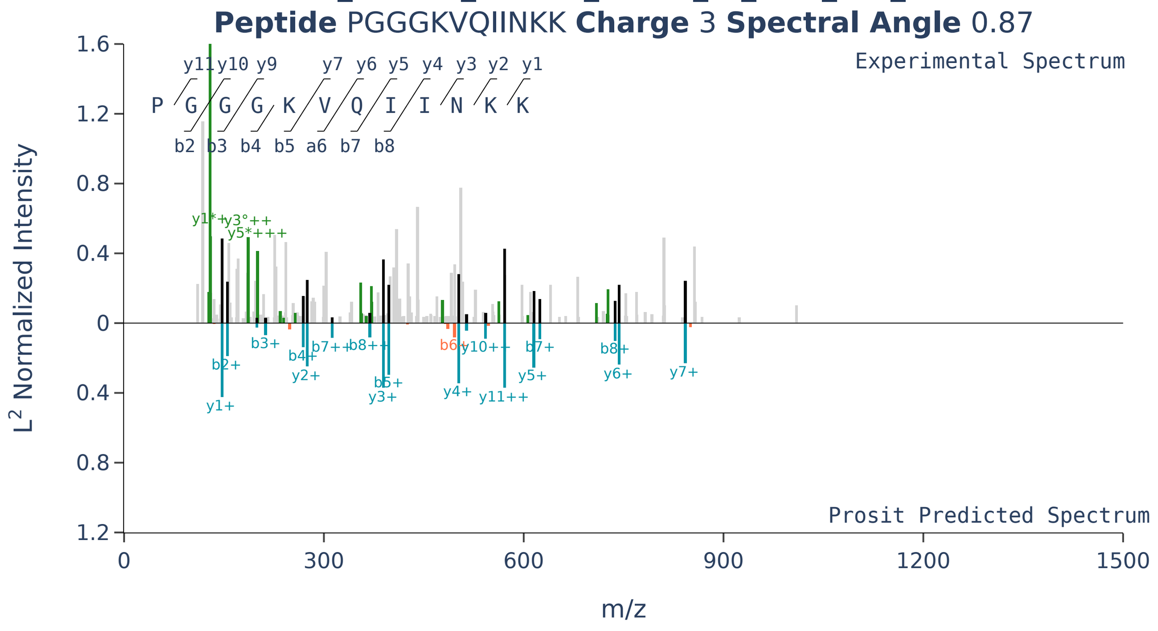


**Source** WSoh_221121_011221_HFGoe_L2_hTau_24h_R1 **Scan** 12447
**Peptide** SRTPSLPTPPTREPKKVA **Charge** 4 **Spectral Angle** 0.87

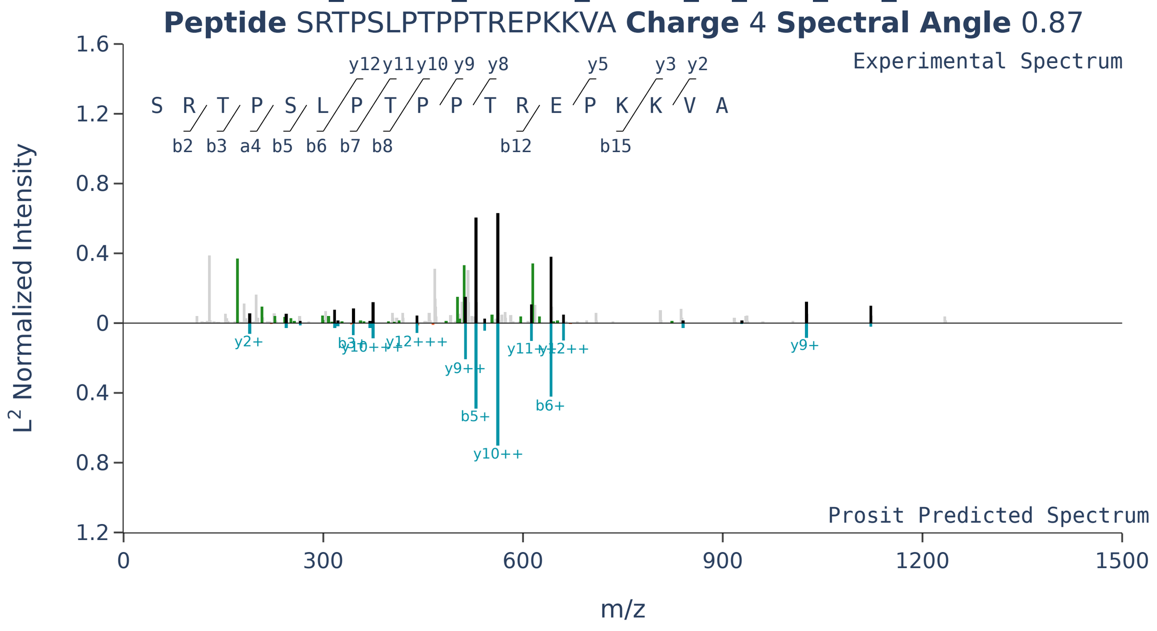


**Source** WSoh_221121_011221_HFGoe_L1_hTau_4h_R1 **Scan** 14238
**Peptide** NLKHVPGGGSVQIVYK **Charge** 4 **Spectral Angle** 0.9

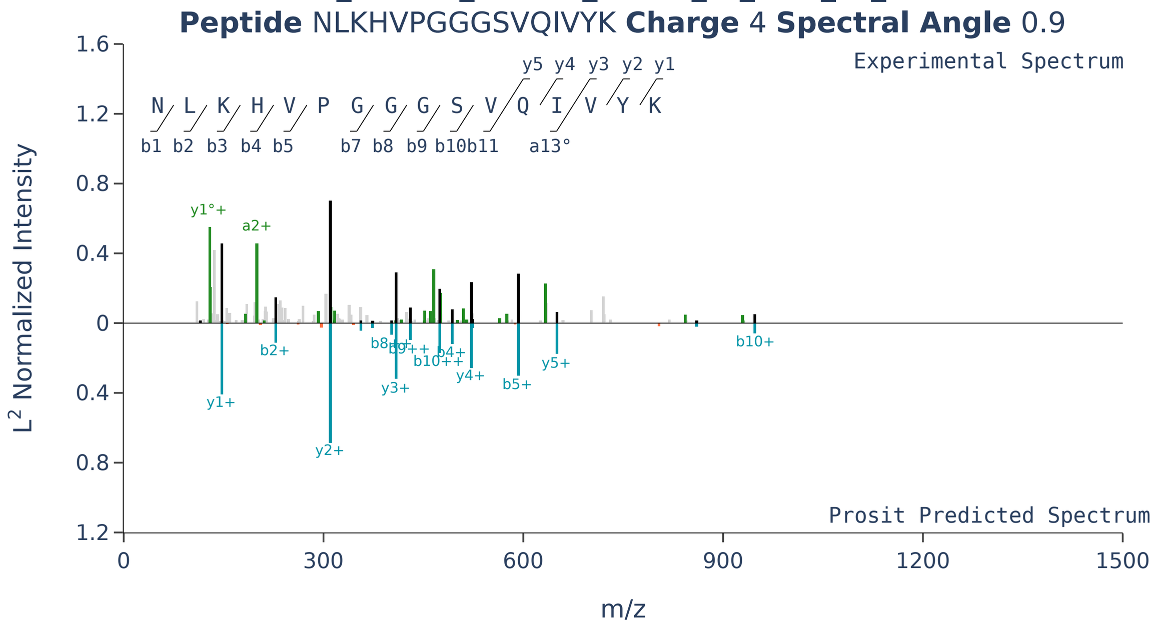


**Source** WSoh_221121_011221_HFGoe_L1_hTau_24h_R2 **Scan** 12260
**Peptide** KESPLQTPTE **Charge** 2 **Spectral Angle** 0.91

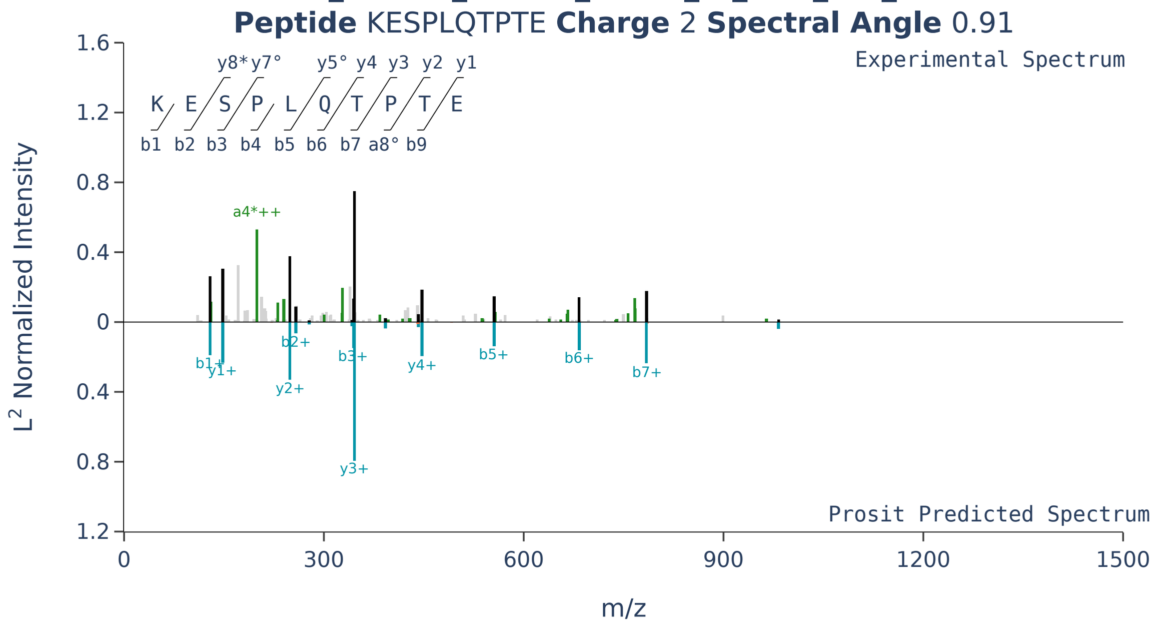


**Source** WSoh_221121_011221_HFGoe_L2_hTau_24h_R1 **Scan** 13082
**Peptide** LVYKPVD **Charge** 2 **Spectral Angle** 0.96

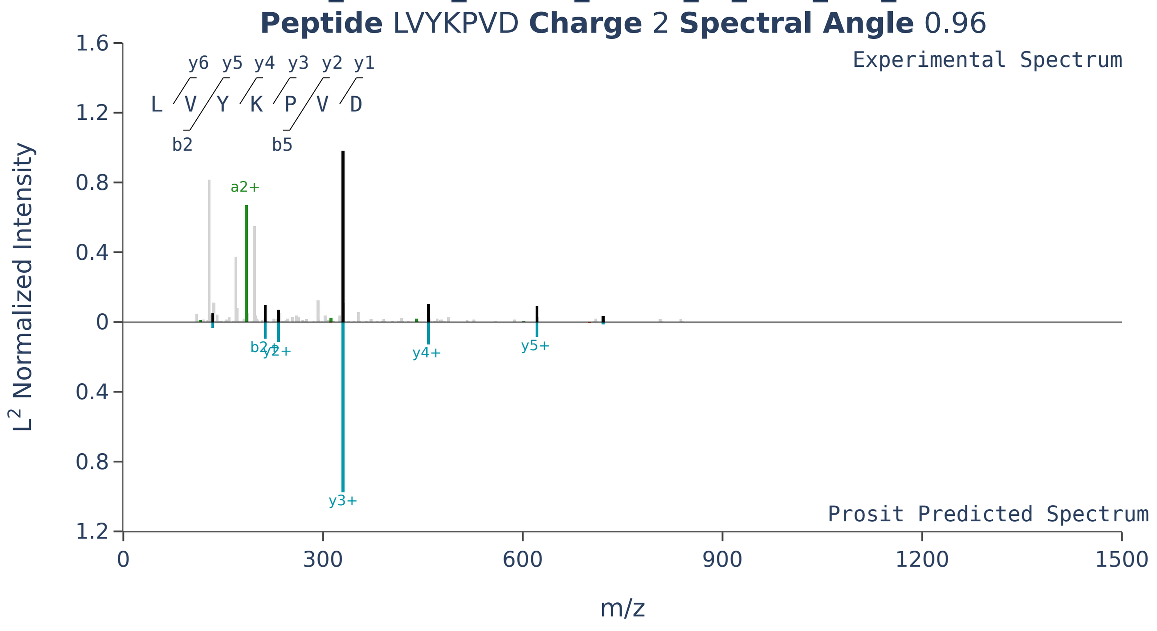


**Source** WSoh_221121_011221_HFGoe_L1_hTau_24h_R2 **Scan** 18851
**Peptide** GIGDTPSLEDEAAGHVTQAR **Charge** 3 **Spectral Angle** 0.84

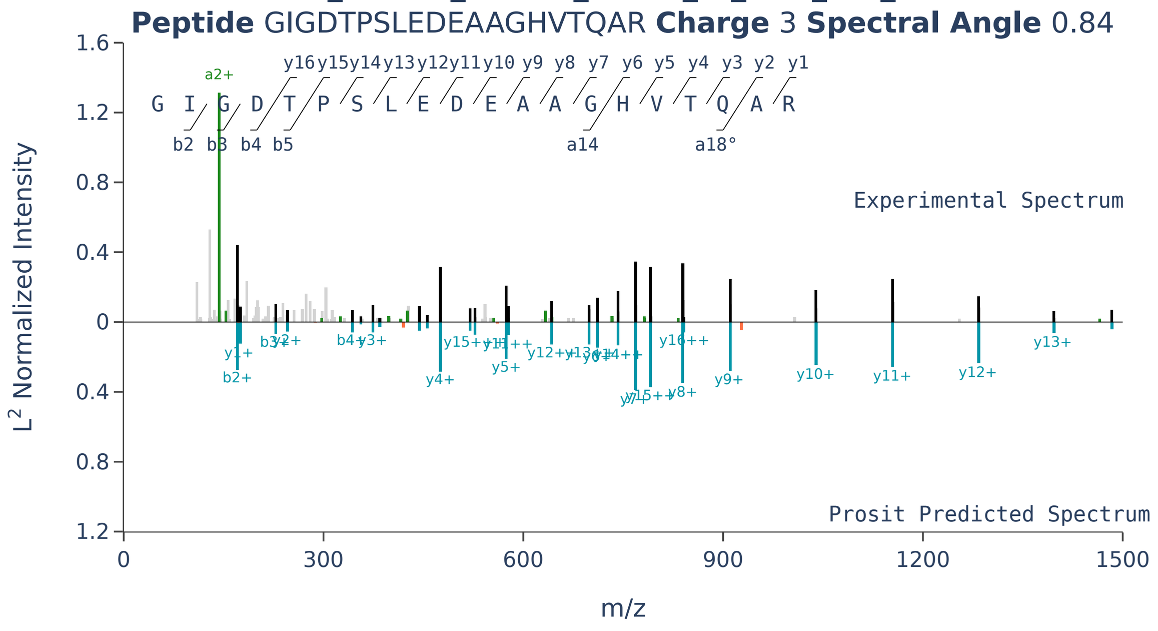


**Source** WSoh_221121_011221_HFGoe_L2_hTau_24h_R1 **Scan** 19754
**Peptide** GIGDTPSLED **Charge** 1 **Spectral Angle** 0.87

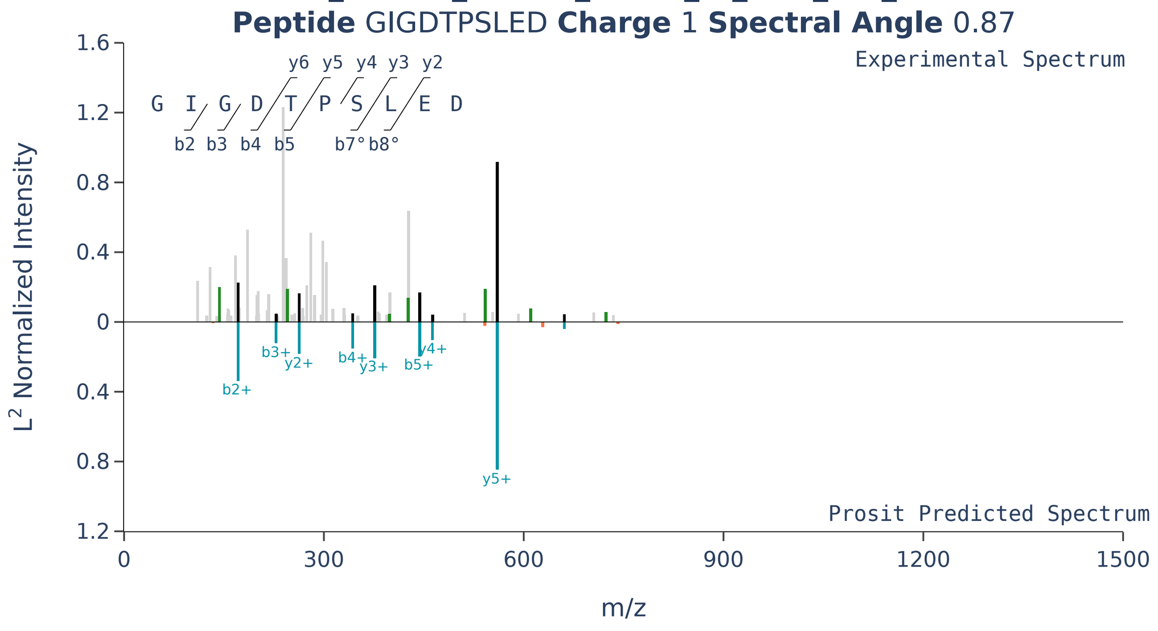


**Source** WSoh_221121_011221_HFGoe_L2_hTau_24h_R2 **Scan** 13780
**Peptide** GSKDNIKHVPGGGSVQIVYK **Charge** 3 **Spectral Angle** 0.89

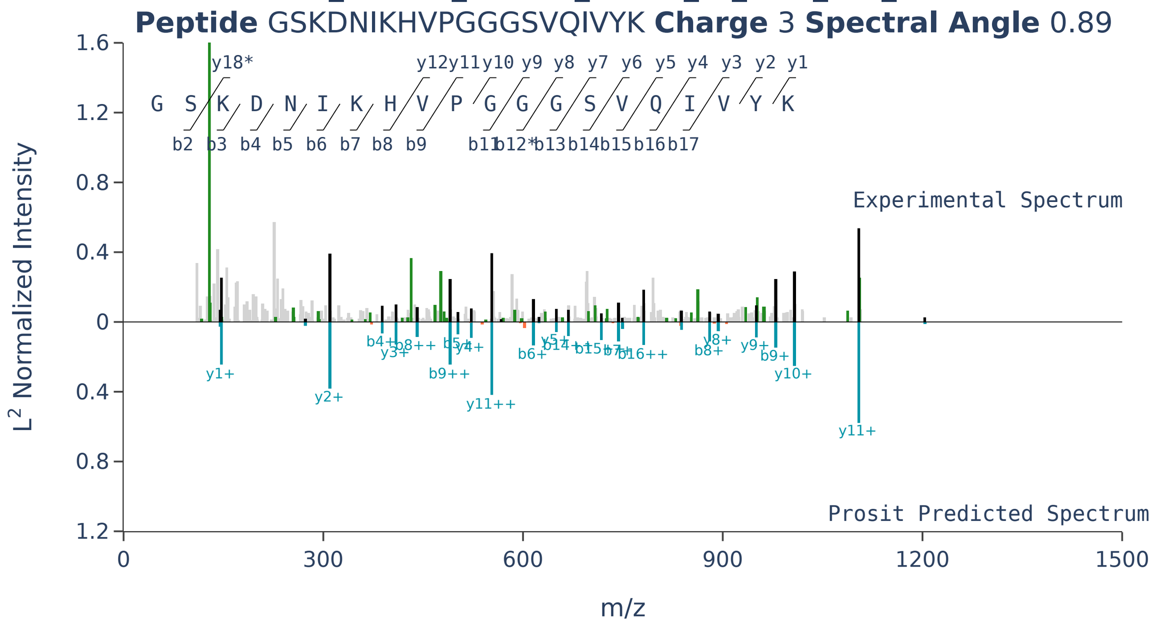


**Source** WSoh_221121_011221_HFGoe_L2_hTau_2h_R1 **Scan** 11608
**Peptide** ADEVSASLAKQG **Charge** 2 **Spectral Angle** 0.88

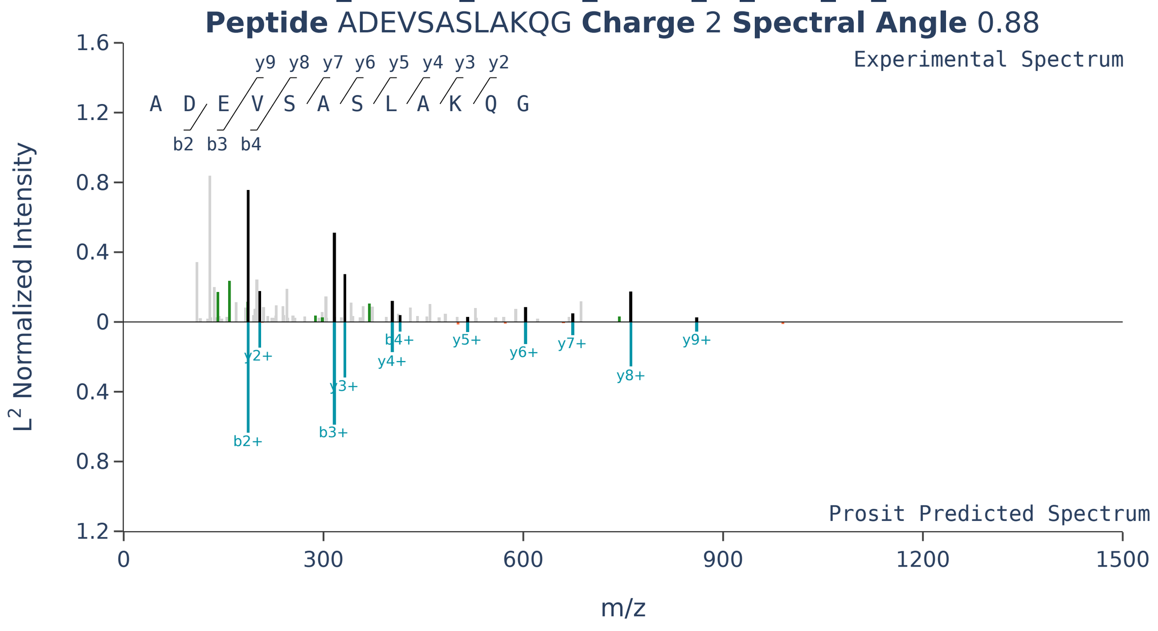

**Source** WSoh_221121_011221_HFGoe_L1_hTau_2h_R1 **Scan** 11565
**Peptide** KLDFKDRV **Charge** 2 **Spectral Angle** 0.85

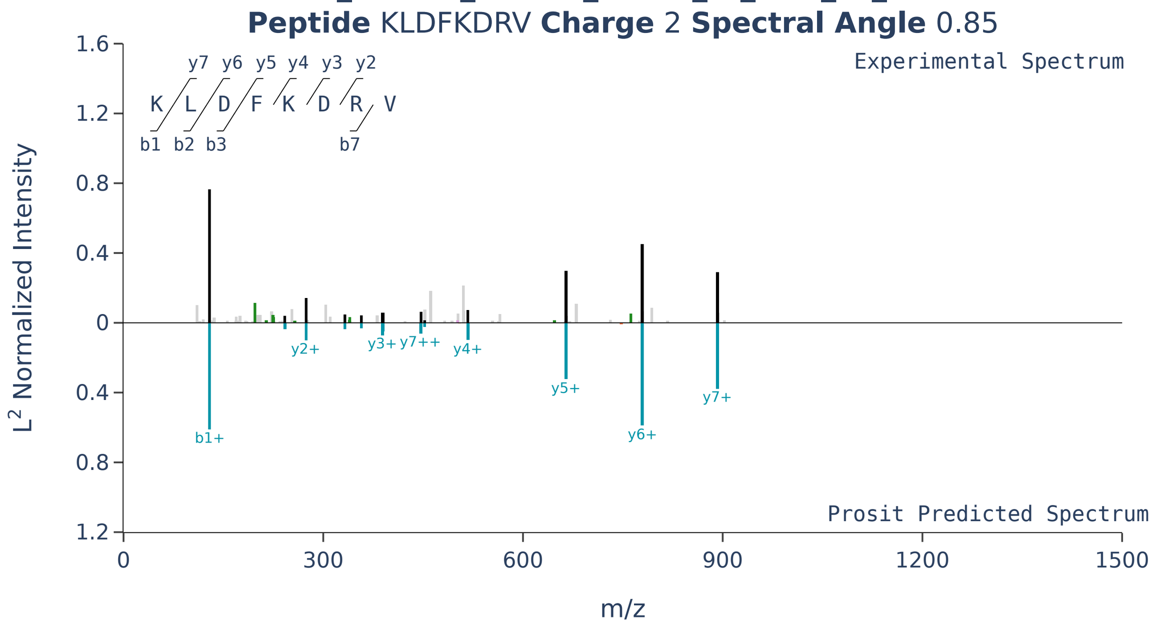


**Source** WSoh_221121_011221_HFGoe_L2_hTau_24h_R2 **Scan** 19723
**Peptide** GLGDTPSLEDEAAGHVTQARMVSK **Charge** 4 **Spectral Angle** 0.86

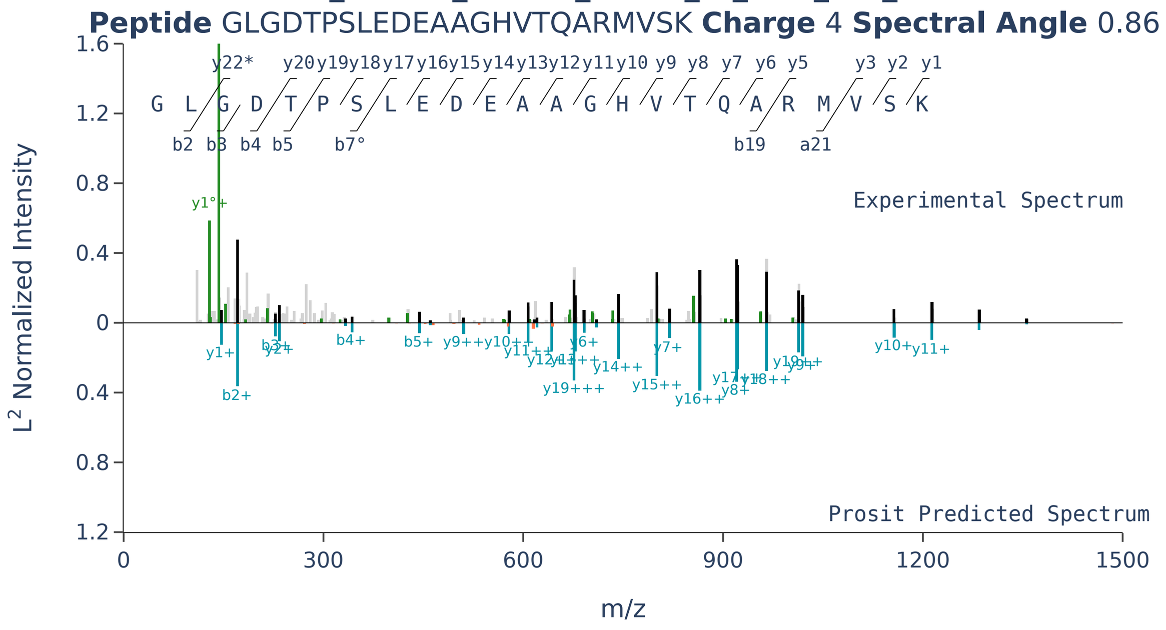


**Source** WSoh_221121_011221_HFGoe_L1_hTau_24h_R1 **Scan** 18423
**Peptide** GAEIVYKSPVVSGDTSPRHL **Charge** 3 **Spectral Angle** 0.84

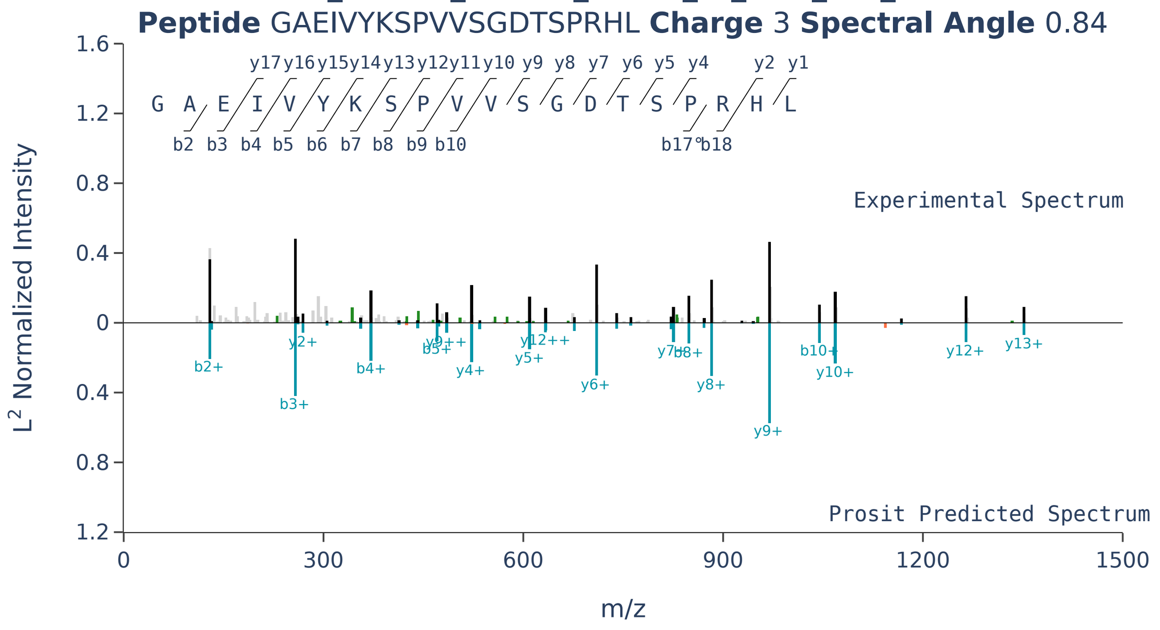


**Source** WSoh_221121_011221_HFGoe_L2_hTau_4h_R1 **Scan** 10572
**Peptide** GKVQIINKKL **Charge** 3 **Spectral Angle** 0.88

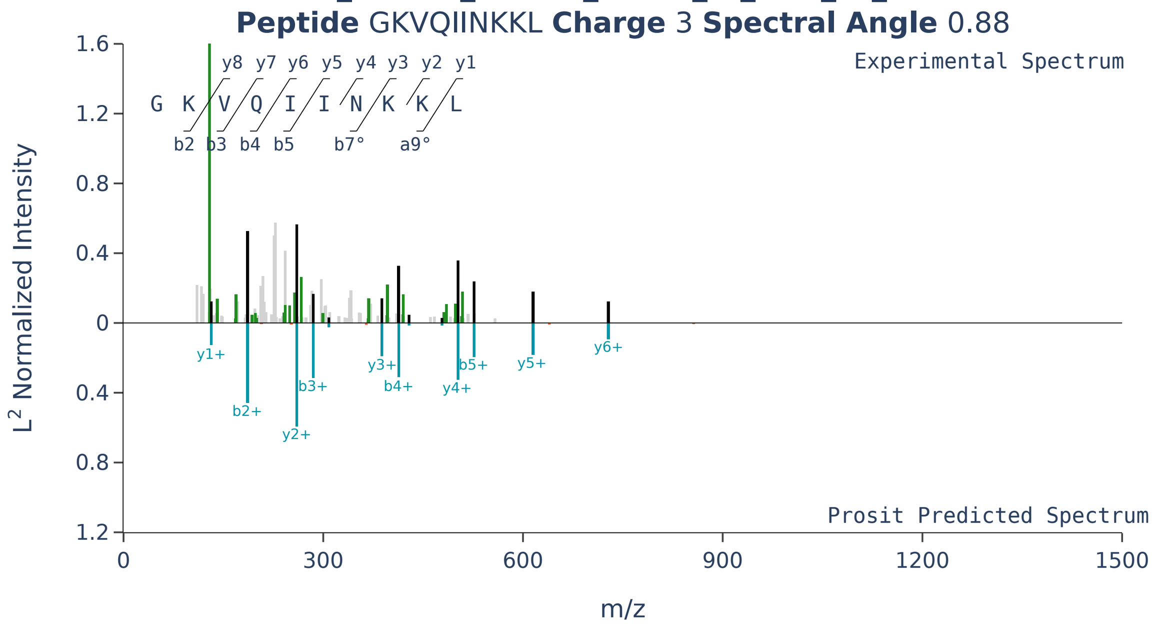


**Source** WSoh_221121_011221_HFGoe_L1_hTau_24h_R1 **Scan** 21187
**Peptide** GLGDTPSLEDEAAGHVTQA **Charge** 2 **Spectral Angle** 0.86

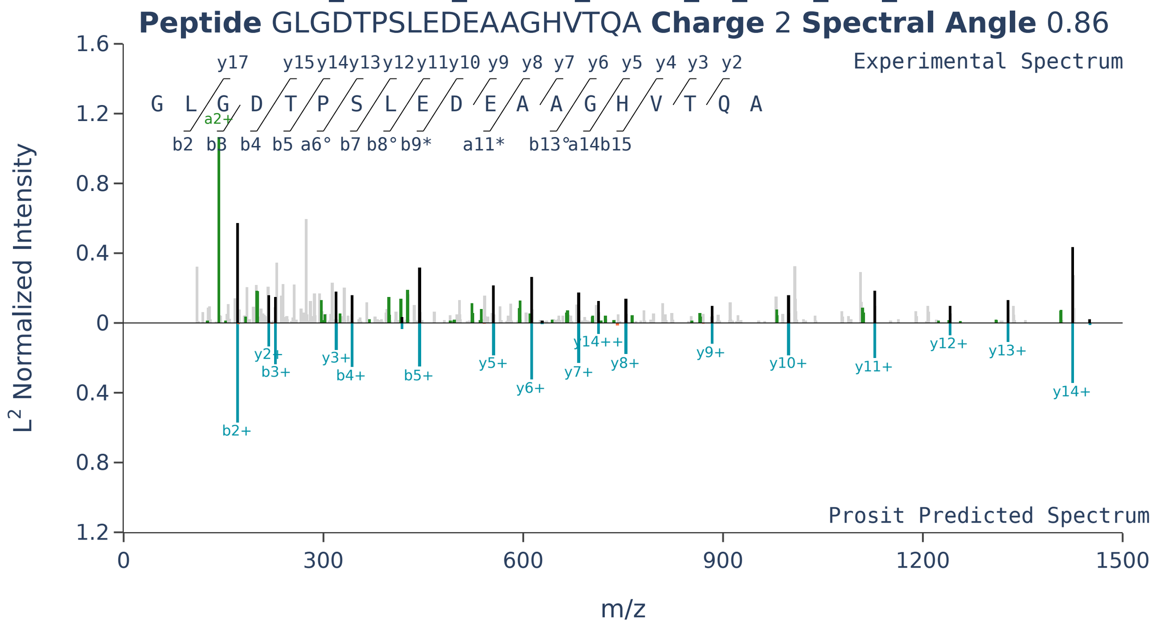


**Source** WSoh_221121_011221_HFGoe_L1_hTau_4h_R1 **Scan** 10192
**Peptide** PTPPTREPKKVAVV **Charge** 2 **Spectral Angle** 0.86

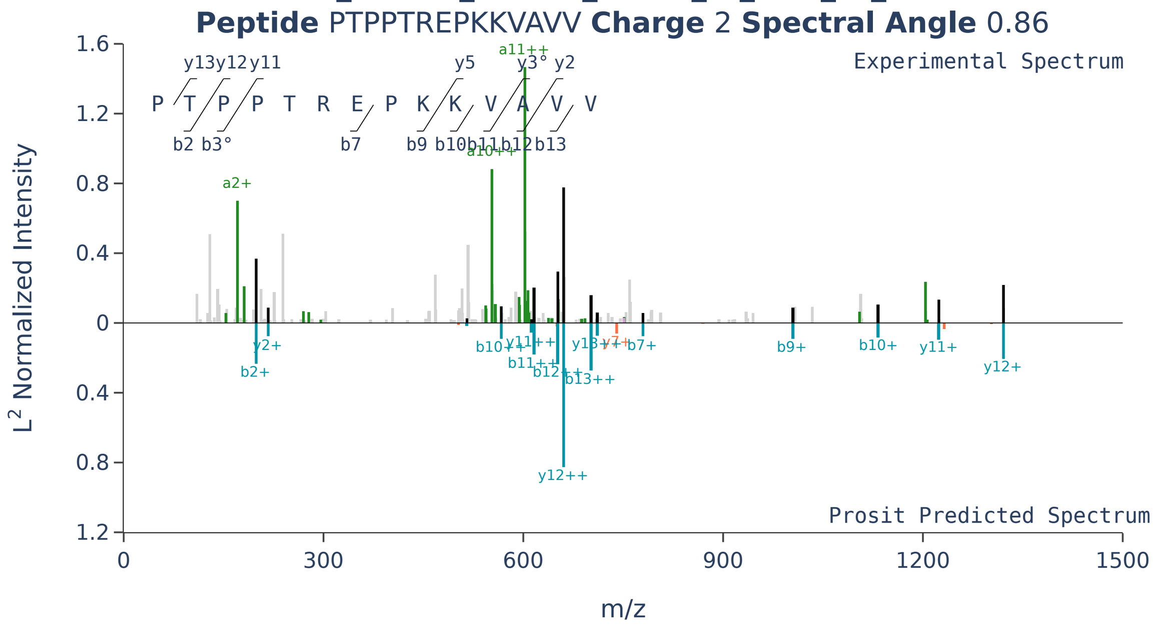


**Source** WSoh_221121_011221_HFGoe_L2_hTau_1h_R1 **Scan** 12311
**Peptide** LDFKDRVQS **Charge** 2 **Spectral Angle** 0.91

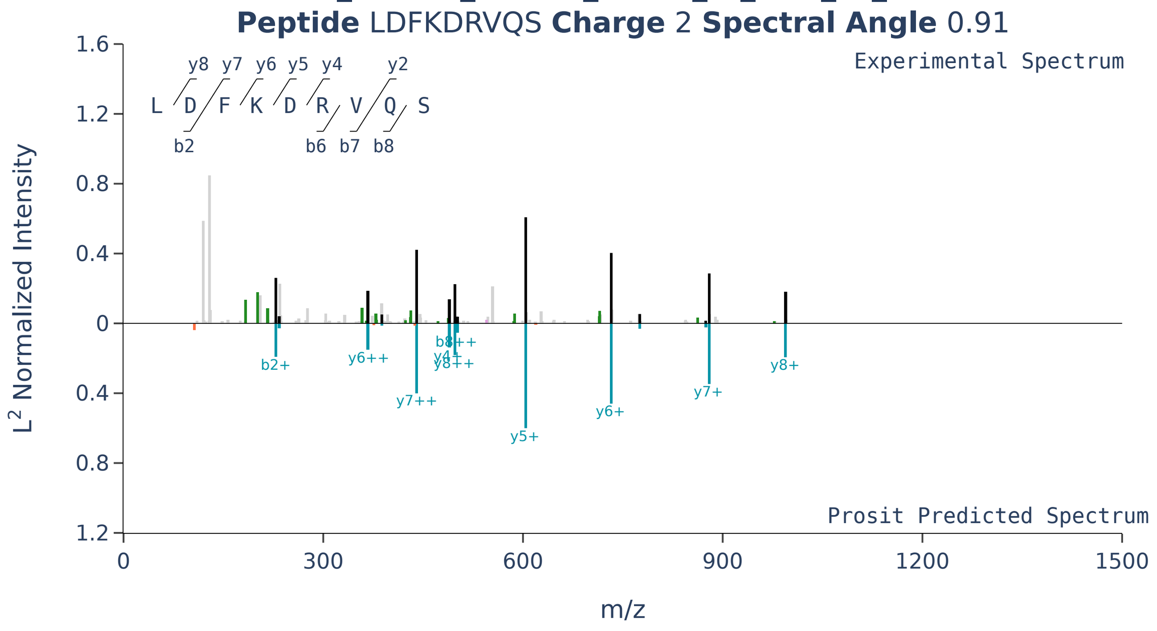


**Source** WSoh_221121_011221_HFGoe_L1_hTau_24h_R2 **Scan** 21990
**Peptide** AGIGDTPSLEDEAAGHVTQARM **Charge** 3 **Spectral Angle** 0.87

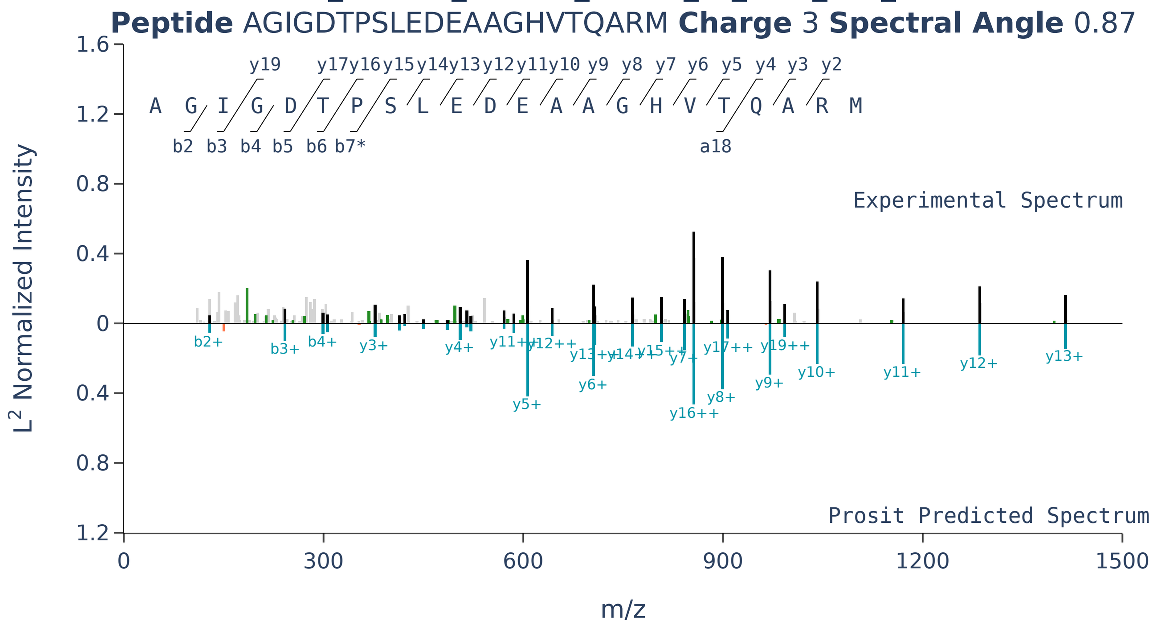


**Source** WSoh_221121_011221_HFGoe_L2_hTau_24h_R1 **Scan** 7248
**Peptide** NITHVPGGGNK **Charge** 3 **Spectral Angle** 0.86

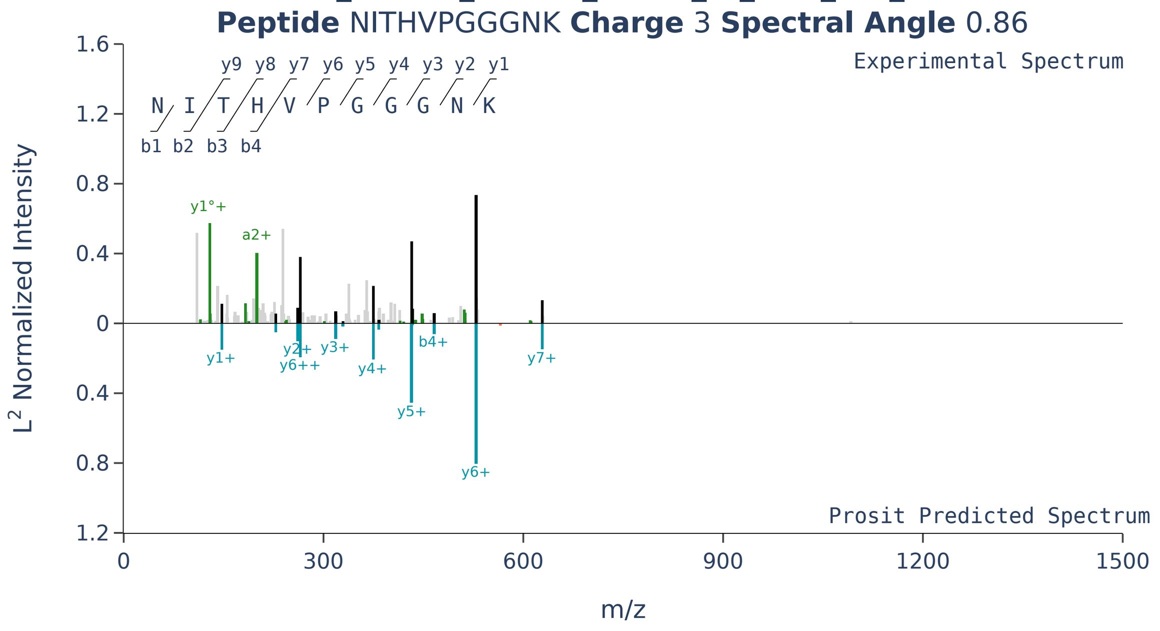


**Source** WSoh_221121_011221_HFGoe_L1_hTau_4h_R2 **Scan** 10898
**Peptide** AQPHTEIPEG **Charge** 2 **Spectral Angle** 0.94

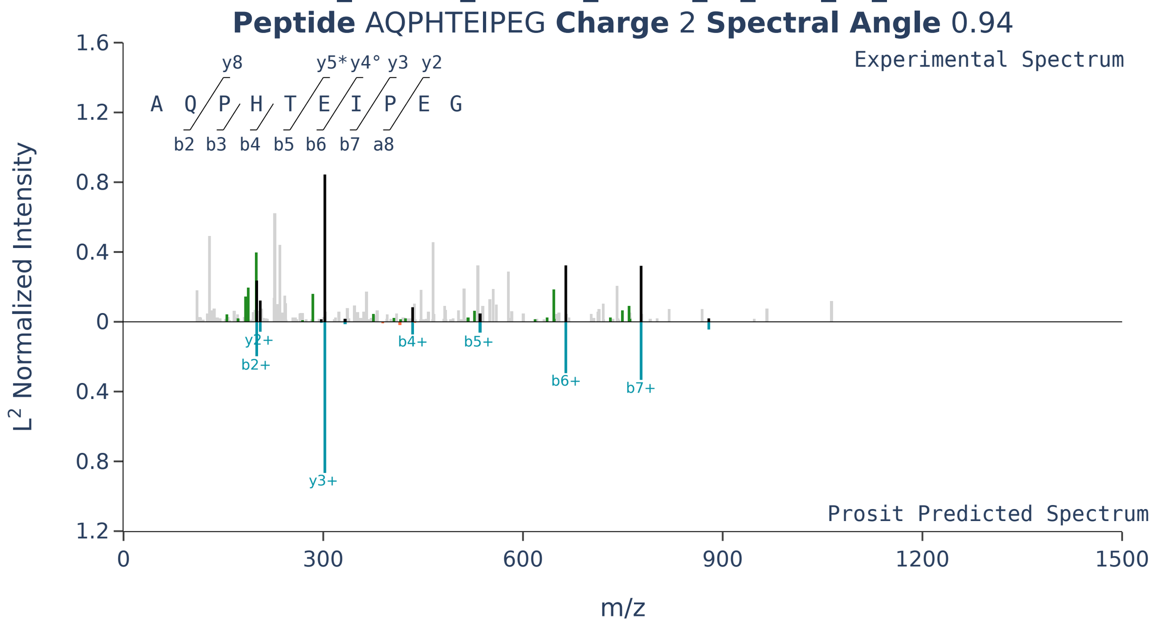


**Source** WSoh_221121_011221_HFGoe_L2_hTau_24h_R1 **Scan** 21070
**Peptide** AKSTPTAEDVTAPLVD **Charge** 2 **Spectral Angle** 0.91

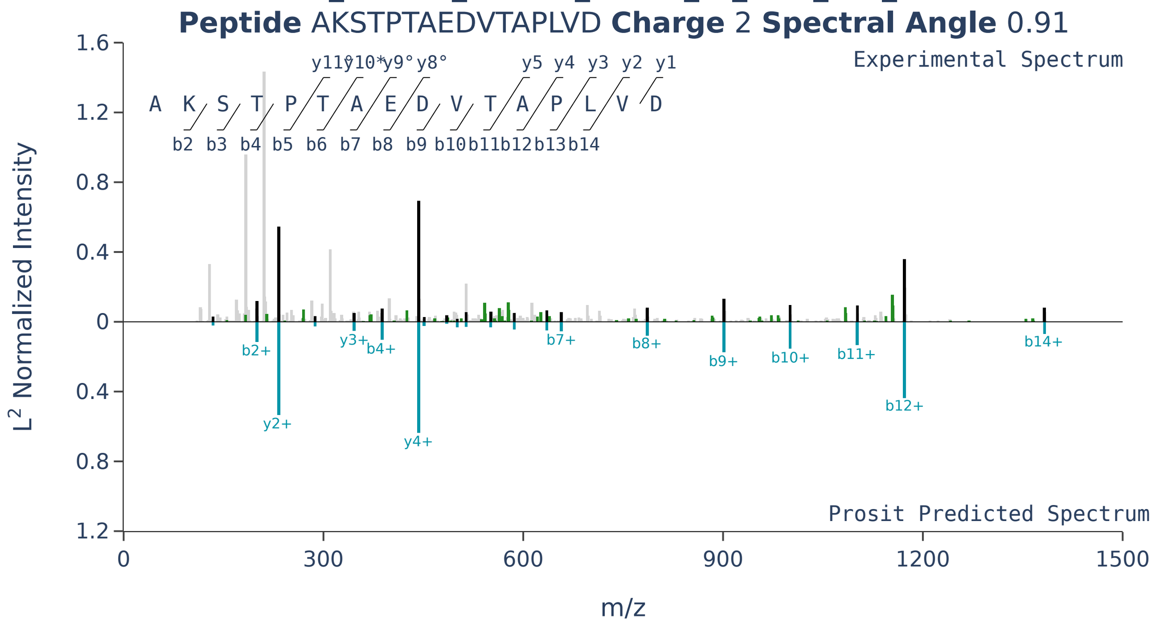


**Source** WSoh_221121_011221_HFGoe_L2_hTau_24h_R1 **Scan** 23459
**Peptide** AGIGDTPSLEDEAAGHVTQARMV **Charge** 3 **Spectral Angle** 0.87

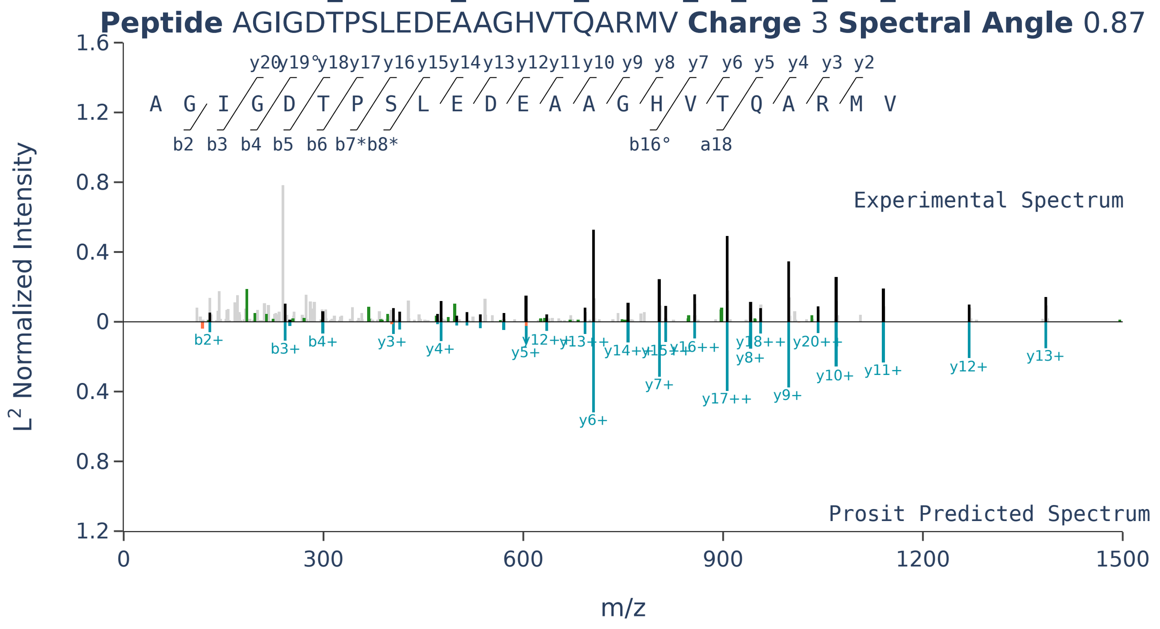


**Source** WSoh_221121_011221_HFGoe_L1_hTau_4h_R1 **Scan** 10405
**Peptide** PGGGNKKIETHKLTF **Charge** 2 **Spectral Angle** 0.9

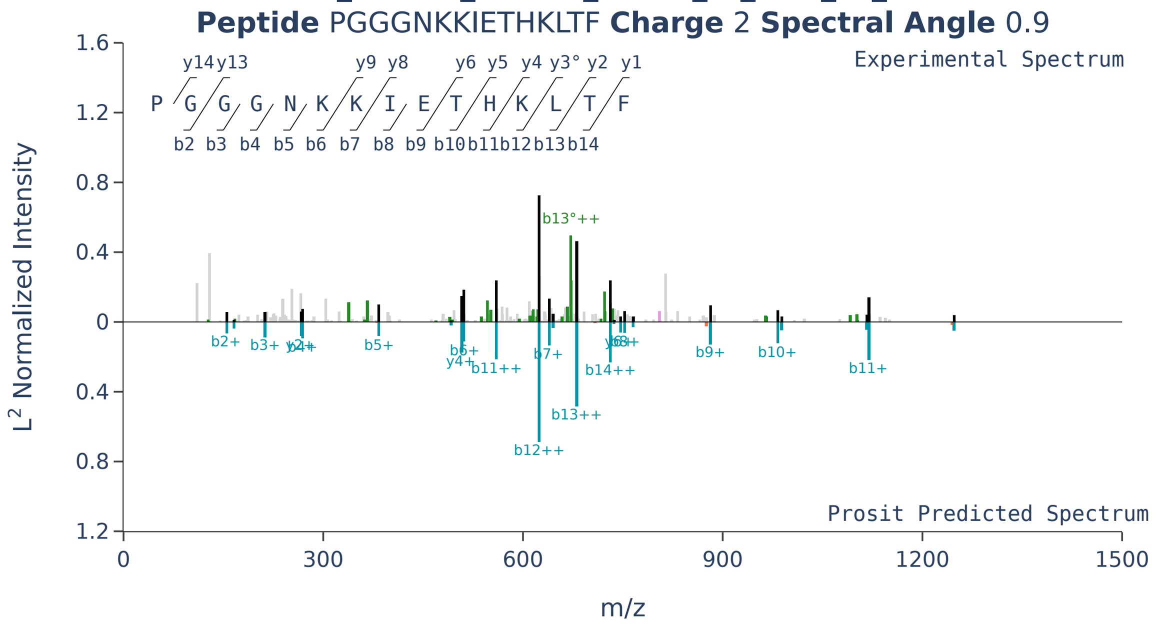


**Source** WSoh_221121_011221_HFGoe_L1_hTau_24h_R1 **Scan** 11979
**Peptide** DEVSASLAKQ **Charge** 2 **Spectral Angle** 0.9

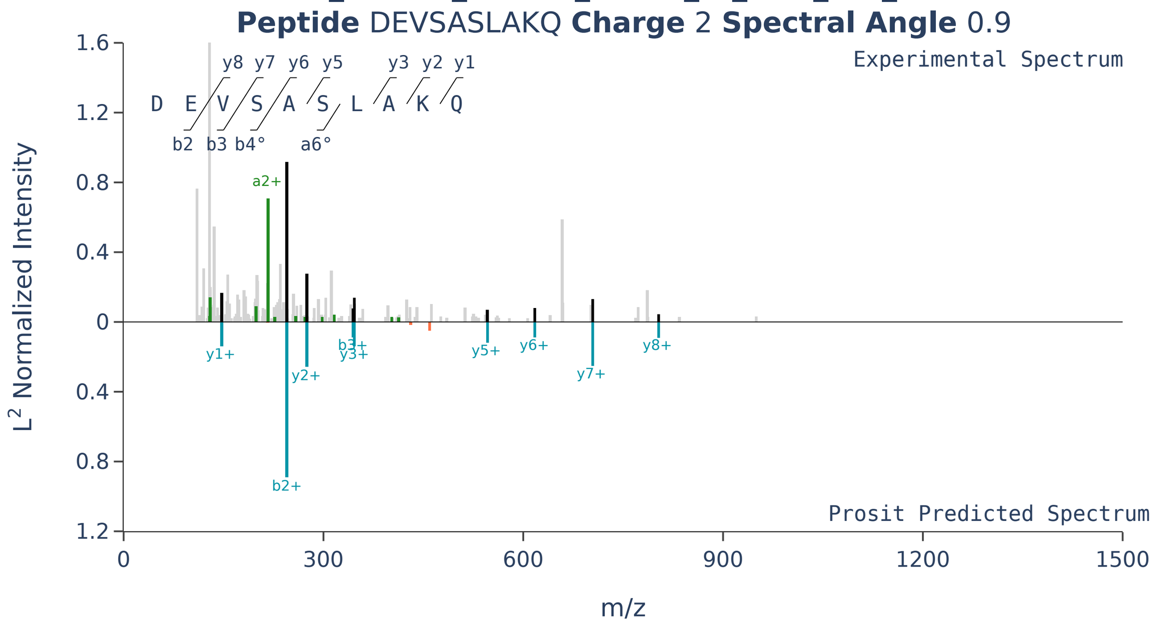


**Source** WSoh_221121_011221_HFGoe_L1_hTau_1h_R1 **Scan** 11491
**Peptide** KPVDLSKV **Charge** 2 **Spectral Angle** 0.95

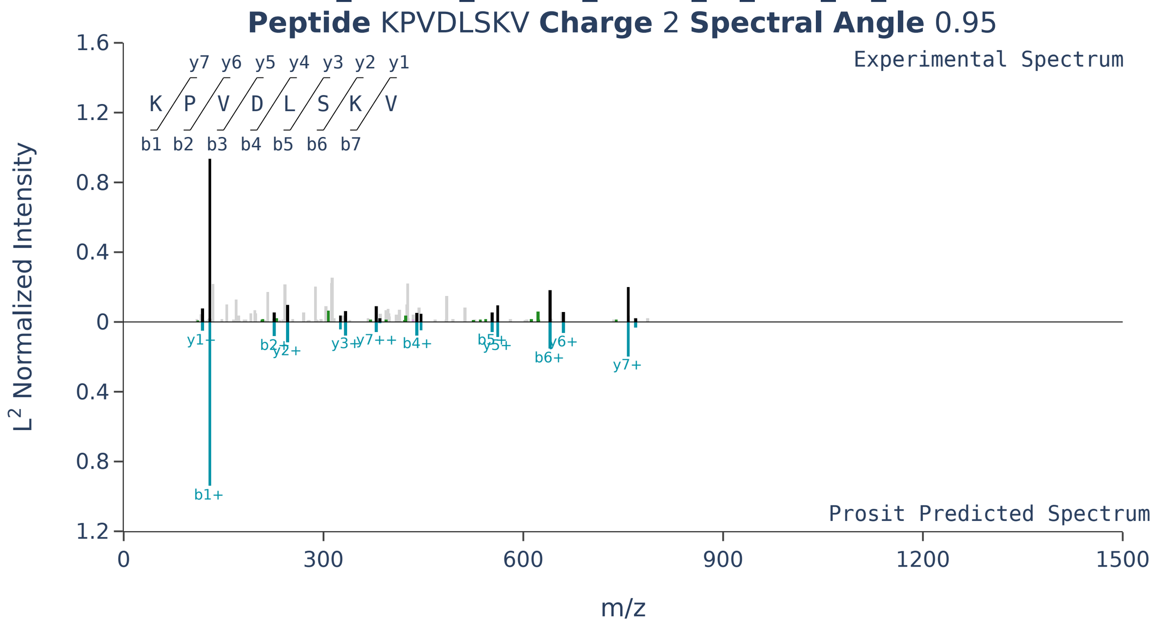

**Source** WSoh_221121_011221_HFGoe_L1_hTau_4h_R1 **Scan** 11685
**Peptide** PVVSGDTSPRHLSNVS **Charge** 2 **Spectral Angle** 0.91

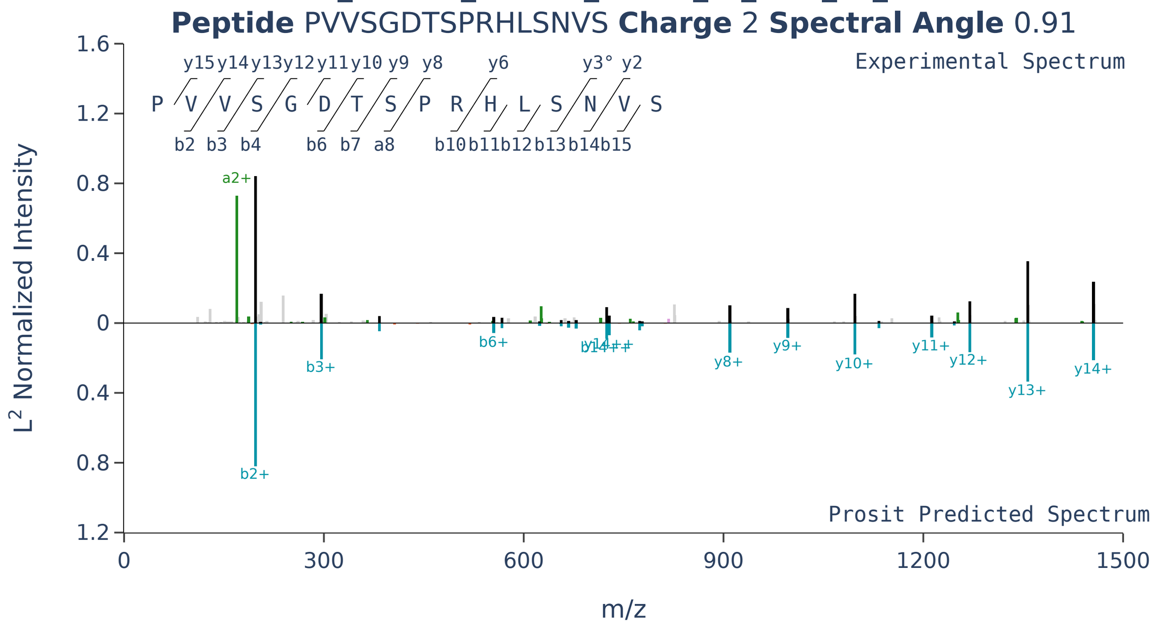


**Source** WSoh_221121_011221_HFGoe_L2_hTau_4h_R2 **Scan** 7629
**Peptide** GDTSPRHLSNVSST **Charge** 2 **Spectral Angle** 0.89

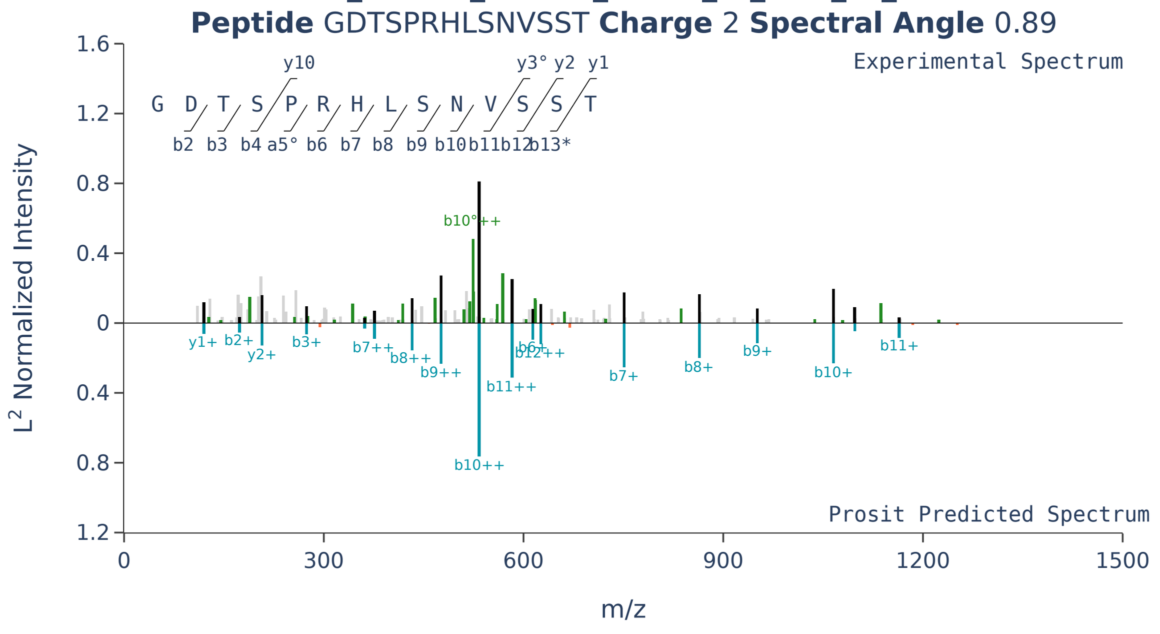


**Source** WSoh_221121_011221_HFGoe_L1_hTau_24h_R1 **Scan** 13925
**Peptide** TPSLPTPPTREPKKVA **Charge** 2 **Spectral Angle** 0.84

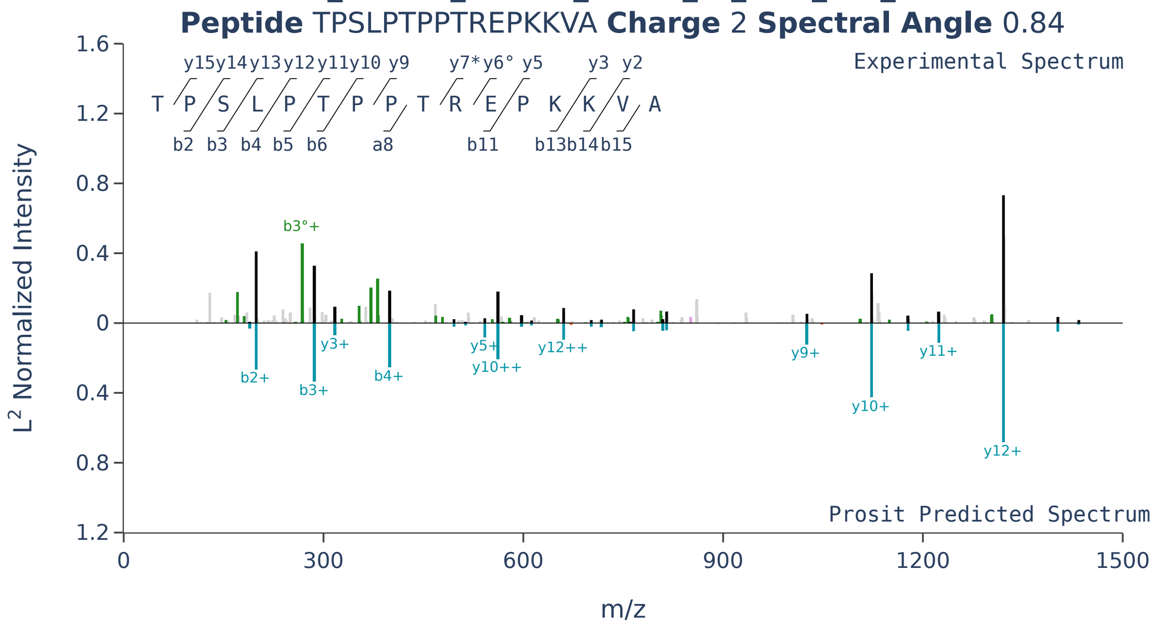


**Source** WSoh_221121_011221_HFGoe_L1_hTau_4h_R2 **Scan** 7980
**Peptide** VSASLAKQ **Charge** 2 **Spectral Angle** 0.89

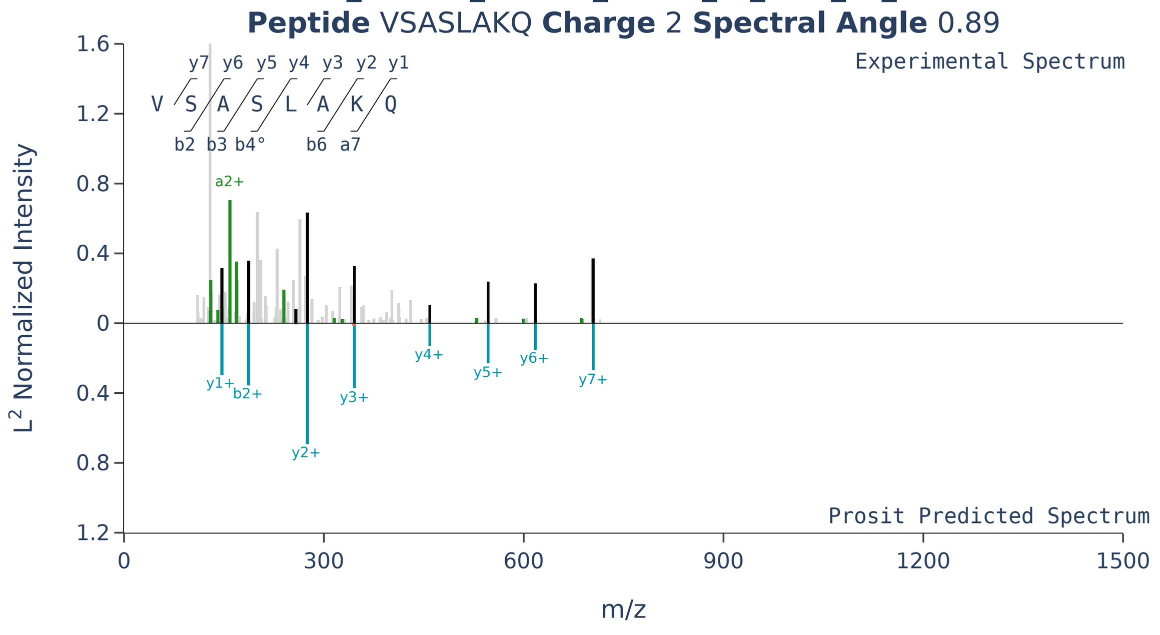


**Source** WSoh_221121_011221_HFGoe_L2_hTau_4h_R1 **Scan** 14568
**Peptide** KTDHGAEIVYKSPVVS **Charge** 2 **Spectral Angle** 0.87

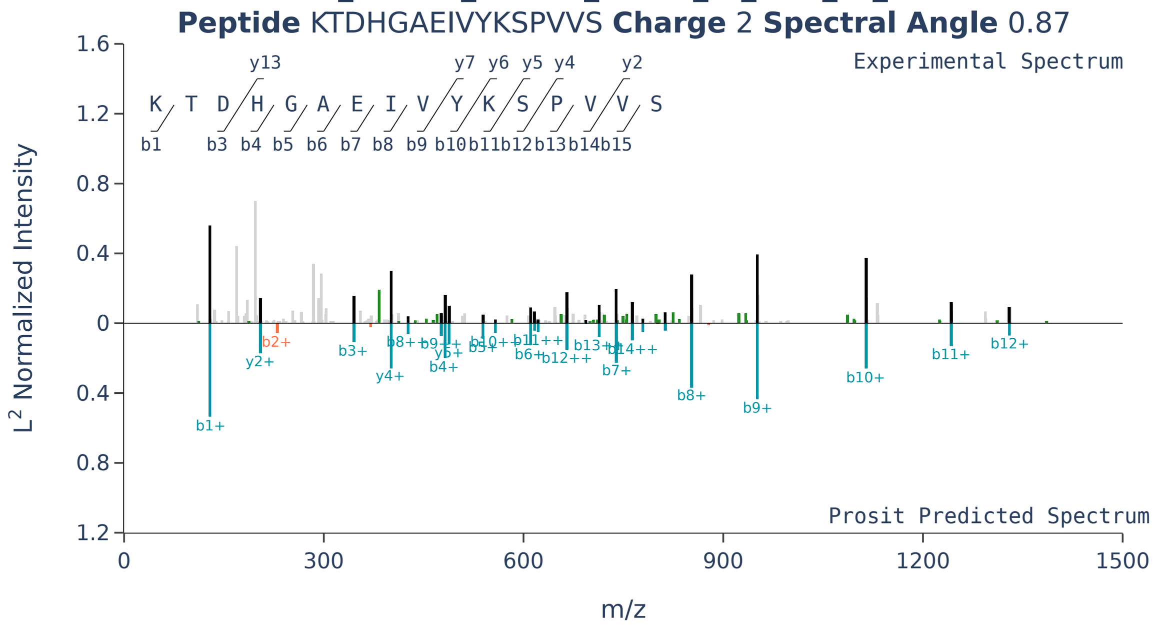


**Source** WSoh_221121_011221_HFGoe_L2_hTau_4h_R1 **Scan** 19261
**Peptide** DTDAGLKESPLQTPTE **Charge** 2 **Spectral Angle** 0.92

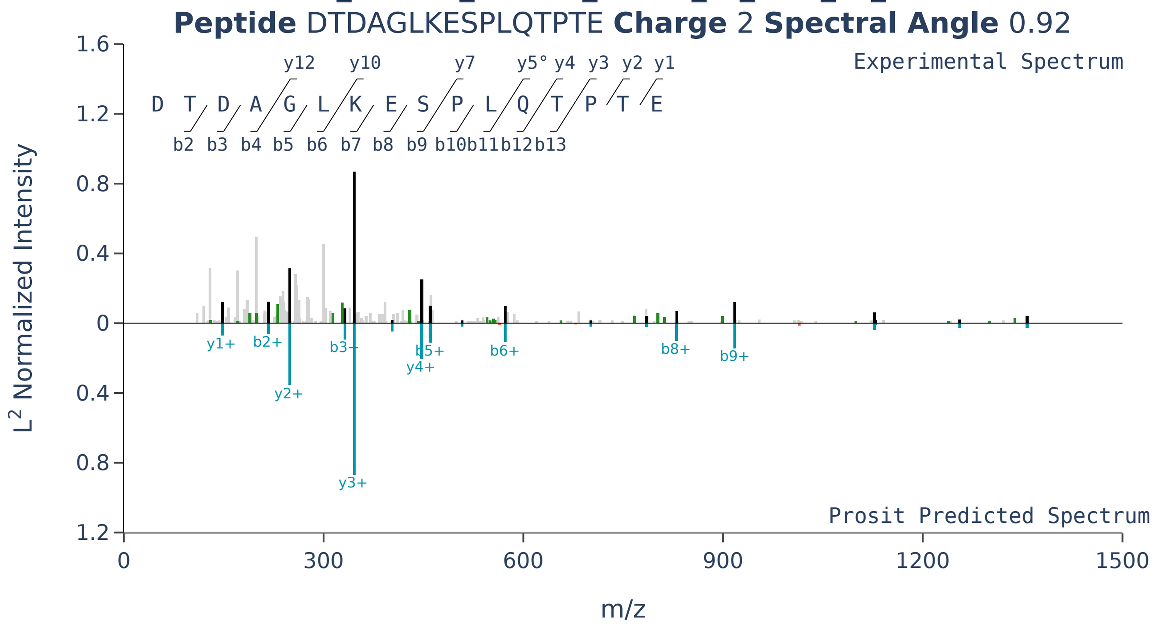


**Source** WSoh_221121_011221_HFGoe_L1_hTau_24h_R1 **Scan** 16335
**Peptide** HGAEIVYKSPVVSG **Charge** 3 **Spectral Angle** 0.88

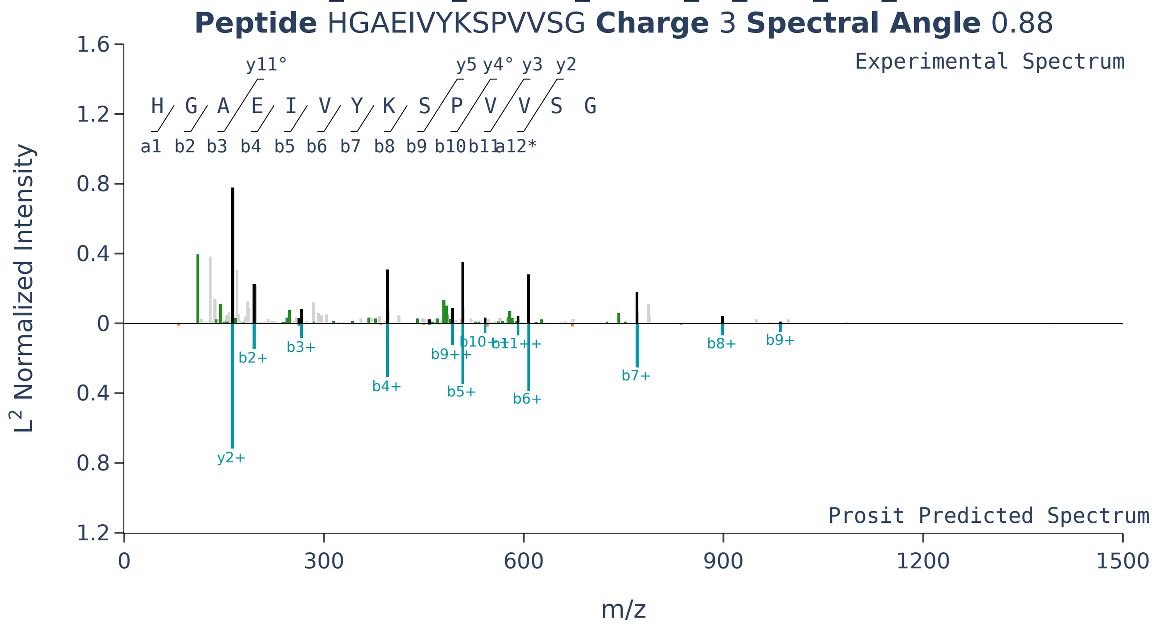


**Source** WSoh_221121_011221_HFGoe_L2_hTau_24h_R2 **Scan** 8632
**Peptide** VVSGDTSPRHL **Charge** 2 **Spectral Angle** 0.91

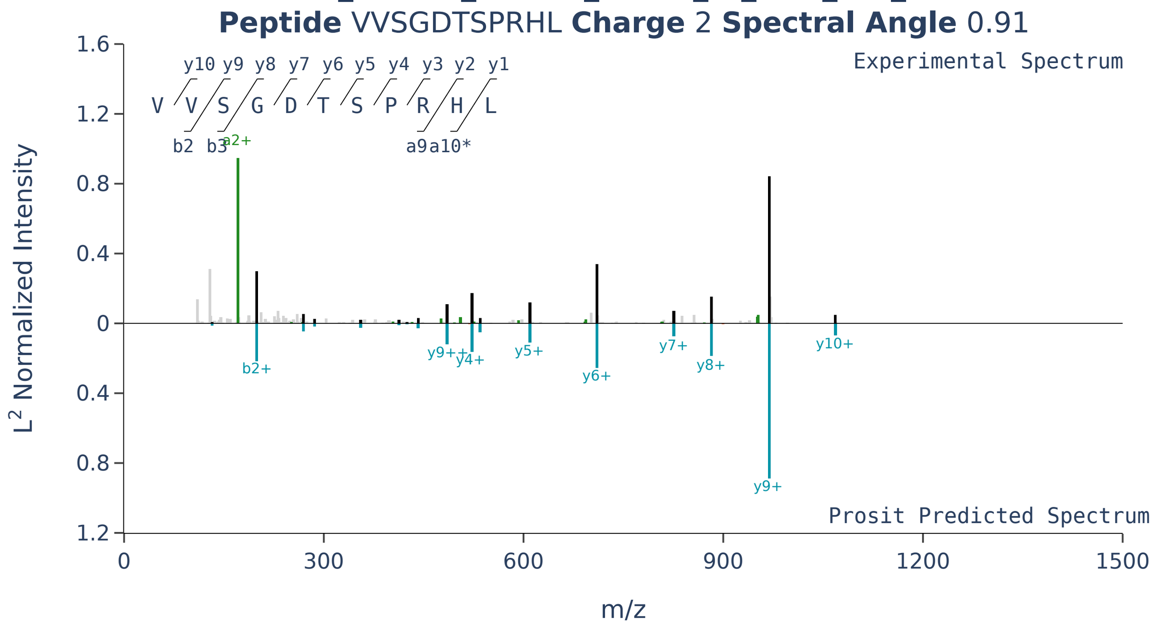


**Source** WSoh_221121_011221_HFGoe_L1_hTau_24h_R1 **Scan** 9144
**Peptide** PVVSGDTSPRHLSN **Charge** 2 **Spectral Angle** 0.89

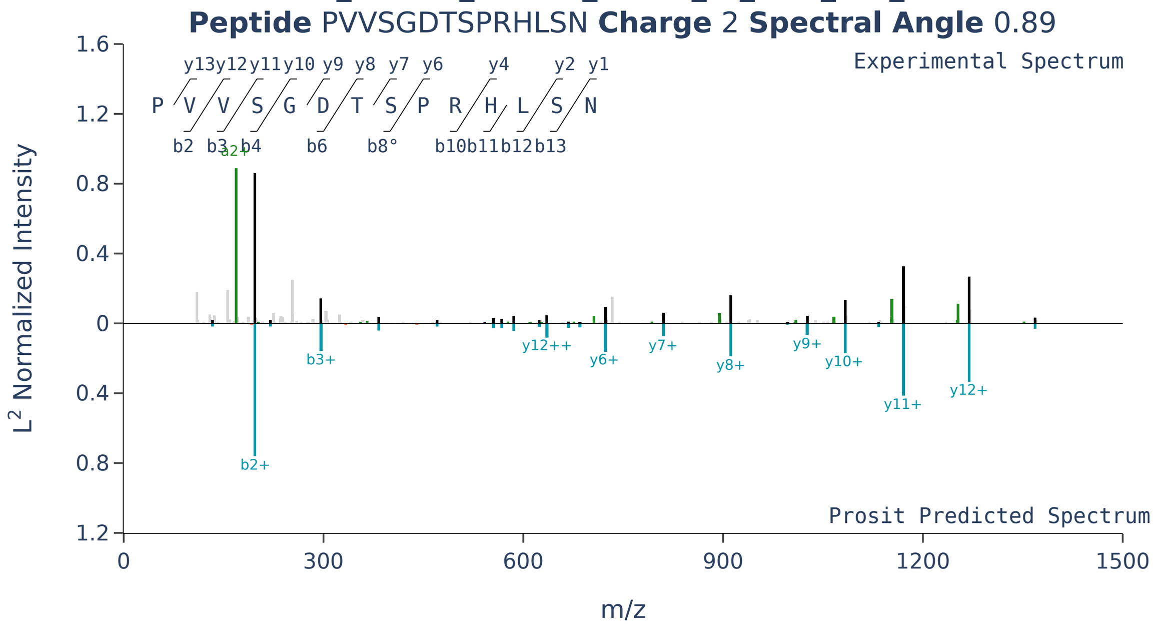


**Source** WSoh_221121_011221_HFGoe_L1_hTau_24h_R2 **Scan** 8658
**Peptide** IATPRGAAPPGQKGQ **Charge** 2 **Spectral Angle** 0.93

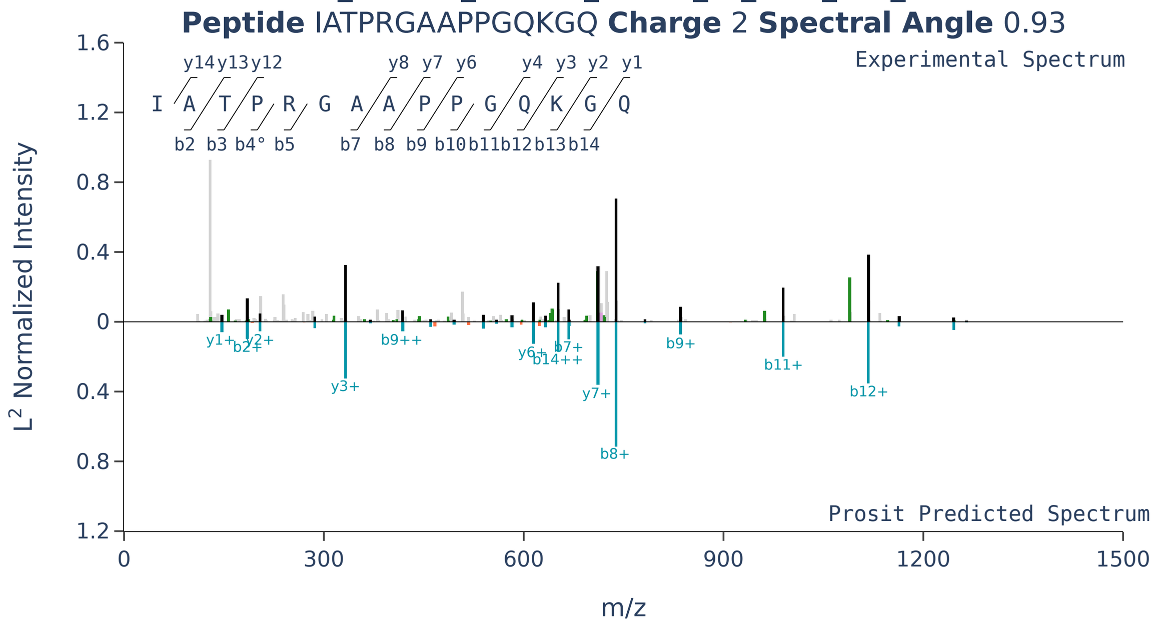


**Source** WSoh_221121_011221_HFGoe_L2_hTau_1h_R1 **Scan** 9654
**Peptide** TDHGAEIVYKS **Charge** 2 **Spectral Angle** 0.9

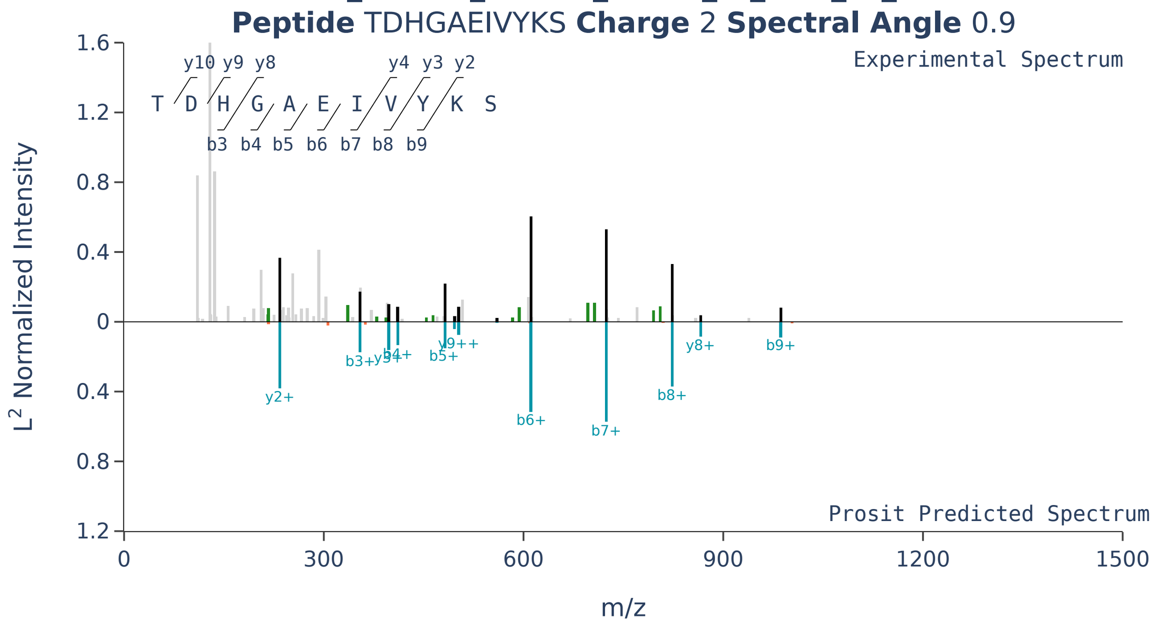


**Source** WSoh_221121_011221_HFGoe_L1_hTau_24h_R1 **Scan** 6434
**Peptide** PGSETSDAKSTPTAED **Charge** 2 **Spectral Angle** 0.85

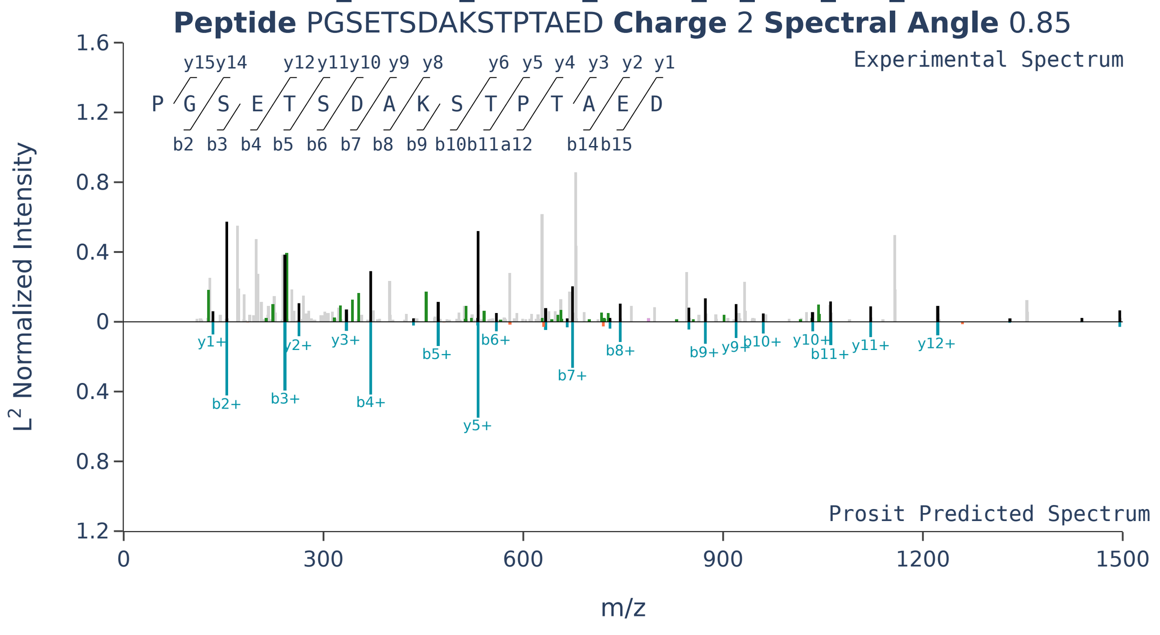


**Source** WSoh_221121_011221_HFGoe_L2_hTau_2h_R1 **Scan** 11538
**Peptide** VYKSPVVSGD **Charge** 2 **Spectral Angle** 0.89

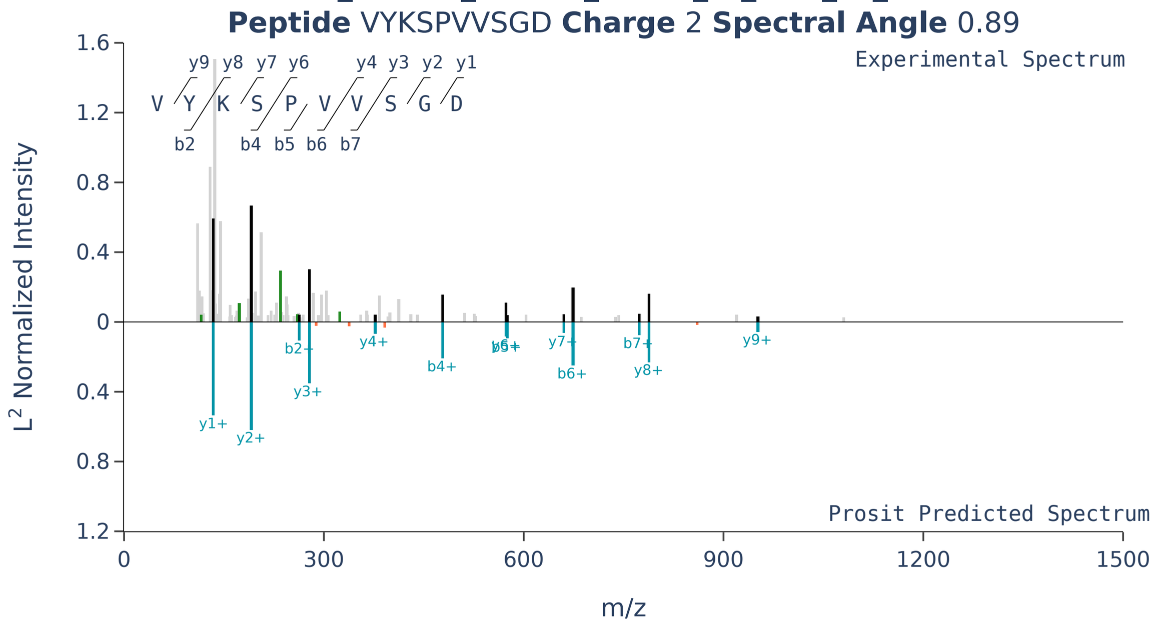


**Source** WSoh_221121_011221_HFGoe_L2_hTau_4h_R2 **Scan** 6970
**Peptide** AKAKTDHGAEIVYKS **Charge** 2 **Spectral Angle** 0.84

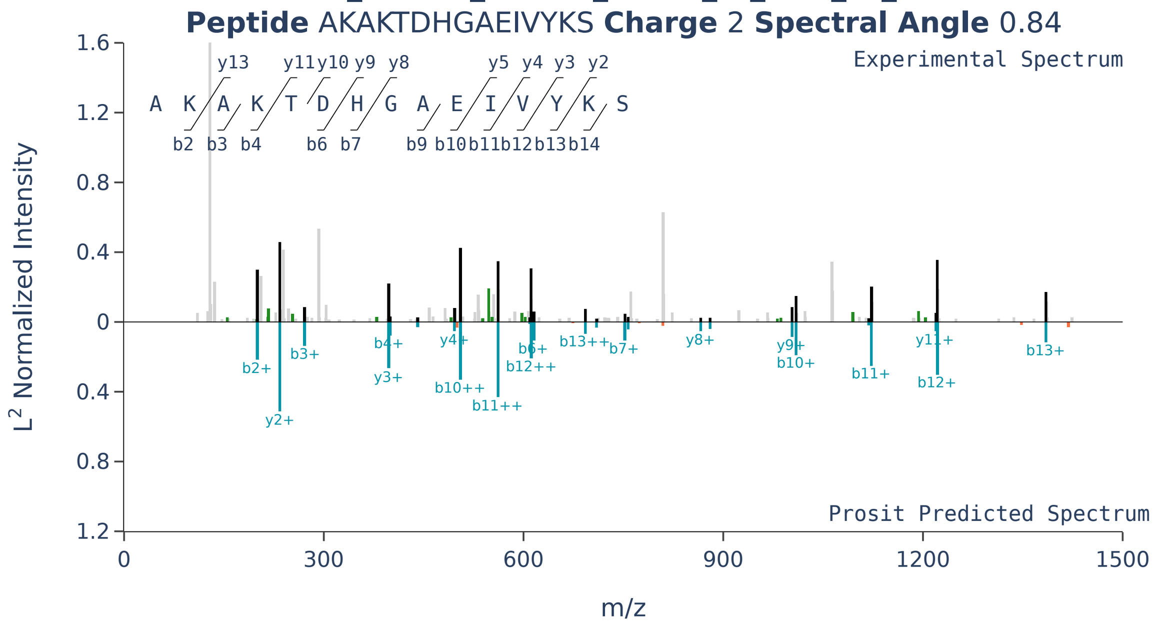


**Source** WSoh_221121_011221_HFGoe_L1_hTau_2h_R2 **Scan** 6102
**Peptide** THVPGGGNKKL **Charge** 3 **Spectral Angle** 0.87

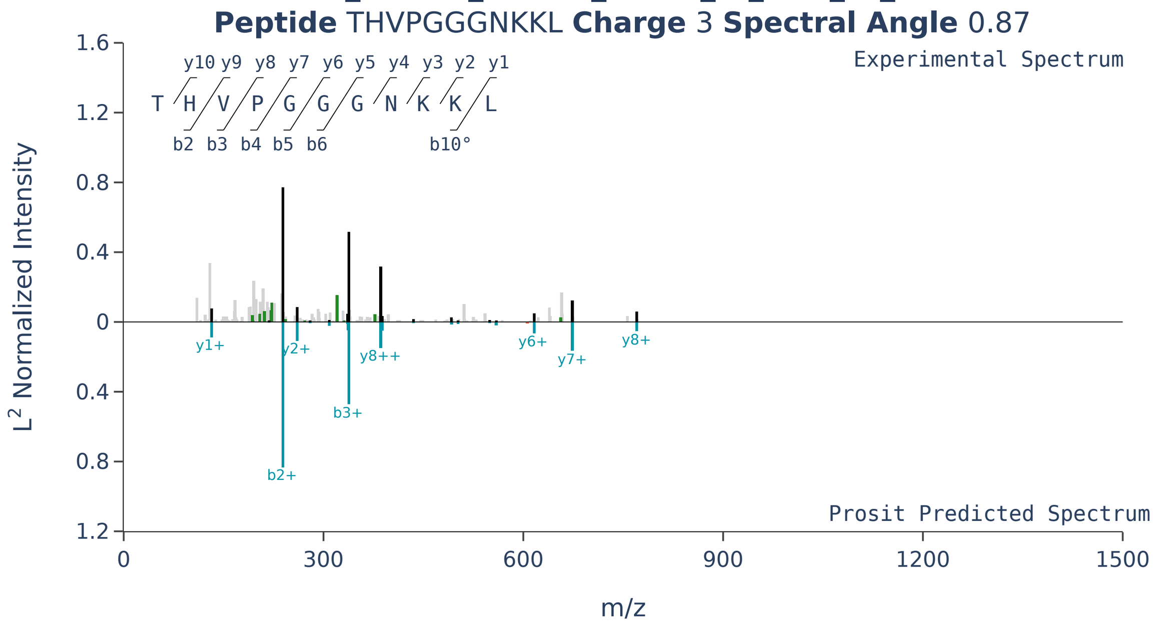

**Source** WSoh_221121_011221_HFGoe_L2_hTau_24h_R2 **Scan** 17478
**Peptide** EIVYKSPVVSGDTSPRHL **Charge** 4 **Spectral Angle** 0.89

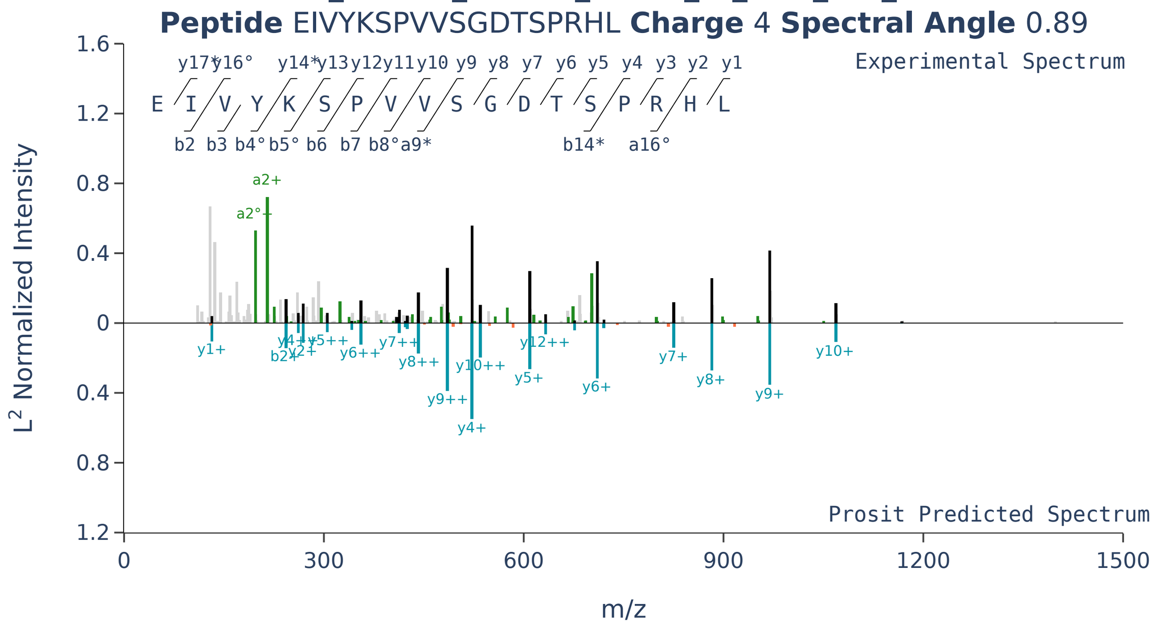


**Source** WSoh_221121_011221_HFGoe_L2_hTau_4h_R1 **Scan** 7166
**Peptide** ENAKAKTDHGAEIVYK **Charge** 3 **Spectral Angle** 0.85

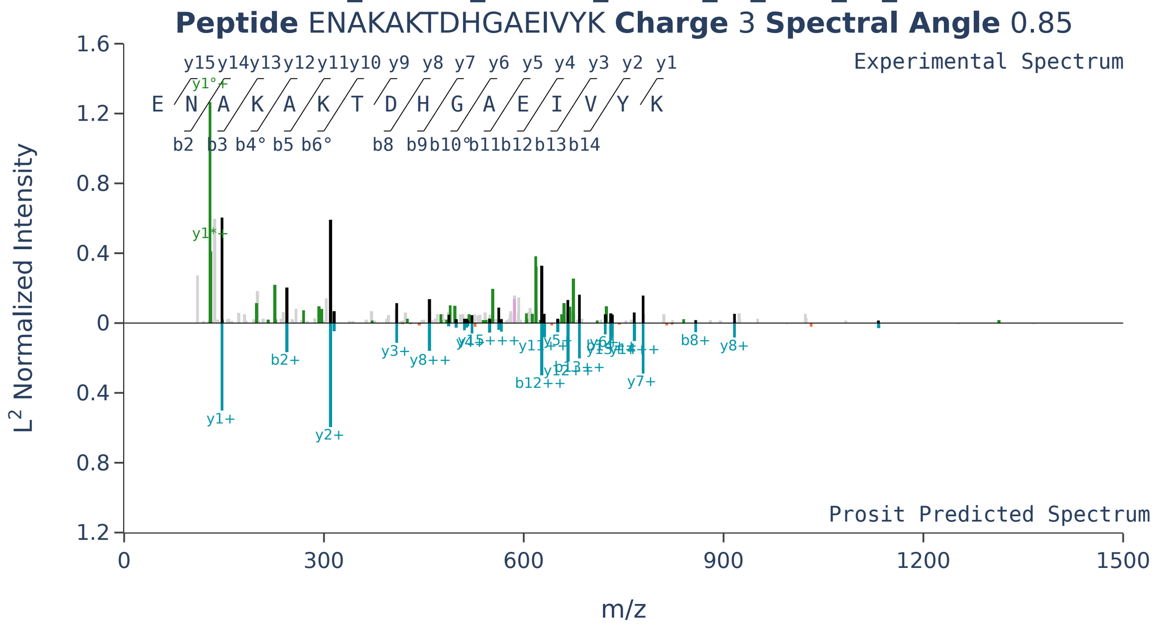


**Source** WSoh_221121_011221_HFGoe_L1_hTau_2h_R2 **Scan** 10232
**Peptide** VKSEKLDFKDRVQS **Charge** 2 **Spectral Angle** 0.85

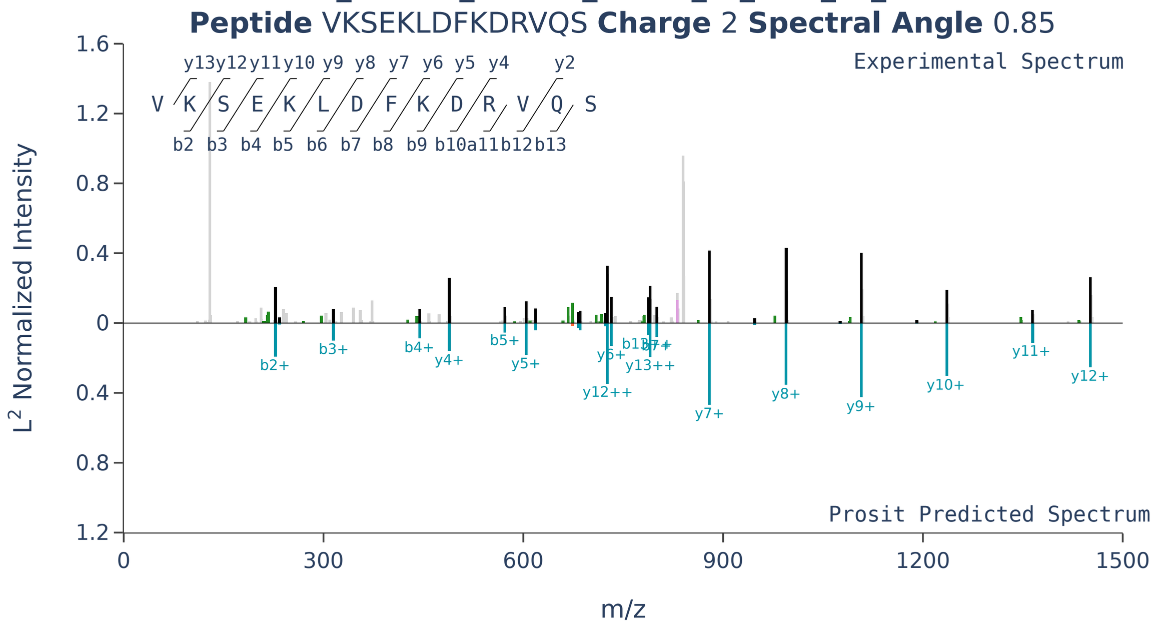


**Source** WSoh_221121_011221_HFGoe_L2_hTau_1h_R1 **Scan** 14349
**Peptide** LINKKLDL **Charge** 2 **Spectral Angle** 0.88

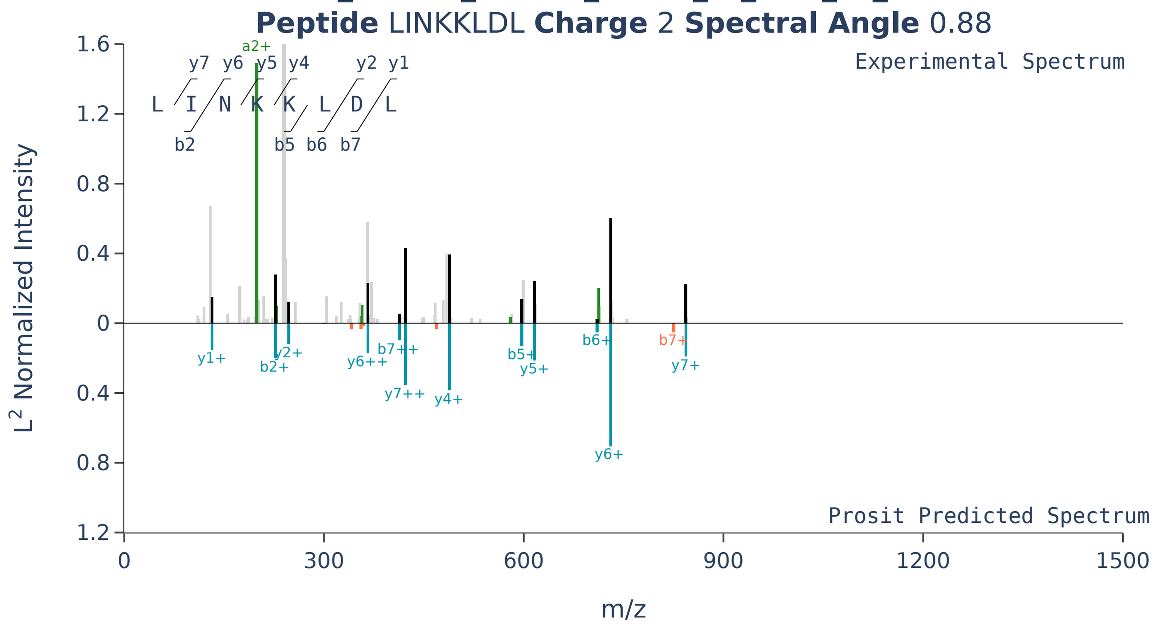


**Source** WSoh_221121_011221_HFGoe_L1_hTau_4h_R1 **Scan** 16996
**Peptide** GKVQIINKKLDL **Charge** 3 **Spectral Angle** 0.88

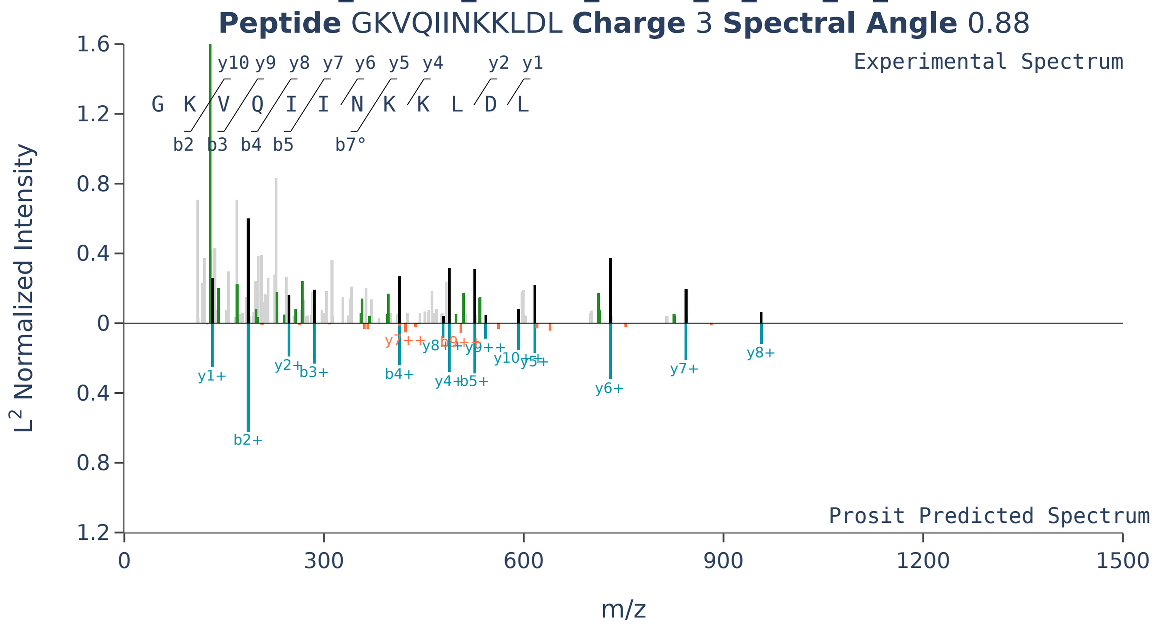


**Source** WSoh_221121_011221_HFGoe_L1_hTau_24h_R1 **Scan** 21679
**Peptide** SNVSSTGSIDMV **Charge** 1 **Spectral Angle** 0.94

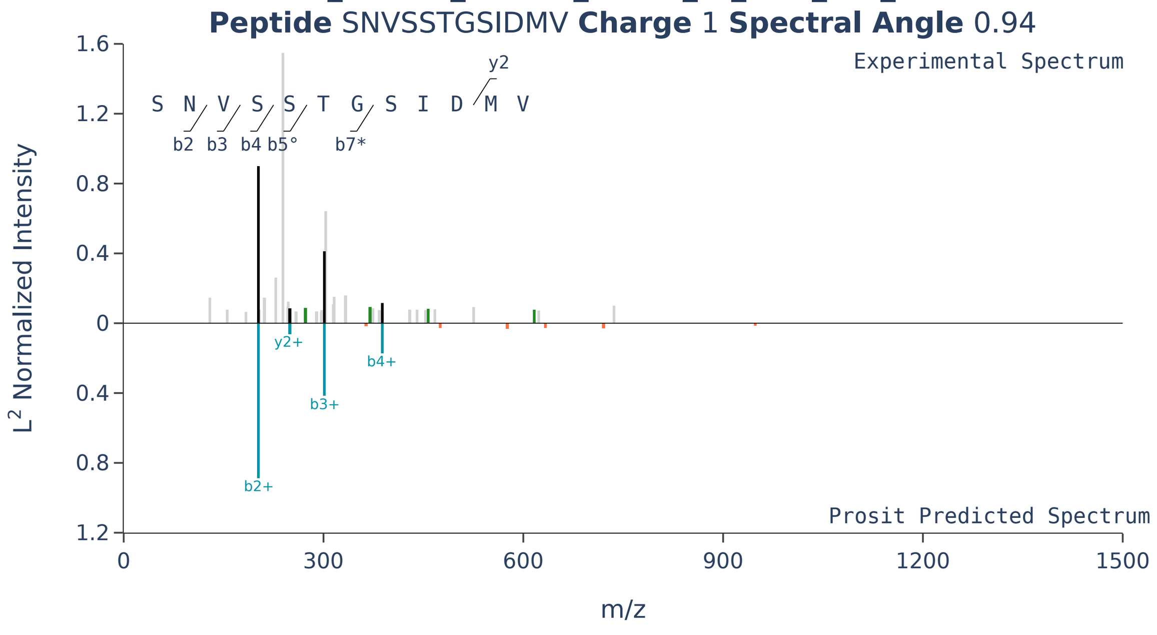


**Source** WSoh_221121_011221_HFGoe_L2_hTau_4h_R2 **Scan** 5952
**Peptide** KLGSTENLKHQ **Charge** 3 **Spectral Angle** 0.91

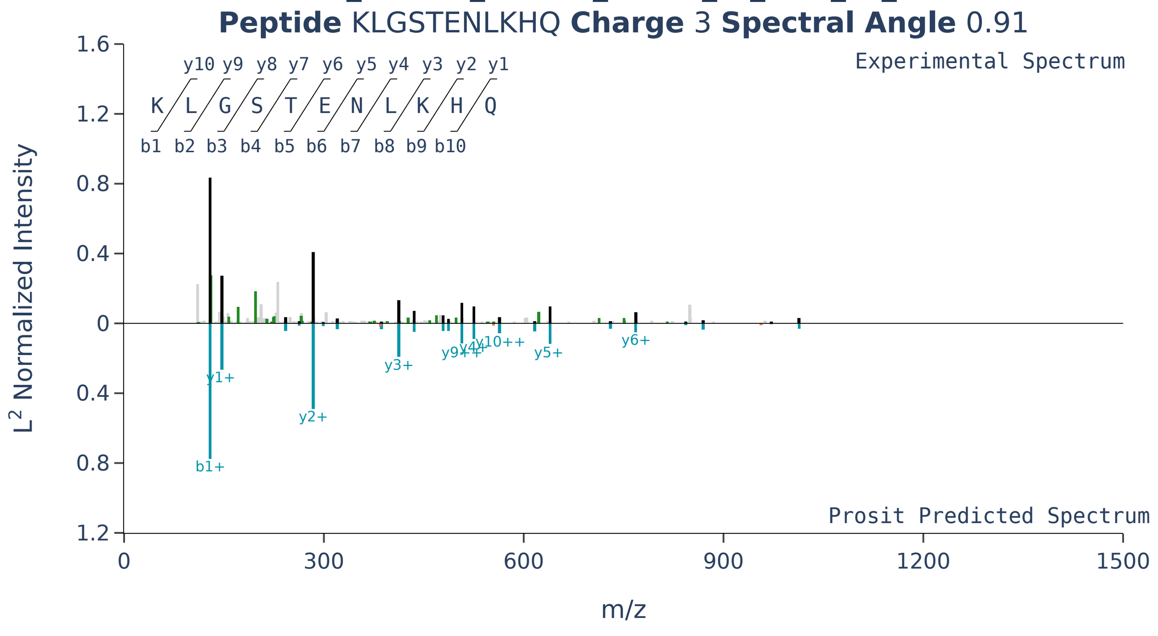


**Source** WSoh_221121_011221_HFGoe_L2_hTau_4h_R2 **Scan** 8754
**Peptide** PGGGNKKIETHKLTFRENA **Charge** 3 **Spectral Angle** 0.86

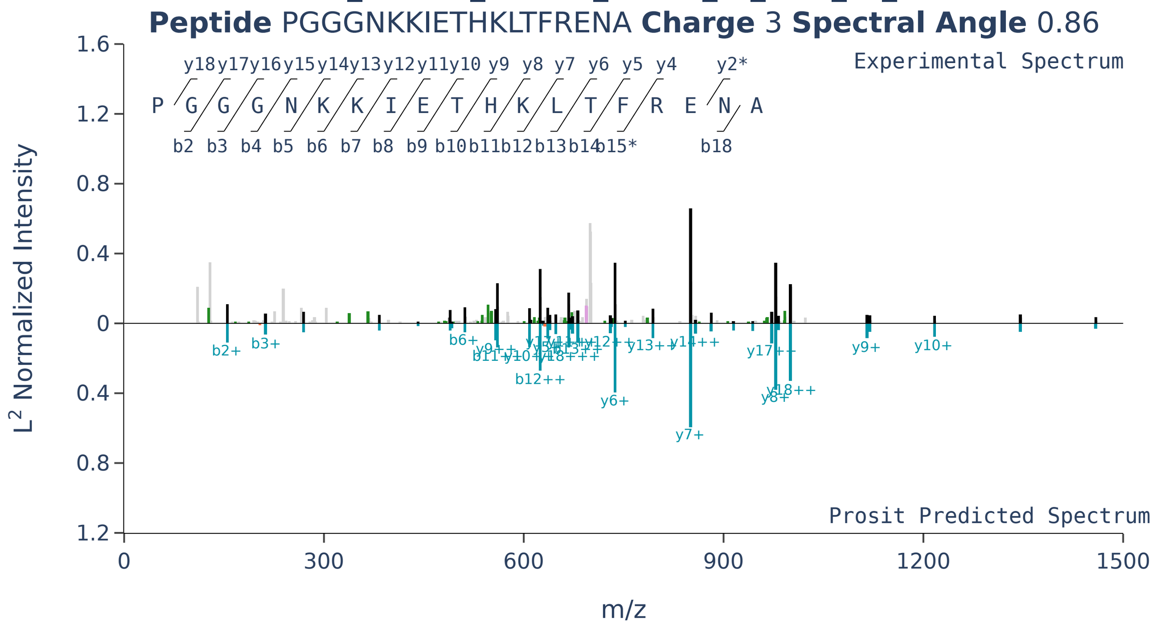


**Source** WSoh_221121_011221_HFGoe_L2_hTau_24h_R1 **Scan** 7563
**Peptide** KLTFRENAK **Charge** 2 **Spectral Angle** 0.82

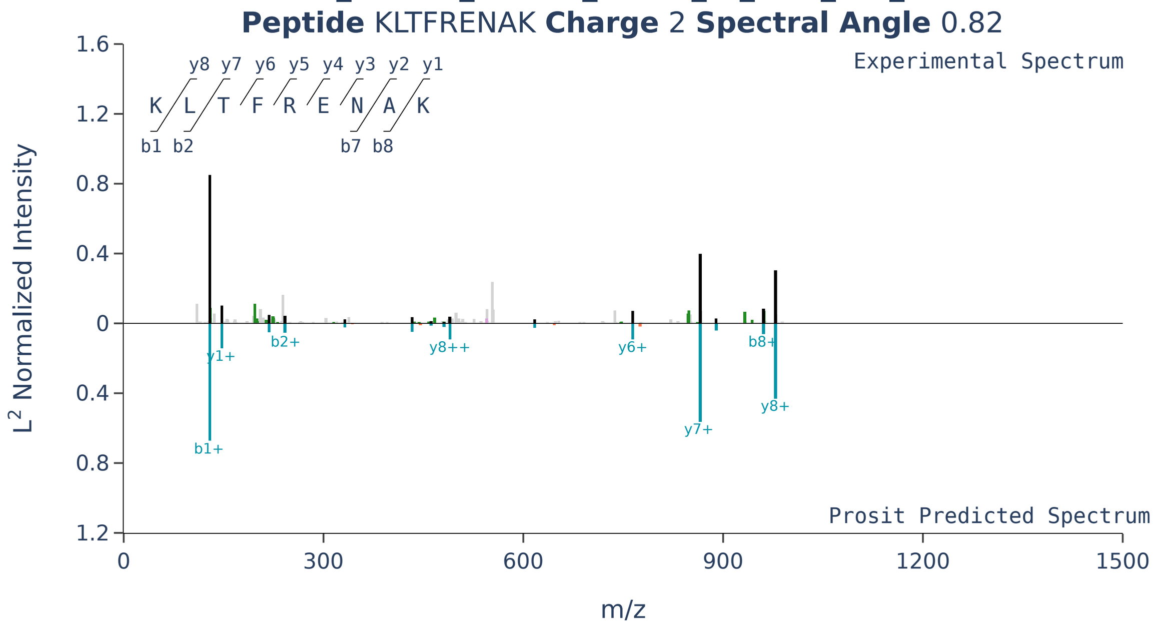


**Source** WSoh_221121_011221_HFGoe_L2_hTau_24h_R2 **Scan** 13215
**Peptide** IINKKLDLSNVQSK **Charge** 3 **Spectral Angle** 0.84

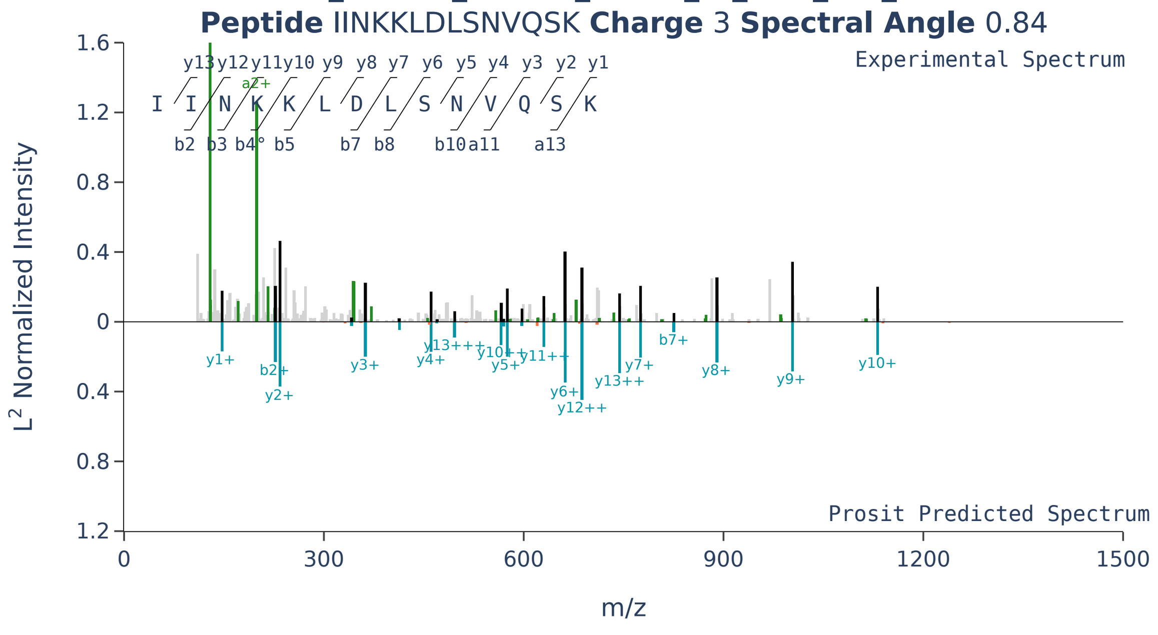


**Source** WSoh_221121_011221_HFGoe_L1_hTau_24h_R2 **Scan** 7304
**Peptide** KSEKLDFKDR **Charge** 2 **Spectral Angle** 0.81

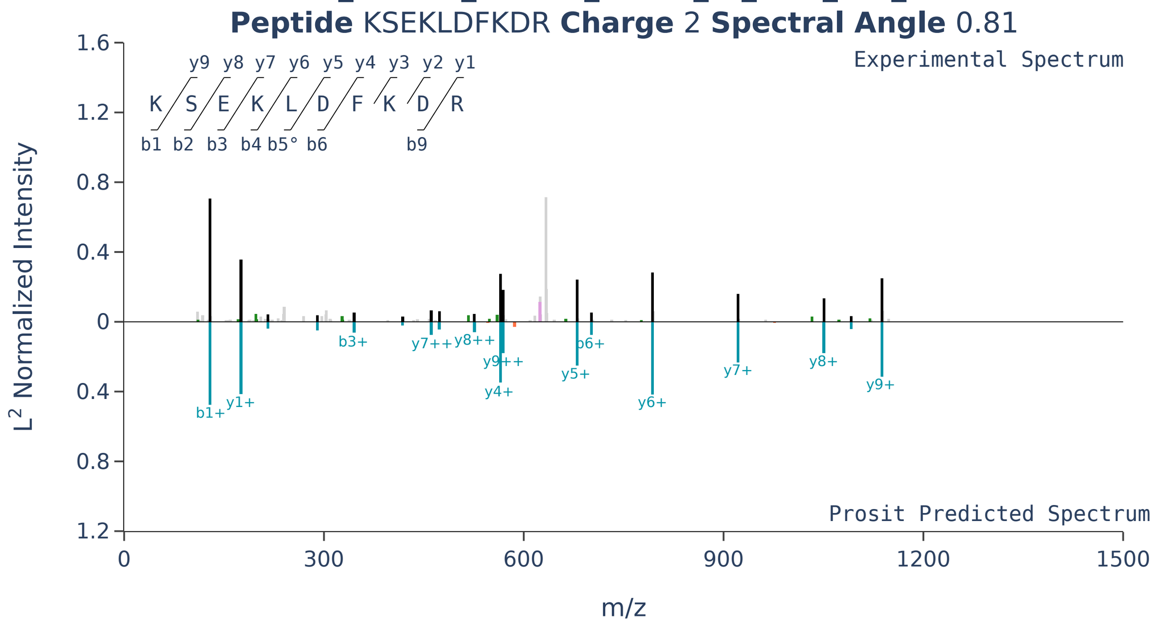


**Source** WSoh_221121_011221_HFGoe_L2_hTau_4h_R2 **Scan** 18419
**Peptide** SKIGSLDNI **Charge** 2 **Spectral Angle** 0.92

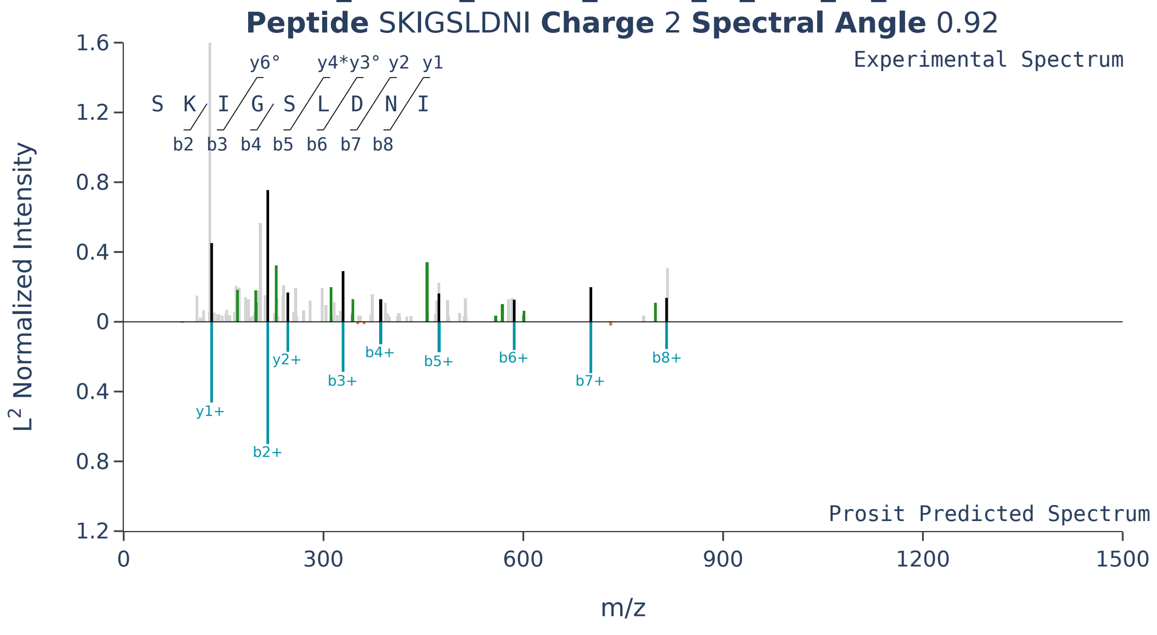


**Source** WSoh_221121_011221_HFGoe_L2_hTau_24h_R1 **Scan** 12390
**Peptide** SPVVSGDTSPRHLSNVSS **Charge** 2 **Spectral Angle** 0.92

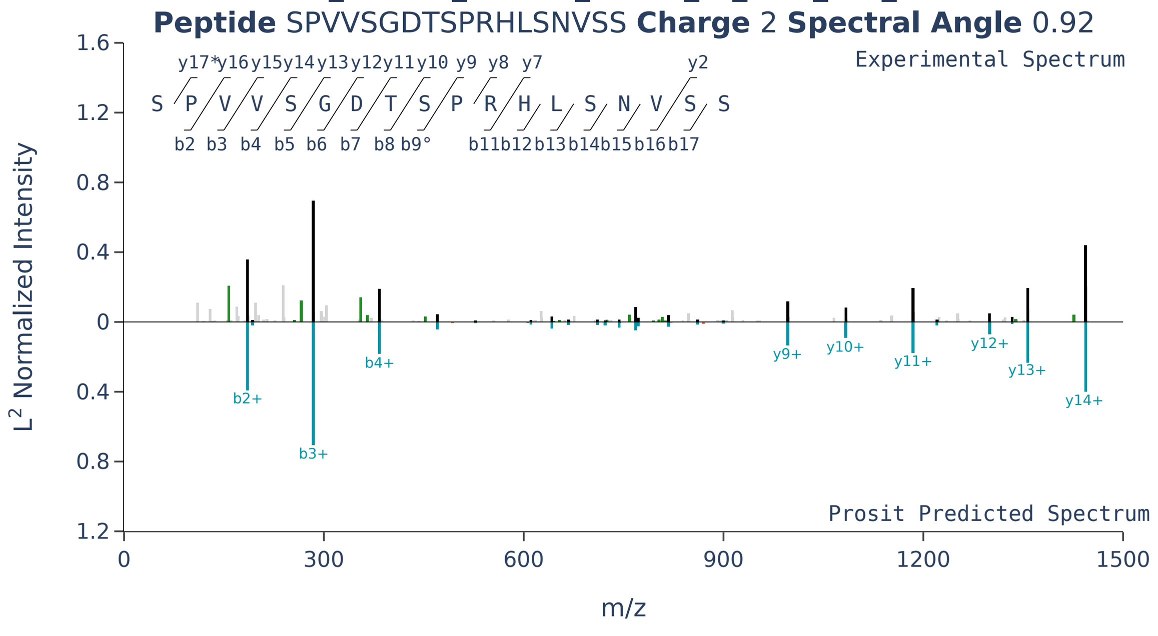


**Source** WSoh_221121_011221_HFGoe_L2_hTau_4h_R2 **Scan** 13030
**Peptide** AAAQPHTEIPEGTTAE **Charge** 2 **Spectral Angle** 0.9

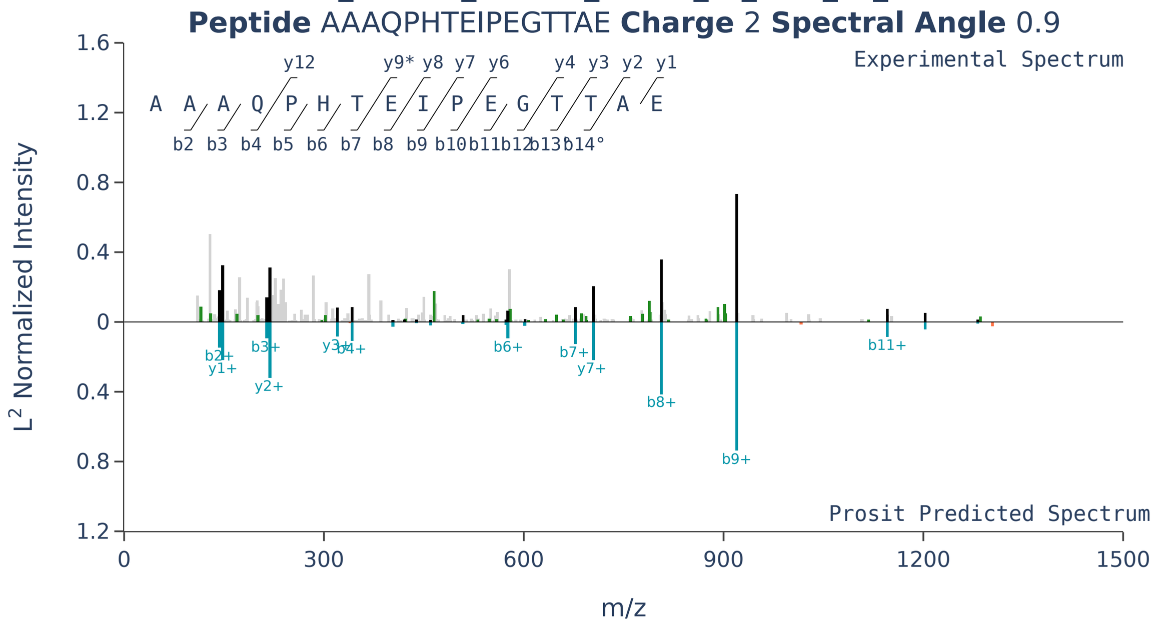


**Source** WSoh_221121_011221_HFGoe_L2_hTau_24h_R1 **Scan** 12534
**Peptide** LDNITHVPGGGNKKL **Charge** 2 **Spectral Angle** 0.89

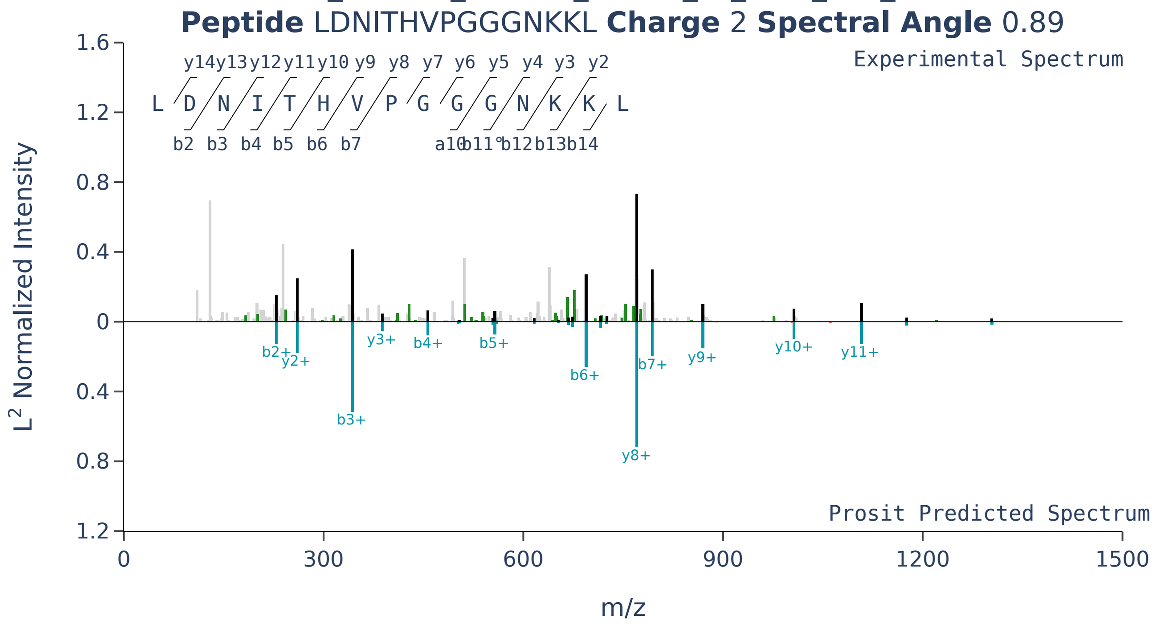

**Source** WSoh_221121_011221_HFGoe_L1_hTau_24h_R1 **Scan** 20952
**Peptide** TAEEAGIGDTPSLED **Charge** 1 **Spectral Angle** 0.85

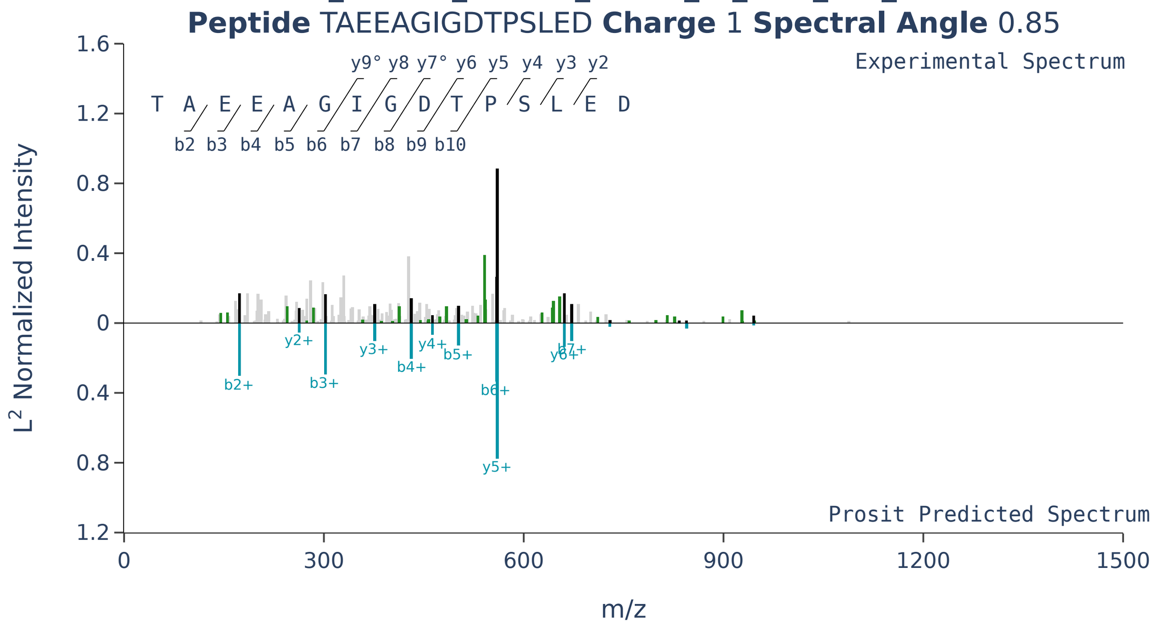


**Source** WSoh_221121_011221_HFGoe_L1_hTau_24h_R2 **Scan** 10996
**Peptide** DHAGTYGLGDRKDQGGY **Charge** 3 **Spectral Angle** 0.95

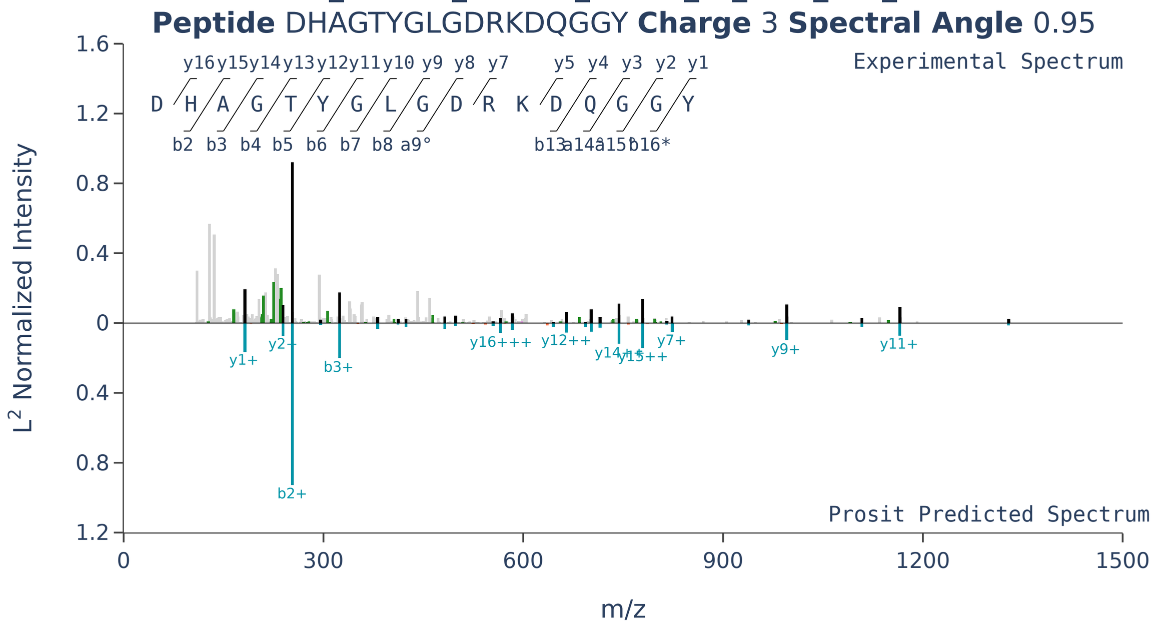


**Source** WSoh_221121_011221_HFGoe_L2_hTau_4h_R2 **Scan** 7572
**Peptide** SLGNIHHKPGGGQVEVKS **Charge** 2 **Spectral Angle** 0.81

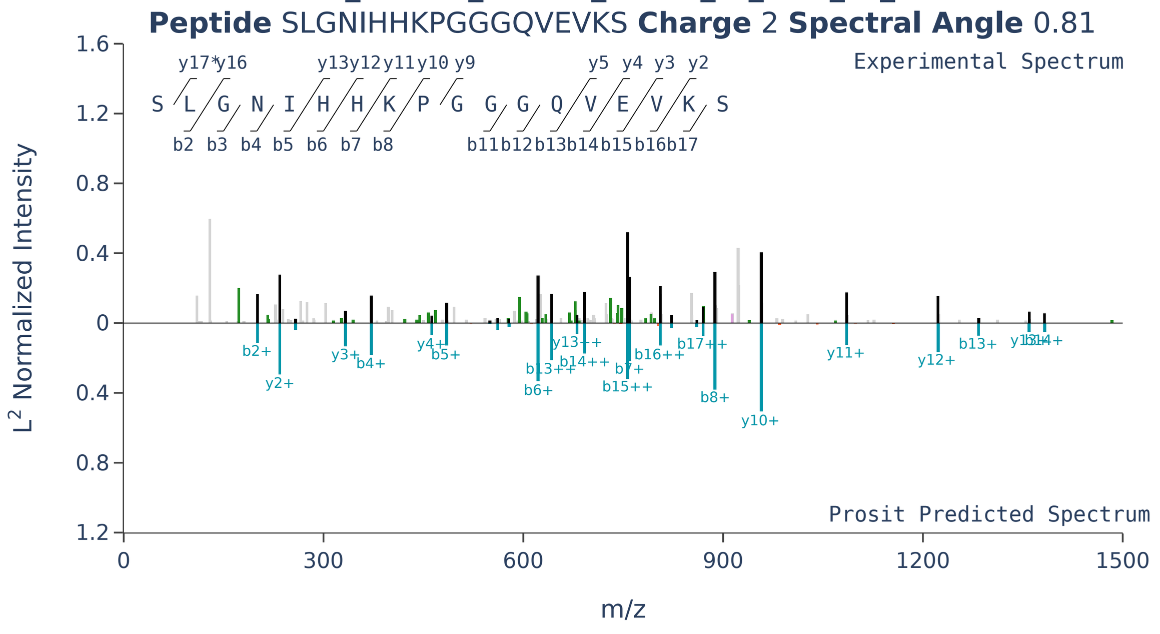


**Source** WSoh_221121_011221_HFGoe_L1_hTau_4h_R2 **Scan** 21682
**Peptide** AKSTPTAEDVTAPLV **Charge** 2 **Spectral Angle** 0.94

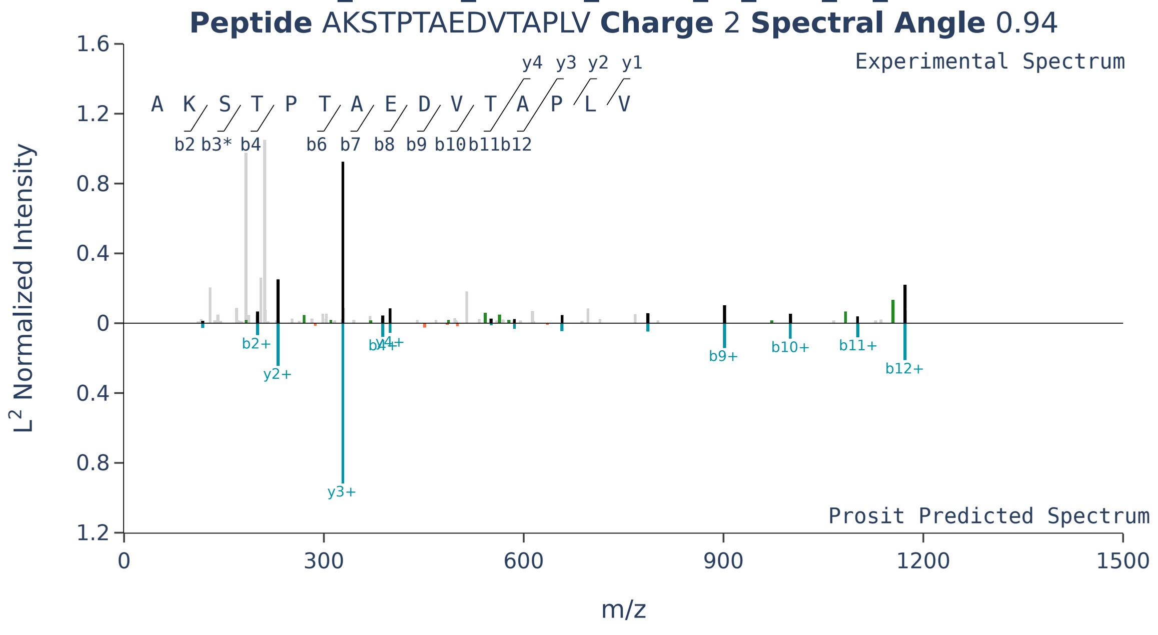


**Source** WSoh_221121_011221_HFGoe_L1_hTau_24h_R2 **Scan** 8957
**Peptide** LDNITHVPGGGNKK **Charge** 3 **Spectral Angle** 0.91

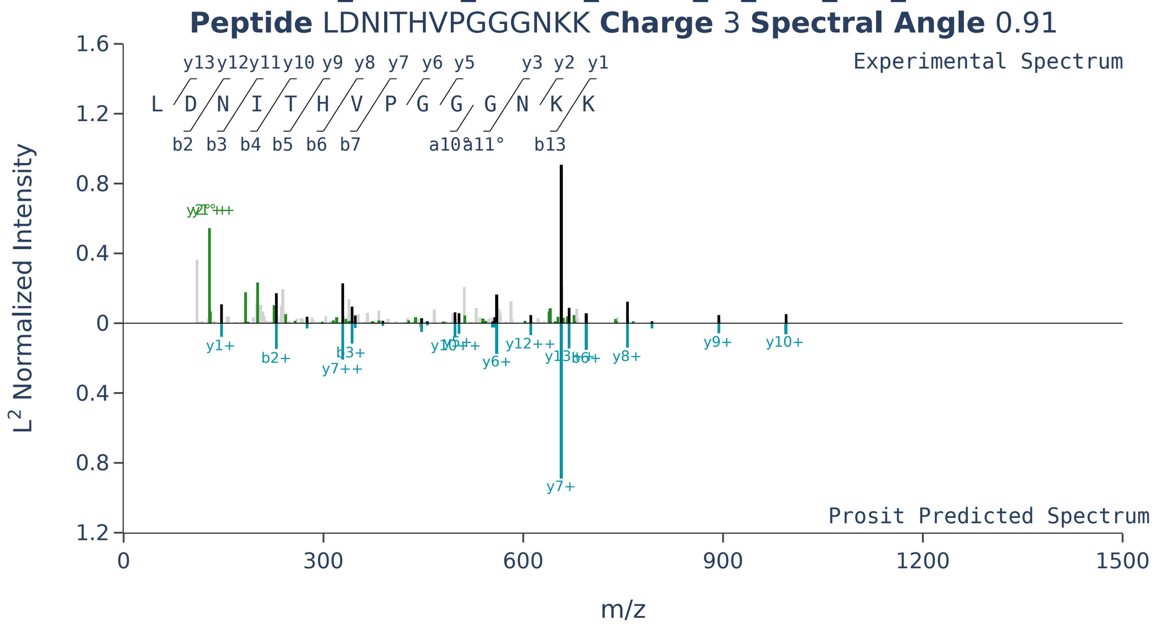


**Source** WSoh_221121_011221_HFGoe_L1_hTau_4h_R2 **Scan** 13383
**Peptide** CGSKDNIKHVPGGGSVQL **Charge** 2 **Spectral Angle** 0.89

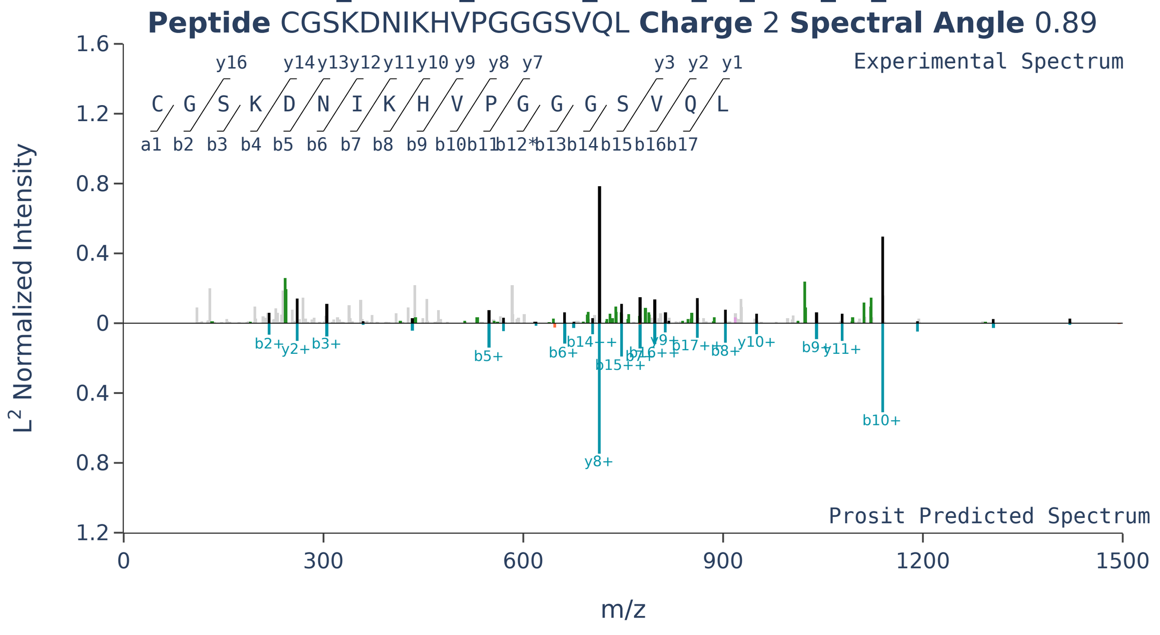


**Source** WSoh_221121_011221_HFGoe_L1_hTau_4h_R2 **Scan** 7126
**Peptide** NITHVPGGGNKKIETH **Charge** 4 **Spectral Angle** 0.88

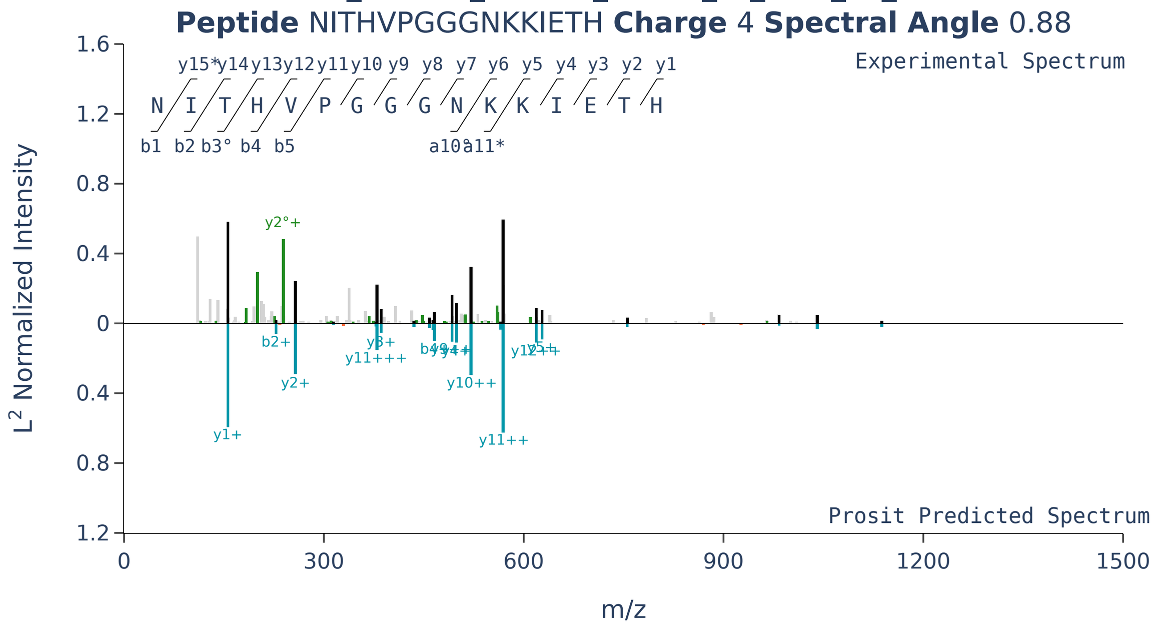


**Source** WSoh_221121_011221_HFGoe_L1_hTau_24h_R2 **Scan** 22373
**Peptide** AGIGDTPSLEDEAAGHV **Charge** 2 **Spectral Angle** 0.88

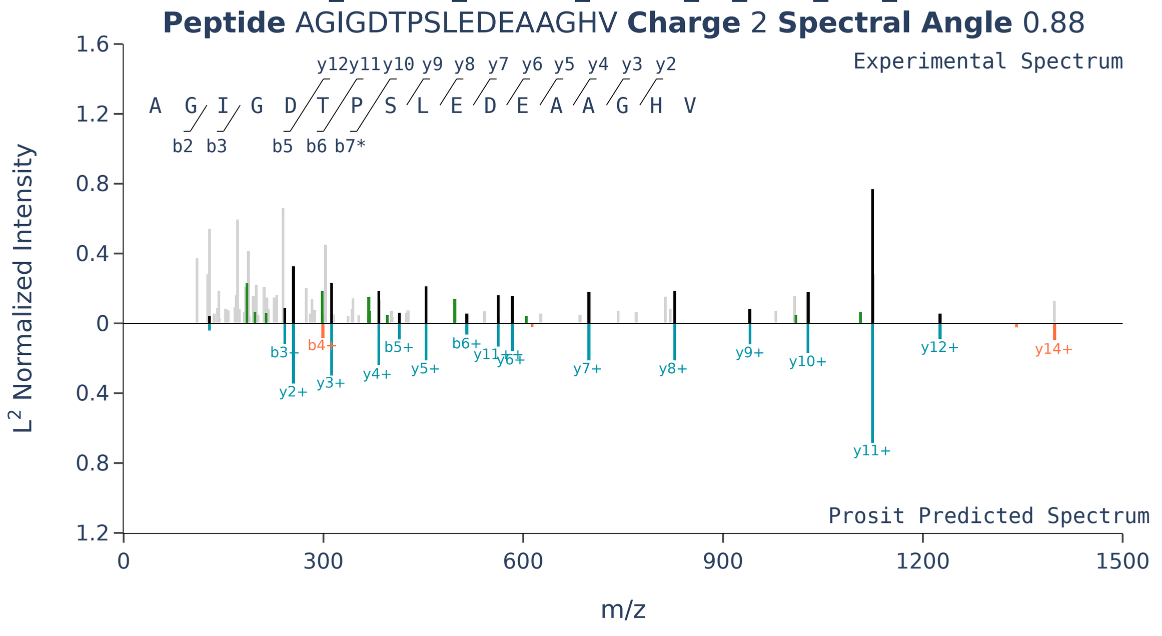


**Source** WSoh_221121_011221_HFGoe_L2_hTau_24h_R2 **Scan** 21273
**Peptide** GIGDTPSLEDEAAGHVTQA **Charge** 2 **Spectral Angle** 0.84

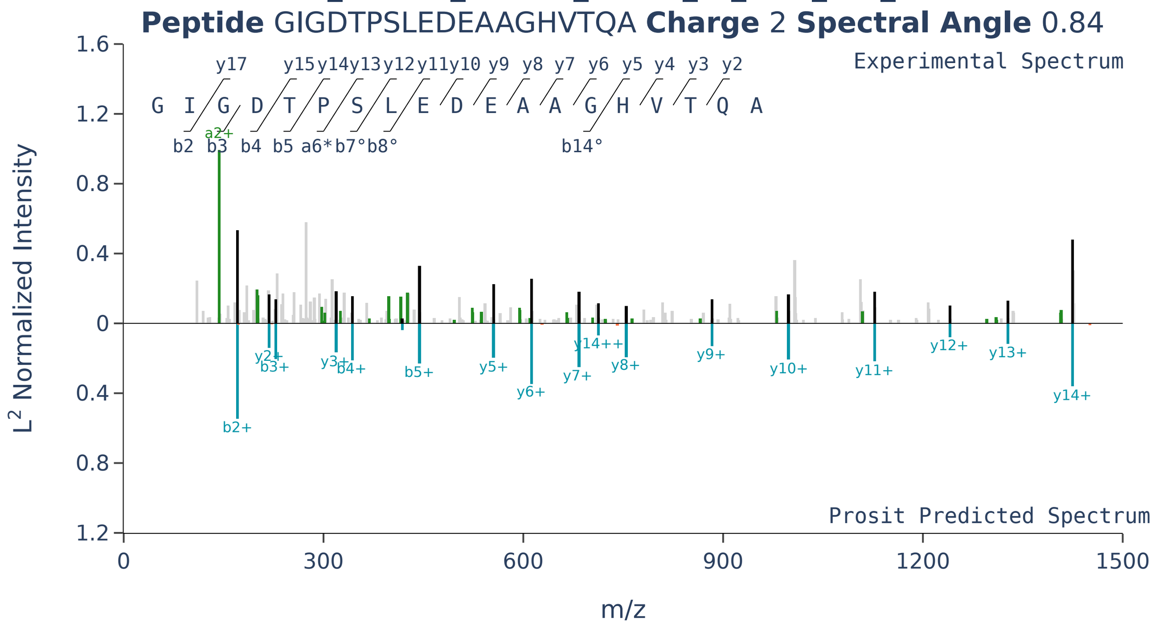


**Source** WSoh_221121_011221_HFGoe_L1_hTau_4h_R2 **Scan** 11231
**Peptide** DHAGTYGLGDRKDQGGYTMHQD **Charge** 3 **Spectral Angle** 0.95

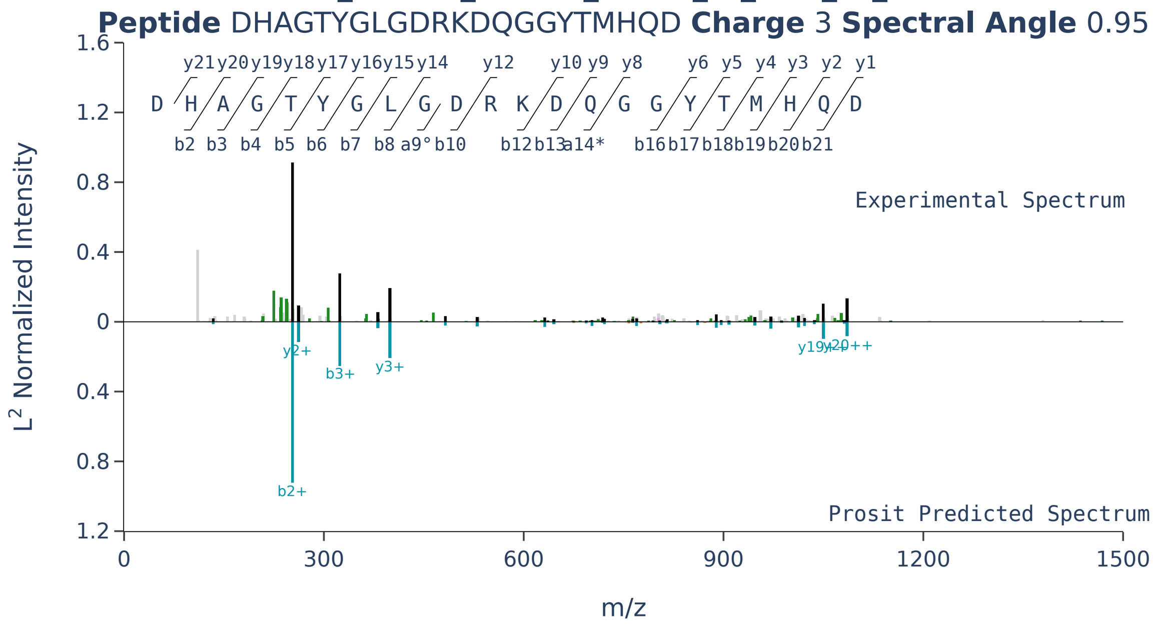


**Source** WSoh_221121_011221_HFGoe_L1_hTau_24h_R2 **Scan** 13648
**Peptide** QPHTEIPEGTTAEEA **Charge** 2 **Spectral Angle** 0.9

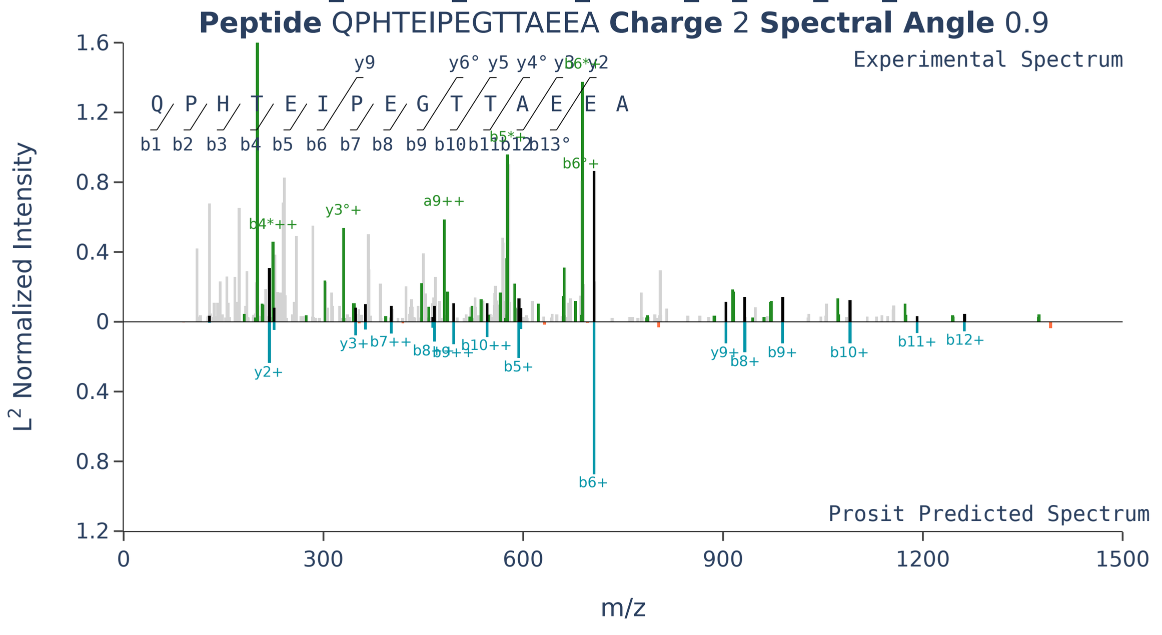


**Source** WSoh_221121_011221_HFGoe_L1_hTau_24h_R1 **Scan** 17269
**Peptide** IGDTPSLED **Charge** 1 **Spectral Angle** 0.82

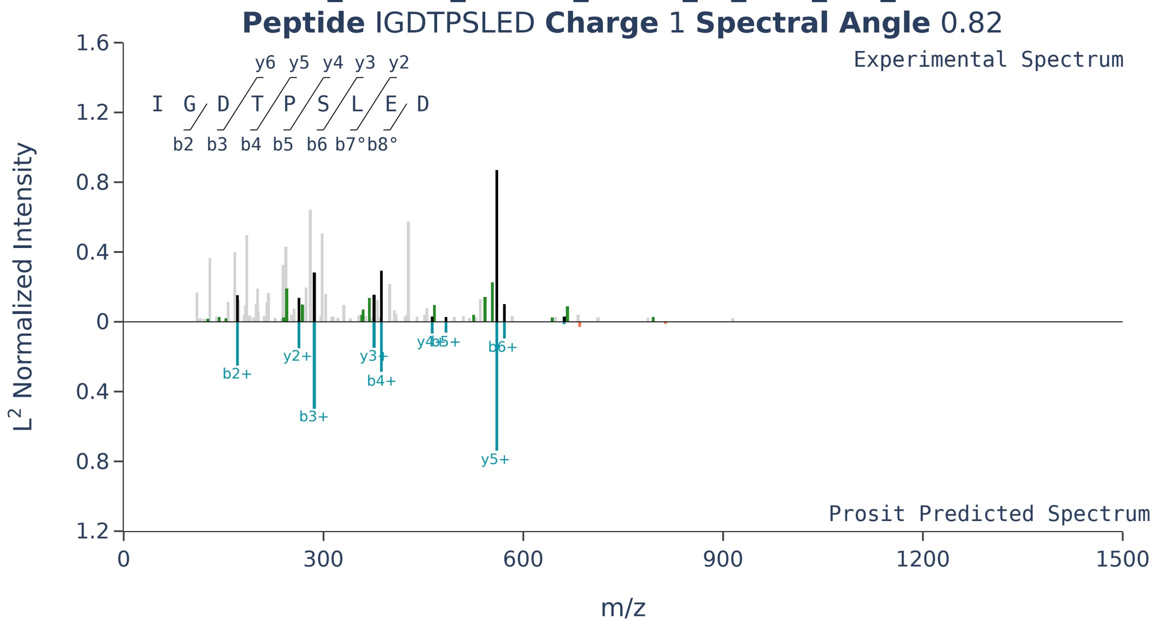


**Source** WSoh_221121_011221_HFGoe_L2_hTau_24h_R1 **Scan** 22861
**Peptide** TSDAKSTPTAEDVTAPLV **Charge** 2 **Spectral Angle** 0.92

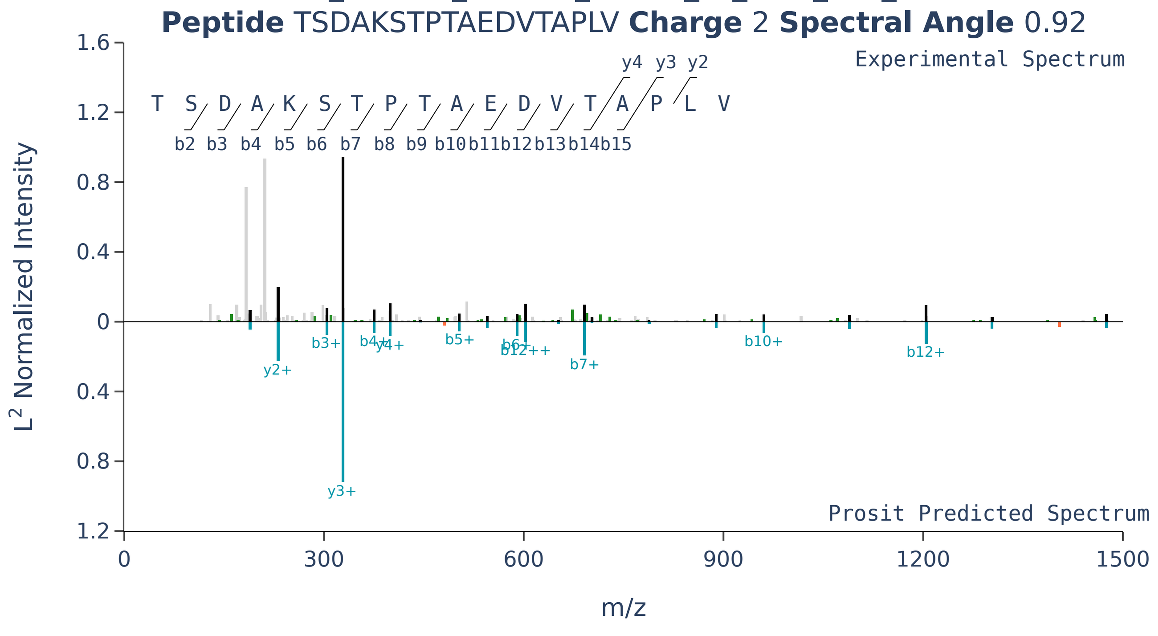


**Source** WSoh_221121_011221_HFGoe_L2_hTau_2h_R1 **Scan** 7132
**Peptide** QANATRIPAKT **Charge** 2 **Spectral Angle** 0.87

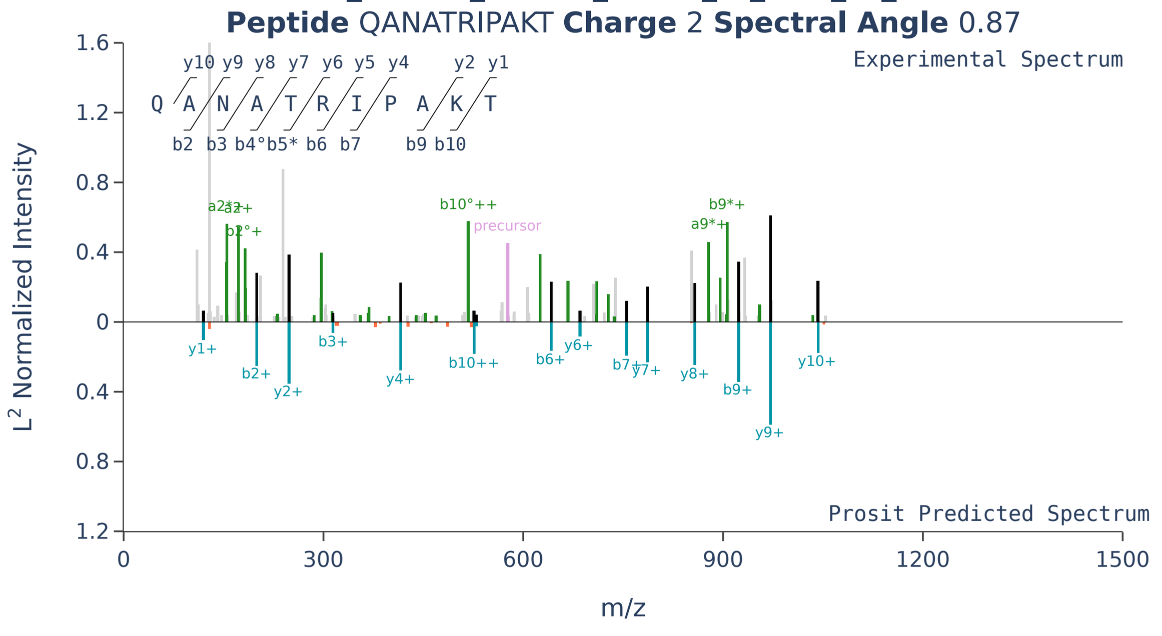


**Source** WSoh_221121_011221_HFGoe_L2_hTau_1h_R2 **Scan** 11794
**Peptide** PLQTPTED **Charge** 2 **Spectral Angle** 0.87

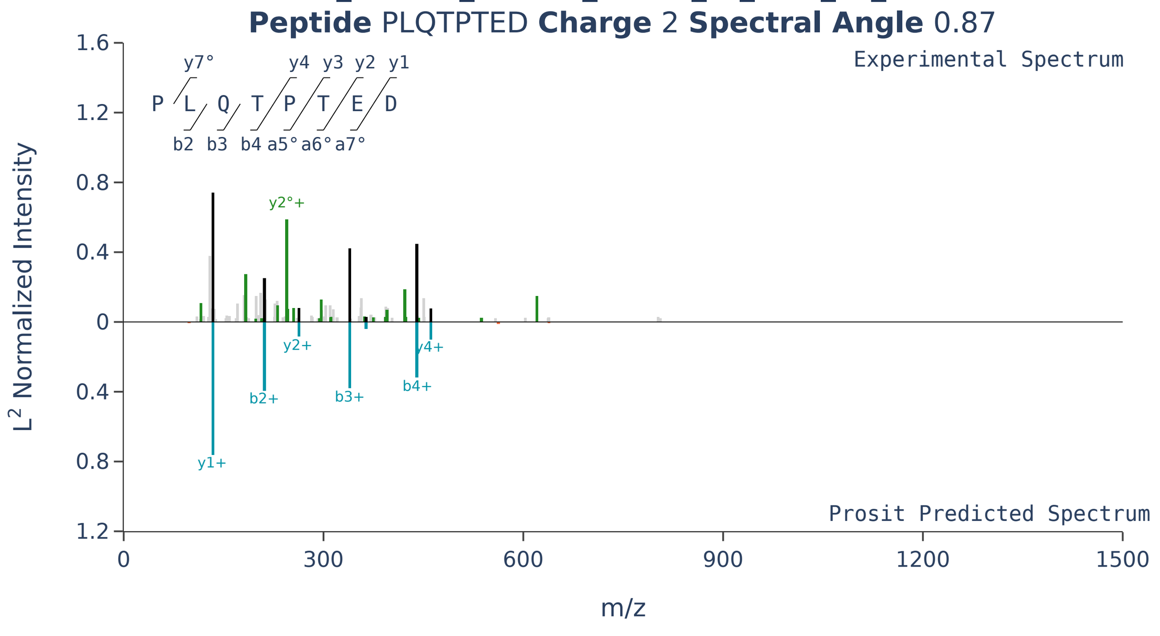

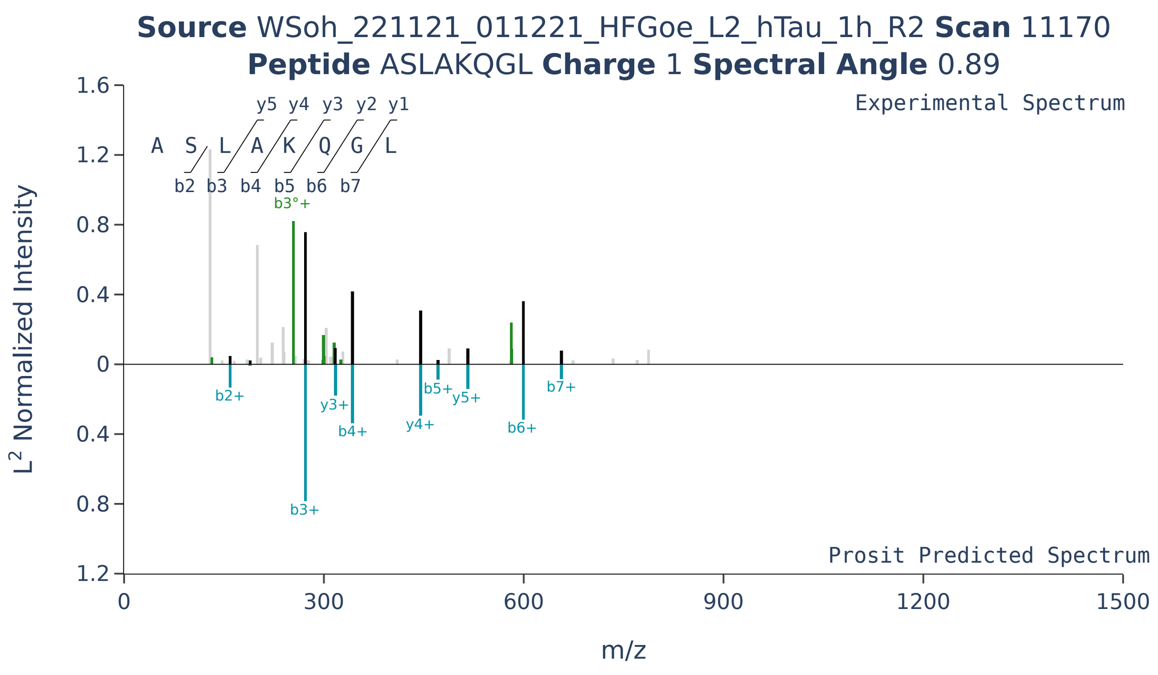
Source WSoh_221121_011221_HFGoe_L2_hTau_1h_R2 Scan 11170
Peptide ASLAKQGL Charge 1 Spectral Angle 0.89
Experimental Spectrum
Prosit Predicted Spectrum
L² Normalized Intensity
m/z

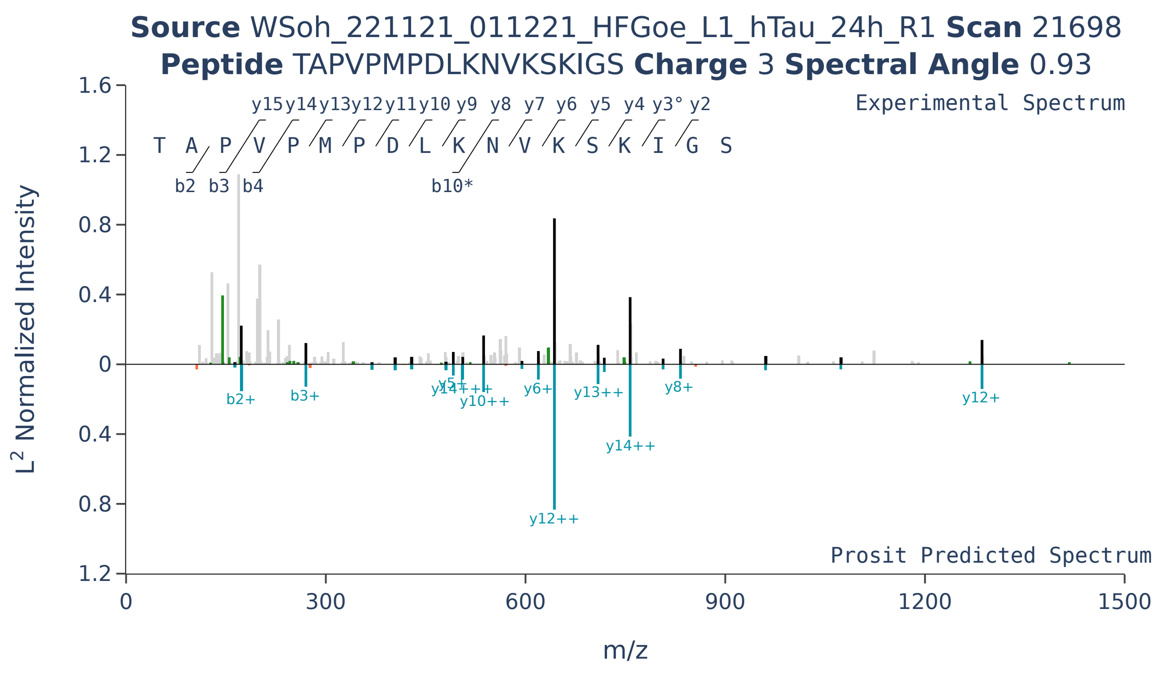
Source WSoh_221121_011221_HFGoe_L1_hTau_24h_R1 Scan 21698
Peptide TAPVPMPDLKNVKSKIGS Charge 3 Spectral Angle 0.93
Experimental Spectrum
Prosit Predicted Spectrum
L² Normalized Intensity
m/z

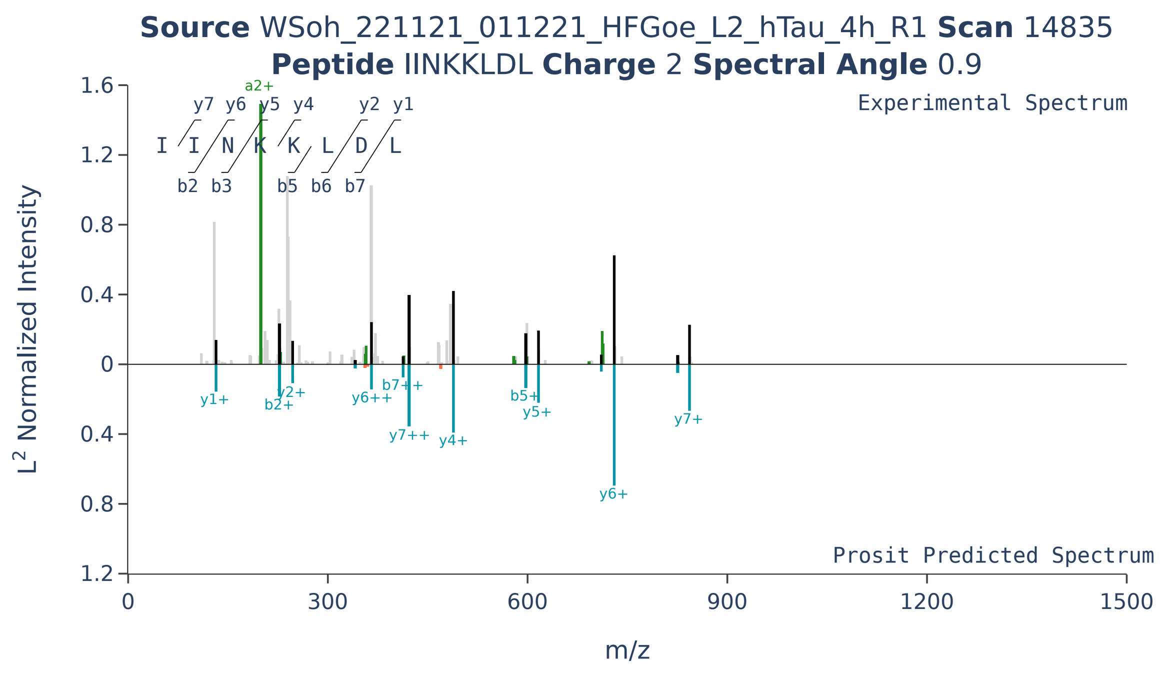
Source WSoh_221121_011221_HFGoe_L2_hTau_4h_R1 Scan 14835
Peptide IINKKLDL Charge 2 Spectral Angle 0.9
Experimental Spectrum
Prosit Predicted Spectrum
L² Normalized Intensity
m/z

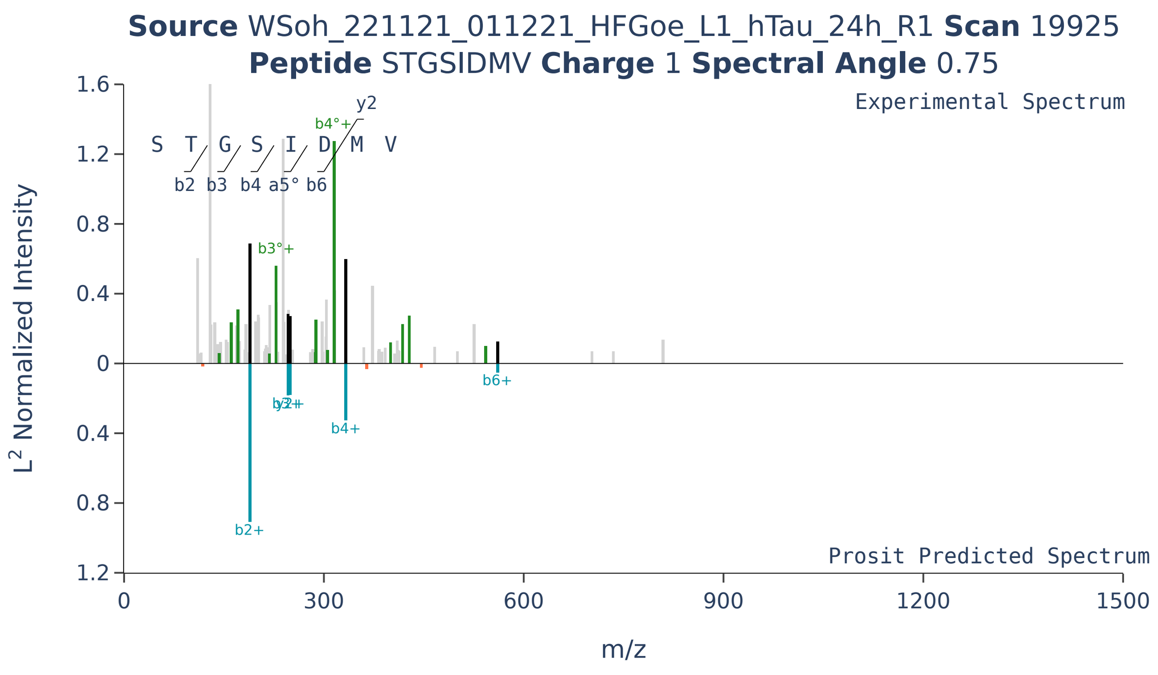
Source WSoh_221121_011221_HFGoe_L1_hTau_24h_R1 Scan 19925
Peptide STGSIDMV Charge 1 Spectral Angle 0.75
Experimental Spectrum
Prosit Predicted Spectrum
L² Normalized Intensity
m/z

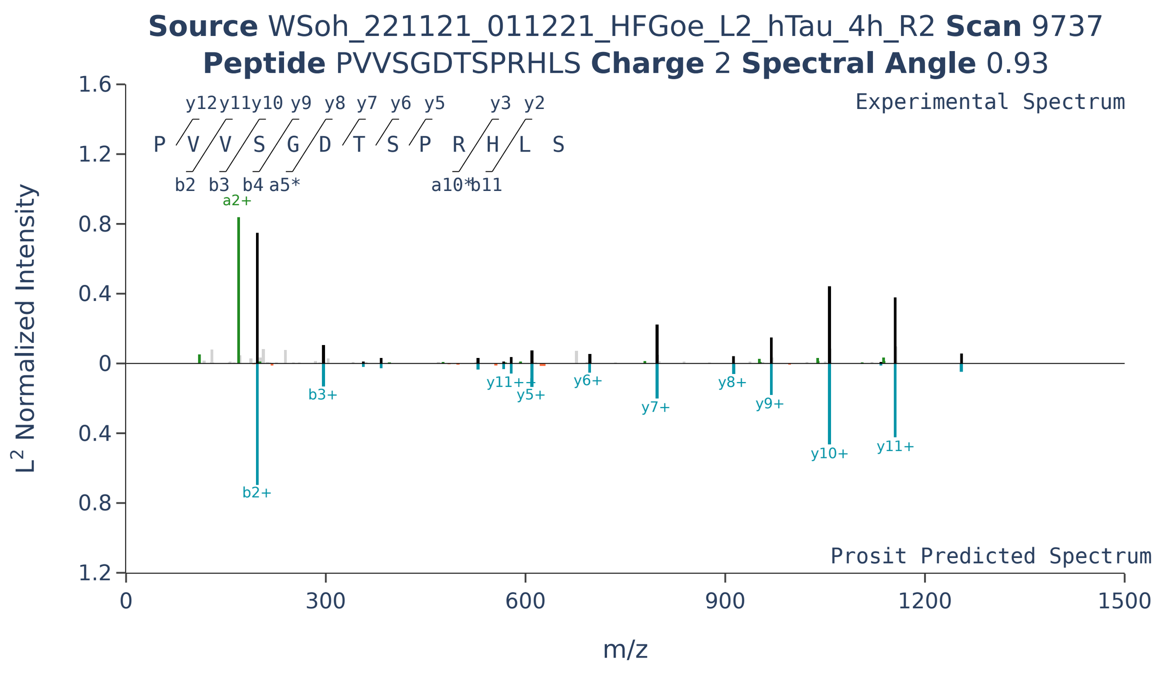
Source WSoh_221121_011221_HFGoe_L2_hTau_4h_R2 Scan 9737
Peptide PVVSGDTSPRHLS Charge 2 Spectral Angle 0.93
Experimental Spectrum
Prosit Predicted Spectrum
L² Normalized Intensity
m/z

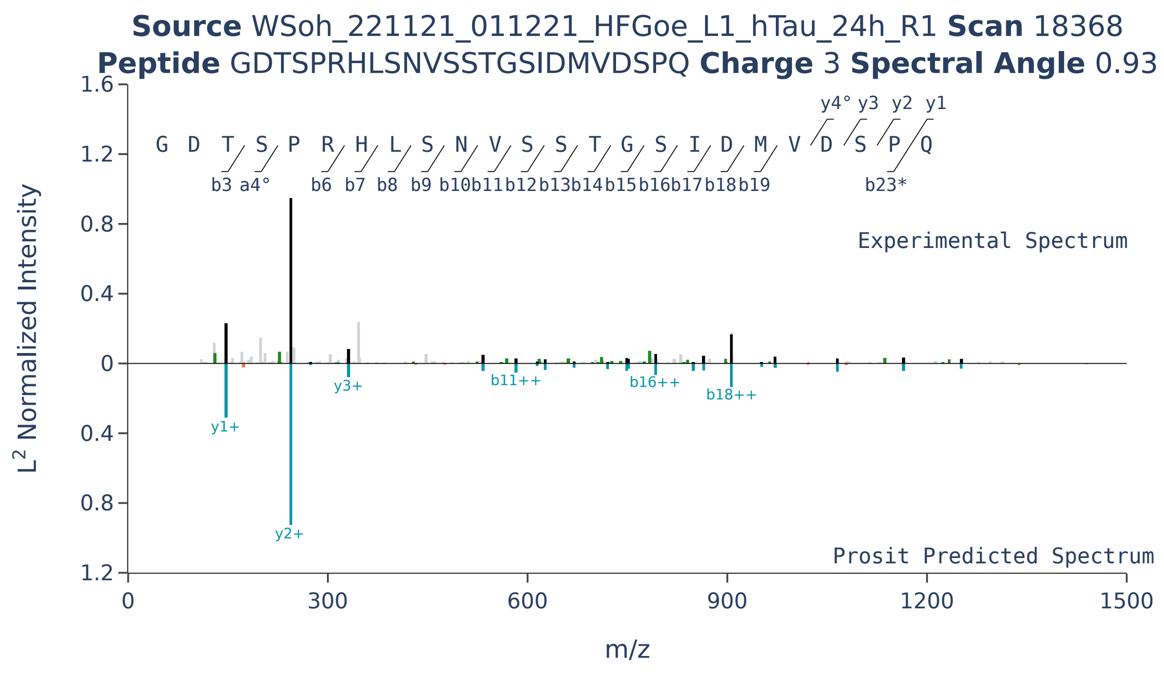
Source WSoh_221121_011221_HFGoe_L1_hTau_24h_R1 Scan 18368
Peptide GDTSPRHLSNVSSTGSIDMVDSPQ Charge 3 Spectral Angle 0.93
Experimental Spectrum
Prosit Predicted Spectrum
L² Normalized Intensity
m/z

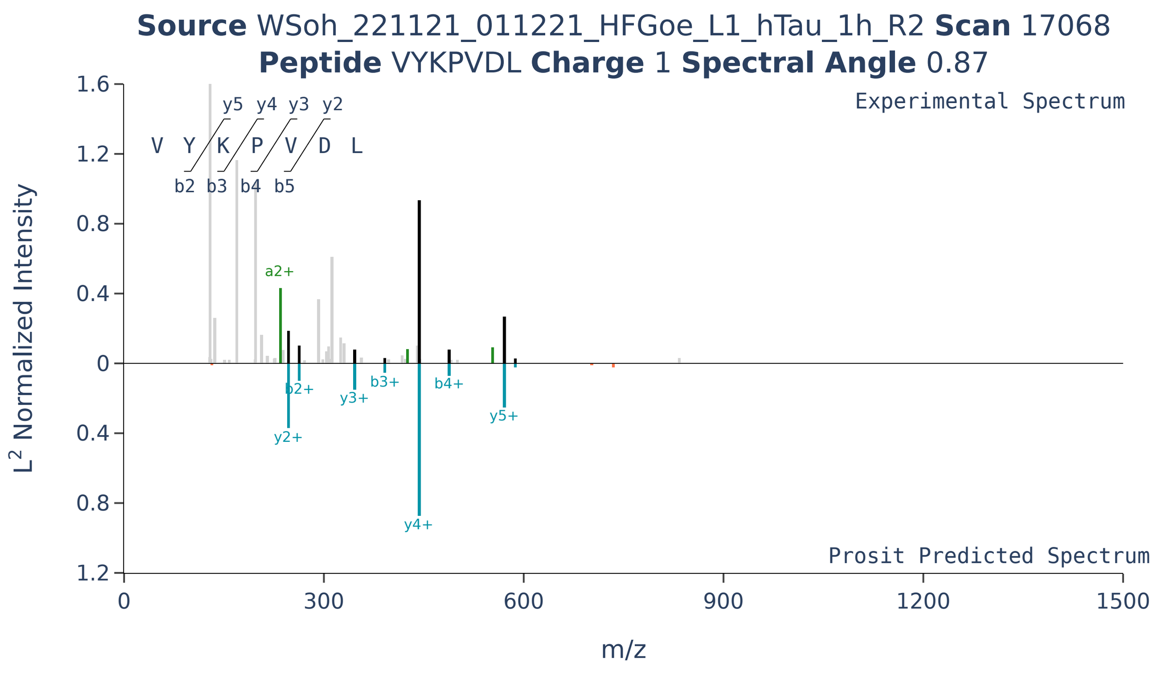
Source WSoh_221121_011221_HFGoe_L1_hTau_1h_R2 Scan 17068
Peptide VYKPVDL Charge 1 Spectral Angle 0.87
Experimental Spectrum
Prosit Predicted Spectrum
L² Normalized Intensity
m/z

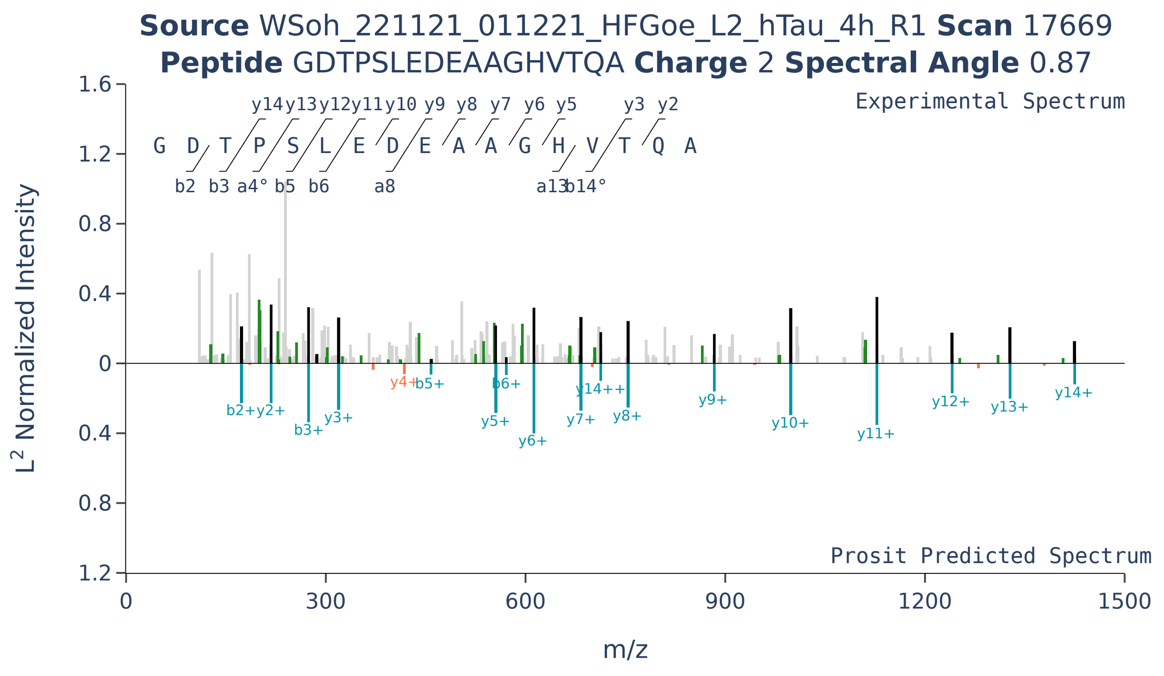
Source WSoh_221121_011221_HFGoe_L2_hTau_4h_R1 Scan 17669
Peptide GDTPSLEDEAAGHVTQA Charge 2 Spectral Angle 0.87
Experimental Spectrum
Prosit Predicted Spectrum
L² Normalized Intensity
m/z

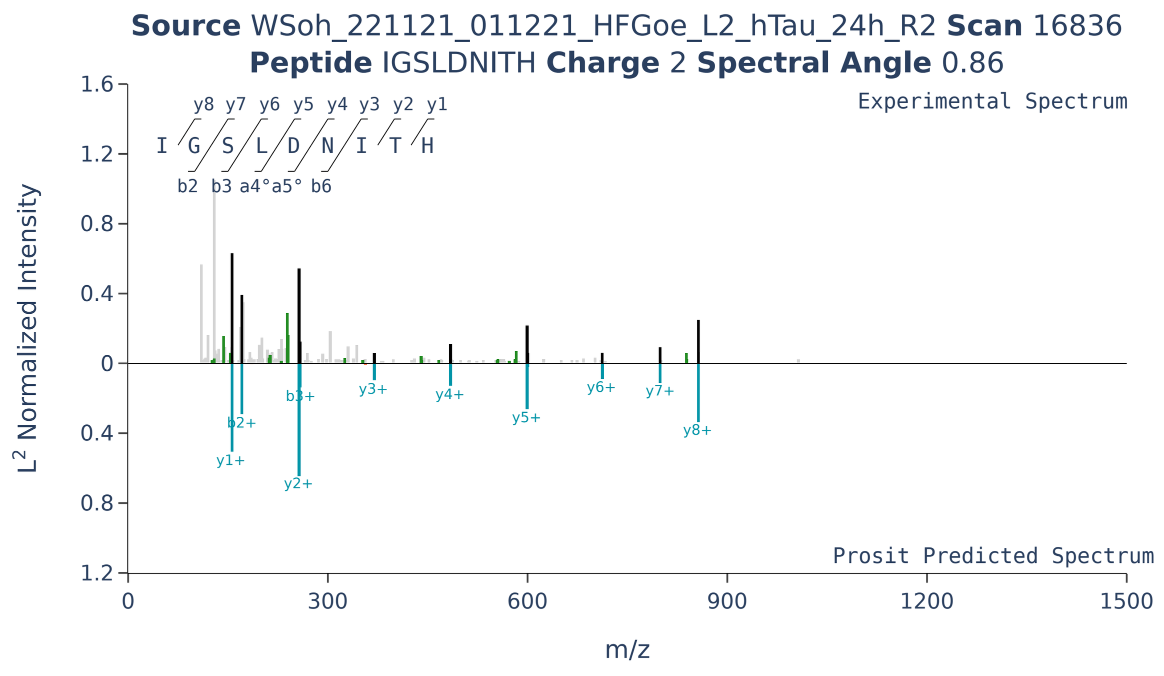
Source WSoh_221121_011221_HFGoe_L2_hTau_24h_R2 Scan 16836
Peptide IGSLDNITH Charge 2 Spectral Angle 0.86
Experimental Spectrum
Prosit Predicted Spectrum
L² Normalized Intensity
m/z

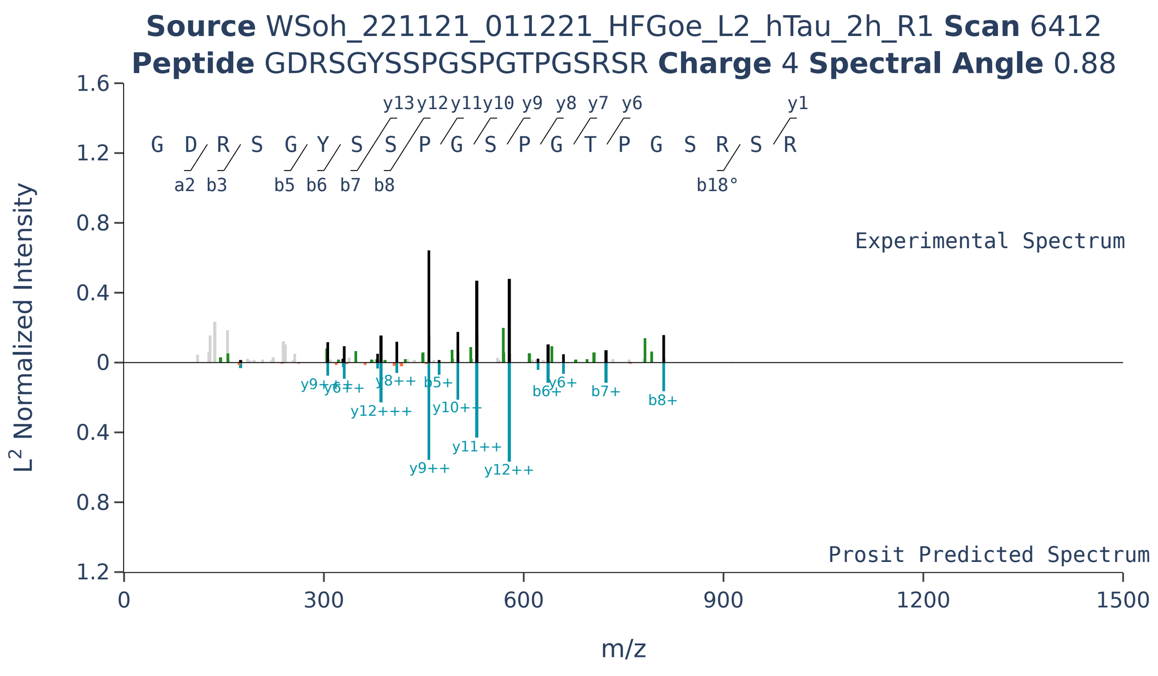
Source WSoh_221121_011221_HFGoe_L2_hTau_2h_R1 Scan 6412
Peptide GDRSGYSSPGSPGTPGSRSR Charge 4 Spectral Angle 0.88
Experimental Spectrum
Prosit Predicted Spectrum
L² Normalized Intensity
m/z

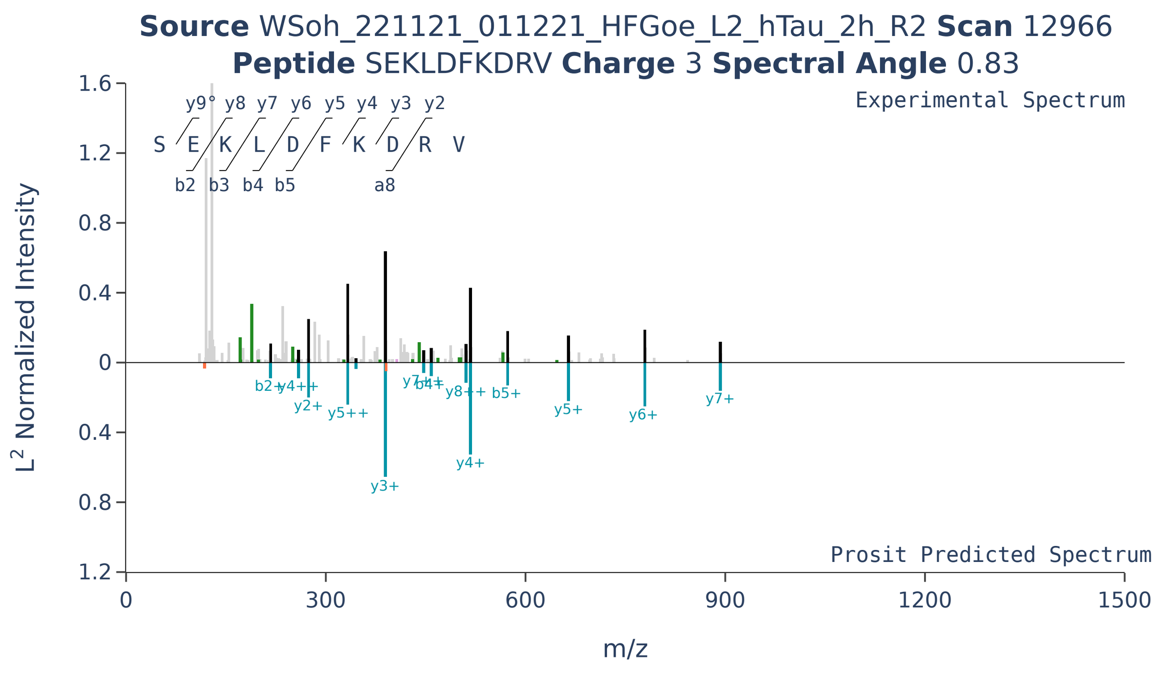
Source WSoh_221121_011221_HFGoe_L2_hTau_2h_R2 Scan 12966
Peptide SEKLDFKDRV Charge 3 Spectral Angle 0.83
Experimental Spectrum
Prosit Predicted Spectrum
L² Normalized Intensity
m/z

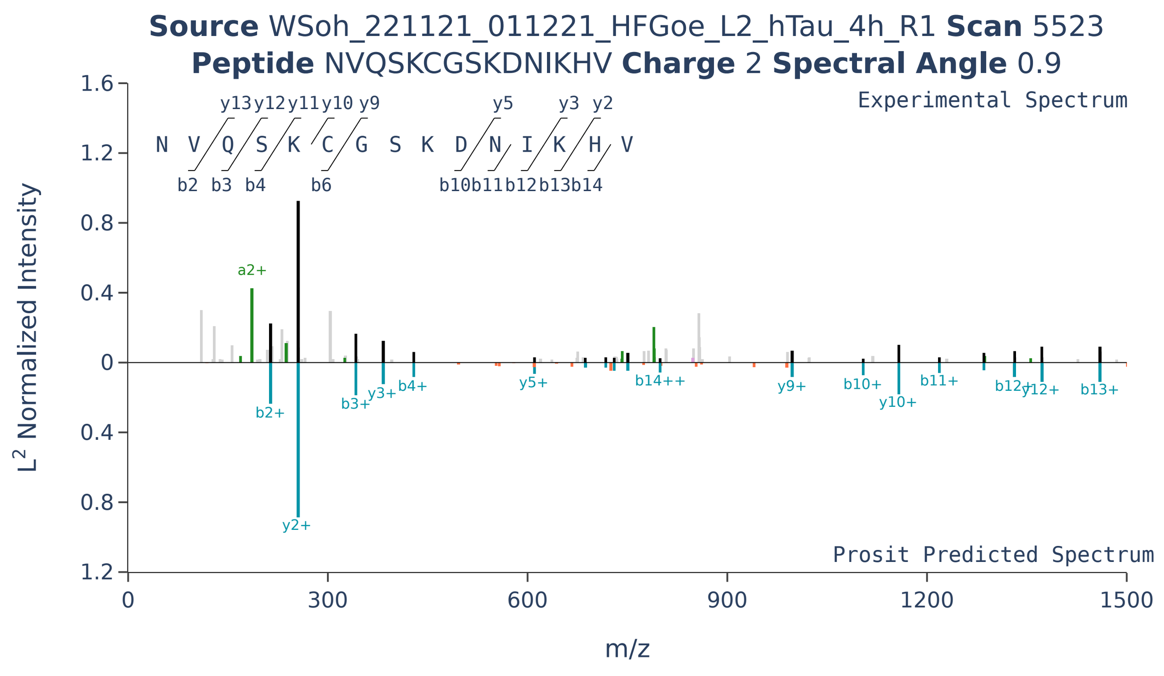
Source WSoh_221121_011221_HFGoe_L2_hTau_4h_R1 Scan 5523
Peptide NVQSKCGSKDNIKHV Charge 2 Spectral Angle 0.9
Experimental Spectrum
Prosit Predicted Spectrum
L² Normalized Intensity
m/z

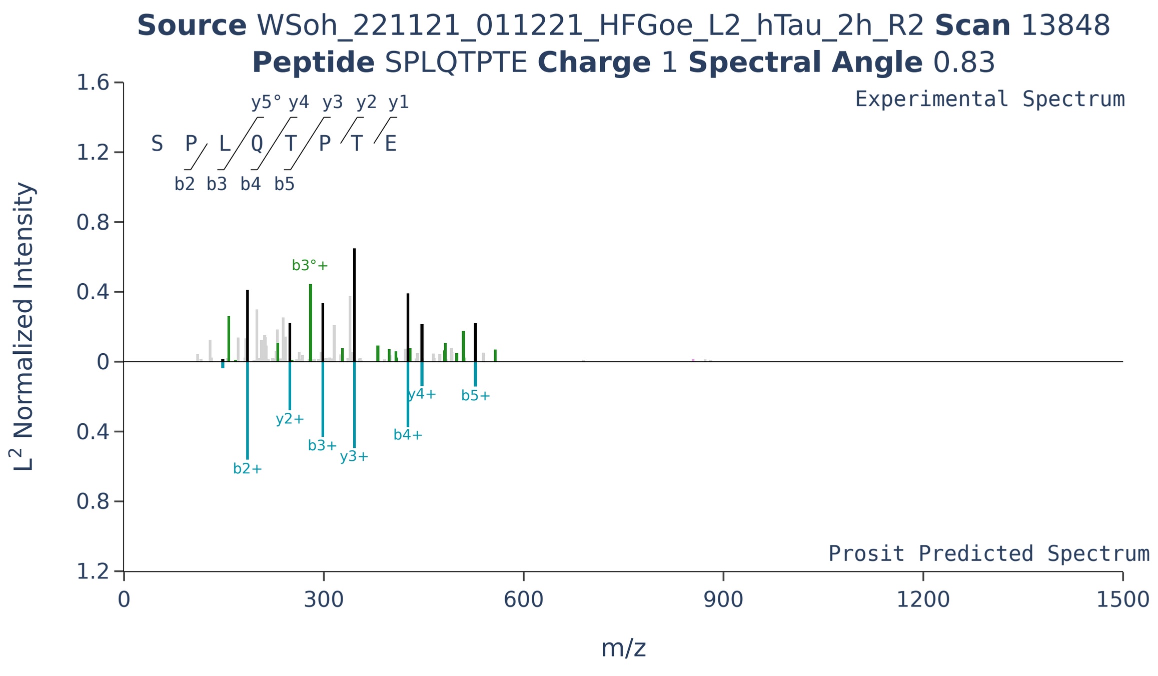
Source WSoh_221121_011221_HFGoe_L2_hTau_2h_R2 Scan 13848
Peptide SPLQTPTE Charge 1 Spectral Angle 0.83
Experimental Spectrum
Prosit Predicted Spectrum
L² Normalized Intensity
m/z

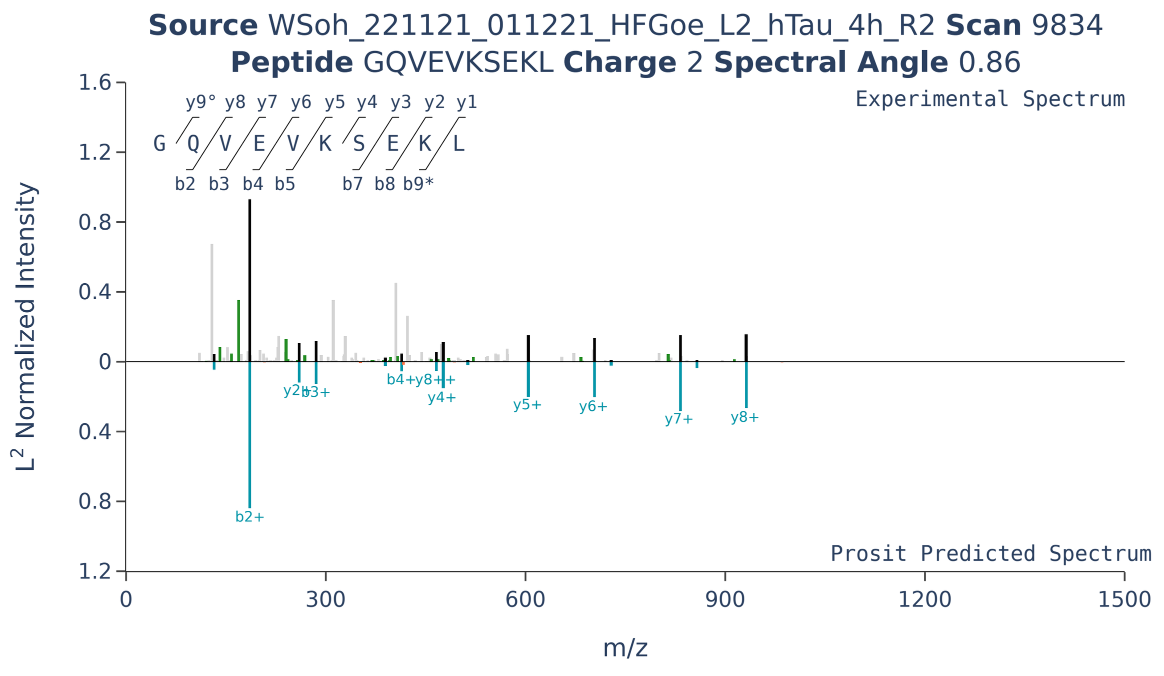
Source WSoh_221121_011221_HFGoe_L2_hTau_4h_R2 Scan 9834
Peptide GQVEVKSEKL Charge 2 Spectral Angle 0.86
Experimental Spectrum
Prosit Predicted Spectrum
L² Normalized Intensity
m/z

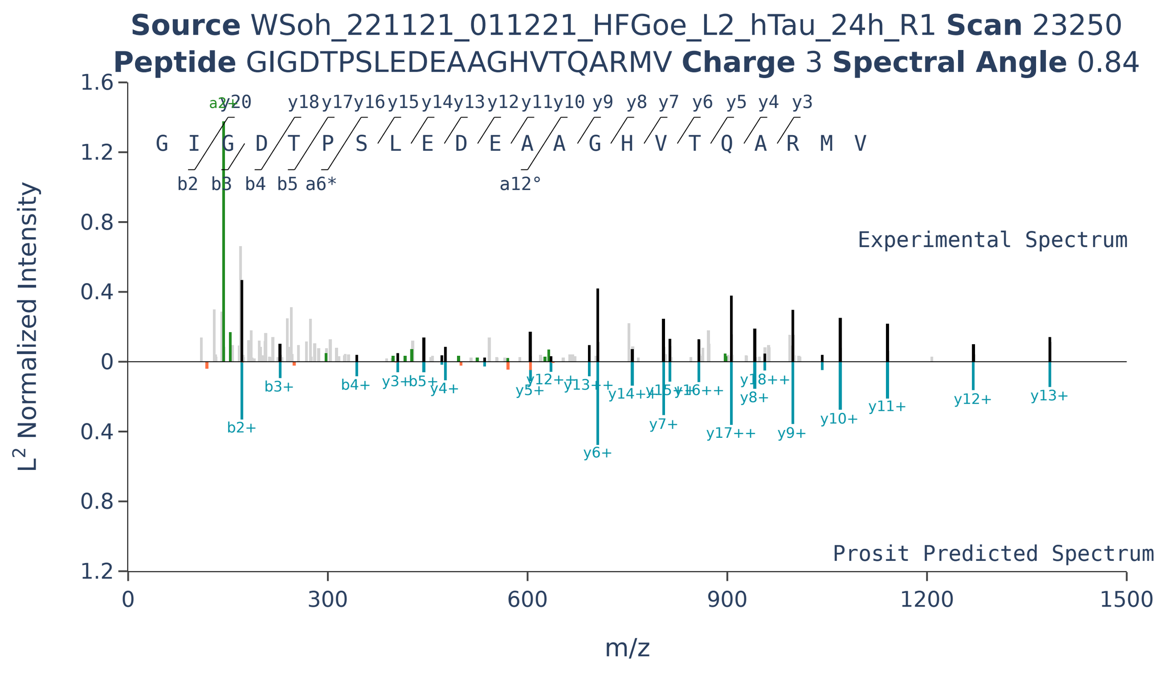
Source WSoh_221121_011221_HFGoe_L2_hTau_24h_R1 Scan 23250
Peptide GIGDTPSLEDEAAGHVTQARMV Charge 3 Spectral Angle 0.84
Experimental Spectrum
Prosit Predicted Spectrum
L² Normalized Intensity
m/z

**Source** WSoh_221121_011221_HFGoe_L2_hTau_4h_R1 **Scan** 11561
**Peptide** QGGYTMHQDQEGDTDAGLKE **Charge** 2 **Spectral Angle** 0.9

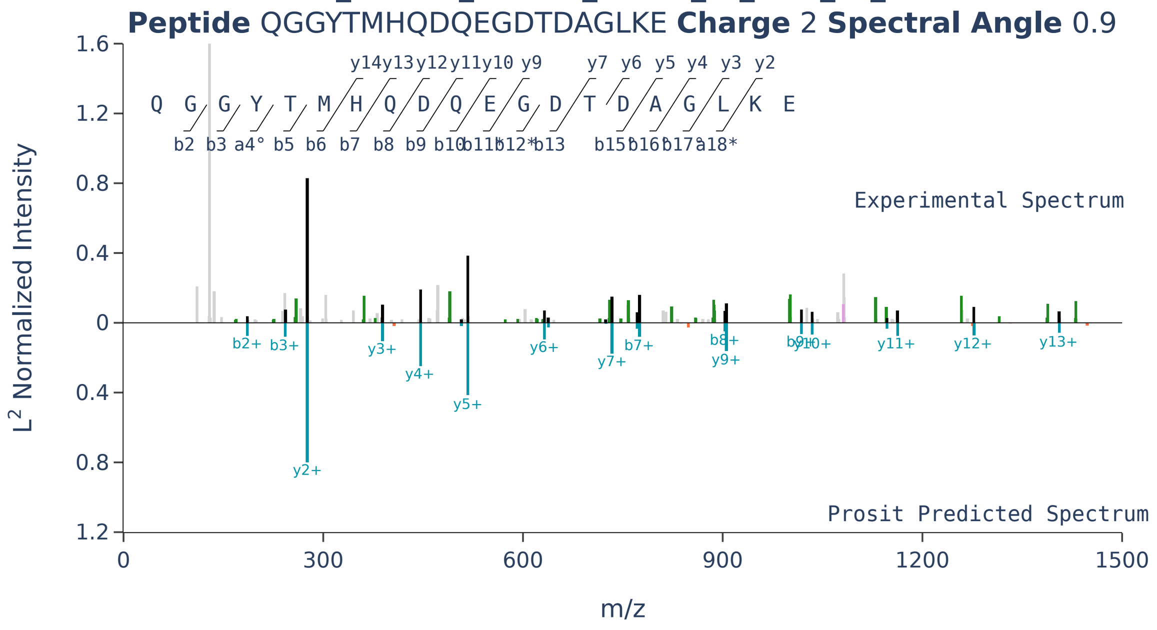


**Source** WSoh_221121_011221_HFGoe_L2_hTau_24h_R1 **Scan** 8078
**Peptide** KTDHGAEIVYKS **Charge** 2 **Spectral Angle** 0.96

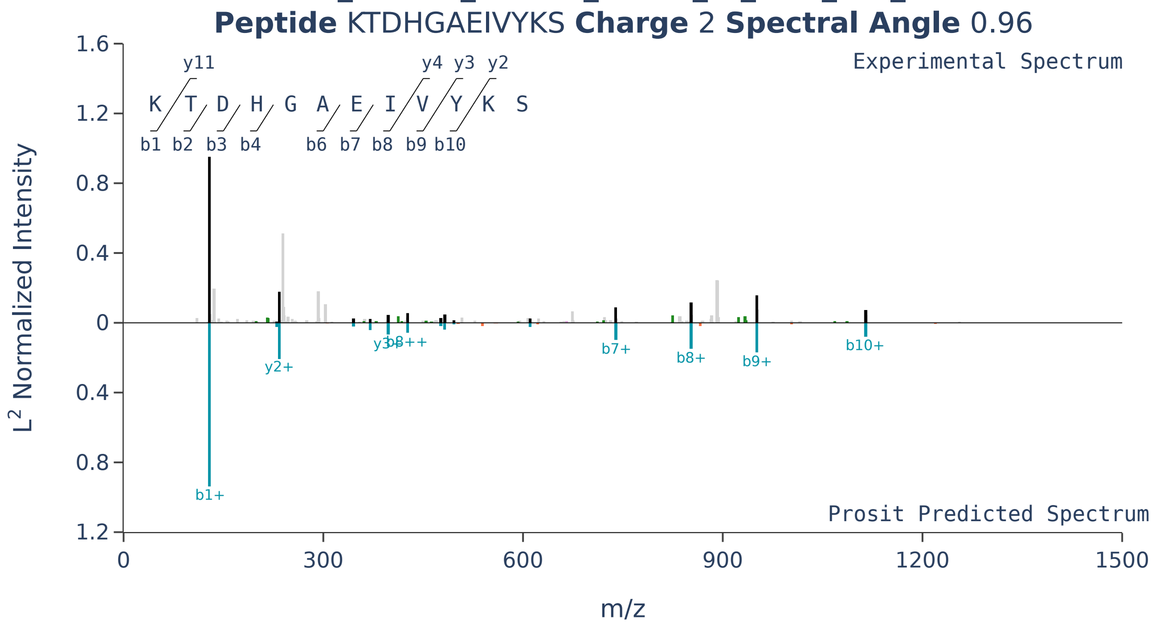


**Source** WSoh_221121_011221_HFGoe_L2_hTau_1h_R1 **Scan** 5920
**Peptide** GSLGNIHHKPGGG **Charge** 3 **Spectral Angle** 0.82

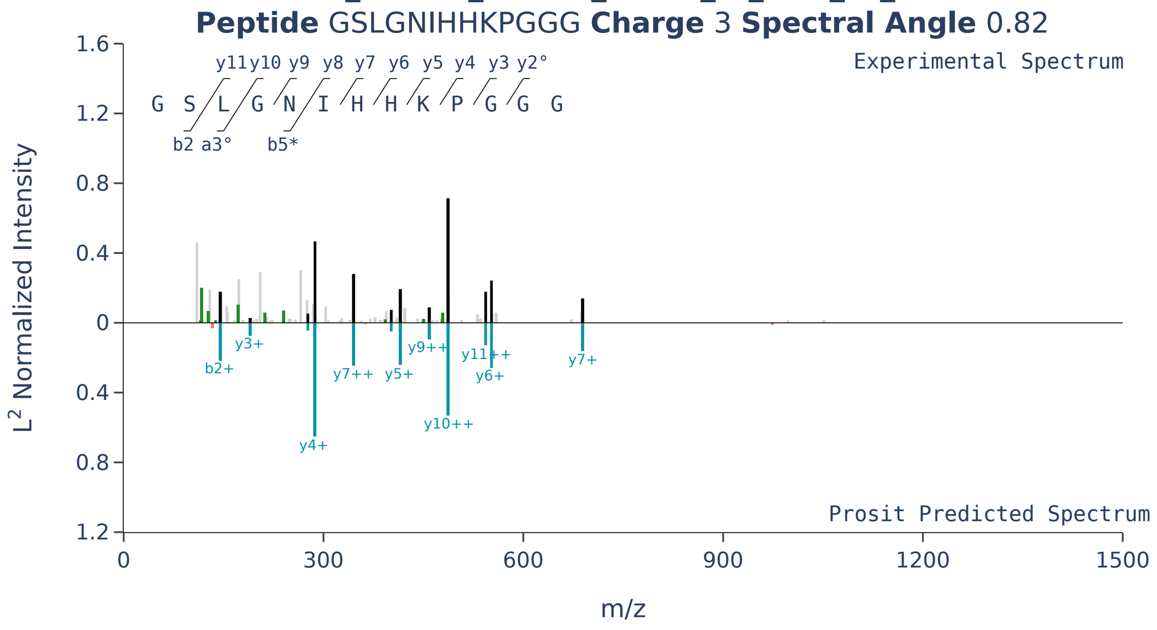


**Source** WSoh_221121_011221_HFGoe_L2_hTau_2h_R1 **Scan** 6283
**Peptide** VRTPPKSPSSAKSRL **Charge** 4 **Spectral Angle** 0.86

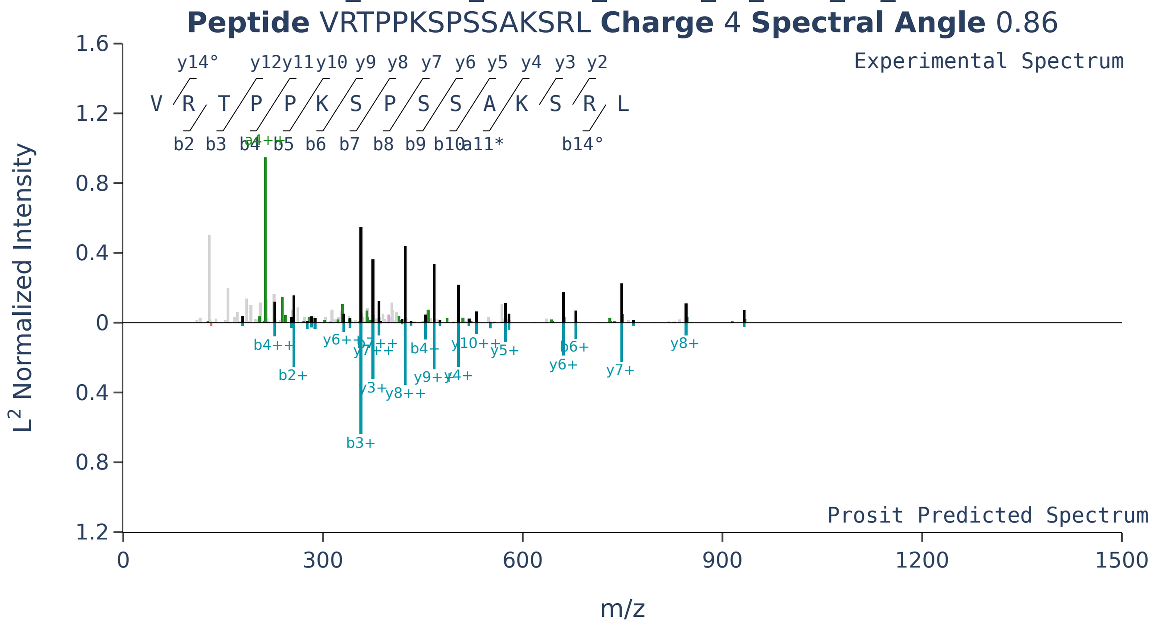


**Source** WSoh_221121_011221_HFGoe_L1_hTau_2h_R2 **Scan** 13015
**Peptide** GSKDNIKHVPGGGSVQI **Charge** 3 **Spectral Angle** 0.78

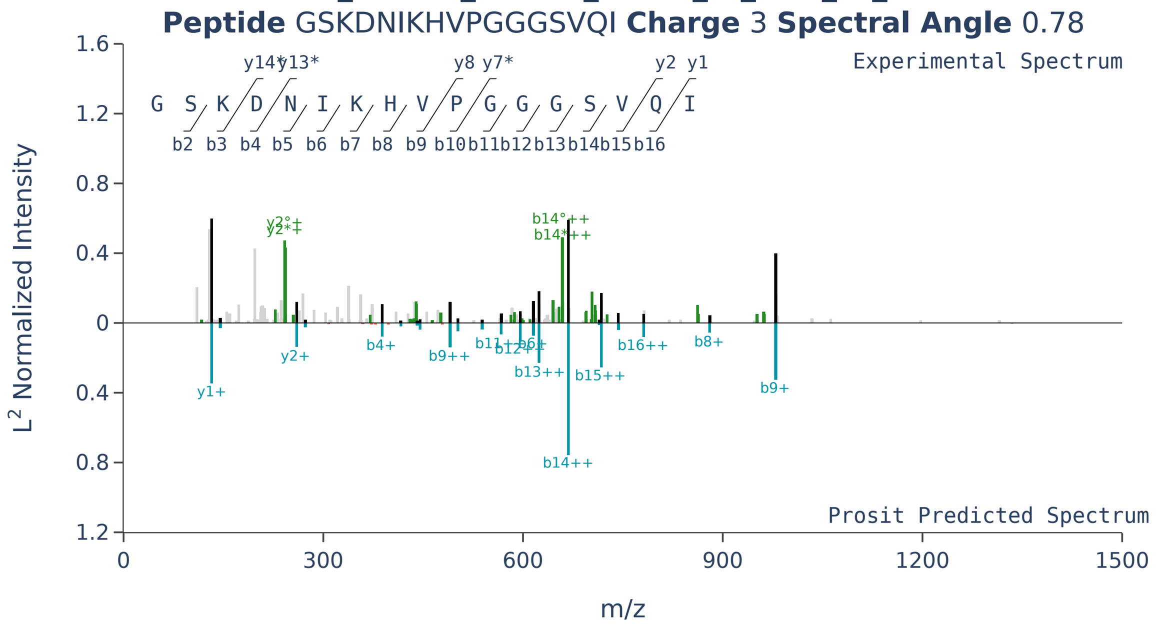


**Source** WSoh_221121_011221_HFGoe_L1_hTau_24h_R2 **Scan** 9492
**Peptide** KAKTDHGAEIVY **Charge** 2 **Spectral Angle** 0.9

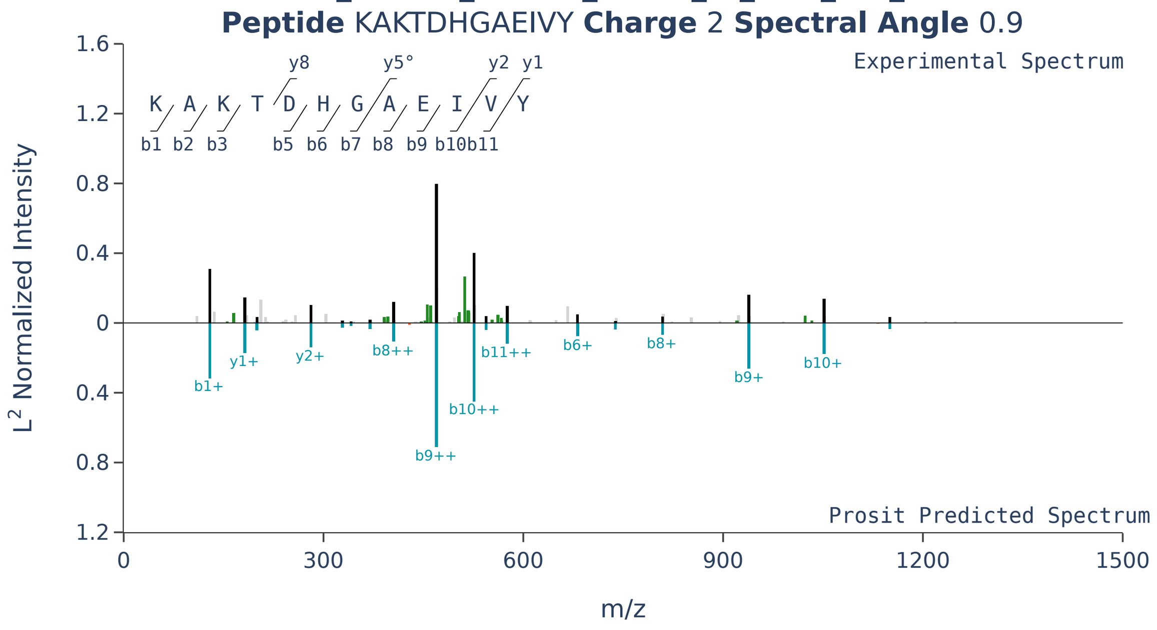


**Source** WSoh_221121_011221_HFGoe_L1_hTau_4h_R1 **Scan** 6716
**Peptide** GNIHHKPGGGQVEV **Charge** 3 **Spectral Angle** 0.83

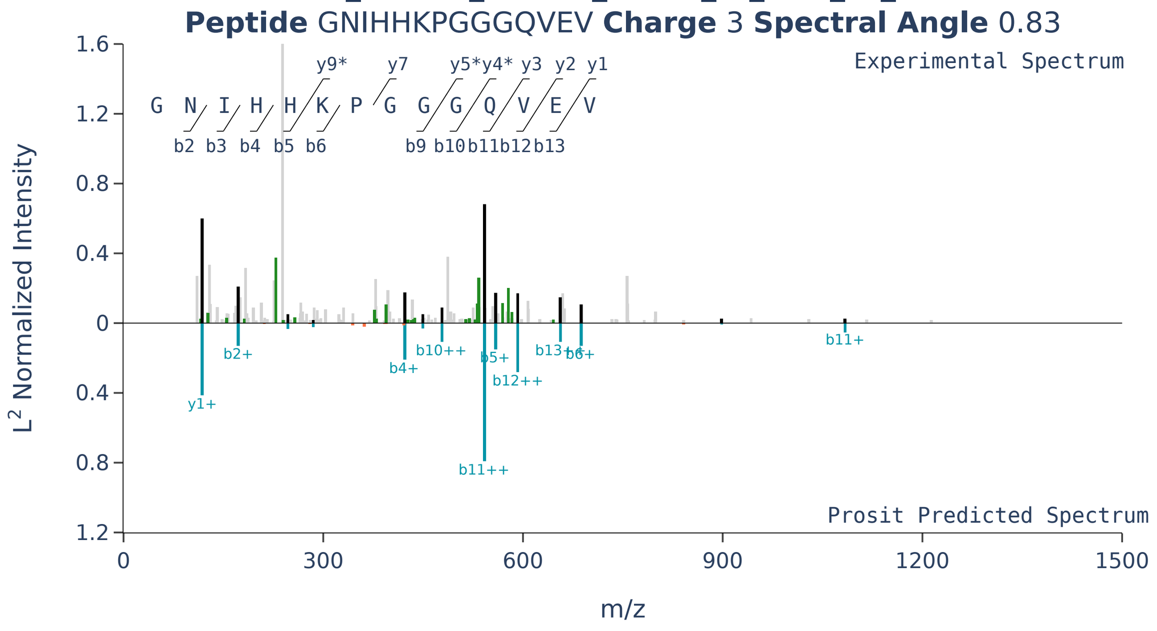


**Source** WSoh_221121_011221_HFGoe_L2_hTau_24h_R1 **Scan** 20761
**Peptide** APVPMPDLKN **Charge** 2 **Spectral Angle** 0.83

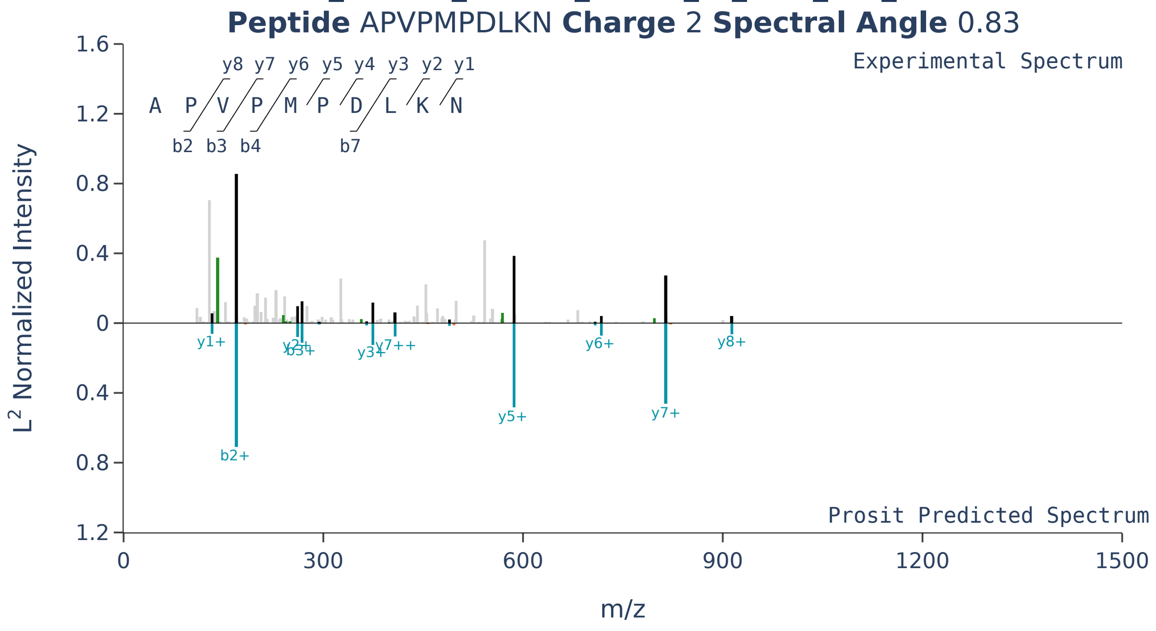


**Source** WSoh_221121_011221_HFGoe_L1_hTau_24h_R1 **Scan** 17626
**Peptide** DHGAEIVYKSPVVSGDTSPRHL **Charge** 4 **Spectral Angle** 0.86

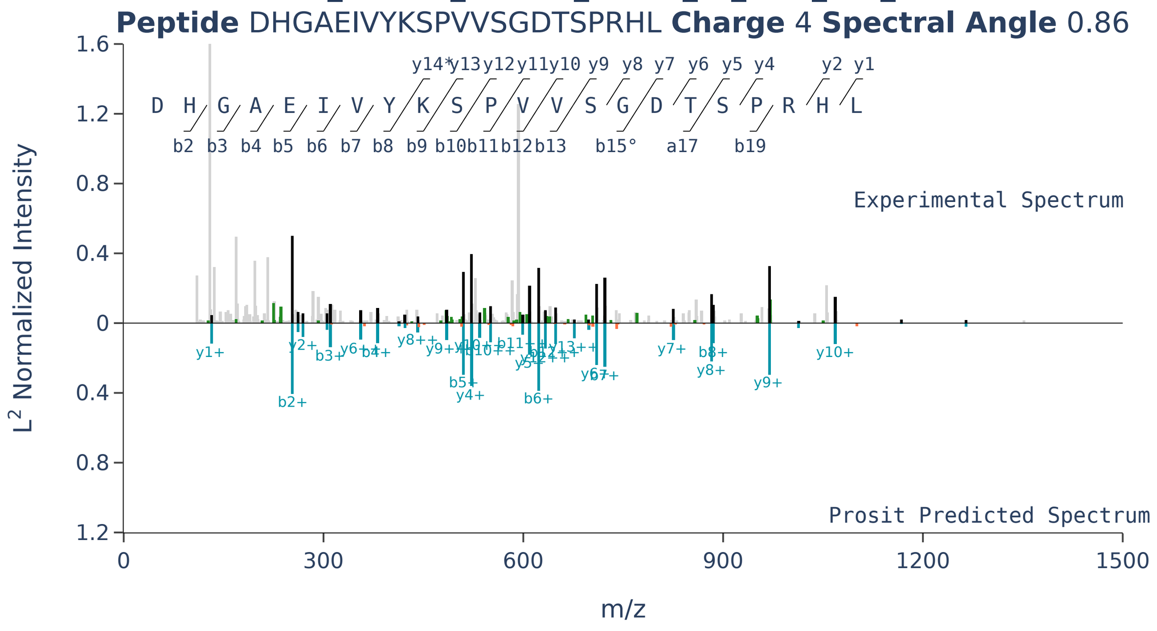


**Source** WSoh_221121_011221_HFGoe_L1_hTau_24h_R1 **Scan** 7036
**Peptide** DEAAGHVTQARMVSKSKD **Charge** 3 **Spectral Angle** 0.88

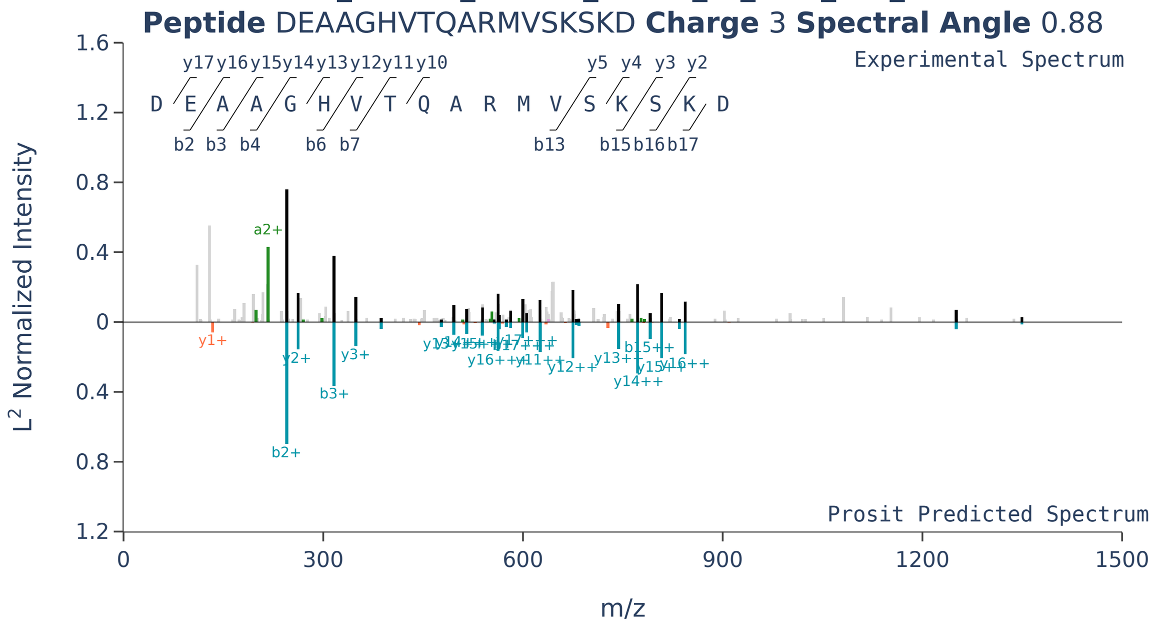


**Source** WSoh_221121_011221_HFGoe_L1_hTau_4h_R2 **Scan** 7021
**Peptide** VVRTPPKSPSSAKSRL **Charge** 4 **Spectral Angle** 0.83

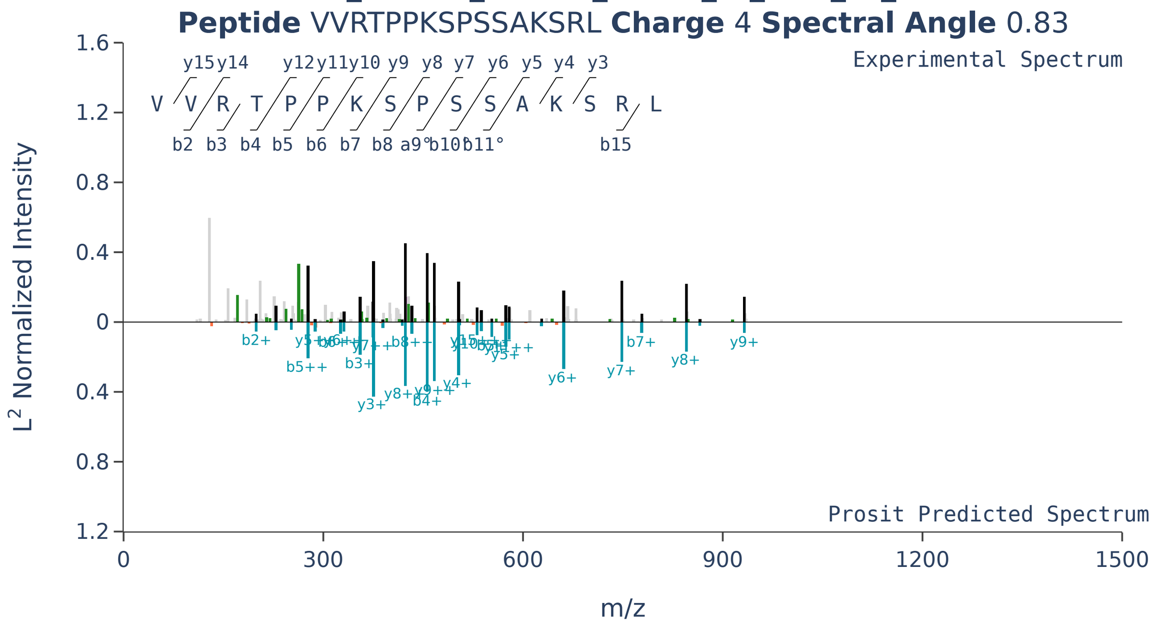


**Source** WSoh_221121_011221_HFGoe_L1_hTau_24h_R1 **Scan** 17500
**Peptide** VPMPDLKNVKS **Charge** 2 **Spectral Angle** 0.87

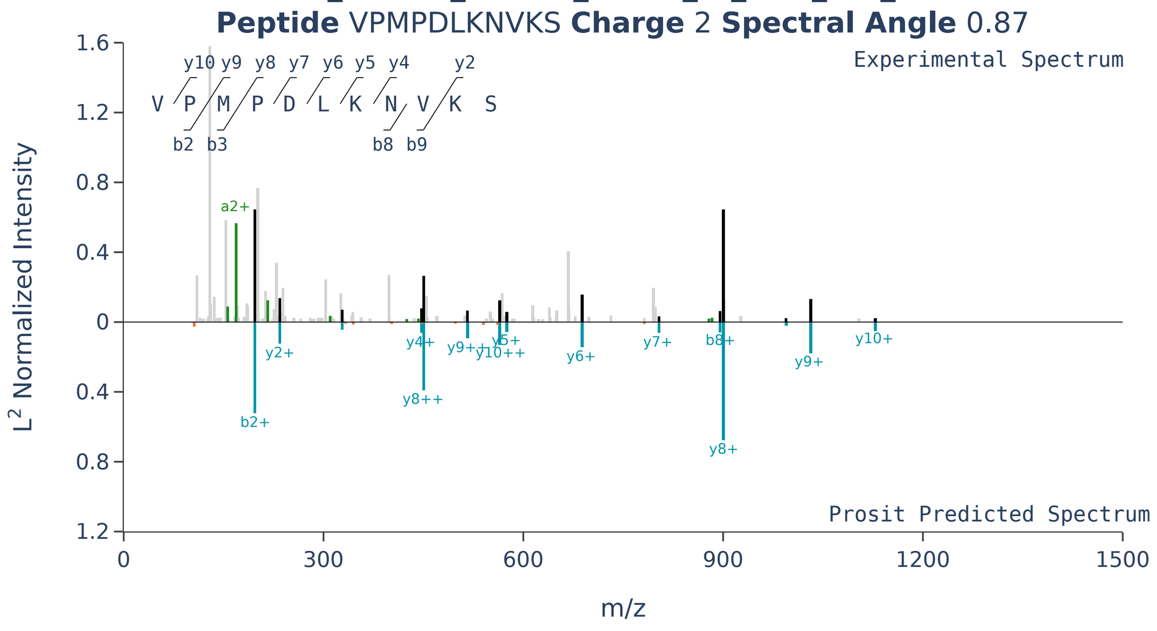


**Source** WSoh_221121_011221_HFGoe_L2_hTau_24h_R1 **Scan** 18063
**Peptide** RLQTAPVPMPDLKNVKSK **Charge** 4 **Spectral Angle** 0.89

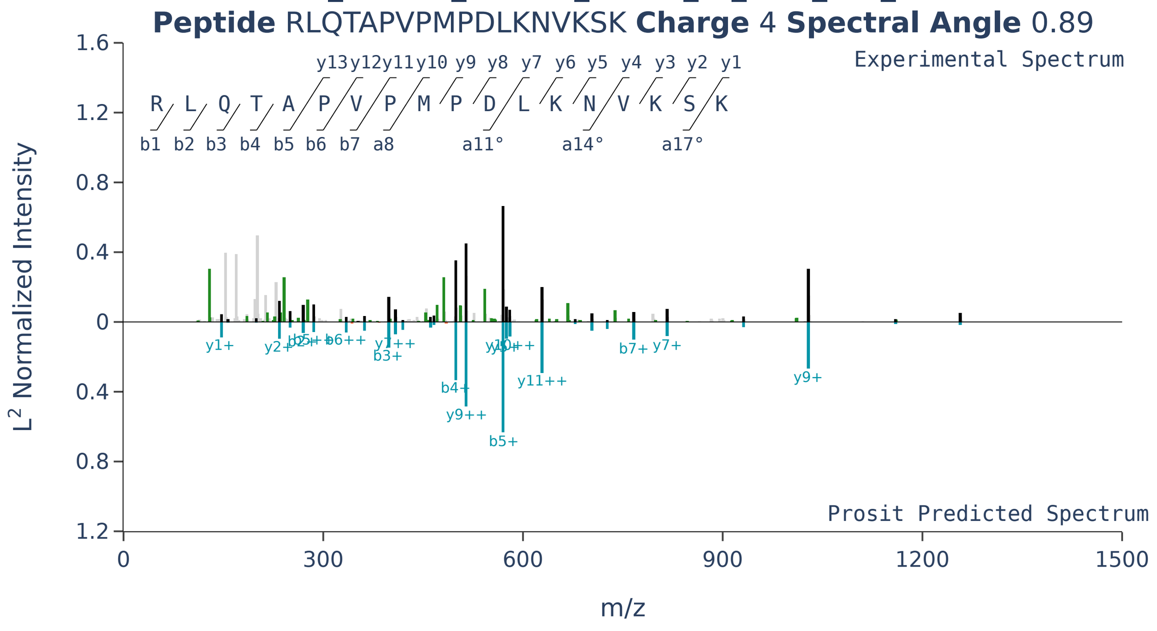


**Source** WSoh_221121_011221_HFGoe_L1_hTau_24h_R1 **Scan** 16261
**Peptide** AEIVYKSPVVSGDTSPRHLSN **Charge** 3 **Spectral Angle** 0.89

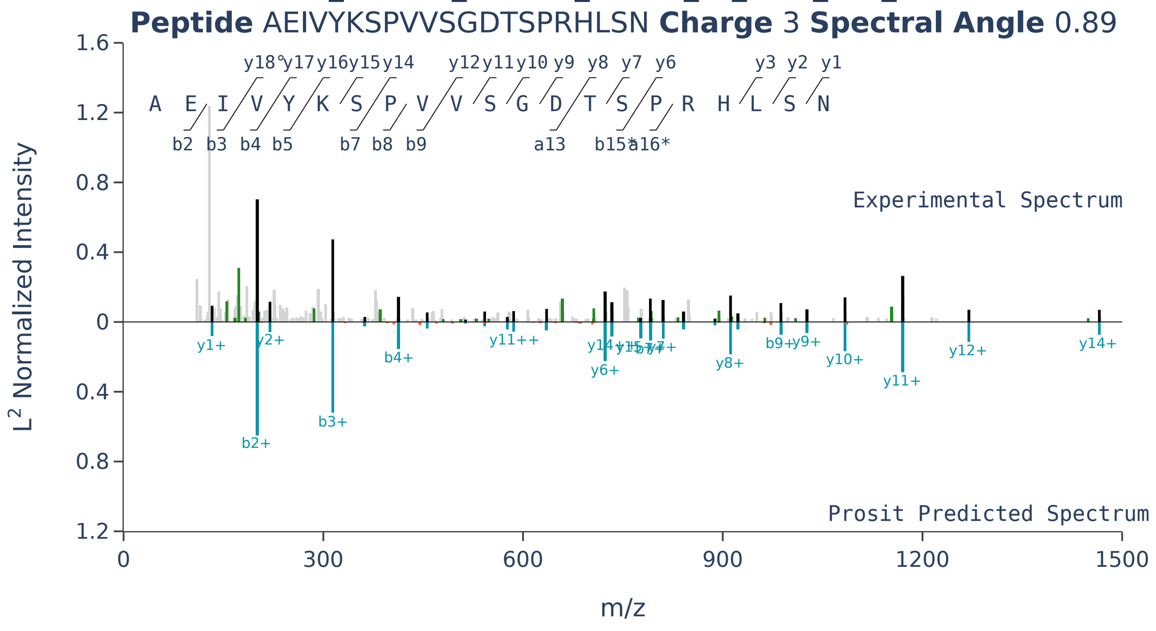


**Source** WSoh_221121_011221_HFGoe_L1_hTau_4h_R2 **Scan** 9252
**Peptide** KSPVVSGDTSPRHL **Charge** 2 **Spectral Angle** 0.93

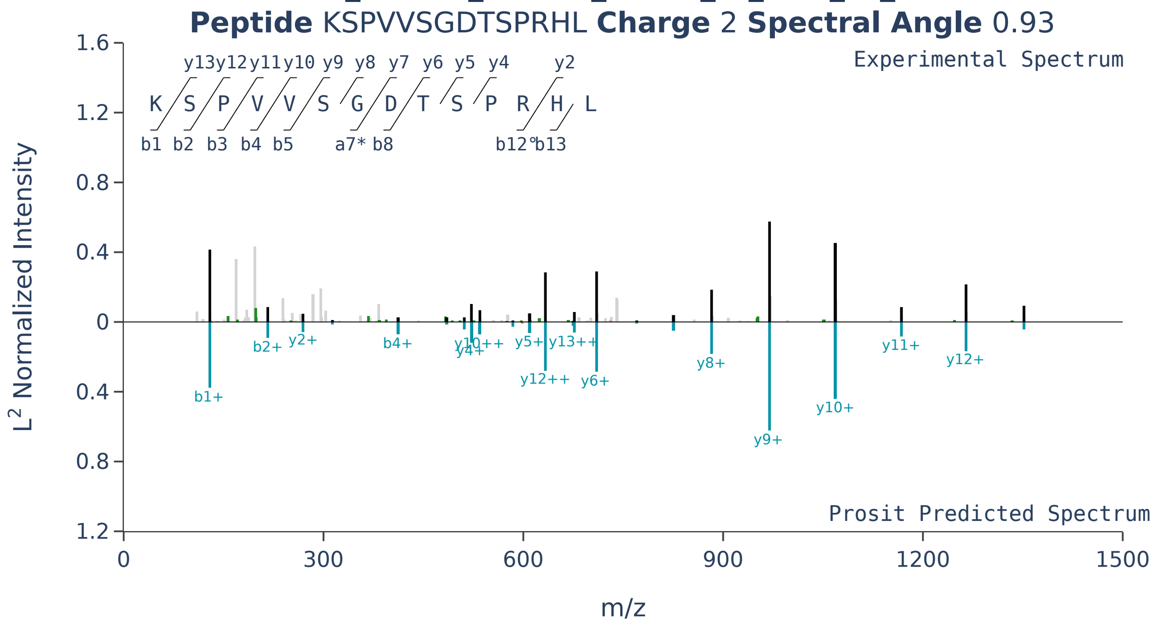

**Source** WSoh_221121_011221_HFGoe_L1_hTau_2h_R2 **Scan** 15433
**Peptide** YKPVDLSKVTSKC **Charge** 3 **Spectral Angle** 0.88

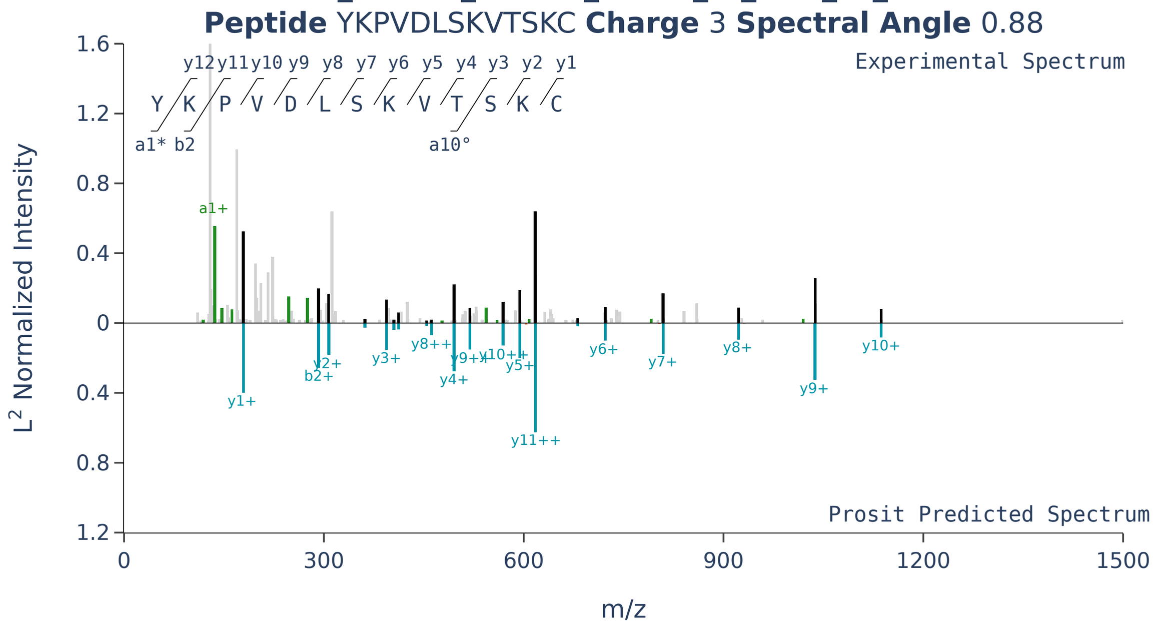


**Source** WSoh_221121_011221_HFGoe_L2_hTau_24h_R2 **Scan** 8078
**Peptide** GSEEPGSETSDAKSTPTA **Charge** 2 **Spectral Angle** 0.88

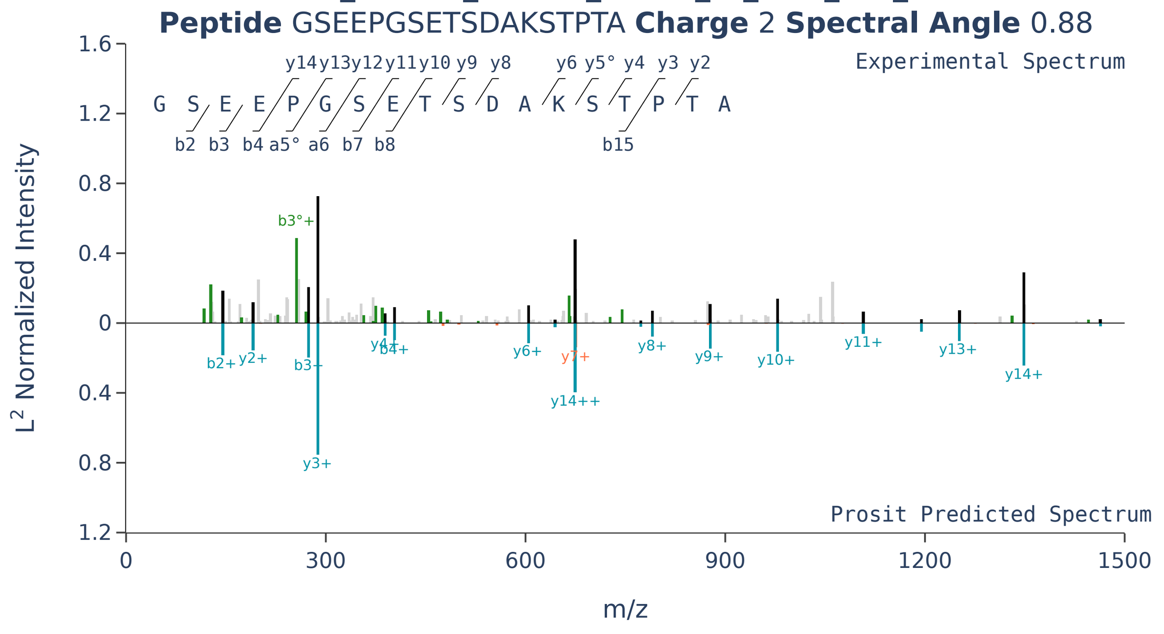


**Source** WSoh_221121_011221_HFGoe_L2_hTau_4h_R1 **Scan** 12000
**Peptide** KSEKLDFKDRVQSKIG **Charge** 3 **Spectral Angle** 0.88

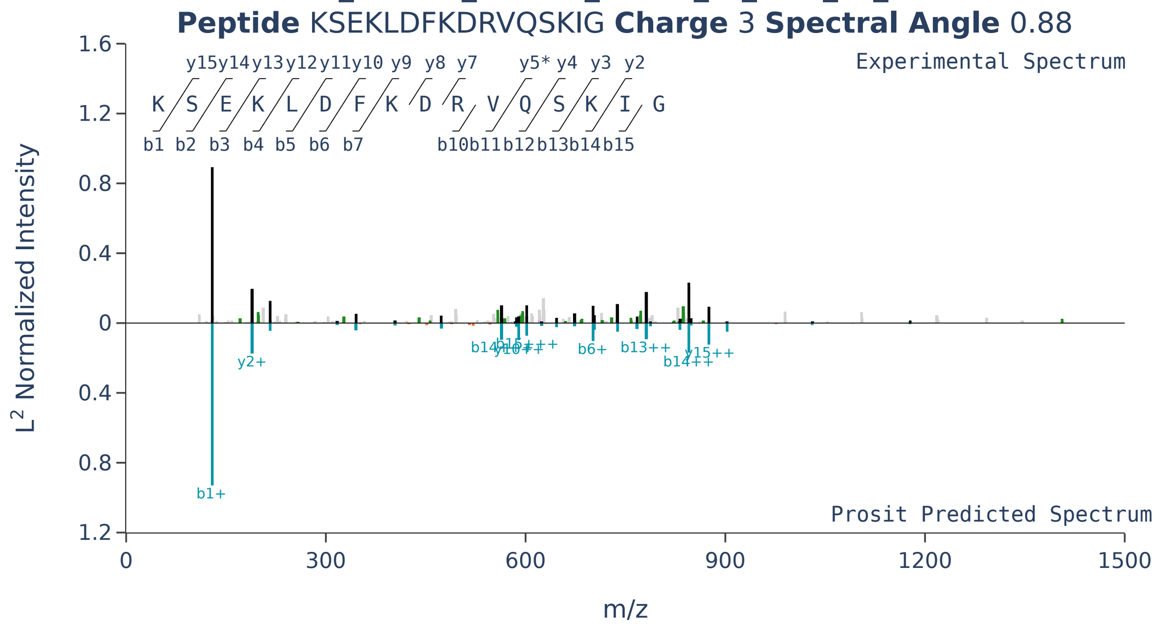


**Source** WSoh_221121_011221_HFGoe_L1_hTau_24h_R2 **Scan** 19267
**Peptide** QLVYKPVDLSKVTSK **Charge** 2 **Spectral Angle** 0.92

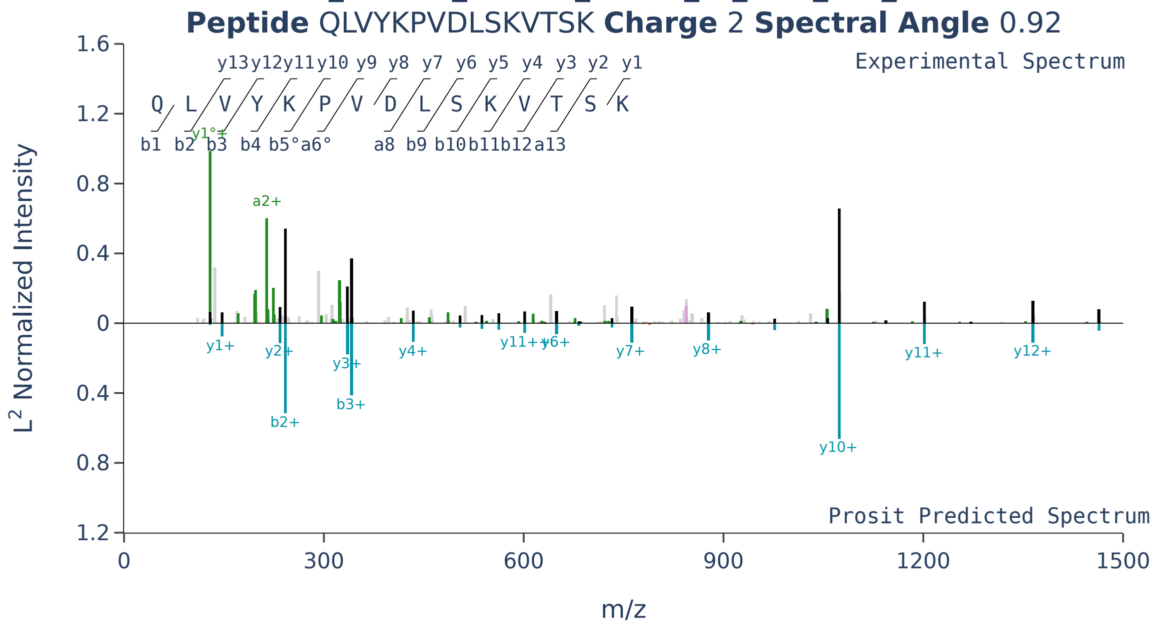


**Source** WSoh_221121_011221_HFGoe_L2_hTau_24h_R1 **Scan** 19020
**Peptide** GDTDAGLKESPLQTPTE **Charge** 2 **Spectral Angle** 0.93

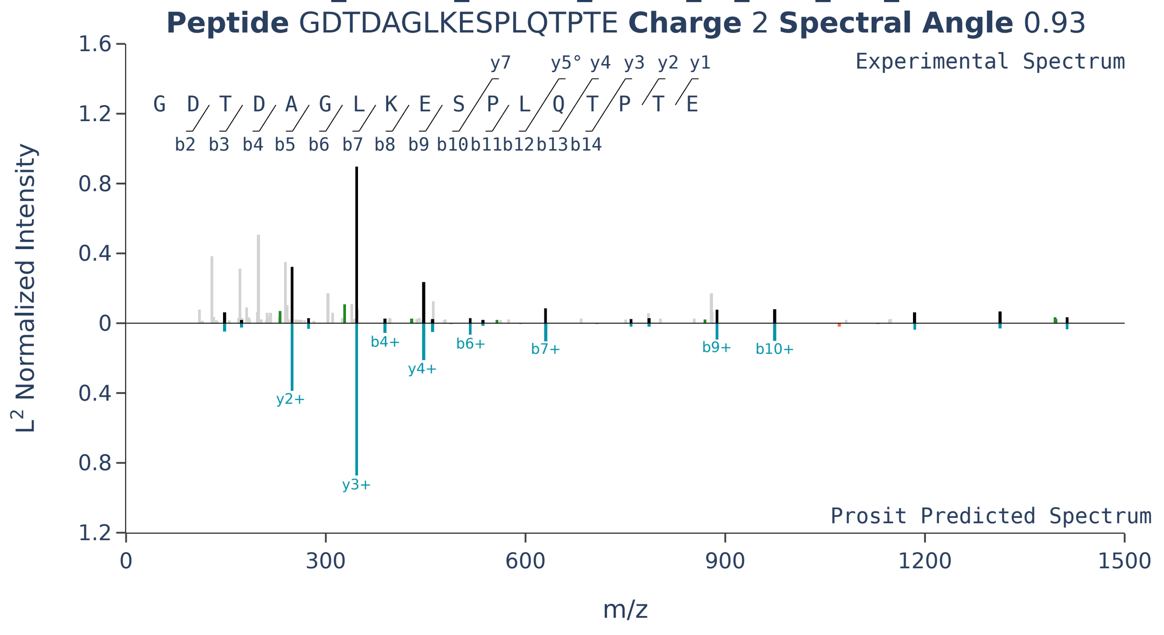


**Source** WSoh_221121_011221_HFGoe_L2_hTau_24h_R1 **Scan** 20810
**Peptide** VQSKIGSLDNITHV **Charge** 3 **Spectral Angle** 0.87

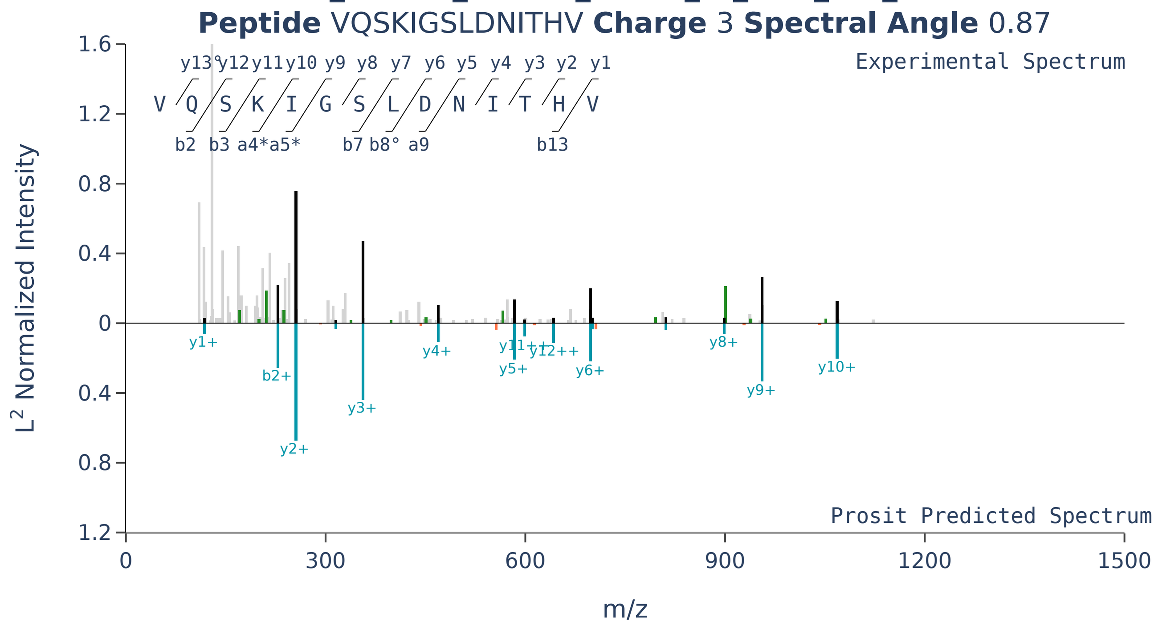


**Source** WSoh_221121_011221_HFGoe_L1_hTau_4h_R2 **Scan** 6654
**Peptide** TSPRHLSNVSSTG **Charge** 2 **Spectral Angle** 0.83

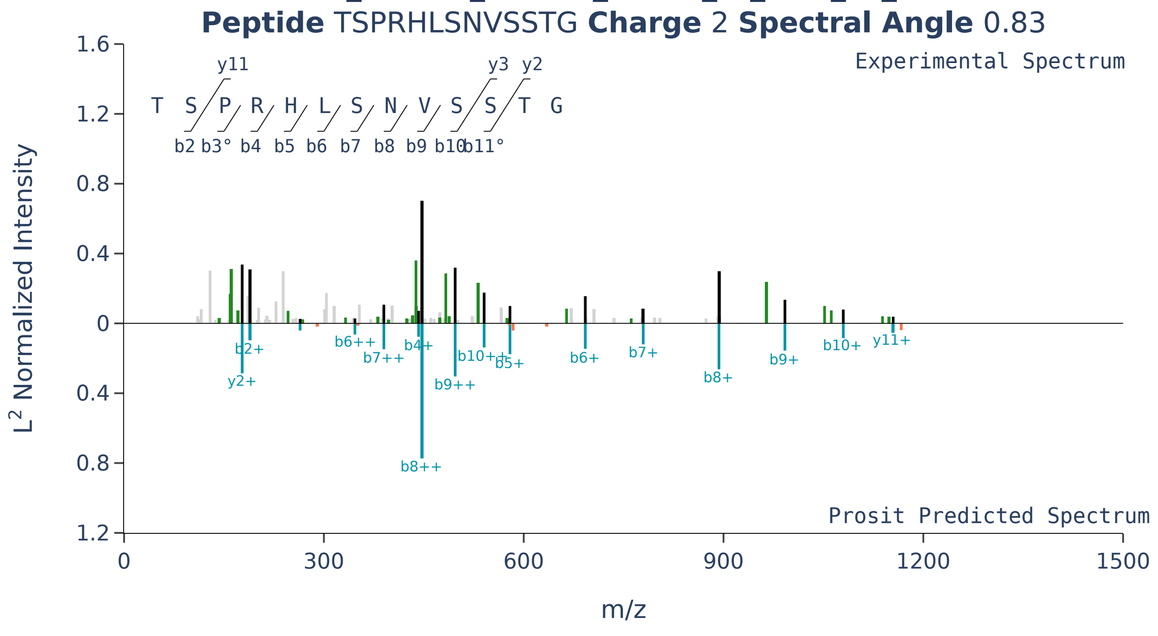


**Source** WSoh_221121_011221_HFGoe_L1_hTau_24h_R1 **Scan** 19657
**Peptide** AAQPHTEIPEGTTAEEAGI **Charge** 2 **Spectral Angle** 0.9

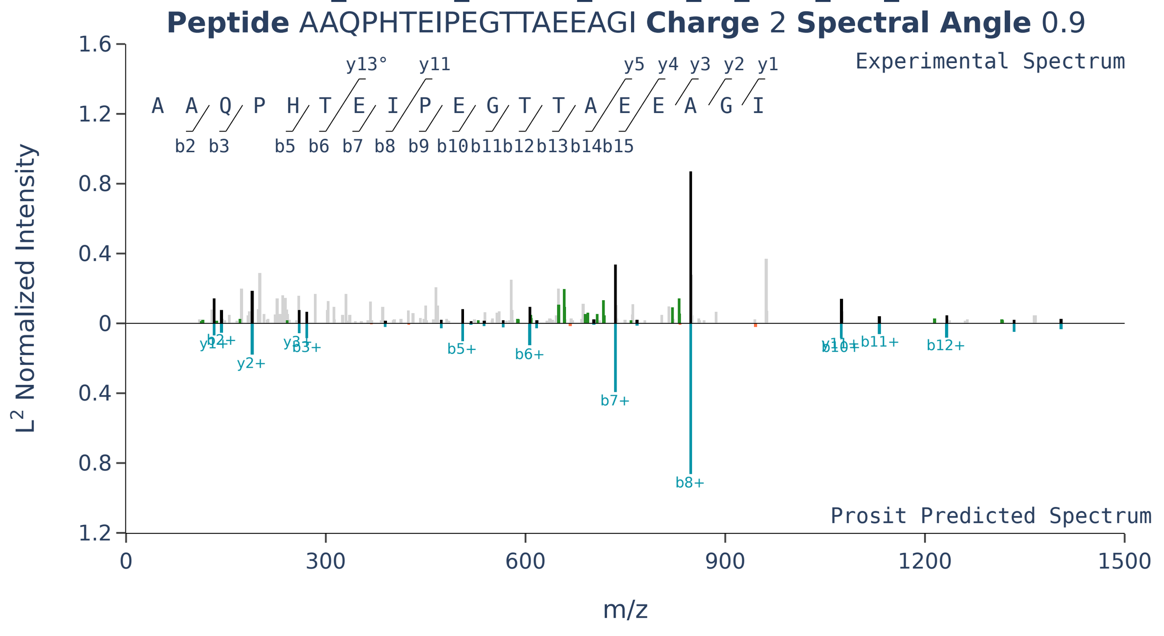


**Source** WSoh_221121_011221_HFGoe_L2_hTau_2h_R2 **Scan** 5988
**Peptide** SLGNIHHKPGGG **Charge** 2 **Spectral Angle** 0.89

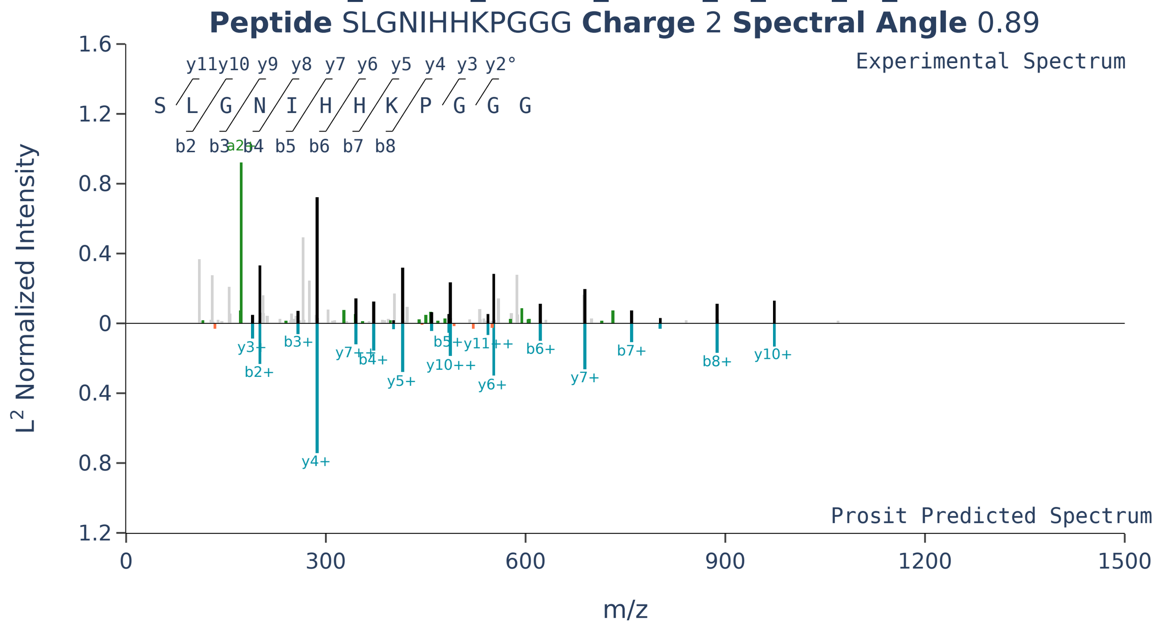


**Source** WSoh_221121_011221_HFGoe_L2_hTau_1h_R2 **Scan** 11190
**Peptide** VKSEKLDFKDRV **Charge** 3 **Spectral Angle** 0.88

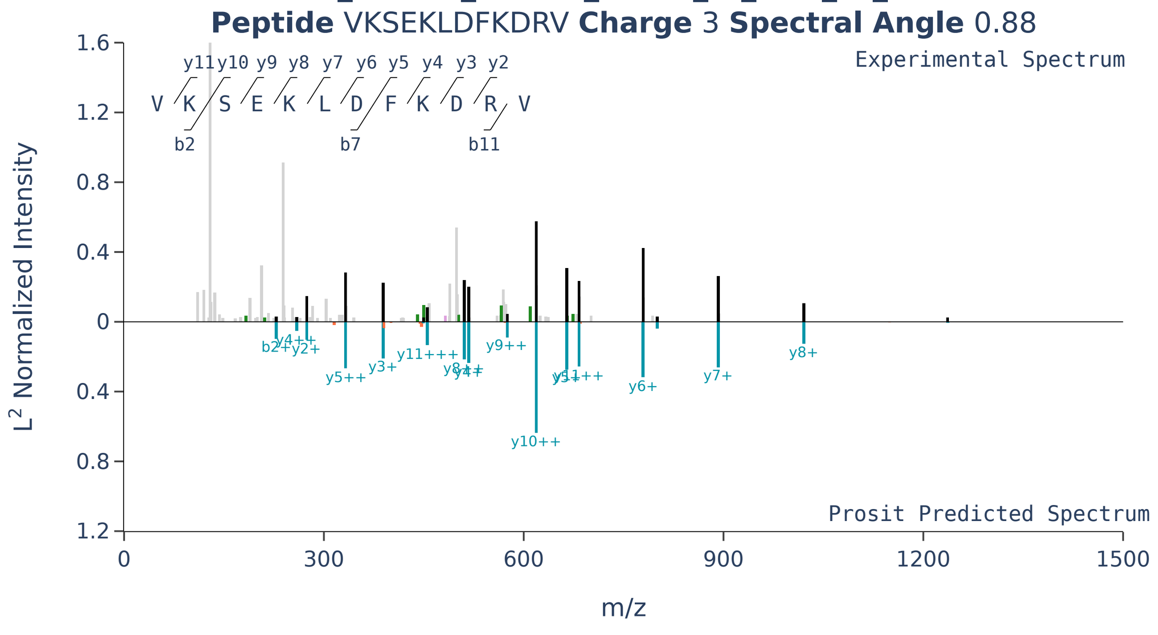


**Source** WSoh_221121_011221_HFGoe_L2_hTau_24h_R2 **Scan** 17251
**Peptide** HQDQEGDTDAGLKESPLQTPTE **Charge** 3 **Spectral Angle** 0.92

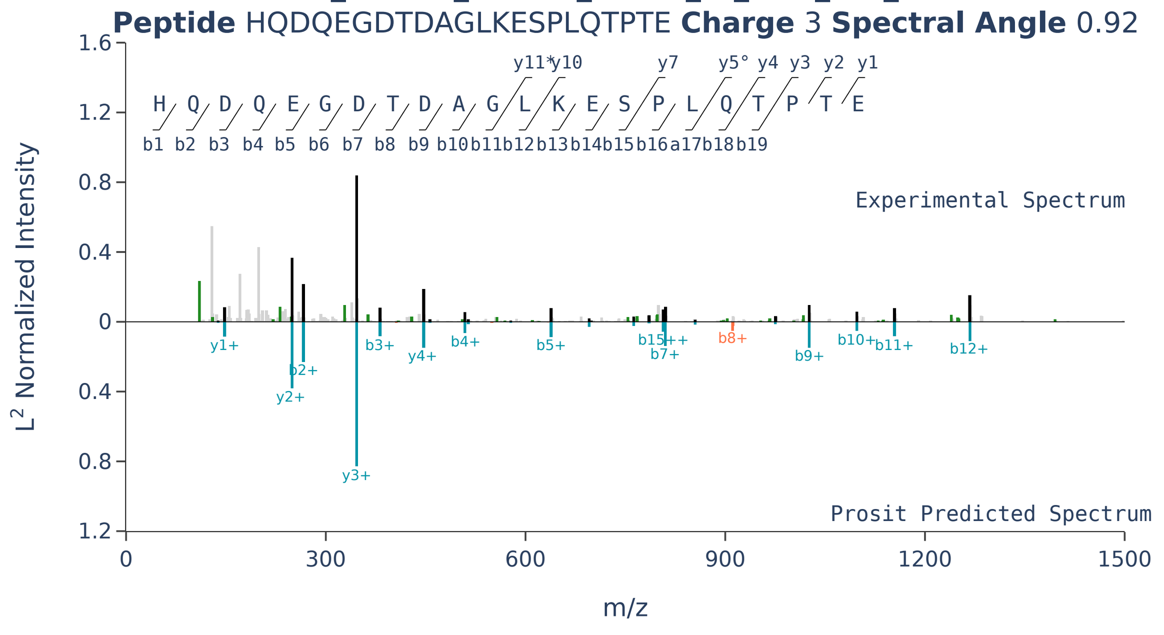


**Source** WSoh_221121_011221_HFGoe_L2_hTau_24h_R1 **Scan** 5825
**Peptide** ENLKHQPGGGKV **Charge** 2 **Spectral Angle** 0.9

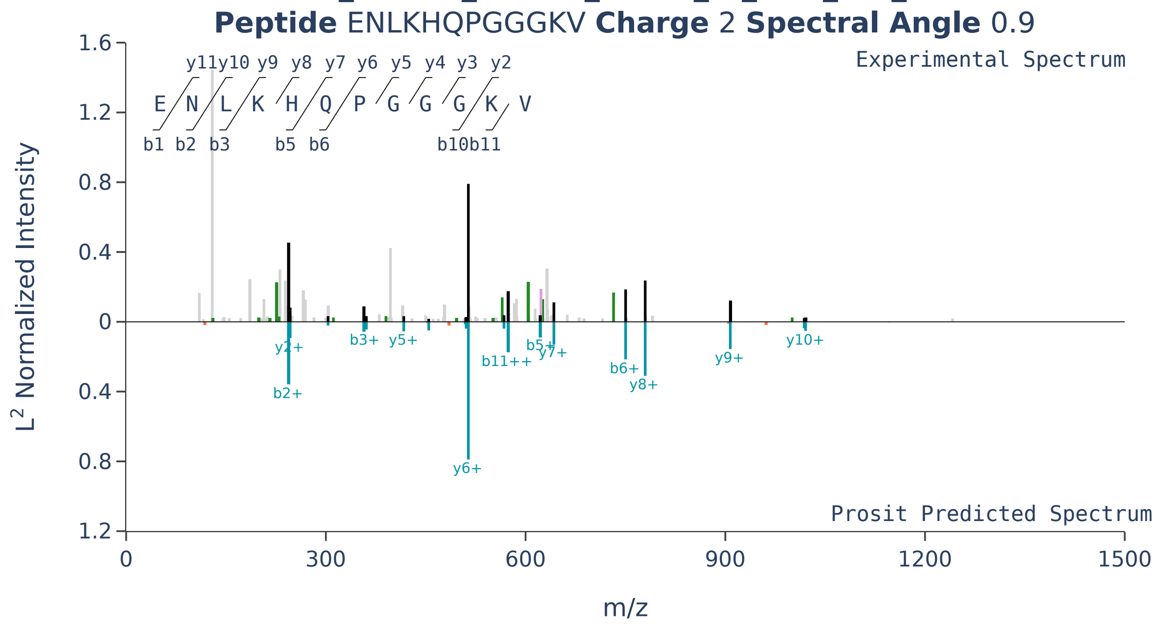


**Source** WSoh_221121_011221_HFGoe_L2_hTau_4h_R2 **Scan** 11850
**Peptide** KPVDLSKVTS **Charge** 1 **Spectral Angle** 0.88

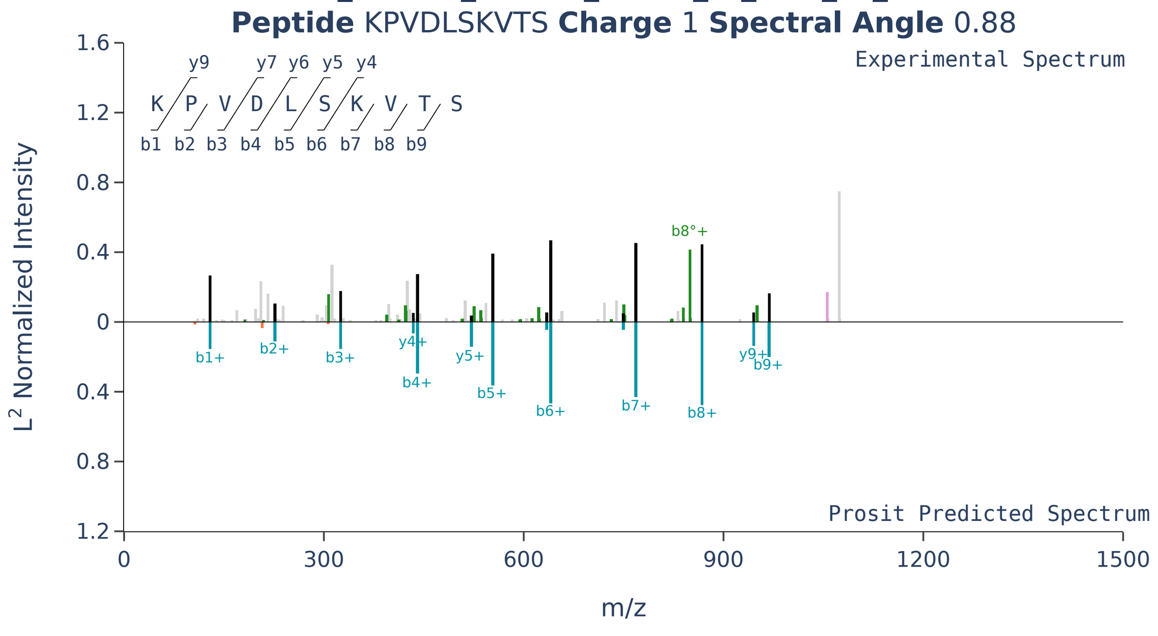


**Source** WSoh_221121_011221_HFGoe_L1_hTau_24h_R2 **Scan** 7788
**Peptide** PGQKGQANATRL **Charge** 2 **Spectral Angle** 0.93

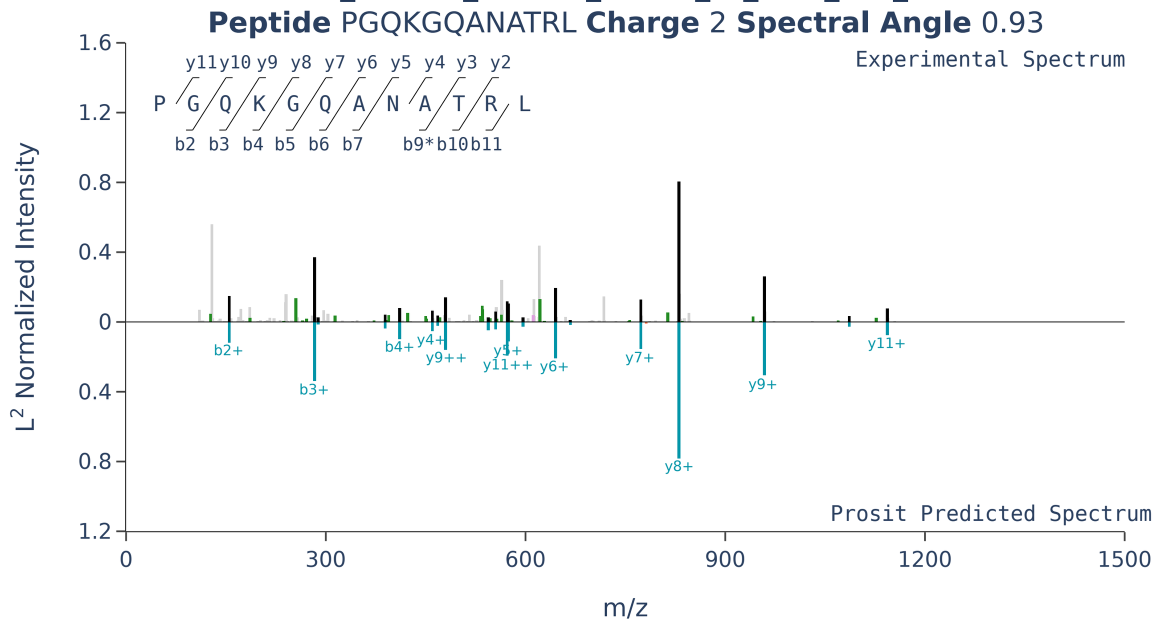


**Source** WSoh_221121_011221_HFGoe_L2_hTau_2h_R1 **Scan** 15250
**Peptide** TLADEVSA **Charge** 1 **Spectral Angle** 0.92

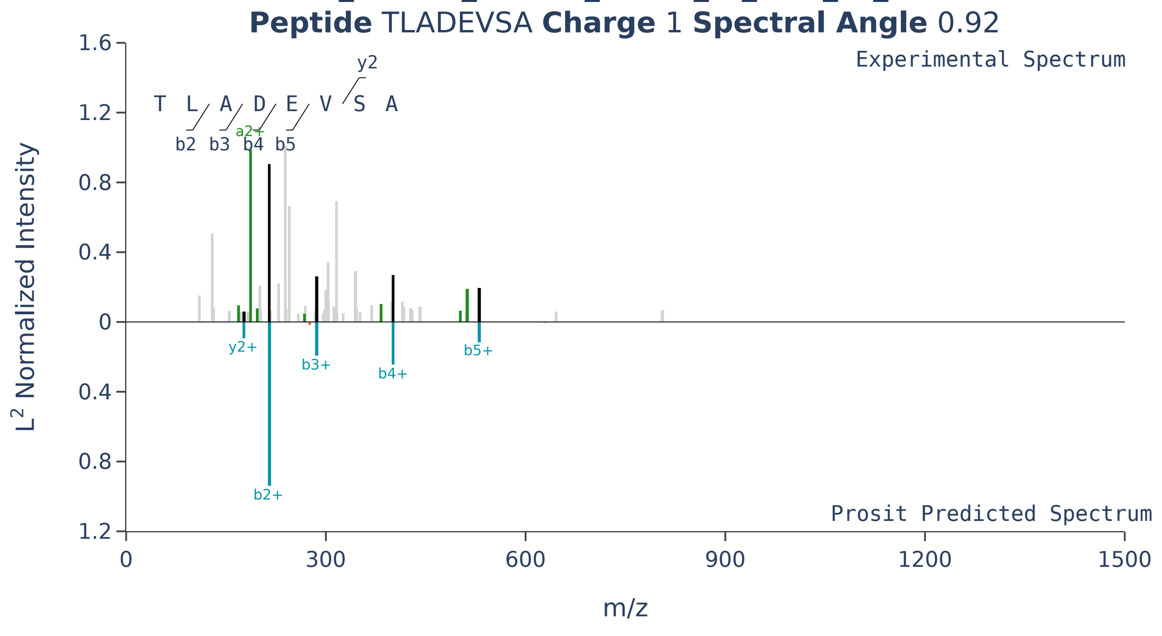

**Source** WSoh_221121_011221_HFGoe_L1_hTau_4h_R2 **Scan** 18088
**Peptide** PGGGKVQIINKKLDLSNVQS **Charge** 3 **Spectral Angle** 0.84

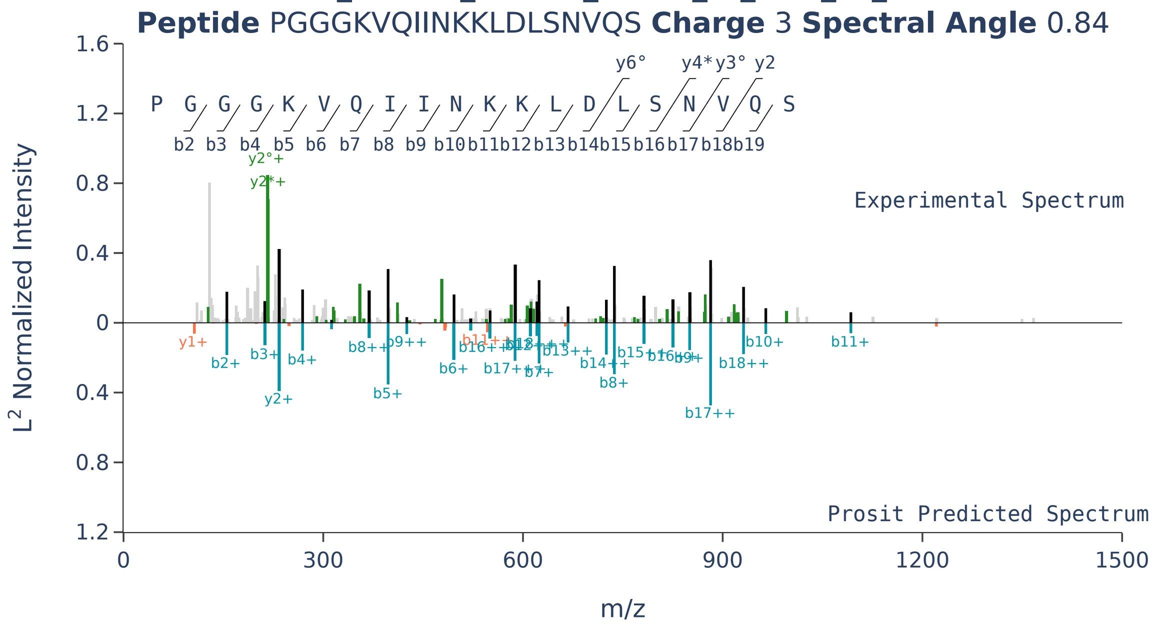


**Source** WSoh_221121_011221_HFGoe_L1_hTau_4h_R2 **Scan** 6075
**Peptide** GSLGNIHHKPGGGQ **Charge** 3 **Spectral Angle** 0.94

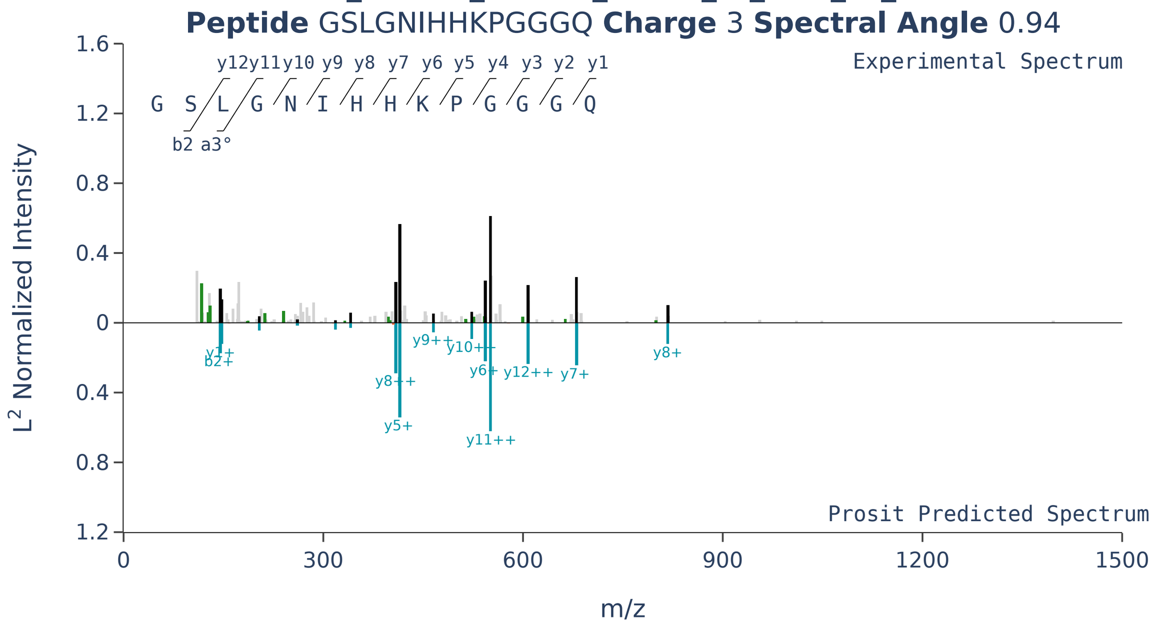


**Source** WSoh_221121_011221_HFGoe_L1_hTau_24h_R1 **Scan** 11133
**Peptide** GAEIVYKS **Charge** 2 **Spectral Angle** 0.88

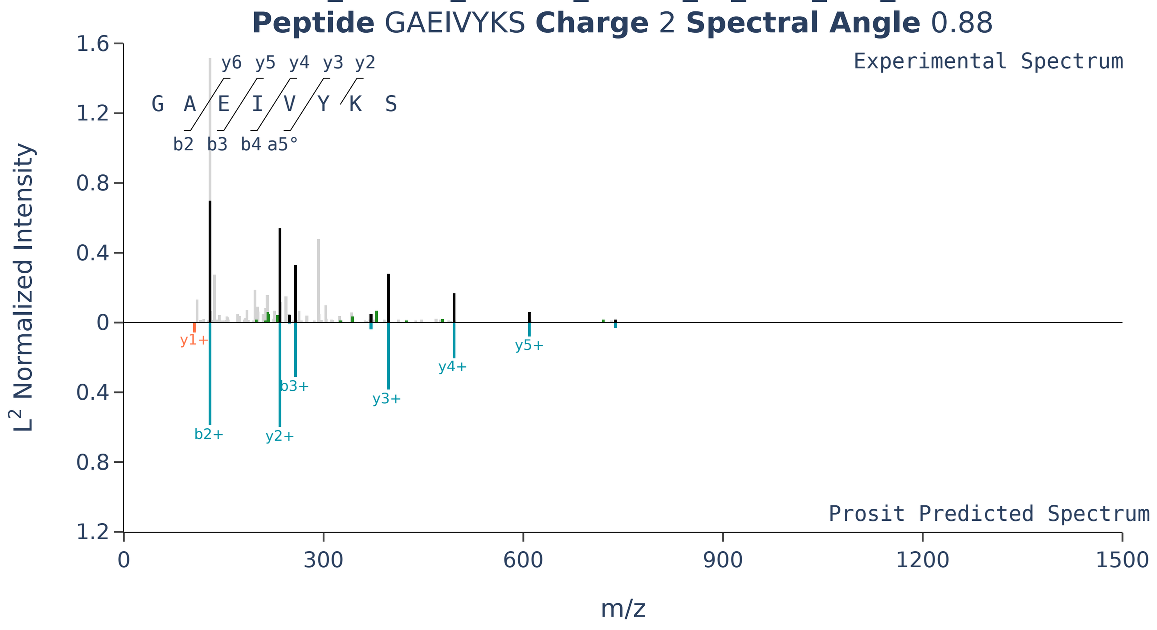


**Source** WSoh_221121_011221_HFGoe_L2_hTau_24h_R2 **Scan** 16530
**Peptide** SPLQTPTEDGSEEPGSETSD **Charge** 2 **Spectral Angle** 0.83

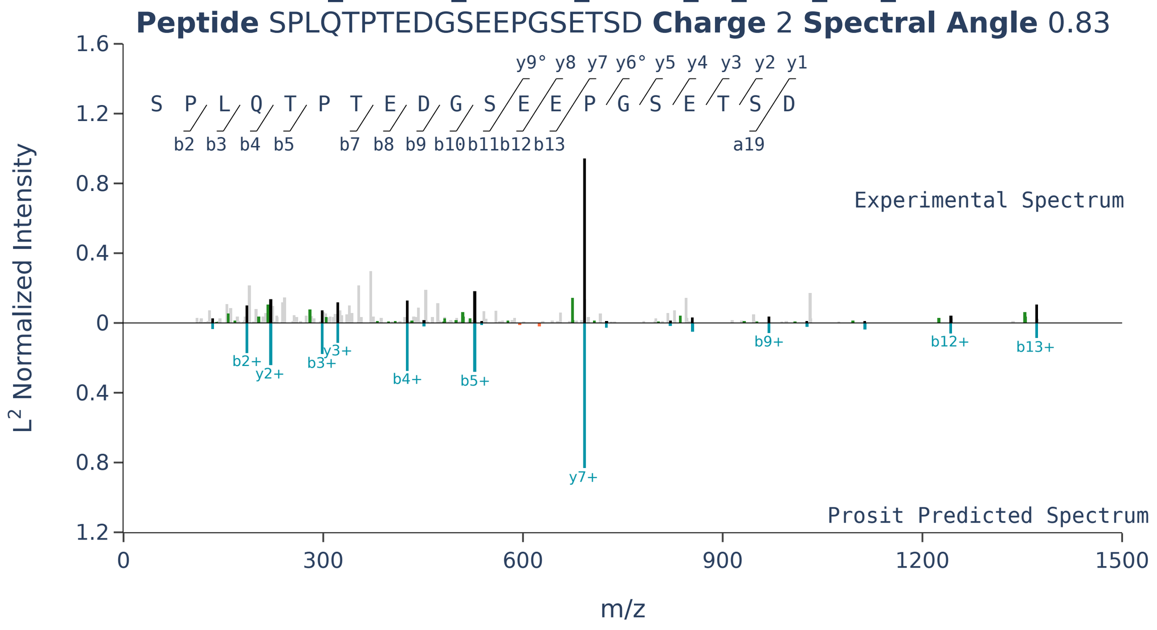


**Source** WSoh_221121_011221_HFGoe_L1_hTau_1h_R2 **Scan** 5563
**Peptide** VPGGGNKKIETH **Charge** 2 **Spectral Angle** 0.93

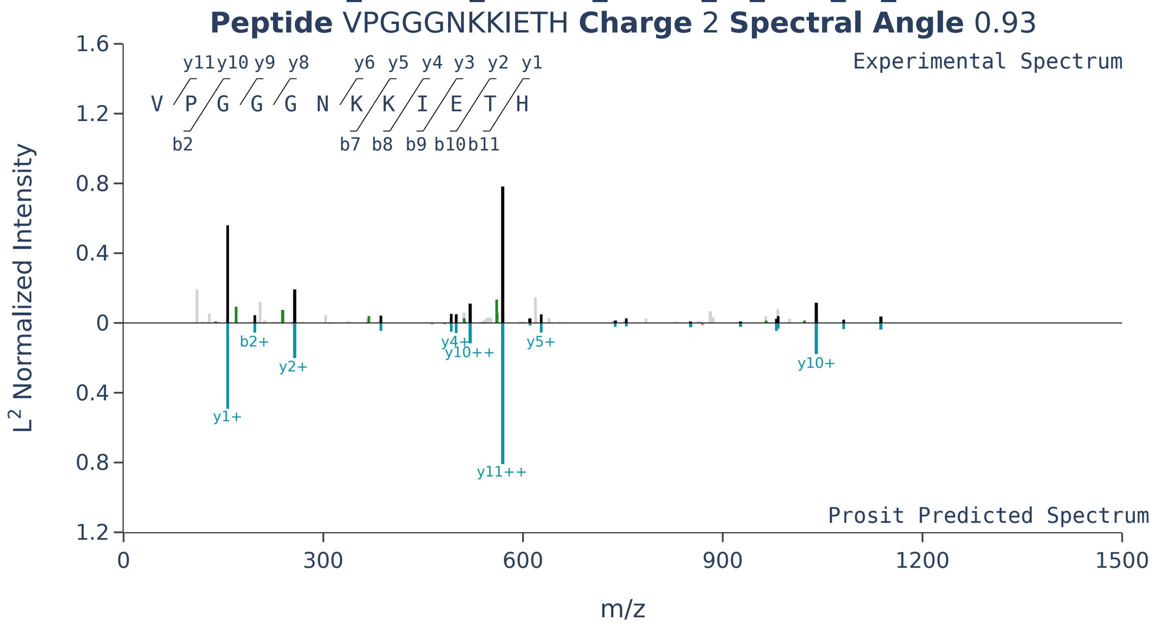


**Source** WSoh_221121_011221_HFGoe_L1_hTau_24h_R1 **Scan** 11999
**Peptide** AGTYGLGDRKDQGGYTMH **Charge** 4 **Spectral Angle** 0.95

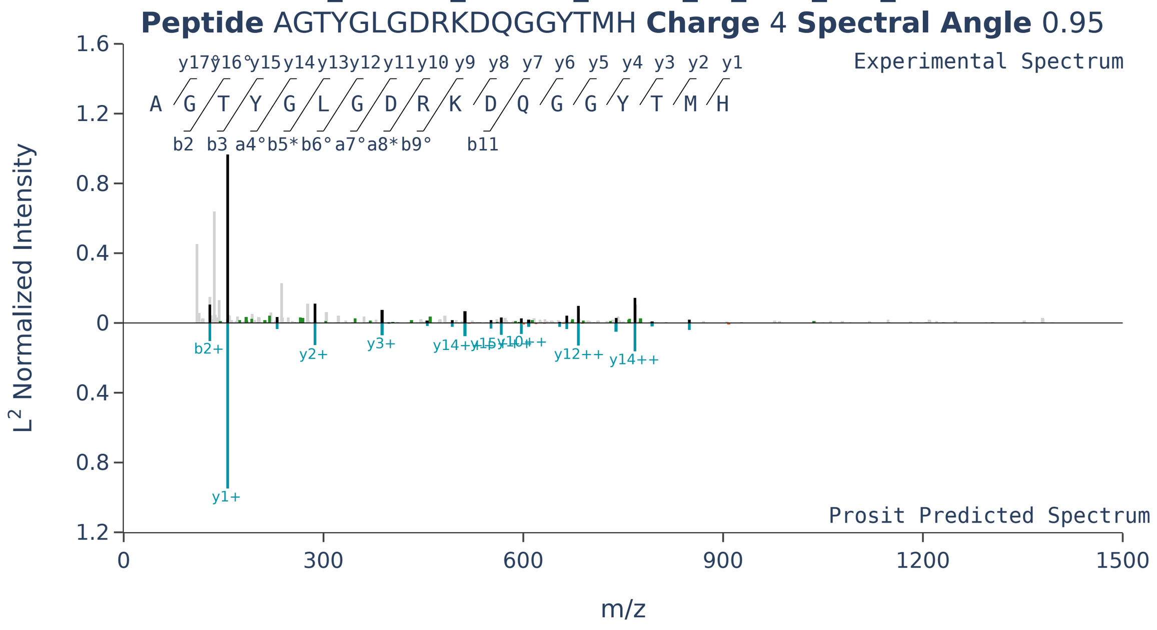


**Source** WSoh_221121_011221_HFGoe_L1_hTau_4h_R2 **Scan** 13120
**Peptide** GSKDNIKHVPGGGSVQL **Charge** 3 **Spectral Angle** 0.83

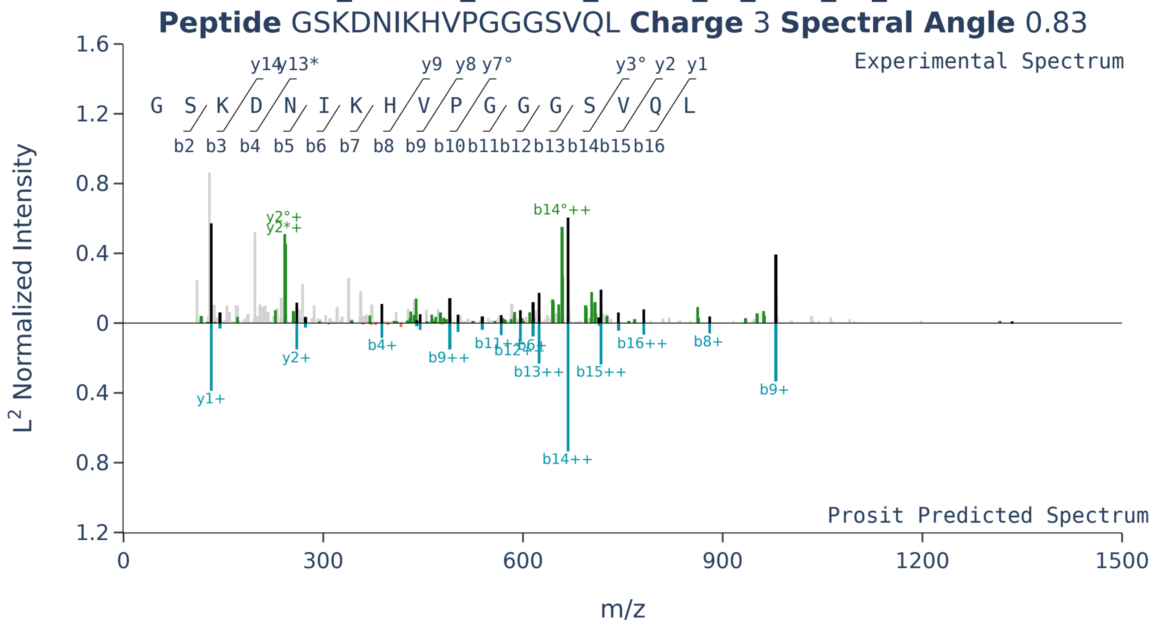


**Source** WSoh_221121_011221_HFGoe_L1_hTau_24h_R1 **Scan** 19392
**Peptide** VPGGGSVQI **Charge** 1 **Spectral Angle** 0.84

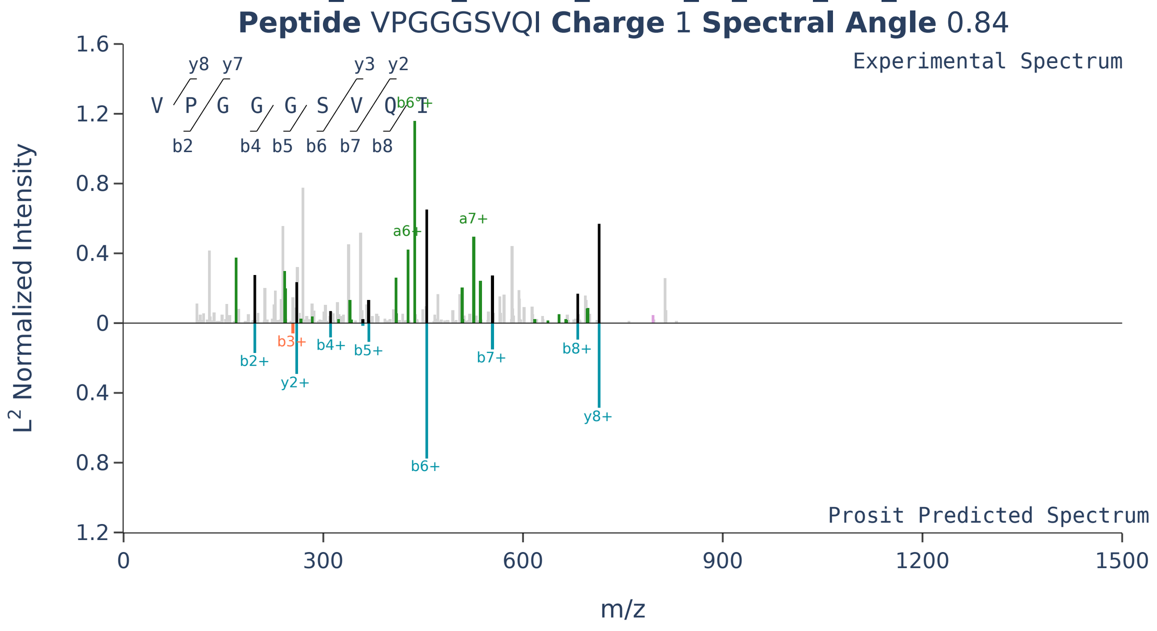


**Source** WSoh_221121_011221_HFGoe_L1_hTau_24h_R1 **Scan** 8061
**Peptide** ATPRGAAPPGQKGQANATRL **Charge** 3 **Spectral Angle** 0.91

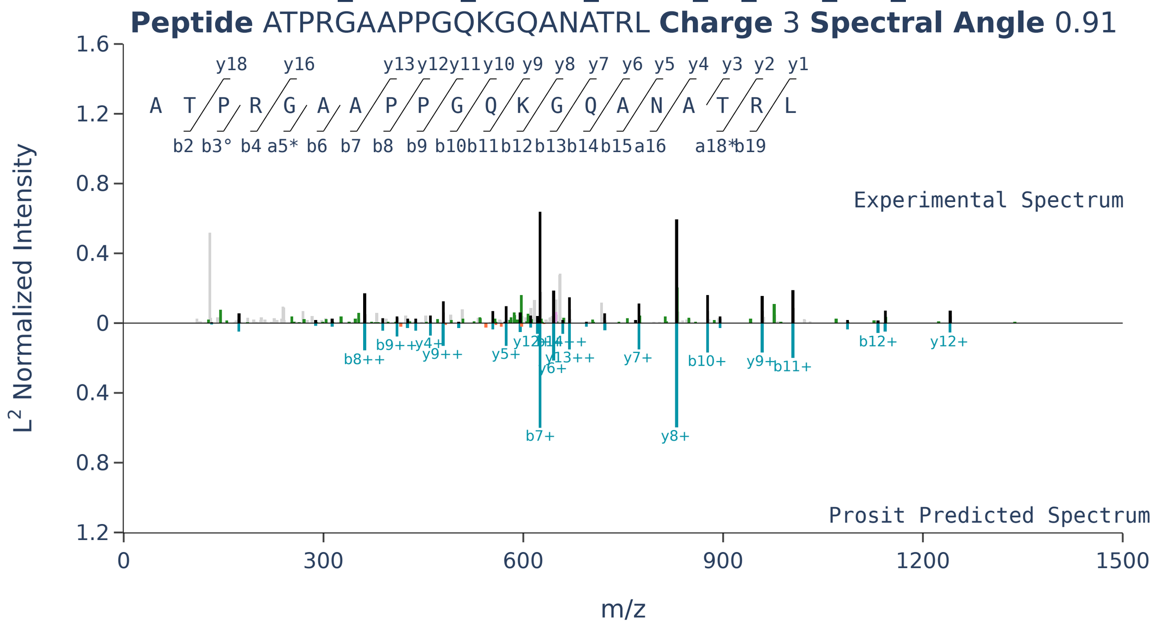


**Source** WSoh_221121_011221_HFGoe_L2_hTau_24h_R2 **Scan** 7232
**Peptide** RSGYSSPGSPGTPGSRS **Charge** 2 **Spectral Angle** 0.88

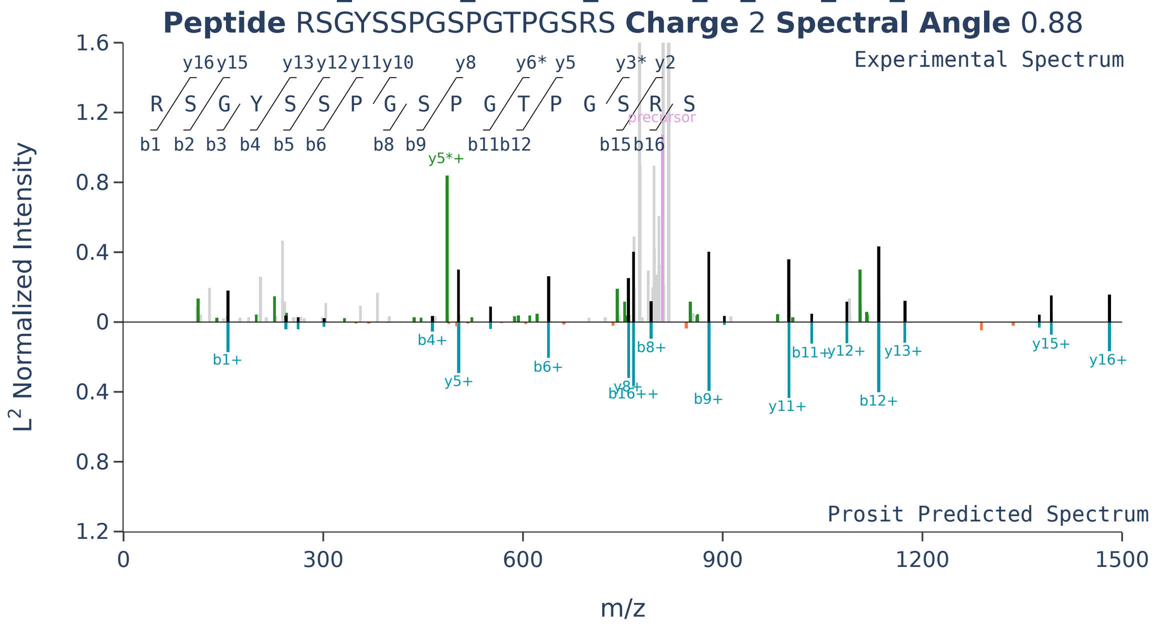


**Source** WSoh_221121_011221_HFGoe_L2_hTau_24h_R1 **Scan** 17439
**Peptide** LGDTPSLED **Charge** 1 **Spectral Angle** 0.8

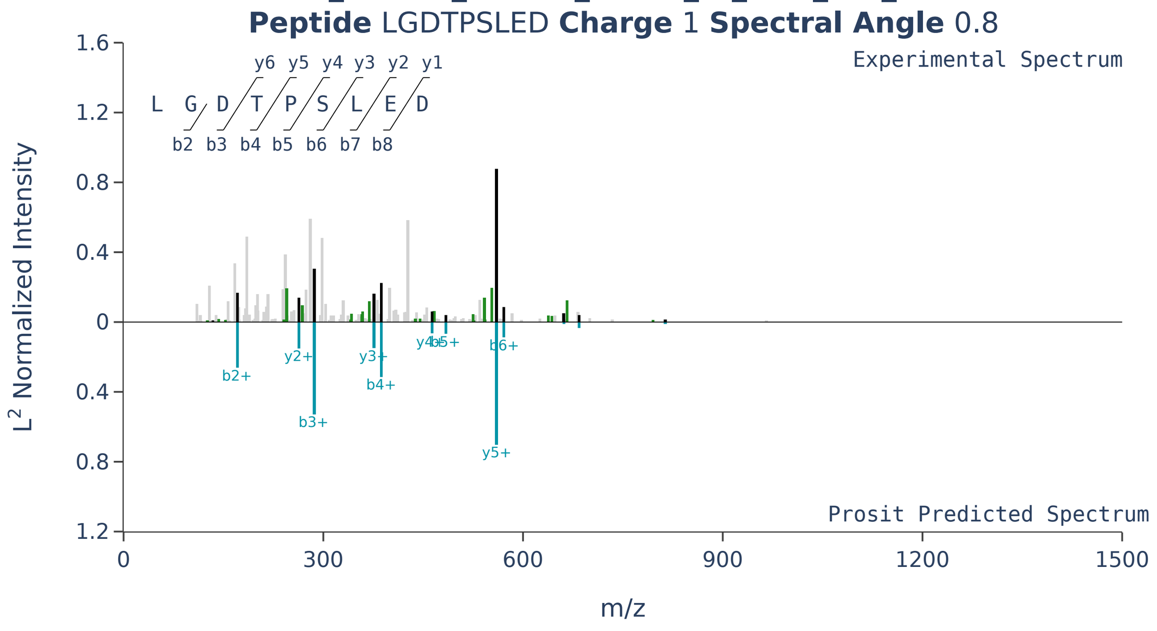


**Source** WSoh_221121_011221_HFGoe_L1_hTau_4h_R2 **Scan** 13063
**Peptide** PVVSGDTSPRHLSNV **Charge** 2 **Spectral Angle** 0.93

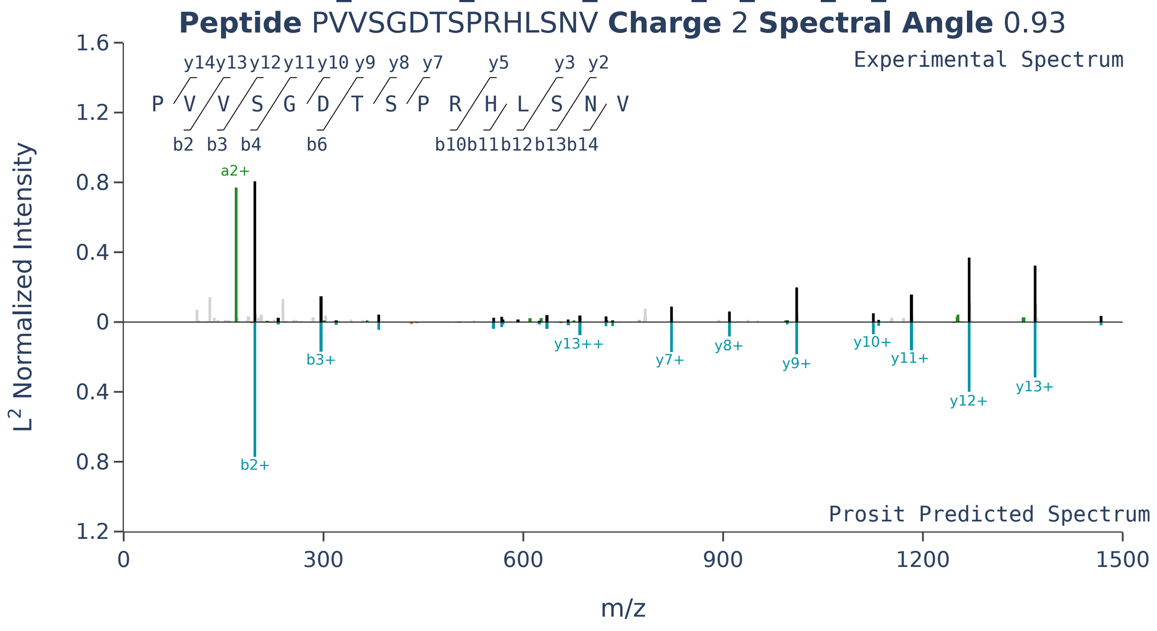


**Source** WSoh_221121_011221_HFGoe_L2_hTau_24h_R1 **Scan** 11630
**Peptide** AQPHTEIPEGTT **Charge** 2 **Spectral Angle** 0.92

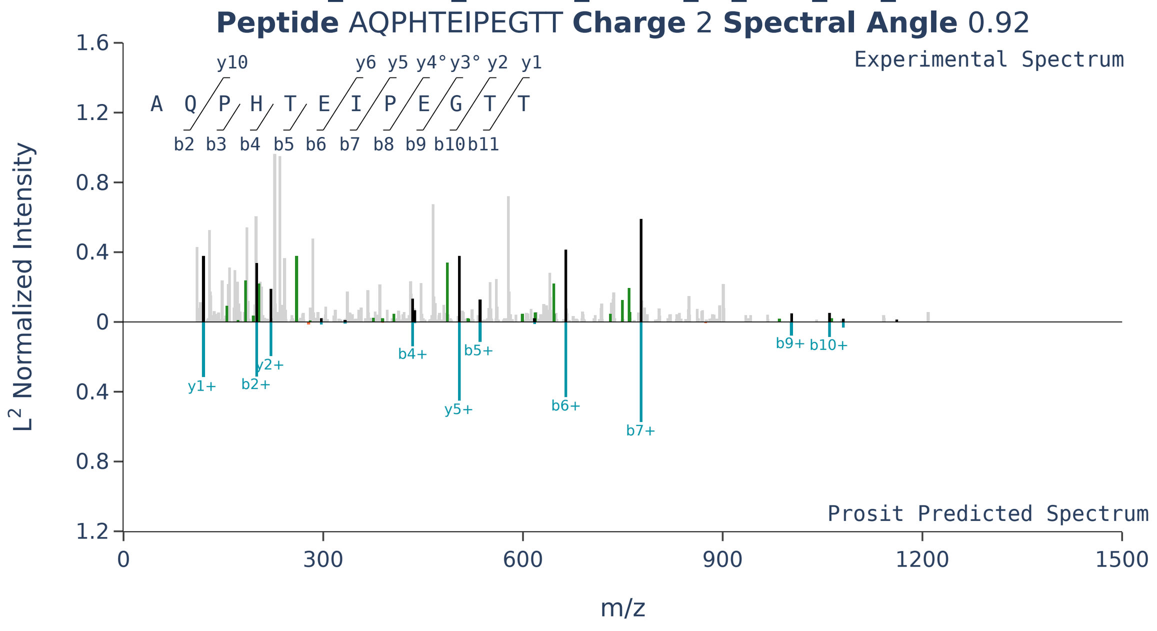


**Source** WSoh_221121_011221_HFGoe_L1_hTau_24h_R1 **Scan** 12223
**Peptide** GSLDNITHVPGGGNKK **Charge** 3 **Spectral Angle** 0.9

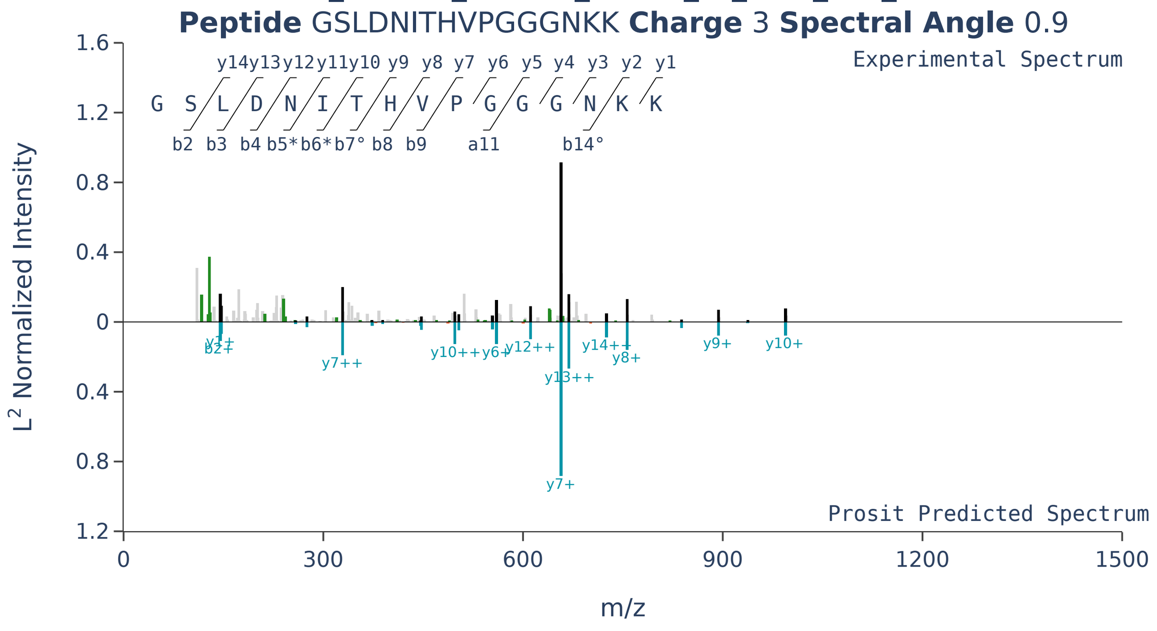


**Source** WSoh_221121_011221_HFGoe_L2_hTau_1h_R1 **Scan** 17882
**Peptide** KLGSLDNITHV **Charge** 2 **Spectral Angle** 0.77

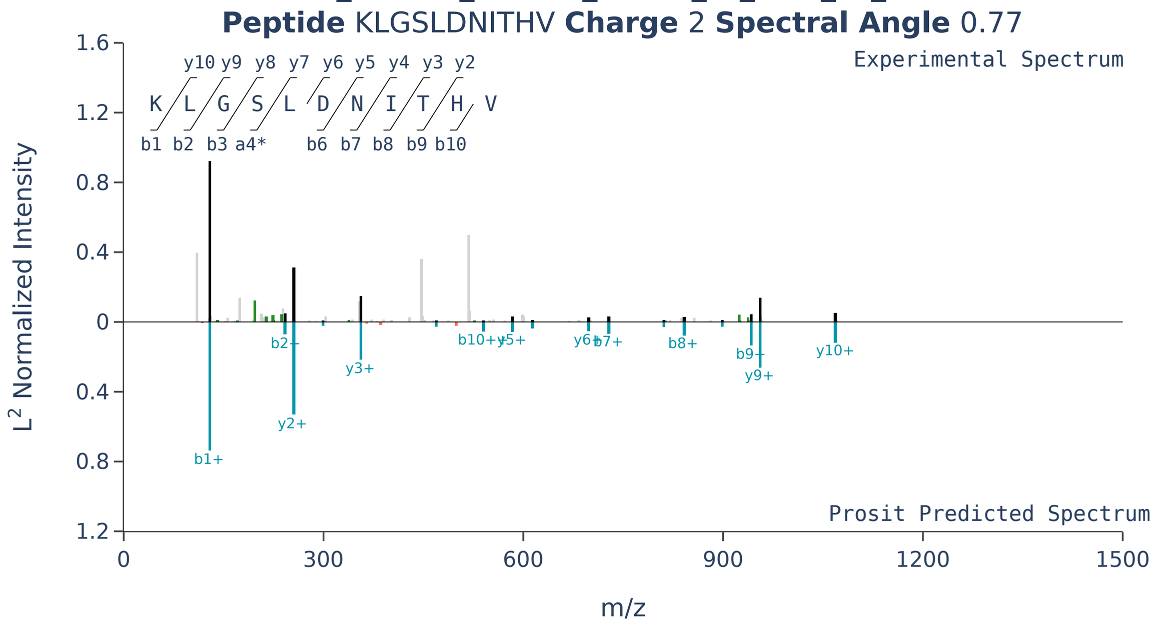

**Source** WSoh_221121_011221_HFGoe_L2_hTau_1h_R2 **Scan** 11319
**Peptide** GTYGLGDRKDQGGY **Charge** 3 **Spectral Angle** 0.87

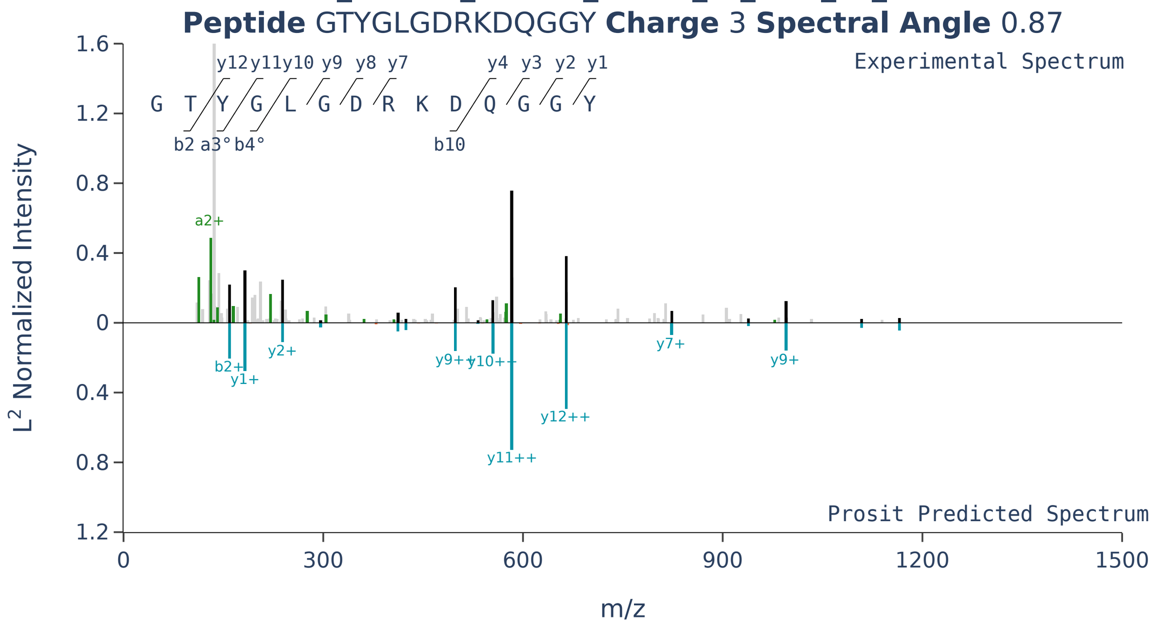


**Source** WSoh_221121_011221_HFGoe_L2_hTau_24h_R1 **Scan** 7772
**Peptide** SGDTSPRHLSNVSSTG **Charge** 2 **Spectral Angle** 0.85

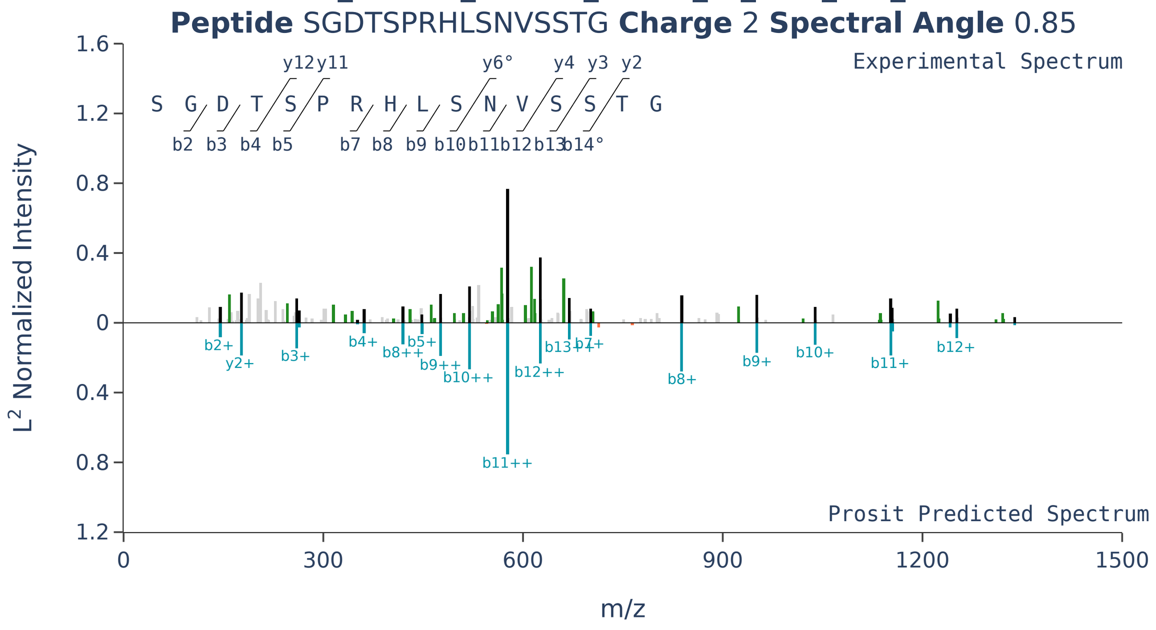


**Source** WSoh_221121_011221_HFGoe_L1_hTau_24h_R1 **Scan** 10571
**Peptide** VMEDHAGTYGLGDRKD **Charge** 2 **Spectral Angle** 0.91

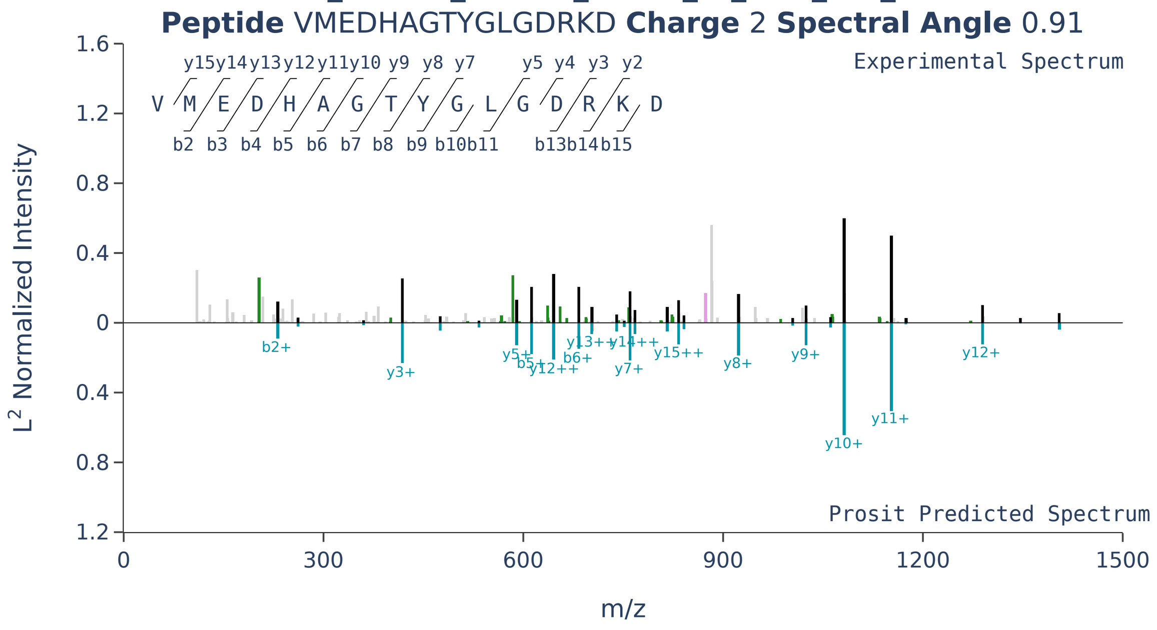


**Source** WSoh_221121_011221_HFGoe_L2_hTau_24h_R1 **Scan** 23258
**Peptide** TTAEEAGIGDTPSL **Charge** 2 **Spectral Angle** 0.96

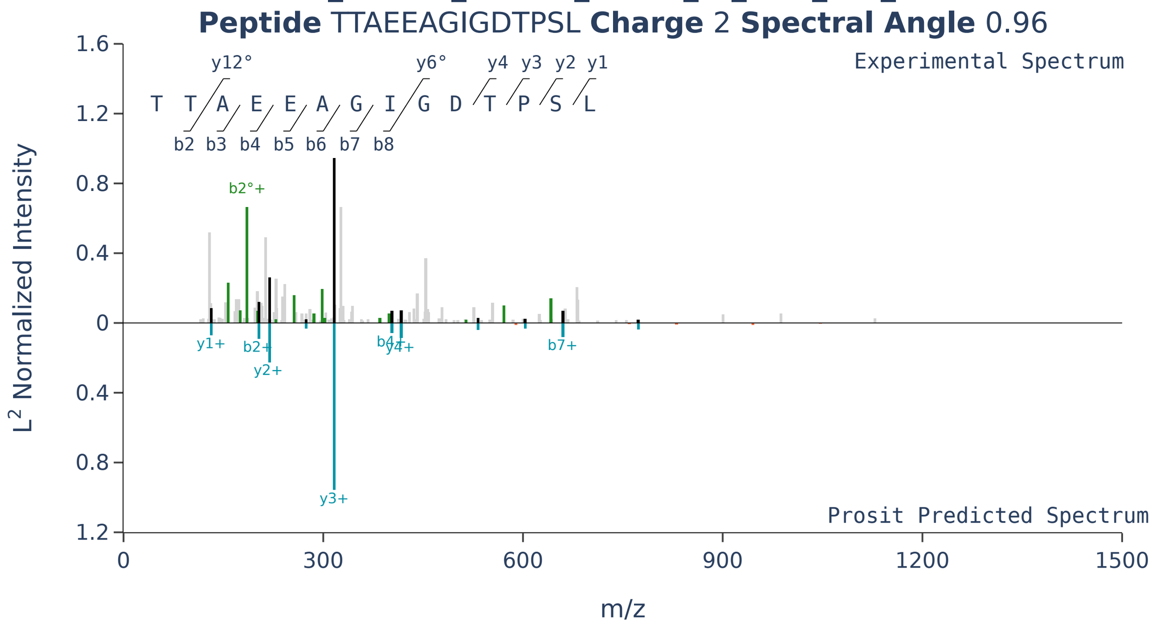


**Source** WSoh_221121_011221_HFGoe_L1_hTau_4h_R2 **Scan** 6949
**Peptide** KLGSTENLKHQPGGGKV **Charge** 3 **Spectral Angle** 0.9

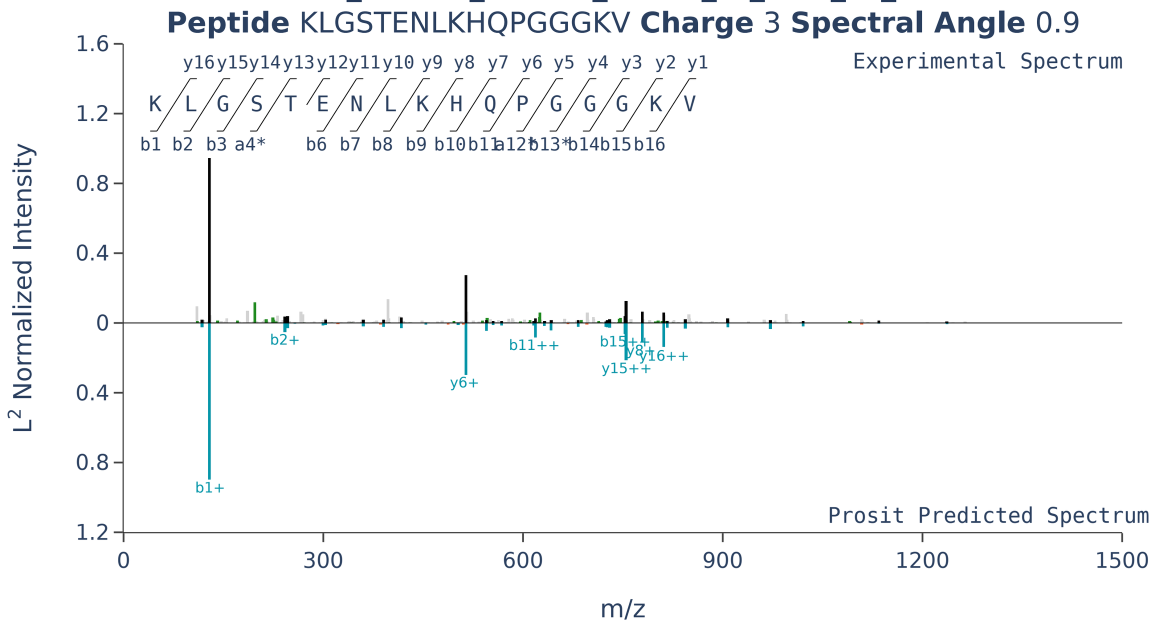


**Source** WSoh_221121_011221_HFGoe_L1_hTau_4h_R2 **Scan** 7847
**Peptide** GSLGNIHHKPGGGQVEVKSE **Charge** 3 **Spectral Angle** 0.86

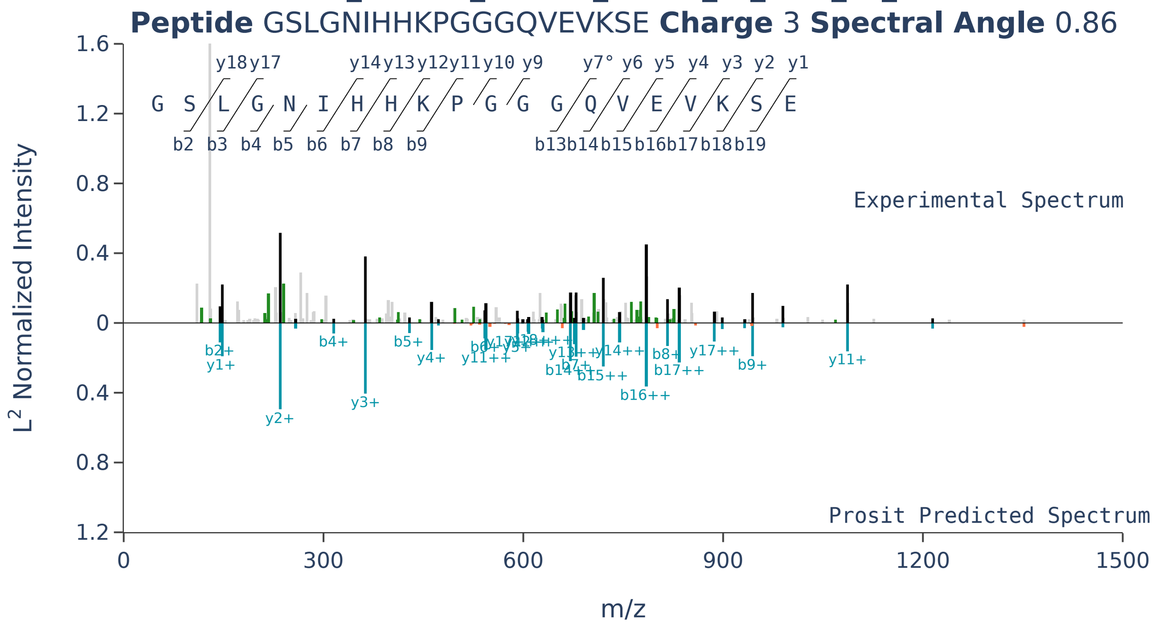


**Source** WSoh_221121_011221_HFGoe_L2_hTau_24h_R2 **Scan** 15863
**Peptide** DLSNVQSKCGSKDNIKHVPGGGSVQL **Charge** 4 **Spectral Angle** 0.88

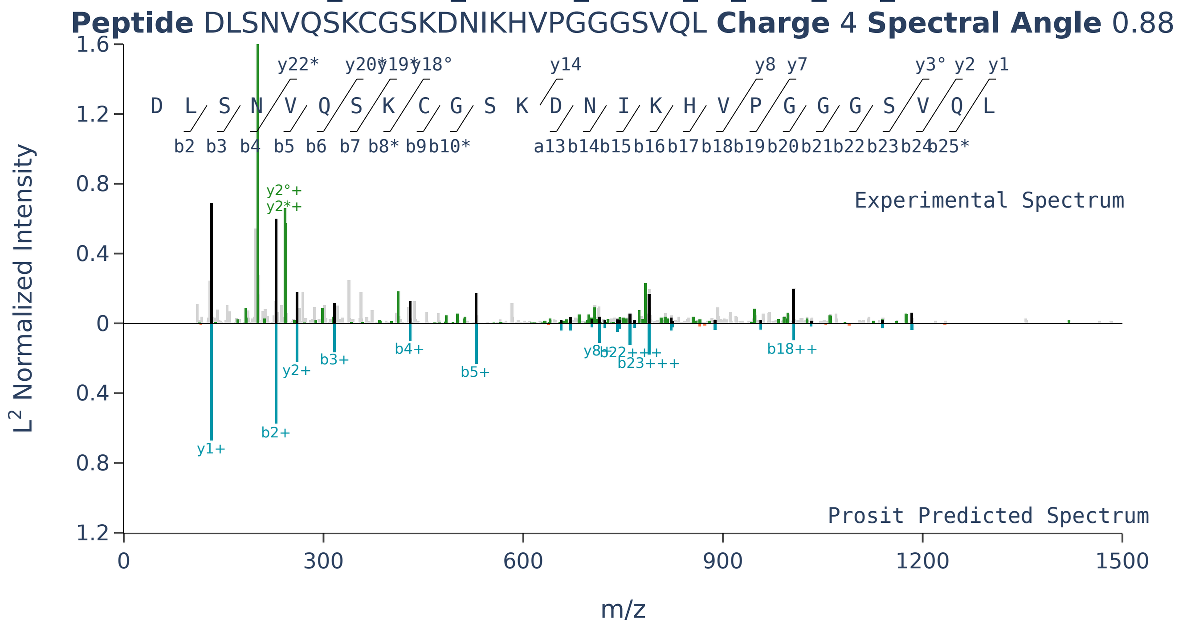


**Source** WSoh_221121_011221_HFGoe_L2_hTau_24h_R1 **Scan** 20204
**Peptide** NVSSTGSIDMVD **Charge** 1 **Spectral Angle** 0.92

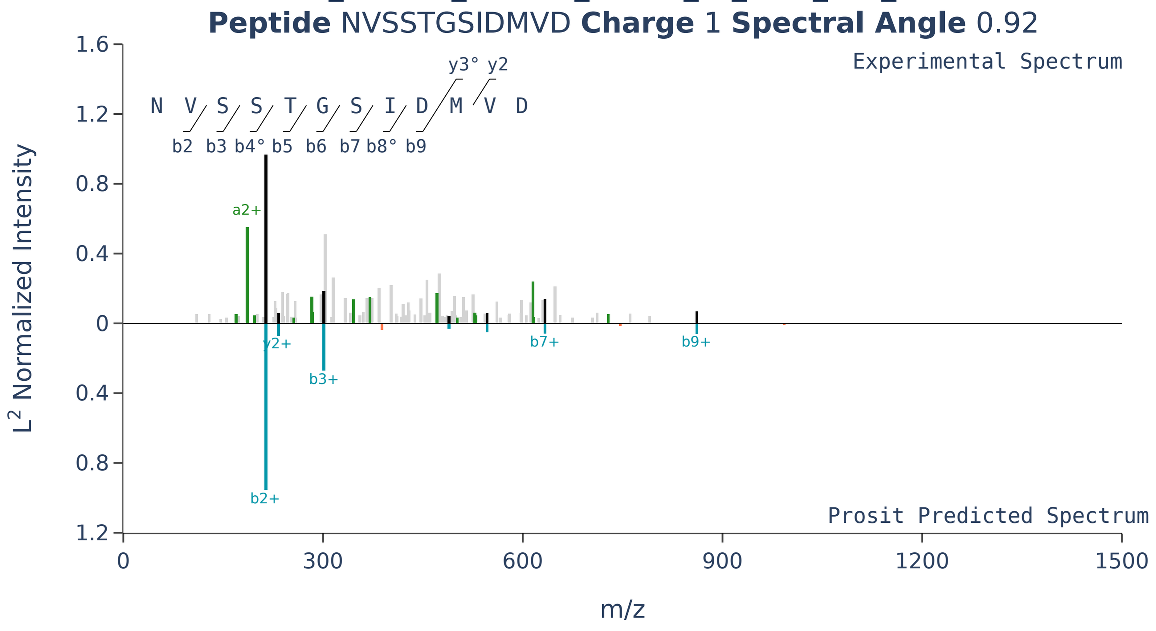


**Source** WSoh_221121_011221_HFGoe_L2_hTau_2h_R1 **Scan** 6477
**Peptide** KHQPGGGKVQIINK **Charge** 4 **Spectral Angle** 0.92

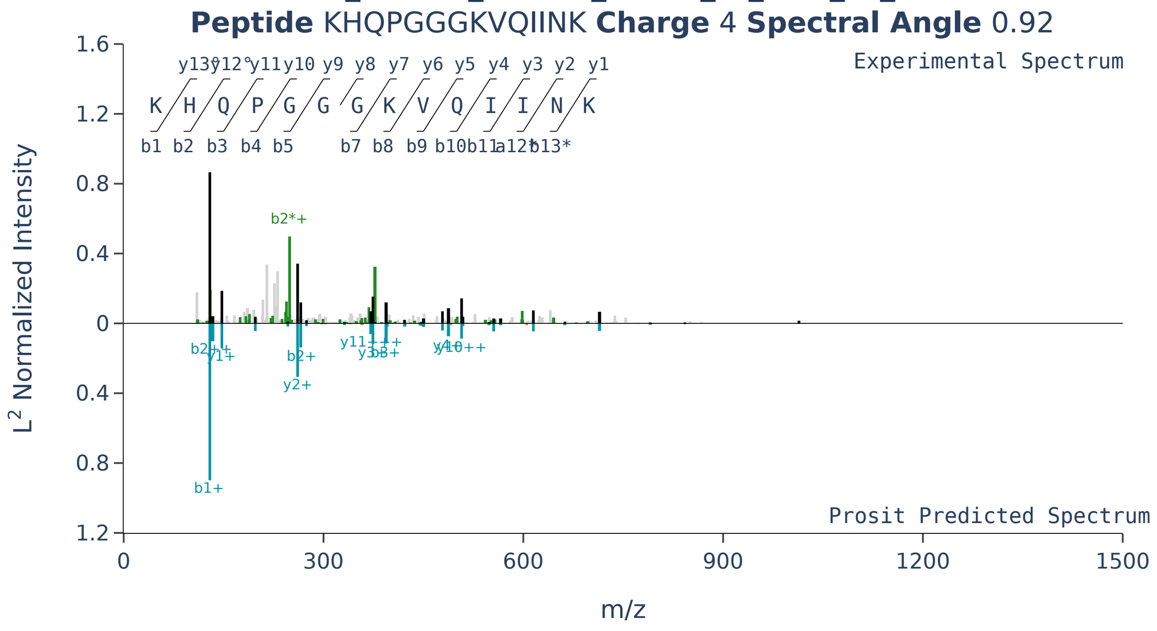


**Source** WSoh_221121_011221_HFGoe_L1_hTau_24h_R1 **Scan** 9095
**Peptide** KSEKLDFKDRVQ **Charge** 2 **Spectral Angle** 0.85

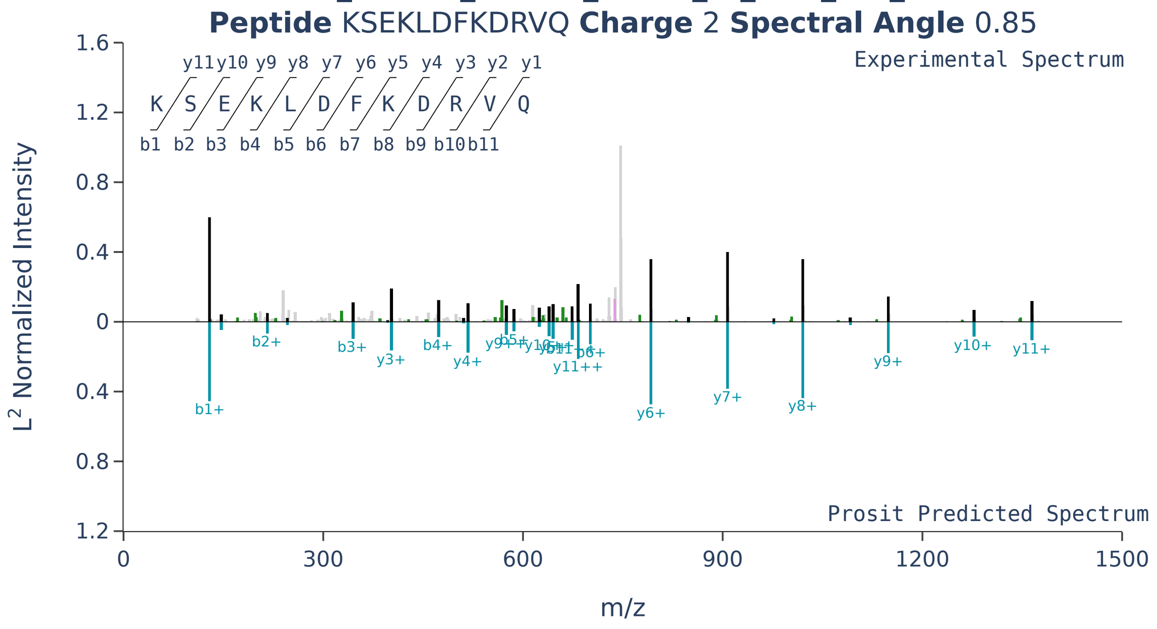


**Source** WSoh_221121_011221_HFGoe_L1_hTau_24h_R2 **Scan** 7677
**Peptide** GDRSGYSSPGSPGTPGSRS **Charge** 3 **Spectral Angle** 0.9

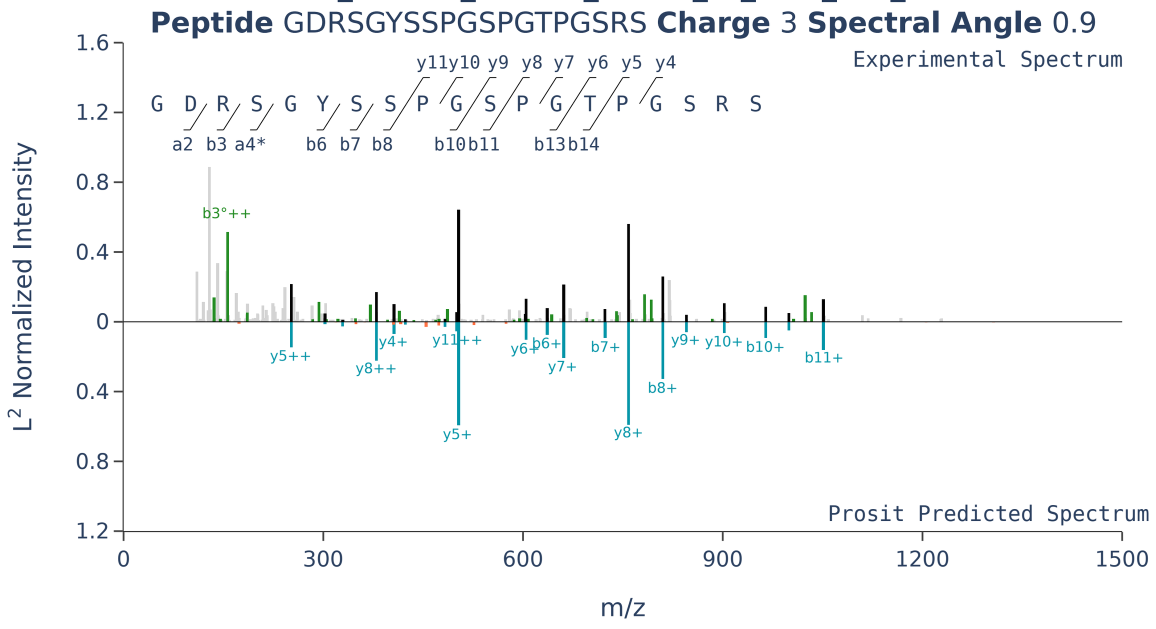


**Source** WSoh_221121_011221_HFGoe_L2_hTau_24h_R2 **Scan** 18097
**Peptide** RVQSKIGSLDNITHV **Charge** 3 **Spectral Angle** 0.9

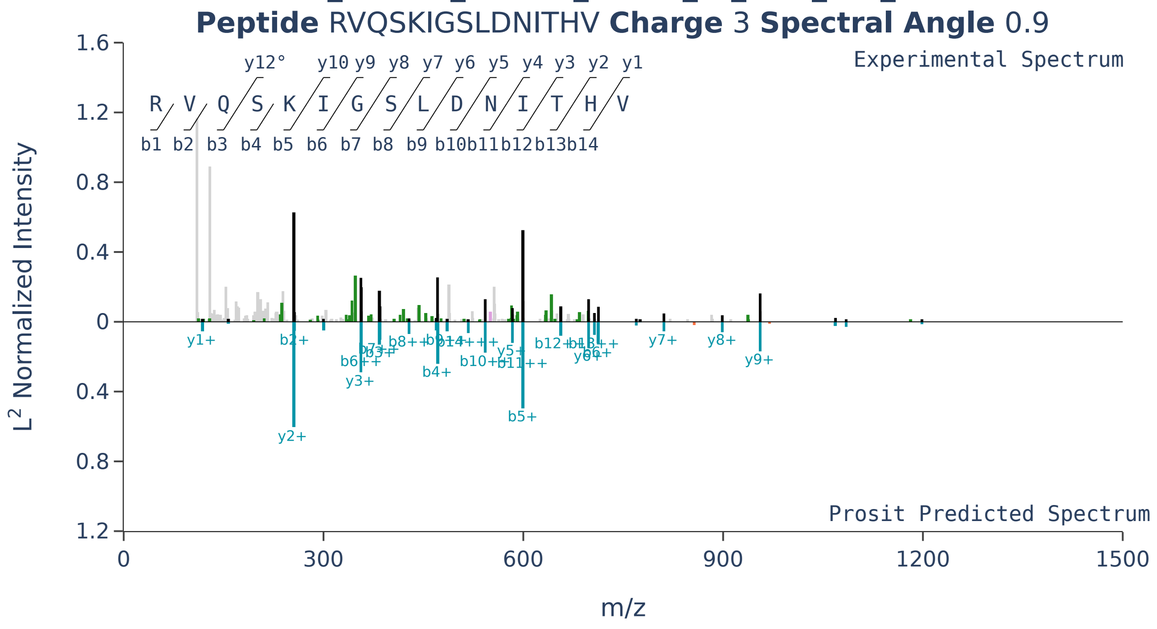


**Source** WSoh_221121_011221_HFGoe_L1_hTau_24h_R2 **Scan** 14475
**Peptide** AAAQPHTEIPEGTTAEEA **Charge** 2 **Spectral Angle** 0.93

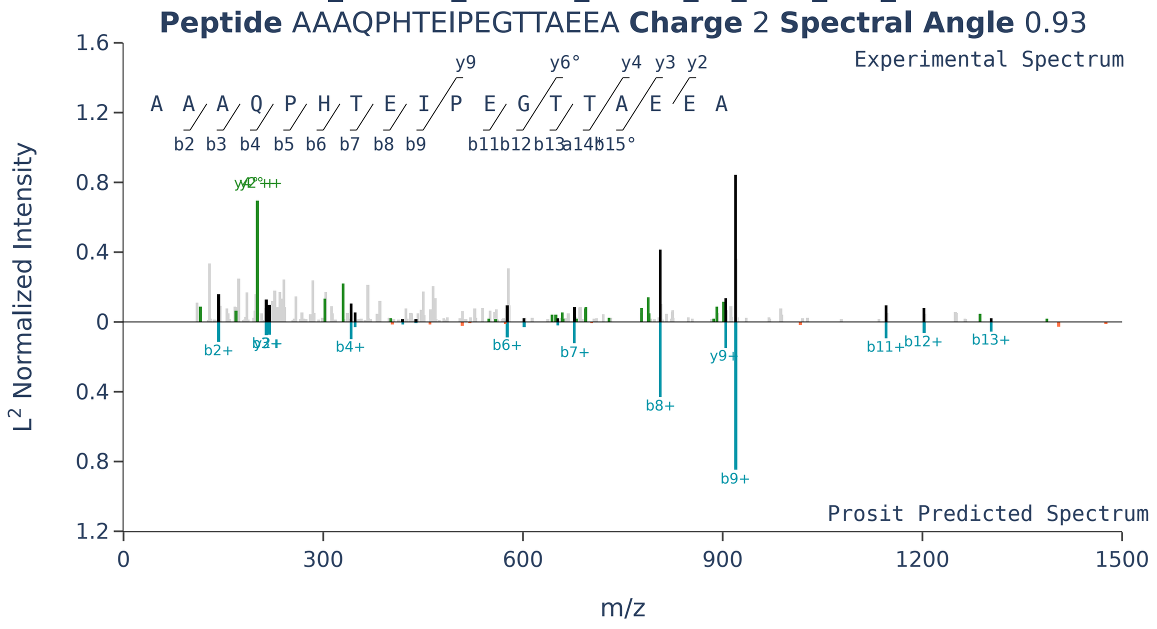


**Source** WSoh_221121_011221_HFGoe_L1_hTau_1h_R2 **Scan** 12530
**Peptide** EKLDFKDRV **Charge** 2 **Spectral Angle** 0.9

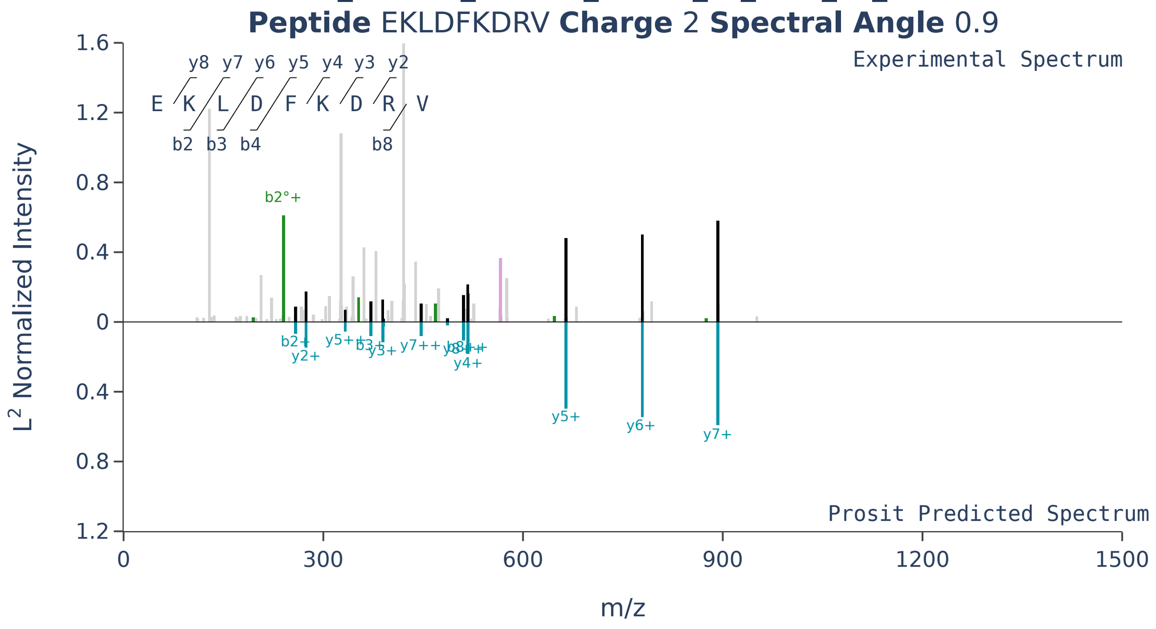


**Source** WSoh_221121_011221_HFGoe_L1_hTau_4h_R1 **Scan** 11064
**Peptide** DHAGTYGLGDRKDQGGYTMH **Charge** 3 **Spectral Angle** 0.94

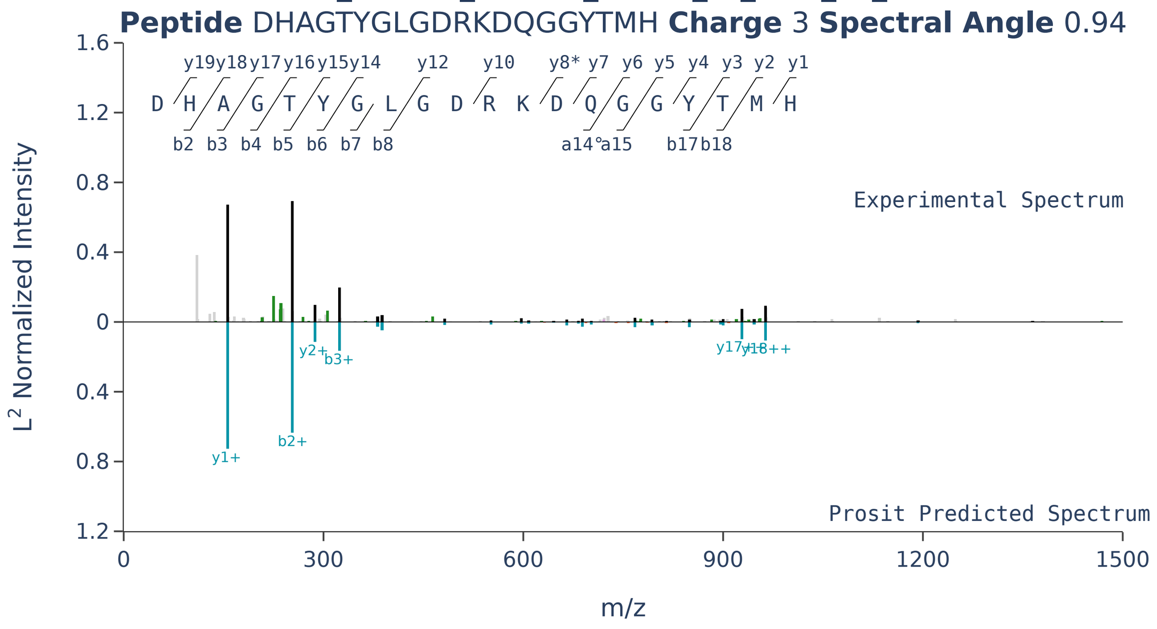

**Source** WSoh_221121_011221_HFGoe_L2_hTau_4h_R1 **Scan** 18244
**Peptide** KLGSLDNI **Charge** 2 **Spectral Angle** 0.88

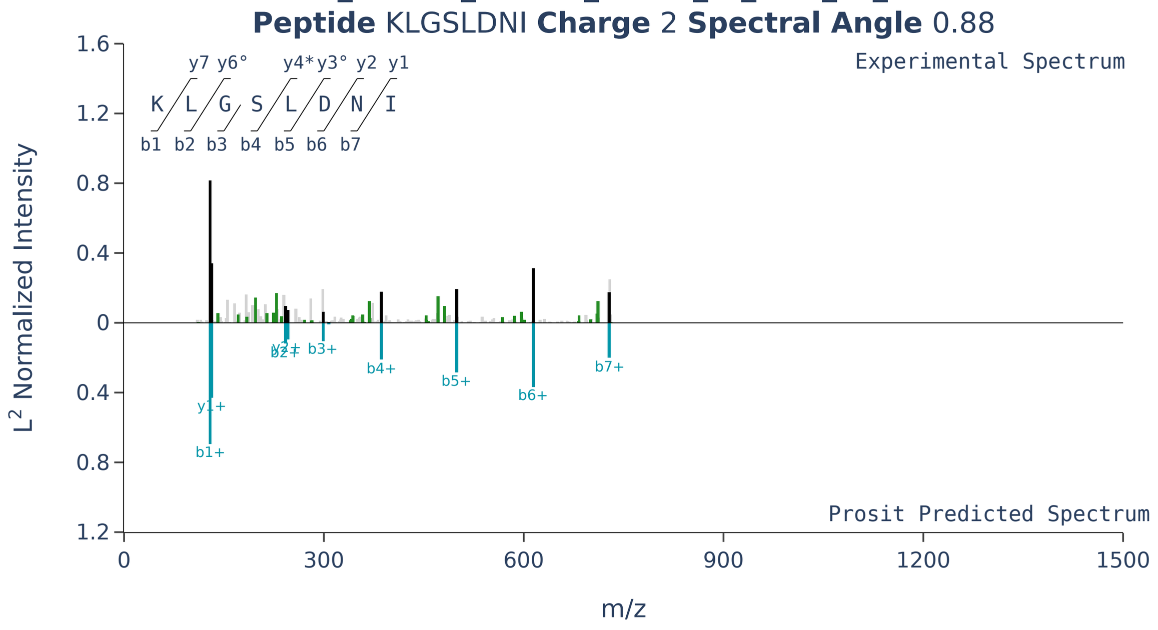

**Source** WSoh_221121_011221_HFGoe_L1_hTau_4h_R1 **Scan** 10484
**Peptide** NATRIPAKTPPAPKTPPSSG **Charge** 3 **Spectral Angle** 0.89

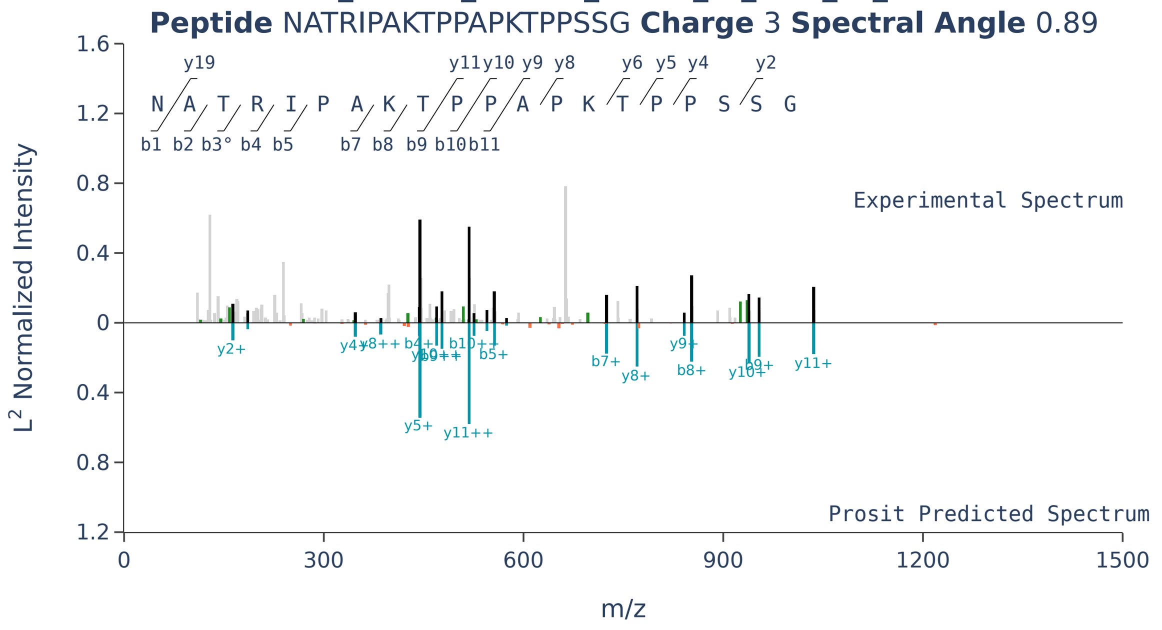

**Source** WSoh_221121_011221_HFGoe_L2_hTau_4h_R1 **Scan** 16297
**Peptide** DNIKHVPGGGSVQI **Charge** 2 **Spectral Angle** 0.83

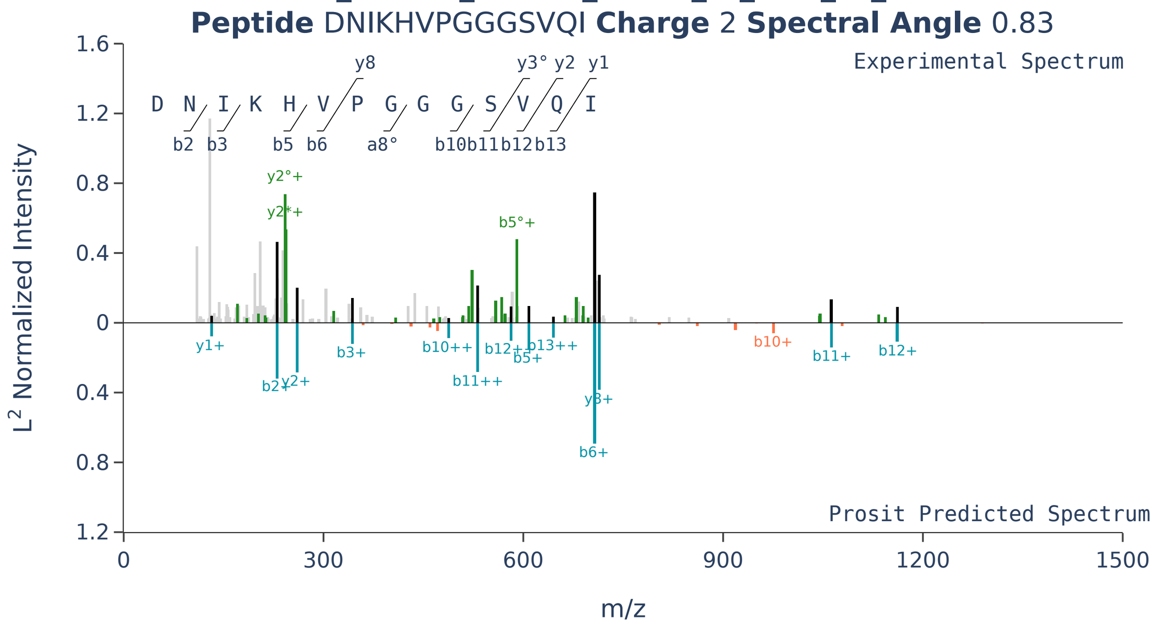

**Source** WSoh_221121_011221_HFGoe_L1_hTau_4h_R1 **Scan** 6910
**Peptide** TSPRHLSNV **Charge** 2 **Spectral Angle** 0.9

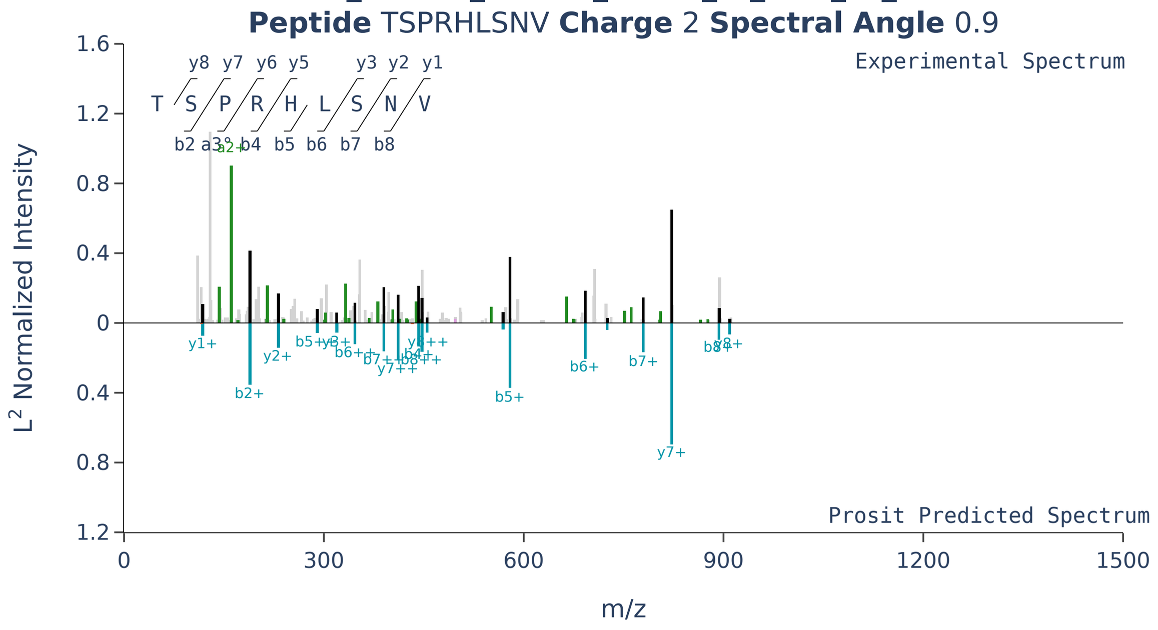

**Source** WSoh_221121_011221_HFGoe_L2_hTau_24h_R1 **Scan** 6482
**Peptide** TSPRHLSNVSS **Charge** 2 **Spectral Angle** 0.87

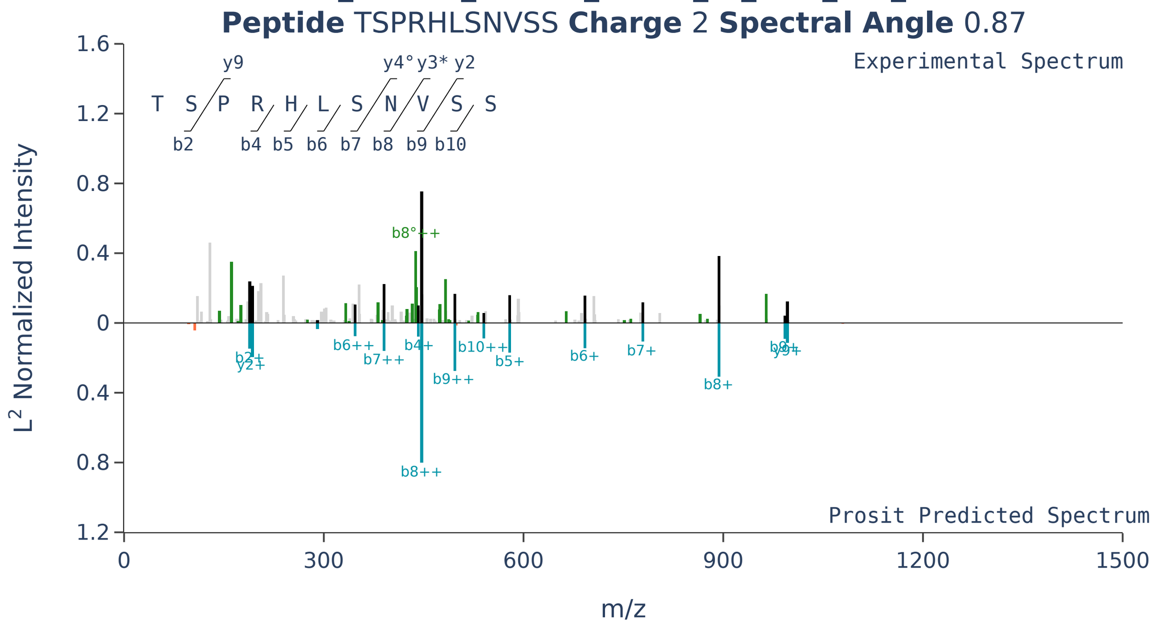

**Source** WSoh_221121_011221_HFGoe_L2_hTau_2h_R1 **Scan** 7437
**Peptide** AAPPGQKGQANATRIPAKT **Charge** 3 **Spectral Angle** 0.88

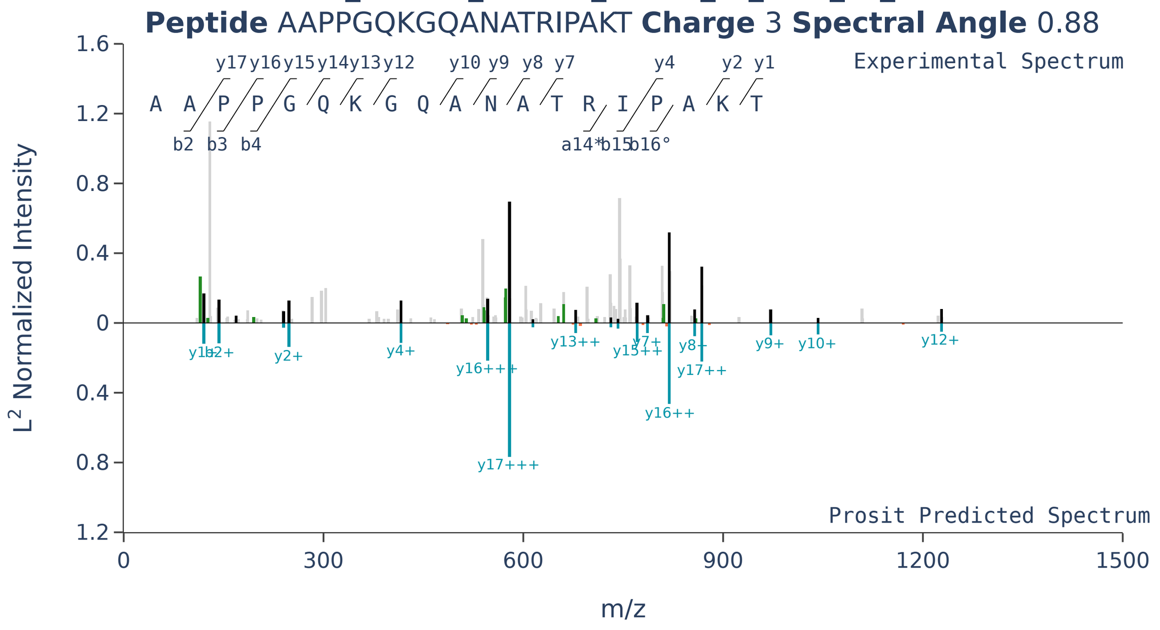

**Source** WSoh_221121_011221_HFGoe_L1_hTau_4h_R1 **Scan** 18426
**Peptide** PVDLSKVTSKCGSL **Charge** 3 **Spectral Angle** 0.84

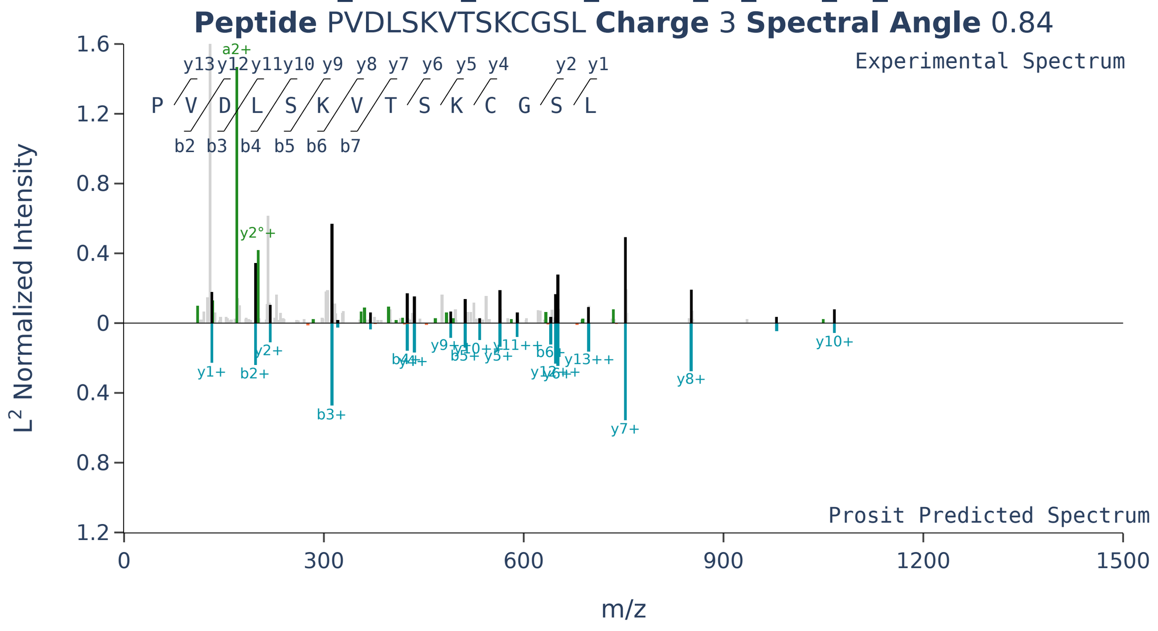

**Source** WSoh_221121_011221_HFGoe_L2_hTau_4h_R1 **Scan** 10088
**Peptide** LGDRKDQGGYTM **Charge** 2 **Spectral Angle** 0.89

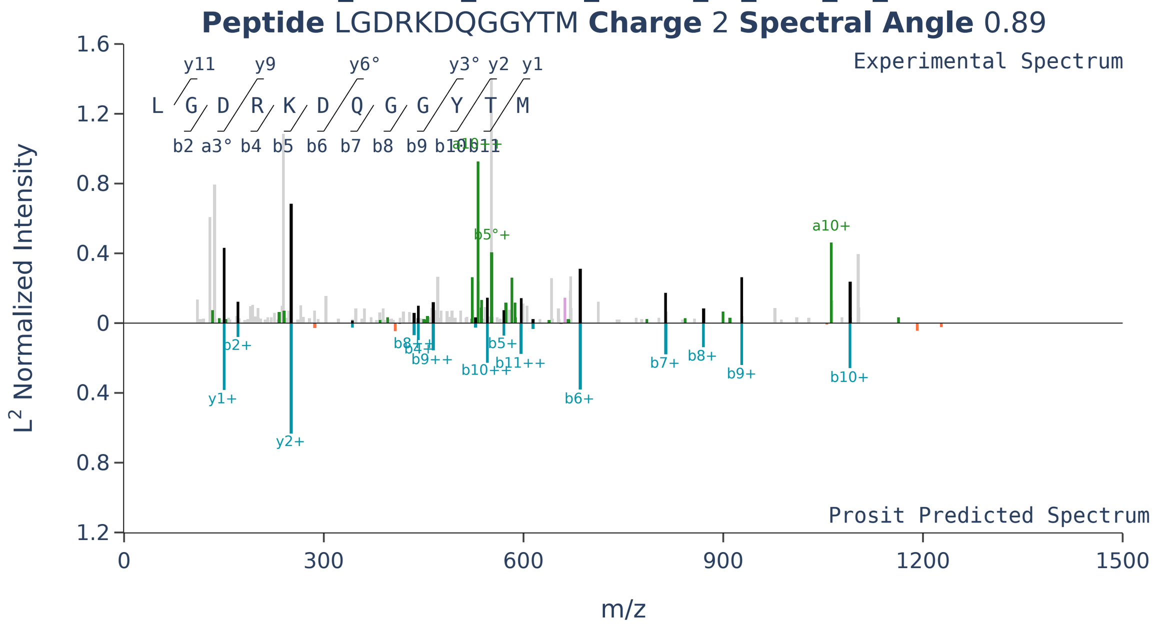

**Source** WSoh_221121_011221_HFGoe_L2_hTau_2h_R1 **Scan** 10247
**Peptide** SLGNIHHKPGGGQVEV **Charge** 2 **Spectral Angle** 0.85

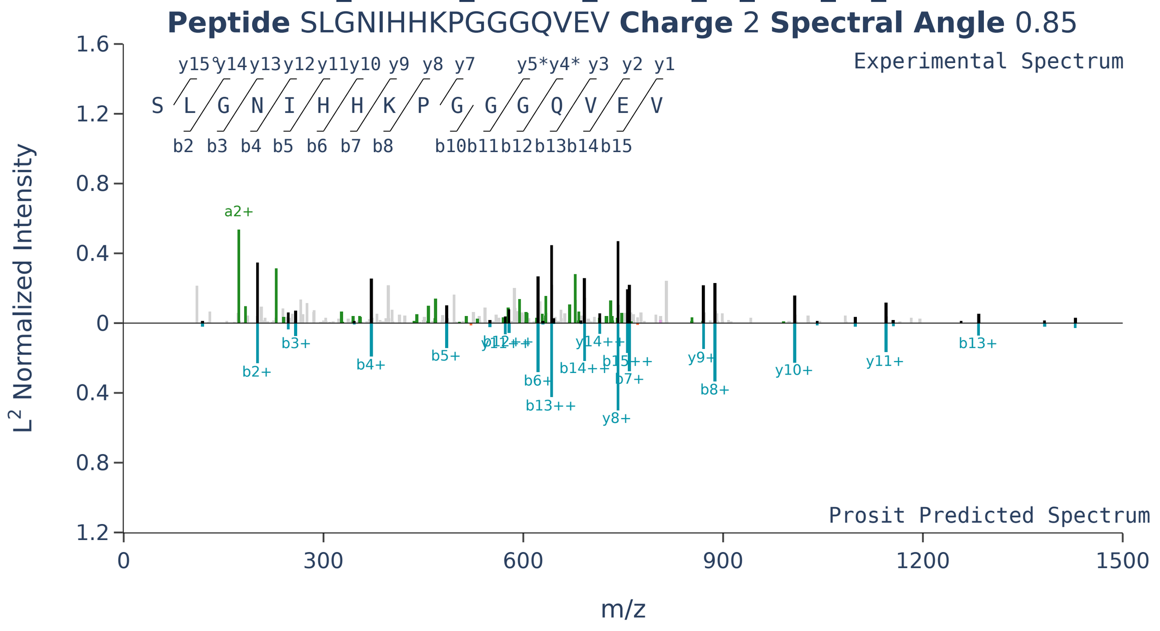

**Source** WSoh_221121_011221_HFGoe_L1_hTau_2h_R1 **Scan** 11246
**Peptide** GDTSPRHLSNVSSTGSID **Charge** 3 **Spectral Angle** 0.89

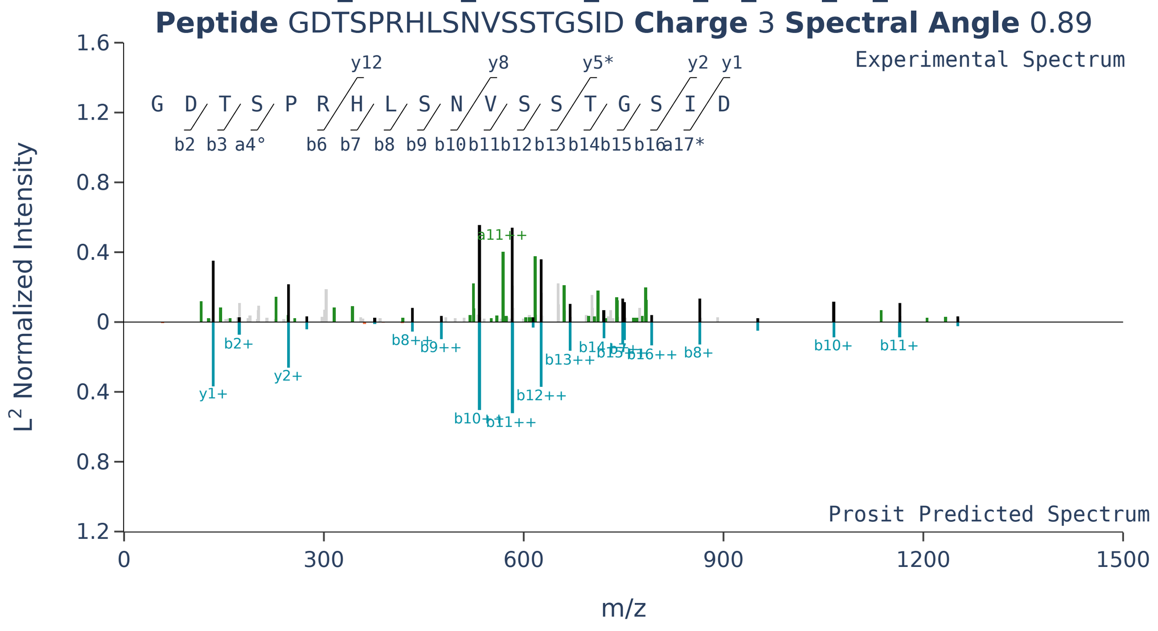

**Source** WSoh_221121_011221_HFGoe_L2_hTau_24h_R1 **Scan** 6986
**Peptide** PPAPKTPPSSGEPPKSGDR **Charge** 4 **Spectral Angle** 0.84

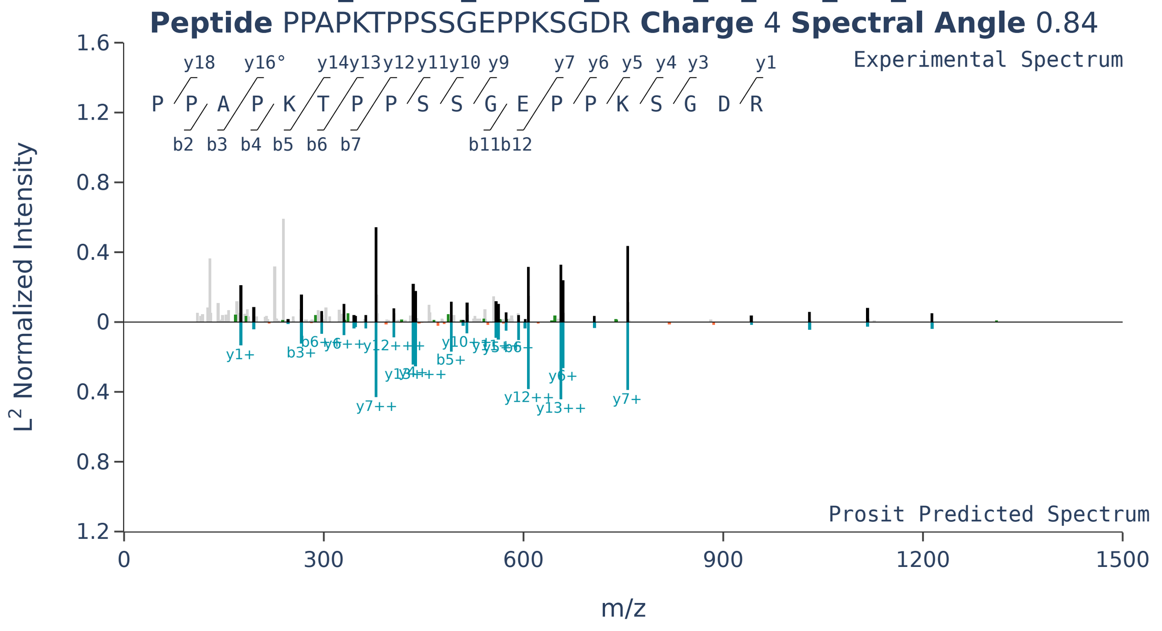

**Source** WSoh_221121_011221_HFGoe_L2_hTau_4h_R1 **Scan** 6639
**Peptide** KLTFRENAKAKT **Charge** 2 **Spectral Angle** 0.87

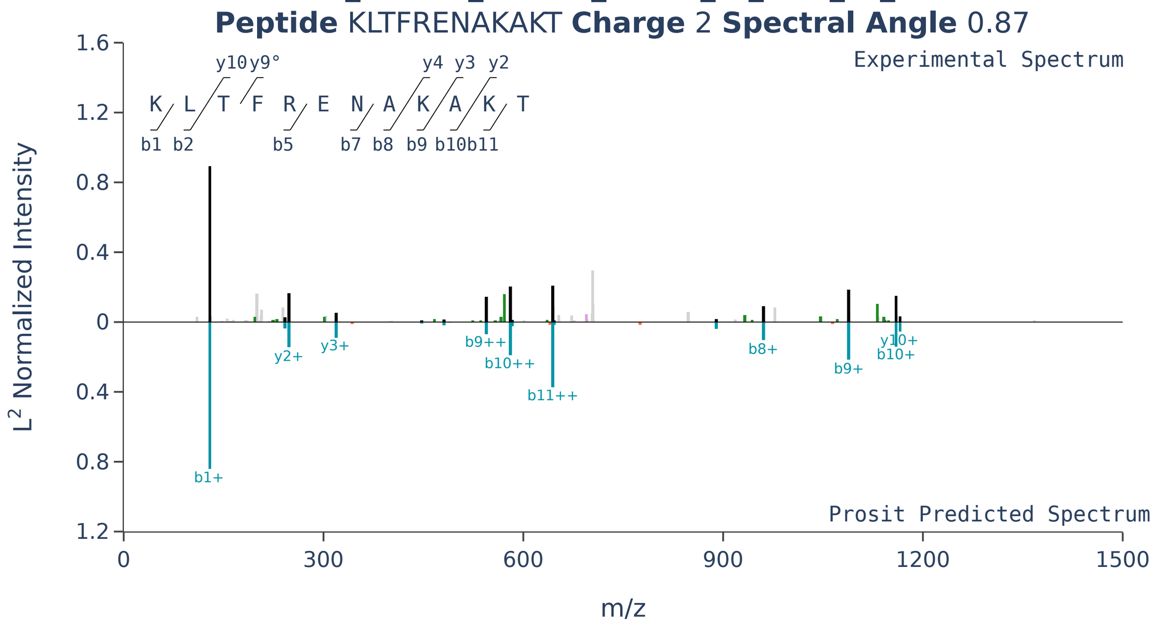

**Source** WSoh_221121_011221_HFGoe_L1_hTau_24h_R2 **Scan** 8813
**Peptide** PGGGNKKIETHKLTFRE **Charge** 3 **Spectral Angle** 0.83

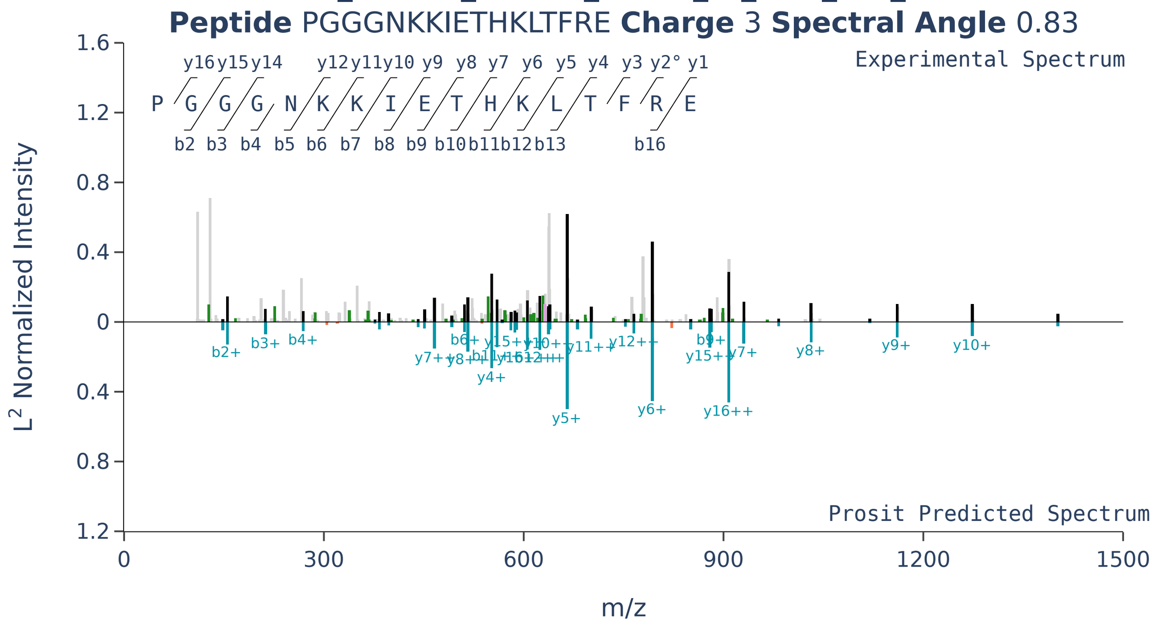

**Source** WSoh_221121_011221_HFGoe_L2_hTau_1h_R2 **Scan** 7660
**Peptide** RIPAKTPPAPKT **Charge** 3 **Spectral Angle** 0.82

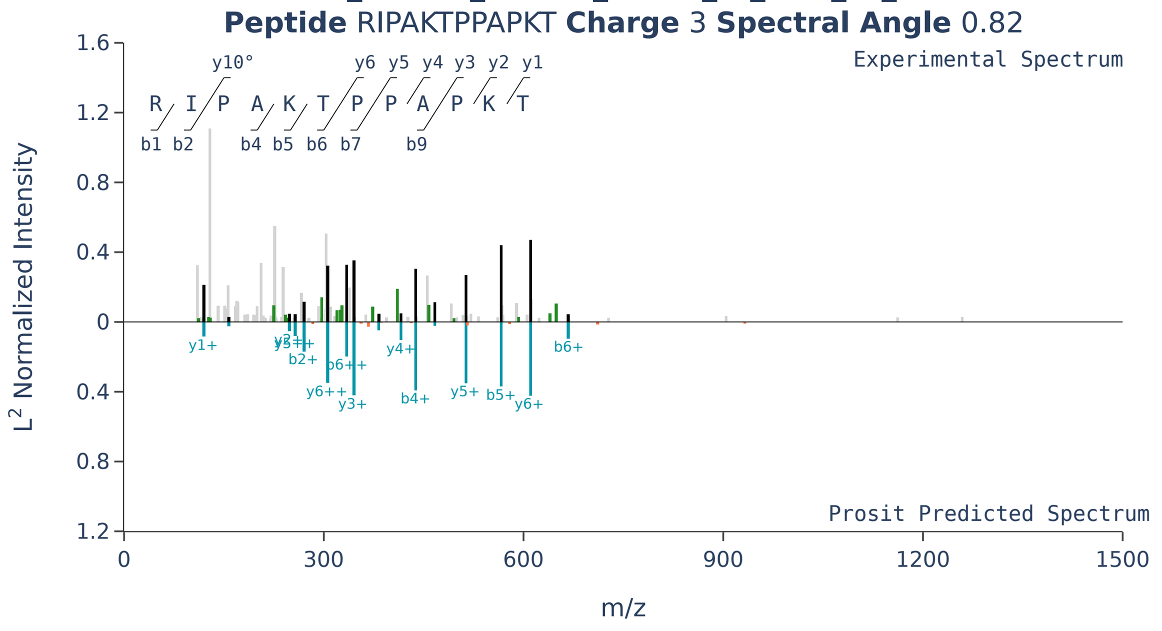

**Source** WSoh_221121_011221_HFGoe_L2_hTau_2h_R2 **Scan** 17711
**Peptide** PGGGKVQIINKKLDL **Charge** 2 **Spectral Angle** 0.84

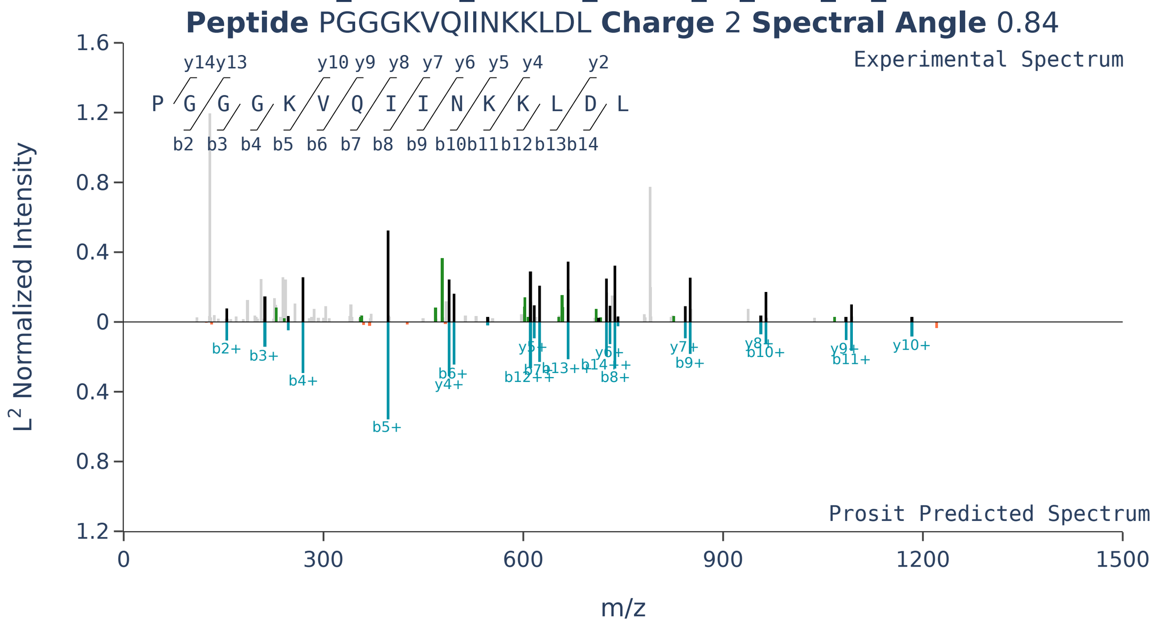

**Source** WSoh_221121_011221_HFGoe_L1_hTau_24h_R1 **Scan** 24187
**Peptide** LQTAPVPMPDLKNVKSKI **Charge** 4 **Spectral Angle** 0.86

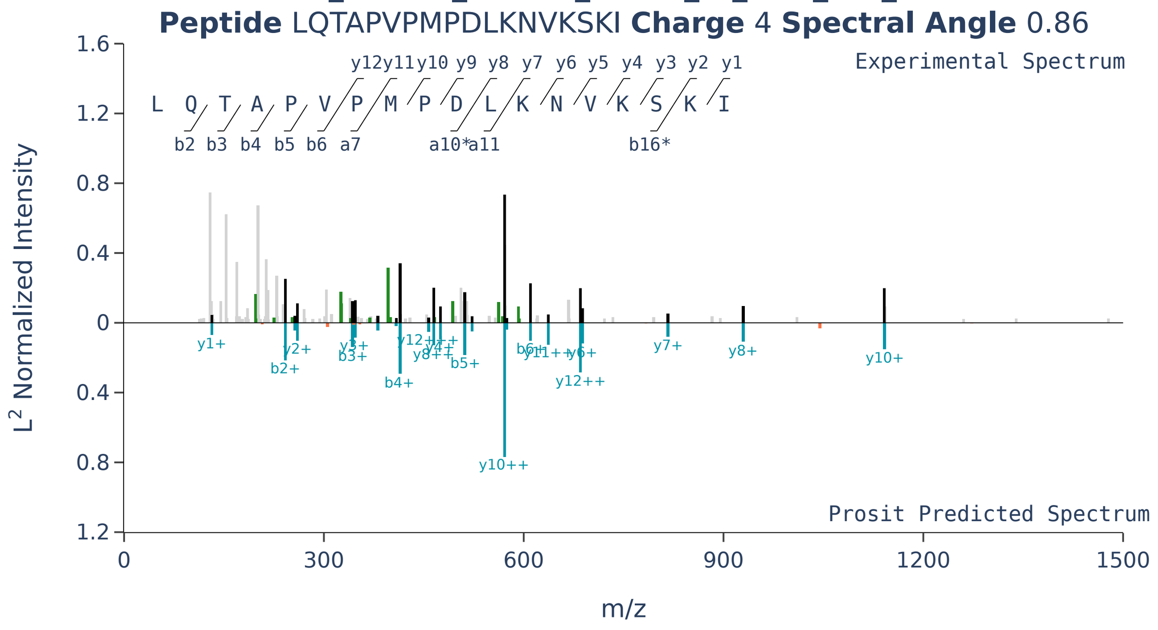


**Source** WSoh_221121_011221_HFGoe_L1_hTau_24h_R1 **Scan** 12239
**Peptide** AKSTPTAEDVTAP **Charge** 2 **Spectral Angle** 0.92

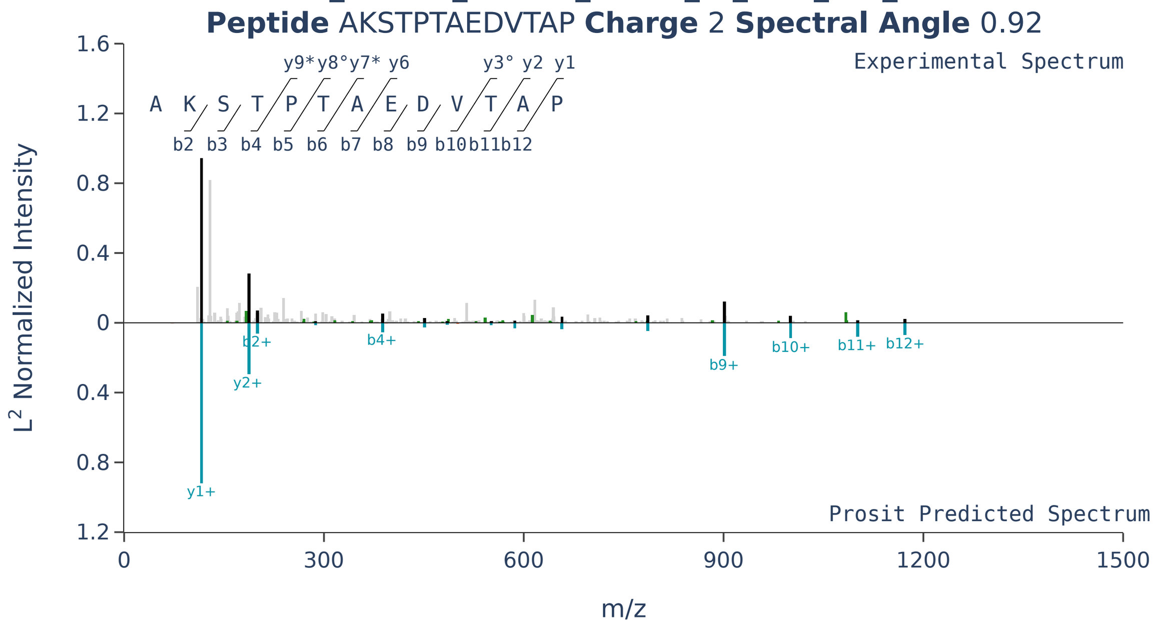


**Source** WSoh_221121_011221_HFGoe_L2_hTau_4h_R2 **Scan** 5954
**Peptide** AGHVTQARMVSKS **Charge** 2 **Spectral Angle** 0.92

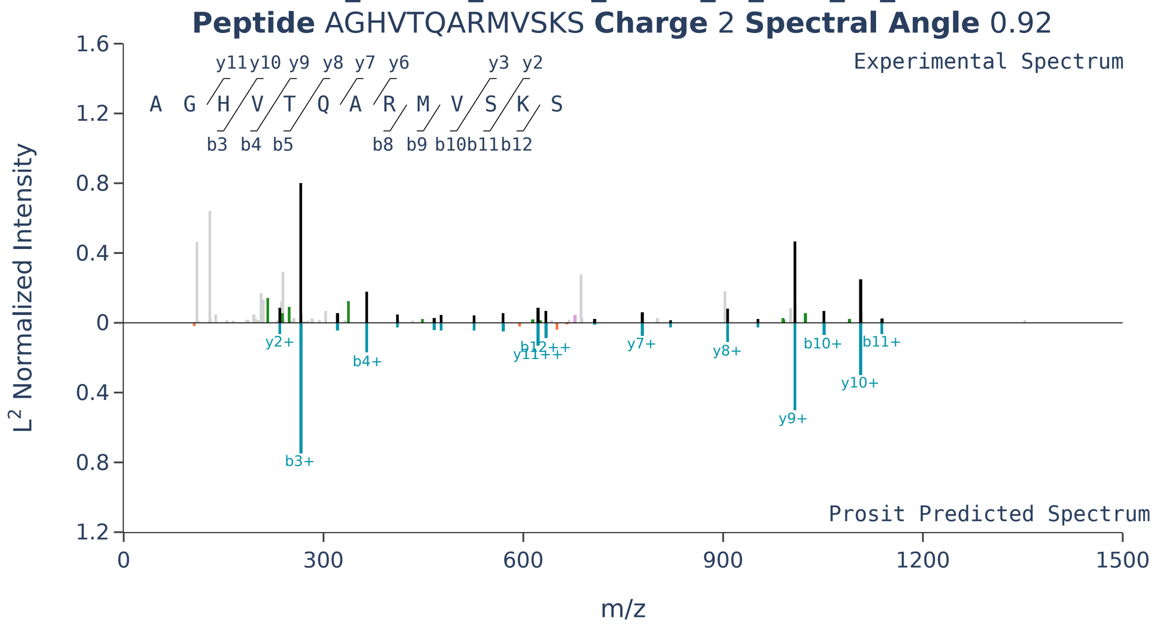


**Source** WSoh_221121_011221_HFGoe_L1_hTau_4h_R1 **Scan** 7260
**Peptide** PRGAAPPGQKGQANATRI **Charge** 4 **Spectral Angle** 0.88

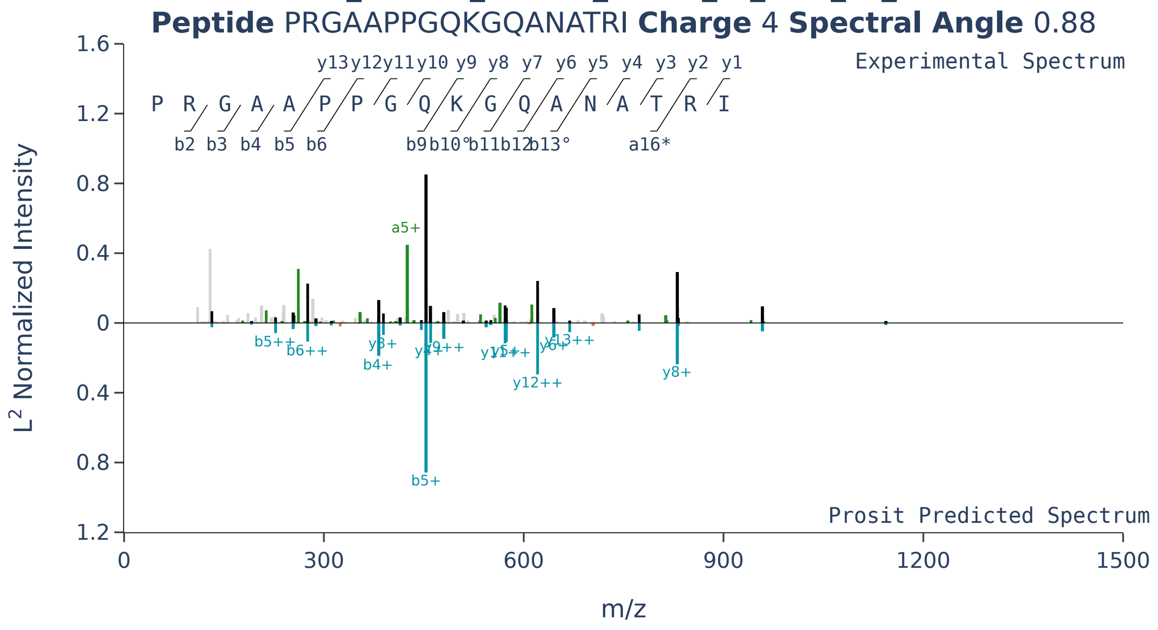


**Source** WSoh_221121_011221_HFGoe_L2_hTau_4h_R1 **Scan** 7610
**Peptide** CGSLGNIHHKPGGGQVE **Charge** 2 **Spectral Angle** 0.86

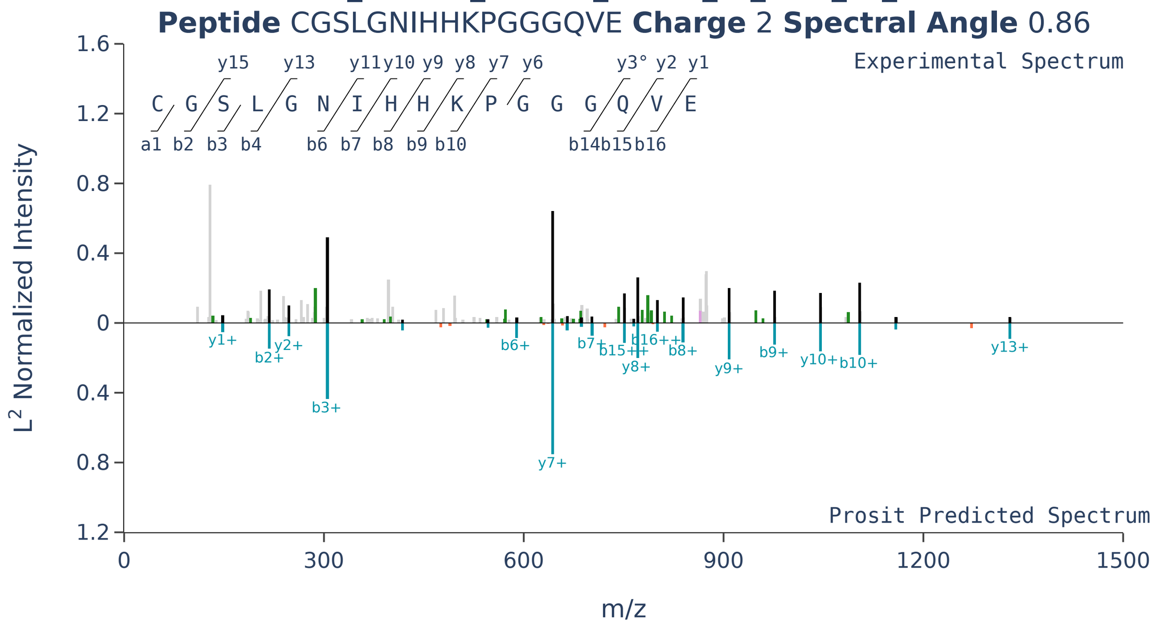


**Source** WSoh_221121_011221_HFGoe_L1_hTau_4h_R1 **Scan** 7298
**Peptide** PPAPKTPPSS **Charge** 1 **Spectral Angle** 0.78

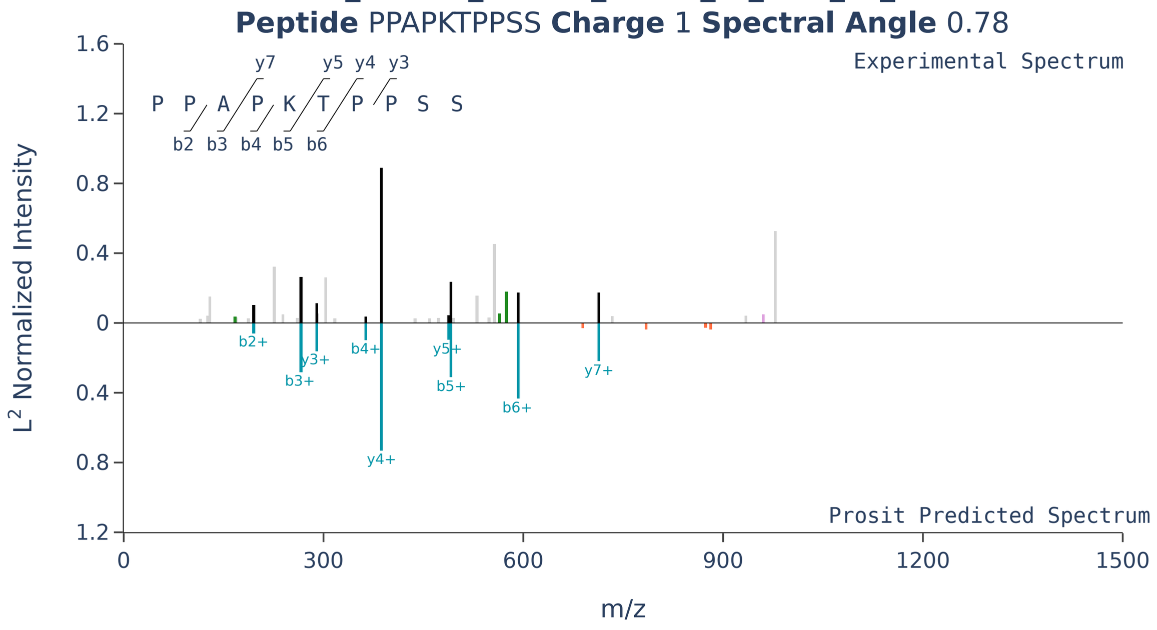


**Source** WSoh_221121_011221_HFGoe_L2_hTau_24h_R2 **Scan** 23179
**Peptide** LQTAPVPMPDLKNVKS **Charge** 2 **Spectral Angle** 0.89

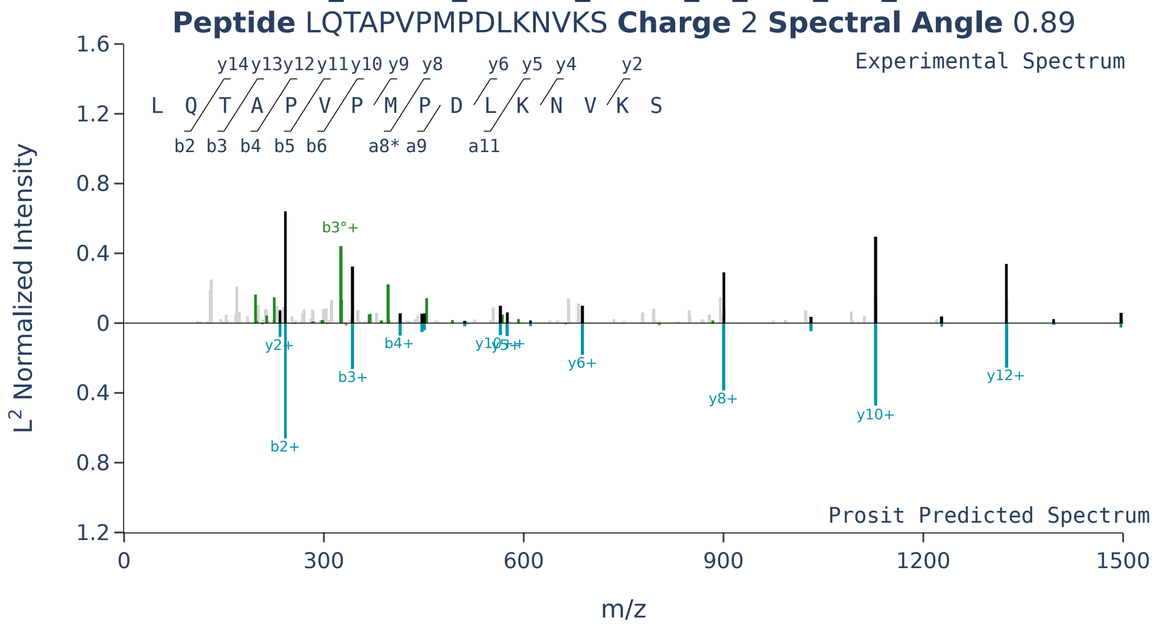


**Source** WSoh_221121_011221_HFGoe_L2_hTau_2h_R2 **Scan** 5810
**Peptide** PPKSPSSAKSRLQ **Charge** 3 **Spectral Angle** 0.91

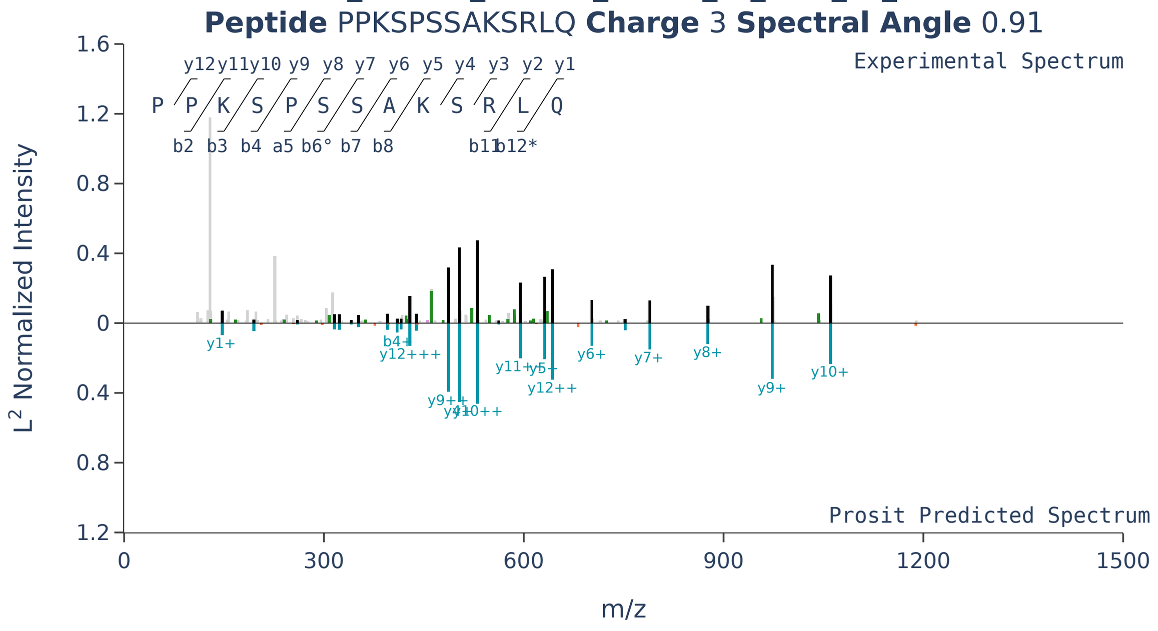


**Source** WSoh_221121_011221_HFGoe_L2_hTau_24h_R2 **Scan** 12685
**Peptide** KLDLSNVQSKC **Charge** 2 **Spectral Angle** 0.93

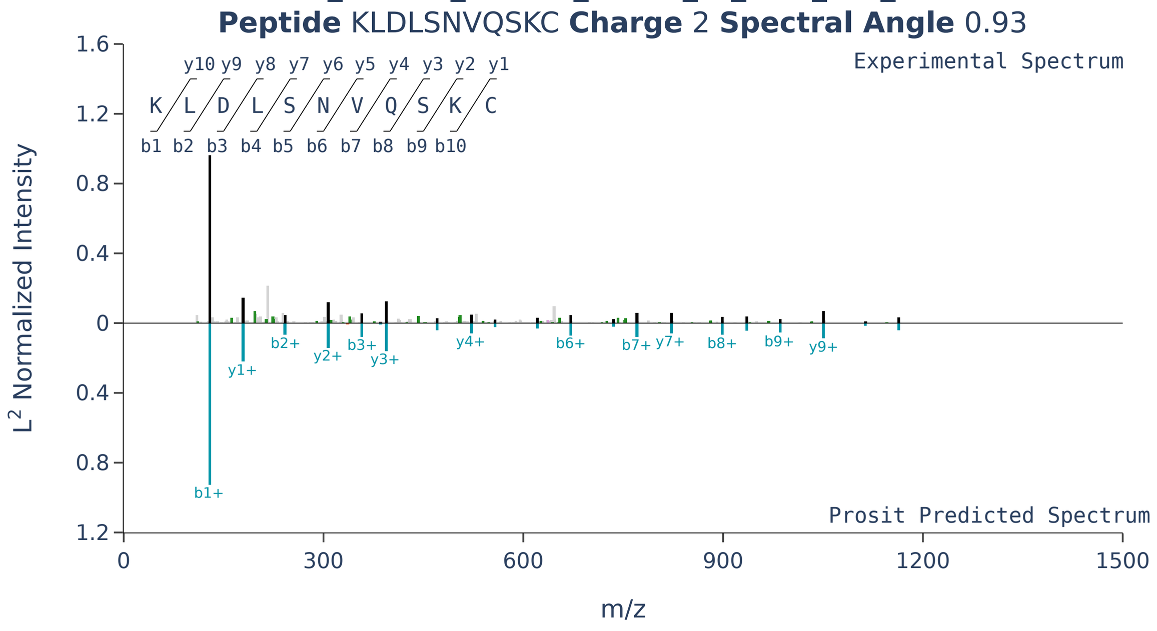


**Source** WSoh_221121_011221_HFGoe_L2_hTau_2h_R2 **Scan** 7934
**Peptide** AAPPGQKGQANATRL **Charge** 3 **Spectral Angle** 0.86

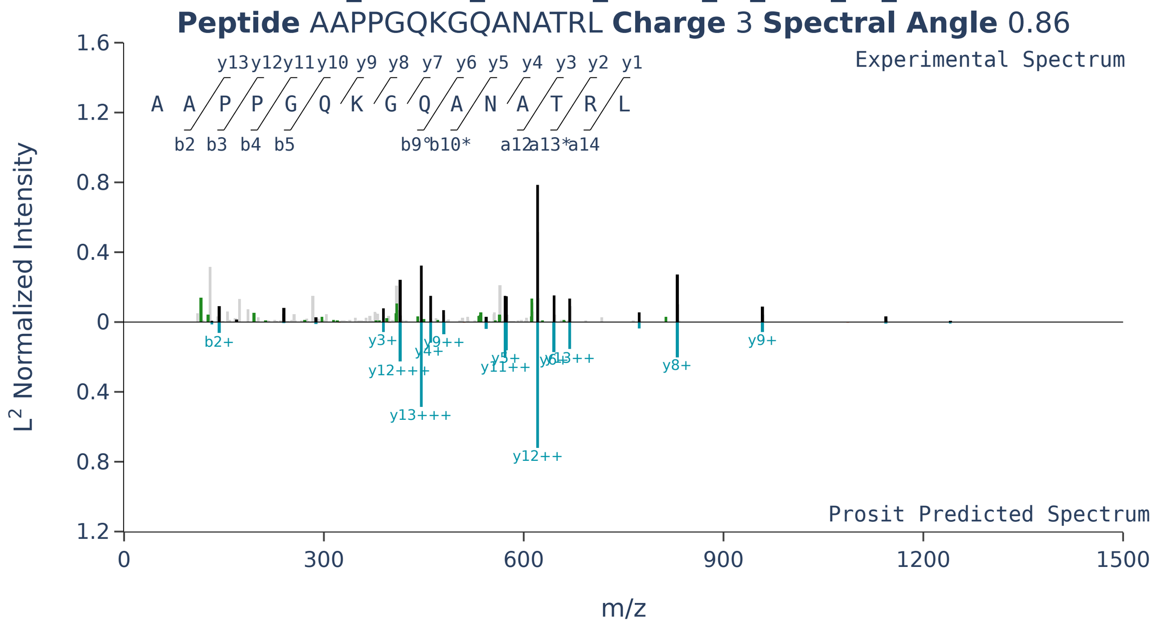


**Source** WSoh_221121_011221_HFGoe_L1_hTau_2h_R1 **Scan** 14920
**Peptide** KPVDLSKVTSKCGSL **Charge** 2 **Spectral Angle** 0.83

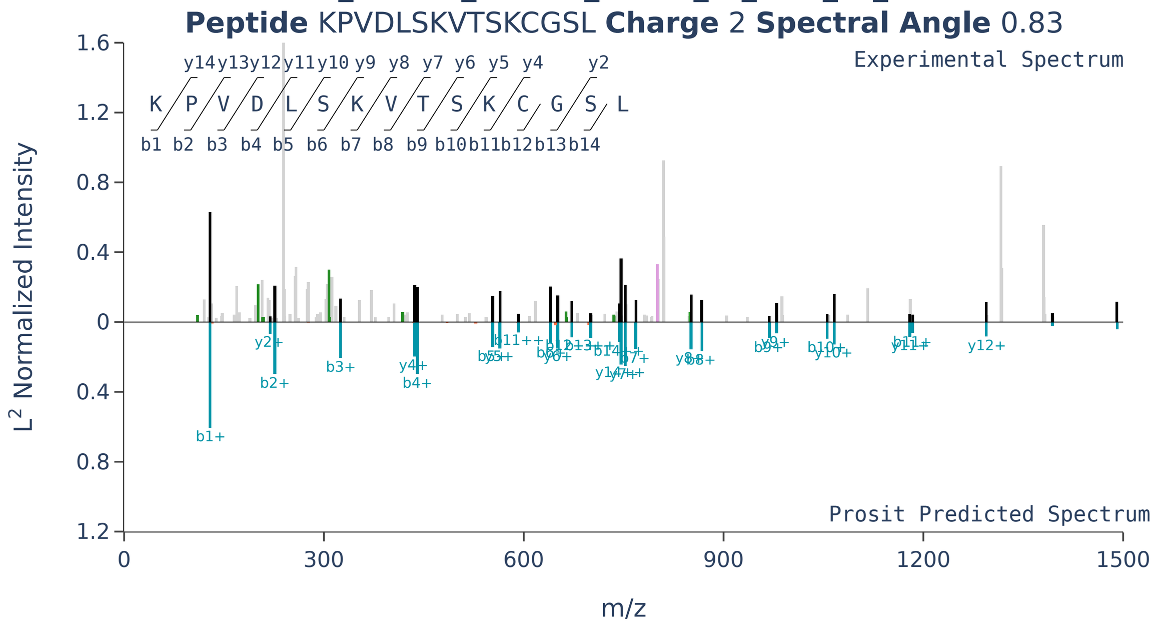


**Source** WSoh_221121_011221_HFGoe_L2_hTau_4h_R1 **Scan** 12352
**Peptide** QPHTEIPEGTTAEE **Charge** 2 **Spectral Angle** 0.89

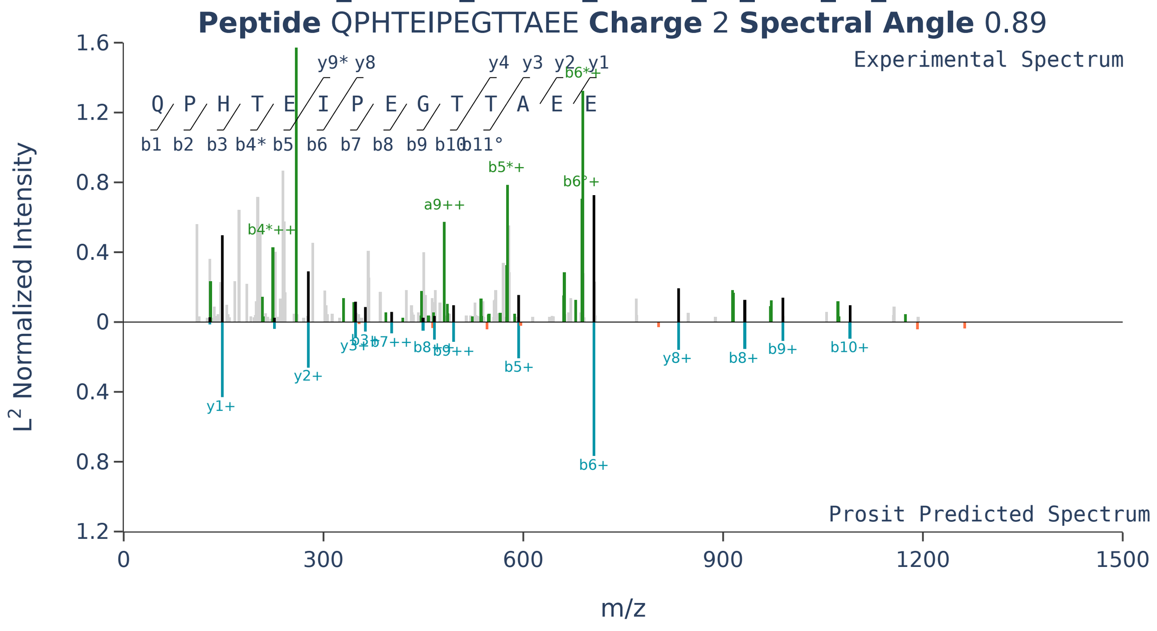


**Source** WSoh_221121_011221_HFGoe_L1_hTau_24h_R2 **Scan** 13705
**Peptide** VYKSPVVSGDTSPRHLSNVS **Charge** 2 **Spectral Angle** 0.82

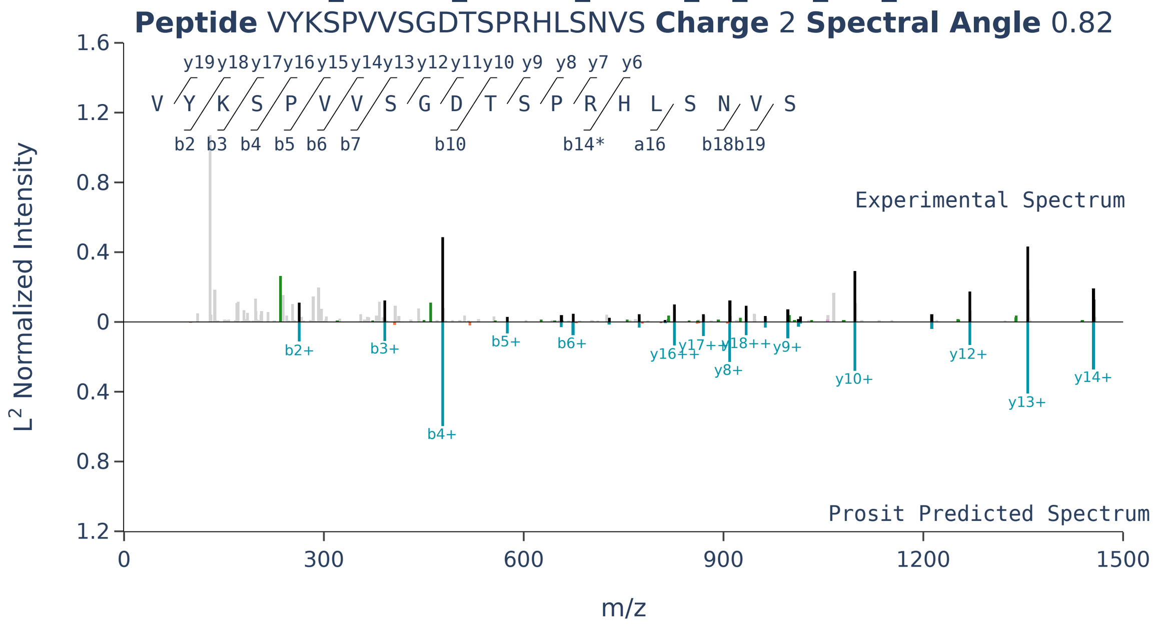


**Source** WSoh_221121_011221_HFGoe_L2_hTau_4h_R1 **Scan** 18509
**Peptide** PRQEFEVMEDHAGTY **Charge** 3 **Spectral Angle** 0.87

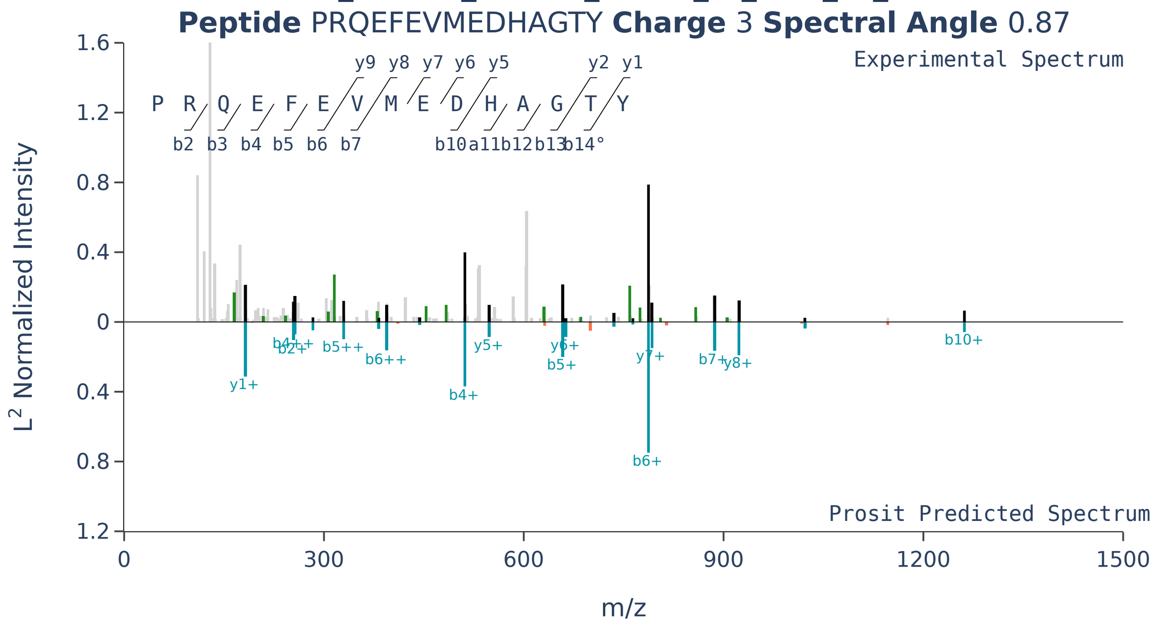


**Source** WSoh_221121_011221_HFGoe_L2_hTau_24h_R2 **Scan** 7903
**Peptide** KNVKSKIGSTENL **Charge** 4 **Spectral Angle** 0.91

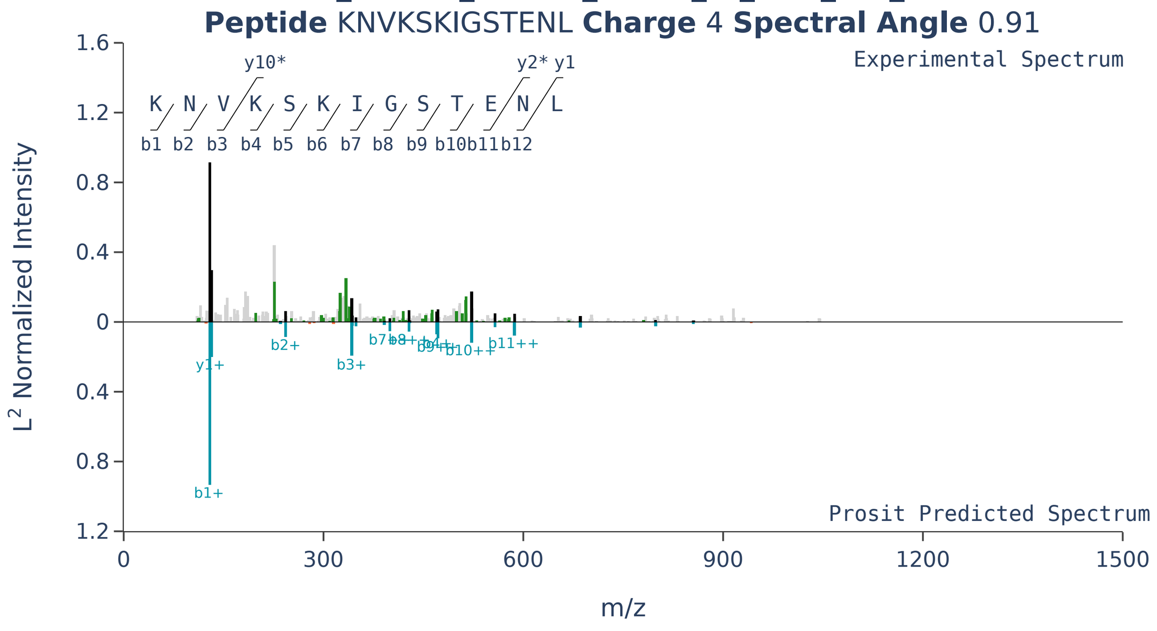

**Source** WSoh_221121_011221_HFGoe_L2_hTau_24h_R2 **Scan** 19020
**Peptide** GDTDAGLKESPLQTPTED **Charge** 2 **Spectral Angle** 0.91

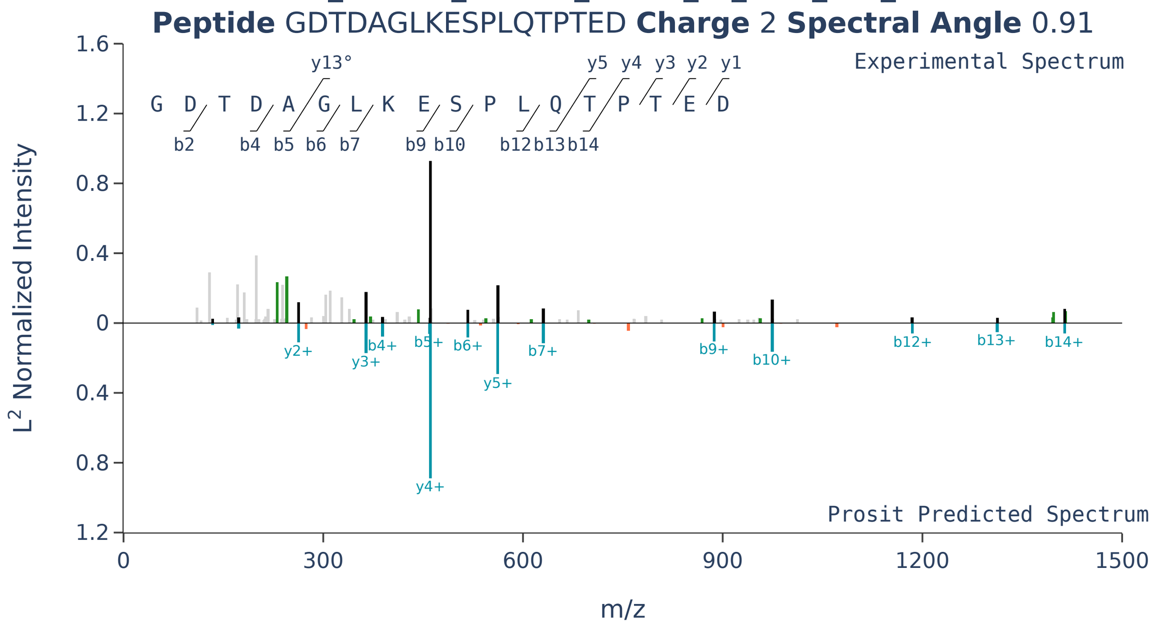


**Source** WSoh_221121_011221_HFGoe_L2_hTau_24h_R1 **Scan** 8032
**Peptide** GSEEPGSETSDAKSTPTAED **Charge** 2 **Spectral Angle** 0.87

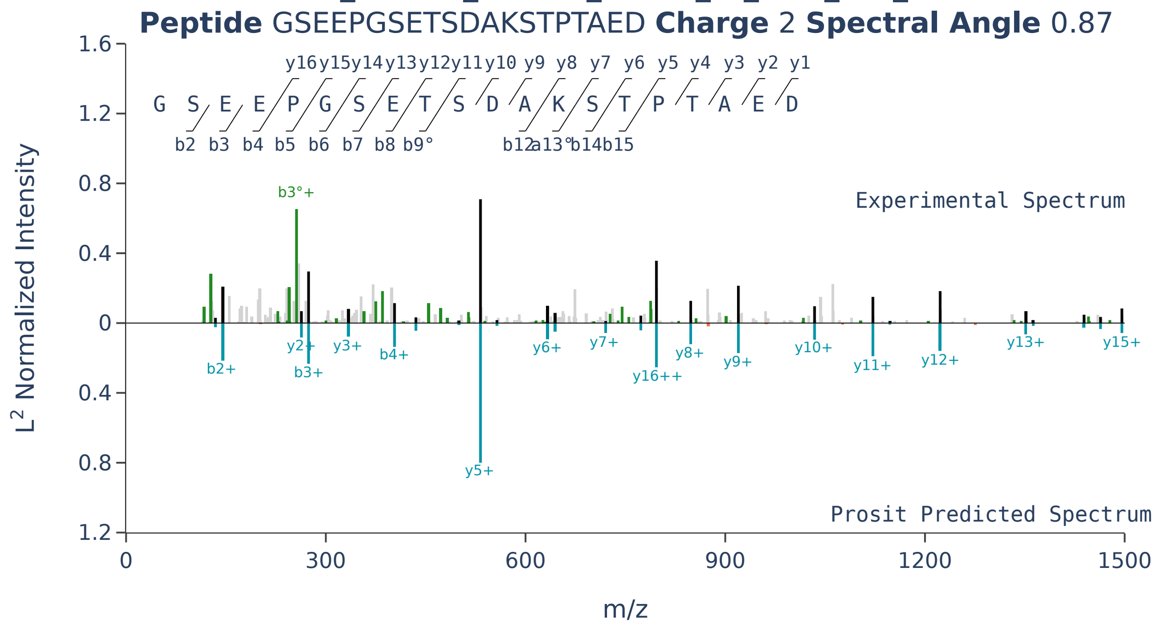


**Source** WSoh_221121_011221_HFGoe_L2_hTau_24h_R1 **Scan** 20742
**Peptide** AGIGDTPSLEDEAAGHVTQ **Charge** 2 **Spectral Angle** 0.84

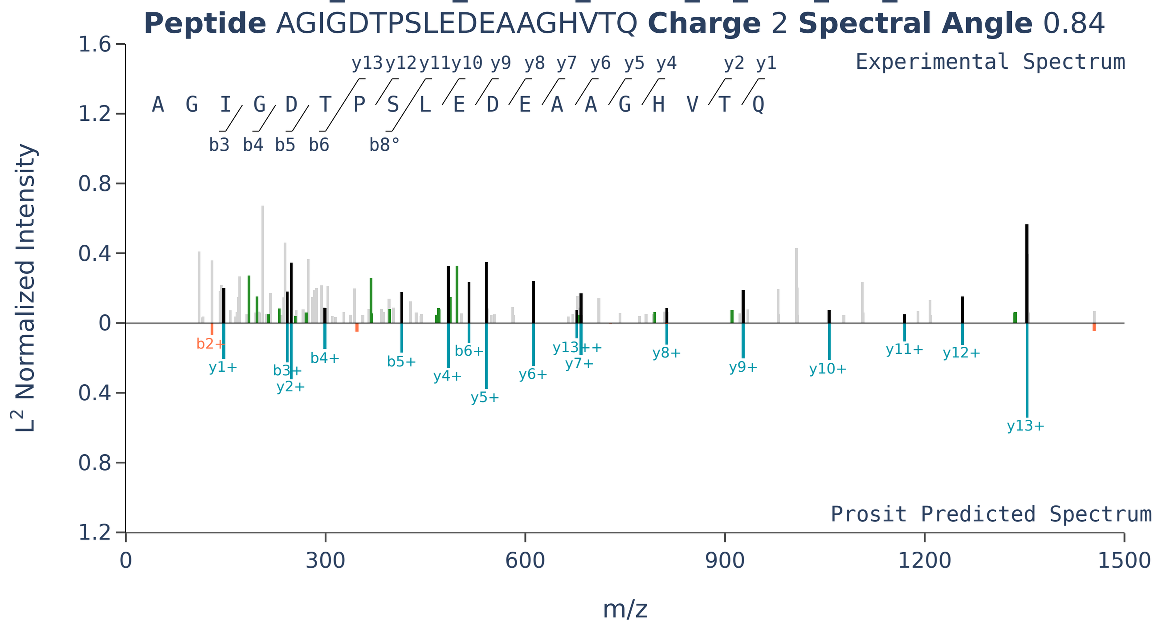


**Source** WSoh_221121_011221_HFGoe_L1_hTau_24h_R1 **Scan** 7138
**Peptide** GNIHHKPGGGQVEVKSEKL **Charge** 2 **Spectral Angle** 0.88

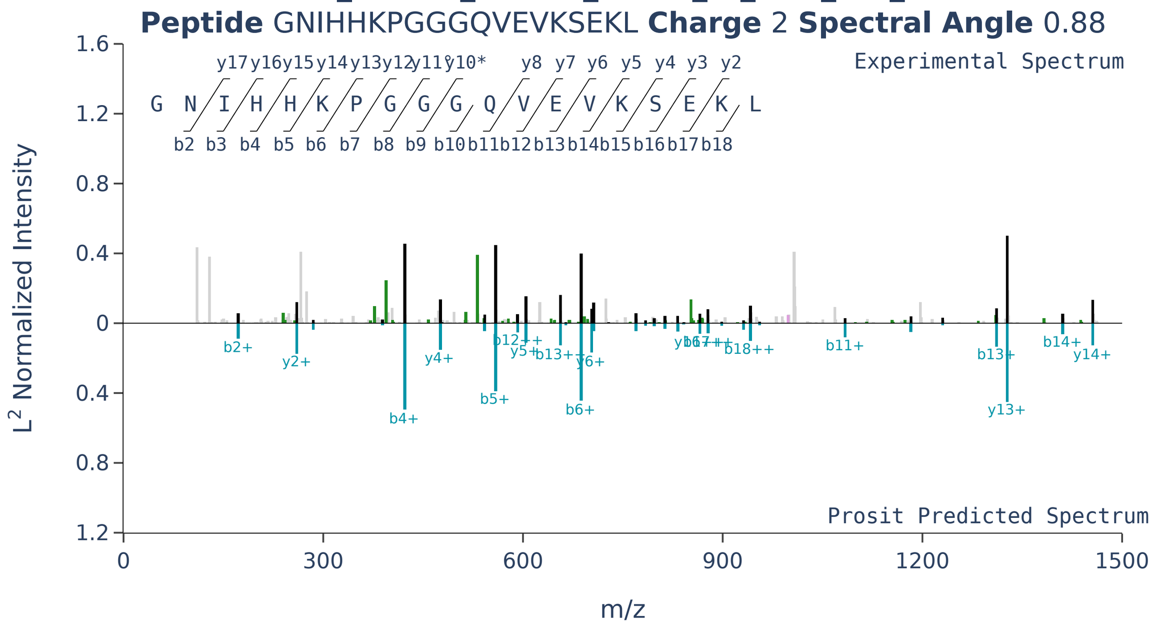


**Source** WSoh_221121_011221_HFGoe_L1_hTau_24h_R2 **Scan** 18211
**Peptide** SSTGSIDMVD **Charge** 1 **Spectral Angle** 0.87

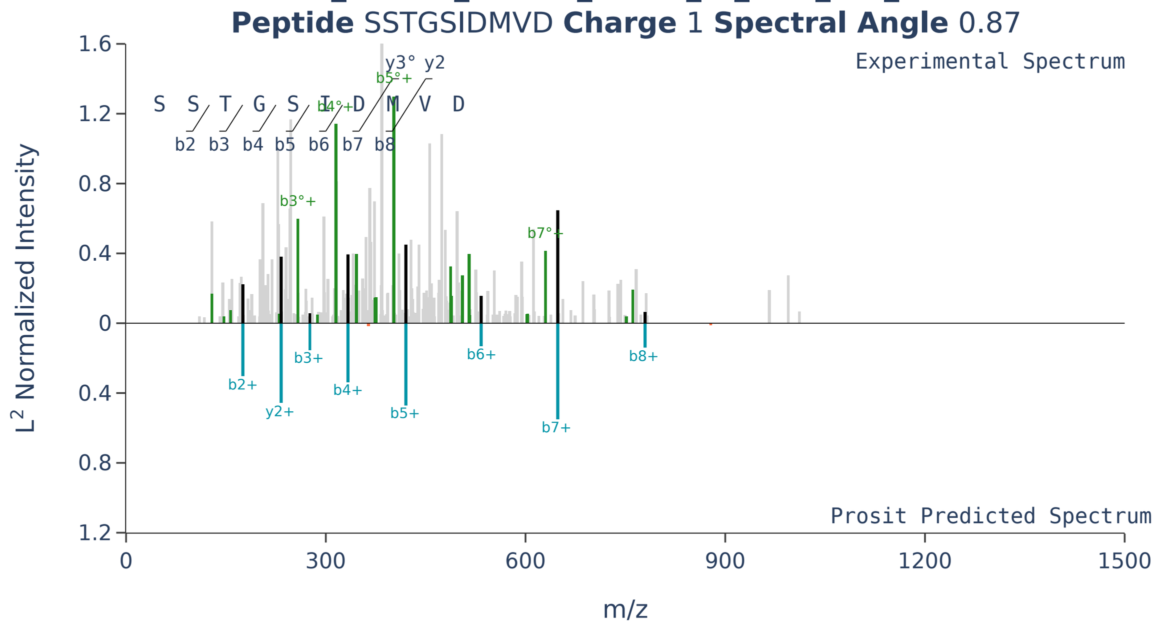


**Source** WSoh_221121_011221_HFGoe_L2_hTau_24h_R2 **Scan** 22221
**Peptide** TAEEAGIGDTPSLEDEAAGHVTQA **Charge** 3 **Spectral Angle** 0.83

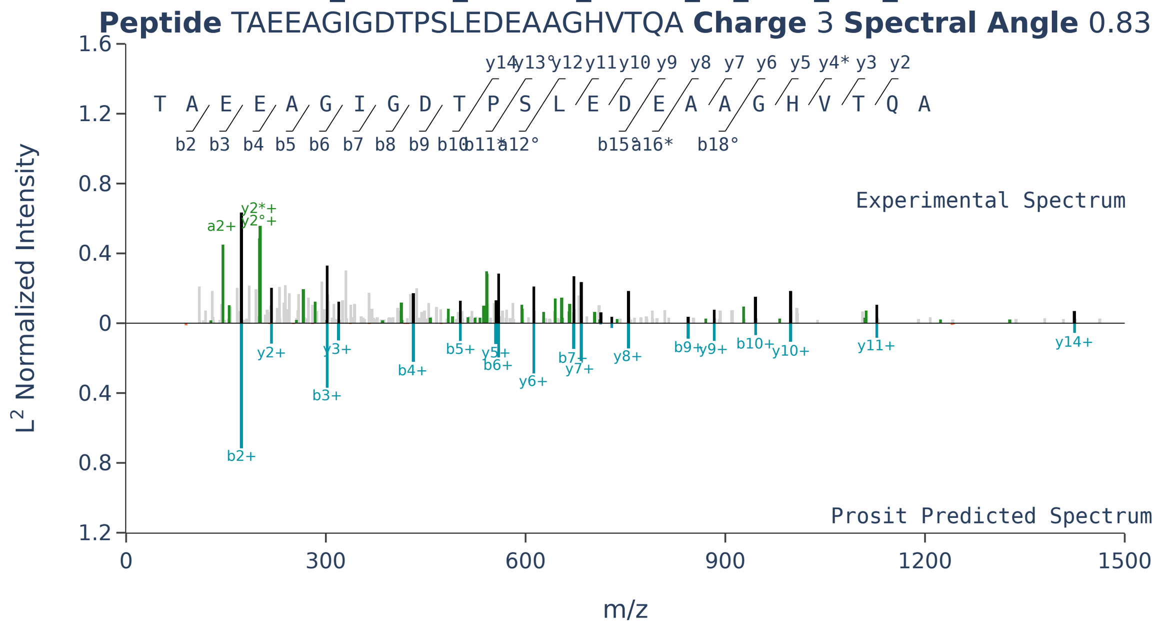


**Source** WSoh_221121_011221_HFGoe_L1_hTau_4h_R1 **Scan** 11555
**Peptide** SEKLDFKDRVQ **Charge** 3 **Spectral Angle** 0.88

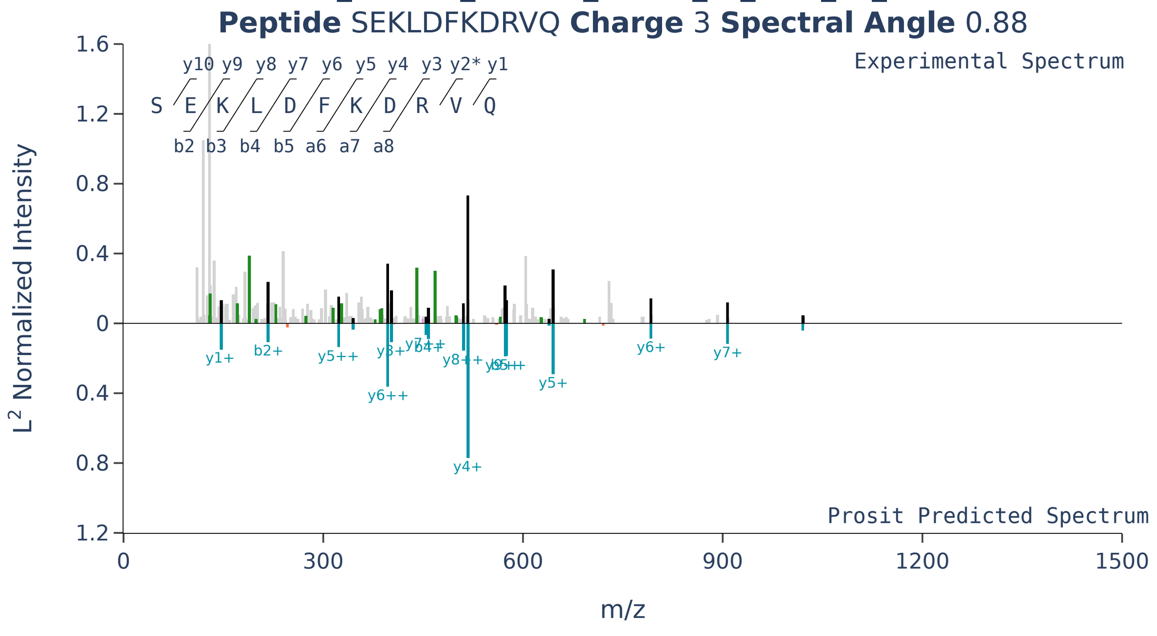


**Source** WSoh_221121_011221_HFGoe_L2_hTau_1h_R2 **Scan** 10840
**Peptide** KHVPGGGSVQI **Charge** 2 **Spectral Angle** 0.85

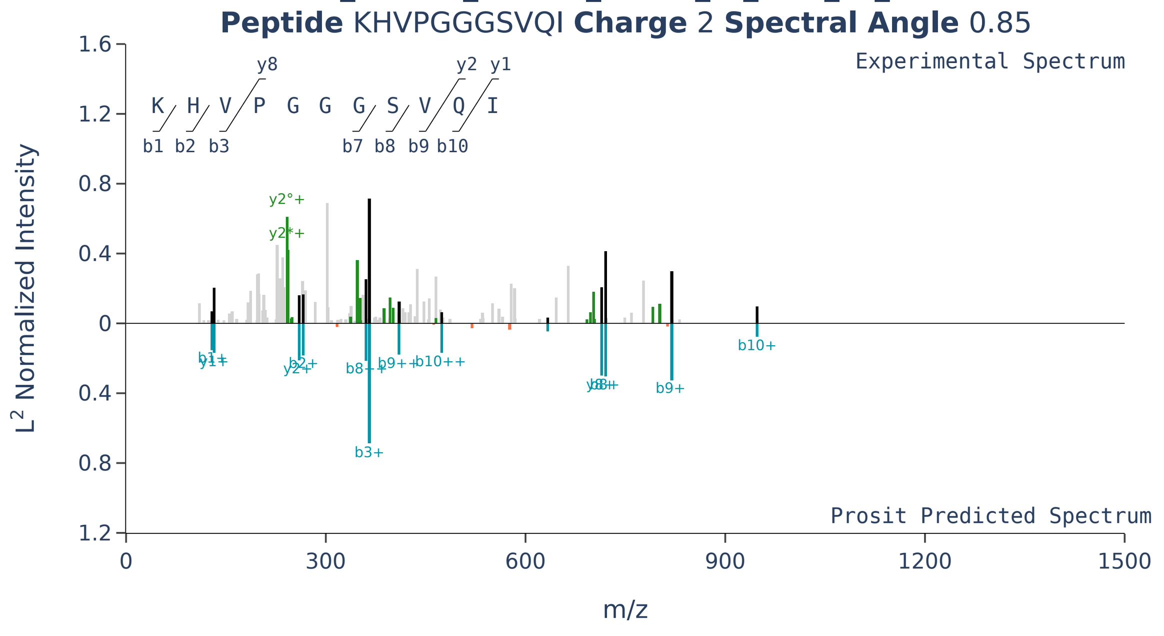


**Source** WSoh_221121_011221_HFGoe_L1_hTau_2h_R2 **Scan** 14828
**Peptide** APVPMPDLKNVKS **Charge** 3 **Spectral Angle** 0.9

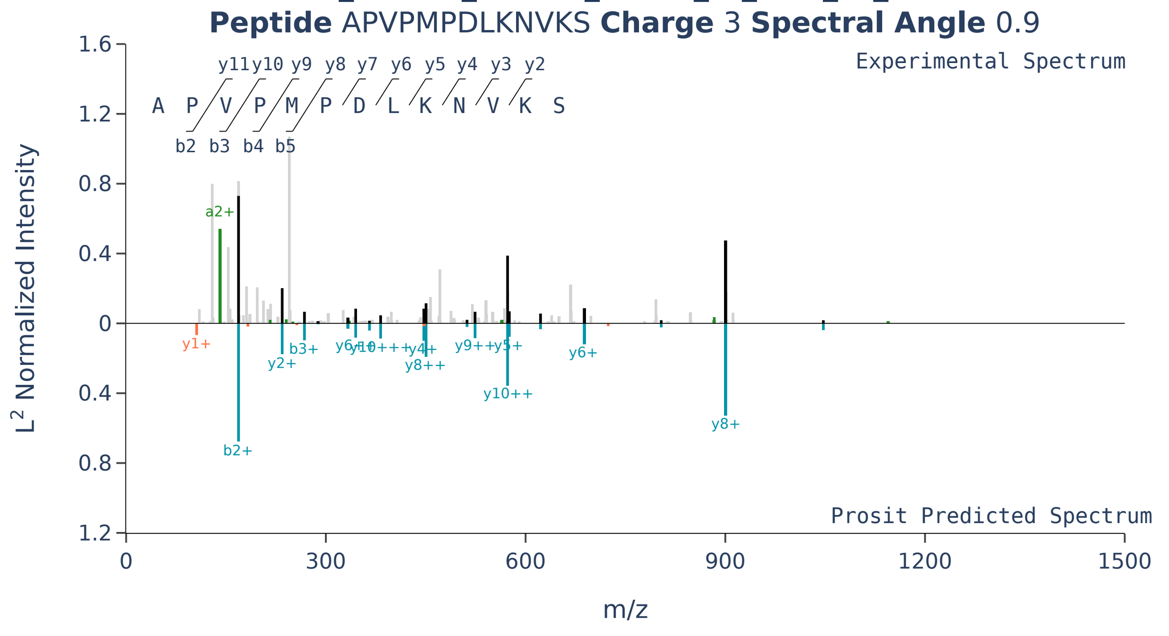


**Source** WSoh_221121_011221_HFGoe_L2_hTau_24h_R2 **Scan** 20020
**Peptide** EDVTAPLVDEGAPGKQA **Charge** 2 **Spectral Angle** 0.9

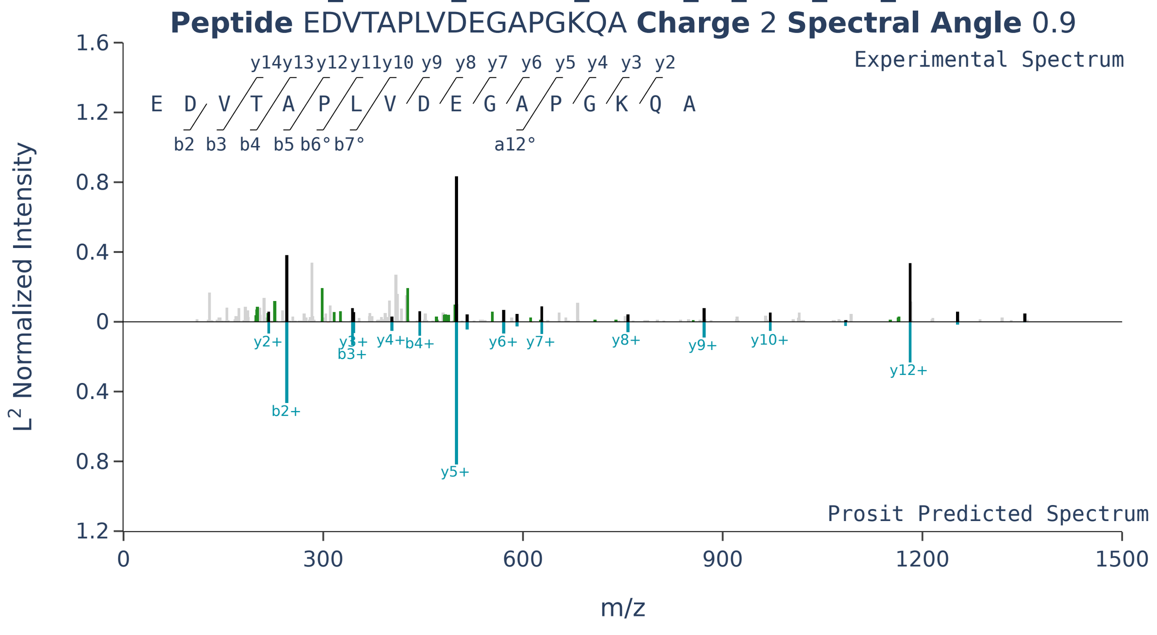


**Source** WSoh_221121_011221_HFGoe_L2_hTau_1h_R2 **Scan** 6465
**Peptide** TPPKSPSSAKSRLQT **Charge** 3 **Spectral Angle** 0.85

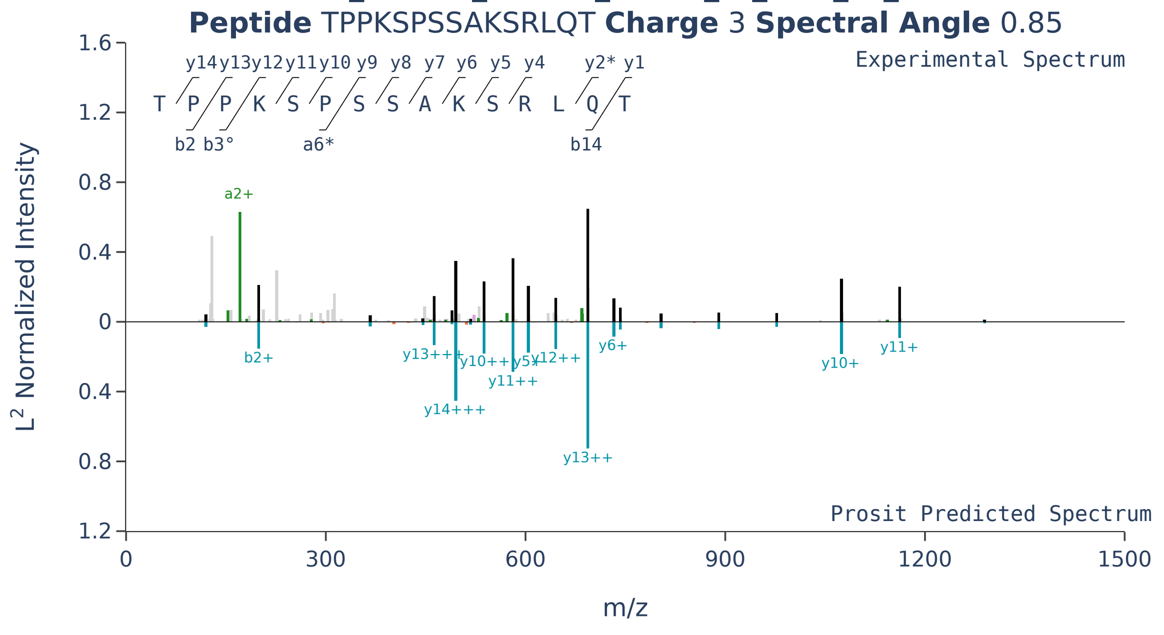


**Source** WSoh_221121_011221_HFGoe_L1_hTau_24h_R1 **Scan** 21503
**Peptide** AGIGDTPSLEDEAAGHVTQA **Charge** 2 **Spectral Angle** 0.82

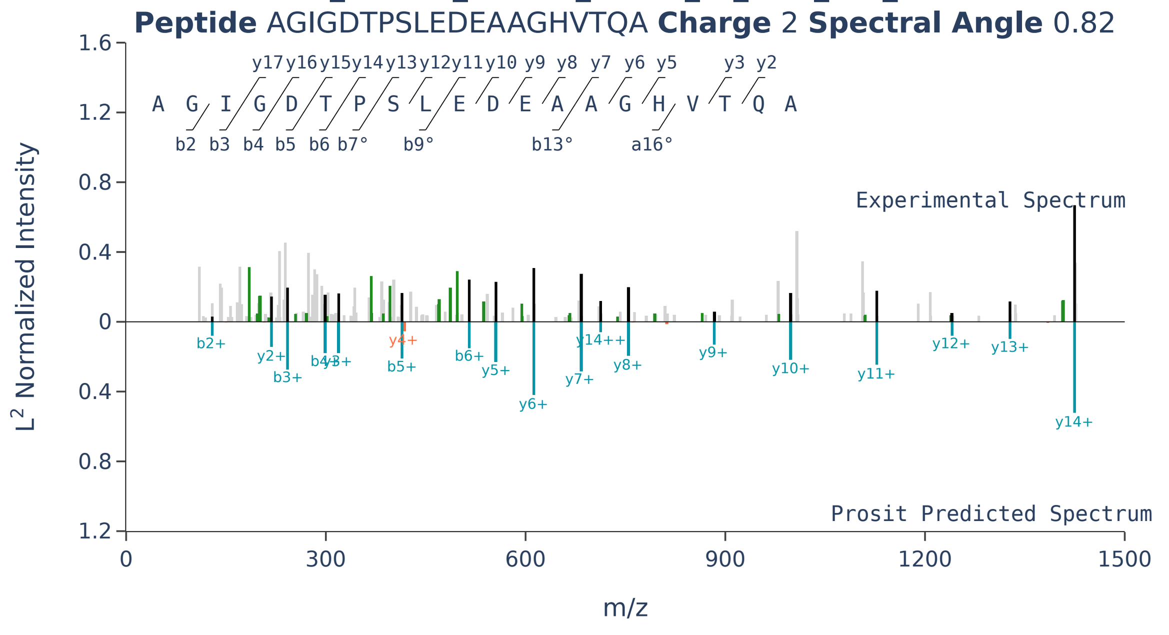


**Source** WSoh_221121_011221_HFGoe_L1_hTau_24h_R2 **Scan** 6983
**Peptide** EAAGHVTQARMVSK **Charge** 3 **Spectral Angle** 0.87

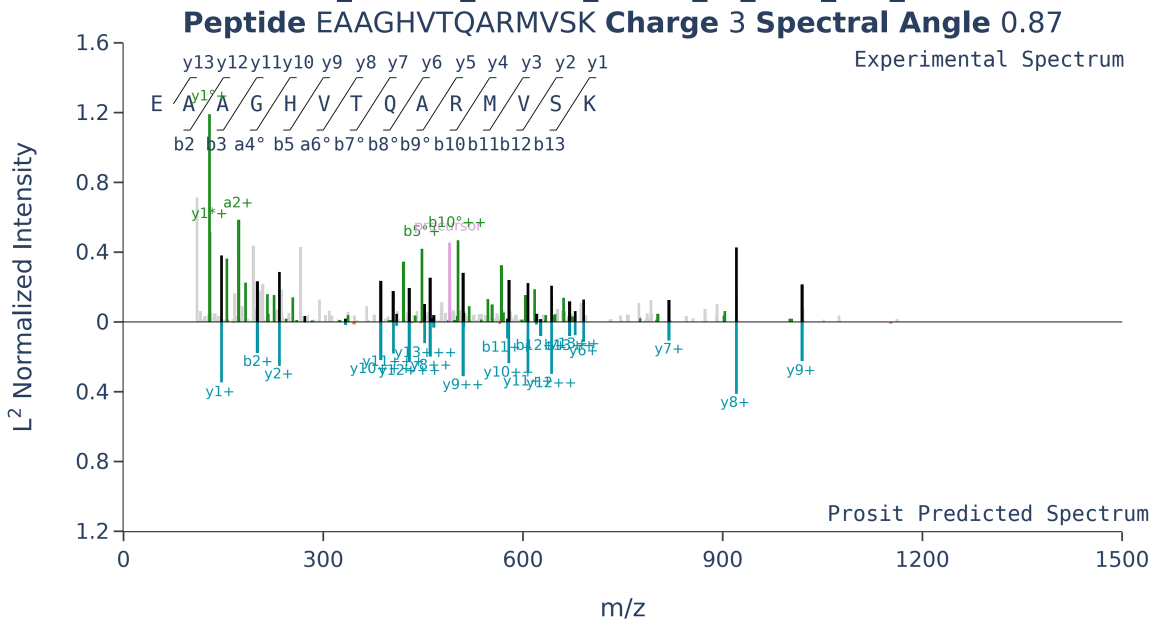


**Source** WSoh_221121_011221_HFGoe_L1_hTau_4h_R2 **Scan** 8232
**Peptide** DNITHVPGGGNKKIETH **Charge** 4 **Spectral Angle** 0.91

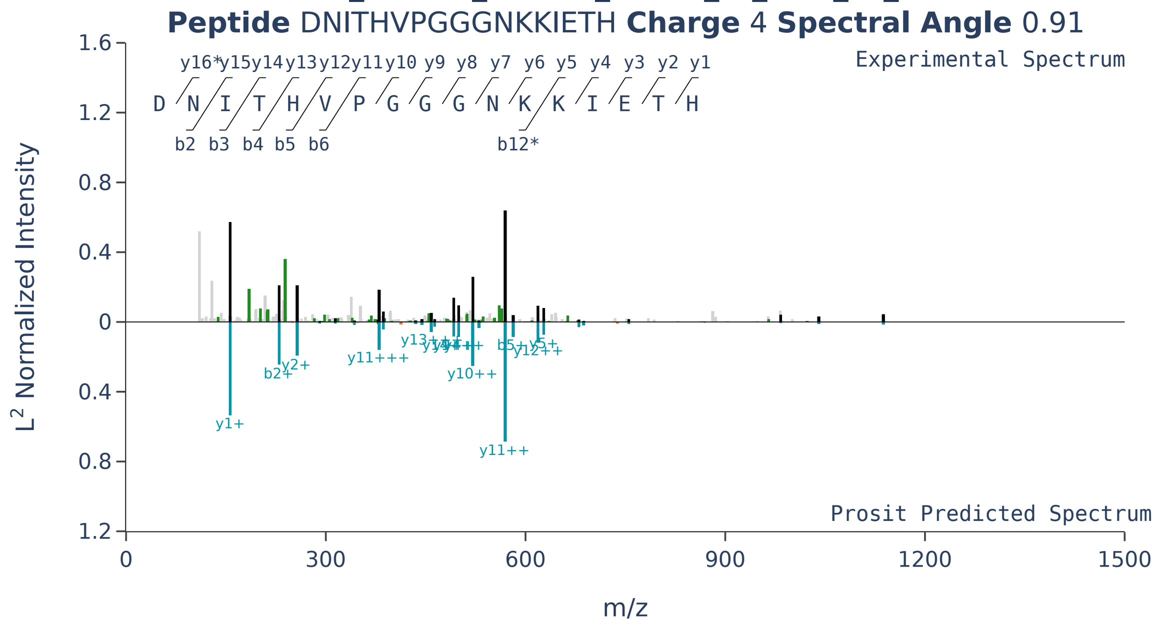


**Source** WSoh_221121_011221_HFGoe_L1_hTau_24h_R2 **Scan** 8797
**Peptide** DGSEEPGSETSDAKSTPTA **Charge** 2 **Spectral Angle** 0.85

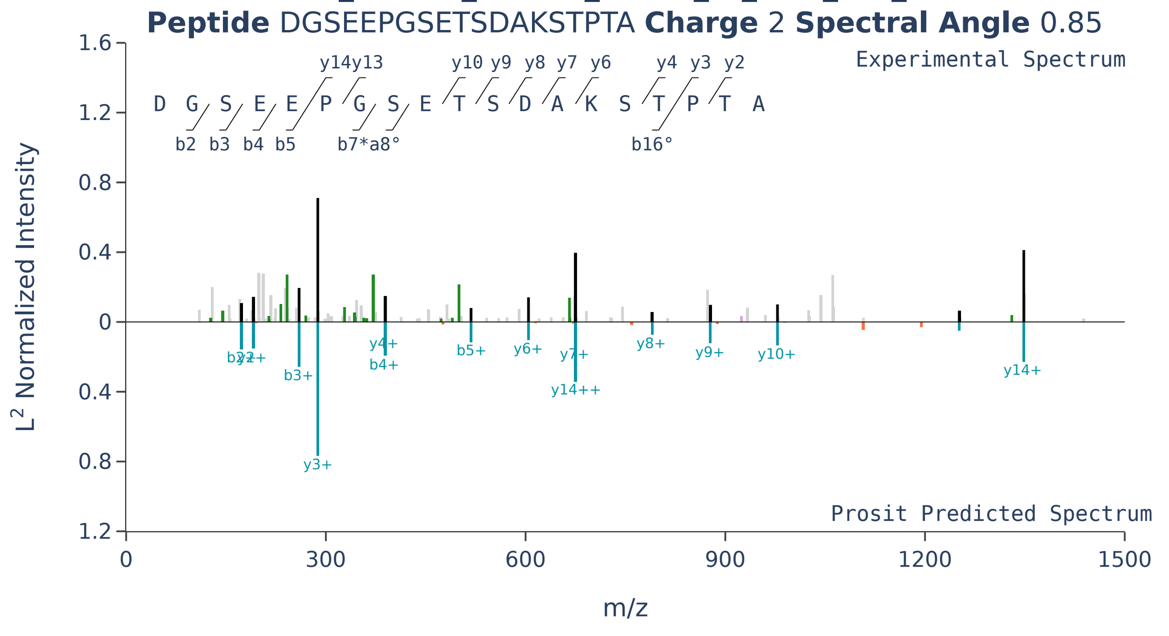

**Source** WSoh_221121_011221_HFGoe_L1_hTau_4h_R2 **Scan** 9303
**Peptide** KDRVQSKIGSLD **Charge** 2 **Spectral Angle** 0.88

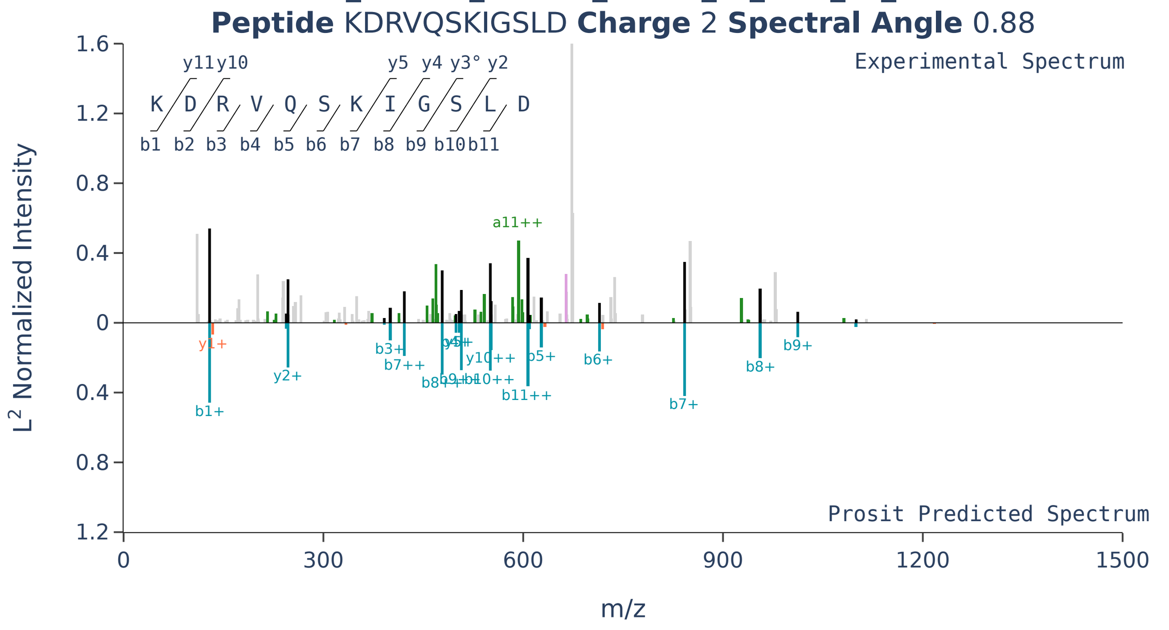


**Source** WSoh_221121_011221_HFGoe_L2_hTau_24h_R2 **Scan** 7778
**Peptide** PGQKGQANATRI **Charge** 2 **Spectral Angle** 0.92

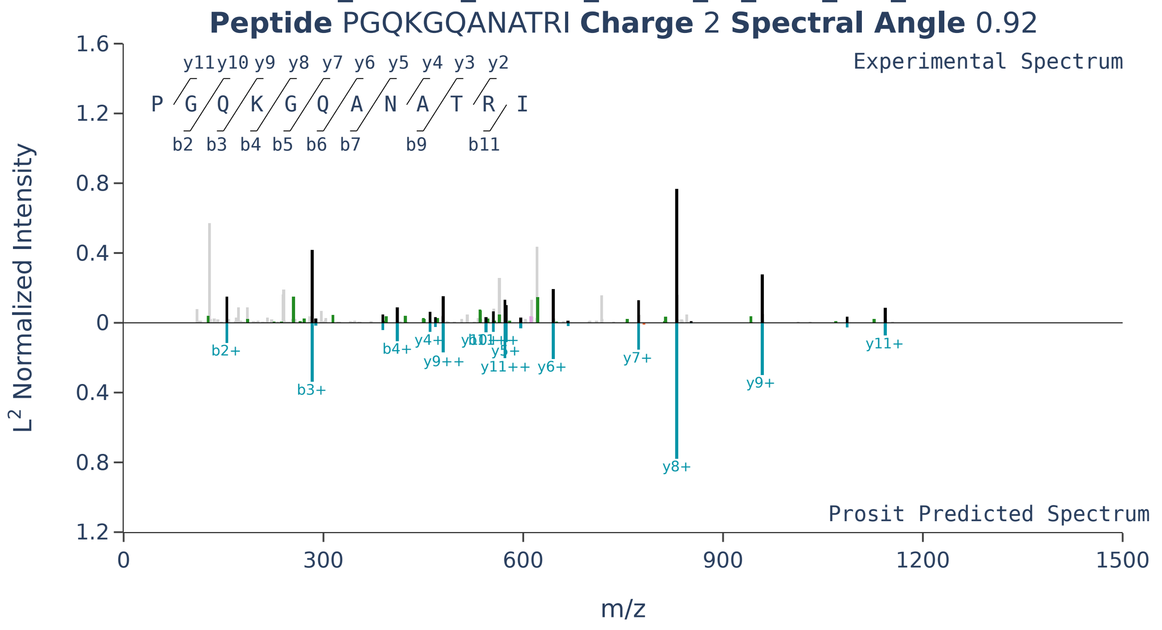


**Source** WSoh_221121_011221_HFGoe_L2_hTau_24h_R2 **Scan** 8237
**Peptide** NITHVPGGGNKKIE **Charge** 3 **Spectral Angle** 0.83

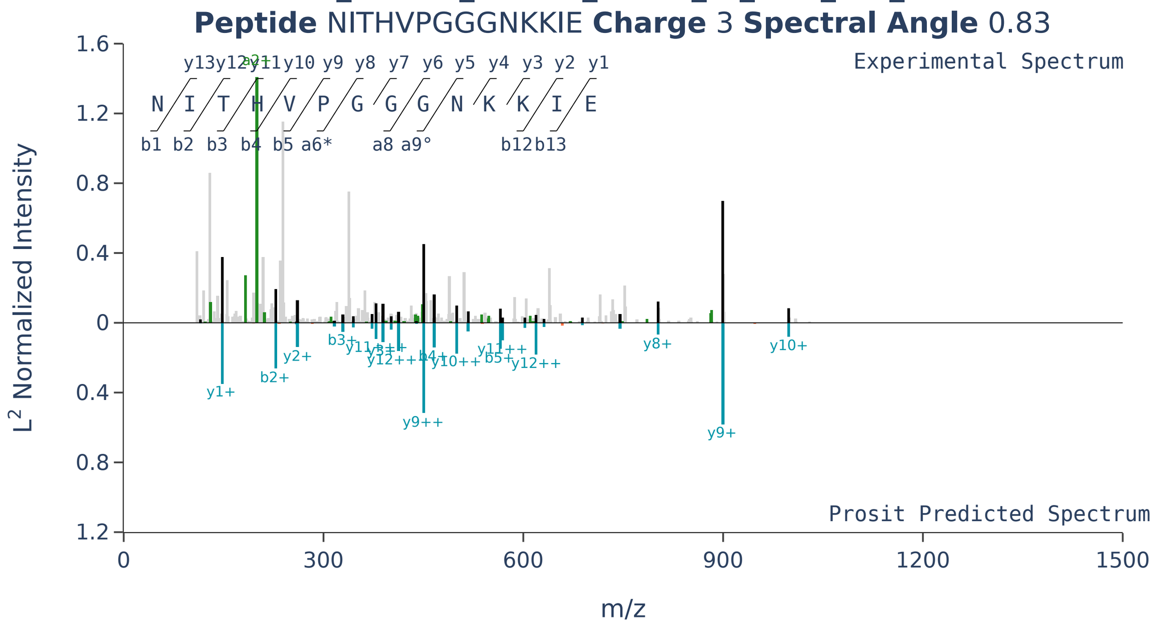


**Source** WSoh_221121_011221_HFGoe_L1_hTau_24h_R1 **Scan** 8351
**Peptide** EDEAAGHVTQARMVSK **Charge** 2 **Spectral Angle** 0.84

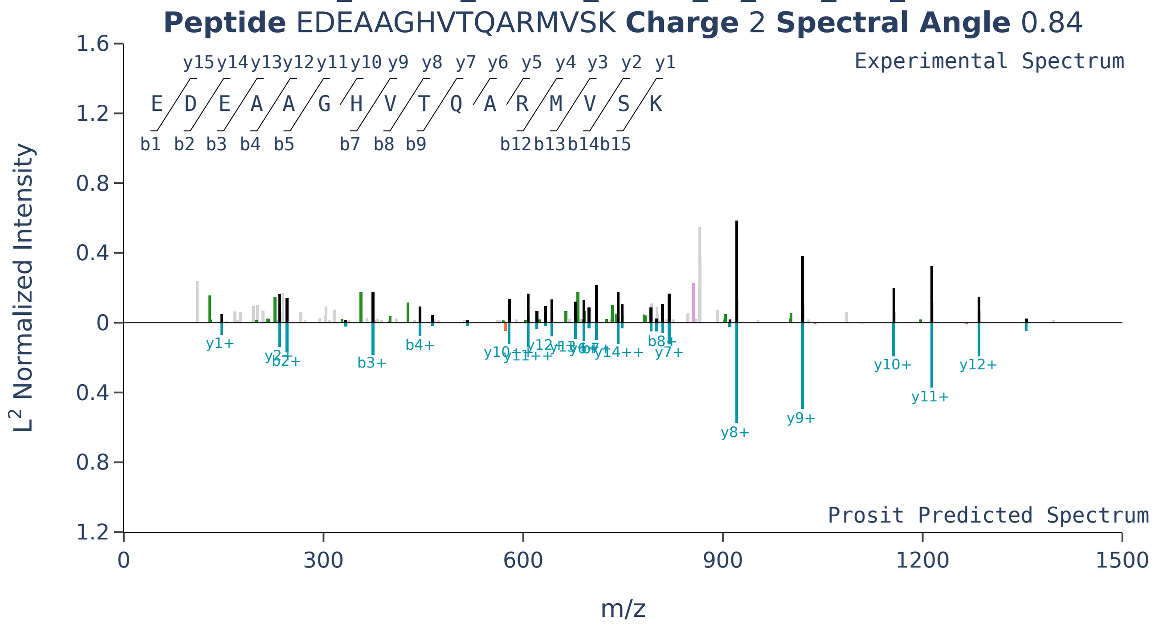


**Source** WSoh_221121_011221_HFGoe_L2_hTau_24h_R2 **Scan** 7383
**Peptide** GSLGNIHHKPGGGQVE **Charge** 2 **Spectral Angle** 0.84

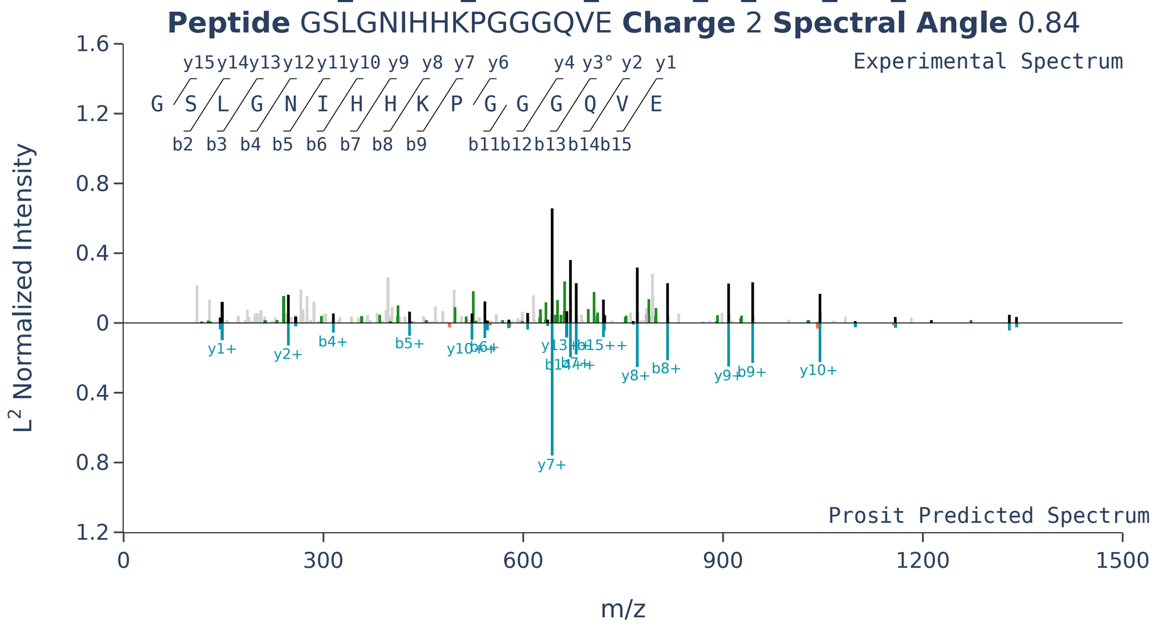


**Source** WSoh_221121_011221_HFGoe_L1_hTau_4h_R1 **Scan** 12019
**Peptide** YKSPVVSGDTSPRHL **Charge** 2 **Spectral Angle** 0.9

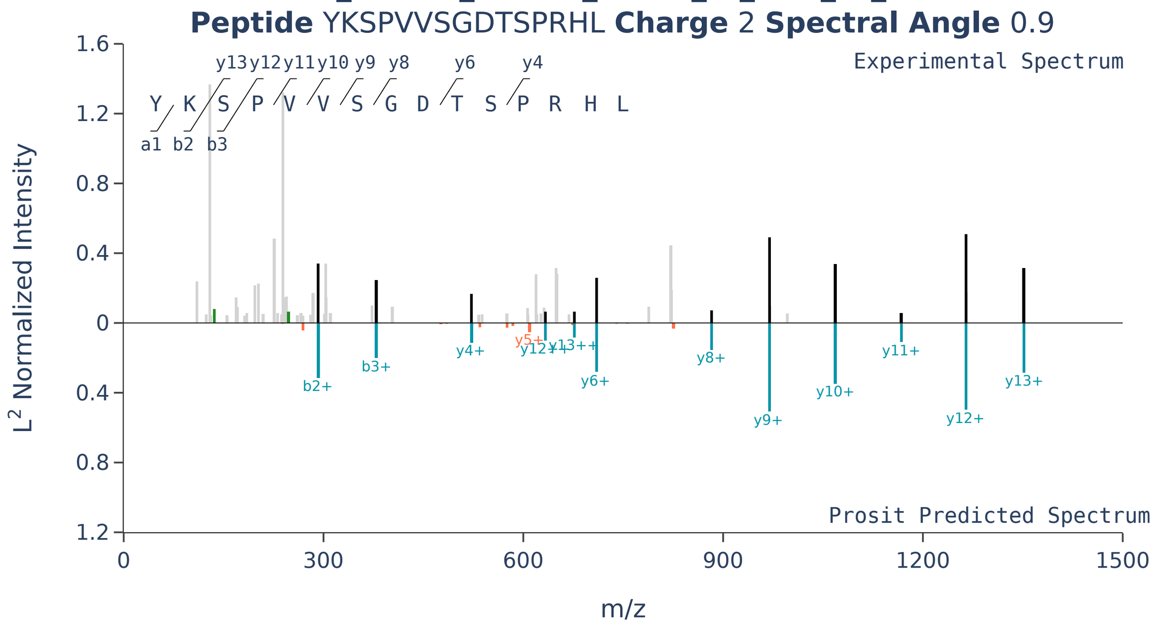


**Source** WSoh_221121_011221_HFGoe_L2_hTau_24h_R2 **Scan** 9500
**Peptide** NKKLDLSNVQSKC **Charge** 2 **Spectral Angle** 0.9

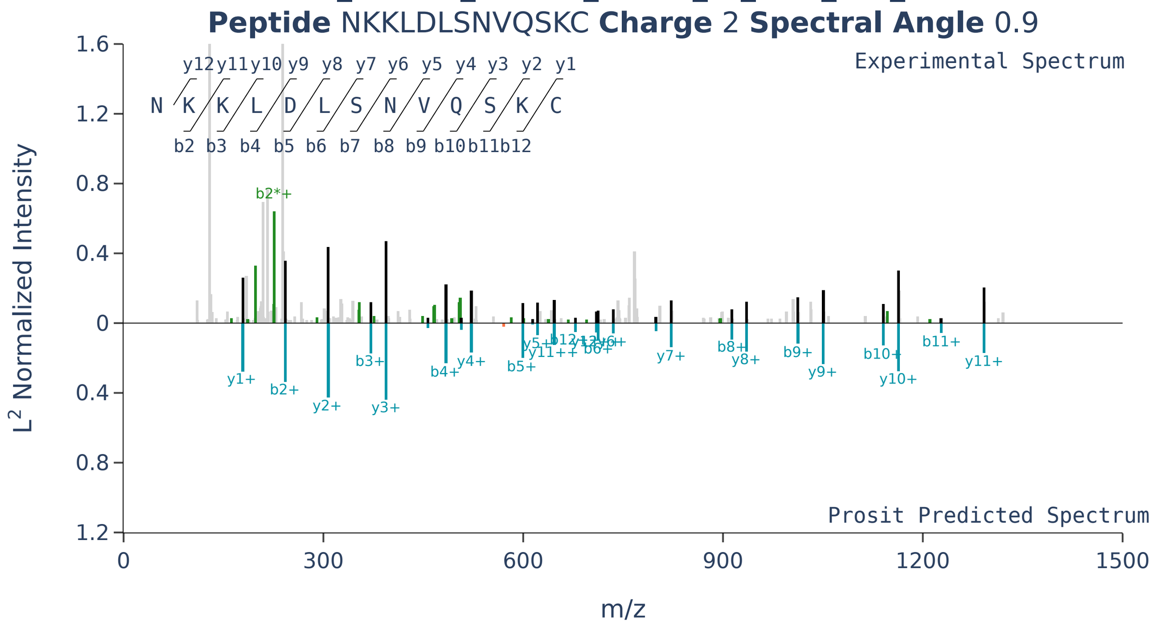


**Source** WSoh_221121_011221_HFGoe_L2_hTau_4h_R2 **Scan** 11138
**Peptide** KCGSKDNIKHVPGGGSVQL **Charge** 3 **Spectral Angle** 0.86

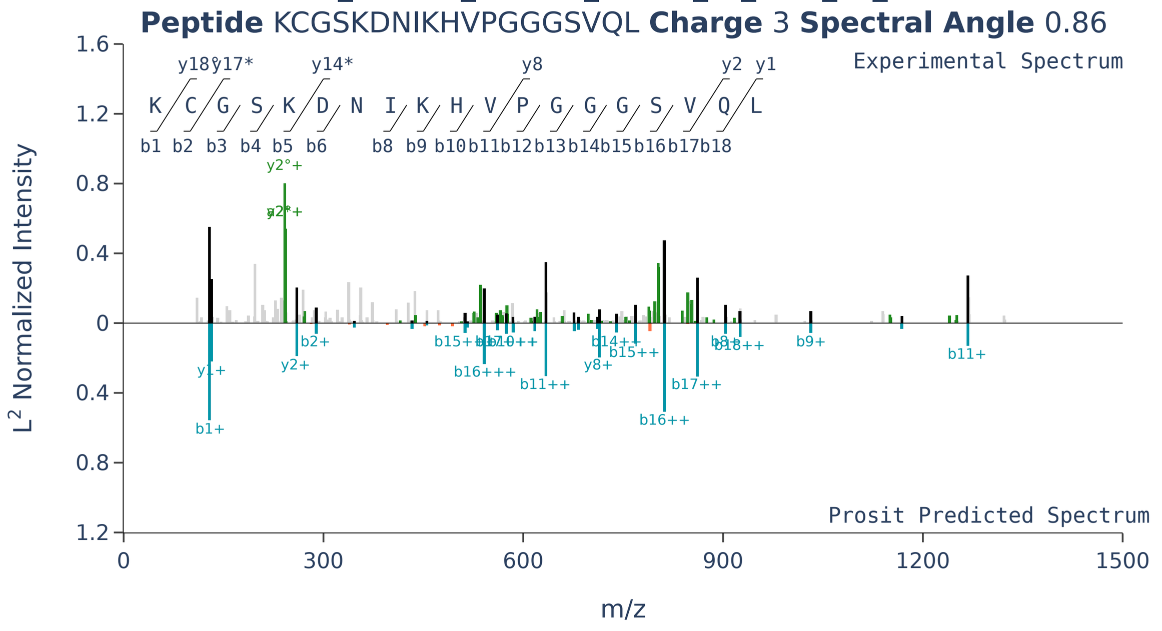


**Source** WSoh_221121_011221_HFGoe_L1_hTau_24h_R2 **Scan** 12594
**Peptide** LDNITHVPGGGNKKI **Charge** 2 **Spectral Angle** 0.88

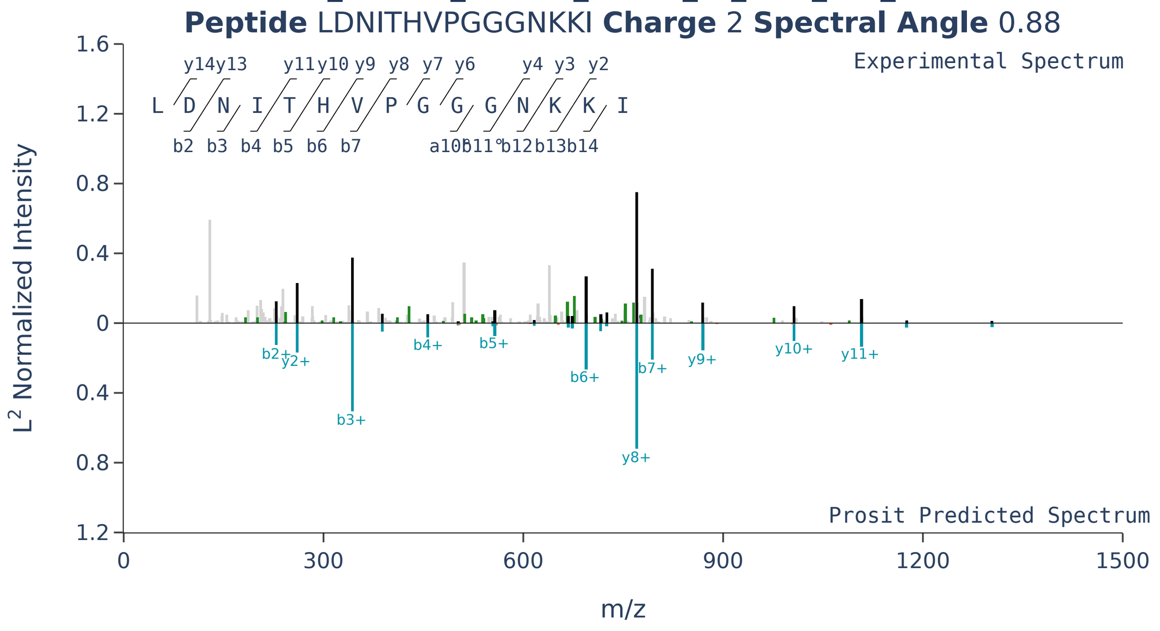


**Source** WSoh_221121_011221_HFGoe_L2_hTau_2h_R2 **Scan** 10750
**Peptide** PGGGQVEVKSEKL **Charge** 2 **Spectral Angle** 0.87

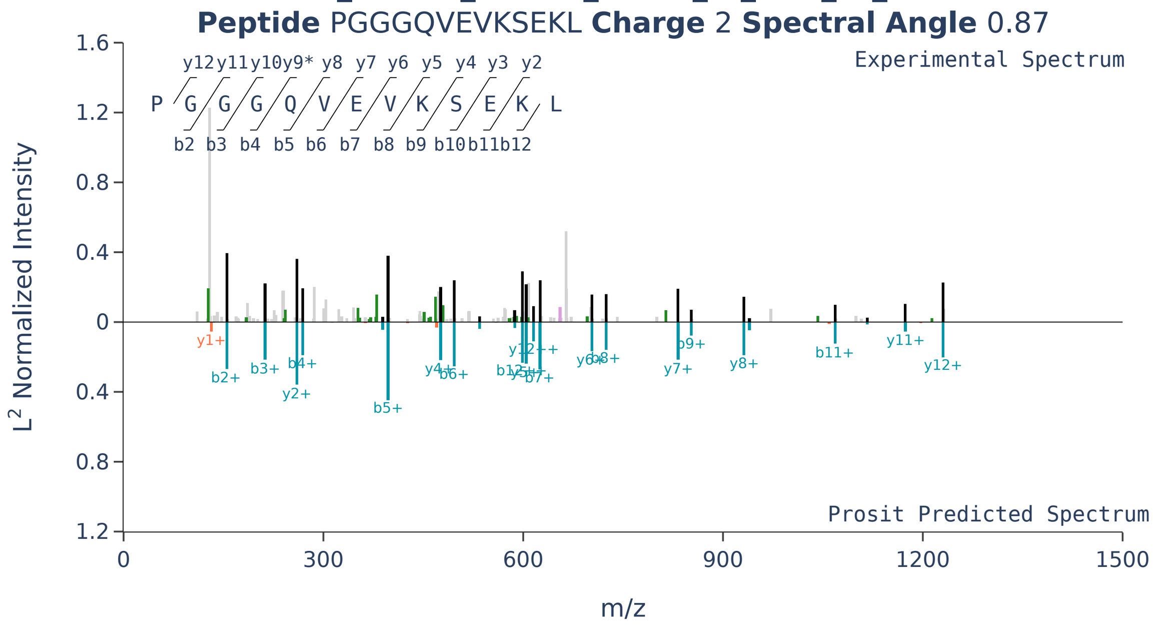


**Source** WSoh_221121_011221_HFGoe_L1_hTau_4h_R2 **Scan** 12774
**Peptide** KDRVQSKL **Charge** 2 **Spectral Angle** 0.91

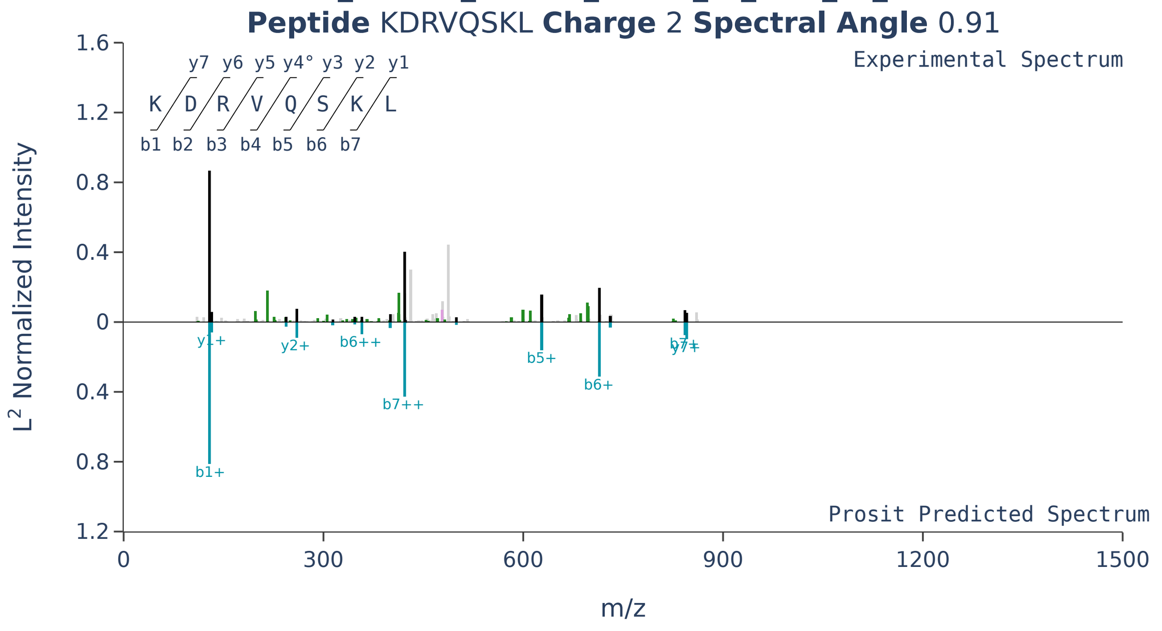


**Source** WSoh_221121_011221_HFGoe_L1_hTau_24h_R1 **Scan** 15081
**Peptide** SGDTSPRHLSNVSSTGSIDMVD **Charge** 3 **Spectral Angle** 0.8

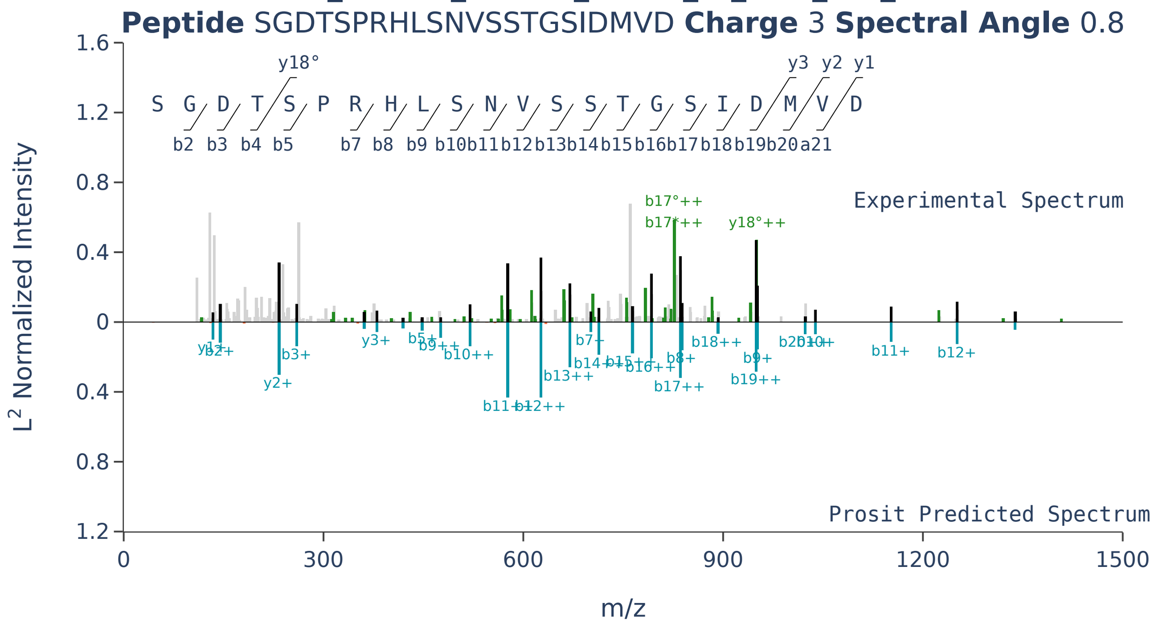


**Source** WSoh_221121_011221_HFGoe_L1_hTau_4h_R1 **Scan** 12040
**Peptide** AQPHTEIPEGTTAE **Charge** 2 **Spectral Angle** 0.91

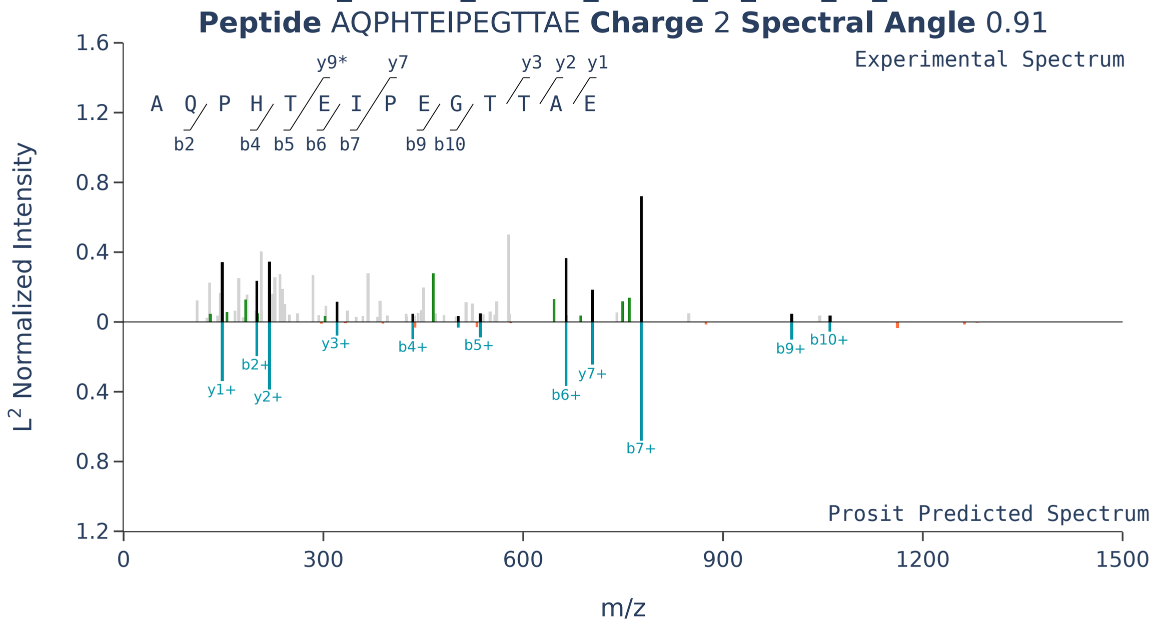


**Source** WSoh_221121_011221_HFGoe_L1_hTau_4h_R2 **Scan** 20132
**Peptide** AEPRQEFEVMEDHAGTY **Charge** 3 **Spectral Angle** 0.83

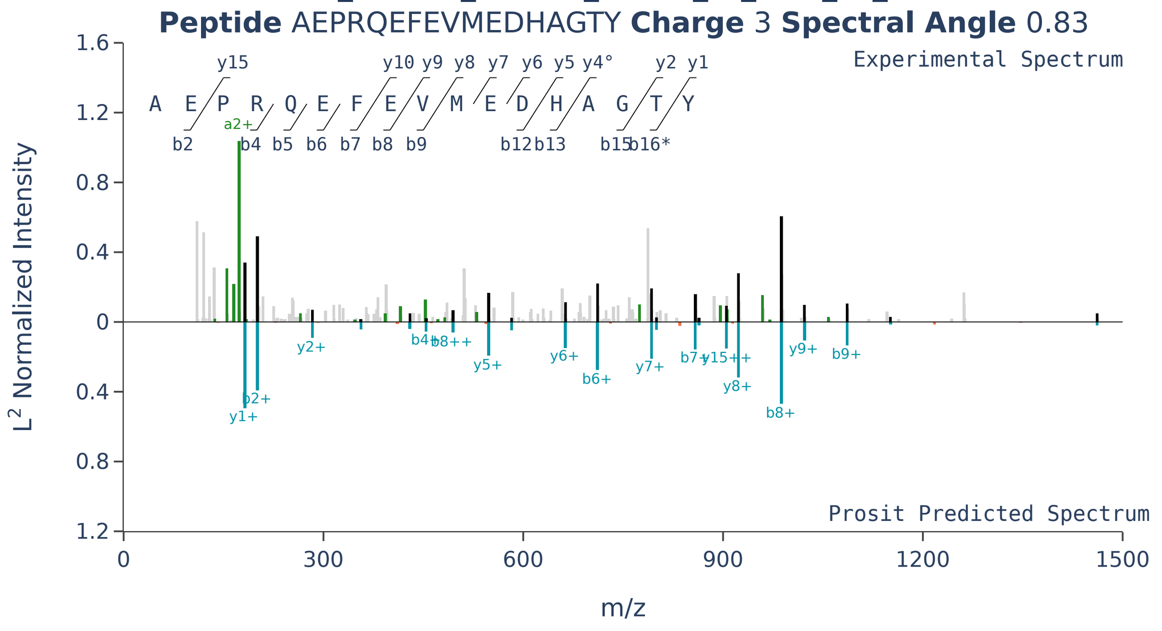


**Source** WSoh_221121_011221_HFGoe_L2_hTau_24h_R2 **Scan** 18288
**Peptide** KSRLQTAPVPMPDLKNVKS **Charge** 3 **Spectral Angle** 0.86

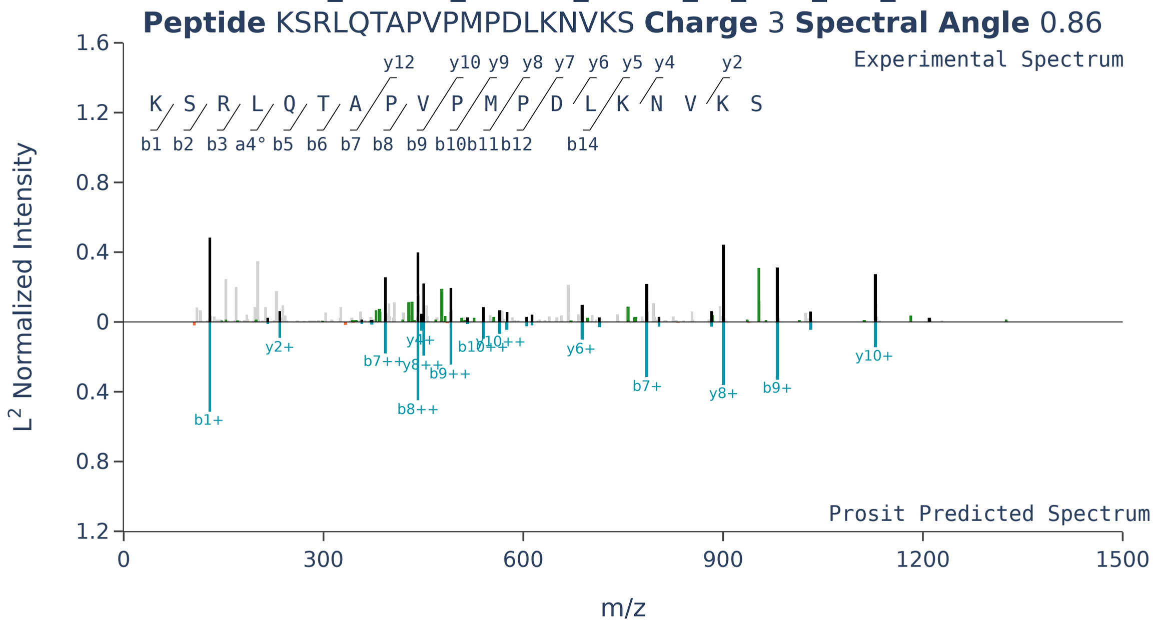

**Source** WSoh_221121_011221_HFGoe_L1_hTau_24h_R2 **Scan** 16747
**Peptide** PRHLSNVSSTGSIDMVD **Charge** 3 **Spectral Angle** 0.89

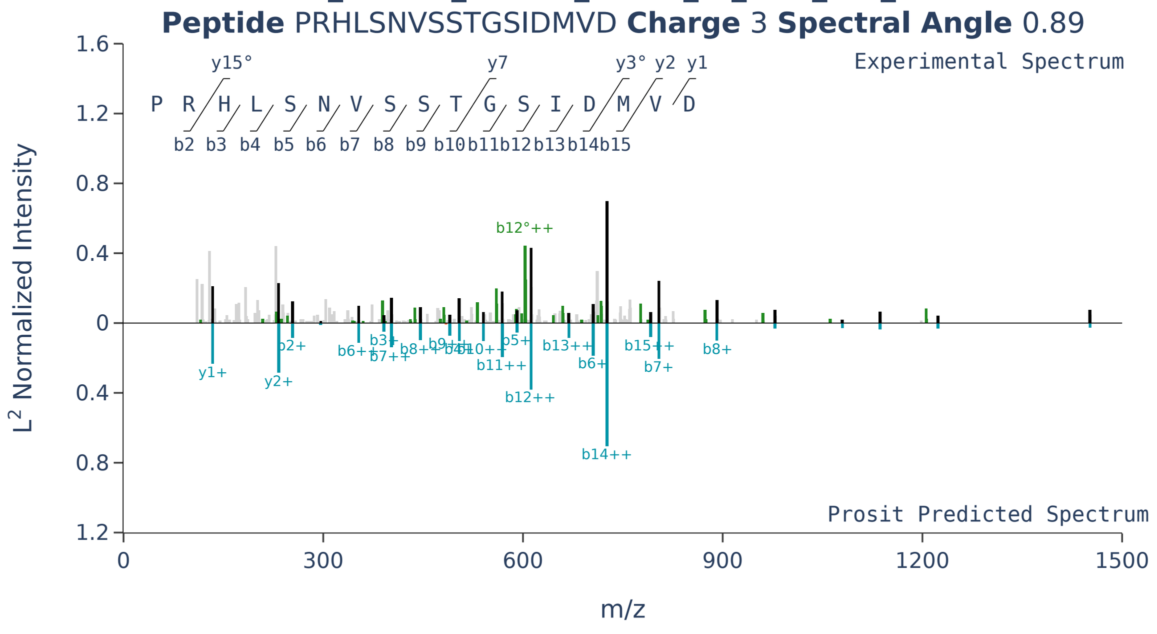


**Source** WSoh_221121_011221_HFGoe_L1_hTau_24h_R1 **Scan** 15165
**Peptide** TDAGLKESPLQ **Charge** 2 **Spectral Angle** 0.88

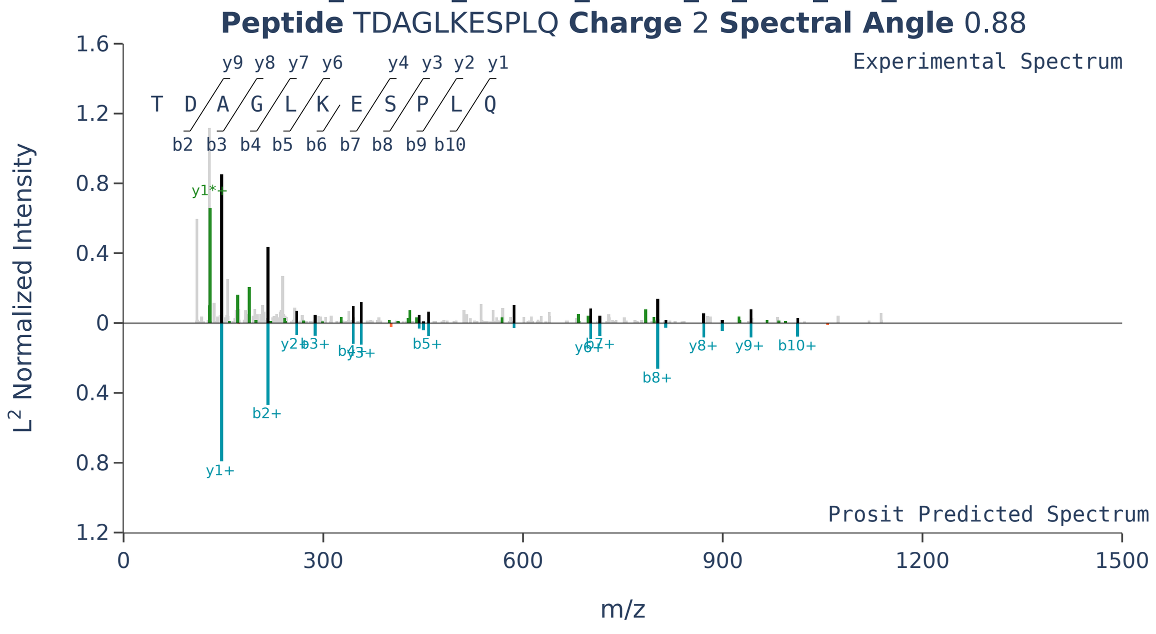


**Source** WSoh_221121_011221_HFGoe_L2_hTau_4h_R1 **Scan** 19671
**Peptide** AEPRQEFEVMEDHAGTYG **Charge** 3 **Spectral Angle** 0.86

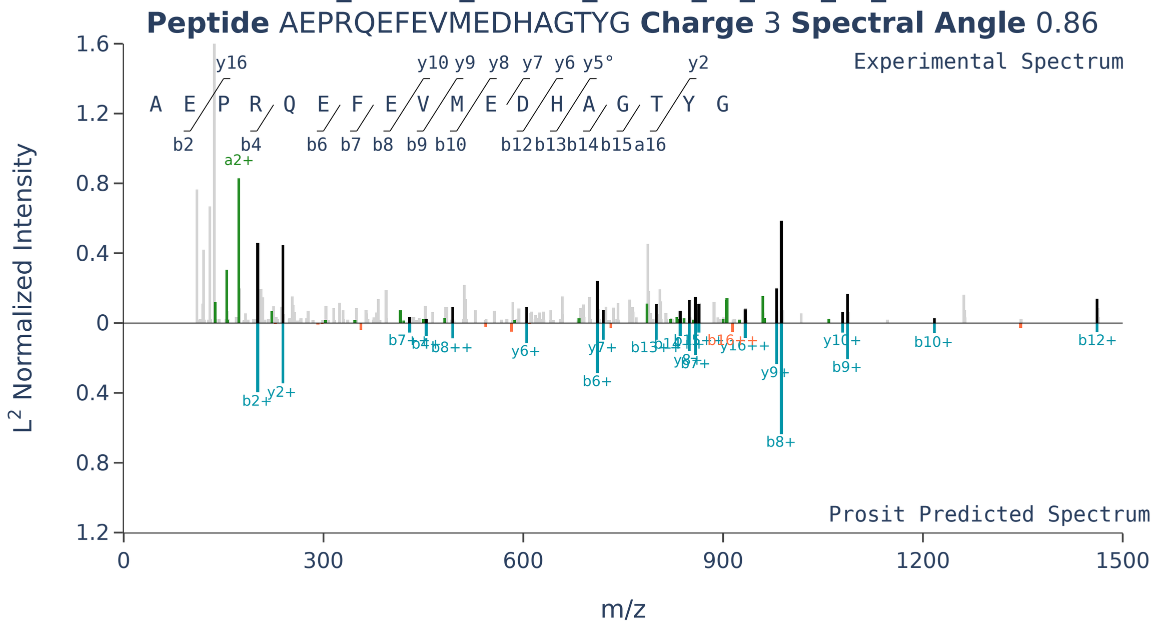


**Source** WSoh_221121_011221_HFGoe_L2_hTau_24h_R2 **Scan** 16749
**Peptide** SKDNIKHVPGGGSVQIVYKPVD **Charge** 3 **Spectral Angle** 0.89

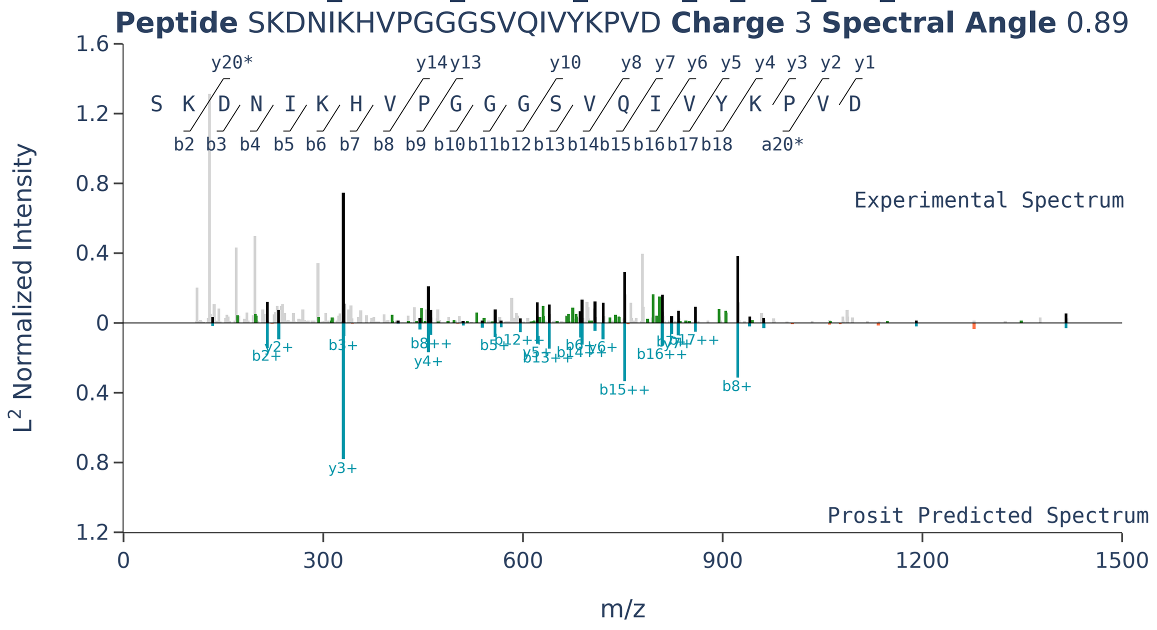


**Source** WSoh_221121_011221_HFGoe_L1_hTau_24h_R1 **Scan** 6271
**Peptide** PPTREPKKV **Charge** 2 **Spectral Angle** 0.84

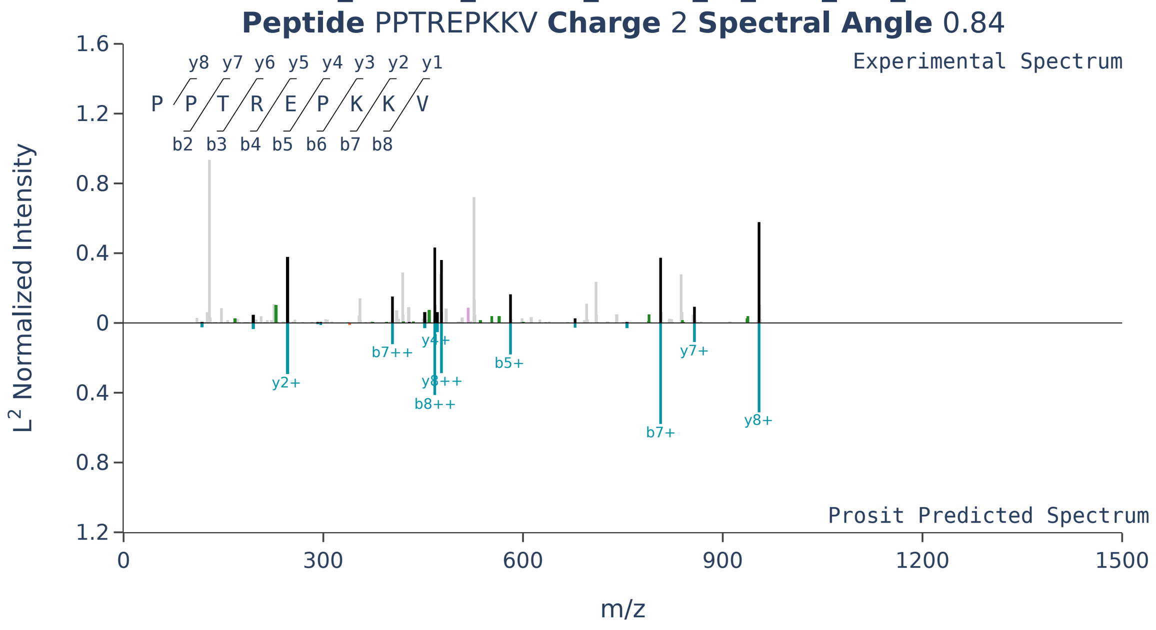


**Source** WSoh_221121_011221_HFGoe_L1_hTau_24h_R1 **Scan** 7962
**Peptide** NLKHQPGGGKVQIINK **Charge** 2 **Spectral Angle** 0.87

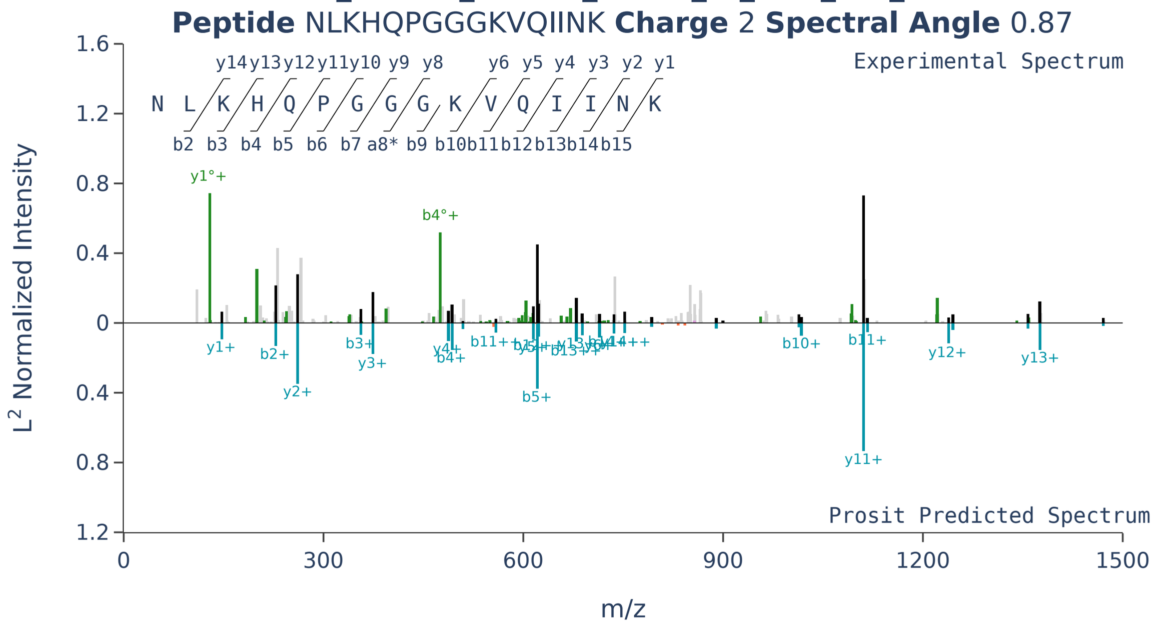


**Source** WSoh_221121_011221_HFGoe_L1_hTau_2h_R2 **Scan** 15750
**Peptide** KIGSLDNITHVPGGGNKKI **Charge** 3 **Spectral Angle** 0.94

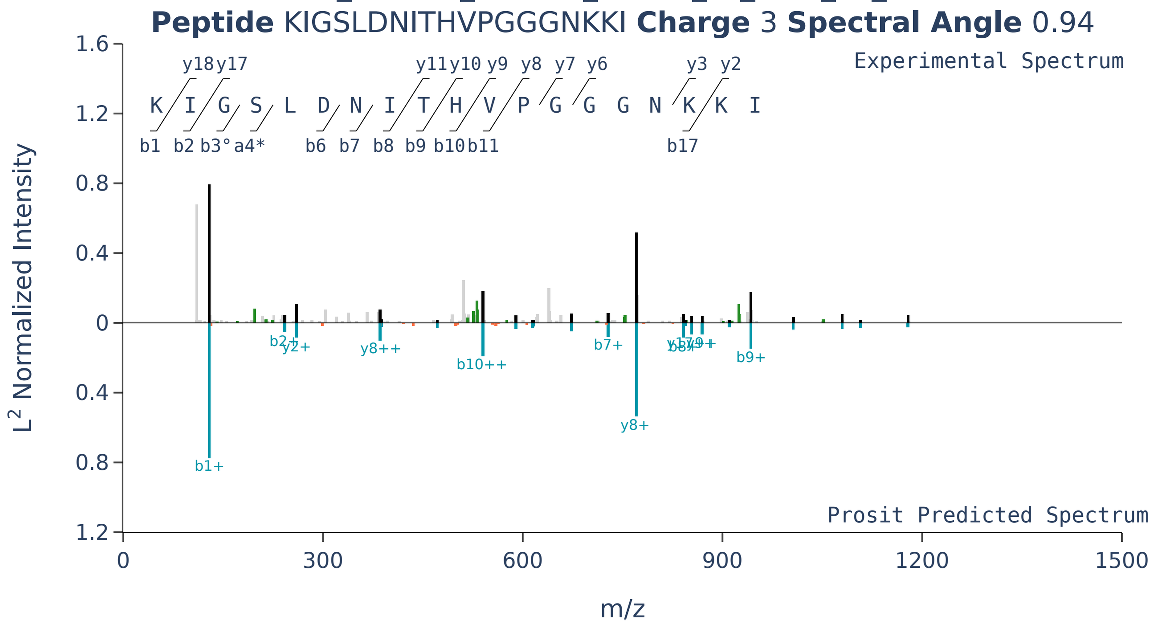


**Source** WSoh_221121_011221_HFGoe_L1_hTau_4h_R2 **Scan** 9769
**Peptide** SPVVSGDTSPRHLSN **Charge** 2 **Spectral Angle** 0.92

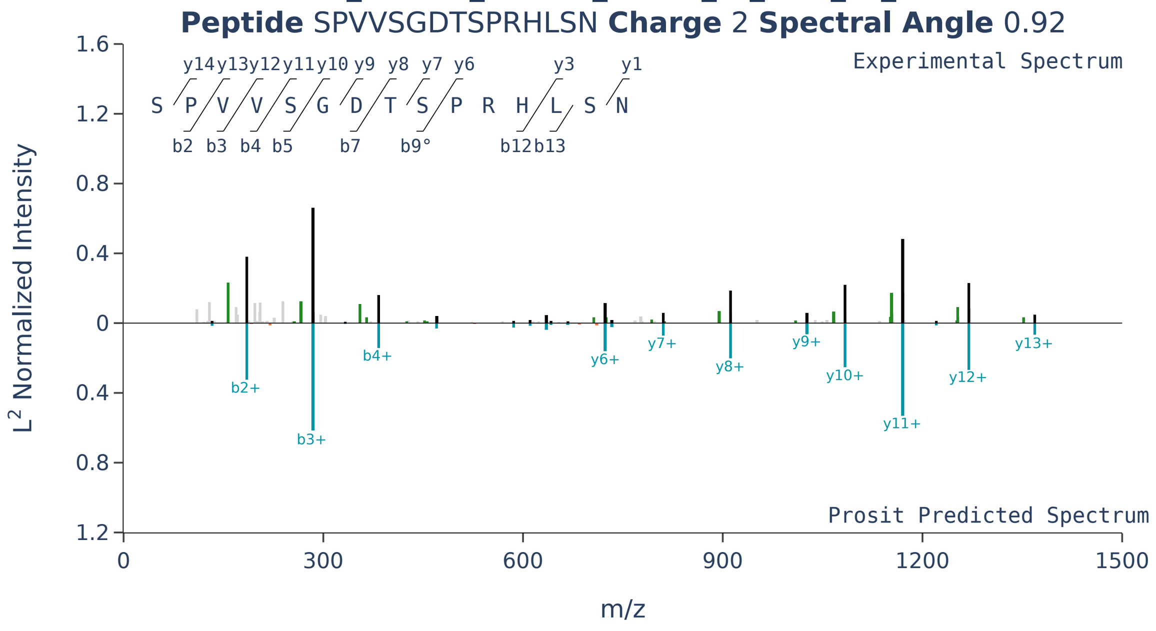


**Source** WSoh_221121_011221_HFGoe_L1_hTau_24h_R1 **Scan** 19531
**Peptide** GAEIVYKSPVVS **Charge** 2 **Spectral Angle** 0.82

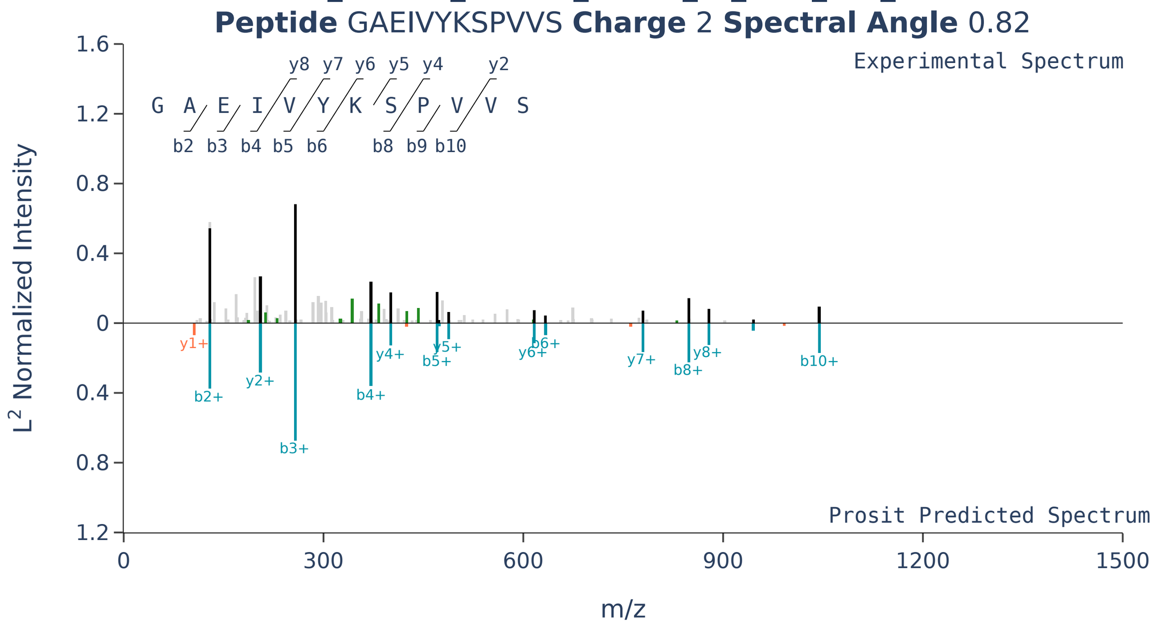


**Source** WSoh_221121_011221_HFGoe_L1_hTau_24h_R1 **Scan** 17180
**Peptide** TDHGAEIVYKSPVVSGDTSPRHL **Charge** 4 **Spectral Angle** 0.86

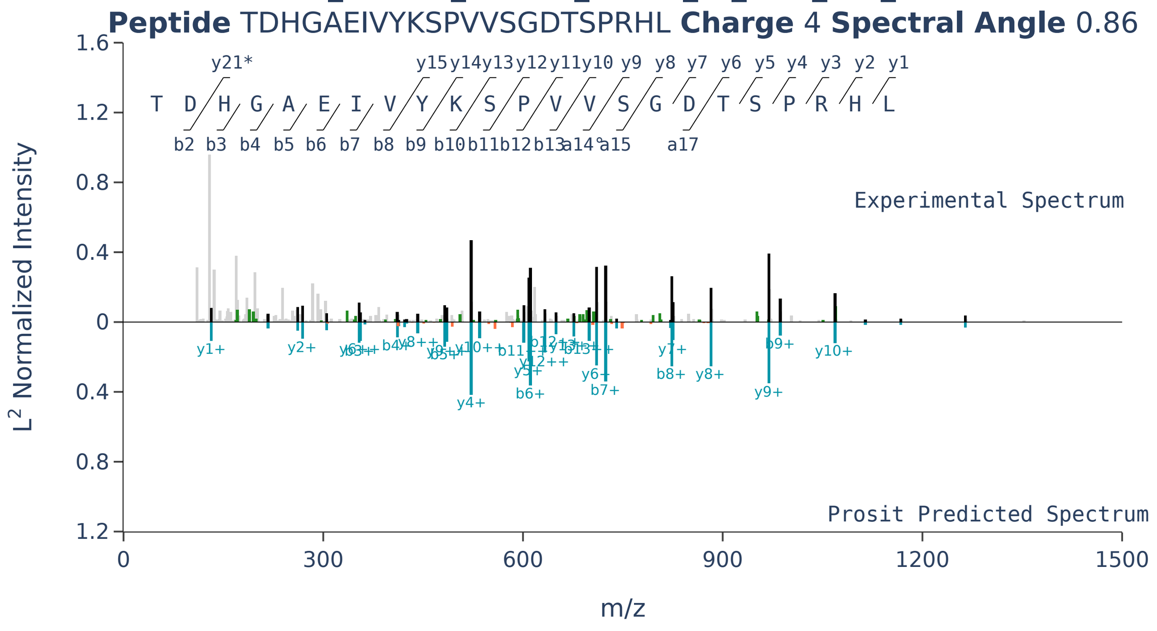


**Source** WSoh_221121_011221_HFGoe_L2_hTau_24h_R1 **Scan** 20328
**Peptide** DVTAPLVDEGAPGKQAA **Charge** 2 **Spectral Angle** 0.9

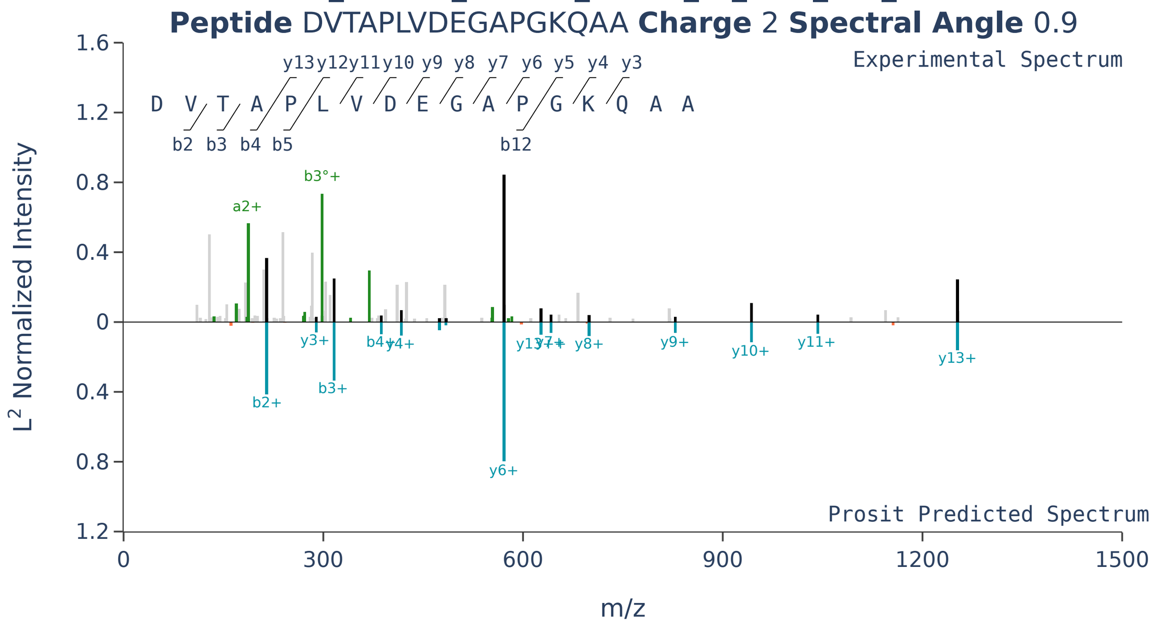


**Source** WSoh_221121_011221_HFGoe_L1_hTau_4h_R2 **Scan** 20969
**Peptide** KHVPGGGSVQIVYKPVDL **Charge** 3 **Spectral Angle** 0.84

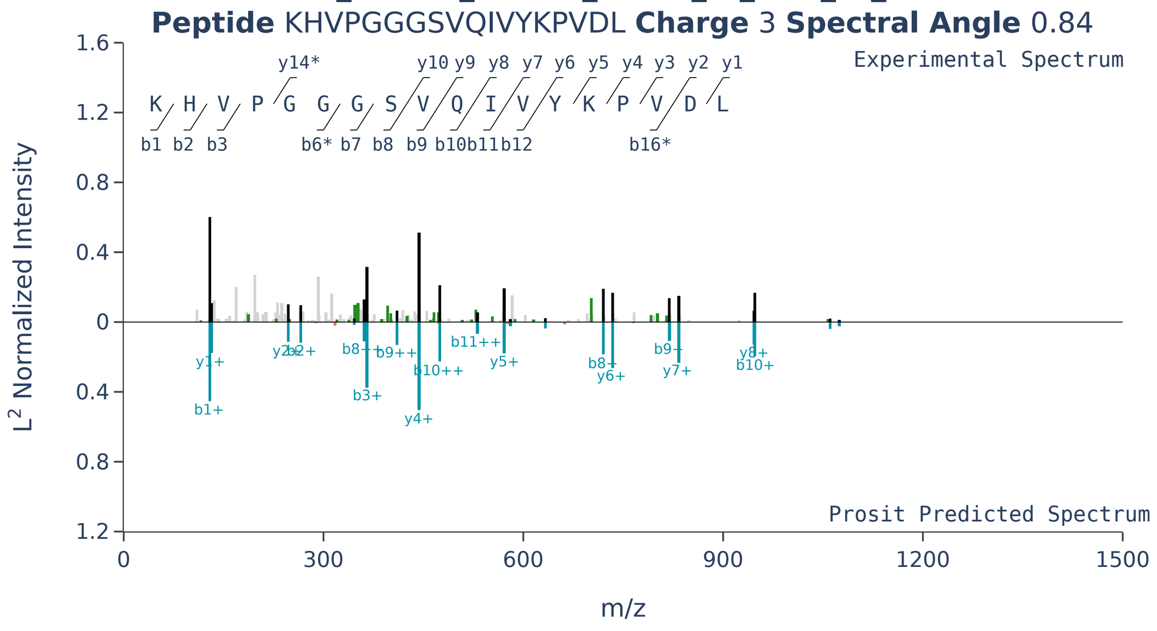


**Source** WSoh_221121_011221_HFGoe_L2_hTau_24h_R1 **Scan** 21977
**Peptide** APVPMPDLKNVKSKI **Charge** 2 **Spectral Angle** 0.89

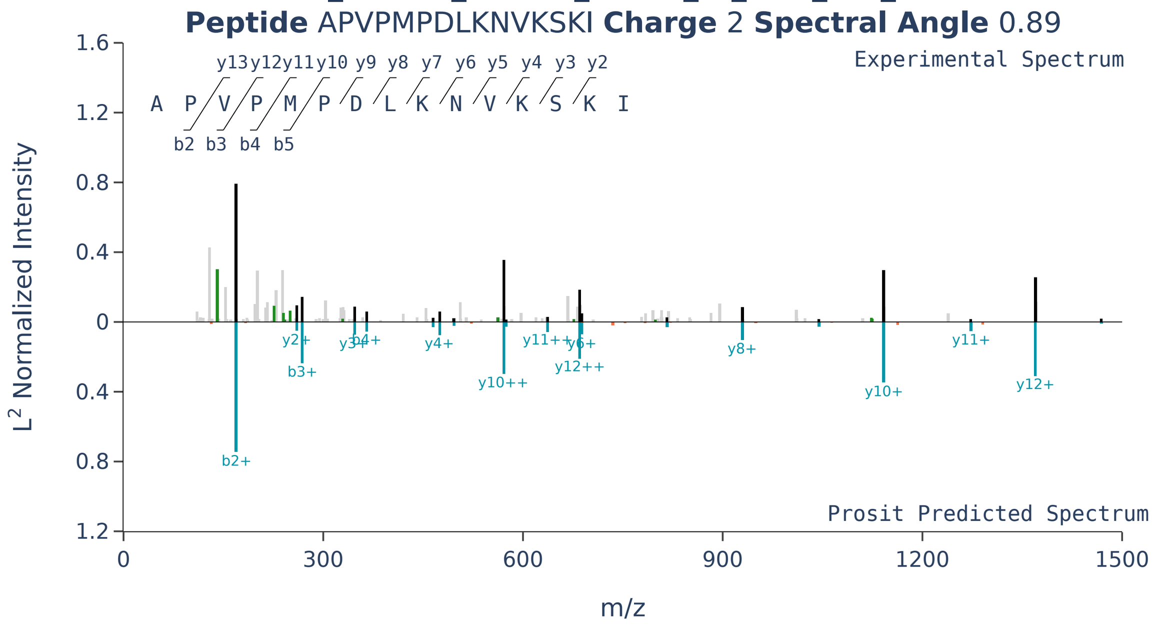


**Source** WSoh_221121_011221_HFGoe_L1_hTau_24h_R1 **Scan** 20673
**Peptide** EAGIGDTPSLE **Charge** 2 **Spectral Angle** 0.94

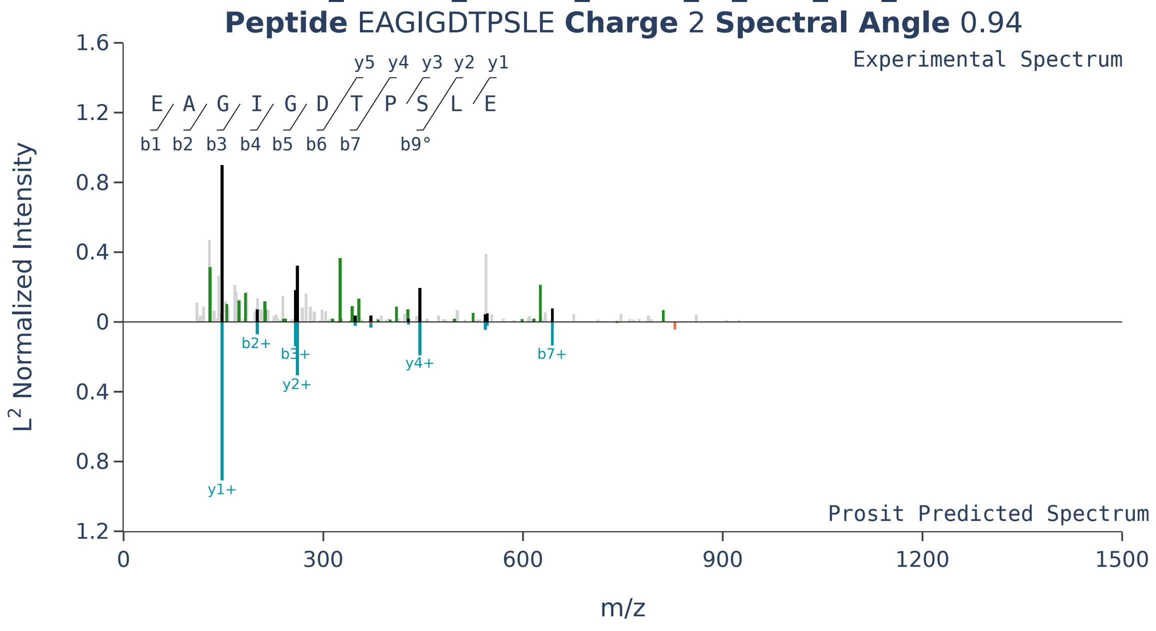


**Source** WSoh_221121_011221_HFGoe_L1_hTau_4h_R1 **Scan** 8053
**Peptide** GSEEPGSETSDAKSTPTAE **Charge** 2 **Spectral Angle** 0.84

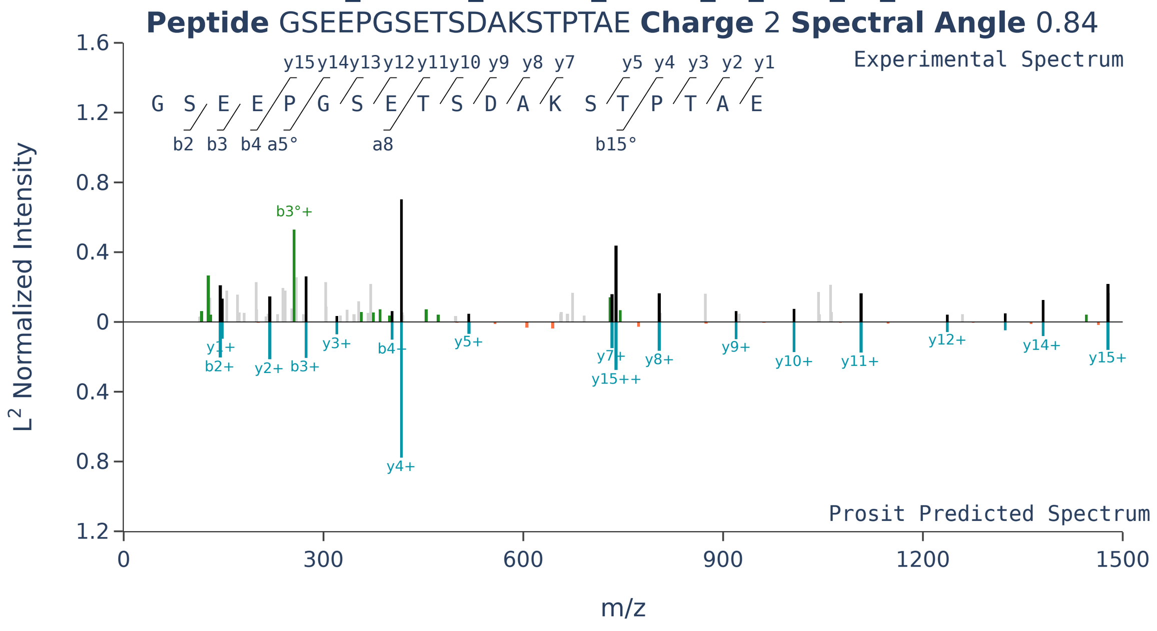

**Source** WSoh_221121_011221_HFGoe_L2_hTau_24h_R2 **Scan** 10895
**Peptide** YGLGDRKDQGGY **Charge** 3 **Spectral Angle** 0.89

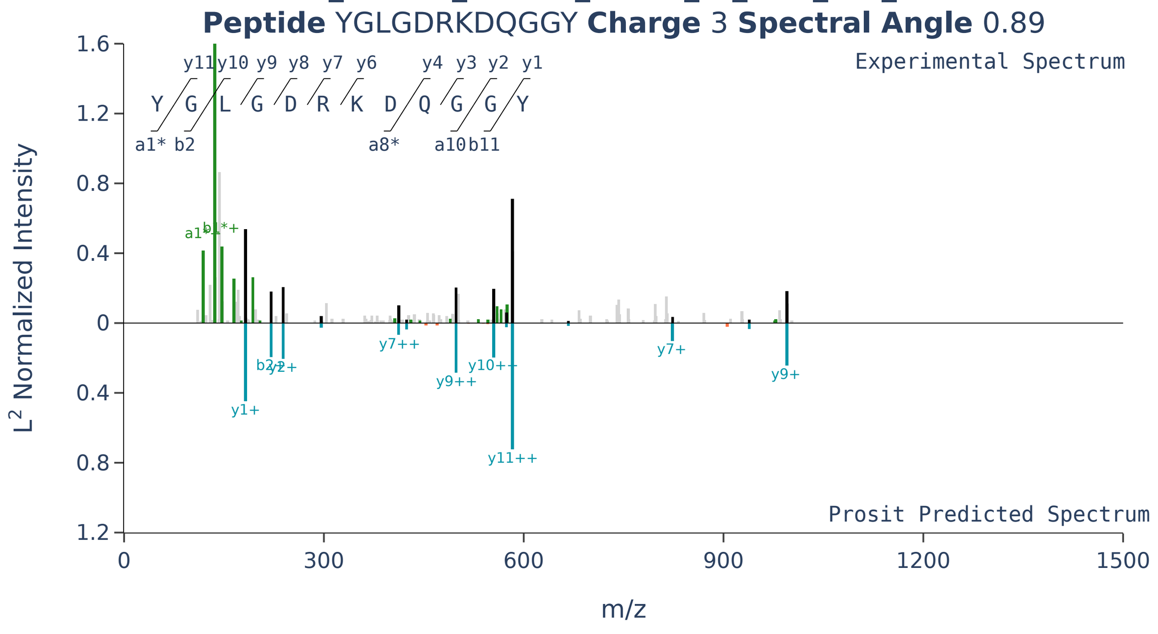


**Source** WSoh_221121_011221_HFGoe_L1_hTau_24h_R2 **Scan** 7790
**Peptide** SKDNIKHVPGGGSV **Charge** 2 **Spectral Angle** 0.93

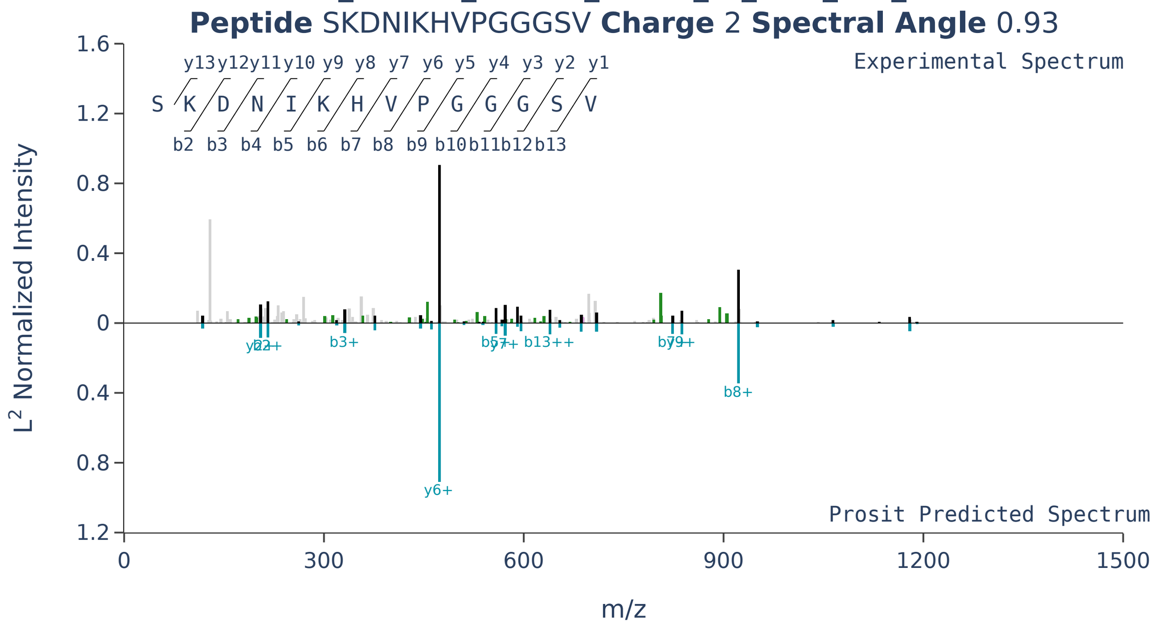


**Source** WSoh_221121_011221_HFGoe_L2_hTau_4h_R2 **Scan** 15131
**Peptide** KTDHGAEIVYKSPVVSGDTSPRHL **Charge** 3 **Spectral Angle** 0.81

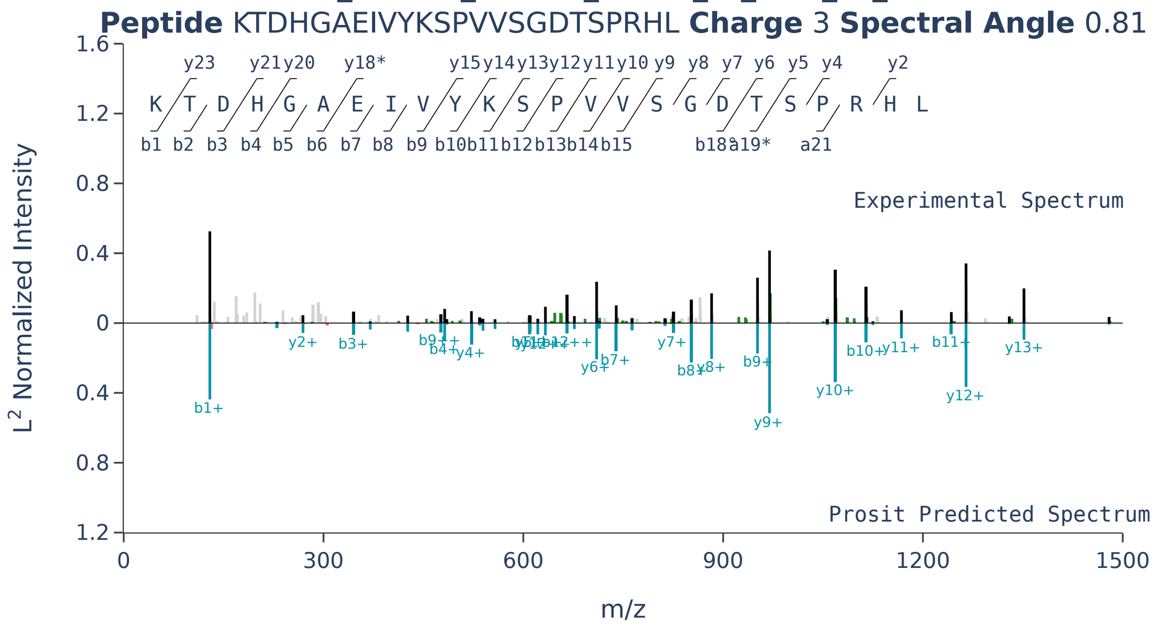


**Source** WSoh_221121_011221_HFGoe_L1_hTau_24h_R2 **Scan** 7998
**Peptide** GSKDNIKHVPGGGSV **Charge** 2 **Spectral Angle** 0.95

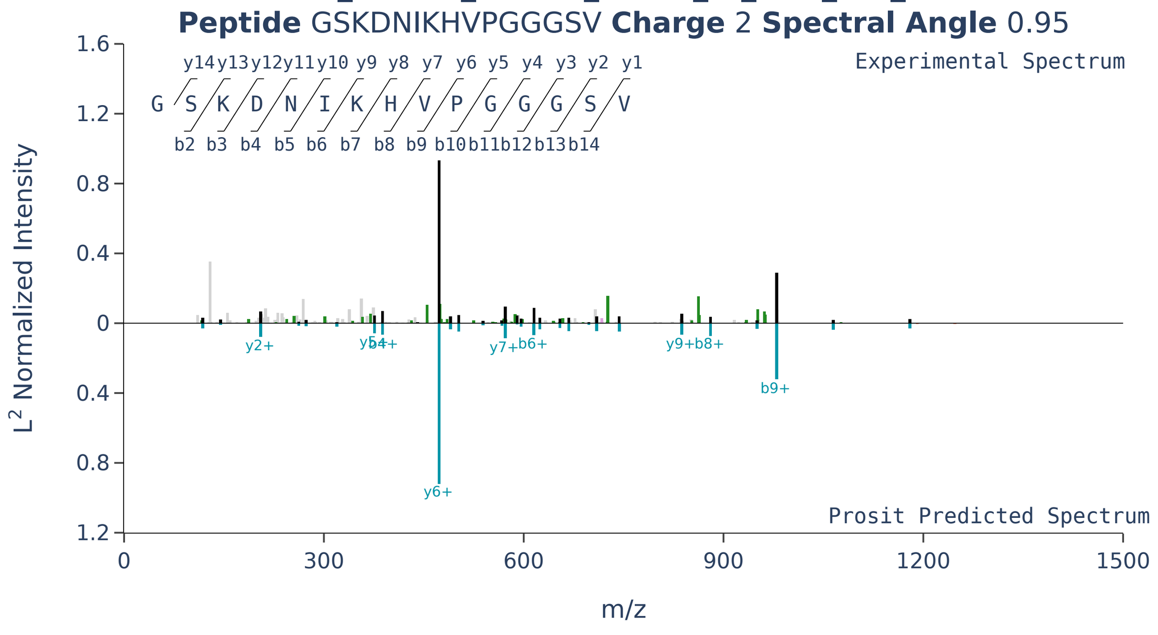


**Source** WSoh_221121_011221_HFGoe_L1_hTau_24h_R2 **Scan** 7723
**Peptide** LKHQPGGGKVQIINK **Charge** 2 **Spectral Angle** 0.9

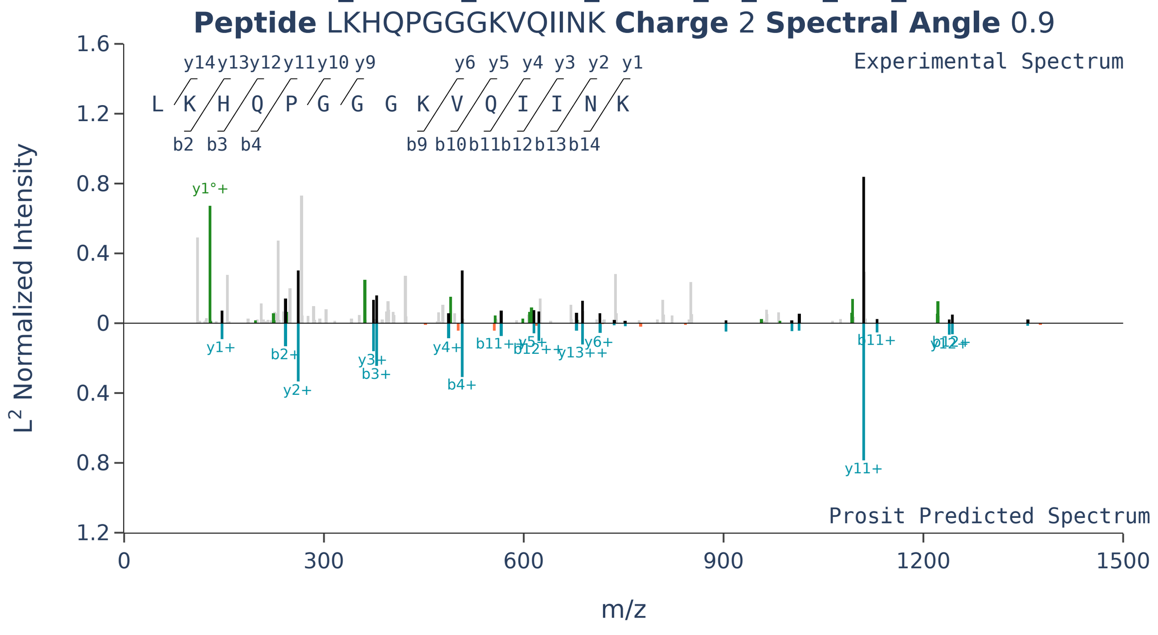


**Source** WSoh_221121_011221_HFGoe_L1_hTau_24h_R1 **Scan** 15088
**Peptide** KTDHGAEIVYKSPVVSGDTS **Charge** 2 **Spectral Angle** 0.82

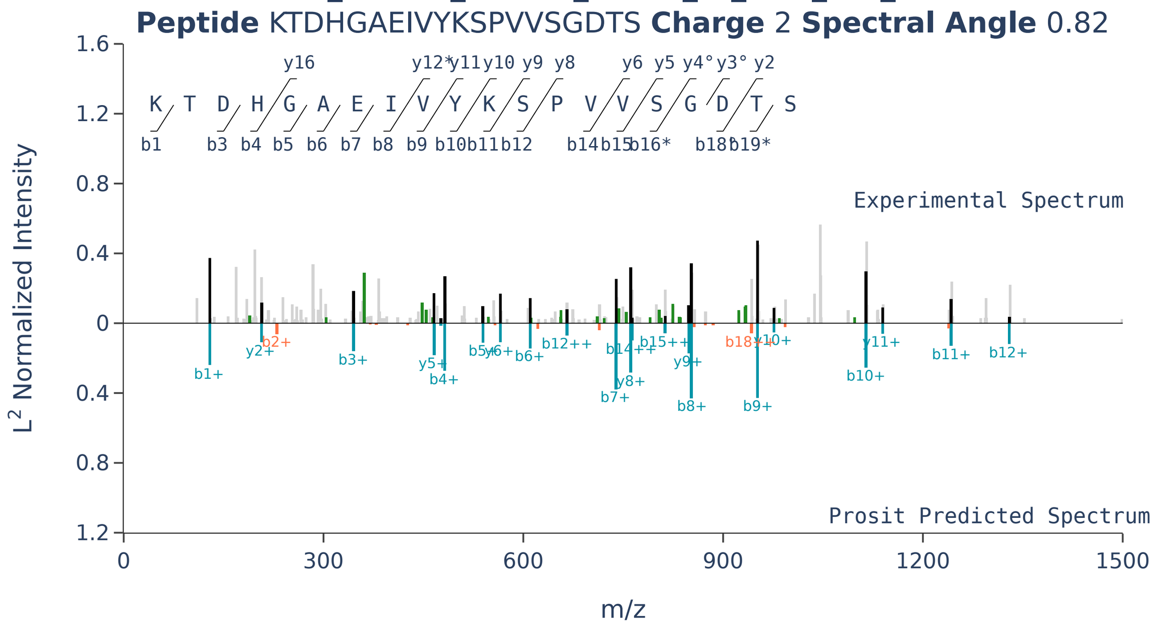


**Source** WSoh_221121_011221_HFGoe_L2_hTau_4h_R2 **Scan** 13714
**Peptide** IKHVPGGGSVQI **Charge** 2 **Spectral Angle** 0.81

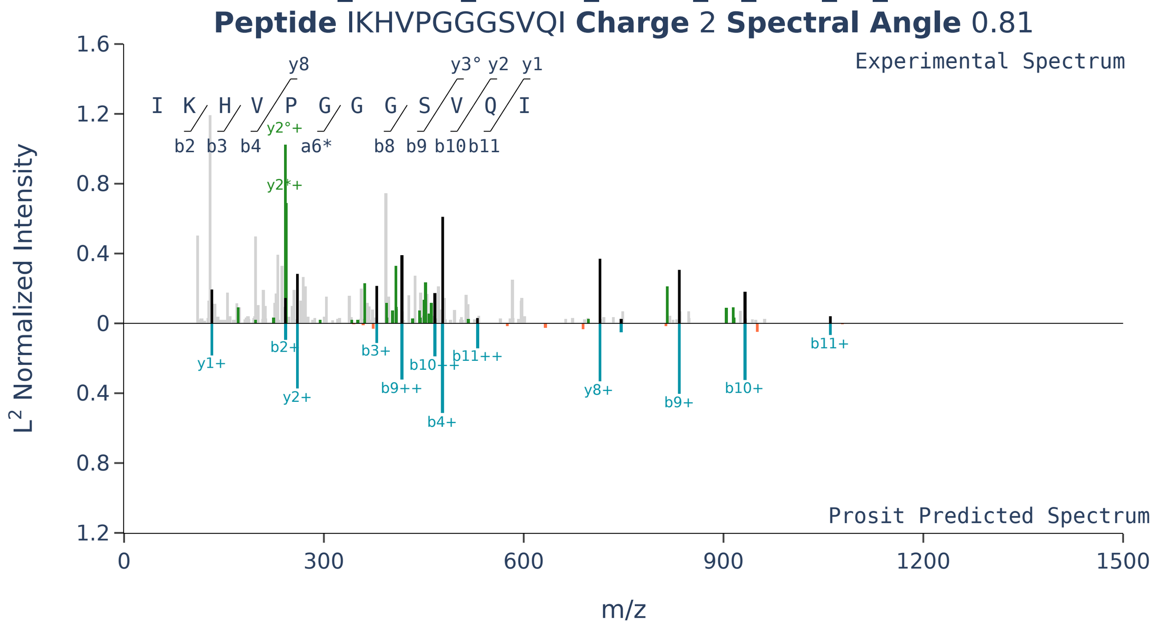


**Source** WSoh_221121_011221_HFGoe_L1_hTau_4h_R2 **Scan** 19789
**Peptide** VRTPPKSPSSAKSRLQTAPVPMPDL **Charge** 3 **Spectral Angle** 0.92

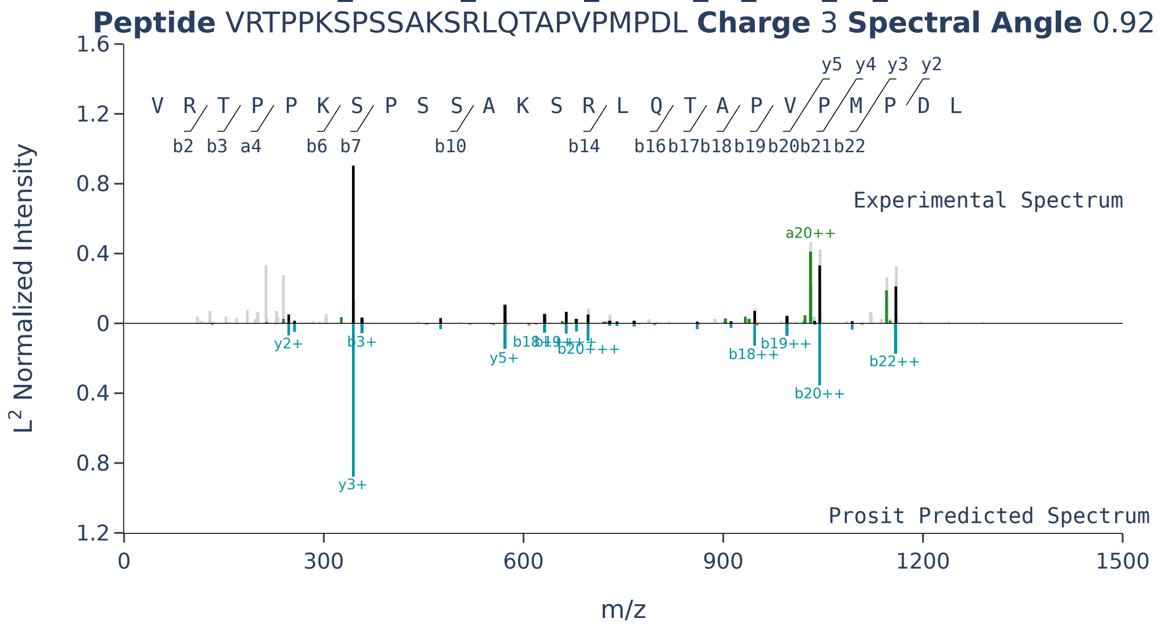


**Source** WSoh_221121_011221_HFGoe_L2_hTau_4h_R1 **Scan** 8189
**Peptide** NITHVPGGGNKKI **Charge** 2 **Spectral Angle** 0.91

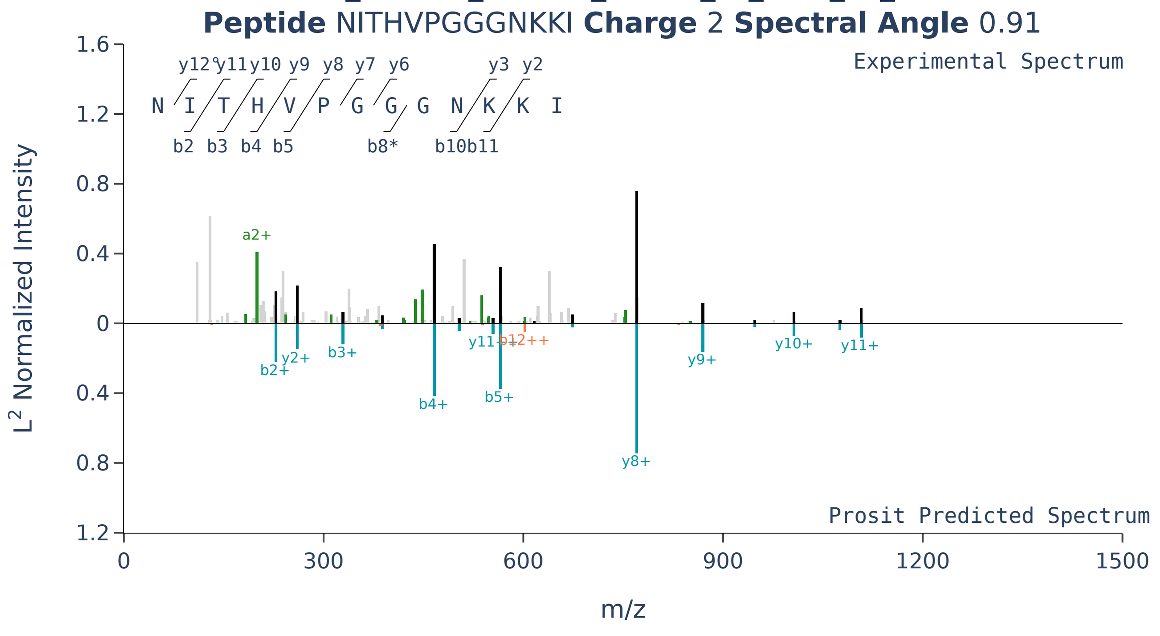


**Source** WSoh_221121_011221_HFGoe_L1_hTau_24h_R1 **Scan** 21200
**Peptide** QTAPVPMPDLKNVK **Charge** 3 **Spectral Angle** 0.88

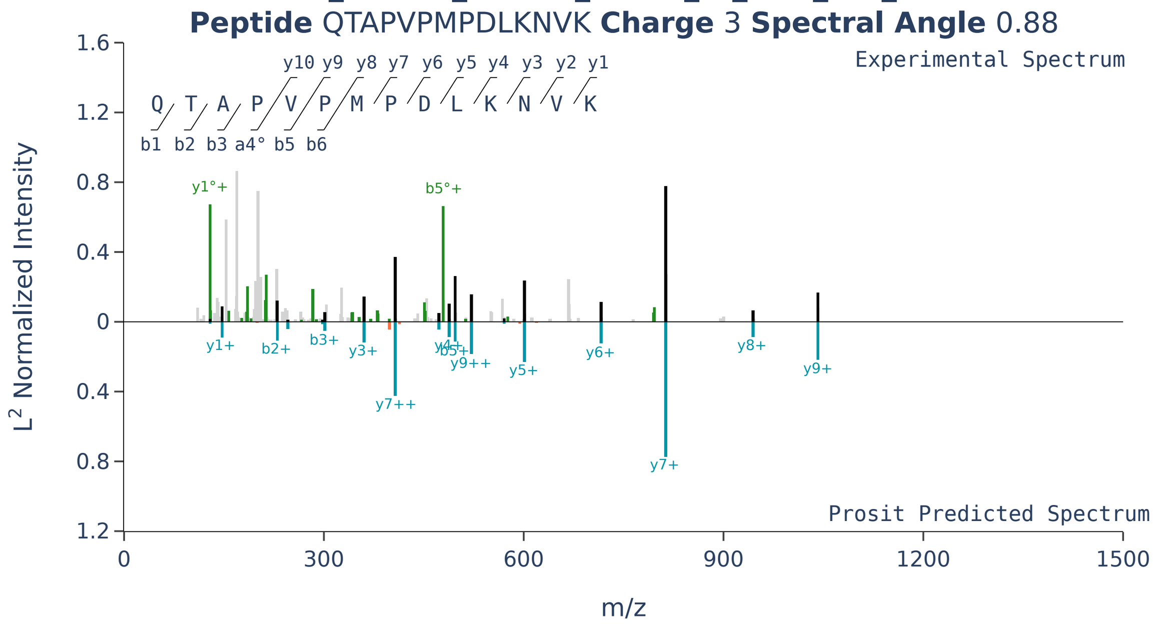


**Source** WSoh_221121_011221_HFGoe_L2_hTau_1h_R1 **Scan** 5689
**Peptide** RTPPKSPSSAKSRL **Charge** 4 **Spectral Angle** 0.86

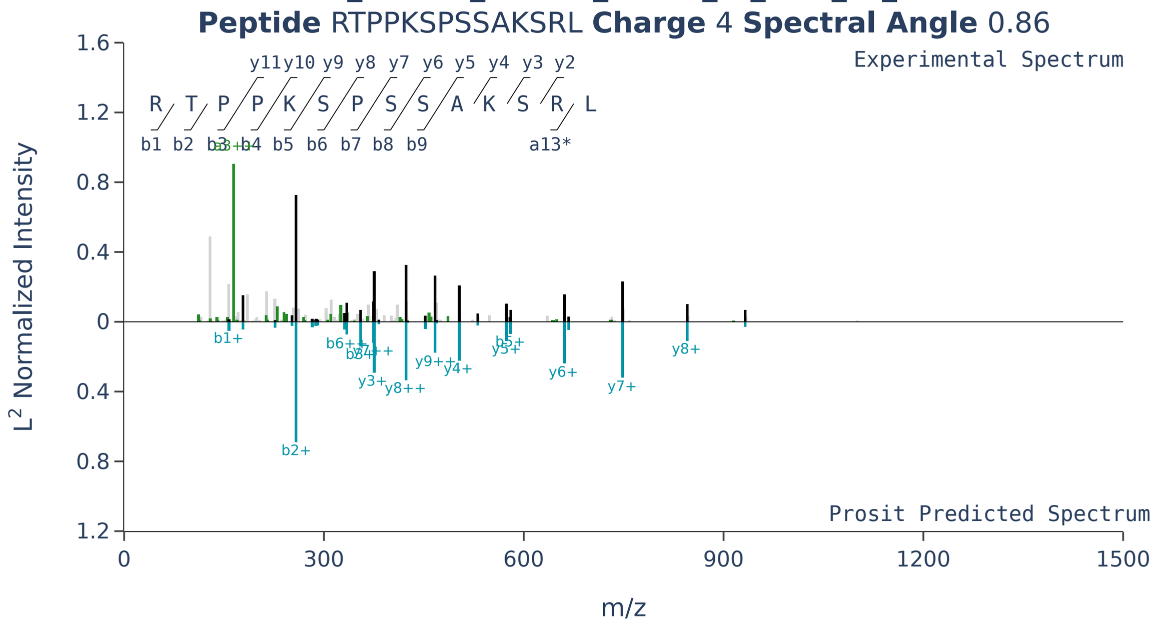


**Source** WSoh_221121_011221_HFGoe_L2_hTau_24h_R1 **Scan** 19926
**Peptide** TAEEAGIGDTPSLEDEAAGHVTQAR **Charge** 3 **Spectral Angle** 0.83

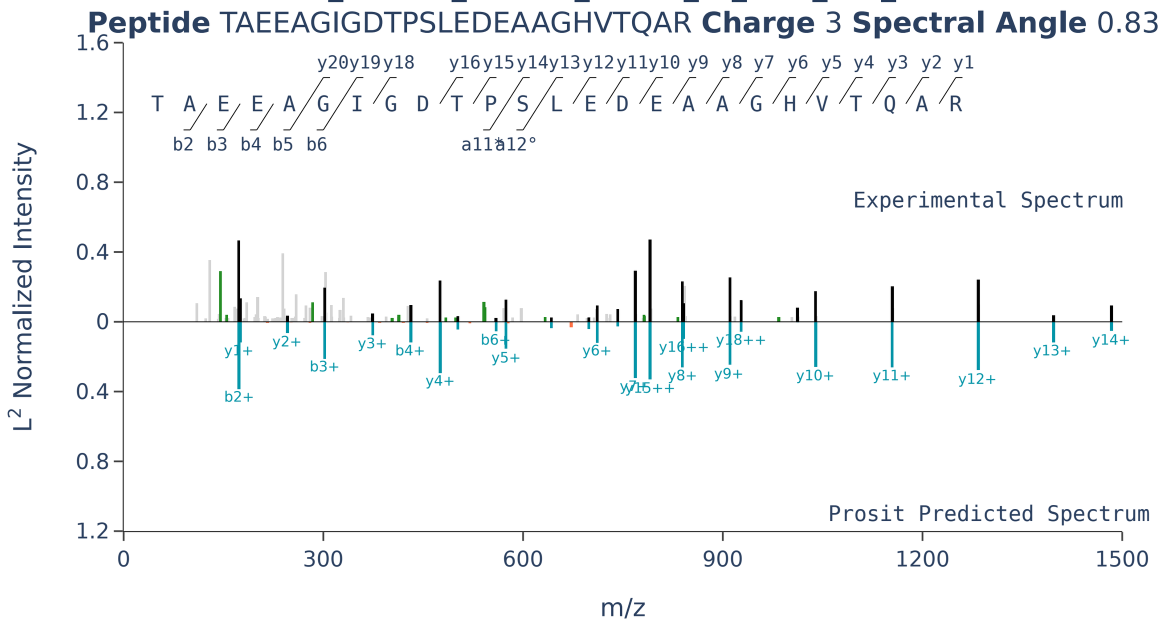


**Source** WSoh_221121_011221_HFGoe_L1_hTau_24h_R1 **Scan** 21507
**Peptide** TAPVPMPDLKN **Charge** 2 **Spectral Angle** 0.83

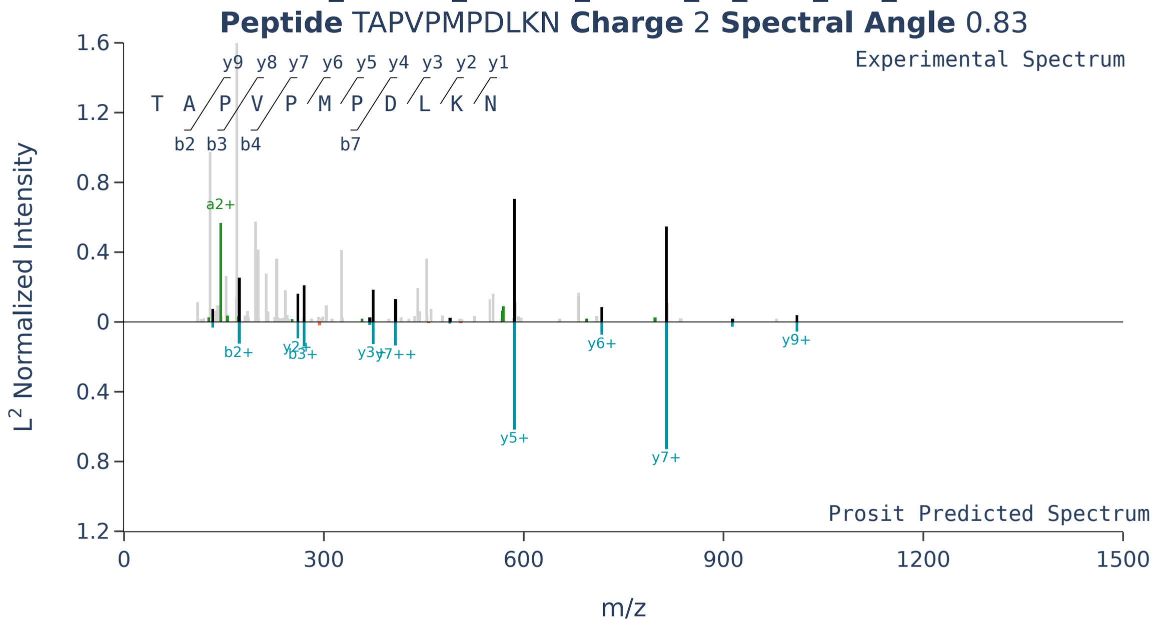


**Source** WSoh_221121_011221_HFGoe_L2_hTau_24h_R2 **Scan** 7731
**Peptide** NIKHVPGGGSVQ **Charge** 2 **Spectral Angle** 0.89

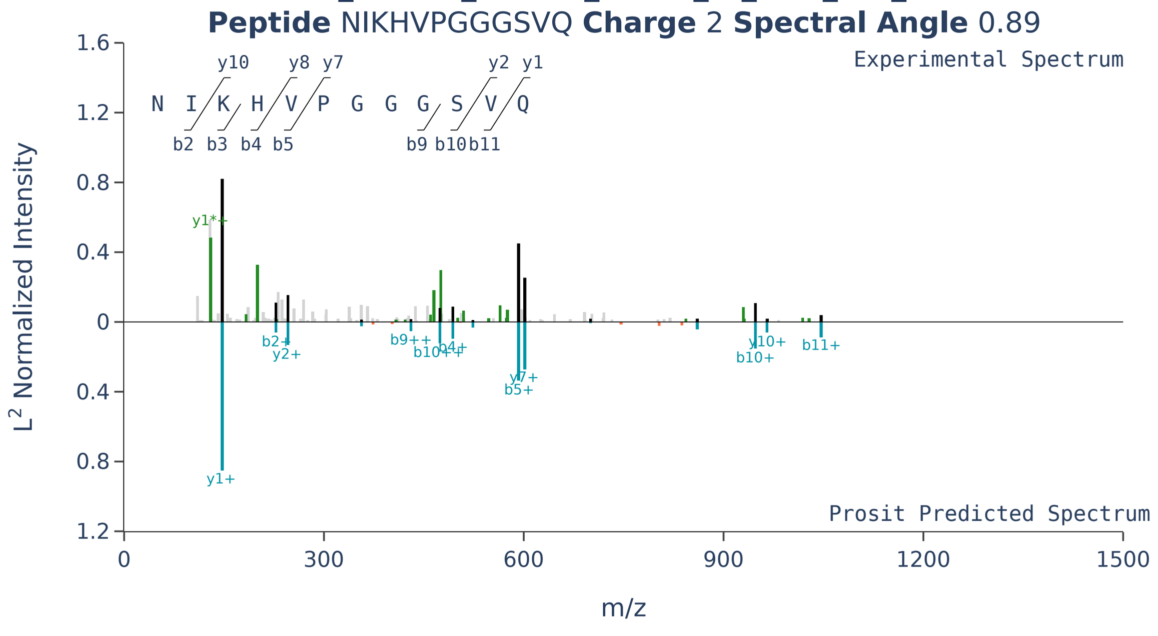


**Source** WSoh_221121_011221_HFGoe_L1_hTau_24h_R1 **Scan** 9916
**Peptide** DNITHVPGGGNKKI **Charge** 2 **Spectral Angle** 0.92

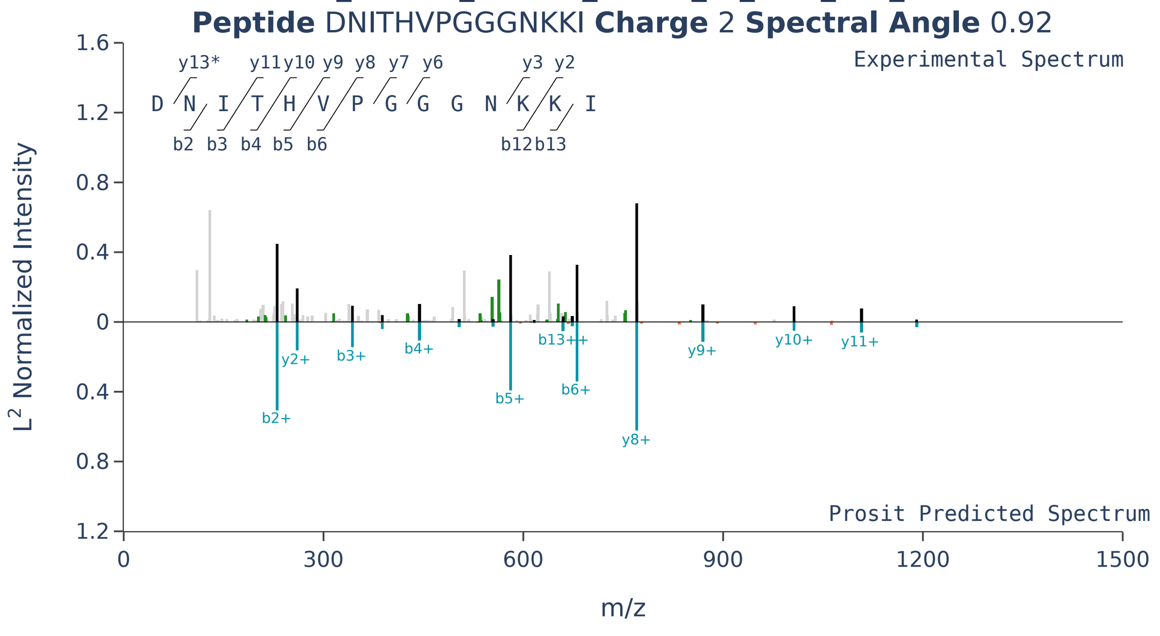

**Source** WSoh_221121_011221_HFGoe_L1_hTau_24h_R1 **Scan** 22509
**Peptide** DSPQLATLAD **Charge** 2 **Spectral Angle** 0.88

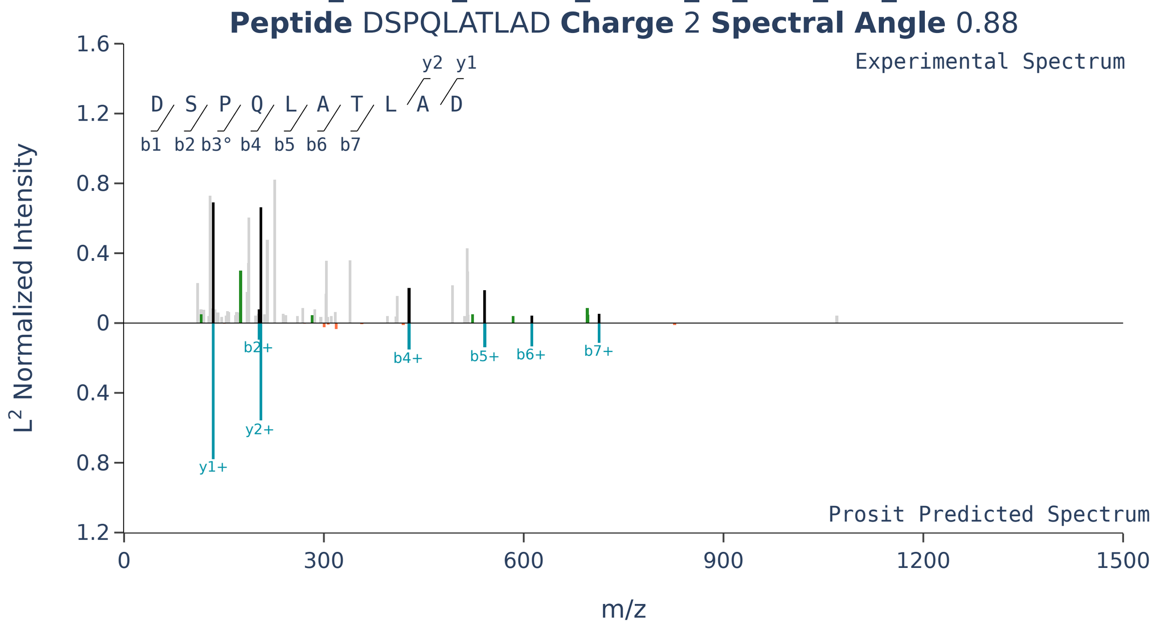


**Source** WSoh_221121_011221_HFGoe_L2_hTau_2h_R2 **Scan** 8836
**Peptide** NITHVPGGGNKKIET **Charge** 2 **Spectral Angle** 0.9

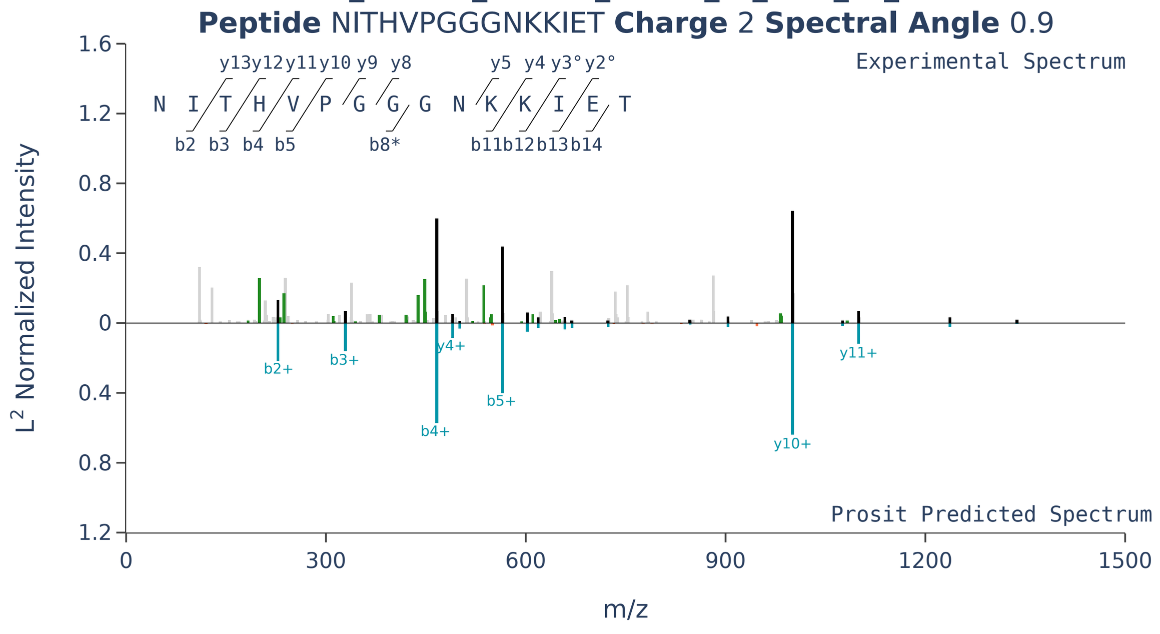


**Source** WSoh_221121_011221_HFGoe_L2_hTau_4h_R1 **Scan** 15251
**Peptide** INKKLDLSNV **Charge** 2 **Spectral Angle** 0.84

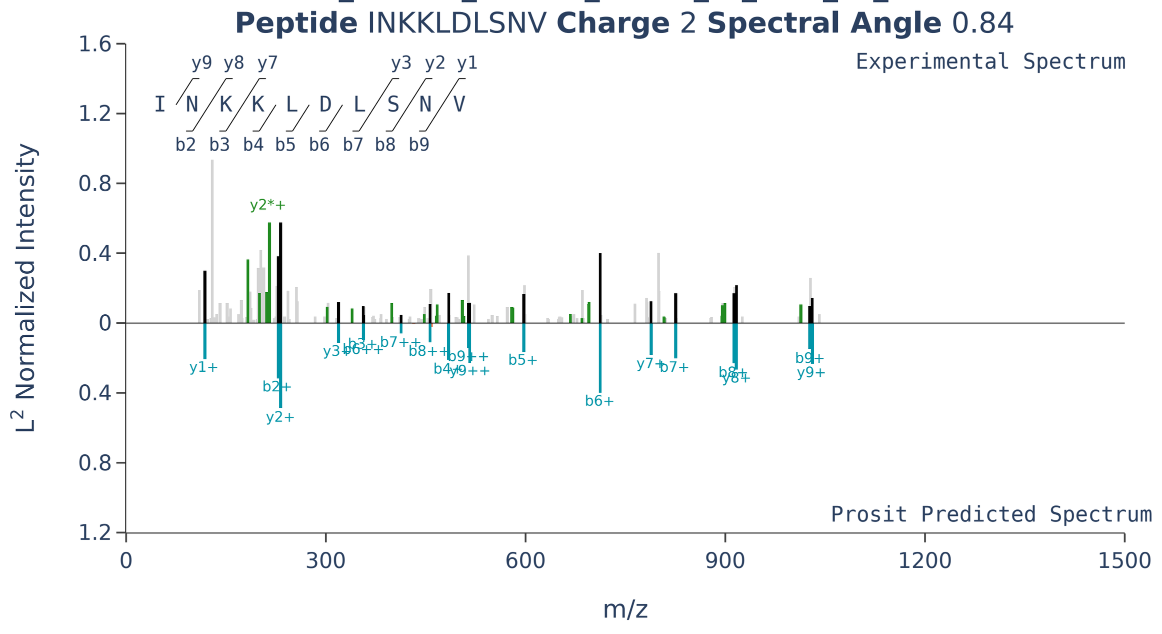


**Source** WSoh_221121_011221_HFGoe_L2_hTau_2h_R1 **Scan** 11060
**Peptide** EGAPGKQAAAQPHTEIPEG **Charge** 3 **Spectral Angle** 0.89

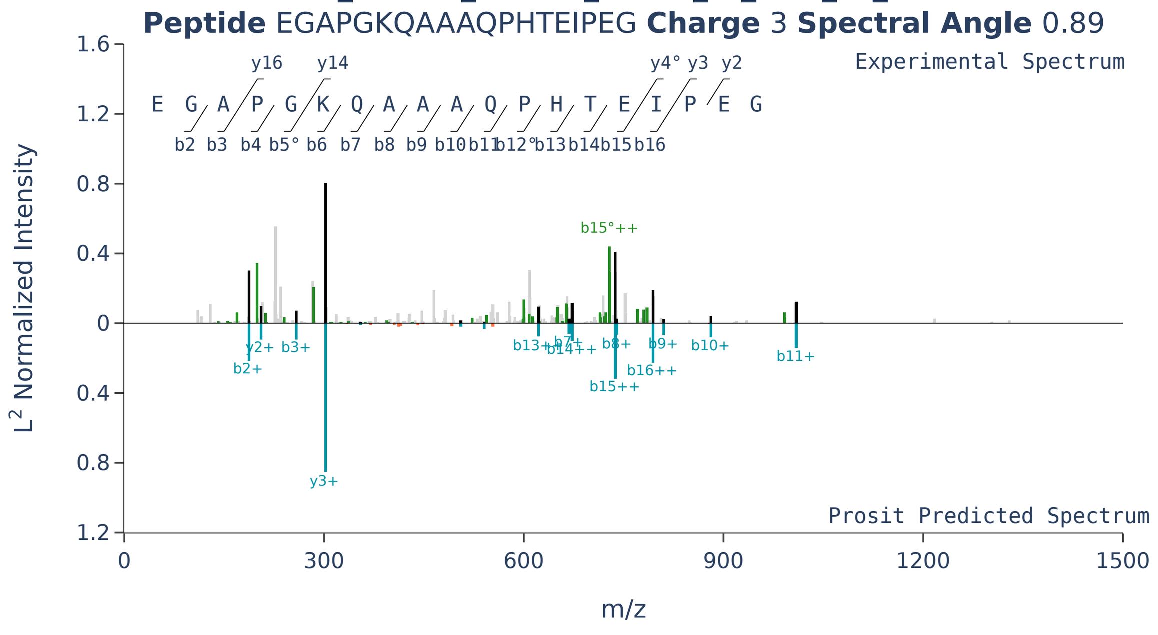


**Source** WSoh_221121_011221_HFGoe_L1_hTau_24h_R1 **Scan** 8007
**Peptide** LATPRGA **Charge** 1 **Spectral Angle** 0.79

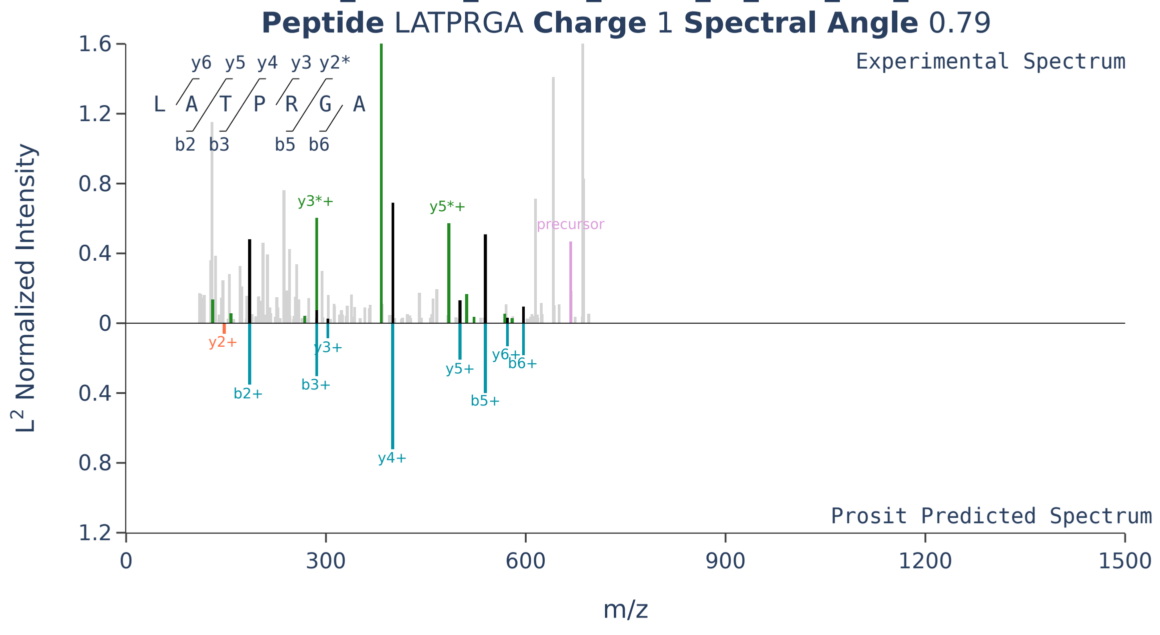


**Source** WSoh_221121_011221_HFGoe_L1_hTau_24h_R1 **Scan** 11848
**Peptide** TYGLGDRKDQGGYTMH **Charge** 4 **Spectral Angle** 0.95

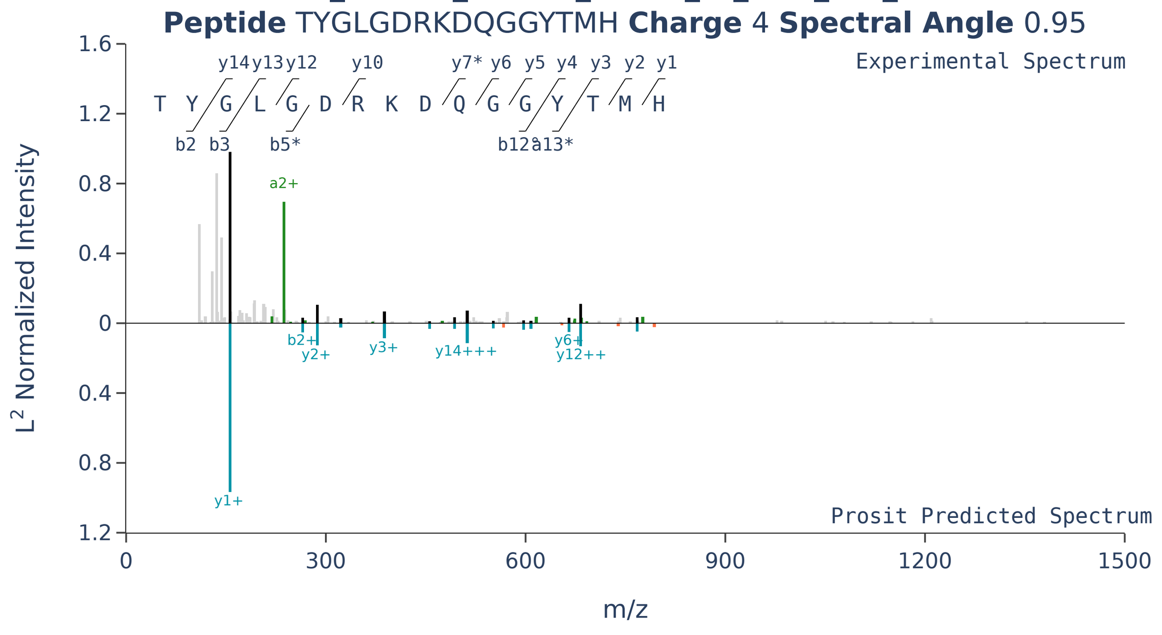


**Source** WSoh_221121_011221_HFGoe_L2_hTau_24h_R2 **Scan** 17953
**Peptide** ATLADEVSA **Charge** 2 **Spectral Angle** 0.95

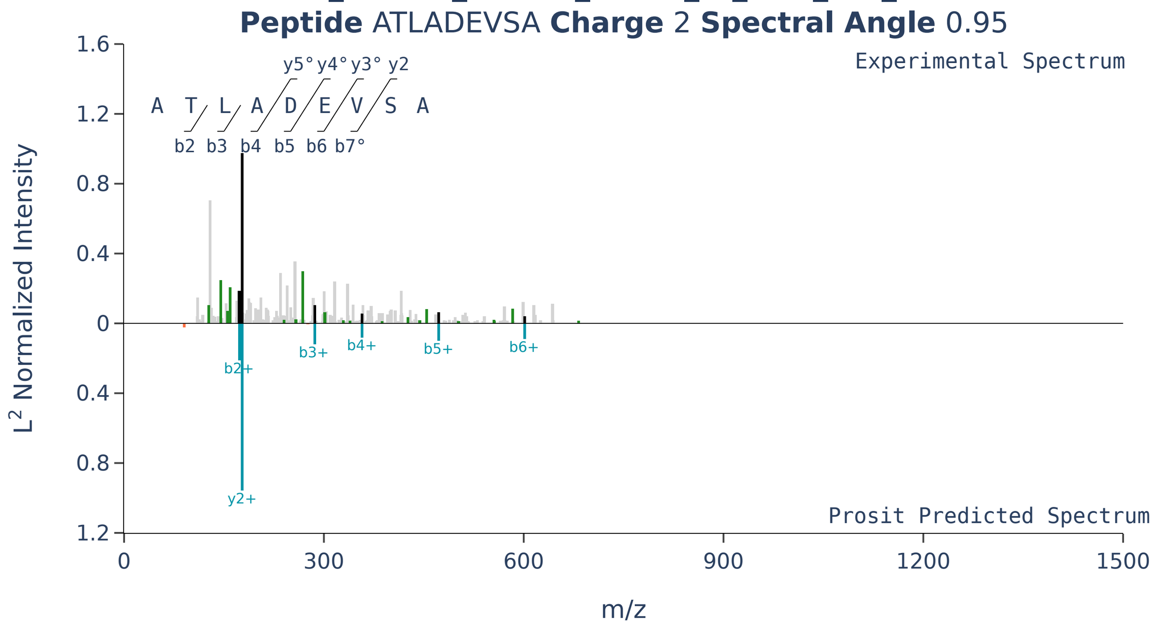


**Source** WSoh_221121_011221_HFGoe_L2_hTau_2h_R2 **Scan** 17067
**Peptide** VYKPVDLSKVTSKCGSLGNIHH **Charge** 4 **Spectral Angle** 0.94

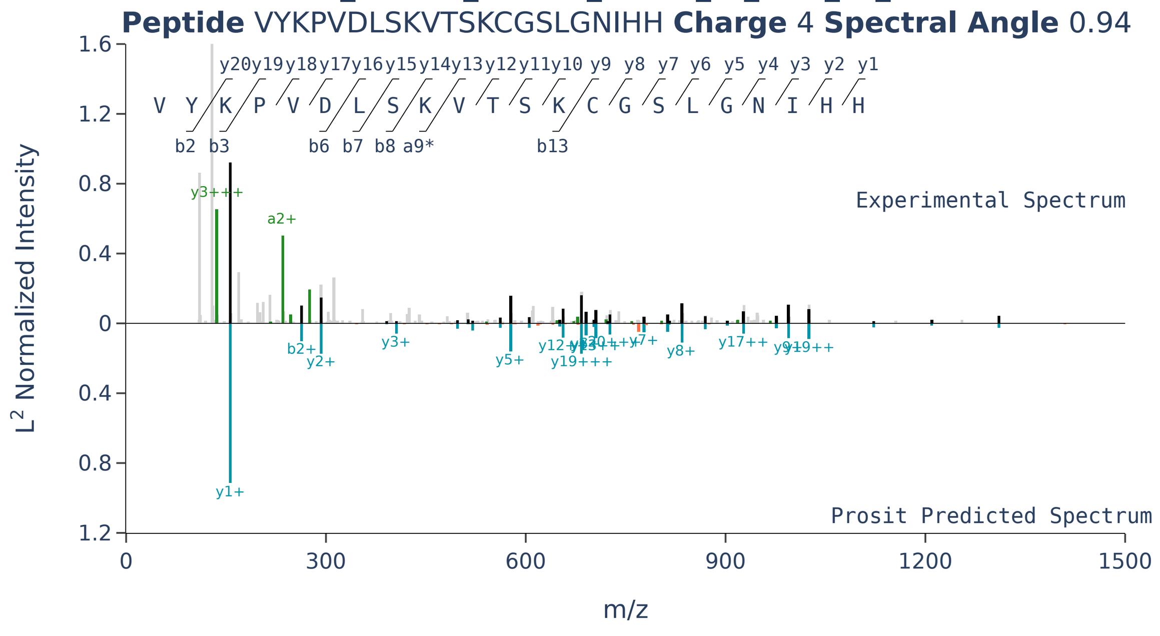


**Source** WSoh_221121_011221_HFGoe_L2_hTau_24h_R2 **Scan** 7934
**Peptide** PPAPKTPPSSGEPPKSG **Charge** 3 **Spectral Angle** 0.87

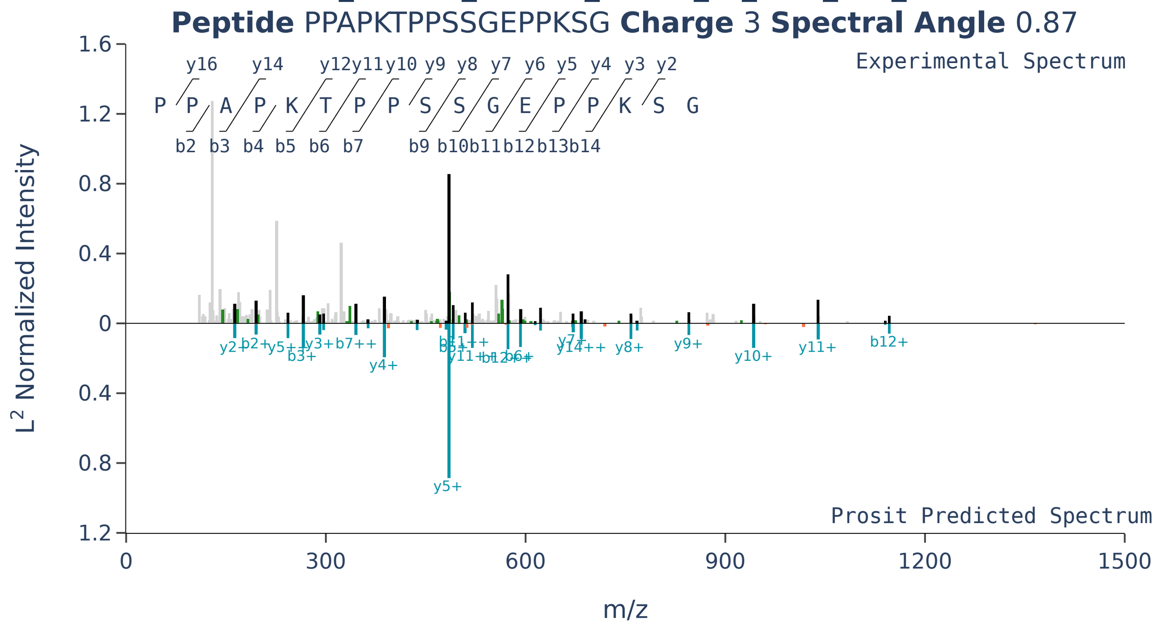


**Source** WSoh_221121_011221_HFGoe_L1_hTau_24h_R2 **Scan** 7794
**Peptide** SGDTSPRHLSNVSSTGS **Charge** 2 **Spectral Angle** 0.85

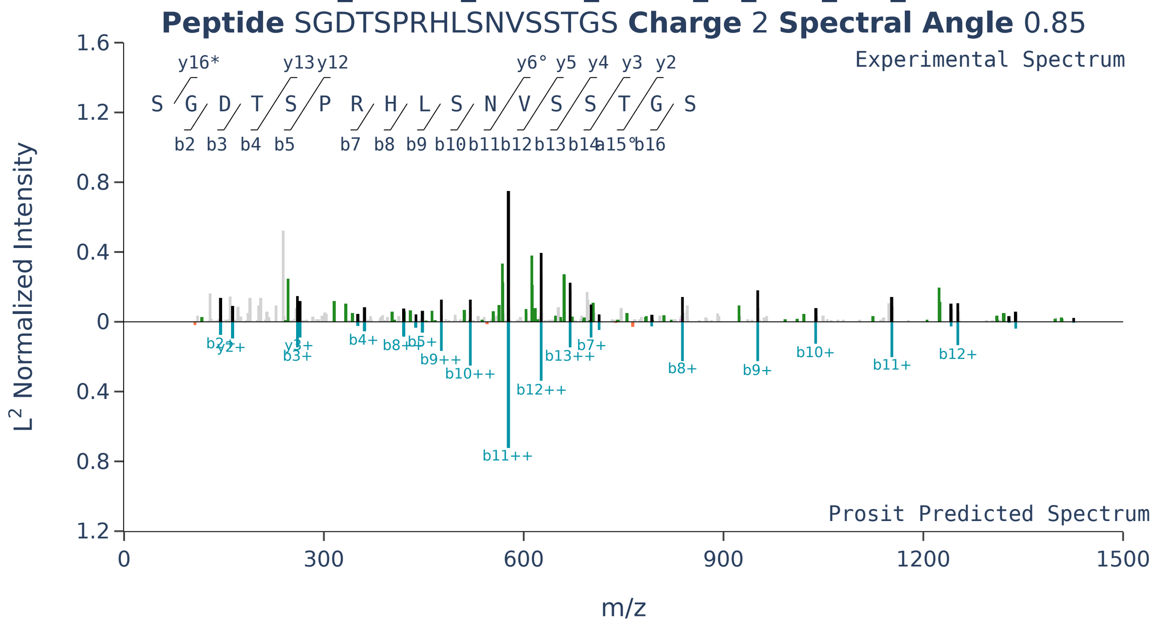


**Source** WSoh_221121_011221_HFGoe_L2_hTau_2h_R1 **Scan** 8584
**Peptide** IATPRGAAPPGQKGQA **Charge** 3 **Spectral Angle** 0.9

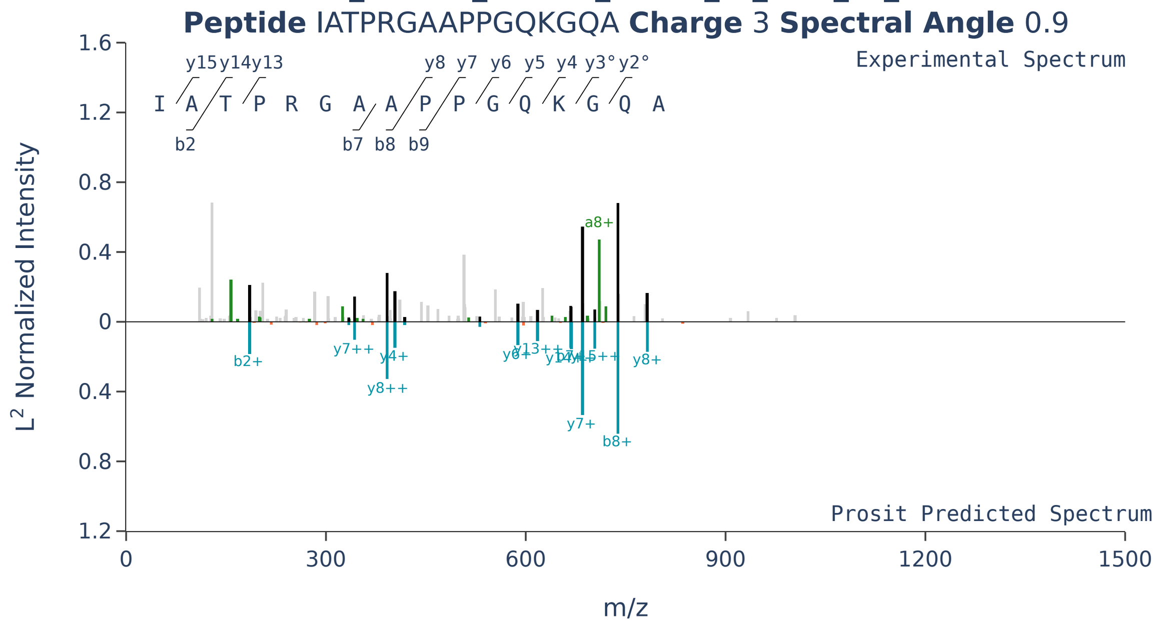


**Source** WSoh_221121_011221_HFGoe_L1_hTau_2h_R2 **Scan** 7782
**Peptide** KSEKLDFKDRVQSK **Charge** 2 **Spectral Angle** 0.79

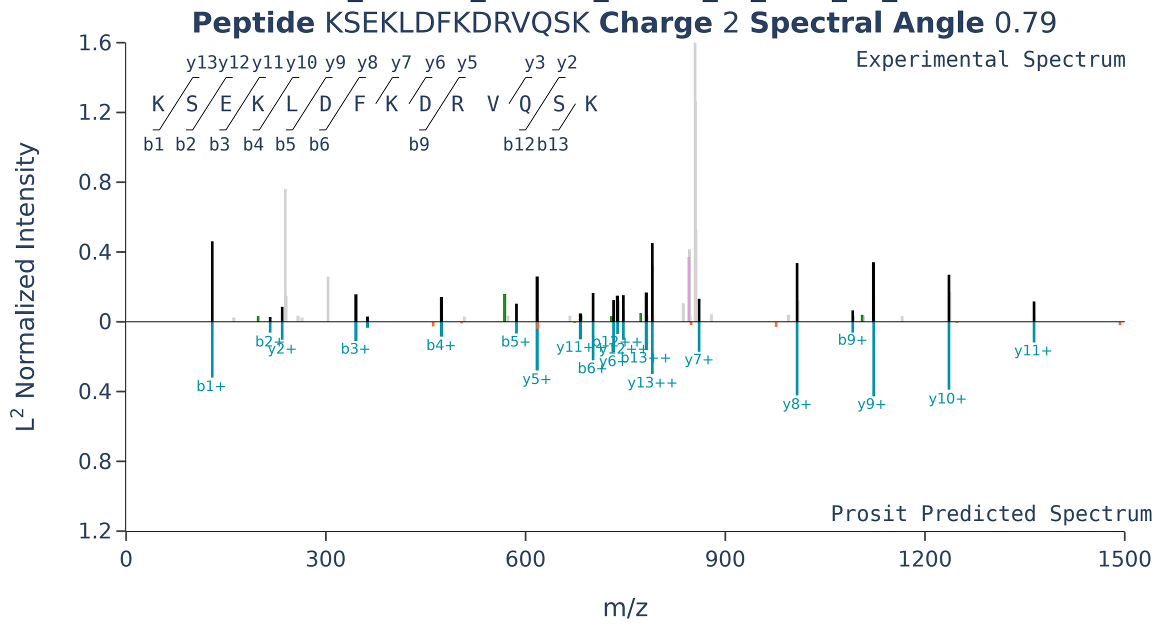


**Source** WSoh_221121_011221_HFGoe_L1_hTau_24h_R1 **Scan** 14456
**Peptide** VYKSPVVSGDTSPRHLSNV **Charge** 2 **Spectral Angle** 0.89

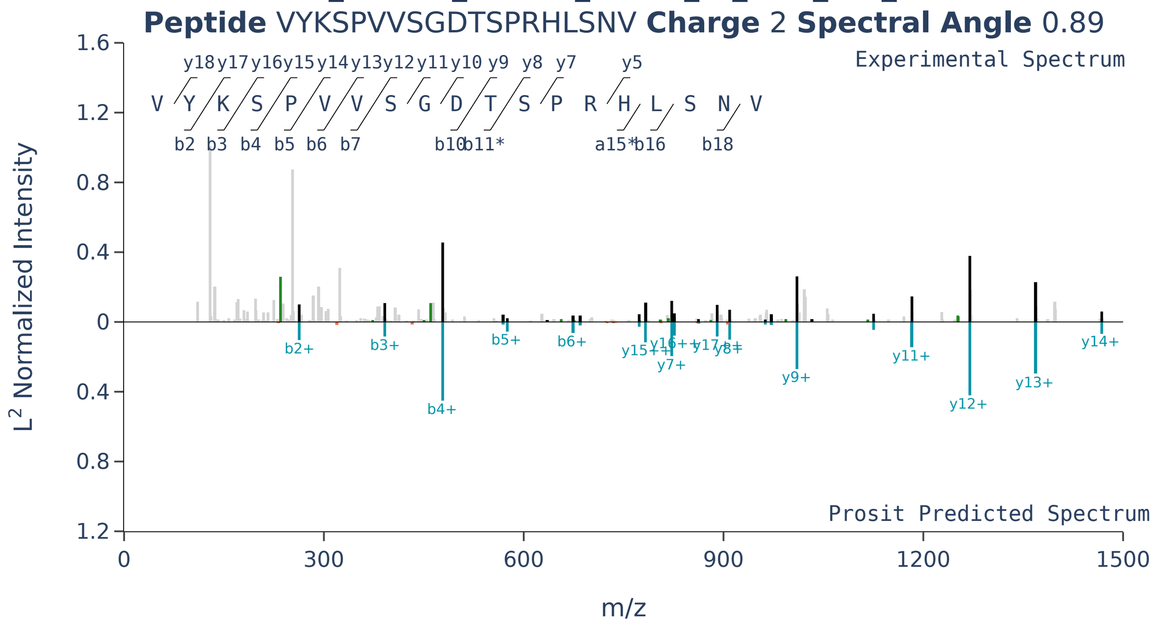


**Source** WSoh_221121_011221_HFGoe_L2_hTau_24h_R2 **Scan** 15911
**Peptide** KHVPGGGSVQIVYKPVD **Charge** 3 **Spectral Angle** 0.82

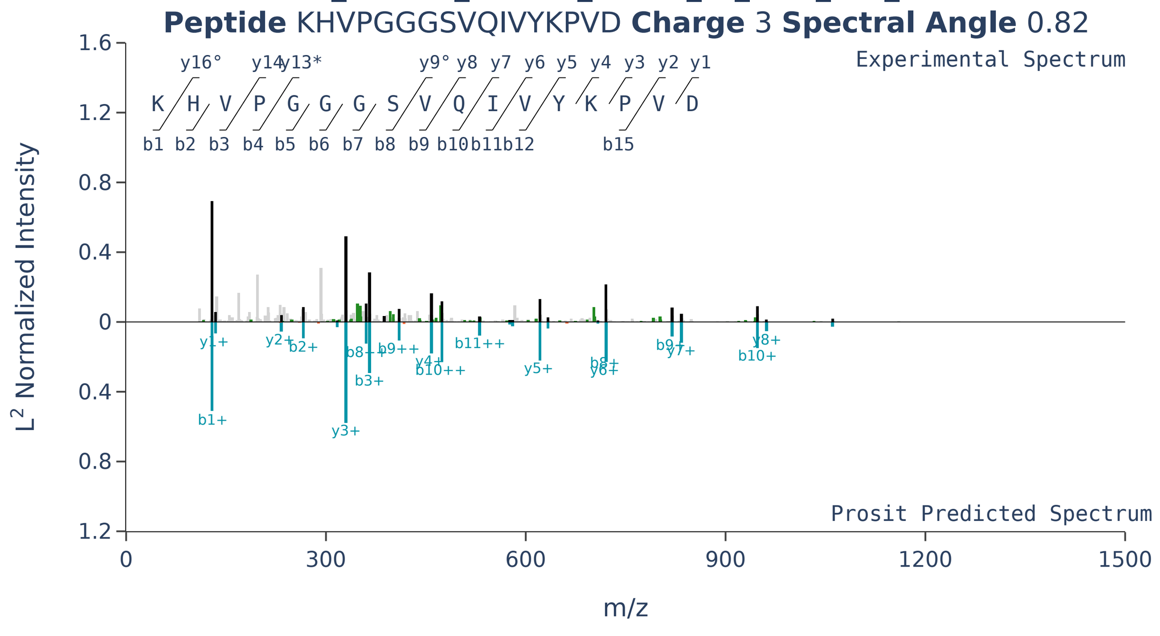


**Source** WSoh_221121_011221_HFGoe_L1_hTau_24h_R2 **Scan** 18471
**Peptide** VYKPVDLSKV **Charge** 3 **Spectral Angle** 0.85

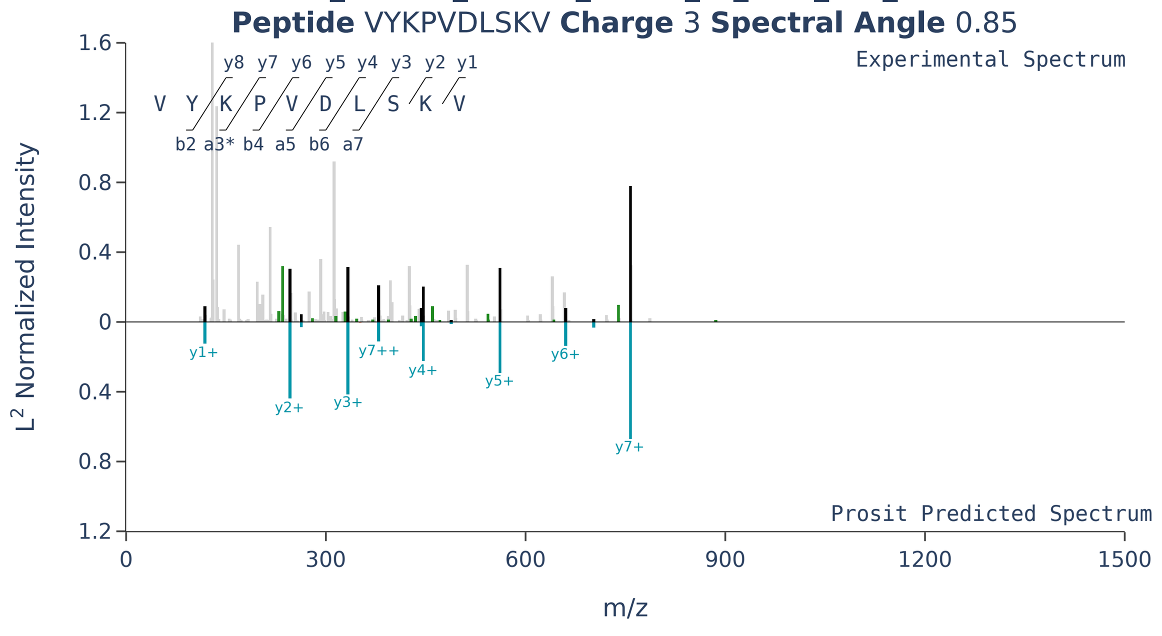

**Source** WSoh_221121_011221_HFGoe_L1_hTau_24h_R1 **Scan** 7555
**Peptide** KDNIKHVPGGGSV **Charge** 2 **Spectral Angle** 0.87

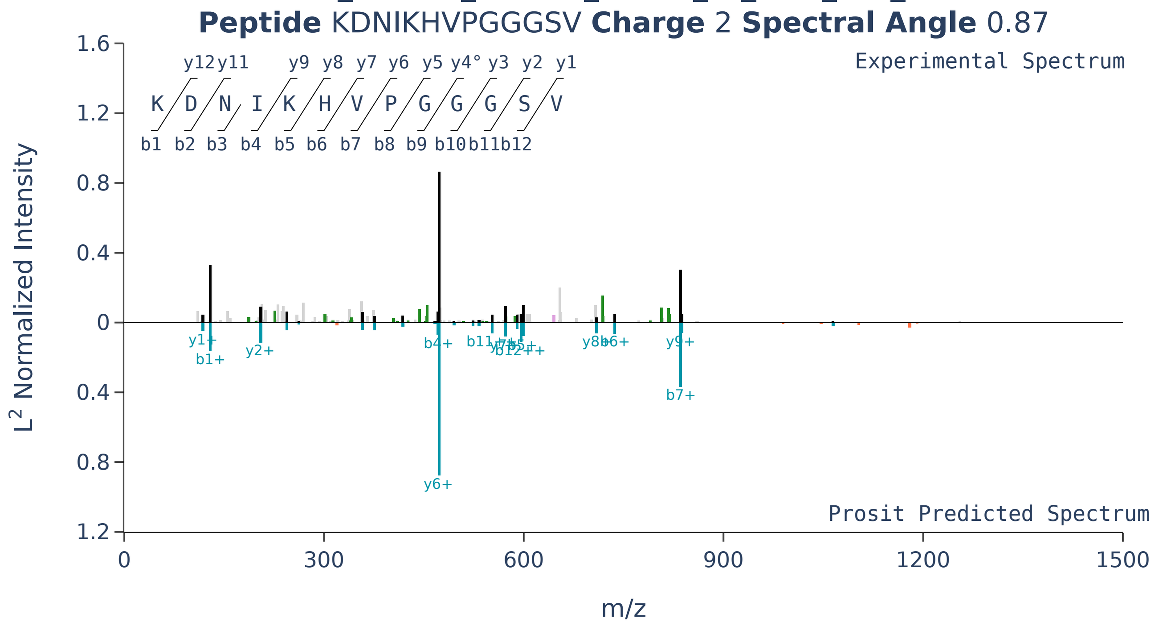


**Source** WSoh_221121_011221_HFGoe_L2_hTau_24h_R1 **Scan** 19544
**Peptide** GLGDTPSLED **Charge** 2 **Spectral Angle** 0.78

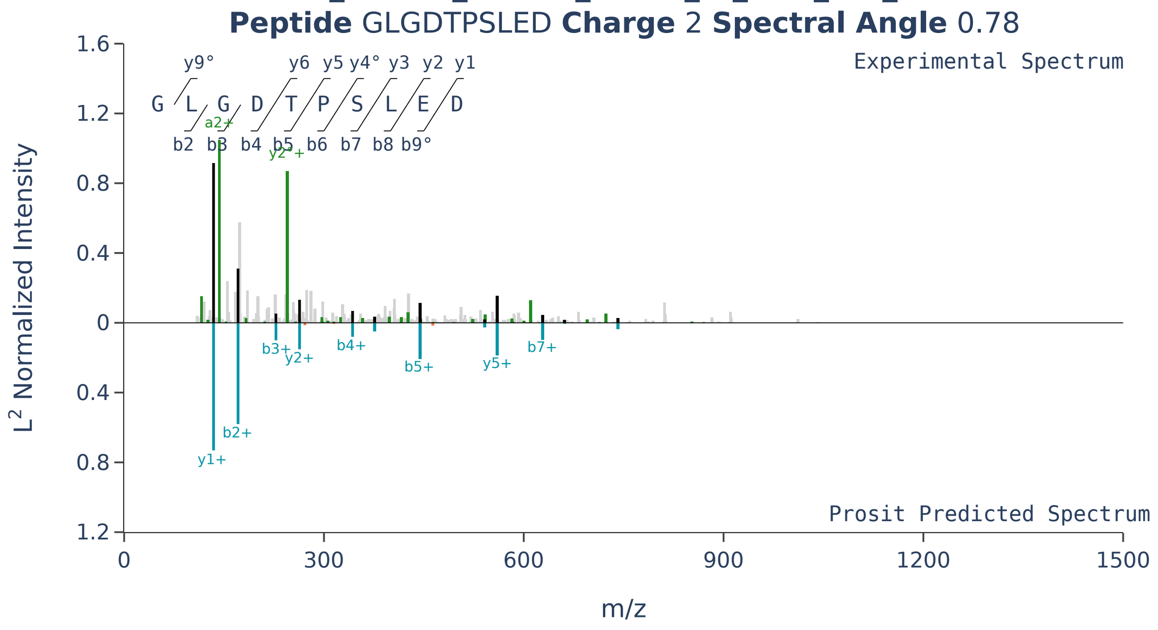


**Source** WSoh_221121_011221_HFGoe_L2_hTau_2h_R2 **Scan** 6034
**Peptide** SLGNIHHKPGG **Charge** 2 **Spectral Angle** 0.88

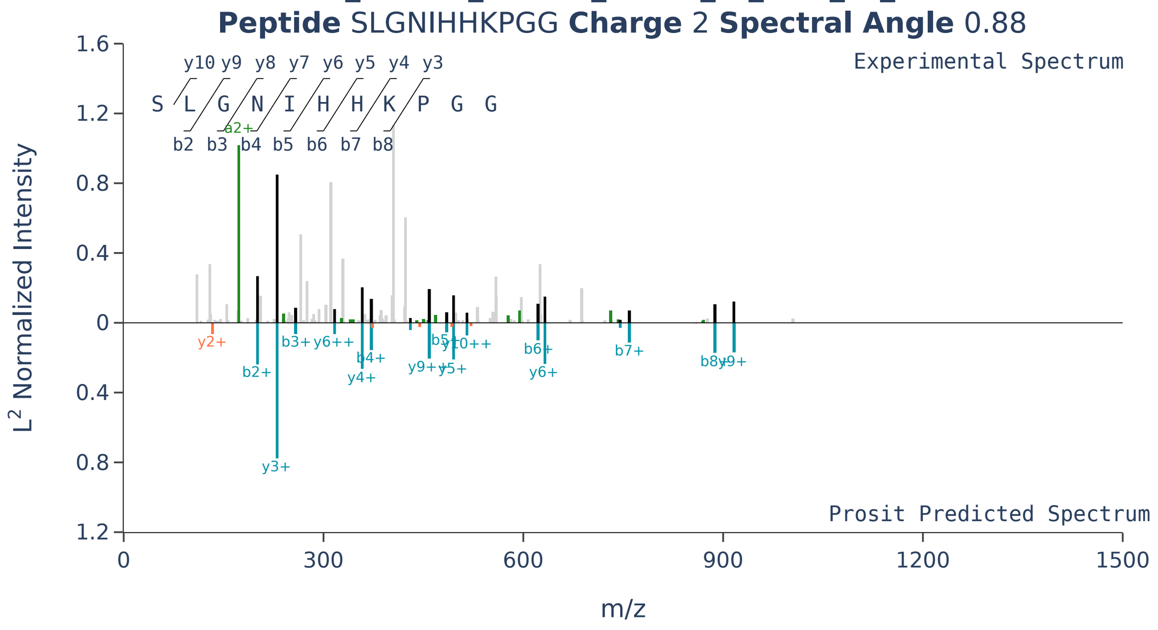


**Source** WSoh_221121_011221_HFGoe_L1_hTau_24h_R1 **Scan** 19023
**Peptide** QIVYKPVDLSKVTSK **Charge** 3 **Spectral Angle** 0.89

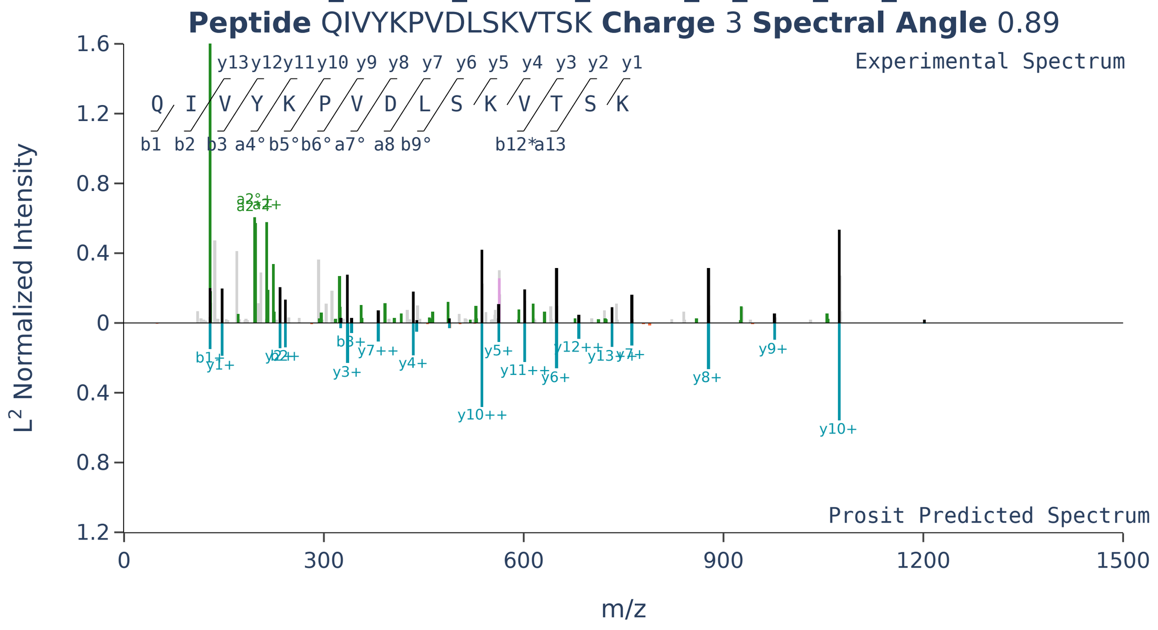


**Source** WSoh_221121_011221_HFGoe_L1_hTau_1h_R1 **Scan** 9254
**Peptide** QIINKKL **Charge** 2 **Spectral Angle** 0.83

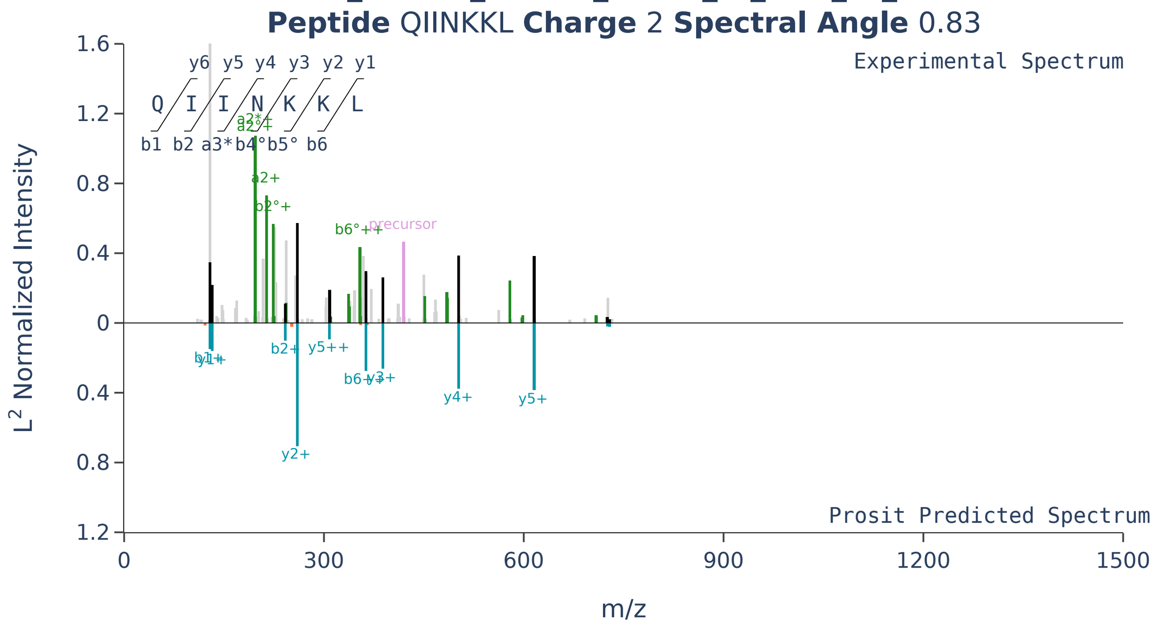


**Source** WSoh_221121_011221_HFGoe_L1_hTau_24h_R1 **Scan** 12164
**Peptide** PVVSGDTSPRHLSNVSSTG **Charge** 2 **Spectral Angle** 0.89

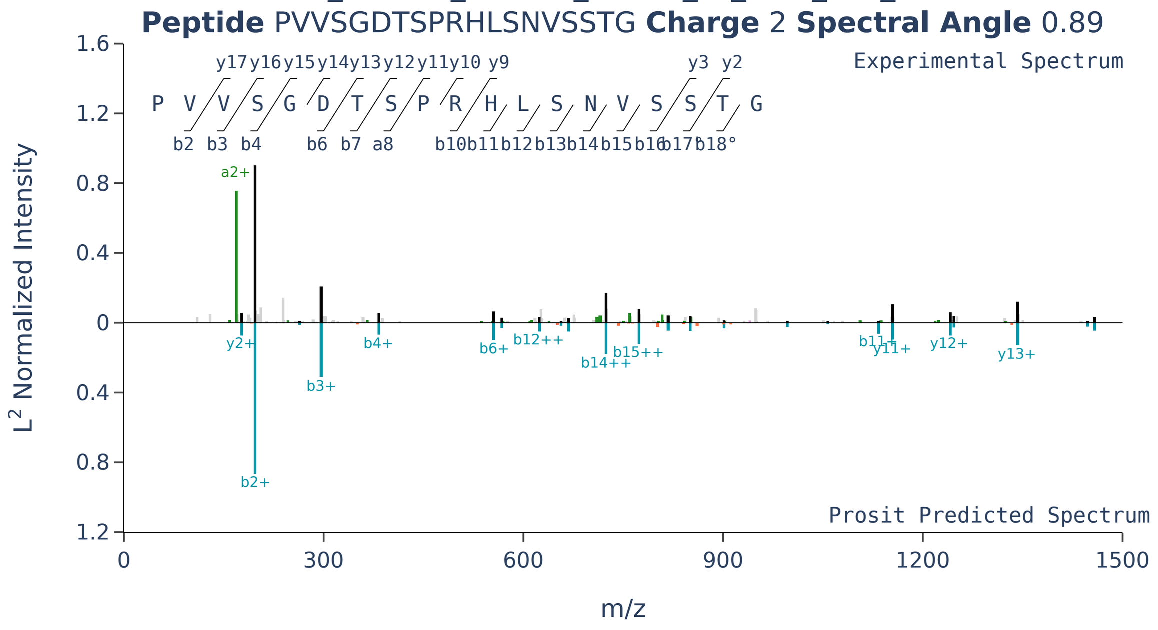


**Source** WSoh_221121_011221_HFGoe_L1_hTau_24h_R1 **Scan** 7226
**Peptide** NIHHKPGGGQVEVKSEKL **Charge** 3 **Spectral Angle** 0.81

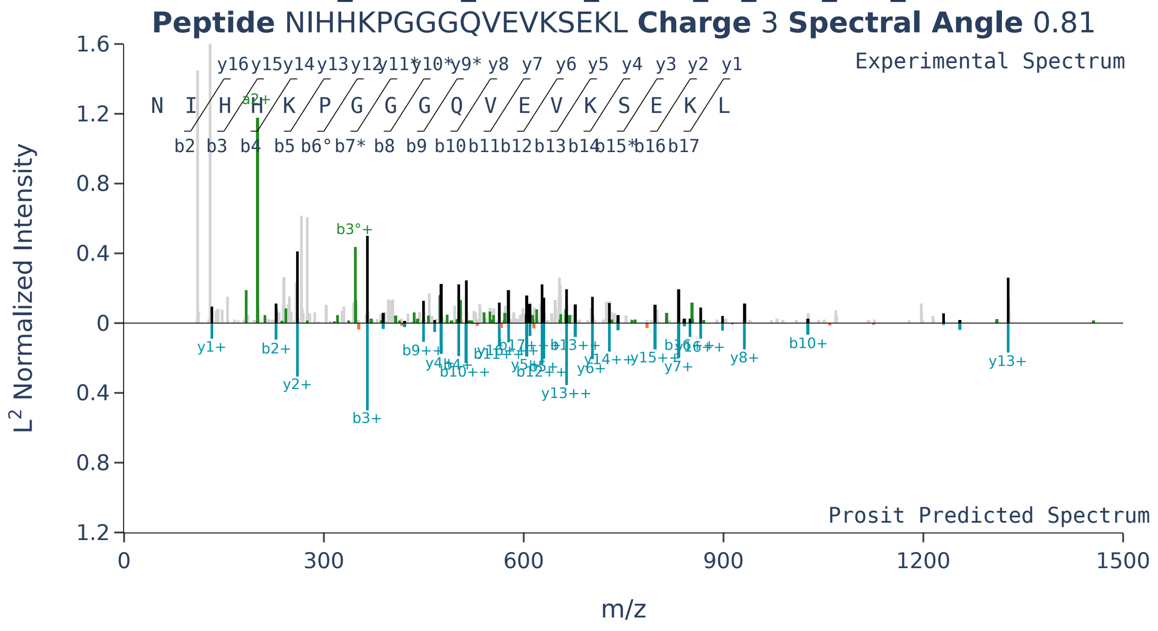


**Source** WSoh_221121_011221_HFGoe_L1_hTau_4h_R2 **Scan** 11175
**Peptide** DEGAPGKQAAAQPHTEI **Charge** 2 **Spectral Angle** 0.91

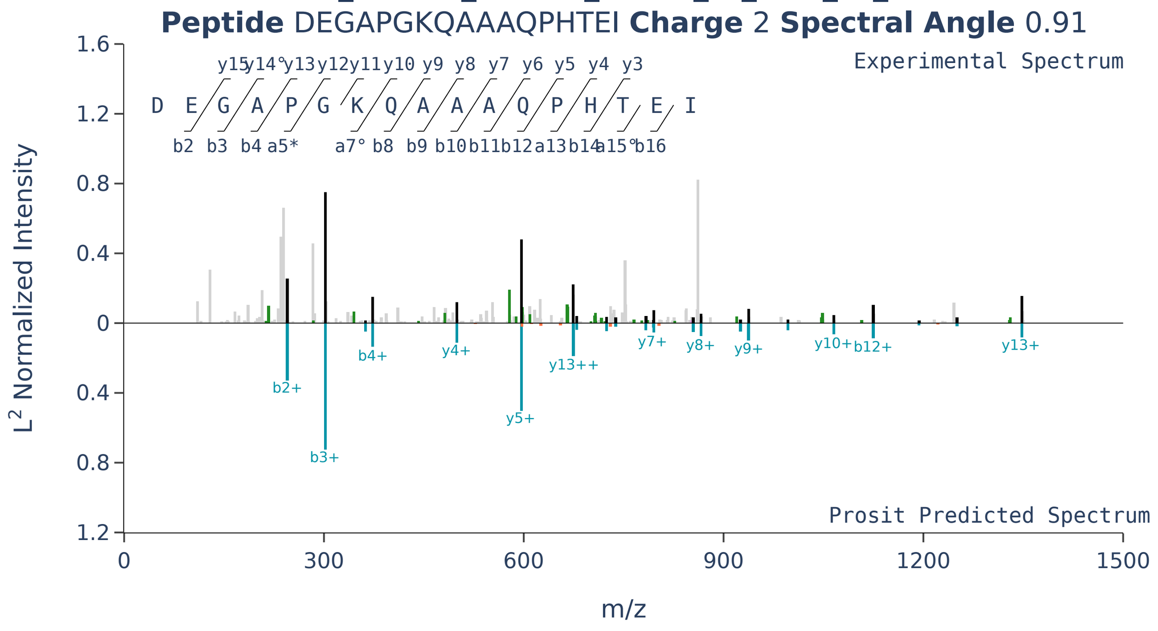


**Source** WSoh_221121_011221_HFGoe_L2_hTau_24h_R1 **Scan** 8201
**Peptide** ATPRGAAPPGQKGQANATRI **Charge** 3 **Spectral Angle** 0.89

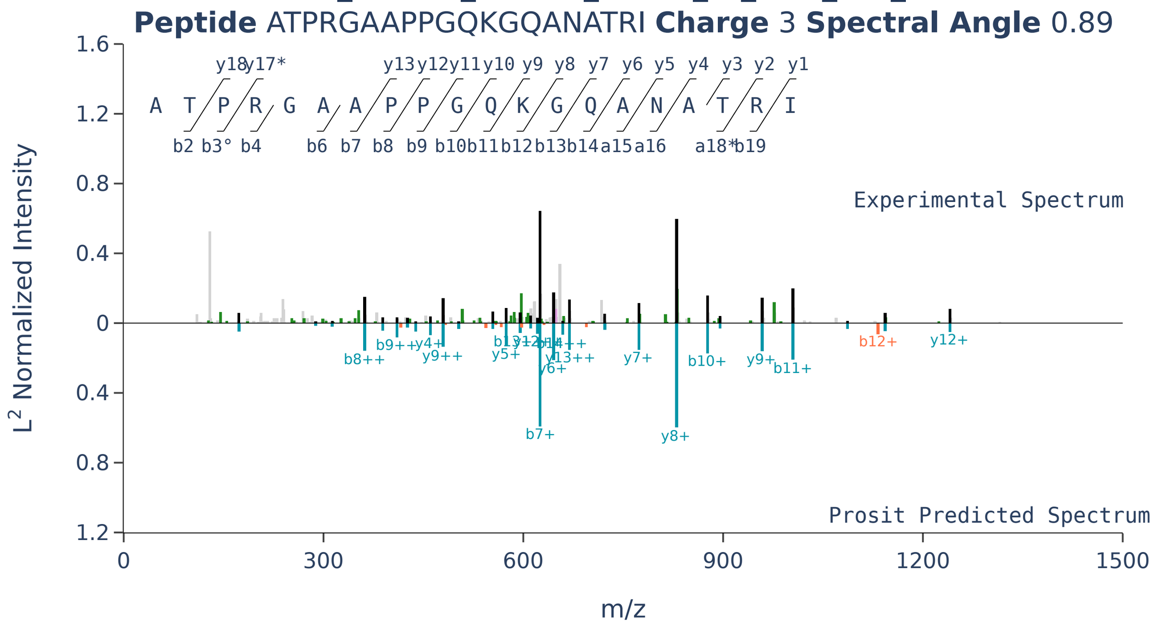


**Source** WSoh_221121_011221_HFGoe_L1_hTau_24h_R1 **Scan** 7466
**Peptide** HKLTFREN **Charge** 1 **Spectral Angle** 0.89

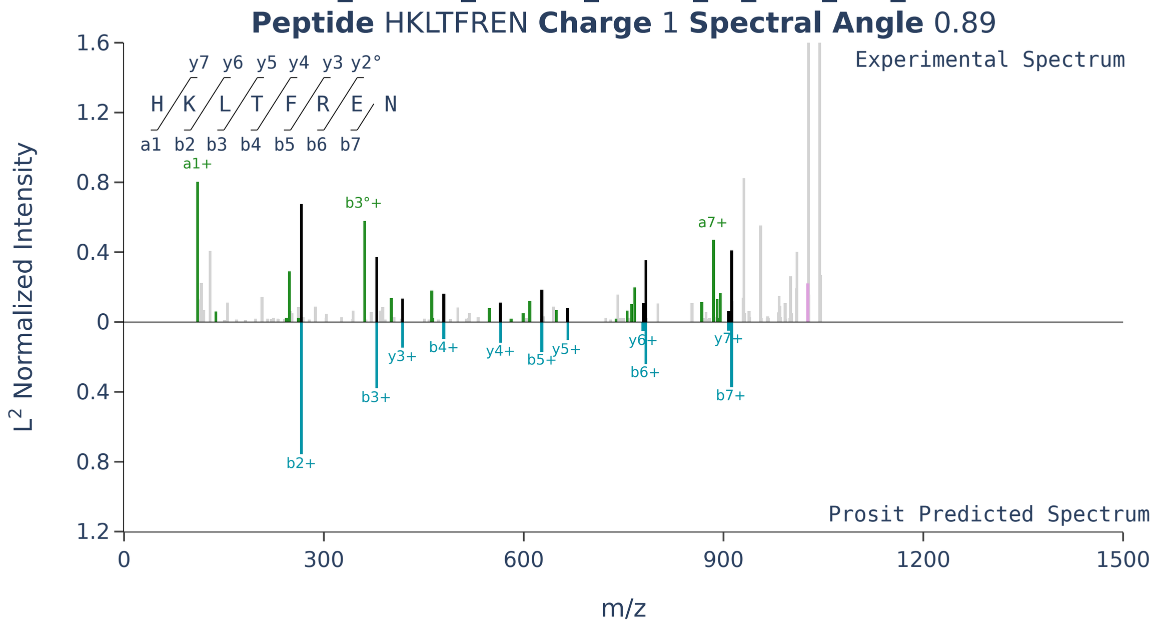


**Source** WSoh_221121_011221_HFGoe_L2_hTau_24h_R1 **Scan** 21214
**Peptide** APVPMPDLKNVKSKIGSTEN **Charge** 3 **Spectral Angle** 0.9

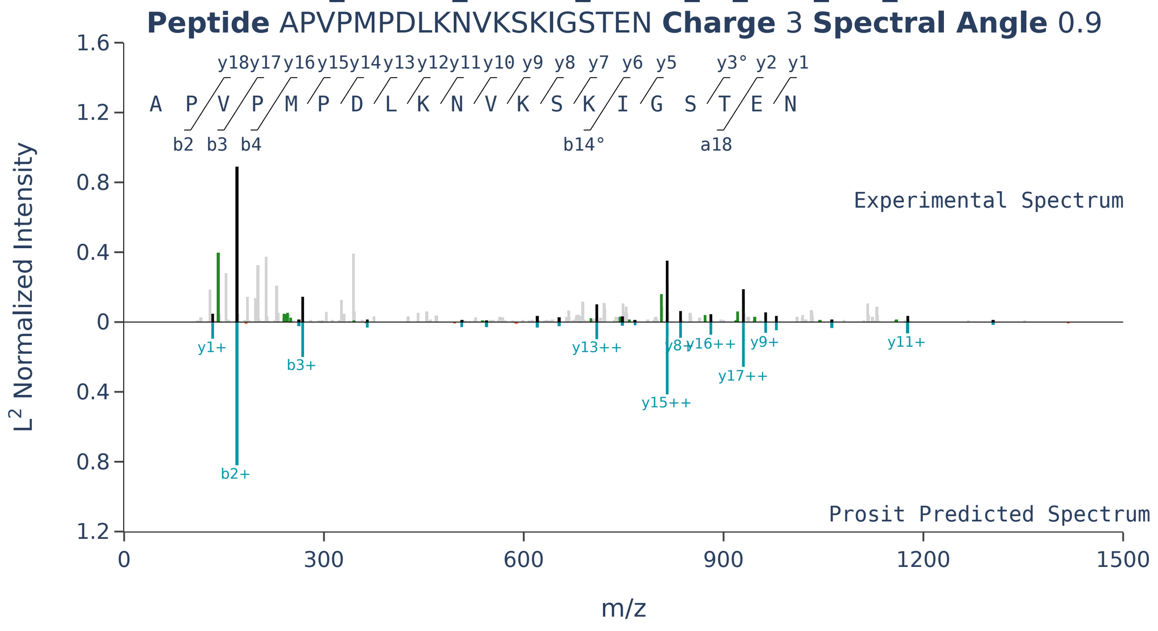


**Source** WSoh_221121_011221_HFGoe_L1_hTau_24h_R2 **Scan** 22114
**Peptide** TAPVPMPDLKNVKSKIGST **Charge** 3 **Spectral Angle** 0.94

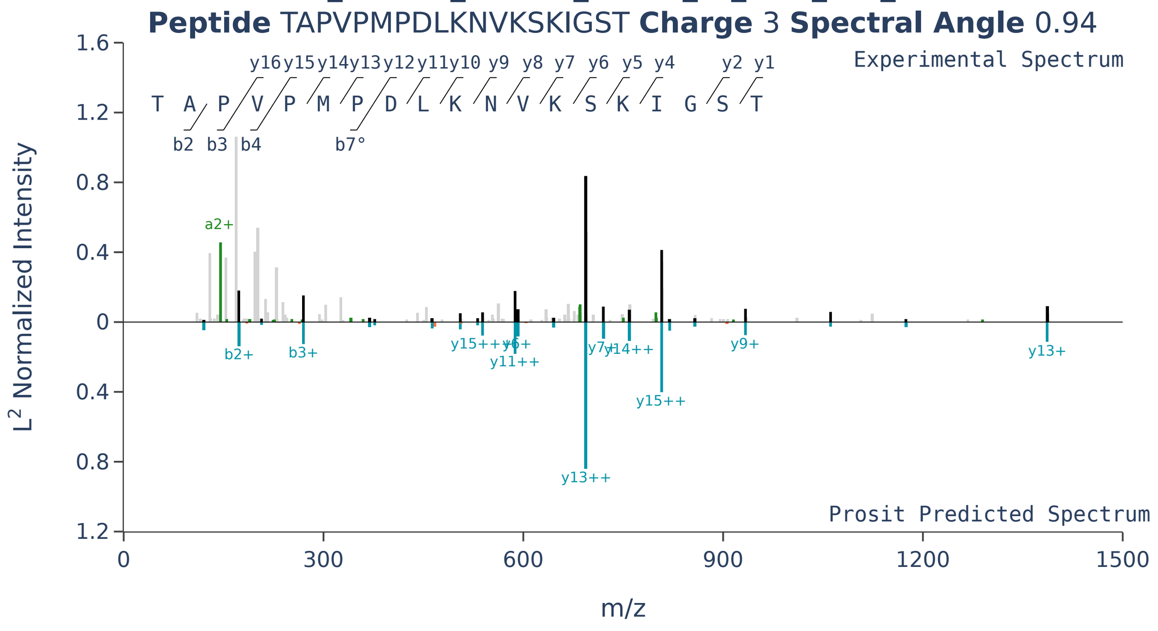


**Source** WSoh_221121_011221_HFGoe_L2_hTau_2h_R1 **Scan** 15057
**Peptide** PVVSGDTSPRHLSNVSSTGSID **Charge** 3 **Spectral Angle** 0.9

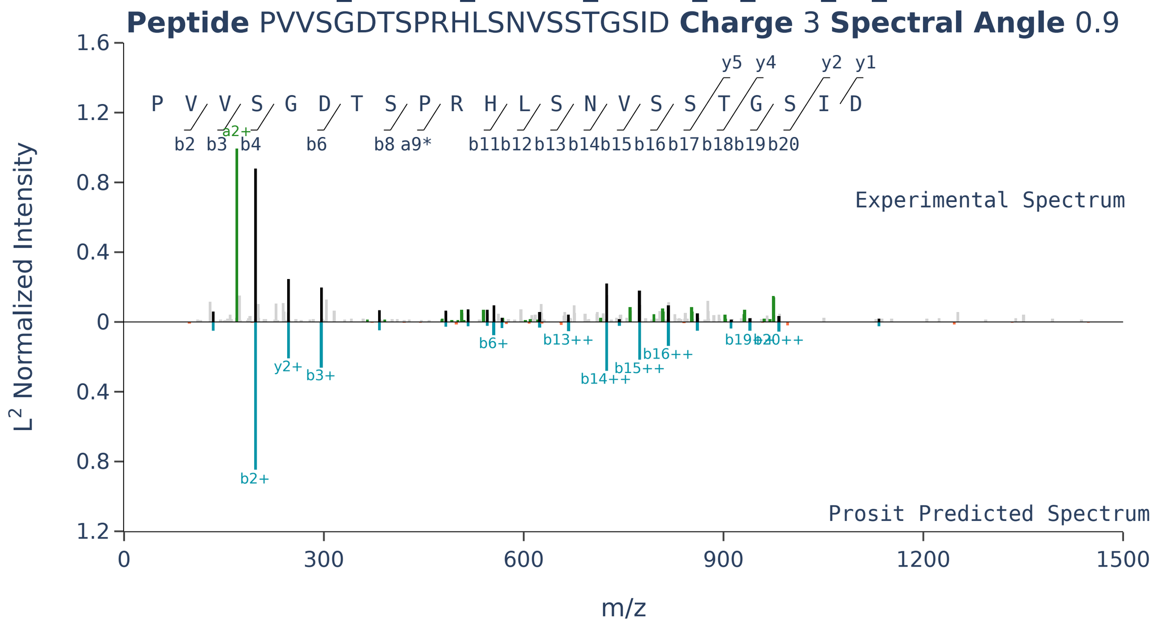


**Source** WSoh_221121_011221_HFGoe_L2_hTau_24h_R2 **Scan** 19476
**Peptide** GAEIVYKSPVVSGDTS **Charge** 2 **Spectral Angle** 0.87

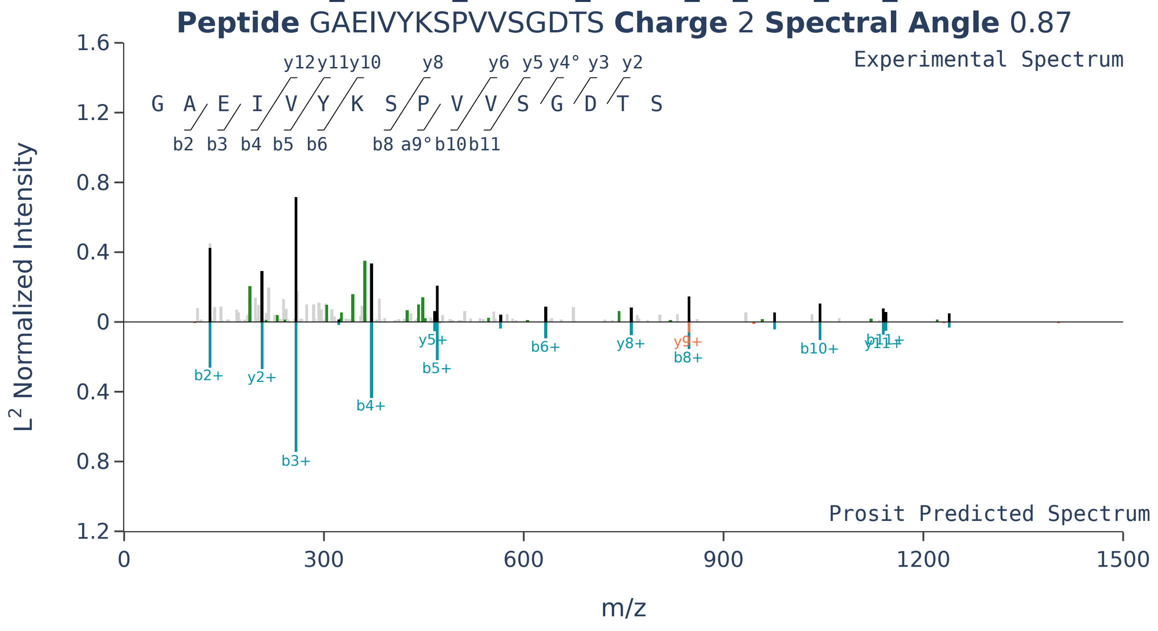


**Source** WSoh_221121_011221_HFGoe_L2_hTau_24h_R1 **Scan** 21642
**Peptide** APVPMPDLKNVKSKIGSTE **Charge** 3 **Spectral Angle** 0.9

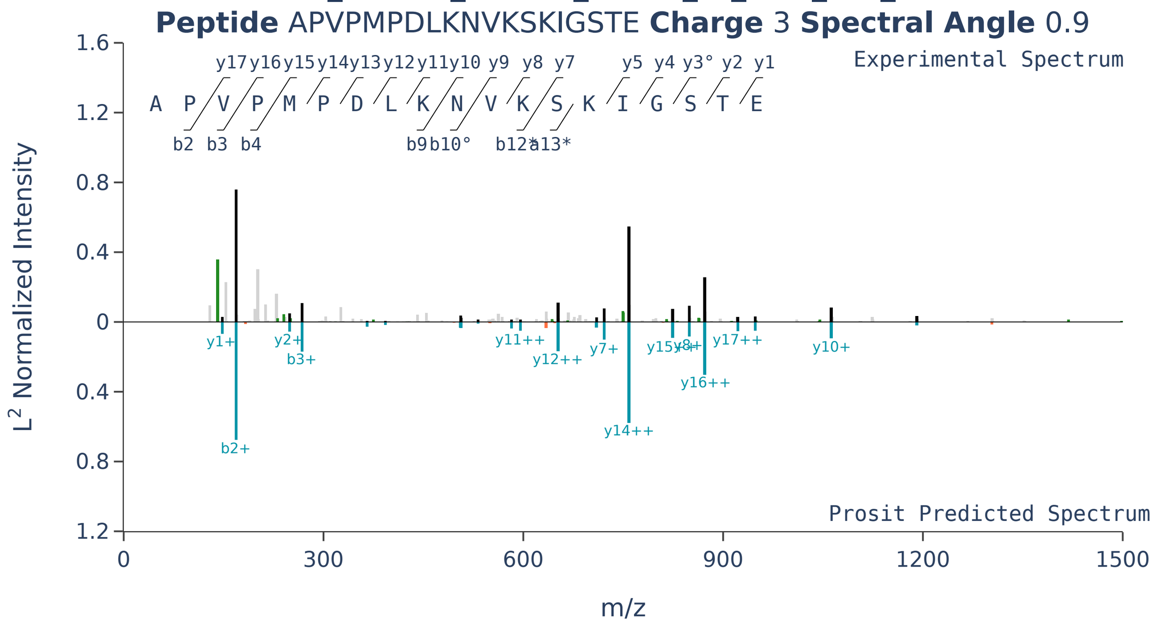

**Source** WSoh_221121_011221_HFGoe_L1_hTau_24h_R1 **Scan** 21253
**Peptide** TAEEAGIGDTPSLE **Charge** 1 **Spectral Angle** 0.87

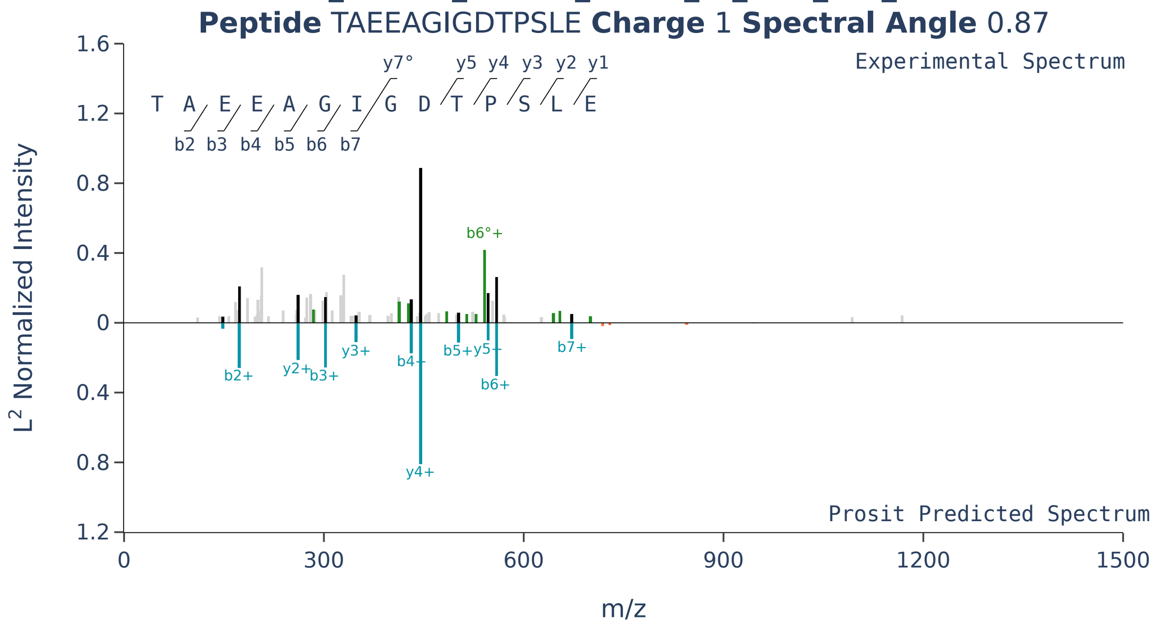


**Source** WSoh_221121_011221_HFGoe_L1_hTau_4h_R2 **Scan** 17119
**Peptide** TPSLEDEAAGHVTQA **Charge** 2 **Spectral Angle** 0.87

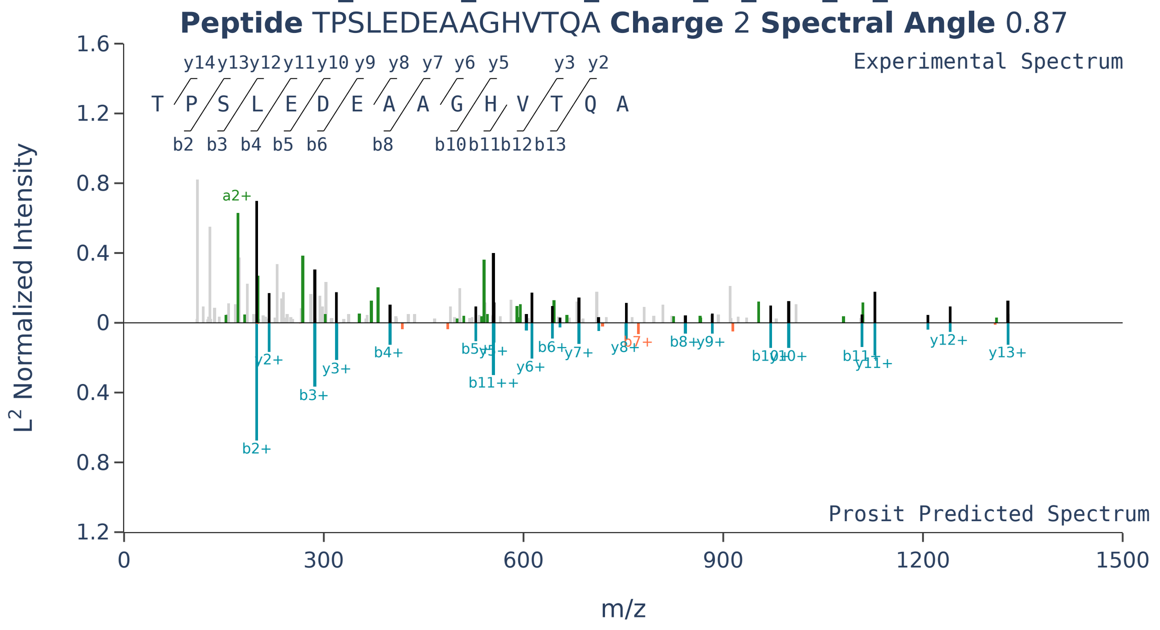


**Source** WSoh_221121_011221_HFGoe_L2_hTau_1h_R2 **Scan** 11800
**Peptide** KESPLQTPTED **Charge** 2 **Spectral Angle** 0.87

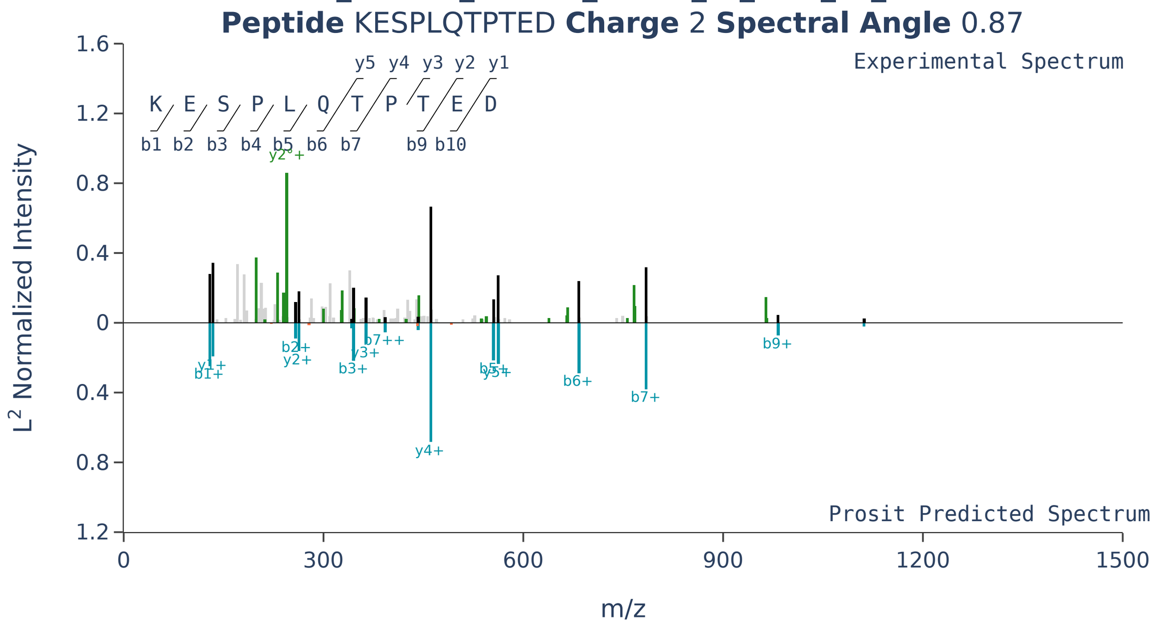


**Source** WSoh_221121_011221_HFGoe_L2_hTau_2h_R2 **Scan** 5764
**Peptide** EGAPGKQAAAQPHTE **Charge** 3 **Spectral Angle** 0.88

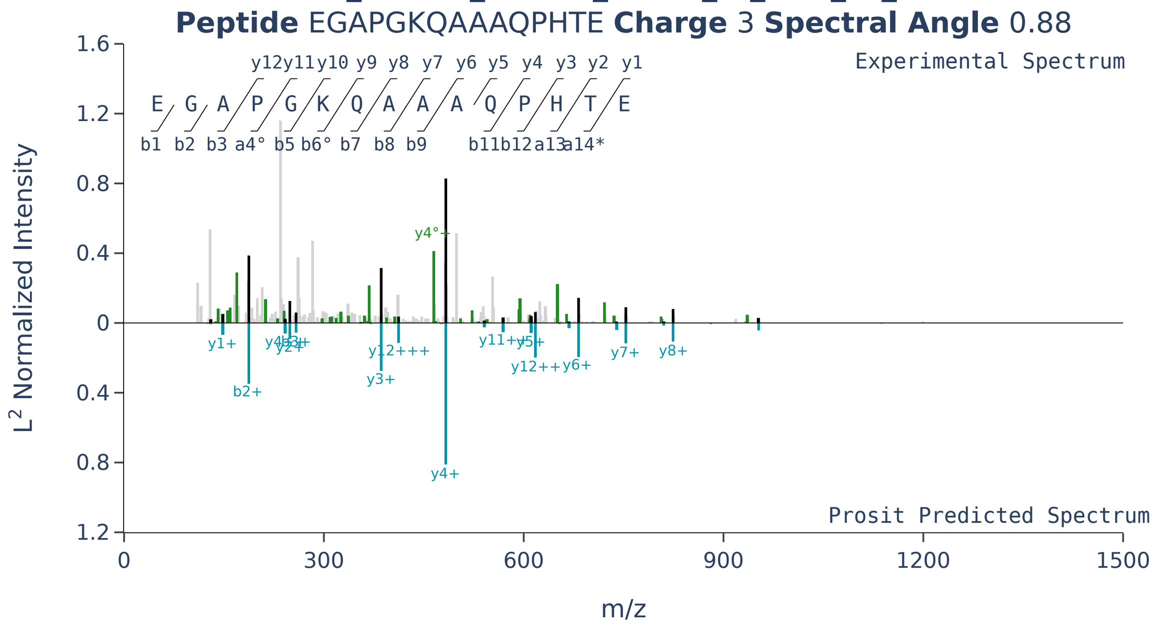


**Source** WSoh_221121_011221_HFGoe_L2_hTau_24h_R1 **Scan** 7221
**Peptide** SPSSAKSRL **Charge** 2 **Spectral Angle** 0.9

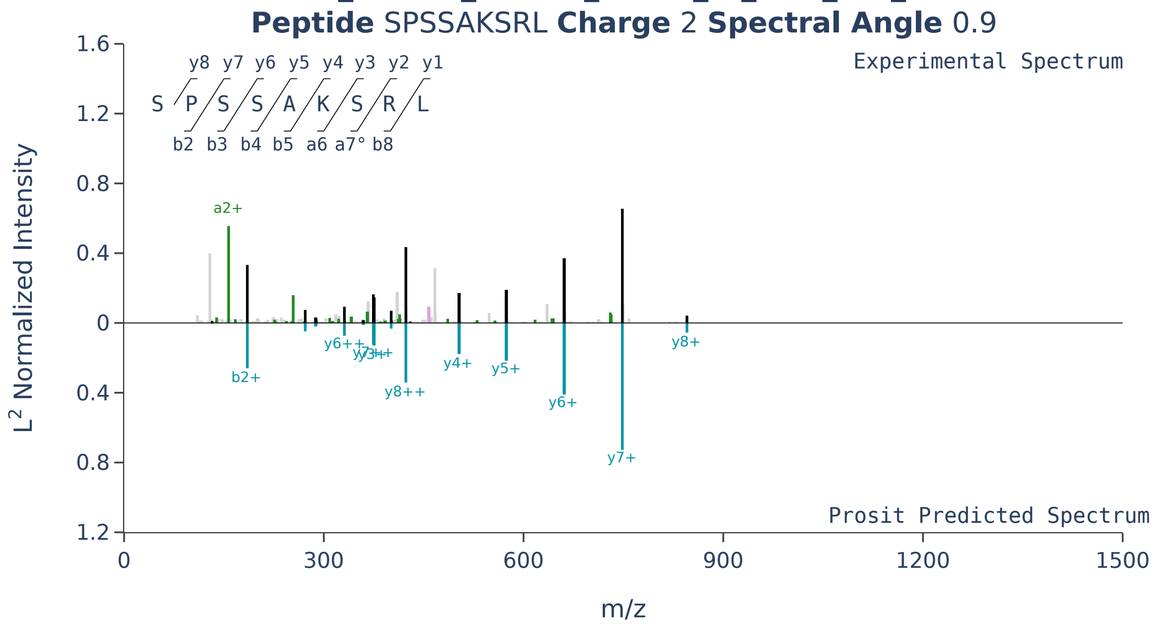


**Source** WSoh_221121_011221_HFGoe_L2_hTau_24h_R1 **Scan** 17053
**Peptide** VPMPDLKNVKSK **Charge** 3 **Spectral Angle** 0.94

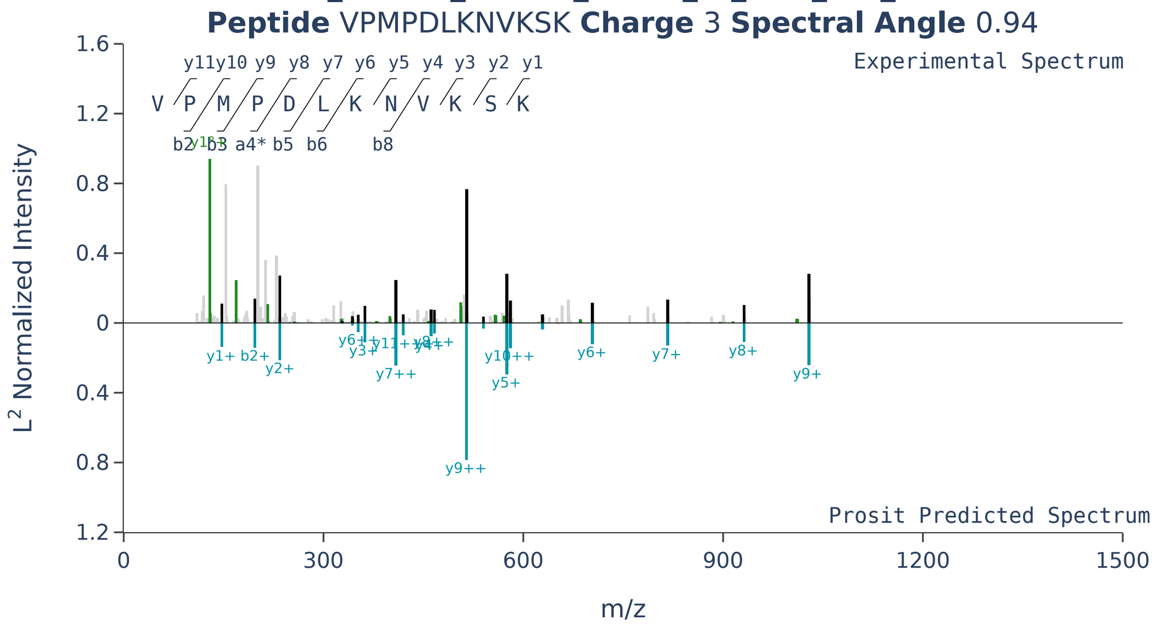


**Source** WSoh_221121_011221_HFGoe_L1_hTau_24h_R1 **Scan** 15602
**Peptide** TYGLGDRKDQGGYTM **Charge** 2 **Spectral Angle** 0.87

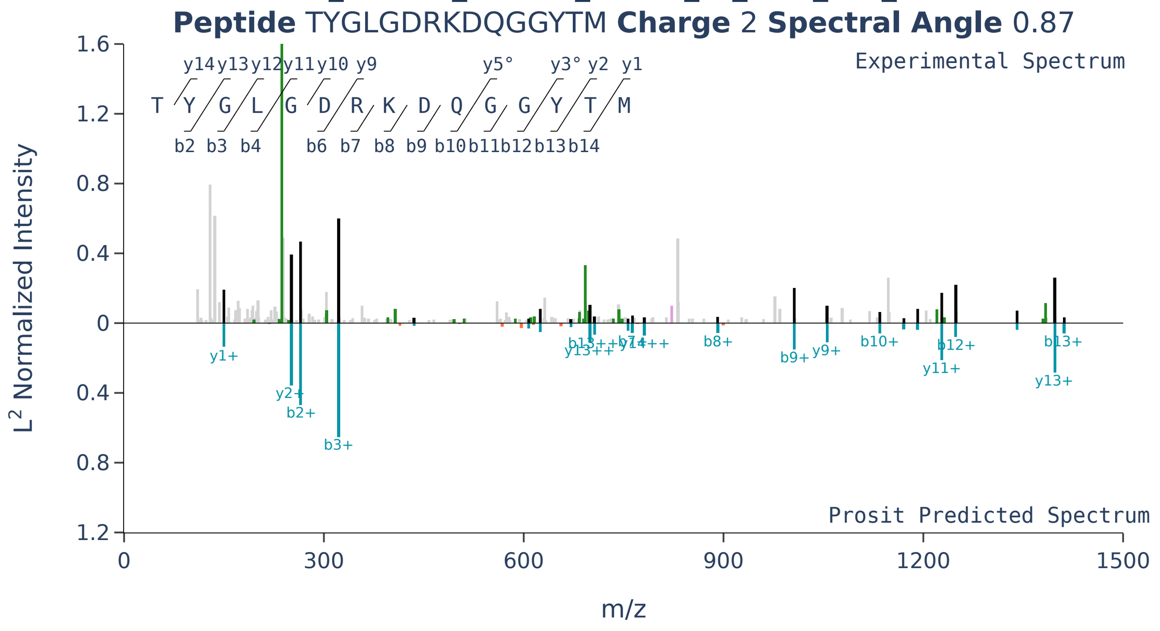


**Source** WSoh_221121_011221_HFGoe_L2_hTau_1h_R2 **Scan** 5604
**Peptide** PRGAAPPGQKGQANAT **Charge** 2 **Spectral Angle** 0.86

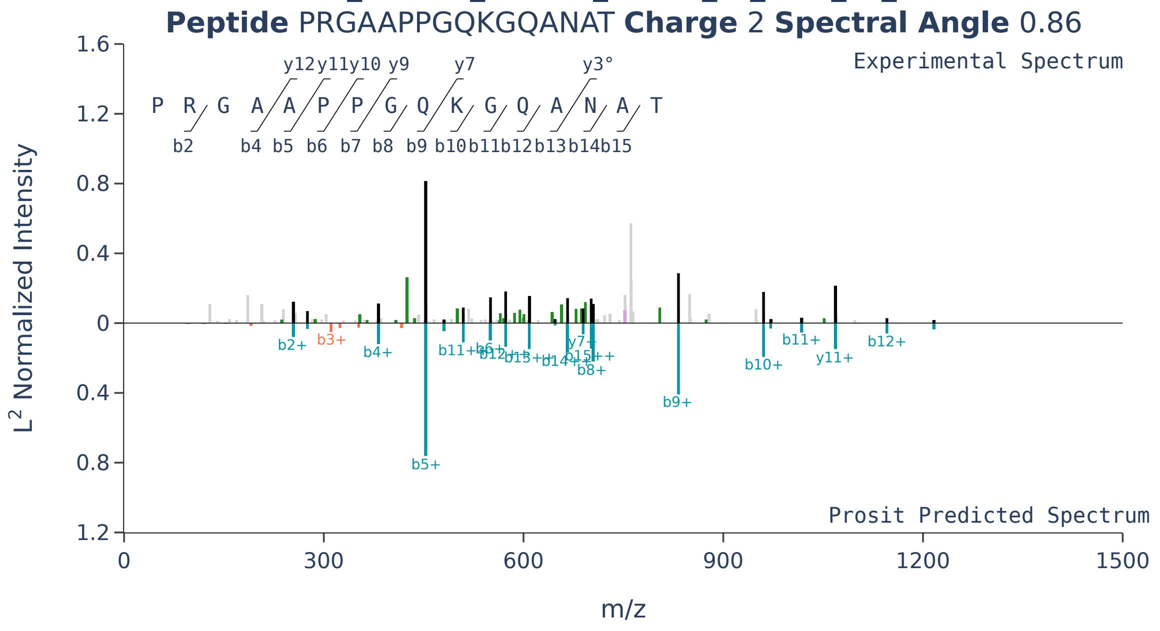


**Source** WSoh_221121_011221_HFGoe_L1_hTau_24h_R2 **Scan** 21298
**Peptide** EAGIGDTPSLEDEAAGHVTQ **Charge** 2 **Spectral Angle** 0.83

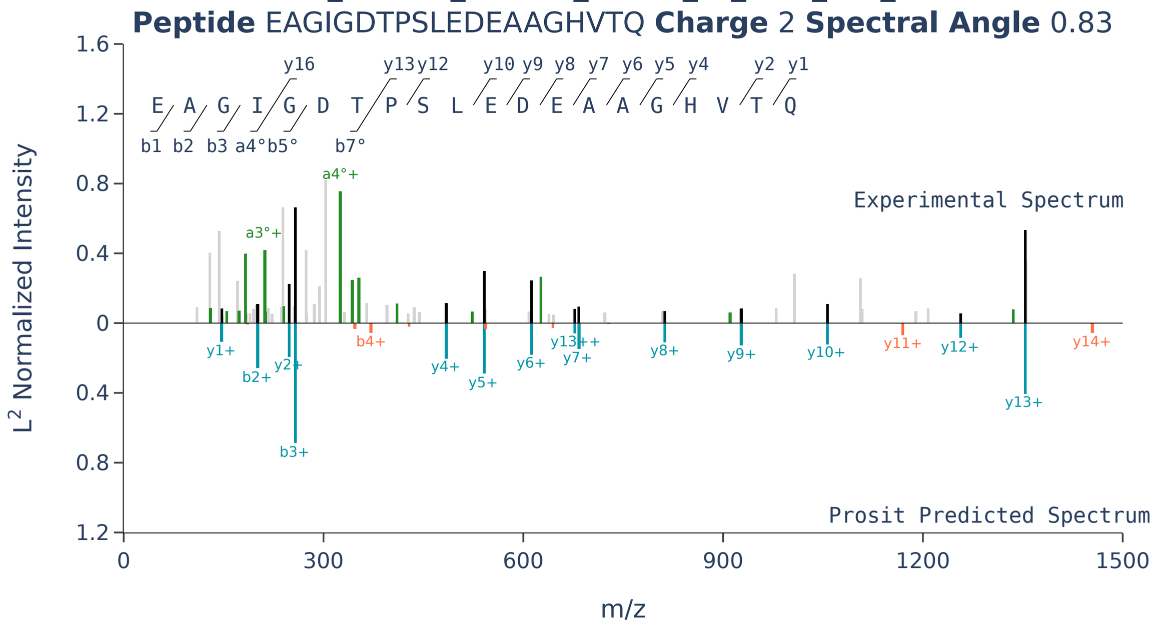


**Source** WSoh_221121_011221_HFGoe_L1_hTau_24h_R1 **Scan** 7008
**Peptide** NIHHKPGGGQVEV **Charge** 2 **Spectral Angle** 0.81

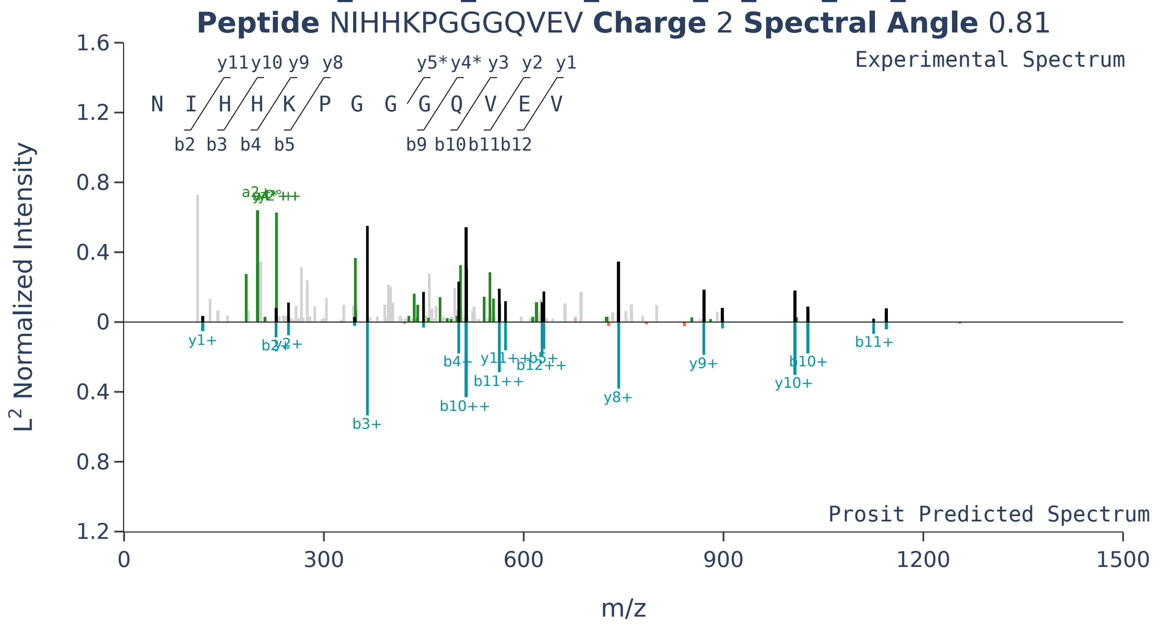


**Source** WSoh_221121_011221_HFGoe_L2_hTau_4h_R1 **Scan** 13352
**Peptide** KPGGGQVEVKSEKLDFKDRV **Charge** 5 **Spectral Angle** 0.85

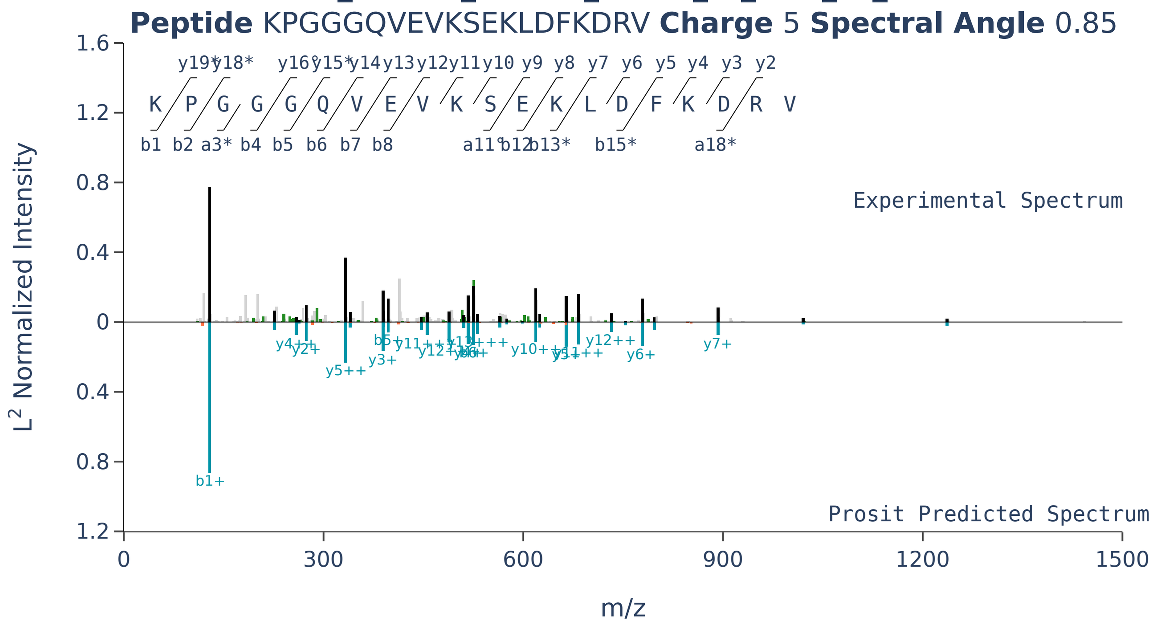


**Source** WSoh_221121_011221_HFGoe_L2_hTau_2h_R1 **Scan** 6422
**Peptide** CGSLGNIHHKPGG **Charge** 3 **Spectral Angle** 0.87

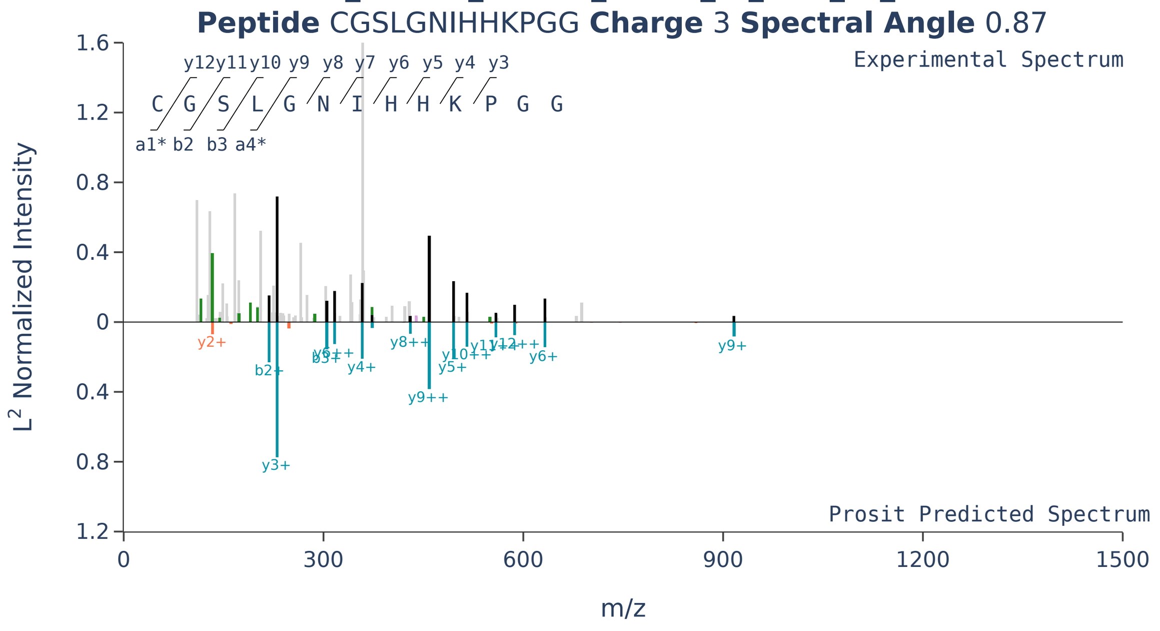


**Source** WSoh_221121_011221_HFGoe_L2_hTau_2h_R1 **Scan** 13379
**Peptide** KAKTDHGAEIVYKSPVVSGDTSPRHL **Charge** 5 **Spectral Angle** 0.87

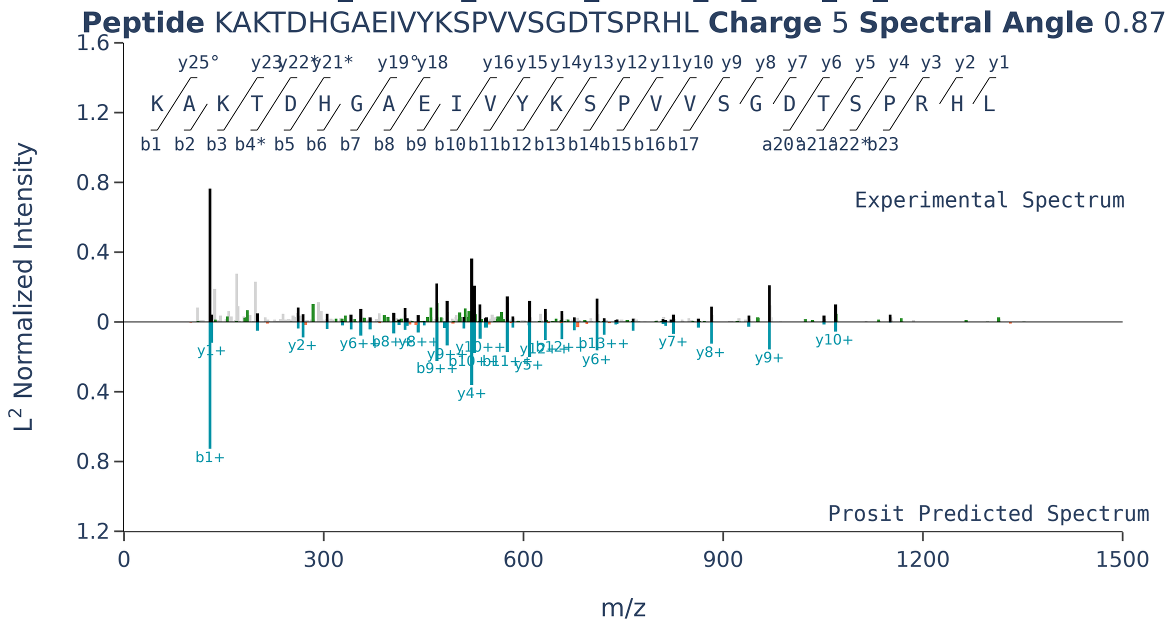


**Source** WSoh_221121_011221_HFGoe_L1_hTau_4h_R2 **Scan** 8067
**Peptide** DFKDRVQ **Charge** 2 **Spectral Angle** 0.94

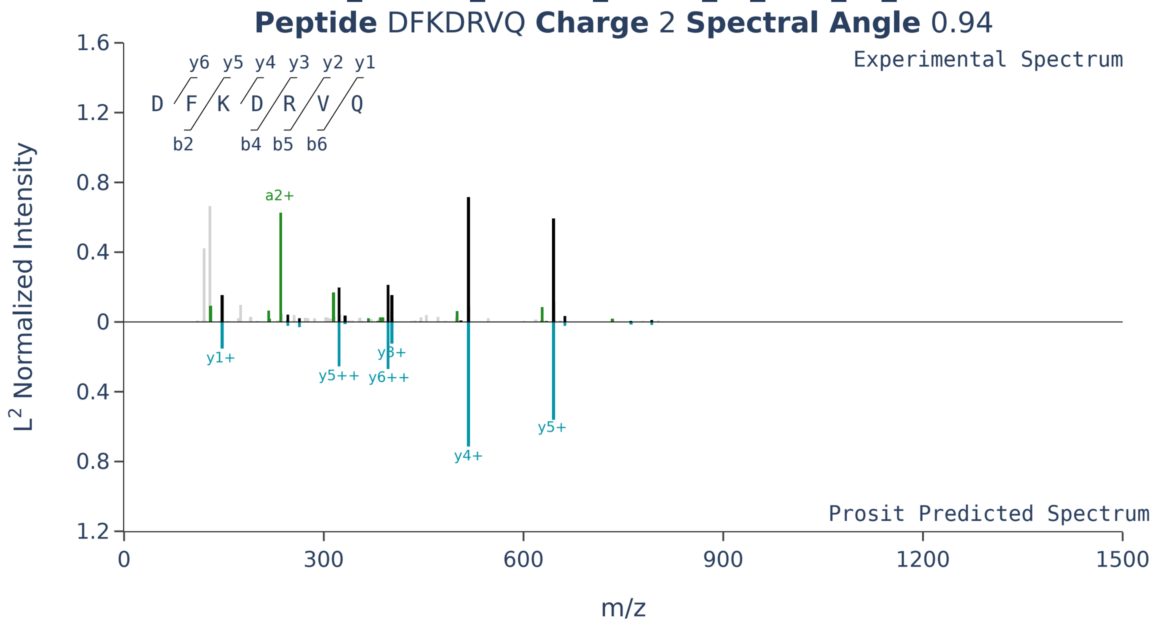


**Source** WSoh_221121_011221_HFGoe_L2_hTau_24h_R2 **Scan** 10053
**Peptide** KIGSTENLKHQPGGGKVQIINK **Charge** 3 **Spectral Angle** 0.91

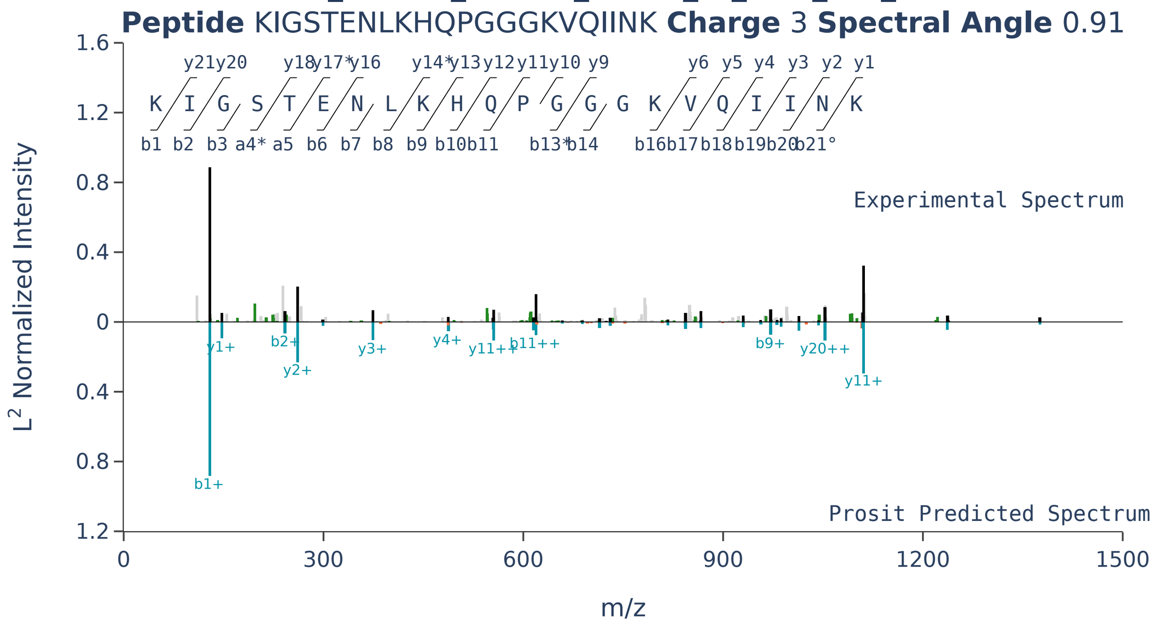

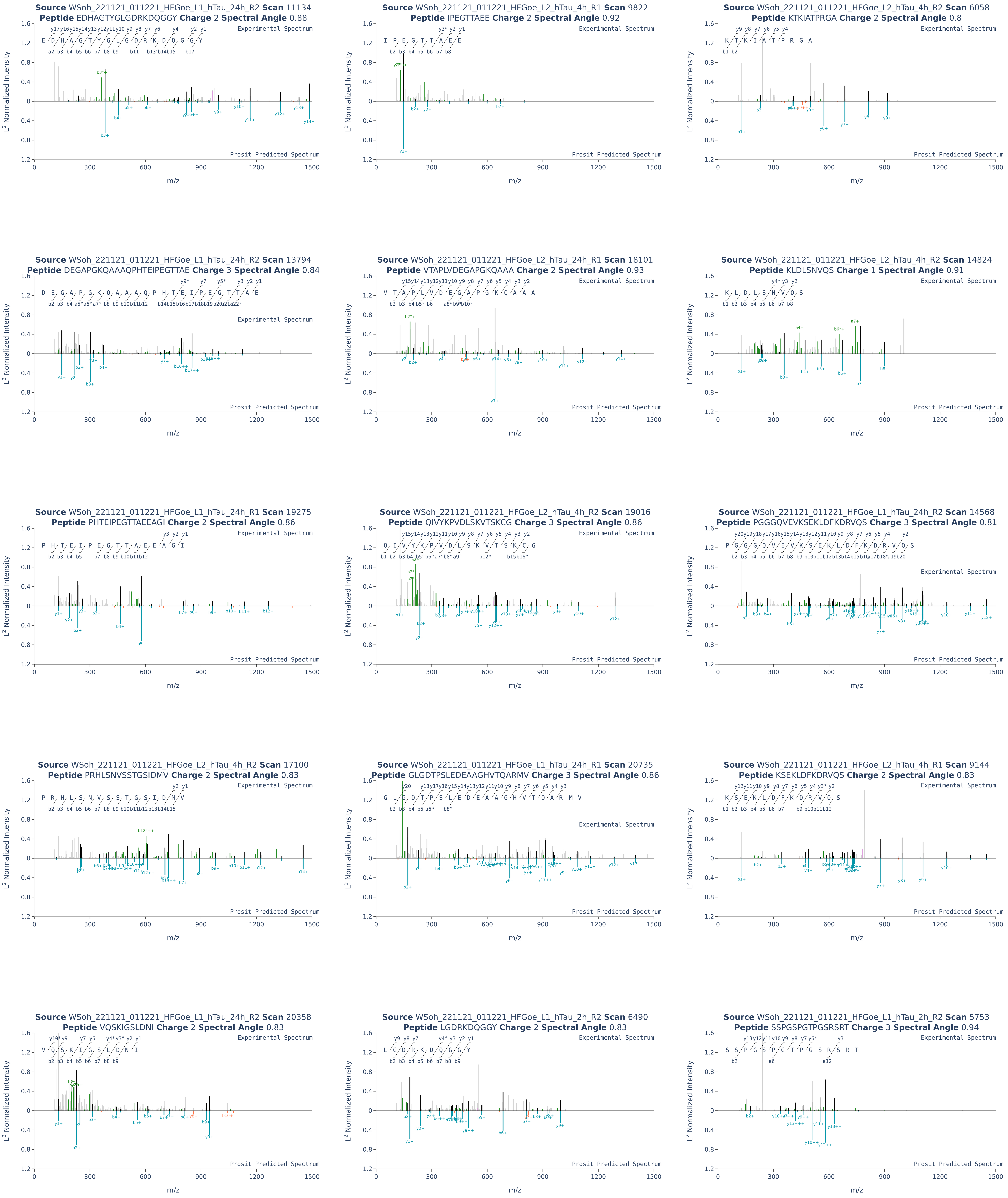

Source WSoh_221121_011221_HFGoe_L1_hTau_24h_R2 Scan 11134
Peptide EDHAGTYGLGDRKDQGGY Charge 2 Spectral Angle 0.88
Source WSoh_221121_011221_HFGoe_L2_hTau_4h_R1 Scan 9822
Peptide IPEGTTAEE Charge 2 Spectral Angle 0.92
Source WSoh_221121_011221_HFGoe_L2_hTau_4h_R2 Scan 6058
Peptide KTKIATPRGA Charge 2 Spectral Angle 0.8
Source WSoh_221121_011221_HFGoe_L1_hTau_24h_R2 Scan 13794
Peptide DEGAPGKQAAAQPHTEIPEGTTAE Charge 3 Spectral Angle 0.84
Source WSoh_221121_011221_HFGoe_L2_hTau_24h_R1 Scan 18101
Peptide VTAPLVDEGAPGKQAAA Charge 2 Spectral Angle 0.93
Source WSoh_221121_011221_HFGoe_L2_hTau_4h_R2 Scan 14824
Peptide KLDLSNVQS Charge 1 Spectral Angle 0.91
Source WSoh_221121_011221_HFGoe_L1_hTau_24h_R1 Scan 19275
Peptide PHTEIPEGTTAEEAGI Charge 2 Spectral Angle 0.86
Source WSoh_221121_011221_HFGoe_L2_hTau_4h_R2 Scan 19016
Peptide QIVYKPVDLSKVTSKCG Charge 3 Spectral Angle 0.86
Source WSoh_221121_011221_HFGoe_L1_hTau_24h_R2 Scan 14568
Peptide PGGGQVEVKSEKLDFKDRVQS Charge 3 Spectral Angle 0.81
Source WSoh_221121_011221_HFGoe_L2_hTau_4h_R2 Scan 17100
Peptide PRHLSNVSSTGSIDMV Charge 2 Spectral Angle 0.83
Source WSoh_221121_011221_HFGoe_L1_hTau_24h_R1 Scan 20735
Peptide GLGDTPSLEDEAAGHVTQARMV Charge 3 Spectral Angle 0.86
Source WSoh_221121_011221_HFGoe_L2_hTau_4h_R1 Scan 9144
Peptide KSEKLDFKDRVQS Charge 2 Spectral Angle 0.83
Source WSoh_221121_011221_HFGoe_L1_hTau_24h_R2 Scan 20358
Peptide VQSKIGSLDNI Charge 2 Spectral Angle 0.83
Source WSoh_221121_011221_HFGoe_L1_hTau_2h_R2 Scan 6490
Peptide LGDRKDQGGY Charge 2 Spectral Angle 0.83
Source WSoh_221121_011221_HFGoe_L1_hTau_2h_R2 Scan 5753
Peptide SSPGSPGTPGSRSRT Charge 3 Spectral Angle 0.94

**Source** WSoh_221121_011221_HFGoe_L2_hTau_2h_R2 **Scan** 6365
**Peptide** RENAKAKTDHGAEIVYK **Charge** 3 **Spectral Angle** 0.86

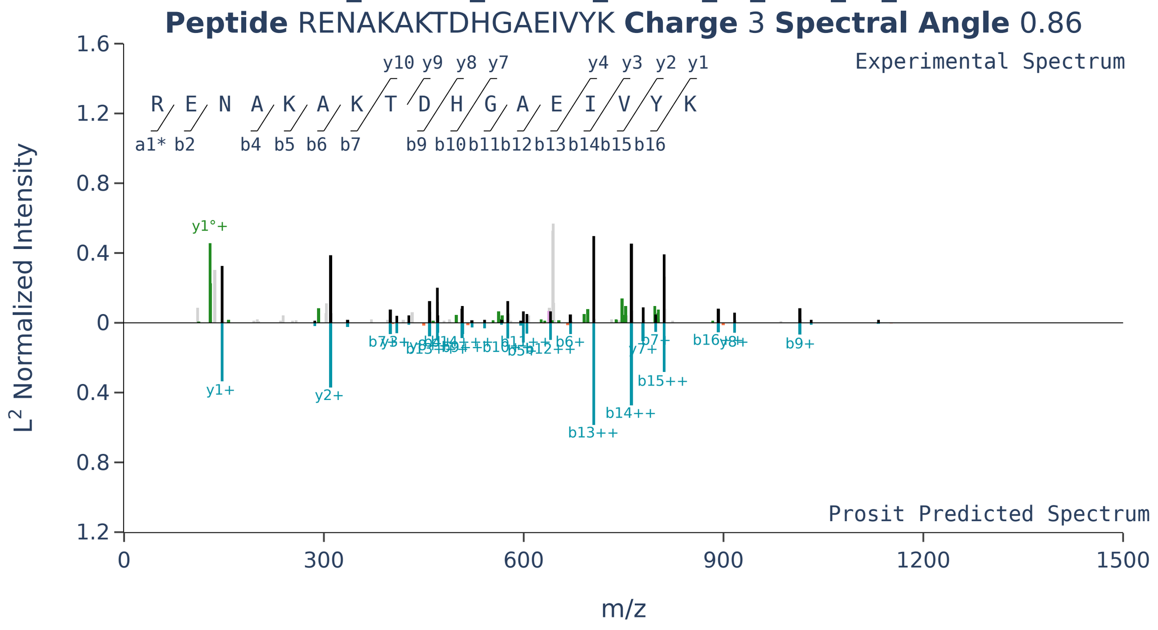


**Source** WSoh_221121_011221_HFGoe_L2_hTau_4h_R1 **Scan** 10545
**Peptide** QSKIGSLD **Charge** 1 **Spectral Angle** 0.93

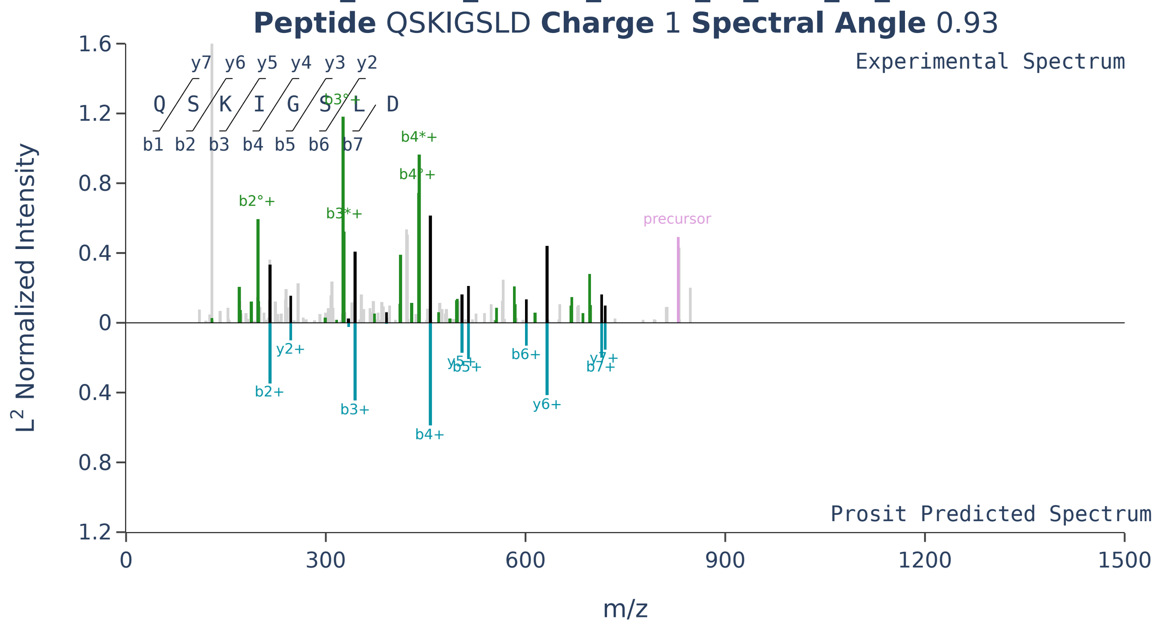


**Source** WSoh_221121_011221_HFGoe_L2_hTau_24h_R2 **Scan** 18042
**Peptide** TPSLPTPPTRE **Charge** 2 **Spectral Angle** 0.9

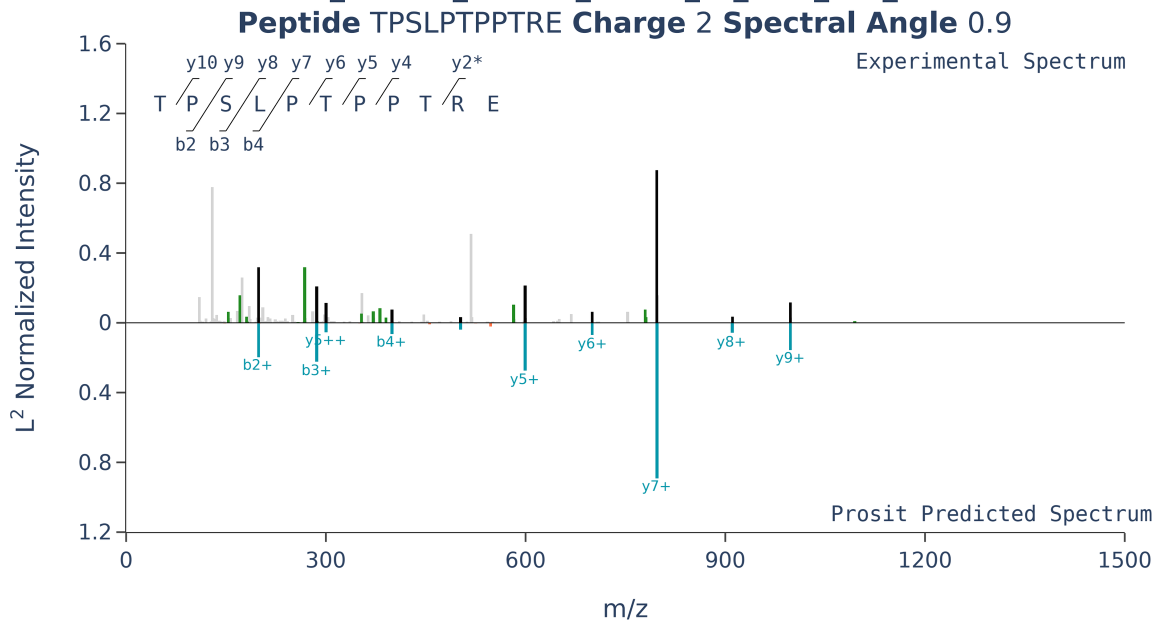


**Source** WSoh_221121_011221_HFGoe_L1_hTau_24h_R1 **Scan** 15789
**Peptide** GSLDNITHVPGGGNKKL **Charge** 3 **Spectral Angle** 0.91

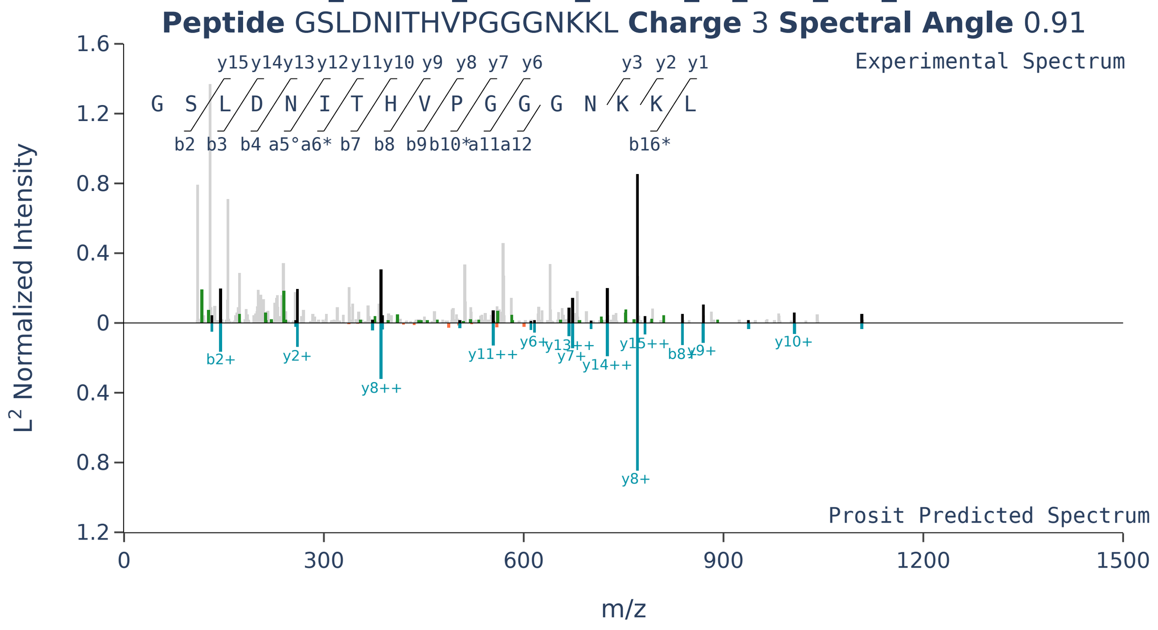


**Source** WSoh_221121_011221_HFGoe_L2_hTau_24h_R2 **Scan** 11881
**Peptide** NLTHVPGGGNKKIETHKLTFRENA **Charge** 3 **Spectral Angle** 0.84

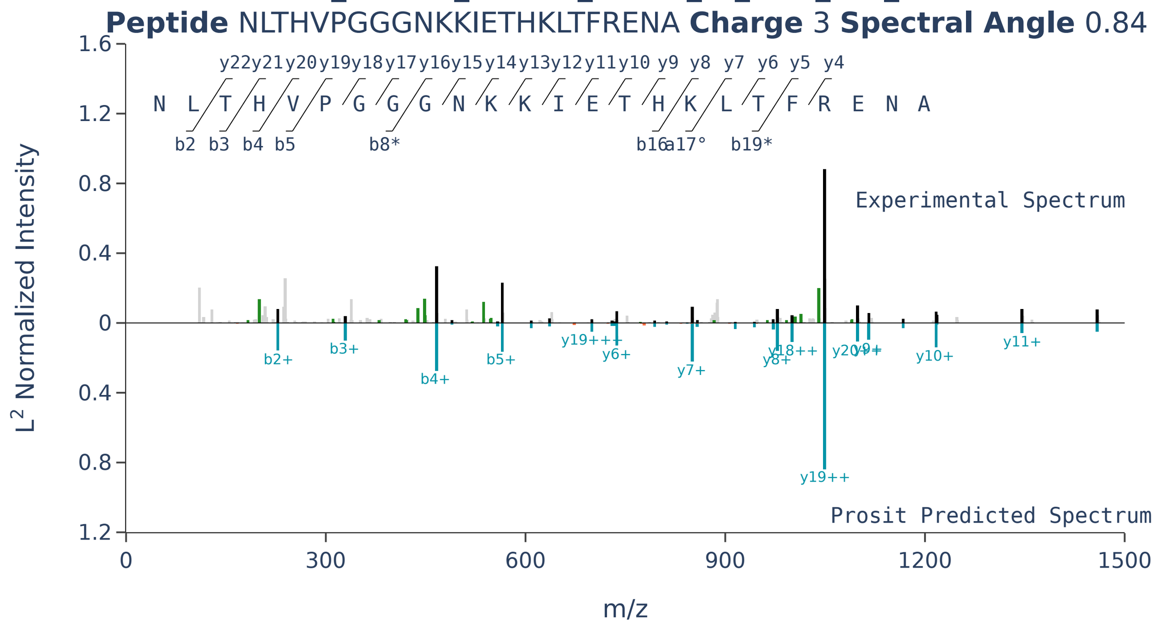


**Source** WSoh_221121_011221_HFGoe_L2_hTau_4h_R1 **Scan** 9129
**Peptide** EVSASLAKQ **Charge** 2 **Spectral Angle** 0.86

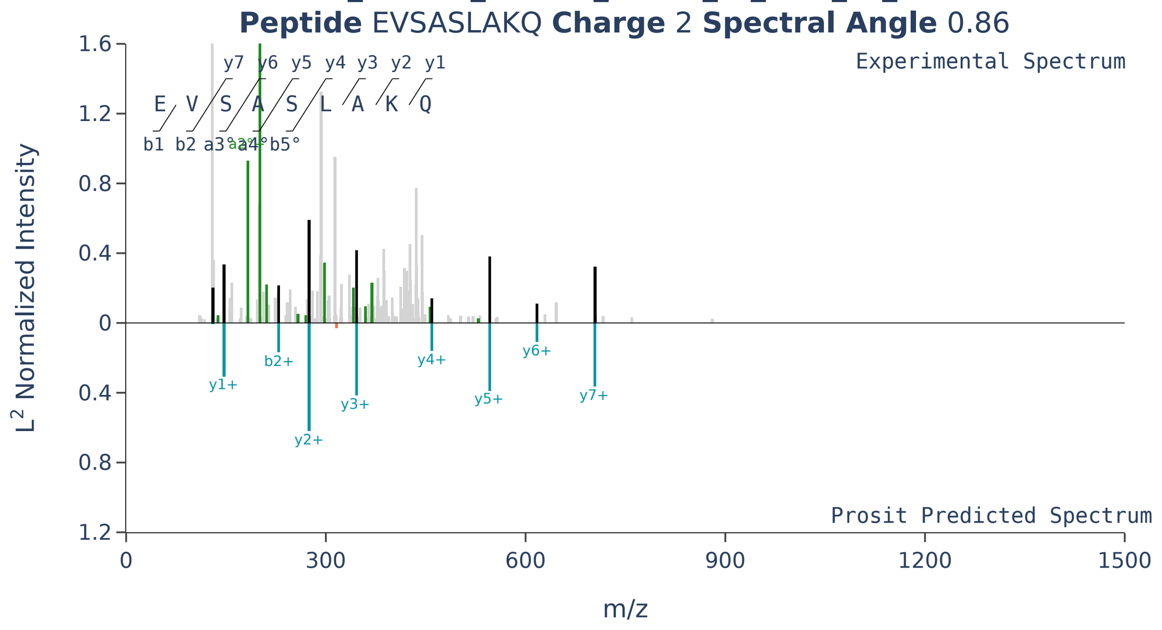


**Source** WSoh_221121_011221_HFGoe_L2_hTau_1h_R1 **Scan** 12804
**Peptide** KLDFKDRVQSKIG **Charge** 3 **Spectral Angle** 0.88

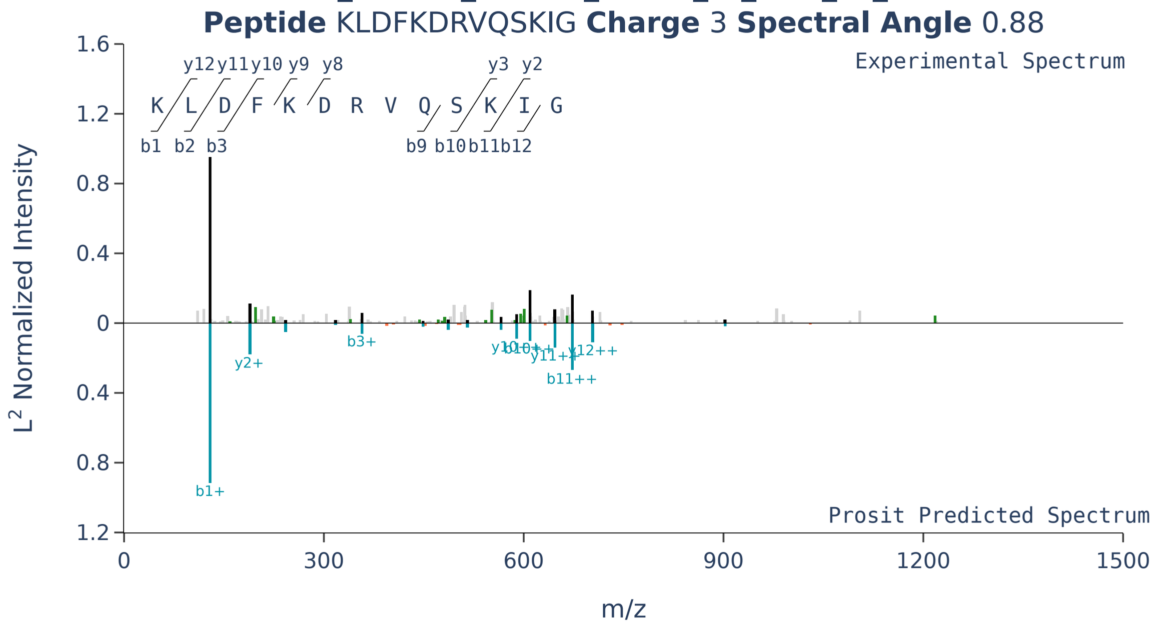


**Source** WSoh_221121_011221_HFGoe_L2_hTau_24h_R2 **Scan** 7428
**Peptide** LTHVPGGGNKKI **Charge** 3 **Spectral Angle** 0.85

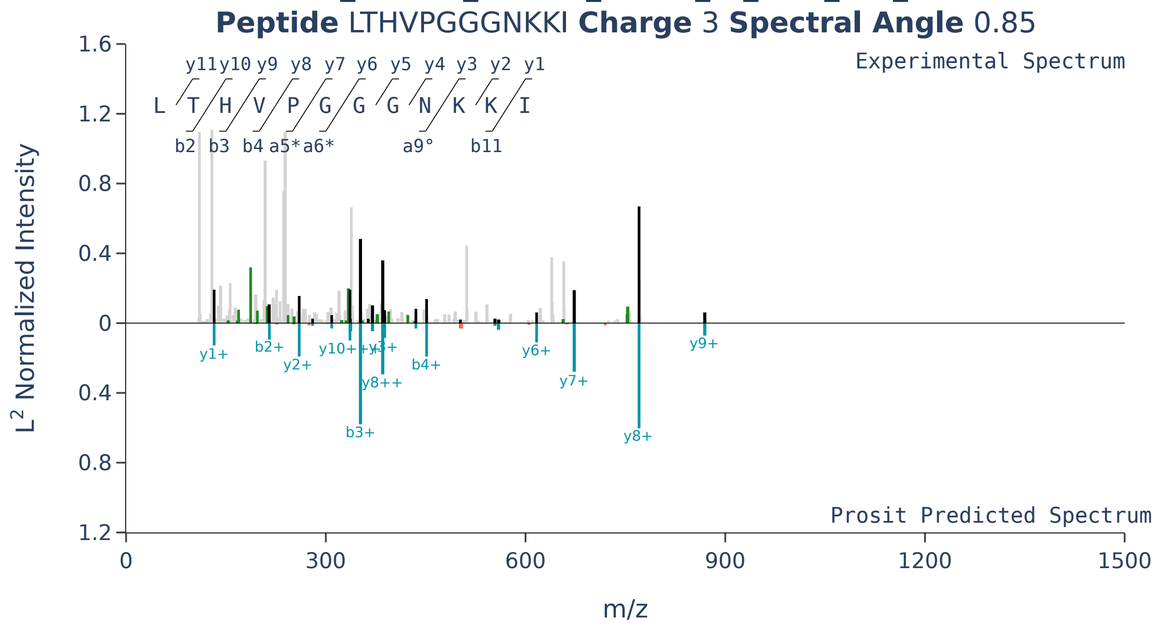


**Source** WSoh_221121_011221_HFGoe_L2_hTau_24h_R2 **Scan** 19443
**Peptide** DFKDRVQSKIGSL **Charge** 3 **Spectral Angle** 0.88

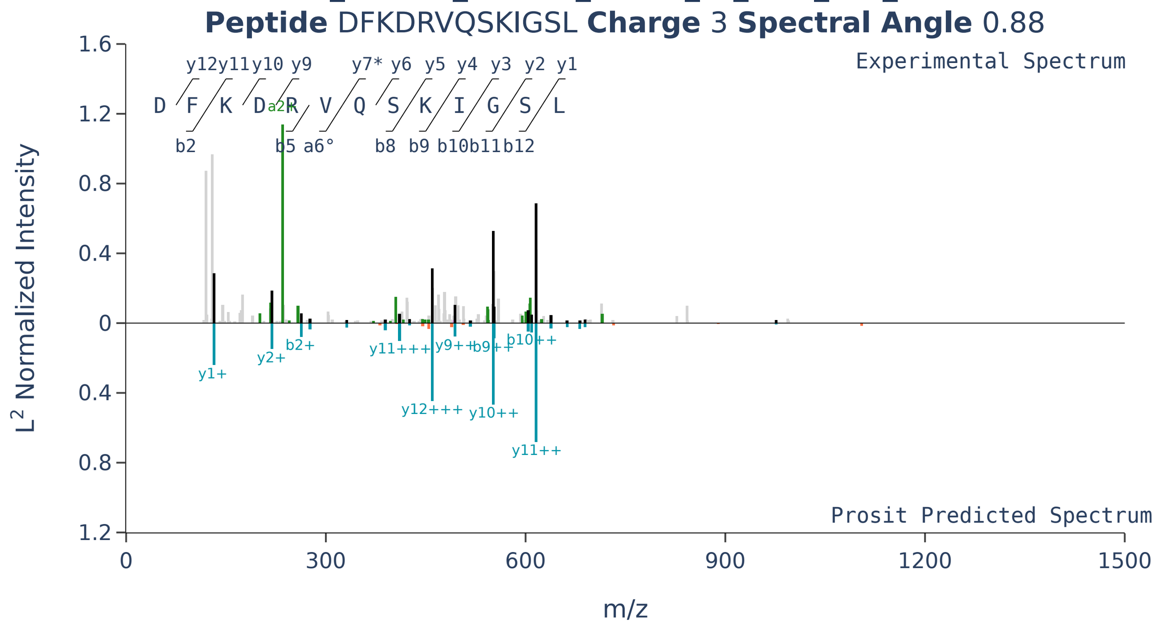


**Source** WSoh_221121_011221_HFGoe_L1_hTau_24h_R1 **Scan** 15100
**Peptide** YKPVDLSKVTSK **Charge** 3 **Spectral Angle** 0.87

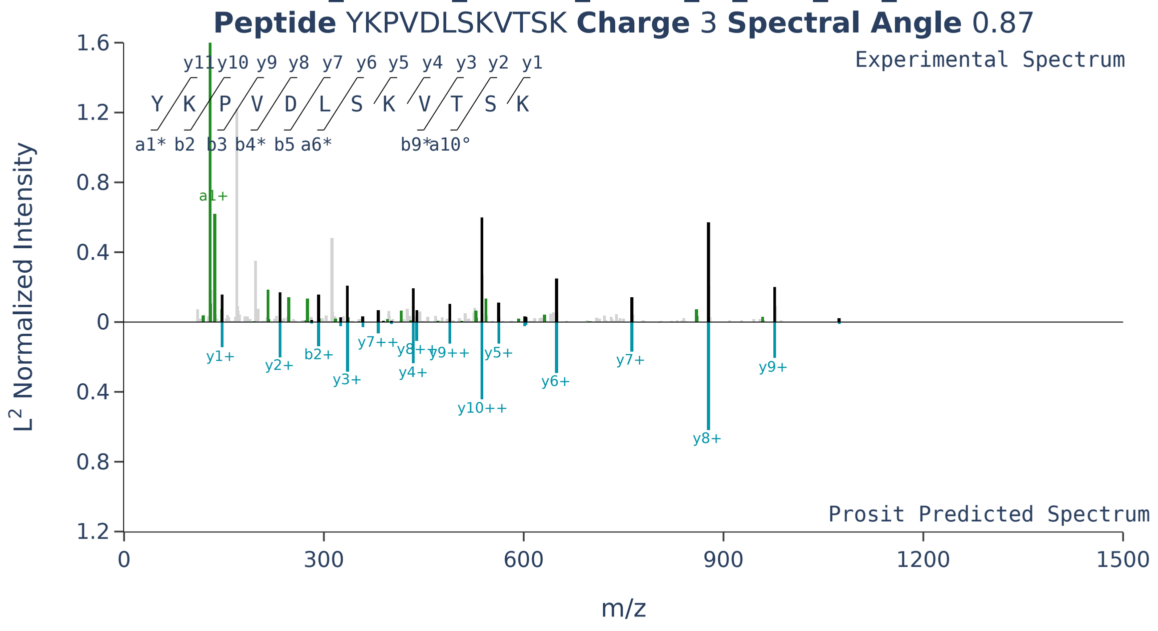


**Source** WSoh_221121_011221_HFGoe_L1_hTau_4h_R1 **Scan** 15774
**Peptide** KLGSLDNITHVPGGGNKKIET **Charge** 3 **Spectral Angle** 0.89

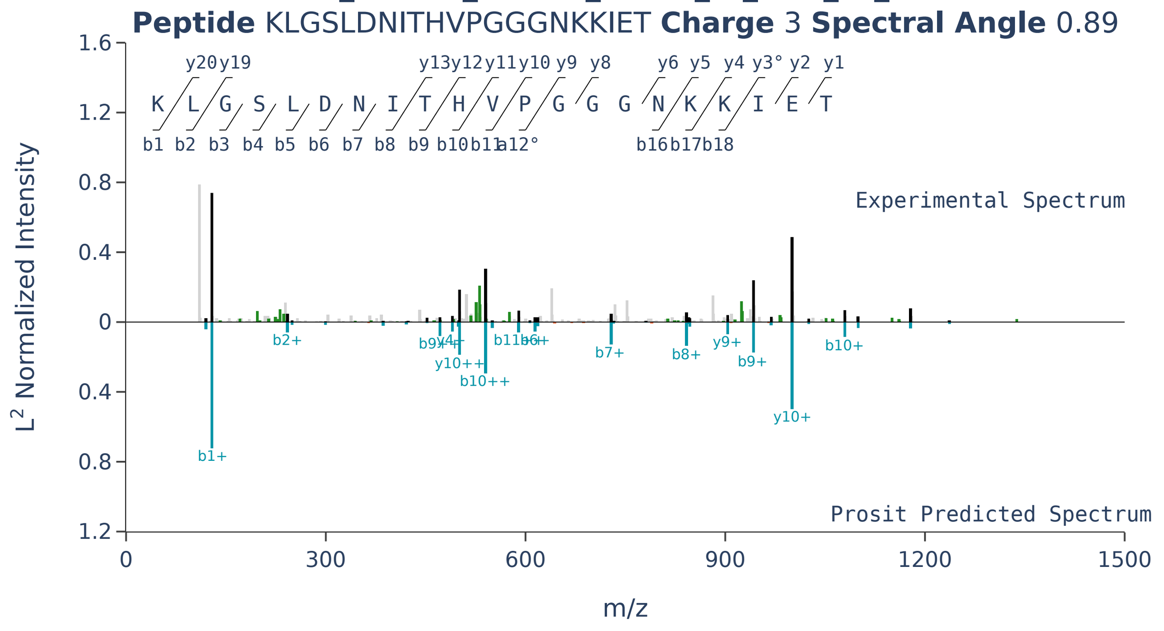


**Source** WSoh_221121_011221_HFGoe_L1_hTau_4h_R1 **Scan** 13405
**Peptide** QVEVKSEKLDFKDRVQS **Charge** 3 **Spectral Angle** 0.83

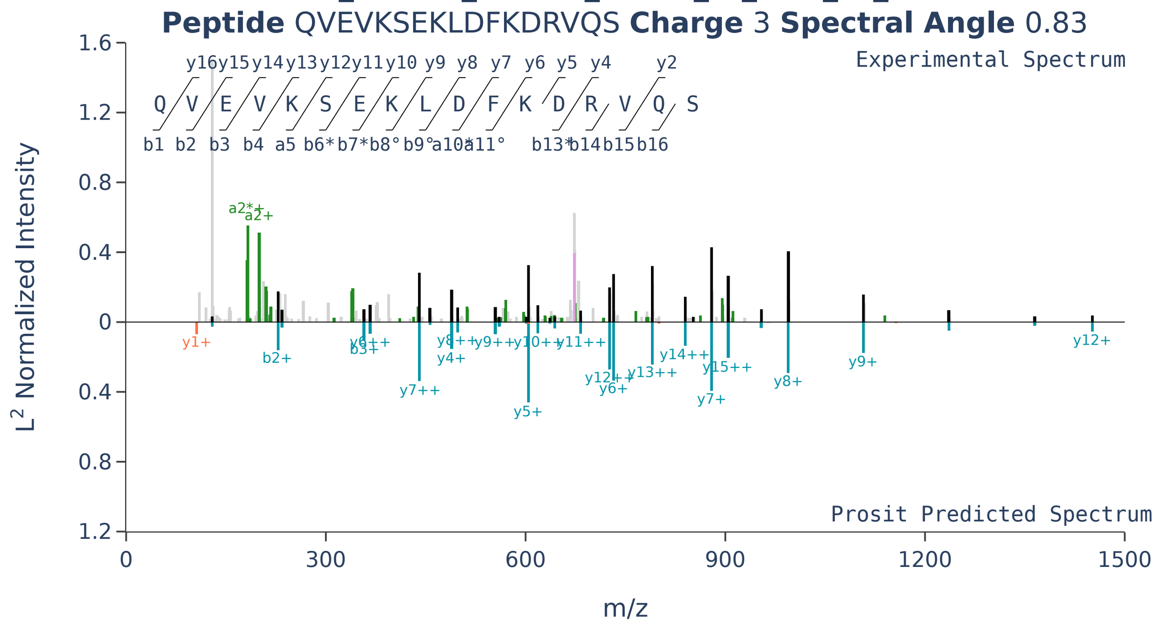


**Source** WSoh_221121_011221_HFGoe_L1_hTau_24h_R1 **Scan** 21802
**Peptide** EAGIGDTPSLEDEAAGHVTQA **Charge** 2 **Spectral Angle** 0.85

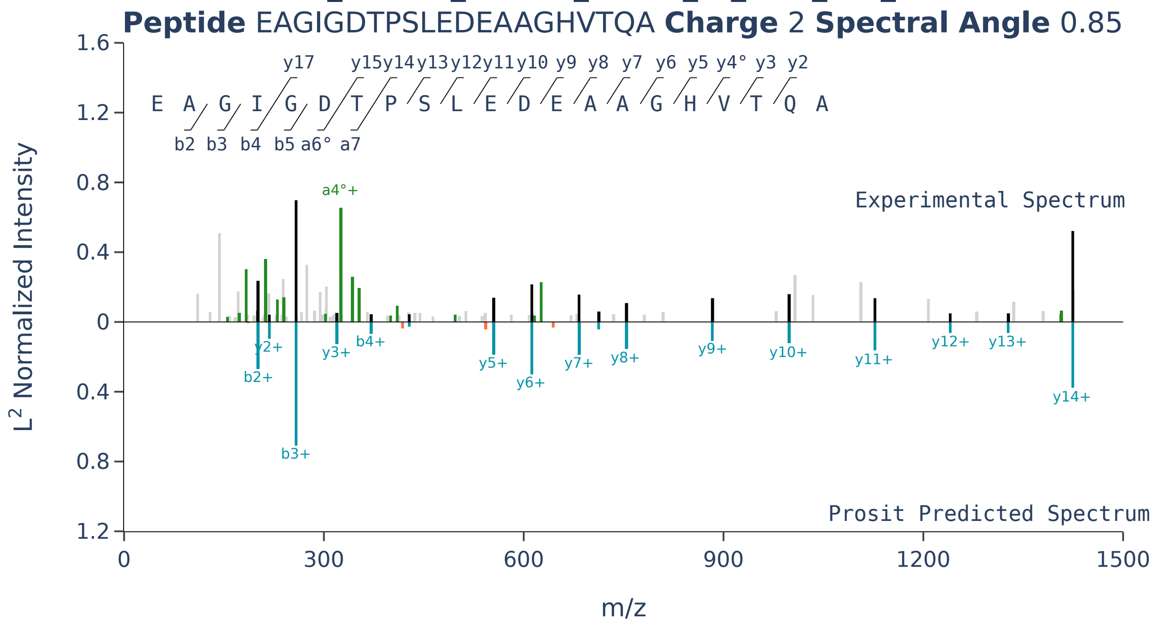


**Source** WSoh_221121_011221_HFGoe_L1_hTau_24h_R2 **Scan** 7281
**Peptide** EPGSETSDAKSTPTAED **Charge** 2 **Spectral Angle** 0.92

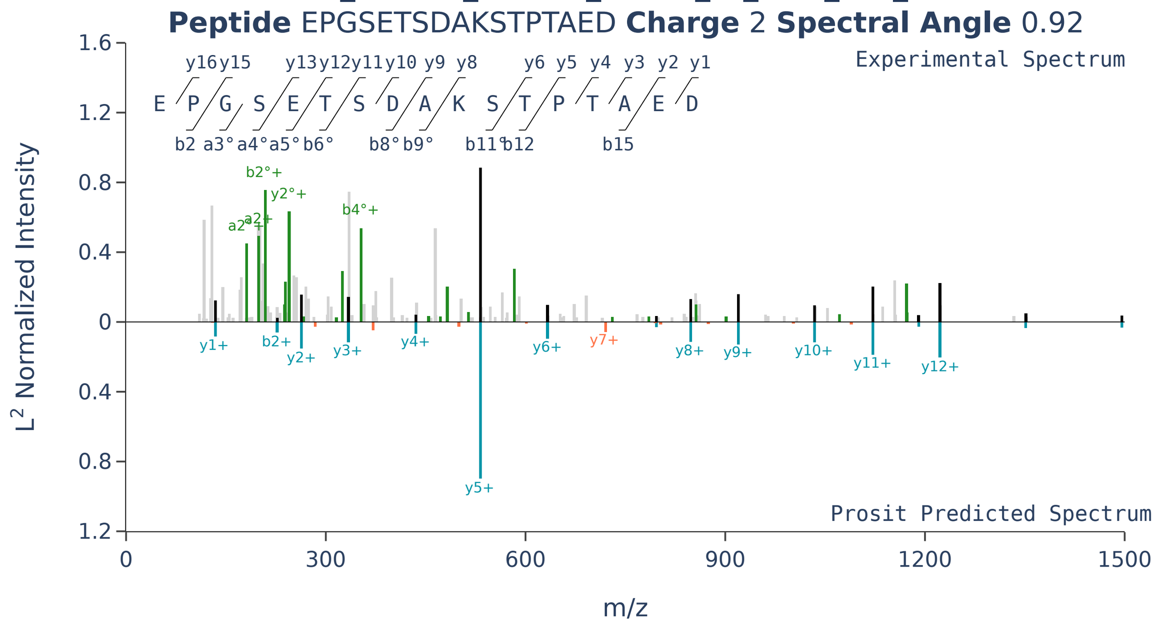


**Source** WSoh_221121_011221_HFGoe_L1_hTau_1h_R2 **Scan** 12334
**Peptide** KDRVQSKI **Charge** 2 **Spectral Angle** 0.9

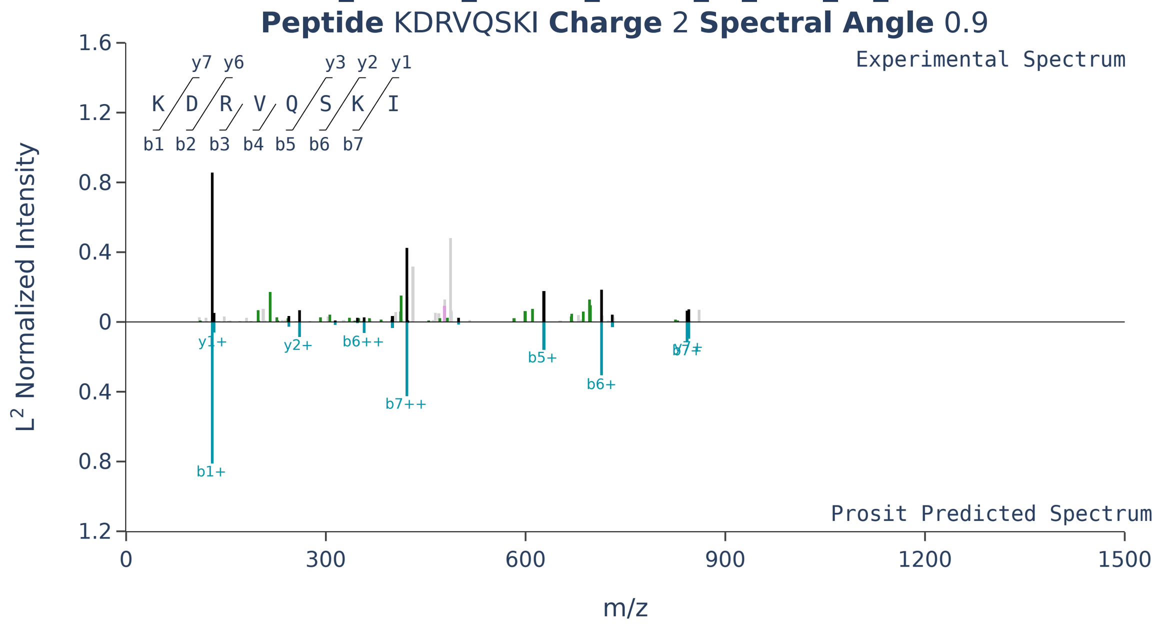

**Source** WSoh_221121_011221_HFGoe_L1_hTau_4h_R1 **Scan** 7551
**Peptide** DTSPRHLSNVS **Charge** 2 **Spectral Angle** 0.82

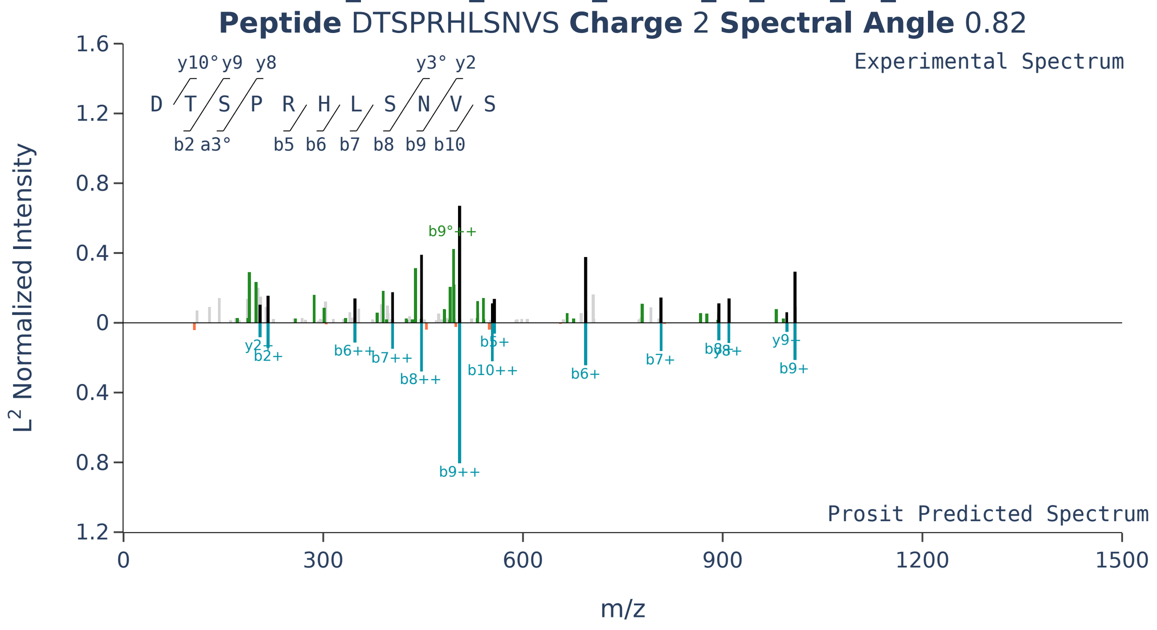


**Source** WSoh_221121_011221_HFGoe_L1_hTau_24h_R2 **Scan** 17334
**Peptide** KVQIINKKLDL **Charge** 3 **Spectral Angle** 0.9

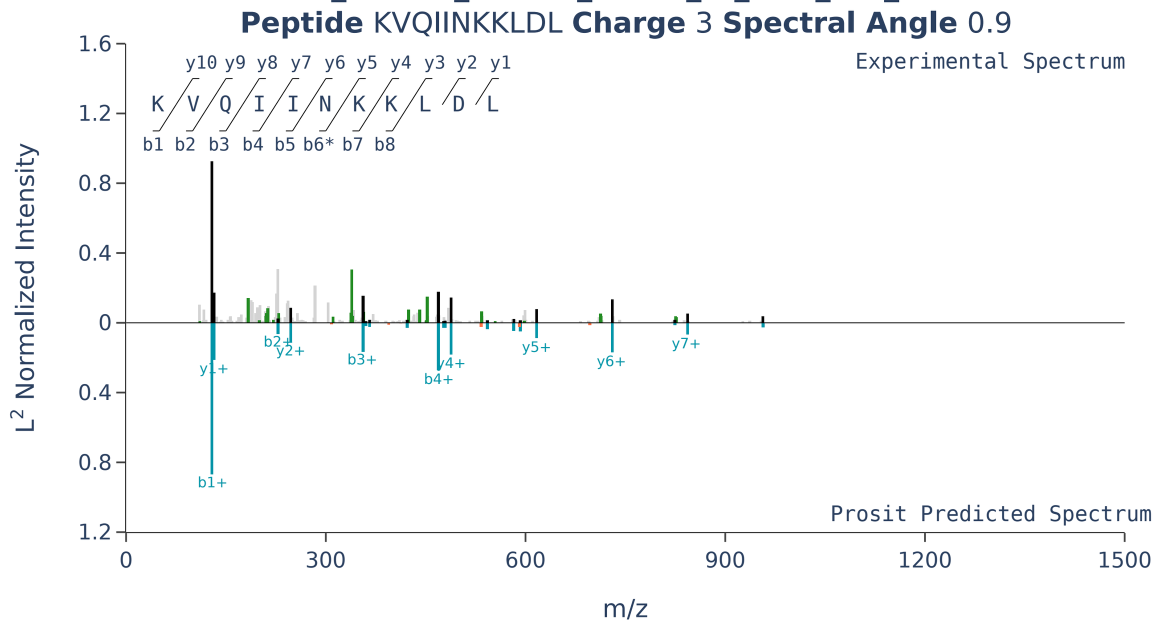


**Source** WSoh_221121_011221_HFGoe_L2_hTau_1h_R2 **Scan** 5697
**Peptide** VRTPPKSPSS **Charge** 2 **Spectral Angle** 0.88

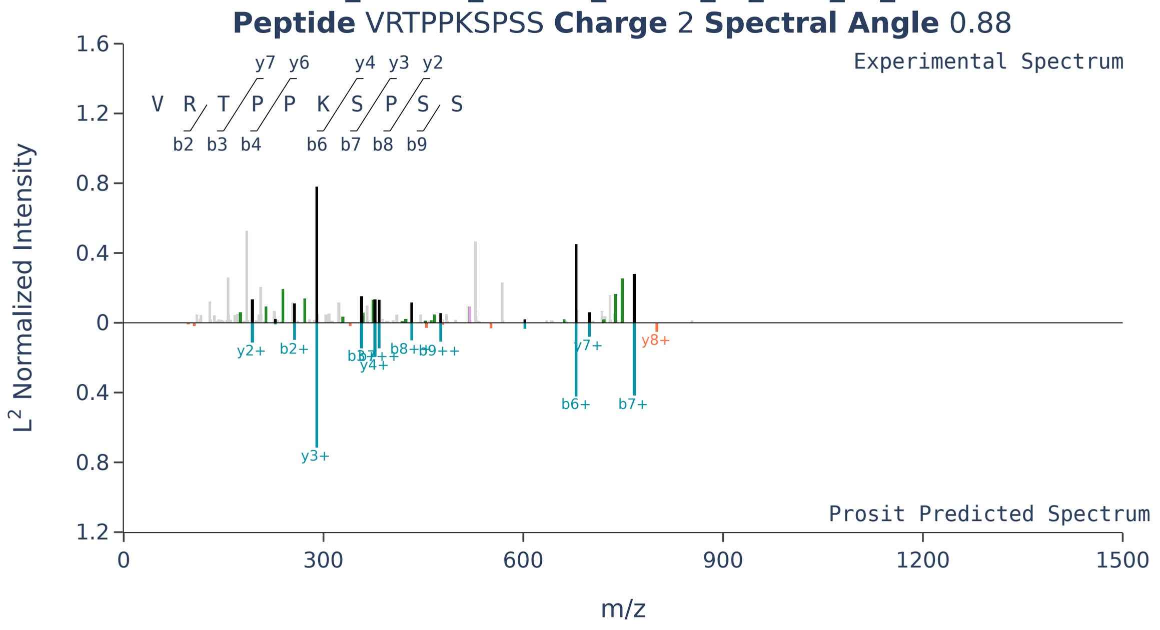


**Source** WSoh_221121_011221_HFGoe_L2_hTau_4h_R1 **Scan** 14194
**Peptide** ENAKAKTDHGAEIVYKSPVV **Charge** 4 **Spectral Angle** 0.89

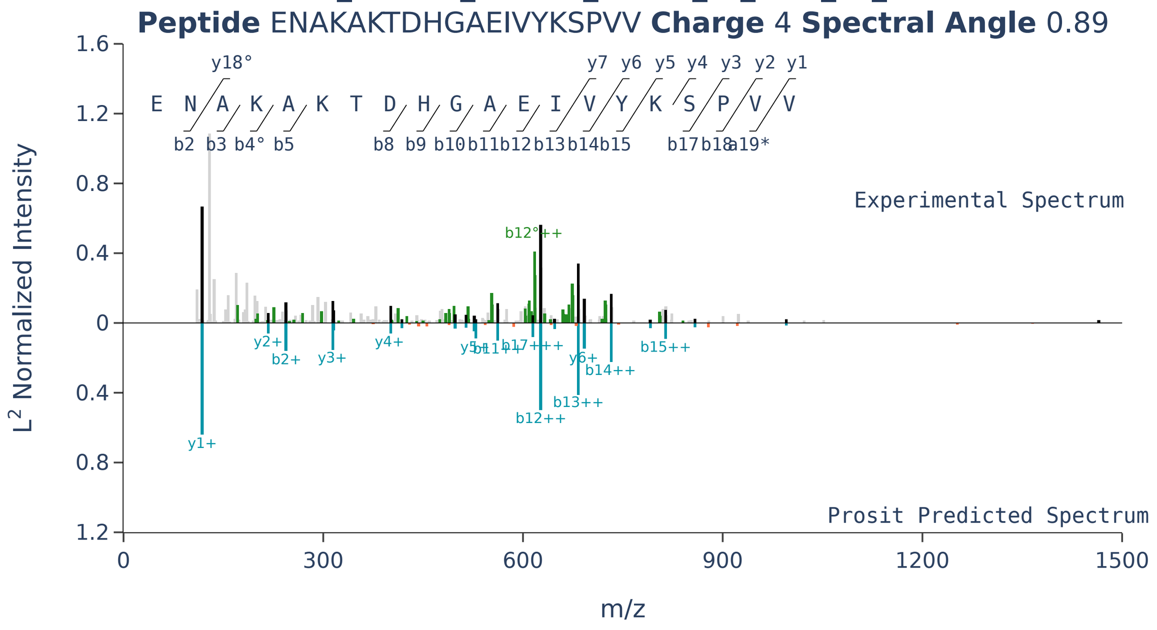


**Source** WSoh_221121_011221_HFGoe_L2_hTau_24h_R1 **Scan** 6890
**Peptide** KCGSLGNIHHKPGGGQV **Charge** 2 **Spectral Angle** 0.86

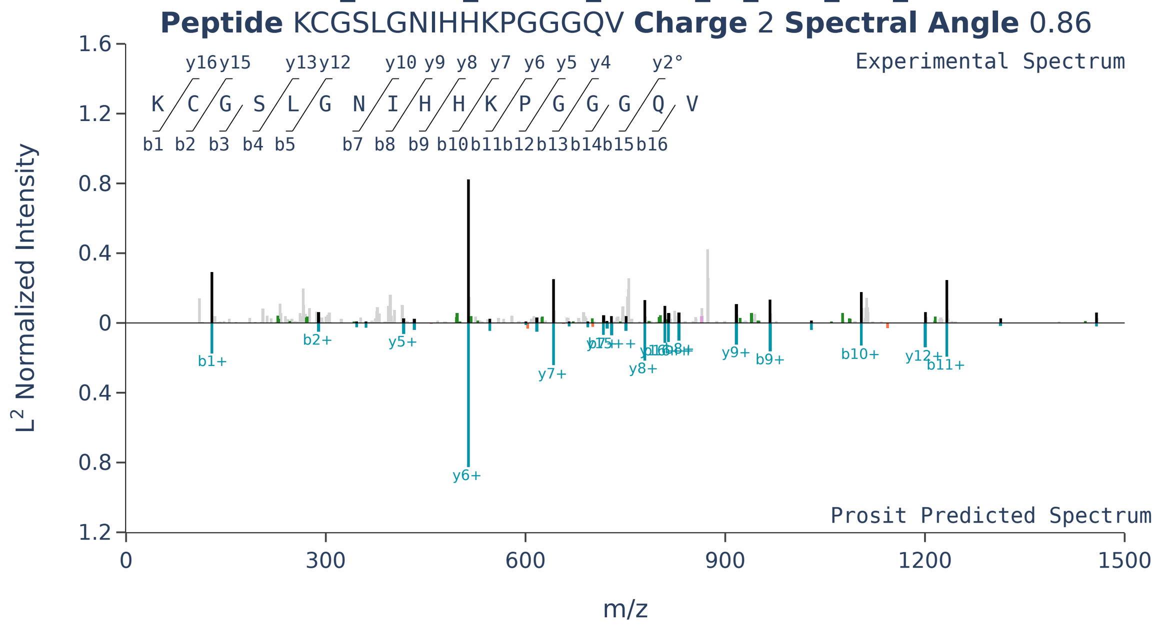


**Source** WSoh_221121_011221_HFGoe_L1_hTau_24h_R1 **Scan** 20725
**Peptide** AEIVYKSPVV **Charge** 2 **Spectral Angle** 0.88

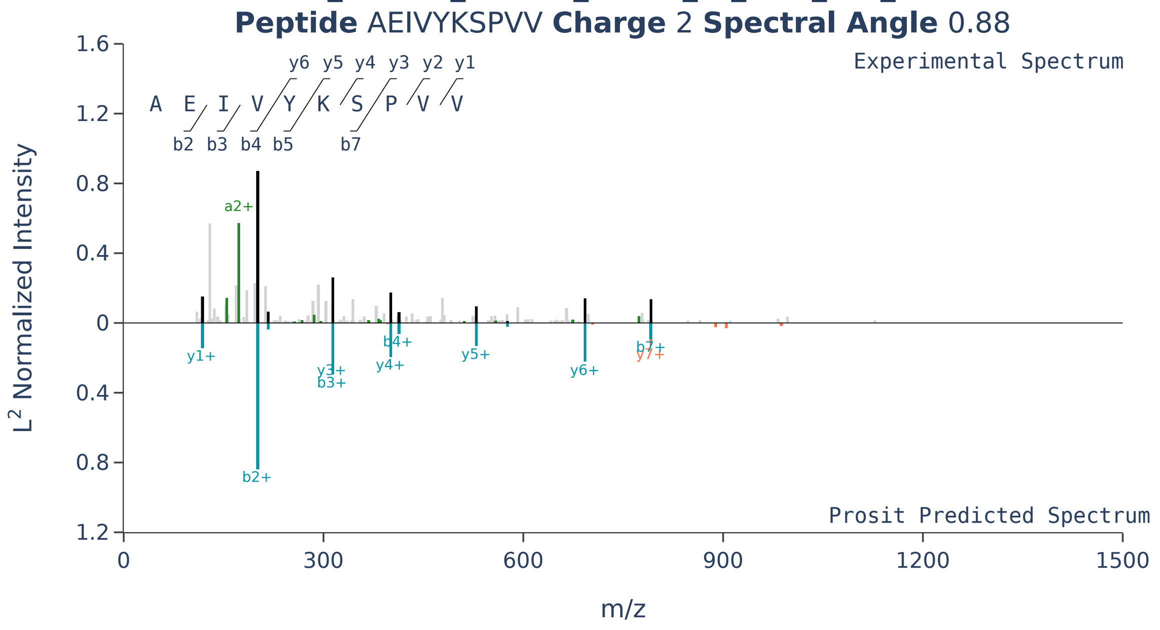


**Source** WSoh_221121_011221_HFGoe_L1_hTau_24h_R1 **Scan** 19609
**Peptide** GIGDTPSLEDEAAGHVTQARMVSK **Charge** 4 **Spectral Angle** 0.79

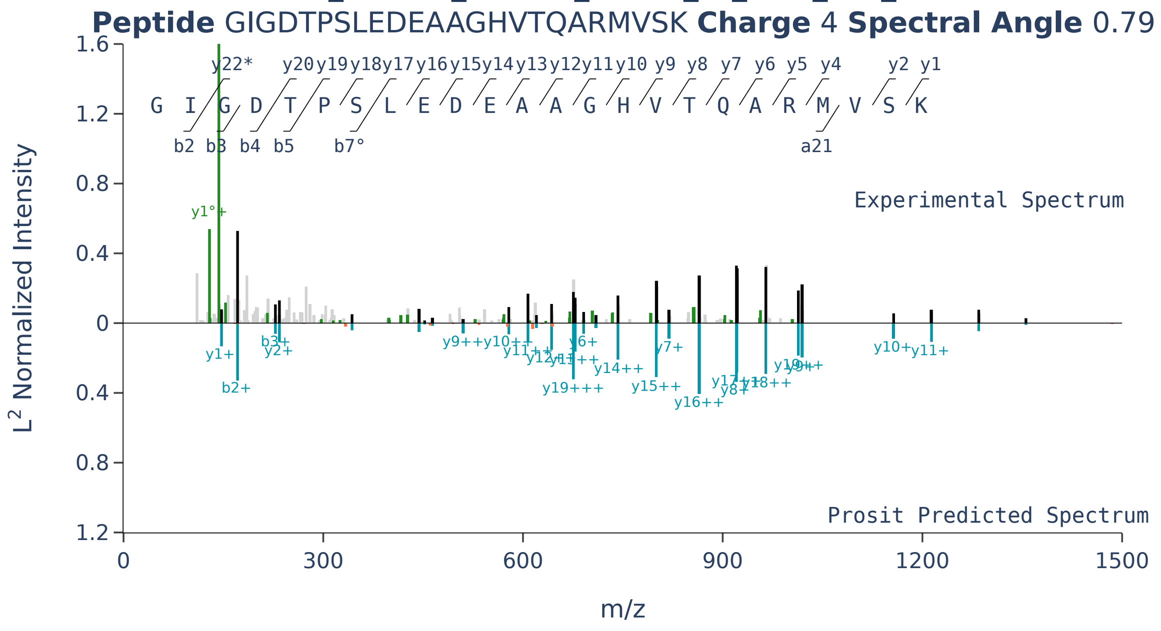


**Source** WSoh_221121_011221_HFGoe_L1_hTau_1h_R2 **Scan** 13969
**Peptide** QIVYKPVD **Charge** 2 **Spectral Angle** 0.86

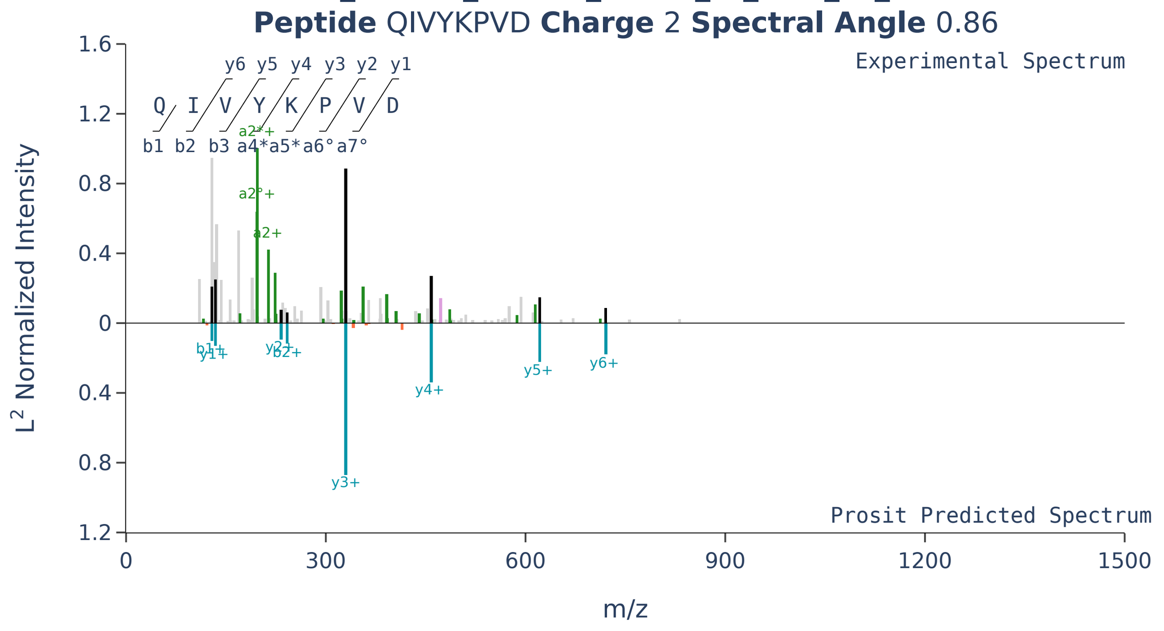


**Source** WSoh_221121_011221_HFGoe_L2_hTau_24h_R2 **Scan** 9242
**Peptide** RIPAKTPPAPKTPPSSG **Charge** 3 **Spectral Angle** 0.82

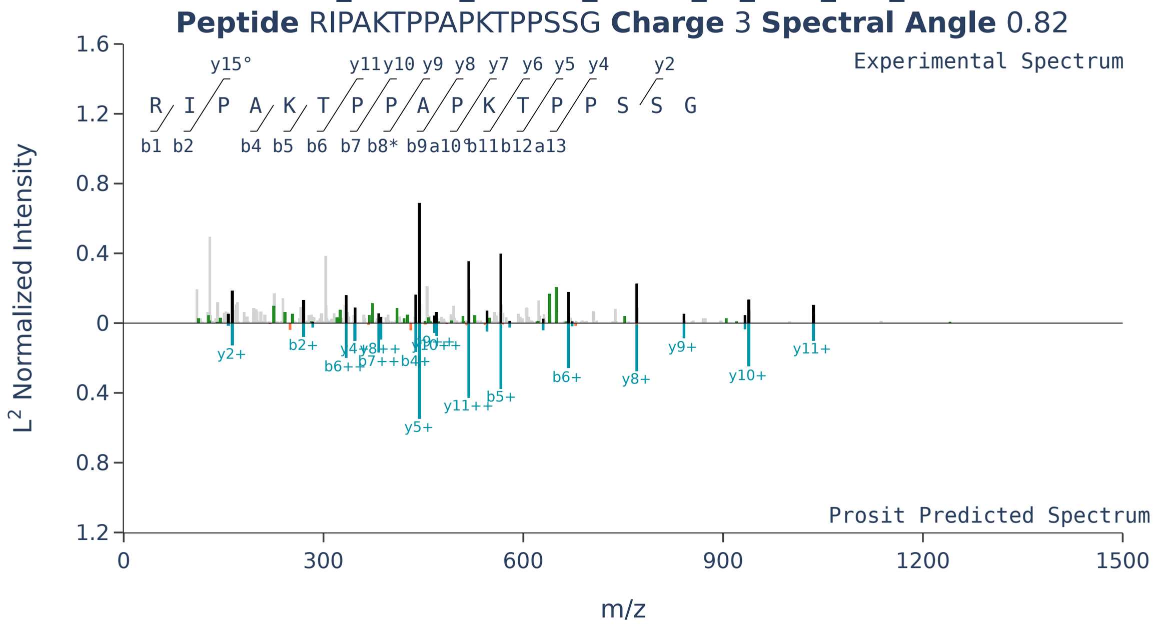


**Source** WSoh_221121_011221_HFGoe_L2_hTau_4h_R1 **Scan** 12375
**Peptide** KPGGGQVEVKSEKLDFKDRVQS **Charge** 4 **Spectral Angle** 0.88

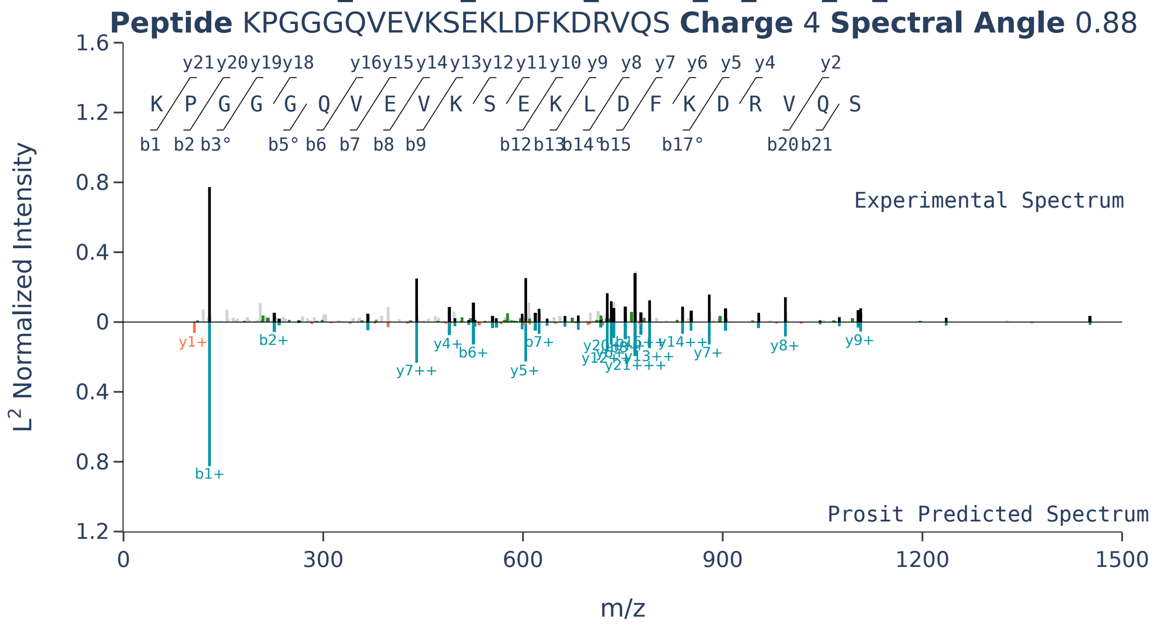


**Source** WSoh_221121_011221_HFGoe_L1_hTau_24h_R1 **Scan** 14788
**Peptide** HGAEIVYKSPVVSGDTSPRHLSN **Charge** 2 **Spectral Angle** 0.84

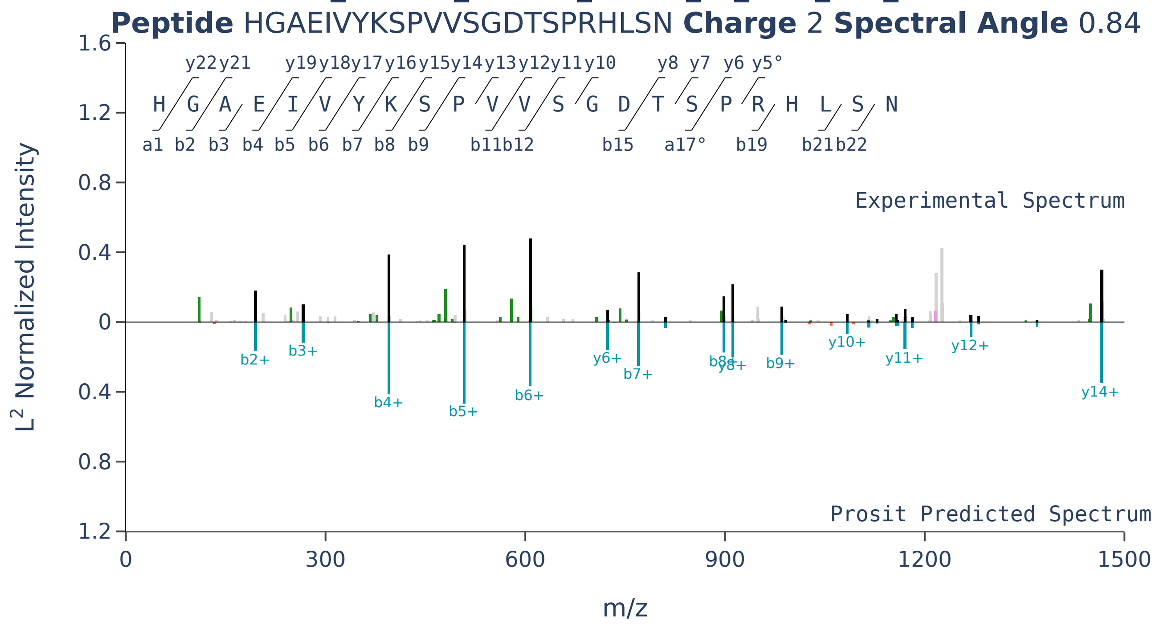


**Source** WSoh_221121_011221_HFGoe_L2_hTau_24h_R1 **Scan** 20015
**Peptide** AGIGDTPSLED **Charge** 2 **Spectral Angle** 0.79

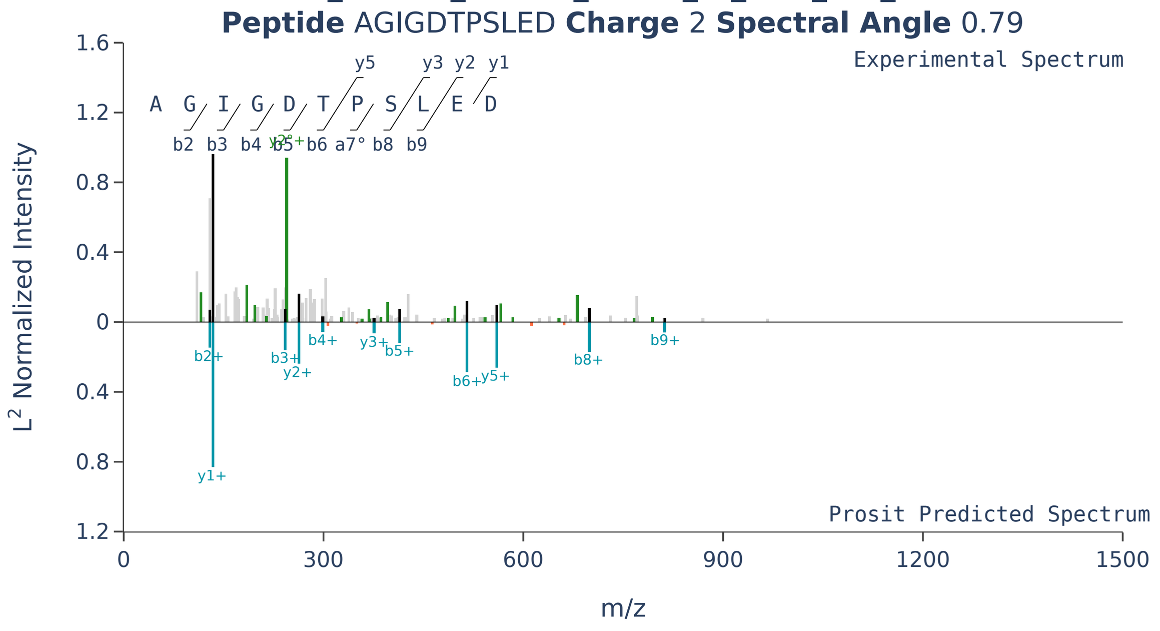


**Source** WSoh_221121_011221_HFGoe_L1_hTau_24h_R2 **Scan** 14034
**Peptide** LSNVSSTGSID **Charge** 1 **Spectral Angle** 0.92

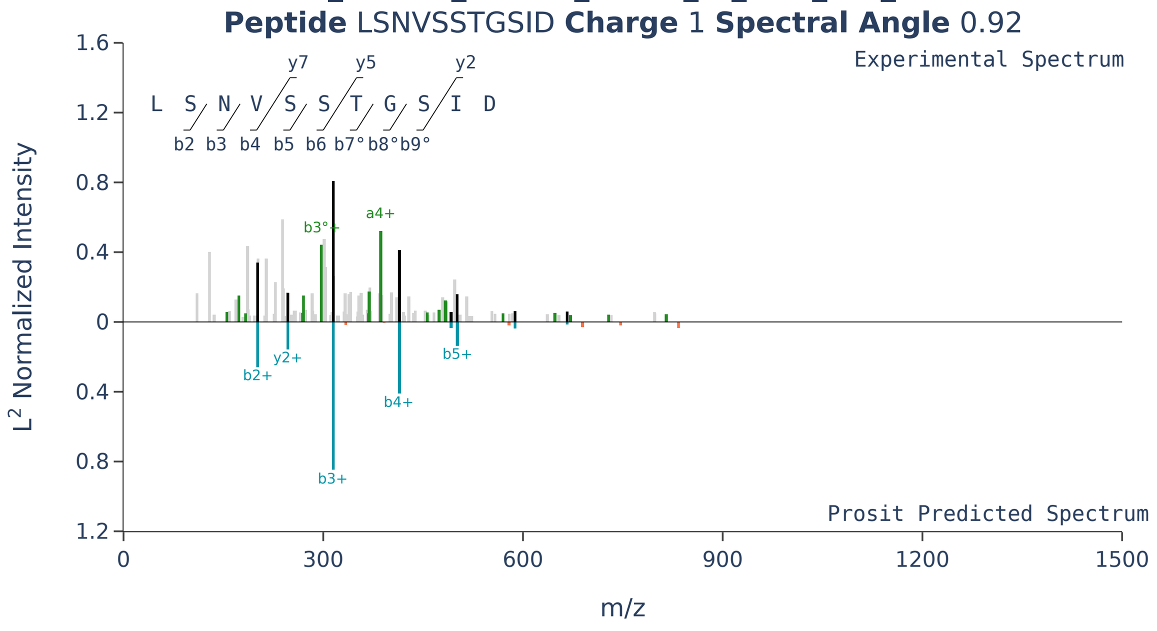


**Source** WSoh_221121_011221_HFGoe_L1_hTau_1h_R2 **Scan** 13992
**Peptide** NIKHVPGGGSVQI **Charge** 3 **Spectral Angle** 0.82

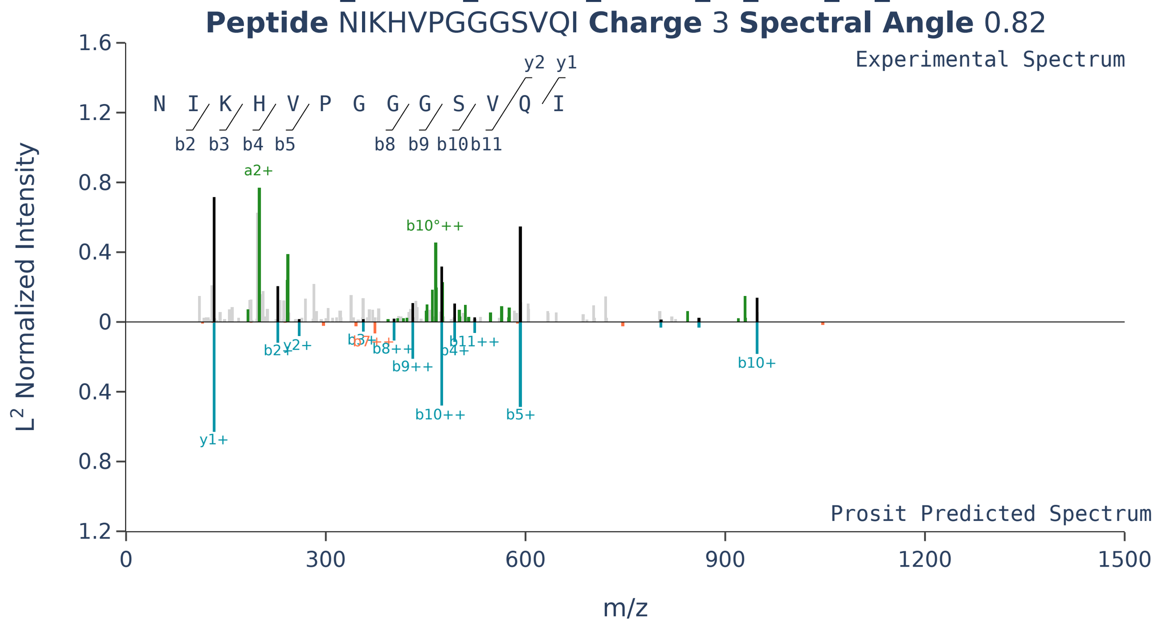


**Source** WSoh_221121_011221_HFGoe_L2_hTau_24h_R1 **Scan** 13534
**Peptide** VMEDHAGTYGLGDRKDQGGYTMH **Charge** 5 **Spectral Angle** 0.93

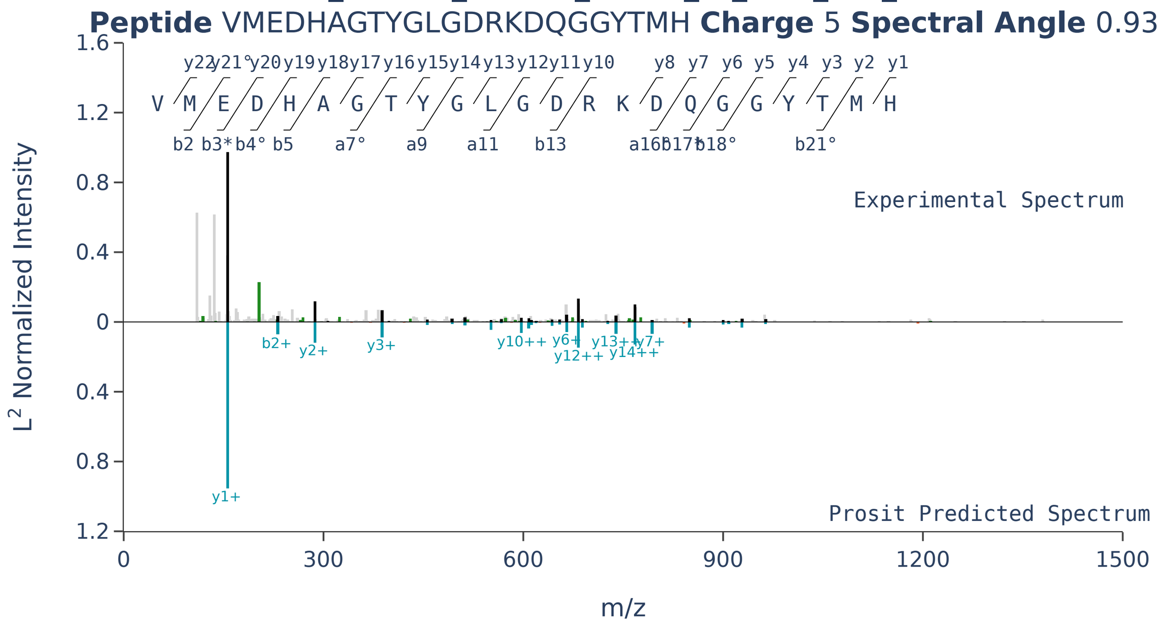

**Source** WSoh_221121_011221_HFGoe_L2_hTau_2h_R2 **Scan** 12710
**Peptide** EDHAGTYGLGD **Charge** 2 **Spectral Angle** 0.82

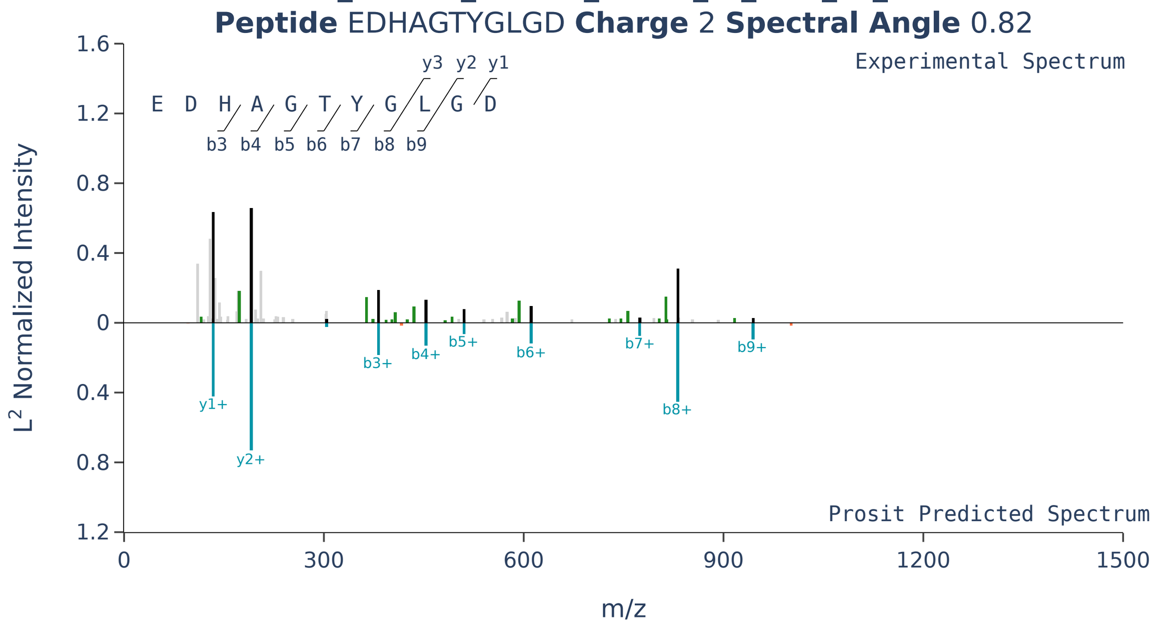


**Source** WSoh_221121_011221_HFGoe_L1_hTau_24h_R2 **Scan** 7405
**Peptide** GDTSPRHLSNVS **Charge** 2 **Spectral Angle** 0.85

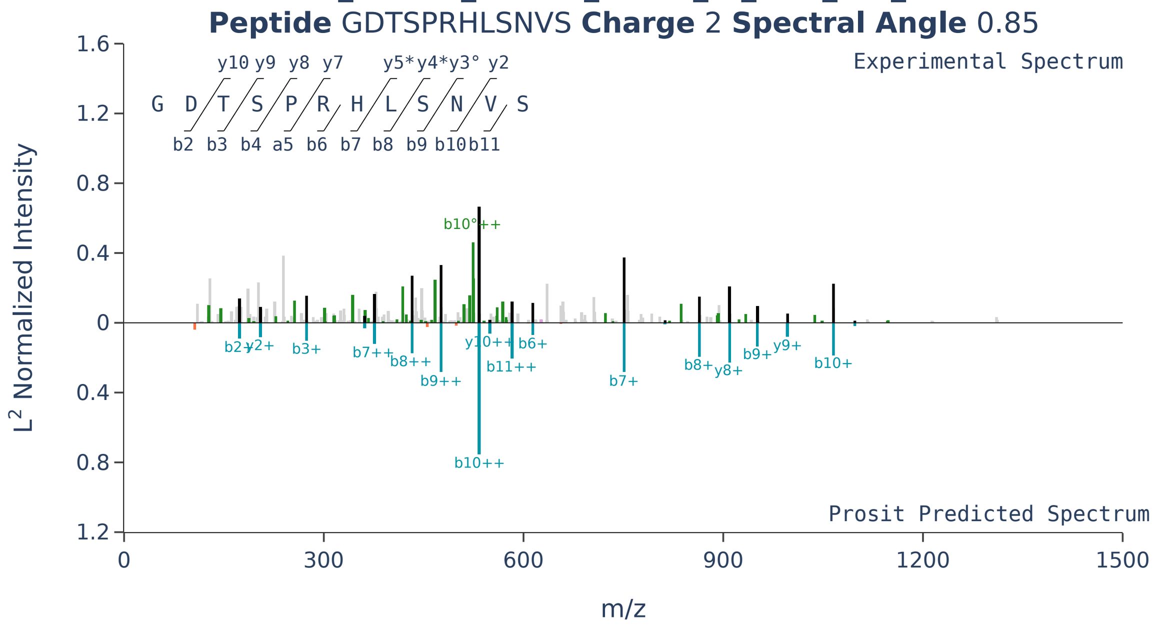


**Source** WSoh_221121_011221_HFGoe_L1_hTau_24h_R2 **Scan** 15168
**Peptide** QIINKKLDLSNVQSK **Charge** 3 **Spectral Angle** 0.91

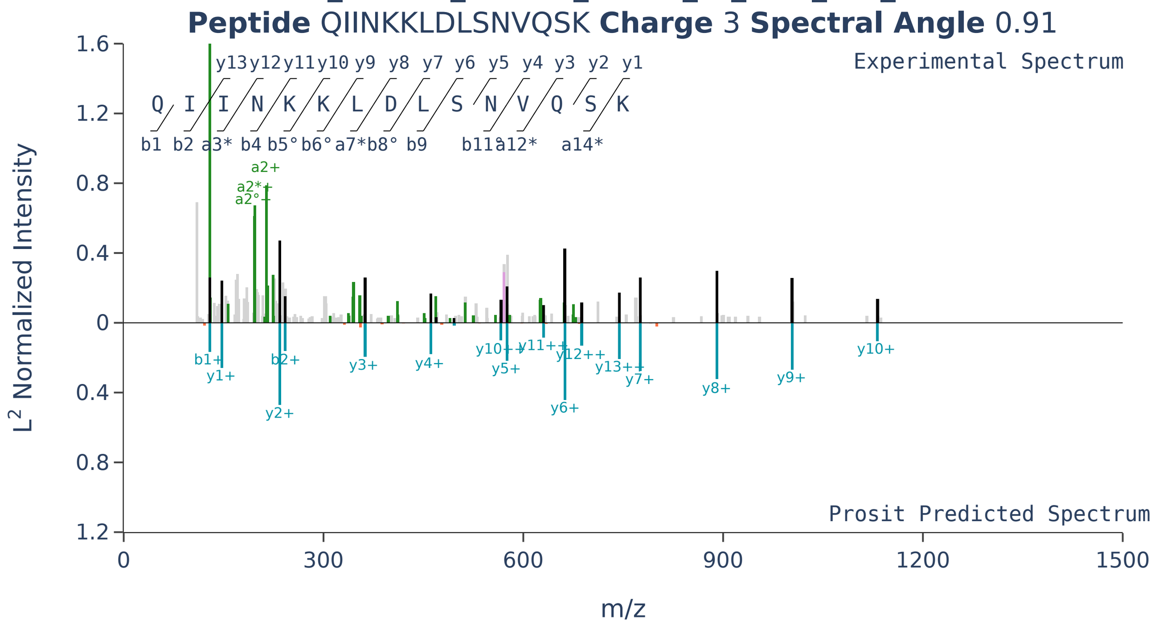


**Source** WSoh_221121_011221_HFGoe_L2_hTau_24h_R1 **Scan** 22442
**Peptide** TAPVPMPDLKNVKSKI **Charge** 3 **Spectral Angle** 0.86

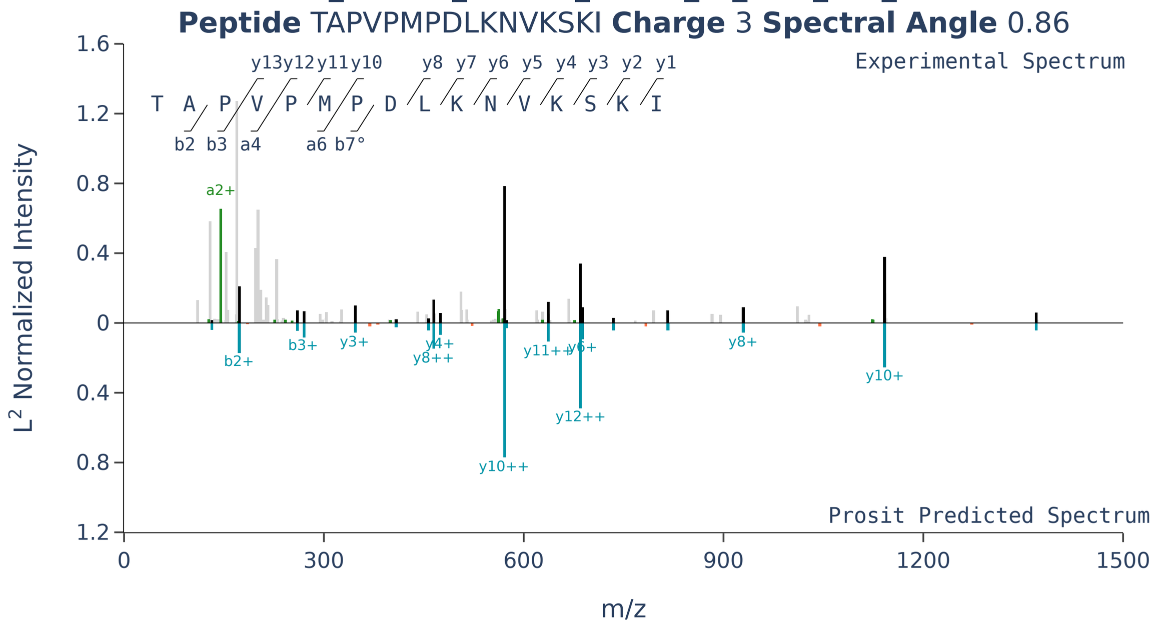


**Source** WSoh_221121_011221_HFGoe_L1_hTau_4h_R2 **Scan** 13688
**Peptide** GQVEVKSEKLDFKDRVQS **Charge** 4 **Spectral Angle** 0.83

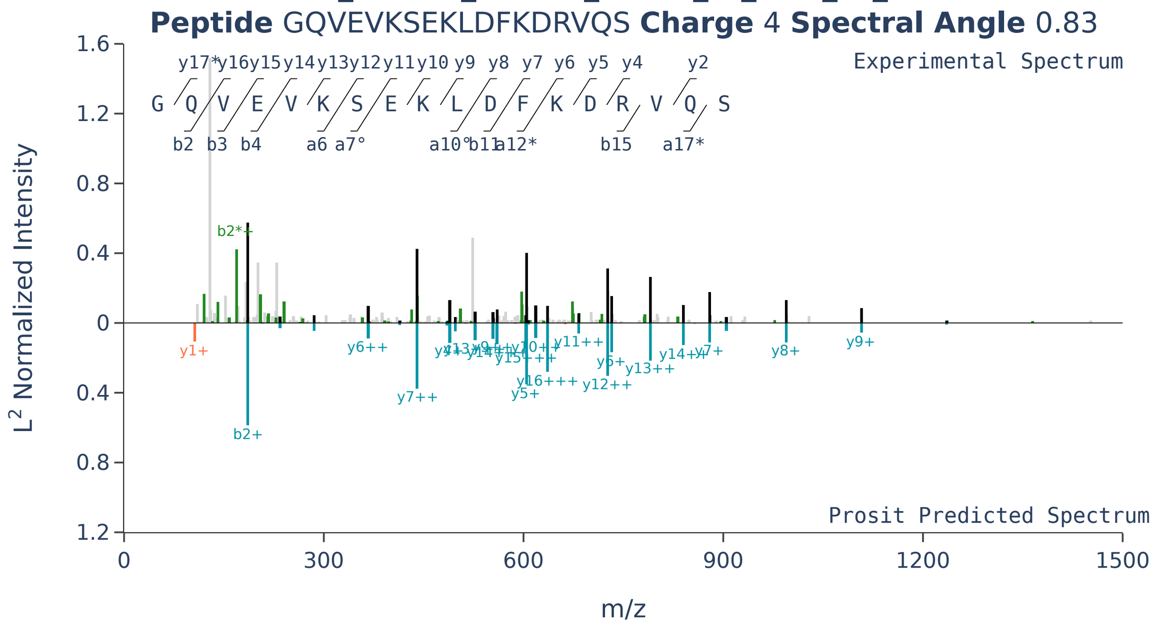


**Source** WSoh_221121_011221_HFGoe_L1_hTau_4h_R1 **Scan** 5693
**Peptide** PGGGNKKIETHKL **Charge** 3 **Spectral Angle** 0.83

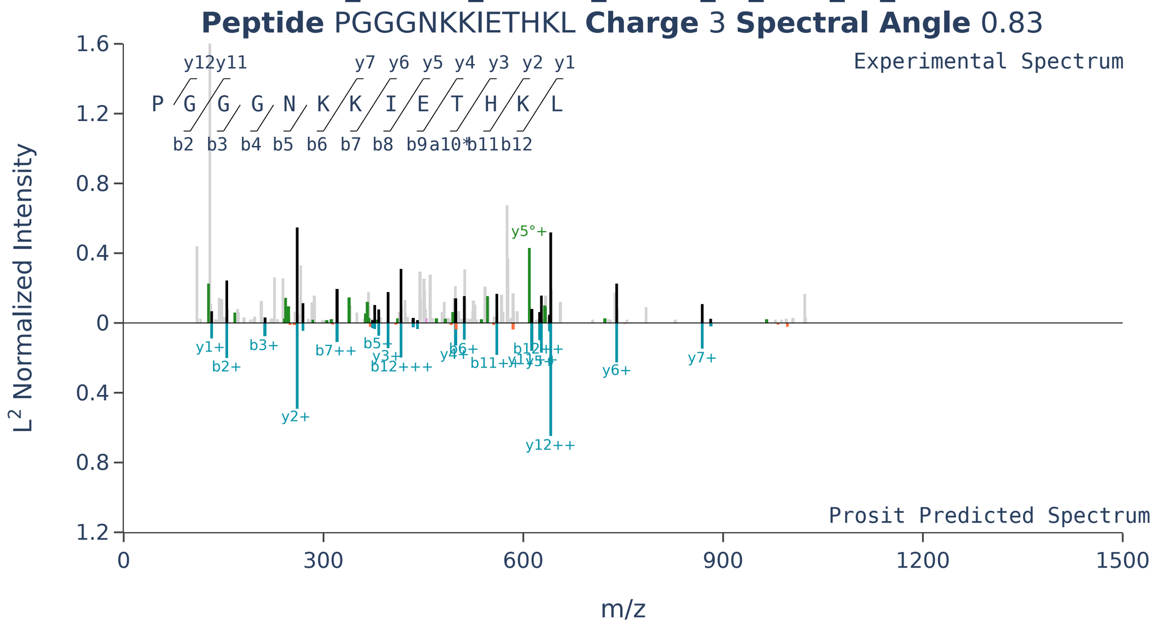


**Source** WSoh_221121_011221_HFGoe_L2_hTau_24h_R1 **Scan** 6122
**Peptide** SLGNIHHKPGGGQ **Charge** 3 **Spectral Angle** 0.95

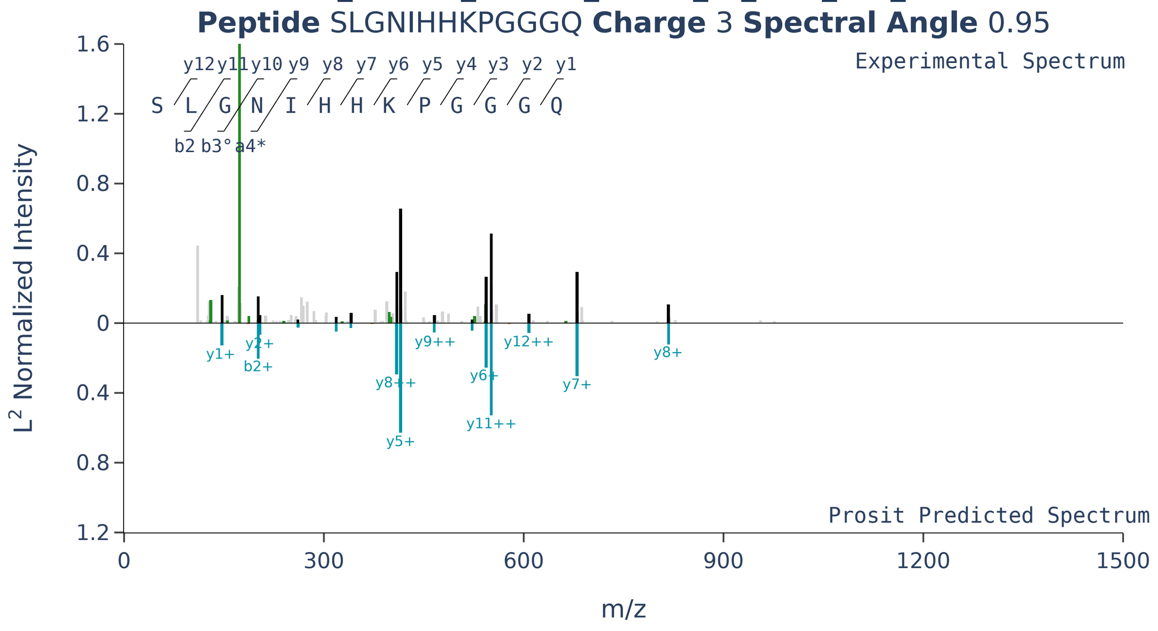


**Source** WSoh_221121_011221_HFGoe_L2_hTau_24h_R1 **Scan** 9731
**Peptide** VPGGGNKKIETHKLTFREN **Charge** 5 **Spectral Angle** 0.91

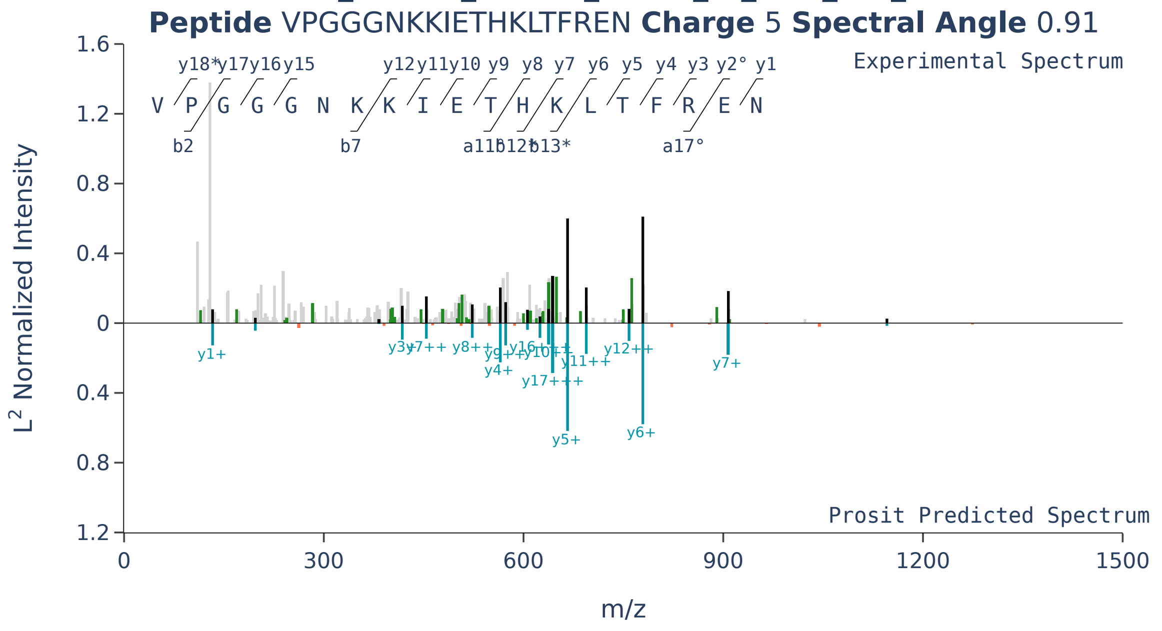


**Source** WSoh_221121_011221_HFGoe_L2_hTau_24h_R2 **Scan** 10195
**Peptide** TRIPAKTPPAPKTPPSSG **Charge** 3 **Spectral Angle** 0.83

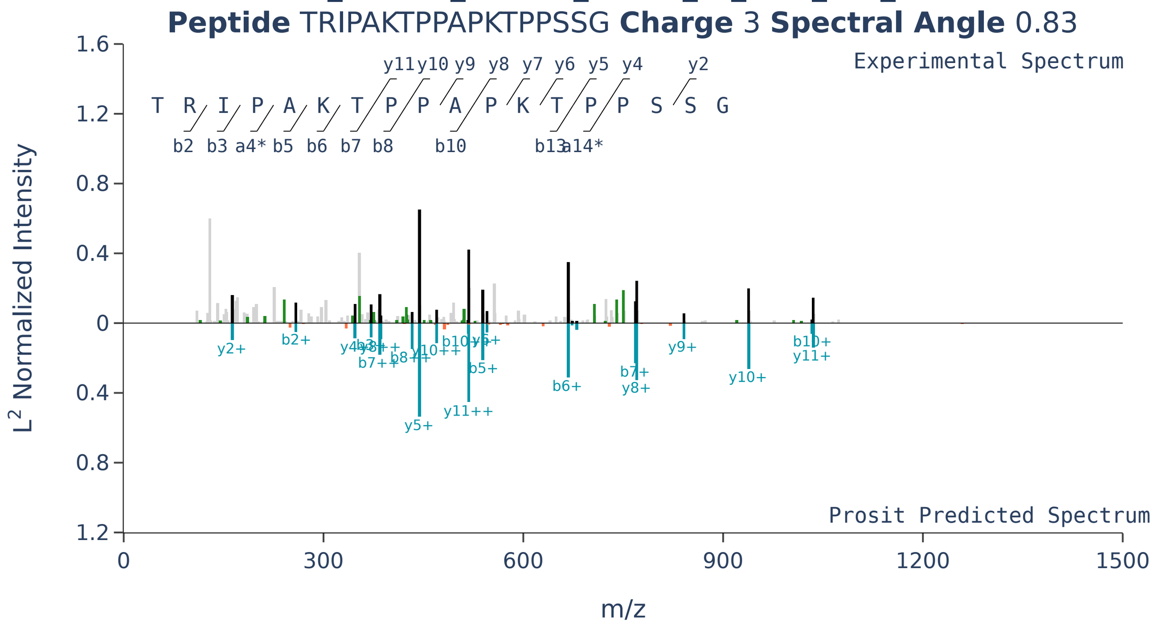


**Source** WSoh_221121_011221_HFGoe_L1_hTau_2h_R2 **Scan** 21885
**Peptide** PGGGSVQIVY **Charge** 2 **Spectral Angle** 0.87

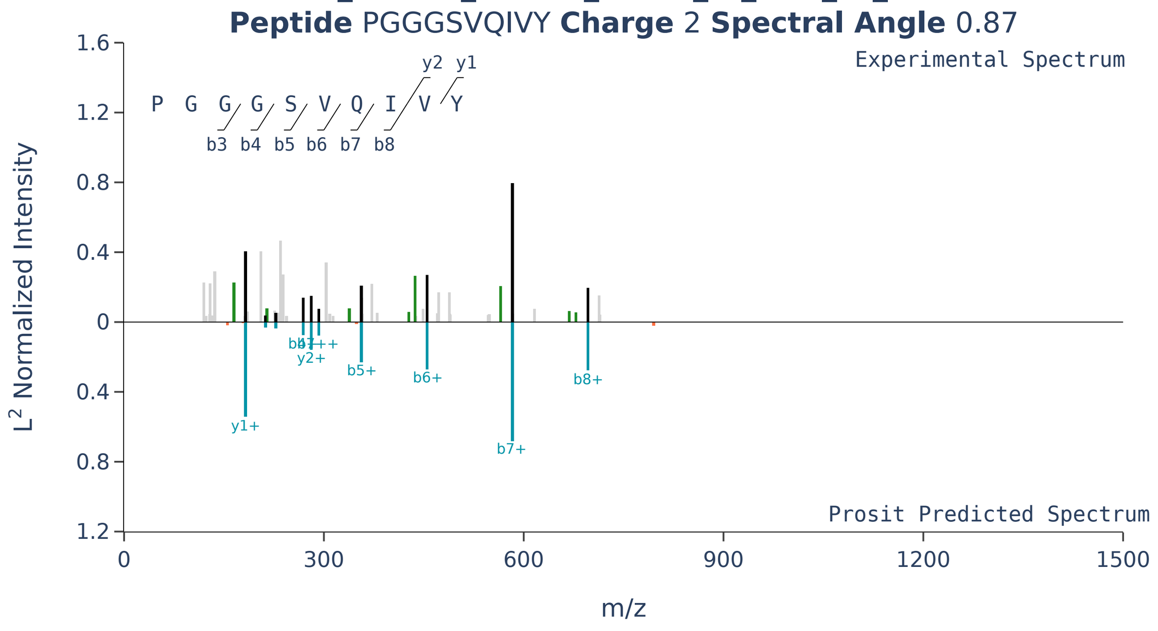


**Source** WSoh_221121_011221_HFGoe_L1_hTau_24h_R1 **Scan** 6562
**Peptide** DLSNVQSKCGSKDNIKH **Charge** 3 **Spectral Angle** 0.94

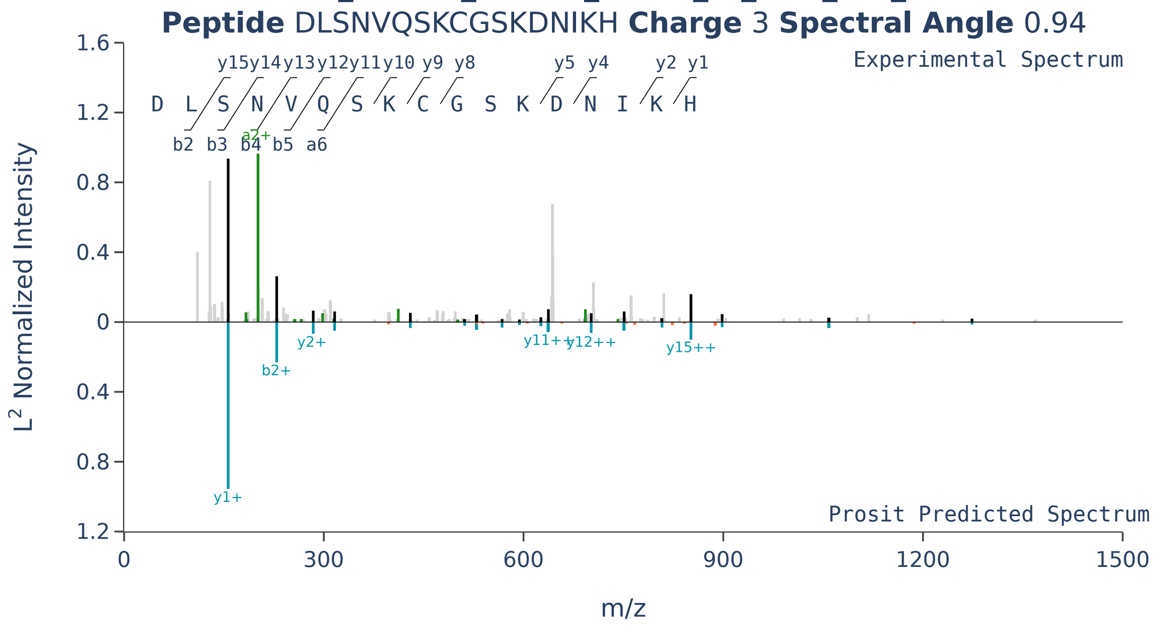


**Source** WSoh_221121_011221_HFGoe_L1_hTau_24h_R1 **Scan** 19836
**Peptide** AKSTPTAEDVTAPLVDEGAPGKQA **Charge** 3 **Spectral Angle** 0.92

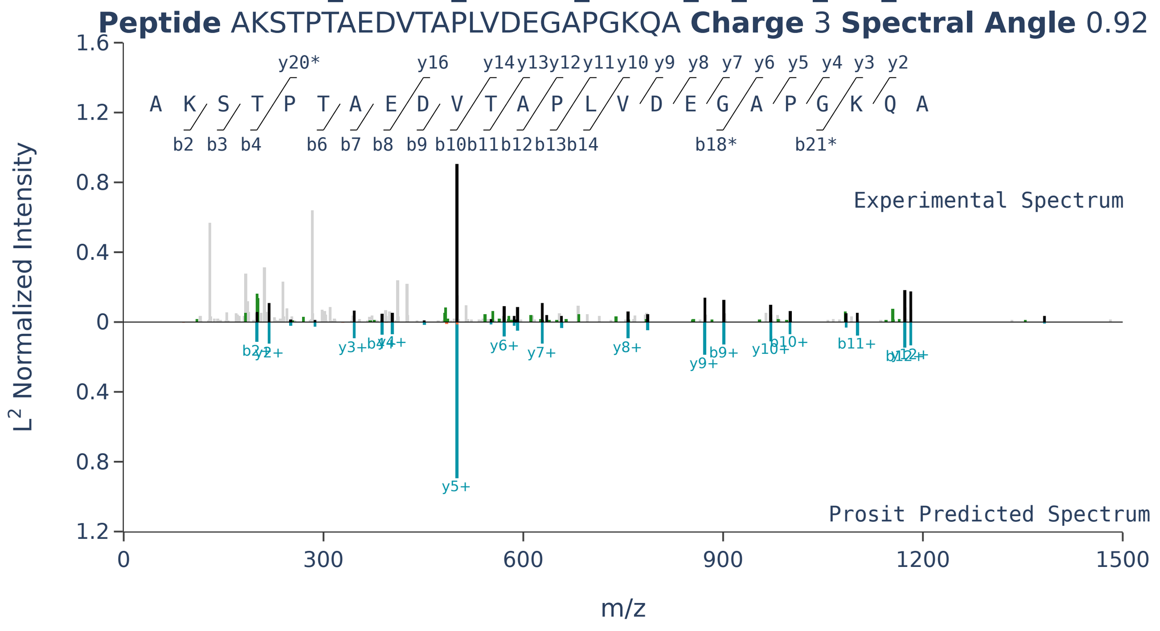


**Source** WSoh_221121_011221_HFGoe_L2_hTau_4h_R1 **Scan** 8046
**Peptide** CGSKDNIKHVPGGGSV **Charge** 2 **Spectral Angle** 0.92

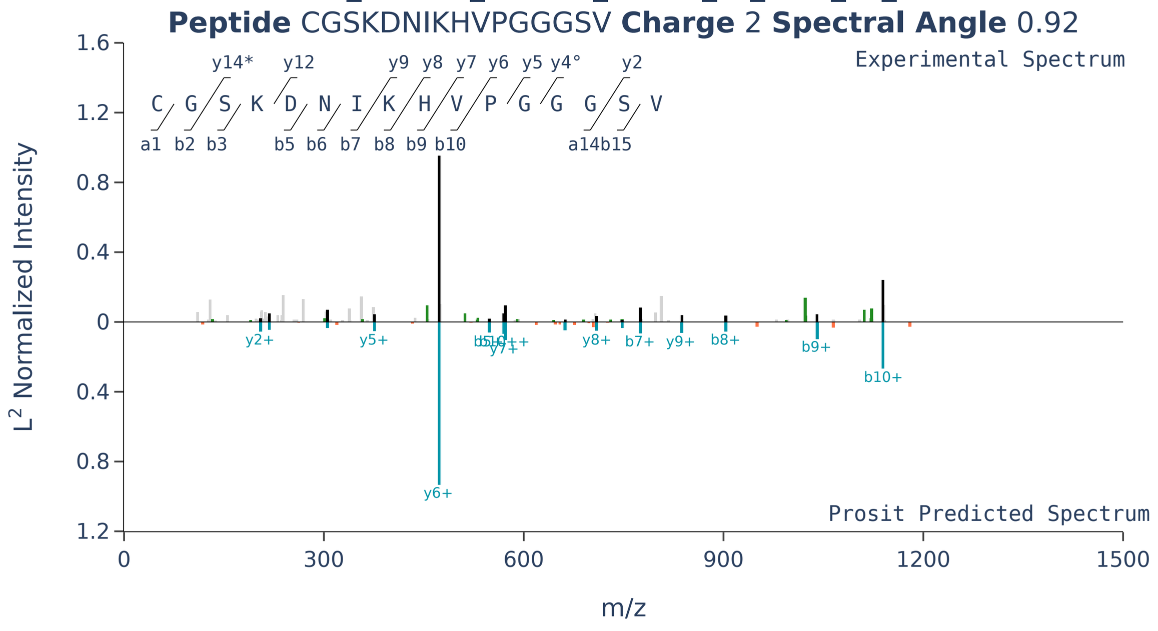


**Source** WSoh_221121_011221_HFGoe_L2_hTau_4h_R2 **Scan** 15476
**Peptide** PGGGSVQL **Charge** 1 **Spectral Angle** 0.77

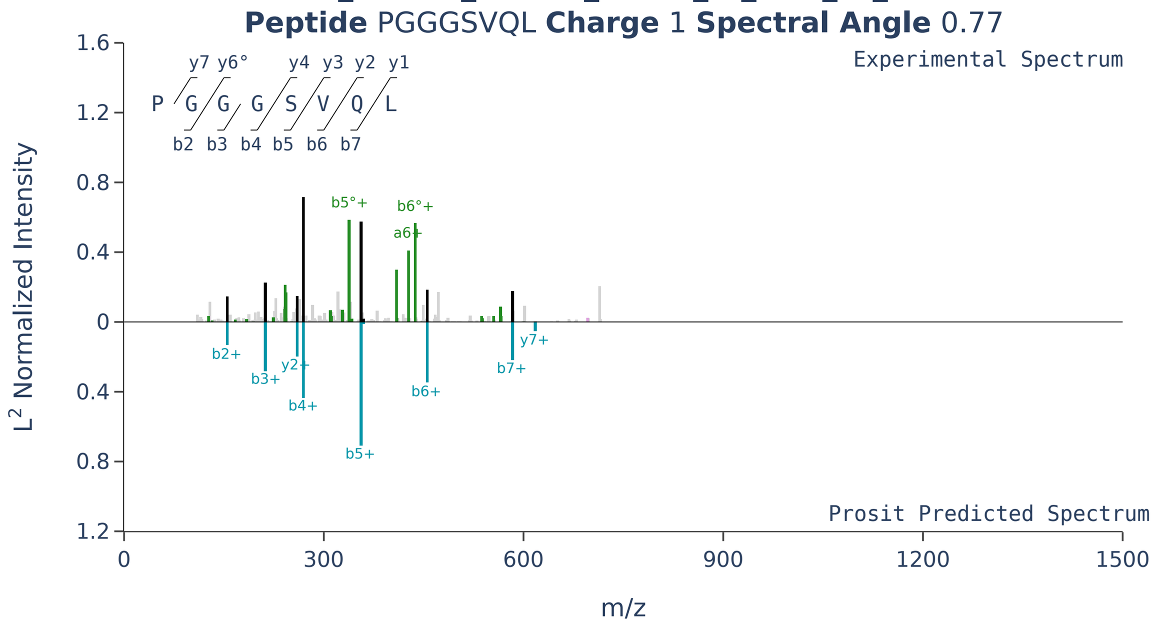


**Source** WSoh_221121_011221_HFGoe_L2_hTau_24h_R2 **Scan** 12577
**Peptide** TDHGAEIVYKSP **Charge** 3 **Spectral Angle** 0.88

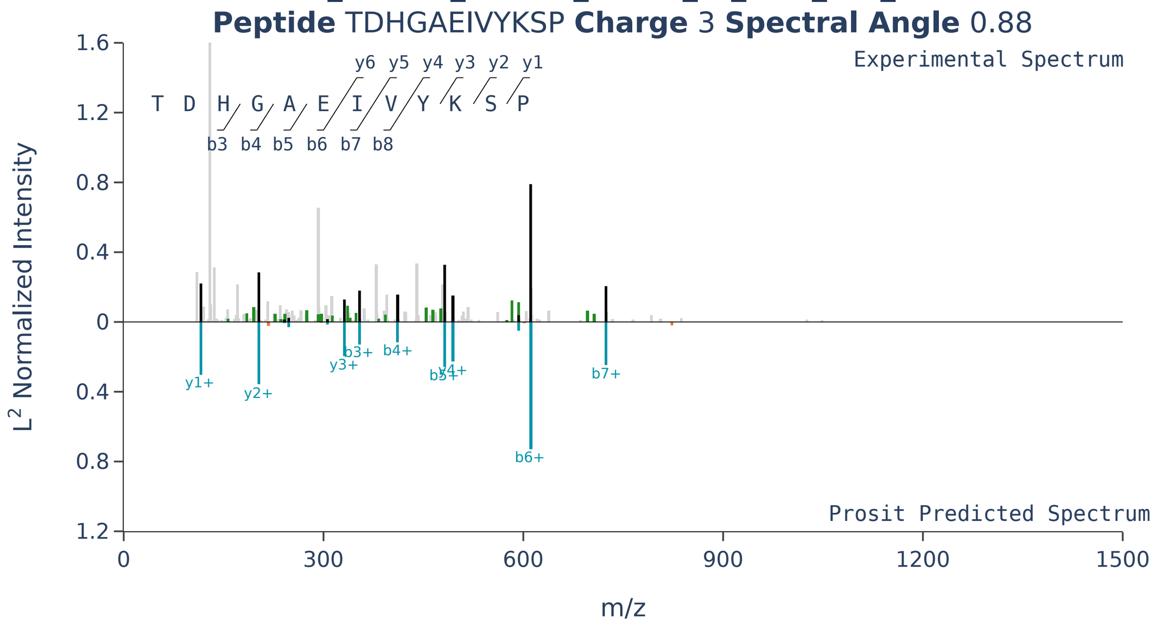

**Source** WSoh_221121_011221_HFGoe_L2_hTau_24h_R2 **Scan** 12899
**Peptide** VQSKIGSLD **Charge** 2 **Spectral Angle** 0.86

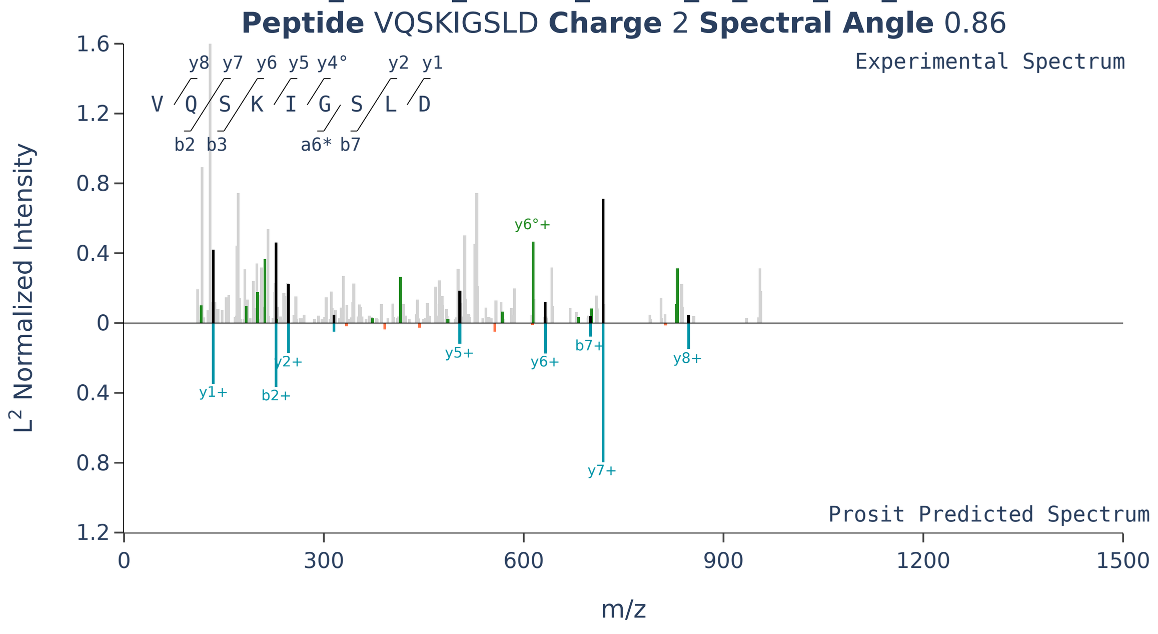


**Source** WSoh_221121_011221_HFGoe_L1_hTau_24h_R1 **Scan** 14338
**Peptide** STGSIDM **Charge** 1 **Spectral Angle** 0.9

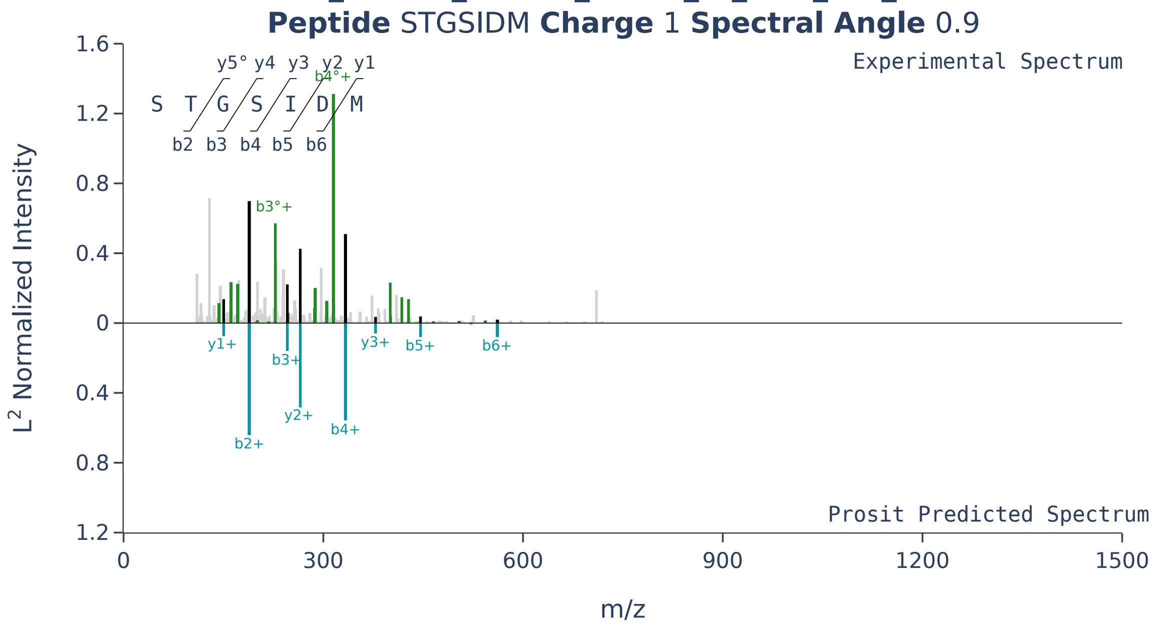


**Source** WSoh_221121_011221_HFGoe_L2_hTau_24h_R1 **Scan** 9864
**Peptide** AEPRQEF **Charge** 2 **Spectral Angle** 0.94

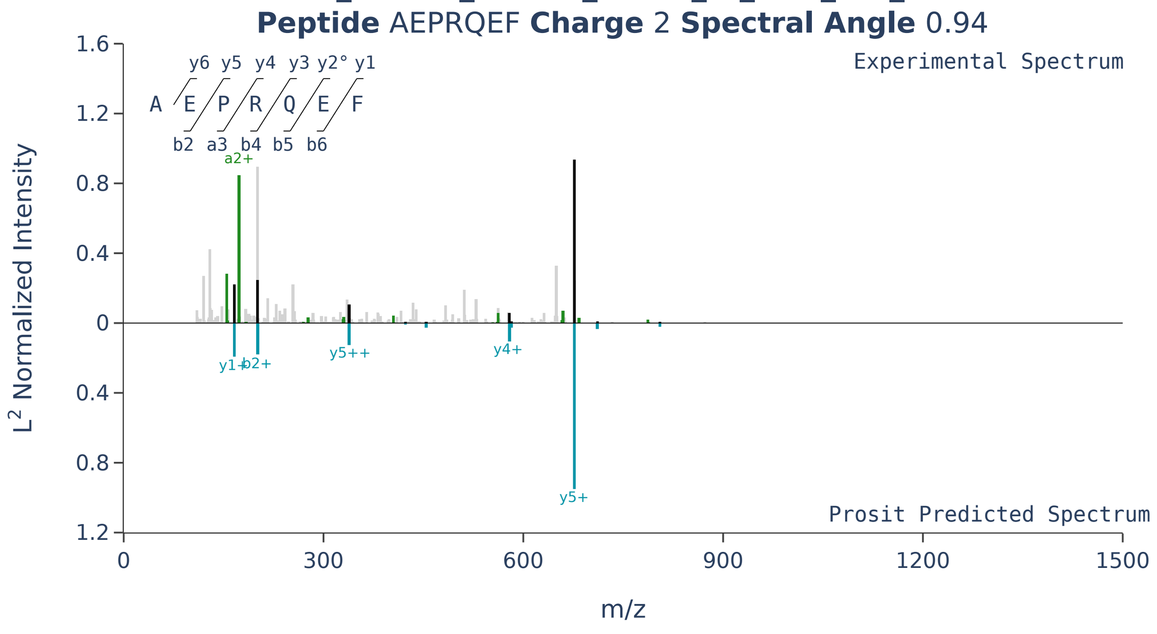


**Source** WSoh_221121_011221_HFGoe_L2_hTau_24h_R1 **Scan** 10039
**Peptide** GGGKVQIINK **Charge** 2 **Spectral Angle** 0.89

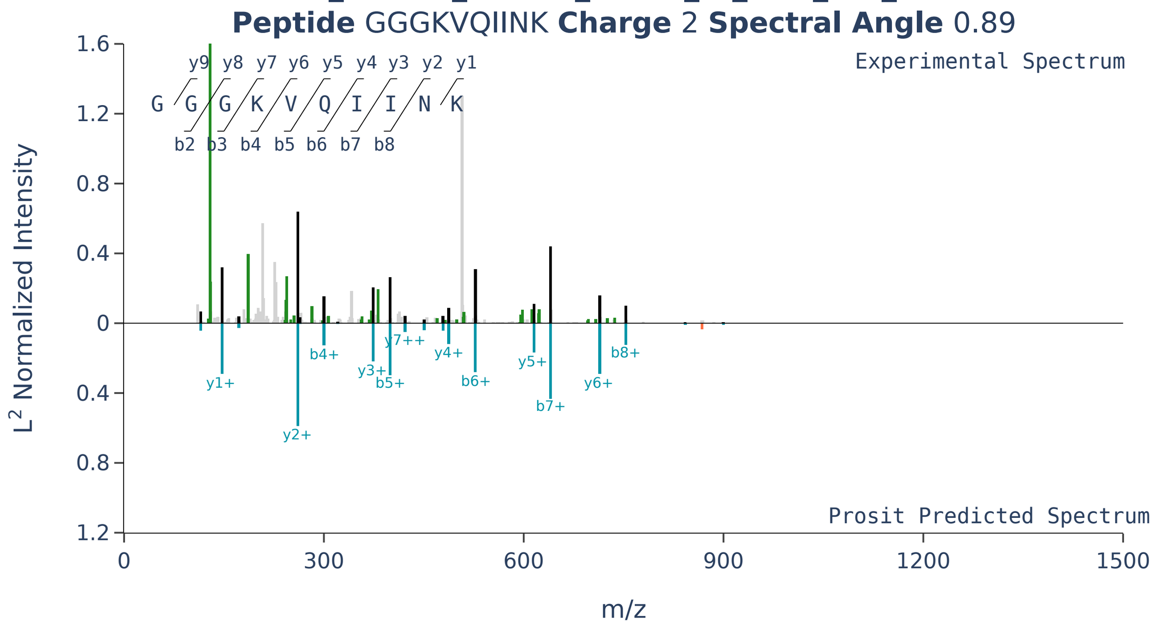


**Source** WSoh_221121_011221_HFGoe_L2_hTau_24h_R2 **Scan** 6096
**Peptide** THVPGGGNKKLE **Charge** 3 **Spectral Angle** 0.88

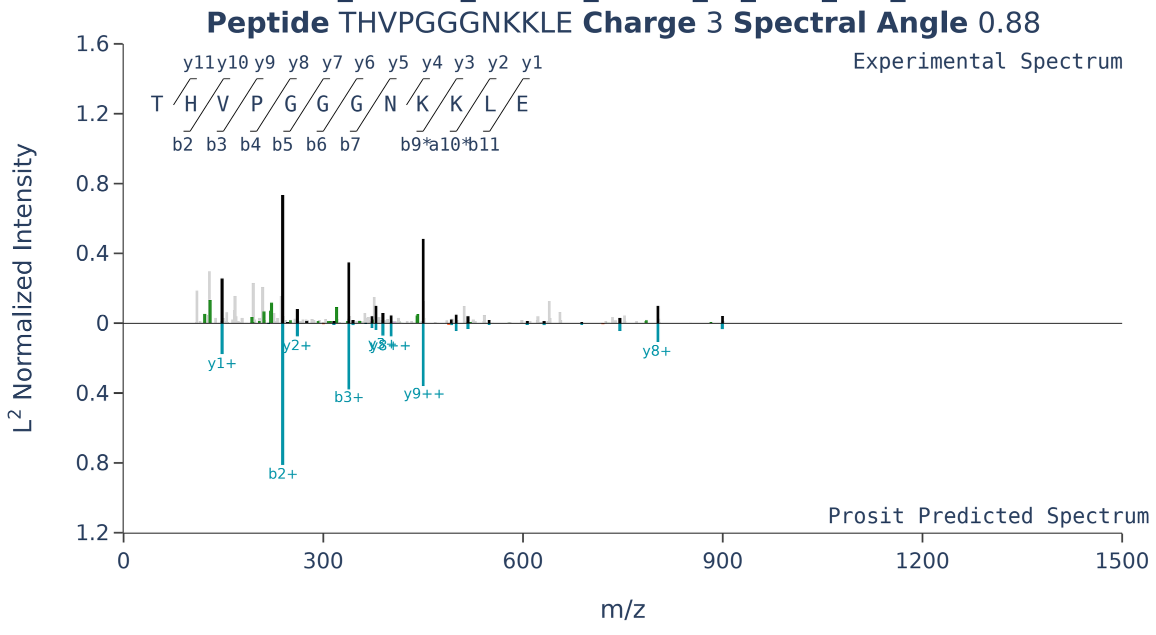


**Source** WSoh_221121_011221_HFGoe_L2_hTau_4h_R1 **Scan** 9445
**Peptide** AKSTPTAEDVTA **Charge** 2 **Spectral Angle** 0.88

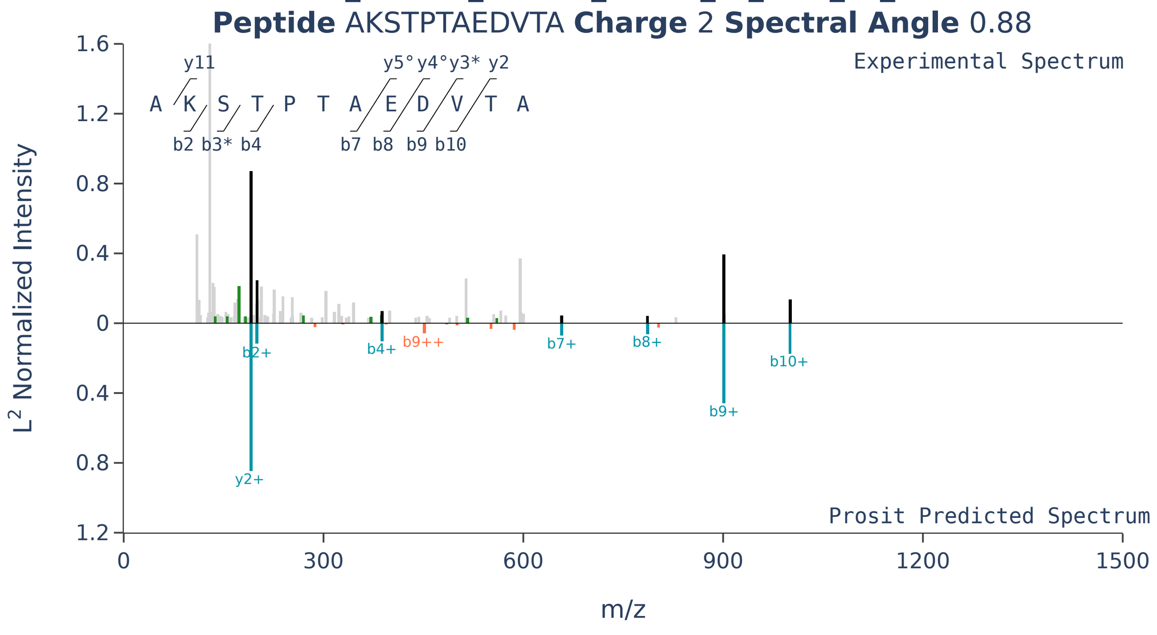


**Source** WSoh_221121_011221_HFGoe_L2_hTau_24h_R2 **Scan** 11525
**Peptide** YGLGDRKDQGGYTMH **Charge** 4 **Spectral Angle** 0.95

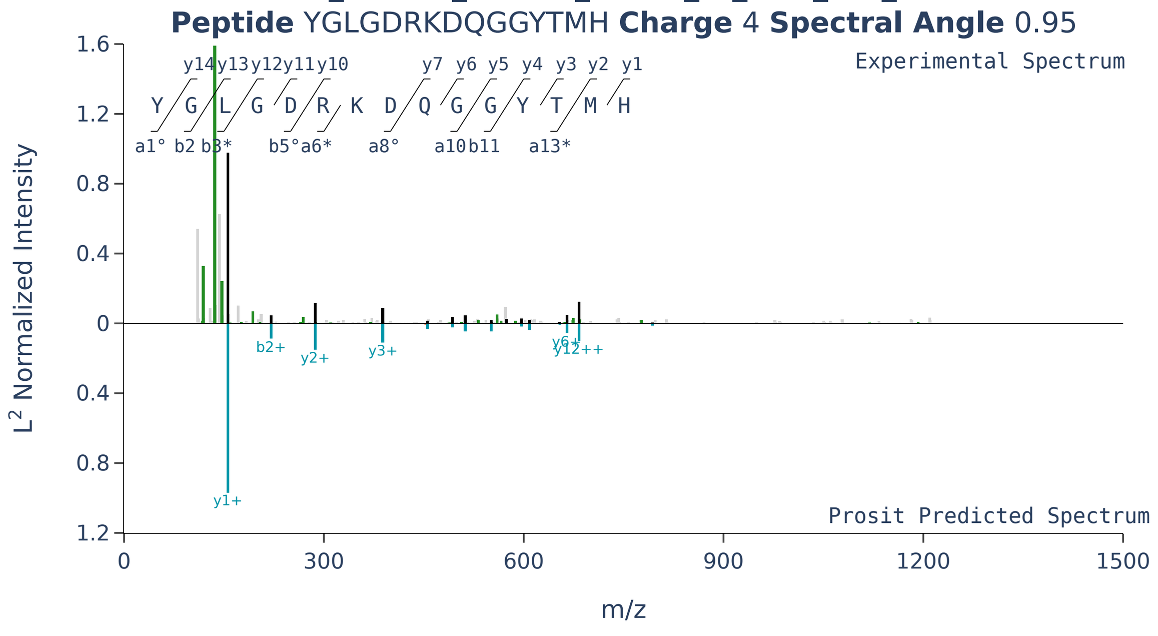


**Source** WSoh_221121_011221_HFGoe_L1_hTau_24h_R2 **Scan** 11918
**Peptide** AGTYGLGDRKDQGGY **Charge** 3 **Spectral Angle** 0.86

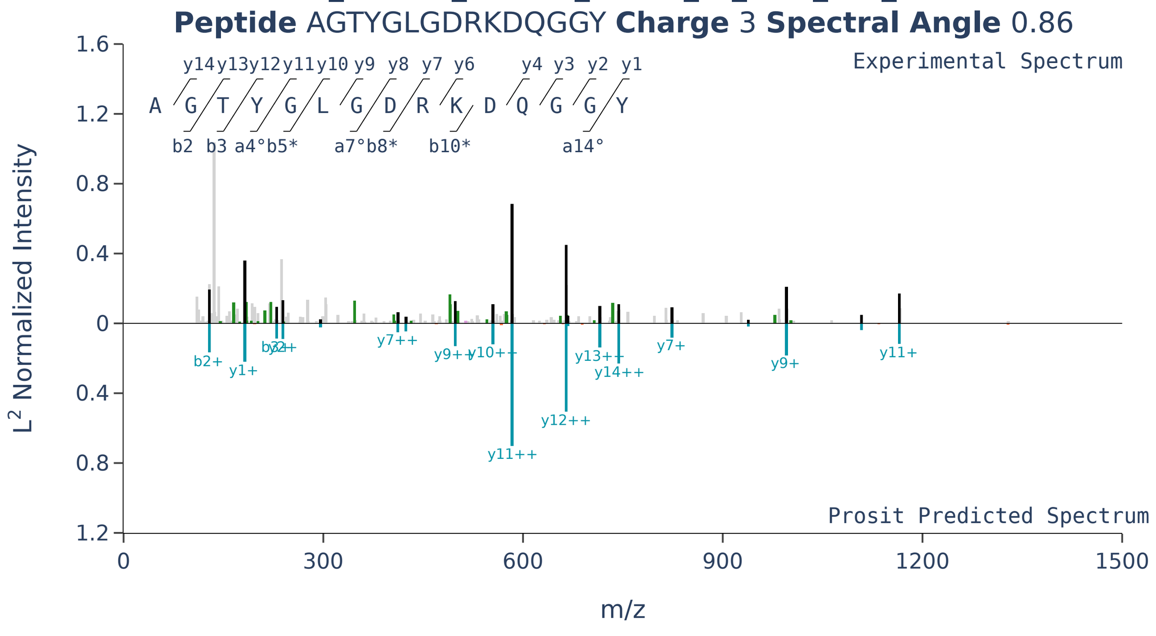


**Source** WSoh_221121_011221_HFGoe_L2_hTau_24h_R2 **Scan** 8442
**Peptide** PGGGNKKIETHKLTFREN **Charge** 2 **Spectral Angle** 0.81

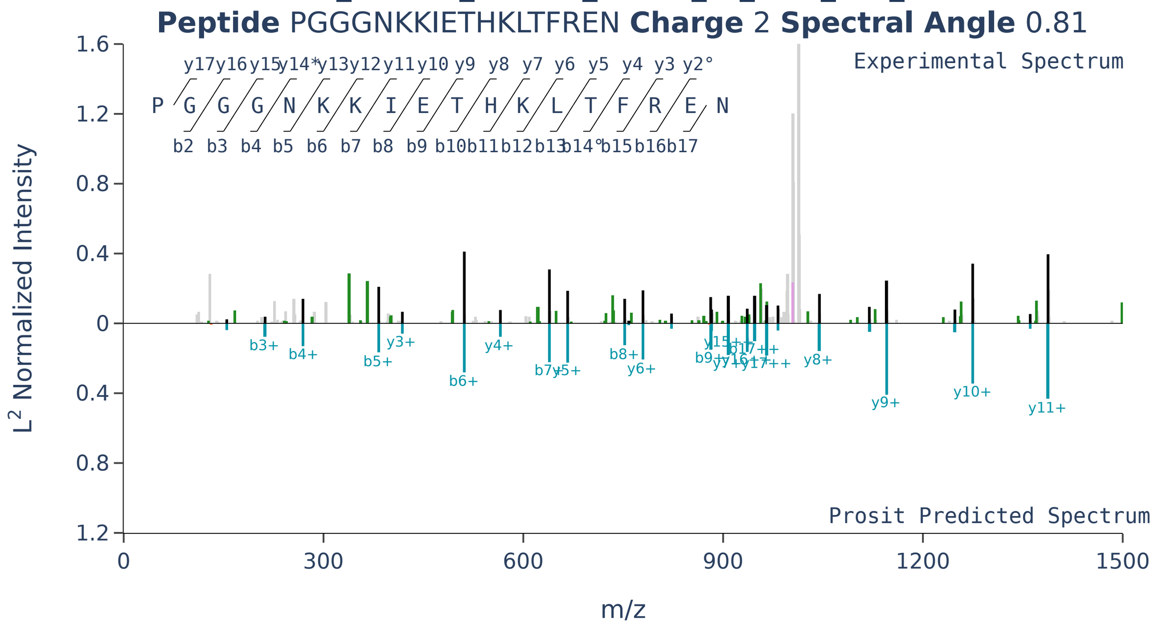


**Source** WSoh_221121_011221_HFGoe_L2_hTau_24h_R2 **Scan** 25139
**Peptide** ATLADEVSASLAKQ **Charge** 2 **Spectral Angle** 0.88

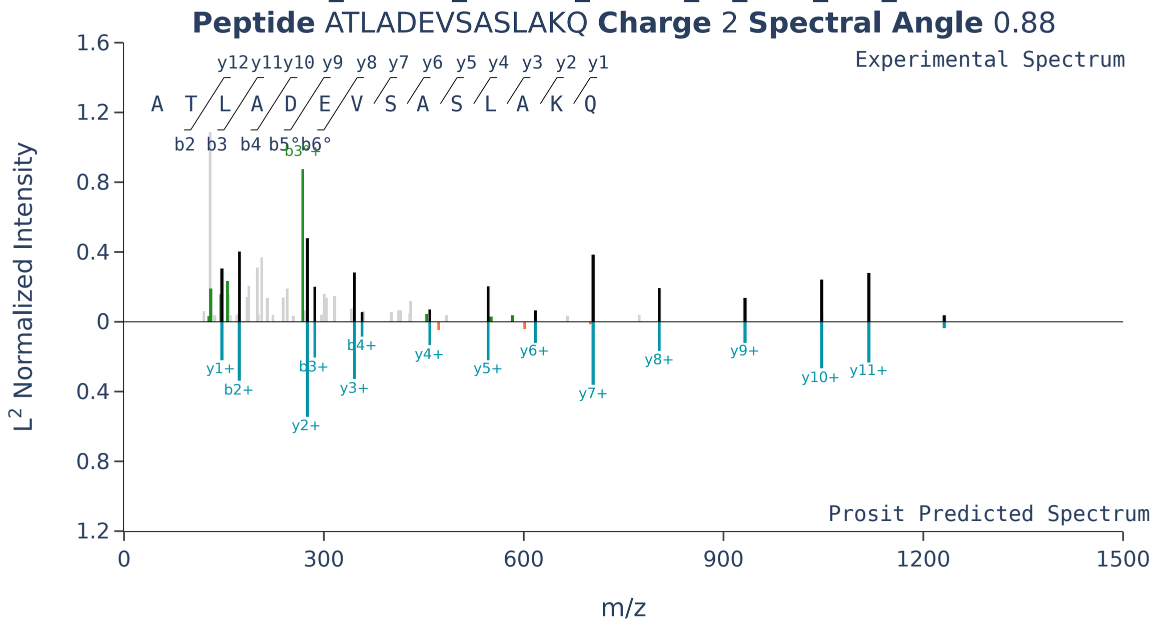


**Source** WSoh_221121_011221_HFGoe_L1_hTau_4h_R2 **Scan** 12522
**Peptide** EVKSEKLDFKDRV **Charge** 4 **Spectral Angle** 0.85

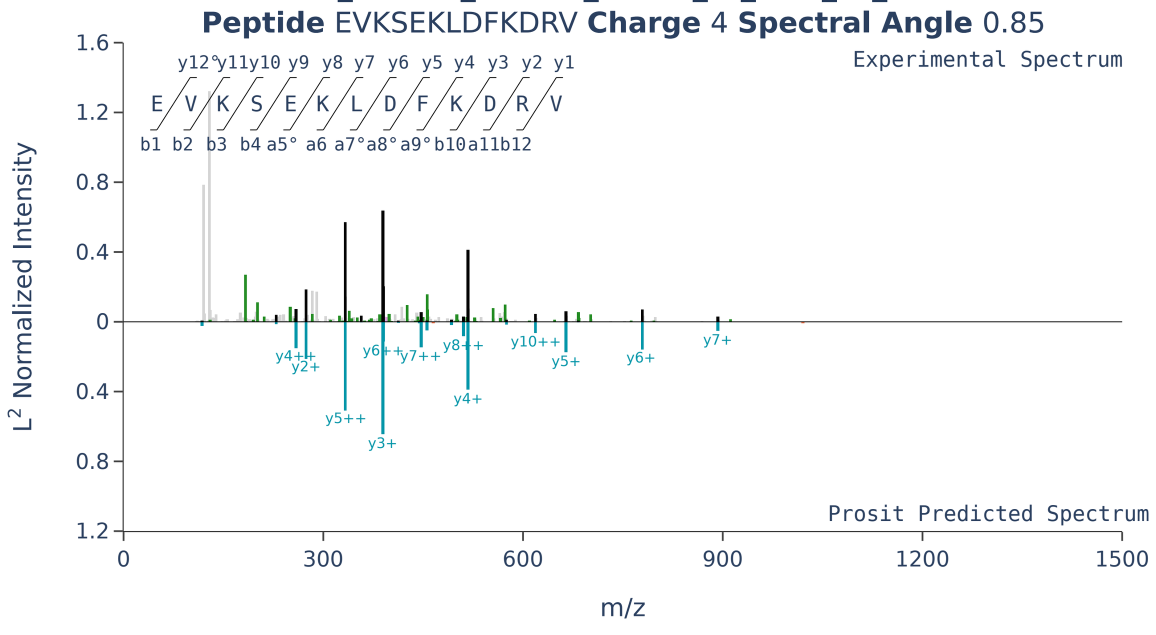


**Source** WSoh_221121_011221_HFGoe_L1_hTau_24h_R2 **Scan** 7208
**Peptide** ANATRIPAKT **Charge** 1 **Spectral Angle** 0.77

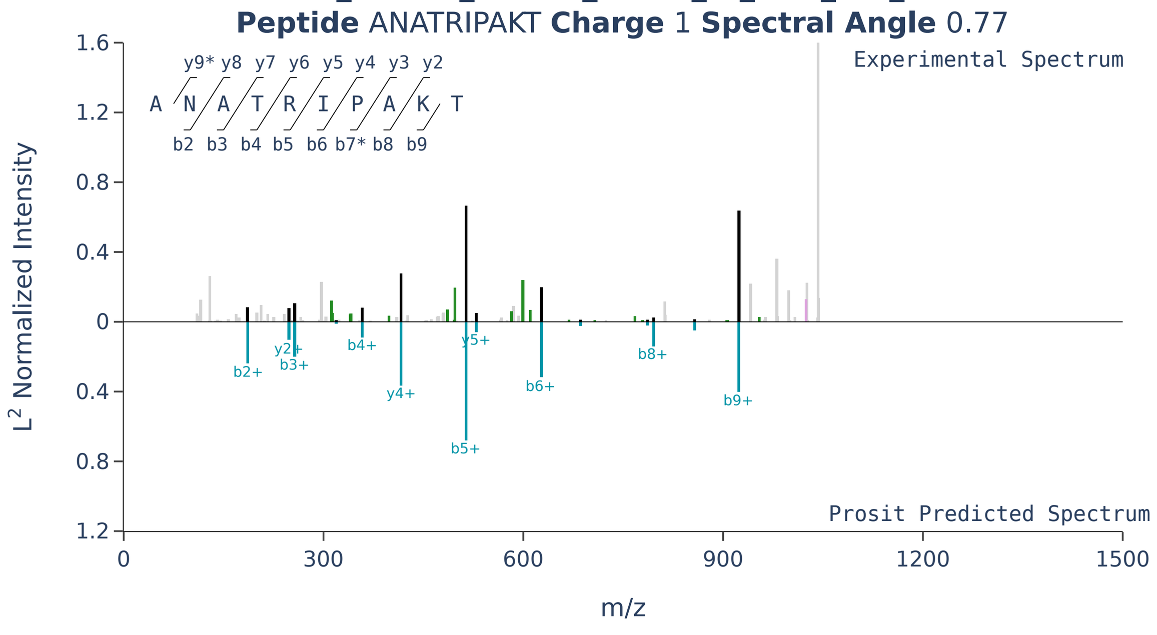


**Source** WSoh_221121_011221_HFGoe_L2_hTau_24h_R1 **Scan** 22215
**Peptide** QTAPVPMPDLKNVKSKIGST **Charge** 4 **Spectral Angle** 0.86

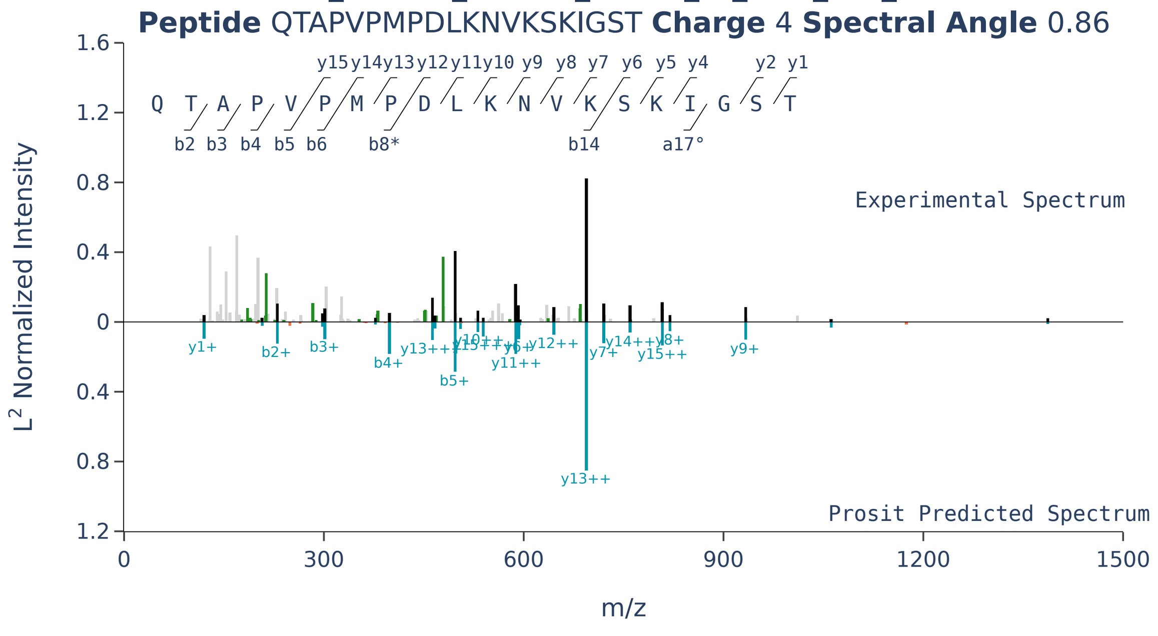


**Source** WSoh_221121_011221_HFGoe_L2_hTau_24h_R1 **Scan** 22421
**Peptide** PEGTTAEEAGIGDTPSLE **Charge** 2 **Spectral Angle** 0.89

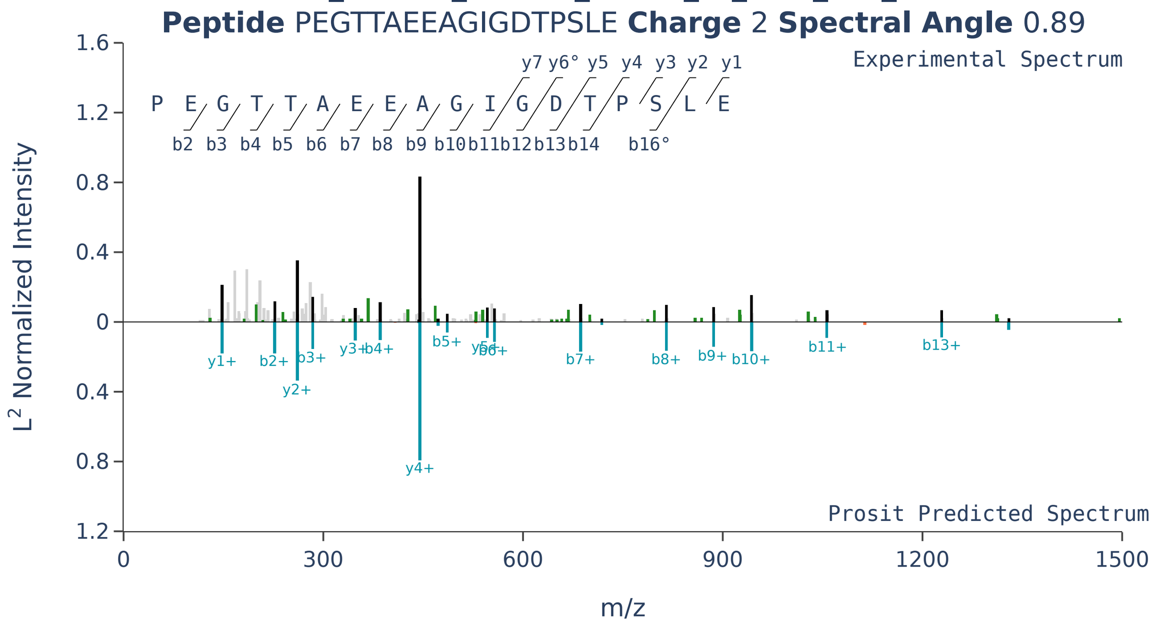


**Source** WSoh_221121_011221_HFGoe_L1_hTau_24h_R1 **Scan** 17993
**Peptide** EIVYKSPVVSGDTS **Charge** 2 **Spectral Angle** 0.9

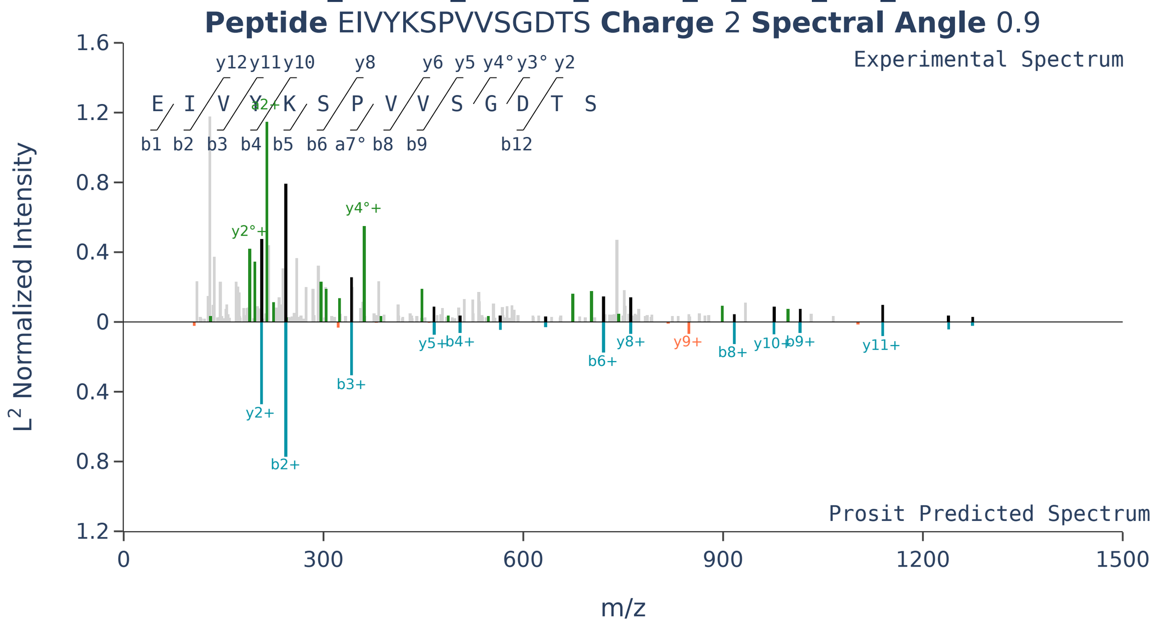

**Source** WSoh_221121_011221_HFGoe_L1_hTau_24h_R1 **Scan** 6094
**Peptide** SNVQSKCGSKDNL **Charge** 2 **Spectral Angle** 0.93

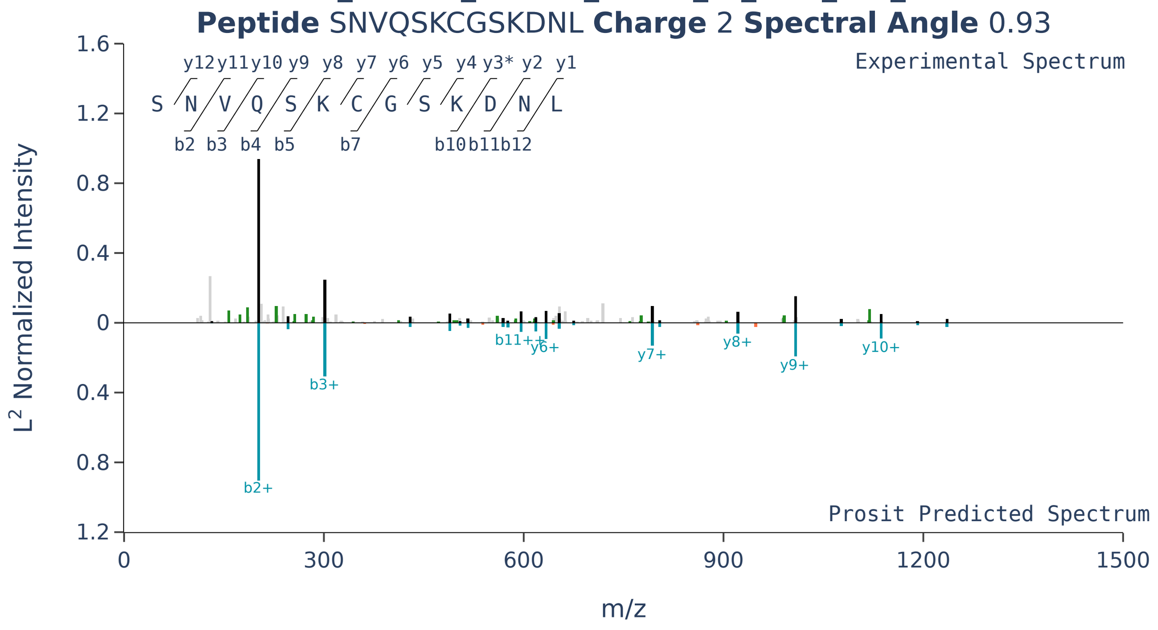


**Source** WSoh_221121_011221_HFGoe_L1_hTau_24h_R2 **Scan** 12990
**Peptide** KLTFRENAKAKTDHGAEIVY **Charge** 3 **Spectral Angle** 0.87

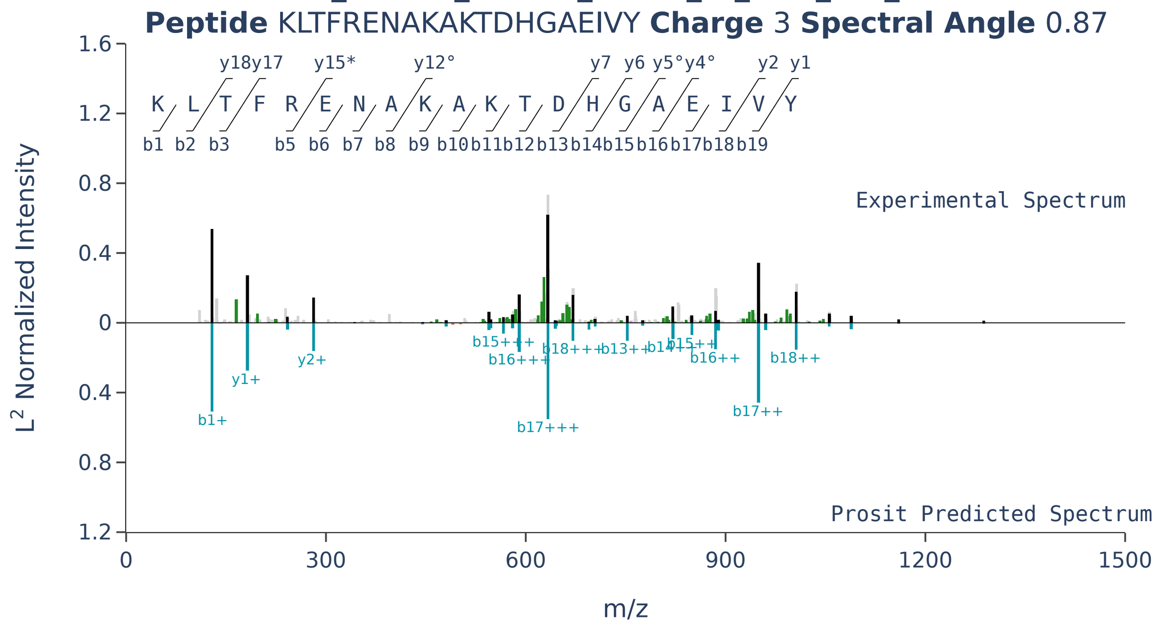


**Source** WSoh_221121_011221_HFGoe_L2_hTau_24h_R1 **Scan** 15524
**Peptide** LKESPLQTPTE **Charge** 2 **Spectral Angle** 0.88

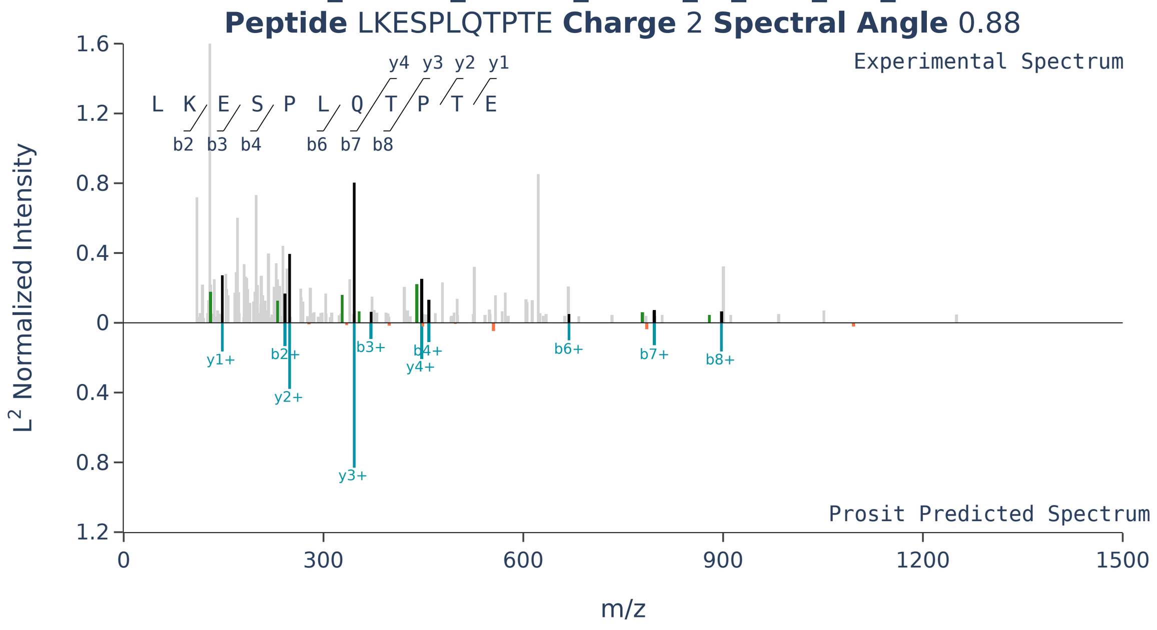


**Source** WSoh_221121_011221_HFGoe_L2_hTau_4h_R1 **Scan** 6431
**Peptide** RENAKAKTDHGAEIVYKS **Charge** 3 **Spectral Angle** 0.89

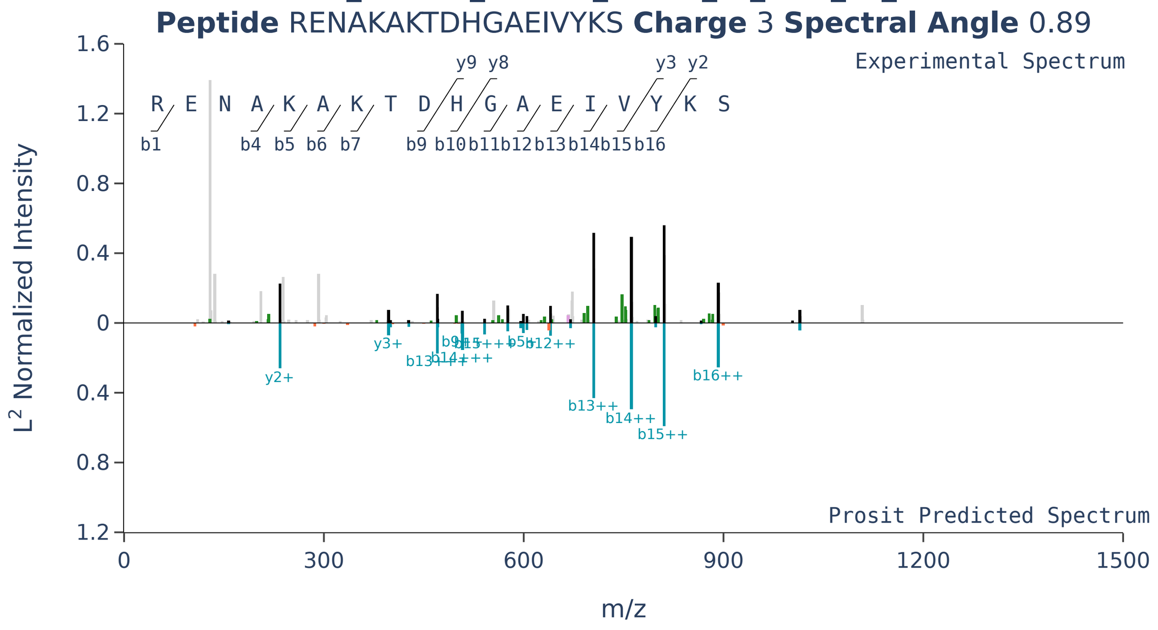


**Source** WSoh_221121_011221_HFGoe_L2_hTau_24h_R2 **Scan** 21952
**Peptide** TAPVPMPDLKNVKSKIG **Charge** 3 **Spectral Angle** 0.92

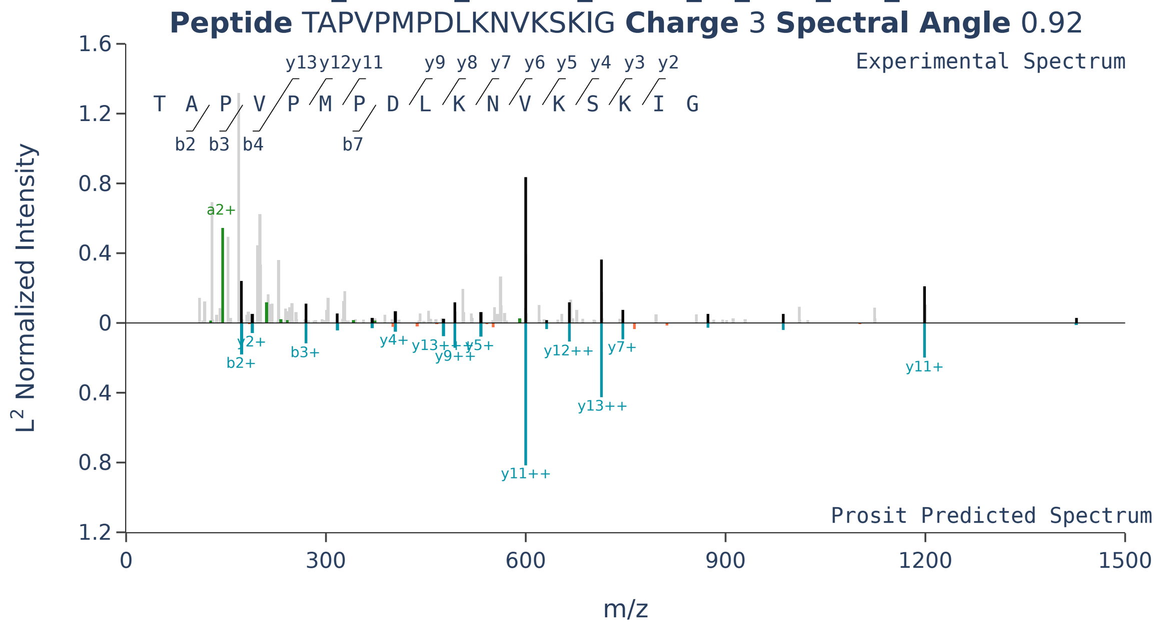


**Source** WSoh_221121_011221_HFGoe_L2_hTau_4h_R1 **Scan** 10208
**Peptide** TSPRHLSNVSSTGSID **Charge** 3 **Spectral Angle** 0.86

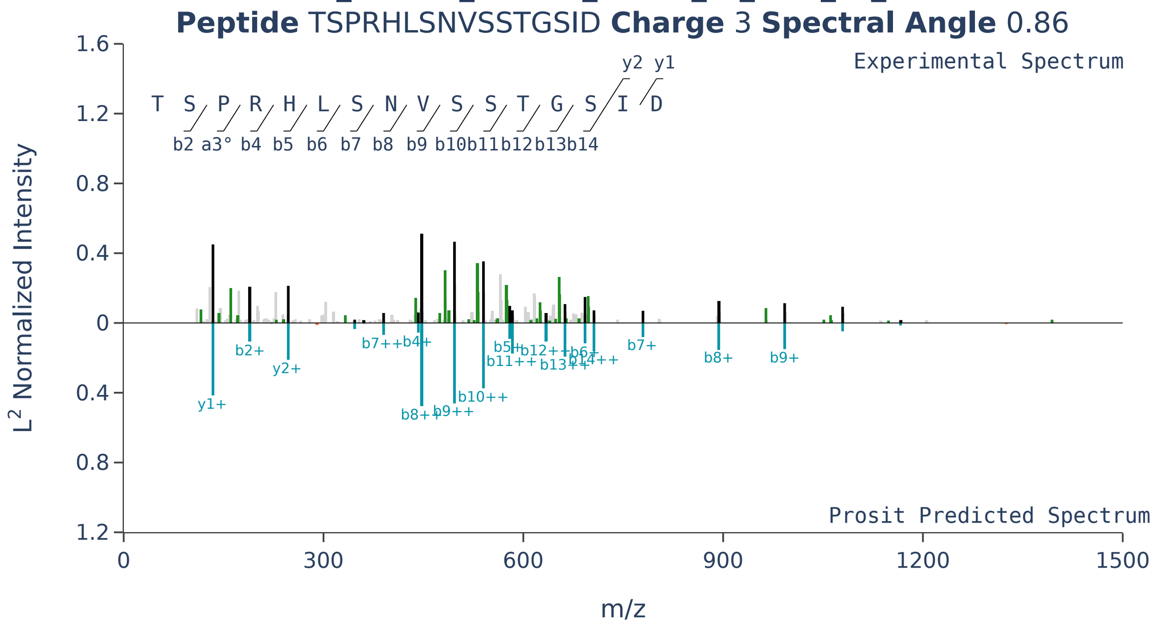


**Source** WSoh_221121_011221_HFGoe_L2_hTau_24h_R1 **Scan** 18941
**Peptide** PGGGSVQIVYKP **Charge** 2 **Spectral Angle** 0.82

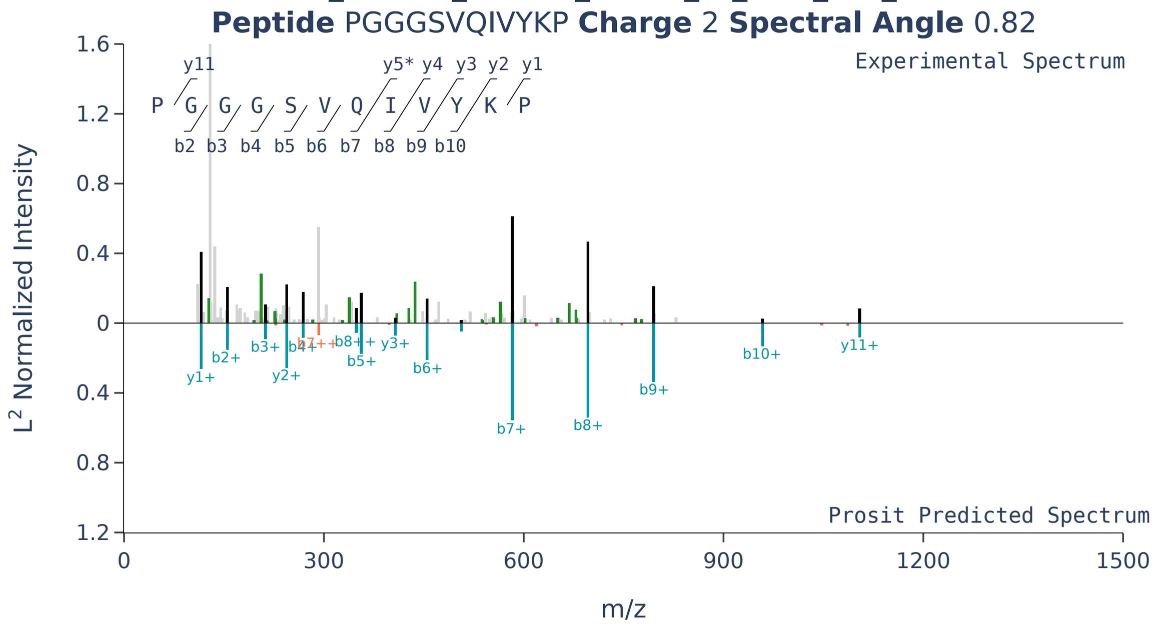


**Source** WSoh_221121_011221_HFGoe_L2_hTau_1h_R2 **Scan** 12342
**Peptide** HAGTYGL **Charge** 2 **Spectral Angle** 0.88

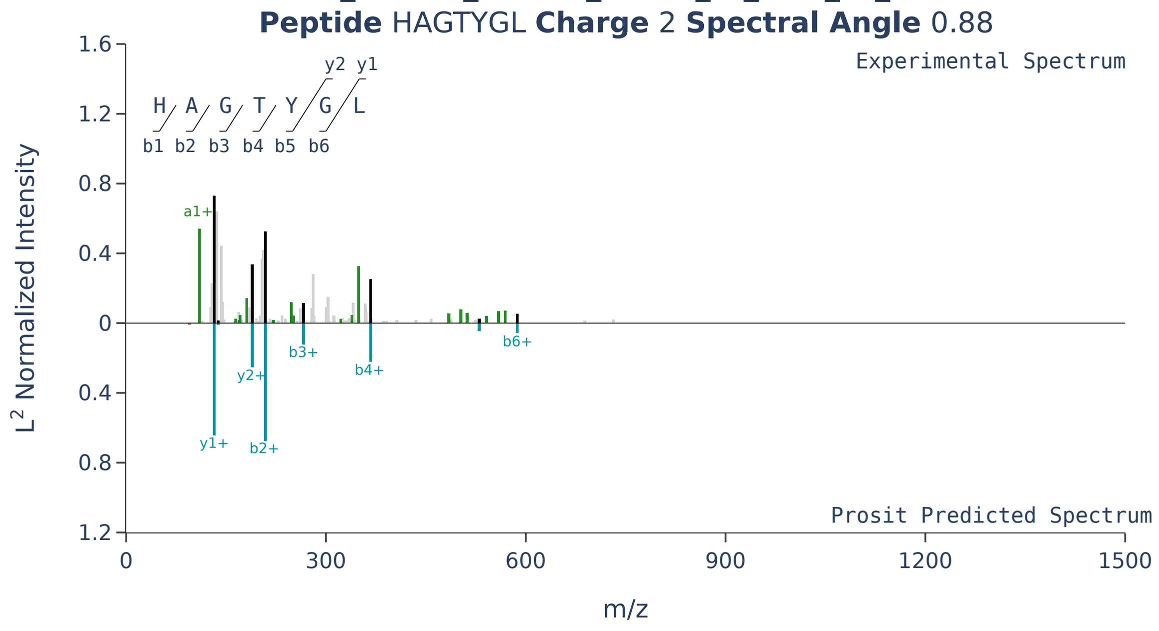


**Source** WSoh_221121_011221_HFGoe_L2_hTau_4h_R2 **Scan** 10078
**Peptide** HAGTYGLGDRKDQGGYTMH **Charge** 3 **Spectral Angle** 0.93

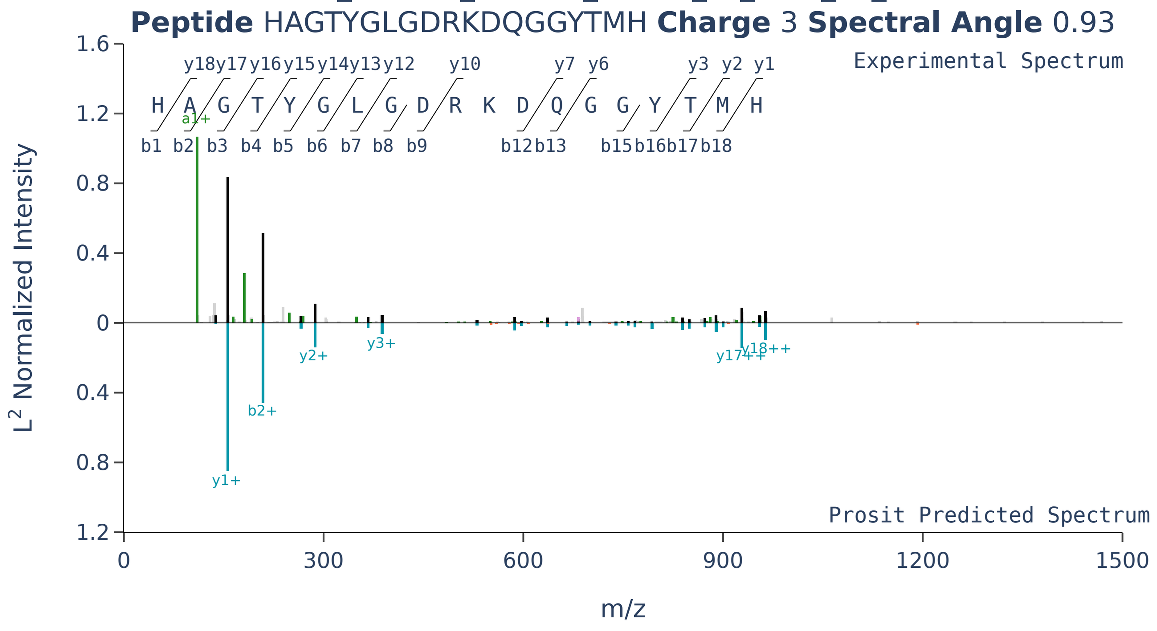


**Source** WSoh_221121_011221_HFGoe_L2_hTau_24h_R2 **Scan** 23739
**Peptide** DFKDRVQSKIGSLDNITHV **Charge** 3 **Spectral Angle** 0.82

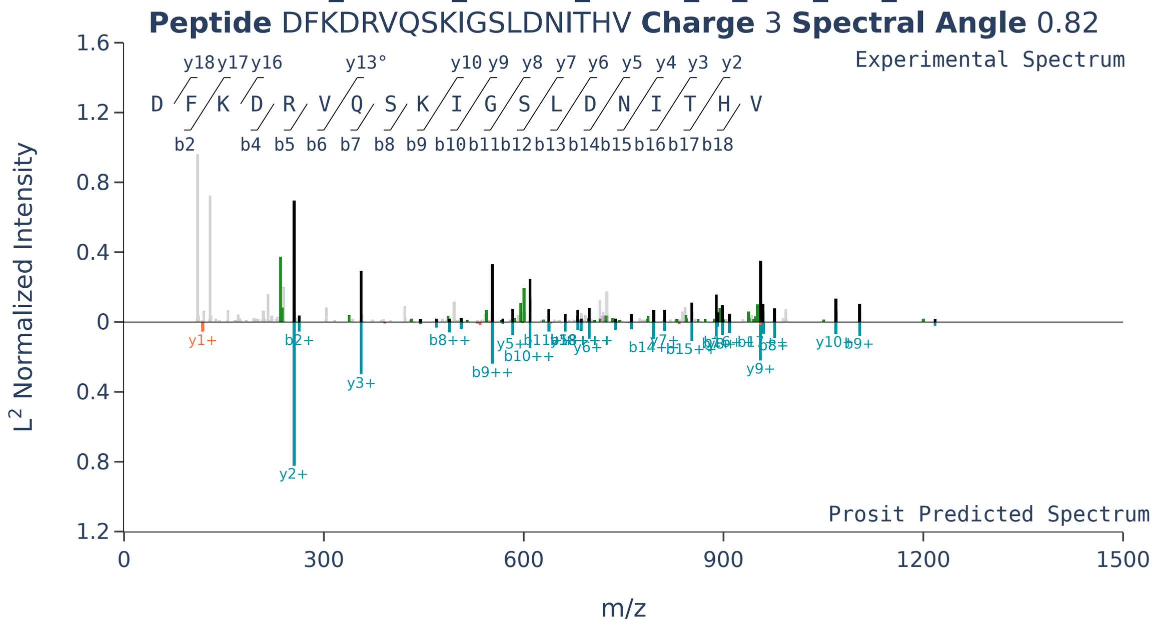


**Source** WSoh_221121_011221_HFGoe_L1_hTau_24h_R1 **Scan** 19149
**Peptide** GAEIVYKSPVVSGDTSPRHLSNV **Charge** 3 **Spectral Angle** 0.88

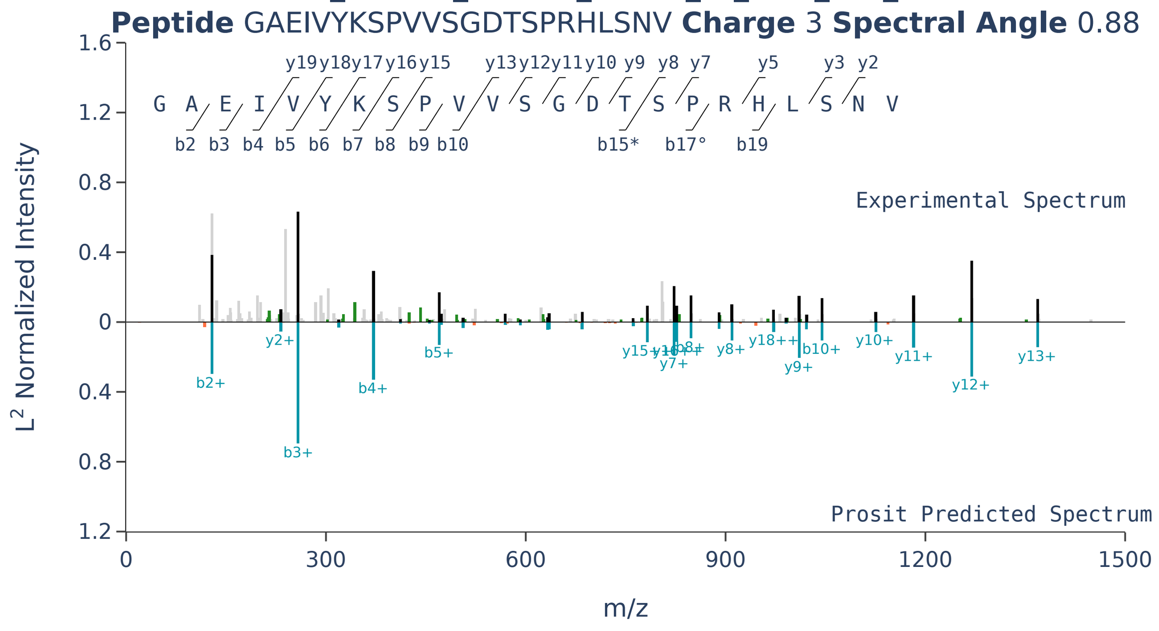


**Source** WSoh_221121_011221_HFGoe_L1_hTau_4h_R2 **Scan** 6342
**Peptide** HHKPGGGQVEV **Charge** 2 **Spectral Angle** 0.8

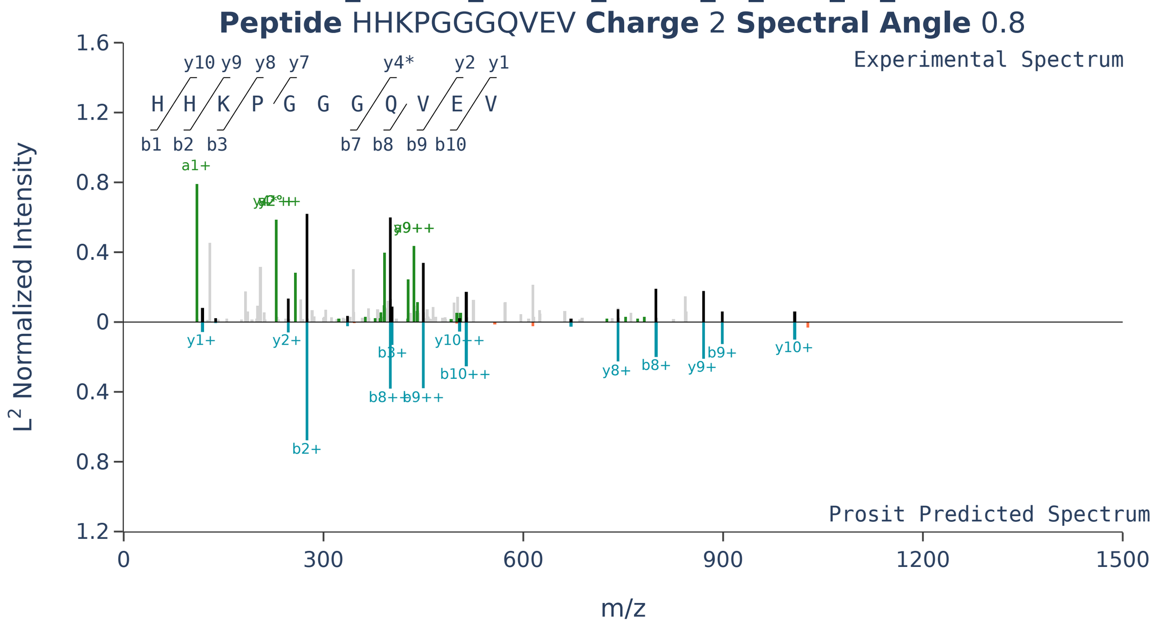


**Source** WSoh_221121_011221_HFGoe_L1_hTau_24h_R2 **Scan** 11808
**Peptide** QEGDTDAGL **Charge** 1 **Spectral Angle** 0.85

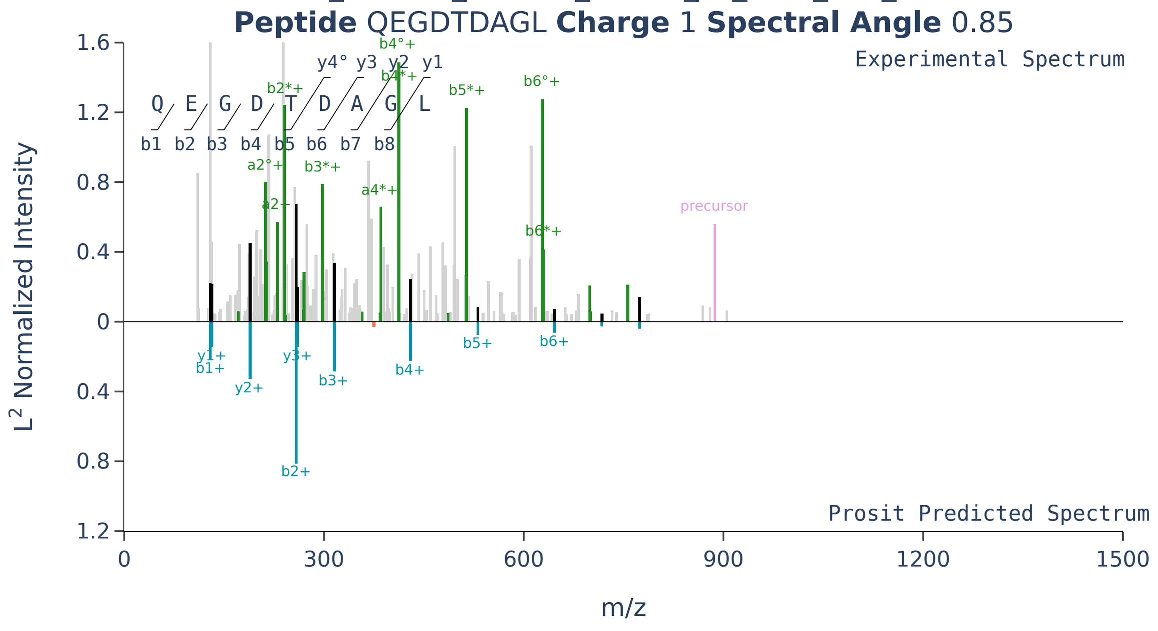


**Source** WSoh_221121_011221_HFGoe_L1_hTau_24h_R1 **Scan** 21910
**Peptide** TAPVPMPDLKNVKSKIGSTE **Charge** 3 **Spectral Angle** 0.86

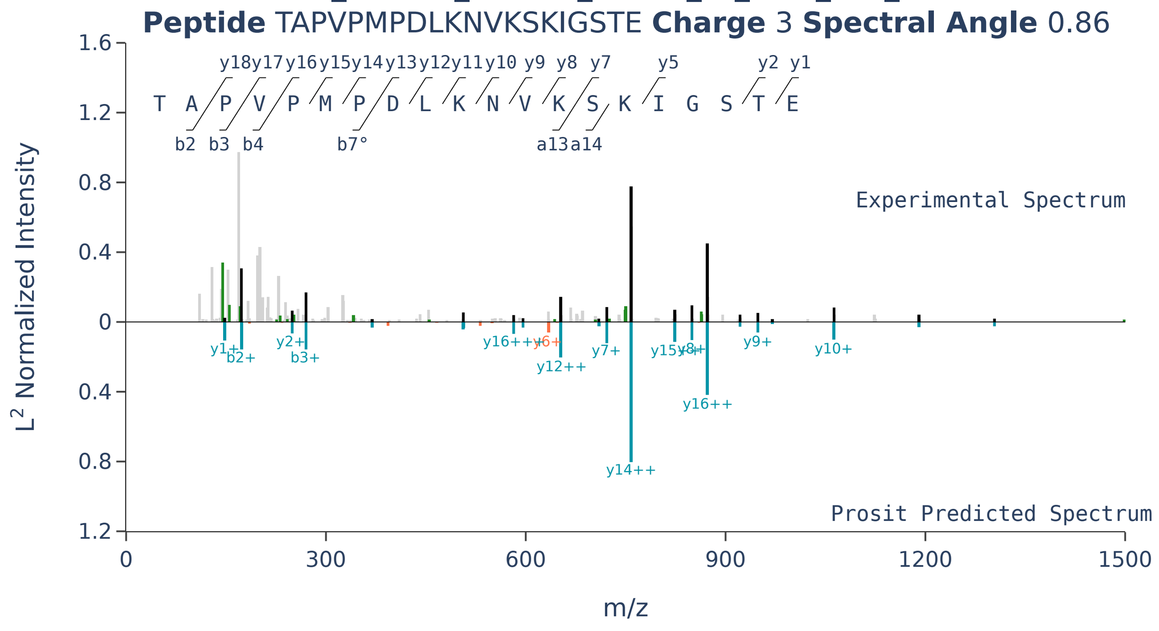


**Source** WSoh_221121_011221_HFGoe_L2_hTau_24h_R1 **Scan** 10253
**Peptide** LDFKDRVQSK **Charge** 3 **Spectral Angle** 0.85

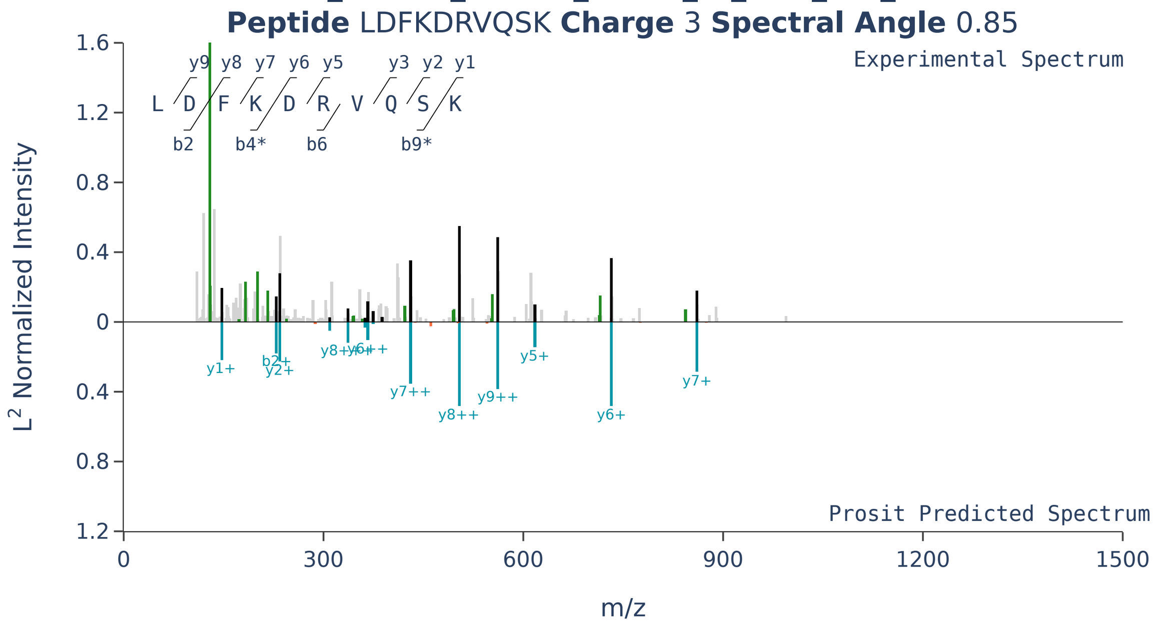

**Source** WSoh_221121_011221_HFGoe_L2_hTau_24h_R1 **Scan** 6963
**Peptide** HAGTYGLGDRKDQ **Charge** 2 **Spectral Angle** 0.82

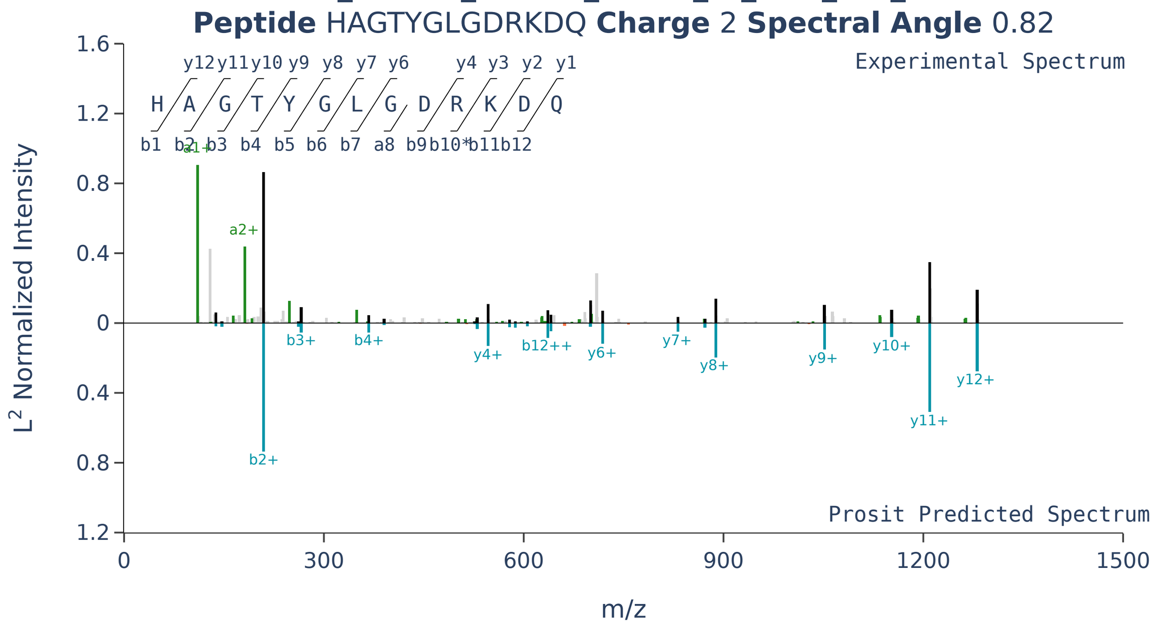


**Source** WSoh_221121_011221_HFGoe_L1_hTau_2h_R1 **Scan** 7650
**Peptide** PPAPKTPPSSGEPPKS **Charge** 2 **Spectral Angle** 0.82

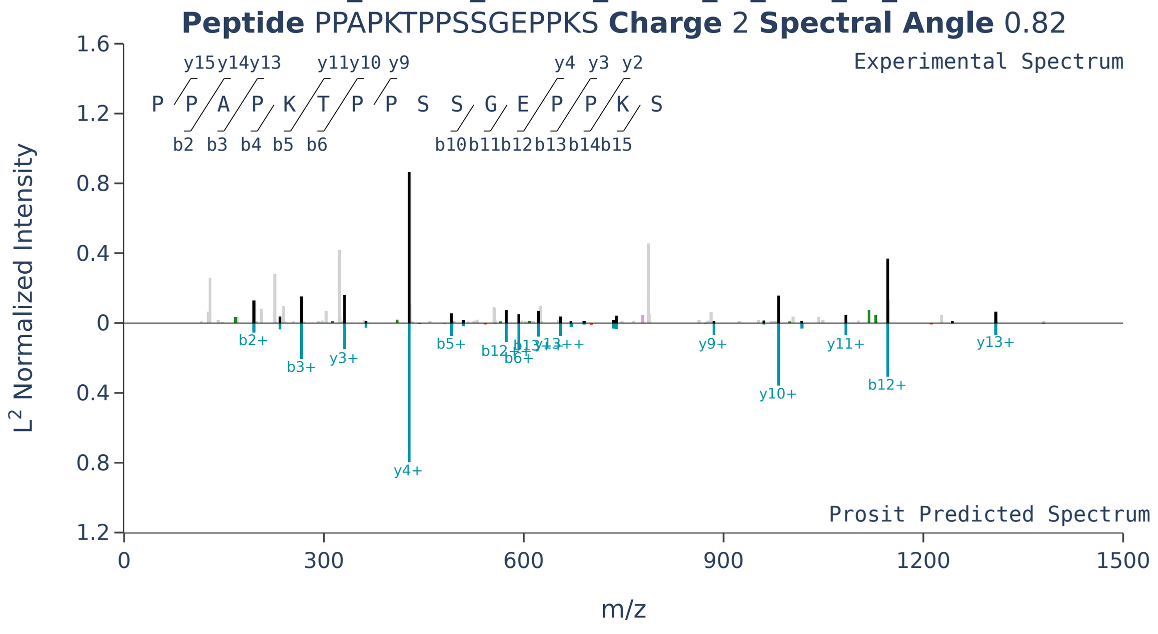


**Source** WSoh_221121_011221_HFGoe_L2_hTau_2h_R2 **Scan** 6669
**Peptide** KLTFRENAKAKTD **Charge** 4 **Spectral Angle** 0.93

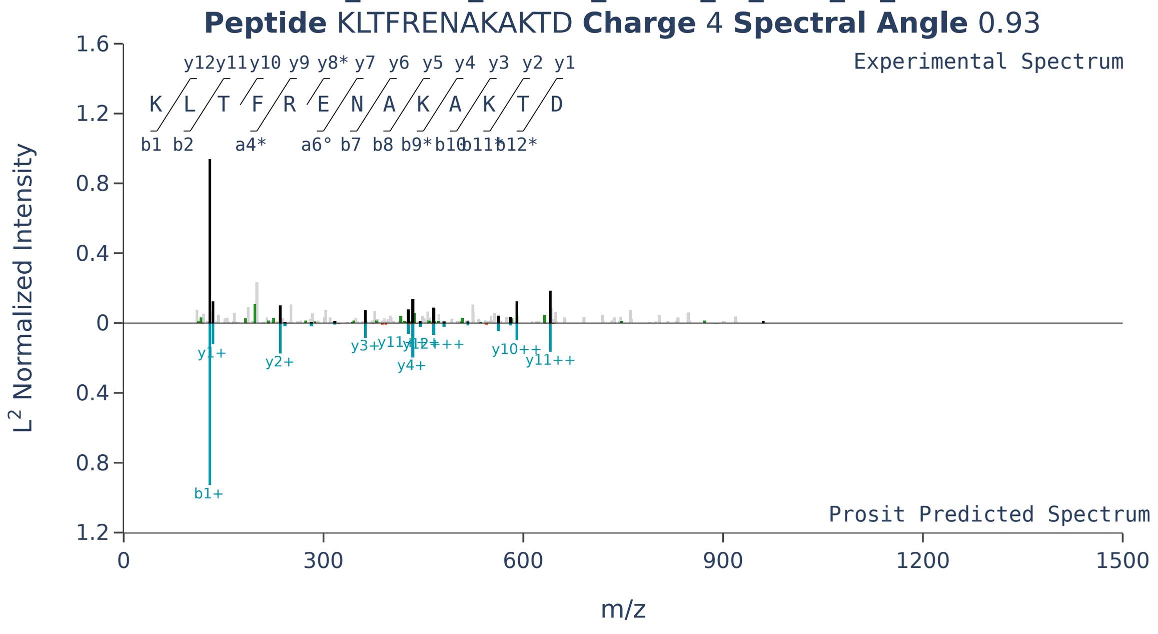


**Source** WSoh_221121_011221_HFGoe_L1_hTau_24h_R2 **Scan** 17490
**Peptide** GAEIVYKSPVVSGDTSPRHLS **Charge** 3 **Spectral Angle** 0.82

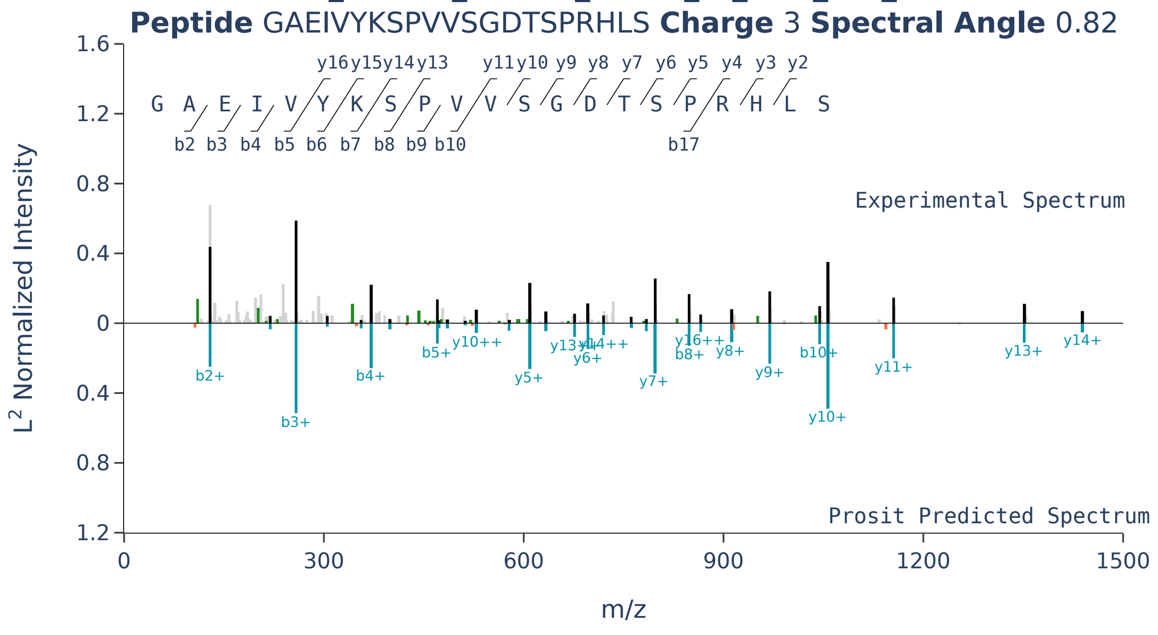


**Source** WSoh_221121_011221_HFGoe_L2_hTau_24h_R1 **Scan** 14931
**Peptide** AKTDHGAEIVYKSPVVS **Charge** 2 **Spectral Angle** 0.83

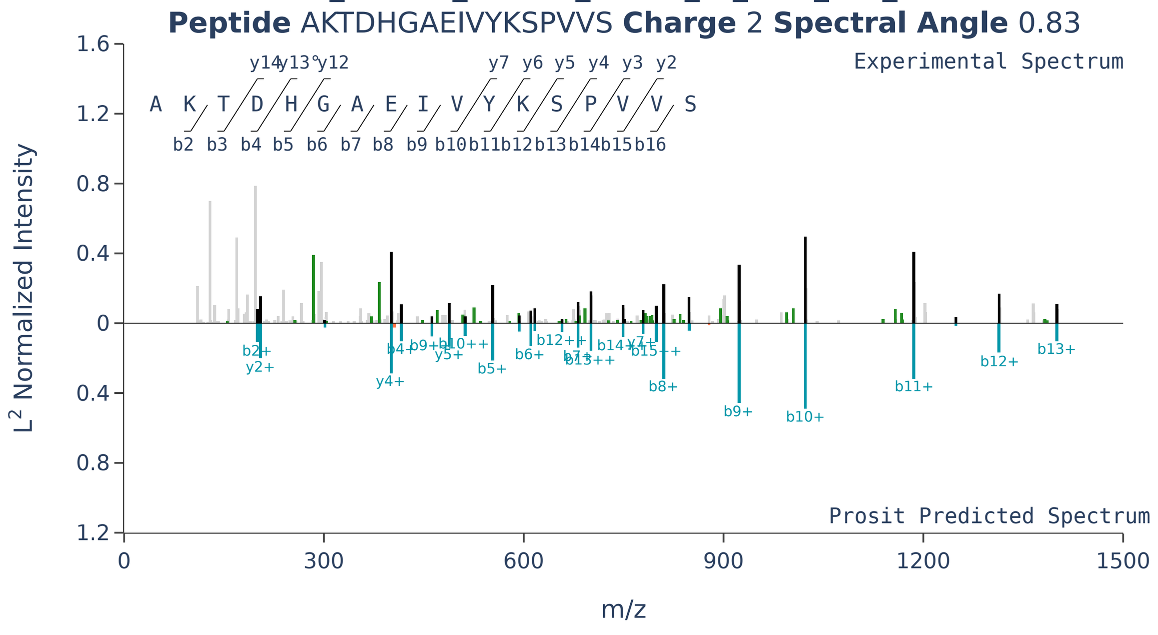


**Source** WSoh_221121_011221_HFGoe_L2_hTau_24h_R1 **Scan** 7446
**Peptide** SLGNIHHKPGGGQV **Charge** 2 **Spectral Angle** 0.85

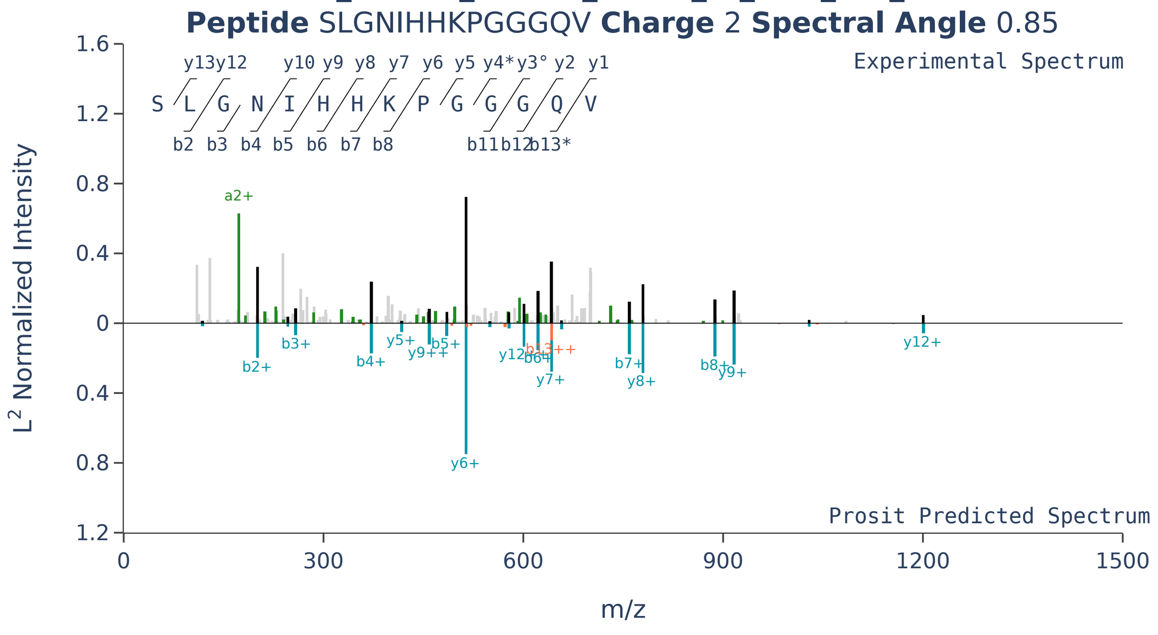


**Source** WSoh_221121_011221_HFGoe_L1_hTau_24h_R1 **Scan** 21302
**Peptide** GAEIVYKSPVV **Charge** 2 **Spectral Angle** 0.85

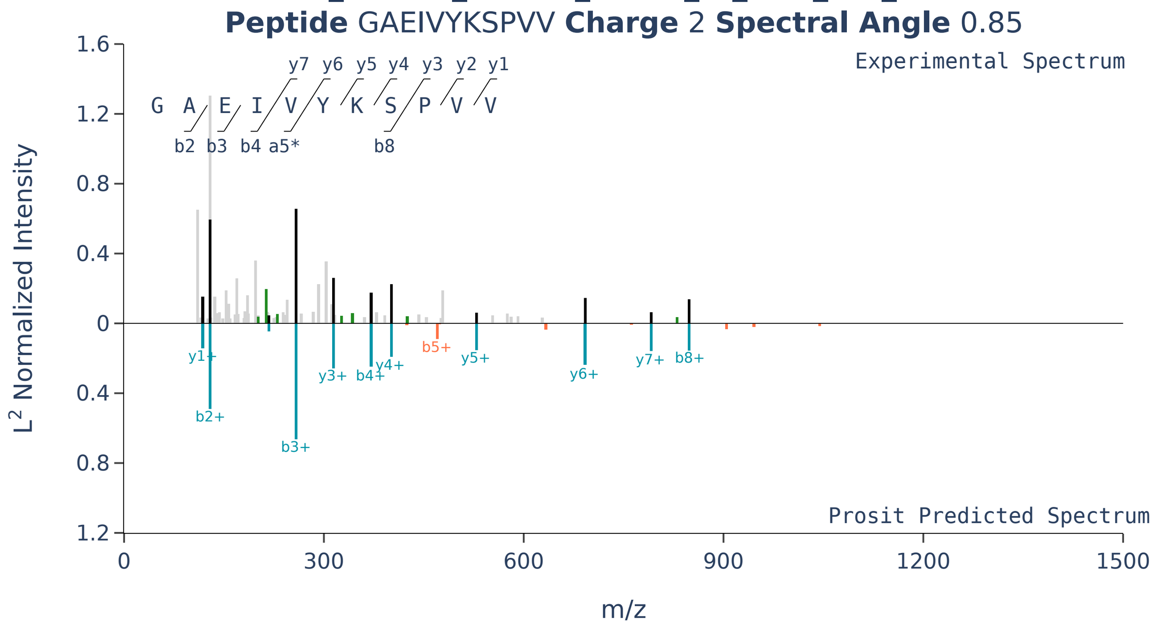


**Source** WSoh_221121_011221_HFGoe_L1_hTau_4h_R1 **Scan** 17634
**Peptide** IGSLDNITHVPGGGNKKI **Charge** 3 **Spectral Angle** 0.85

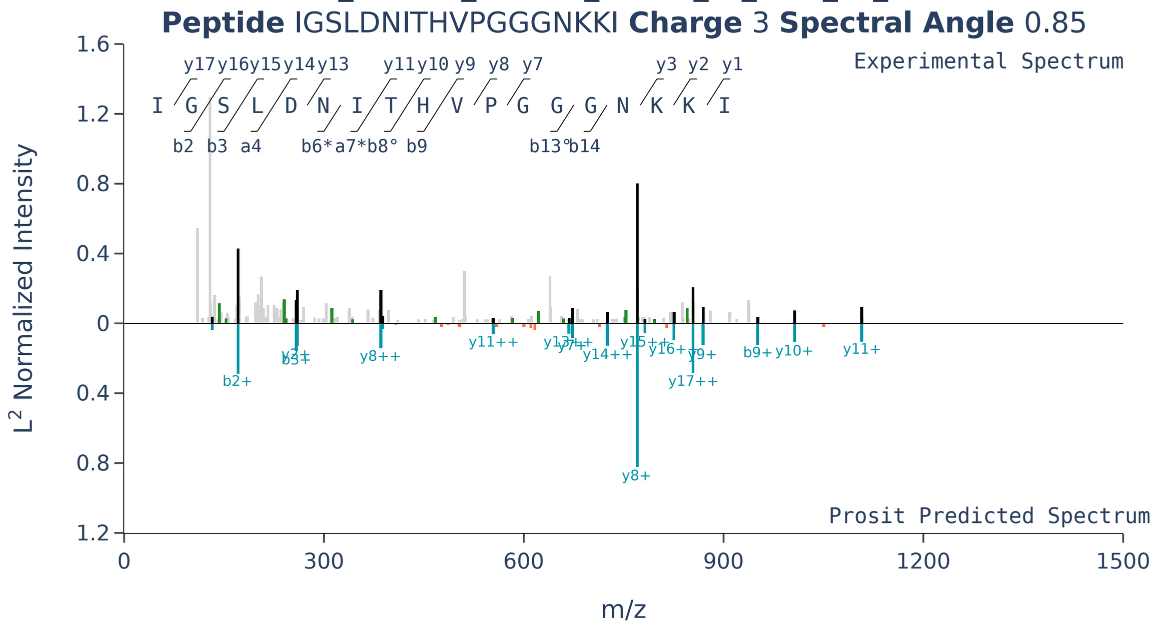


**Source** WSoh_221121_011221_HFGoe_L1_hTau_4h_R2 **Scan** 16142
**Peptide** VMEDHAGTYGLGD **Charge** 2 **Spectral Angle** 0.86

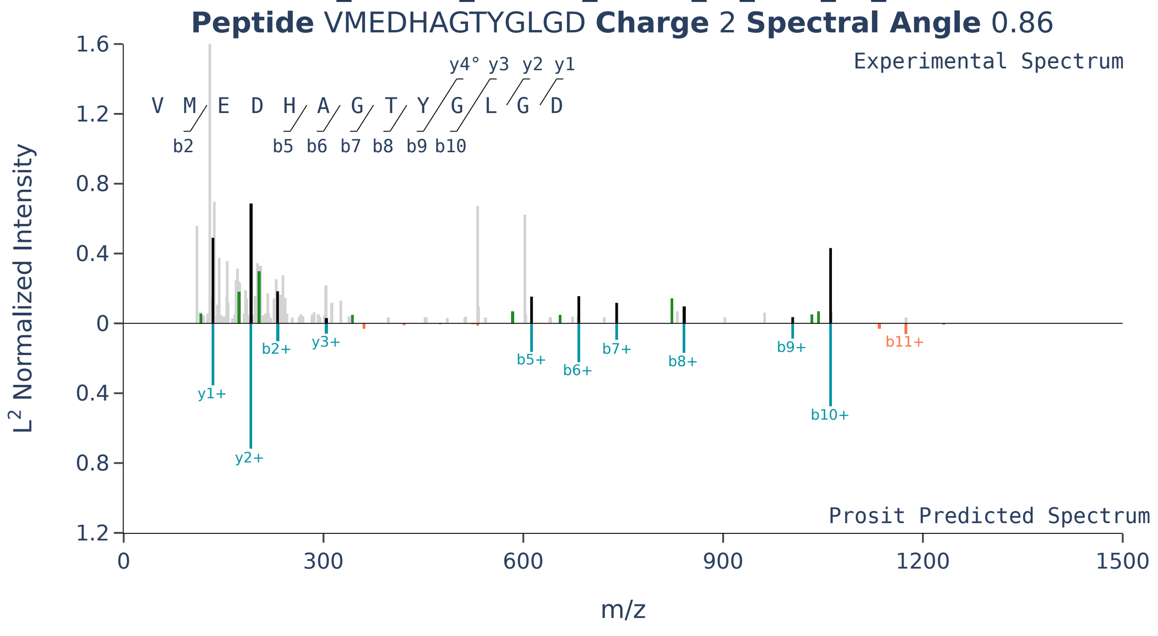


**Source** WSoh_221121_011221_HFGoe_L2_hTau_1h_R1 **Scan** 5638
**Peptide** ATPRGAAPPGQKG **Charge** 3 **Spectral Angle** 0.89

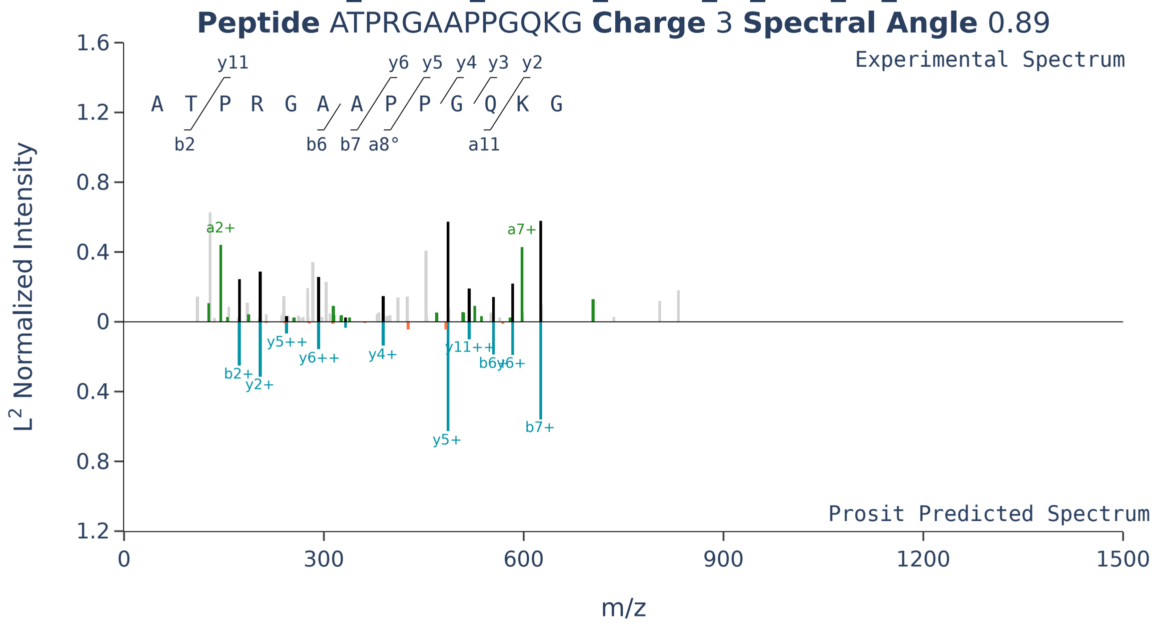


**Source** WSoh_221121_011221_HFGoe_L2_hTau_1h_R1 **Scan** 10638
**Peptide** QPHTEIPEGT **Charge** 2 **Spectral Angle** 0.89

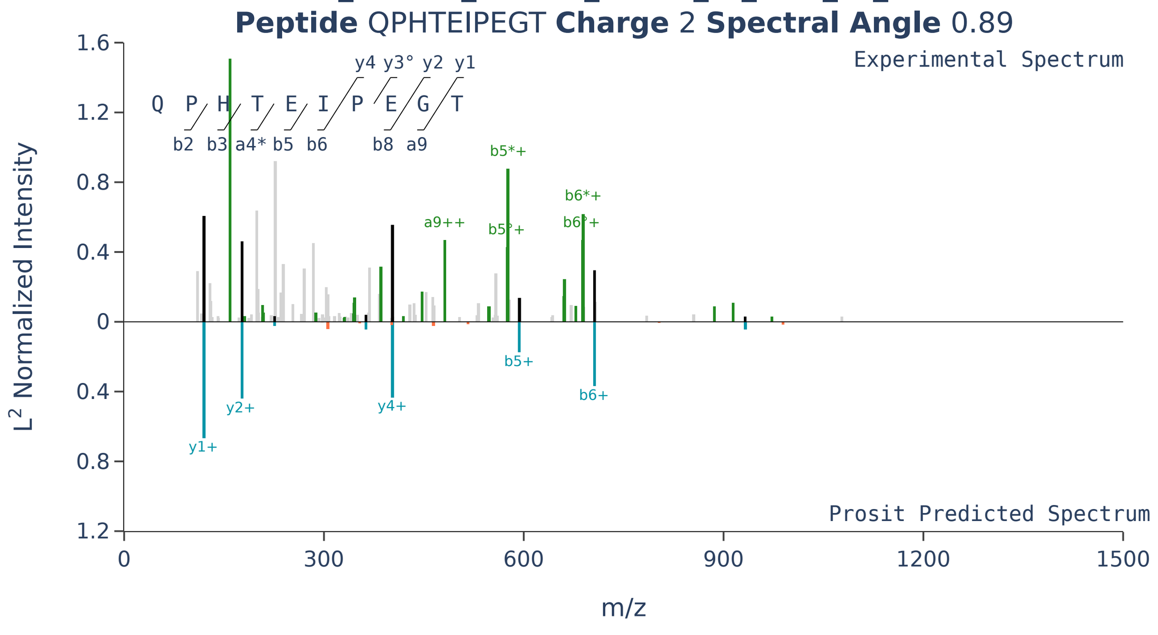


**Source** WSoh_221121_011221_HFGoe_L1_hTau_24h_R1 **Scan** 7238
**Peptide** NLHHKPGGGQVEVKSEKL **Charge** 2 **Spectral Angle** 0.91

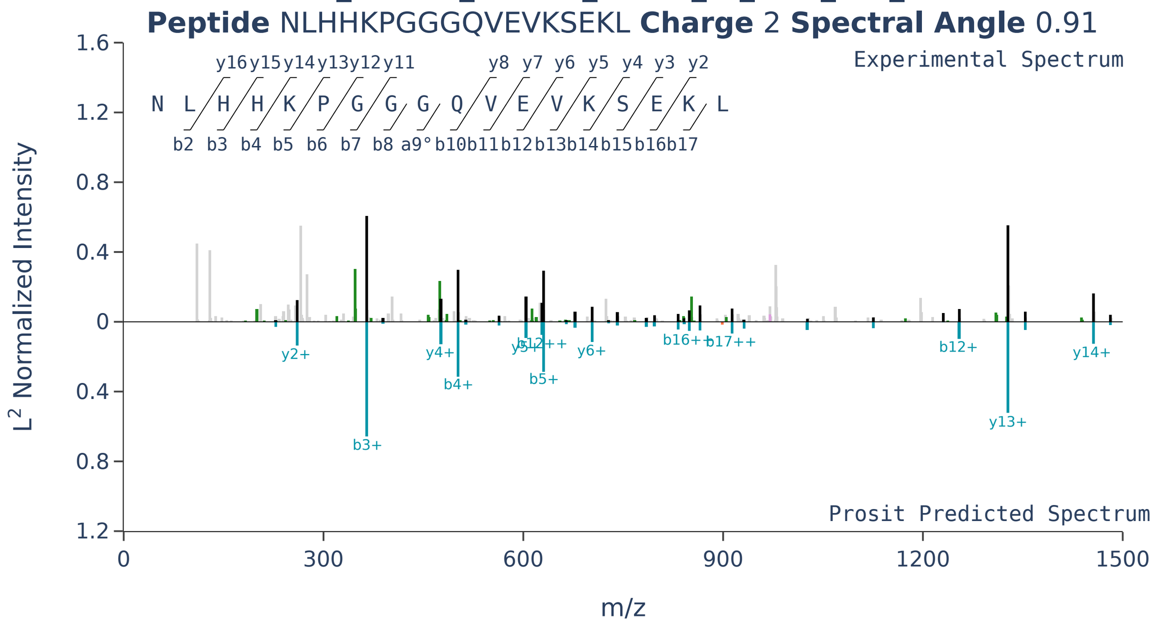


**Source** WSoh_221121_011221_HFGoe_L2_hTau_24h_R1 **Scan** 6768
**Peptide** TSKCGSLGNIHH **Charge** 3 **Spectral Angle** 0.91

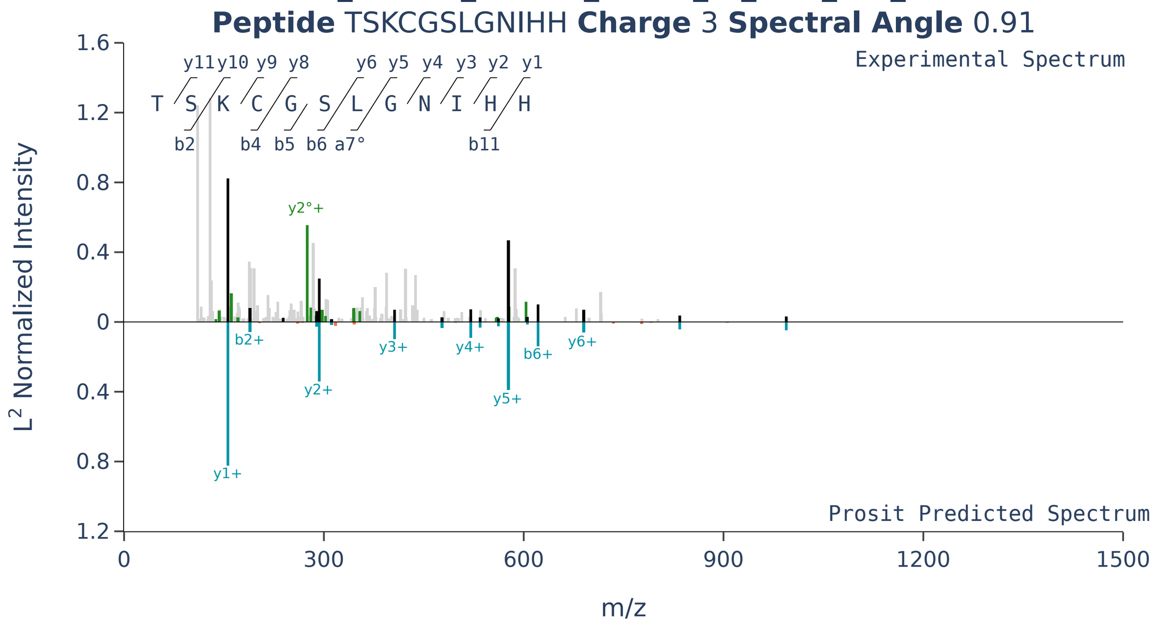


**Source** WSoh_221121_011221_HFGoe_L2_hTau_2h_R1 **Scan** 18742
**Peptide** QIVYKPVDLSKVTSKC **Charge** 3 **Spectral Angle** 0.91

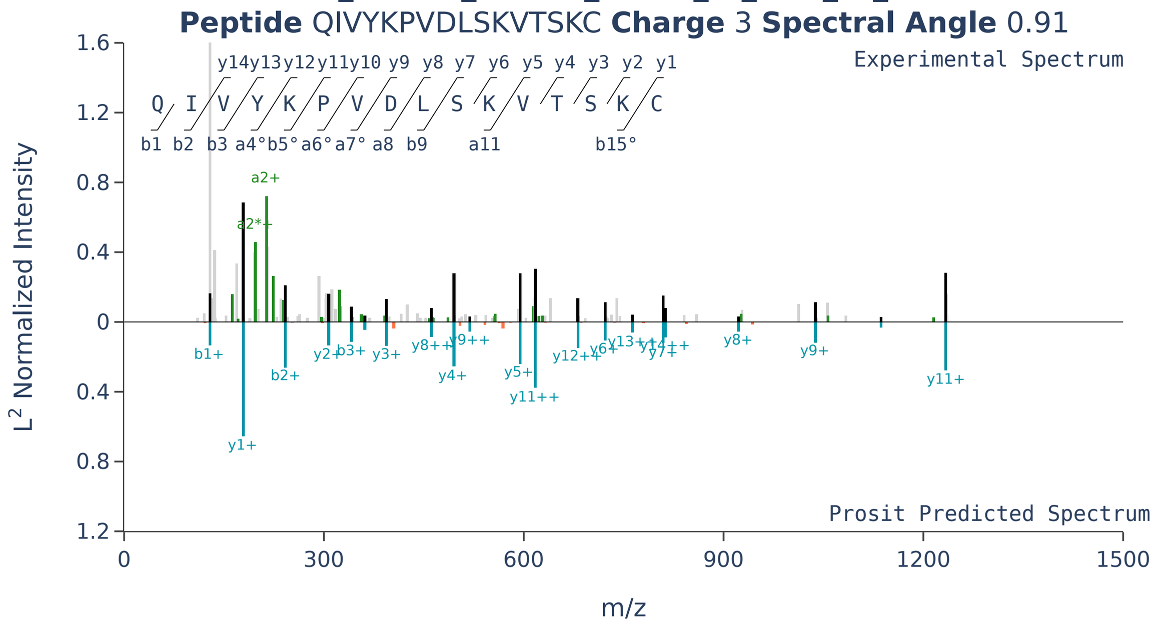


**Source** WSoh_221121_011221_HFGoe_L2_hTau_1h_R1 **Scan** 5664
**Peptide** KGADGKTKIATPRG **Charge** 3 **Spectral Angle** 0.86

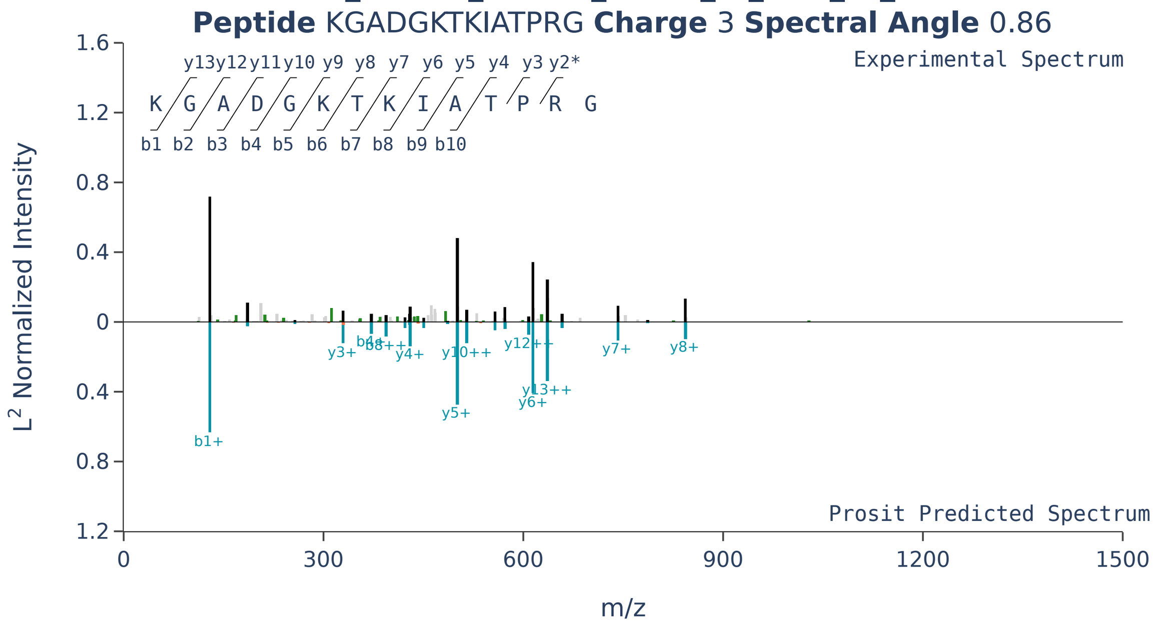

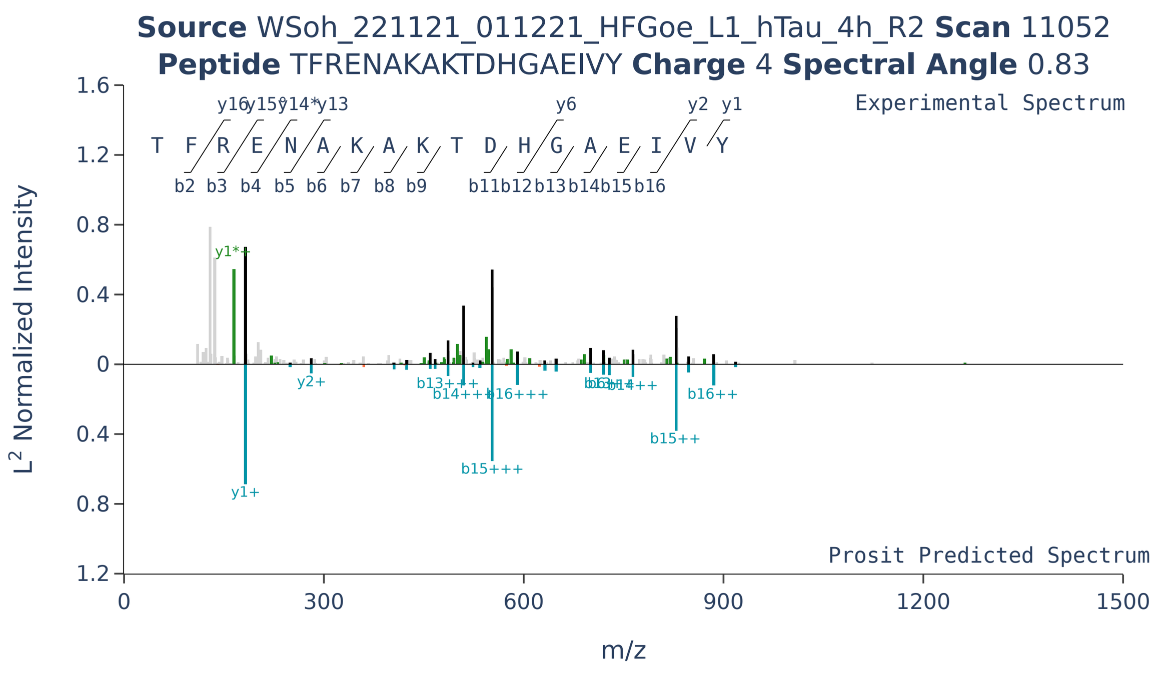
**Source** WSoh_221121_011221_HFGoe_L1_hTau_4h_R2 **Scan** 11052
**Peptide** TFRENAKAKTDHGAEIVY **Charge** 4 **Spectral Angle** 0.83
Experimental Spectrum
Prosit Predicted Spectrum
m/z
L² Normalized Intensity

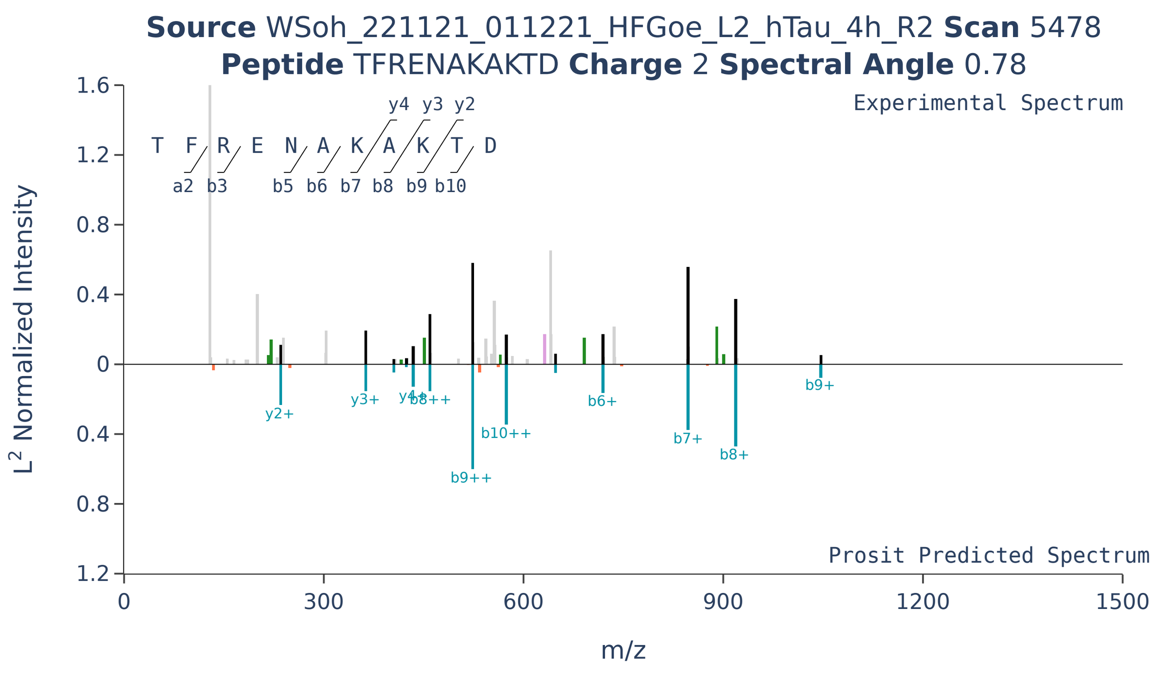
**Source** WSoh_221121_011221_HFGoe_L2_hTau_4h_R2 **Scan** 5478
**Peptide** TFRENAKAKTD **Charge** 2 **Spectral Angle** 0.78
Experimental Spectrum
Prosit Predicted Spectrum
m/z
L² Normalized Intensity

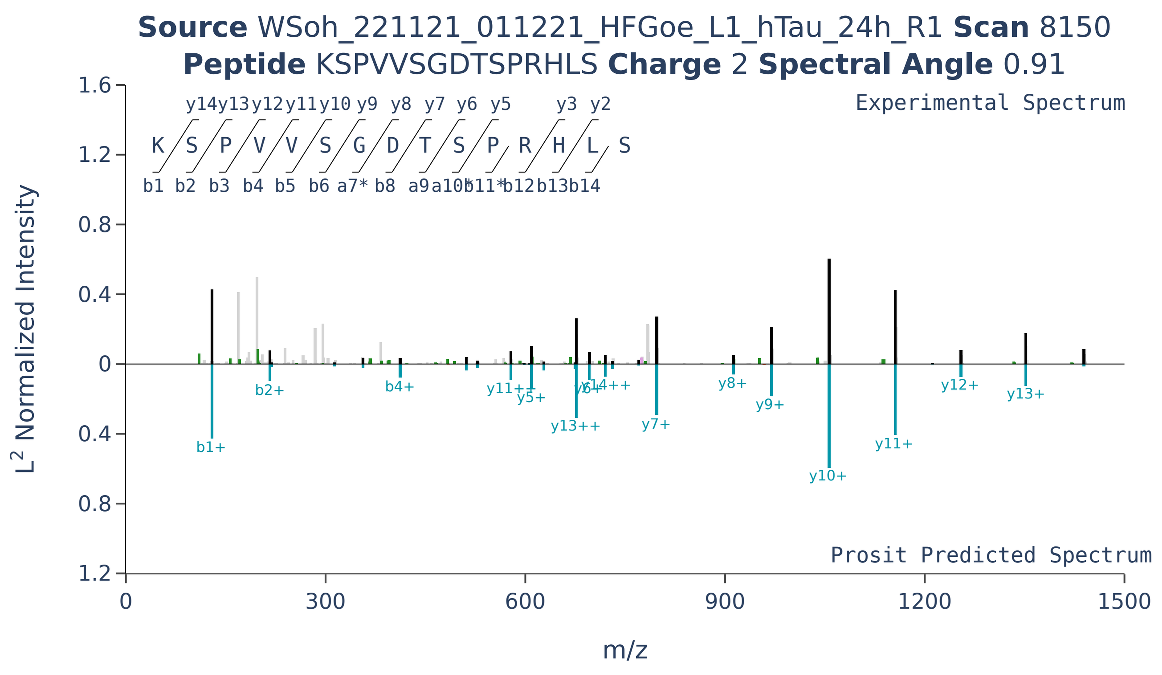
**Source** WSoh_221121_011221_HFGoe_L1_hTau_24h_R1 **Scan** 8150
**Peptide** KSPVVSGDTSPRHLS **Charge** 2 **Spectral Angle** 0.91
Experimental Spectrum
Prosit Predicted Spectrum
m/z
L² Normalized Intensity

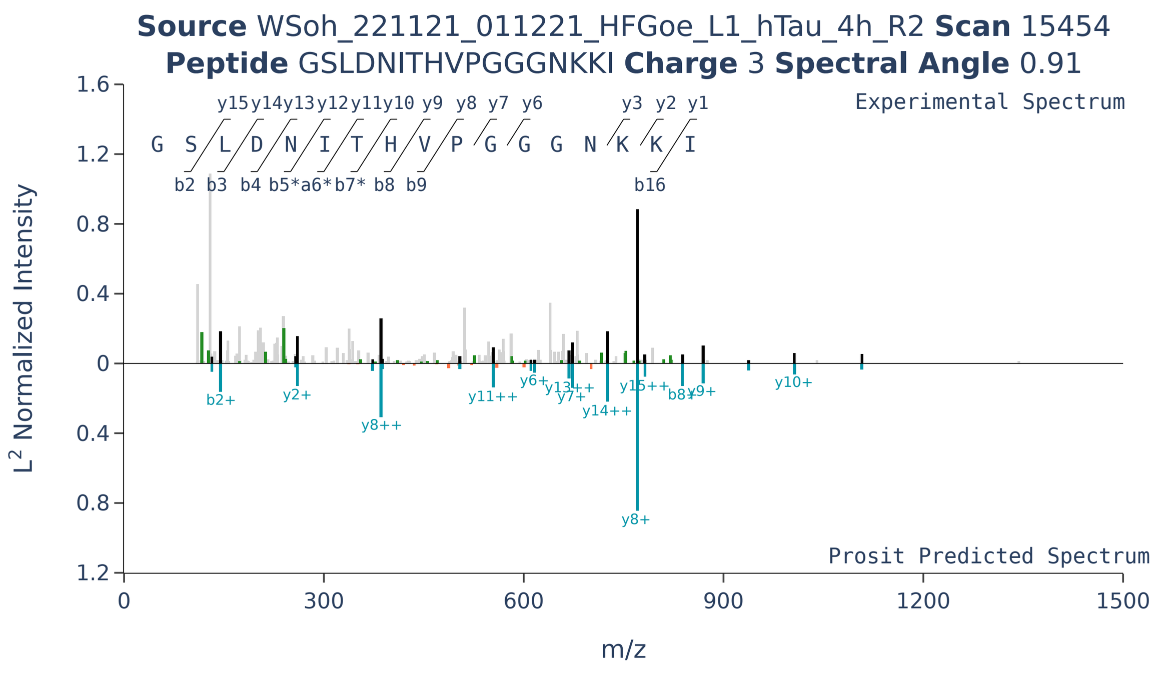
**Source** WSoh_221121_011221_HFGoe_L1_hTau_4h_R2 **Scan** 15454
**Peptide** GSLDNITHVPGGGNKKI **Charge** 3 **Spectral Angle** 0.91
Experimental Spectrum
Prosit Predicted Spectrum
m/z
L² Normalized Intensity

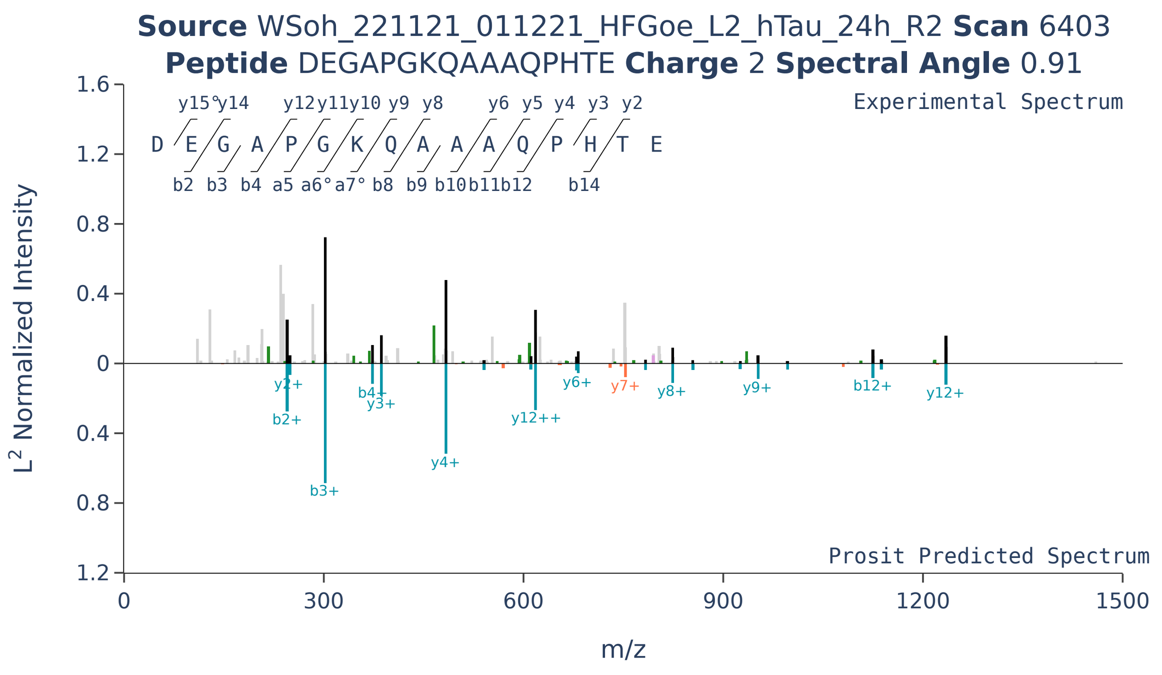
**Source** WSoh_221121_011221_HFGoe_L2_hTau_24h_R2 **Scan** 6403
**Peptide** DEGAPGKQAAAQPHTE **Charge** 2 **Spectral Angle** 0.91
Experimental Spectrum
Prosit Predicted Spectrum
m/z
L² Normalized Intensity

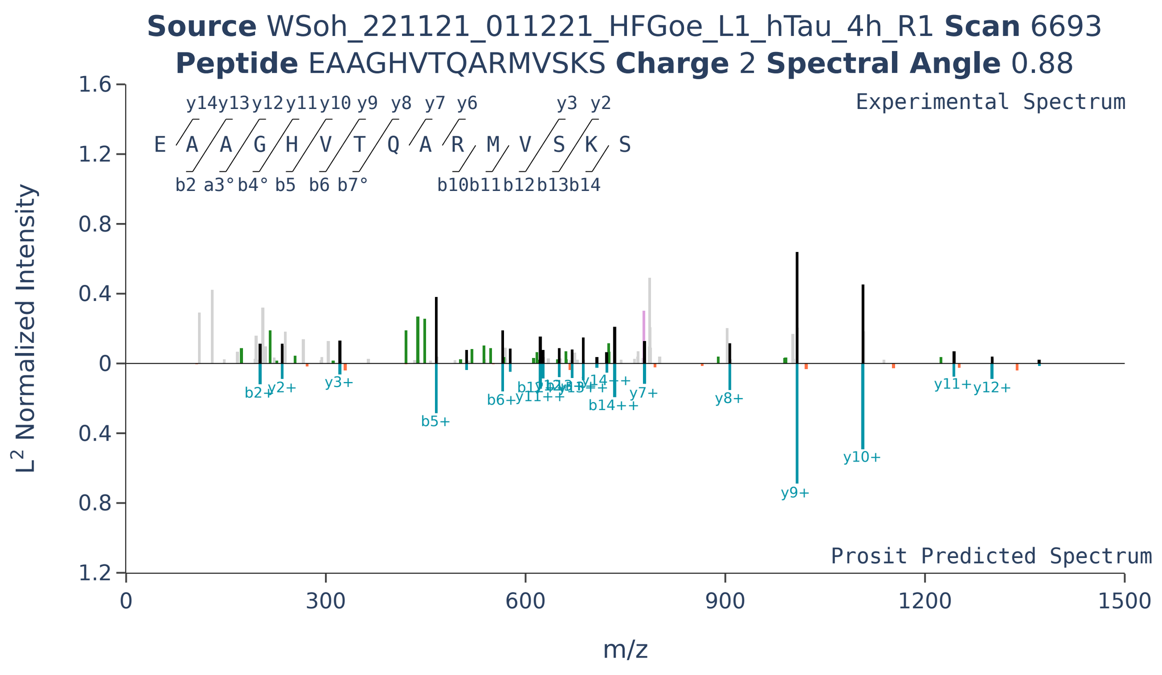
**Source** WSoh_221121_011221_HFGoe_L1_hTau_4h_R1 **Scan** 6693
**Peptide** EAAGHVTQARMVSKS **Charge** 2 **Spectral Angle** 0.88
Experimental Spectrum
Prosit Predicted Spectrum
m/z
L² Normalized Intensity

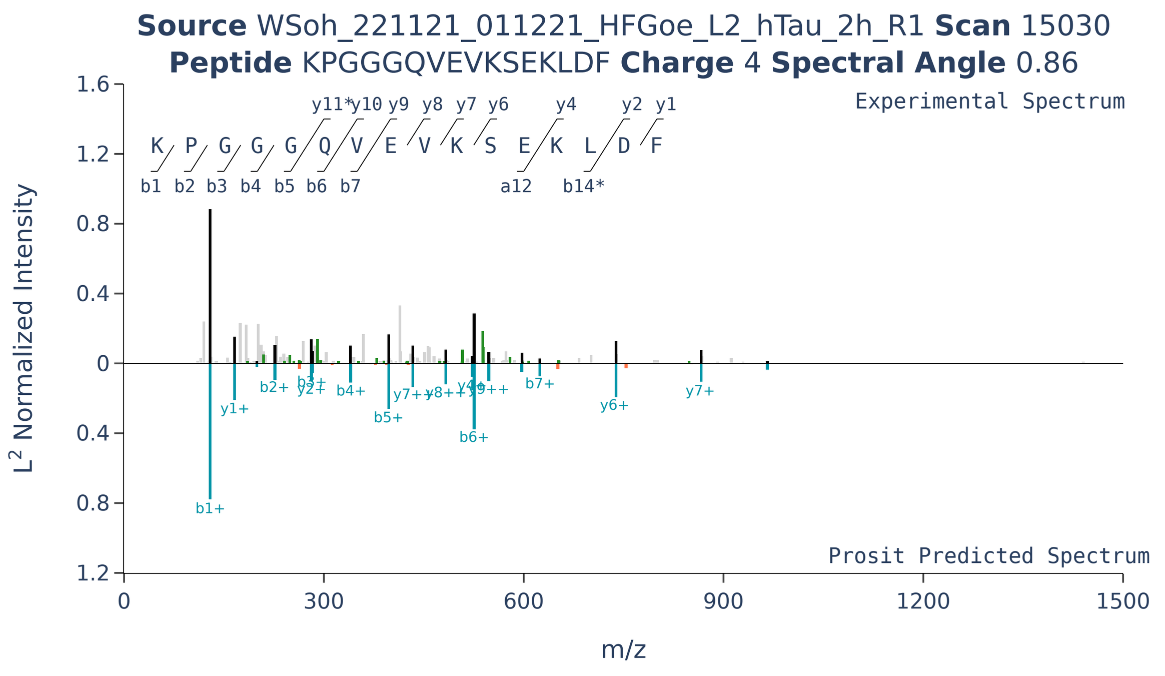
**Source** WSoh_221121_011221_HFGoe_L2_hTau_2h_R1 **Scan** 15030
**Peptide** KPGGGQVEVKSEKLDF **Charge** 4 **Spectral Angle** 0.86
Experimental Spectrum
Prosit Predicted Spectrum
m/z
L² Normalized Intensity

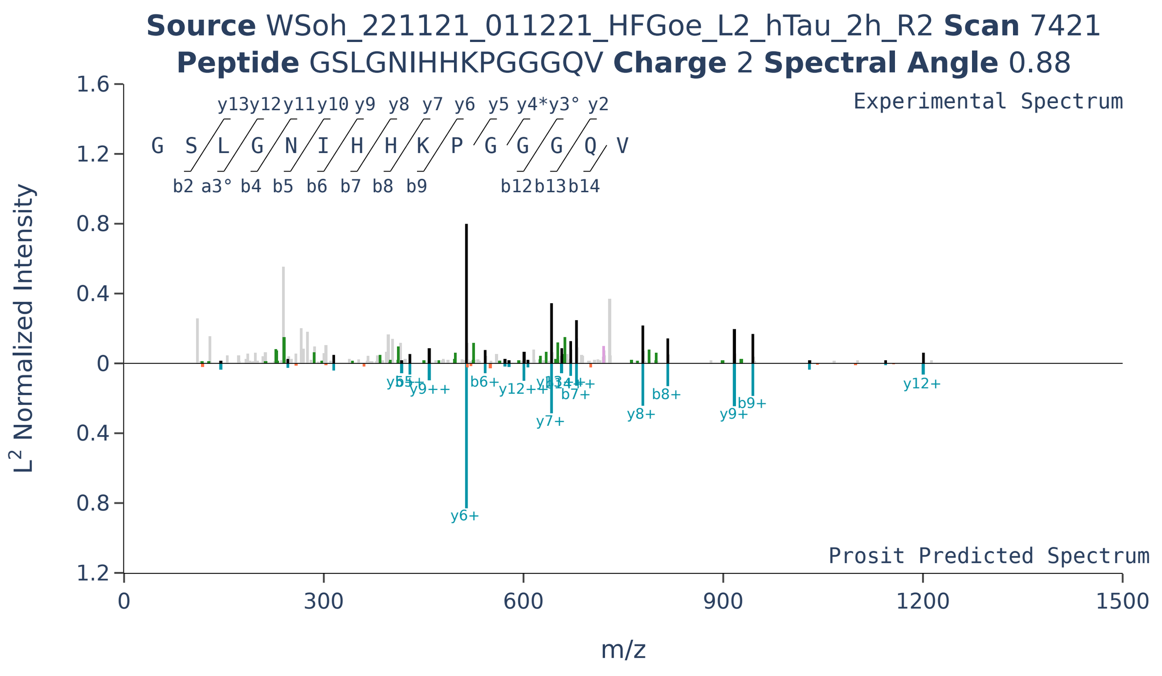
**Source** WSoh_221121_011221_HFGoe_L2_hTau_2h_R2 **Scan** 7421
**Peptide** GSLGNIHHKPGGGQV **Charge** 2 **Spectral Angle** 0.88
Experimental Spectrum
Prosit Predicted Spectrum
m/z
L² Normalized Intensity

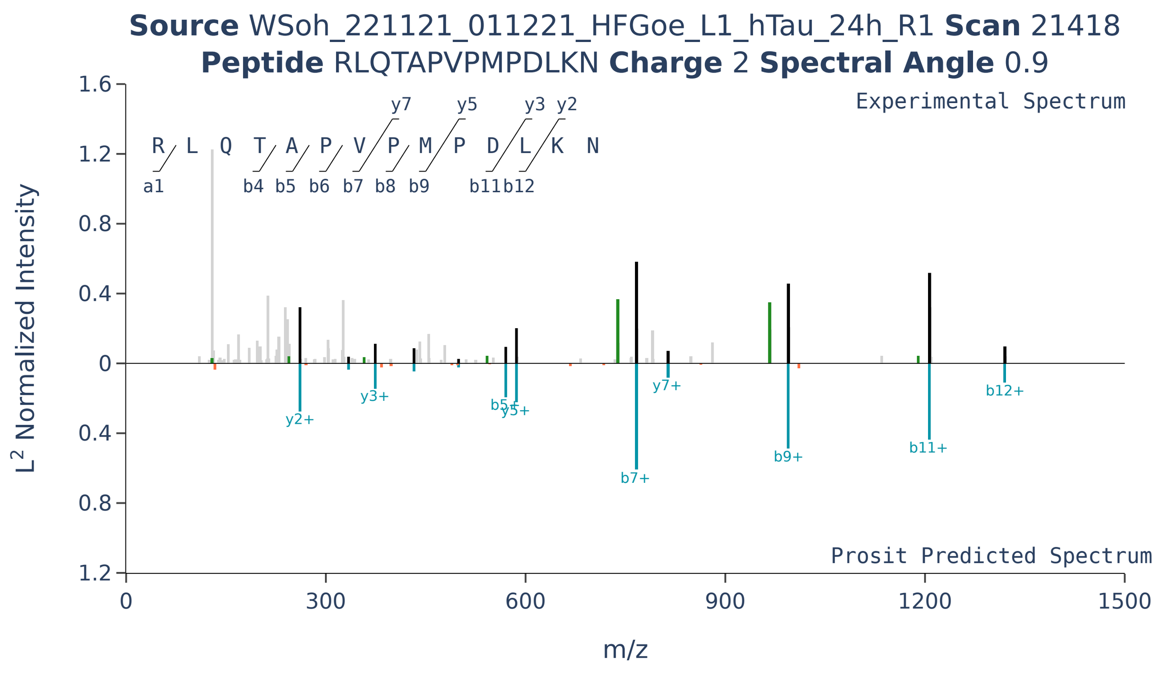
**Source** WSoh_221121_011221_HFGoe_L1_hTau_24h_R1 **Scan** 21418
**Peptide** RLQTAPVPMPDLKN **Charge** 2 **Spectral Angle** 0.9
Experimental Spectrum
Prosit Predicted Spectrum
m/z
L² Normalized Intensity

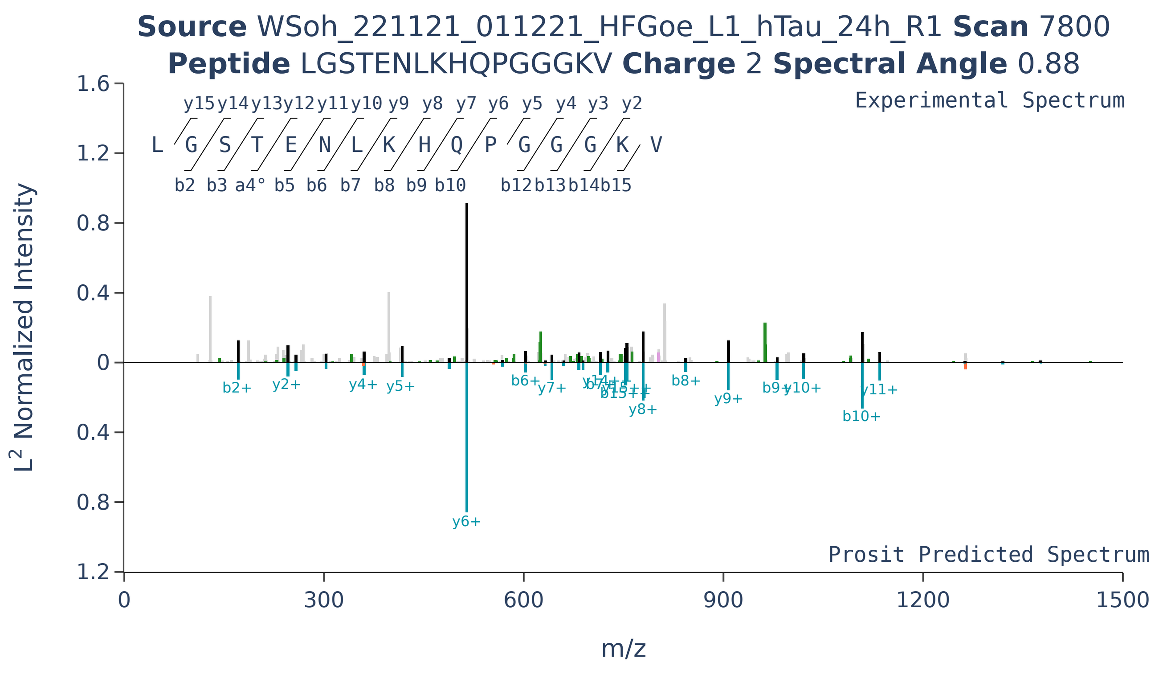
**Source** WSoh_221121_011221_HFGoe_L1_hTau_24h_R1 **Scan** 7800
**Peptide** LGSTENLKHQPGGGKV **Charge** 2 **Spectral Angle** 0.88
Experimental Spectrum
Prosit Predicted Spectrum
m/z
L² Normalized Intensity

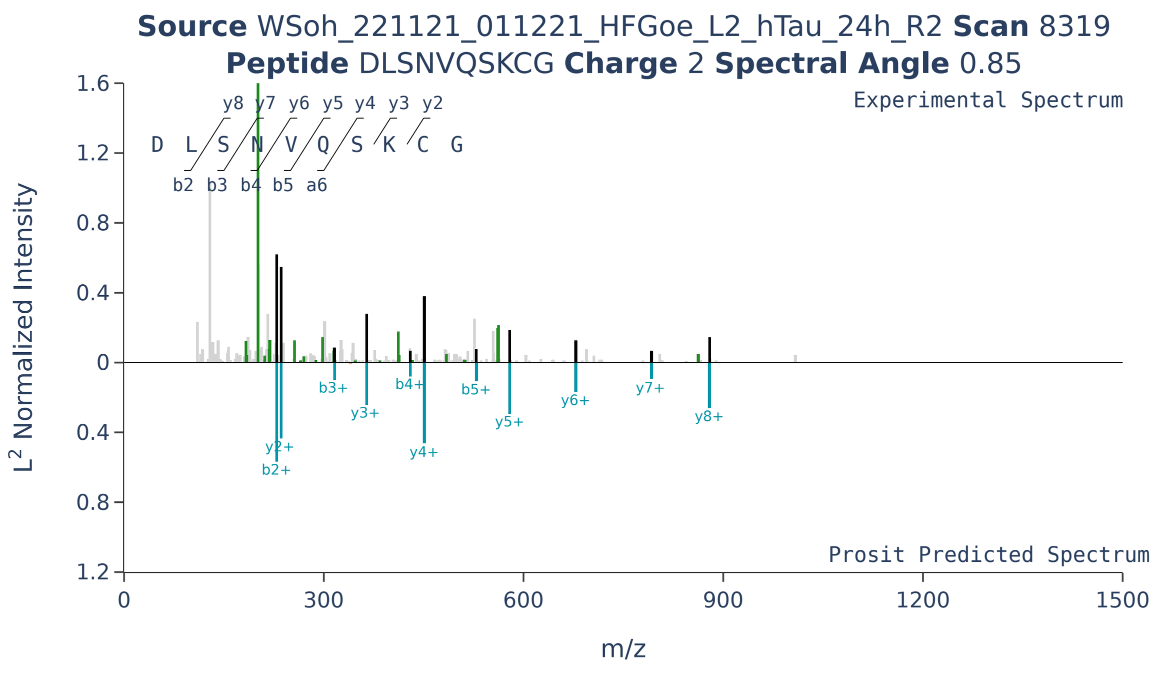
**Source** WSoh_221121_011221_HFGoe_L2_hTau_24h_R2 **Scan** 8319
**Peptide** DLSNVQSKCG **Charge** 2 **Spectral Angle** 0.85
Experimental Spectrum
Prosit Predicted Spectrum
m/z
L² Normalized Intensity

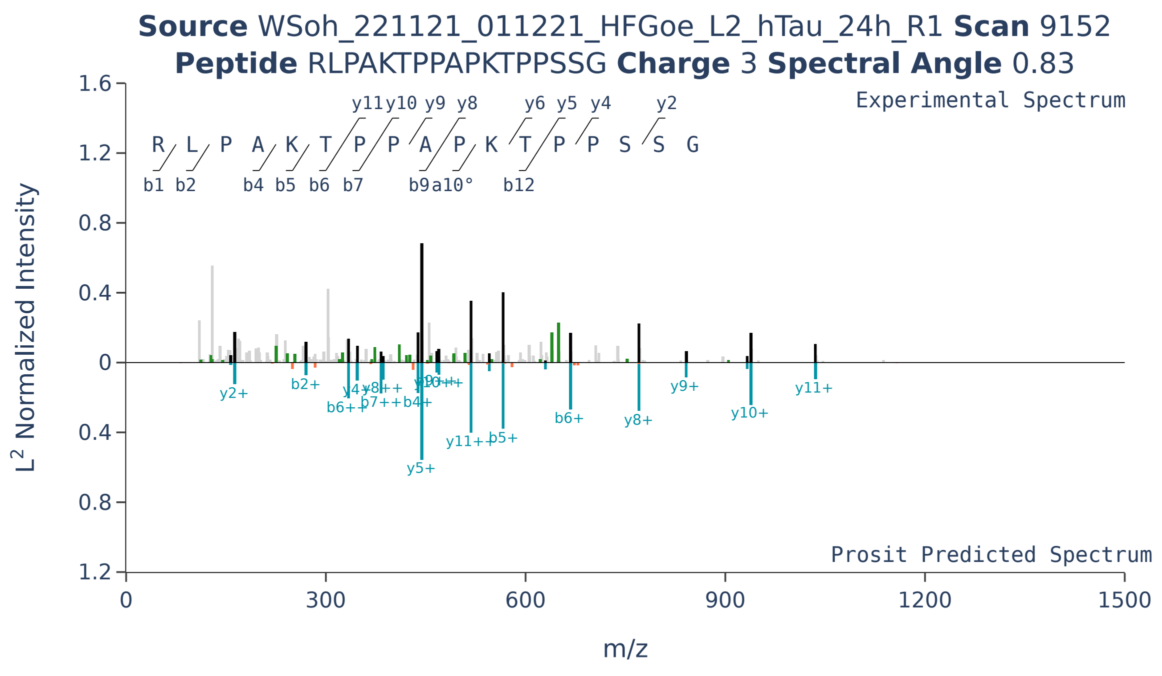
**Source** WSoh_221121_011221_HFGoe_L2_hTau_24h_R1 **Scan** 9152
**Peptide** RLPAKTPPAPKTPPSSG **Charge** 3 **Spectral Angle** 0.83
Experimental Spectrum
Prosit Predicted Spectrum
m/z
L² Normalized Intensity

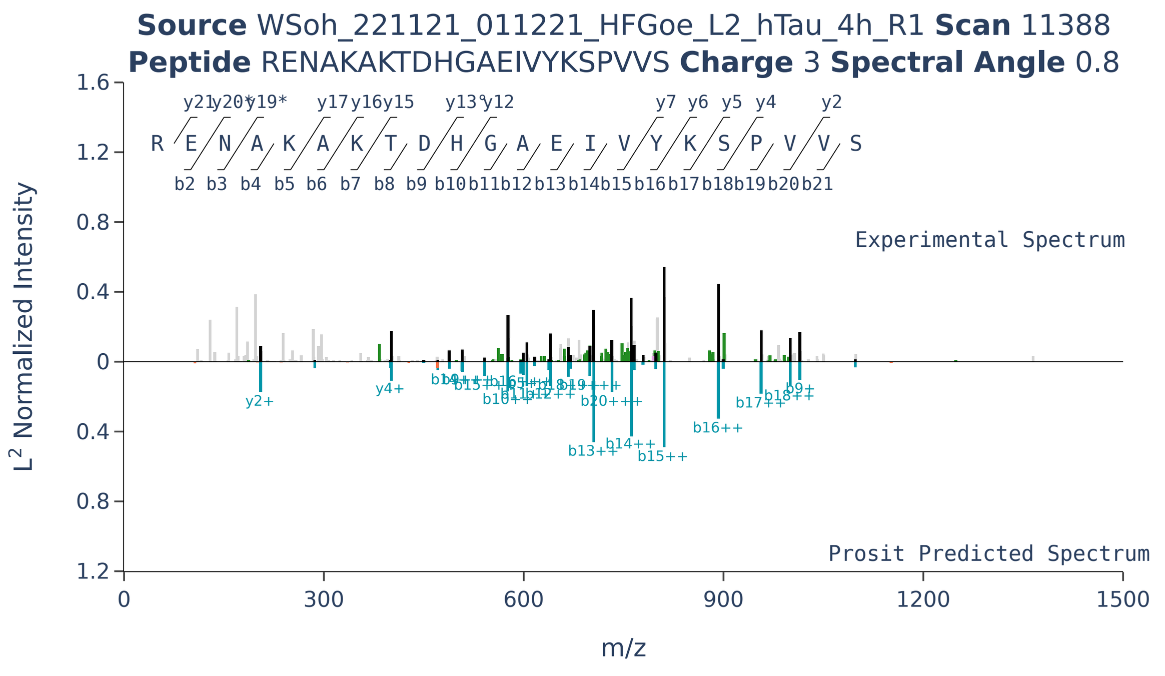
**Source** WSoh_221121_011221_HFGoe_L2_hTau_4h_R1 **Scan** 11388
**Peptide** RENAKAKTDHGAEIVYKSPVVS **Charge** 3 **Spectral Angle** 0.8
Experimental Spectrum
Prosit Predicted Spectrum
m/z
L² Normalized Intensity

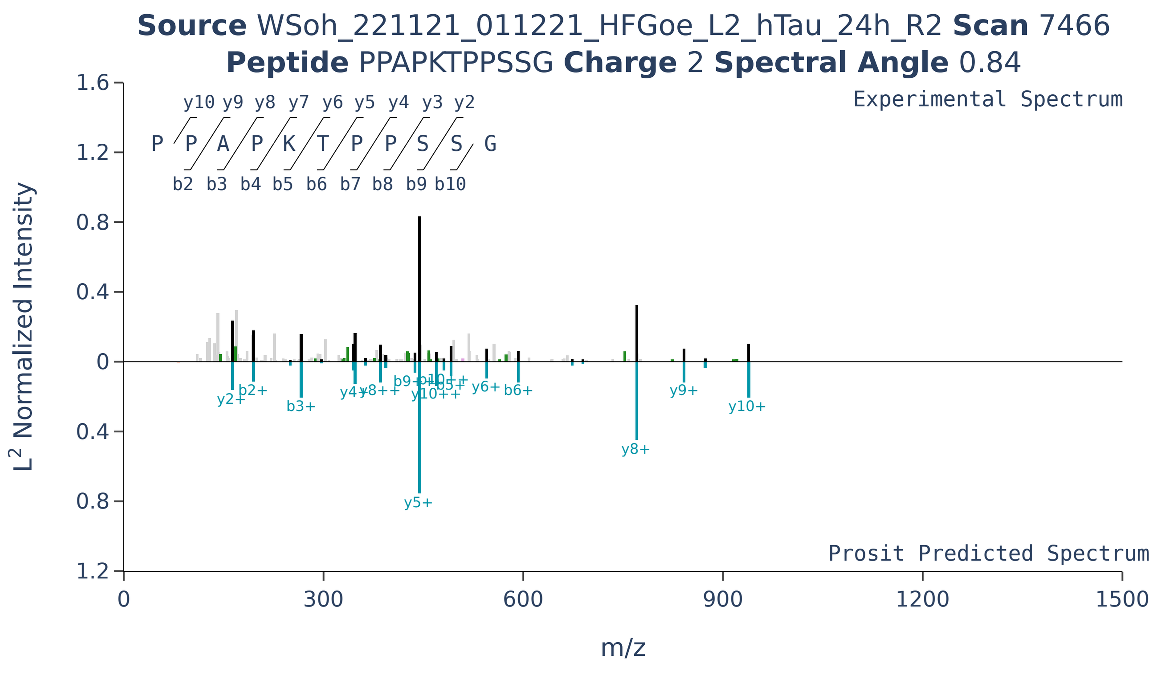
**Source** WSoh_221121_011221_HFGoe_L2_hTau_24h_R2 **Scan** 7466
**Peptide** PPAPKTPPSSG **Charge** 2 **Spectral Angle** 0.84
Experimental Spectrum
Prosit Predicted Spectrum
m/z
L² Normalized Intensity

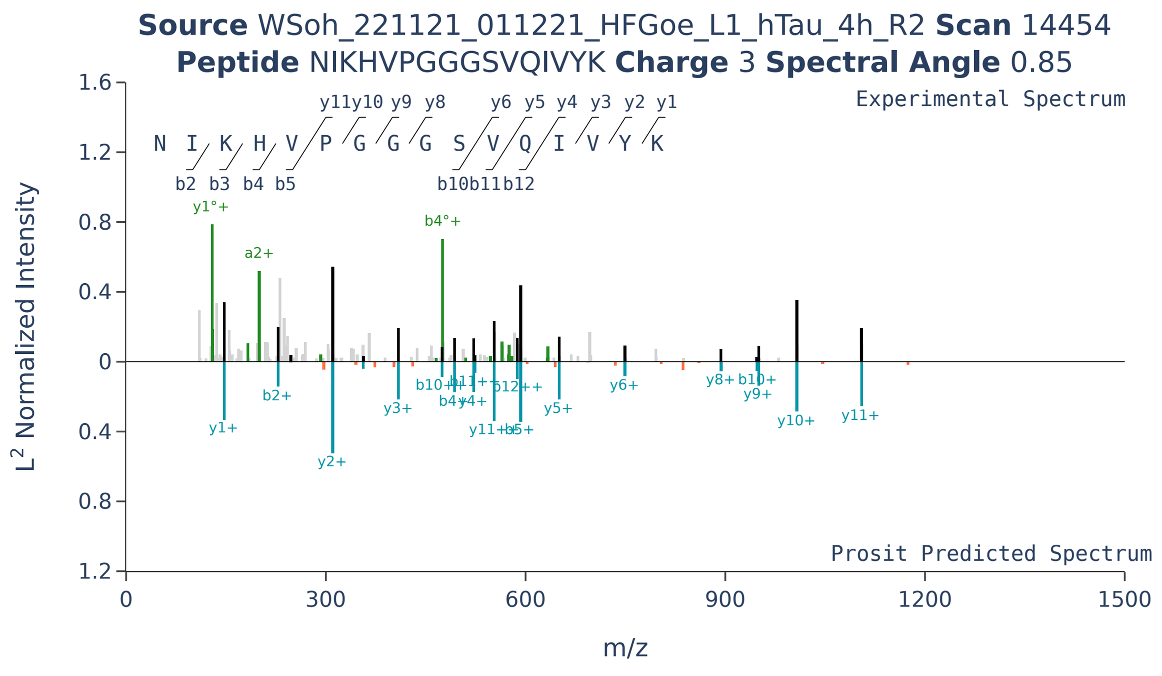
**Source** WSoh_221121_011221_HFGoe_L1_hTau_4h_R2 **Scan** 14454
**Peptide** NIKHVPGGGSVQIVYK **Charge** 3 **Spectral Angle** 0.85
Experimental Spectrum
Prosit Predicted Spectrum
m/z
L² Normalized Intensity

**Source** WSoh_221121_011221_HFGoe_L1_hTau_4h_R2 **Scan** 12292
**Peptide** EGAPGKQAAAQPHTEIPEGTTA **Charge** 3 **Spectral Angle** 0.86

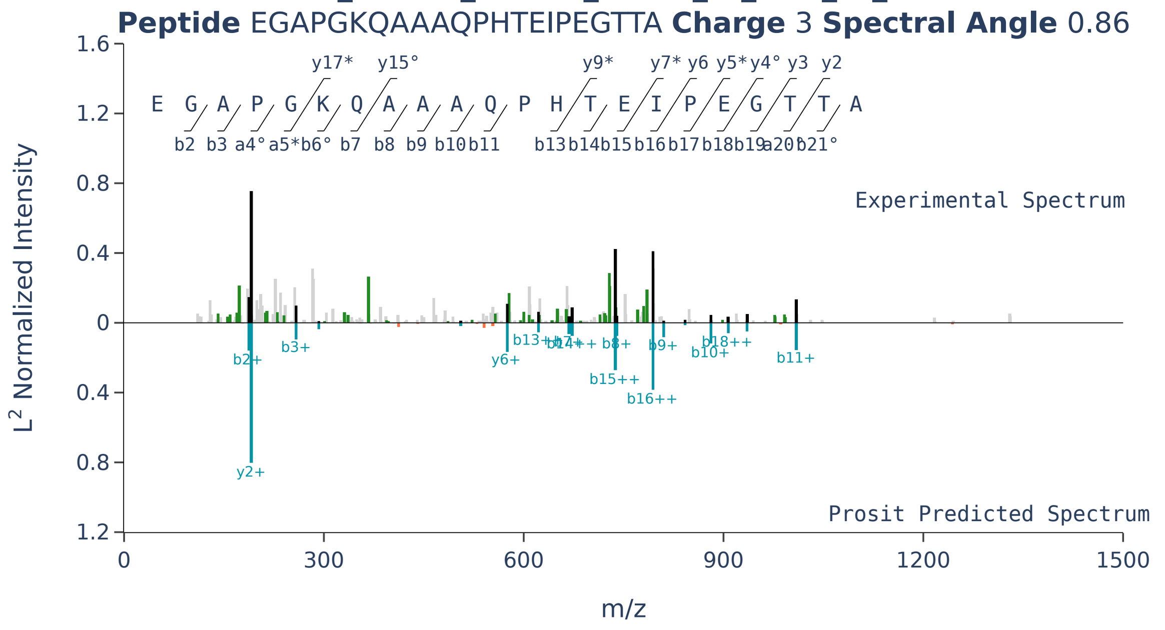

**Source** WSoh_221121_011221_HFGoe_L1_hTau_24h_R2 **Scan** 14531
**Peptide** KTDHGAEIVYKSPVVSGDTSPRHLS **Charge** 3 **Spectral Angle** 0.82

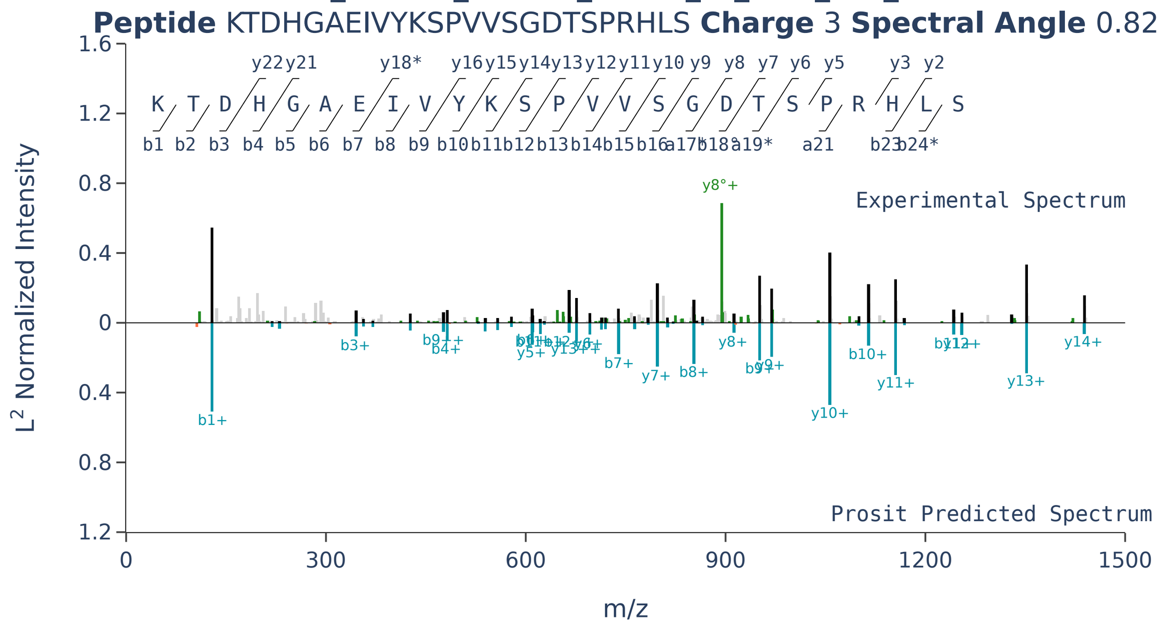

**Source** WSoh_221121_011221_HFGoe_L2_hTau_24h_R2 **Scan** 11371
**Peptide** CGSLGNIHHKPGGGQVEV **Charge** 2 **Spectral Angle** 0.88

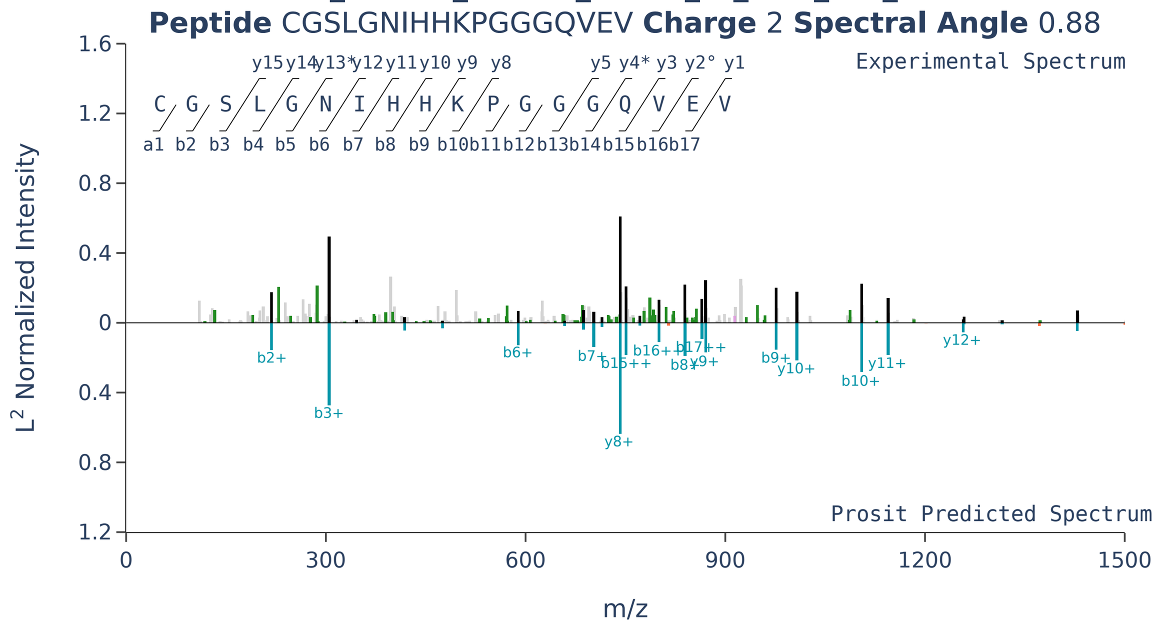

**Source** WSoh_221121_011221_HFGoe_L1_hTau_4h_R2 **Scan** 6181
**Peptide** VRTPPKSPSSAKSRLQ **Charge** 4 **Spectral Angle** 0.88

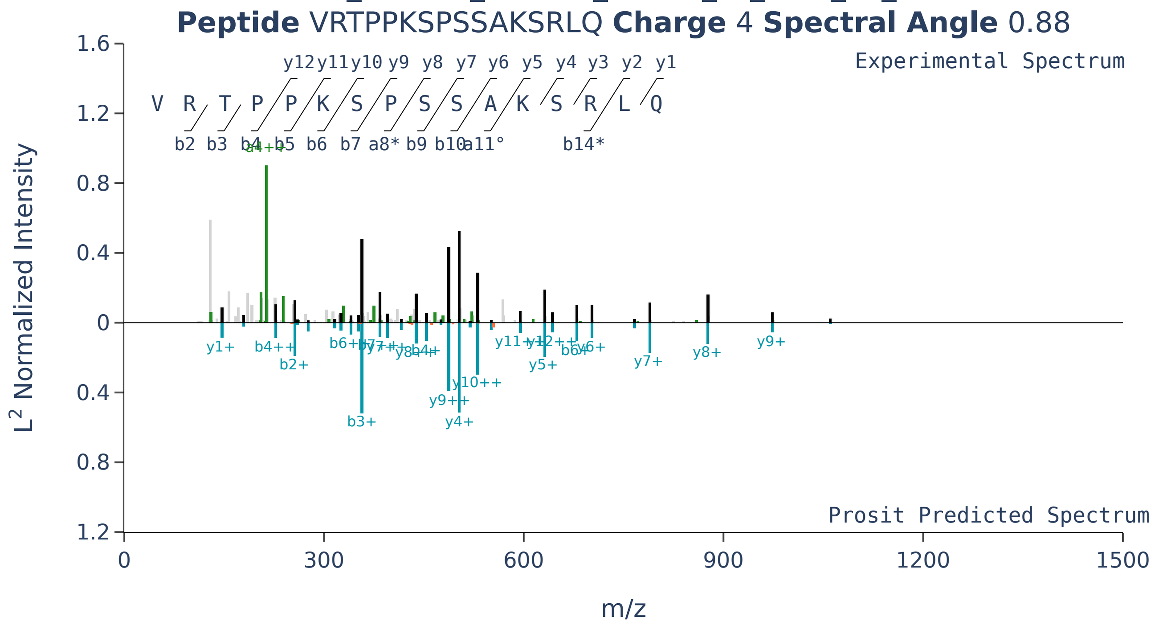

**Source** WSoh_221121_011221_HFGoe_L2_hTau_24h_R2 **Scan** 8006
**Peptide** TPRGAAPPGQKGQANATRI **Charge** 3 **Spectral Angle** 0.88

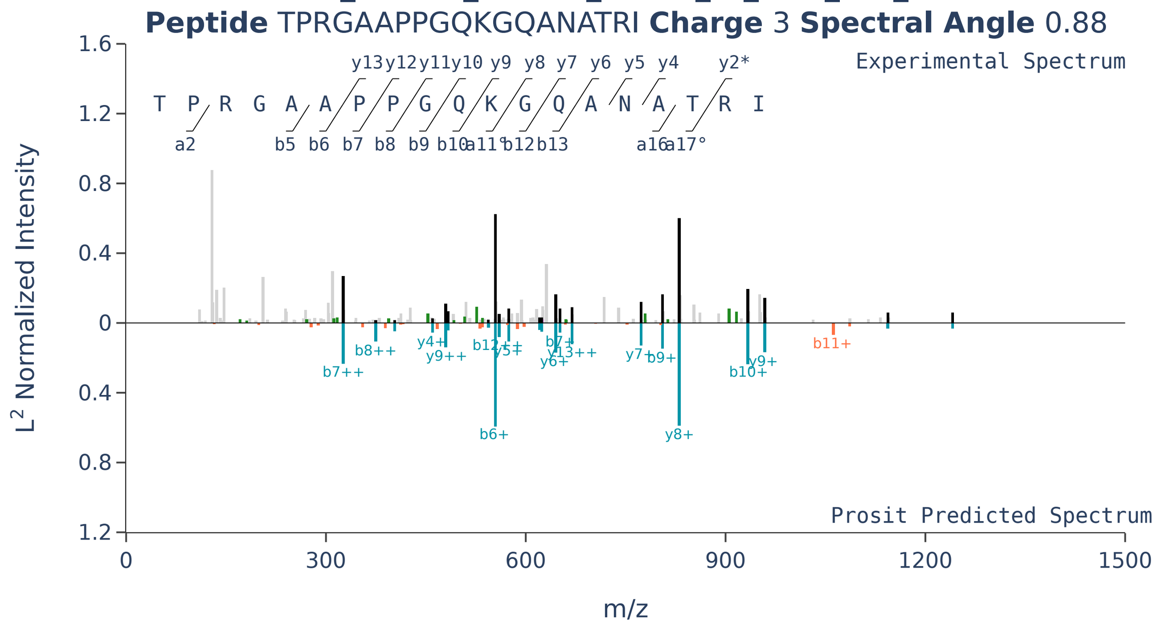

**Source** WSoh_221121_011221_HFGoe_L2_hTau_24h_R1 **Scan** 15729
**Peptide** VYKPVDLSKVTSKCGSLGNIHHK **Charge** 5 **Spectral Angle** 0.85

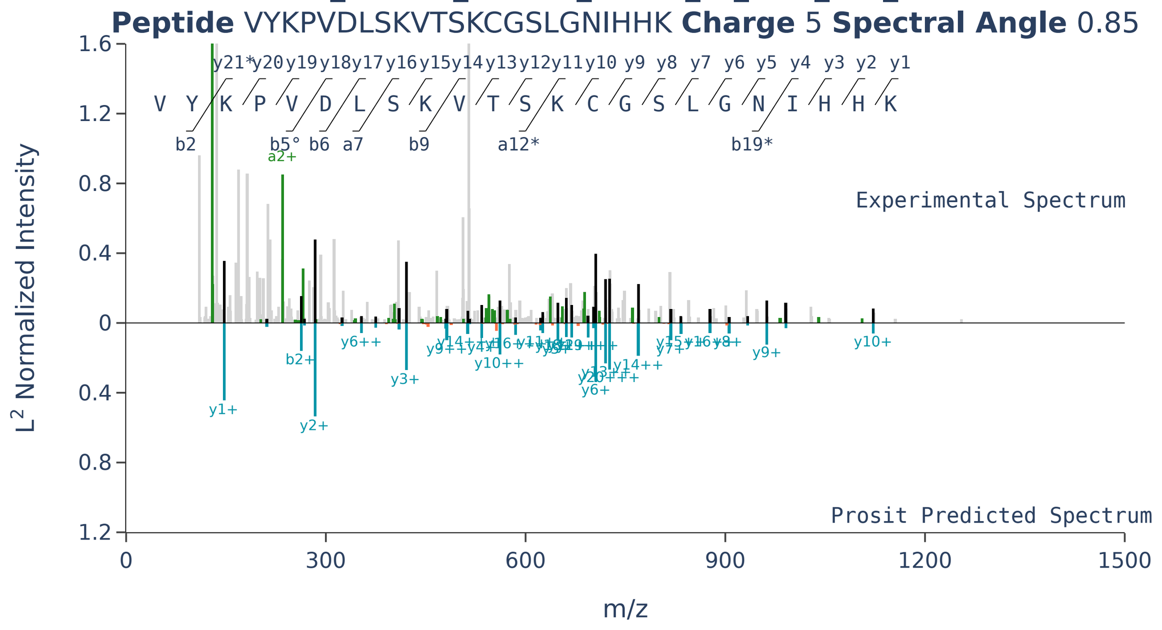

**Source** WSoh_221121_011221_HFGoe_L1_hTau_4h_R2 **Scan** 19181
**Peptide** GLGDTPSLE **Charge** 1 **Spectral Angle** 0.84

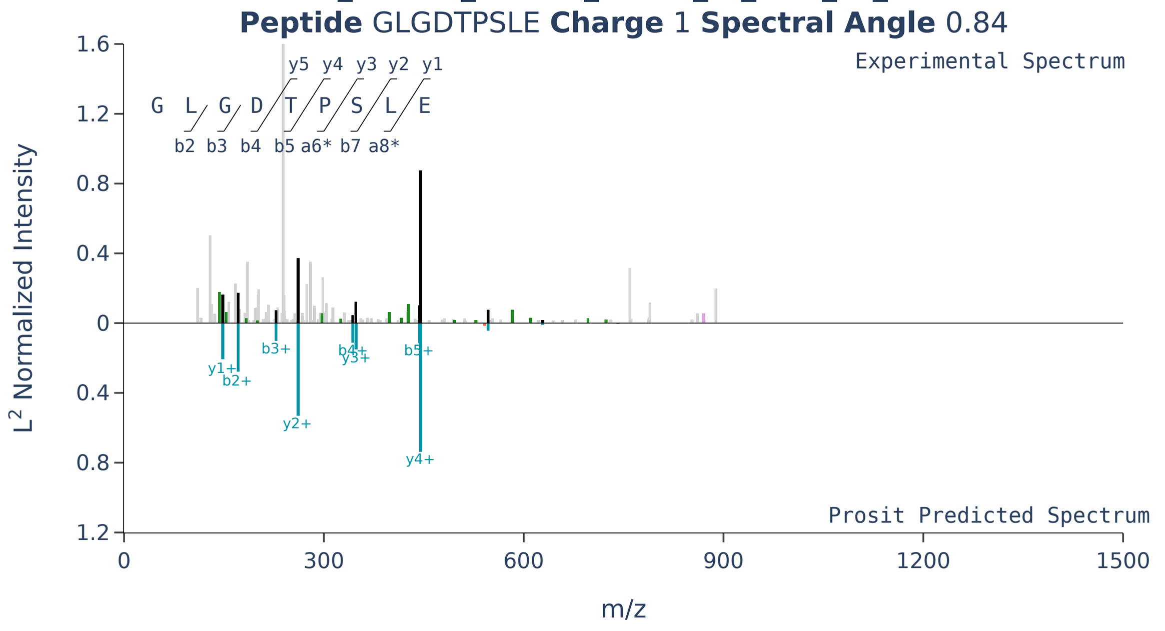

**Source** WSoh_221121_011221_HFGoe_L1_hTau_4h_R2 **Scan** 8222
**Peptide** PGGGNKKIETHKLTFR **Charge** 4 **Spectral Angle** 0.84

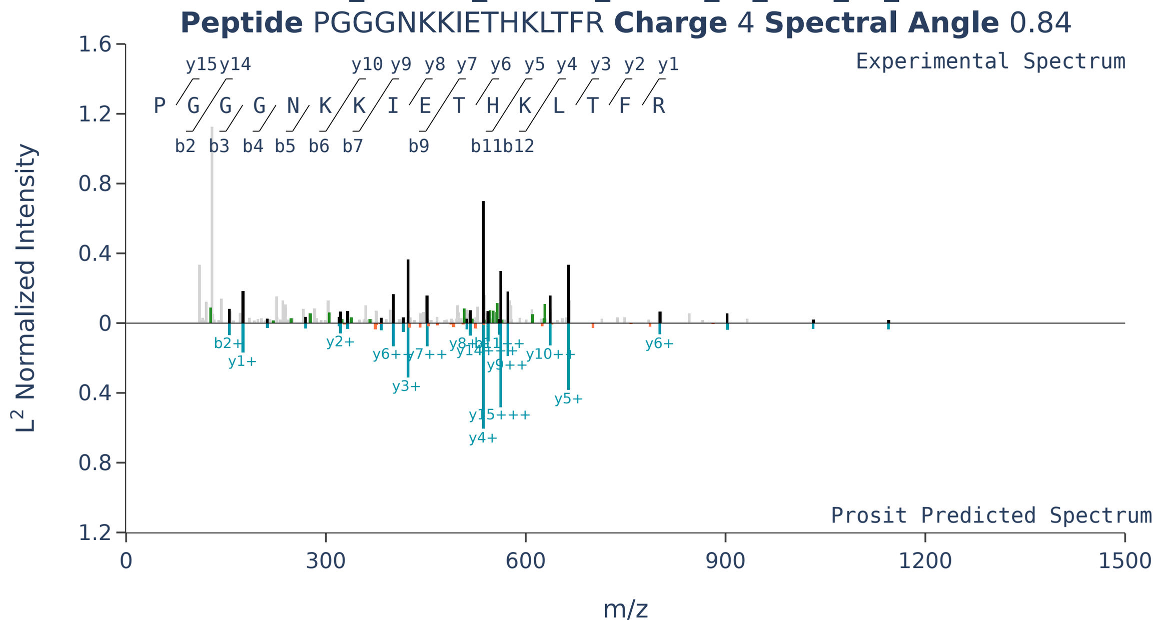

**Source** WSoh_221121_011221_HFGoe_L2_hTau_4h_R1 **Scan** 13624
**Peptide** KIGSTENLKHQPGGGKVQII **Charge** 2 **Spectral Angle** 0.85

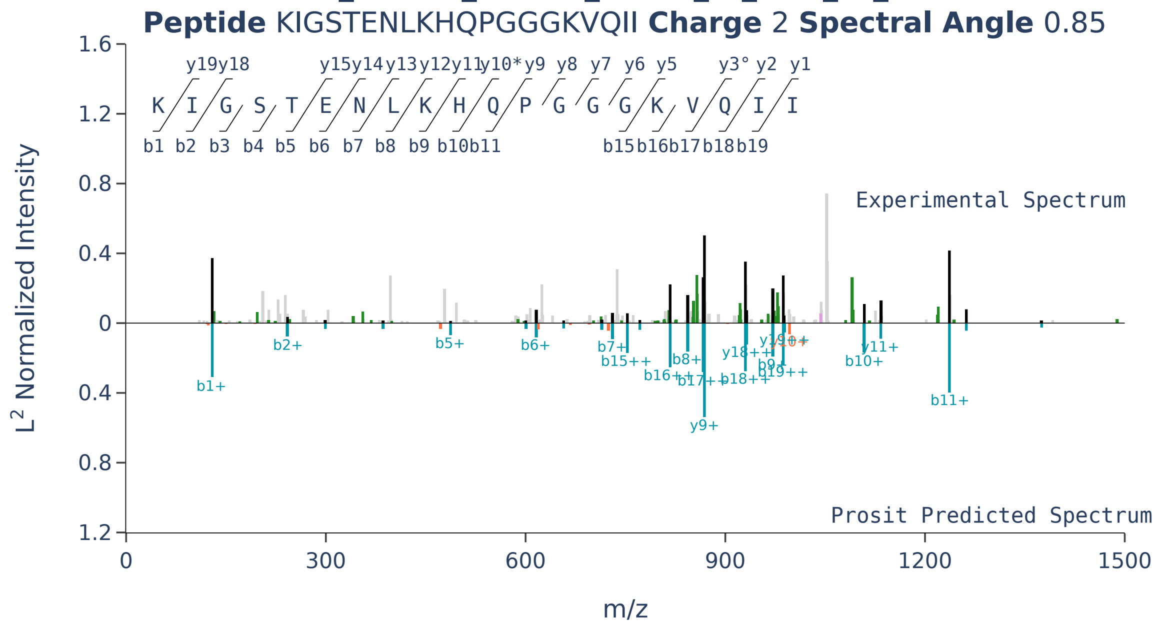

**Source** WSoh_221121_011221_HFGoe_L1_hTau_24h_R1 **Scan** 18970
**Peptide** NIKHVPGGGSVQIVY **Charge** 3 **Spectral Angle** 0.82

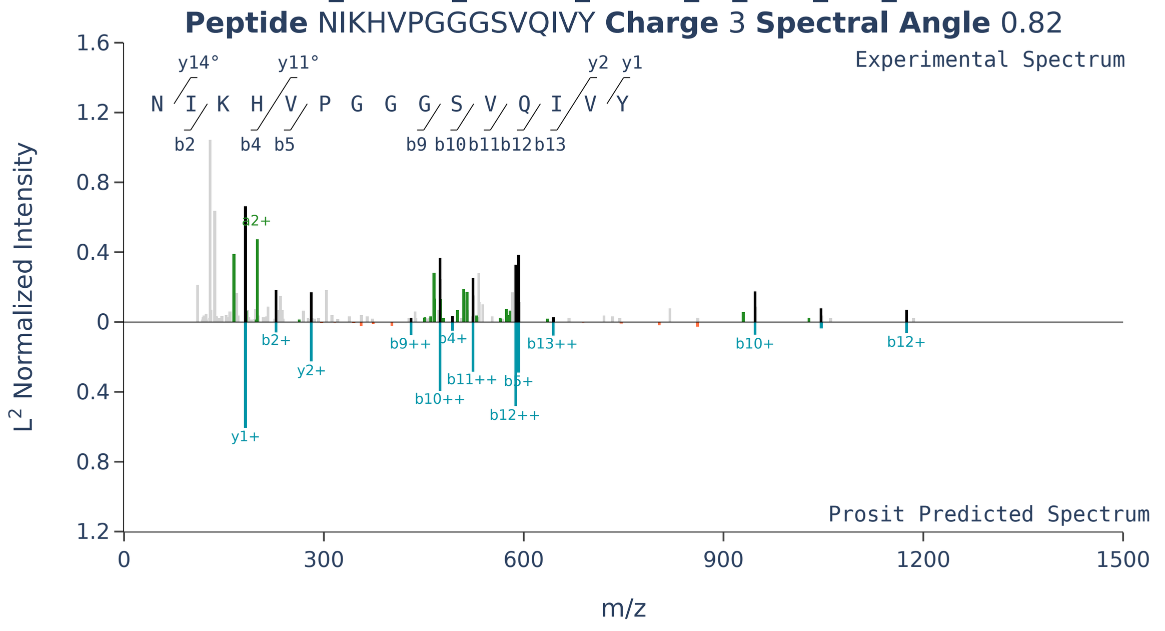

**Source** WSoh_221121_011221_HFGoe_L2_hTau_24h_R2 **Scan** 17060
**Peptide** GAEIVYKSPVVSGDTSPRHLSN **Charge** 3 **Spectral Angle** 0.86

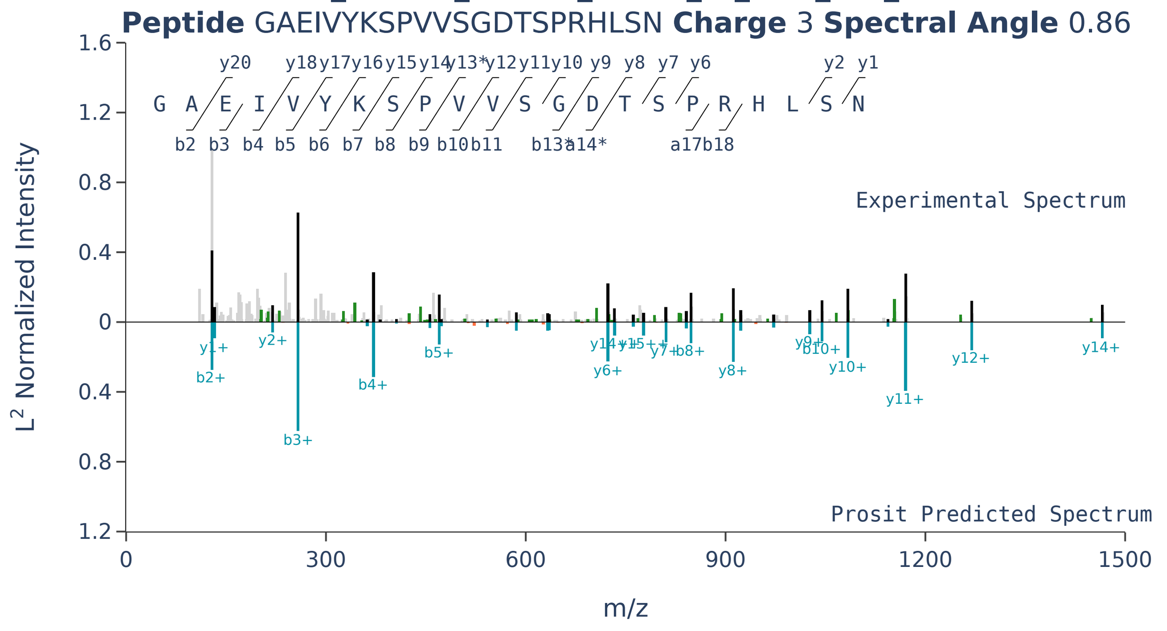

**Source** WSoh_221121_011221_HFGoe_L2_hTau_24h_R1 **Scan** 16746
**Peptide** TPSLPTPPTREPKKVAV **Charge** 4 **Spectral Angle** 0.87

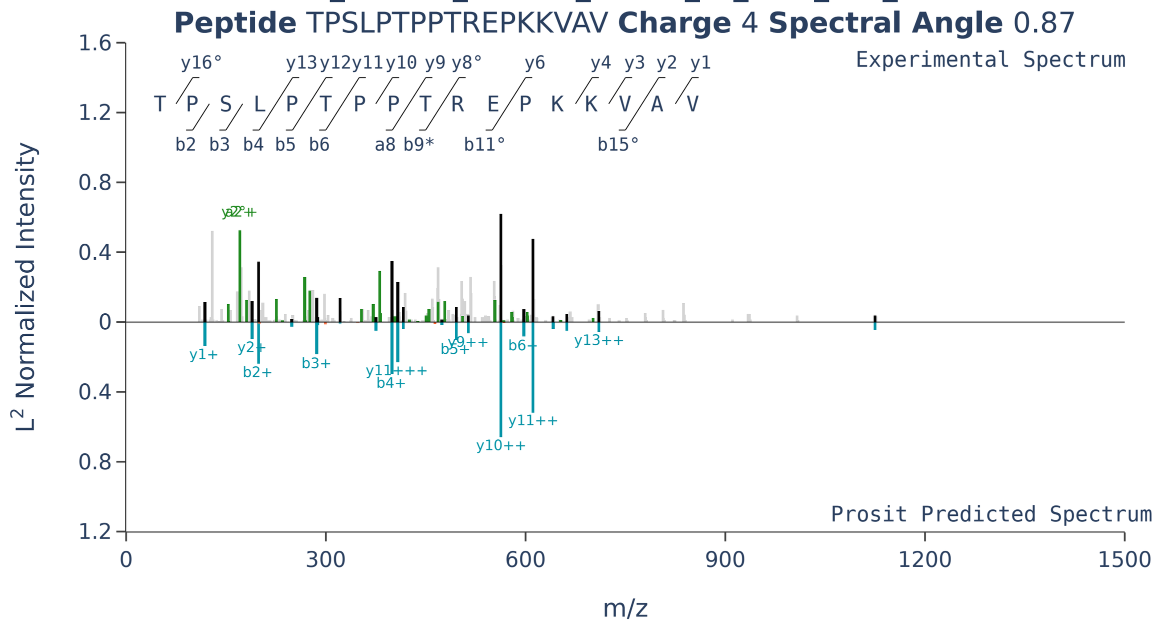

**Source** WSoh_221121_011221_HFGoe_L2_hTau_24h_R2 **Scan** 11006
**Peptide** NATRIPAKTPPAPKTPPSS **Charge** 3 **Spectral Angle** 0.86

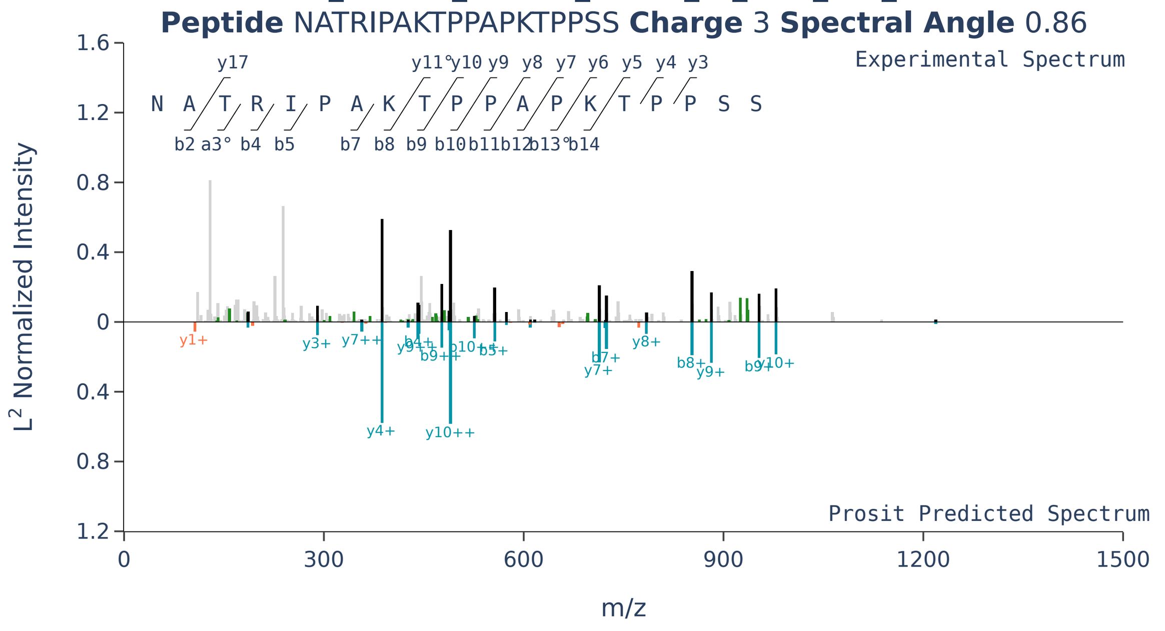

**Source** WSoh_221121_011221_HFGoe_L2_hTau_24h_R2 **Scan** 20652
**Peptide** TPSLEDEAAGHVTQARMV **Charge** 3 **Spectral Angle** 0.87

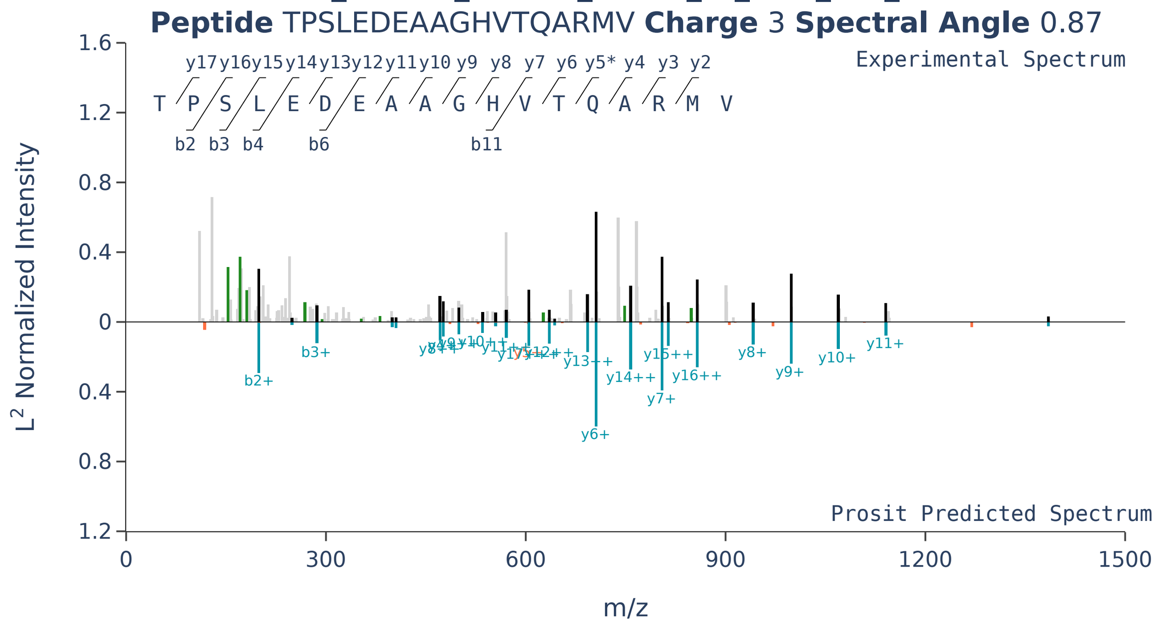

**Source** WSoh_221121_011221_HFGoe_L1_hTau_4h_R2 **Scan** 17176
**Peptide** MHQDQEGDTDAGLKESPLQTPTED **Charge** 3 **Spectral Angle** 0.9

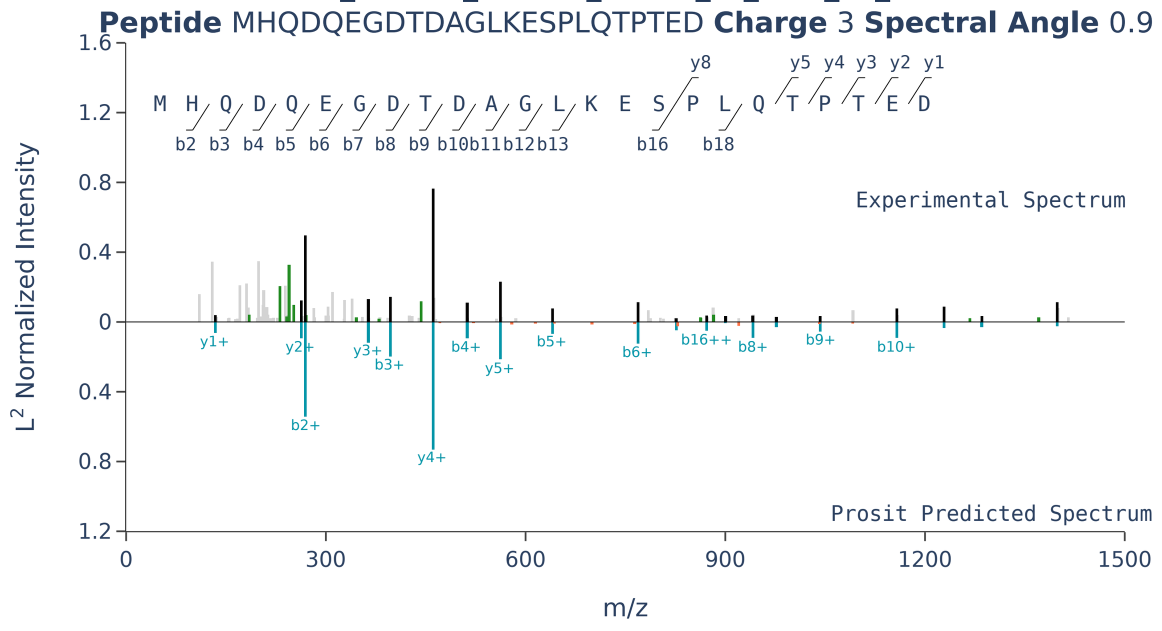

**Source** WSoh_221121_011221_HFGoe_L1_hTau_24h_R2 **Scan** 10469
**Peptide** AGIGDTPS **Charge** 1 **Spectral Angle** 0.96

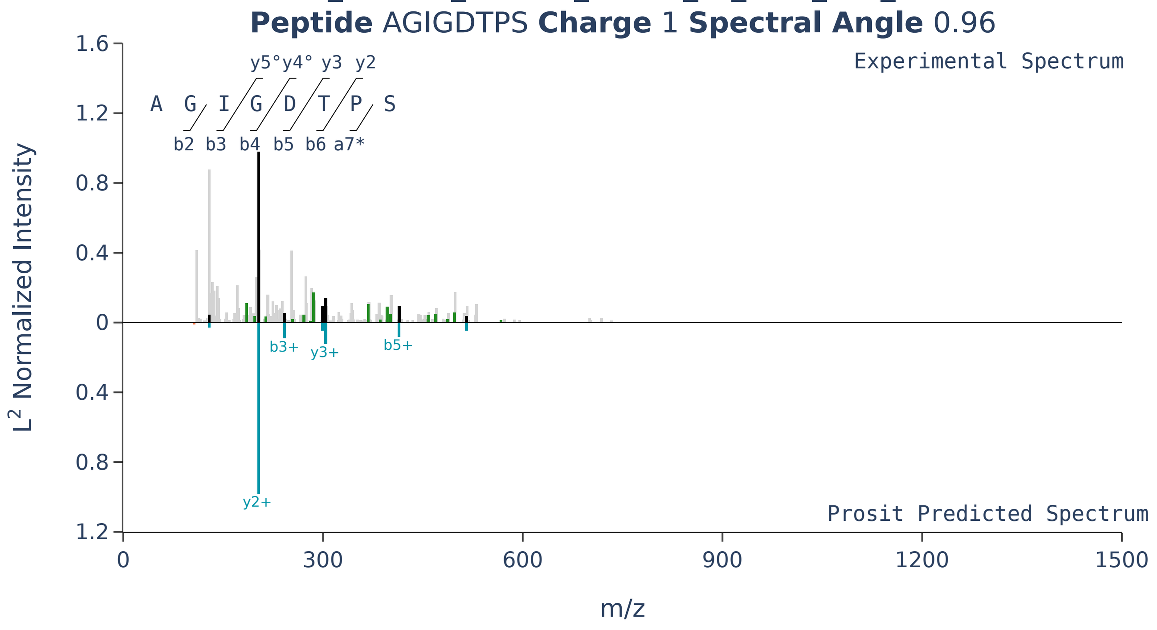


**Source** WSoh_221121_011221_HFGoe_L1_hTau_24h_R1 **Scan** 10288
**Peptide** DNITHVPGGGNKKIET **Charge** 2 **Spectral Angle** 0.86

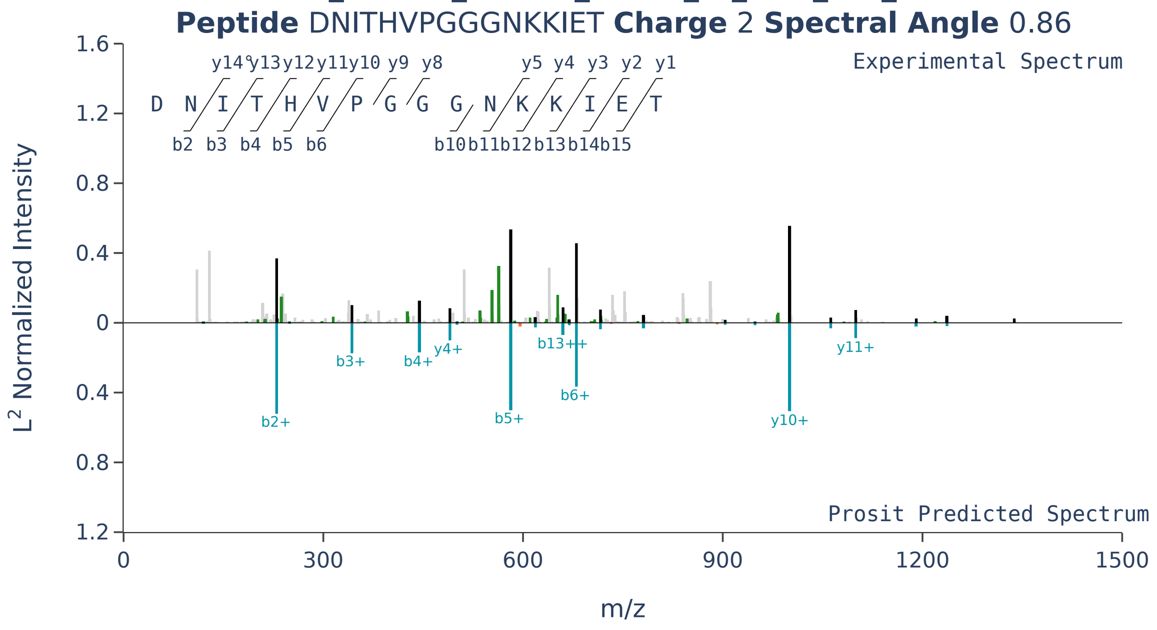


**Source** WSoh_221121_011221_HFGoe_L1_hTau_4h_R2 **Scan** 21448
**Peptide** TSDAKSTPTAEDVTAPLVD **Charge** 2 **Spectral Angle** 0.87

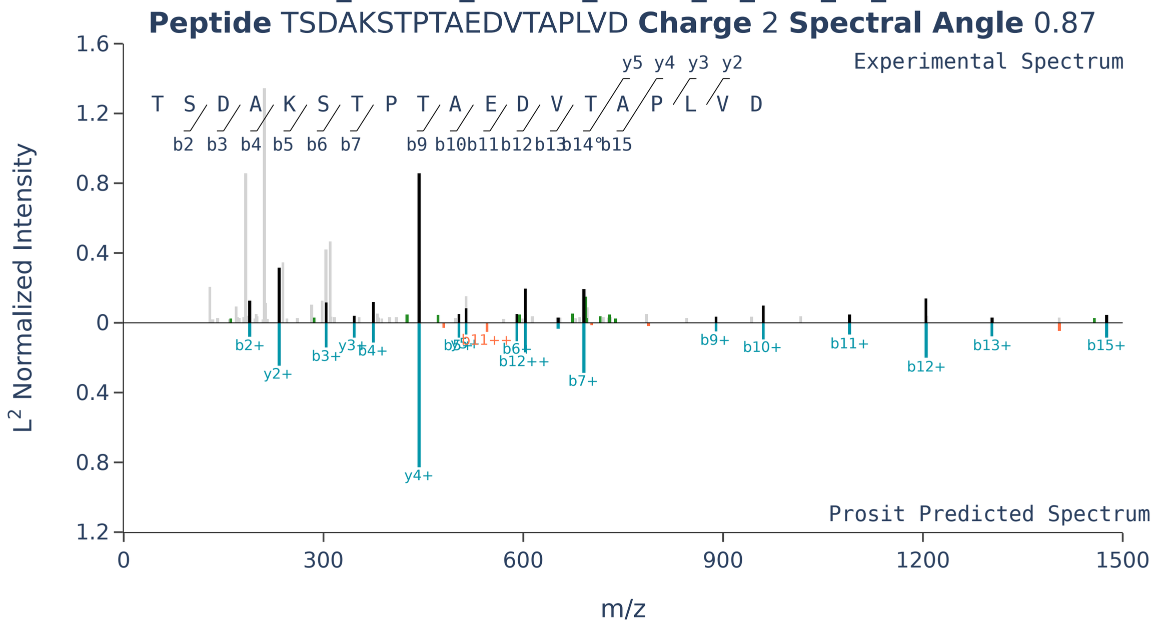


**Source** WSoh_221121_011221_HFGoe_L1_hTau_24h_R2 **Scan** 9852
**Peptide** AKAKTDHGAEIVY **Charge** 2 **Spectral Angle** 0.81

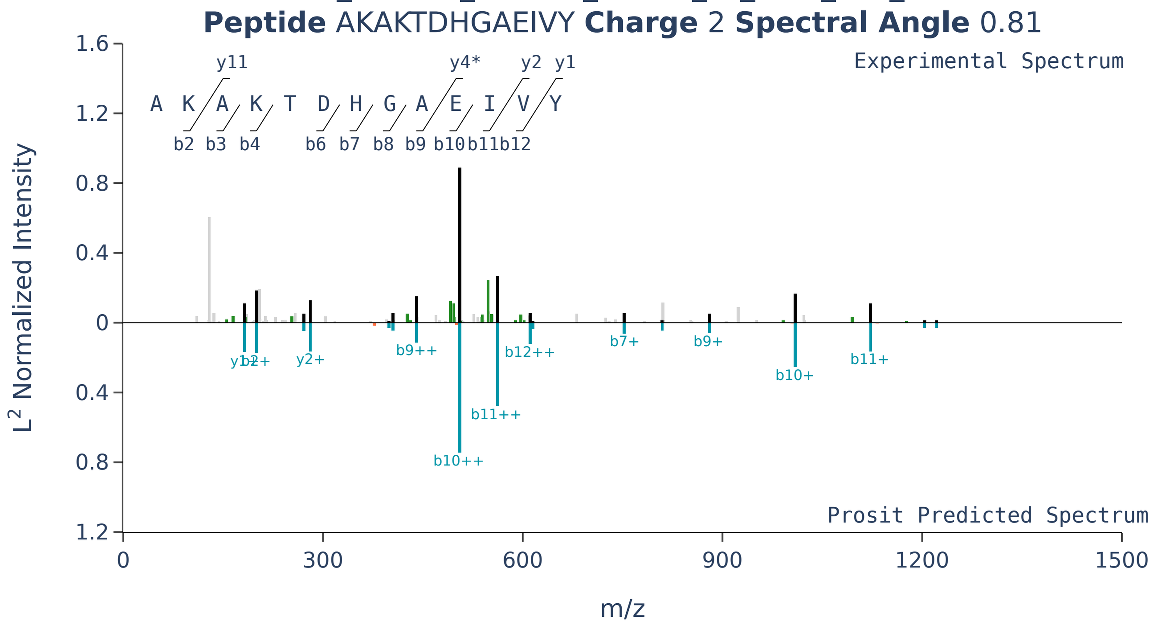


**Source** WSoh_221121_011221_HFGoe_L1_hTau_24h_R1 **Scan** 9217
**Peptide** SKCGSLGNIHHKPGGGQVEV **Charge** 2 **Spectral Angle** 0.83

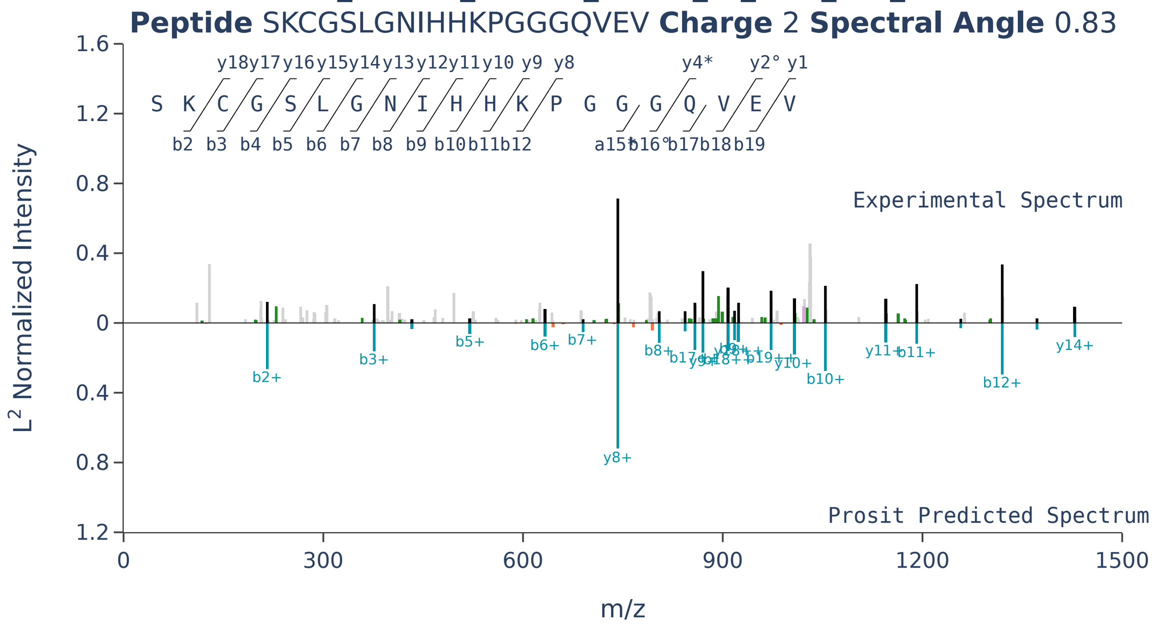


**Source** WSoh_221121_011221_HFGoe_L1_hTau_1h_R2 **Scan** 12838
**Peptide** VYKSPVV **Charge** 2 **Spectral Angle** 0.86

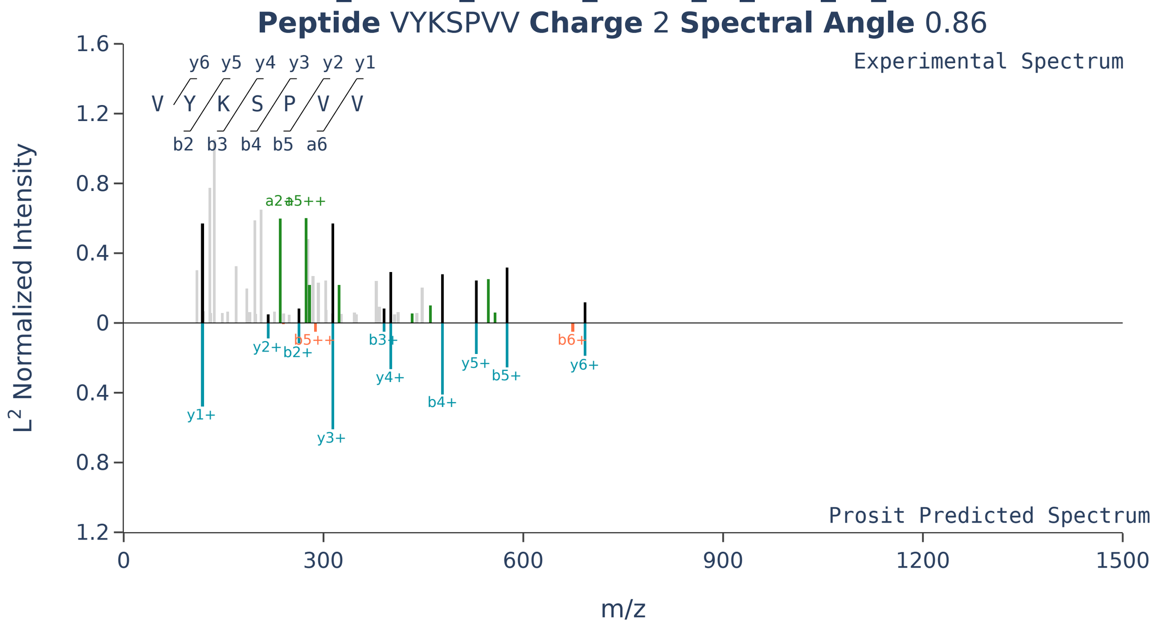


**Source** WSoh_221121_011221_HFGoe_L1_hTau_24h_R1 **Scan** 19967
**Peptide** RTPPKSPSSAKSRLQTAPVPMPDL **Charge** 3 **Spectral Angle** 0.9

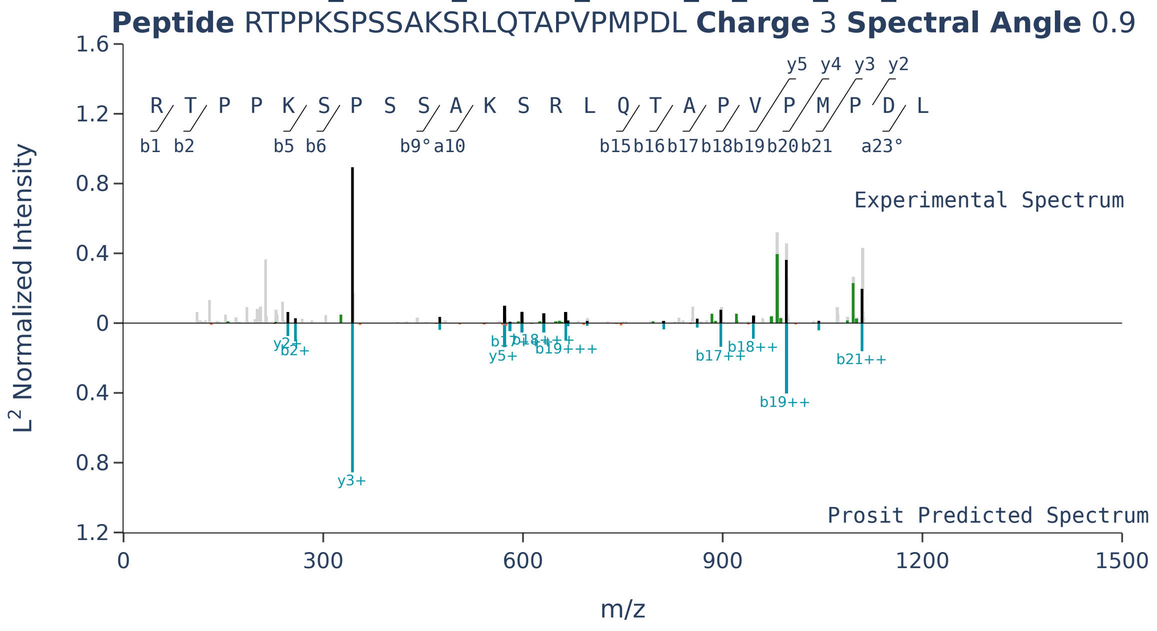


**Source** WSoh_221121_011221_HFGoe_L1_hTau_24h_R1 **Scan** 17473
**Peptide** STGSIDMVDS **Charge** 1 **Spectral Angle** 0.73

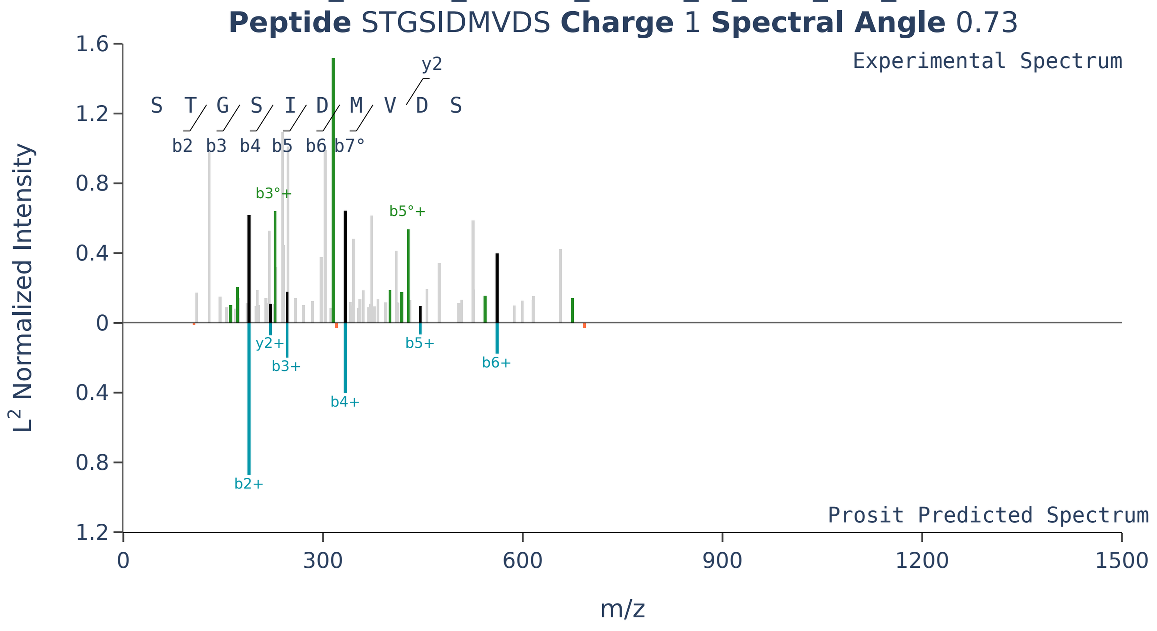


**Source** WSoh_221121_011221_HFGoe_L2_hTau_24h_R2 **Scan** 19340
**Peptide** PRQEFEVM **Charge** 2 **Spectral Angle** 0.91

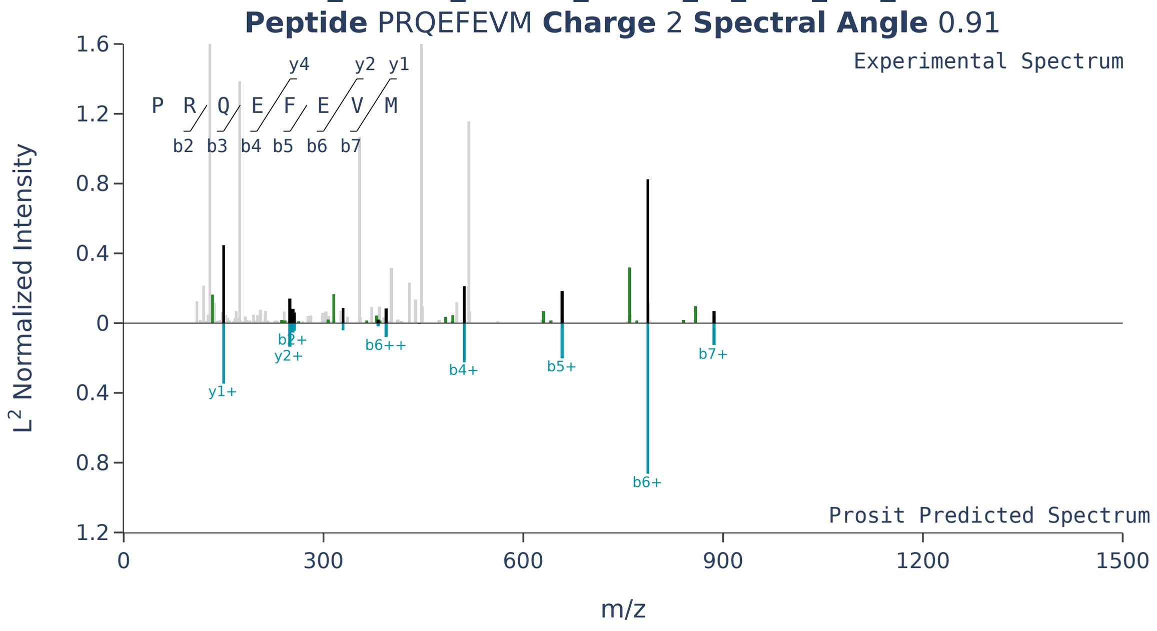


**Source** WSoh_221121_011221_HFGoe_L1_hTau_24h_R1 **Scan** 6898
**Peptide** YSSPGSPGTPGSRSR **Charge** 2 **Spectral Angle** 0.85

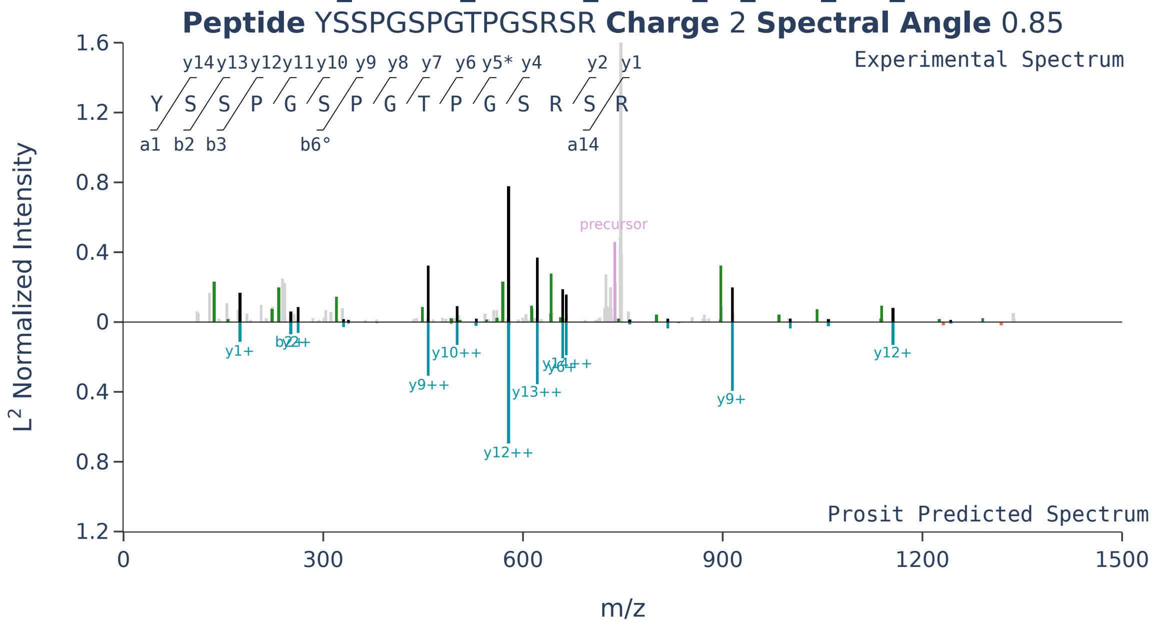


**Source** WSoh_221121_011221_HFGoe_L1_hTau_2h_R1 **Scan** 10563
**Peptide** GSLGNIHHKPGGGQVEV **Charge** 2 **Spectral Angle** 0.83

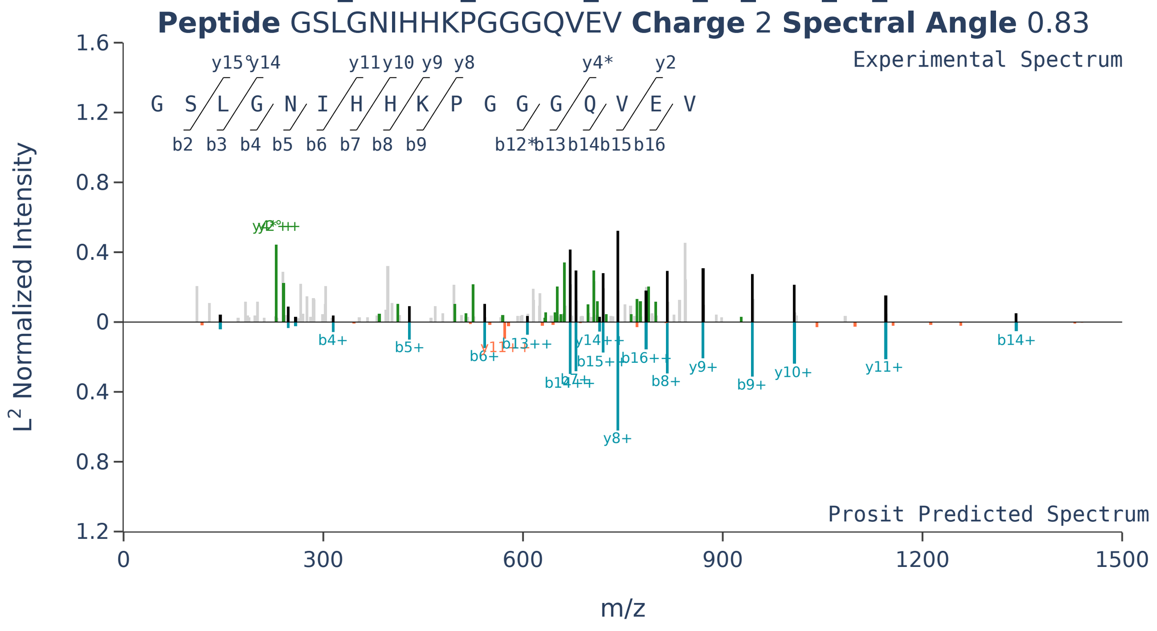


**Source** WSoh_221121_011221_HFGoe_L1_hTau_4h_R1 **Scan** 6689
**Peptide** PPSSGEPPKSGDRSGY **Charge** 2 **Spectral Angle** 0.86

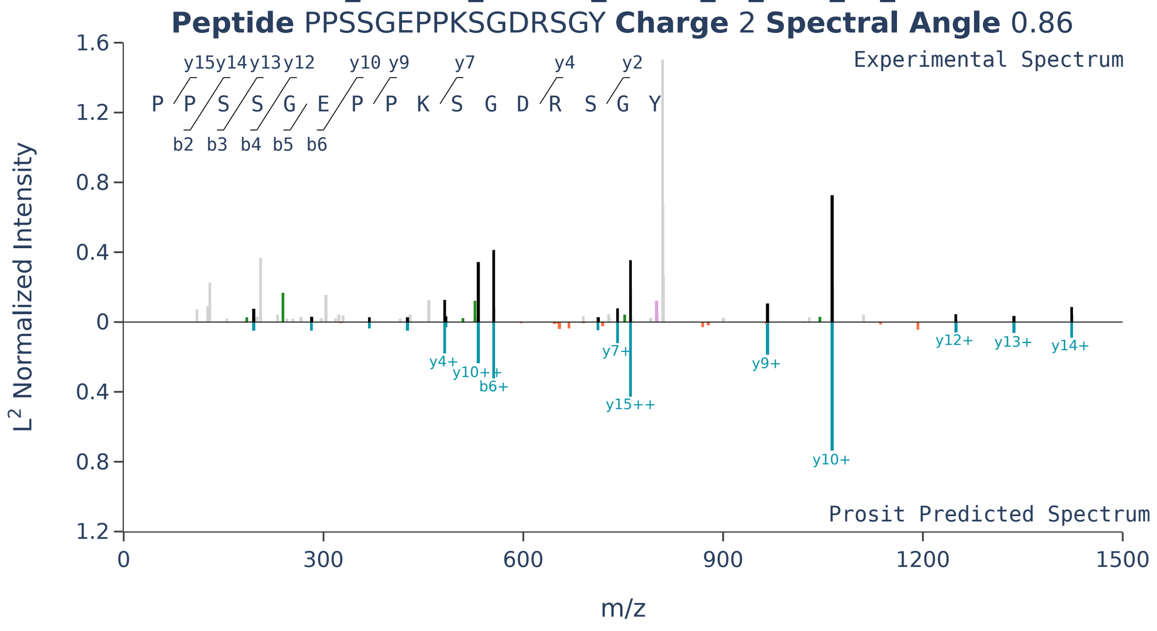


**Source** WSoh_221121_011221_HFGoe_L2_hTau_2h_R2 **Scan** 11393
**Peptide** SSPGSPGTPGSRSRTPSL **Charge** 3 **Spectral Angle** 0.95

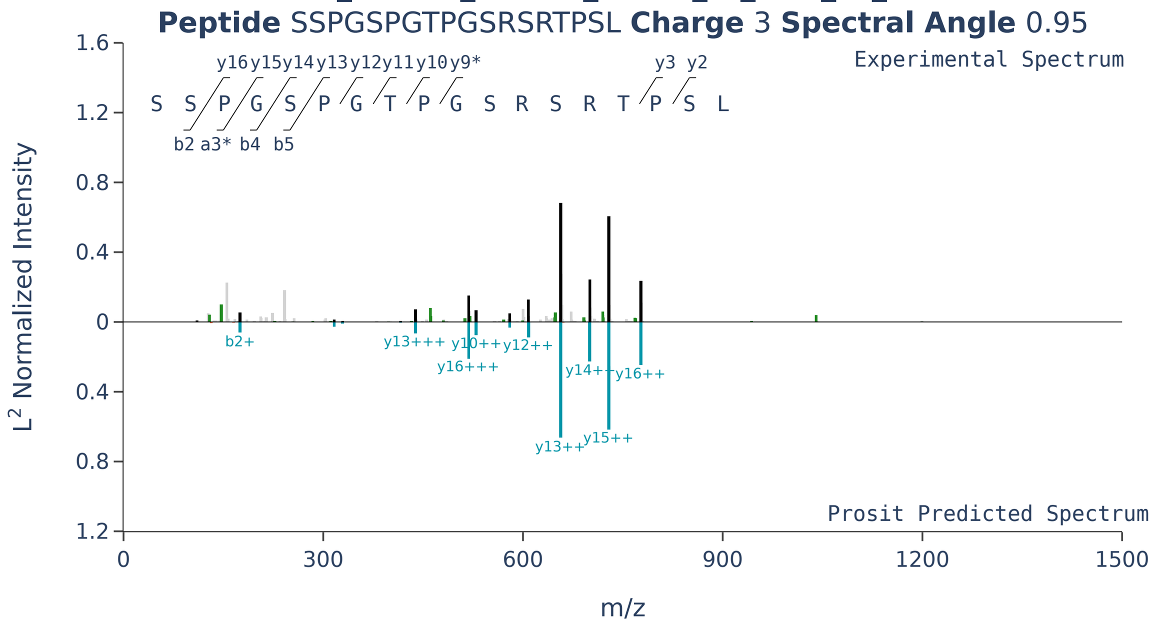


**Source** WSoh_221121_011221_HFGoe_L1_hTau_24h_R2 **Scan** 7284
**Peptide** NLTHVPGGGNKKIETH **Charge** 3 **Spectral Angle** 0.84

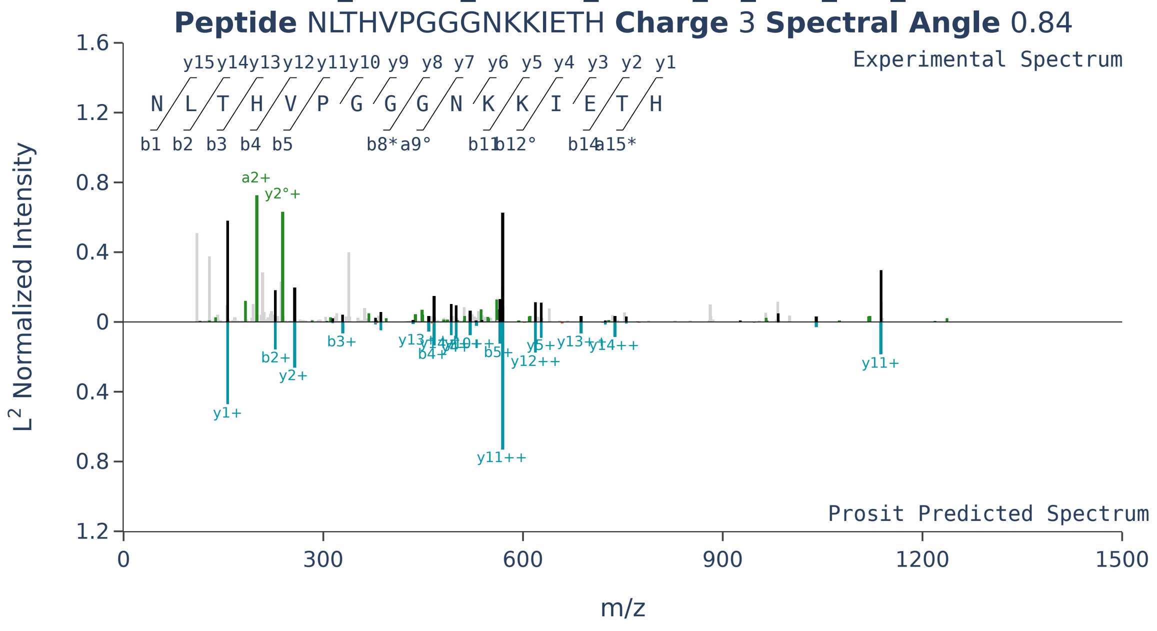


**Source** WSoh_221121_011221_HFGoe_L2_hTau_4h_R2 **Scan** 6568
**Peptide** VPGGGNKKIET **Charge** 2 **Spectral Angle** 0.93

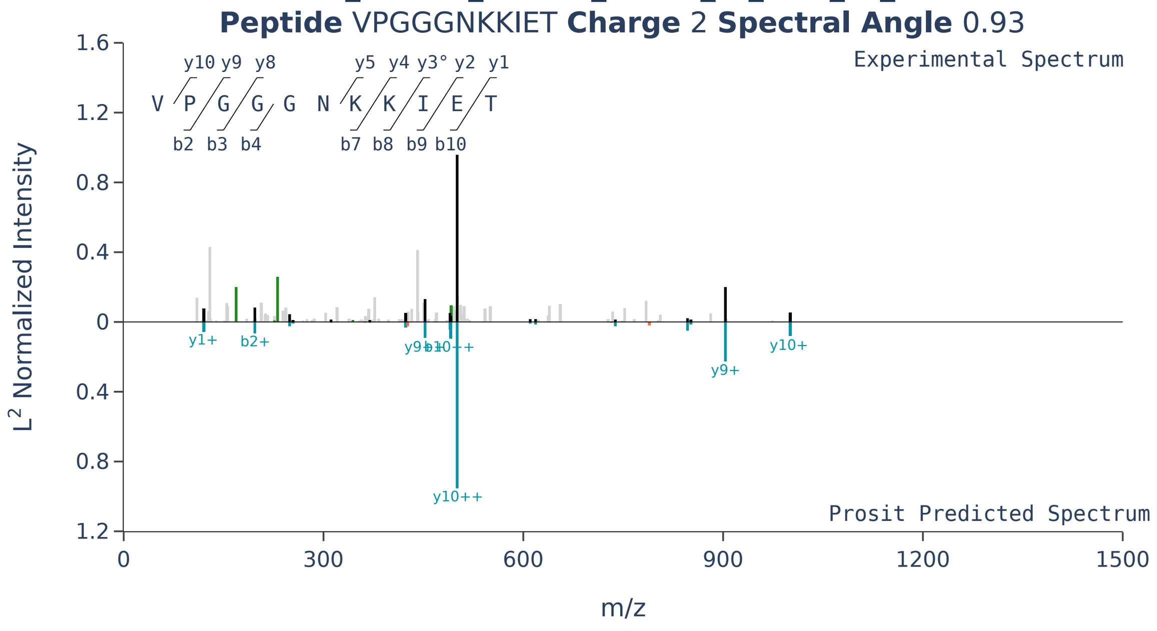

**Source** WSoh_221121_011221_HFGoe_L1_hTau_24h_R1 **Scan** 8286
**Peptide** TFRENAKAKTDHGAEIVYK **Charge** 3 **Spectral Angle** 0.83

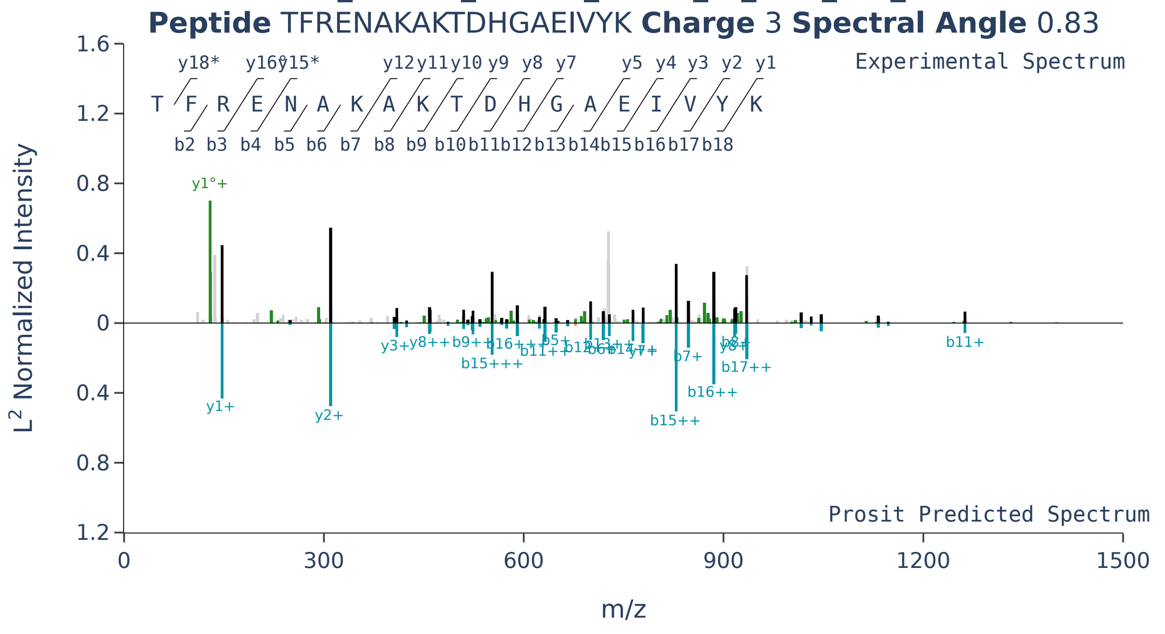


**Source** WSoh_221121_011221_HFGoe_L2_hTau_24h_R2 **Scan** 11637
**Peptide** EDHAGTYGLGDRKDQGGYTMH **Charge** 5 **Spectral Angle** 0.91

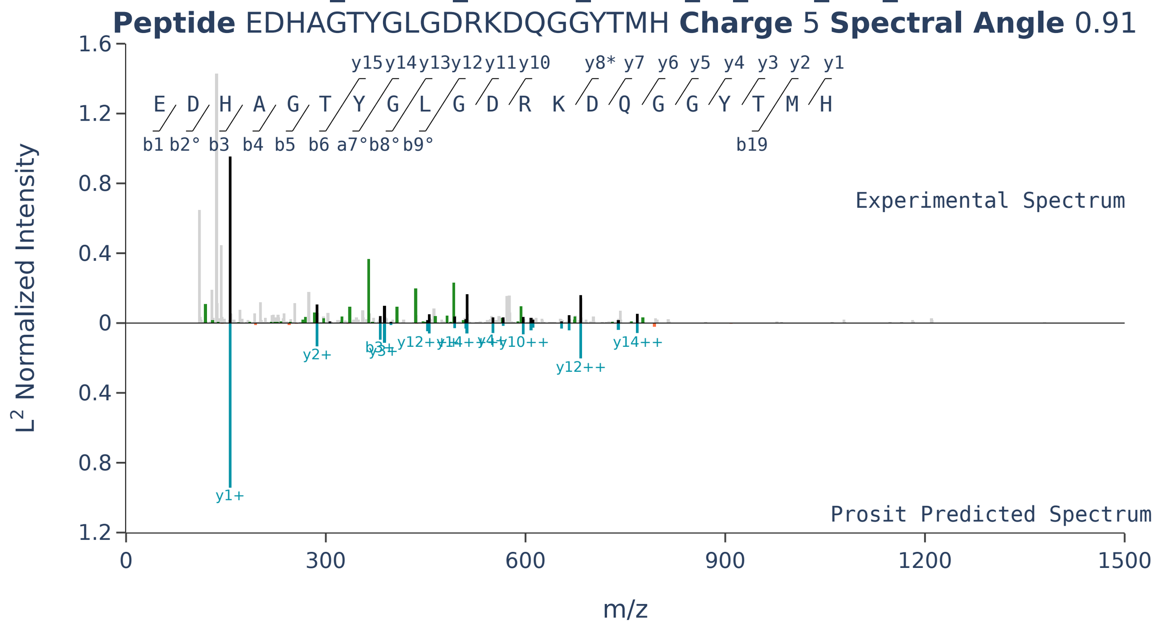


**Source** WSoh_221121_011221_HFGoe_L2_hTau_24h_R1 **Scan** 7598
**Peptide** GLGDRKDQGGYT **Charge** 2 **Spectral Angle** 0.81

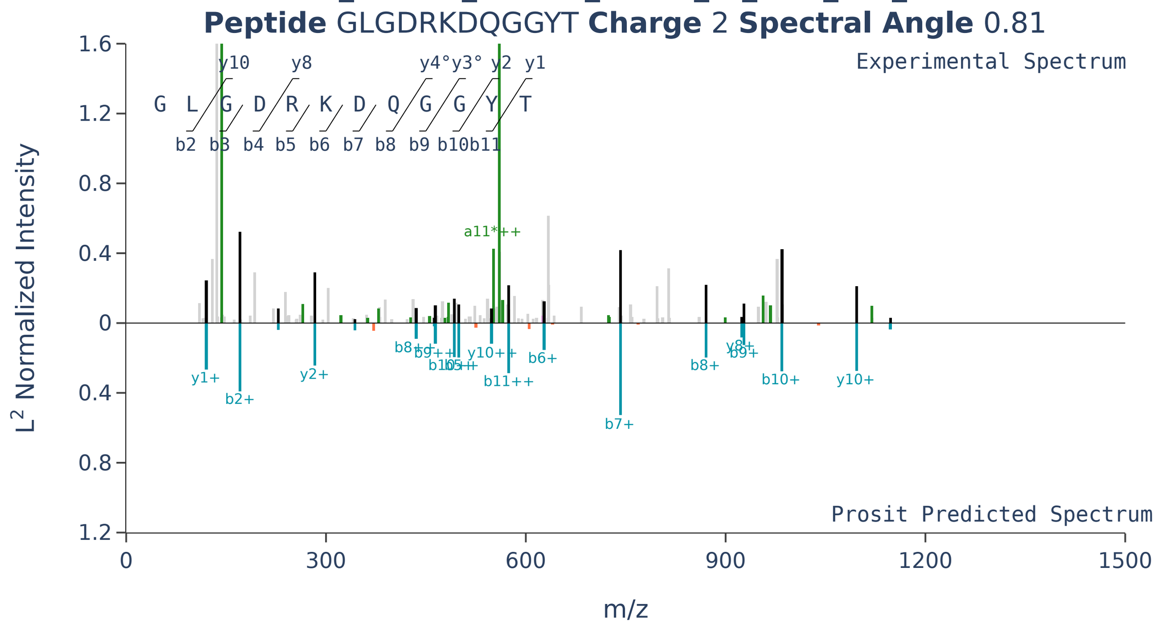


**Source** WSoh_221121_011221_HFGoe_L2_hTau_4h_R2 **Scan** 13200
**Peptide** INKKLDLSNVQS **Charge** 2 **Spectral Angle** 0.78

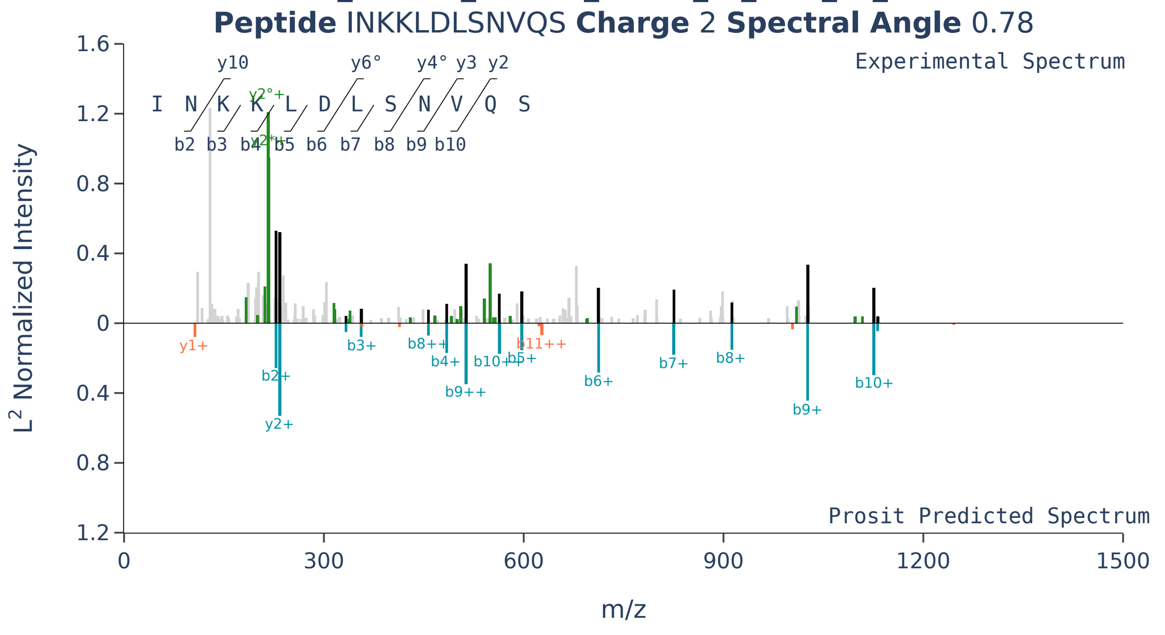


**Source** WSoh_221121_011221_HFGoe_L1_hTau_24h_R1 **Scan** 21231
**Peptide** TTAEEAGIGDTPSLED **Charge** 2 **Spectral Angle** 0.8

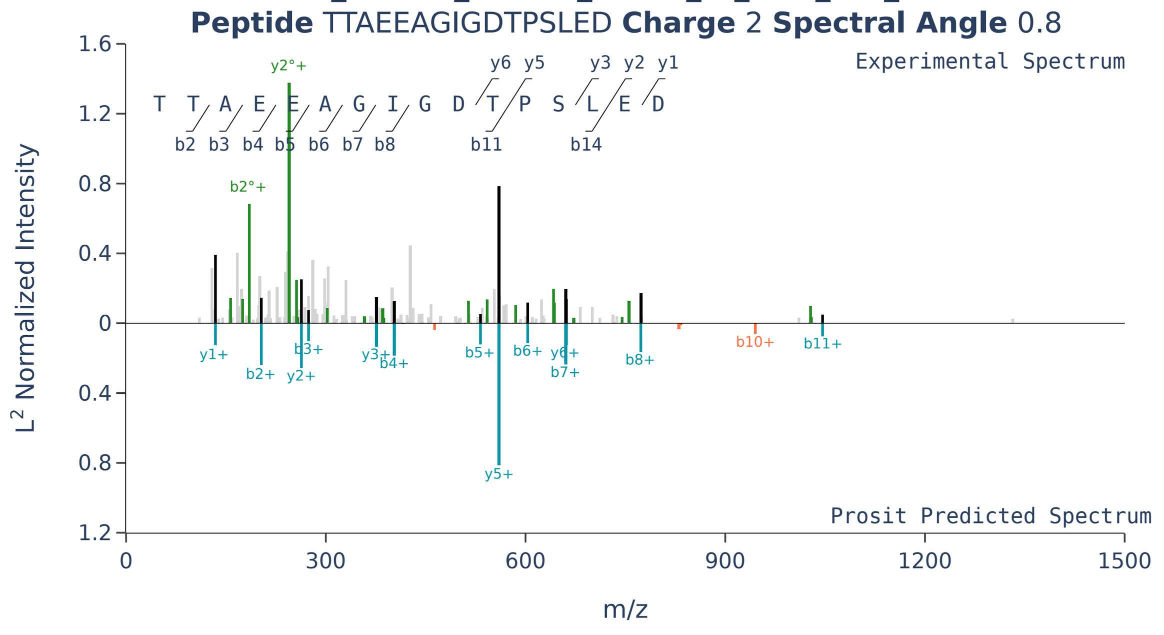


**Source** WSoh_221121_011221_HFGoe_L2_hTau_2h_R1 **Scan** 5989
**Peptide** THVPGGGNKKI **Charge** 3 **Spectral Angle** 0.85

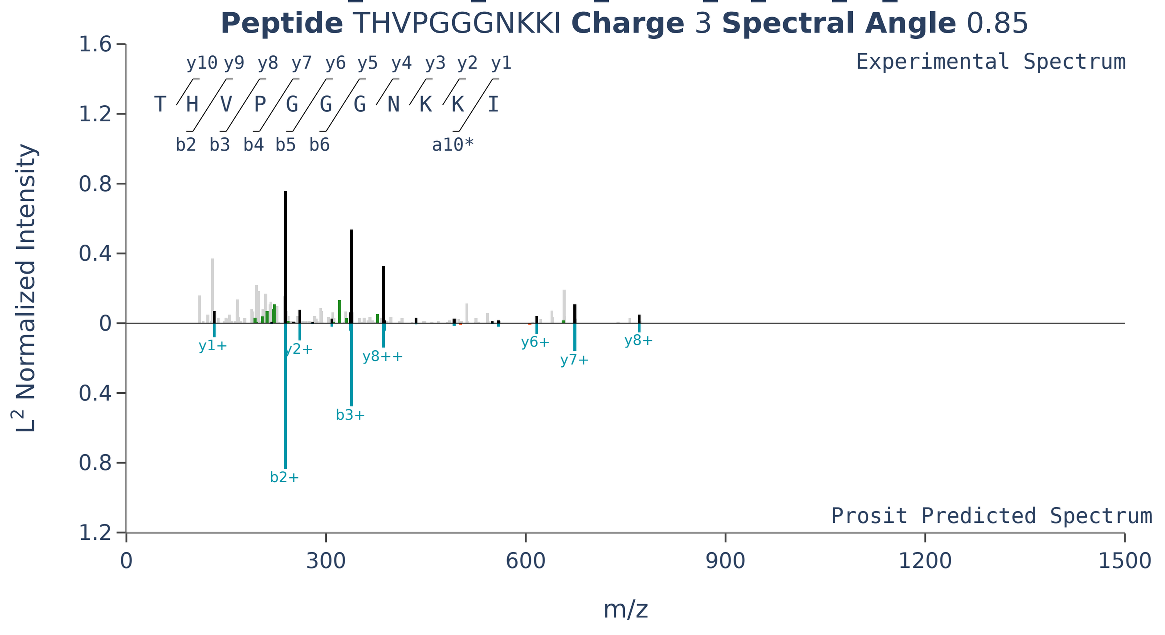


**Source** WSoh_221121_011221_HFGoe_L1_hTau_24h_R1 **Scan** 18641
**Peptide** STGSIDMVDSPQ **Charge** 2 **Spectral Angle** 0.95

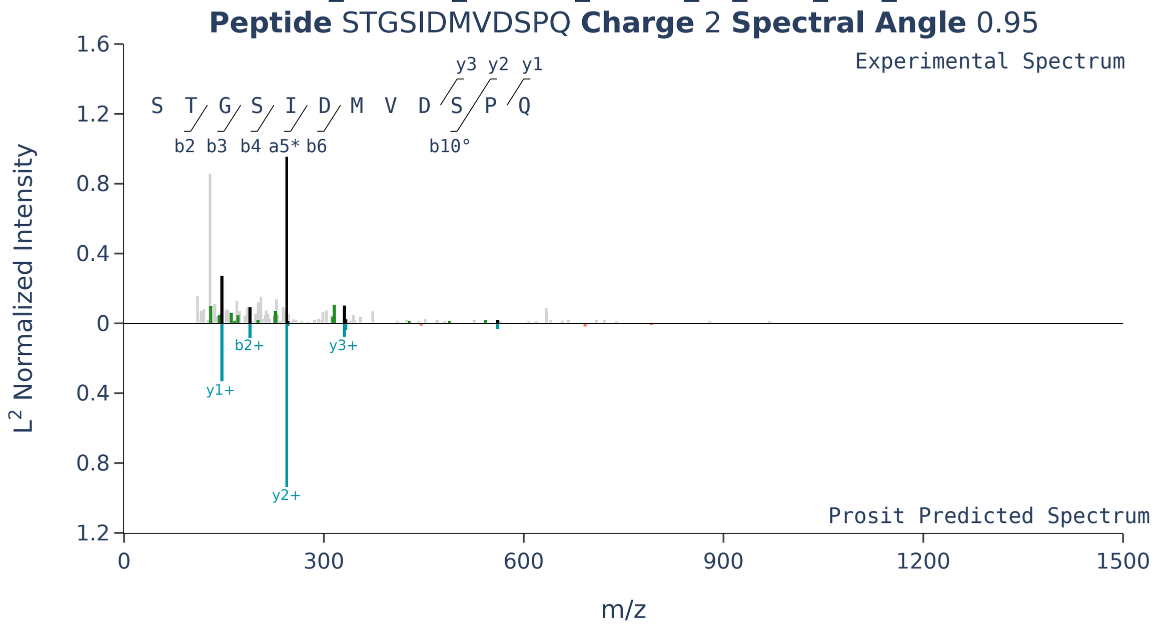


**Source** WSoh_221121_011221_HFGoe_L2_hTau_24h_R1 **Scan** 12351
**Peptide** RTPSLPTPPTREPKKVA **Charge** 4 **Spectral Angle** 0.89

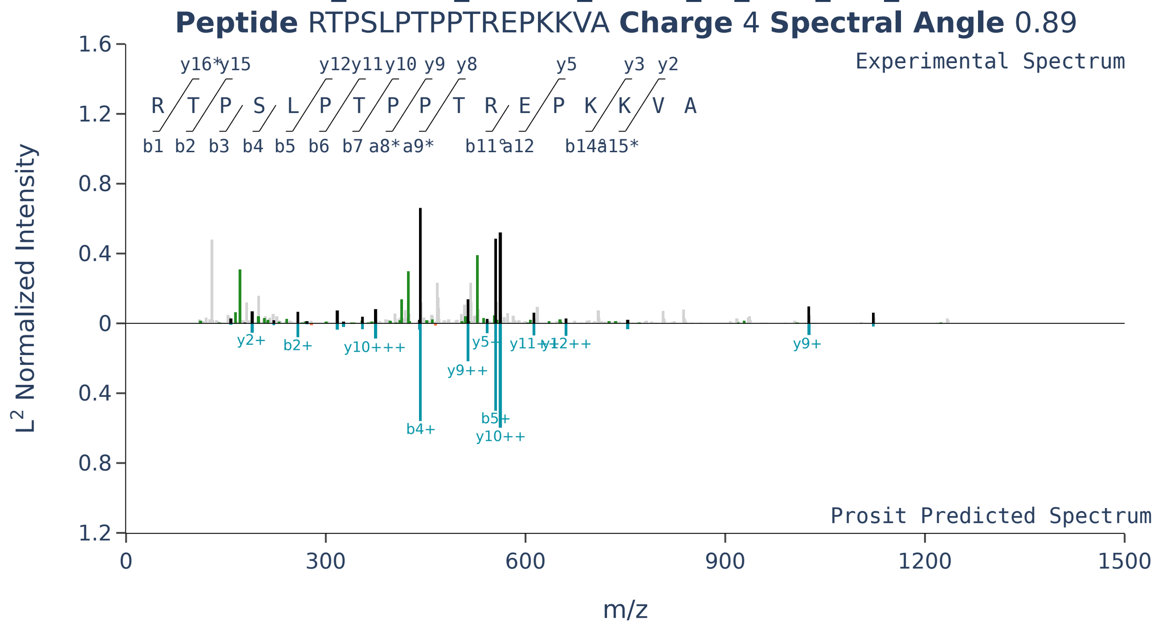


**Source** WSoh_221121_011221_HFGoe_L1_hTau_4h_R1 **Scan** 6237
**Peptide** AVVRTPPKSPSSAKS **Charge** 2 **Spectral Angle** 0.8

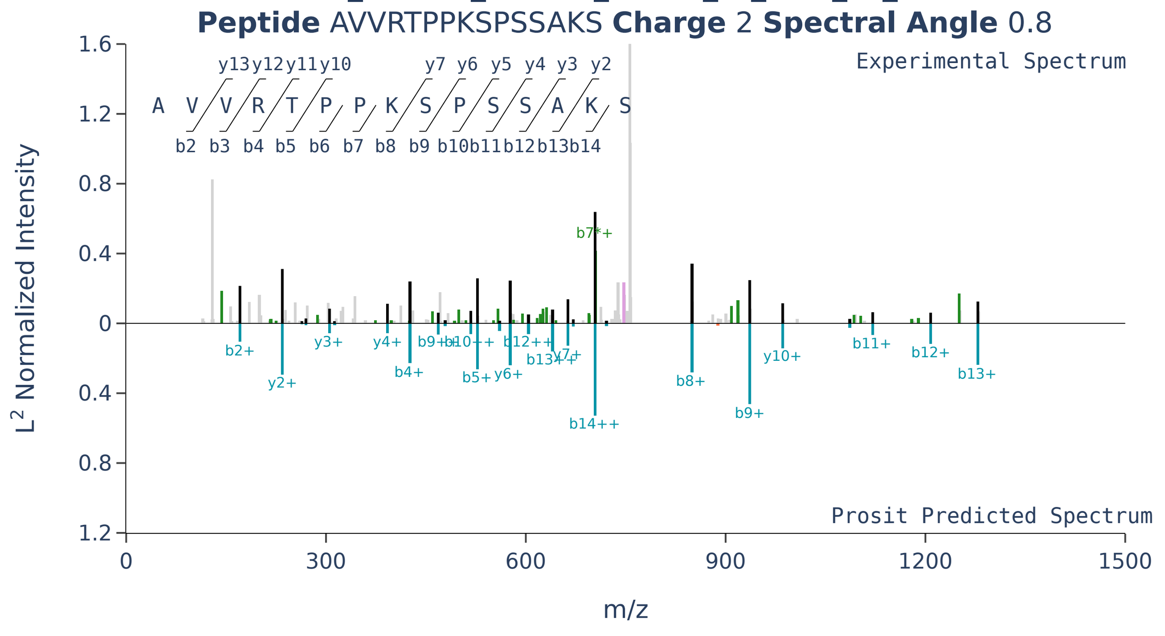


**Source** WSoh_221121_011221_HFGoe_L1_hTau_24h_R2 **Scan** 8073
**Peptide** GTYGLGDRKDQGG **Charge** 2 **Spectral Angle** 0.87

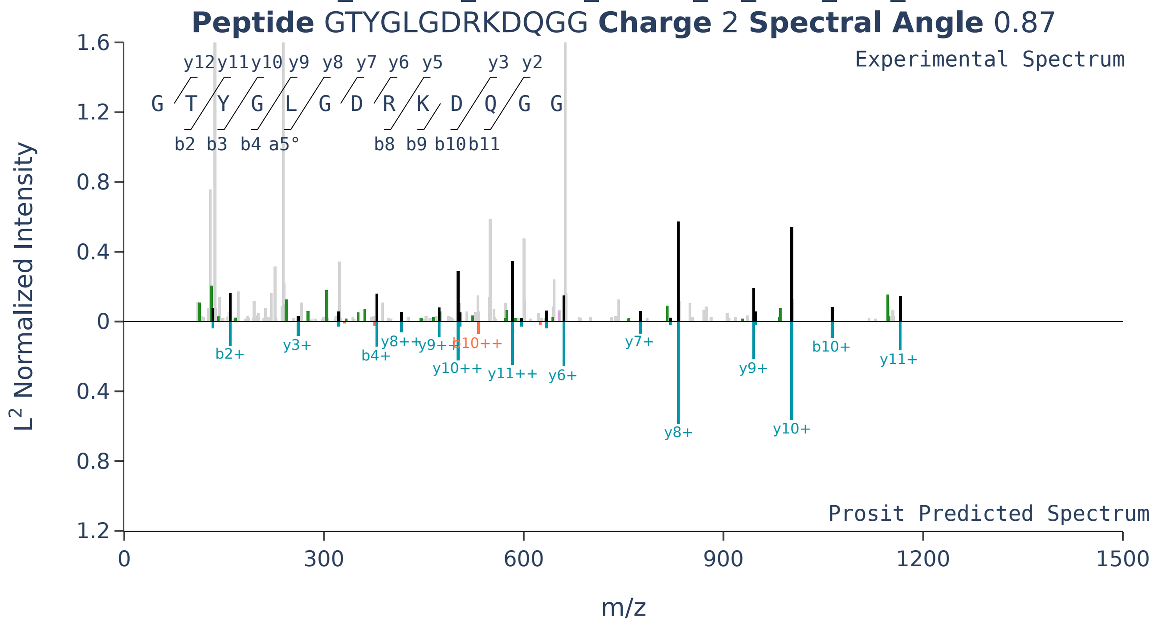


**Source** WSoh_221121_011221_HFGoe_L2_hTau_24h_R1 **Scan** 19748
**Peptide** GIGDTPSLE **Charge** 1 **Spectral Angle** 0.85

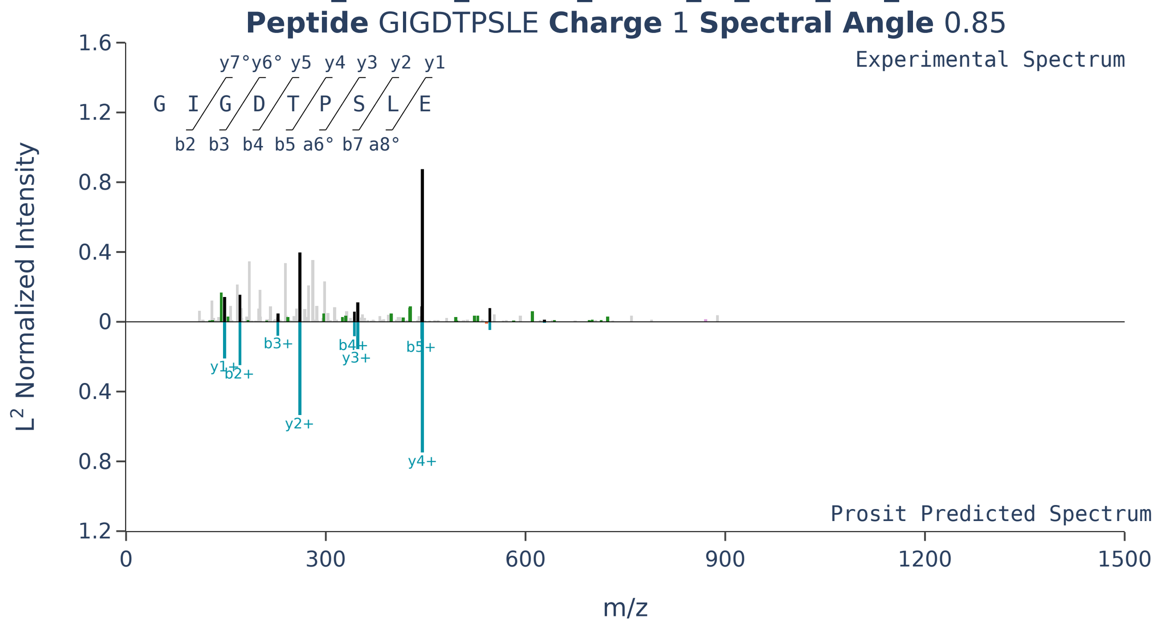


**Source** WSoh_221121_011221_HFGoe_L1_hTau_24h_R2 **Scan** 6798
**Peptide** LGNIHHKPGGGQV **Charge** 2 **Spectral Angle** 0.84

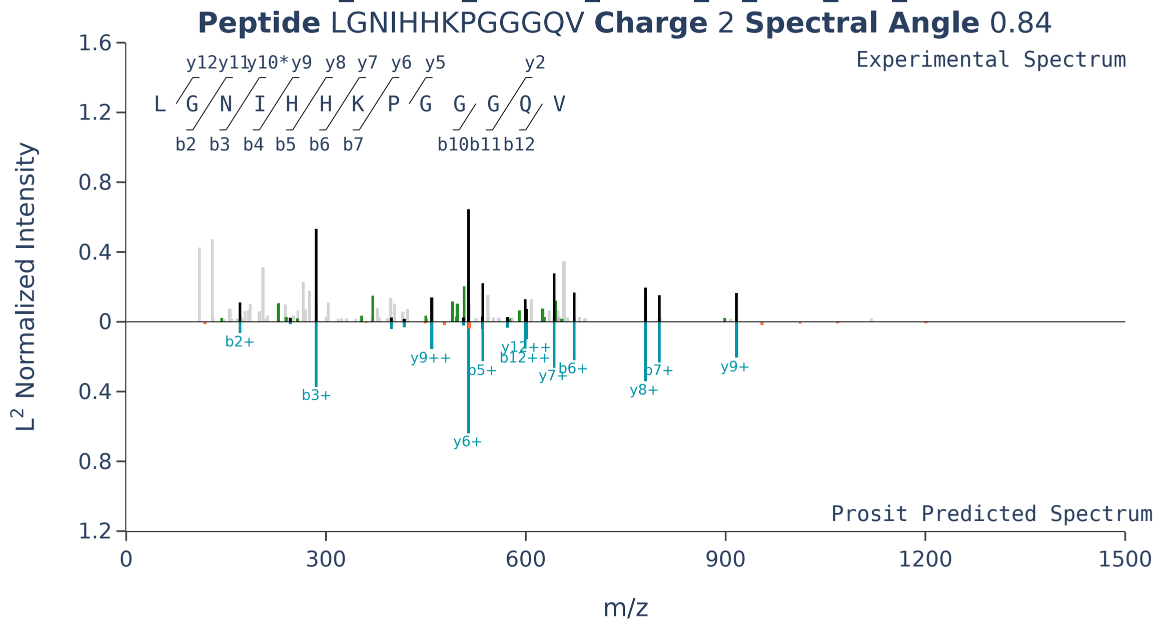


**Source** WSoh_221121_011221_HFGoe_L1_hTau_4h_R1 **Scan** 6633
**Peptide** VVRTPPKSPSSA **Charge** 3 **Spectral Angle** 0.76

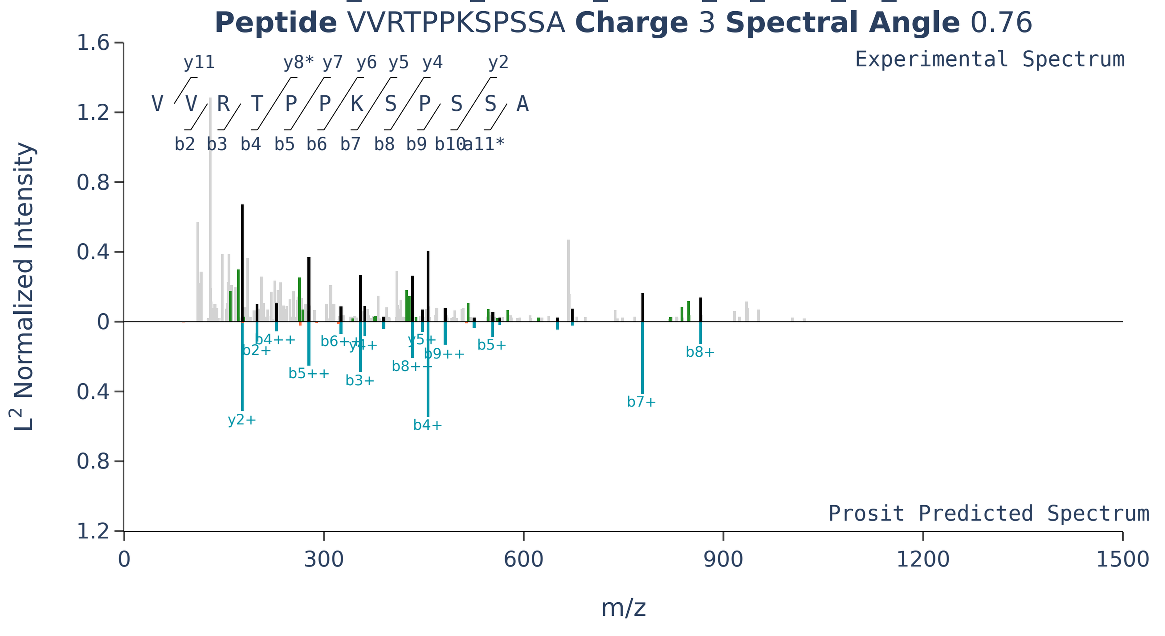


**Source** WSoh_221121_011221_HFGoe_L2_hTau_24h_R1 **Scan** 6911
**Peptide** TPPKSPSSAKSRLQTA **Charge** 3 **Spectral Angle** 0.87

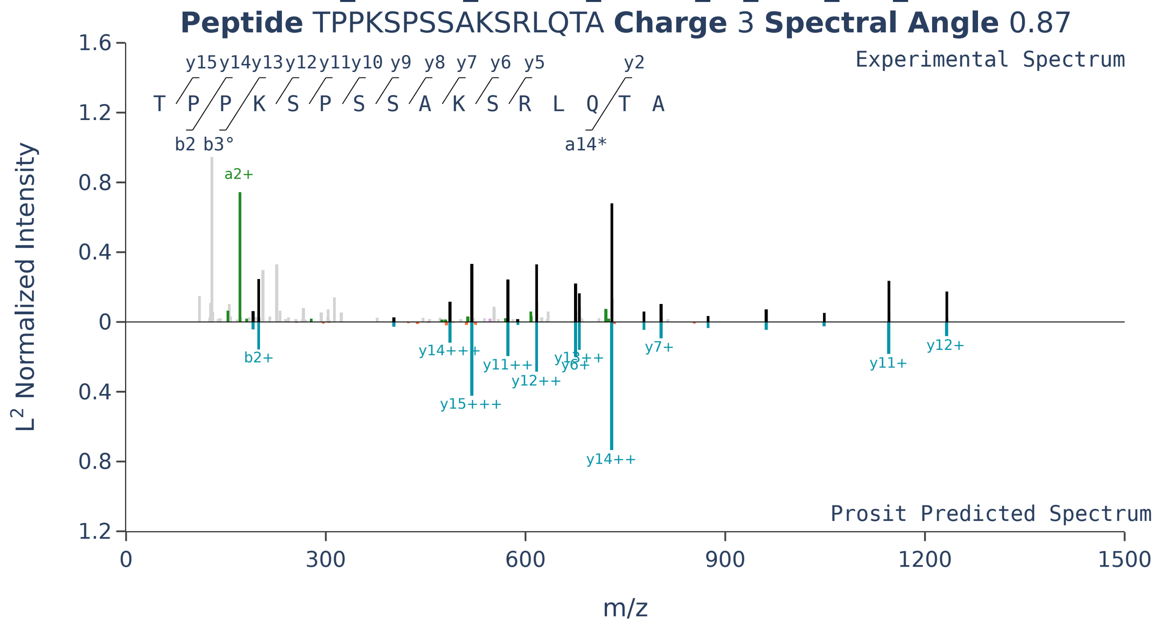


**Source** WSoh_221121_011221_HFGoe_L2_hTau_4h_R2 **Scan** 16266
**Peptide** GLKESPLQTPTE **Charge** 2 **Spectral Angle** 0.8

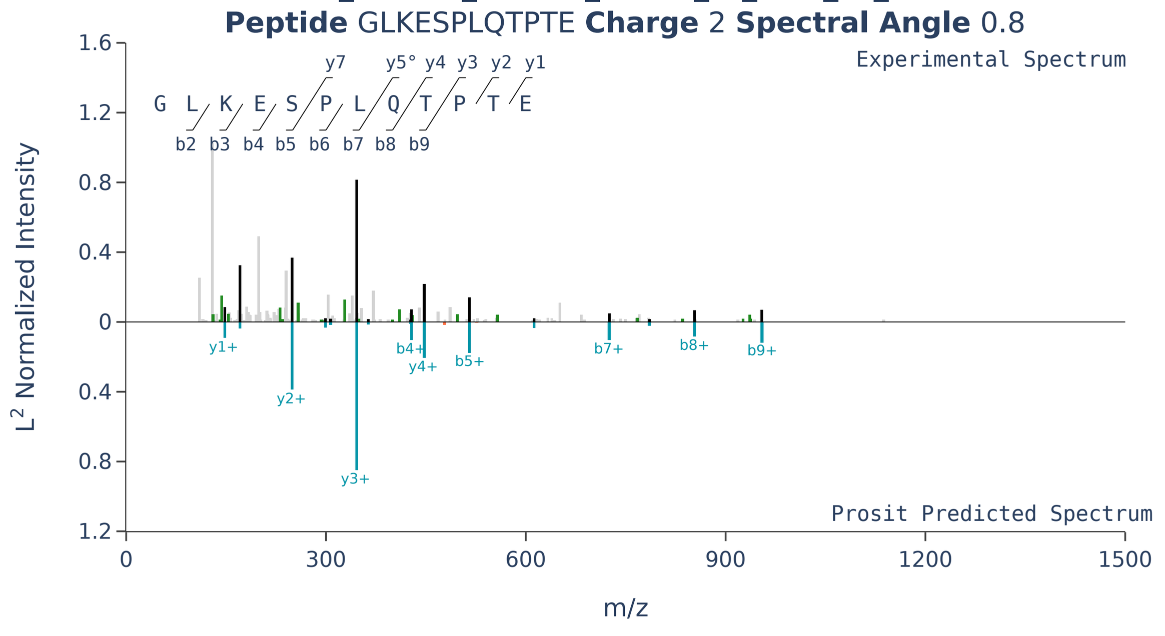

**Source** WSoh_221121_011221_HFGoe_L1_hTau_4h_R2 **Scan** 21656
**Peptide** AGIGDTPSL **Charge** 2 **Spectral Angle** 0.9

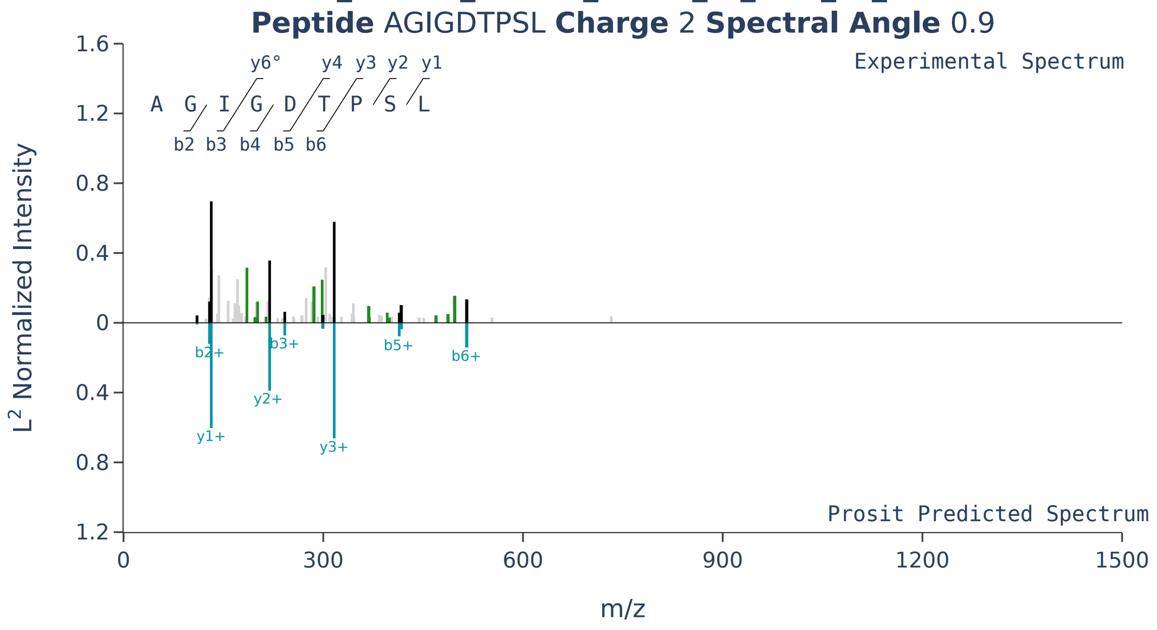


**Source** WSoh_221121_011221_HFGoe_L2_hTau_24h_R1 **Scan** 6966
**Peptide** SKVTSKCGSLGNIHHKPGGGQVE **Charge** 3 **Spectral Angle** 0.88

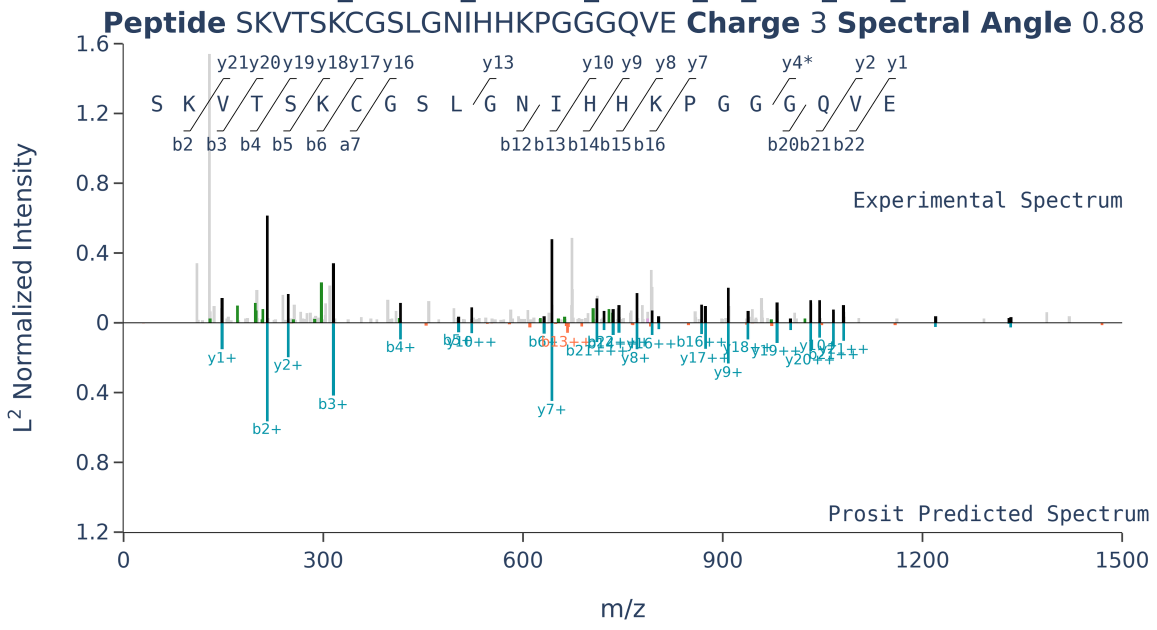


**Source** WSoh_221121_011221_HFGoe_L2_hTau_24h_R2 **Scan** 9623
**Peptide** HAGTYGLGDRKDQGGYT **Charge** 2 **Spectral Angle** 0.88

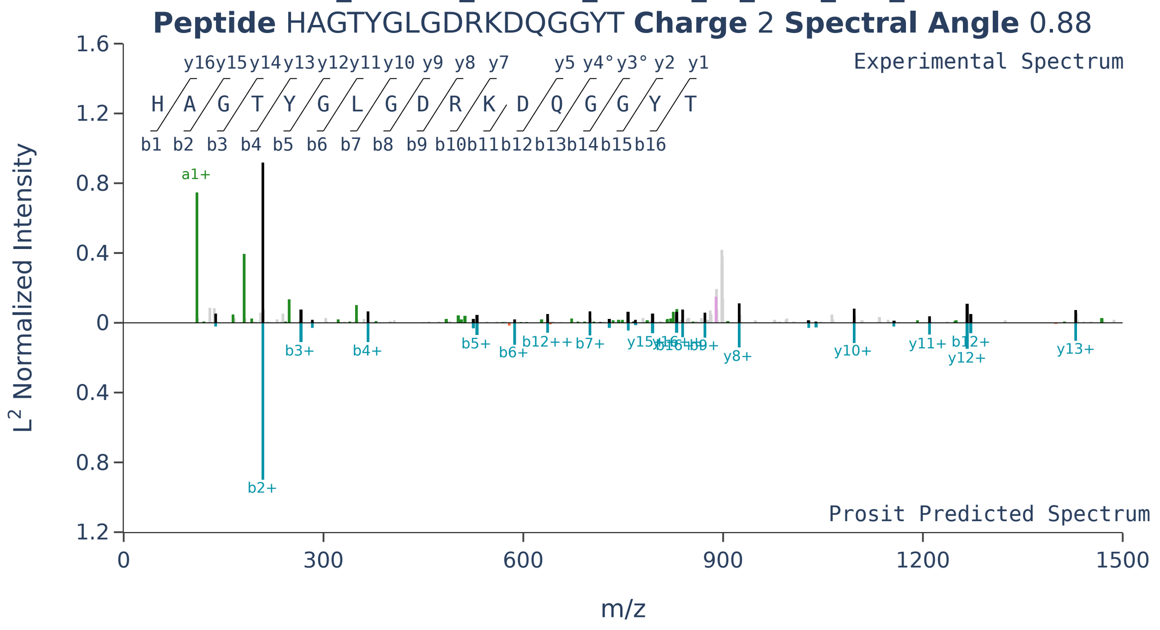


**Source** WSoh_221121_011221_HFGoe_L1_hTau_24h_R1 **Scan** 8364
**Peptide** LATPRGAAPPGQKG **Charge** 3 **Spectral Angle** 0.79

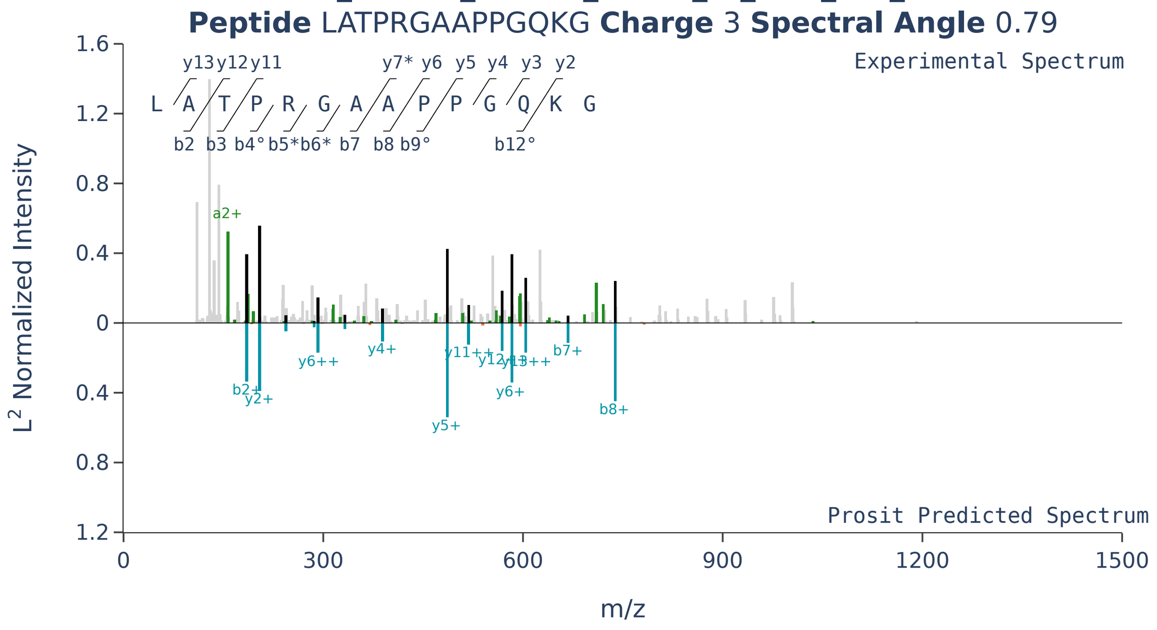


**Source** WSoh_221121_011221_HFGoe_L1_hTau_24h_R1 **Scan** 17374
**Peptide** VTAPLVDEGAPGKQAAAQ **Charge** 2 **Spectral Angle** 0.93

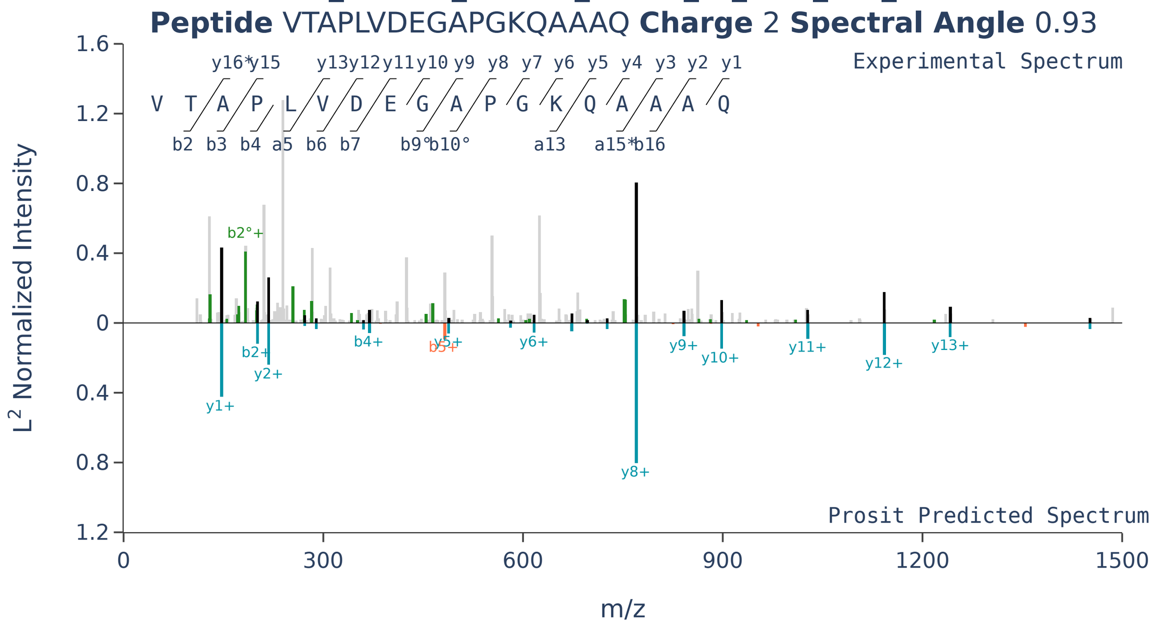


**Source** WSoh_221121_011221_HFGoe_L2_hTau_24h_R2 **Scan** 7996
**Peptide** IGSTENLKHQPGGGKV **Charge** 2 **Spectral Angle** 0.87

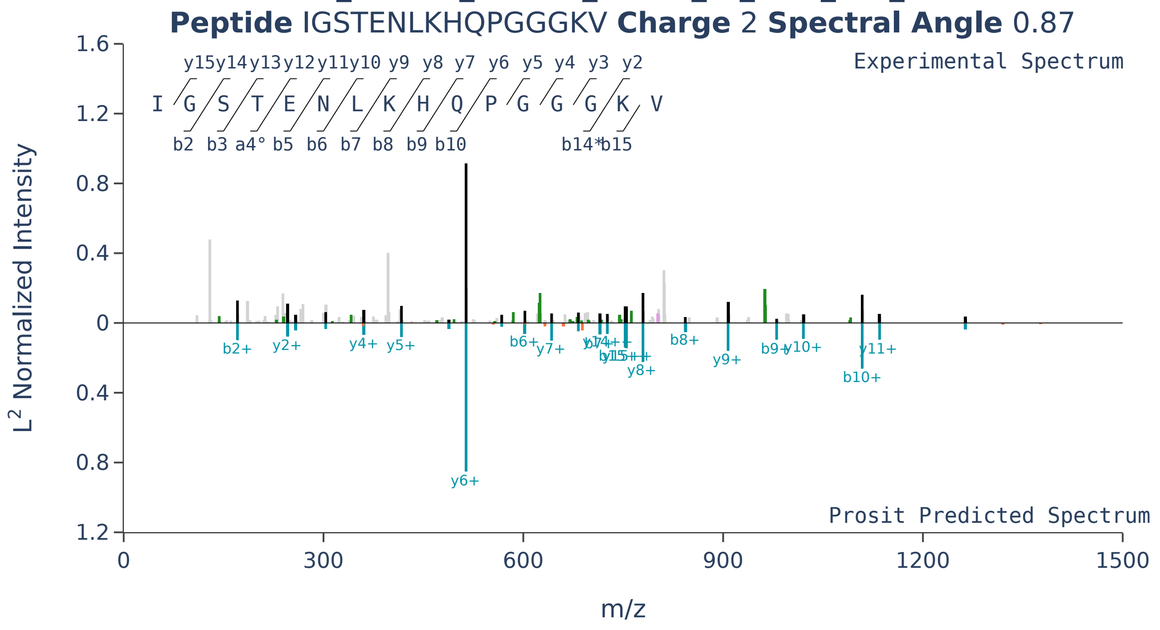


**Source** WSoh_221121_011221_HFGoe_L1_hTau_24h_R1 **Scan** 24342
**Peptide** KSRLQTAPVPMPDL **Charge** 2 **Spectral Angle** 0.9

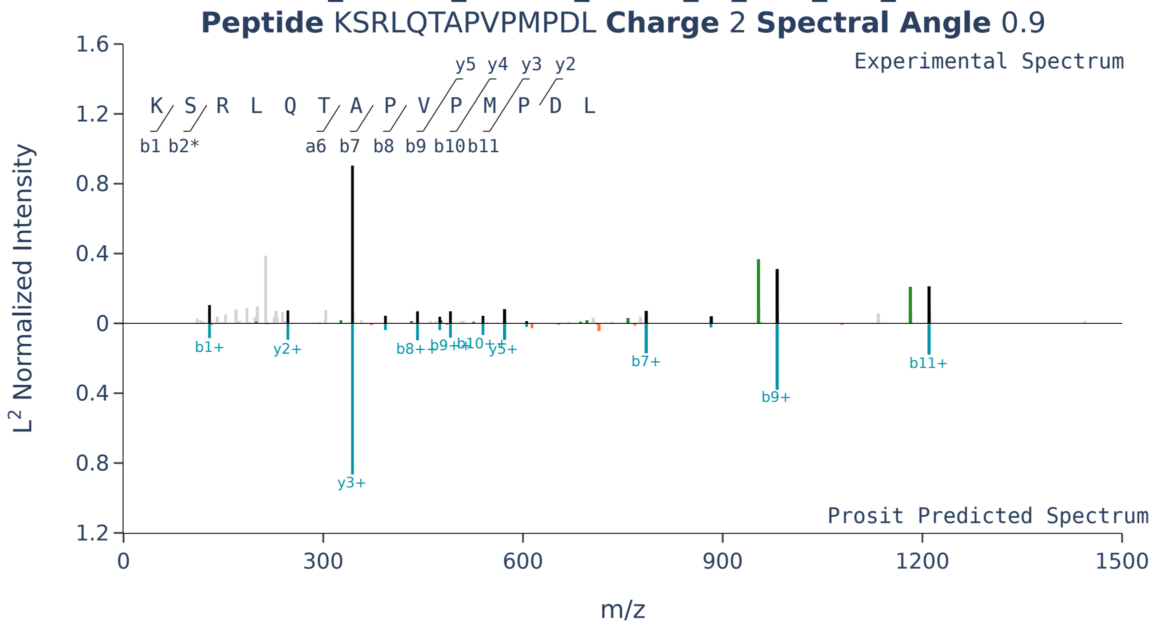


**Source** WSoh_221121_011221_HFGoe_L1_hTau_24h_R2 **Scan** 17784
**Peptide** TMHQDQEGDTDAGLKESPLQTPTE **Charge** 3 **Spectral Angle** 0.92

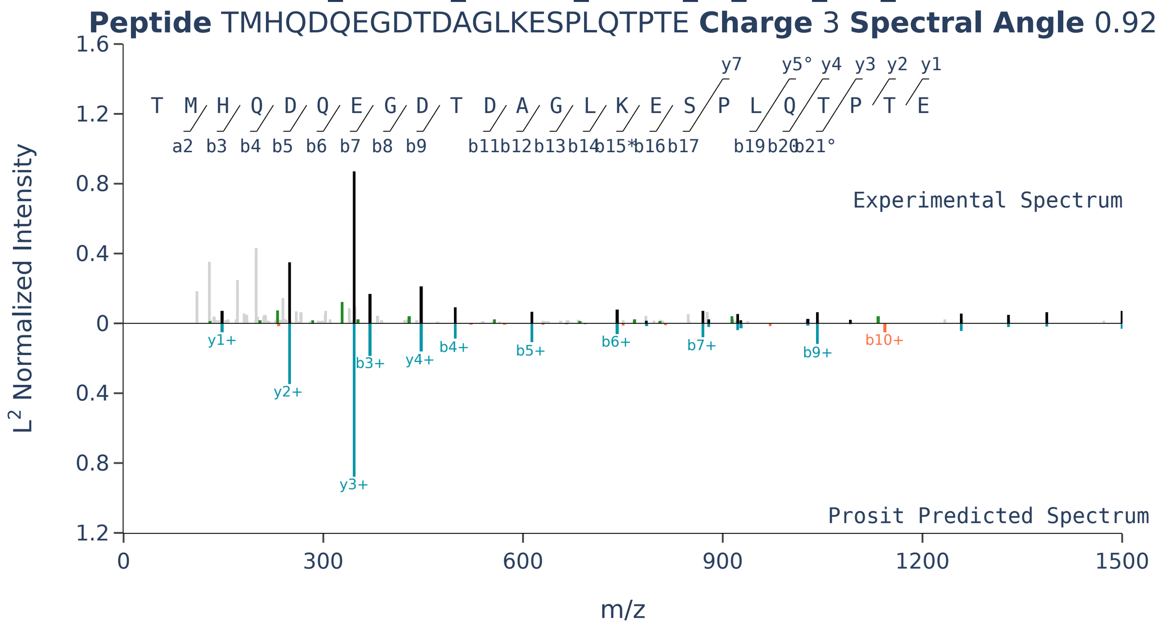


**Source** WSoh_221121_011221_HFGoe_L1_hTau_24h_R1 **Scan** 12923
**Peptide** LINKKLDLSNVQSK **Charge** 3 **Spectral Angle** 0.81

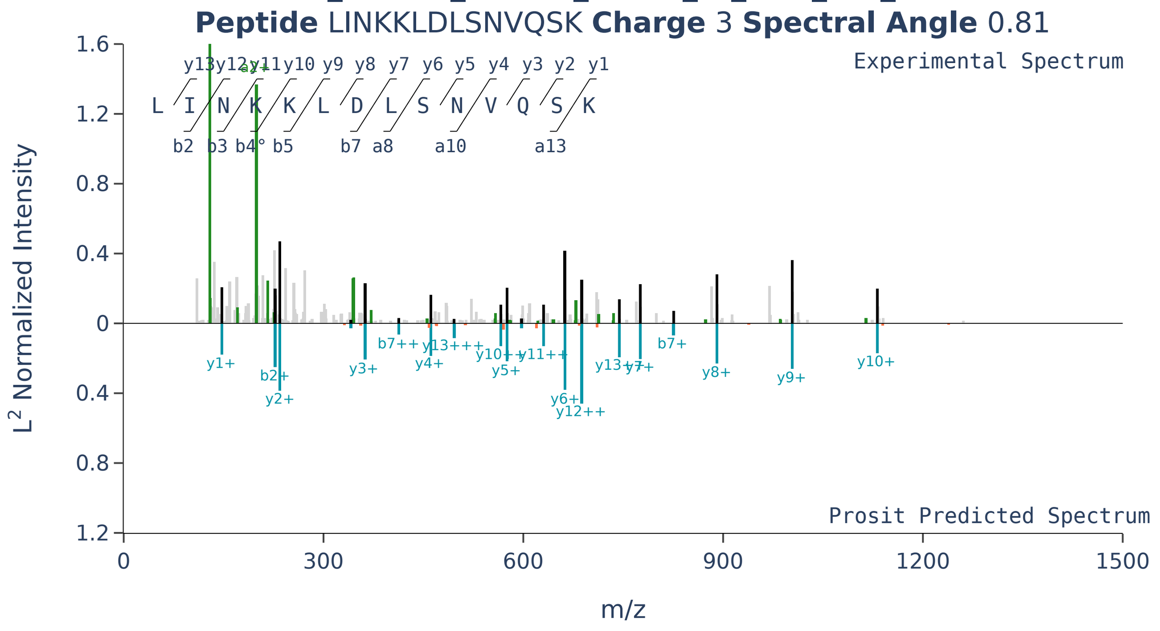


**Source** WSoh_221121_011221_HFGoe_L1_hTau_24h_R2 **Scan** 18067
**Peptide** NLKHVPGGGSVQIVYKPVD **Charge** 3 **Spectral Angle** 0.87

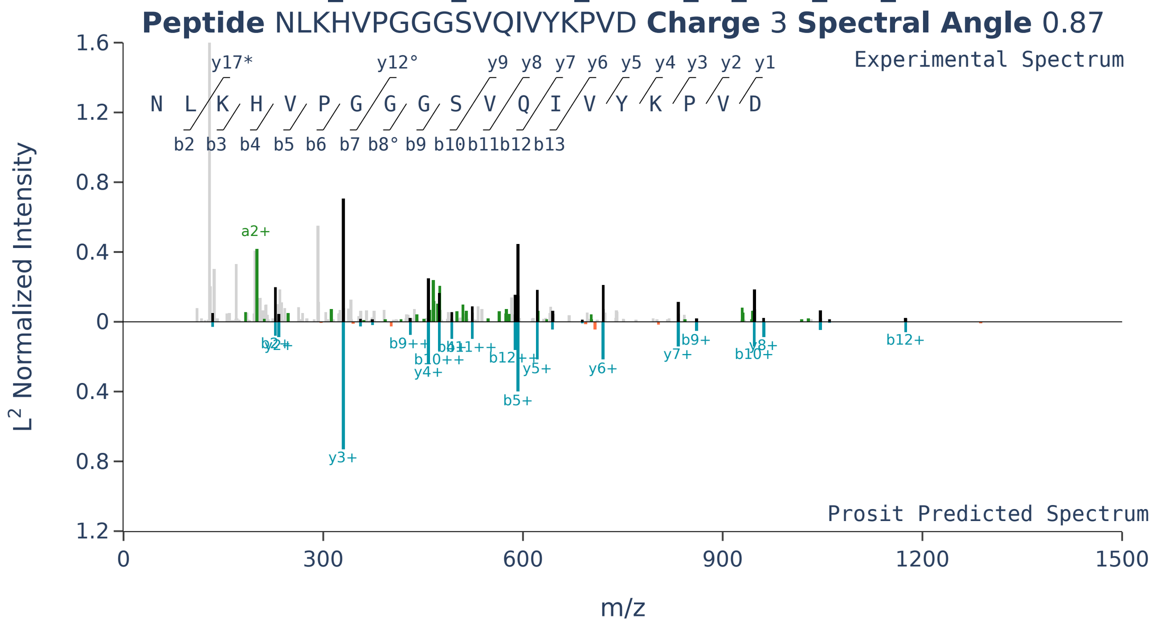


**Source** WSoh_221121_011221_HFGoe_L2_hTau_2h_R1 **Scan** 12260
**Peptide** KSEKLDFKDRVQSKI **Charge** 3 **Spectral Angle** 0.86

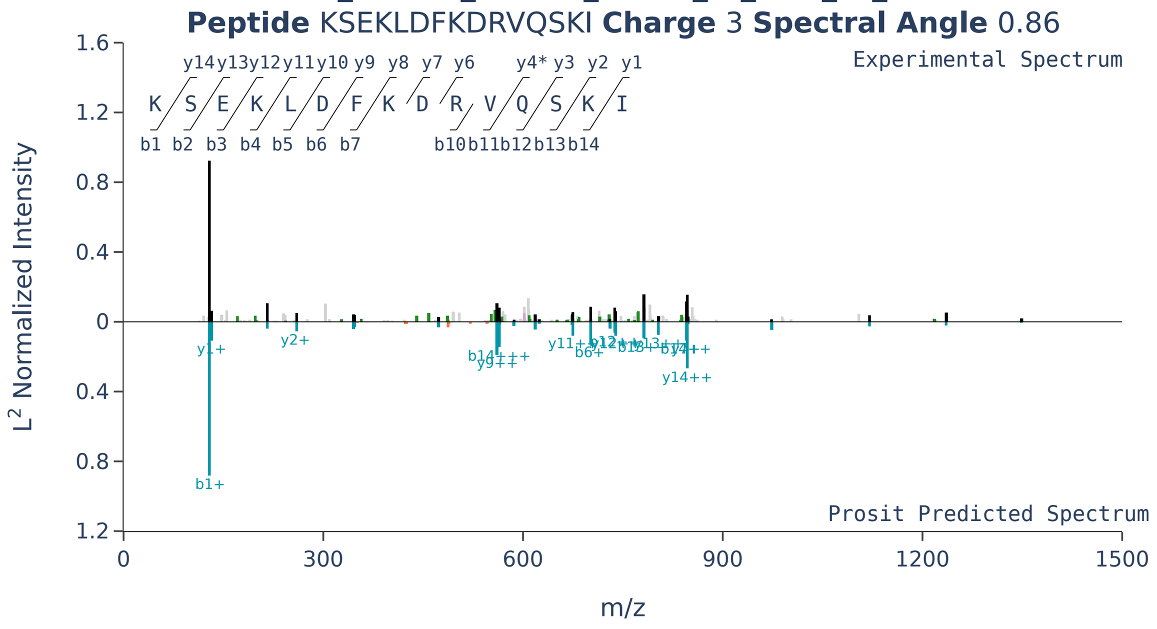


**Source** WSoh_221121_011221_HFGoe_L1_hTau_24h_R2 **Scan** 12663
**Peptide** KPGGGQVEVKSEKLDFKDRVQ **Charge** 4 **Spectral Angle** 0.84

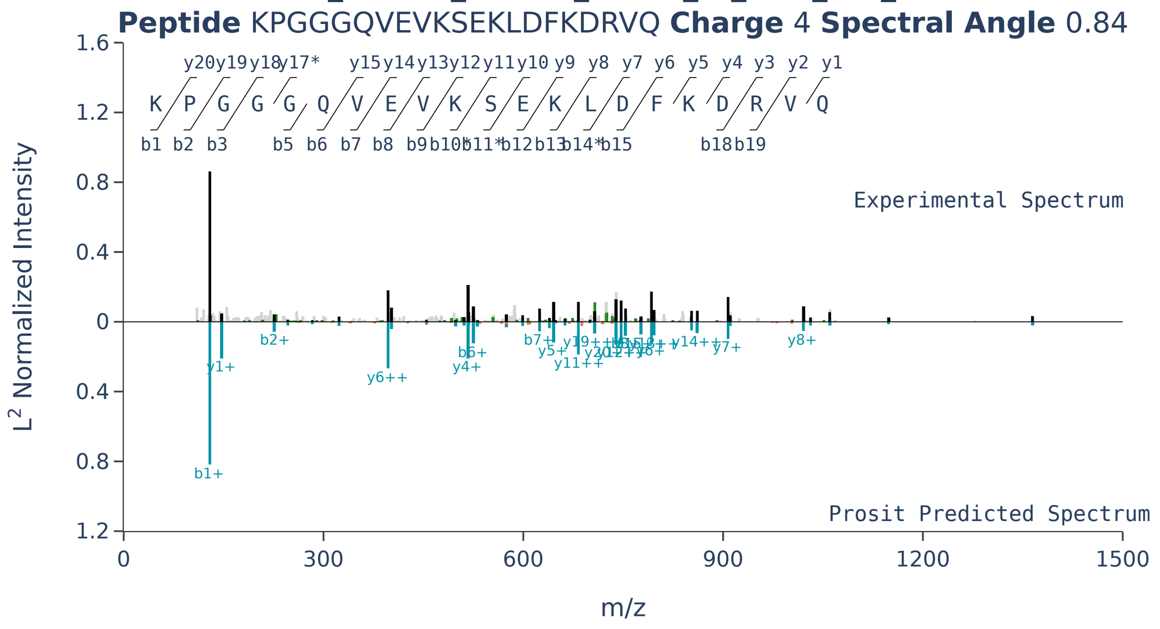


**Source** WSoh_221121_011221_HFGoe_L2_hTau_4h_R2 **Scan** 6951
**Peptide** IHHKPGGGQVEVKSEKL **Charge** 3 **Spectral Angle** 0.81

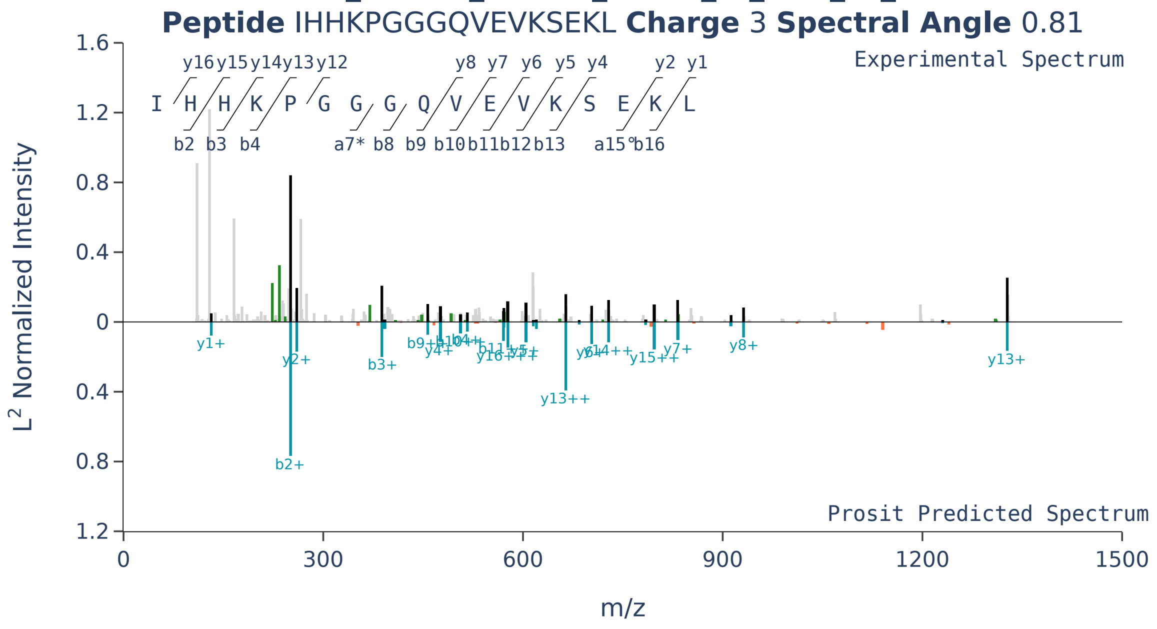


**Source** WSoh_221121_011221_HFGoe_L2_hTau_4h_R2 **Scan** 13826
**Peptide** KLDFKDRVQSKI **Charge** 4 **Spectral Angle** 0.91

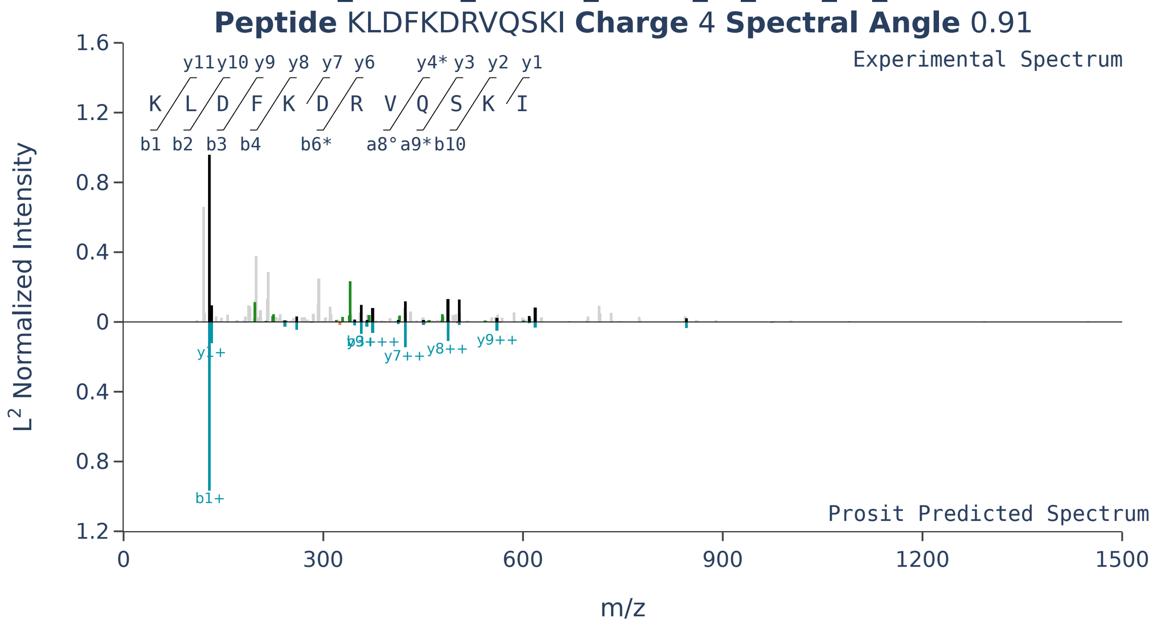


**Source** WSoh_221121_011221_HFGoe_L1_hTau_4h_R1 **Scan** 6418
**Peptide** VRTPPKSPSSAKSRLQT **Charge** 4 **Spectral Angle** 0.86

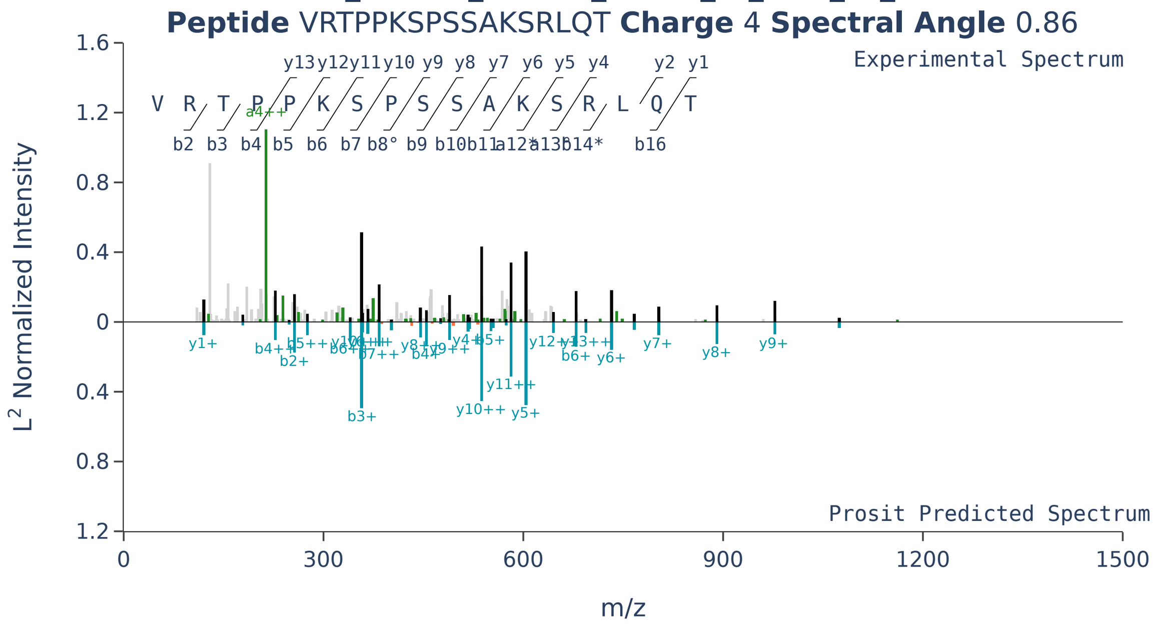

**Source** WSoh_221121_011221_HFGoe_L1_hTau_24h_R1 **Scan** 23969
**Peptide** TAEEAGIGDTPSLEDEAAGHVTQARMV **Charge** 3 **Spectral Angle** 0.81

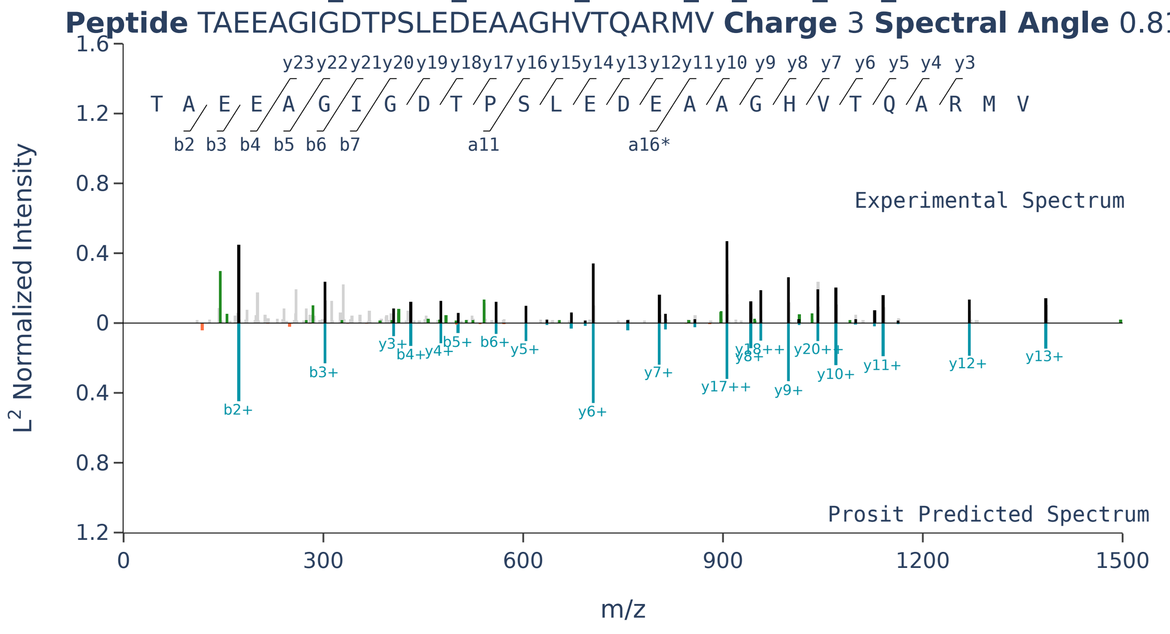


**Source** WSoh_221121_011221_HFGoe_L1_hTau_24h_R2 **Scan** 7561
**Peptide** VVRTPPKSPSSAKSRLQTA **Charge** 3 **Spectral Angle** 0.77

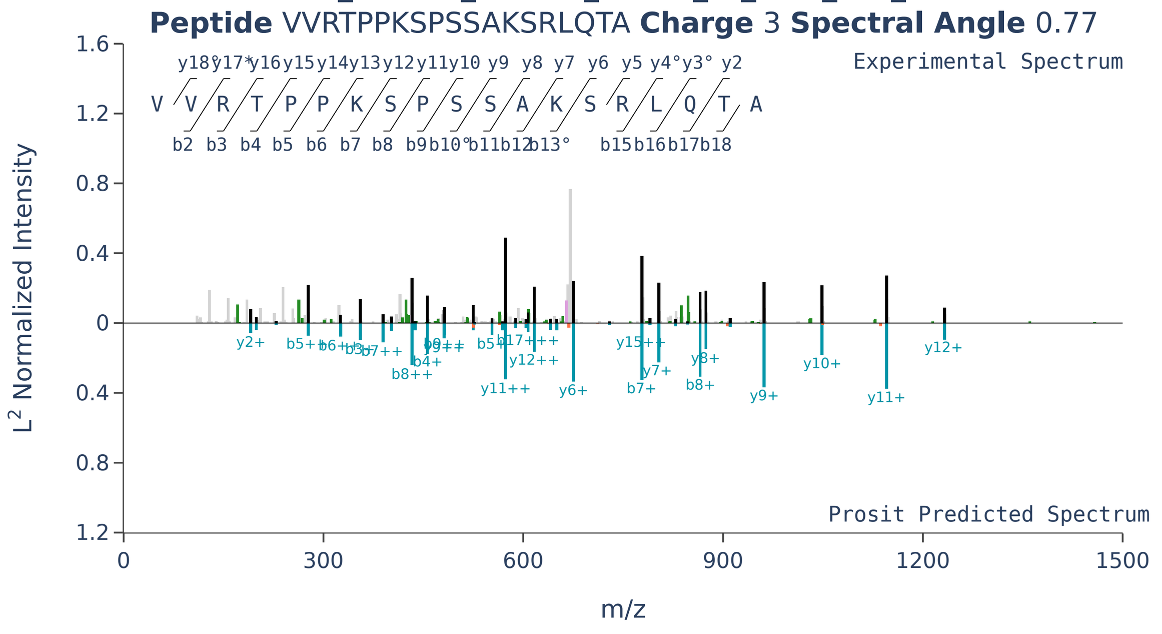


**Source** WSoh_221121_011221_HFGoe_L1_hTau_24h_R1 **Scan** 8637
**Peptide** LSKVTSKCGSL **Charge** 2 **Spectral Angle** 0.89

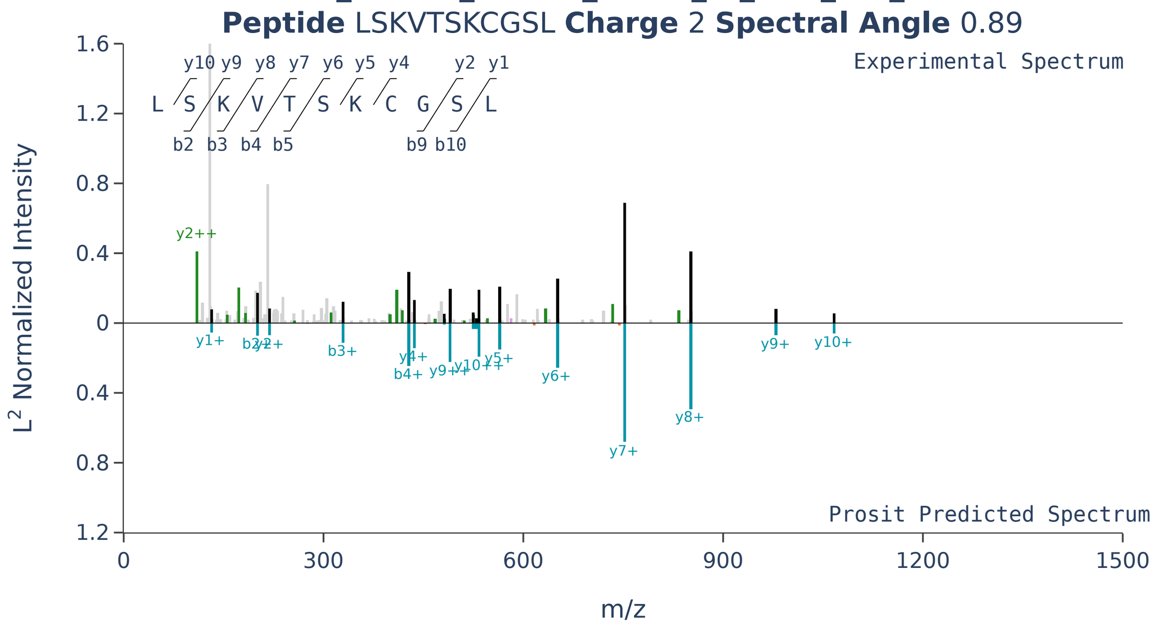


**Source** WSoh_221121_011221_HFGoe_L1_hTau_24h_R1 **Scan** 8046
**Peptide** NLTHVPGGGNKKIE **Charge** 3 **Spectral Angle** 0.86

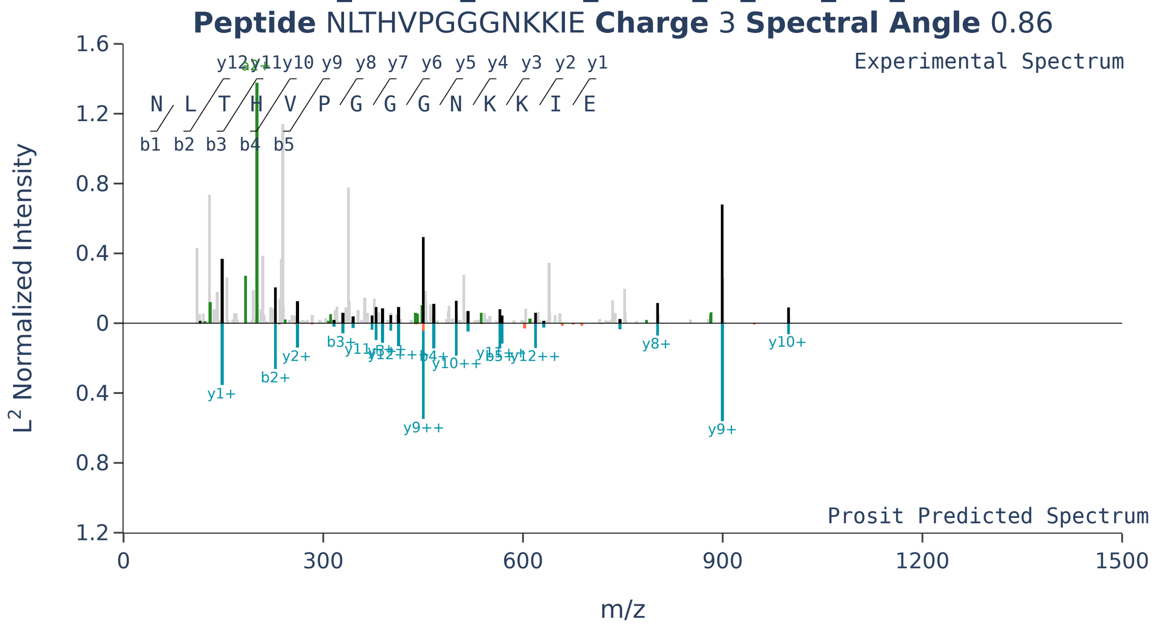


**Source** WSoh_221121_011221_HFGoe_L1_hTau_24h_R1 **Scan** 17304
**Peptide** QEGDTDAGLKESPLQT **Charge** 2 **Spectral Angle** 0.87

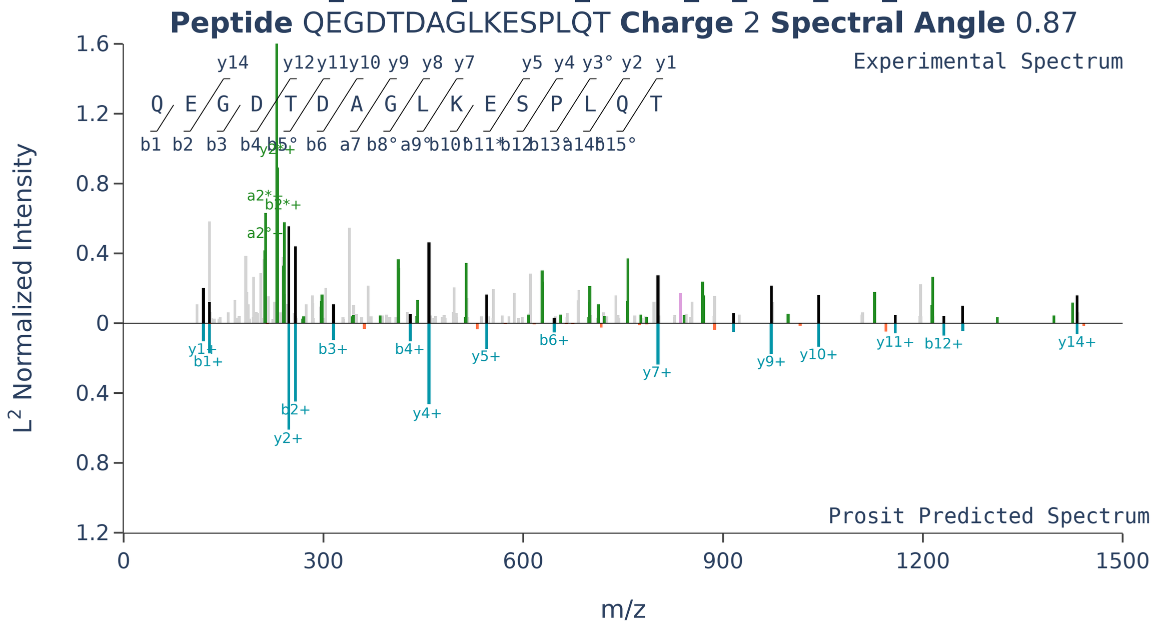


**Source** WSoh_221121_011221_HFGoe_L1_hTau_24h_R1 **Scan** 16673
**Peptide** GSKDNIKHVPGGGSVQIVYKPVD **Charge** 3 **Spectral Angle** 0.87

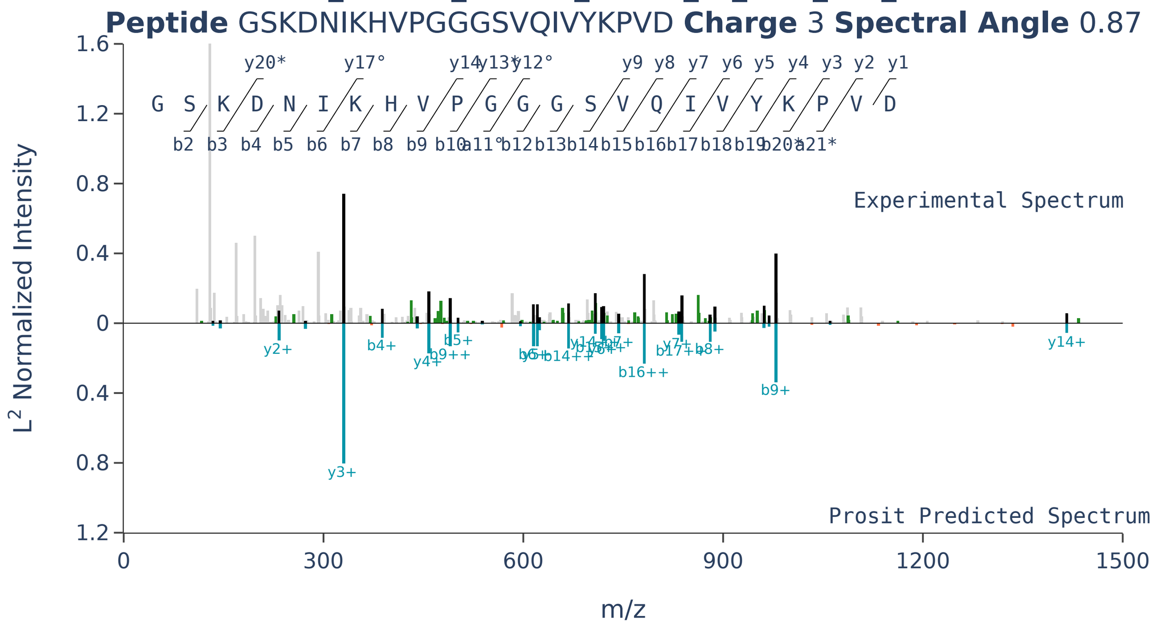


**Source** WSoh_221121_011221_HFGoe_L1_hTau_24h_R1 **Scan** 6777
**Peptide** HQPGGGKVQIINK **Charge** 2 **Spectral Angle** 0.96

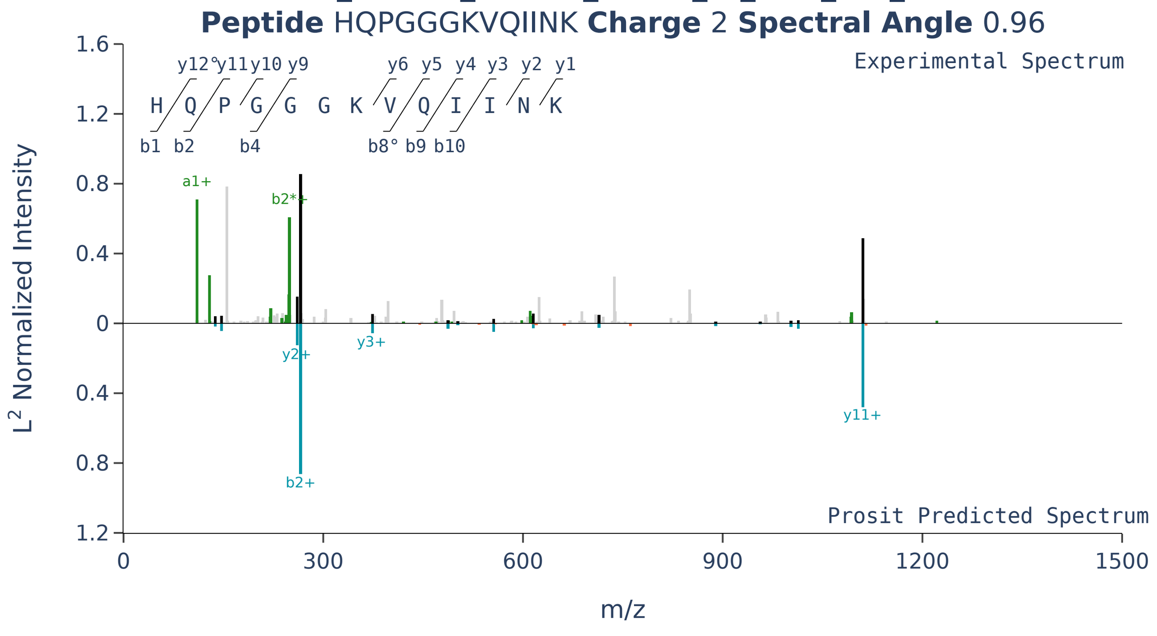


**Source** WSoh_221121_011221_HFGoe_L1_hTau_24h_R2 **Scan** 20408
**Peptide** EAGIGDTPSLEDEAAGHVTQARMVSK **Charge** 4 **Spectral Angle** 0.86

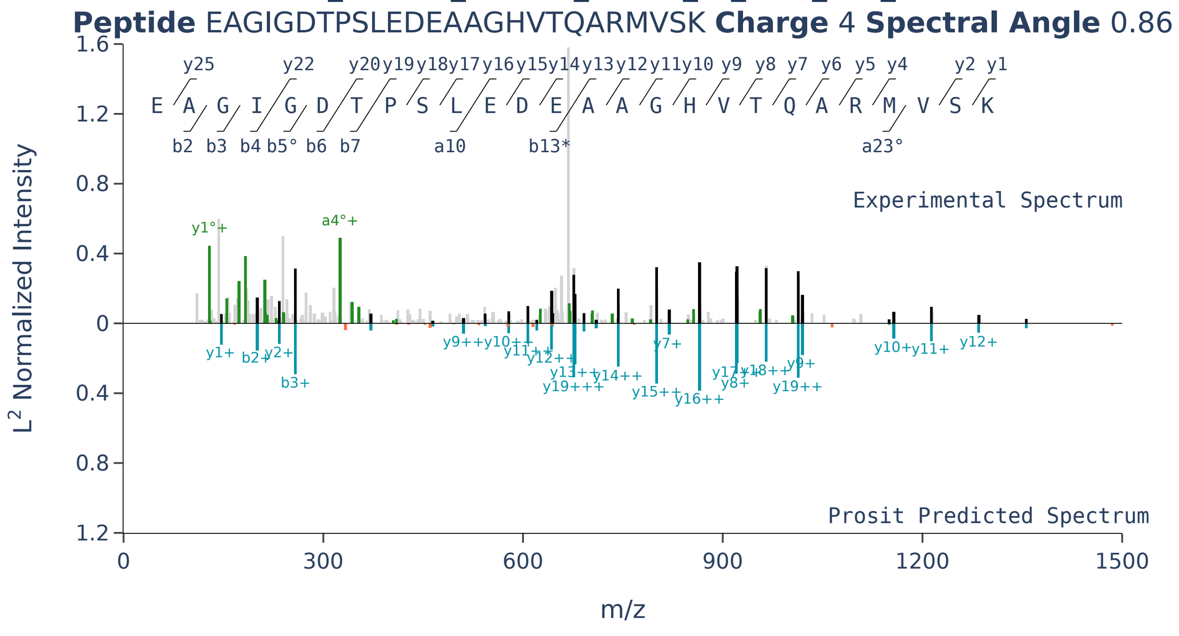


**Source** WSoh_221121_011221_HFGoe_L2_hTau_24h_R2 **Scan** 9436
**Peptide** RLPAKTPPAPKTPPSS **Charge** 2 **Spectral Angle** 0.87

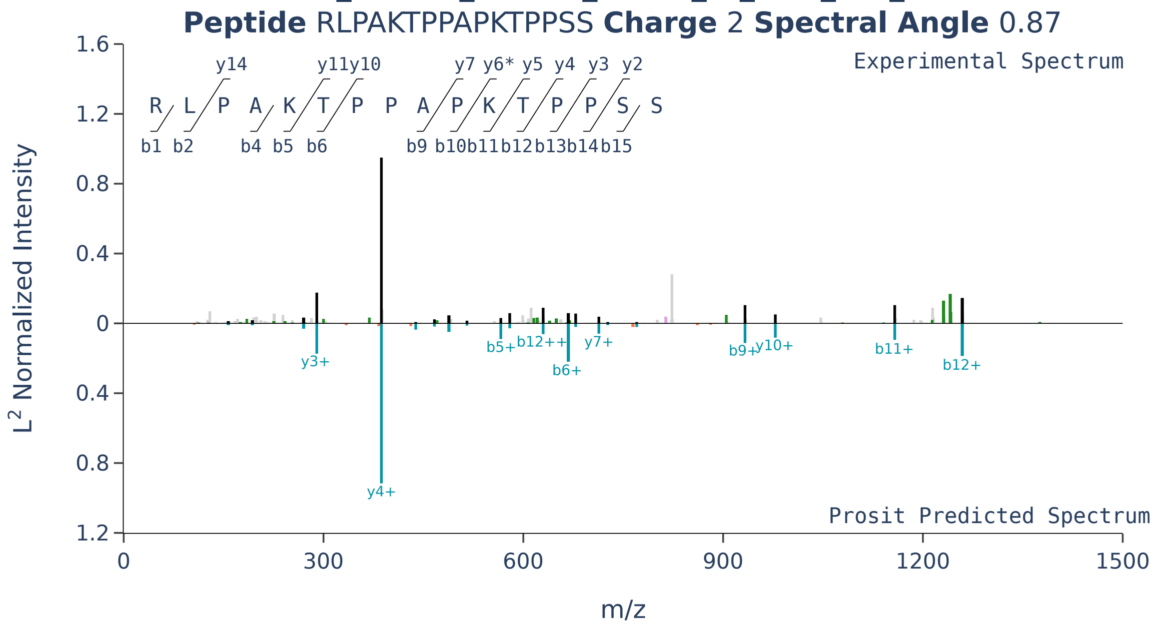


**Source** WSoh_221121_011221_HFGoe_L1_hTau_24h_R1 **Scan** 23761
**Peptide** LADEVSASLAKQGL **Charge** 2 **Spectral Angle** 0.9

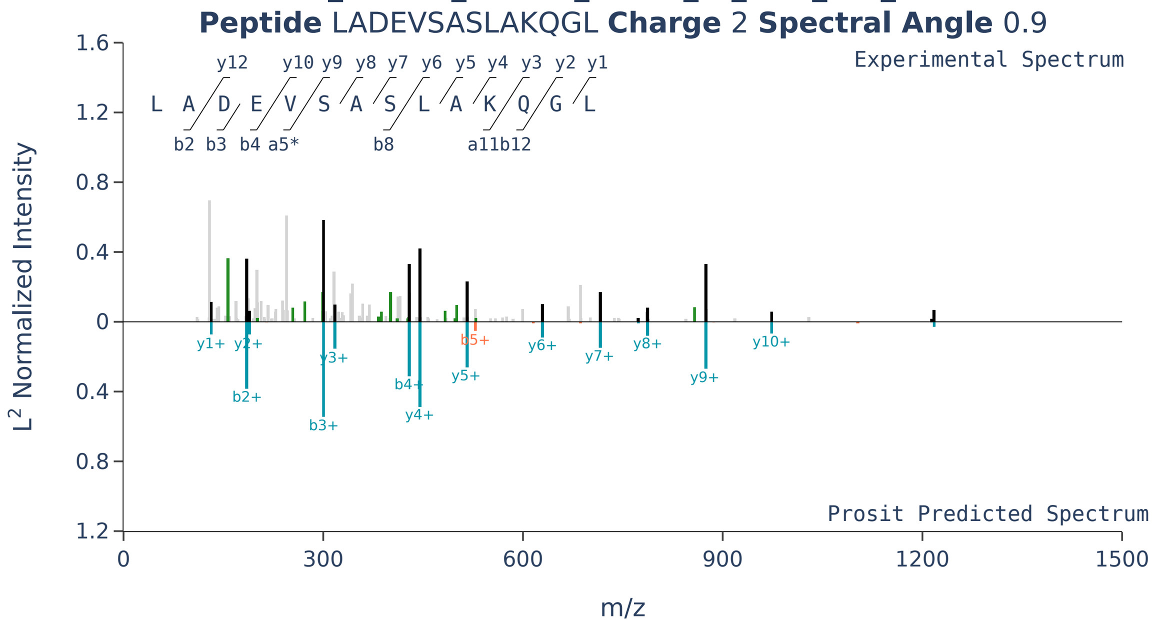


**Source** WSoh_221121_011221_HFGoe_L2_hTau_24h_R1 **Scan** 13779
**Peptide** LKHVPGGGSVQI **Charge** 3 **Spectral Angle** 0.86

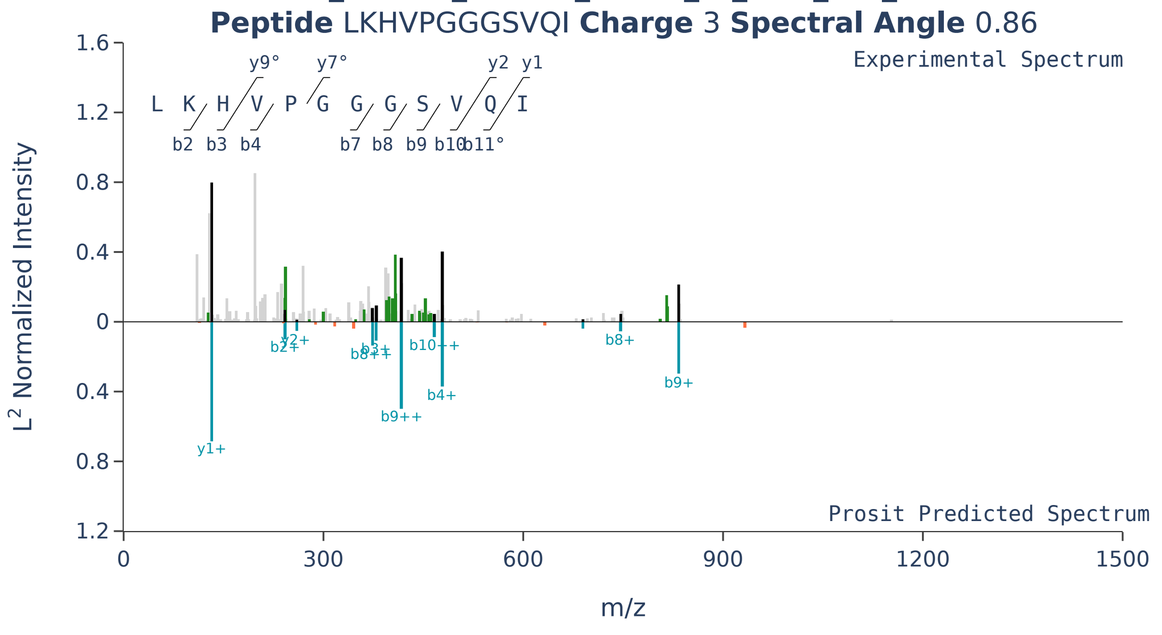


**Source** WSoh_221121_011221_HFGoe_L2_hTau_4h_R2 **Scan** 11146
**Peptide** KSPVVSGDTSPRHLSNV **Charge** 2 **Spectral Angle** 0.88

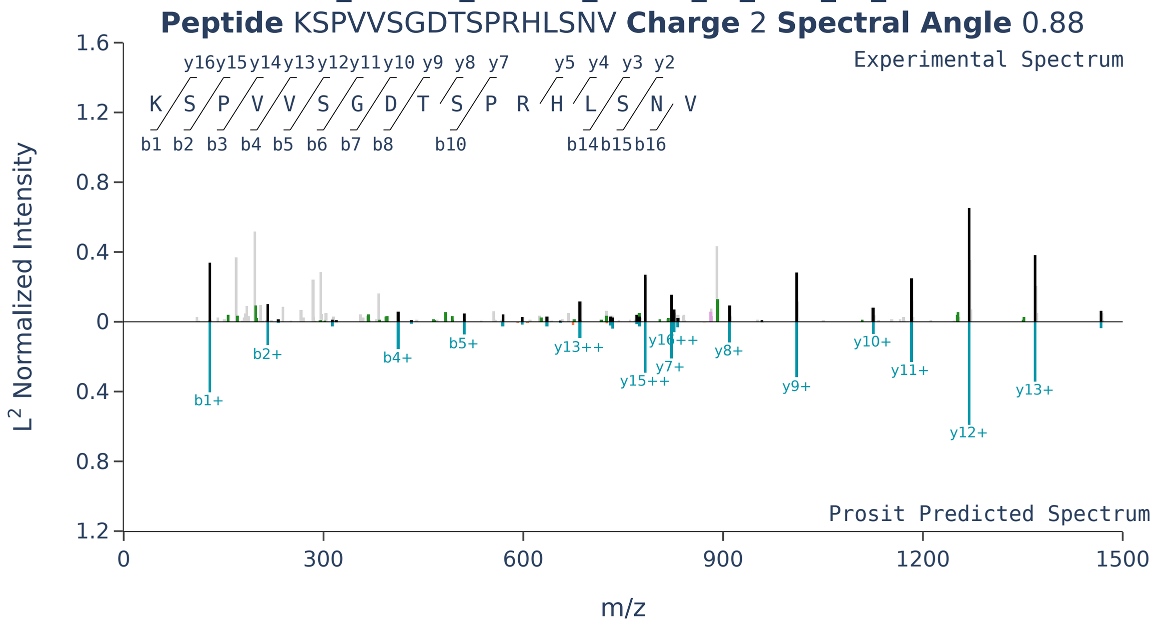


**Source** WSoh_221121_011221_HFGoe_L1_hTau_24h_R2 **Scan** 20749
**Peptide** TAEEAGIGDTPSLEDEAAGHVTQARMVSK **Charge** 4 **Spectral Angle** 0.83

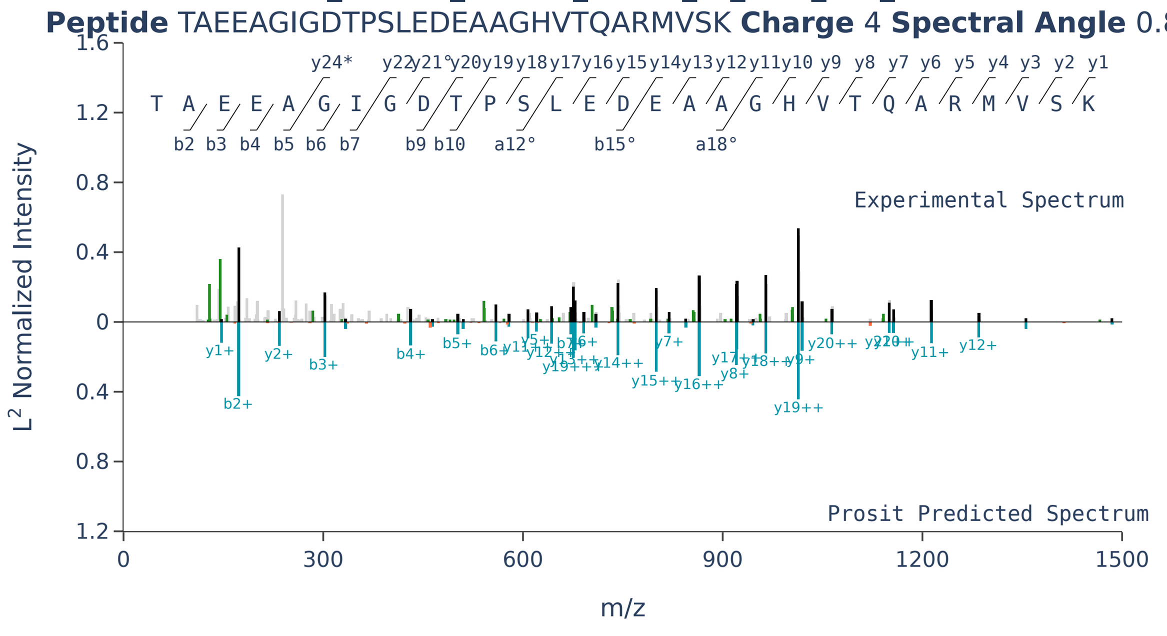


**Source** WSoh_221121_011221_HFGoe_L2_hTau_24h_R2 **Scan** 21121
**Peptide** PVPMPDLKNVKSKIGSTE **Charge** 3 **Spectral Angle** 0.87

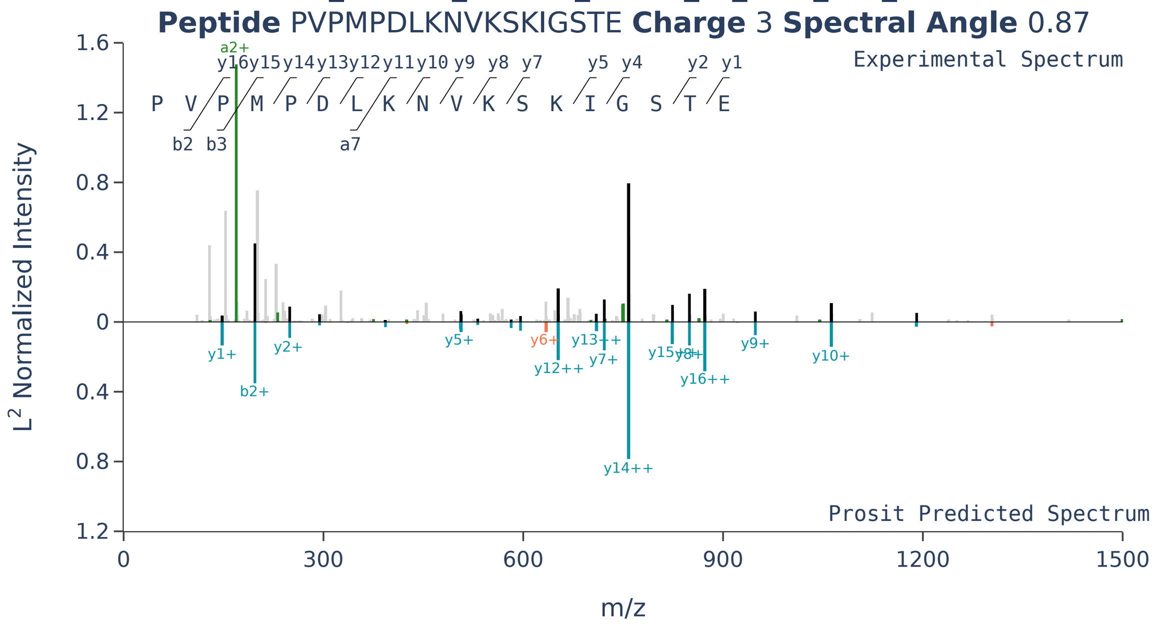


**Source** WSoh_221121_011221_HFGoe_L2_hTau_24h_R2 **Scan** 16793
**Peptide** DNIKHVPGGGSVQL **Charge** 2 **Spectral Angle** 0.81

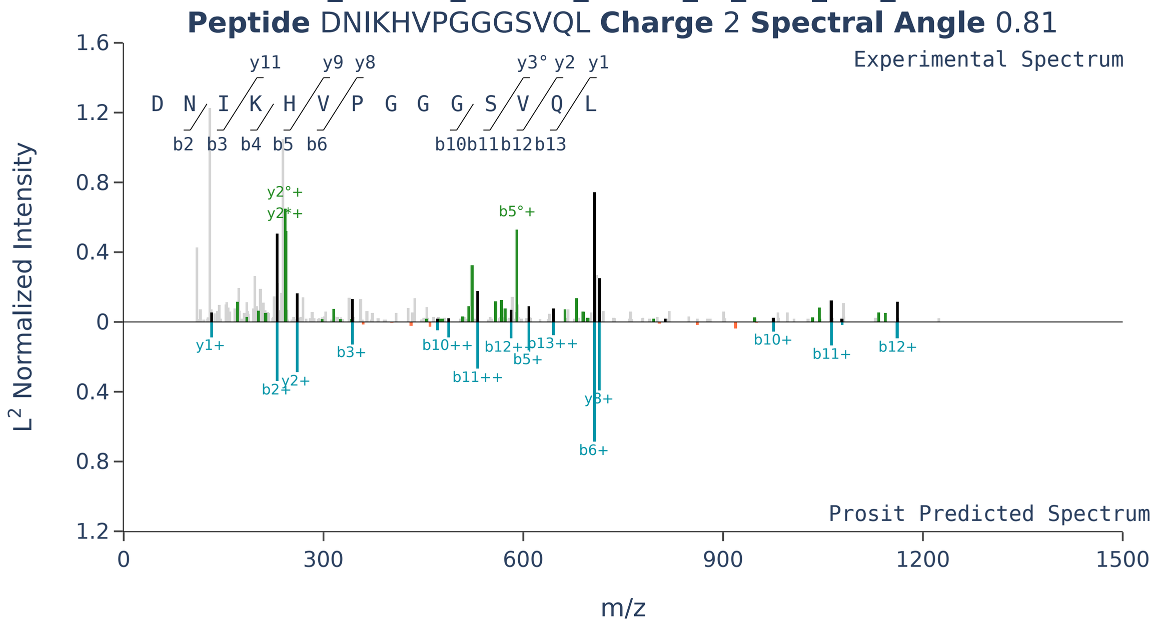

**Source** WSoh_221121_011221_HFGoe_L1_hTau_4h_R2 **Scan** 12798
**Peptide** DEGAPGKQAAAQPHTEIPEGT **Charge** 3 **Spectral Angle** 0.85

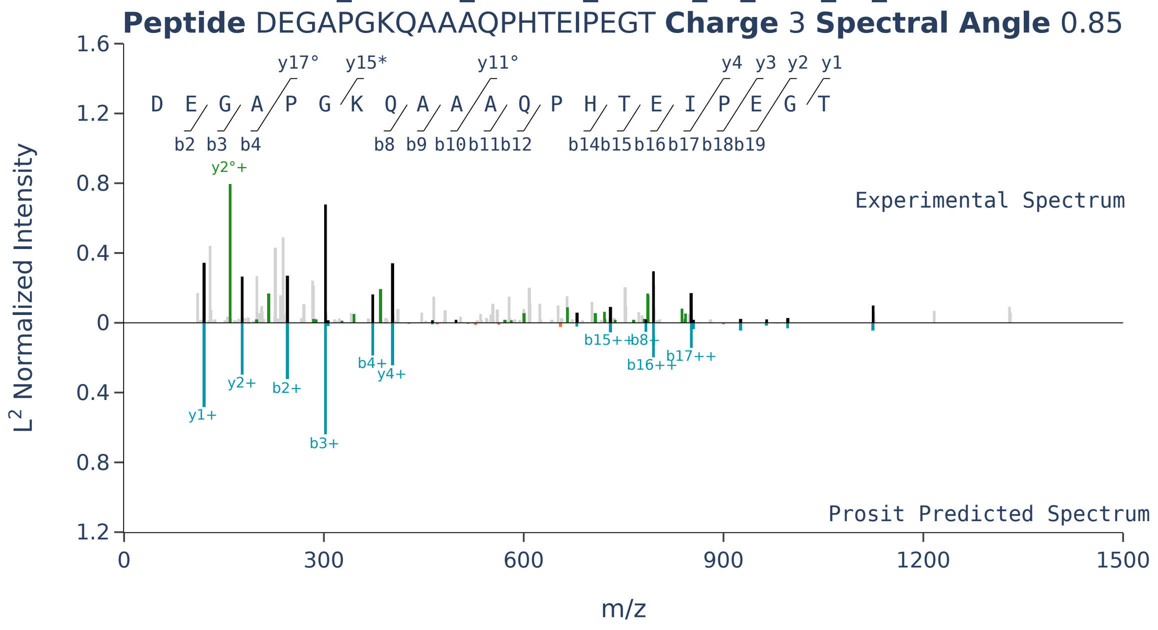


**Source** WSoh_221121_011221_HFGoe_L1_hTau_24h_R1 **Scan** 19833
**Peptide** GIGDTPSLEDE **Charge** 2 **Spectral Angle** 0.88

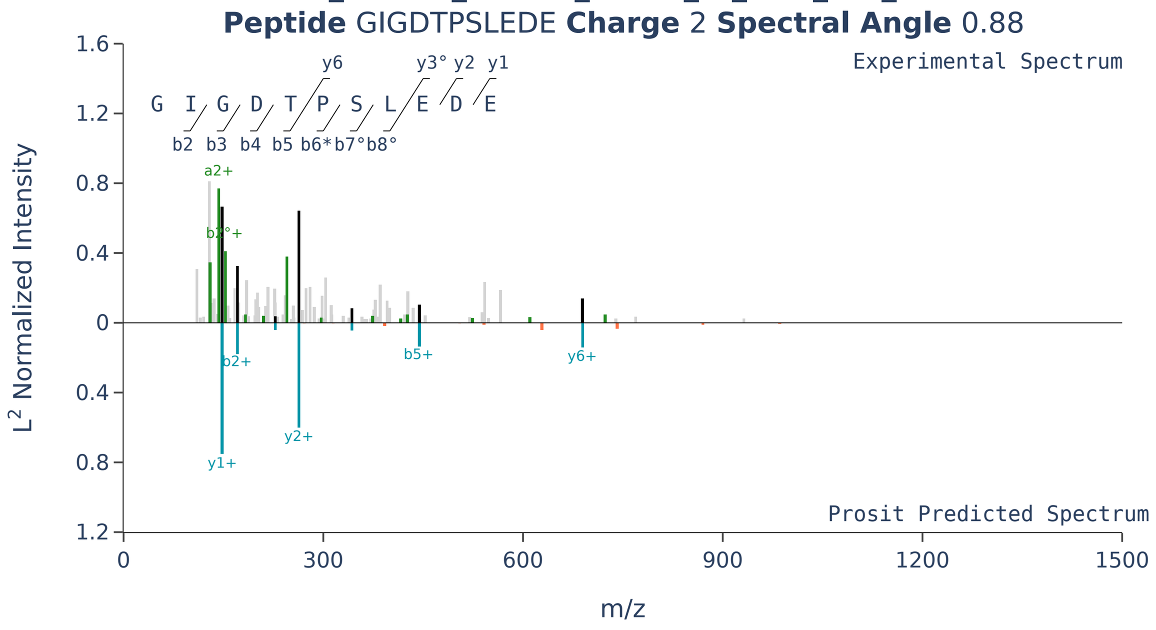


**Source** WSoh_221121_011221_HFGoe_L2_hTau_24h_R1 **Scan** 21886
**Peptide** RLQTAPVPMPDLKNVKSKIGSTE **Charge** 4 **Spectral Angle** 0.89

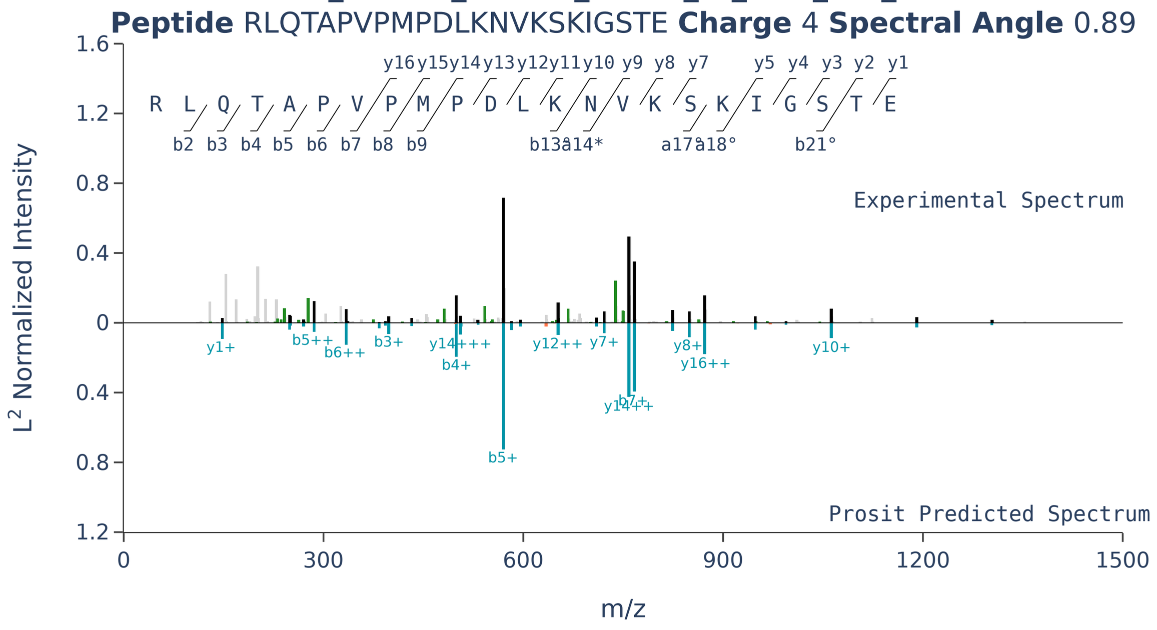


**Source** WSoh_221121_011221_HFGoe_L2_hTau_24h_R1 **Scan** 8328
**Peptide** AKAKTDHGAEIVYKSP **Charge** 2 **Spectral Angle** 0.83

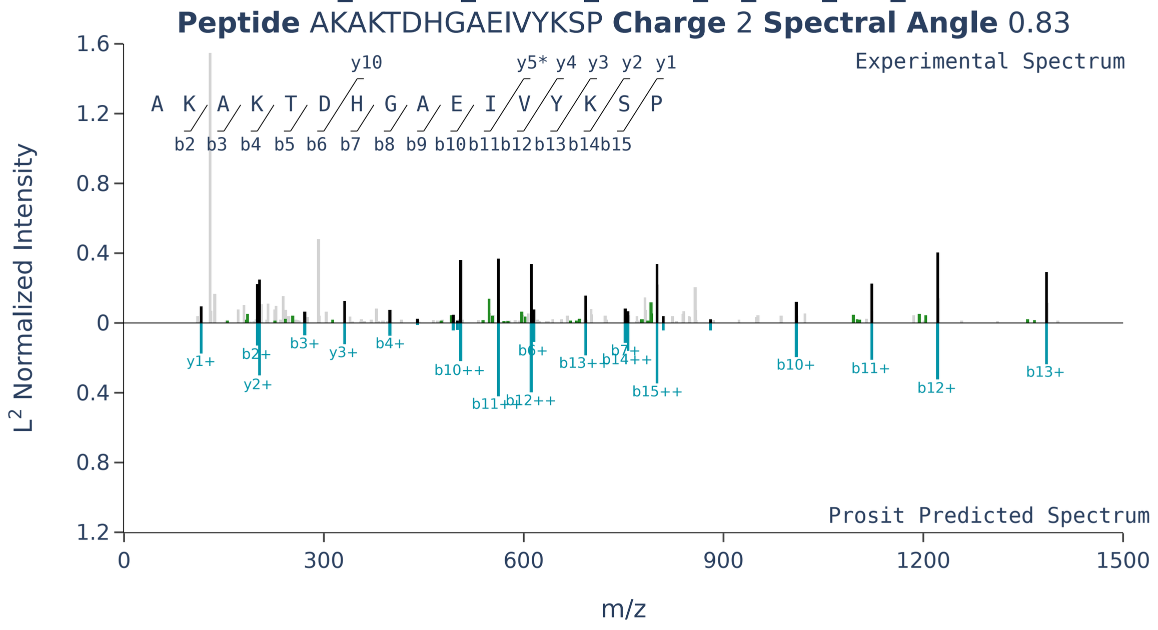


**Source** WSoh_221121_011221_HFGoe_L2_hTau_24h_R2 **Scan** 18287
**Peptide** VYKPVDLSKVT **Charge** 2 **Spectral Angle** 0.82

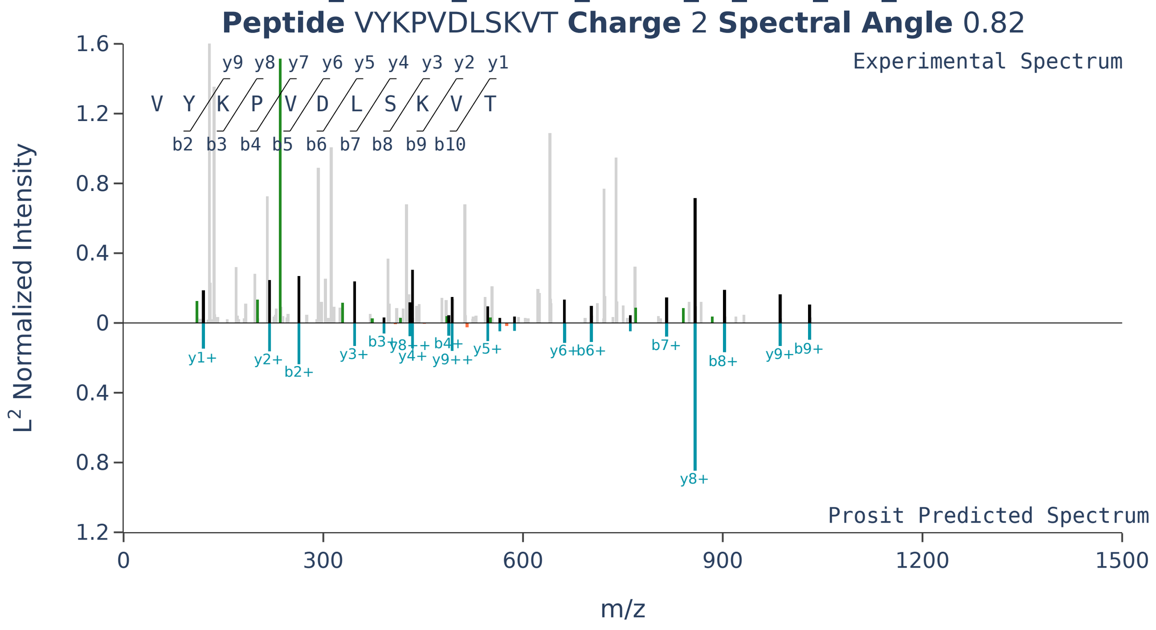


**Source** WSoh_221121_011221_HFGoe_L2_hTau_24h_R1 **Scan** 7472
**Peptide** LGDRKDQGGYTMHQD **Charge** 2 **Spectral Angle** 0.86

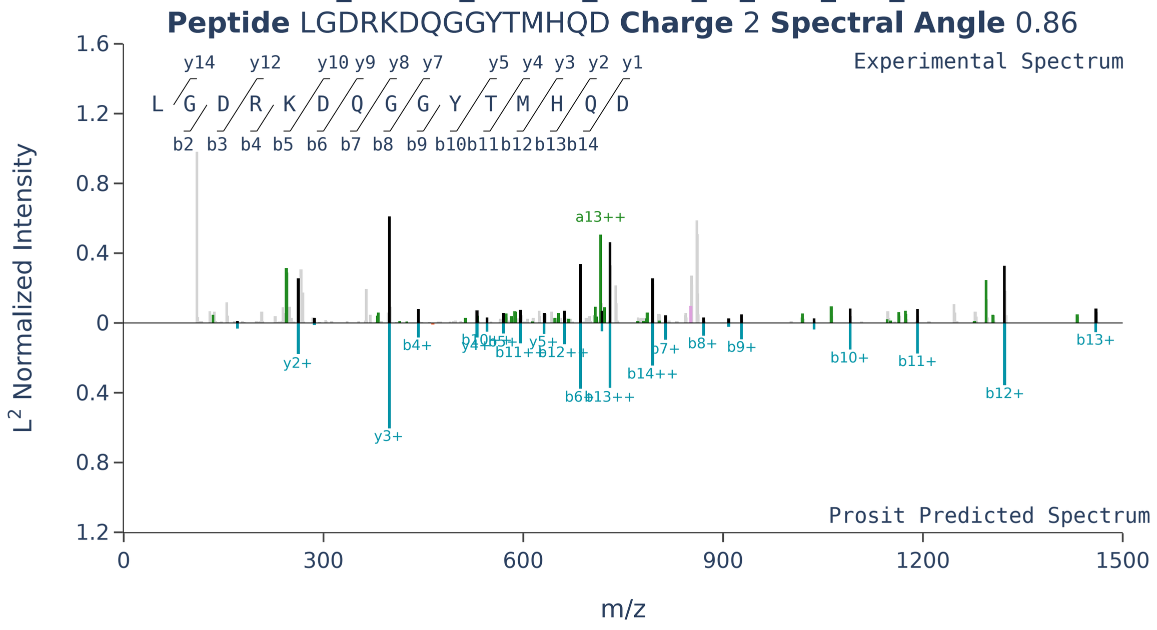


**Source** WSoh_221121_011221_HFGoe_L1_hTau_24h_R1 **Scan** 15031
**Peptide** TPSLPTPPTREPKKVAVVR **Charge** 4 **Spectral Angle** 0.85

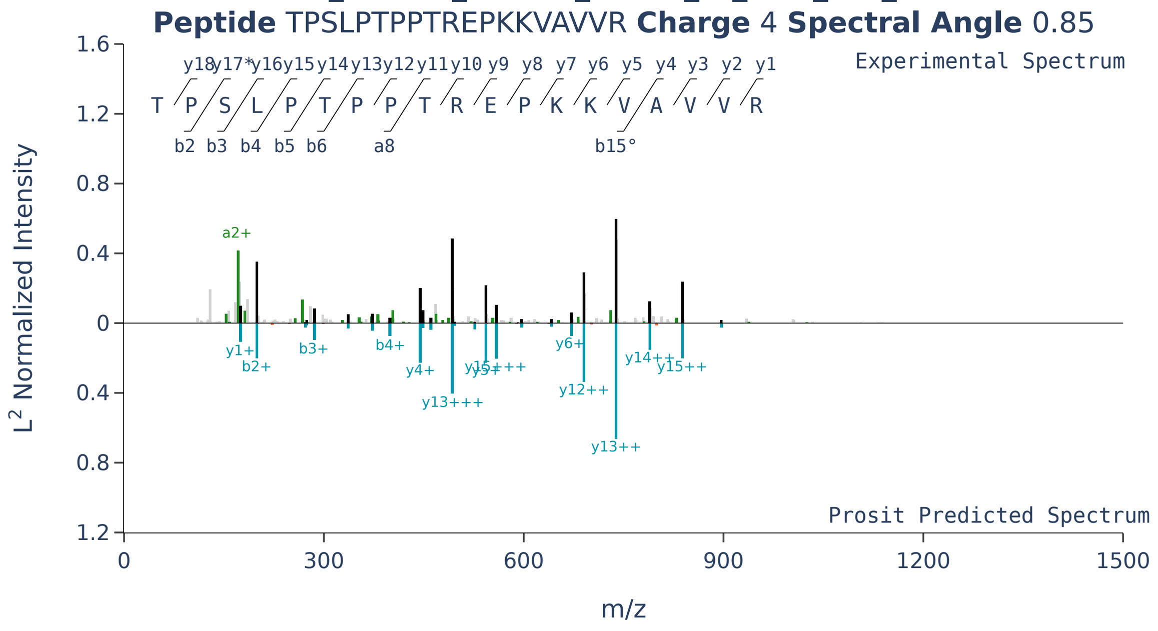


**Source** WSoh_221121_011221_HFGoe_L2_hTau_24h_R2 **Scan** 6764
**Peptide** THVPGGGNKKIETHKL **Charge** 2 **Spectral Angle** 0.92

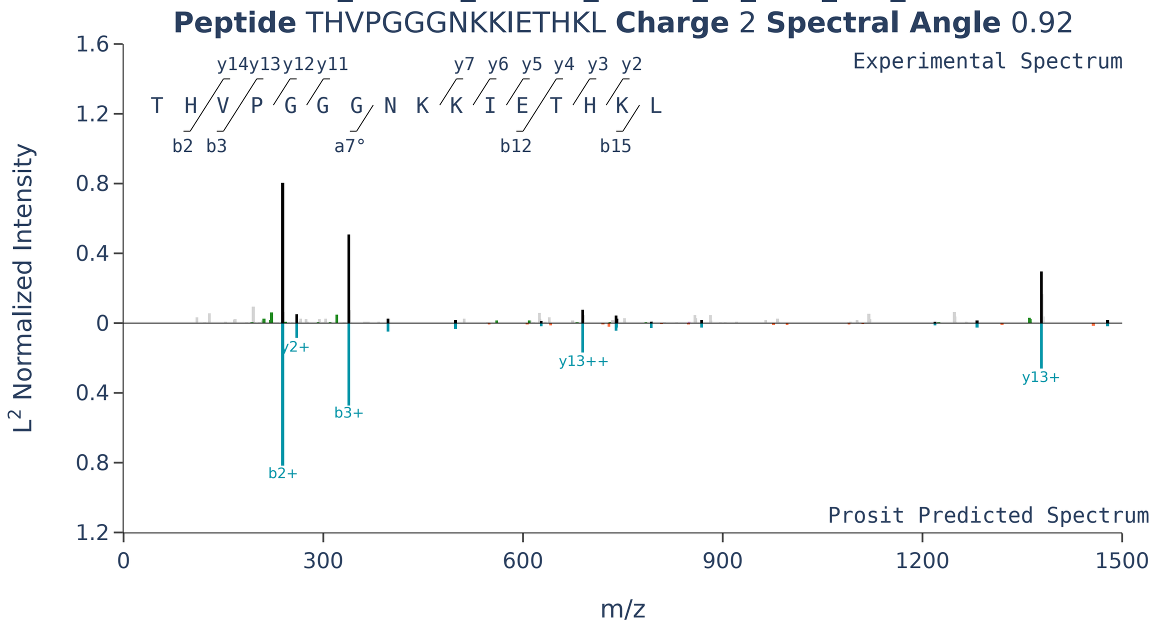


**Source** WSoh_221121_011221_HFGoe_L1_hTau_24h_R2 **Scan** 14739
**Peptide** DHAGTYGLGDRKDQGGYTM **Charge** 3 **Spectral Angle** 0.94

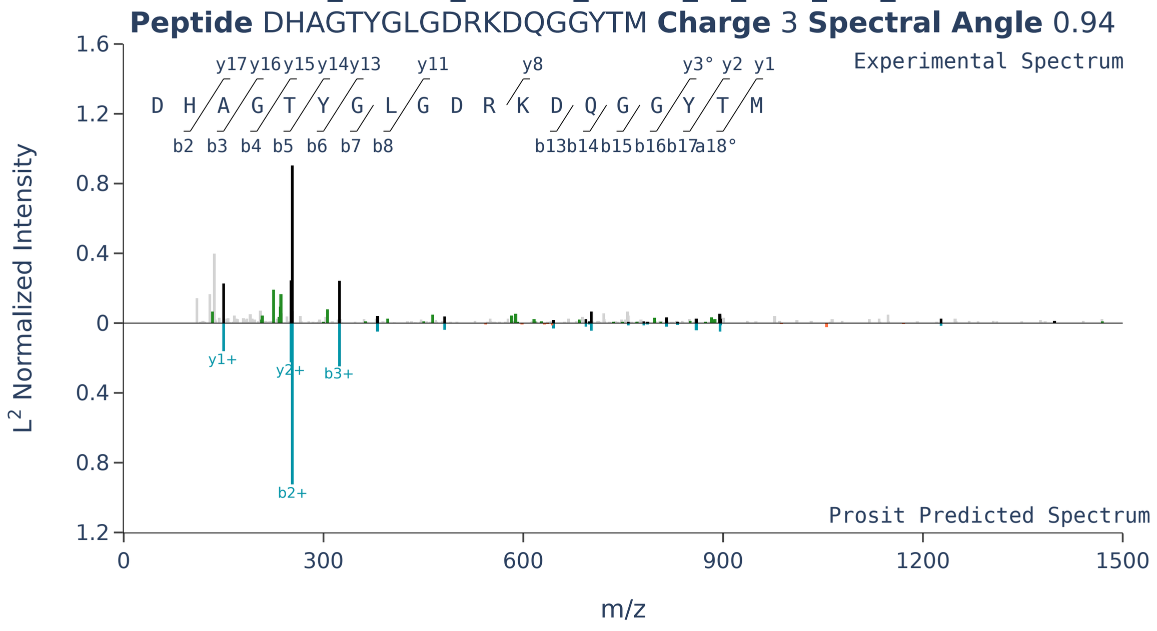


**Source** WSoh_221121_011221_HFGoe_L1_hTau_4h_R2 **Scan** 17377
**Peptide** AEIVYKSPVVSGDTSPRHLSNVS **Charge** 3 **Spectral Angle** 0.88

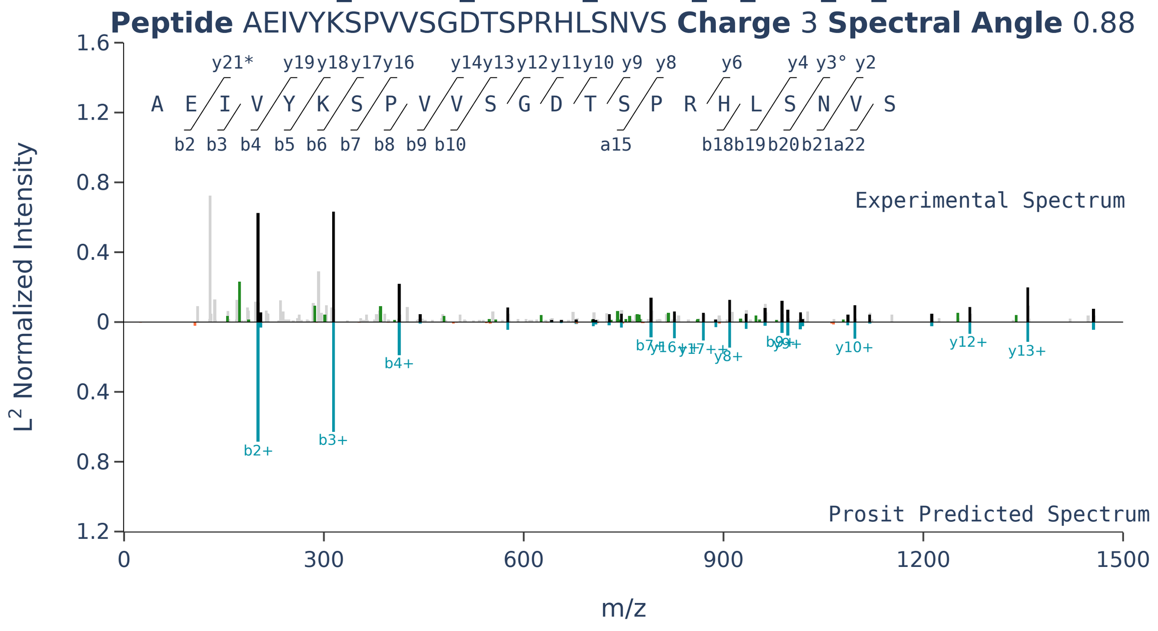


**Source** WSoh_221121_011221_HFGoe_L2_hTau_24h_R2 **Scan** 9737
**Peptide** THVPGGGNKKIETHKLTFRENA **Charge** 3 **Spectral Angle** 0.92

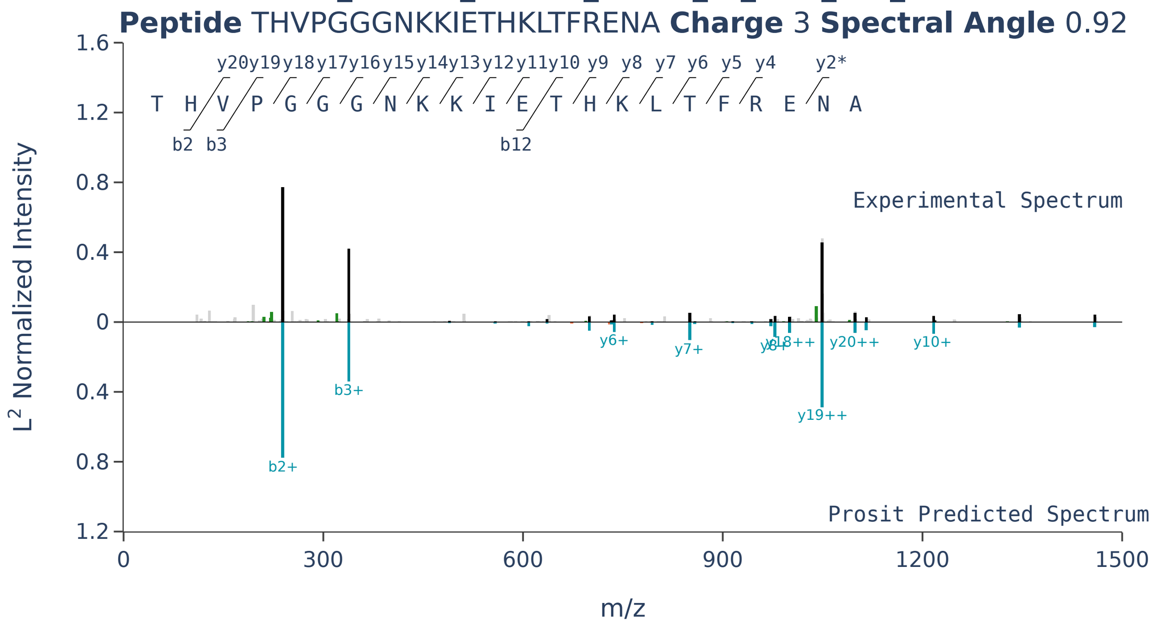


**Source** WSoh_221121_011221_HFGoe_L1_hTau_24h_R1 **Scan** 15873
**Peptide** SPLQTPTEDGSEEPGSETSDAKSTPTA **Charge** 2 **Spectral Angle** 0.83

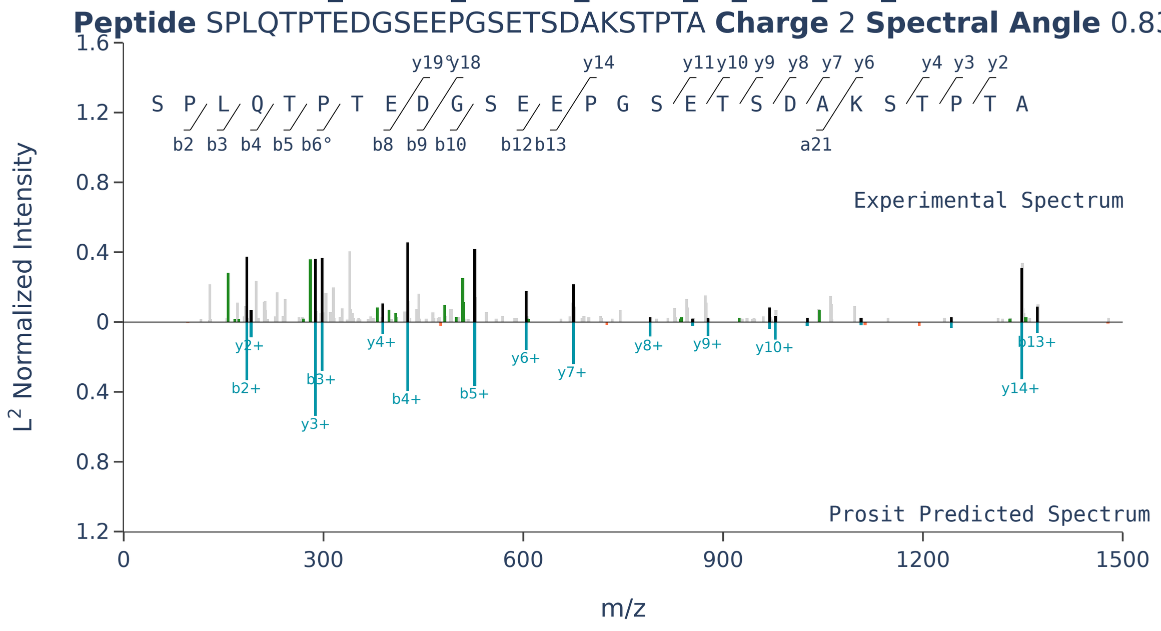


**Source** WSoh_221121_011221_HFGoe_L2_hTau_4h_R1 **Scan** 18507
**Peptide** DTSPRHLSNVSSTGSIDMVD **Charge** 3 **Spectral Angle** 0.83

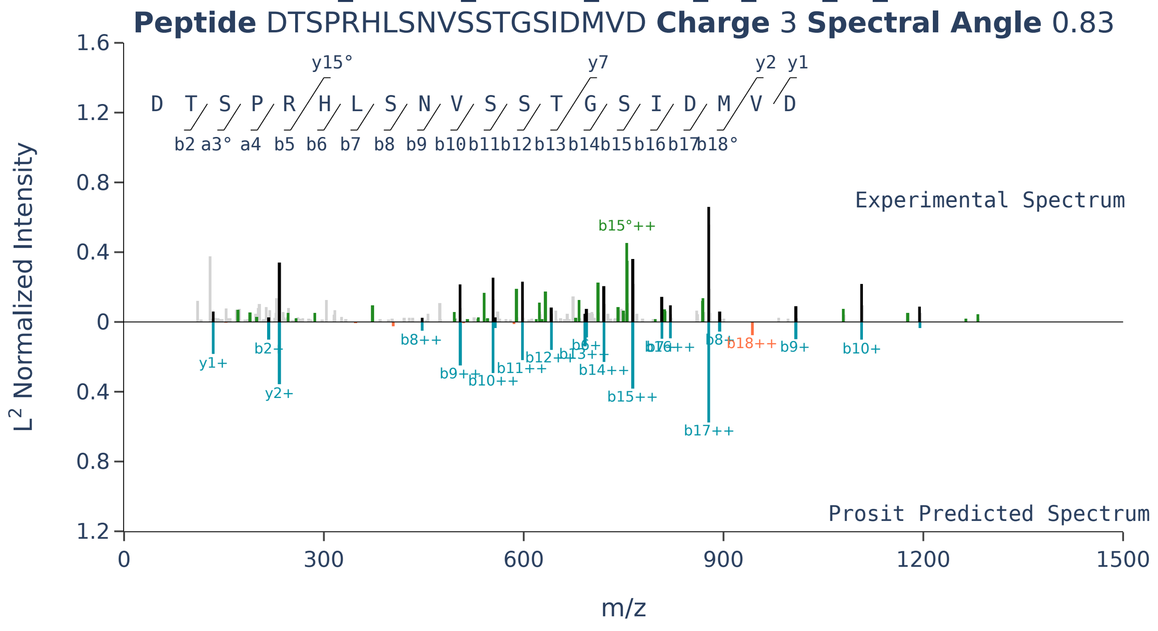


**Source** WSoh_221121_011221_HFGoe_L1_hTau_24h_R1 **Scan** 24237
**Peptide** LQTAPVPMPDLKNVKSKL **Charge** 3 **Spectral Angle** 0.83

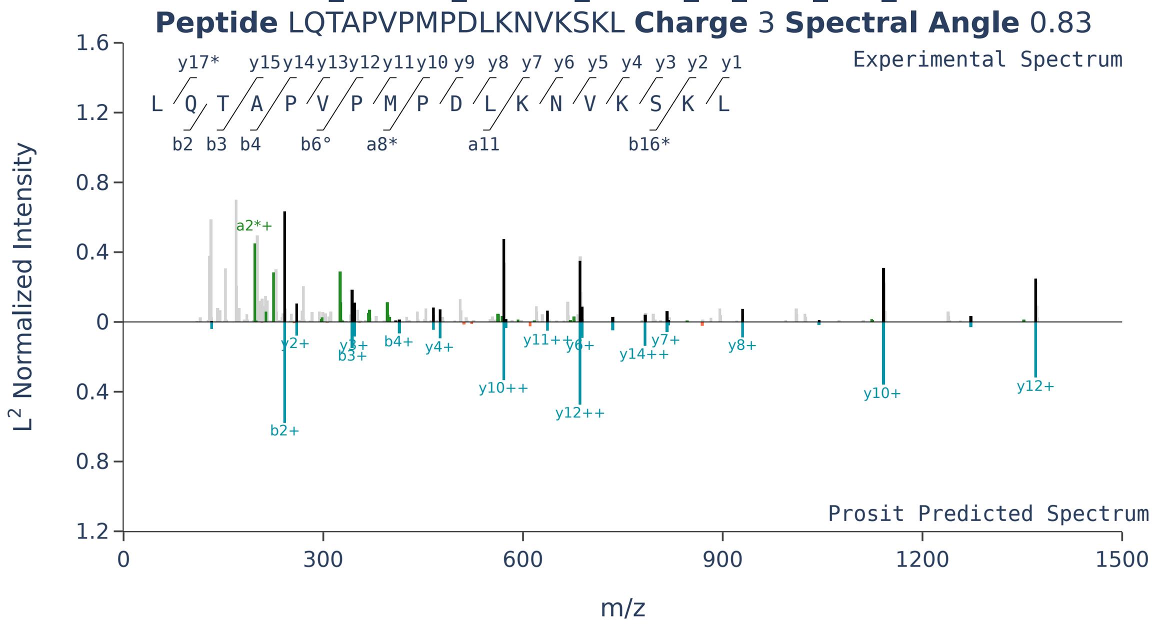


**Source** WSoh_221121_011221_HFGoe_L2_hTau_2h_R2 **Scan** 5662
**Peptide** GNIHHKPGGGQVEVKS **Charge** 2 **Spectral Angle** 0.84

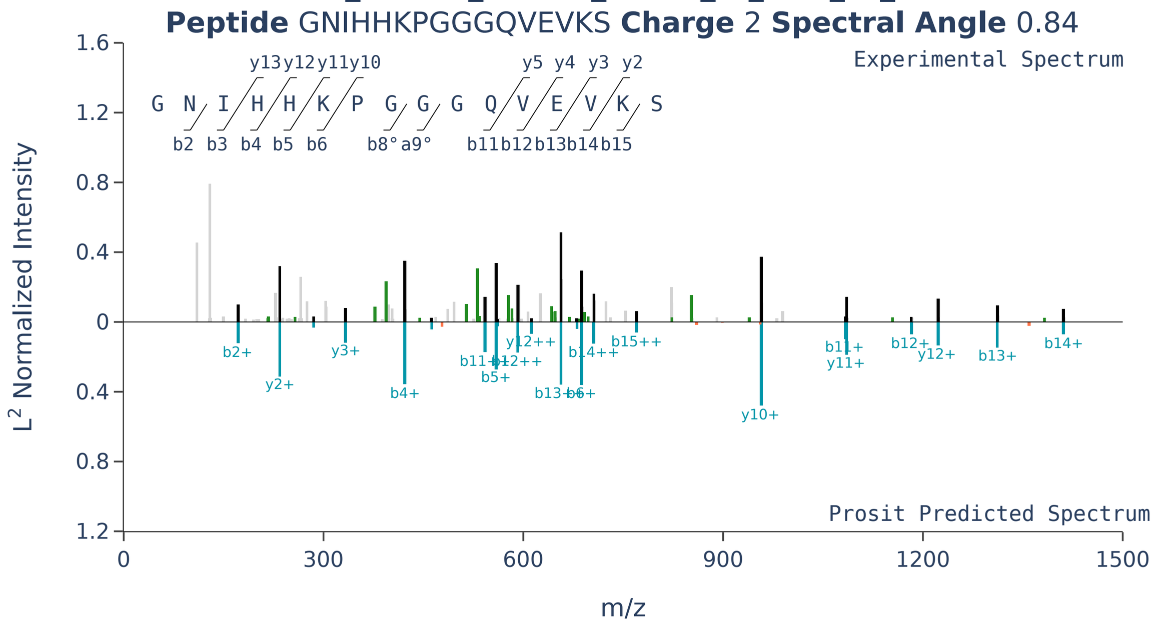

**Source** WSoh_221121_011221_HFGoe_L1_hTau_4h_R2 **Scan** 7327
**Peptide** APPGQKGQANATRIPAKT **Charge** 2 **Spectral Angle** 0.85

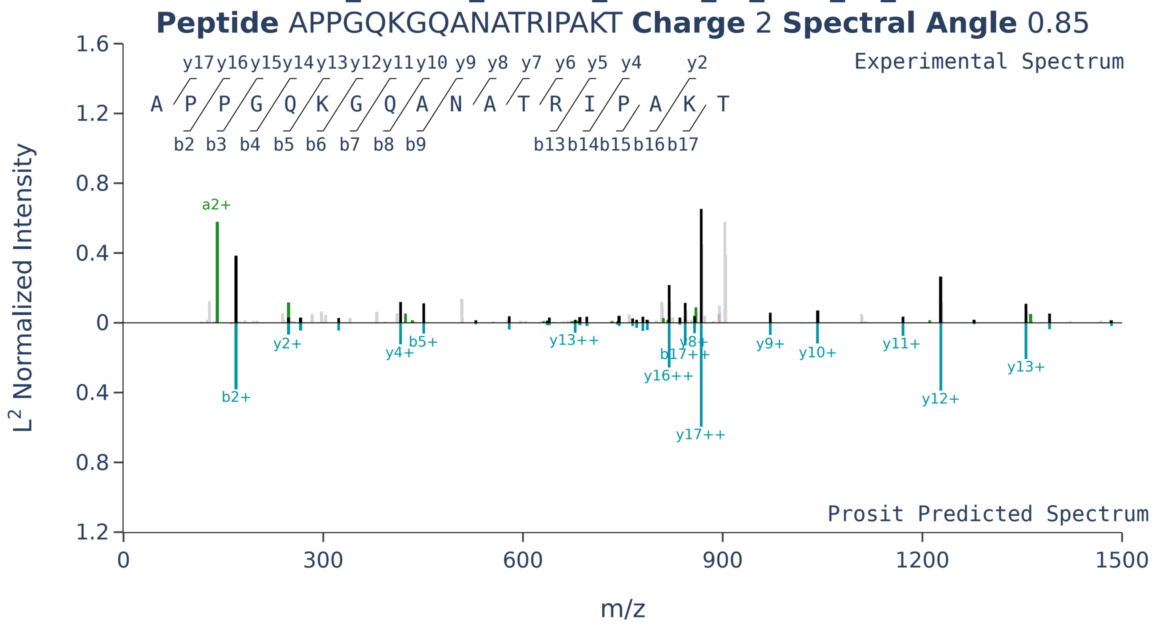


**Source** WSoh_221121_011221_HFGoe_L2_hTau_24h_R2 **Scan** 7980
**Peptide** EGDTDAGLKE **Charge** 2 **Spectral Angle** 0.87

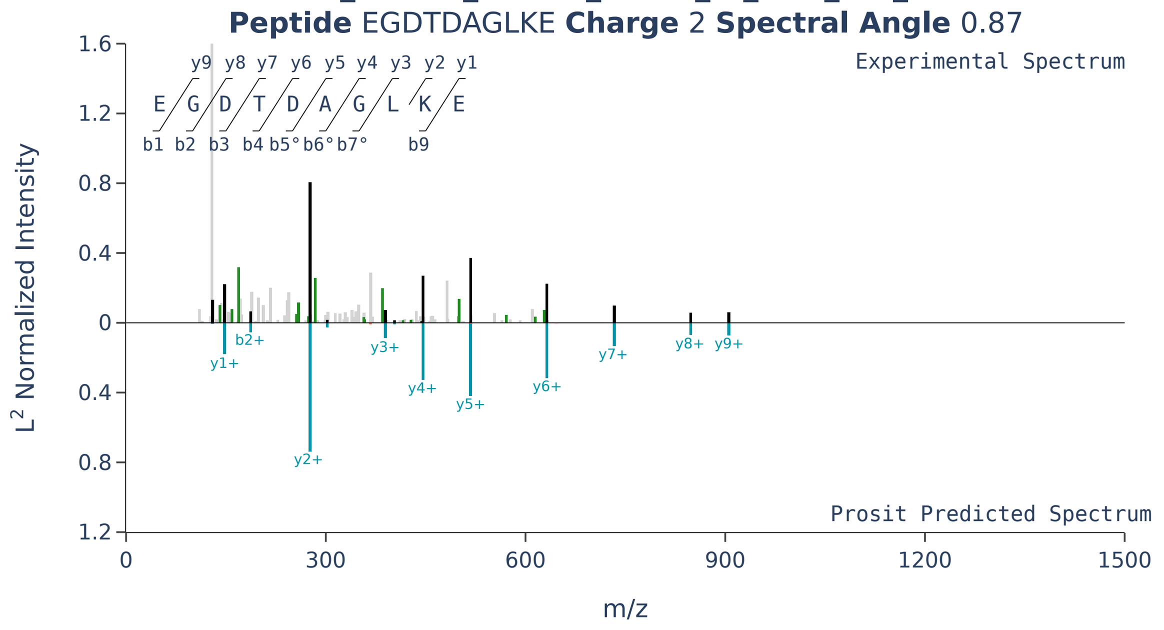


**Source** WSoh_221121_011221_HFGoe_L1_hTau_4h_R1 **Scan** 6440
**Peptide** RENAKAKTDHGAEIV **Charge** 3 **Spectral Angle** 0.82

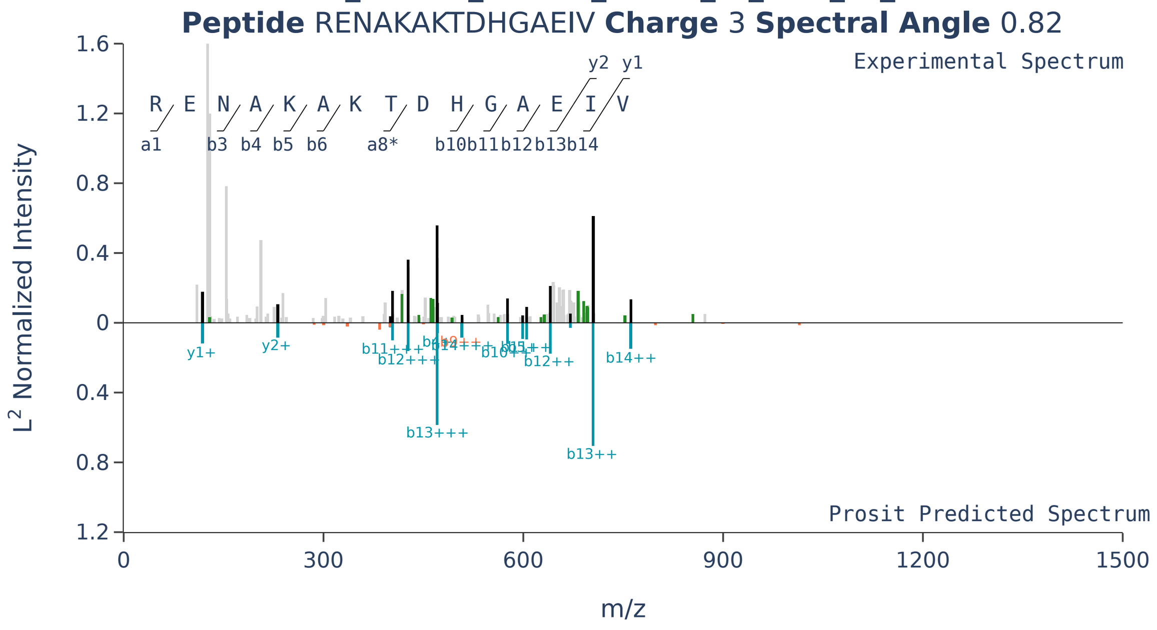


**Source** WSoh_221121_011221_HFGoe_L2_hTau_24h_R2 **Scan** 5997
**Peptide** GSKDNIKHVPGGGS **Charge** 2 **Spectral Angle** 0.93

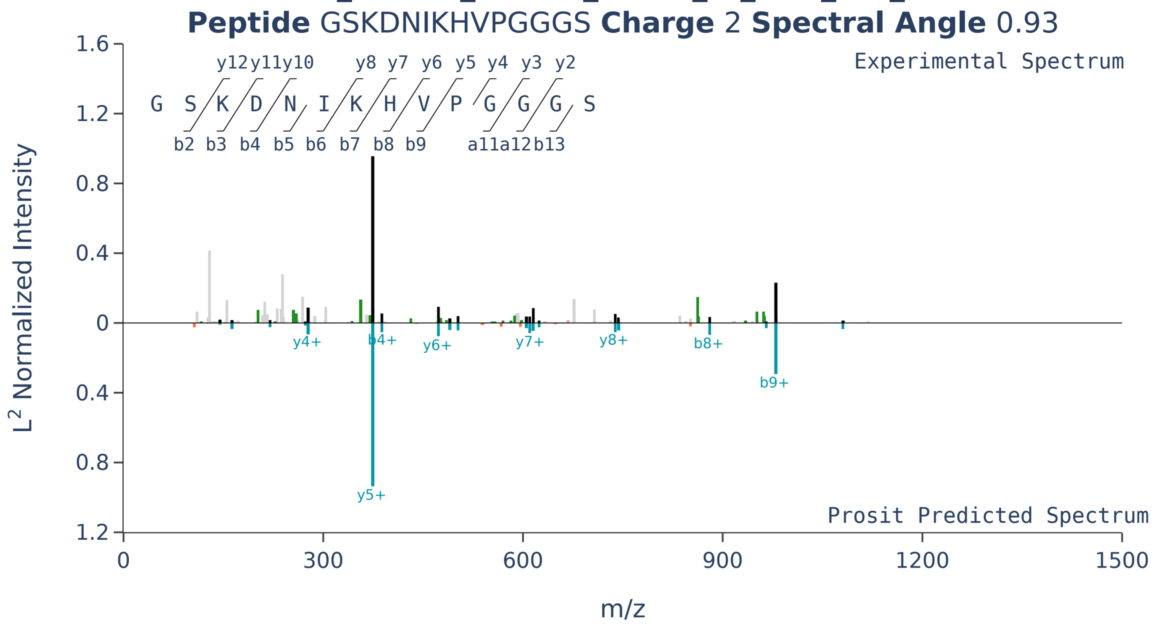


**Source** WSoh_221121_011221_HFGoe_L1_hTau_2h_R1 **Scan** 12877
**Peptide** CGSKDNIKHVPGGGSVQI **Charge** 3 **Spectral Angle** 0.78

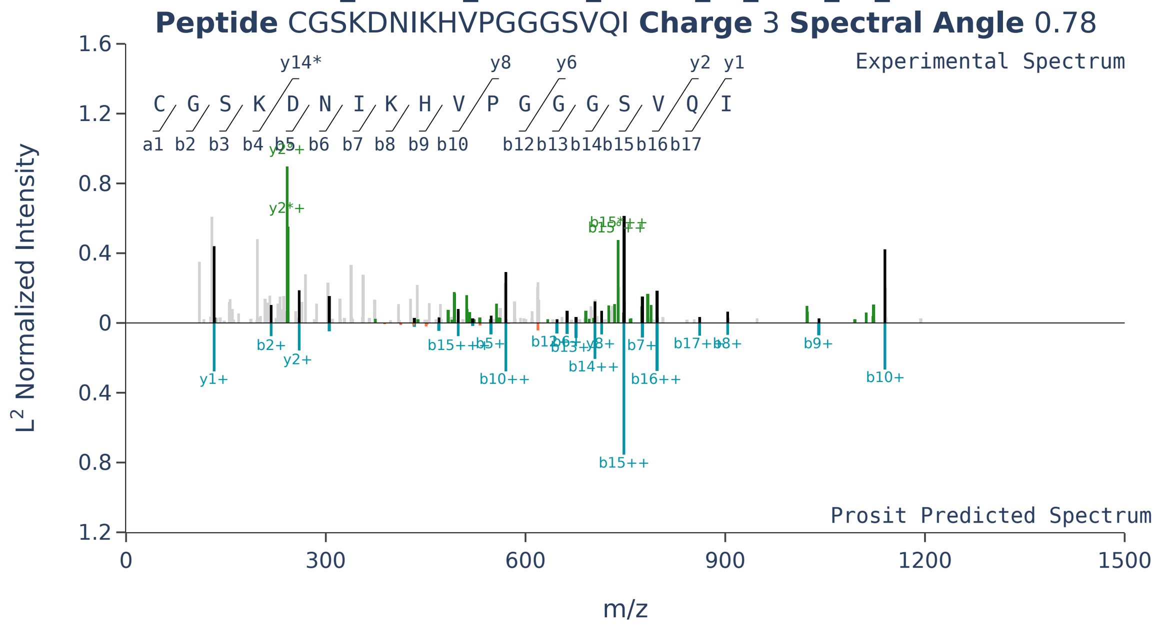


**Source** WSoh_221121_011221_HFGoe_L1_hTau_24h_R1 **Scan** 22989
**Peptide** SDAKSTPTAEDVTAPLV **Charge** 2 **Spectral Angle** 0.92

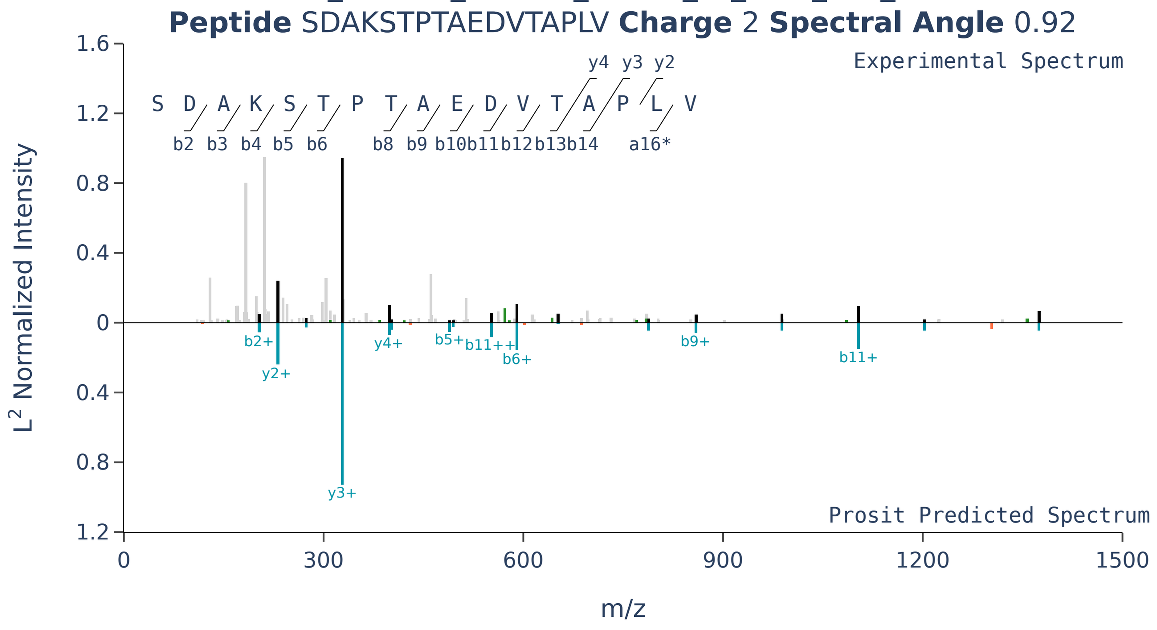


**Source** WSoh_221121_011221_HFGoe_L1_hTau_24h_R1 **Scan** 18528
**Peptide** LDLSNVQS **Charge** 1 **Spectral Angle** 0.8

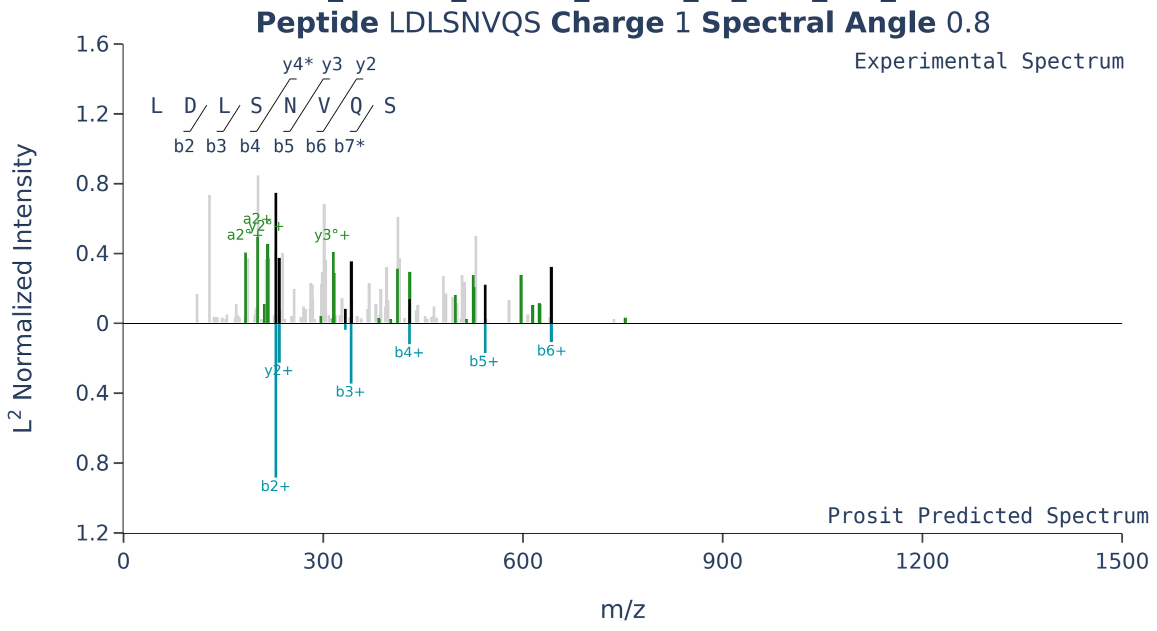


**Source** WSoh_221121_011221_HFGoe_L1_hTau_24h_R1 **Scan** 19917
**Peptide** AGIGDTPSLEDEAAGHVTQARMVSK **Charge** 4 **Spectral Angle** 0.85

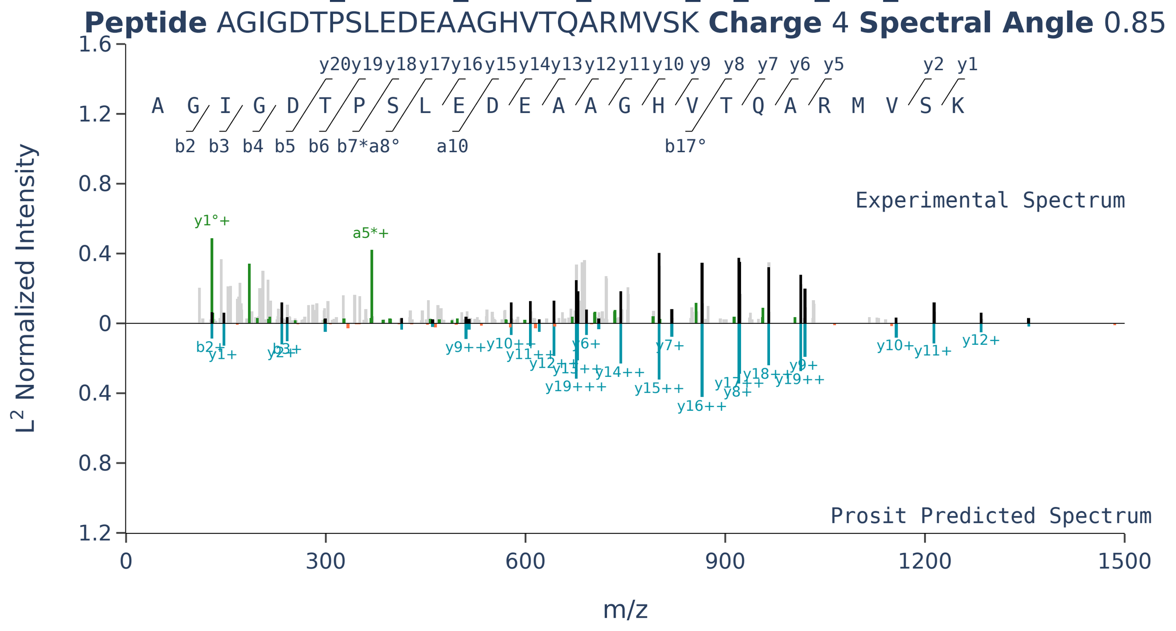


**Source** WSoh_221121_011221_HFGoe_L1_hTau_4h_R1 **Scan** 13550
**Peptide** KLDFKDRVQSKL **Charge** 4 **Spectral Angle** 0.91

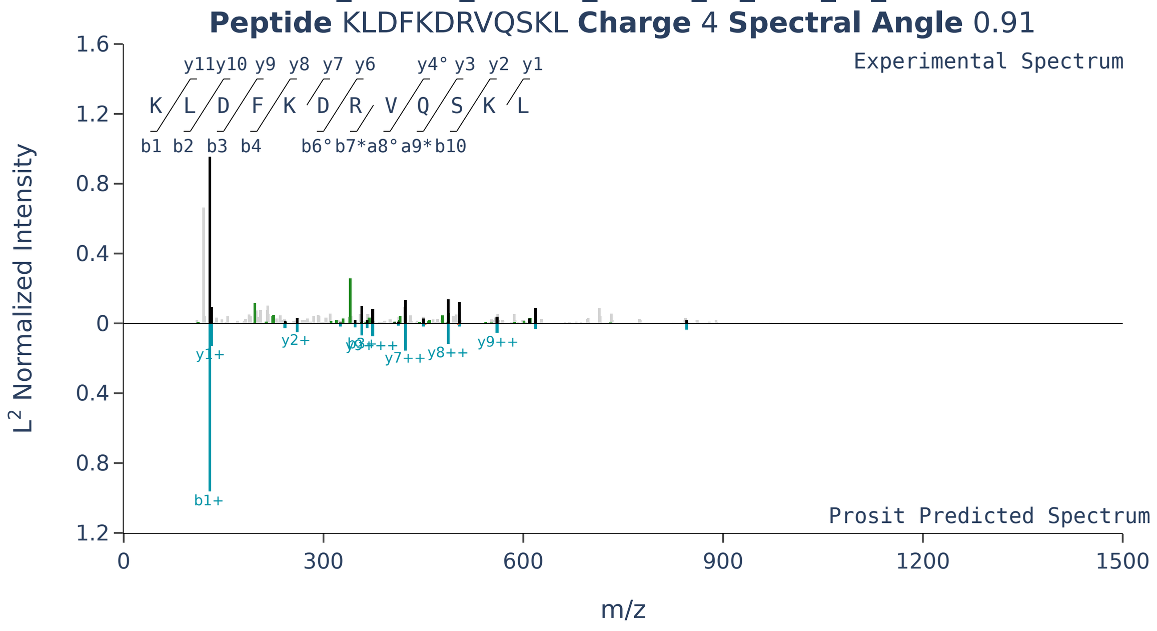


**Source** WSoh_221121_011221_HFGoe_L1_hTau_24h_R1 **Scan** 8813
**Peptide** SEKLDFKDR **Charge** 3 **Spectral Angle** 0.79

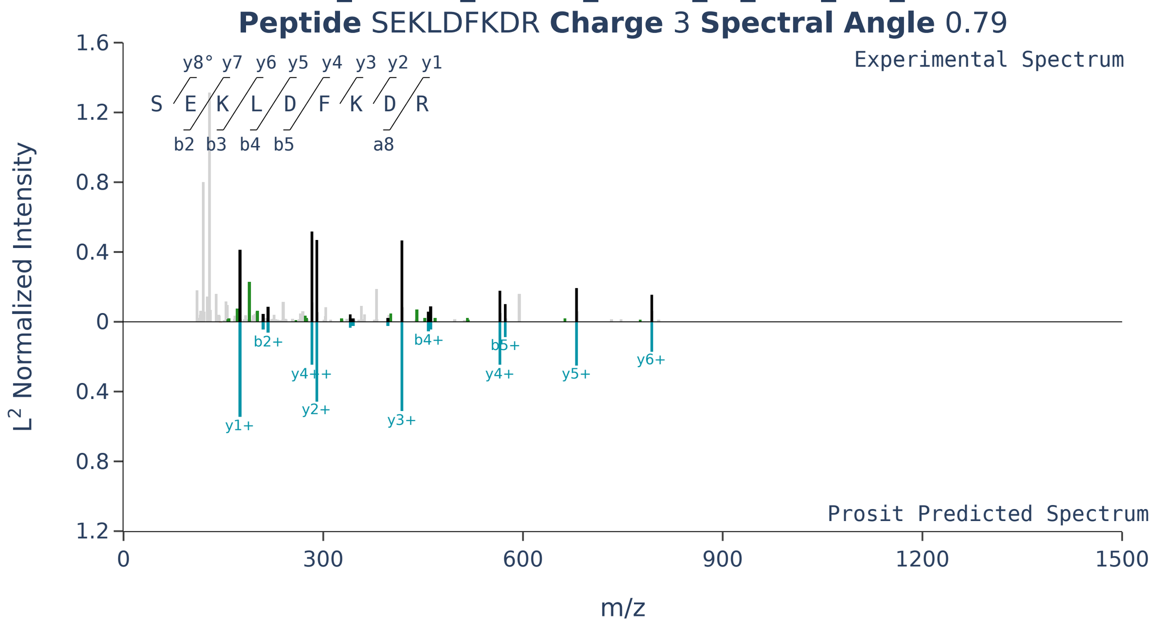


**Source** WSoh_221121_011221_HFGoe_L1_hTau_24h_R2 **Scan** 6302
**Peptide** SPSSAKSRLQT **Charge** 2 **Spectral Angle** 0.85

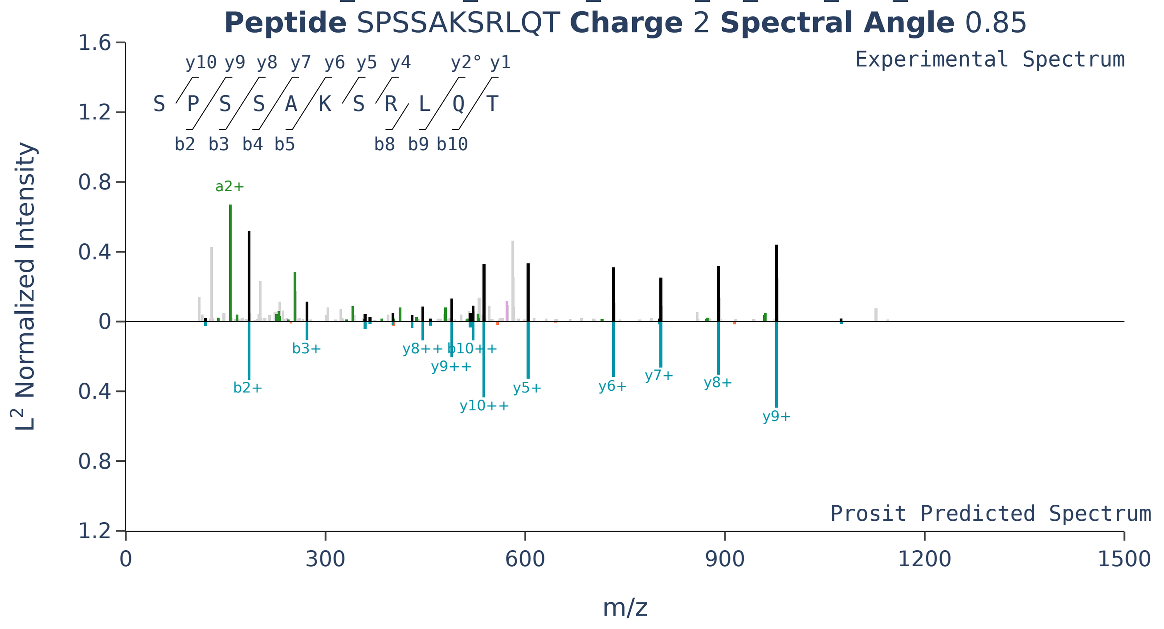


**Source** WSoh_221121_011221_HFGoe_L2_hTau_2h_R1 **Scan** 8451
**Peptide** AQPHTEI **Charge** 1 **Spectral Angle** 0.88

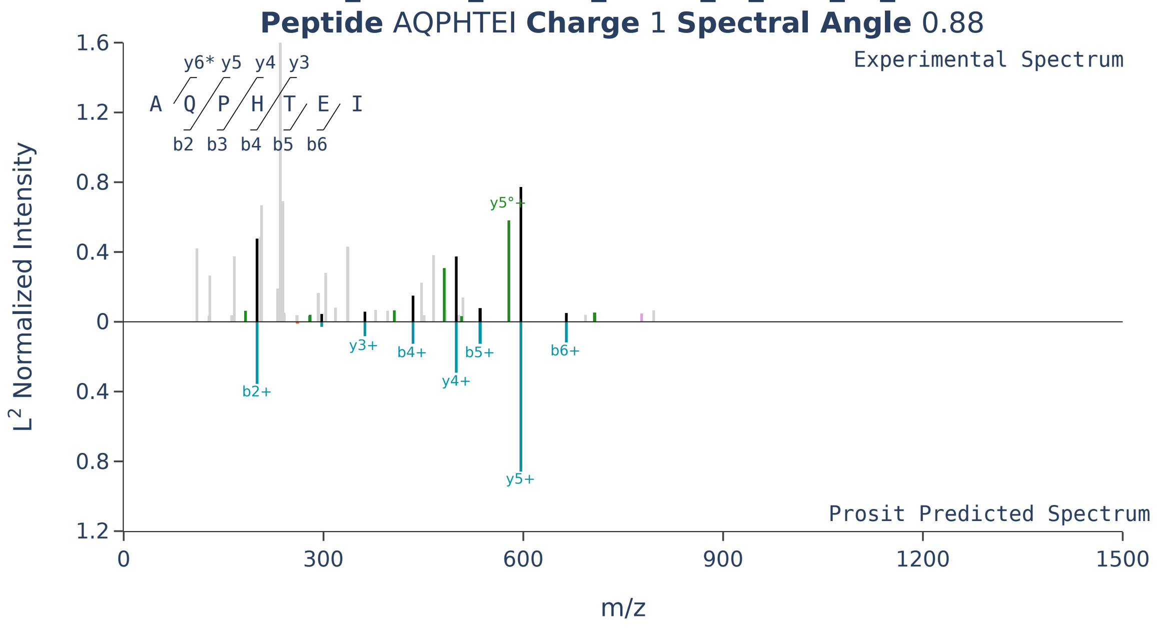


**Source** WSoh_221121_011221_HFGoe_L2_hTau_24h_R1 **Scan** 15822
**Peptide** SPLQTPTEDGSEEPGSETSDAKSTPTAE **Charge** 2 **Spectral Angle** 0.87

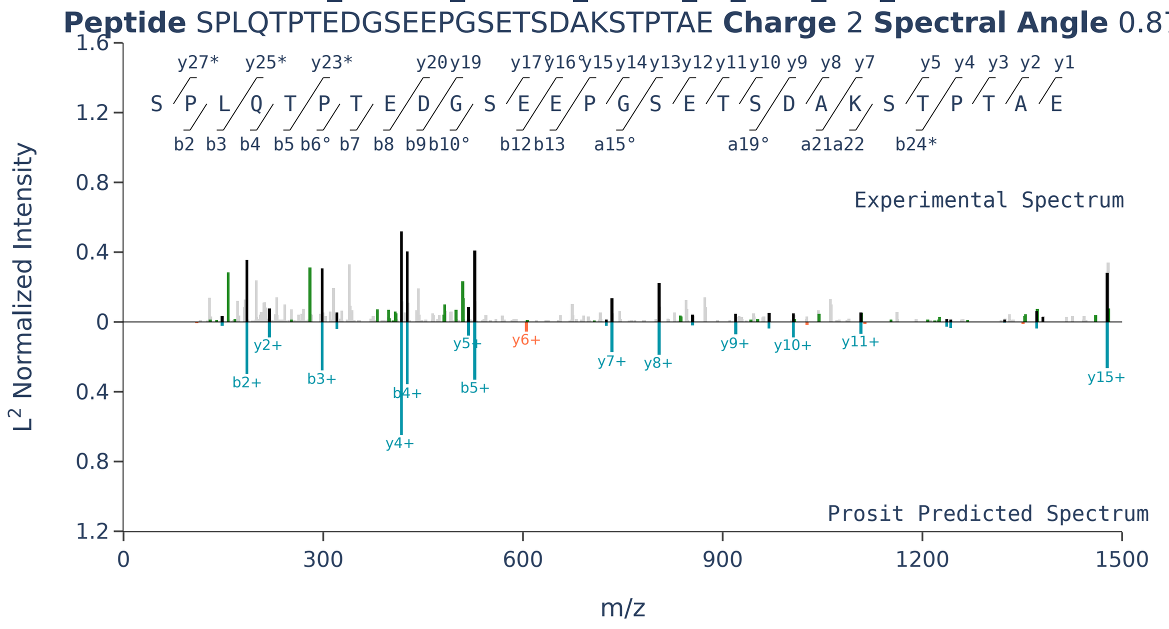


**Source** WSoh_221121_011221_HFGoe_L1_hTau_4h_R1 **Scan** 6265
**Peptide** LTHVPGGGNKKIETH **Charge** 3 **Spectral Angle** 0.89

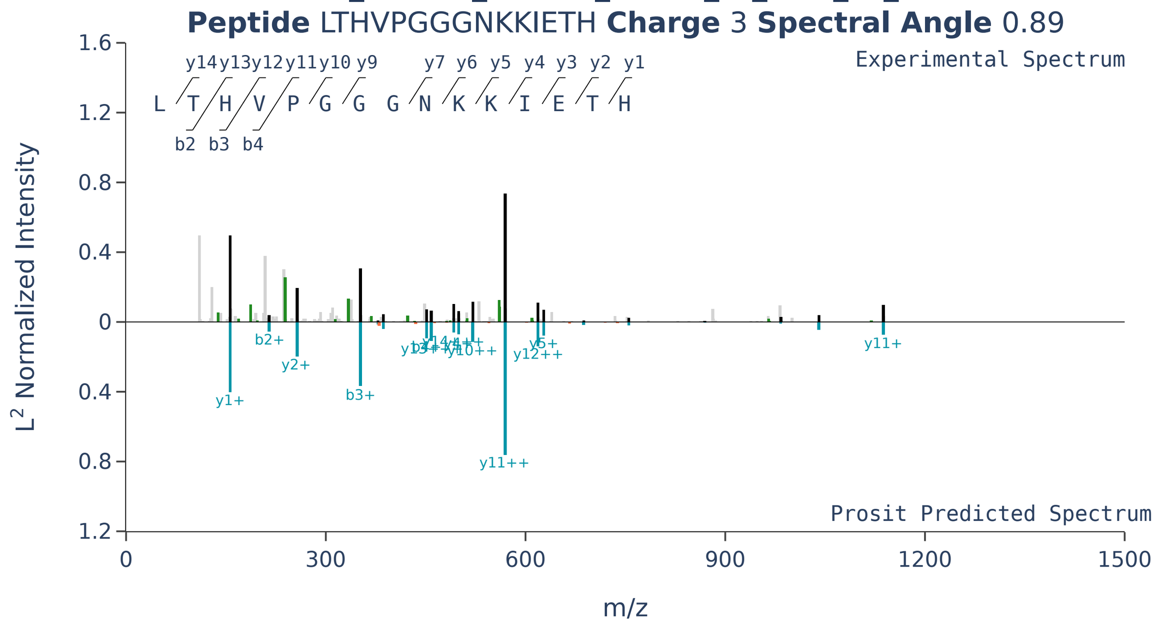


**Source** WSoh_221121_011221_HFGoe_L1_hTau_24h_R2 **Scan** 18215
**Peptide** LVYKSPVV **Charge** 2 **Spectral Angle** 0.82

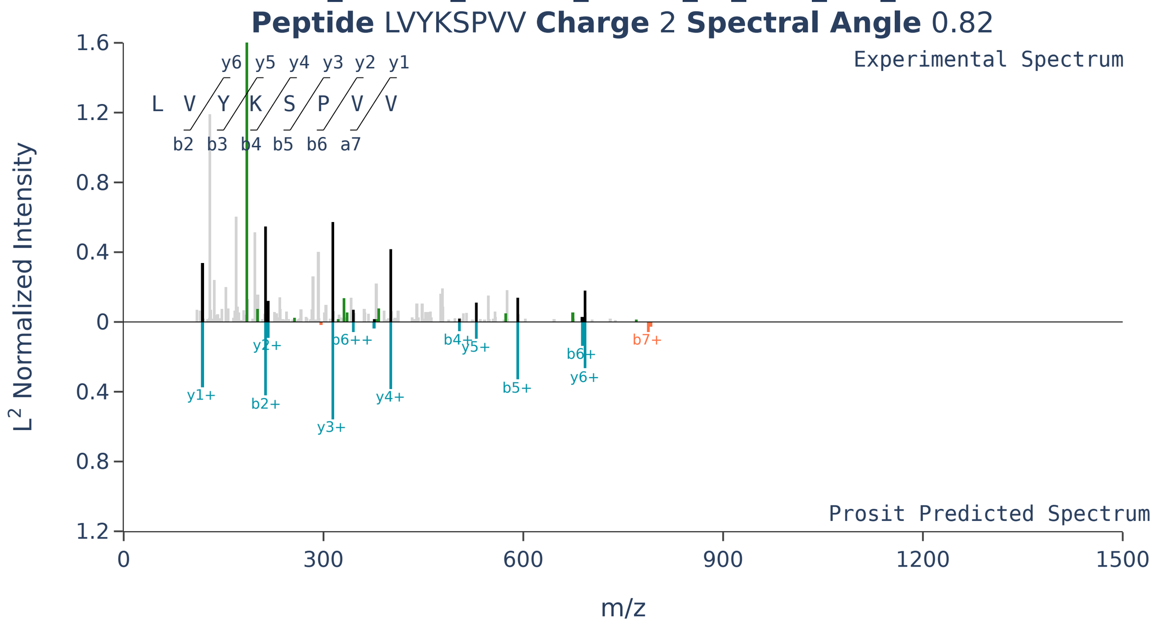

**Source** WSoh_221121_011221_HFGoe_L1_hTau_24h_R2 **Scan** 21530
**Peptide** APVPMPDLKNVKSKIG **Charge** 4 **Spectral Angle** 0.88

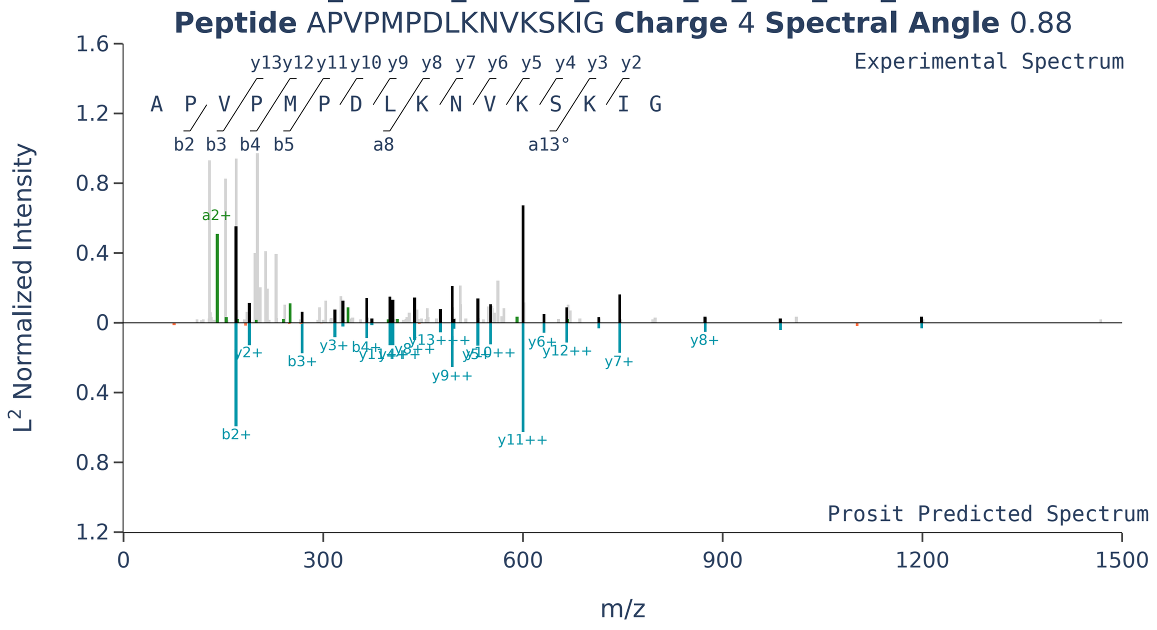


**Source** WSoh_221121_011221_HFGoe_L1_hTau_4h_R1 **Scan** 12248
**Peptide** RENAKAKTDHGAEIVYKSPVV **Charge** 3 **Spectral Angle** 0.85

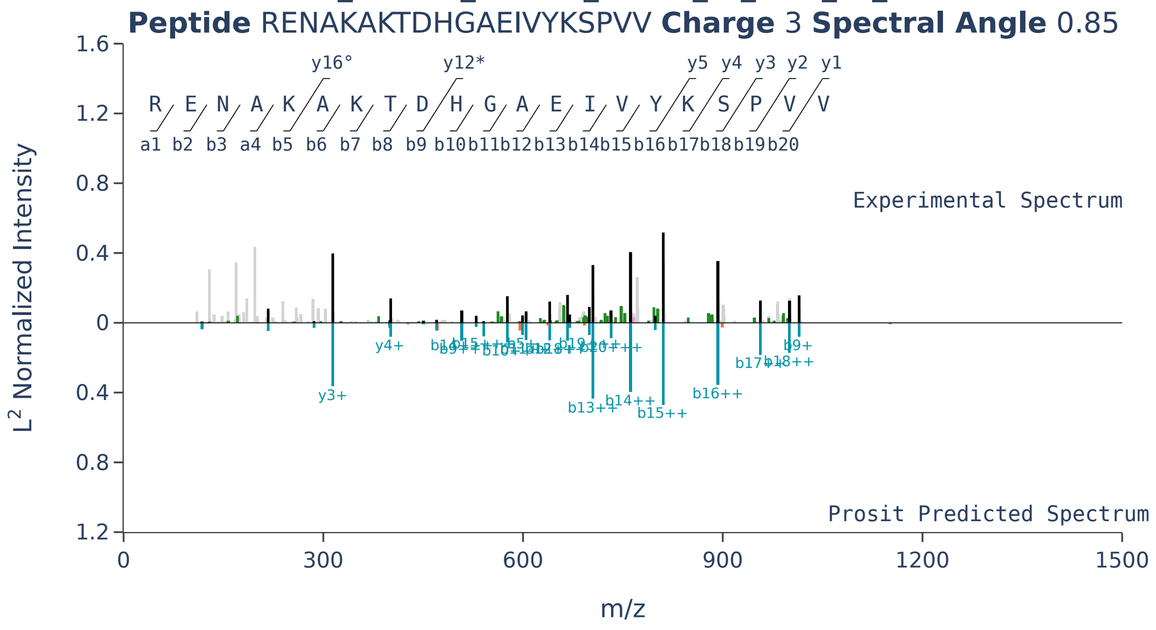


**Source** WSoh_221121_011221_HFGoe_L2_hTau_24h_R2 **Scan** 6382
**Peptide** PKSPSSAKSRLQ **Charge** 3 **Spectral Angle** 0.87

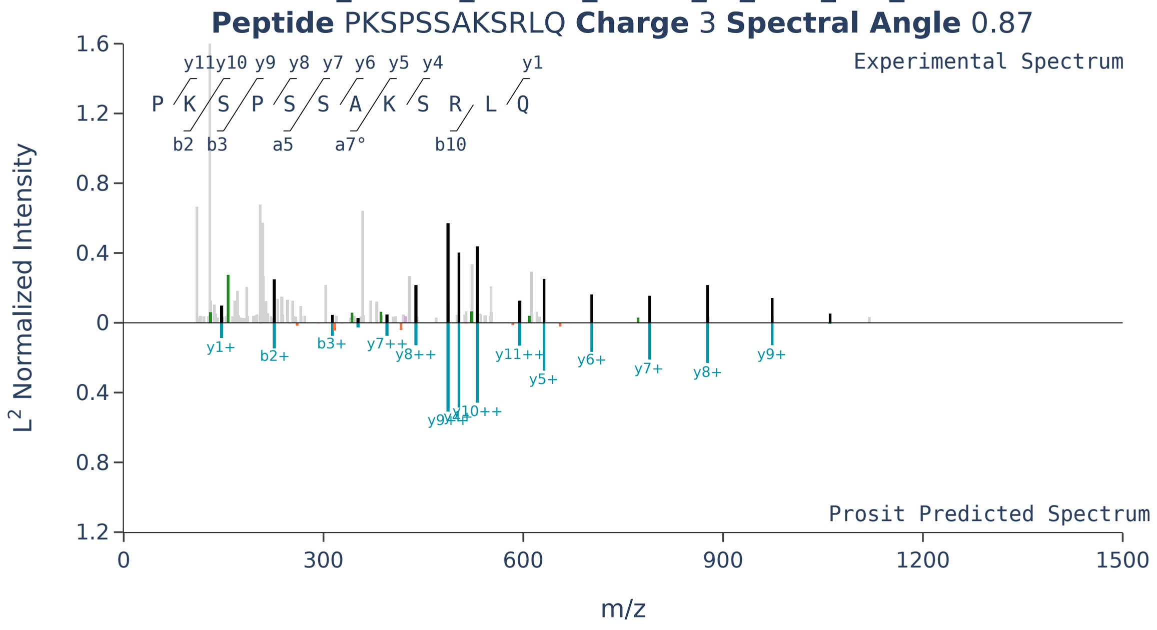


**Source** WSoh_221121_011221_HFGoe_L2_hTau_24h_R2 **Scan** 9210
**Peptide** KCGSLGNIHHKPGGGQVEV **Charge** 2 **Spectral Angle** 0.85

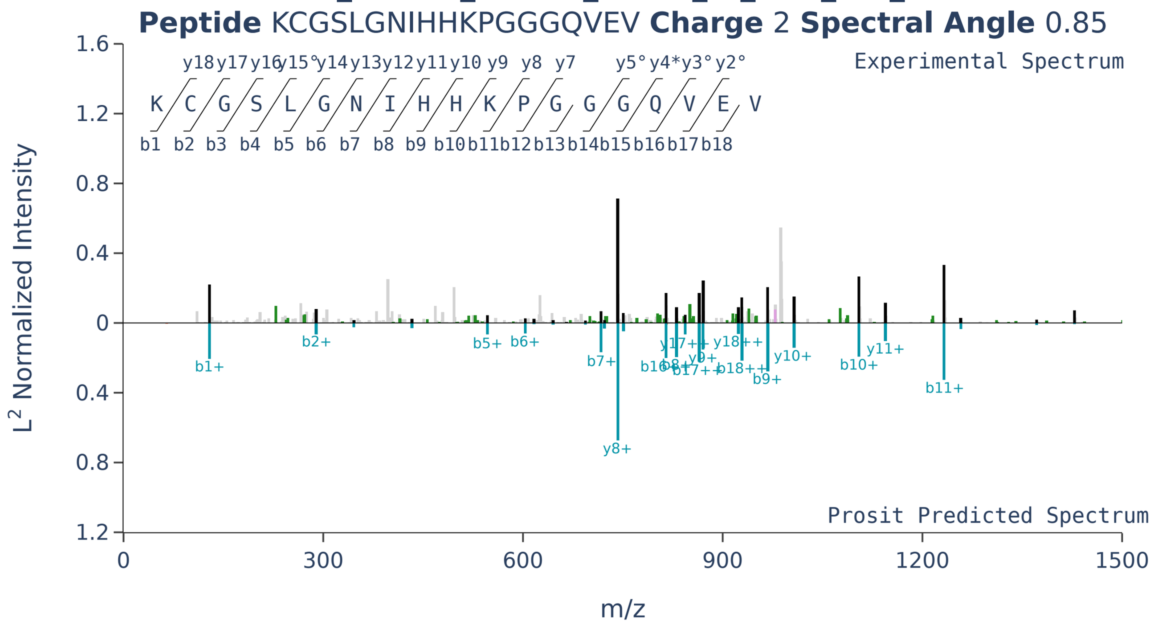


**Source** WSoh_221121_011221_HFGoe_L2_hTau_24h_R2 **Scan** 19869
**Peptide** DTDAGLKESPLQTPTED **Charge** 2 **Spectral Angle** 0.88

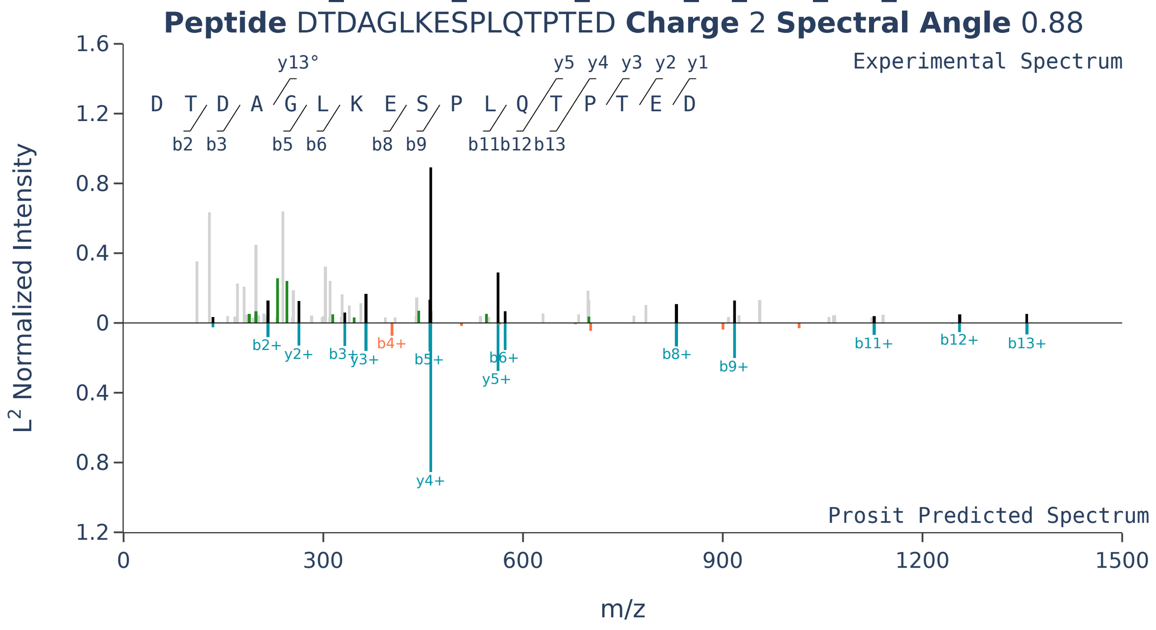


**Source** WSoh_221121_011221_HFGoe_L2_hTau_24h_R1 **Scan** 23947
**Peptide** PEGTTAEEAGIGDTPSL **Charge** 2 **Spectral Angle** 0.91

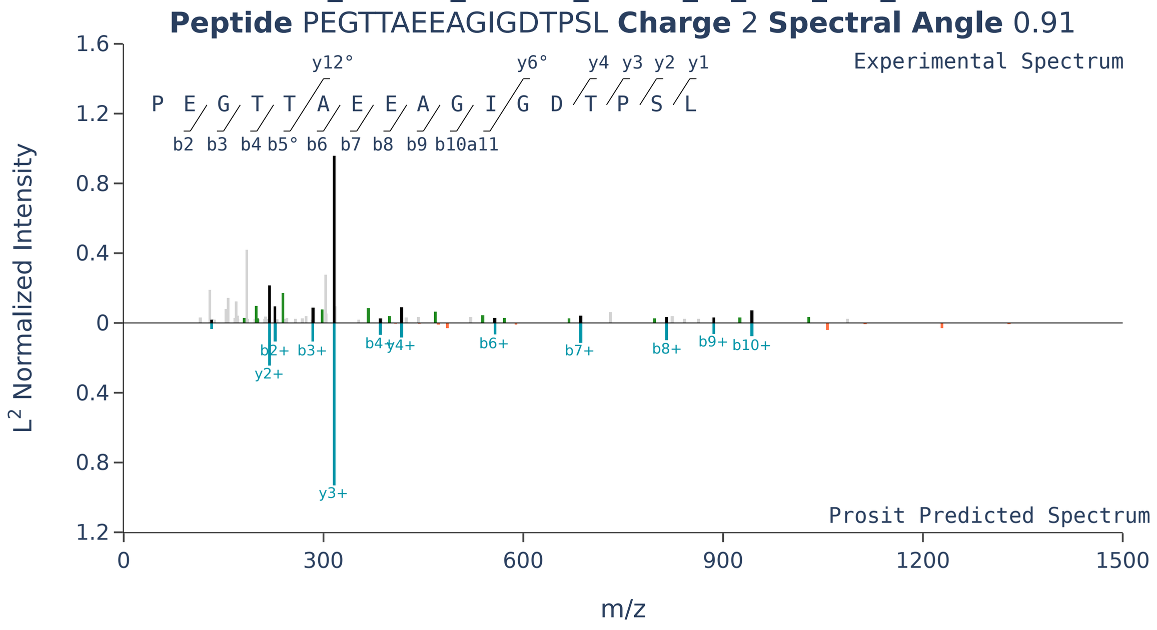


**Source** WSoh_221121_011221_HFGoe_L1_hTau_24h_R2 **Scan** 8535
**Peptide** IATPRGAAPPGQKGQAN **Charge** 2 **Spectral Angle** 0.89

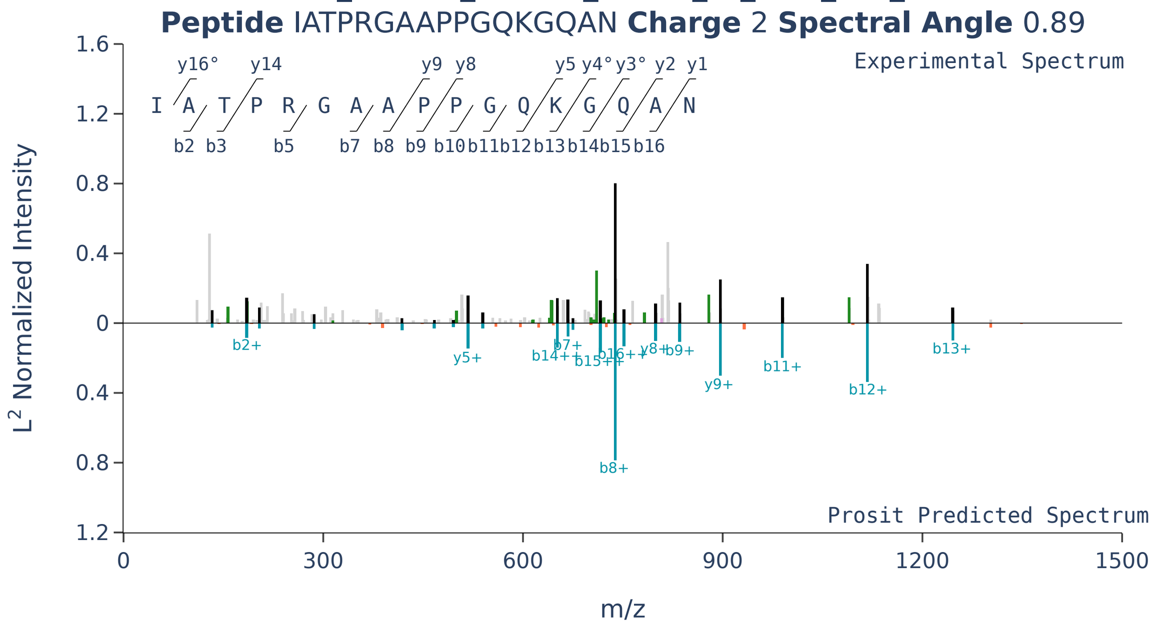


**Source** WSoh_221121_011221_HFGoe_L2_hTau_4h_R1 **Scan** 11619
**Peptide** SEKLDFKDRVQS **Charge** 3 **Spectral Angle** 0.87

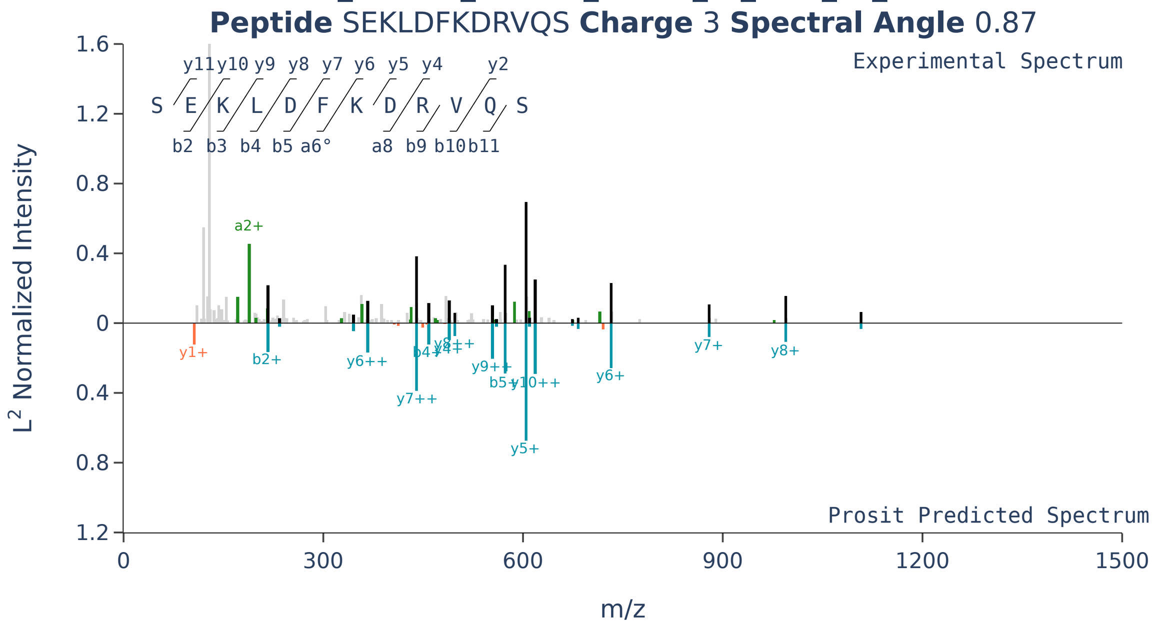


**Source** WSoh_221121_011221_HFGoe_L2_hTau_4h_R1 **Scan** 12355
**Peptide** DTSPRHLSNVSSTGSID **Charge** 2 **Spectral Angle** 0.87

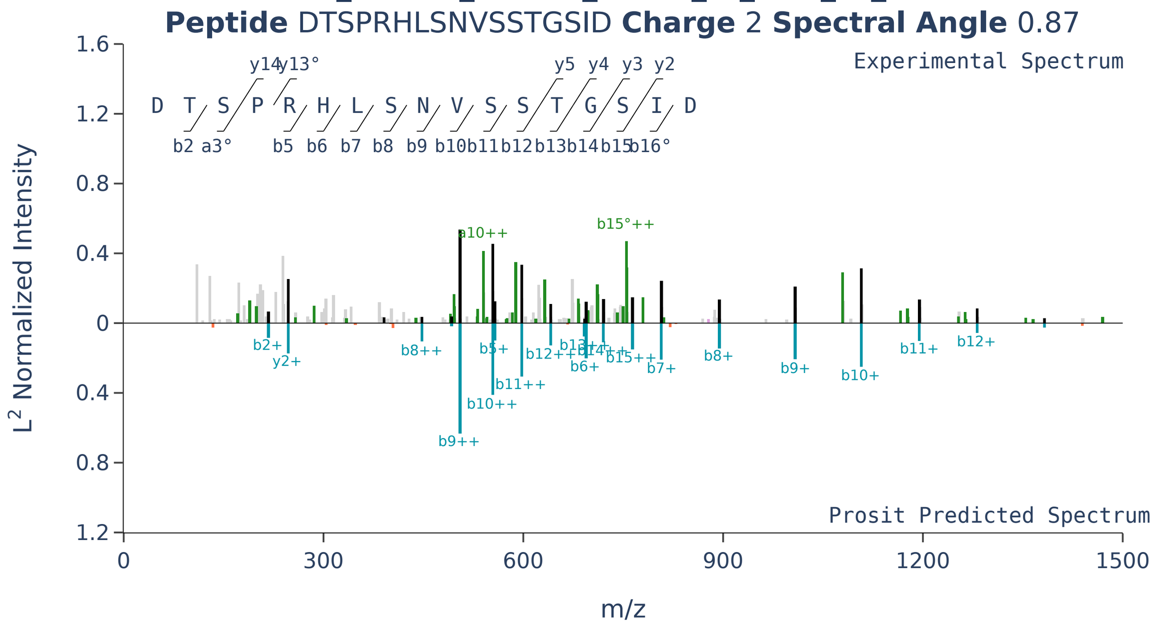


**Source** WSoh_221121_011221_HFGoe_L2_hTau_24h_R2 **Scan** 7257
**Peptide** ITHVPGGGNKKIE **Charge** 3 **Spectral Angle** 0.89

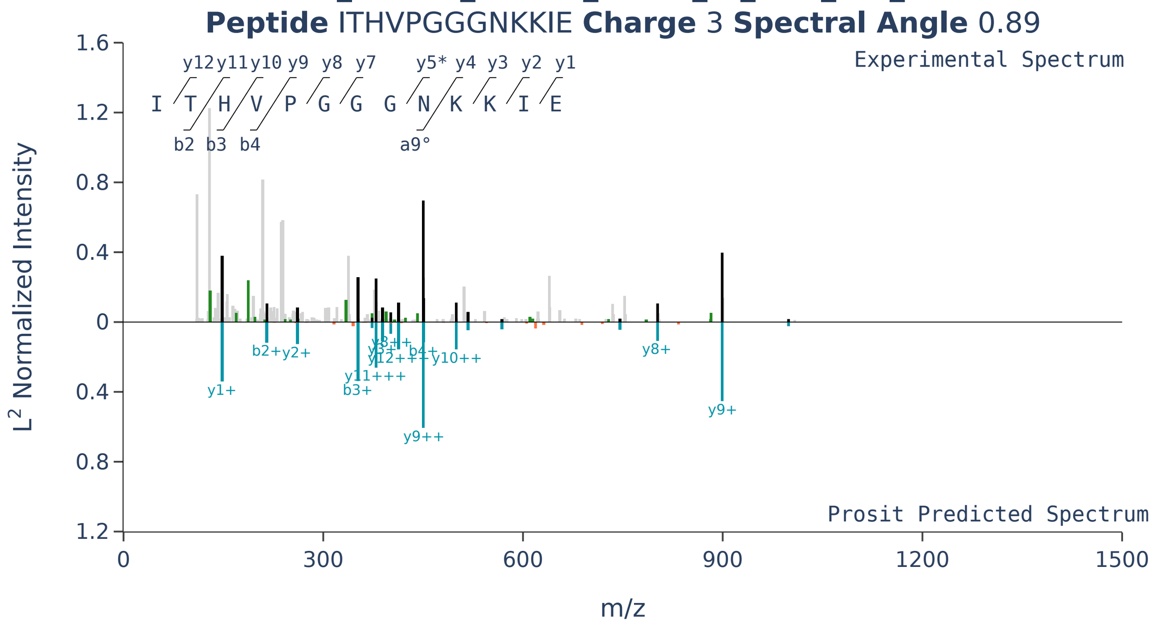


**Source** WSoh_221121_011221_HFGoe_L2_hTau_24h_R1 **Scan** 6941
**Peptide** KCGSKDNIKHVPGGGSV **Charge** 2 **Spectral Angle** 0.92

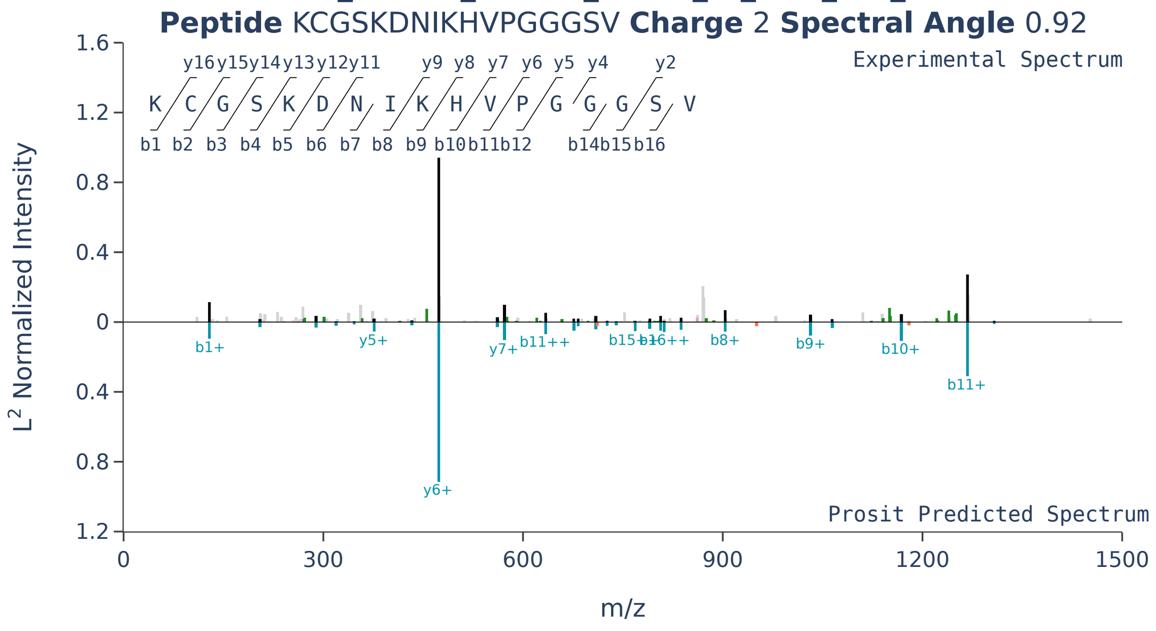


**Source** WSoh_221121_011221_HFGoe_L2_hTau_24h_R1 **Scan** 22638
**Peptide** QTAPVPMPDLKNVKSKI **Charge** 3 **Spectral Angle** 0.87

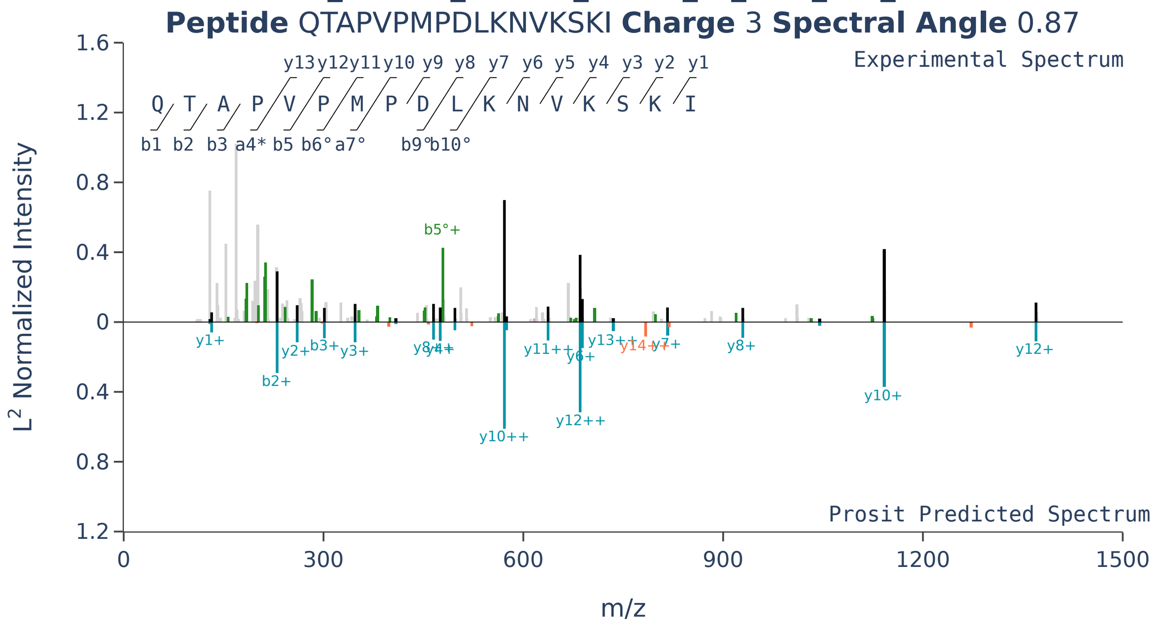


**Source** WSoh_221121_011221_HFGoe_L2_hTau_4h_R2 **Scan** 13949
**Peptide** RVQSKIGSLDNITHVPGGGNKKIETH **Charge** 5 **Spectral Angle** 0.89

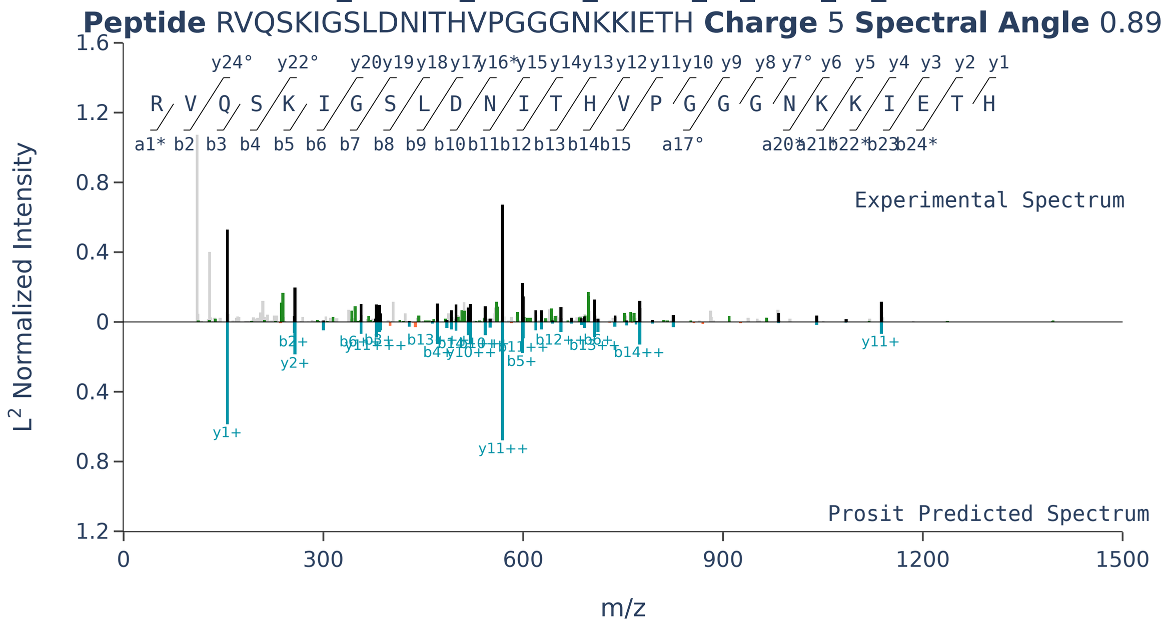


**Source** WSoh_221121_011221_HFGoe_L2_hTau_24h_R2 **Scan** 21574
**Peptide** PGGGSVQIVYKPVDLSK **Charge** 3 **Spectral Angle** 0.89

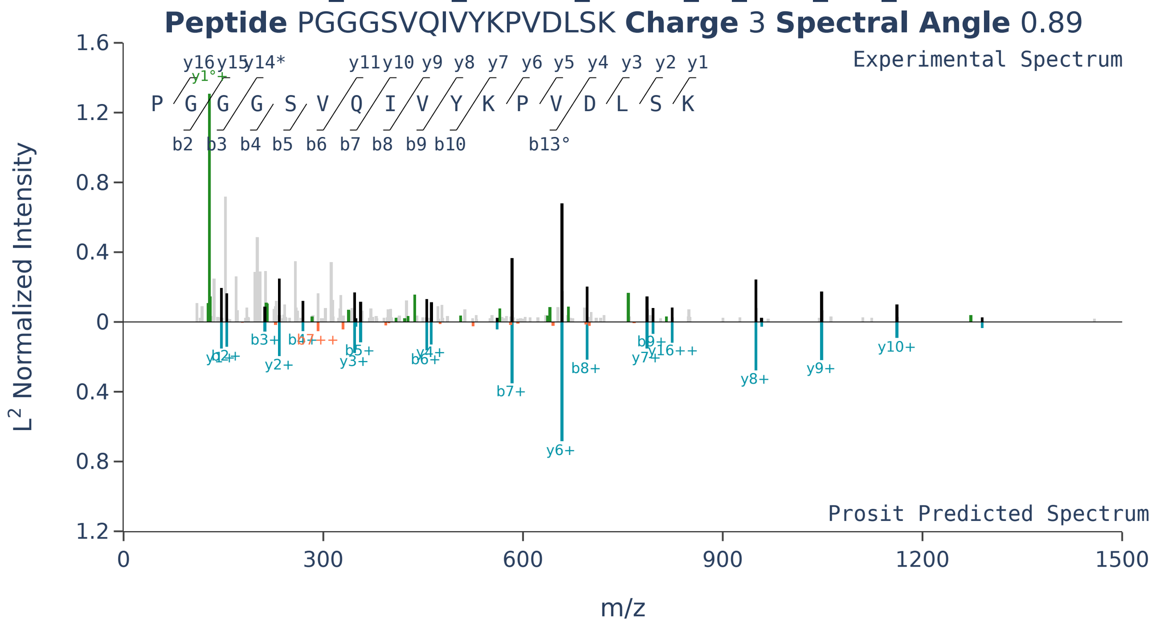


**Source** WSoh_221121_011221_HFGoe_L1_hTau_2h_R2 **Scan** 6392
**Peptide** ITHVPGGGNKKIETH **Charge** 4 **Spectral Angle** 0.87

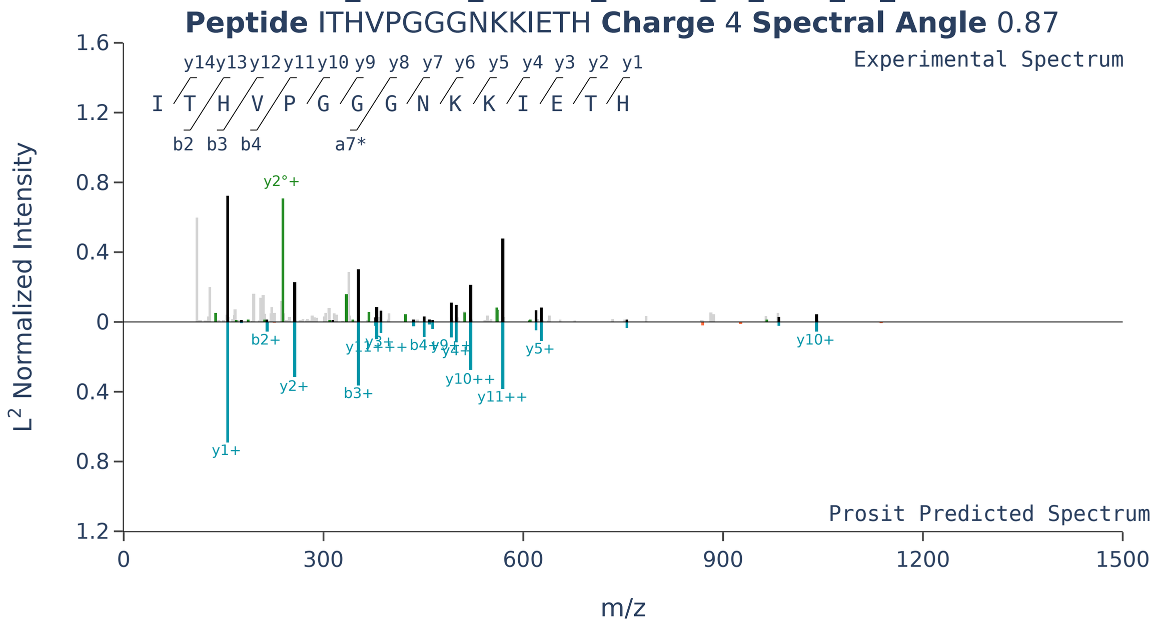

**Source** WSoh_221121_011221_HFGoe_L1_hTau_24h_R1 **Scan** 8632
**Peptide** DGSEEPGSETSDAKSTPTAE **Charge** 2 **Spectral Angle** 0.8

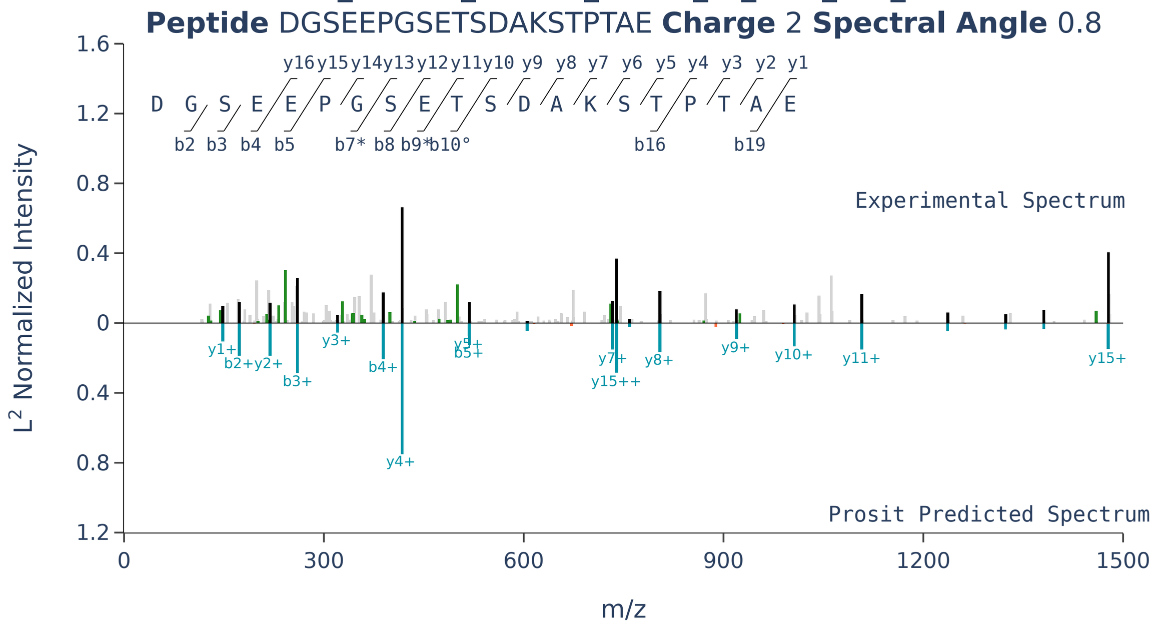


**Source** WSoh_221121_011221_HFGoe_L1_hTau_24h_R1 **Scan** 19831
**Peptide** EFEVMEDHAGTYGLGDRKD **Charge** 3 **Spectral Angle** 0.88

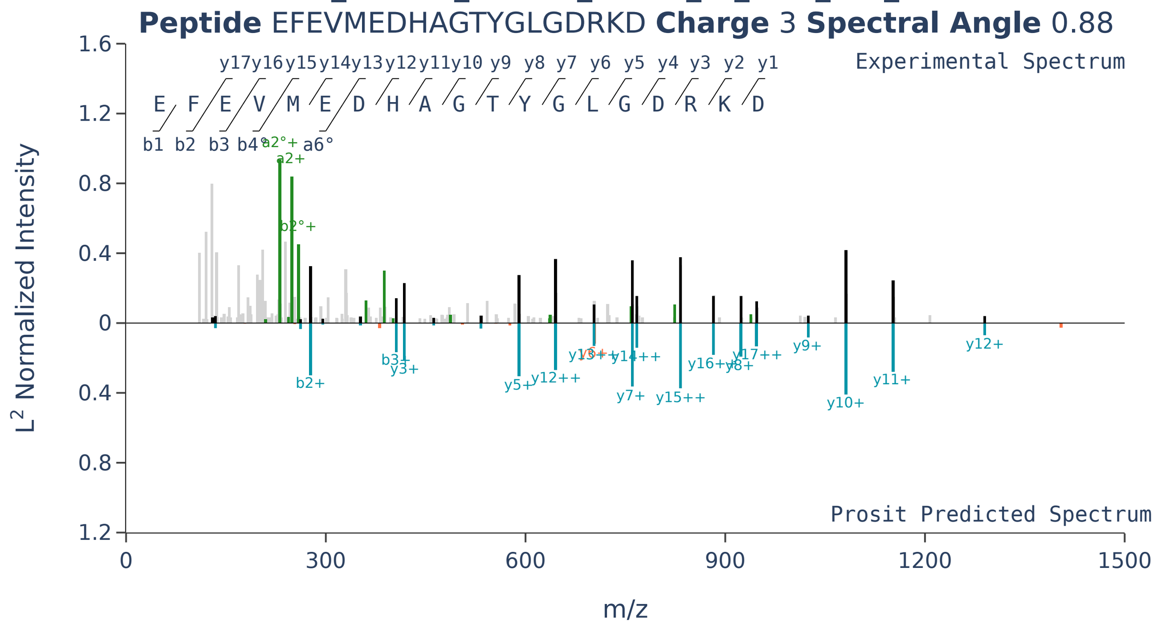


**Source** WSoh_221121_011221_HFGoe_L1_hTau_24h_R1 **Scan** 9610
**Peptide** ANATRIPAKTPPAPKT **Charge** 3 **Spectral Angle** 0.89

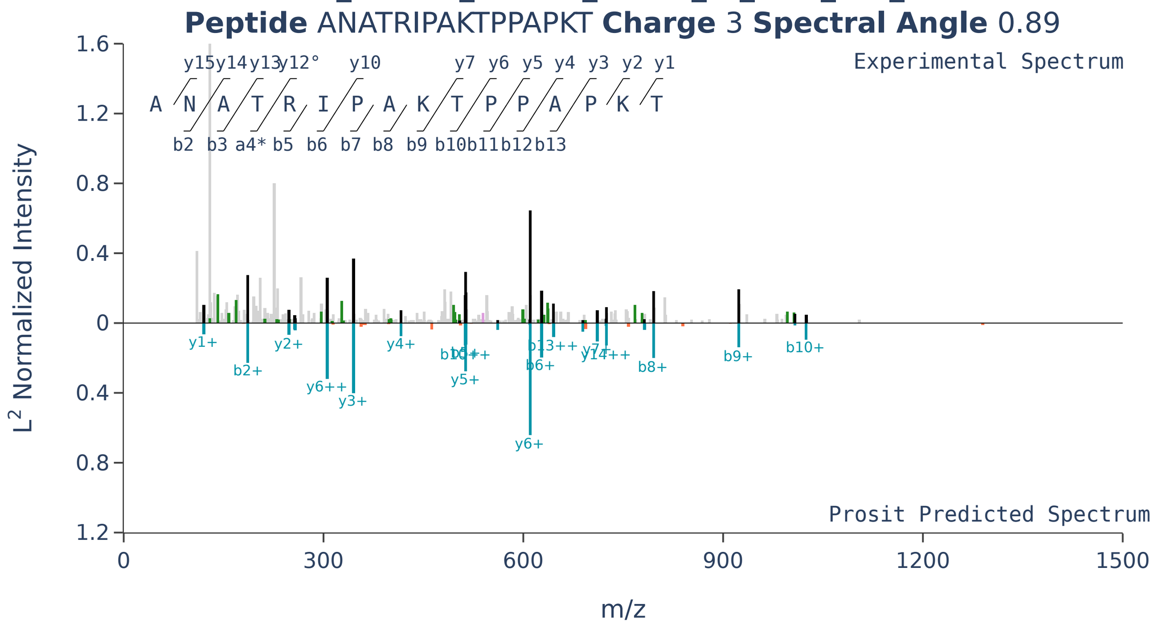


**Source** WSoh_221121_011221_HFGoe_L2_hTau_24h_R1 **Scan** 7980
**Peptide** PPAPKTPPSSGEPPKSGD **Charge** 2 **Spectral Angle** 0.86

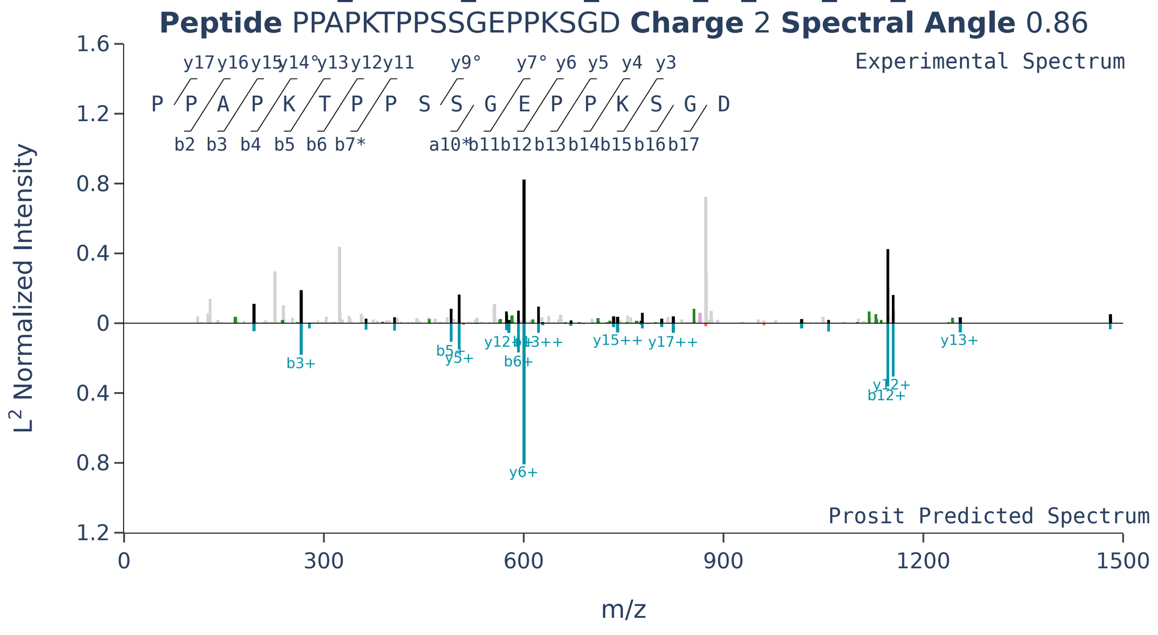


**Source** WSoh_221121_011221_HFGoe_L2_hTau_24h_R2 **Scan** 18361
**Peptide** TDAGLKESPLQTPTEDGSE **Charge** 2 **Spectral Angle** 0.85

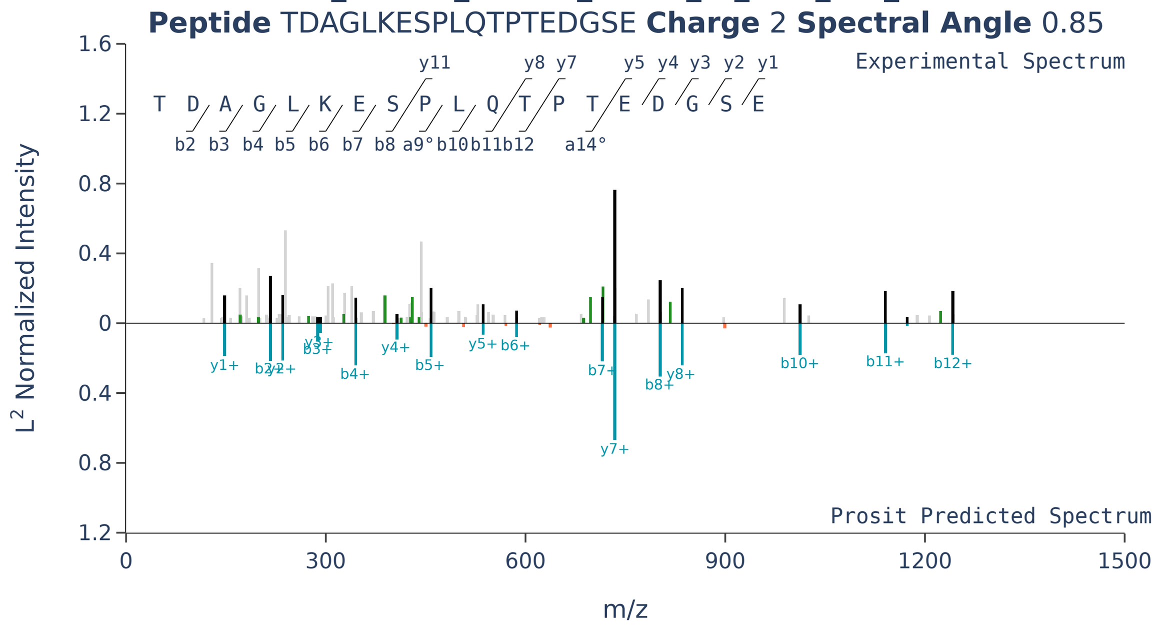


**Source** WSoh_221121_011221_HFGoe_L1_hTau_2h_R1 **Scan** 17000
**Peptide** NIKHVPGGGSVQIVYKPVD **Charge** 3 **Spectral Angle** 0.88

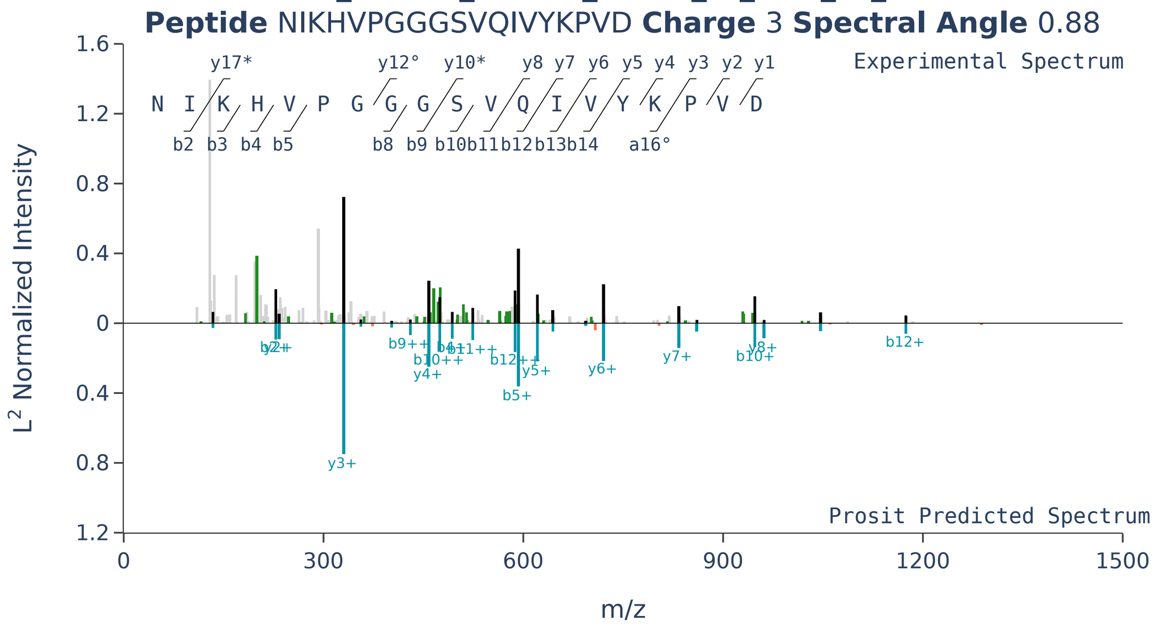


**Source** WSoh_221121_011221_HFGoe_L1_hTau_4h_R1 **Scan** 7759
**Peptide** AVVRTPPKSPSSA **Charge** 2 **Spectral Angle** 0.86

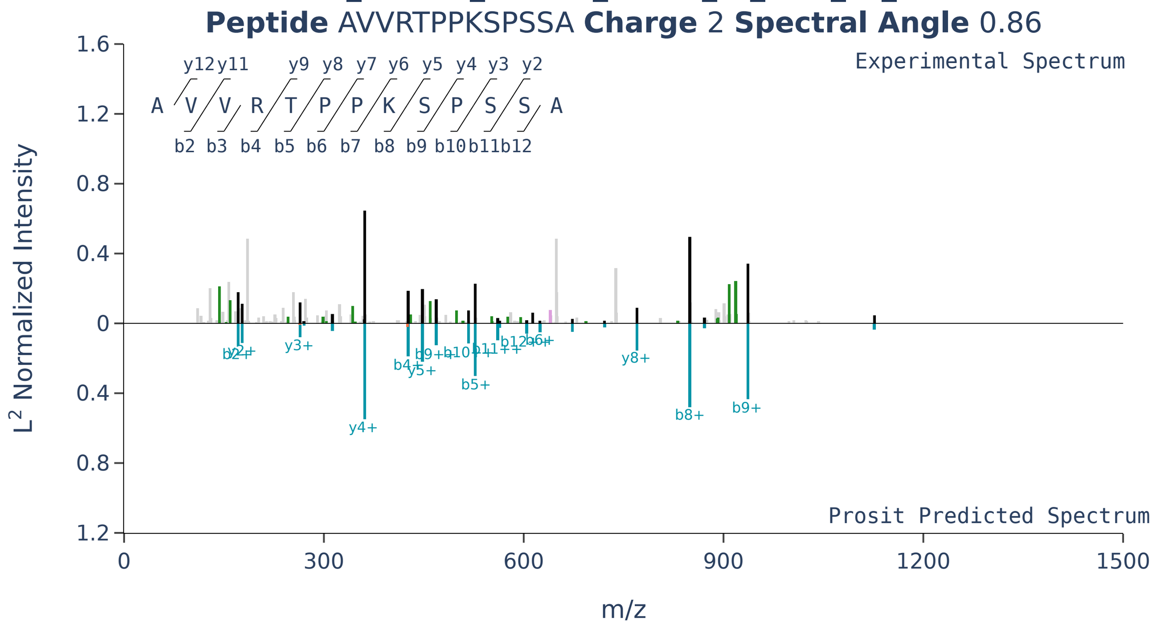


**Source** WSoh_221121_011221_HFGoe_L2_hTau_24h_R1 **Scan** 22558
**Peptide** AGIGDTPSLEDEAAGHVTQARMVS **Charge** 3 **Spectral Angle** 0.85

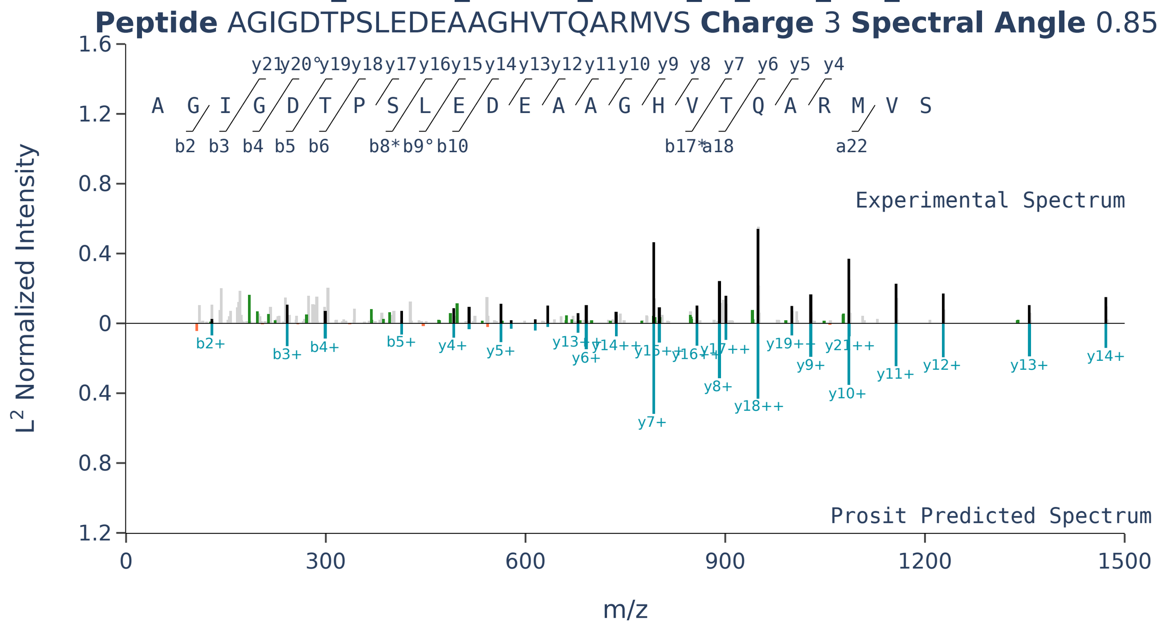


**Source** WSoh_221121_011221_HFGoe_L1_hTau_4h_R2 **Scan** 9228
**Peptide** KLTFRENAKAKTDHGAEI **Charge** 3 **Spectral Angle** 0.87

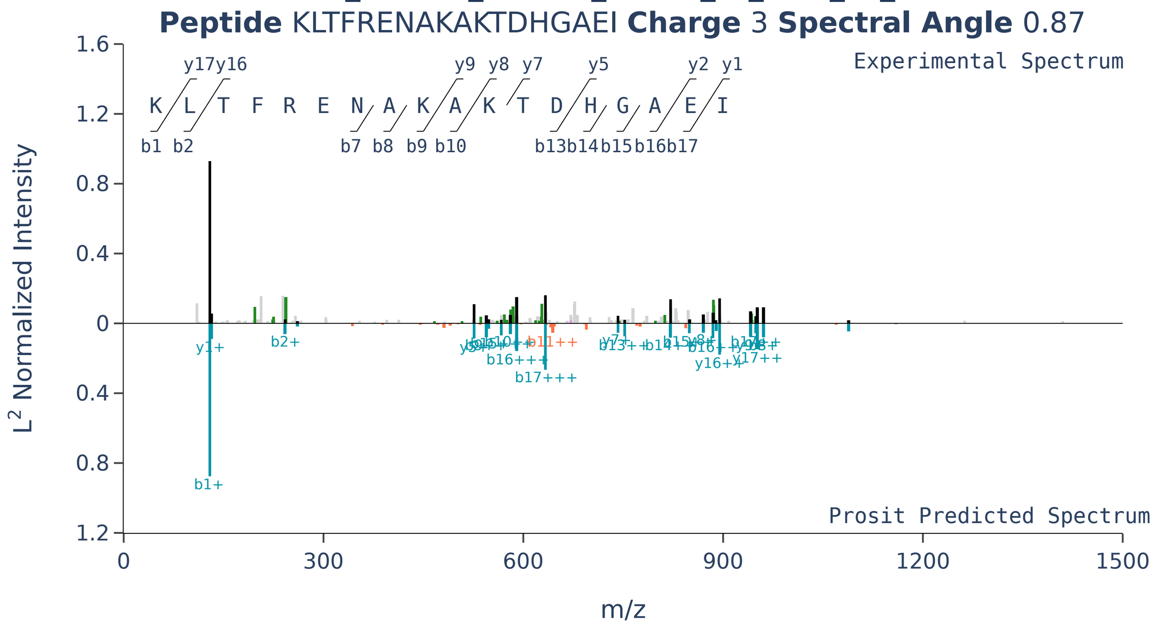


**Source** WSoh_221121_011221_HFGoe_L2_hTau_4h_R2 **Scan** 18985
**Peptide** EPRQEFEVME **Charge** 2 **Spectral Angle** 0.79

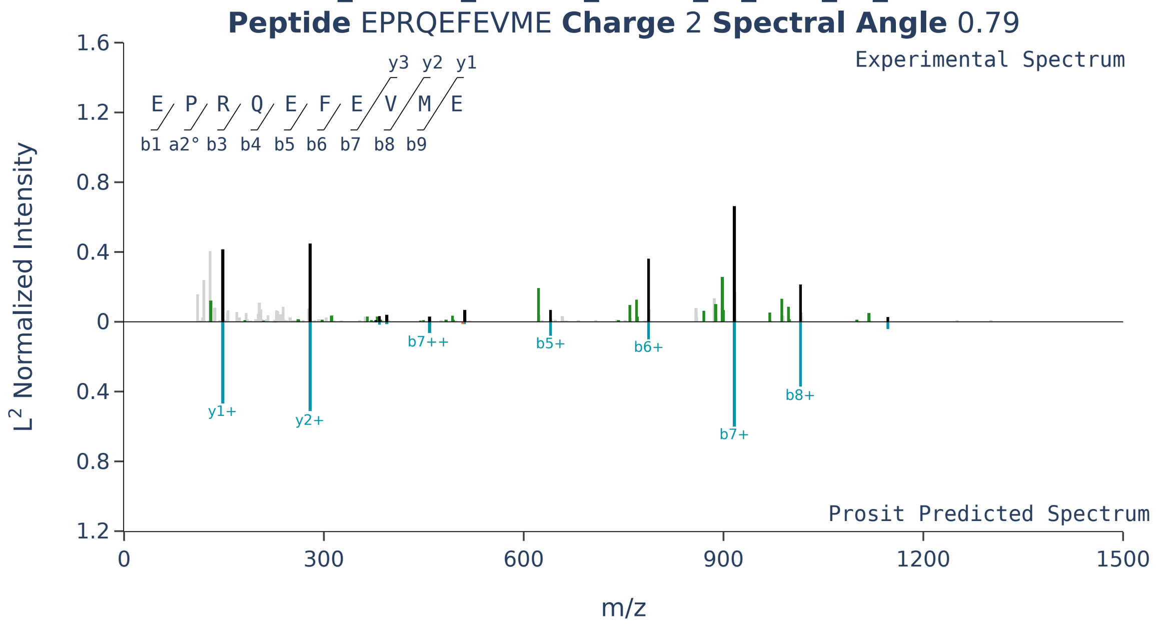


**Source** WSoh_221121_011221_HFGoe_L1_hTau_24h_R1 **Scan** 8131
**Peptide** TMHQDQEGDTDAGLKE **Charge** 2 **Spectral Angle** 0.88

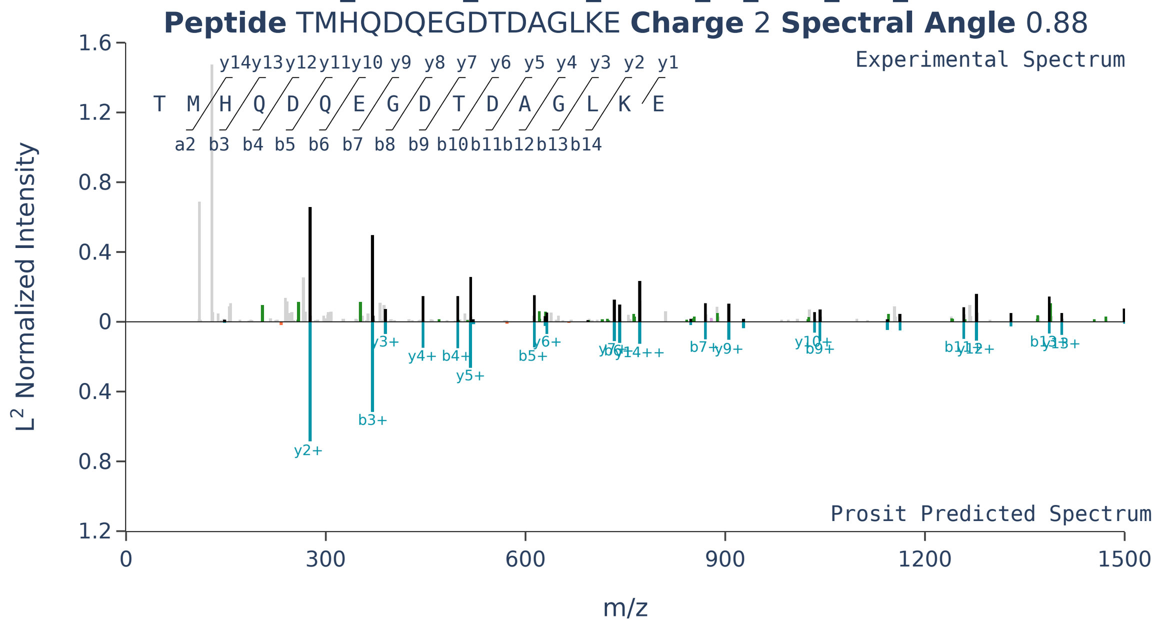


**Source** WSoh_221121_011221_HFGoe_L2_hTau_4h_R2 **Scan** 8612
**Peptide** NITHVPGGGNKKIETHKL **Charge** 2 **Spectral Angle** 0.8

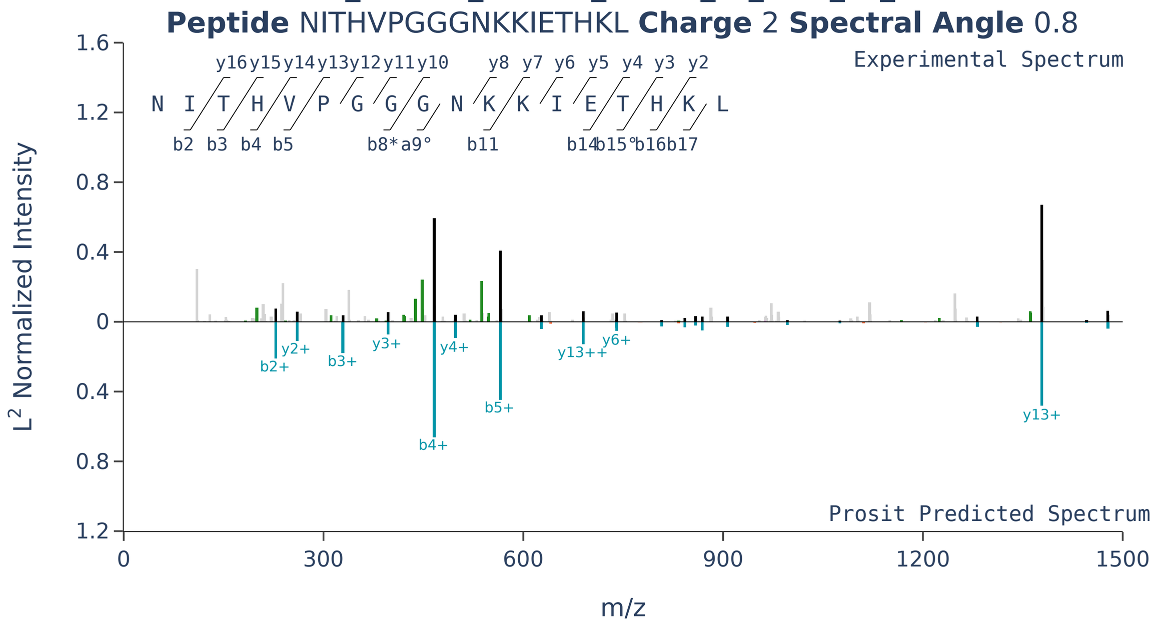


**Source** WSoh_221121_011221_HFGoe_L2_hTau_2h_R1 **Scan** 6129
**Peptide** RSGYSSPGSPGTPGSRSR **Charge** 4 **Spectral Angle** 0.87

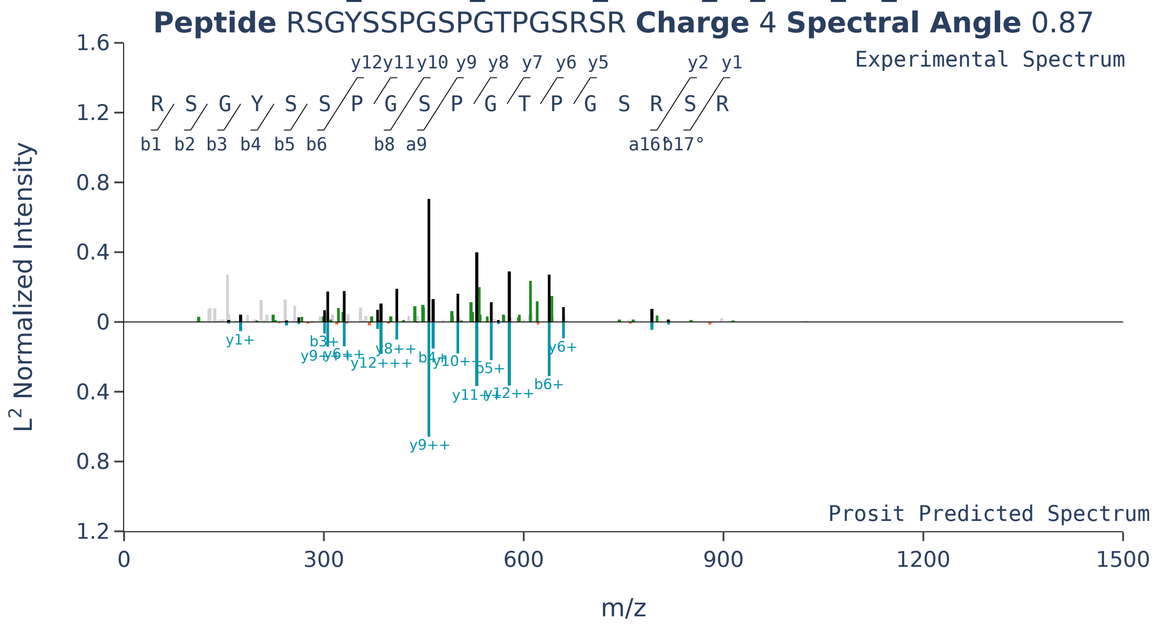


**Source** WSoh_221121_011221_HFGoe_L1_hTau_24h_R1 **Scan** 8672
**Peptide** ADGKTKIATPRGAAPPGQ **Charge** 3 **Spectral Angle** 0.92

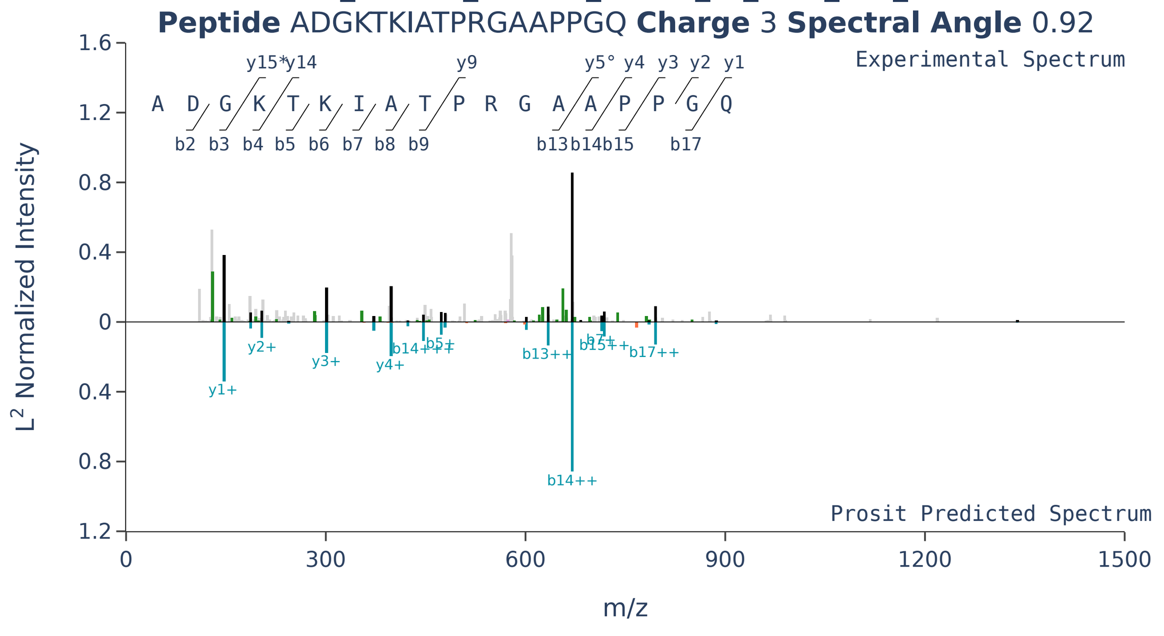


**Source** WSoh_221121_011221_HFGoe_L2_hTau_24h_R1 **Scan** 7755
**Peptide** PGGGNKKIETHKLTFRENAKA **Charge** 3 **Spectral Angle** 0.81

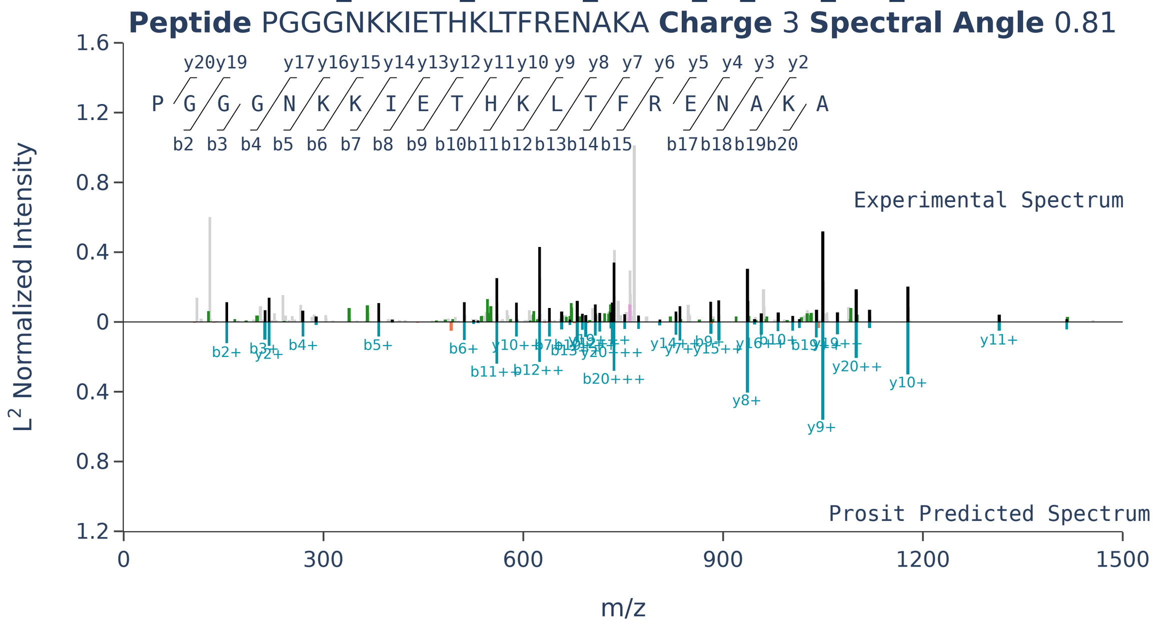

**Source** WSoh_221121_011221_HFGoe_L1_hTau_24h_R1 **Scan** 10579
**Peptide** DHAGTYGLGDRKDQGGYT **Charge** 3 **Spectral Angle** 0.95

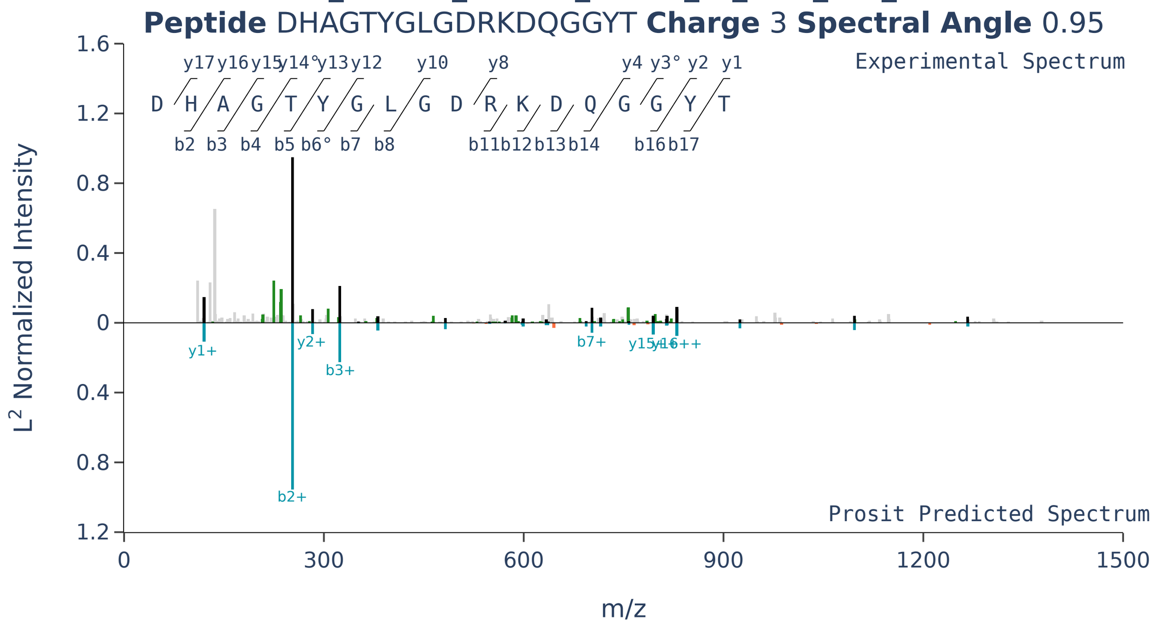


**Source** WSoh_221121_011221_HFGoe_L1_hTau_24h_R2 **Scan** 7206
**Peptide** LGNIHHKPGGGQVEVKSE **Charge** 2 **Spectral Angle** 0.84

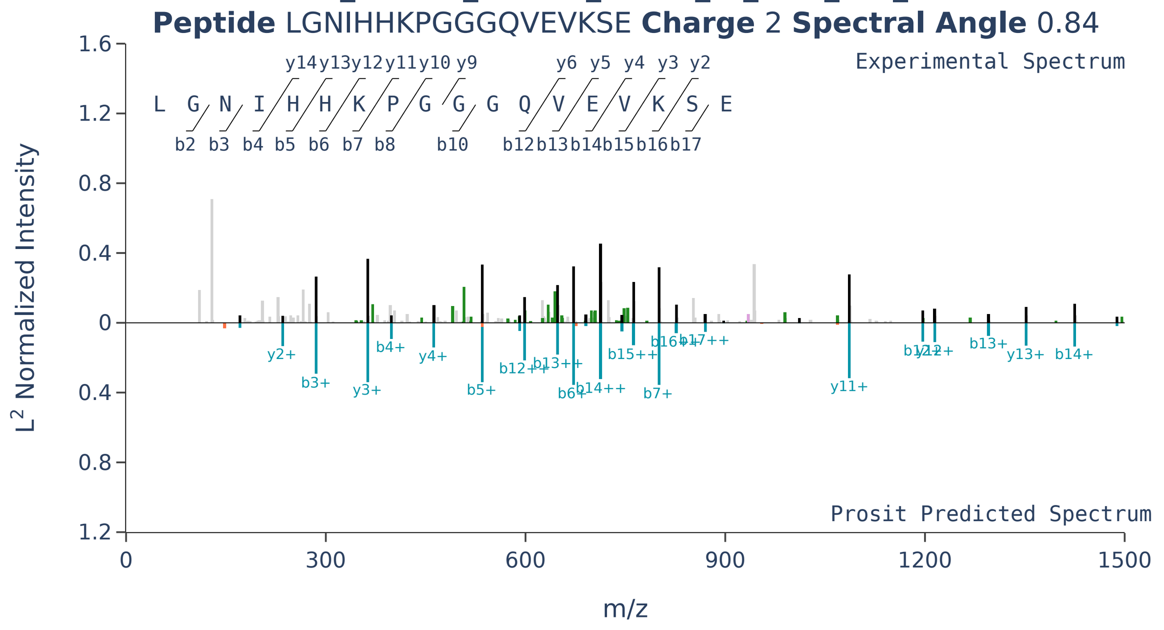


**Source** WSoh_221121_011221_HFGoe_L1_hTau_24h_R1 **Scan** 10353
**Peptide** ATRIPAKTPPAPKTPPSSG **Charge** 3 **Spectral Angle** 0.81

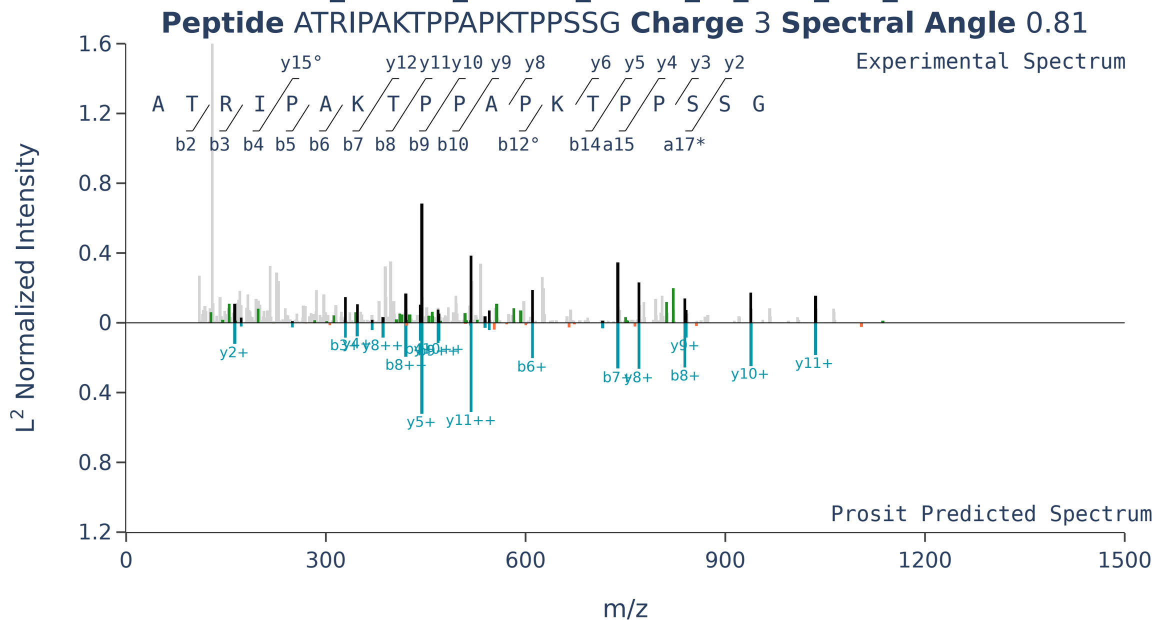


**Source** WSoh_221121_011221_HFGoe_L1_hTau_4h_R2 **Scan** 22717
**Peptide** QIVYKPVDL **Charge** 2 **Spectral Angle** 0.85

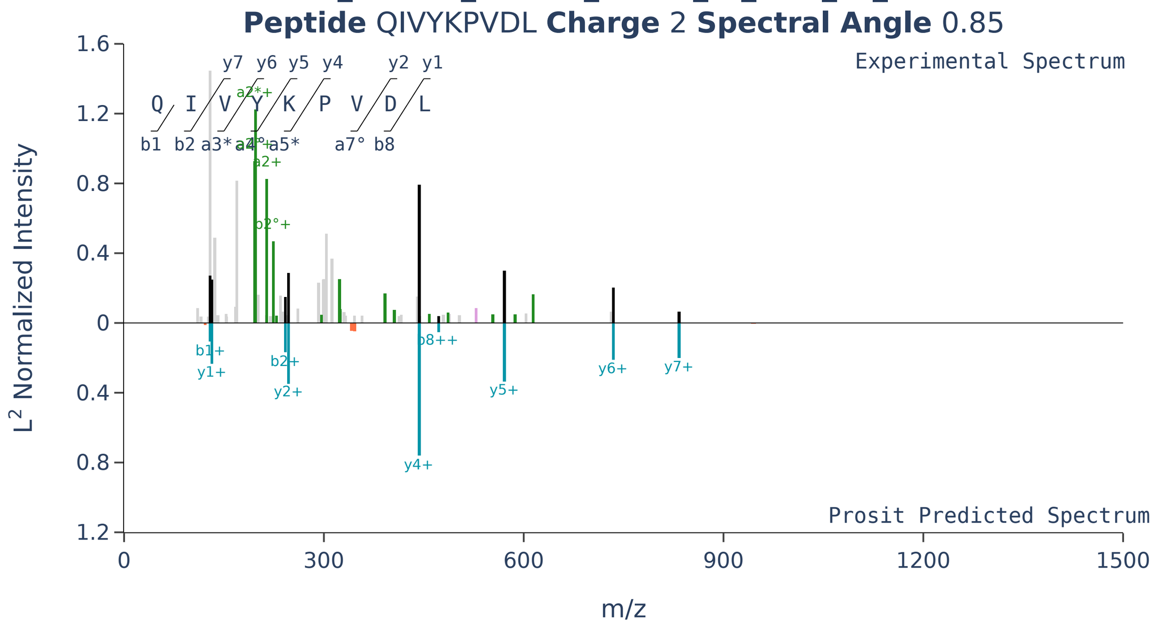


**Source** WSoh_221121_011221_HFGoe_L1_hTau_4h_R1 **Scan** 11976
**Peptide** GDRSGYSSPGSPGTPGSRSRTPSL **Charge** 4 **Spectral Angle** 0.84

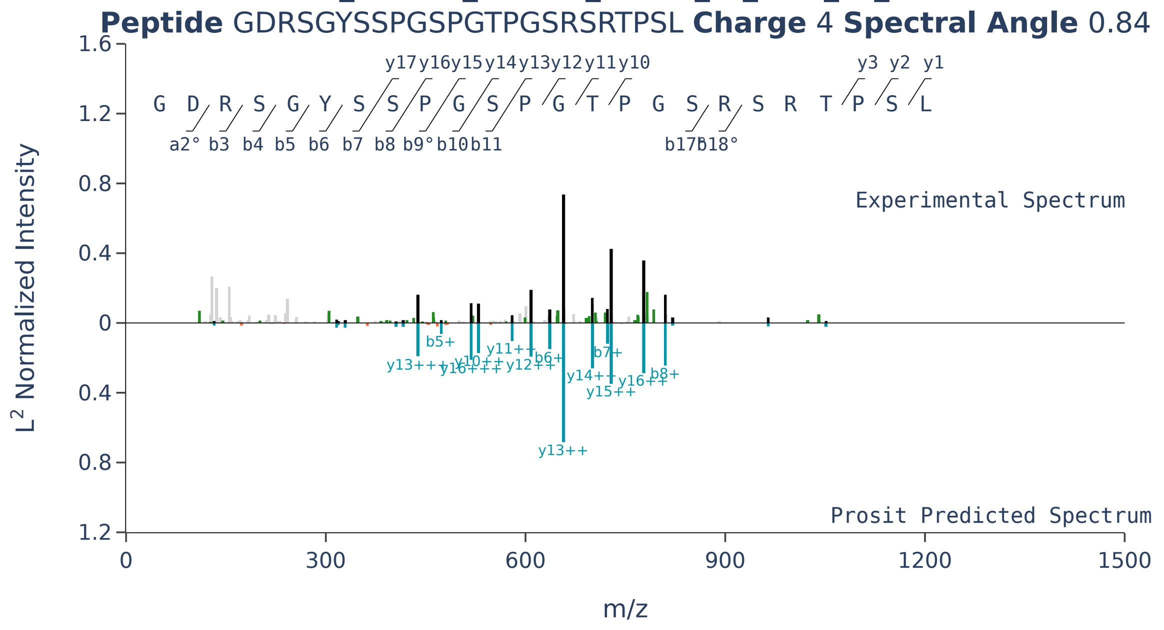


**Source** WSoh_221121_011221_HFGoe_L2_hTau_2h_R1 **Scan** 5532
**Peptide** PRGAAPPGQKGQANA **Charge** 2 **Spectral Angle** 0.88

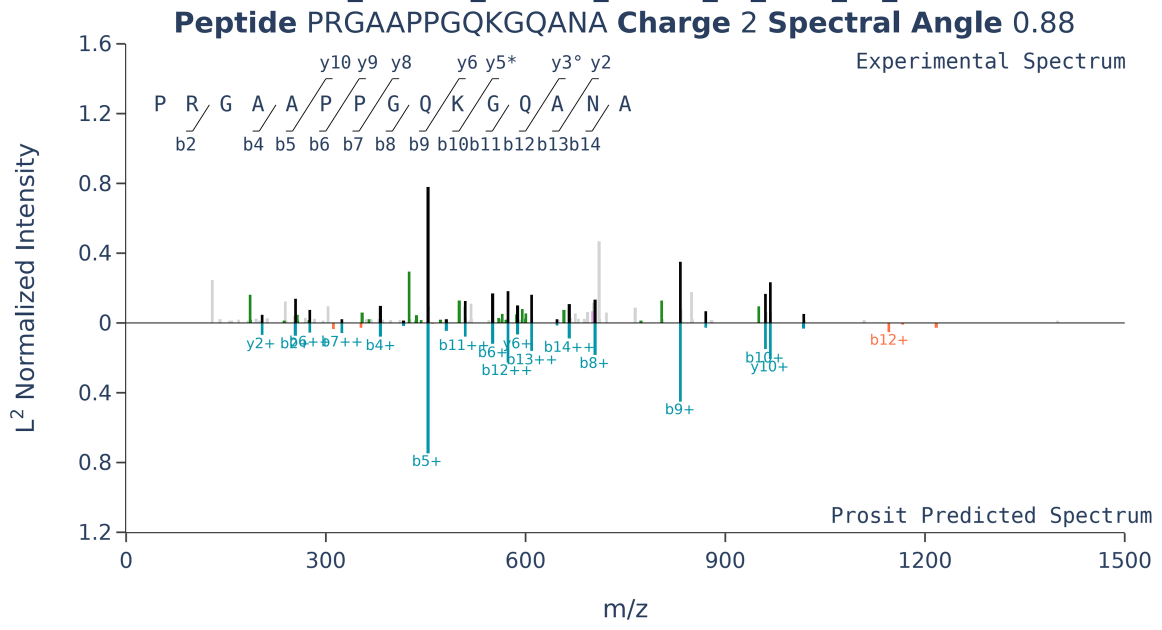


**Source** WSoh_221121_011221_HFGoe_L1_hTau_24h_R1 **Scan** 11352
**Peptide** SLAKQGL **Charge** 2 **Spectral Angle** 0.78

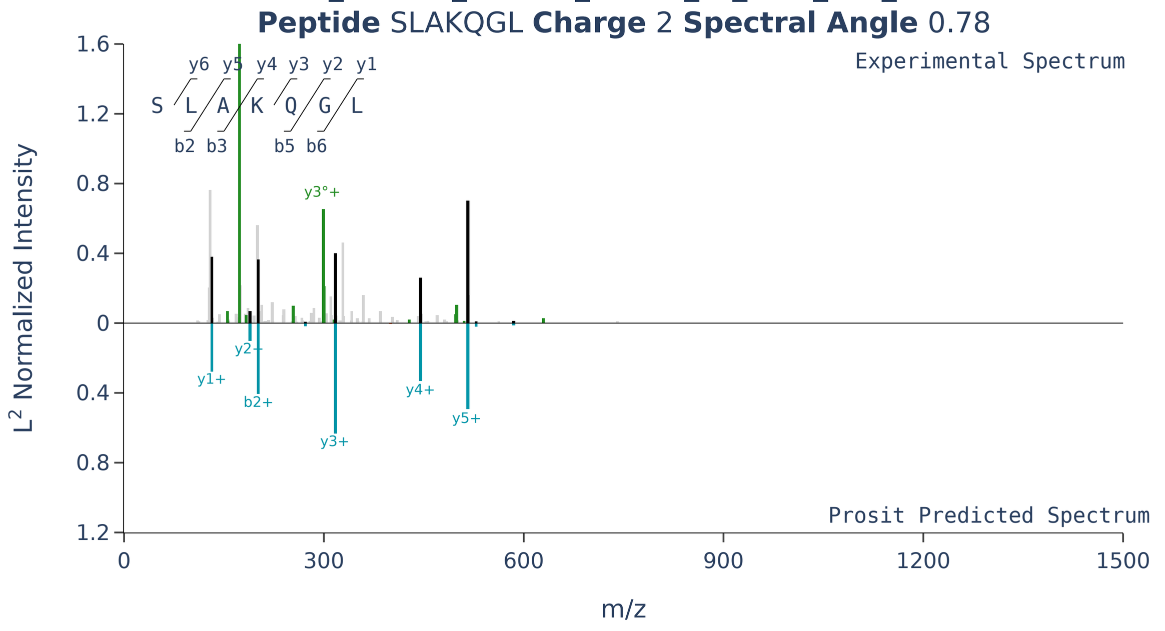


**Source** WSoh_221121_011221_HFGoe_L2_hTau_24h_R1 **Scan** 6107
**Peptide** GSKDNIKHVPGG **Charge** 3 **Spectral Angle** 0.88

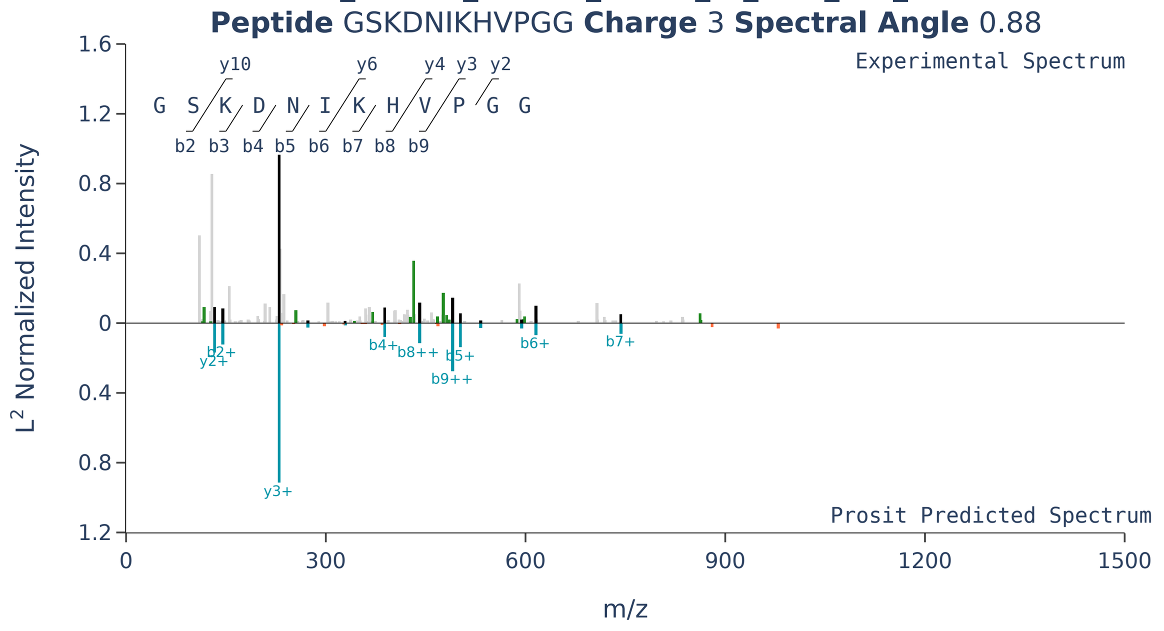


**Source** WSoh_221121_011221_HFGoe_L1_hTau_4h_R2 **Scan** 12953
**Peptide** SKDNIKHVPGGGSVQL **Charge** 4 **Spectral Angle** 0.77

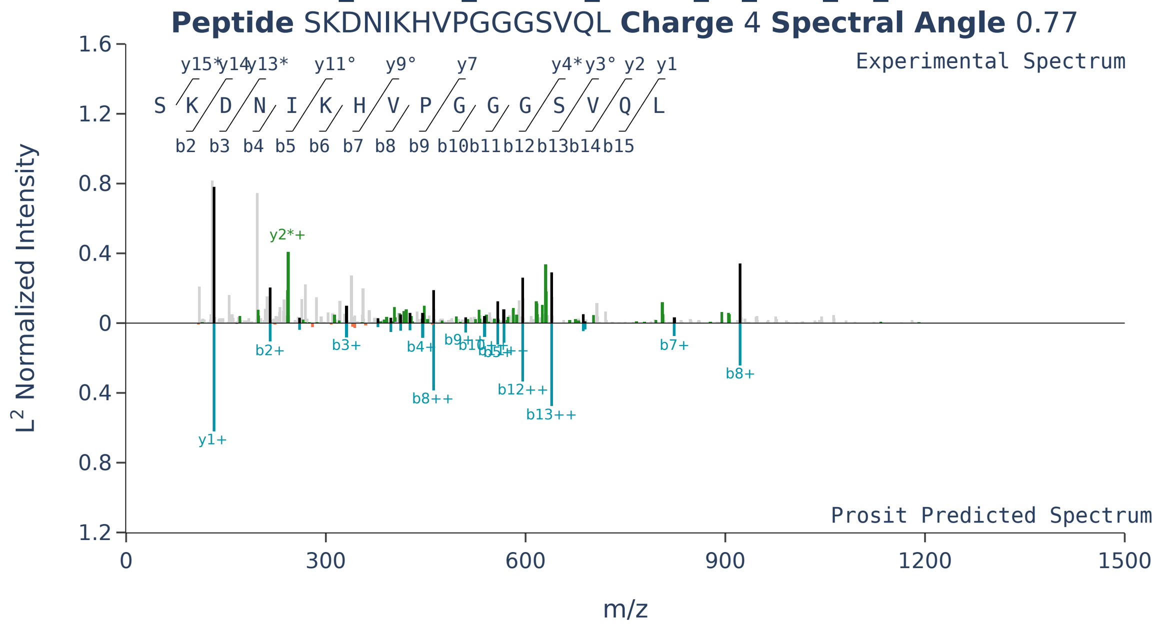


**Source** WSoh_221121_011221_HFGoe_L1_hTau_2h_R1 **Scan** 13198
**Peptide** KSPVVSGDTSPRHLSNVSSTGSID **Charge** 4 **Spectral Angle** 0.87

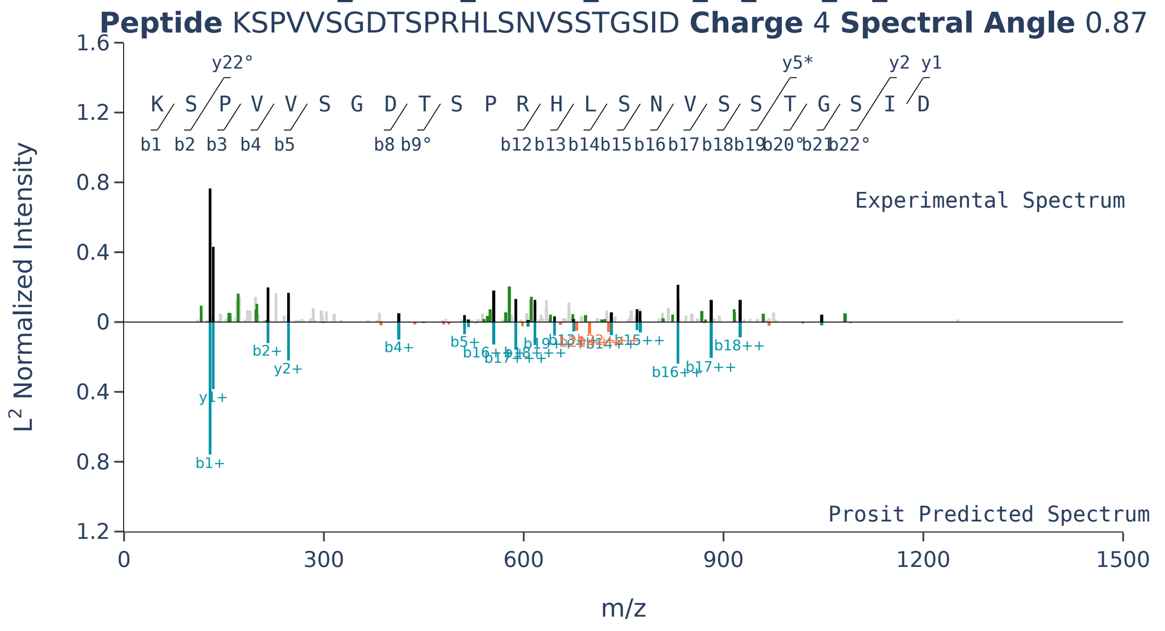


**Source** WSoh_221121_011221_HFGoe_L2_hTau_24h_R2 **Scan** 12417
**Peptide** LEDEAAGHVTQARM **Charge** 3 **Spectral Angle** 0.82

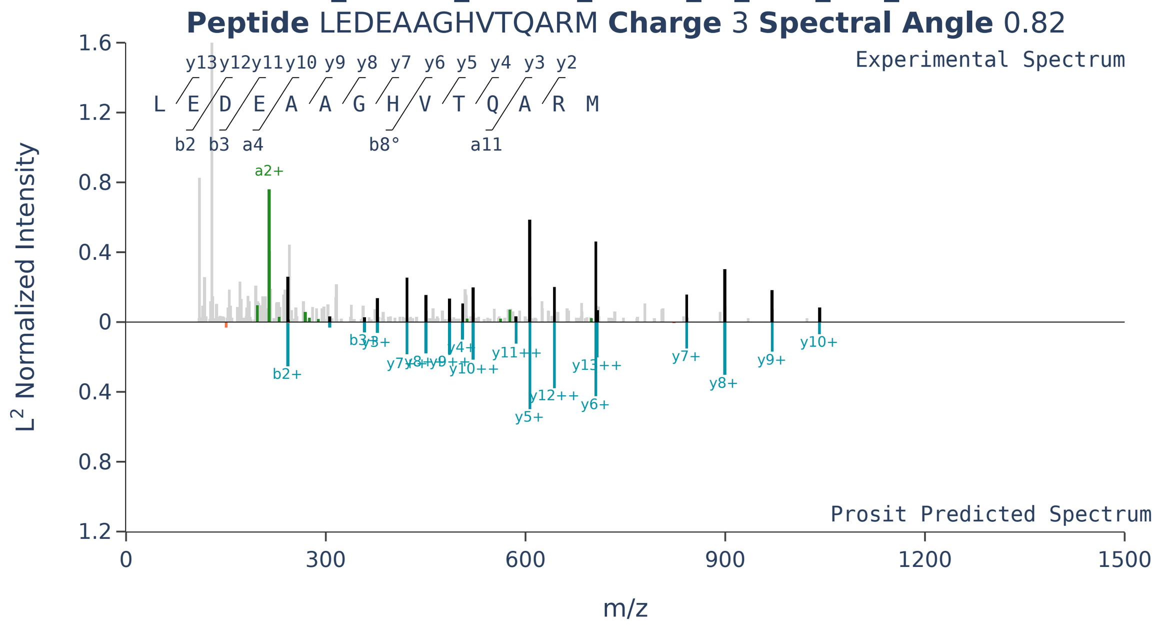


**Source** WSoh_221121_011221_HFGoe_L1_hTau_24h_R2 **Scan** 6516
**Peptide** GDRKDQGGYTMHQDQEGD **Charge** 2 **Spectral Angle** 0.77

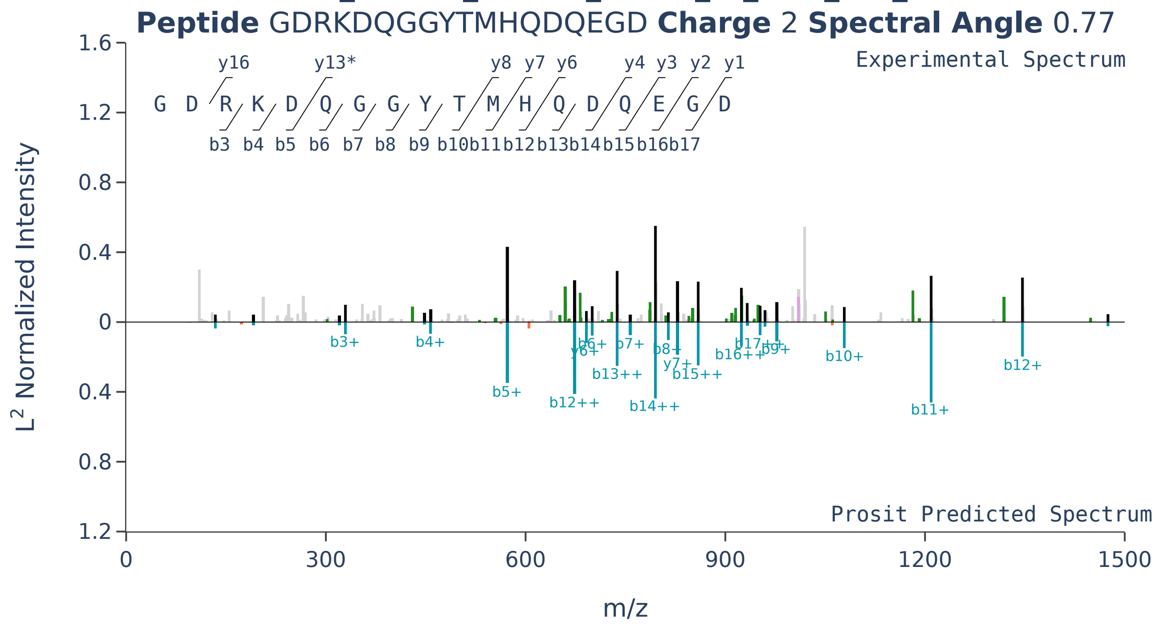


**Source** WSoh_221121_011221_HFGoe_L2_hTau_24h_R1 **Scan** 22316
**Peptide** GIGDTPSLEDEAAGHVTQARMVS **Charge** 3 **Spectral Angle** 0.84

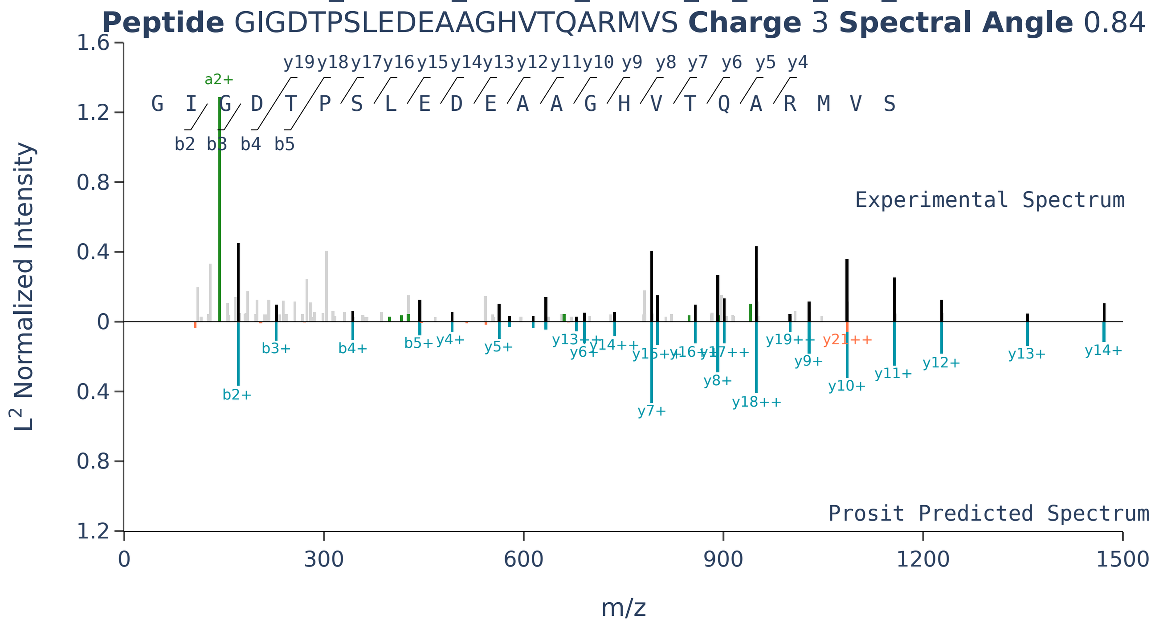


**Source** WSoh_221121_011221_HFGoe_L2_hTau_24h_R1 **Scan** 19107
**Peptide** AGLGDTPSLEDEAAGHVTQAR **Charge** 3 **Spectral Angle** 0.86

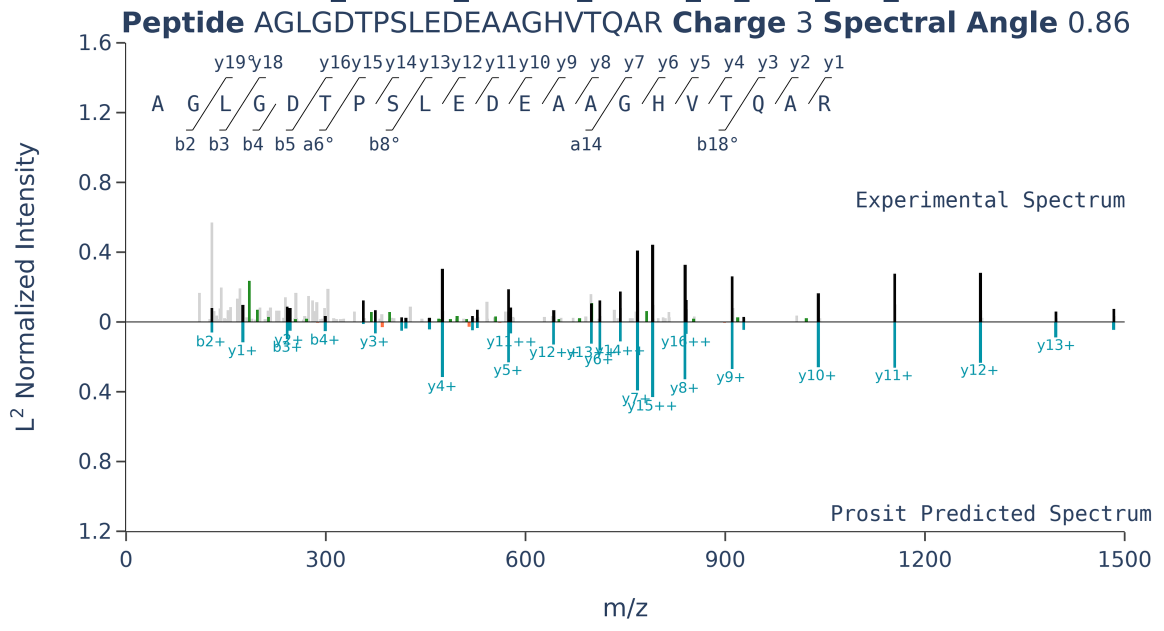


**Source** WSoh_221121_011221_HFGoe_L2_hTau_24h_R2 **Scan** 23718
**Peptide** EAGIGDTPSLEDEAAGHVTQARMV **Charge** 3 **Spectral Angle** 0.83

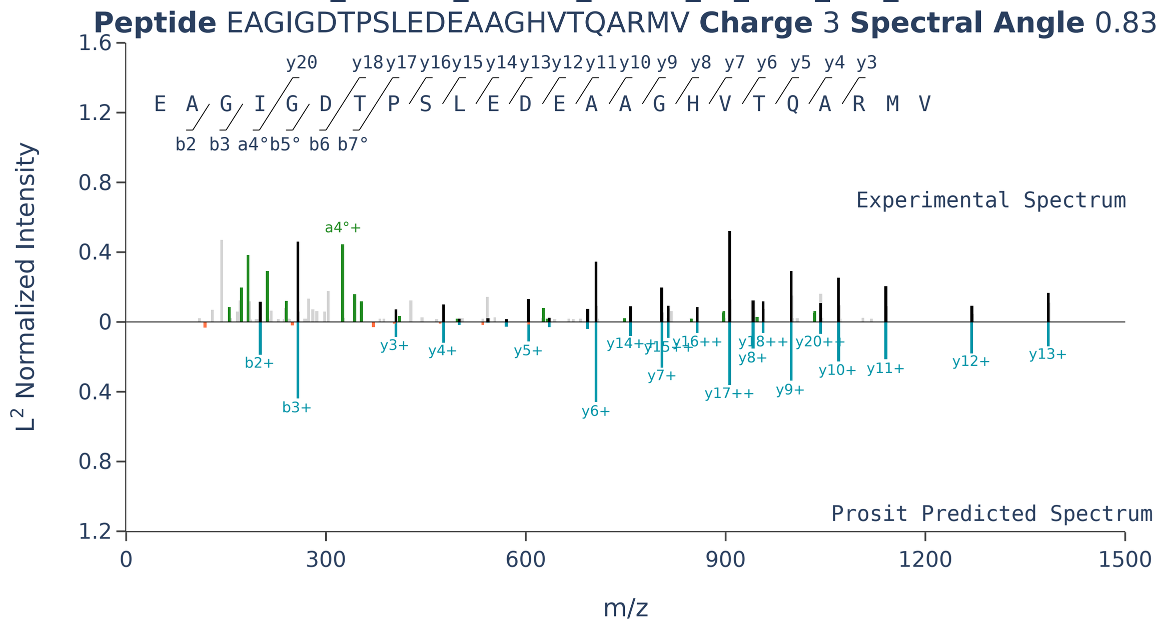

**Source** WSoh_221121_011221_HFGoe_L1_hTau_24h_R1 **Scan** 24491
**Peptide** LQTAPVPMPDLKN **Charge** 2 **Spectral Angle** 0.87

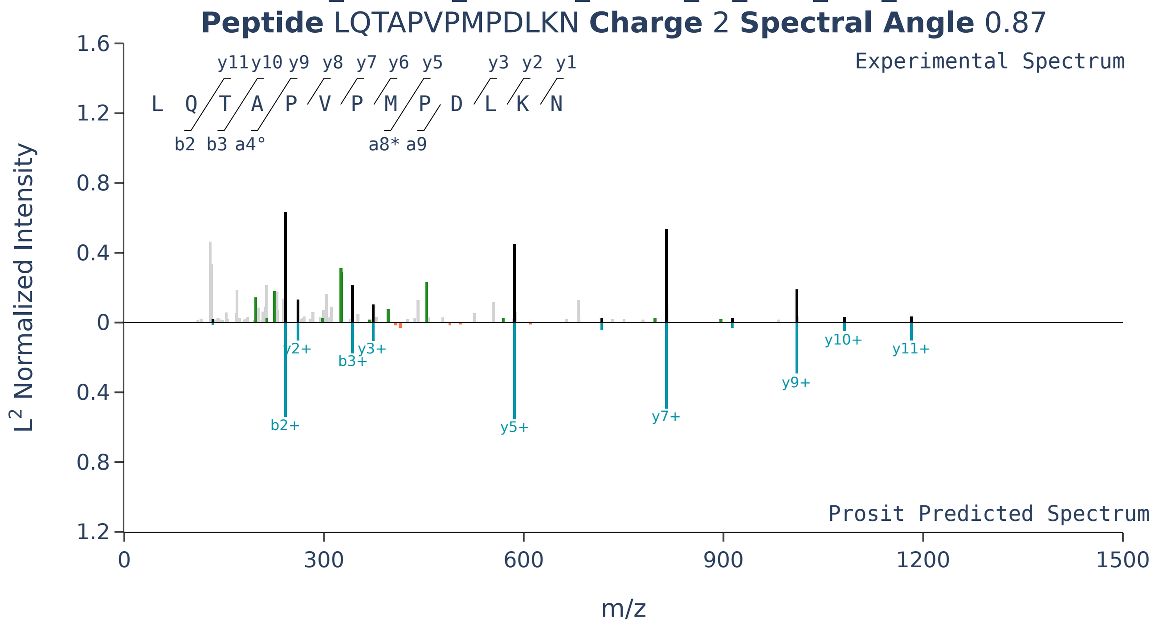


**Source** WSoh_221121_011221_HFGoe_L1_hTau_24h_R1 **Scan** 18418
**Peptide** TDAGLKESPLQTPTEDGSEE **Charge** 2 **Spectral Angle** 0.82

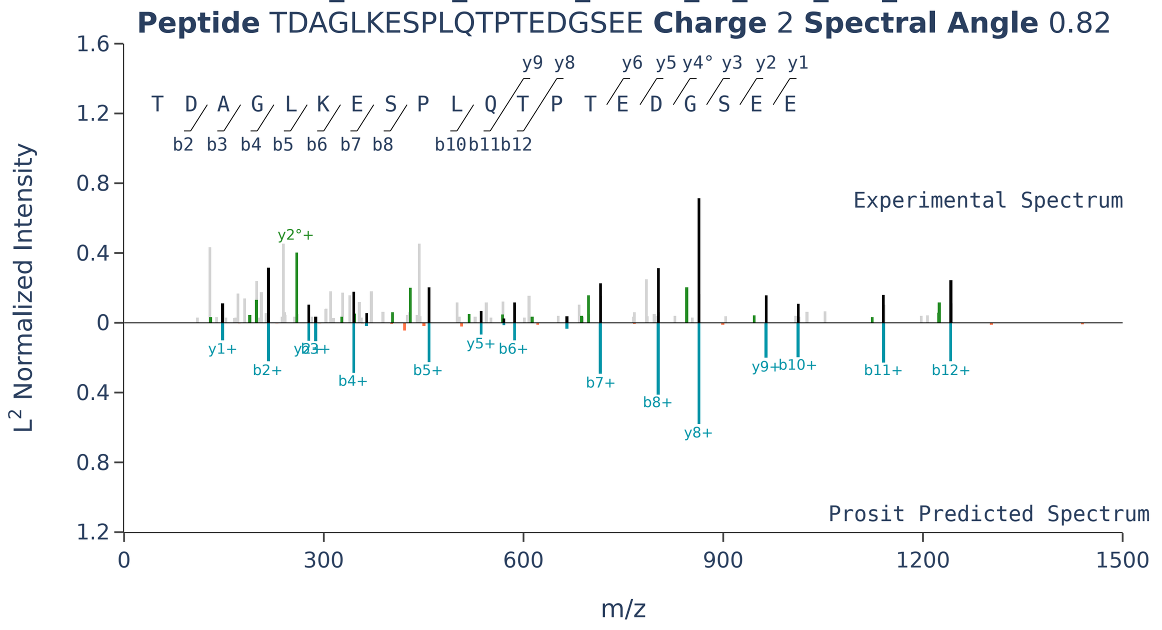


**Source** WSoh_221121_011221_HFGoe_L2_hTau_1h_R2 **Scan** 6370
**Peptide** THVPGGGNKKIET **Charge** 3 **Spectral Angle** 0.91

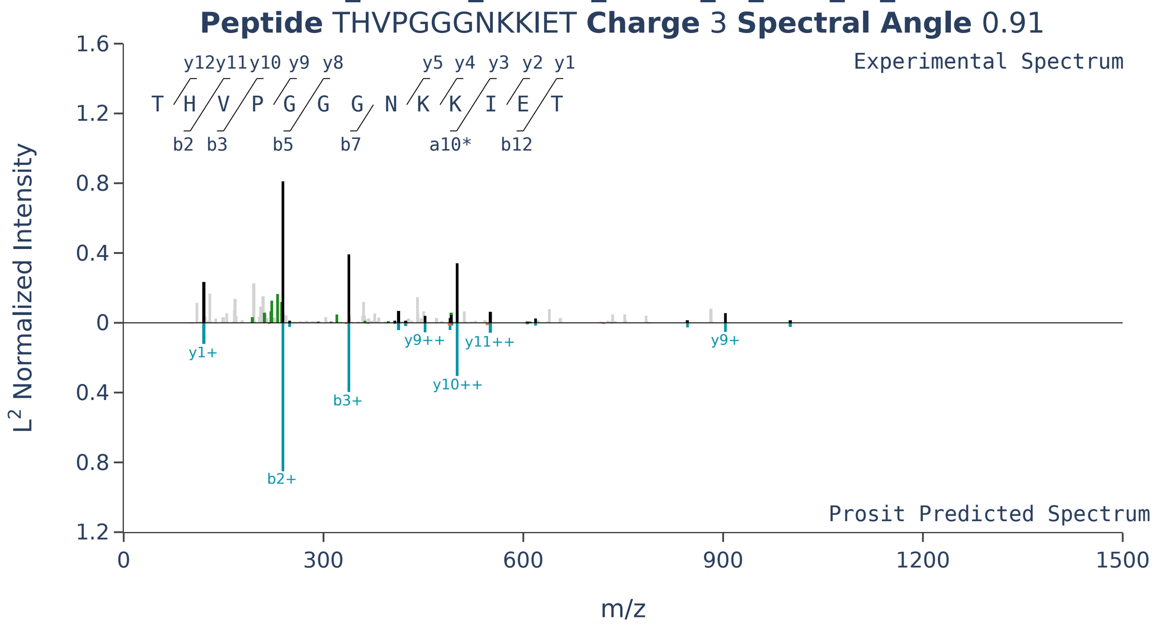


**Source** WSoh_221121_011221_HFGoe_L2_hTau_2h_R2 **Scan** 15462
**Peptide** SPVVSGDTSPRHLSNVSSTGSID **Charge** 3 **Spectral Angle** 0.91

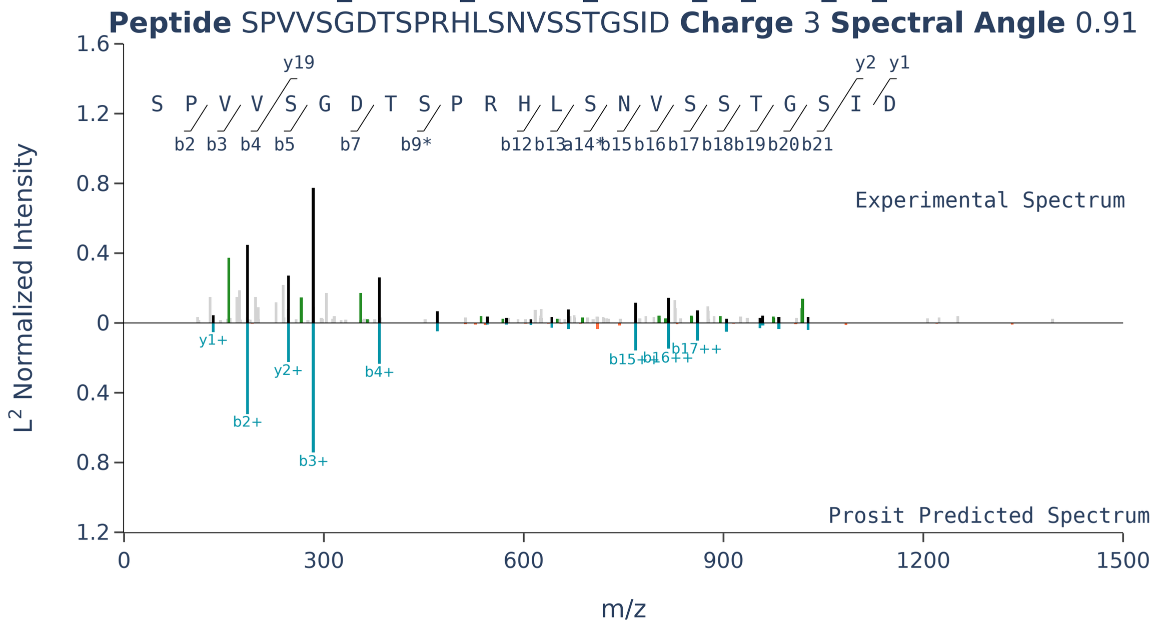


**Source** WSoh_221121_011221_HFGoe_L2_hTau_24h_R2 **Scan** 11910
**Peptide** GGYTMHQDQEGDTDAGLKE **Charge** 3 **Spectral Angle** 0.89

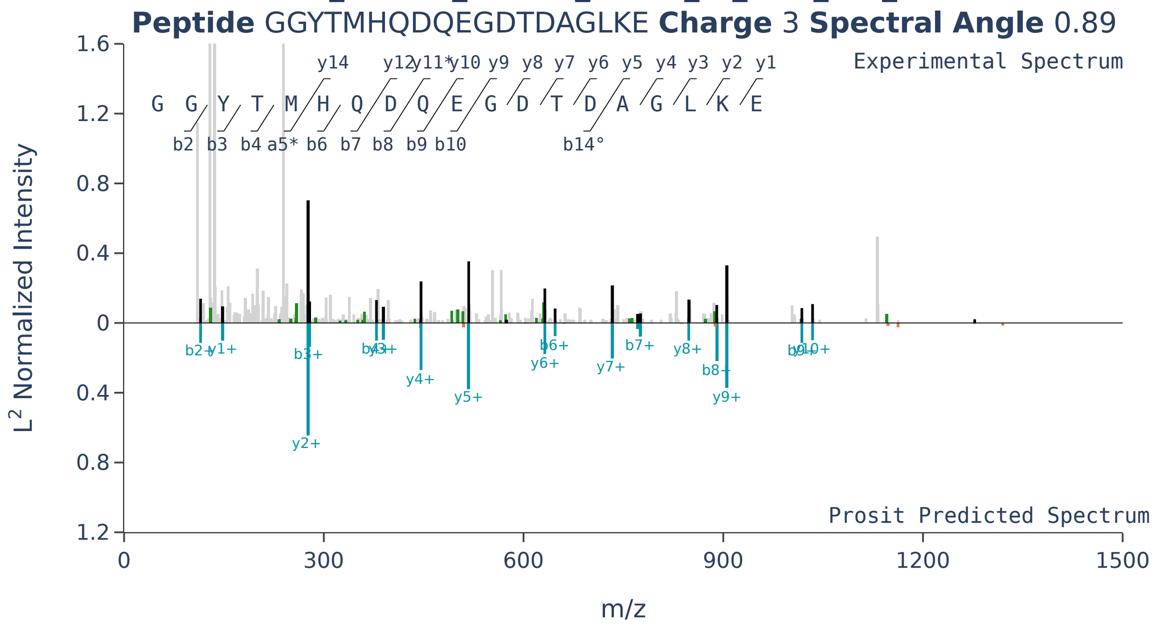


**Source** WSoh_221121_011221_HFGoe_L1_hTau_2h_R1 **Scan** 17432
**Peptide** YKPVDLSKV **Charge** 2 **Spectral Angle** 0.93

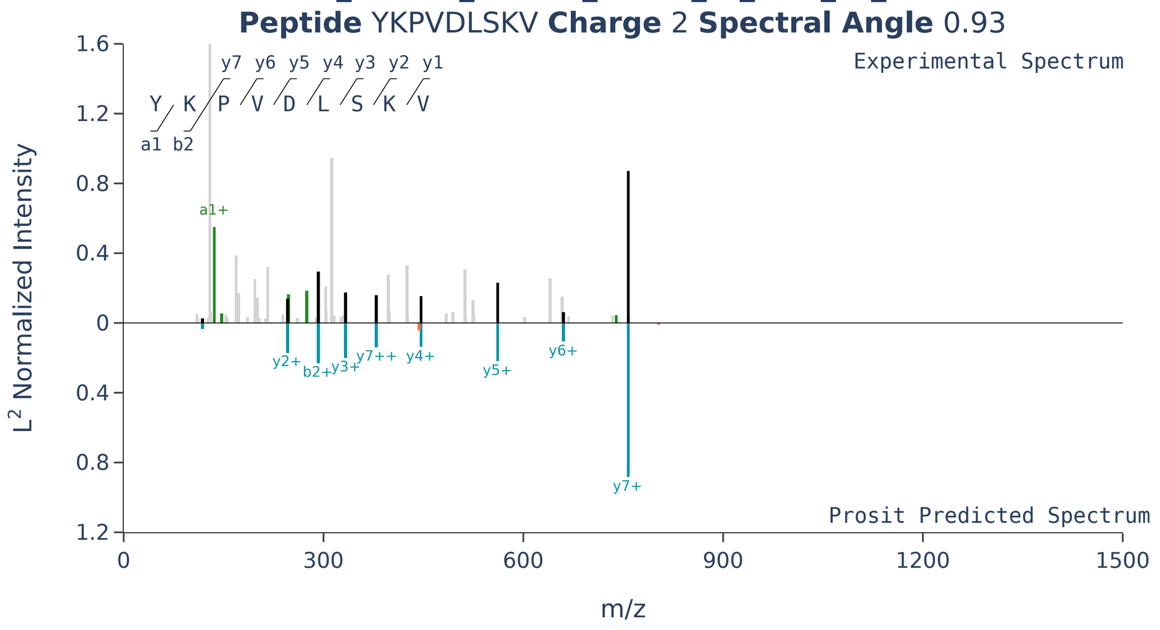


**Source** WSoh_221121_011221_HFGoe_L1_hTau_24h_R1 **Scan** 18406
**Peptide** AKSRLQTAPVPMPDLKNVKS **Charge** 3 **Spectral Angle** 0.87

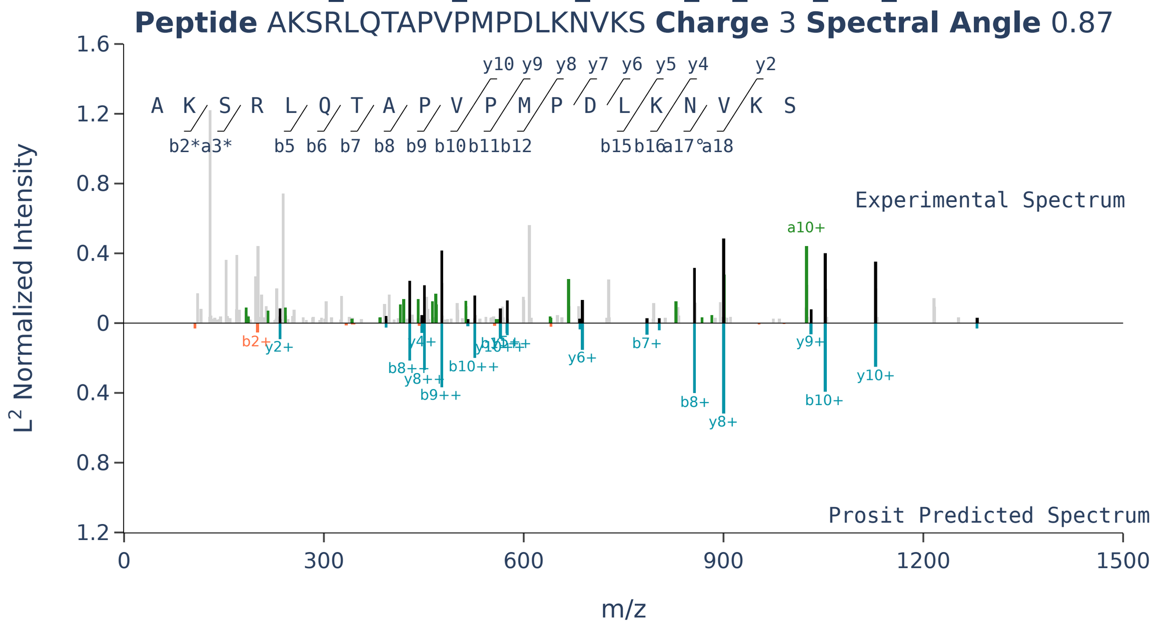


**Source** WSoh_221121_011221_HFGoe_L2_hTau_24h_R1 **Scan** 16524
**Peptide** GSLDNITHVPGGGNKKIETHKLTFR **Charge** 5 **Spectral Angle** 0.82

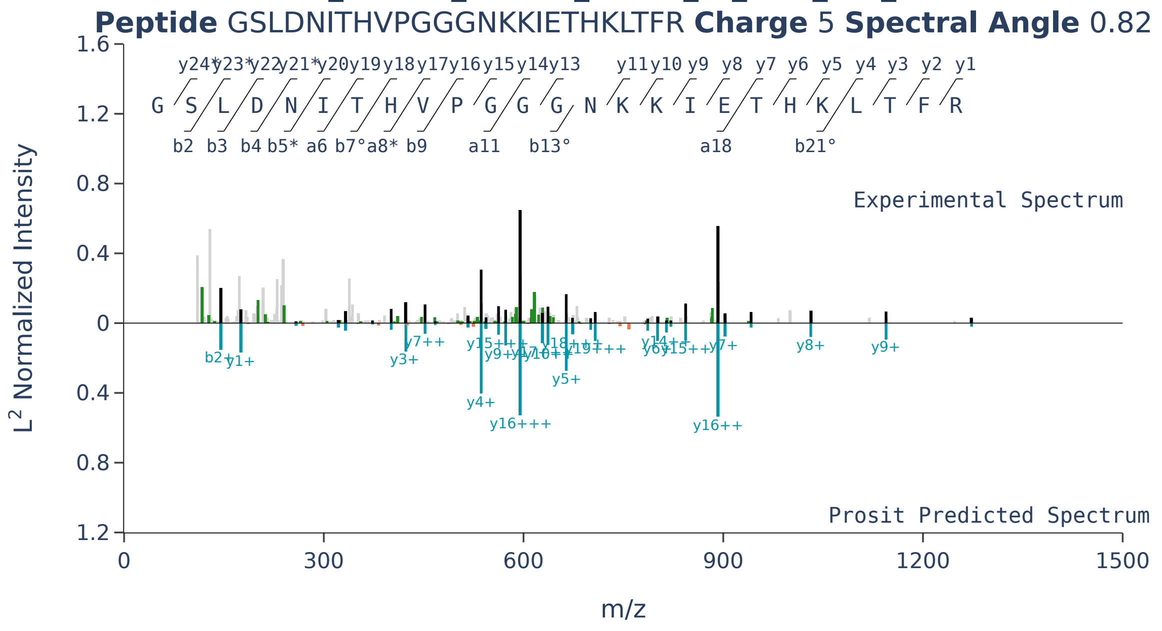


**Source** WSoh_221121_011221_HFGoe_L1_hTau_24h_R1 **Scan** 14977
**Peptide** GSKDNIKHVPGGGSVQIV **Charge** 3 **Spectral Angle** 0.84

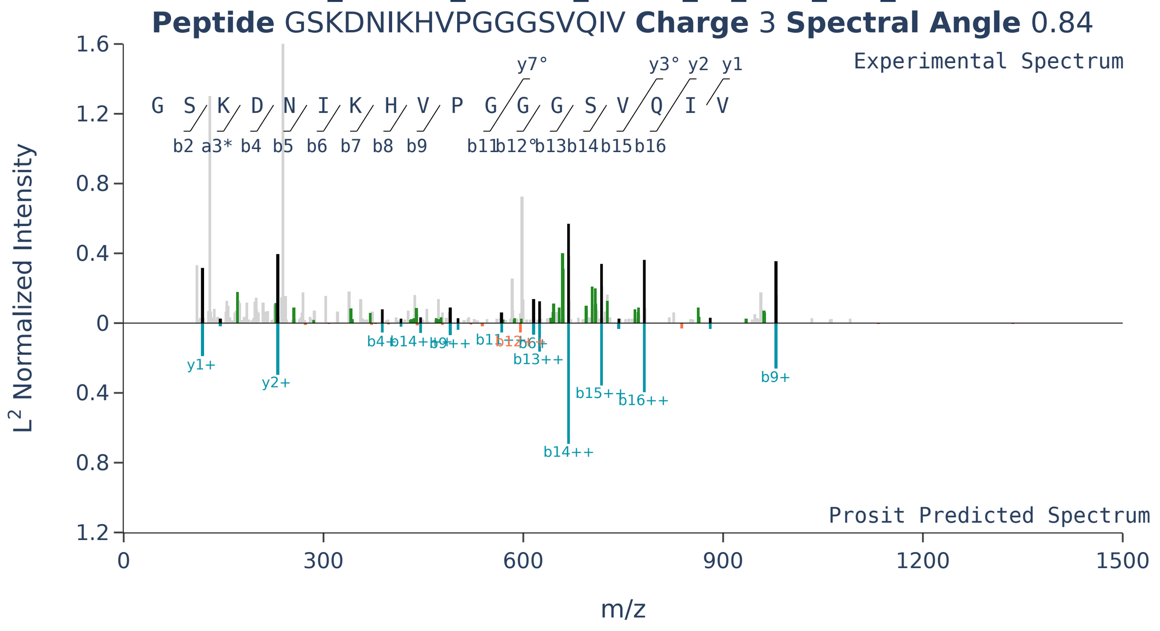


**Source** WSoh_221121_011221_HFGoe_L2_hTau_24h_R2 **Scan** 21228
**Peptide** APVPMPDLKNVKSKIGS **Charge** 4 **Spectral Angle** 0.89

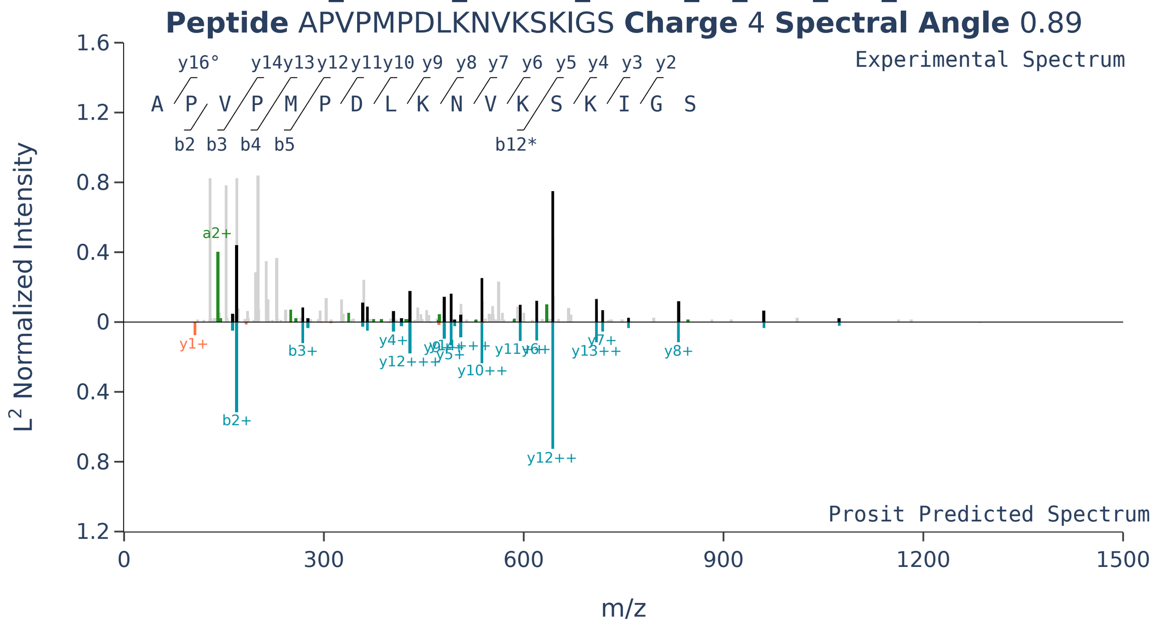


**Source** WSoh_221121_011221_HFGoe_L2_hTau_24h_R1 **Scan** 10089
**Peptide** KDRVQSKIGSL **Charge** 2 **Spectral Angle** 0.87

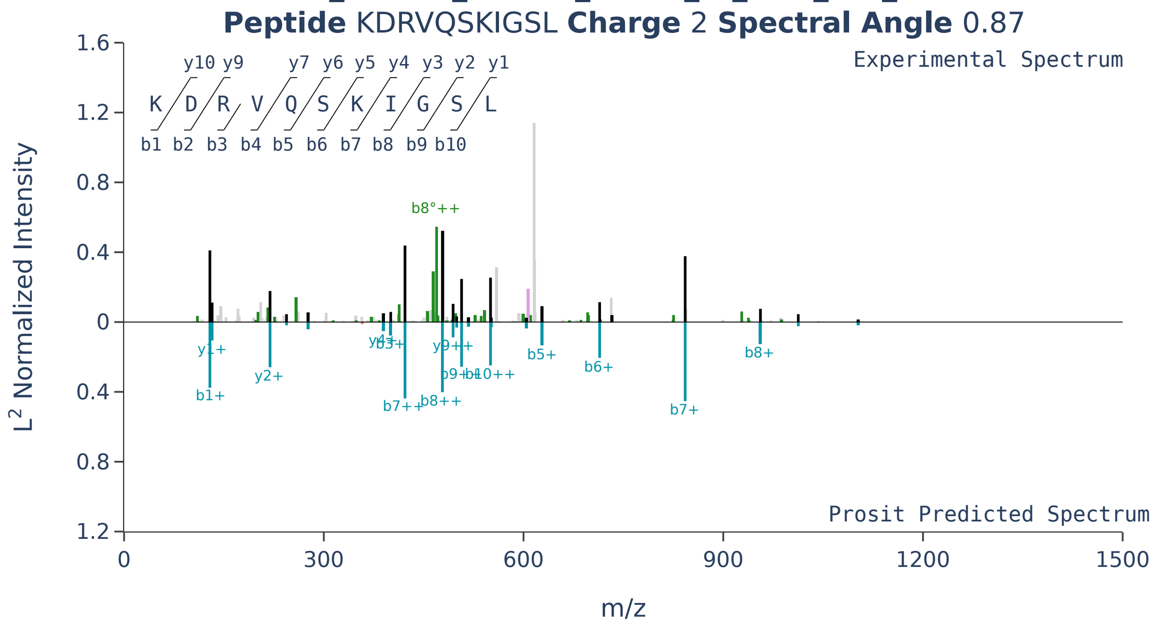


**Source** WSoh_221121_011221_HFGoe_L1_hTau_24h_R2 **Scan** 5475
**Peptide** VRTPPKSPSSAKS **Charge** 2 **Spectral Angle** 0.89

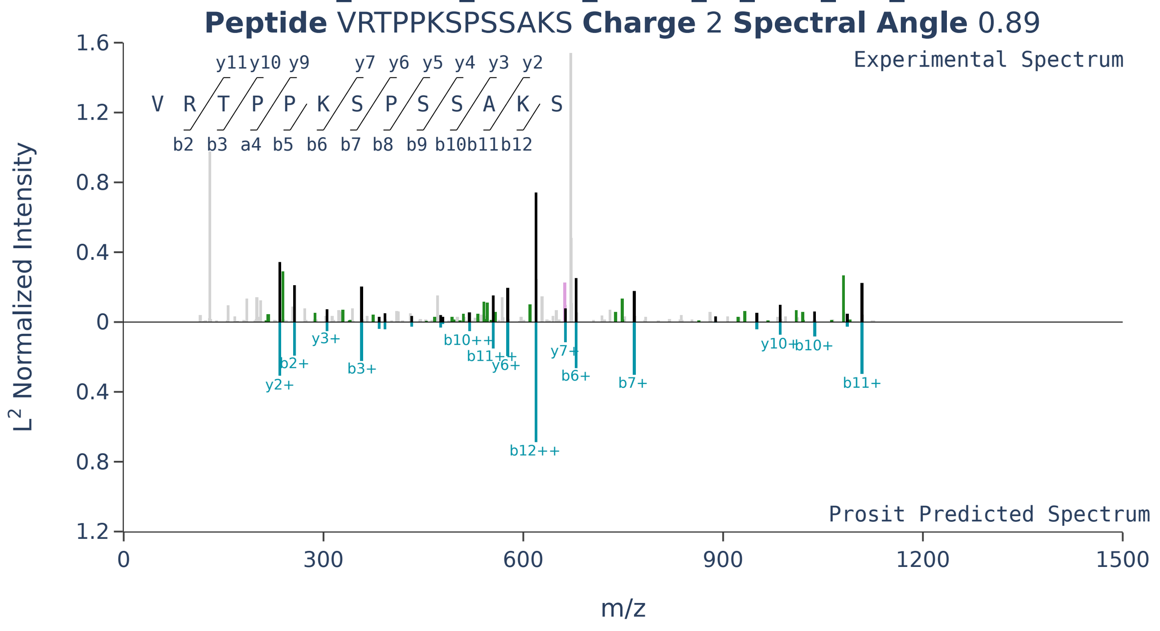


**Source** WSoh_221121_011221_HFGoe_L2_hTau_24h_R2 **Scan** 6607
**Peptide** LGDRKDQGGYT **Charge** 2 **Spectral Angle** 0.83

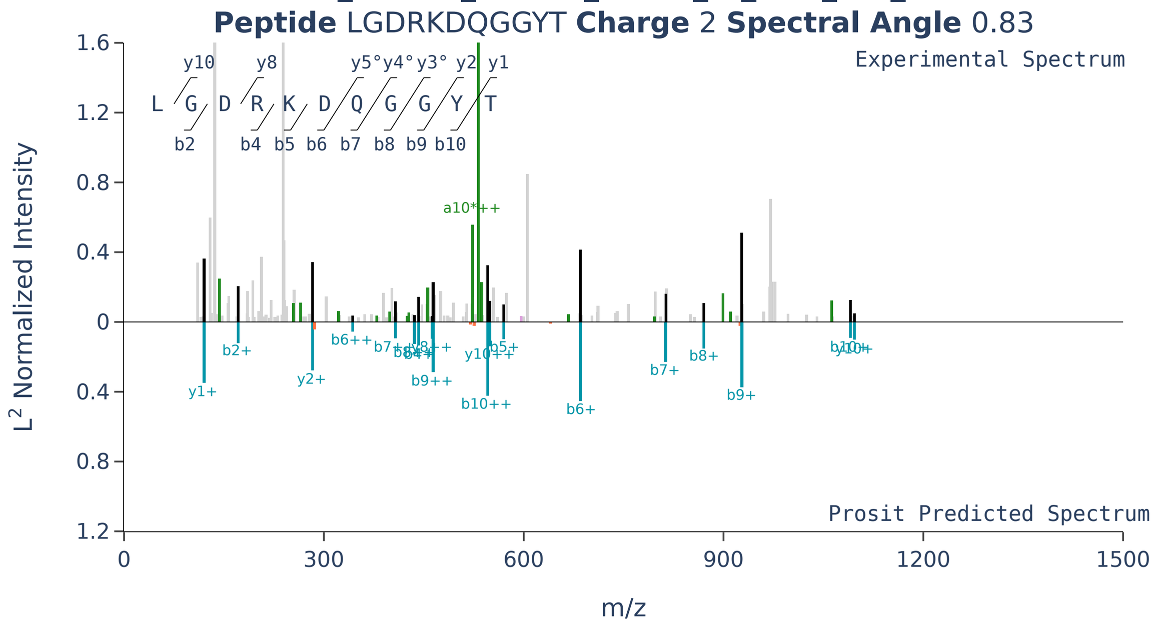


**Source** WSoh_221121_011221_HFGoe_L1_hTau_24h_R1 **Scan** 15917
**Peptide** TDHGAEIVYKSPVVSGDTSPRHLSN **Charge** 3 **Spectral Angle** 0.88

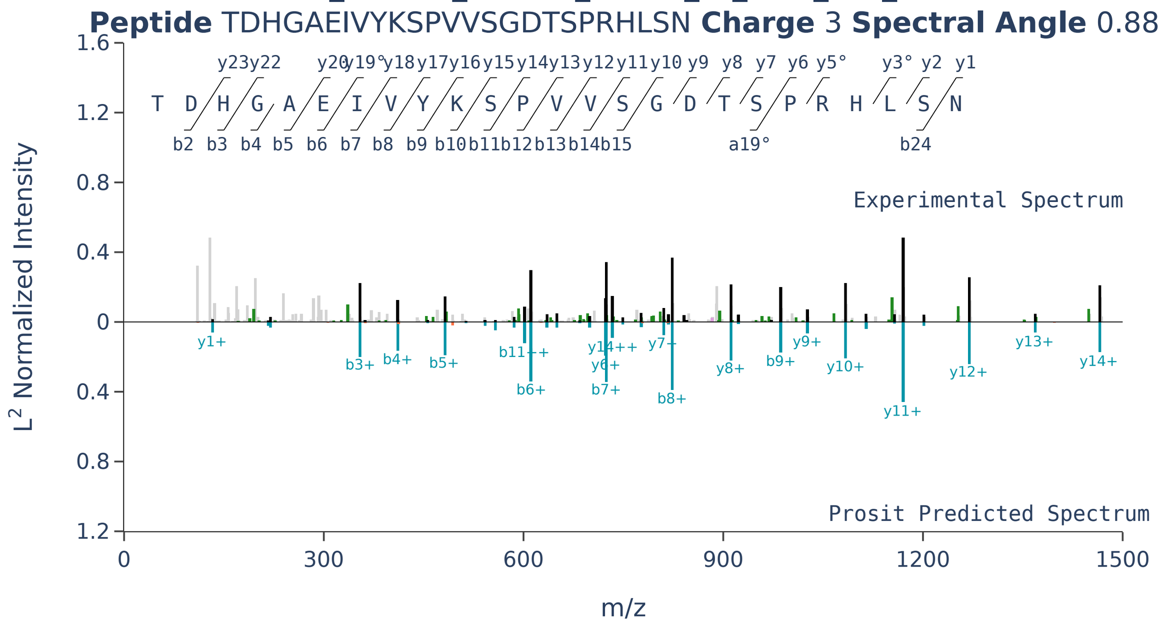


**Source** WSoh_221121_011221_HFGoe_L2_hTau_24h_R1 **Scan** 7841
**Peptide** LTHVPGGGNKKIET **Charge** 2 **Spectral Angle** 0.92

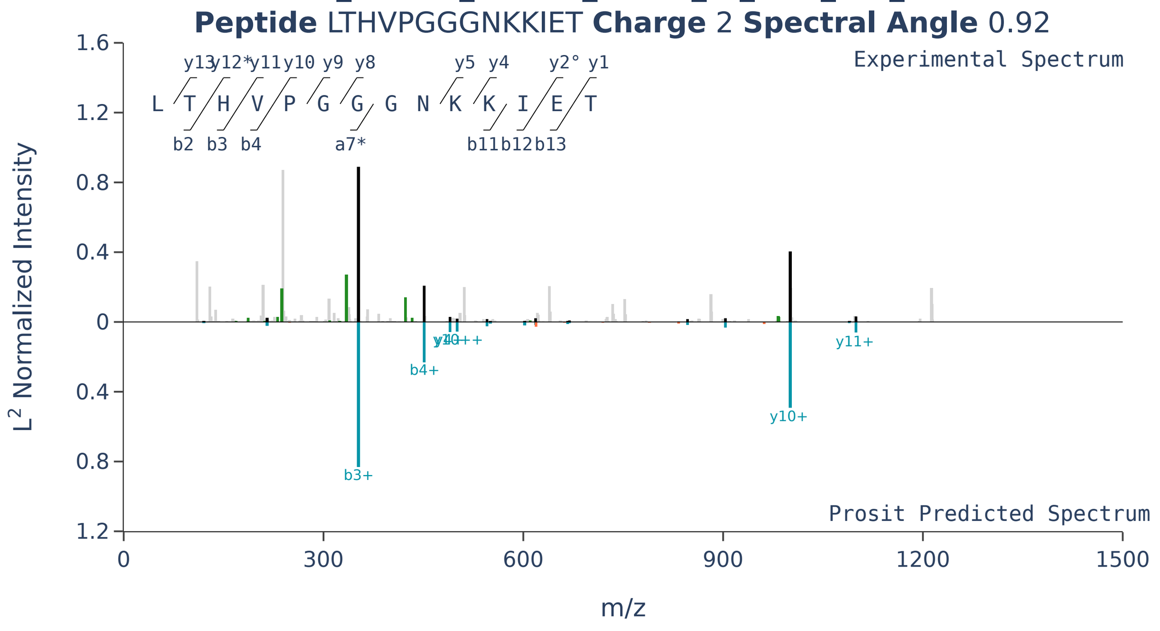

**Source** WSoh_221121_011221_HFGoe_L1_hTau_24h_R2 **Scan** 8723
**Peptide** KKLDLSNVQSK **Charge** 2 **Spectral Angle** 0.73

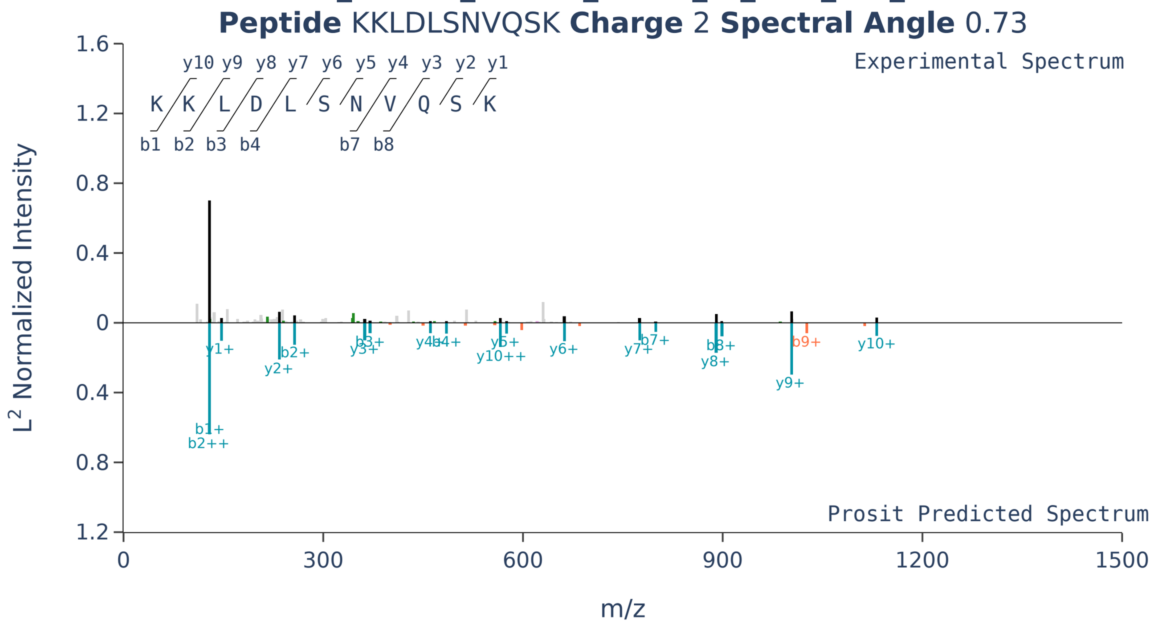


**Source** WSoh_221121_011221_HFGoe_L2_hTau_24h_R2 **Scan** 22106
**Peptide** PTAEDVTAPLVDEGAPGKQA **Charge** 2 **Spectral Angle** 0.93

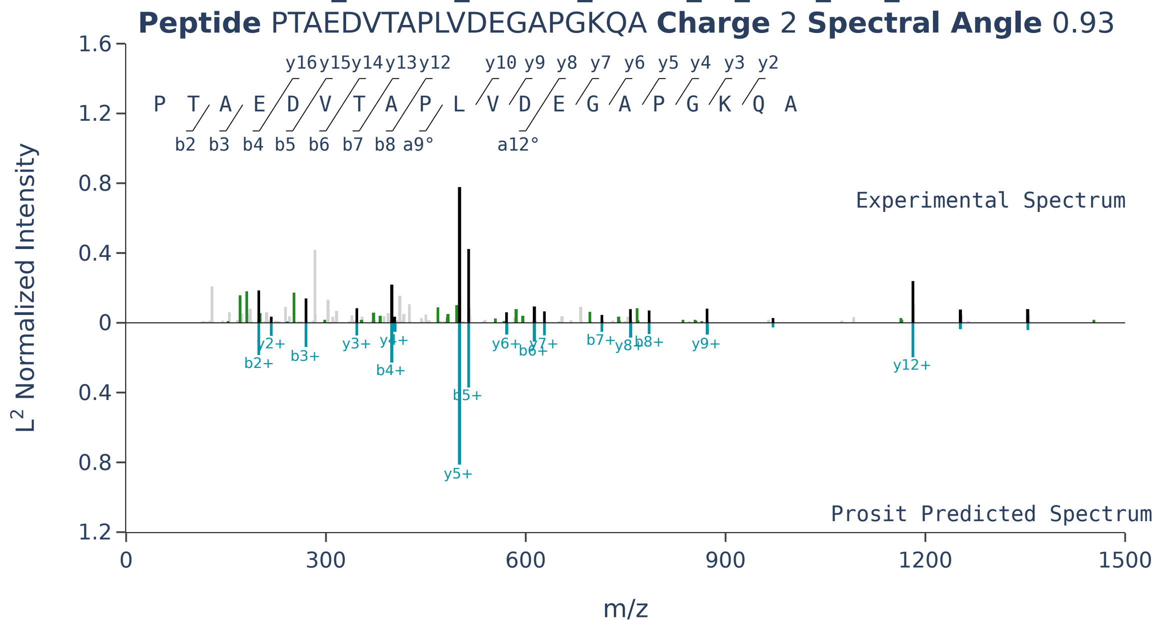


**Source** WSoh_221121_011221_HFGoe_L2_hTau_4h_R2 **Scan** 15481
**Peptide** PVVSGDTSPRHLSNVSSTGSLD **Charge** 3 **Spectral Angle** 0.93

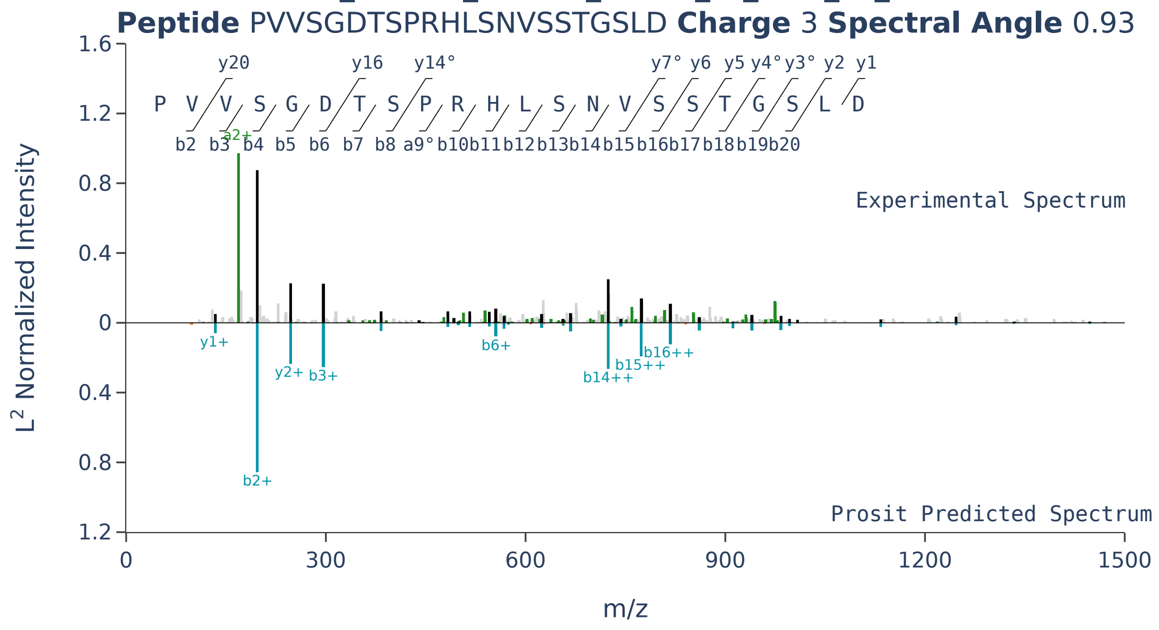


**Source** WSoh_221121_011221_HFGoe_L2_hTau_24h_R1 **Scan** 18159
**Peptide** IINKKLDLSNV **Charge** 3 **Spectral Angle** 0.85

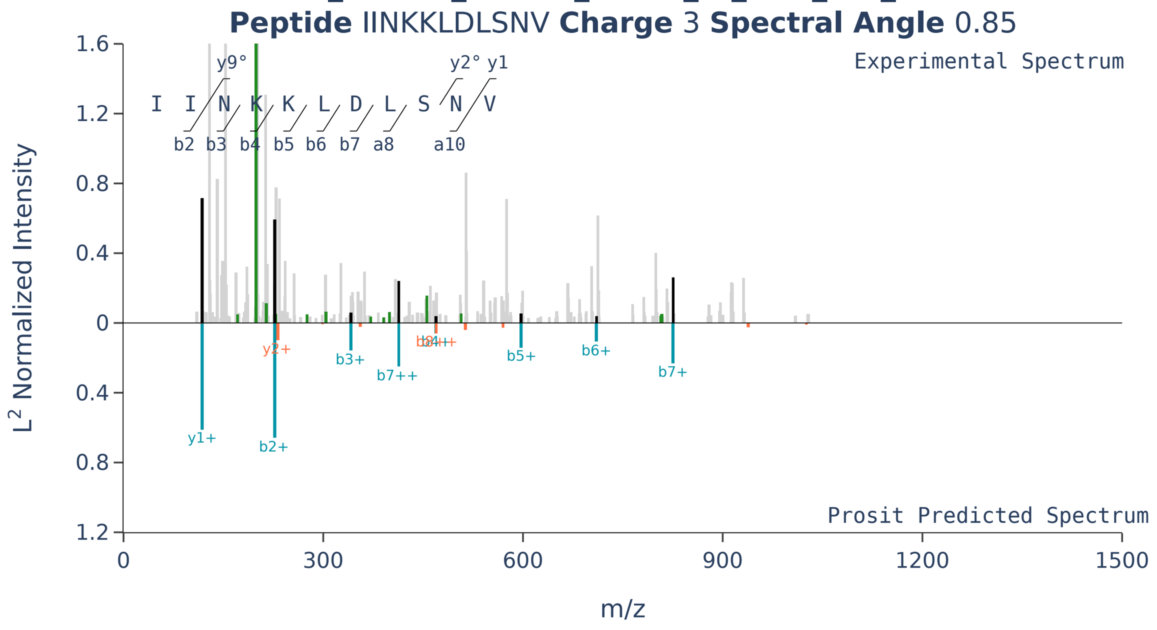


**Source** WSoh_221121_011221_HFGoe_L1_hTau_4h_R2 **Scan** 18949
**Peptide** VSSTGSIDMVD **Charge** 2 **Spectral Angle** 0.9

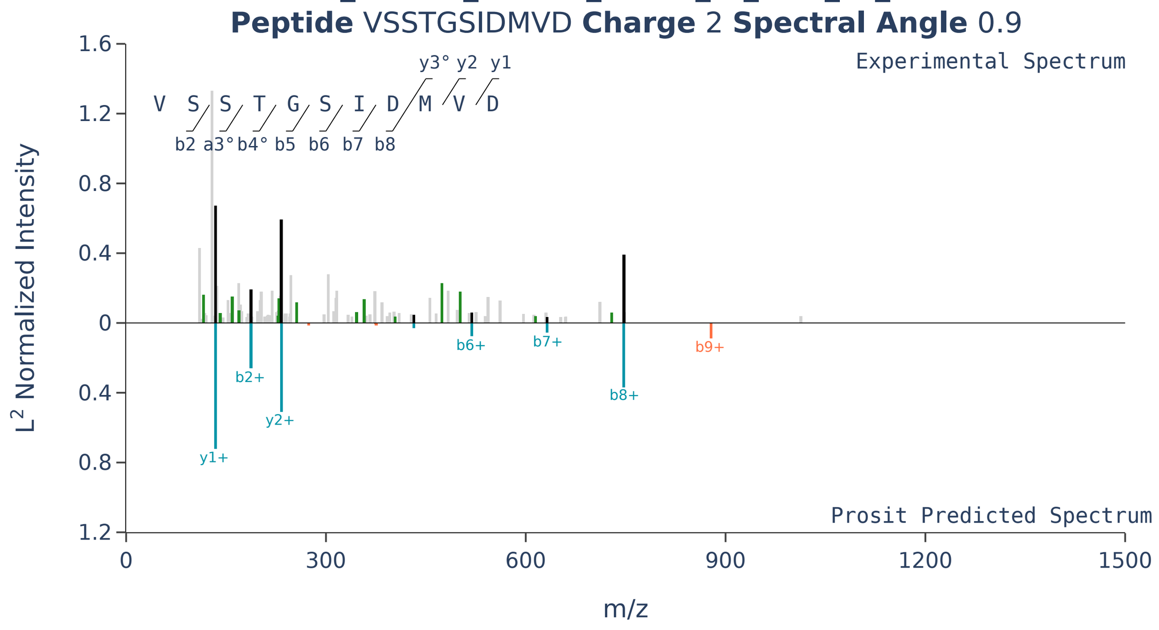


**Source** WSoh_221121_011221_HFGoe_L1_hTau_4h_R2 **Scan** 16664
**Peptide** AEPRQEFEV **Charge** 2 **Spectral Angle** 0.87

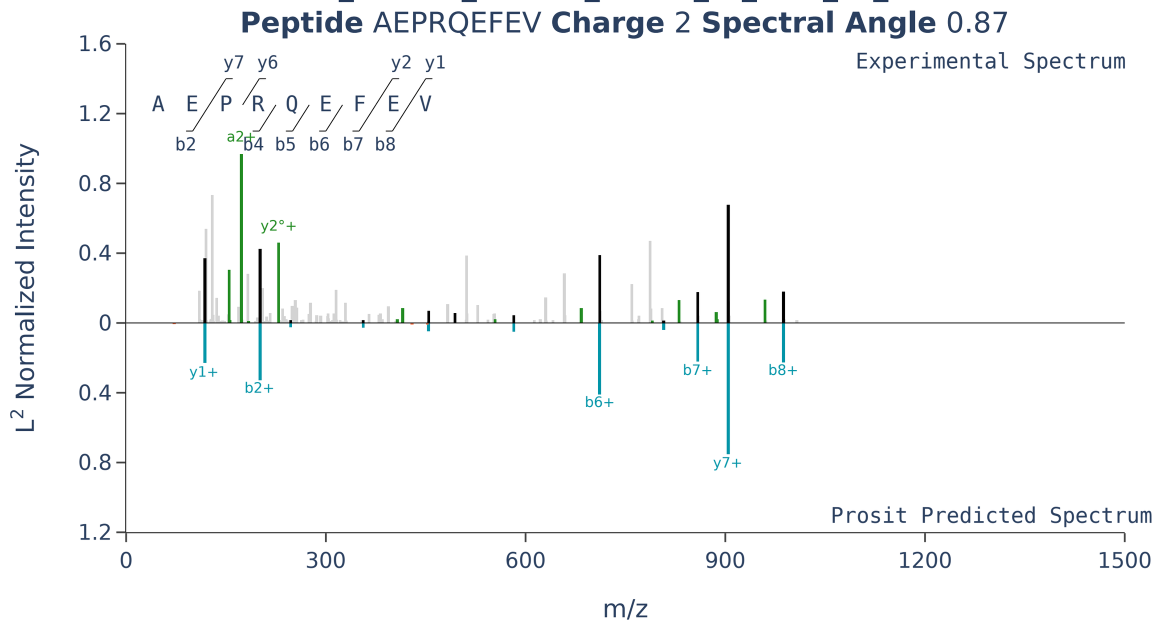


**Source** WSoh_221121_011221_HFGoe_L2_hTau_24h_R1 **Scan** 7397
**Peptide** ITHVPGGGNKKI **Charge** 3 **Spectral Angle** 0.85

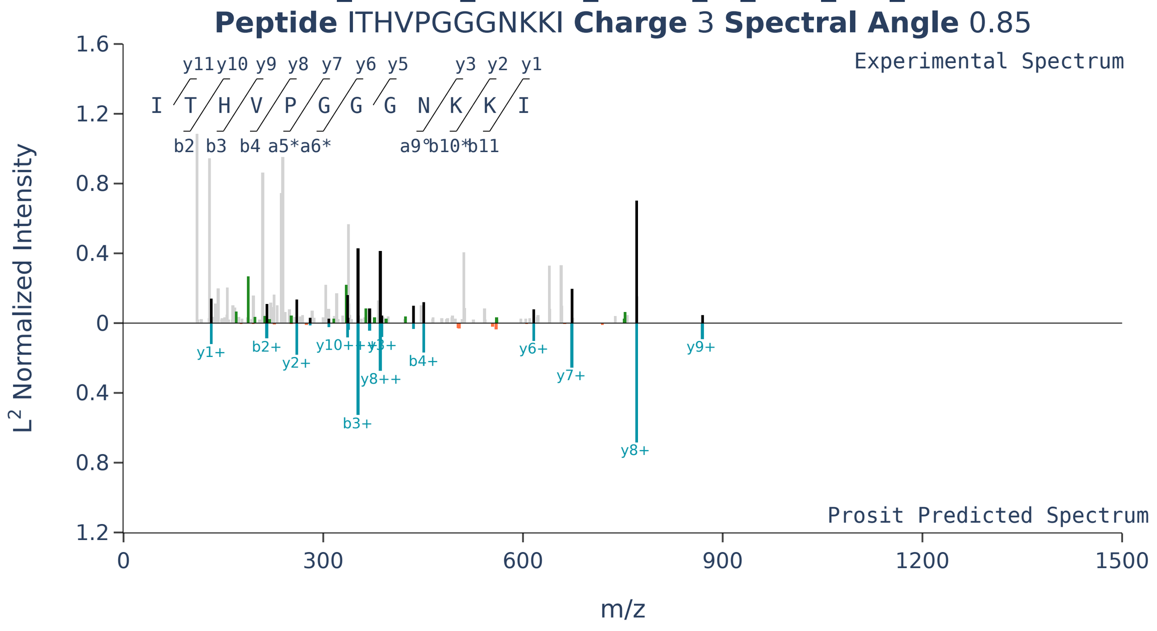


**Source** WSoh_221121_011221_HFGoe_L2_hTau_24h_R2 **Scan** 11984
**Peptide** EGAPGKQAAAQPHTEIPEGTT **Charge** 3 **Spectral Angle** 0.83

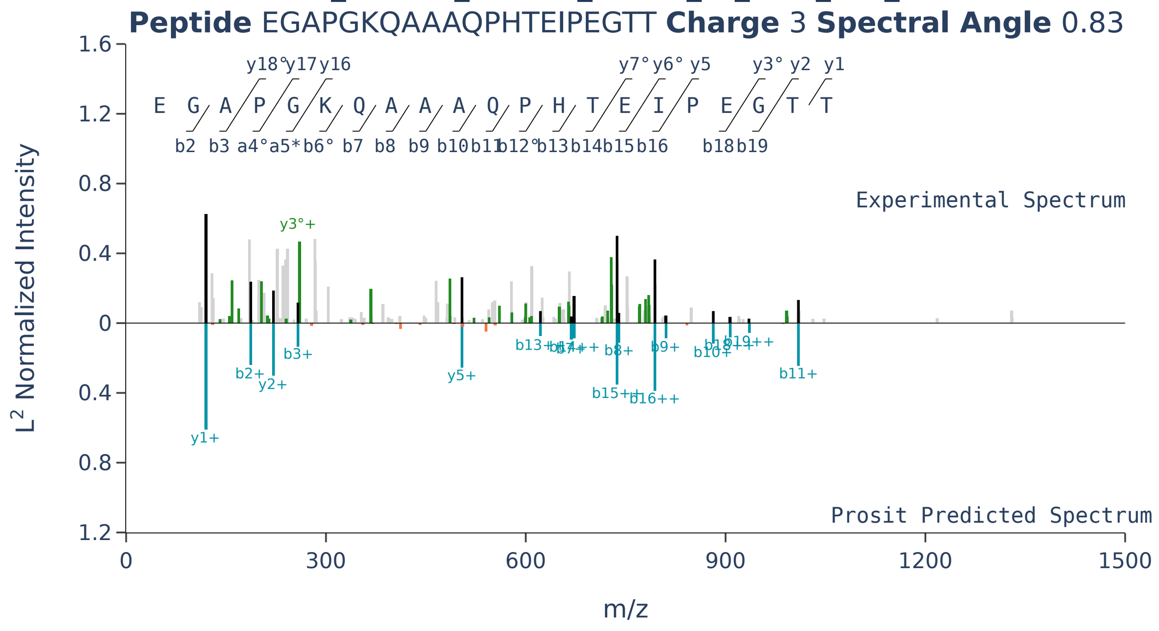


**Source** WSoh_221121_011221_HFGoe_L2_hTau_4h_R2 **Scan** 16991
**Peptide** KDRVQSKIGSLDNITHV **Charge** 3 **Spectral Angle** 0.79

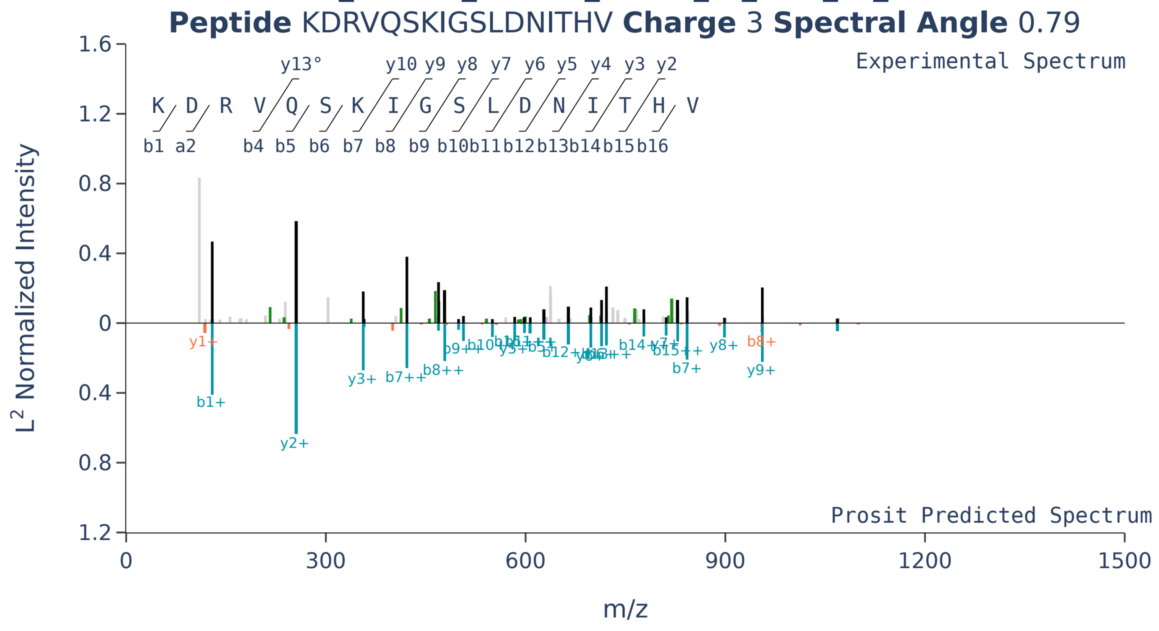


**Source** WSoh_221121_011221_HFGoe_L1_hTau_2h_R2 **Scan** 7871
**Peptide** AVVRTPPKSPSSAKSRL **Charge** 4 **Spectral Angle** 0.83

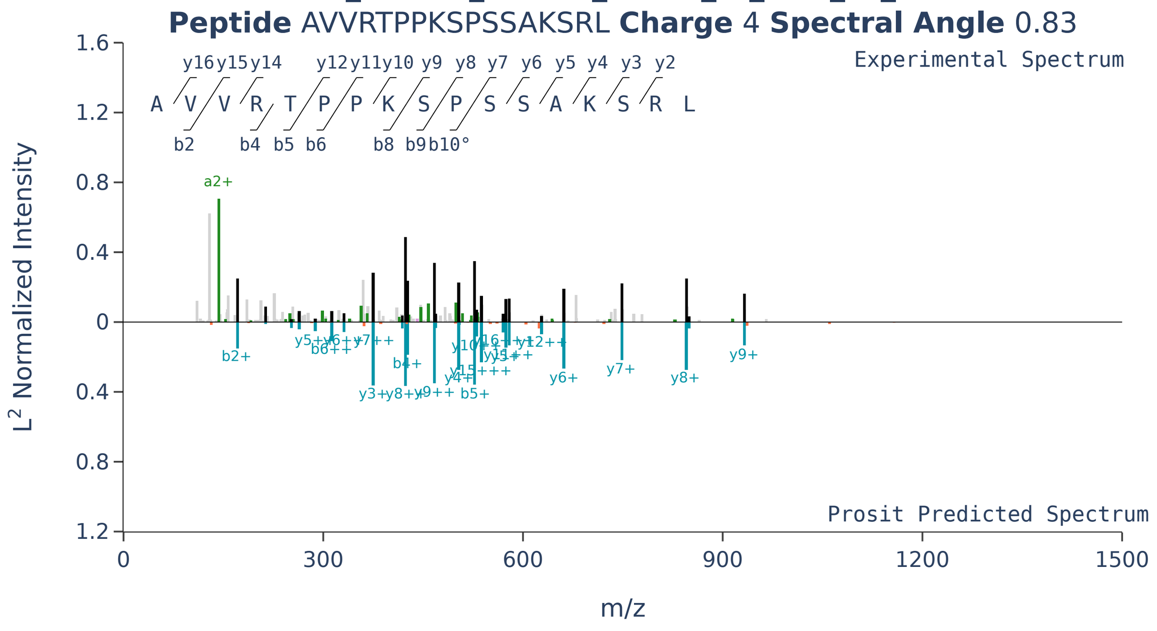


**Source** WSoh_221121_011221_HFGoe_L2_hTau_1h_R1 **Scan** 5919
**Peptide** KGADGKTKIATPRGA **Charge** 3 **Spectral Angle** 0.85

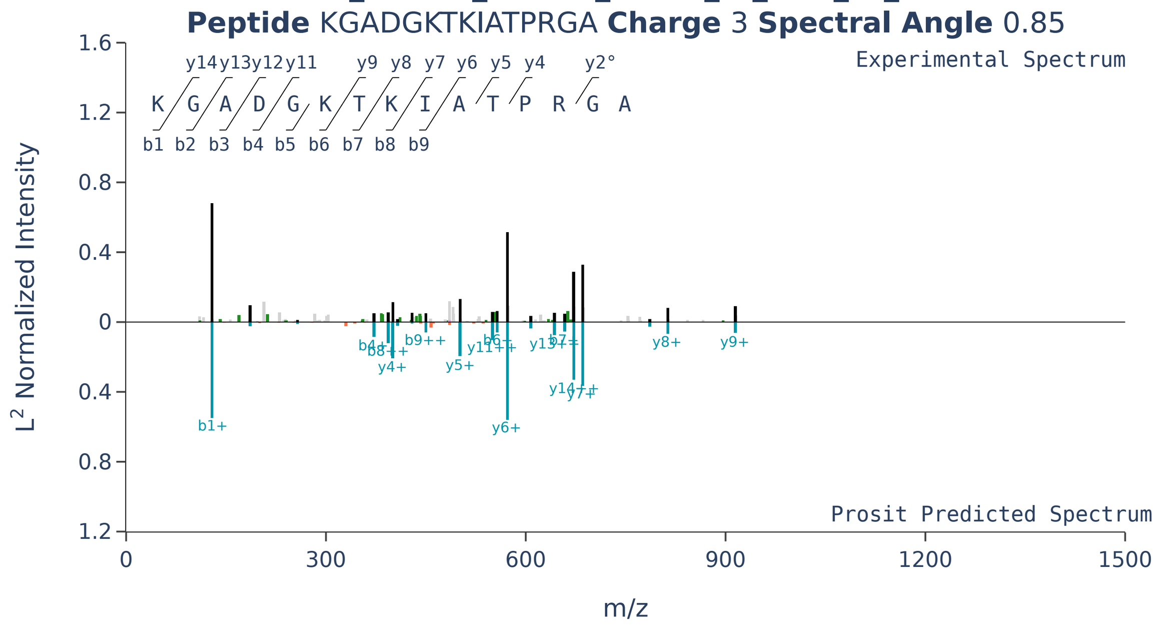


**Source** WSoh_221121_011221_HFGoe_L2_hTau_4h_R2 **Scan** 13818
**Peptide** KLGSLDNITH **Charge** 3 **Spectral Angle** 0.89

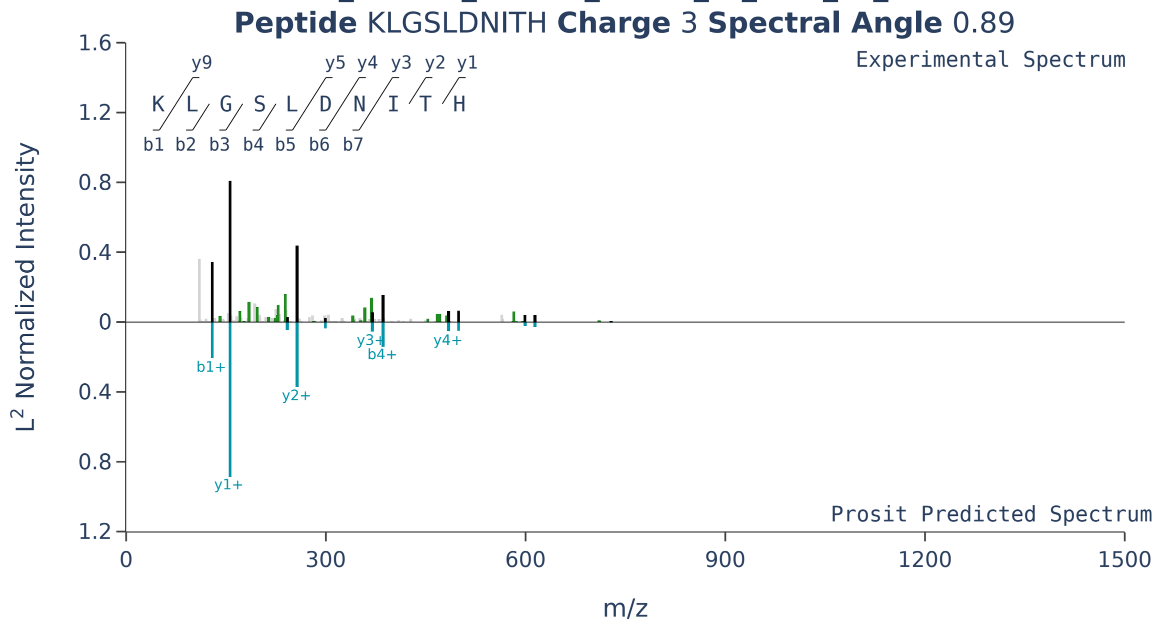


**Source** WSoh_221121_011221_HFGoe_L2_hTau_24h_R2 **Scan** 15669
**Peptide** AKTDHGAEIVYKSPVVSGDTSPRHL **Charge** 5 **Spectral Angle** 0.83

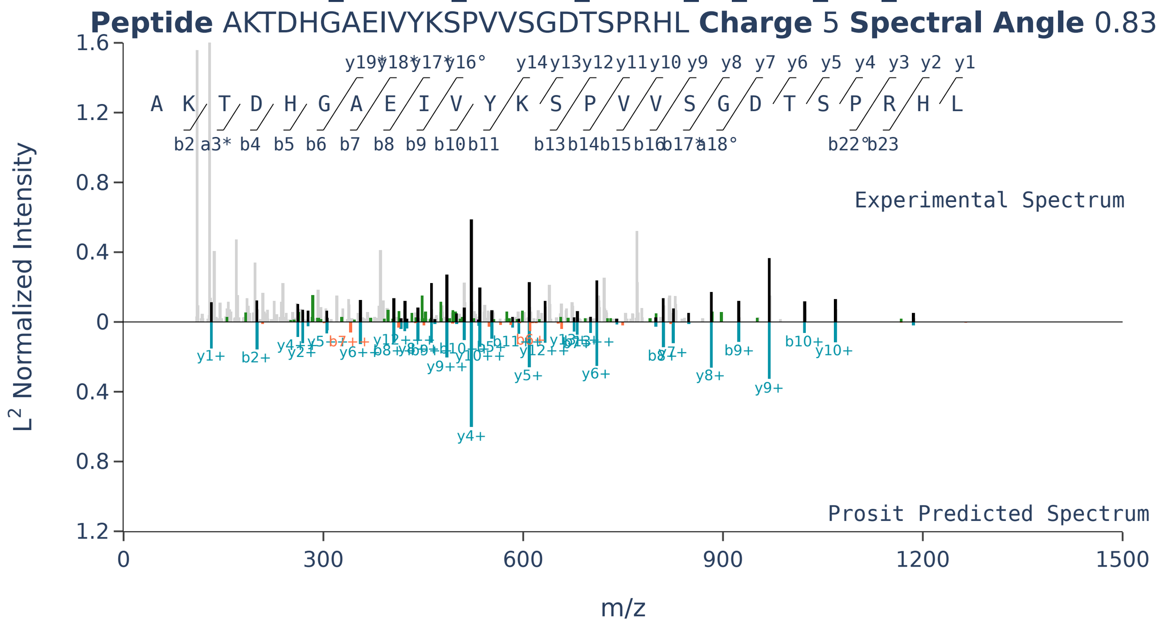


**Source** WSoh_221121_011221_HFGoe_L1_hTau_24h_R2 **Scan** 19589
**Peptide** QLVYKPVDLSKVTSKCG **Charge** 3 **Spectral Angle** 0.79

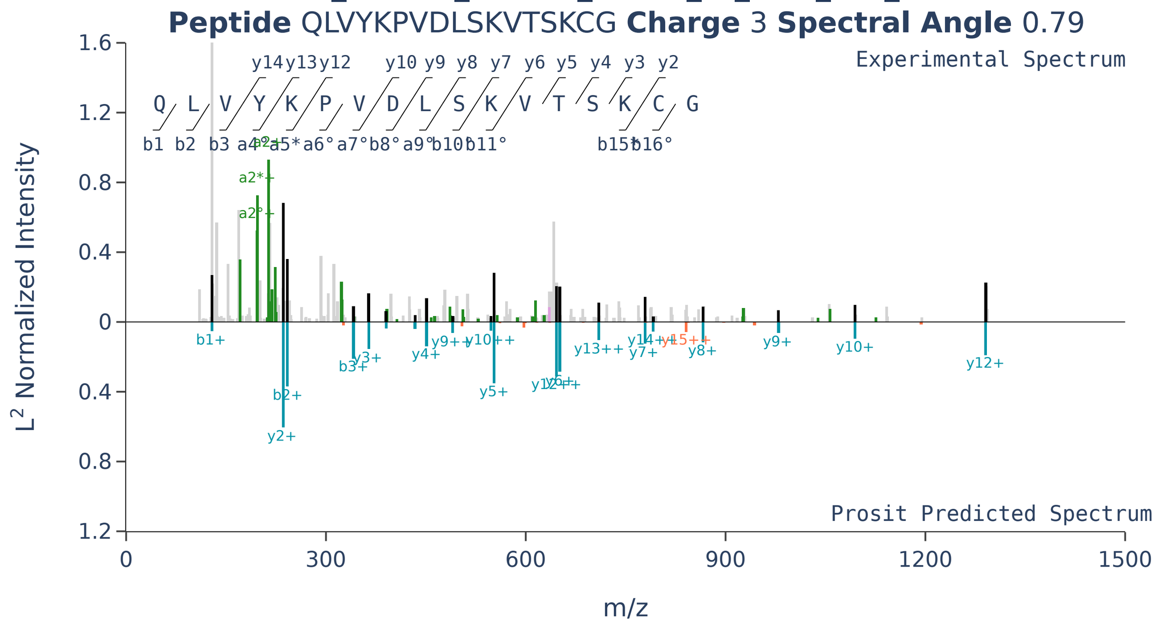


**Source** WSoh_221121_011221_HFGoe_L2_hTau_24h_R2 **Scan** 19138
**Peptide** TDAGLKESPLQTPTEDGSEEPGSE **Charge** 2 **Spectral Angle** 0.92

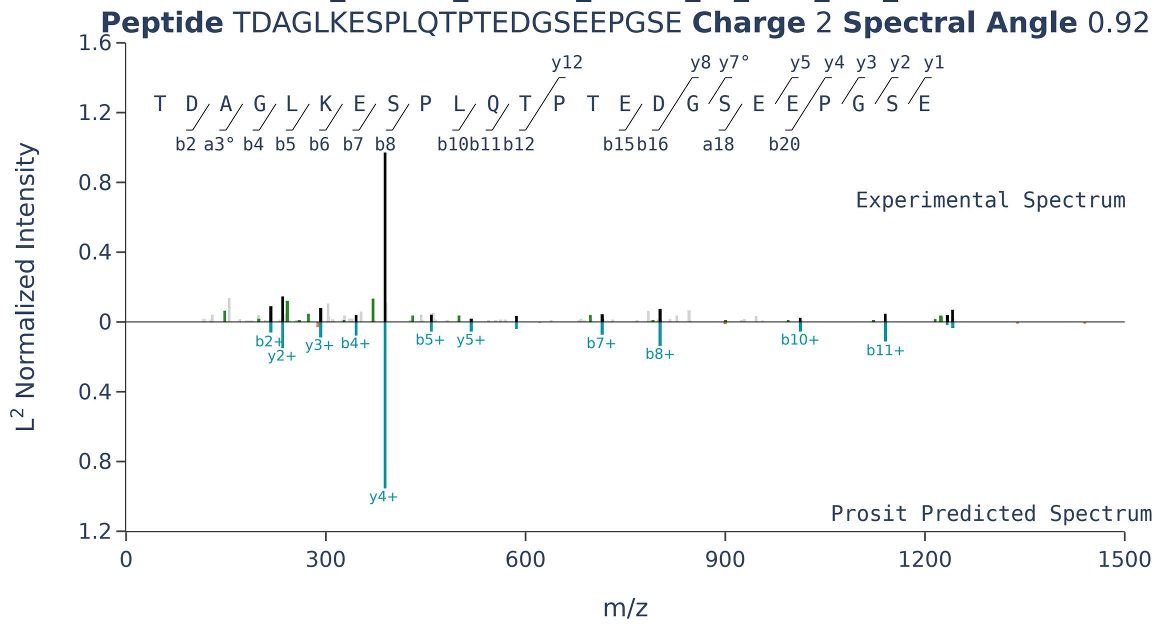

**Source** WSoh_221121_011221_HFGoe_L1_hTau_4h_R2 **Scan** 13522
**Peptide** AAQPHTEIPEGTTAEEAG **Charge** 2 **Spectral Angle** 0.89

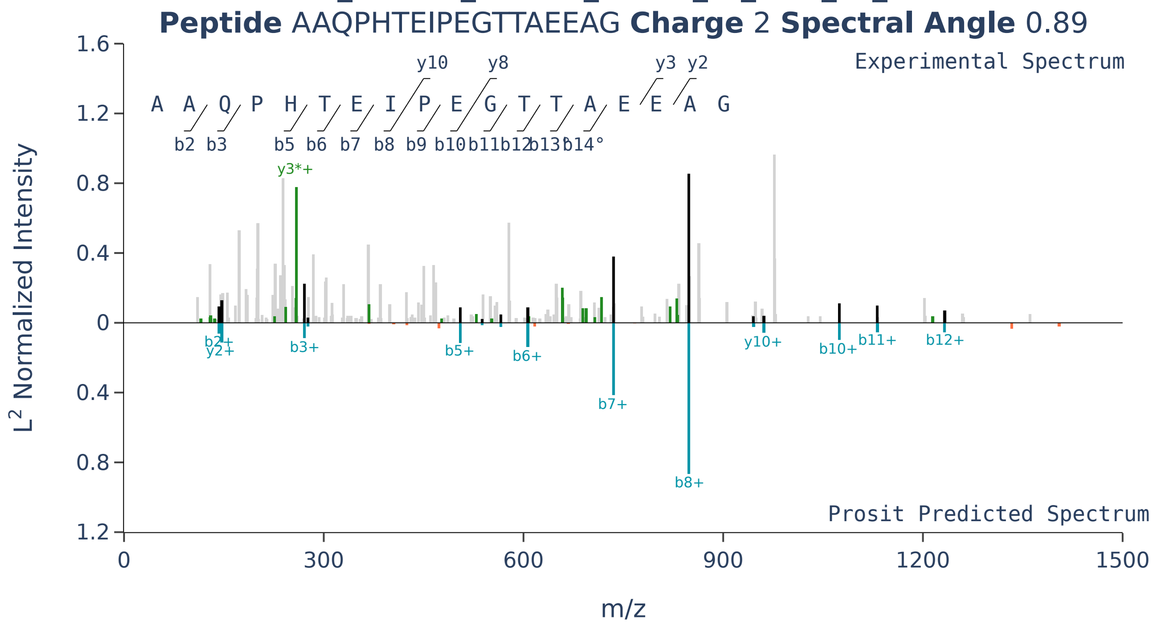


**Source** WSoh_221121_011221_HFGoe_L1_hTau_24h_R2 **Scan** 11242
**Peptide** PGSPGTPGSRSRTPSL **Charge** 3 **Spectral Angle** 0.9

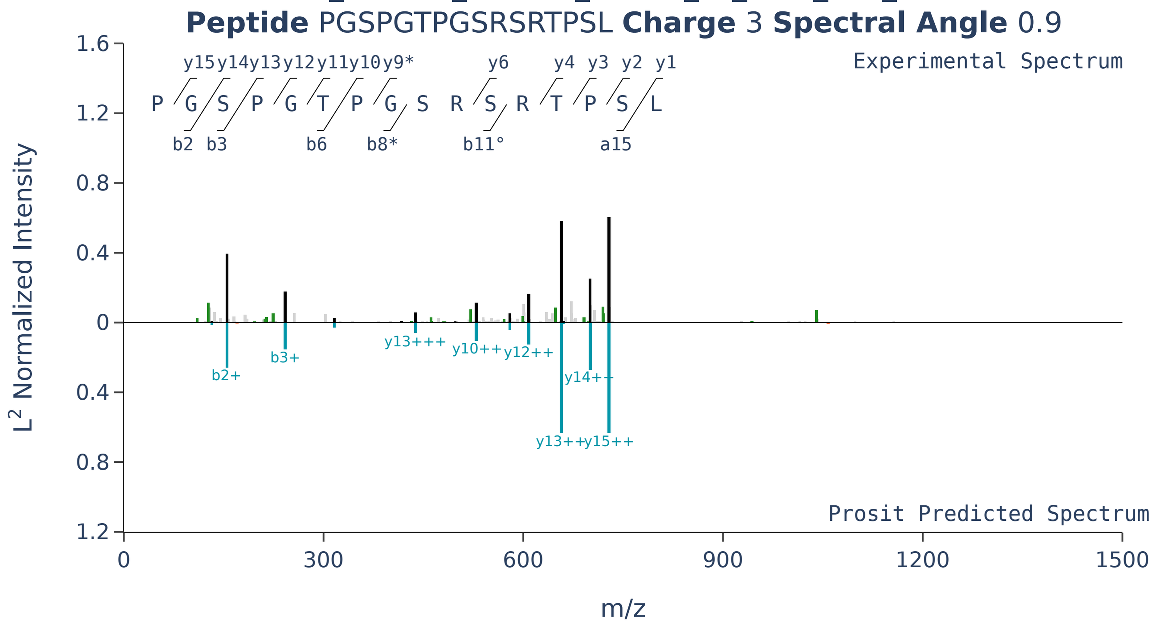


**Source** WSoh_221121_011221_HFGoe_L2_hTau_2h_R1 **Scan** 6577
**Peptide** VVRTPPKSPSSAKSRLQ **Charge** 4 **Spectral Angle** 0.85

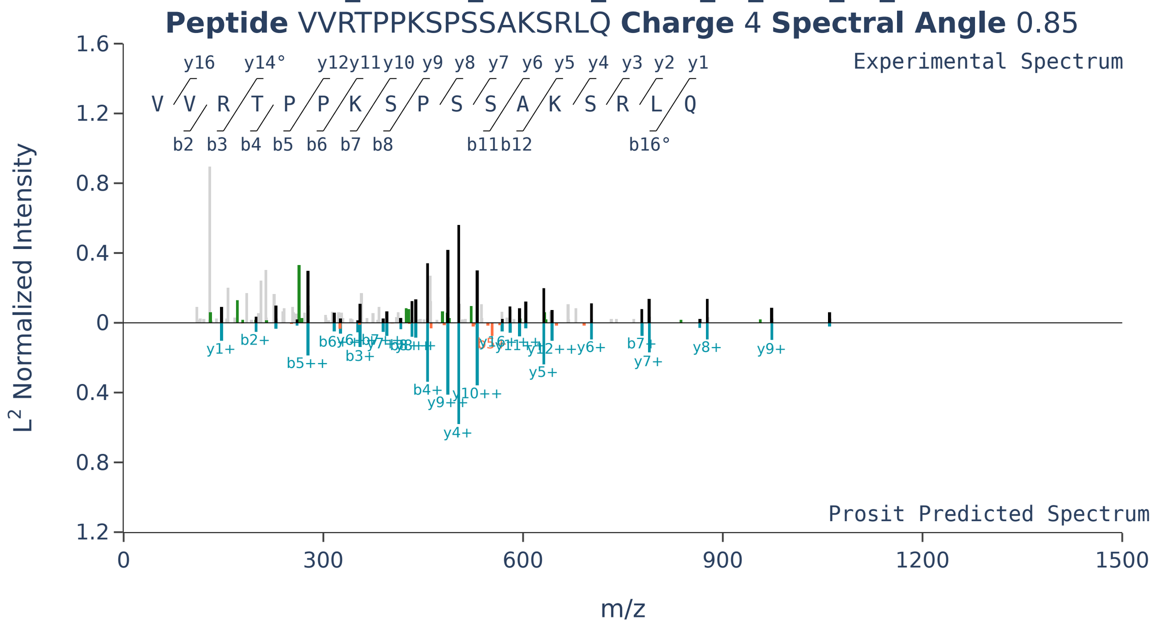


**Source** WSoh_221121_011221_HFGoe_L2_hTau_24h_R2 **Scan** 15132
**Peptide** SRTPSLPTPPTREPKKVAV **Charge** 4 **Spectral Angle** 0.86

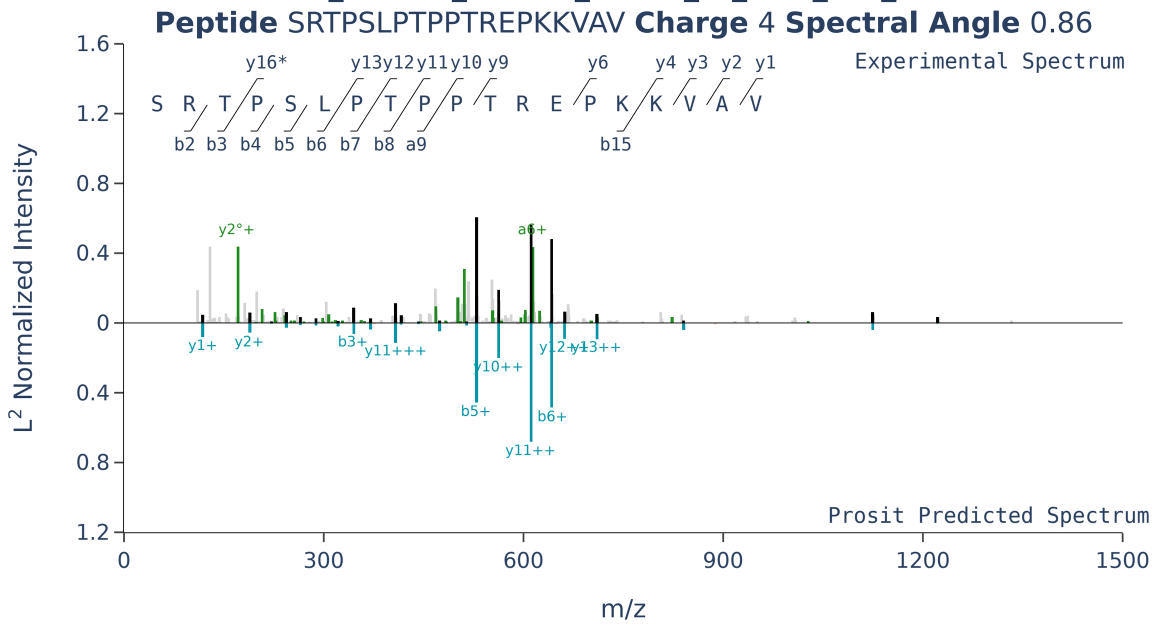


**Source** WSoh_221121_011221_HFGoe_L2_hTau_1h_R2 **Scan** 10248
**Peptide** KSEKLDFKDRV **Charge** 2 **Spectral Angle** 0.85

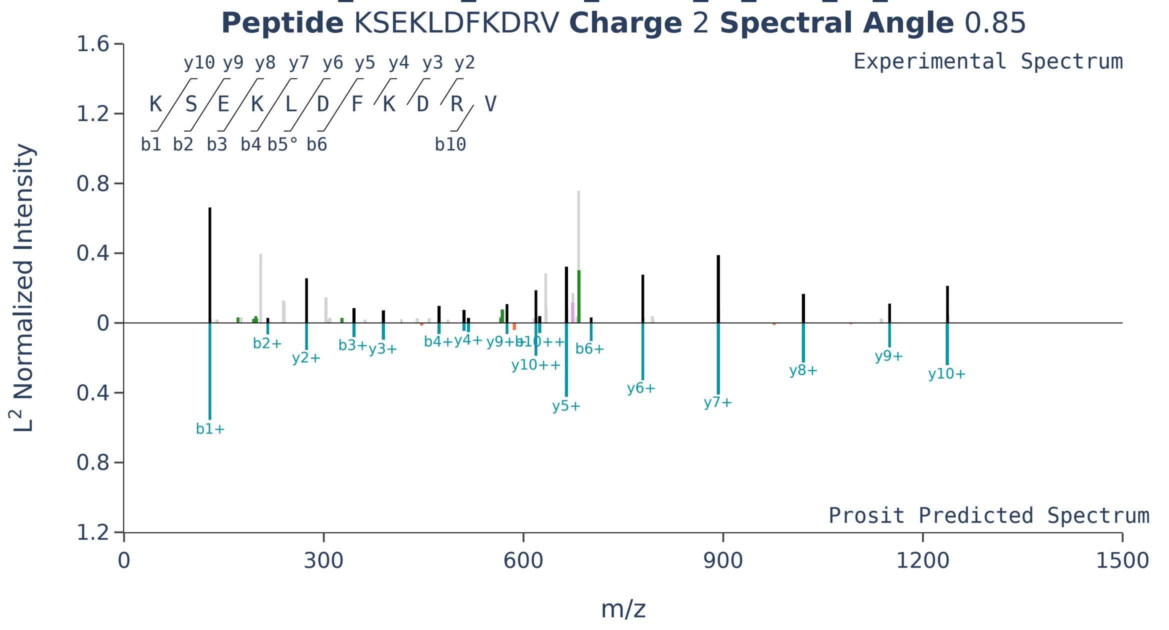


**Source** WSoh_221121_011221_HFGoe_L2_hTau_24h_R2 **Scan** 15990
**Peptide** LGSLDNITHVPGGGNKKIETH **Charge** 5 **Spectral Angle** 0.87

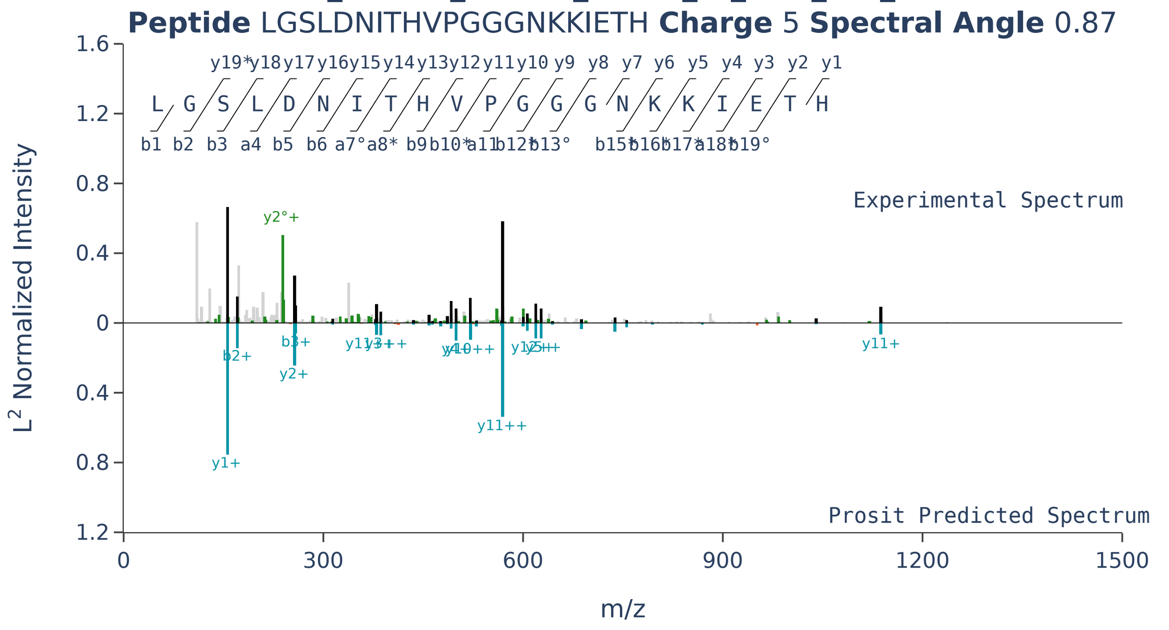


**Source** WSoh_221121_011221_HFGoe_L1_hTau_24h_R2 **Scan** 15674
**Peptide** IGSLDNITHVPGGGNKK **Charge** 4 **Spectral Angle** 0.89

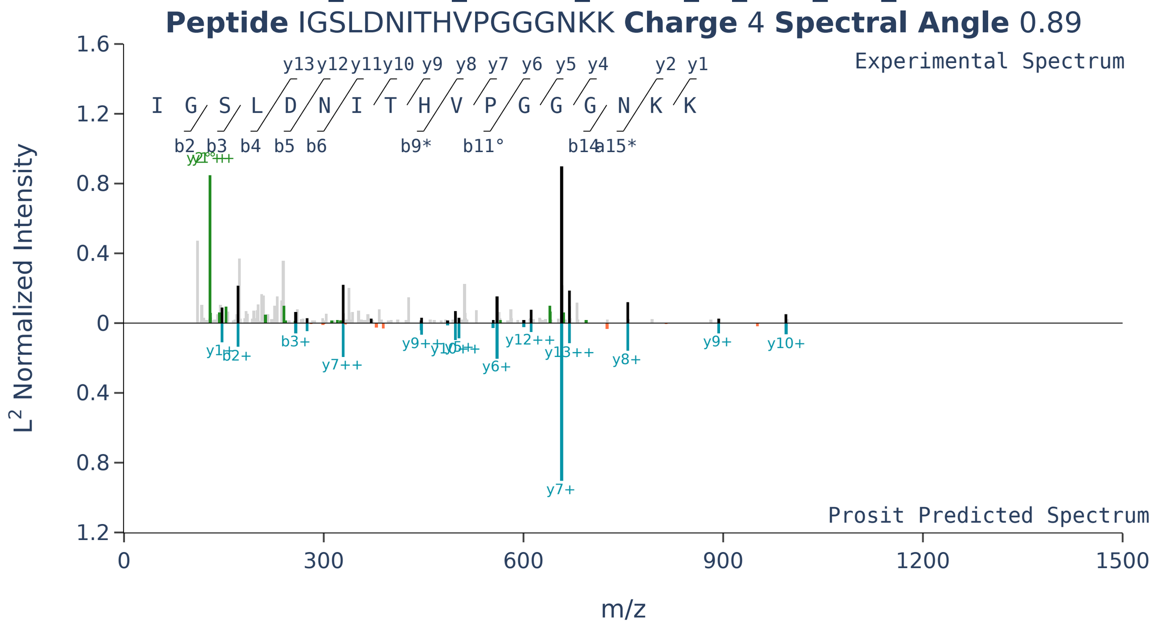


**Source** WSoh_221121_011221_HFGoe_L2_hTau_4h_R1 **Scan** 17500
**Peptide** GIGDTPSLEDEAAGHVTQARMVSKSKD **Charge** 4 **Spectral Angle** 0.85

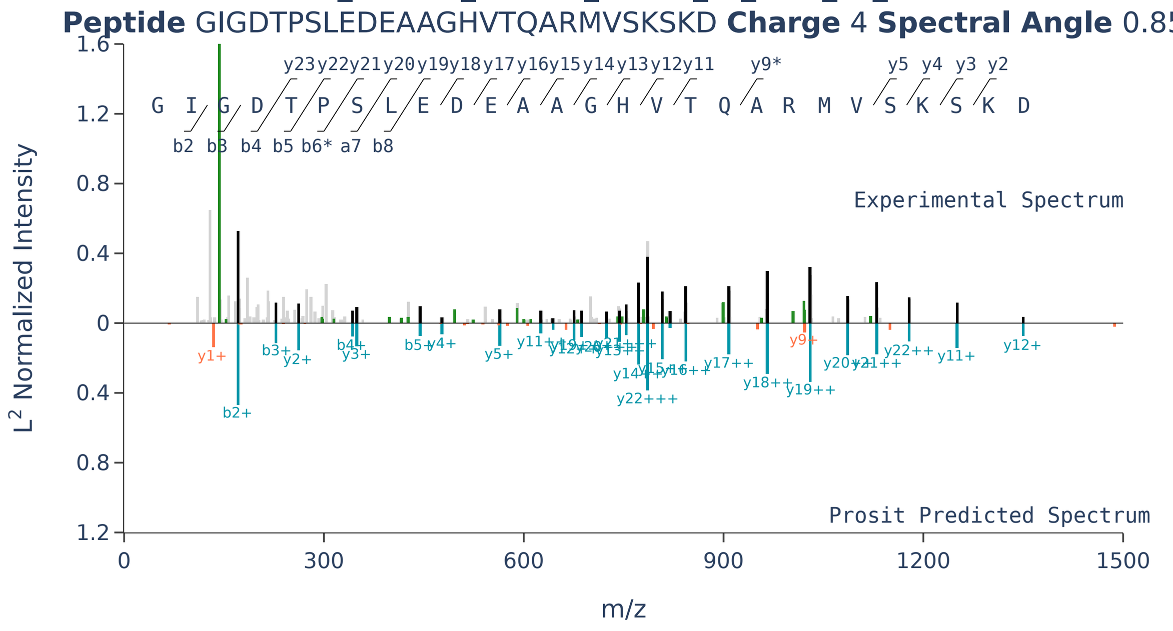


**Source** WSoh_221121_011221_HFGoe_L2_hTau_24h_R2 **Scan** 9440
**Peptide** THVPGGGNKKIETHKLTFREN **Charge** 4 **Spectral Angle** 0.89

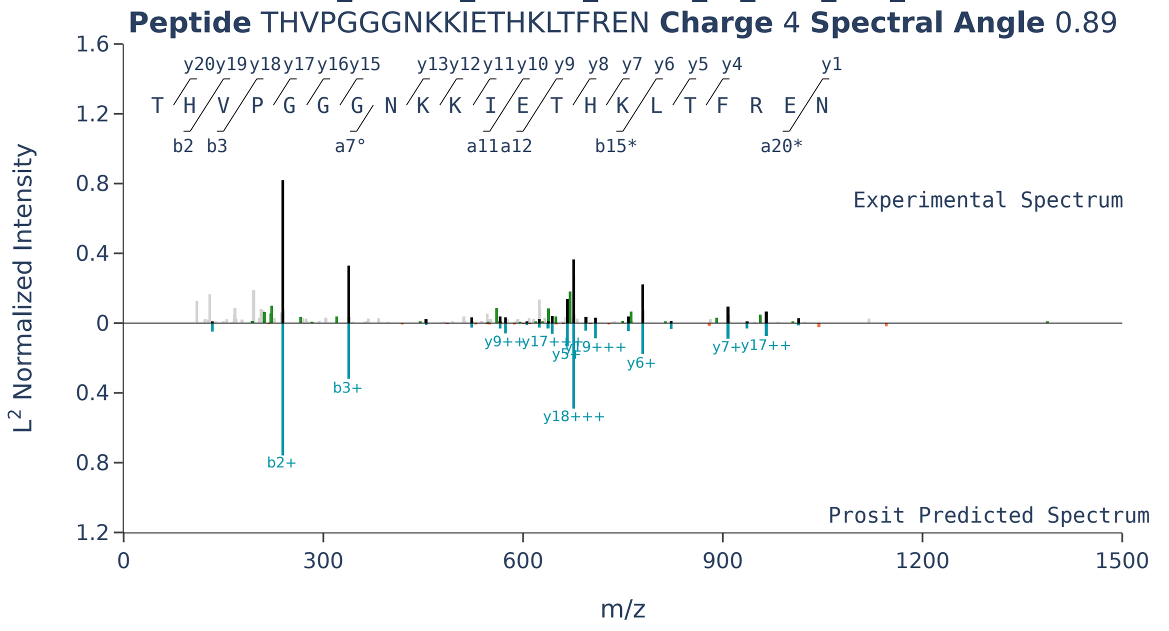


**Source** WSoh_221121_011221_HFGoe_L2_hTau_24h_R2 **Scan** 6642
**Peptide** PPKSPSSAKSRLQTA **Charge** 2 **Spectral Angle** 0.8

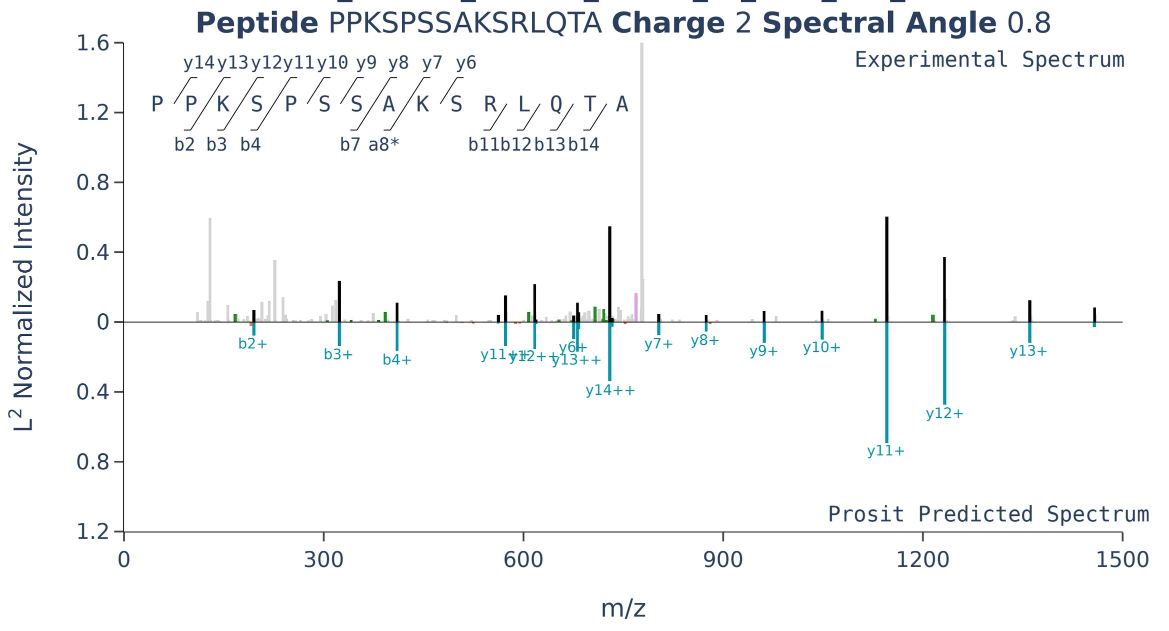


**Source** WSoh_221121_011221_HFGoe_L2_hTau_4h_R1 **Scan** 13607
**Peptide** KLGSTENLKHQPGGGKVQII **Charge** 3 **Spectral Angle** 0.92

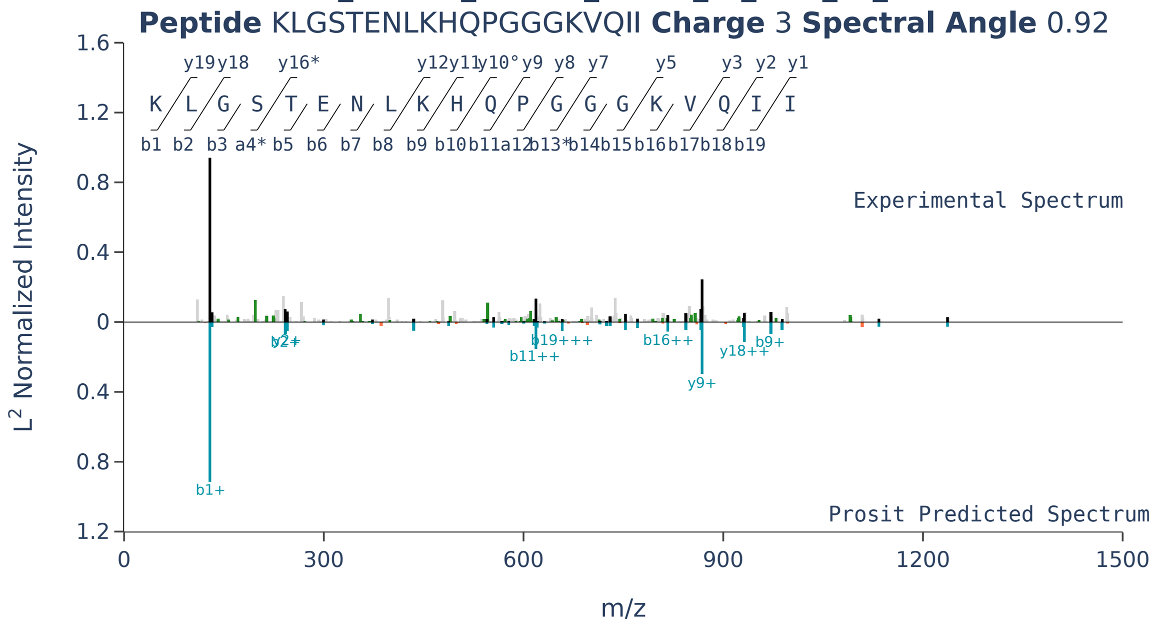


**Source** WSoh_221121_011221_HFGoe_L1_hTau_4h_R1 **Scan** 10088
**Peptide** LDNITHVPGGGNKKIETH **Charge** 2 **Spectral Angle** 0.82

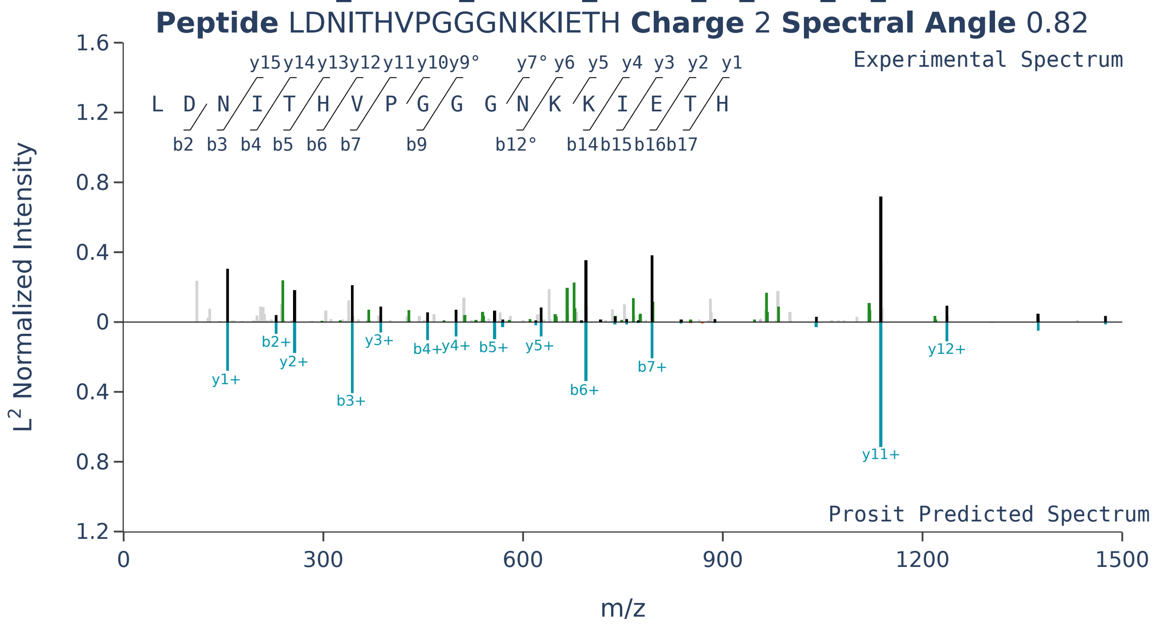


**Source** WSoh_221121_011221_HFGoe_L1_hTau_24h_R1 **Scan** 15636
**Peptide** AGTYGLGDRKDQGGYTM **Charge** 2 **Spectral Angle** 0.84

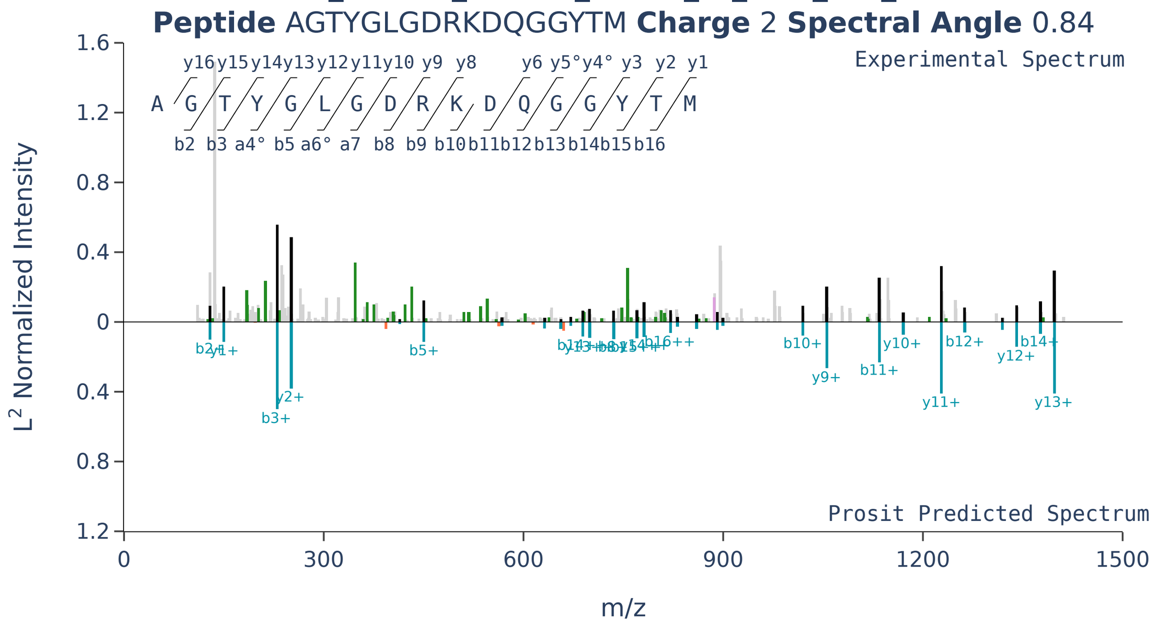


**Source** WSoh_221121_011221_HFGoe_L1_hTau_24h_R2 **Scan** 19494
**Peptide** EPRQEFEVMED **Charge** 2 **Spectral Angle** 0.79

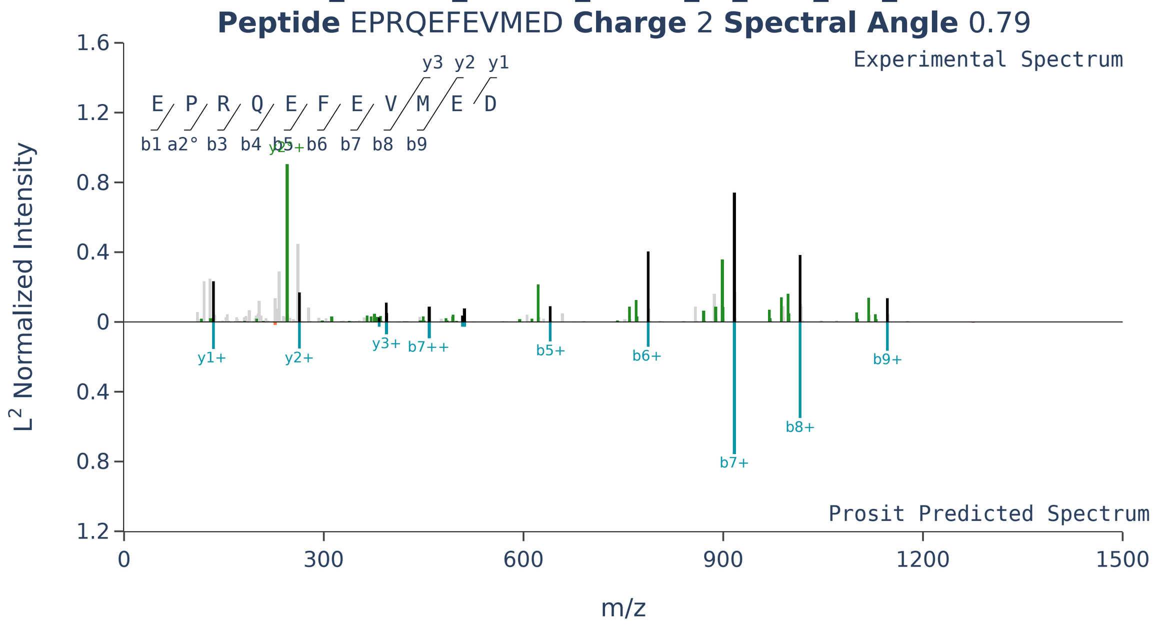


**Source** WSoh_221121_011221_HFGoe_L1_hTau_24h_R1 **Scan** 17871
**Peptide** SPVVSGDTSPRHLSNVSSTGSIDMVD **Charge** 3 **Spectral Angle** 0.9

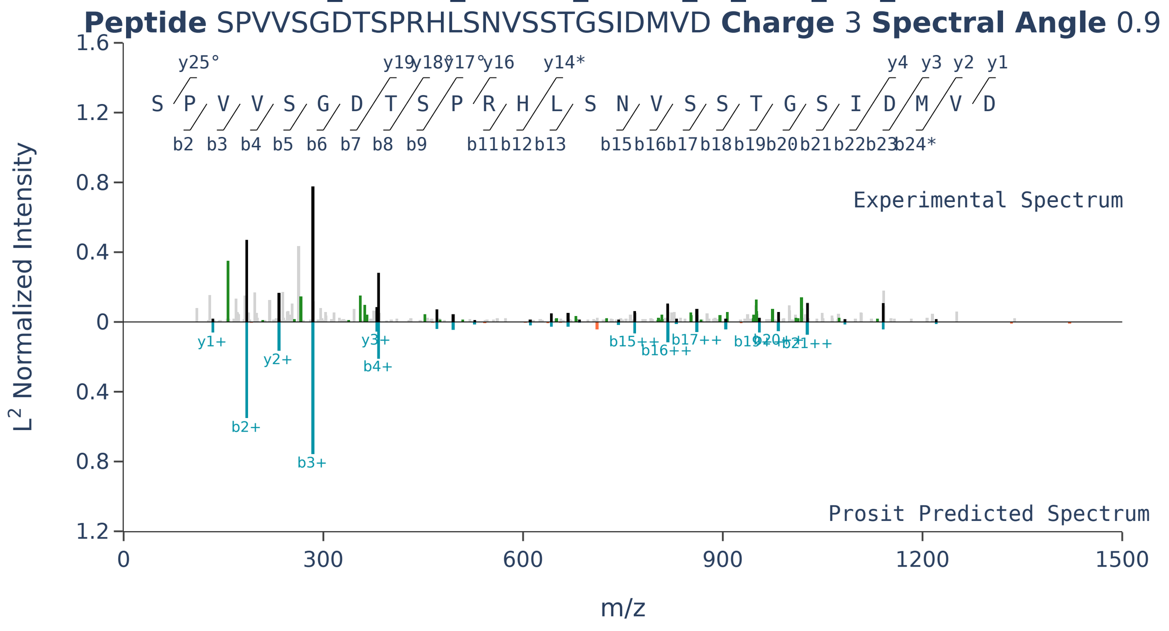

**Source** WSoh_221121_011221_HFGoe_L1_hTau_24h_R2 **Scan** 11530
**Peptide** NITHVPGGGNKKIETHKLTFR **Charge** 5 **Spectral Angle** 0.83

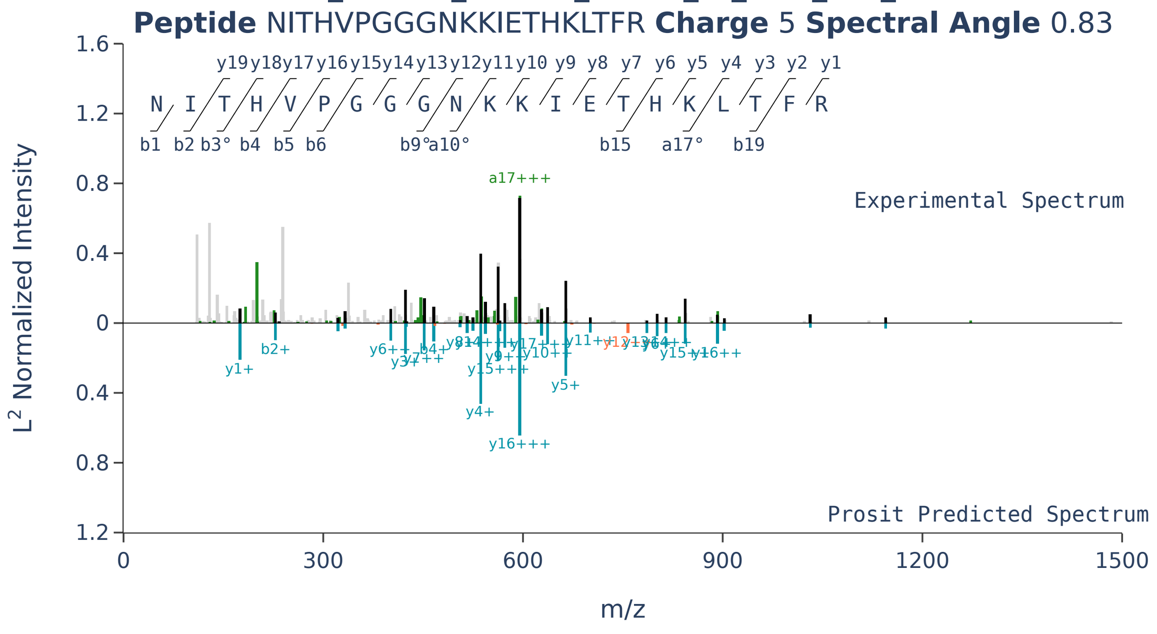


**Source** WSoh_221121_011221_HFGoe_L1_hTau_24h_R2 **Scan** 11999
**Peptide** KCGSKDNIKHVPGGGSVQIVYK **Charge** 3 **Spectral Angle** 0.78

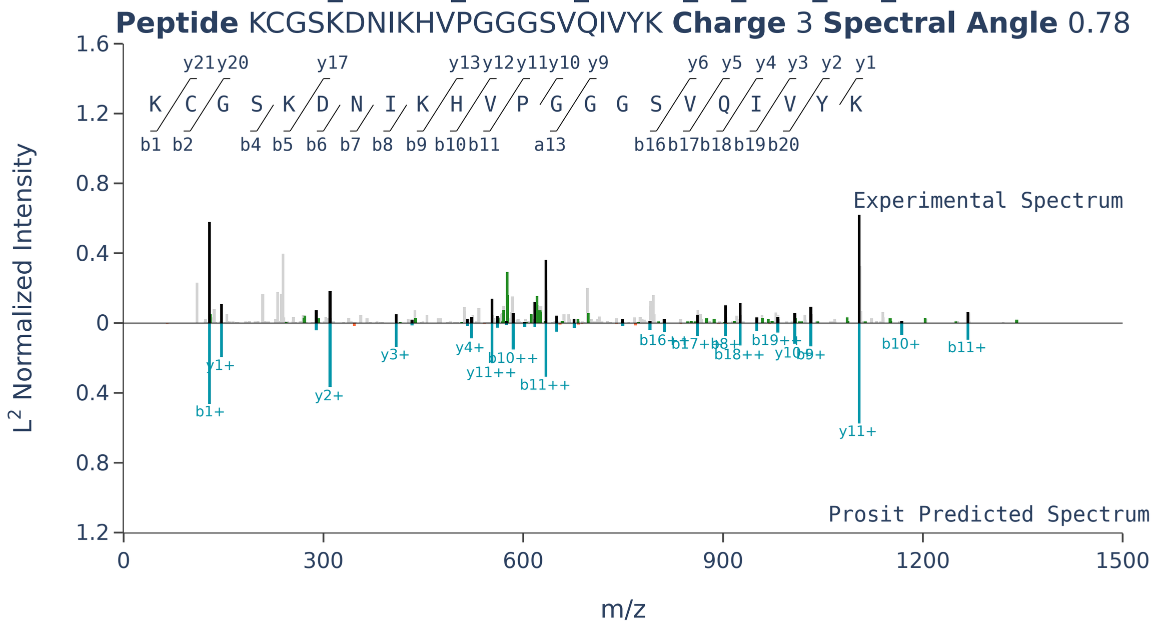


**Source** WSoh_221121_011221_HFGoe_L1_hTau_24h_R2 **Scan** 11267
**Peptide** DHAGTYGLGDRKDQGGYTMHQDQ **Charge** 3 **Spectral Angle** 0.92

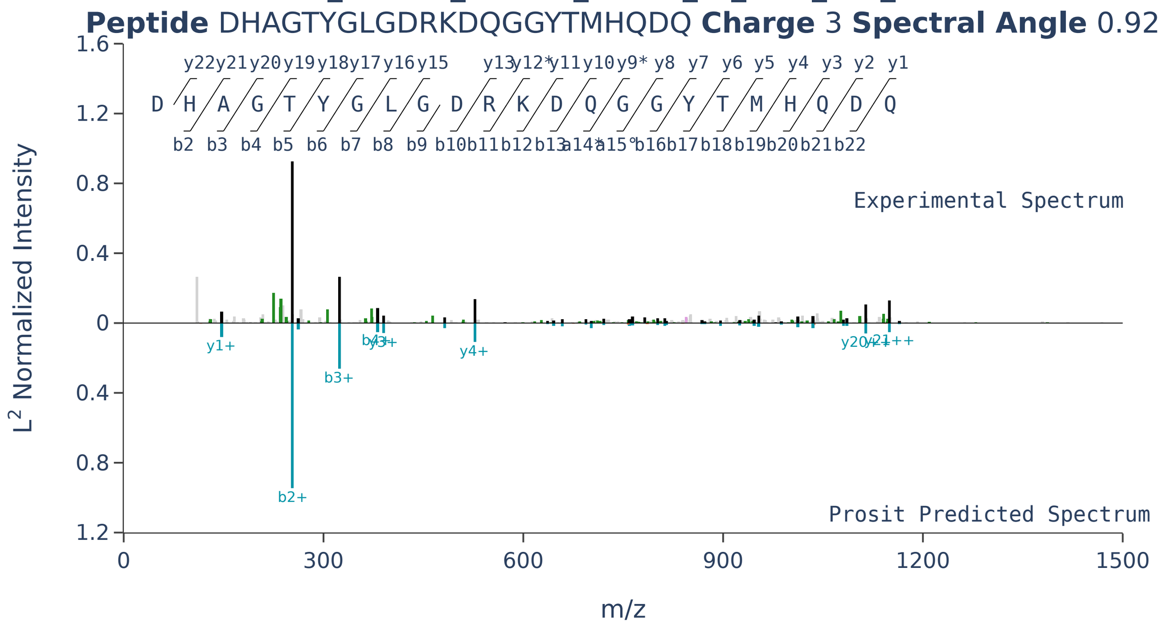


**Source** WSoh_221121_011221_HFGoe_L1_hTau_24h_R1 **Scan** 26227
**Peptide** TAPVPMPDLKNV **Charge** 2 **Spectral Angle** 0.89

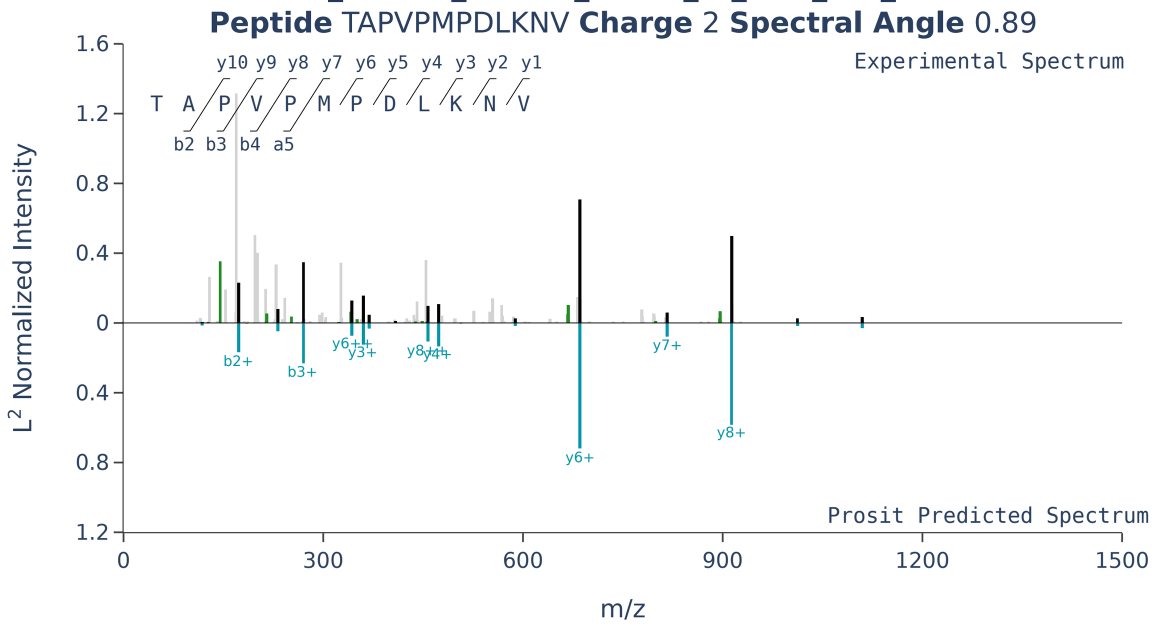


**Source** WSoh_221121_011221_HFGoe_L2_hTau_24h_R1 **Scan** 24417
**Peptide** AQPHTEIPEGTTAEEAGIGDTPSL **Charge** 3 **Spectral Angle** 0.92

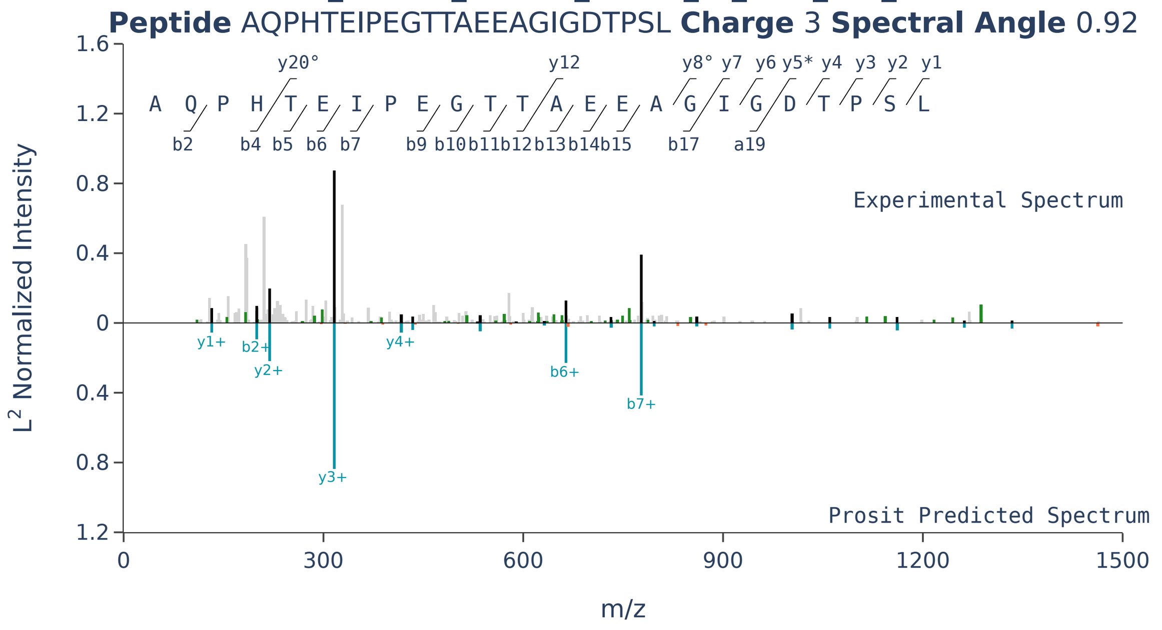


**Source** WSoh_221121_011221_HFGoe_L1_hTau_24h_R1 **Scan** 8206
**Peptide** KSEKLDFKD **Charge** 3 **Spectral Angle** 0.96

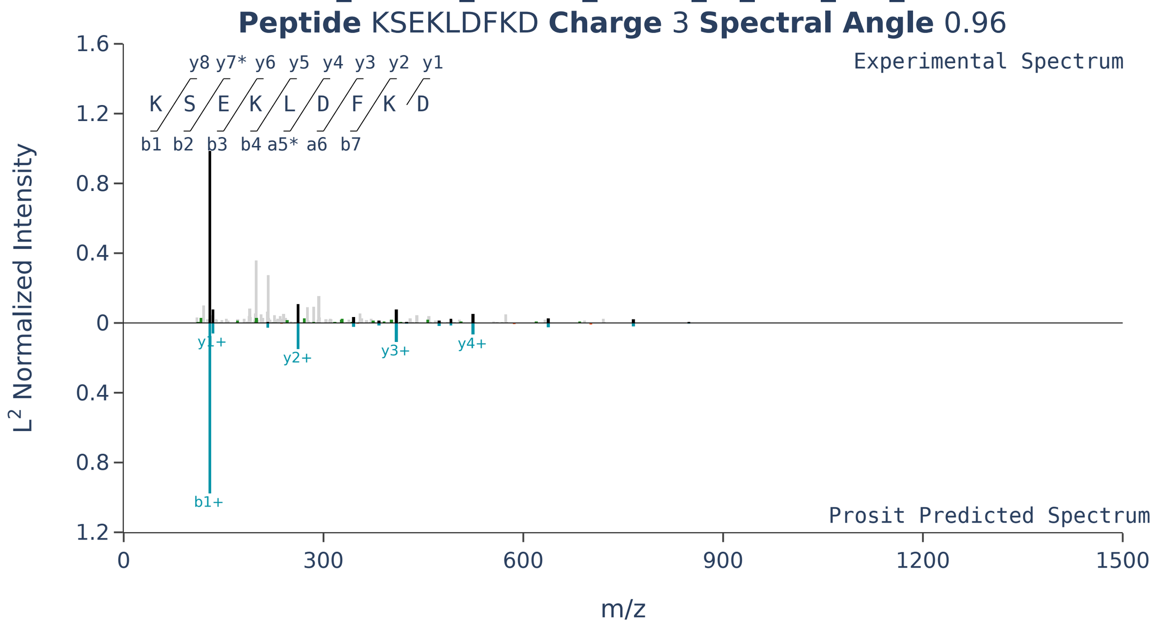


**Source** WSoh_221121_011221_HFGoe_L2_hTau_24h_R1 **Scan** 14996
**Peptide** SLDNITHVPGGGNKKIET **Charge** 3 **Spectral Angle** 0.86

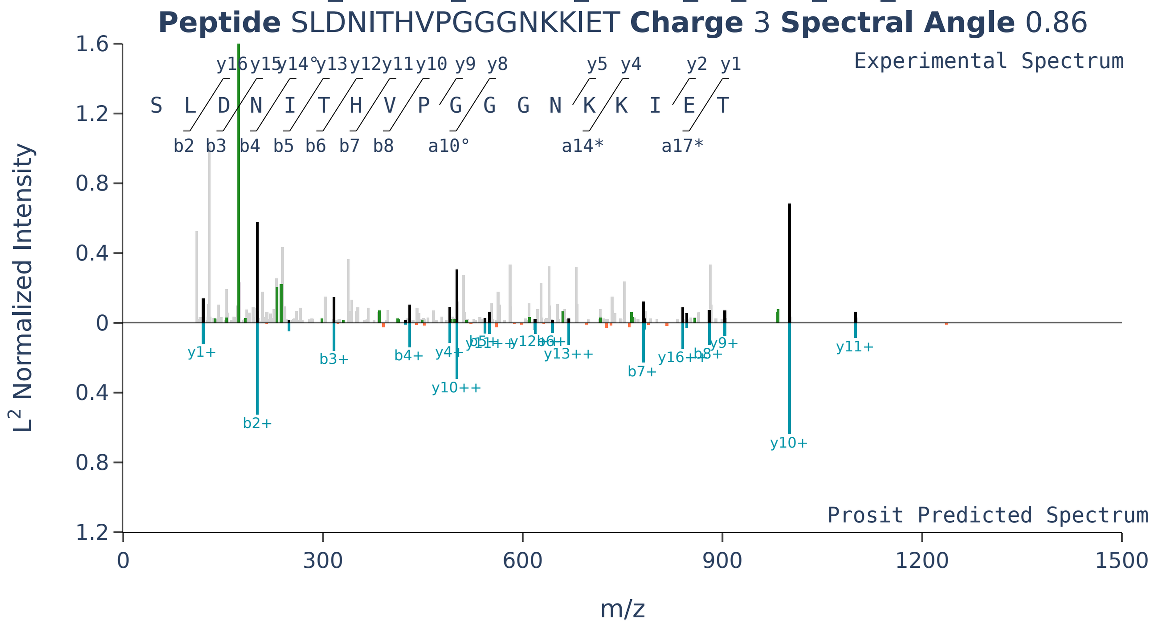


**Source** WSoh_221121_011221_HFGoe_L2_hTau_4h_R1 **Scan** 15884
**Peptide** KLGSLDNITHVPGGGNKKI **Charge** 3 **Spectral Angle** 0.83

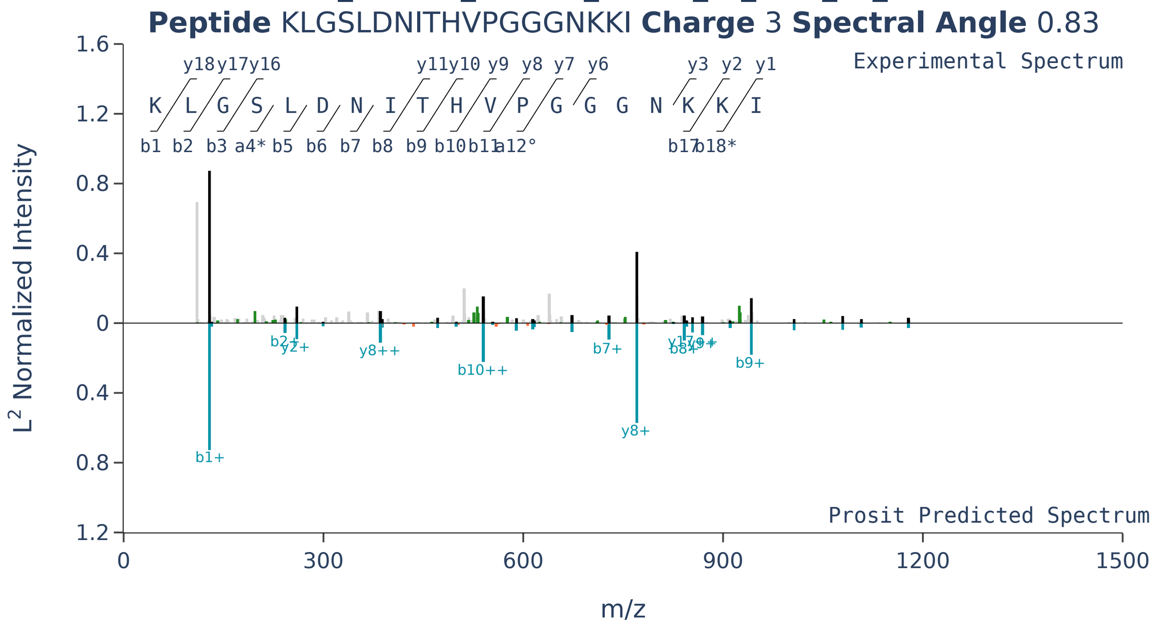


**Source** WSoh_221121_011221_HFGoe_L1_hTau_24h_R2 **Scan** 8775
**Peptide** TFRENAKAKTDHGAEIVYKS **Charge** 3 **Spectral Angle** 0.89

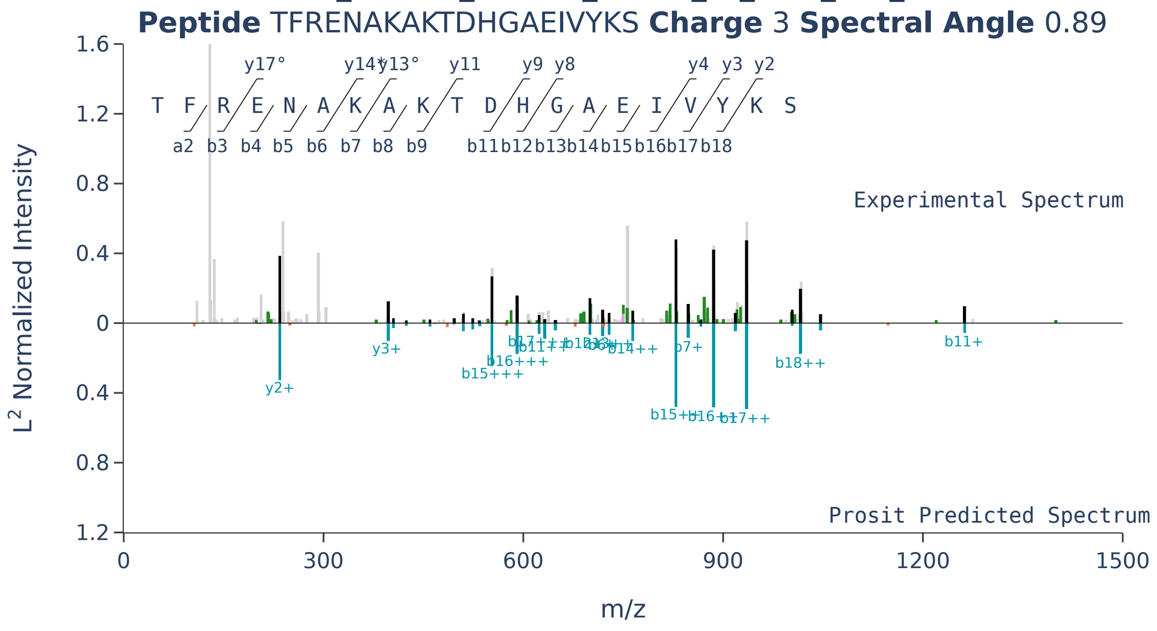


**Source** WSoh_221121_011221_HFGoe_L1_hTau_24h_R2 **Scan** 5980
**Peptide** PGGGNKKIETHKLT **Charge** 2 **Spectral Angle** 0.84

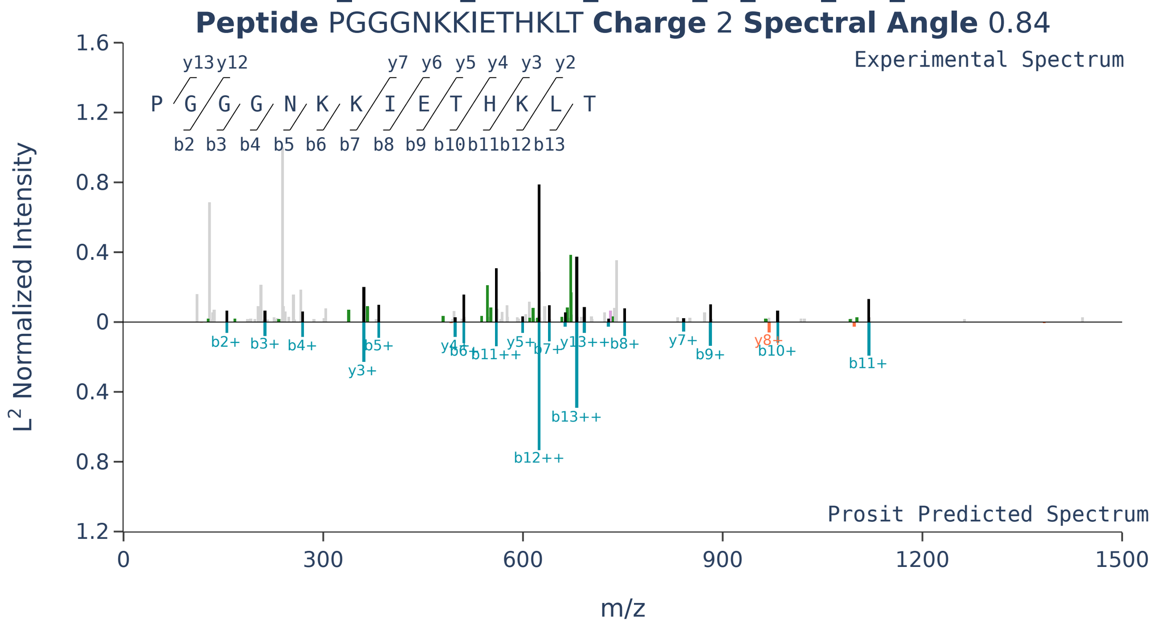


**Source** WSoh_221121_011221_HFGoe_L2_hTau_2h_R2 **Scan** 5955
**Peptide** THVPGGGNKKIE **Charge** 3 **Spectral Angle** 0.86

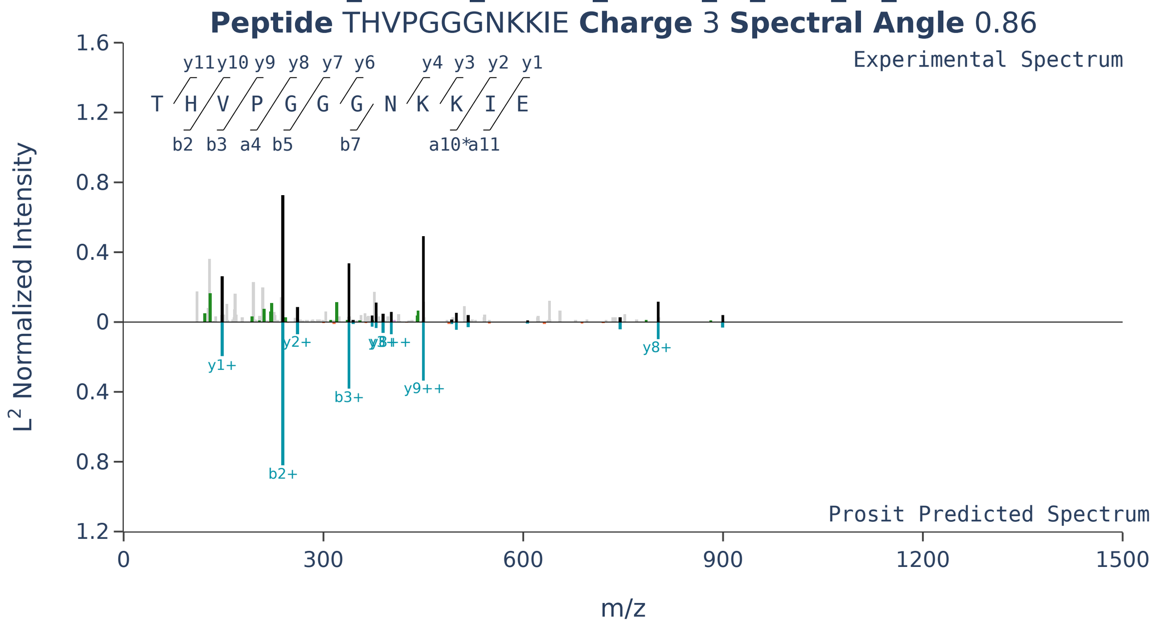


**Source** WSoh_221121_011221_HFGoe_L1_hTau_24h_R2 **Scan** 12361
**Peptide** KPVDLSKVTSKCGSLGNIHHK **Charge** 4 **Spectral Angle** 0.91

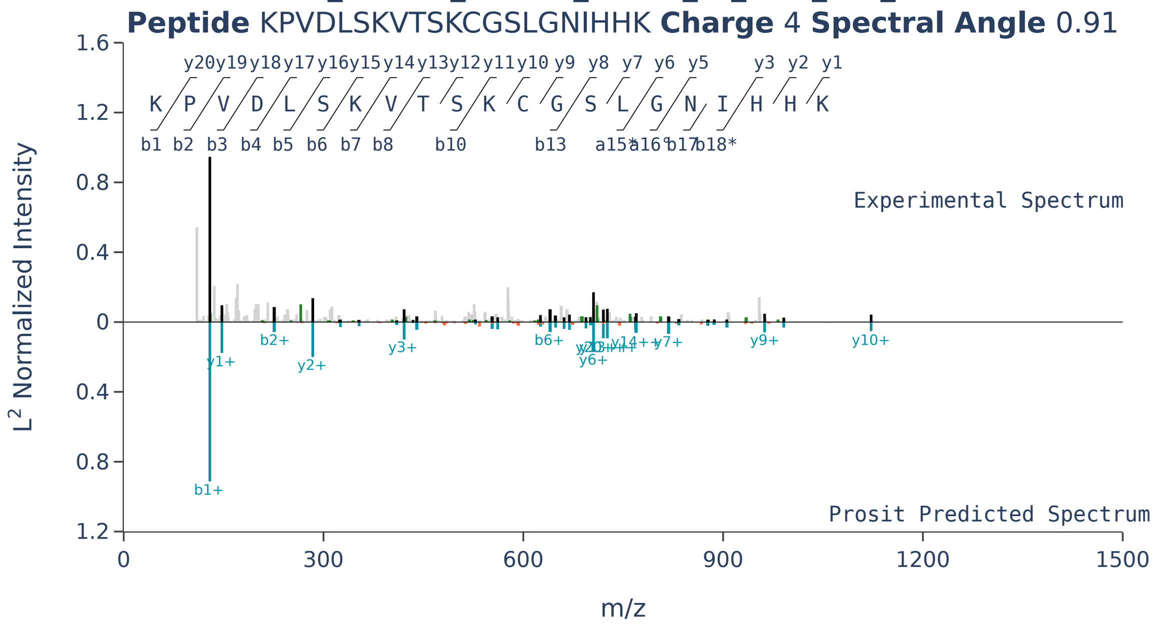


**Source** WSoh_221121_011221_HFGoe_L2_hTau_2h_R1 **Scan** 9104
**Peptide** RIPAKTPPAPKTPPSS **Charge** 3 **Spectral Angle** 0.84

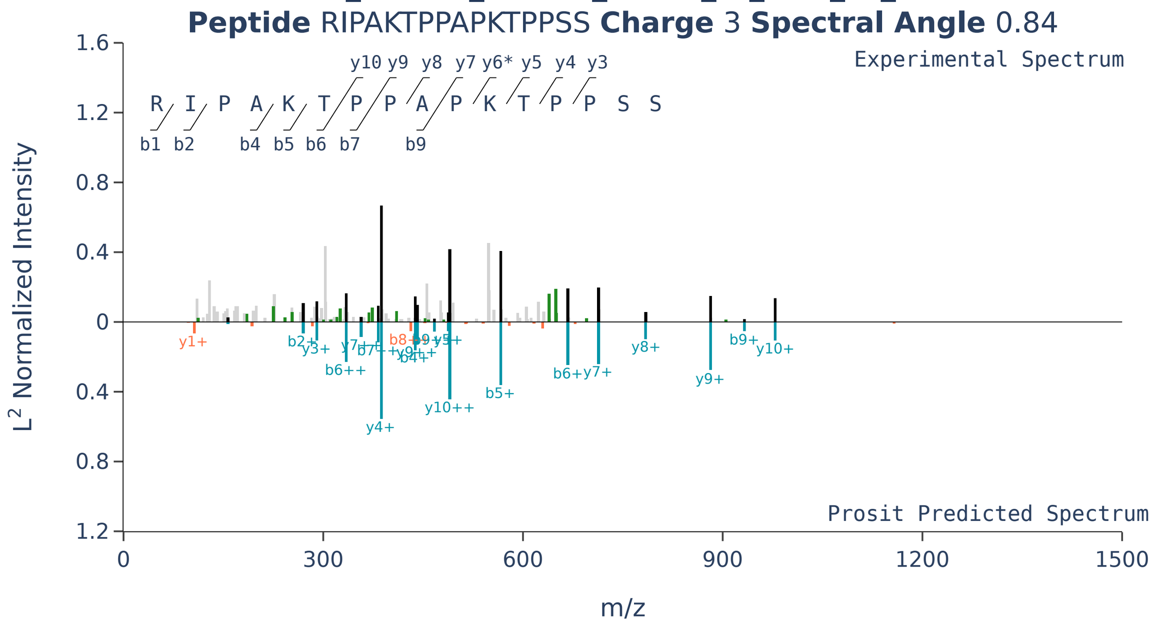


**Source** WSoh_221121_011221_HFGoe_L1_hTau_24h_R1 **Scan** 23721
**Peptide** LQTAPVPMPDLKNVKSKIG **Charge** 3 **Spectral Angle** 0.88

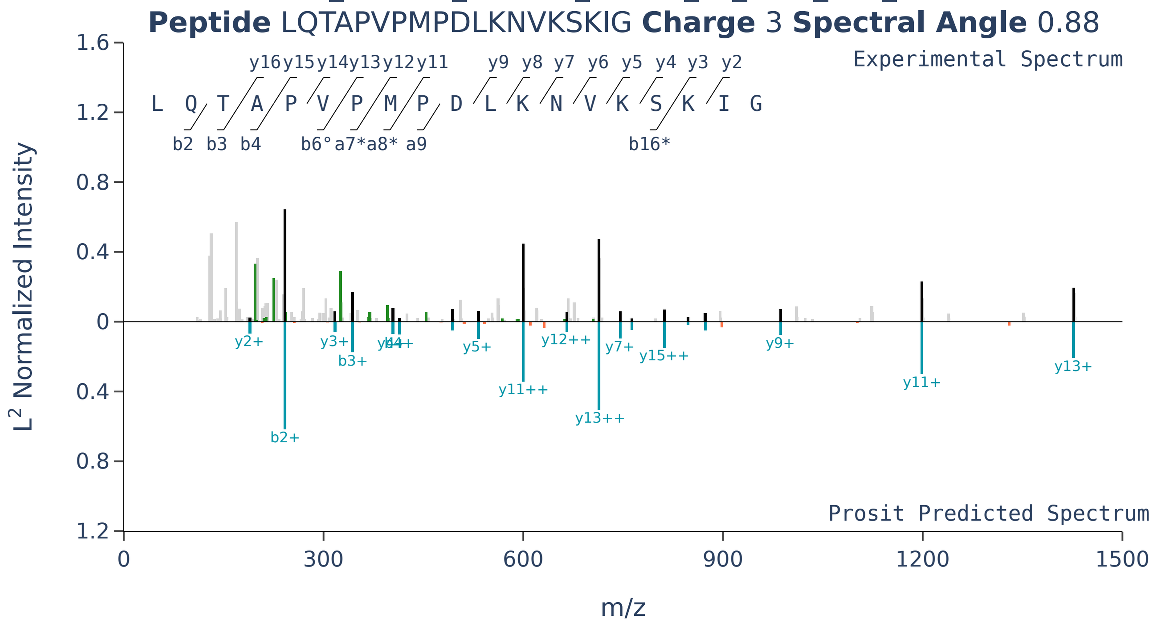


**Source** WSoh_221121_011221_HFGoe_L2_hTau_4h_R1 **Scan** 8942
**Peptide** PPAPKTPPSSGEPPKSGDRSGY **Charge** 4 **Spectral Angle** 0.75

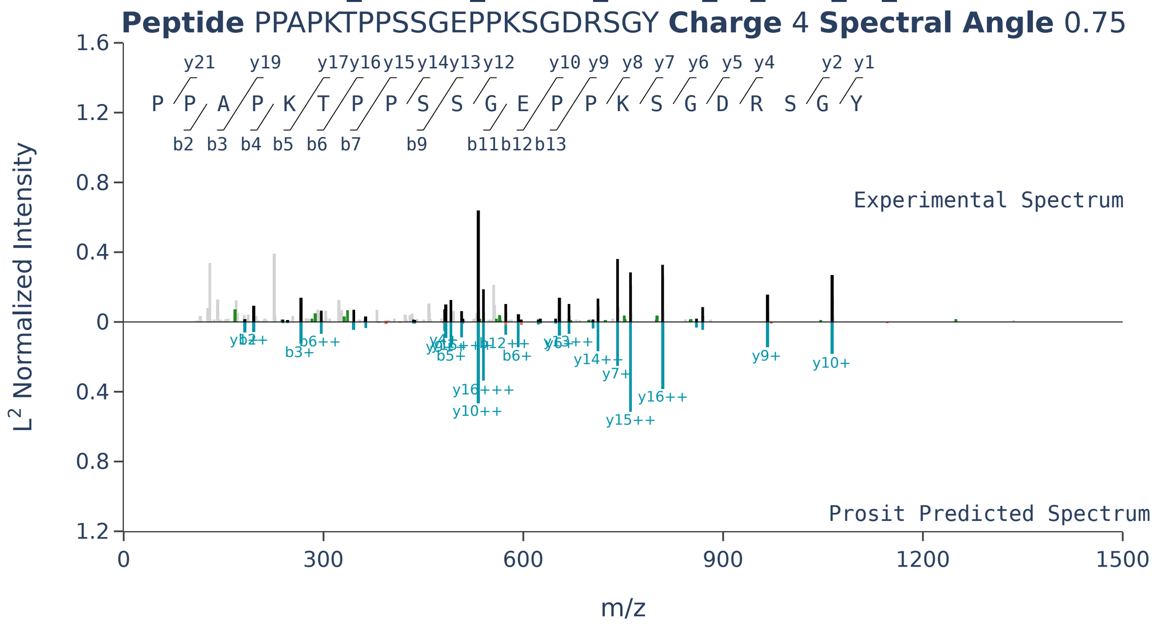

**Source** WSoh_221121_011221_HFGoe_L1_hTau_24h_R2 **Scan** 11837
**Peptide** NITHVPGGGNKKIETHKLTFRENA **Charge** 3 **Spectral Angle** 0.83

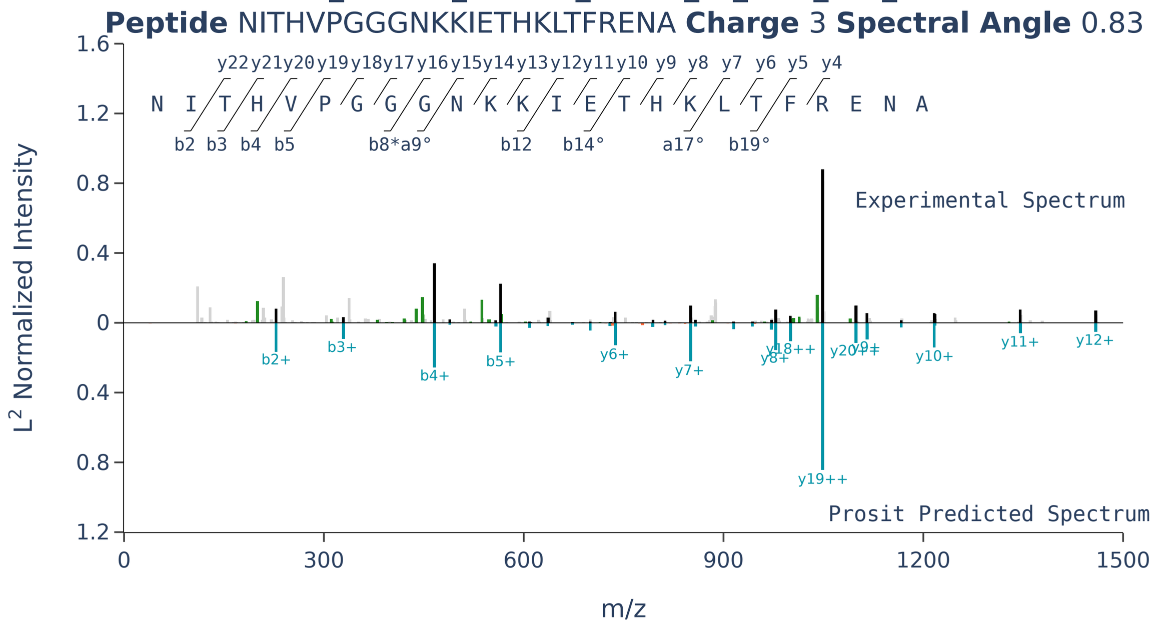


**Source** WSoh_221121_011221_HFGoe_L1_hTau_24h_R2 **Scan** 6963
**Peptide** PPAPKTPPSSGEPPKSGDRS **Charge** 3 **Spectral Angle** 0.8

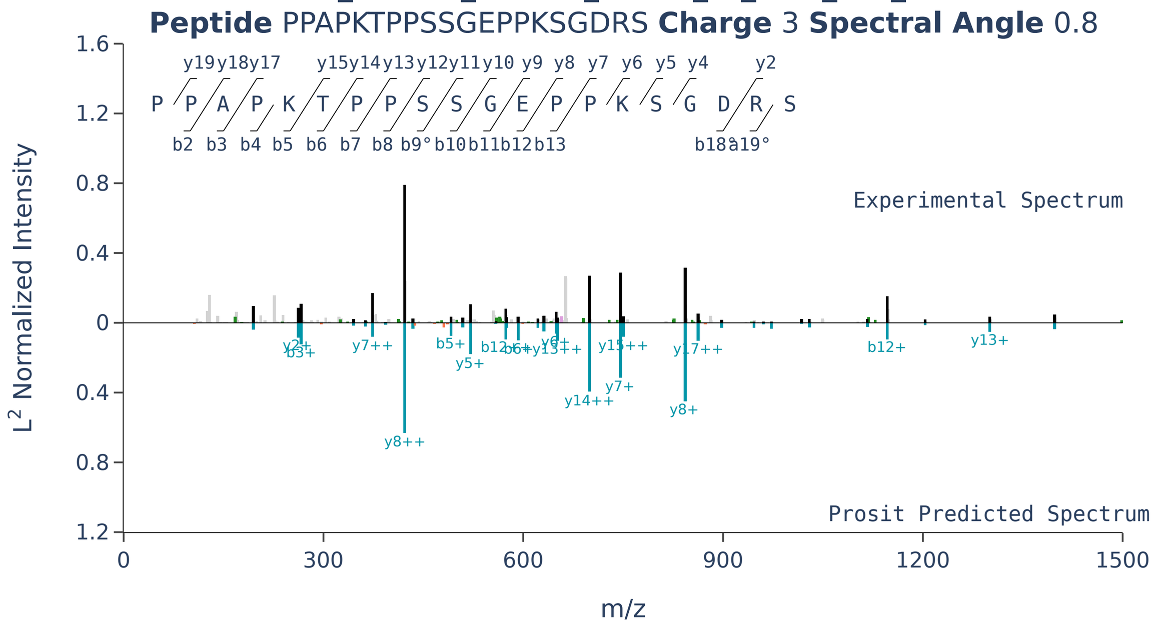


**Source** WSoh_221121_011221_HFGoe_L1_hTau_24h_R1 **Scan** 23491
**Peptide** LQTAPVPMPDLKNVK **Charge** 3 **Spectral Angle** 0.92

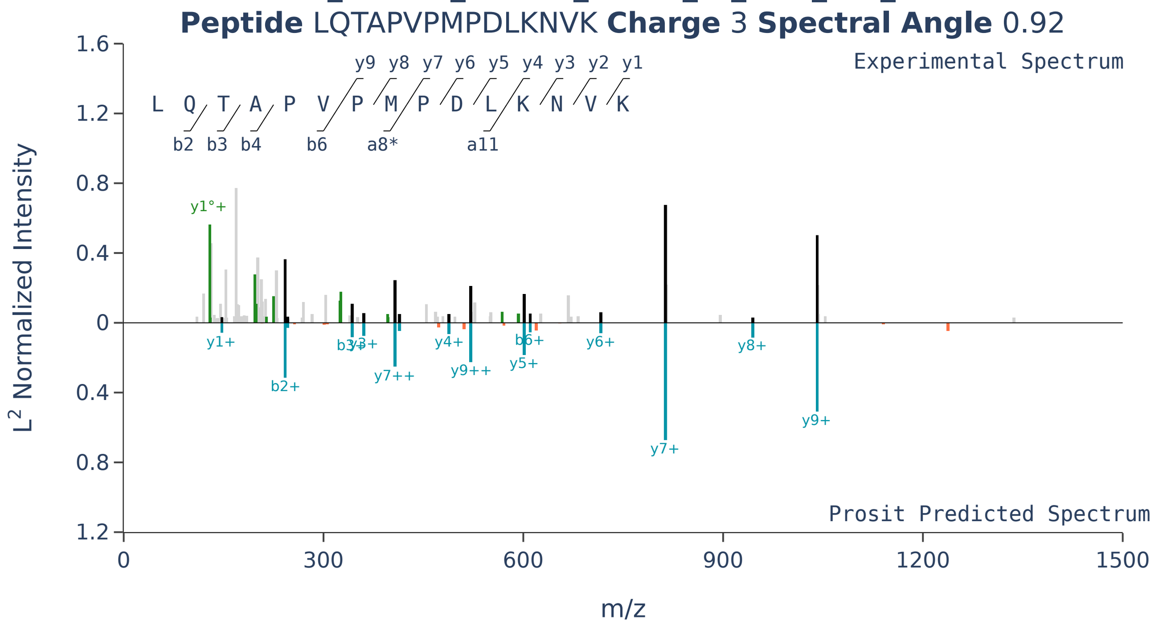


**Source** WSoh_221121_011221_HFGoe_L1_hTau_24h_R1 **Scan** 8881
**Peptide** VEVKSEKL **Charge** 2 **Spectral Angle** 0.89

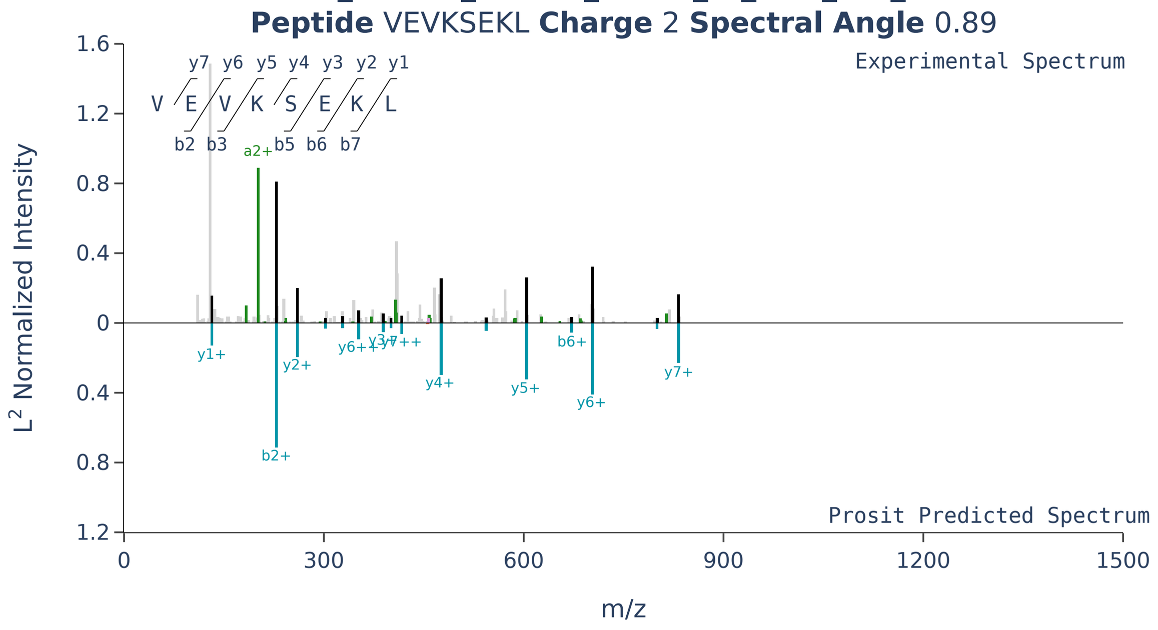


**Source** WSoh_221121_011221_HFGoe_L1_hTau_24h_R2 **Scan** 12161
**Peptide** LDNITHVPGGGNKKIE **Charge** 2 **Spectral Angle** 0.83

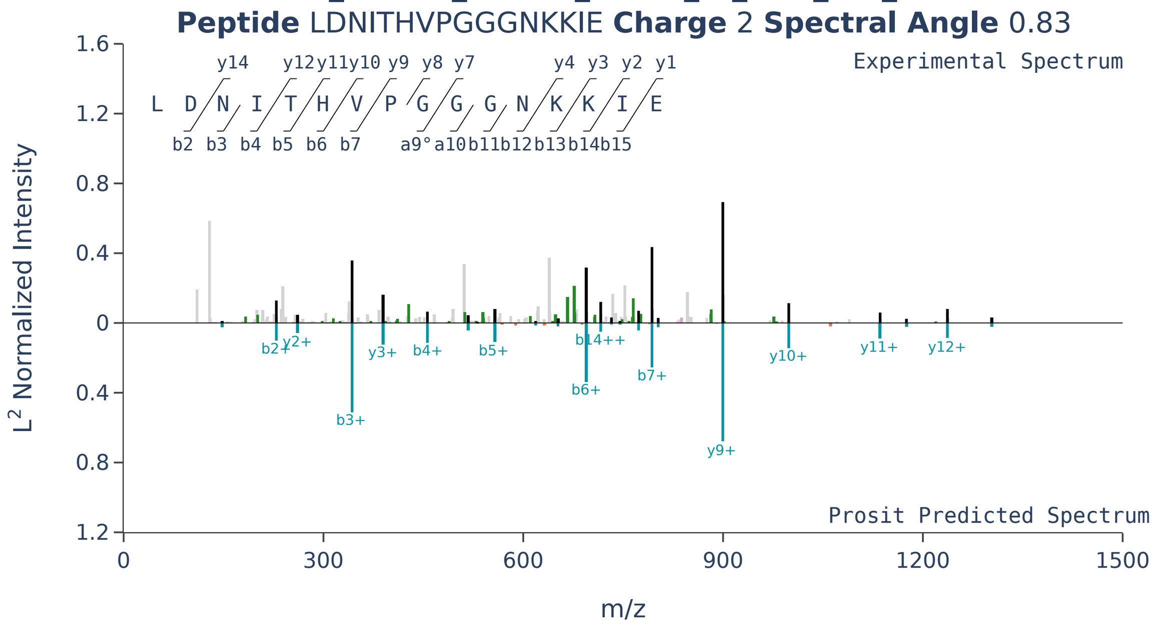


**Source** WSoh_221121_011221_HFGoe_L1_hTau_24h_R2 **Scan** 16771
**Peptide** GLKESPLQTPTED **Charge** 2 **Spectral Angle** 0.76

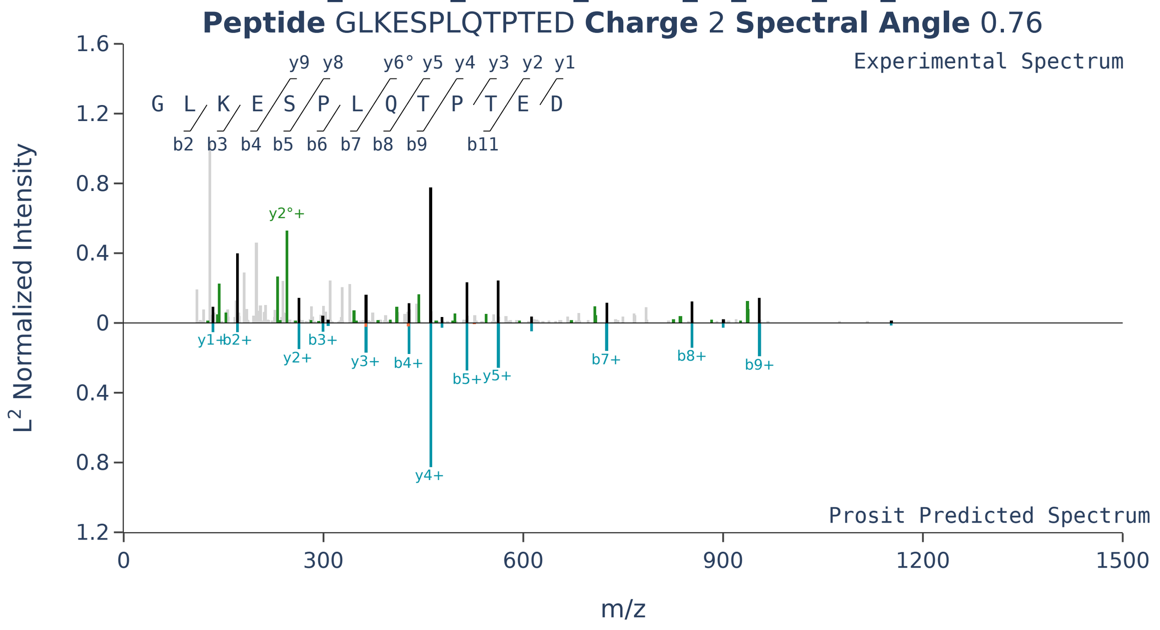


**Source** WSoh_221121_011221_HFGoe_L1_hTau_24h_R1 **Scan** 8141
**Peptide** DLSNVQSKC **Charge** 1 **Spectral Angle** 0.85

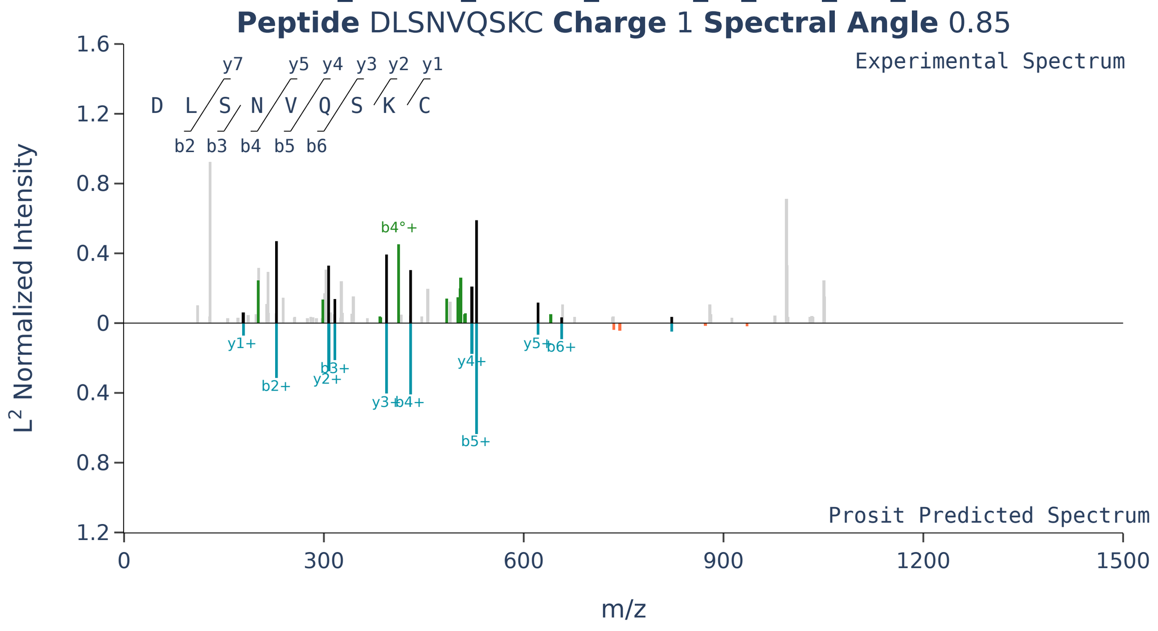


**Source** WSoh_221121_011221_HFGoe_L1_hTau_24h_R1 **Scan** 6182
**Peptide** KPGGGQVEVKSE **Charge** 2 **Spectral Angle** 0.82

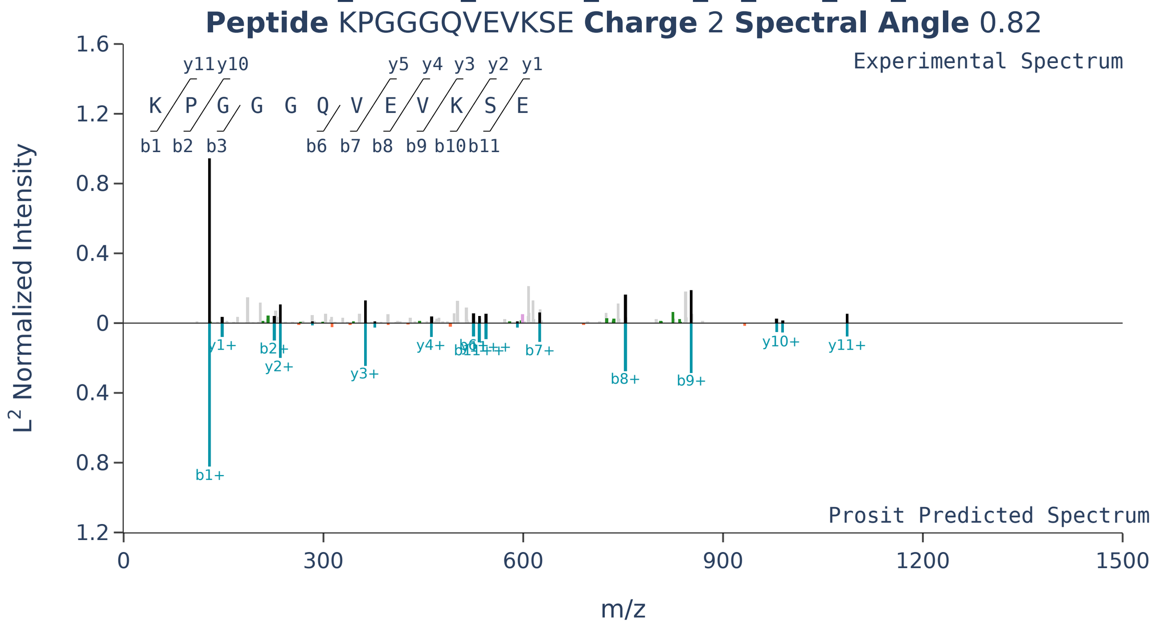


**Source** WSoh_221121_011221_HFGoe_L2_hTau_24h_R1 **Scan** 17759
**Peptide** TDHGAEIVYKSPVVSGDTS **Charge** 2 **Spectral Angle** 0.84

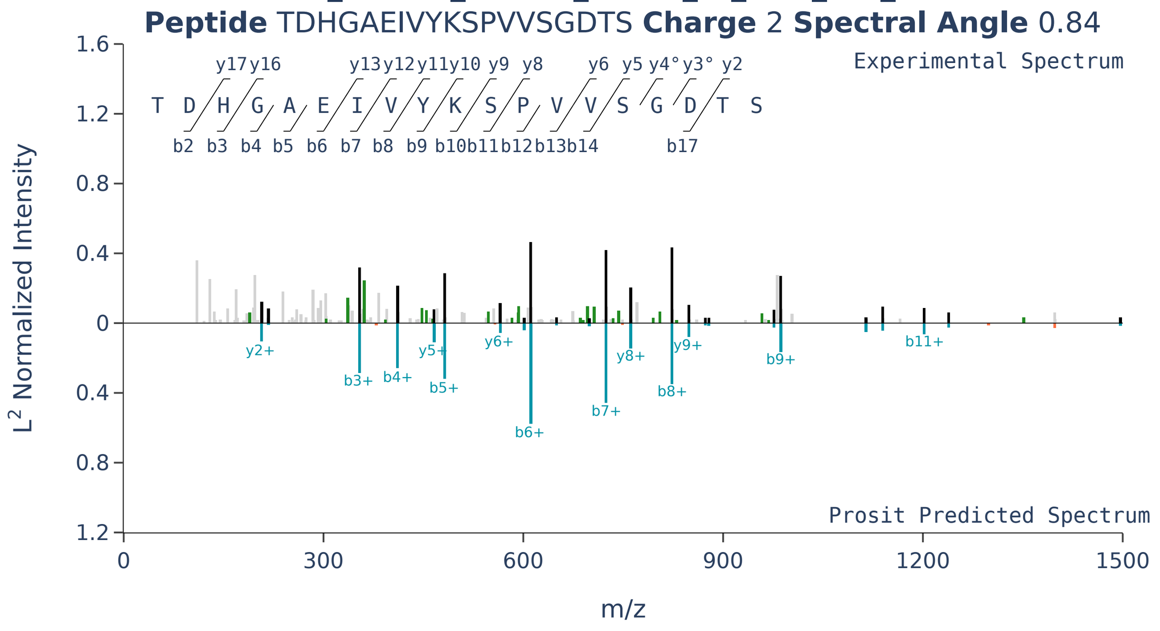


**Source** WSoh_221121_011221_HFGoe_L2_hTau_24h_R1 **Scan** 14768
**Peptide** RTPSLPTPPTREPKKVAV **Charge** 4 **Spectral Angle** 0.9

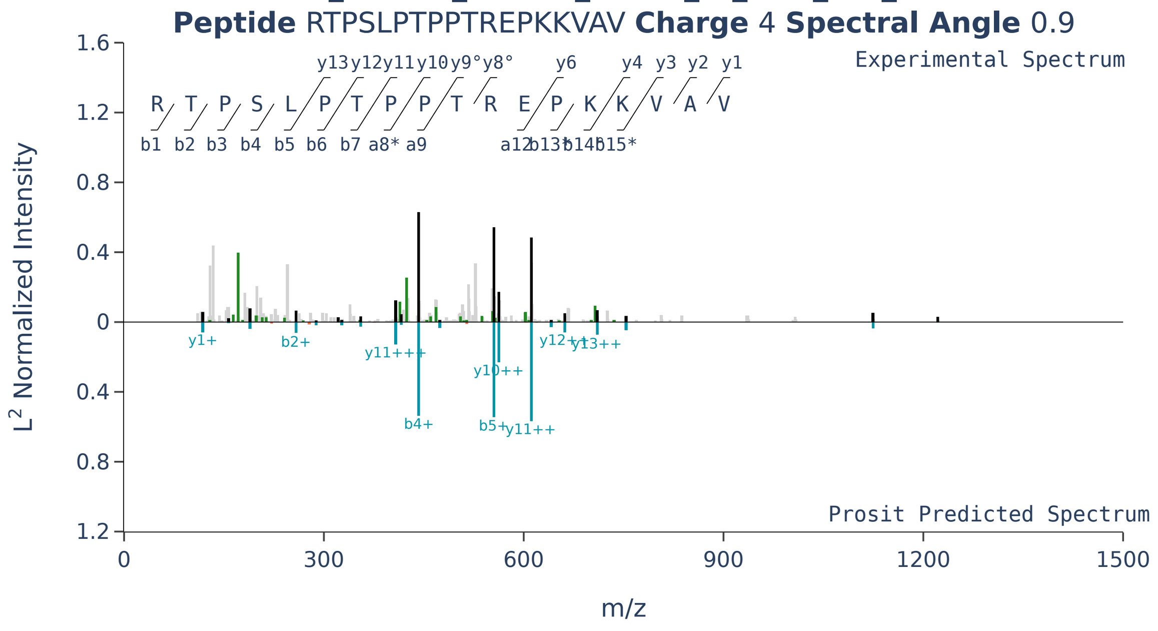


**Source** WSoh_221121_011221_HFGoe_L2_hTau_4h_R2 **Scan** 21080
**Peptide** GIGDTPSL **Charge** 1 **Spectral Angle** 0.97

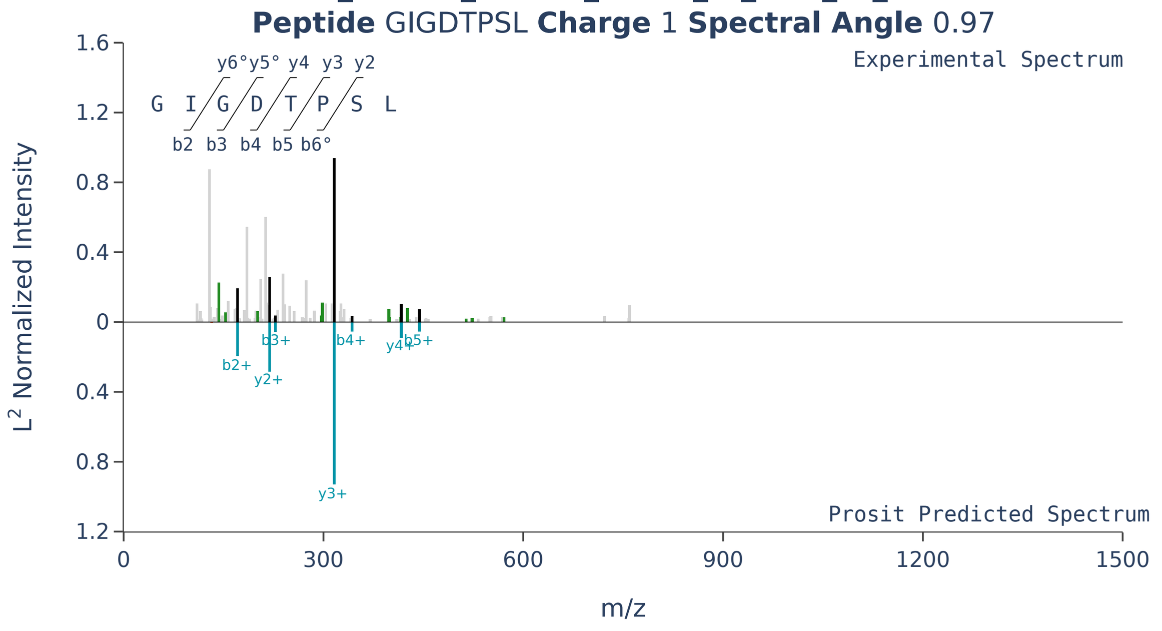


**Source** WSoh_221121_011221_HFGoe_L2_hTau_2h_R2 **Scan** 5974
**Peptide** ATPRGAAPPGQKGQANA **Charge** 2 **Spectral Angle** 0.88

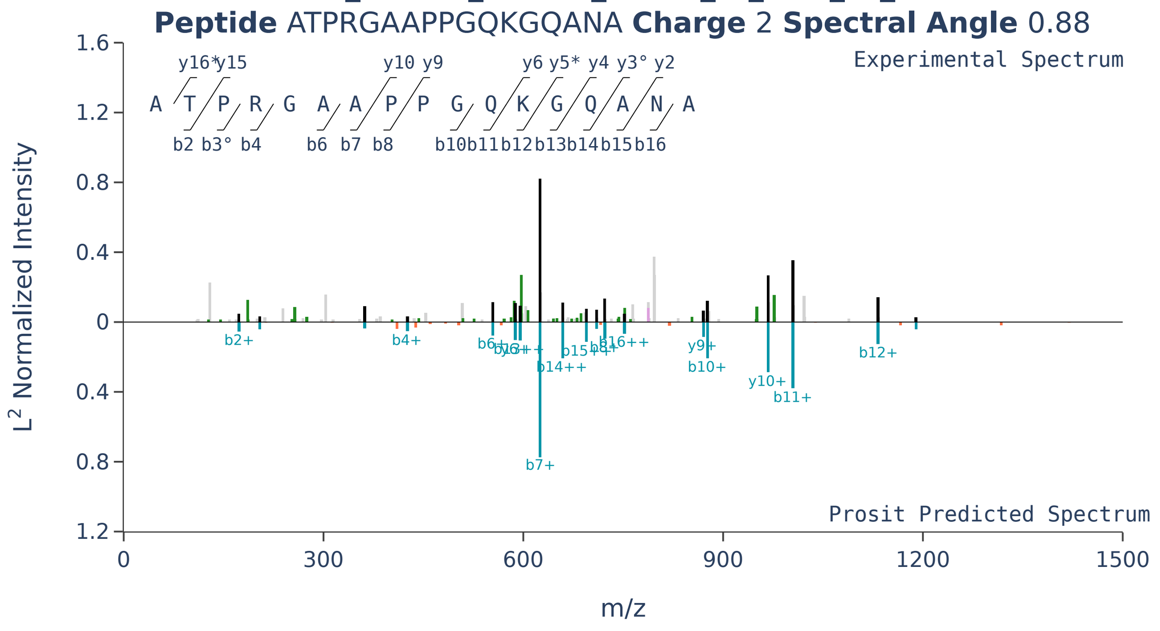


**Source** WSoh_221121_011221_HFGoe_L2_hTau_1h_R2 **Scan** 5766
**Peptide** AVVRTPPKSPSSAKSR **Charge** 4 **Spectral Angle** 0.77

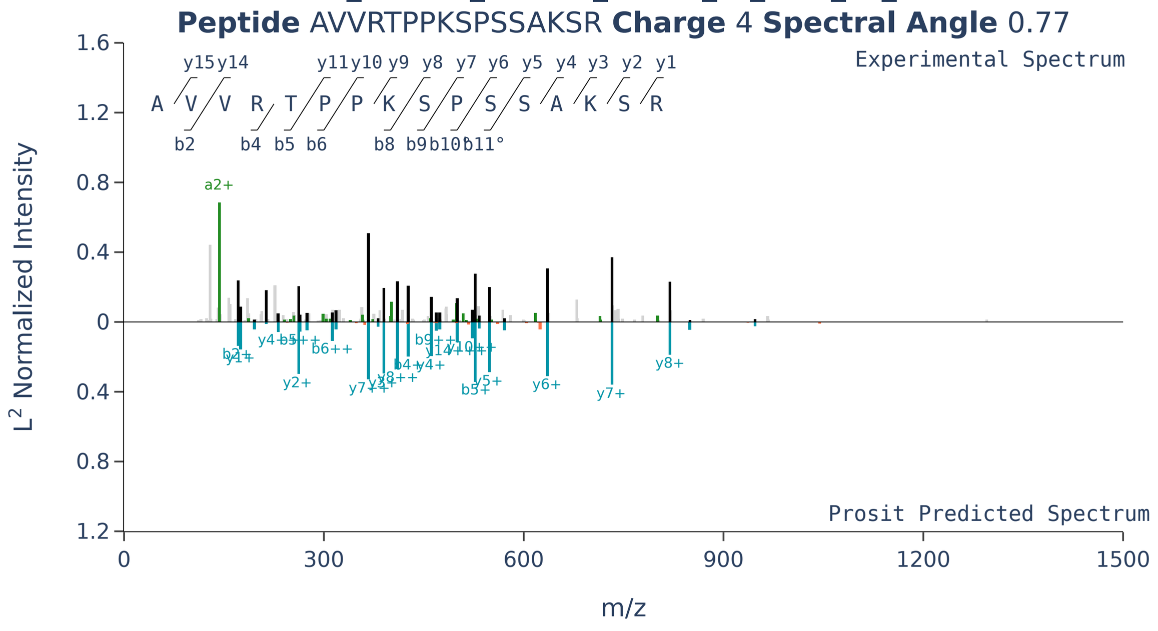


**Source** WSoh_221121_011221_HFGoe_L2_hTau_24h_R1 **Scan** 27696
**Peptide** PVPMPDL **Charge** 1 **Spectral Angle** 0.93

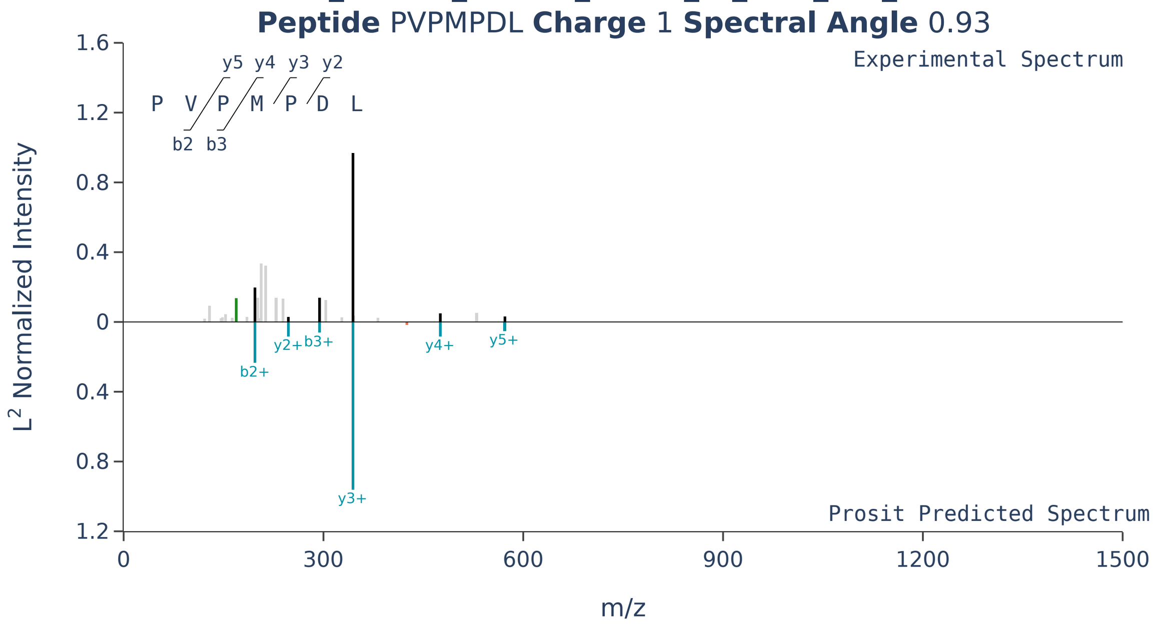


**Source** WSoh_221121_011221_HFGoe_L2_hTau_4h_R1 **Scan** 11613
**Peptide** INKKLDL **Charge** 2 **Spectral Angle** 0.86

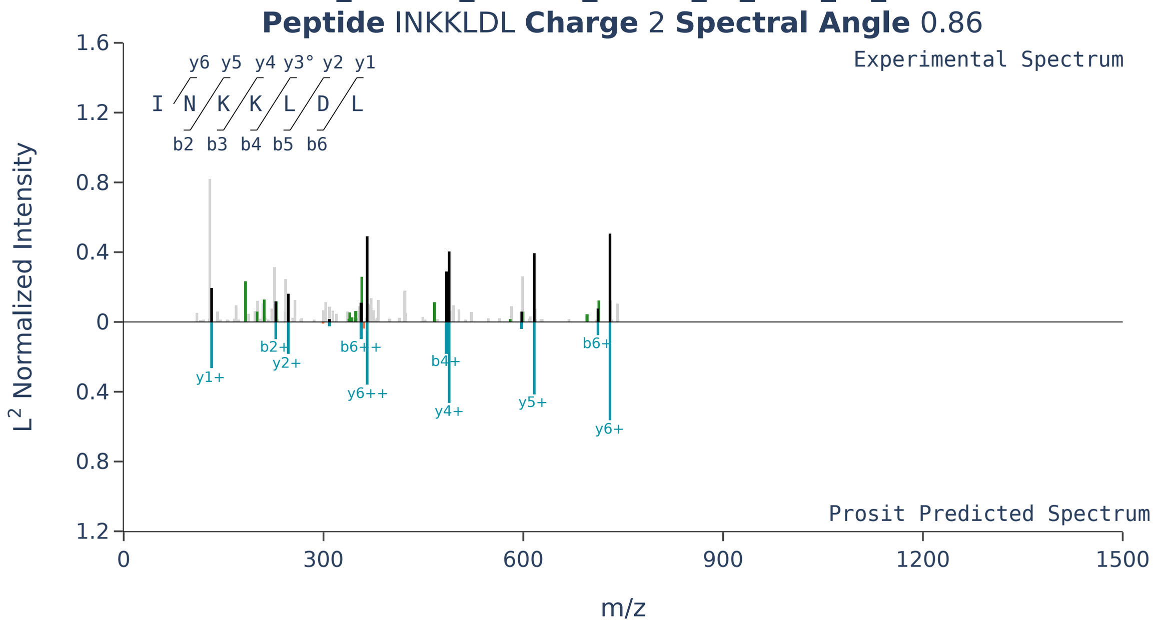

**Source** WSoh_221121_011221_HFGoe_L2_hTau_24h_R1 **Scan** 23774
**Peptide** LQTAPVPMPDLKNVKSKIGSTE **Charge** 3 **Spectral Angle** 0.88

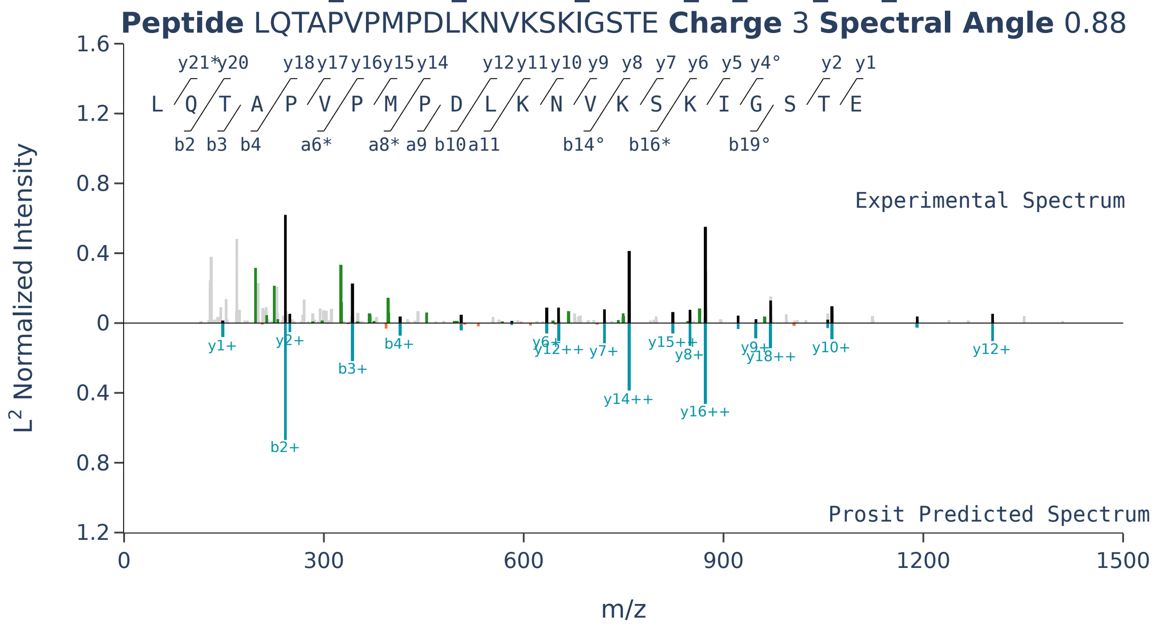


**Source** WSoh_221121_011221_HFGoe_L1_hTau_24h_R1 **Scan** 6800
**Peptide** HHKPGGGQVEVKSEKL **Charge** 2 **Spectral Angle** 0.89

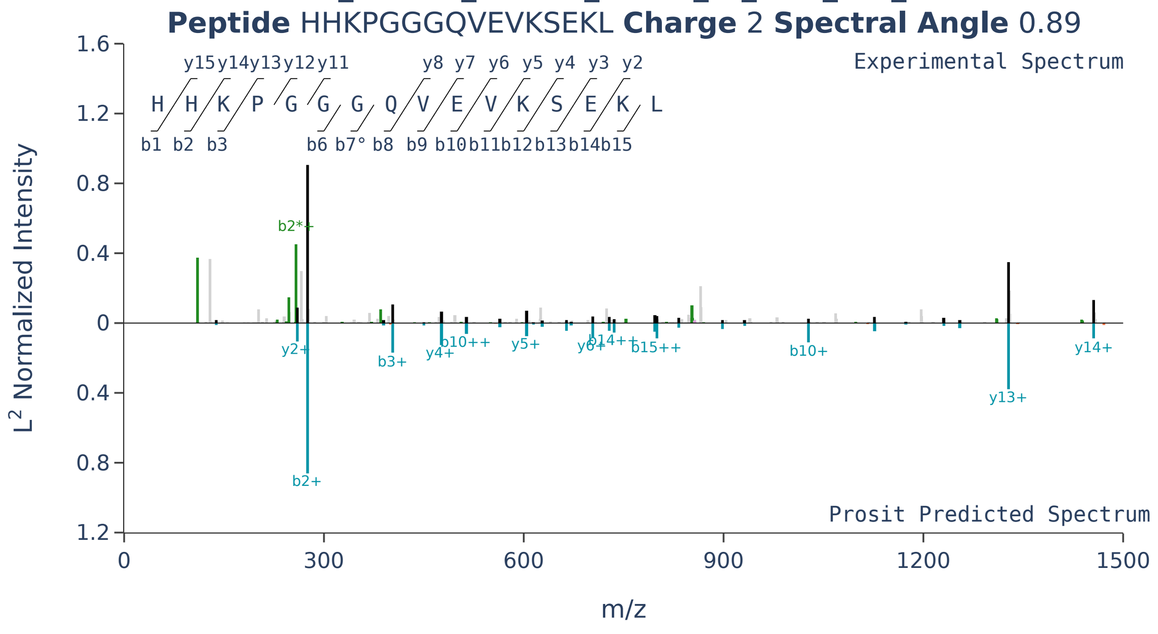


**Source** WSoh_221121_011221_HFGoe_L2_hTau_24h_R1 **Scan** 7832
**Peptide** ITHVPGGGNKKIETHKL **Charge** 4 **Spectral Angle** 0.86

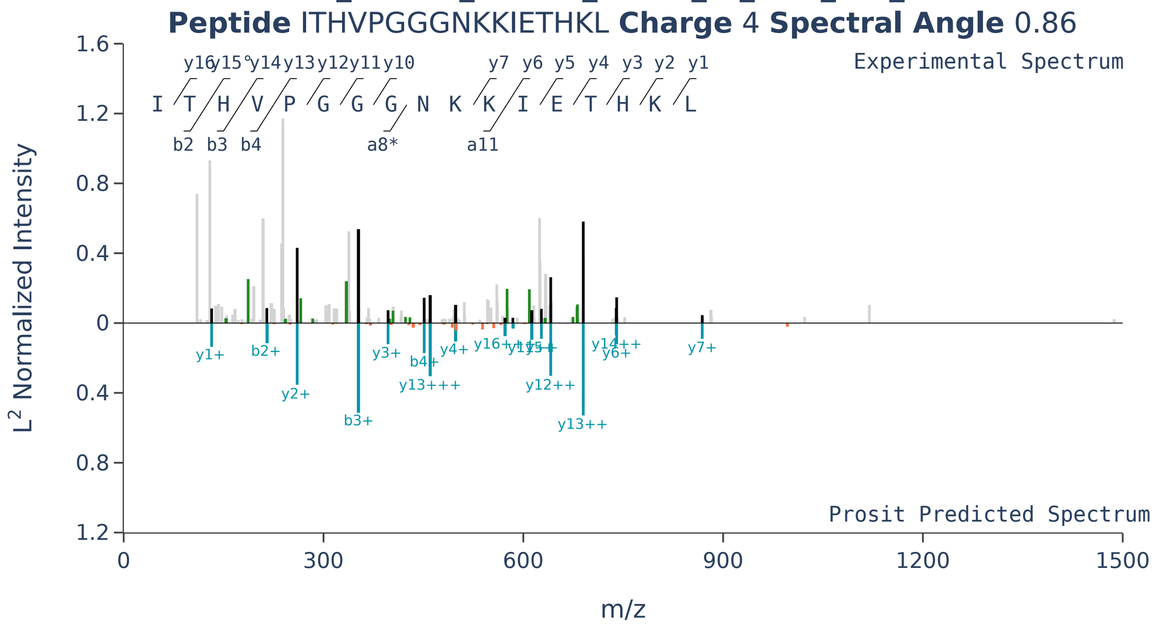


**Source** WSoh_221121_011221_HFGoe_L2_hTau_2h_R1 **Scan** 6799
**Peptide** KHQPGGGKVQI **Charge** 3 **Spectral Angle** 0.85

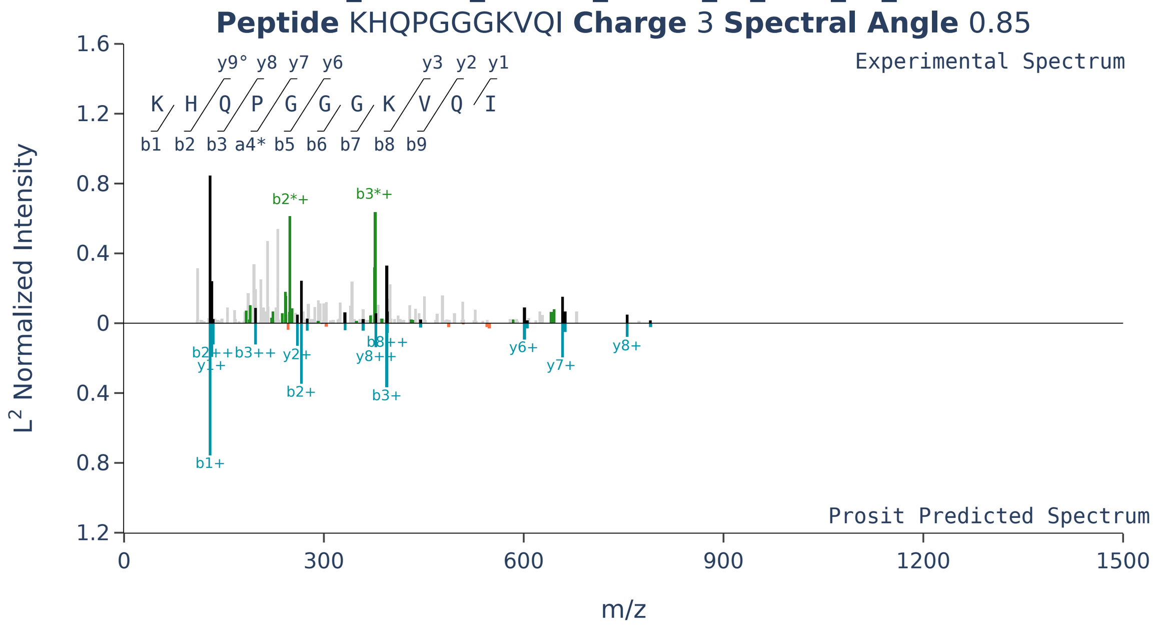


**Source** WSoh_221121_011221_HFGoe_L1_hTau_24h_R1 **Scan** 6892
**Peptide** DEAAGHVTQARMVSKSK **Charge** 3 **Spectral Angle** 0.86

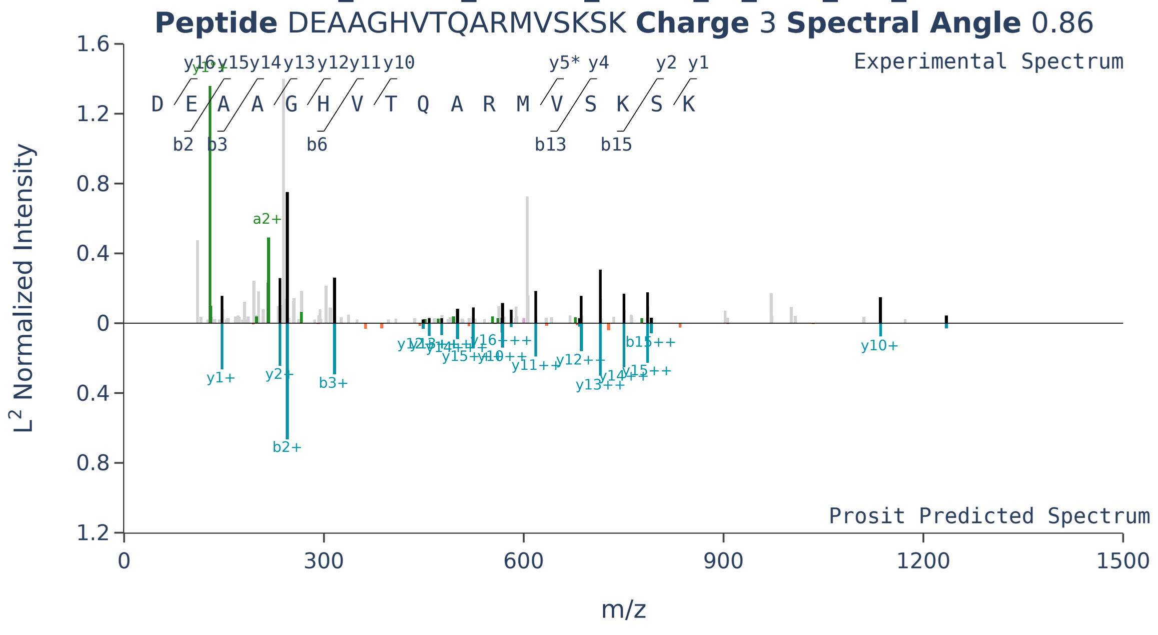


**Source** WSoh_221121_011221_HFGoe_L2_hTau_24h_R2 **Scan** 11118
**Peptide** KIGSTENLKHQPGGGKVQI **Charge** 2 **Spectral Angle** 0.88

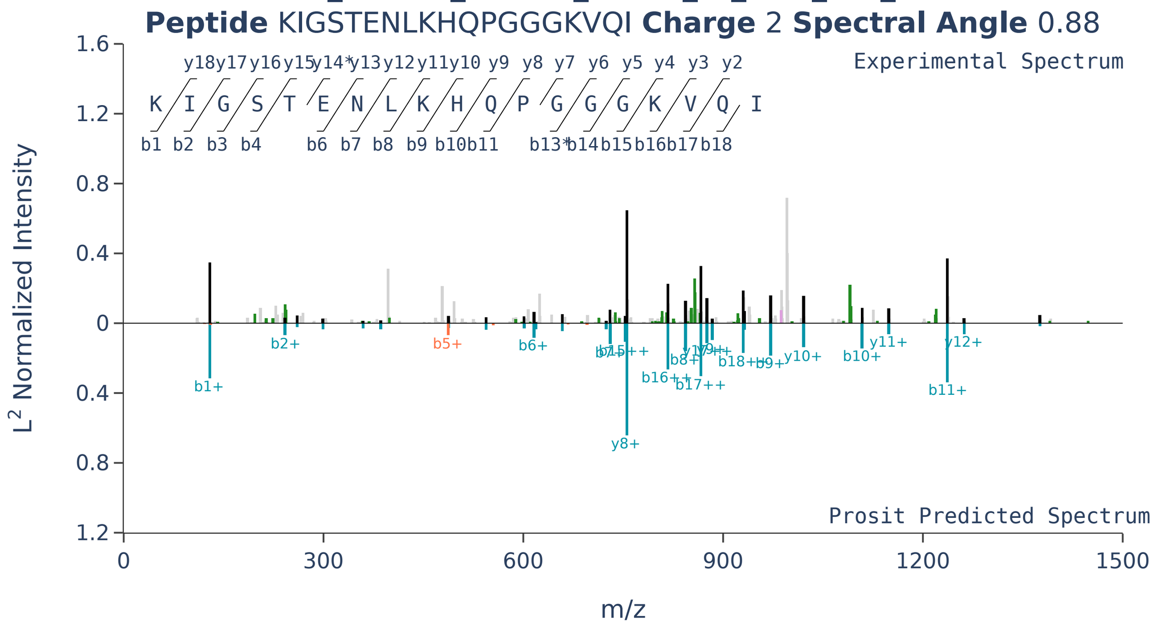


**Source** WSoh_221121_011221_HFGoe_L2_hTau_2h_R2 **Scan** 14515
**Peptide** EKLDFKDRVQSKI **Charge** 2 **Spectral Angle** 0.82

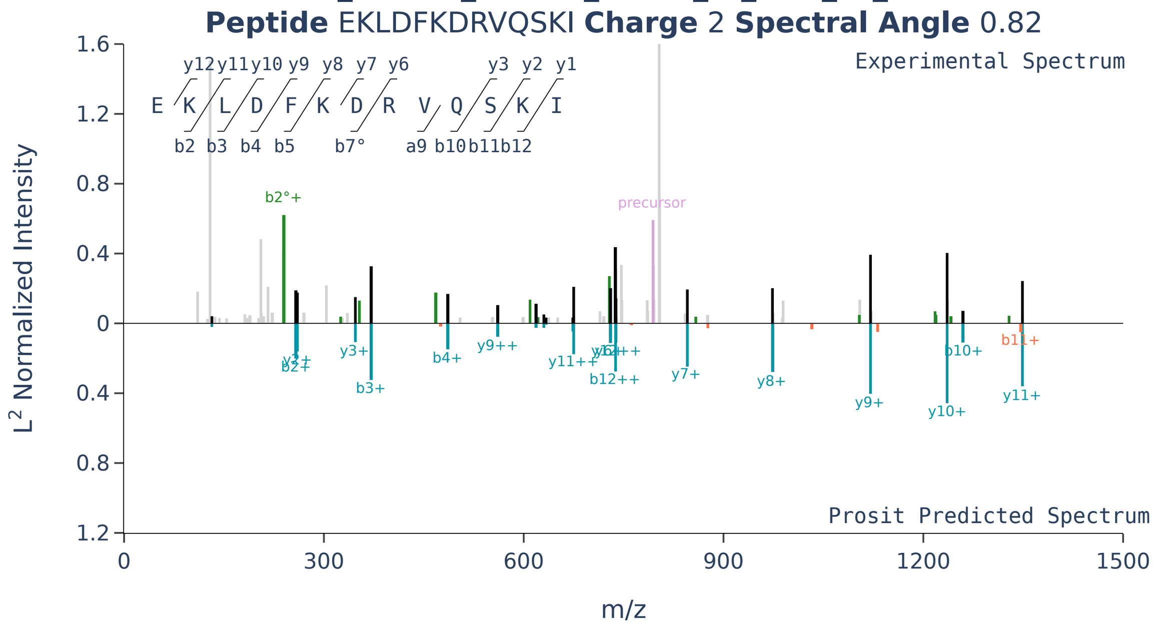


**Source** WSoh_221121_011221_HFGoe_L1_hTau_24h_R1 **Scan** 19554
**Peptide** AQPHTEIPEGTTAEEAGI **Charge** 2 **Spectral Angle** 0.88

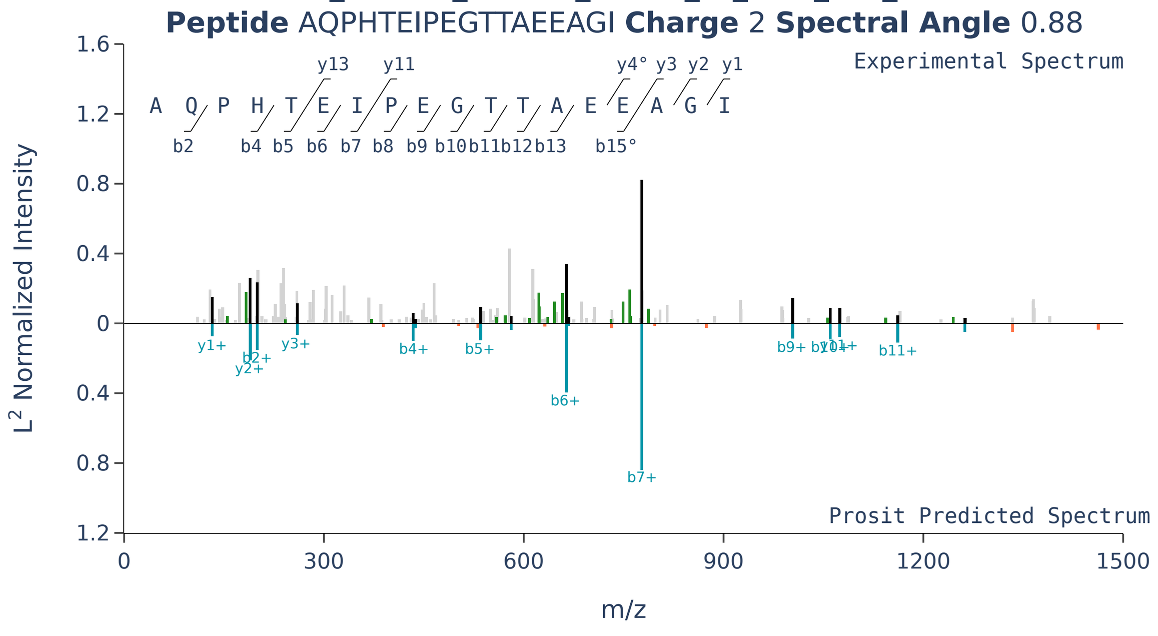


**Source** WSoh_221121_011221_HFGoe_L2_hTau_24h_R2 **Scan** 11516
**Peptide** SLDNITHVPGGGNKK **Charge** 3 **Spectral Angle** 0.85

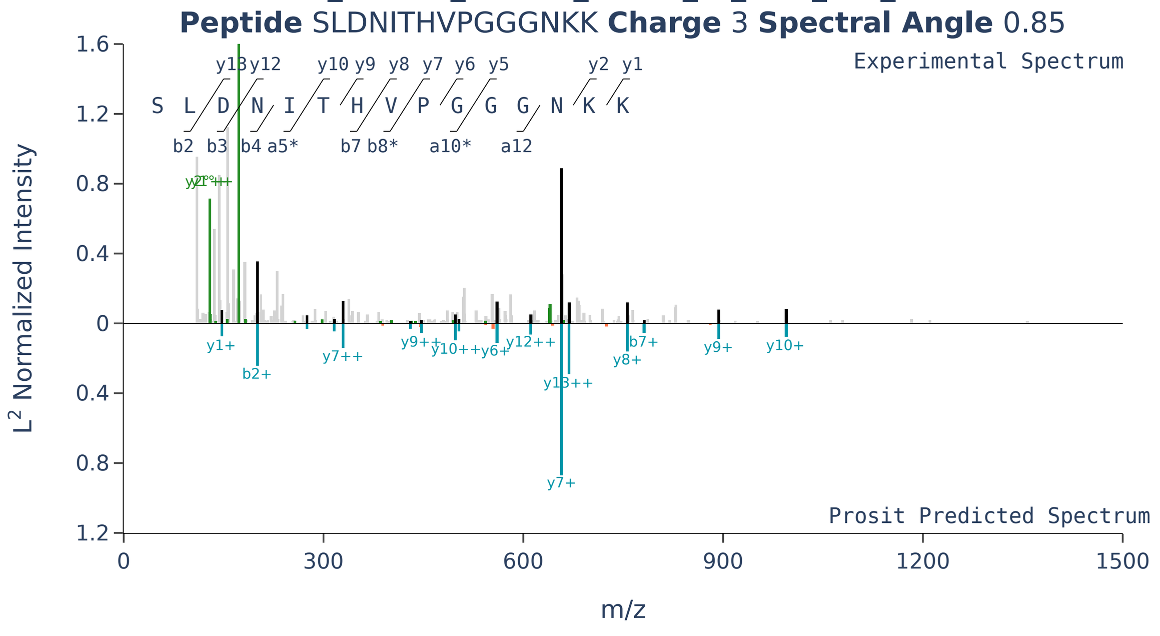


**Source** WSoh_221121_011221_HFGoe_L2_hTau_4h_R2 **Scan** 5674
**Peptide** NIHHKPGGGQVEVKS **Charge** 2 **Spectral Angle** 0.82

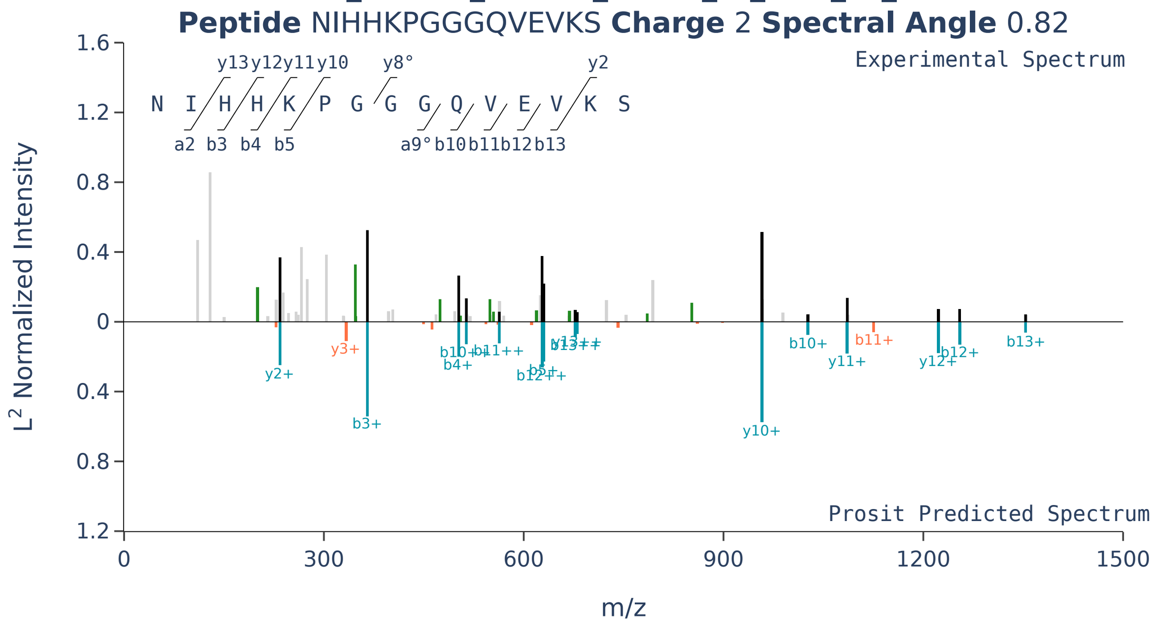


**Source** WSoh_221121_011221_HFGoe_L2_hTau_24h_R2 **Scan** 16014
**Peptide** IGSLDNITHVPGGGNKKIETH **Charge** 3 **Spectral Angle** 0.87

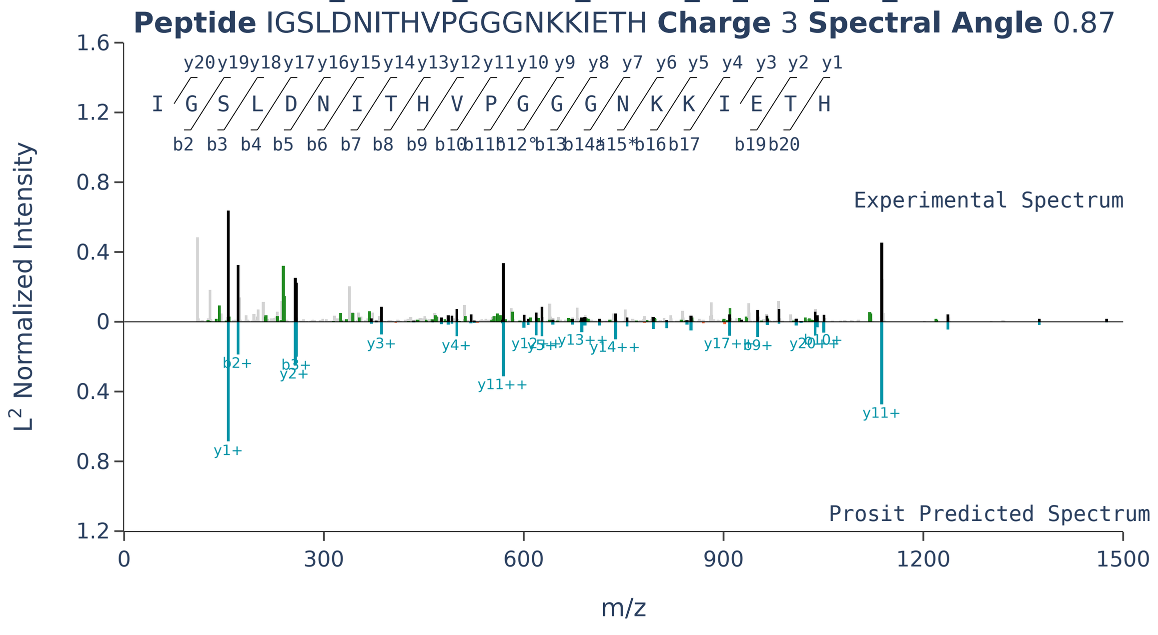


**Source** WSoh_221121_011221_HFGoe_L2_hTau_24h_R2 **Scan** 7787
**Peptide** PPGQKGQANATRL **Charge** 3 **Spectral Angle** 0.88

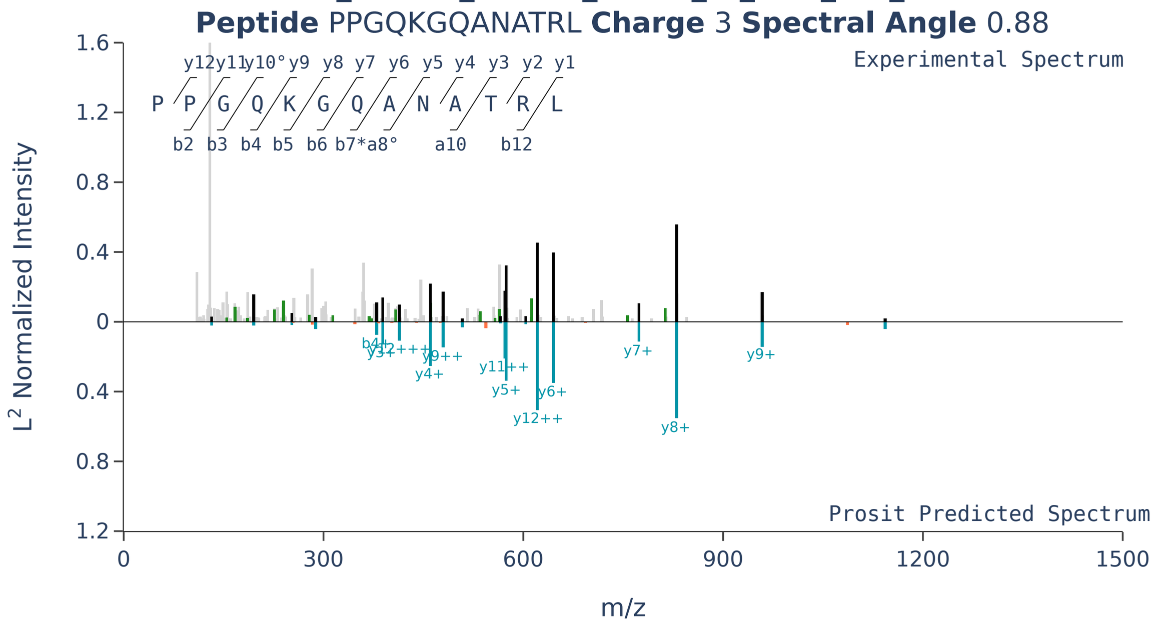


**Source** WSoh_221121_011221_HFGoe_L1_hTau_24h_R1 **Scan** 9306
**Peptide** GIGDTPS **Charge** 1 **Spectral Angle** 0.95

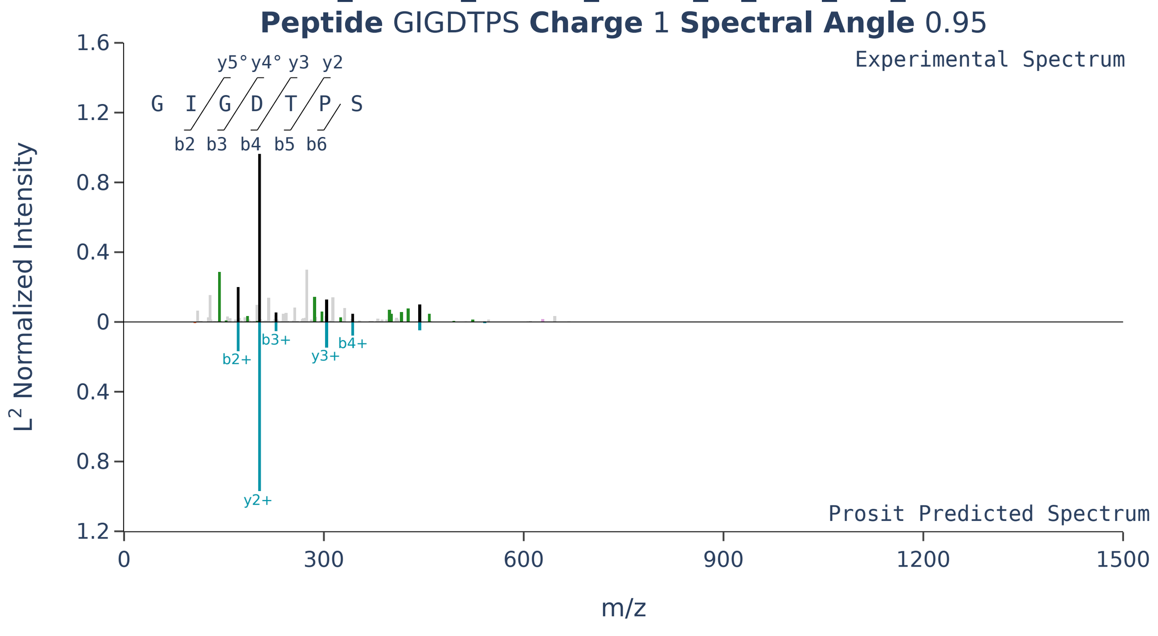


**Source** WSoh_221121_011221_HFGoe_L2_hTau_4h_R1 **Scan** 7383
**Peptide** AVVRTPPKSPSSAKSRLQ **Charge** 4 **Spectral Angle** 0.85

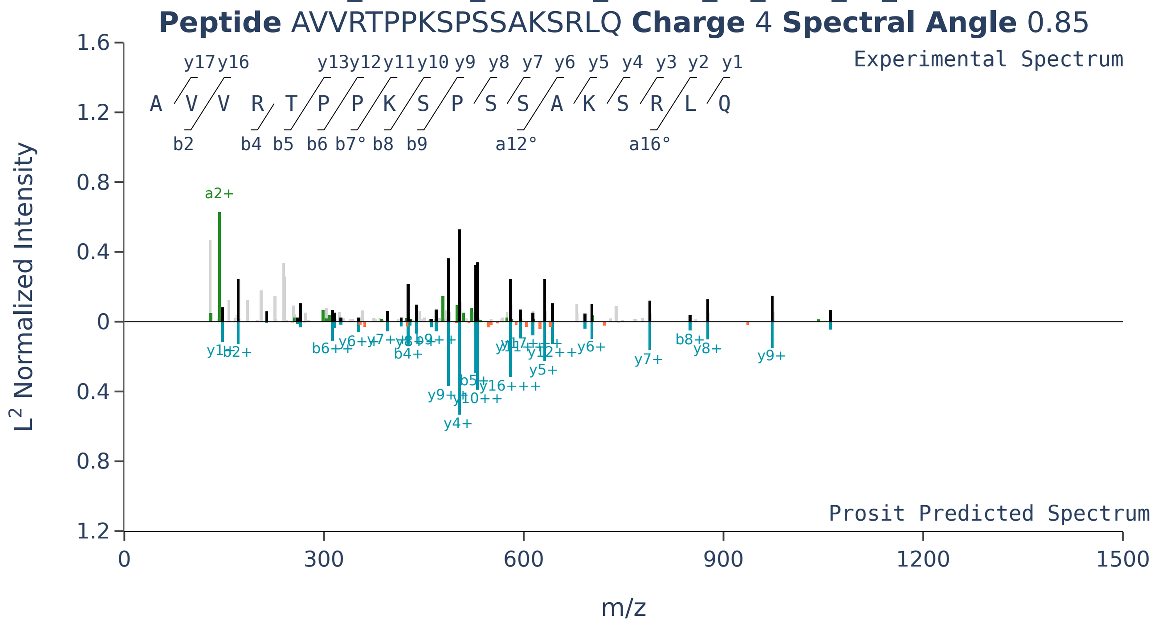


**Source** WSoh_221121_011221_HFGoe_L1_hTau_24h_R1 **Scan** 11228
**Peptide** NLTHVPGGGNKKIETHKLTFREN **Charge** 3 **Spectral Angle** 0.85

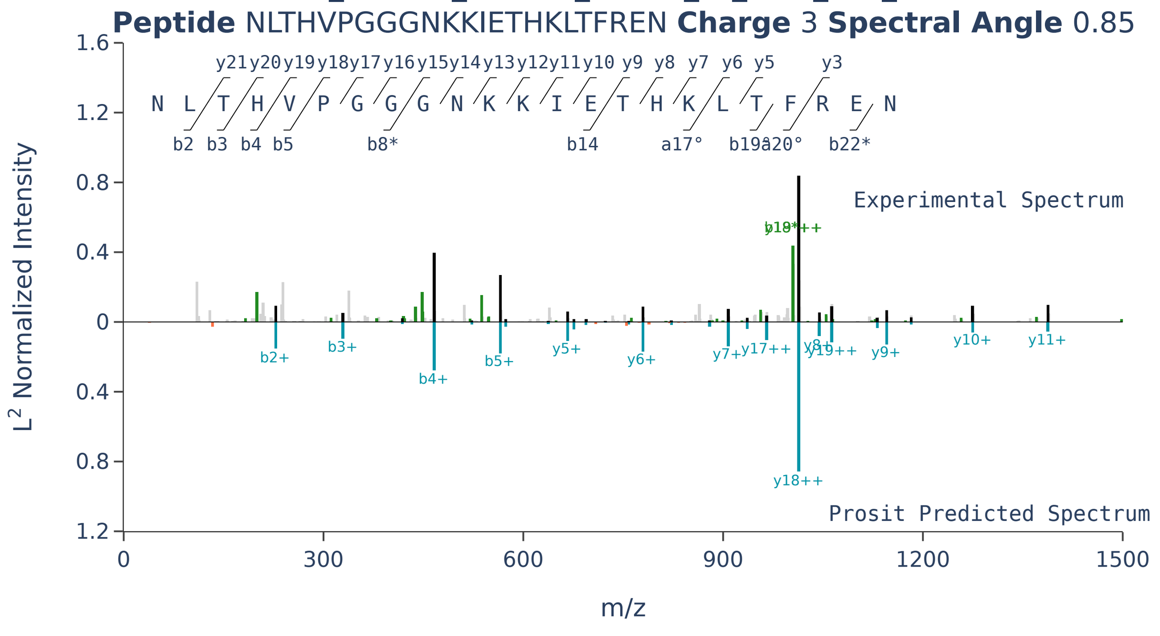

**Source** WSoh_221121_011221_HFGoe_L1_hTau_24h_R1 **Scan** 19882
**Peptide** APVPMPDLKNVKSKIGSTENLKHQ **Charge** 4 **Spectral Angle** 0.89

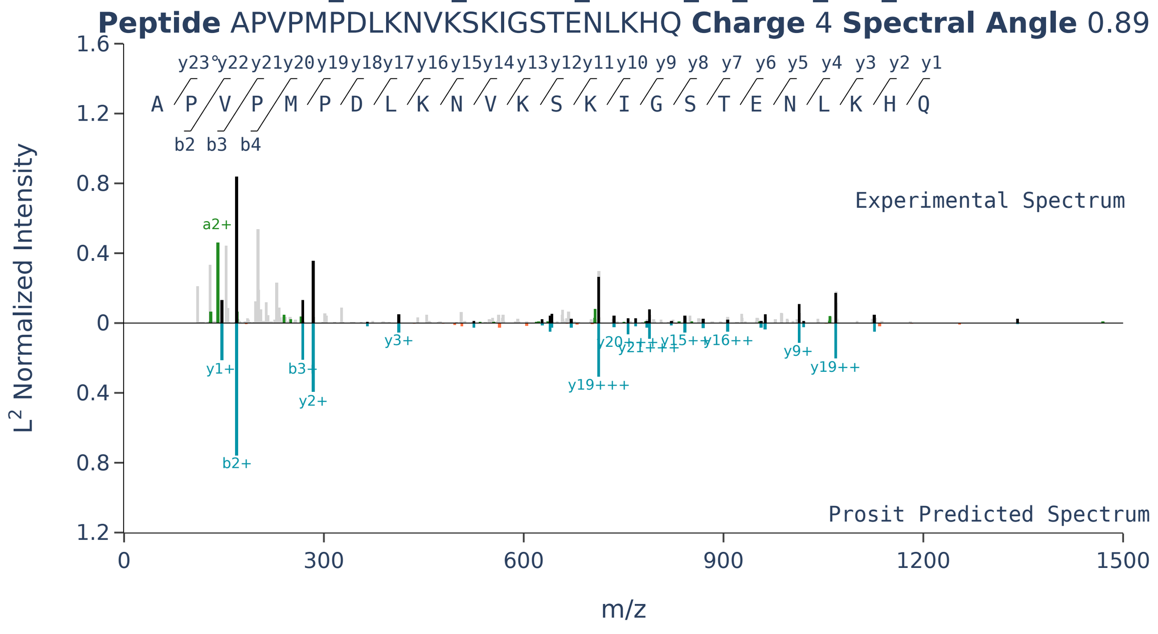


**Source** WSoh_221121_011221_HFGoe_L2_hTau_24h_R2 **Scan** 7563
**Peptide** GLGDRKDQGGY **Charge** 2 **Spectral Angle** 0.79

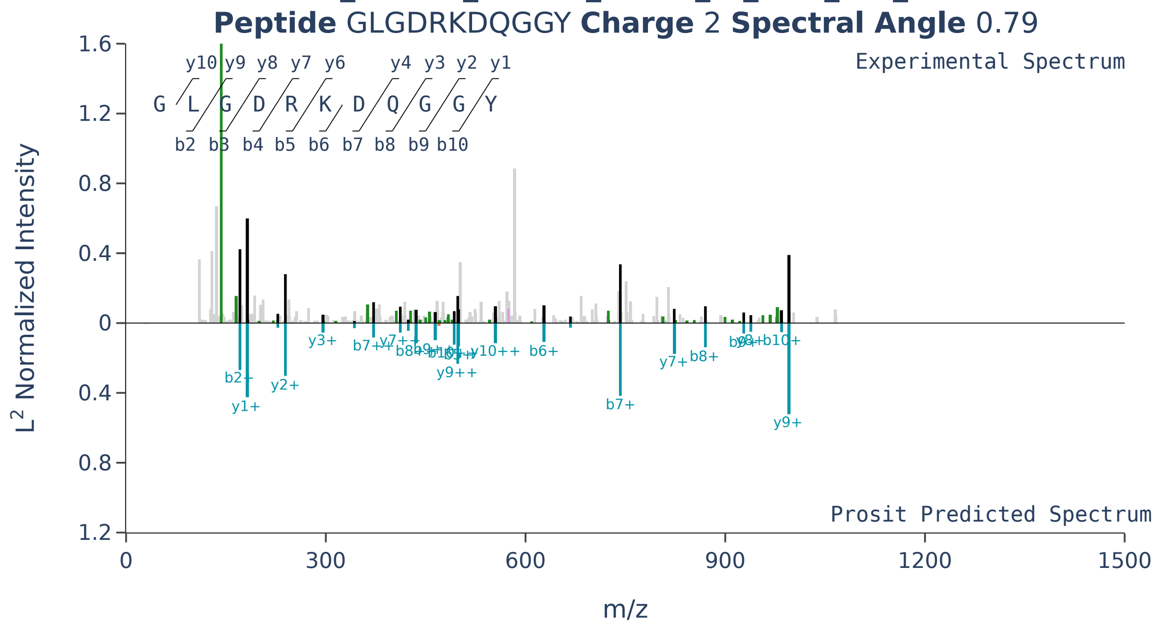


**Source** WSoh_221121_011221_HFGoe_L2_hTau_24h_R1 **Scan** 6464
**Peptide** KCGSKDNIKHVPGGGSVQ **Charge** 2 **Spectral Angle** 0.89

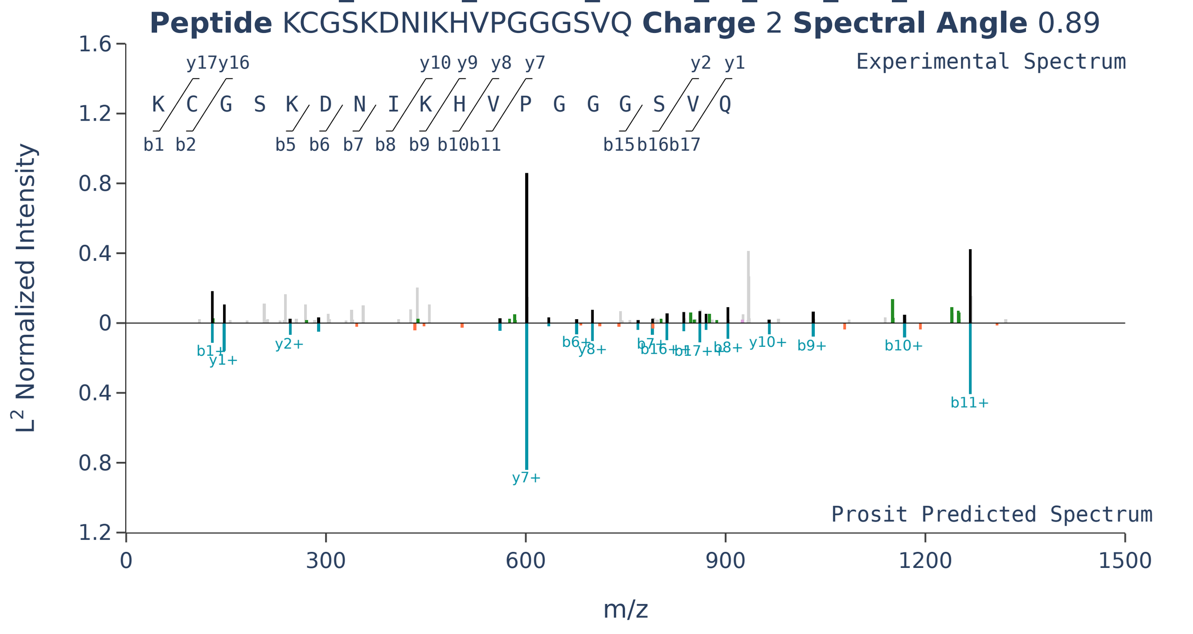


**Source** WSoh_221121_011221_HFGoe_L2_hTau_24h_R1 **Scan** 12071
**Peptide** KSEKLDFKDRVQSKIGS **Charge** 2 **Spectral Angle** 0.83

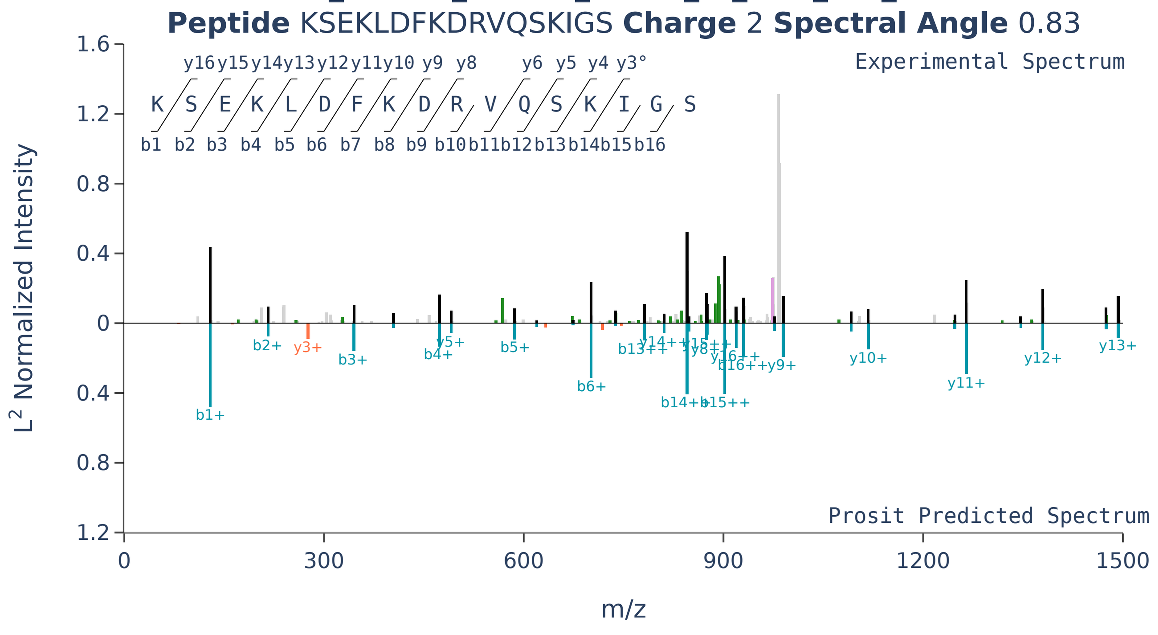


**Source** WSoh_221121_011221_HFGoe_L1_hTau_24h_R1 **Scan** 15139
**Peptide** GSLDNITHVPGGGNKKIE **Charge** 3 **Spectral Angle** 0.86

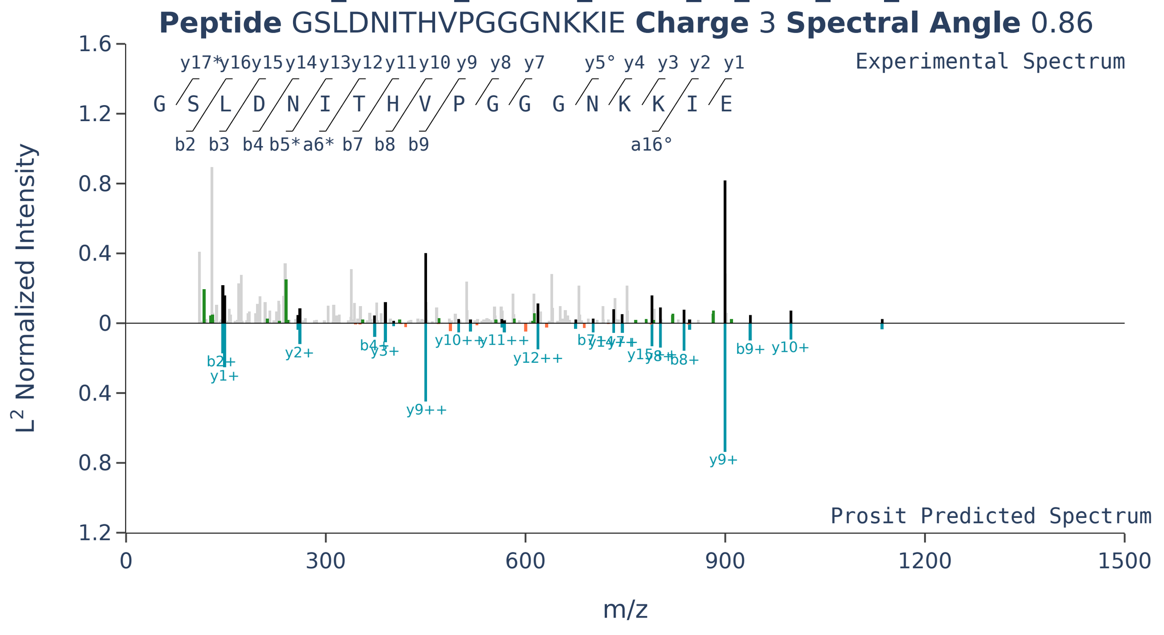


**Source** WSoh_221121_011221_HFGoe_L1_hTau_24h_R1 **Scan** 9453
**Peptide** NAKAKTDHGAEIVY **Charge** 3 **Spectral Angle** 0.85

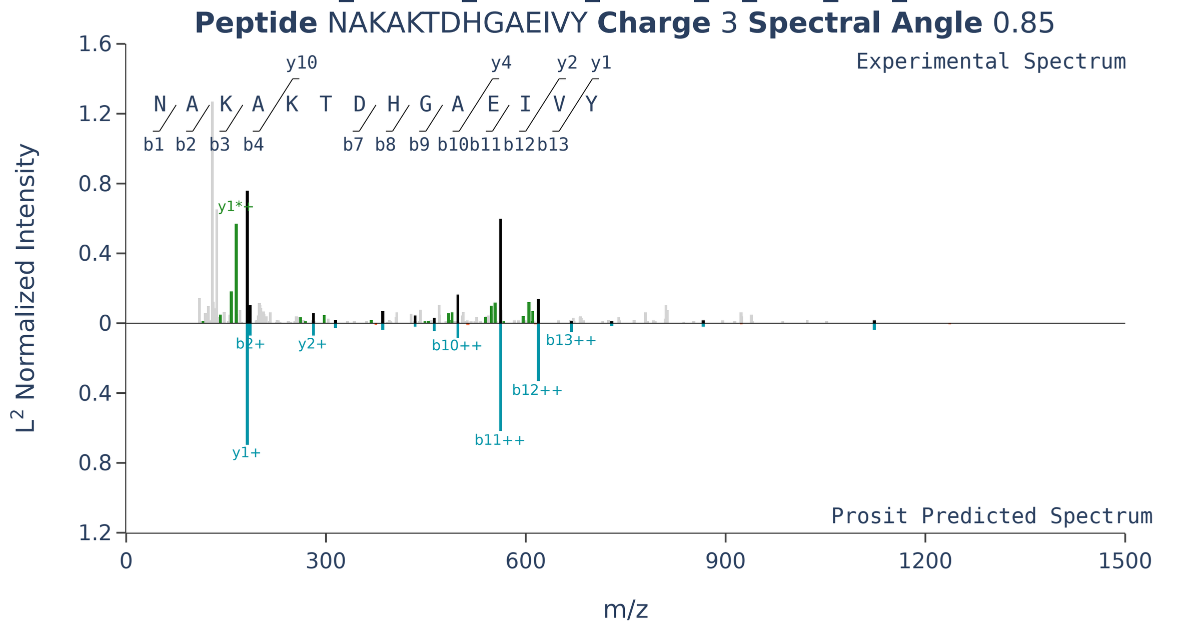


**Source** WSoh_221121_011221_HFGoe_L2_hTau_24h_R2 **Scan** 6563
**Peptide** PKSPSSAKS **Charge** 2 **Spectral Angle** 0.88

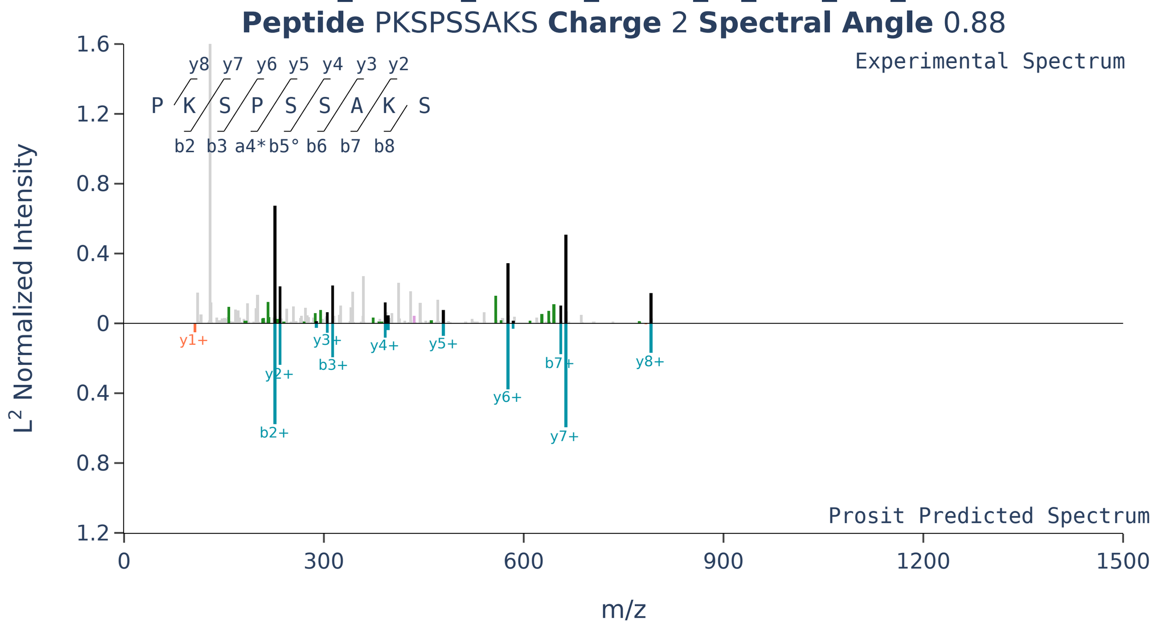


**Source** WSoh_221121_011221_HFGoe_L1_hTau_24h_R1 **Scan** 9404
**Peptide** HAGTYGLGDRKDQGGY **Charge** 2 **Spectral Angle** 0.87

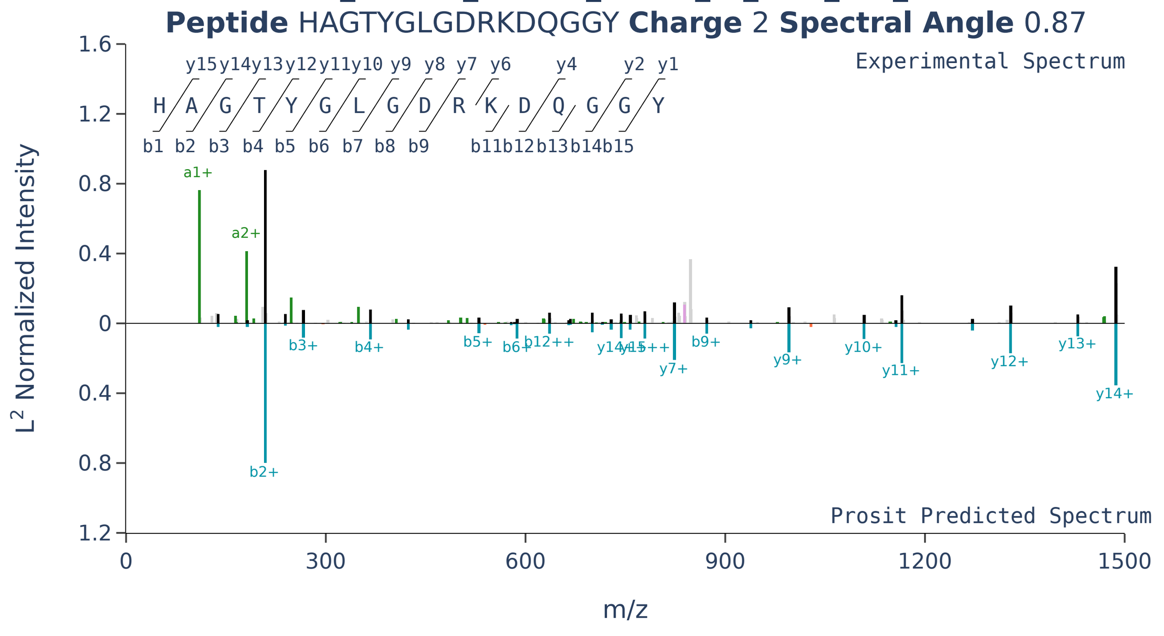


**Source** WSoh_221121_011221_HFGoe_L1_hTau_24h_R1 **Scan** 19866
**Peptide** AEPRQEFEVMEDHAGTYGLGDRKD **Charge** 4 **Spectral Angle** 0.85

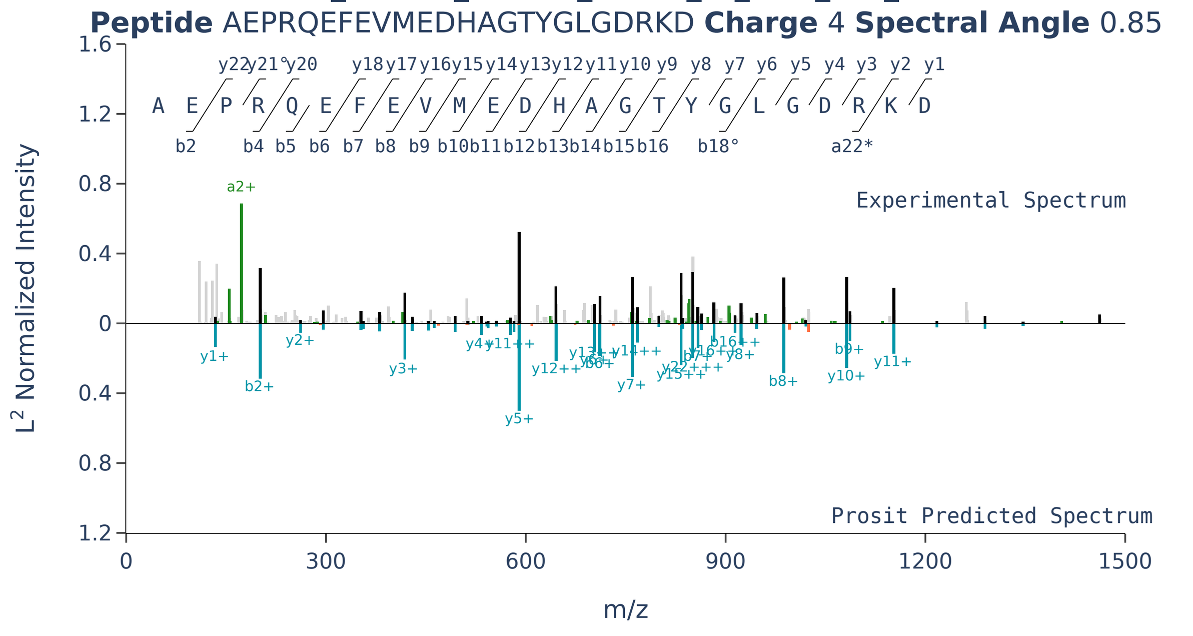


**Source** WSoh_221121_011221_HFGoe_L1_hTau_24h_R2 **Scan** 6606
**Peptide** GEPPKSGDRSGYSSPGSPGTPGSRSR **Charge** 4 **Spectral Angle** 0.9

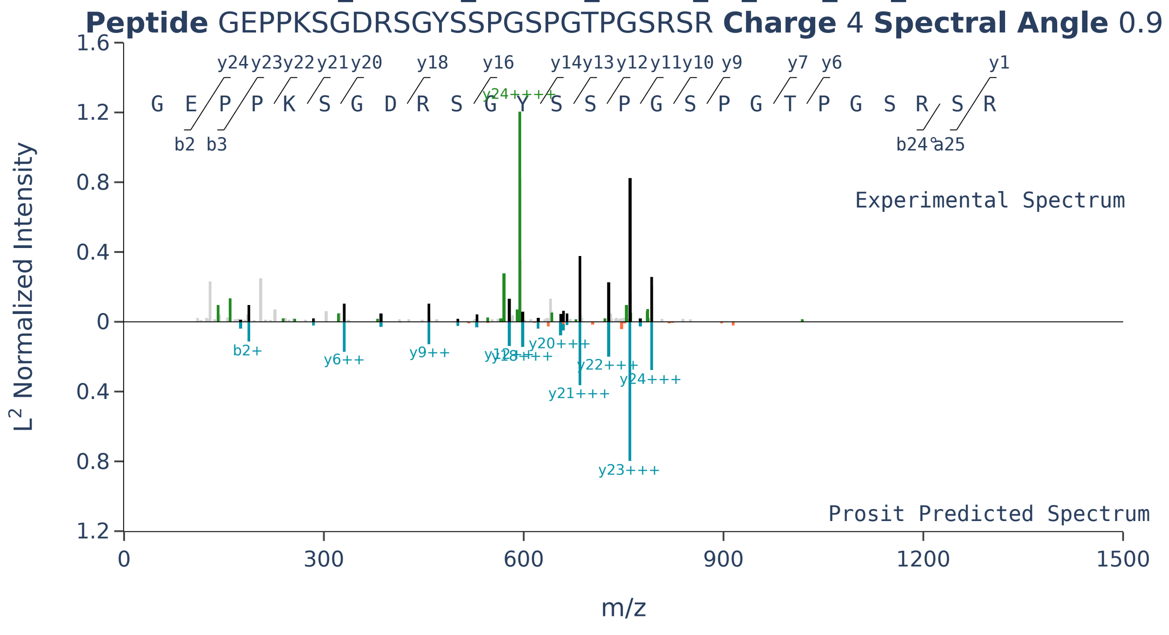


**Source** WSoh_221121_011221_HFGoe_L1_hTau_24h_R1 **Scan** 6332
**Peptide** PKSPSSAKSRLQT **Charge** 3 **Spectral Angle** 0.89

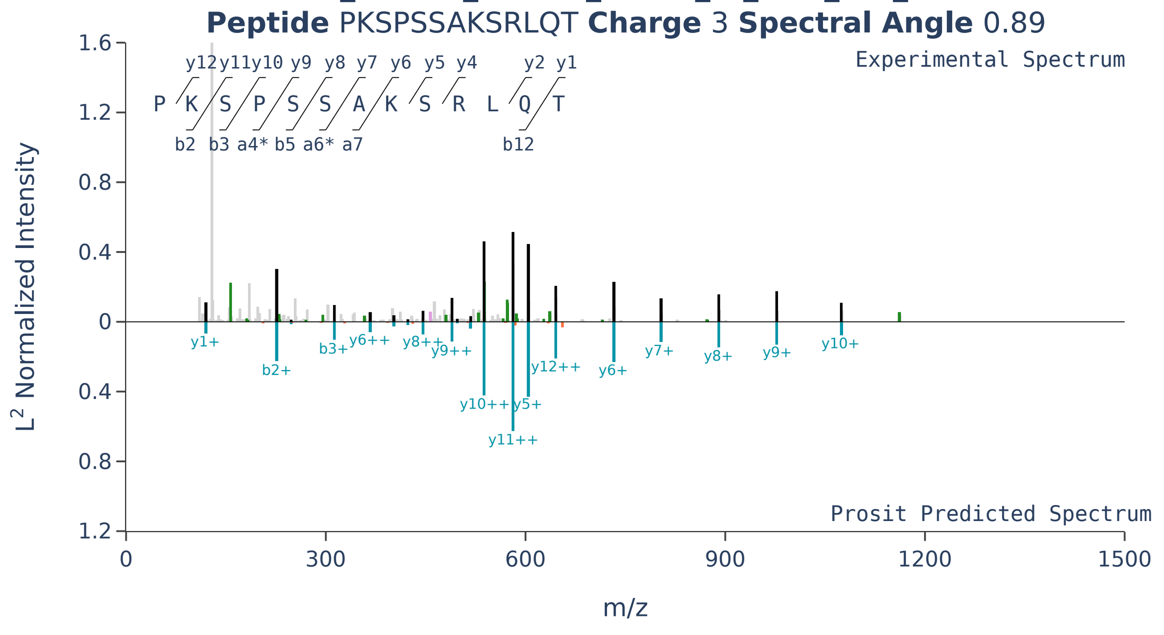


**Source** WSoh_221121_011221_HFGoe_L1_hTau_24h_R2 **Scan** 18090
**Peptide** GLGDTPSLEDEAAGHVTQARMVSKSKD **Charge** 4 **Spectral Angle** 0.84

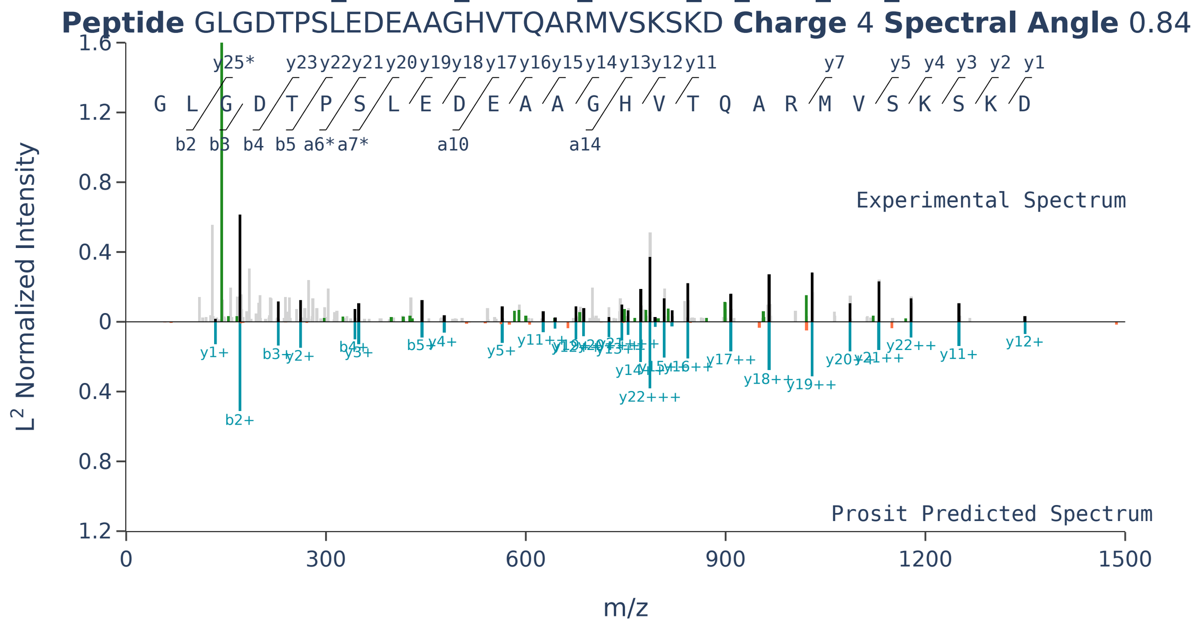


**Source** WSoh_221121_011221_HFGoe_L2_hTau_4h_R2 **Scan** 6034
**Peptide** SNVQSKCGSKDNI **Charge** 2 **Spectral Angle** 0.9

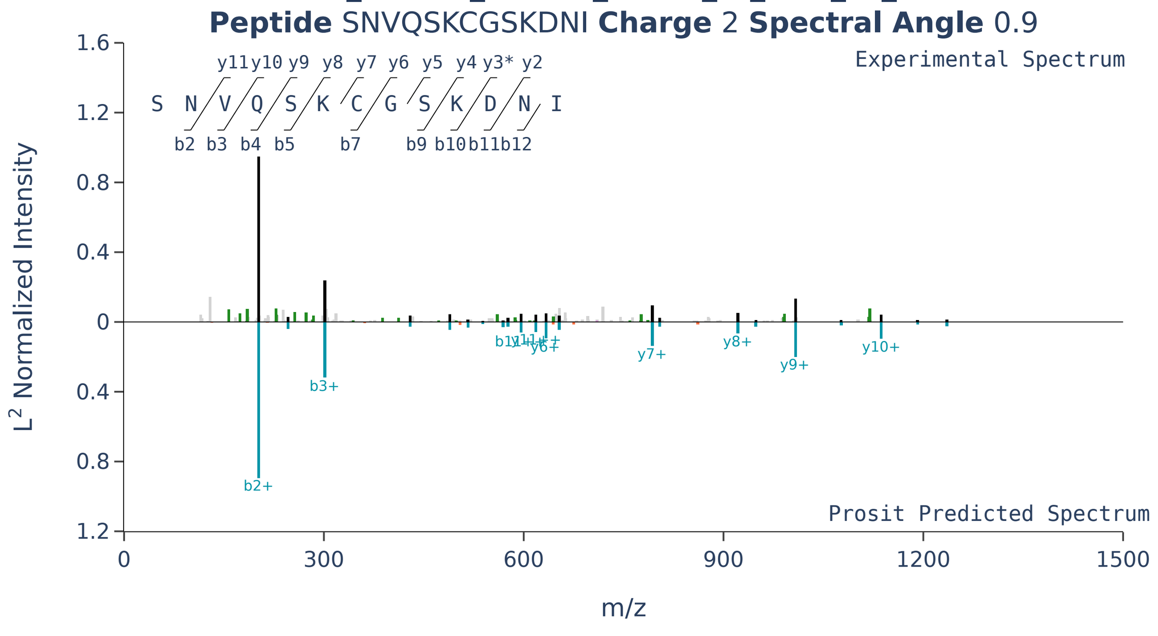


**Source** WSoh_221121_011221_HFGoe_L2_hTau_24h_R1 **Scan** 26274
**Peptide** QTAPVPMPDLKNV **Charge** 2 **Spectral Angle** 0.9

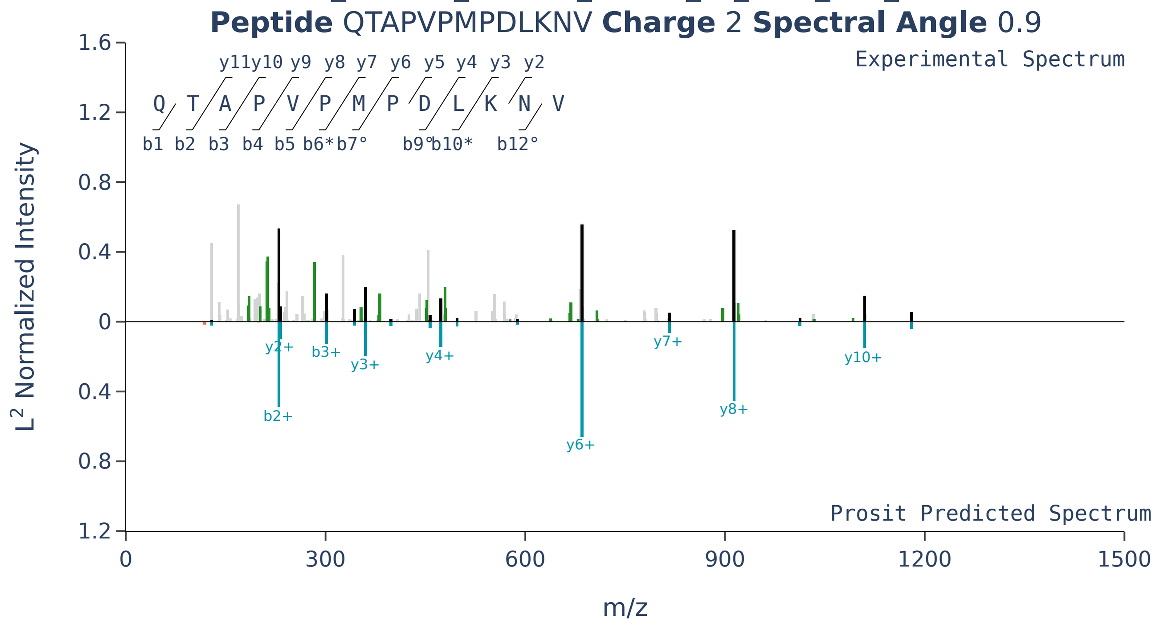


**Source** WSoh_221121_011221_HFGoe_L2_hTau_24h_R1 **Scan** 8878
**Peptide** PAKTPPAPKTPPSSGEPPKSGDRSGY **Charge** 5 **Spectral Angle** 0.84

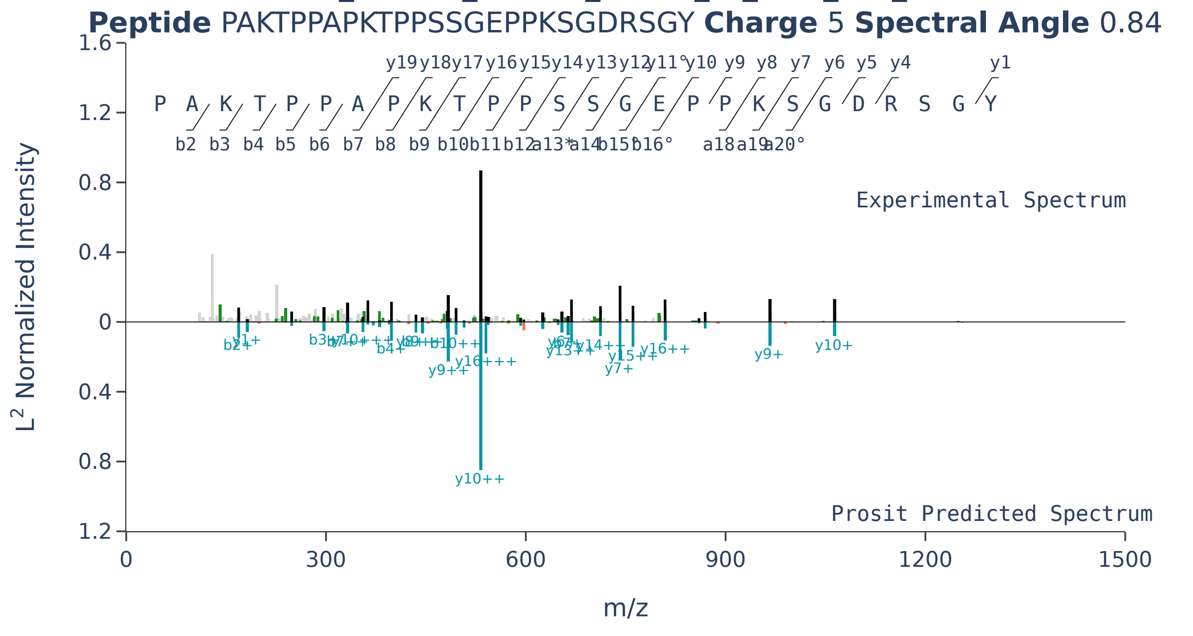

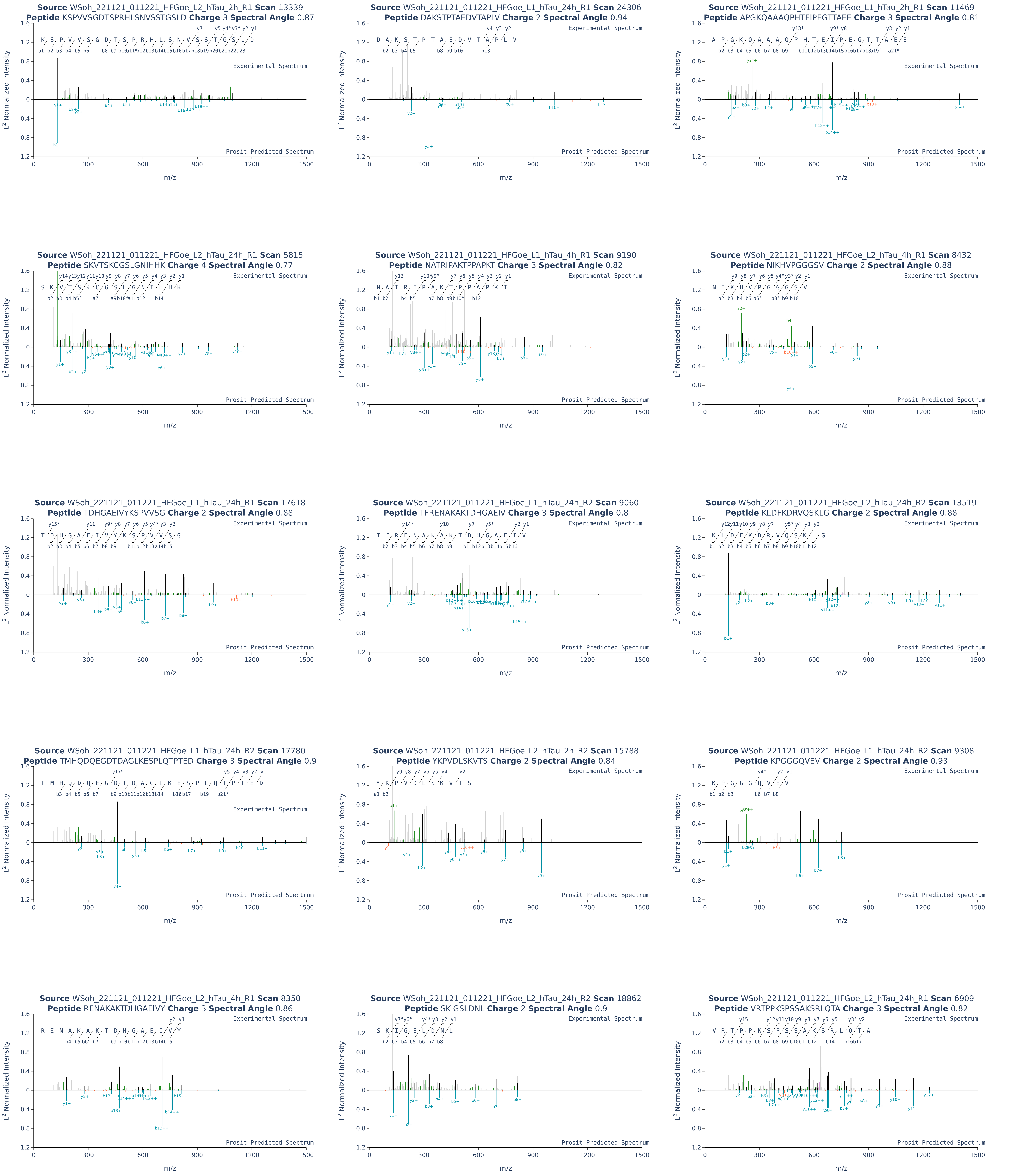
Source WSoh_221121_011221_HFGoe_L2_hTau_2h_R1 Scan 13339
Peptide KSPVVSGDTSPRHLSNVSSTGSLD Charge 3 Spectral Angle 0.87
Source WSoh_221121_011221_HFGoe_L1_hTau_24h_R1 Scan 24306
Peptide DAKSTPTAEDVTAPLV Charge 2 Spectral Angle 0.94
Source WSoh_221121_011221_HFGoe_L1_hTau_2h_R1 Scan 11469
Peptide APGKQAAAQPHTEIPEGTTAEE Charge 3 Spectral Angle 0.81
Source WSoh_221121_011221_HFGoe_L2_hTau_24h_R1 Scan 5815
Peptide SKVTSKCGSLGNIHHK Charge 4 Spectral Angle 0.77
Source WSoh_221121_011221_HFGoe_L1_hTau_4h_R1 Scan 9190
Peptide NATRIPAKTPPAPKT Charge 3 Spectral Angle 0.82
Source WSoh_221121_011221_HFGoe_L2_hTau_4h_R1 Scan 8432
Peptide NIKHVPGGGSV Charge 2 Spectral Angle 0.88
Source WSoh_221121_011221_HFGoe_L1_hTau_24h_R1 Scan 17618
Peptide TDHGAEIVYKSPVVSG Charge 2 Spectral Angle 0.88
Source WSoh_221121_011221_HFGoe_L1_hTau_24h_R2 Scan 9060
Peptide TFRENAKAKTDHGAEIV Charge 3 Spectral Angle 0.8
Source WSoh_221121_011221_HFGoe_L2_hTau_24h_R2 Scan 13519
Peptide KLDFKDRVQSKLG Charge 2 Spectral Angle 0.88
Source WSoh_221121_011221_HFGoe_L1_hTau_24h_R2 Scan 17780
Peptide TMHQDQEGDTDAGLKESPLQTPTED Charge 3 Spectral Angle 0.9
Source WSoh_221121_011221_HFGoe_L2_hTau_2h_R2 Scan 15788
Peptide YKPVDLSKVTS Charge 2 Spectral Angle 0.84
Source WSoh_221121_011221_HFGoe_L1_hTau_24h_R2 Scan 9308
Peptide KPGGGQVEV Charge 2 Spectral Angle 0.93
Source WSoh_221121_011221_HFGoe_L2_hTau_4h_R1 Scan 8350
Peptide RENAKAKTDHGAEIVY Charge 3 Spectral Angle 0.86
Source WSoh_221121_011221_HFGoe_L2_hTau_24h_R2 Scan 18862
Peptide SKIGSLDNL Charge 2 Spectral Angle 0.9
Source WSoh_221121_011221_HFGoe_L2_hTau_24h_R1 Scan 6909
Peptide VRTPPKSPSSAKSRLQTA Charge 3 Spectral Angle 0.82
Experimental Spectrum
Prosit Predicted Spectrum
L² Normalized Intensity
m/z

**Source** WSoh_221121_011221_HFGoe_L2_hTau_24h_R2 **Scan** 16206
**Peptide** KIGSLDNITHVPGGGNK **Charge** 4 **Spectral Angle** 0.84

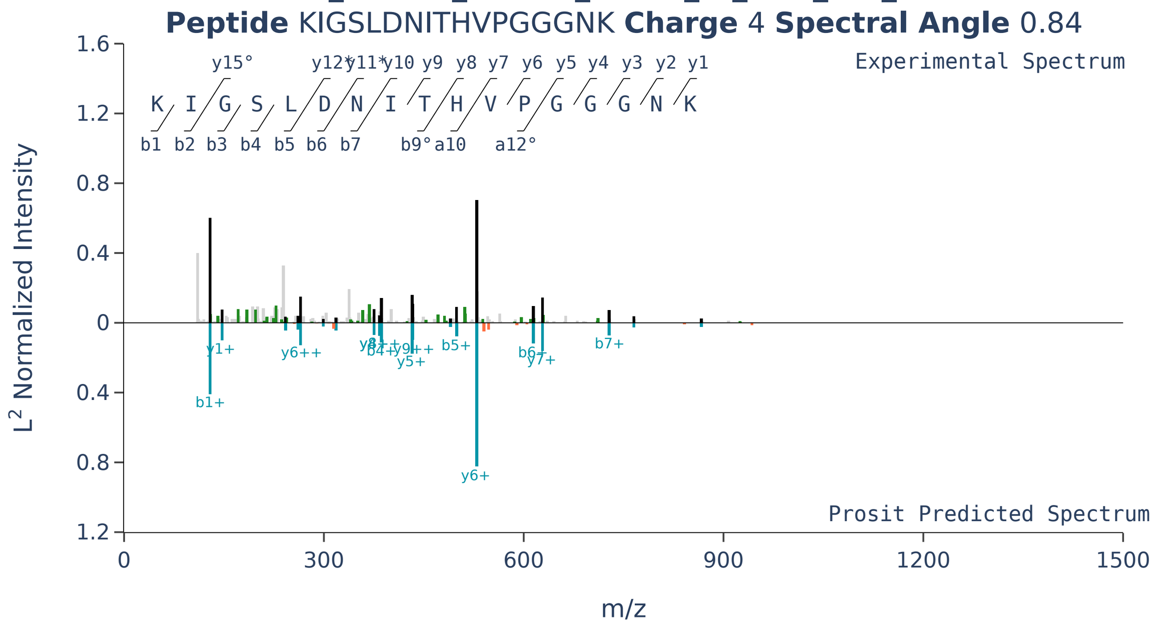


**Source** WSoh_221121_011221_HFGoe_L1_hTau_4h_R1 **Scan** 15503
**Peptide** LVYKSPVVSGDTSPRHL **Charge** 4 **Spectral Angle** 0.84

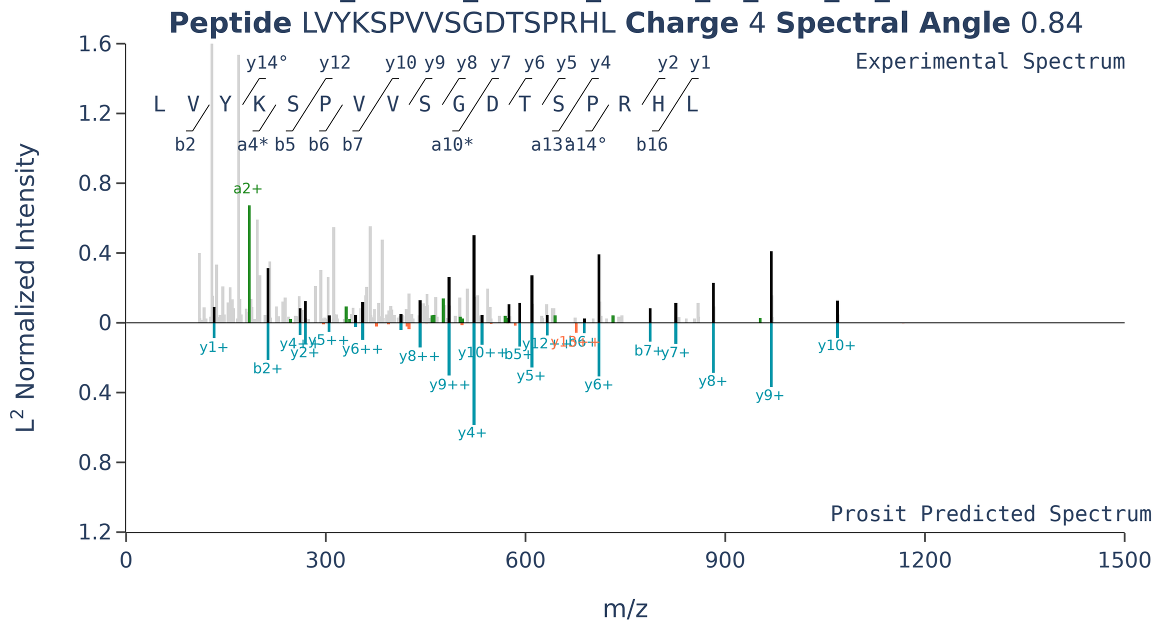


**Source** WSoh_221121_011221_HFGoe_L2_hTau_24h_R2 **Scan** 14140
**Peptide** KTDHGAEIVYKSPVVSGDTSPRHLSN **Charge** 3 **Spectral Angle** 0.8

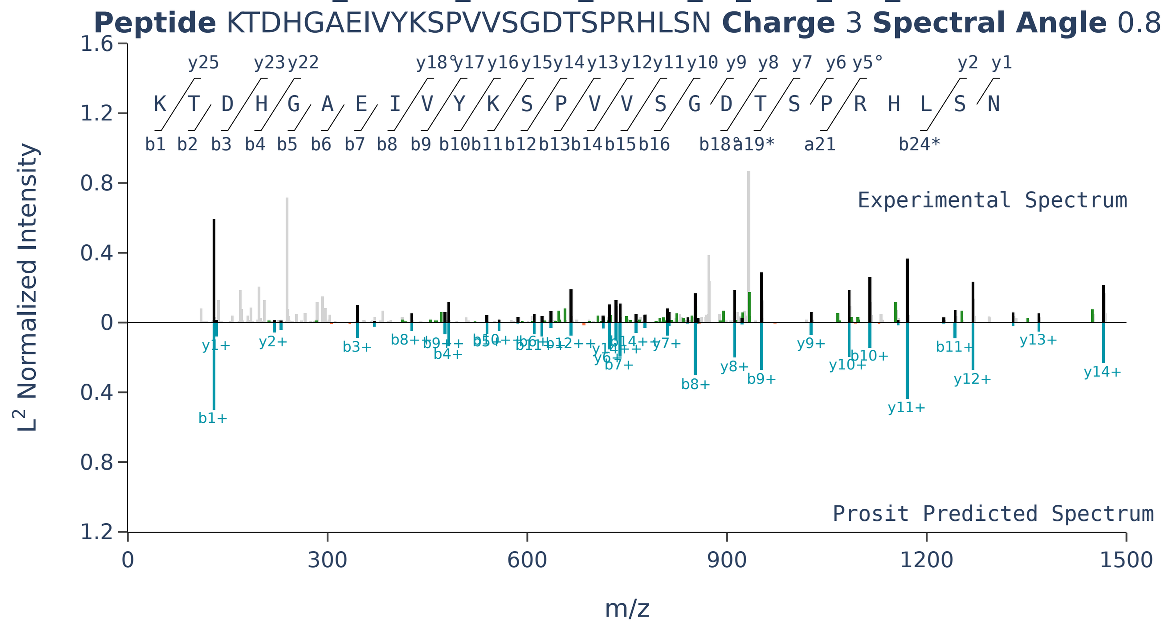


**Source** WSoh_221121_011221_HFGoe_L1_hTau_24h_R1 **Scan** 10104
**Peptide** KLTFRENAKAKTDHGAEIVYK **Charge** 3 **Spectral Angle** 0.82

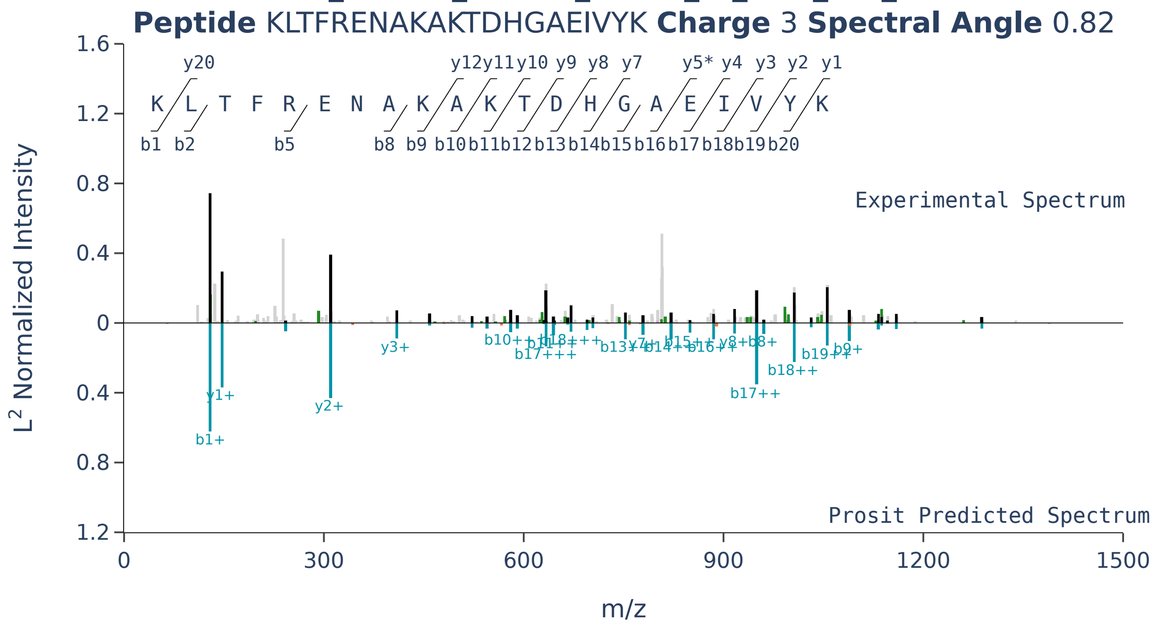


**Source** WSoh_221121_011221_HFGoe_L2_hTau_4h_R2 **Scan** 6371
**Peptide** PPKSPSSAKSRLQT **Charge** 3 **Spectral Angle** 0.86

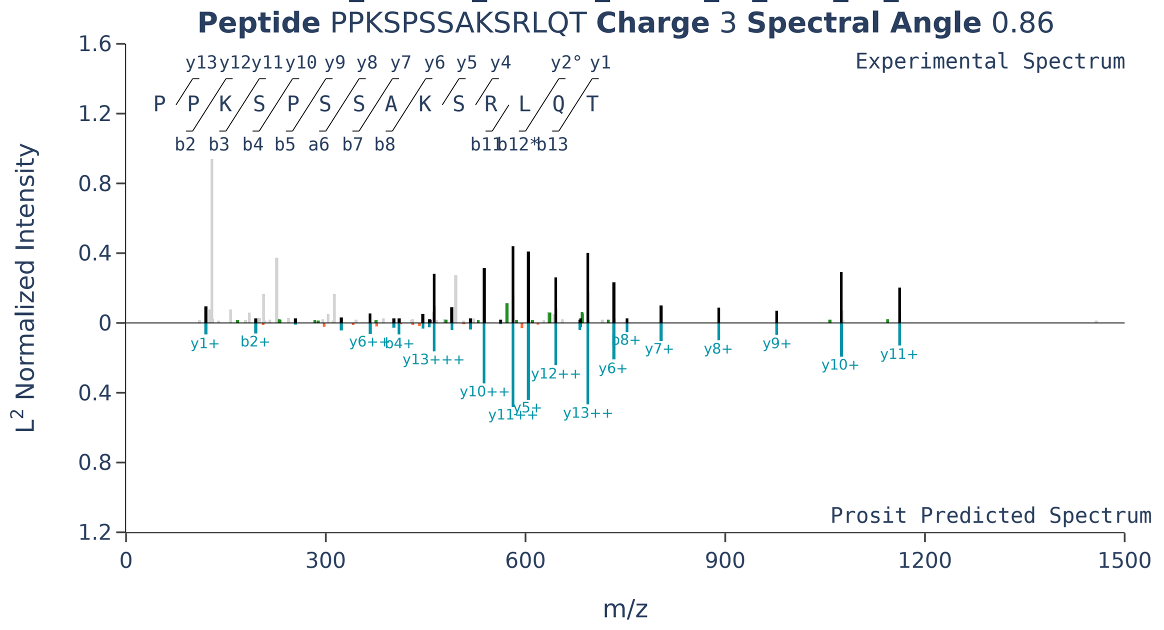


**Source** WSoh_221121_011221_HFGoe_L1_hTau_24h_R1 **Scan** 7190
**Peptide** EDEAAGHVTQARMVSKSKD **Charge** 3 **Spectral Angle** 0.82

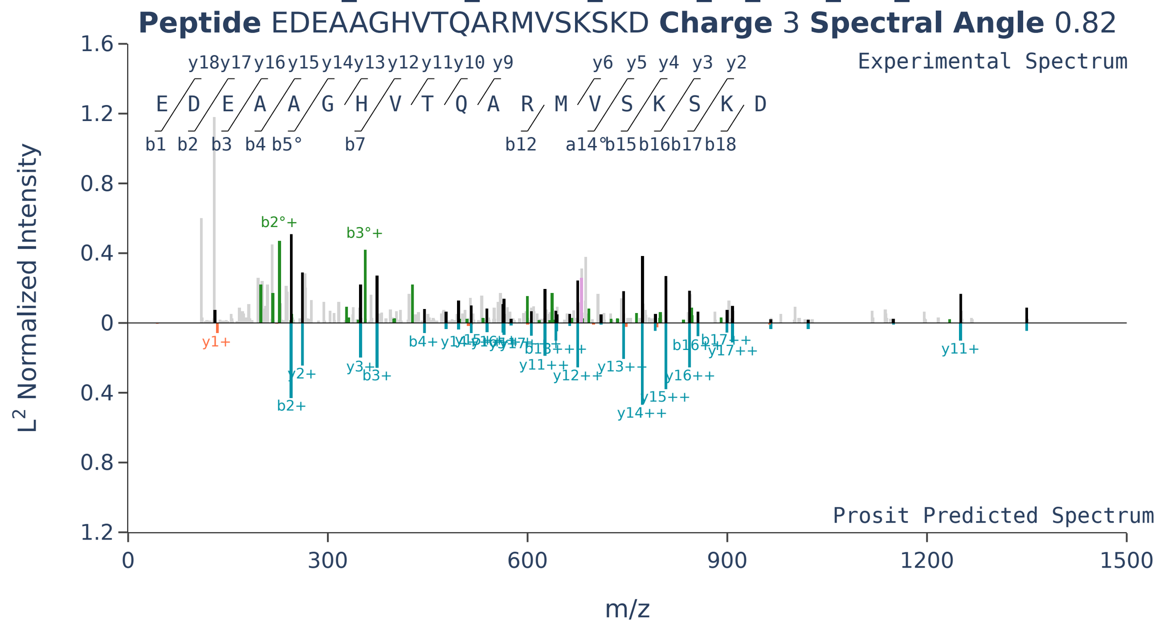


**Source** WSoh_221121_011221_HFGoe_L1_hTau_2h_R1 **Scan** 16087
**Peptide** TSPRHLSNVSSTGSIDMVD **Charge** 3 **Spectral Angle** 0.81

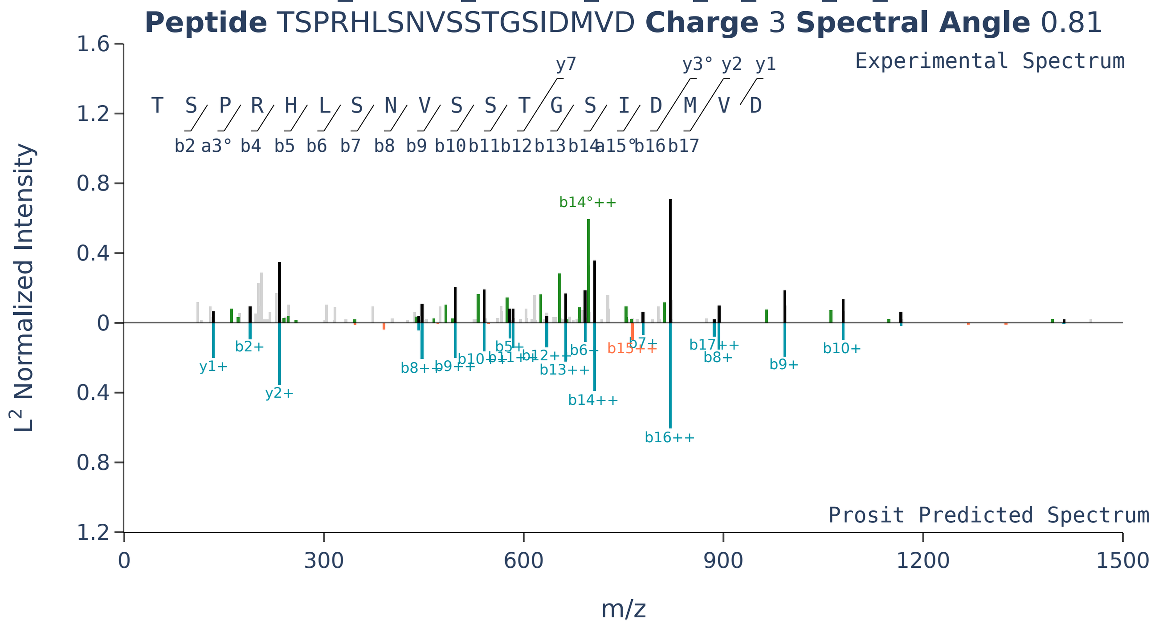


**Source** WSoh_221121_011221_HFGoe_L1_hTau_24h_R1 **Scan** 15013
**Peptide** YGLGDRKDQGGYTM **Charge** 2 **Spectral Angle** 0.84

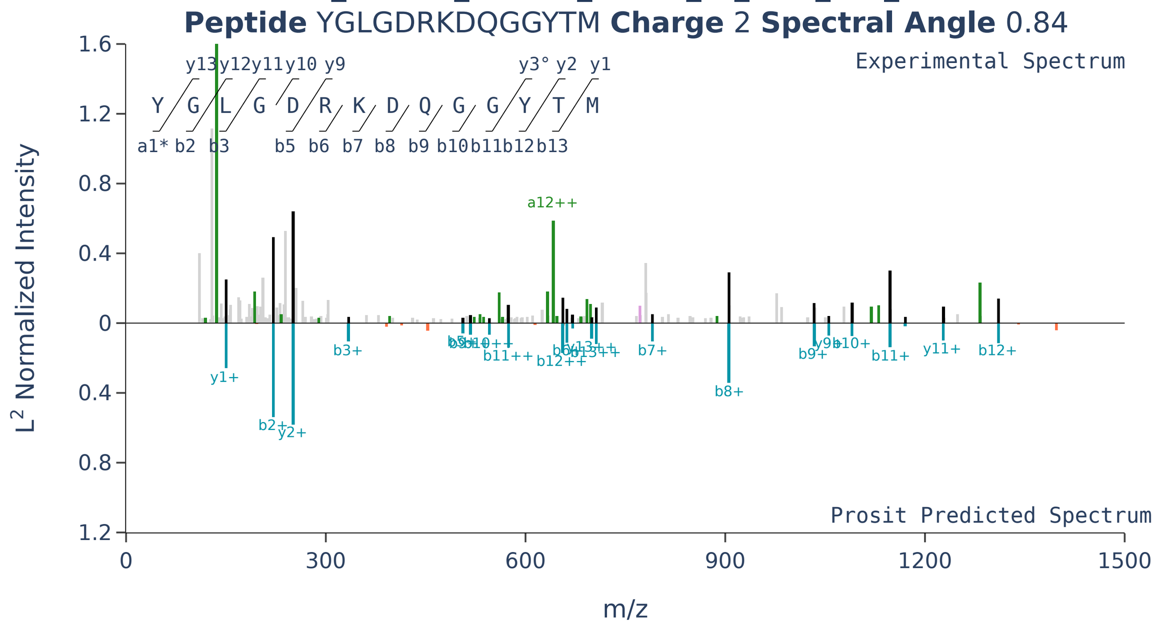


**Source** WSoh_221121_011221_HFGoe_L2_hTau_24h_R1 **Scan** 14799
**Peptide** PLQTPTEDGSEEPGSETSDAKSTPTAED **Charge** 2 **Spectral Angle** 0.79

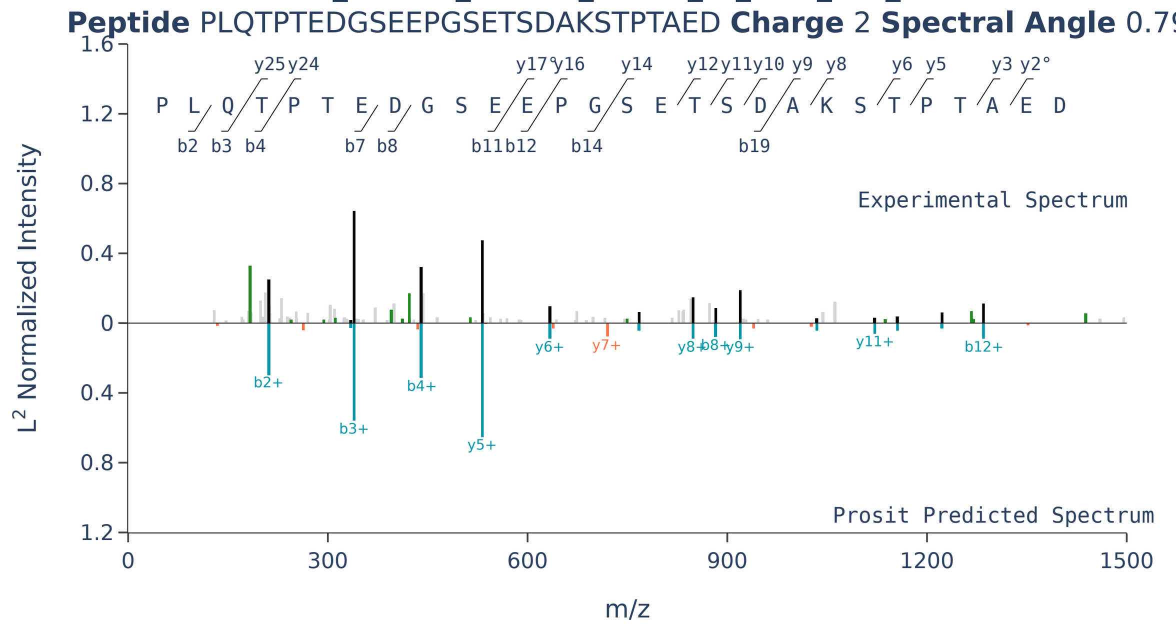


**Source** WSoh_221121_011221_HFGoe_L2_hTau_24h_R1 **Scan** 12658
**Peptide** DTSPRHLSNVSSTGSLD **Charge** 2 **Spectral Angle** 0.86

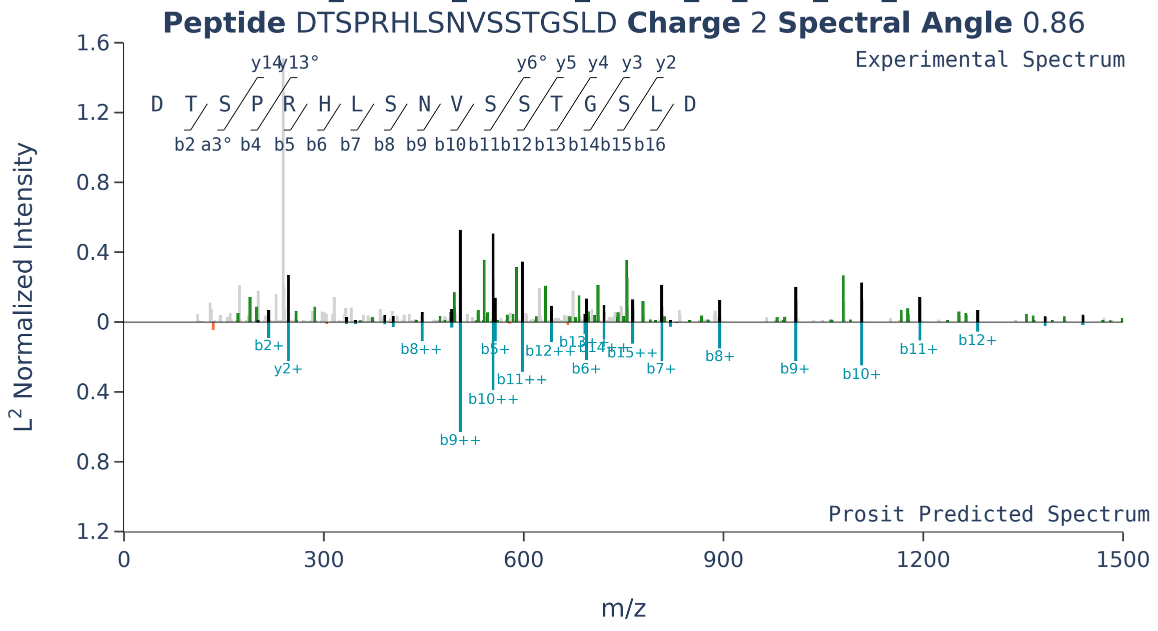


**Source** WSoh_221121_011221_HFGoe_L1_hTau_4h_R1 **Scan** 23157
**Peptide** EEAGIGDTPSLEDEAAGHVTQARMV **Charge** 3 **Spectral Angle** 0.86

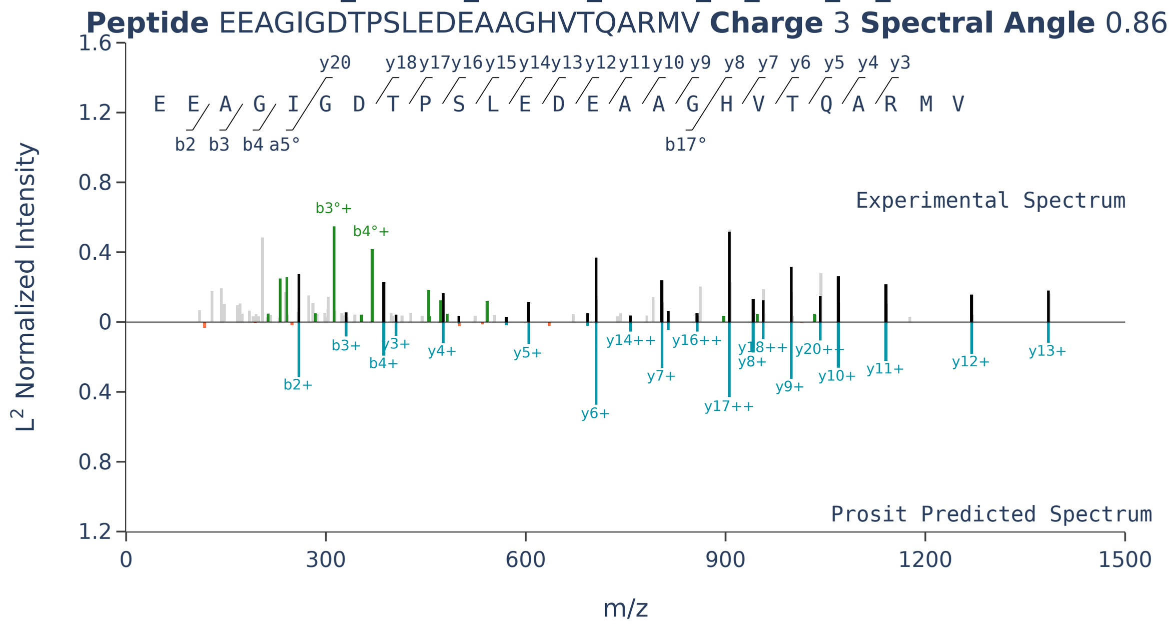


**Source** WSoh_221121_011221_HFGoe_L2_hTau_24h_R1 **Scan** 15120
**Peptide** PLQTPTEDGSEEPGSETSD **Charge** 2 **Spectral Angle** 0.86

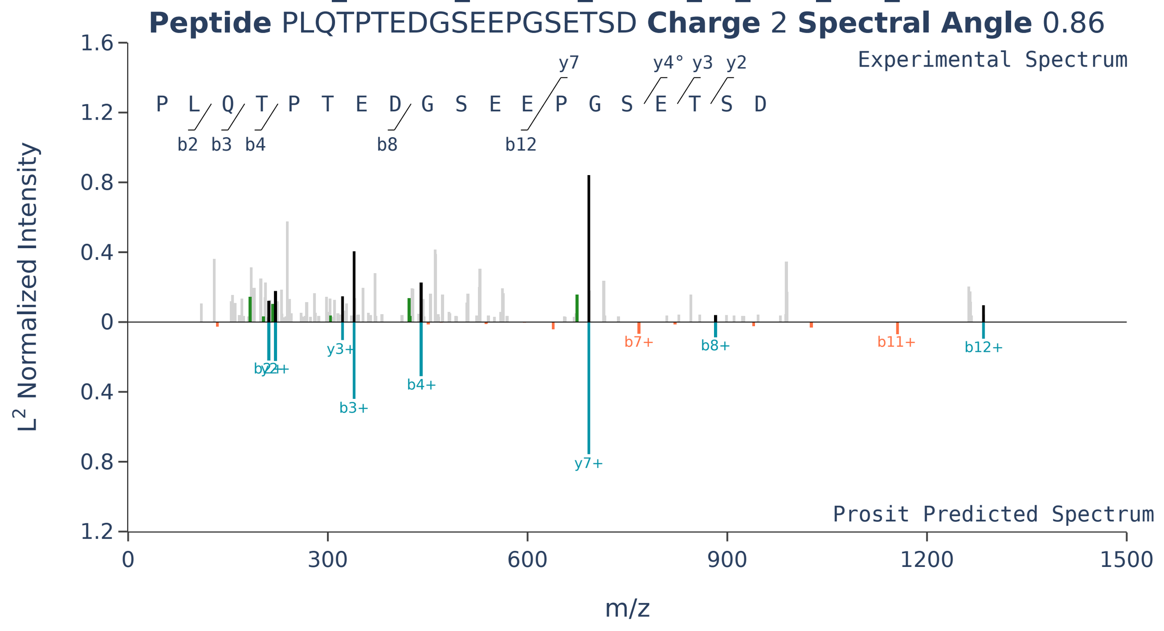


**Source** WSoh_221121_011221_HFGoe_L2_hTau_24h_R2 **Scan** 7909
**Peptide** LGDRKDQGGYTMHQDQEGD **Charge** 4 **Spectral Angle** 0.87

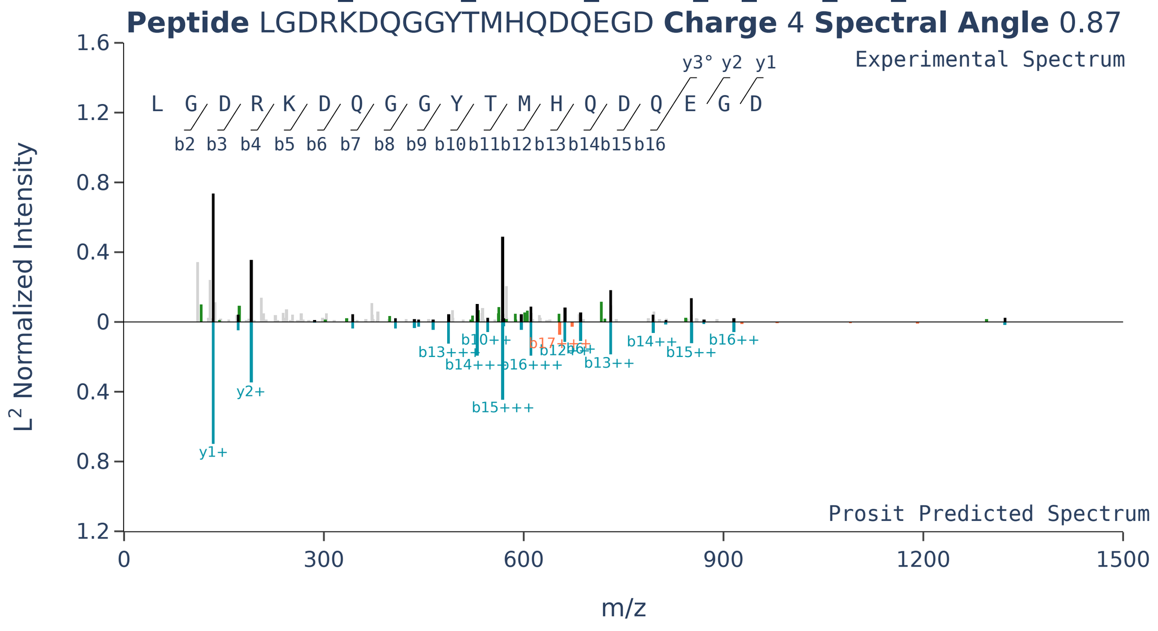


**Source** WSoh_221121_011221_HFGoe_L2_hTau_24h_R2 **Scan** 7163
**Peptide** SKVTSKCGSLGNIHHKPGGGQV **Charge** 3 **Spectral Angle** 0.9

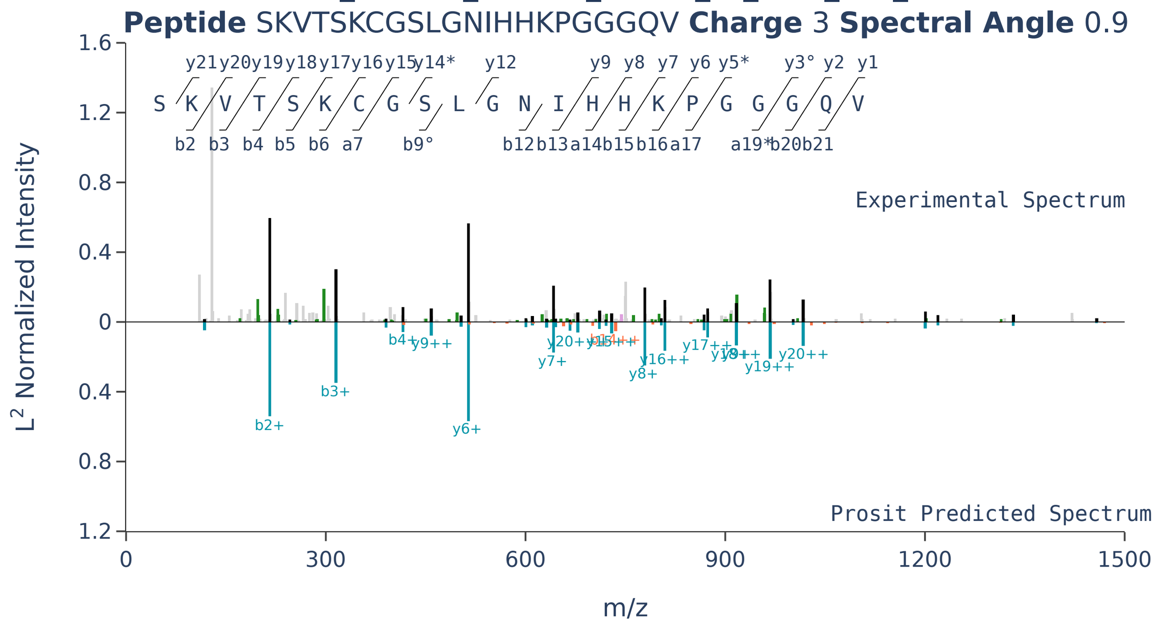


**Source** WSoh_221121_011221_HFGoe_L1_hTau_24h_R1 **Scan** 18048
**Peptide** RQEFEVMED **Charge** 2 **Spectral Angle** 0.84

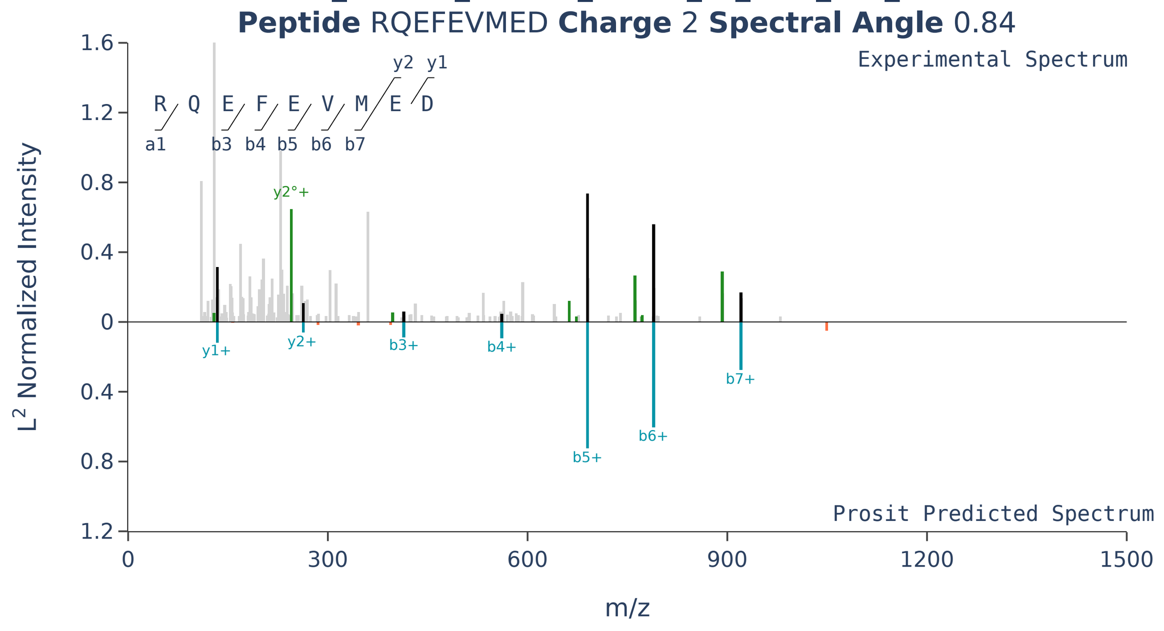

**Source** WSoh_221121_011221_HFGoe_L1_hTau_4h_R1 **Scan** 9468
**Peptide** VPGGGNKKIETHKLTFR **Charge** 4 **Spectral Angle** 0.8

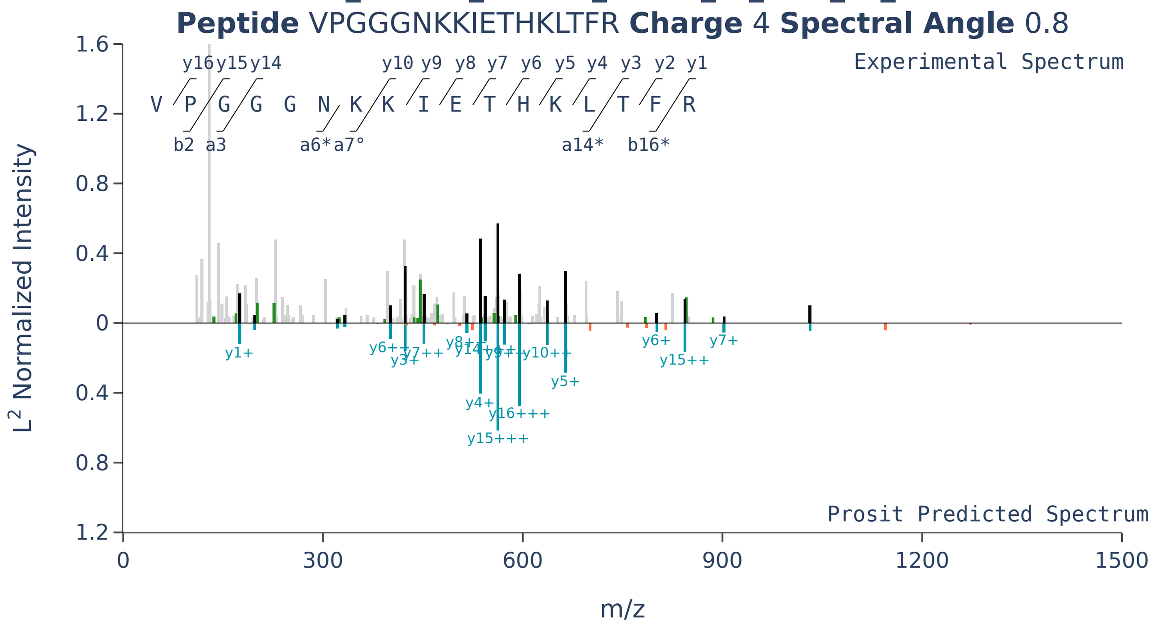


**Source** WSoh_221121_011221_HFGoe_L2_hTau_24h_R1 **Scan** 21455
**Peptide** AGIGDTPSLEDEA **Charge** 2 **Spectral Angle** 0.83

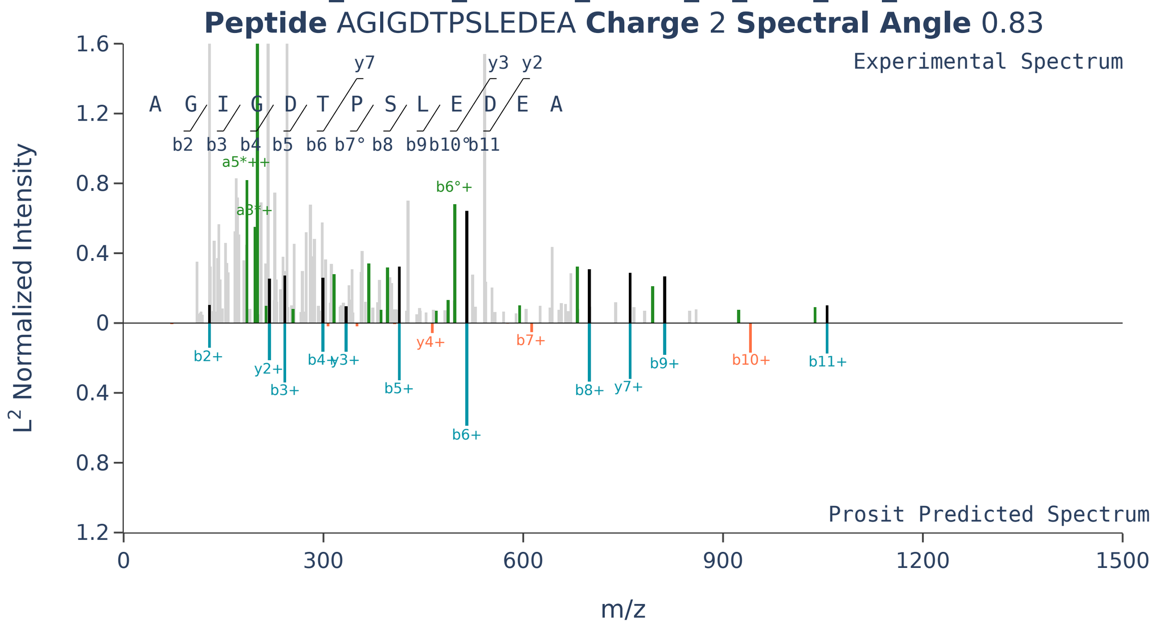


**Source** WSoh_221121_011221_HFGoe_L1_hTau_4h_R1 **Scan** 12932
**Peptide** VKSEKLDFKDRVQSKL **Charge** 3 **Spectral Angle** 0.85

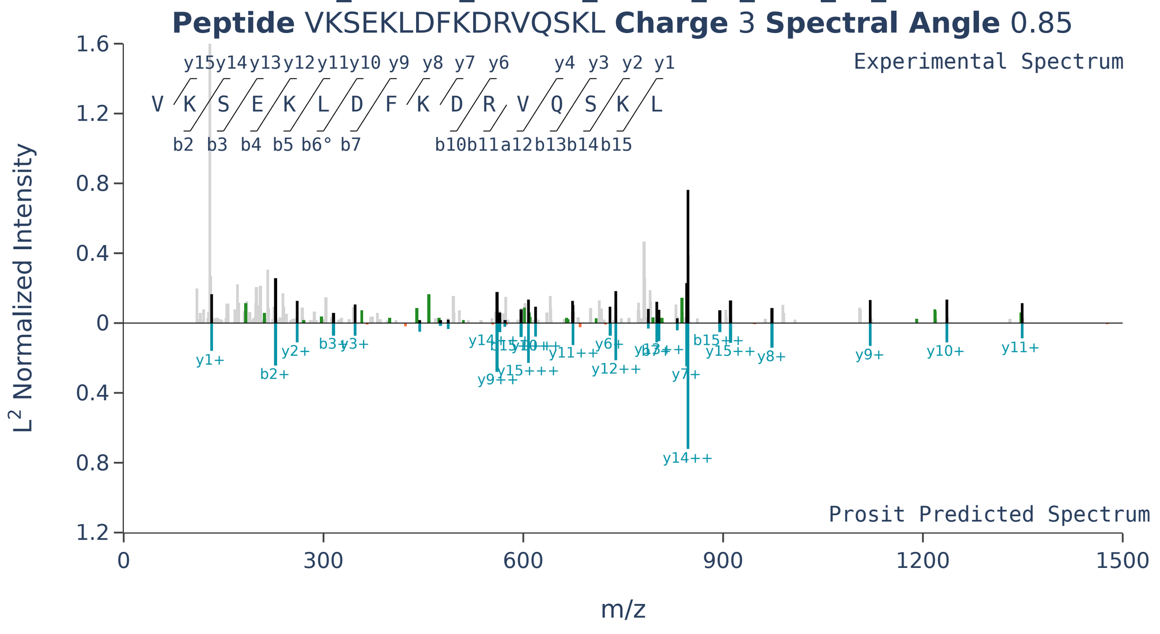


**Source** WSoh_221121_011221_HFGoe_L1_hTau_24h_R2 **Scan** 15449
**Peptide** KLTFRENAKAKTDHGAEIVYKSPVV **Charge** 4 **Spectral Angle** 0.86

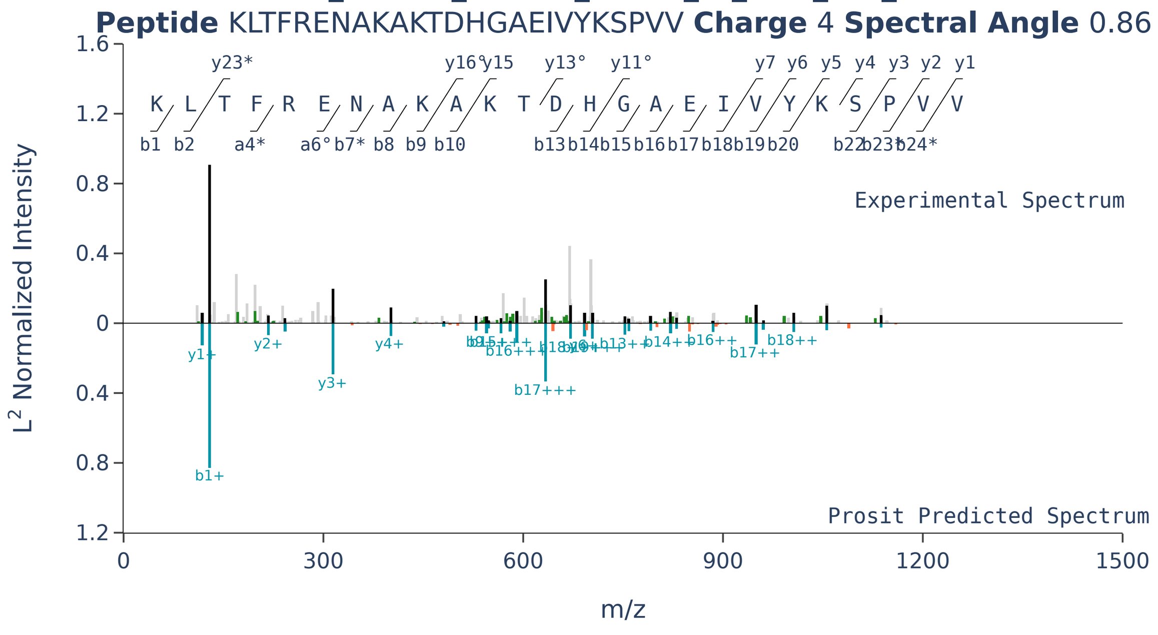


**Source** WSoh_221121_011221_HFGoe_L1_hTau_4h_R2 **Scan** 21655
**Peptide** KLDFKDRVQSKIGSLDNI **Charge** 3 **Spectral Angle** 0.91

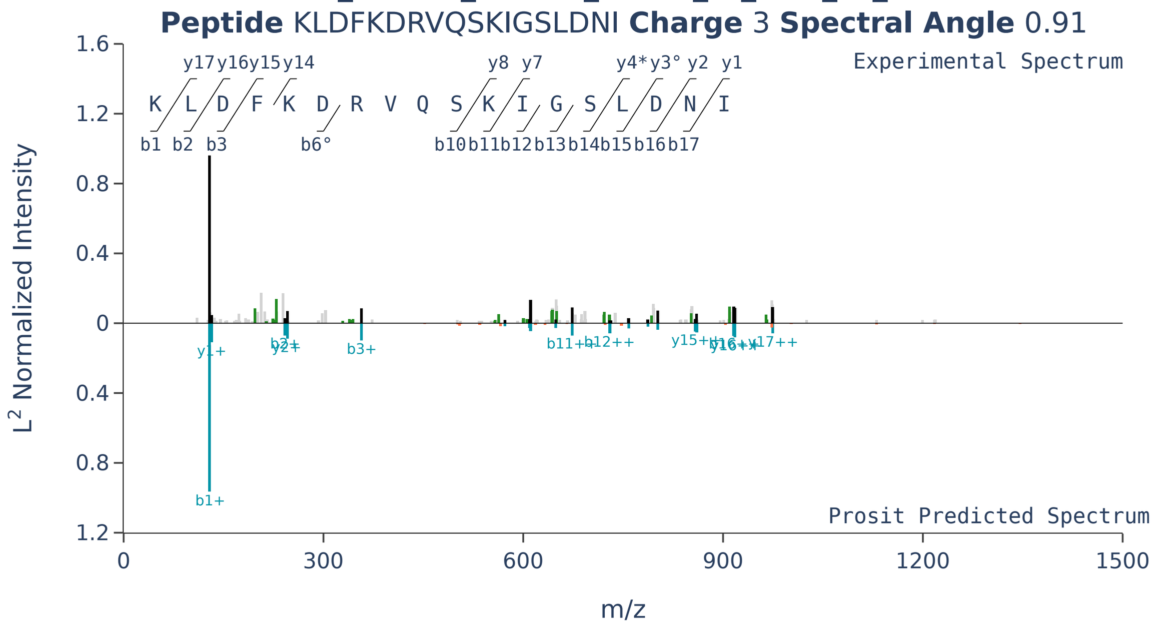


**Source** WSoh_221121_011221_HFGoe_L1_hTau_24h_R1 **Scan** 18019
**Peptide** LINKKLDLSNV **Charge** 2 **Spectral Angle** 0.83

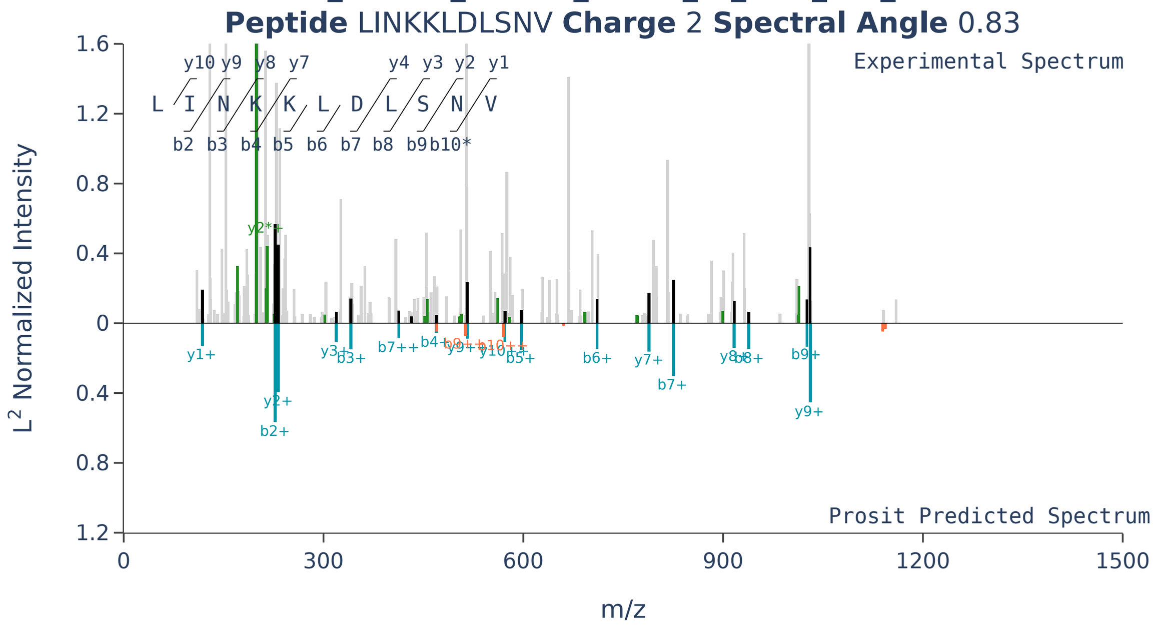


**Source** WSoh_221121_011221_HFGoe_L2_hTau_24h_R2 **Scan** 15264
**Peptide** GSKDNIKHVPGGGSVQLV **Charge** 3 **Spectral Angle** 0.84

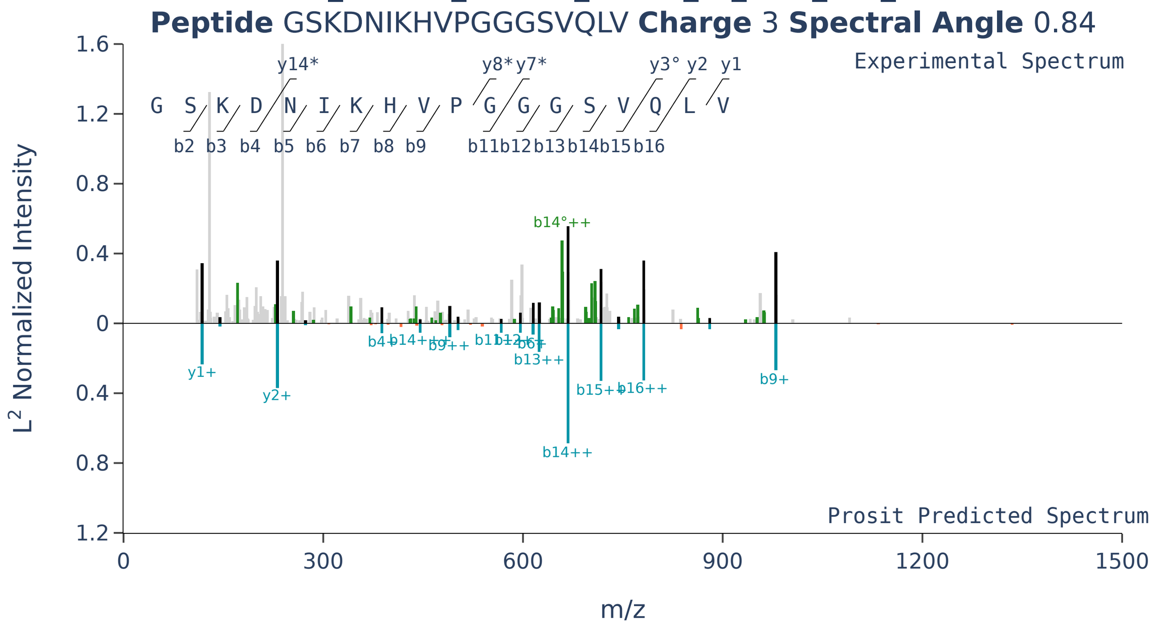


**Source** WSoh_221121_011221_HFGoe_L2_hTau_2h_R2 **Scan** 13302
**Peptide** YSSPGSPGTPGSRSRTPSL **Charge** 3 **Spectral Angle** 0.91

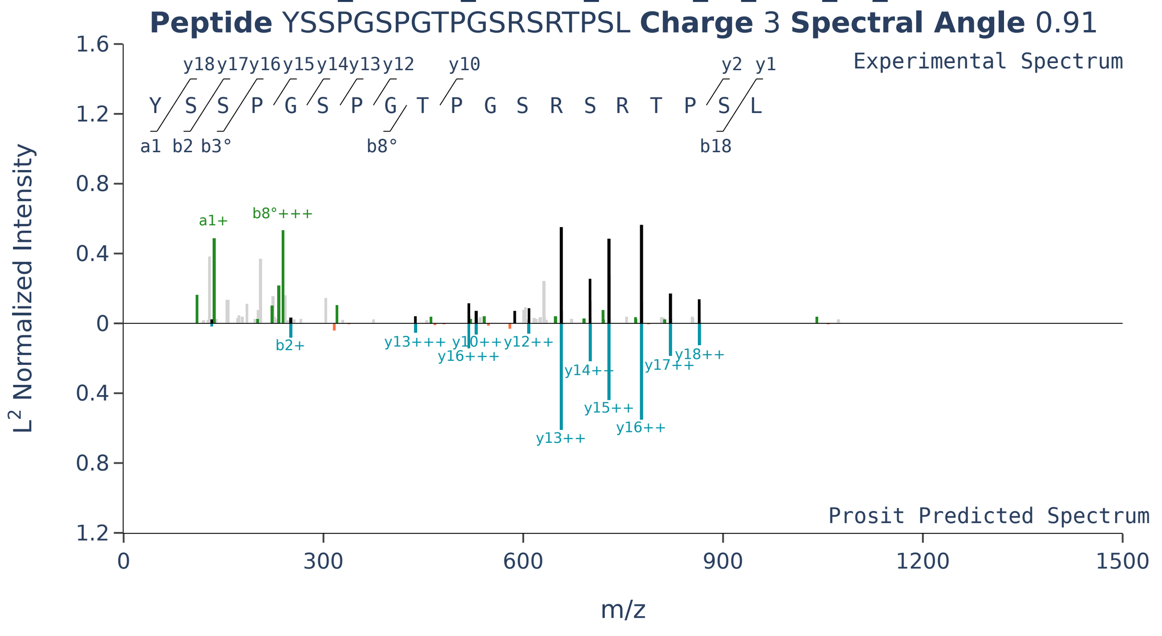


**Source** WSoh_221121_011221_HFGoe_L2_hTau_24h_R1 **Scan** 7387
**Peptide** TFRENAKAKTDHGAEL **Charge** 4 **Spectral Angle** 0.79

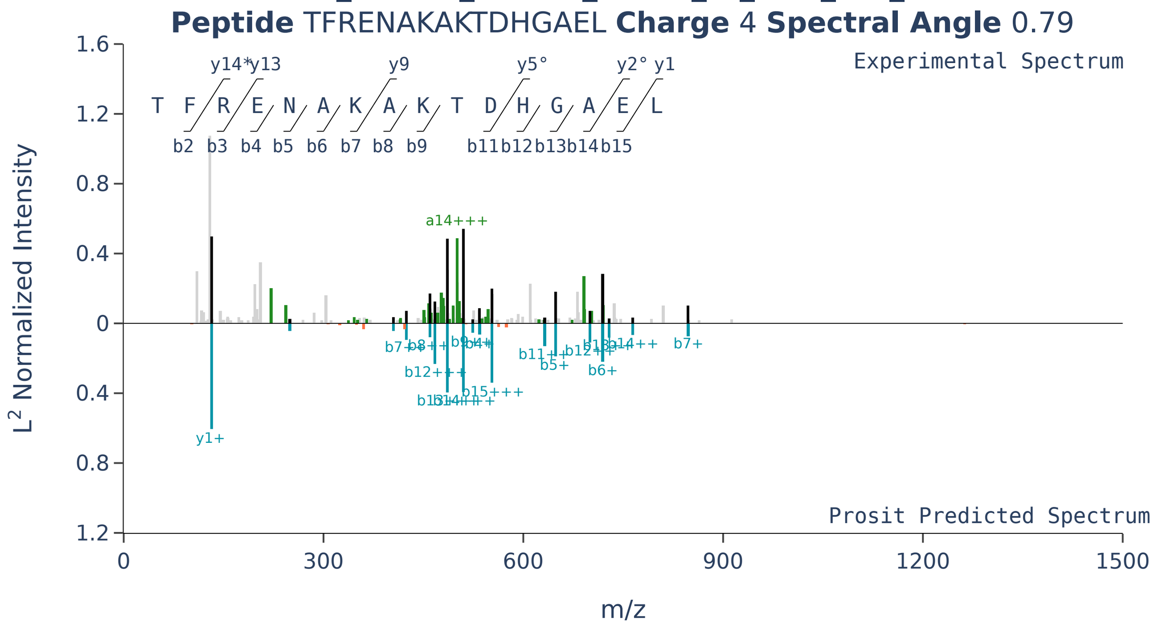


**Source** WSoh_221121_011221_HFGoe_L1_hTau_4h_R1 **Scan** 10615
**Peptide** IATPRGAAPPGQ **Charge** 2 **Spectral Angle** 0.89

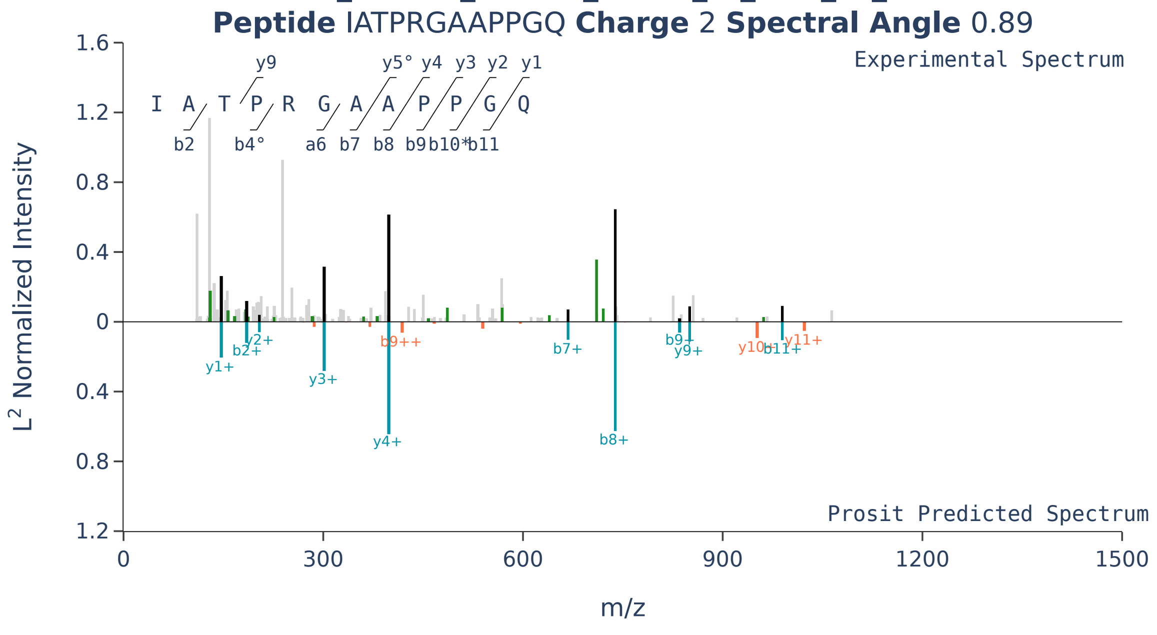


**Source** WSoh_221121_011221_HFGoe_L2_hTau_24h_R1 **Scan** 7126
**Peptide** KCGSLGNIHHKPGGGQVEVKS **Charge** 3 **Spectral Angle** 0.94

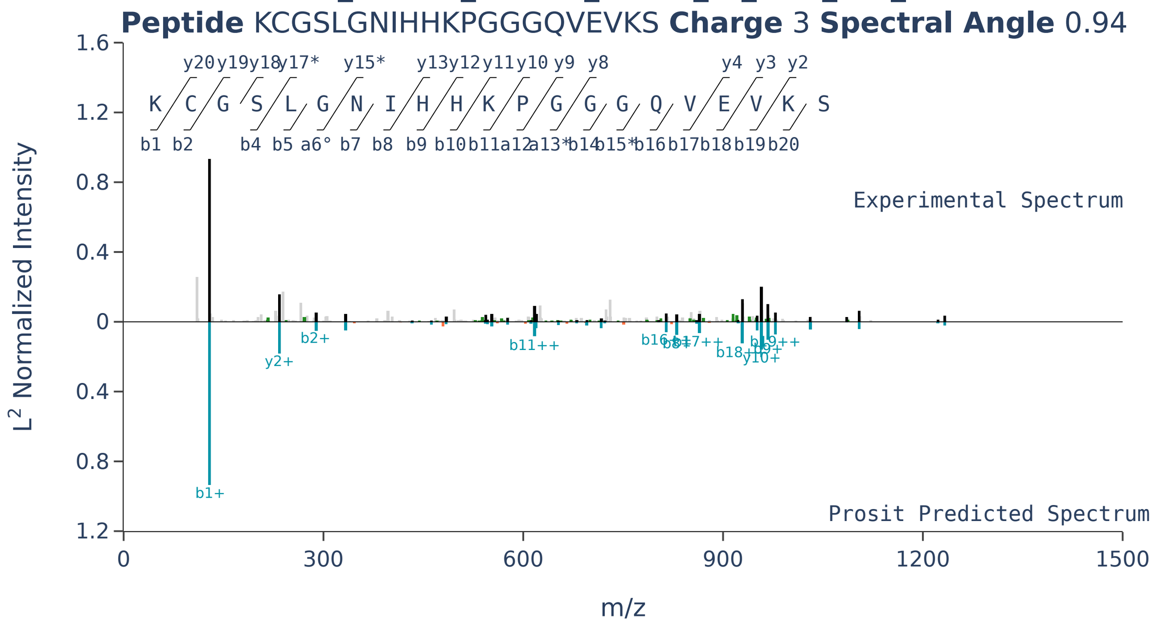


**Source** WSoh_221121_011221_HFGoe_L1_hTau_2h_R1 **Scan** 12112
**Peptide** KSEKLDFKDRVQSKL **Charge** 3 **Spectral Angle** 0.88

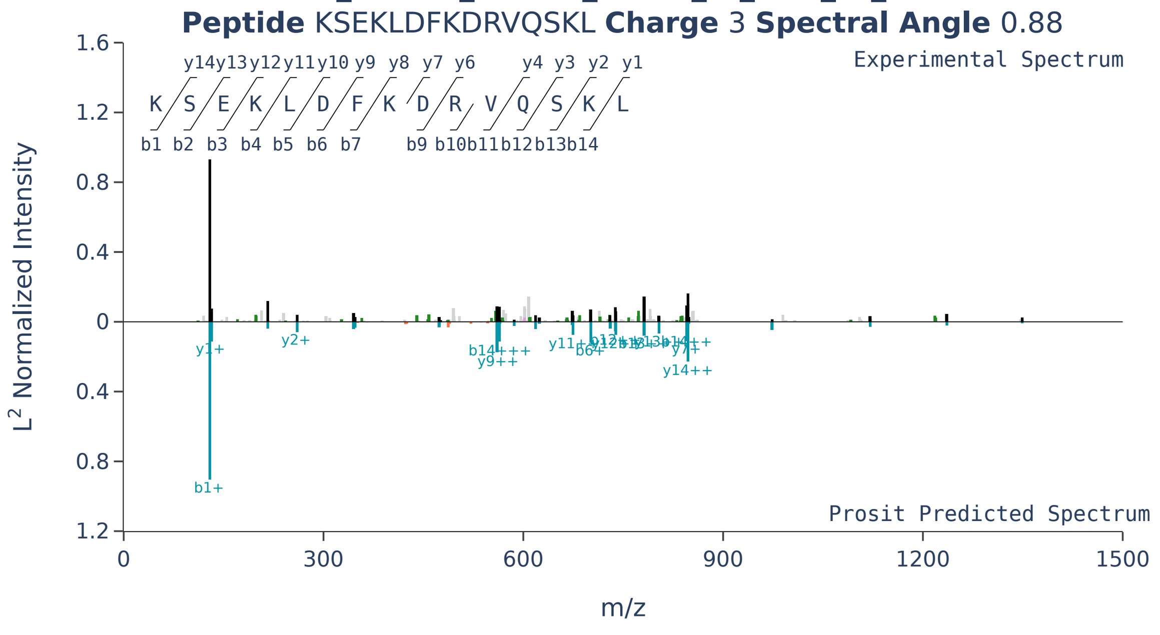


**Source** WSoh_221121_011221_HFGoe_L1_hTau_24h_R2 **Scan** 6457
**Peptide** VVRTPPKSPSS **Charge** 2 **Spectral Angle** 0.87

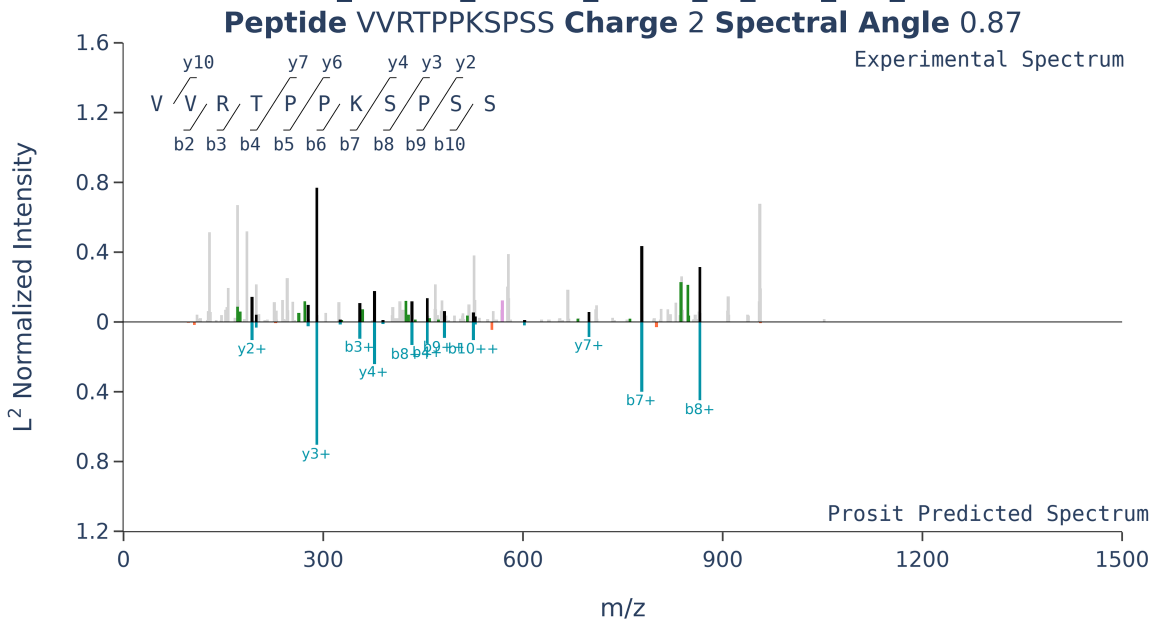


**Source** WSoh_221121_011221_HFGoe_L2_hTau_24h_R1 **Scan** 6695
**Peptide** AVVRTPPK **Charge** 2 **Spectral Angle** 0.83

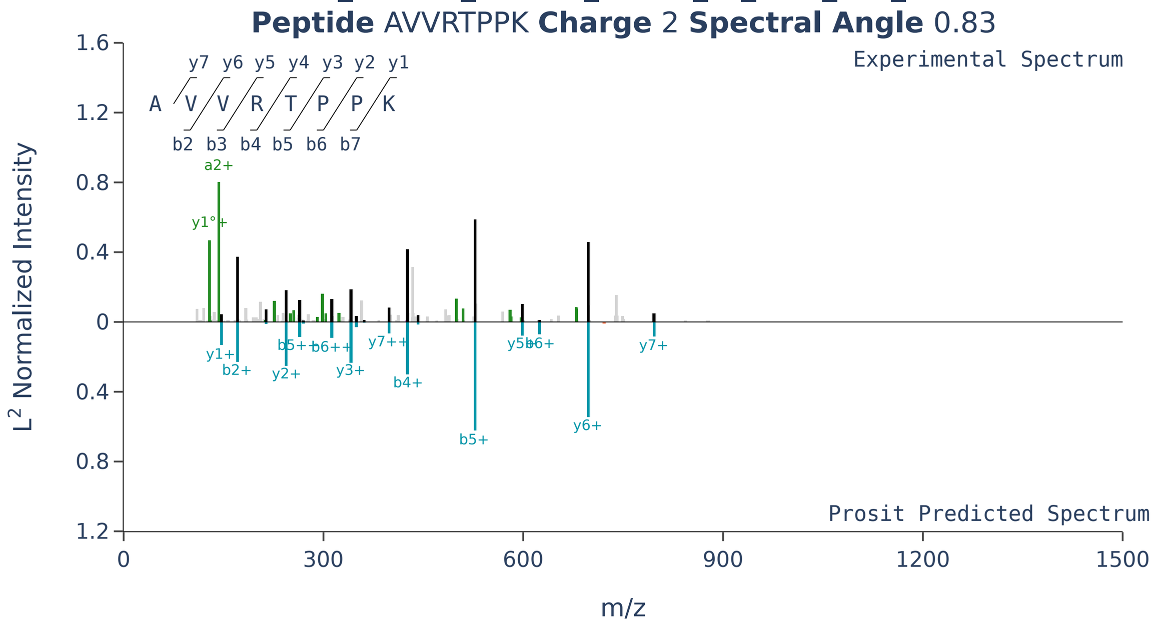


**Source** WSoh_221121_011221_HFGoe_L1_hTau_24h_R2 **Scan** 15860
**Peptide** TENLKHQPGGGKVQIINKKLDL **Charge** 5 **Spectral Angle** 0.87

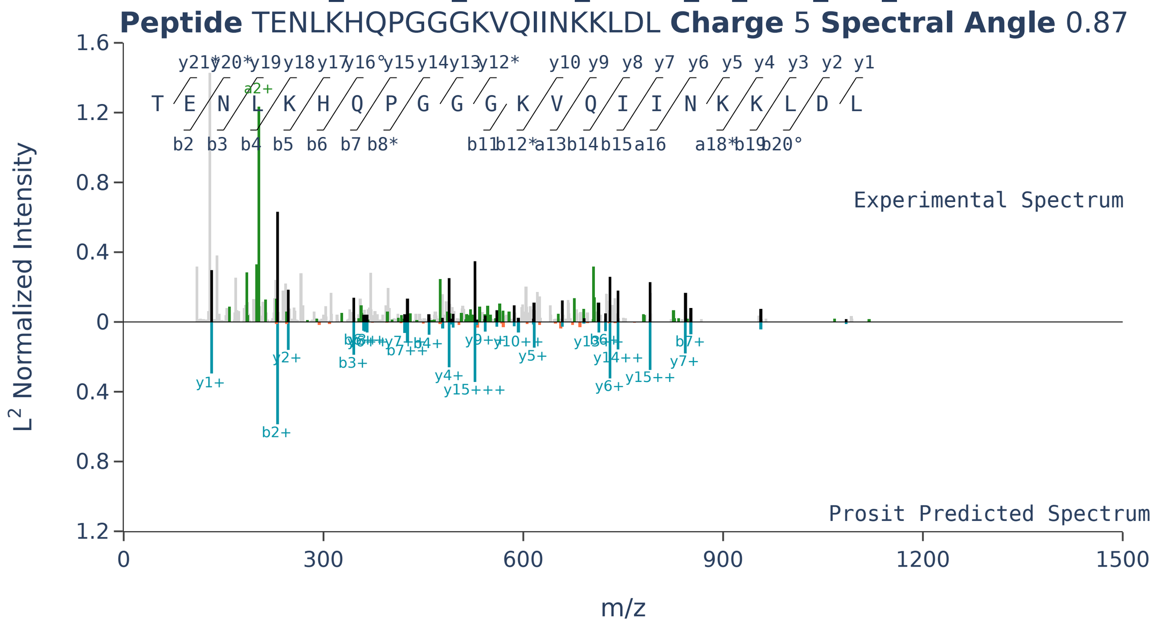

**Source** WSoh_221121_011221_HFGoe_L1_hTau_4h_R2 **Scan** 11802
**Peptide** GAPGKQAAAQPHTEIPEGTTAE **Charge** 3 **Spectral Angle** 0.81

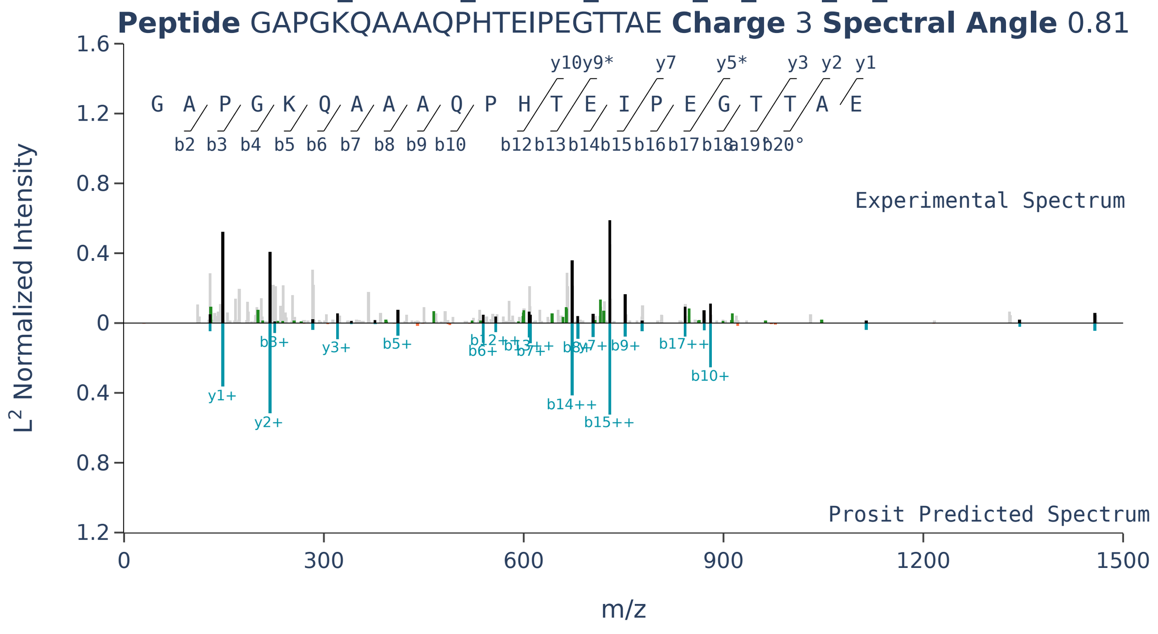


**Source** WSoh_221121_011221_HFGoe_L1_hTau_4h_R1 **Scan** 15725
**Peptide** LDFKDRVQSKI **Charge** 3 **Spectral Angle** 0.79

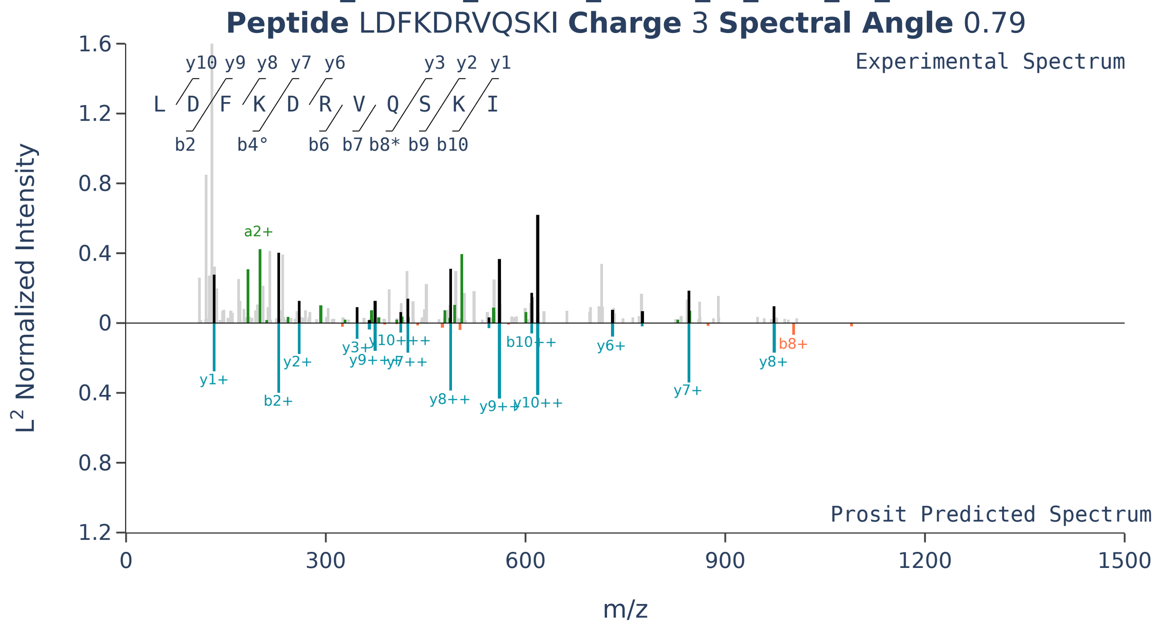


**Source** WSoh_221121_011221_HFGoe_L2_hTau_24h_R2 **Scan** 15168
**Peptide** SEKLDFKDRVQSKI **Charge** 2 **Spectral Angle** 0.76

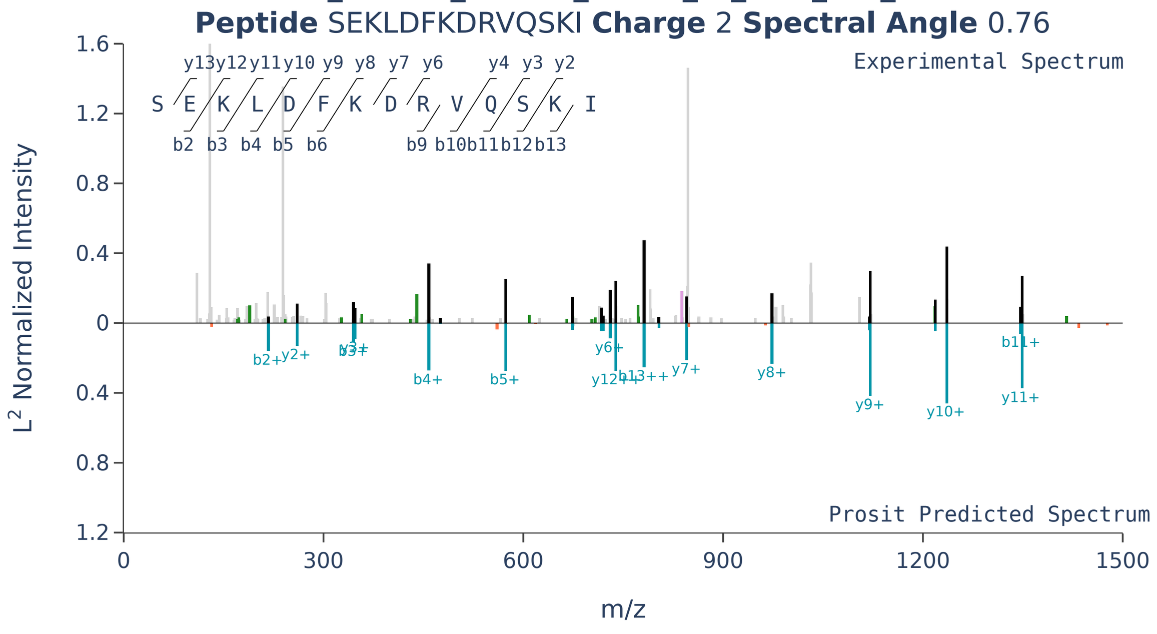


**Source** WSoh_221121_011221_HFGoe_L1_hTau_24h_R2 **Scan** 19146
**Peptide** FEVMEDHAGTYGLGDRKDQGGYTMH **Charge** 4 **Spectral Angle** 0.9

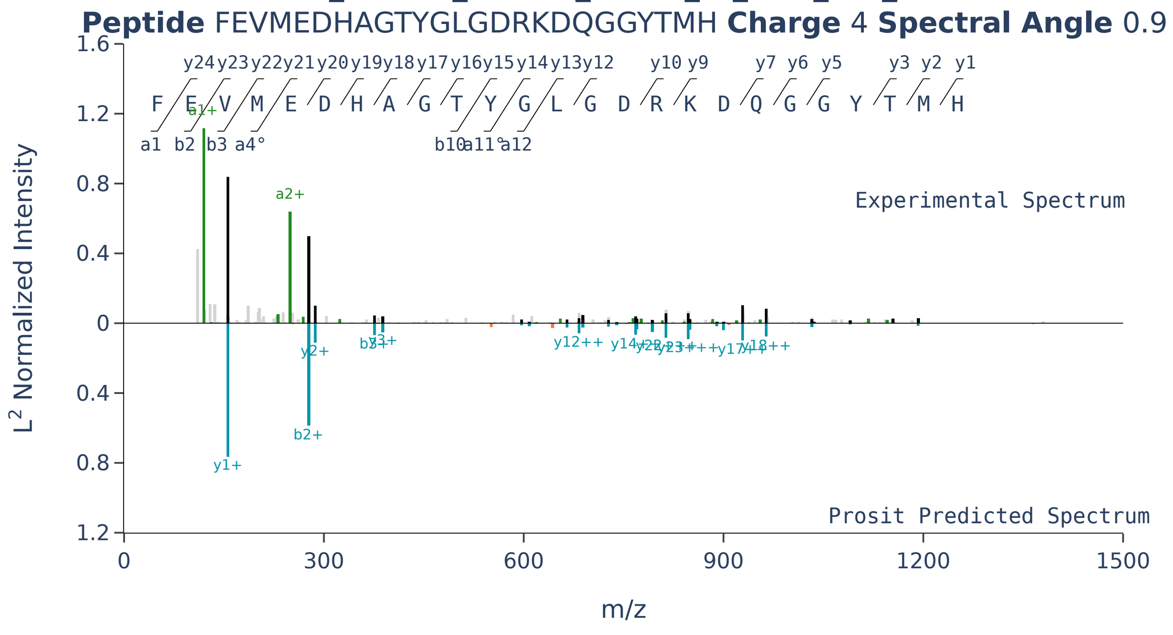


**Source** WSoh_221121_011221_HFGoe_L2_hTau_24h_R2 **Scan** 7680
**Peptide** NKKLDLSNVQSKCGSKDNLKH **Charge** 3 **Spectral Angle** 0.92

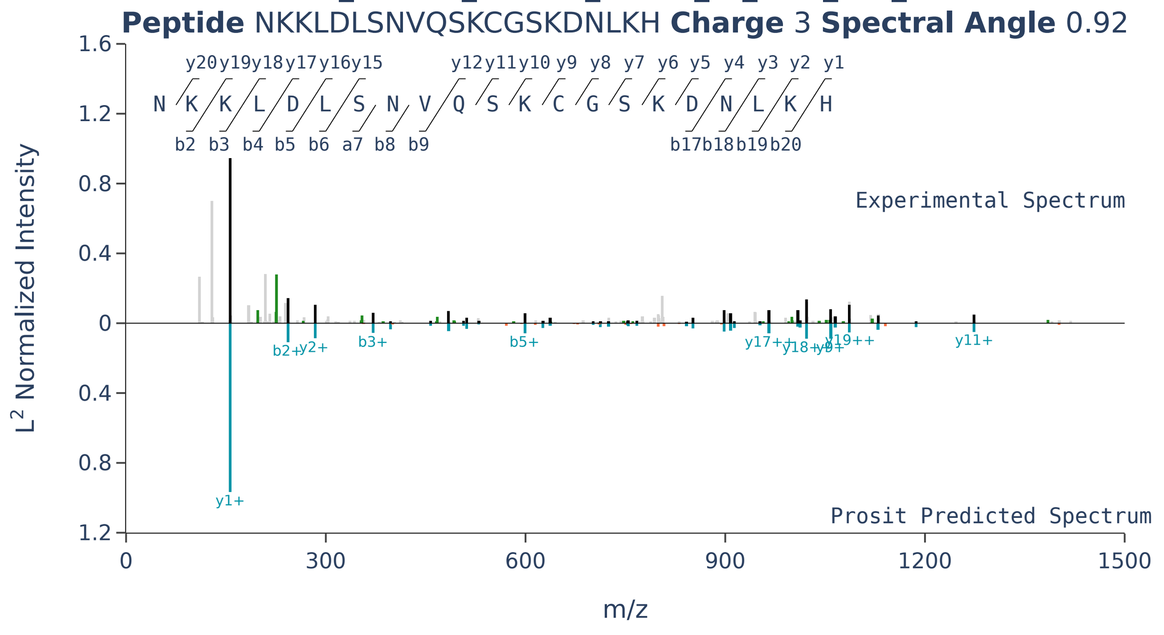


**Source** WSoh_221121_011221_HFGoe_L2_hTau_24h_R1 **Scan** 21891
**Peptide** QTAPVPMPDLKNVKSKIGS **Charge** 3 **Spectral Angle** 0.9

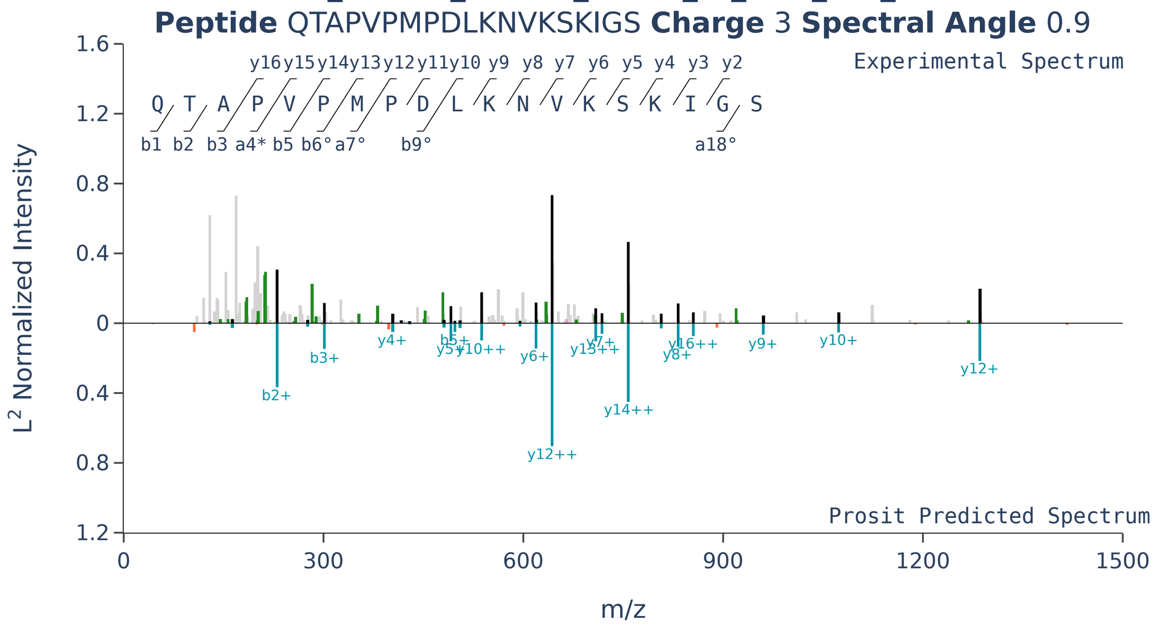


**Source** WSoh_221121_011221_HFGoe_L2_hTau_24h_R2 **Scan** 24636
**Peptide** AAQPHTEIPEGTTAEEAGIGDTPSL **Charge** 3 **Spectral Angle** 0.91

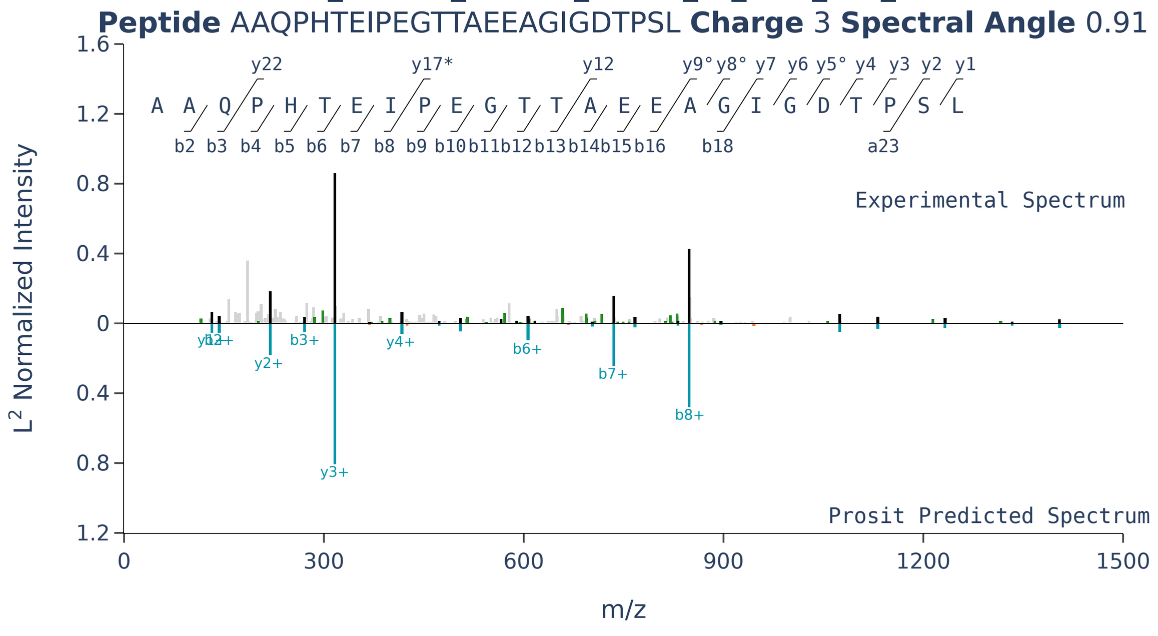


**Source** WSoh_221121_011221_HFGoe_L1_hTau_24h_R2 **Scan** 5373
**Peptide** KCGSKDNIKHV **Charge** 2 **Spectral Angle** 0.92

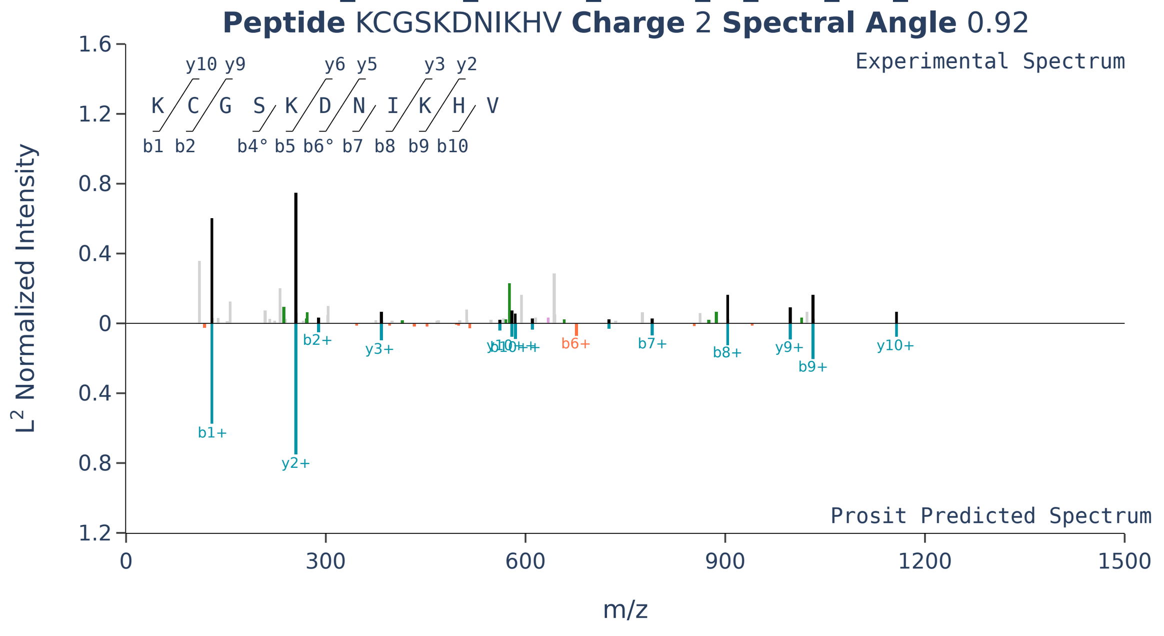


**Source** WSoh_221121_011221_HFGoe_L2_hTau_4h_R1 **Scan** 7003
**Peptide** AKAKTDHGAEIV **Charge** 2 **Spectral Angle** 0.83

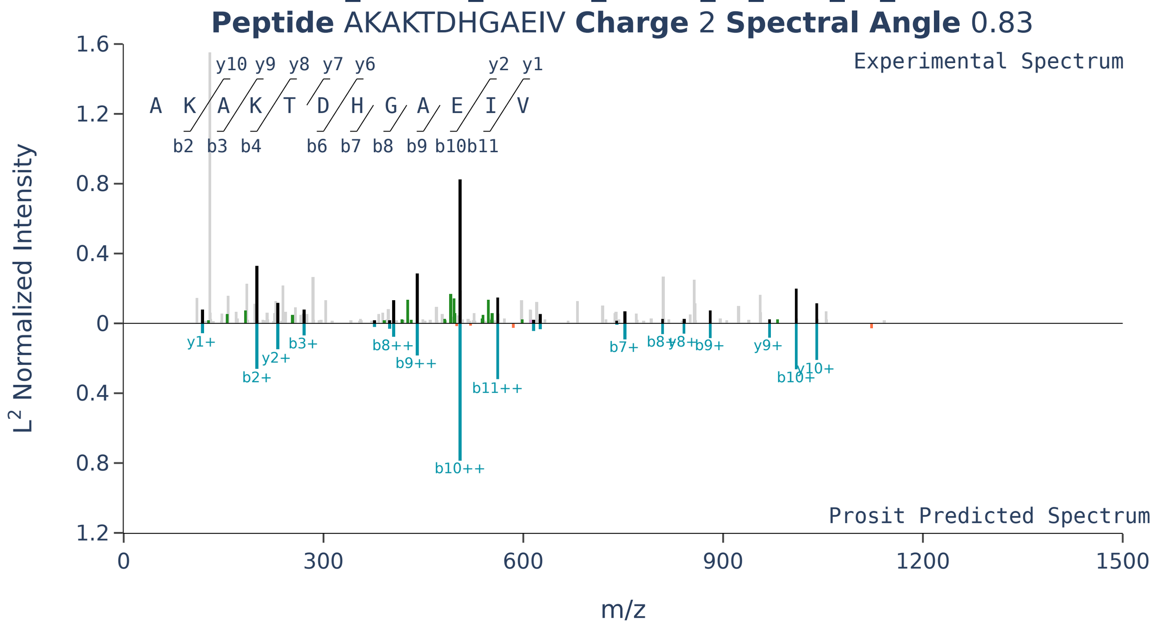


**Source** WSoh_221121_011221_HFGoe_L2_hTau_24h_R1 **Scan** 7584
**Peptide** SGYSSPGSPGTPGSRSRT **Charge** 3 **Spectral Angle** 0.9

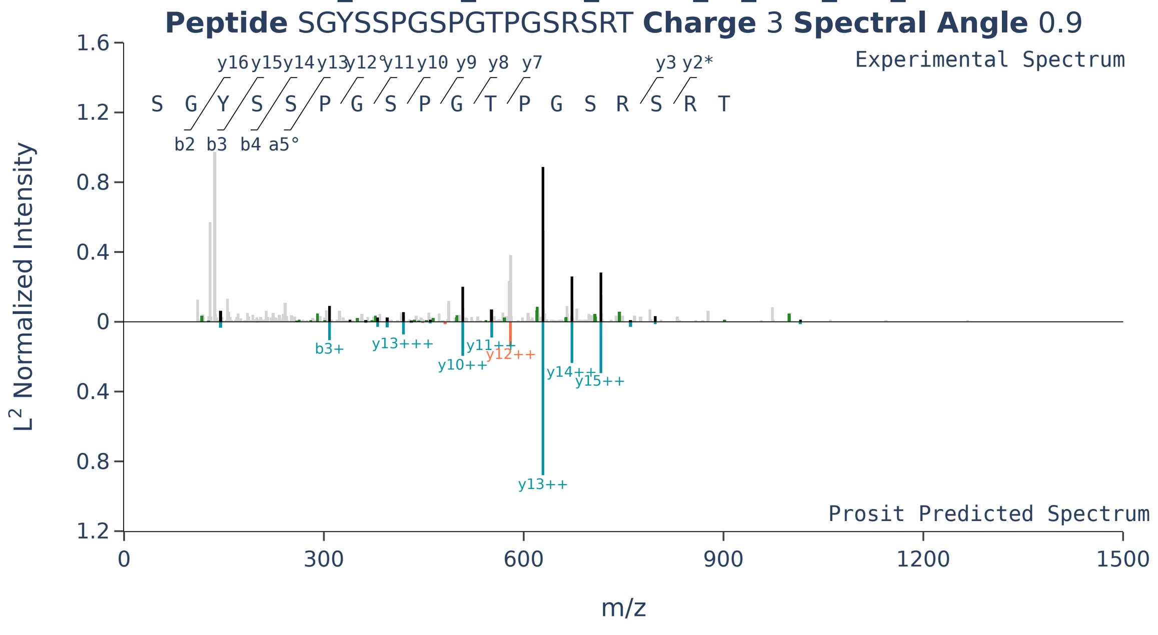


**Source** WSoh_221121_011221_HFGoe_L2_hTau_4h_R1 **Scan** 7804
**Peptide** KLDFKDR **Charge** 2 **Spectral Angle** 0.86

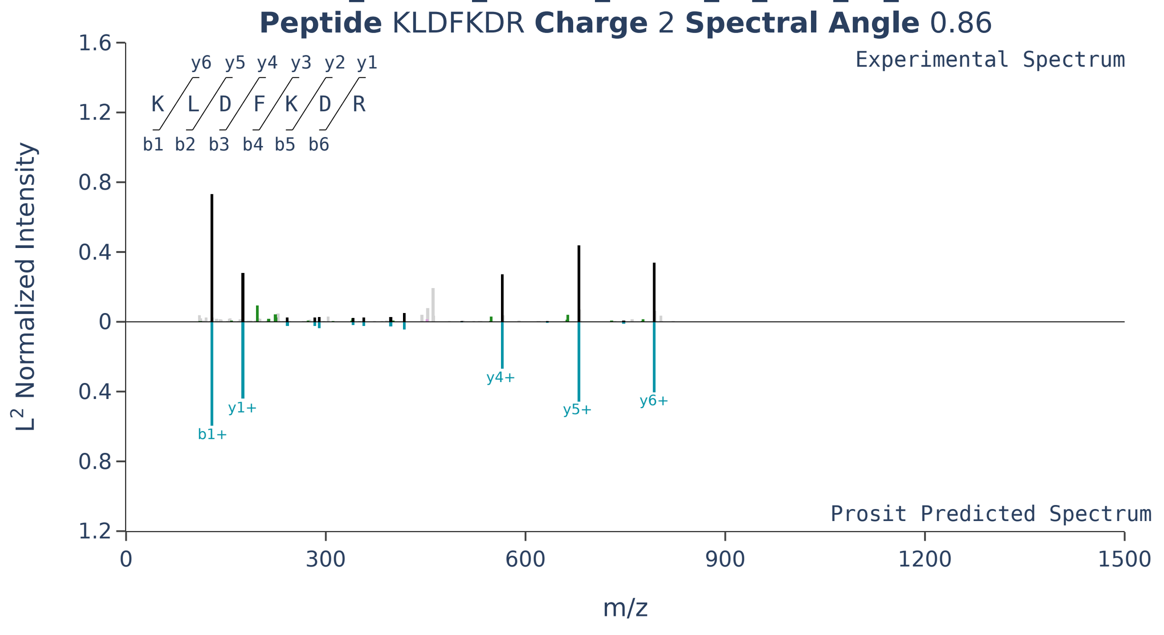


**Source** WSoh_221121_011221_HFGoe_L1_hTau_2h_R2 **Scan** 7402
**Peptide** PRGAAPPGQKGQANATRL **Charge** 4 **Spectral Angle** 0.88

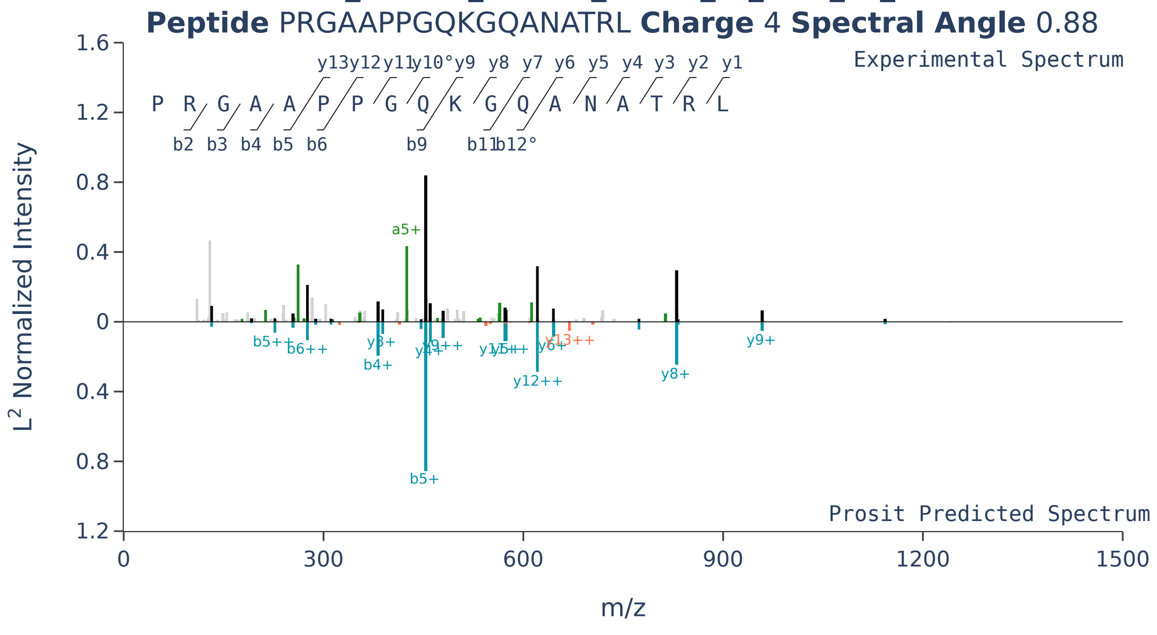


**Source** WSoh_221121_011221_HFGoe_L2_hTau_2h_R1 **Scan** 5903
**Peptide** EAAGHVTQARMVSKSKD **Charge** 4 **Spectral Angle** 0.82

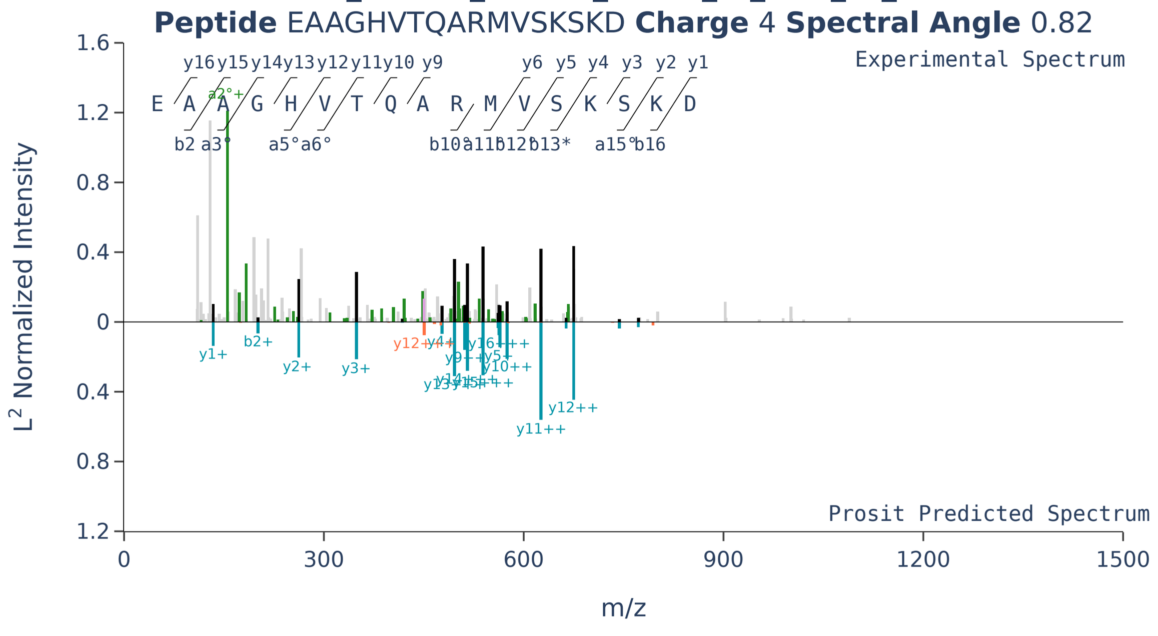


**Source** WSoh_221121_011221_HFGoe_L2_hTau_24h_R1 **Scan** 16038
**Peptide** IVYKSPVVSGDTSPRHL **Charge** 4 **Spectral Angle** 0.86

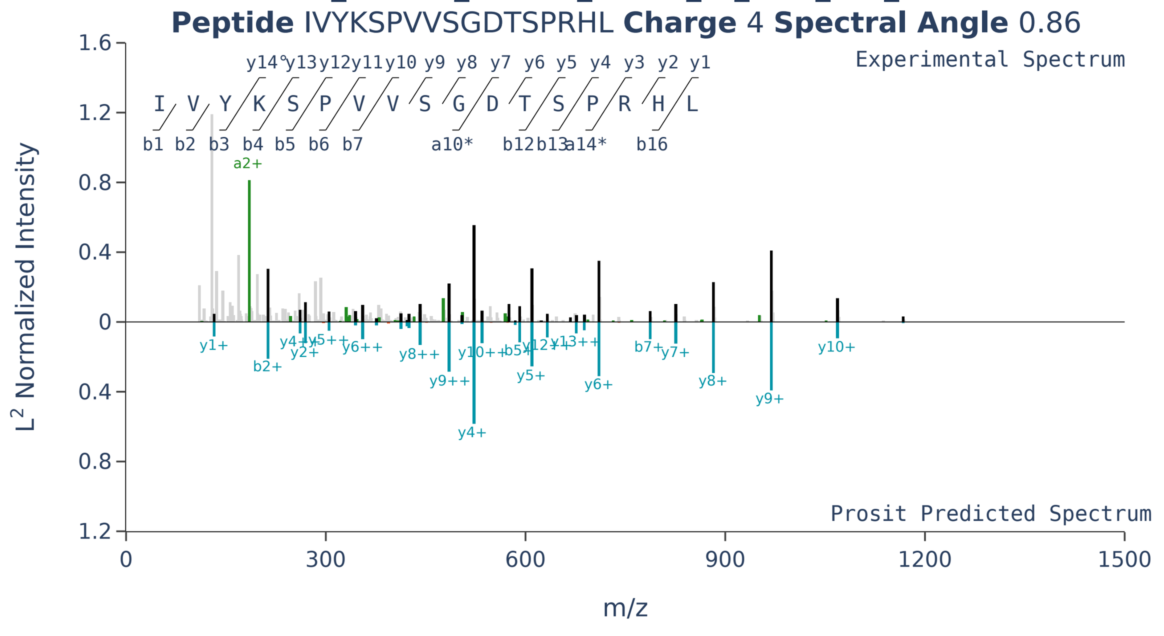


**Source** WSoh_221121_011221_HFGoe_L1_hTau_4h_R2 **Scan** 8061
**Peptide** LSKVTSKCGSLG **Charge** 2 **Spectral Angle** 0.89

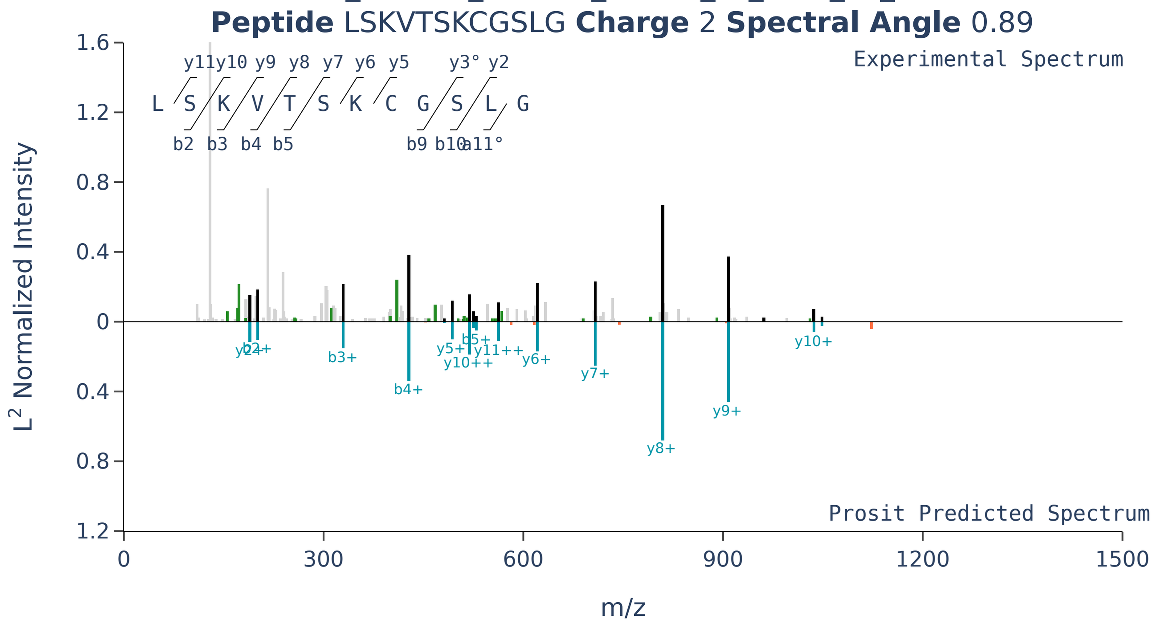

**Source** WSoh_221121_011221_HFGoe_L2_hTau_24h_R1 **Scan** 24632
**Peptide** TAPVPMPDLKNVKSKIGSTENL **Charge** 2 **Spectral Angle** 0.82

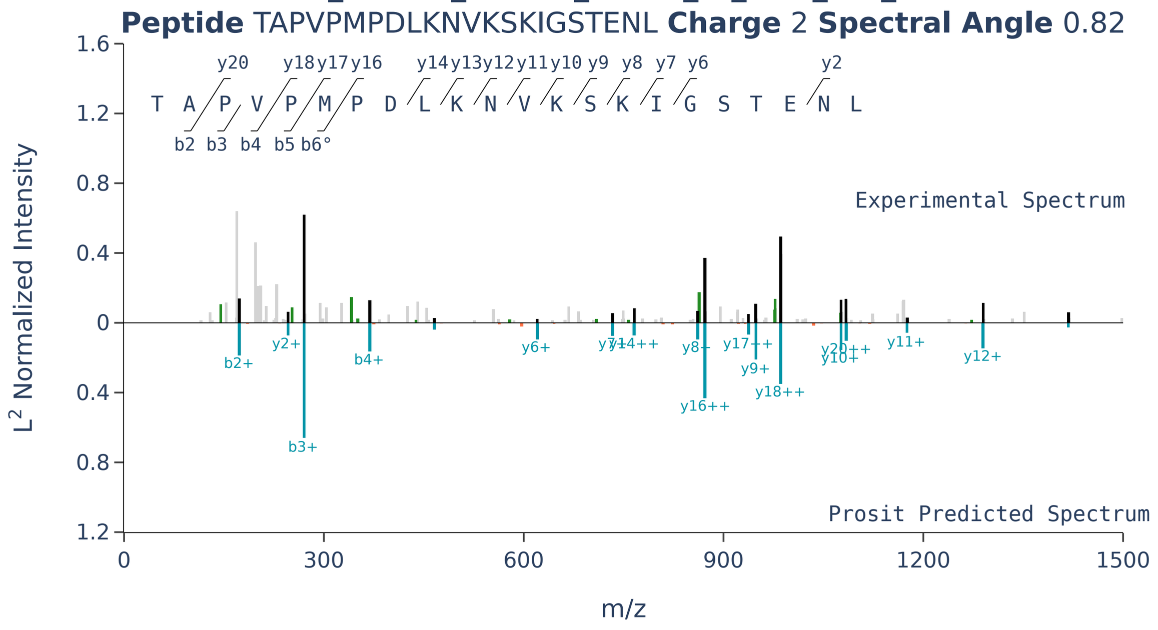

**Source** WSoh_221121_011221_HFGoe_L2_hTau_24h_R2 **Scan** 24533
**Peptide** AKSRLQTAPVPMPDL **Charge** 3 **Spectral Angle** 0.89

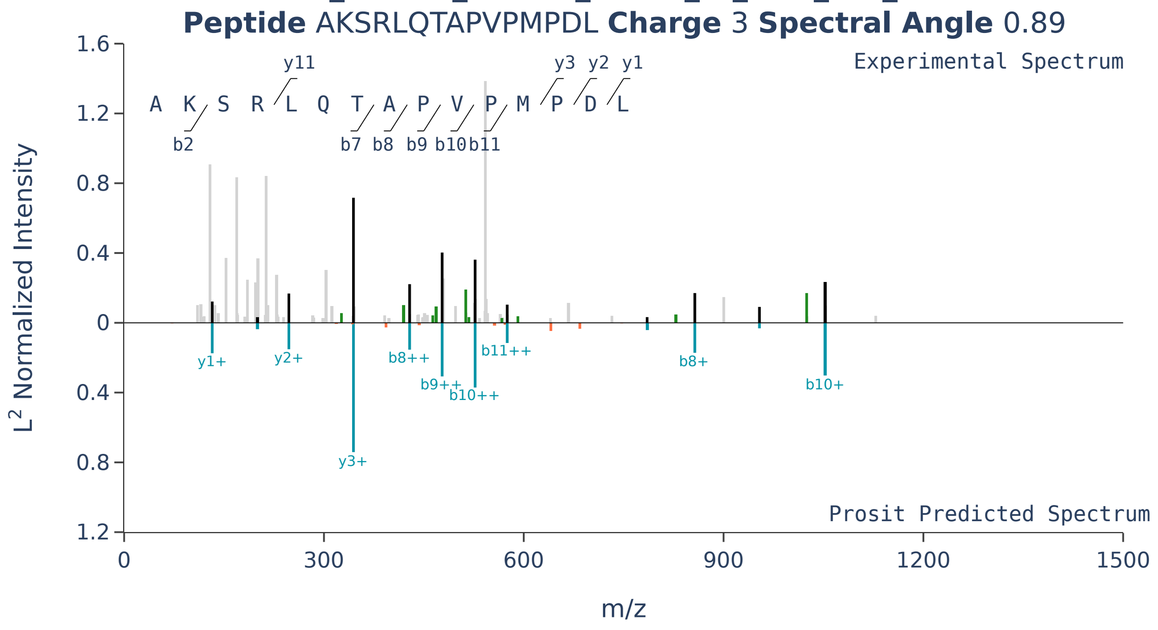

**Source** WSoh_221121_011221_HFGoe_L2_hTau_24h_R2 **Scan** 11882
**Peptide** GLGDRKDQGGYTMHQDQEGDTDAGLKE **Charge** 5 **Spectral Angle** 0.78

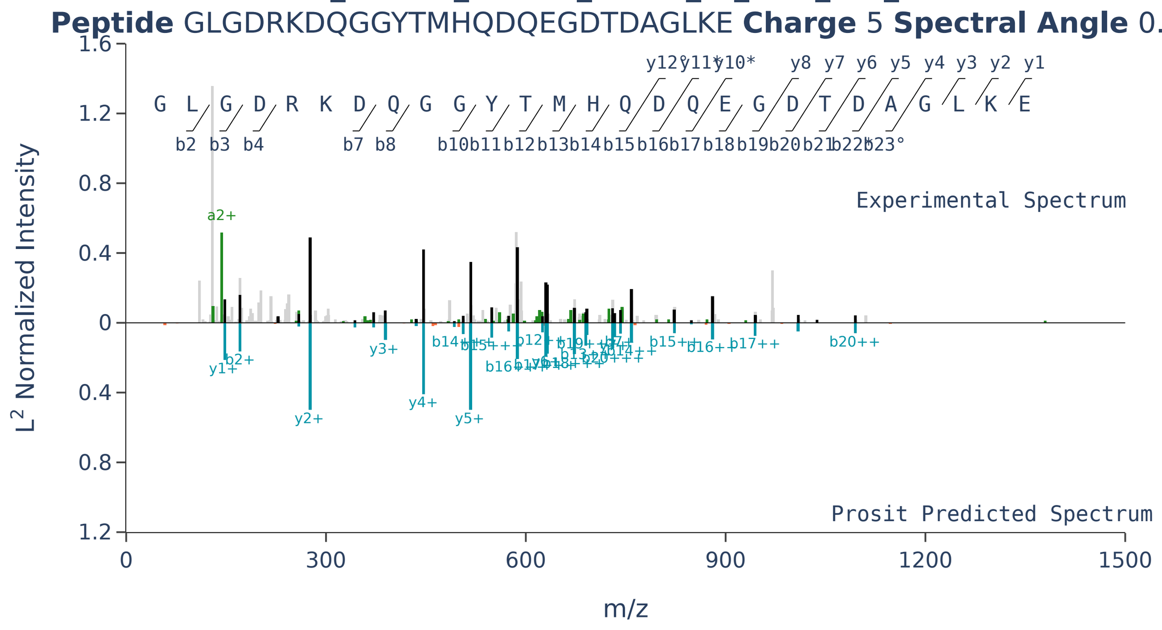

**Source** WSoh_221121_011221_HFGoe_L2_hTau_24h_R2 **Scan** 6677
**Peptide** KIGSTENLKHQPGGGKVQ **Charge** 2 **Spectral Angle** 0.87

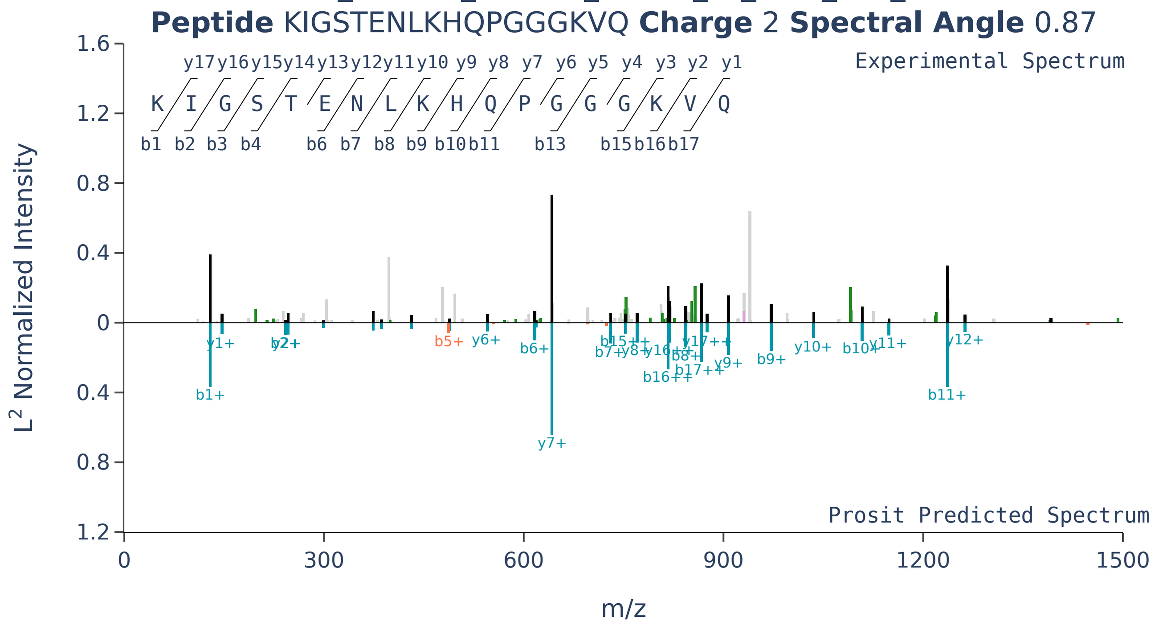

**Source** WSoh_221121_011221_HFGoe_L1_hTau_24h_R2 **Scan** 6124
**Peptide** TENLKHQPGGGKV **Charge** 2 **Spectral Angle** 0.89

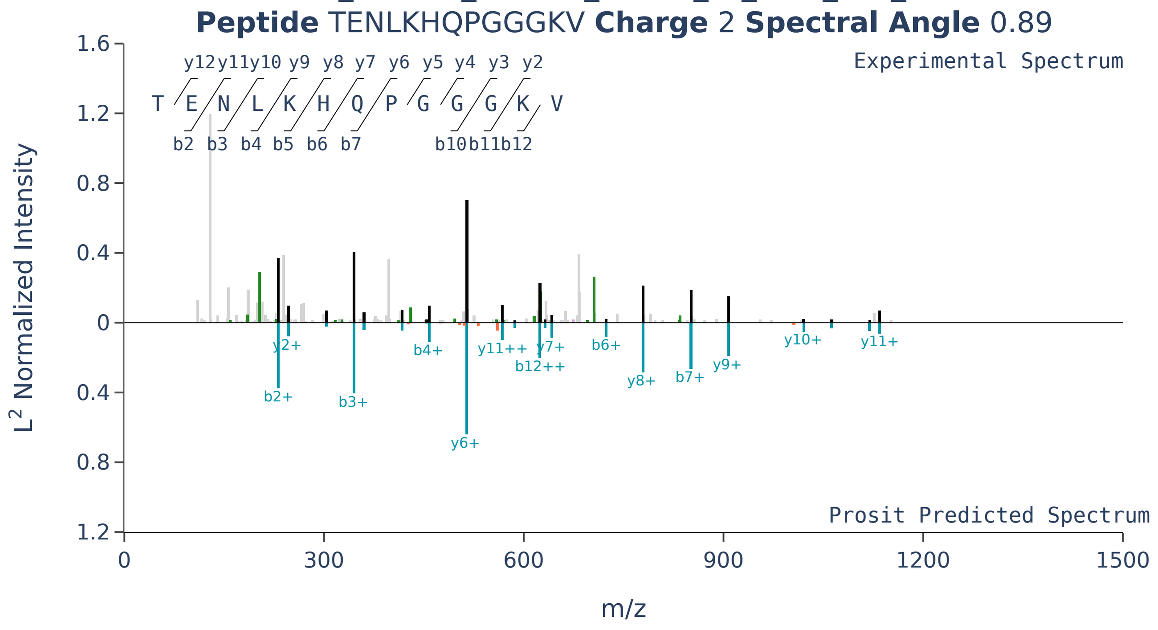

**Source** WSoh_221121_011221_HFGoe_L2_hTau_24h_R1 **Scan** 12782
**Peptide** LGSTENLKHQPGGGKVQIINKKL **Charge** 3 **Spectral Angle** 0.84

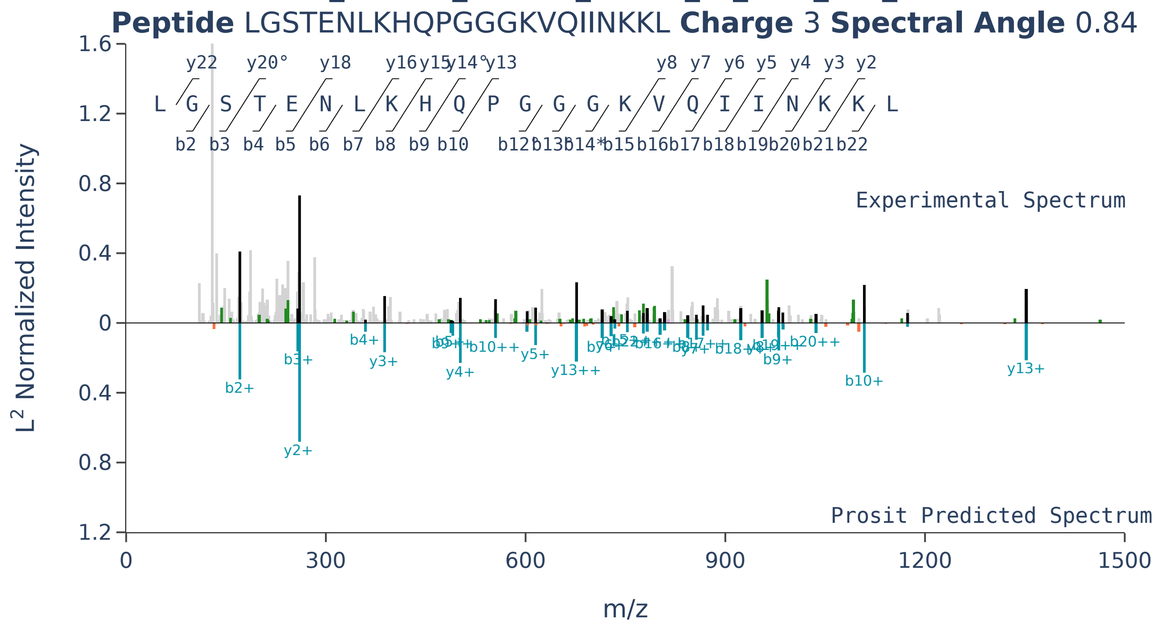

**Source** WSoh_221121_011221_HFGoe_L1_hTau_24h_R1 **Scan** 22386
**Peptide** EDVTAPLVD **Charge** 2 **Spectral Angle** 0.78

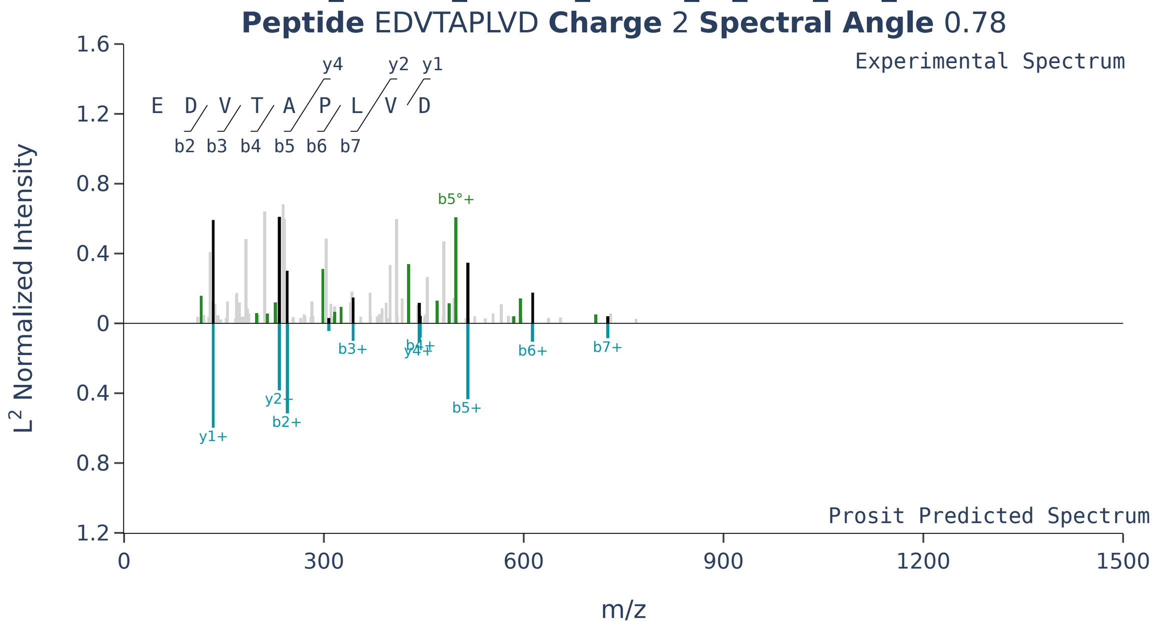

**Source** WSoh_221121_011221_HFGoe_L2_hTau_2h_R2 **Scan** 6065
**Peptide** HKLTFRENAKAKTD **Charge** 4 **Spectral Angle** 0.8

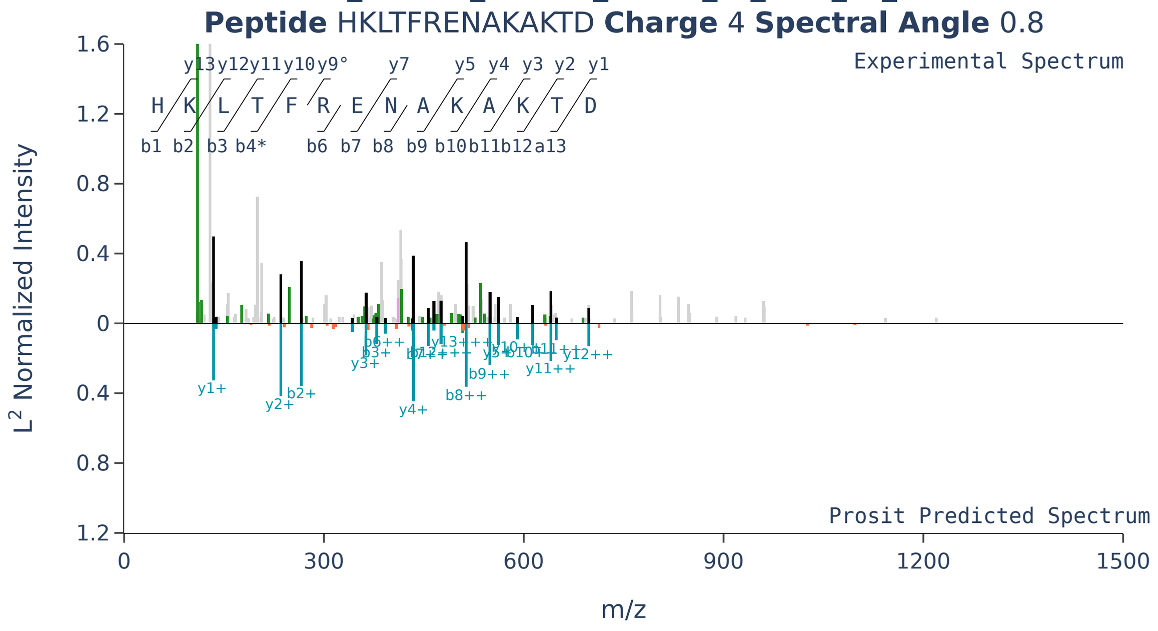

**Source** WSoh_221121_011221_HFGoe_L1_hTau_4h_R2 **Scan** 7485
**Peptide** KIATPRGAAPPGQKGQANA **Charge** 2 **Spectral Angle** 0.78

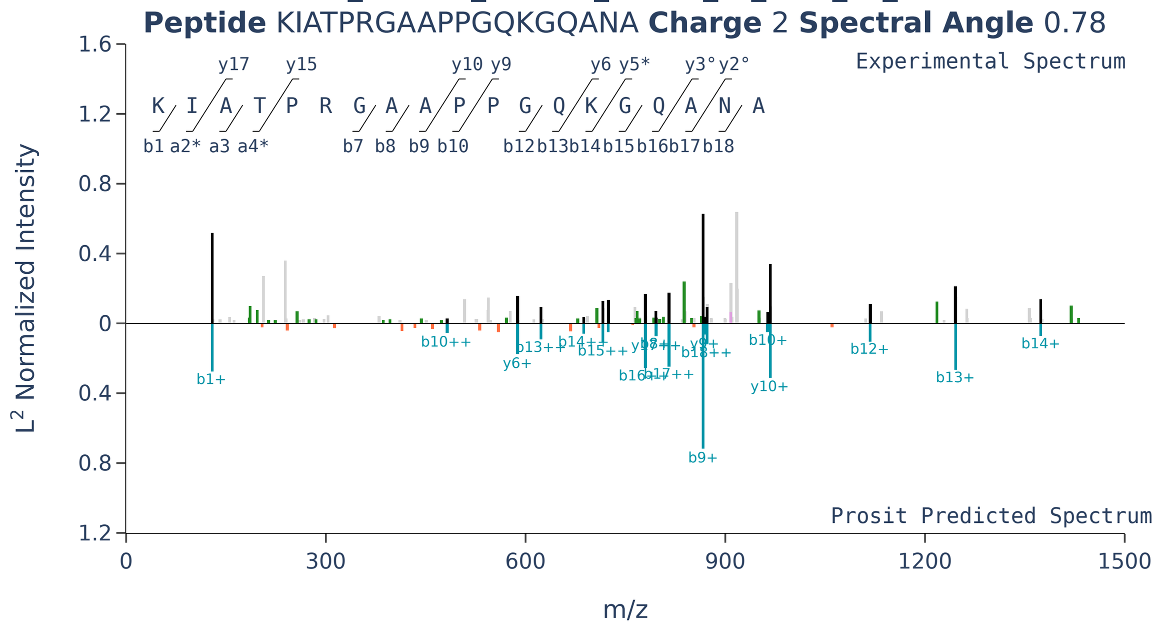

**Source** WSoh_221121_011221_HFGoe_L1_hTau_4h_R1 **Scan** 11897
**Peptide** PHTEIPEGTTAEE **Charge** 2 **Spectral Angle** 0.81

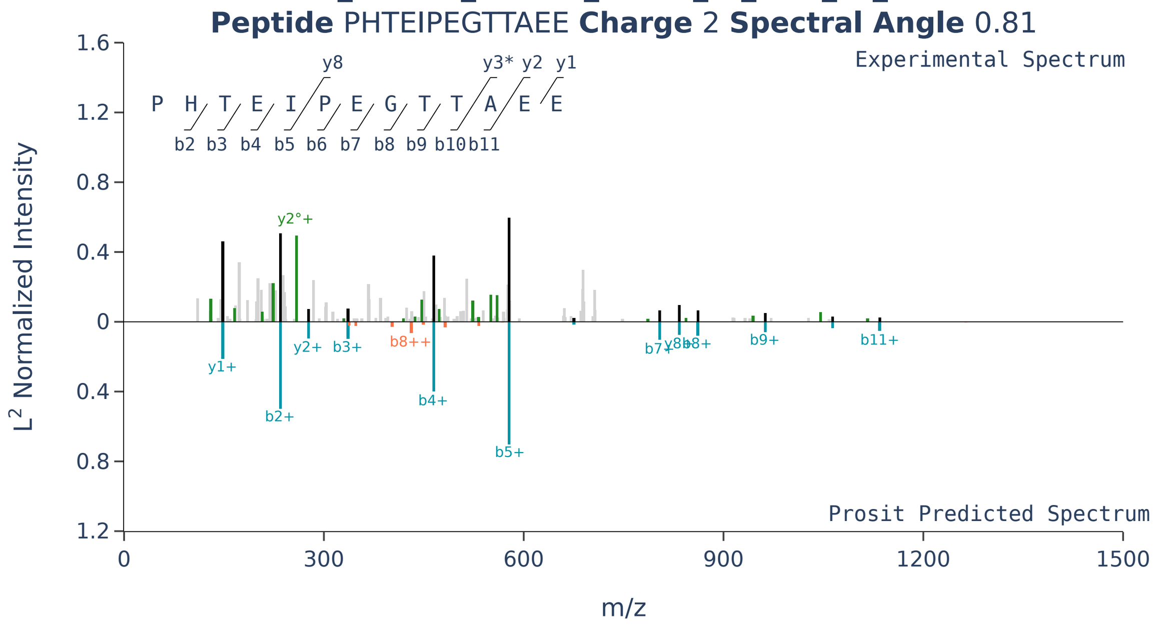

**Source** WSoh_221121_011221_HFGoe_L1_hTau_4h_R1 **Scan** 7326
**Peptide** ADGKTKIATPRGAAPPGQKGQANA **Charge** 4 **Spectral Angle** 0.91

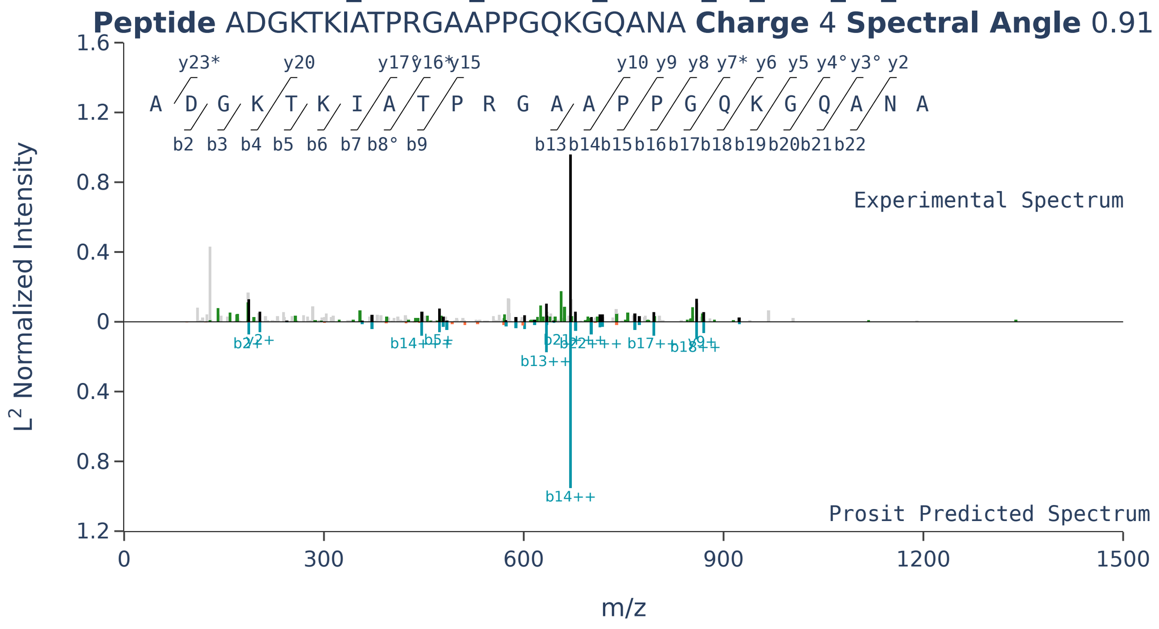

**Source** WSoh_221121_011221_HFGoe_L2_hTau_4h_R1 **Scan** 13904
**Peptide** KPVDLSKVTSKCGSLGNIHH **Charge** 3 **Spectral Angle** 0.83

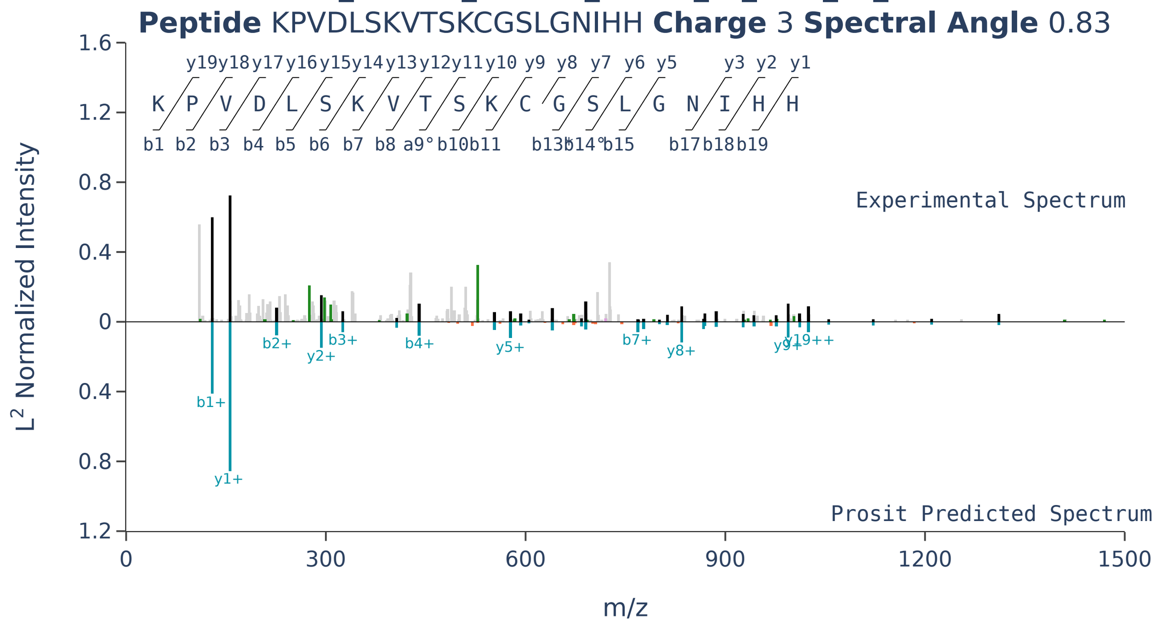

**Source** WSoh_221121_011221_HFGoe_L1_hTau_4h_R1 **Scan** 10897
**Peptide** KCGSKDNIKHVPGGGSVQI **Charge** 3 **Spectral Angle** 0.83

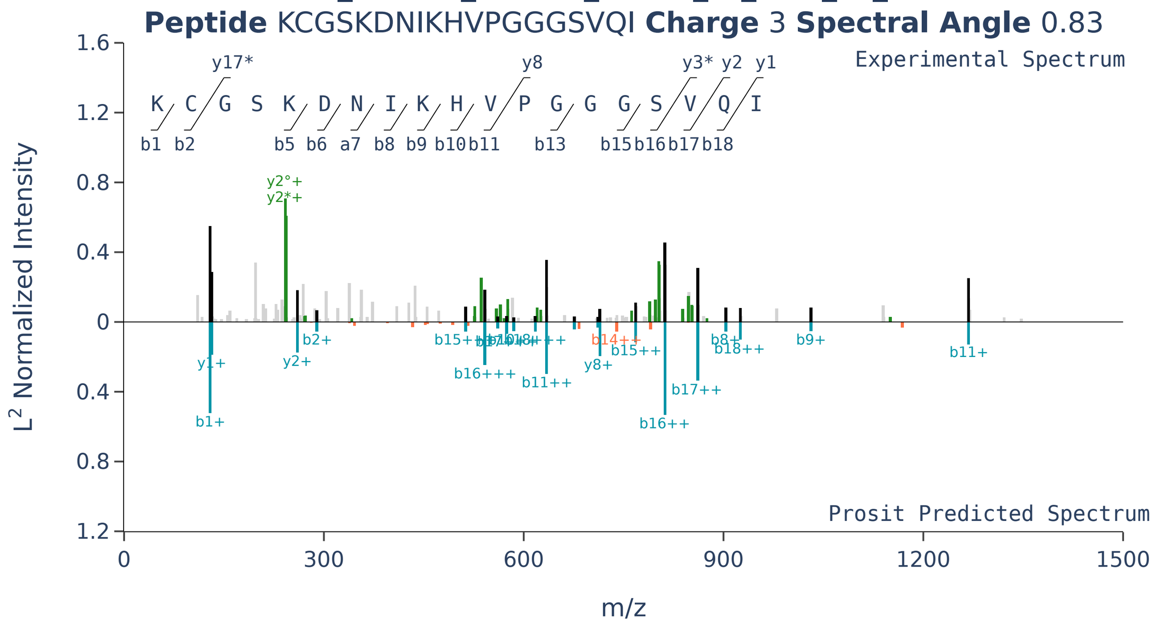

**Source** WSoh_221121_011221_HFGoe_L2_hTau_2h_R2 **Scan** 13268
**Peptide** KIGSLDNITHVPGGGNKK **Charge** 4 **Spectral Angle** 0.87

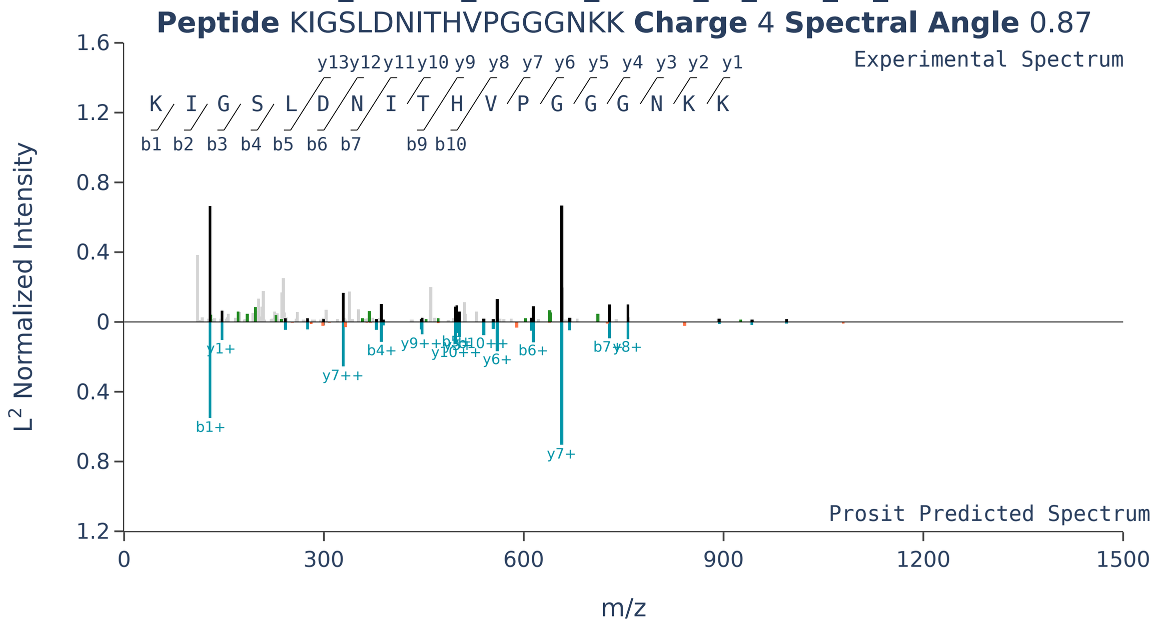

**Source** WSoh_221121_011221_HFGoe_L2_hTau_1h_R1 **Scan** 5869
**Peptide** ATPRGAAPPGQKGQANAT **Charge** 2 **Spectral Angle** 0.83

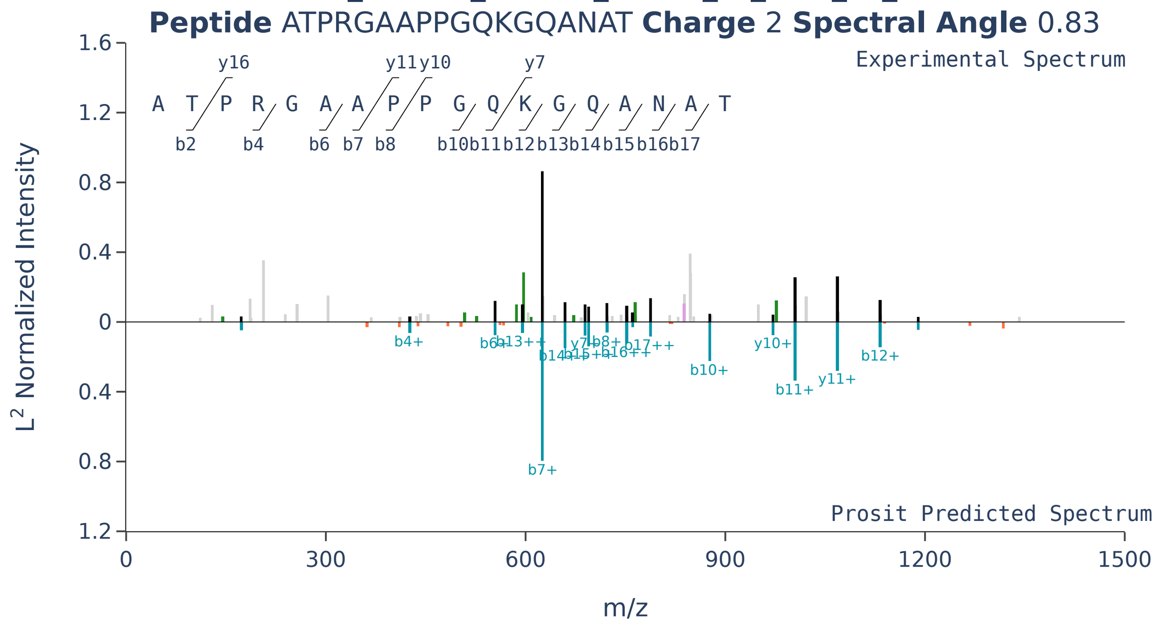

**Source** WSoh_221121_011221_HFGoe_L1_hTau_4h_R1 **Scan** 9301
**Peptide** VMEDHAGTY **Charge** 2 **Spectral Angle** 0.82

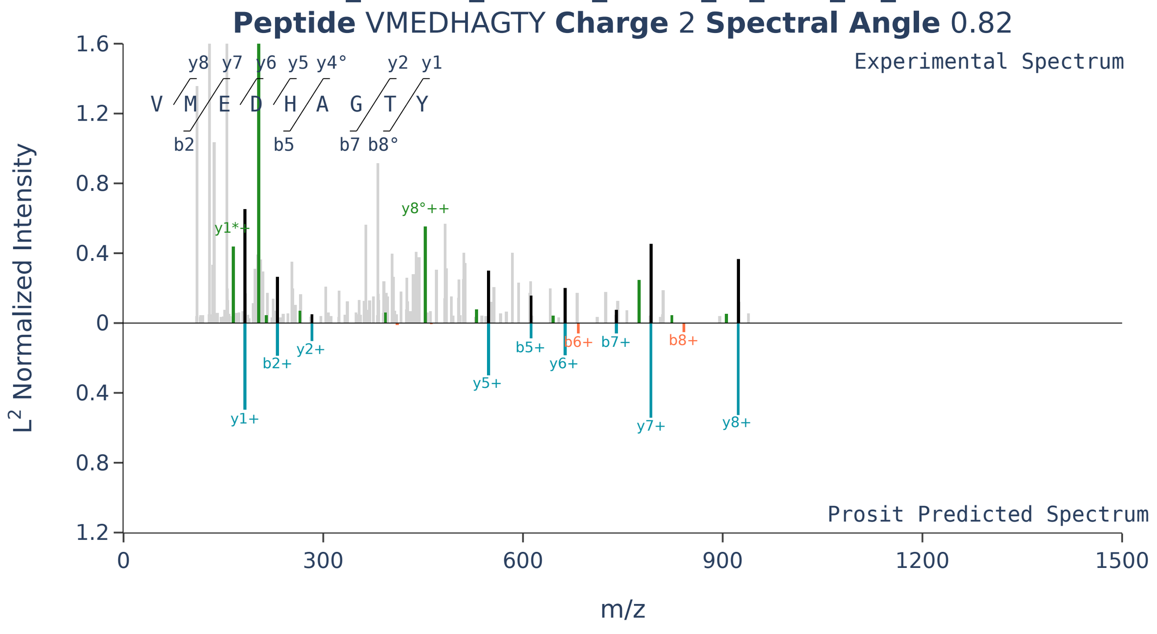


**Source** WSoh_221121_011221_HFGoe_L1_hTau_24h_R2 **Scan** 15973
**Peptide** KIGSLDNITHVPGGGNKKIE **Charge** 3 **Spectral Angle** 0.89

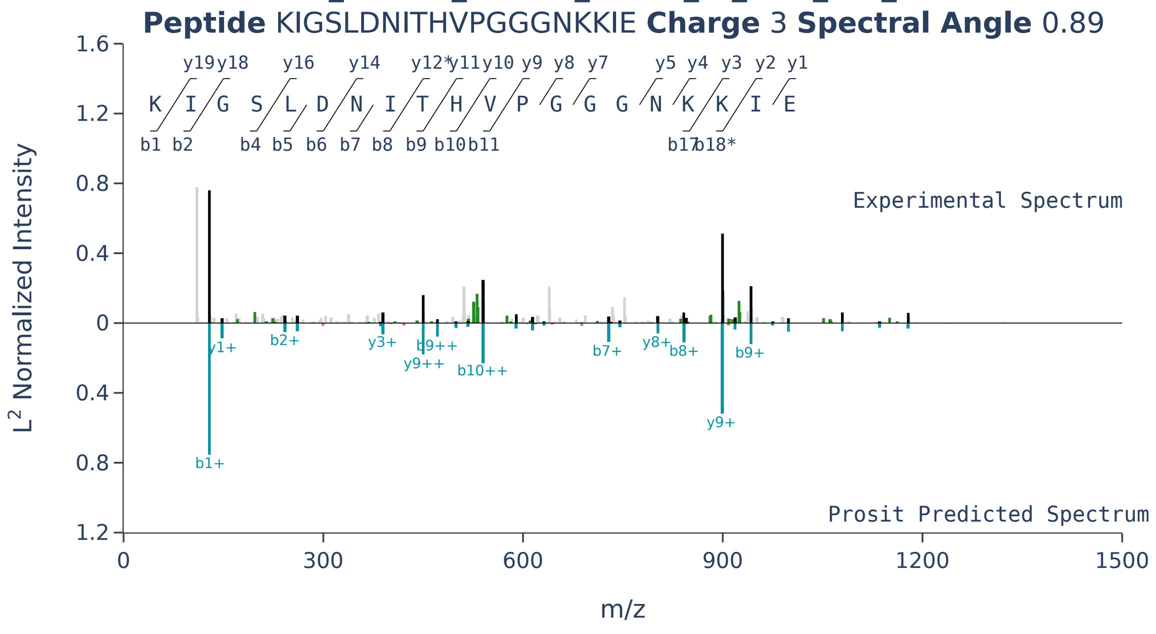


**Source** WSoh_221121_011221_HFGoe_L1_hTau_24h_R1 **Scan** 11180
**Peptide** SKCGSKDNIKHVPGGGSVQI **Charge** 2 **Spectral Angle** 0.9

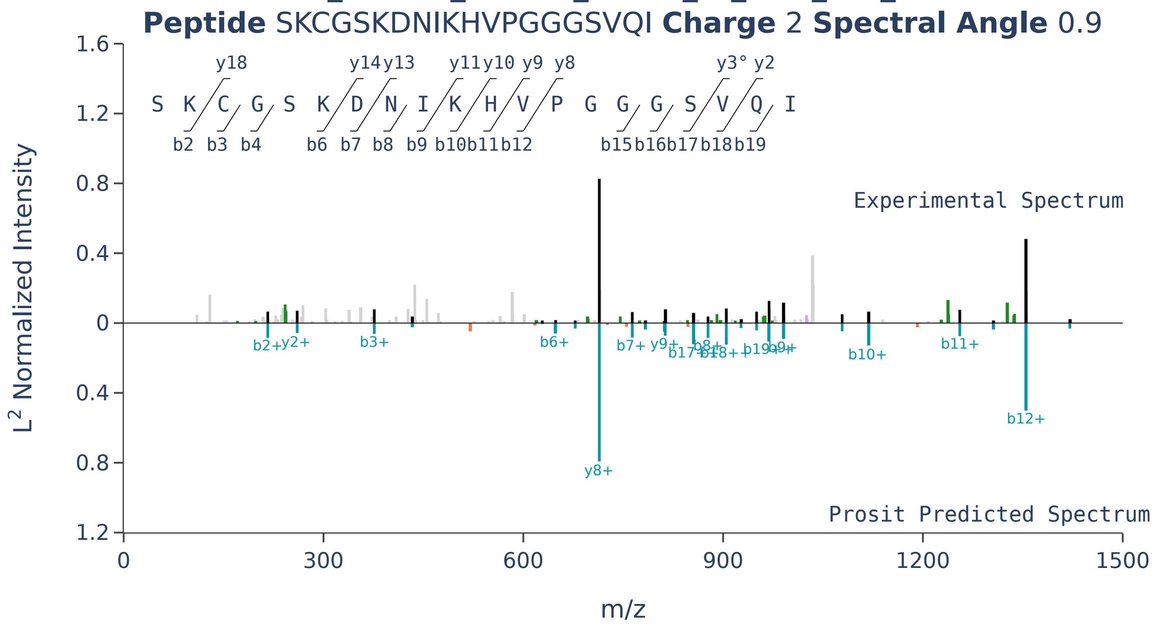


**Source** WSoh_221121_011221_HFGoe_L2_hTau_4h_R2 **Scan** 9119
**Peptide** AAQPHTEI **Charge** 1 **Spectral Angle** 0.9

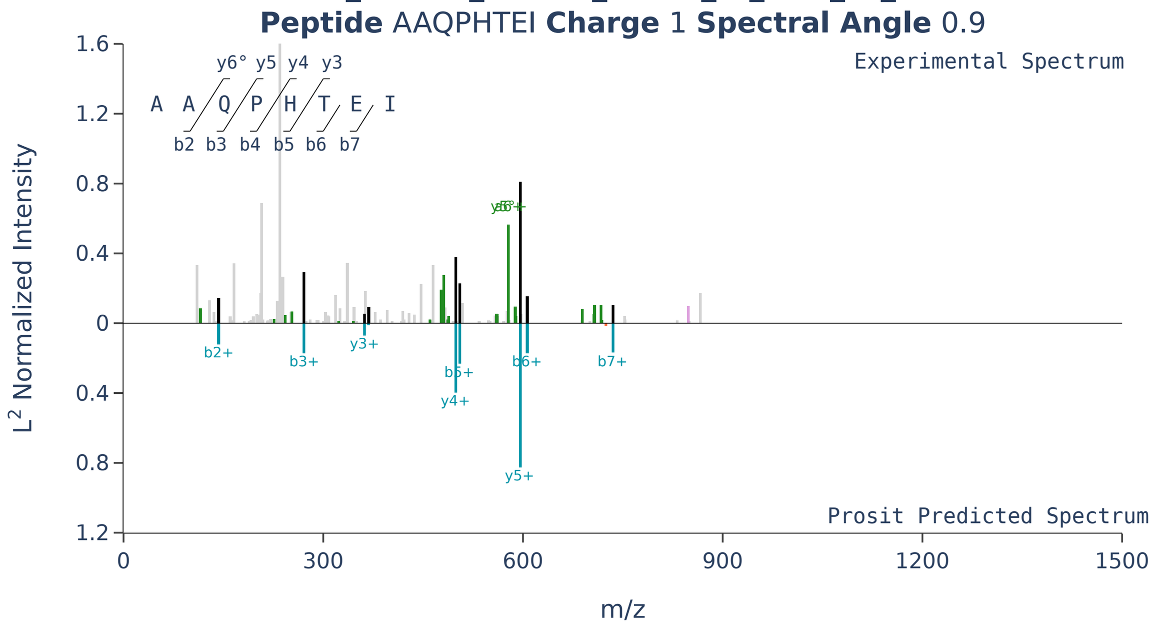


**Source** WSoh_221121_011221_HFGoe_L1_hTau_24h_R1 **Scan** 9763
**Peptide** HAGTYGLGDRKDQGGYTMHQDQ **Charge** 3 **Spectral Angle** 0.87

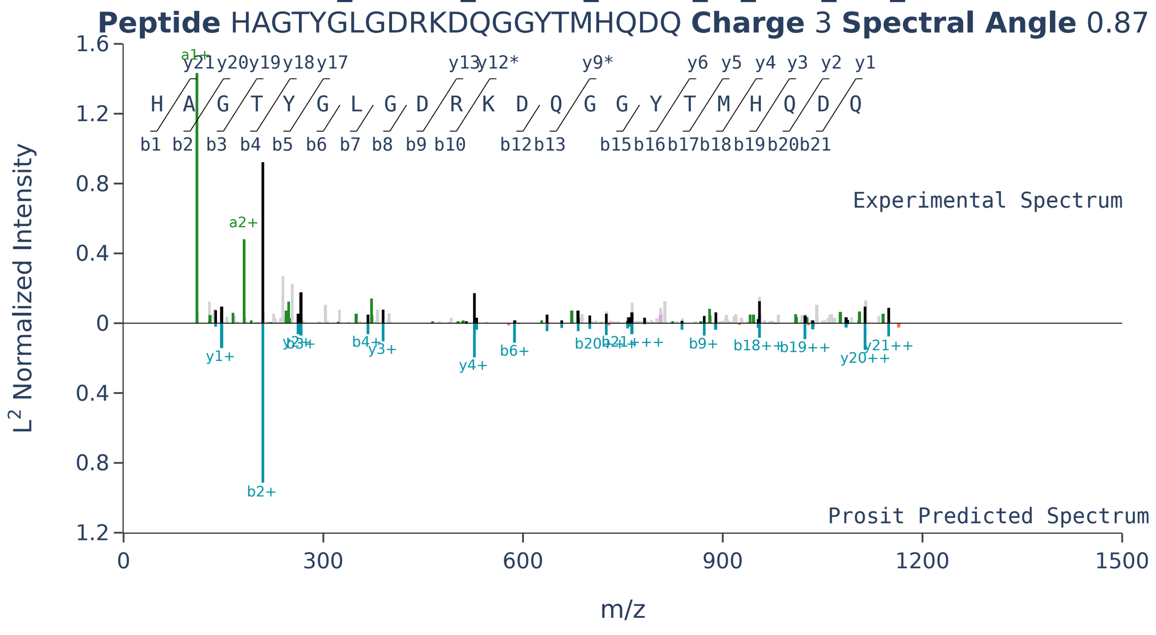


**Source** WSoh_221121_011221_HFGoe_L1_hTau_24h_R2 **Scan** 10033
**Peptide** KLGSTENLKHQPGGGKVQIINK **Charge** 3 **Spectral Angle** 0.91

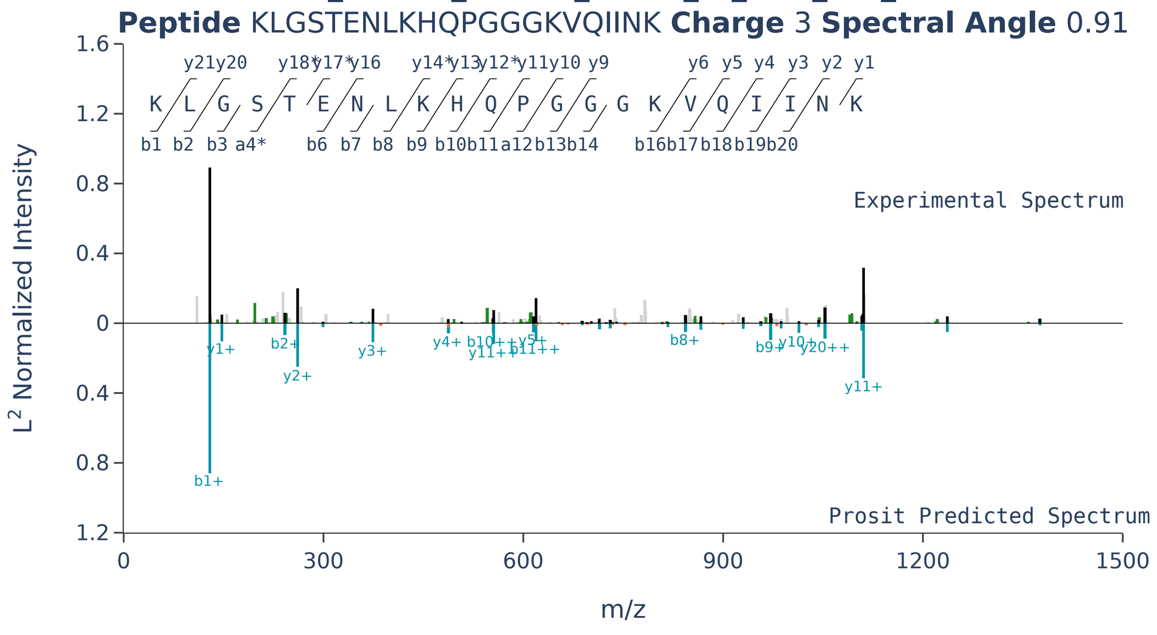


**Source** WSoh_221121_011221_HFGoe_L2_hTau_24h_R2 **Scan** 14853
**Peptide** QSKIGSLDNITH **Charge** 2 **Spectral Angle** 0.9

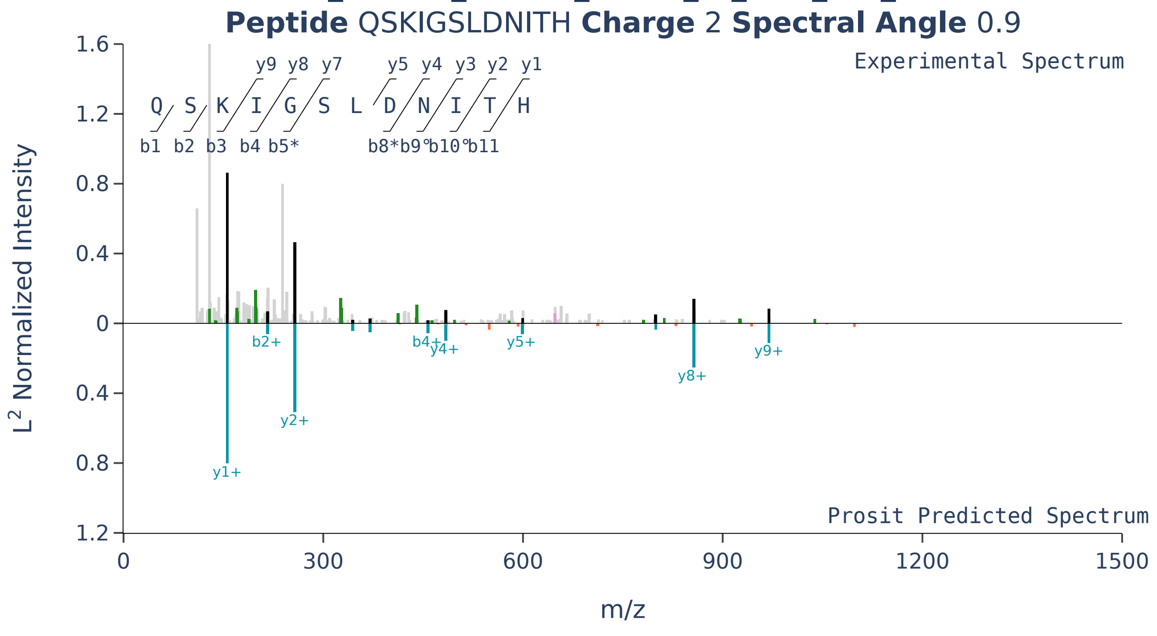


**Source** WSoh_221121_011221_HFGoe_L2_hTau_1h_R2 **Scan** 5482
**Peptide** VVRTPPKSPSSAKSR **Charge** 4 **Spectral Angle** 0.84

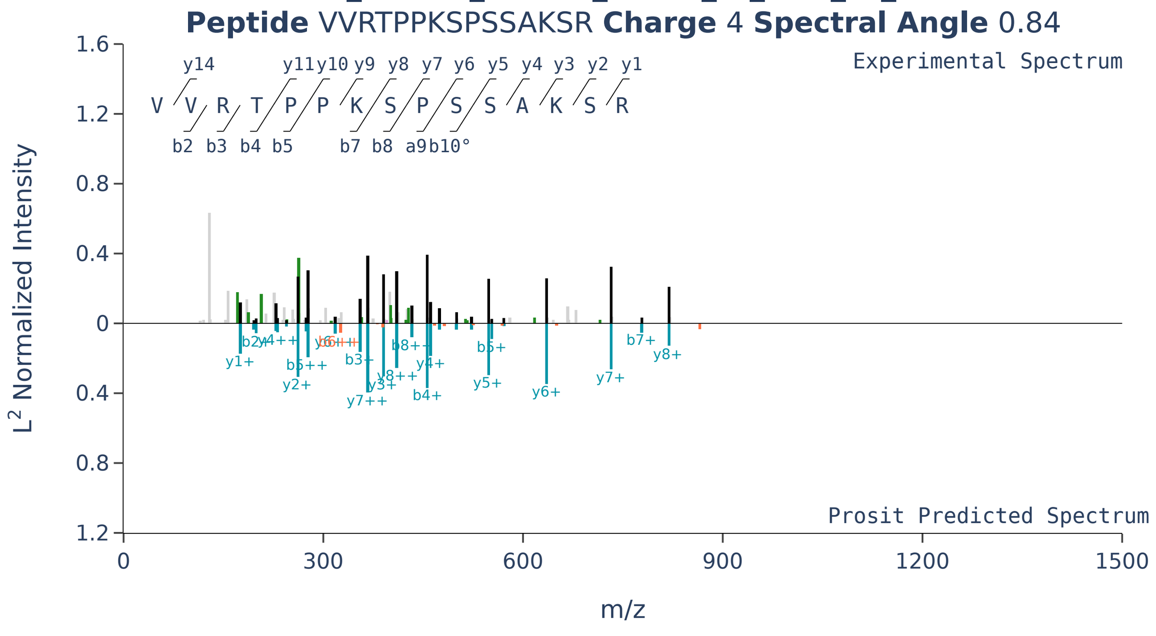


**Source** WSoh_221121_011221_HFGoe_L2_hTau_2h_R2 **Scan** 17173
**Peptide** TAPVPMPDLKNVKSK **Charge** 4 **Spectral Angle** 0.81

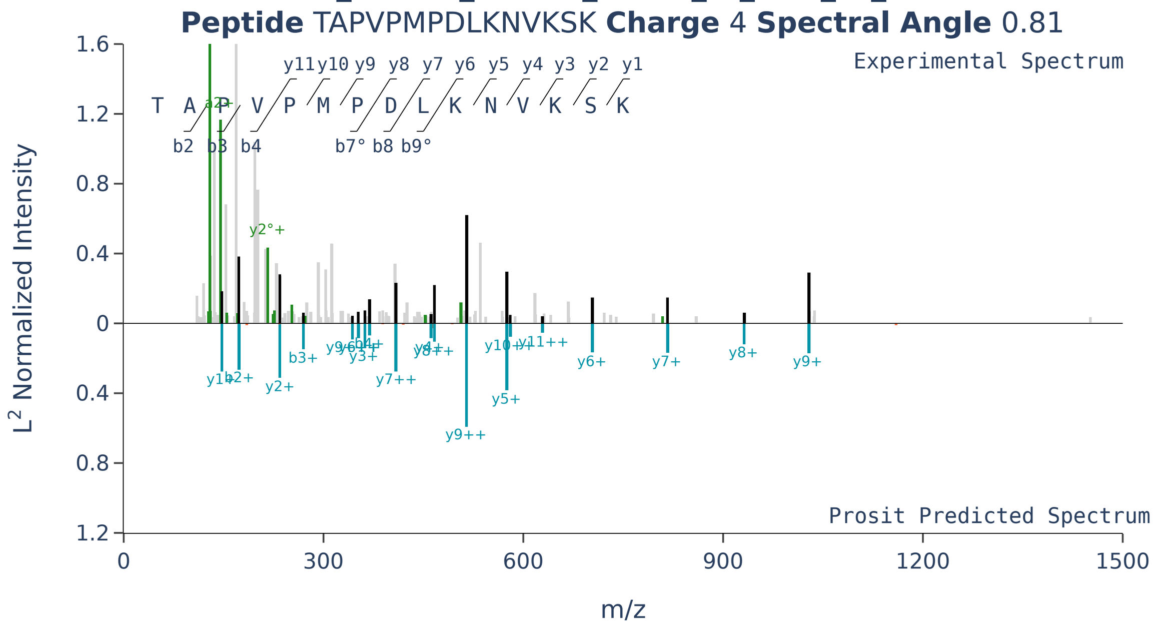


**Source** WSoh_221121_011221_HFGoe_L2_hTau_2h_R2 **Scan** 10583
**Peptide** GAPGKQAAAQPHTEIPEG **Charge** 3 **Spectral Angle** 0.88

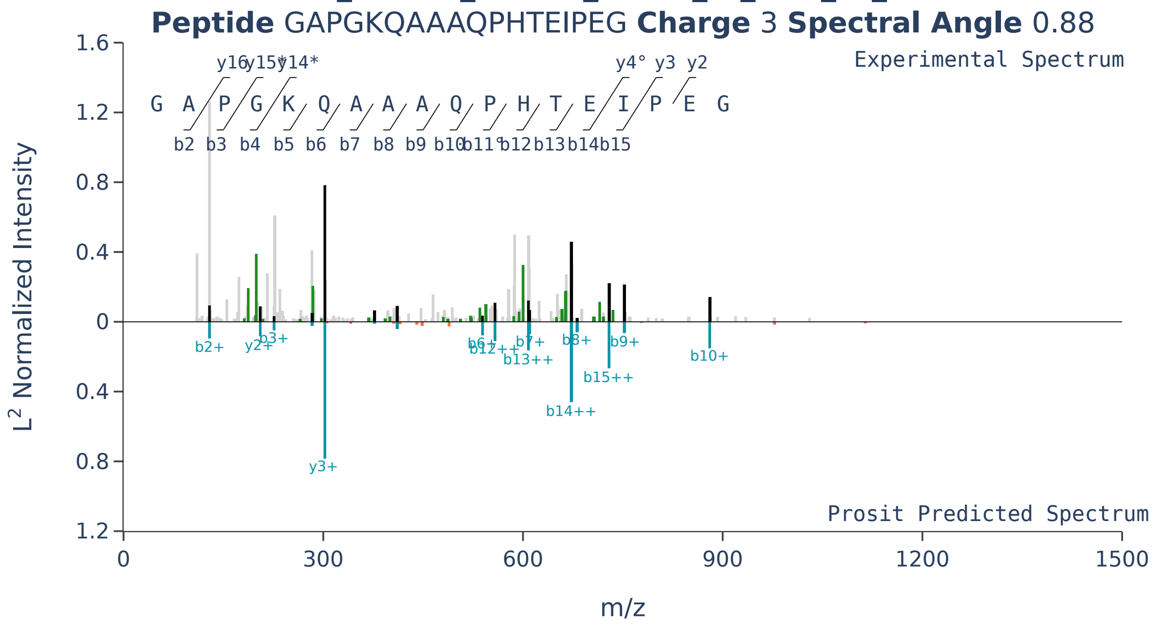


**Source** WSoh_221121_011221_HFGoe_L1_hTau_4h_R2 **Scan** 12989
**Peptide** AKAKTDHGAEIVYKSPVVSGD **Charge** 3 **Spectral Angle** 0.8

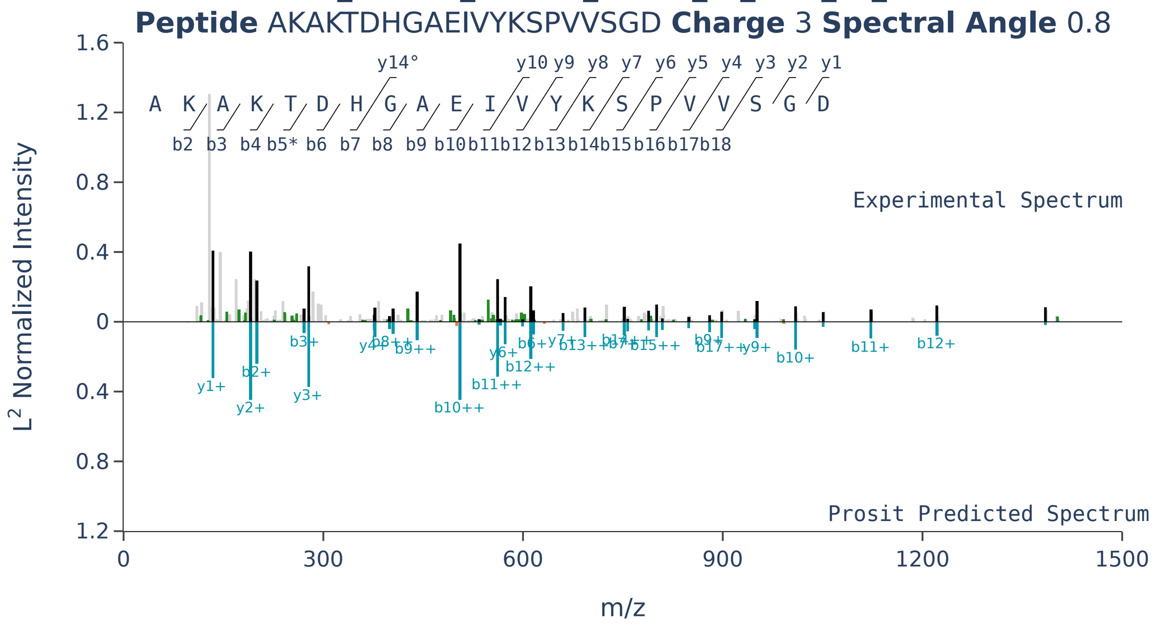


**Source** WSoh_221121_011221_HFGoe_L1_hTau_4h_R2 **Scan** 14093
**Peptide** TFRENAKAKTDHGAEIVYKSPVV **Charge** 3 **Spectral Angle** 0.77

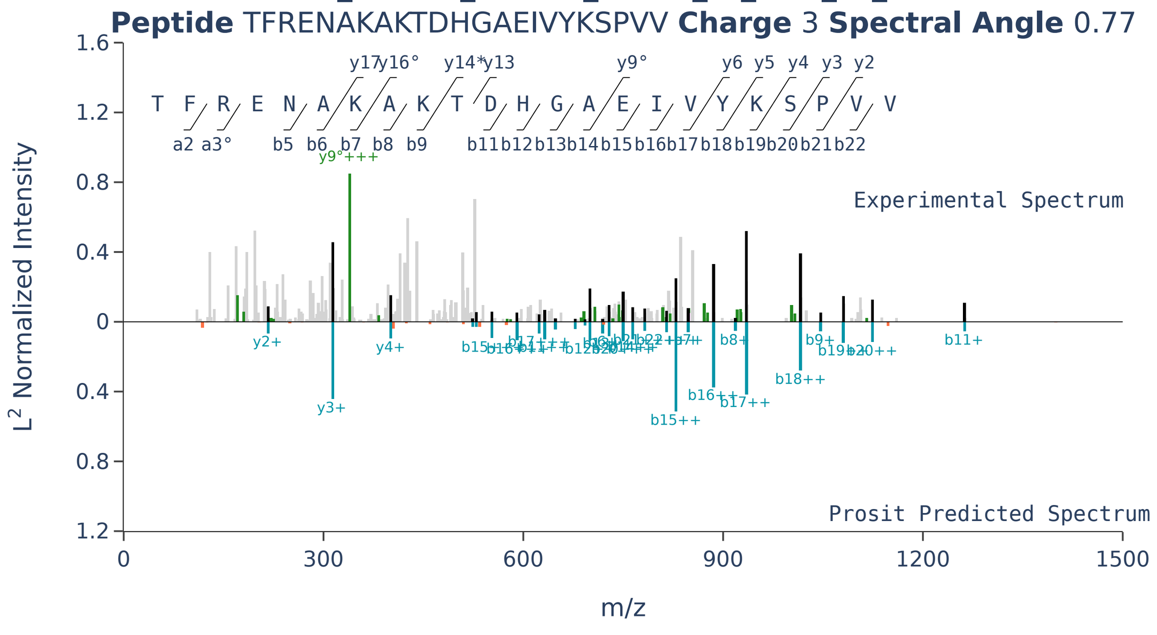


**Source** WSoh_221121_011221_HFGoe_L1_hTau_4h_R2 **Scan** 22325
**Peptide** DFKDRVQSKIGSLDNI **Charge** 3 **Spectral Angle** 0.78

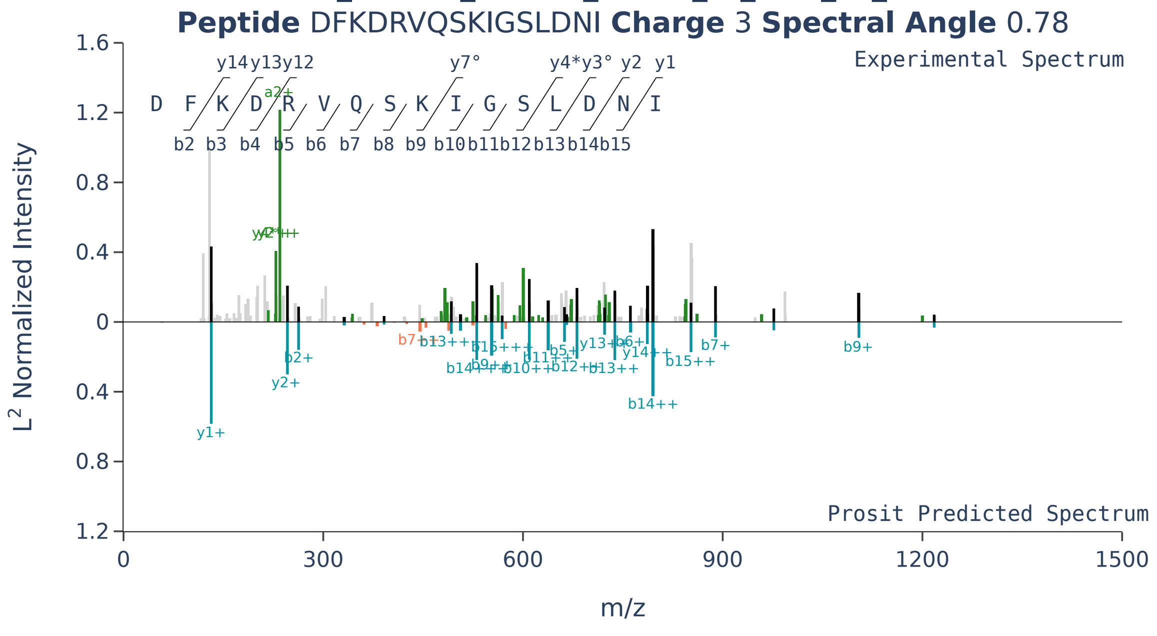


**Source** WSoh_221121_011221_HFGoe_L1_hTau_24h_R2 **Scan** 11361
**Peptide** IATPRGAAPPGQKGQANATRI **Charge** 3 **Spectral Angle** 0.88

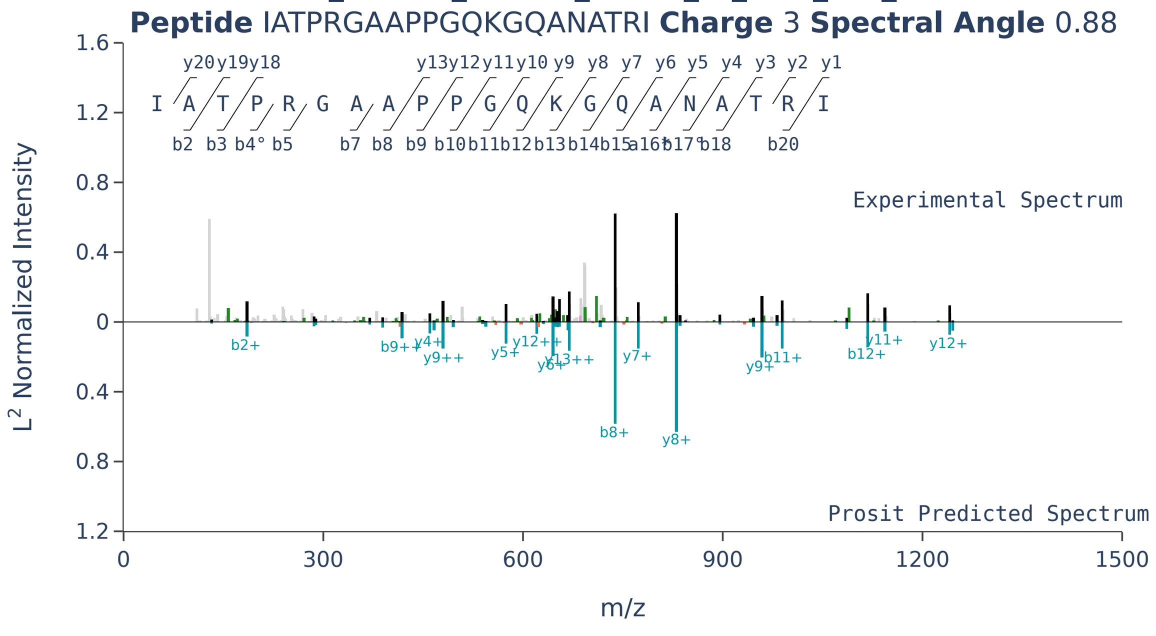


**Source** WSoh_221121_011221_HFGoe_L2_hTau_24h_R2 **Scan** 11644
**Peptide** DHAGTYGLGDRKDQGGYTMHQDQEGD **Charge** 4 **Spectral Angle** 0.86

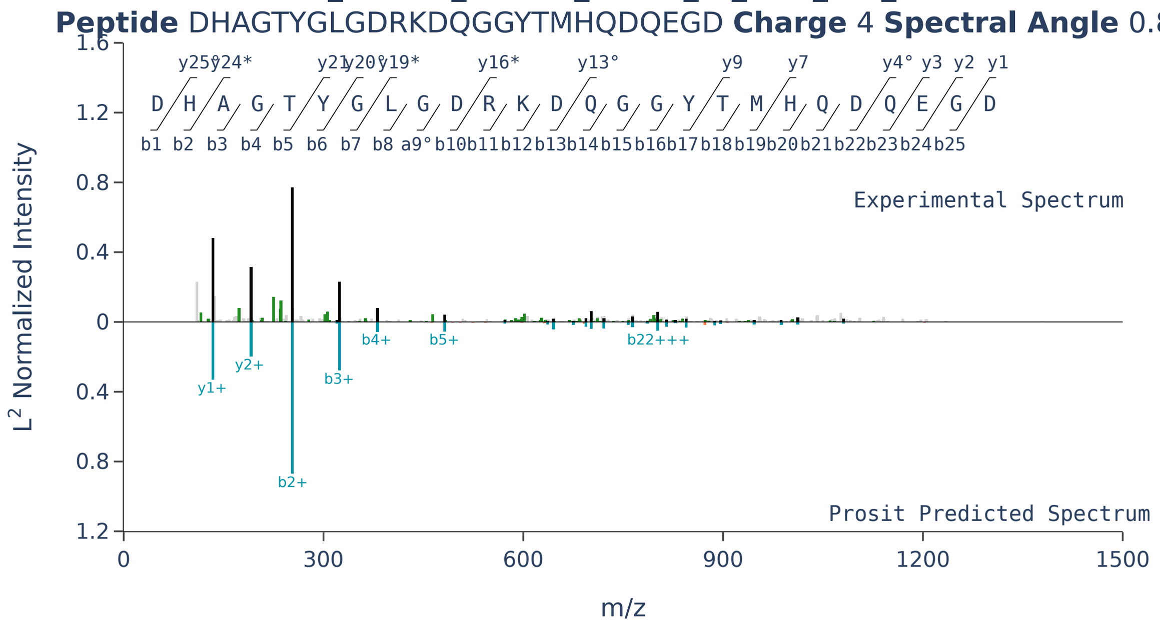

**Source** WSoh_221121_011221_HFGoe_L1_hTau_2h_R1 **Scan** 10168
**Peptide** KHQPGGGKVQII **Charge** 3 **Spectral Angle** 0.89

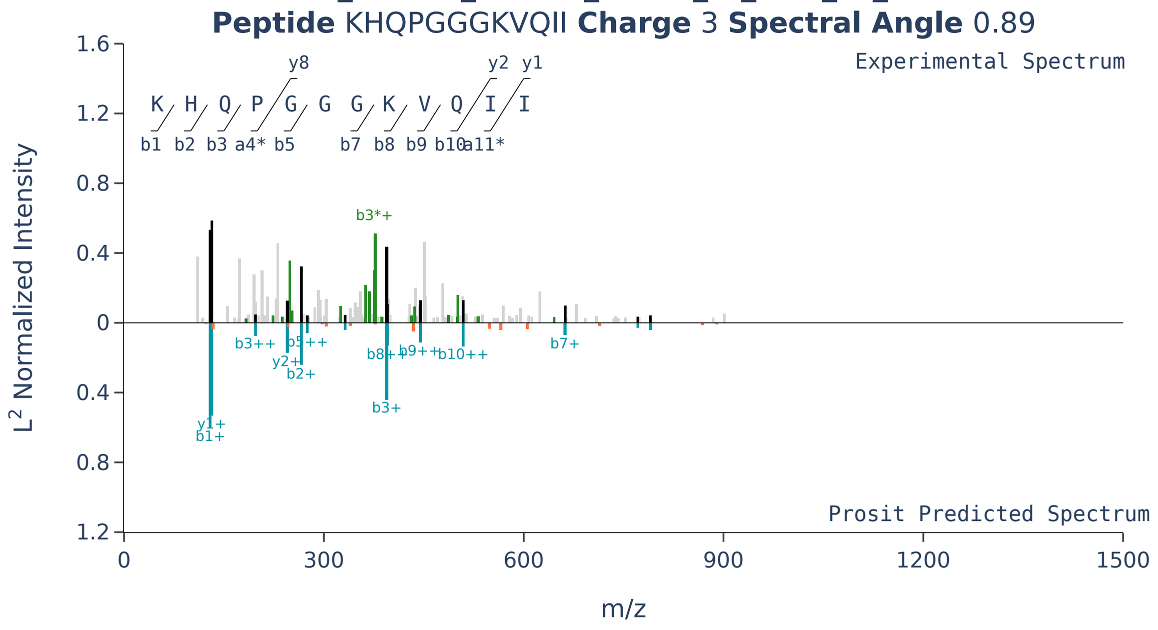


**Source** WSoh_221121_011221_HFGoe_L2_hTau_24h_R2 **Scan** 11231
**Peptide** GGGKVQIINKKL **Charge** 3 **Spectral Angle** 0.85

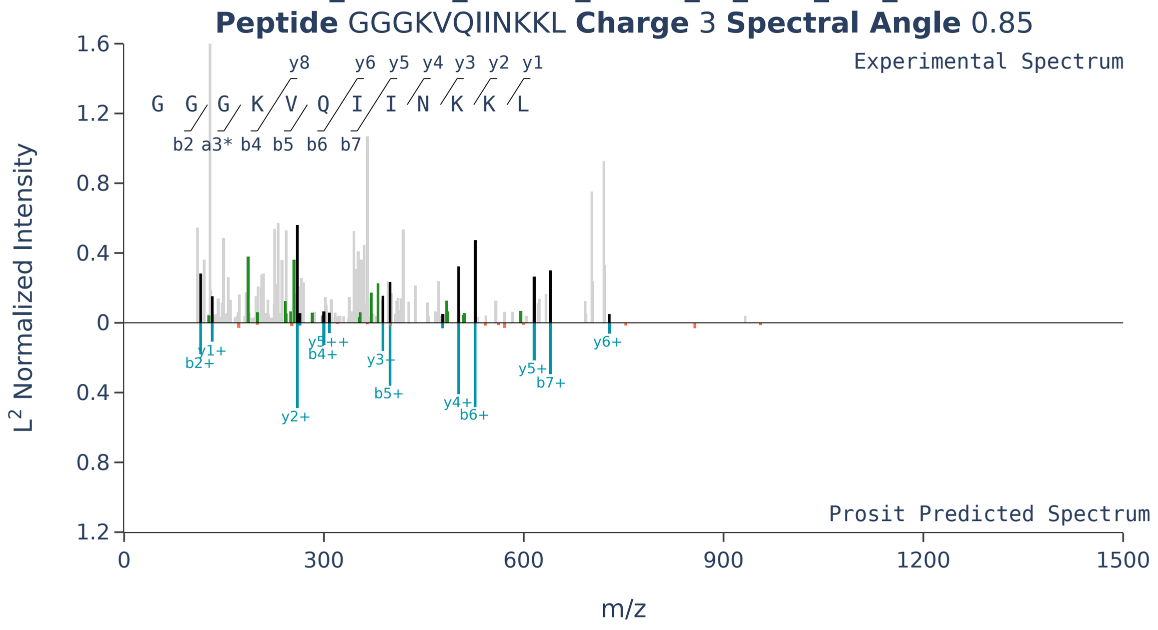


**Source** WSoh_221121_011221_HFGoe_L1_hTau_4h_R1 **Scan** 10288
**Peptide** KSPVVSGDTSPRHLSNVSSTG **Charge** 3 **Spectral Angle** 0.92

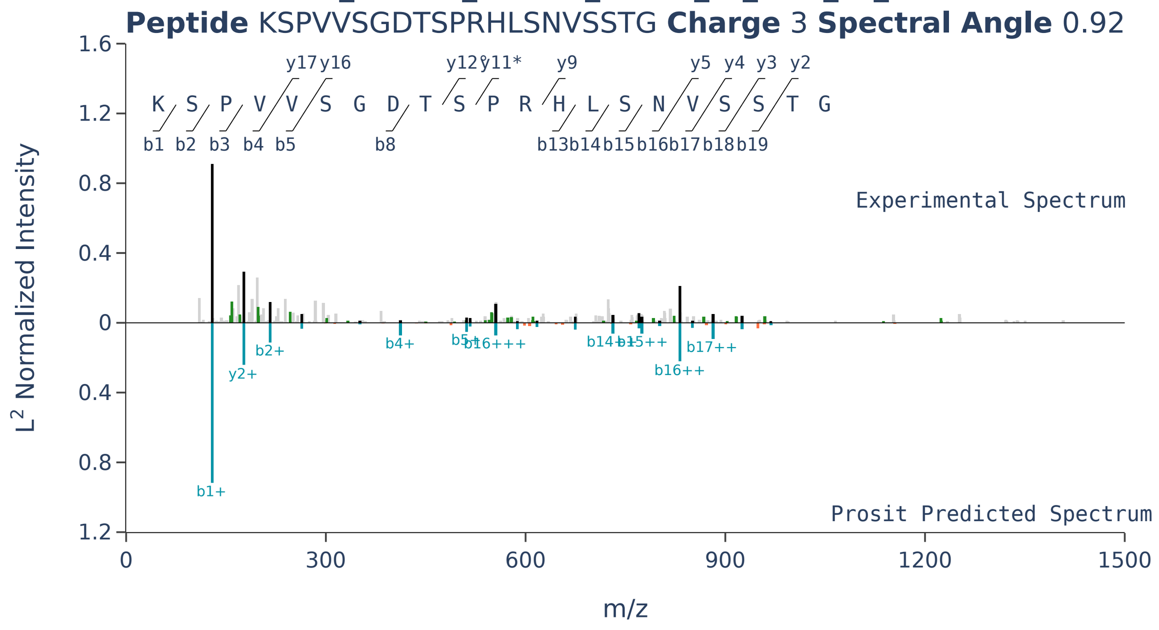


**Source** WSoh_221121_011221_HFGoe_L2_hTau_4h_R2 **Scan** 9469
**Peptide** HAGTYGLGDRKDQGGYTM **Charge** 3 **Spectral Angle** 0.9

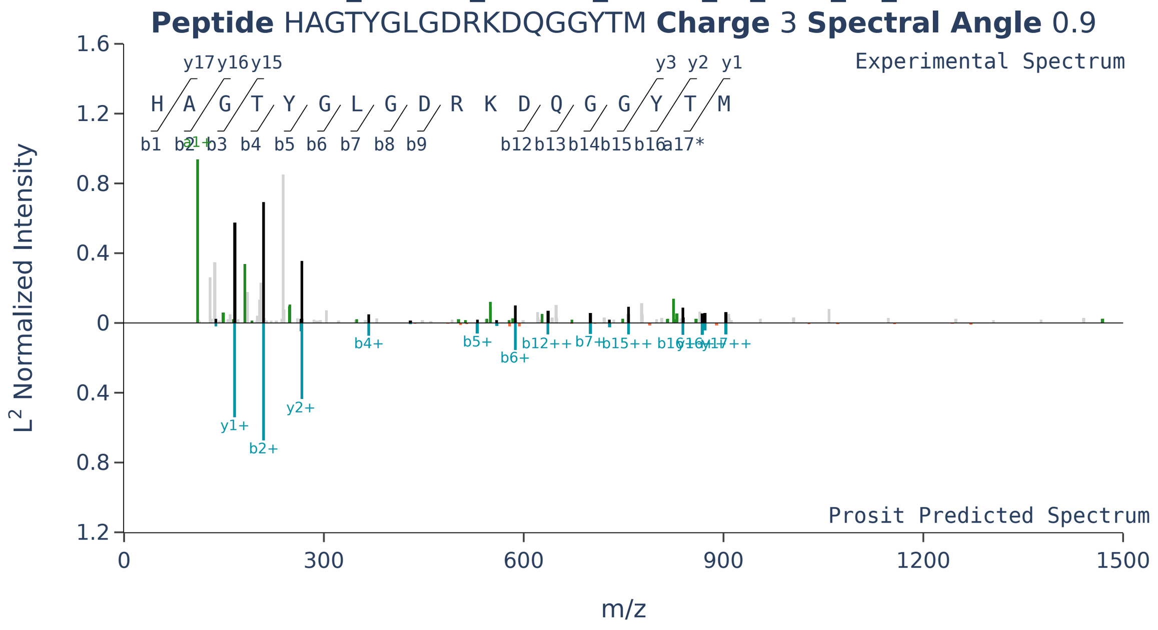


**Source** WSoh_221121_011221_HFGoe_L1_hTau_24h_R2 **Scan** 7128
**Peptide** NLHHKPGGGQVEV **Charge** 3 **Spectral Angle** 0.8

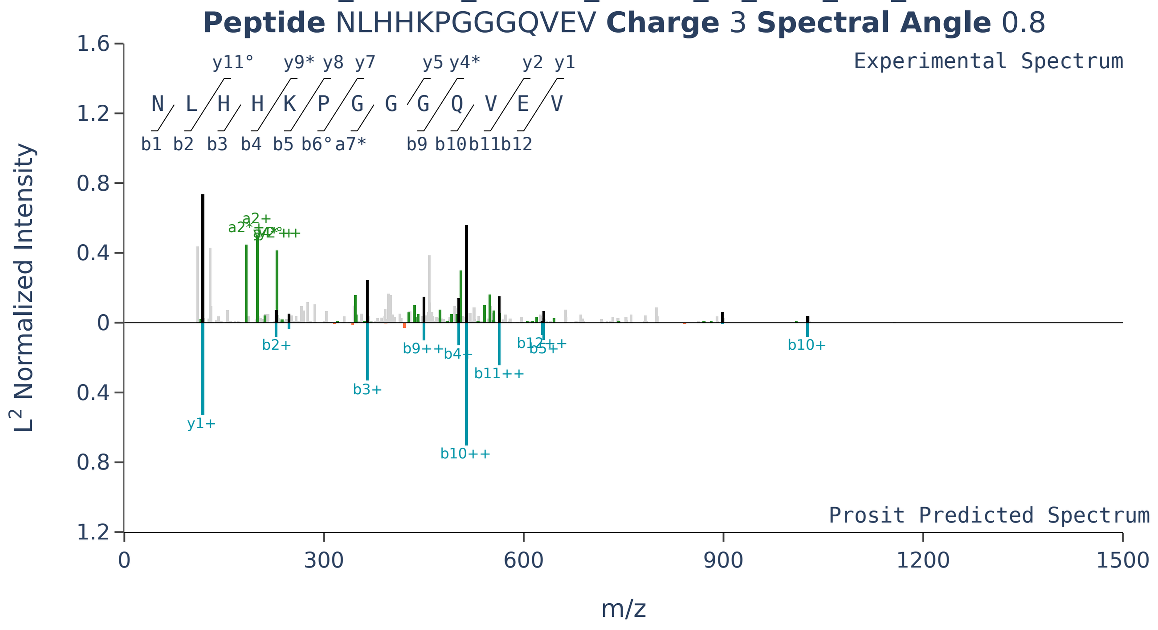


**Source** WSoh_221121_011221_HFGoe_L1_hTau_24h_R1 **Scan** 6762
**Peptide** LHHKPGGGQVEV **Charge** 3 **Spectral Angle** 0.87

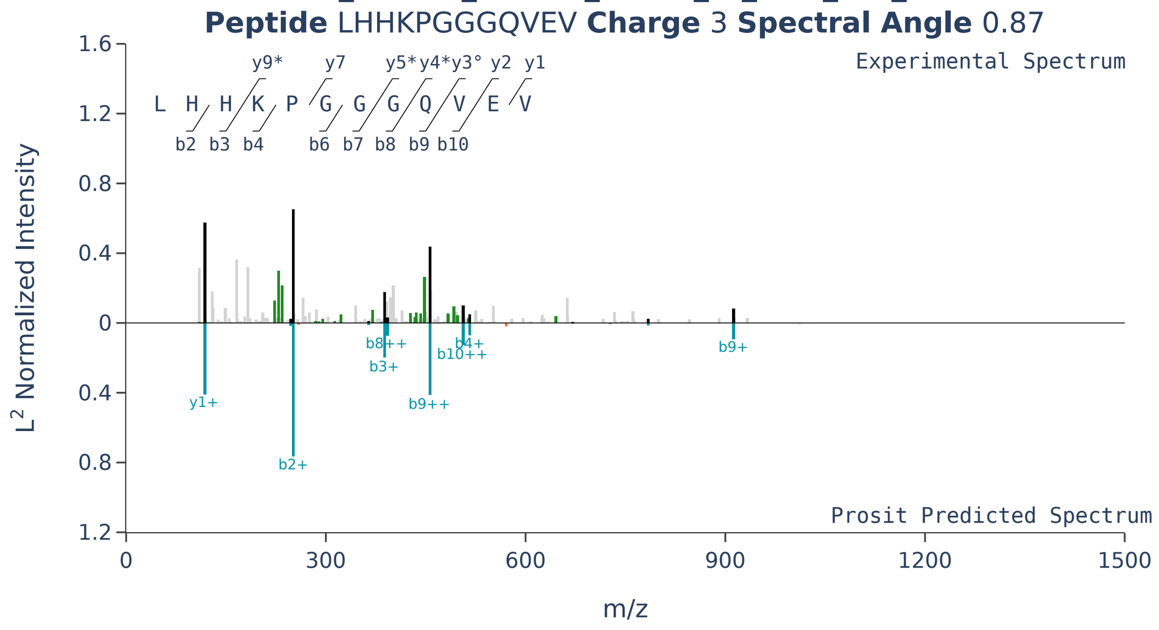


**Source** WSoh_221121_011221_HFGoe_L2_hTau_24h_R1 **Scan** 20085
**Peptide** APVPMPDLKNVKSKIGSTENLKH **Charge** 4 **Spectral Angle** 0.87

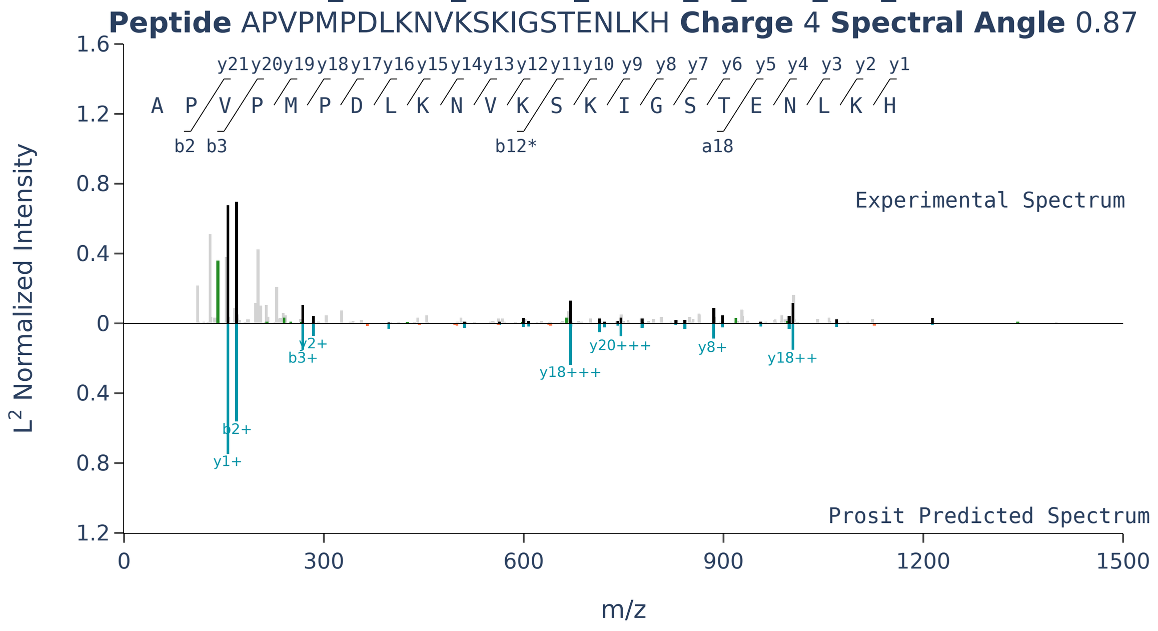


**Source** WSoh_221121_011221_HFGoe_L1_hTau_24h_R1 **Scan** 14568
**Peptide** EDHAGTYGLGDRKDQGGYTM **Charge** 3 **Spectral Angle** 0.9

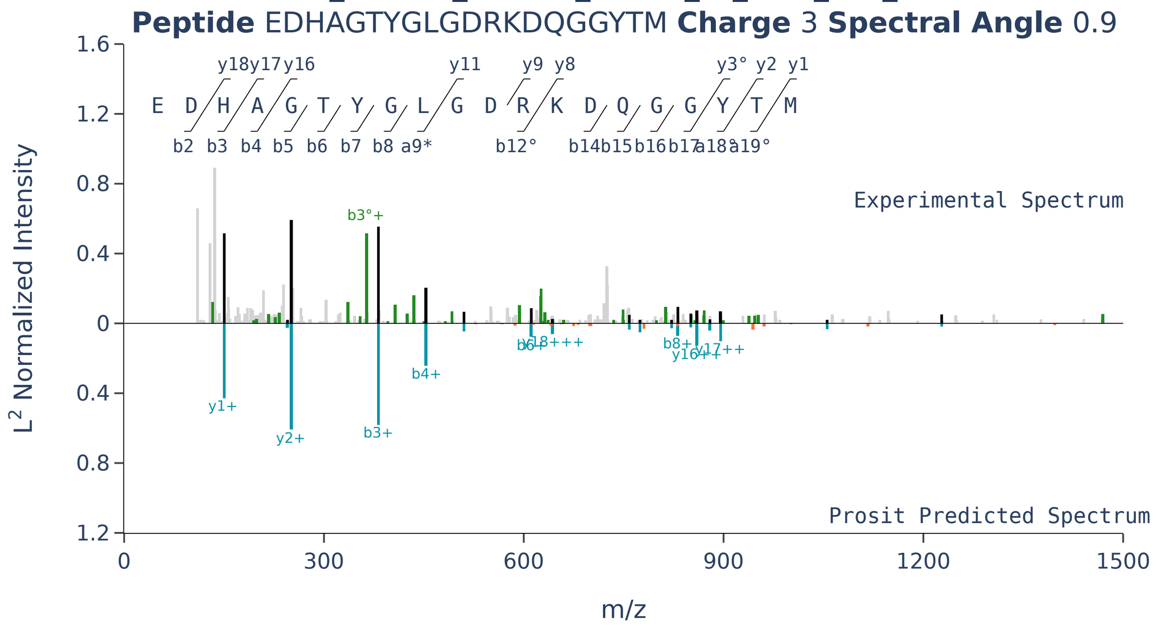


**Source** WSoh_221121_011221_HFGoe_L2_hTau_2h_R1 **Scan** 13349
**Peptide** KHQPGGGKVQIINKKLDL **Charge** 4 **Spectral Angle** 0.89

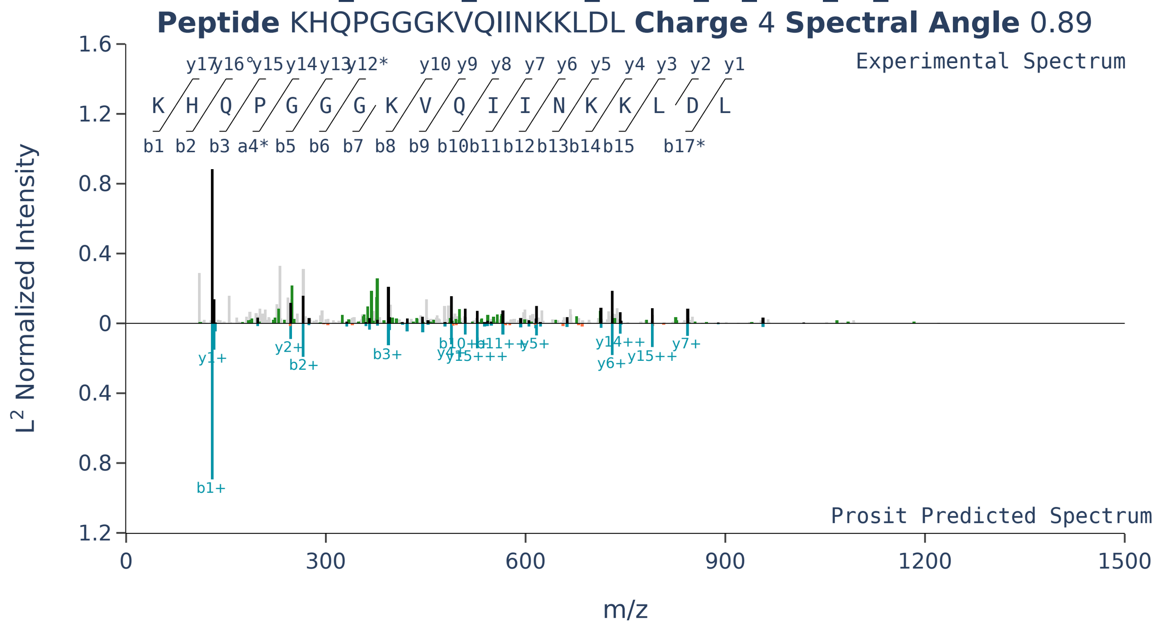


**Source** WSoh_221121_011221_HFGoe_L1_hTau_24h_R1 **Scan** 22358
**Peptide** KSRLQTAPVPMPDLKNV **Charge** 4 **Spectral Angle** 0.87

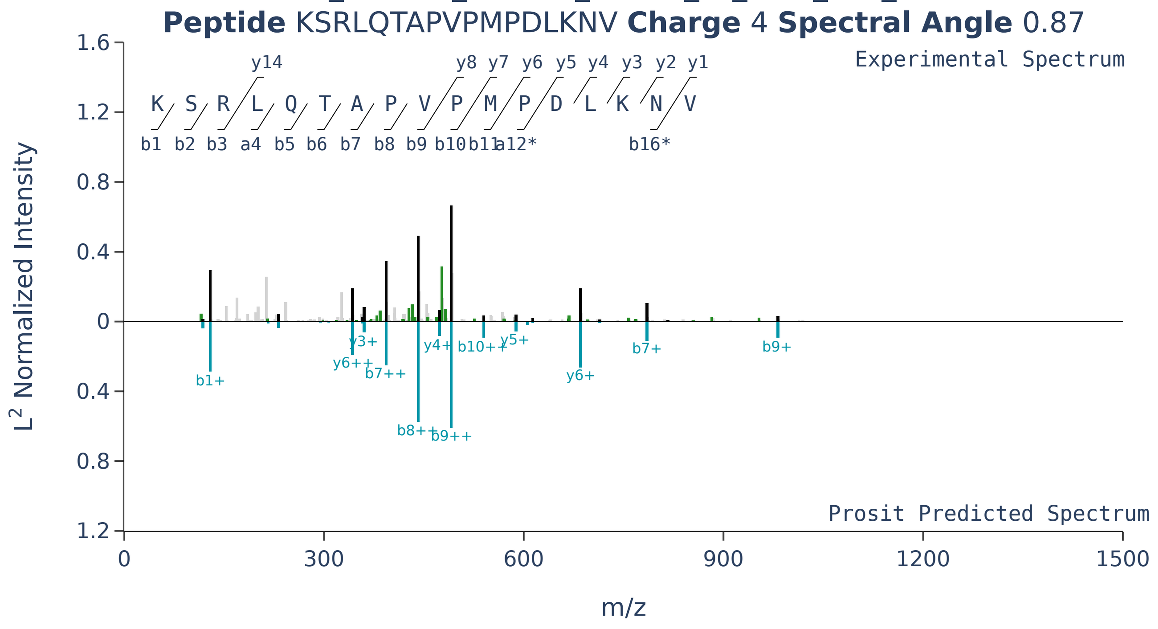


**Source** WSoh_221121_011221_HFGoe_L1_hTau_24h_R1 **Scan** 7287
**Peptide** TFRENAKAKTDHGAEI **Charge** 4 **Spectral Angle** 0.76

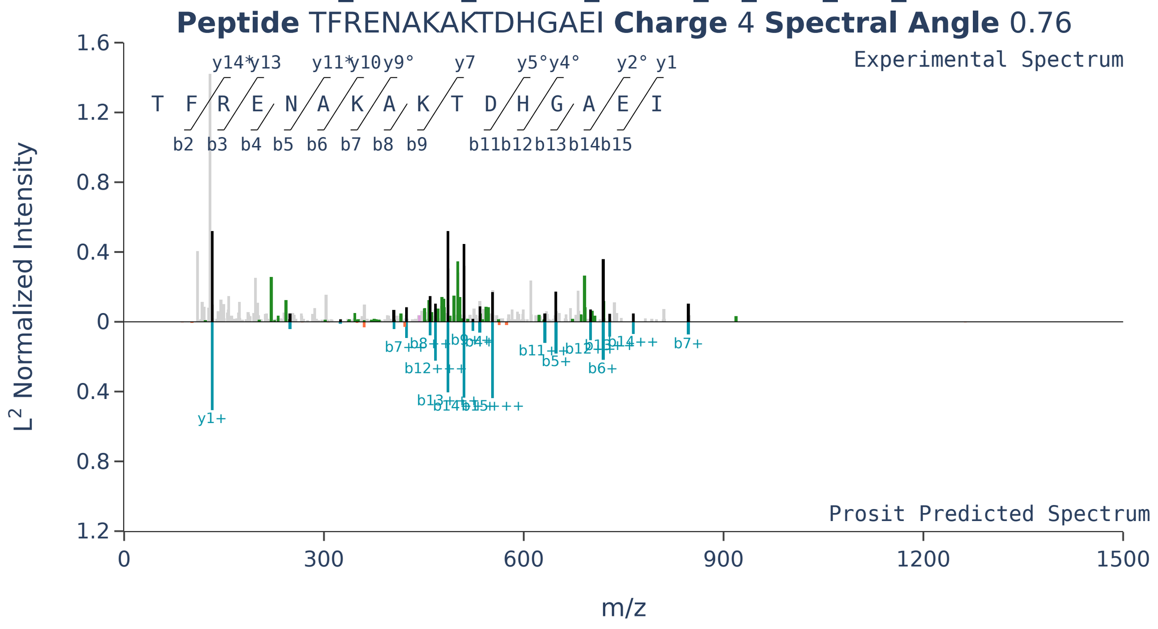


**Source** WSoh_221121_011221_HFGoe_L2_hTau_4h_R2 **Scan** 9673
**Peptide** RLPAKTPPAPKTPPSSGEPPKSGD **Charge** 4 **Spectral Angle** 0.88

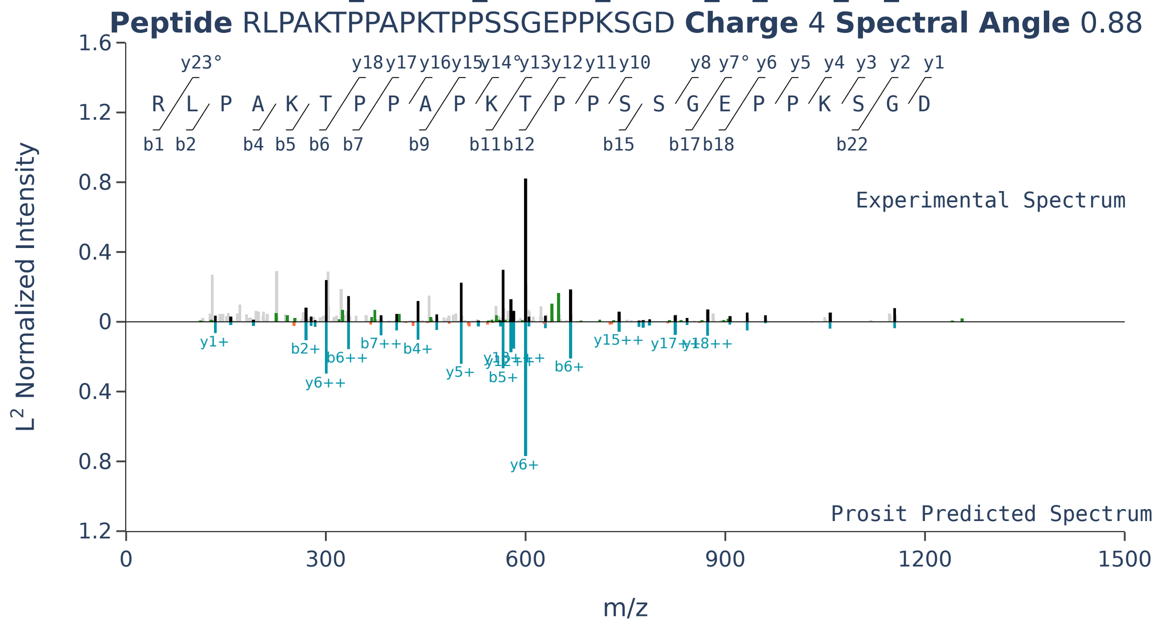


**Source** WSoh_221121_011221_HFGoe_L1_hTau_4h_R2 **Scan** 6963
**Peptide** KIGSTENLKHQPGGGKV **Charge** 2 **Spectral Angle** 0.84

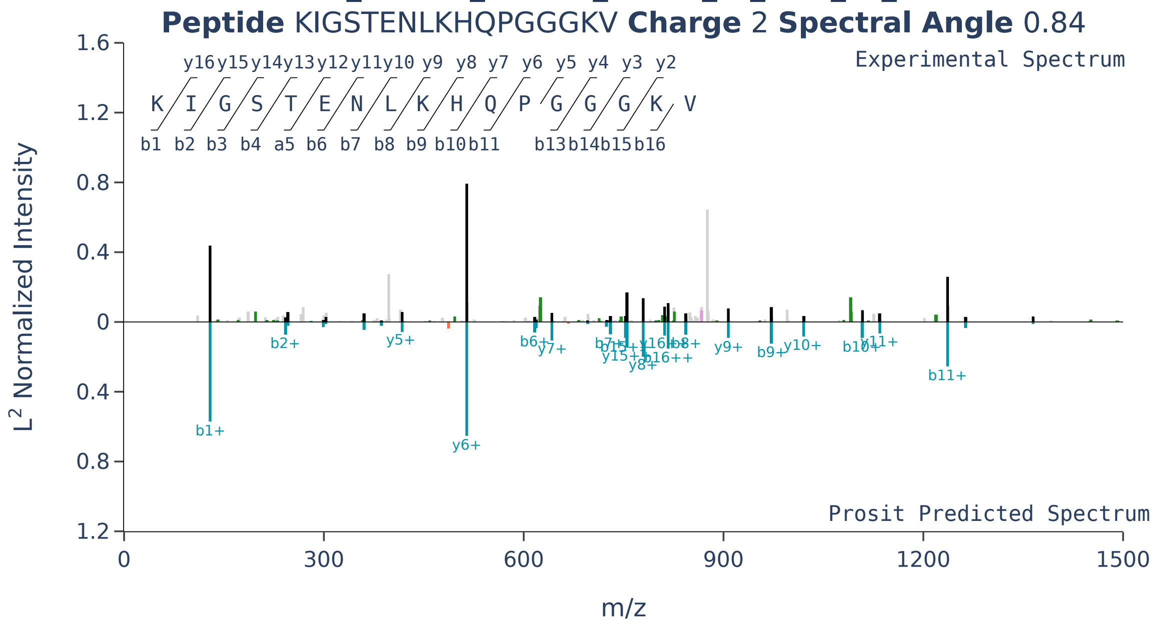


**Source** WSoh_221121_011221_HFGoe_L2_hTau_24h_R2 **Scan** 16592
**Peptide** SRTPSLPTPPTREPKKVAVV **Charge** 4 **Spectral Angle** 0.85

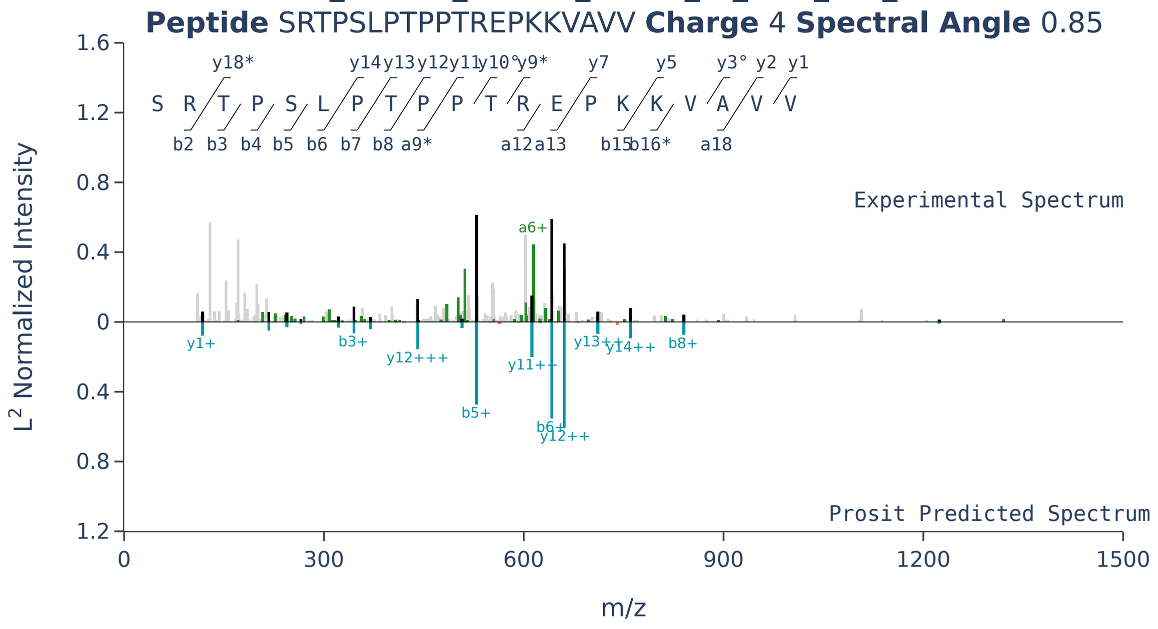


**Source** WSoh_221121_011221_HFGoe_L1_hTau_24h_R1 **Scan** 19413
**Peptide** QDQEGDTDAGLKESPLQTPTE **Charge** 2 **Spectral Angle** 0.9

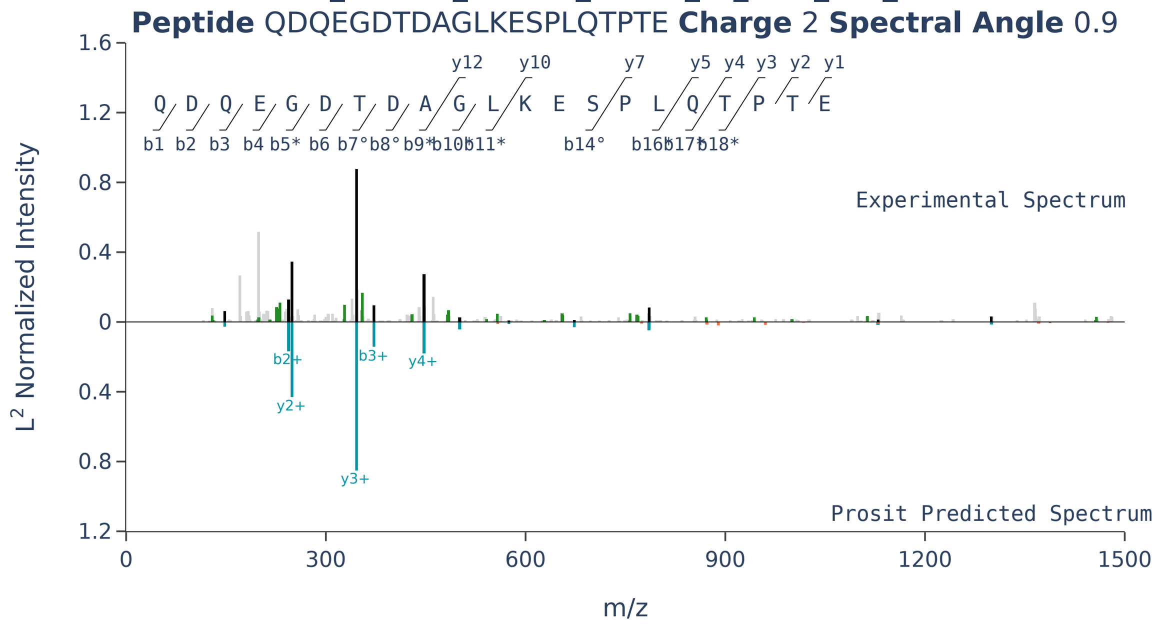

**Source** WSoh_221121_011221_HFGoe_L2_hTau_24h_R2 **Scan** 7530
**Peptide** AVVRTPPKSPS **Charge** 3 **Spectral Angle** 0.88

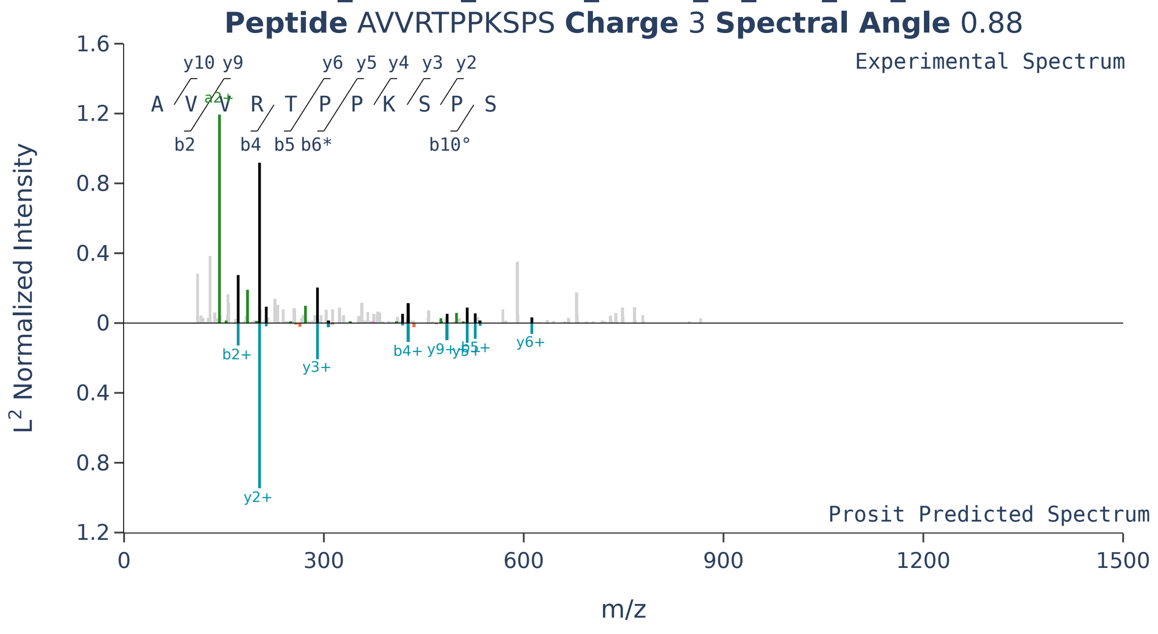


**Source** WSoh_221121_011221_HFGoe_L2_hTau_4h_R2 **Scan** 11512
**Peptide** RSGYSSPGSPGTPGSRSRTPSL **Charge** 4 **Spectral Angle** 0.84

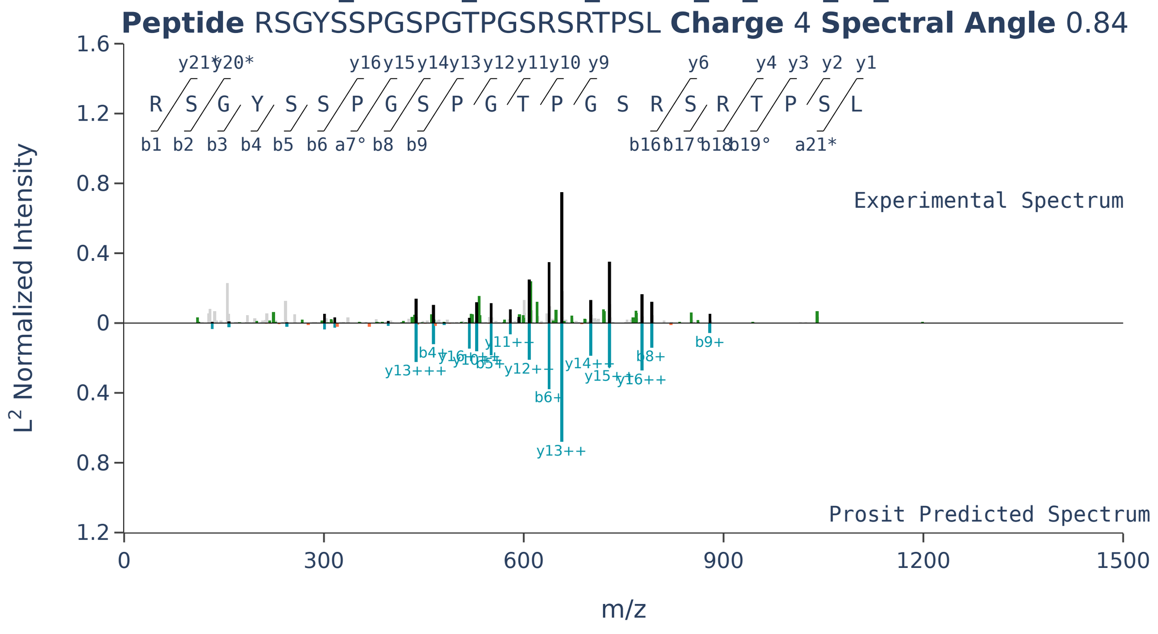


**Source** WSoh_221121_011221_HFGoe_L2_hTau_24h_R1 **Scan** 16799
**Peptide** RLQTAPVPMPDLKNVKS **Charge** 2 **Spectral Angle** 0.85

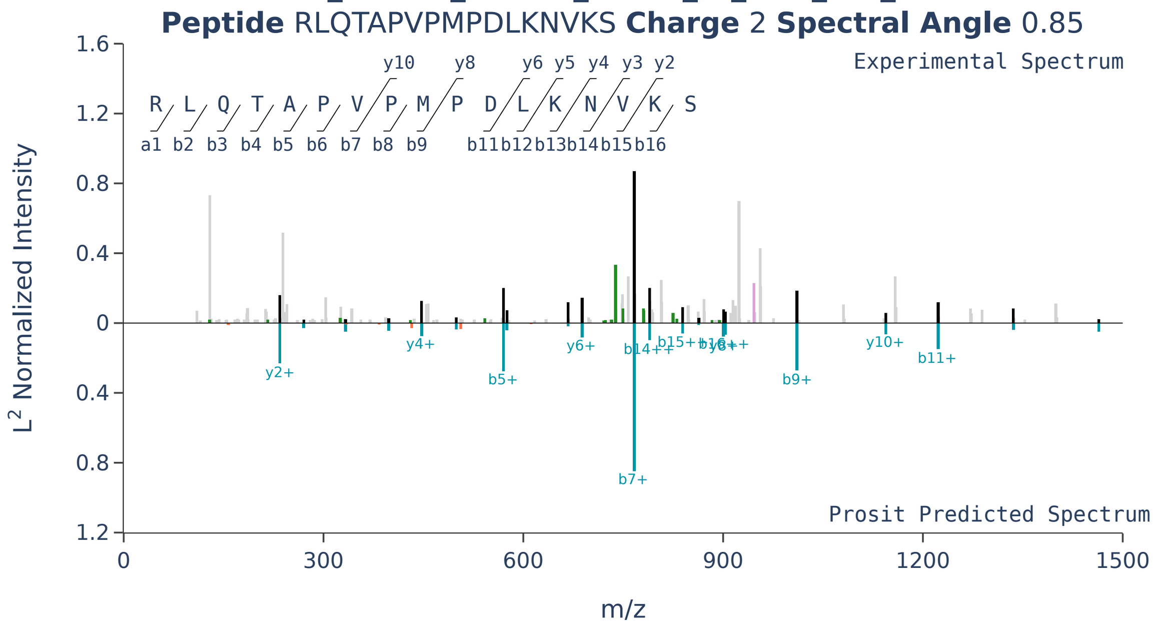


**Source** WSoh_221121_011221_HFGoe_L2_hTau_24h_R2 **Scan** 12155
**Peptide** AGTYGLGDRKDQGGYTMHQD **Charge** 4 **Spectral Angle** 0.85

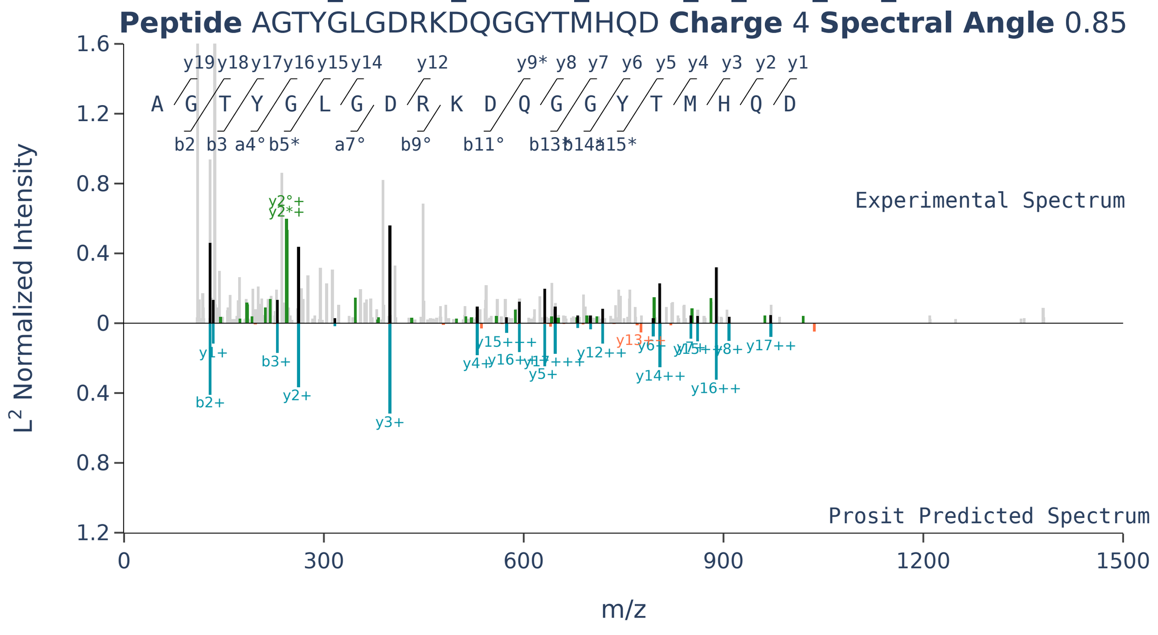


**Source** WSoh_221121_011221_HFGoe_L2_hTau_24h_R1 **Scan** 22704
**Peptide** EAGIGDTPSL **Charge** 1 **Spectral Angle** 0.9

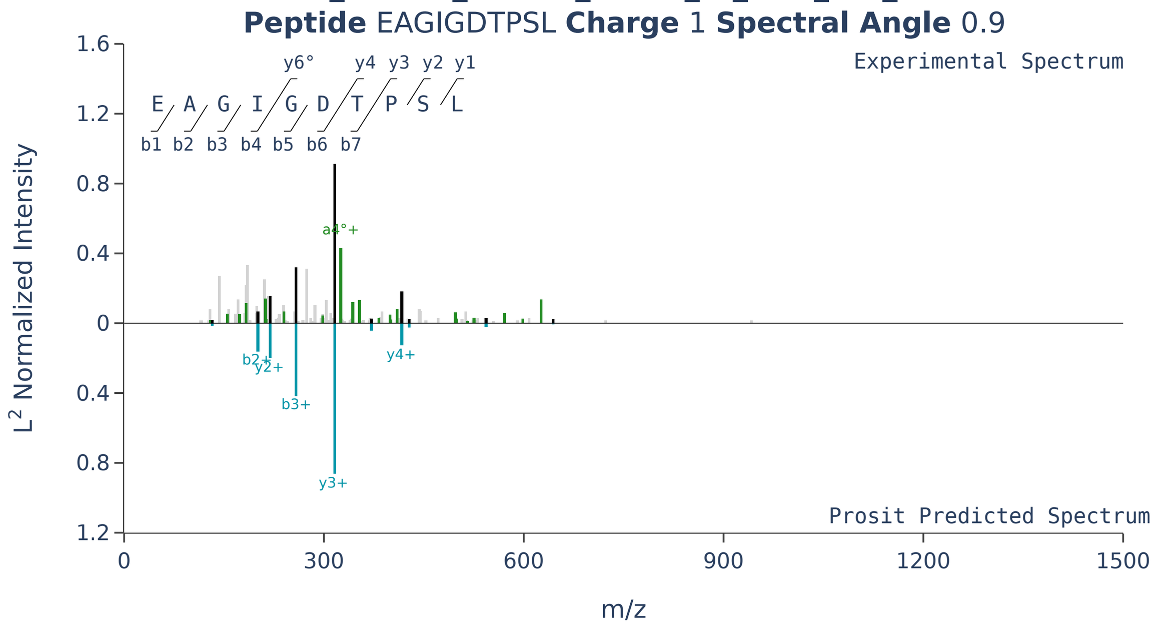


**Source** WSoh_221121_011221_HFGoe_L1_hTau_24h_R2 **Scan** 13569
**Peptide** HQPGGGKVQII **Charge** 3 **Spectral Angle** 0.92

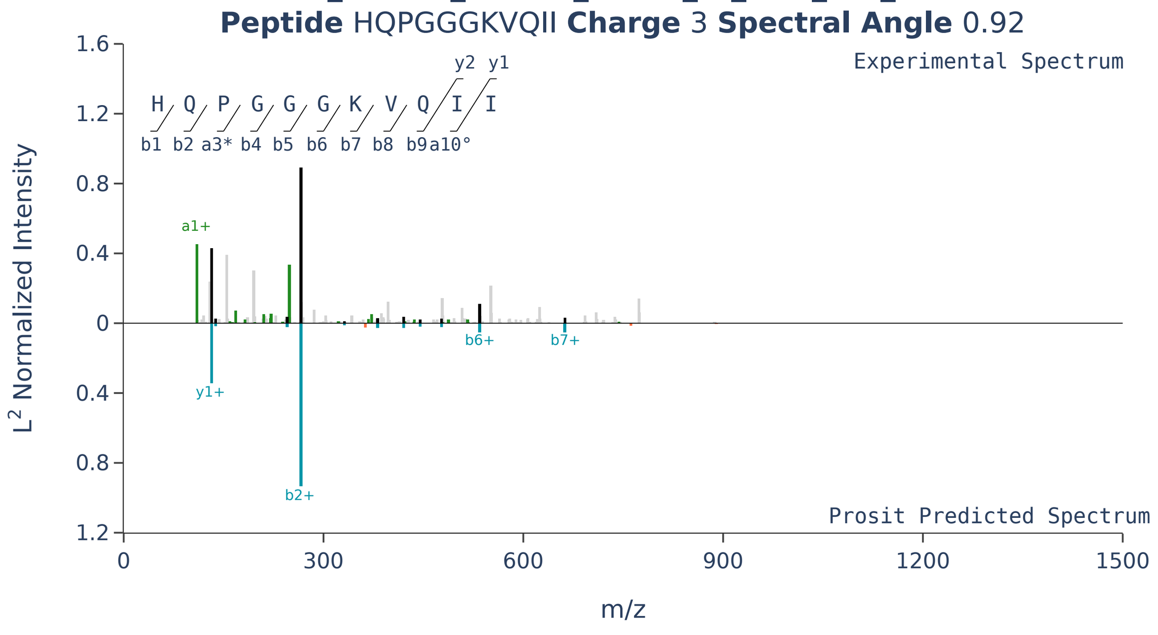


**Source** WSoh_221121_011221_HFGoe_L1_hTau_4h_R2 **Scan** 18438
**Peptide** GSLDNITHVPGGGNKKIETHKLTF **Charge** 4 **Spectral Angle** 0.81

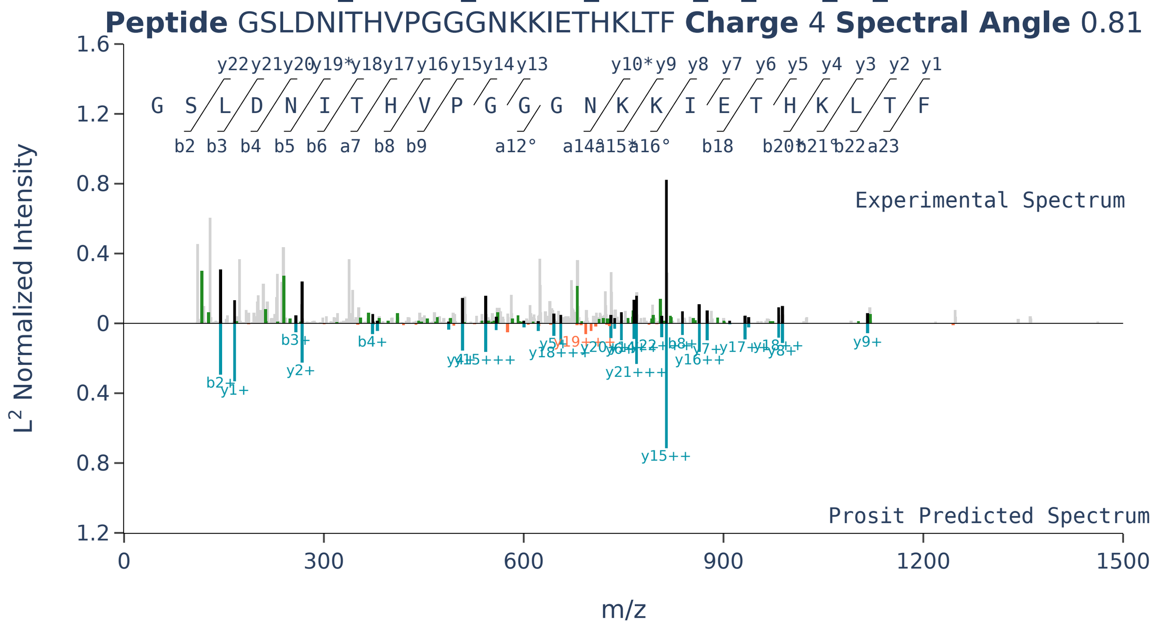


**Source** WSoh_221121_011221_HFGoe_L1_hTau_24h_R1 **Scan** 10865
**Peptide** KPGGGQVEVKSEKLDFKDRVQSK **Charge** 4 **Spectral Angle** 0.87

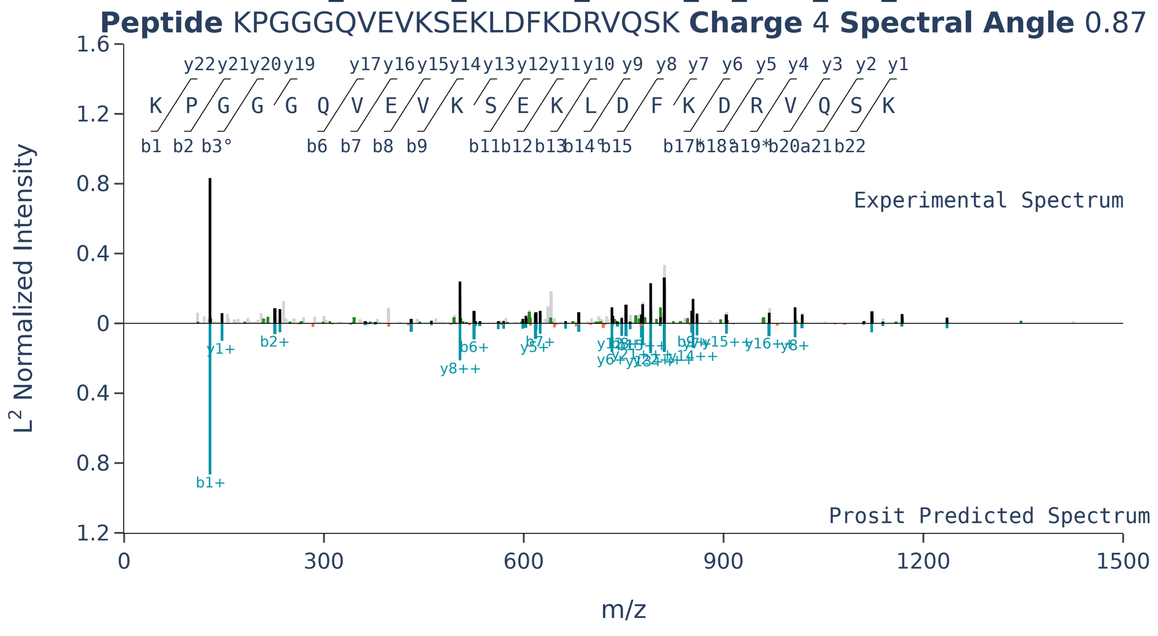


**Source** WSoh_221121_011221_HFGoe_L2_hTau_2h_R1 **Scan** 15595
**Peptide** KIGSLDNITHVPGGGNKKIET **Charge** 3 **Spectral Angle** 0.86

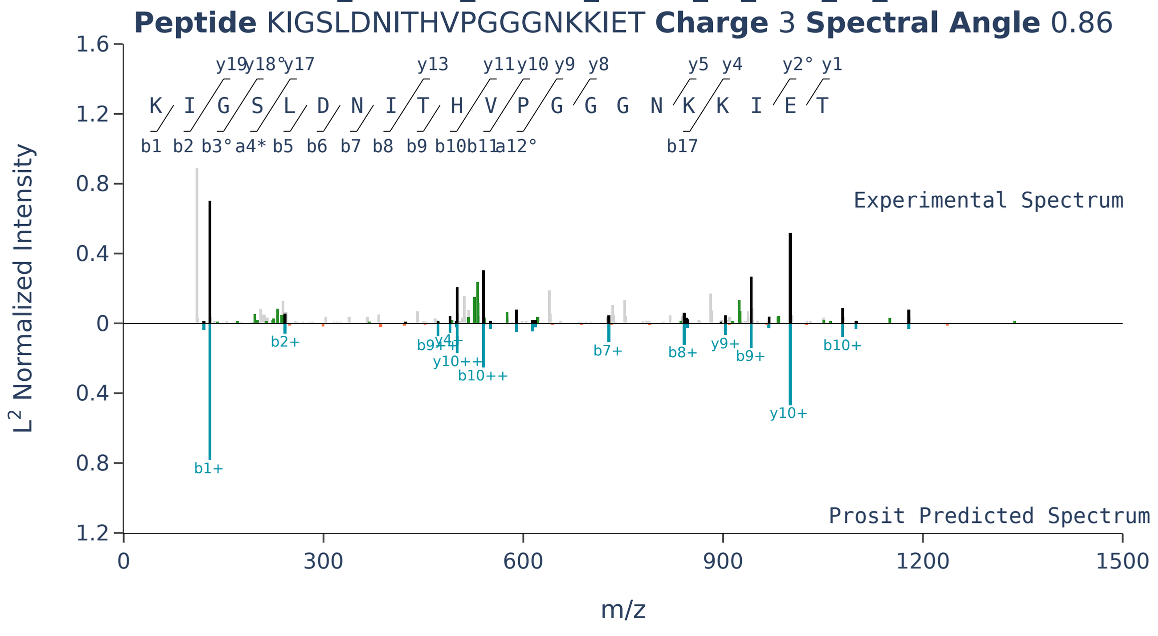


**Source** WSoh_221121_011221_HFGoe_L2_hTau_24h_R2 **Scan** 8004
**Peptide** PAKTPPAPKTPPSS **Charge** 2 **Spectral Angle** 0.86

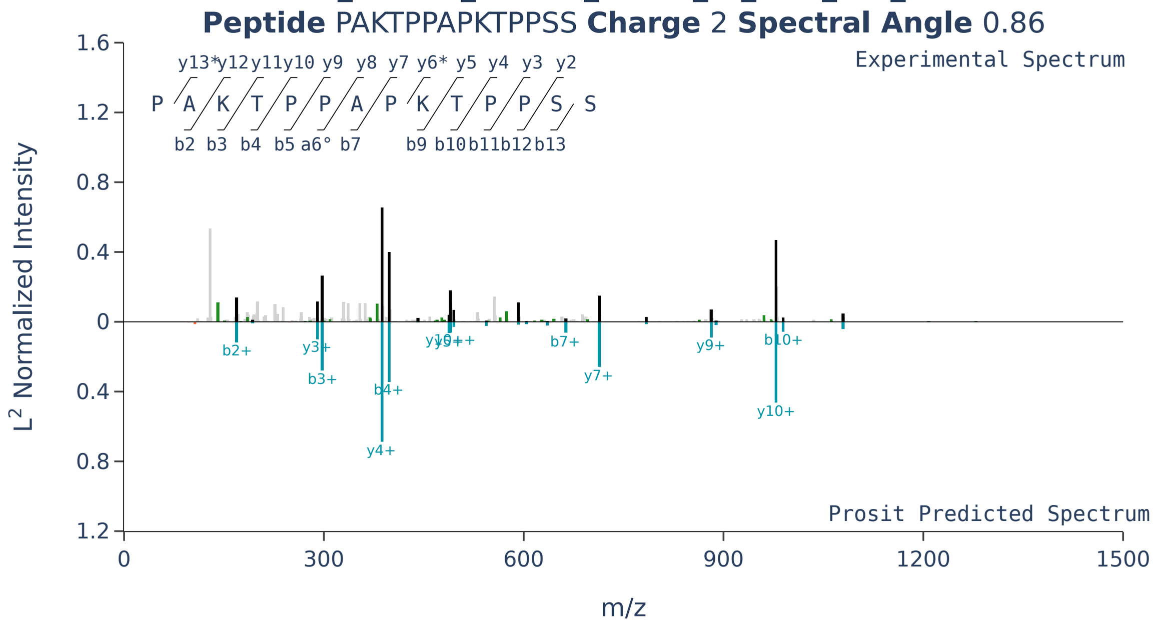


**Source** WSoh_221121_011221_HFGoe_L2_hTau_24h_R2 **Scan** 17133
**Peptide** GDTPSLEDEAAGHVTQ **Charge** 2 **Spectral Angle** 0.85

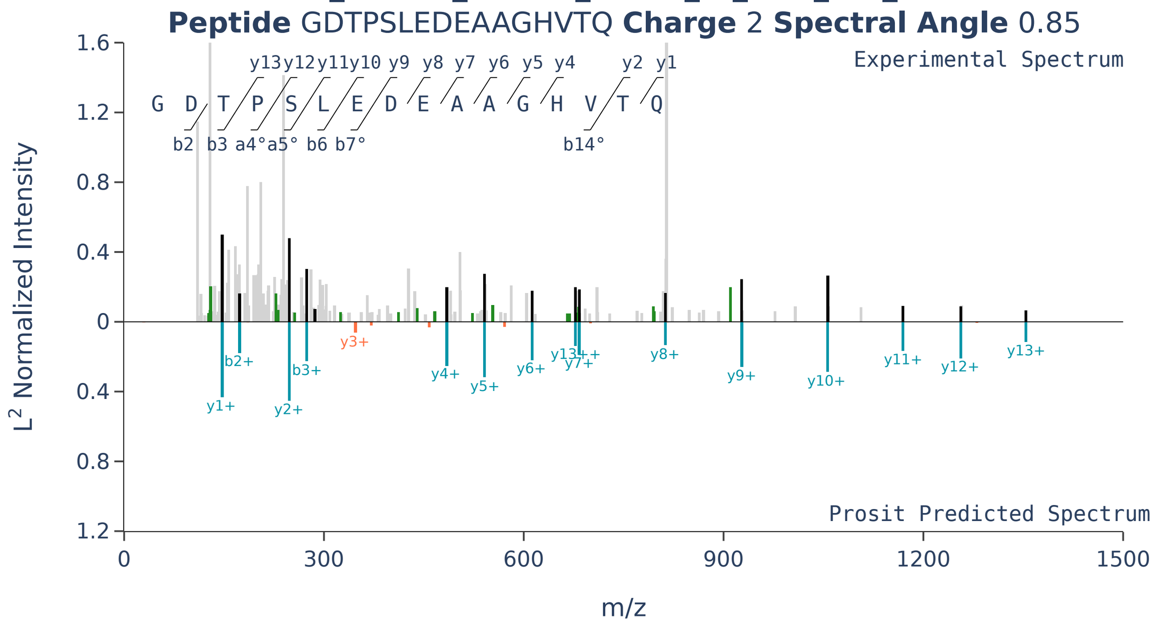


**Source** WSoh_221121_011221_HFGoe_L2_hTau_4h_R2 **Scan** 14667
**Peptide** SLDNITHVPGGGNKKL **Charge** 3 **Spectral Angle** 0.86

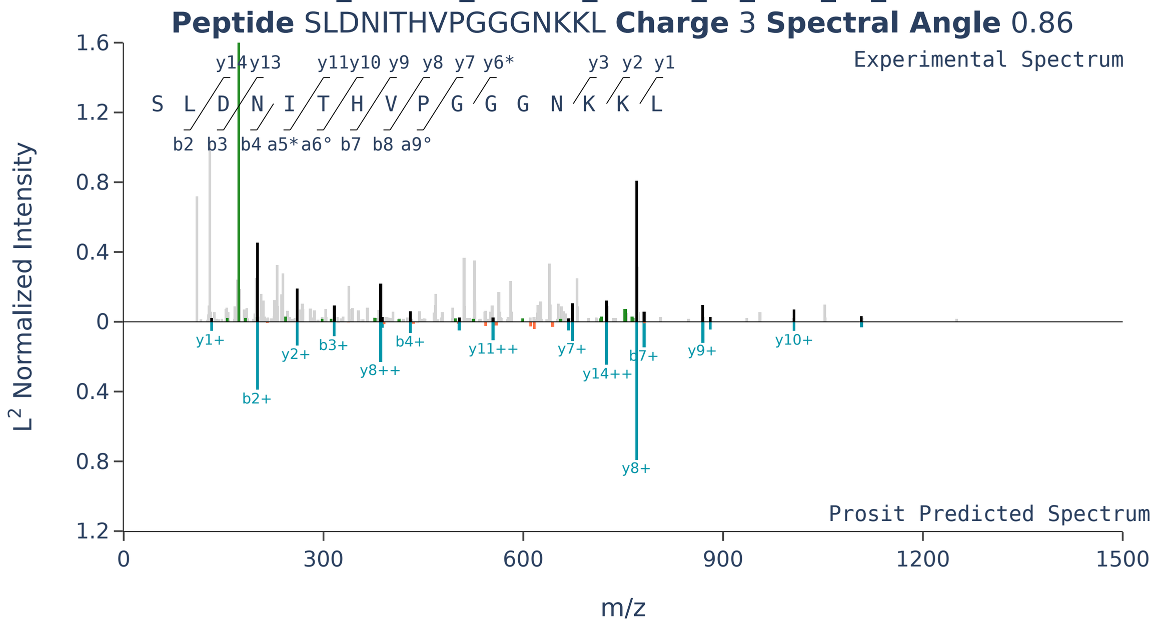


**Source** WSoh_221121_011221_HFGoe_L1_hTau_24h_R1 **Scan** 15482
**Peptide** TPSLPTPPTREPKKVAVVRT **Charge** 4 **Spectral Angle** 0.8

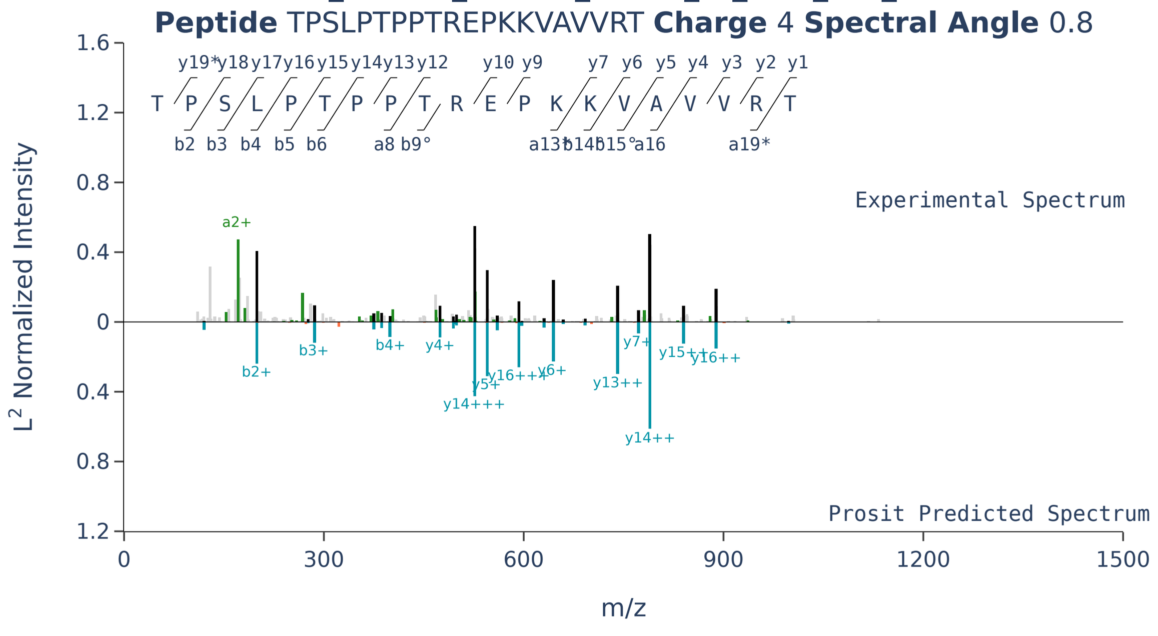


**Source** WSoh_221121_011221_HFGoe_L2_hTau_24h_R1 **Scan** 22136
**Peptide** EEAGIGDTPSLEDEAAGHVTQA **Charge** 3 **Spectral Angle** 0.73

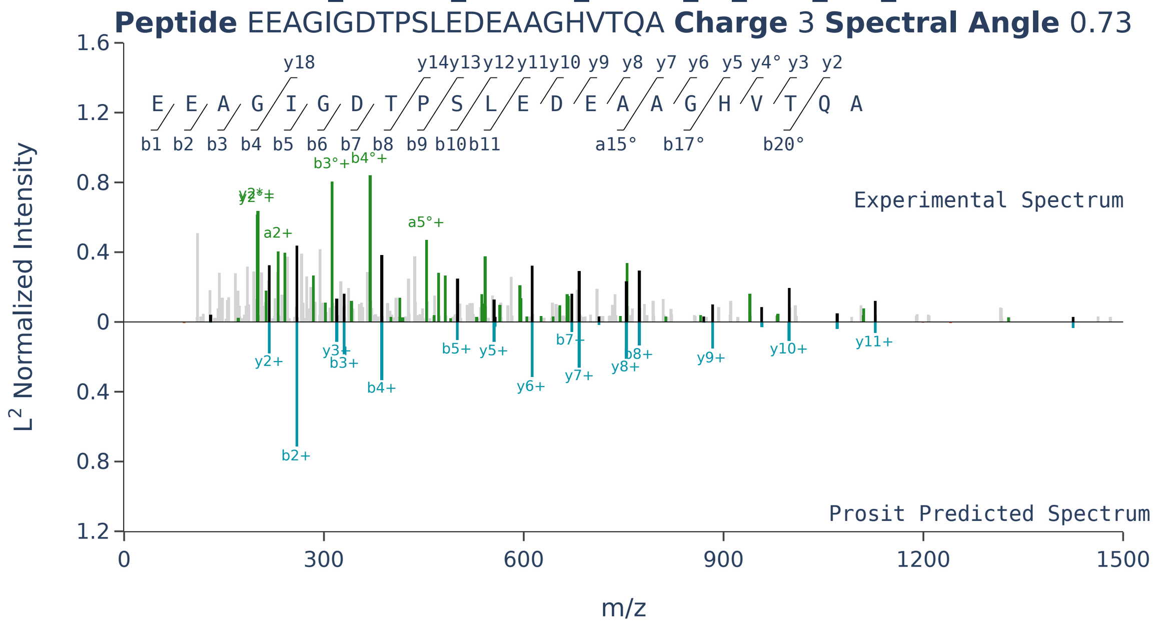


**Source** WSoh_221121_011221_HFGoe_L1_hTau_24h_R2 **Scan** 16528
**Peptide** KSEKLDFKDRVQSKIGSLDN **Charge** 3 **Spectral Angle** 0.89

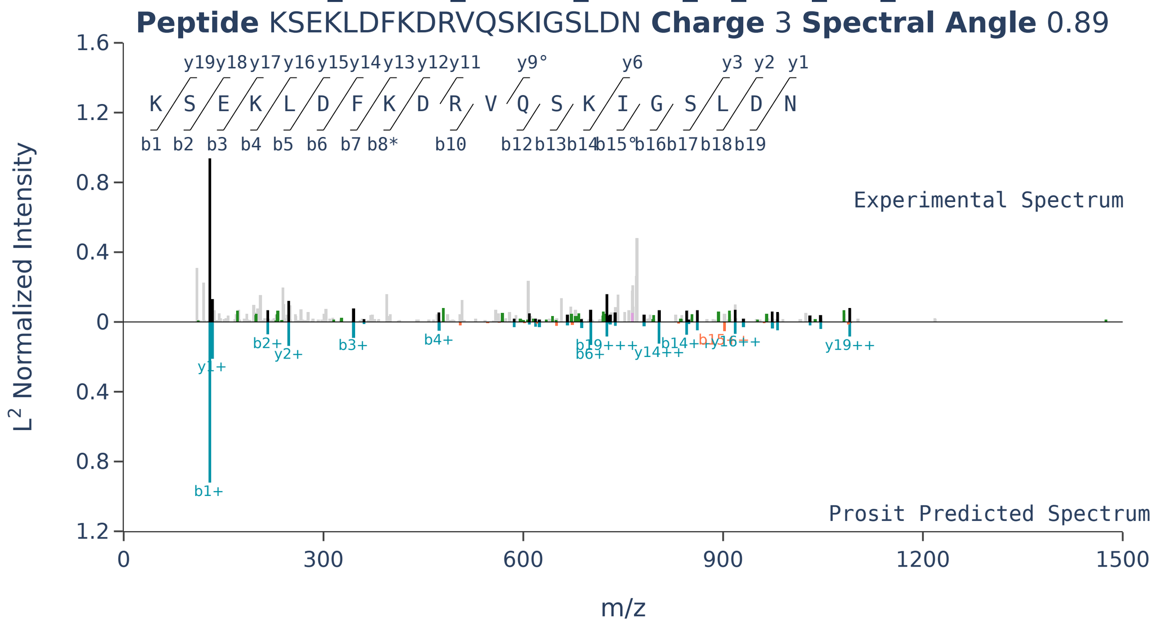

**Source** WSoh_221121_011221_HFGoe_L2_hTau_4h_R1 **Scan** 16697
**Peptide** PGGGKVQII **Charge** 2 **Spectral Angle** 0.76

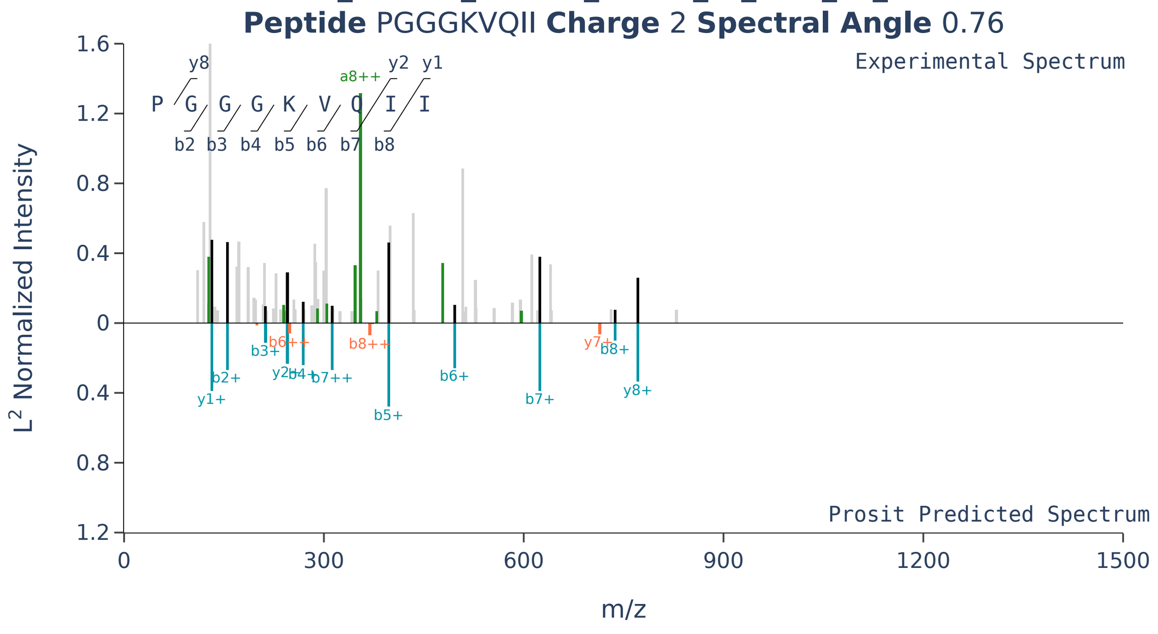


**Source** WSoh_221121_011221_HFGoe_L1_hTau_4h_R2 **Scan** 17789
**Peptide** KLDFKDRVQSKIGSLD **Charge** 3 **Spectral Angle** 0.83

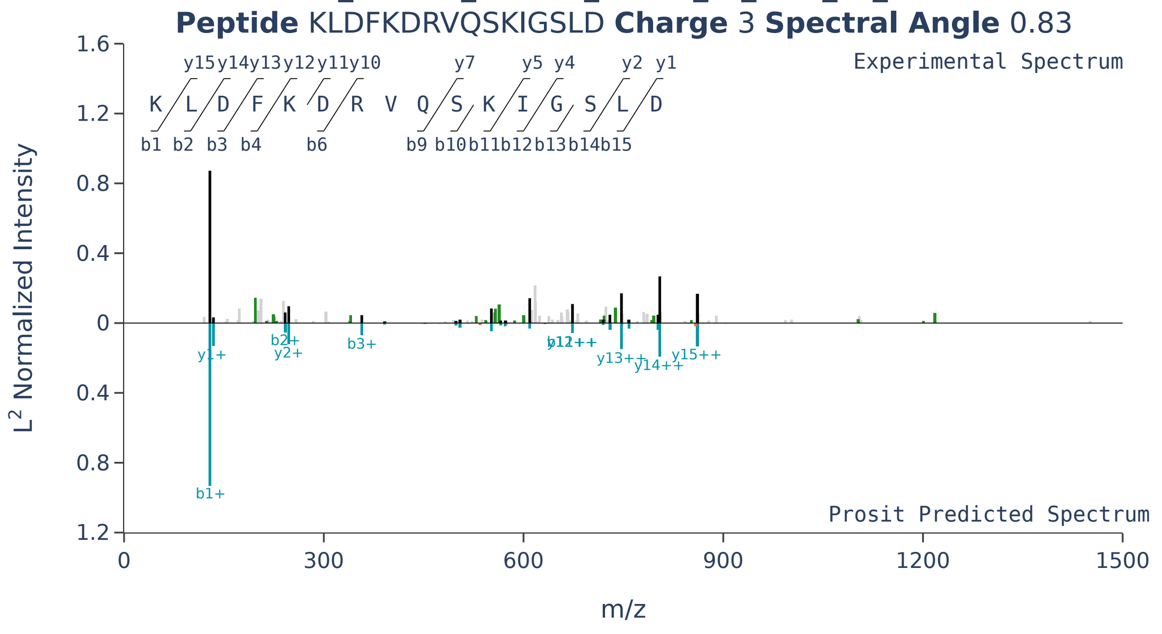


**Source** WSoh_221121_011221_HFGoe_L2_hTau_24h_R2 **Scan** 9199
**Peptide** STENLKHQPGGGKVQIINK **Charge** 3 **Spectral Angle** 0.86

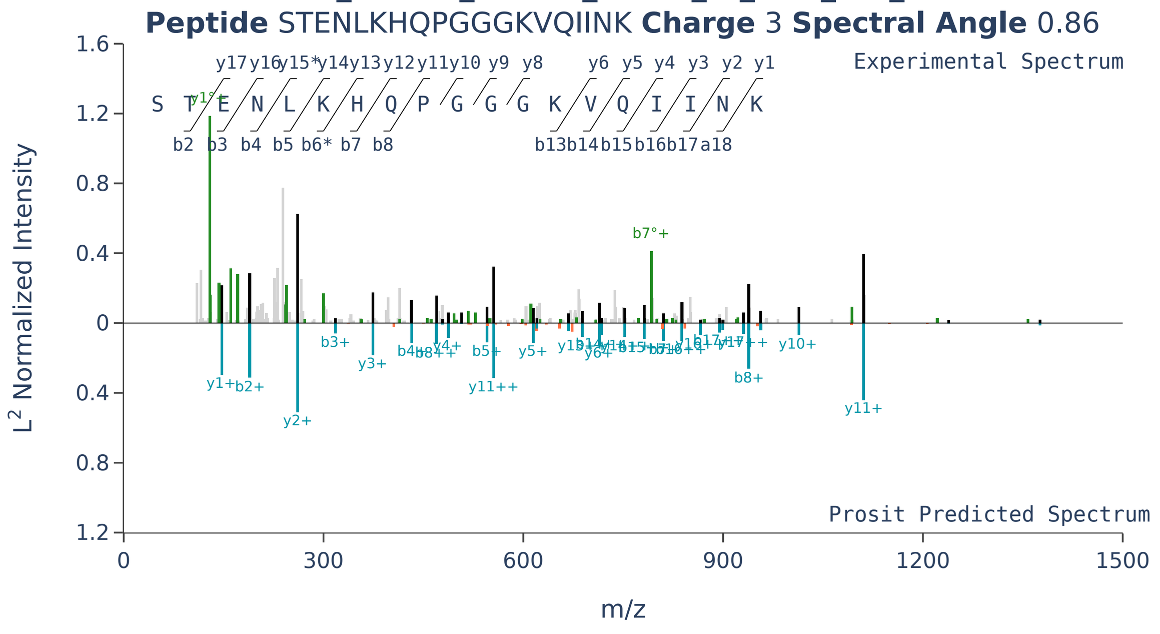


**Source** WSoh_221121_011221_HFGoe_L2_hTau_24h_R2 **Scan** 12669
**Peptide** KAKTDHGAEIVYKSPVVSGDTSPRHLSN **Charge** 4 **Spectral Angle** 0.83

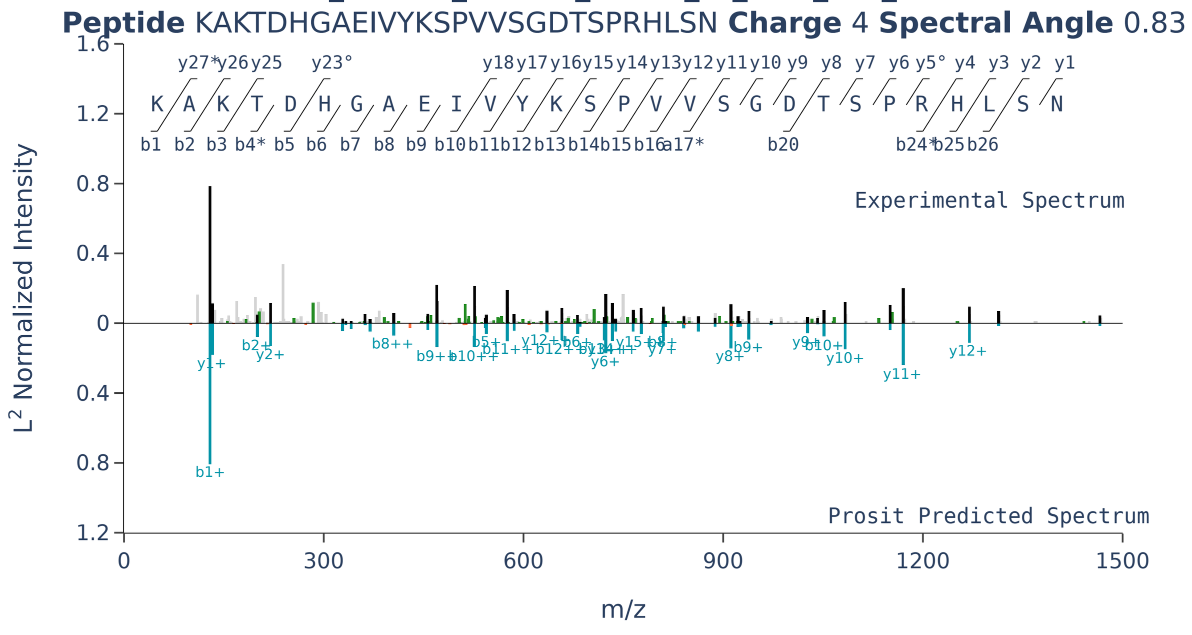


**Source** WSoh_221121_011221_HFGoe_L2_hTau_24h_R2 **Scan** 10205
**Peptide** KHQPGGGKVQIL **Charge** 3 **Spectral Angle** 0.89

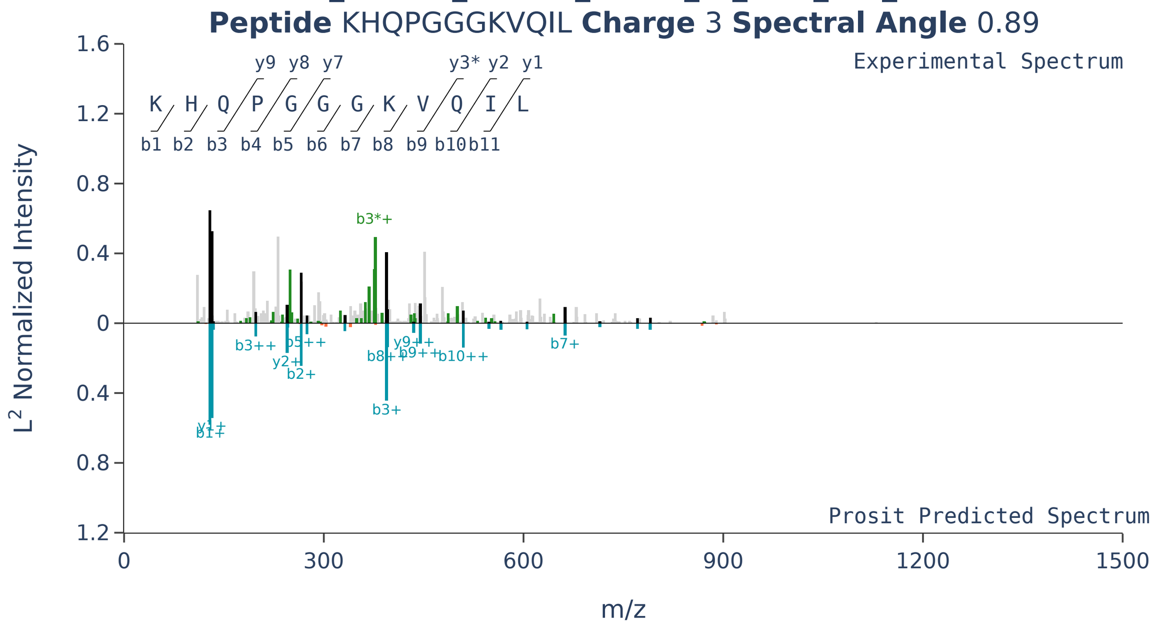


**Source** WSoh_221121_011221_HFGoe_L1_hTau_4h_R2 **Scan** 7414
**Peptide** AVVRTPPKSPSS **Charge** 2 **Spectral Angle** 0.89

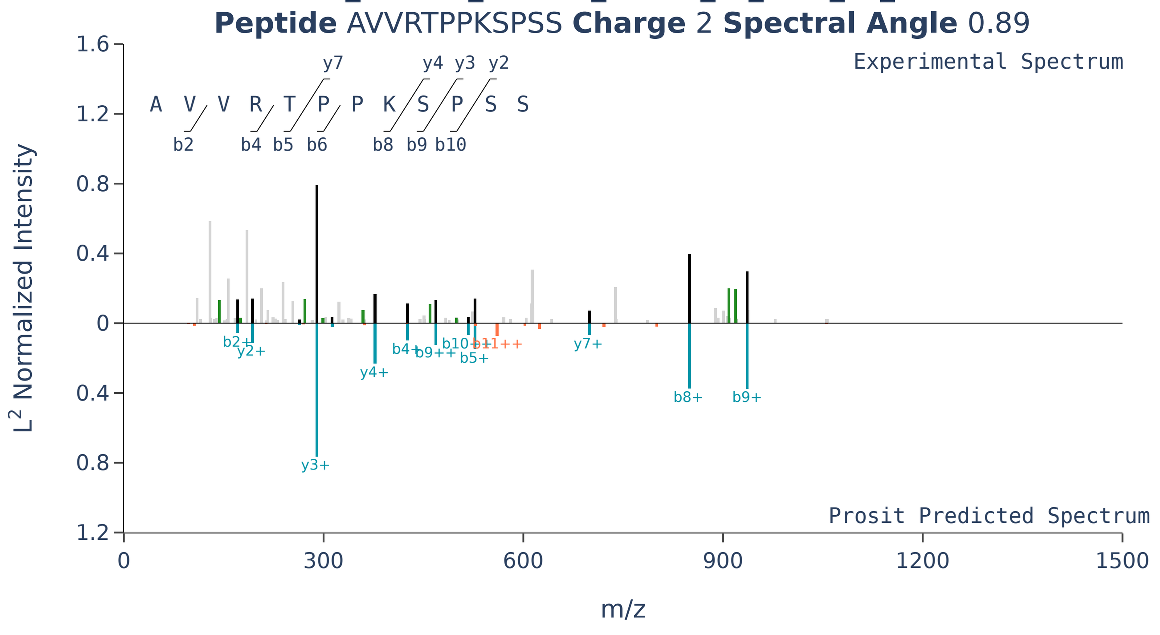


**Source** WSoh_221121_011221_HFGoe_L2_hTau_24h_R1 **Scan** 8039
**Peptide** LTFRENAKAKTD **Charge** 2 **Spectral Angle** 0.72

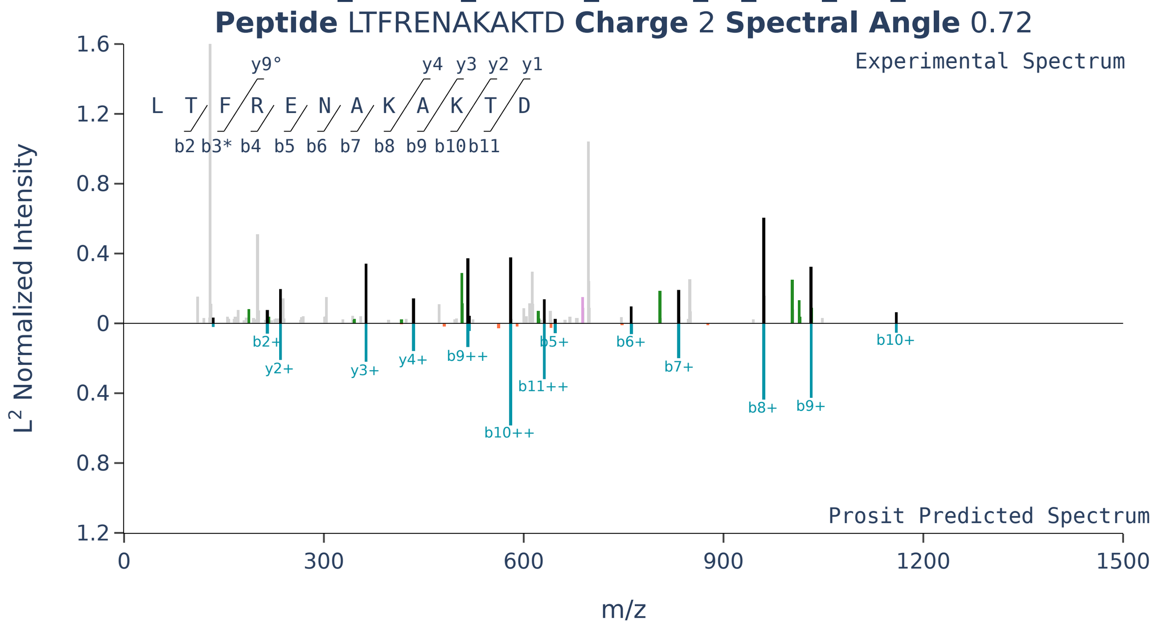


**Source** WSoh_221121_011221_HFGoe_L2_hTau_24h_R1 **Scan** 6600
**Peptide** EPPKSGDRSGYSSPGSPGTPGSRSR **Charge** 4 **Spectral Angle** 0.88

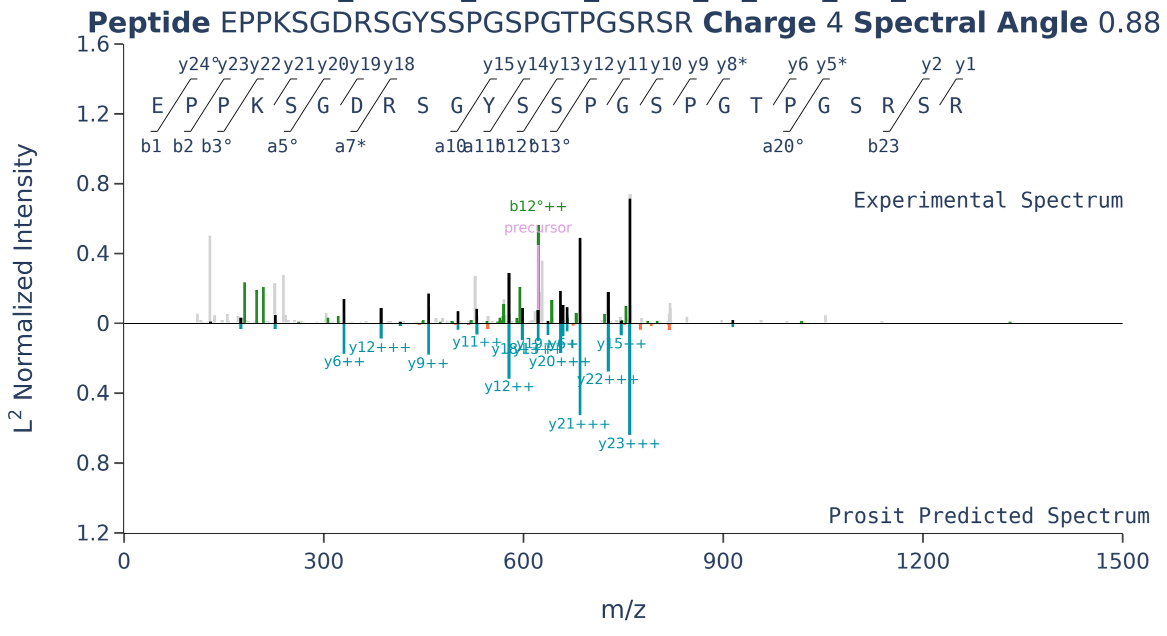


**Source** WSoh_221121_011221_HFGoe_L2_hTau_24h_R2 **Scan** 15832
**Peptide** KDRVQSKIGSLDNI **Charge** 3 **Spectral Angle** 0.8

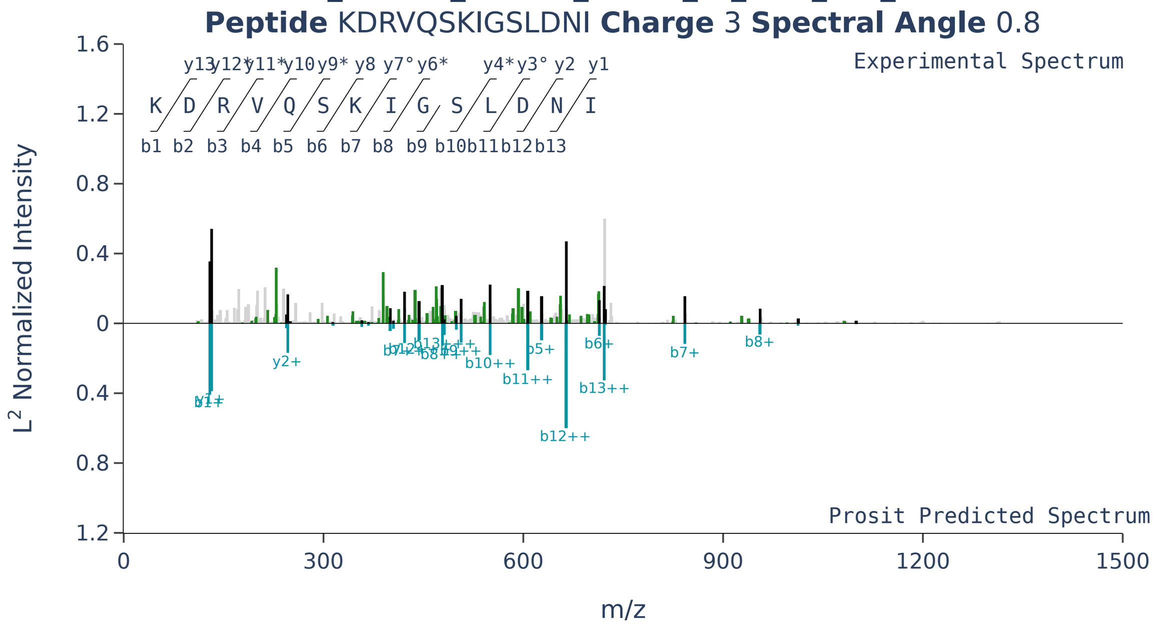


**Source** WSoh_221121_011221_HFGoe_L1_hTau_24h_R2 **Scan** 8907
**Peptide** GLGDRKDQGGYTMHQDQEGD **Charge** 2 **Spectral Angle** 0.76

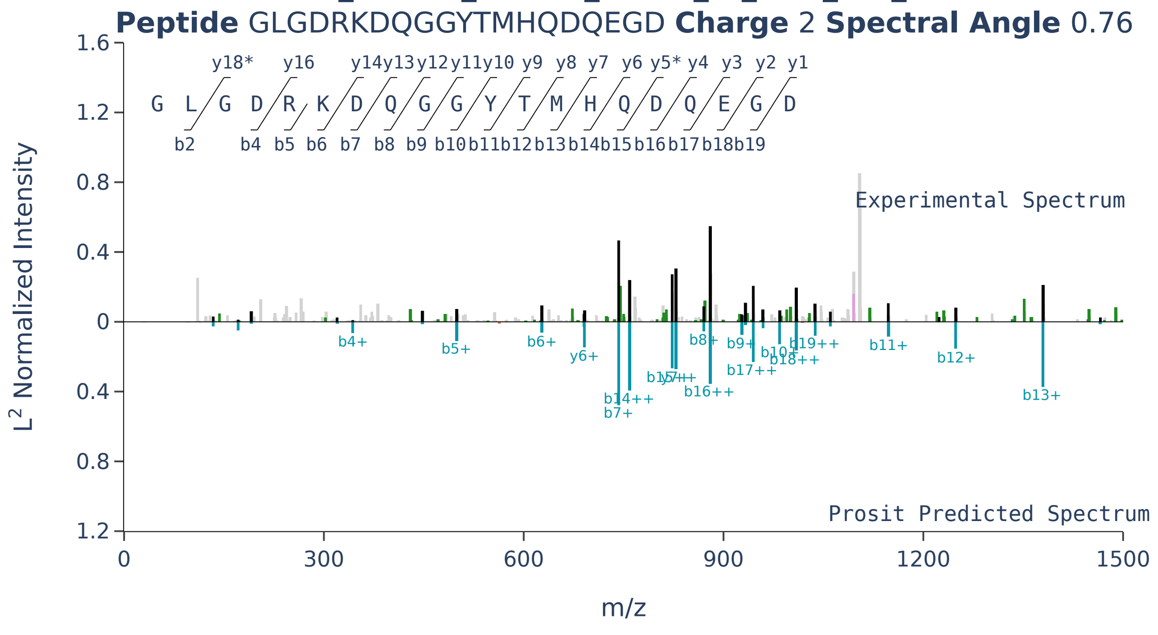


**Source** WSoh_221121_011221_HFGoe_L2_hTau_4h_R2 **Scan** 15510
**Peptide** GSLDNITHVPGGGNKKIET **Charge** 3 **Spectral Angle** 0.83

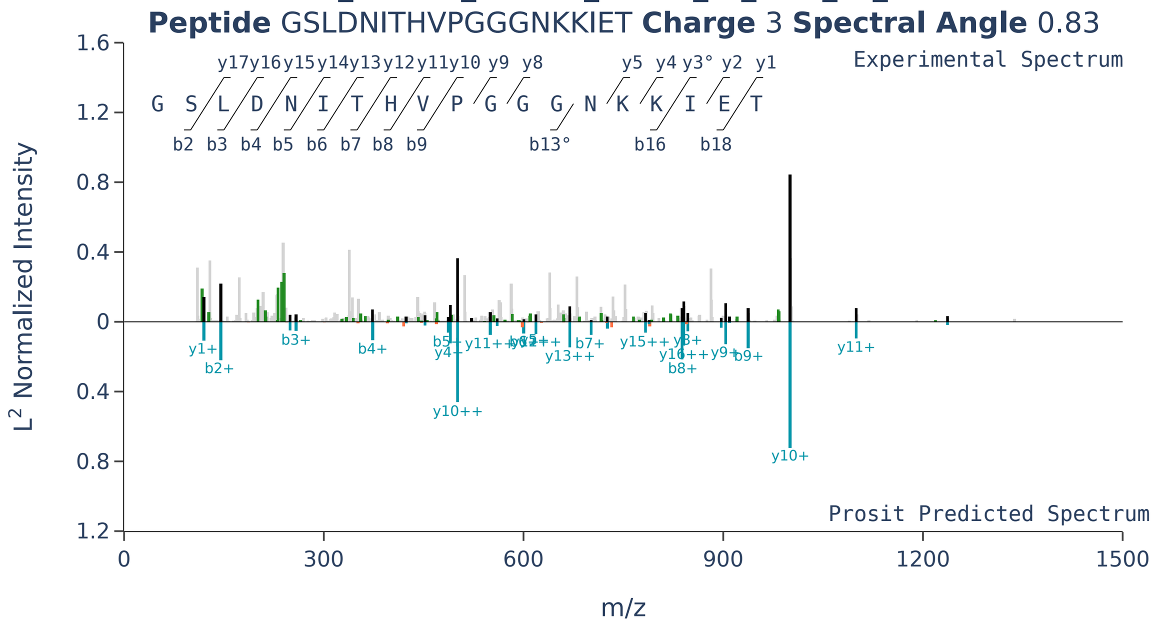


**Source** WSoh_221121_011221_HFGoe_L1_hTau_24h_R2 **Scan** 7140
**Peptide** KHQPGGGKVQL **Charge** 2 **Spectral Angle** 0.78

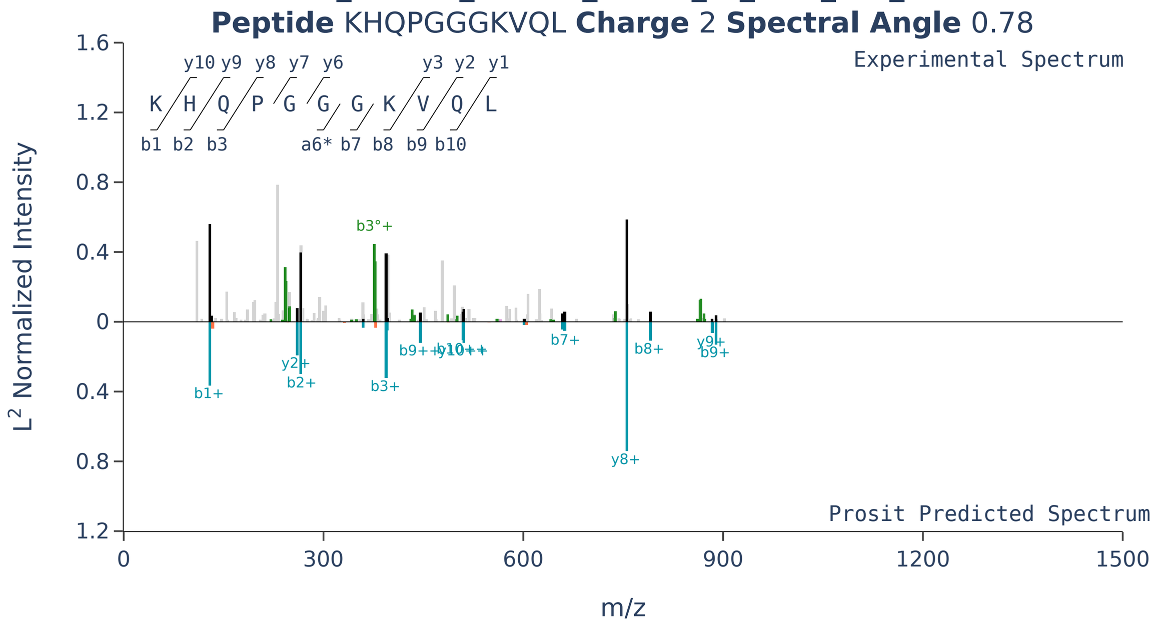


**Source** WSoh_221121_011221_HFGoe_L2_hTau_4h_R2 **Scan** 7303
**Peptide** GYSSPGSPGTPGSRSR **Charge** 2 **Spectral Angle** 0.8

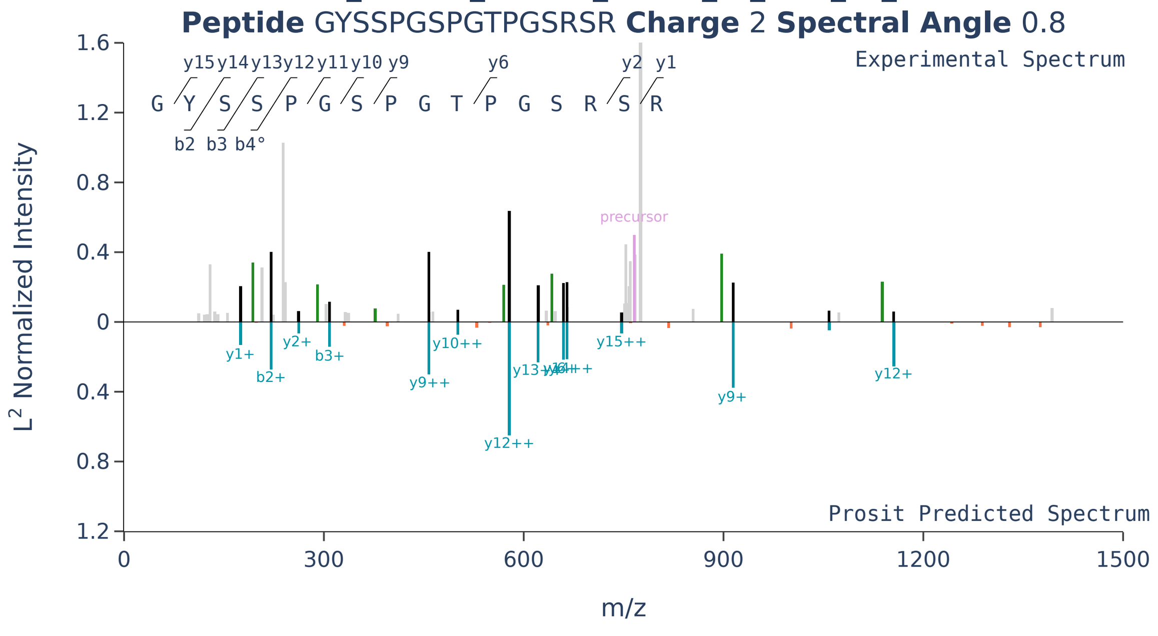


**Source** WSoh_221121_011221_HFGoe_L1_hTau_24h_R2 **Scan** 12373
**Peptide** SNVQSKCGSKDNIKHVPGGGSVQIVYK **Charge** 4 **Spectral Angle** 0.82

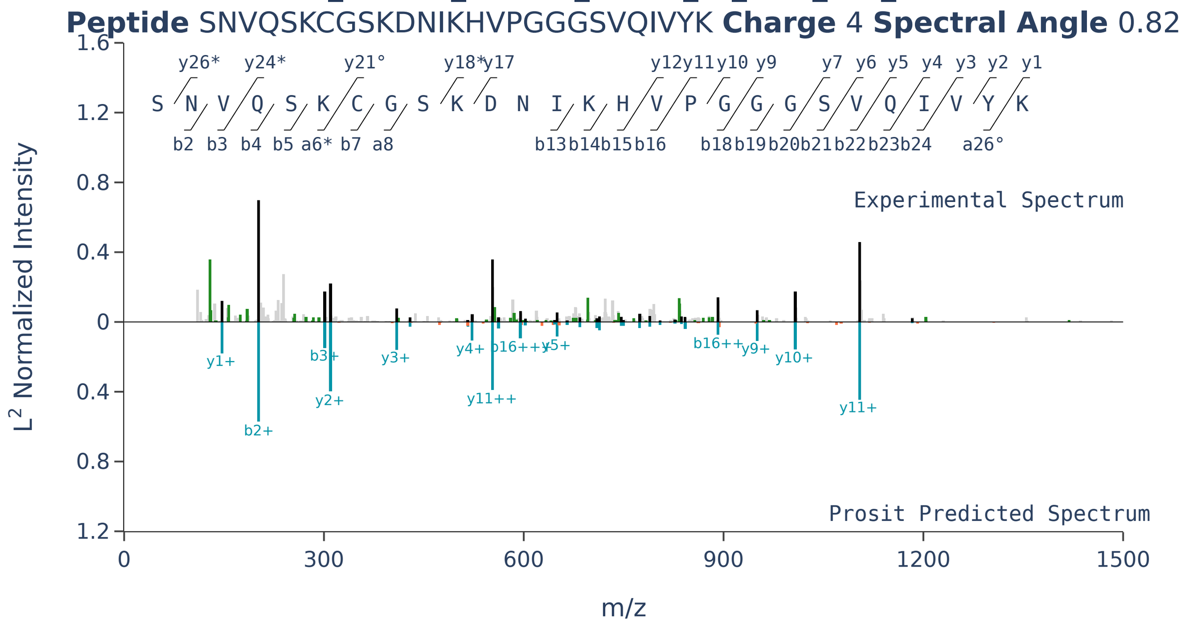


**Source** WSoh_221121_011221_HFGoe_L2_hTau_4h_R2 **Scan** 14461
**Peptide** SKIGSLDNITHVPGGGNKKIETH **Charge** 4 **Spectral Angle** 0.85

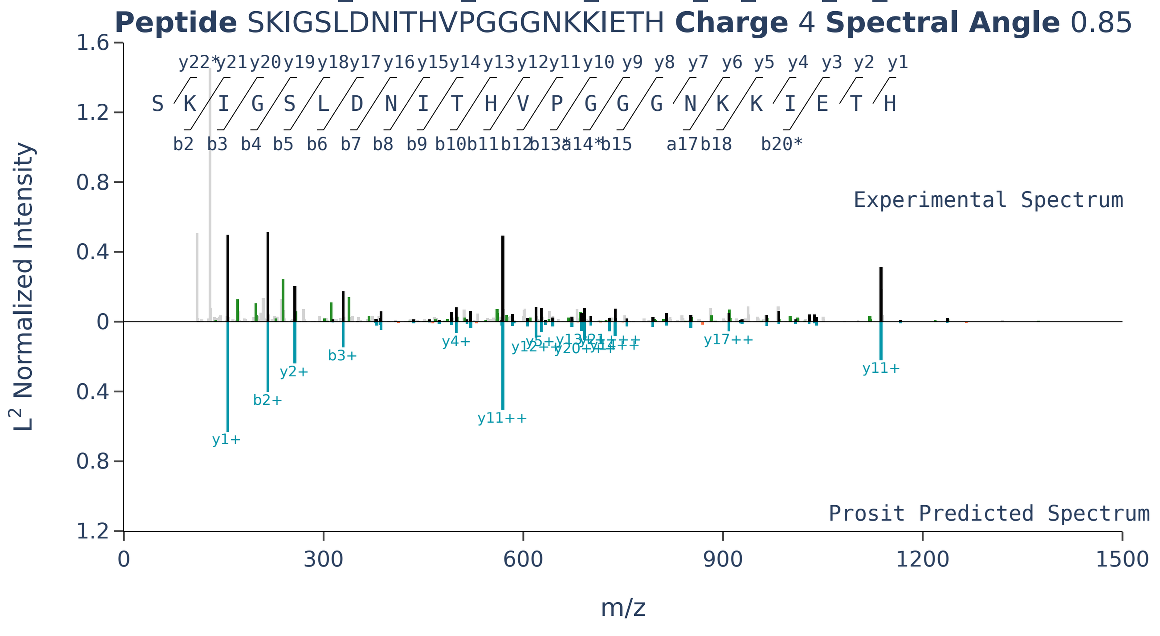

**Source** WSoh_221121_011221_HFGoe_L2_hTau_4h_R1 **Scan** 18190
**Peptide** PGGGQVEVKSEKLDF **Charge** 3 **Spectral Angle** 0.79

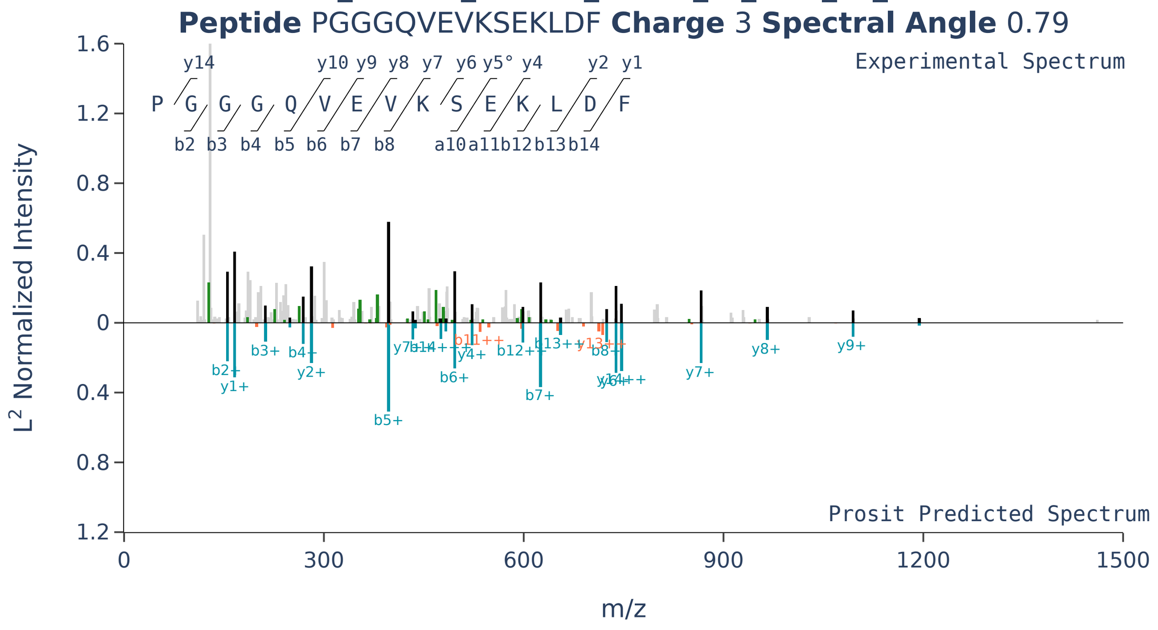


**Source** WSoh_221121_011221_HFGoe_L2_hTau_4h_R1 **Scan** 5719
**Peptide** TPRGAAPPGQKGQA **Charge** 3 **Spectral Angle** 0.91

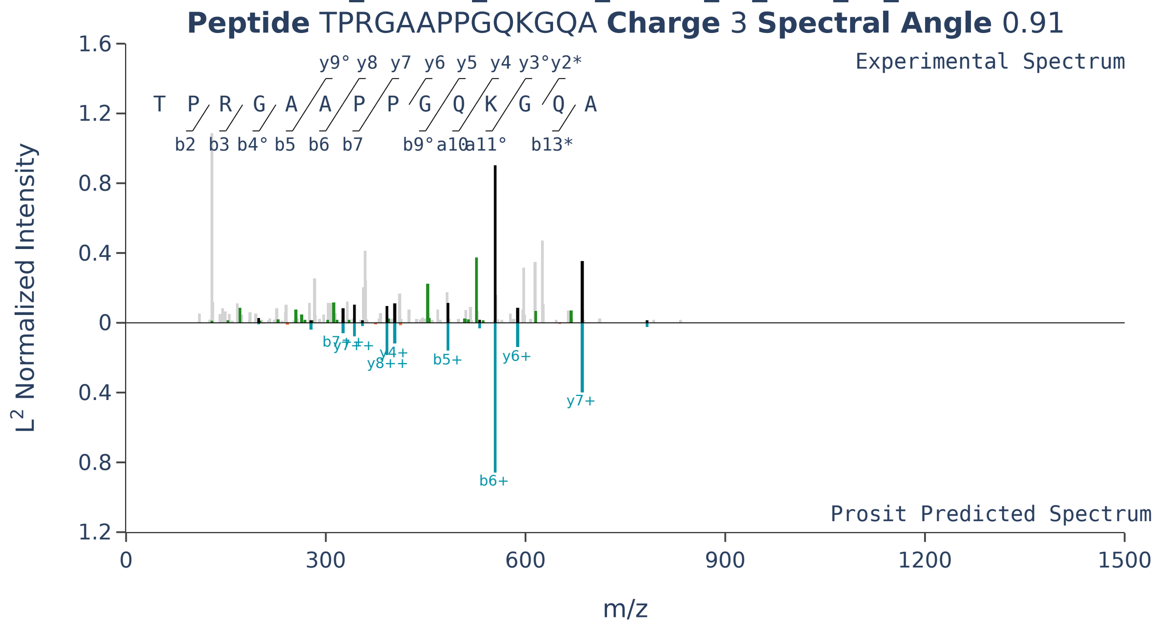


**Source** WSoh_221121_011221_HFGoe_L2_hTau_24h_R1 **Scan** 22756
**Peptide** RLQTAPVPMPDL **Charge** 2 **Spectral Angle** 0.92

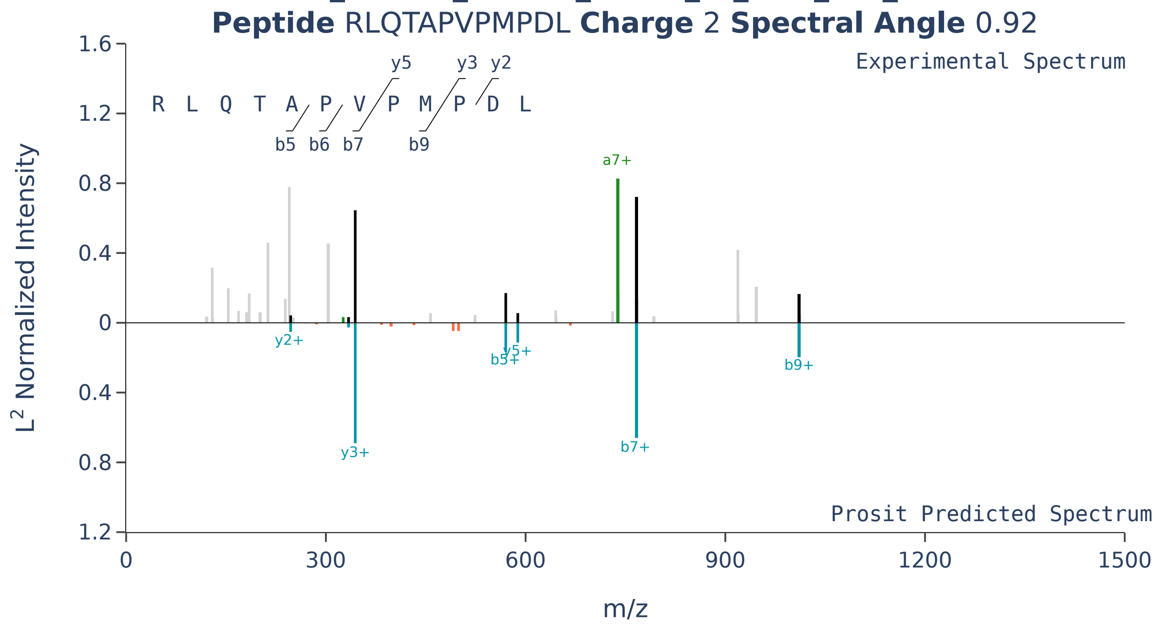


**Source** WSoh_221121_011221_HFGoe_L1_hTau_24h_R1 **Scan** 8561
**Peptide** ENLKHQPGGGKVQIINK **Charge** 2 **Spectral Angle** 0.81

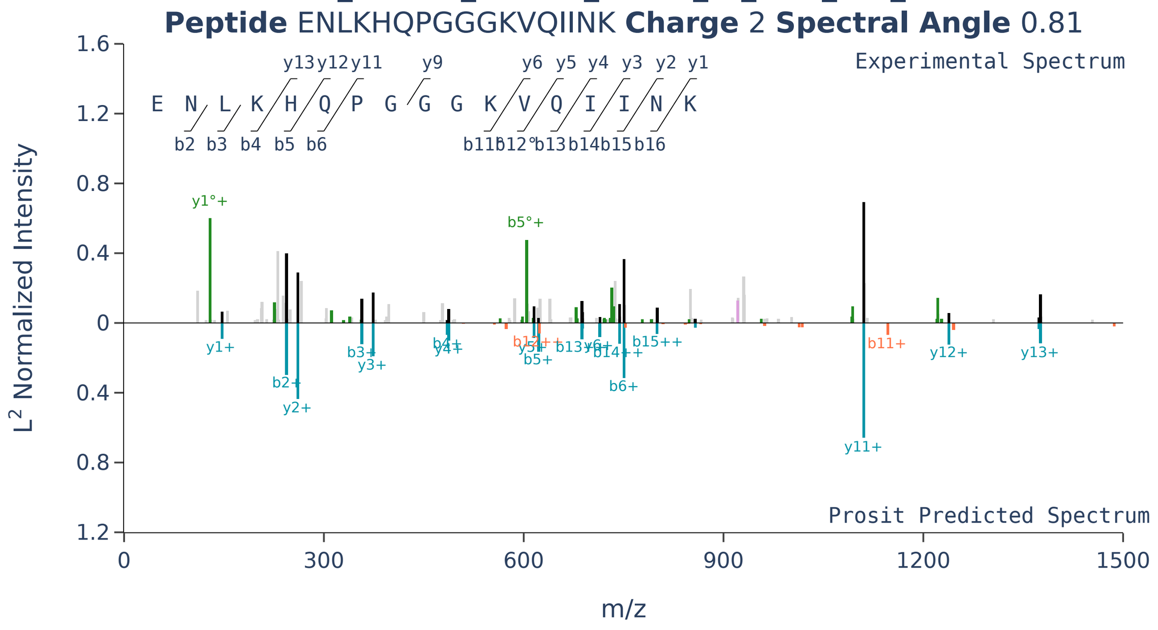


**Source** WSoh_221121_011221_HFGoe_L2_hTau_24h_R1 **Scan** 9230
**Peptide** PTEDGSEEPGSETSDAKSTPTAE **Charge** 2 **Spectral Angle** 0.8

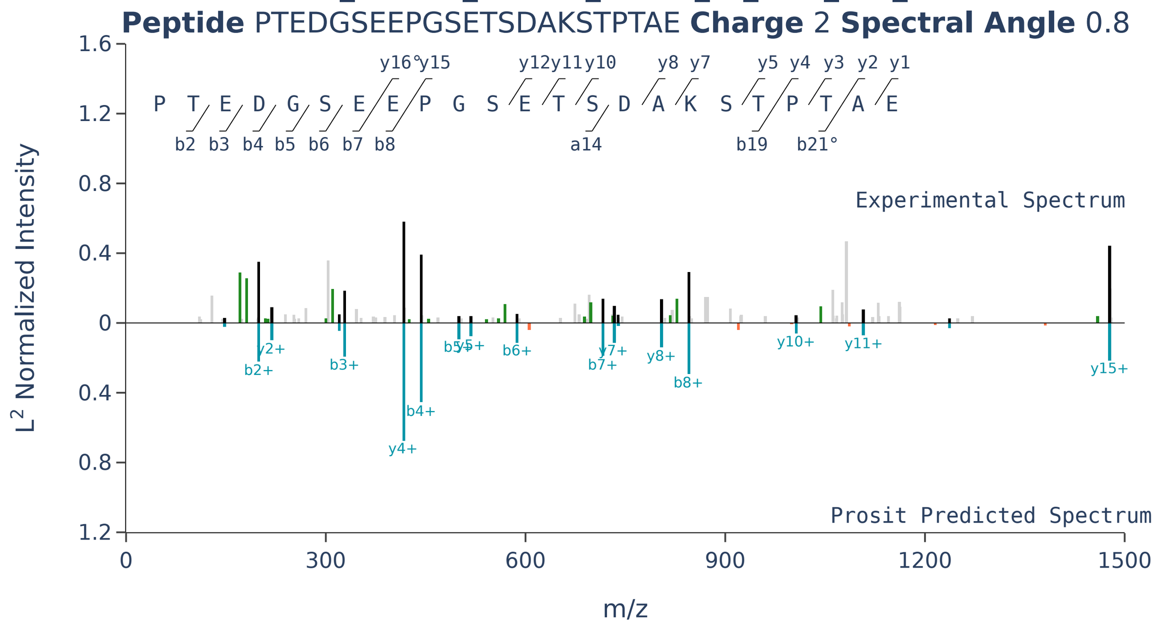


**Source** WSoh_221121_011221_HFGoe_L1_hTau_24h_R2 **Scan** 19553
**Peptide** QDQEGDTDAGLKESPLQTPTED **Charge** 3 **Spectral Angle** 0.87

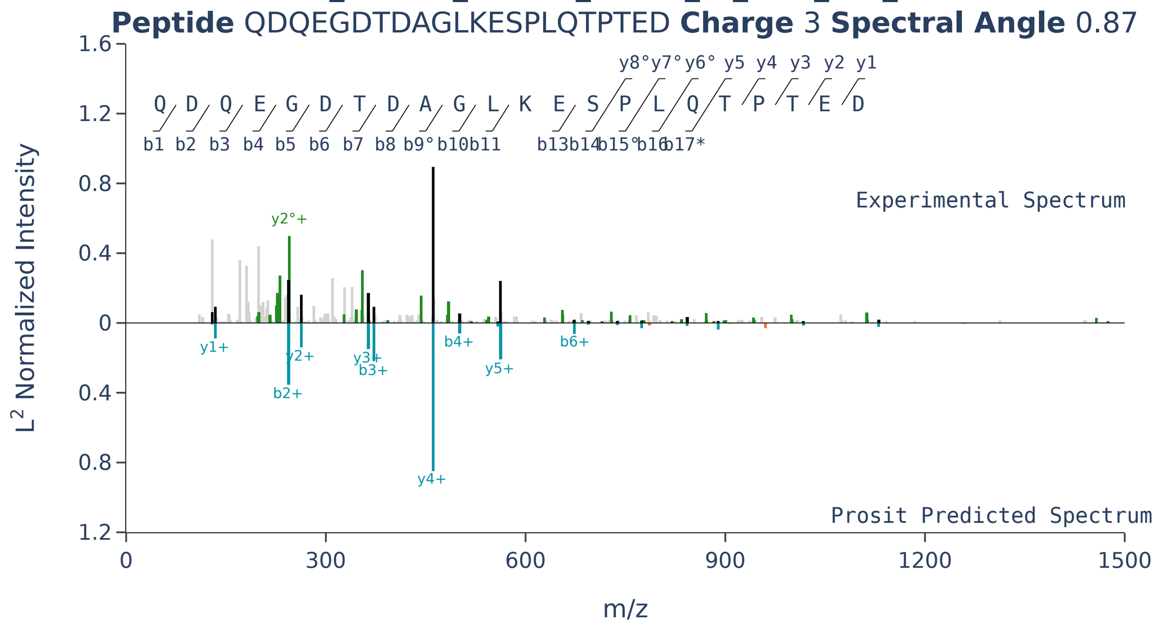


**Source** WSoh_221121_011221_HFGoe_L1_hTau_4h_R2 **Scan** 11806
**Peptide** VYKSPVVSGDTS **Charge** 2 **Spectral Angle** 0.84

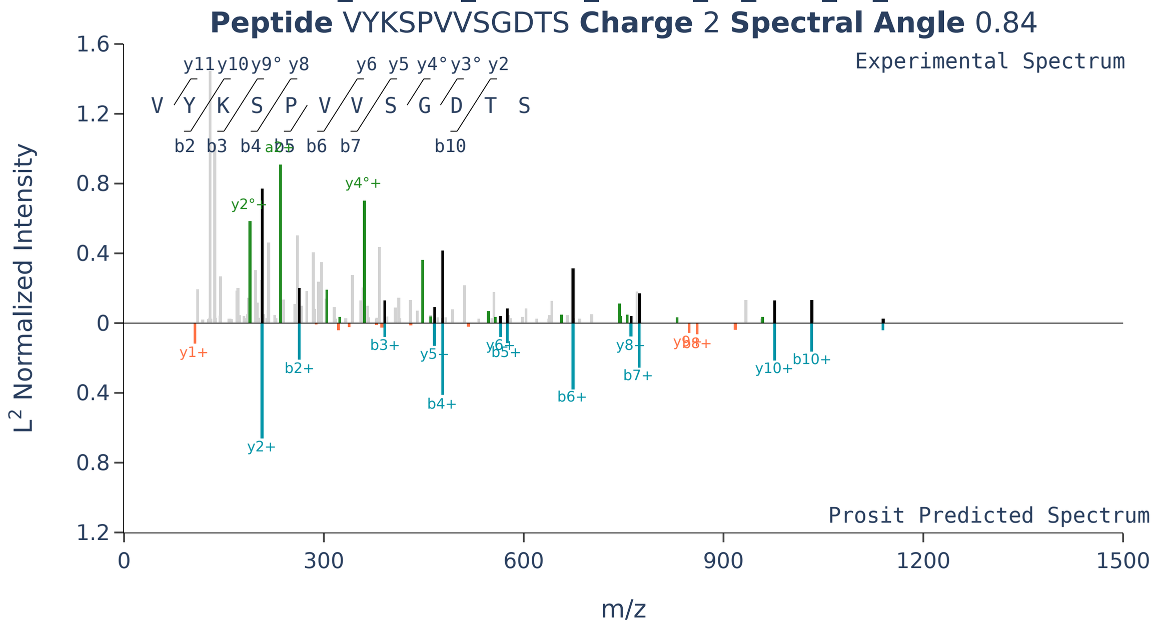


**Source** WSoh_221121_011221_HFGoe_L1_hTau_24h_R1 **Scan** 8287
**Peptide** KHQPGGGKVQIIN **Charge** 2 **Spectral Angle** 0.81

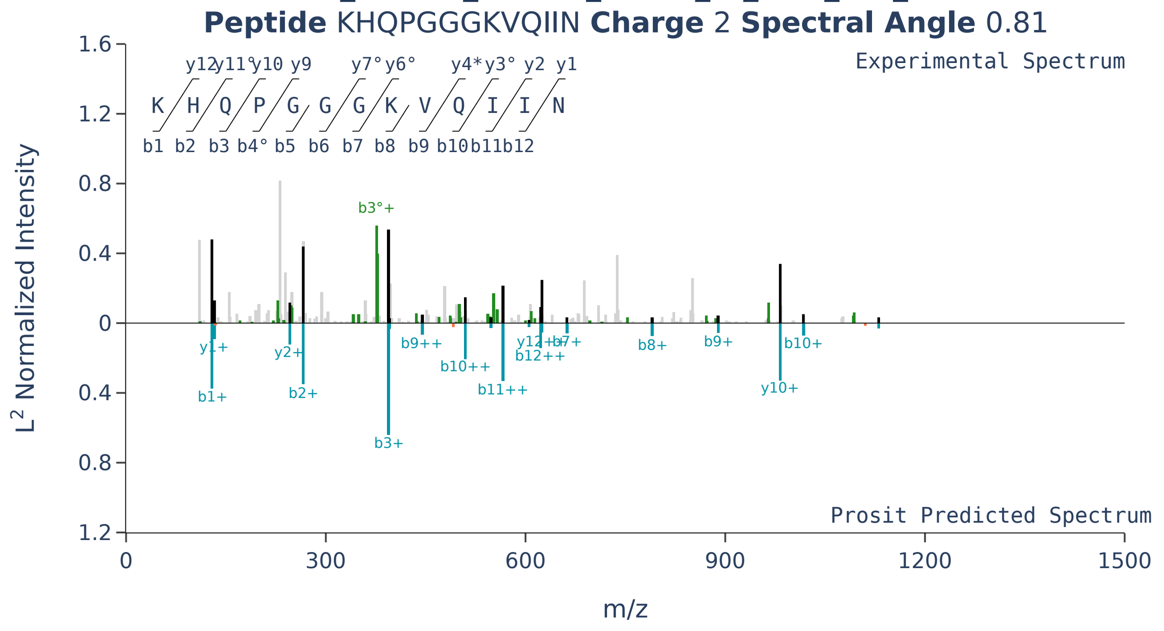


**Source** WSoh_221121_011221_HFGoe_L2_hTau_24h_R1 **Scan** 10339
**Peptide** ENAKAKTDHGAEIVY **Charge** 2 **Spectral Angle** 0.82

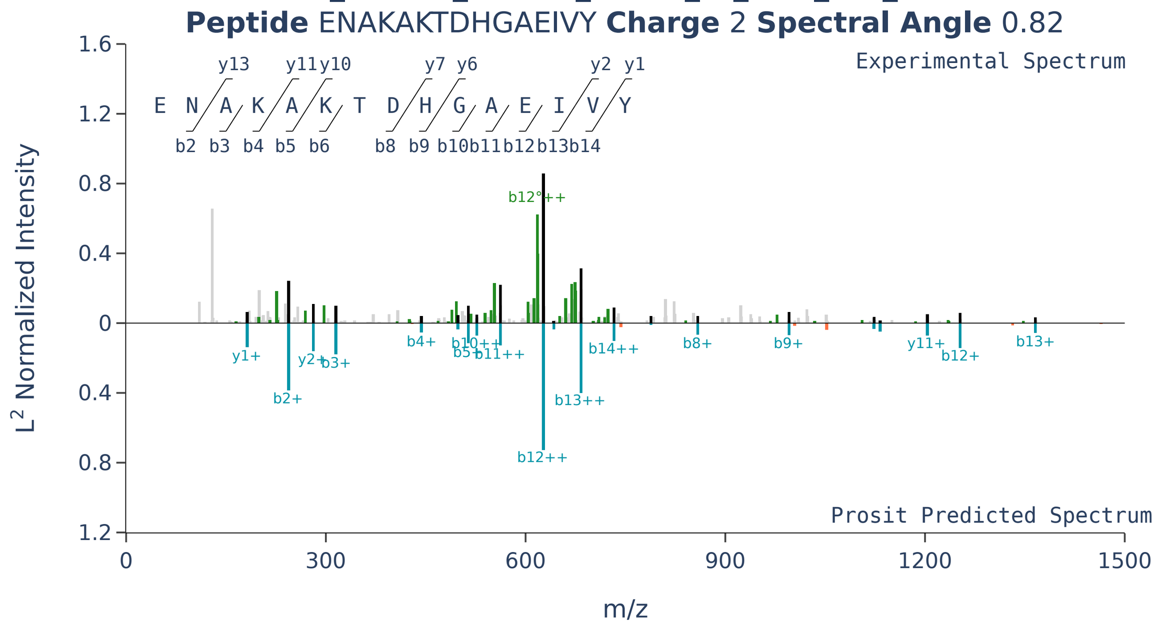


**Source** WSoh_221121_011221_HFGoe_L1_hTau_24h_R1 **Scan** 14503
**Peptide** PGGGQVEVKSEKLDFKDRVQ **Charge** 5 **Spectral Angle** 0.79

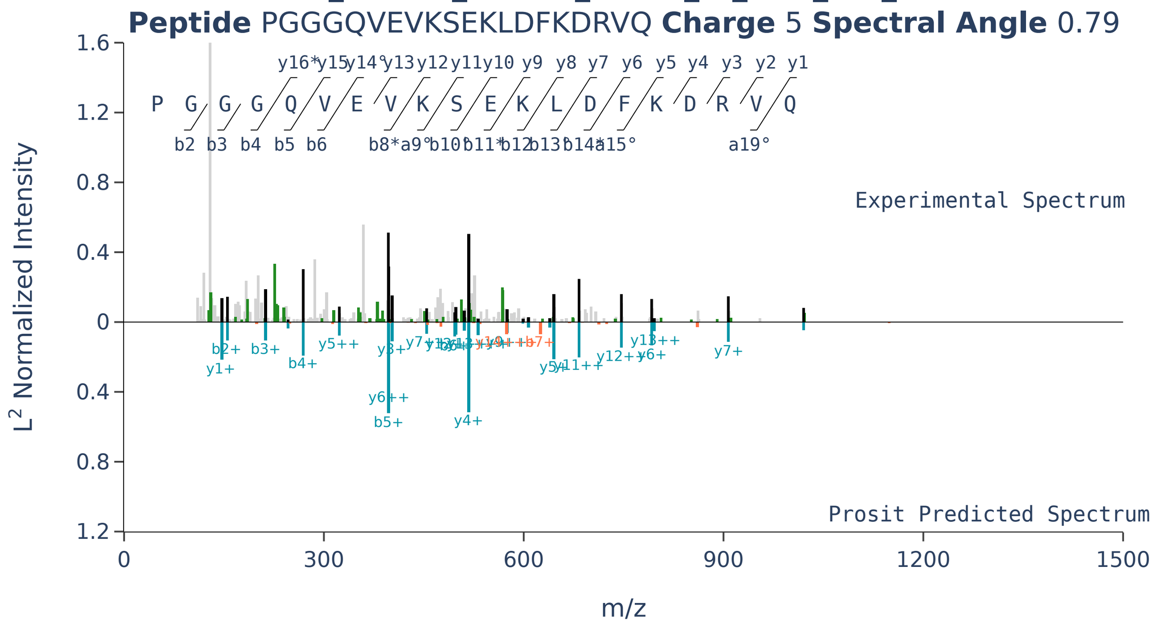


**Source** WSoh_221121_011221_HFGoe_L1_hTau_4h_R2 **Scan** 9703
**Peptide** EGAPGKQAAAQPHTEI **Charge** 2 **Spectral Angle** 0.86

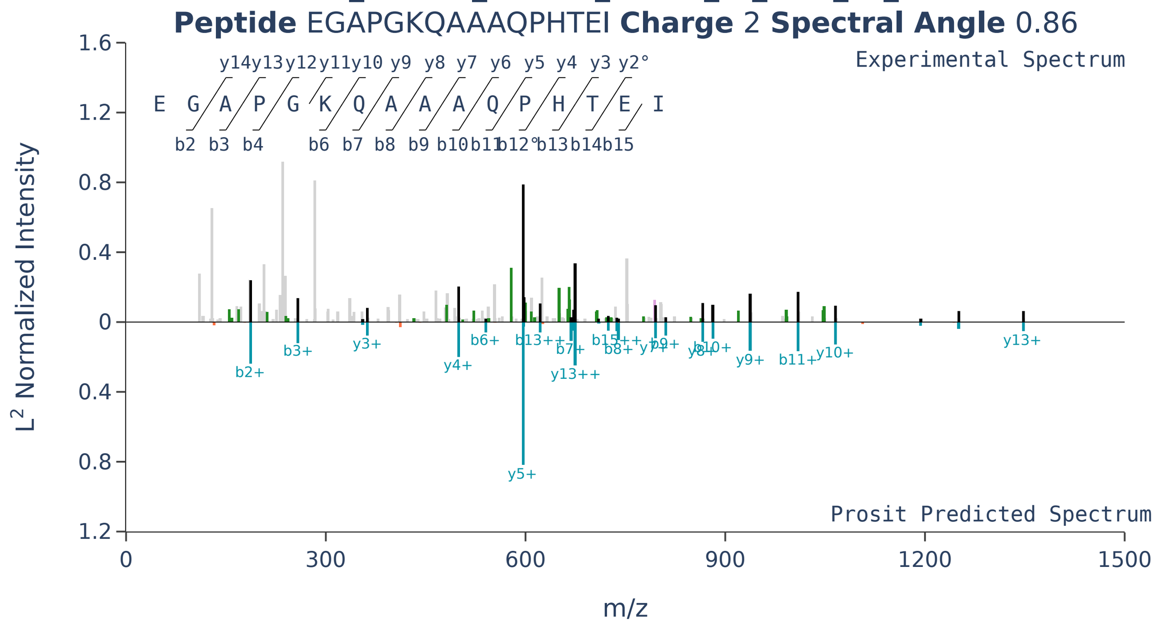


**Source** WSoh_221121_011221_HFGoe_L2_hTau_24h_R2 **Scan** 6361
**Peptide** AAGHVTQARMVSK **Charge** 3 **Spectral Angle** 0.82

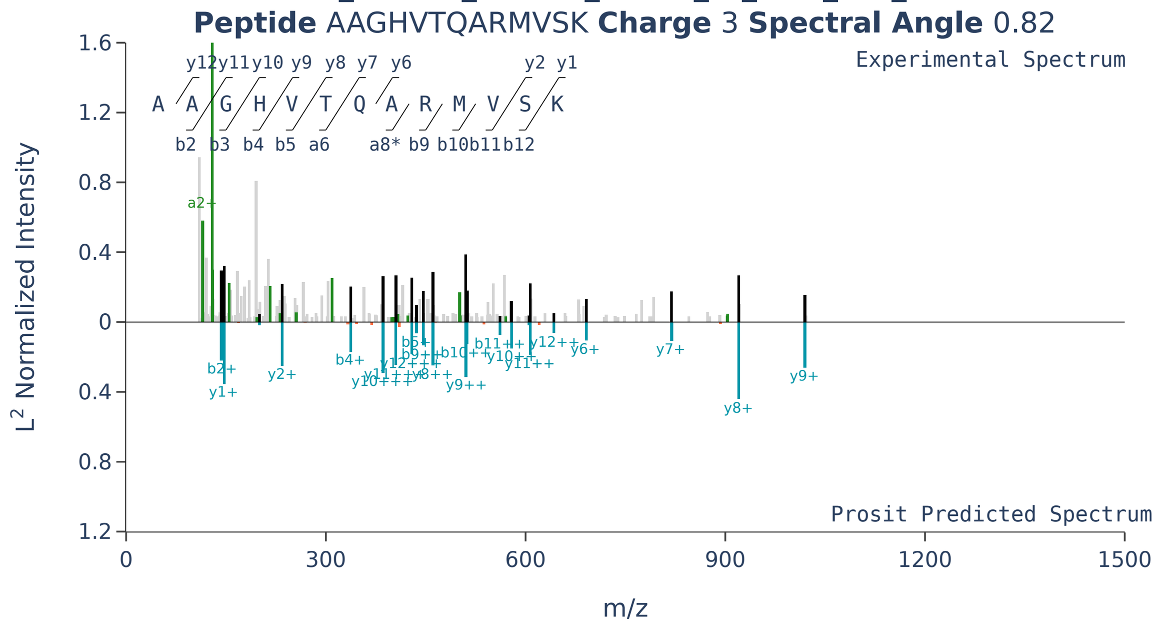


**Source** WSoh_221121_011221_HFGoe_L1_hTau_24h_R1 **Scan** 22066
**Peptide** PEGTTAEEAGIGDTPSLED **Charge** 2 **Spectral Angle** 0.85

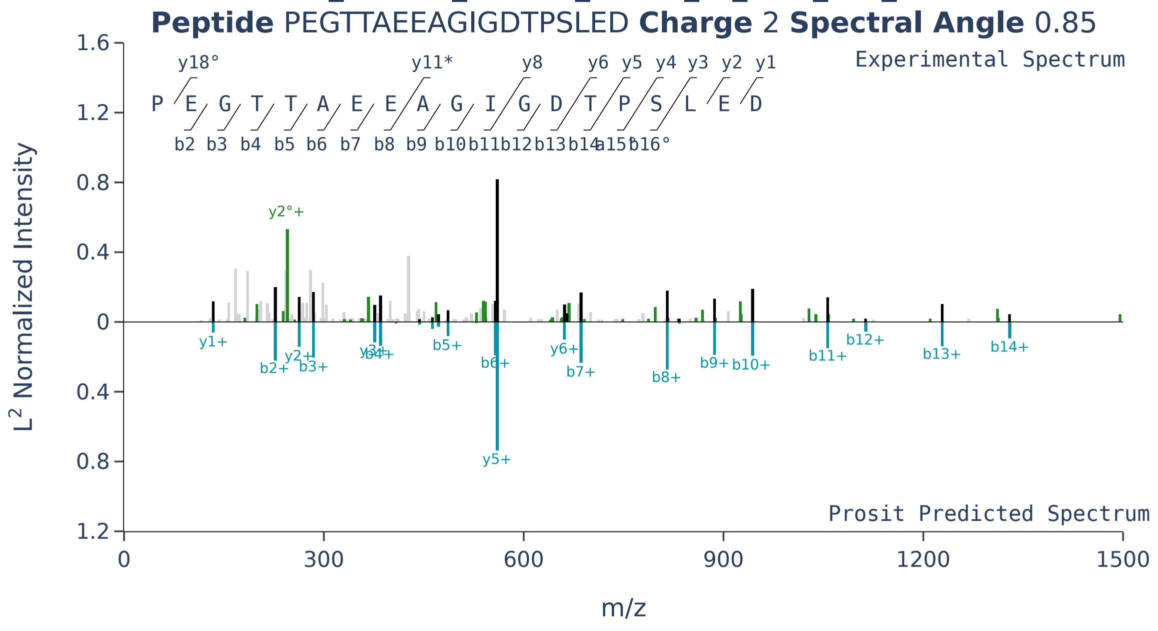


**Source** WSoh_221121_011221_HFGoe_L2_hTau_1h_R1 **Scan** 12054
**Peptide** SLDNITHVPGGGNKKIETH **Charge** 3 **Spectral Angle** 0.84

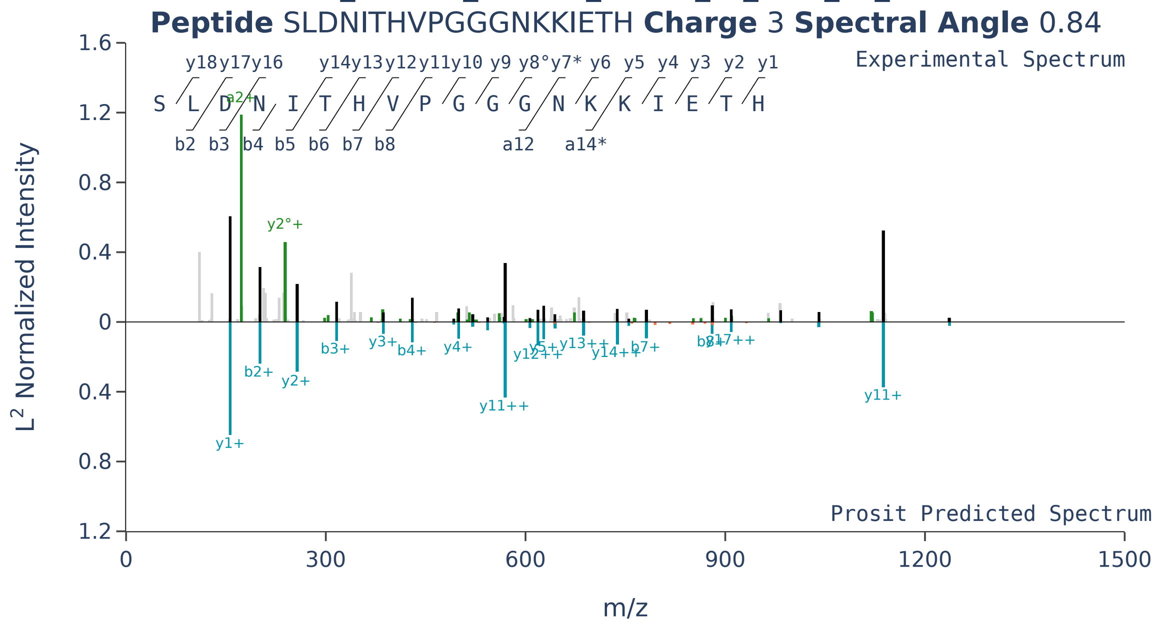


**Source** WSoh_221121_011221_HFGoe_L2_hTau_24h_R2 **Scan** 8219
**Peptide** GTYGLGDRKD **Charge** 2 **Spectral Angle** 0.9

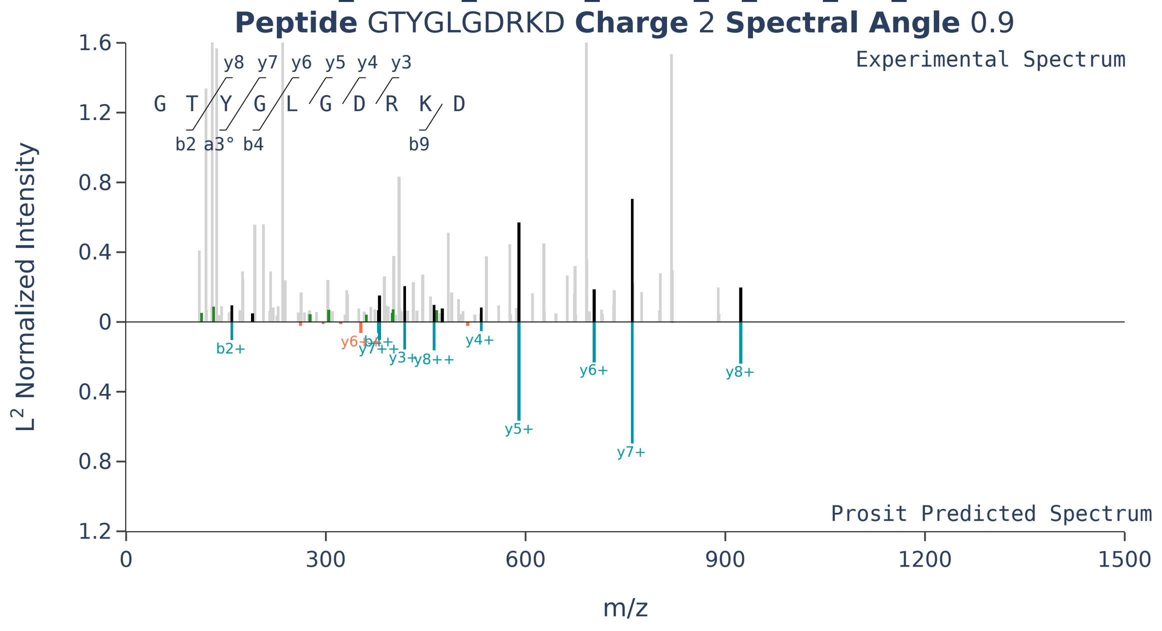

**Source** WSoh_221121_011221_HFGoe_L1_hTau_1h_R1 **Scan** 16190
**Peptide** QIINKKLDL **Charge** 2 **Spectral Angle** 0.77

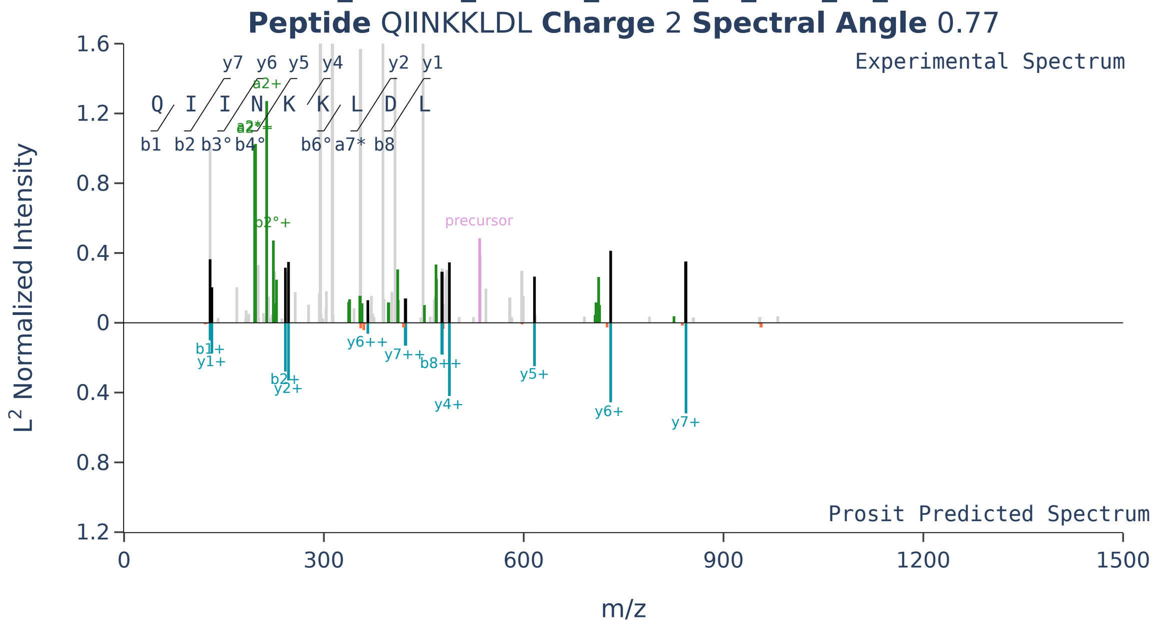


**Source** WSoh_221121_011221_HFGoe_L1_hTau_24h_R1 **Scan** 14770
**Peptide** EKLDFKDRVQSKL **Charge** 2 **Spectral Angle** 0.79

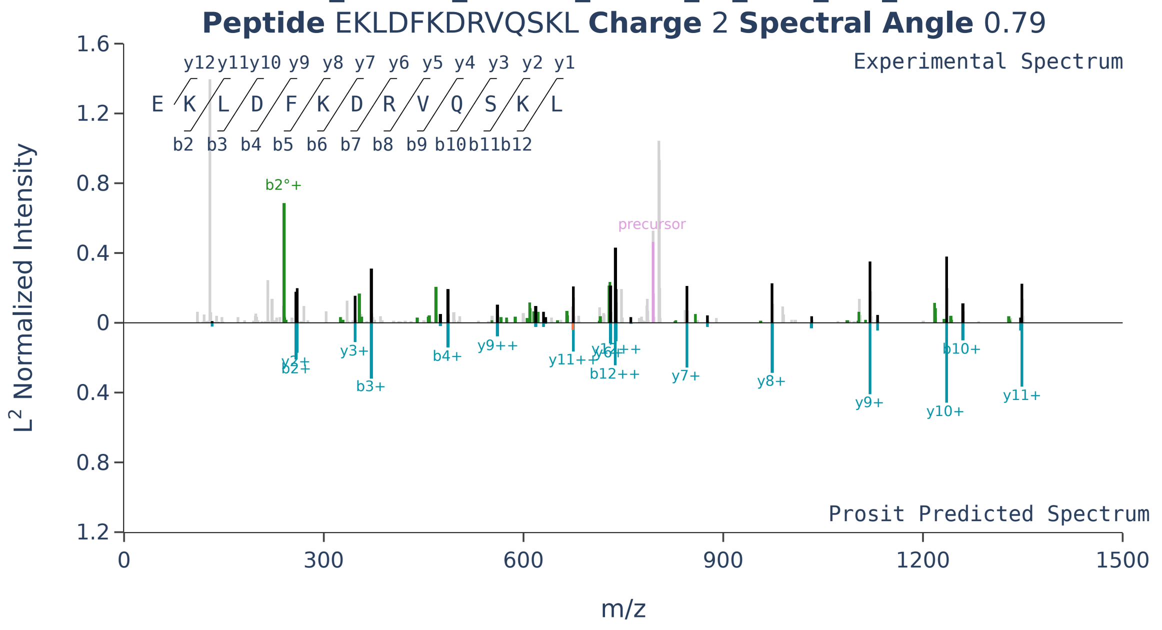


**Source** WSoh_221121_011221_HFGoe_L2_hTau_1h_R2 **Scan** 11258
**Peptide** SPGSPGTPGSRSRTPSL **Charge** 3 **Spectral Angle** 0.89

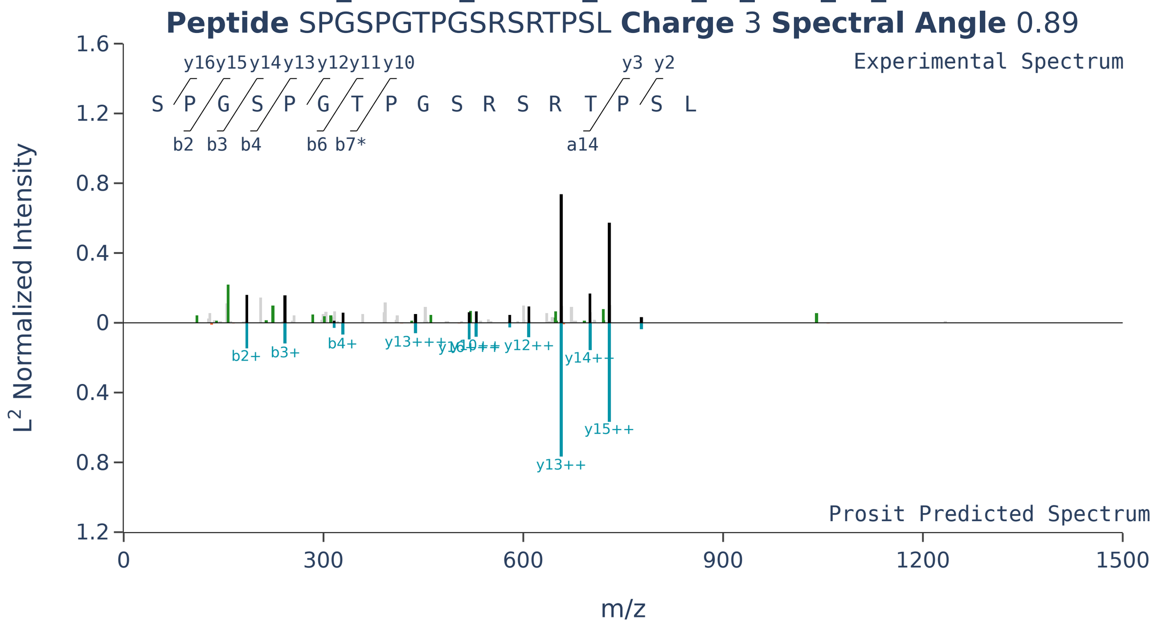


**Source** WSoh_221121_011221_HFGoe_L2_hTau_24h_R2 **Scan** 10072
**Peptide** QEGDTDAGLKESP **Charge** 2 **Spectral Angle** 0.85

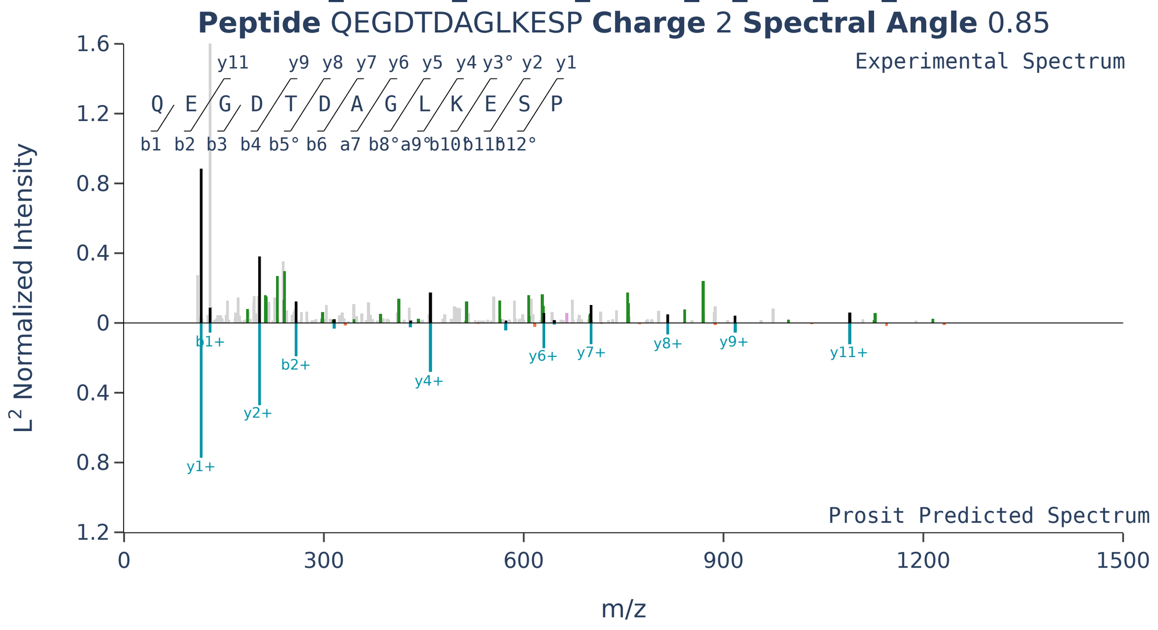


**Source** WSoh_221121_011221_HFGoe_L2_hTau_24h_R1 **Scan** 15552
**Peptide** KDRVQSKIGSLDNITHVPGGGNKKIET **Charge** 4 **Spectral Angle** 0.8

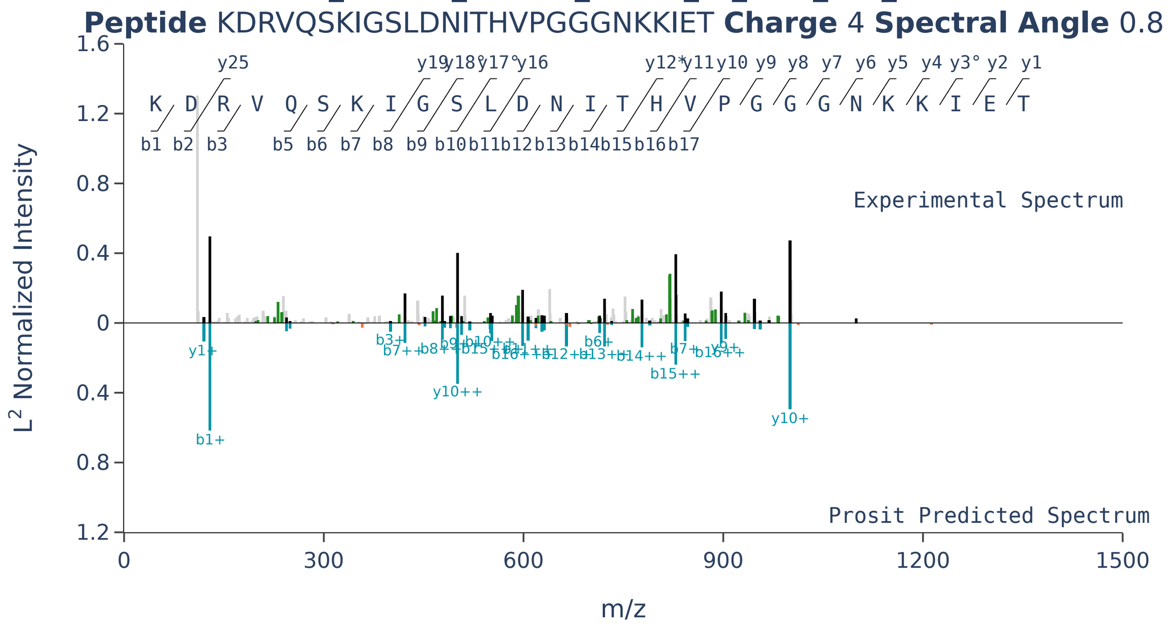


**Source** WSoh_221121_011221_HFGoe_L1_hTau_4h_R1 **Scan** 11153
**Peptide** KNVKSKIGSTENLKHQPGGGKVQII **Charge** 4 **Spectral Angle** 0.89

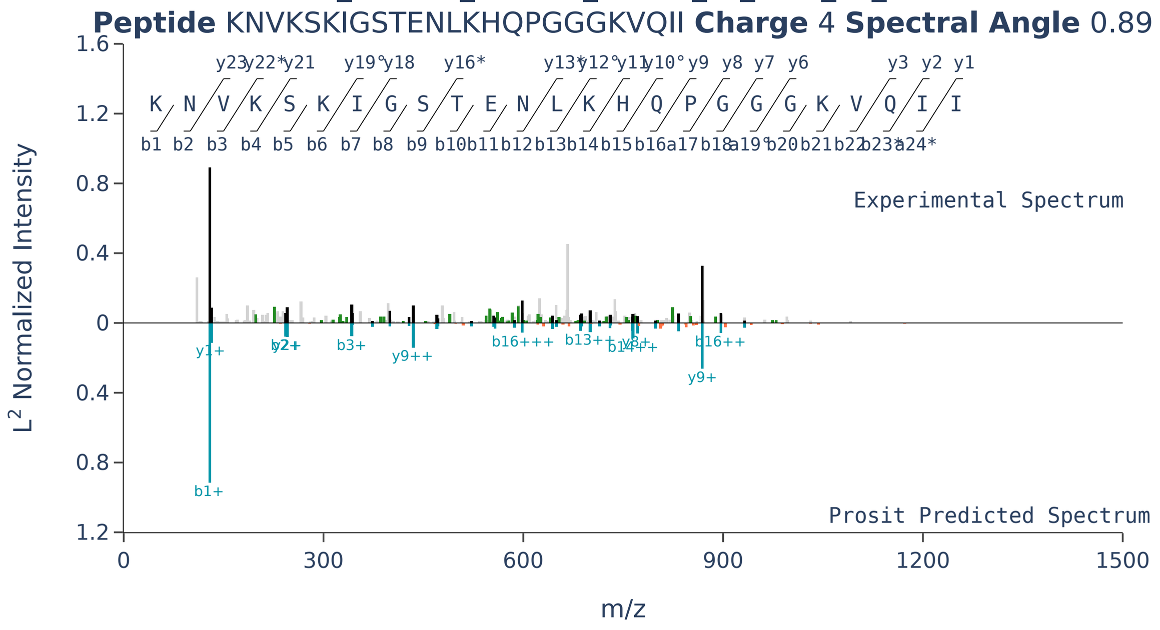


**Source** WSoh_221121_011221_HFGoe_L2_hTau_2h_R1 **Scan** 13832
**Peptide** EVKSEKLDFKDRVQSKL **Charge** 3 **Spectral Angle** 0.82

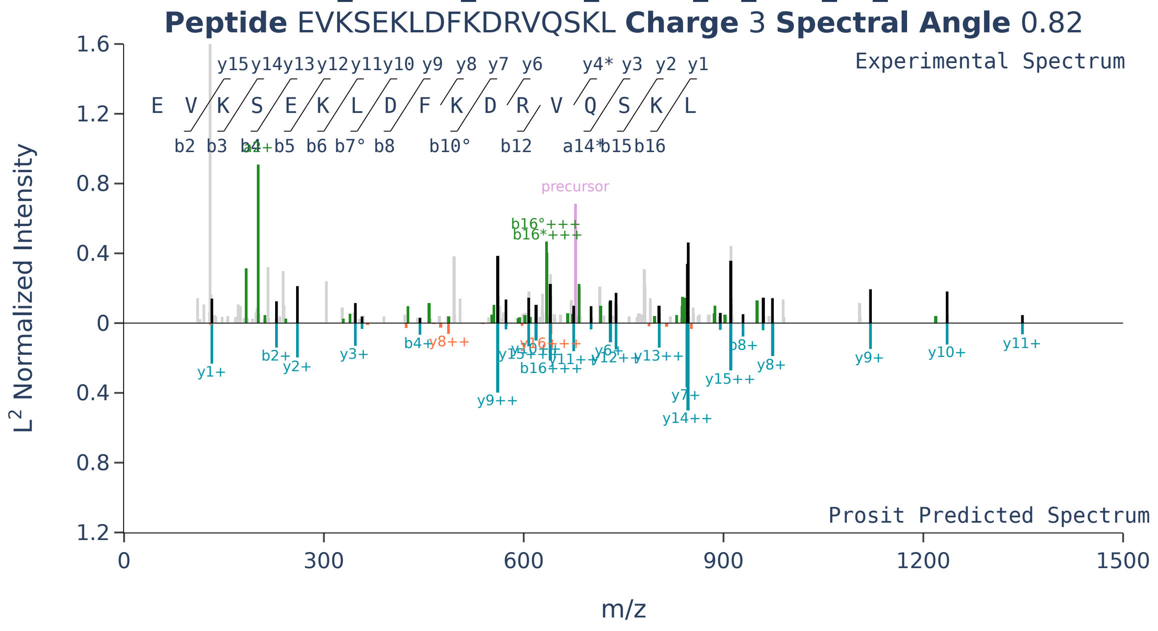


**Source** WSoh_221121_011221_HFGoe_L2_hTau_0h_R1 **Scan** 7077
**Peptide** DFKDRVQS **Charge** 2 **Spectral Angle** 0.91

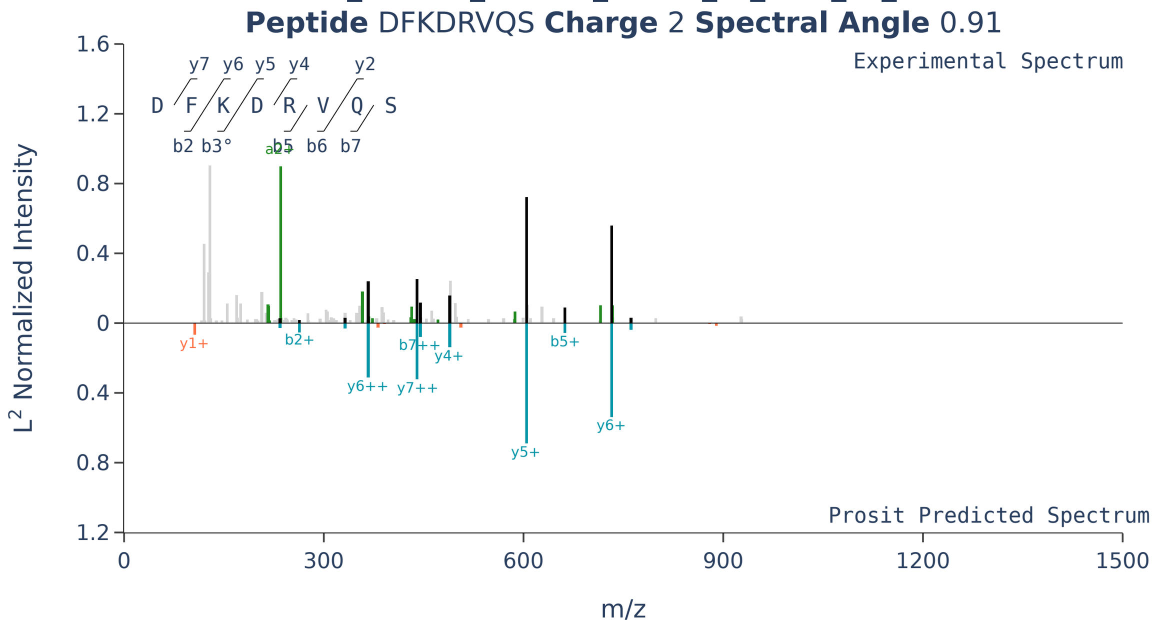


**Source** WSoh_221121_011221_HFGoe_L2_hTau_24h_R1 **Scan** 21523
**Peptide** APVPMPDLKNVKSKIGST **Charge** 3 **Spectral Angle** 0.84

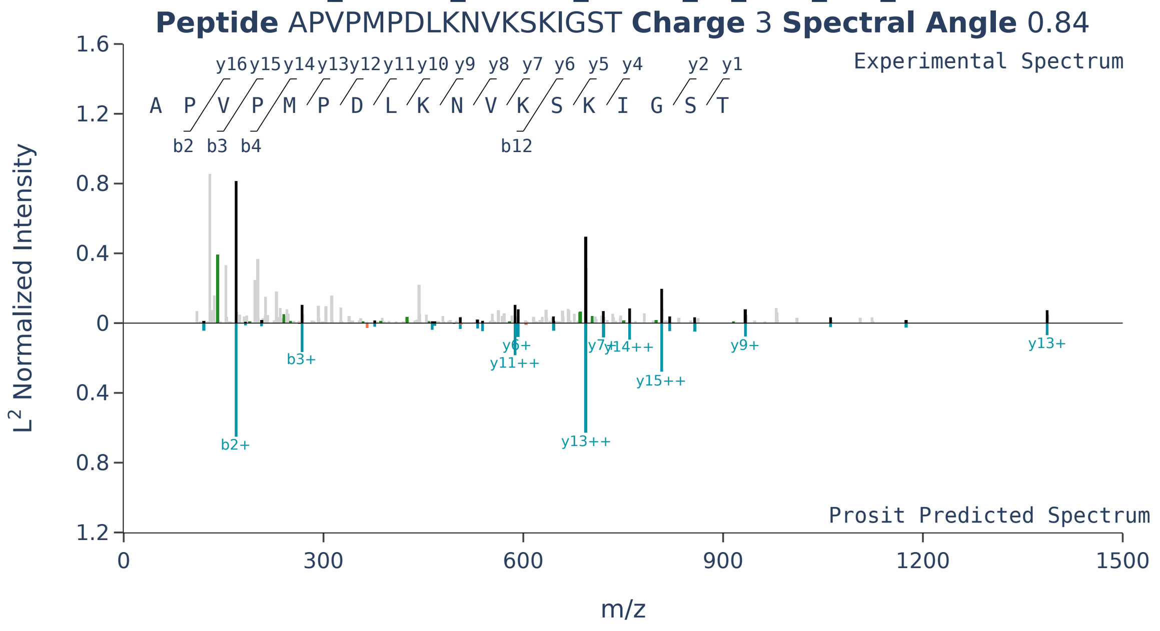


**Source** WSoh_221121_011221_HFGoe_L1_hTau_24h_R1 **Scan** 28510
**Peptide** APVPMPDL **Charge** 1 **Spectral Angle** 0.89

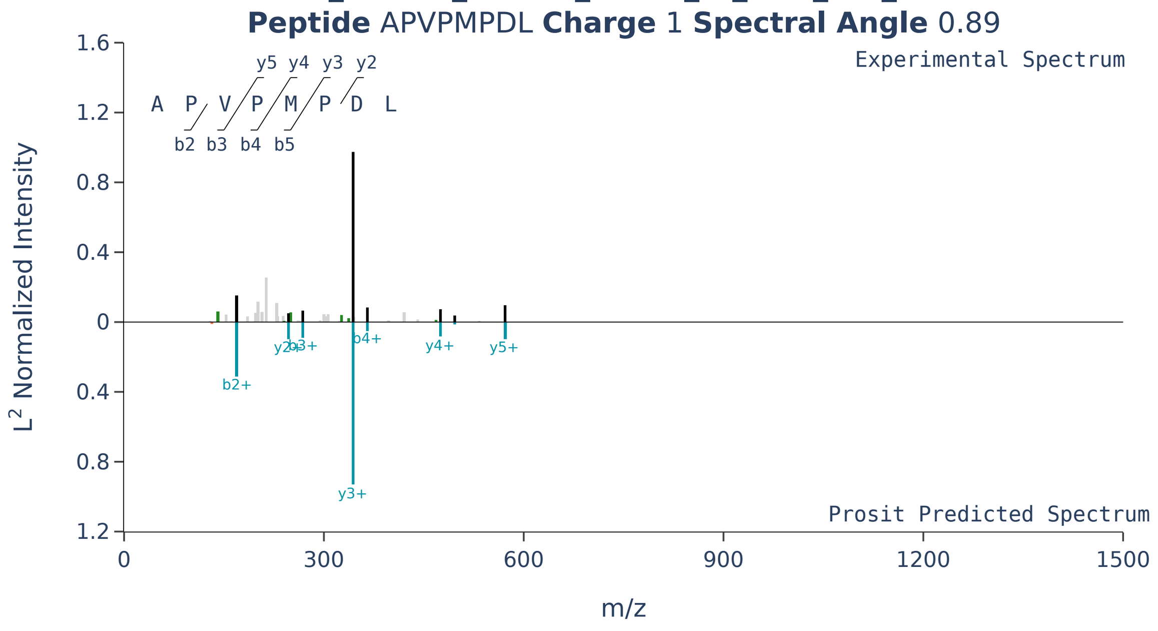


**Source** WSoh_221121_011221_HFGoe_L1_hTau_24h_R1 **Scan** 16609
**Peptide** KSEKLDFKDRVQSKIGSLD **Charge** 3 **Spectral Angle** 0.86

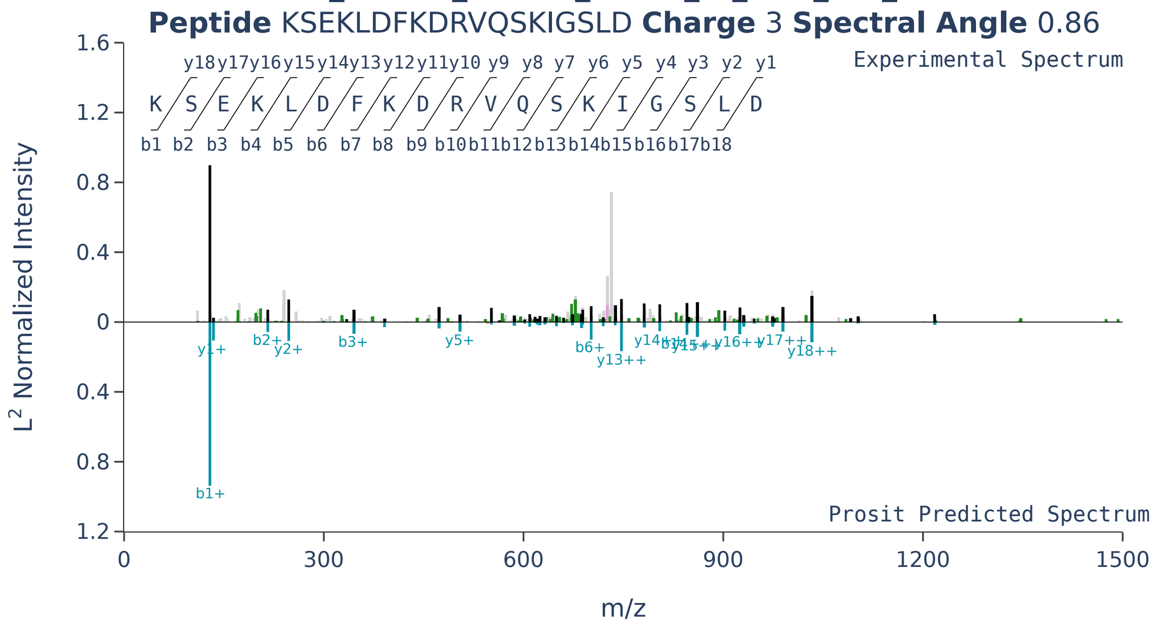


**Source** WSoh_221121_011221_HFGoe_L2_hTau_24h_R1 **Scan** 6412
**Peptide** NITHVPGGGNKKIETHK **Charge** 4 **Spectral Angle** 0.89

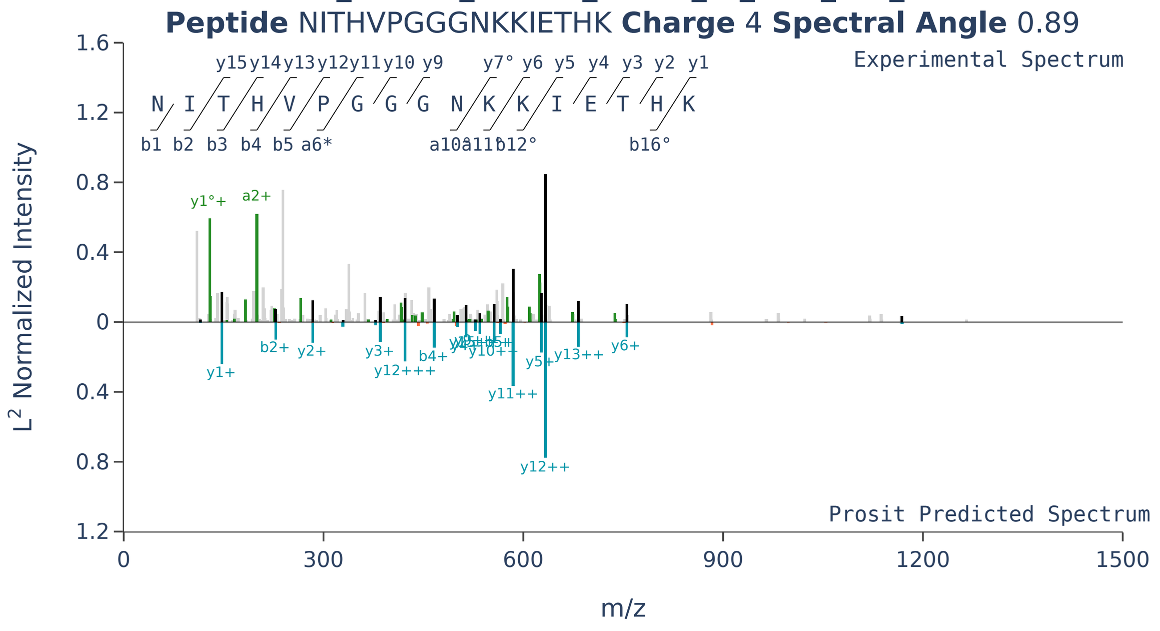


**Source** WSoh_221121_011221_HFGoe_L1_hTau_24h_R1 **Scan** 6668
**Peptide** PTEDGSEEPGSETSDAKST **Charge** 2 **Spectral Angle** 0.88

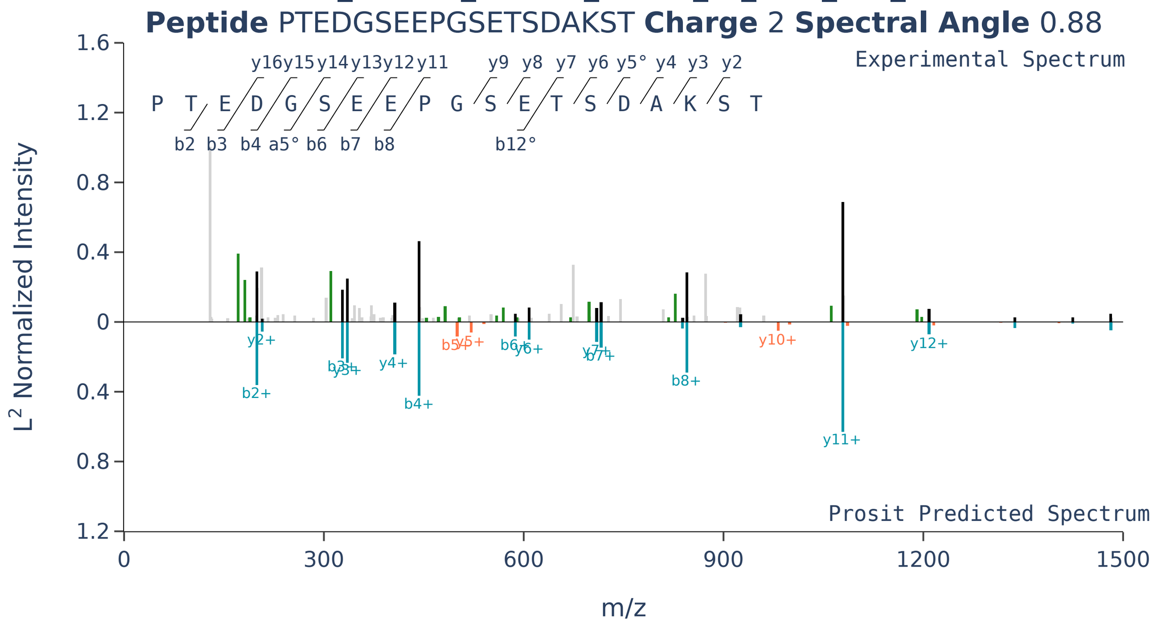


**Source** WSoh_221121_011221_HFGoe_L1_hTau_24h_R2 **Scan** 15258
**Peptide** KCGSKDNIKHVPGGGSVQIVY **Charge** 3 **Spectral Angle** 0.82

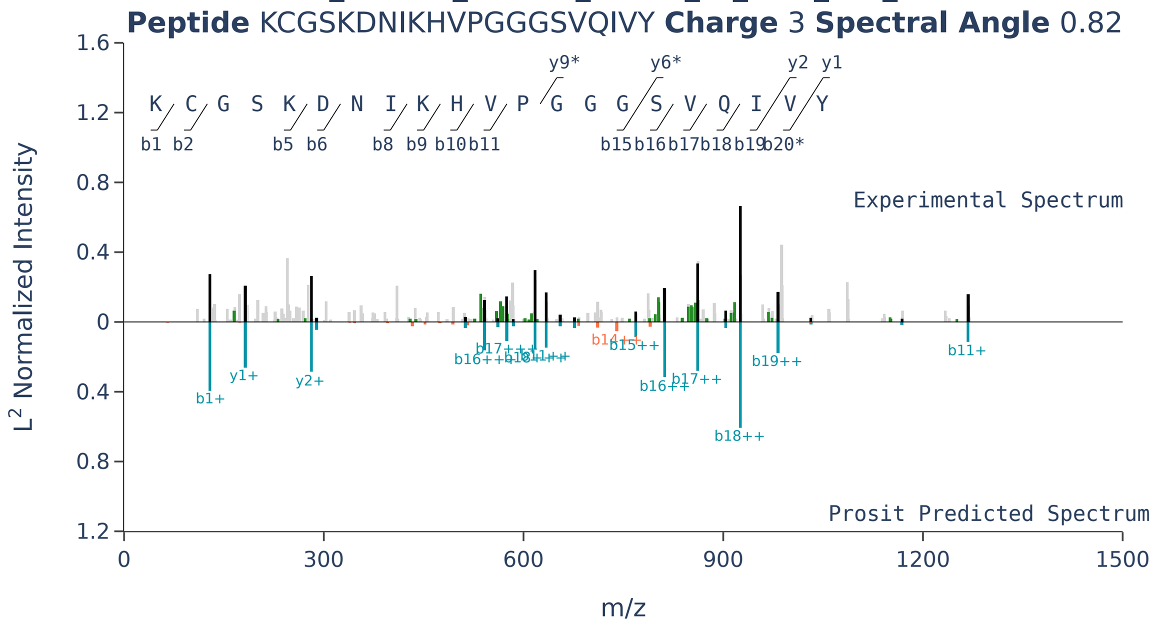


**Source** WSoh_221121_011221_HFGoe_L2_hTau_1h_R2 **Scan** 11624
**Peptide** VYKSPVVSGDTSPRHLS **Charge** 4 **Spectral Angle** 0.81

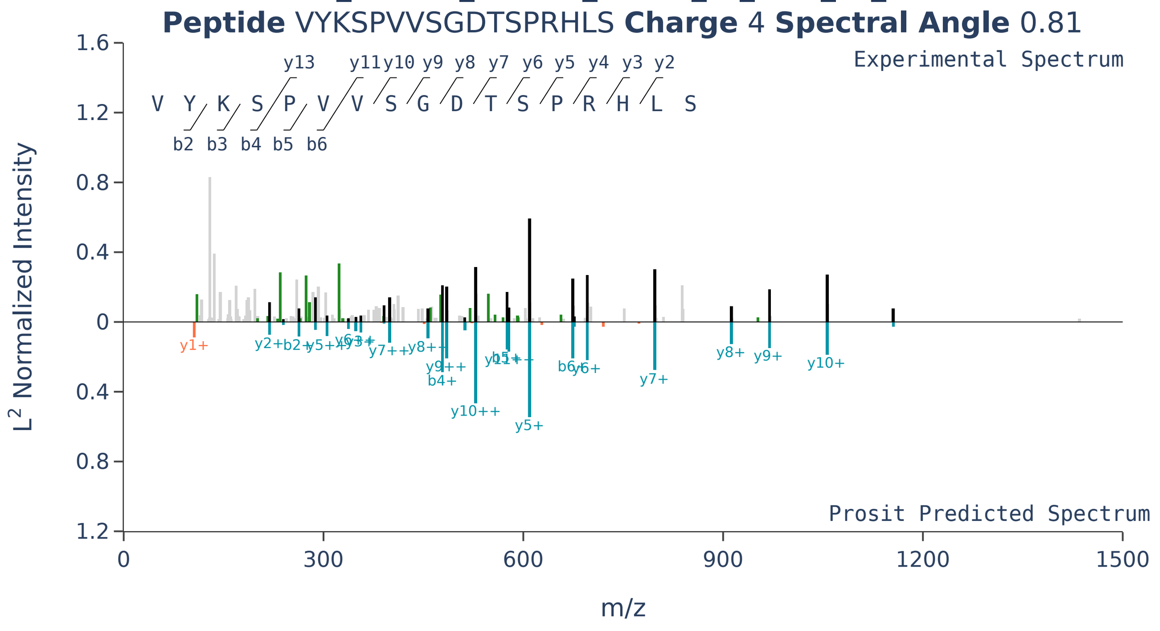

**Source** WSoh_221121_011221_HFGoe_L1_hTau_4h_R1 **Scan** 13153
**Peptide** TFRENAKAKTDHGAEIVYKSPVVSG **Charge** 4 **Spectral Angle** 0.84

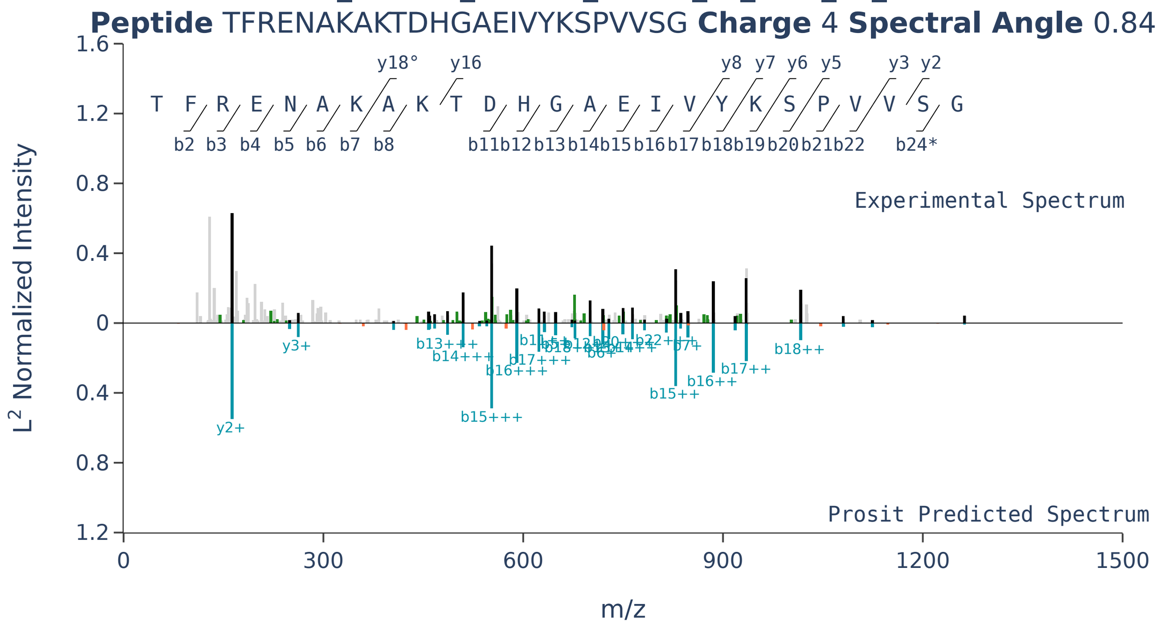


**Source** WSoh_221121_011221_HFGoe_L1_hTau_4h_R2 **Scan** 6505
**Peptide** SKCGSLGNIHH **Charge** 2 **Spectral Angle** 0.83

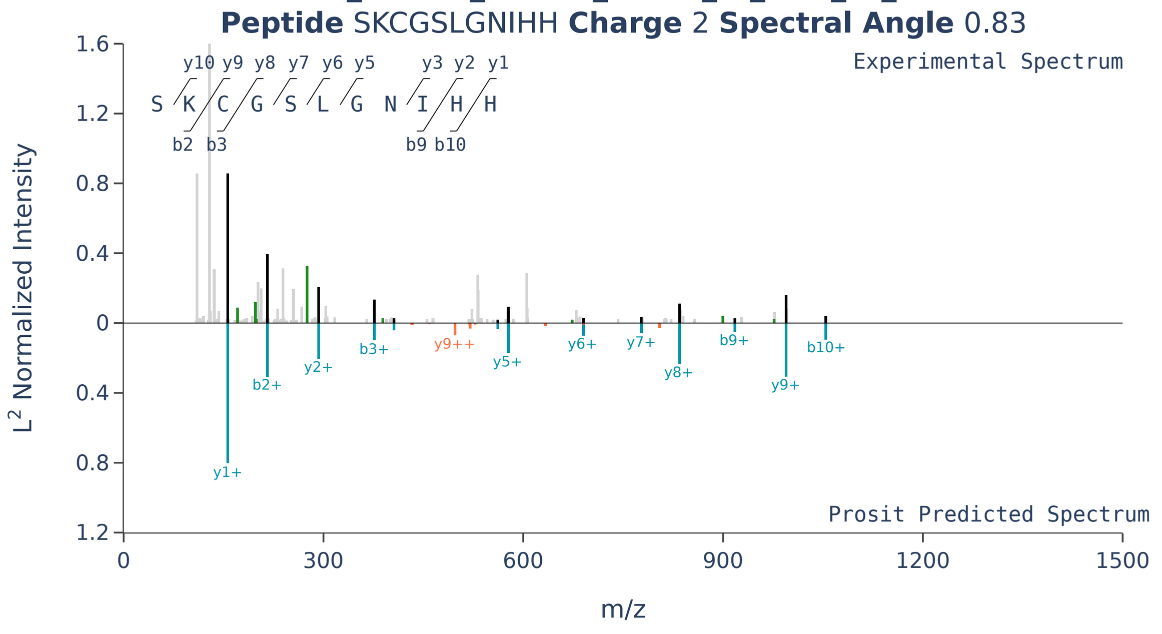


**Source** WSoh_221121_011221_HFGoe_L2_hTau_1h_R2 **Scan** 11969
**Peptide** KSEKLDF **Charge** 2 **Spectral Angle** 0.9

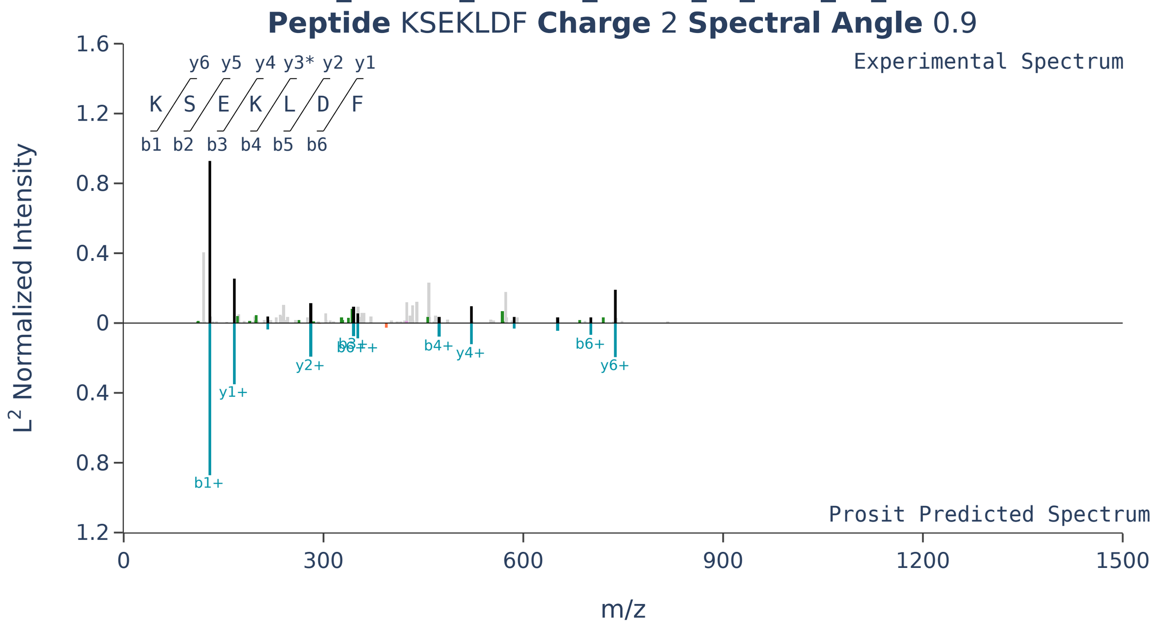


**Source** WSoh_221121_011221_HFGoe_L1_hTau_24h_R2 **Scan** 5697
**Peptide** TENLKHQPGGGKVQ **Charge** 3 **Spectral Angle** 0.87

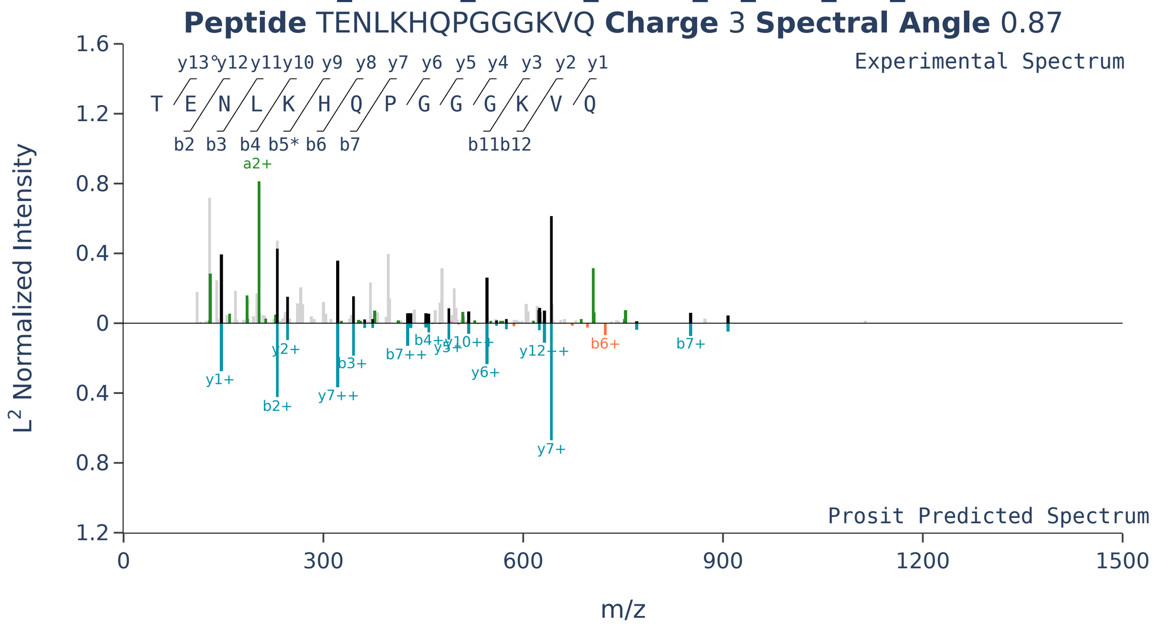


**Source** WSoh_221121_011221_HFGoe_L1_hTau_2h_R1 **Scan** 14993
**Peptide** YKPVDLSKVTSKCG **Charge** 3 **Spectral Angle** 0.89

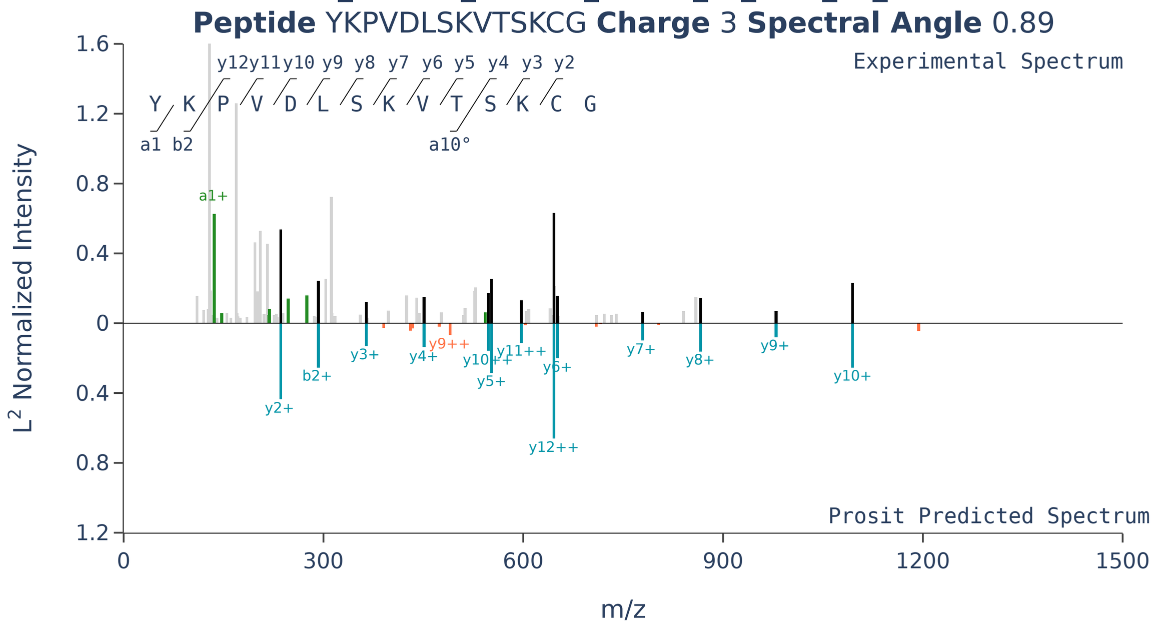


**Source** WSoh_221121_011221_HFGoe_L2_hTau_4h_R1 **Scan** 12132
**Peptide** PVVSGDTSPRHLSNVSST **Charge** 3 **Spectral Angle** 0.88

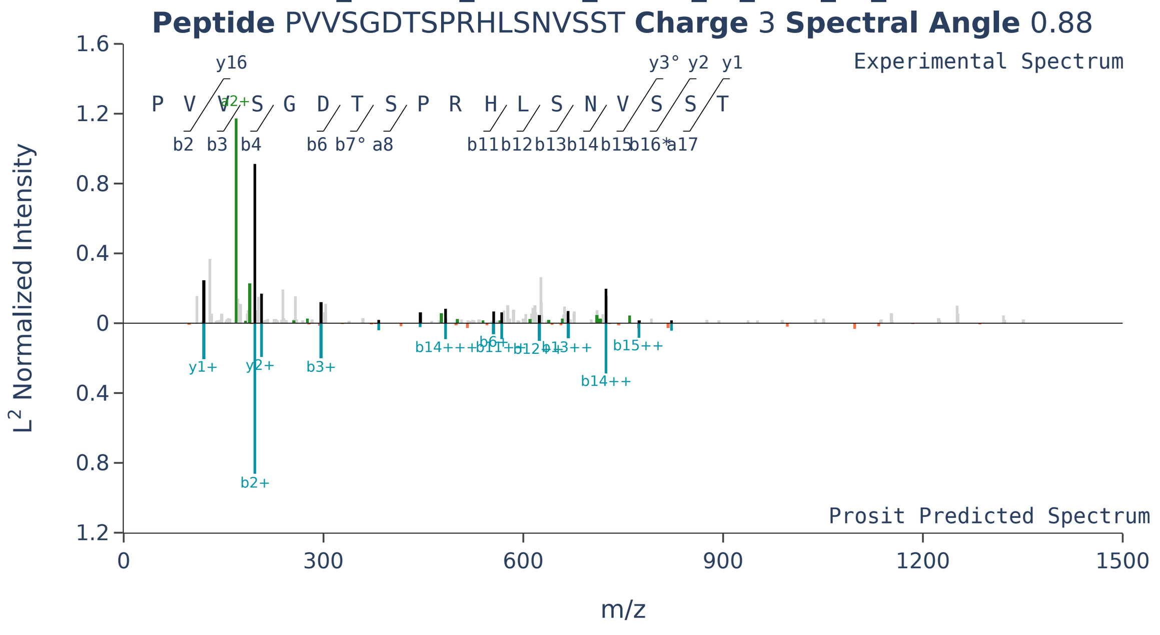


**Source** WSoh_221121_011221_HFGoe_L1_hTau_24h_R2 **Scan** 10670
**Peptide** GNIHHKPGGGQVEVKSEKLDFKDRVQS **Charge** 3 **Spectral Angle** 0.77

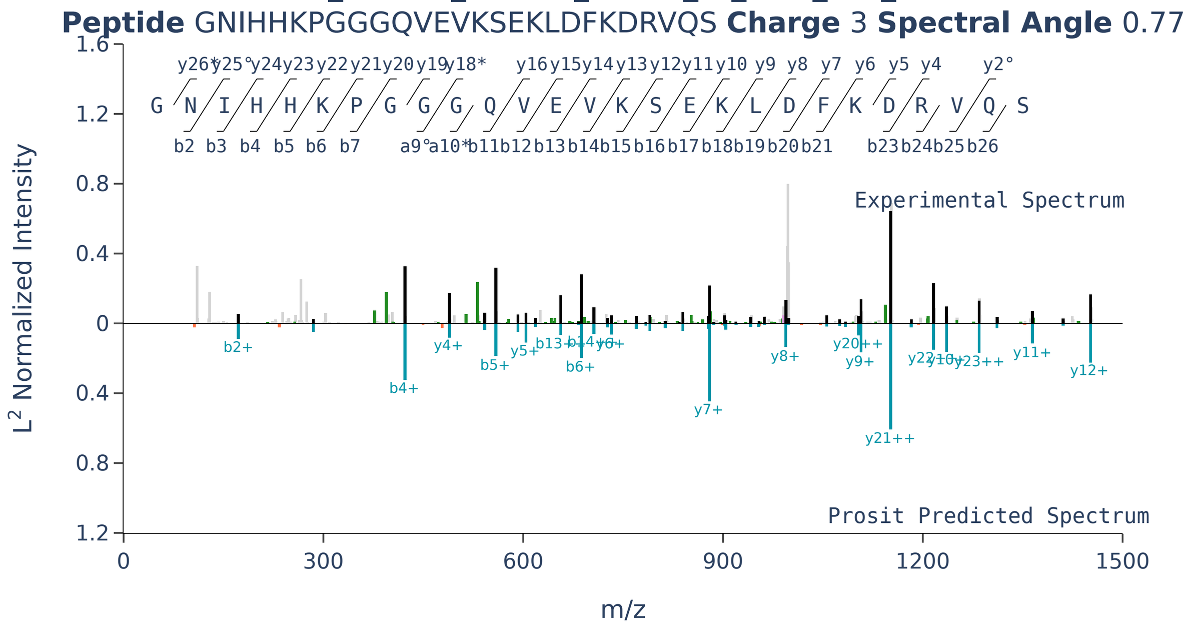


**Source** WSoh_221121_011221_HFGoe_L1_hTau_24h_R2 **Scan** 6233
**Peptide** PRHLSNVSSTGS **Charge** 2 **Spectral Angle** 0.82

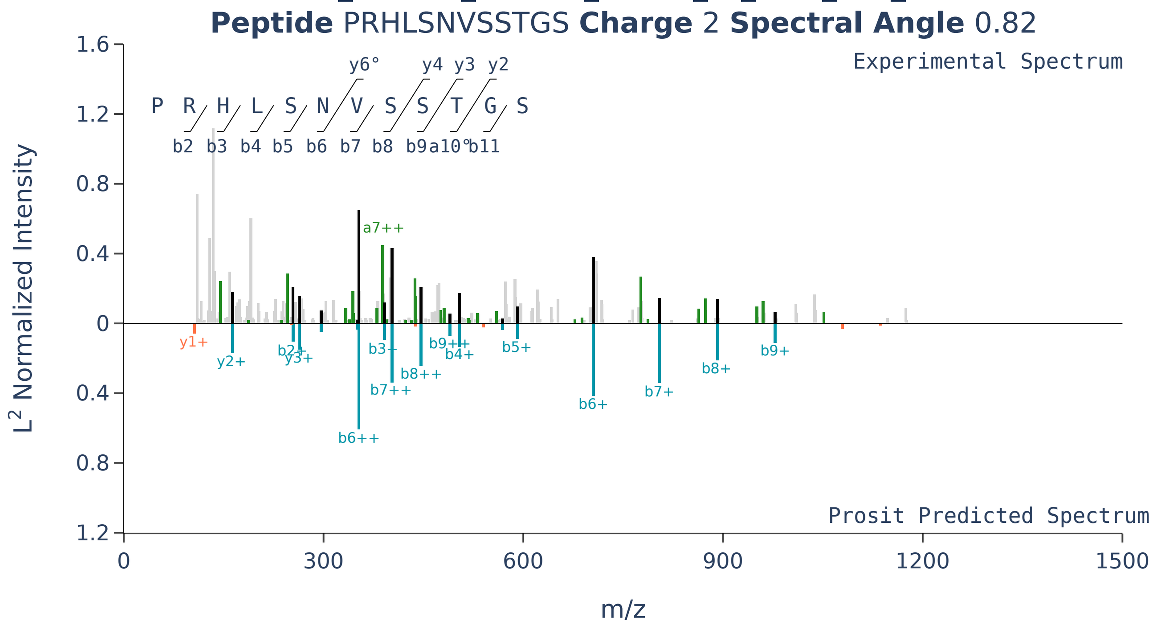


**Source** WSoh_221121_011221_HFGoe_L2_hTau_24h_R2 **Scan** 21724
**Peptide** QIVYKPVDLSKVTS **Charge** 2 **Spectral Angle** 0.9

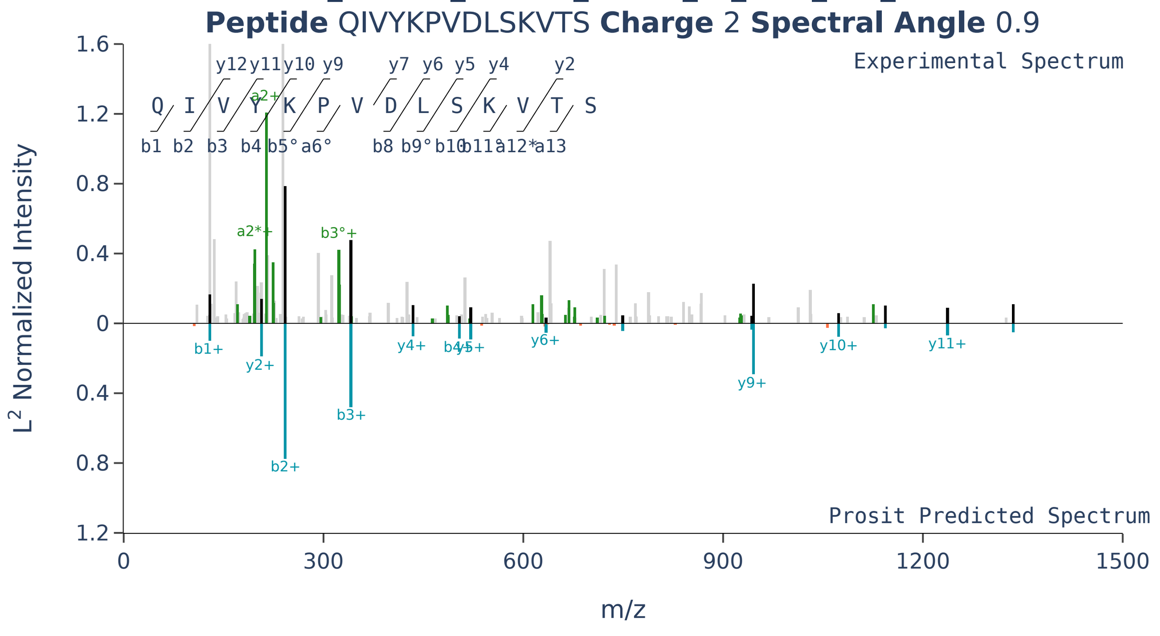


**Source** WSoh_221121_011221_HFGoe_L2_hTau_2h_R2 **Scan** 10499
**Peptide** KSPVVSGDTSPRHLSNVSST **Charge** 3 **Spectral Angle** 0.92

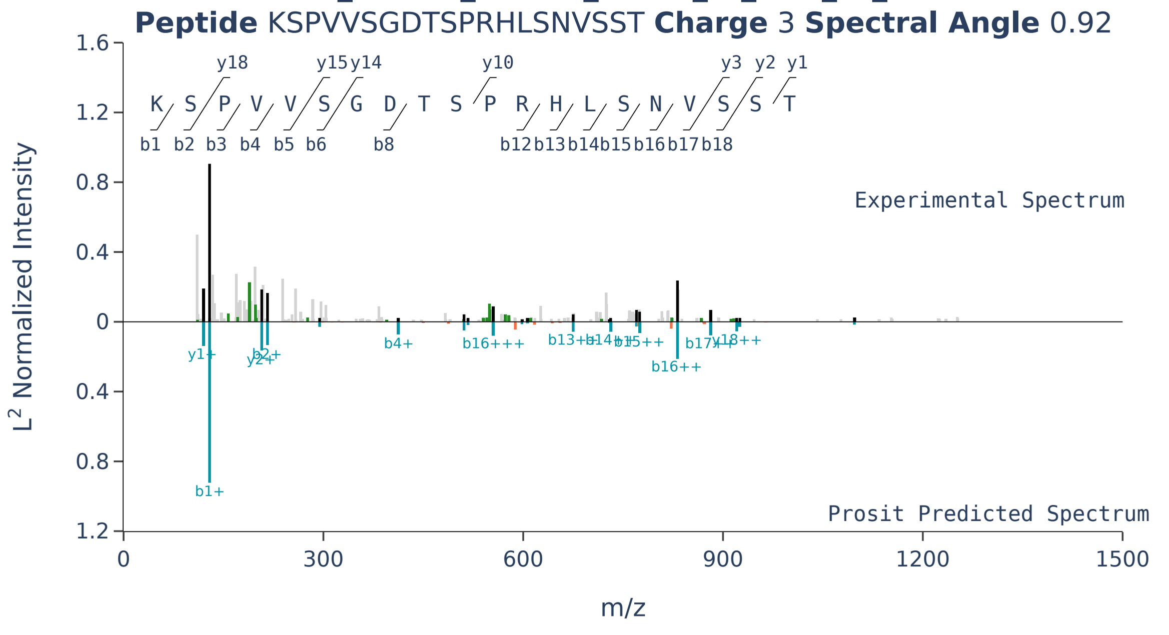


**Source** WSoh_221121_011221_HFGoe_L1_hTau_24h_R1 **Scan** 21571
**Peptide** SPVVSGDTSPRHLSNVSSTGSIDMV **Charge** 3 **Spectral Angle** 0.89

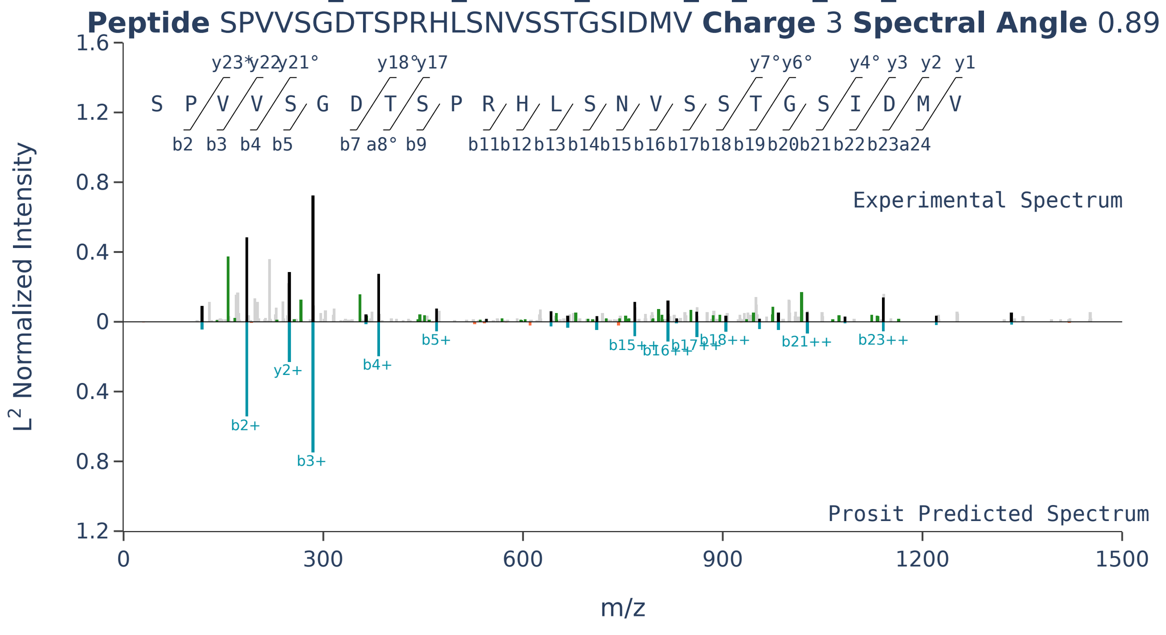


**Source** WSoh_221121_011221_HFGoe_L1_hTau_4h_R1 **Scan** 13372
**Peptide** CGSKDNIKHVPGGGSVQIVYK **Charge** 4 **Spectral Angle** 0.81

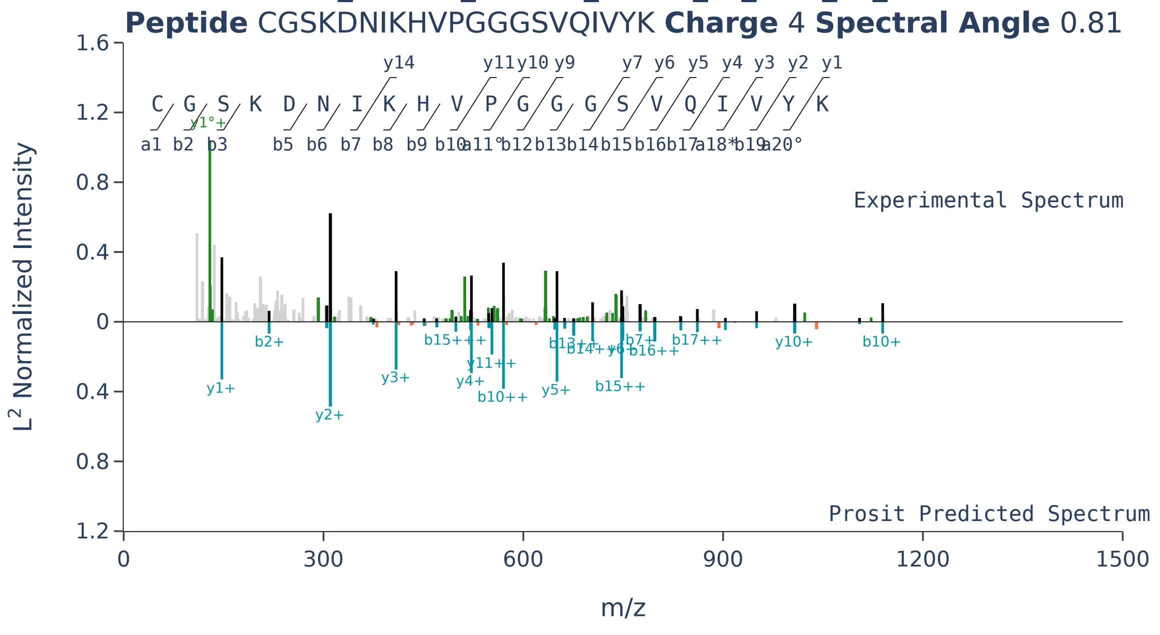


**Source** WSoh_221121_011221_HFGoe_L2_hTau_24h_R1 **Scan** 18062
**Peptide** TGSIDMVDS **Charge** 1 **Spectral Angle** 0.76

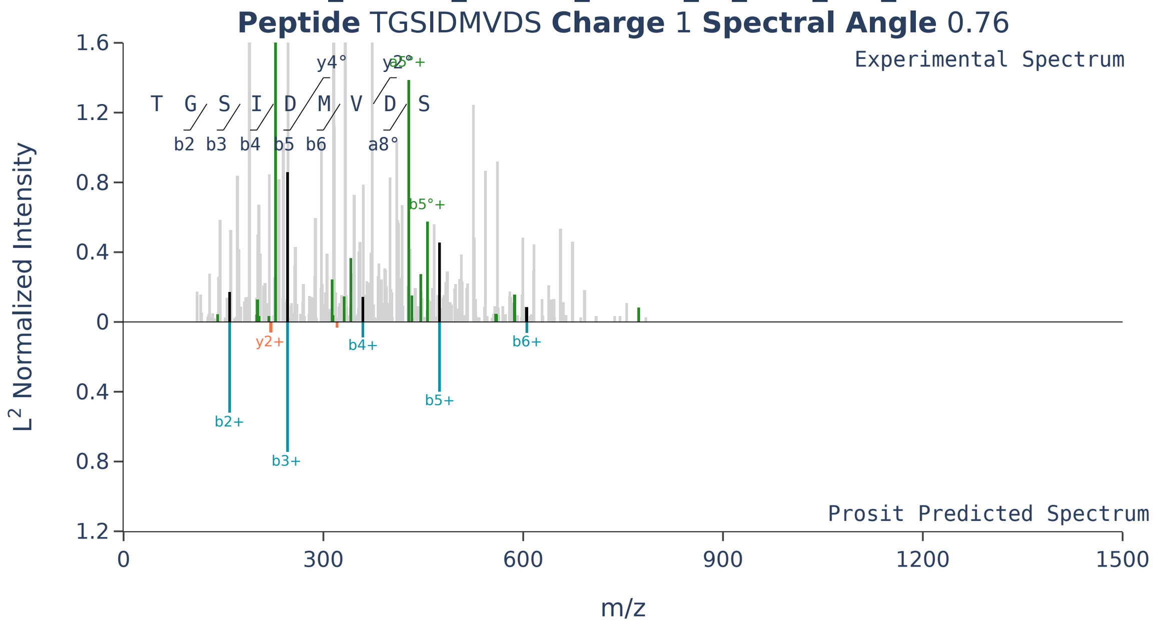


**Source** WSoh_221121_011221_HFGoe_L2_hTau_4h_R2 **Scan** 14558
**Peptide** KPGGGQVEVKSEKLDFKDRVQSKL **Charge** 4 **Spectral Angle** 0.86

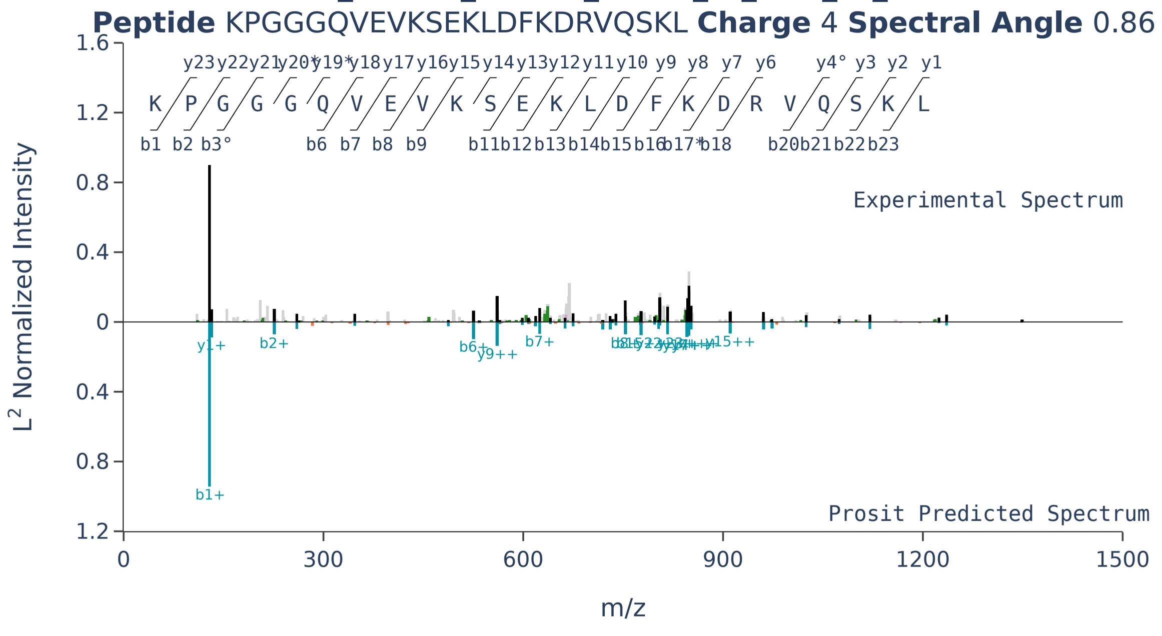


**Source** WSoh_221121_011221_HFGoe_L1_hTau_4h_R1 **Scan** 7159
**Peptide** EPPKSGDRSGYSSPGSPGTPGSRS **Charge** 3 **Spectral Angle** 0.84

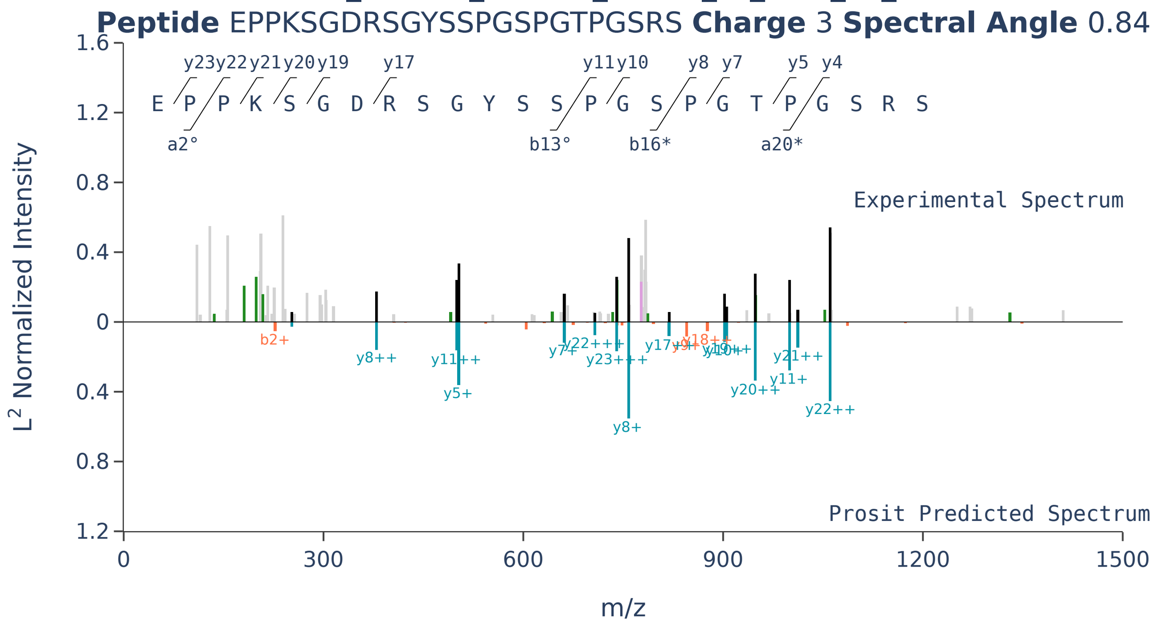

**Source** WSoh_221121_011221_HFGoe_L2_hTau_24h_R1 **Scan** 21685
**Peptide** RLQTAPVPMPDLKNVKSKIG **Charge** 4 **Spectral Angle** 0.86

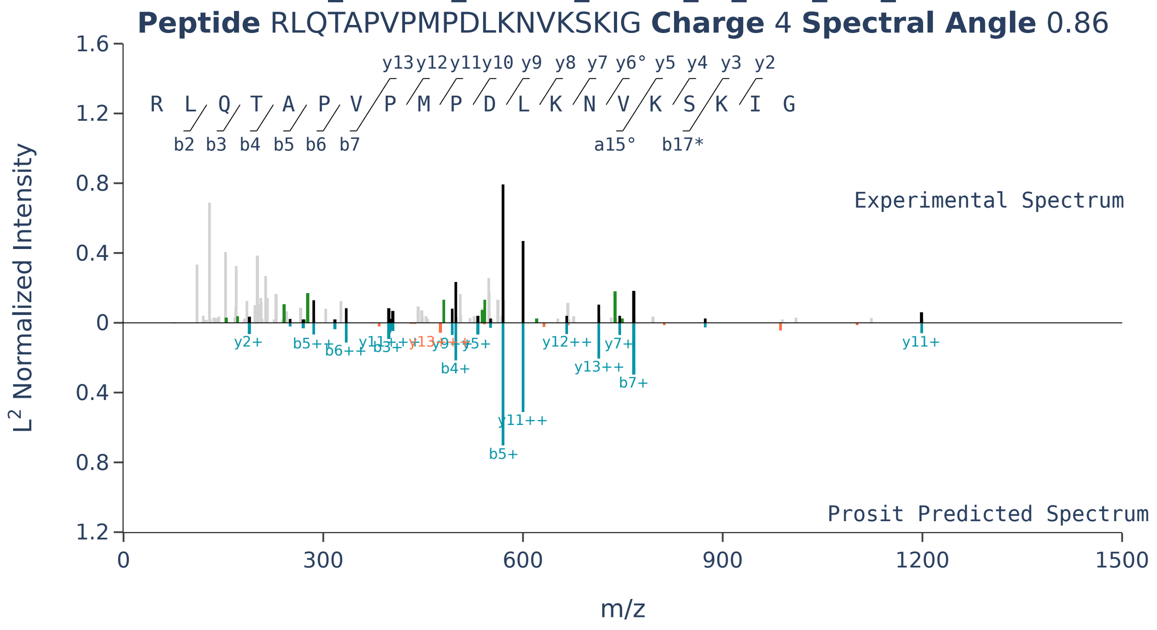


**Source** WSoh_221121_011221_HFGoe_L2_hTau_1h_R1 **Scan** 5634
**Peptide** VVRTPPKSPSSAKS **Charge** 2 **Spectral Angle** 0.77

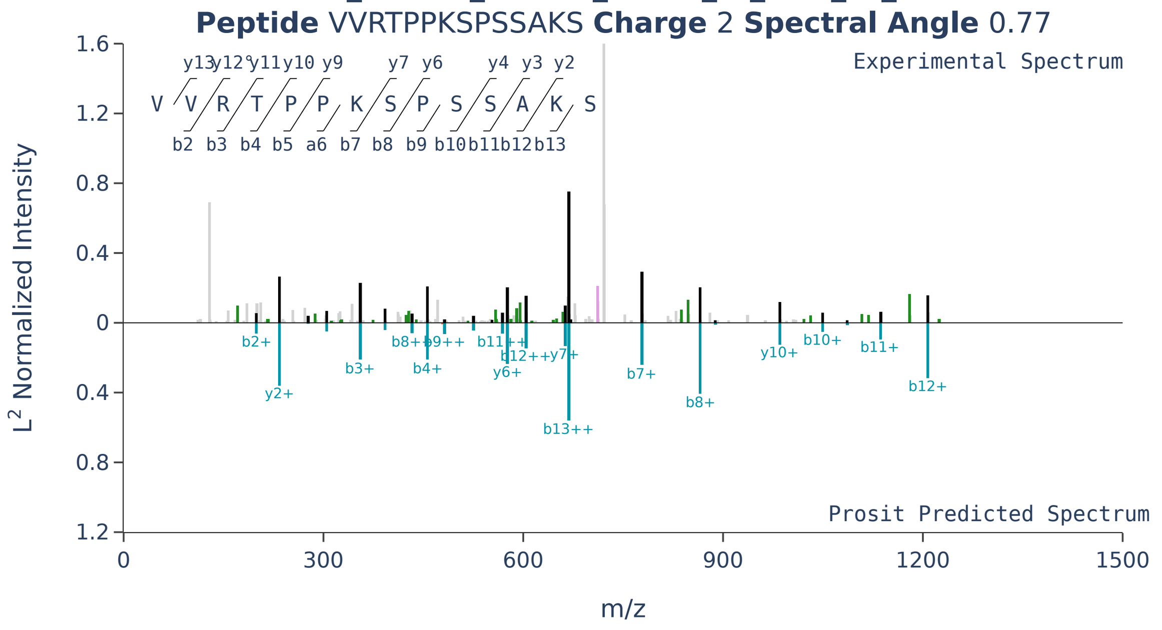


**Source** WSoh_221121_011221_HFGoe_L2_hTau_24h_R1 **Scan** 24228
**Peptide** PVPMPDLKNVKSKIGSTENL **Charge** 3 **Spectral Angle** 0.89

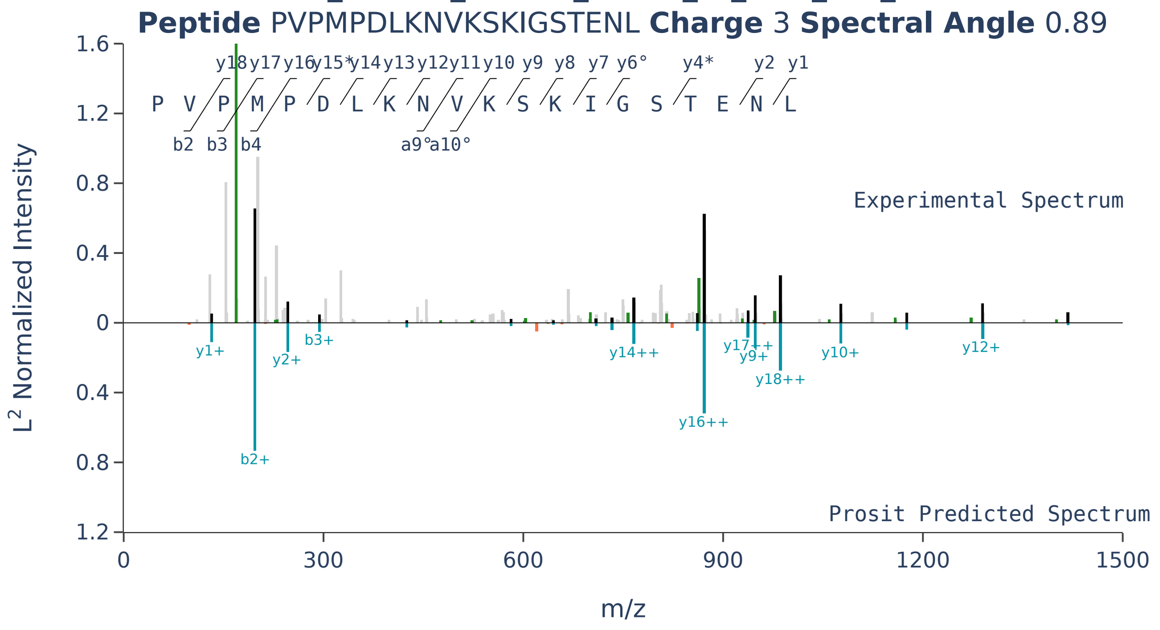


**Source** WSoh_221121_011221_HFGoe_L2_hTau_24h_R1 **Scan** 9590
**Peptide** PRHLSNVSSTGSLD **Charge** 2 **Spectral Angle** 0.81

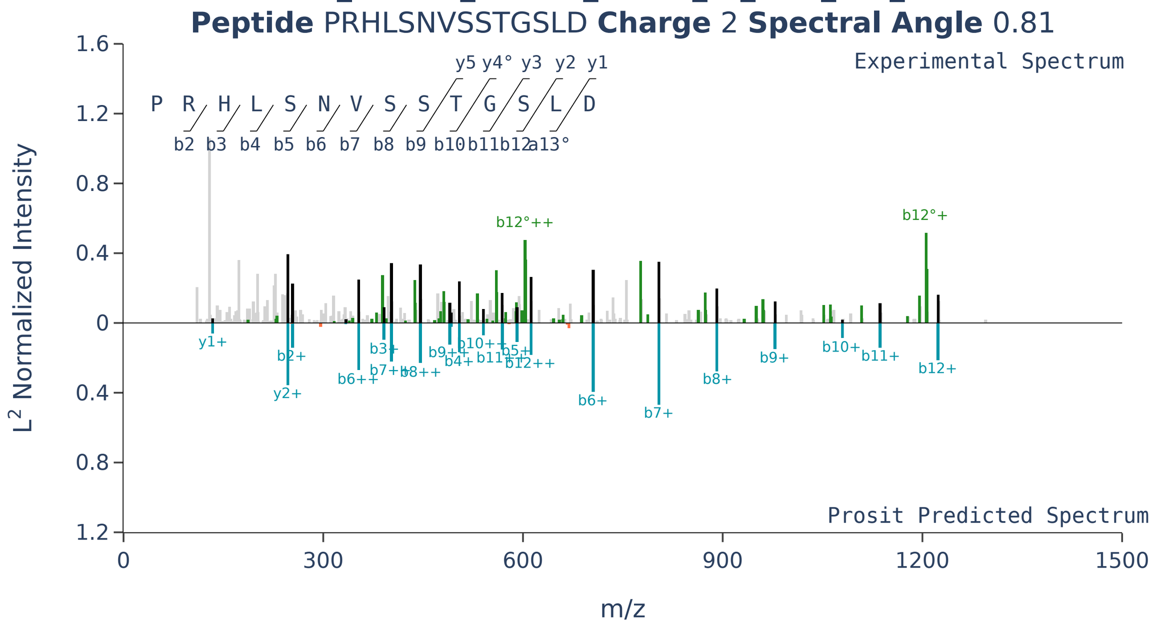


**Source** WSoh_221121_011221_HFGoe_L1_hTau_24h_R1 **Scan** 9545
**Peptide** VKSKIGSTENL **Charge** 2 **Spectral Angle** 0.87

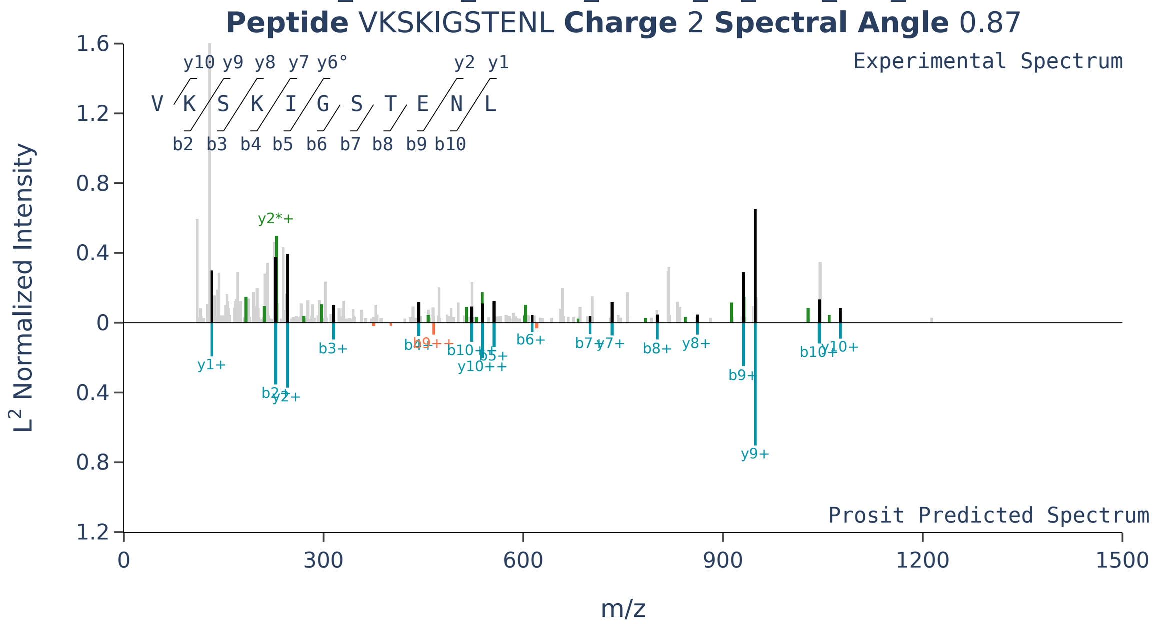


**Source** WSoh_221121_011221_HFGoe_L1_hTau_24h_R1 **Scan** 8078
**Peptide** AKTDHGAEIVYKS **Charge** 3 **Spectral Angle** 0.79

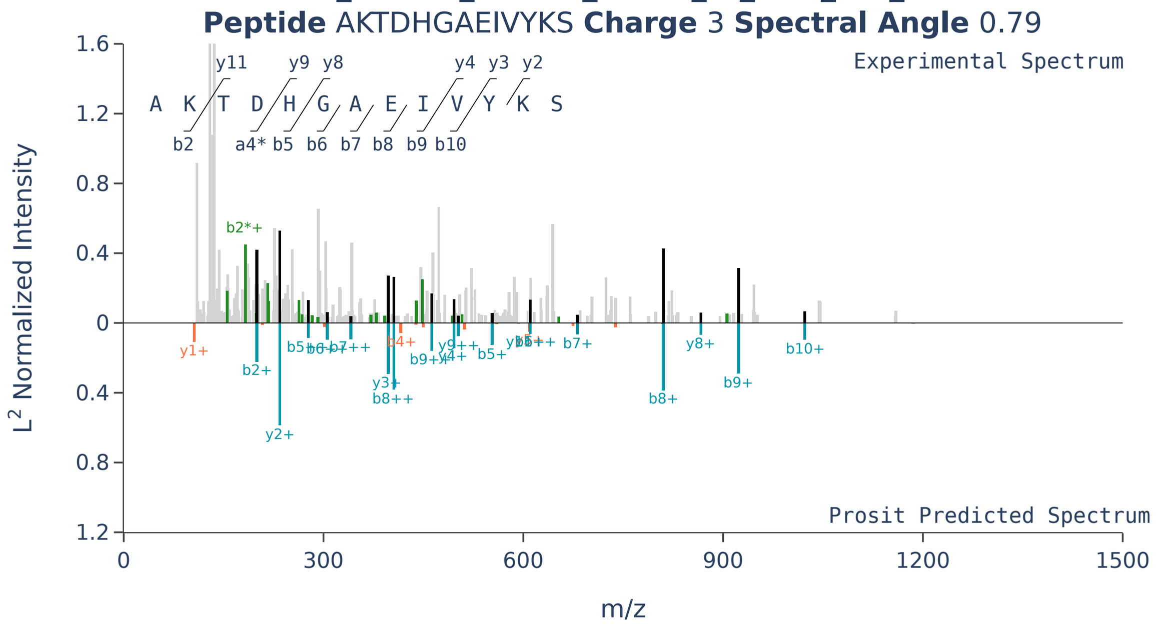


**Source** WSoh_221121_011221_HFGoe_L1_hTau_24h_R1 **Scan** 12419
**Peptide** DNITHVPGGGNKKIETHKLTFREN **Charge** 5 **Spectral Angle** 0.8

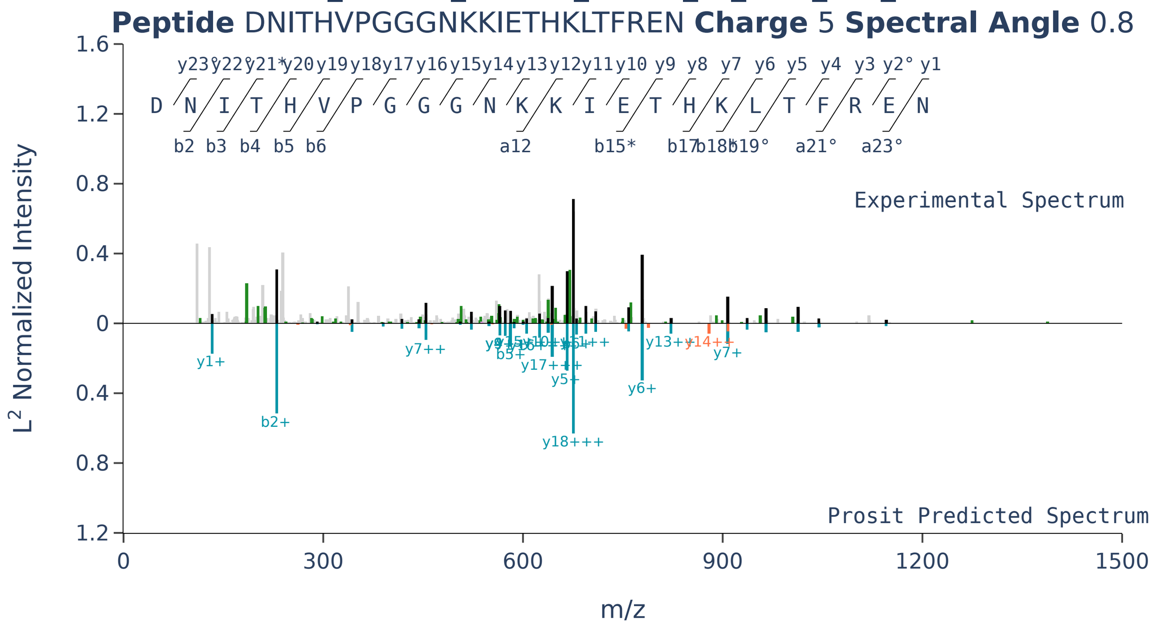


**Source** WSoh_221121_011221_HFGoe_L1_hTau_4h_R1 **Scan** 6955
**Peptide** KAKTDHGAEIV **Charge** 3 **Spectral Angle** 0.86

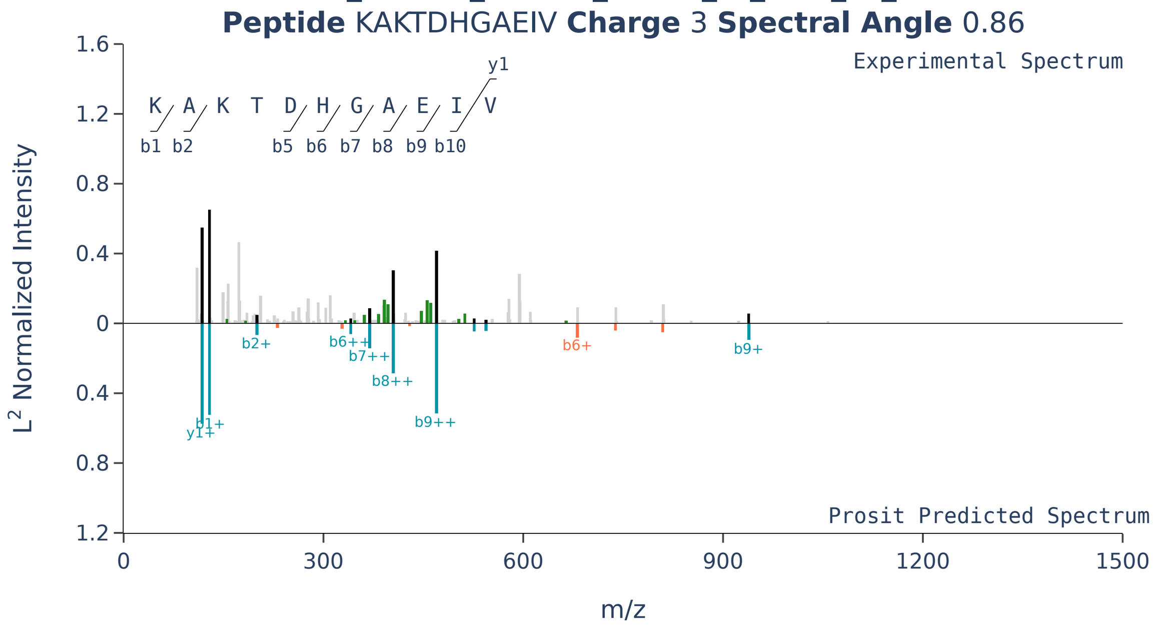


**Source** WSoh_221121_011221_HFGoe_L2_hTau_24h_R1 **Scan** 18164
**Peptide** PVDLSKVTSKCGSLG **Charge** 3 **Spectral Angle** 0.81

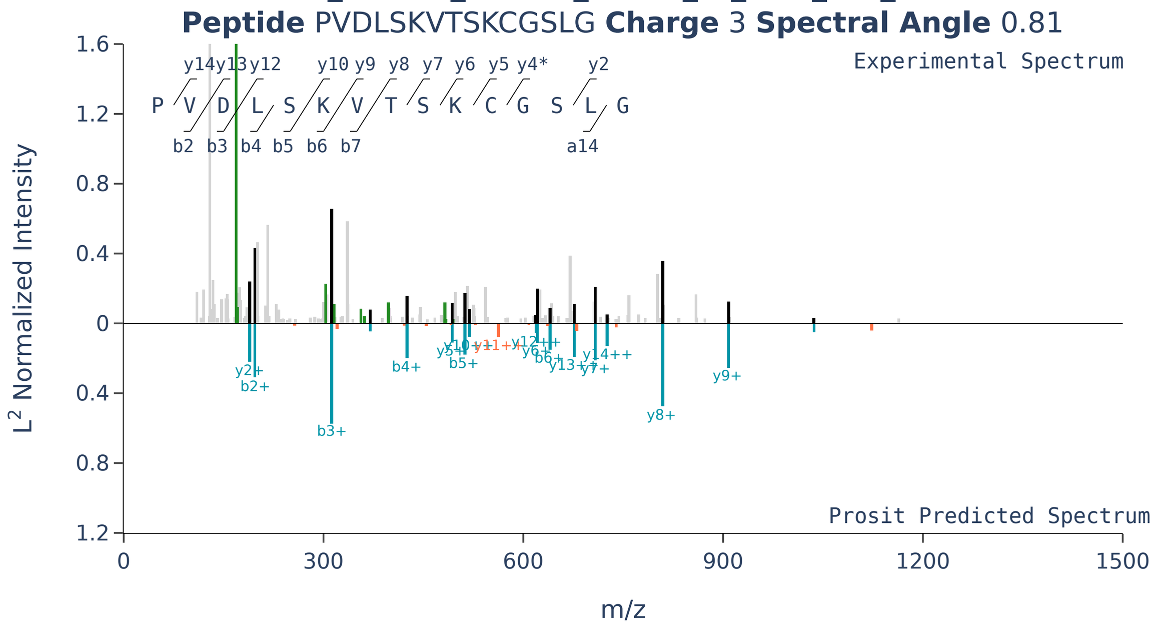


**Source** WSoh_221121_011221_HFGoe_L2_hTau_24h_R1 **Scan** 9352
**Peptide** GSTENLKHQPGGGKVQIINK **Charge** 3 **Spectral Angle** 0.86

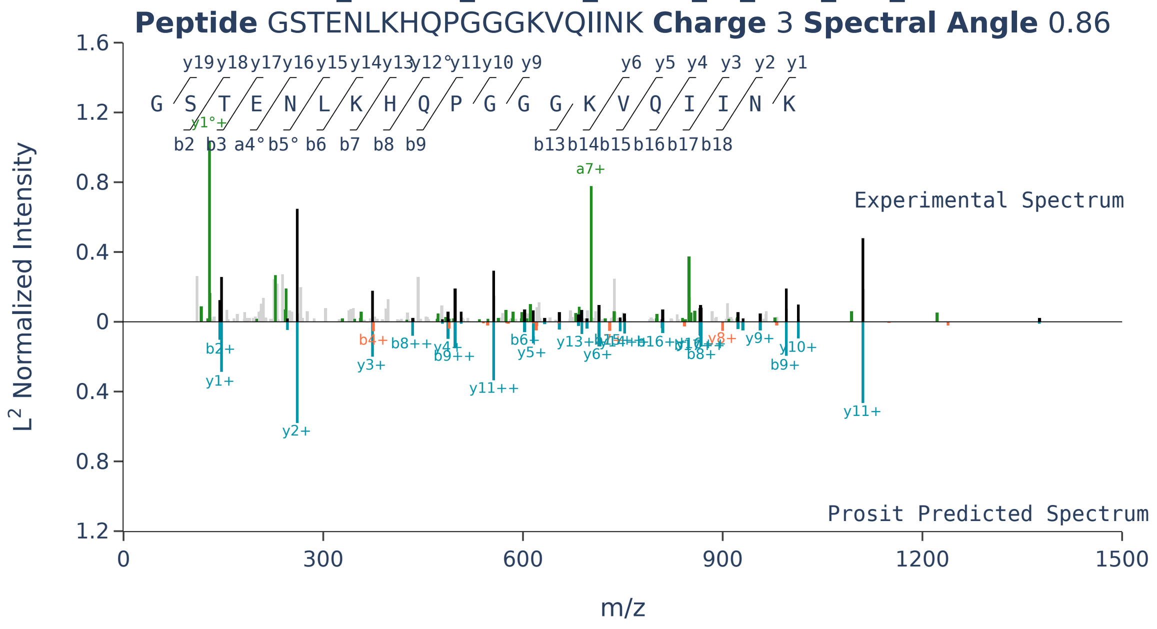


**Source** WSoh_221121_011221_HFGoe_L2_hTau_24h_R2 **Scan** 6710
**Peptide** DLSNVQSKCGSKDNLKH **Charge** 3 **Spectral Angle** 0.95

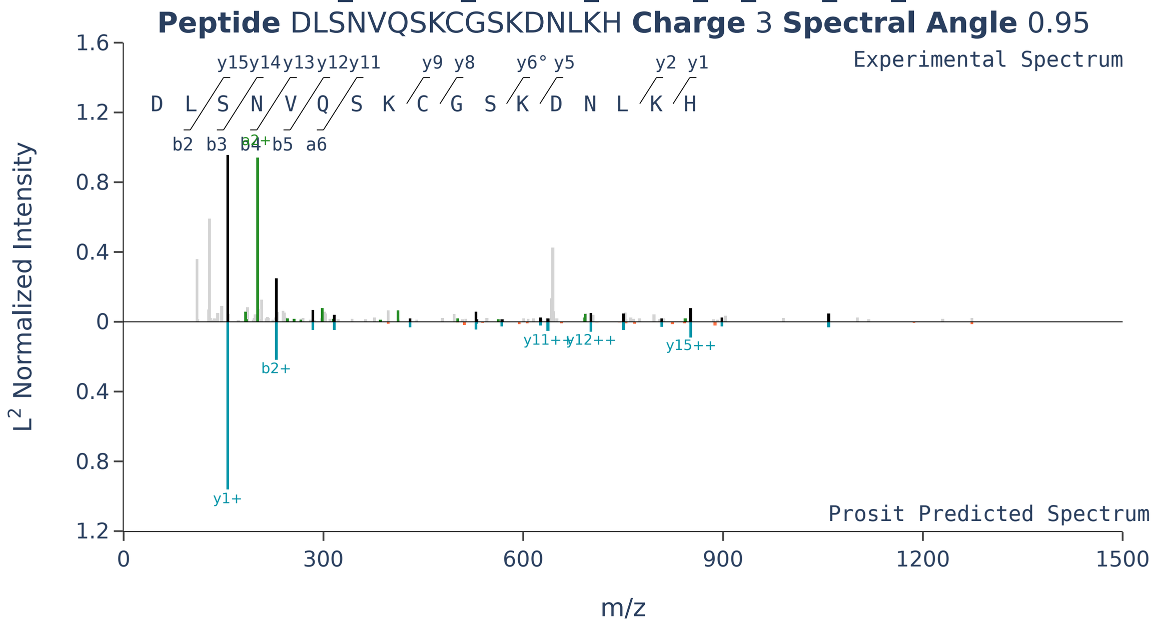


**Source** WSoh_221121_011221_HFGoe_L1_hTau_24h_R2 **Scan** 18517
**Peptide** TPSLPTPPTREPKKVAVV **Charge** 4 **Spectral Angle** 0.8

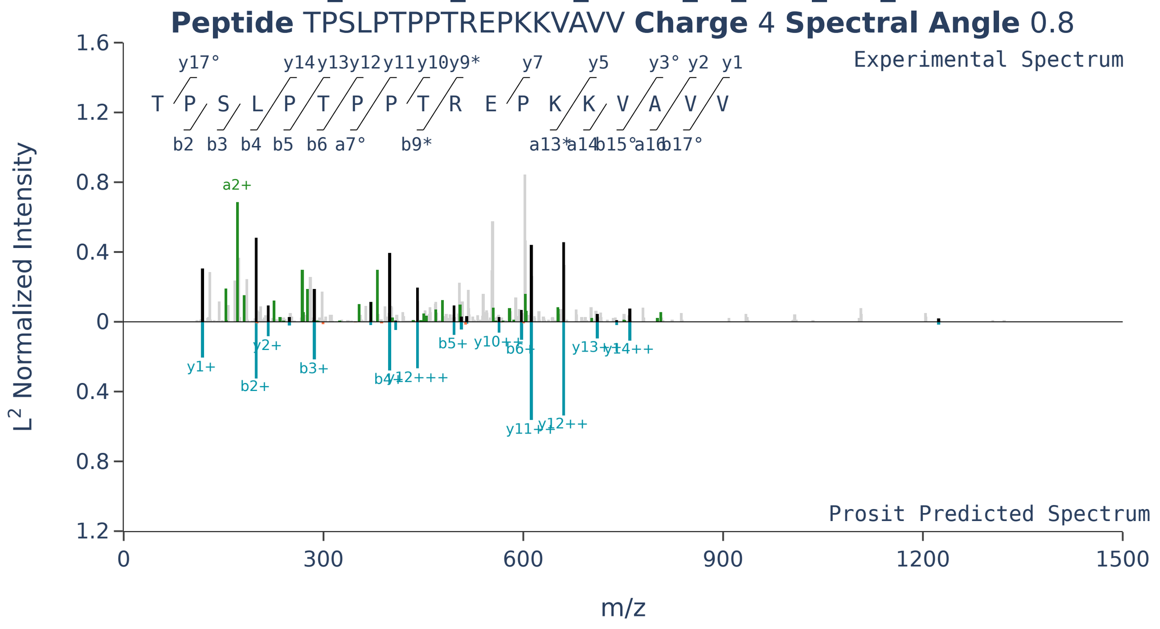


**Source** WSoh_221121_011221_HFGoe_L1_hTau_4h_R2 **Scan** 20906
**Peptide** SKDNIKHVPGGGSVQIVYKPVDL **Charge** 3 **Spectral Angle** 0.85

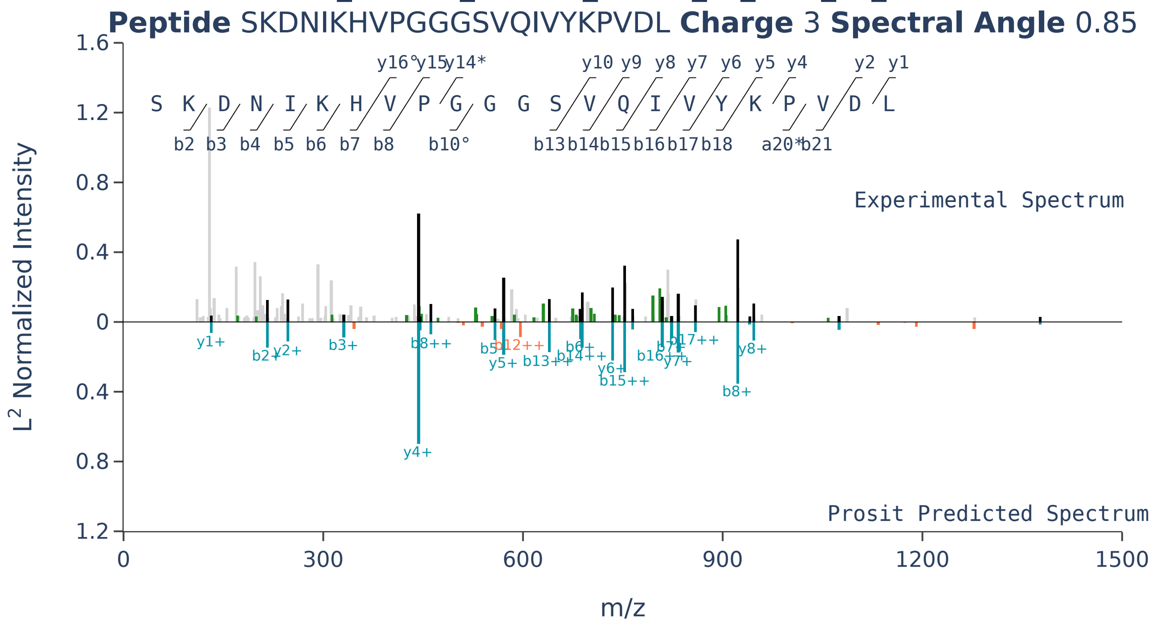


**Source** WSoh_221121_011221_HFGoe_L1_hTau_24h_R2 **Scan** 8950
**Peptide** IATPRGAAPPGQKGQANA **Charge** 2 **Spectral Angle** 0.86

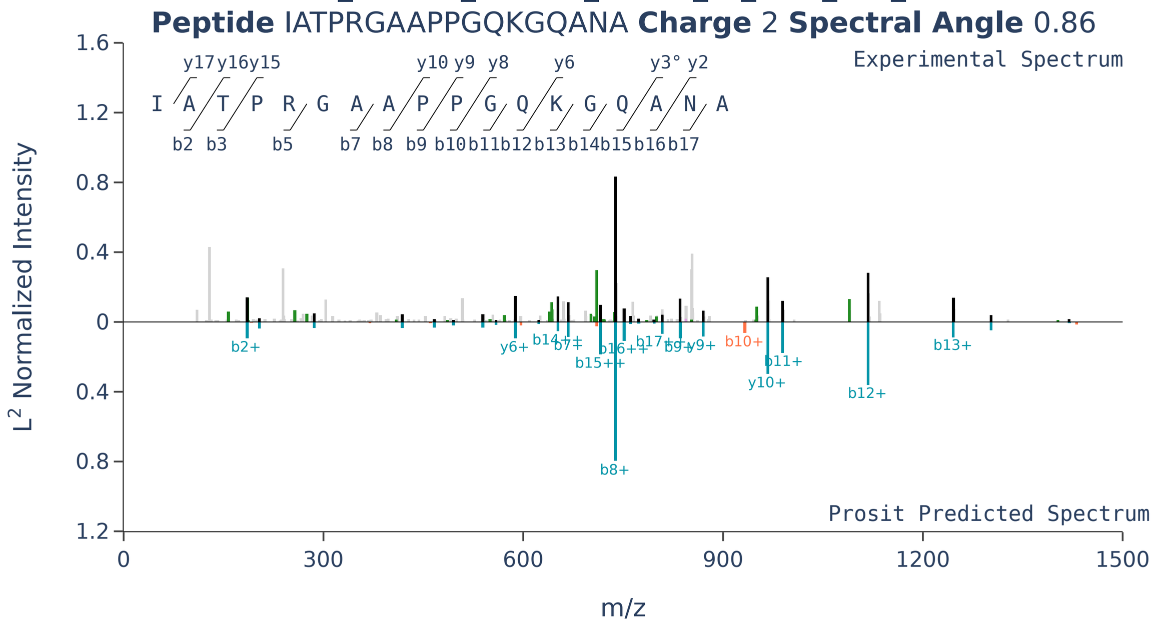


**Source** WSoh_221121_011221_HFGoe_L1_hTau_4h_R2 **Scan** 7700
**Peptide** SKVTSKCGSLGNIH **Charge** 3 **Spectral Angle** 0.92

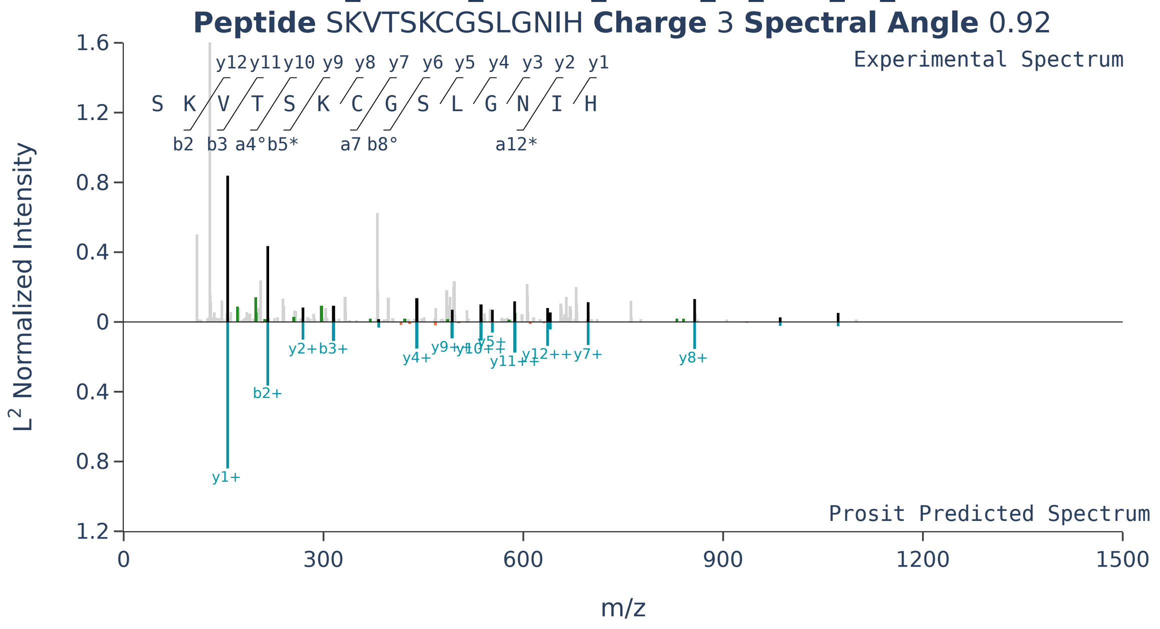

**Source** WSoh_221121_011221_HFGoe_L1_hTau_4h_R2 **Scan** 21010
**Peptide** PVVSGDTSPRHLSNVSSTGSIDMV **Charge** 3 **Spectral Angle** 0.89

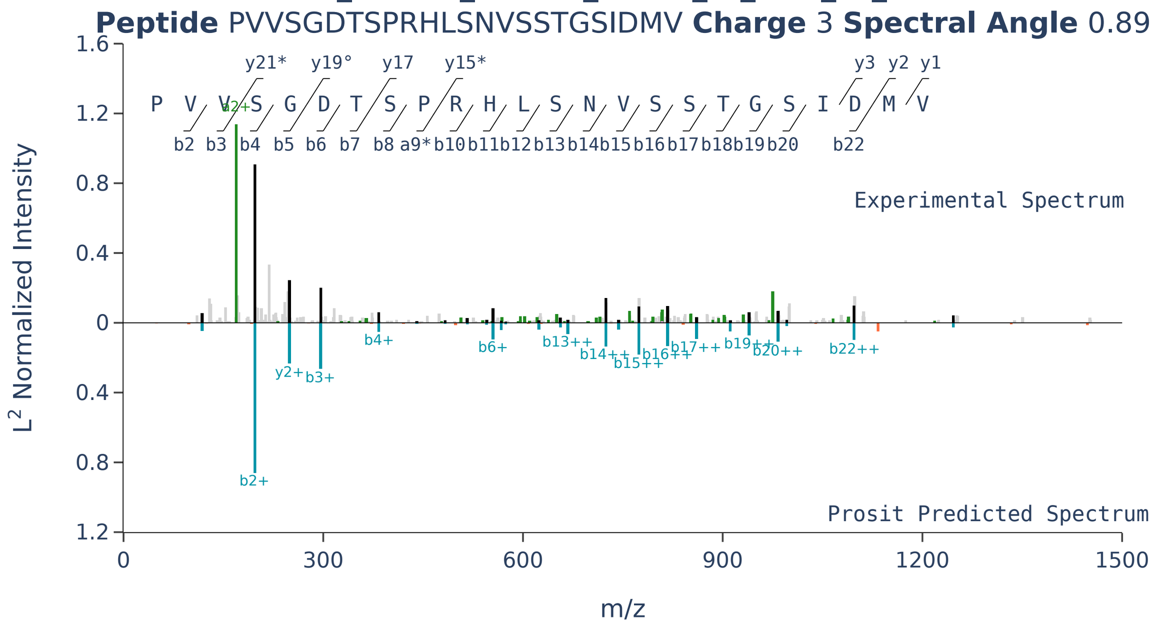


**Source** WSoh_221121_011221_HFGoe_L2_hTau_24h_R1 **Scan** 6198
**Peptide** RKDQGGYTMHQDQEGD **Charge** 3 **Spectral Angle** 0.76

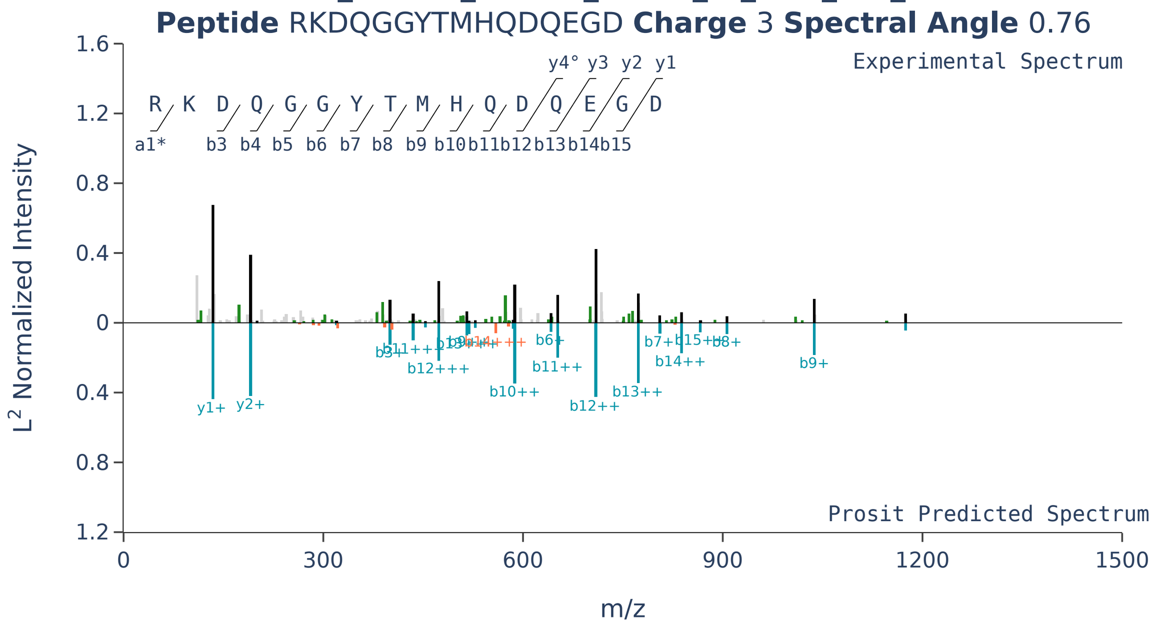


**Source** WSoh_221121_011221_HFGoe_L2_hTau_24h_R2 **Scan** 9238
**Peptide** AAQPHTEL **Charge** 1 **Spectral Angle** 0.88

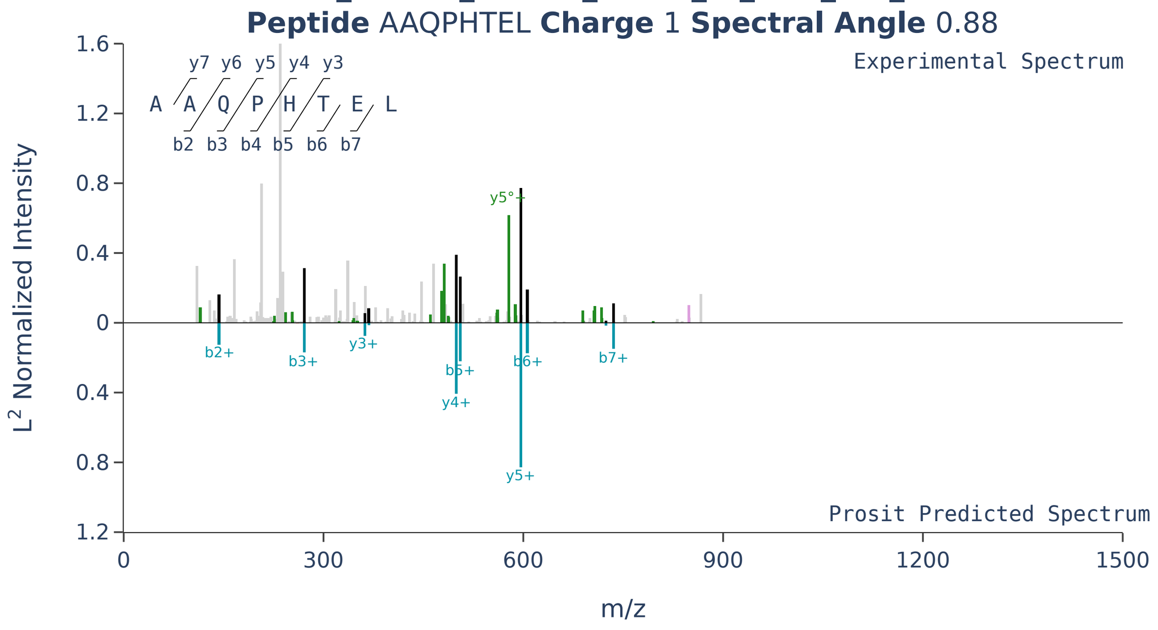


**Source** WSoh_221121_011221_HFGoe_L1_hTau_2h_R1 **Scan** 7841
**Peptide** GDTSPRHLSNV **Charge** 2 **Spectral Angle** 0.86

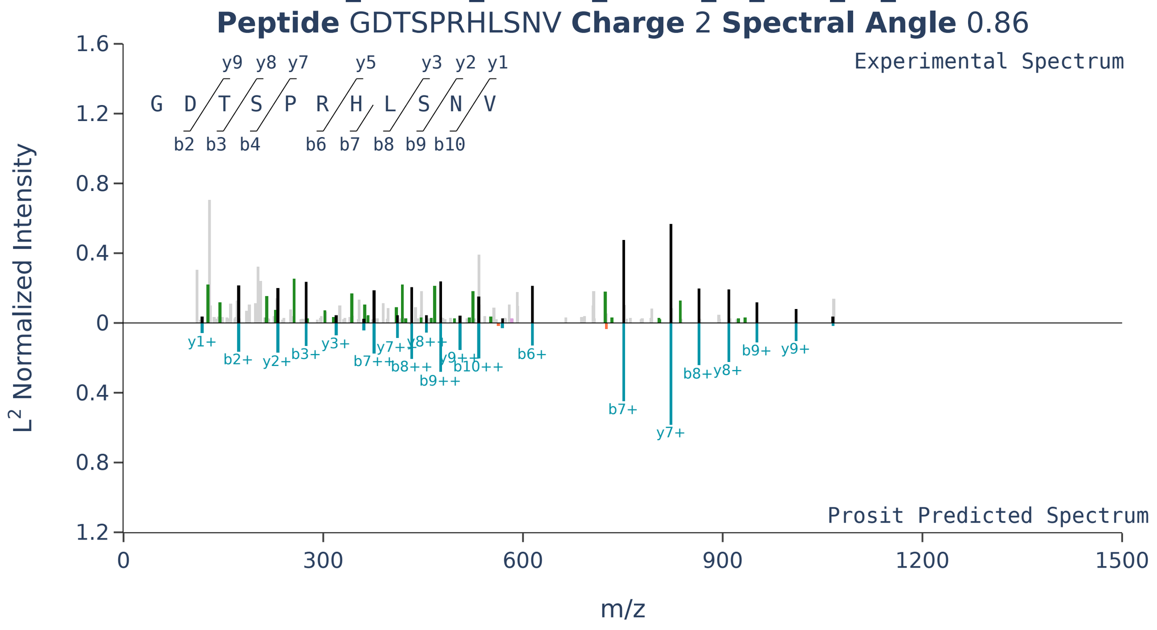


**Source** WSoh_221121_011221_HFGoe_L2_hTau_24h_R2 **Scan** 8555
**Peptide** HAGTYGLGDRKDQGGYTMHQD **Charge** 3 **Spectral Angle** 0.89

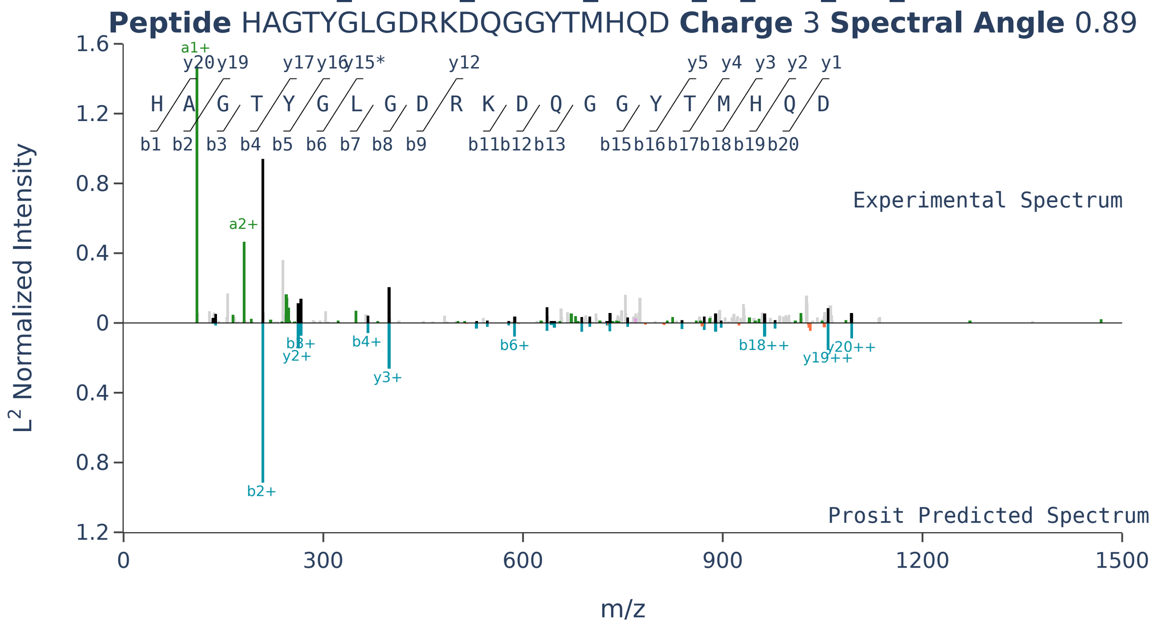


**Source** WSoh_221121_011221_HFGoe_L1_hTau_2h_R1 **Scan** 15564
**Peptide** PGGGQVEVKSEKLDFKDRVQSKI **Charge** 4 **Spectral Angle** 0.78

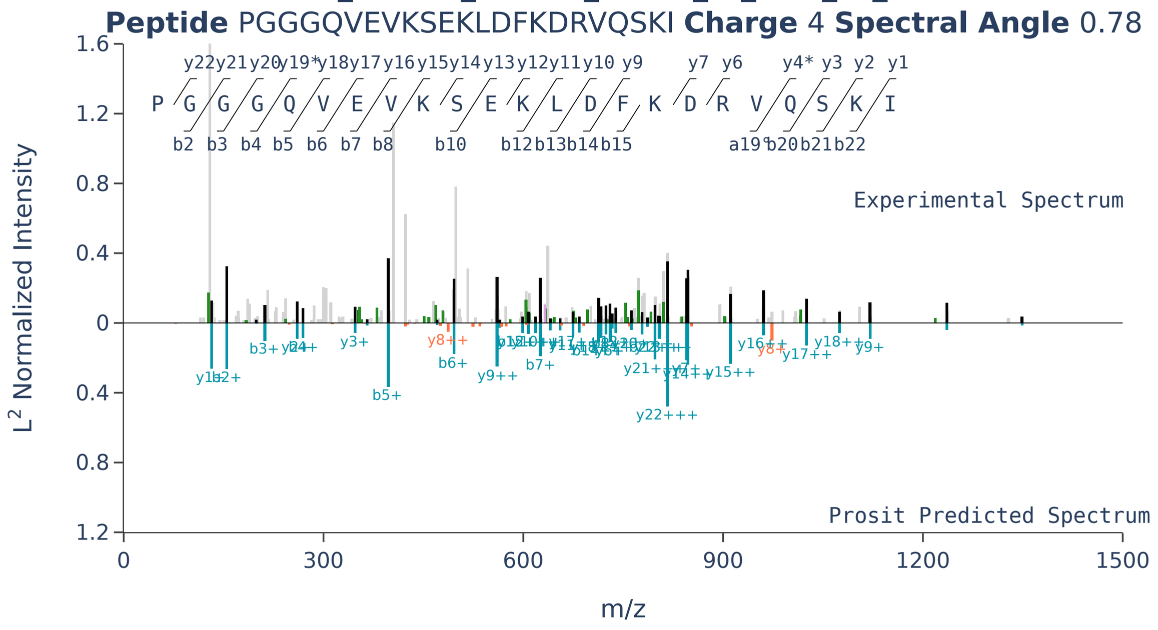


**Source** WSoh_221121_011221_HFGoe_L2_hTau_24h_R2 **Scan** 20532
**Peptide** RLQTAPVPMPDLKNVKSKIGSTENLKHQ **Charge** 5 **Spectral Angle** 0.81

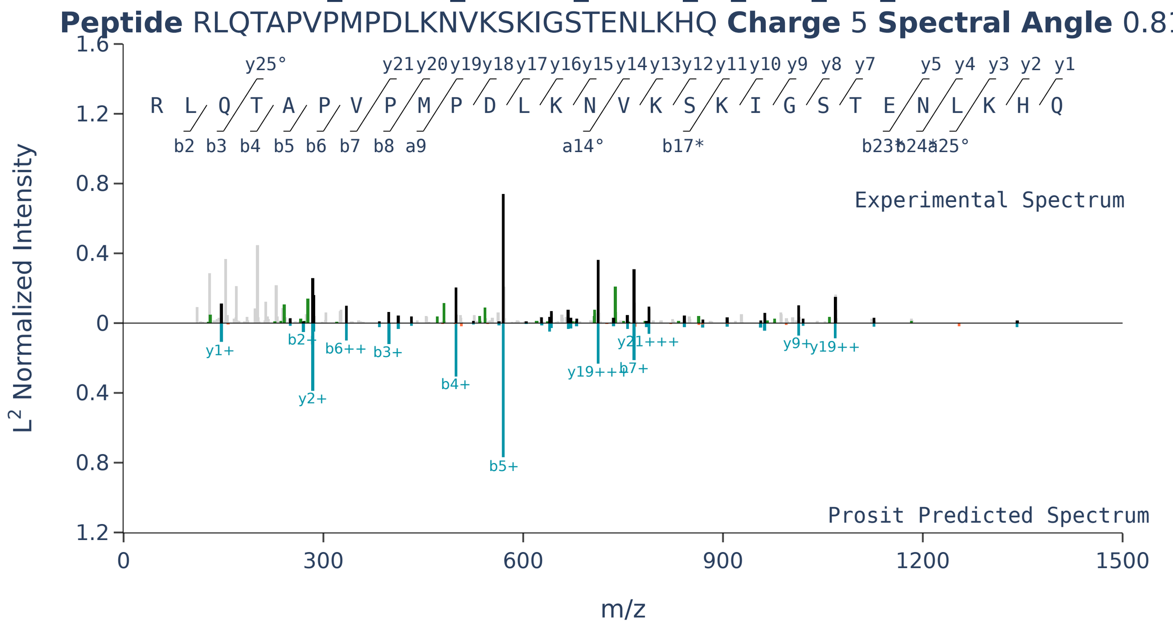


**Source** WSoh_221121_011221_HFGoe_L1_hTau_4h_R1 **Scan** 8432
**Peptide** NLTHVPGGGNKKIETHKL **Charge** 2 **Spectral Angle** 0.78

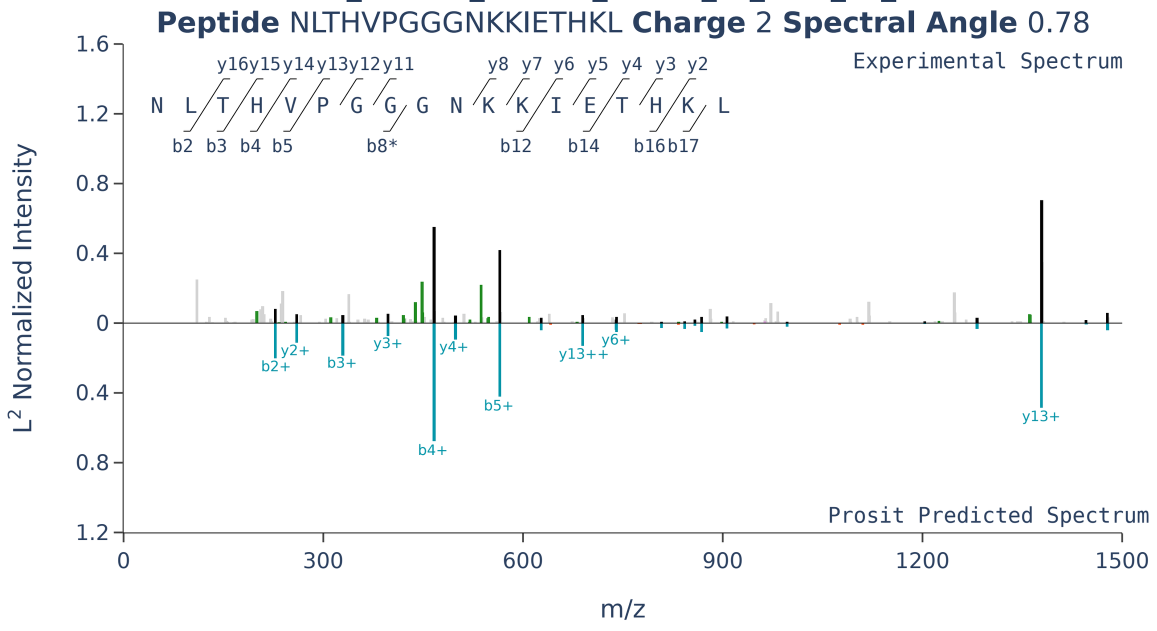


**Source** WSoh_221121_011221_HFGoe_L1_hTau_2h_R1 **Scan** 8519
**Peptide** DTSPRHLSNV **Charge** 3 **Spectral Angle** 0.85

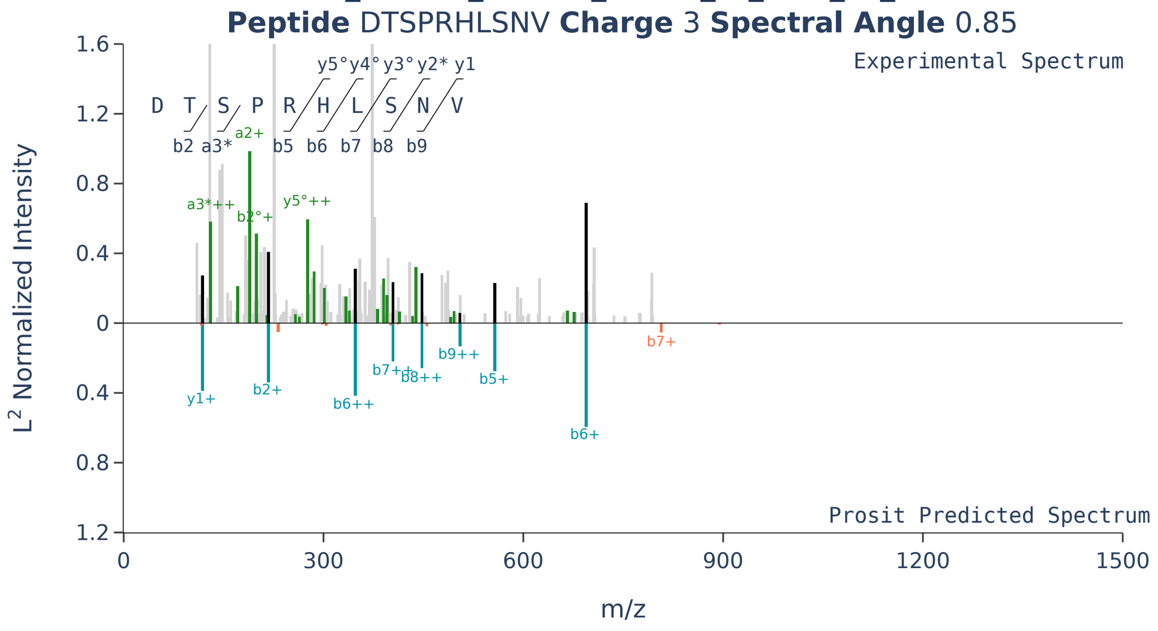


**Source** WSoh_221121_011221_HFGoe_L2_hTau_24h_R1 **Scan** 5302
**Peptide** EGAPGKQA **Charge** 2 **Spectral Angle** 0.82

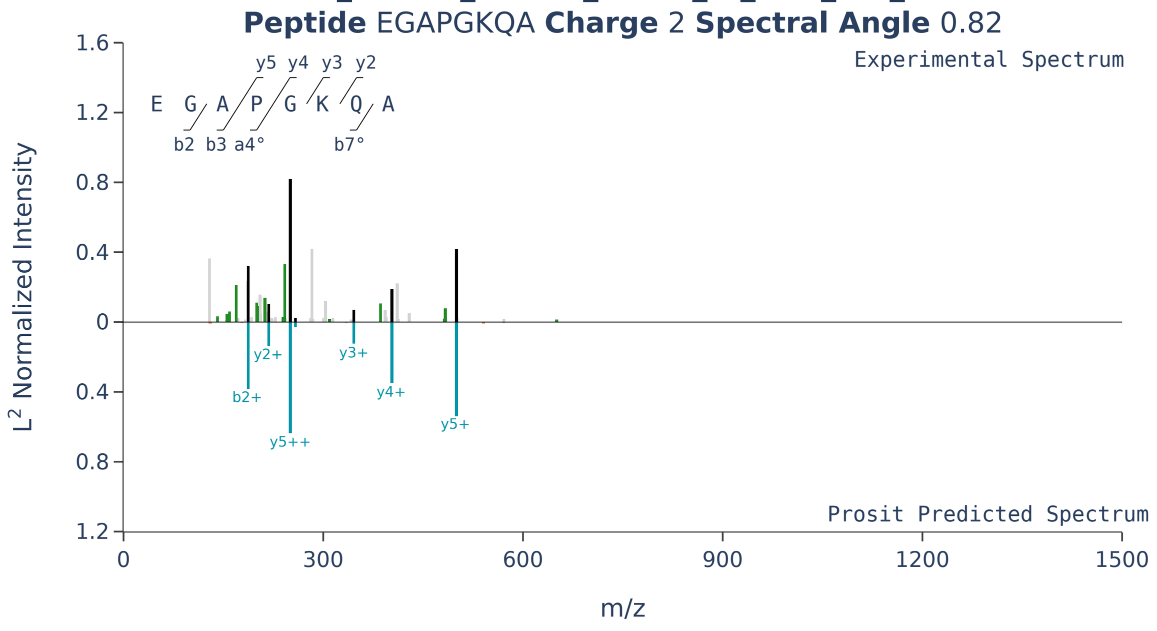


**Source** WSoh_221121_011221_HFGoe_L2_hTau_4h_R2 **Scan** 5735
**Peptide** ATPRGAAPPGQKGQ **Charge** 3 **Spectral Angle** 0.82

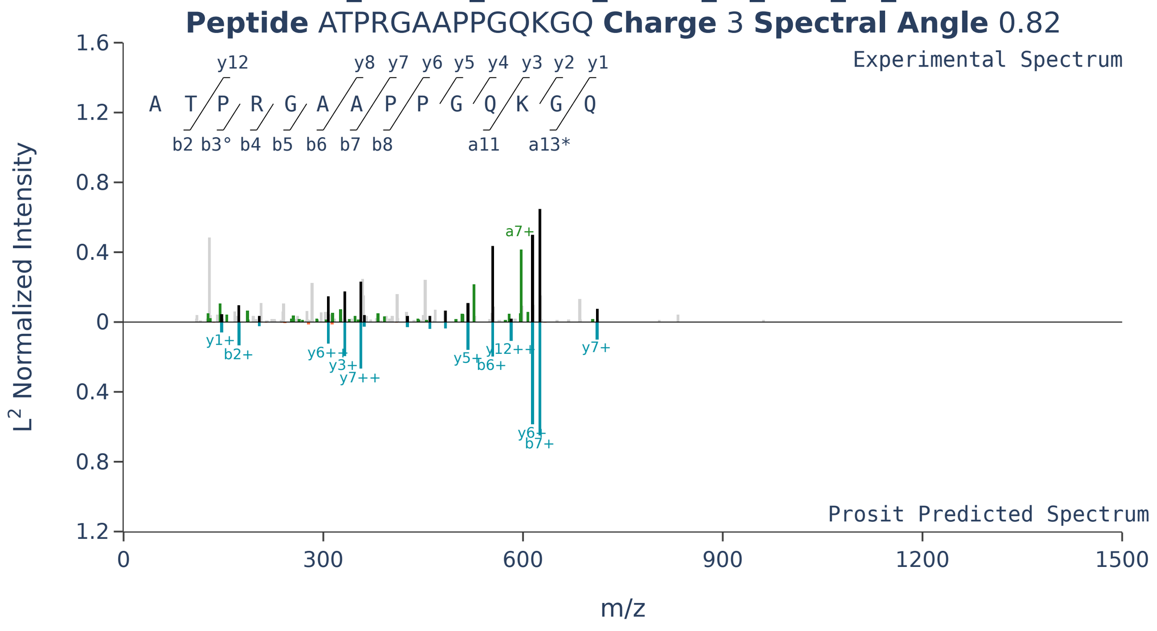


**Source** WSoh_221121_011221_HFGoe_L1_hTau_24h_R1 **Scan** 12718
**Peptide** KAKTDHGAEIVYKSPVVSGDTSPRHLS **Charge** 4 **Spectral Angle** 0.87

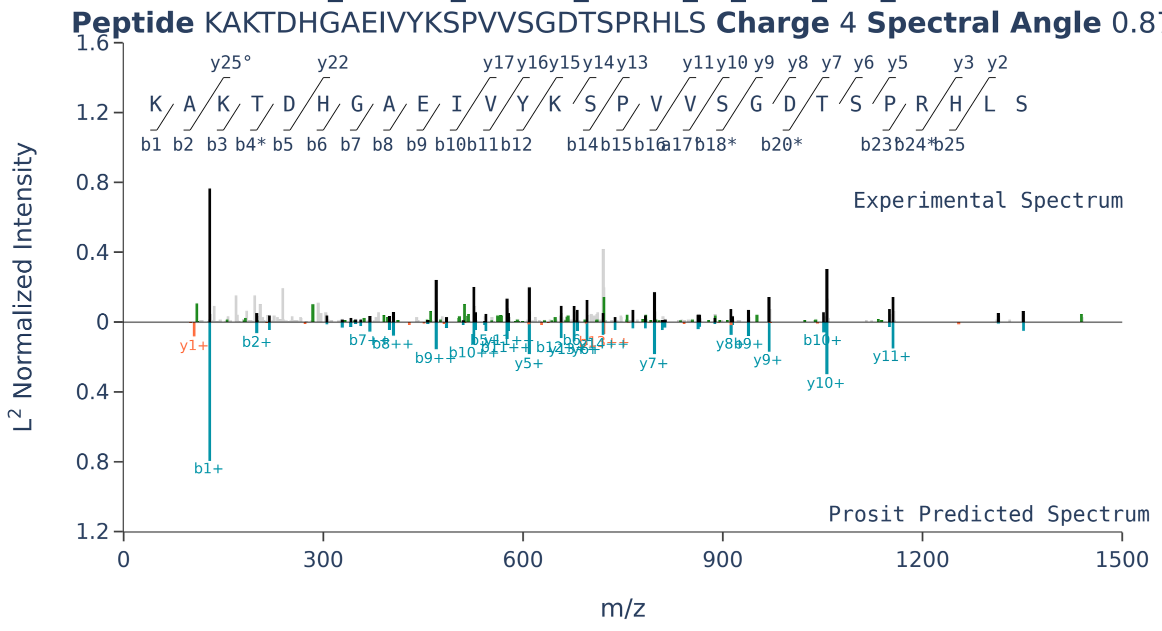


**Source** WSoh_221121_011221_HFGoe_L2_hTau_24h_R1 **Scan** 7104
**Peptide** LHHKPGGGQVEVKSEKL **Charge** 2 **Spectral Angle** 0.72

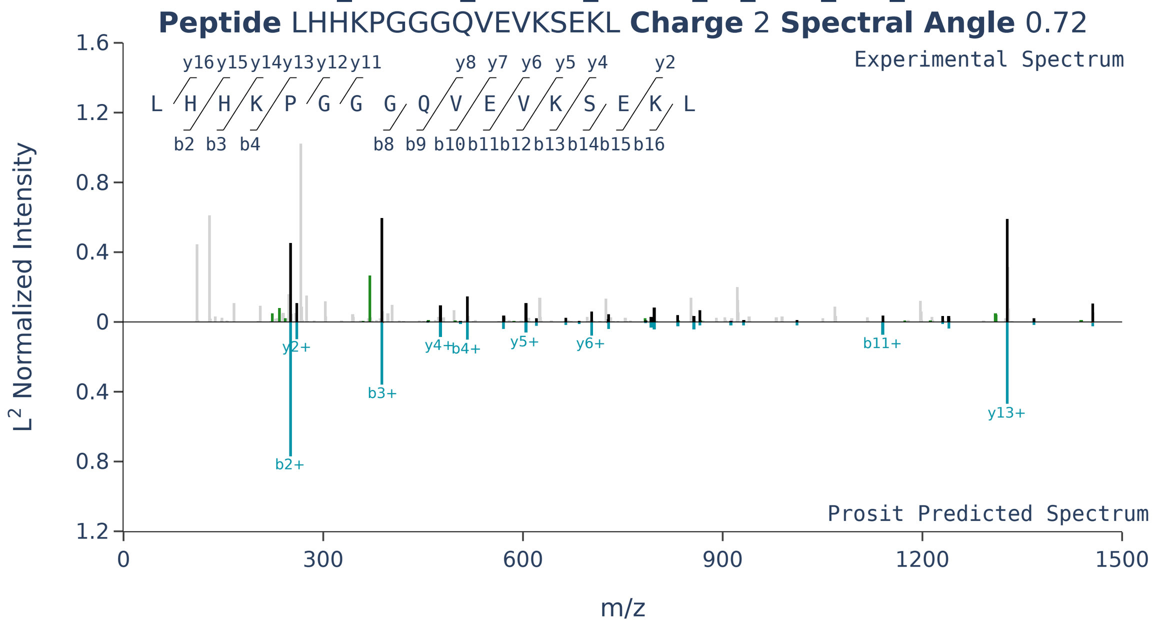


**Source** WSoh_221121_011221_HFGoe_L2_hTau_24h_R1 **Scan** 21358
**Peptide** TAEEAGIGDTPSLEDE **Charge** 2 **Spectral Angle** 0.82

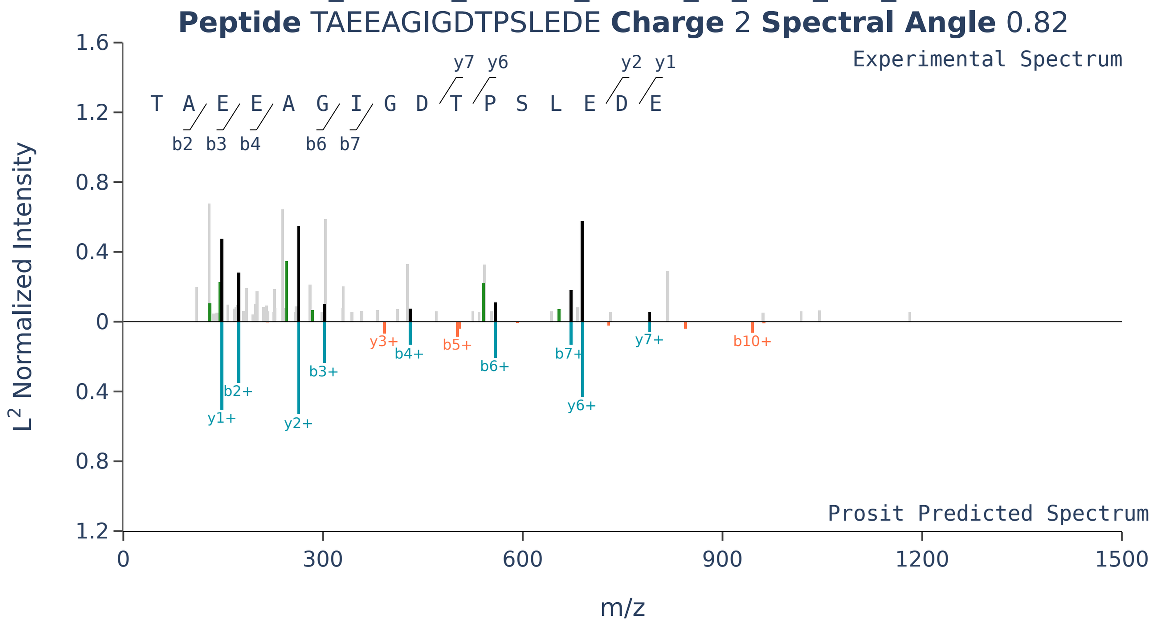


**Source** WSoh_221121_011221_HFGoe_L1_hTau_4h_R1 **Scan** 7787
**Peptide** CGSLGNIHHKPGGGQV **Charge** 2 **Spectral Angle** 0.88

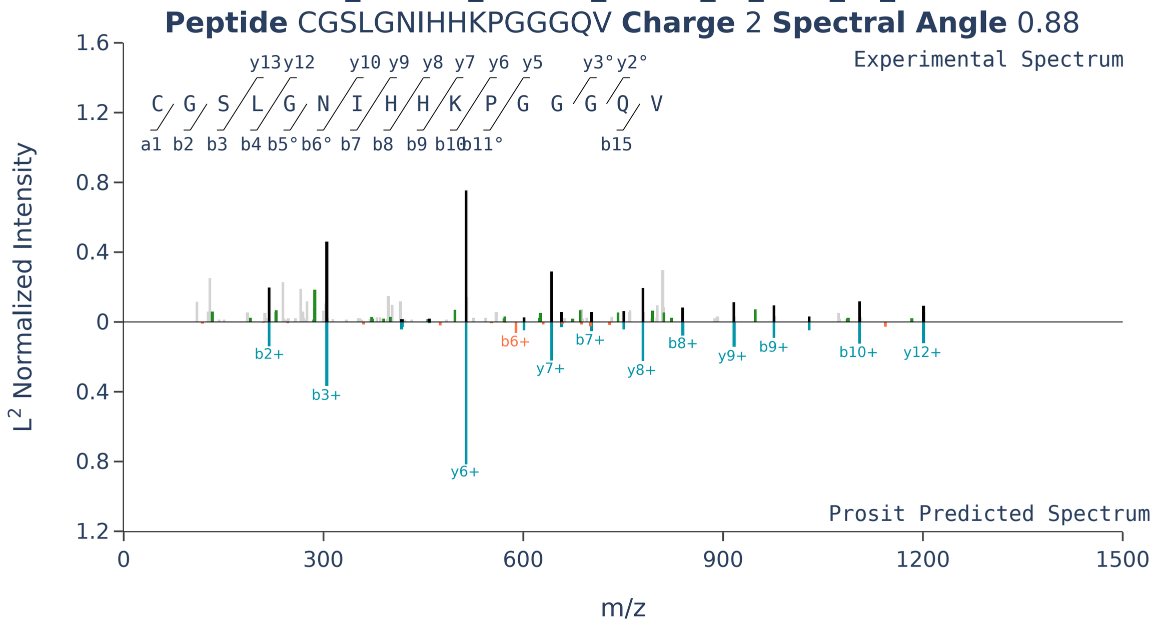

**Source** WSoh_221121_011221_HFGoe_L2_hTau_24h_R2 **Scan** 9308
**Peptide** KLTFRENAKAKTDHGAEL **Charge** 3 **Spectral Angle** 0.87

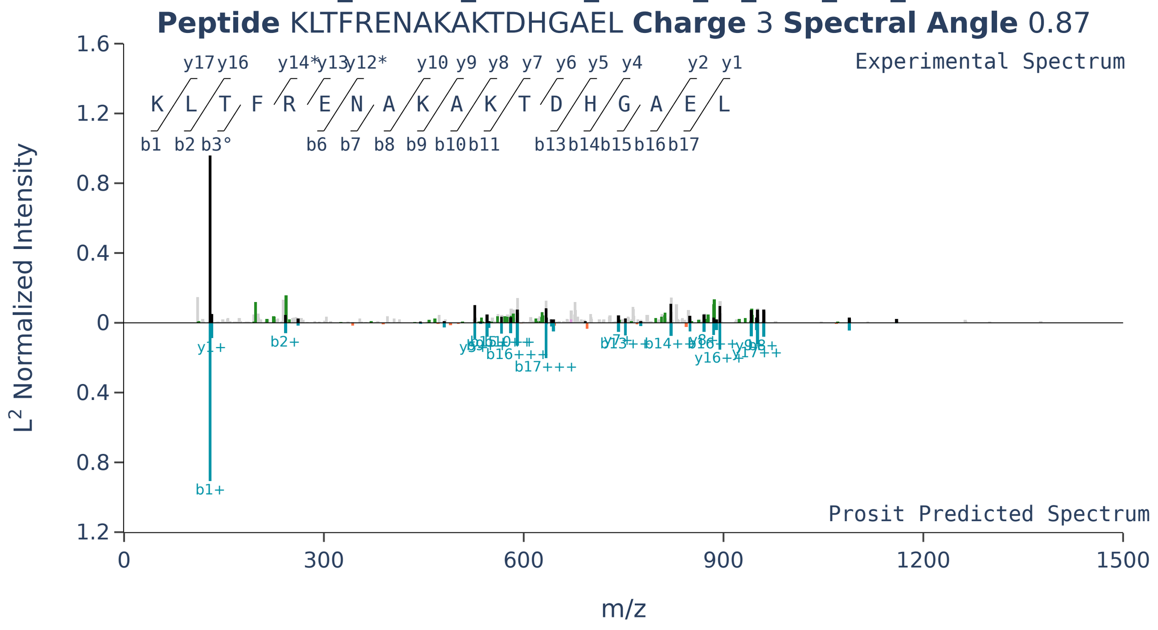


**Source** WSoh_221121_011221_HFGoe_L2_hTau_24h_R1 **Scan** 24124
**Peptide** RLQTAPVPMPDLKNVKSKIGSTENL **Charge** 4 **Spectral Angle** 0.88

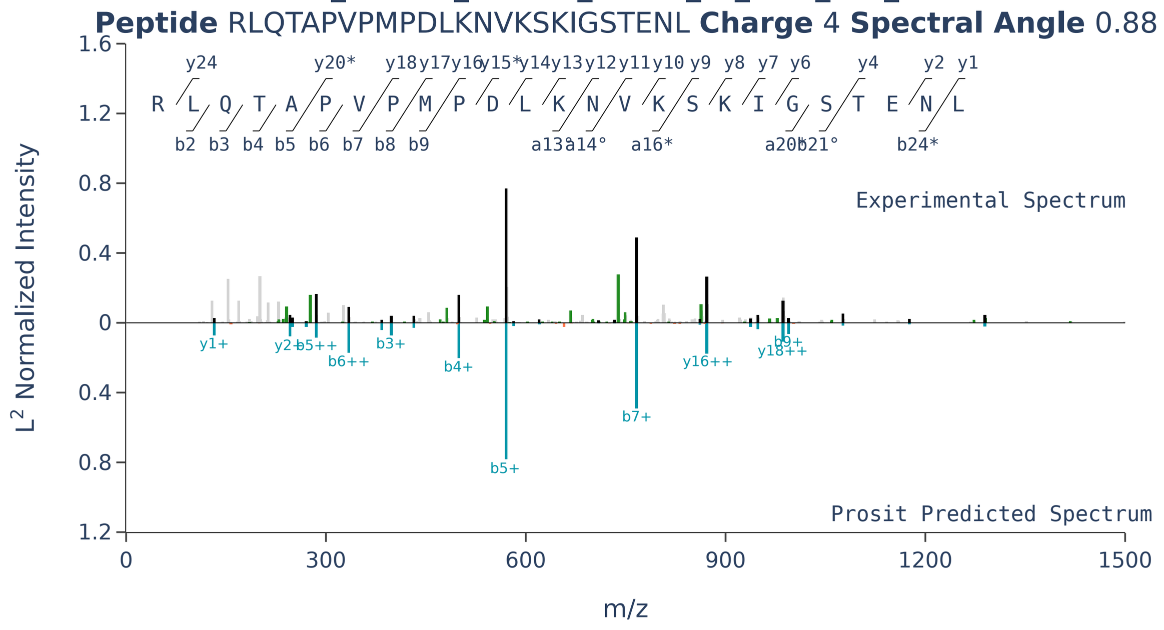


**Source** WSoh_221121_011221_HFGoe_L2_hTau_1h_R1 **Scan** 5783
**Peptide** RTPPKSPSSAKSRLQT **Charge** 4 **Spectral Angle** 0.83

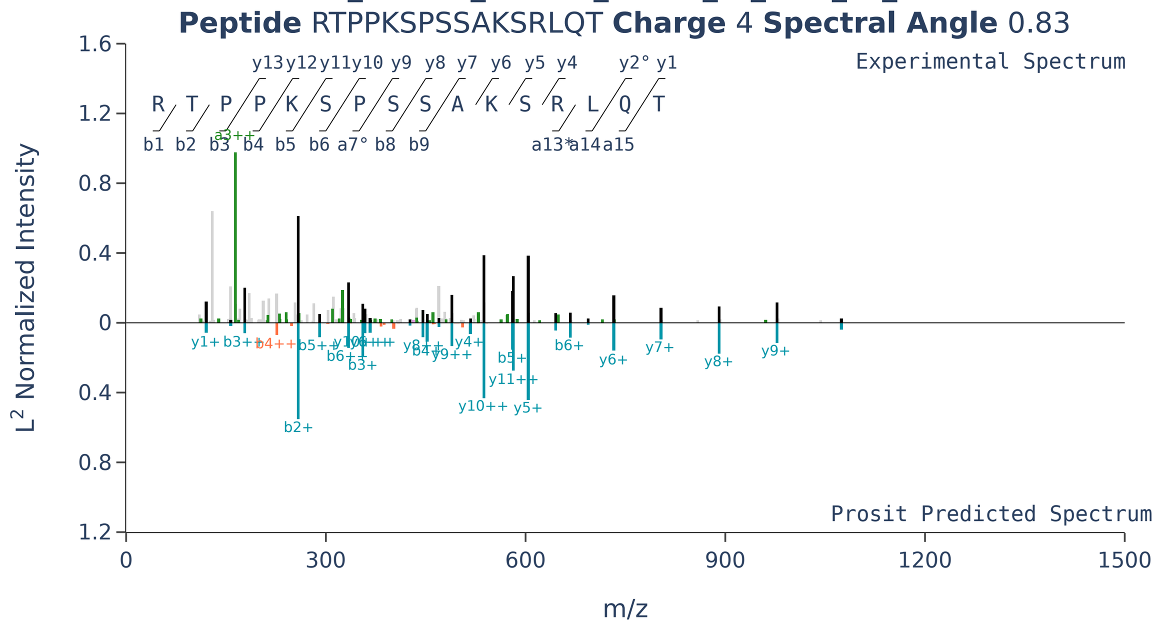


**Source** WSoh_221121_011221_HFGoe_L1_hTau_4h_R1 **Scan** 11308
**Peptide** KLGSTENLKHQPGGGKVQIINKKL **Charge** 4 **Spectral Angle** 0.93

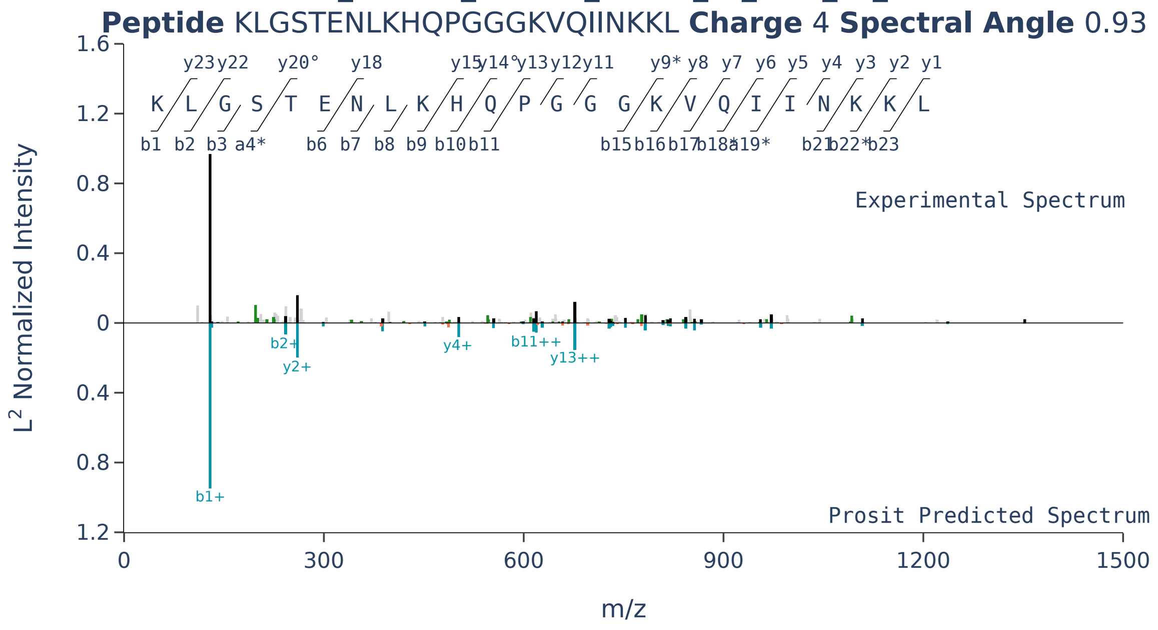


**Source** WSoh_221121_011221_HFGoe_L2_hTau_24h_R2 **Scan** 12477
**Peptide** LDLSNVQSKCGSKD **Charge** 2 **Spectral Angle** 0.86

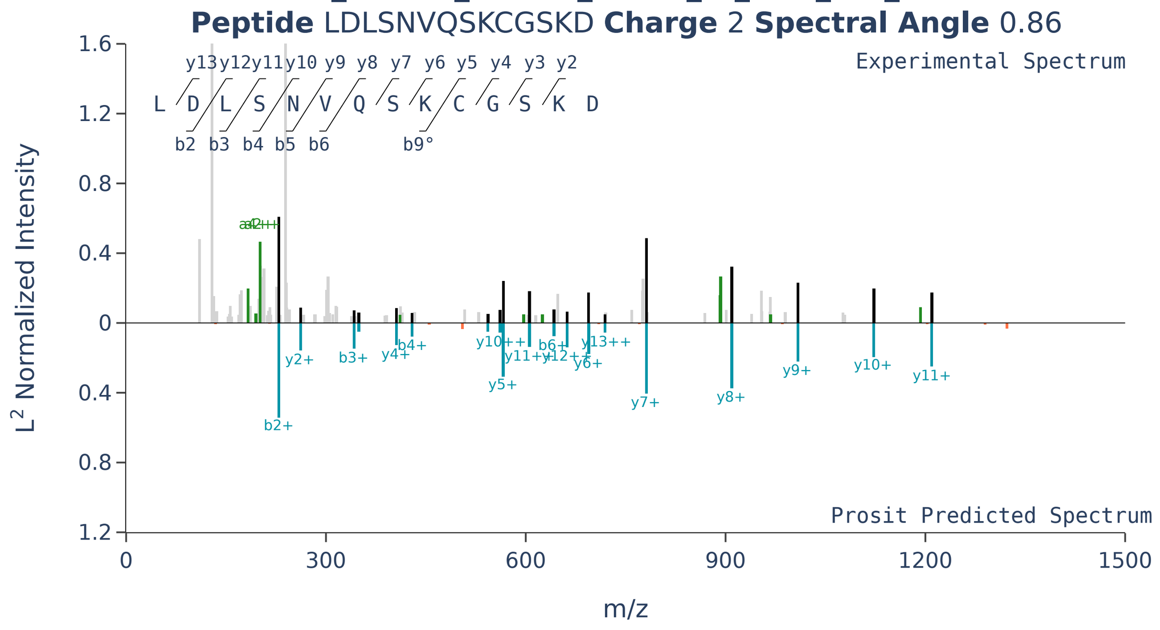


**Source** WSoh_221121_011221_HFGoe_L1_hTau_24h_R1 **Scan** 11903
**Peptide** LDNITHVPGGGNKKLE **Charge** 2 **Spectral Angle** 0.82

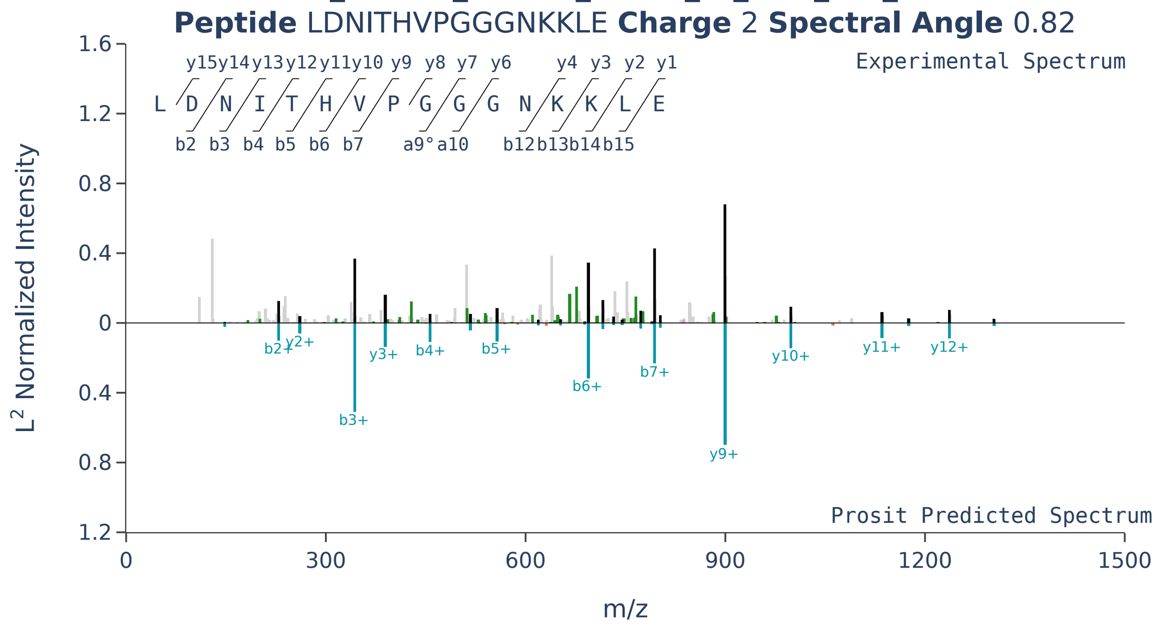


**Source** WSoh_221121_011221_HFGoe_L2_hTau_24h_R2 **Scan** 23455
**Peptide** LQTAPVPMPDLKNVKSKIGSTEN **Charge** 4 **Spectral Angle** 0.86

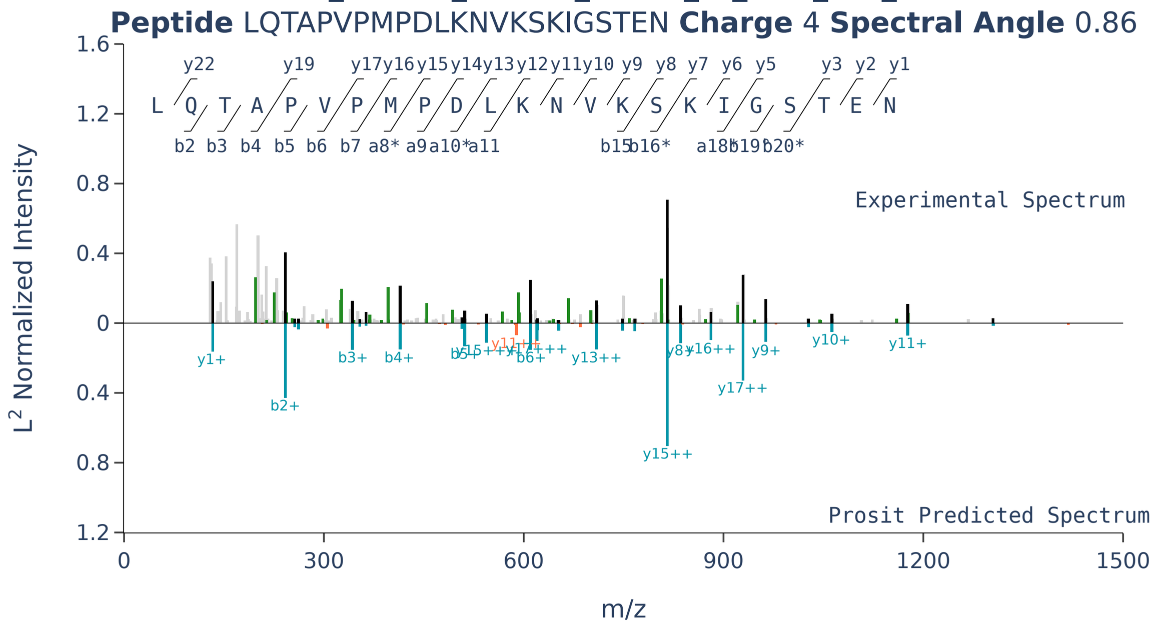


**Source** WSoh_221121_011221_HFGoe_L1_hTau_24h_R1 **Scan** 14510
**Peptide** NLKHVPGGGSVQI **Charge** 3 **Spectral Angle** 0.79

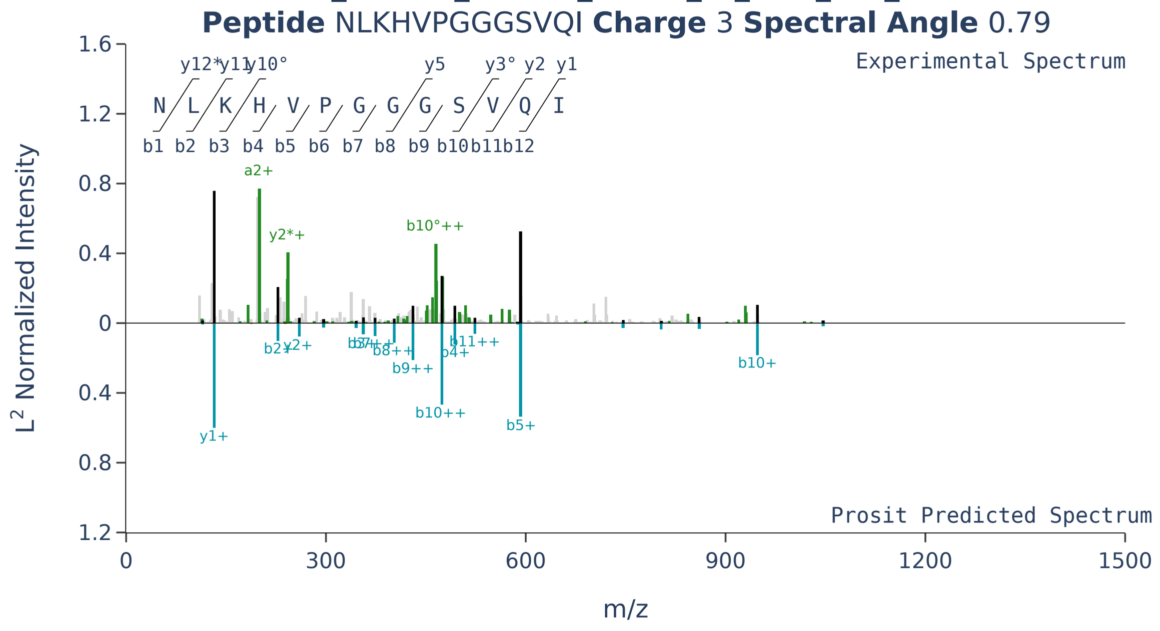


**Source** WSoh_221121_011221_HFGoe_L2_hTau_24h_R2 **Scan** 14194
**Peptide** SLPTPPTREPKKVAV **Charge** 3 **Spectral Angle** 0.8

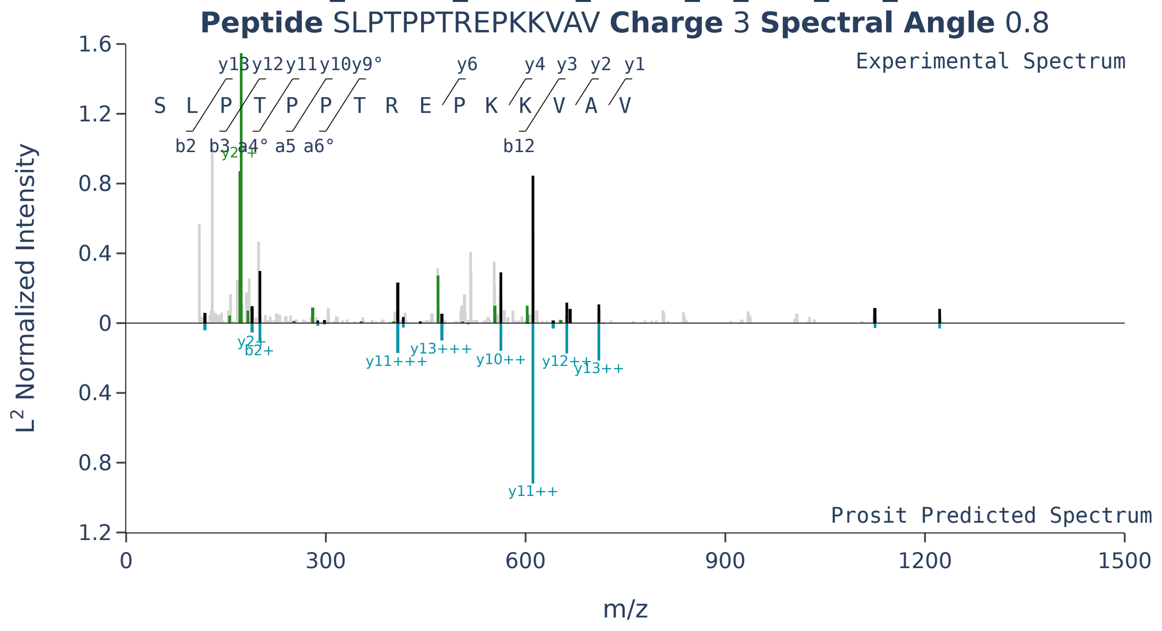


**Source** WSoh_221121_011221_HFGoe_L1_hTau_24h_R1 **Scan** 14929
**Peptide** LTFRENAKAKTDHGAEIVY **Charge** 3 **Spectral Angle** 0.82

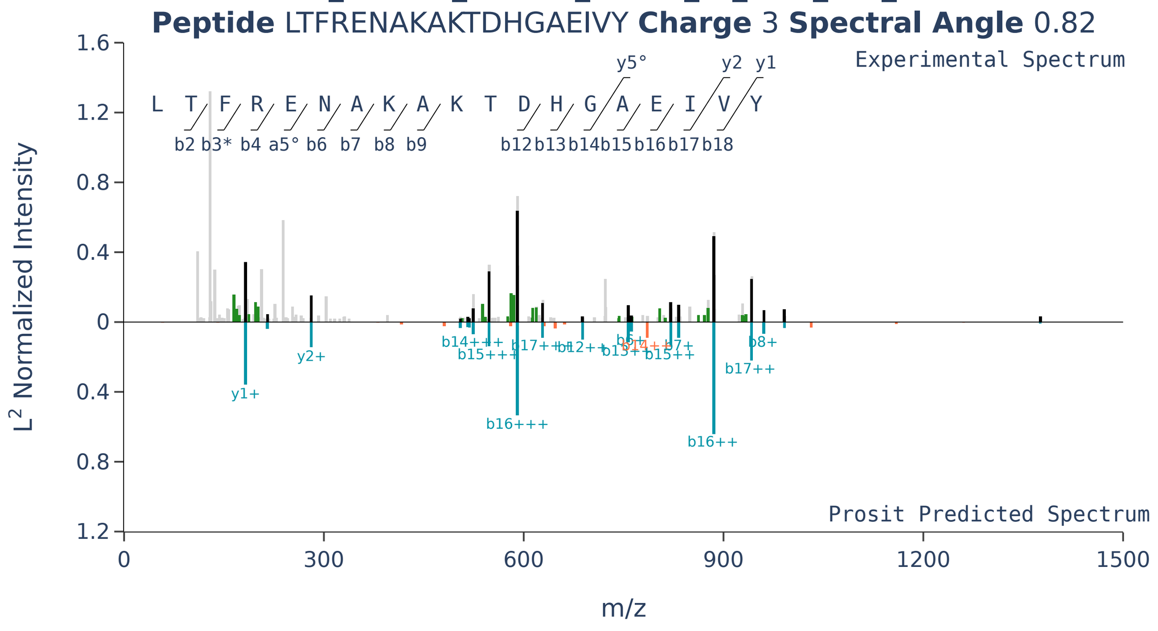


**Source** WSoh_221121_011221_HFGoe_L1_hTau_24h_R2 **Scan** 15741
**Peptide** KDRVQSKIGSLDNL **Charge** 3 **Spectral Angle** 0.85

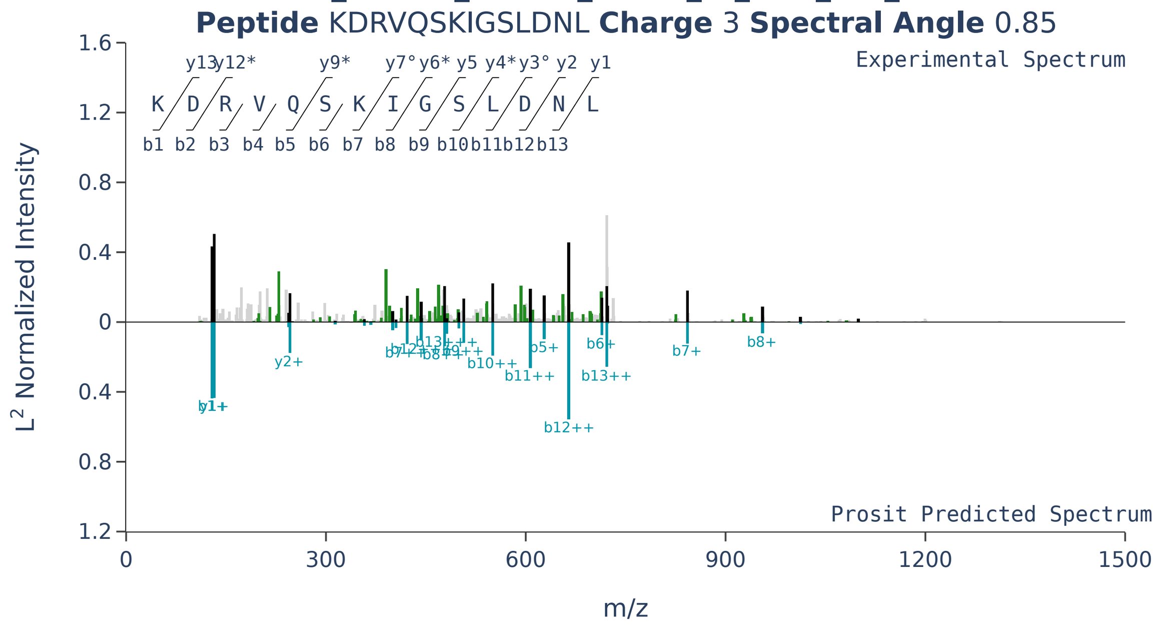


**Source** WSoh_221121_011221_HFGoe_L1_hTau_24h_R2 **Scan** 8352
**Peptide** KHQPGGGKVQIINKKLD **Charge** 4 **Spectral Angle** 0.88

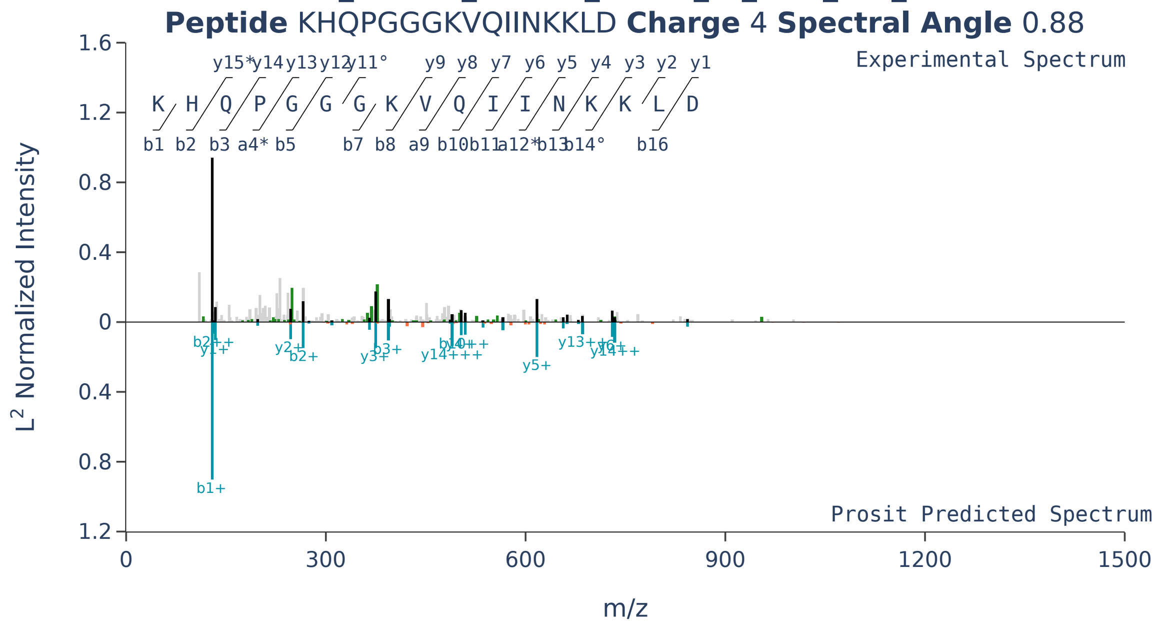


**Source** WSoh_221121_011221_HFGoe_L2_hTau_24h_R1 **Scan** 12561
**Peptide** LDLSNVQSKCGSKDNIKHV **Charge** 5 **Spectral Angle** 0.91

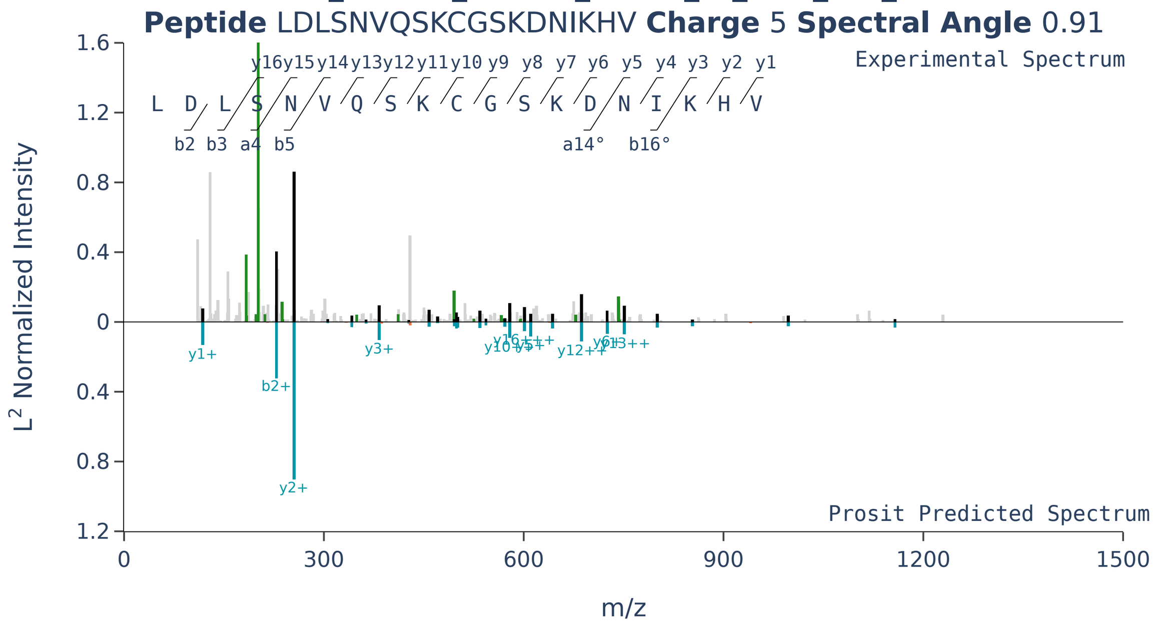


**Source** WSoh_221121_011221_HFGoe_L1_hTau_24h_R1 **Scan** 17429
**Peptide** VEVKSEKLDF **Charge** 3 **Spectral Angle** 0.85

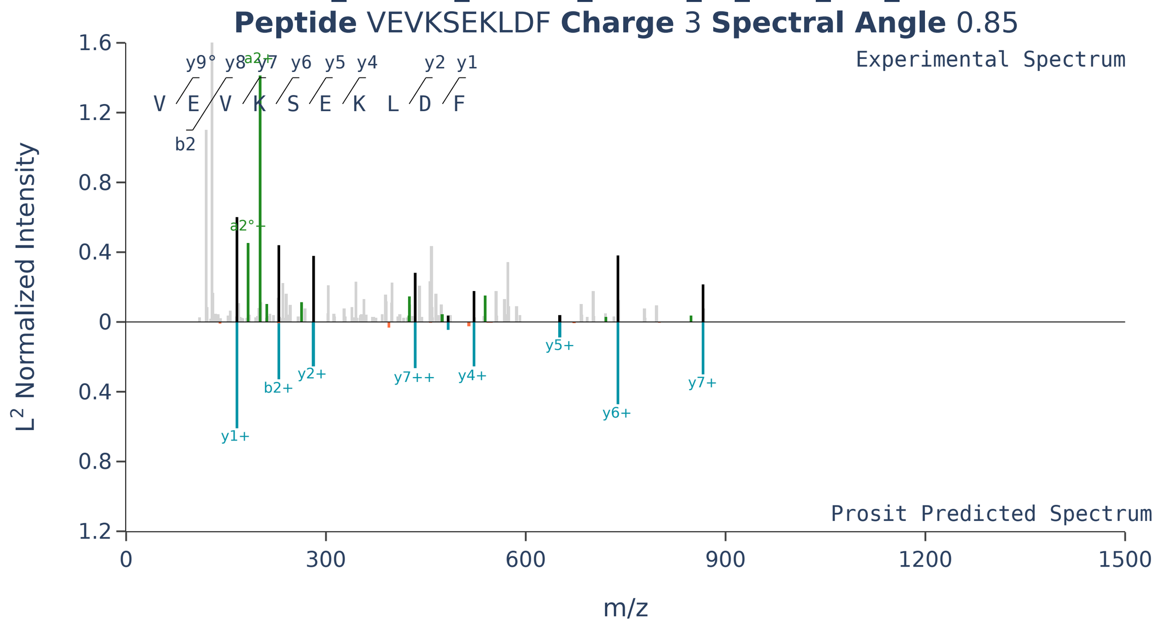


**Source** WSoh_221121_011221_HFGoe_L1_hTau_24h_R1 **Scan** 12897
**Peptide** KAKTDHGAEIVYKSPVVSGD **Charge** 3 **Spectral Angle** 0.76

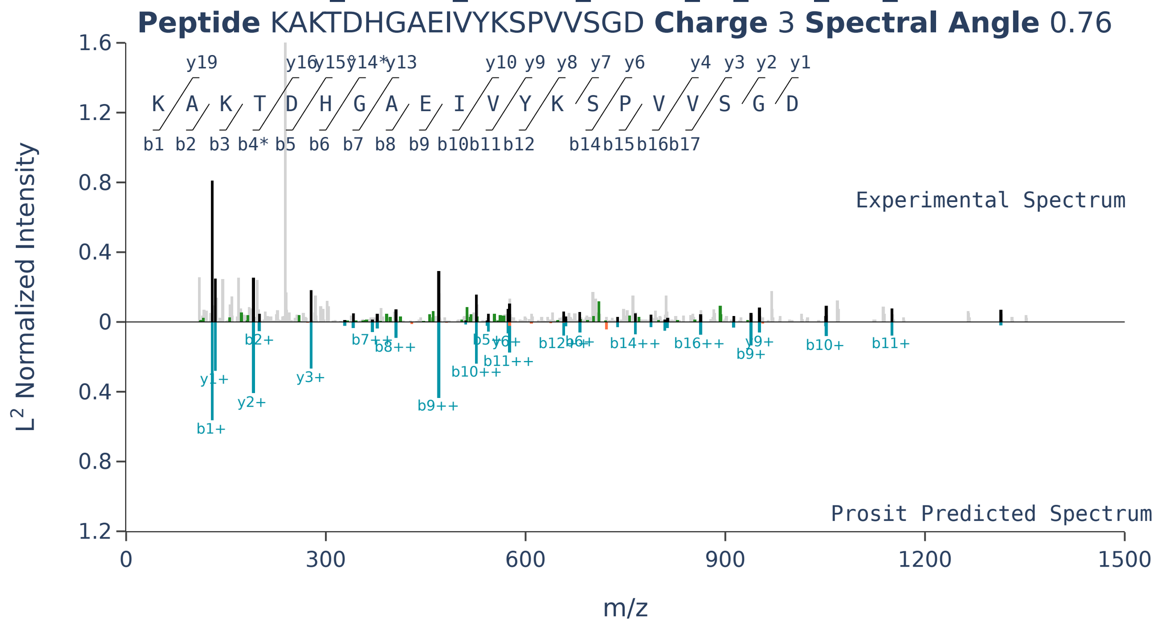

**Source** WSoh_221121_011221_HFGoe_L1_hTau_2h_R1 **Scan** 21582
**Peptide** EEAGIGDTPSL **Charge** 2 **Spectral Angle** 0.91

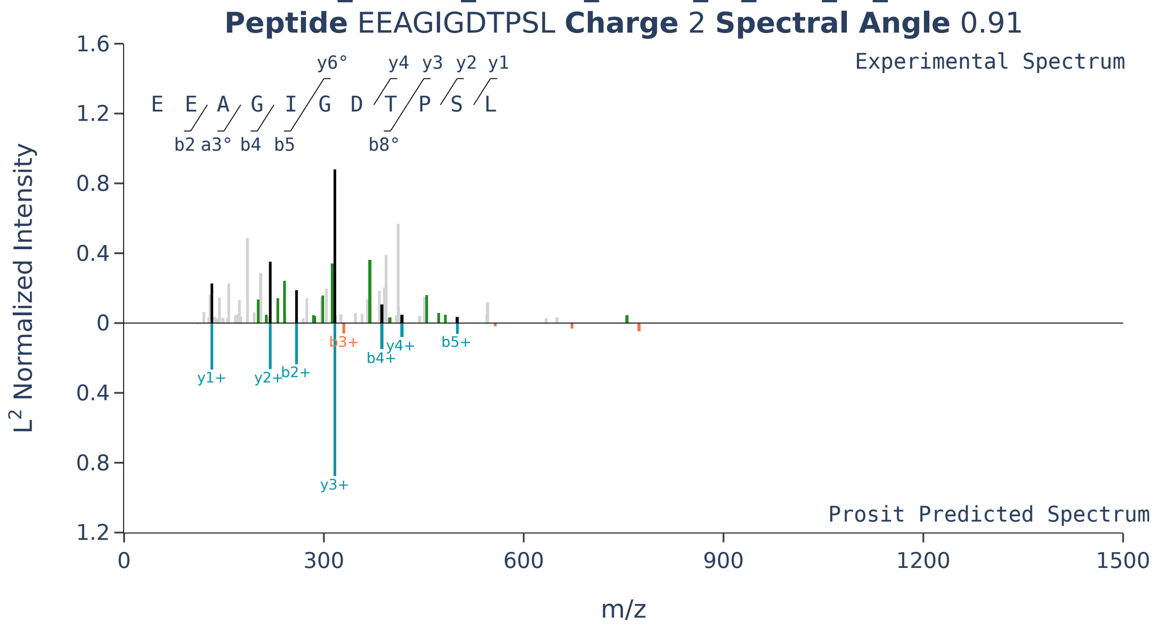


**Source** WSoh_221121_011221_HFGoe_L1_hTau_24h_R1 **Scan** 12673
**Peptide** KHQPGGGKVQIINKKLDLSNVQSK **Charge** 5 **Spectral Angle** 0.85

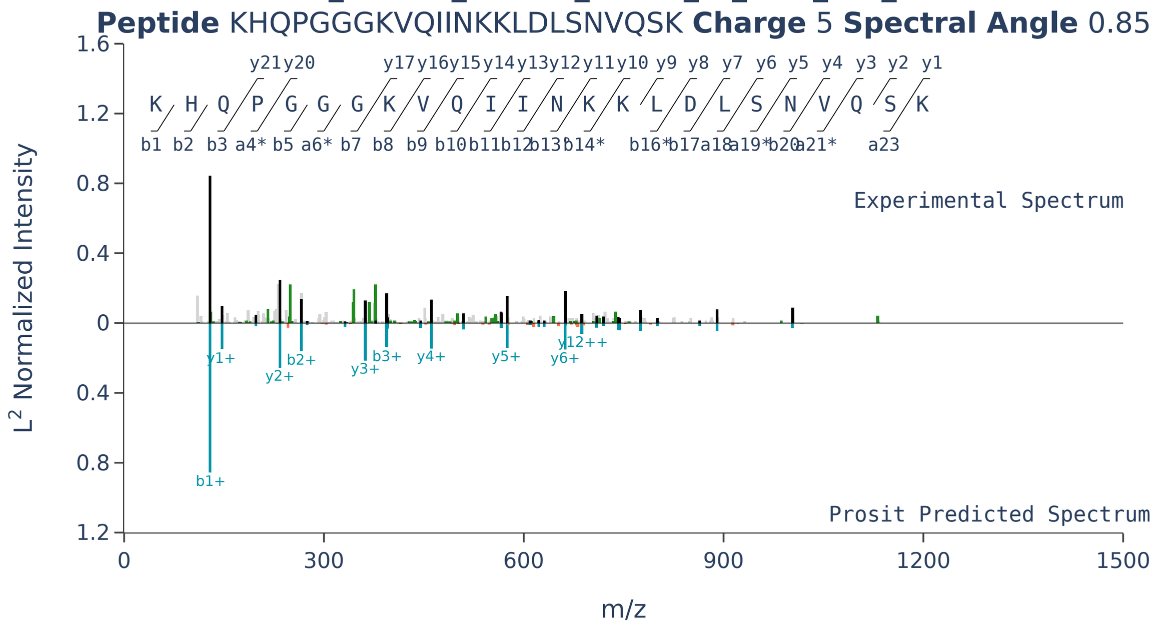


**Source** WSoh_221121_011221_HFGoe_L2_hTau_24h_R1 **Scan** 19903
**Peptide** VTAPLVDEGAPGKQAAAQPHTEI **Charge** 3 **Spectral Angle** 0.82

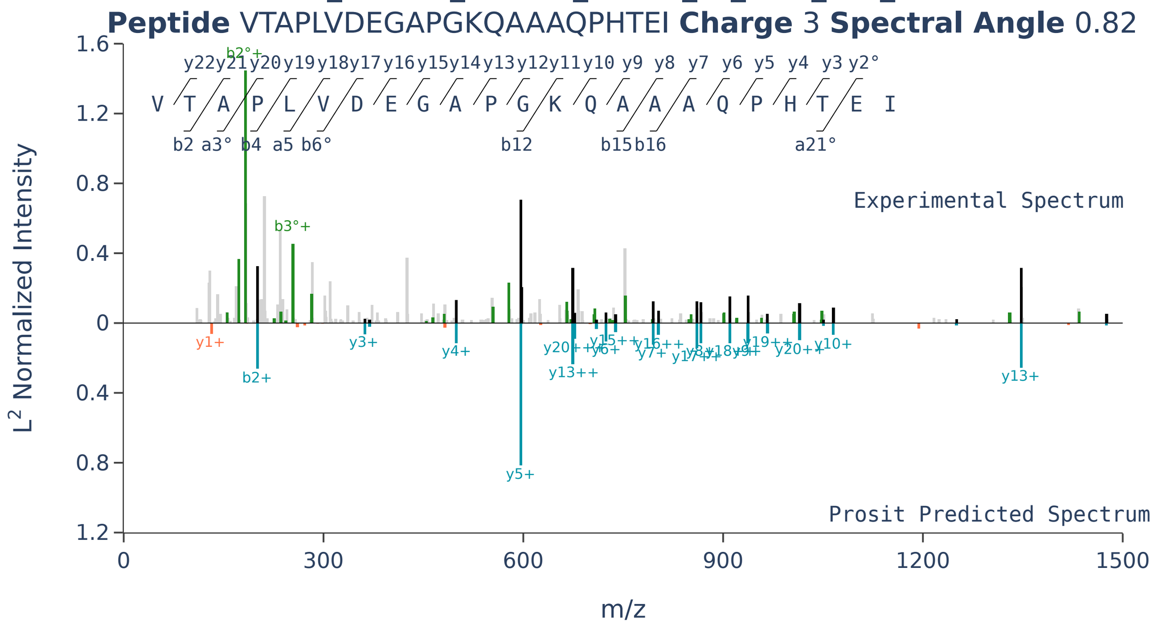


**Source** WSoh_221121_011221_HFGoe_L1_hTau_24h_R1 **Scan** 10265
**Peptide** KLTFRENAKAKTDHGAEIVYKS **Charge** 4 **Spectral Angle** 0.93

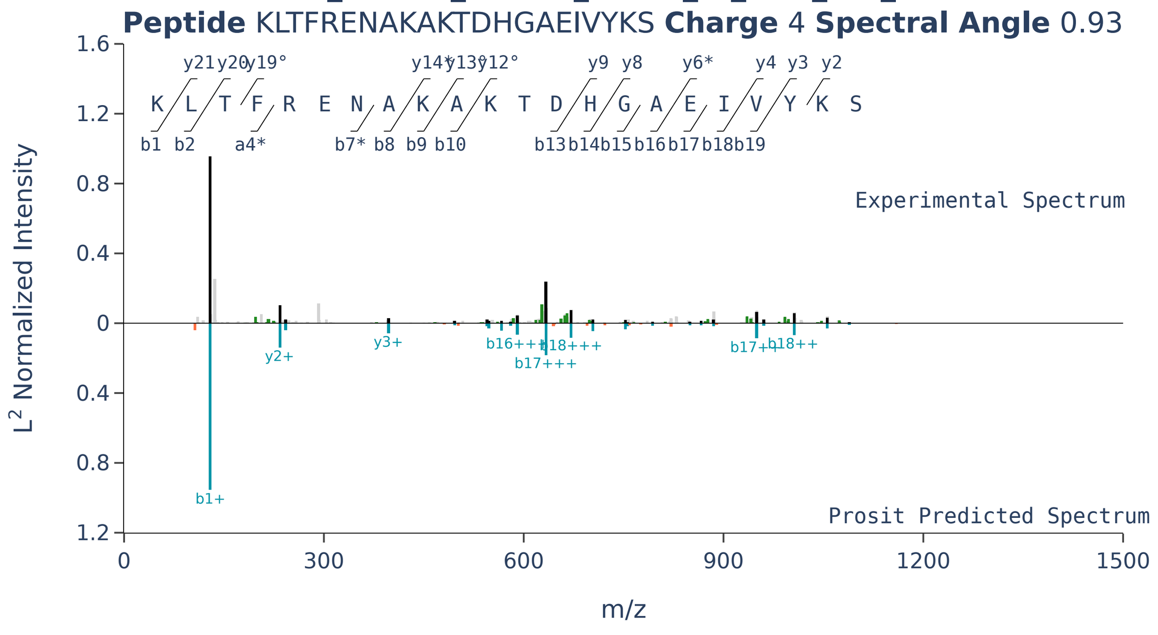


**Source** WSoh_221121_011221_HFGoe_L2_hTau_24h_R1 **Scan** 14440
**Peptide** ENAKAKTDHGAEIVYKSPVVSGDTSPRHL **Charge** 5 **Spectral Angle** 0.8

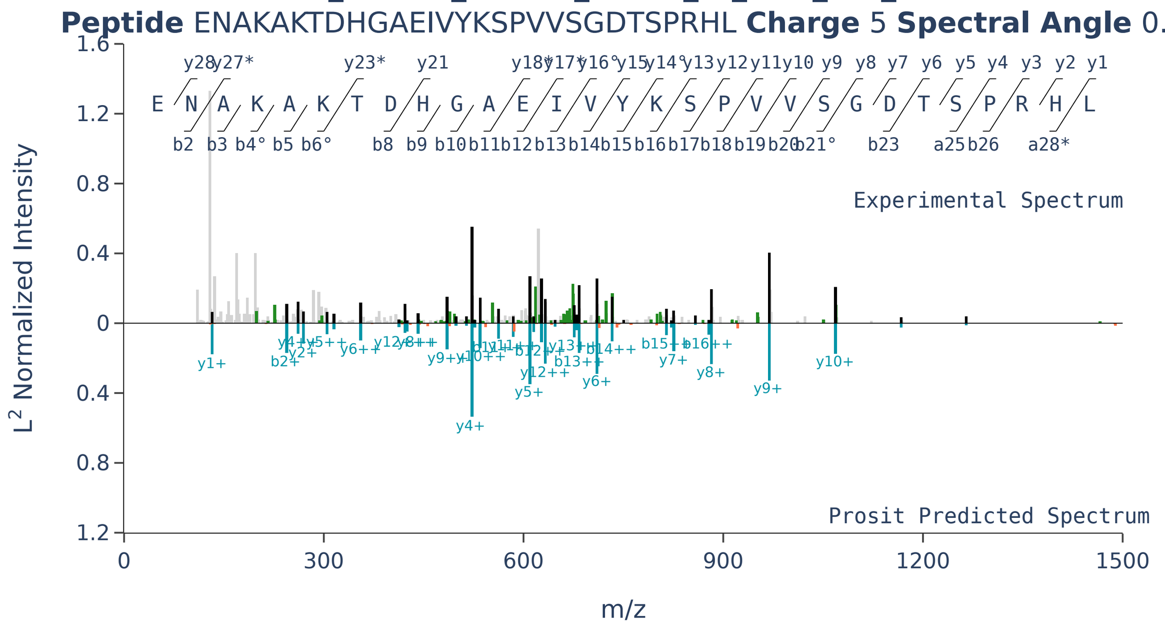


**Source** WSoh_221121_011221_HFGoe_L1_hTau_24h_R1 **Scan** 20723
**Peptide** EEAGIGDTPSLED **Charge** 1 **Spectral Angle** 0.73

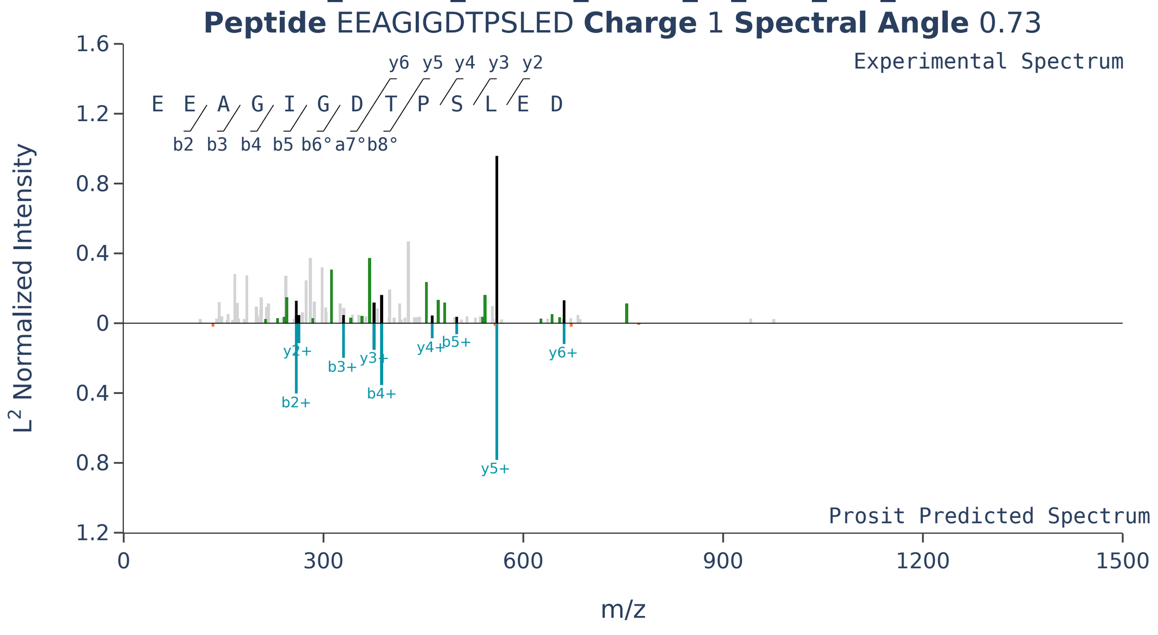


**Source** WSoh_221121_011221_HFGoe_L2_hTau_4h_R1 **Scan** 7071
**Peptide** KCGSLGNIHHKPGGGQVEVKSE **Charge** 3 **Spectral Angle** 0.84

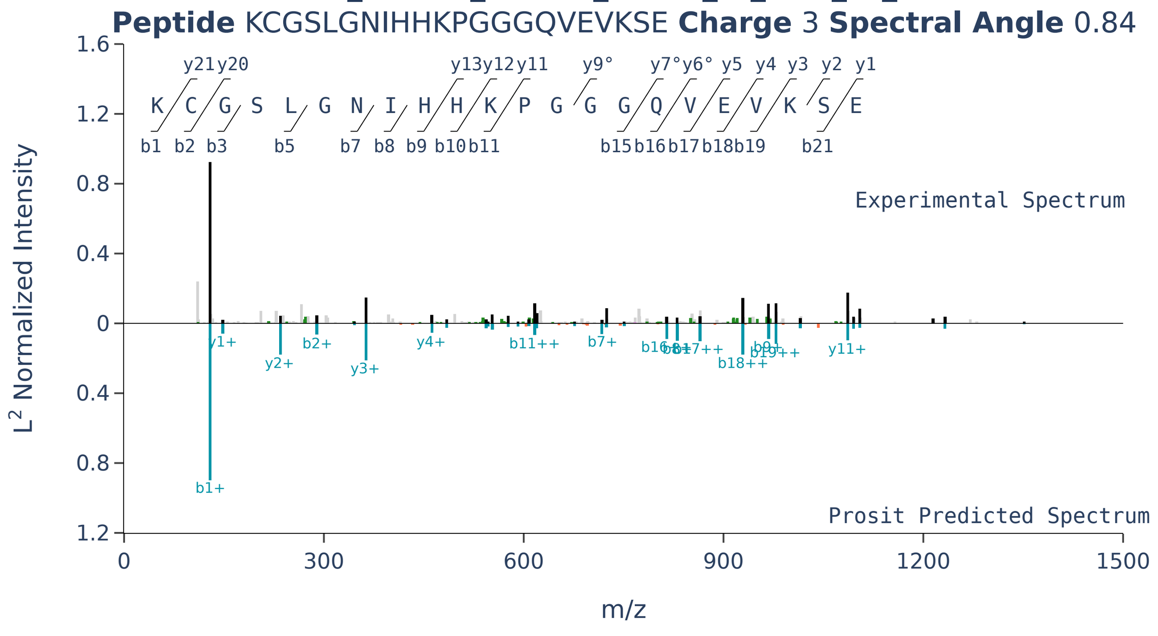


**Source** WSoh_221121_011221_HFGoe_L1_hTau_24h_R1 **Scan** 5661
**Peptide** SGDTSPRHLSN **Charge** 2 **Spectral Angle** 0.82

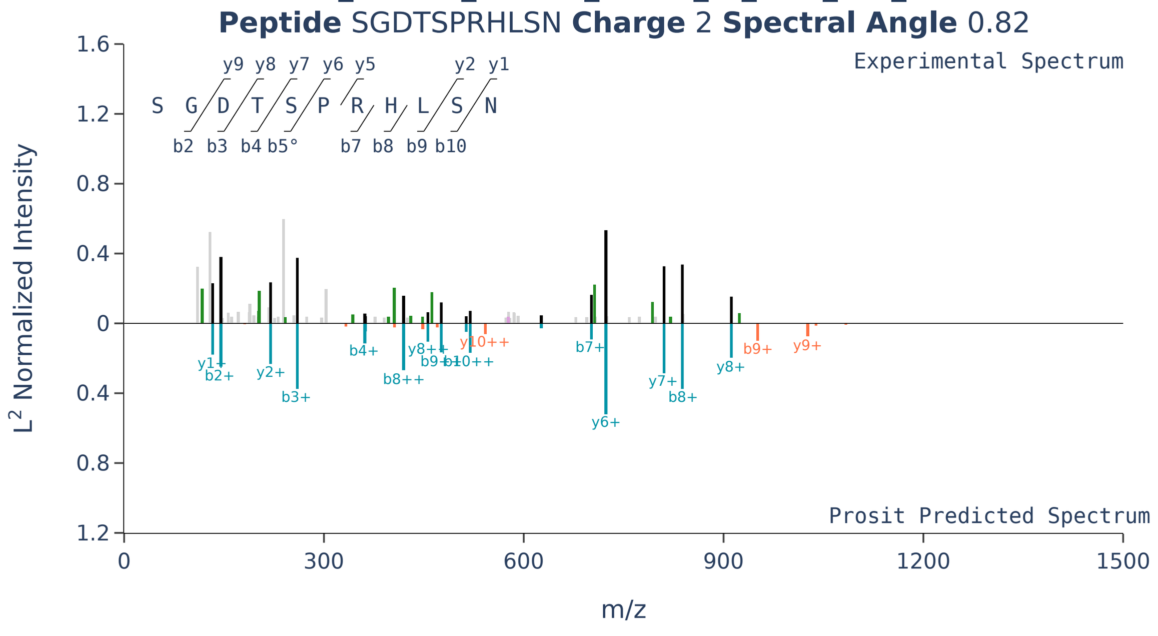


**Source** WSoh_221121_011221_HFGoe_L1_hTau_24h_R2 **Scan** 20252
**Peptide** EPRQEFEVM **Charge** 2 **Spectral Angle** 0.72

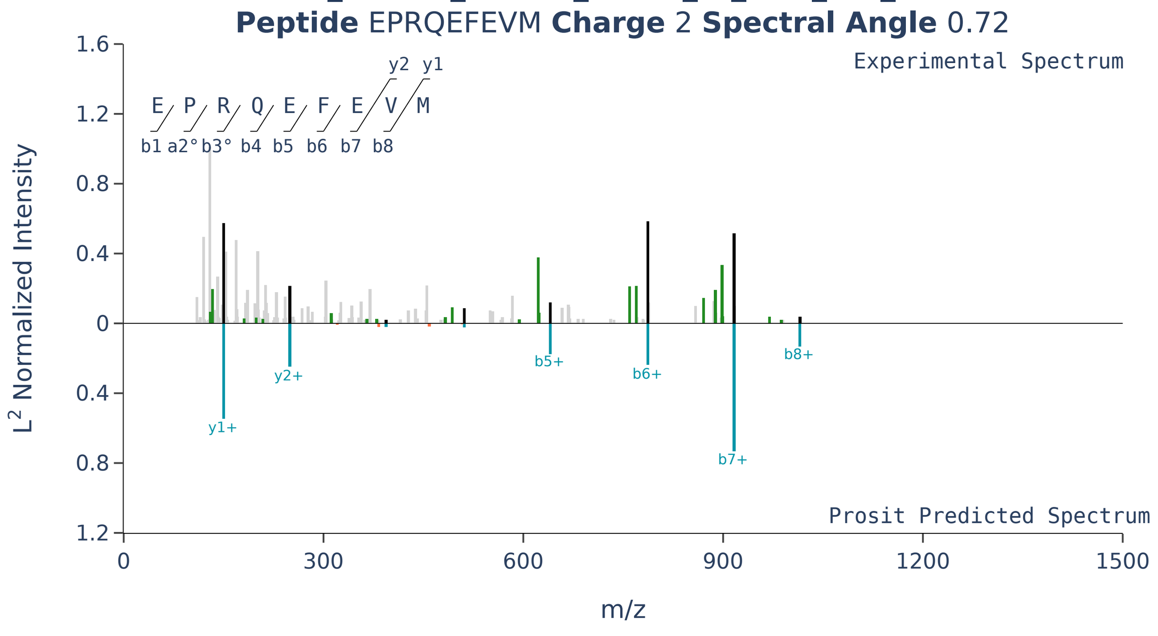


**Source** WSoh_221121_011221_HFGoe_L2_hTau_24h_R1 **Scan** 10196
**Peptide** HQPGGGKVQIINKKL **Charge** 4 **Spectral Angle** 0.92

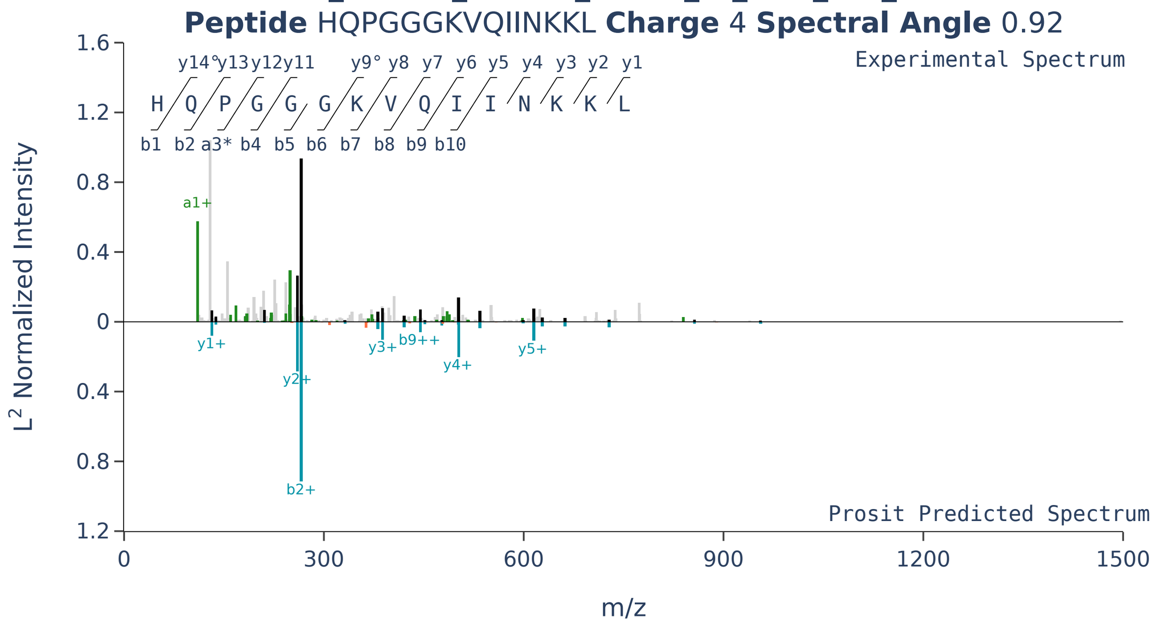


**Source** WSoh_221121_011221_HFGoe_L1_hTau_24h_R2 **Scan** 20775
**Peptide** DVTAPLVDEGAPGKQAAA **Charge** 2 **Spectral Angle** 0.9

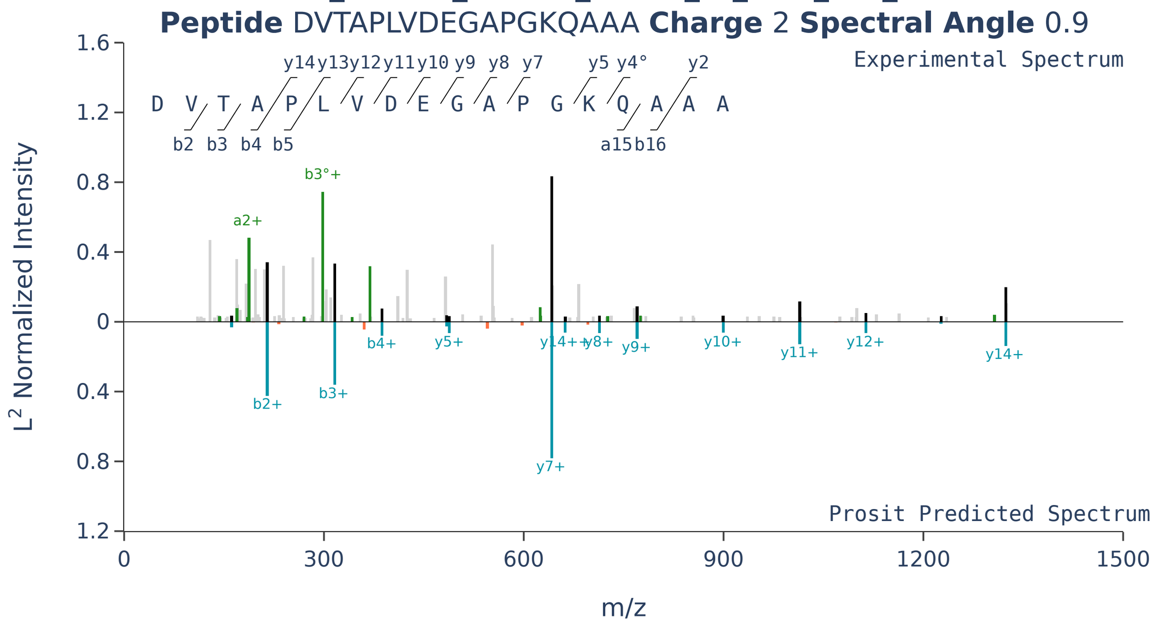


**Source** WSoh_221121_011221_HFGoe_L2_hTau_24h_R1 **Scan** 8141
**Peptide** LSKVTSKCGSLGNIHHKPGGGQVE **Charge** 3 **Spectral Angle** 0.88

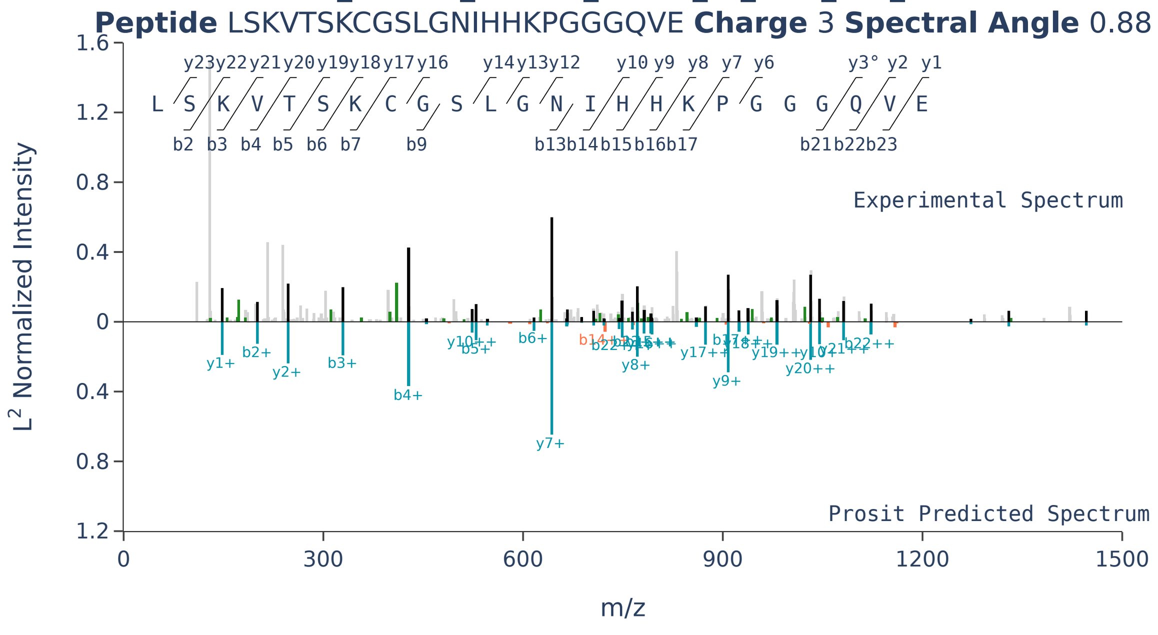


**Source** WSoh_221121_011221_HFGoe_L1_hTau_24h_R1 **Scan** 22462
**Peptide** PGGGSVQIVYKPVDLSKVTSKCG **Charge** 3 **Spectral Angle** 0.85

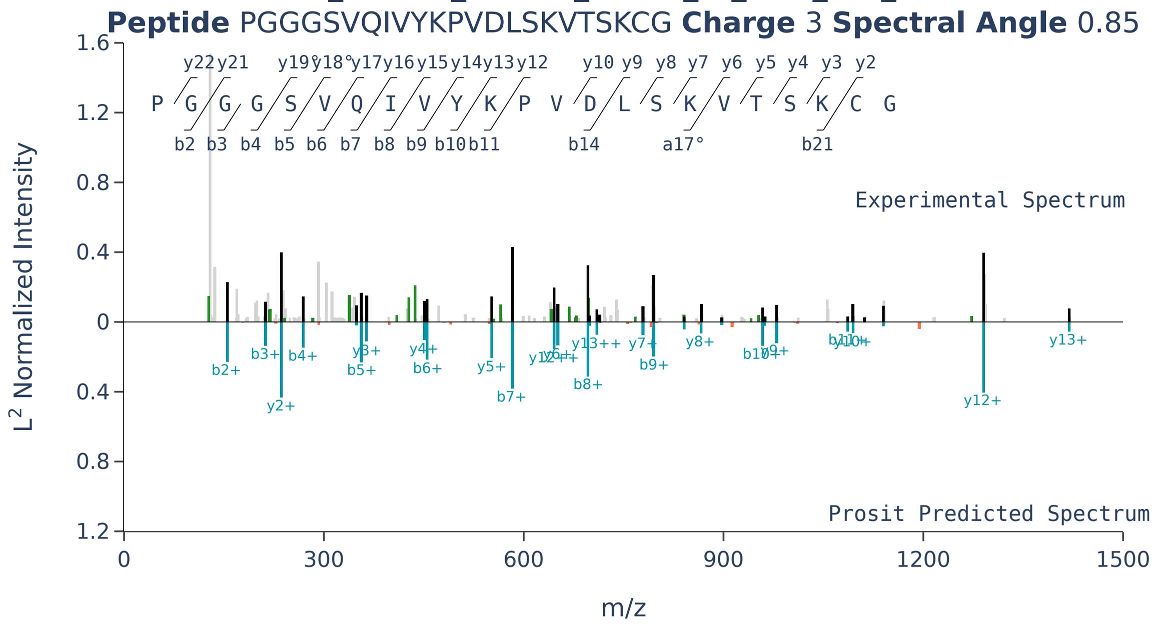


**Source** WSoh_221121_011221_HFGoe_L2_hTau_1h_R2 **Scan** 5621
**Peptide** THVPGGGNKKIETH **Charge** 4 **Spectral Angle** 0.84

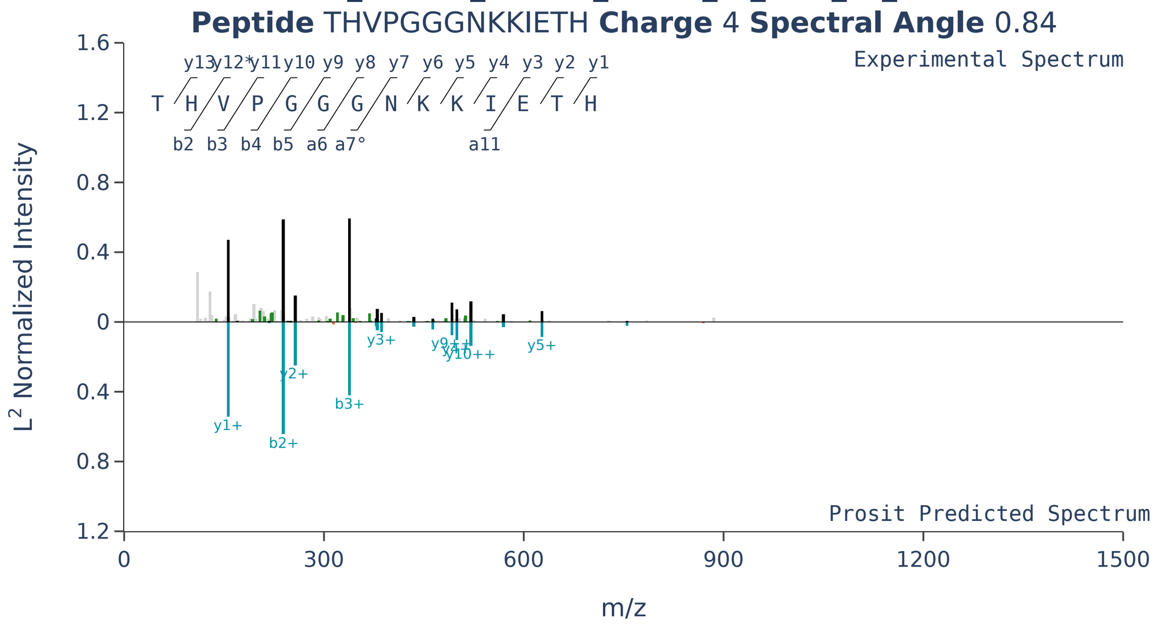


**Source** WSoh_221121_011221_HFGoe_L1_hTau_2h_R1 **Scan** 12607
**Peptide** GDTPSLE **Charge** 1 **Spectral Angle** 0.83

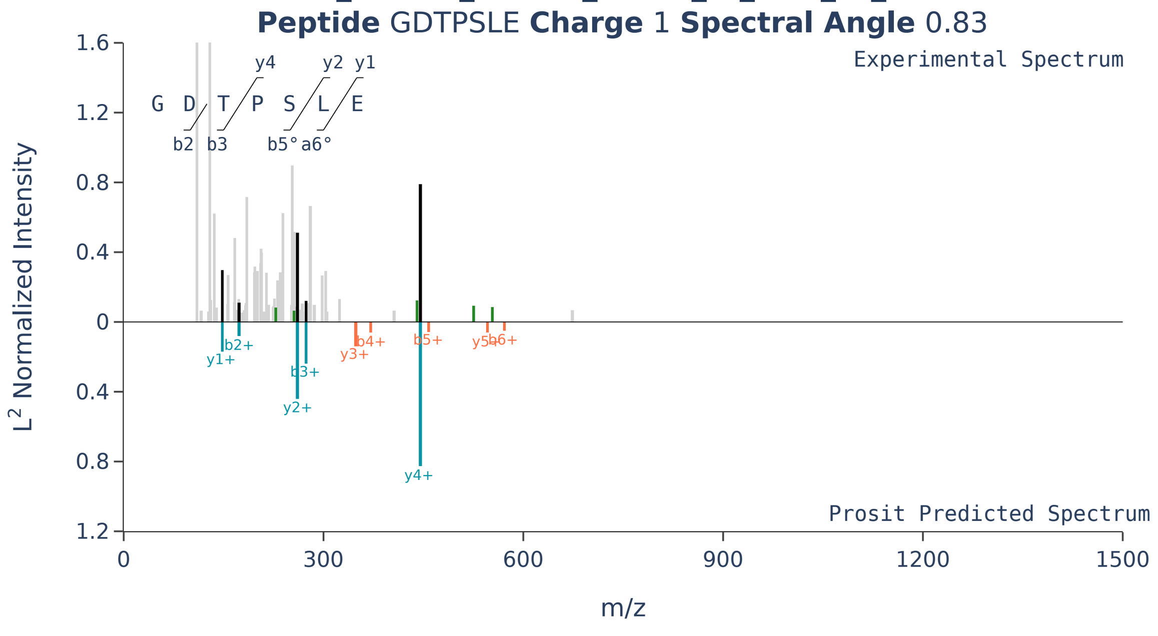

**Source** WSoh_221121_011221_HFGoe_L2_hTau_24h_R2 **Scan** 10535
**Peptide** HAGTYGLGDRKDQGGYTMHQDQEGD **Charge** 4 **Spectral Angle** 0.84

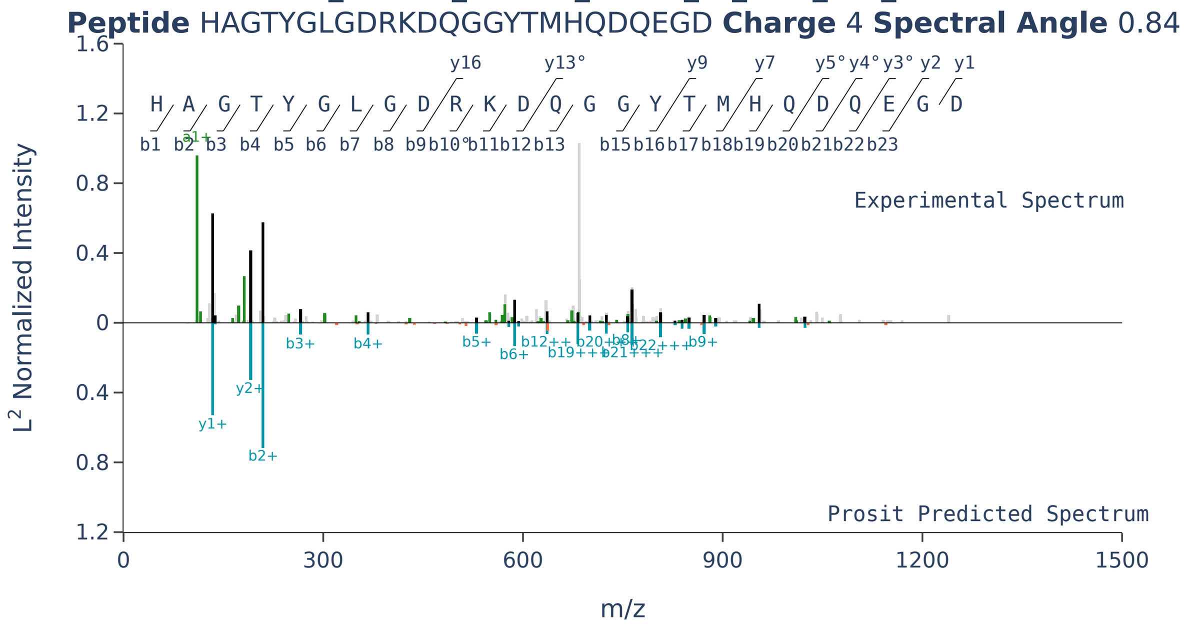


**Source** WSoh_221121_011221_HFGoe_L1_hTau_4h_R2 **Scan** 14149
**Peptide** EVKSEKLDFKDRVQSKI **Charge** 3 **Spectral Angle** 0.81

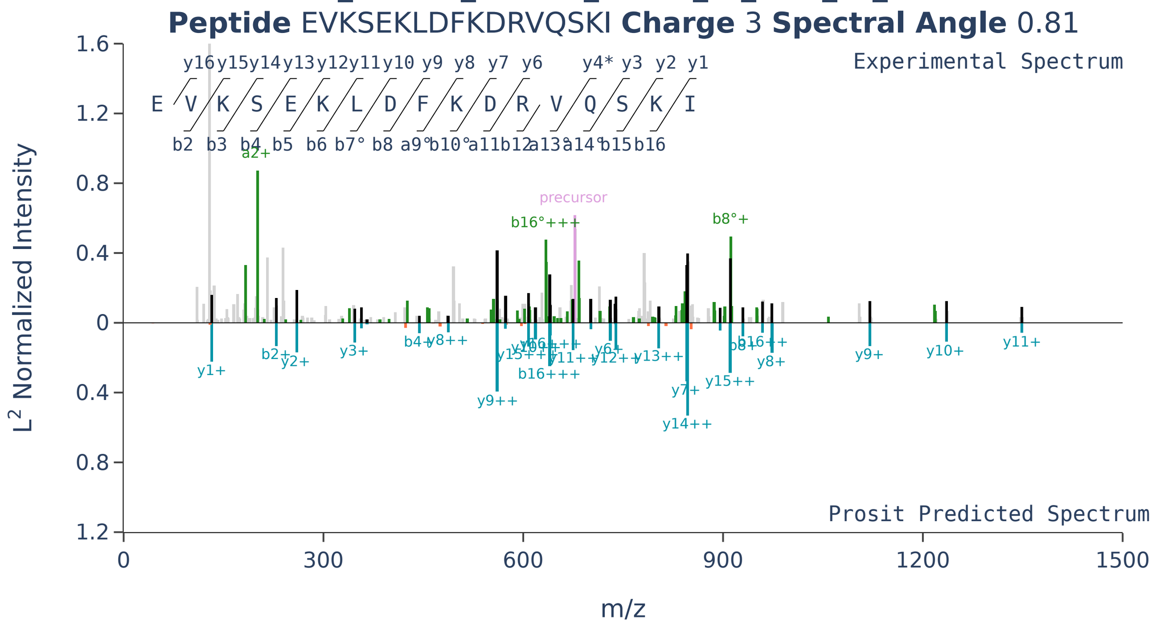


**Source** WSoh_221121_011221_HFGoe_L2_hTau_1h_R1 **Scan** 5251
**Peptide** VRTPPKSPSSAKSR **Charge** 3 **Spectral Angle** 0.87

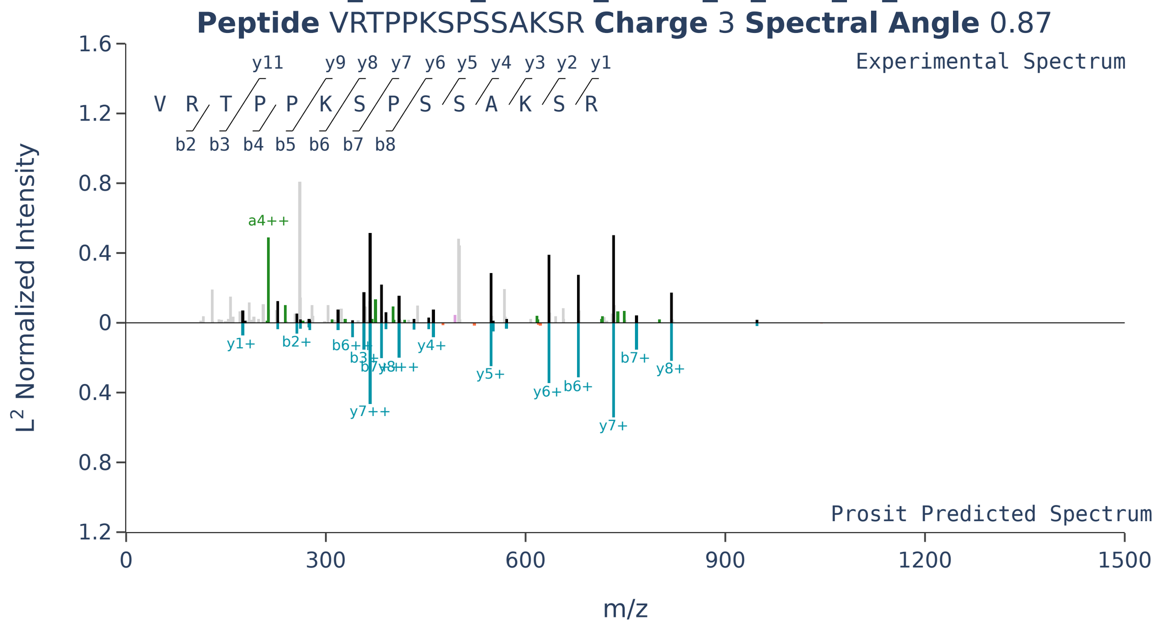


**Source** WSoh_221121_011221_HFGoe_L1_hTau_24h_R1 **Scan** 18091
**Peptide** TDHGAEIVYKSPVVSGDTSPRHLSNV **Charge** 4 **Spectral Angle** 0.82

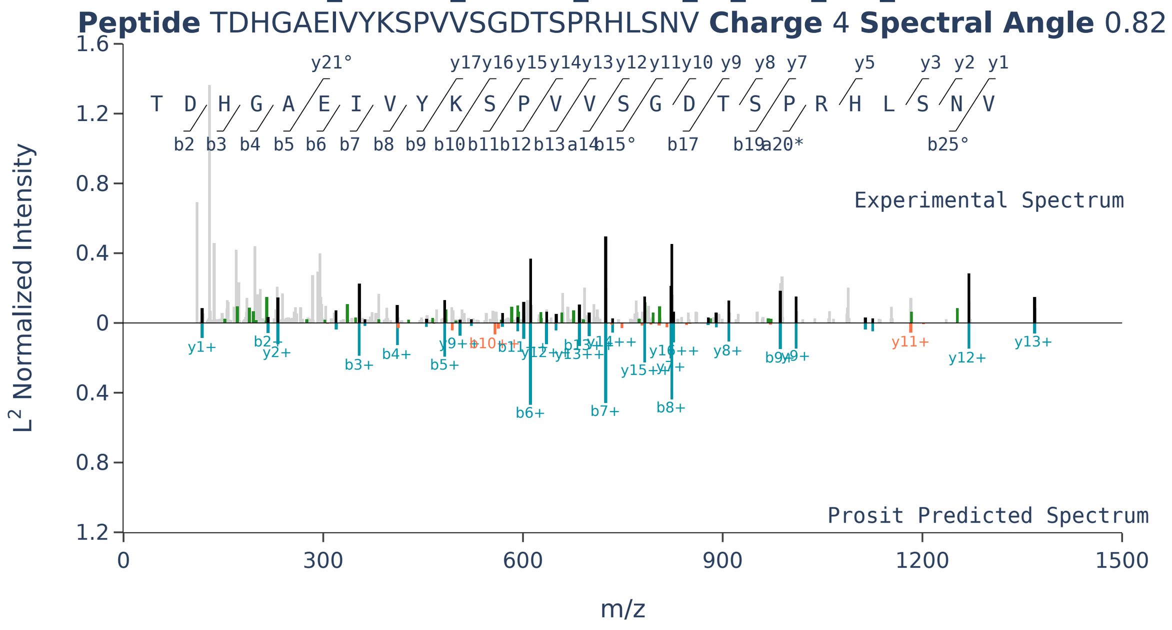


**Source** WSoh_221121_011221_HFGoe_L2_hTau_4h_R1 **Scan** 18553
**Peptide** TDAGLKESPLQTPTEDGSEEPGSETSD **Charge** 3 **Spectral Angle** 0.88

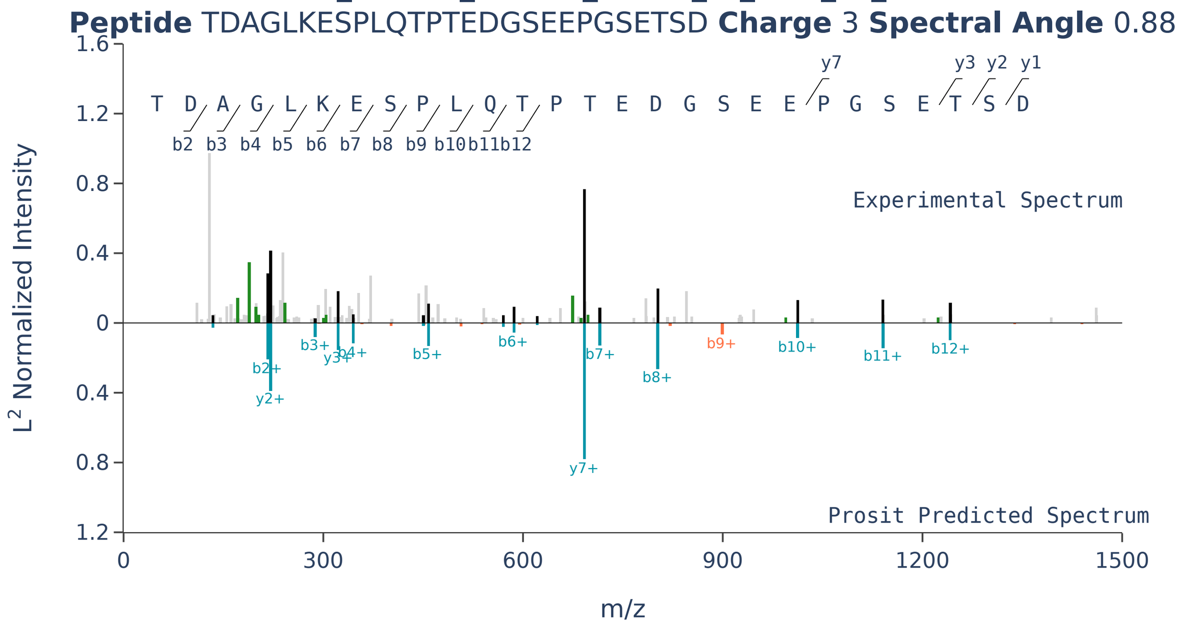


**Source** WSoh_221121_011221_HFGoe_L1_hTau_24h_R2 **Scan** 11382
**Peptide** YGLGDRKDQGGYTMHQD **Charge** 2 **Spectral Angle** 0.85

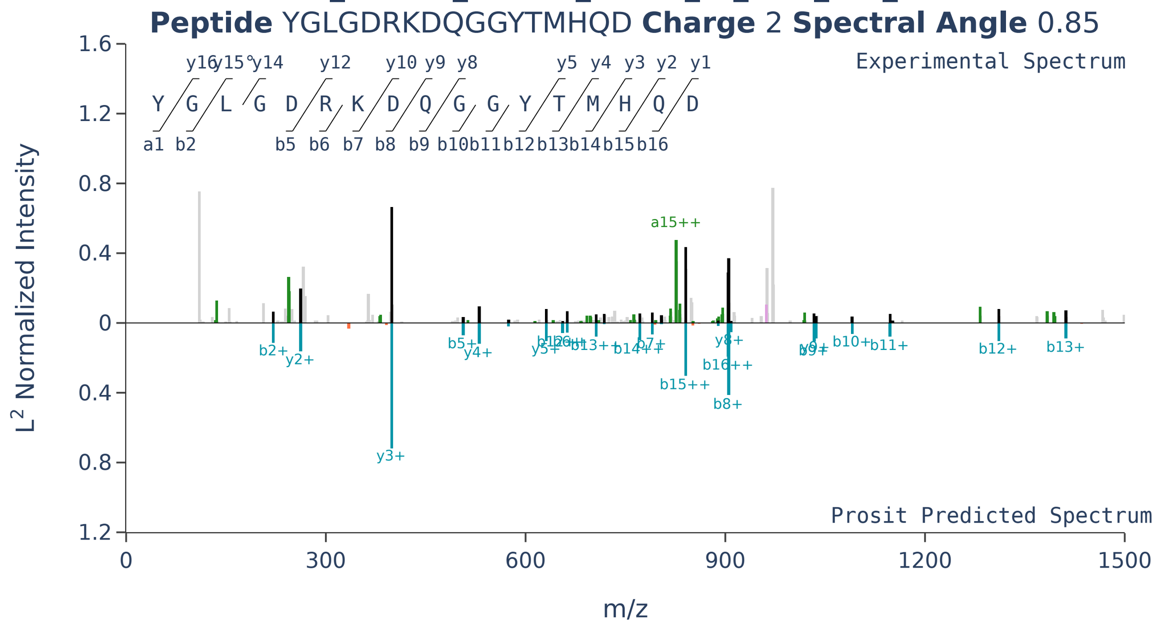


**Source** WSoh_221121_011221_HFGoe_L2_hTau_24h_R2 **Scan** 11535
**Peptide** GNIHHKPGGGQVEVKSEKLDFKDRV **Charge** 3 **Spectral Angle** 0.77

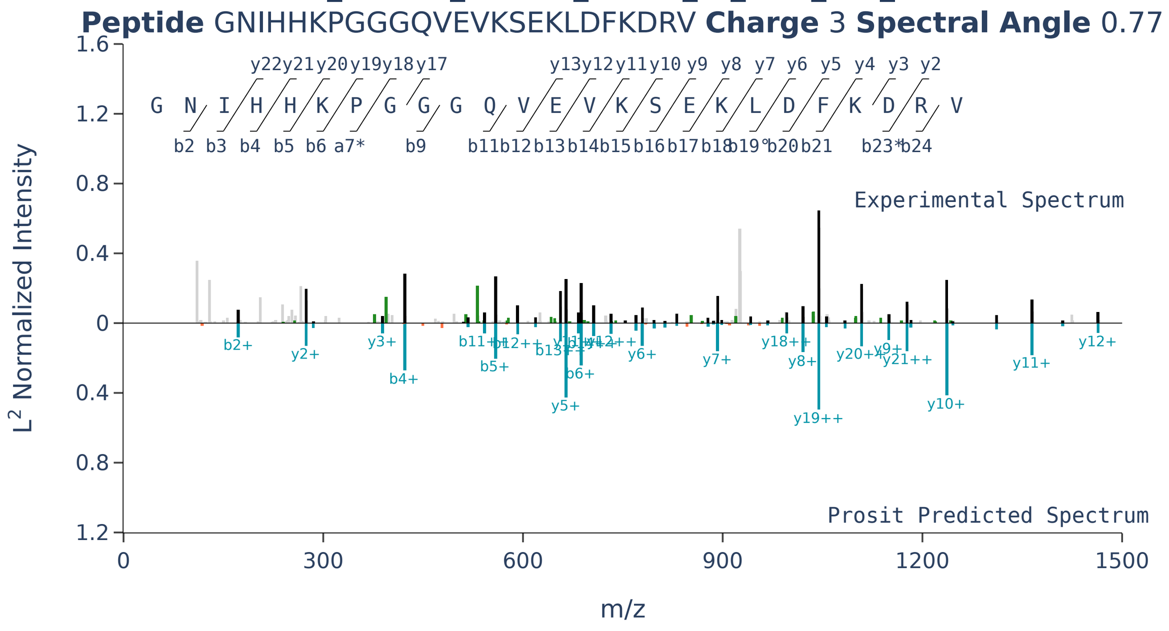


**Source** WSoh_221121_011221_HFGoe_L1_hTau_24h_R1 **Scan** 16277
**Peptide** PGGGQVEVKSEKLDFKDRVQSKL **Charge** 5 **Spectral Angle** 0.83

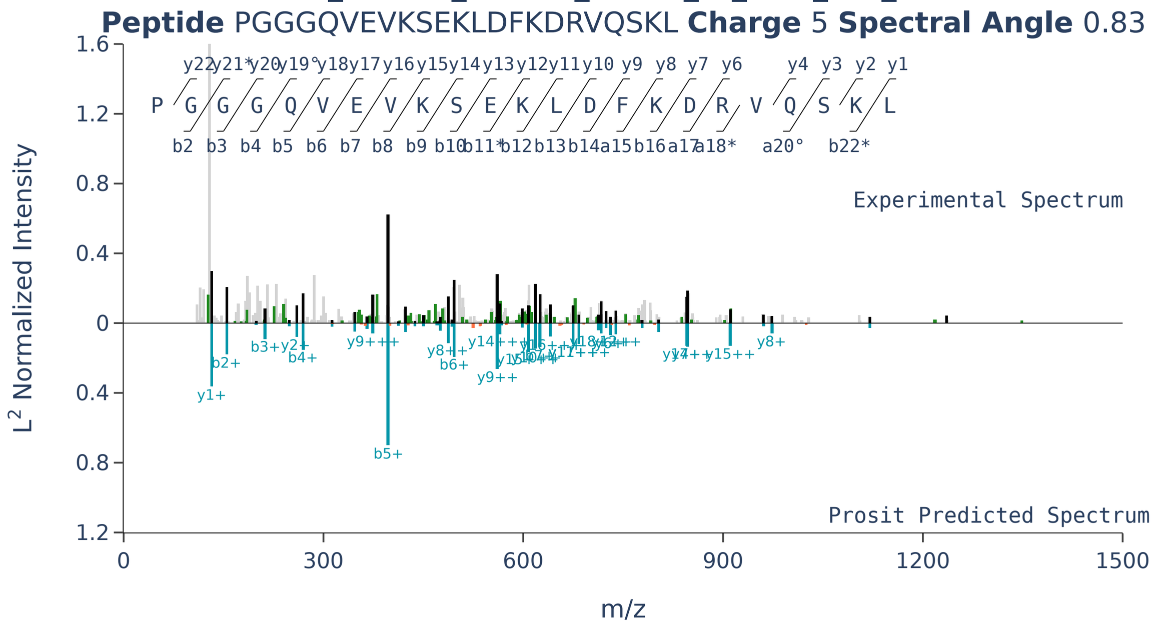


**Source** WSoh_221121_011221_HFGoe_L1_hTau_24h_R1 **Scan** 14117
**Peptide** KESPLQTPTEDGSEEPGSE **Charge** 2 **Spectral Angle** 0.91

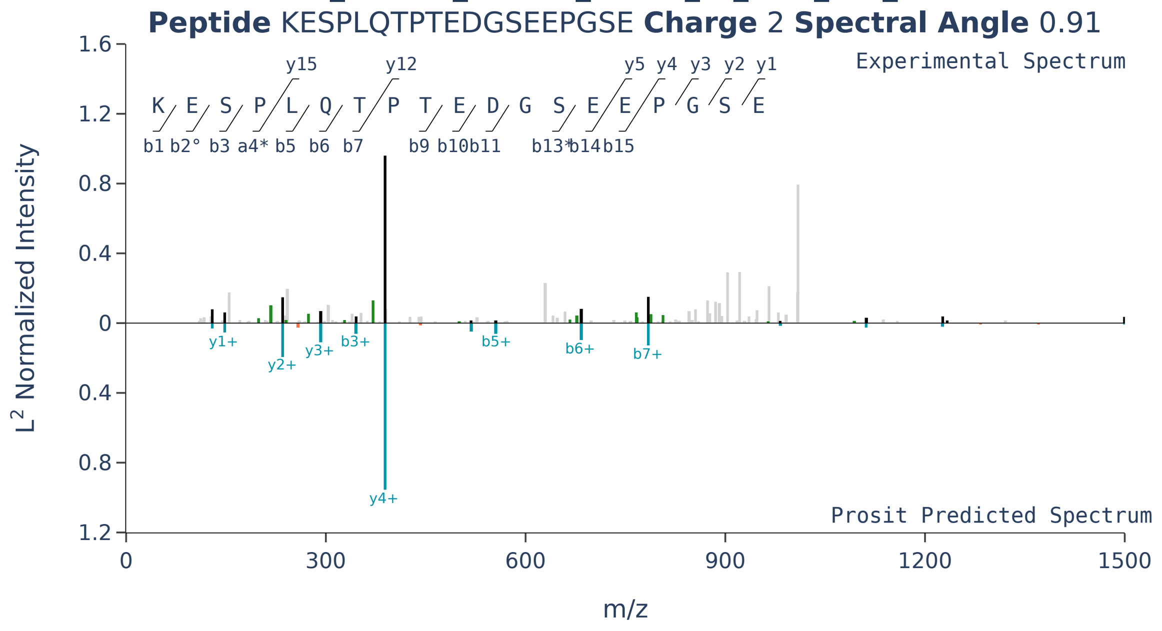


**Source** WSoh_221121_011221_HFGoe_L1_hTau_24h_R2 **Scan** 12704
**Peptide** IGSTENLKHQPGGGKVQI **Charge** 2 **Spectral Angle** 0.79

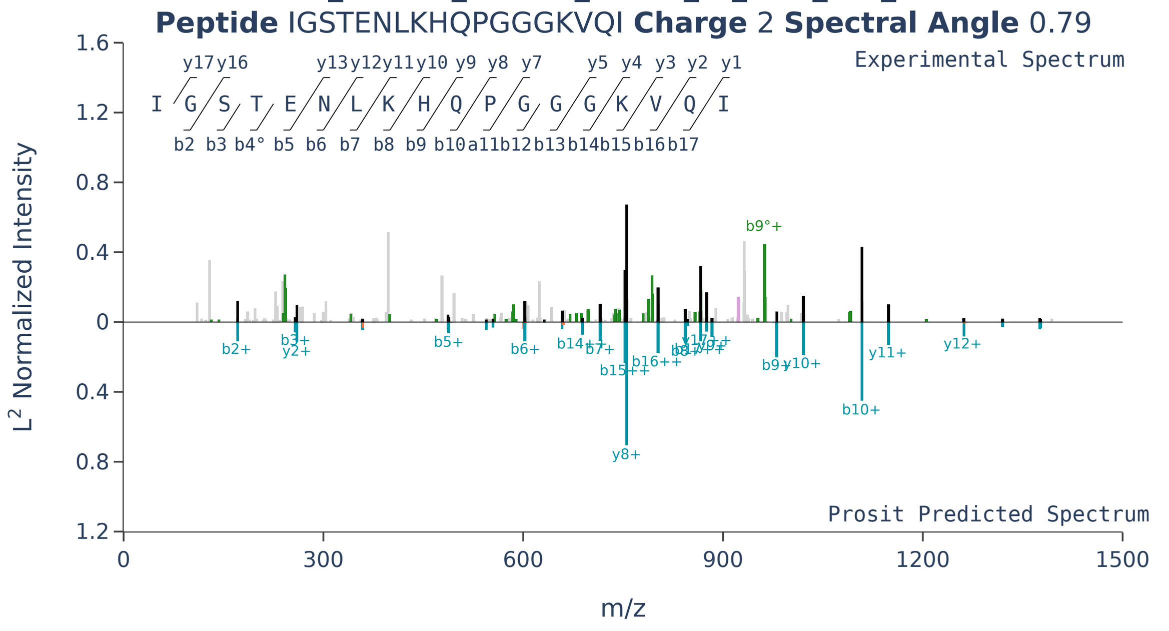


**Source** WSoh_221121_011221_HFGoe_L2_hTau_24h_R1 **Scan** 23655
**Peptide** LQTAPVPMPDLKNVKSKIGS **Charge** 3 **Spectral Angle** 0.87

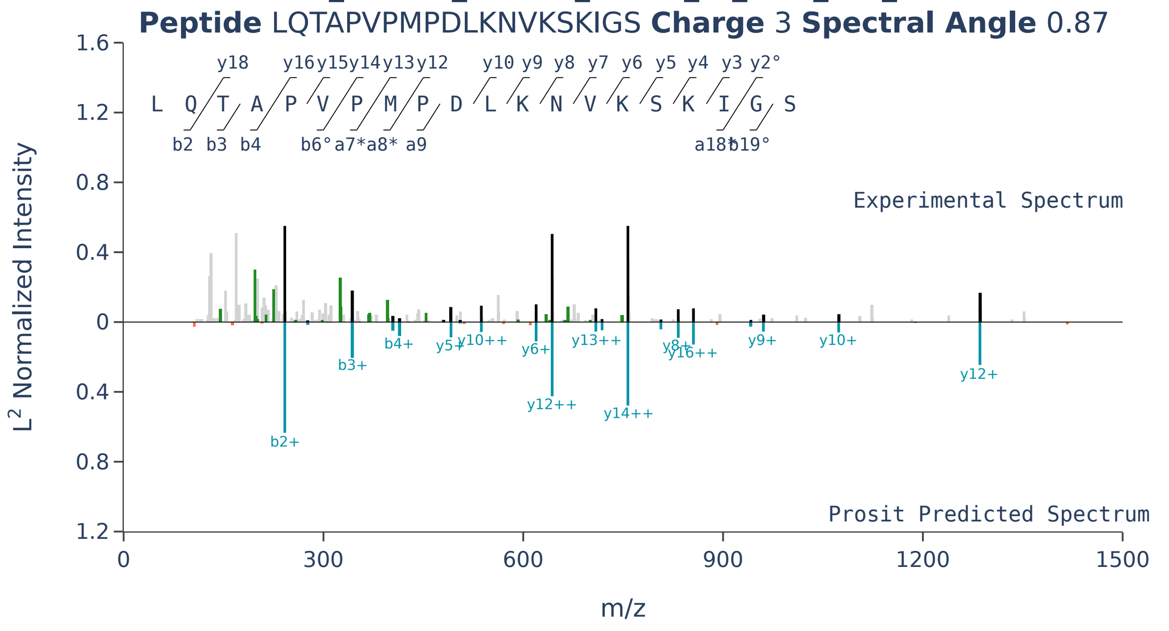


**Source** WSoh_221121_011221_HFGoe_L1_hTau_24h_R2 **Scan** 9197
**Peptide** KIGSLDN **Charge** 2 **Spectral Angle** 0.97

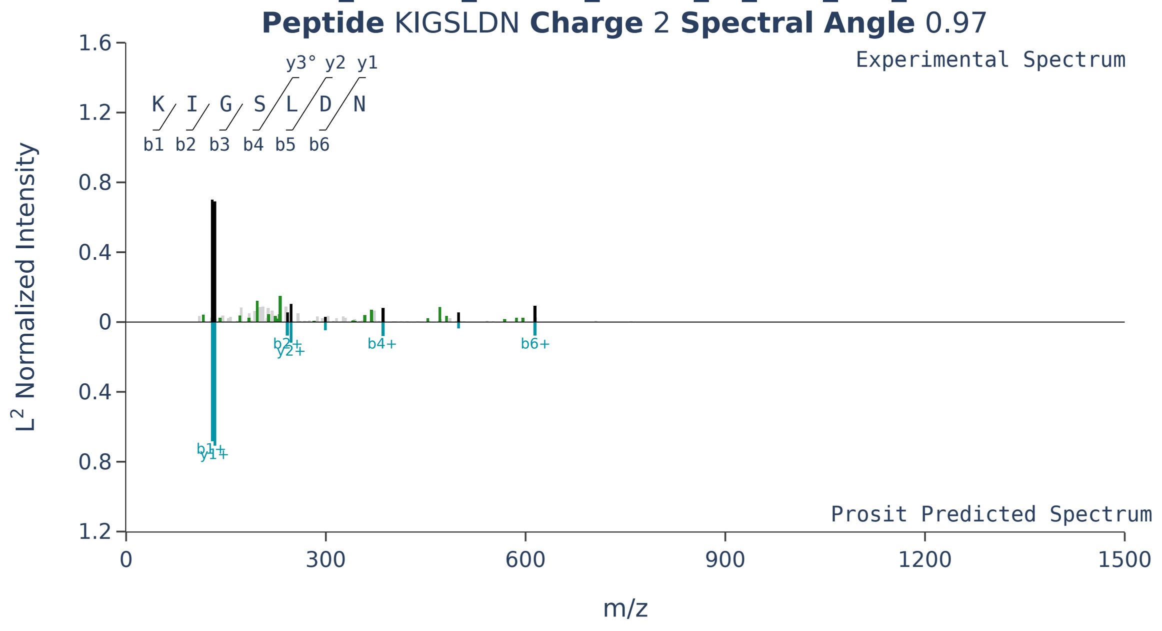


**Source** WSoh_221121_011221_HFGoe_L2_hTau_24h_R1 **Scan** 16591
**Peptide** HGAEIVYKSPVVSGDTS **Charge** 3 **Spectral Angle** 0.79

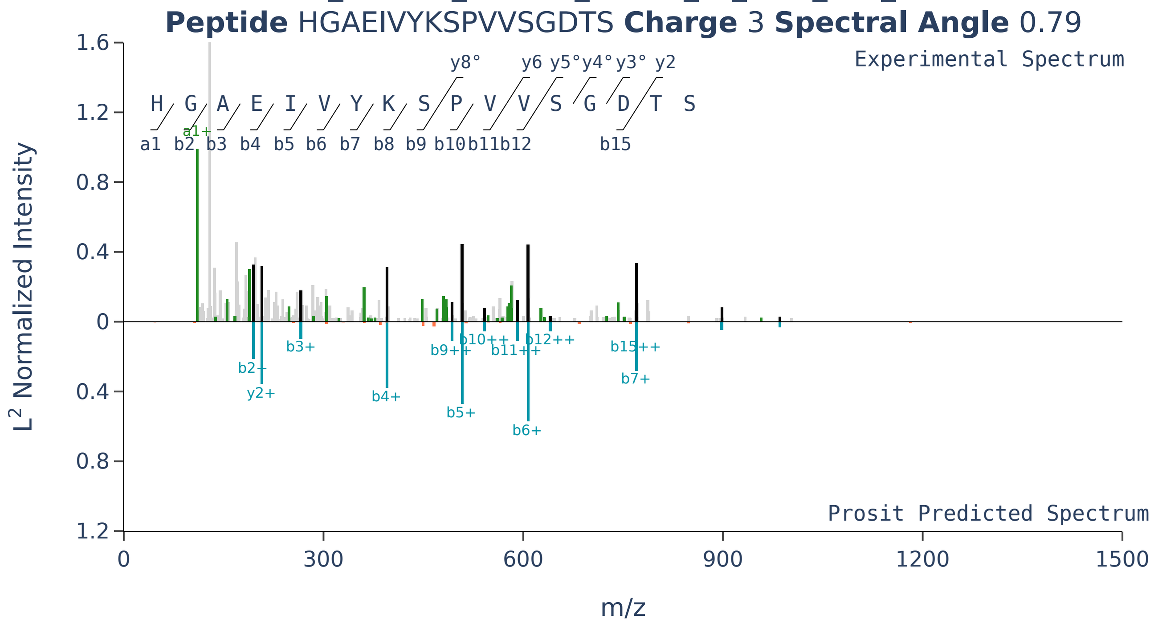


**Source** WSoh_221121_011221_HFGoe_L1_hTau_24h_R2 **Scan** 15867
**Peptide** SPLQTPTEDGSEEPGSETSDAKSTPTAED **Charge** 2 **Spectral Angle** 0.84

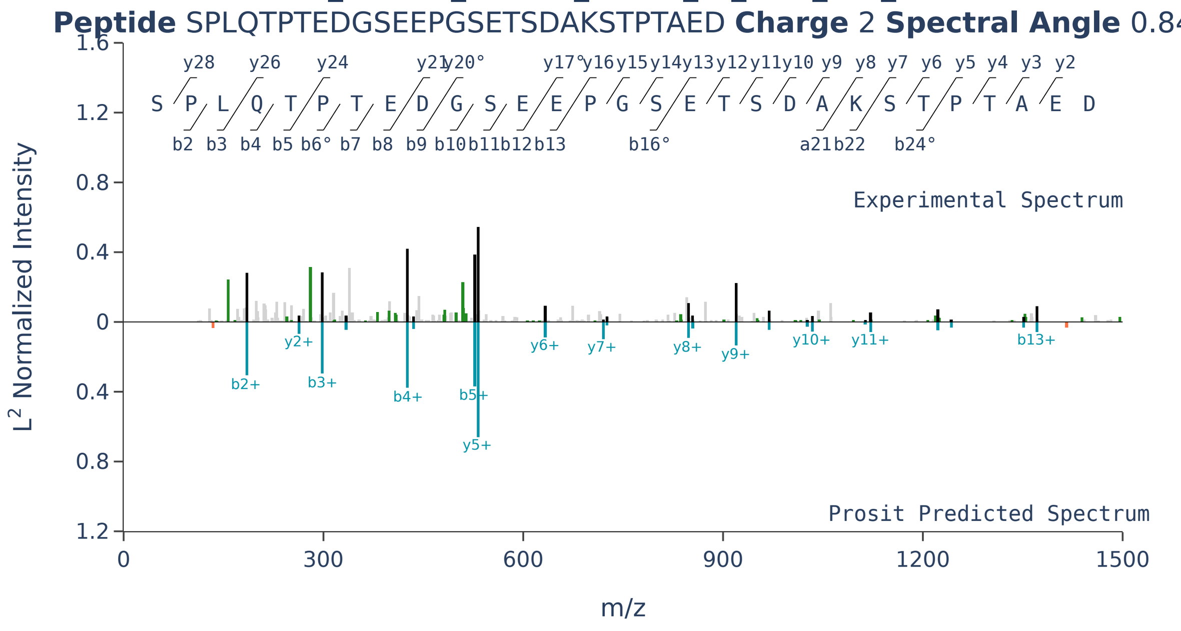


**Source** WSoh_221121_011221_HFGoe_L1_hTau_24h_R1 **Scan** 12816
**Peptide** HGAEIVYKSPVVSGDTSPRH **Charge** 4 **Spectral Angle** 0.85

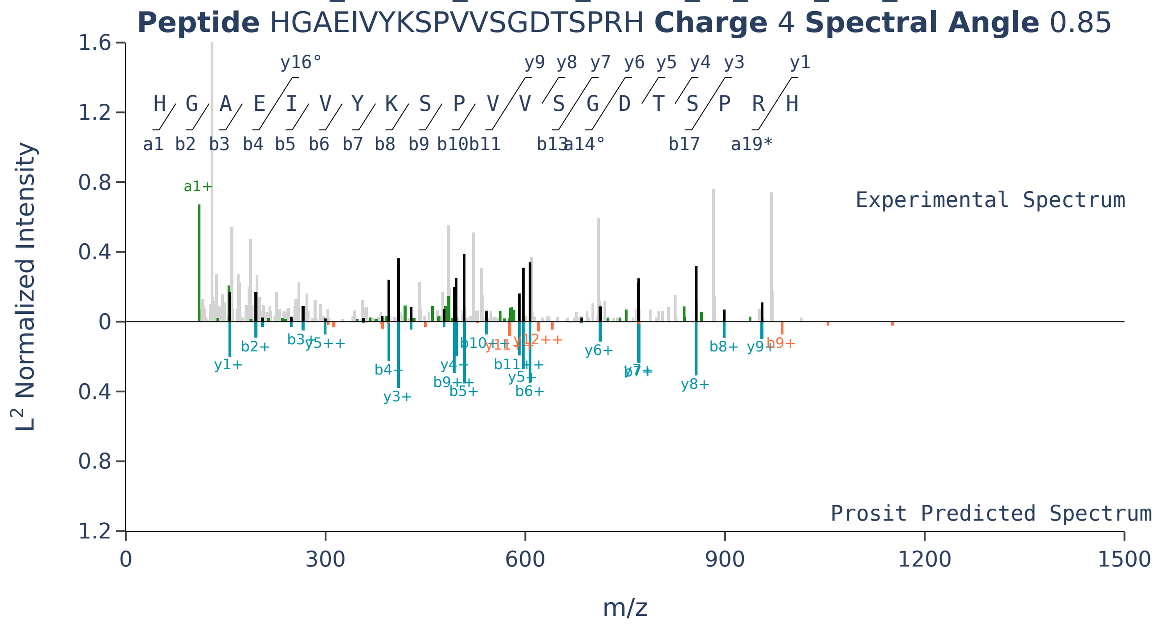

**Source** WSoh_221121_011221_HFGoe_L1_hTau_24h_R1 **Scan** 24776
**Peptide** QTAPVPMPDLKNVKSKIGSTENL **Charge** 2 **Spectral Angle** 0.81

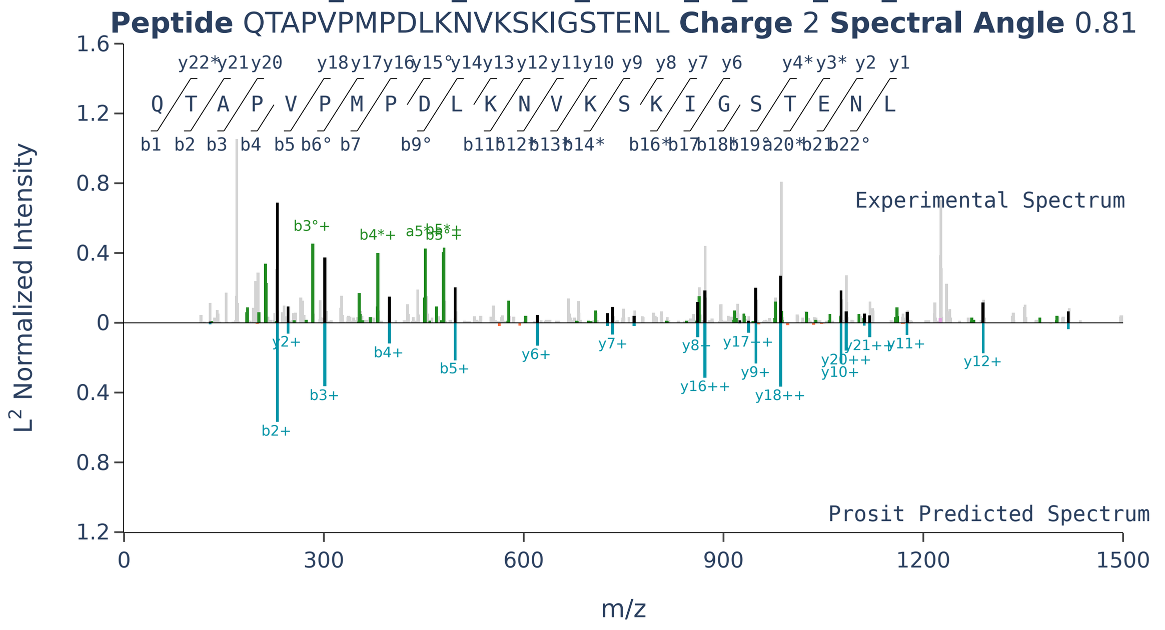


**Source** WSoh_221121_011221_HFGoe_L2_hTau_24h_R1 **Scan** 7781
**Peptide** LTHVPGGGNKKIETHKL **Charge** 5 **Spectral Angle** 0.76

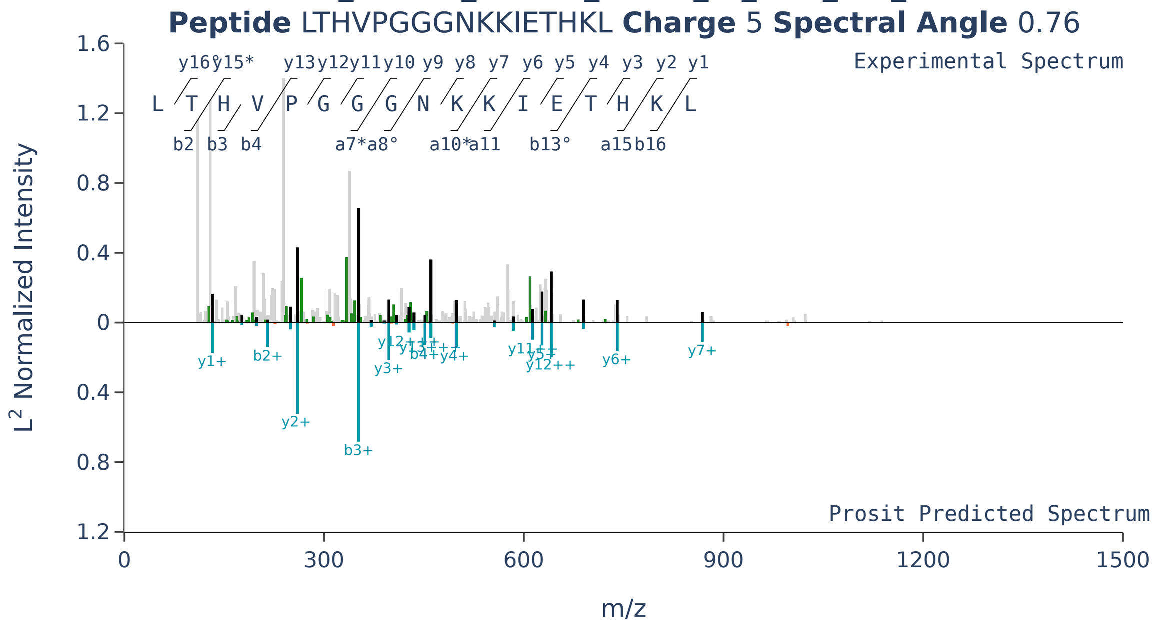


**Source** WSoh_221121_011221_HFGoe_L2_hTau_24h_R2 **Scan** 10435
**Peptide** TRIPAKTPPAPKTPPSSGEPPKSGD **Charge** 2 **Spectral Angle** 0.89

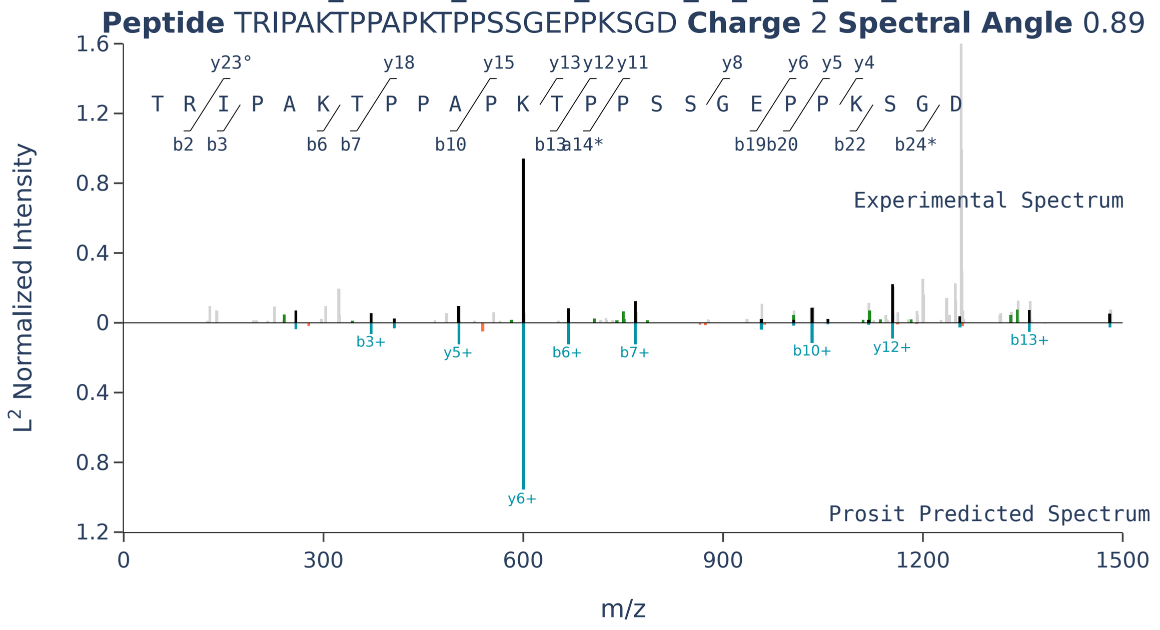


**Source** WSoh_221121_011221_HFGoe_L1_hTau_24h_R2 **Scan** 9061
**Peptide** RKDQGGYTMHQDQEGDTDAGLKE **Charge** 5 **Spectral Angle** 0.81

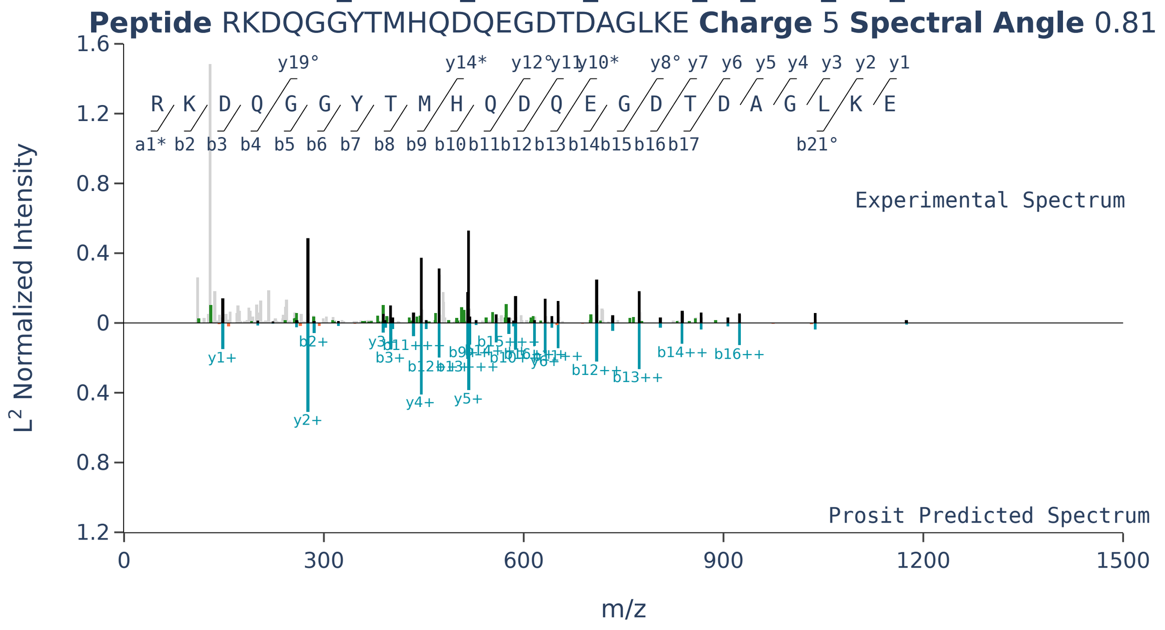


**Source** WSoh_221121_011221_HFGoe_L1_hTau_24h_R2 **Scan** 5662
**Peptide** IHHKPGGGQVEVKS **Charge** 2 **Spectral Angle** 0.88

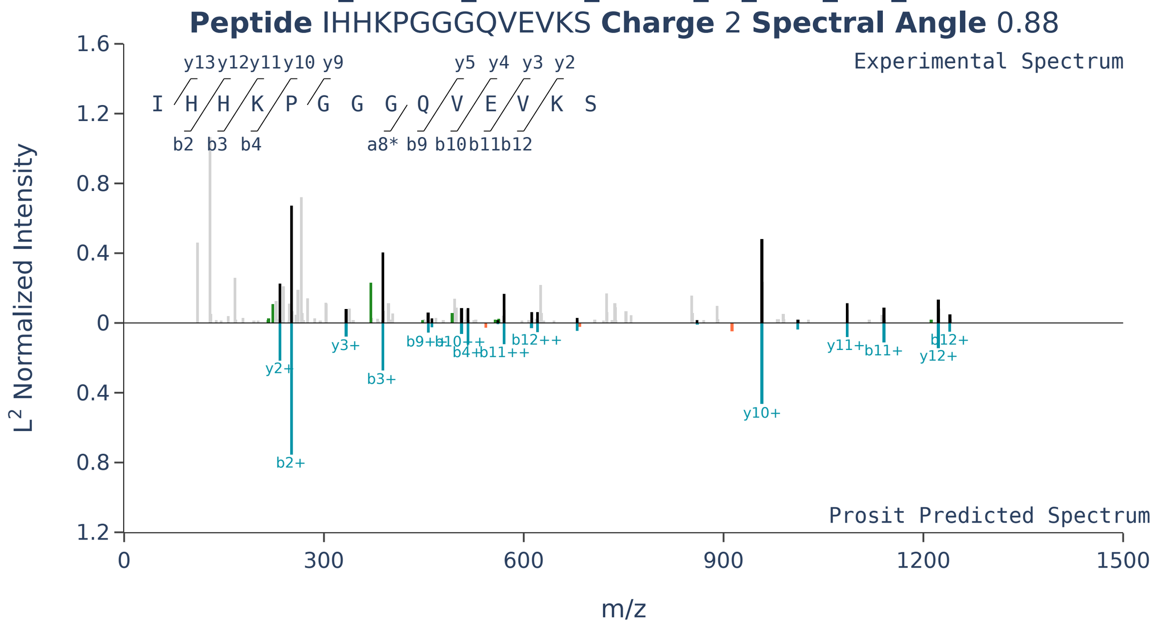


**Source** WSoh_221121_011221_HFGoe_L2_hTau_24h_R2 **Scan** 21688
**Peptide** DAKSTPTAEDVTAPLVDEGAPGKQA **Charge** 3 **Spectral Angle** 0.9

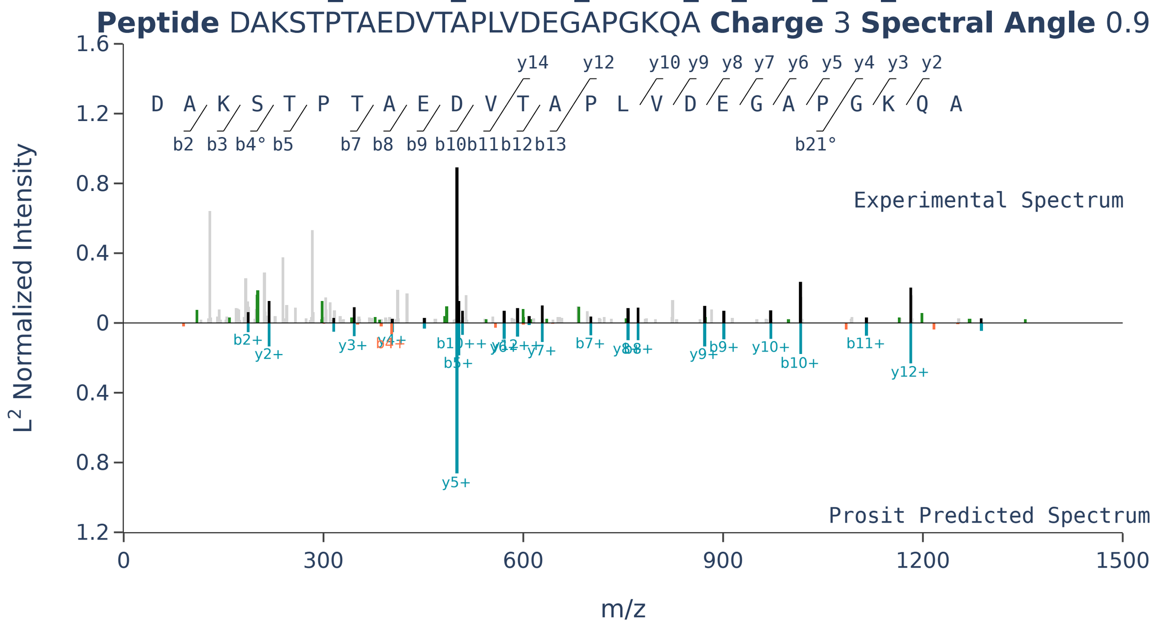


**Source** WSoh_221121_011221_HFGoe_L2_hTau_24h_R1 **Scan** 6060
**Peptide** EAAGHVTQARMVSKSK **Charge** 3 **Spectral Angle** 0.84

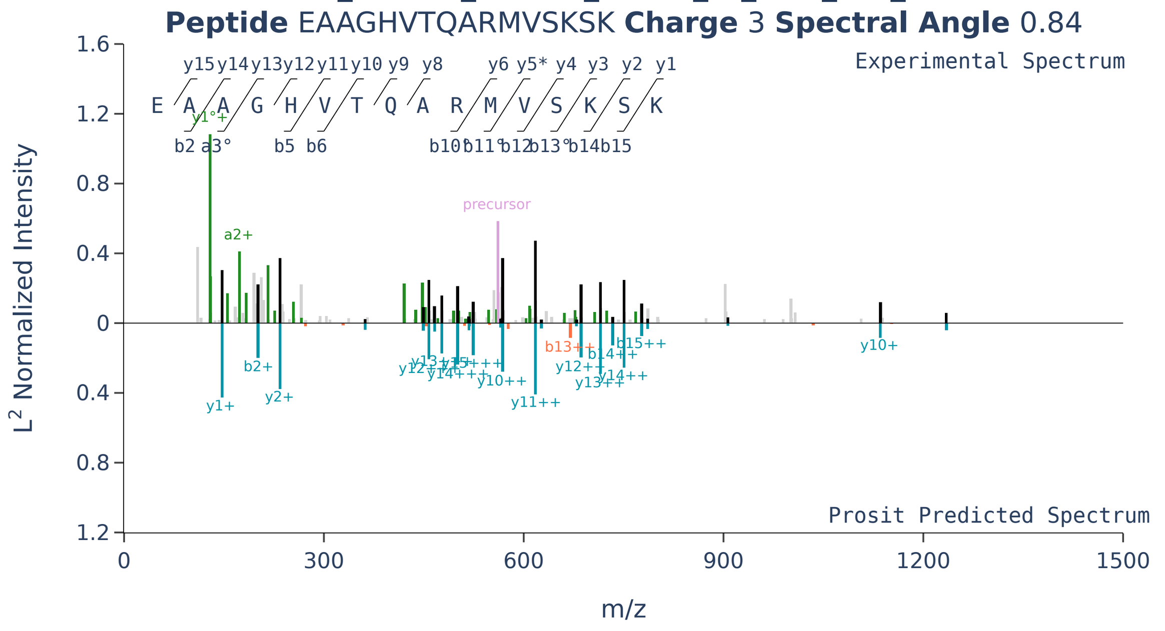


**Source** WSoh_221121_011221_HFGoe_L2_hTau_24h_R1 **Scan** 17803
**Peptide** VYKPVDLSKVTS **Charge** 2 **Spectral Angle** 0.74

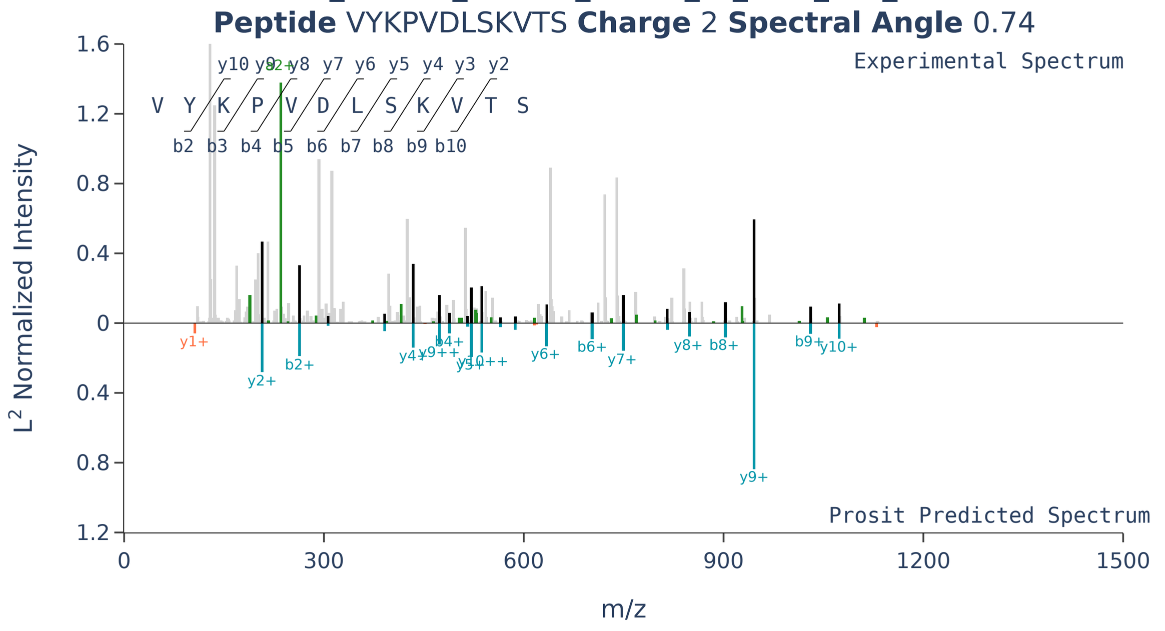


**Source** WSoh_221121_011221_HFGoe_L2_hTau_24h_R1 **Scan** 20465
**Peptide** GDTSPRHLSNVSSTGSIDMVDSPQL **Charge** 3 **Spectral Angle** 0.83

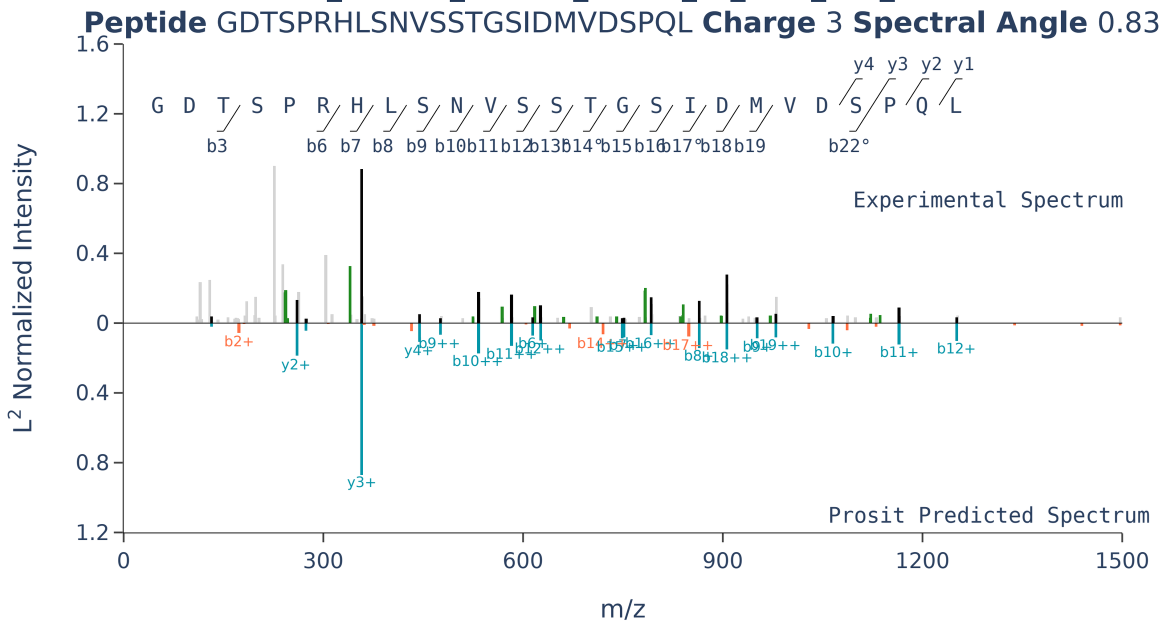


**Source** WSoh_221121_011221_HFGoe_L1_hTau_24h_R2 **Scan** 8437
**Peptide** RLPAKTPPAPKTPPSSGEPPKSGDR **Charge** 5 **Spectral Angle** 0.81

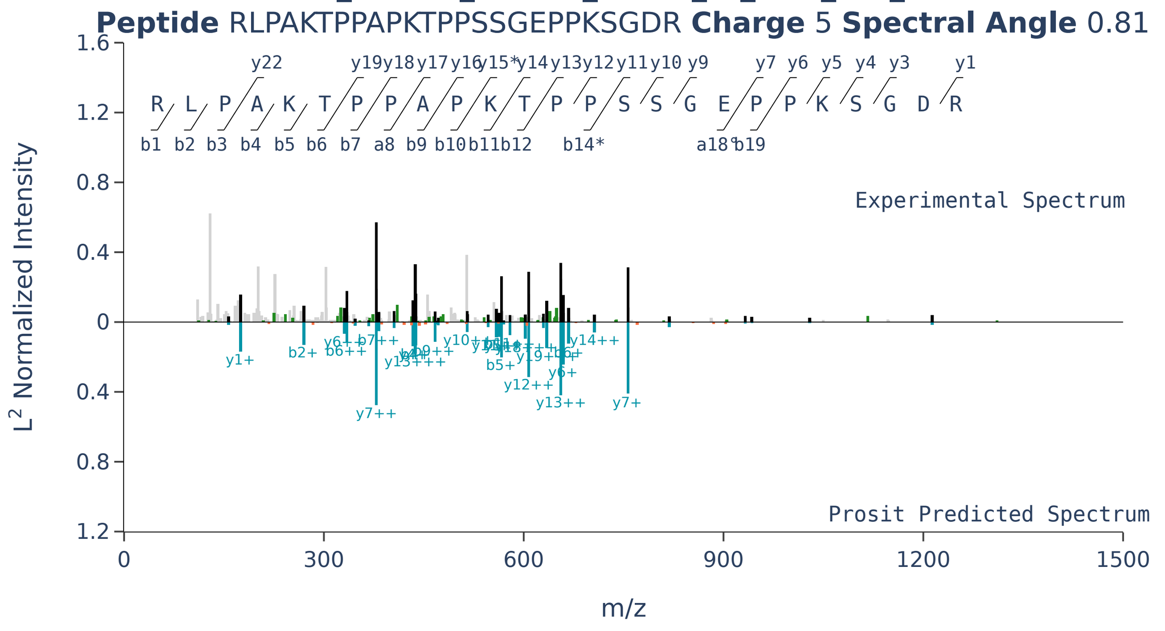


**Source** WSoh_221121_011221_HFGoe_L1_hTau_24h_R1 **Scan** 20874
**Peptide** KSTPTAEDVTAPLVD **Charge** 2 **Spectral Angle** 0.78

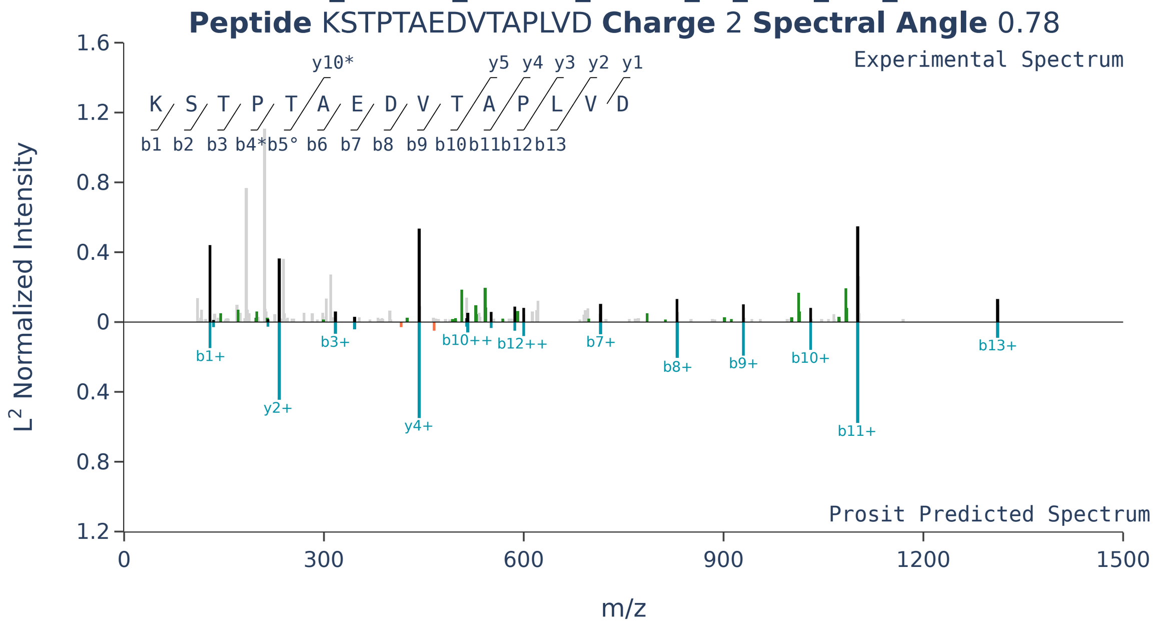


**Source** WSoh_221121_011221_HFGoe_L1_hTau_24h_R1 **Scan** 8949
**Peptide** DGSEEPGSETSDAKSTPTAED **Charge** 2 **Spectral Angle** 0.79

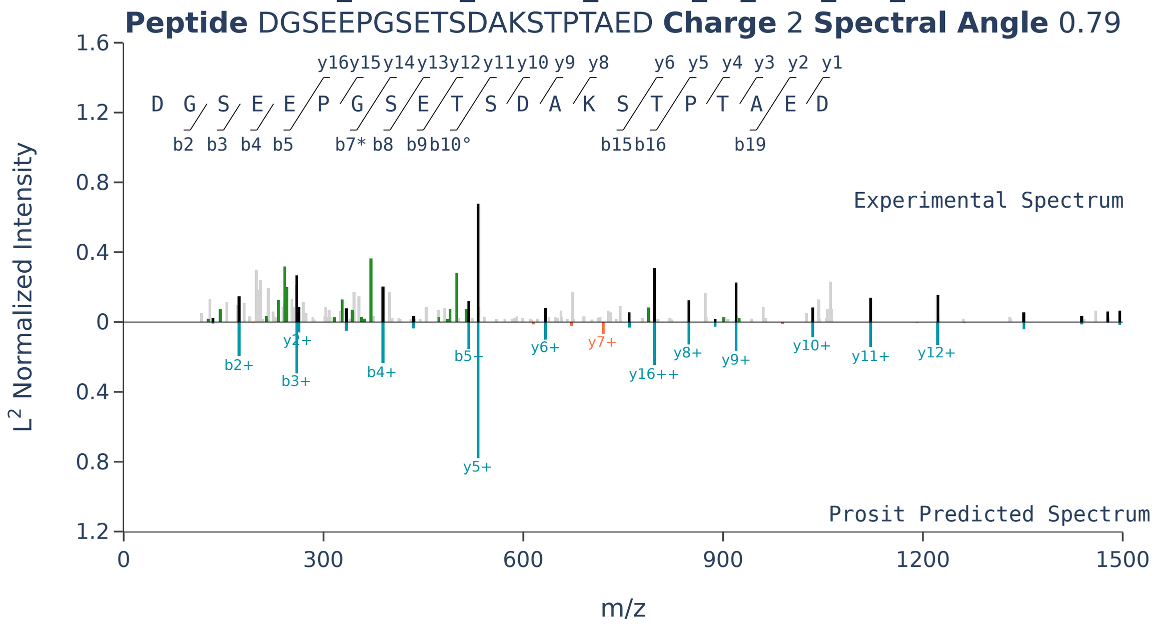


**Source** WSoh_221121_011221_HFGoe_L2_hTau_2h_R2 **Scan** 17183
**Peptide** KIGSLDNIT **Charge** 2 **Spectral Angle** 0.74

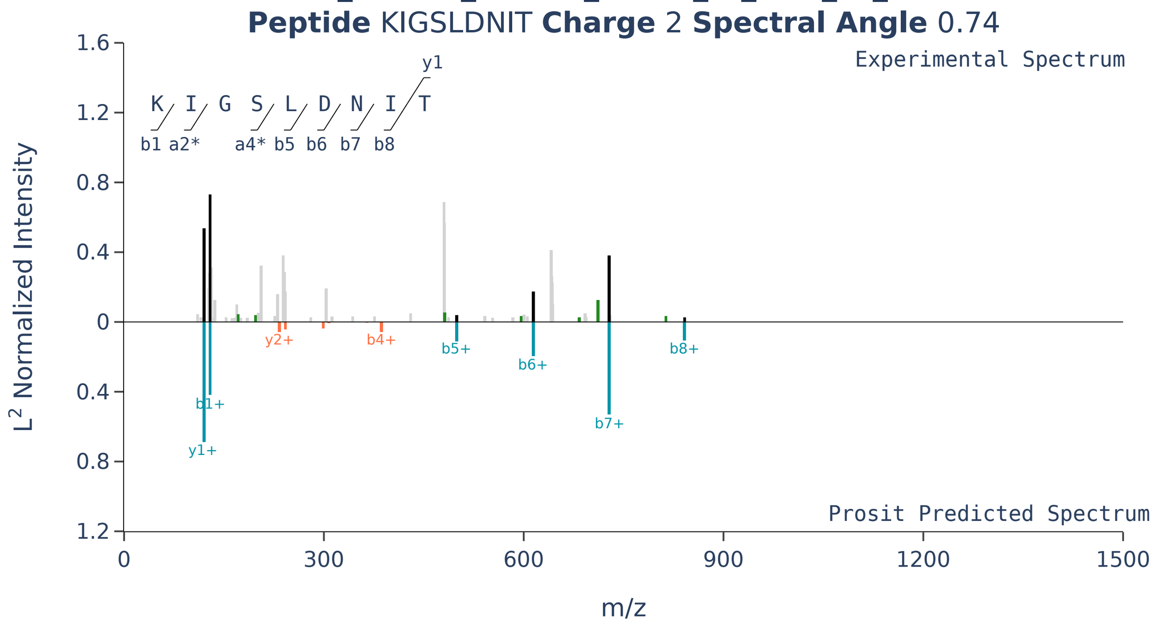


**Source** WSoh_221121_011221_HFGoe_L1_hTau_24h_R2 **Scan** 9994
**Peptide** KTDHGAEIVYKSP **Charge** 3 **Spectral Angle** 0.85

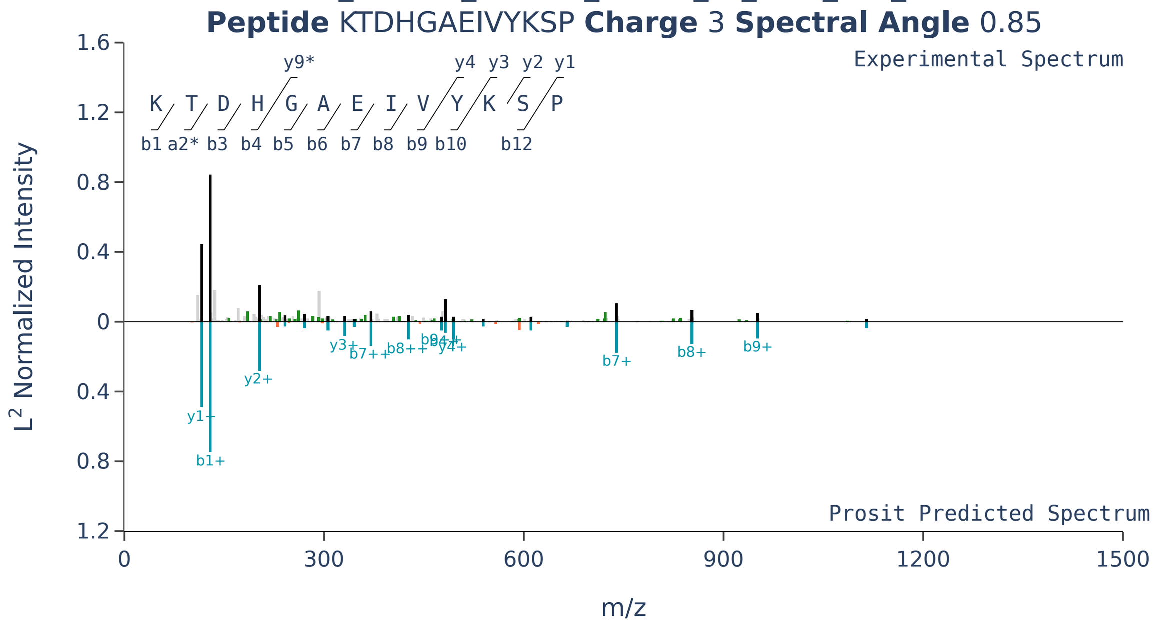


**Source** WSoh_221121_011221_HFGoe_L1_hTau_4h_R1 **Scan** 18379
**Peptide** NLKHVPGGGSVQIVY **Charge** 3 **Spectral Angle** 0.82

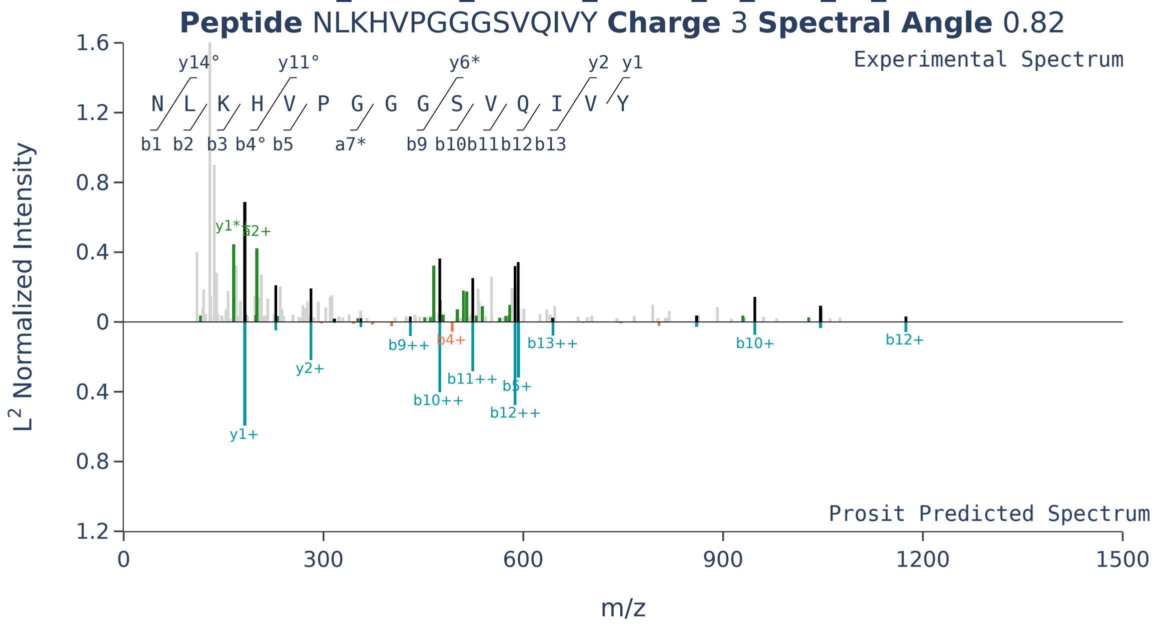

**Source** WSoh_221121_011221_HFGoe_L1_hTau_4h_R2 **Scan** 12136
**Peptide** KSEKLDFKDRVQSKLG **Charge** 4 **Spectral Angle** 0.87

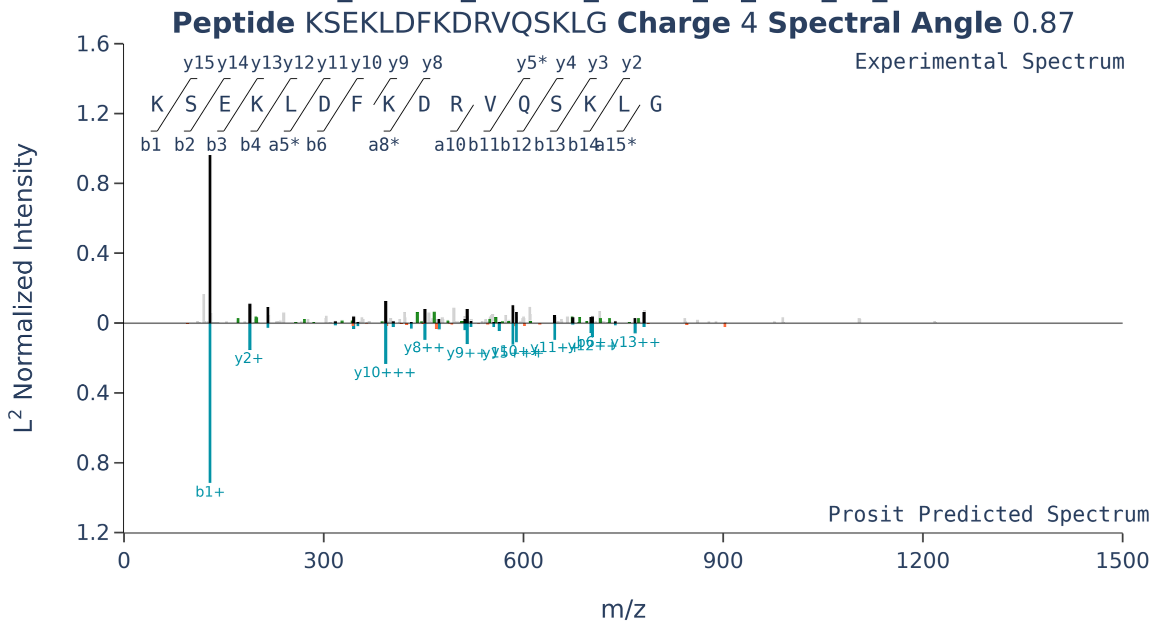


**Source** WSoh_221121_011221_HFGoe_L2_hTau_4h_R2 **Scan** 24699
**Peptide** AEPRQEFEVMEDHAGTYGL **Charge** 3 **Spectral Angle** 0.84

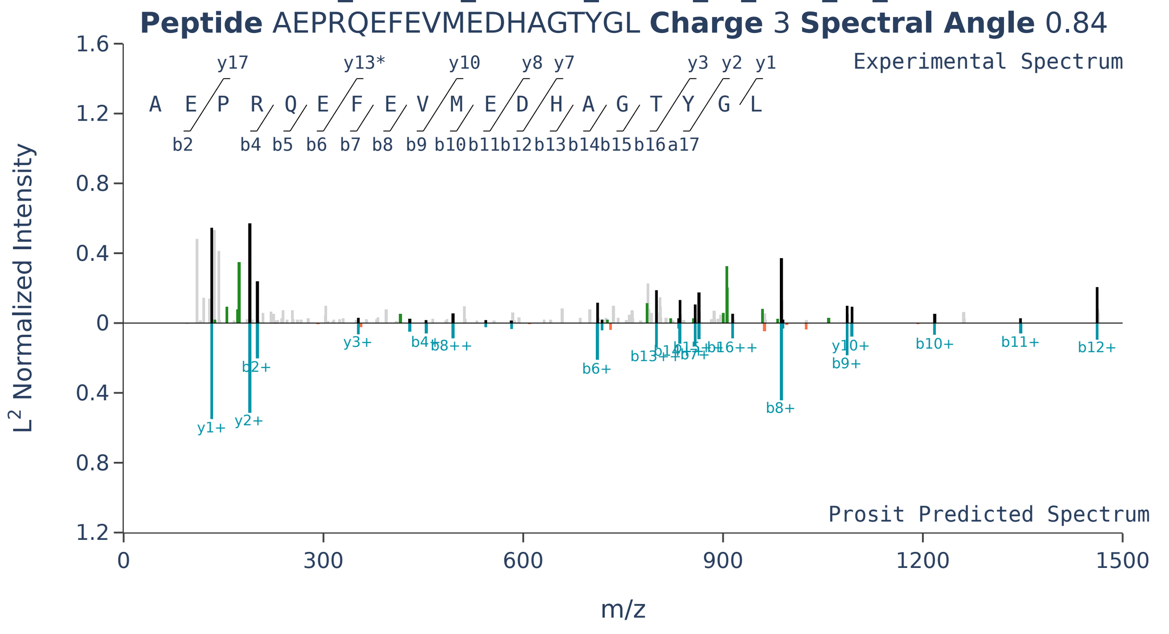


**Source** WSoh_221121_011221_HFGoe_L1_hTau_24h_R1 **Scan** 22073
**Peptide** PPKSPSSAKSRLQTAPVPMPDL **Charge** 3 **Spectral Angle** 0.86

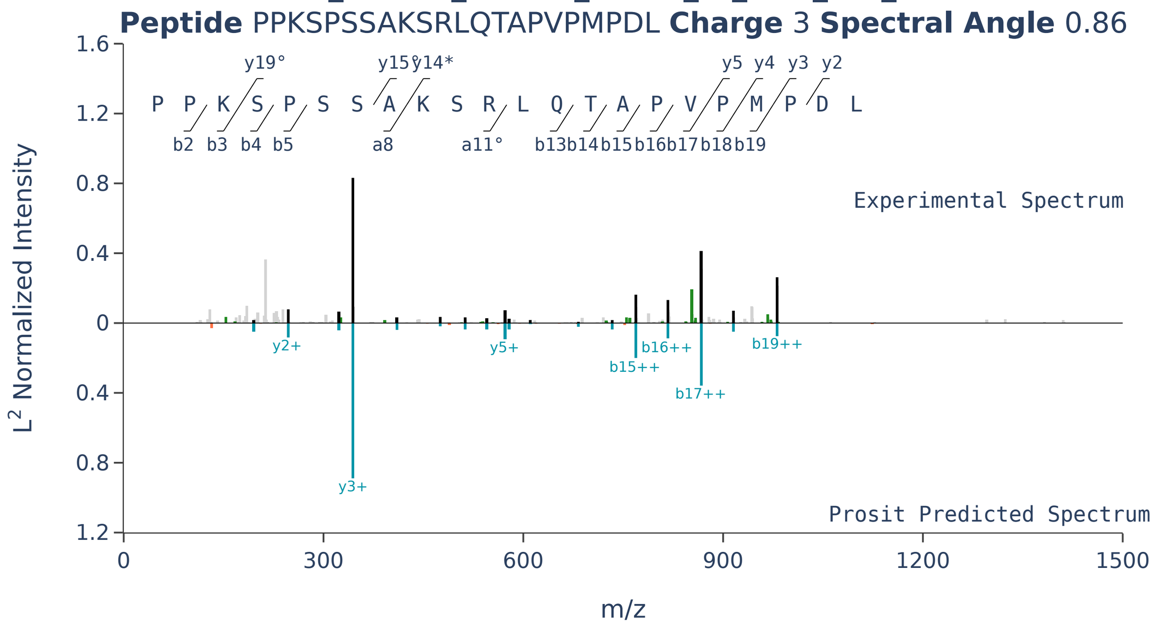


**Source** WSoh_221121_011221_HFGoe_L1_hTau_24h_R1 **Scan** 19822
**Peptide** VTAPLVDEGAPGKQAAAQPHTEL **Charge** 3 **Spectral Angle** 0.82

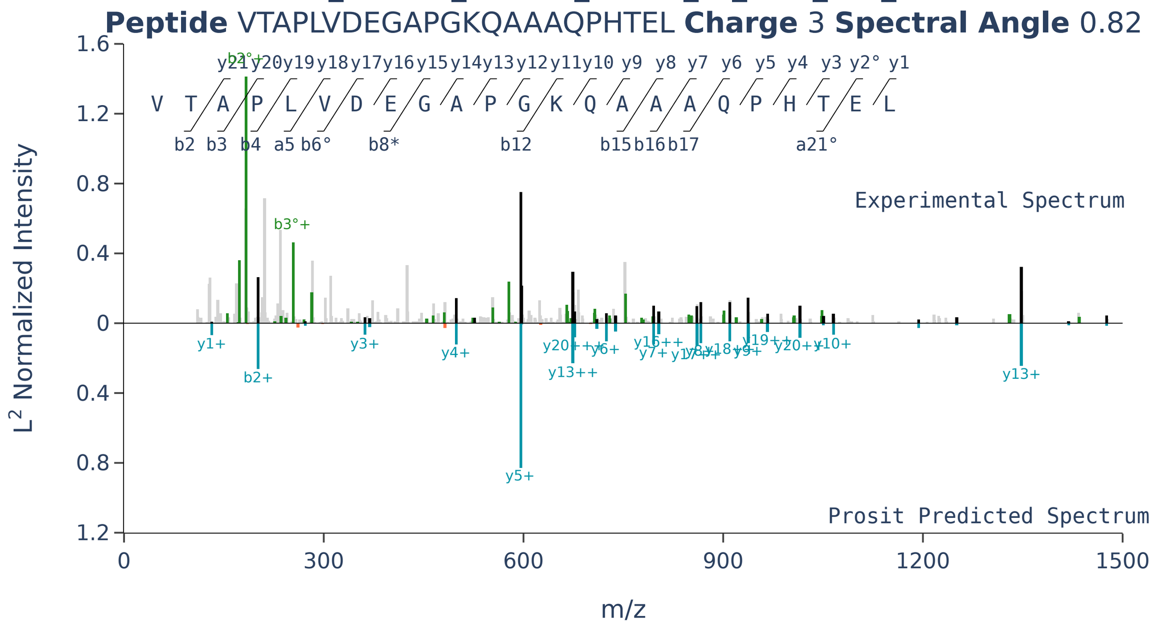


**Source** WSoh_221121_011221_HFGoe_L1_hTau_24h_R1 **Scan** 18102
**Peptide** GAEIVYKSPVVSGDTSPRHLSNVSS **Charge** 3 **Spectral Angle** 0.85

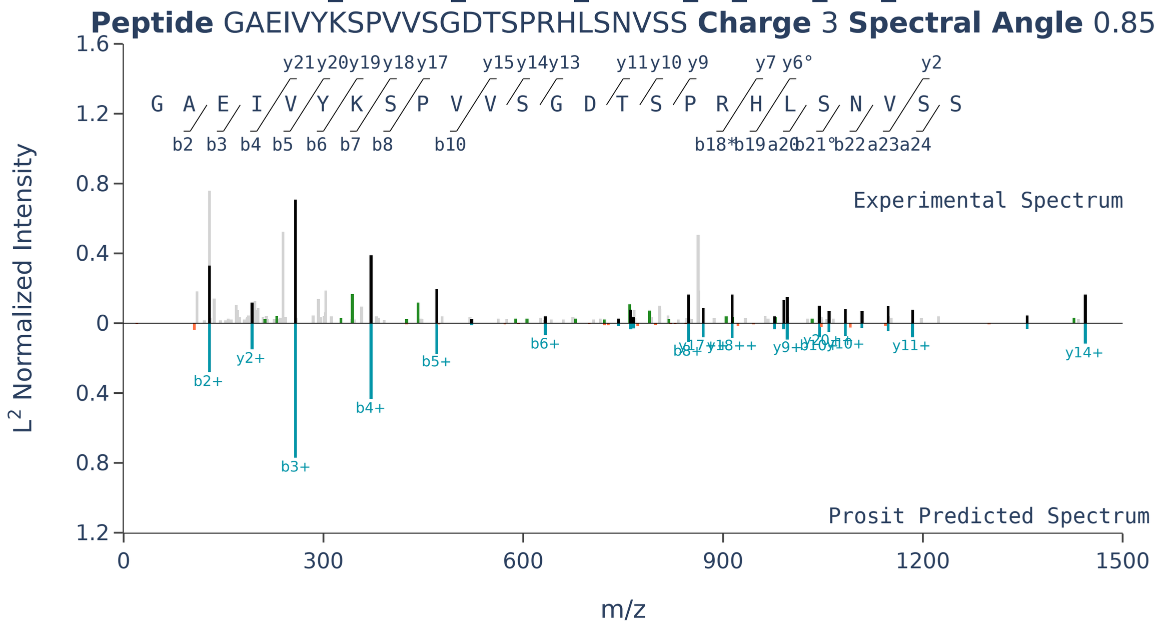


**Source** WSoh_221121_011221_HFGoe_L1_hTau_2h_R2 **Scan** 8437
**Peptide** AKSTPTAEDV **Charge** 2 **Spectral Angle** 0.88

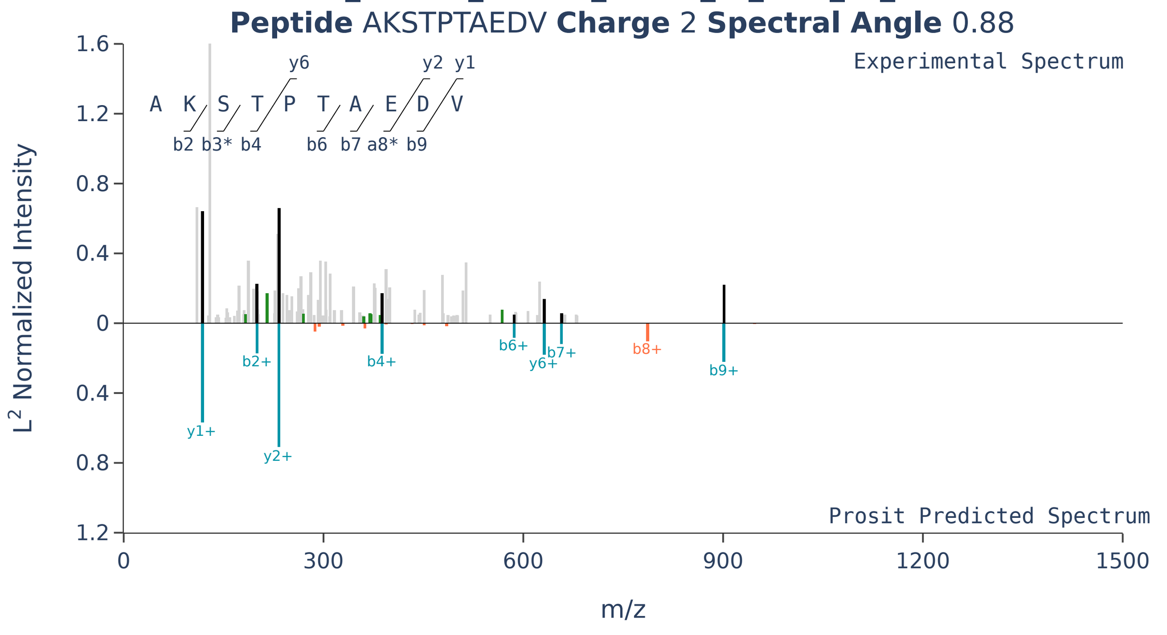


**Source** WSoh_221121_011221_HFGoe_L1_hTau_2h_R1 **Scan** 10371
**Peptide** EKLDFKD **Charge** 2 **Spectral Angle** 0.89

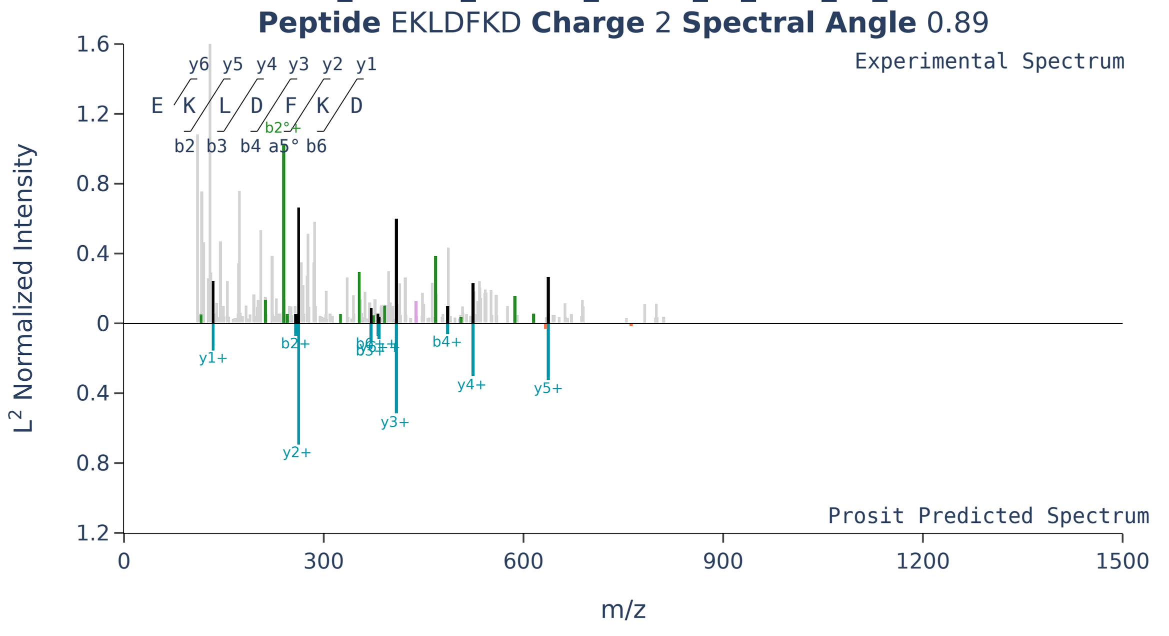


**Source** WSoh_221121_011221_HFGoe_L2_hTau_24h_R2 **Scan** 9214
**Peptide** KLDLSNVQSKCGSKDNIKH **Charge** 3 **Spectral Angle** 0.93

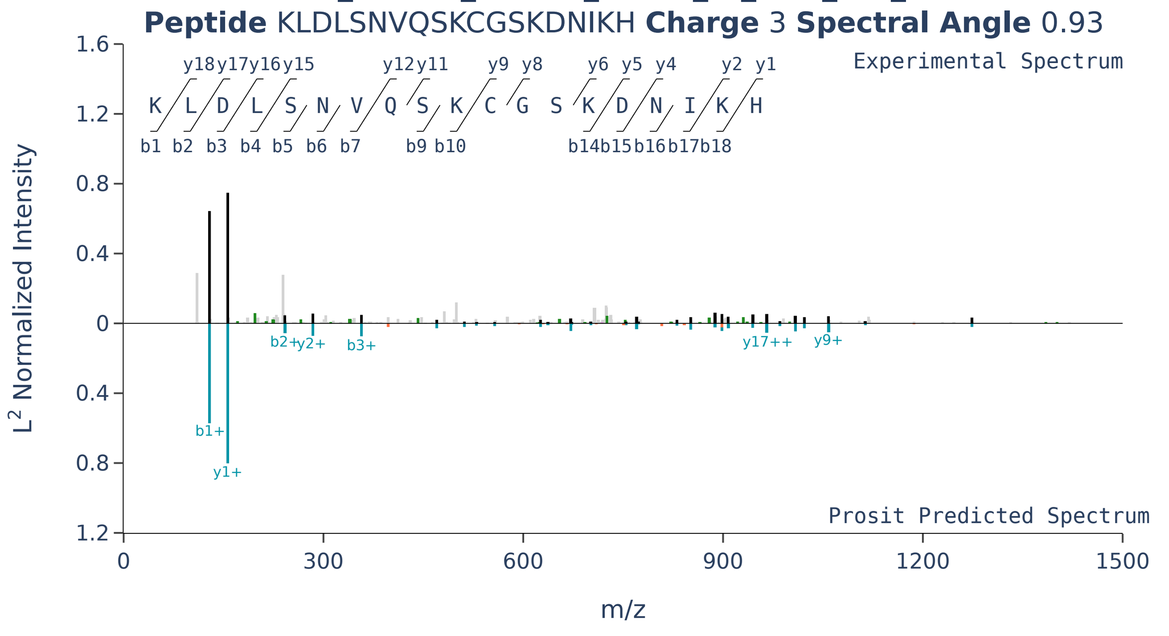


**Source** WSoh_221121_011221_HFGoe_L1_hTau_4h_R2 **Scan** 8675
**Peptide** EKLDFKDR **Charge** 3 **Spectral Angle** 0.78

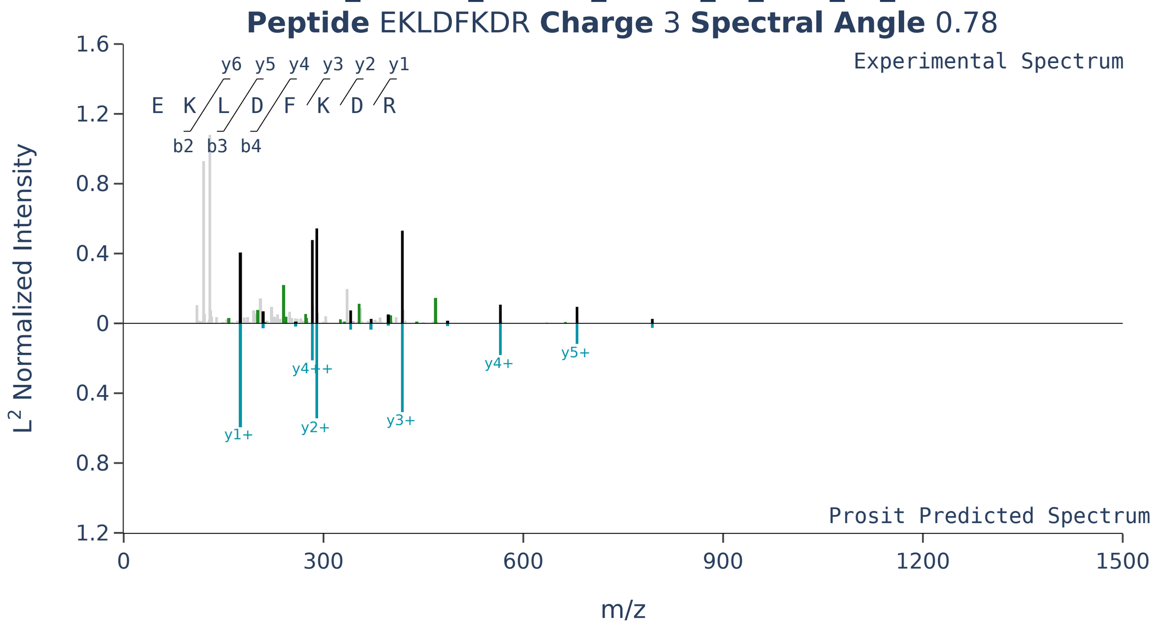


**Source** WSoh_221121_011221_HFGoe_L1_hTau_4h_R2 **Scan** 7926
**Peptide** NKKLDLSNVQSKCGSKD **Charge** 3 **Spectral Angle** 0.84

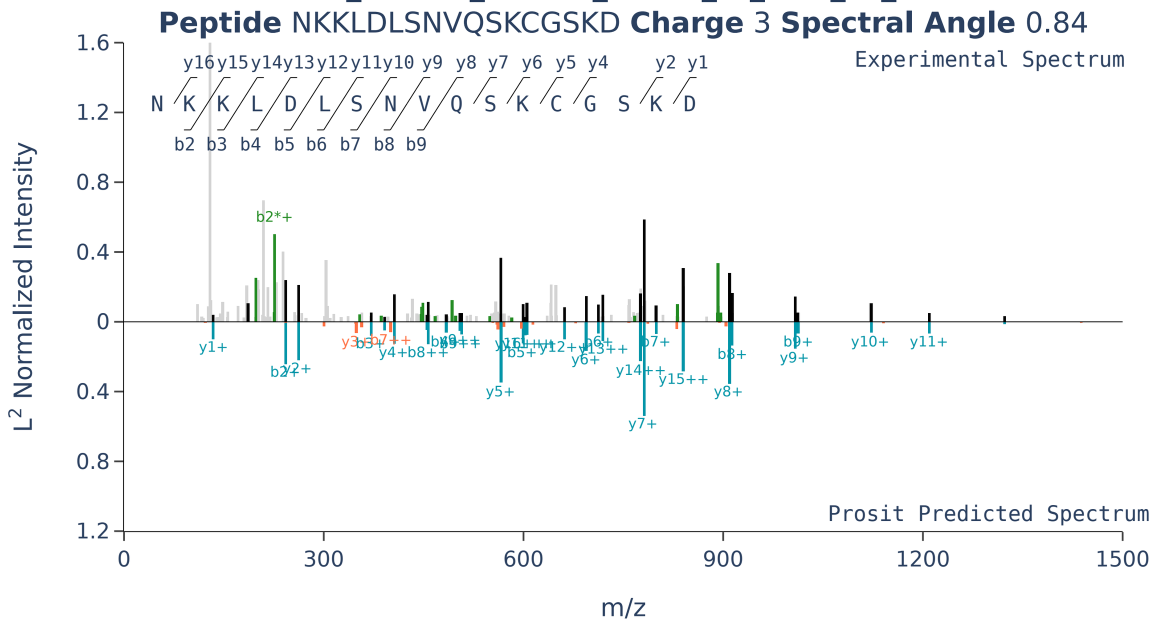


**Source** WSoh_221121_011221_HFGoe_L1_hTau_24h_R2 **Scan** 21536
**Peptide** APVPMPDLKNVKSKIGSTENL **Charge** 3 **Spectral Angle** 0.93

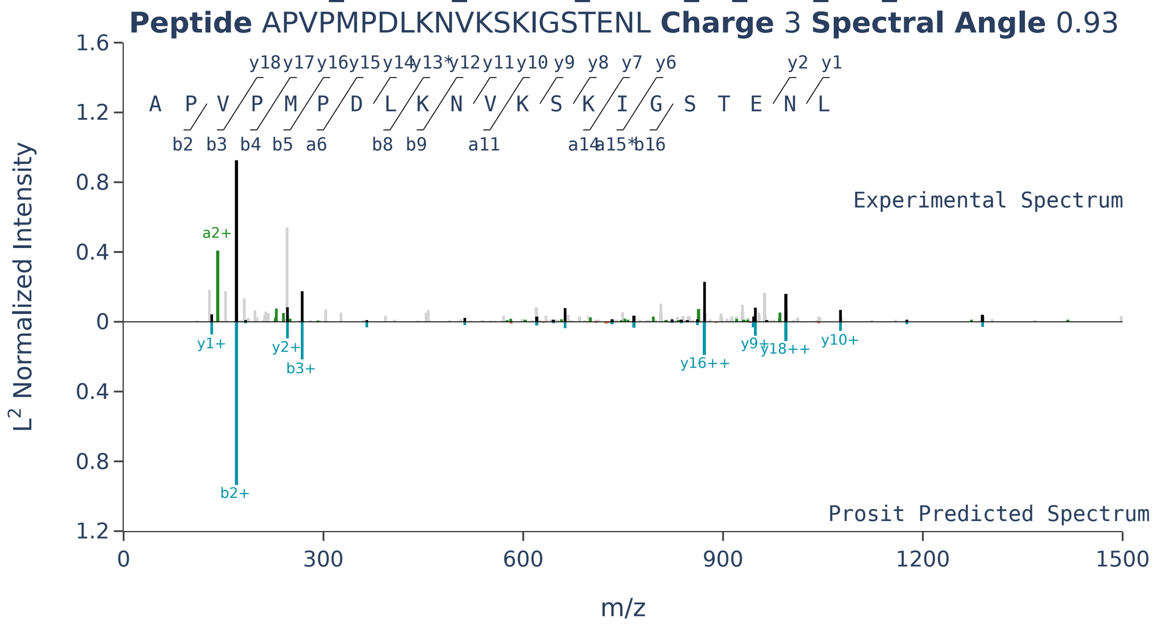


**Source** WSoh_221121_011221_HFGoe_L1_hTau_24h_R1 **Scan** 7535
**Peptide** NKKLDLSNVQSKCGSKDNIKH **Charge** 3 **Spectral Angle** 0.89

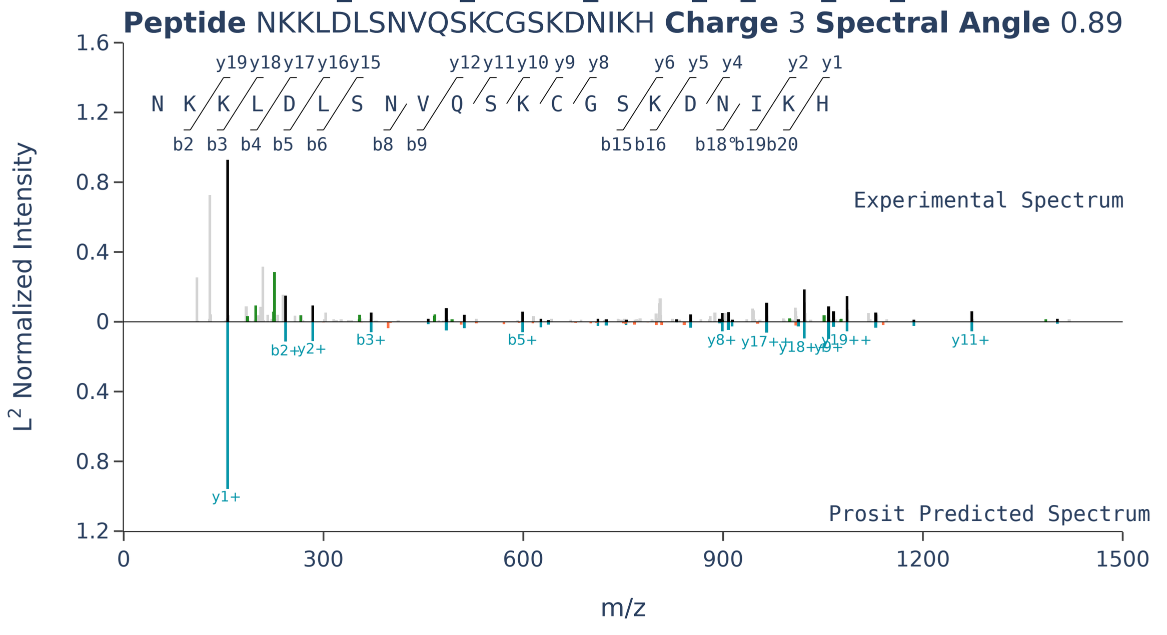


**Source** WSoh_221121_011221_HFGoe_L1_hTau_24h_R1 **Scan** 19627
**Peptide** GLGDTPSLEDEAAGHVTQARMVSKS **Charge** 4 **Spectral Angle** 0.78

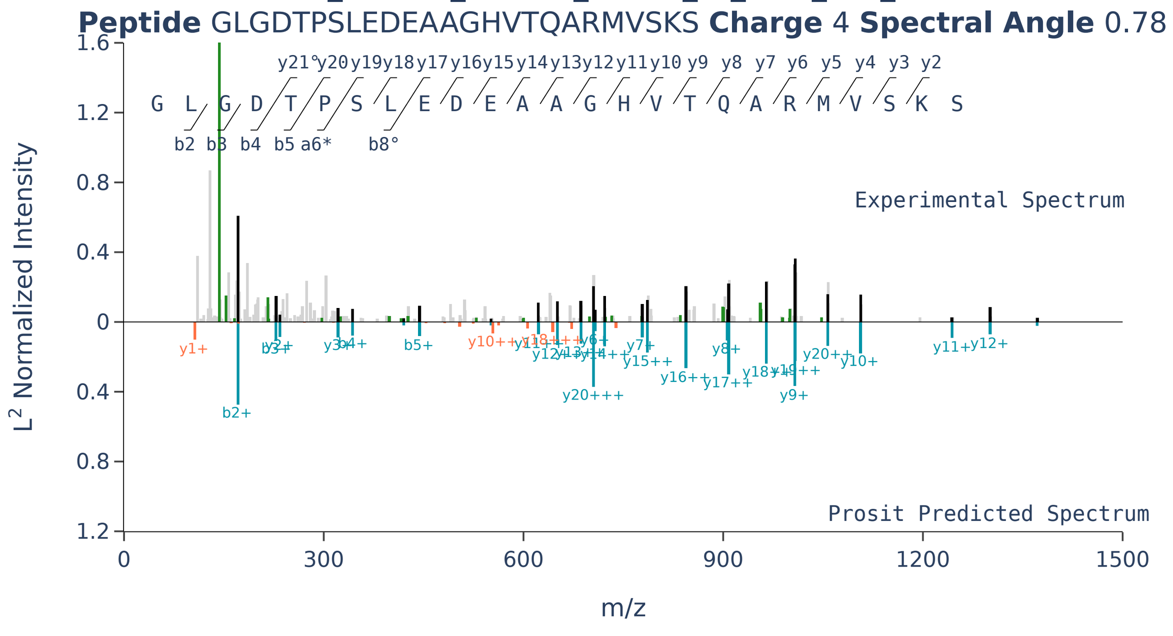


**Source** WSoh_221121_011221_HFGoe_L2_hTau_24h_R1 **Scan** 7580
**Peptide** DGKTKIATPRGAAPPGQKG **Charge** 3 **Spectral Angle** 0.82

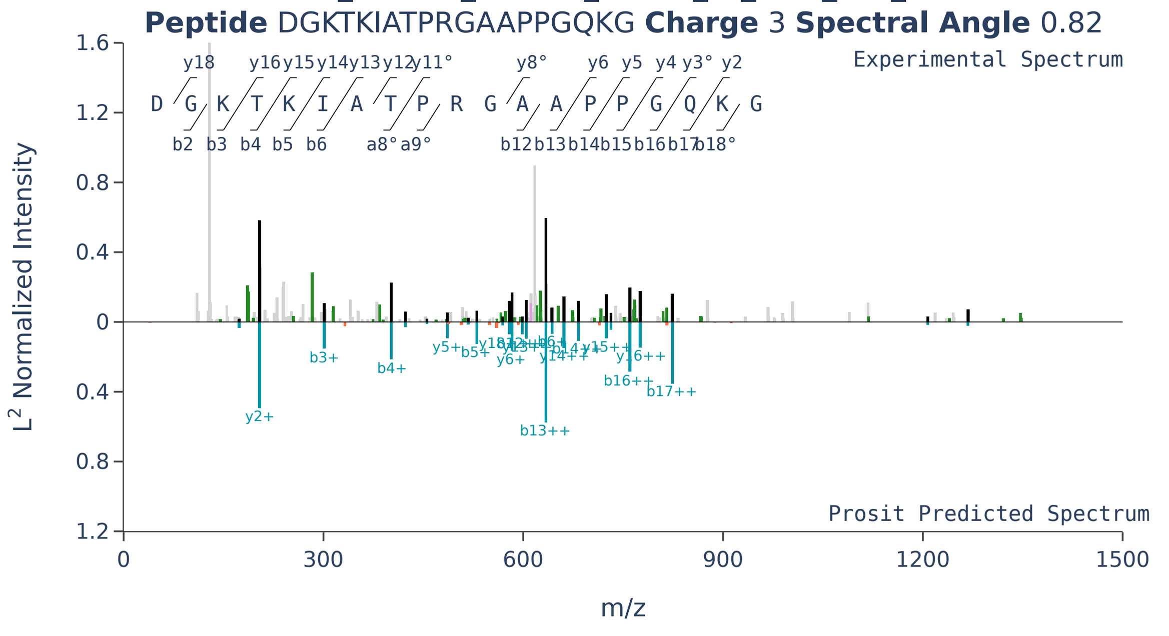


**Source** WSoh_221121_011221_HFGoe_L2_hTau_24h_R2 **Scan** 20346
**Peptide** AGLGDTPSLED **Charge** 1 **Spectral Angle** 0.86

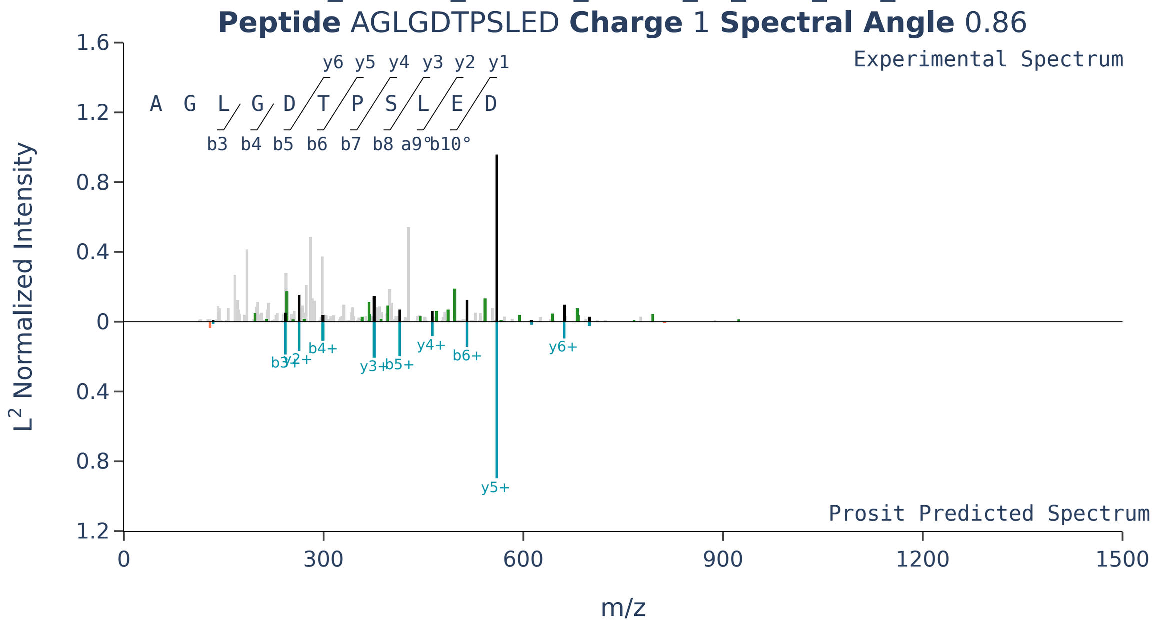

**Source** WSoh_221121_011221_HFGoe_L1_hTau_24h_R1 **Scan** 8764
**Peptide** LGNIHHKPGGGQVEVKSEKL **Charge** 3 **Spectral Angle** 0.76

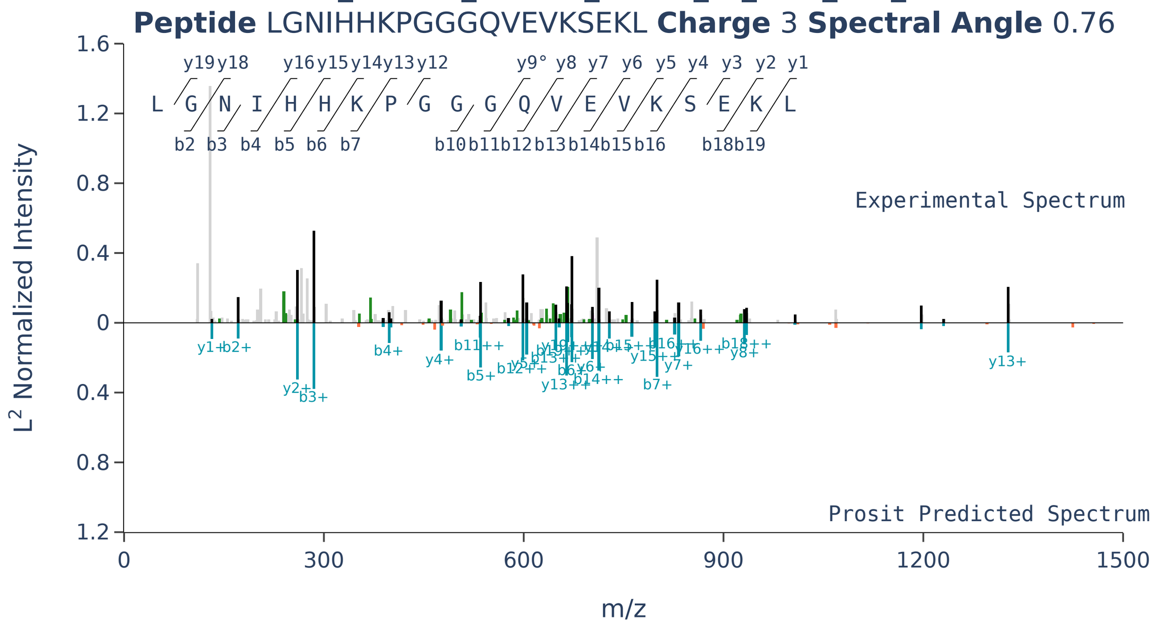


**Source** WSoh_221121_011221_HFGoe_L2_hTau_2h_R2 **Scan** 6066
**Peptide** KLTFRENAKAKTDHG **Charge** 4 **Spectral Angle** 0.85

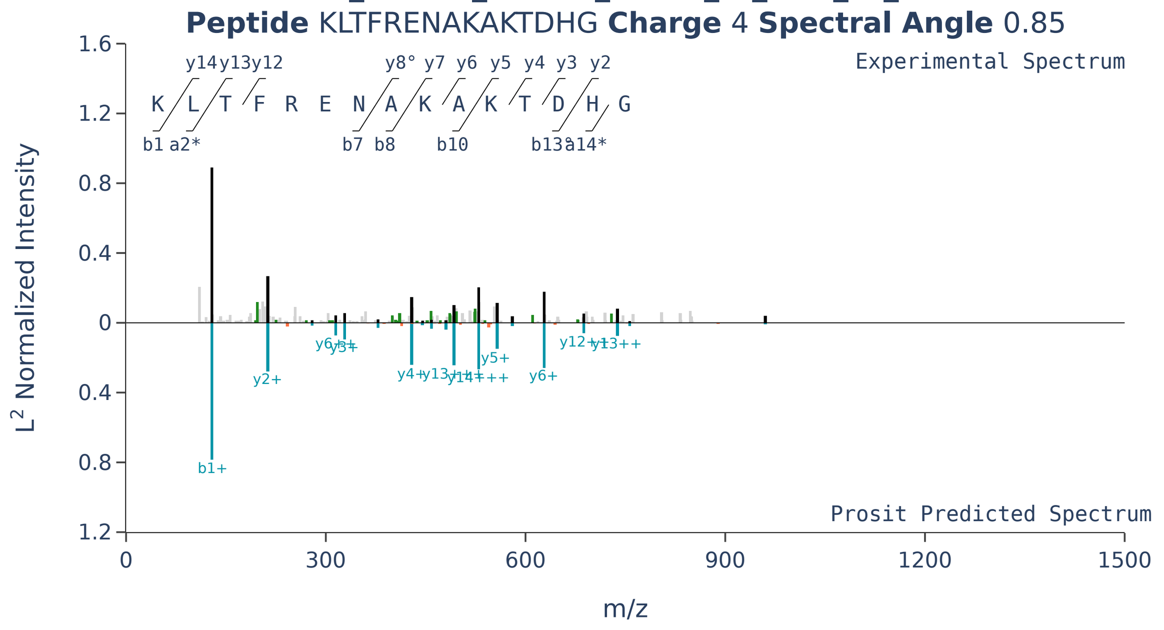


**Source** WSoh_221121_011221_HFGoe_L2_hTau_24h_R1 **Scan** 9001
**Peptide** LATPRGAAPPGQKGQANAT **Charge** 2 **Spectral Angle** 0.89

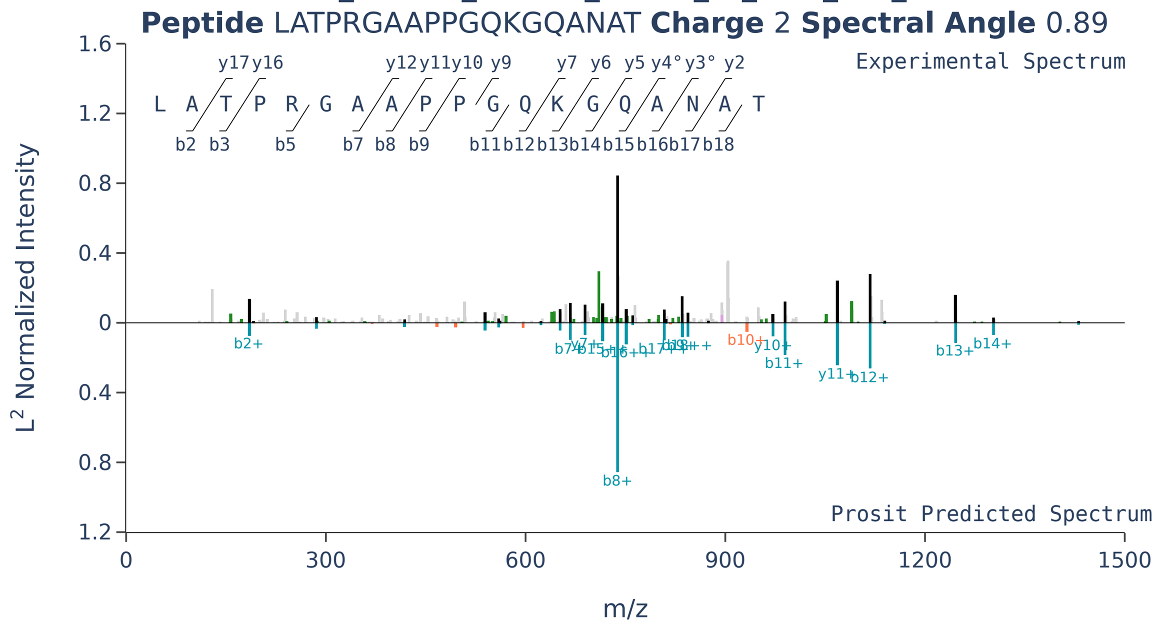


**Source** WSoh_221121_011221_HFGoe_L2_hTau_4h_R1 **Scan** 8030
**Peptide** LSKVTSKCGSLGNIHH **Charge** 4 **Spectral Angle** 0.9

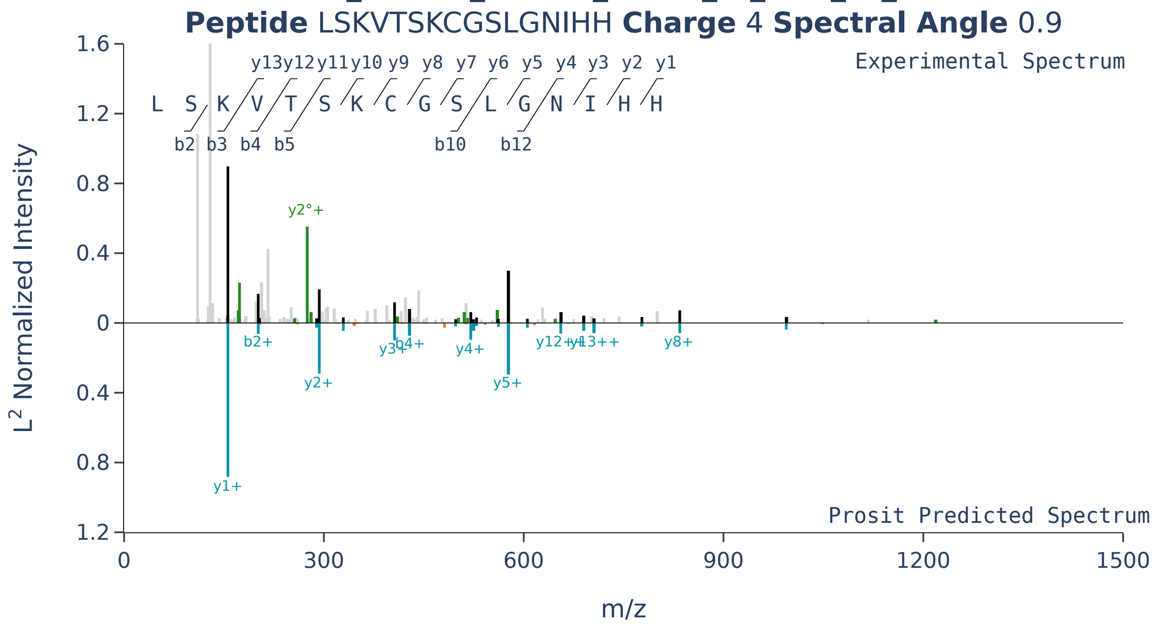


**Source** WSoh_221121_011221_HFGoe_L1_hTau_24h_R1 **Scan** 9213
**Peptide** VKSEKLDFKD **Charge** 3 **Spectral Angle** 0.85

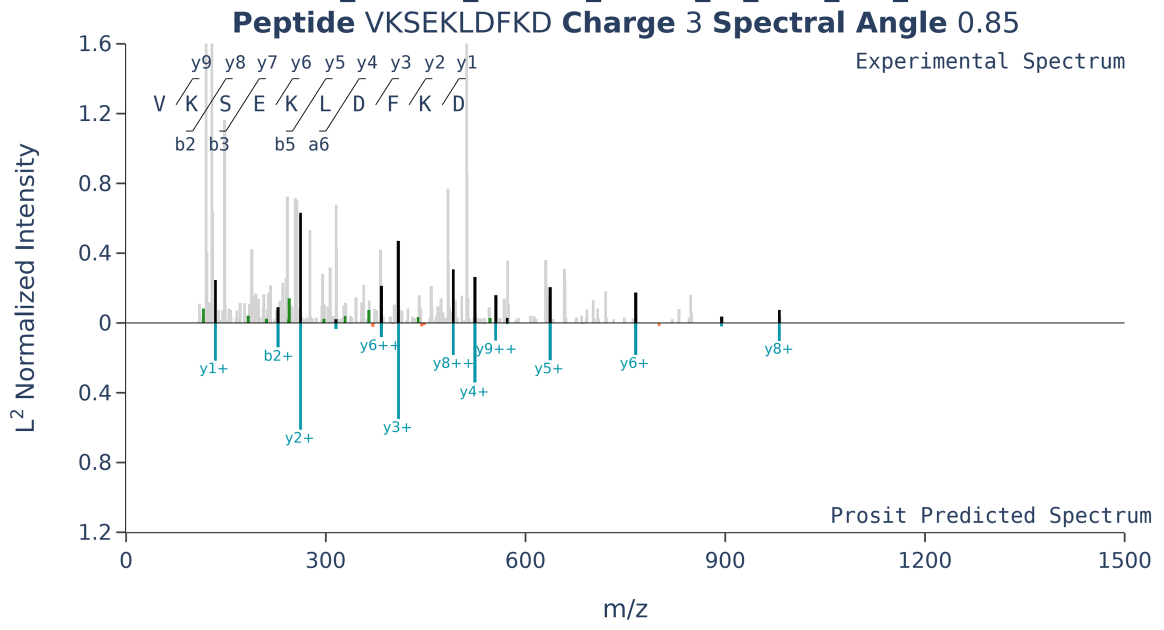


**Source** WSoh_221121_011221_HFGoe_L2_hTau_4h_R2 **Scan** 10180
**Peptide** KSPVVSGDTSPRHLSNVSS **Charge** 2 **Spectral Angle** 0.75

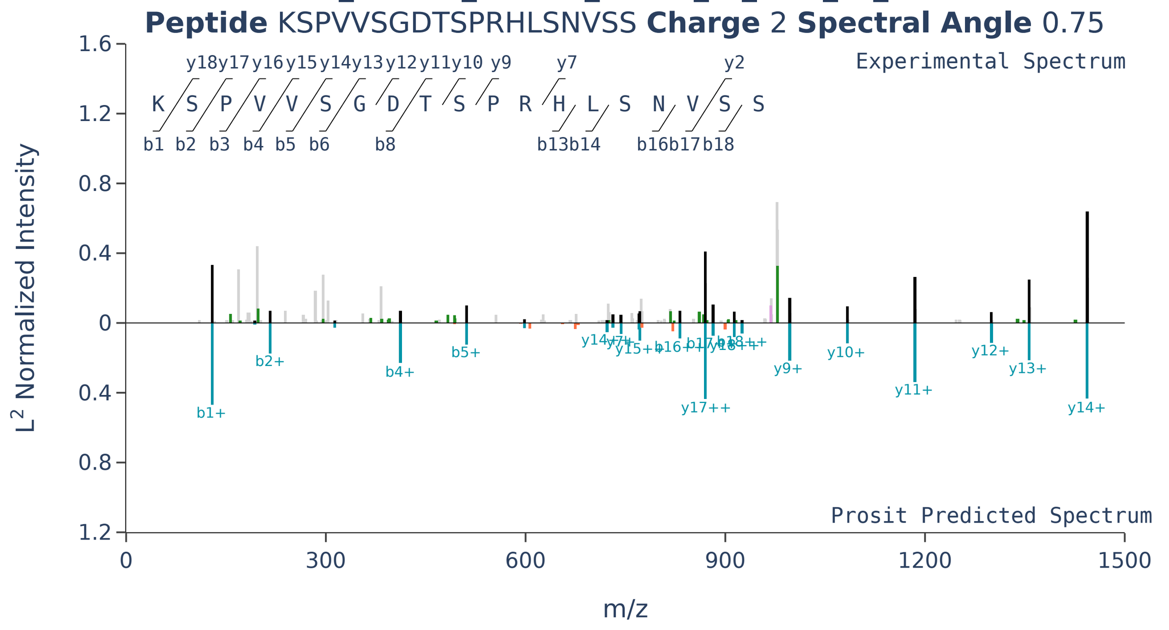


**Source** WSoh_221121_011221_HFGoe_L2_hTau_4h_R1 **Scan** 8840
**Peptide** IATPRGAAPPGQKGQANAT **Charge** 2 **Spectral Angle** 0.86

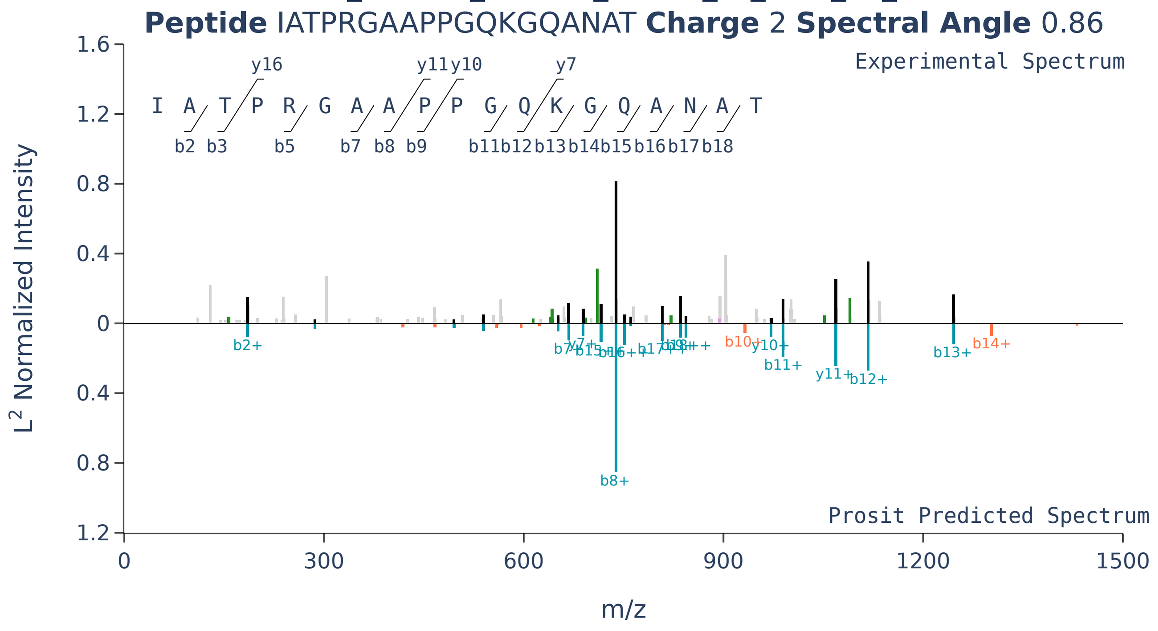


**Source** WSoh_221121_011221_HFGoe_L1_hTau_4h_R2 **Scan** 16356
**Peptide** HGAEIVYKSPVVSGD **Charge** 3 **Spectral Angle** 0.74

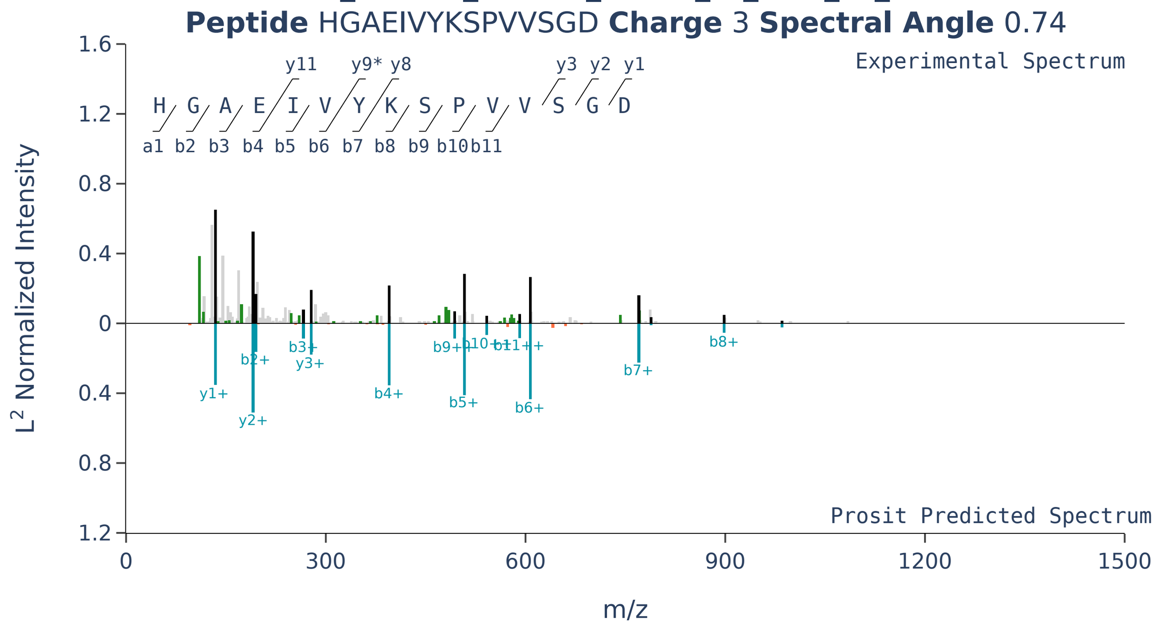


**Source** WSoh_221121_011221_HFGoe_L2_hTau_24h_R2 **Scan** 16295
**Peptide** KHQPGGGKVQIINKKLDLSNV **Charge** 5 **Spectral Angle** 0.9

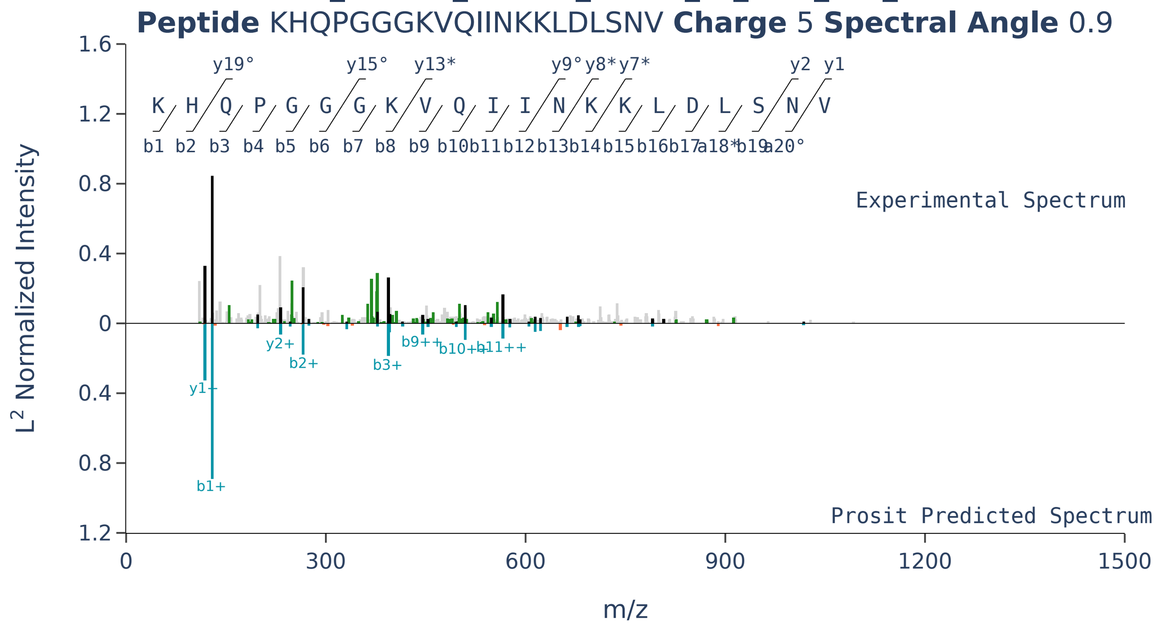


**Source** WSoh_221121_011221_HFGoe_L1_hTau_24h_R1 **Scan** 8211
**Peptide** NLTHVPGGGNKKI **Charge** 2 **Spectral Angle** 0.9

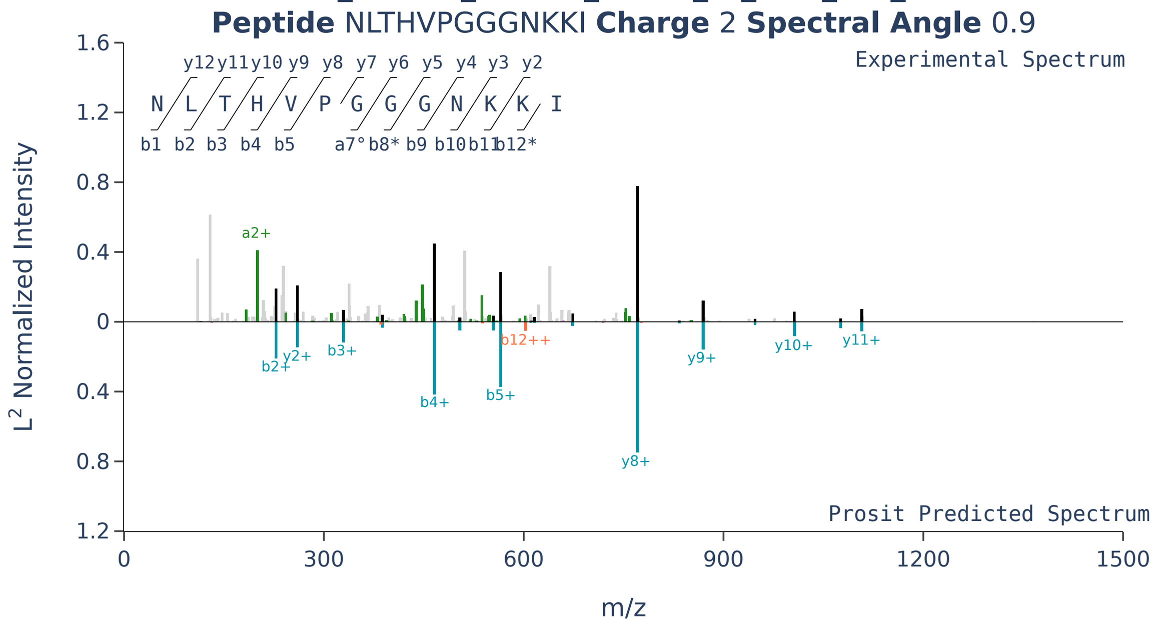


**Source** WSoh_221121_011221_HFGoe_L2_hTau_24h_R2 **Scan** 5903
**Peptide** KHQPGGGKVQIINKK **Charge** 4 **Spectral Angle** 0.89

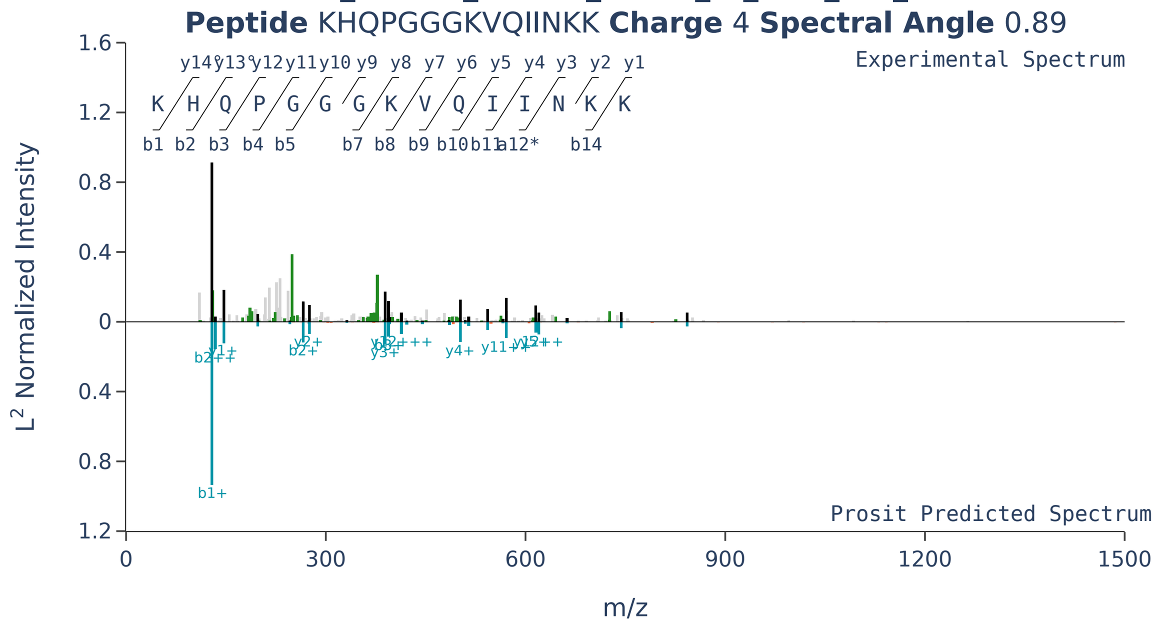


**Source** WSoh_221121_011221_HFGoe_L1_hTau_24h_R2 **Scan** 19305
**Peptide** KSPVVSGDTSPRHLSNVSSTGSIDMV **Charge** 3 **Spectral Angle** 0.86

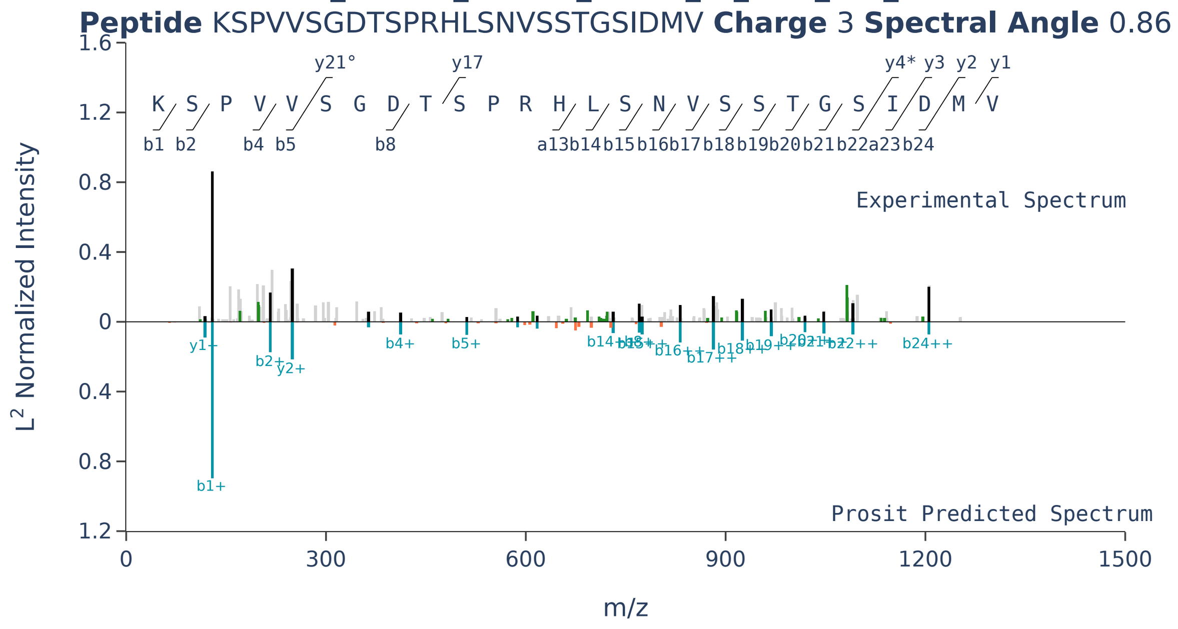


**Source** WSoh_221121_011221_HFGoe_L2_hTau_24h_R1 **Scan** 19307
**Peptide** EGDTDAGLKESPLQTPTE **Charge** 2 **Spectral Angle** 0.92

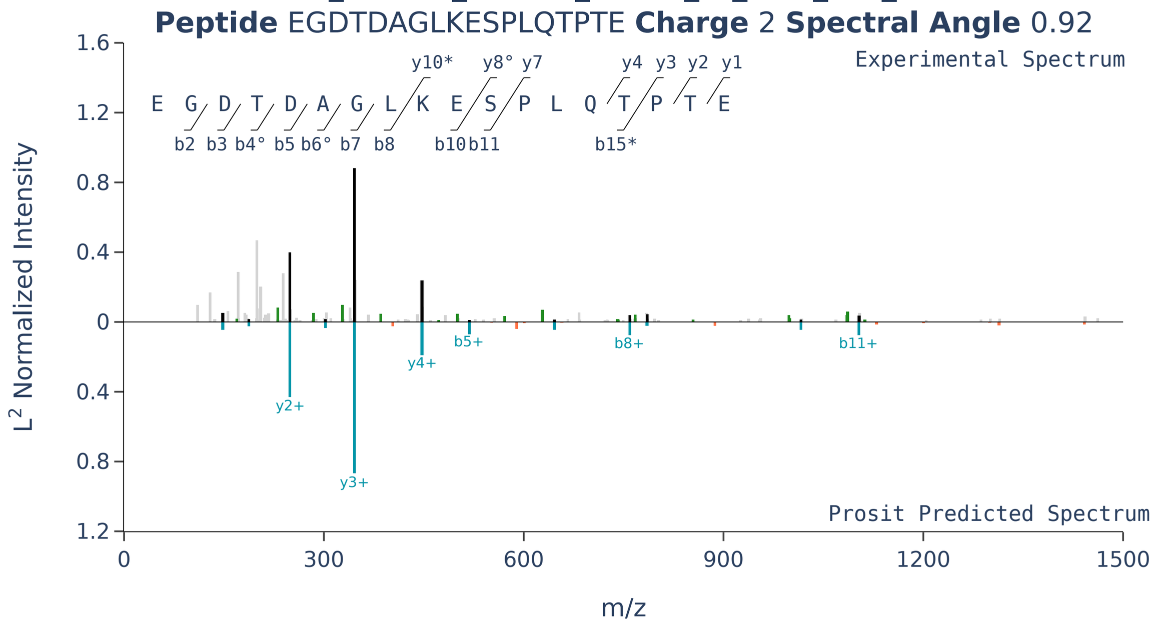


**Source** WSoh_221121_011221_HFGoe_L2_hTau_4h_R1 **Scan** 5601
**Peptide** RENAKAKTDHGAEI **Charge** 4 **Spectral Angle** 0.73

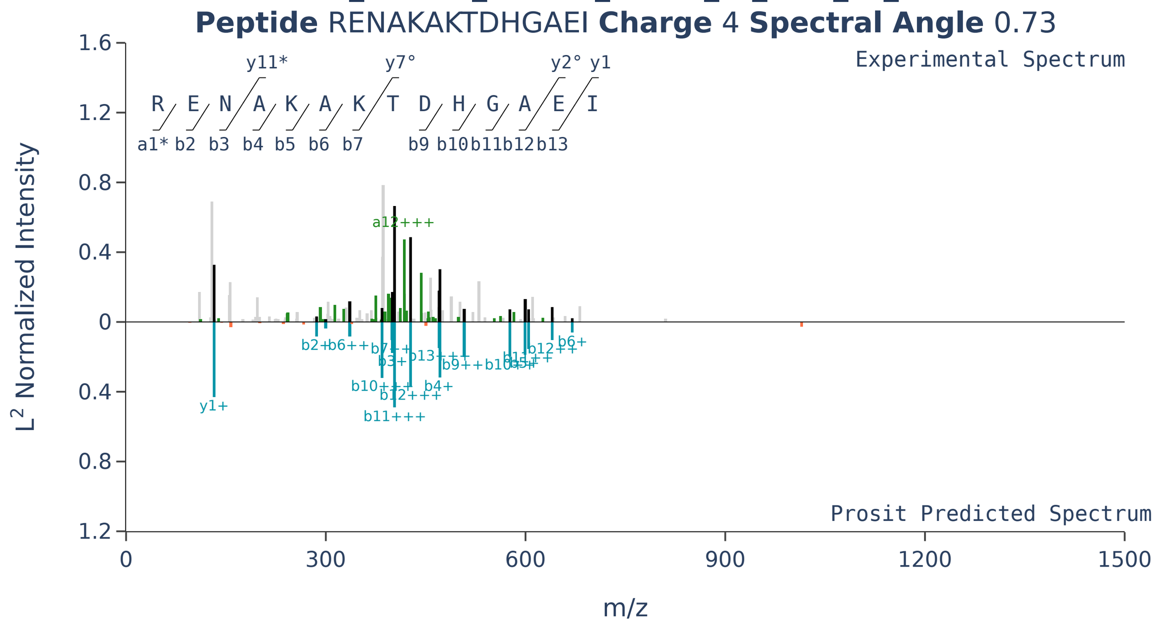


**Source** WSoh_221121_011221_HFGoe_L2_hTau_2h_R1 **Scan** 19832
**Peptide** SRLQTAPVPMPDLKNVKS **Charge** 3 **Spectral Angle** 0.9

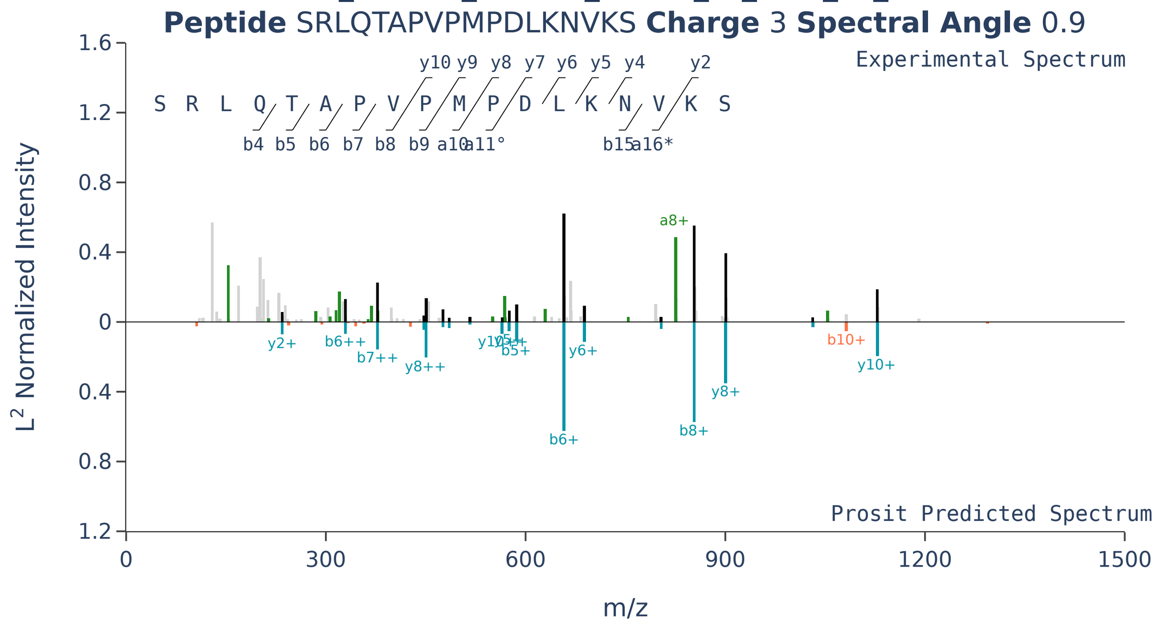

**Source** WSoh_221121_011221_HFGoe_L1_hTau_4h_R2 **Scan** 13871
**Peptide** HKLTFRENAKAKTDHGAEIVYKSPVV **Charge** 5 **Spectral Angle** 0.78

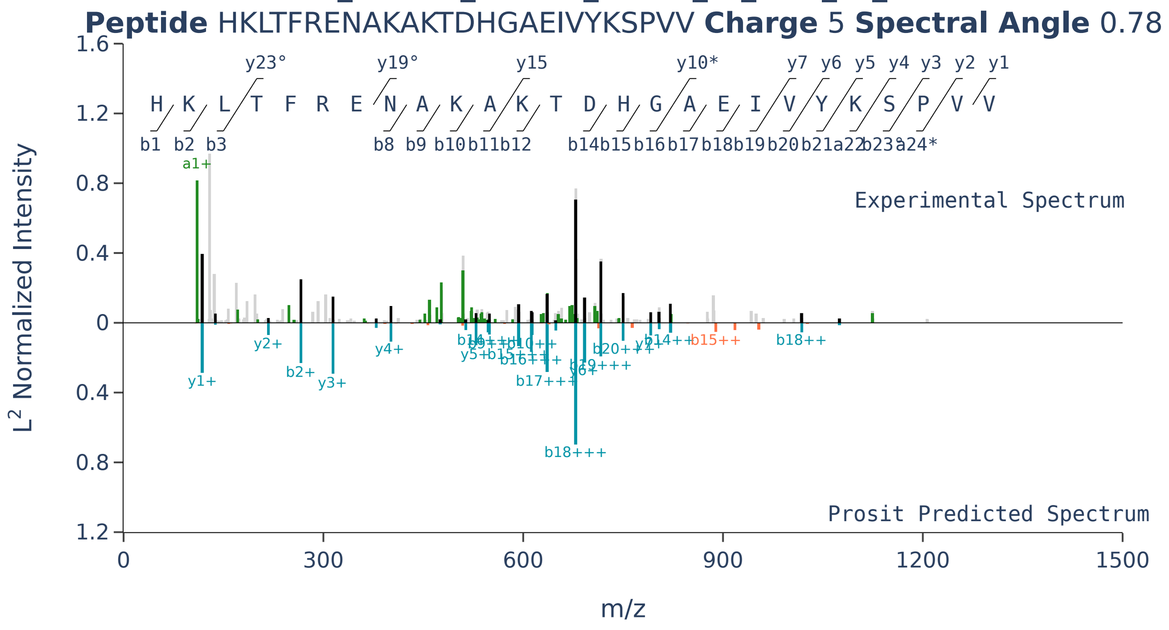


**Source** WSoh_221121_011221_HFGoe_L2_hTau_4h_R1 **Scan** 11530
**Peptide** KHVPGGGSVQIVYK **Charge** 2 **Spectral Angle** 0.68

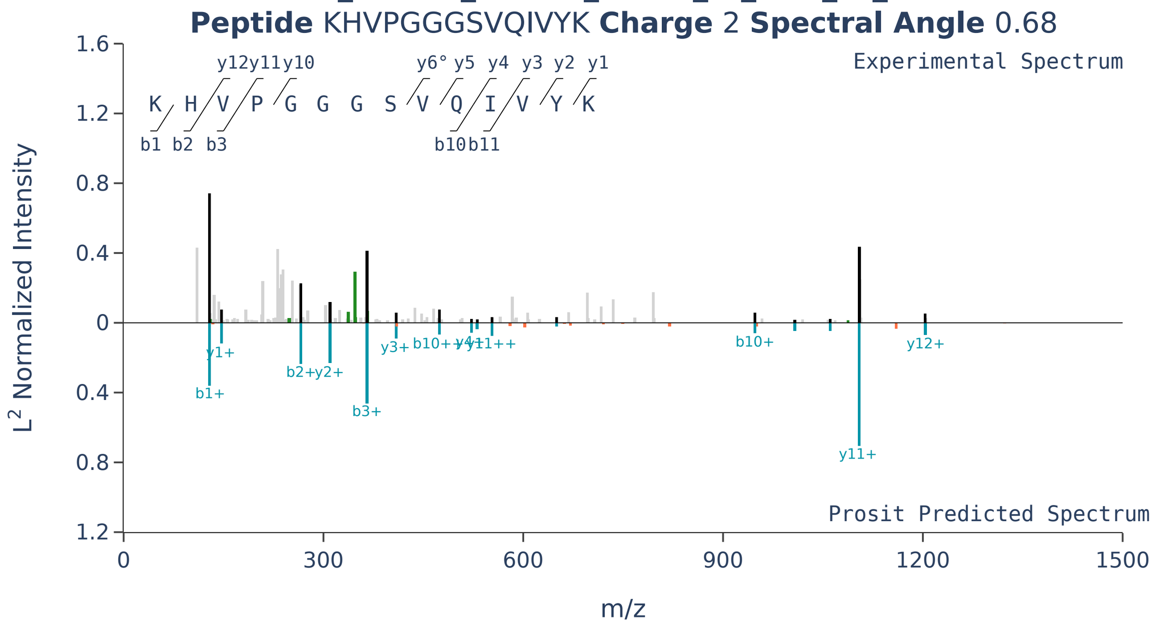


**Source** WSoh_221121_011221_HFGoe_L1_hTau_24h_R2 **Scan** 8283
**Peptide** LKHQPGGGKVQI **Charge** 2 **Spectral Angle** 0.85

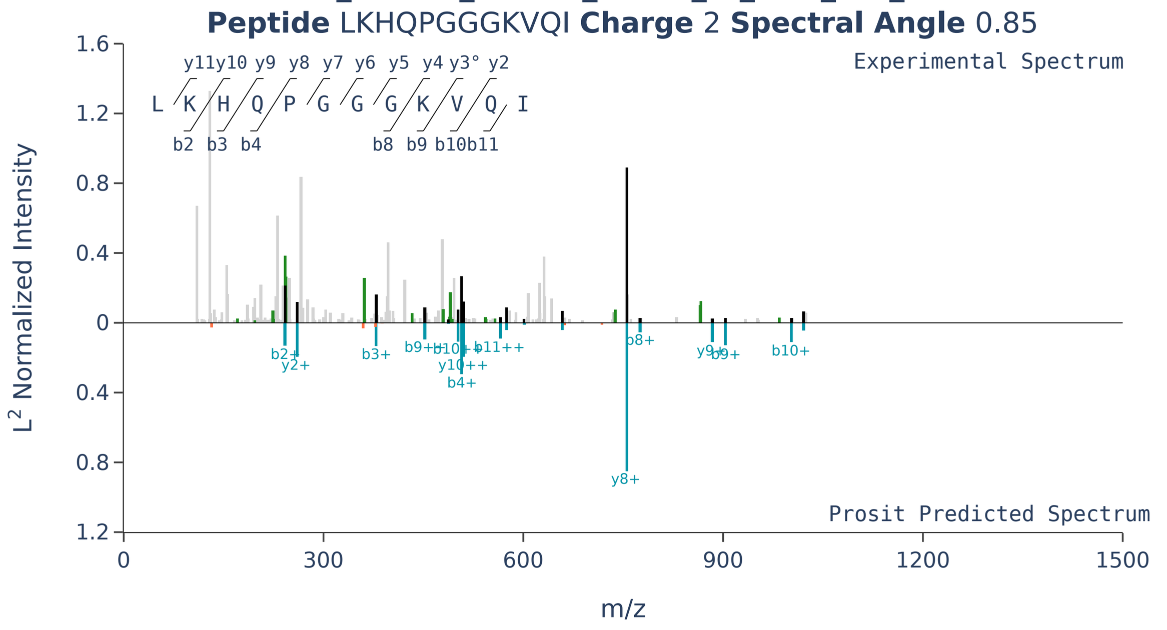


**Source** WSoh_221121_011221_HFGoe_L1_hTau_4h_R1 **Scan** 5650
**Peptide** NIHHKPGGGQVEVKSE **Charge** 2 **Spectral Angle** 0.82

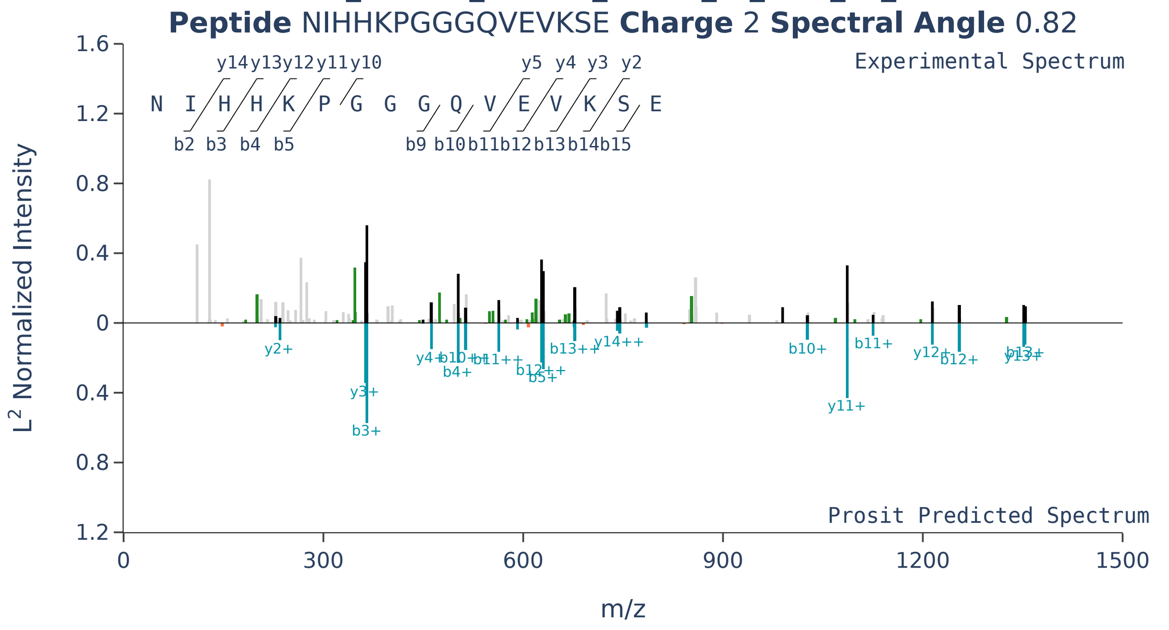


**Source** WSoh_221121_011221_HFGoe_L1_hTau_24h_R1 **Scan** 18330
**Peptide** GAEIVYKSPVVSGDTSPRHLSNVS **Charge** 3 **Spectral Angle** 0.87

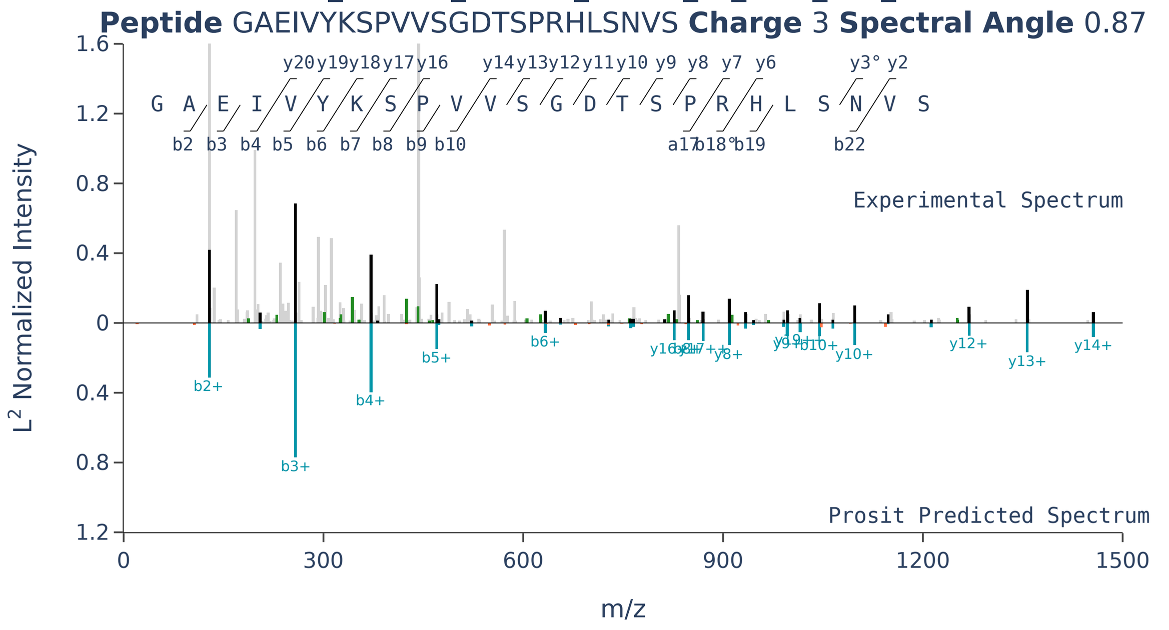


**Source** WSoh_221121_011221_HFGoe_L1_hTau_4h_R2 **Scan** 13711
**Peptide** DEGAPGKQAAAQPHTEIPEGTTAEE **Charge** 3 **Spectral Angle** 0.78

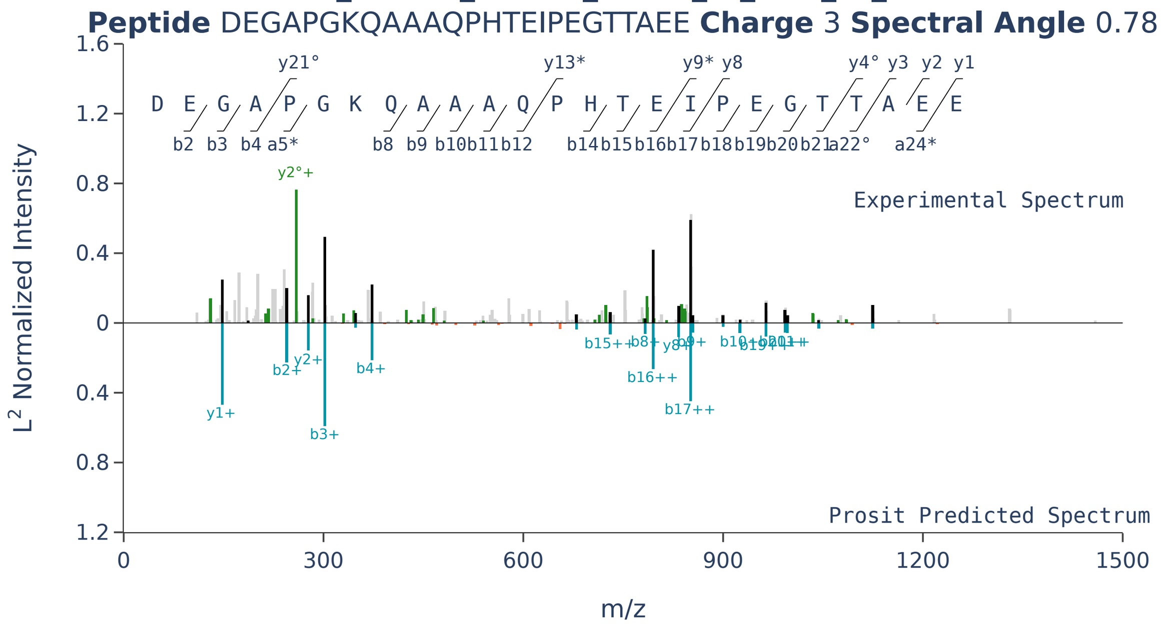


**Source** WSoh_221121_011221_HFGoe_L1_hTau_24h_R1 **Scan** 10276
**Peptide** ATRIPAKTPPAPKTPPSSGEPPKSGD **Charge** 4 **Spectral Angle** 0.87

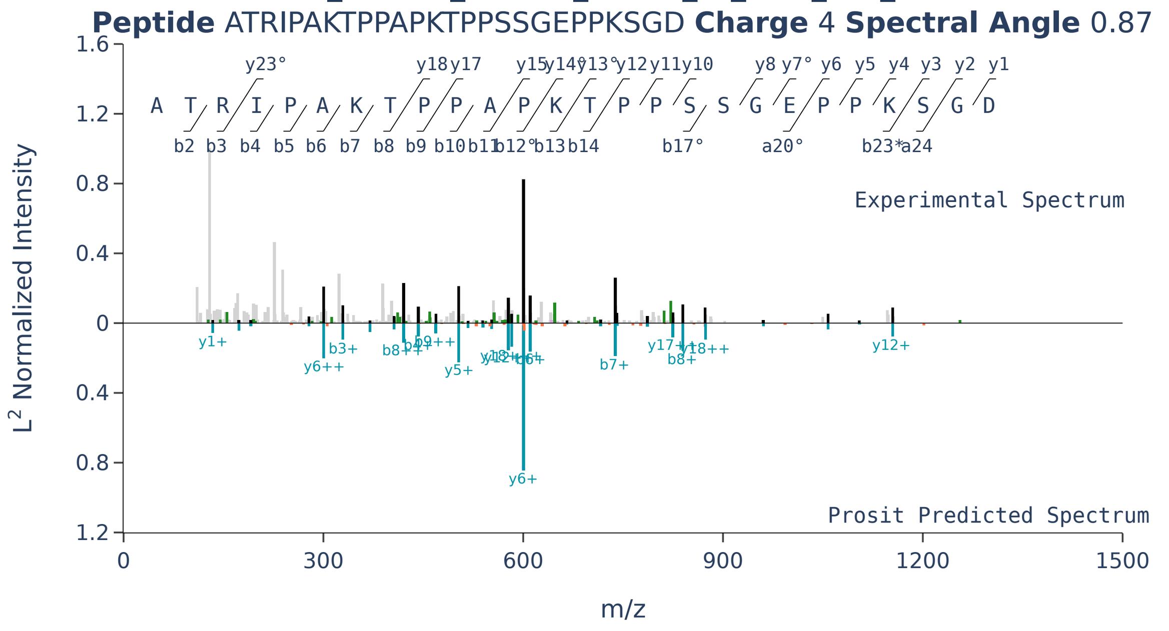


**Source** WSoh_221121_011221_HFGoe_L1_hTau_2h_R2 **Scan** 9642
**Peptide** QVEVKSEKL **Charge** 2 **Spectral Angle** 0.84

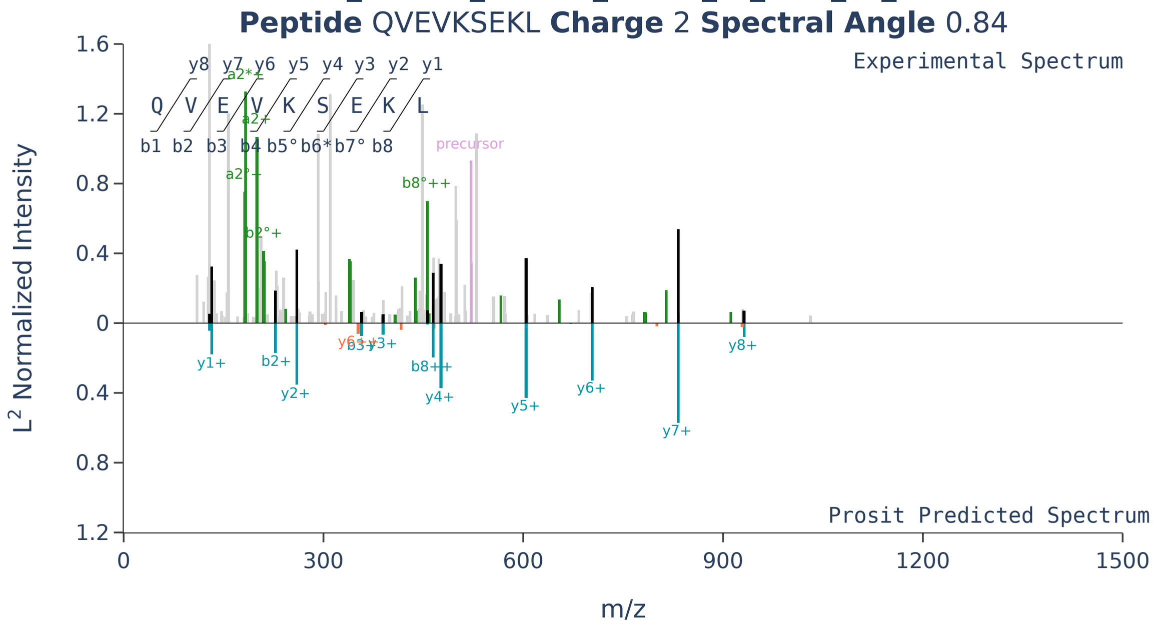


**Source** WSoh_221121_011221_HFGoe_L2_hTau_4h_R2 **Scan** 14868
**Peptide** KIGSLDNITHVPGGGNKKIETHKL **Charge** 4 **Spectral Angle** 0.89

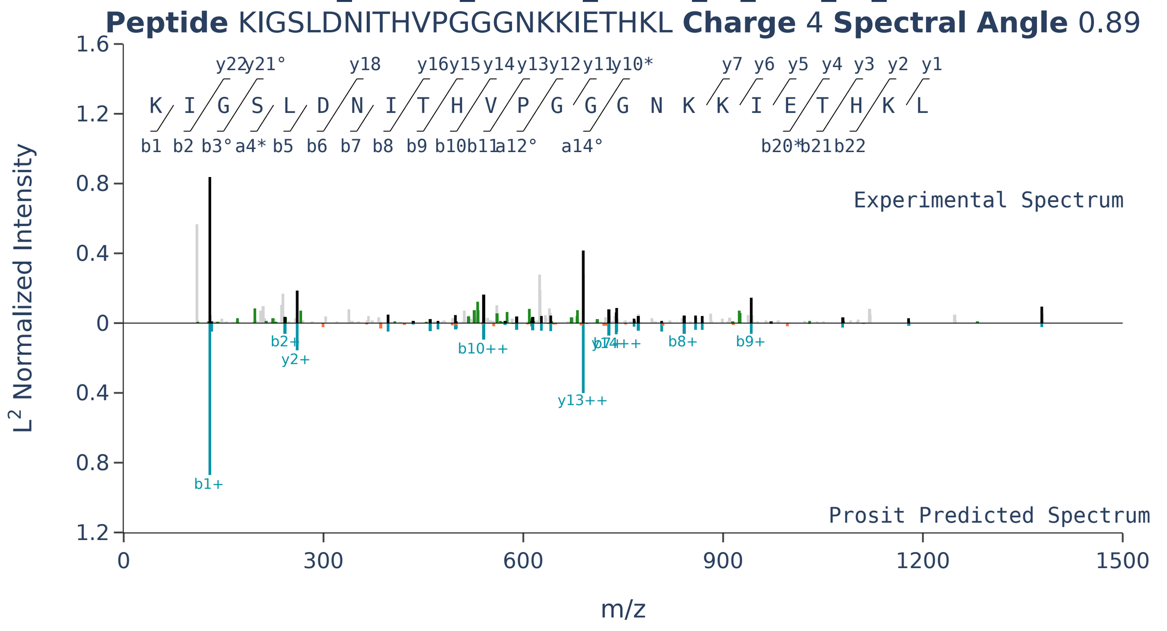


**Source** WSoh_221121_011221_HFGoe_L1_hTau_2h_R1 **Scan** 12765
**Peptide** VKSEKLDF **Charge** 2 **Spectral Angle** 0.85

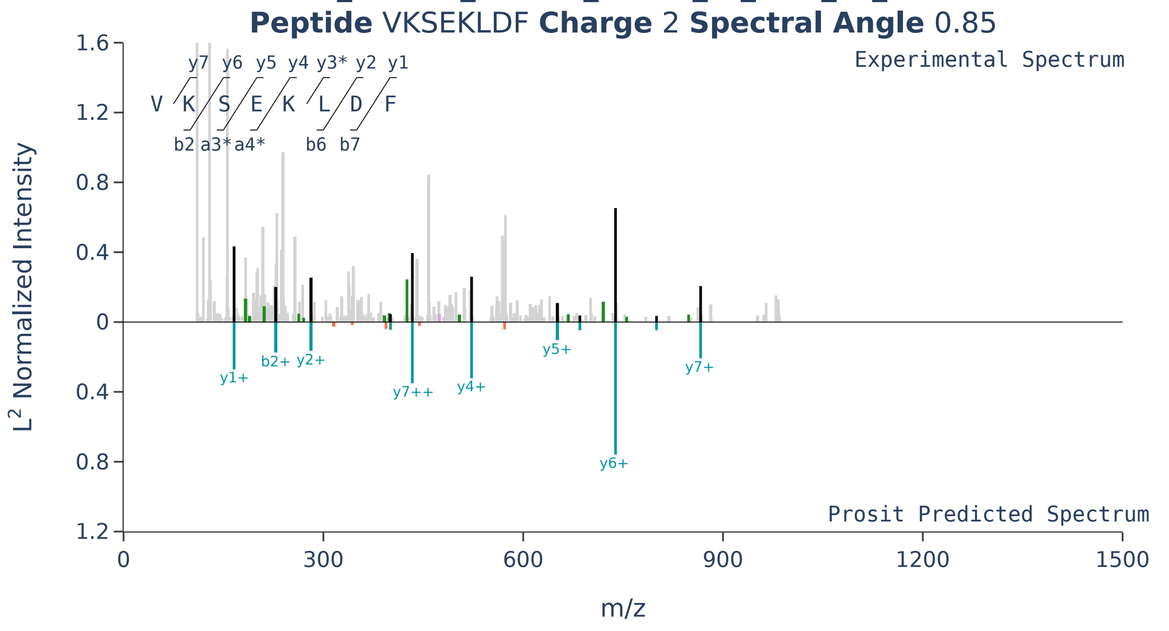


**Source** WSoh_221121_011221_HFGoe_L2_hTau_4h_R2 **Scan** 9750
**Peptide** SLGNIHHKPGGGQVEVKSEKL **Charge** 3 **Spectral Angle** 0.76

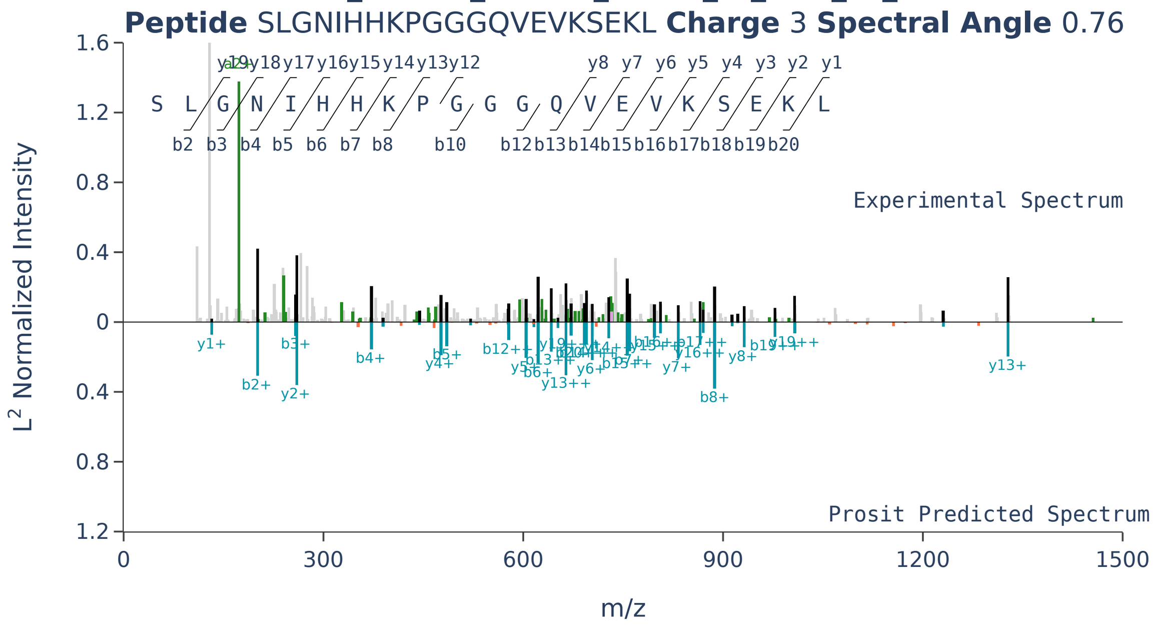


**Source** WSoh_221121_011221_HFGoe_L1_hTau_4h_R1 **Scan** 11124
**Peptide** NITHVPGGGNKKIETHKLTFREN **Charge** 3 **Spectral Angle** 0.84

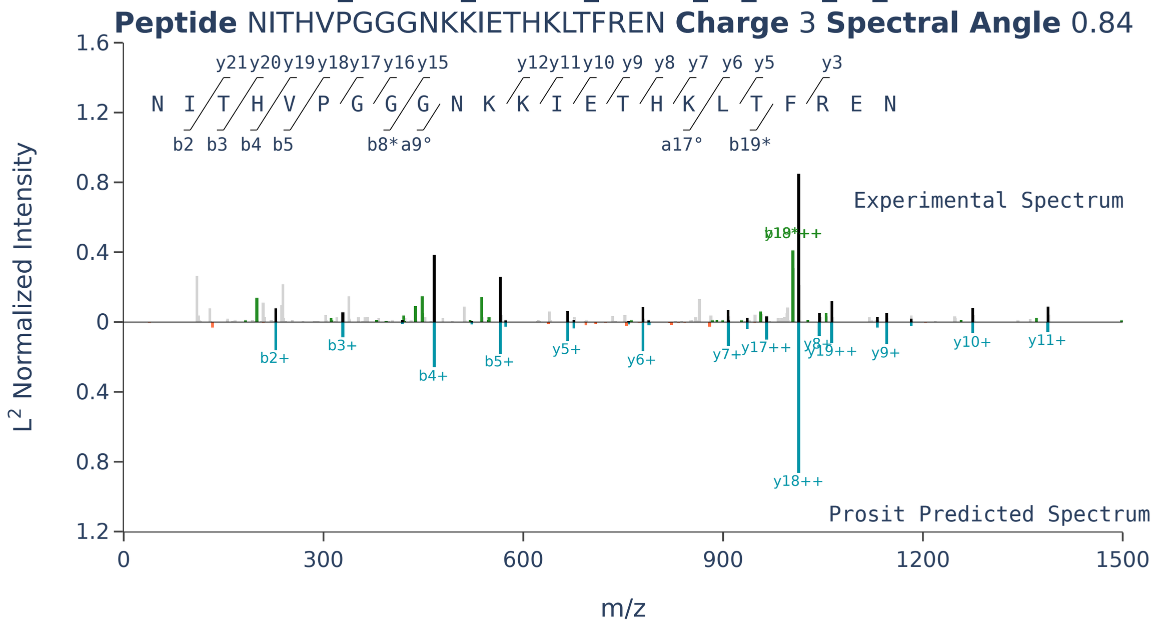


**Source** WSoh_221121_011221_HFGoe_L1_hTau_24h_R2 **Scan** 11886
**Peptide** GDTSPRHLSNVSSTGSLD **Charge** 2 **Spectral Angle** 0.85

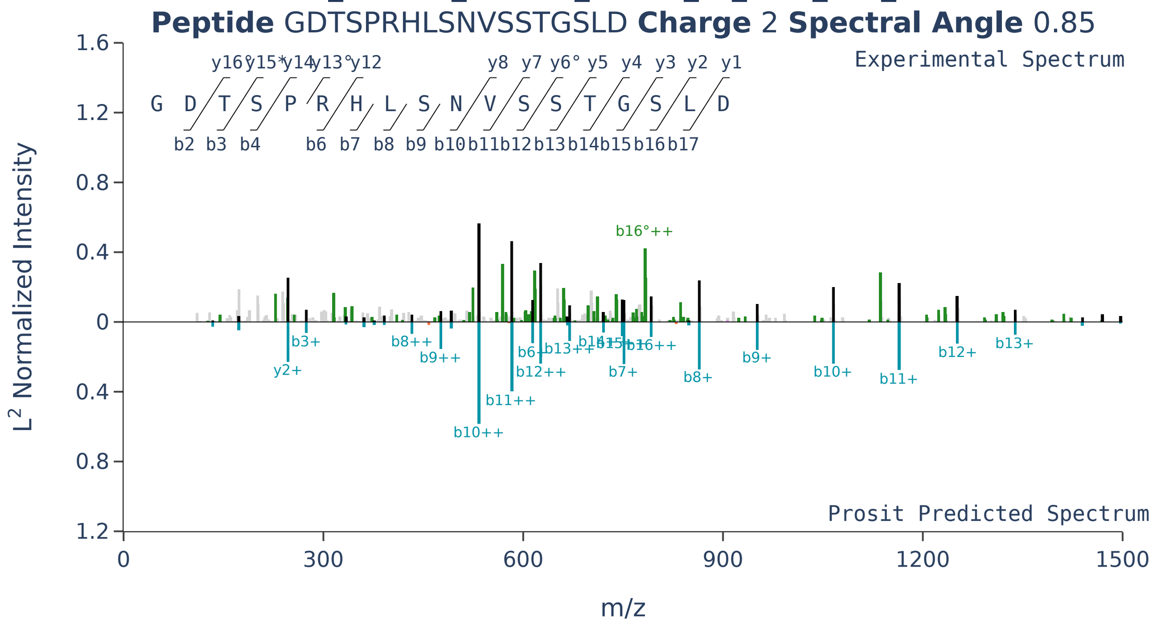


**Source** WSoh_221121_011221_HFGoe_L2_hTau_24h_R2 **Scan** 22184
**Peptide** AGLGDTPSL **Charge** 1 **Spectral Angle** 0.87

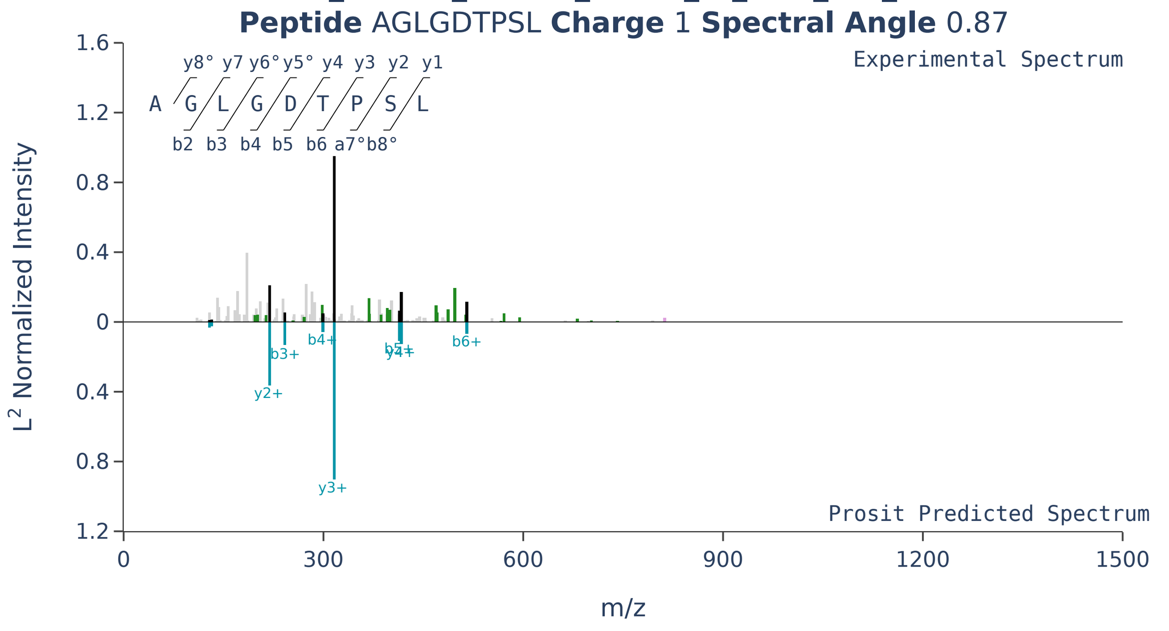


**Source** WSoh_221121_011221_HFGoe_L2_hTau_4h_R2 **Scan** 12706
**Peptide** IGSTENLKHQPGGGKVQIINKKL **Charge** 3 **Spectral Angle** 0.8

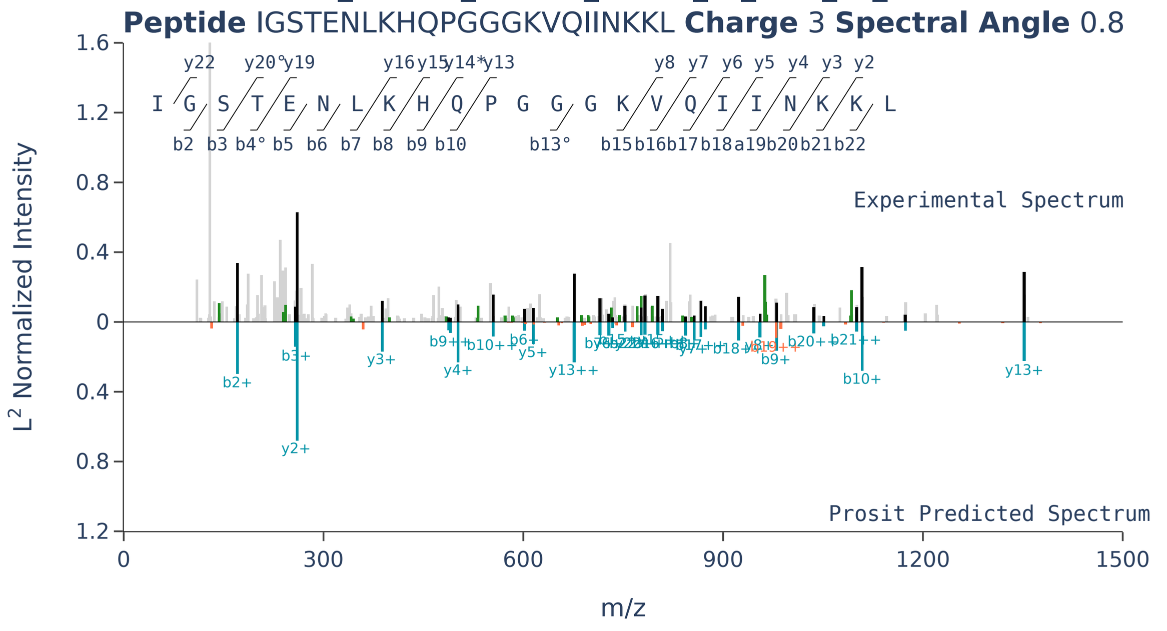

**Source** WSoh_221121_011221_HFGoe_L2_hTau_4h_R2 **Scan** 14688
**Peptide** KPVDLSKVTSKCGSLG **Charge** 3 **Spectral Angle** 0.79

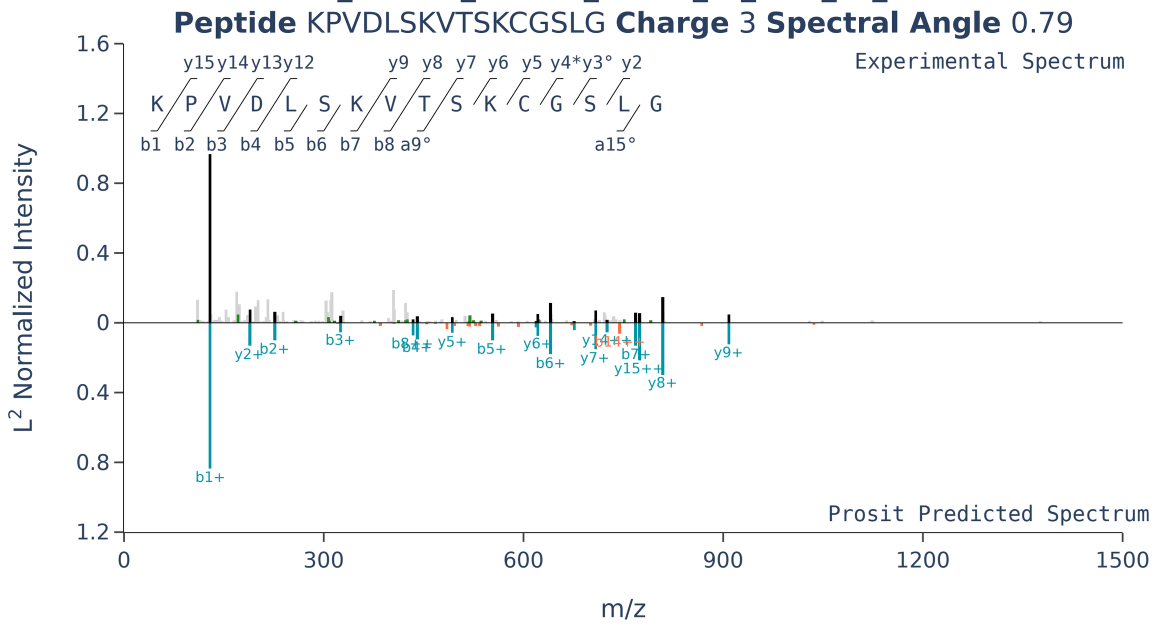


**Source** WSoh_221121_011221_HFGoe_L2_hTau_24h_R1 **Scan** 9121
**Peptide** ATRIPAKTPPAPKT **Charge** 3 **Spectral Angle** 0.74

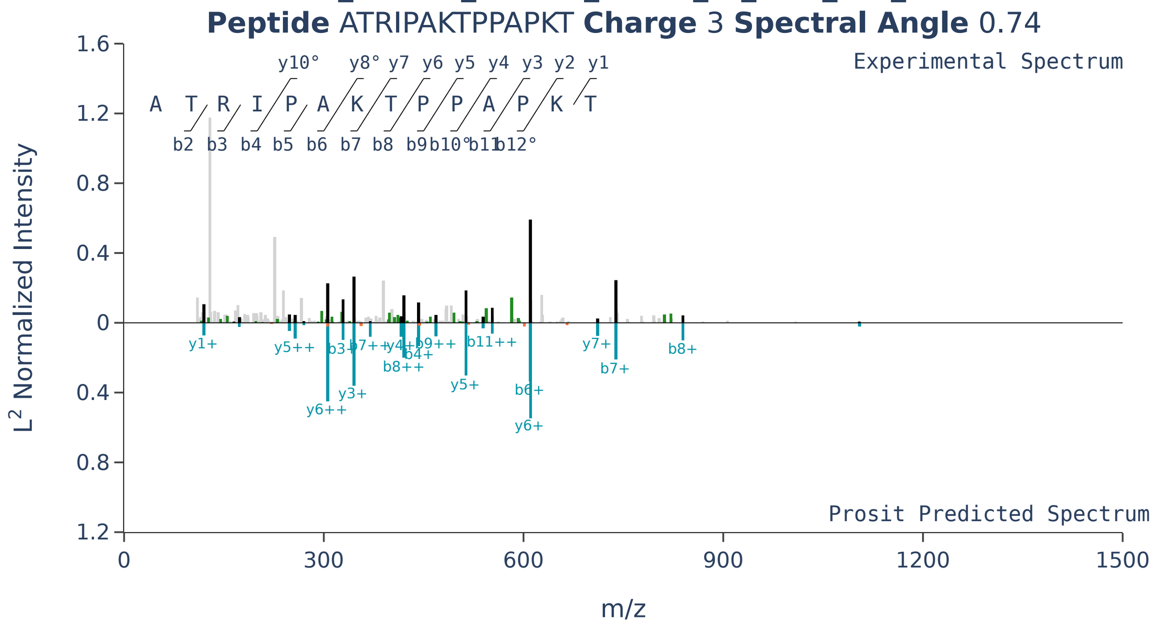


**Source** WSoh_221121_011221_HFGoe_L1_hTau_2h_R2 **Scan** 12481
**Peptide** KAKTDHGAEIVYKSPVVS **Charge** 3 **Spectral Angle** 0.85

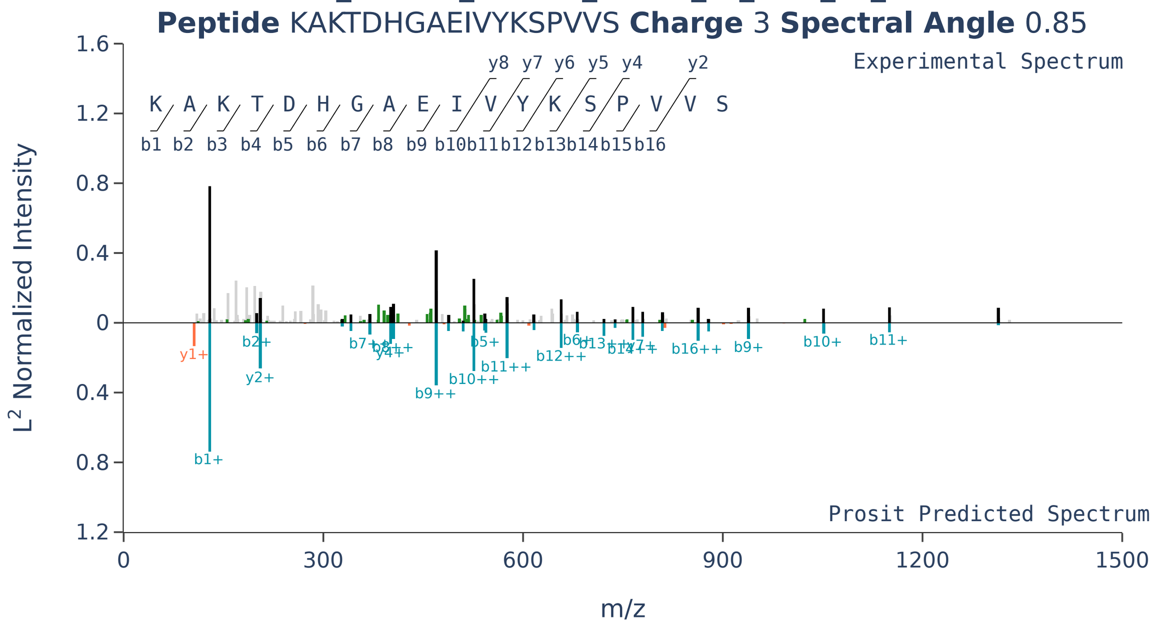


**Source** WSoh_221121_011221_HFGoe_L1_hTau_4h_R2 **Scan** 13247
**Peptide** KHQPGGGKVQIINKKLDLSNVQSKC **Charge** 5 **Spectral Angle** 0.89

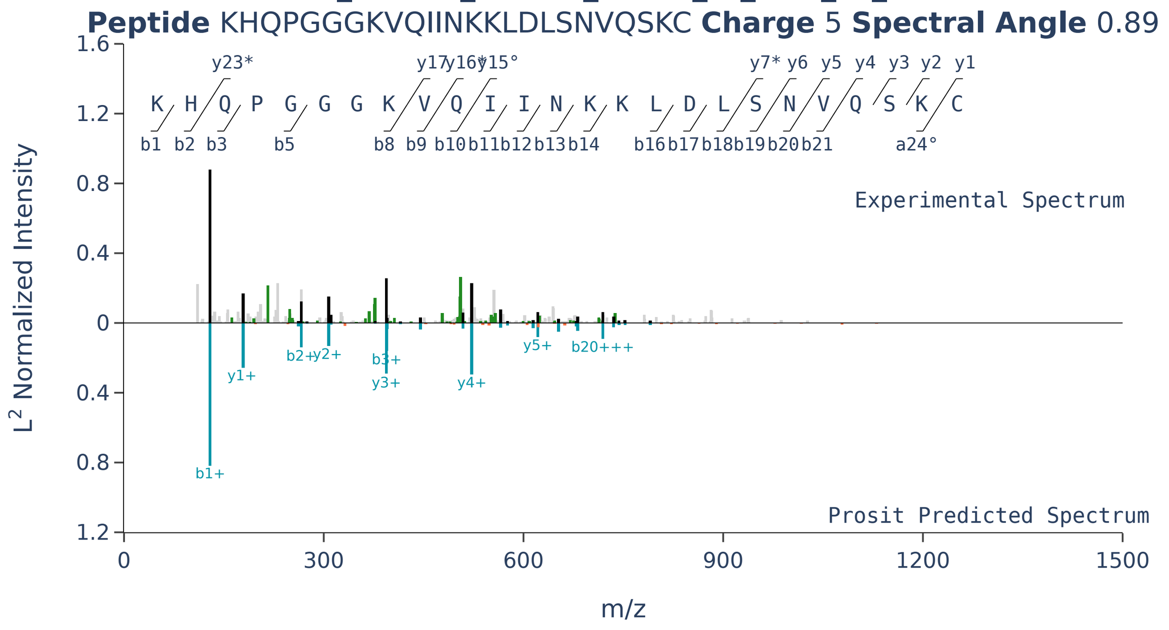


**Source** WSoh_221121_011221_HFGoe_L1_hTau_4h_R1 **Scan** 14787
**Peptide** HGAEIVYKSPVVSGDTSPRHLS **Charge** 3 **Spectral Angle** 0.79

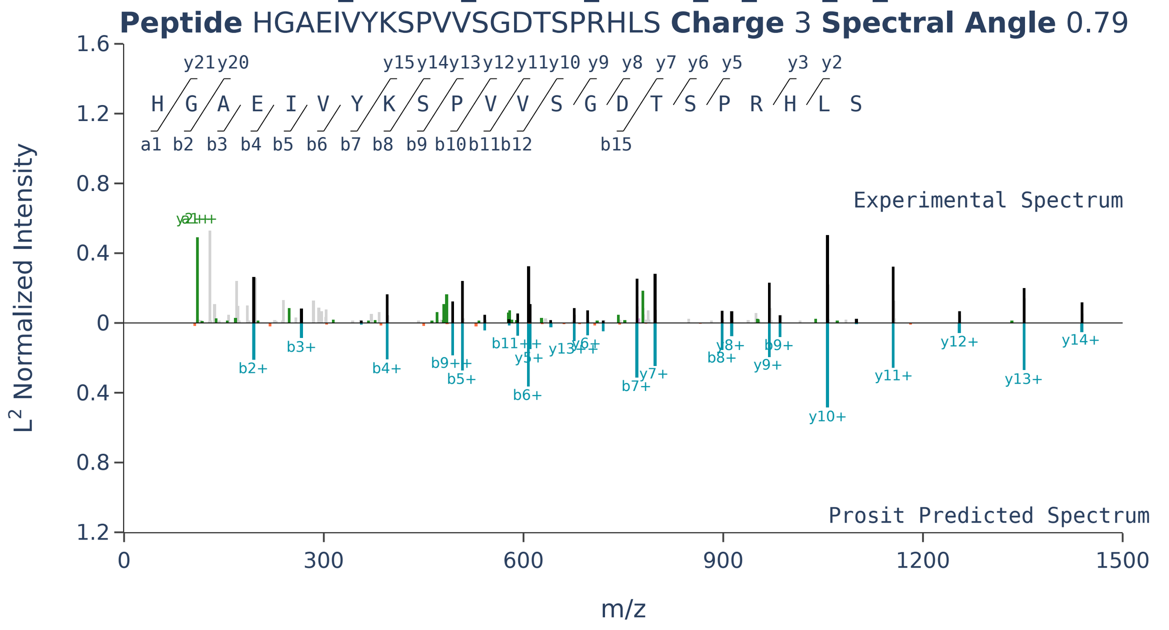


**Source** WSoh_221121_011221_HFGoe_L1_hTau_24h_R2 **Scan** 5947
**Peptide** TPRGAAPPGQKGQANAT **Charge** 2 **Spectral Angle** 0.85

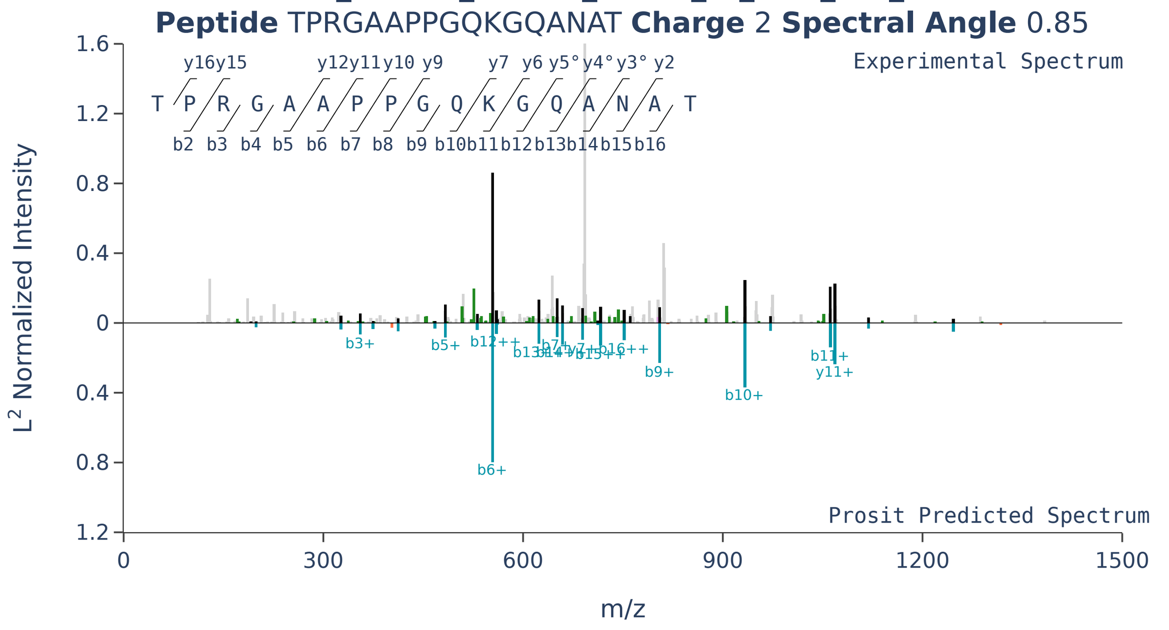


**Source** WSoh_221121_011221_HFGoe_L2_hTau_4h_R1 **Scan** 10026
**Peptide** KLDLSNVQSKCGSKD **Charge** 3 **Spectral Angle** 0.87

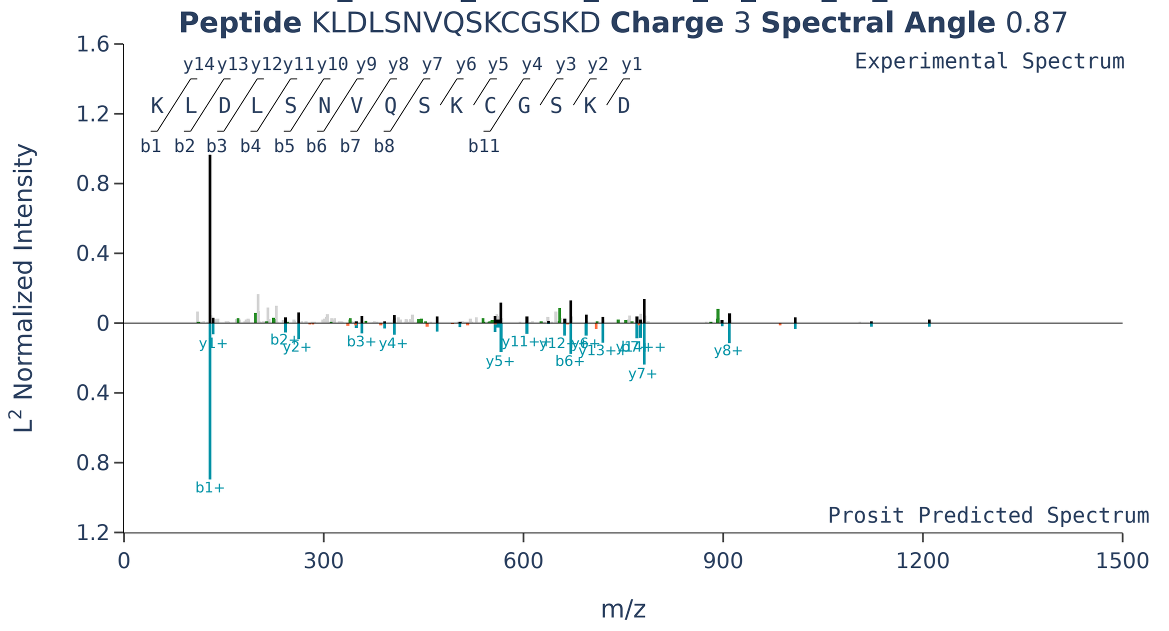


**Source** WSoh_221121_011221_HFGoe_L2_hTau_24h_R1 **Scan** 20602
**Peptide** APVPMPDLKNV **Charge** 2 **Spectral Angle** 0.85

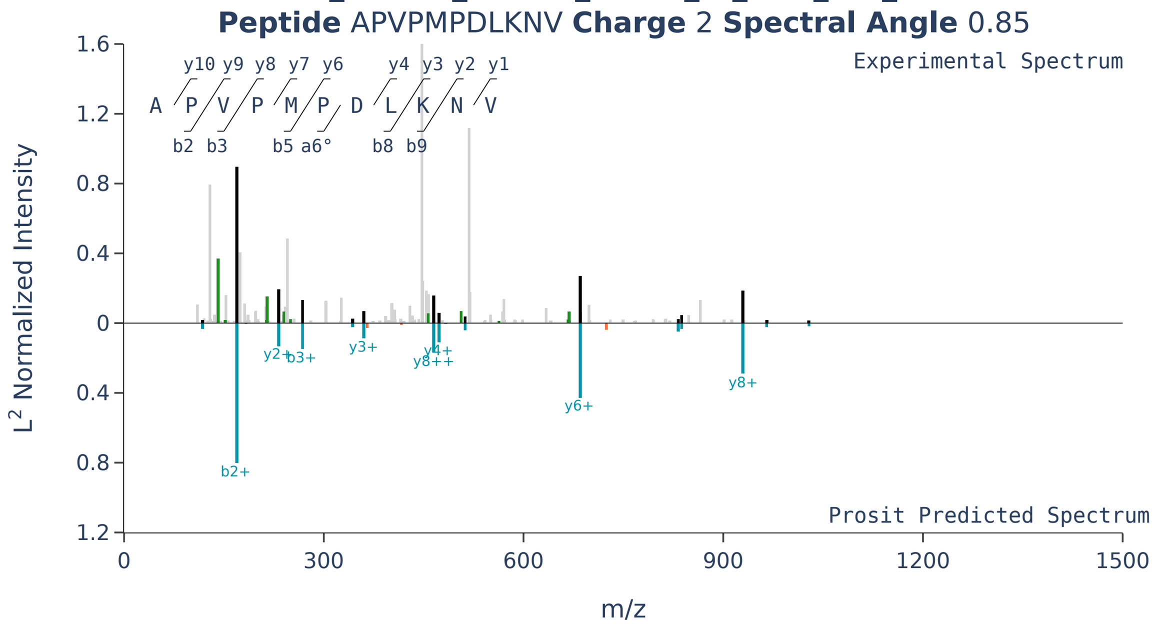


**Source** WSoh_221121_011221_HFGoe_L2_hTau_24h_R1 **Scan** 9007
**Peptide** KHQPGGGKVQIINKKL **Charge** 4 **Spectral Angle** 0.9

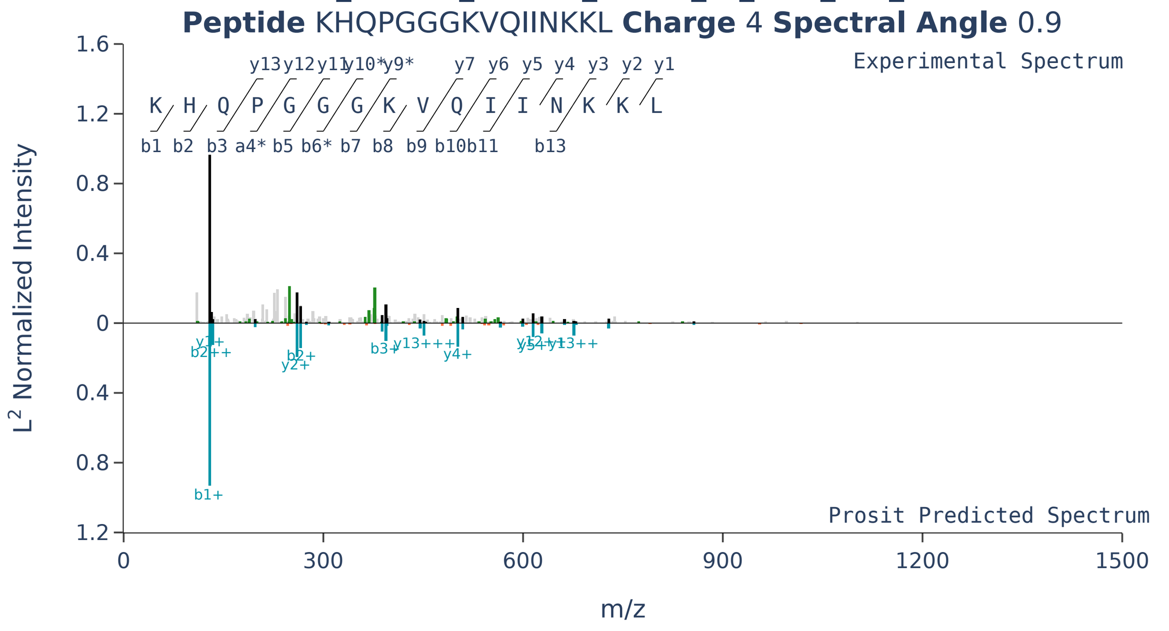


**Source** WSoh_221121_011221_HFGoe_L1_hTau_24h_R2 **Scan** 16496
**Peptide** TDAGLKESPLQT **Charge** 2 **Spectral Angle** 0.84

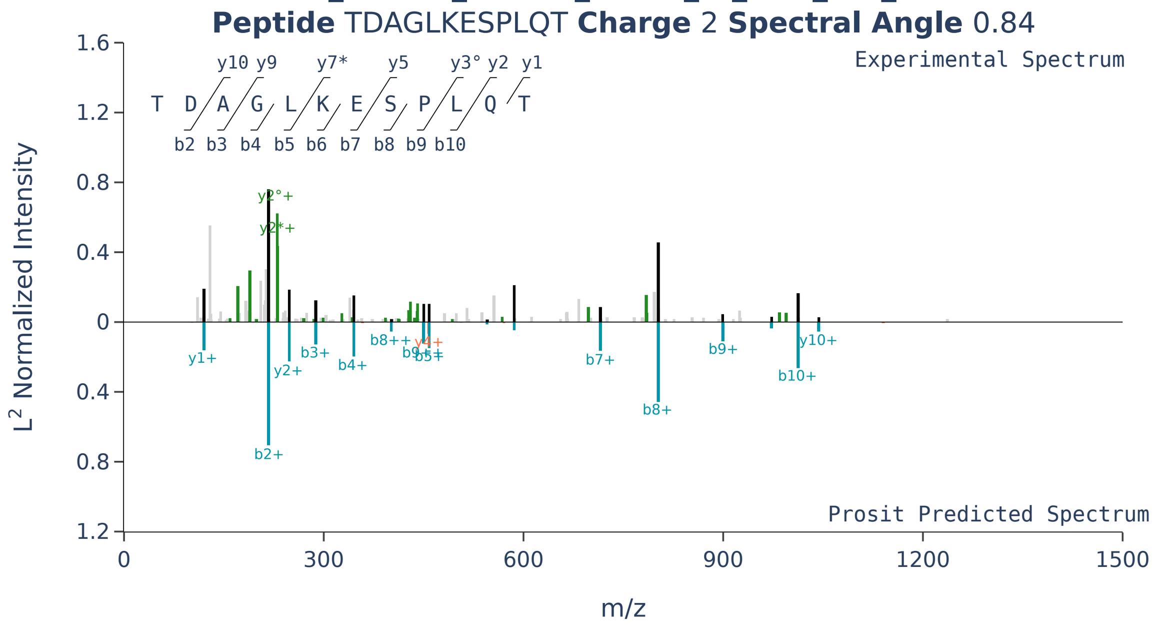


**Source** WSoh_221121_011221_HFGoe_L1_hTau_4h_R2 **Scan** 13186
**Peptide** GSLDNITHVPGGGNKKIETH **Charge** 3 **Spectral Angle** 0.84

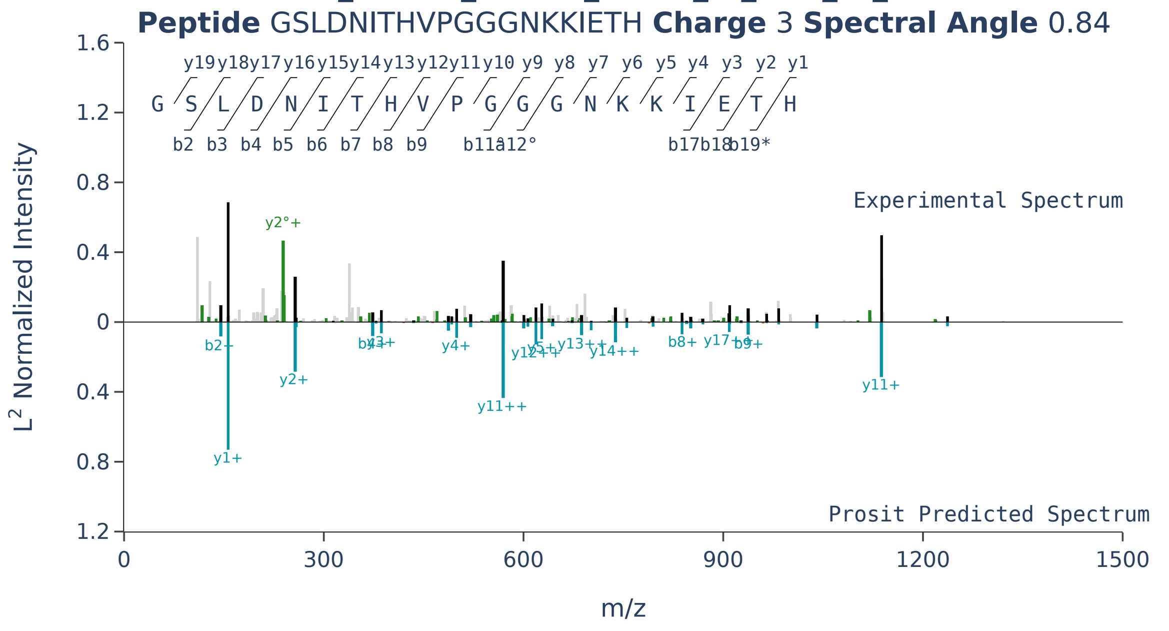


**Source** WSoh_221121_011221_HFGoe_L2_hTau_24h_R2 **Scan** 13284
**Peptide** KKLDLSNV **Charge** 2 **Spectral Angle** 0.77

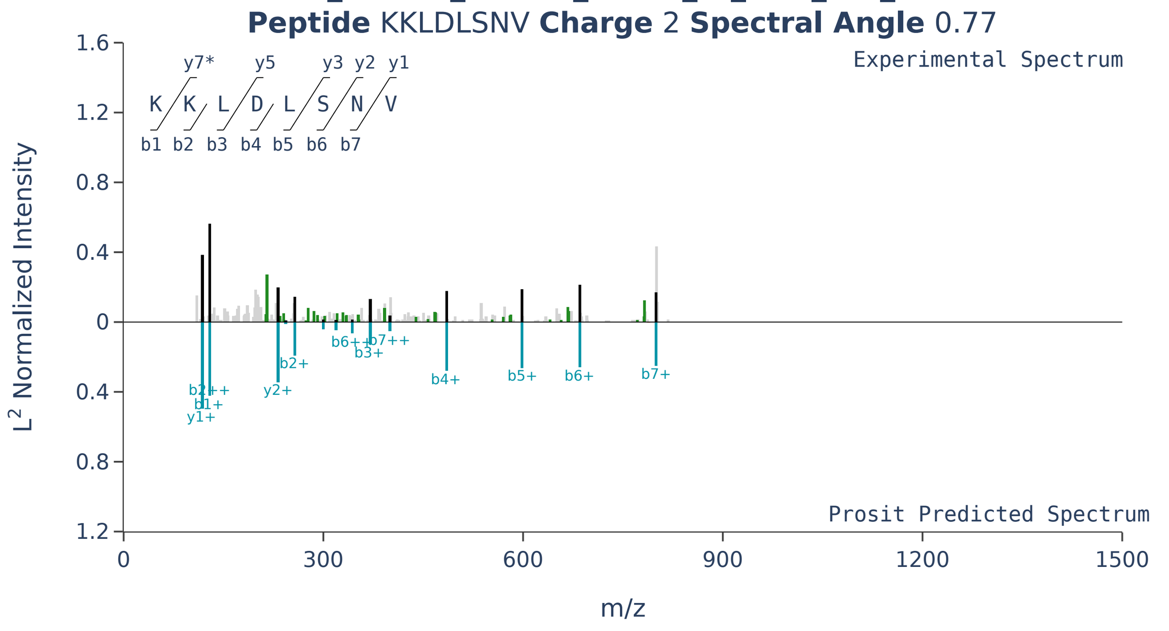


**Source** WSoh_221121_011221_HFGoe_L1_hTau_24h_R2 **Scan** 11118
**Peptide** HGAEIVYKSP **Charge** 3 **Spectral Angle** 0.87

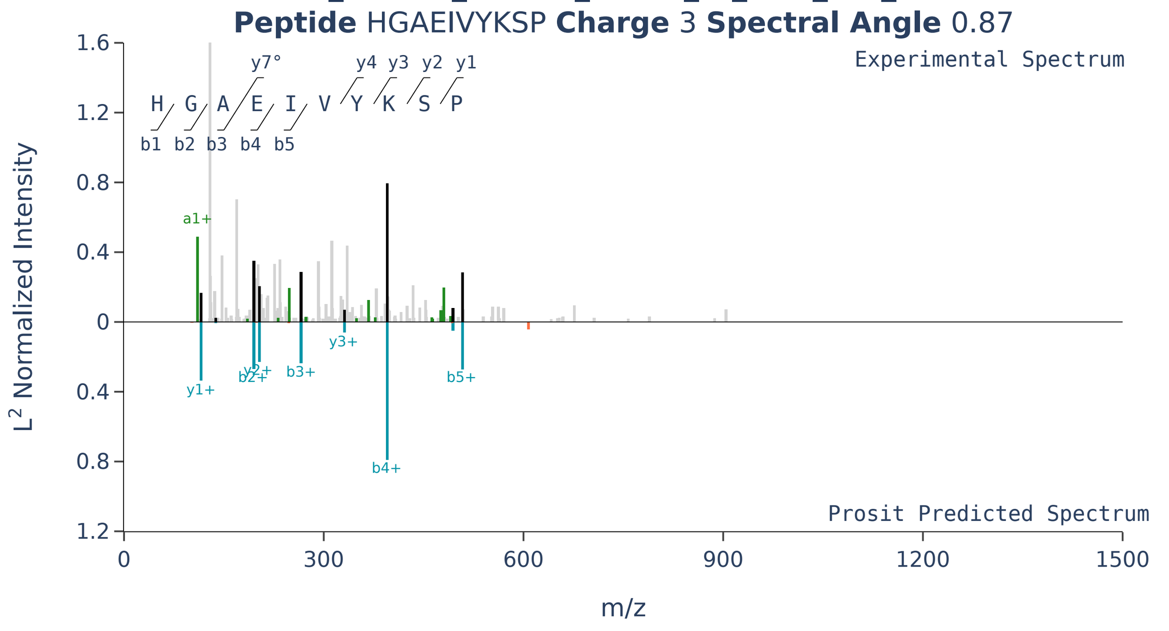


**Source** WSoh_221121_011221_HFGoe_L1_hTau_4h_R2 **Scan** 14567
**Peptide** LKHQPGGGKVQIINKKLDL **Charge** 3 **Spectral Angle** 0.81

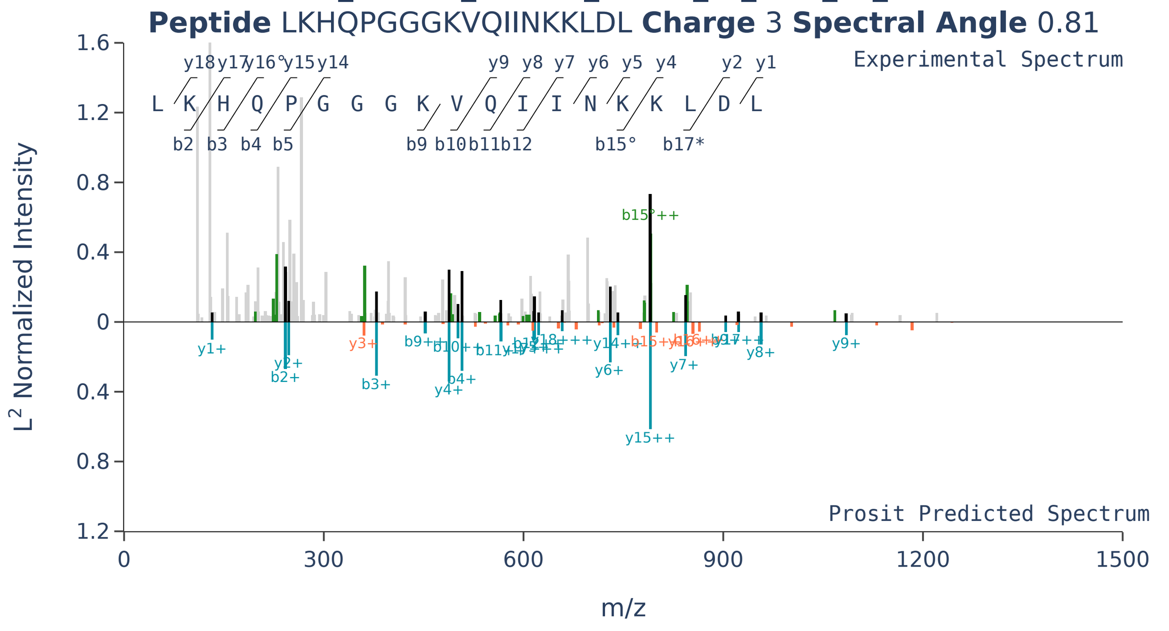


**Source** WSoh_221121_011221_HFGoe_L2_hTau_24h_R1 **Scan** 8408
**Peptide** CGSLGNIHHKPGGGQVEVKSE **Charge** 4 **Spectral Angle** 0.84

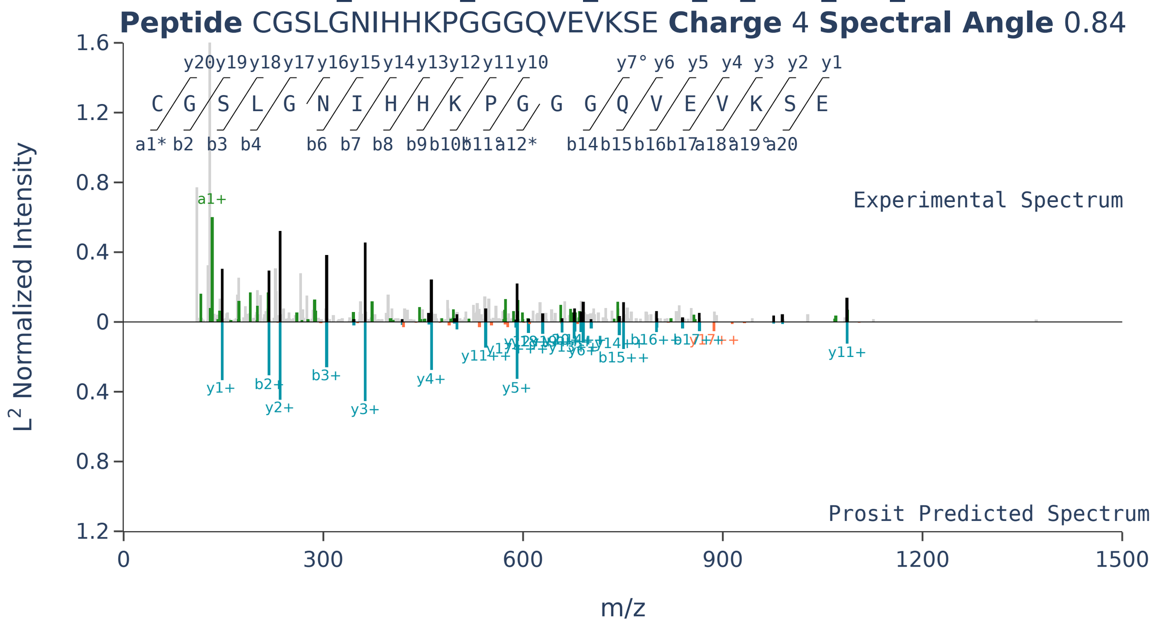

**Source** WSoh_221121_011221_HFGoe_L1_hTau_4h_R2 **Scan** 8141
**Peptide** CGSLGNIHHKPGGGQVEVKS **Charge** 2 **Spectral Angle** 0.76

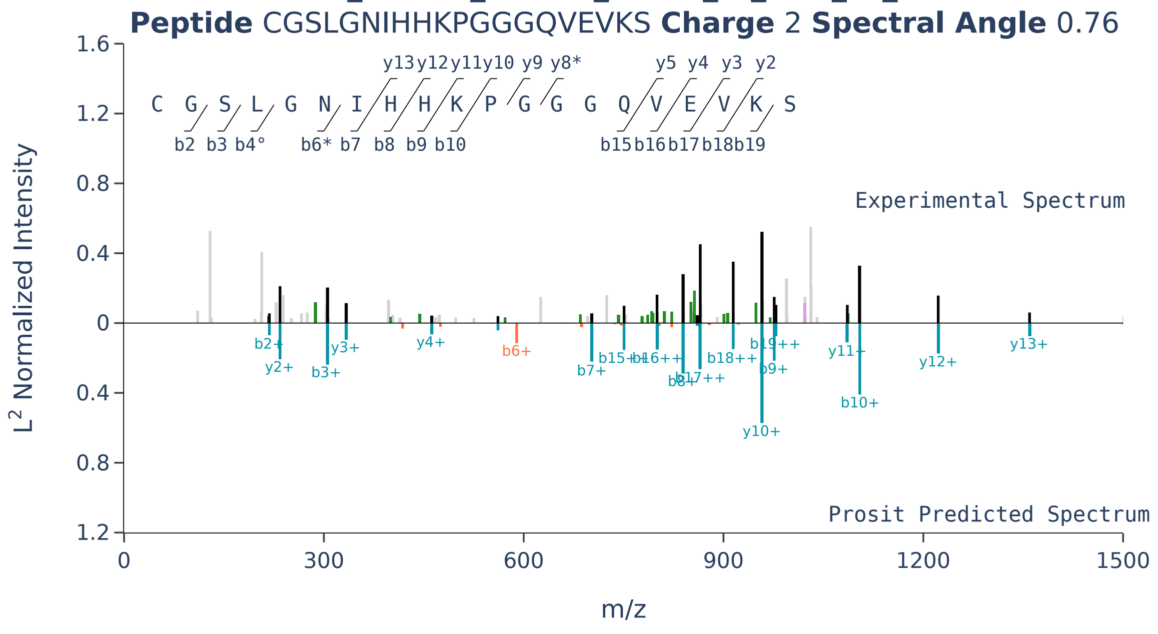


**Source** WSoh_221121_011221_HFGoe_L2_hTau_24h_R2 **Scan** 11315
**Peptide** SLPTPPTREPKKVA **Charge** 3 **Spectral Angle** 0.77

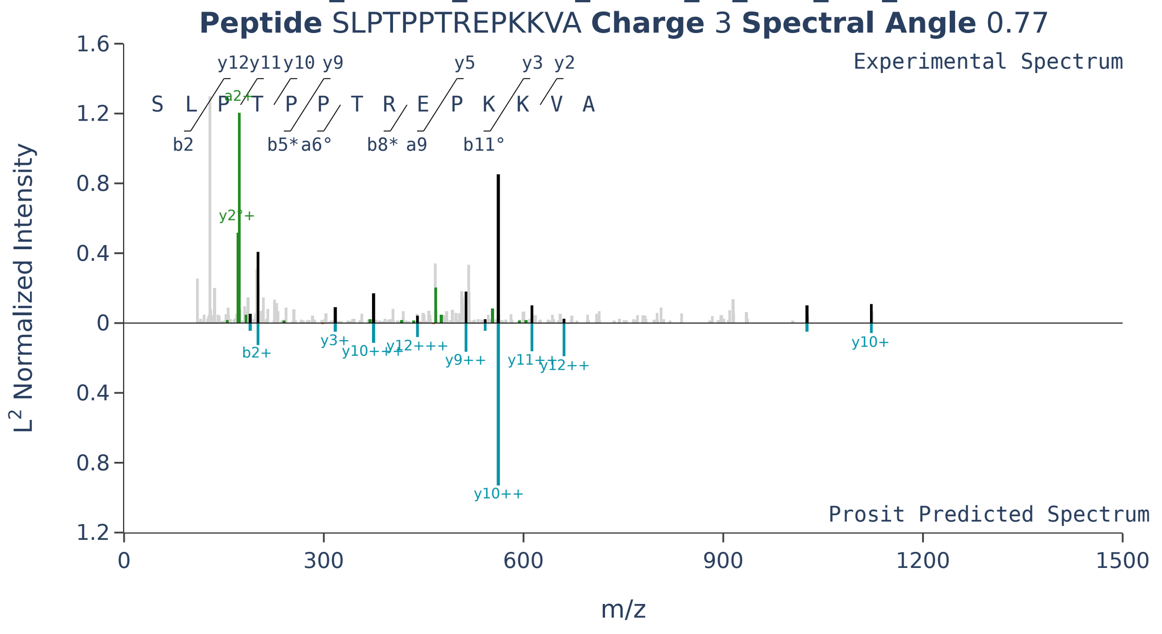


**Source** WSoh_221121_011221_HFGoe_L2_hTau_1h_R1 **Scan** 7508
**Peptide** DGKTKIATPRGAAPPGQKGQANA **Charge** 4 **Spectral Angle** 0.9

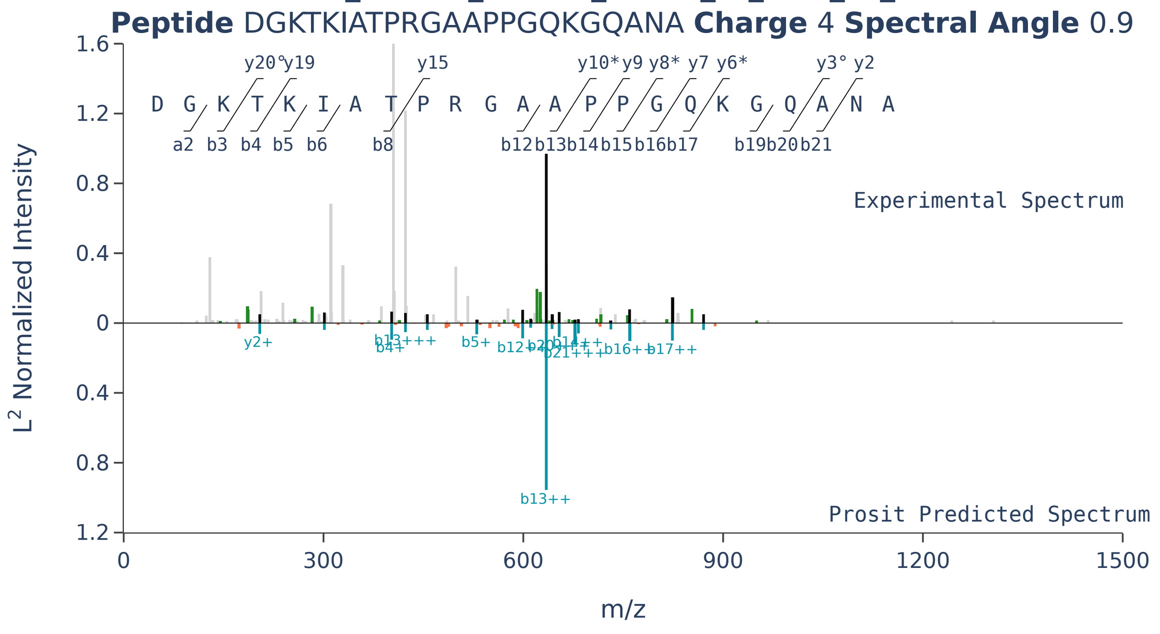


**Source** WSoh_221121_011221_HFGoe_L2_hTau_4h_R1 **Scan** 8553
**Peptide** AQPHTEL **Charge** 1 **Spectral Angle** 0.85

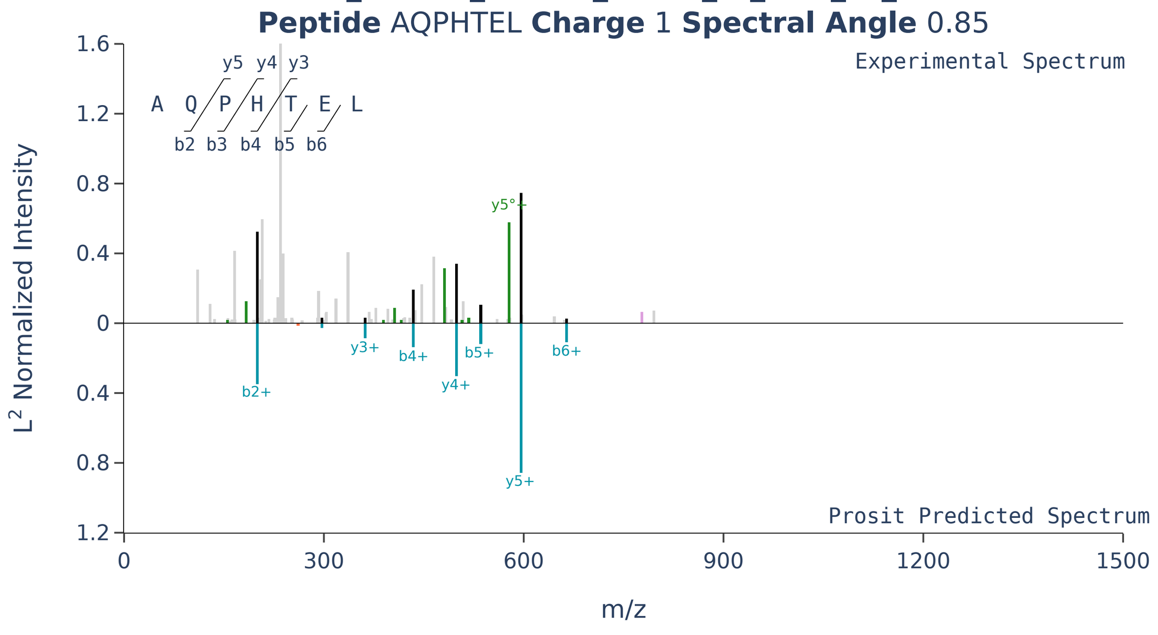


**Source** WSoh_221121_011221_HFGoe_L2_hTau_24h_R2 **Scan** 18483
**Peptide** AAQPHTEIPEGTTAEEAGIGD **Charge** 2 **Spectral Angle** 0.9

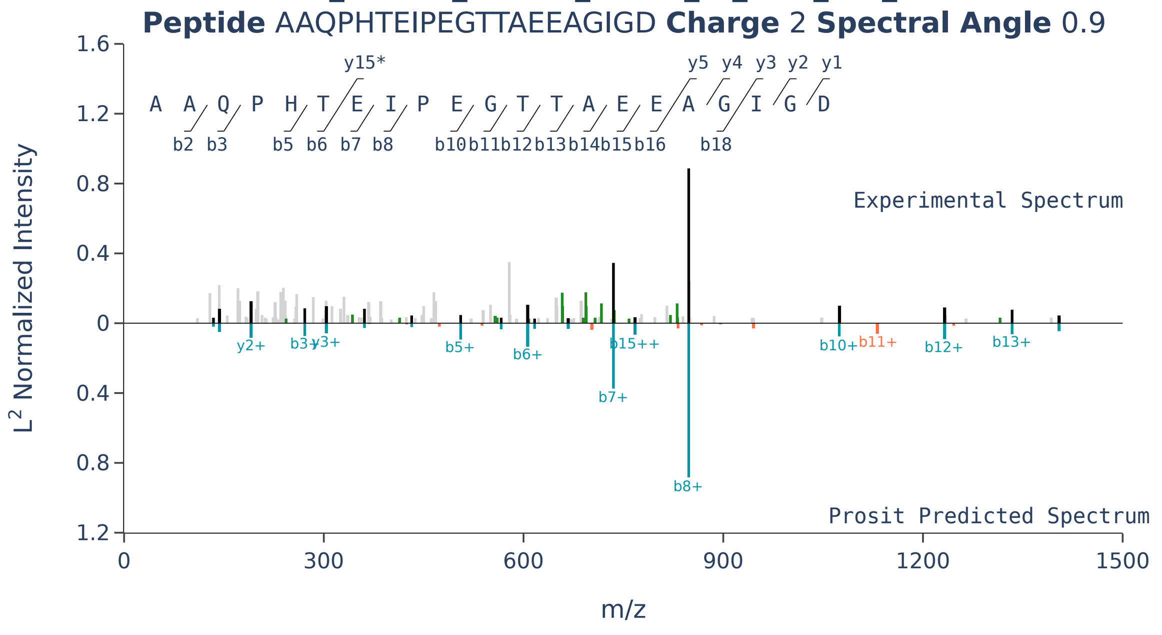


**Source** WSoh_221121_011221_HFGoe_L1_hTau_24h_R1 **Scan** 15822
**Peptide** PSLPTPPTREPKKVAV **Charge** 2 **Spectral Angle** 0.8

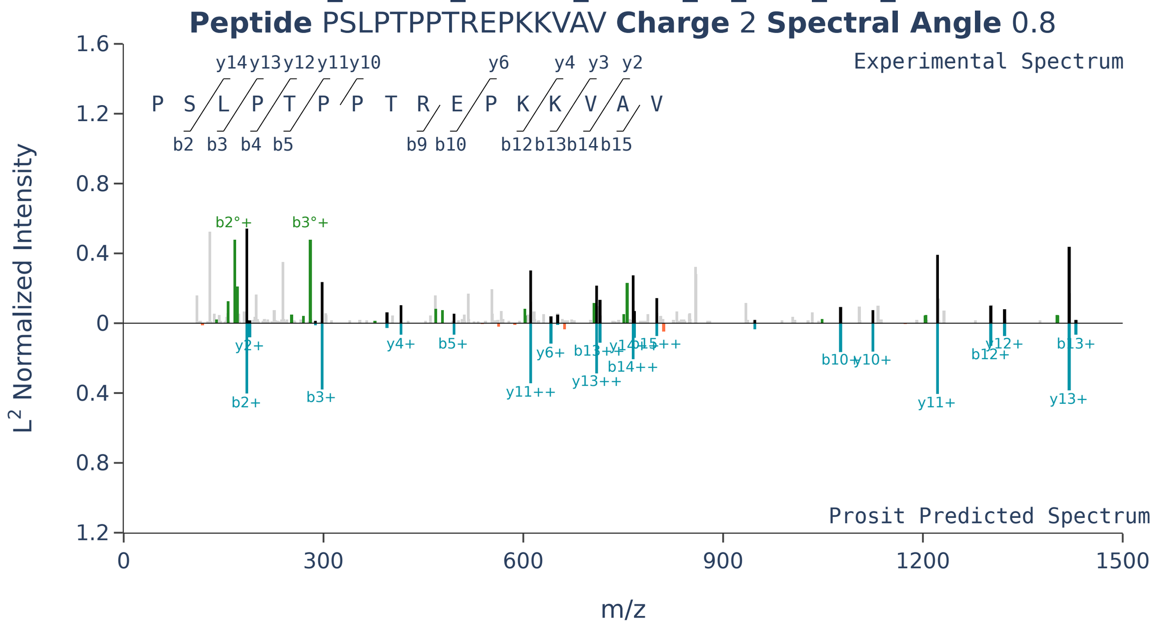


**Source** WSoh_221121_011221_HFGoe_L1_hTau_24h_R1 **Scan** 7284
**Peptide** YGLGDRKDQGG **Charge** 2 **Spectral Angle** 0.76

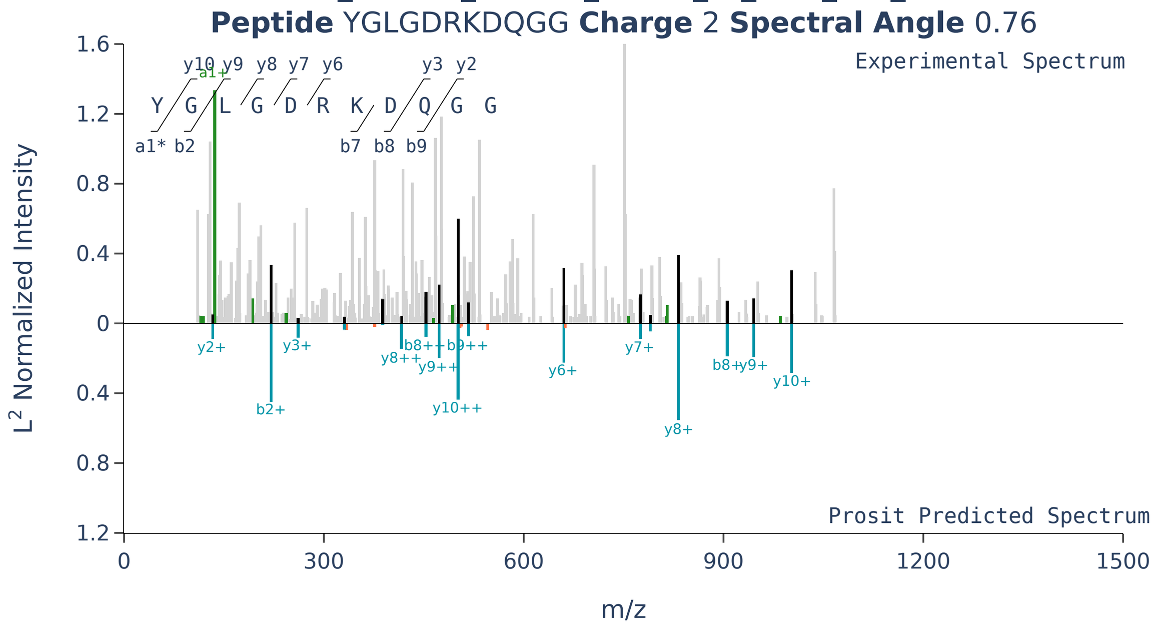


**Source** WSoh_221121_011221_HFGoe_L1_hTau_24h_R2 **Scan** 7986
**Peptide** GGYTMHQDQEGD **Charge** 2 **Spectral Angle** 0.77

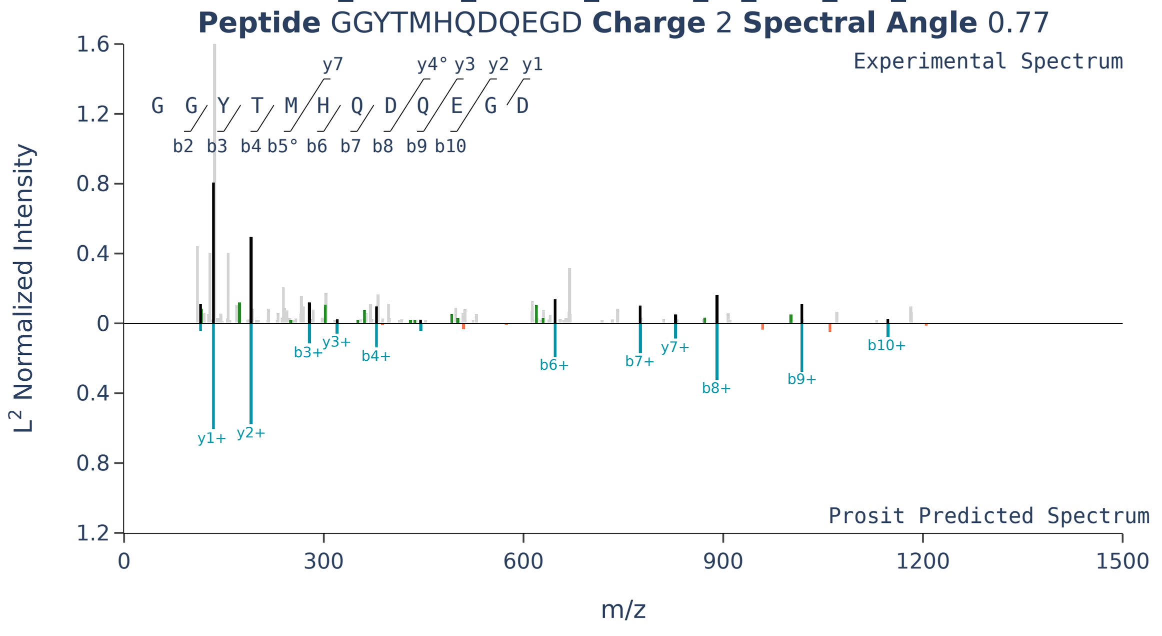


**Source** WSoh_221121_011221_HFGoe_L1_hTau_24h_R2 **Scan** 11608
**Peptide** QANATRIPAKTPPAPKTPPSS **Charge** 3 **Spectral Angle** 0.83

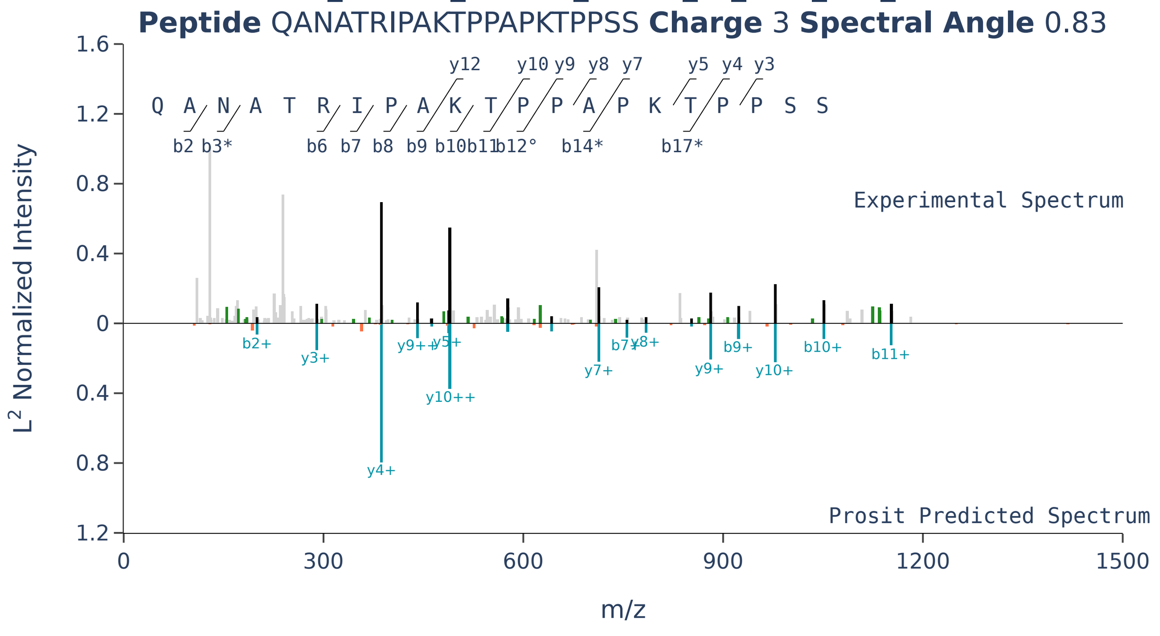


**Source** WSoh_221121_011221_HFGoe_L2_hTau_24h_R1 **Scan** 6696
**Peptide** KIGSTENLKHQPGGG **Charge** 3 **Spectral Angle** 0.77

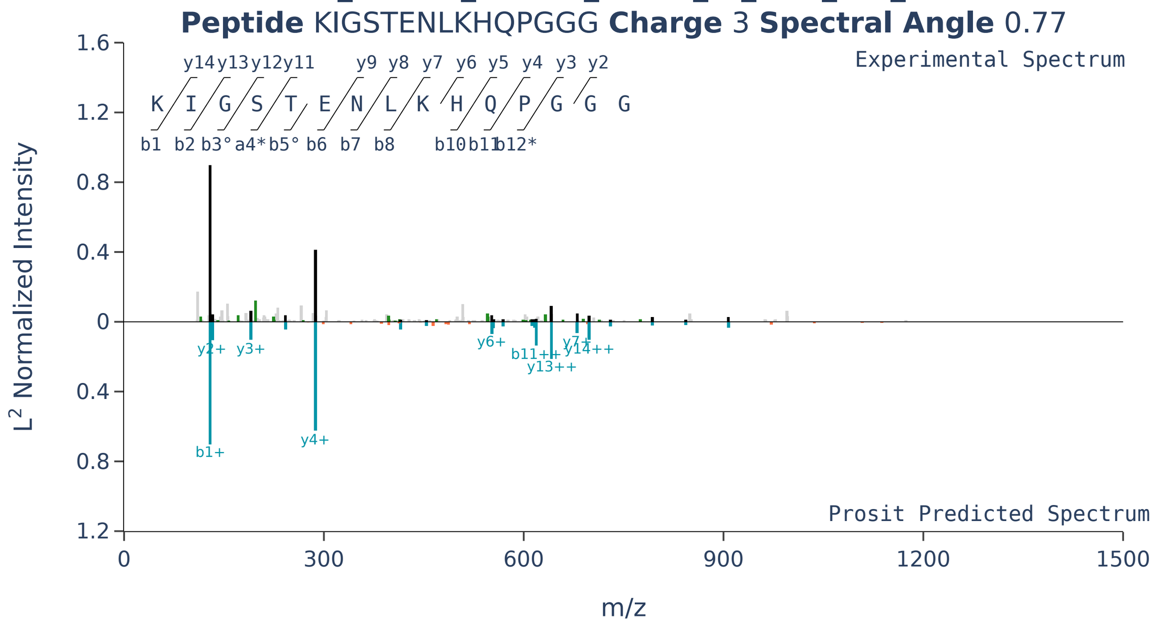


**Source** WSoh_221121_011221_HFGoe_L2_hTau_24h_R2 **Scan** 20550
**Peptide** QTAPVPMPDLKNVKSKIGSTENLKHQ **Charge** 4 **Spectral Angle** 0.87

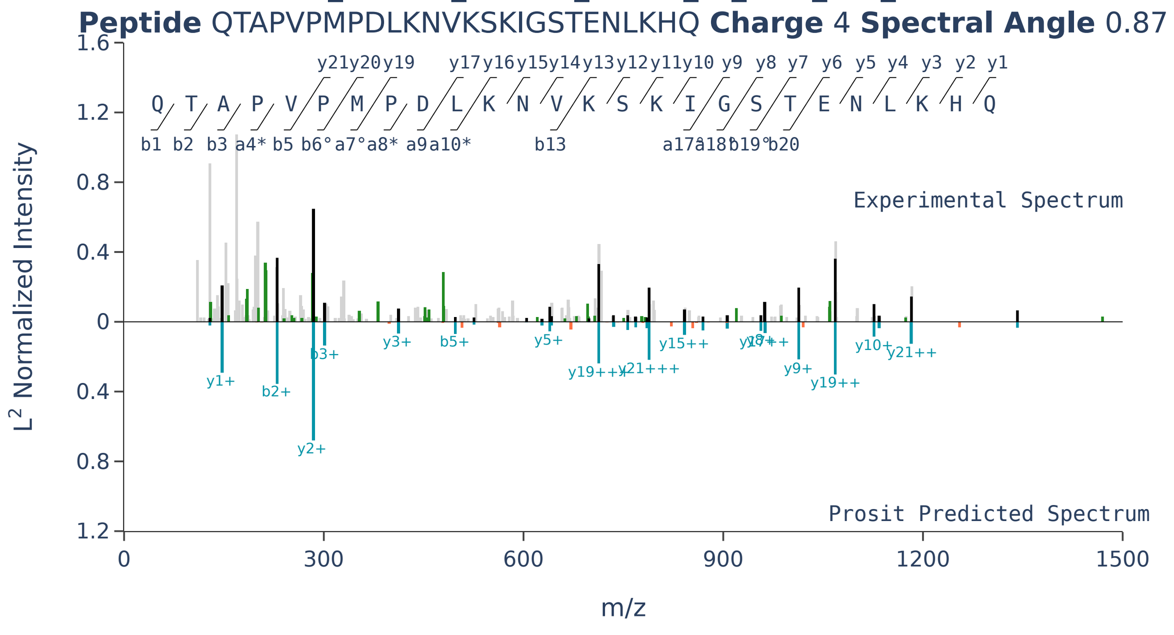


**Source** WSoh_221121_011221_HFGoe_L2_hTau_2h_R1 **Scan** 14203
**Peptide** KPGGGQVEVKSEKLDFKDRVQSKI **Charge** 4 **Spectral Angle** 0.83

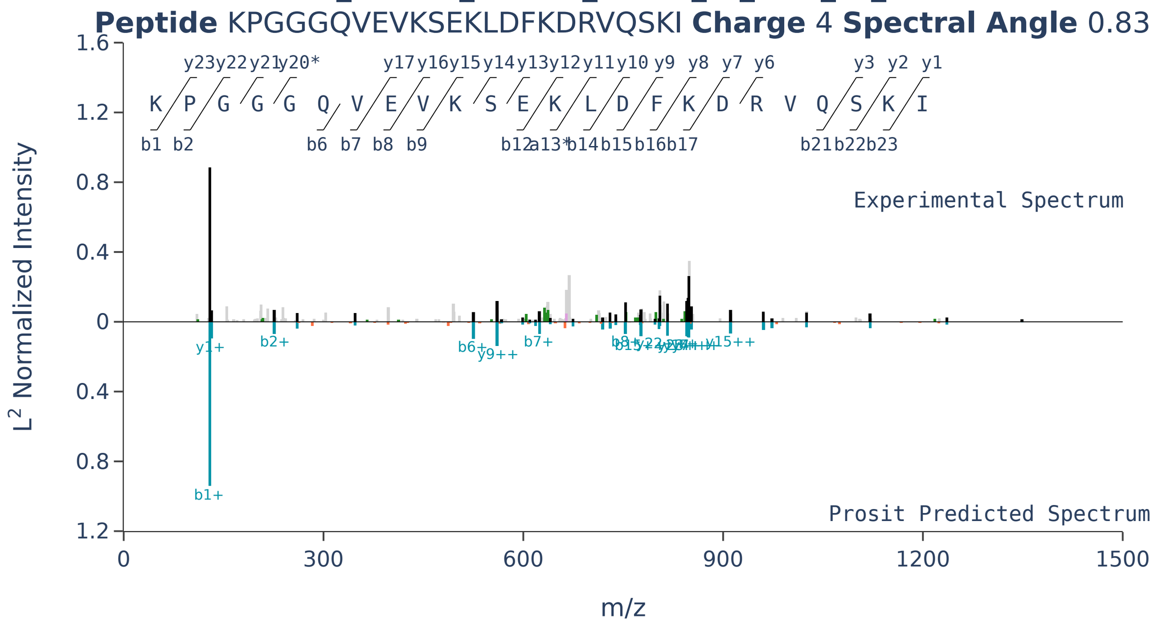


**Source** WSoh_221121_011221_HFGoe_L2_hTau_4h_R2 **Scan** 6051
**Peptide** KSPVVSGDTSPRH **Charge** 3 **Spectral Angle** 0.77

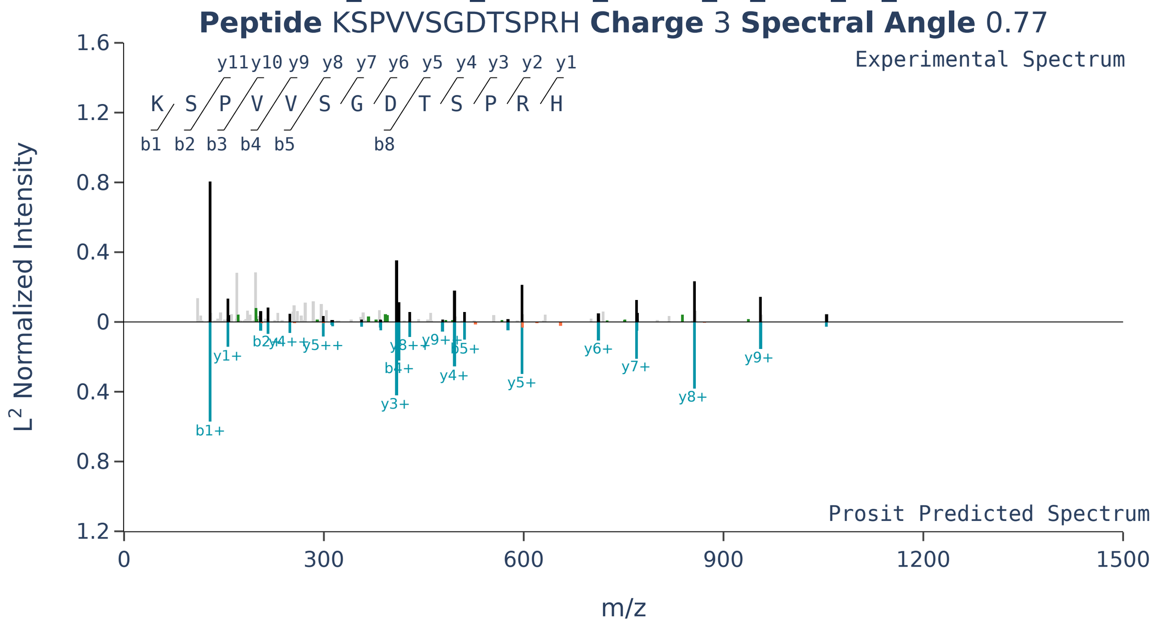


**Source** WSoh_221121_011221_HFGoe_L1_hTau_24h_R2 **Scan** 19871
**Peptide** AKSTPTAEDVTAPLVDEGAPGKQ **Charge** 3 **Spectral Angle** 0.89

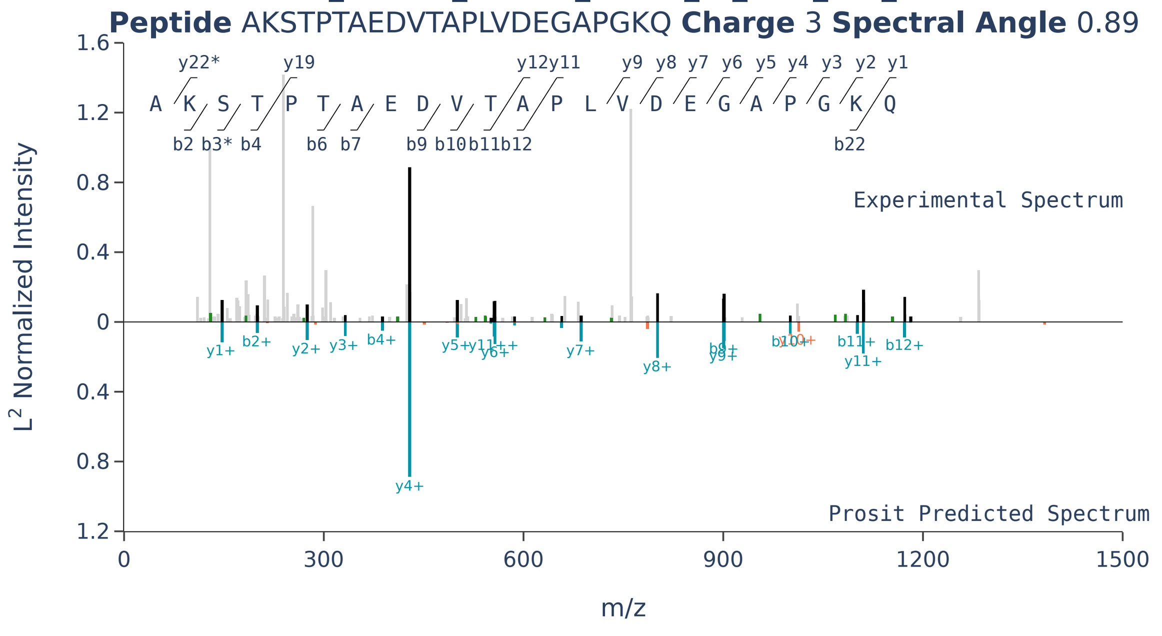


**Source** WSoh_221121_011221_HFGoe_L1_hTau_24h_R1 **Scan** 21476
**Peptide** GSKDNIKHVPGGGSVQIVYKPVDL **Charge** 4 **Spectral Angle** 0.8

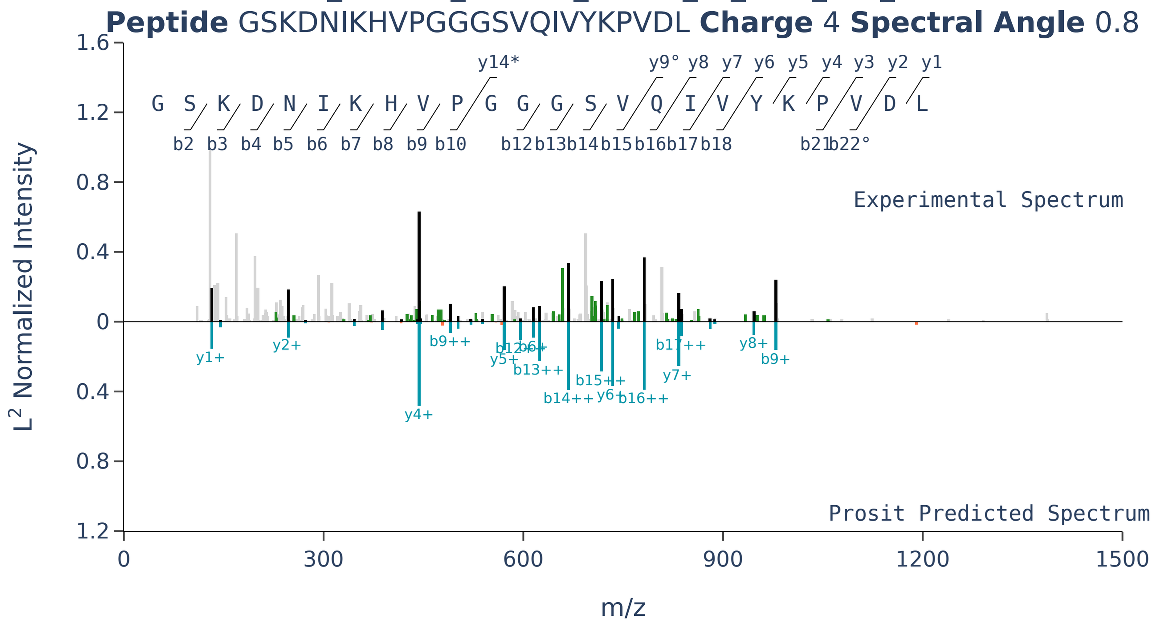

**Source** WSoh_221121_011221_HFGoe_L2_hTau_24h_R2 **Scan** 7531
**Peptide** GEPPKSGDRSGYSSPGSPGTPGSR **Charge** 3 **Spectral Angle** 0.78

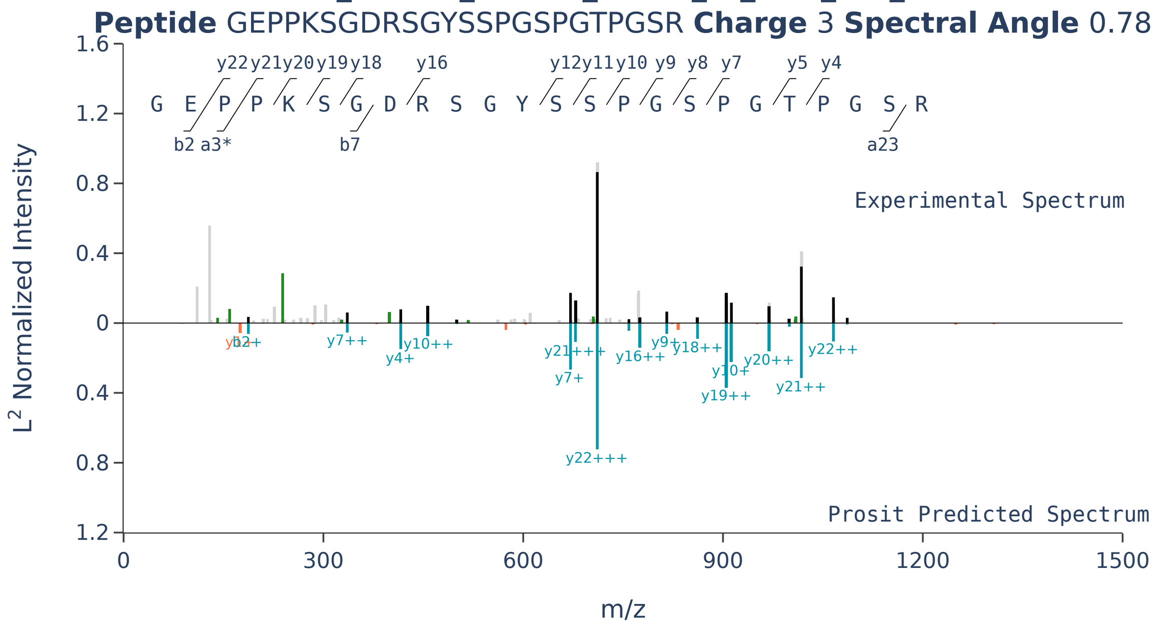


**Source** WSoh_221121_011221_HFGoe_L1_hTau_4h_R1 **Scan** 9891
**Peptide** TRIPAKTPPAPKTPPSSGEPPKS **Charge** 3 **Spectral Angle** 0.8

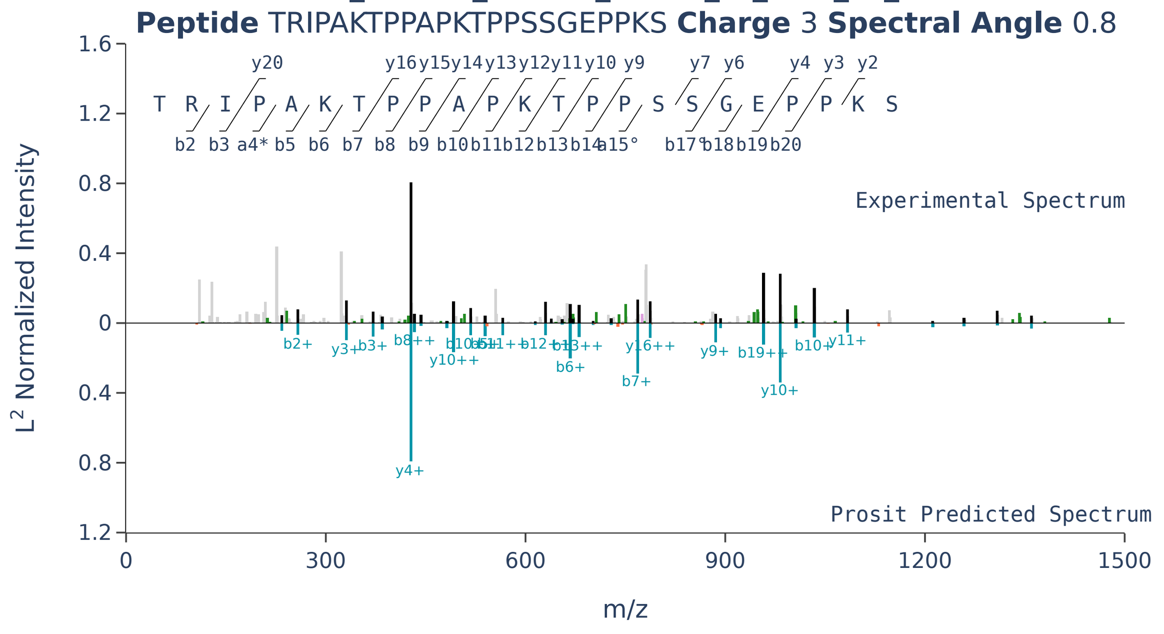


**Source** WSoh_221121_011221_HFGoe_L2_hTau_4h_R1 **Scan** 18475
**Peptide** KLGSLDNITHVPGGGNKKIETHKLTF **Charge** 4 **Spectral Angle** 0.85

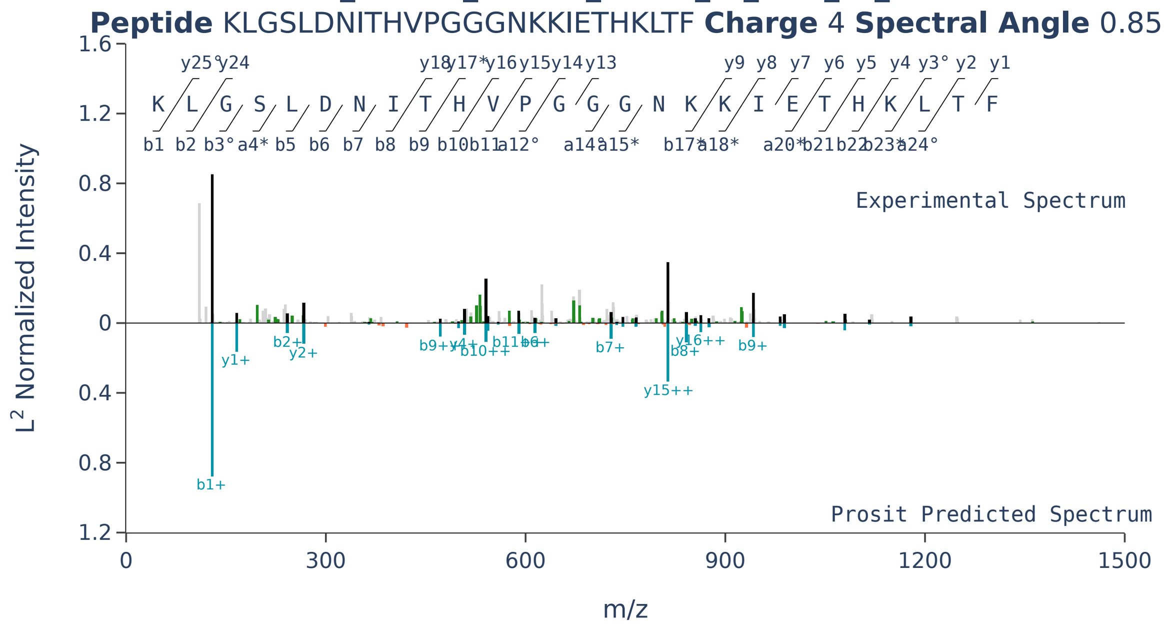


**Source** WSoh_221121_011221_HFGoe_L1_hTau_24h_R1 **Scan** 18678
**Peptide** PGGGKVQIINKKLDLSNVQ **Charge** 3 **Spectral Angle** 0.87

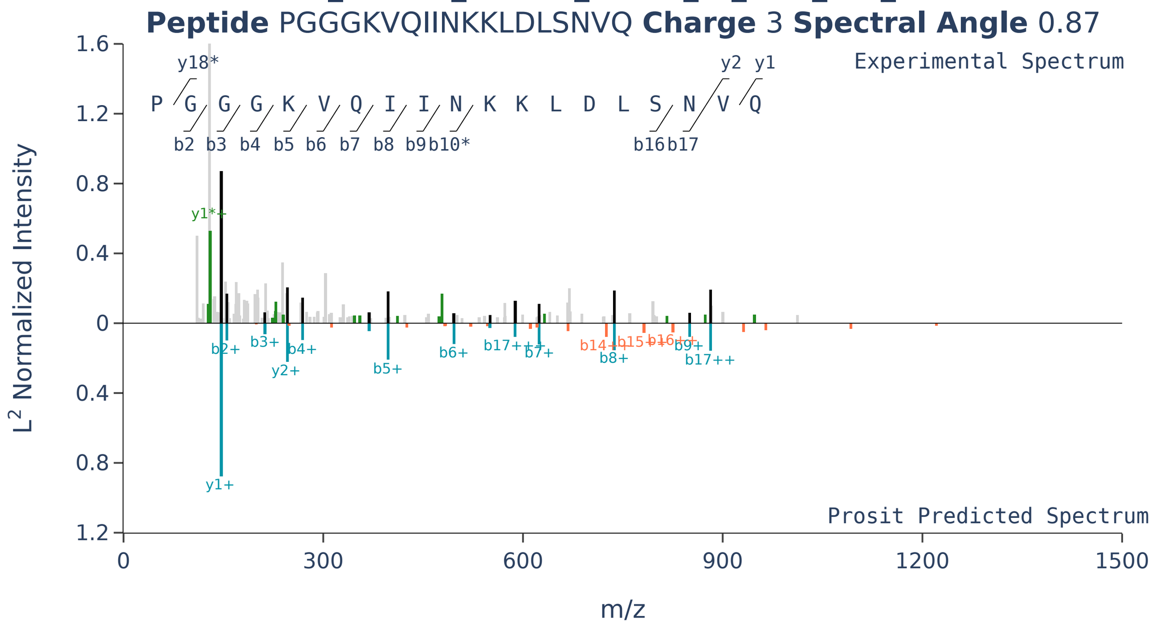


**Source** WSoh_221121_011221_HFGoe_L1_hTau_4h_R1 **Scan** 6464
**Peptide** KCGSLGNIHHKPGGGQVE **Charge** 2 **Spectral Angle** 0.82

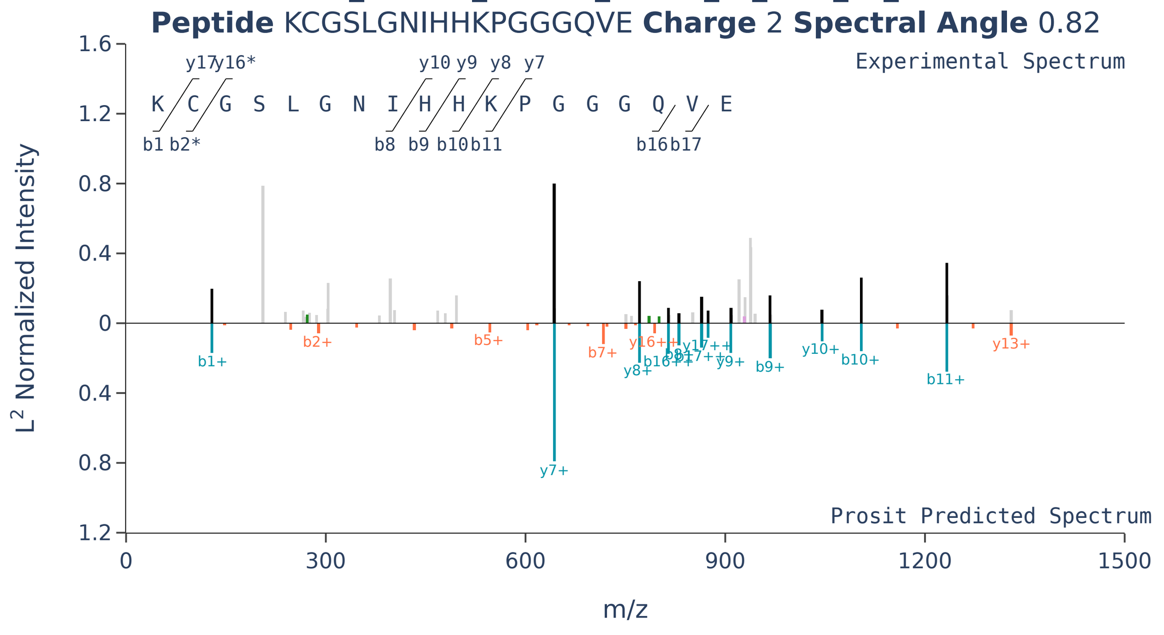


**Source** WSoh_221121_011221_HFGoe_L2_hTau_2h_R1 **Scan** 6207
**Peptide** KGQANATRIPAKT **Charge** 2 **Spectral Angle** 0.79

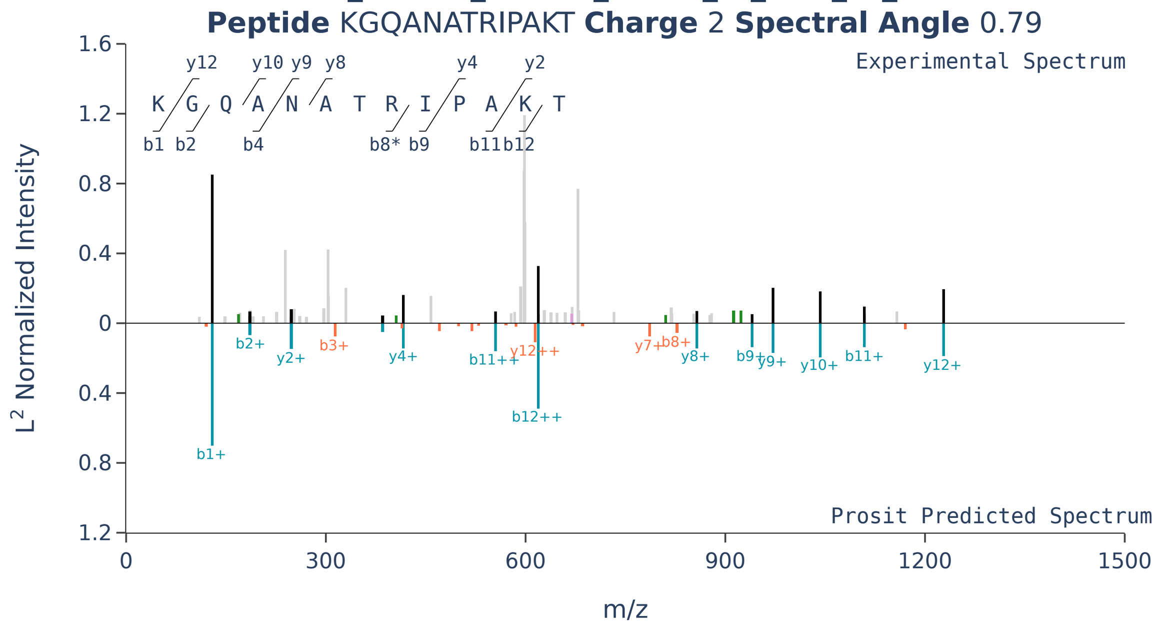


**Source** WSoh_221121_011221_HFGoe_L1_hTau_24h_R1 **Scan** 15942
**Peptide** KSRLQTAPVPMPDLKNVKSK **Charge** 5 **Spectral Angle** 0.72

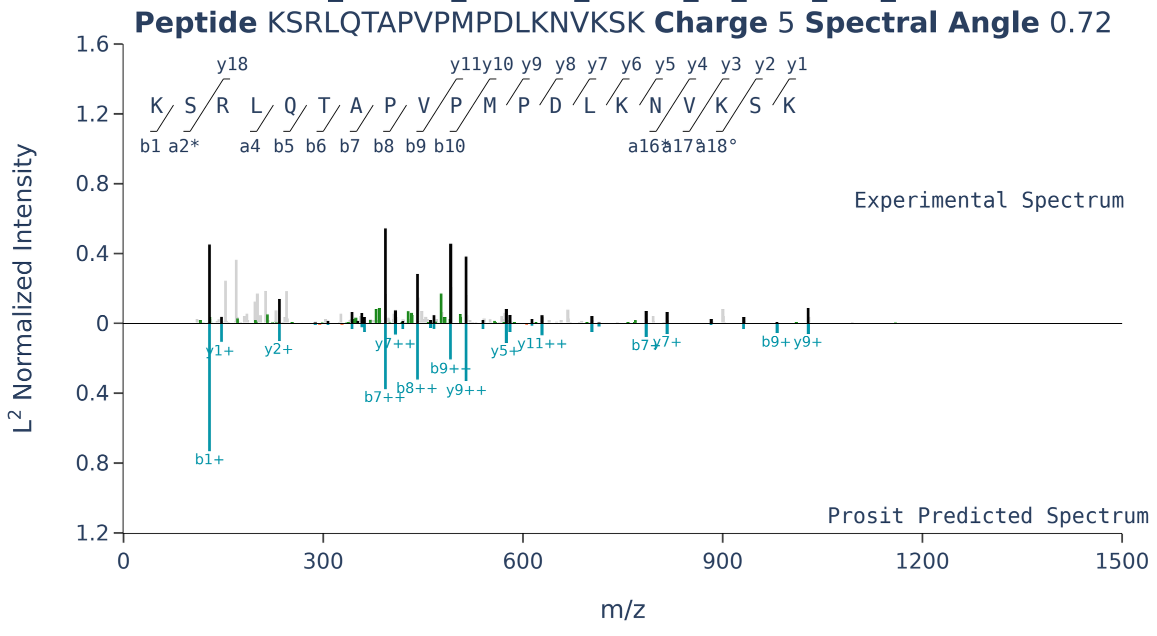


**Source** WSoh_221121_011221_HFGoe_L1_hTau_24h_R2 **Scan** 8789
**Peptide** KESPLQT **Charge** 2 **Spectral Angle** 0.8

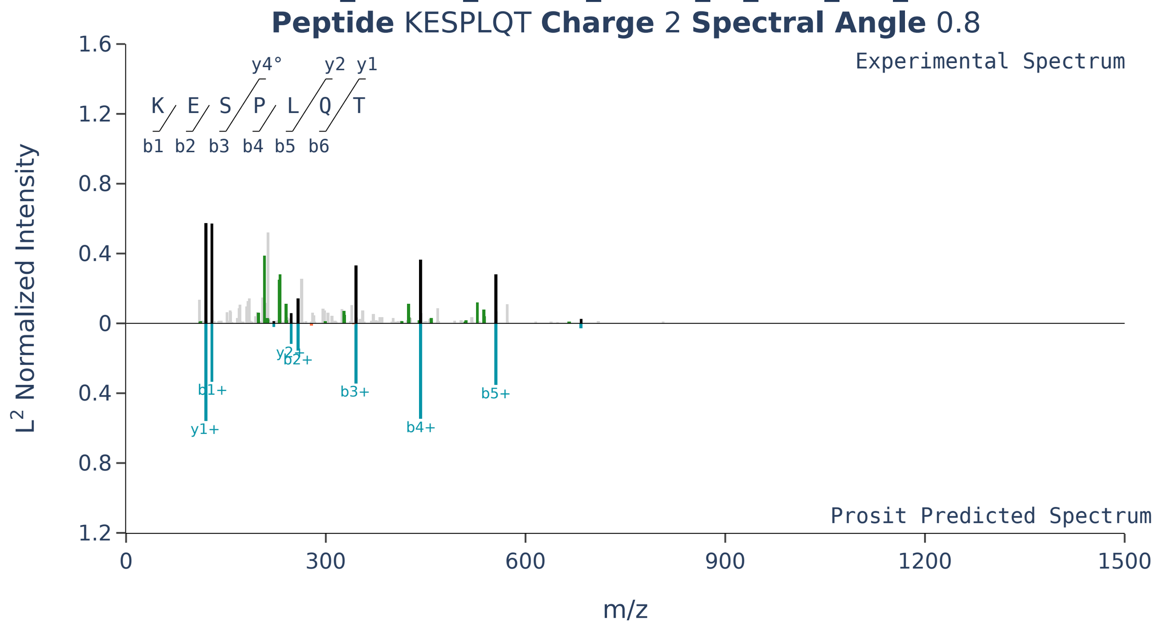


**Source** WSoh_221121_011221_HFGoe_L2_hTau_24h_R2 **Scan** 16470
**Peptide** KTDHGAEIVYKSPVVSGDTSPRHLSNV **Charge** 3 **Spectral Angle** 0.78

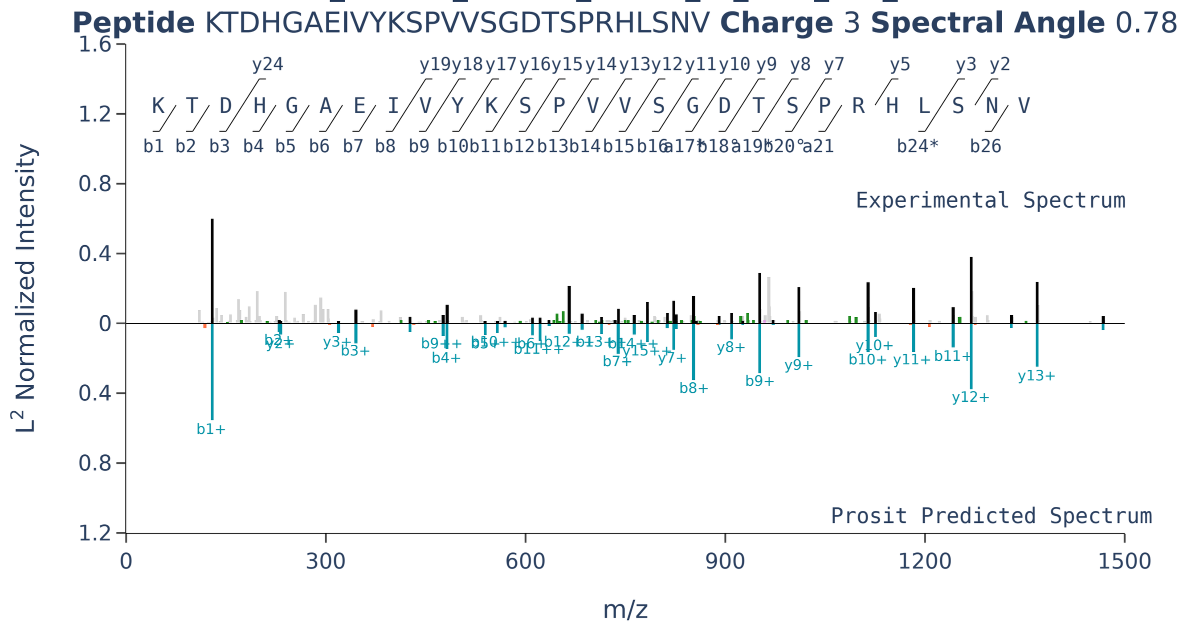


**Source** WSoh_221121_011221_HFGoe_L1_hTau_24h_R2 **Scan** 11937
**Peptide** NLTHVPGGGNKKIETHKLTFRE **Charge** 3 **Spectral Angle** 0.86

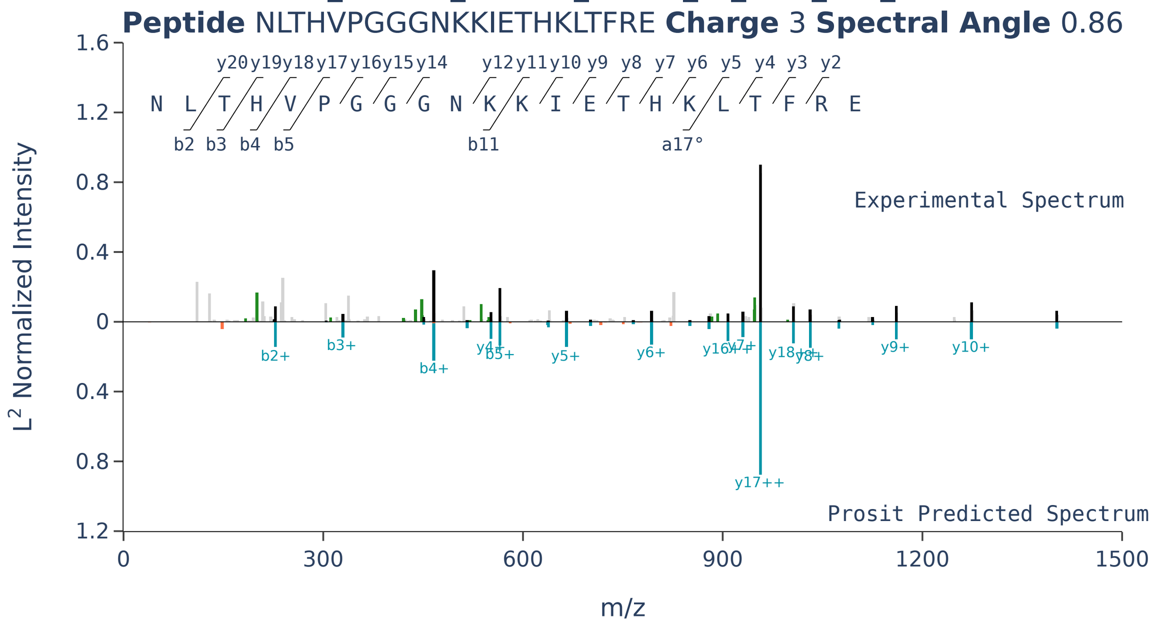


**Source** WSoh_221121_011221_HFGoe_L1_hTau_24h_R1 **Scan** 18645
**Peptide** IGDTPSLEDEAAGHVTQARMVSK **Charge** 4 **Spectral Angle** 0.79

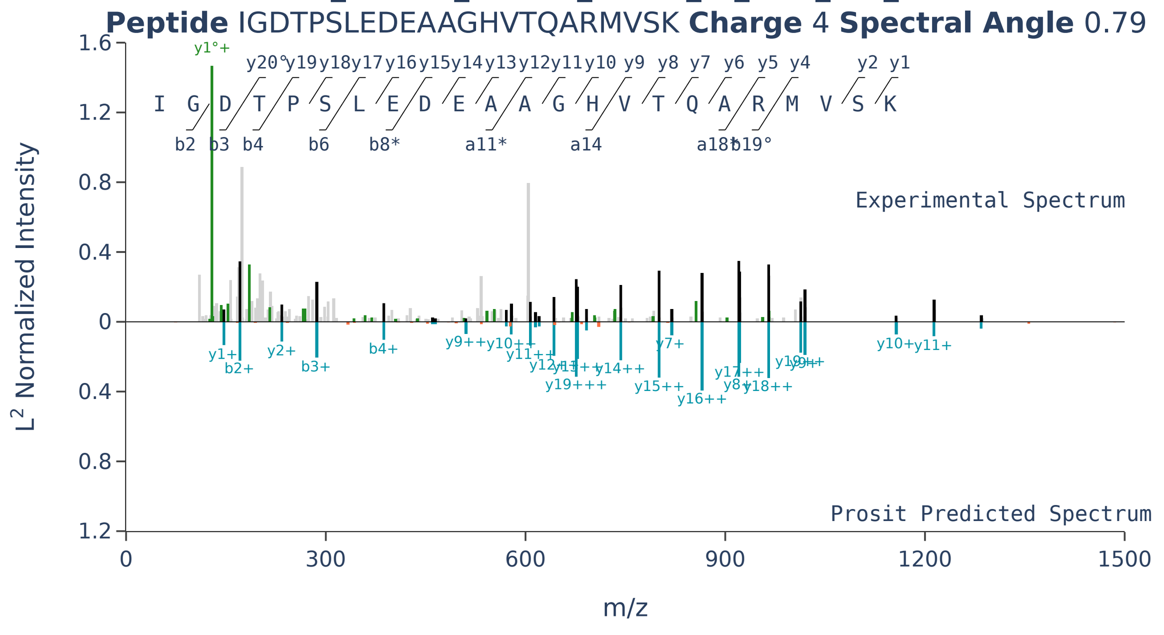


**Source** WSoh_221121_011221_HFGoe_L2_hTau_4h_R1 **Scan** 17185
**Peptide** KLDFKDRVQSKIGSLDN **Charge** 3 **Spectral Angle** 0.84

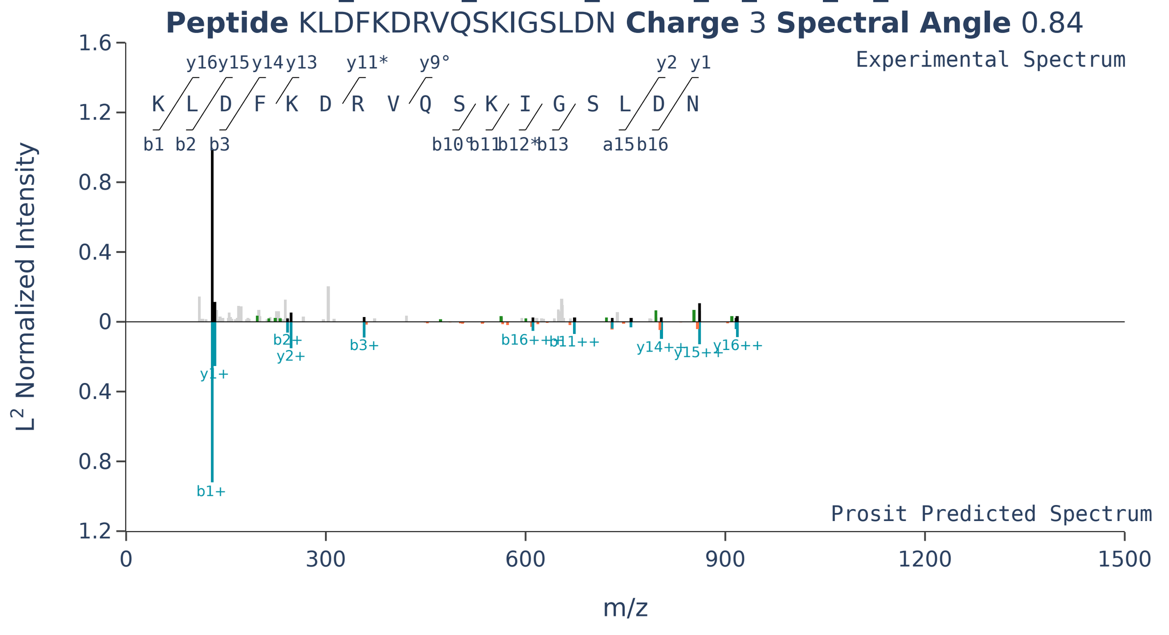


**Source** WSoh_221121_011221_HFGoe_L1_hTau_24h_R2 **Scan** 16762
**Peptide** SKIGSLDNITHVPGGGNKKIET **Charge** 3 **Spectral Angle** 0.86

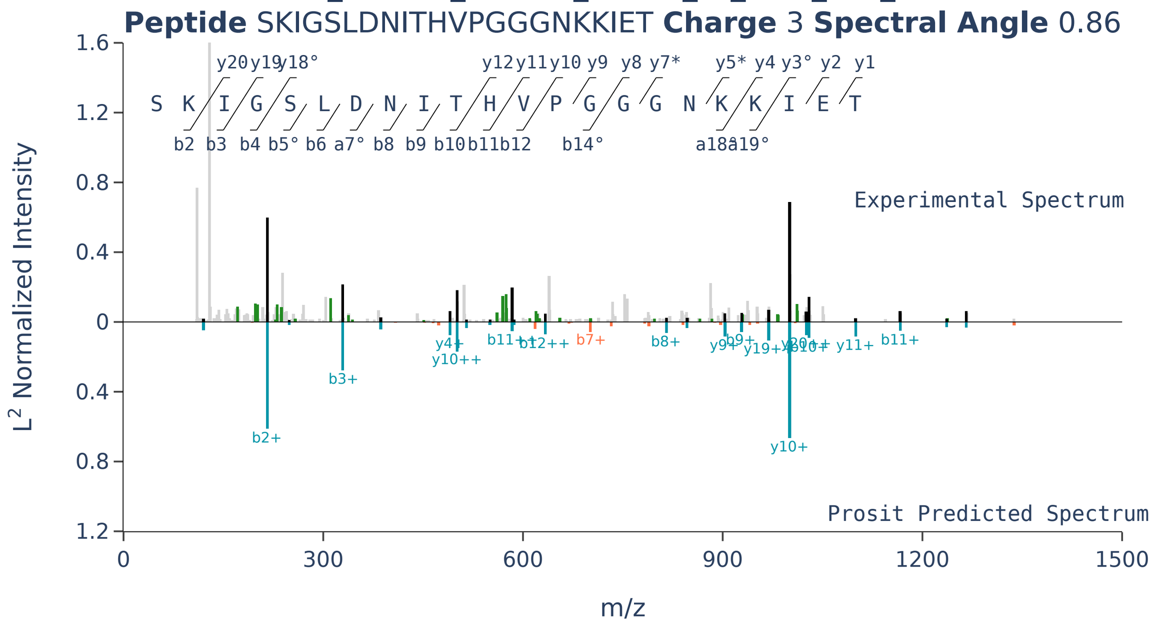


**Source** WSoh_221121_011221_HFGoe_L1_hTau_24h_R1 **Scan** 9430
**Peptide** TSKCGSLGNIHHKPGGGQVEV **Charge** 2 **Spectral Angle** 0.83

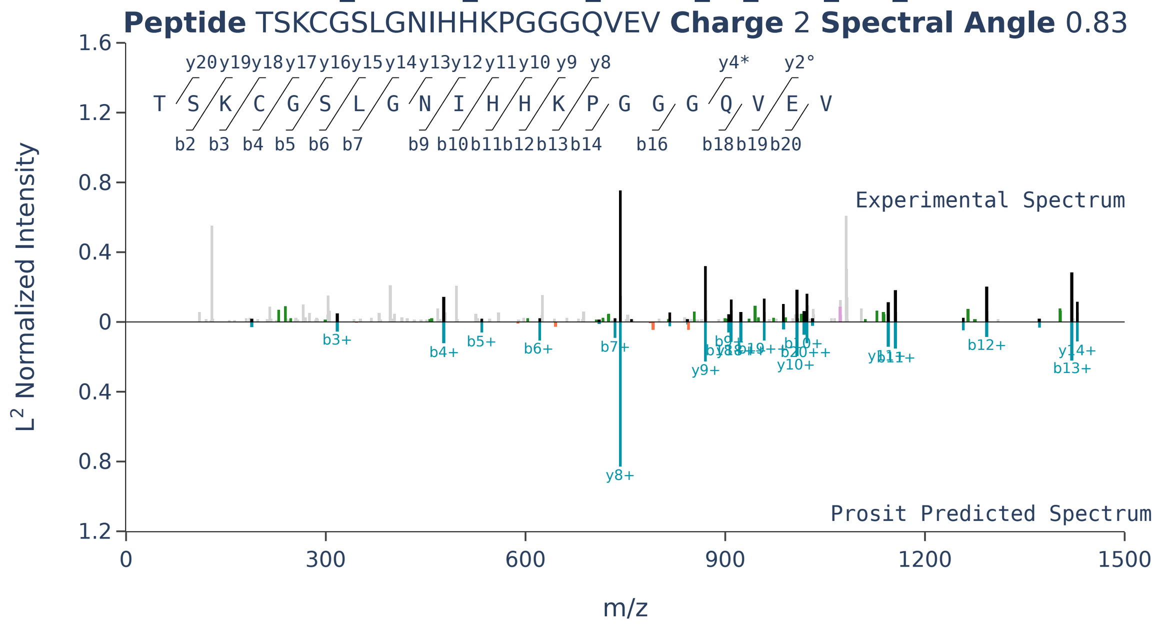


**Source** WSoh_221121_011221_HFGoe_L2_hTau_4h_R2 **Scan** 6324
**Peptide** VRTPPKSPSSA **Charge** 2 **Spectral Angle** 0.83

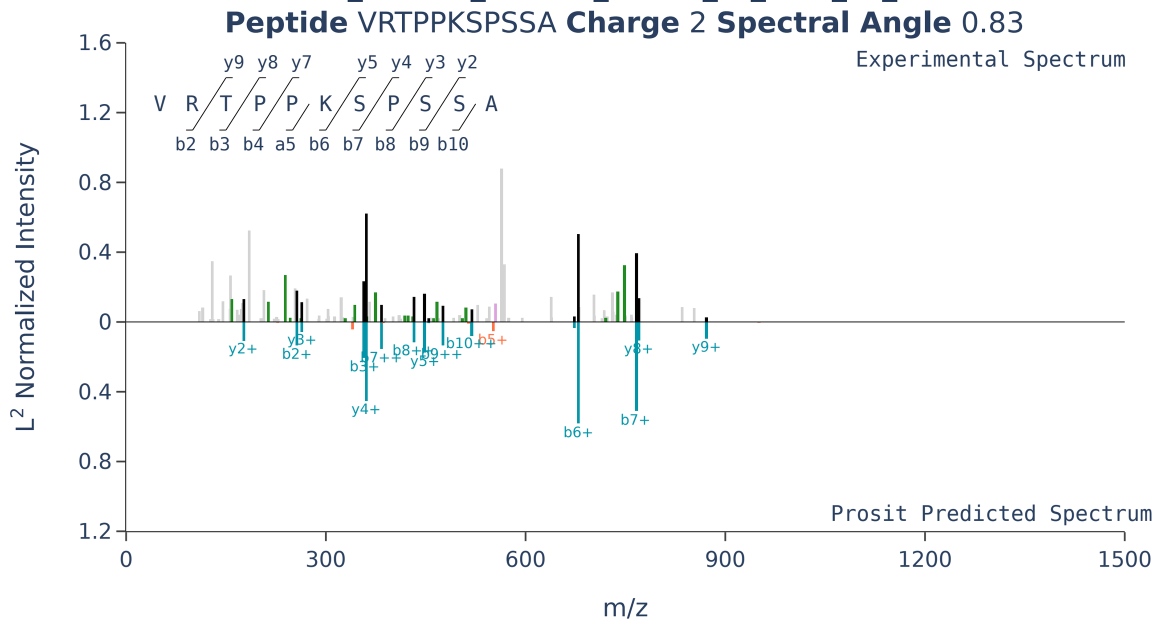

**Source** WSoh_221121_011221_HFGoe_L1_hTau_24h_R2 **Scan** 16860
**Peptide** PLVDEGAPGKQAAAQPHTEL **Charge** 3 **Spectral Angle** 0.86

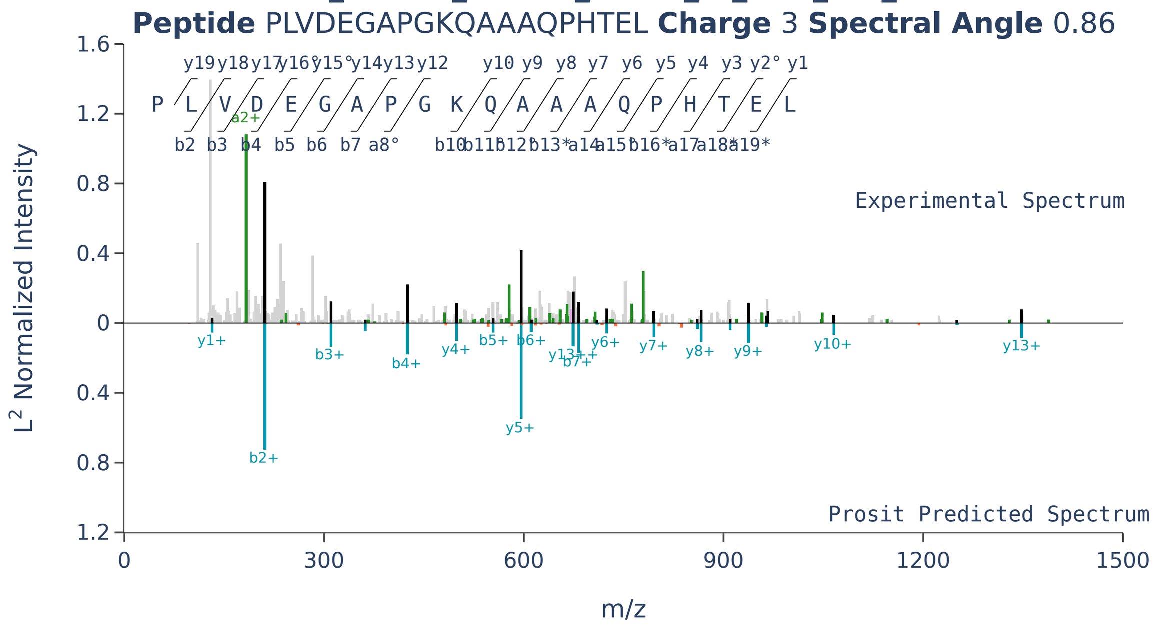


**Source** WSoh_221121_011221_HFGoe_L1_hTau_24h_R2 **Scan** 9440
**Peptide** RIPAKTPPAPKTPPSSGEPPKSG **Charge** 4 **Spectral Angle** 0.84

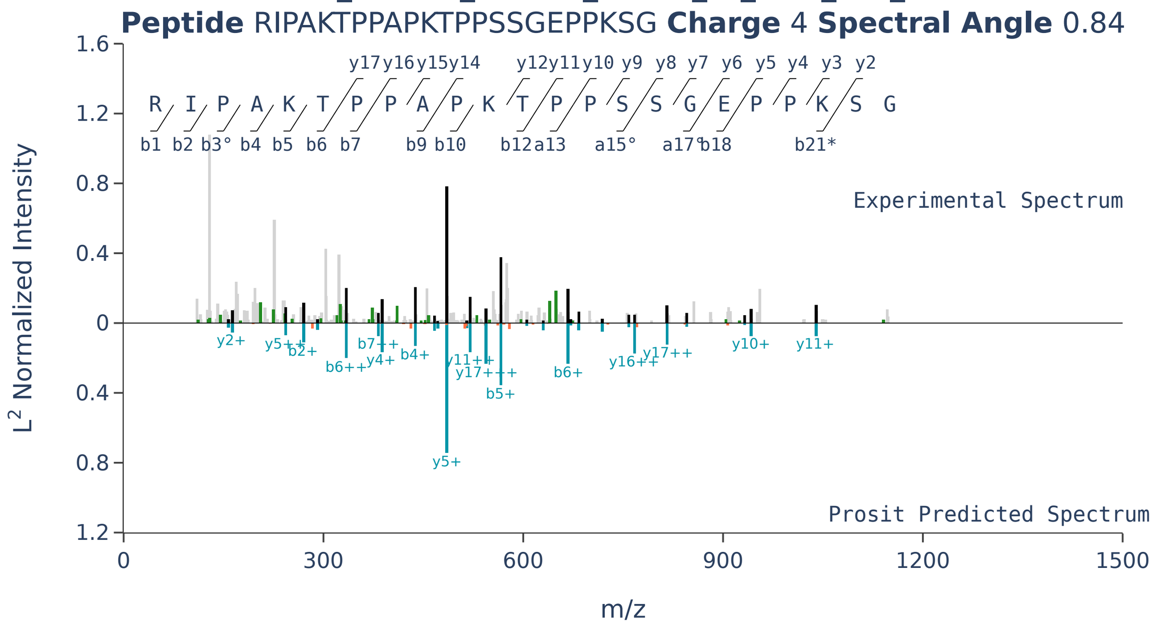


**Source** WSoh_221121_011221_HFGoe_L2_hTau_24h_R2 **Scan** 9041
**Peptide** KKLDLSNVQSKCGSKDNIKHV **Charge** 3 **Spectral Angle** 0.9

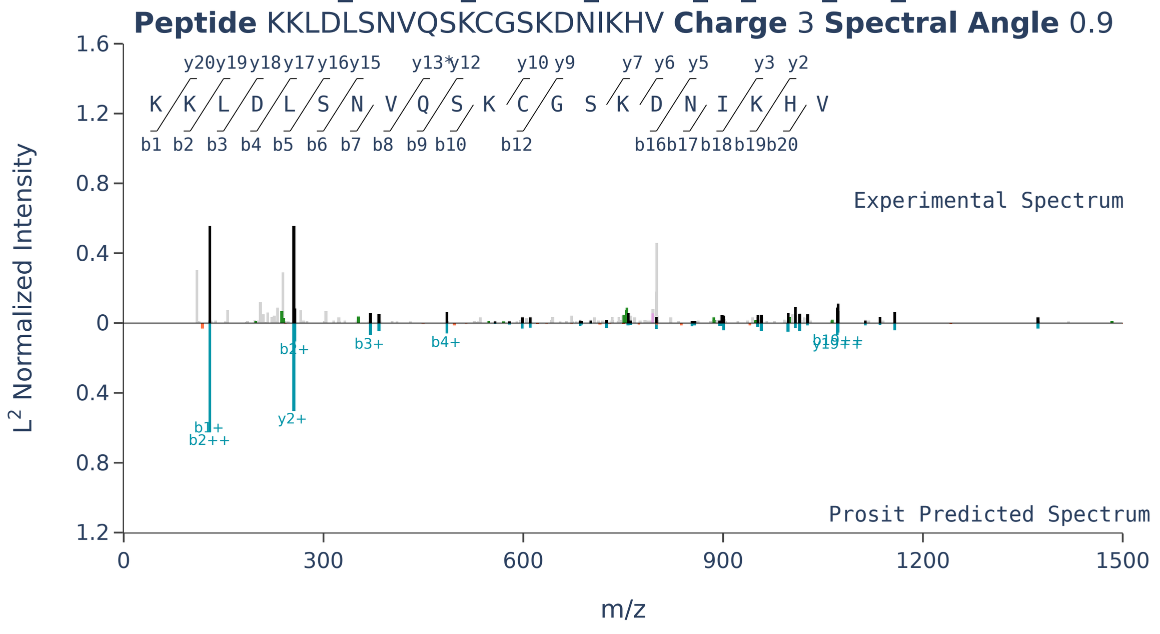


**Source** WSoh_221121_011221_HFGoe_L2_hTau_24h_R1 **Scan** 14812
**Peptide** PSLPTPPTREPKKVAVVRT **Charge** 4 **Spectral Angle** 0.84

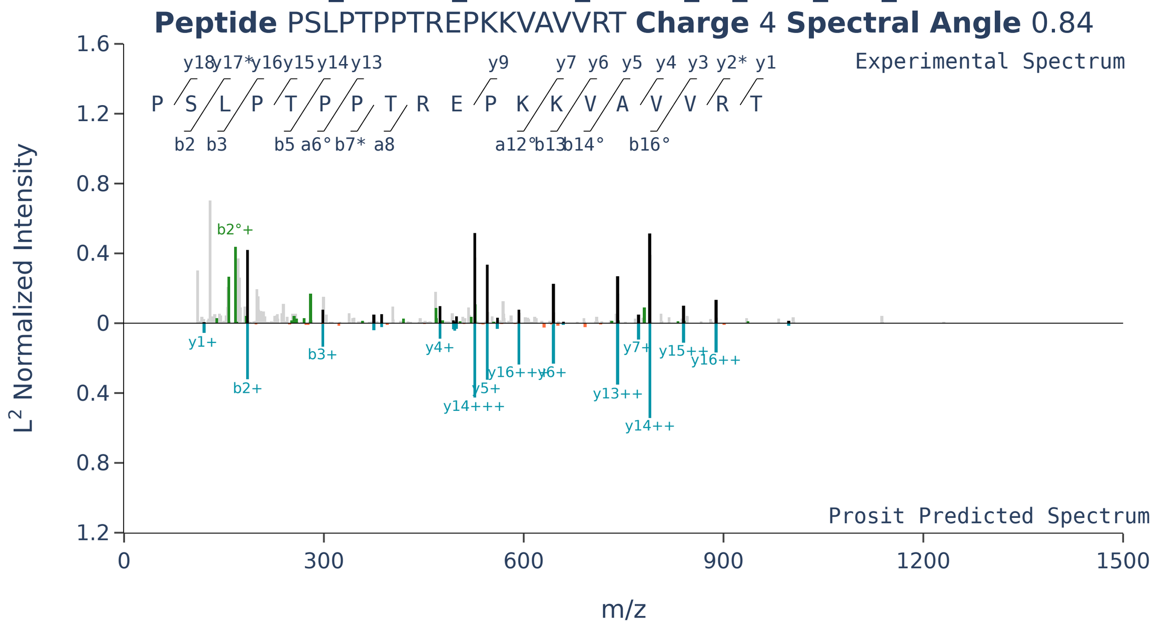


**Source** WSoh_221121_011221_HFGoe_L2_hTau_24h_R1 **Scan** 7754
**Peptide** PPGQKGQANATRI **Charge** 3 **Spectral Angle** 0.85

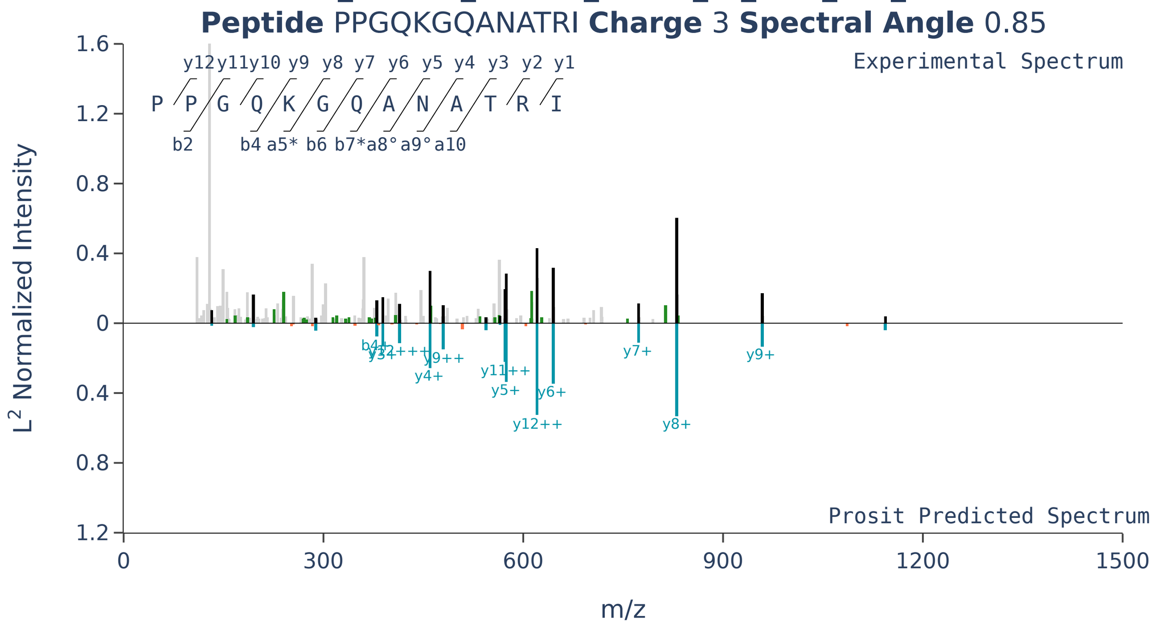


**Source** WSoh_221121_011221_HFGoe_L2_hTau_24h_R1 **Scan** 20571
**Peptide** DRVQSKIGSLDNITHV **Charge** 3 **Spectral Angle** 0.85

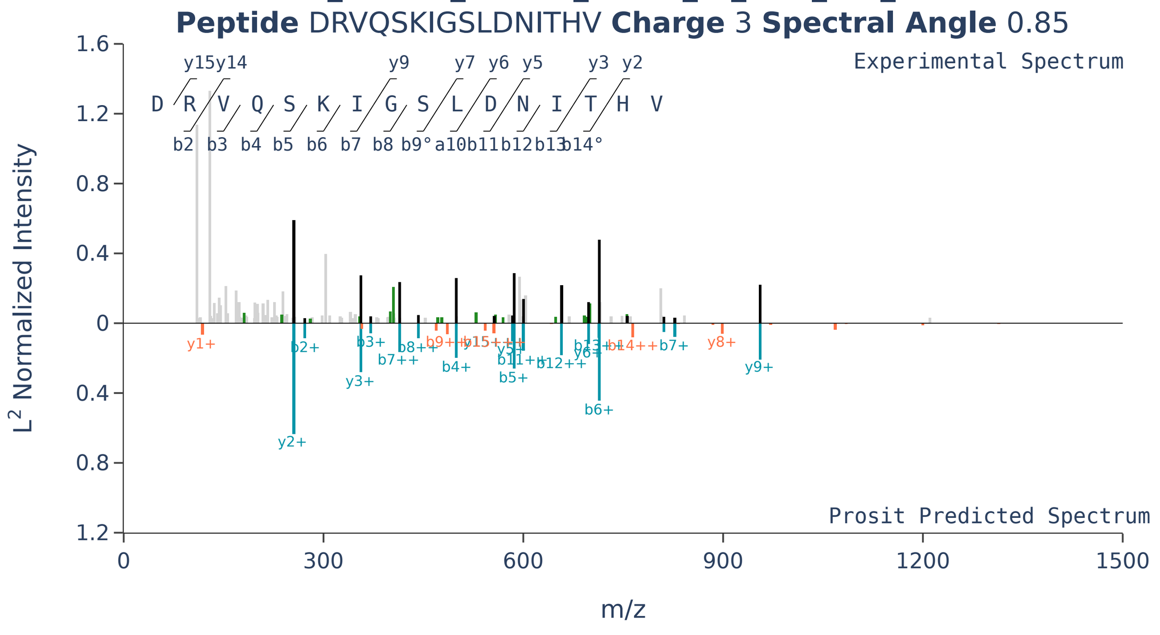


**Source** WSoh_221121_011221_HFGoe_L2_hTau_24h_R2 **Scan** 20564
**Peptide** TAPVPMPDLKNVKSKIGSTENLKH **Charge** 5 **Spectral Angle** 0.82

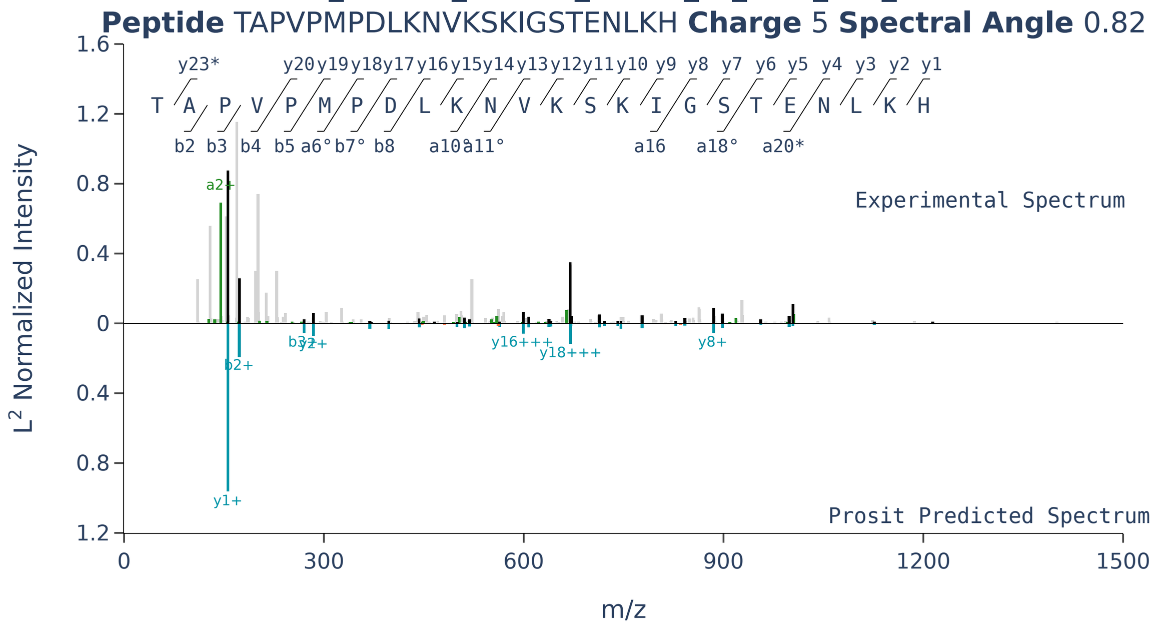


**Source** WSoh_221121_011221_HFGoe_L2_hTau_24h_R1 **Scan** 7657
**Peptide** ADGKTKIATPRGAAPPGQKGQANAT **Charge** 4 **Spectral Angle** 0.82

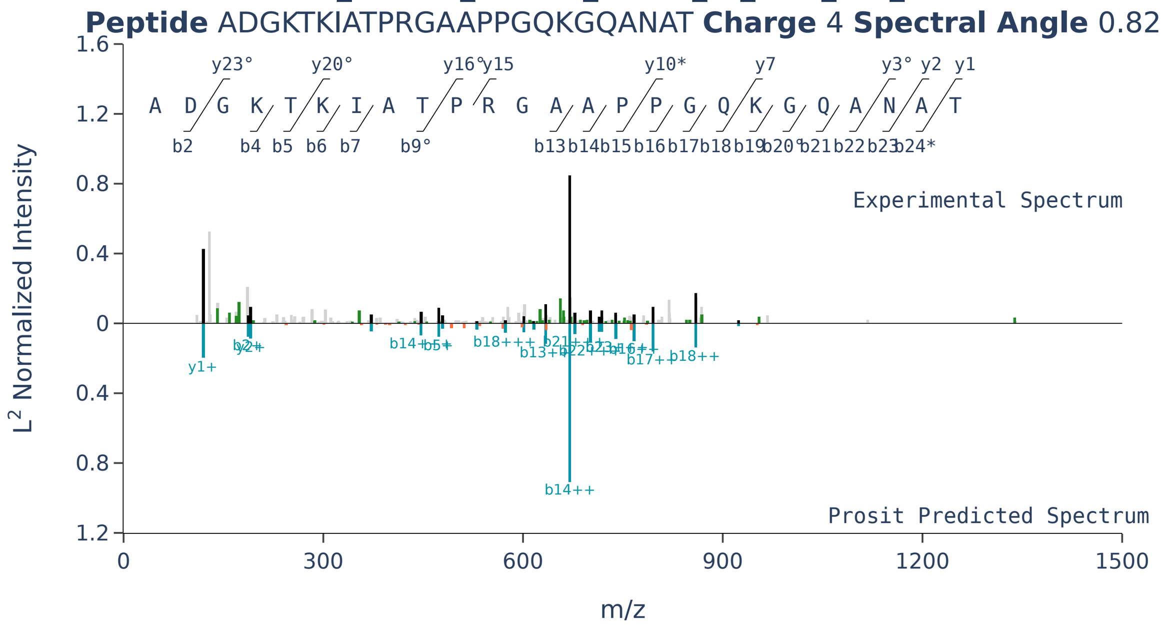


**Source** WSoh_221121_011221_HFGoe_L1_hTau_24h_R1 **Scan** 15080
**Peptide** KLGSLDNITHVPGGGNKKIETHKL **Charge** 5 **Spectral Angle** 0.87

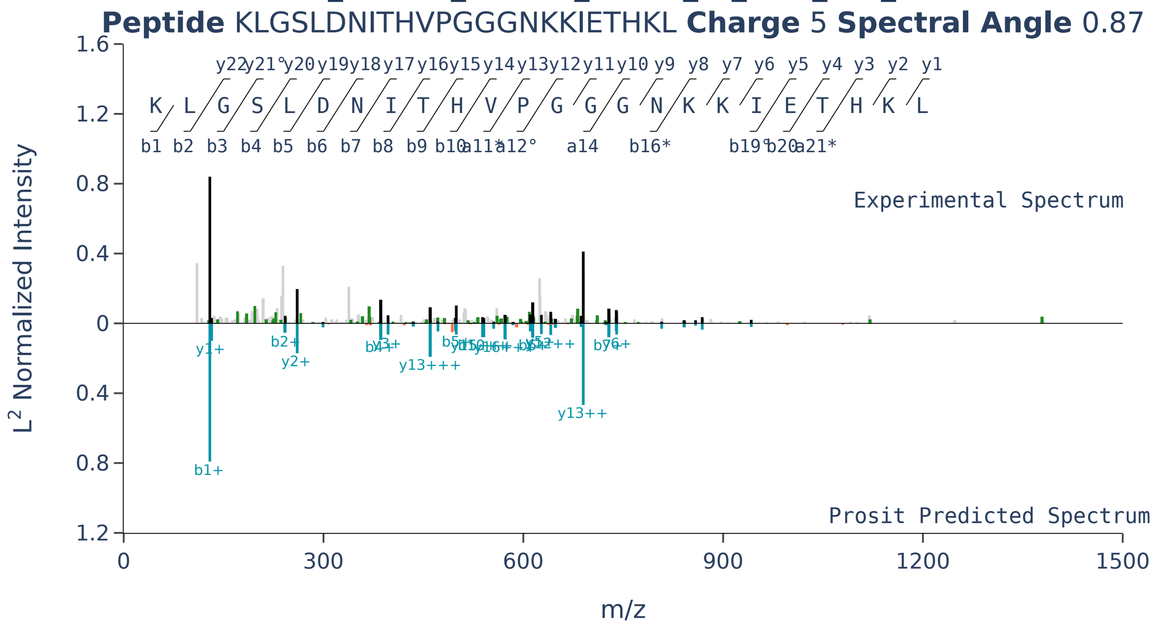


**Source** WSoh_221121_011221_HFGoe_L1_hTau_24h_R1 **Scan** 25842
**Peptide** PTAEDVTAPLV **Charge** 1 **Spectral Angle** 0.77

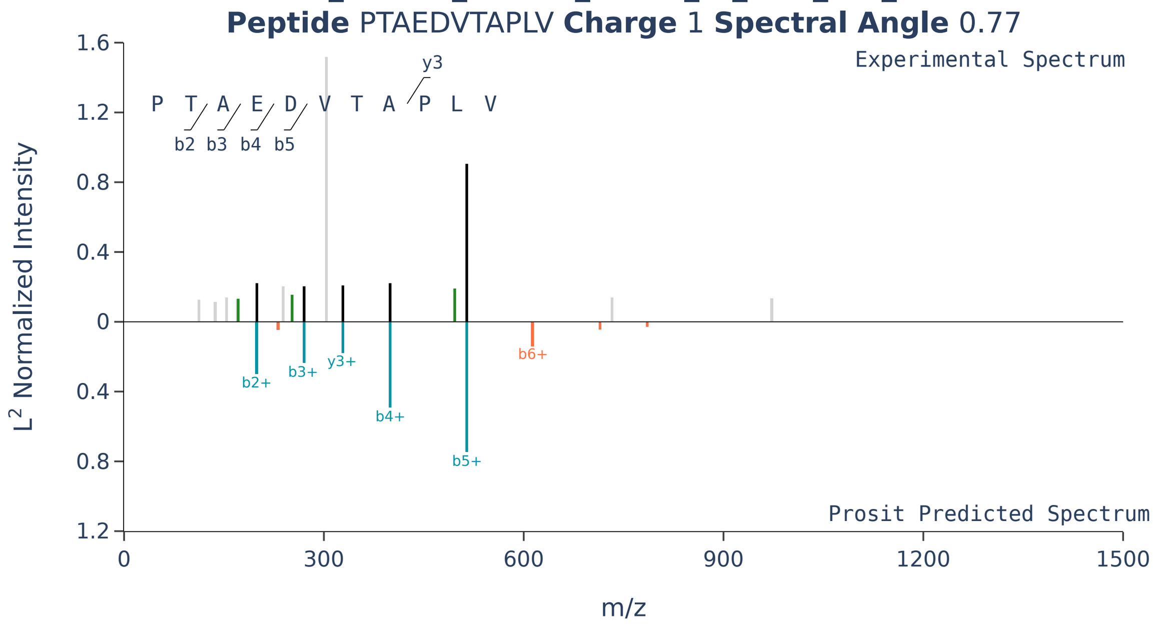


**Source** WSoh_221121_011221_HFGoe_L1_hTau_4h_R2 **Scan** 9052
**Peptide** GAPGKQAAAQPHTEL **Charge** 2 **Spectral Angle** 0.77

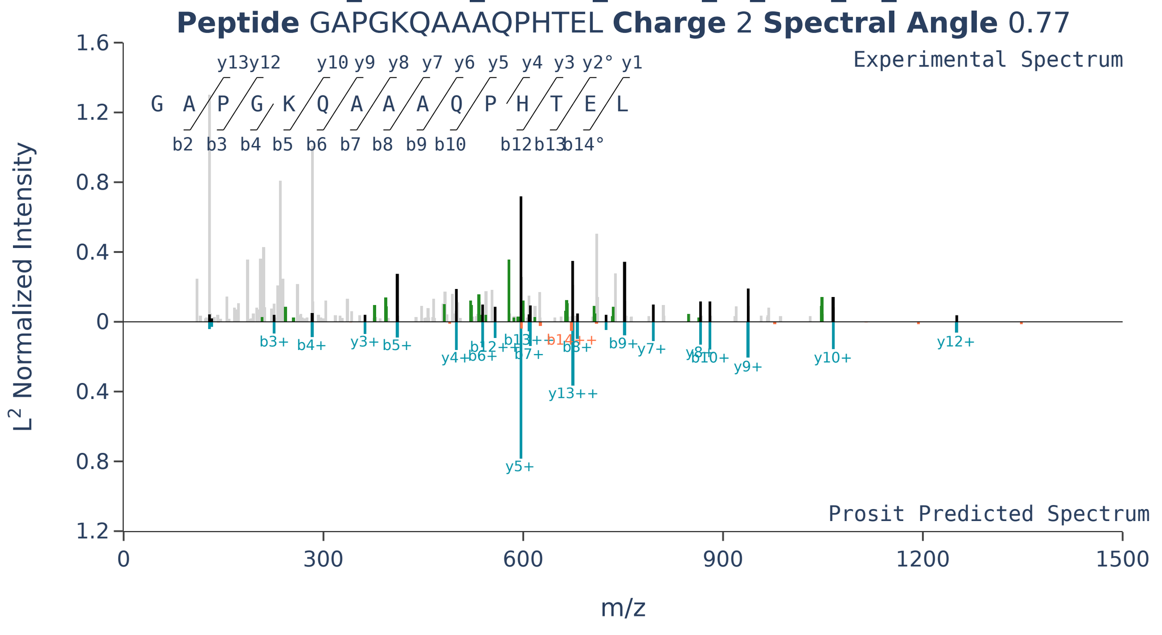


**Source** WSoh_221121_011221_HFGoe_L1_hTau_24h_R1 **Scan** 7418
**Peptide** ADGKTKIATPRGAAPPGQKGQA **Charge** 3 **Spectral Angle** 0.82

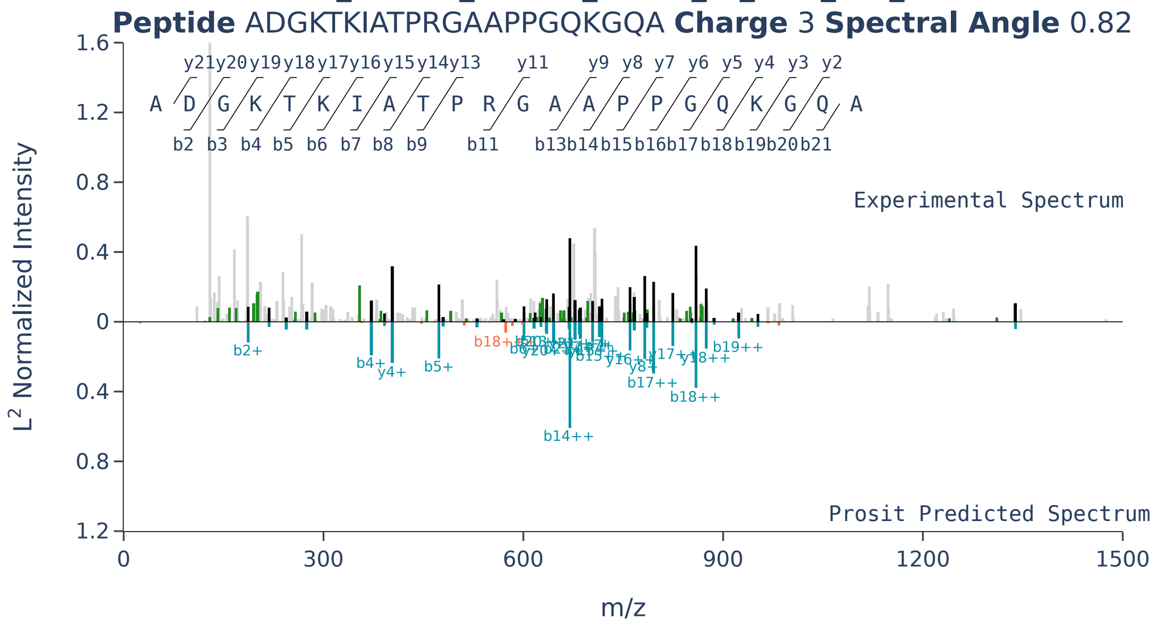


**Source** WSoh_221121_011221_HFGoe_L2_hTau_24h_R2 **Scan** 22988
**Peptide** QTAPVPMPDLK **Charge** 2 **Spectral Angle** 0.91

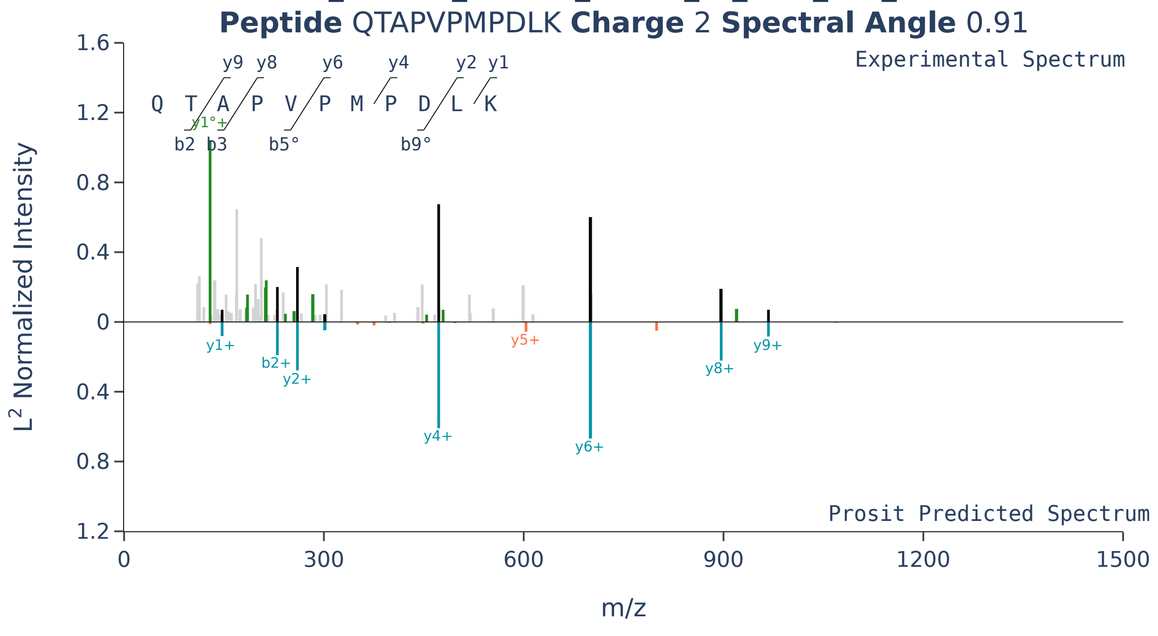


**Source** WSoh_221121_011221_HFGoe_L2_hTau_24h_R1 **Scan** 17888
**Peptide** QDQEGDTDAGLKESPLQT **Charge** 2 **Spectral Angle** 0.82

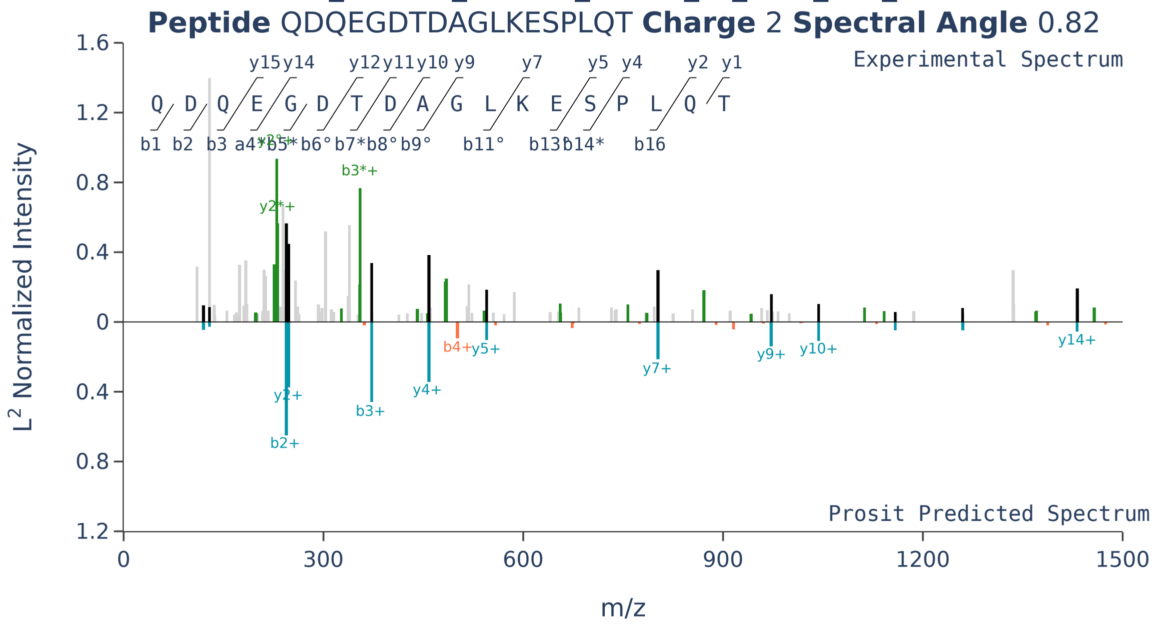


**Source** WSoh_221121_011221_HFGoe_L2_hTau_4h_R2 **Scan** 10806
**Peptide** TENLKHQPGGGKVQIINKKL **Charge** 3 **Spectral Angle** 0.82

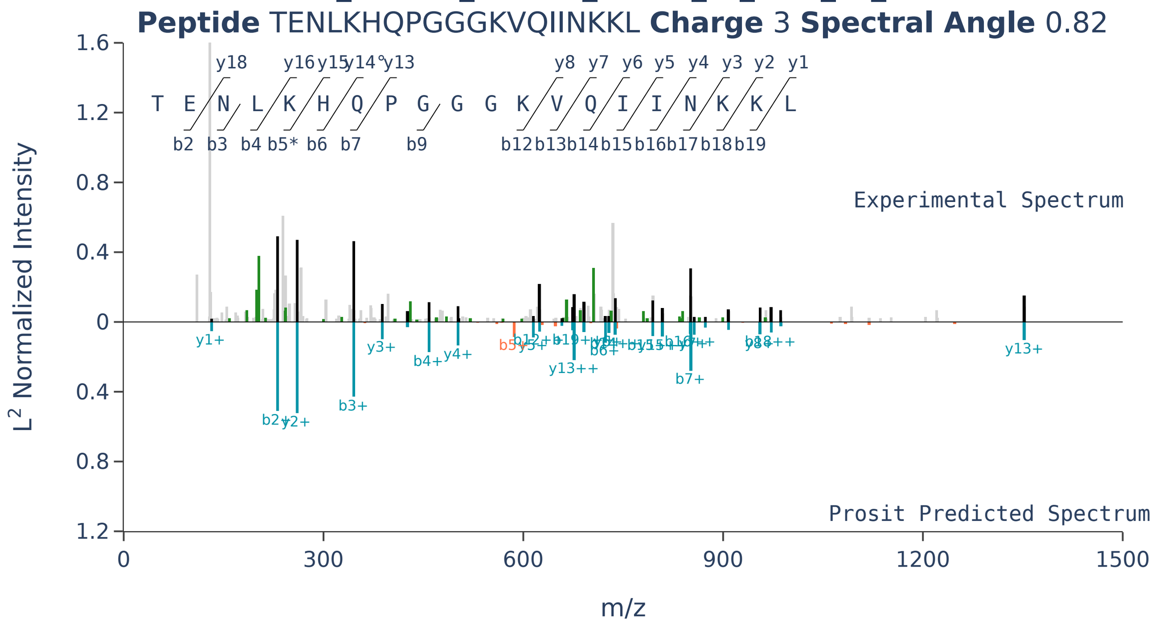

**Source** WSoh_221121_011221_HFGoe_L1_hTau_24h_R1 **Scan** 24160
**Peptide** PTAEDVTAPLVD **Charge** 1 **Spectral Angle** 0.83

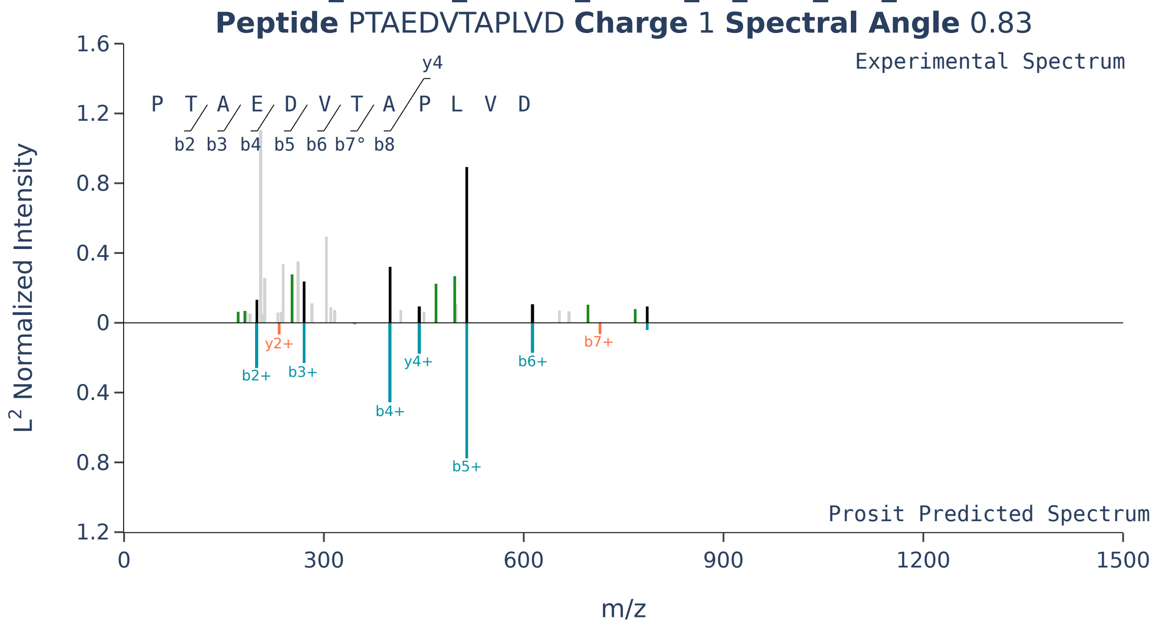


**Source** WSoh_221121_011221_HFGoe_L1_hTau_4h_R1 **Scan** 21024
**Peptide** AEPRQEFEVMEDHAGTYGLGDR **Charge** 4 **Spectral Angle** 0.85

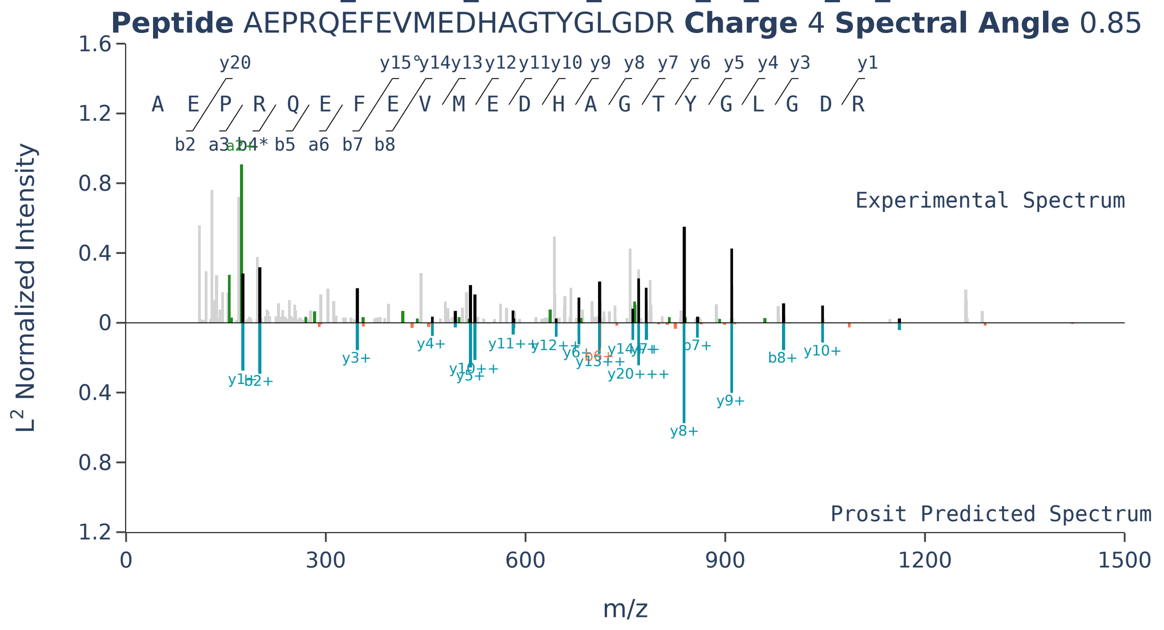


**Source** WSoh_221121_011221_HFGoe_L1_hTau_24h_R2 **Scan** 18305
**Peptide** AGLGDTPSLEDEAAGHVTQARMVSKSK **Charge** 5 **Spectral Angle** 0.82

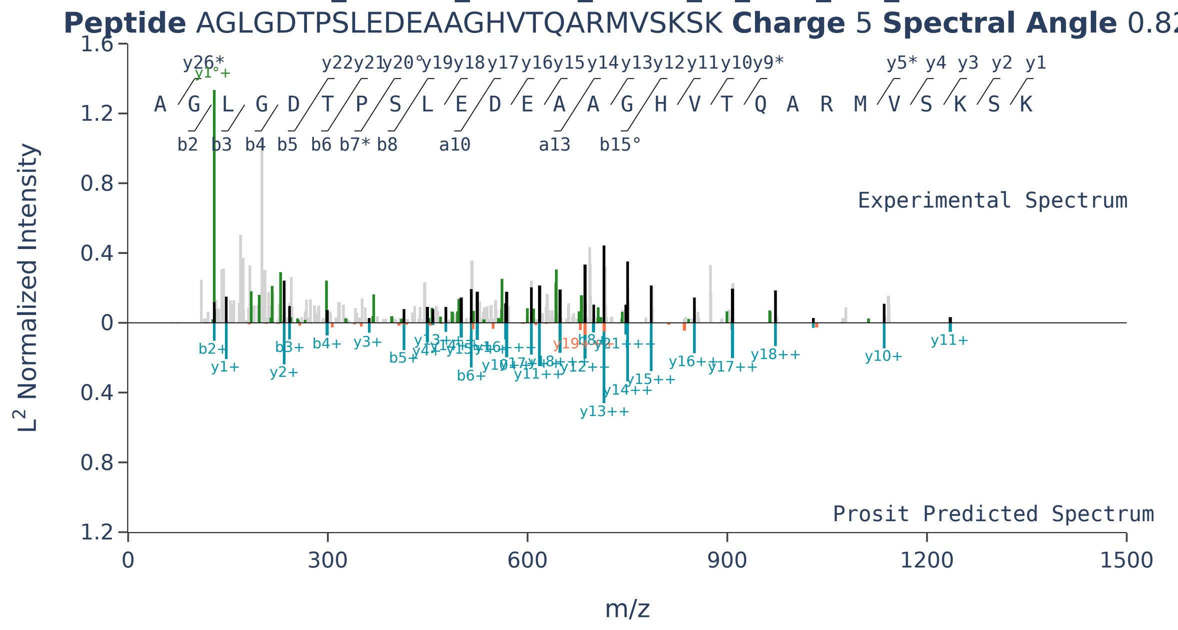


**Source** WSoh_221121_011221_HFGoe_L1_hTau_24h_R1 **Scan** 7496
**Peptide** DGKTKIATPRGAAPPGQKGQ **Charge** 3 **Spectral Angle** 0.84

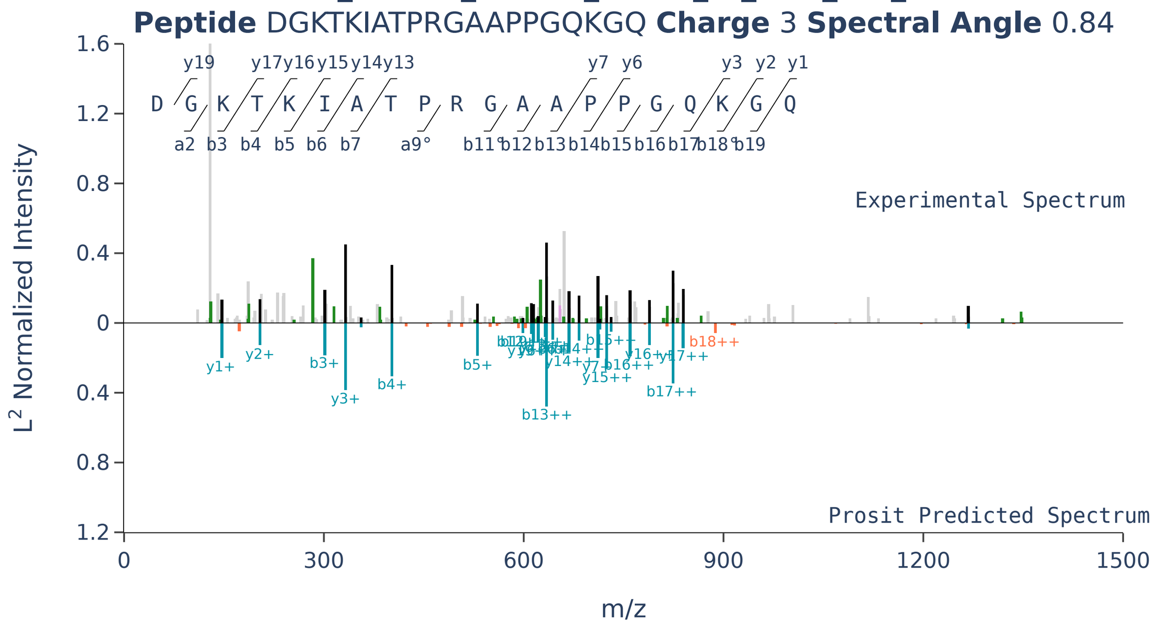


**Source** WSoh_221121_011221_HFGoe_L1_hTau_24h_R2 **Scan** 8592
**Peptide** KLGSTENLKHQPGGGKVQIINKK **Charge** 5 **Spectral Angle** 0.84

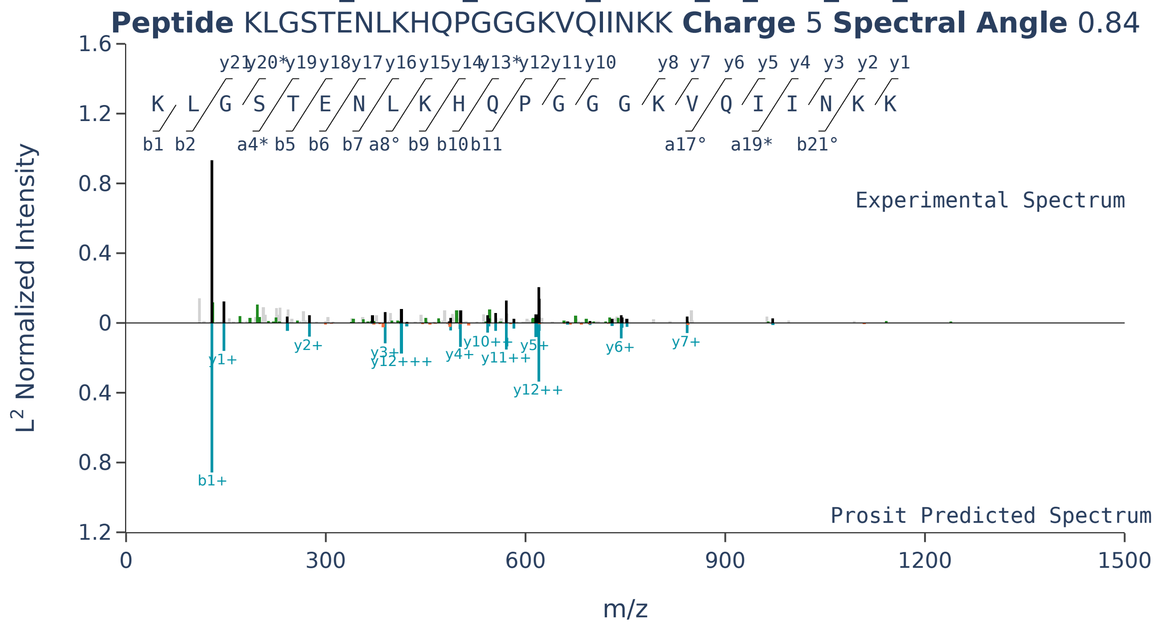


**Source** WSoh_221121_011221_HFGoe_L2_hTau_24h_R1 **Scan** 22362
**Peptide** PGGGSVQIVYKPVDLSKVTSK **Charge** 3 **Spectral Angle** 0.84

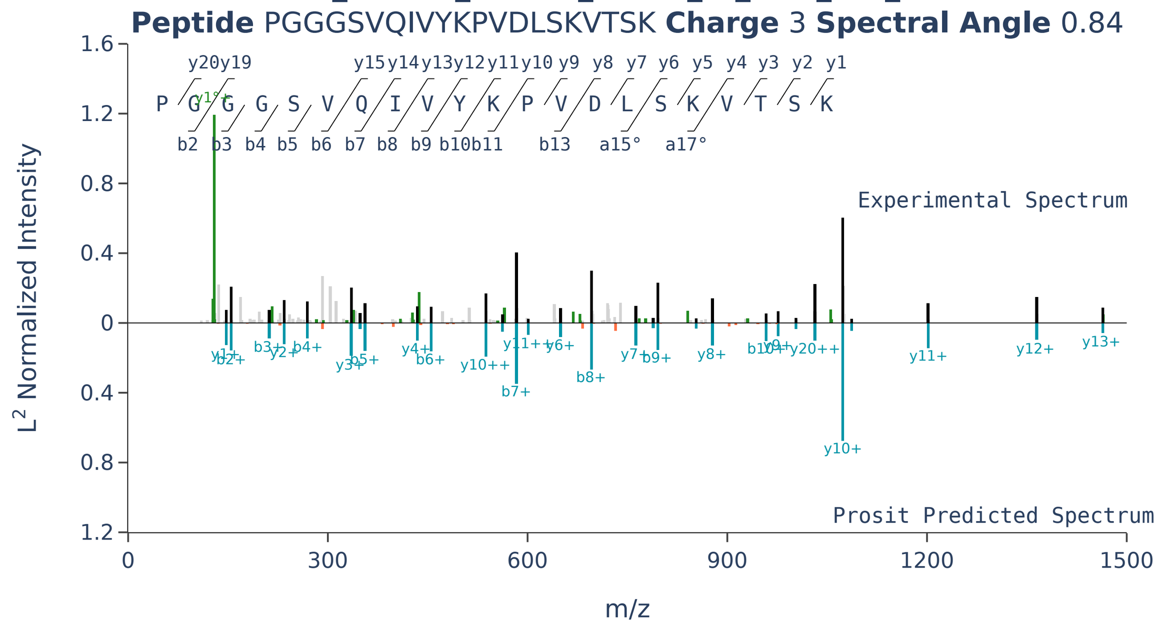


**Source** WSoh_221121_011221_HFGoe_L2_hTau_24h_R1 **Scan** 8604
**Peptide** NLKHVPGGGSV **Charge** 2 **Spectral Angle** 0.9

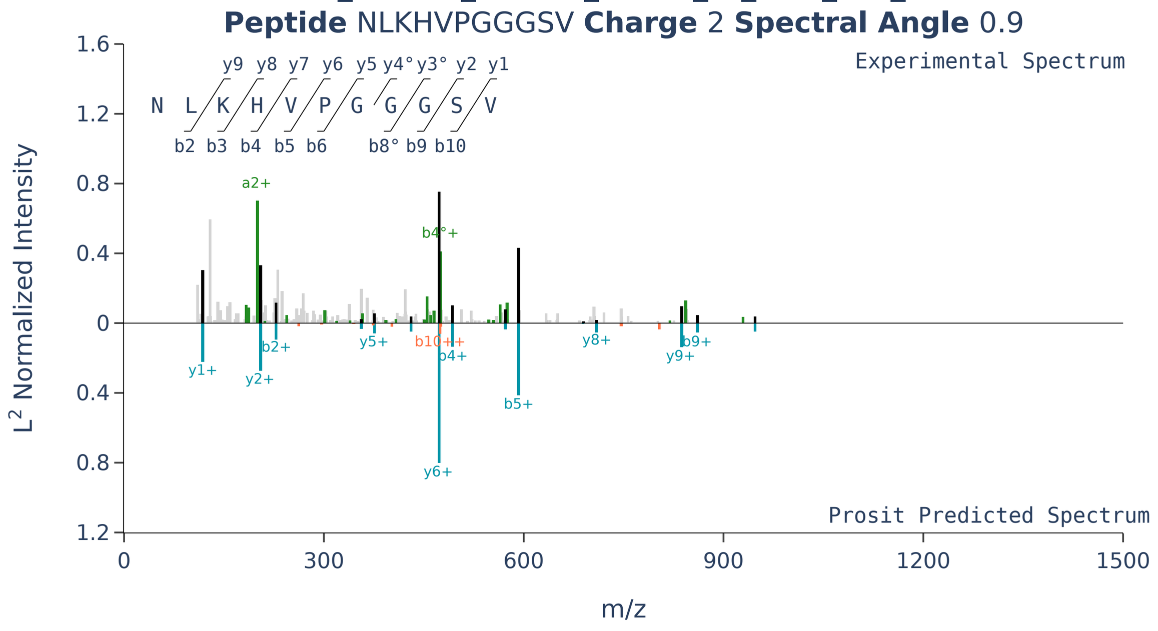


**Source** WSoh_221121_011221_HFGoe_L2_hTau_1h_R1 **Scan** 5600
**Peptide** GNIHHKPGGGQVEVKSE **Charge** 3 **Spectral Angle** 0.78

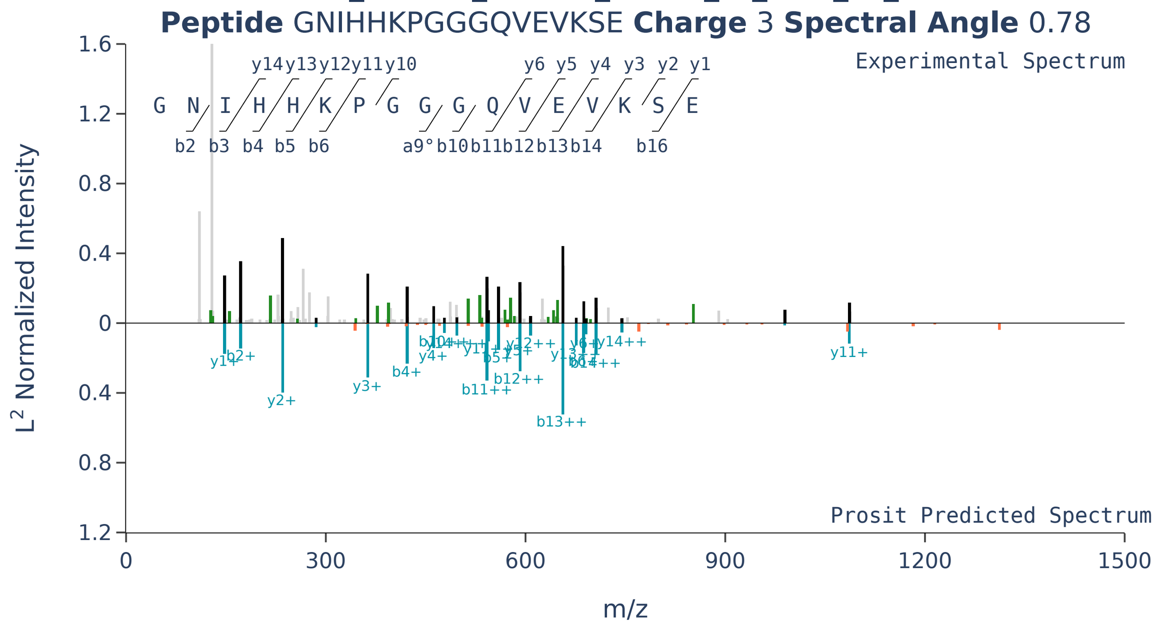


**Source** WSoh_221121_011221_HFGoe_L2_hTau_4h_R2 **Scan** 6509
**Peptide** KLGSTENLKHQPGGGKVQ **Charge** 3 **Spectral Angle** 0.89

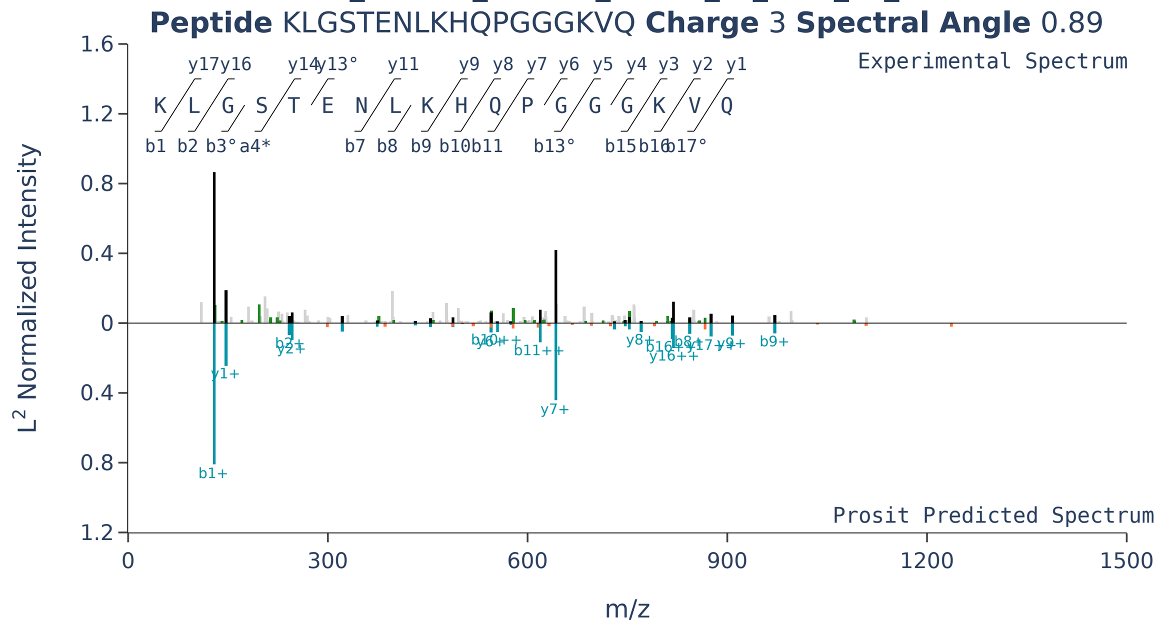


**Source** WSoh_221121_011221_HFGoe_L2_hTau_24h_R2 **Scan** 15393
**Peptide** DNITHVPGGGNKKIETHKLTF **Charge** 4 **Spectral Angle** 0.88

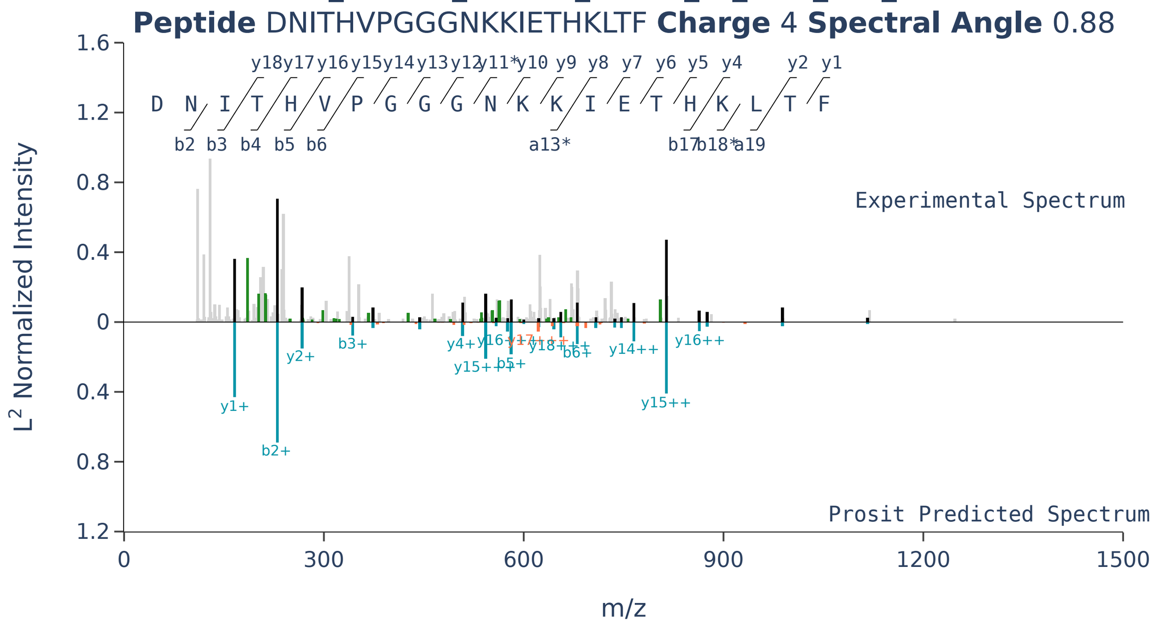


**Source** WSoh_221121_011221_HFGoe_L2_hTau_4h_R2 **Scan** 5615
**Peptide** IHHKPGGGQVEVKSE **Charge** 3 **Spectral Angle** 0.88

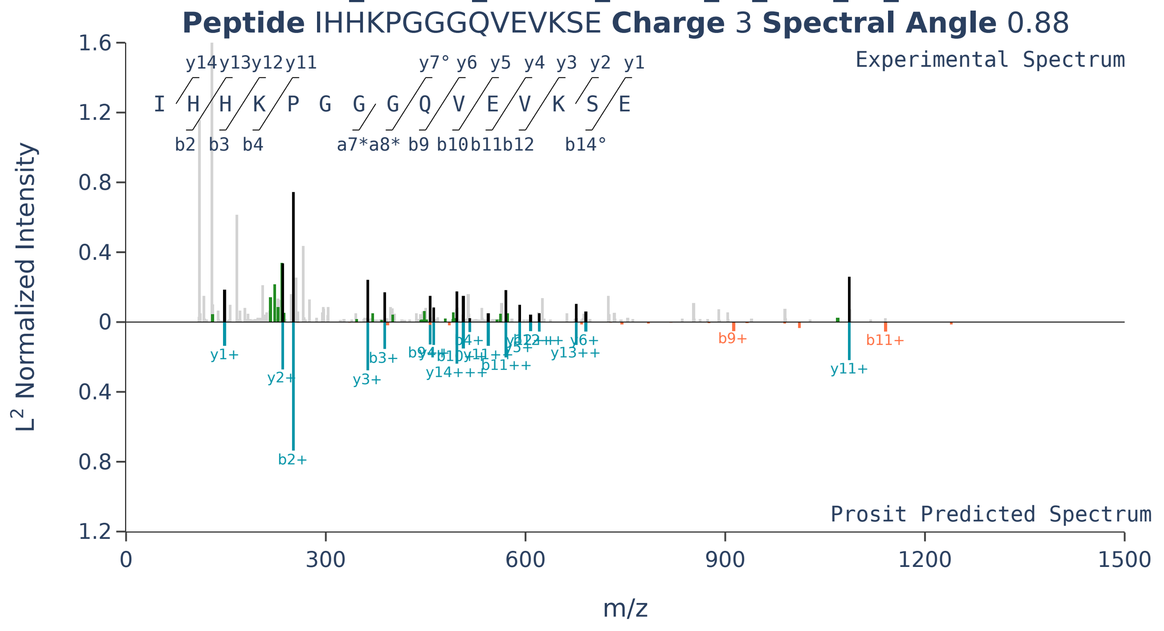


**Source** WSoh_221121_011221_HFGoe_L1_hTau_24h_R2 **Scan** 15831
**Peptide** SLDNITHVPGGGNKKIETHKLTFREN **Charge** 4 **Spectral Angle** 0.79

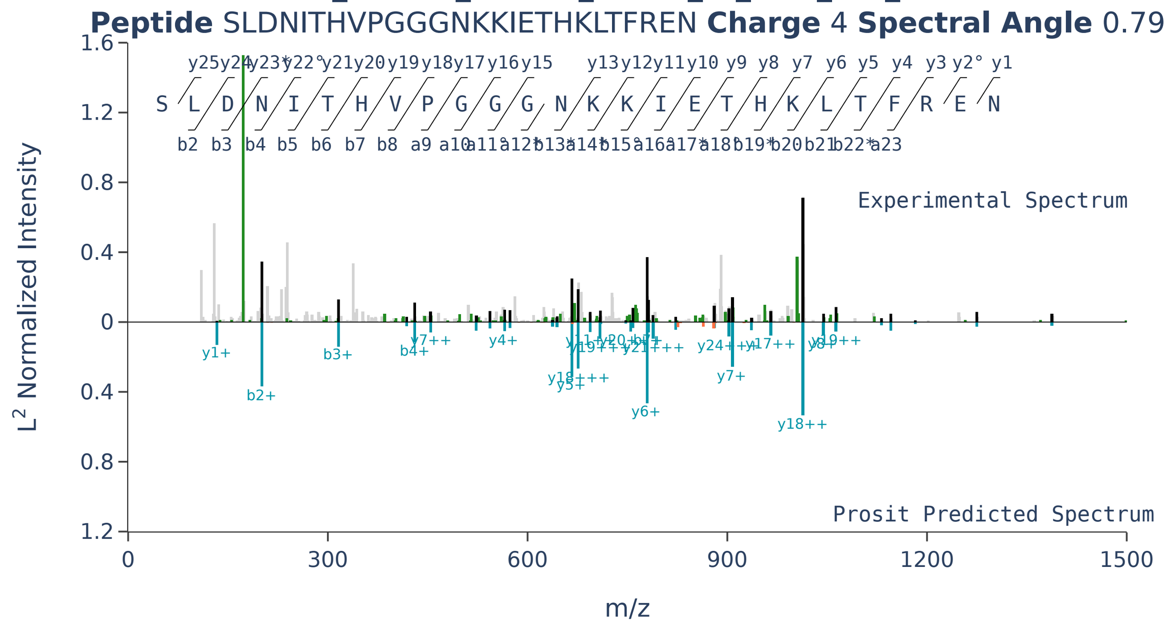


**Source** WSoh_221121_011221_HFGoe_L2_hTau_24h_R1 **Scan** 17729
**Peptide** KSEKLDFKDRVQSKIGSL **Charge** 3 **Spectral Angle** 0.84

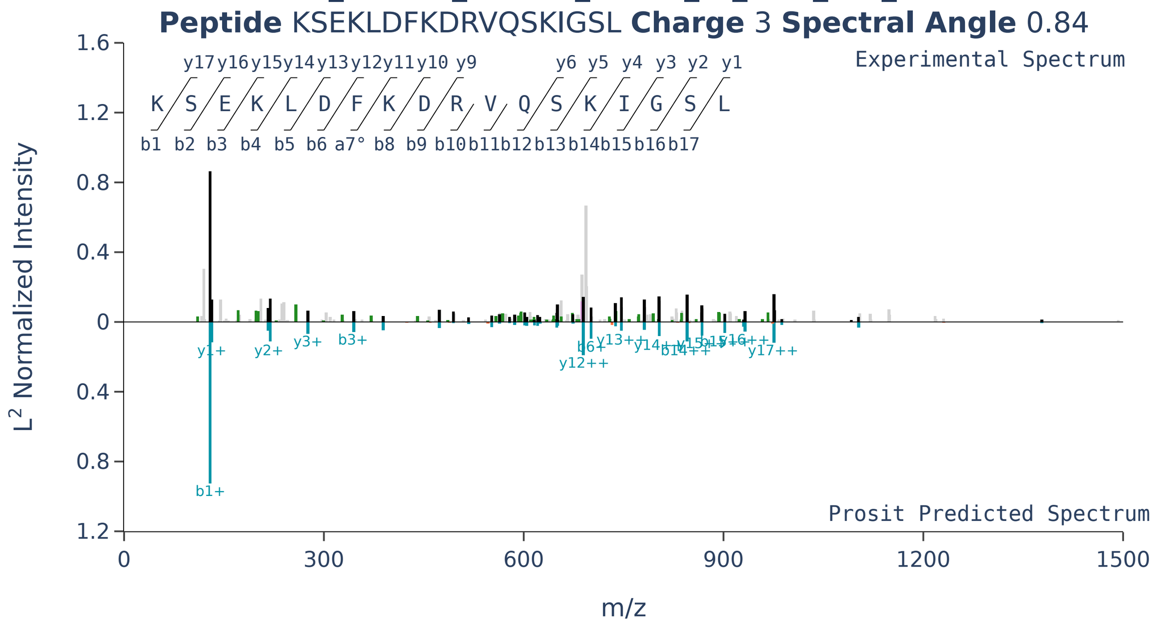


**Source** WSoh_221121_011221_HFGoe_L1_hTau_24h_R2 **Scan** 12396
**Peptide** KPVDLSKVT **Charge** 2 **Spectral Angle** 0.8

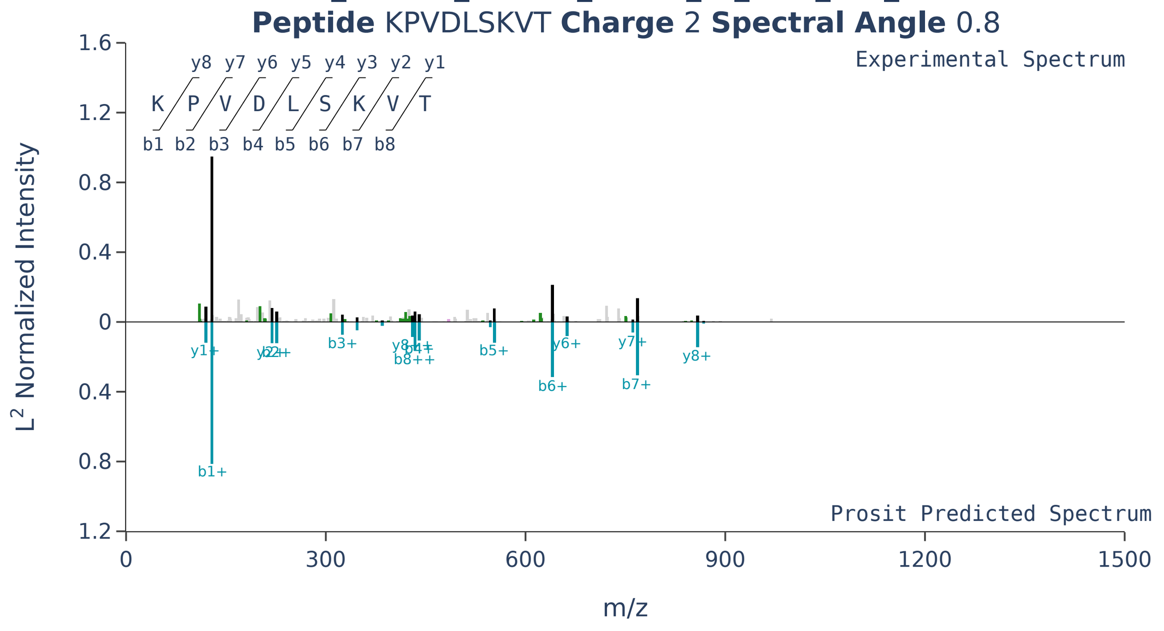


**Source** WSoh_221121_011221_HFGoe_L1_hTau_24h_R1 **Scan** 20839
**Peptide** DNIKHVPGGGSVQIVY **Charge** 3 **Spectral Angle** 0.81

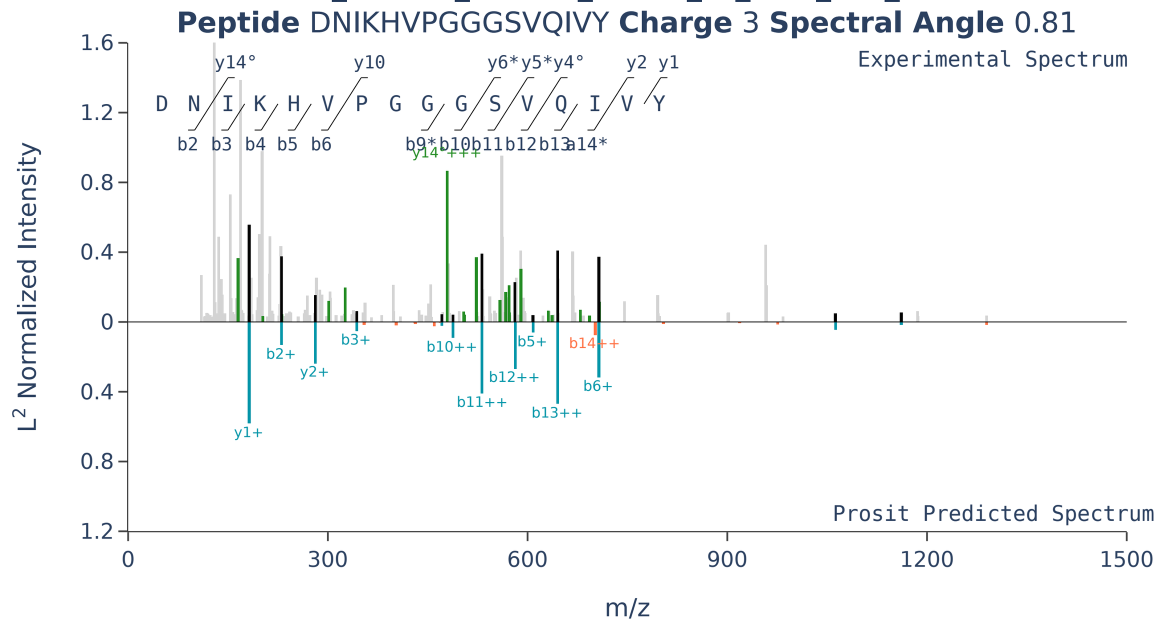

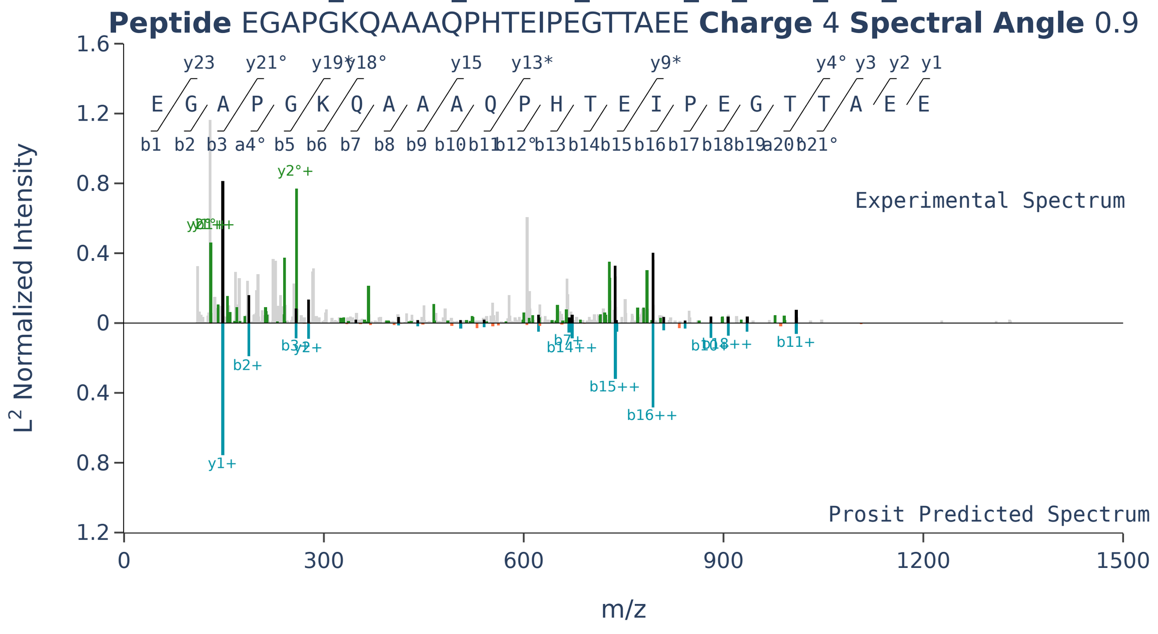

**Source** WSoh_221121_011221_HFGoe_L1_hTau_24h_R2 **Scan** 12947
**Peptide** EGAPGKQAAAQPHTEIPEGTTAEE **Charge** 4 **Spectral Angle** 0.9

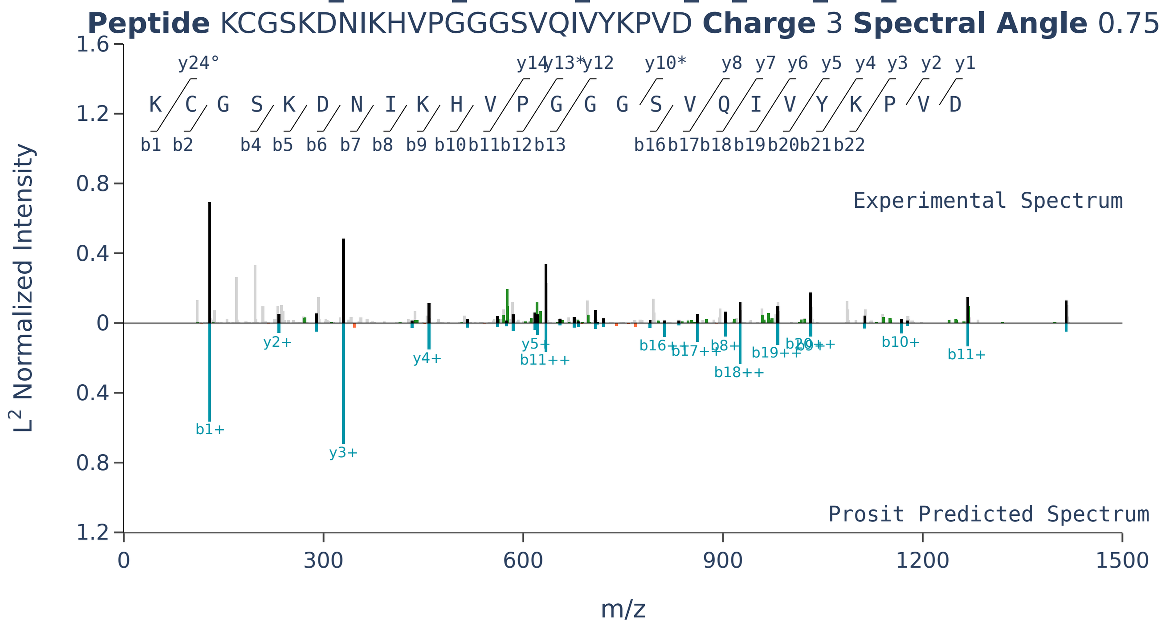

**Source** WSoh_221121_011221_HFGoe_L2_hTau_24h_R2 **Scan** 15136
**Peptide** KCGSKDNIKHVPGGGSVQIVYKPVD **Charge** 3 **Spectral Angle** 0.75

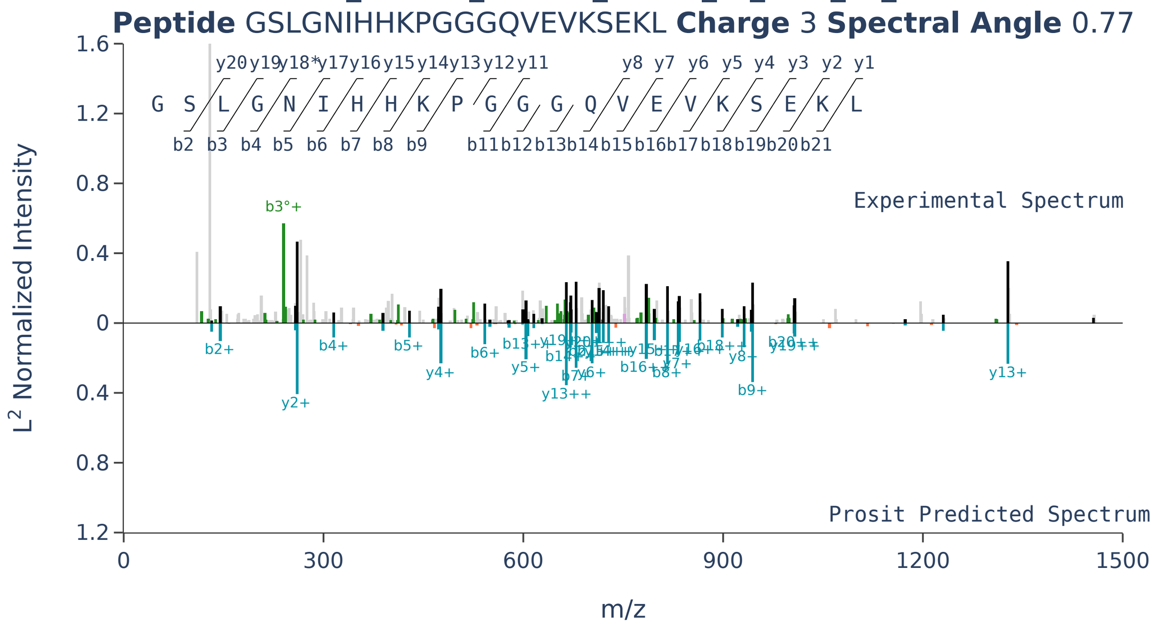

**Source** WSoh_221121_011221_HFGoe_L1_hTau_4h_R1 **Scan** 9614
**Peptide** GSLGNIHHKPGGGQVEVKSEKL **Charge** 3 **Spectral Angle** 0.77

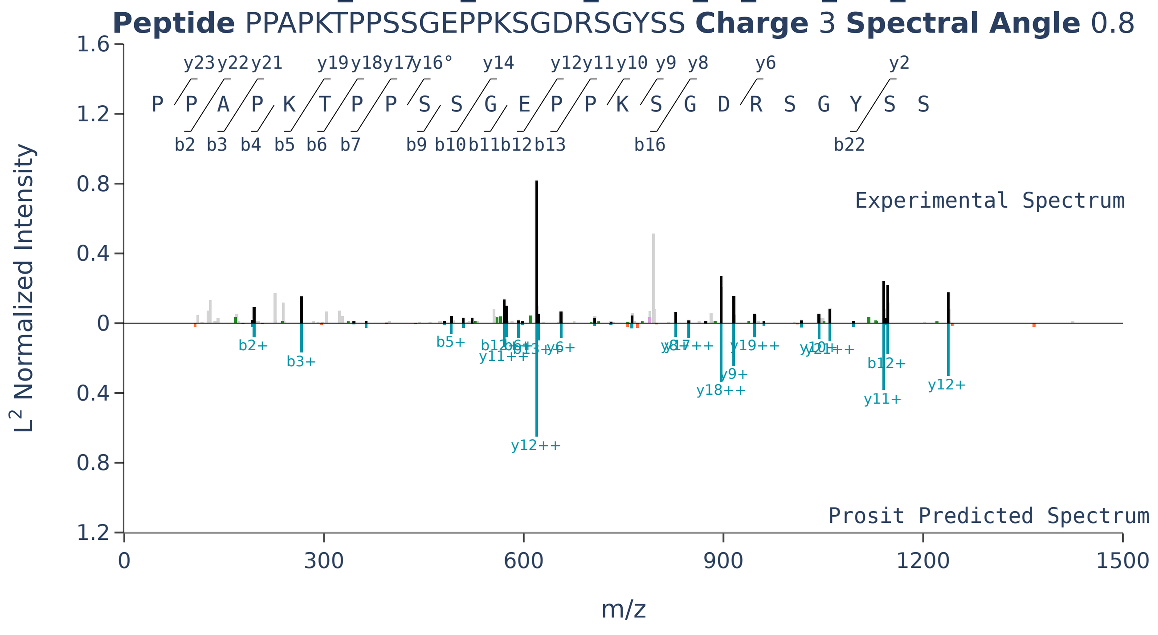

**Source** WSoh_221121_011221_HFGoe_L2_hTau_24h_R2 **Scan** 8234
**Peptide** PPAPKTPPSSGEPPKSGDRSGYSS **Charge** 3 **Spectral Angle** 0.8

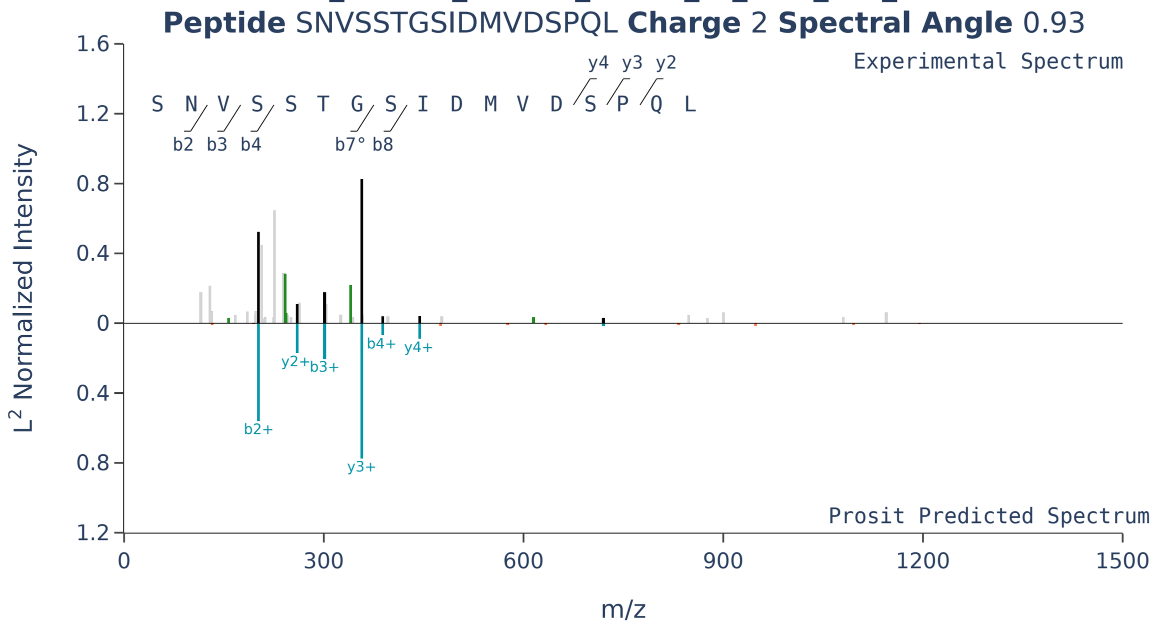

**Source** WSoh_221121_011221_HFGoe_L2_hTau_24h_R1 **Scan** 23298
**Peptide** SNVSSTGSIDMVDSPQL **Charge** 2 **Spectral Angle** 0.93

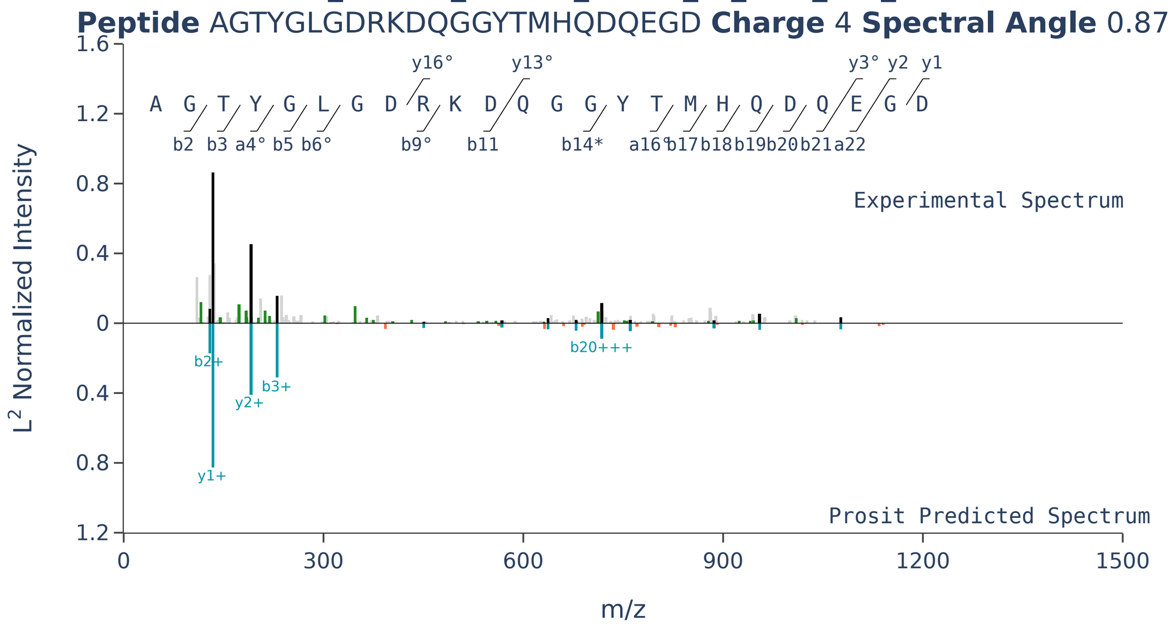

**Source** WSoh_221121_011221_HFGoe_L1_hTau_24h_R2 **Scan** 12328
**Peptide** AGTYGLGDRKDQGGYTMHQDQEGD **Charge** 4 **Spectral Angle** 0.87

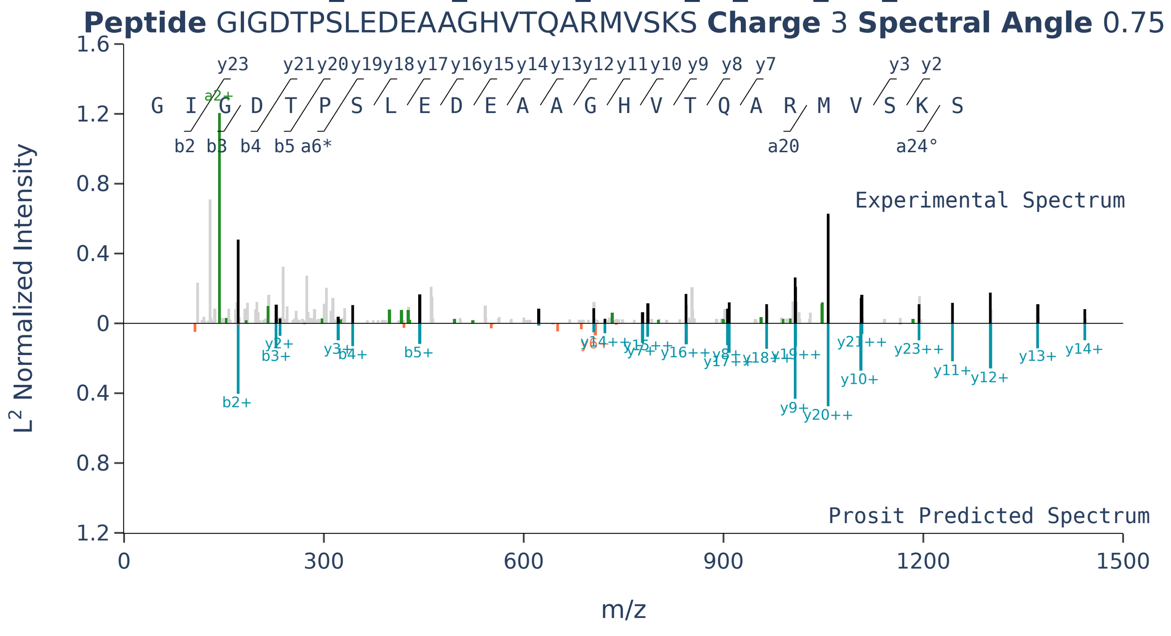

**Source** WSoh_221121_011221_HFGoe_L1_hTau_24h_R2 **Scan** 19850
**Peptide** GIGDTPSLEDEAAGHVTQARMVSKS **Charge** 3 **Spectral Angle** 0.75

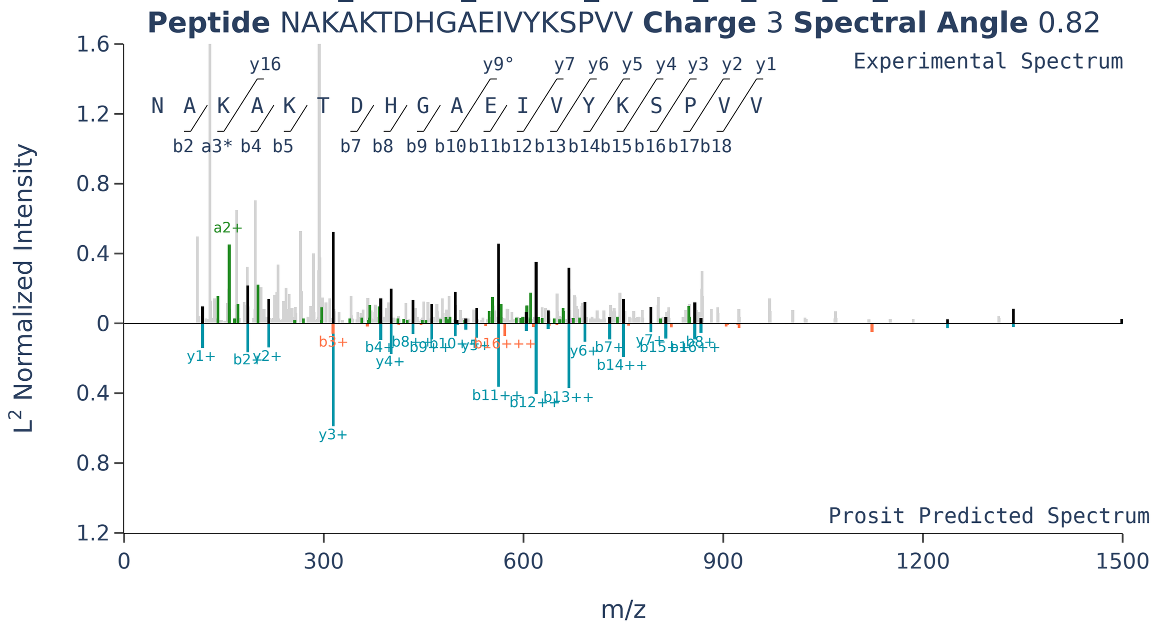

**Source** WSoh_221121_011221_HFGoe_L2_hTau_4h_R1 **Scan** 13582
**Peptide** NAKAKTDHGAEIVYKSPVV **Charge** 3 **Spectral Angle** 0.82

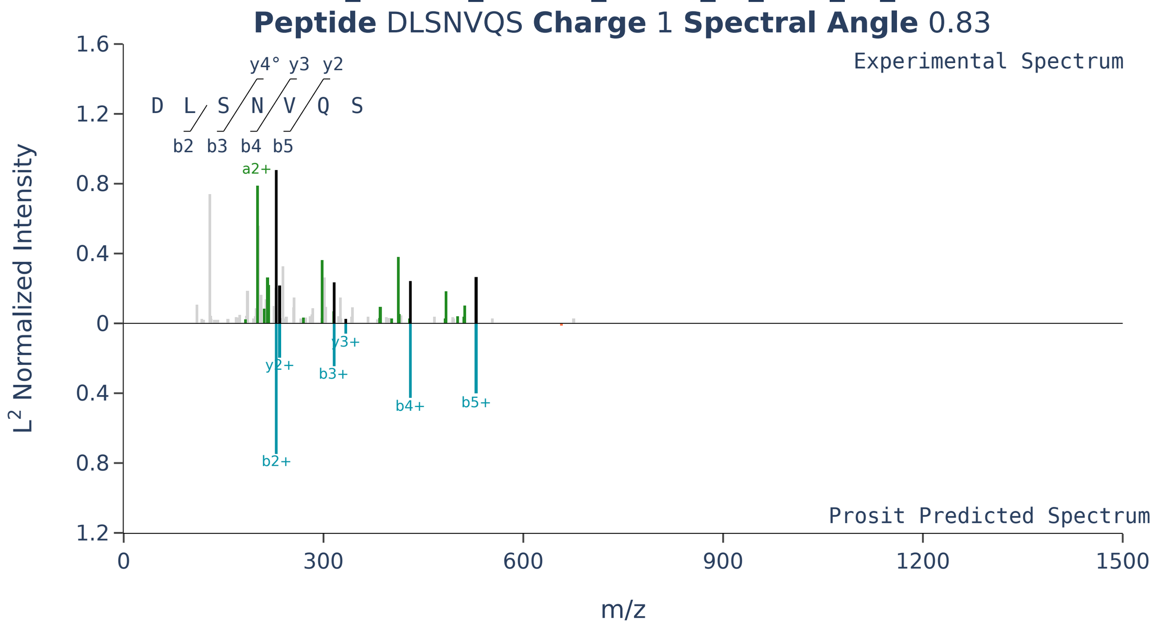

**Source** WSoh_221121_011221_HFGoe_L2_hTau_4h_R2 **Scan** 9989
**Peptide** DLSNVQS **Charge** 1 **Spectral Angle** 0.83

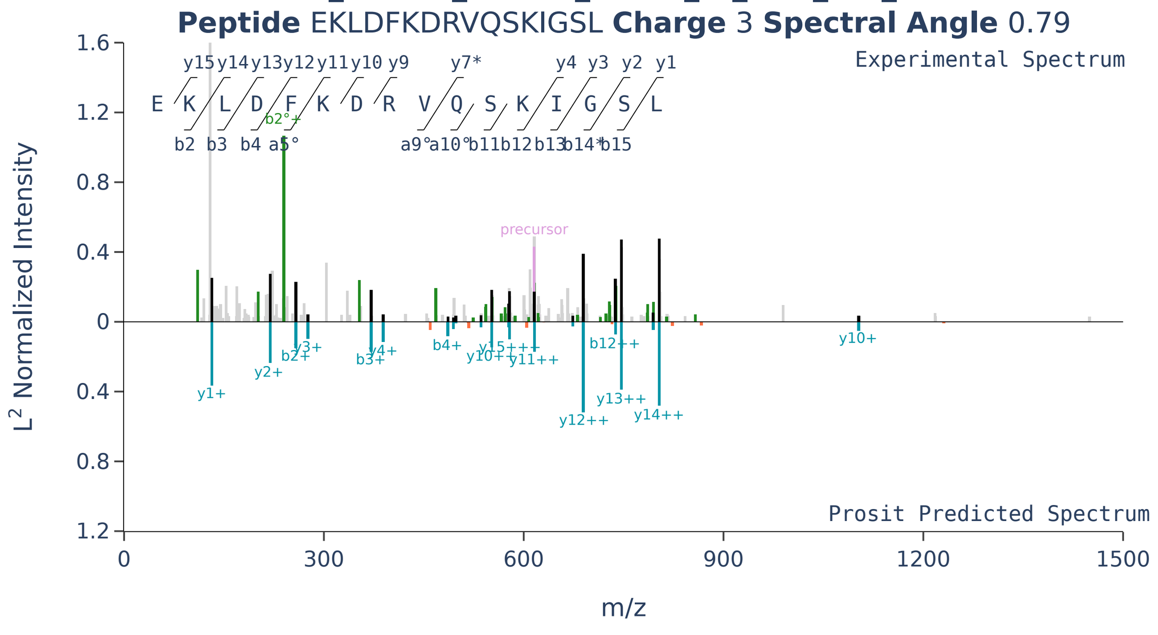

**Source** WSoh_221121_011221_HFGoe_L1_hTau_24h_R1 **Scan** 19890
**Peptide** EKLDFKDRVQSKIGSL **Charge** 3 **Spectral Angle** 0.79

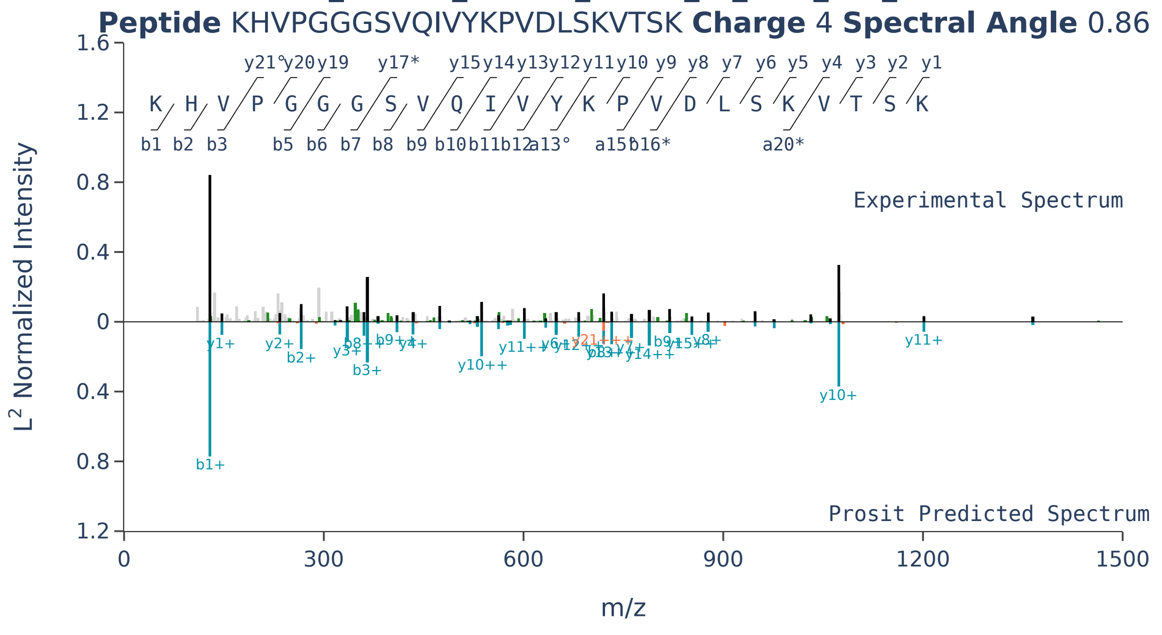

**Source** WSoh_221121_011221_HFGoe_L2_hTau_24h_R1 **Scan** 19016
**Peptide** KHVPGGGSVQIVYKPVDLSKVTSK **Charge** 4 **Spectral Angle** 0.86

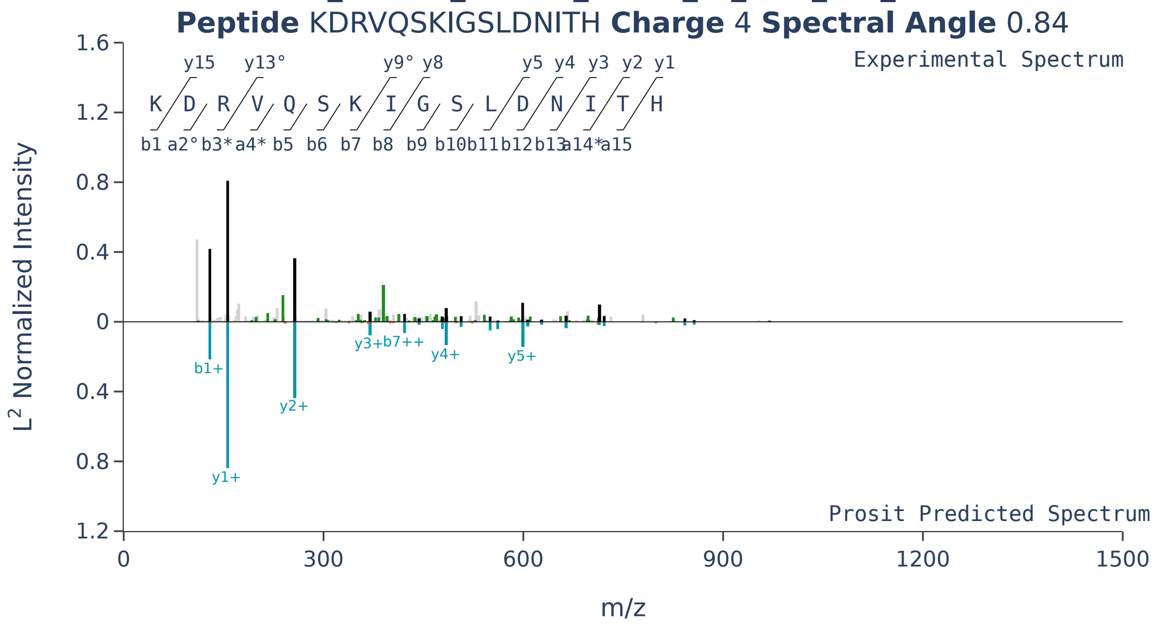

**Source** WSoh_221121_011221_HFGoe_L2_hTau_24h_R1 **Scan** 13011
**Peptide** KDRVQSKIGSLDNITH **Charge** 4 **Spectral Angle** 0.84

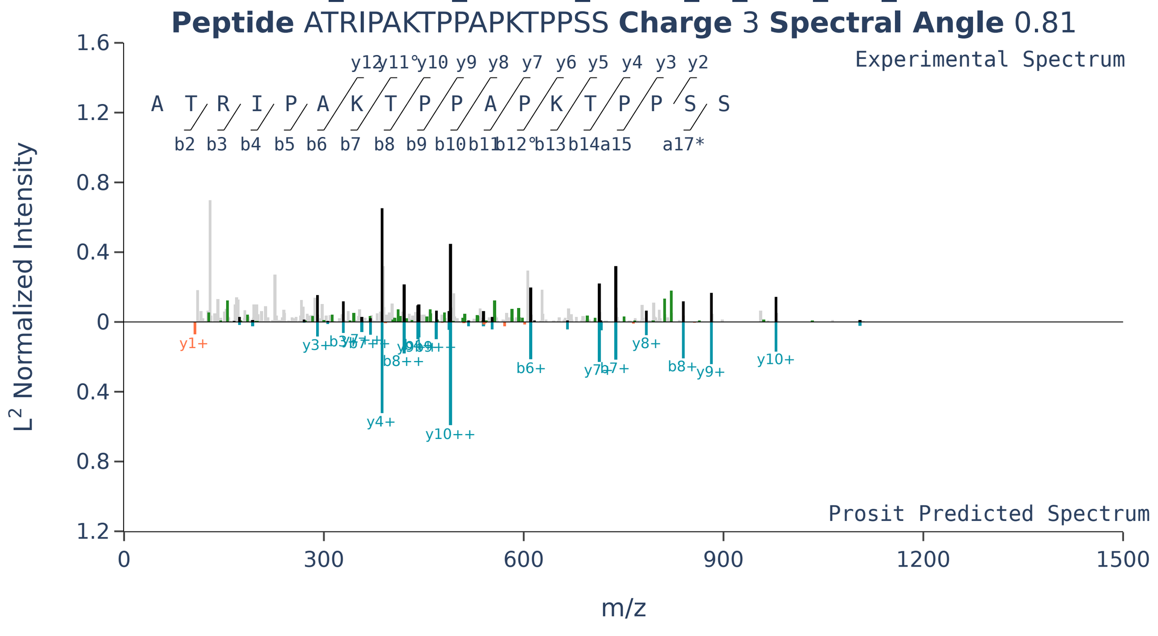

**Source** WSoh_221121_011221_HFGoe_L1_hTau_24h_R1 **Scan** 10459
**Peptide** ATRIPAKTPPAPKTPPSS **Charge** 3 **Spectral Angle** 0.81

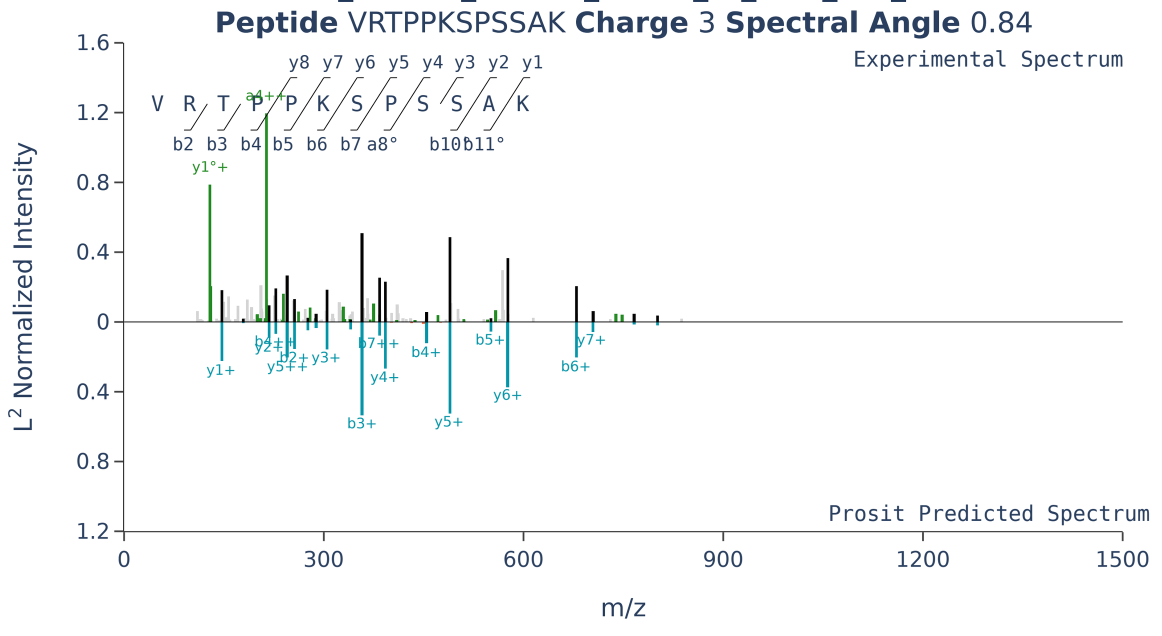

**Source** WSoh_221121_011221_HFGoe_L2_hTau_24h_R1 **Scan** 5467
**Peptide** VRTPPKSPSSAK **Charge** 3 **Spectral Angle** 0.84

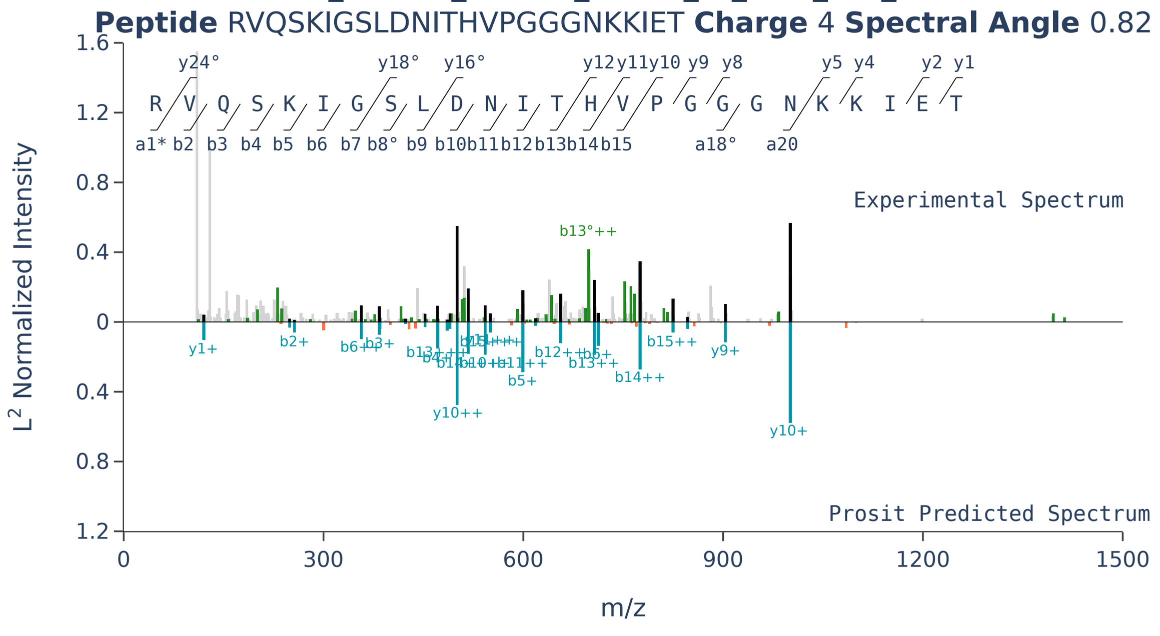

**Source** WSoh_221121_011221_HFGoe_L2_hTau_24h_R1 **Scan** 15880
**Peptide** RVQSKIGSLDNITHVPGGGNKKIET **Charge** 4 **Spectral Angle** 0.82

**Source** WSoh_221121_011221_HFGoe_L1_hTau_4h_R2 **Scan** 8312
**Peptide** AVVRTPPKSPSSAKSRLQTA **Charge** 3 **Spectral Angle** 0.73

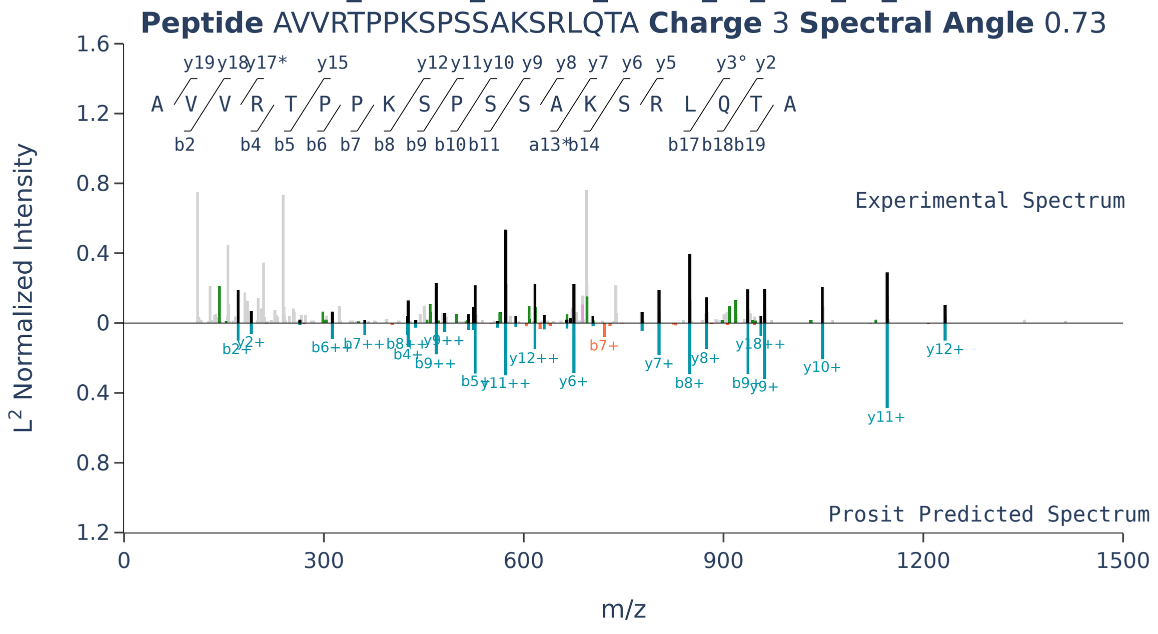


**Source** WSoh_221121_011221_HFGoe_L1_hTau_24h_R1 **Scan** 11513
**Peptide** RENAKAKTDHGAEIVYKSPVVSG **Charge** 4 **Spectral Angle** 0.83

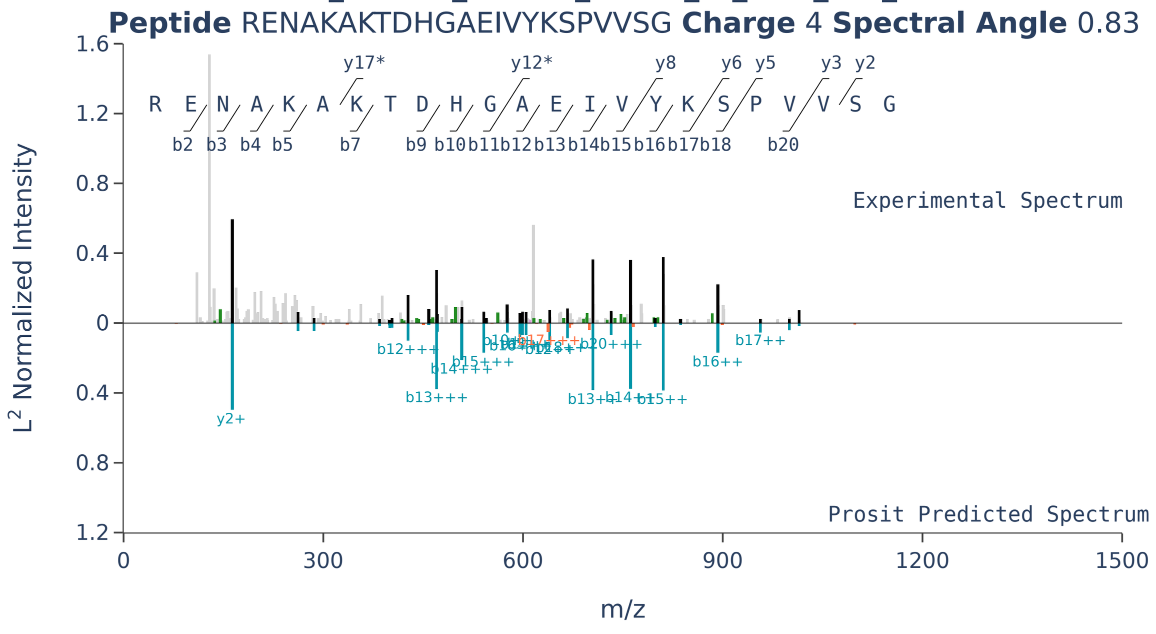


**Source** WSoh_221121_011221_HFGoe_L1_hTau_24h_R2 **Scan** 16185
**Peptide** SPVVSGDTSPRHLSNVSSTGSLD **Charge** 2 **Spectral Angle** 0.86

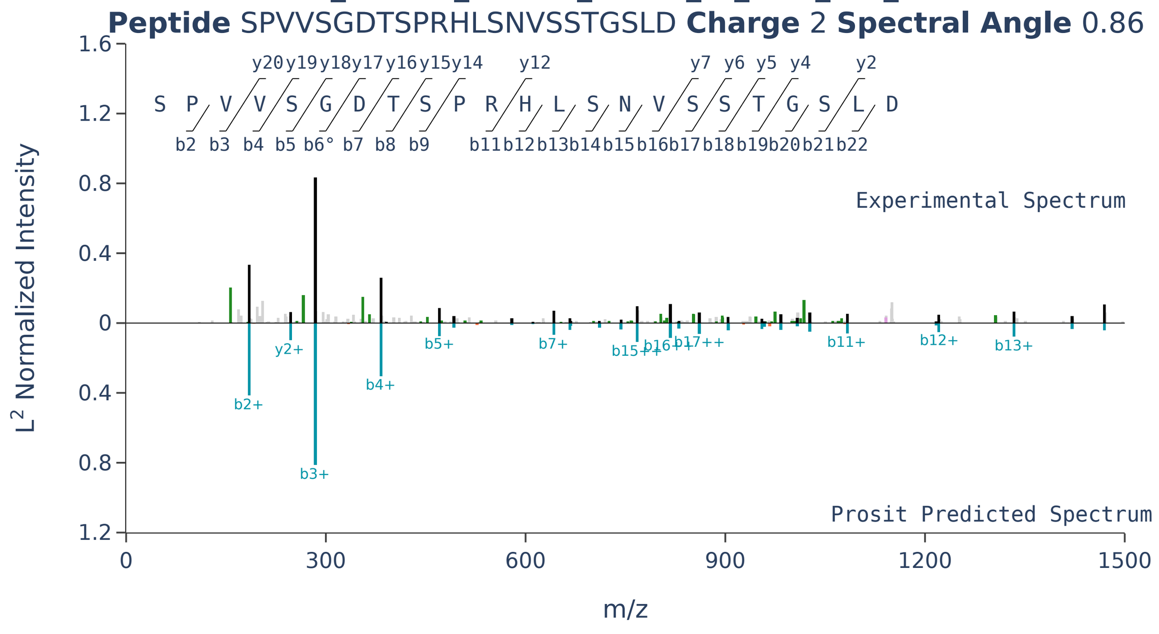


**Source** WSoh_221121_011221_HFGoe_L1_hTau_2h_R1 **Scan** 8050
**Peptide** KSKIGSTENL **Charge** 2 **Spectral Angle** 0.79

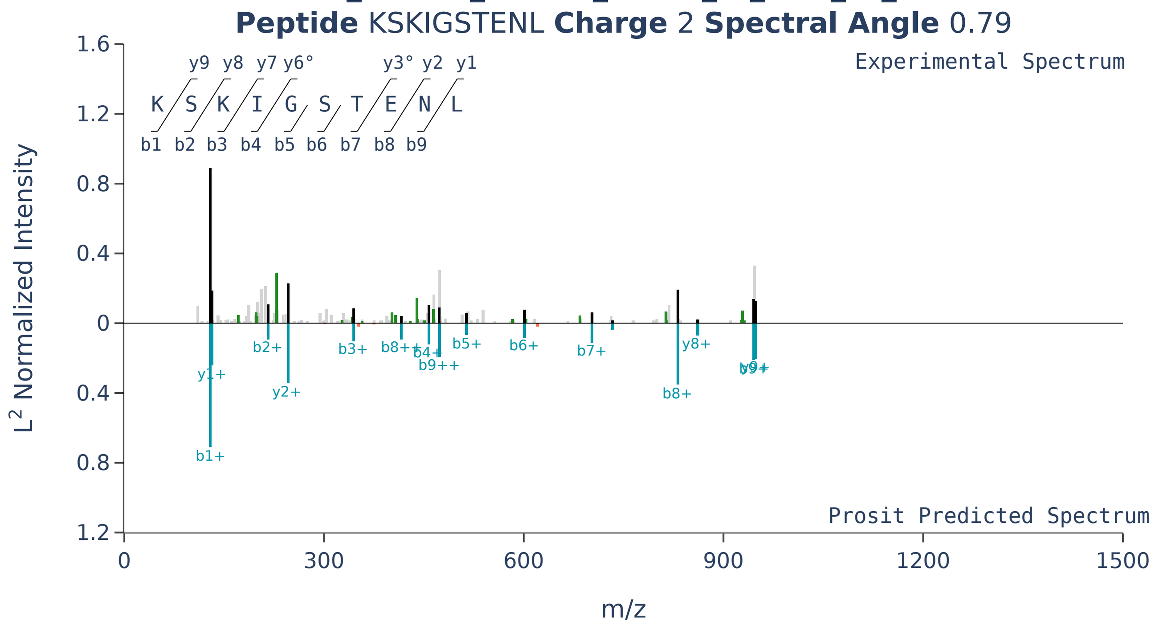


**Source** WSoh_221121_011221_HFGoe_L1_hTau_24h_R2 **Scan** 8057
**Peptide** PTPPTREPKKVAVVR **Charge** 4 **Spectral Angle** 0.79

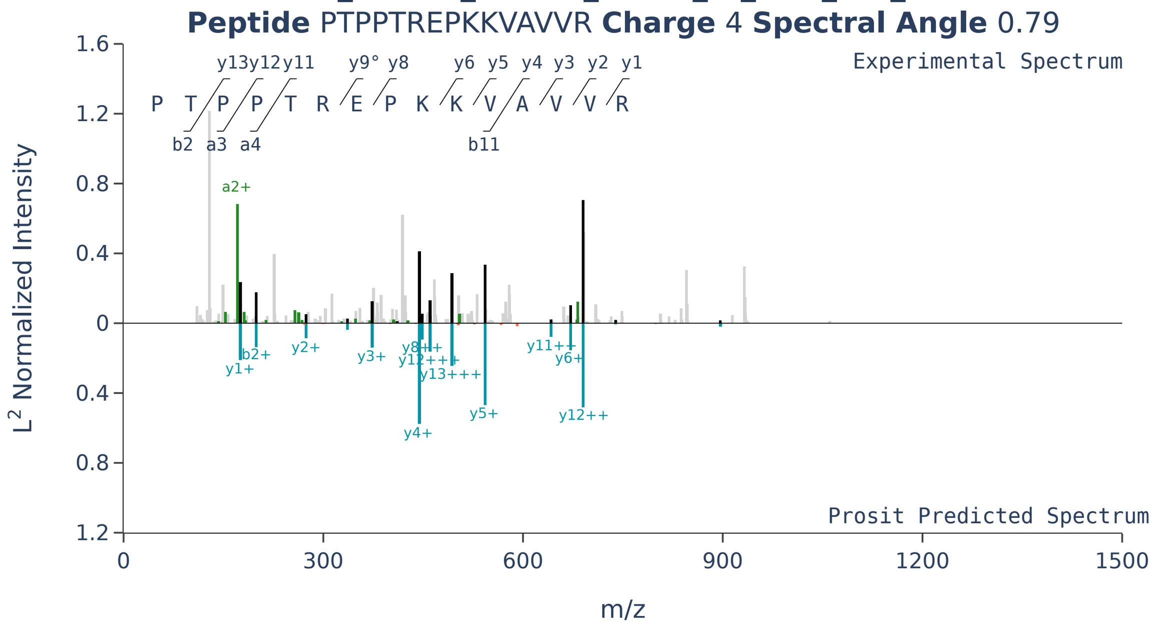


**Source** WSoh_221121_011221_HFGoe_L1_hTau_4h_R2 **Scan** 16700
**Peptide** KSPVVSGDTSPRHLSNVSSTGSIDM **Charge** 3 **Spectral Angle** 0.86

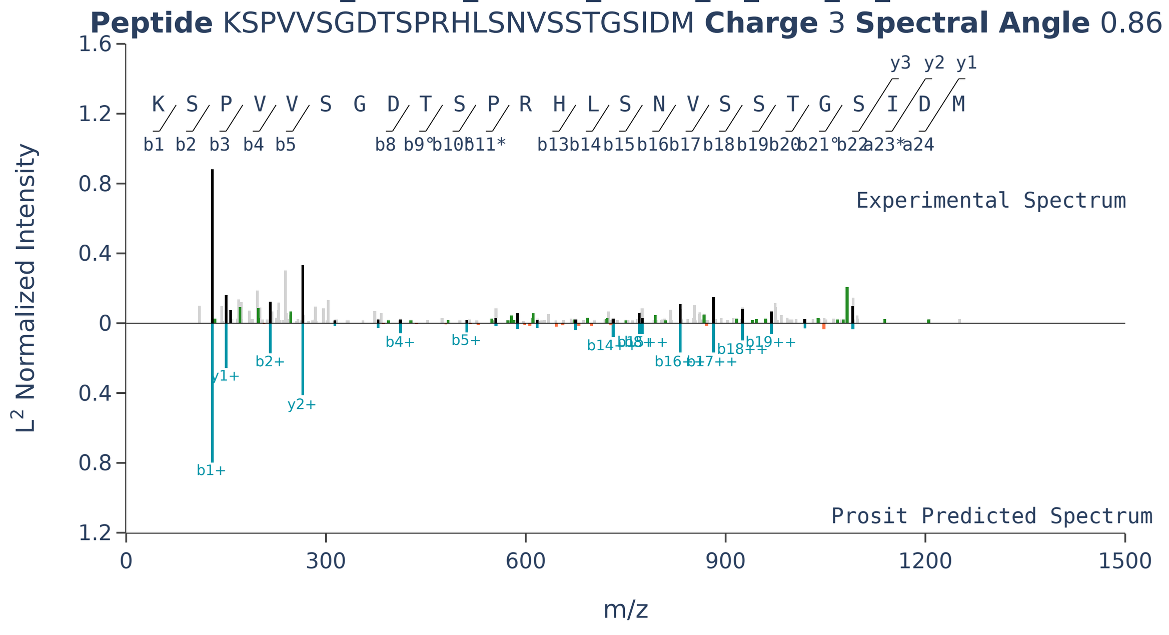


**Source** WSoh_221121_011221_HFGoe_L2_hTau_4h_R1 **Scan** 13744
**Peptide** GSLDNITHVPGGGNKKIETHKL **Charge** 5 **Spectral Angle** 0.88

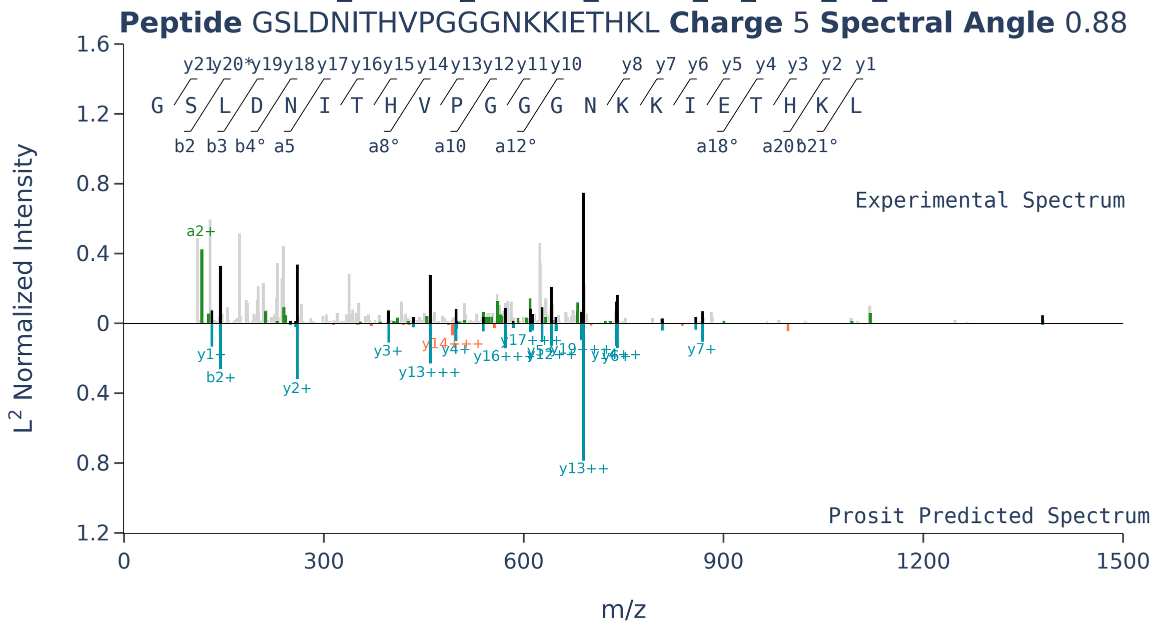


**Source** WSoh_221121_011221_HFGoe_L1_hTau_24h_R1 **Scan** 11249
**Peptide** KNVKSKIGSTENLKHQPGGGKVQIL **Charge** 4 **Spectral Angle** 0.89

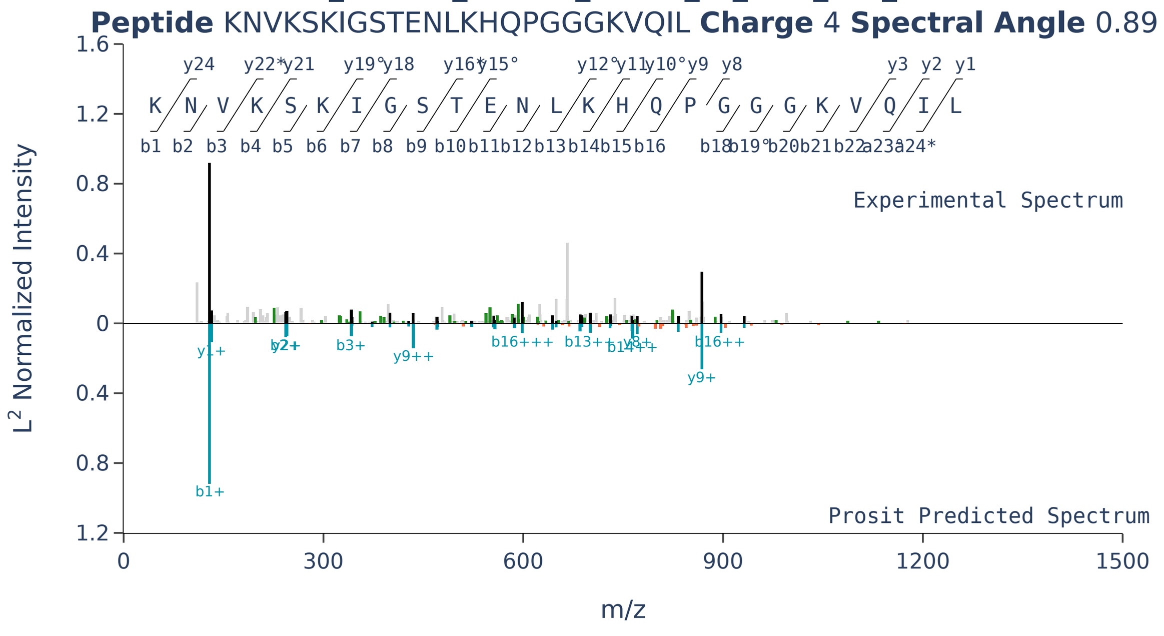


**Source** WSoh_221121_011221_HFGoe_L2_hTau_4h_R2 **Scan** 16309
**Peptide** KDNIKHVPGGGSVQIVYKPVD **Charge** 3 **Spectral Angle** 0.8

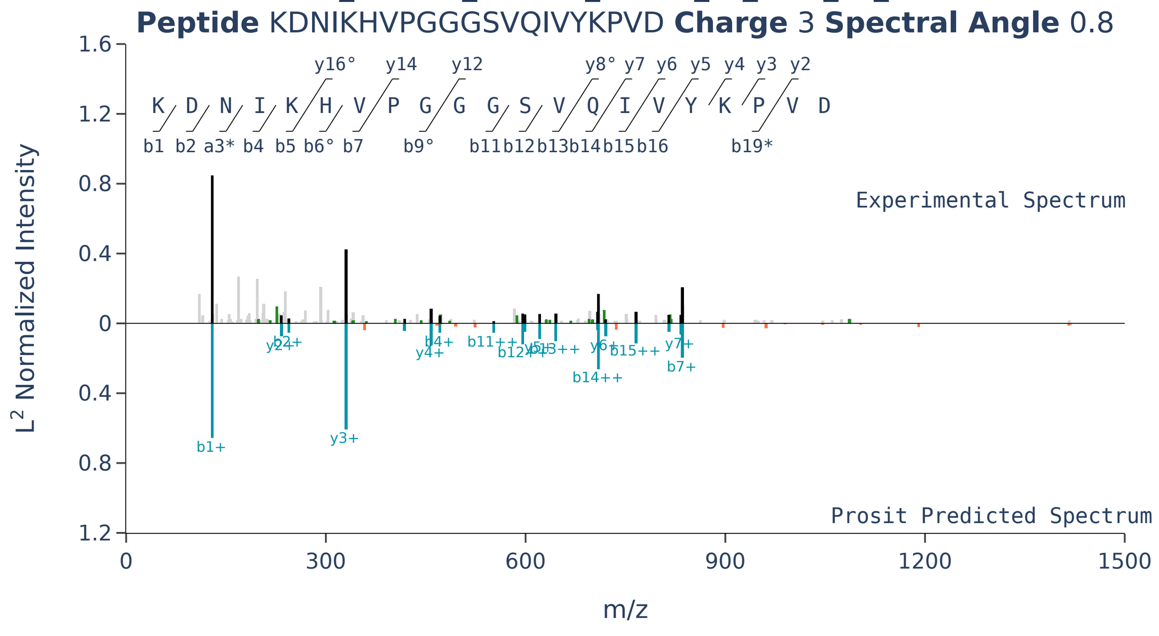


**Source** WSoh_221121_011221_HFGoe_L2_hTau_24h_R2 **Scan** 7609
**Peptide** LTFRENAKAK **Charge** 3 **Spectral Angle** 0.83

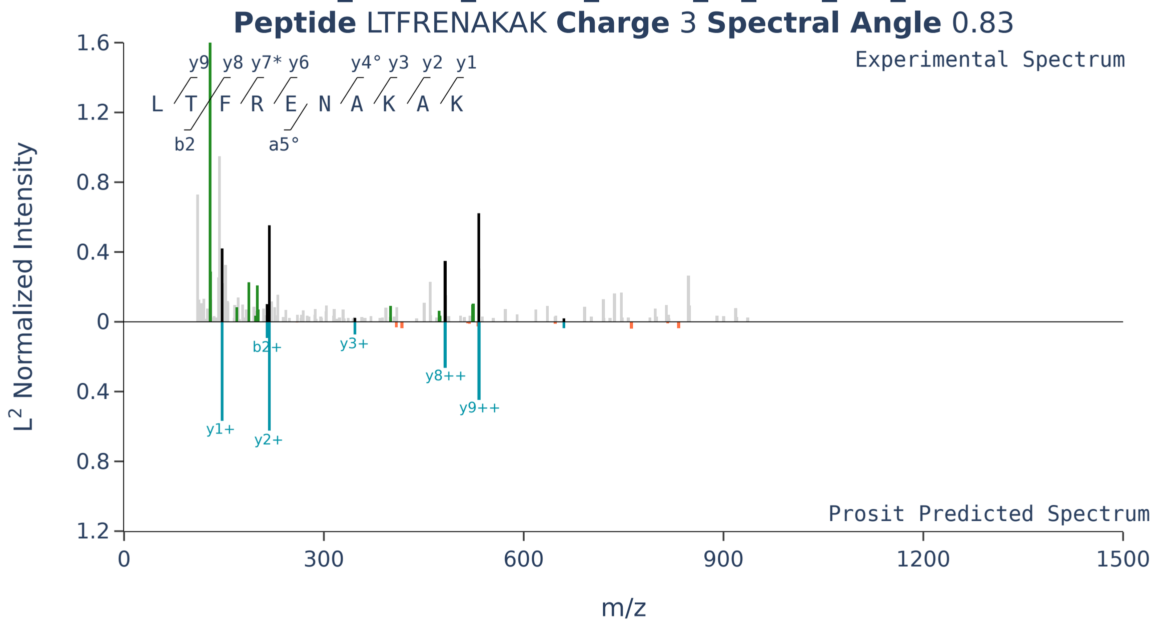


**Source** WSoh_221121_011221_HFGoe_L1_hTau_24h_R2 **Scan** 14292
**Peptide** SRTPSLPTPPTREPKKVAVVRT **Charge** 5 **Spectral Angle** 0.82

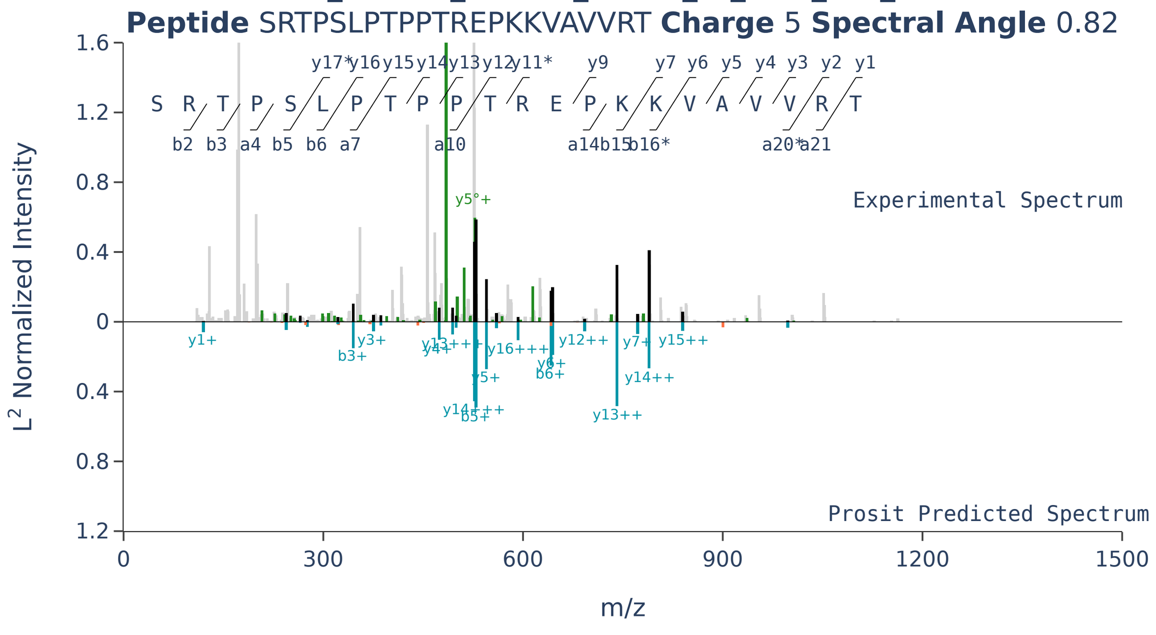


**Source** WSoh_221121_011221_HFGoe_L1_hTau_24h_R1 **Scan** 23006
**Peptide** KLDFKDRVQSKIGSLDNITHV **Charge** 4 **Spectral Angle** 0.9

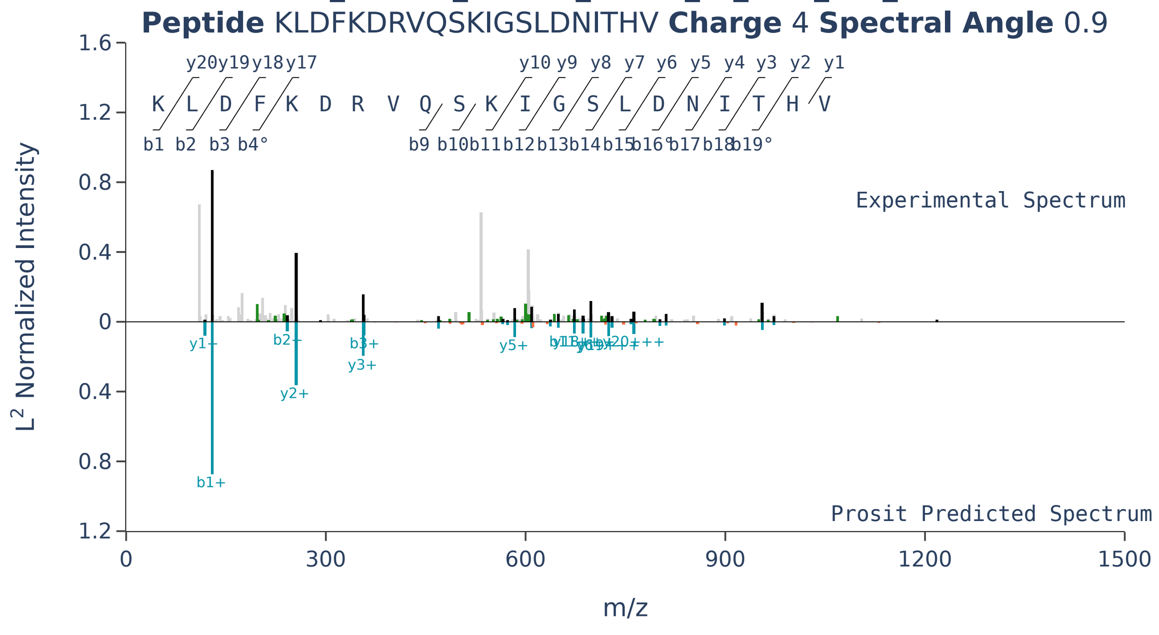


**Source** WSoh_221121_011221_HFGoe_L1_hTau_4h_R1 **Scan** 18473
**Peptide** VYKPVDLSKVTSKCGSLG **Charge** 4 **Spectral Angle** 0.88

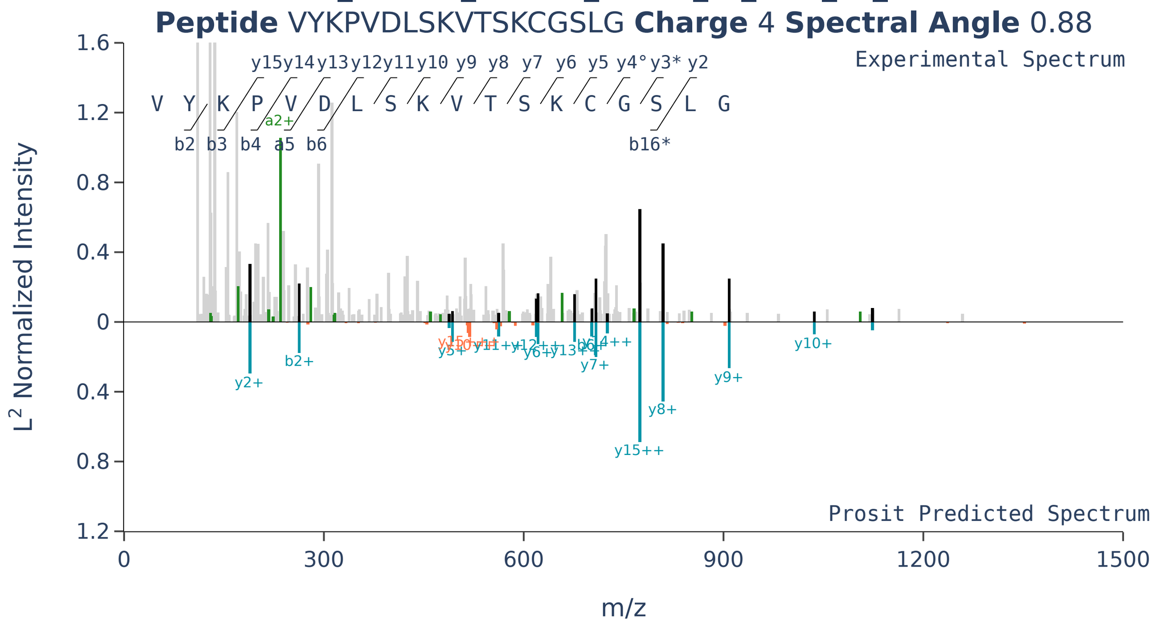


**Source** WSoh_221121_011221_HFGoe_L1_hTau_4h_R1 **Scan** 8864
**Peptide** SKVTSKCGSLGNIHHKPGGGQVEV **Charge** 3 **Spectral Angle** 0.81

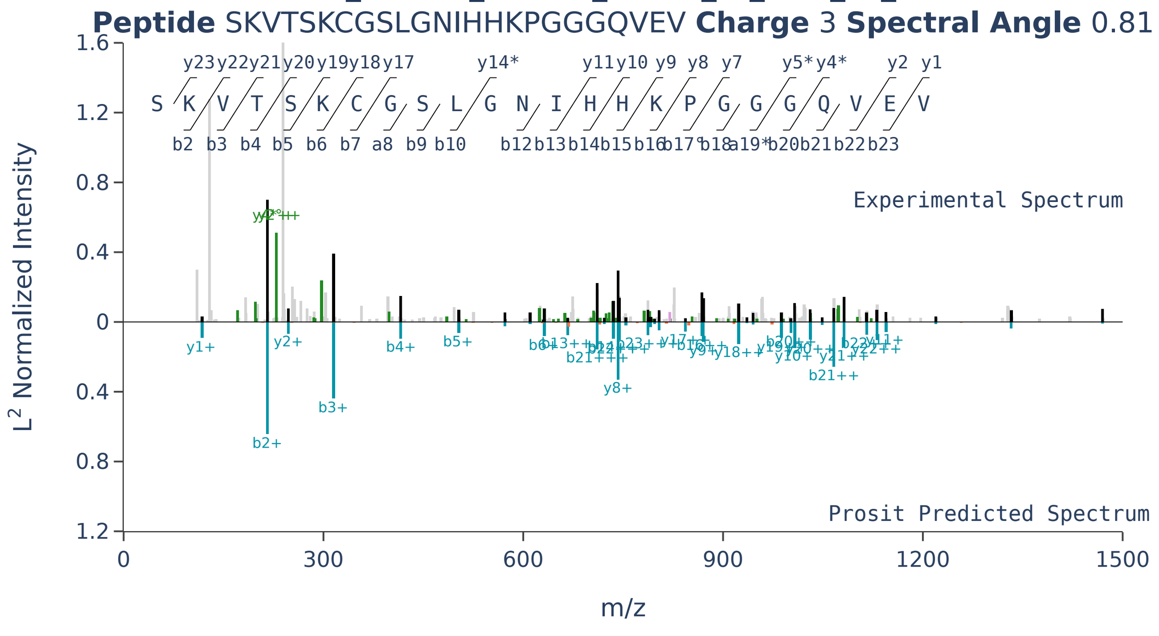


**Source** WSoh_221121_011221_HFGoe_L1_hTau_4h_R2 **Scan** 12744
**Peptide** AKAKTDHGAEIVYKSPVVSG **Charge** 3 **Spectral Angle** 0.78

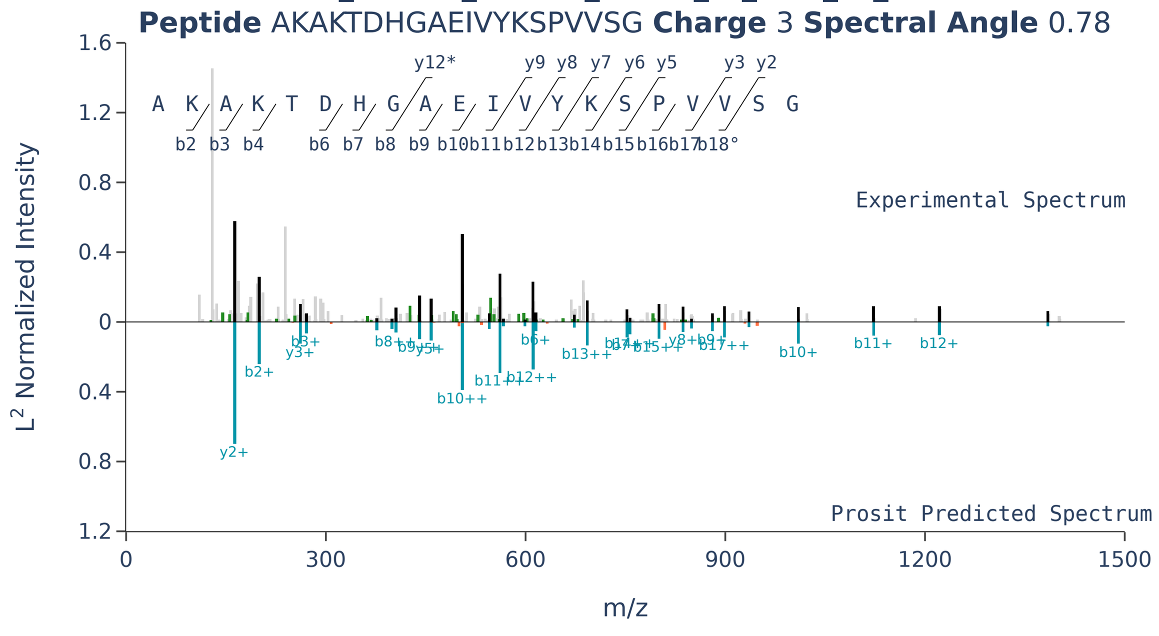

**Source** WSoh_221121_011221_HFGoe_L2_hTau_24h_R1 **Scan** 18594
**Peptide** VYKPVDLSKVTSKCGSLGN **Charge** 3 **Spectral Angle** 0.85

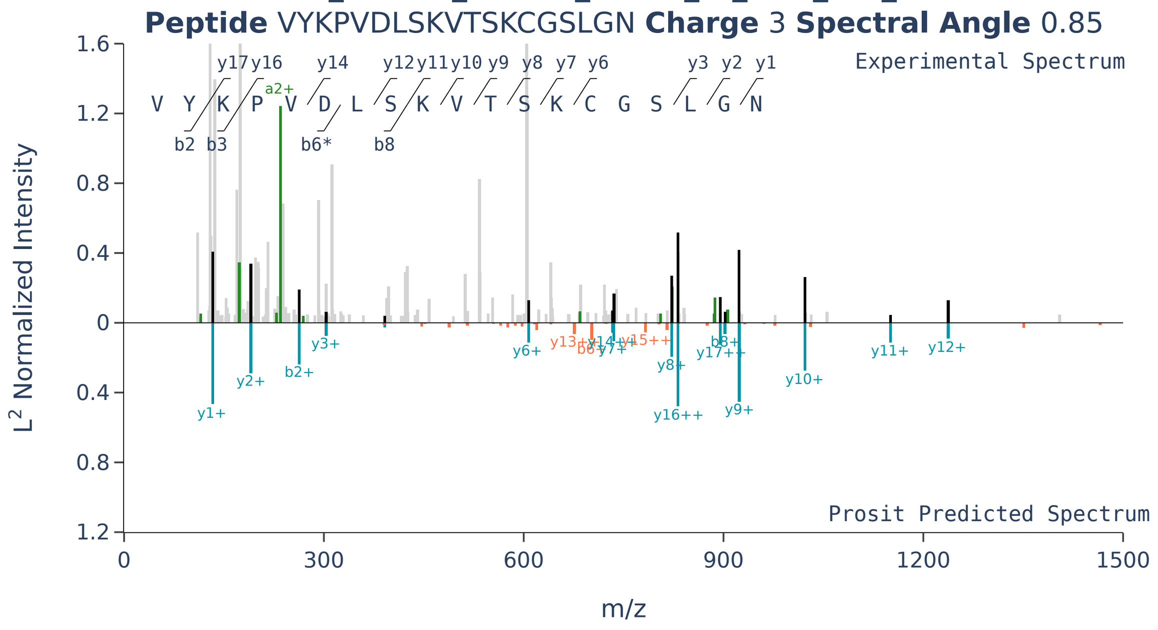


**Source** WSoh_221121_011221_HFGoe_L1_hTau_24h_R2 **Scan** 22165
**Peptide** QTAPVPMPDLKNVKSKIG **Charge** 3 **Spectral Angle** 0.88

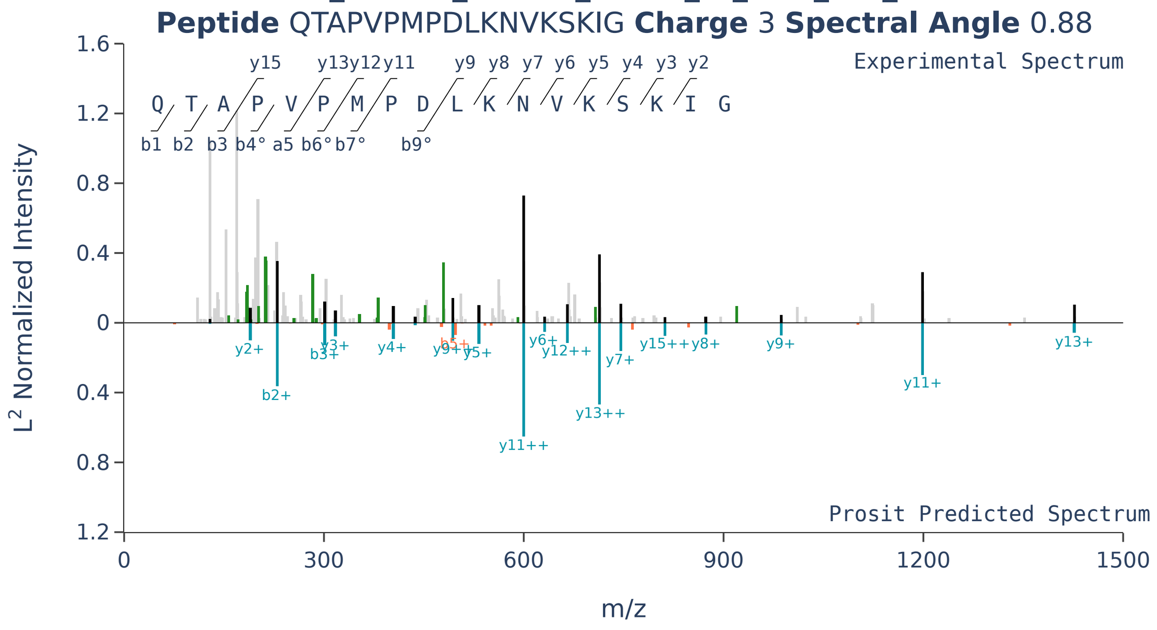


**Source** WSoh_221121_011221_HFGoe_L2_hTau_24h_R1 **Scan** 8443
**Peptide** RIPAKTPPAPKTPPSSGEPPKSGDR **Charge** 3 **Spectral Angle** 0.77

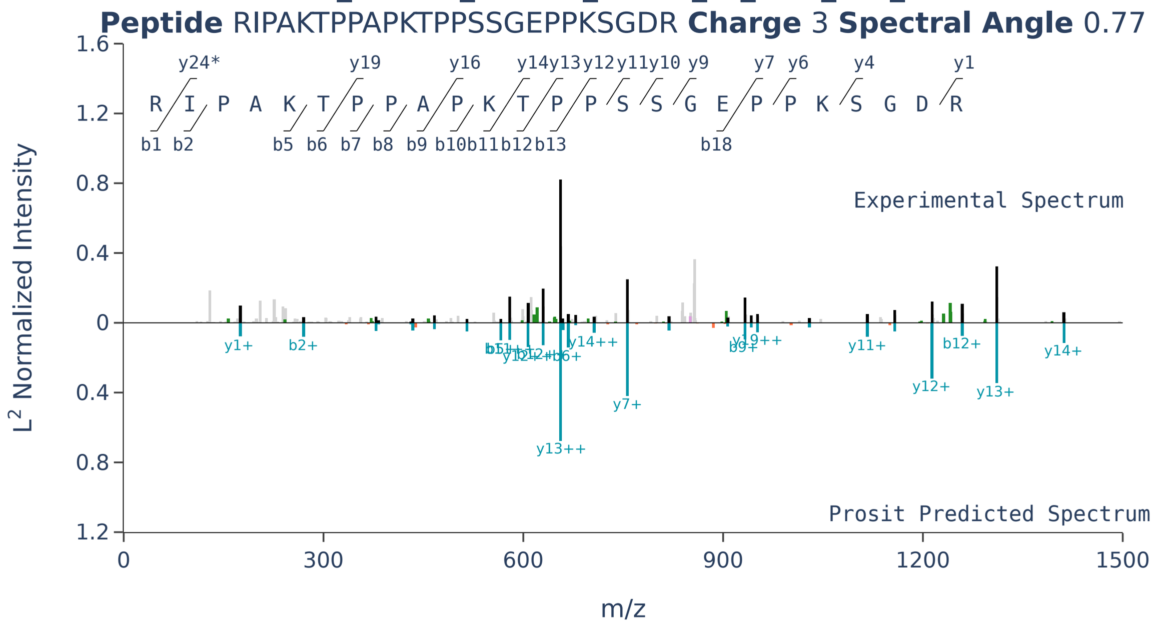


**Source** WSoh_221121_011221_HFGoe_L2_hTau_24h_R2 **Scan** 21932
**Peptide** LQTAPVPMPDLKNVKSKIGSTENLKHQ **Charge** 5 **Spectral Angle** 0.74

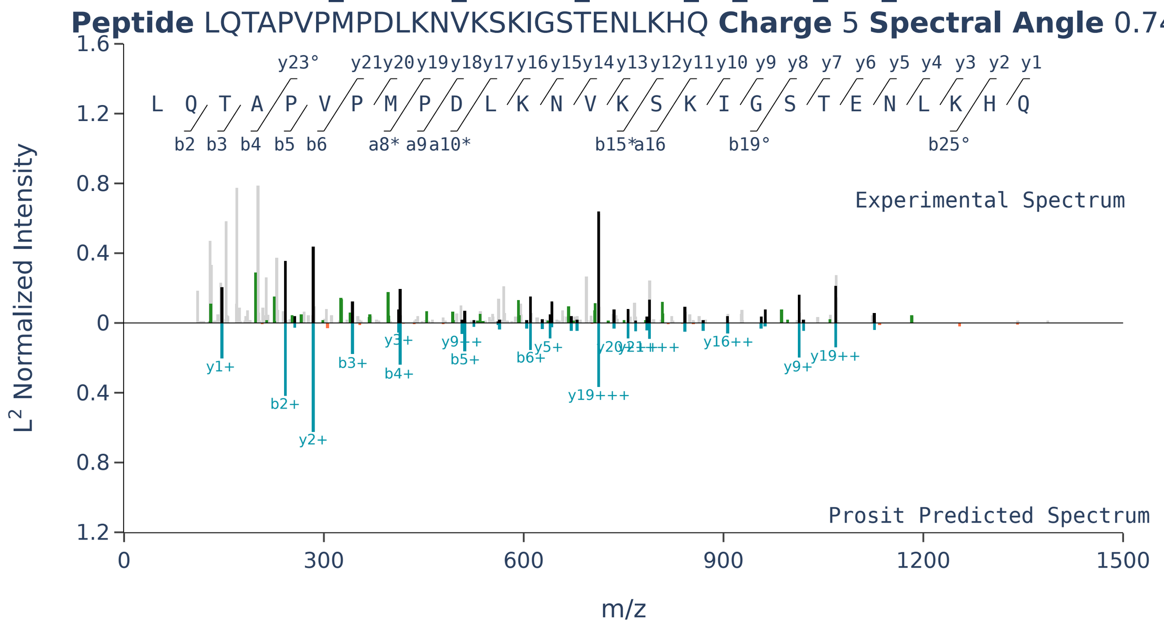


**Source** WSoh_221121_011221_HFGoe_L1_hTau_4h_R2 **Scan** 15239
**Peptide** KDRVQSKIGSLDNITHVPGGGNKKI **Charge** 4 **Spectral Angle** 0.82

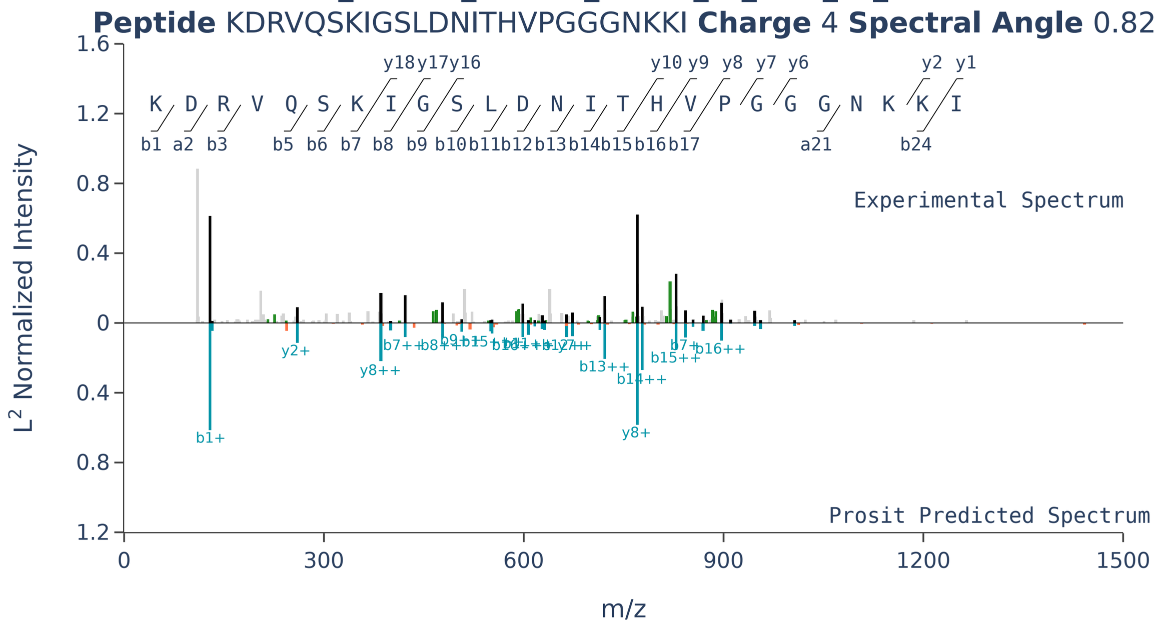


**Source** WSoh_221121_011221_HFGoe_L1_hTau_4h_R2 **Scan** 12821
**Peptide** KAKTDHGAEIVYKSPVVSG **Charge** 3 **Spectral Angle** 0.76

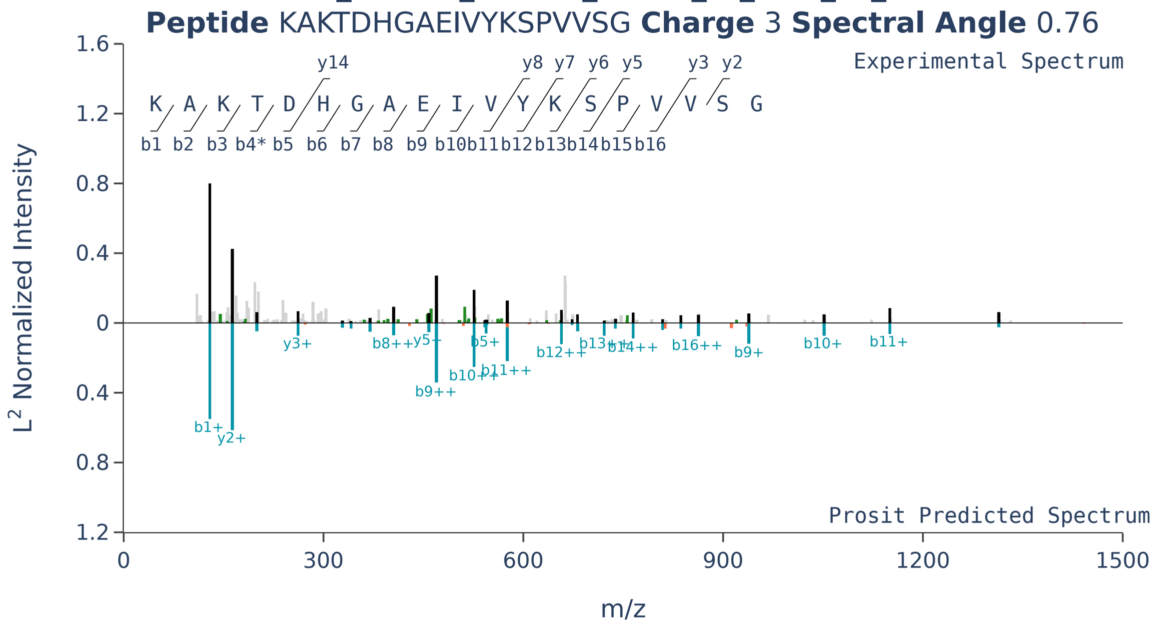


**Source** WSoh_221121_011221_HFGoe_L1_hTau_24h_R1 **Scan** 22185
**Peptide** RLQTAPVPMPDLKNVKSKI **Charge** 3 **Spectral Angle** 0.87

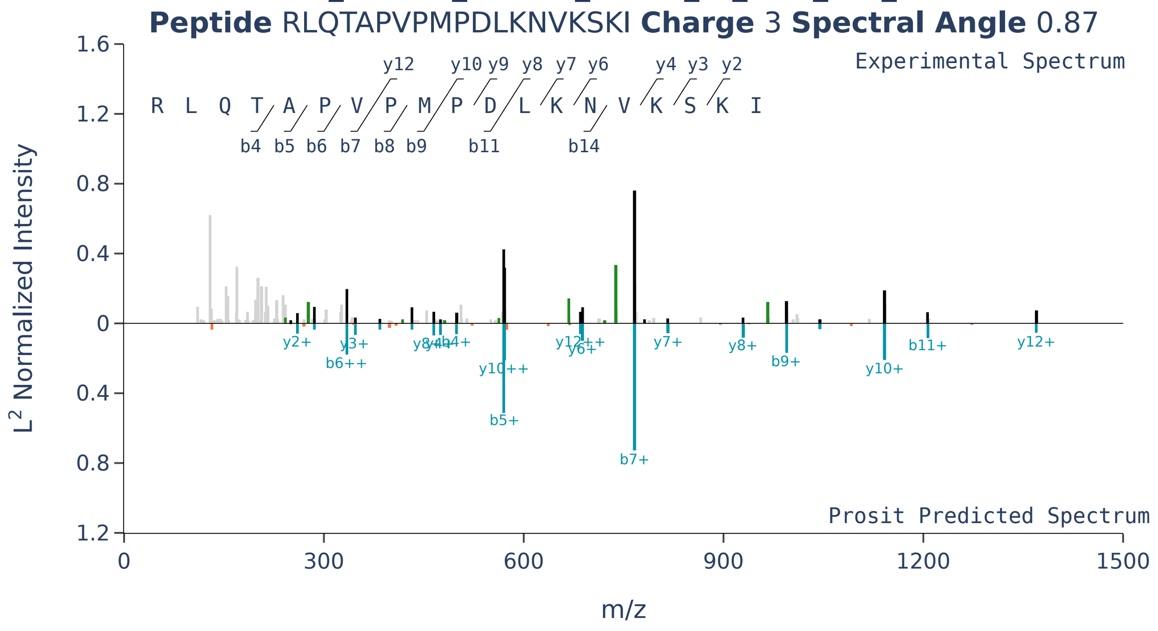


**Source** WSoh_221121_011221_HFGoe_L2_hTau_2h_R1 **Scan** 12116
**Peptide** KPGGGQVEVKSEKLDFKD **Charge** 4 **Spectral Angle** 0.87

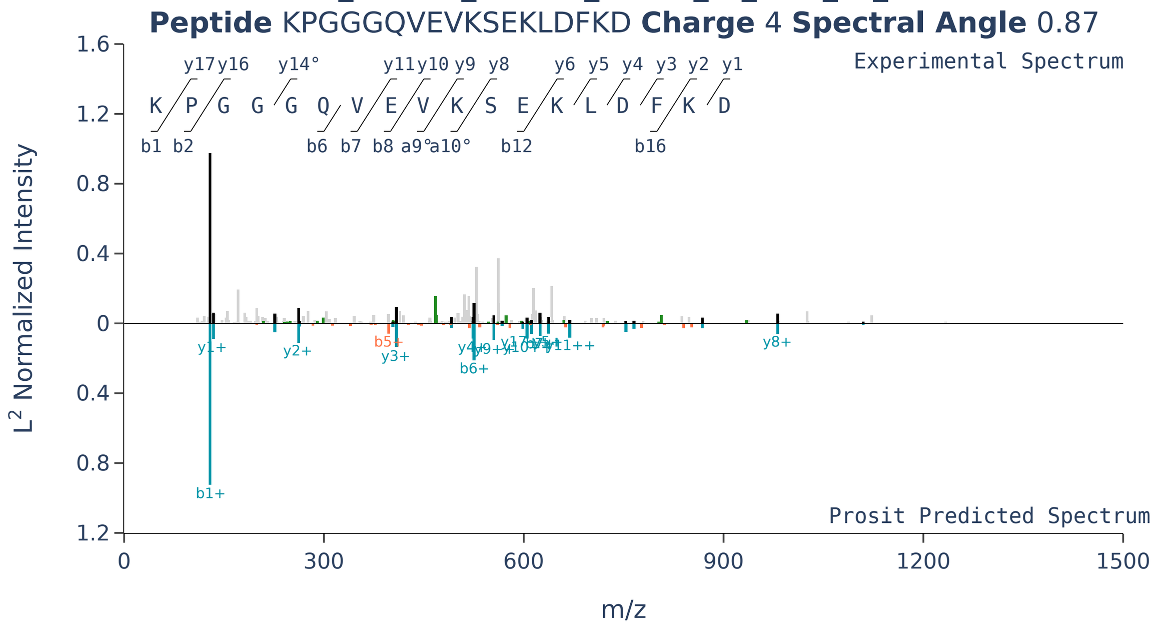


**Source** WSoh_221121_011221_HFGoe_L2_hTau_2h_R1 **Scan** 9582
**Peptide** RIPAKTPPAPKTPPSSGEPPKSGDRSGY **Charge** 5 **Spectral Angle** 0.8

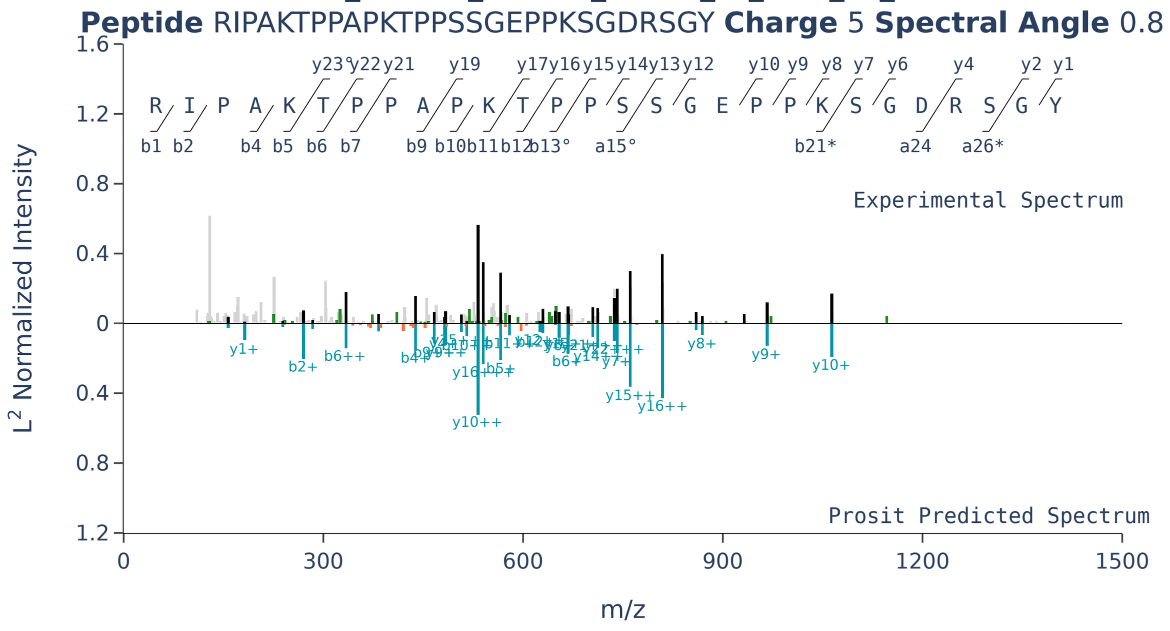


**Source** WSoh_221121_011221_HFGoe_L1_hTau_24h_R1 **Scan** 7585
**Peptide** QPGGGKVQIINKK **Charge** 3 **Spectral Angle** 0.74

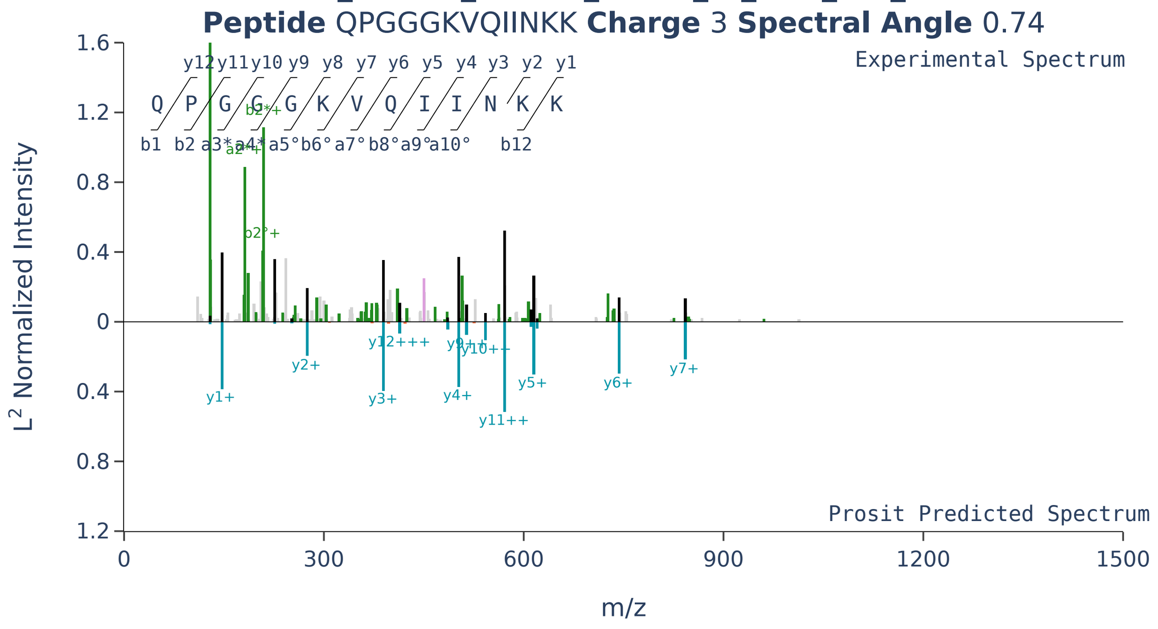


**Source** WSoh_221121_011221_HFGoe_L1_hTau_24h_R1 **Scan** 22704
**Peptide** GDTSPRHLSNVSSTGSIDMVDSPQLA **Charge** 3 **Spectral Angle** 0.8

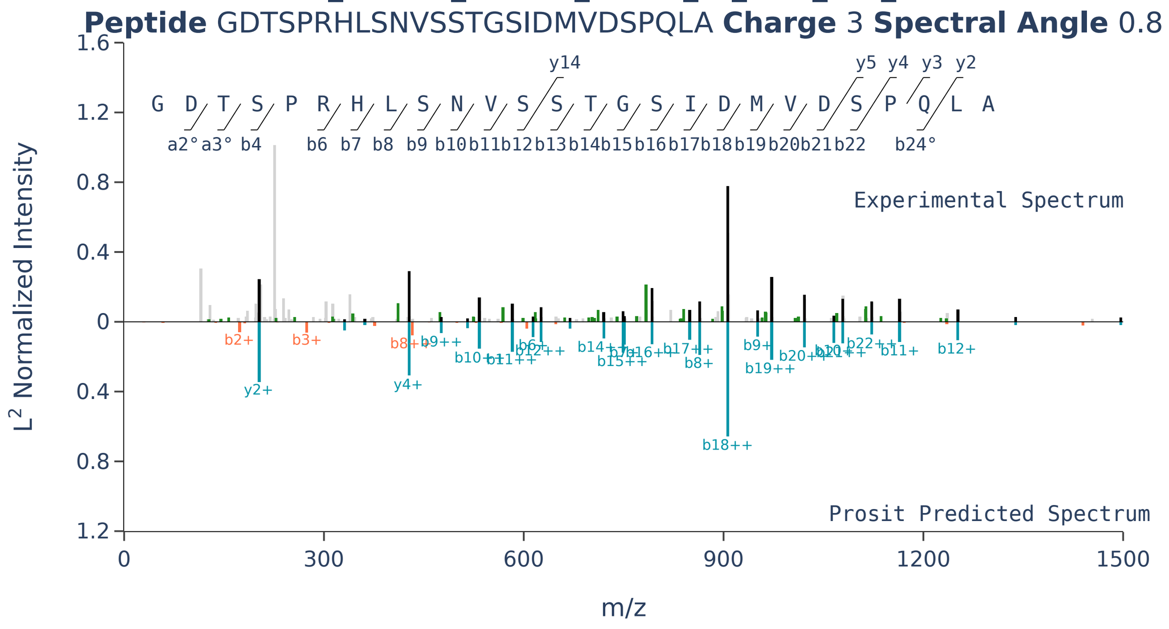


**Source** WSoh_221121_011221_HFGoe_L1_hTau_4h_R1 **Scan** 5568
**Peptide** AAGHVTQARMVSKSKD **Charge** 2 **Spectral Angle** 0.8

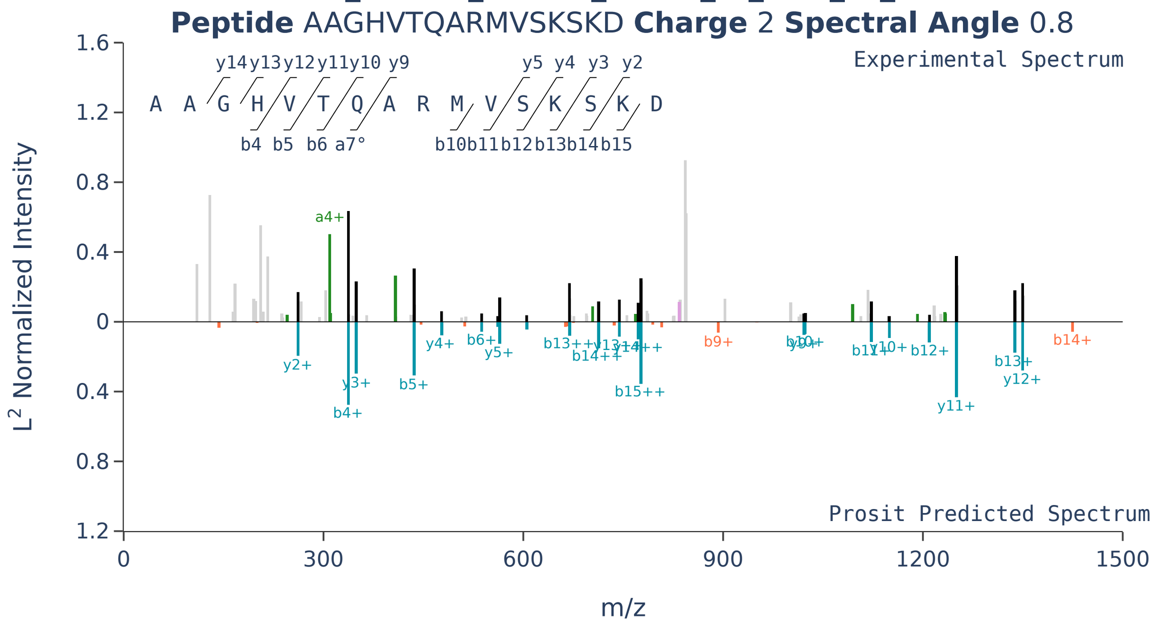


**Source** WSoh_221121_011221_HFGoe_L1_hTau_4h_R1 **Scan** 15385
**Peptide** KLGSLDNITHVPGGGNKKIE **Charge** 3 **Spectral Angle** 0.88

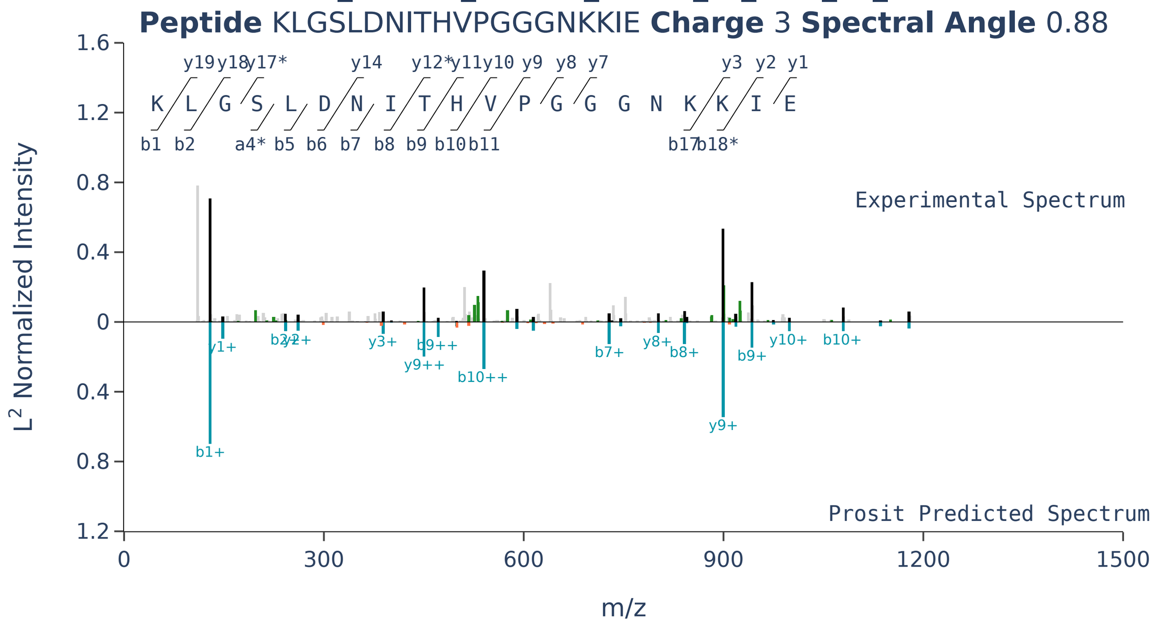


**Source** WSoh_221121_011221_HFGoe_L1_hTau_4h_R1 **Scan** 10312
**Peptide** LTHVPGGGNKKIETHKLTFREN **Charge** 4 **Spectral Angle** 0.82

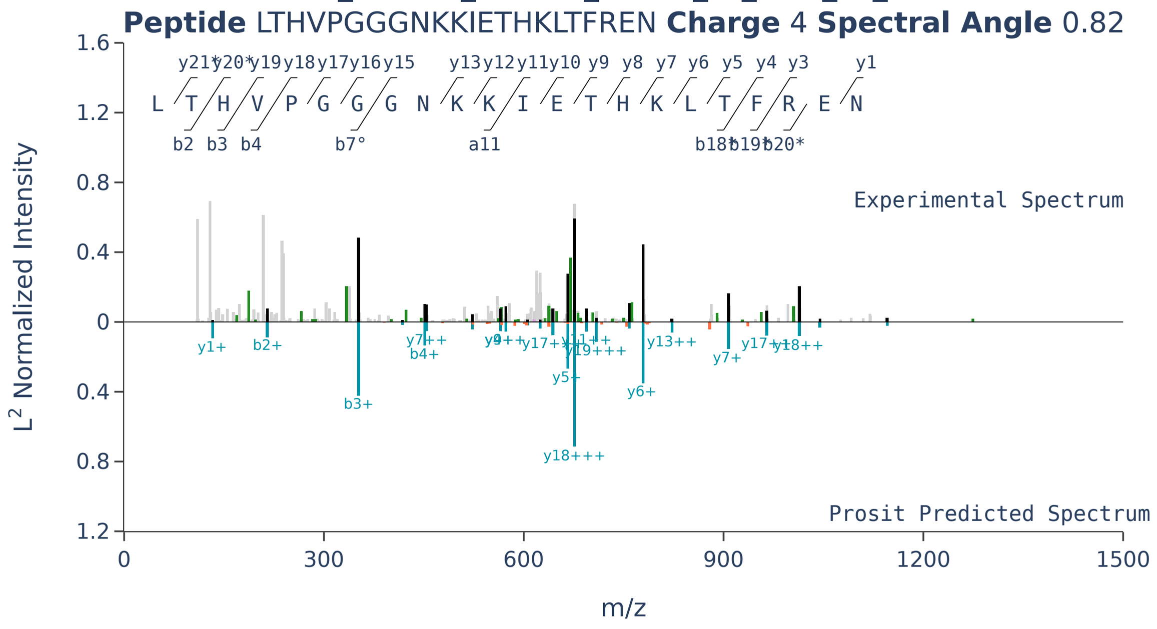


**Source** WSoh_221121_011221_HFGoe_L2_hTau_4h_R1 **Scan** 17067
**Peptide** CGSKDNIKHVPGGGSVQIVY **Charge** 4 **Spectral Angle** 0.82

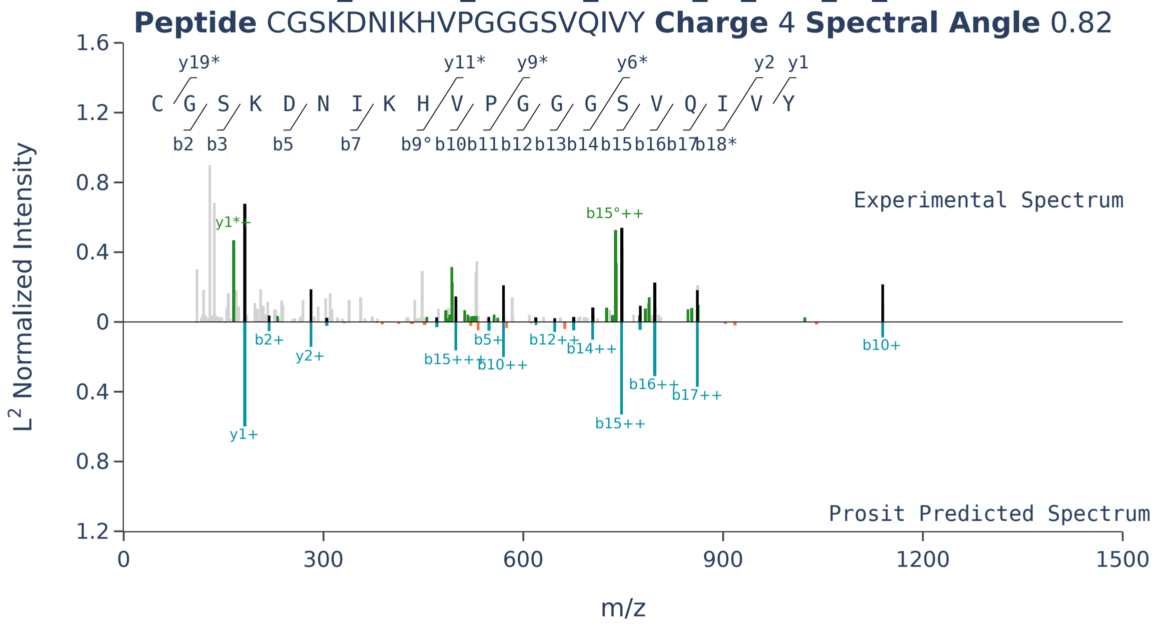

**Source** WSoh_221121_011221_HFGoe_L1_hTau_24h_R1 **Scan** 12421
**Peptide** EGAPGKQAAAQPHTEIPEGTTAE **Charge** 3 **Spectral Angle** 0.81

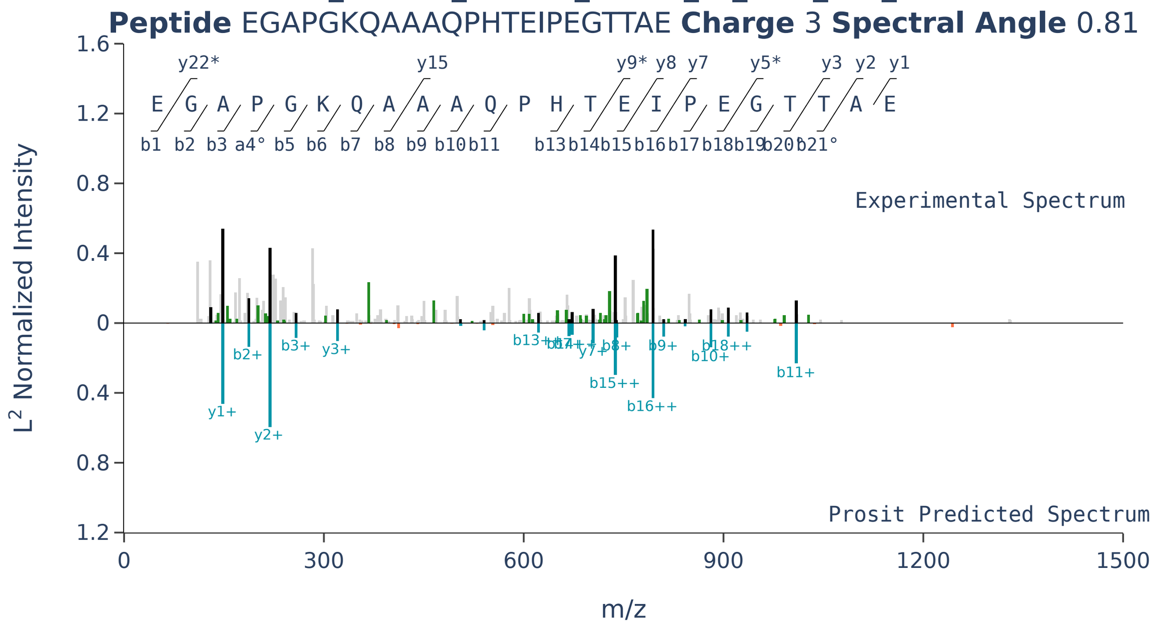


**Source** WSoh_221121_011221_HFGoe_L1_hTau_4h_R2 **Scan** 24960
**Peptide** STGSIDMVDSPQLA **Charge** 2 **Spectral Angle** 0.78

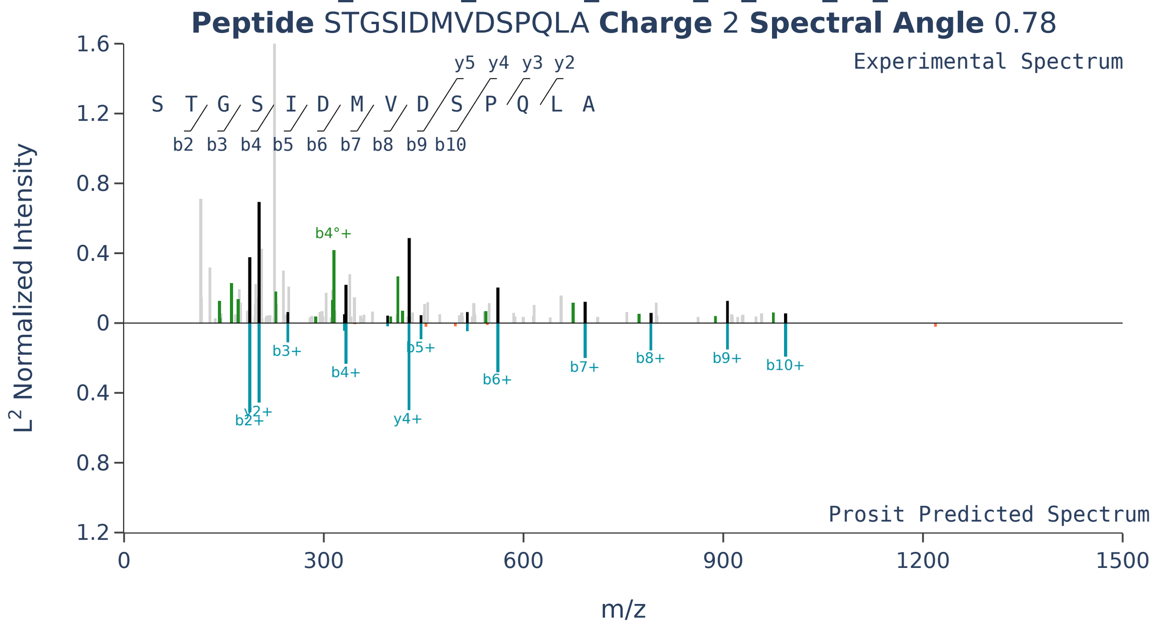


**Source** WSoh_221121_011221_HFGoe_L1_hTau_24h_R1 **Scan** 23302
**Peptide** AAQPHTEIPEGTTAEEAGIGDTPSLE **Charge** 3 **Spectral Angle** 0.86

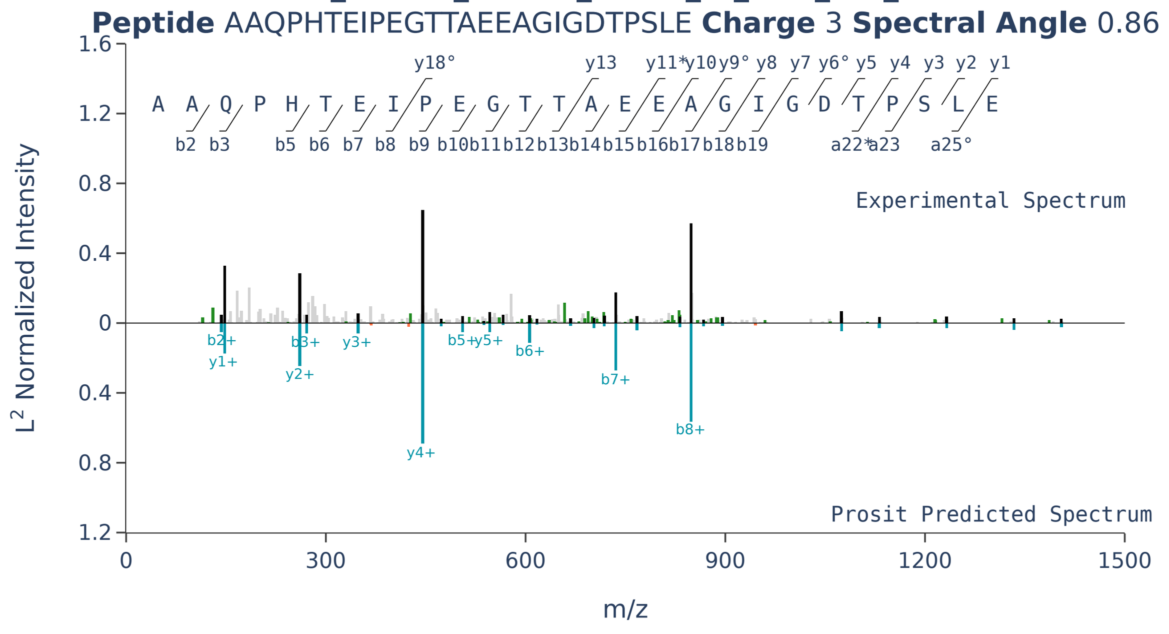


**Source** WSoh_221121_011221_HFGoe_L1_hTau_24h_R1 **Scan** 24632
**Peptide** DGSEEPGSETSDAKSTPTAEDVTAPLV **Charge** 3 **Spectral Angle** 0.95

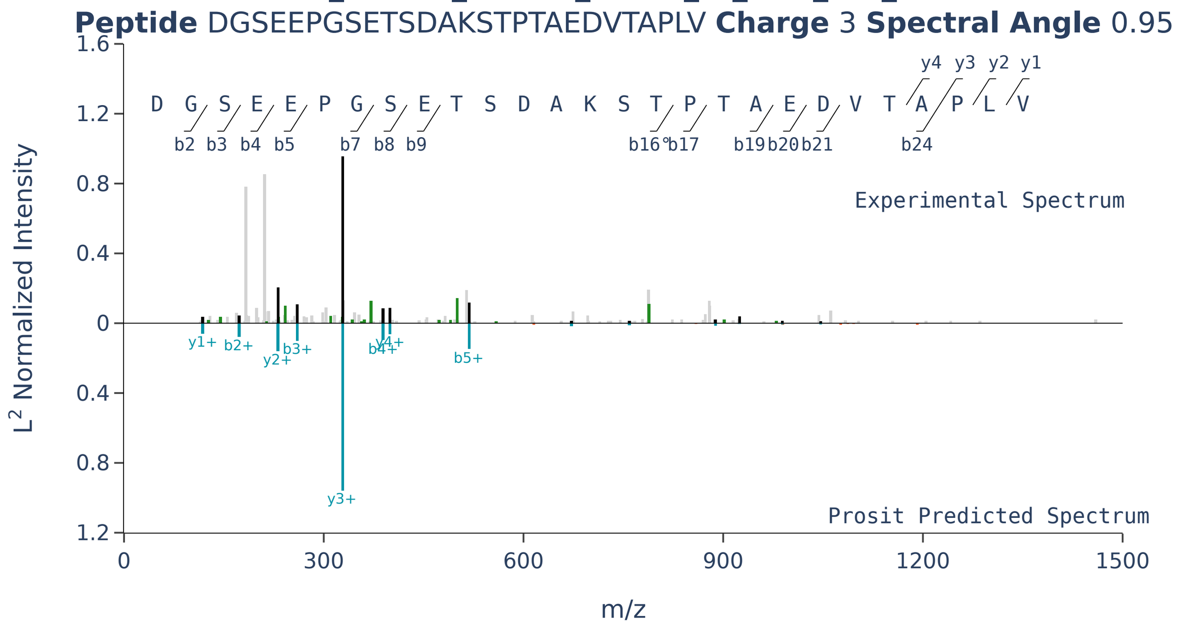


**Source** WSoh_221121_011221_HFGoe_L2_hTau_4h_R1 **Scan** 11647
**Peptide** NITHVPGGGNKKIETHKLTFRE **Charge** 3 **Spectral Angle** 0.86

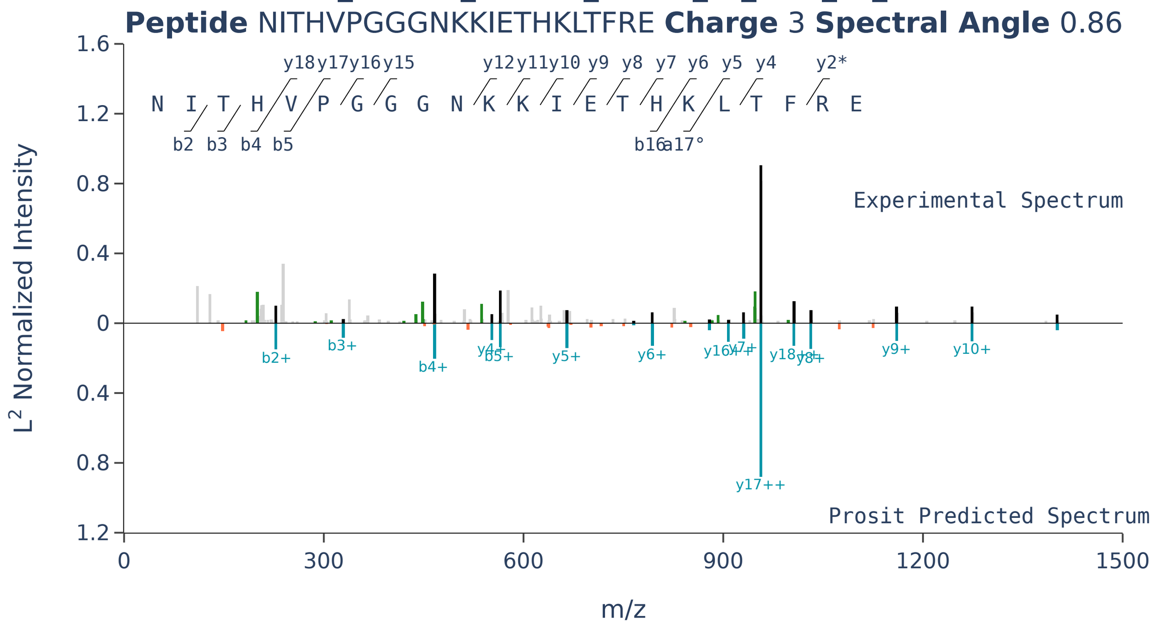


**Source** WSoh_221121_011221_HFGoe_L1_hTau_2h_R1 **Scan** 7486
**Peptide** PAKTPPAPKTPPSSG **Charge** 3 **Spectral Angle** 0.75

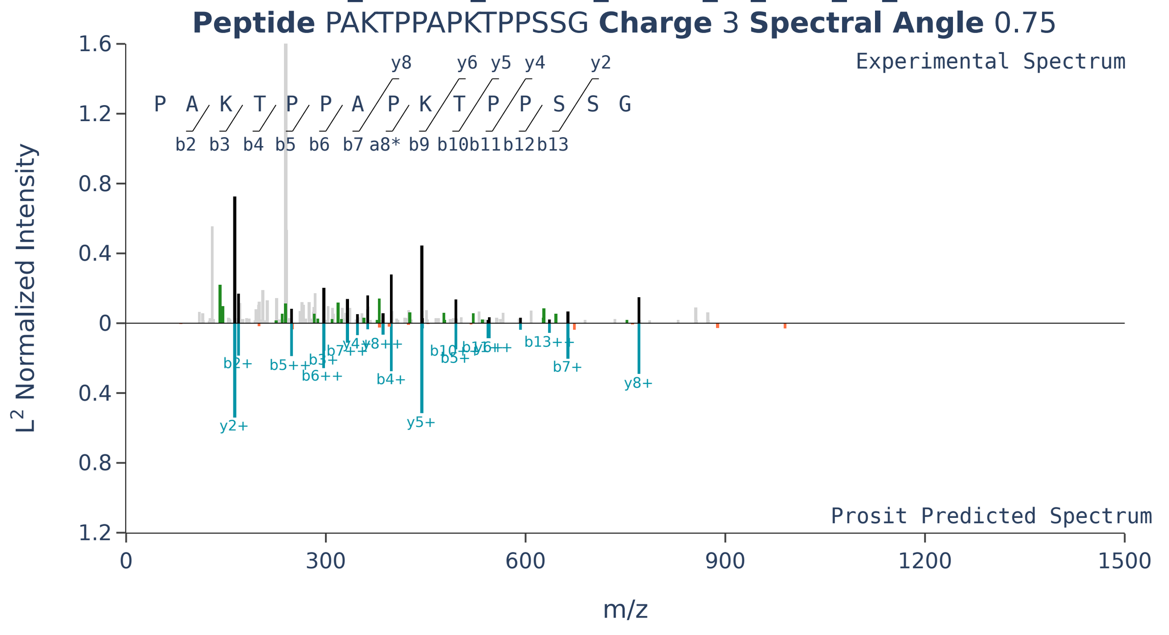


**Source** WSoh_221121_011221_HFGoe_L1_hTau_24h_R2 **Scan** 6083
**Peptide** HKLTFRENAKAK **Charge** 2 **Spectral Angle** 0.84

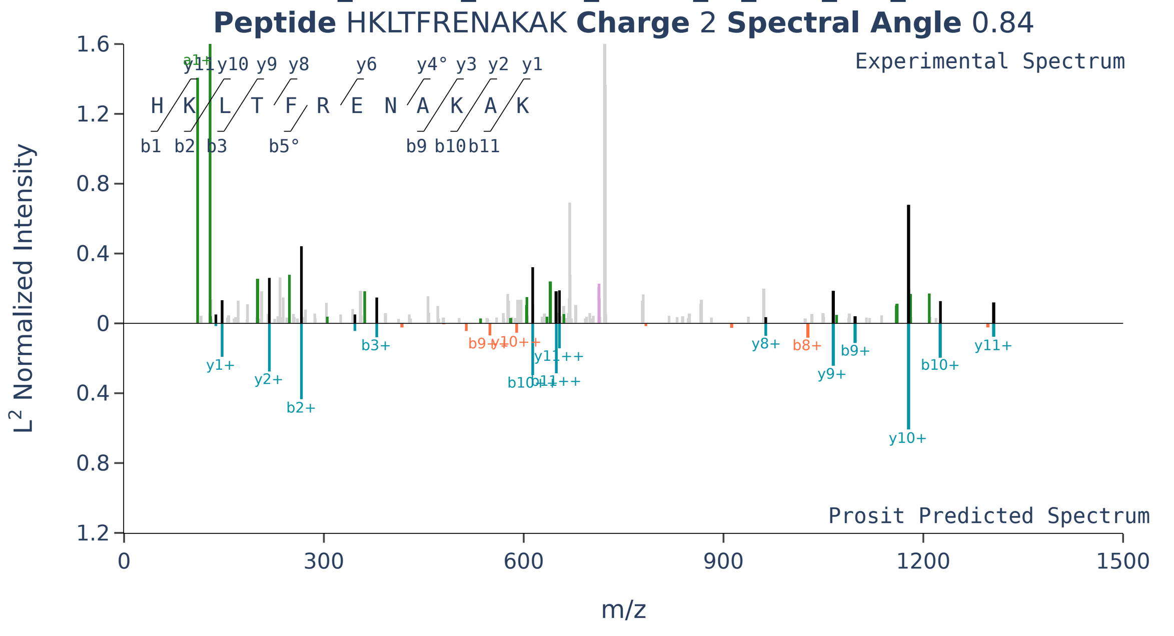


**Source** WSoh_221121_011221_HFGoe_L2_hTau_24h_R1 **Scan** 23807
**Peptide** LQTAPVPMPDLKNVKSKIGST **Charge** 3 **Spectral Angle** 0.86

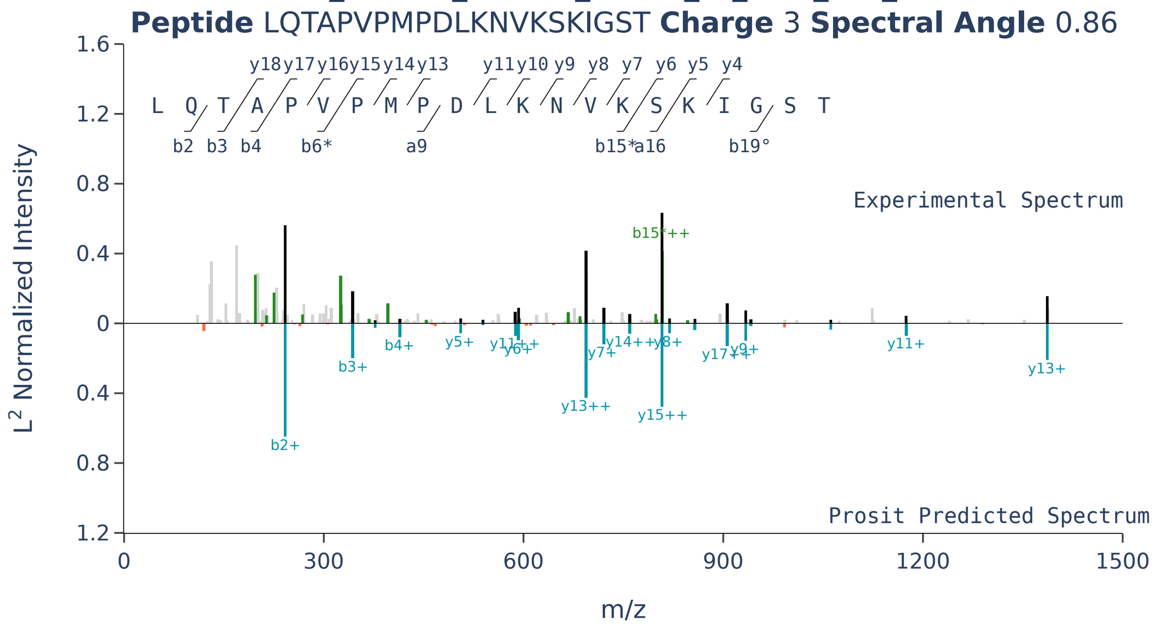


**Source** WSoh_221121_011221_HFGoe_L1_hTau_24h_R2 **Scan** 9747
**Peptide** THVPGGGNKKIETHKLTFRE **Charge** 4 **Spectral Angle** 0.86

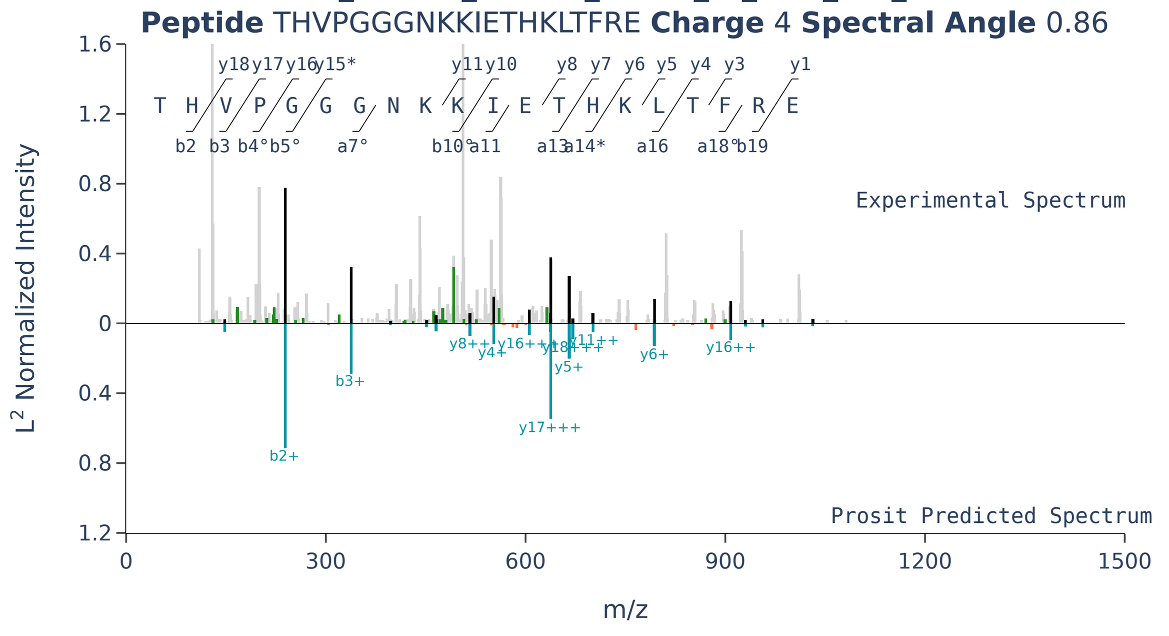


**Source** WSoh_221121_011221_HFGoe_L1_hTau_24h_R1 **Scan** 9852
**Peptide** VPGGGNKKIETHKLTFRENA **Charge** 4 **Spectral Angle** 0.8

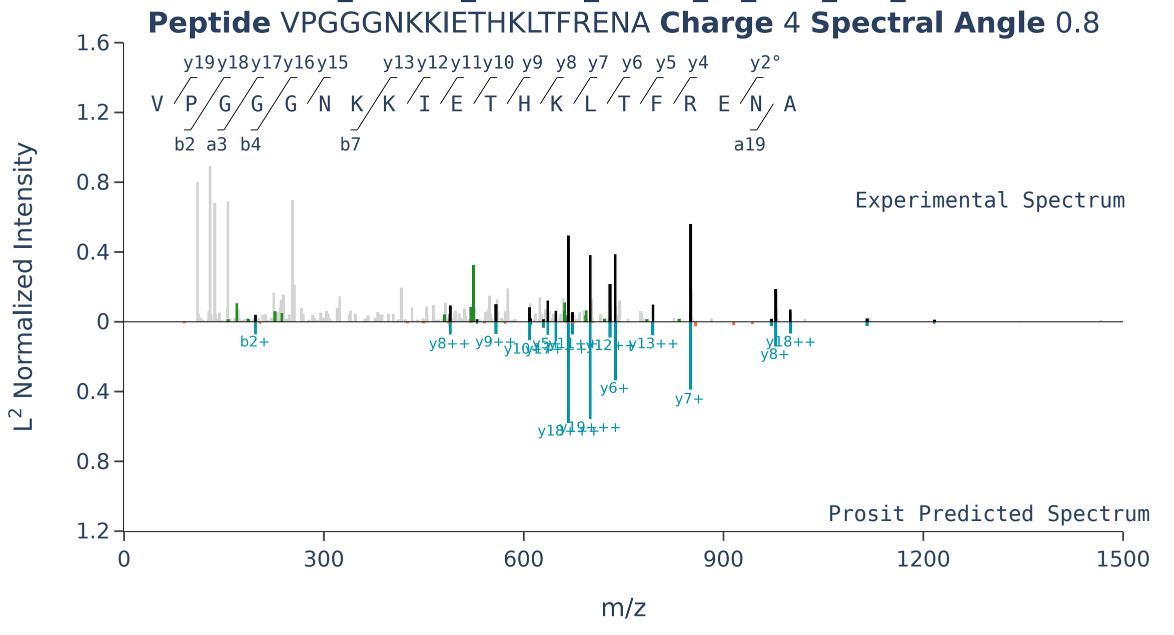


**Source** WSoh_221121_011221_HFGoe_L1_hTau_24h_R1 **Scan** 5433
**Peptide** KNVKSKIGSTE **Charge** 2 **Spectral Angle** 0.76

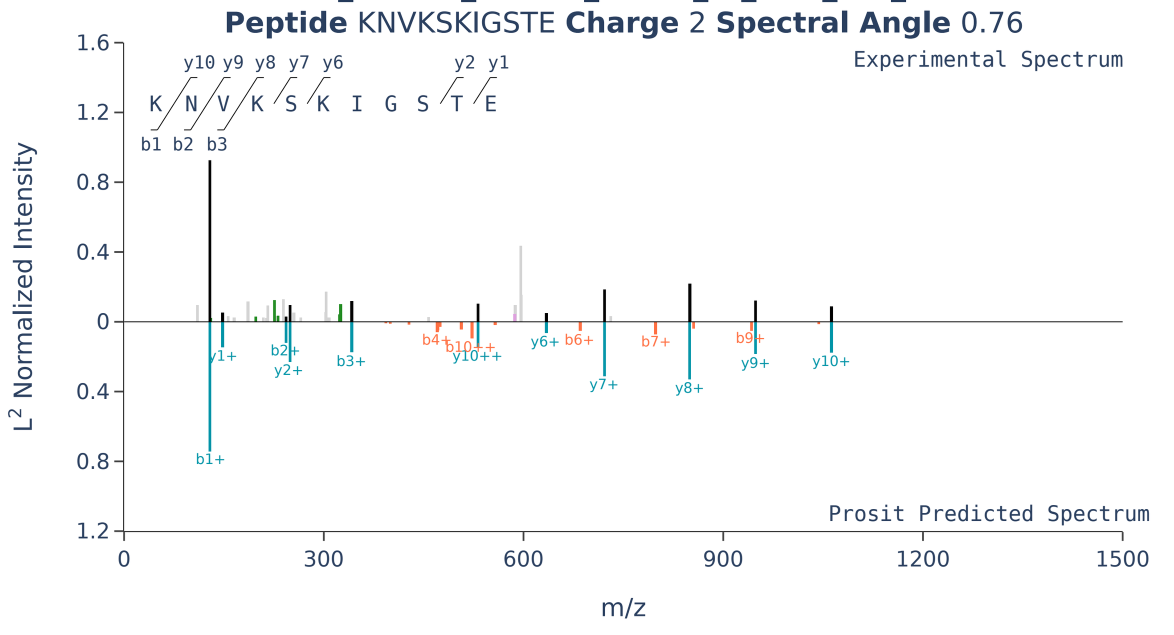


**Source** WSoh_221121_011221_HFGoe_L2_hTau_24h_R1 **Scan** 18617
**Peptide** DHGAEIVYKSPVVSGDTSPRHLSNV **Charge** 4 **Spectral Angle** 0.83

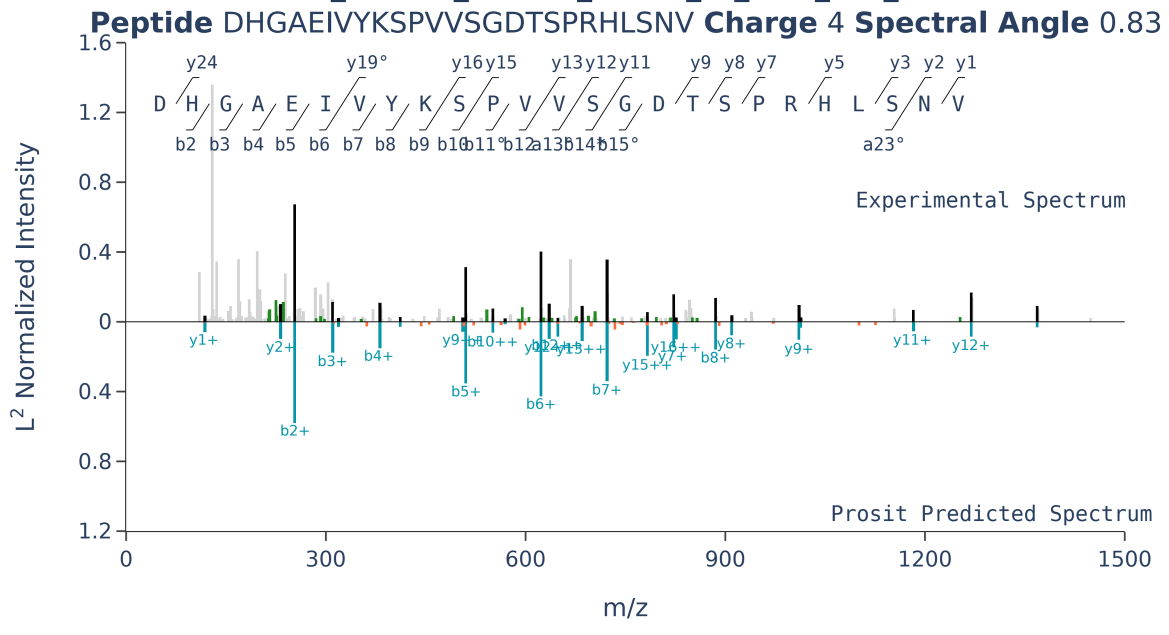


**Source** WSoh_221121_011221_HFGoe_L2_hTau_24h_R1 **Scan** 9150
**Peptide** KLGSLDN **Charge** 2 **Spectral Angle** 0.95

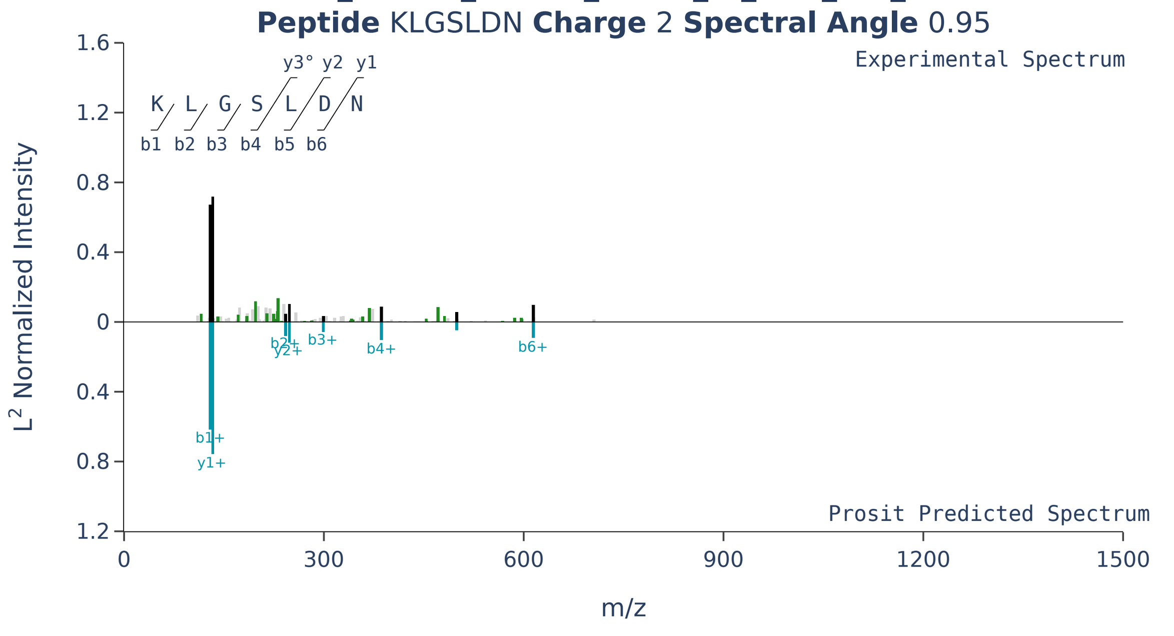


**Source** WSoh_221121_011221_HFGoe_L2_hTau_4h_R2 **Scan** 9337
**Peptide** RIPAKTPPAPKTPPSSGEPPKSGD **Charge** 4 **Spectral Angle** 0.85

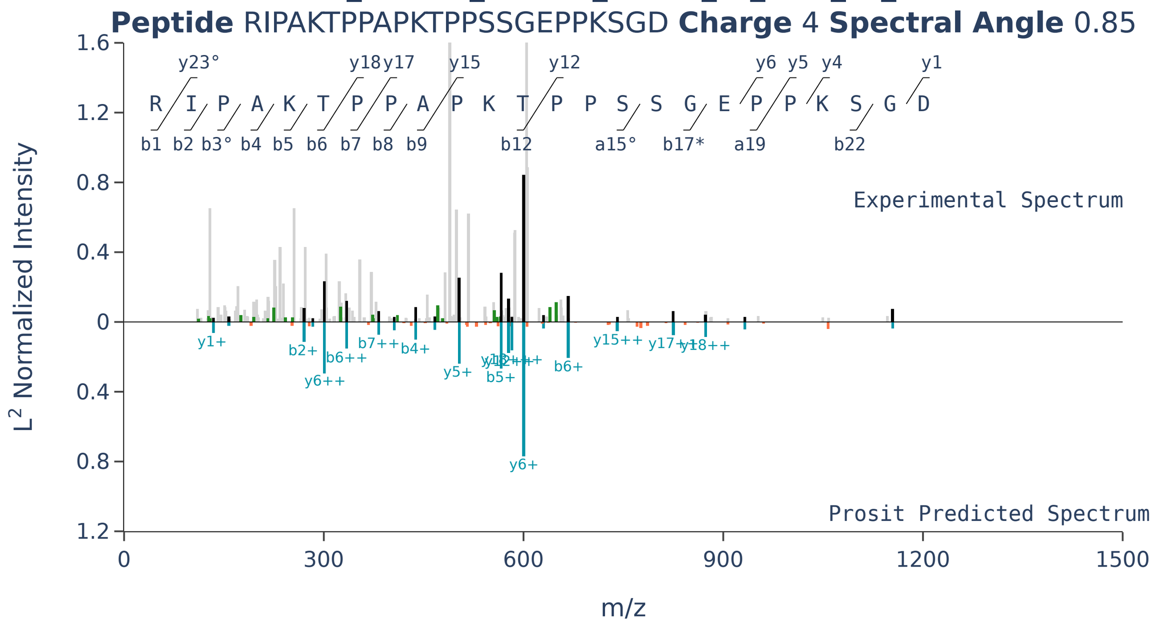


**Source** WSoh_221121_011221_HFGoe_L1_hTau_24h_R2 **Scan** 8576
**Peptide** KIGSTENLKHQPGGGKVQIINKK **Charge** 3 **Spectral Angle** 0.86

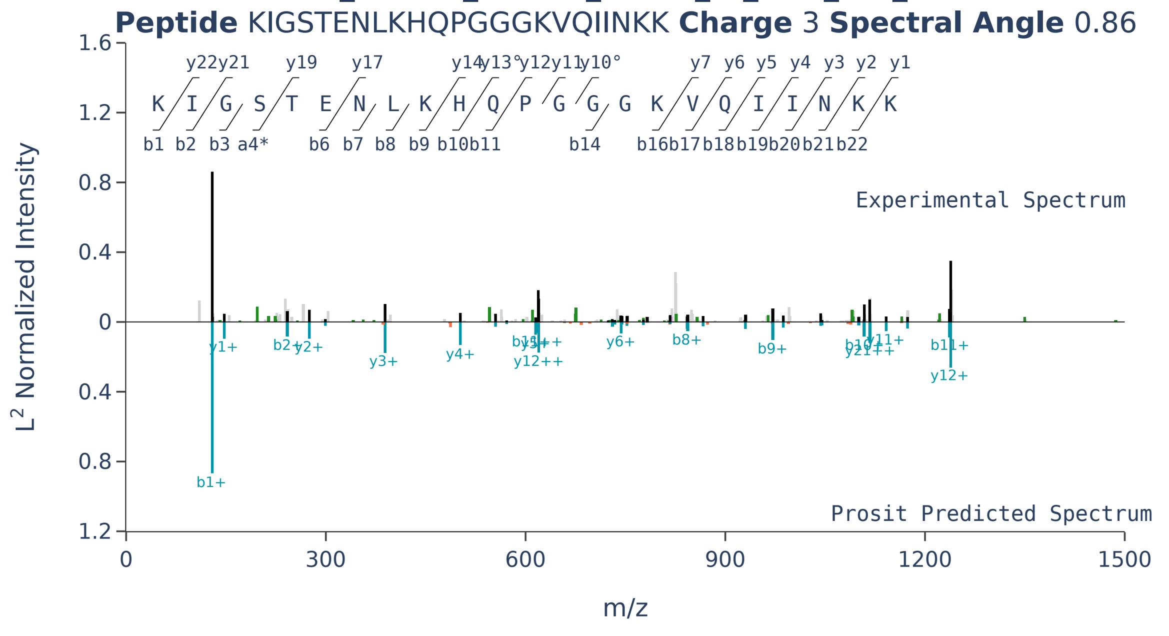

**Source** WSoh_221121_011221_HFGoe_L1_hTau_24h_R1 **Scan** 11897
**Peptide** GTYGLGDRKDQGGYTMHQDQEGD **Charge** 3 **Spectral Angle** 0.79

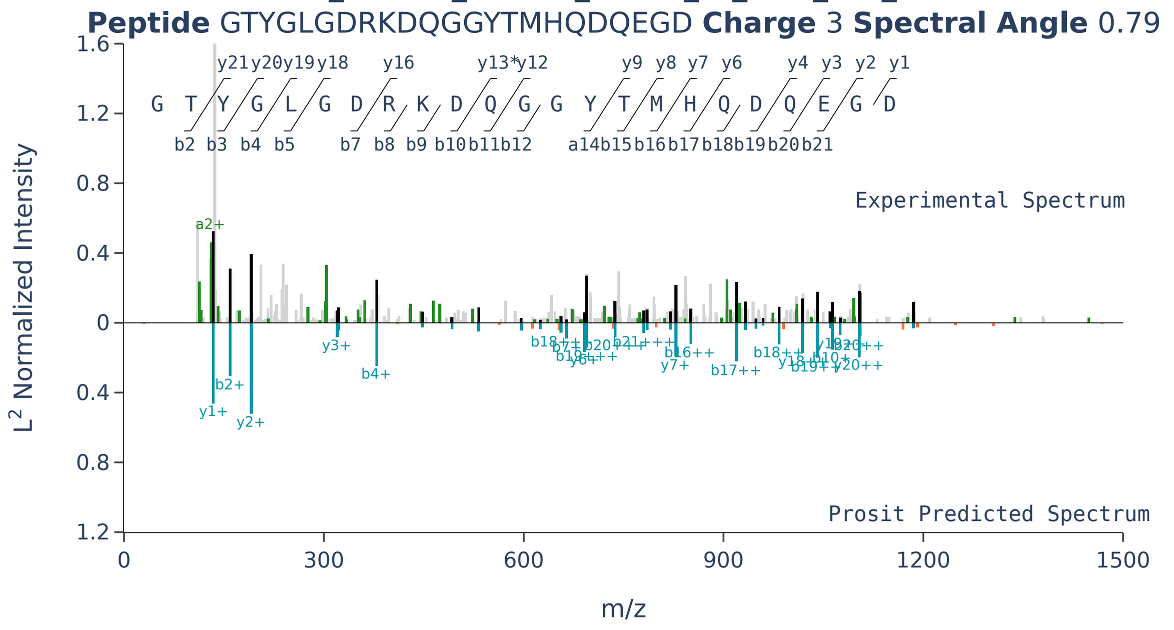


**Source** WSoh_221121_011221_HFGoe_L1_hTau_4h_R2 **Scan** 18532
**Peptide** EKLDFKDRVQSKIGSLD **Charge** 3 **Spectral Angle** 0.76

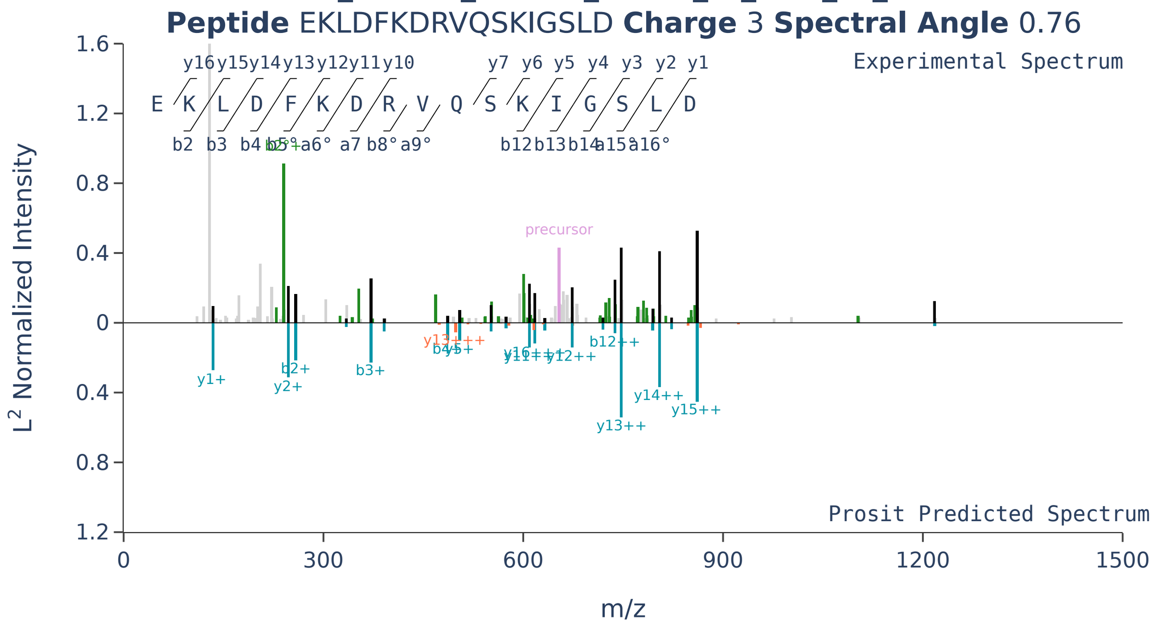


**Source** WSoh_221121_011221_HFGoe_L2_hTau_2h_R2 **Scan** 7971
**Peptide** AVVRTPPKSPSSAKSRLQT **Charge** 3 **Spectral Angle** 0.73

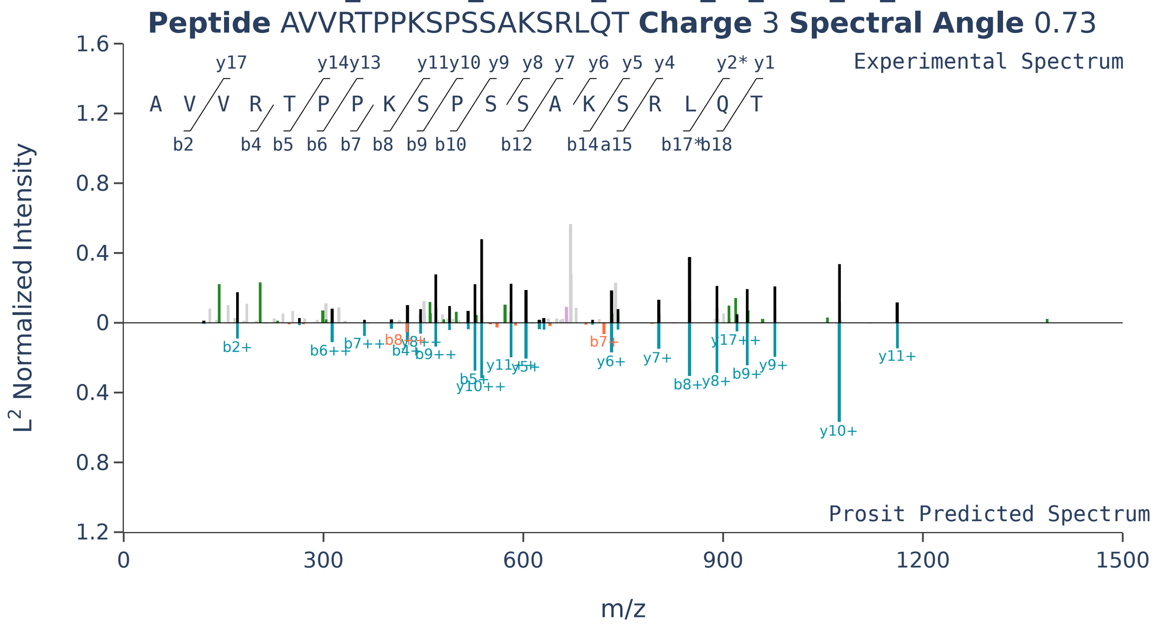


**Source** WSoh_221121_011221_HFGoe_L2_hTau_24h_R1 **Scan** 13415
**Peptide** ENAKAKTDHGAEIVYKSPVVS **Charge** 2 **Spectral Angle** 0.77

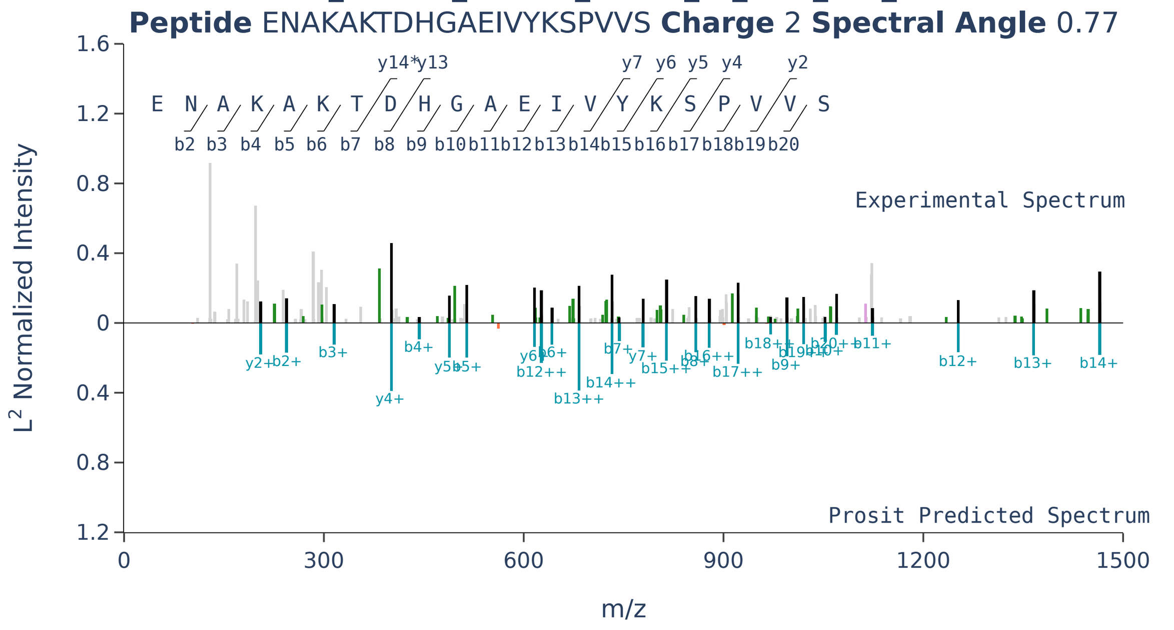


**Source** WSoh_221121_011221_HFGoe_L2_hTau_24h_R2 **Scan** 6512
**Peptide** AVVRTPPKSPSSAK **Charge** 3 **Spectral Angle** 0.77

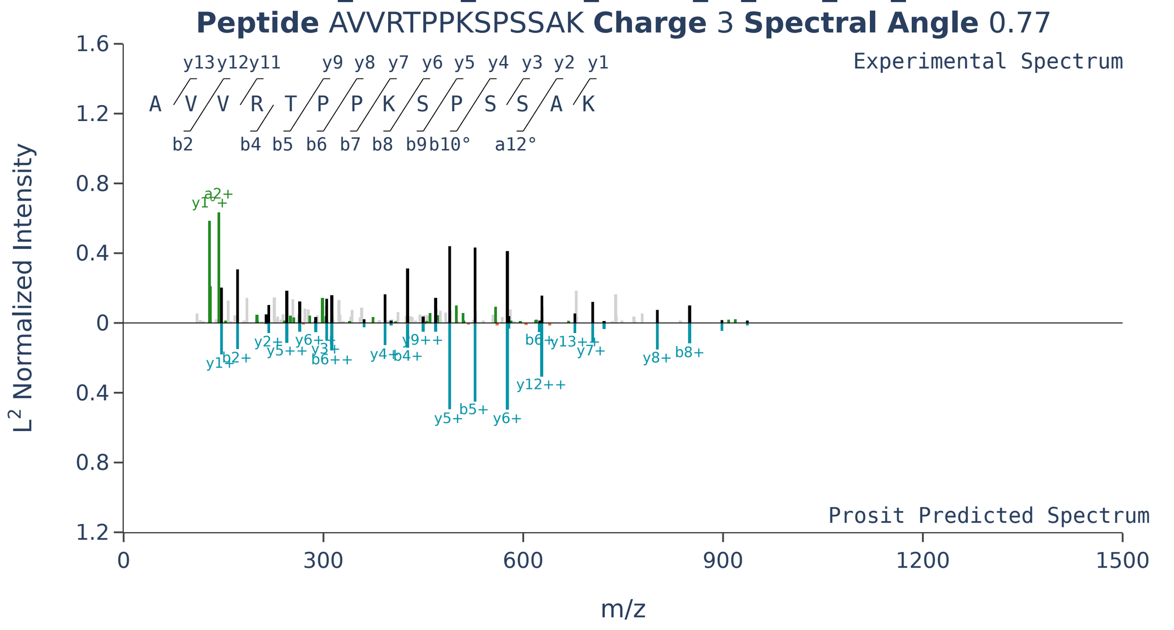


**Source** WSoh_221121_011221_HFGoe_L1_hTau_24h_R1 **Scan** 22554
**Peptide** LSNVSSTGSIDMVD **Charge** 2 **Spectral Angle** 0.81

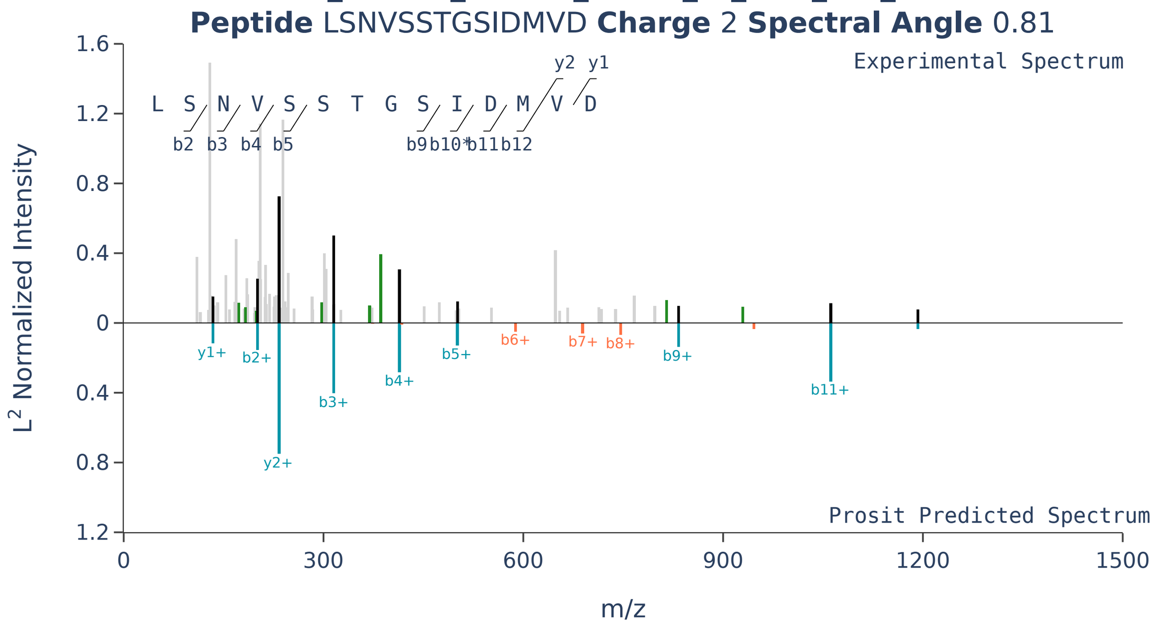


**Source** WSoh_221121_011221_HFGoe_L1_hTau_4h_R1 **Scan** 17680
**Peptide** AGLGDTPSLEDEAAGHVTQARMVSKSKD **Charge** 5 **Spectral Angle** 0.74

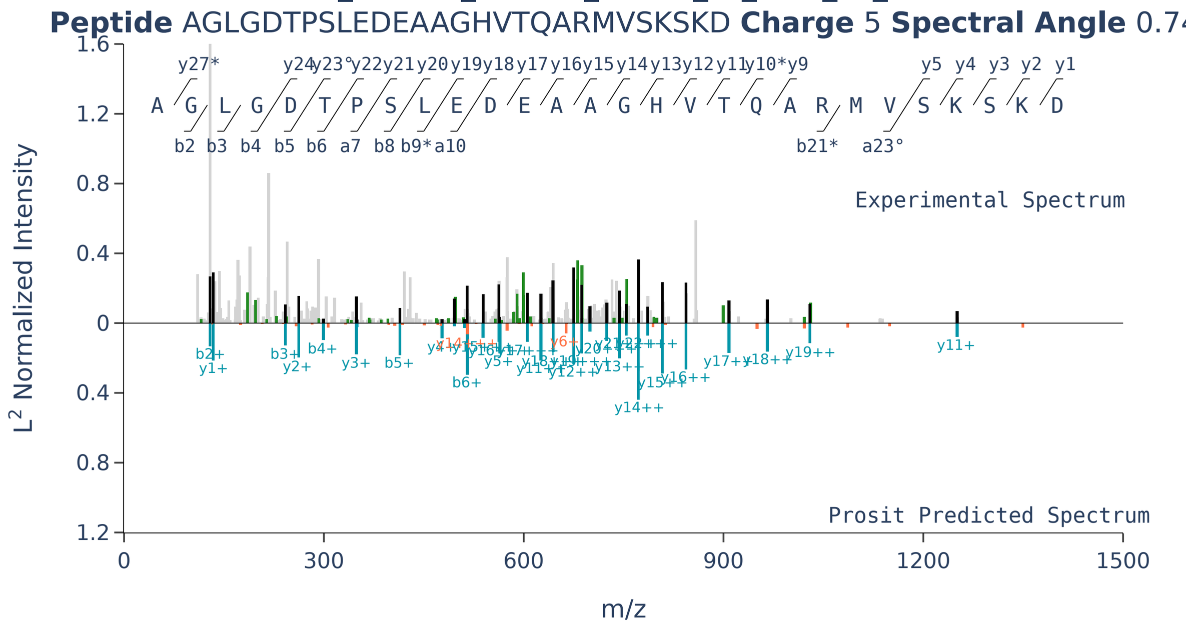


**Source** WSoh_221121_011221_HFGoe_L2_hTau_24h_R2 **Scan** 18319
**Peptide** AGIGDTPSLEDEAAGHVTQARMVSKSKD **Charge** 4 **Spectral Angle** 0.78

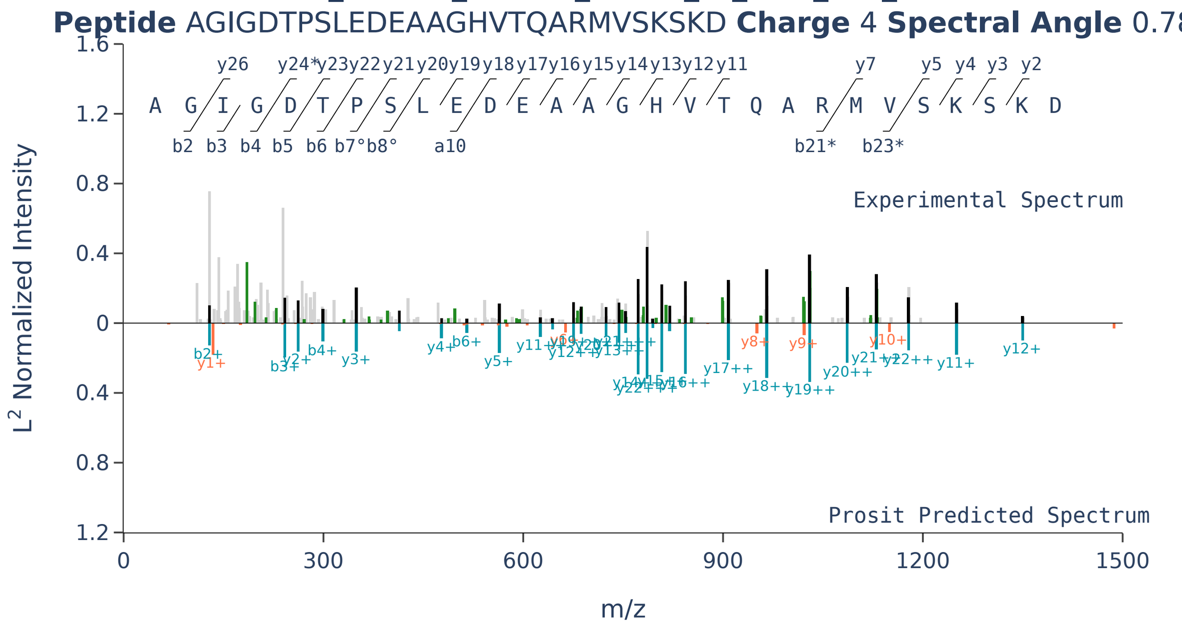


**Source** WSoh_221121_011221_HFGoe_L1_hTau_24h_R2 **Scan** 16358
**Peptide** IGSLDNITHVPGGGNKKIETHKL **Charge** 5 **Spectral Angle** 0.86

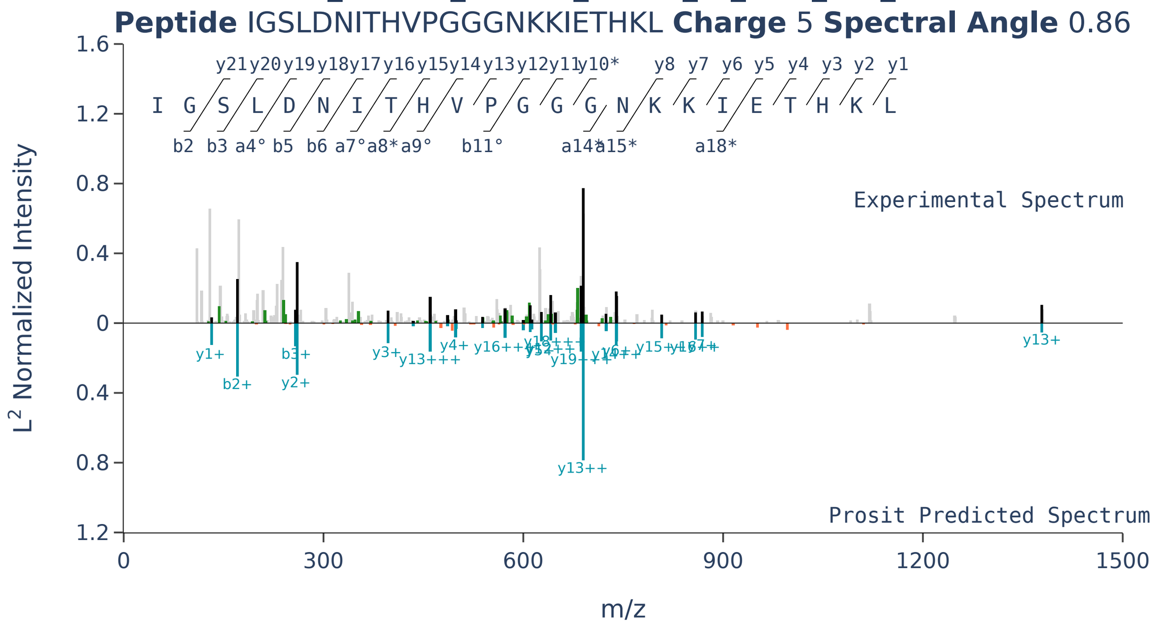


**Source** WSoh_221121_011221_HFGoe_L2_hTau_24h_R2 **Scan** 8296
**Peptide** PAKTPPAPKTPPSSGEPPKSGD **Charge** 3 **Spectral Angle** 0.83

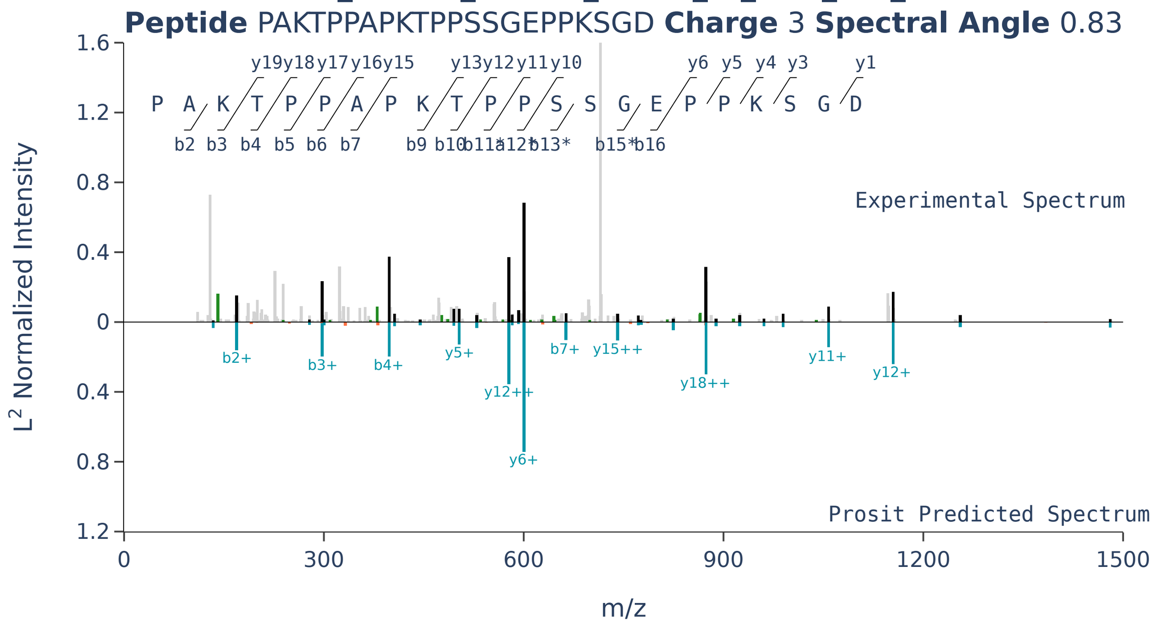


**Source** WSoh_221121_011221_HFGoe_L2_hTau_4h_R1 **Scan** 13318
**Peptide** SLDNITHVPGGGNKKIETHKL **Charge** 3 **Spectral Angle** 0.82

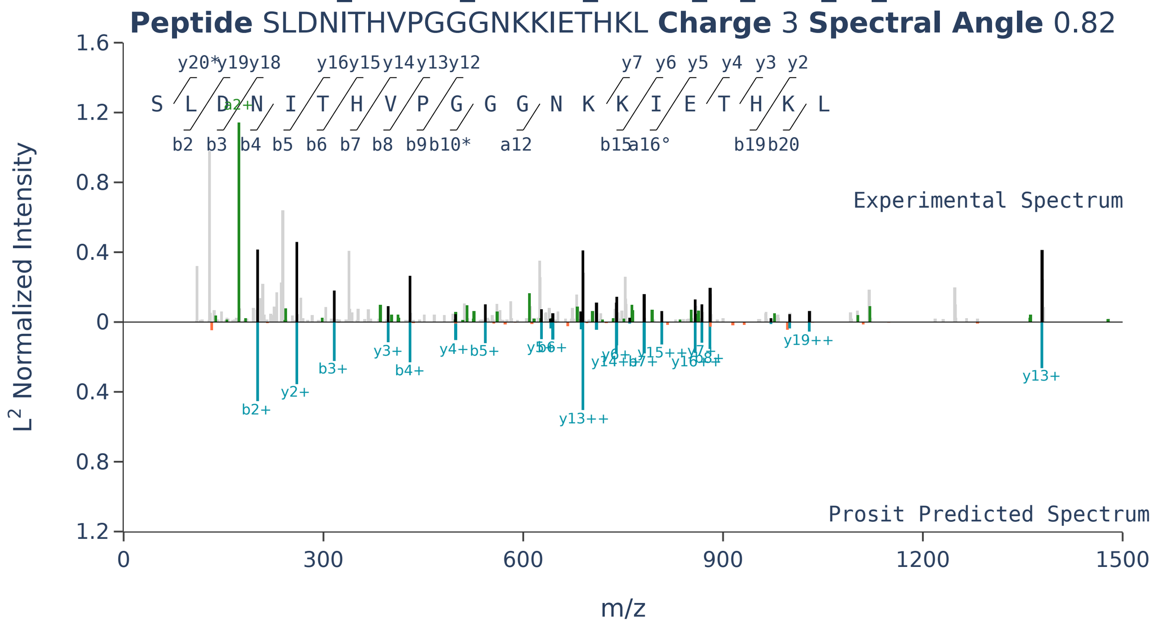


**Source** WSoh_221121_011221_HFGoe_L1_hTau_24h_R2 **Scan** 8692
**Peptide** THVPGGGNKKIETHKLTFRENAKA **Charge** 4 **Spectral Angle** 0.96

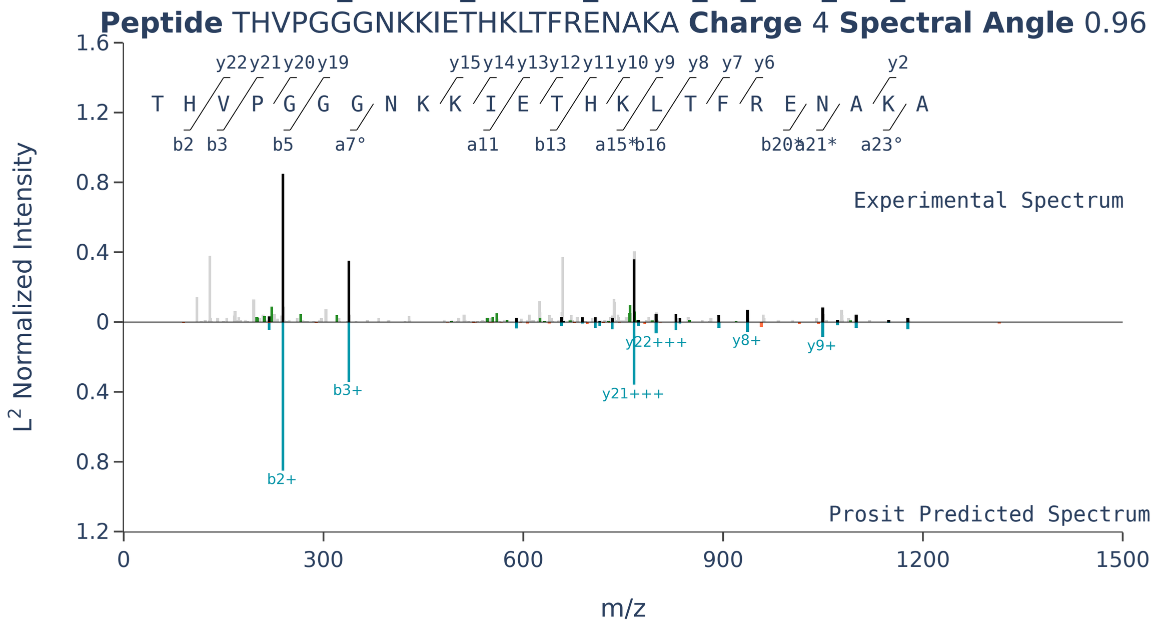


**Source** WSoh_221121_011221_HFGoe_L2_hTau_4h_R2 **Scan** 6345
**Peptide** KSKIGSTENLKHQPGGGKV **Charge** 3 **Spectral Angle** 0.9

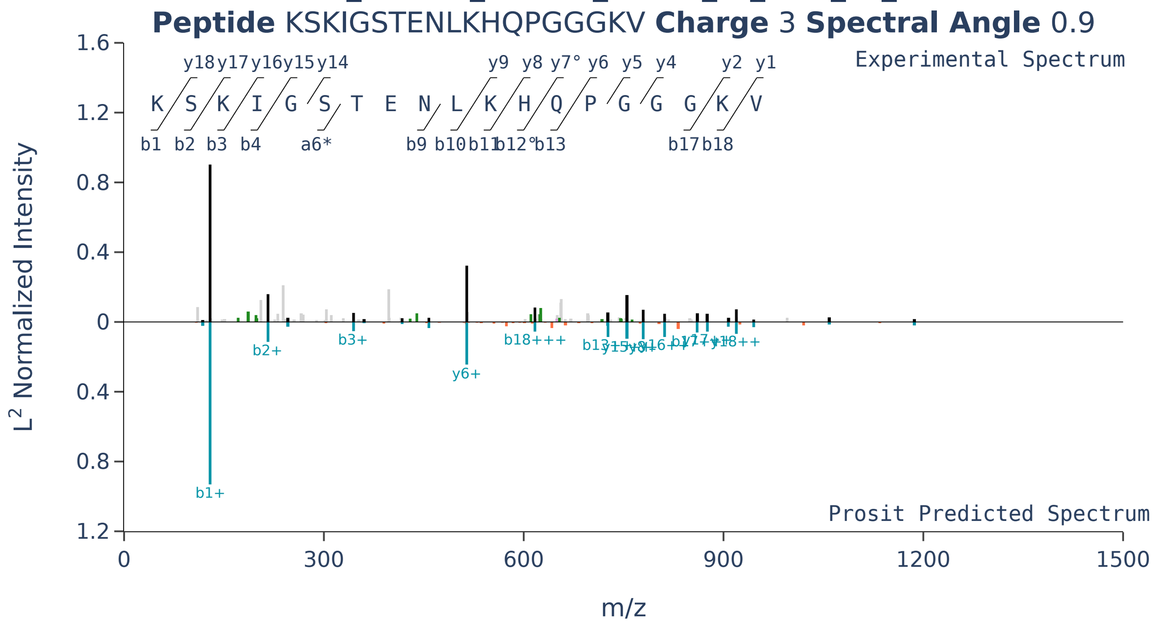


**Source** WSoh_221121_011221_HFGoe_L2_hTau_4h_R1 **Scan** 15209
**Peptide** QIINKKLDLSNVQSKC **Charge** 3 **Spectral Angle** 0.86

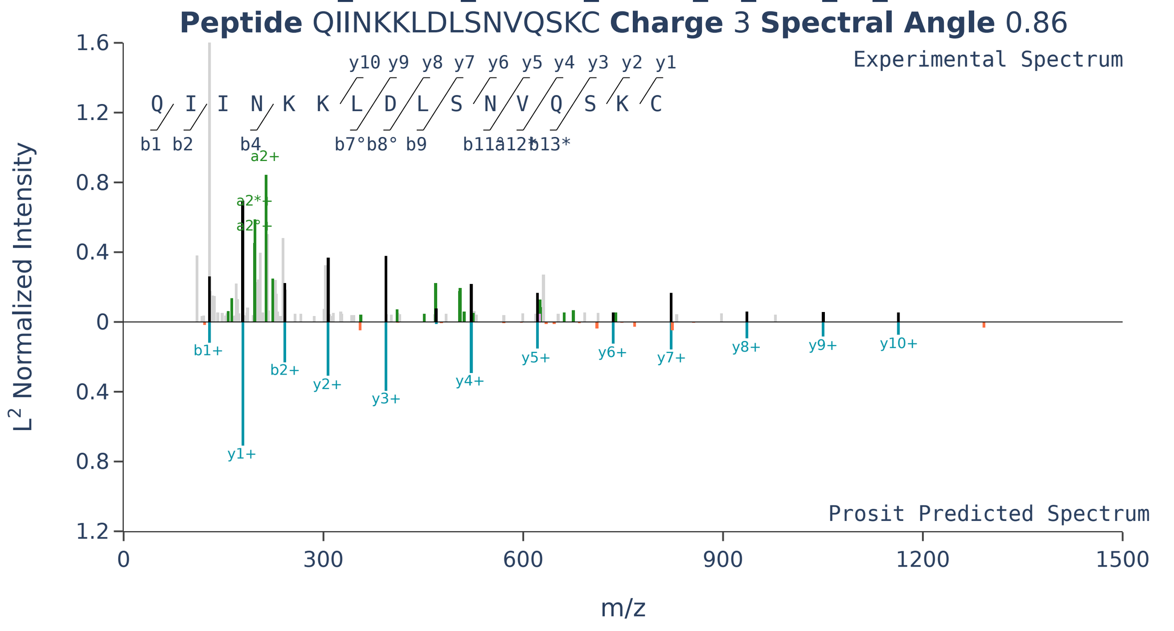


**Source** WSoh_221121_011221_HFGoe_L1_hTau_24h_R2 **Scan** 13475
**Peptide** VMEDHAGTYGLGDRKDQGGYTMHQD **Charge** 3 **Spectral Angle** 0.85

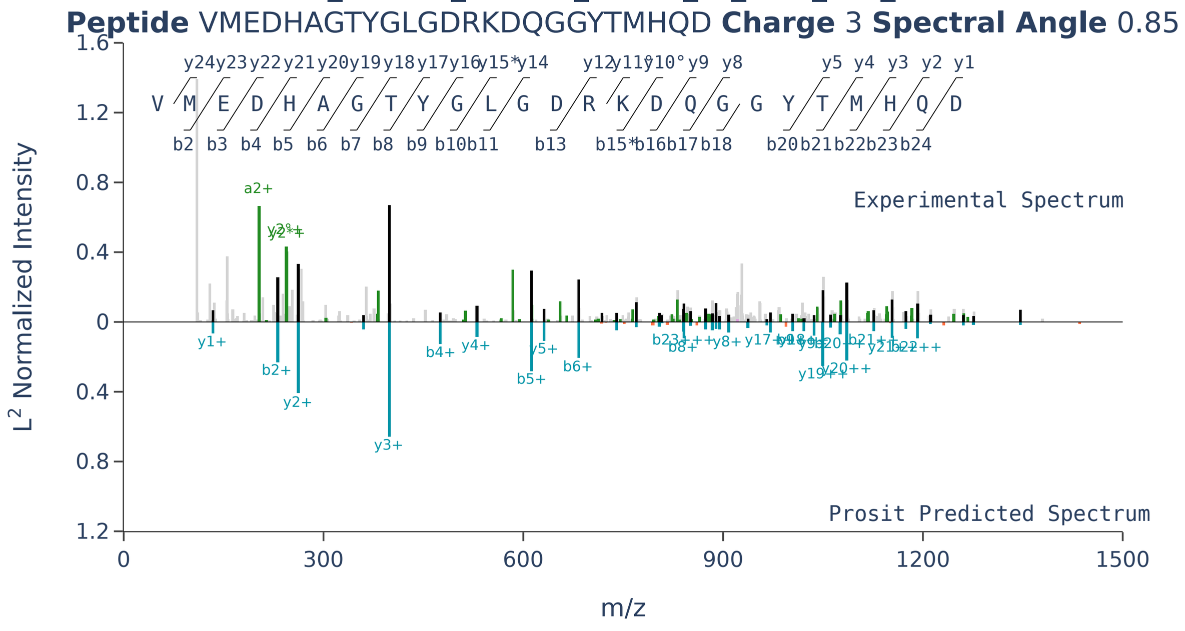

**Source** WSoh_221121_011221_HFGoe_L2_hTau_24h_R2 **Scan** 7265
**Peptide** EPGSETSDAKSTPTAE **Charge** 2 **Spectral Angle** 0.85

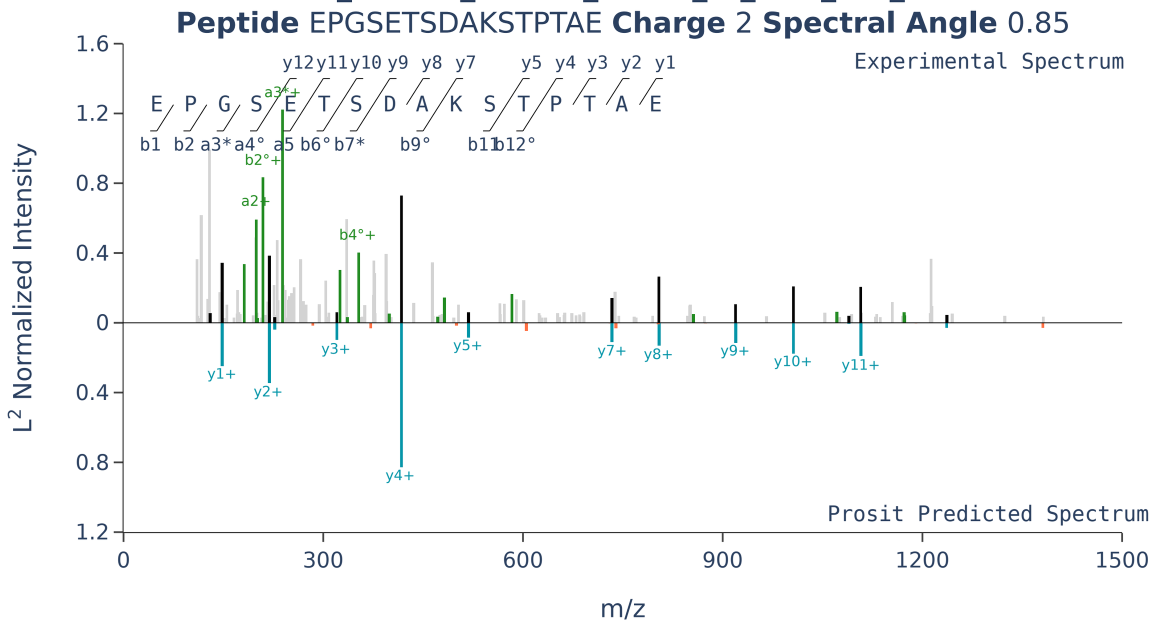


**Source** WSoh_221121_011221_HFGoe_L1_hTau_4h_R2 **Scan** 16535
**Peptide** PTREPKKVAV **Charge** 2 **Spectral Angle** 0.86

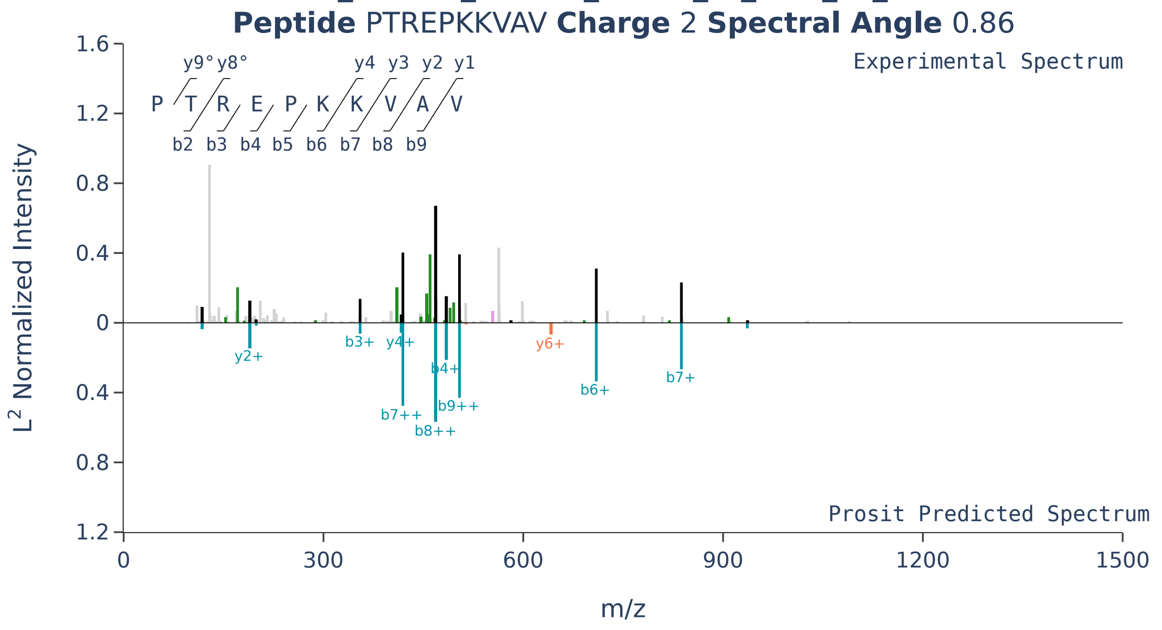


**Source** WSoh_221121_011221_HFGoe_L1_hTau_24h_R1 **Scan** 9278
**Peptide** IGSTENLKHQPGGGKVQIINKK **Charge** 3 **Spectral Angle** 0.79

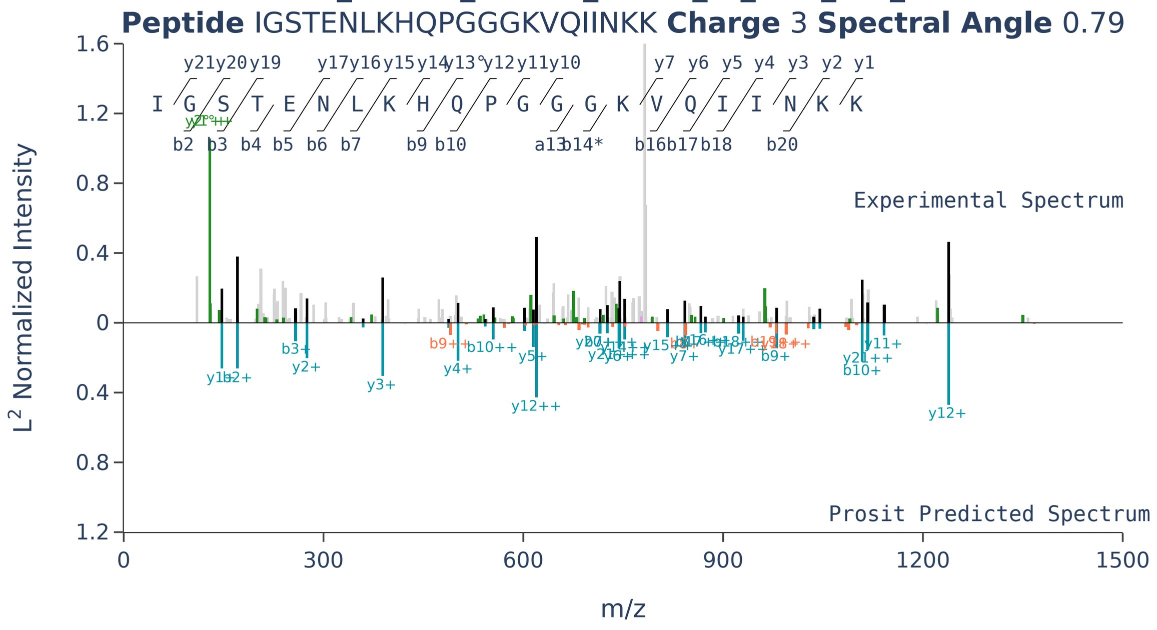


**Source** WSoh_221121_011221_HFGoe_L1_hTau_24h_R1 **Scan** 23022
**Peptide** AQPHTEIPEGTTAEEAGIGDTPSLED **Charge** 3 **Spectral Angle** 0.81

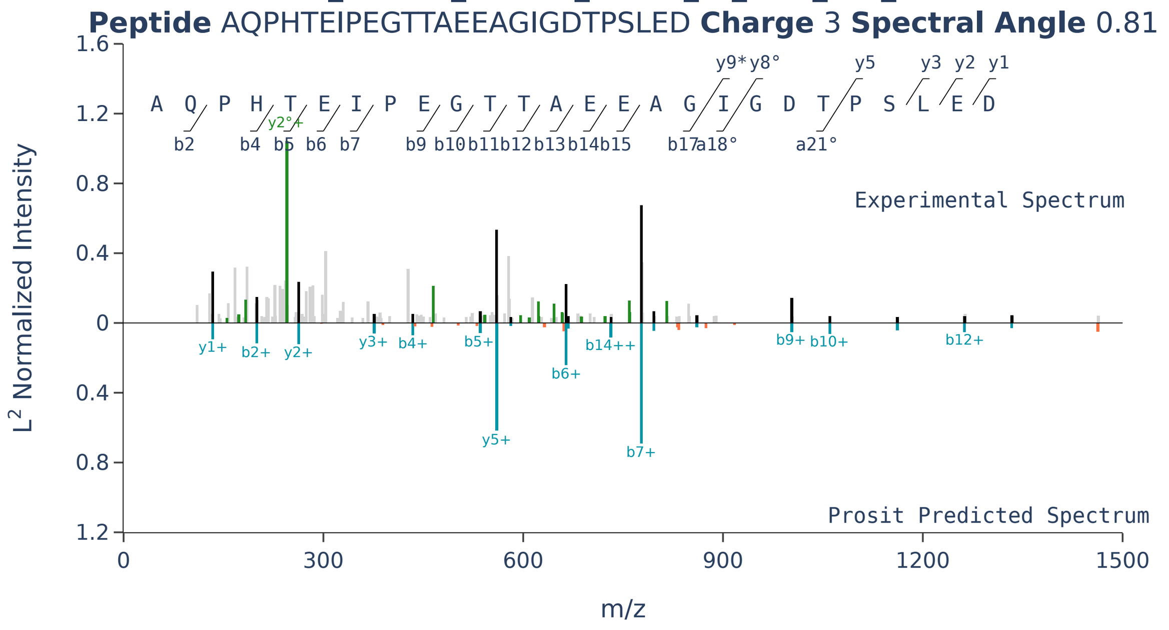


**Source** WSoh_221121_011221_HFGoe_L1_hTau_2h_R2 **Scan** 17475
**Peptide** GQVEVKSEKLDF **Charge** 3 **Spectral Angle** 0.84

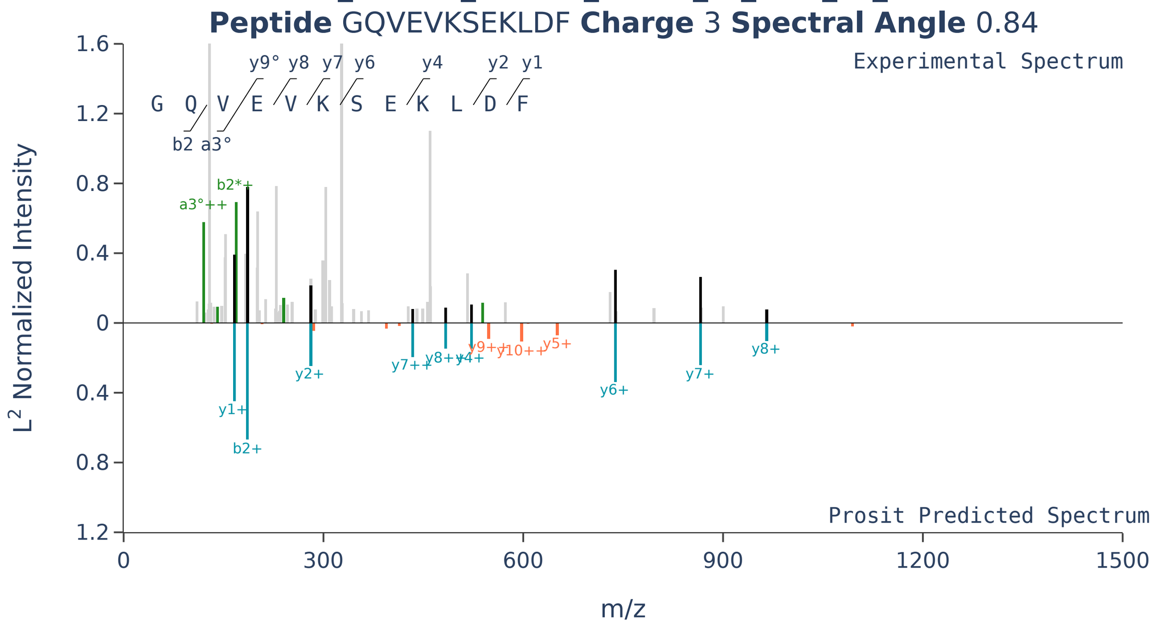


**Source** WSoh_221121_011221_HFGoe_L1_hTau_4h_R1 **Scan** 9654
**Peptide** DNITHVPGGGNKKIETHKL **Charge** 5 **Spectral Angle** 0.83

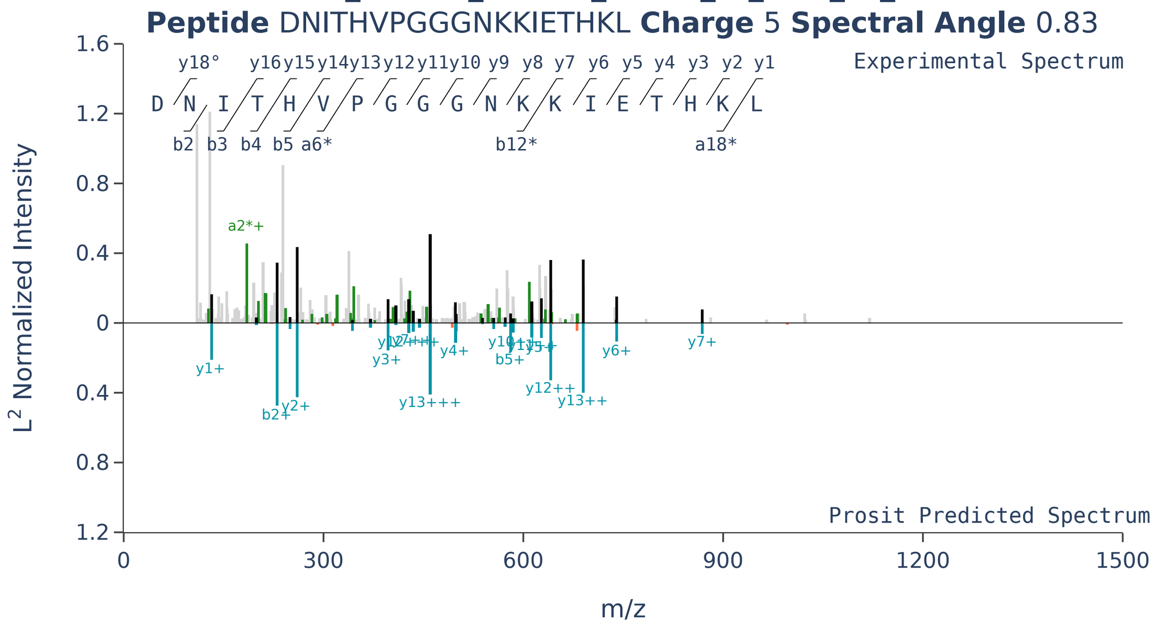


**Source** WSoh_221121_011221_HFGoe_L1_hTau_4h_R2 **Scan** 22243
**Peptide** NIKHVPGGGSVQIVYKPVDL **Charge** 3 **Spectral Angle** 0.83

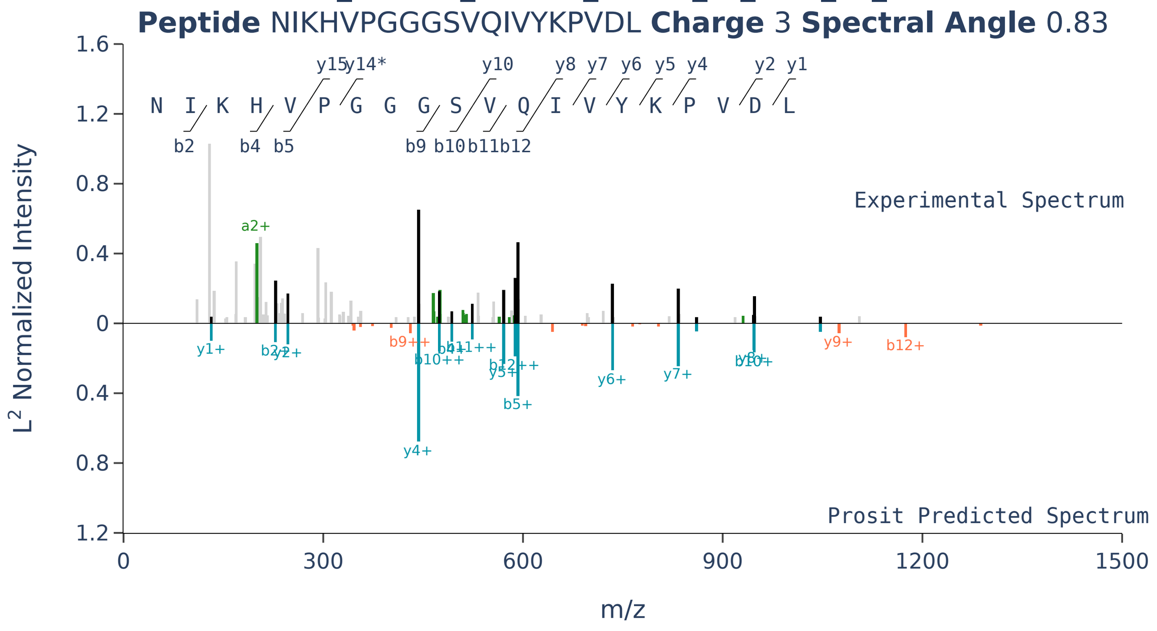


**Source** WSoh_221121_011221_HFGoe_L1_hTau_4h_R1 **Scan** 10384
**Peptide** ENLKHQPGGGKVQIINKKL **Charge** 3 **Spectral Angle** 0.79

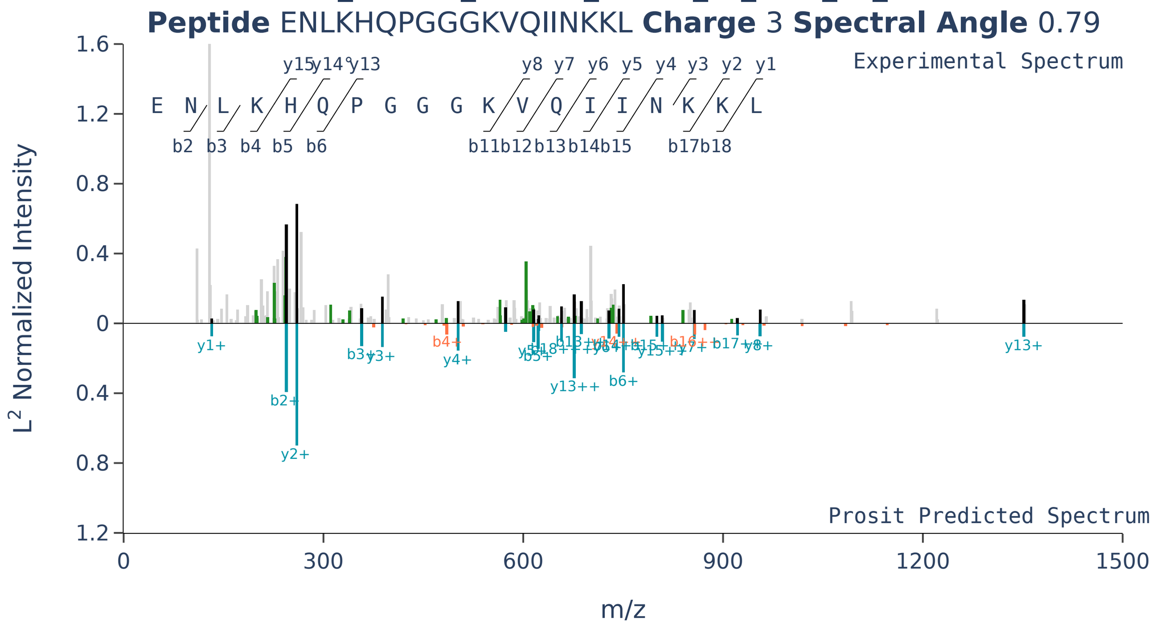


**Source** WSoh_221121_011221_HFGoe_L1_hTau_24h_R1 **Scan** 18974
**Peptide** QEGDTDAGLKESPLQTPTE **Charge** 2 **Spectral Angle** 0.91

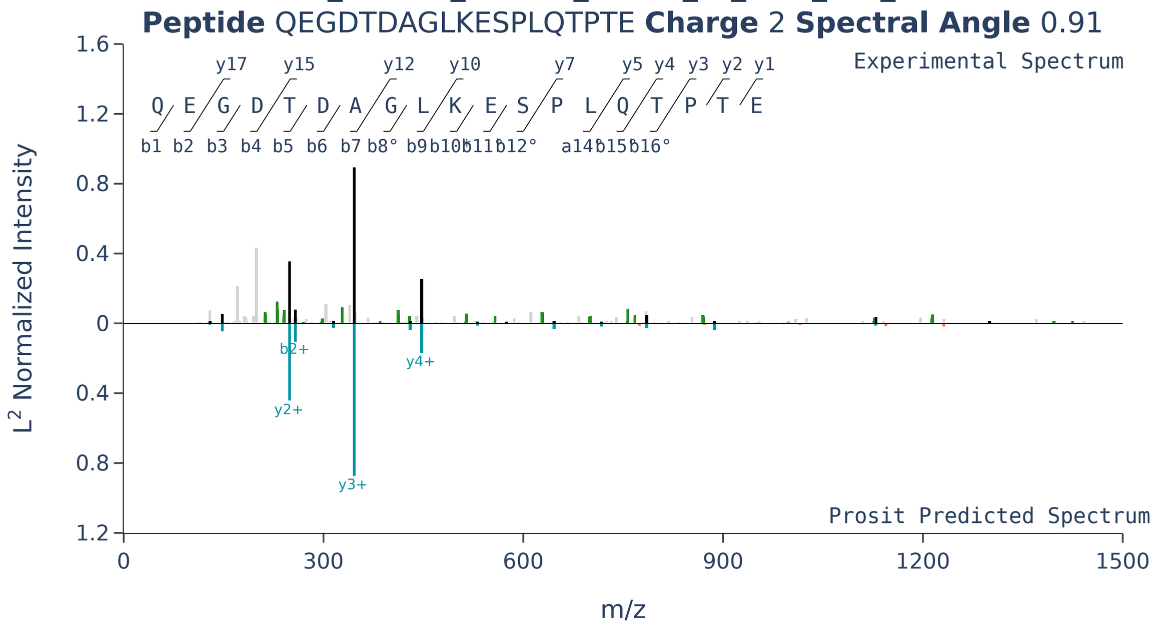


**Source** WSoh_221121_011221_HFGoe_L2_hTau_4h_R1 **Scan** 6119
**Peptide** GSTENLKHQPGGGKV **Charge** 3 **Spectral Angle** 0.84

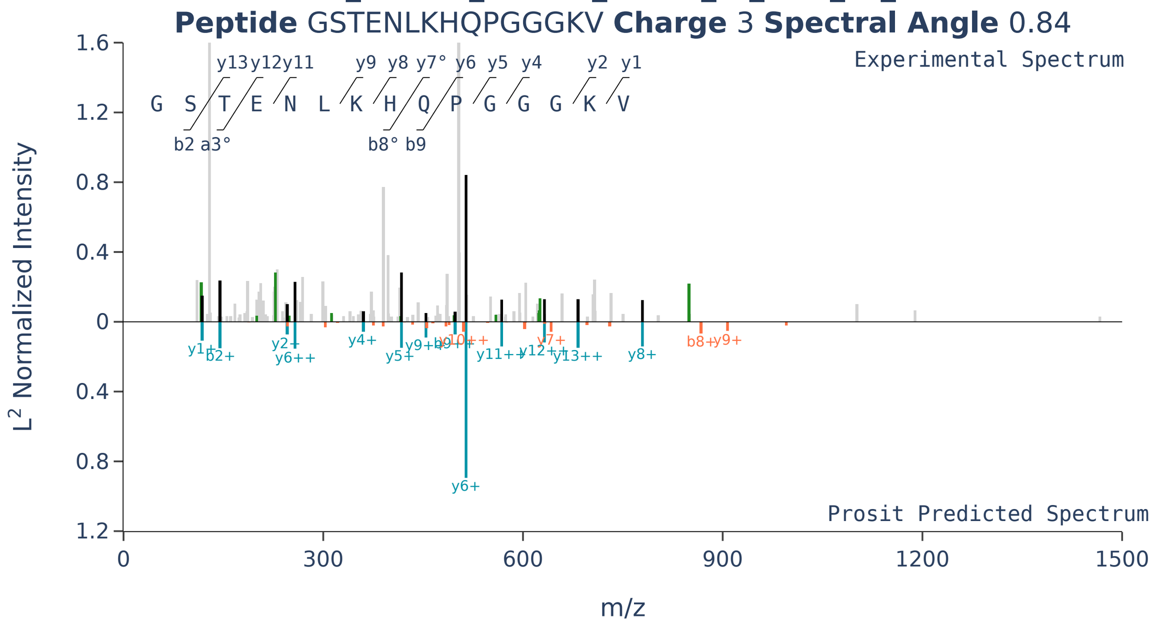


**Source** WSoh_221121_011221_HFGoe_L1_hTau_2h_R2 **Scan** 11004
**Peptide** PGGGKVQL **Charge** 2 **Spectral Angle** 0.78

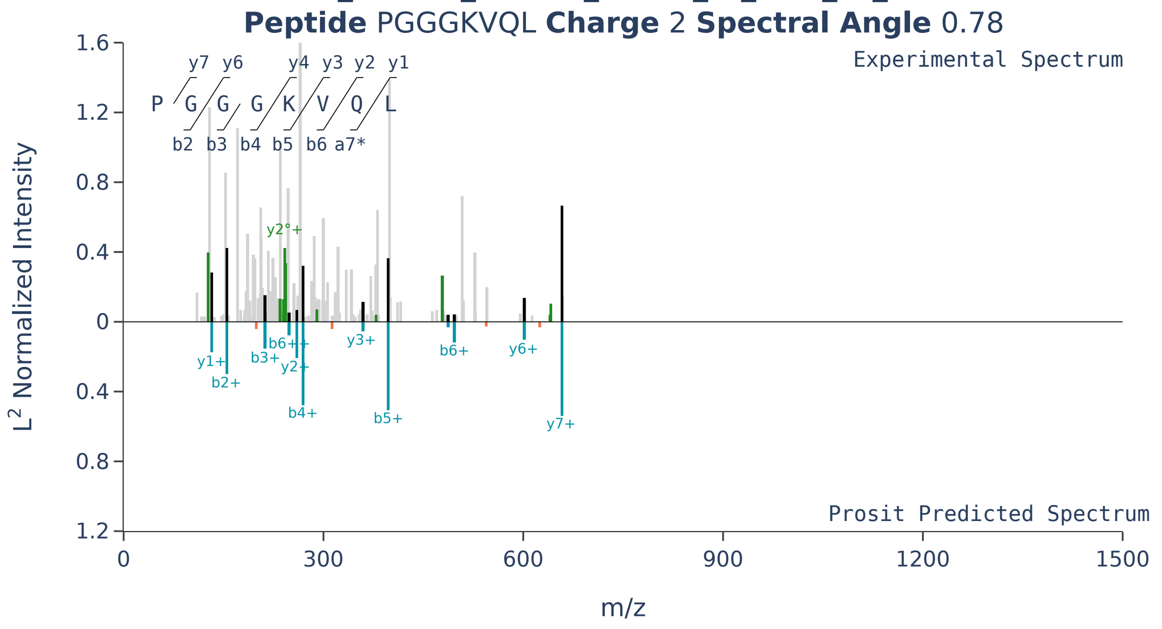


**Source** WSoh_221121_011221_HFGoe_L1_hTau_4h_R2 **Scan** 6773
**Peptide** HQDQEGDTDAGLKES **Charge** 2 **Spectral Angle** 0.8

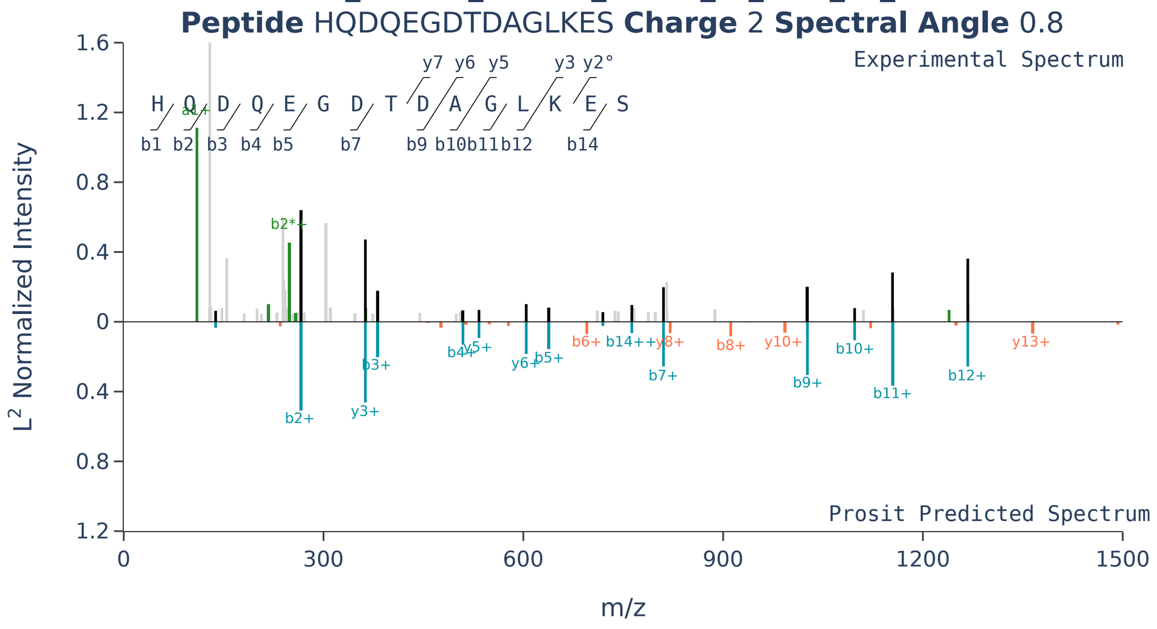


**Source** WSoh_221121_011221_HFGoe_L1_hTau_4h_R1 **Scan** 8844
**Peptide** GAPGKQAAAQPHTEI **Charge** 2 **Spectral Angle** 0.76

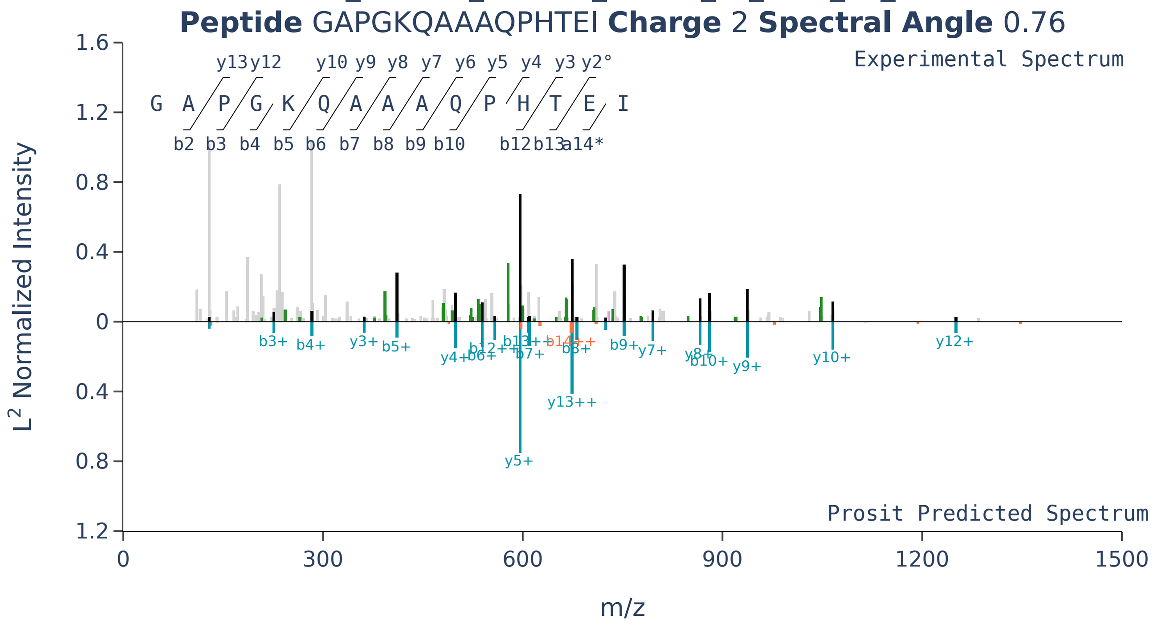


**Source** WSoh_221121_011221_HFGoe_L2_hTau_4h_R1 **Scan** 11413
**Peptide** HKLTFRENAKAKTDHGAEIVY **Charge** 4 **Spectral Angle** 0.88

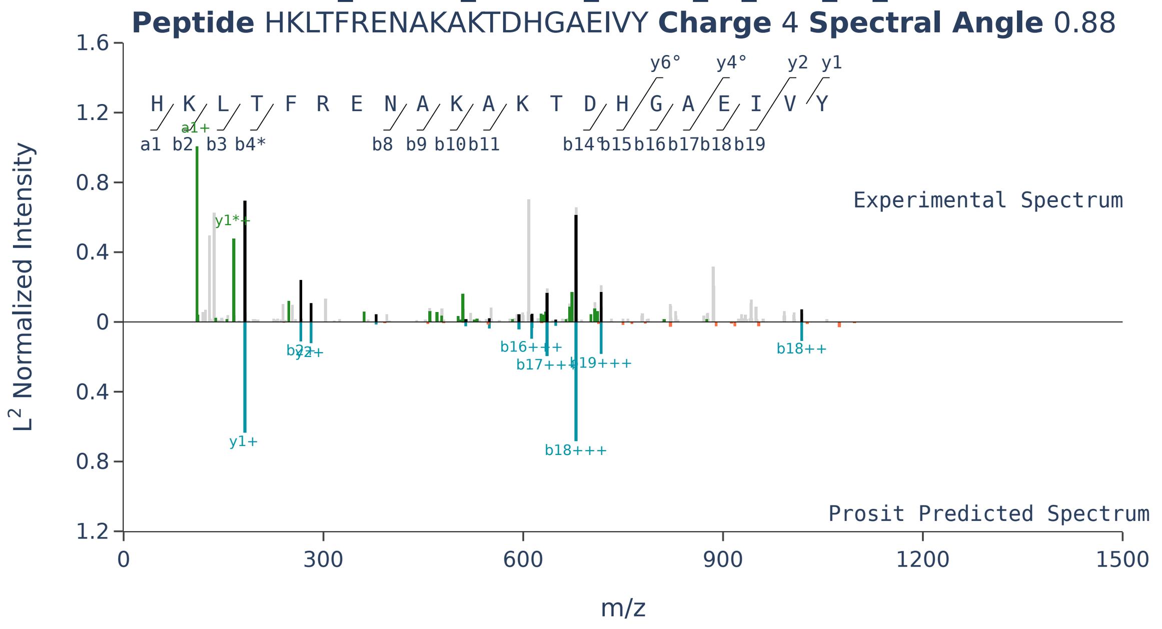


**Source** WSoh_221121_011221_HFGoe_L2_hTau_24h_R1 **Scan** 17241
**Peptide** VKSEKLDFKDRVQSKIGSLD **Charge** 3 **Spectral Angle** 0.78

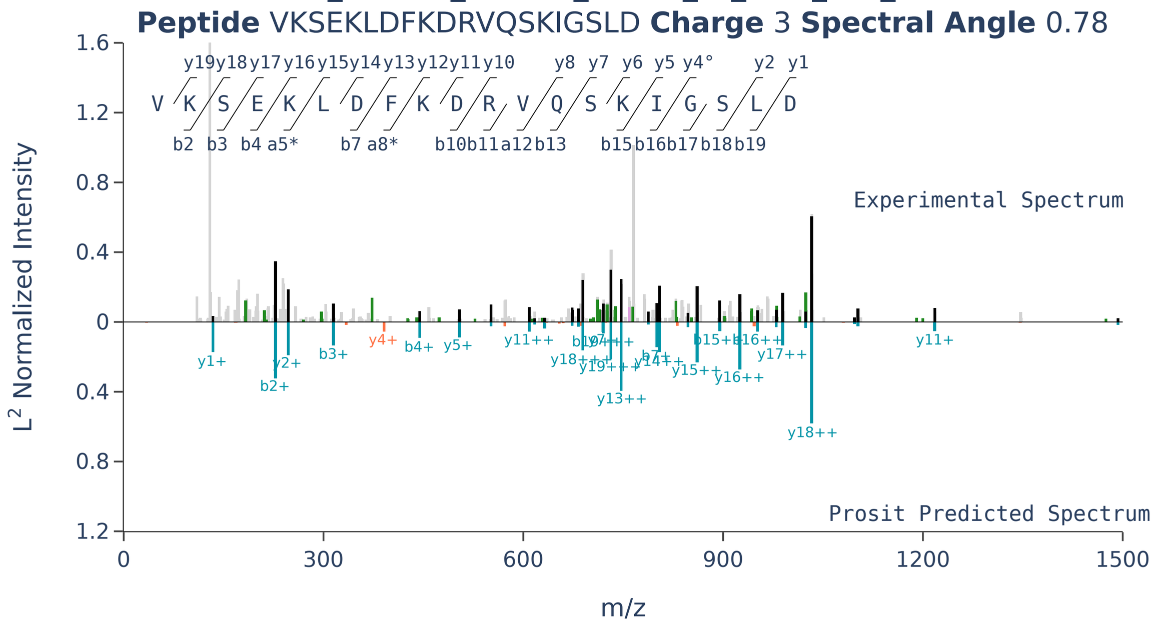

**Source** WSoh_221121_011221_HFGoe_L1_hTau_4h_R2 **Scan** 16014
**Peptide** RTPSLPTPPTREPKKVAVV **Charge** 4 **Spectral Angle** 0.9

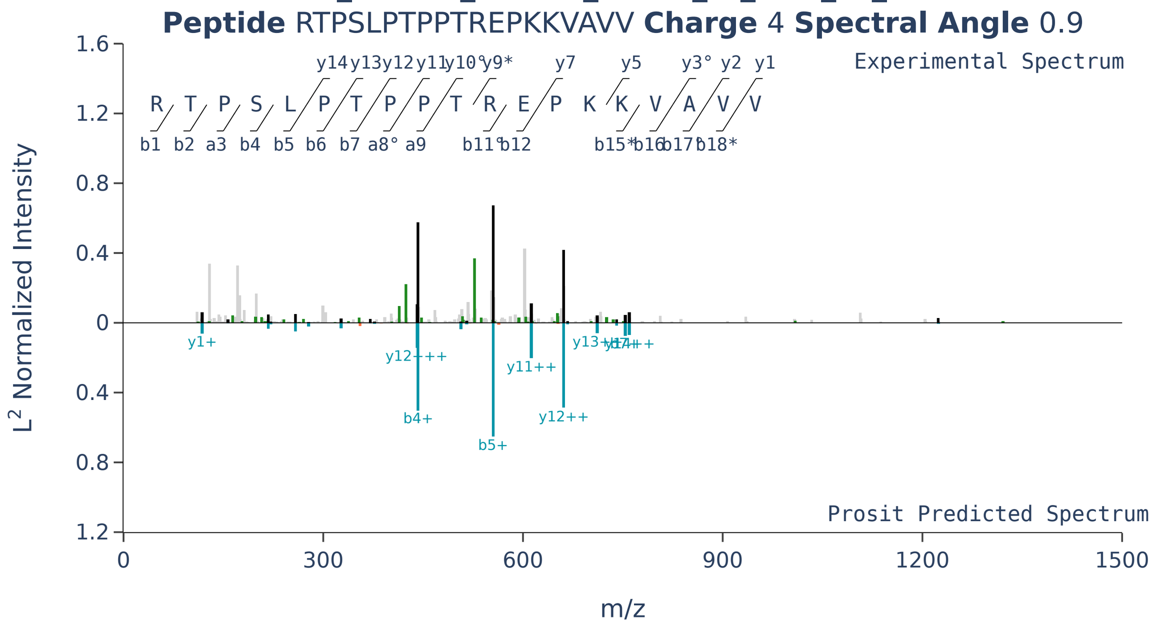


**Source** WSoh_221121_011221_HFGoe_L2_hTau_24h_R2 **Scan** 20801
**Peptide** QTAPVPMPDLKNVKSKIGSTENLKH **Charge** 6 **Spectral Angle** 0.89

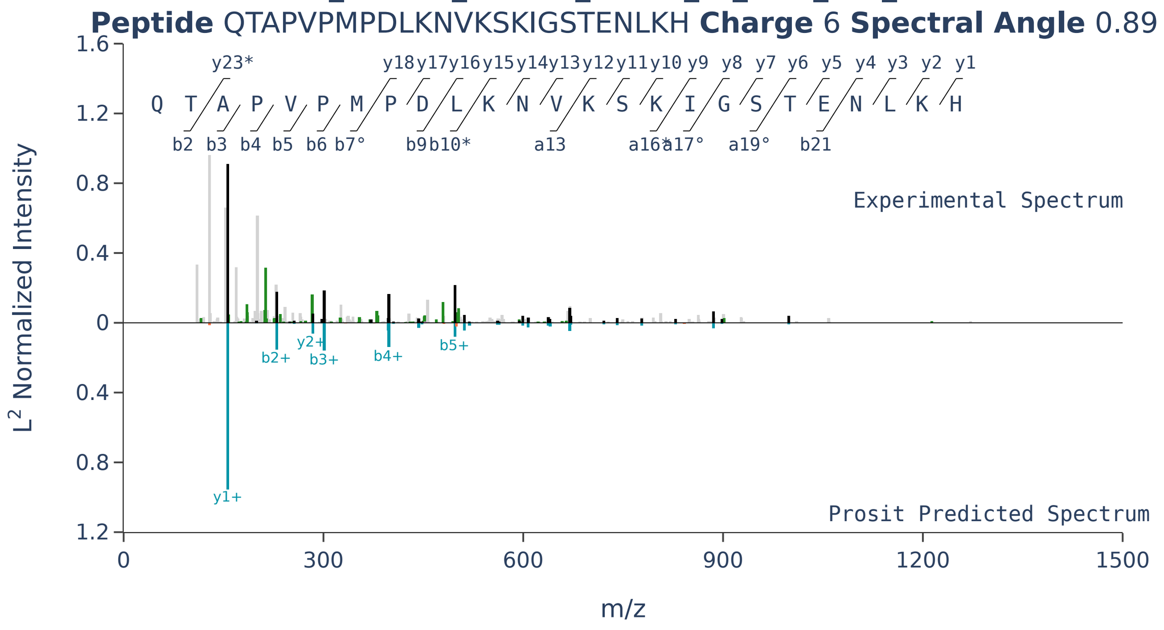


**Source** WSoh_221121_011221_HFGoe_L2_hTau_1h_R1 **Scan** 12275
**Peptide** DFKDRVQSK **Charge** 2 **Spectral Angle** 0.91

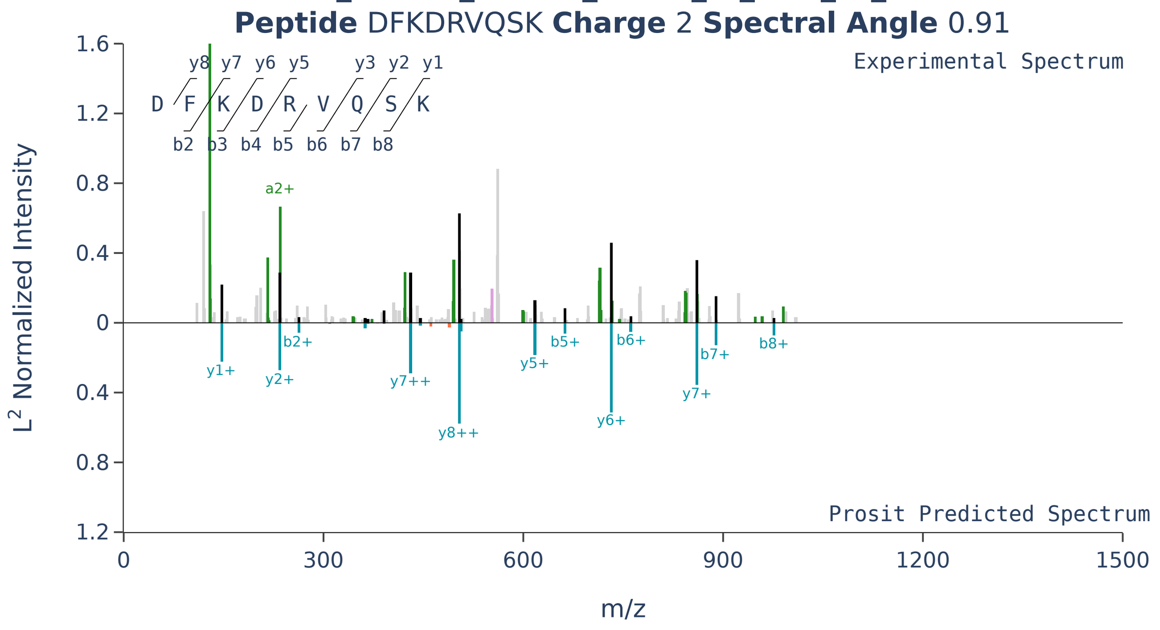


**Source** WSoh_221121_011221_HFGoe_L2_hTau_4h_R1 **Scan** 5729
**Peptide** VVRTPPKSPSSAK **Charge** 3 **Spectral Angle** 0.78

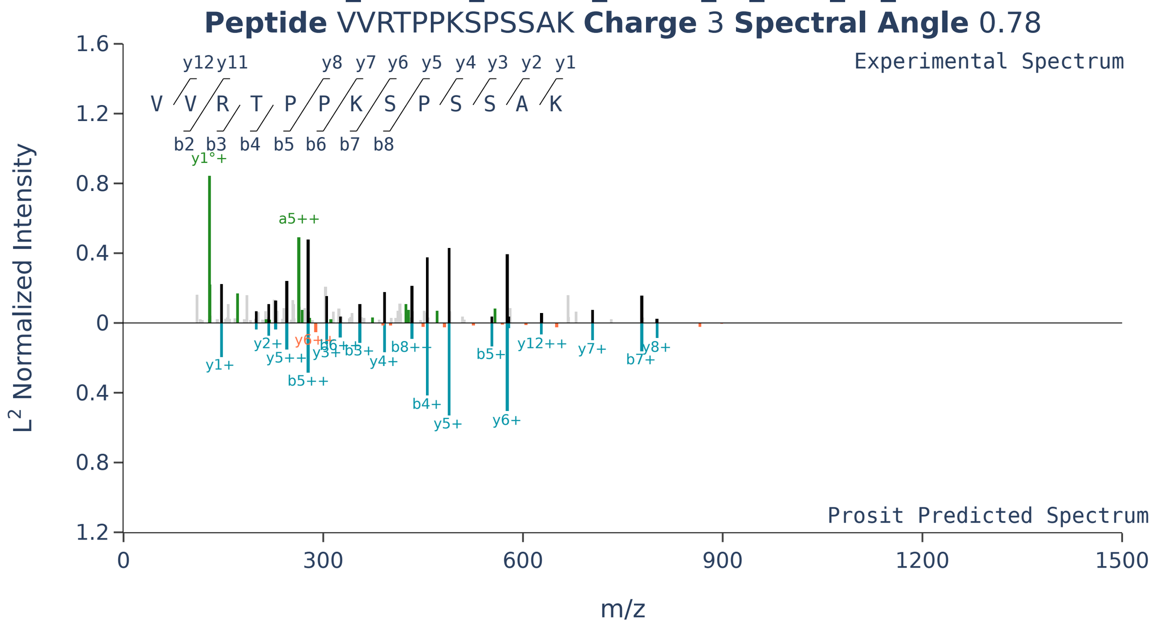


**Source** WSoh_221121_011221_HFGoe_L2_hTau_4h_R2 **Scan** 14654
**Peptide** SLGNIHHKPGGGQVEVKSEKLDF **Charge** 3 **Spectral Angle** 0.72

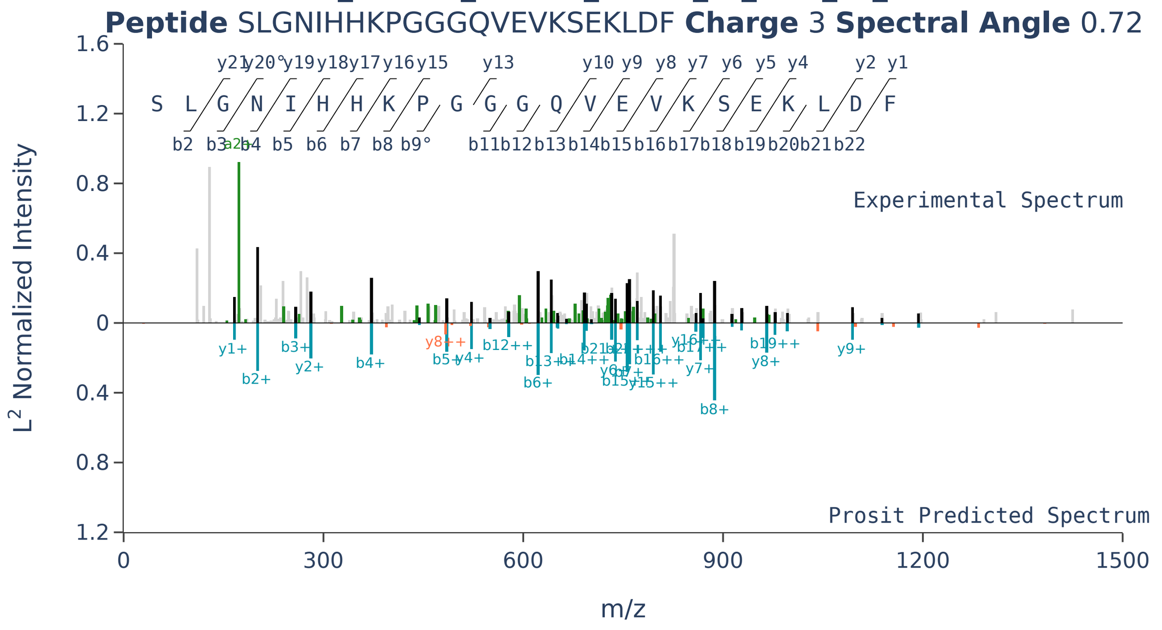


**Source** WSoh_221121_011221_HFGoe_L1_hTau_4h_R1 **Scan** 8399
**Peptide** NITHVPGGGNKKIETHKLT **Charge** 4 **Spectral Angle** 0.81

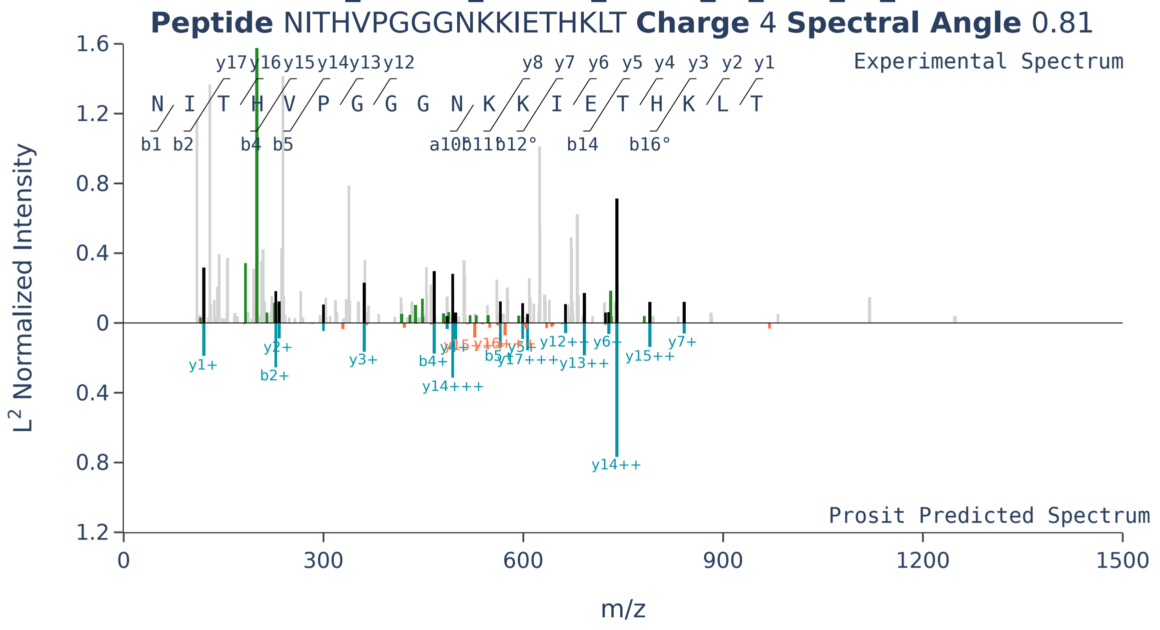


**Source** WSoh_221121_011221_HFGoe_L2_hTau_24h_R2 **Scan** 22792
**Peptide** PGGGSVQIVYKPVDLSKVTSKC **Charge** 3 **Spectral Angle** 0.8

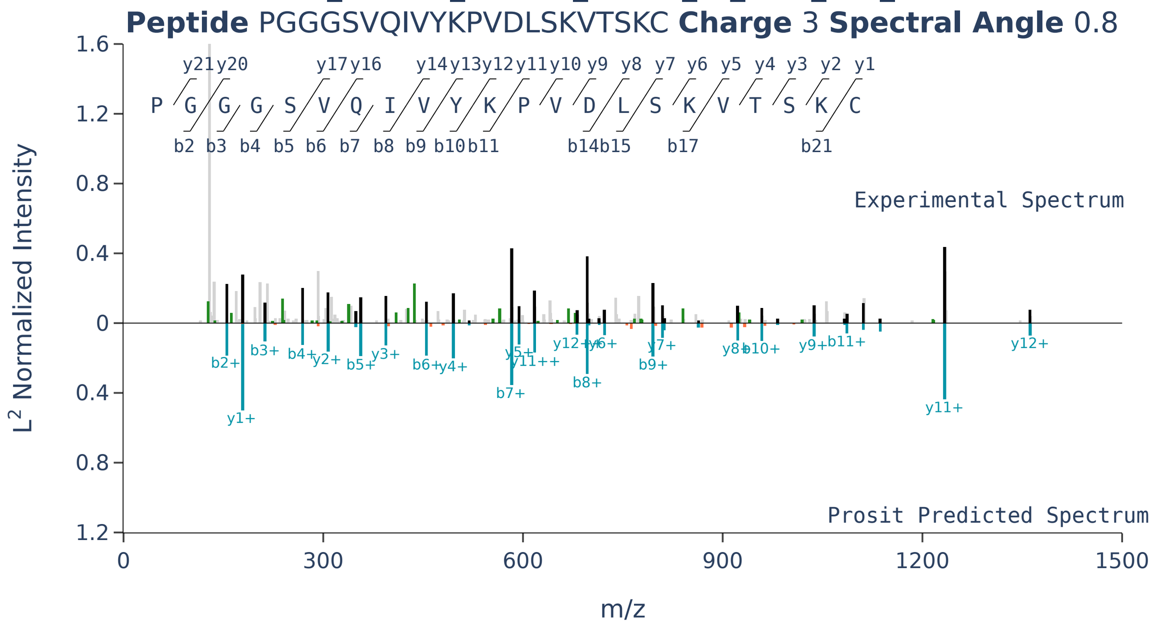


**Source** WSoh_221121_011221_HFGoe_L1_hTau_24h_R1 **Scan** 19175
**Peptide** EGDTDAGLKESPLQTPTED **Charge** 2 **Spectral Angle** 0.9

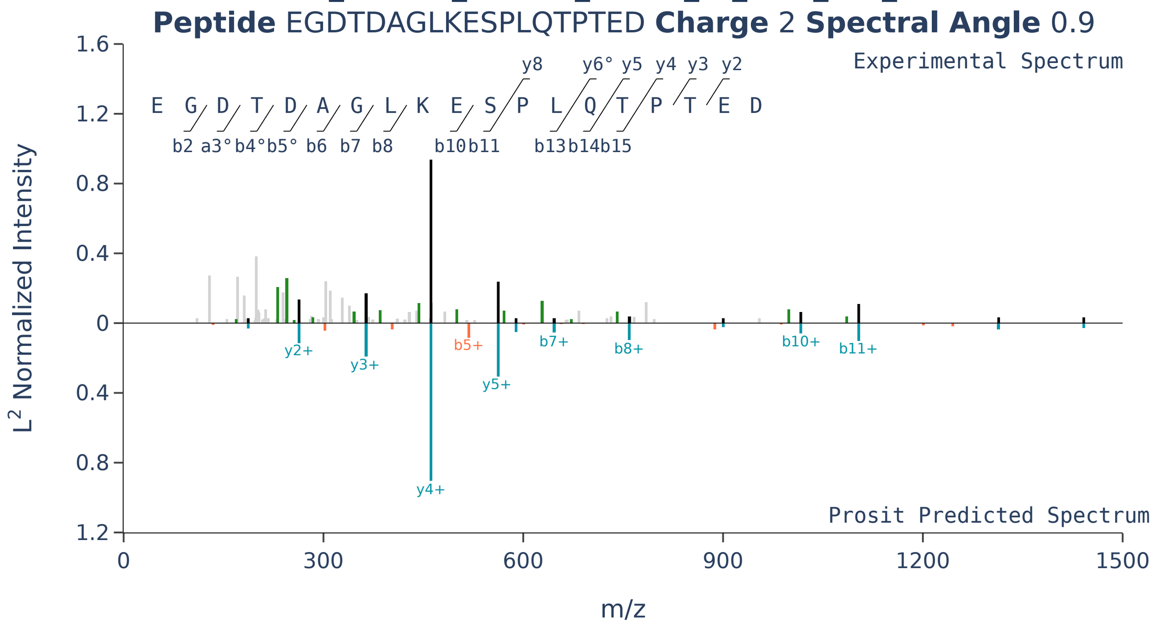


**Source** WSoh_221121_011221_HFGoe_L2_hTau_4h_R2 **Scan** 15449
**Peptide** DLSNVQSKCGSKDNIKHVPGGGSVQI **Charge** 4 **Spectral Angle** 0.81

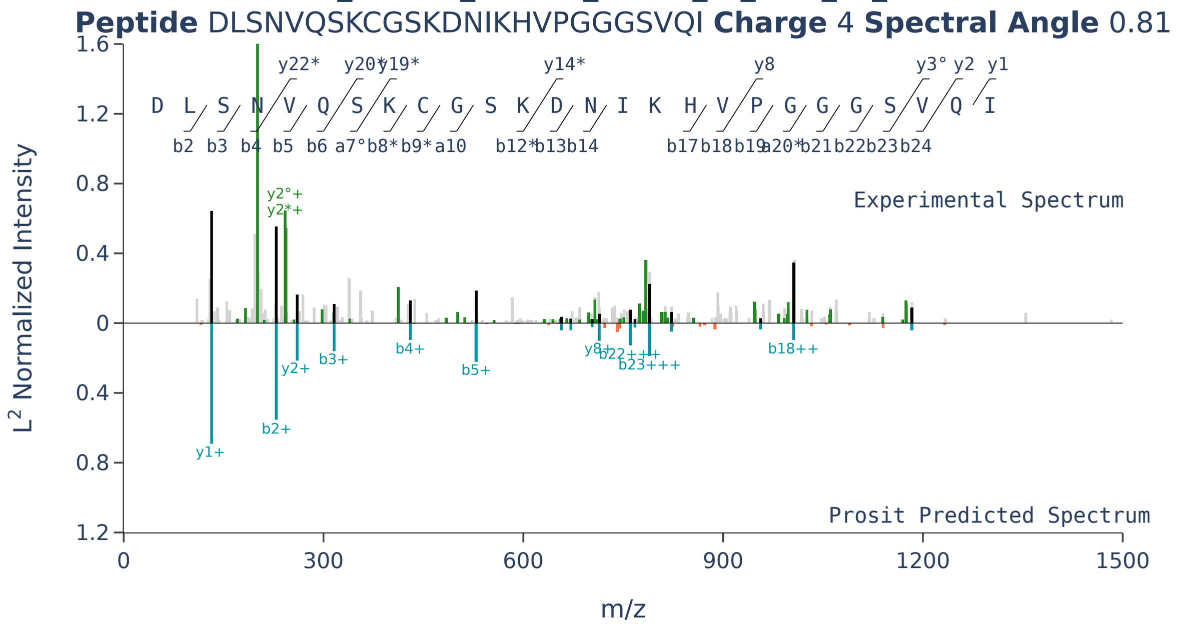


**Source** WSoh_221121_011221_HFGoe_L1_hTau_24h_R2 **Scan** 12303
**Peptide** GAPGKQAAAQPHTEIPEGTTAEE **Charge** 3 **Spectral Angle** 0.81

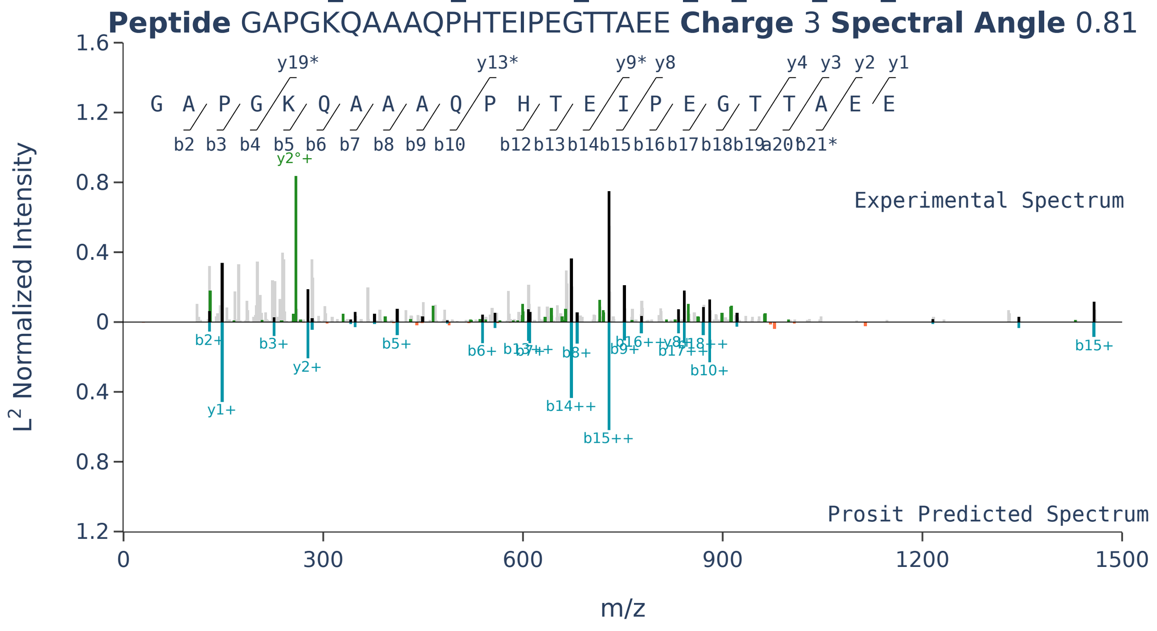


**Source** WSoh_221121_011221_HFGoe_L1_hTau_2h_R2 **Scan** 8868
**Peptide** NLTHVPGGGNKKIET **Charge** 3 **Spectral Angle** 0.78

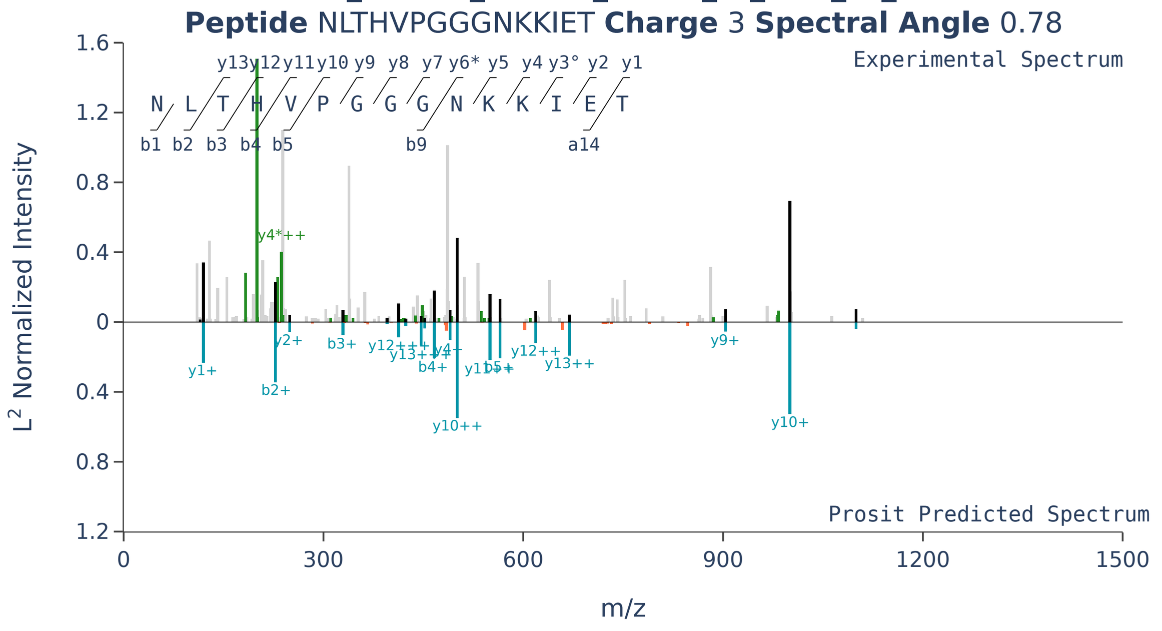


**Source** WSoh_221121_011221_HFGoe_L2_hTau_24h_R1 **Scan** 5701
**Peptide** RTPPKSPSSAKSRLQ **Charge** 4 **Spectral Angle** 0.85

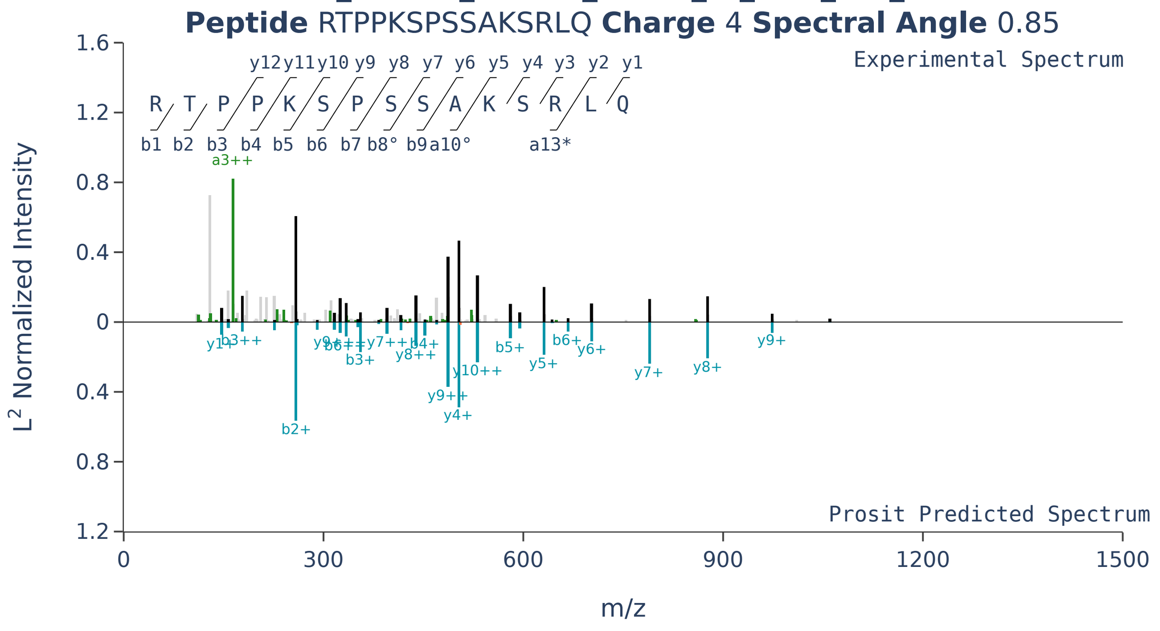


**Source** WSoh_221121_011221_HFGoe_L1_hTau_24h_R1 **Scan** 20821
**Peptide** PPKSPSSAKSRLQTAPVPMPDLKNV **Charge** 4 **Spectral Angle** 0.83

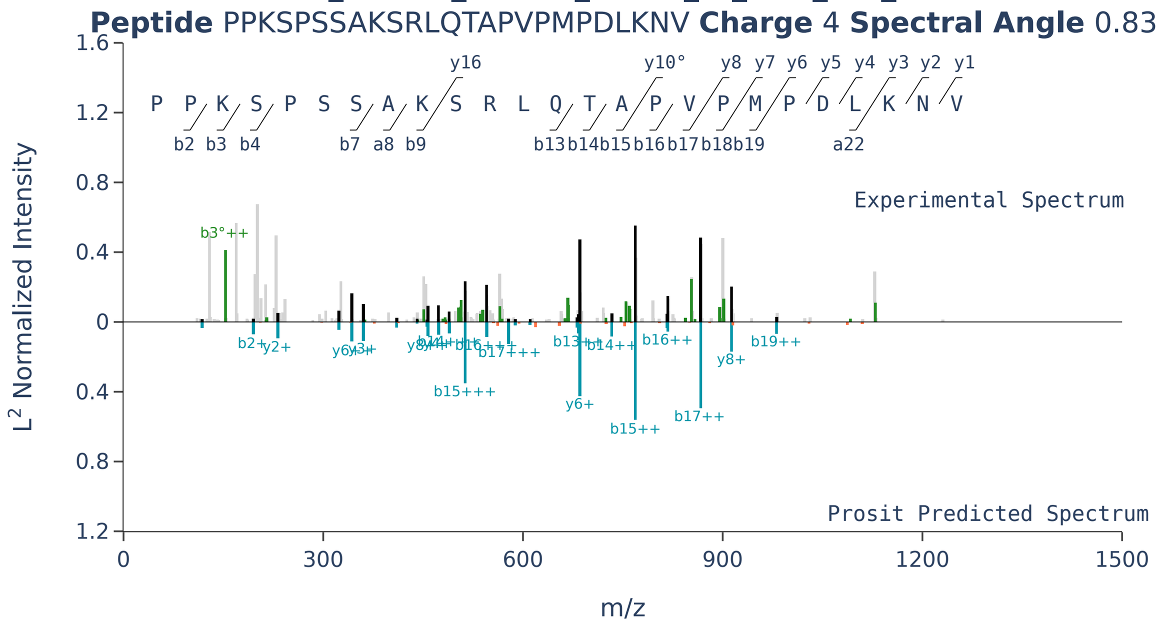


**Source** WSoh_221121_011221_HFGoe_L2_hTau_24h_R2 **Scan** 5955
**Peptide** AKAKTDHGAEI **Charge** 2 **Spectral Angle** 0.71

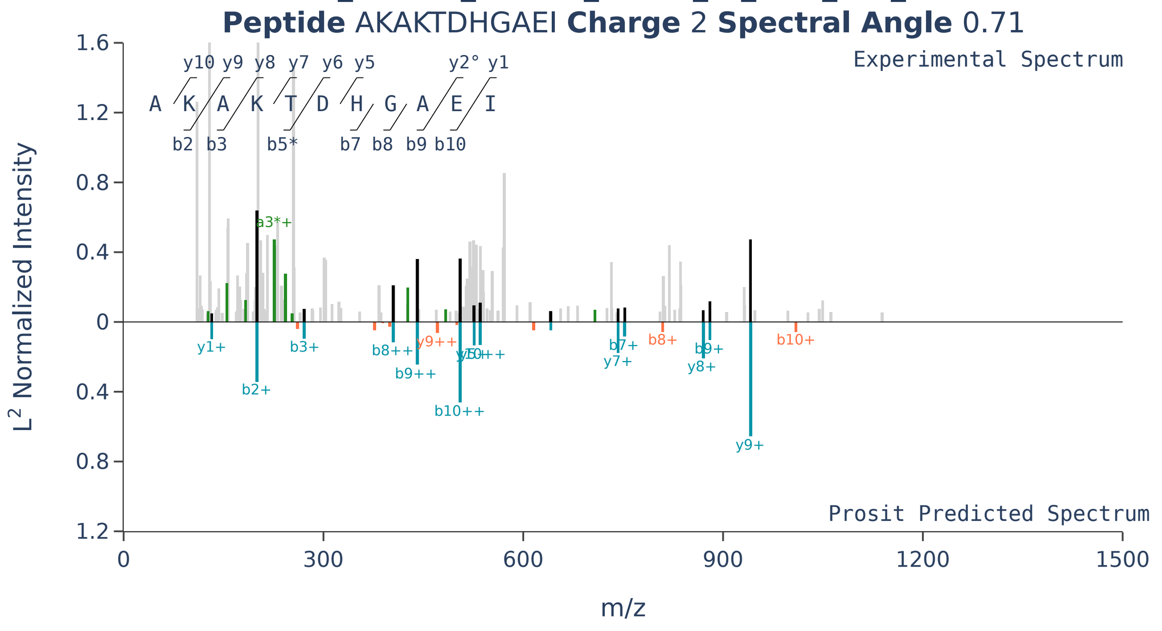


**Source** WSoh_221121_011221_HFGoe_L2_hTau_4h_R1 **Scan** 17892
**Peptide** VPGGGSVQIVYK **Charge** 2 **Spectral Angle** 0.86

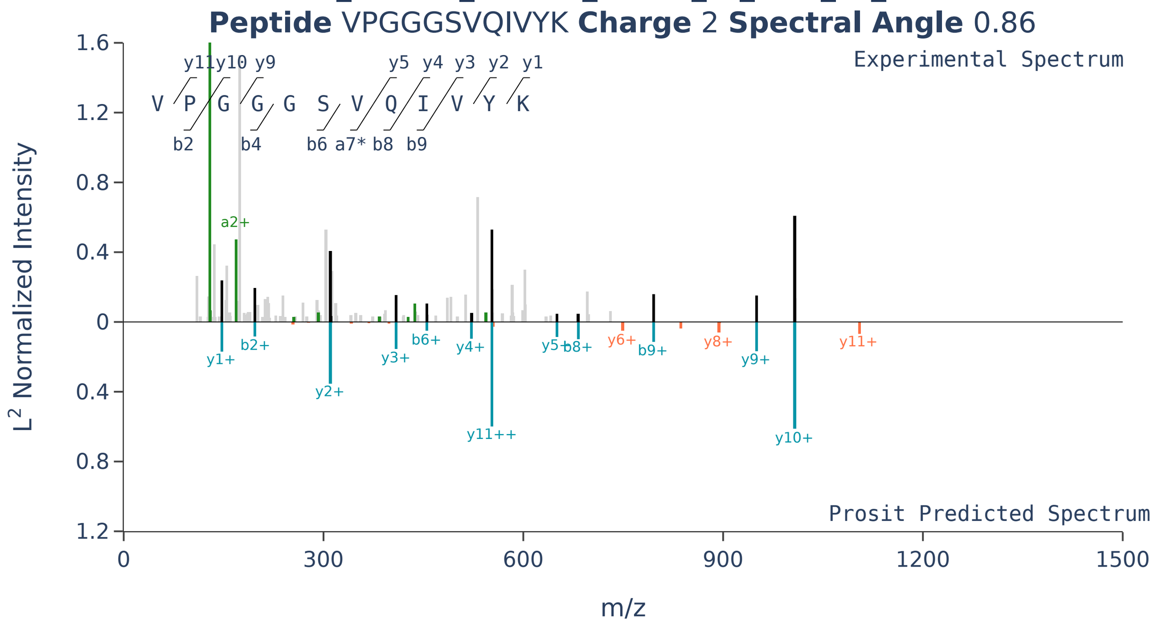

**Source** WSoh_221121_011221_HFGoe_L1_hTau_24h_R2 **Scan** 20518
**Peptide** TAPVPMPDLKNVKSKIGSTENLKHQ **Charge** 3 **Spectral Angle** 0.79

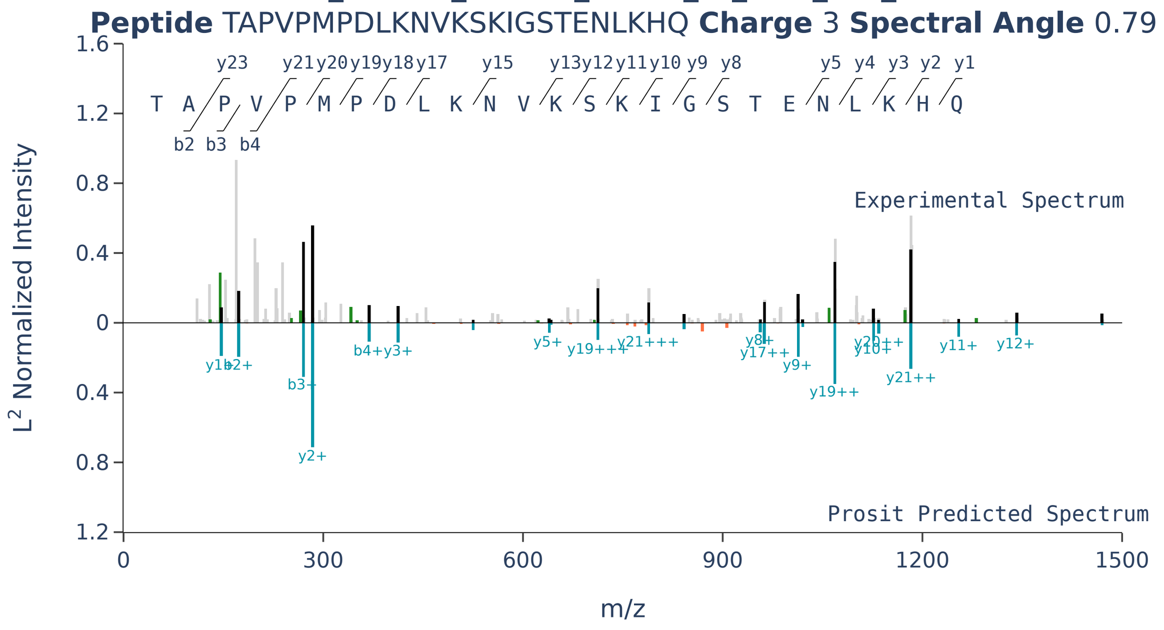


**Source** WSoh_221121_011221_HFGoe_L1_hTau_24h_R2 **Scan** 15689
**Peptide** GTYGLGDRKDQGGYTM **Charge** 2 **Spectral Angle** 0.82

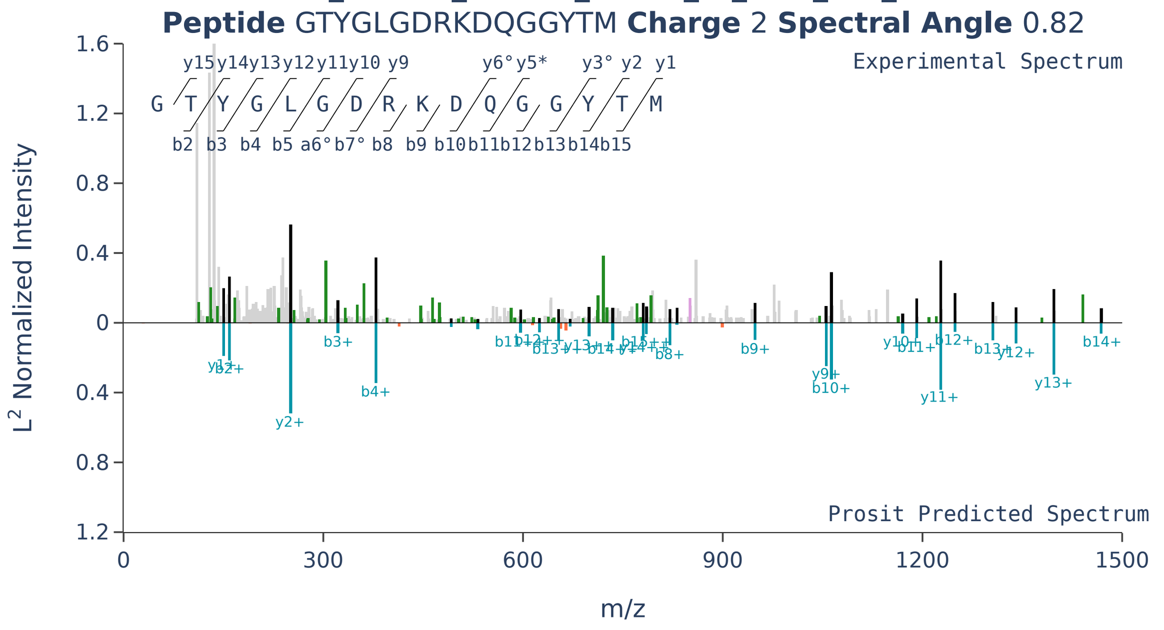


**Source** WSoh_221121_011221_HFGoe_L2_hTau_24h_R2 **Scan** 7061
**Peptide** LGNIHHKPGGGQVEVKS **Charge** 3 **Spectral Angle** 0.78

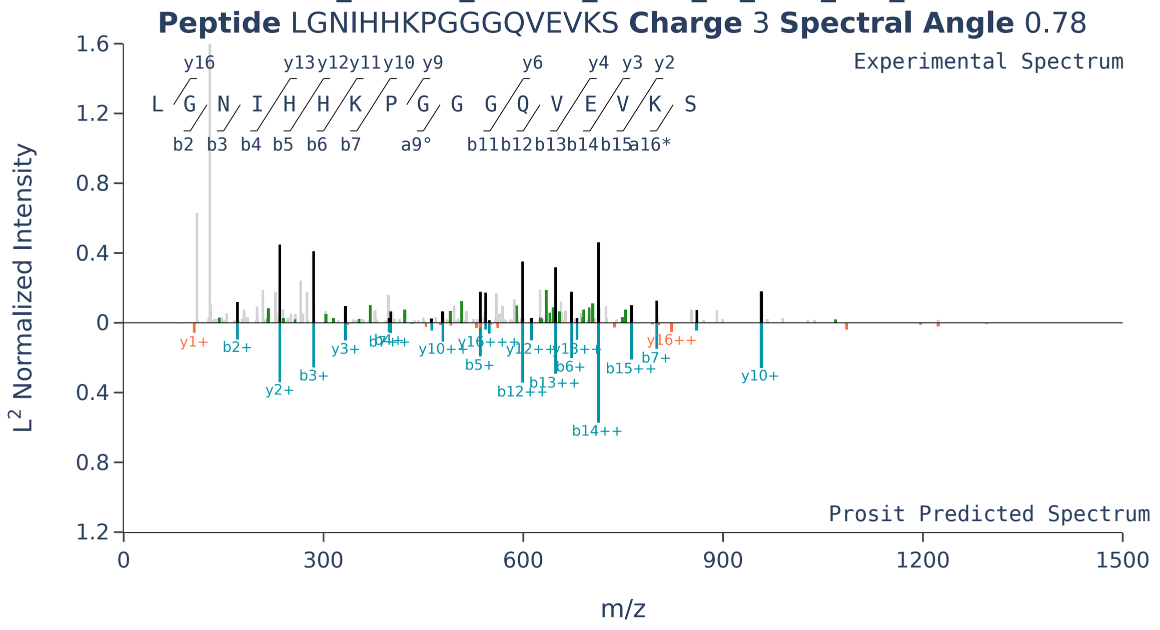


**Source** WSoh_221121_011221_HFGoe_L1_hTau_24h_R2 **Scan** 13033
**Peptide** SNVQSKCGSKDNIKHVPGGGSVQI **Charge** 4 **Spectral Angle** 0.85

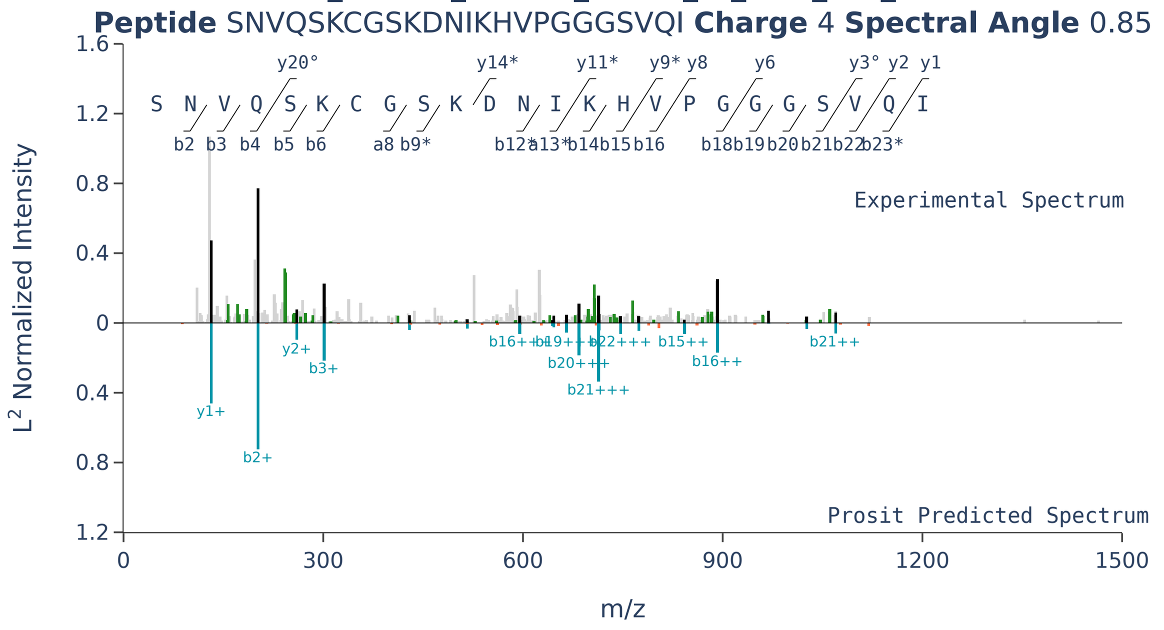


**Source** WSoh_221121_011221_HFGoe_L1_hTau_24h_R1 **Scan** 22589
**Peptide** TGSIDMVDSPQL **Charge** 2 **Spectral Angle** 0.81

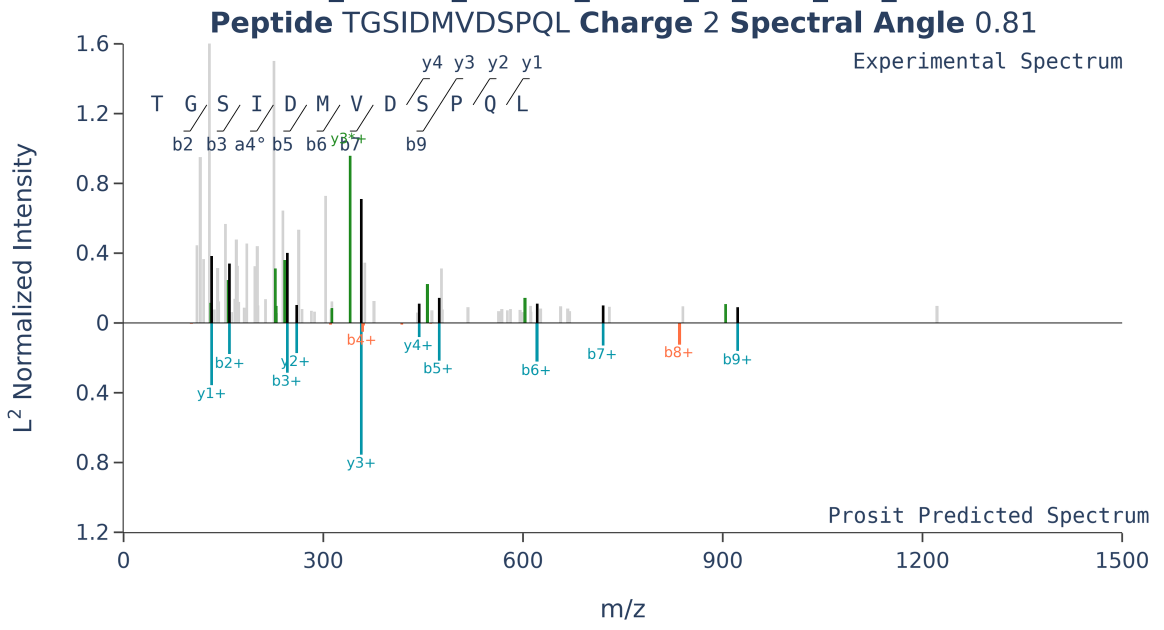


**Source** WSoh_221121_011221_HFGoe_L2_hTau_24h_R2 **Scan** 11388
**Peptide** DEGAPGKQAAAQPHTEL **Charge** 2 **Spectral Angle** 0.83

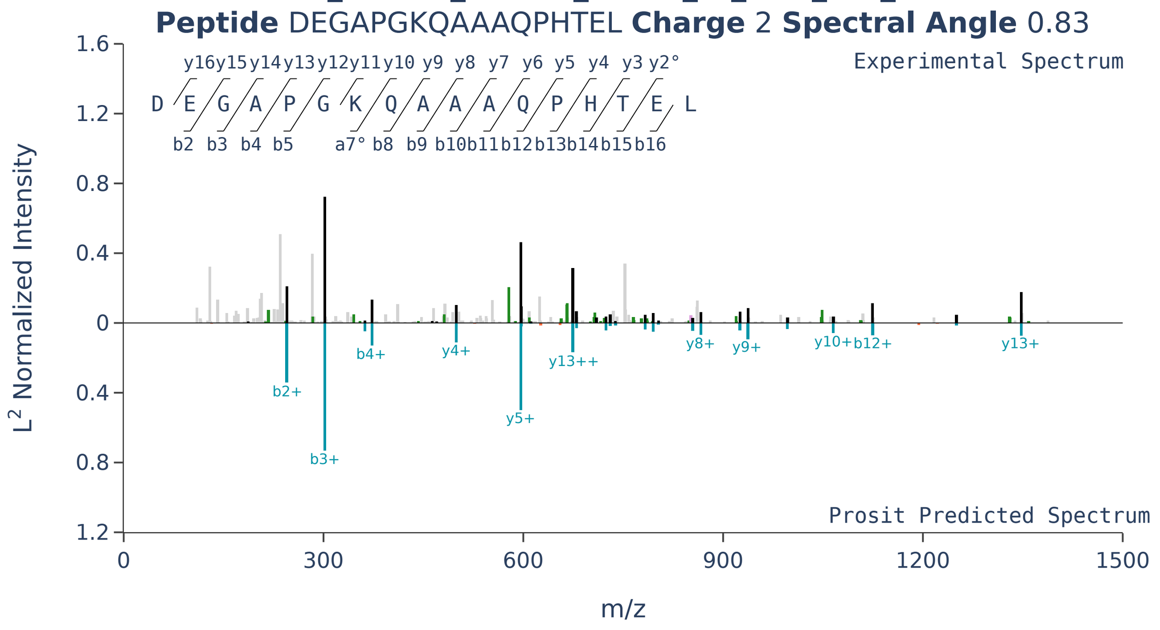


**Source** WSoh_221121_011221_HFGoe_L1_hTau_24h_R1 **Scan** 21800
**Peptide** SDAKSTPTAEDVTAPLVD **Charge** 2 **Spectral Angle** 0.85

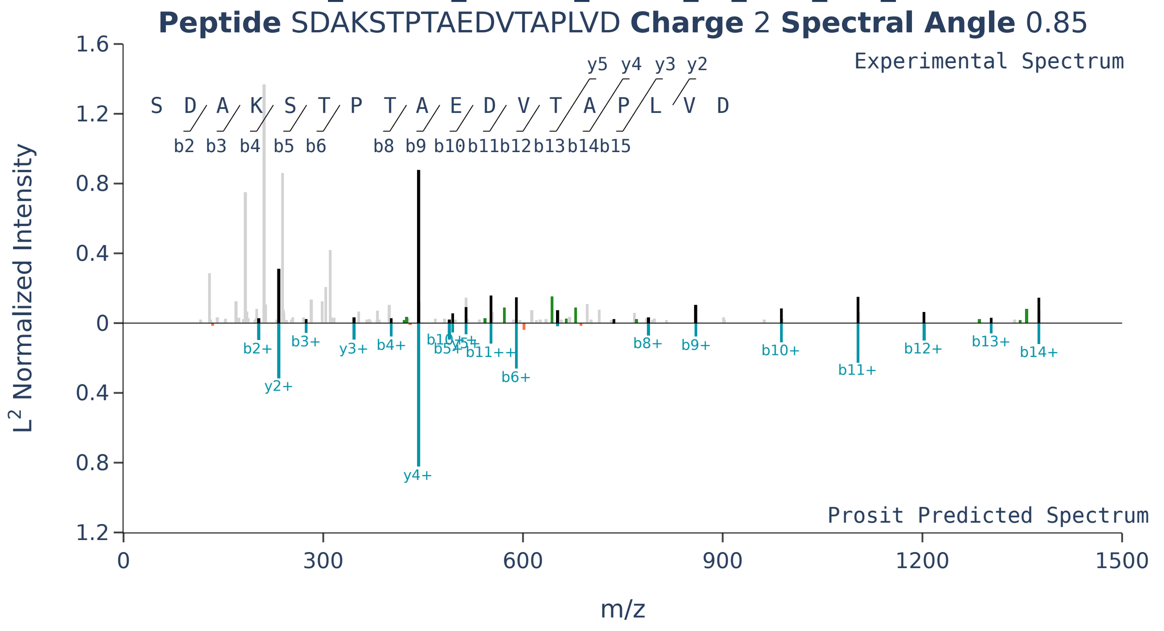


**Source** WSoh_221121_011221_HFGoe_L2_hTau_1h_R2 **Scan** 10761
**Peptide** RVQSKIGSL **Charge** 2 **Spectral Angle** 0.85

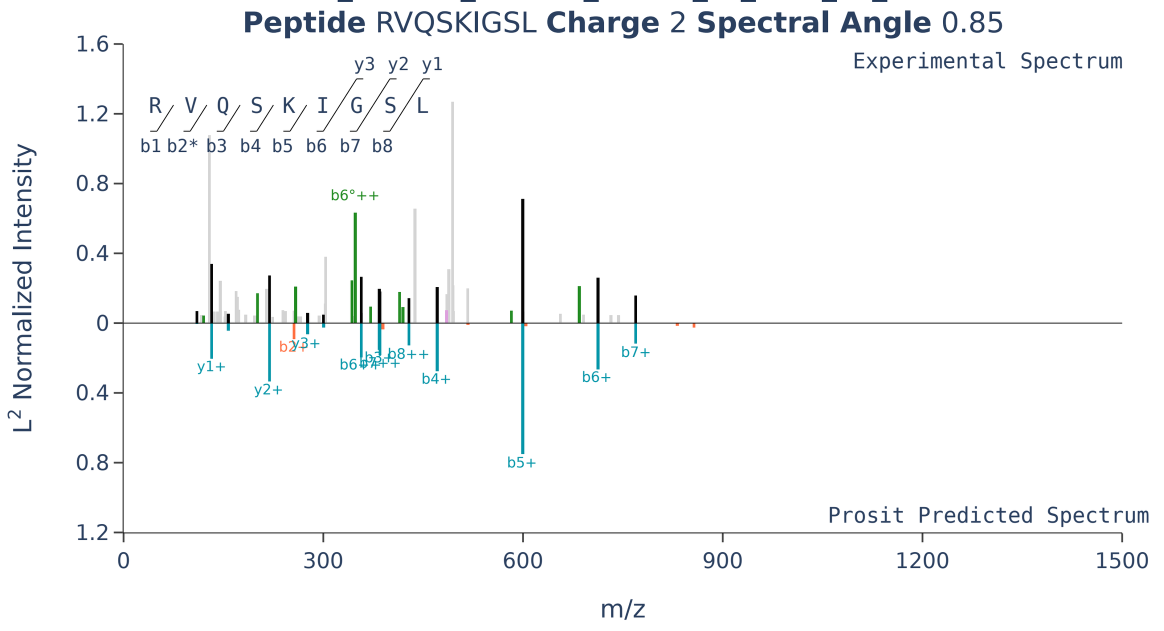


**Source** WSoh_221121_011221_HFGoe_L2_hTau_4h_R1 **Scan** 7323
**Peptide** HKPGGGQVEVKSEKL **Charge** 4 **Spectral Angle** 0.77

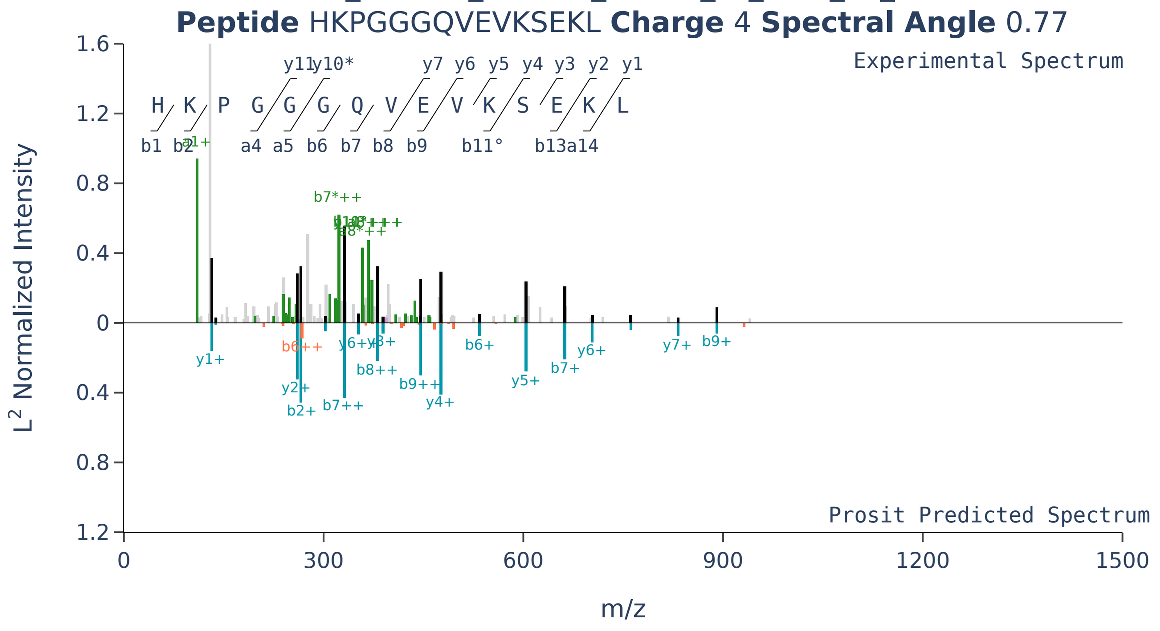


**Source** WSoh_221121_011221_HFGoe_L1_hTau_24h_R2 **Scan** 9408
**Peptide** PTEDGSEEPGSETSDAKSTPTAED **Charge** 2 **Spectral Angle** 0.77

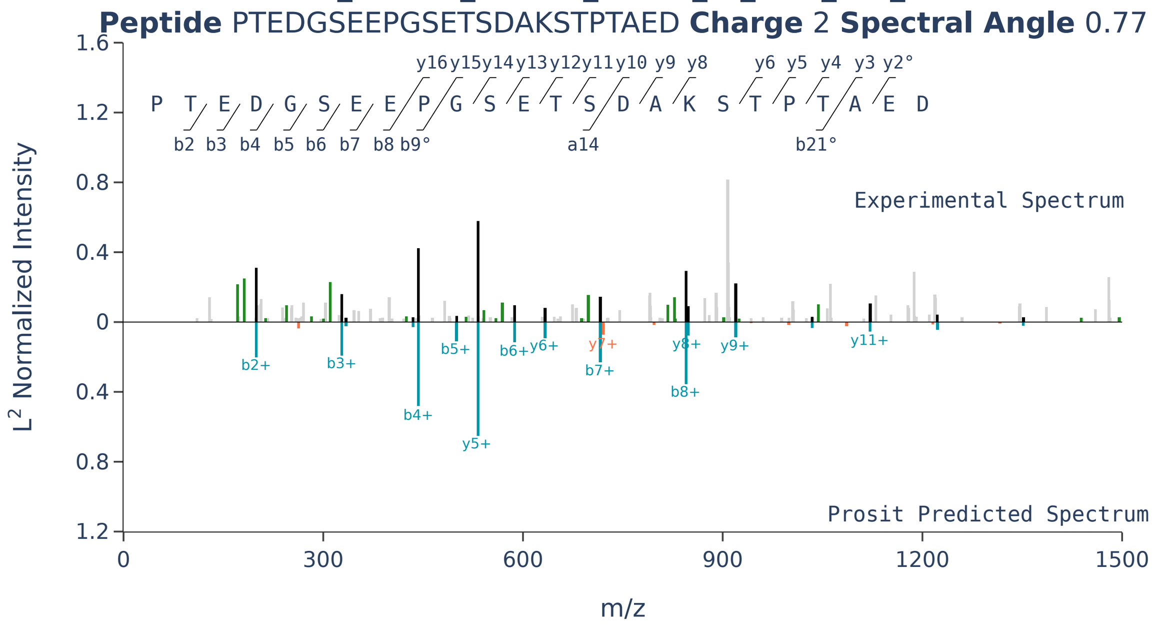


**Source** WSoh_221121_011221_HFGoe_L1_hTau_24h_R2 **Scan** 16108
**Peptide** GSTENLKHQPGGGKVQIINKKLDL **Charge** 5 **Spectral Angle** 0.81

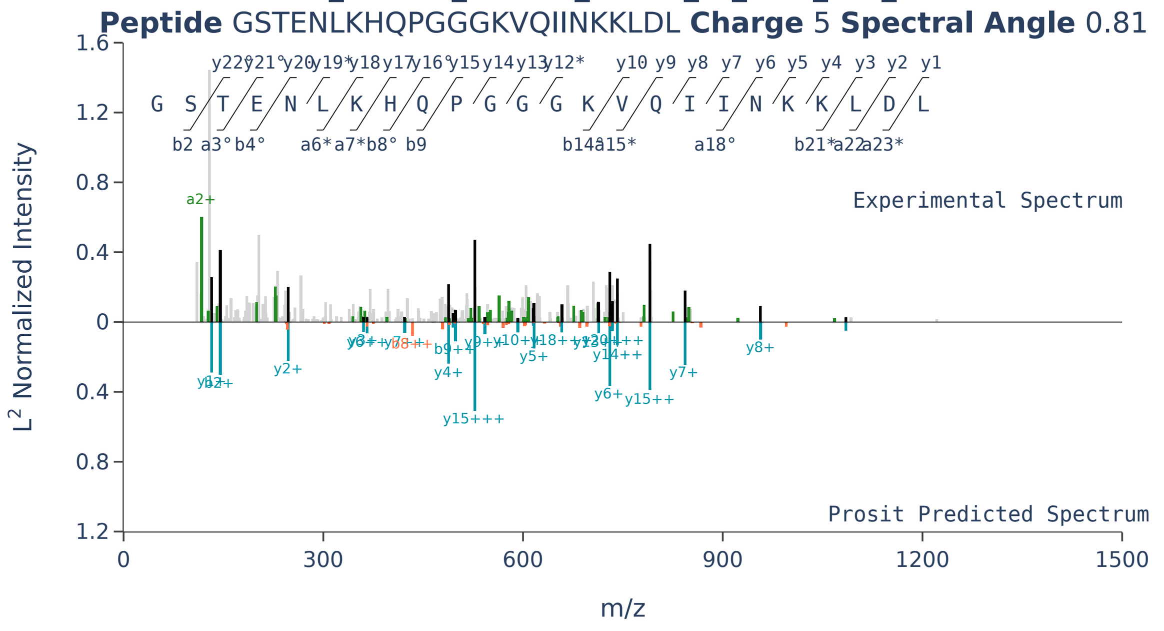


**Source** WSoh_221121_011221_HFGoe_L1_hTau_2h_R2 **Scan** 11605
**Peptide** LDNITHVPGGGNKKIETHKL **Charge** 3 **Spectral Angle** 0.83

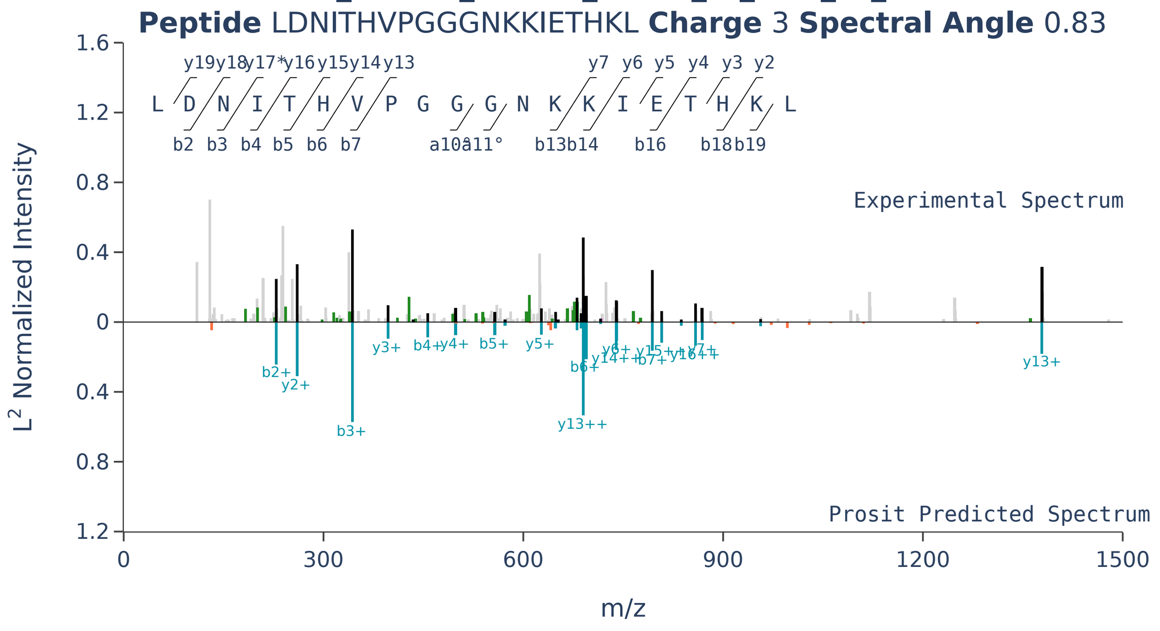


**Source** WSoh_221121_011221_HFGoe_L1_hTau_4h_R2 **Scan** 13621
**Peptide** AKAKTDHGAEIVYKSPVVSGDTSPRHL **Charge** 5 **Spectral Angle** 0.78

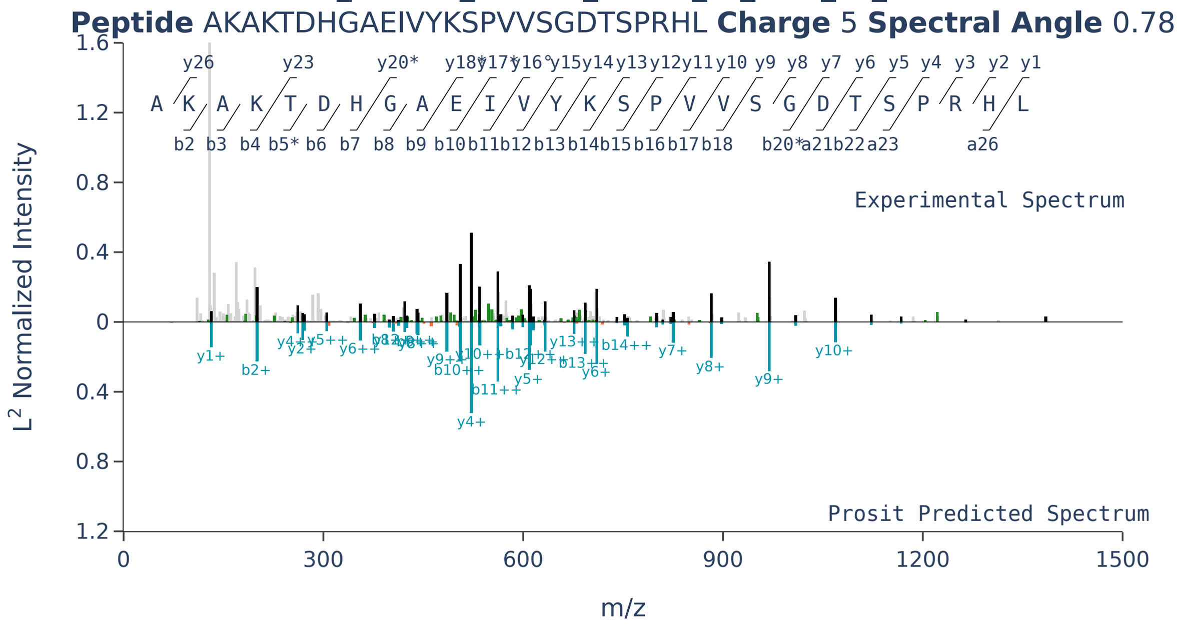


**Source** WSoh_221121_011221_HFGoe_L1_hTau_2h_R2 **Scan** 11864
**Peptide** QPHTEIPEGTTA **Charge** 2 **Spectral Angle** 0.81

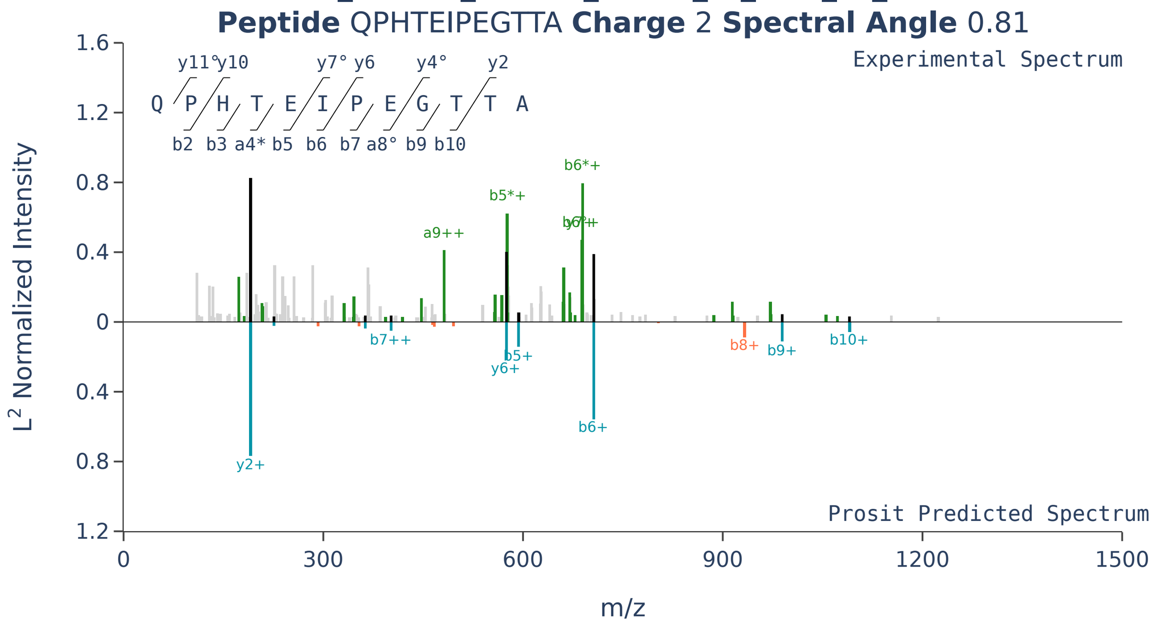


**Source** WSoh_221121_011221_HFGoe_L2_hTau_4h_R1 **Scan** 10830
**Peptide** KLDLSNVQSKCGSKDNIKHV **Charge** 3 **Spectral Angle** 0.9

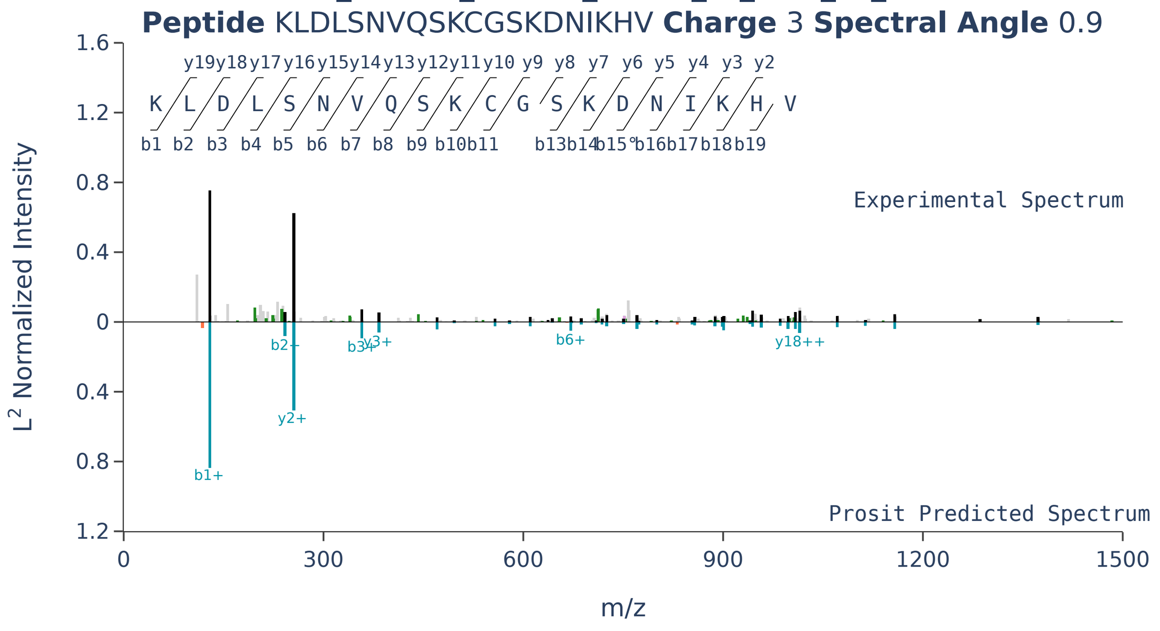

**Source** WSoh_221121_011221_HFGoe_L1_hTau_24h_R2 **Scan** 13058
**Peptide** DNITHVPGGGNKKIETHKLTFRENA **Charge** 3 **Spectral Angle** 0.76

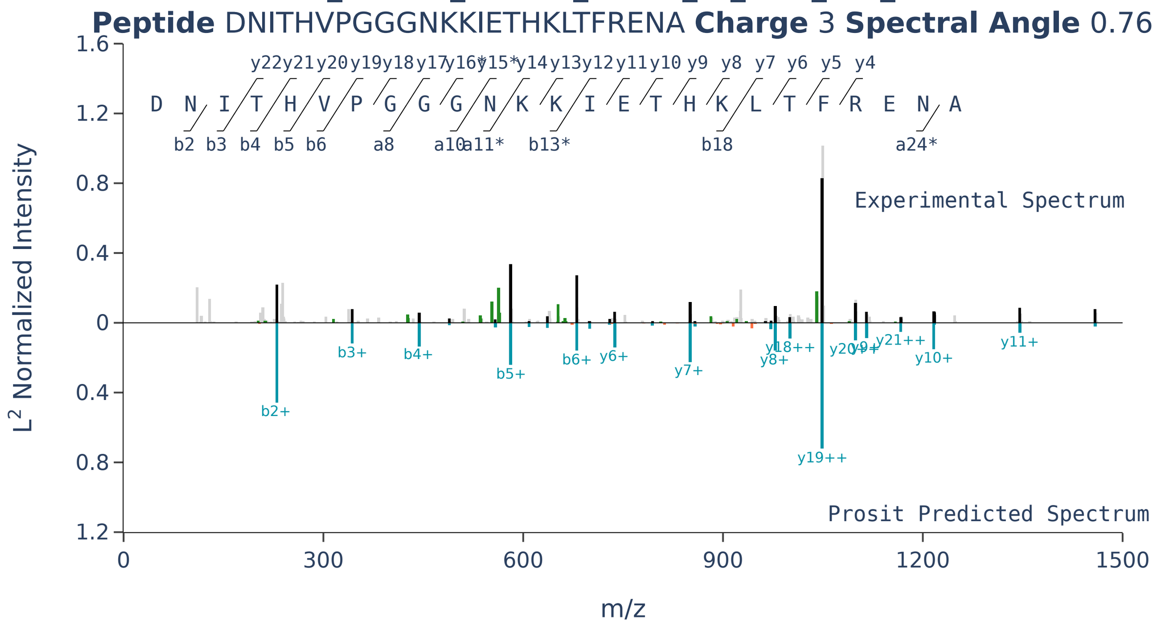


**Source** WSoh_221121_011221_HFGoe_L1_hTau_1h_R1 **Scan** 6897
**Peptide** VVRTPPKSPSSAKSRLQT **Charge** 3 **Spectral Angle** 0.73

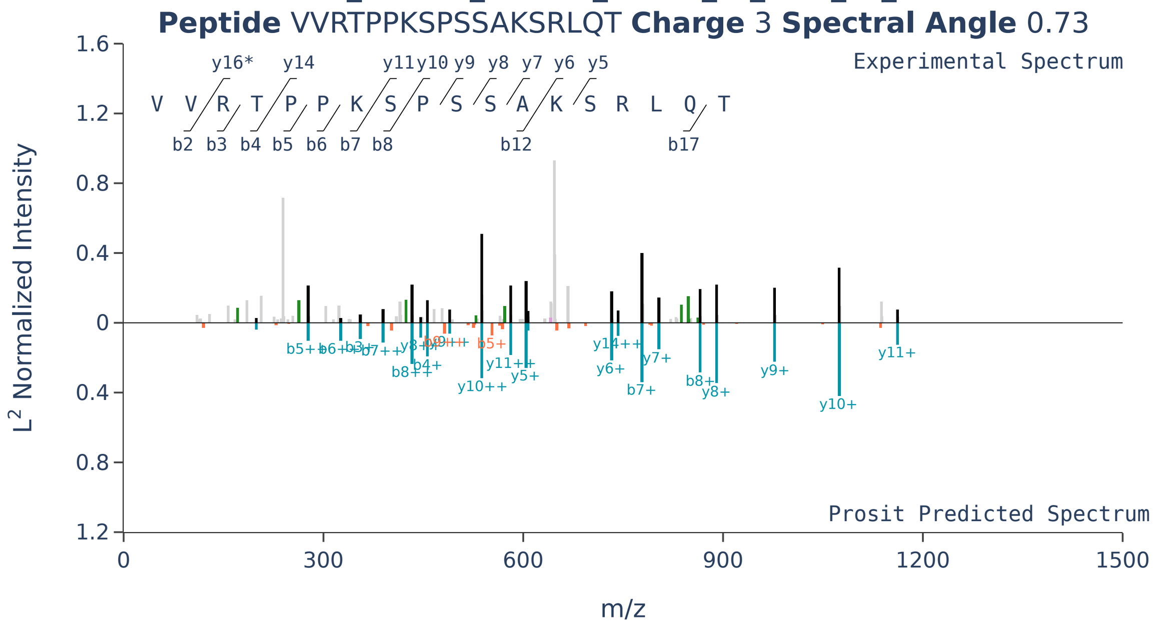


**Source** WSoh_221121_011221_HFGoe_L1_hTau_4h_R1 **Scan** 10858
**Peptide** GSTENLKHQPGGGKVQIINKKL **Charge** 4 **Spectral Angle** 0.82

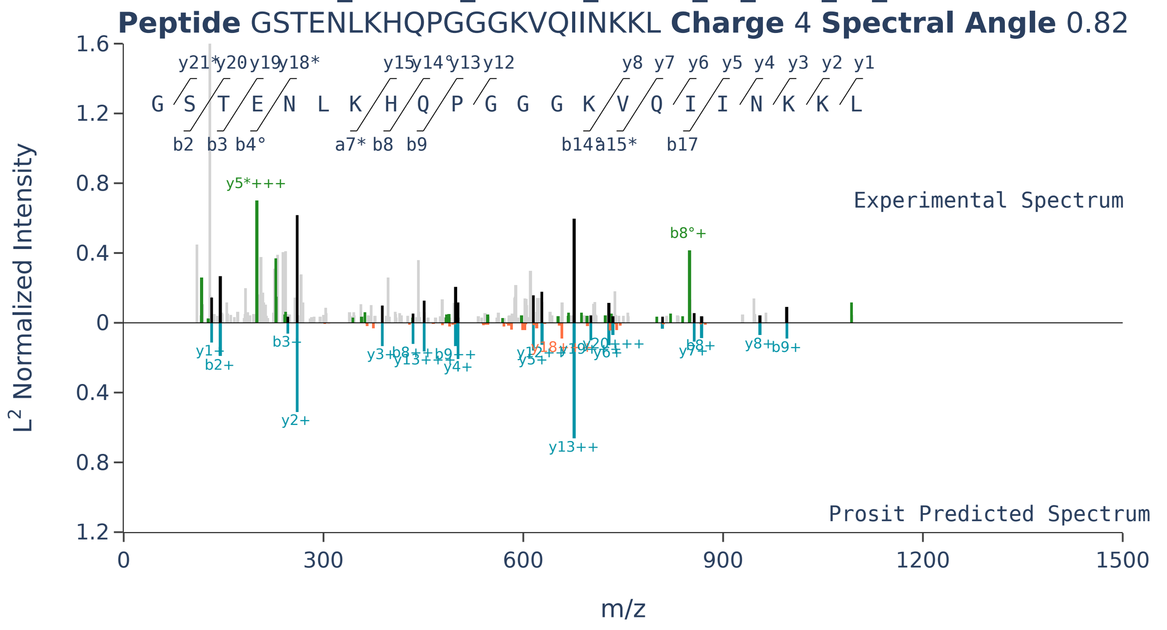


**Source** WSoh_221121_011221_HFGoe_L2_hTau_24h_R2 **Scan** 15583
**Peptide** SNVQSKCGSKDNIKHVPGGGSVQIVY **Charge** 4 **Spectral Angle** 0.79

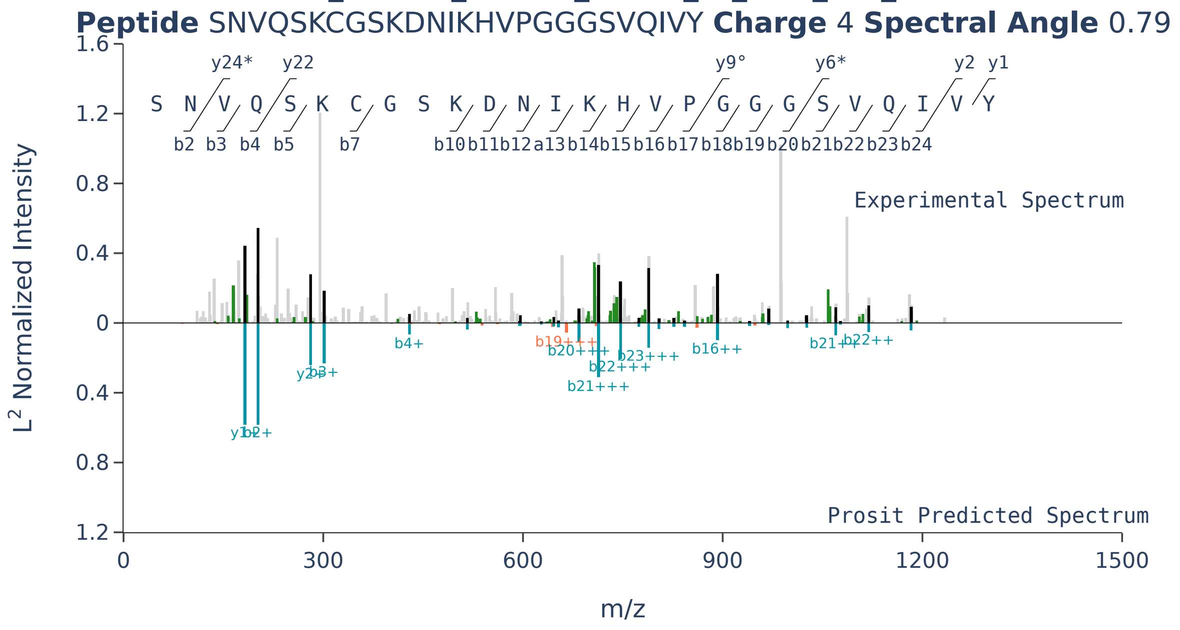


**Source** WSoh_221121_011221_HFGoe_L1_hTau_4h_R1 **Scan** 12351
**Peptide** LDNITHVPGGGNKKIET **Charge** 3 **Spectral Angle** 0.83

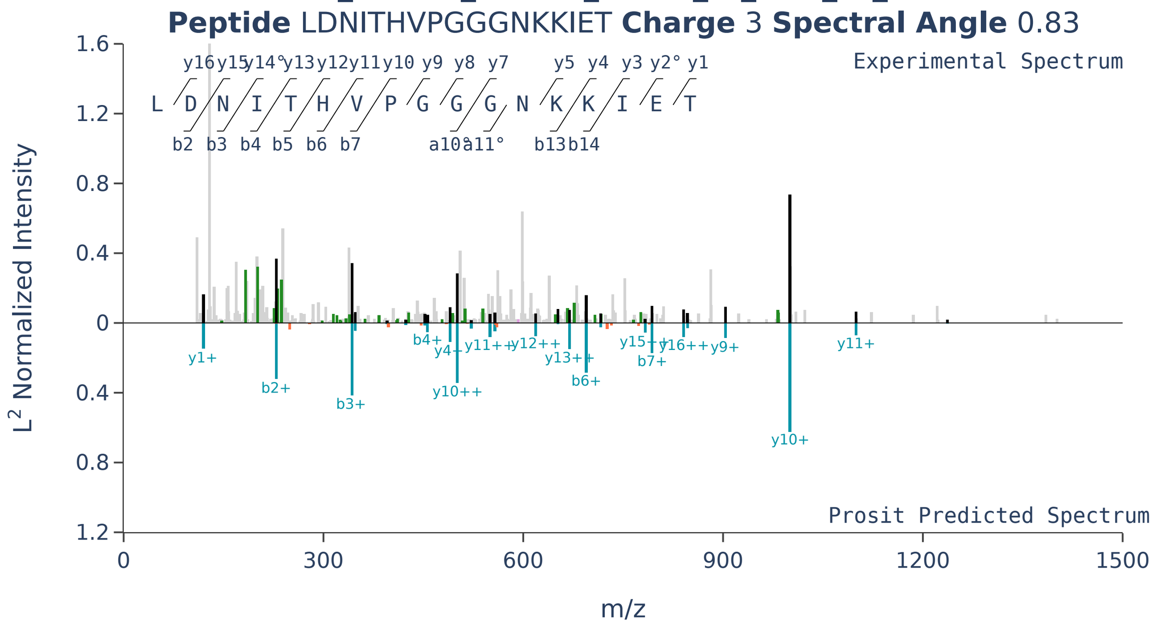


**Source** WSoh_221121_011221_HFGoe_L1_hTau_4h_R2 **Scan** 21237
**Peptide** AKSTPTAEDVTAPLVDE **Charge** 2 **Spectral Angle** 0.85

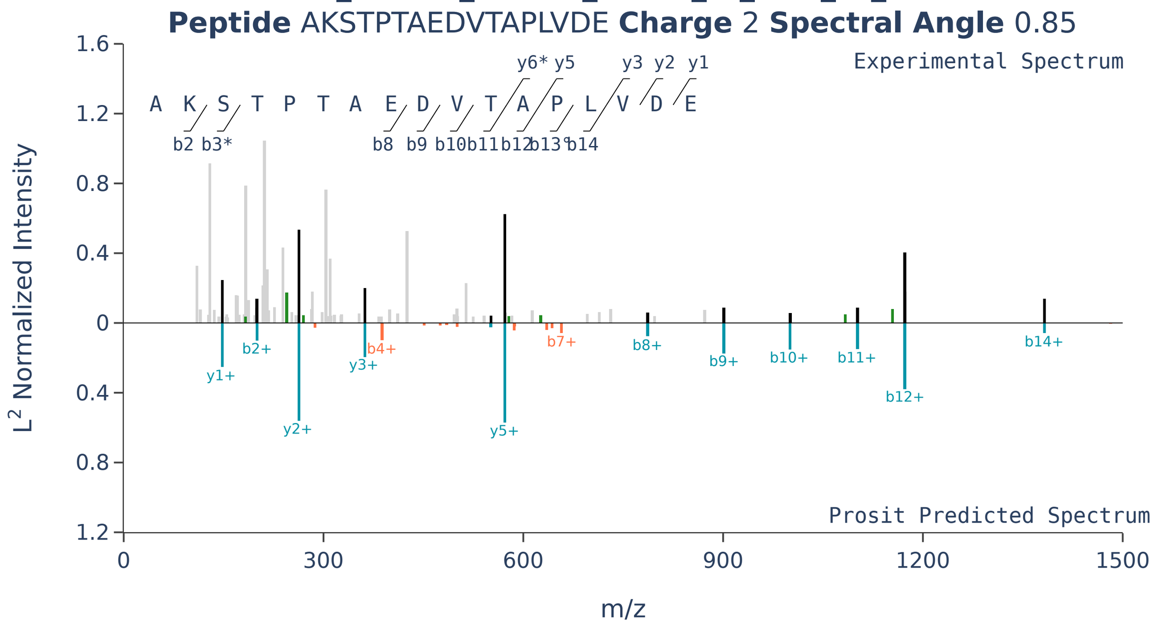


**Source** WSoh_221121_011221_HFGoe_L2_hTau_24h_R1 **Scan** 11846
**Peptide** SGDTSPRHLSNVSSTGSLD **Charge** 2 **Spectral Angle** 0.81

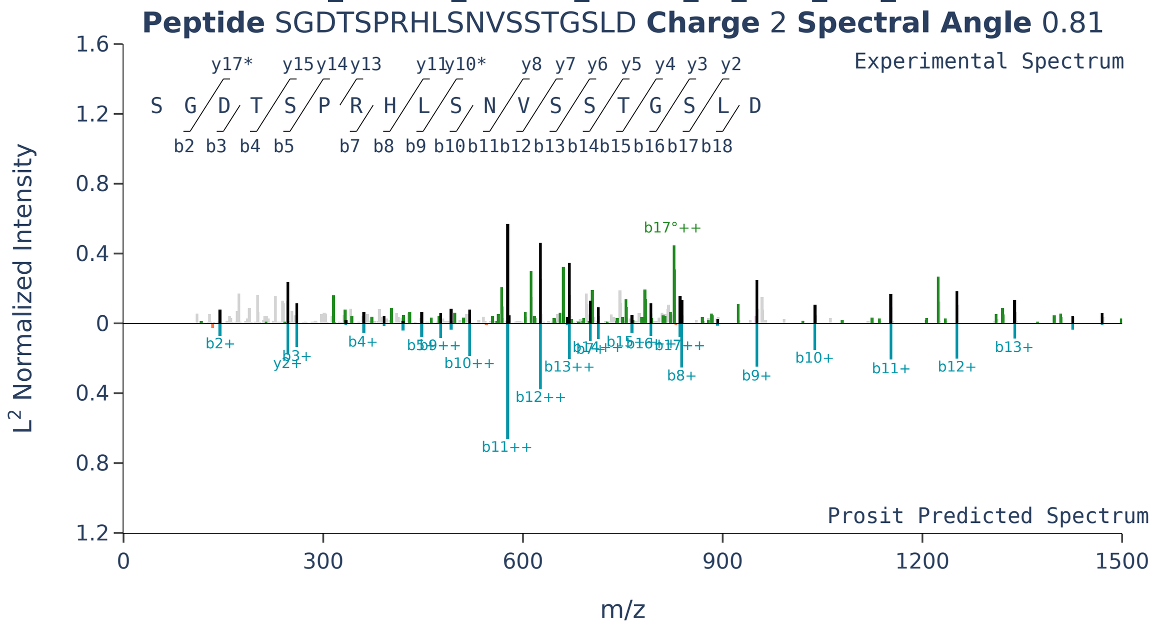


**Source** WSoh_221121_011221_HFGoe_L1_hTau_4h_R2 **Scan** 11050
**Peptide** GAPGKQAAAQPHTEIPEGT **Charge** 3 **Spectral Angle** 0.75

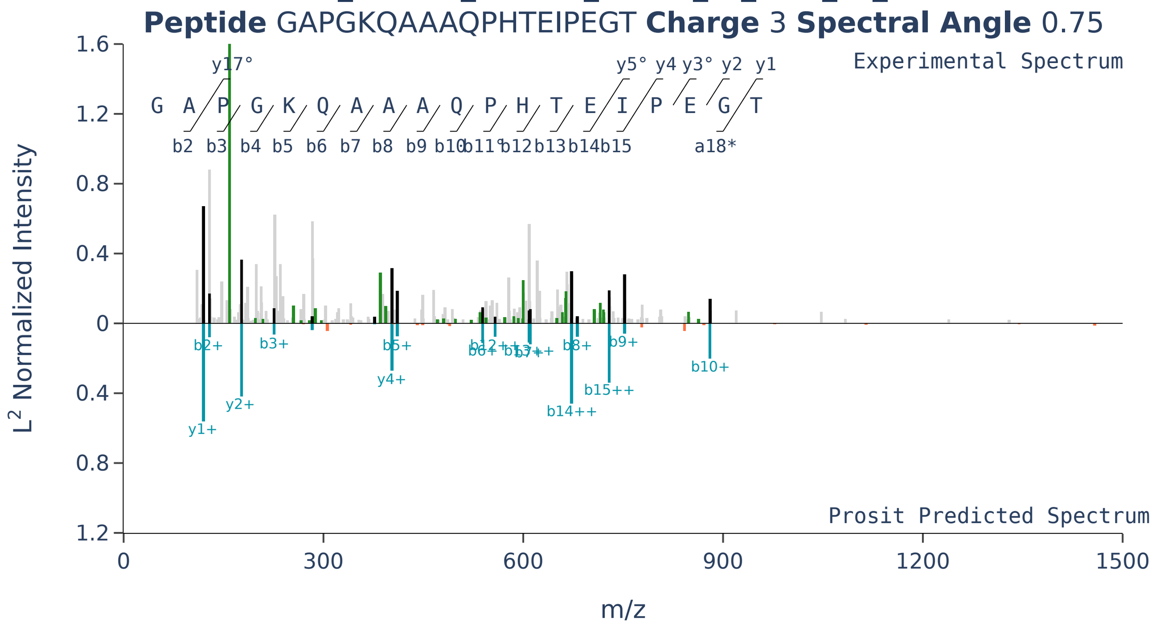


**Source** WSoh_221121_011221_HFGoe_L1_hTau_24h_R1 **Scan** 7672
**Peptide** GADGKTKIATPRGAAPPGQKGQANA **Charge** 4 **Spectral Angle** 0.89

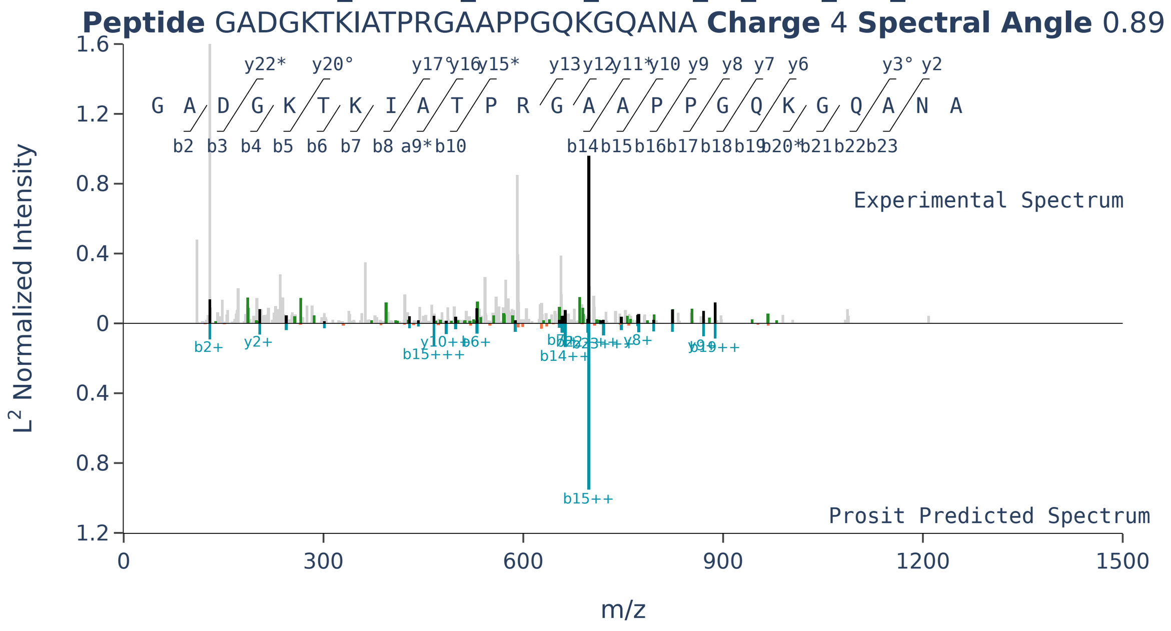


**Source** WSoh_221121_011221_HFGoe_L2_hTau_24h_R1 **Scan** 12452
**Peptide** GSEEPGSETSDAKSTPTAEDV **Charge** 2 **Spectral Angle** 0.81

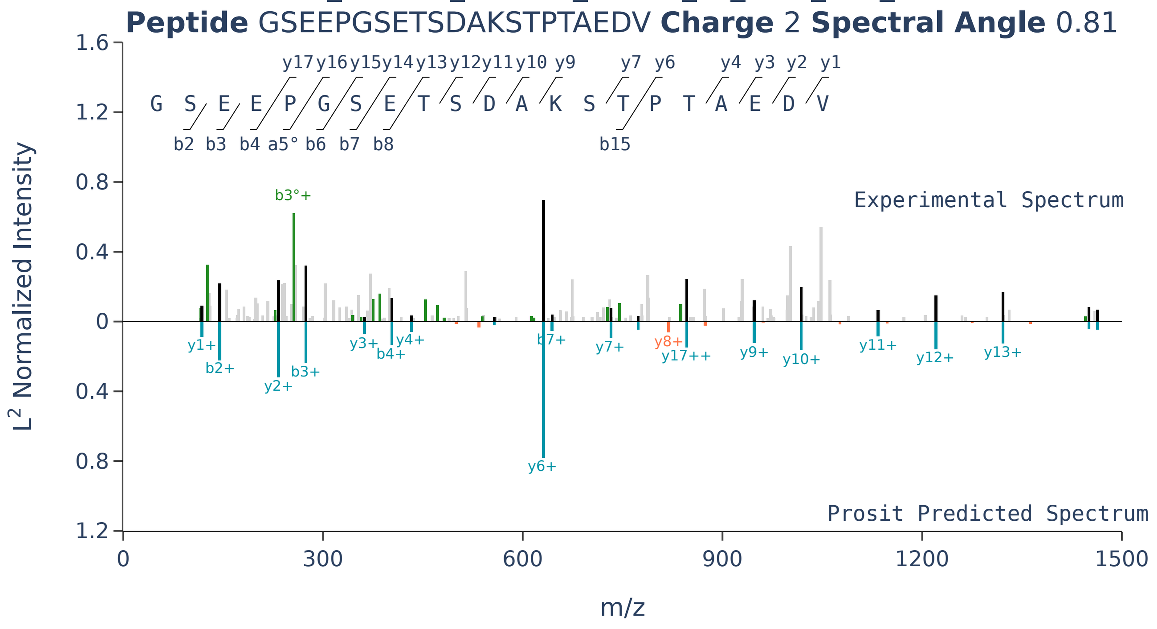


**Source** WSoh_221121_011221_HFGoe_L1_hTau_24h_R1 **Scan** 20857
**Peptide** LVYKPVDLSKVTS **Charge** 3 **Spectral Angle** 0.8

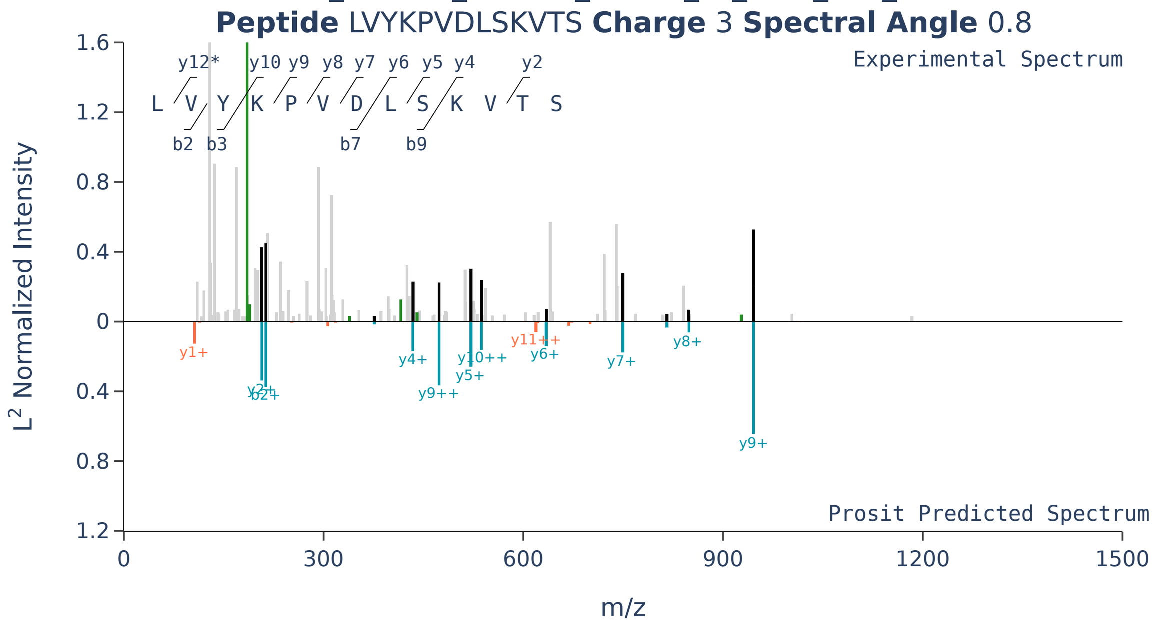


**Source** WSoh_221121_011221_HFGoe_L2_hTau_24h_R1 **Scan** 20589
**Peptide** VVRTPPKSPSSAKSRLQTAPVPMPDL **Charge** 3 **Spectral Angle** 0.88

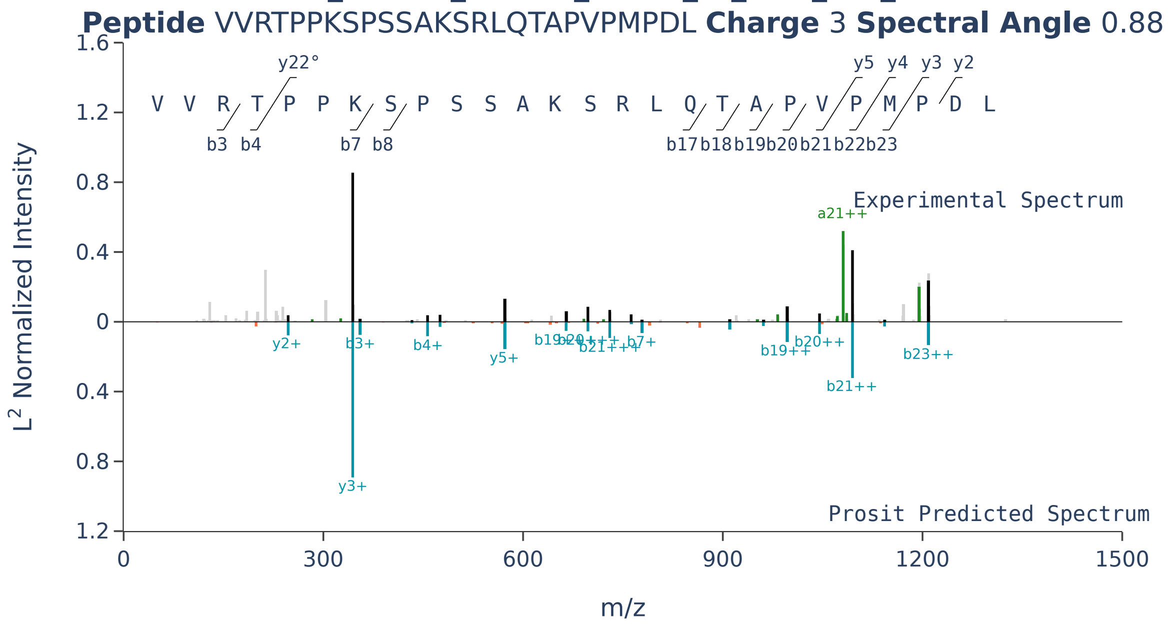


**Source** WSoh_221121_011221_HFGoe_L1_hTau_24h_R1 **Scan** 8751
**Peptide** KCGSLGNIHHKPGGGQVEVKSEKL **Charge** 5 **Spectral Angle** 0.9

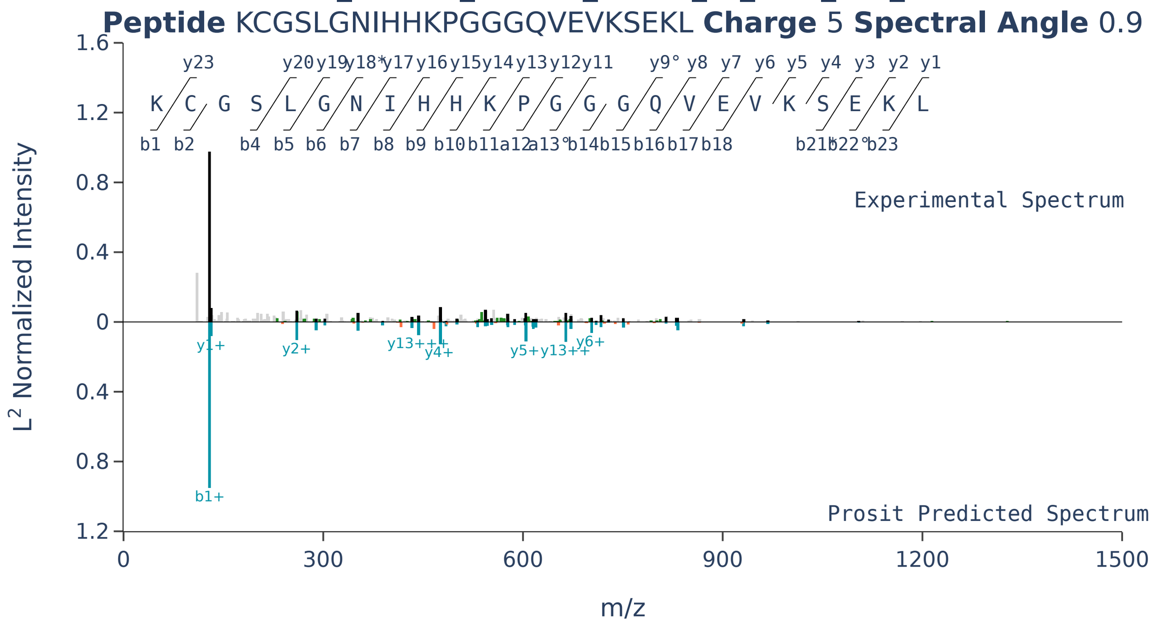


**Source** WSoh_221121_011221_HFGoe_L1_hTau_4h_R2 **Scan** 7229
**Peptide** ADGKTKIATPRGAAPPGQKG **Charge** 4 **Spectral Angle** 0.82

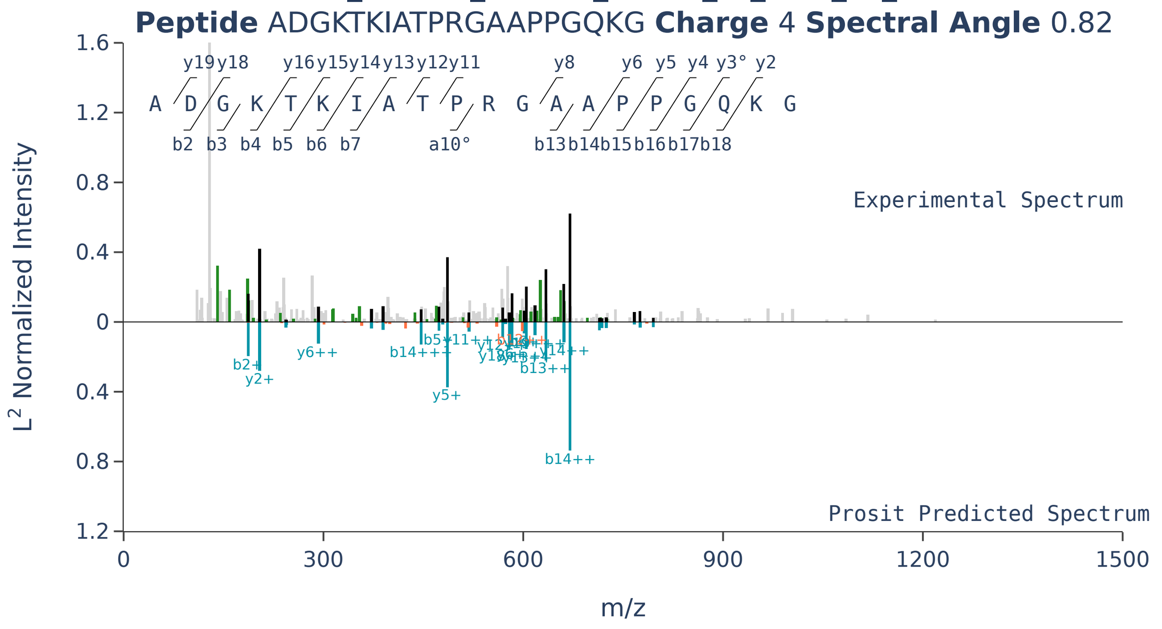


**Source** WSoh_221121_011221_HFGoe_L1_hTau_24h_R2 **Scan** 18658
**Peptide** QPGGGKVQIINKKLDL **Charge** 4 **Spectral Angle** 0.72

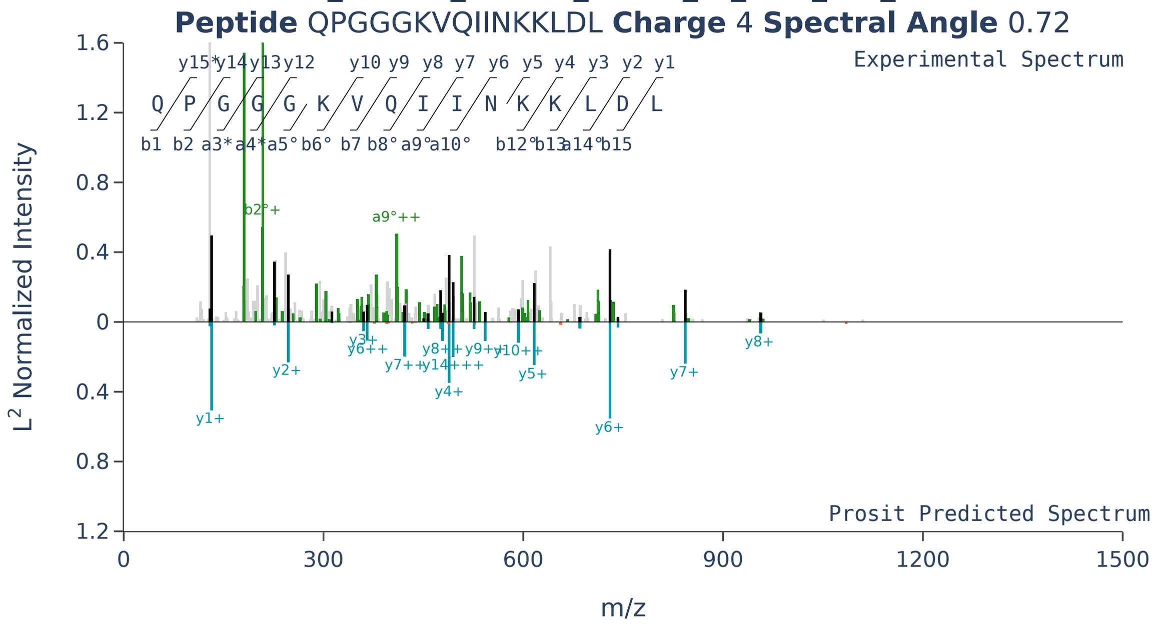

**Source** WSoh_221121_011221_HFGoe_L2_hTau_24h_R2 **Scan** 12029
**Peptide** DFKDRVQSKIGS **Charge** 2 **Spectral Angle** 0.77

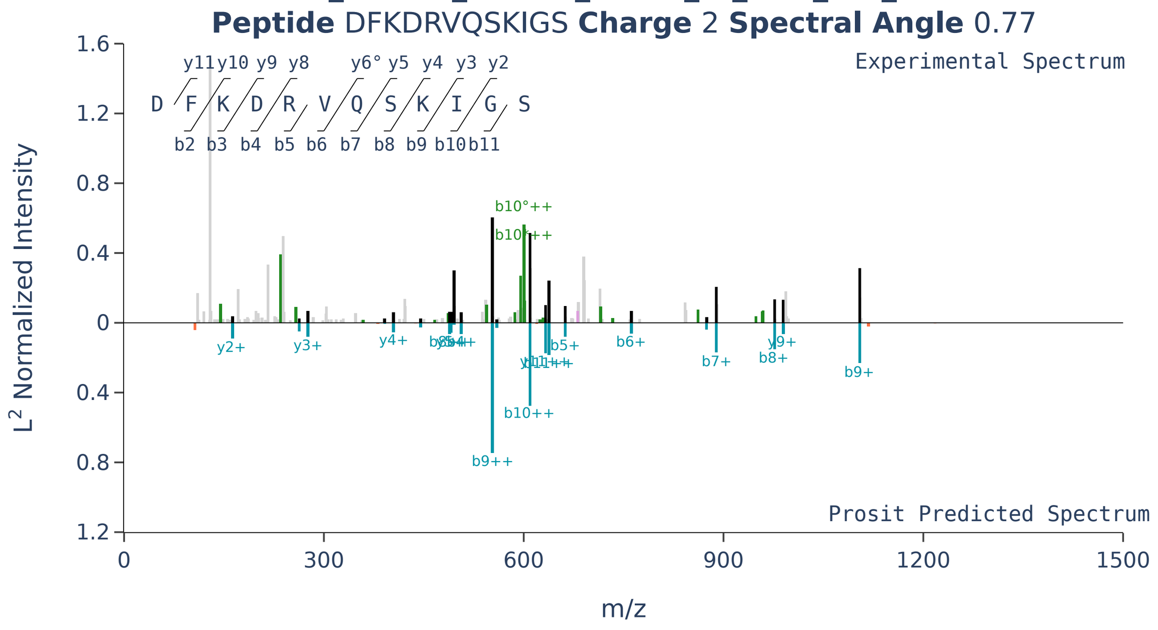


**Source** WSoh_221121_011221_HFGoe_L2_hTau_4h_R1 **Scan** 25198
**Peptide** LQTAPVPMPDLKNVKSKIGSTENL **Charge** 3 **Spectral Angle** 0.82

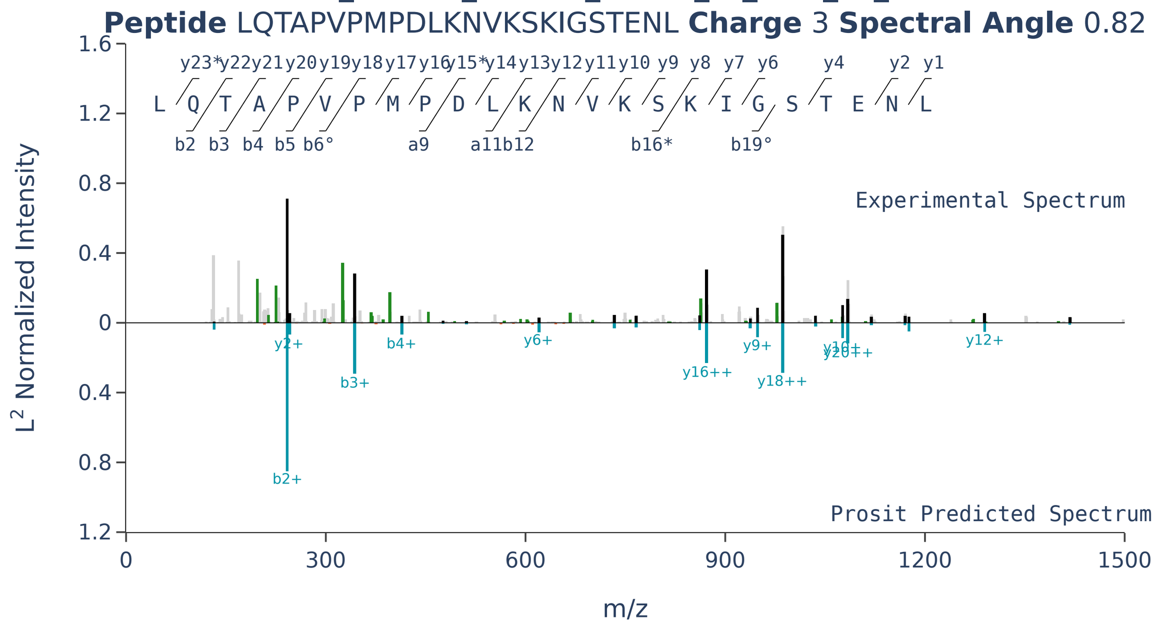


**Source** WSoh_221121_011221_HFGoe_L2_hTau_2h_R1 **Scan** 10665
**Peptide** KKLDLSNVQS **Charge** 2 **Spectral Angle** 0.69

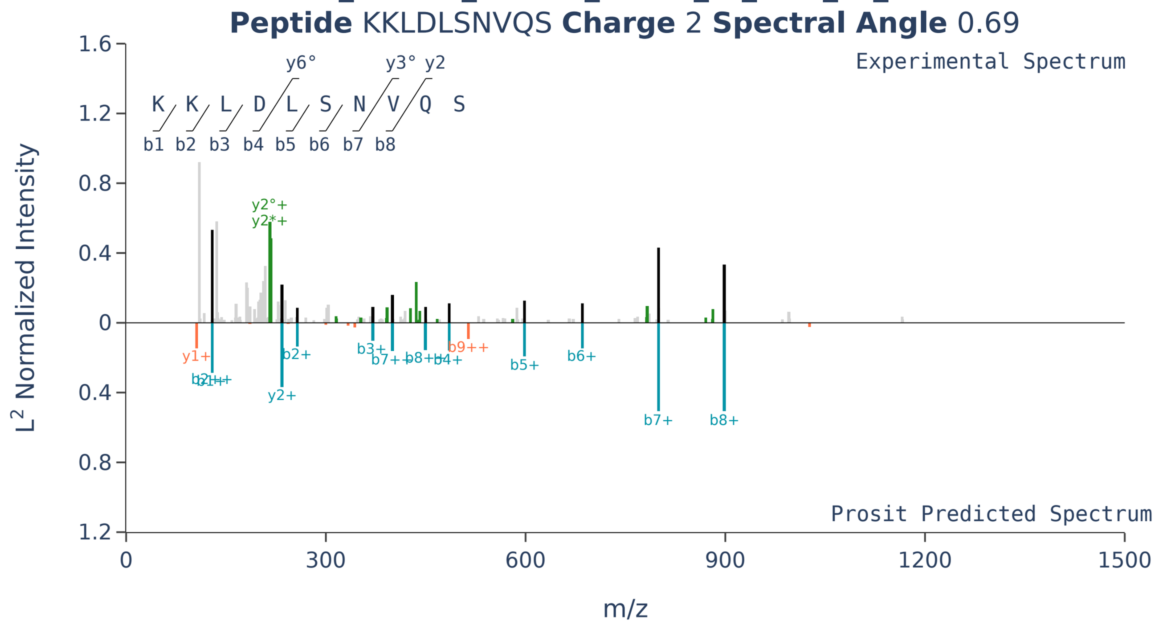


**Source** WSoh_221121_011221_HFGoe_L1_hTau_24h_R2 **Scan** 10130
**Peptide** DNITHVPGGGNKKL **Charge** 3 **Spectral Angle** 0.84

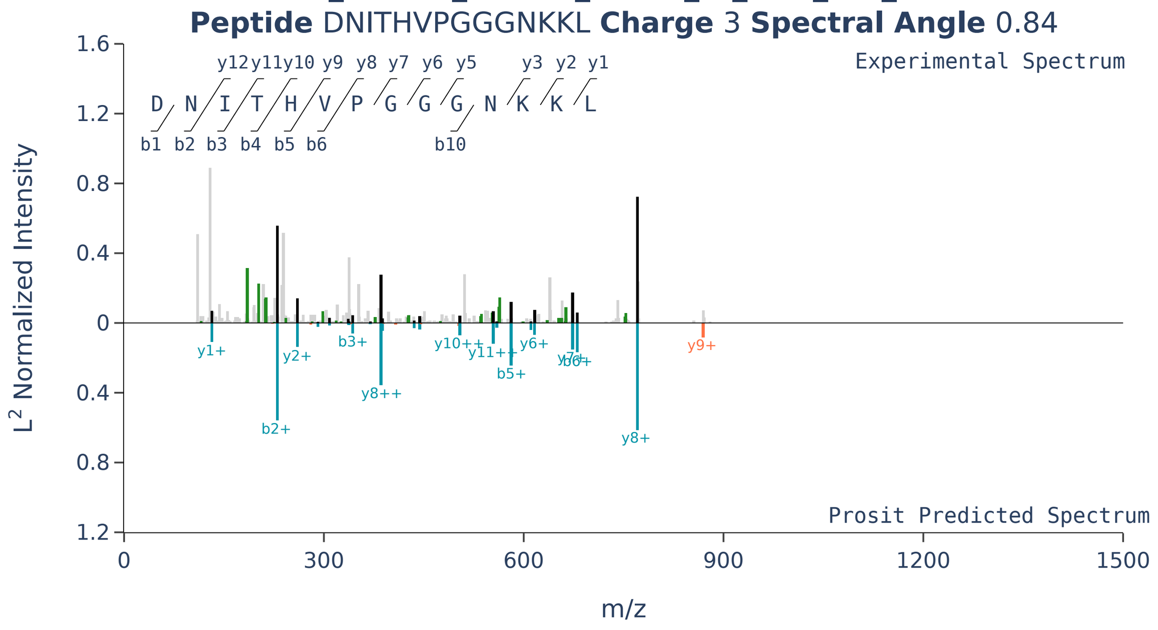


**Source** WSoh_221121_011221_HFGoe_L1_hTau_24h_R2 **Scan** 22632
**Peptide** TTAEEAGIGDTPSLEDEAAGHVTQA **Charge** 3 **Spectral Angle** 0.76

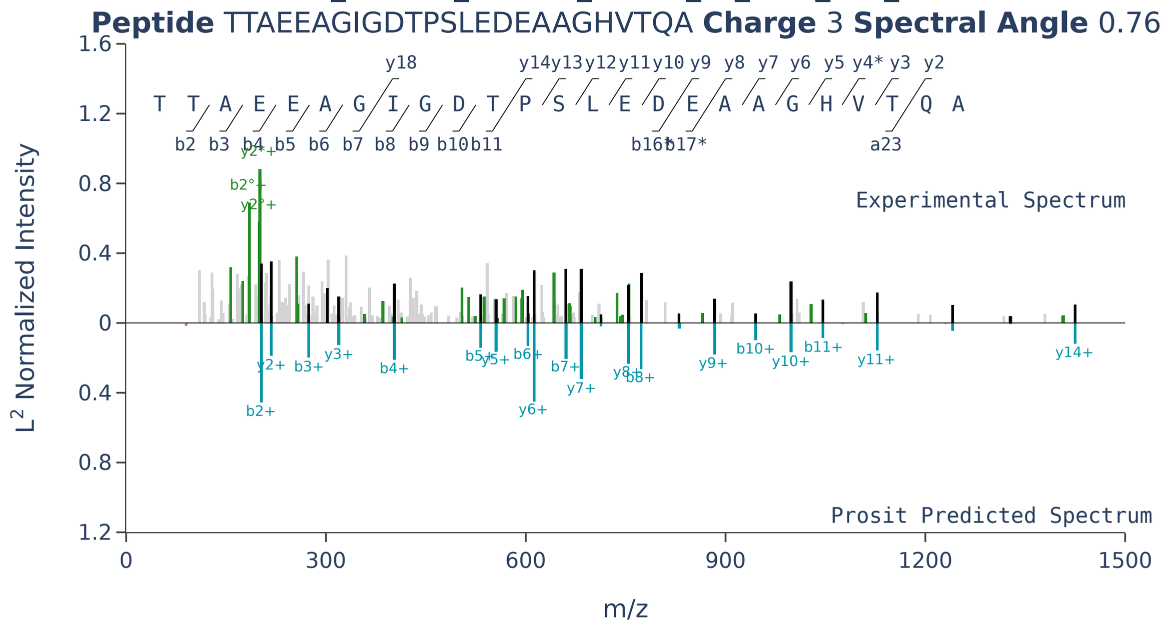


**Source** WSoh_221121_011221_HFGoe_L1_hTau_24h_R1 **Scan** 20531
**Peptide** VQIINKKLDLSNVQS **Charge** 2 **Spectral Angle** 0.94

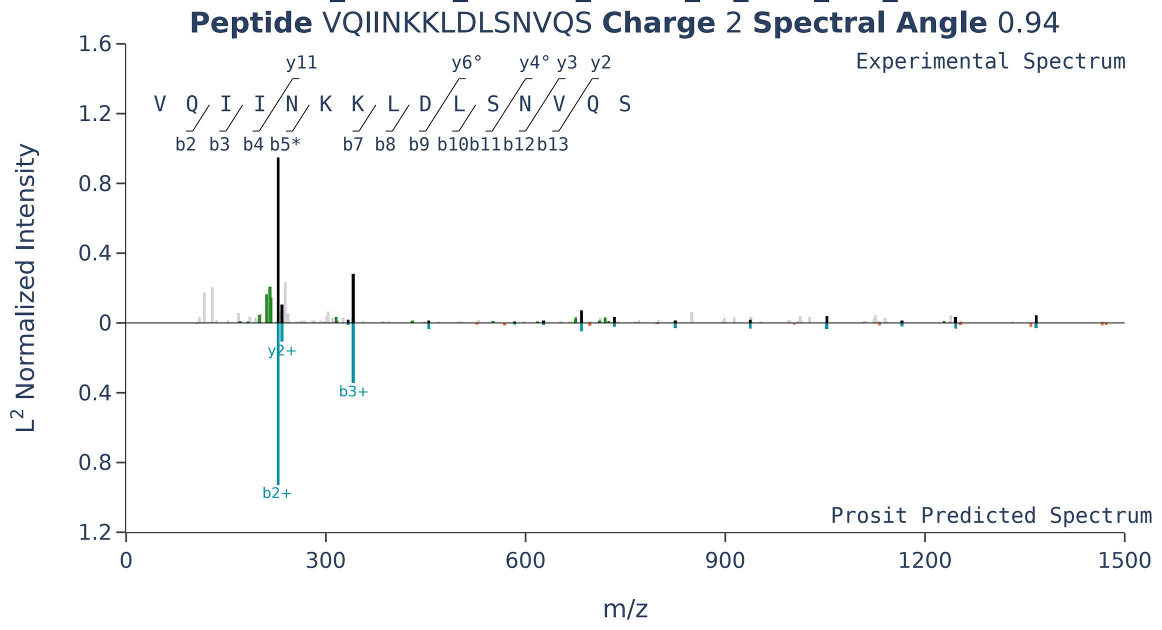


**Source** WSoh_221121_011221_HFGoe_L2_hTau_24h_R2 **Scan** 14787
**Peptide** KIGSLDNITHVPGGGNKKIETH **Charge** 5 **Spectral Angle** 0.75

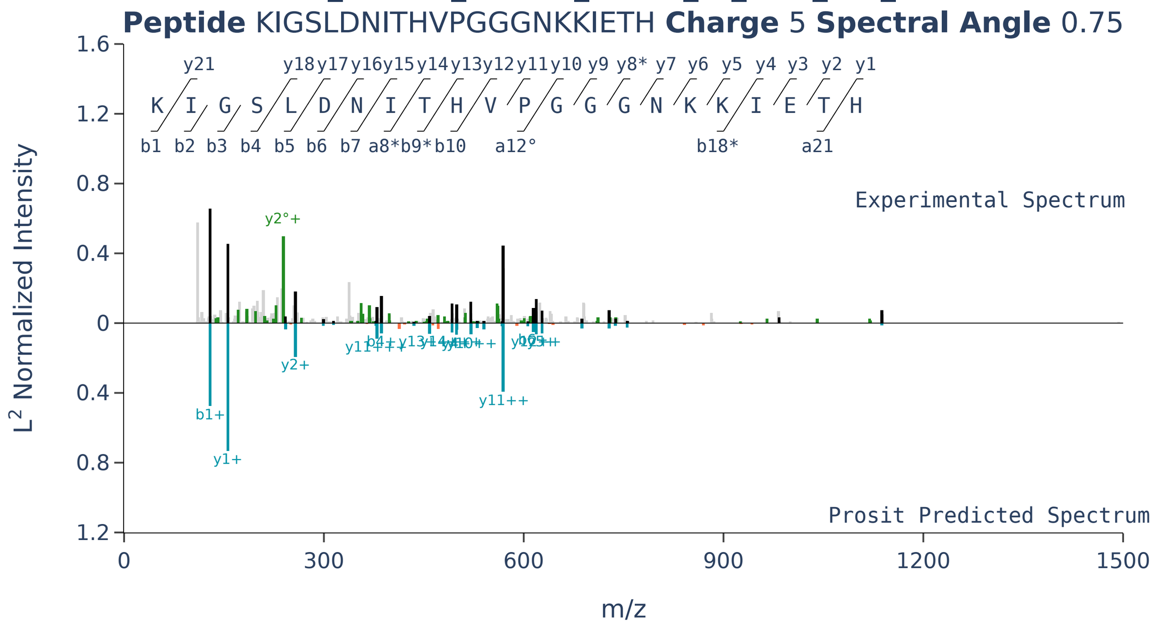


**Source** WSoh_221121_011221_HFGoe_L2_hTau_24h_R2 **Scan** 8217
**Peptide** QDQEGDTDAGLKES **Charge** 2 **Spectral Angle** 0.83

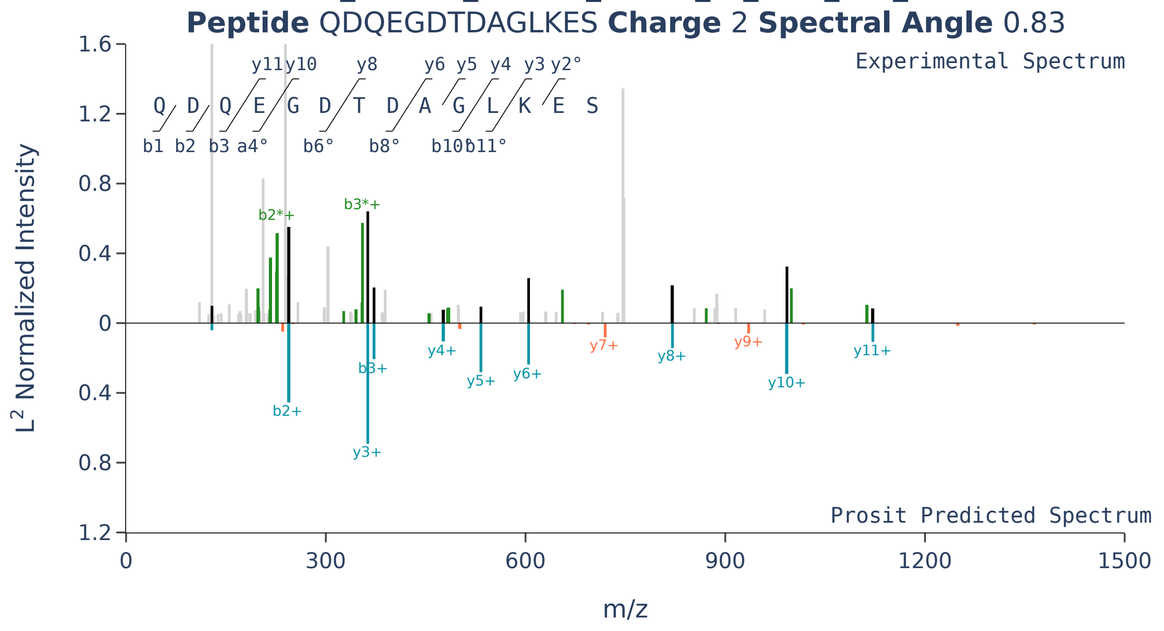


**Source** WSoh_221121_011221_HFGoe_L1_hTau_4h_R2 **Scan** 14525
**Peptide** QSKIGSLDNITHVPGGGNKKIETH **Charge** 5 **Spectral Angle** 0.86

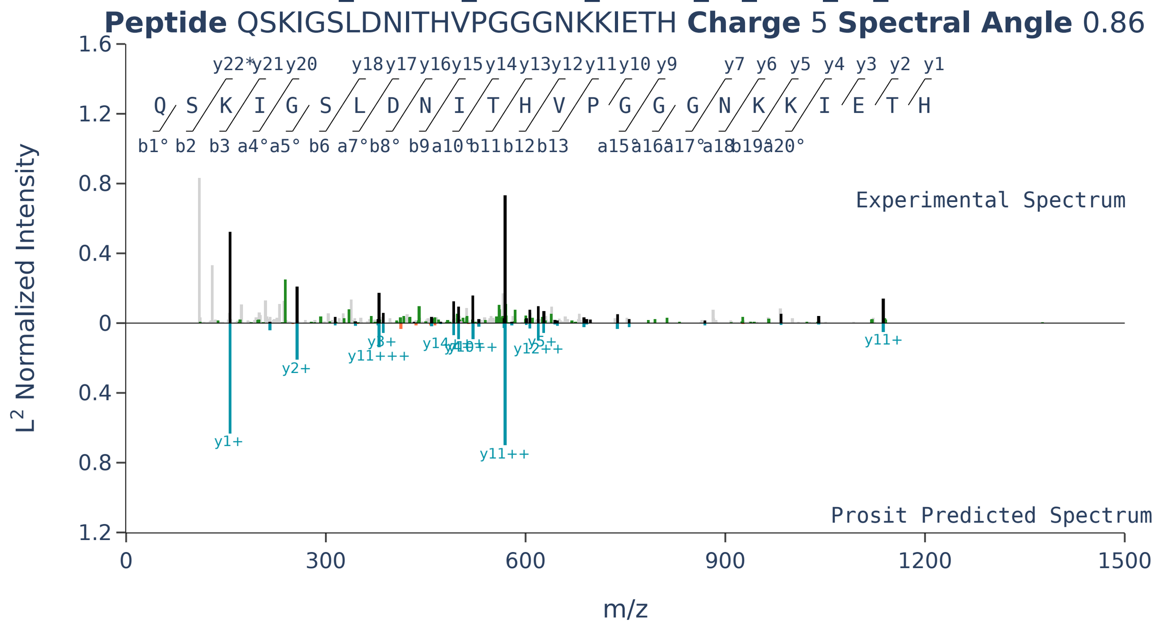


**Source** WSoh_221121_011221_HFGoe_L2_hTau_24h_R2 **Scan** 23209
**Peptide** DAKSTPTAEDVTAPLVD **Charge** 2 **Spectral Angle** 0.91

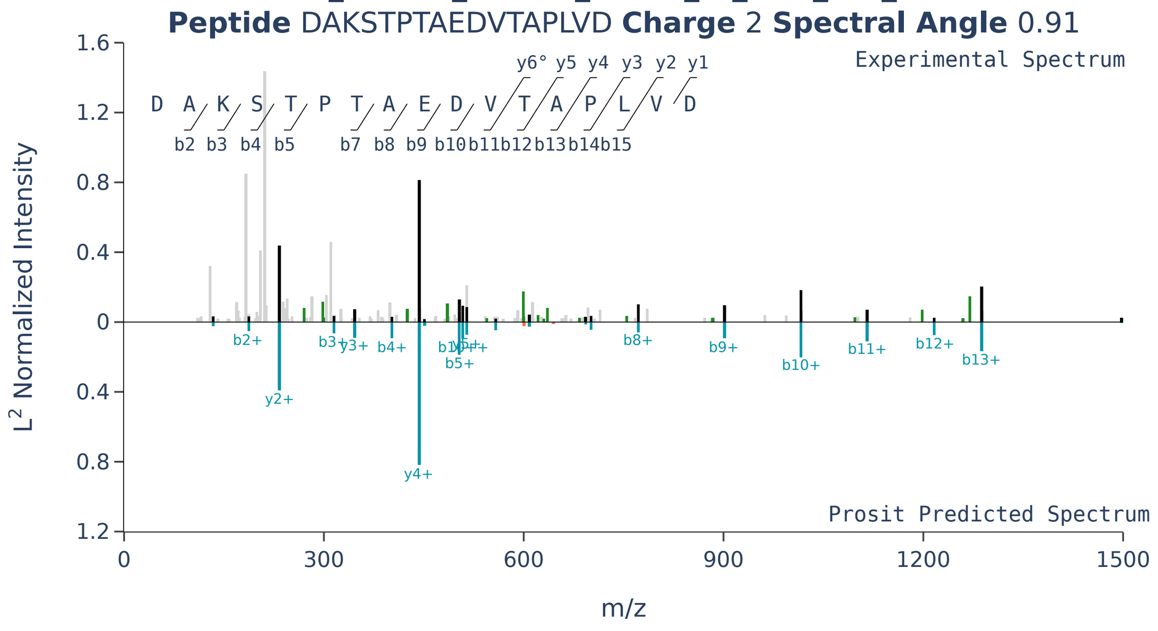


**Source** WSoh_221121_011221_HFGoe_L1_hTau_24h_R1 **Scan** 11681
**Peptide** TYGLGDRKDQGGYTMHQD **Charge** 3 **Spectral Angle** 0.87

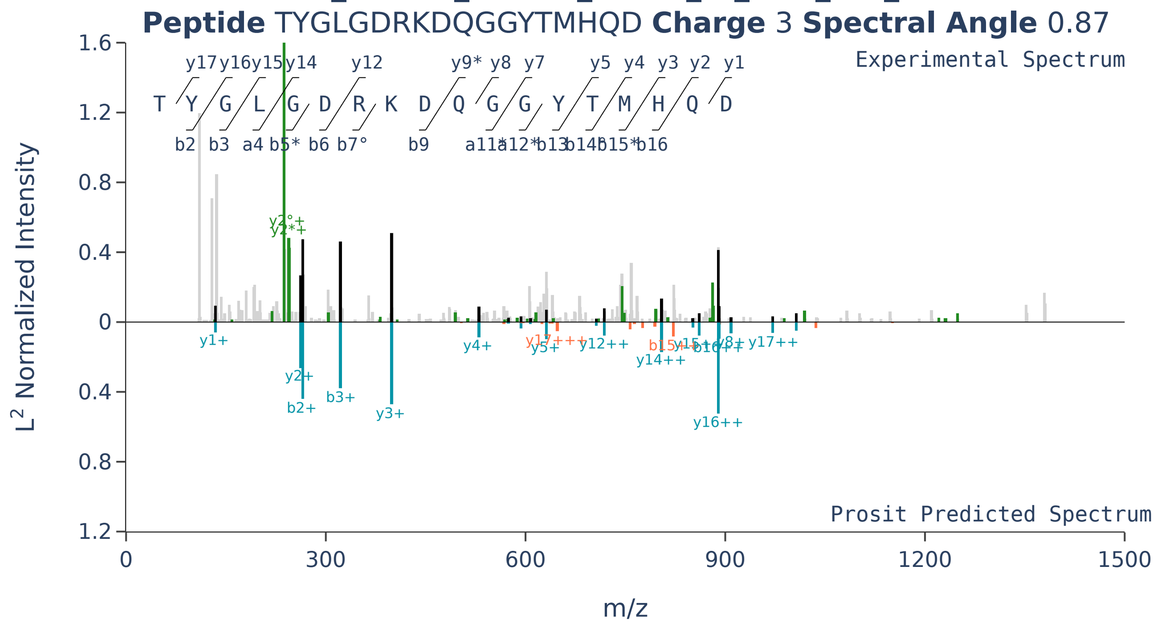


**Source** WSoh_221121_011221_HFGoe_L1_hTau_4h_R2 **Scan** 5387
**Peptide** TFRENAKAKTDHGA **Charge** 3 **Spectral Angle** 0.81

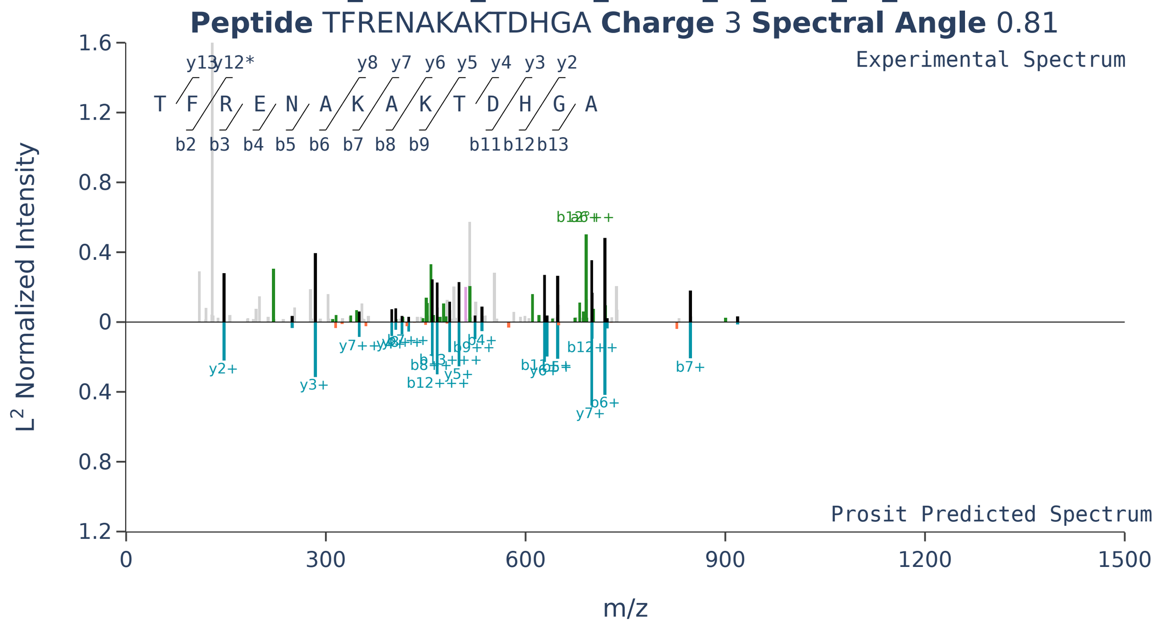


**Source** WSoh_221121_011221_HFGoe_L2_hTau_4h_R1 **Scan** 8995
**Peptide** NKKLDLSNVQSKCGSKDNIKHV **Charge** 3 **Spectral Angle** 0.9

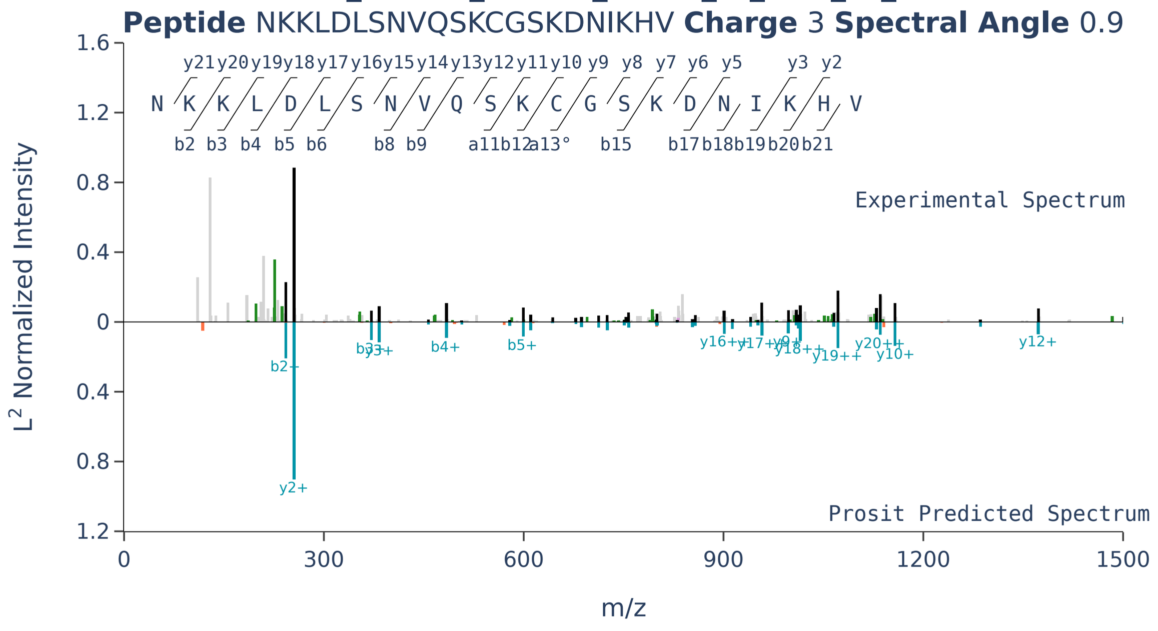


**Source** WSoh_221121_011221_HFGoe_L2_hTau_24h_R1 **Scan** 13554
**Peptide** KLGSLDNITHVPGGGNKK **Charge** 3 **Spectral Angle** 0.74

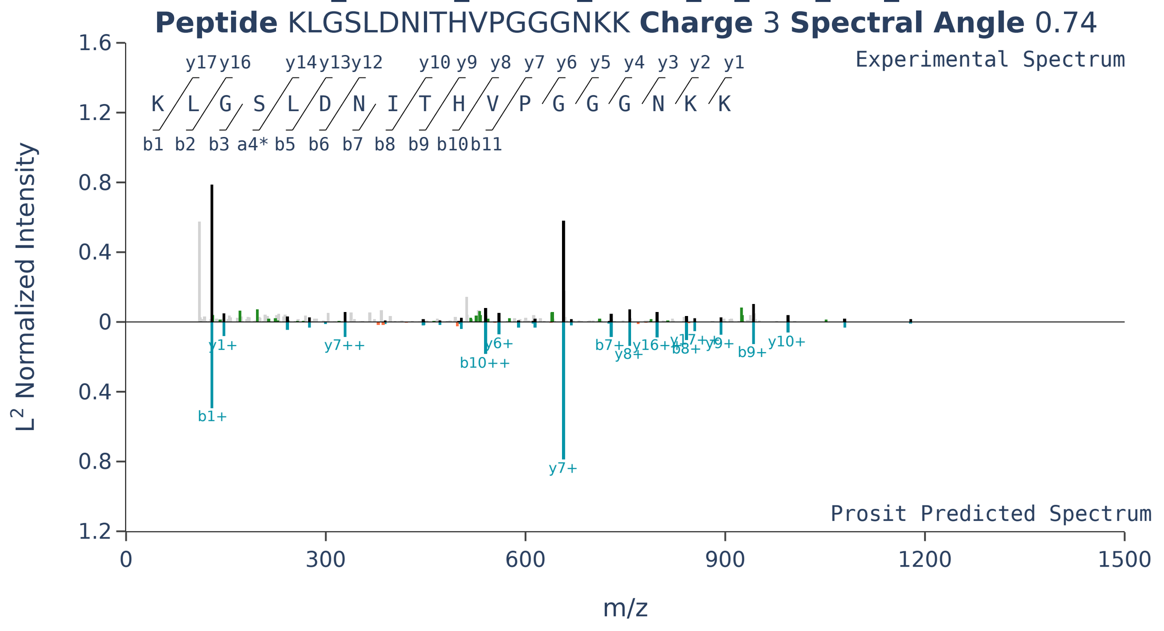


**Source** WSoh_221121_011221_HFGoe_L1_hTau_24h_R2 **Scan** 13522
**Peptide** SLGNIHHKPGGGQVEVKSEKLDFKDRV **Charge** 4 **Spectral Angle** 0.77

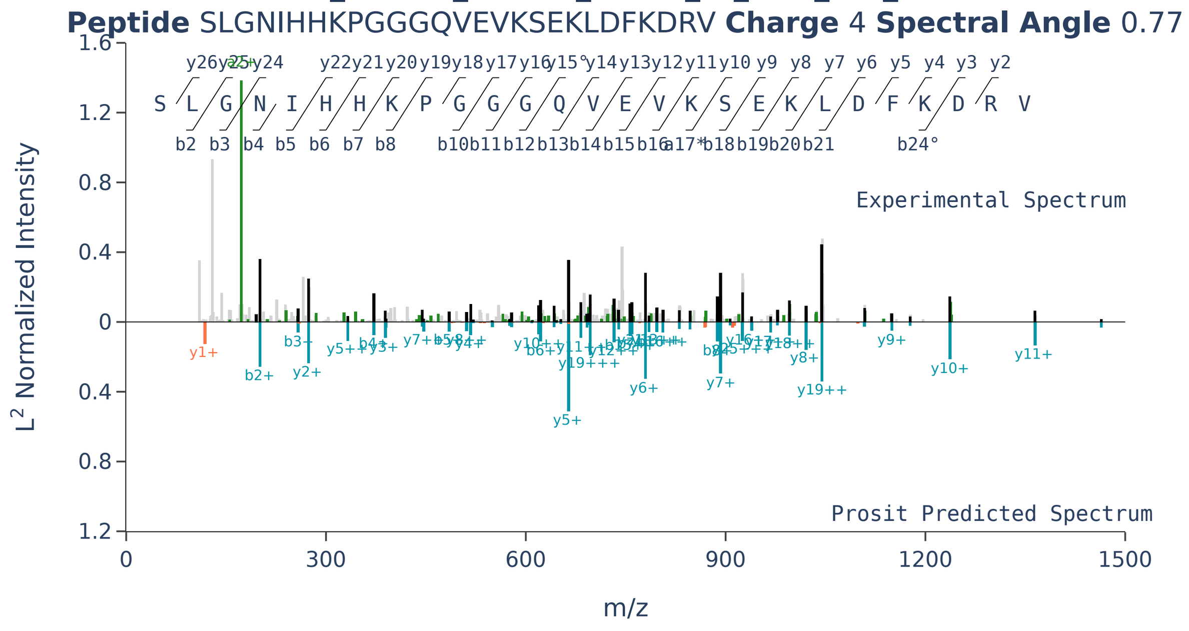

**Source** WSoh_221121_011221_HFGoe_L1_hTau_24h_R1 **Scan** 17286
**Peptide** EIVYKSPVVSGDTSPRHLSNVS **Charge** 3 **Spectral Angle** 0.87

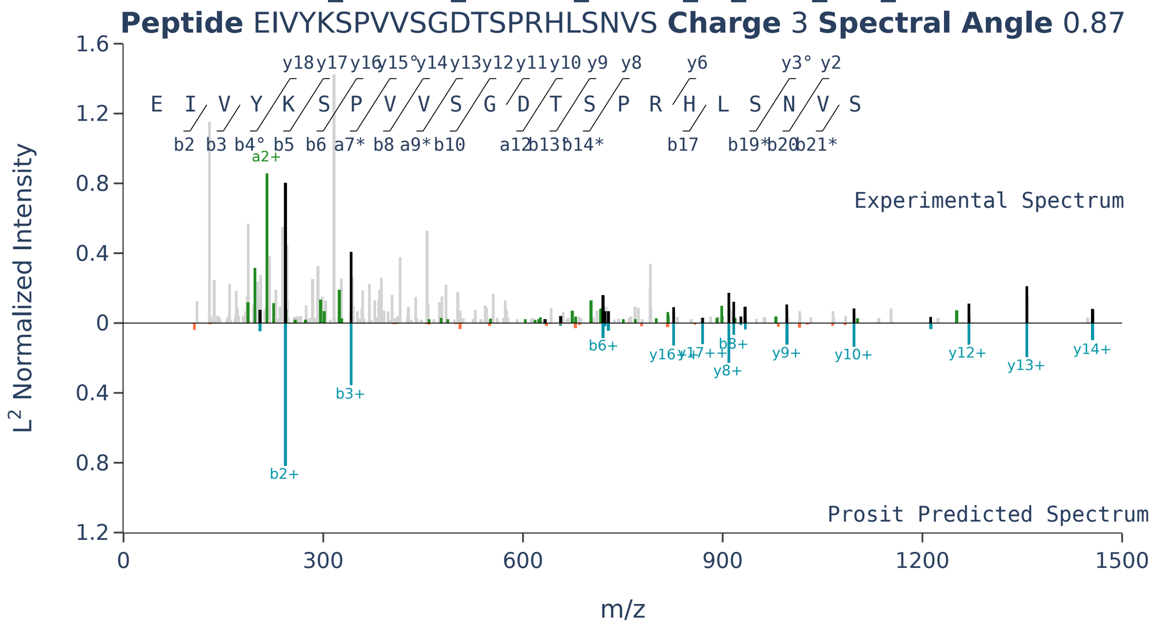


**Source** WSoh_221121_011221_HFGoe_L1_hTau_4h_R1 **Scan** 15049
**Peptide** KDRVQSKIGSLDNITHVPGGGNKKL **Charge** 5 **Spectral Angle** 0.82

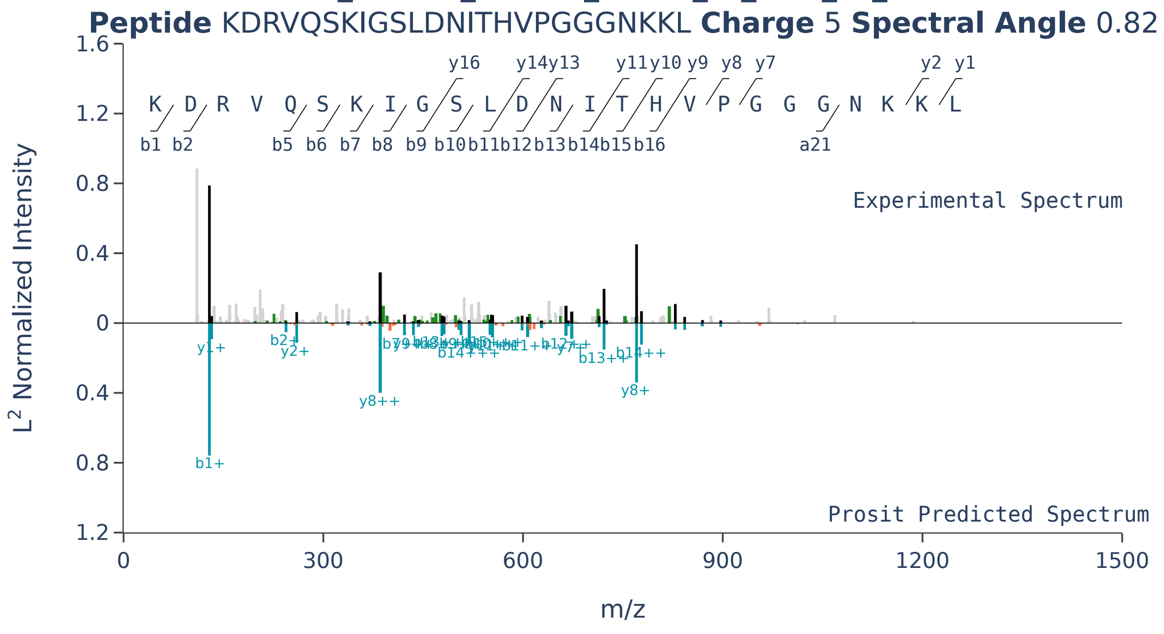


**Source** WSoh_221121_011221_HFGoe_L2_hTau_2h_R2 **Scan** 13987
**Peptide** EKLDFKDRVQSKIG **Charge** 3 **Spectral Angle** 0.78

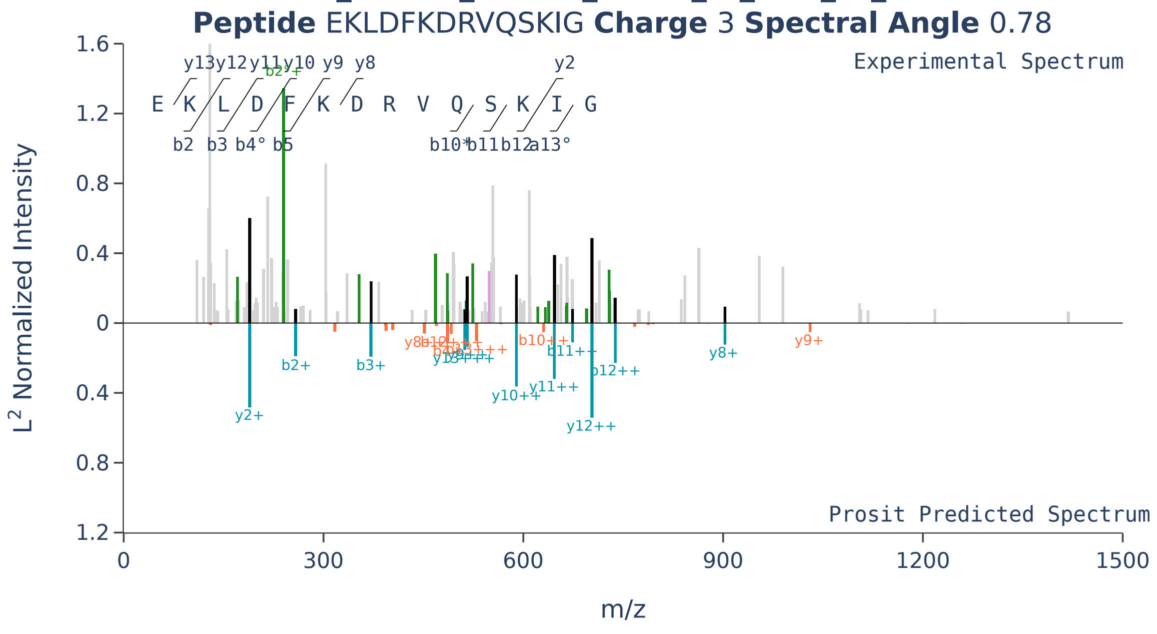


**Source** WSoh_221121_011221_HFGoe_L2_hTau_24h_R2 **Scan** 18366
**Peptide** EIVYKSPVVSGDTSPRHLSNV **Charge** 3 **Spectral Angle** 0.86

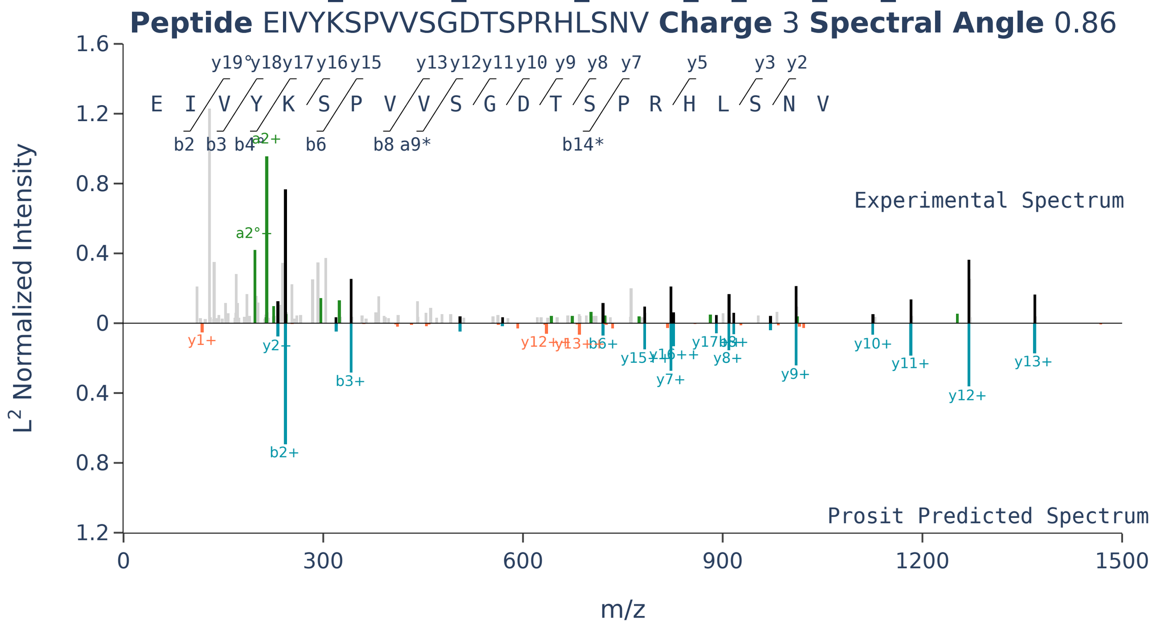


**Source** WSoh_221121_011221_HFGoe_L1_hTau_24h_R2 **Scan** 10629
**Peptide** NVKSKIGSTENL **Charge** 2 **Spectral Angle** 0.8

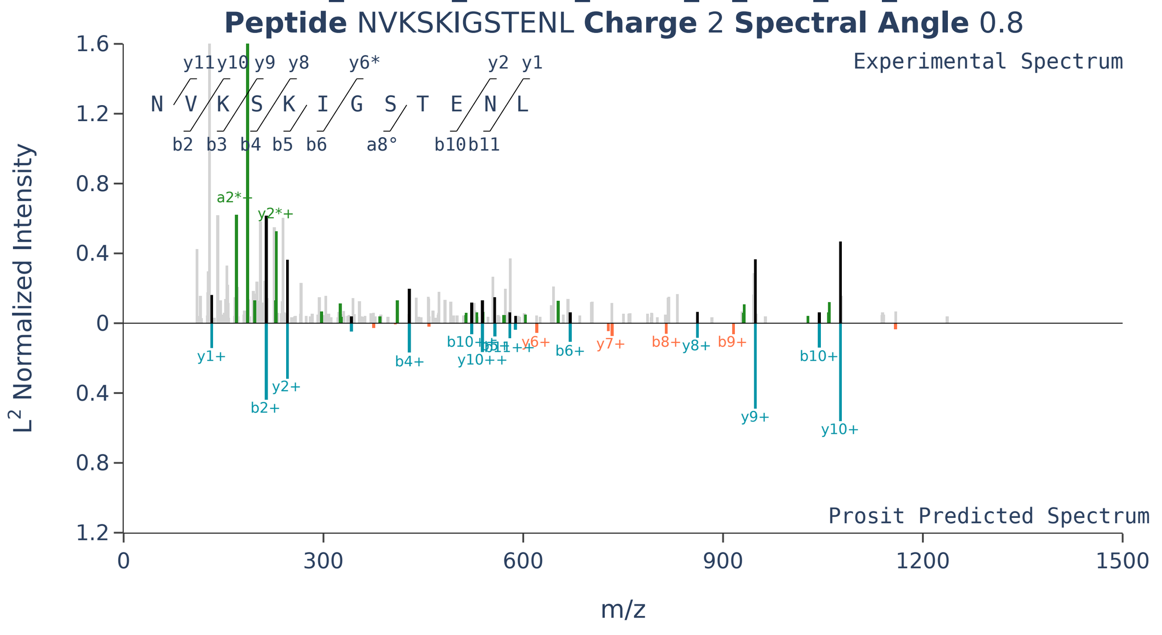


**Source** WSoh_221121_011221_HFGoe_L2_hTau_24h_R1 **Scan** 16290
**Peptide** LGSLDNITHVPGGGNKKIETHKL **Charge** 5 **Spectral Angle** 0.85

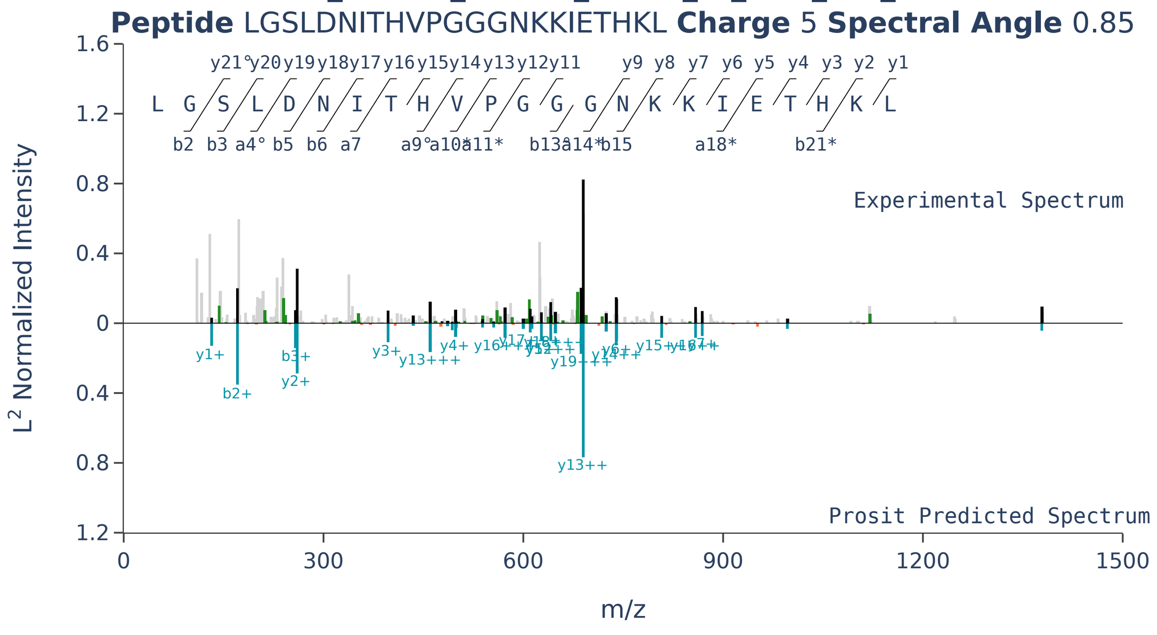


**Source** WSoh_221121_011221_HFGoe_L1_hTau_4h_R1 **Scan** 14712
**Peptide** NLKHQPGGGKVQIINKKLDL **Charge** 4 **Spectral Angle** 0.77

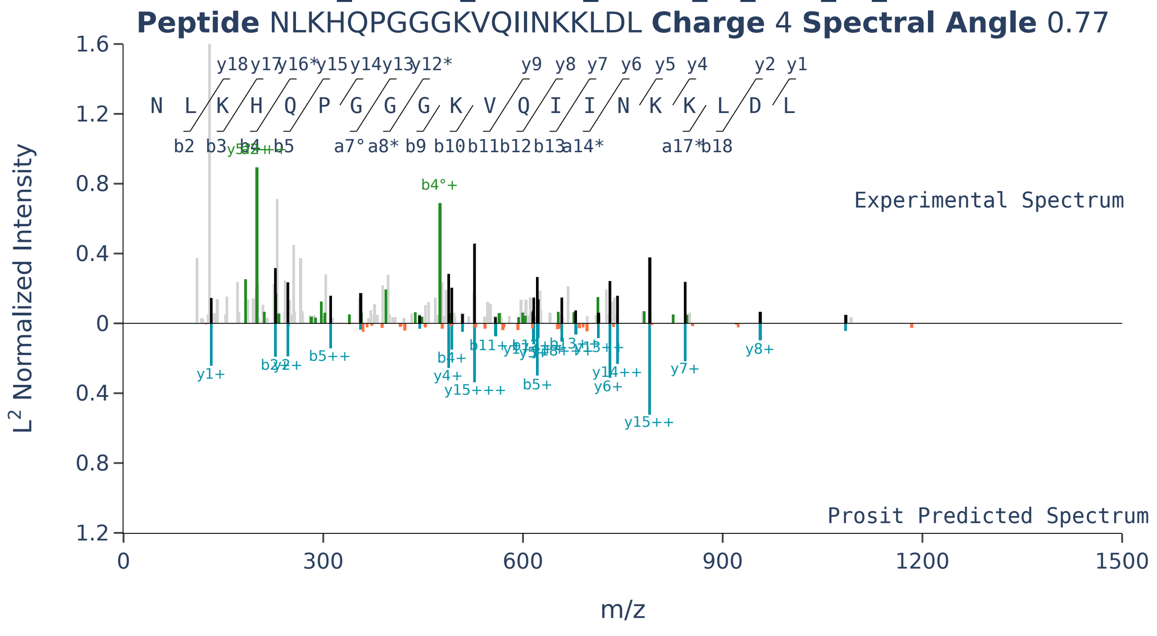


**Source** WSoh_221121_011221_HFGoe_L1_hTau_24h_R1 **Scan** 22012
**Peptide** LQTAPVPMPDLKNVKSKIGSTENLKH **Charge** 5 **Spectral Angle** 0.88

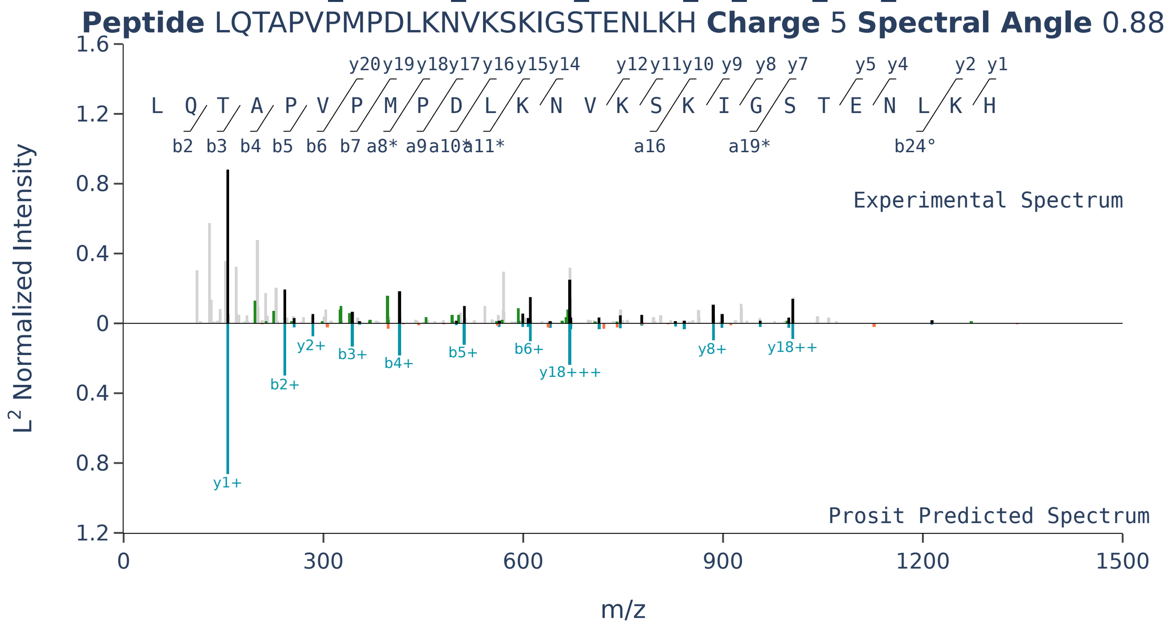


**Source** WSoh_221121_011221_HFGoe_L2_hTau_4h_R2 **Scan** 12316
**Peptide** KIGSLDNITHVPGGGNKKIETHK **Charge** 5 **Spectral Angle** 0.86

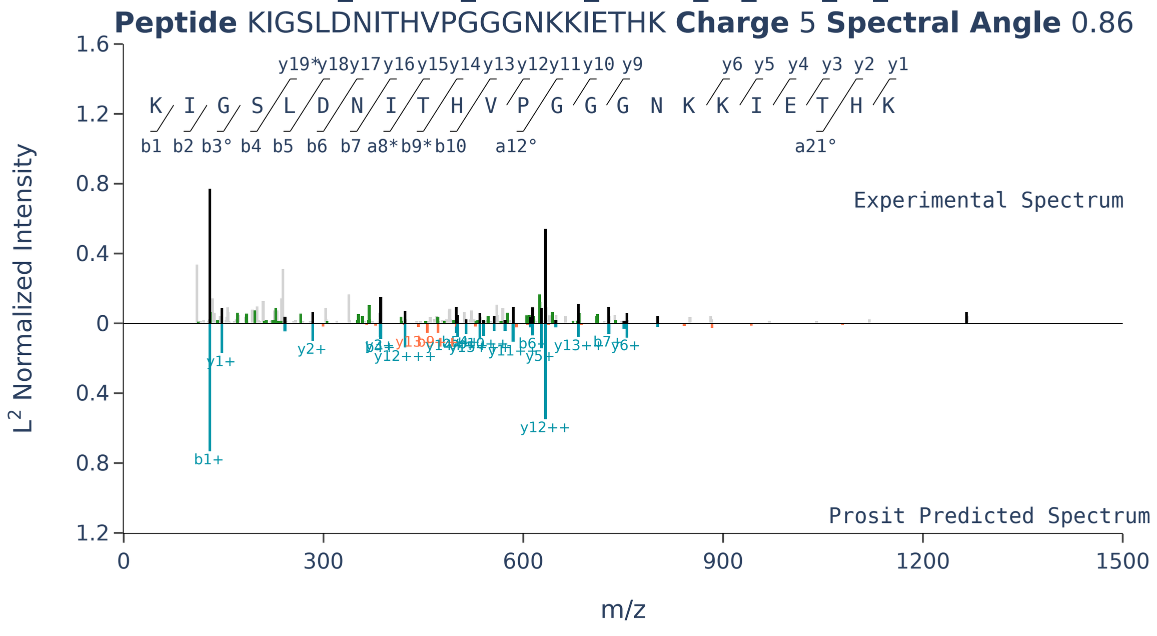


**Source** WSoh_221121_011221_HFGoe_L1_hTau_24h_R1 **Scan** 22755
**Peptide** EAGIGDTPSLEDEAAGHVTQARMVS **Charge** 3 **Spectral Angle** 0.82

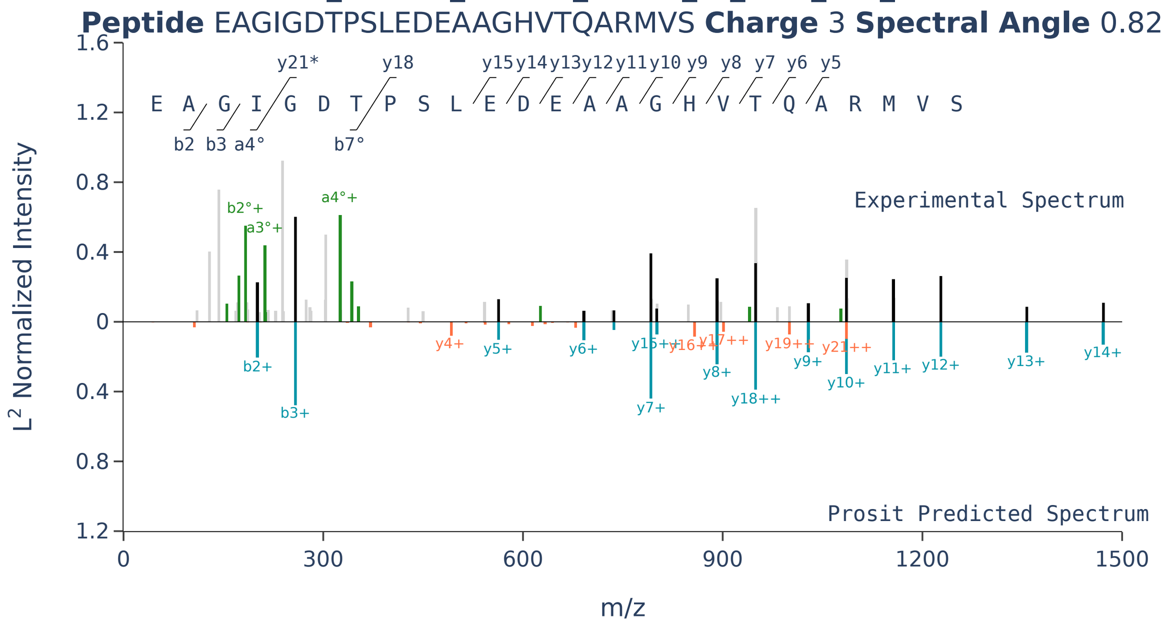


**Source** WSoh_221121_011221_HFGoe_L2_hTau_24h_R2 **Scan** 13461
**Peptide** TFRENAKAKTDHGAEIVYKSPVVS **Charge** 5 **Spectral Angle** 0.71

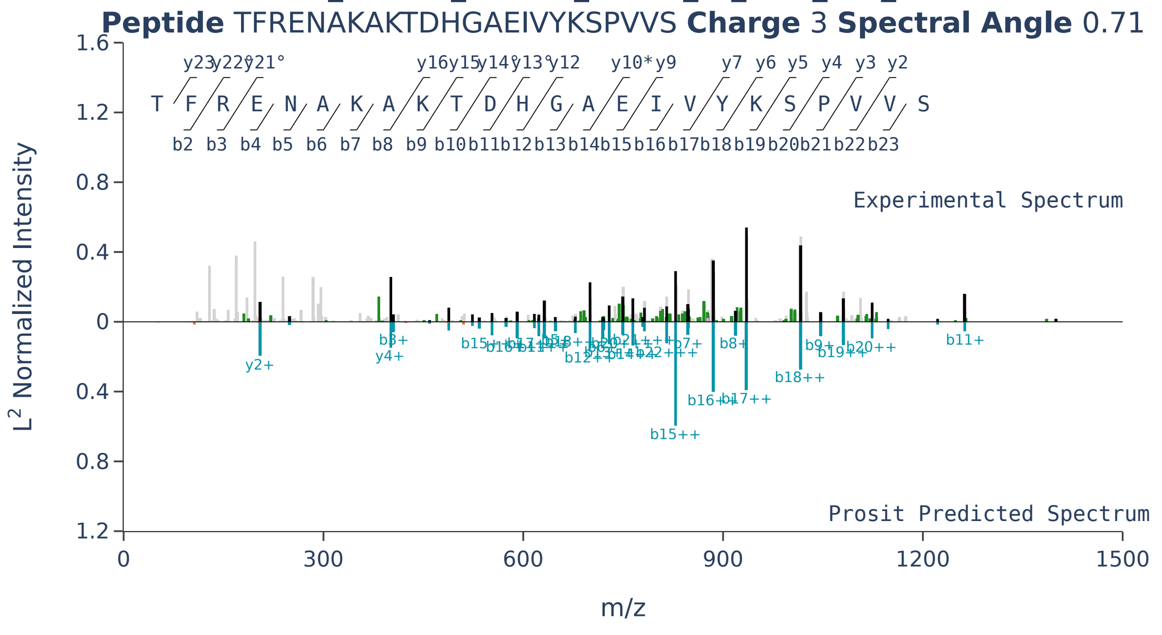


**Source** WSoh_221121_011221_HFGoe_L1_hTau_24h_R2 **Scan** 13383
**Peptide** TENLKHQPGGGKVQII **Charge** 2 **Spectral Angle** 0.8

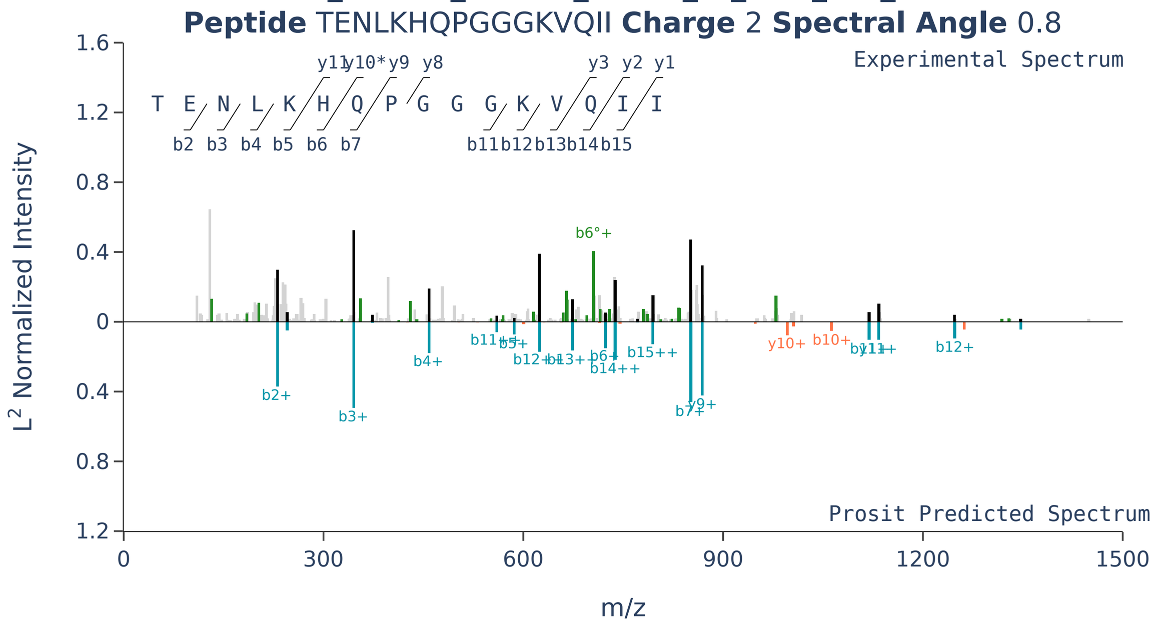


**Source** WSoh_221121_011221_HFGoe_L1_hTau_2h_R2 **Scan** 5991
**Peptide** GSKDNIKHVPGGG **Charge** 3 **Spectral Angle** 0.88

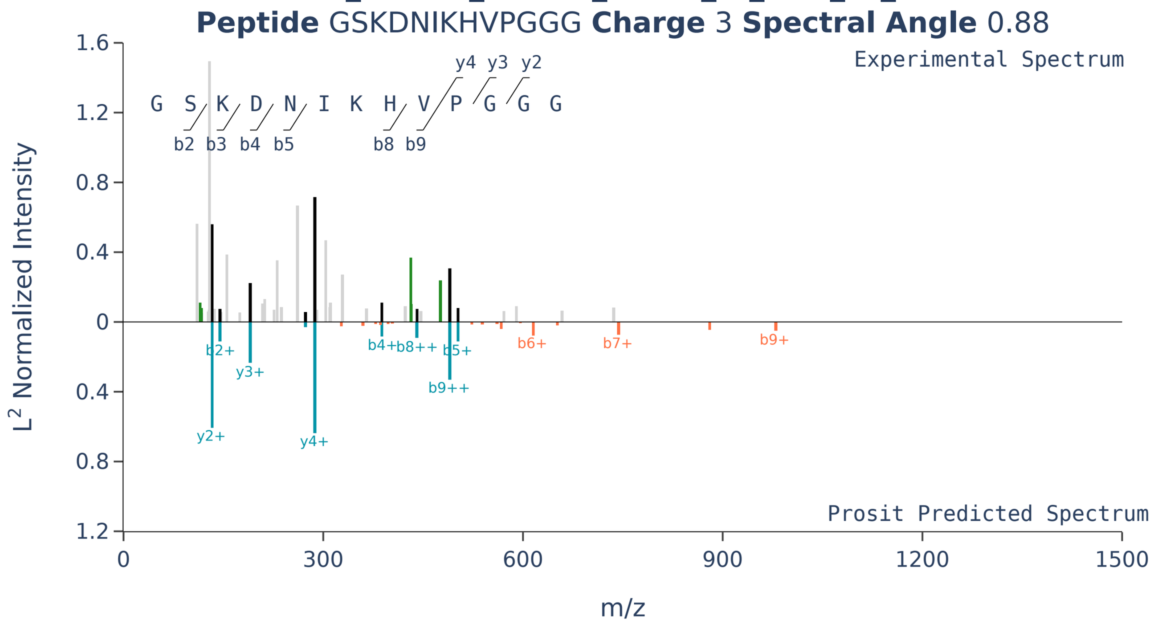


**Source** WSoh_221121_011221_HFGoe_L1_hTau_2h_R2 **Scan** 6812
**Peptide** HKLTFRENAKA **Charge** 3 **Spectral Angle** 0.83

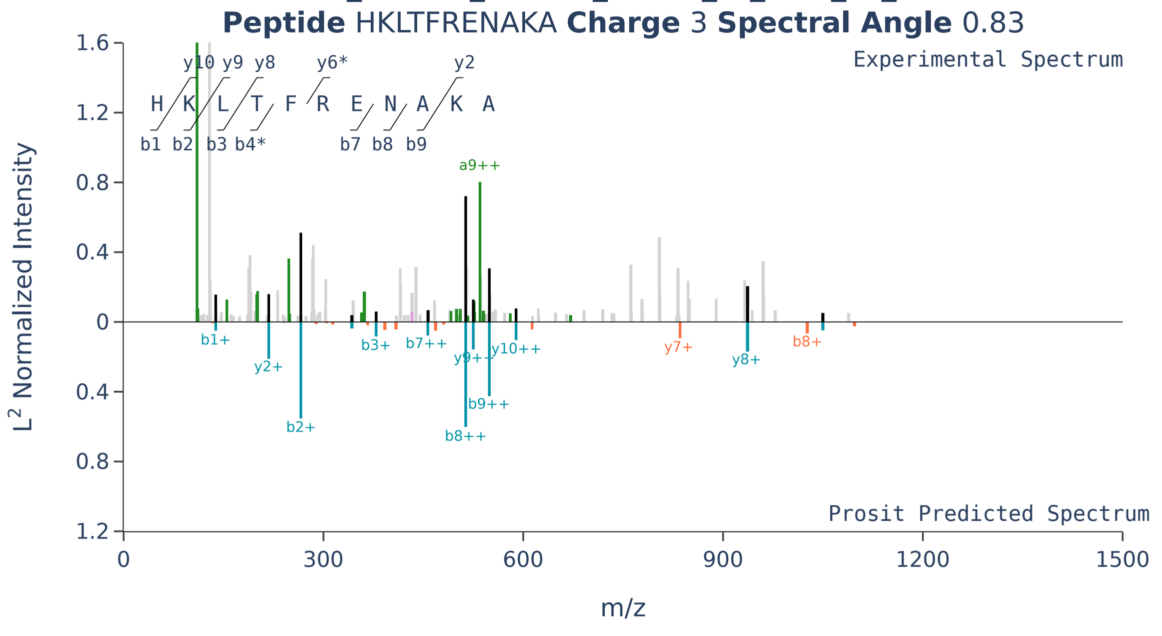


**Source** WSoh_221121_011221_HFGoe_L2_hTau_24h_R1 **Scan** 17165
**Peptide** HGAEIVYKSPVVSGDTSPRHLSNV **Charge** 3 **Spectral Angle** 0.76

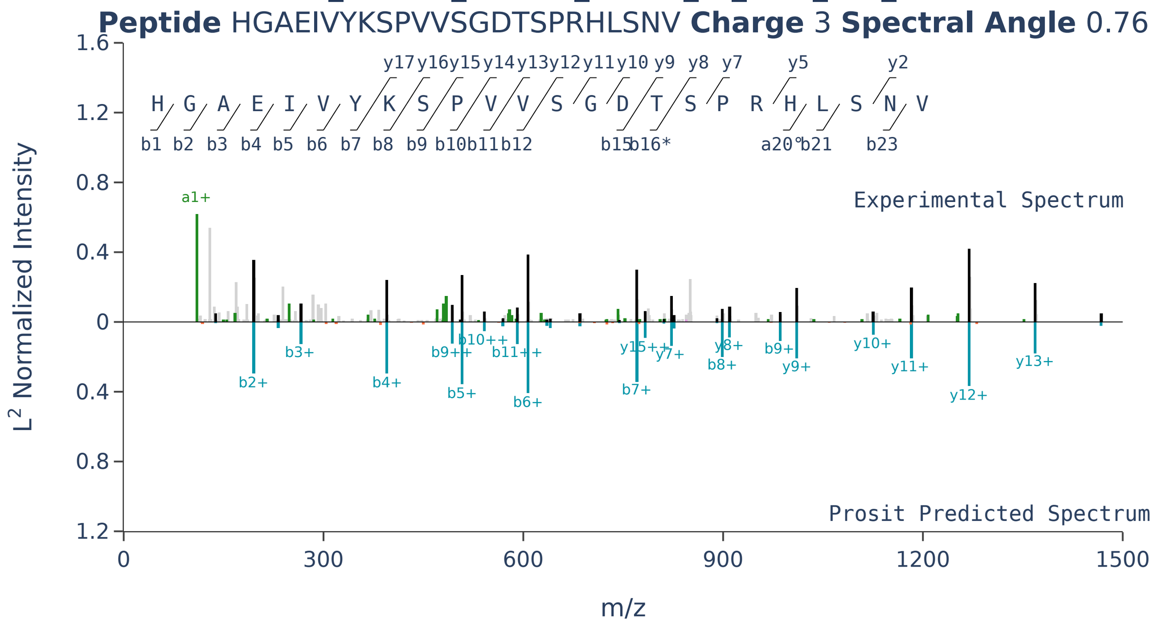

**Source** WSoh_221121_011221_HFGoe_L2_hTau_24h_R2 **Scan** 5928
**Peptide** EGAPGKQAAAQPHT **Charge** 3 **Spectral Angle** 0.9

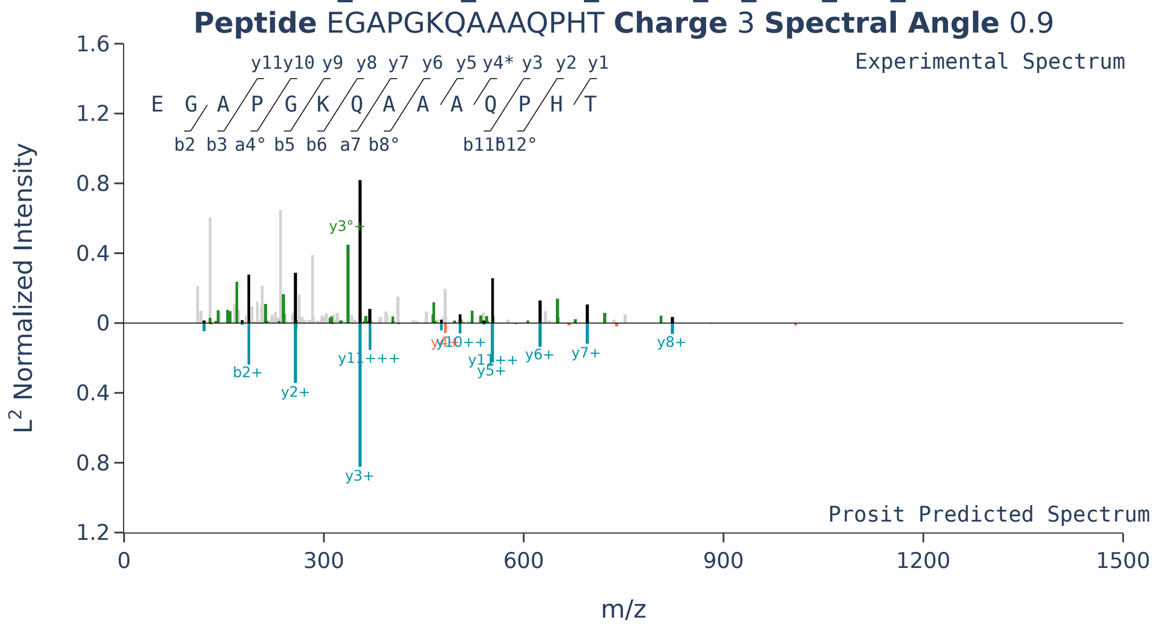


**Source** WSoh_221121_011221_HFGoe_L1_hTau_4h_R1 **Scan** 16308
**Peptide** CGSKDNIKHVPGGGSVQIVYKPVD **Charge** 4 **Spectral Angle** 0.75

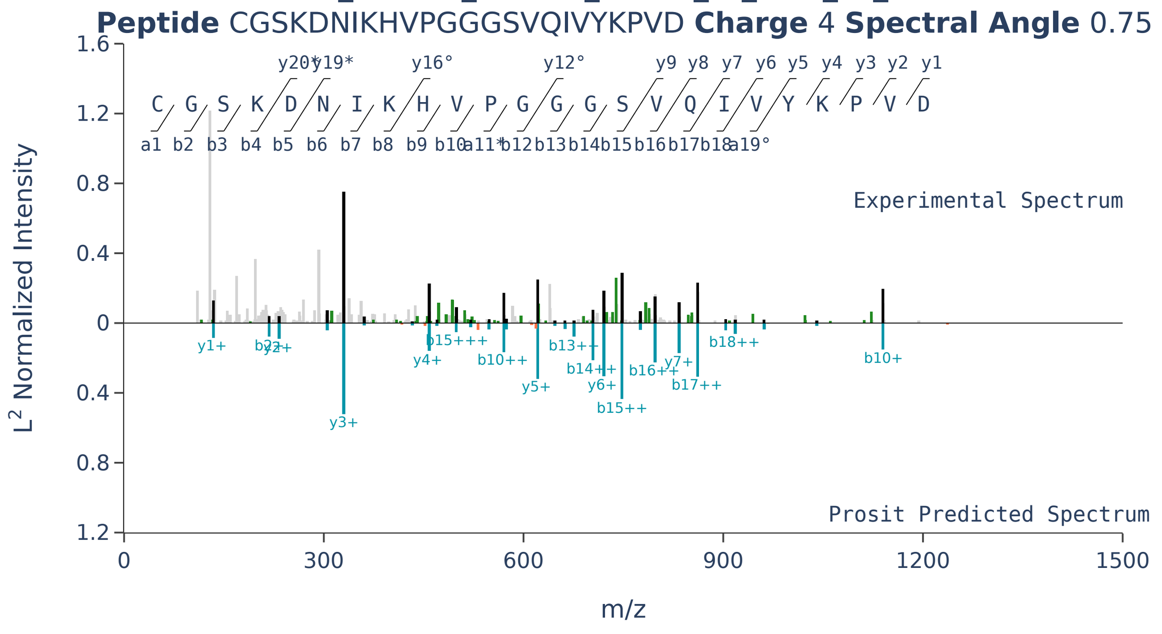


**Source** WSoh_221121_011221_HFGoe_L2_hTau_4h_R2 **Scan** 13154
**Peptide** KHQPGGGKVQIINKKLDLSNVQSKCG **Charge** 4 **Spectral Angle** 0.88

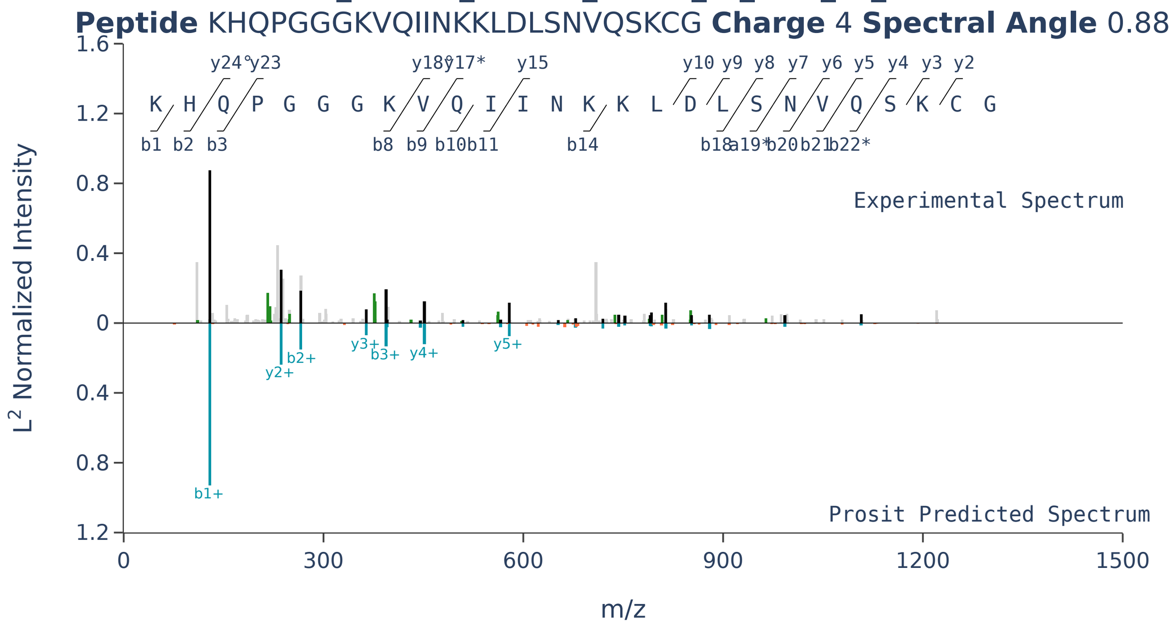


**Source** WSoh_221121_011221_HFGoe_L2_hTau_4h_R2 **Scan** 15975
**Peptide** GSLDNITHVPGGGNKKIETHKLTFREN **Charge** 6 **Spectral Angle** 0.77

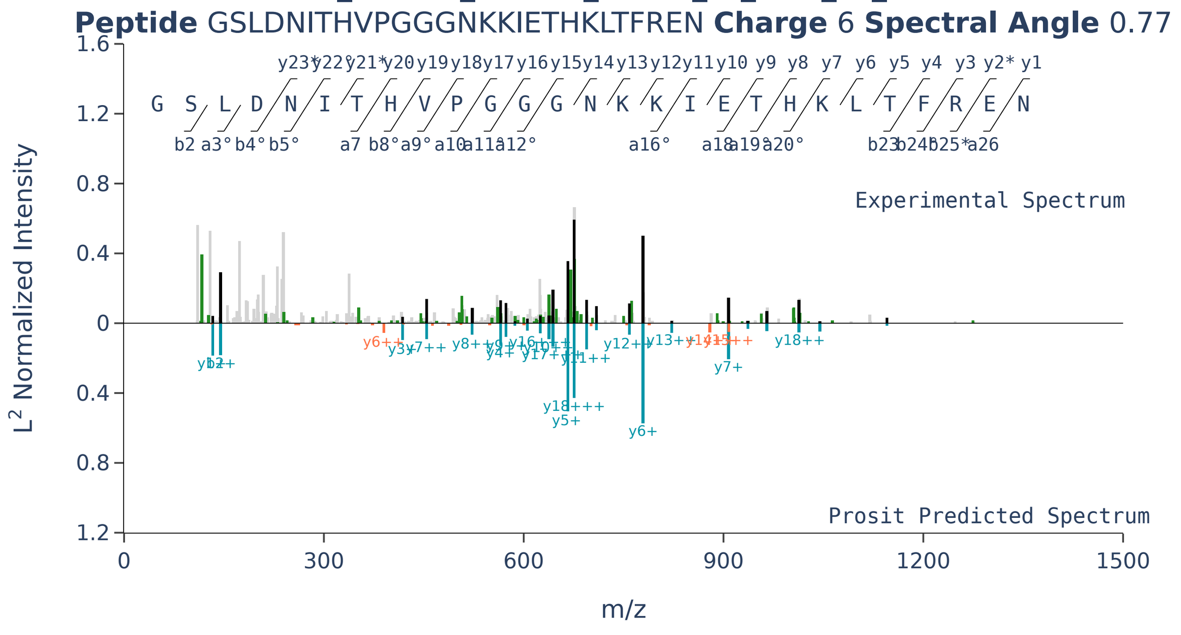


**Source** WSoh_221121_011221_HFGoe_L1_hTau_4h_R1 **Scan** 16190
**Peptide** PVDLSKVTSKCGSLGNIHH **Charge** 3 **Spectral Angle** 0.83

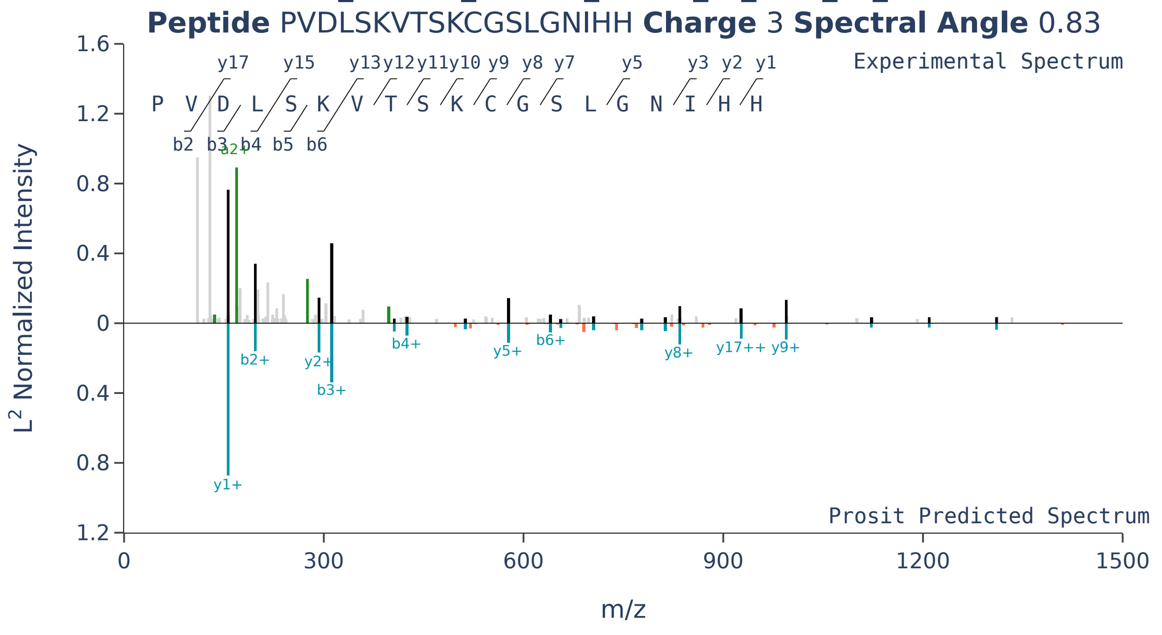


**Source** WSoh_221121_011221_HFGoe_L2_hTau_24h_R1 **Scan** 5746
**Peptide** KNVKSKIGSTENLKH **Charge** 2 **Spectral Angle** 0.88

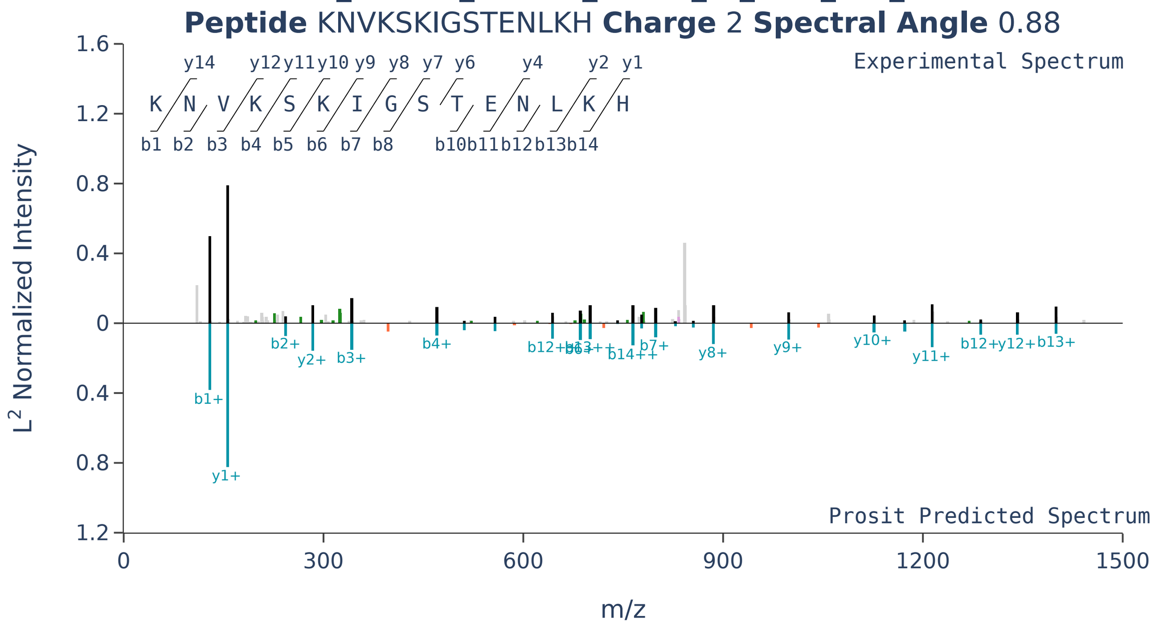


**Source** WSoh_221121_011221_HFGoe_L1_hTau_4h_R2 **Scan** 8309
**Peptide** SGDTSPRHLS **Charge** 2 **Spectral Angle** 0.85

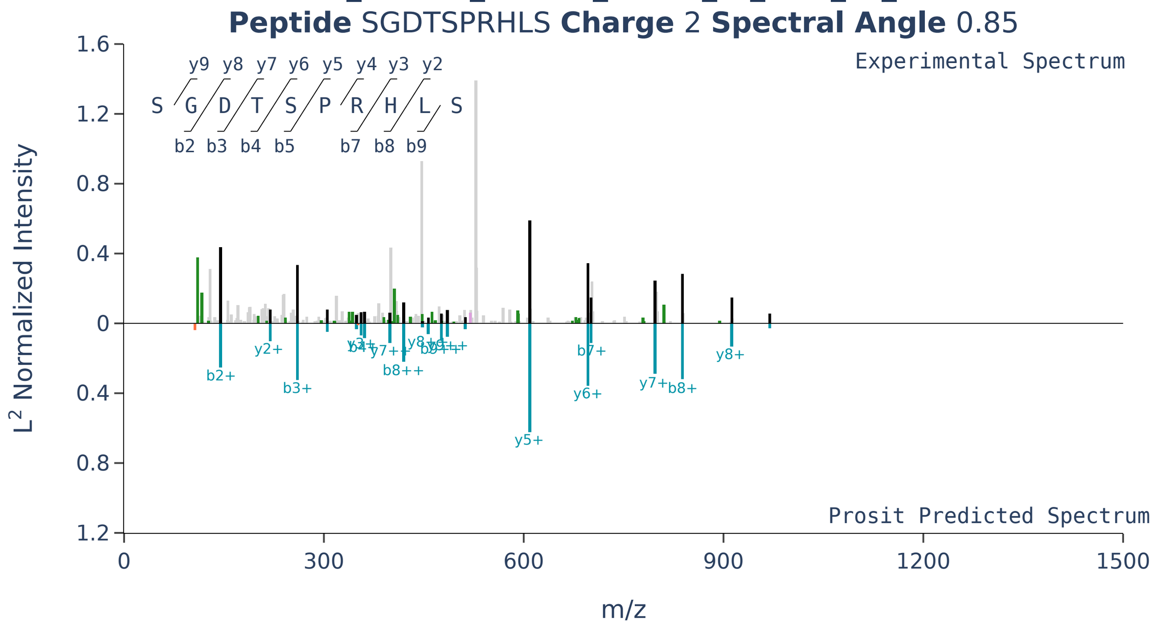


**Source** WSoh_221121_011221_HFGoe_L1_hTau_24h_R2 **Scan** 11349
**Peptide** LGSTENLKHQPGGGKVQIINK **Charge** 3 **Spectral Angle** 0.81

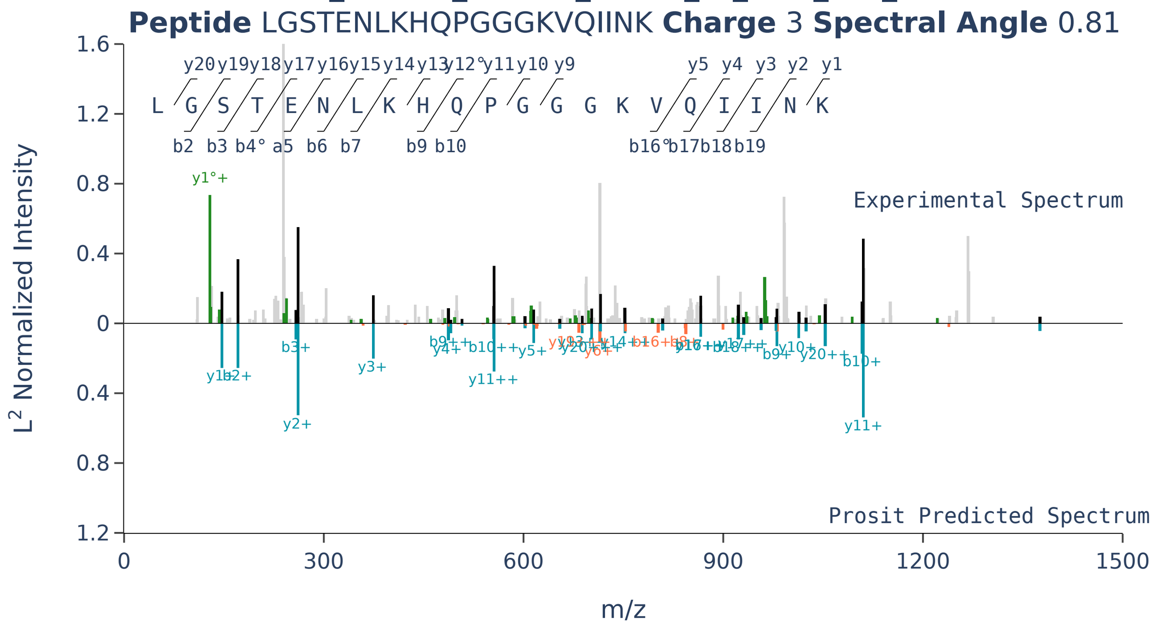


**Source** WSoh_221121_011221_HFGoe_L2_hTau_24h_R2 **Scan** 9020
**Peptide** KSKIGSTENLKHQPGGGKVQIINK **Charge** 3 **Spectral Angle** 0.87

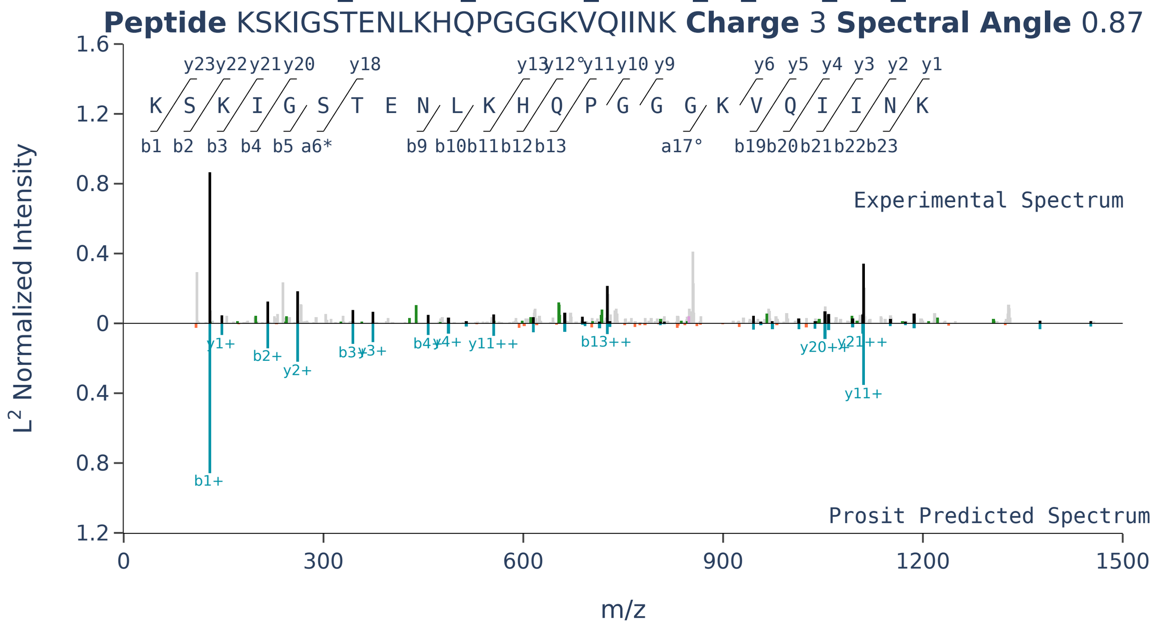


**Source** WSoh_221121_011221_HFGoe_L1_hTau_4h_R2 **Scan** 6056
**Peptide** SKVTSKCGSLGNIHHKPGGG **Charge** 3 **Spectral Angle** 0.85

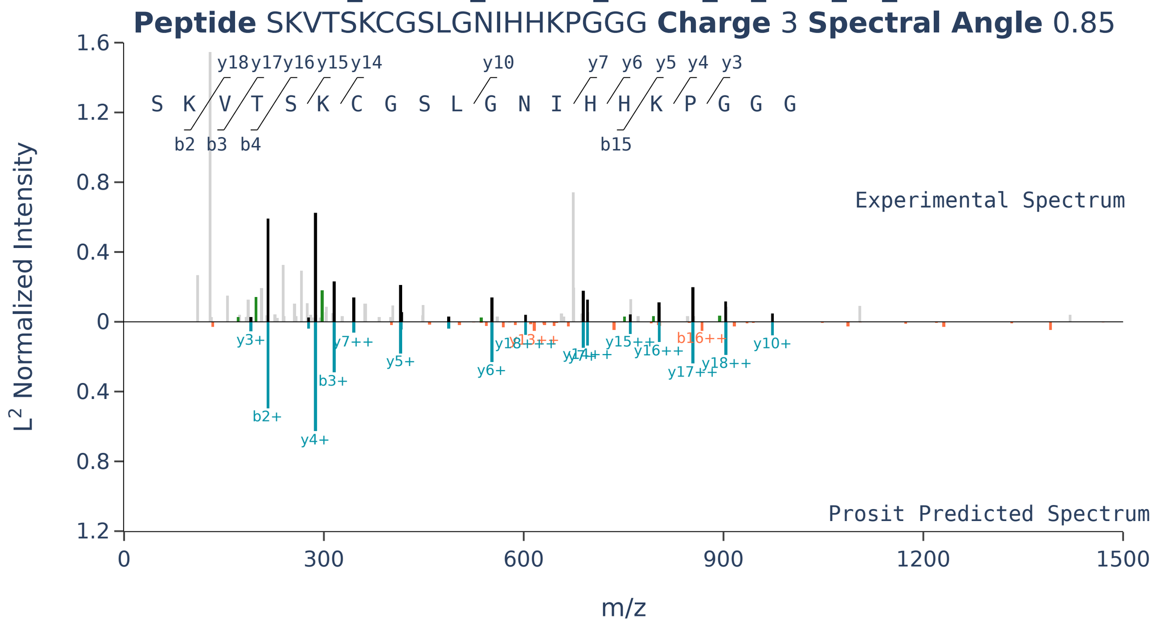


**Source** WSoh_221121_011221_HFGoe_L1_hTau_24h_R1 **Scan** 14855
**Peptide** GSLGNIHHKPGGGQVEVKSEKLDF **Charge** 3 **Spectral Angle** 0.76

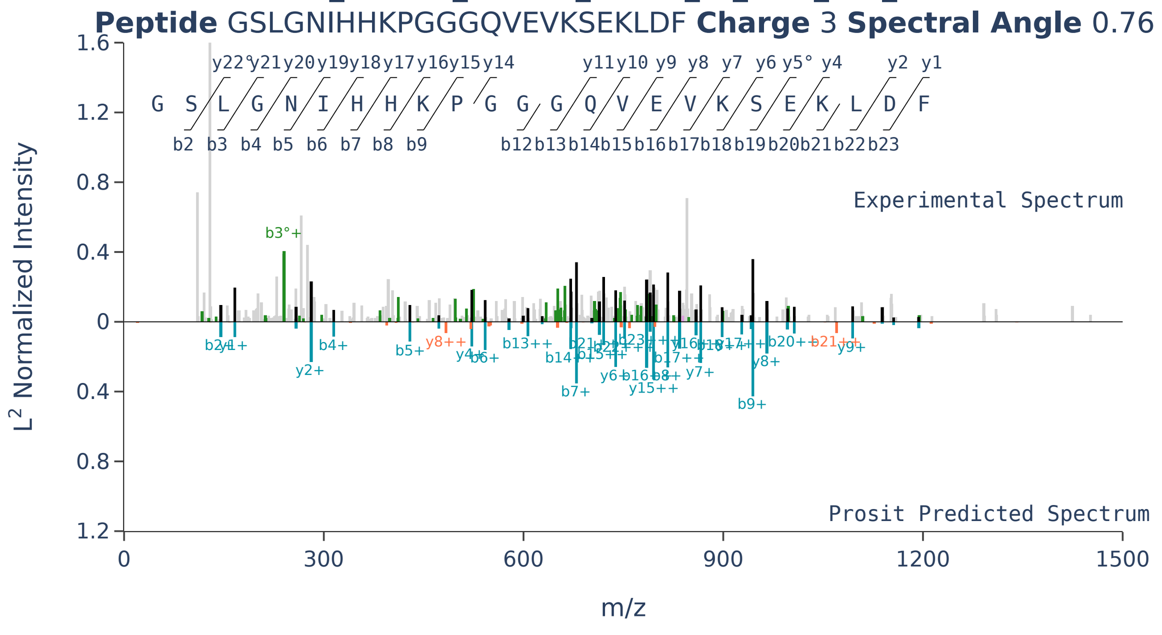


**Source** WSoh_221121_011221_HFGoe_L2_hTau_24h_R1 **Scan** 17632
**Peptide** AEIVYKSPVVSGDTSPRHLSNVSS **Charge** 3 **Spectral Angle** 0.89

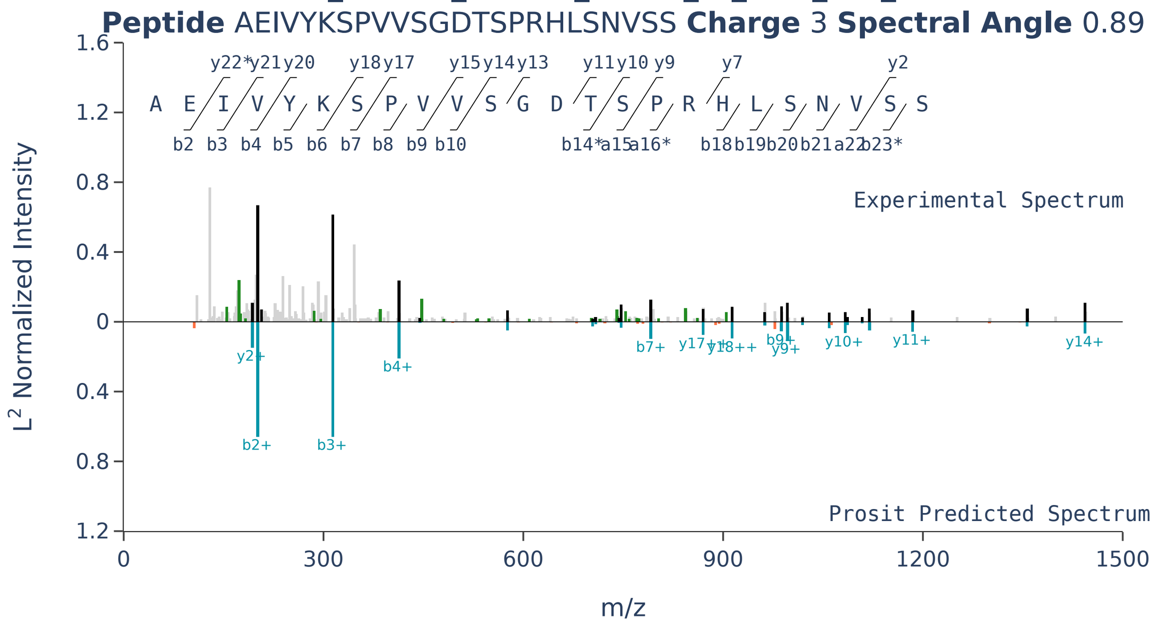


**Source** WSoh_221121_011221_HFGoe_L2_hTau_24h_R2 **Scan** 11915
**Peptide** GTYGLGDRKDQGGYTMHQD **Charge** 4 **Spectral Angle** 0.82

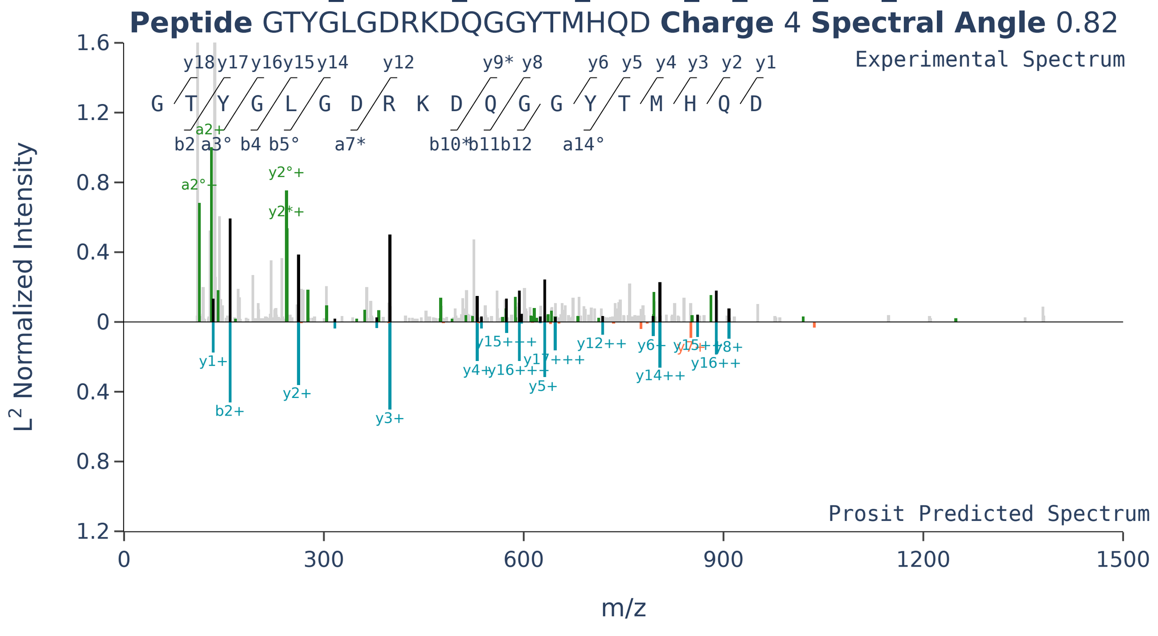


**Source** WSoh_221121_011221_HFGoe_L1_hTau_24h_R1 **Scan** 16497
**Peptide** QEGDTDAGLKESPLQ **Charge** 2 **Spectral Angle** 0.84

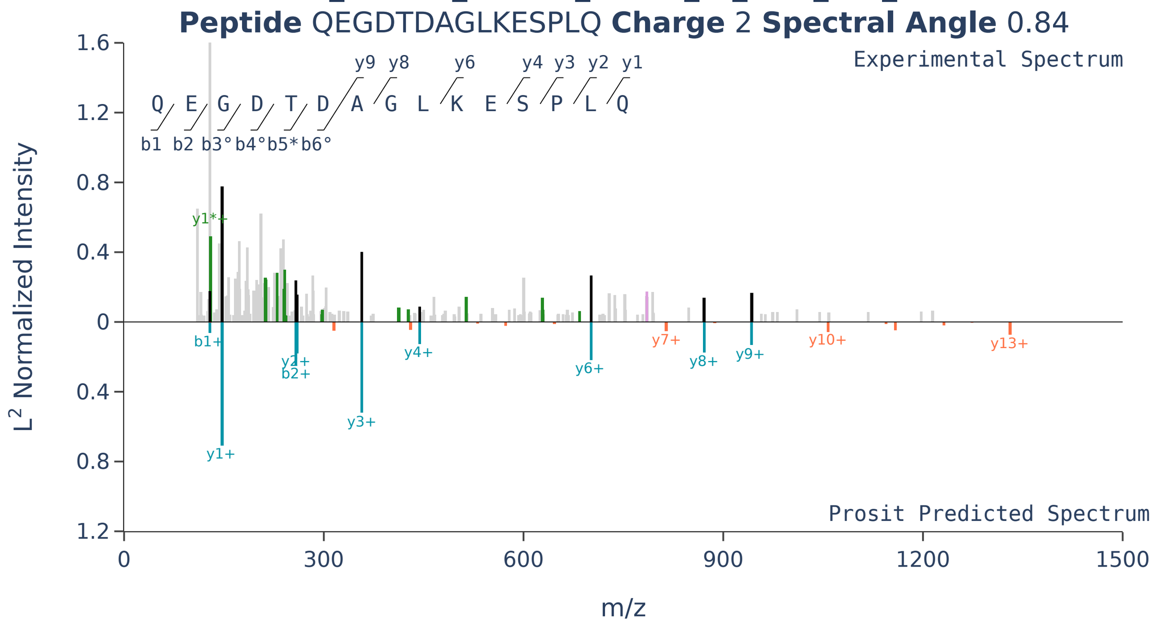


**Source** WSoh_221121_011221_HFGoe_L1_hTau_24h_R1 **Scan** 20471
**Peptide** TSDAKSTPTAEDVTAPLVDEGAPGKQA **Charge** 4 **Spectral Angle** 0.88

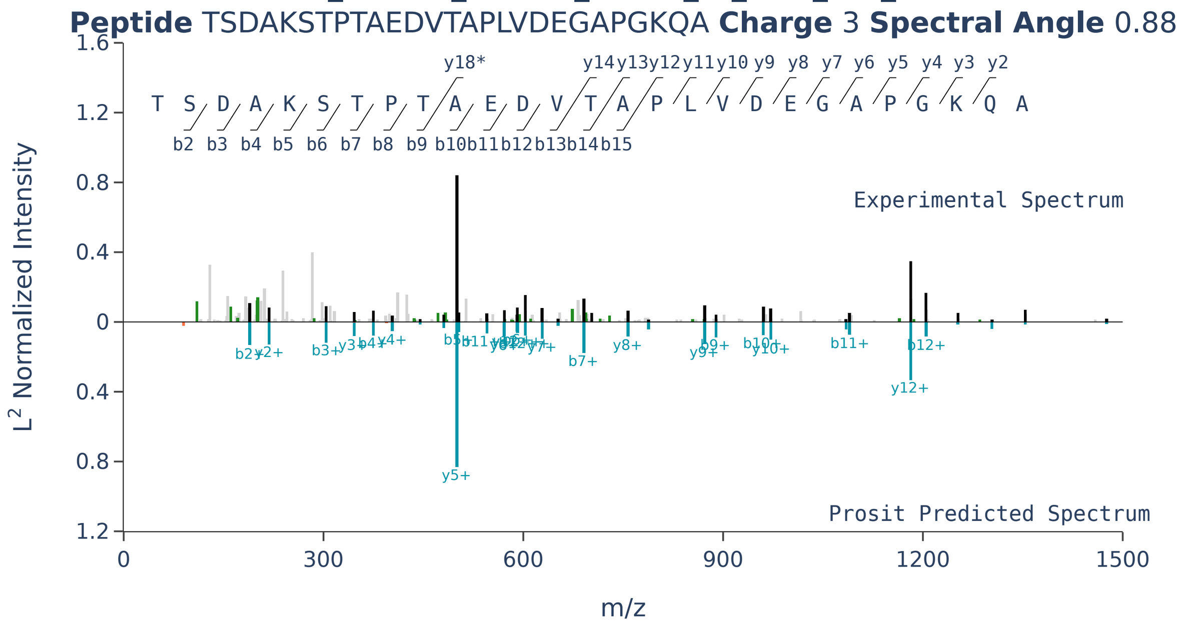

**Source** WSoh_221121_011221_HFGoe_L2_hTau_24h_R1 **Scan** 13673
**Peptide** SRTPSLPTPPTREPKKVAVVR **Charge** 5 **Spectral Angle** 0.78

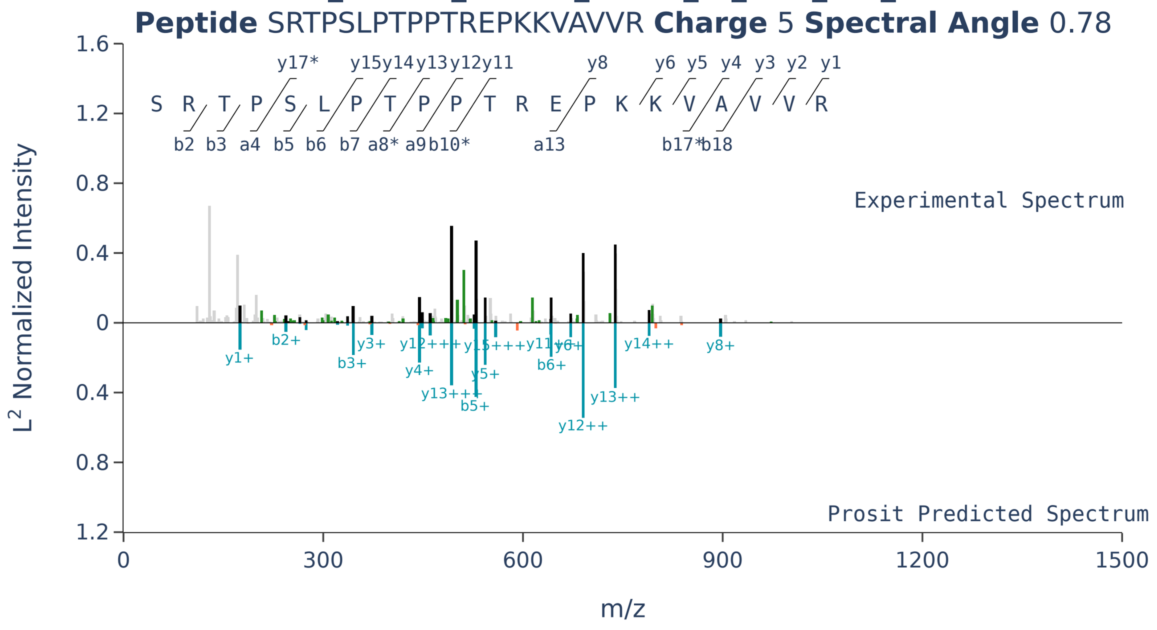


**Source** WSoh_221121_011221_HFGoe_L2_hTau_4h_R2 **Scan** 13883
**Peptide** TAPLVDEGAPGKQA **Charge** 2 **Spectral Angle** 0.86

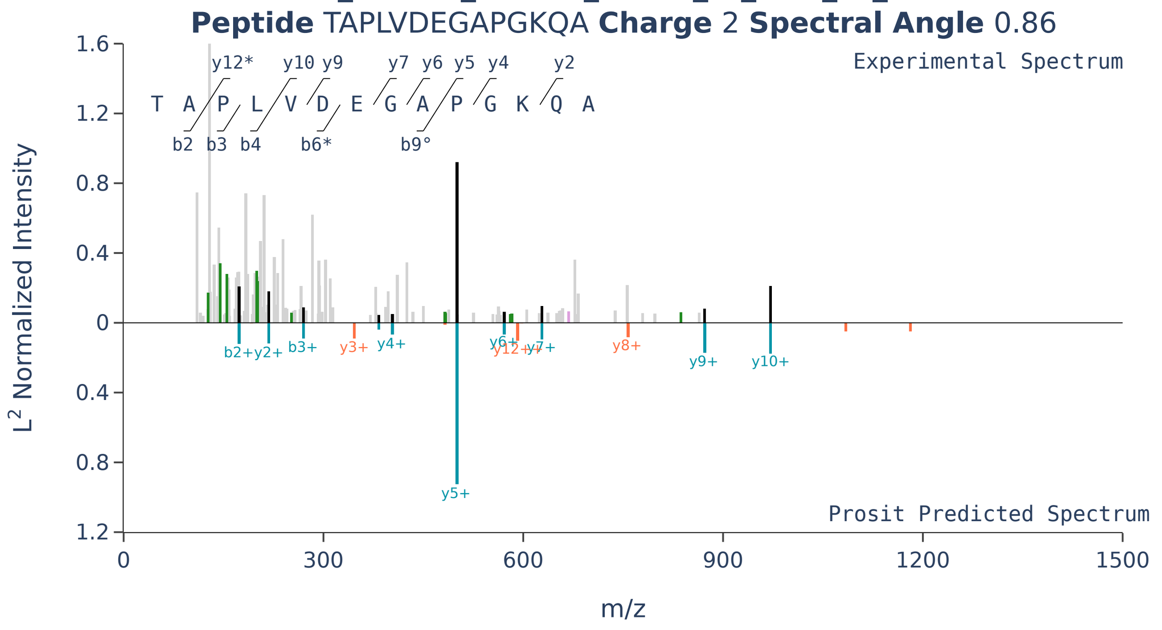


**Source** WSoh_221121_011221_HFGoe_L1_hTau_24h_R1 **Scan** 18665
**Peptide** FEVMEDHAGTYGLGDRKDQGGYTMHQD **Charge** 4 **Spectral Angle** 0.9

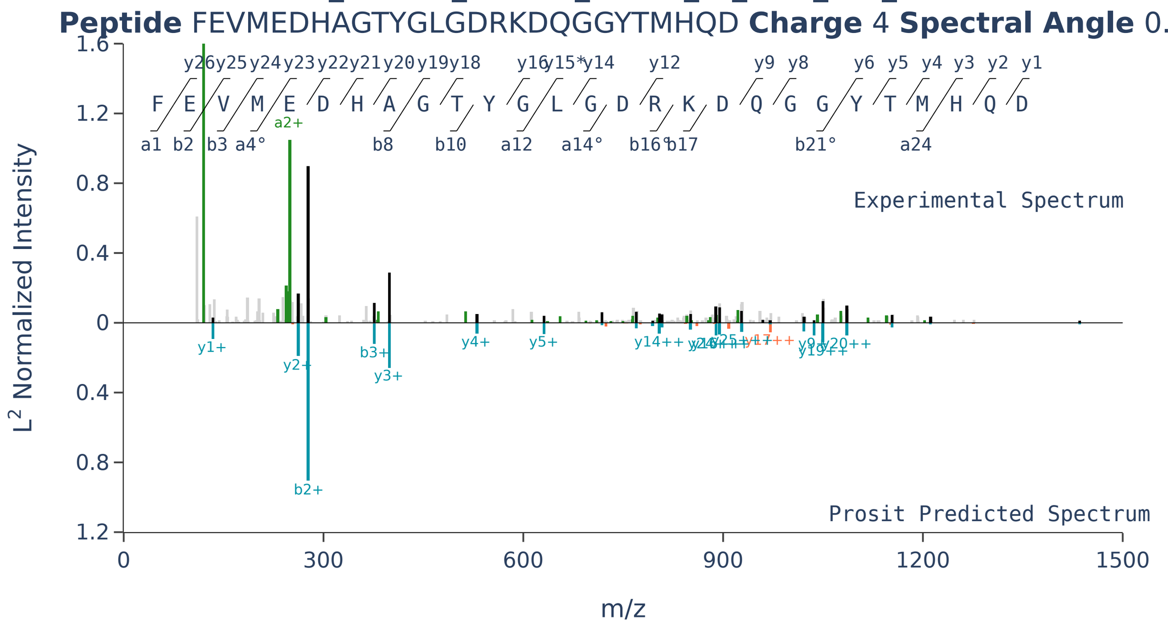


**Source** WSoh_221121_011221_HFGoe_L2_hTau_24h_R1 **Scan** 18304
**Peptide** GAEIVYKSPVVSGDTSPRHLSNVSSTG **Charge** 3 **Spectral Angle** 0.86

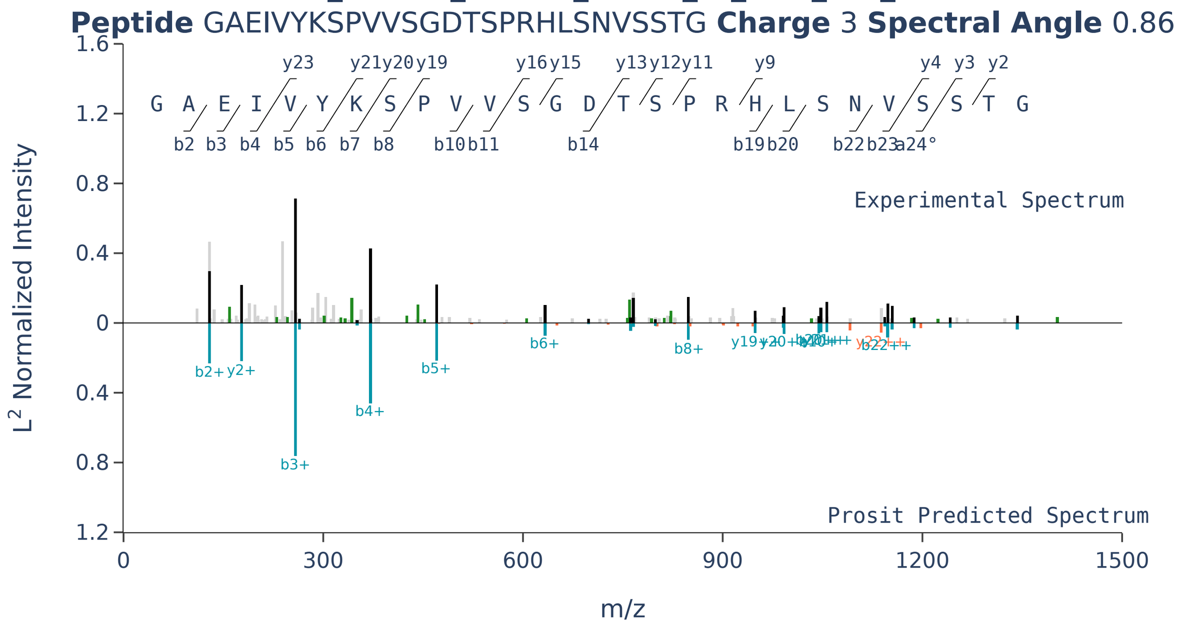


**Source** WSoh_221121_011221_HFGoe_L1_hTau_24h_R1 **Scan** 8313
**Peptide** PGSRSRTPSL **Charge** 2 **Spectral Angle** 0.81

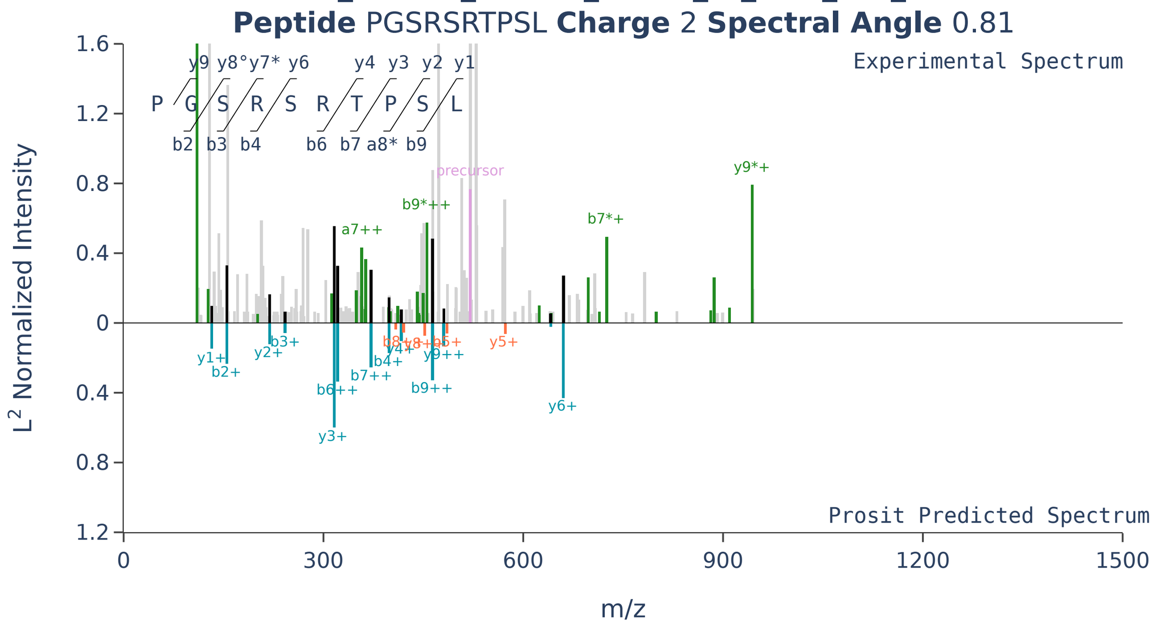


**Source** WSoh_221121_011221_HFGoe_L1_hTau_2h_R1 **Scan** 7239
**Peptide** QGGYTMHQD **Charge** 2 **Spectral Angle** 0.84

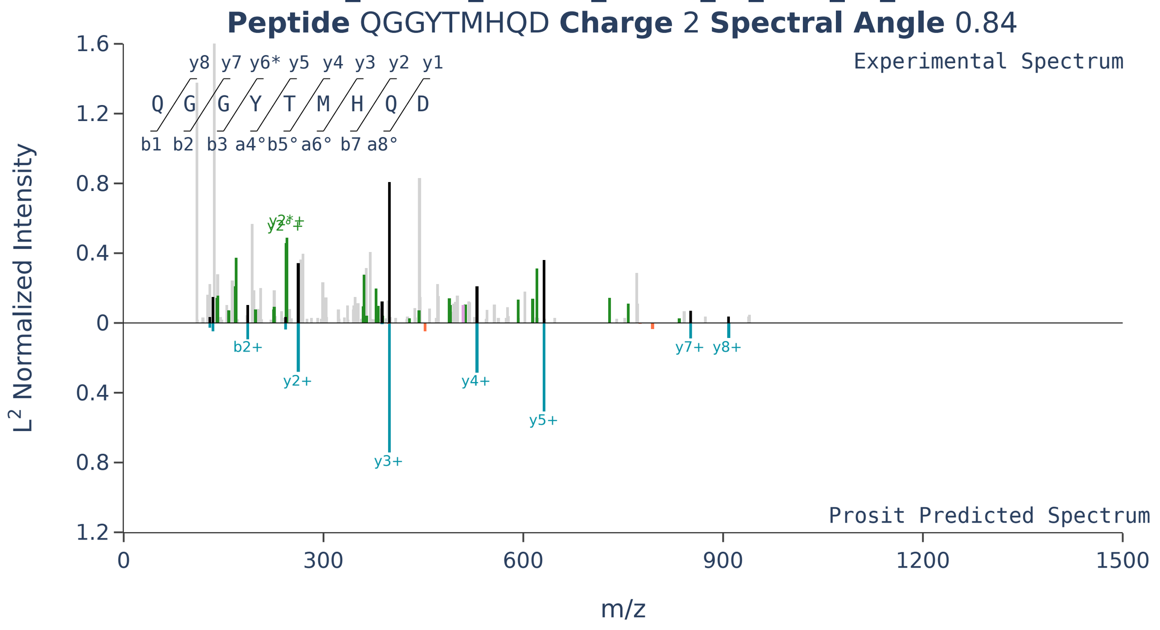


**Source** WSoh_221121_011221_HFGoe_L1_hTau_24h_R2 **Scan** 7012
**Peptide** KTKIATPRGAAPPGQKGQANA **Charge** 3 **Spectral Angle** 0.83

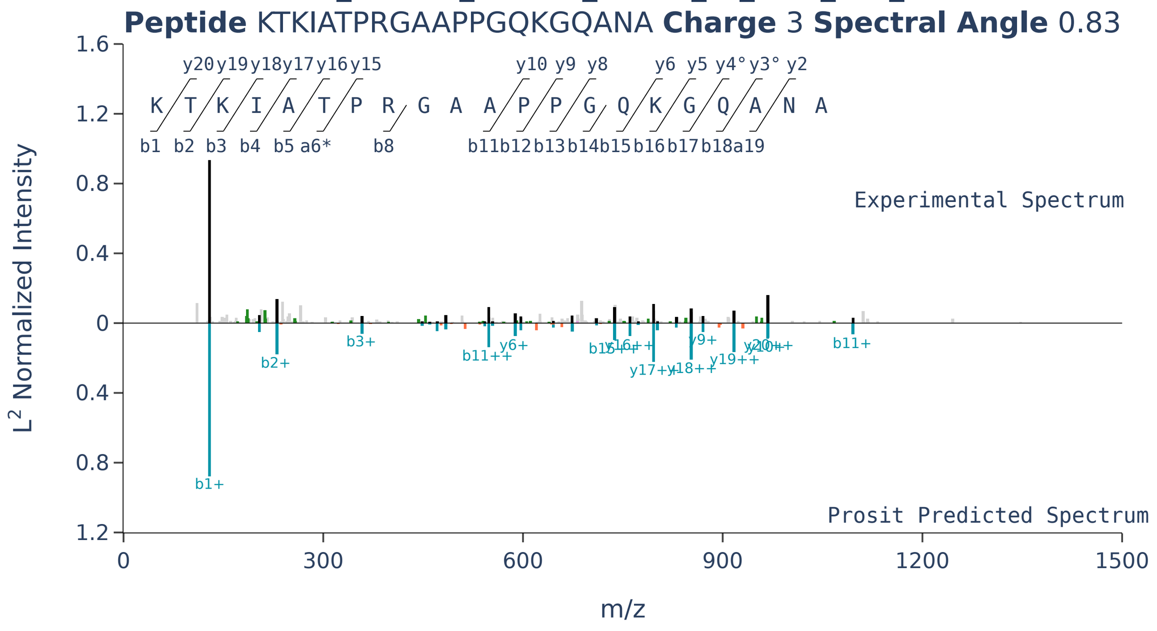


**Source** WSoh_221121_011221_HFGoe_L1_hTau_24h_R1 **Scan** 10368
**Peptide** LHHKPGGGQVEVKSEKLDFKDRVQS **Charge** 4 **Spectral Angle** 0.81

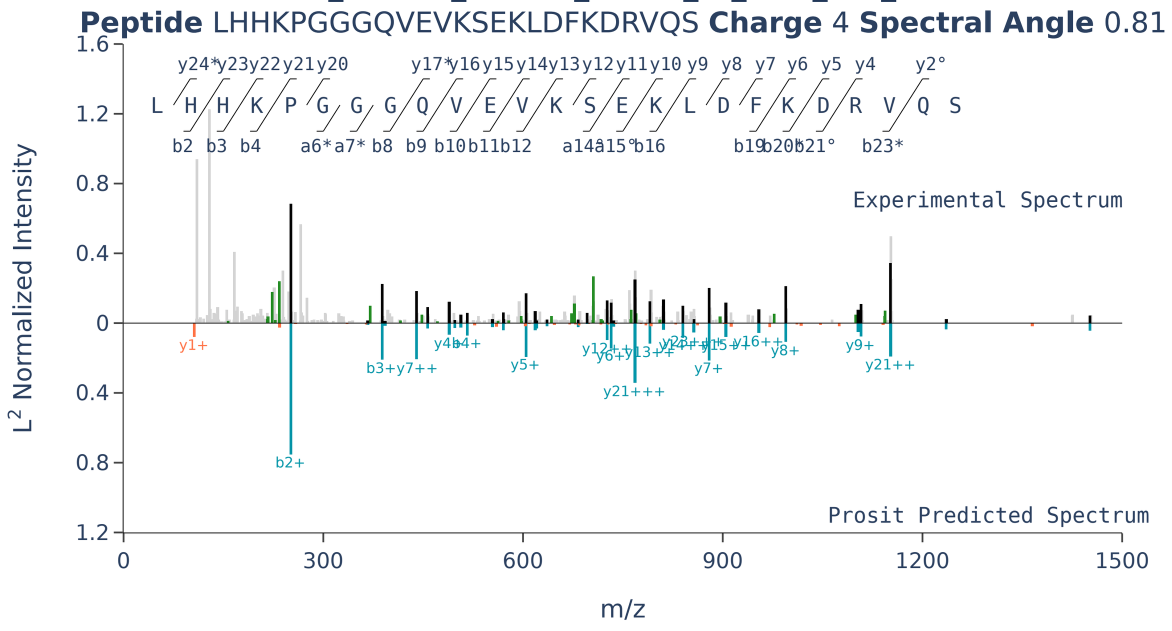


**Source** WSoh_221121_011221_HFGoe_L2_hTau_24h_R1 **Scan** 10391
**Peptide** LNKKLDLSNVQSKCGSKDNIKHV **Charge** 5 **Spectral Angle** 0.89

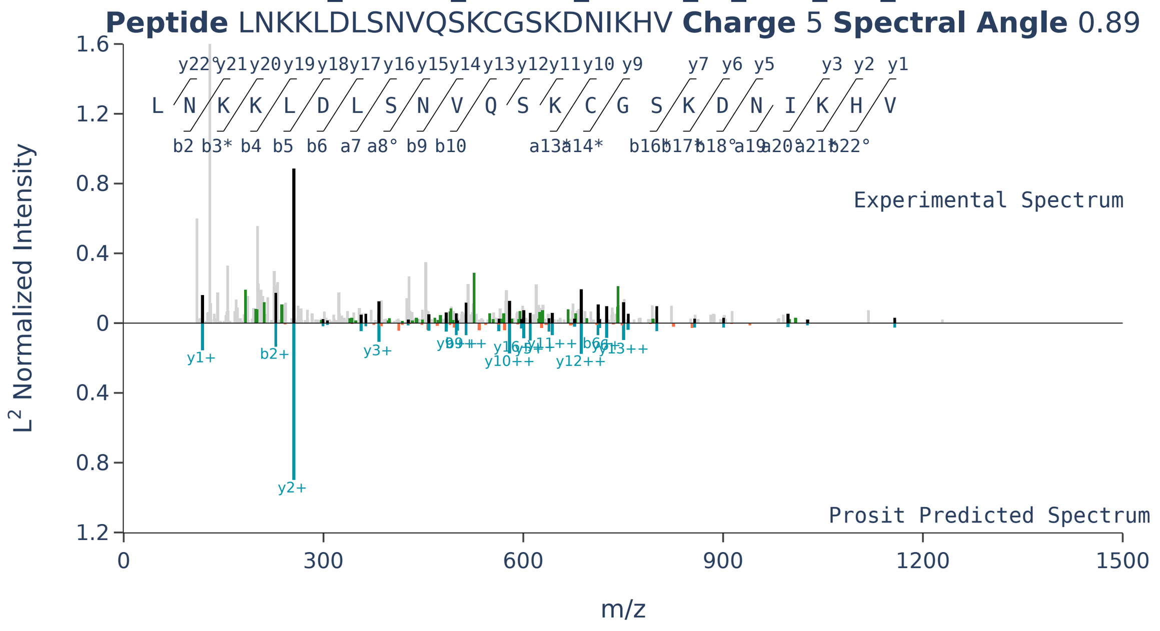


**Source** WSoh_221121_011221_HFGoe_L1_hTau_24h_R2 **Scan** 7716
**Peptide** KIATPRGAAPPGQKGQANAT **Charge** 2 **Spectral Angle** 0.8

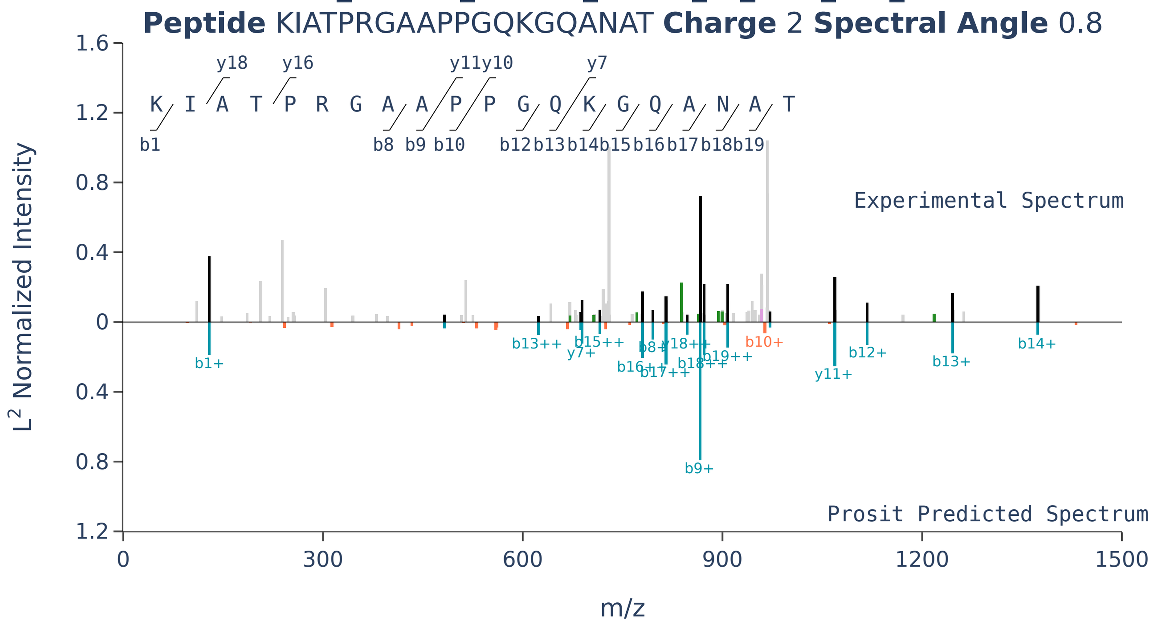


**Source** WSoh_221121_011221_HFGoe_L2_hTau_24h_R1 **Scan** 7578
**Peptide** DGKTKIATPRGAAPPGQKGQAN **Charge** 3 **Spectral Angle** 0.83

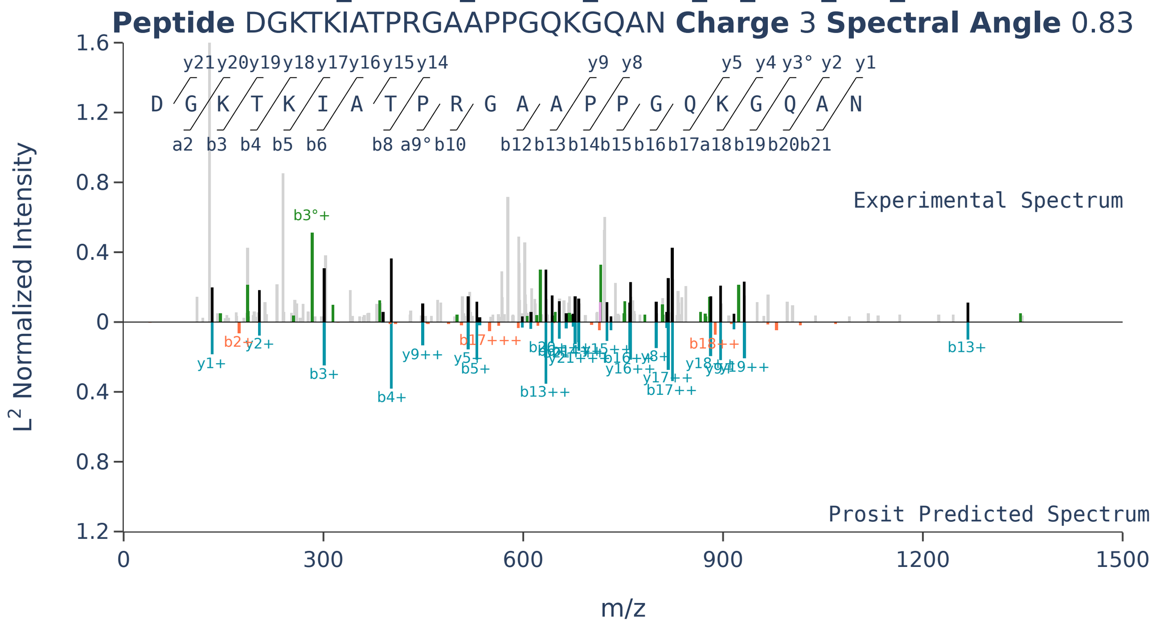


**Source** WSoh_221121_011221_HFGoe_L1_hTau_24h_R2 **Scan** 9365
**Peptide** RLPAKTPPAPKTPPSSGEPPKS **Charge** 4 **Spectral Angle** 0.79

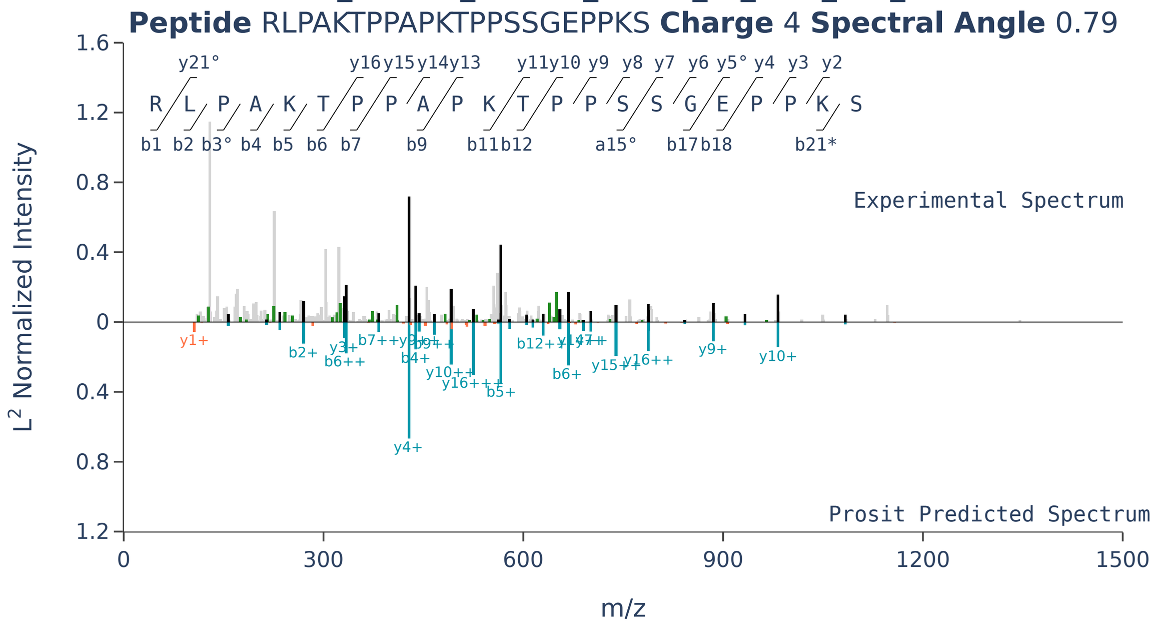


**Source** WSoh_221121_011221_HFGoe_L1_hTau_1h_R2 **Scan** 13320
**Peptide** IGSTENL **Charge** 1 **Spectral Angle** 0.81

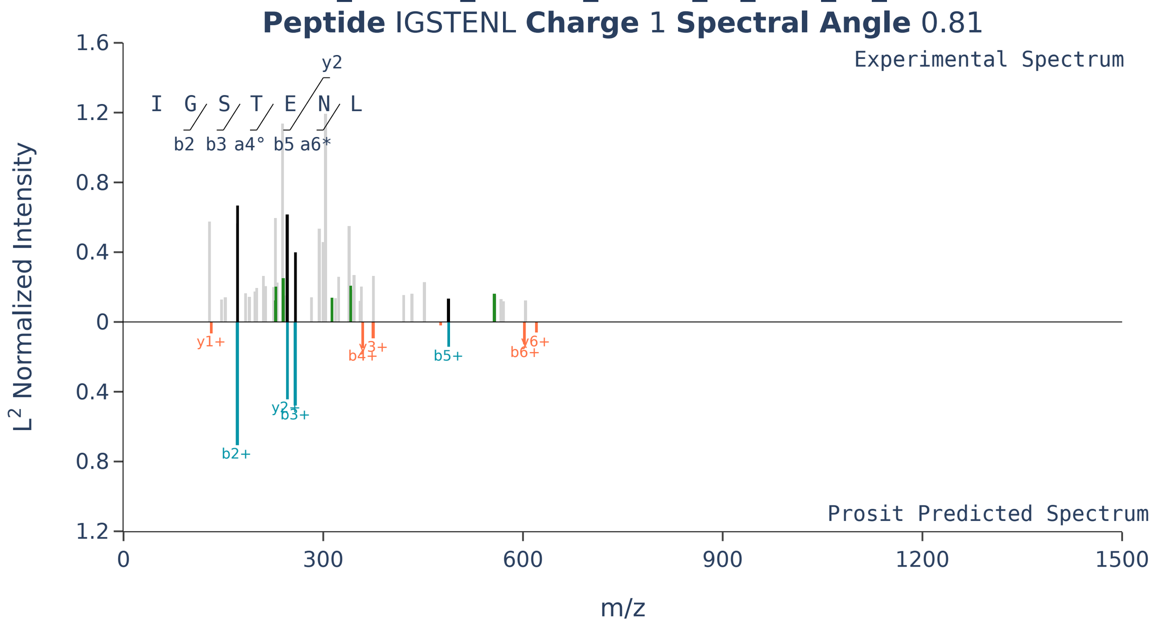


**Source** WSoh_221121_011221_HFGoe_L2_hTau_24h_R2 **Scan** 13363
**Peptide** QIINKKLDLSNVQSKCGSKDNIKHV **Charge** 4 **Spectral Angle** 0.88

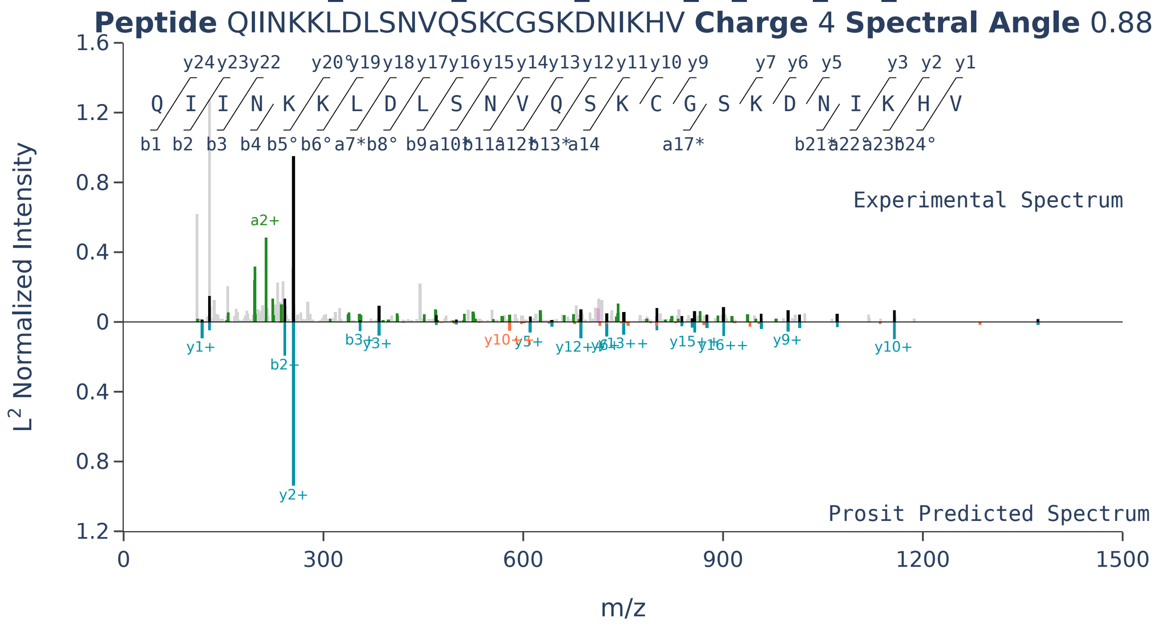


**Source** WSoh_221121_011221_HFGoe_L1_hTau_24h_R1 **Scan** 7756
**Peptide** DGKTKIATPRGAAPPGQKGQANAT **Charge** 3 **Spectral Angle** 0.77

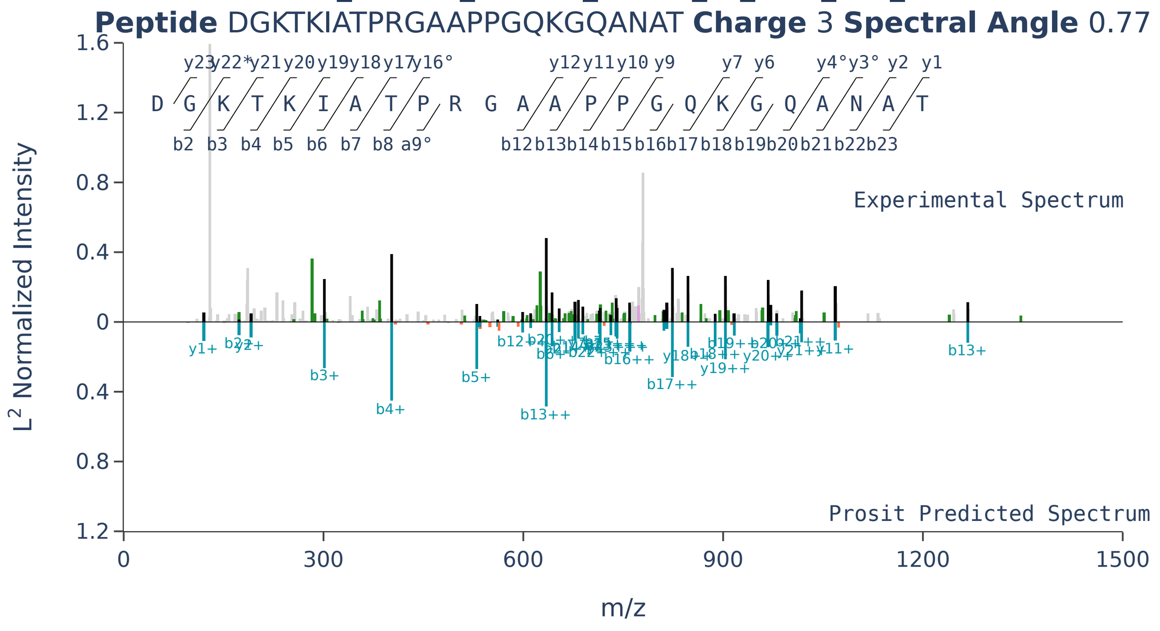

**Source** WSoh_221121_011221_HFGoe_L1_hTau_24h_R2 **Scan** 13936
**Peptide** NAKAKTDHGAEIVYKSPVVSGDTSPRHL **Charge** 4 **Spectral Angle** 0.81

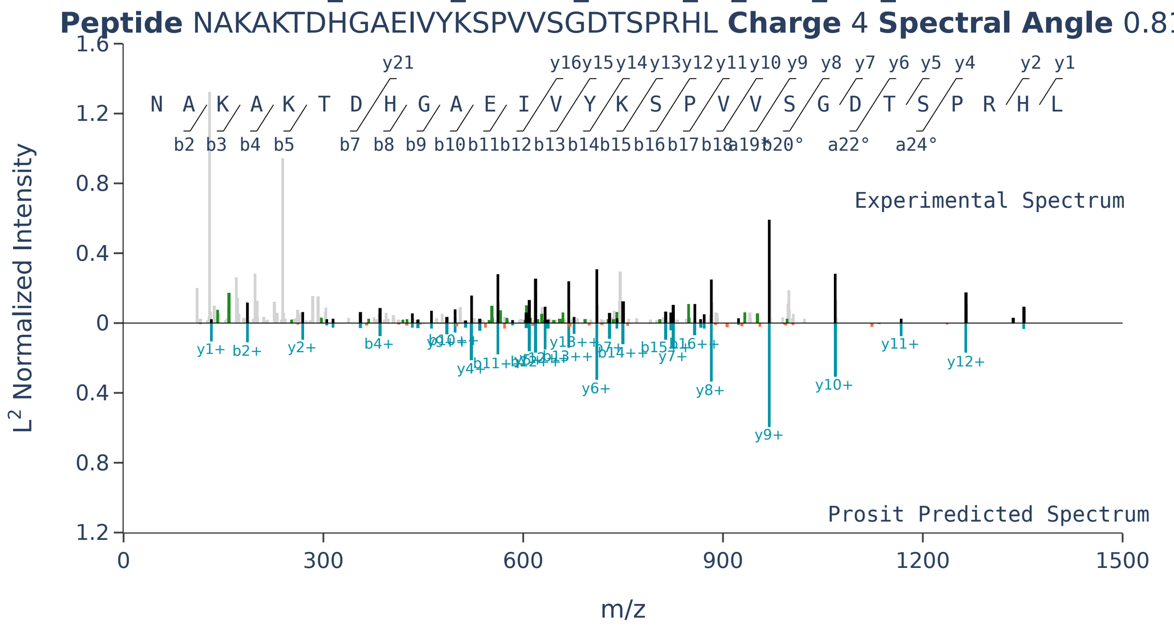


**Source** WSoh_221121_011221_HFGoe_L1_hTau_24h_R2 **Scan** 18183
**Peptide** IGSLDNITHVPGGGNKKIETHKLTFR **Charge** 5 **Spectral Angle** 0.76

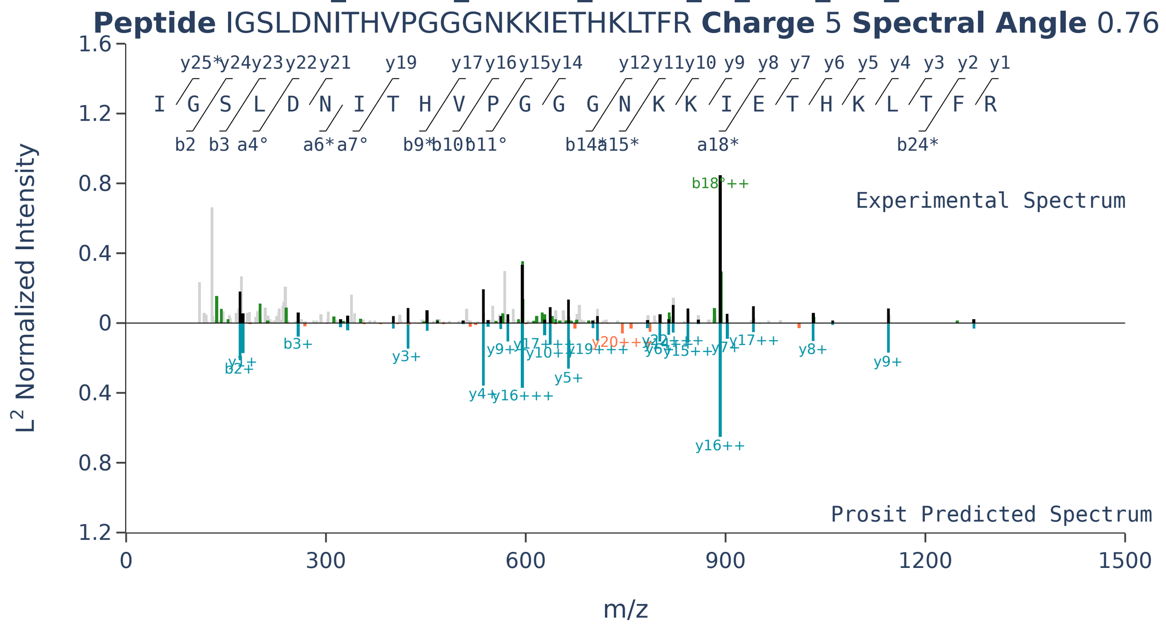


**Source** WSoh_221121_011221_HFGoe_L1_hTau_4h_R2 **Scan** 17122
**Peptide** PVDLSKVTSKCGSLGNIHHKPGGGQVEV **Charge** 3 **Spectral Angle** 0.78

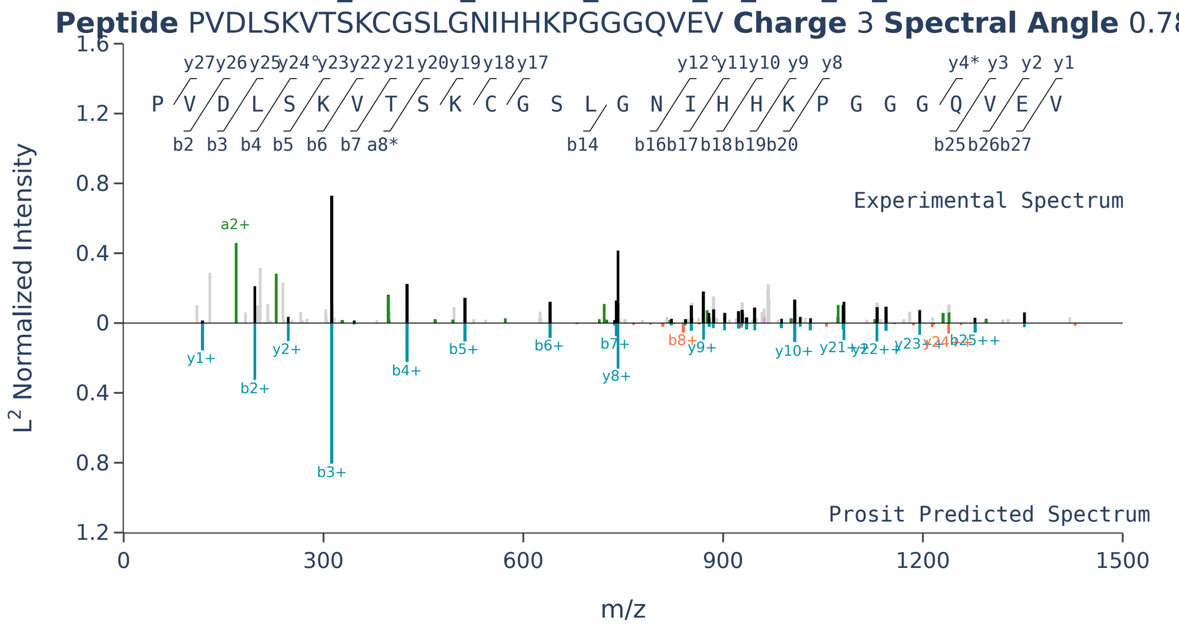


**Source** WSoh_221121_011221_HFGoe_L2_hTau_4h_R1 **Scan** 11597
**Peptide** LKHQPGGGKVQII **Charge** 3 **Spectral Angle** 0.76

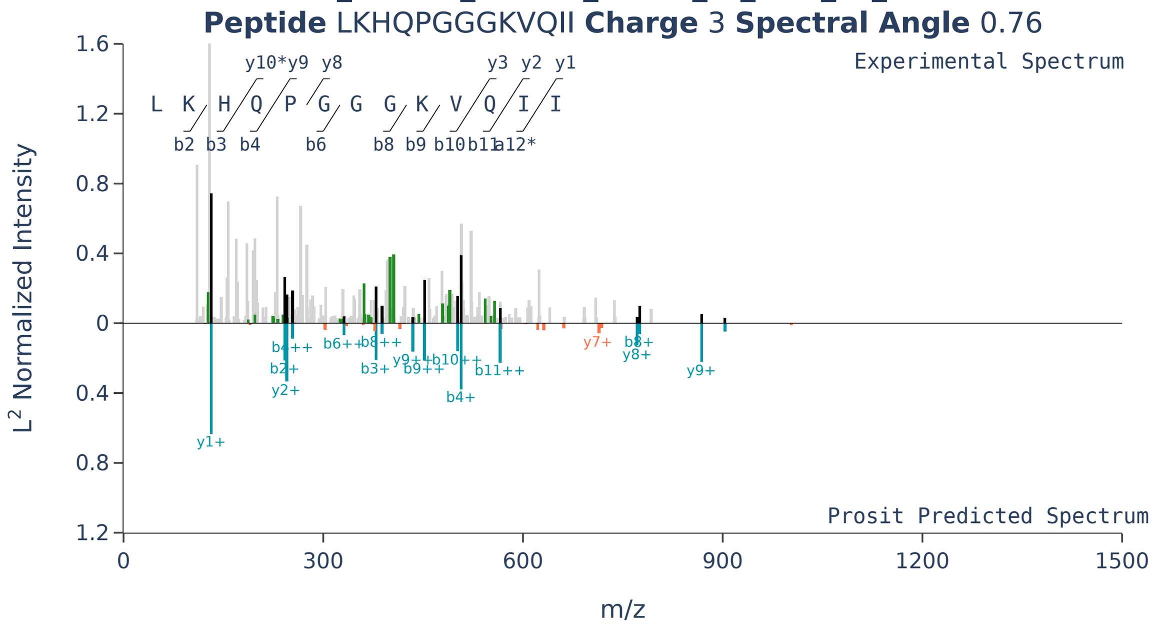


**Source** WSoh_221121_011221_HFGoe_L2_hTau_4h_R2 **Scan** 22263
**Peptide** NLKHVPGGGSVQIVYKPVDL **Charge** 3 **Spectral Angle** 0.82

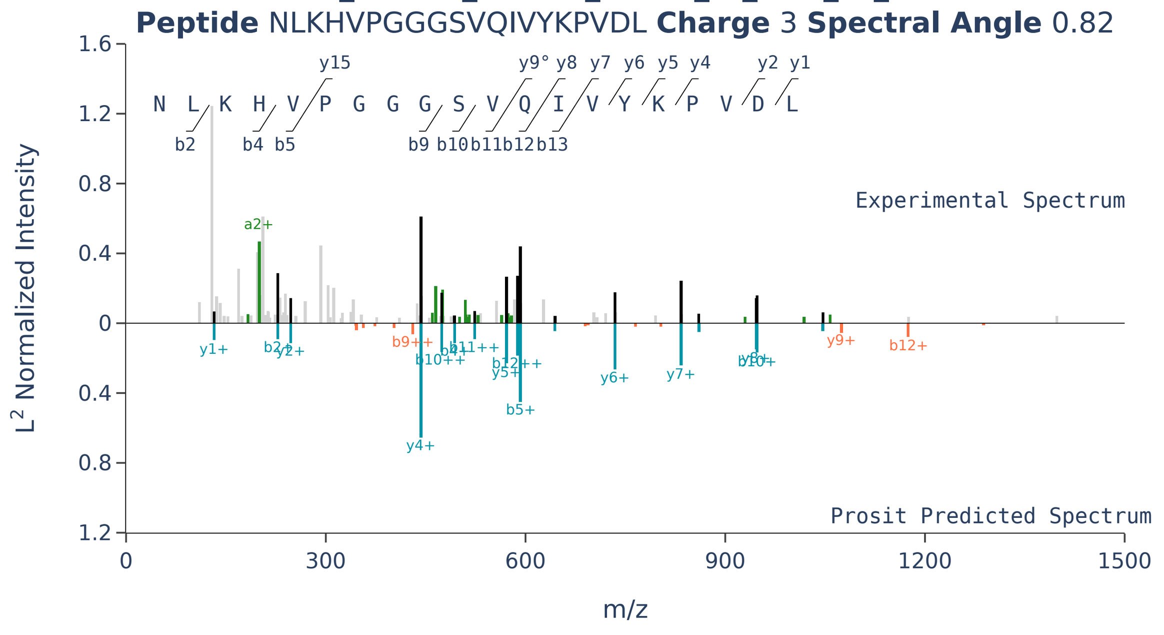


**Source** WSoh_221121_011221_HFGoe_L2_hTau_4h_R1 **Scan** 20483
**Peptide** AGLGDTPSLEDEAAGHVTQARMV **Charge** 3 **Spectral Angle** 0.75

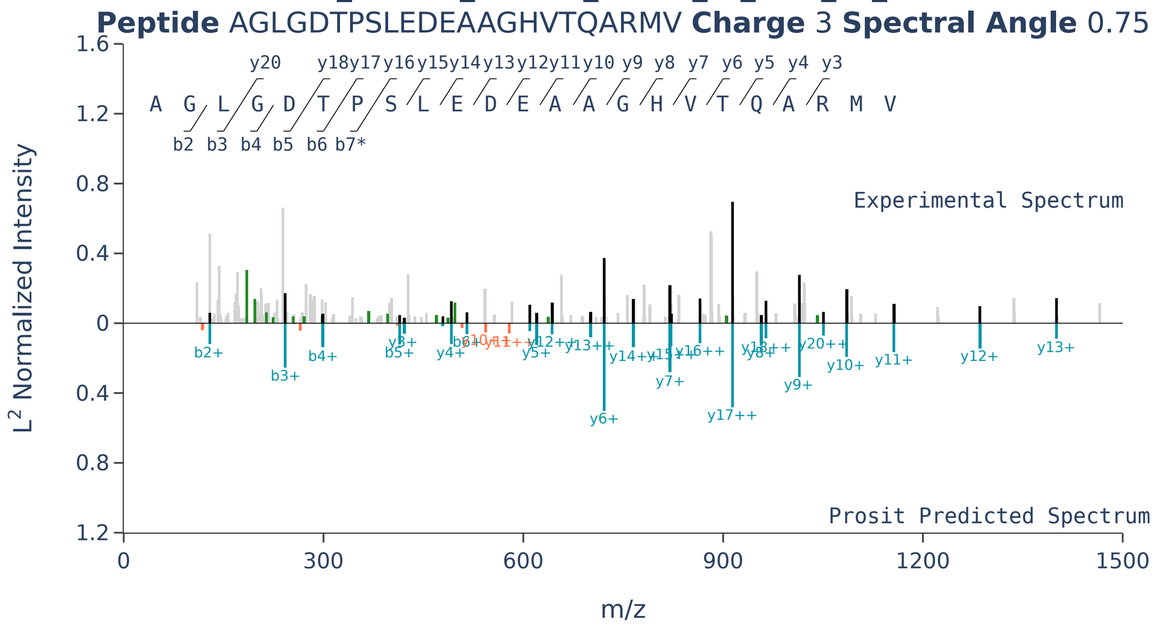


**Source** WSoh_221121_011221_HFGoe_L1_hTau_4h_R1 **Scan** 6211
**Peptide** KLTFRENAKAKTDHGA **Charge** 3 **Spectral Angle** 0.85

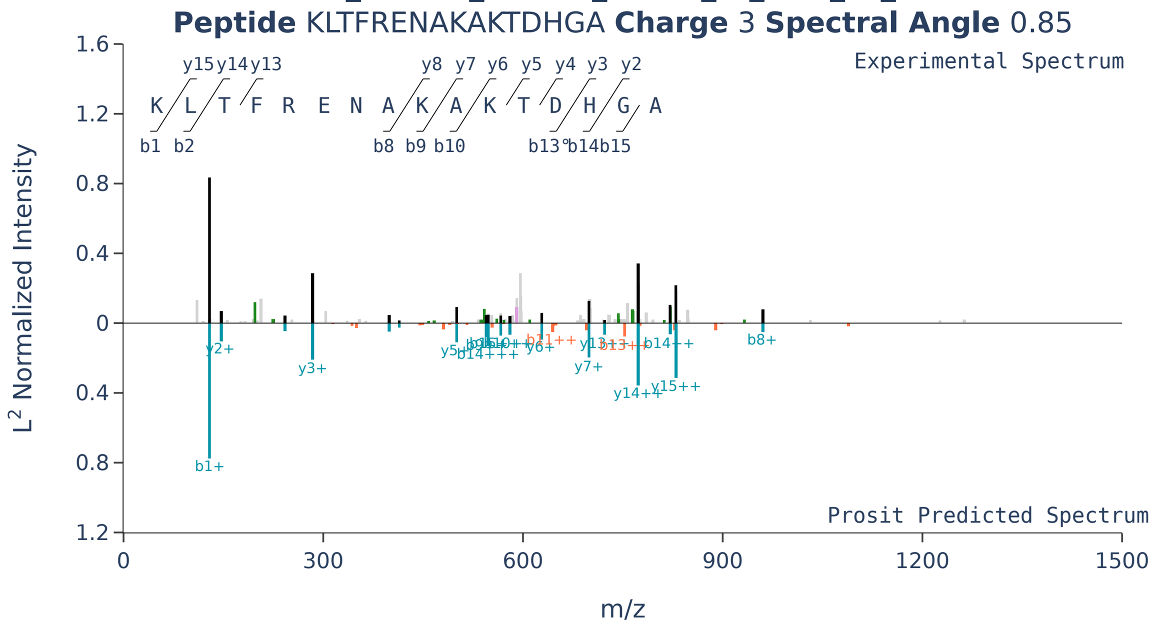


**Source** WSoh_221121_011221_HFGoe_L1_hTau_4h_R1 **Scan** 17482
**Peptide** IGSLDNITHVPGGGNKKIET **Charge** 3 **Spectral Angle** 0.87

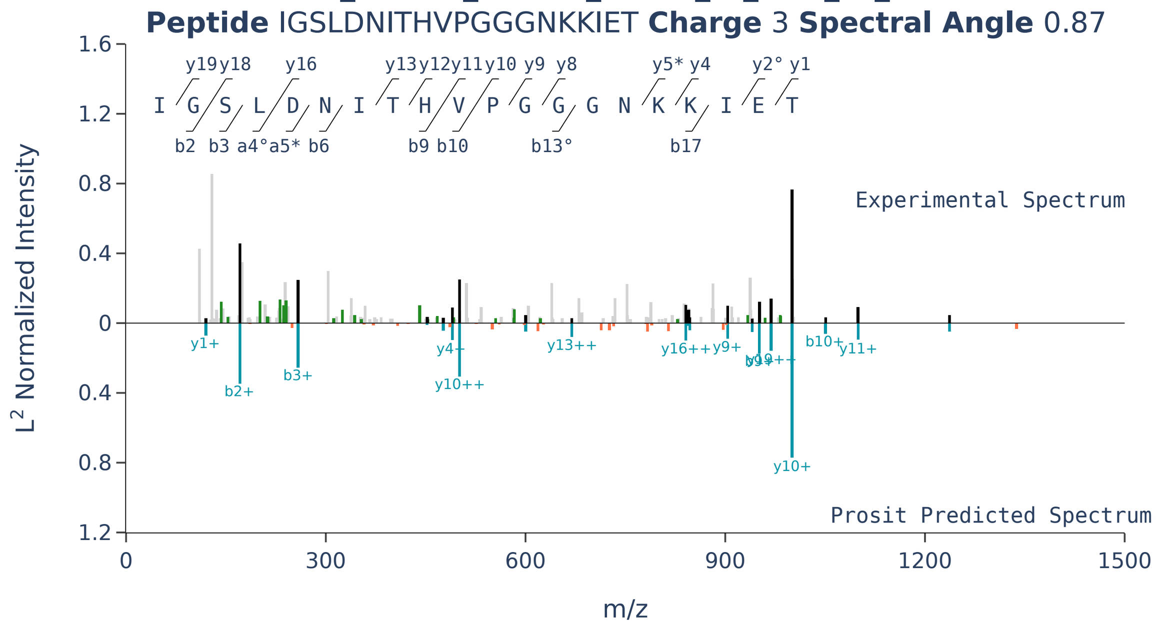


**Source** WSoh_221121_011221_HFGoe_L1_hTau_2h_R1 **Scan** 14133
**Peptide** SLDNITHVPGGGNKKI **Charge** 4 **Spectral Angle** 0.91

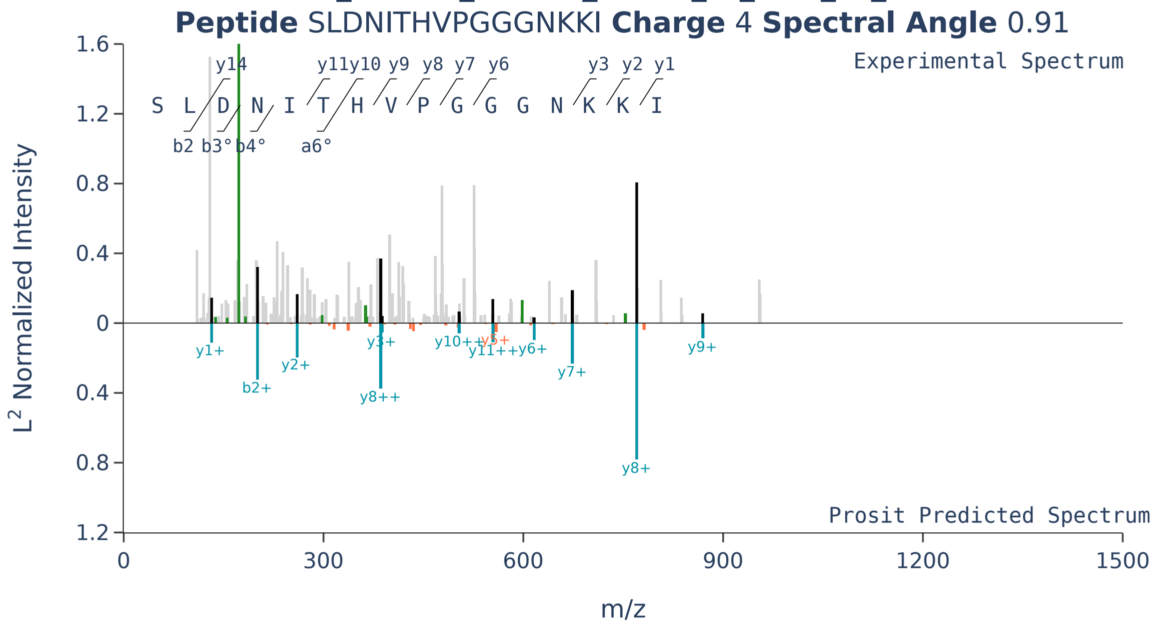


**Source** WSoh_221121_011221_HFGoe_L2_hTau_4h_R1 **Scan** 18506
**Peptide** QEGDTDAGLKESPLQTPTED **Charge** 3 **Spectral Angle** 0.9

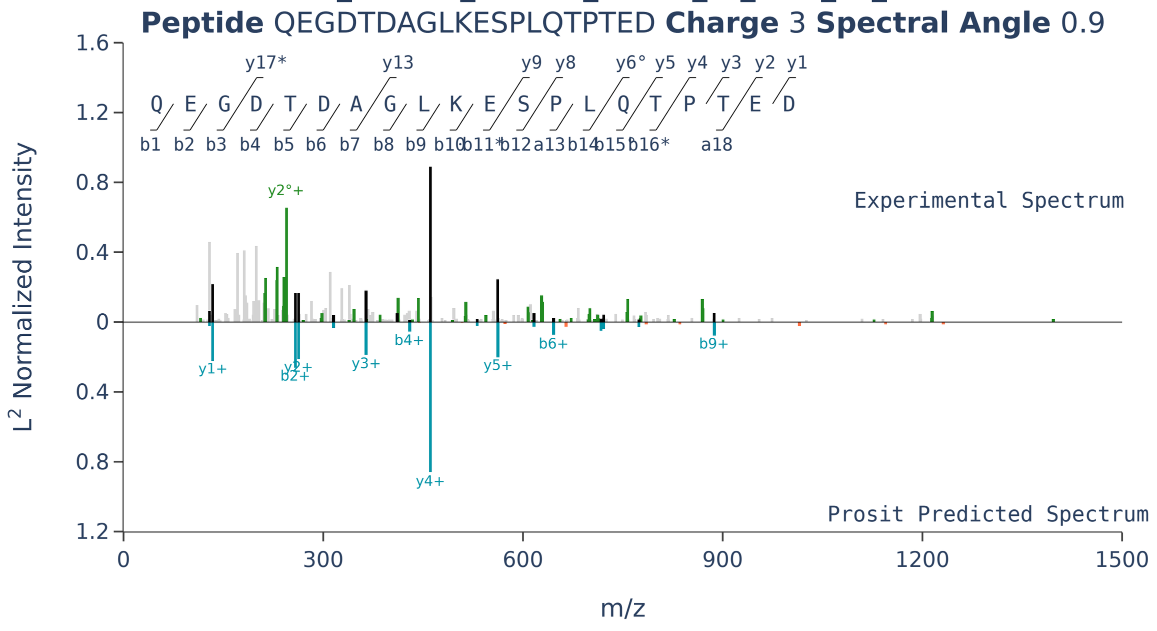


**Source** WSoh_221121_011221_HFGoe_L1_hTau_24h_R1 **Scan** 23356
**Peptide** GSEEPGSETSDAKSTPTAEDVTAPLV **Charge** 3 **Spectral Angle** 0.94

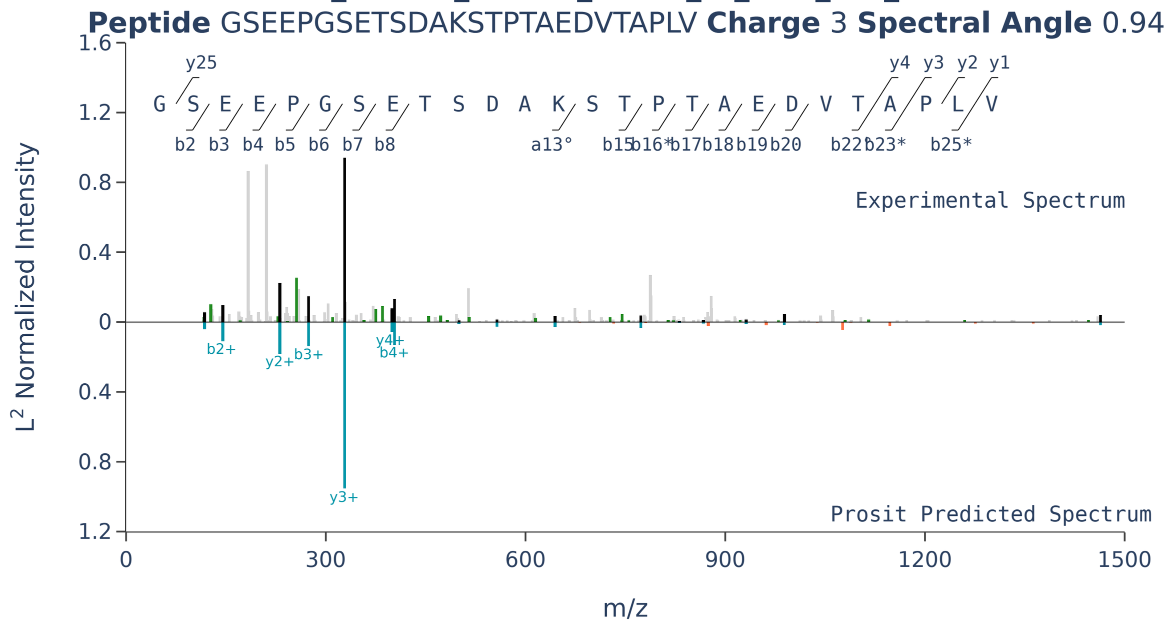


**Source** WSoh_221121_011221_HFGoe_L2_hTau_24h_R1 **Scan** 17911
**Peptide** GIGDTPSLEDEAAGHVTQARMVSKSK **Charge** 4 **Spectral Angle** 0.77

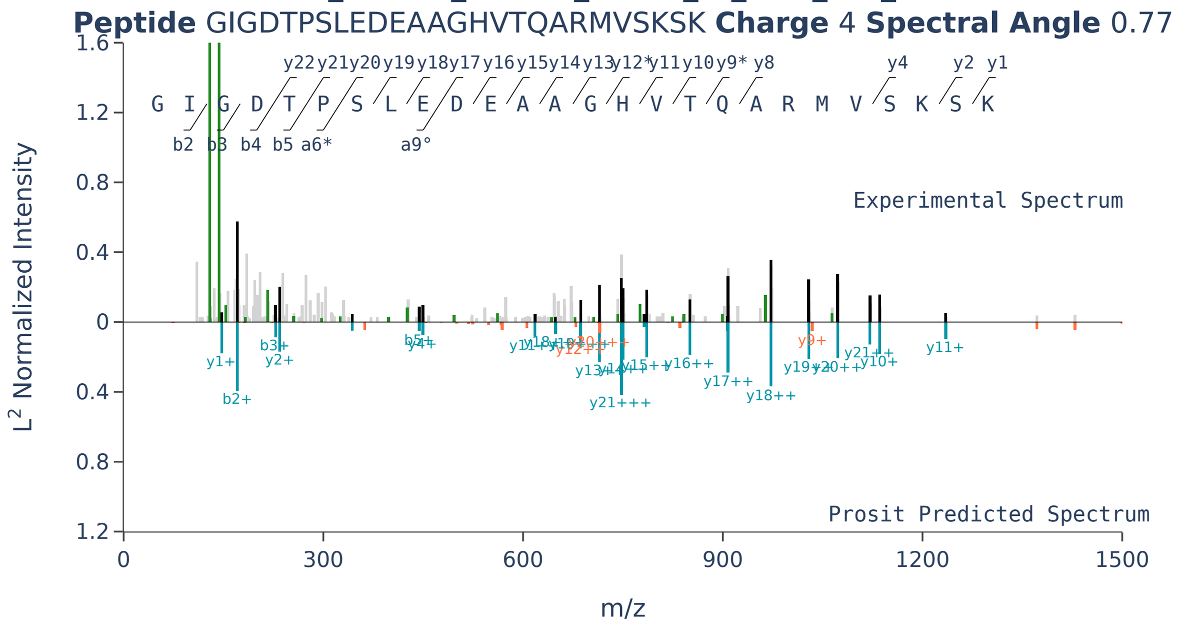


**Source** WSoh_221121_011221_HFGoe_L2_hTau_2h_R1 **Scan** 9301
**Peptide** LKHQPGGGKVQIINKKL **Charge** 4 **Spectral Angle** 0.81

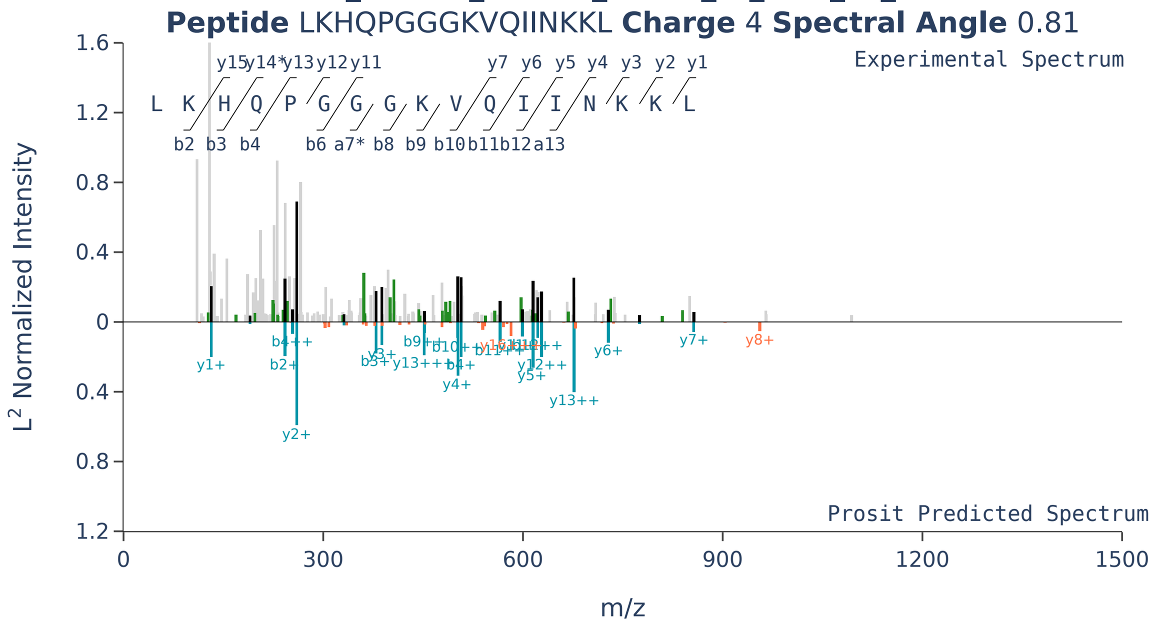


**Source** WSoh_221121_011221_HFGoe_L1_hTau_24h_R2 **Scan** 15786
**Peptide** VQSKIGSLDNITHVPGGGNKKIETH **Charge** 5 **Spectral Angle** 0.86

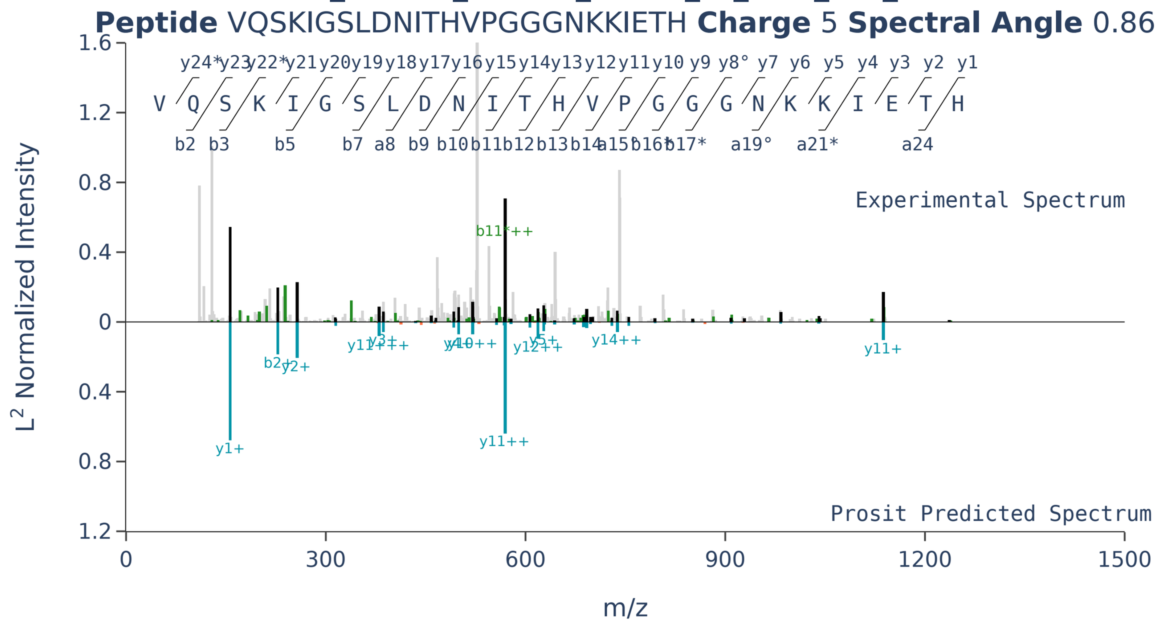


**Source** WSoh_221121_011221_HFGoe_L2_hTau_2h_R2 **Scan** 14660
**Peptide** GQVEVKSEKLDFKDRV **Charge** 4 **Spectral Angle** 0.73

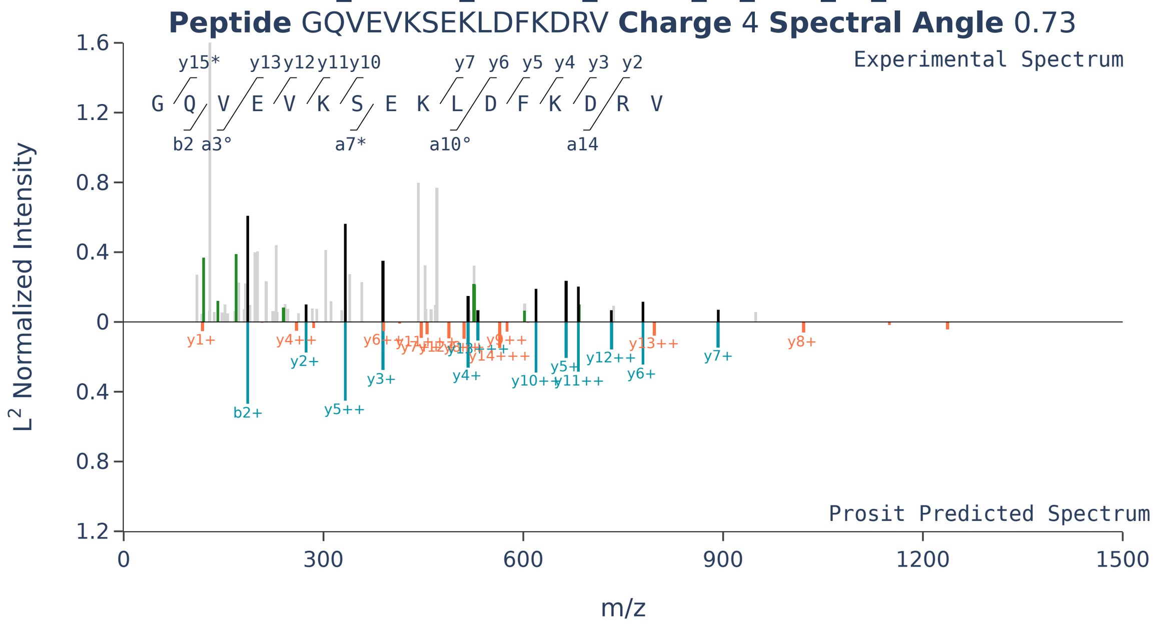

**Source** WSoh_221121_011221_HFGoe_L2_hTau_1h_R1 **Scan** 12468
**Peptide** PSLPTPPTREPKKVA **Charge** 4 **Spectral Angle** 0.86

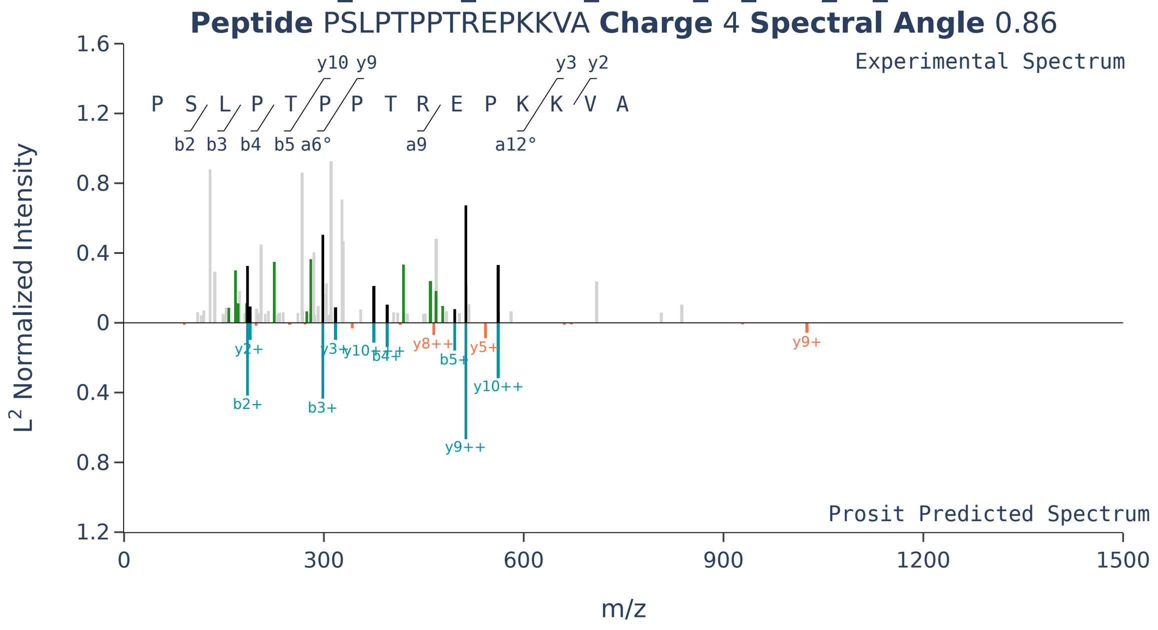


**Source** WSoh_221121_011221_HFGoe_L2_hTau_24h_R1 **Scan** 11056
**Peptide** STENLKHQPGGGKVQIINKKL **Charge** 4 **Spectral Angle** 0.83

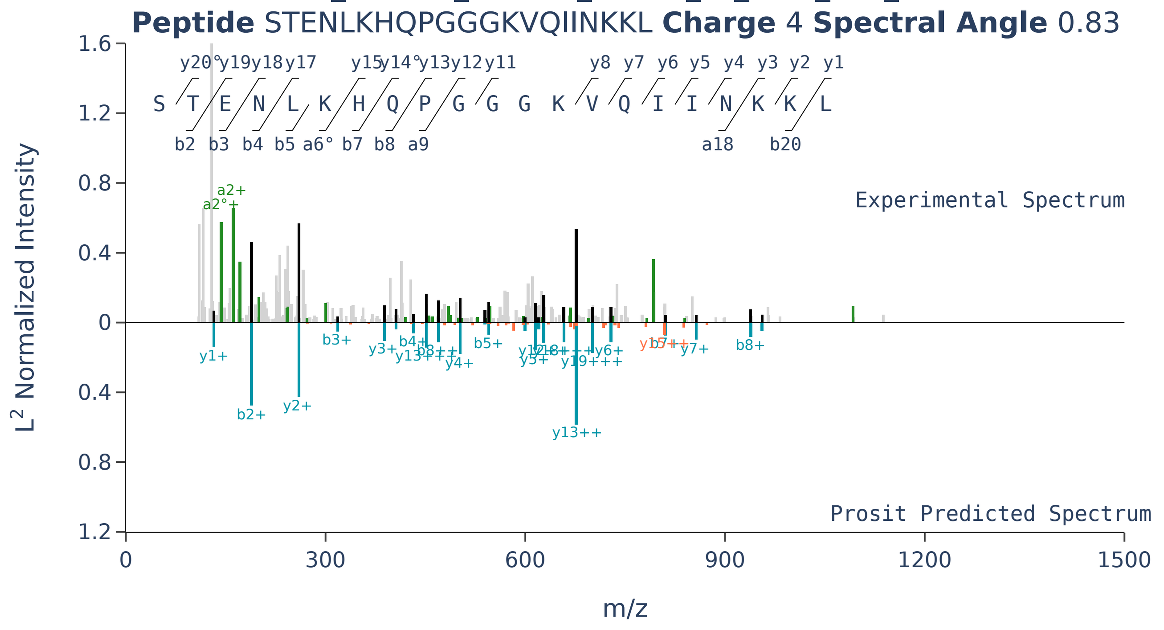


**Source** WSoh_221121_011221_HFGoe_L1_hTau_4h_R1 **Scan** 7568
**Peptide** ITHVPGGGNKKIET **Charge** 2 **Spectral Angle** 0.91

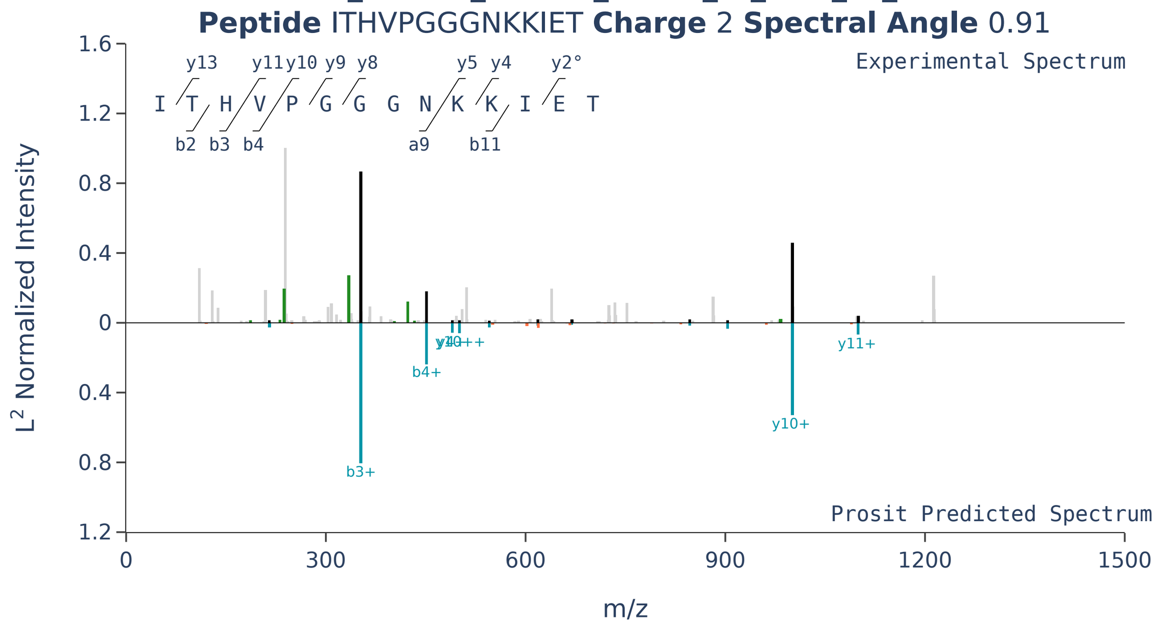


**Source** WSoh_221121_011221_HFGoe_L1_hTau_24h_R1 **Scan** 7662
**Peptide** THVPGGGNKKIETHKLTFRENAKAKTD **Charge** 4 **Spectral Angle** 0.85

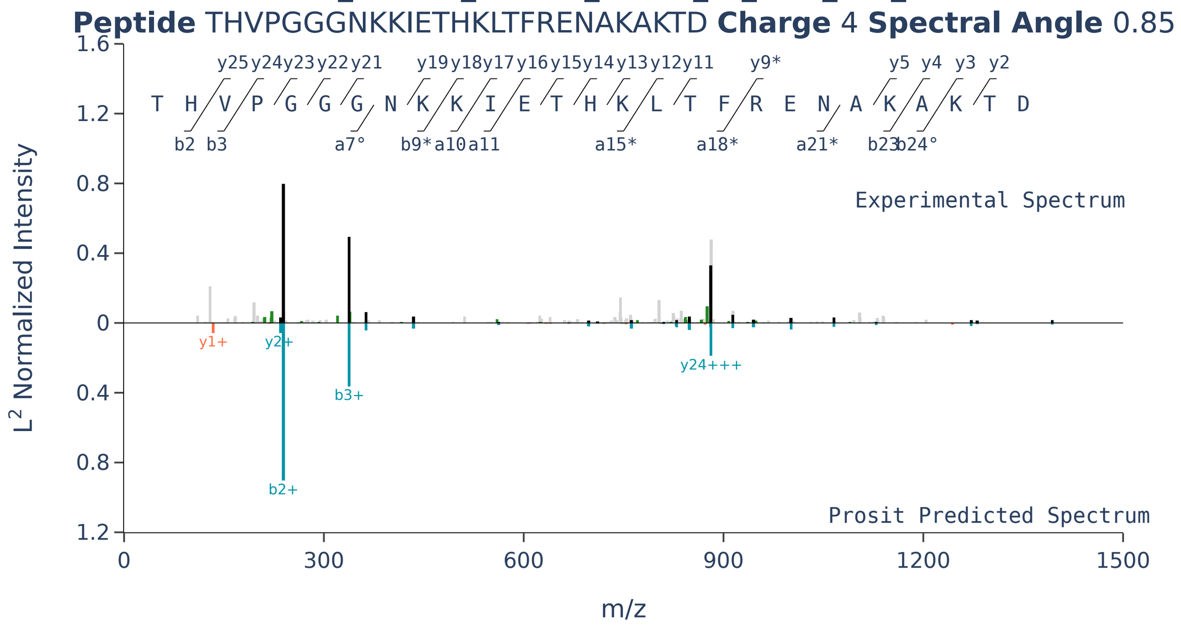


**Source** WSoh_221121_011221_HFGoe_L2_hTau_4h_R1 **Scan** 19050
**Peptide** VYKPVDLSKVTSKCGSLGNIH **Charge** 4 **Spectral Angle** 0.89

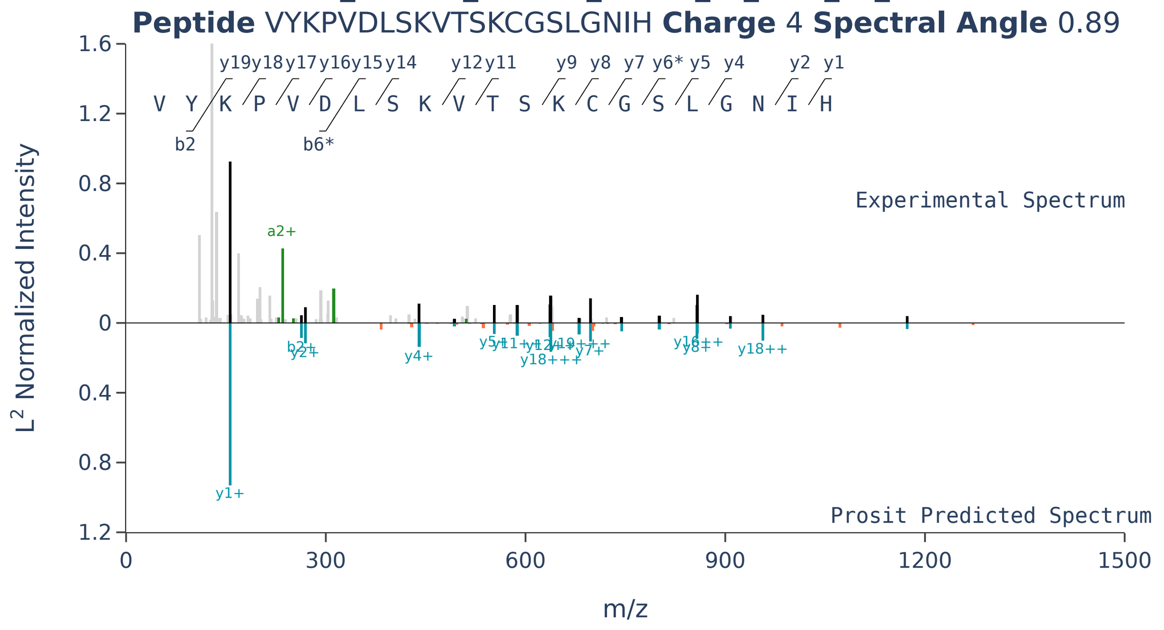


**Source** WSoh_221121_011221_HFGoe_L1_hTau_4h_R2 **Scan** 11552
**Peptide** KIGSTENLKHQPGGGKVQIINKKL **Charge** 4 **Spectral Angle** 0.93

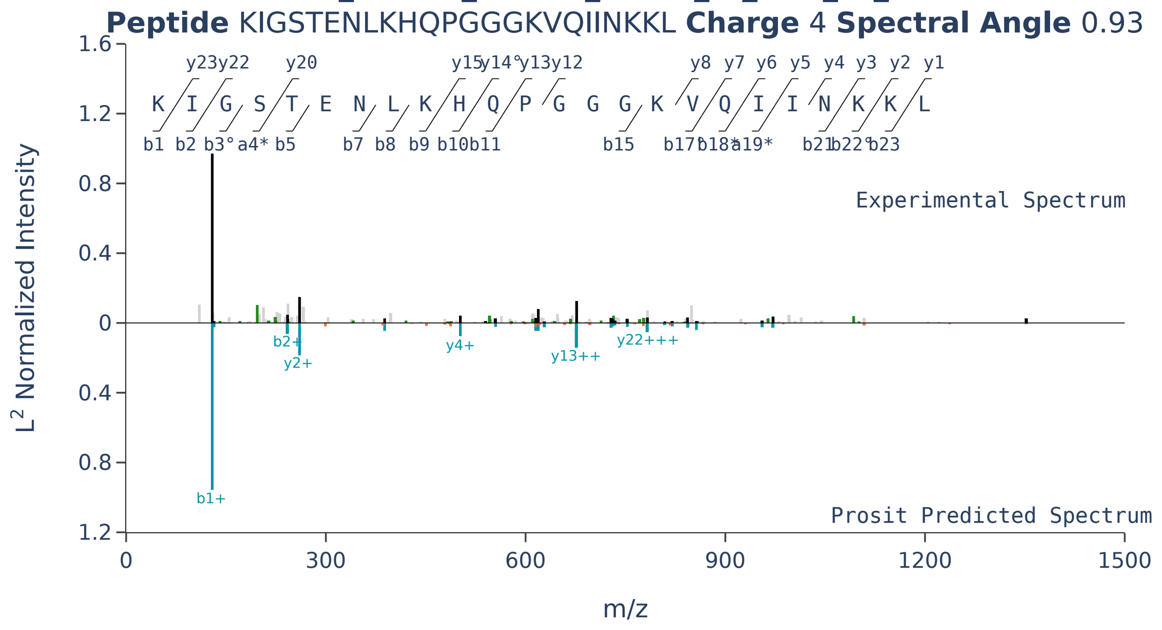


**Source** WSoh_221121_011221_HFGoe_L1_hTau_24h_R2 **Scan** 9648
**Peptide** ADGKTKIATPRGAAPPGQKGQANATRL **Charge** 4 **Spectral Angle** 0.82

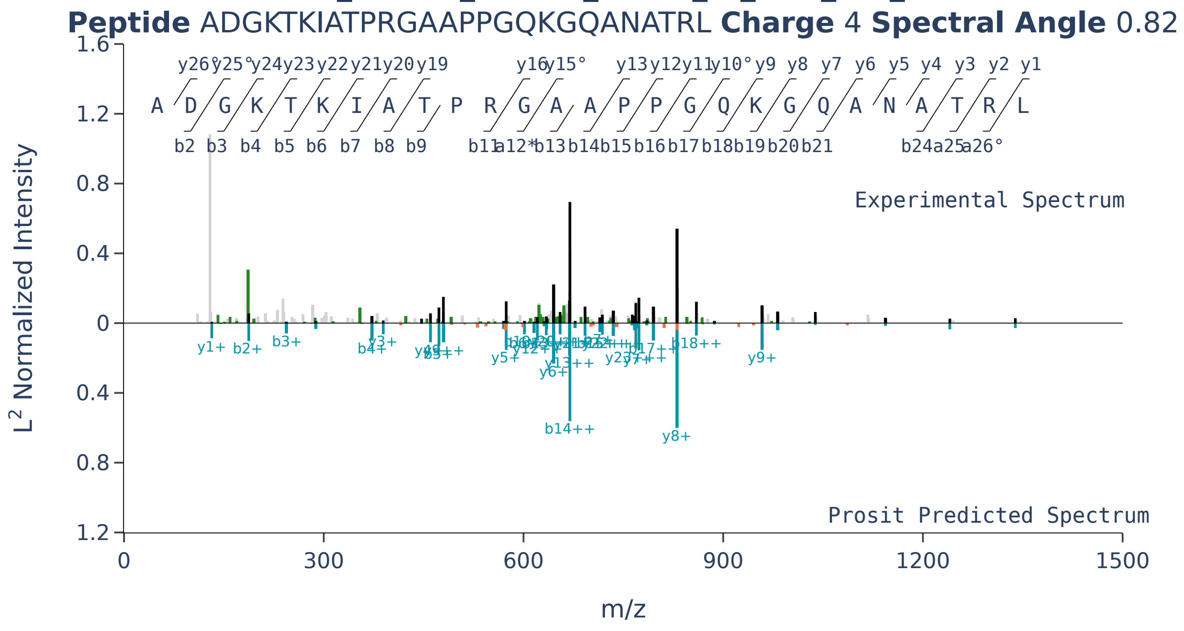


**Source** WSoh_221121_011221_HFGoe_L2_hTau_24h_R1 **Scan** 7878
**Peptide** ATPRGAAPPGQKGQANATRIPAKT **Charge** 3 **Spectral Angle** 0.76

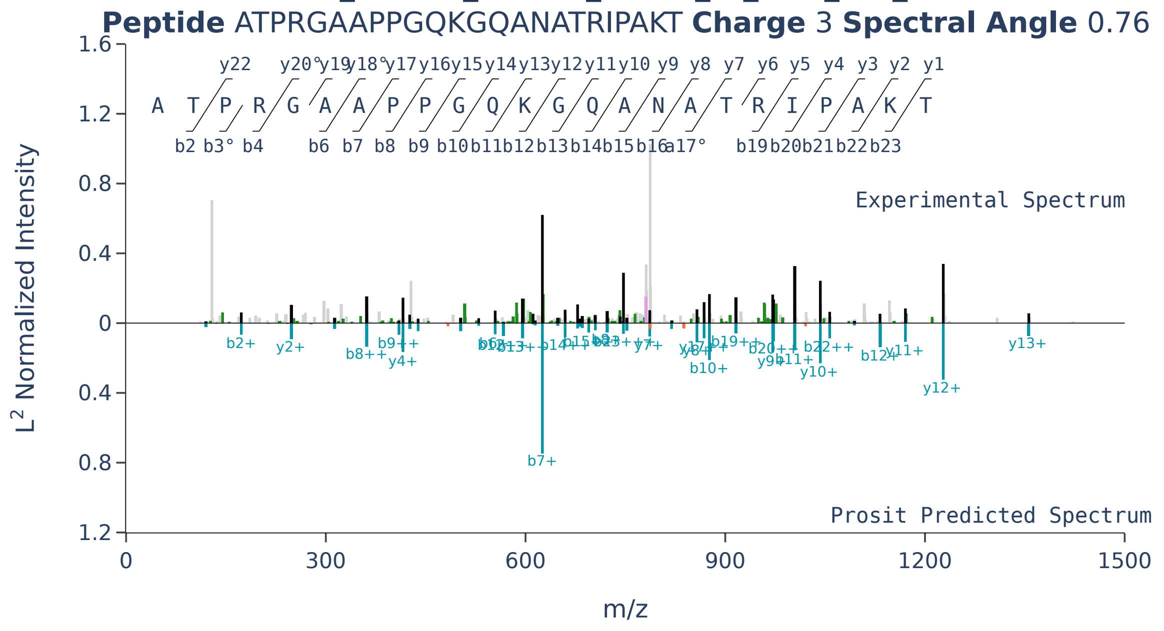


**Source** WSoh_221121_011221_HFGoe_L2_hTau_24h_R1 **Scan** 11341
**Peptide** THVPGGGNKKIETHKLTF **Charge** 3 **Spectral Angle** 0.93

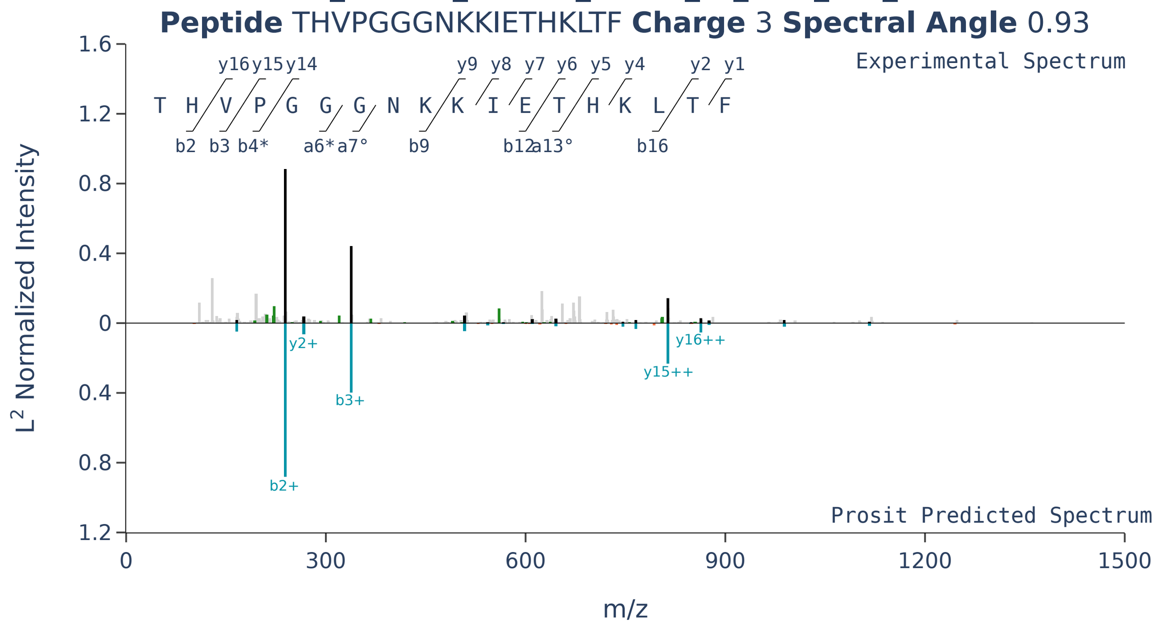


**Source** WSoh_221121_011221_HFGoe_L2_hTau_24h_R2 **Scan** 22090
**Peptide** DVTAPLVDEGAPGKQAAAQPHTEL **Charge** 3 **Spectral Angle** 0.86

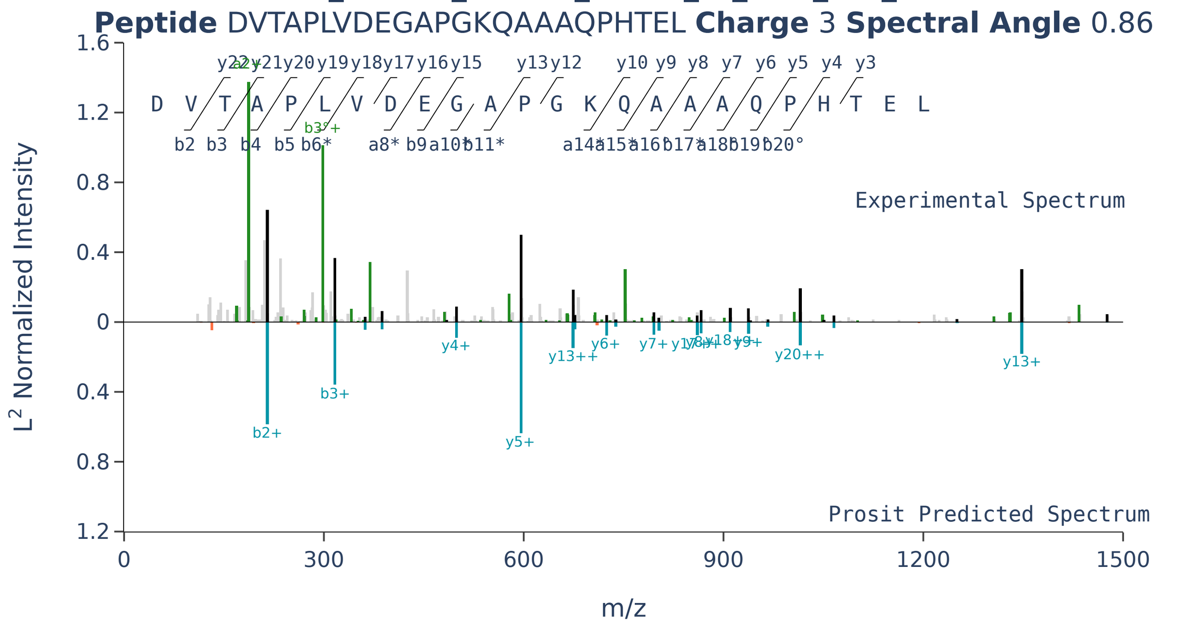


**Source** WSoh_221121_011221_HFGoe_L2_hTau_2h_R1 **Scan** 10454
**Peptide** NATRIPAKTPPAPKTPPSSGEPPKSGD **Charge** 4 **Spectral Angle** 0.84

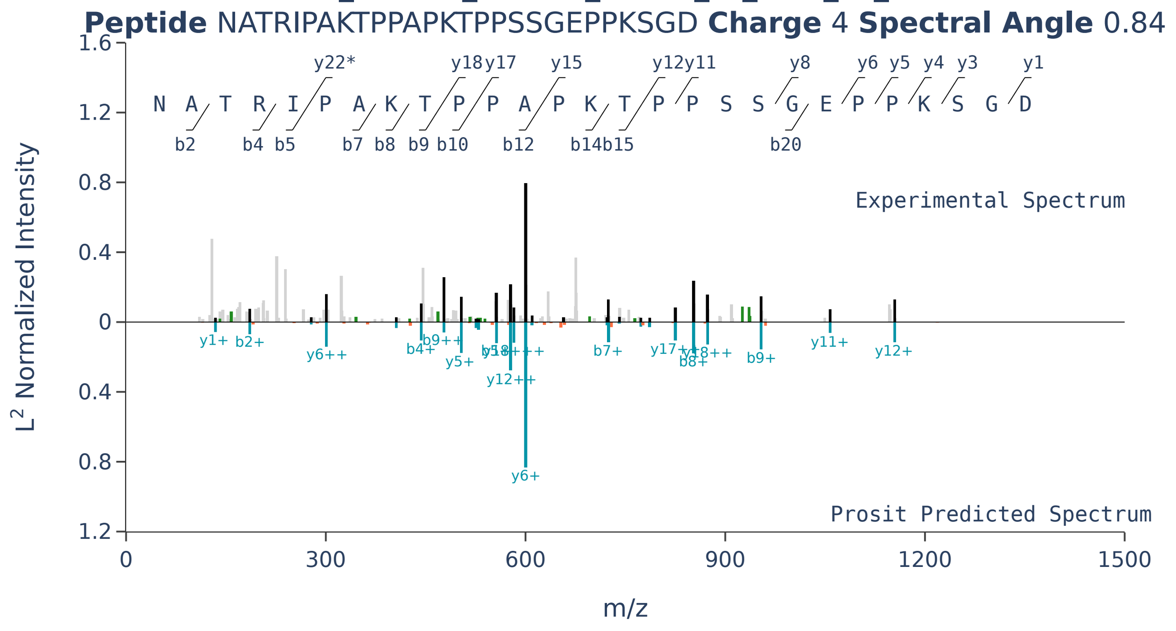


**Source** WSoh_221121_011221_HFGoe_L2_hTau_24h_R1 **Scan** 5584
**Peptide** AGHVTQARMVSKSKD **Charge** 2 **Spectral Angle** 0.79

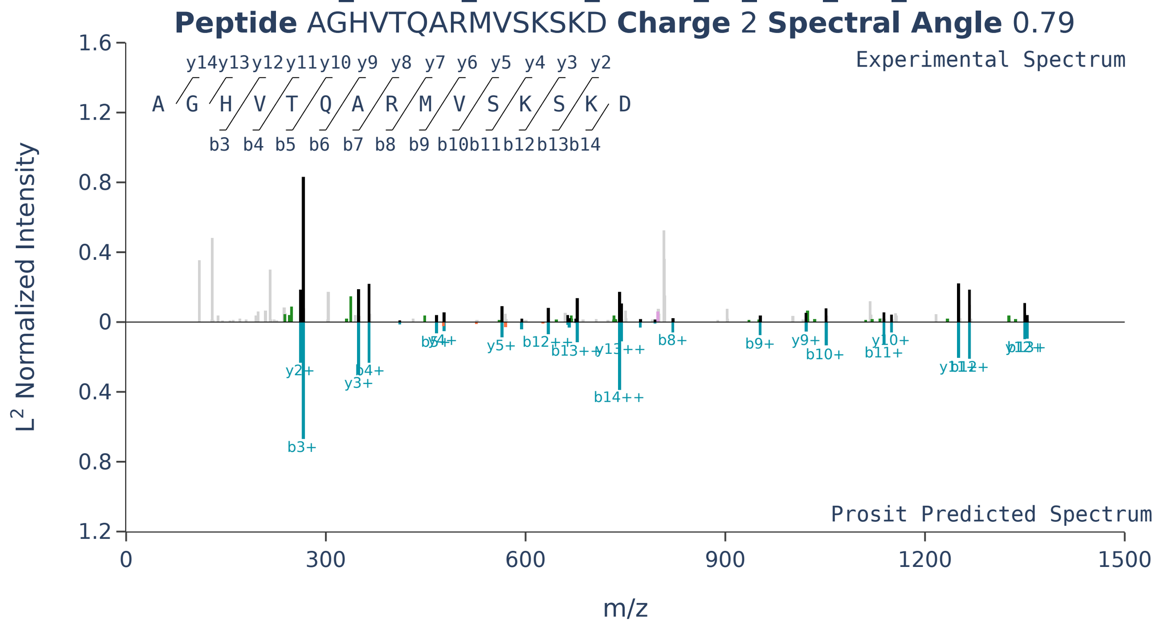


**Source** WSoh_221121_011221_HFGoe_L1_hTau_24h_R2 **Scan** 9211
**Peptide** TRIPAKTPPAPKTPPSSGEPPKSGDR **Charge** 3 **Spectral Angle** 0.75

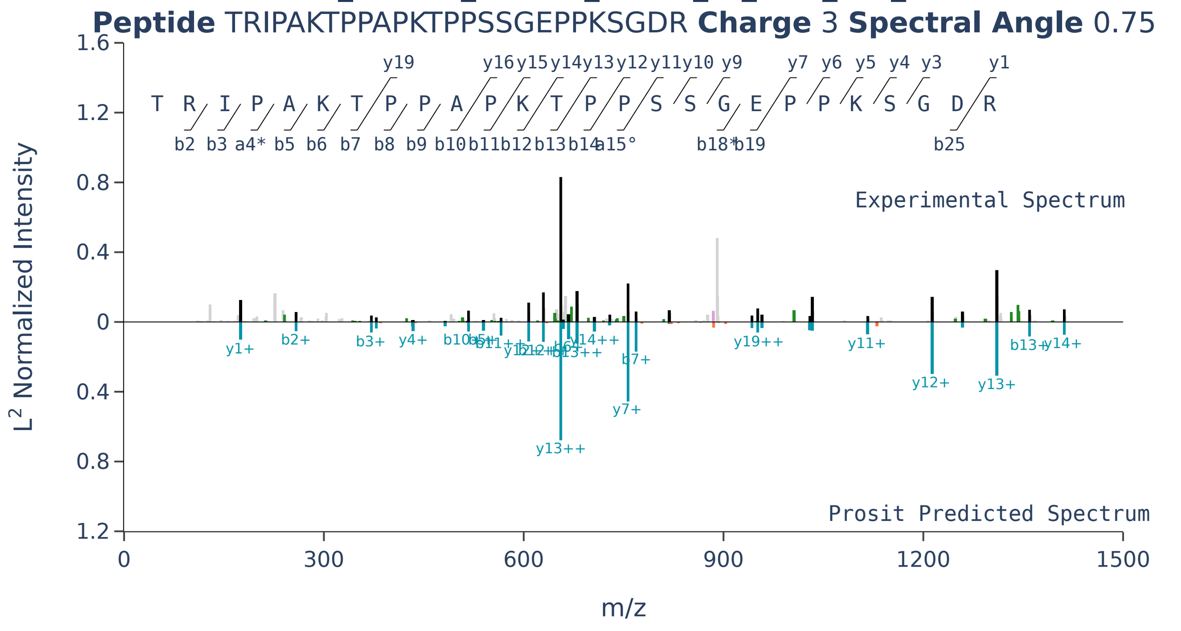


**Source** WSoh_221121_011221_HFGoe_L2_hTau_24h_R1 **Scan** 18700
**Peptide** KSRLQTAPVPMPDLKN **Charge** 4 **Spectral Angle** 0.74

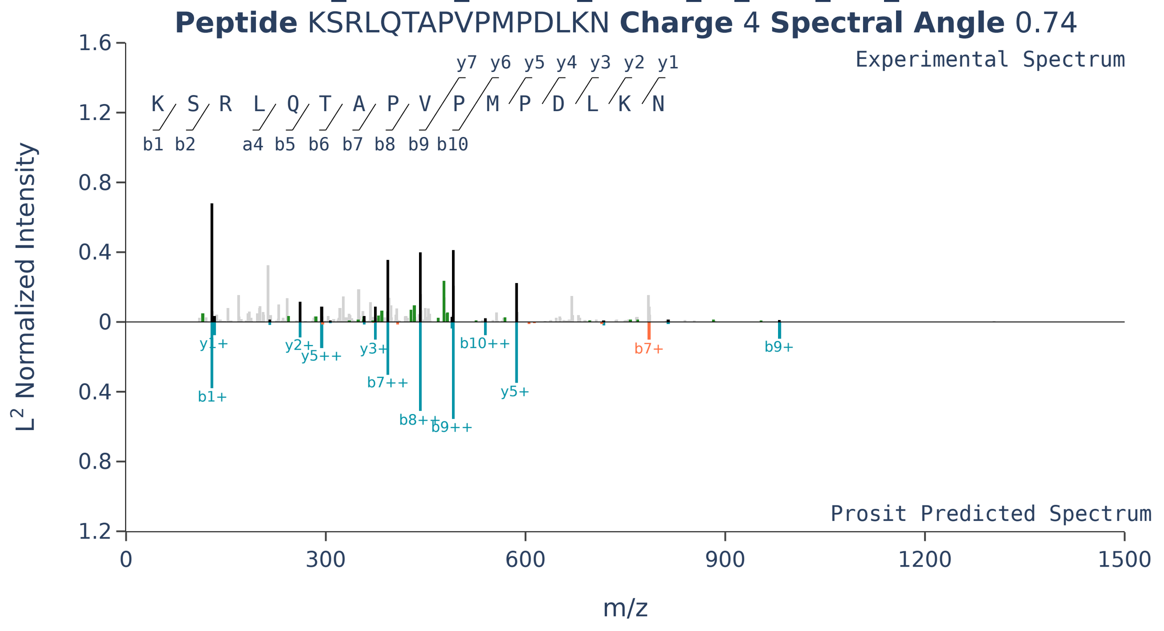


**Source** WSoh_221121_011221_HFGoe_L1_hTau_1h_R1 **Scan** 16892
**Peptide** EGAPGKQAAAQPHTEIPEGTTAEEAGI **Charge** 3 **Spectral Angle** 0.75

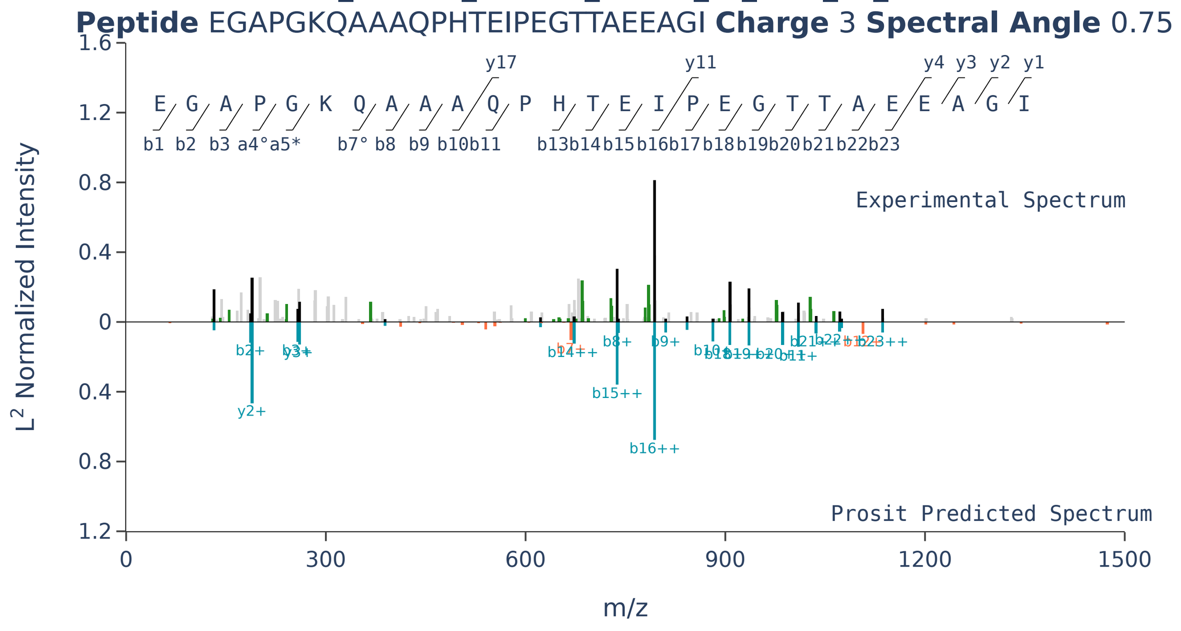

**Source** WSoh_221121_011221_HFGoe_L1_hTau_24h_R1 **Scan** 21978
**Peptide** PGSETSDAKSTPTAEDVTAPLVD **Charge** 2 **Spectral Angle** 0.77

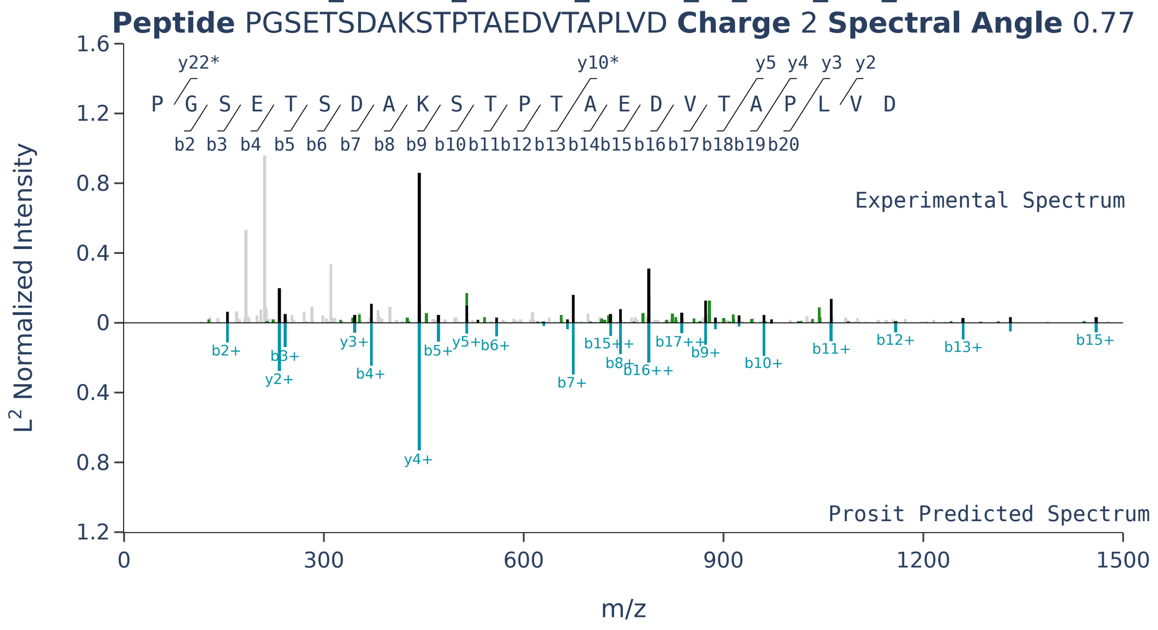


**Source** WSoh_221121_011221_HFGoe_L2_hTau_4h_R2 **Scan** 5609
**Peptide** TPRGAAPPGQKGQAN **Charge** 3 **Spectral Angle** 0.88

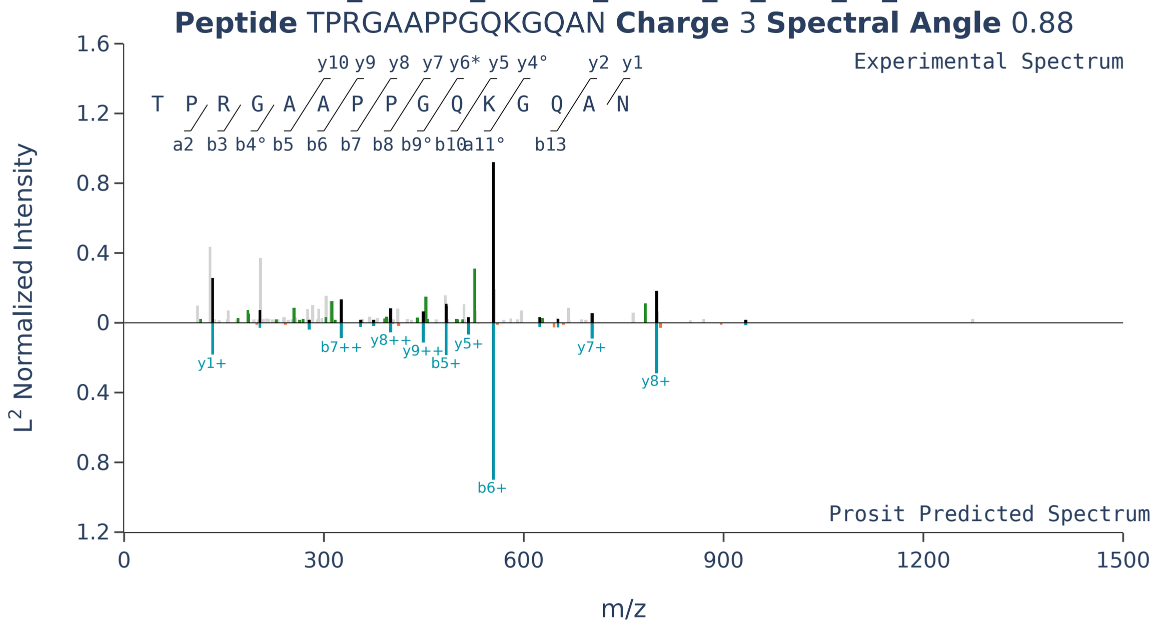


**Source** WSoh_221121_011221_HFGoe_L1_hTau_24h_R2 **Scan** 20982
**Peptide** TTAEEAGIGDTPSLEDEAAGHVTQARMVSK **Charge** 4 **Spectral Angle** 0.76

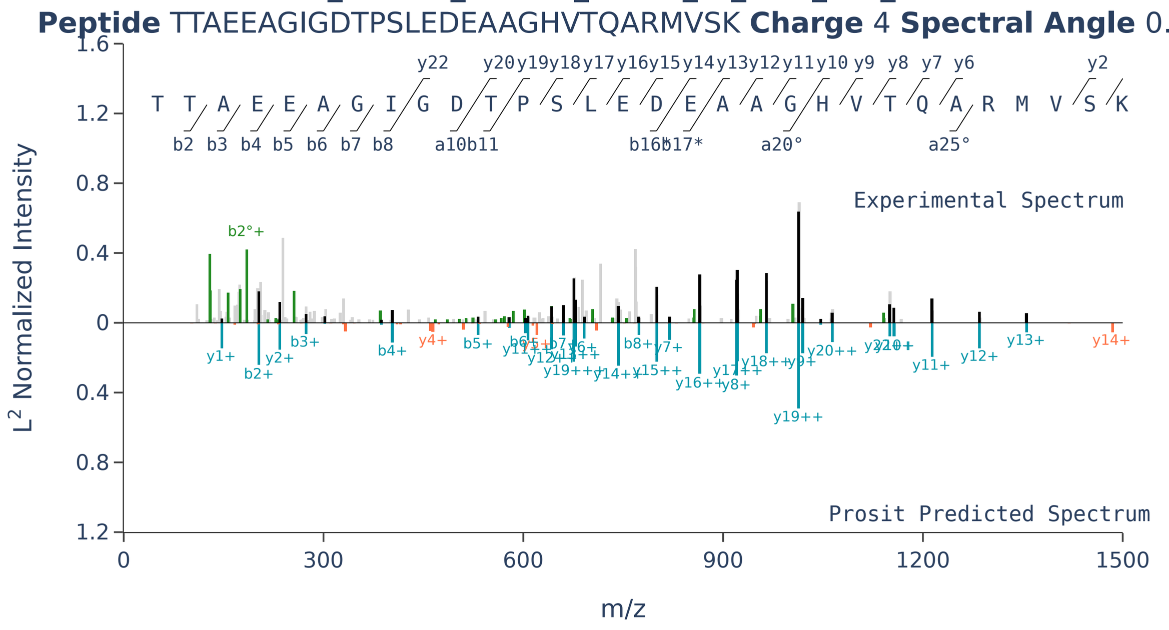


**Source** WSoh_221121_011221_HFGoe_L2_hTau_2h_R1 **Scan** 8042
**Peptide** AKSTPTAEDVT **Charge** 2 **Spectral Angle** 0.88

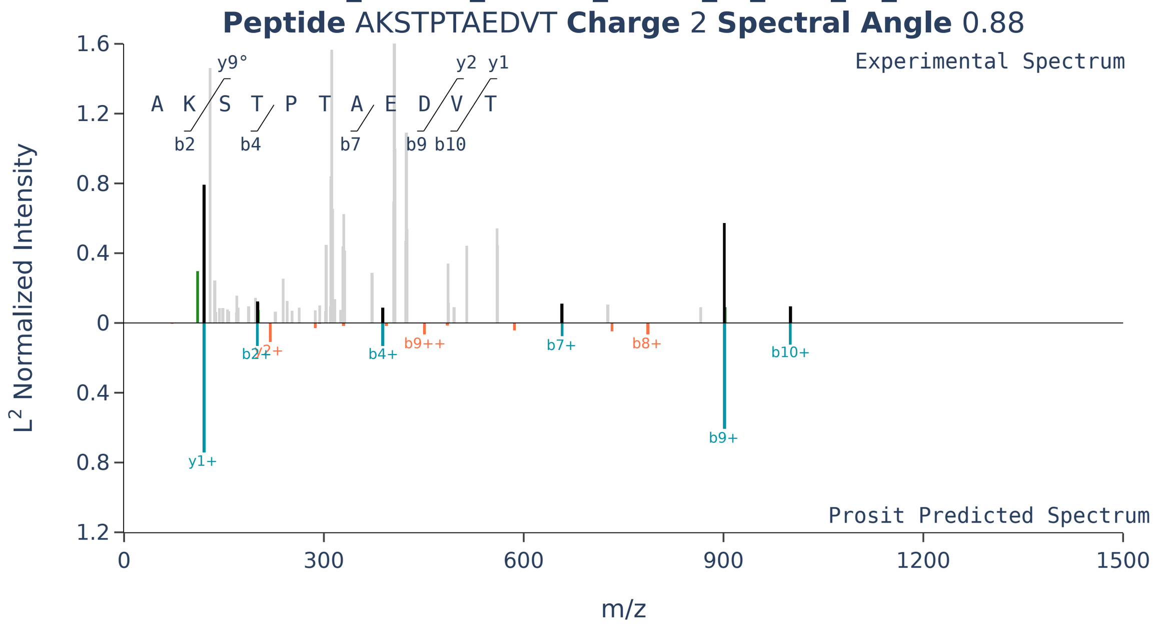


**Source** WSoh_221121_011221_HFGoe_L1_hTau_4h_R1 **Scan** 16590
**Peptide** LGSTENLKHQPGGGKVQIINKKLDL **Charge** 4 **Spectral Angle** 0.79

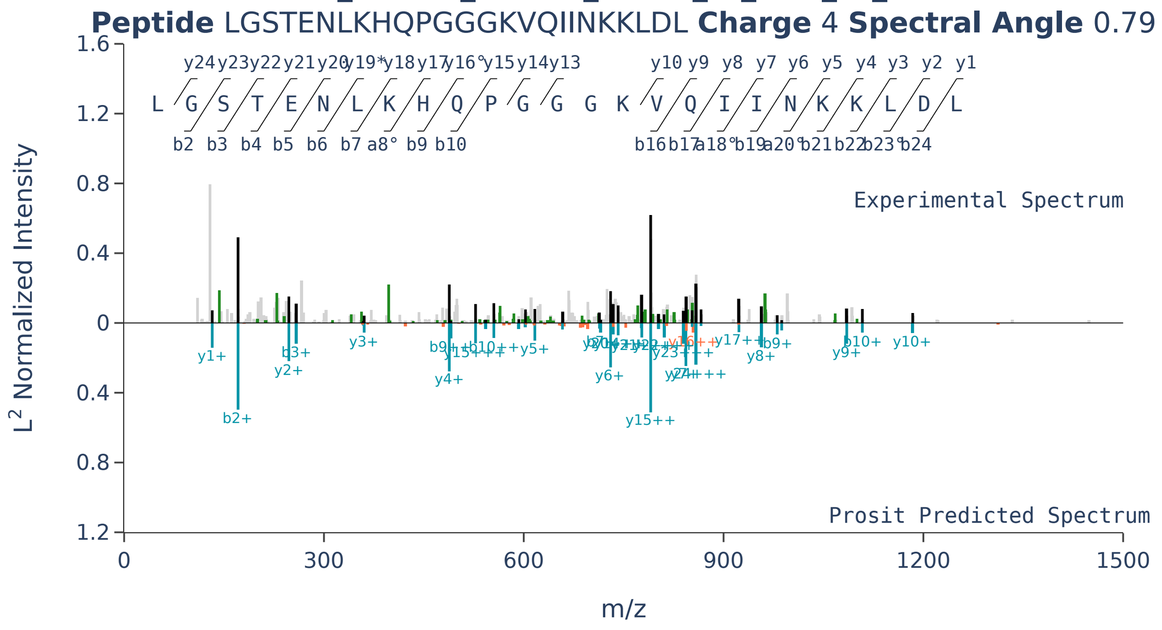


**Source** WSoh_221121_011221_HFGoe_L1_hTau_24h_R2 **Scan** 9843
**Peptide** RIPAKTPPAPKTPPSSGEPPKS **Charge** 3 **Spectral Angle** 0.82

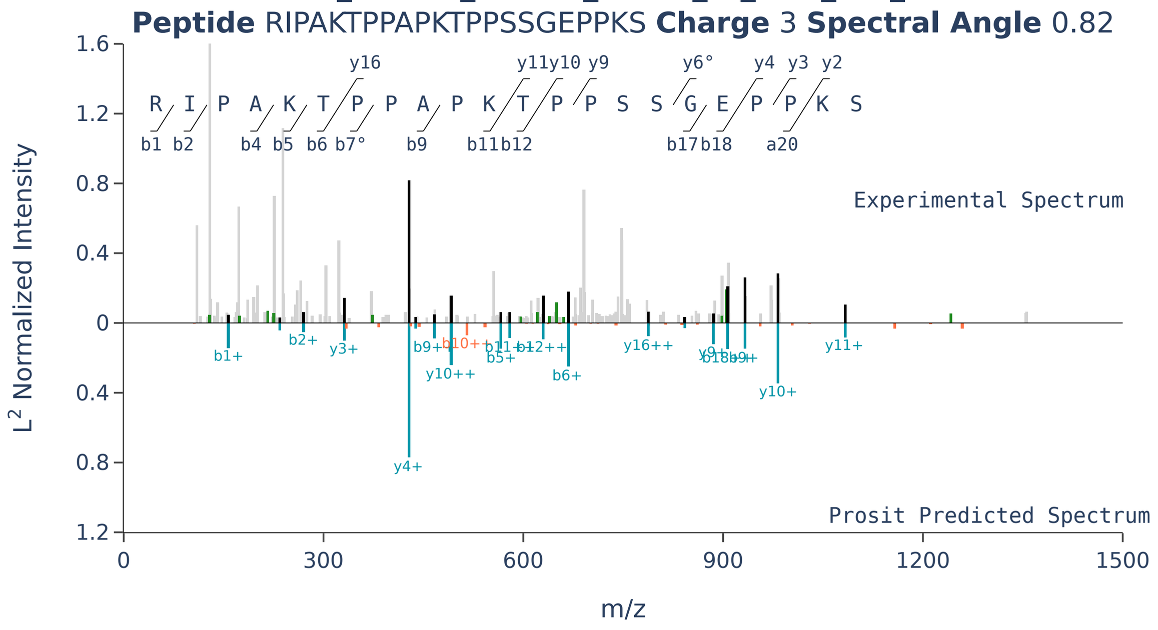


**Source** WSoh_221121_011221_HFGoe_L1_hTau_24h_R1 **Scan** 14665
**Peptide** KHQPGGGKVQIINKKLDLSNVQS **Charge** 3 **Spectral Angle** 0.72

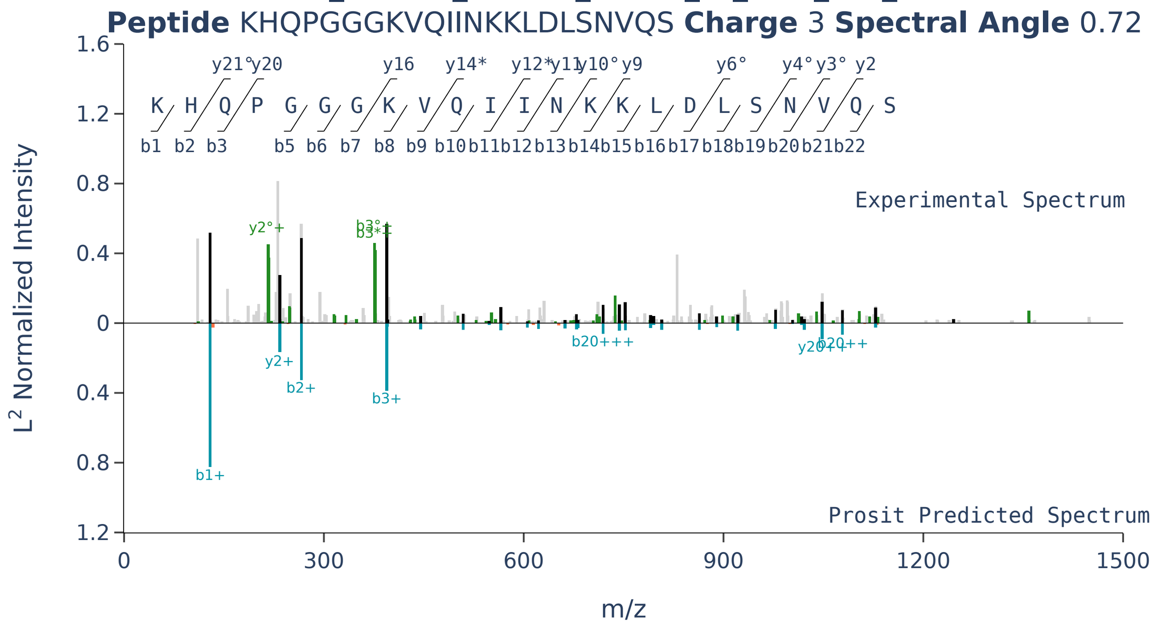


**Source** WSoh_221121_011221_HFGoe_L1_hTau_2h_R2 **Scan** 6165
**Peptide** KLTFRENAKAKTDH **Charge** 3 **Spectral Angle** 0.74

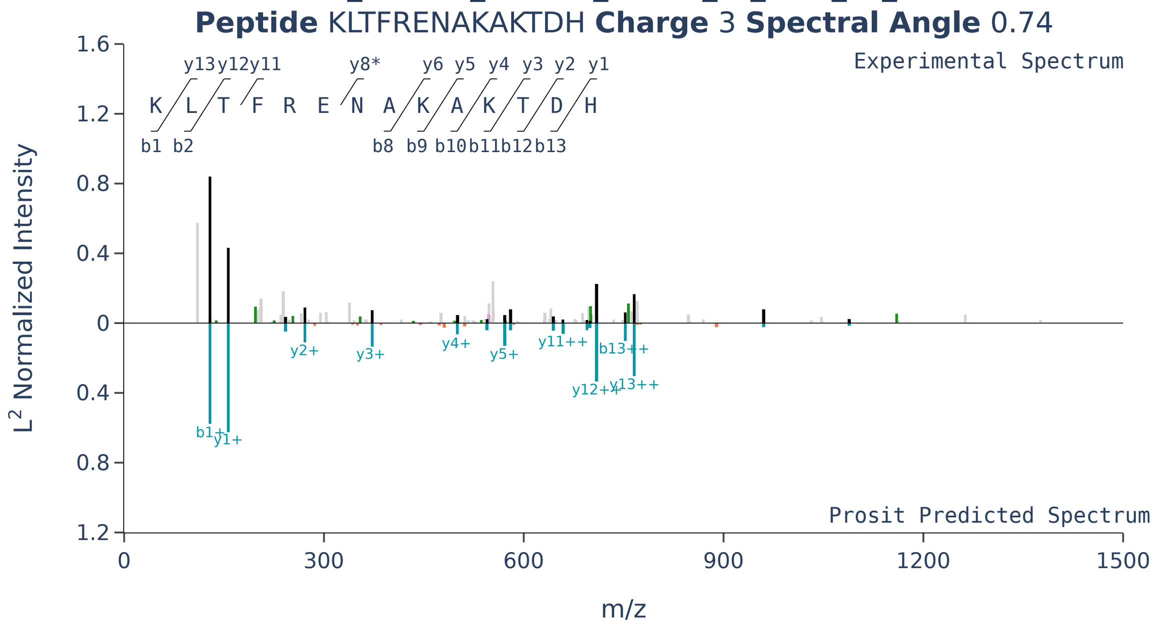


**Source** WSoh_221121_011221_HFGoe_L2_hTau_4h_R2 **Scan** 15037
**Peptide** SKIGSLDNITHVPGGGNKKIETHKL **Charge** 5 **Spectral Angle** 0.85

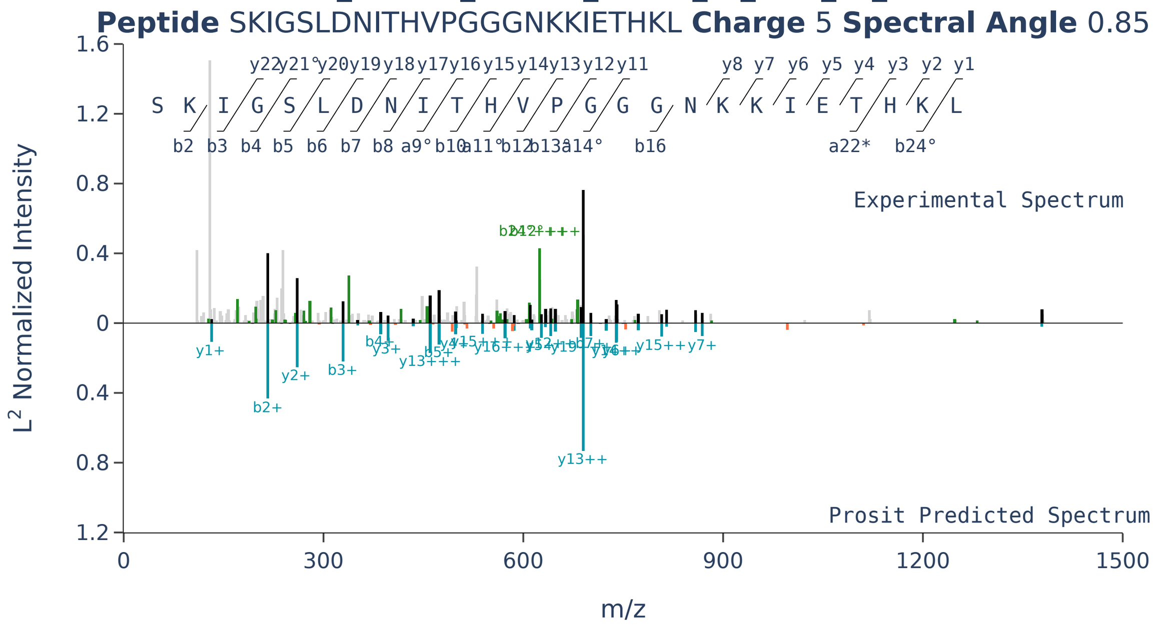


**Source** WSoh_221121_011221_HFGoe_L1_hTau_24h_R2 **Scan** 6478
**Peptide** VVRTPPKSPS **Charge** 2 **Spectral Angle** 0.87

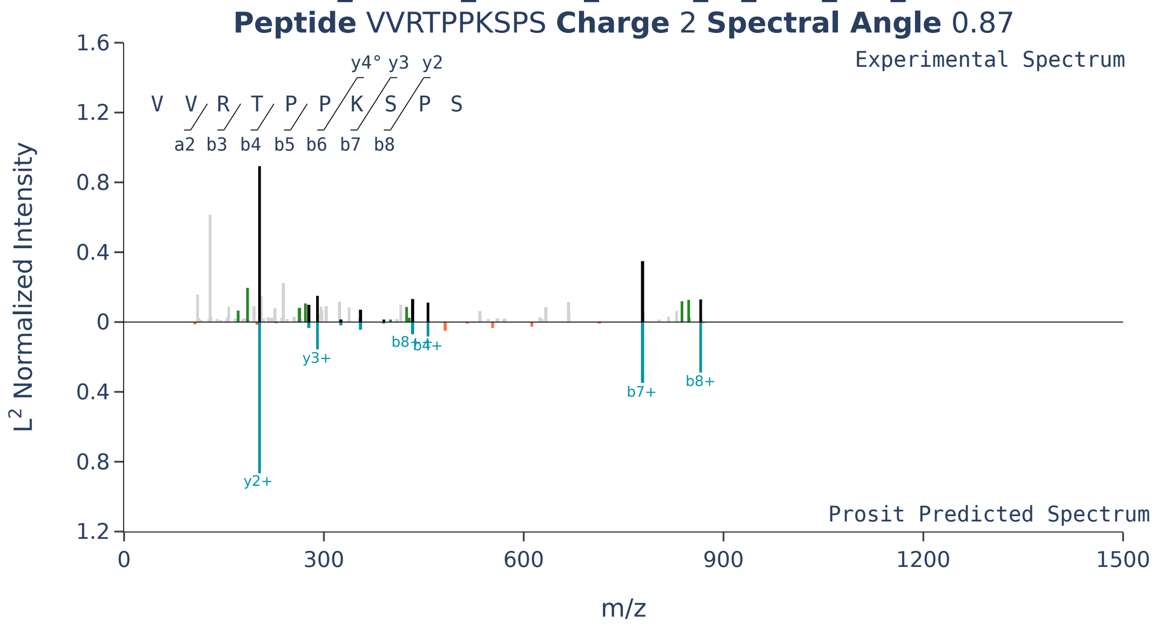


**Source** WSoh_221121_011221_HFGoe_L1_hTau_24h_R1 **Scan** 21937
**Peptide** GSVQIVYKPVDLSKVTSK **Charge** 3 **Spectral Angle** 0.84

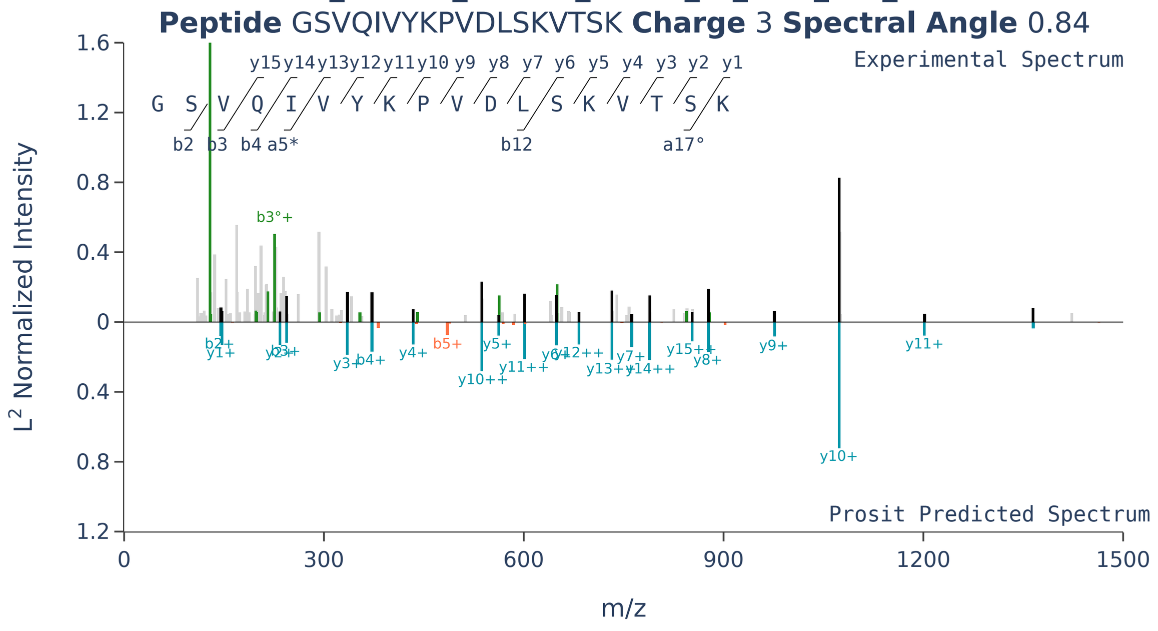


**Source** WSoh_221121_011221_HFGoe_L2_hTau_24h_R2 **Scan** 9459
**Peptide** RLPAKTPPAPKTPPSSGEPPKSG **Charge** 4 **Spectral Angle** 0.81

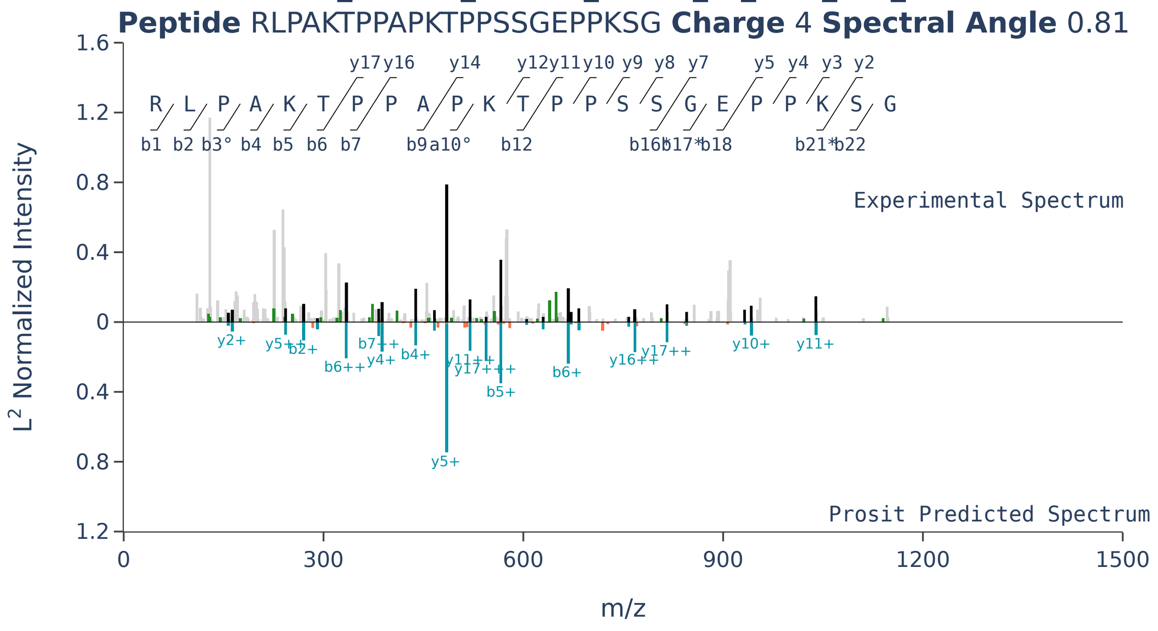


**Source** WSoh_221121_011221_HFGoe_L2_hTau_24h_R2 **Scan** 10761
**Peptide** LSKVTSKCGSLGNIHHKPGGGQVEV **Charge** 3 **Spectral Angle** 0.82

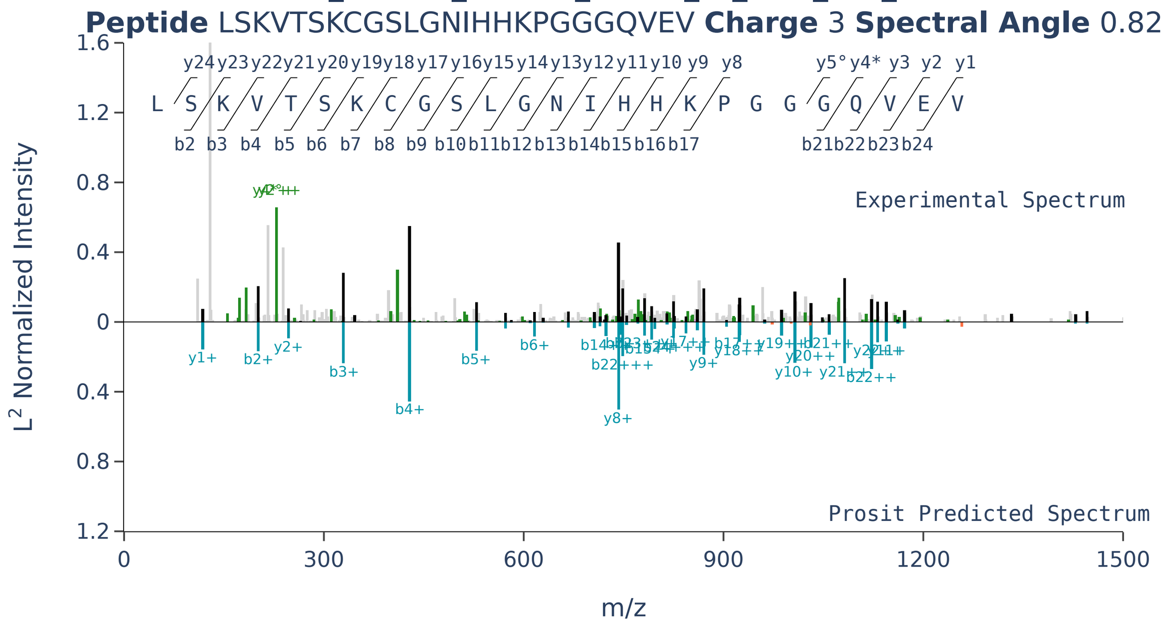


**Source** WSoh_221121_011221_HFGoe_L1_hTau_24h_R1 **Scan** 13060
**Peptide** KAKTDHGAEIVYKSPVVSGDTS **Charge** 3 **Spectral Angle** 0.83

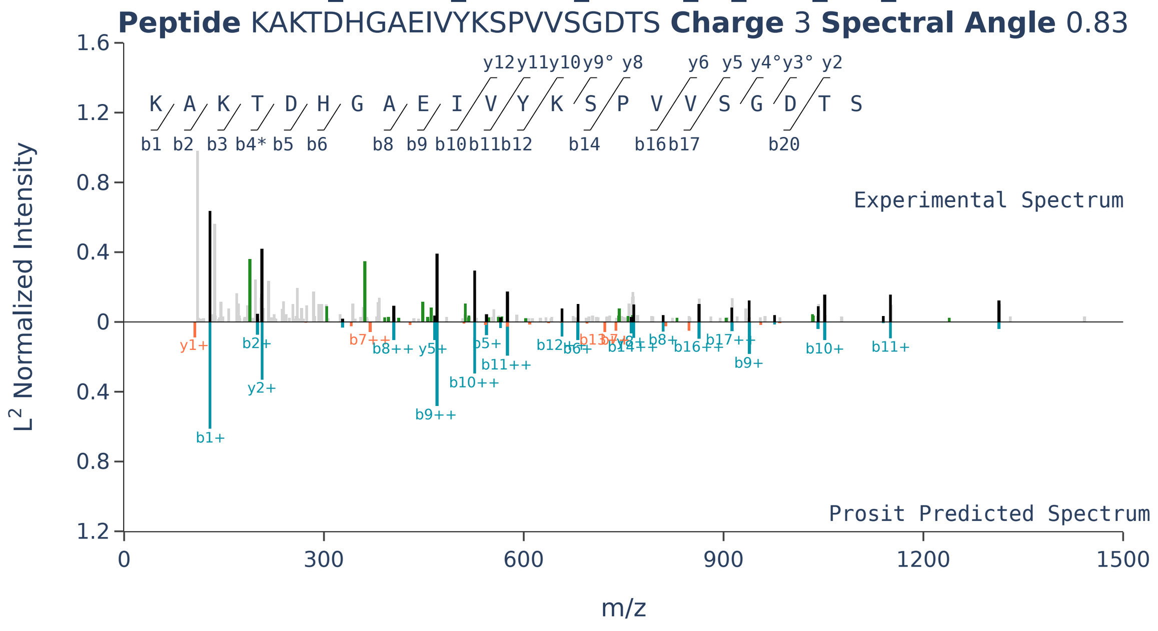


**Source** WSoh_221121_011221_HFGoe_L2_hTau_24h_R1 **Scan** 20518
**Peptide** EEAGIGDTPSLEDEAAGHVTQARMVSK **Charge** 4 **Spectral Angle** 0.79

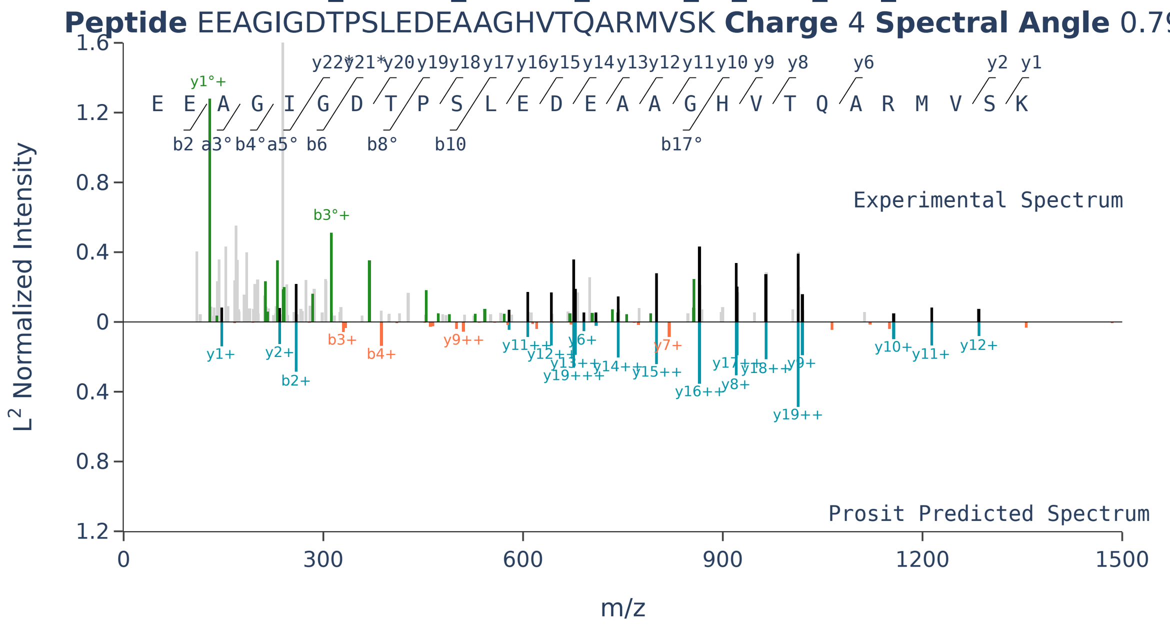

**Source** WSoh_221121_011221_HFGoe_L1_hTau_24h_R2 **Scan** 11027
**Peptide** KHVPGGGSVQL **Charge** 2 **Spectral Angle** 0.82

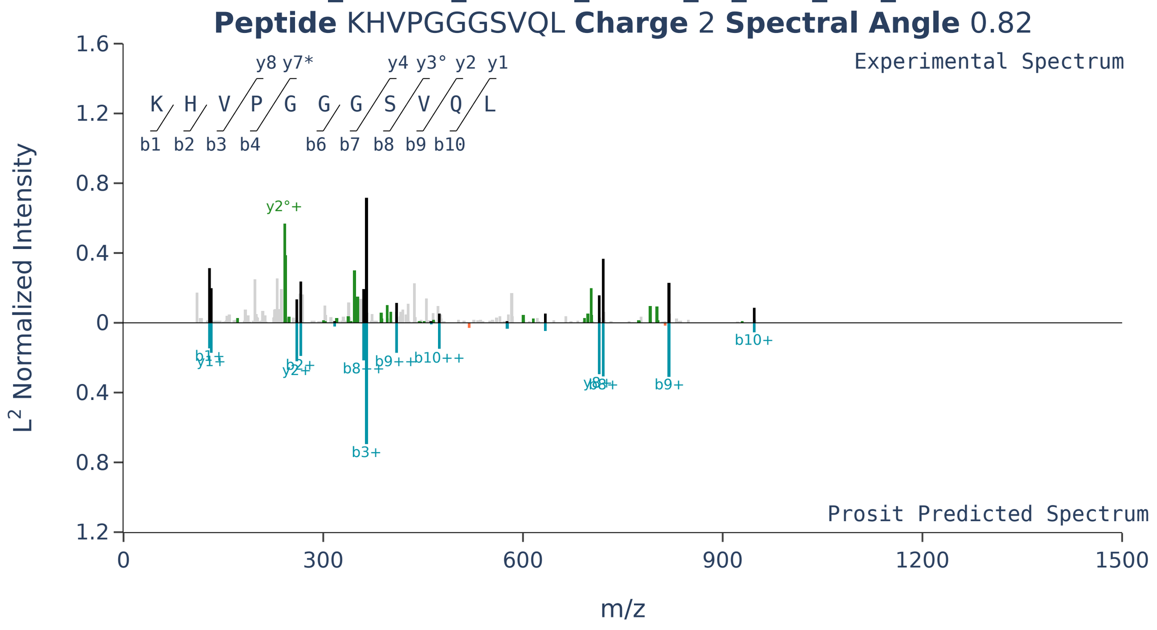


**Source** WSoh_221121_011221_HFGoe_L2_hTau_24h_R2 **Scan** 22749
**Peptide** SGDTSPRHLSNVSSTGSIDMVDSPQLA **Charge** 3 **Spectral Angle** 0.8

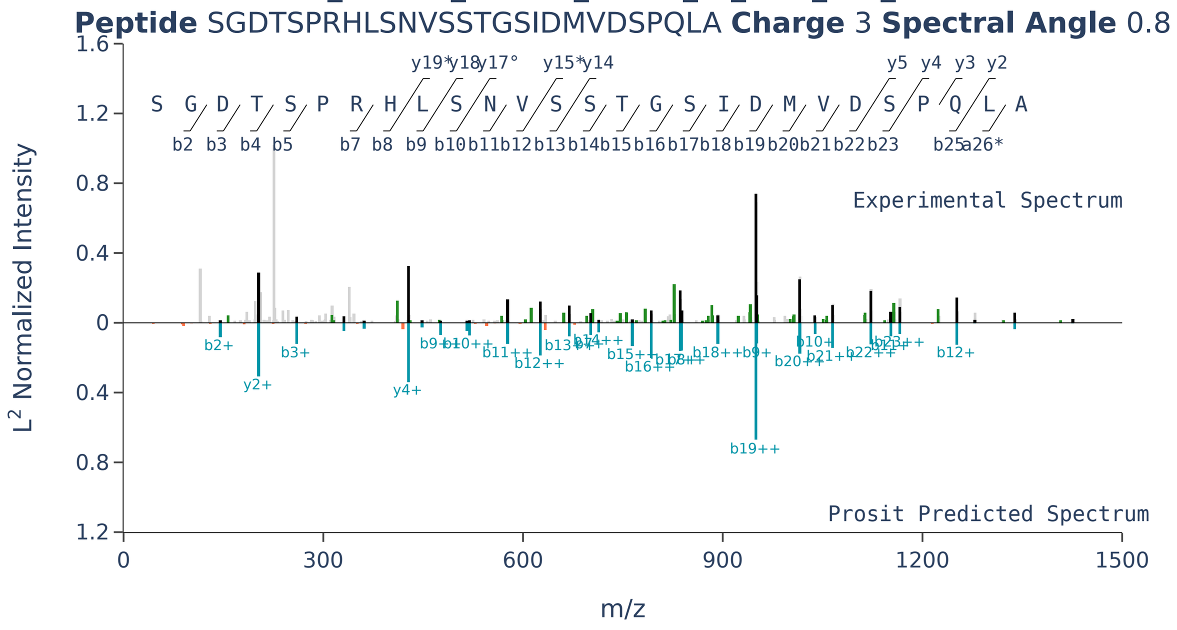


**Source** WSoh_221121_011221_HFGoe_L1_hTau_24h_R2 **Scan** 7747
**Peptide** TPRGAAPPGQKGQANATRIPAKT **Charge** 4 **Spectral Angle** 0.84

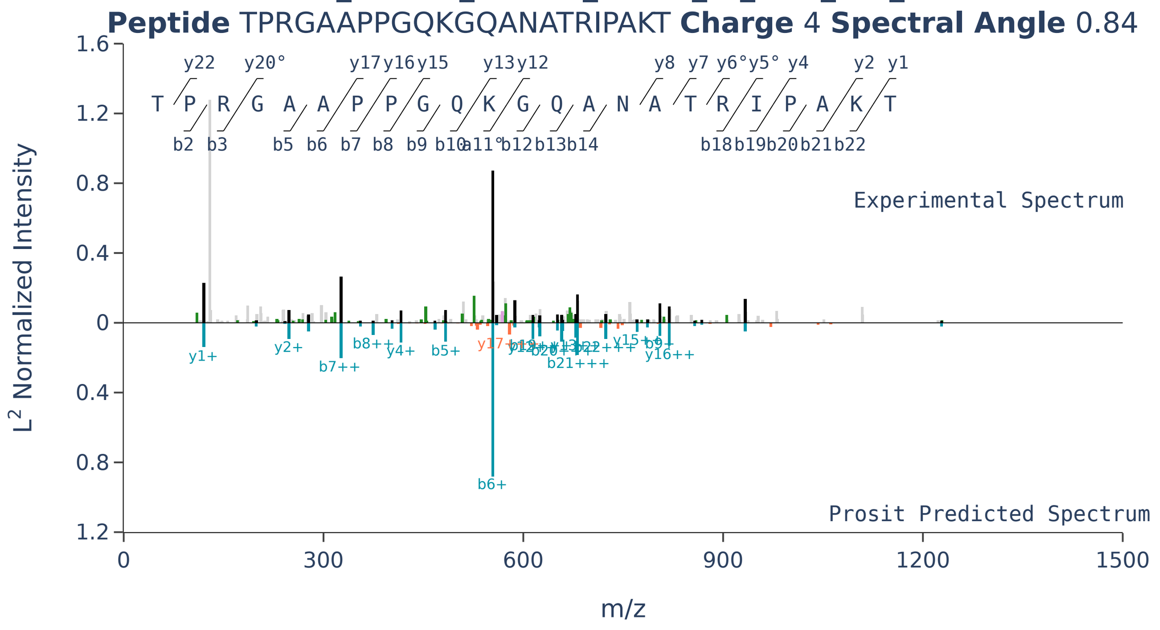


**Source** WSoh_221121_011221_HFGoe_L1_hTau_24h_R2 **Scan** 11676
**Peptide** EGAPGKQAAAQPHTEIPE **Charge** 3 **Spectral Angle** 0.96

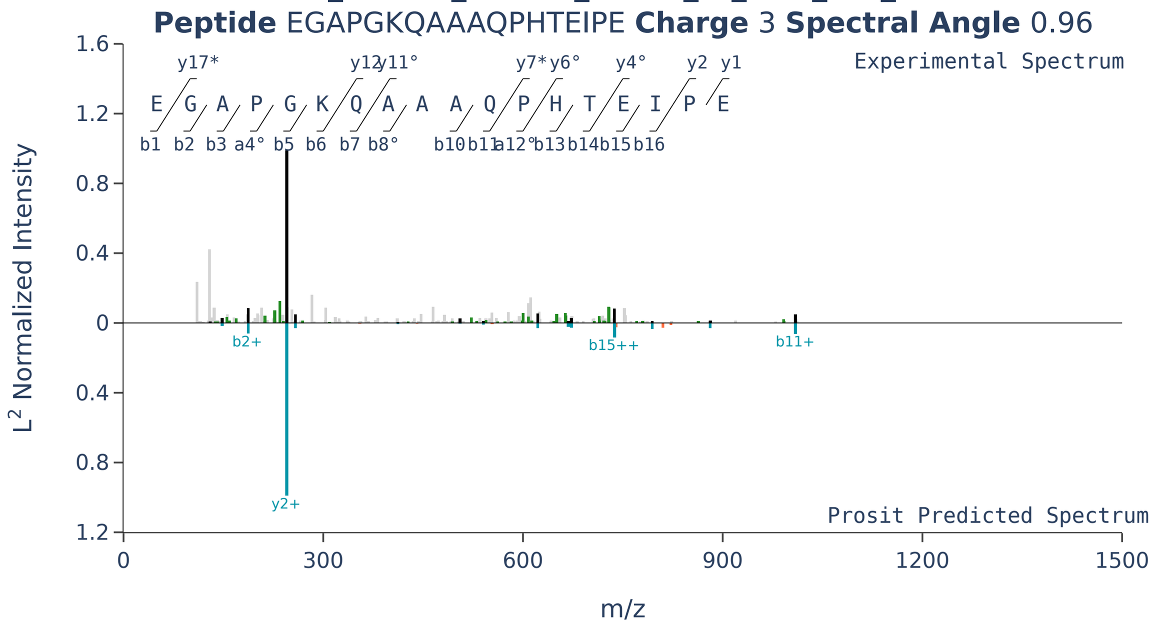


**Source** WSoh_221121_011221_HFGoe_L1_hTau_24h_R1 **Scan** 20345
**Peptide** LDFKDRVQSKIGSLD **Charge** 3 **Spectral Angle** 0.66

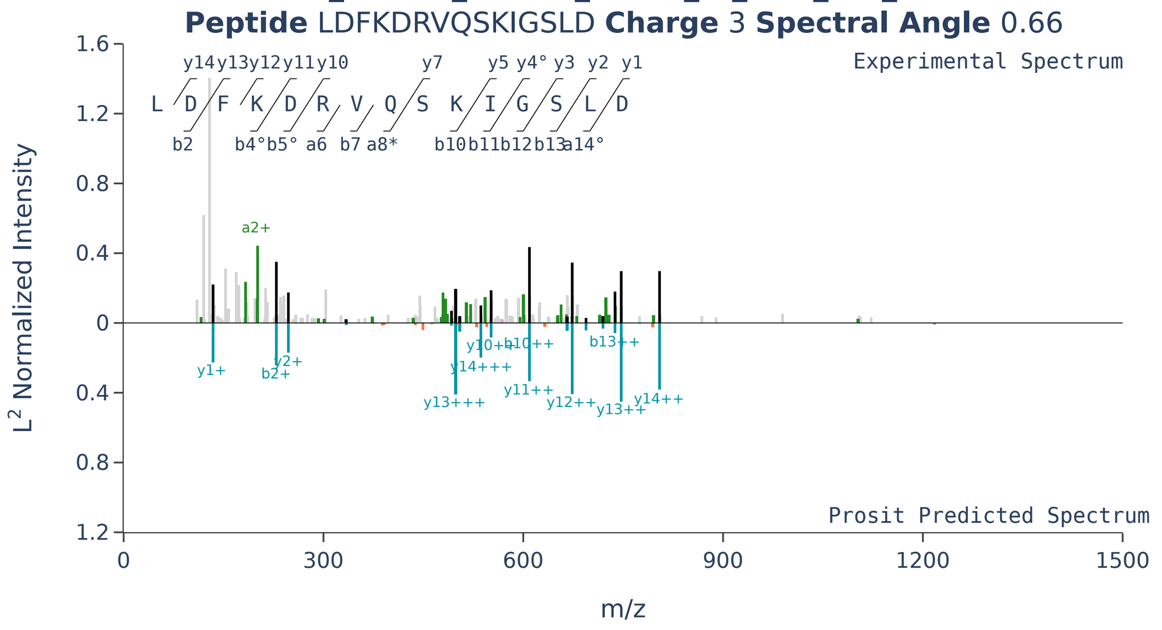


**Source** WSoh_221121_011221_HFGoe_L2_hTau_4h_R1 **Scan** 6826
**Peptide** SKCGSKDNIKHVPGGGSV **Charge** 3 **Spectral Angle** 0.79

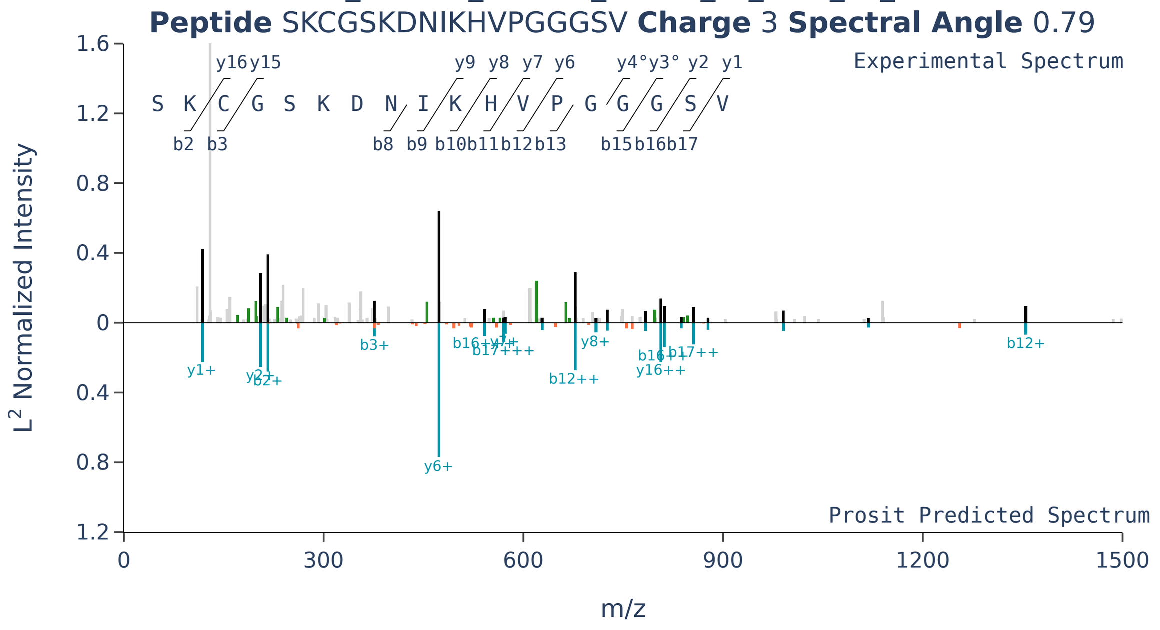


**Source** WSoh_221121_011221_HFGoe_L1_hTau_4h_R1 **Scan** 7933
**Peptide** PAKTPPAPKTPPSSGEPPKS **Charge** 3 **Spectral Angle** 0.84

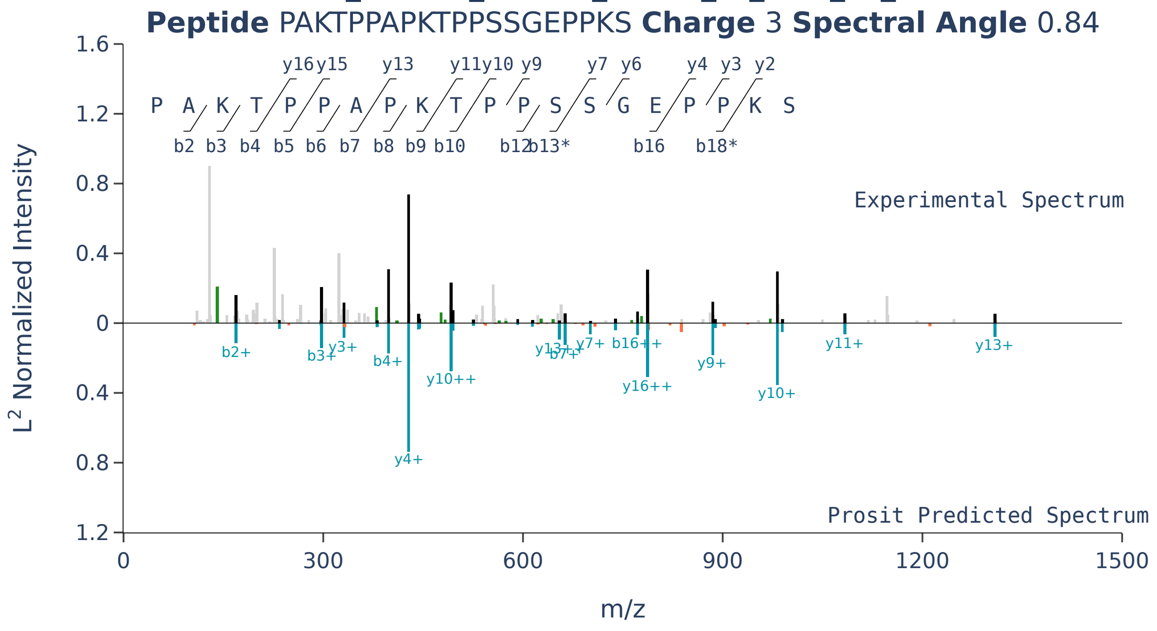


**Source** WSoh_221121_011221_HFGoe_L2_hTau_24h_R2 **Scan** 18275
**Peptide** LGSLDNITHVPGGGNKKI **Charge** 4 **Spectral Angle** 0.92

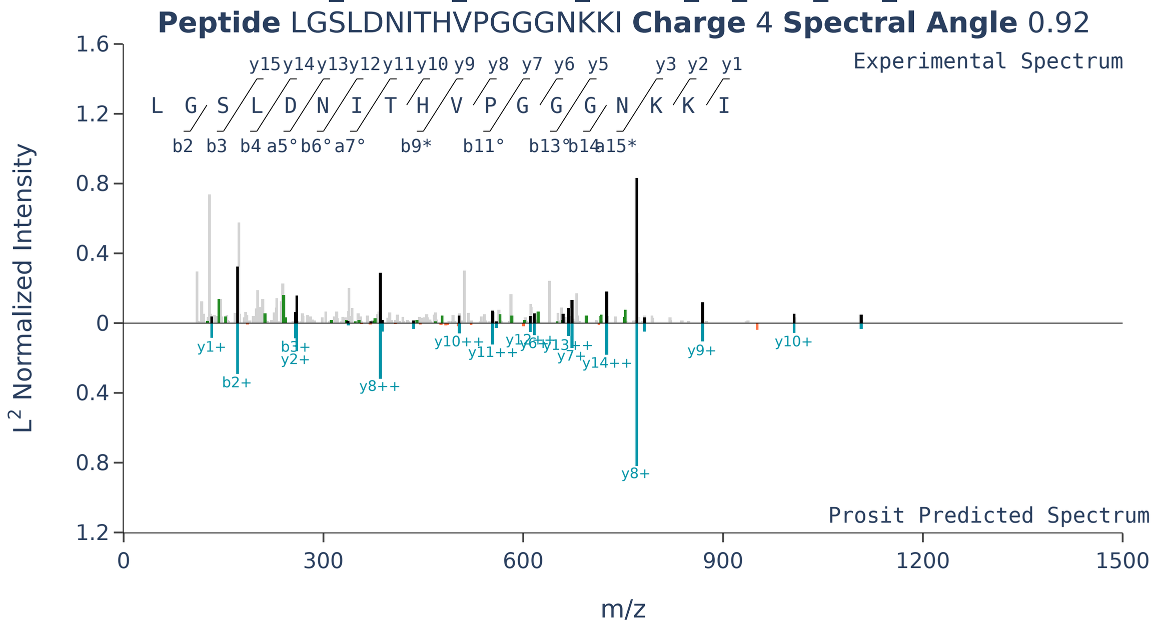


**Source** WSoh_221121_011221_HFGoe_L1_hTau_24h_R2 **Scan** 11852
**Peptide** LKHQPGGGKVQIL **Charge** 2 **Spectral Angle** 0.83

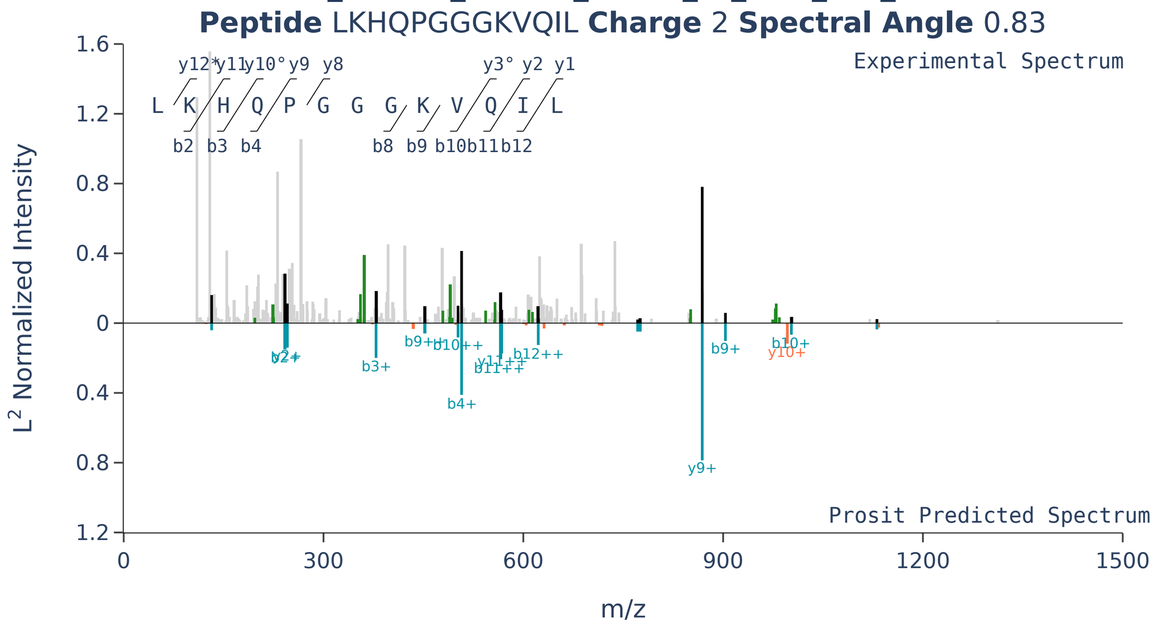


**Source** WSoh_221121_011221_HFGoe_L2_hTau_24h_R1 **Scan** 6195
**Peptide** STENLKHQPGGGKV **Charge** 3 **Spectral Angle** 0.85

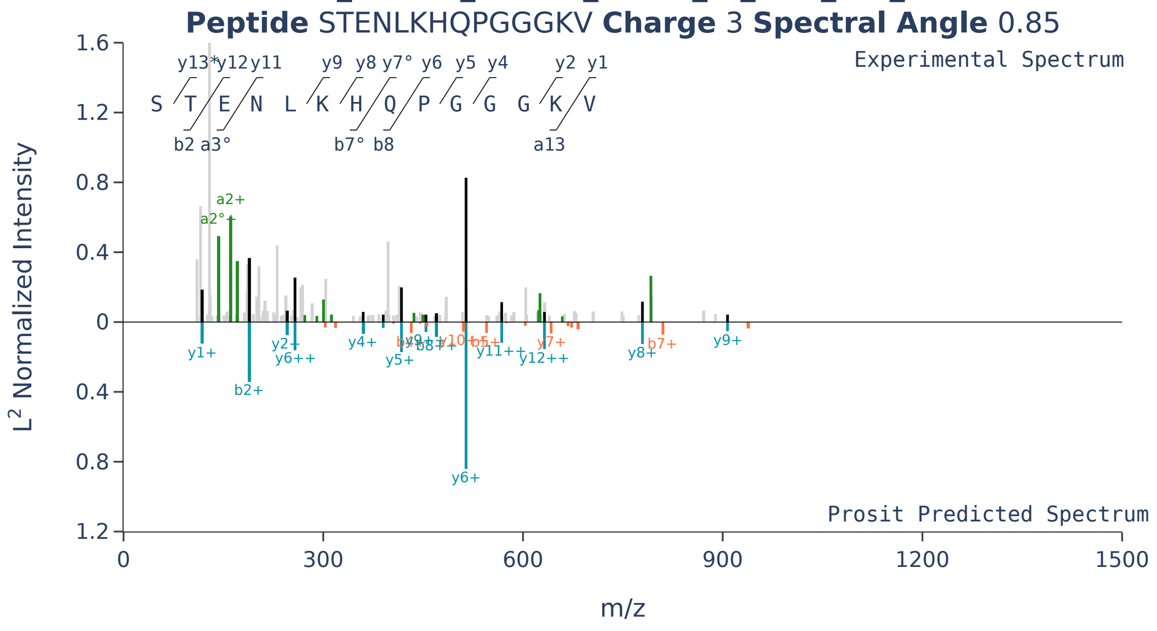


**Source** WSoh_221121_011221_HFGoe_L2_hTau_1h_R1 **Scan** 16328
**Peptide** QLINKKLDL **Charge** 2 **Spectral Angle** 0.75

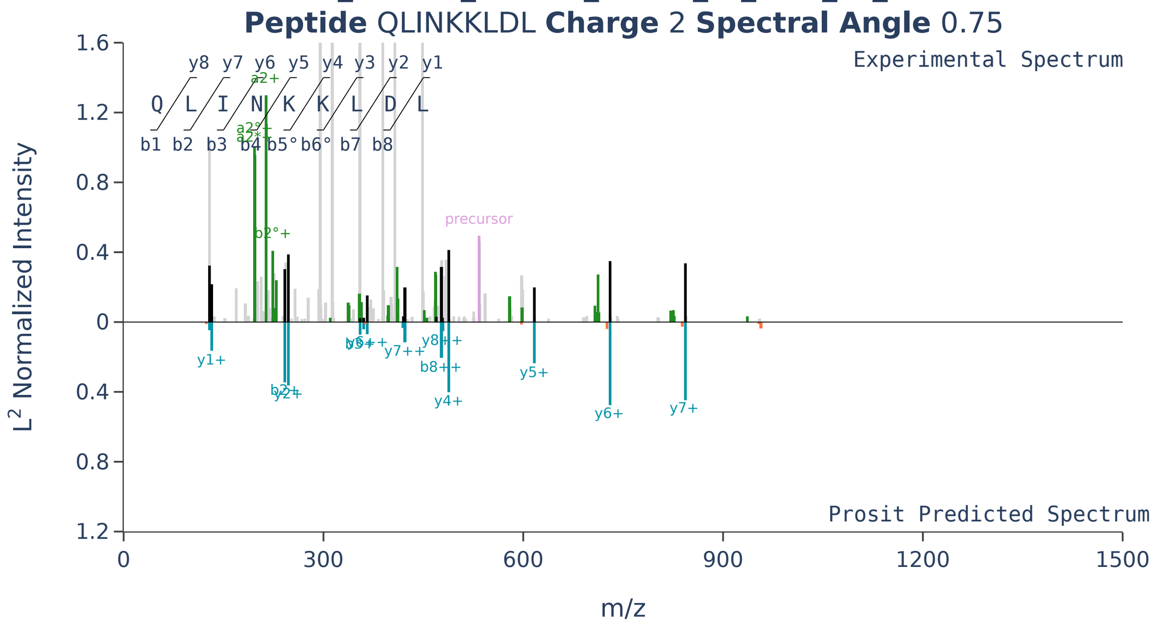


**Source** WSoh_221121_011221_HFGoe_L2_hTau_24h_R2 **Scan** 8619
**Peptide** TDAGLKESP **Charge** 2 **Spectral Angle** 0.86

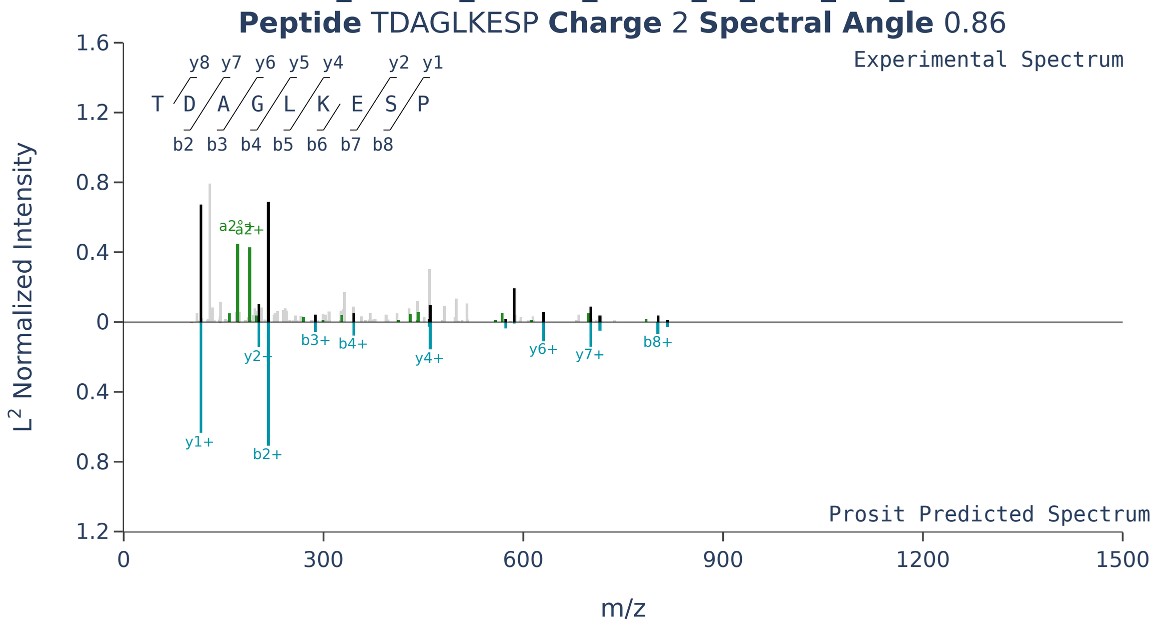


**Source** WSoh_221121_011221_HFGoe_L2_hTau_4h_R1 **Scan** 11267
**Peptide** EDHAGTYGLGDRKDQGGYTMHQD **Charge** 3 **Spectral Angle** 0.79

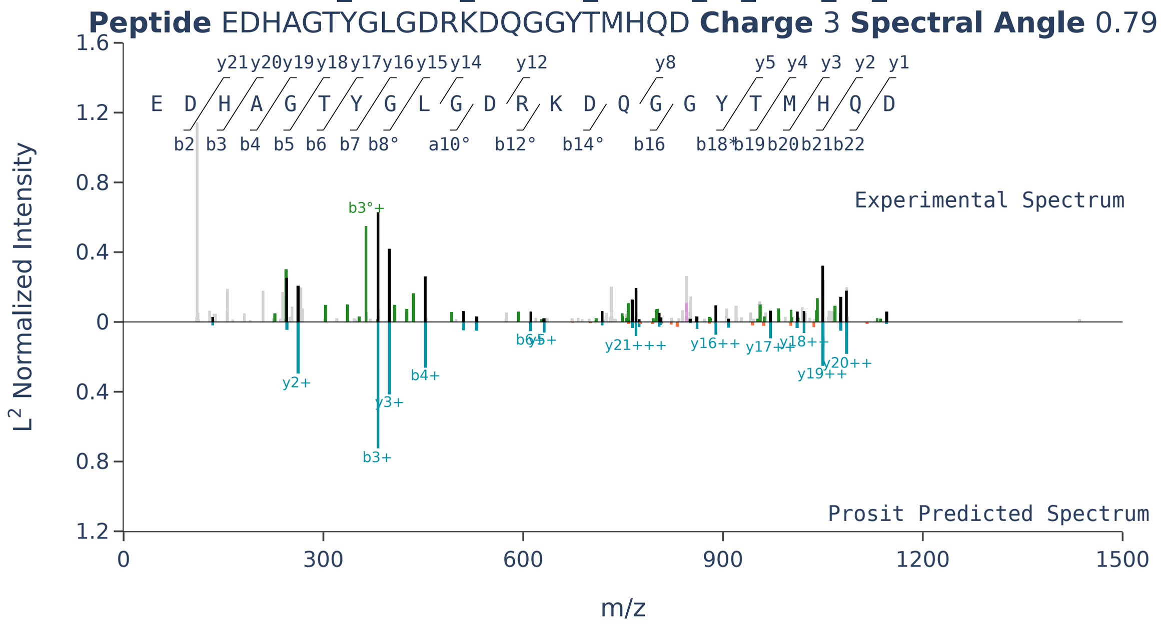


**Source** WSoh_221121_011221_HFGoe_L2_hTau_4h_R1 **Scan** 7230
**Peptide** PAKTPPAPKTPPSSGEPPKSGDR **Charge** 4 **Spectral Angle** 0.84

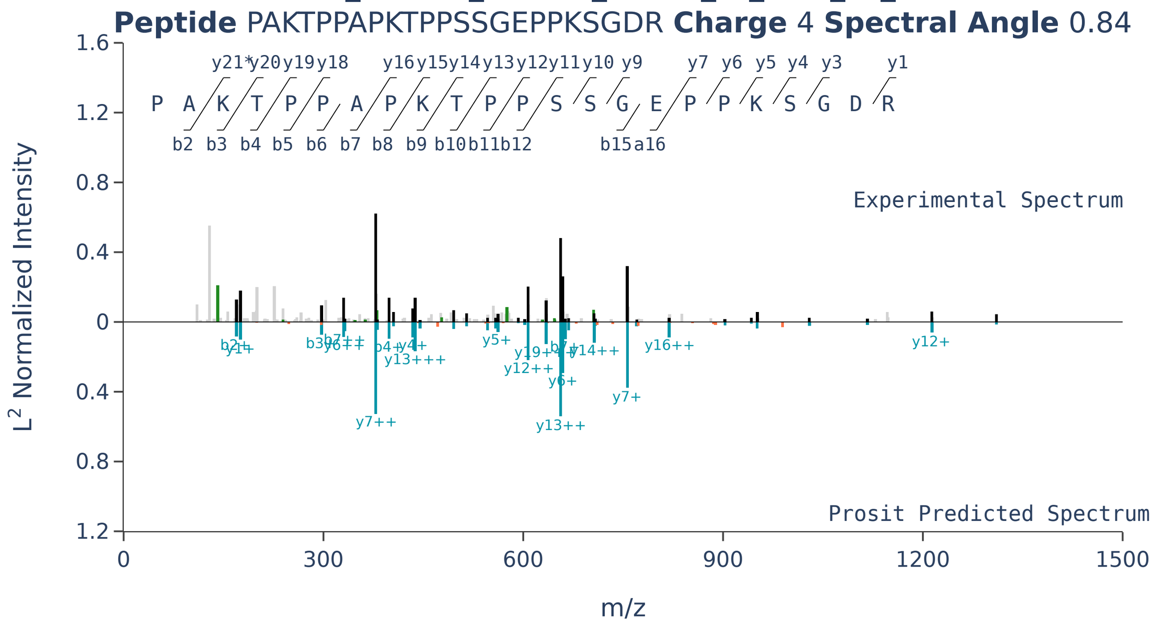


**Source** WSoh_221121_011221_HFGoe_L2_hTau_4h_R1 **Scan** 21178
**Peptide** PRHLSNVSSTGSIDMVDSPQLA **Charge** 3 **Spectral Angle** 0.79

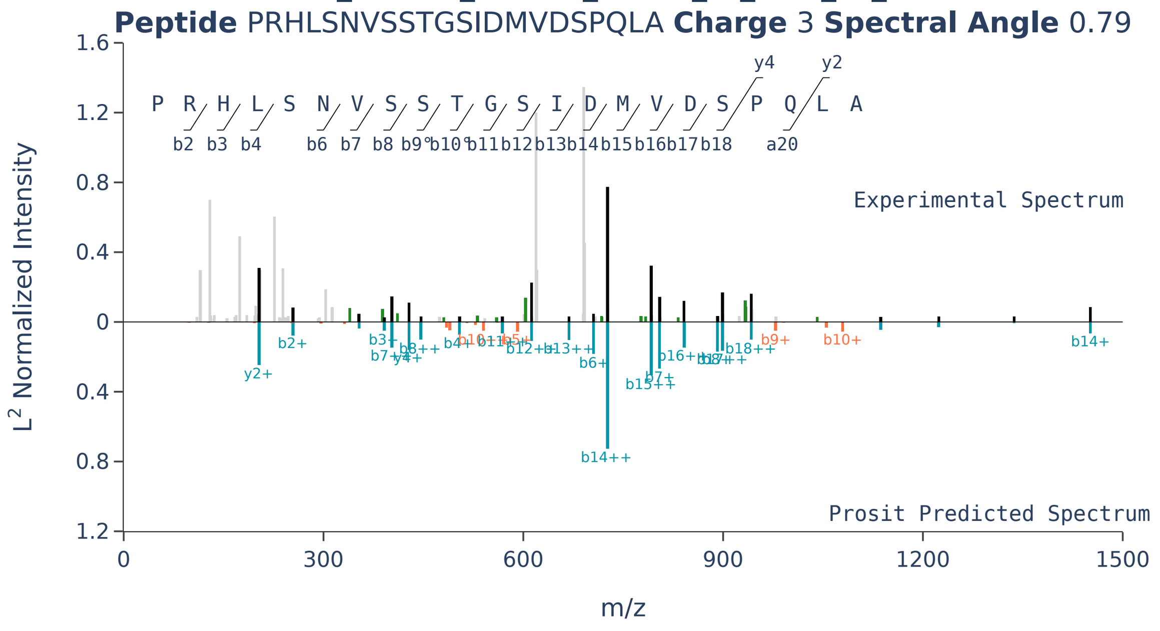

**Source** WSoh_221121_011221_HFGoe_L2_hTau_24h_R1 **Scan** 6212
**Peptide** GSLGNIHHKPG **Charge** 3 **Spectral Angle** 0.74

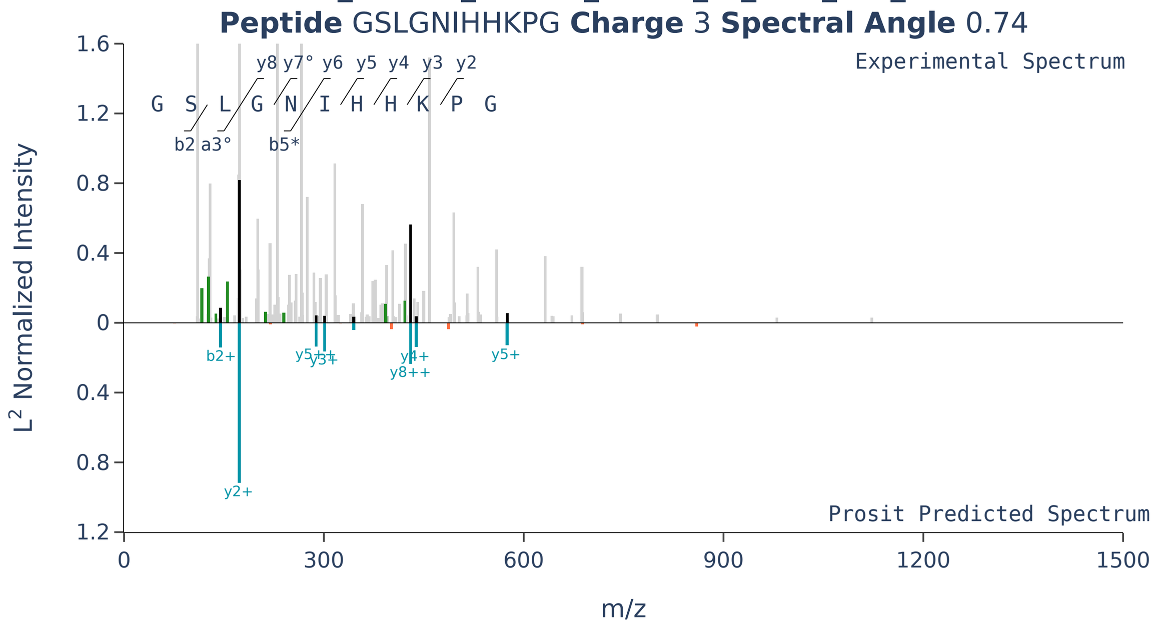


**Source** WSoh_221121_011221_HFGoe_L1_hTau_24h_R2 **Scan** 10495
**Peptide** DLSNVQSKCGSKDNIKHV **Charge** 3 **Spectral Angle** 0.88

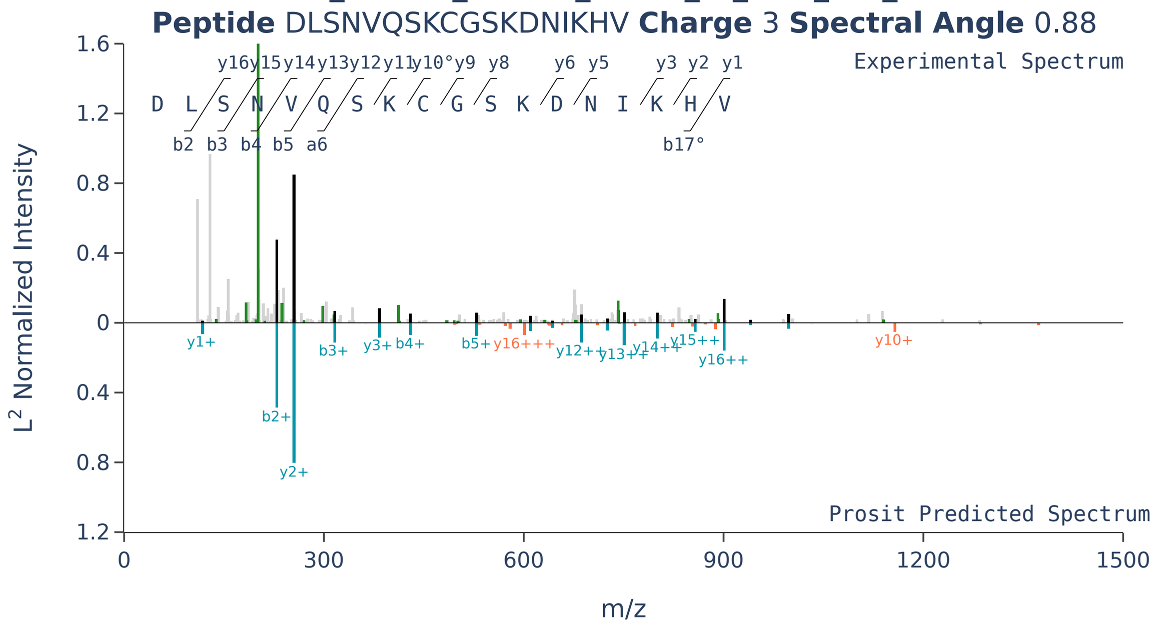


**Source** WSoh_221121_011221_HFGoe_L1_hTau_24h_R2 **Scan** 16201
**Peptide** KLGSLDNITHVPGGGNK **Charge** 4 **Spectral Angle** 0.81

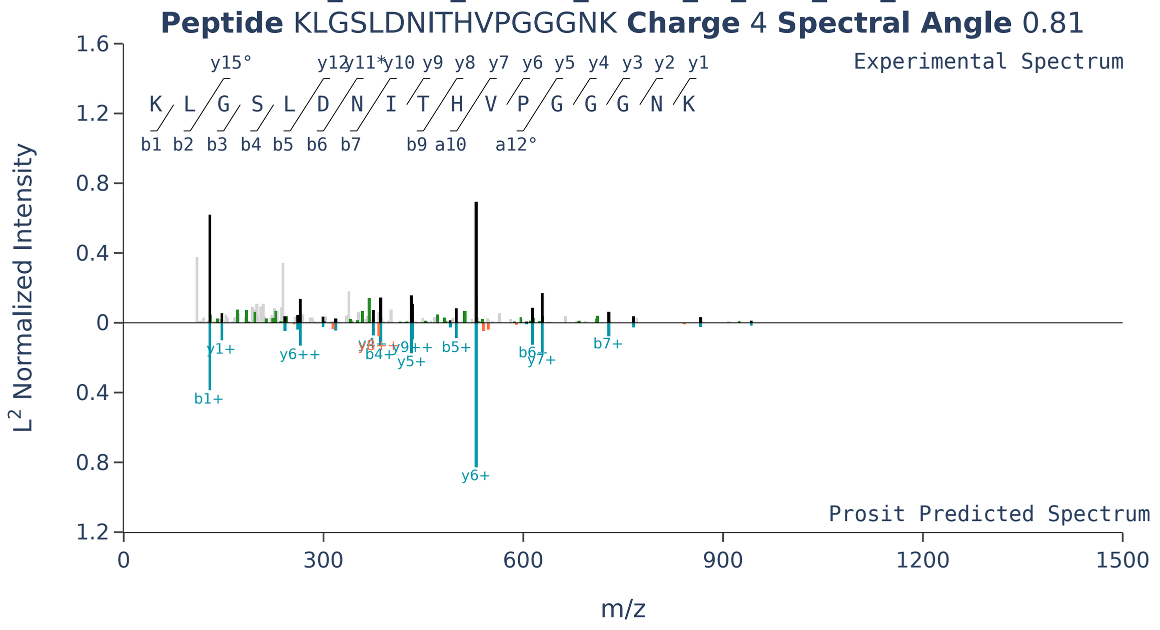


**Source** WSoh_221121_011221_HFGoe_L1_hTau_24h_R1 **Scan** 13372
**Peptide** AQPHTEIPEGTTAEEAG **Charge** 2 **Spectral Angle** 0.69

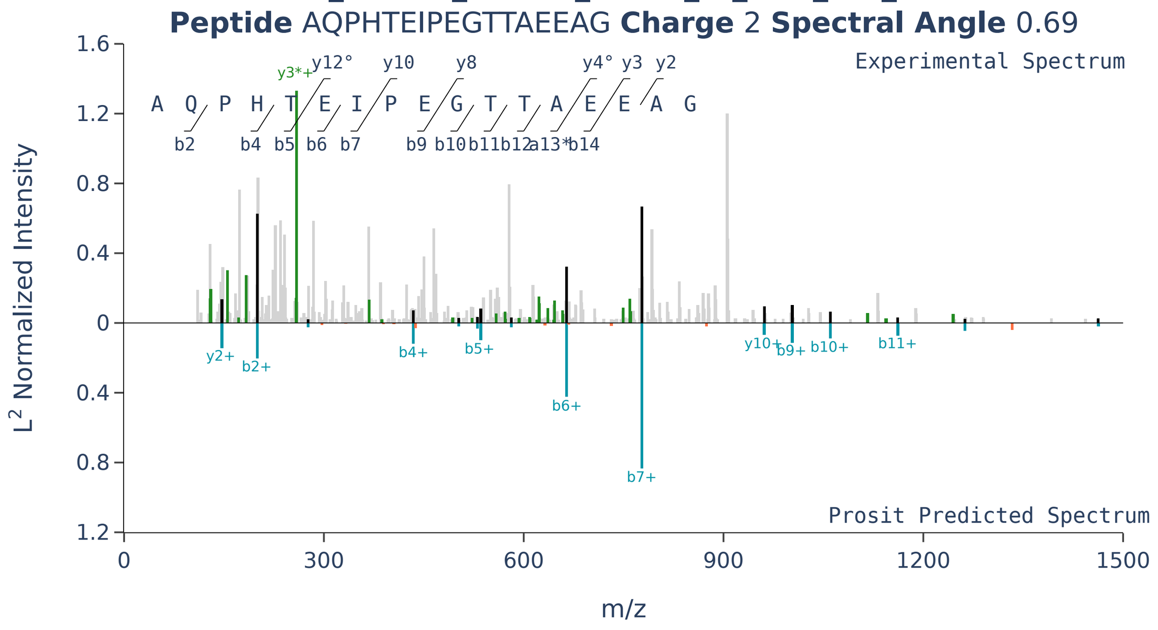


**Source** WSoh_221121_011221_HFGoe_L1_hTau_24h_R1 **Scan** 22063
**Peptide** ETSDAKSTPTAEDVTAPLVD **Charge** 2 **Spectral Angle** 0.89

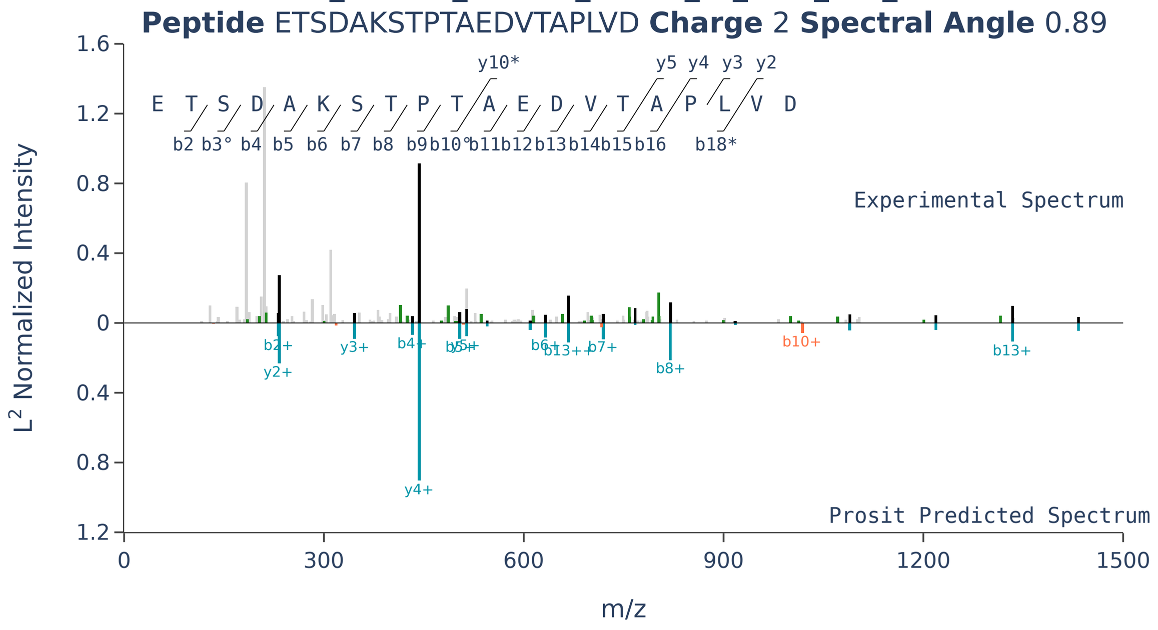


**Source** WSoh_221121_011221_HFGoe_L2_hTau_24h_R2 **Scan** 21547
**Peptide** PVPMPDLKNVKSKI **Charge** 4 **Spectral Angle** 0.74

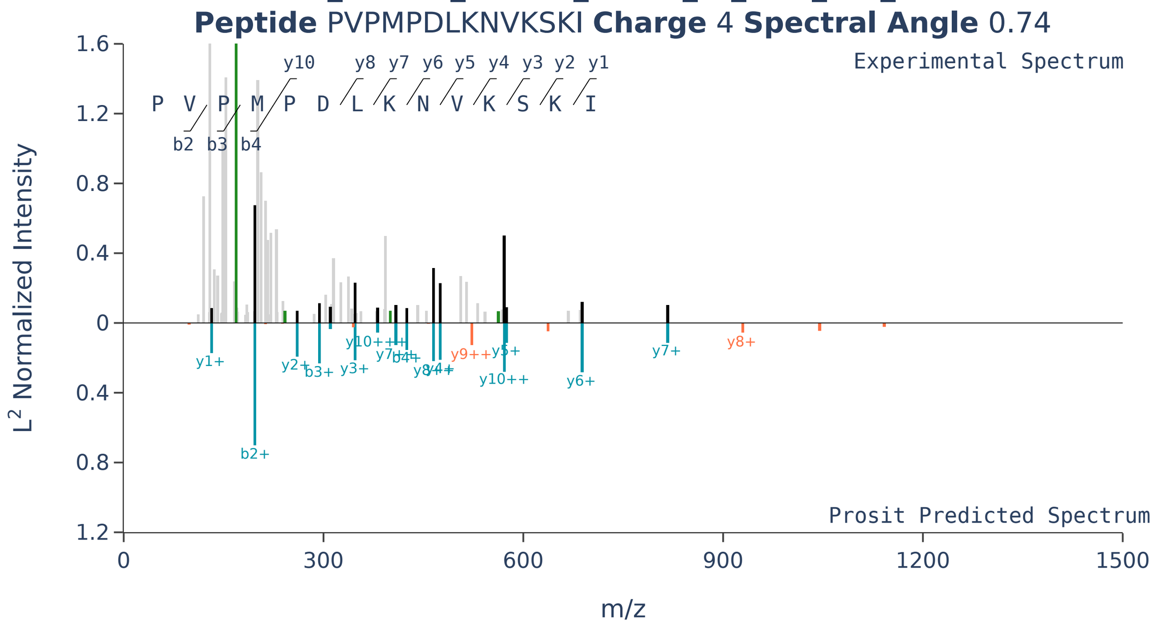


**Source** WSoh_221121_011221_HFGoe_L1_hTau_2h_R1 **Scan** 9845
**Peptide** PHTEIPEG **Charge** 2 **Spectral Angle** 0.9

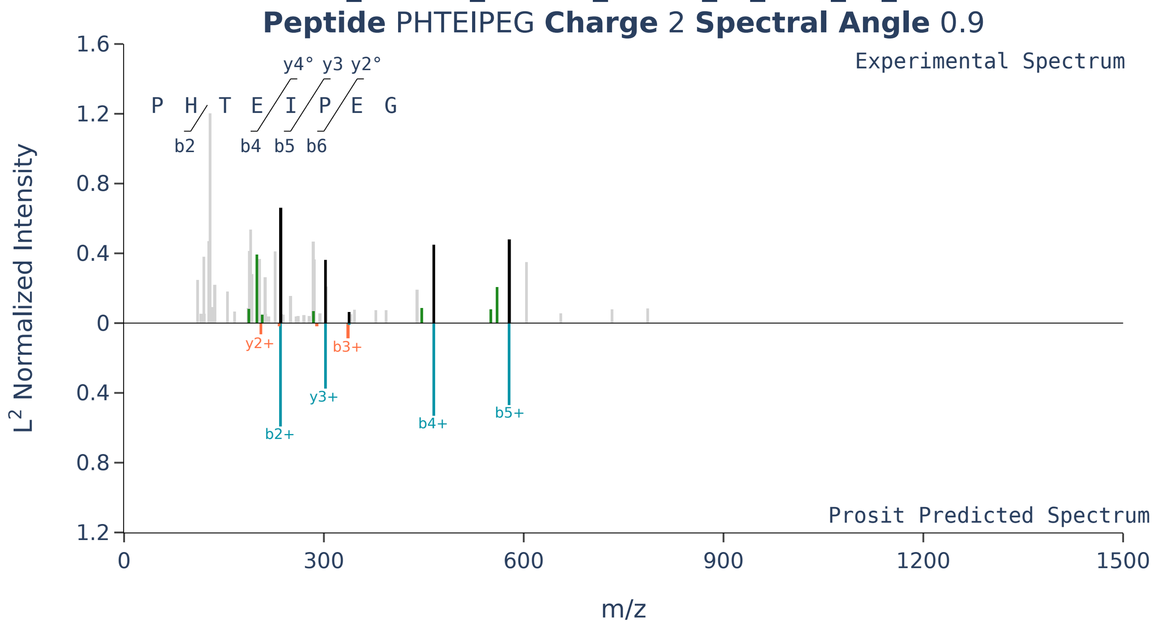


**Source** WSoh_221121_011221_HFGoe_L2_hTau_24h_R2 **Scan** 24672
**Peptide** PSSAKSRLQTAPVPMPDL **Charge** 3 **Spectral Angle** 0.83

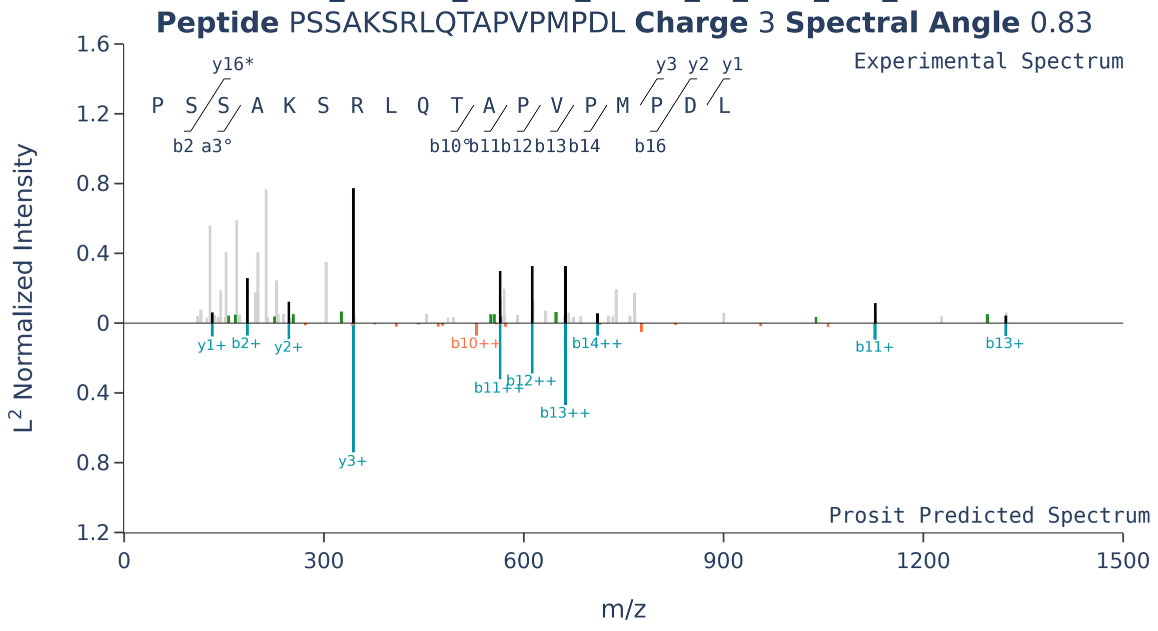


**Source** WSoh_221121_011221_HFGoe_L2_hTau_1h_R1 **Scan** 17540
**Peptide** QSKIGSLDNI **Charge** 2 **Spectral Angle** 0.8

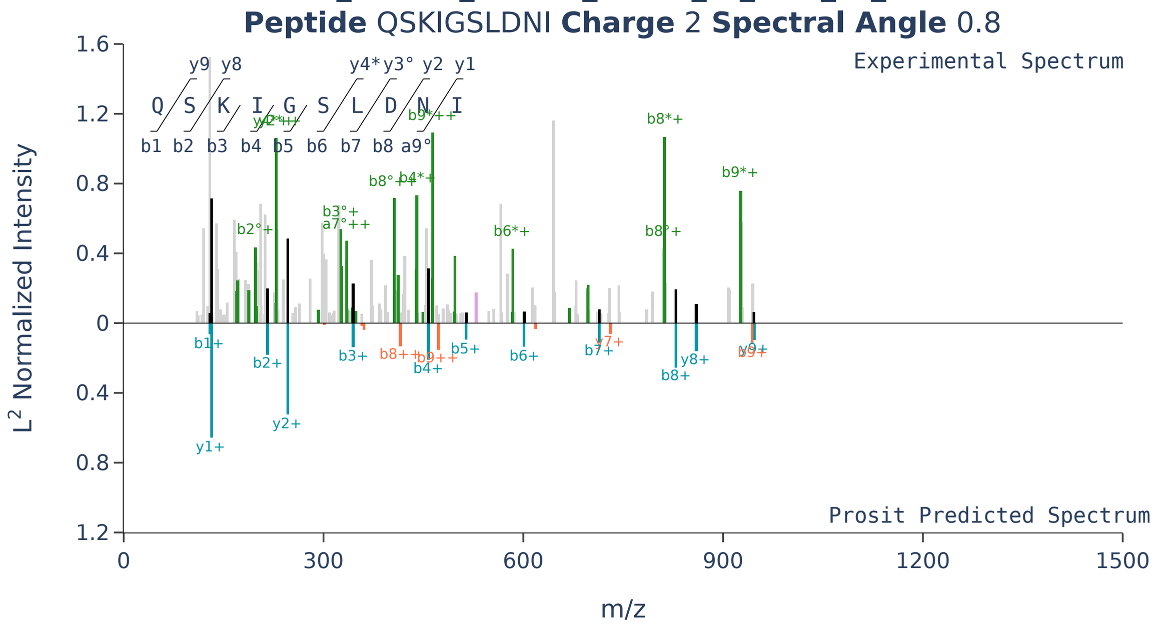


**Source** WSoh_221121_011221_HFGoe_L1_hTau_4h_R2 **Scan** 23387
**Peptide** AEPRQEFEVMEDHAGTYGLGD **Charge** 3 **Spectral Angle** 0.84

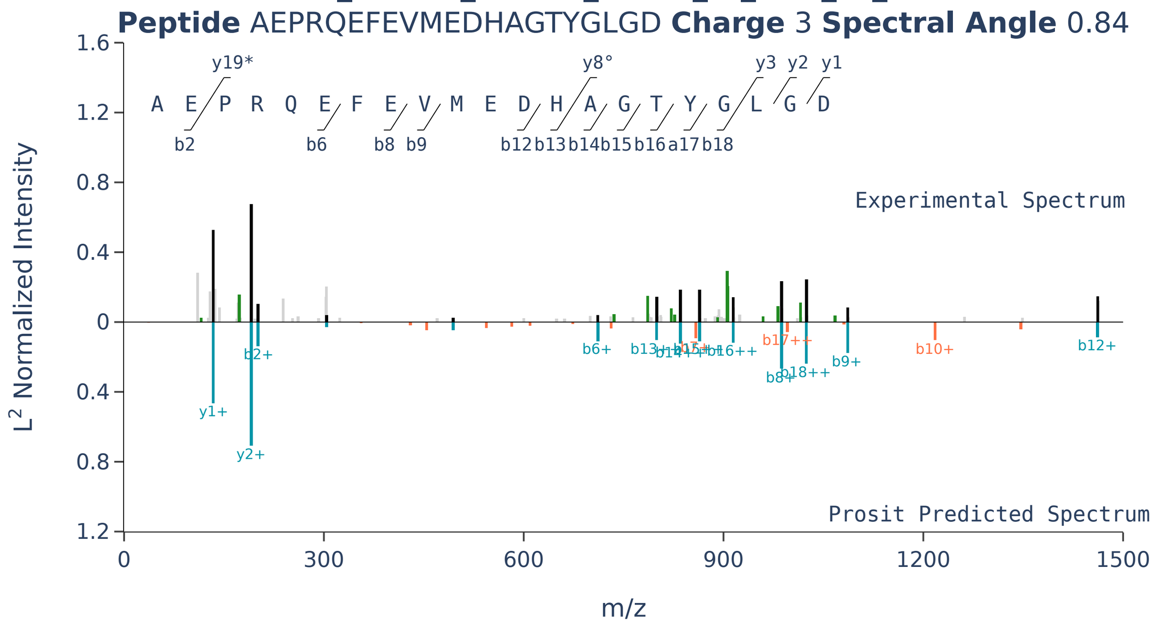


**Source** WSoh_221121_011221_HFGoe_L2_hTau_24h_R1 **Scan** 15017
**Peptide** HAGTYGLGDRKDQGGYTMHQDQEGDTDAGL **Charge** 4 **Spectral Angle** 0.83

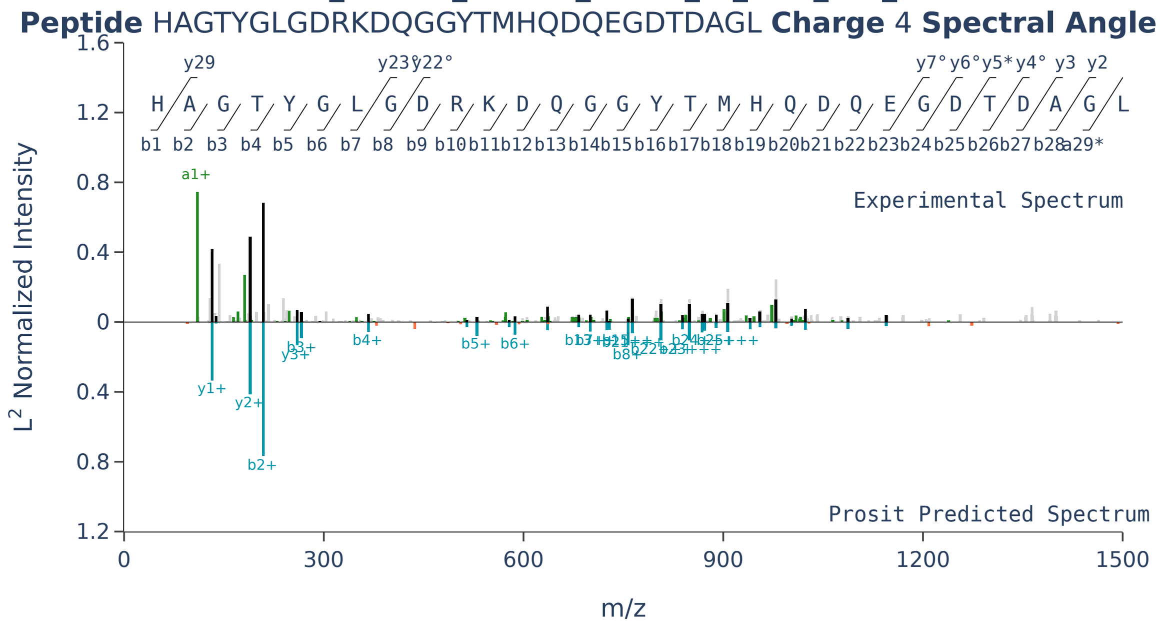


**Source** WSoh_221121_011221_HFGoe_L1_hTau_24h_R2 **Scan** 7857
**Peptide** DGKTKIATPRGAAPPGQKGQA **Charge** 3 **Spectral Angle** 0.77

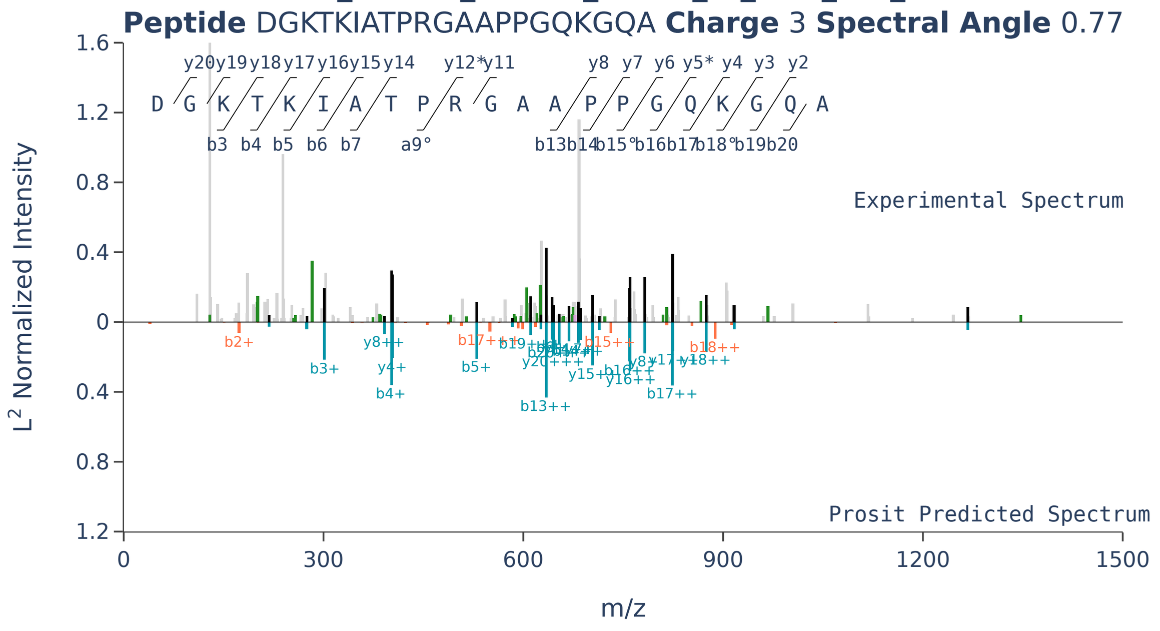


**Source** WSoh_221121_011221_HFGoe_L1_hTau_4h_R2 **Scan** 12770
**Peptide** ETHKLTF **Charge** 2 **Spectral Angle** 0.92

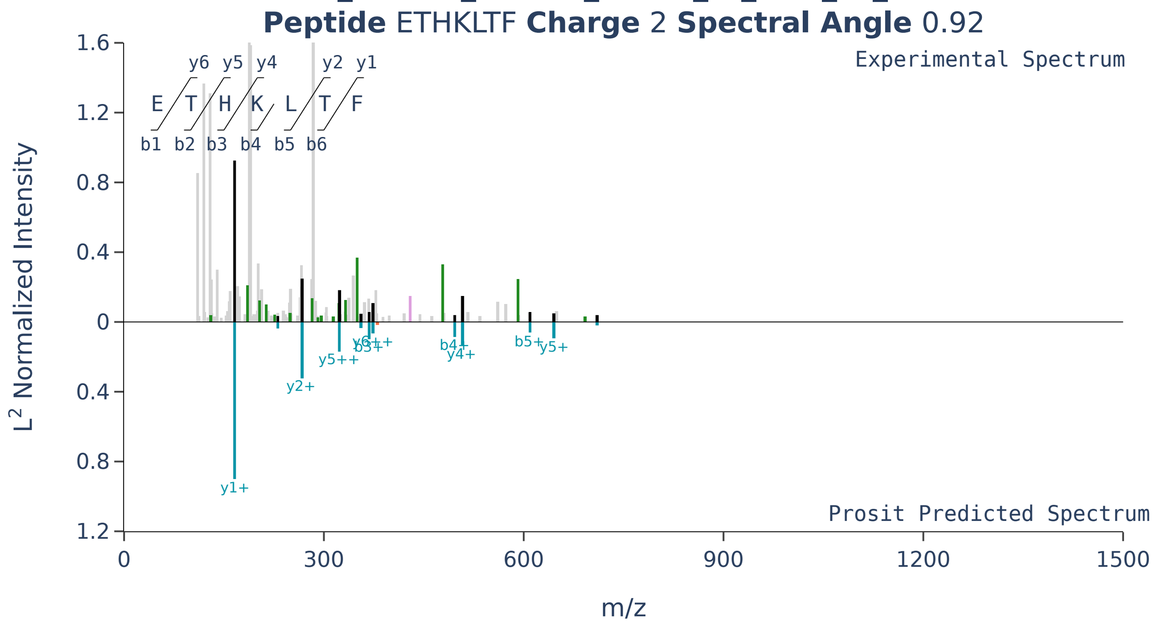


**Source** WSoh_221121_011221_HFGoe_L1_hTau_2h_R1 **Scan** 7242
**Peptide** GDRKDQGGY **Charge** 2 **Spectral Angle** 0.87

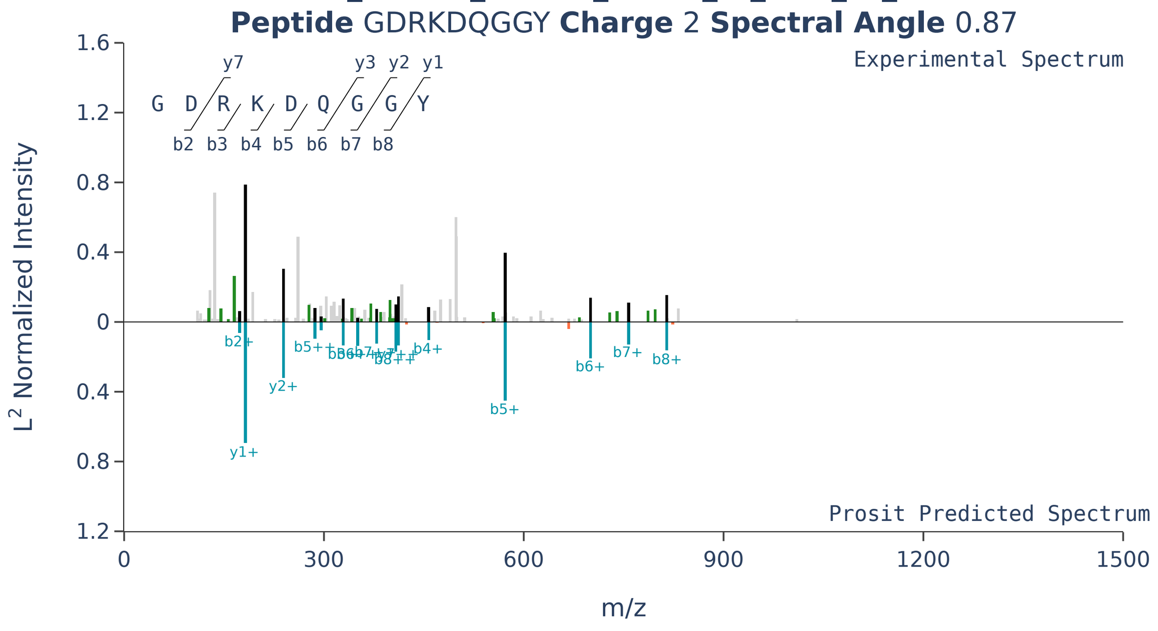


**Source** WSoh_221121_011221_HFGoe_L1_hTau_24h_R1 **Scan** 7488
**Peptide** NVQSKCGSKDNIKHVPGGGSV **Charge** 3 **Spectral Angle** 0.92

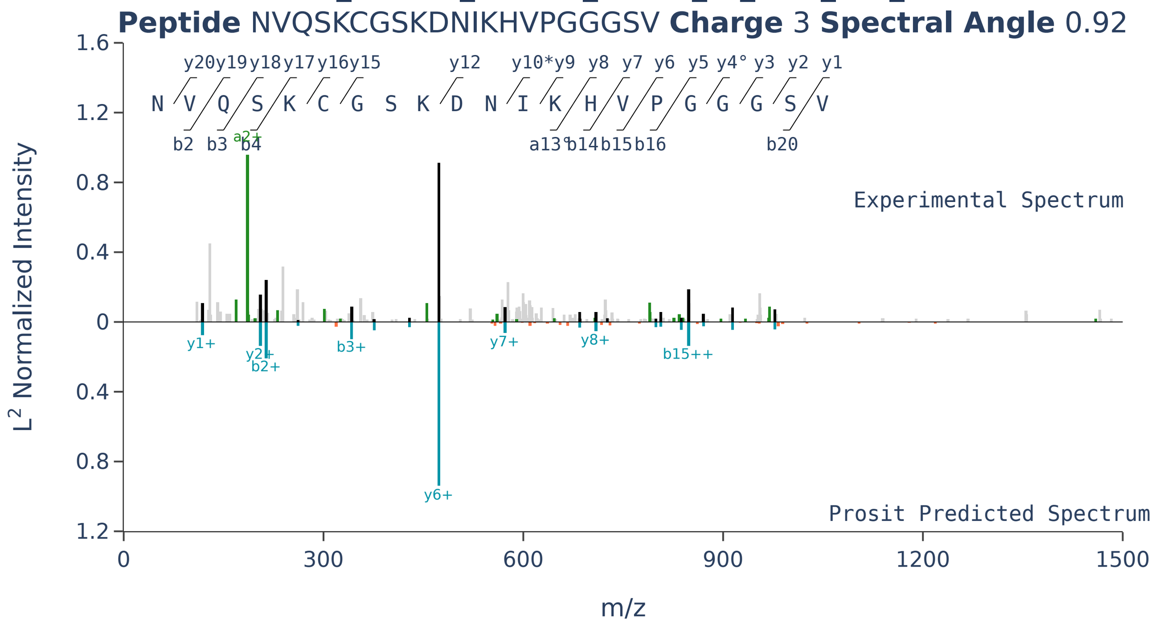

**Source** WSoh_221121_011221_HFGoe_L1_hTau_24h_R2 **Scan** 23081
**Peptide** DFKDRVQSKIGSLDNL **Charge** 2 **Spectral Angle** 0.74

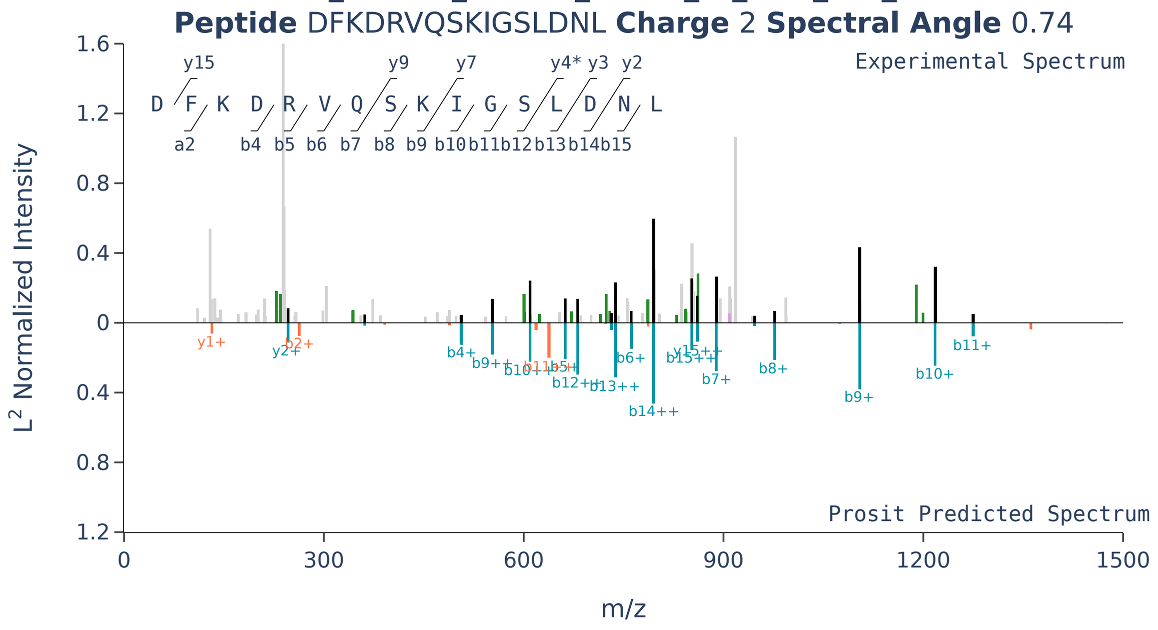


**Source** WSoh_221121_011221_HFGoe_L2_hTau_4h_R1 **Scan** 9696
**Peptide** RLPAKTPPAPKTPPSSGEPPKSGDRSGY **Charge** 5 **Spectral Angle** 0.8

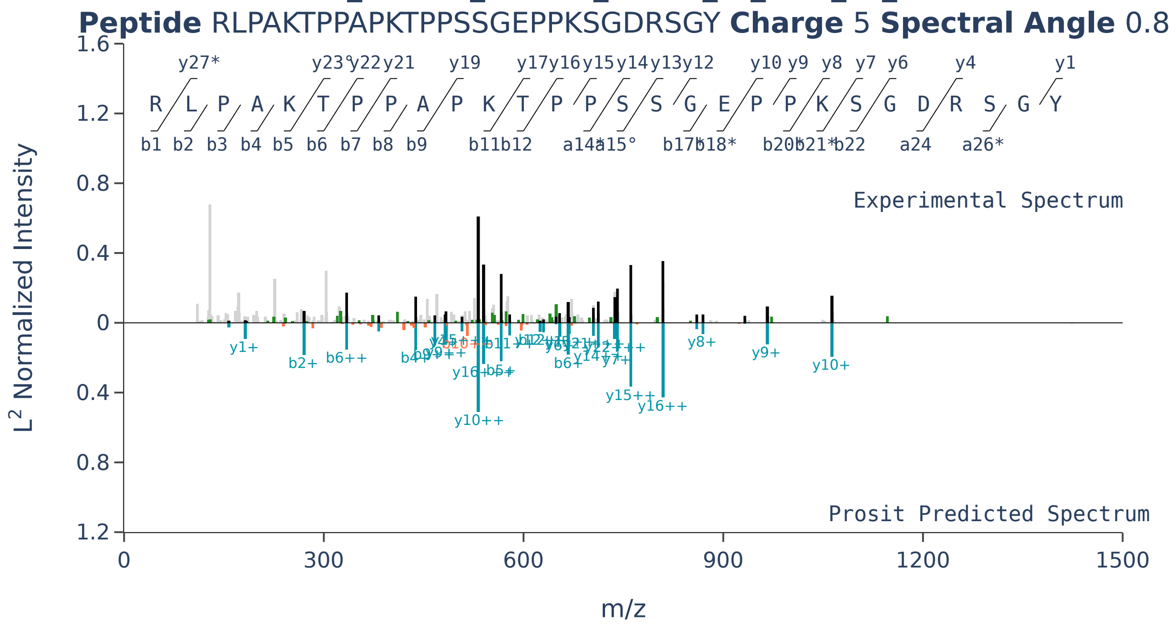


**Source** WSoh_221121_011221_HFGoe_L1_hTau_24h_R2 **Scan** 16685
**Peptide** QPGGGKVQIINKKLDLSNVQSK **Charge** 4 **Spectral Angle** 0.8

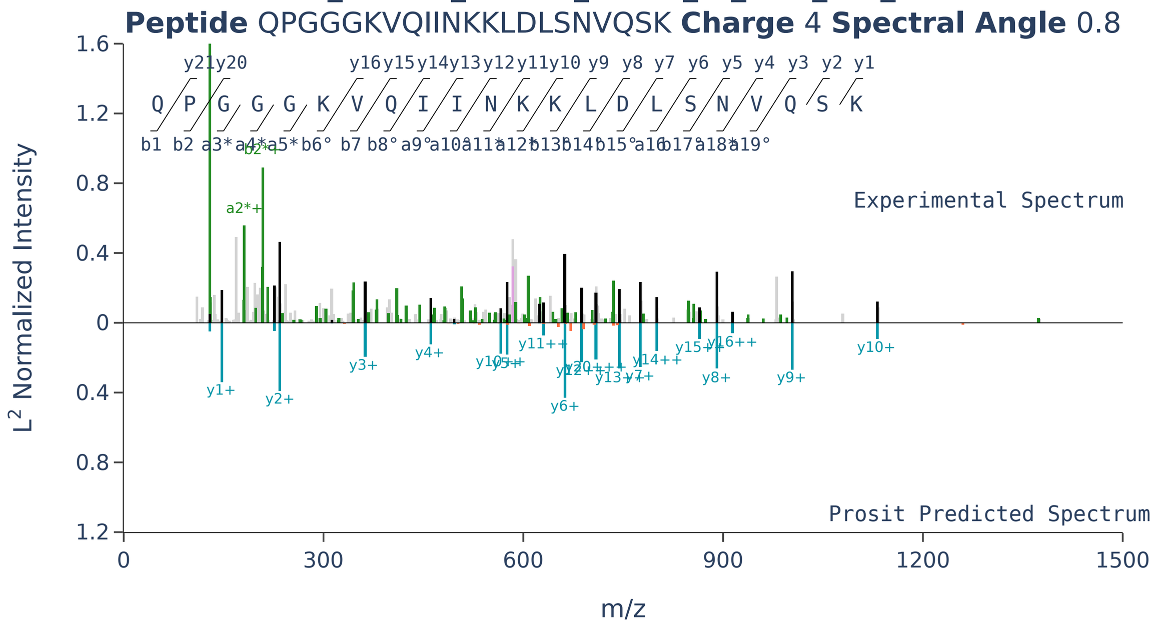


**Source** WSoh_221121_011221_HFGoe_L1_hTau_4h_R2 **Scan** 13104
**Peptide** EGAPGKQAAAQPHTEIPEGTTAEEAG **Charge** 3 **Spectral Angle** 0.74

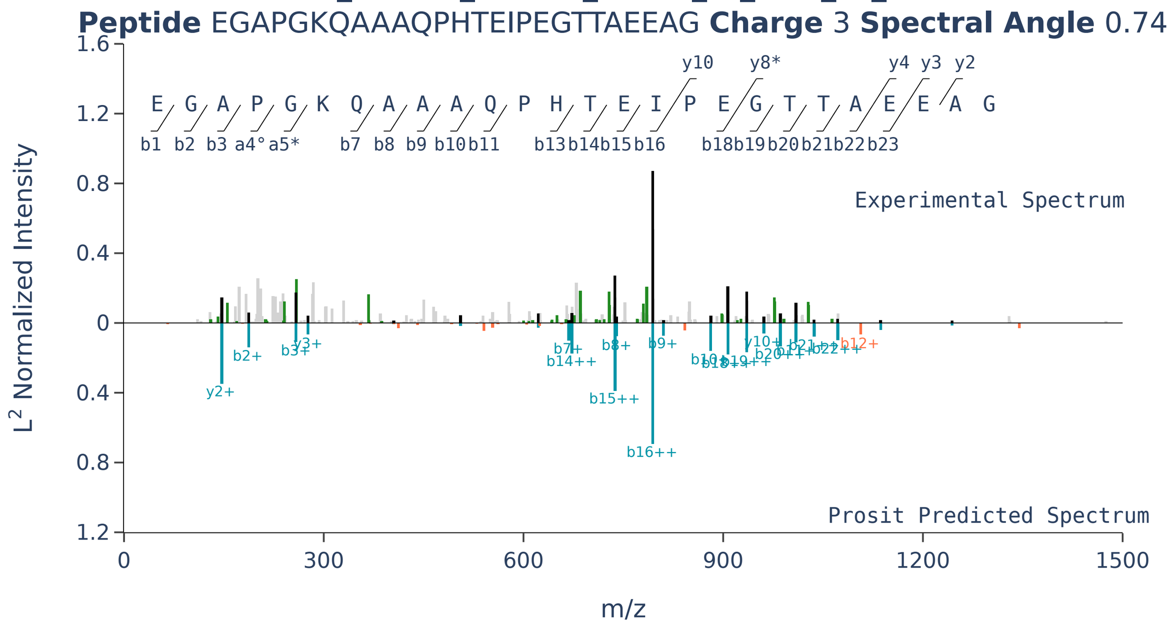


**Source** WSoh_221121_011221_HFGoe_L1_hTau_24h_R1 **Scan** 11977
**Peptide** KSKIGSTENLKHQPGGGKVQII **Charge** 3 **Spectral Angle** 0.86

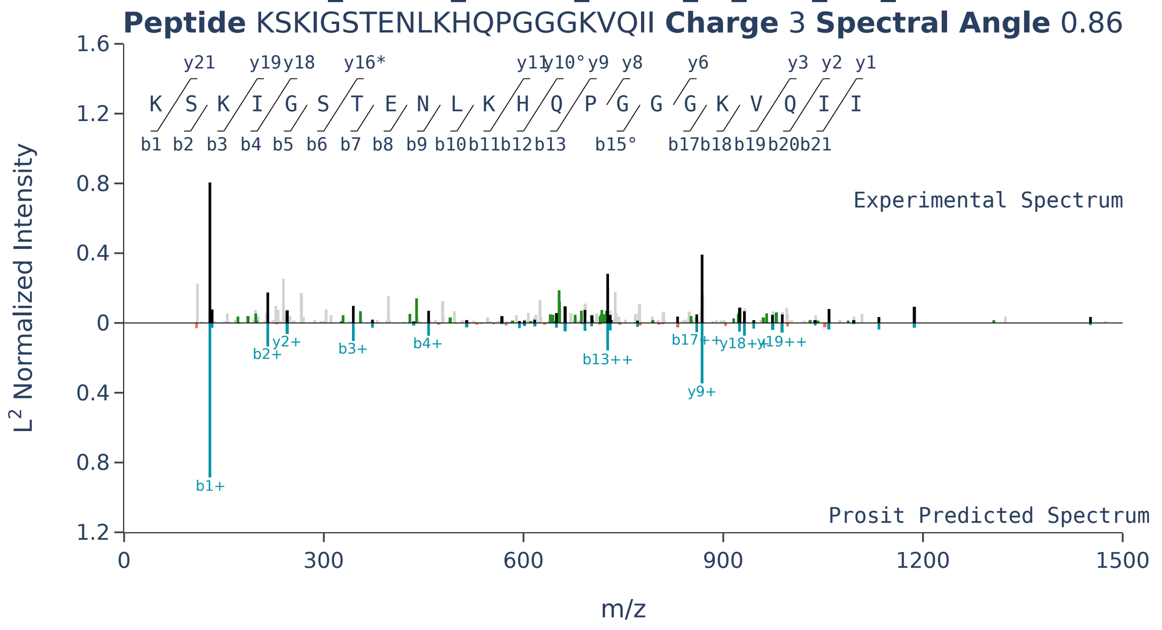


**Source** WSoh_221121_011221_HFGoe_L1_hTau_24h_R2 **Scan** 15900
**Peptide** TPPKSPSSAKSRLQTAPVPMPDLKNVKSK **Charge** 6 **Spectral Angle** 0.81

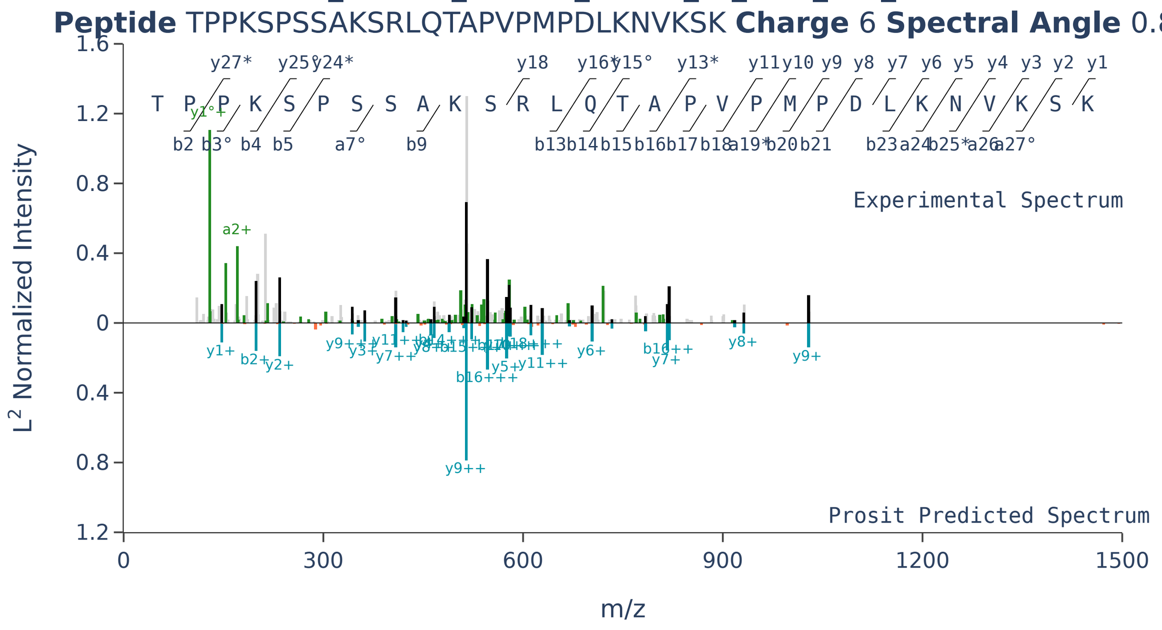


**Source** WSoh_221121_011221_HFGoe_L2_hTau_24h_R1 **Scan** 6959
**Peptide** AKTDHGAEIVYK **Charge** 3 **Spectral Angle** 0.84

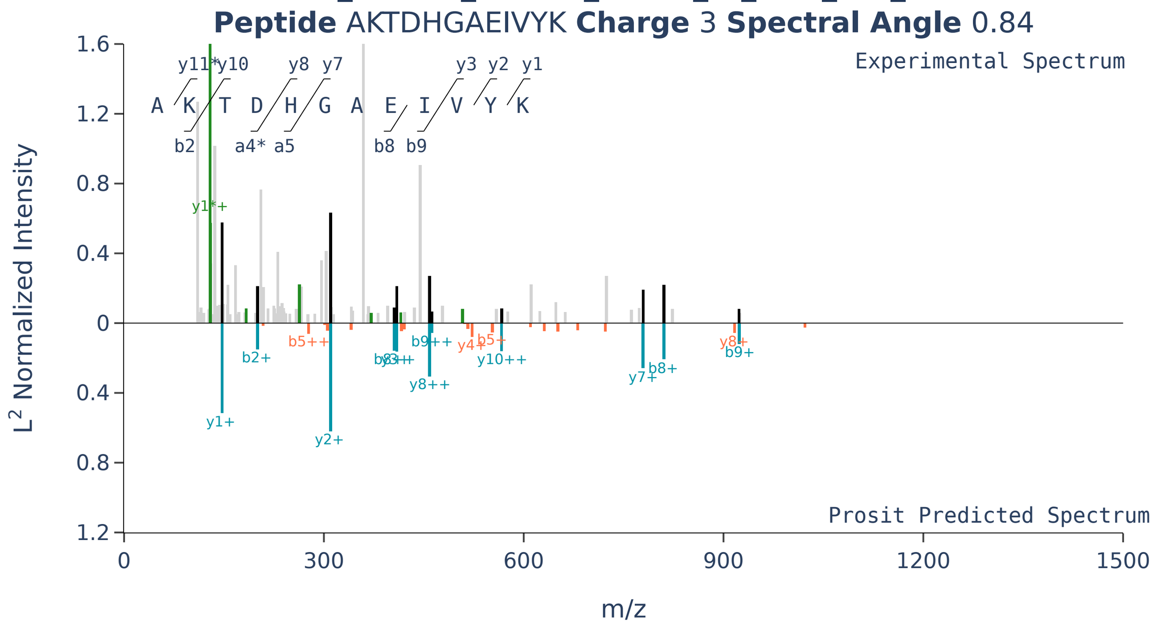


**Source** WSoh_221121_011221_HFGoe_L2_hTau_4h_R2 **Scan** 18569
**Peptide** KIGSLDNITHVPGGGNKKIETHKLTF **Charge** 4 **Spectral Angle** 0.85

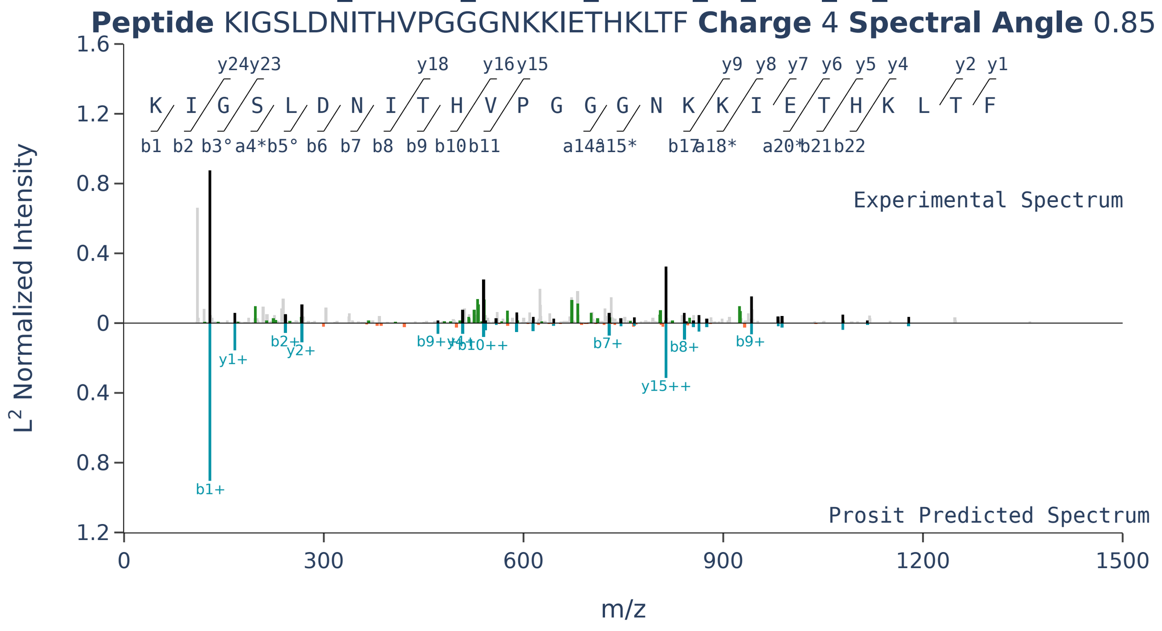


**Source** WSoh_221121_011221_HFGoe_L1_hTau_24h_R1 **Scan** 19077
**Peptide** KLDFKDRVQSKIGSL **Charge** 4 **Spectral Angle** 0.9

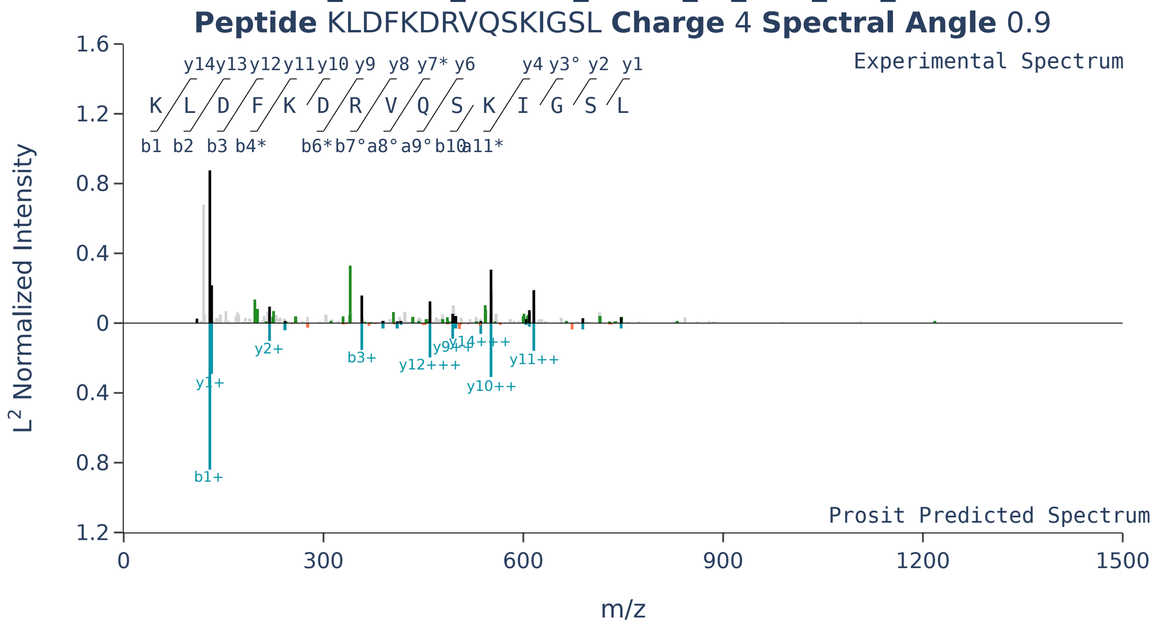


**Source** WSoh_221121_011221_HFGoe_L1_hTau_4h_R1 **Scan** 5370
**Peptide** TFRENAKAKTDHGAE **Charge** 3 **Spectral Angle** 0.78

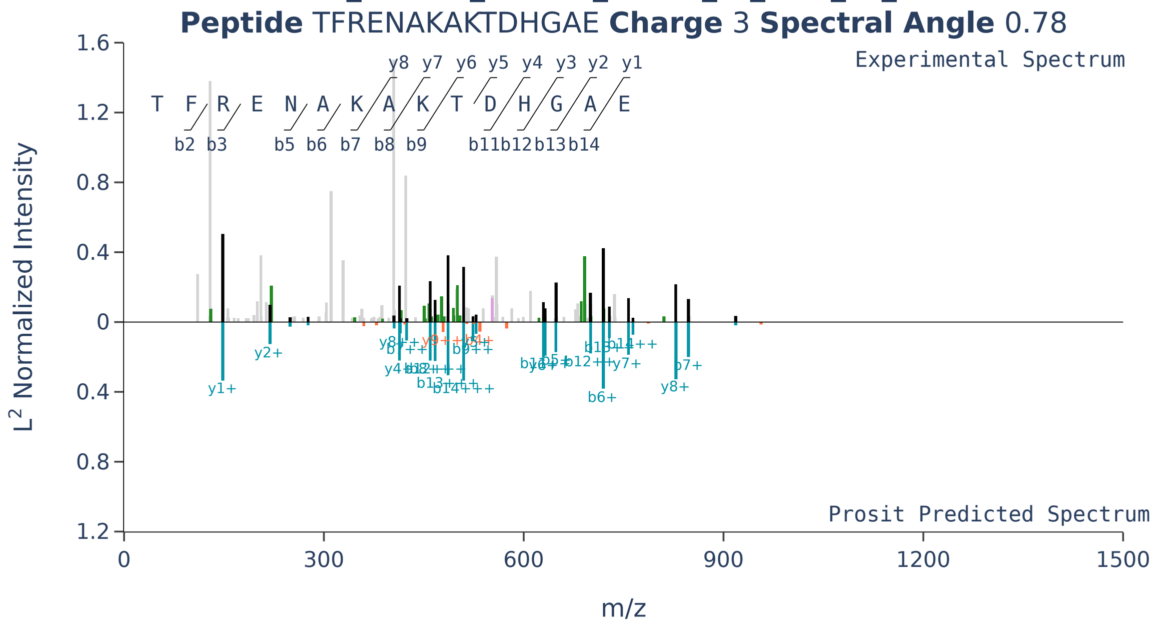


**Source** WSoh_221121_011221_HFGoe_L1_hTau_24h_R1 **Scan** 12105
**Peptide** GNIHHKPGGGQVEVKSEKLDF **Charge** 3 **Spectral Angle** 0.8

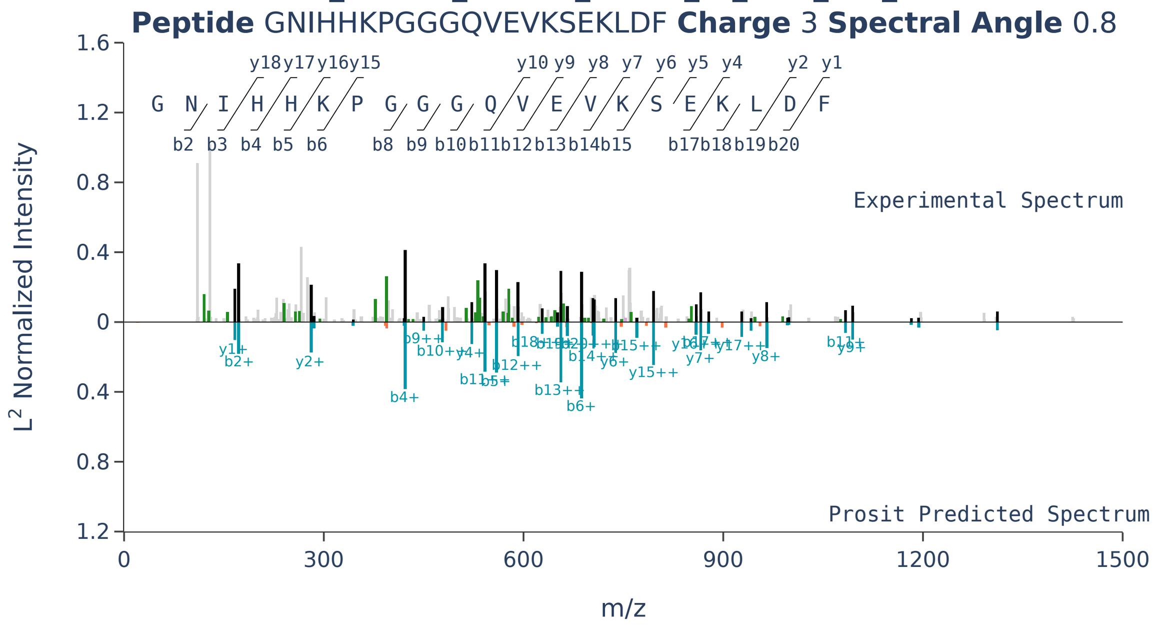


**Source** WSoh_221121_011221_HFGoe_L1_hTau_24h_R2 **Scan** 7729
**Peptide** NLKHVPGGGSVQ **Charge** 3 **Spectral Angle** 0.91

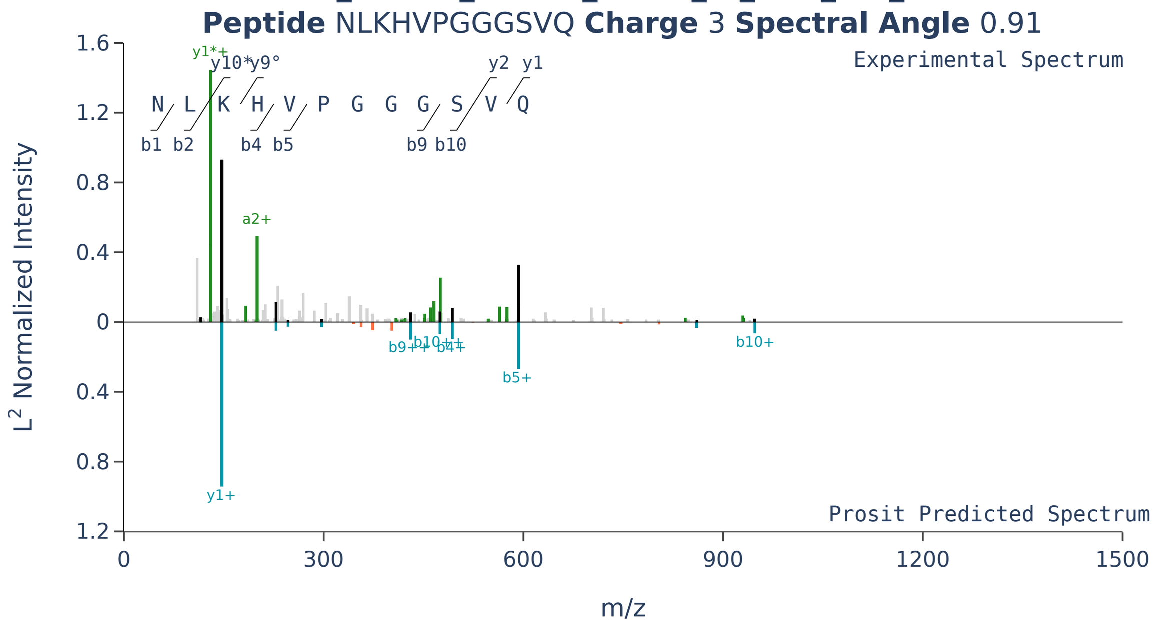


**Source** WSoh_221121_011221_HFGoe_L2_hTau_24h_R2 **Scan** 5438
**Peptide** THVPGGGNKKIETHK **Charge** 3 **Spectral Angle** 0.9

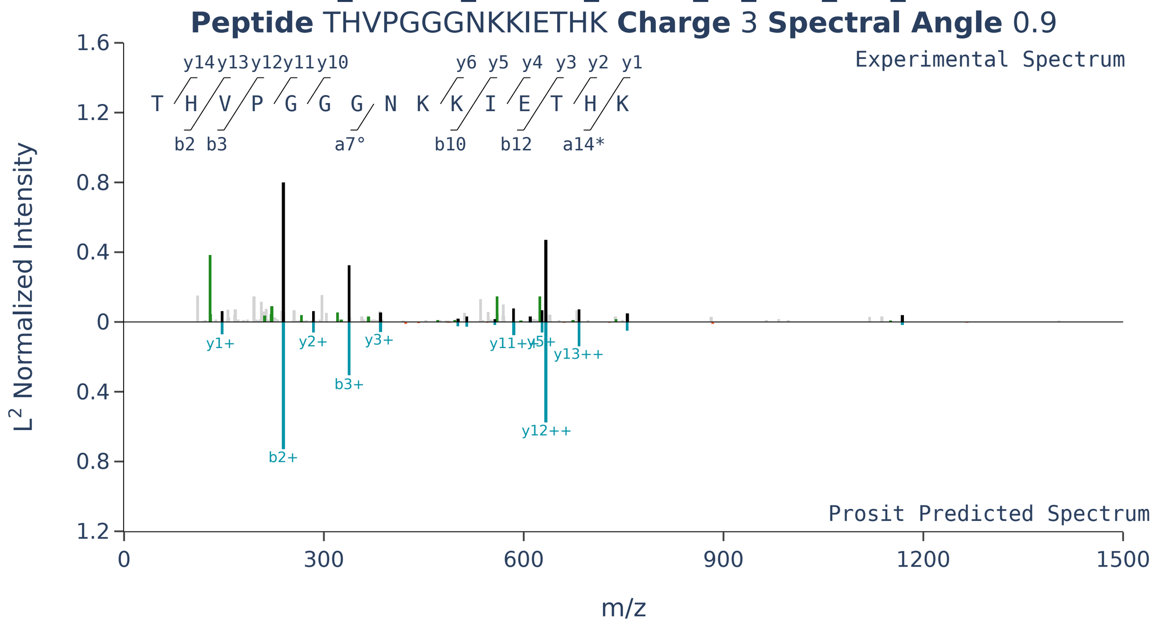


**Source** WSoh_221121_011221_HFGoe_L1_hTau_4h_R1 **Scan** 6724
**Peptide** NAKAKTDHGAEIVYK **Charge** 3 **Spectral Angle** 0.75

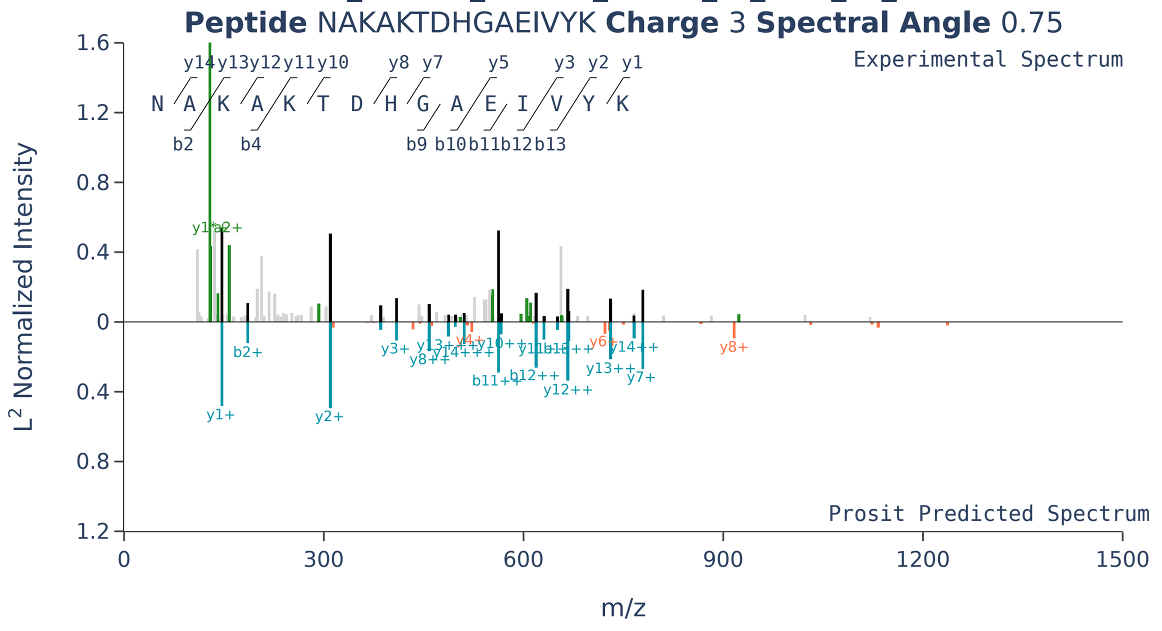


**Source** WSoh_221121_011221_HFGoe_L2_hTau_24h_R2 **Scan** 6478
**Peptide** LSNVQSKCGSKDNIKHV **Charge** 3 **Spectral Angle** 0.84

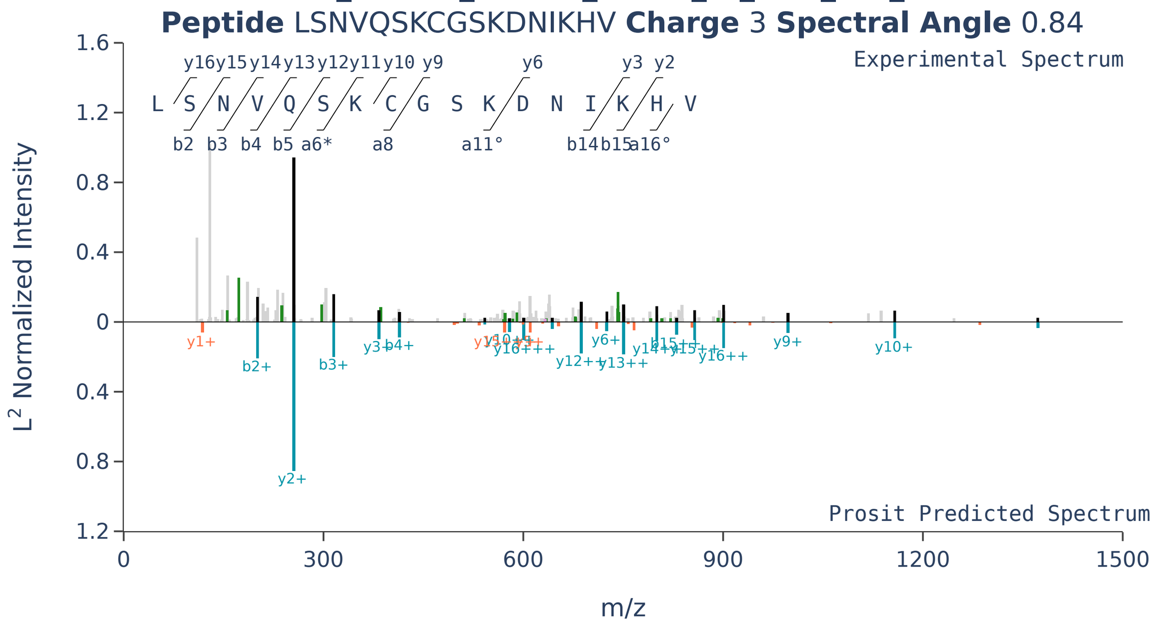

**Source** WSoh_221121_011221_HFGoe_L1_hTau_4h_R1 **Scan** 21982
**Peptide** QIVYKPVDLSKVTSKCGSL **Charge** 3 **Spectral Angle** 0.79

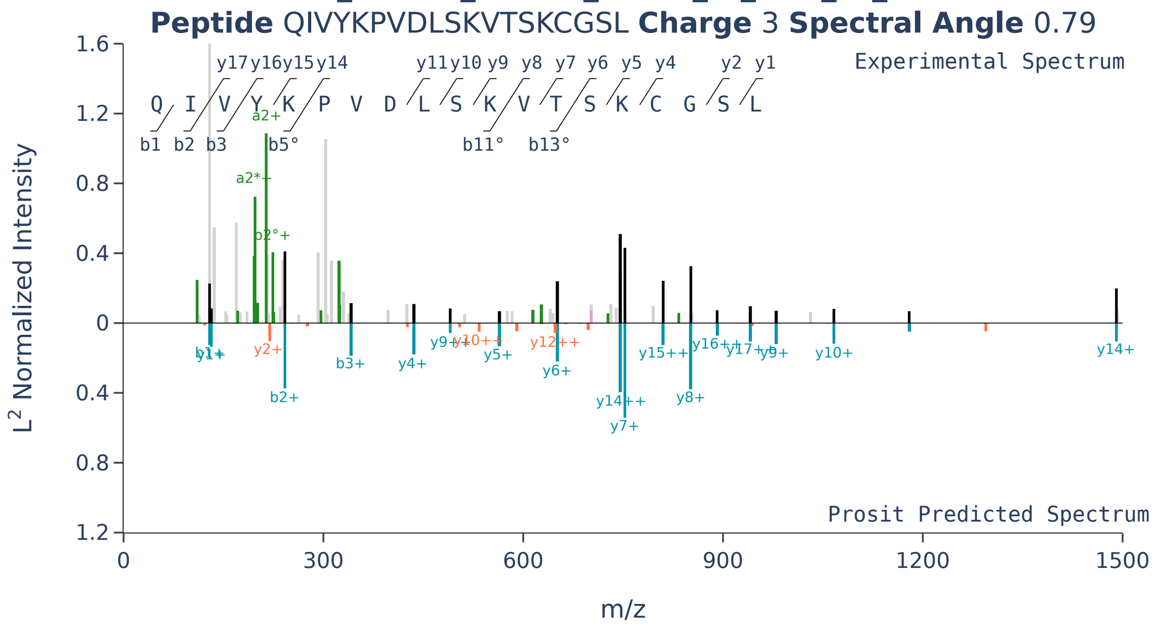


**Source** WSoh_221121_011221_HFGoe_L1_hTau_4h_R2 **Scan** 11829
**Peptide** QPGGGKVQL **Charge** 2 **Spectral Angle** 0.64

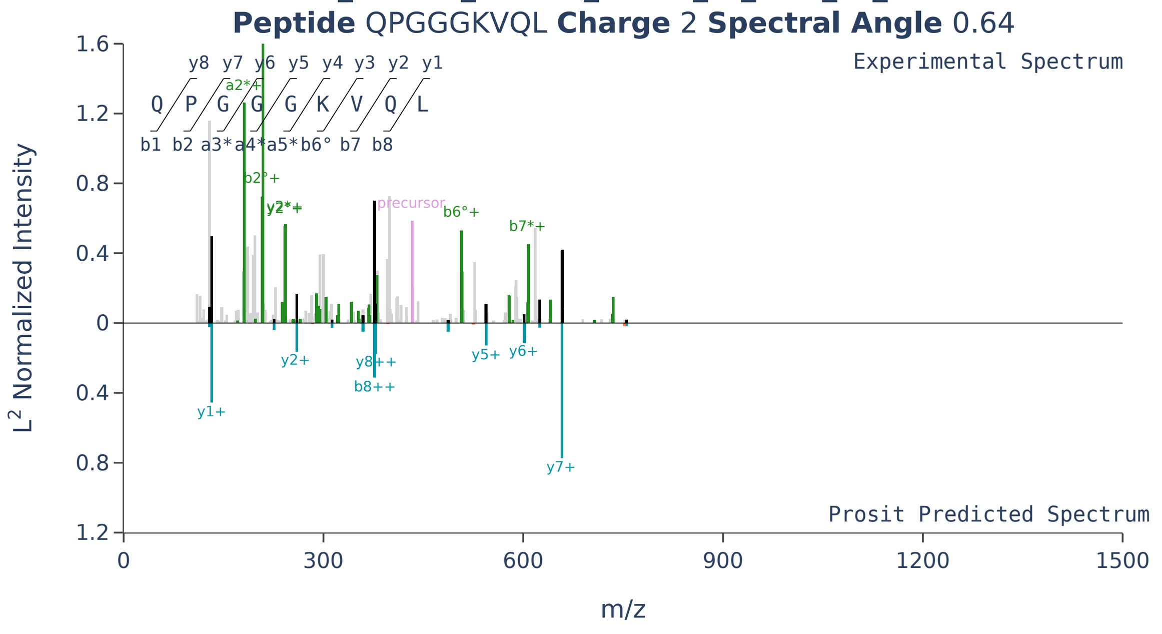


**Source** WSoh_221121_011221_HFGoe_L1_hTau_24h_R1 **Scan** 16350
**Peptide** GSLDNITHVPGGGNKKIETHKLTFRENA **Charge** 3 **Spectral Angle** 0.7

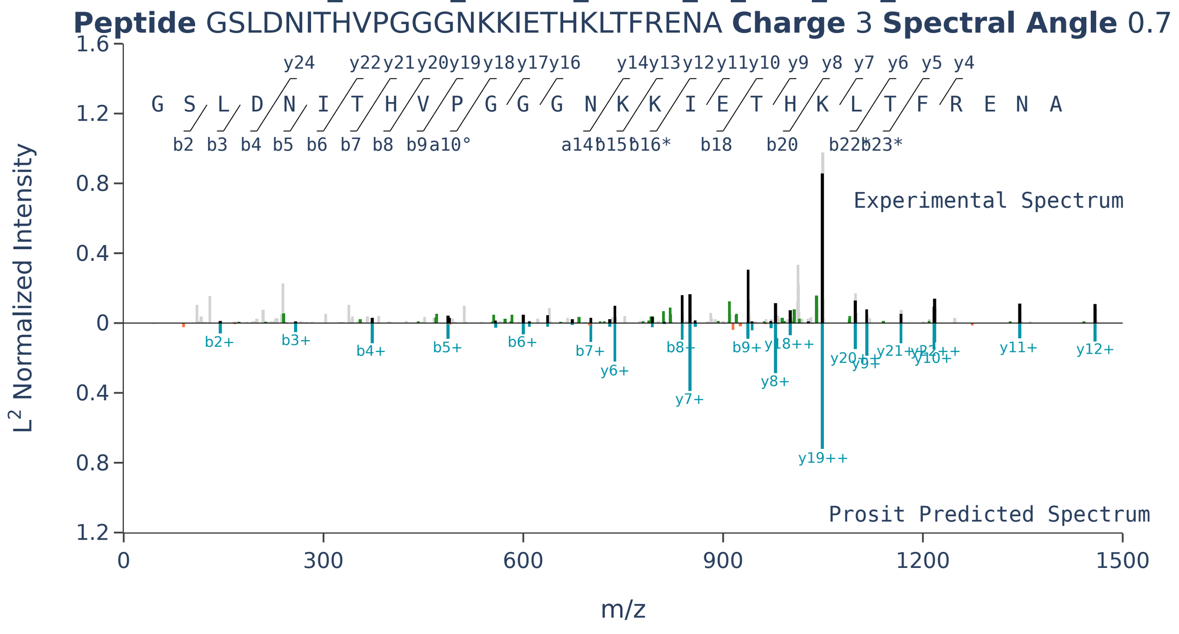


**Source** WSoh_221121_011221_HFGoe_L1_hTau_24h_R2 **Scan** 12261
**Peptide** DFKDRVQSKLG **Charge** 3 **Spectral Angle** 0.87

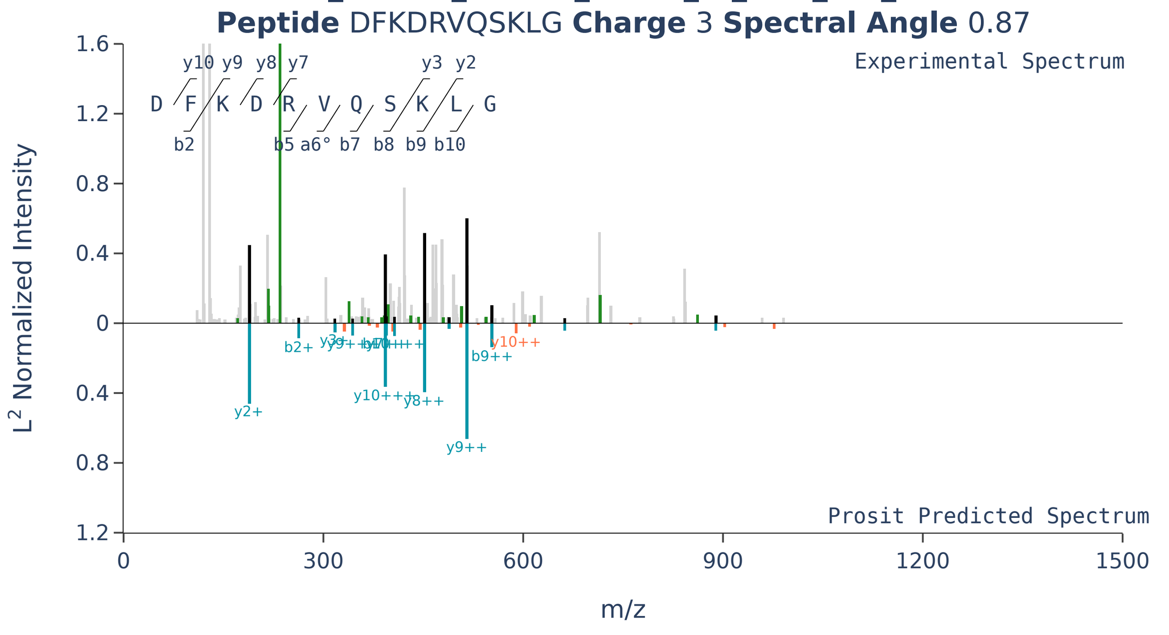


**Source** WSoh_221121_011221_HFGoe_L2_hTau_1h_R2 **Scan** 5855
**Peptide** GGGNKKIETHKL **Charge** 2 **Spectral Angle** 0.79

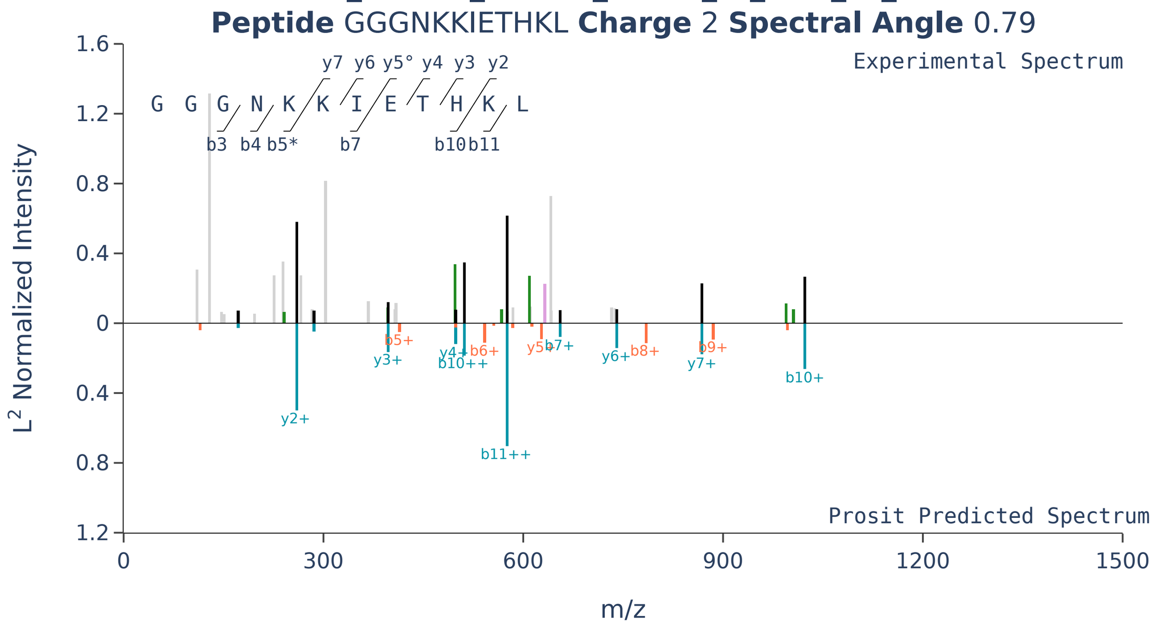


**Source** WSoh_221121_011221_HFGoe_L2_hTau_4h_R2 **Scan** 20752
**Peptide** KHVPGGGSVQIVYKPVDLSKV **Charge** 4 **Spectral Angle** 0.83

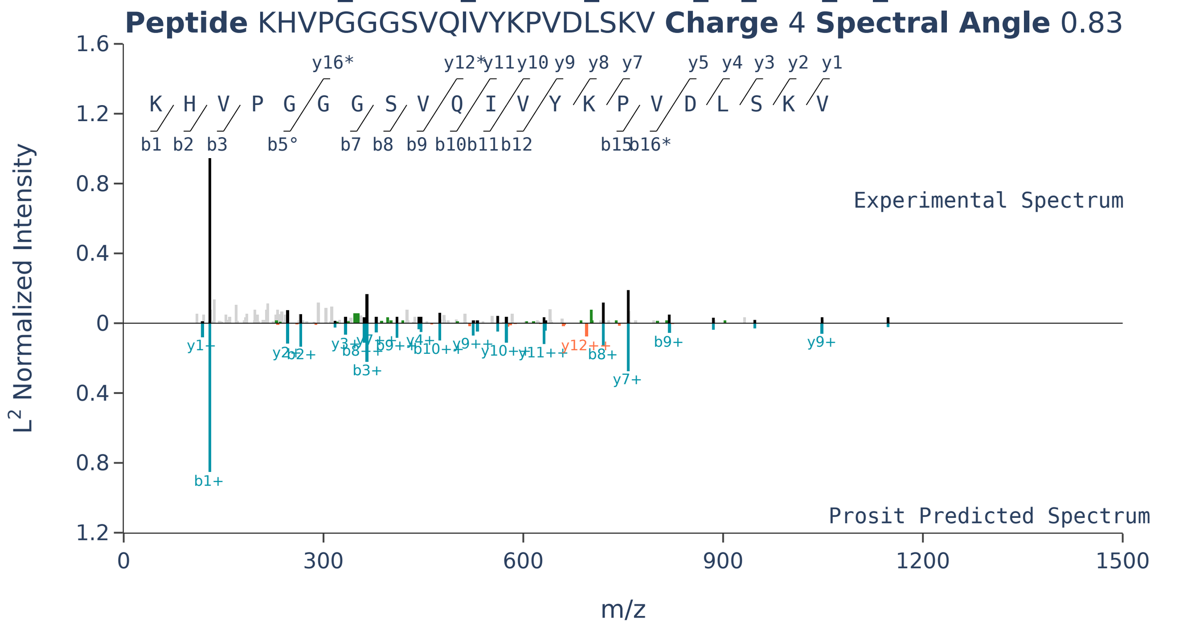


**Source** WSoh_221121_011221_HFGoe_L2_hTau_24h_R1 **Scan** 15637
**Peptide** KTDHGAEIVYKSPVVSGDTSPRHLSNVS **Charge** 3 **Spectral Angle** 0.78

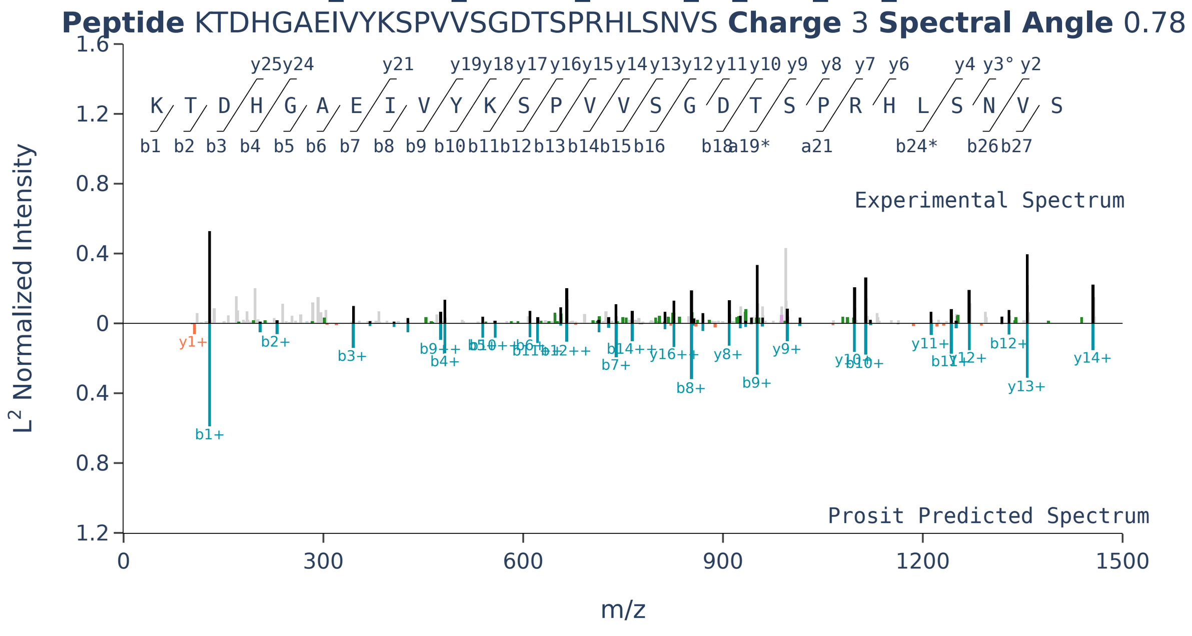


**Source** WSoh_221121_011221_HFGoe_L1_hTau_24h_R2 **Scan** 8549
**Peptide** RLPAKTPPAPKTPPSSGEPPKSGDRS **Charge** 3 **Spectral Angle** 0.77

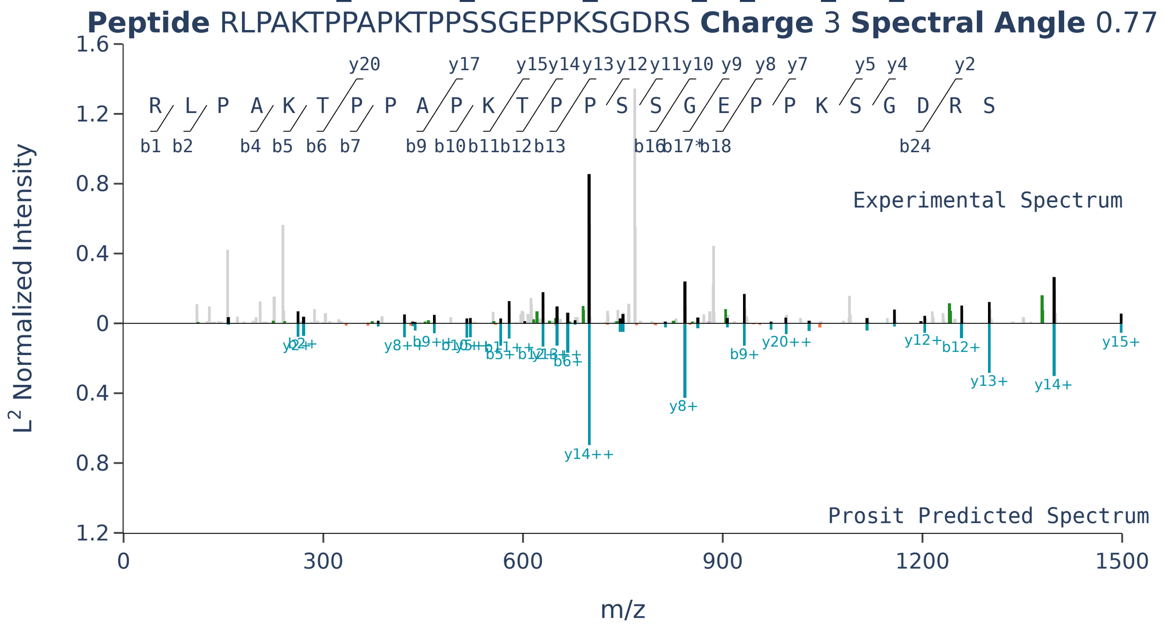


**Source** WSoh_221121_011221_HFGoe_L1_hTau_24h_R2 **Scan** 19066
**Peptide** SPSSAKSRLQTAPVPMPDLKNVKS **Charge** 4 **Spectral Angle** 0.85

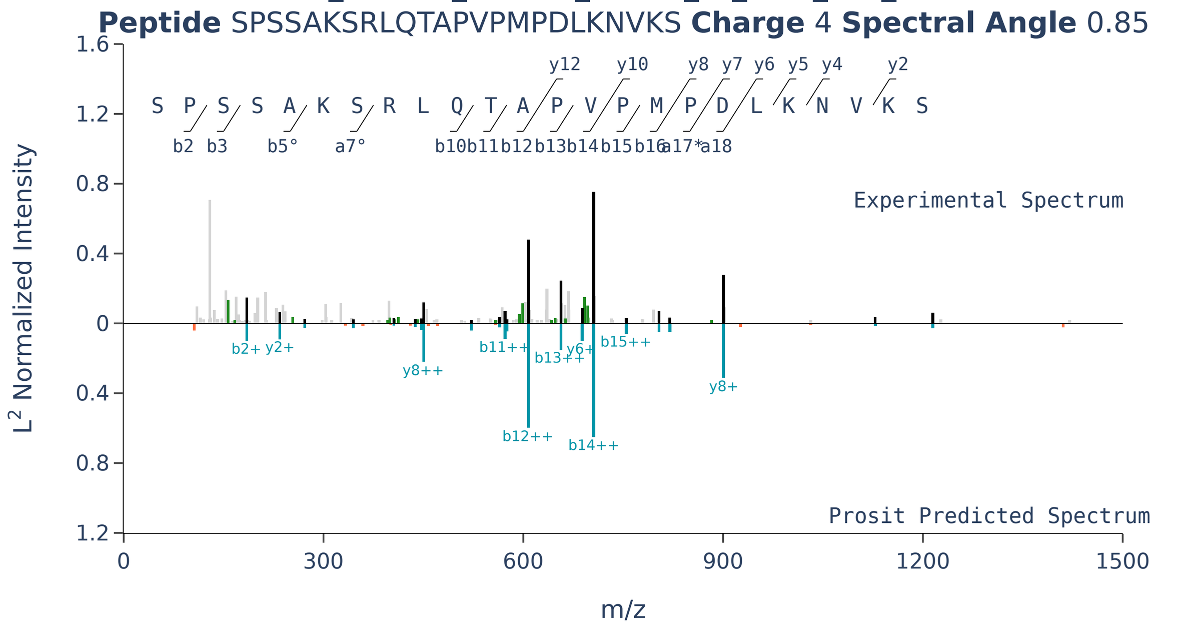


**Source** WSoh_221121_011221_HFGoe_L2_hTau_2h_R1 **Scan** 13383
**Peptide** KDRVQSKIGSLDNITHVPGGGNKKIETH **Charge** 6 **Spectral Angle** 0.85

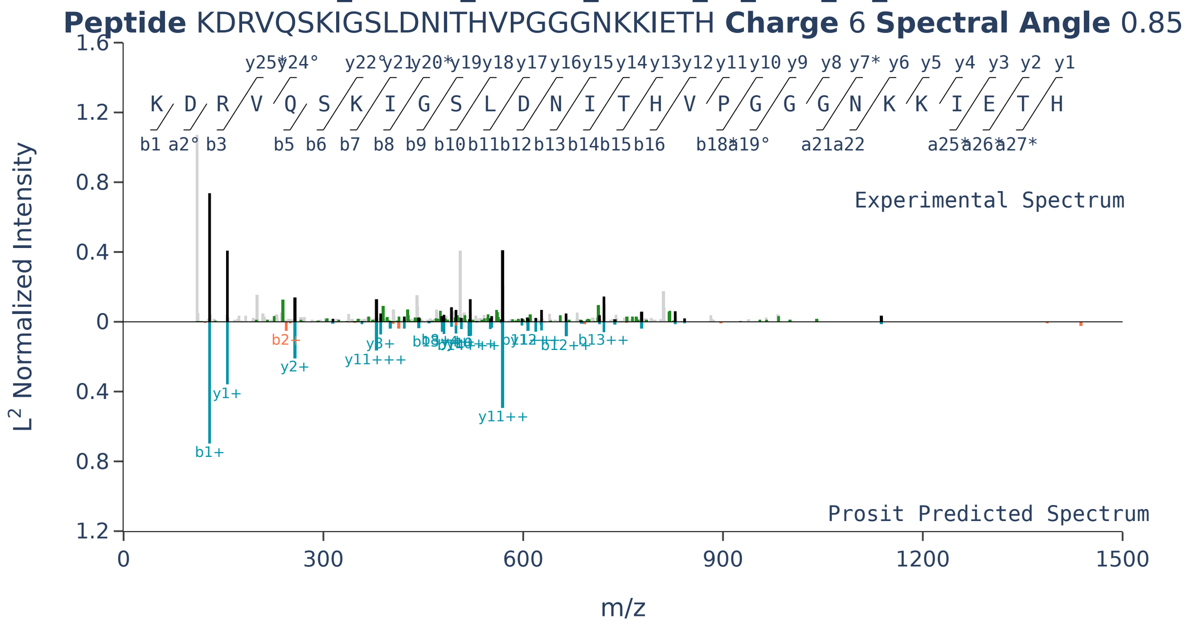


**Source** WSoh_221121_011221_HFGoe_L2_hTau_24h_R2 **Scan** 5890
**Peptide** GSTENLKHQPGGGKVQ **Charge** 2 **Spectral Angle** 0.81

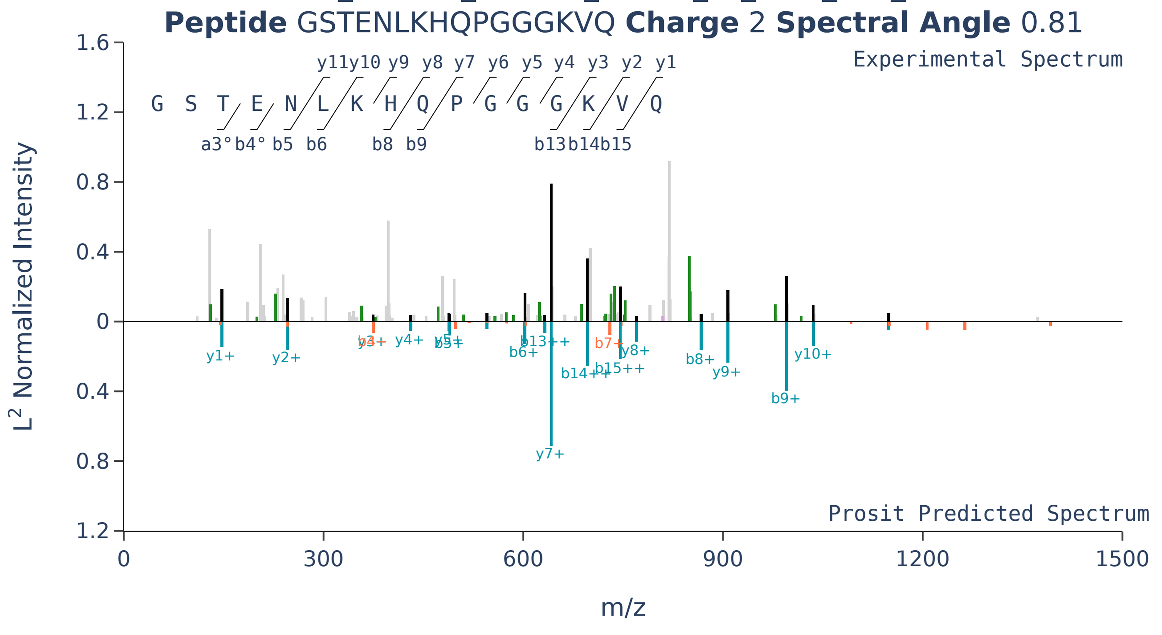


**Source** WSoh_221121_011221_HFGoe_L1_hTau_24h_R1 **Scan** 23481
**Peptide** VPGGGSVQIVYKPVDLSKVTSK **Charge** 3 **Spectral Angle** 0.85

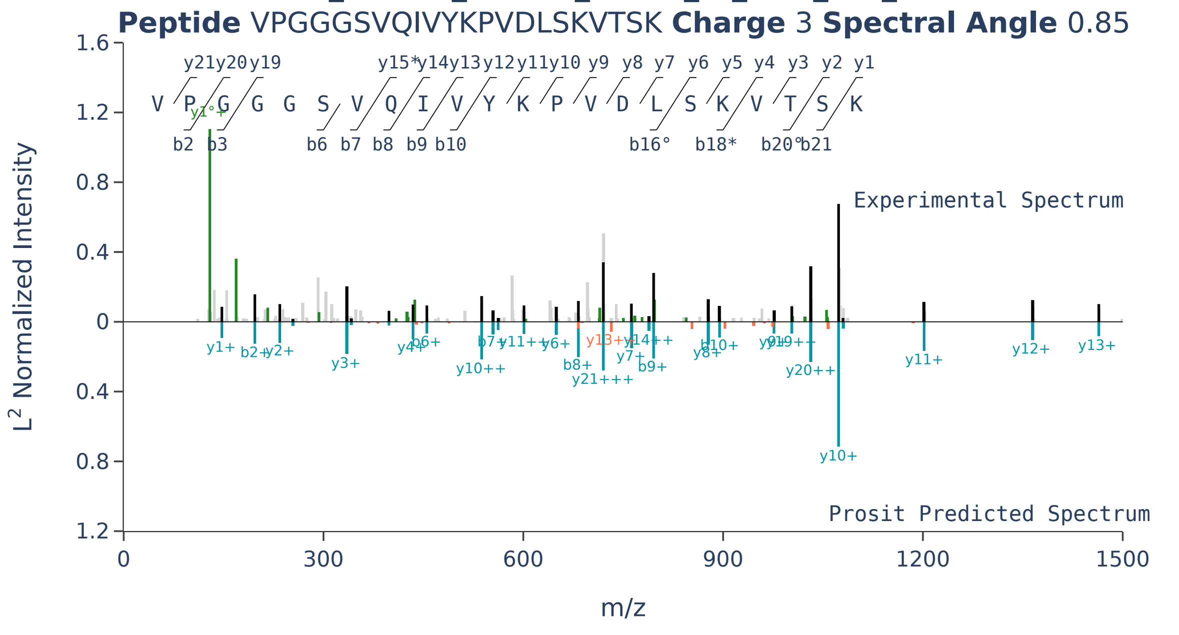


**Source** WSoh_221121_011221_HFGoe_L2_hTau_24h_R2 **Scan** 16744
**Peptide** SKIGSLDNITHVPGGGNKKL **Charge** 4 **Spectral Angle** 0.86

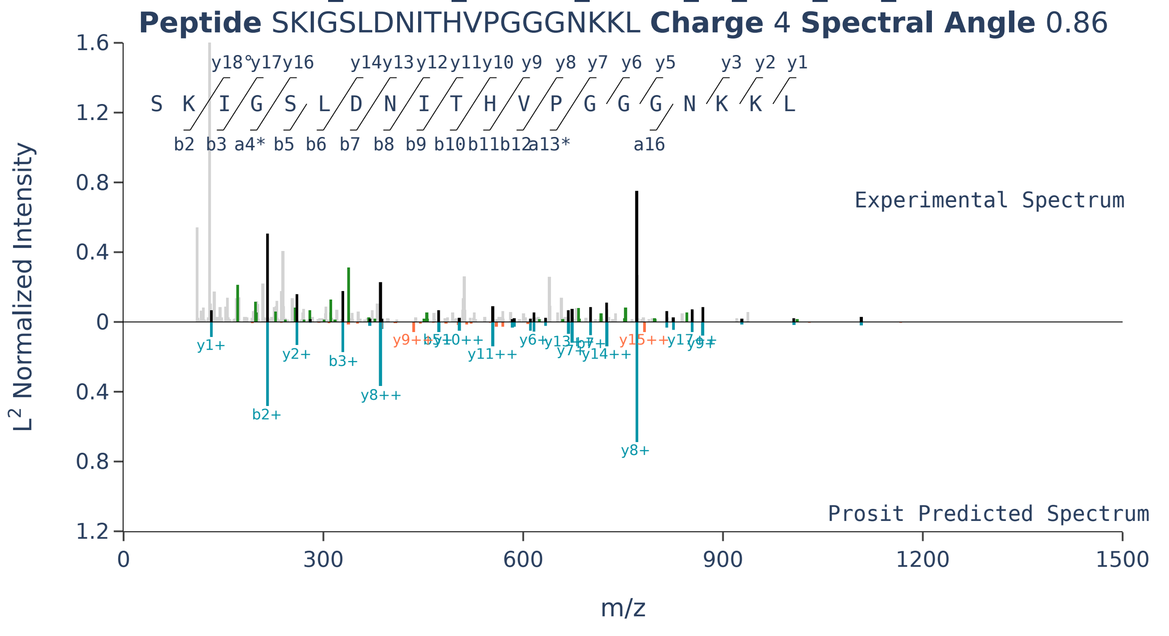


**Source** WSoh_221121_011221_HFGoe_L1_hTau_4h_R2 **Scan** 12316
**Peptide** KLGSLDNITHVPGGGNKKIETHK **Charge** 5 **Spectral Angle** 0.85

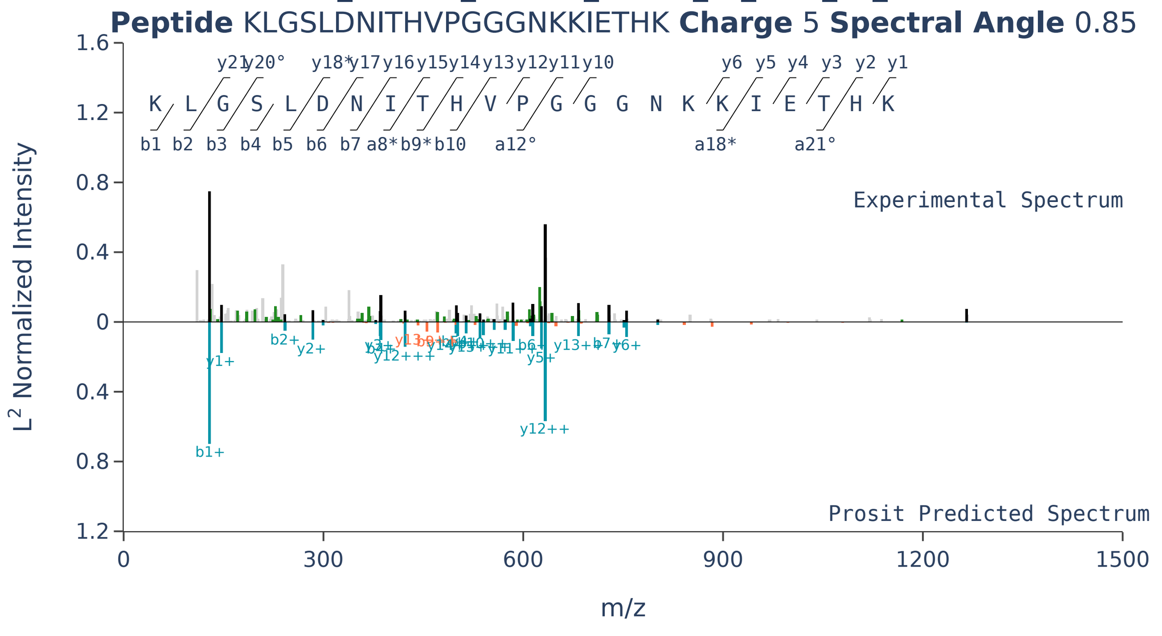


**Source** WSoh_221121_011221_HFGoe_L1_hTau_4h_R2 **Scan** 10765
**Peptide** DEAAGHVTQARMVS **Charge** 2 **Spectral Angle** 0.82

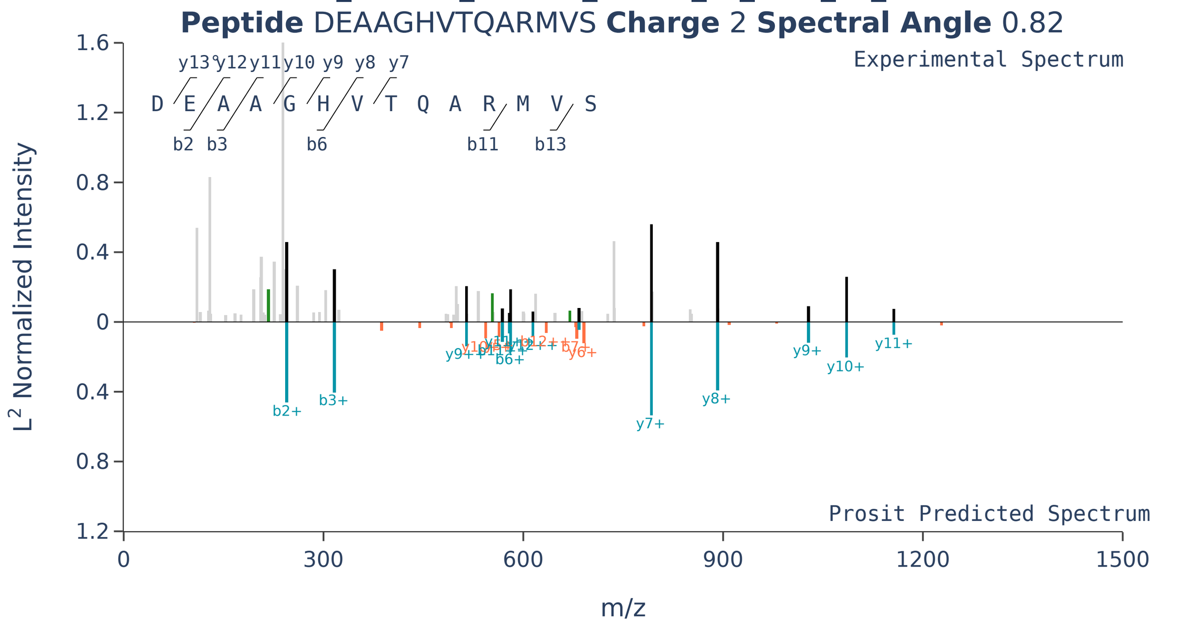

**Source** WSoh_221121_011221_HFGoe_L1_hTau_24h_R1 **Scan** 10613
**Peptide** HAGTYGLGDRKDQGGYTMHQDQEGDTD **Charge** 4 **Spectral Angle** 0.83

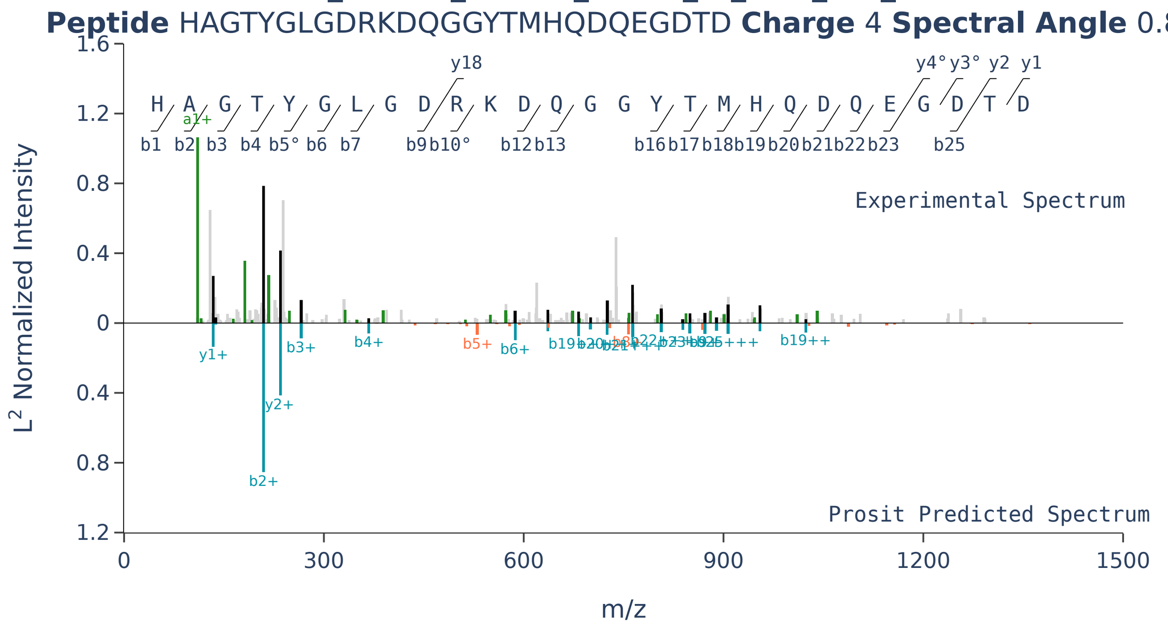


**Source** WSoh_221121_011221_HFGoe_L2_hTau_24h_R1 **Scan** 10356
**Peptide** IATPRGAAPPGQKGQANATRIPAKT **Charge** 3 **Spectral Angle** 0.74

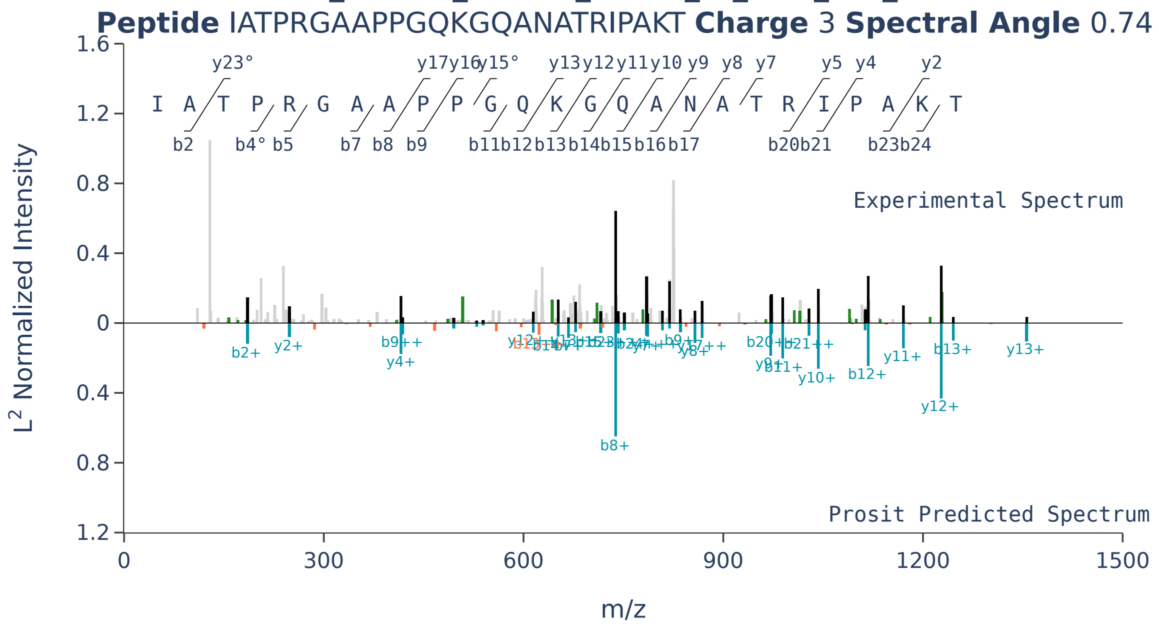


**Source** WSoh_221121_011221_HFGoe_L2_hTau_24h_R1 **Scan** 11054
**Peptide** KLGSTENLKHQPGGGKVQI **Charge** 3 **Spectral Angle** 0.9

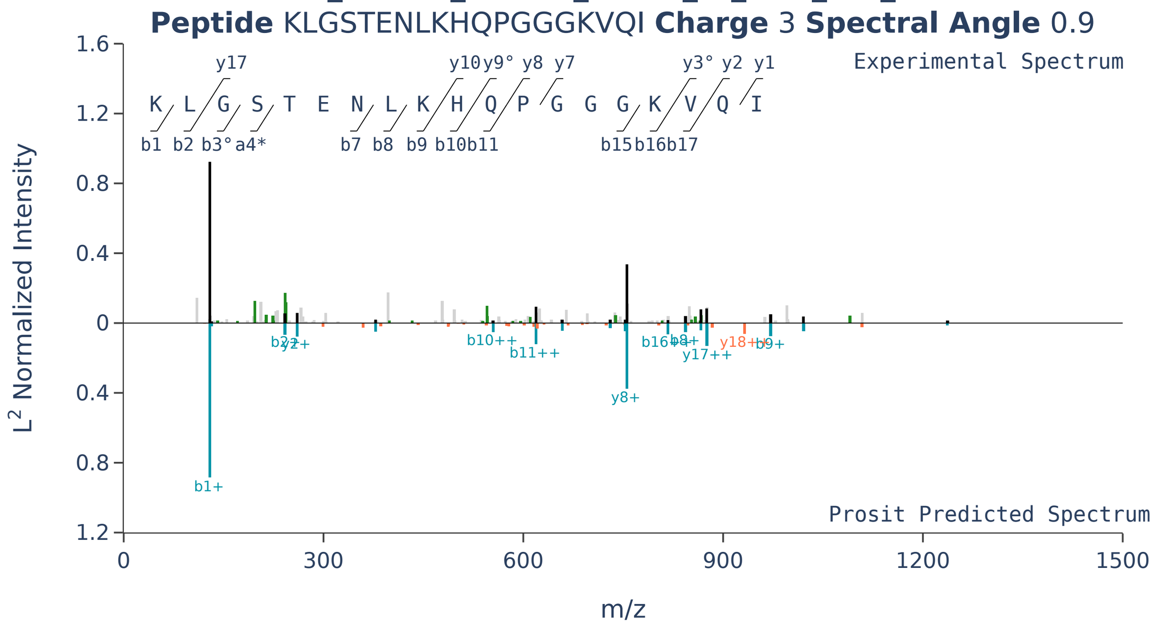


**Source** WSoh_221121_011221_HFGoe_L1_hTau_24h_R1 **Scan** 9302
**Peptide** SNVSSTGSLD **Charge** 2 **Spectral Angle** 0.82

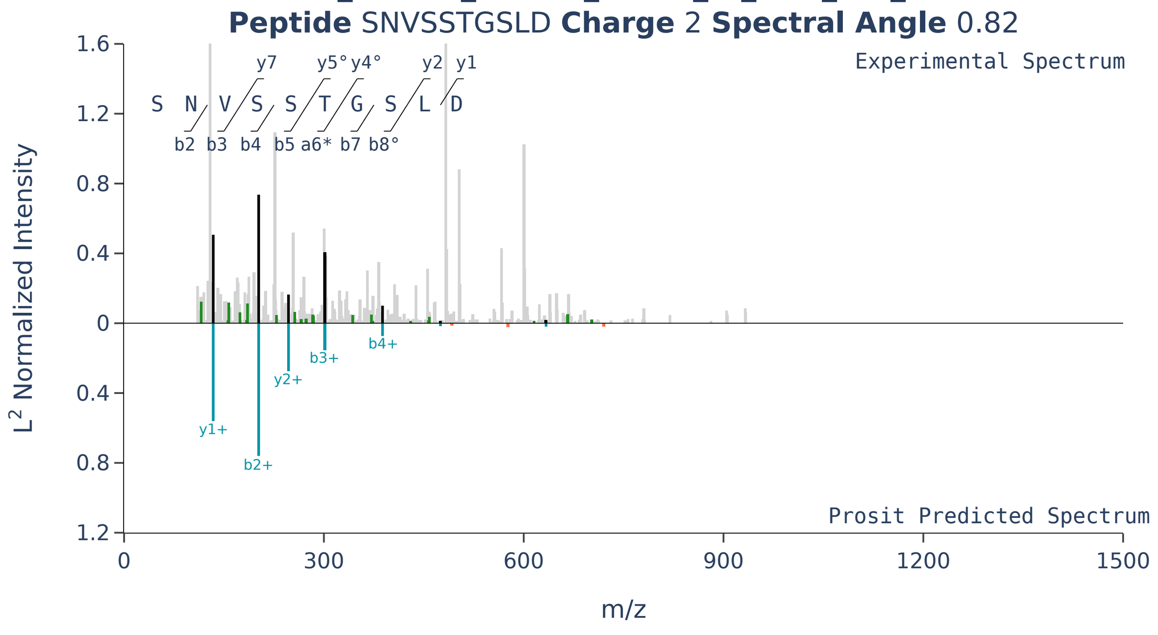


**Source** WSoh_221121_011221_HFGoe_L2_hTau_24h_R2 **Scan** 5633
**Peptide** LHHKPGGGQVEVKS **Charge** 3 **Spectral Angle** 0.86

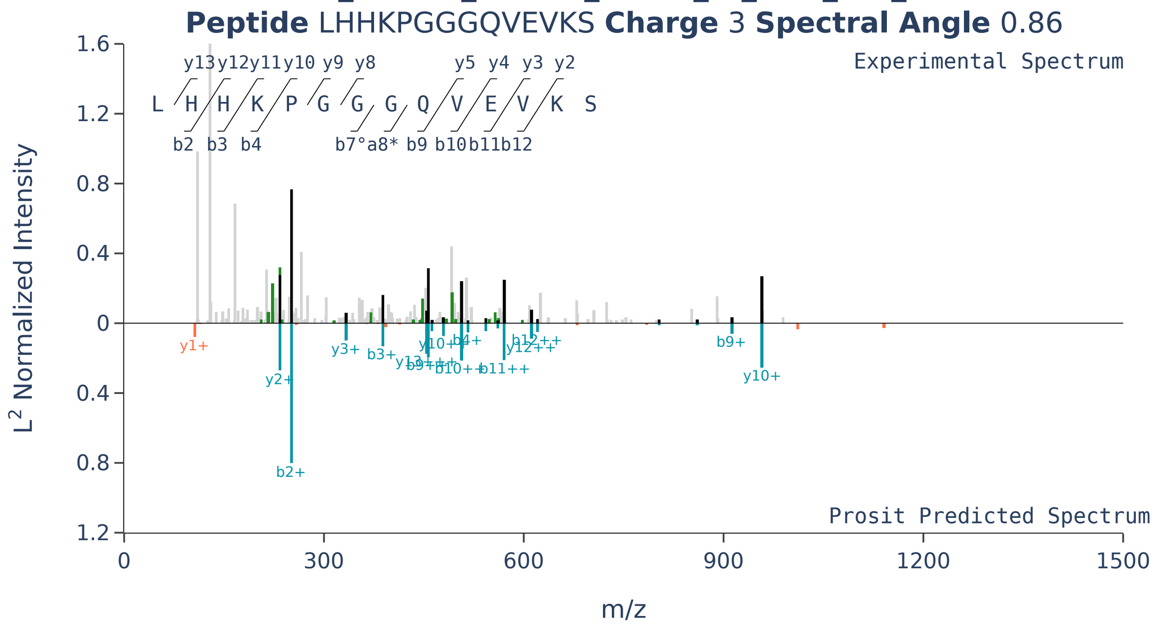


**Source** WSoh_221121_011221_HFGoe_L2_hTau_24h_R1 **Scan** 12846
**Peptide** KHQPGGGKVQIINKKLDLS **Charge** 3 **Spectral Angle** 0.9

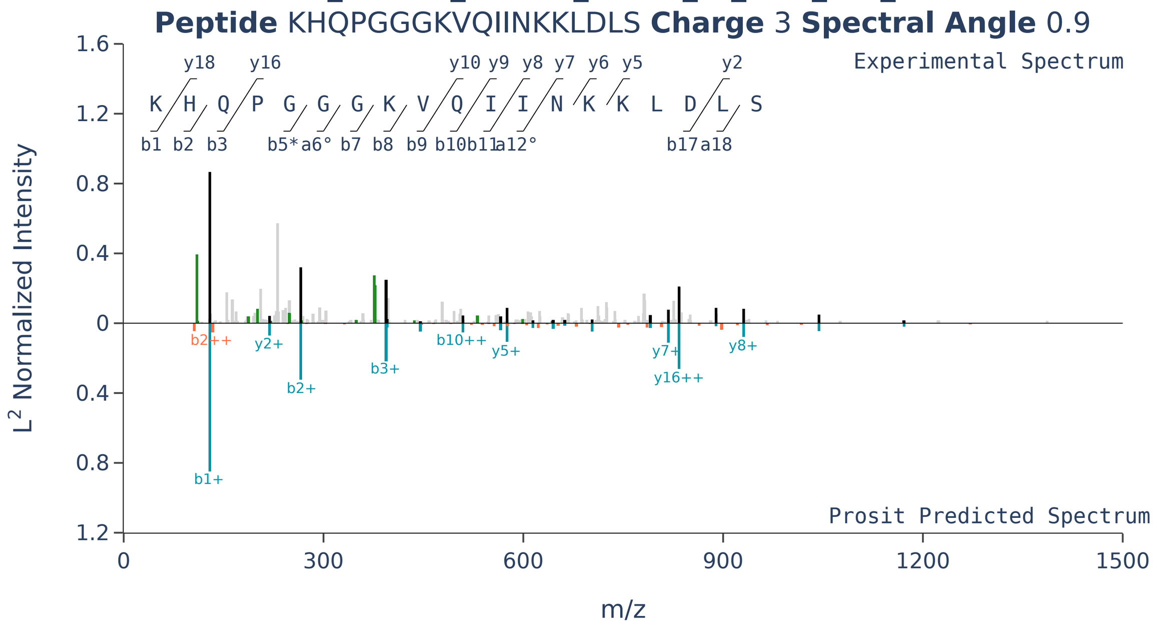


**Source** WSoh_221121_011221_HFGoe_L2_hTau_24h_R1 **Scan** 9893
**Peptide** DGKTKIATPRGAAPPGQKGQANATRL **Charge** 5 **Spectral Angle** 0.81

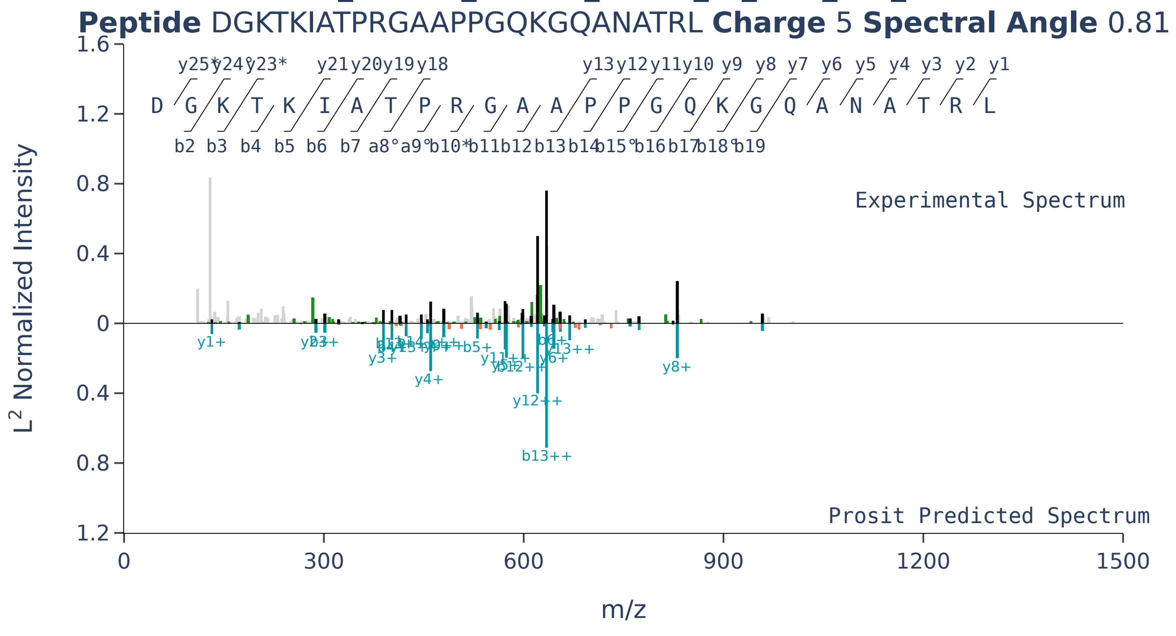


**Source** WSoh_221121_011221_HFGoe_L1_hTau_24h_R1 **Scan** 15525
**Peptide** DLSNVQSKCGSKDNIKHVPGGGSVQIVYK **Charge** 4 **Spectral Angle** 0.85

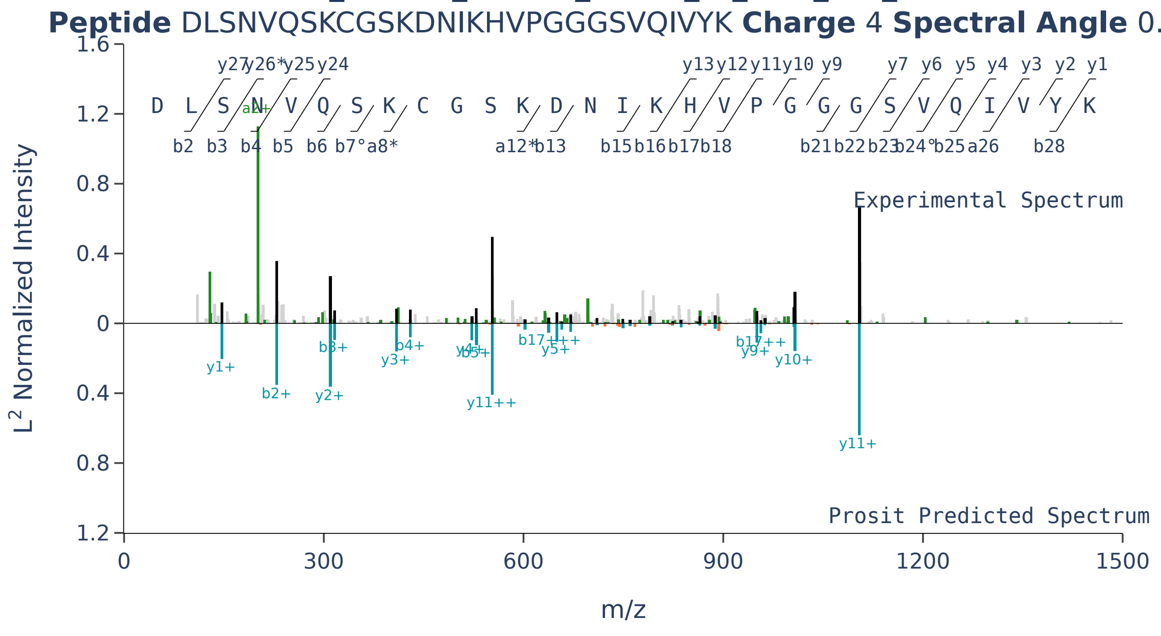


**Source** WSoh_221121_011221_HFGoe_L2_hTau_4h_R2 **Scan** 13686
**Peptide** AKAKTDHGAEIVYKSPVV **Charge** 2 **Spectral Angle** 0.8

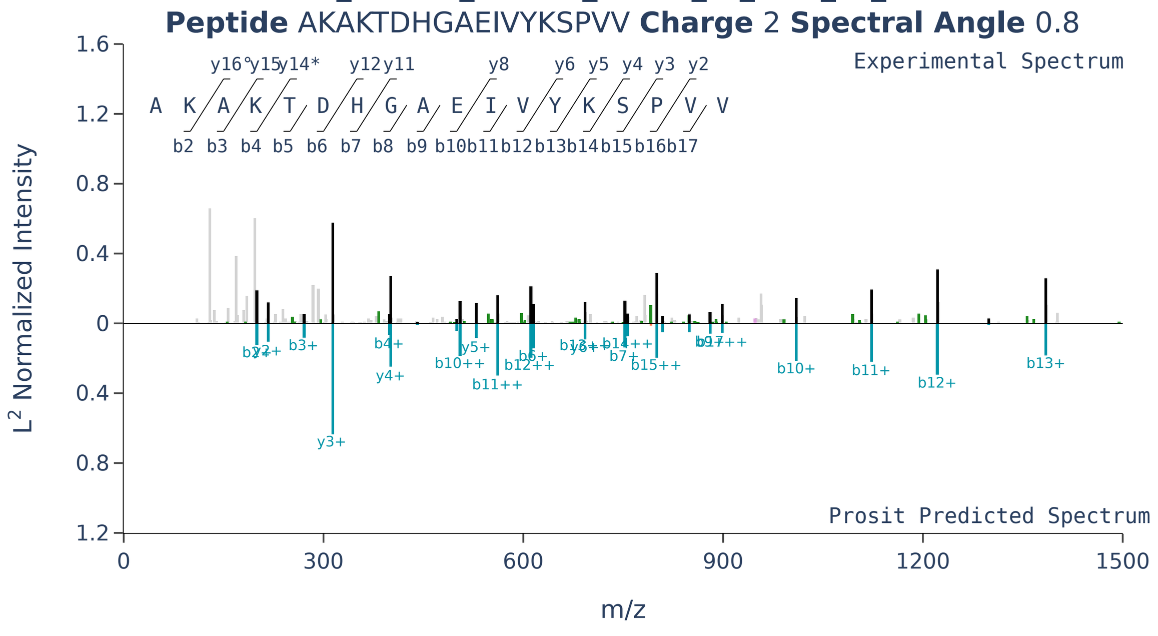


**Source** WSoh_221121_011221_HFGoe_L1_hTau_4h_R1 **Scan** 11936
**Peptide** QDQEGDTDAGL **Charge** 2 **Spectral Angle** 0.91

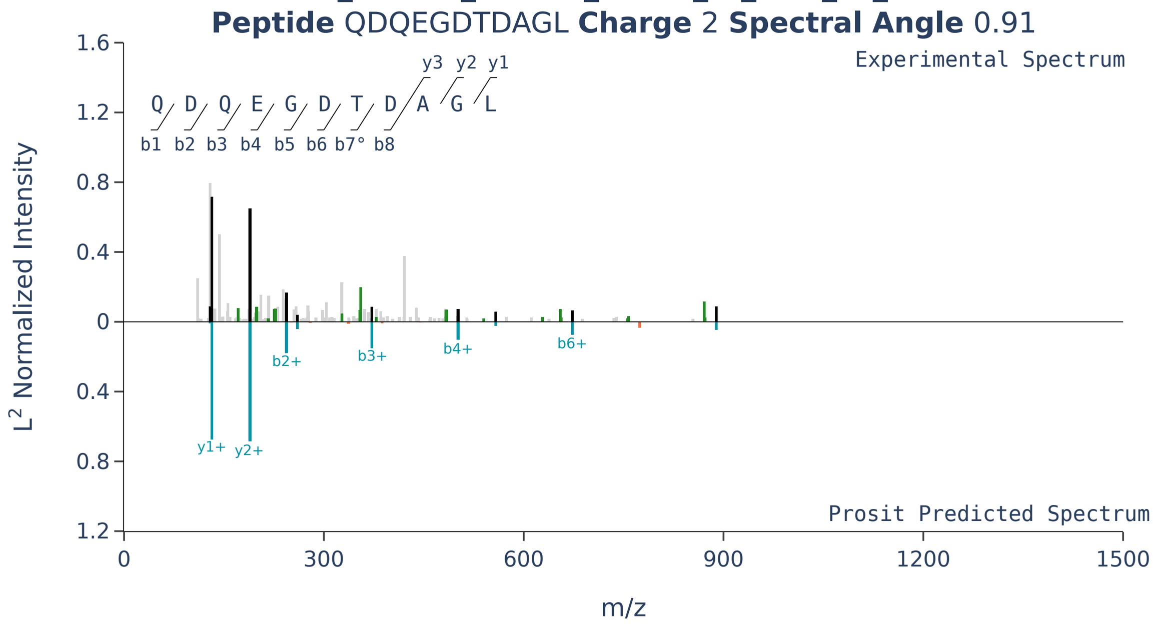


**Source** WSoh_221121_011221_HFGoe_L1_hTau_24h_R2 **Scan** 15853
**Peptide** ENLKHQPGGGKVQIINKKLDL **Charge** 3 **Spectral Angle** 0.76

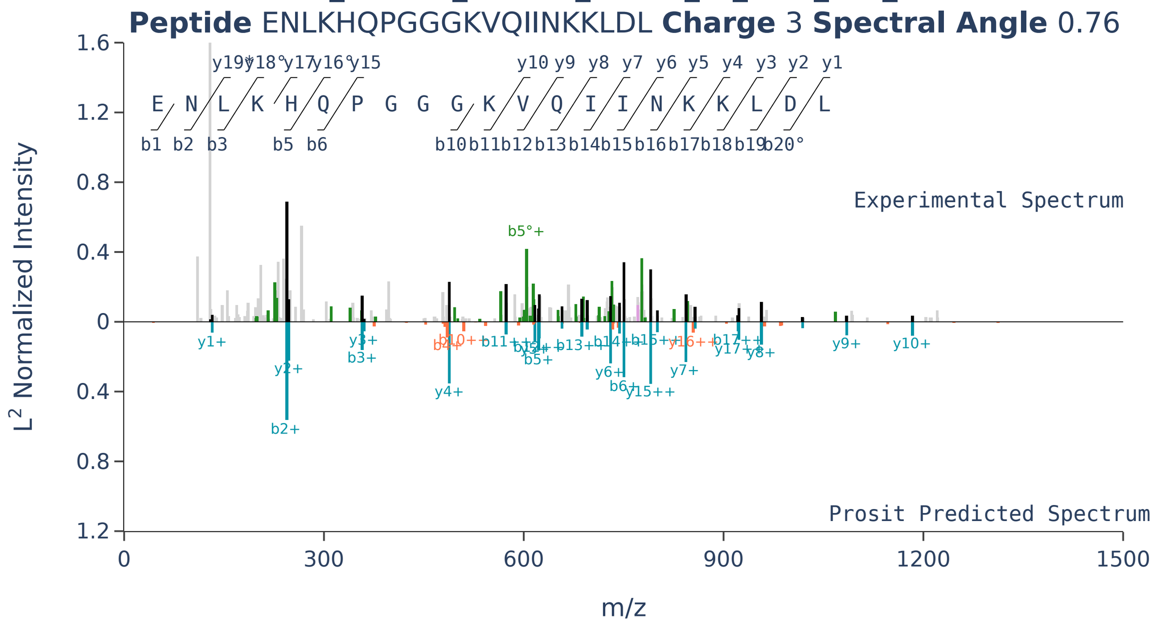


**Source** WSoh_221121_011221_HFGoe_L2_hTau_24h_R1 **Scan** 11279
**Peptide** ANATRIPAKTPPAPKTPPSS **Charge** 3 **Spectral Angle** 0.84

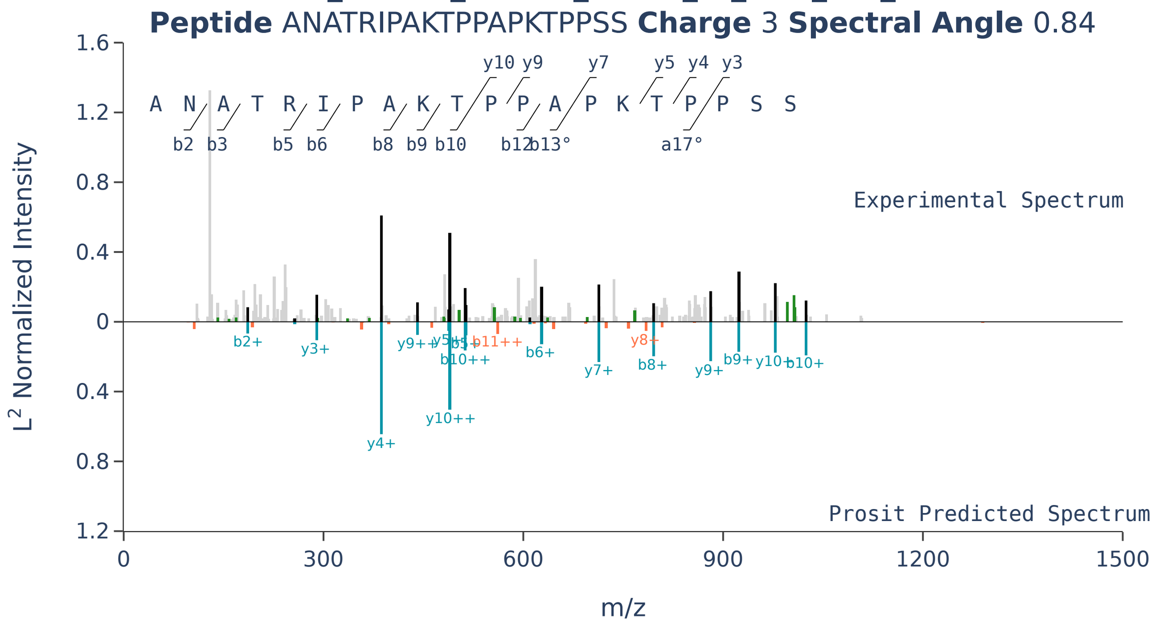


**Source** WSoh_221121_011221_HFGoe_L1_hTau_24h_R1 **Scan** 20467
**Peptide** LGSLDNITHVPGGGNKKIETHKLTF **Charge** 4 **Spectral Angle** 0.82

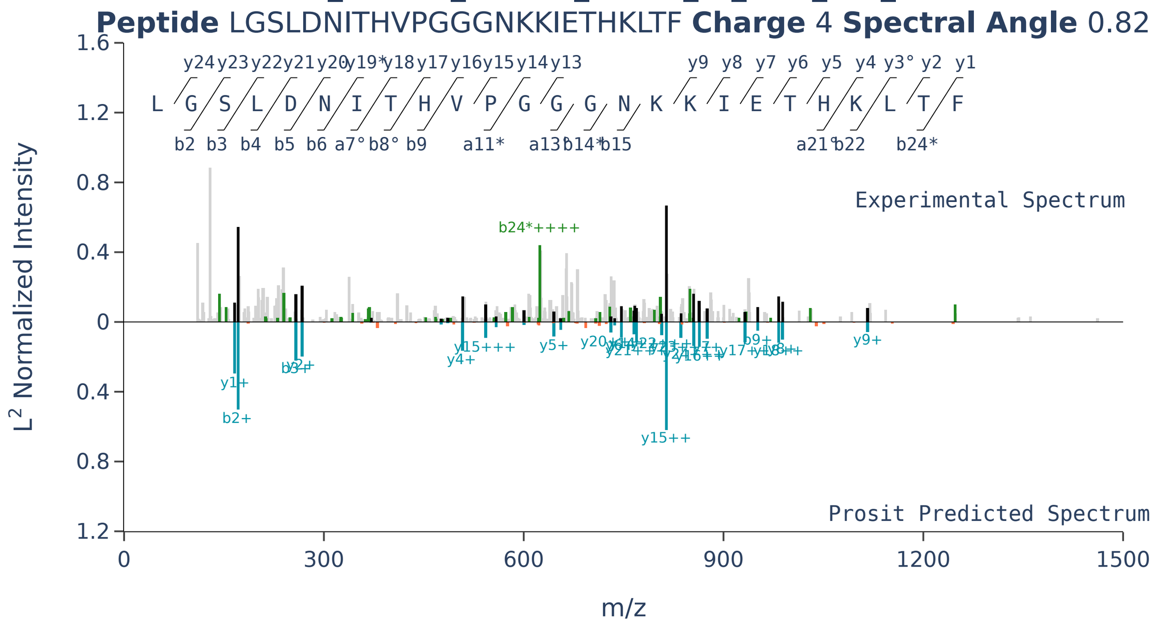


**Source** WSoh_221121_011221_HFGoe_L2_hTau_24h_R1 **Scan** 18047
**Peptide** QVEVKSEKLDF **Charge** 3 **Spectral Angle** 0.82

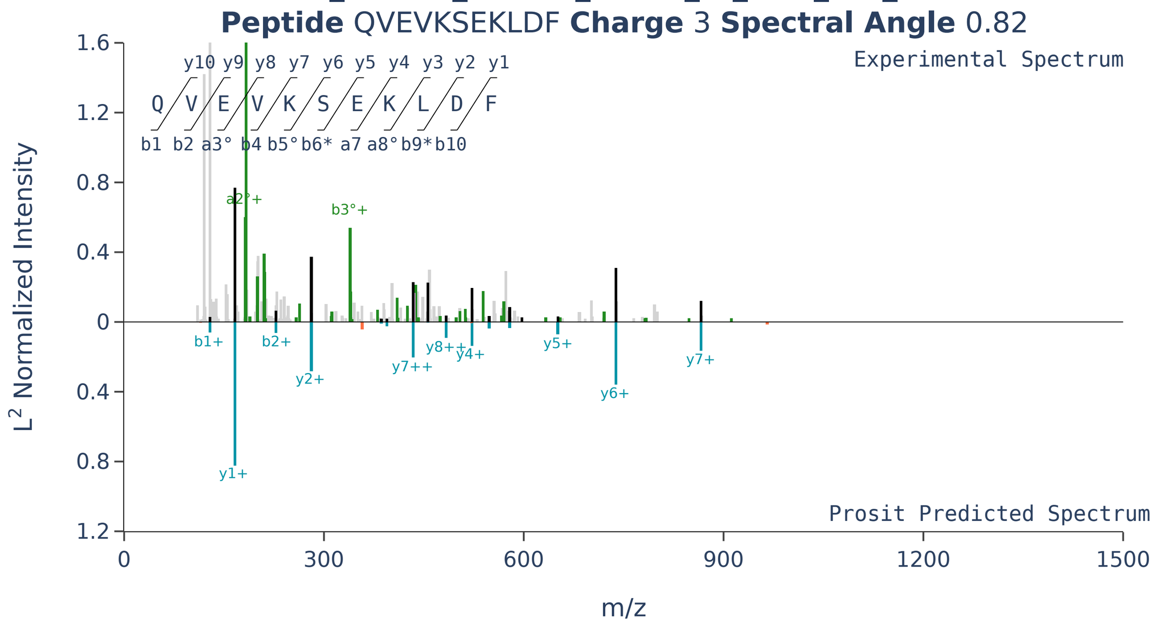


**Source** WSoh_221121_011221_HFGoe_L2_hTau_4h_R1 **Scan** 10654
**Peptide** KLGSTENL **Charge** 1 **Spectral Angle** 0.77

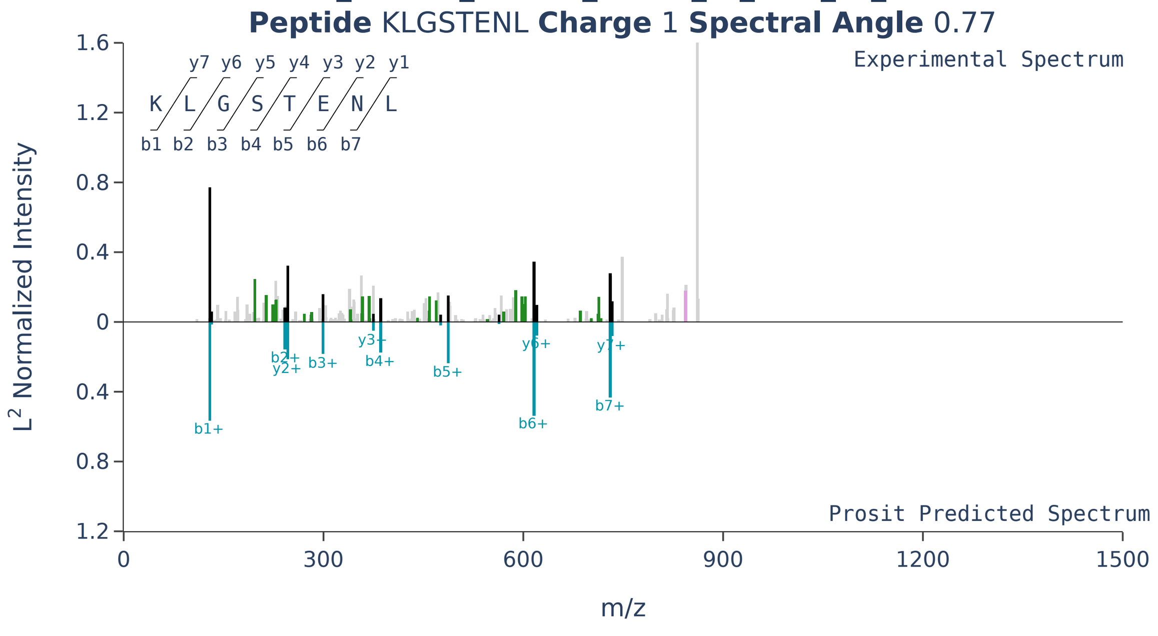

**Source** WSoh_221121_011221_HFGoe_L1_hTau_24h_R1 **Scan** 8402
**Peptide** EDEAAGHVTQARMVSKS **Charge** 2 **Spectral Angle** 0.76

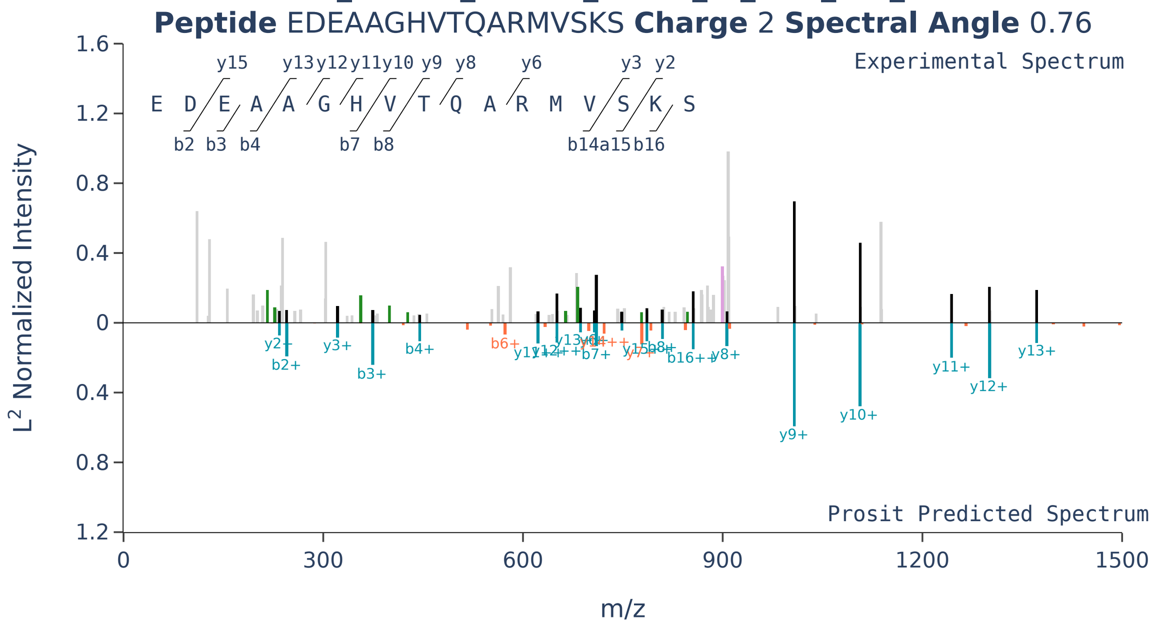


**Source** WSoh_221121_011221_HFGoe_L1_hTau_4h_R2 **Scan** 7227
**Peptide** IGSTENLKHQPGGGKVQ **Charge** 4 **Spectral Angle** 0.84

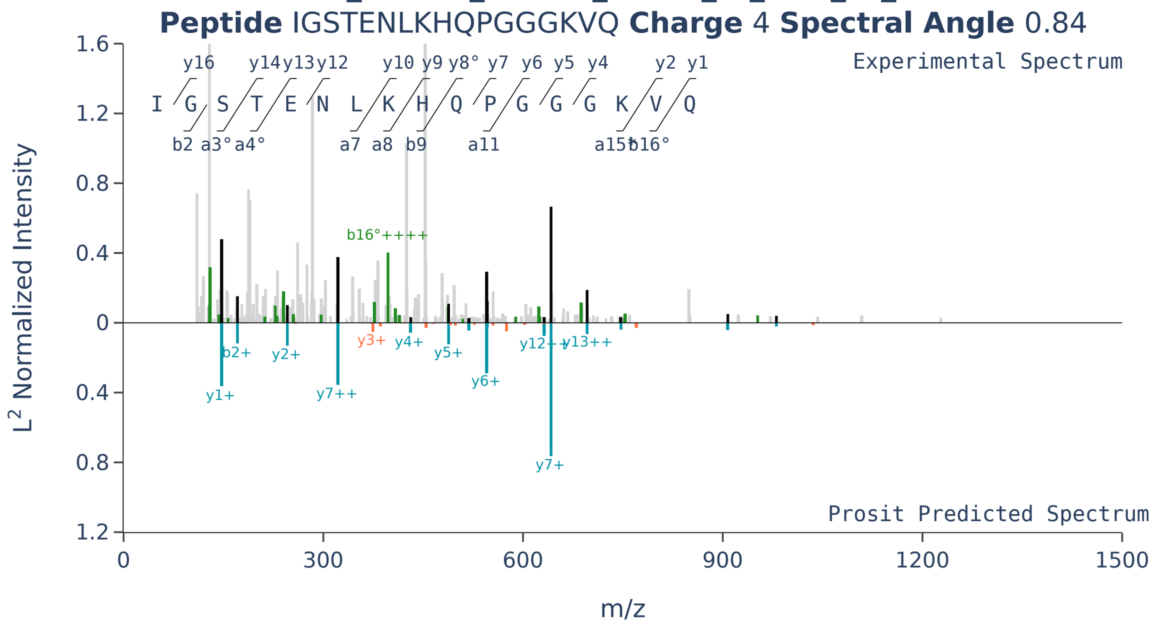


**Source** WSoh_221121_011221_HFGoe_L2_hTau_24h_R2 **Scan** 11139
**Peptide** EDEAAGHVTQARMVS **Charge** 3 **Spectral Angle** 0.81

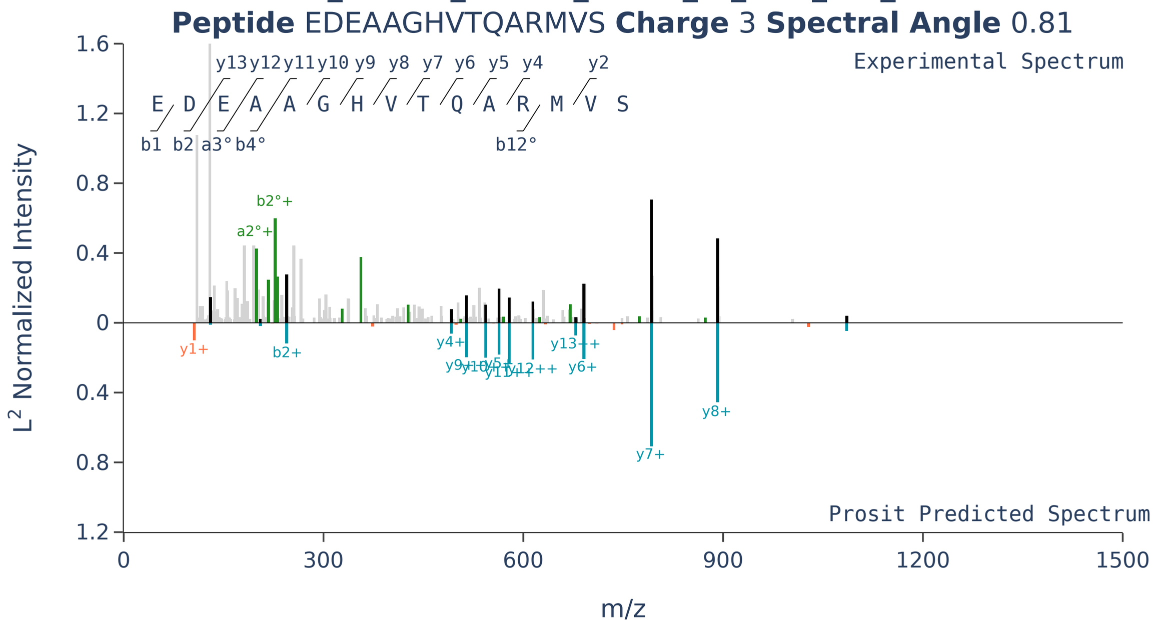


**Source** WSoh_221121_011221_HFGoe_L1_hTau_4h_R1 **Scan** 7289
**Peptide** SNVQSKCGSKDNIKHVPGGGSV **Charge** 3 **Spectral Angle** 0.93

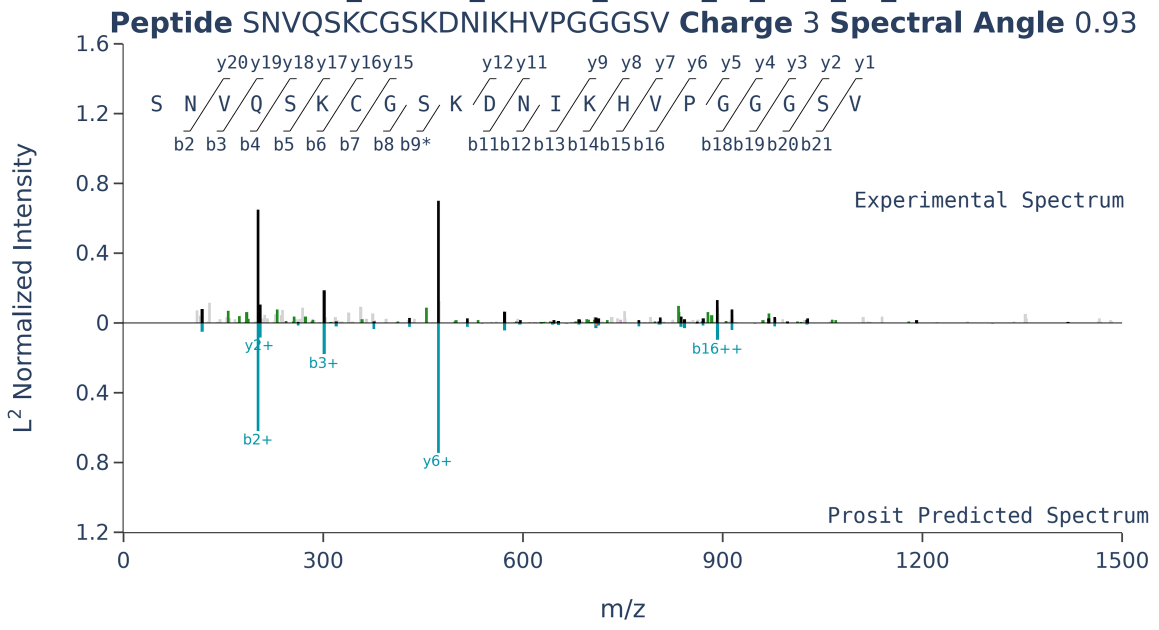


**Source** WSoh_221121_011221_HFGoe_L1_hTau_1h_R2 **Scan** 5568
**Peptide** PGSPGTPGSRSR **Charge** 2 **Spectral Angle** 0.82

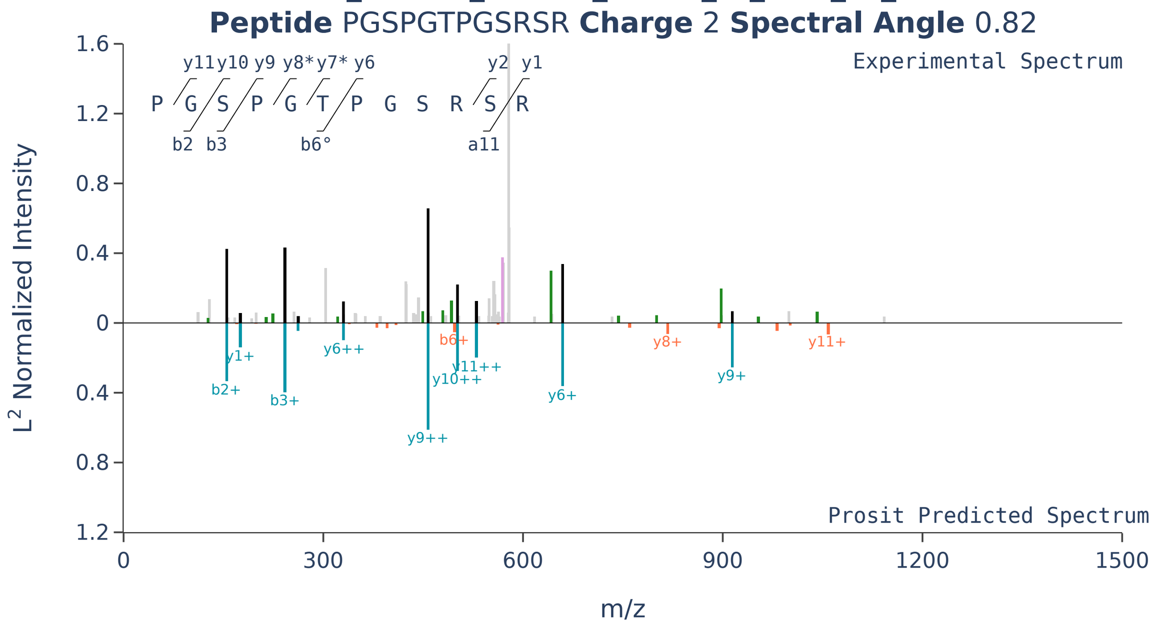


**Source** WSoh_221121_011221_HFGoe_L1_hTau_1h_R2 **Scan** 8907
**Peptide** TRIPAKTPPAPKTPPSSGEPPKSGDRS **Charge** 5 **Spectral Angle** 0.82

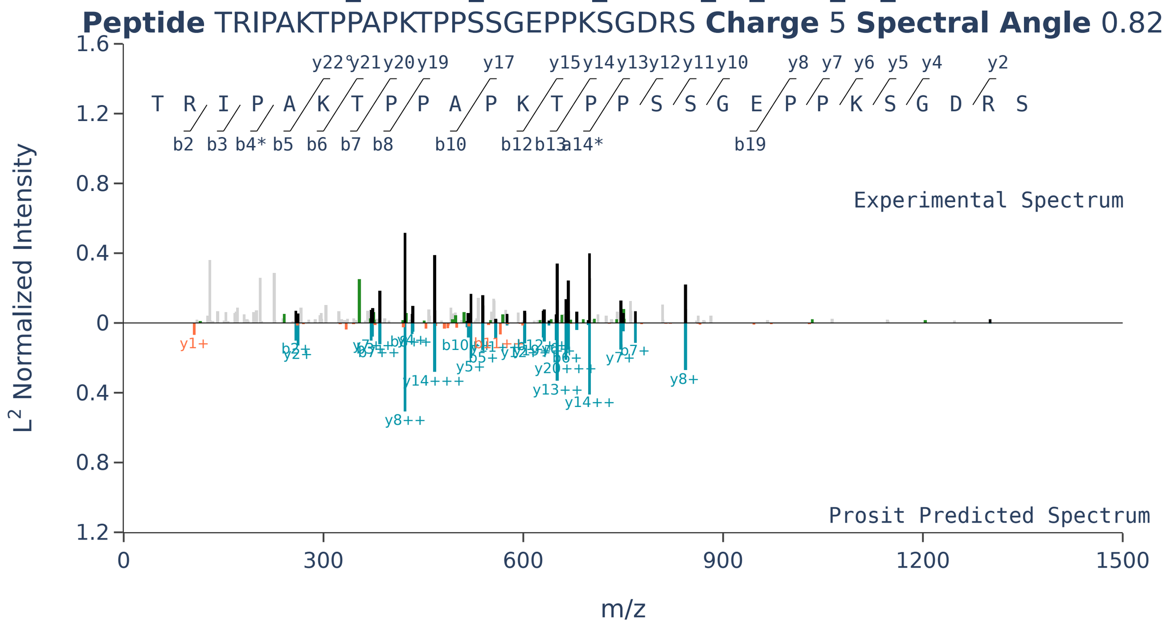


**Source** WSoh_221121_011221_HFGoe_L2_hTau_24h_R2 **Scan** 23367
**Peptide** SGDTSPRHLSNVSSTGSIDMVDSPQL **Charge** 3 **Spectral Angle** 0.8

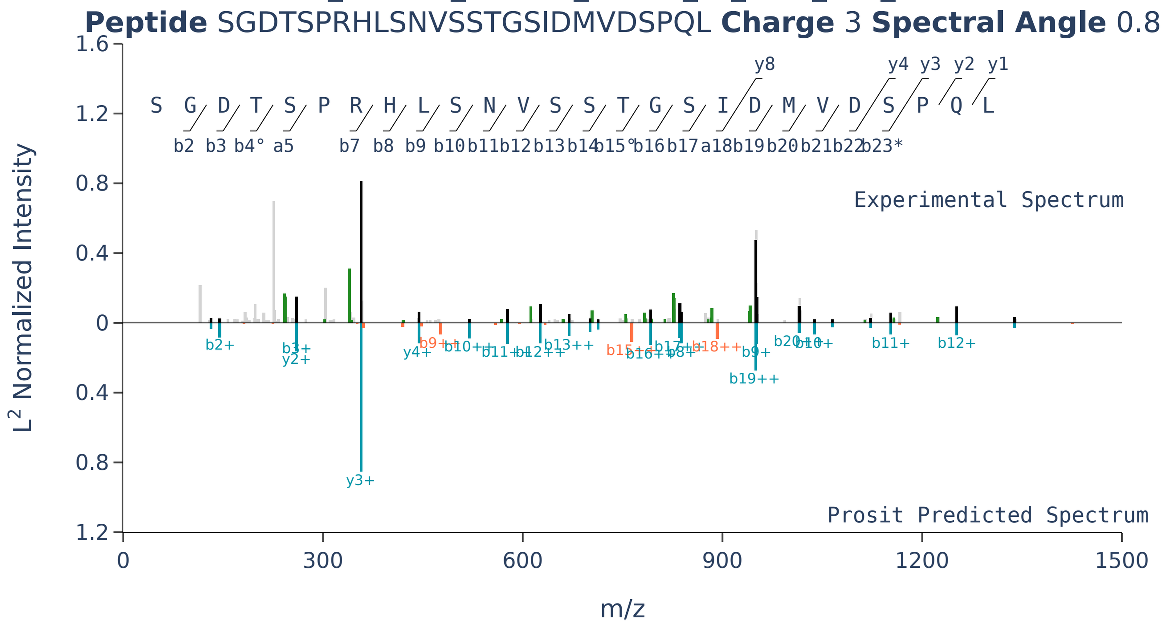


**Source** WSoh_221121_011221_HFGoe_L1_hTau_4h_R1 **Scan** 7067
**Peptide** ADGKTKIATPRGAAPPGQKGQAN **Charge** 3 **Spectral Angle** 0.78

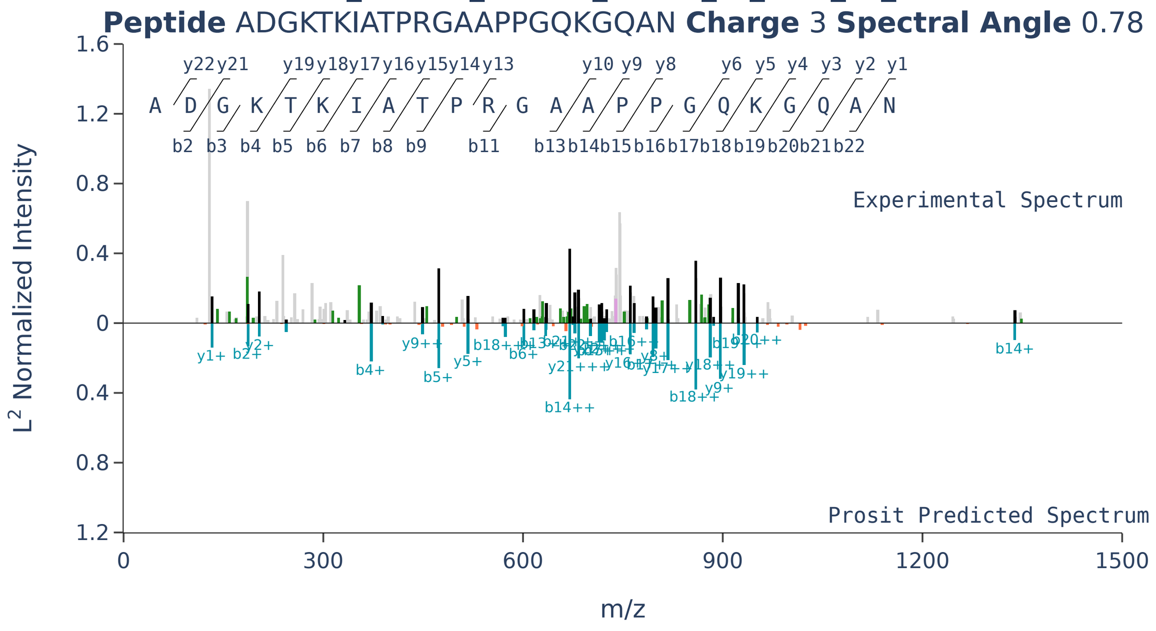


**Source** WSoh_221121_011221_HFGoe_L2_hTau_24h_R1 **Scan** 10574
**Peptide** IHHKPGGGQVEVKSEKLDFKDRVQS **Charge** 4 **Spectral Angle** 0.81

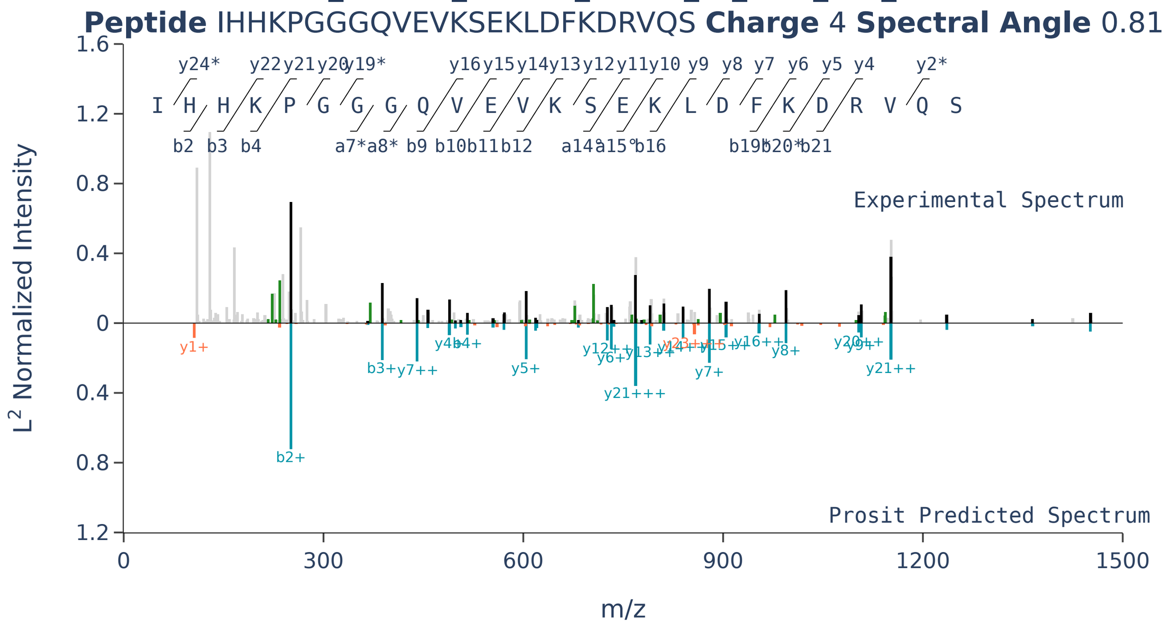


**Source** WSoh_221121_011221_HFGoe_L1_hTau_24h_R1 **Scan** 14448
**Peptide** KLTFRENAKAKTDHGAEIVYKSPVVS **Charge** 4 **Spectral Angle** 0.84

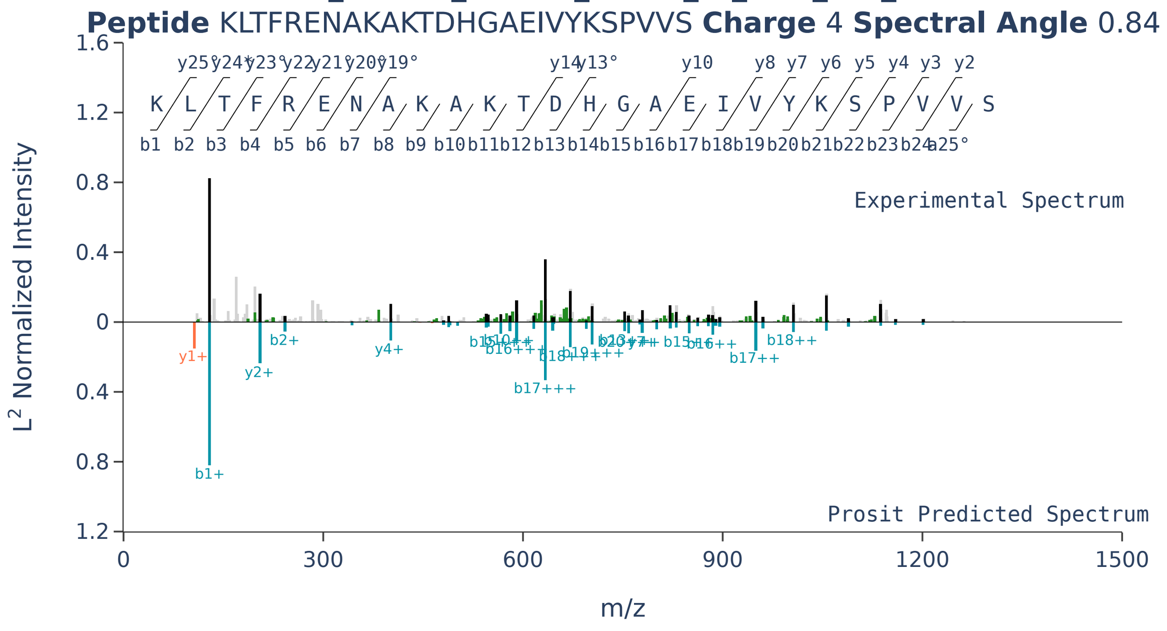


**Source** WSoh_221121_011221_HFGoe_L1_hTau_24h_R2 **Scan** 12235
**Peptide** KSKIGSTENLKHQPGGGKVQIL **Charge** 4 **Spectral Angle** 0.87

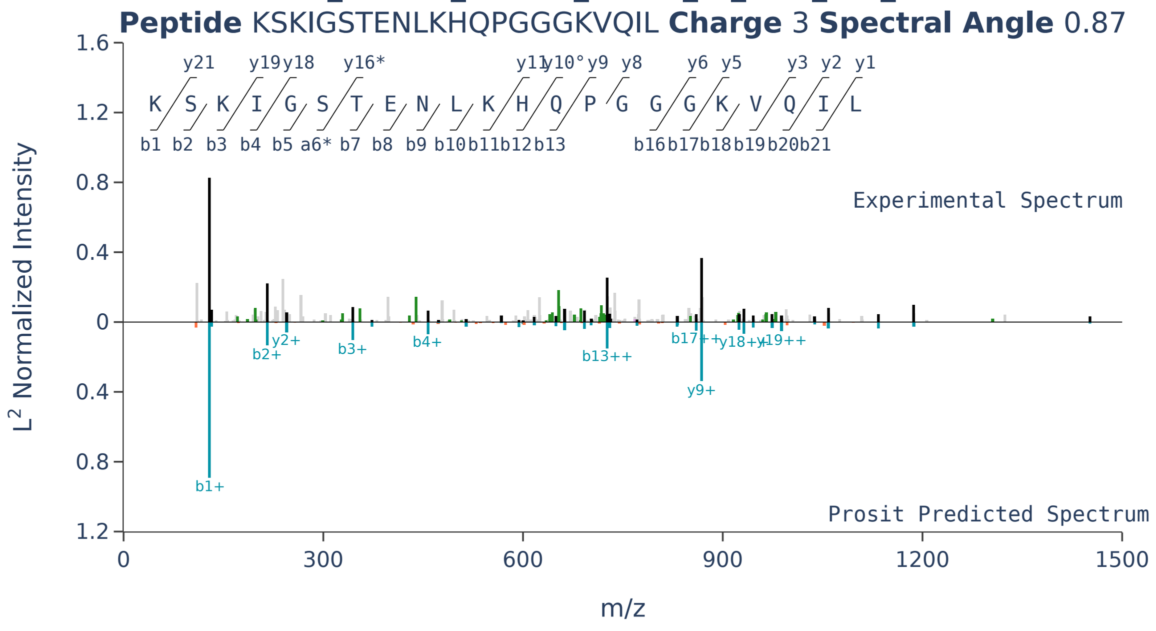


**Source** WSoh_221121_011221_HFGoe_L1_hTau_24h_R1 **Scan** 16207
**Peptide** VTAPLVDEGAPGKQAAAQPHT **Charge** 3 **Spectral Angle** 0.81

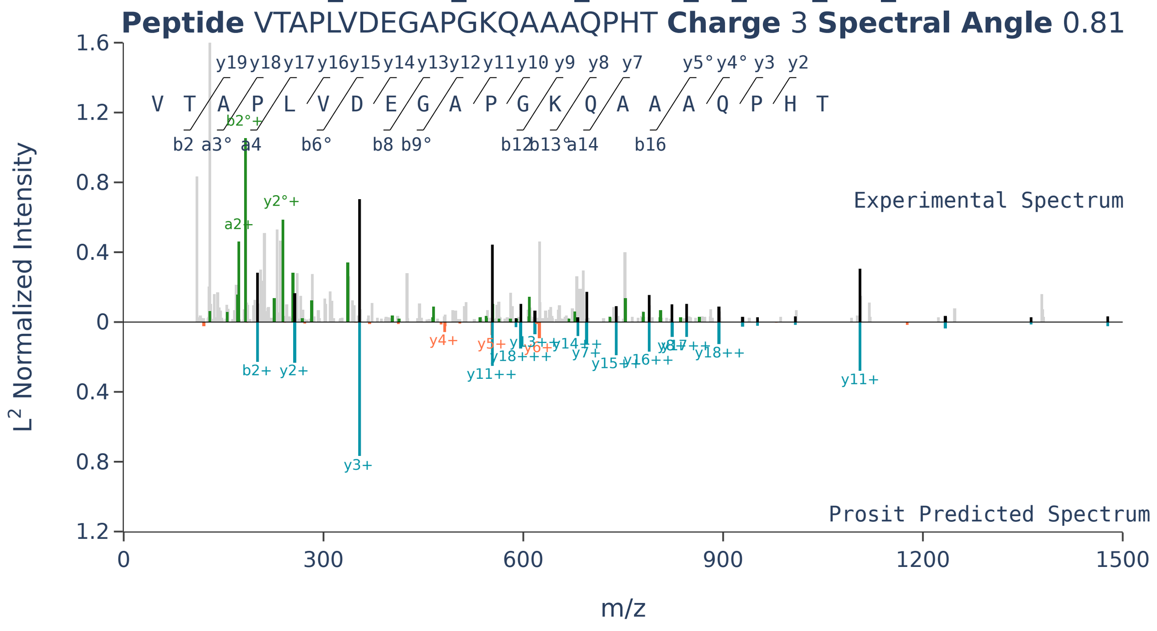


**Source** WSoh_221121_011221_HFGoe_L2_hTau_24h_R1 **Scan** 9939
**Peptide** NLKHQPGGGKVQIINKKL **Charge** 2 **Spectral Angle** 0.81

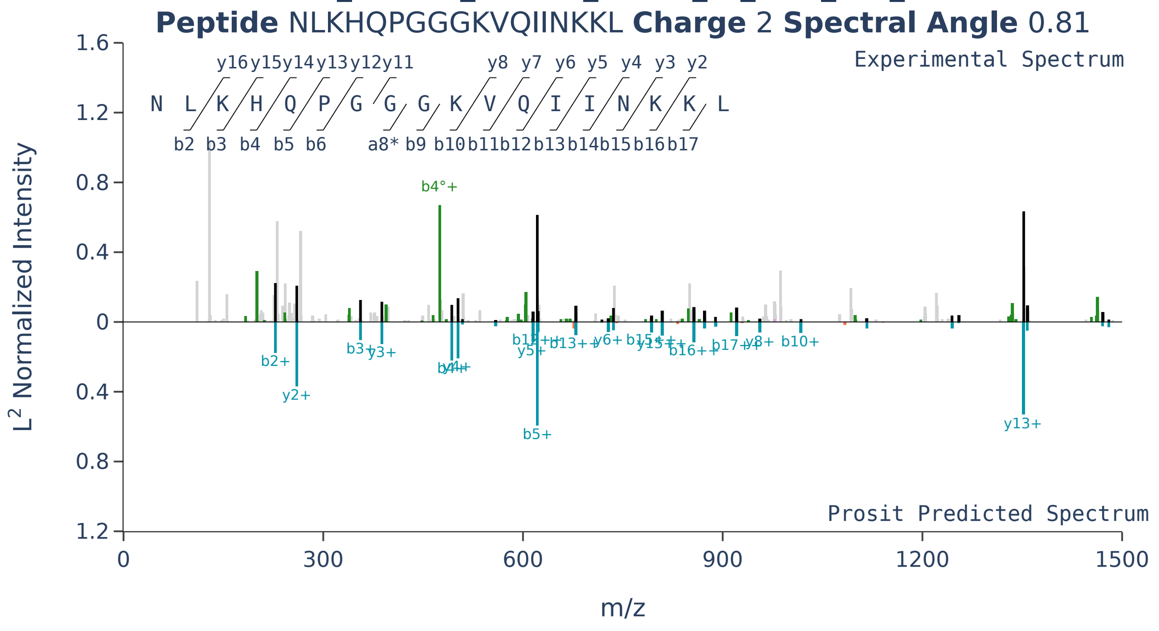


**Source** WSoh_221121_011221_HFGoe_L1_hTau_24h_R2 **Scan** 7625
**Peptide** VVSGDTSPRHLS **Charge** 3 **Spectral Angle** 0.82

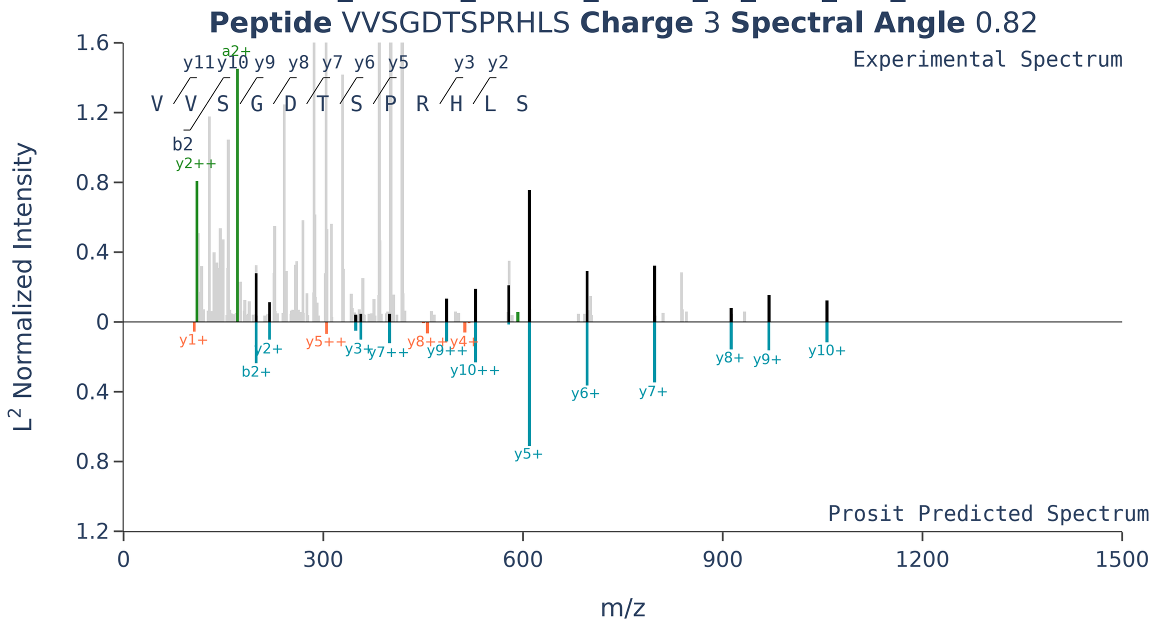


**Source** WSoh_221121_011221_HFGoe_L2_hTau_4h_R1 **Scan** 13437
**Peptide** NITHVPGGGNKKIETHKLTF **Charge** 5 **Spectral Angle** 0.83

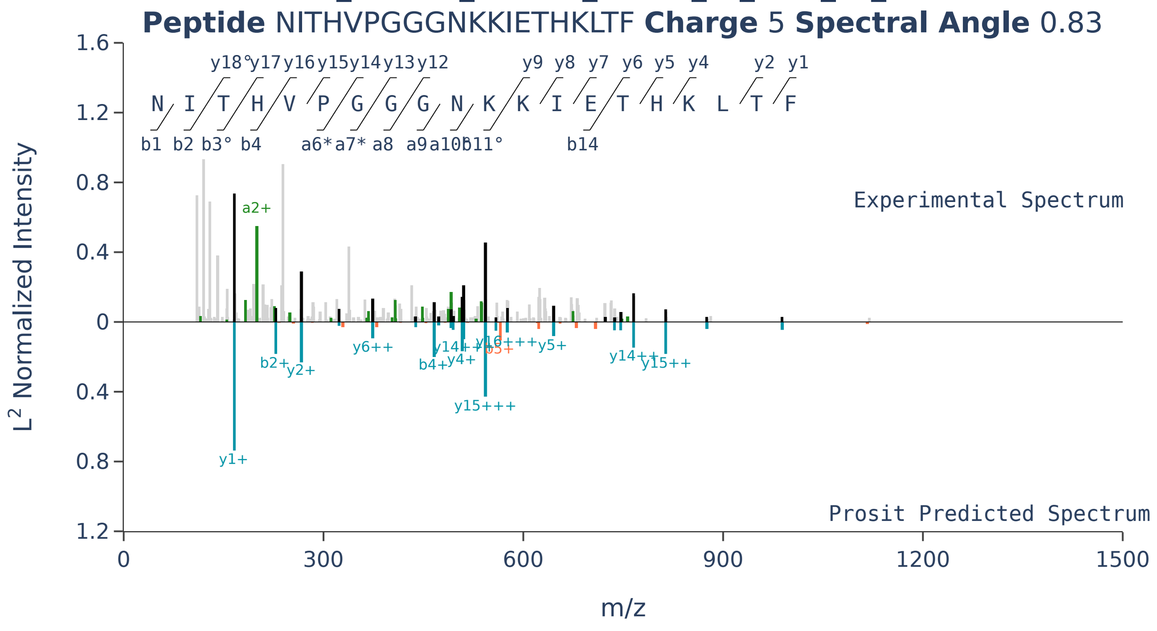

**Source** WSoh_221121_011221_HFGoe_L2_hTau_2h_R1 **Scan** 13279
**Peptide** NLTHVPGGGNKKIETHKLTF **Charge** 5 **Spectral Angle** 0.78

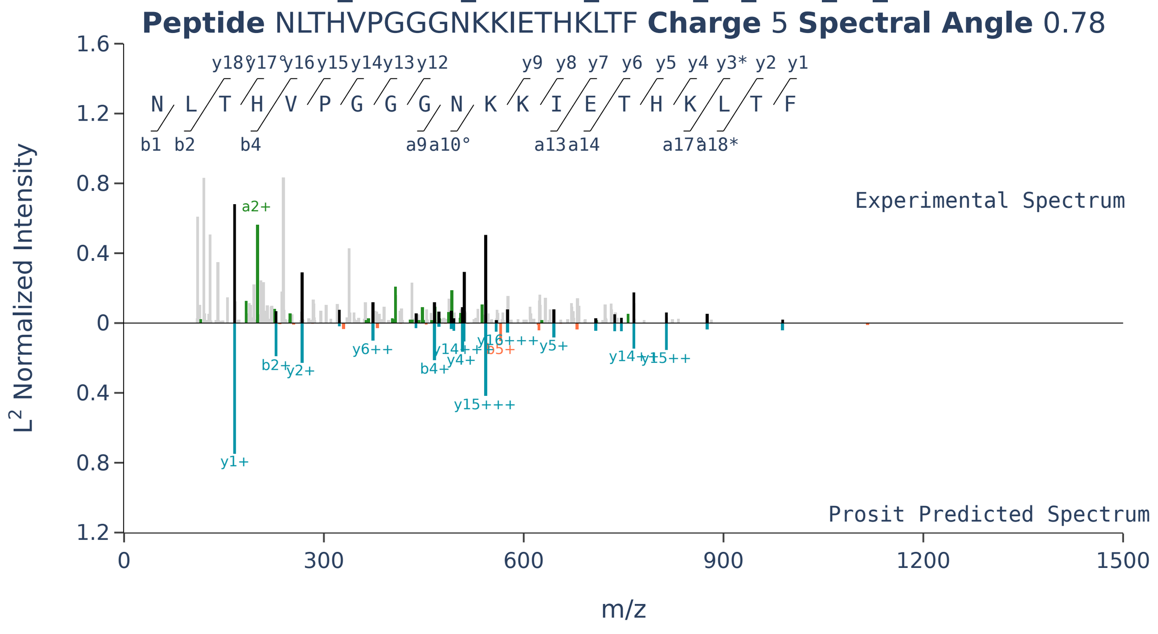


**Source** WSoh_221121_011221_HFGoe_L1_hTau_24h_R2 **Scan** 14494
**Peptide** KPGGGQVEVKSEKLDFKDRVQSKLG **Charge** 5 **Spectral Angle** 0.89

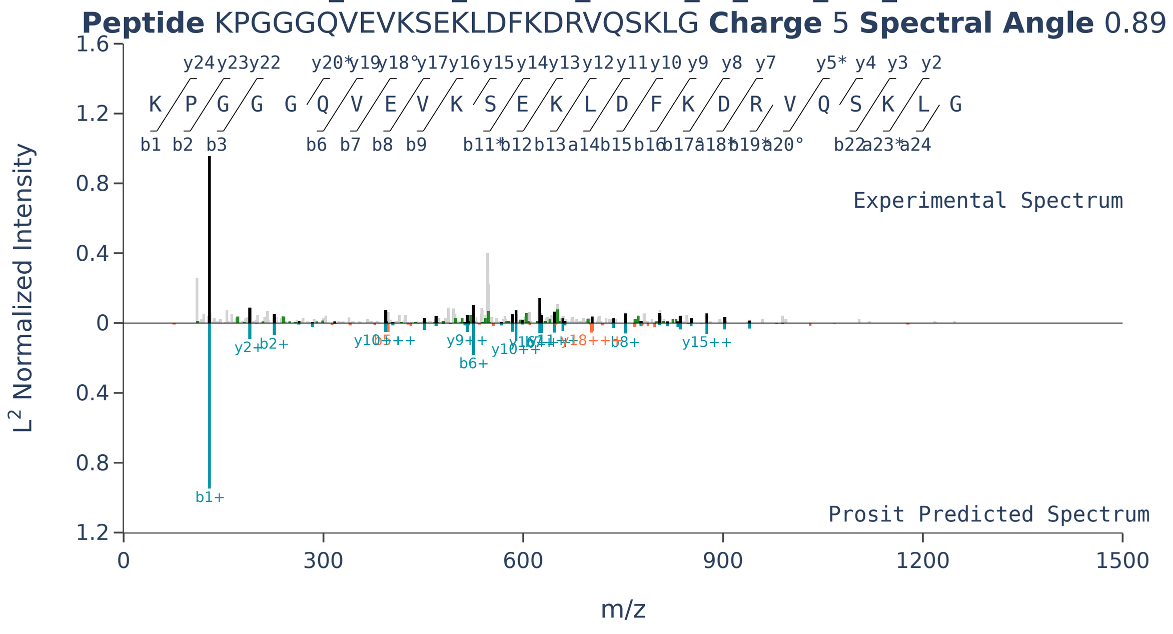


**Source** WSoh_221121_011221_HFGoe_L2_hTau_2h_R1 **Scan** 5502
**Peptide** GTGSDDKKAKGADGKTKIATPRG **Charge** 5 **Spectral Angle** 0.85

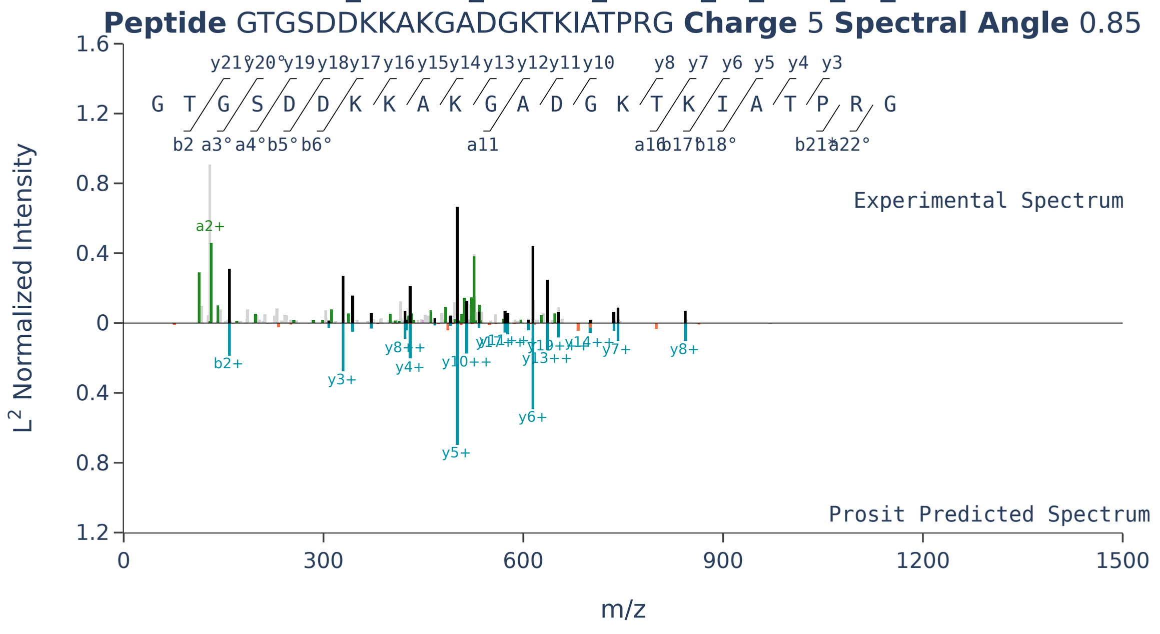


**Source** WSoh_221121_011221_HFGoe_L2_hTau_24h_R1 **Scan** 12648
**Peptide** LGSTENLKHQPGGGKVQI **Charge** 2 **Spectral Angle** 0.8

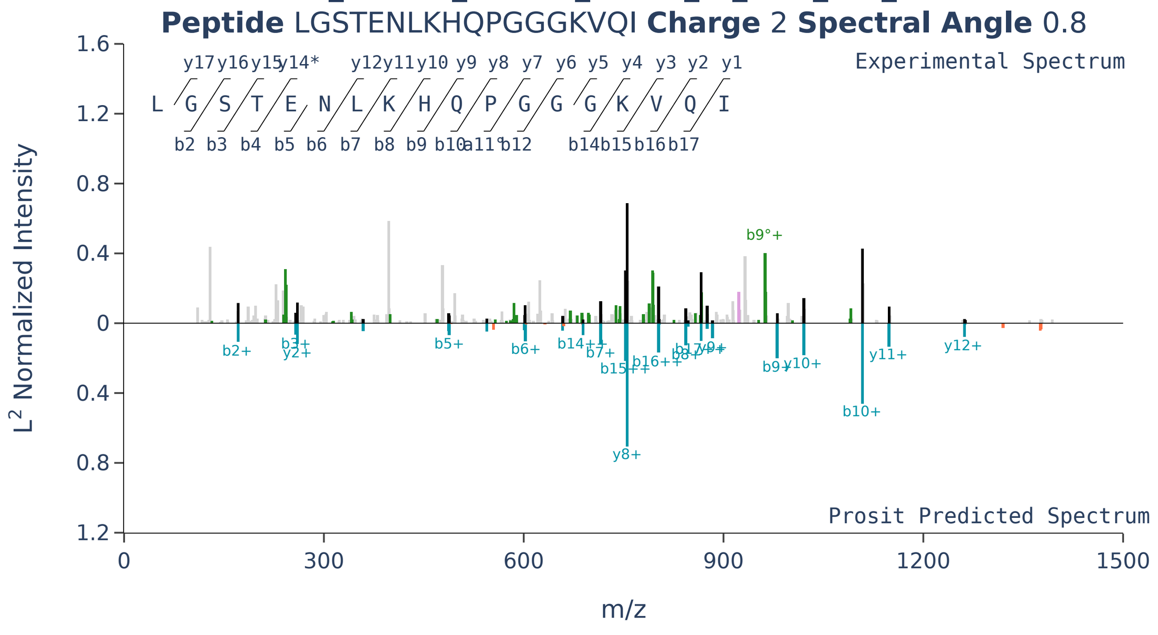


**Source** WSoh_221121_011221_HFGoe_L1_hTau_4h_R1 **Scan** 15352
**Peptide** EIVYKSPVVSGDTSPRHLSN **Charge** 3 **Spectral Angle** 0.83

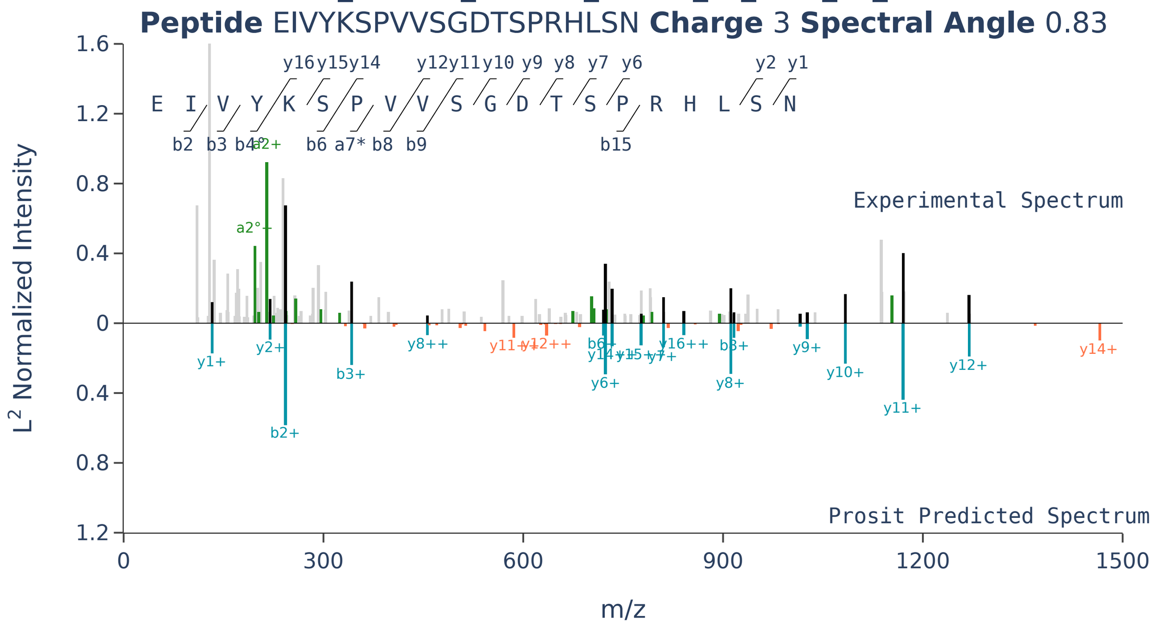


**Source** WSoh_221121_011221_HFGoe_L1_hTau_24h_R1 **Scan** 20306
**Peptide** EAGIGDTPSLED **Charge** 2 **Spectral Angle** 0.83

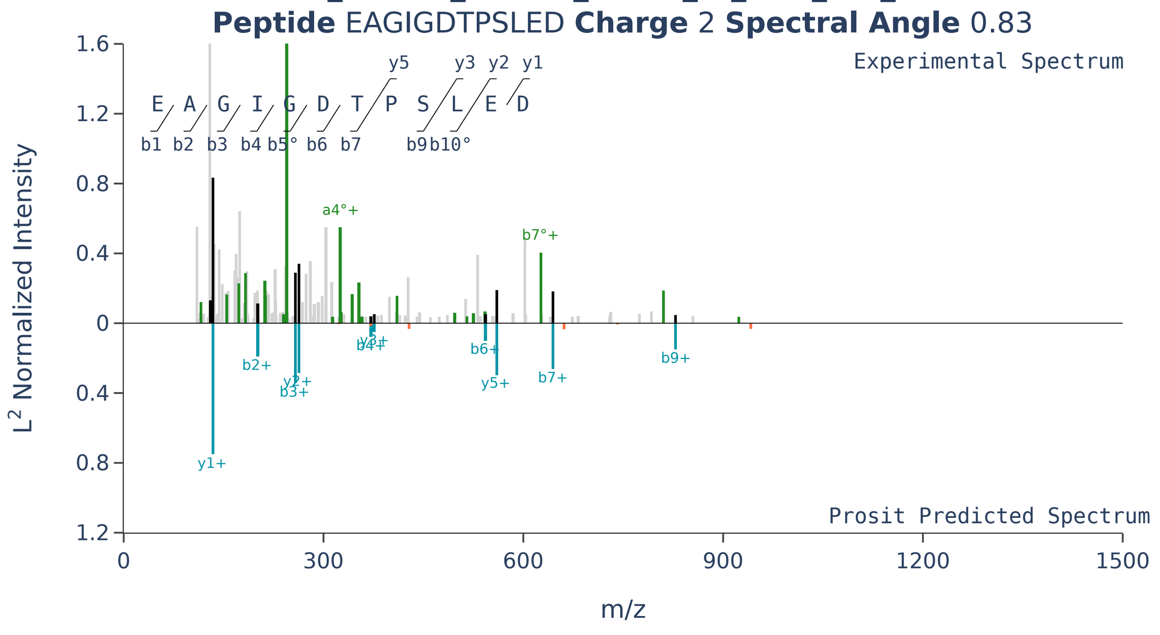


**Source** WSoh_221121_011221_HFGoe_L1_hTau_24h_R2 **Scan** 7376
**Peptide** LGSTENLKHQPGGGKVQ **Charge** 4 **Spectral Angle** 0.9

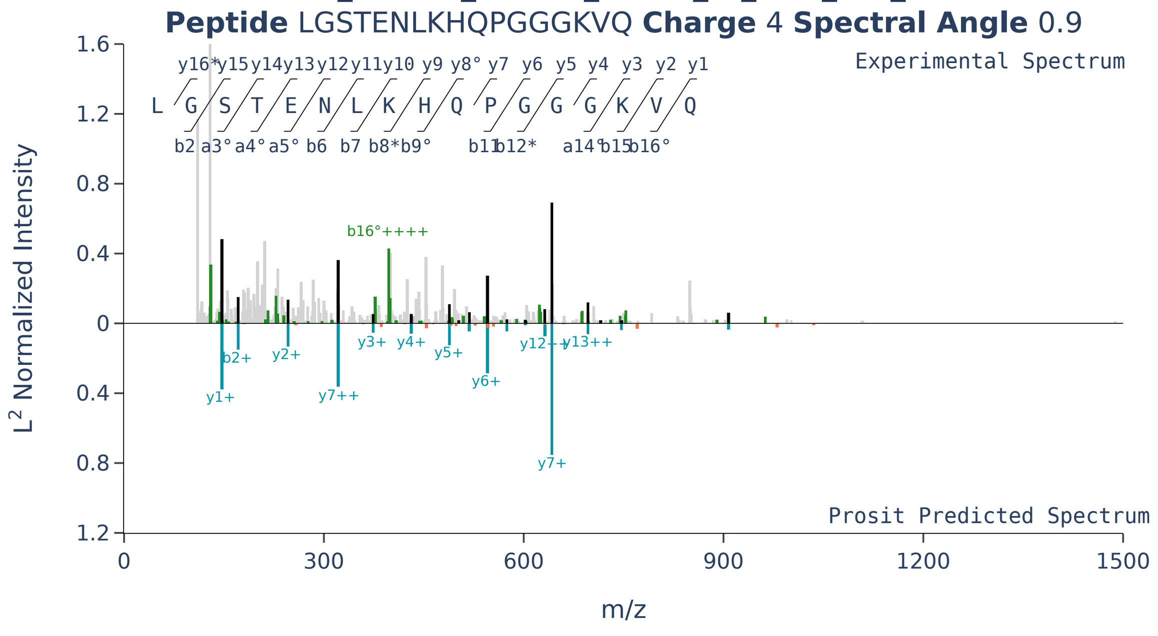


**Source** WSoh_221121_011221_HFGoe_L2_hTau_24h_R1 **Scan** 19716
**Peptide** PVPMPDLKNVKSKIGSTENLKH **Charge** 5 **Spectral Angle** 0.9

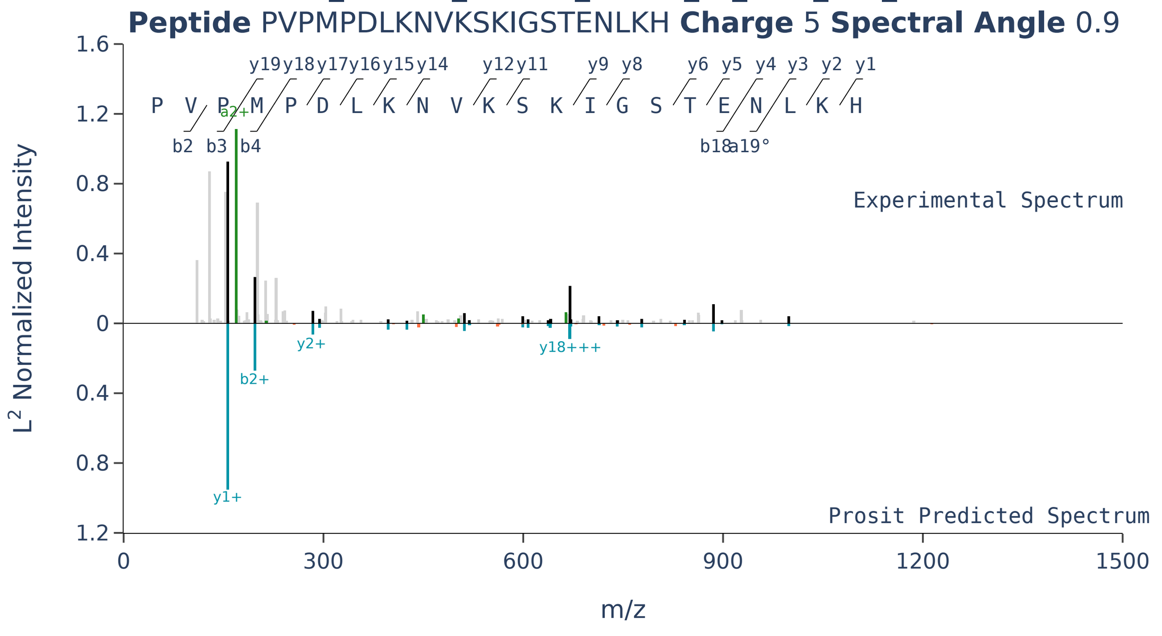


**Source** WSoh_221121_011221_HFGoe_L1_hTau_24h_R1 **Scan** 7239
**Peptide** ADGKTKIATPRGAAPPGQKGQ **Charge** 4 **Spectral Angle** 0.85

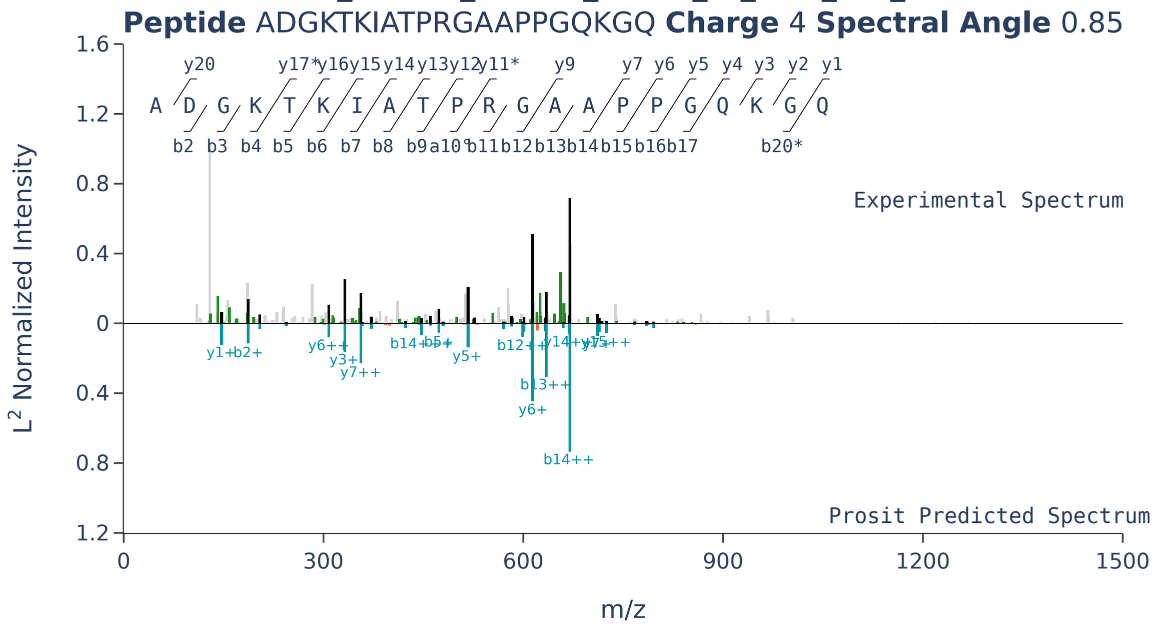


**Source** WSoh_221121_011221_HFGoe_L1_hTau_2h_R1 **Scan** 11404
**Peptide** QPGGGKVQI **Charge** 2 **Spectral Angle** 0.66

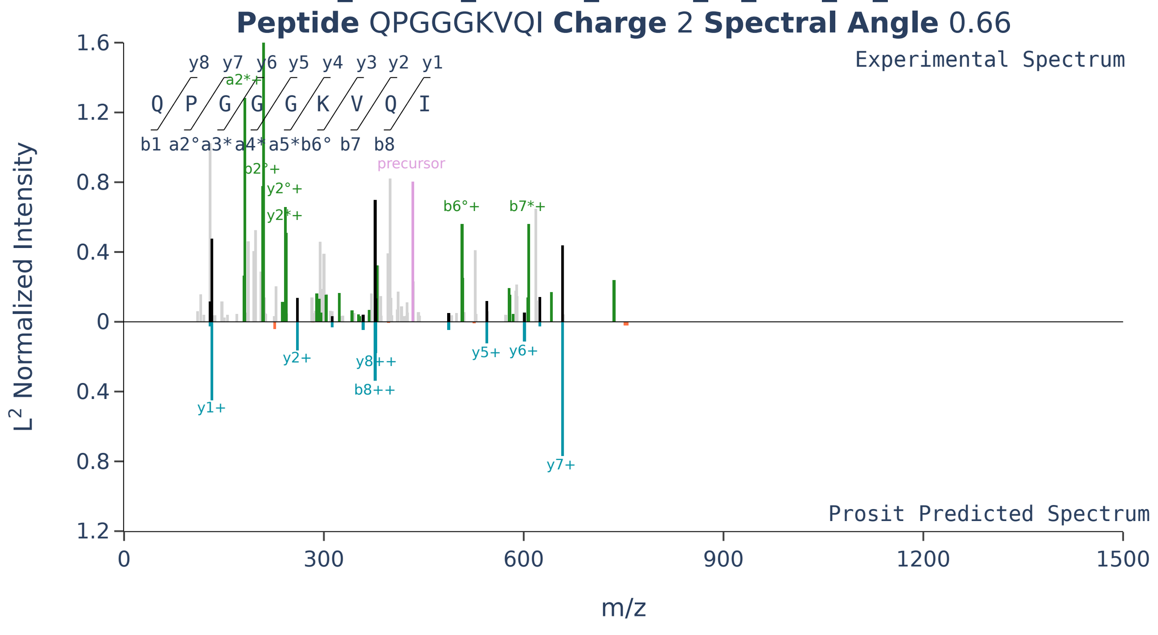


**Source** WSoh_221121_011221_HFGoe_L2_hTau_24h_R2 **Scan** 10546
**Peptide** ATRIPAKTPPAPKTPPSSGEPPKS **Charge** 4 **Spectral Angle** 0.8

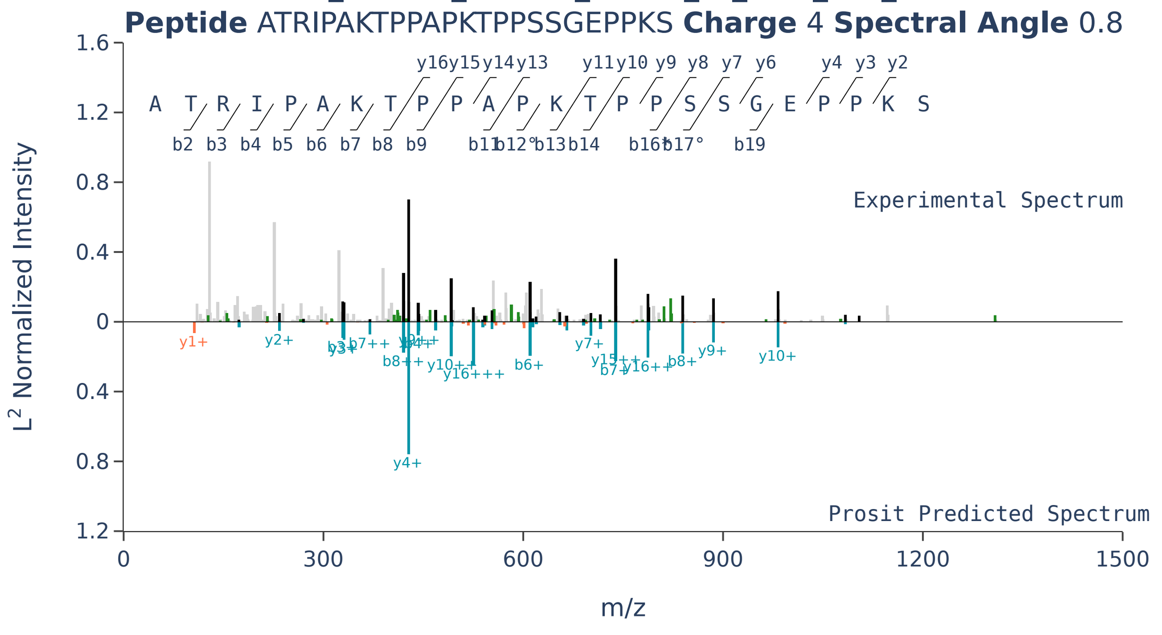


**Source** WSoh_221121_011221_HFGoe_L2_hTau_4h_R2 **Scan** 11589
**Peptide** EGAPGKQAAAQPHTEIPEGT **Charge** 2 **Spectral Angle** 0.85

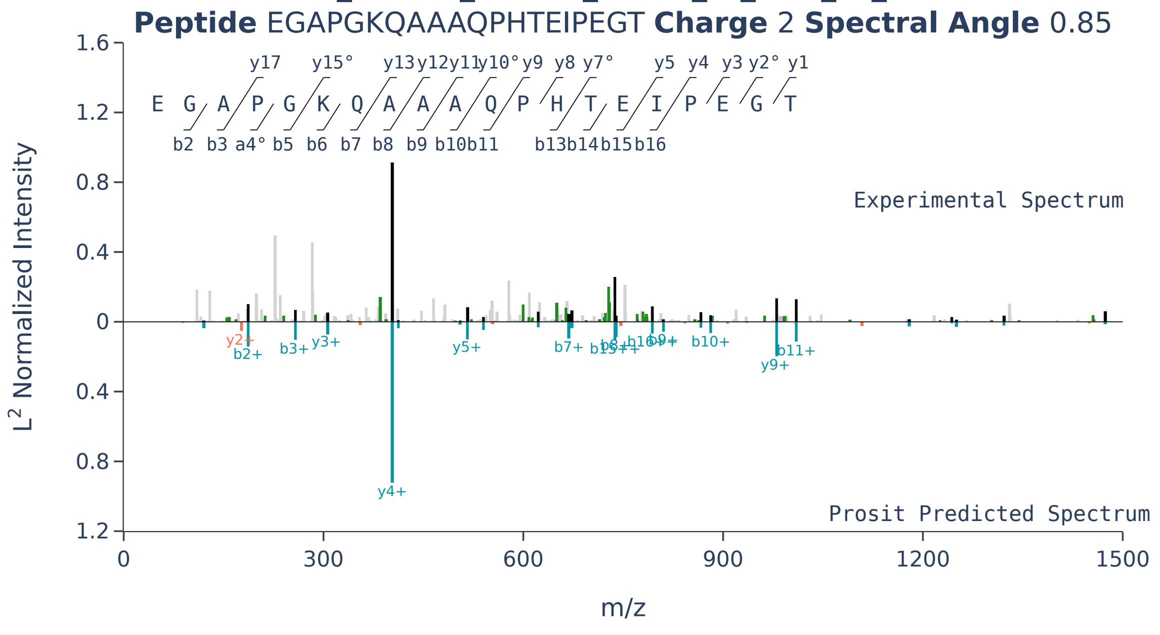


**Source** WSoh_221121_011221_HFGoe_L1_hTau_24h_R1 **Scan** 17223
**Peptide** TDHGAEIVYKSPVVSGDTSPRHLSNVS **Charge** 3 **Spectral Angle** 0.83

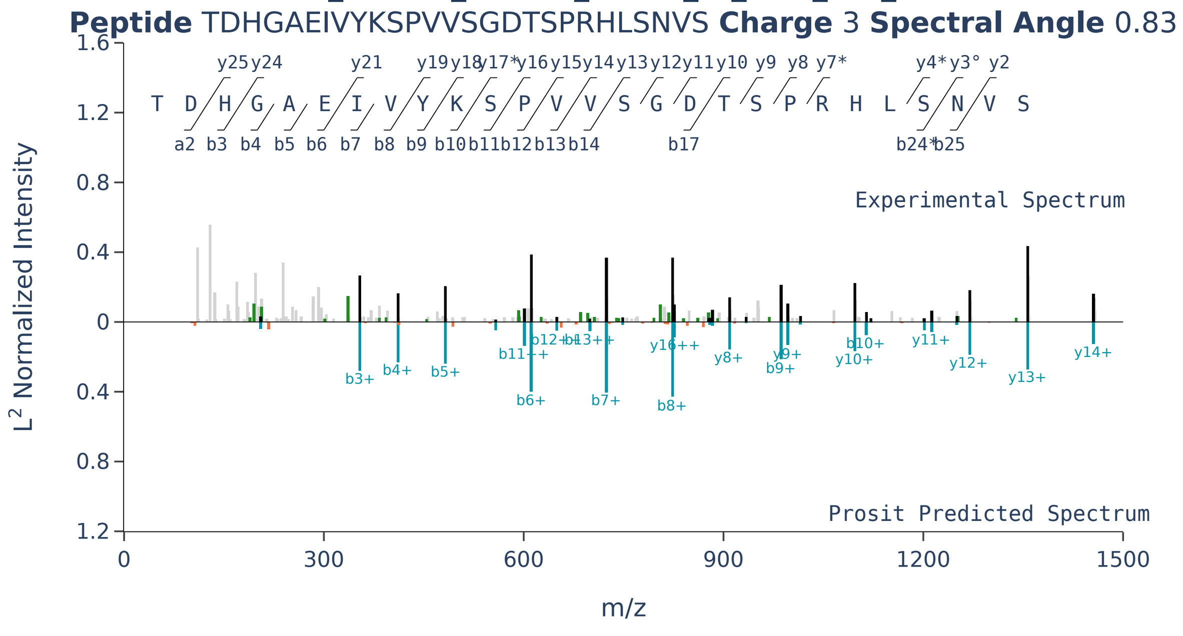


**Source** WSoh_221121_011221_HFGoe_L2_hTau_2h_R2 **Scan** 11641
**Peptide** SNVQSKCGSKDNIKHVPGGGSVQL **Charge** 3 **Spectral Angle** 0.82

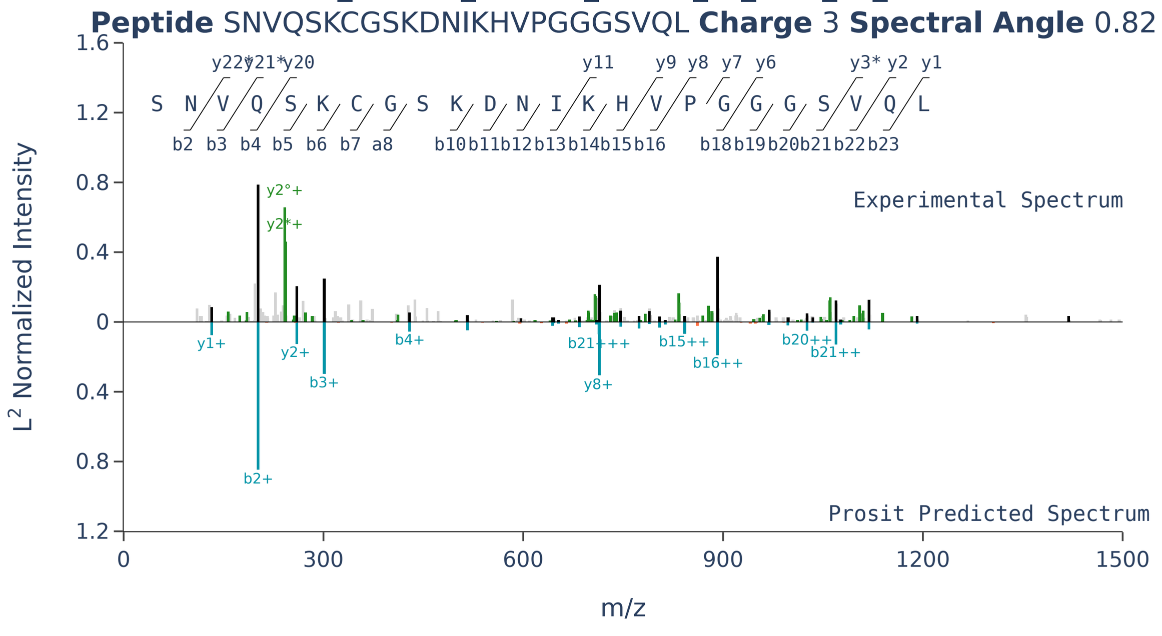


**Source** WSoh_221121_011221_HFGoe_L1_hTau_4h_R2 **Scan** 7085
**Peptide** AKAKTDHGAELV **Charge** 2 **Spectral Angle** 0.75

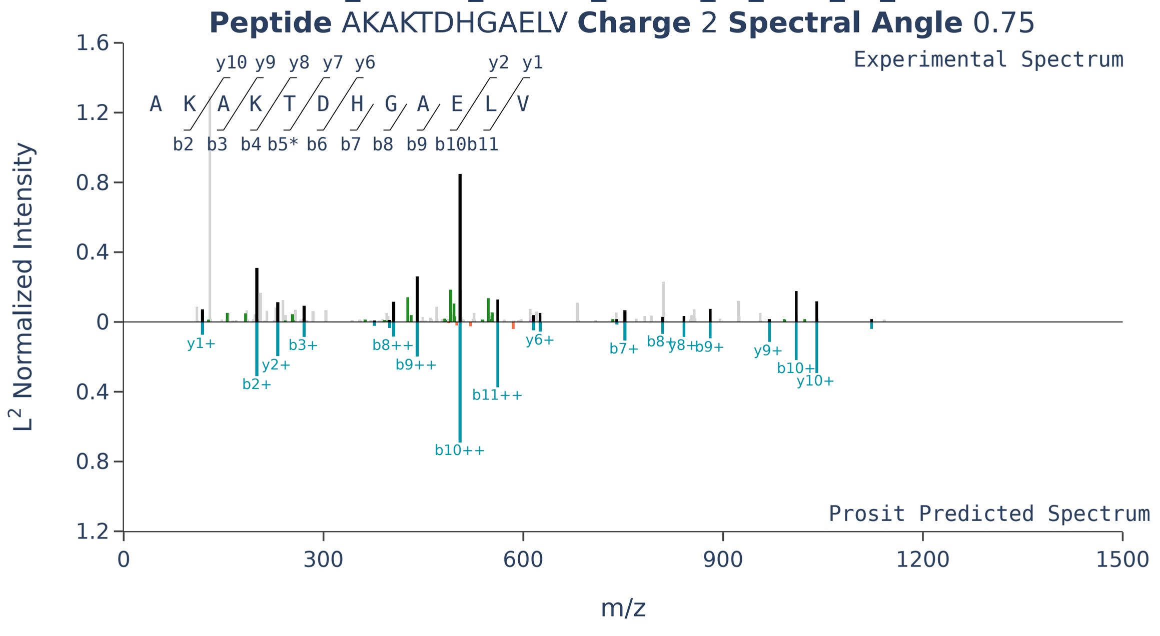

**Source** WSoh_221121_011221_HFGoe_L2_hTau_24h_R1 **Scan** 10565
**Peptide** KSKIGSTENLKHQPGGGKVQIINKKL **Charge** 4 **Spectral Angle** 0.94

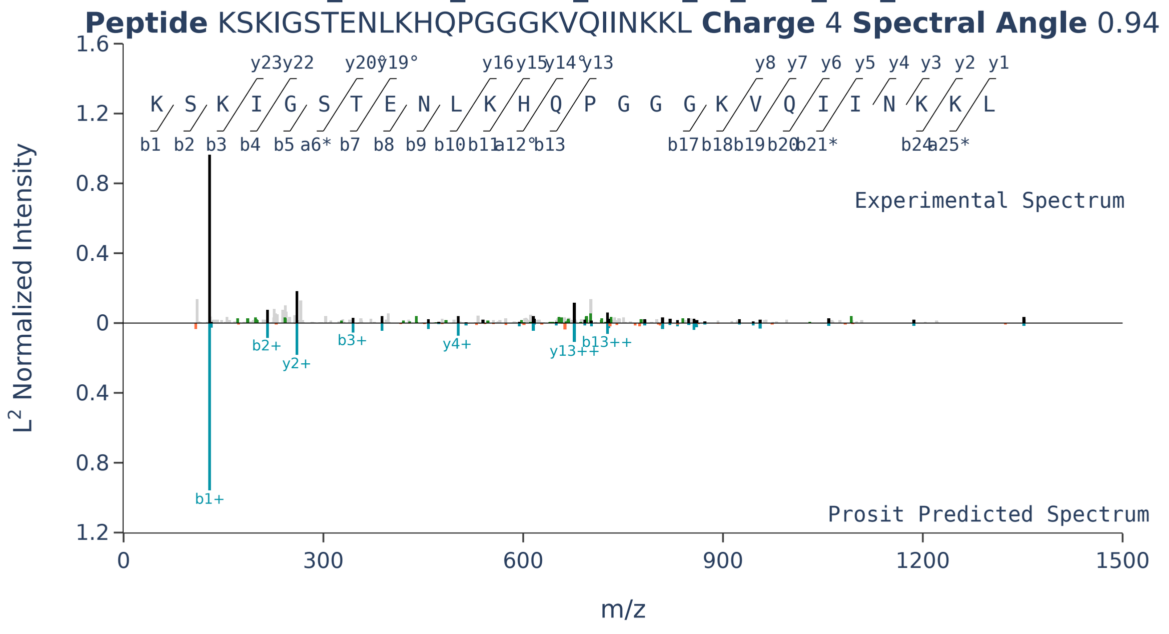


**Source** WSoh_221121_011221_HFGoe_L1_hTau_4h_R1 **Scan** 12215
**Peptide** KESPLQTPTEDGSEEPGSETSDAKST **Charge** 3 **Spectral Angle** 0.74

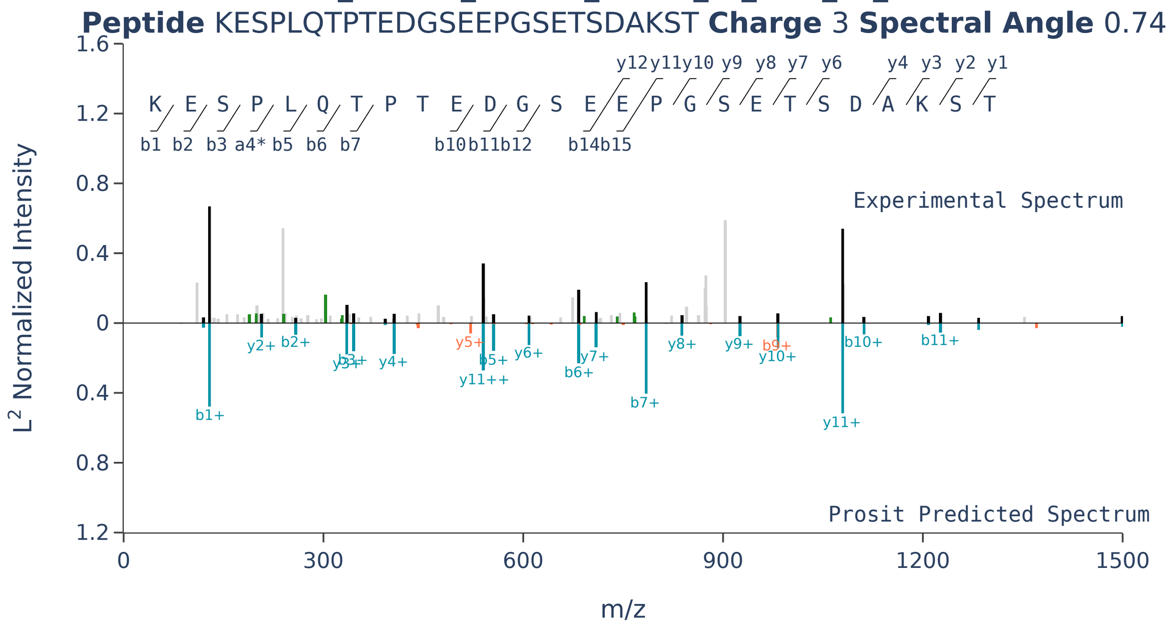


**Source** WSoh_221121_011221_HFGoe_L2_hTau_24h_R1 **Scan** 17008
**Peptide** KLDLSNVQSKCGSKDNIKHVPGGGSVQI **Charge** 4 **Spectral Angle** 0.78

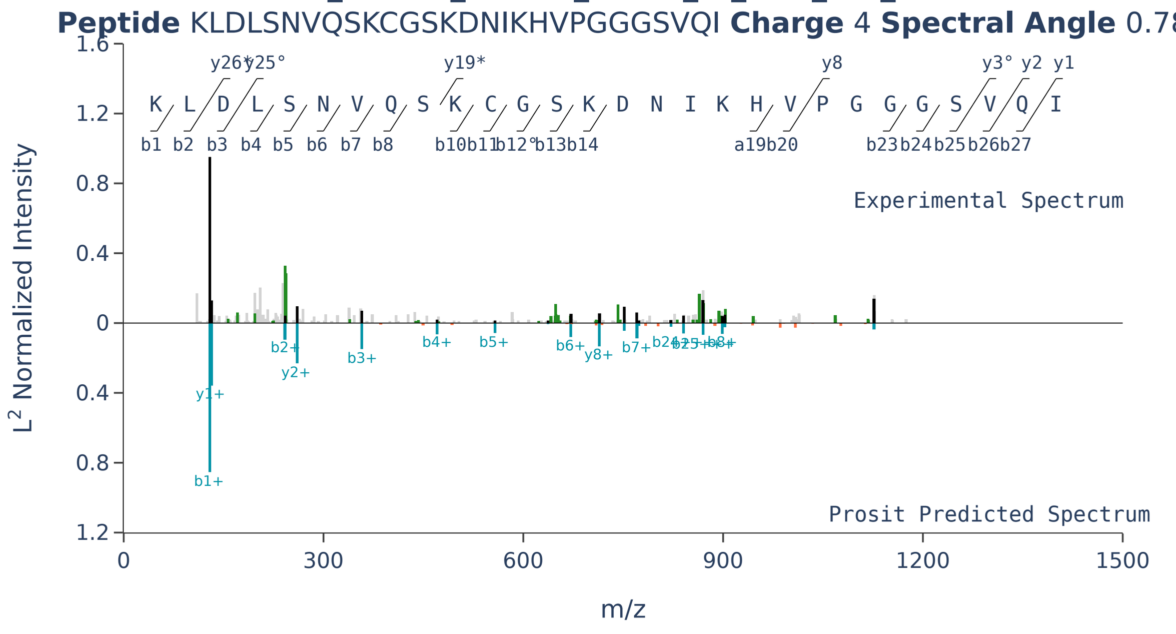


**Source** WSoh_221121_011221_HFGoe_L1_hTau_24h_R1 **Scan** 16709
**Peptide** KLGSLDNITHVPGGGNKKIETHKLTFR **Charge** 6 **Spectral Angle** 0.76

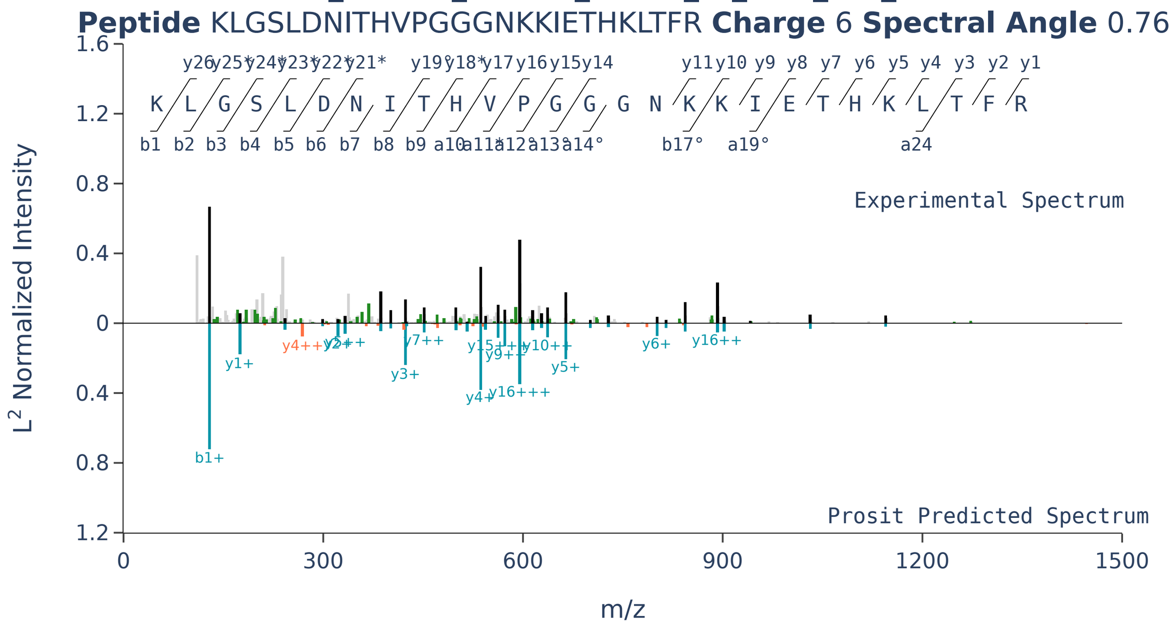


**Source** WSoh_221121_011221_HFGoe_L1_hTau_24h_R1 **Scan** 19989
**Peptide** AGIGDTPSLEDEAAGHVTQARMVSKS **Charge** 3 **Spectral Angle** 0.67

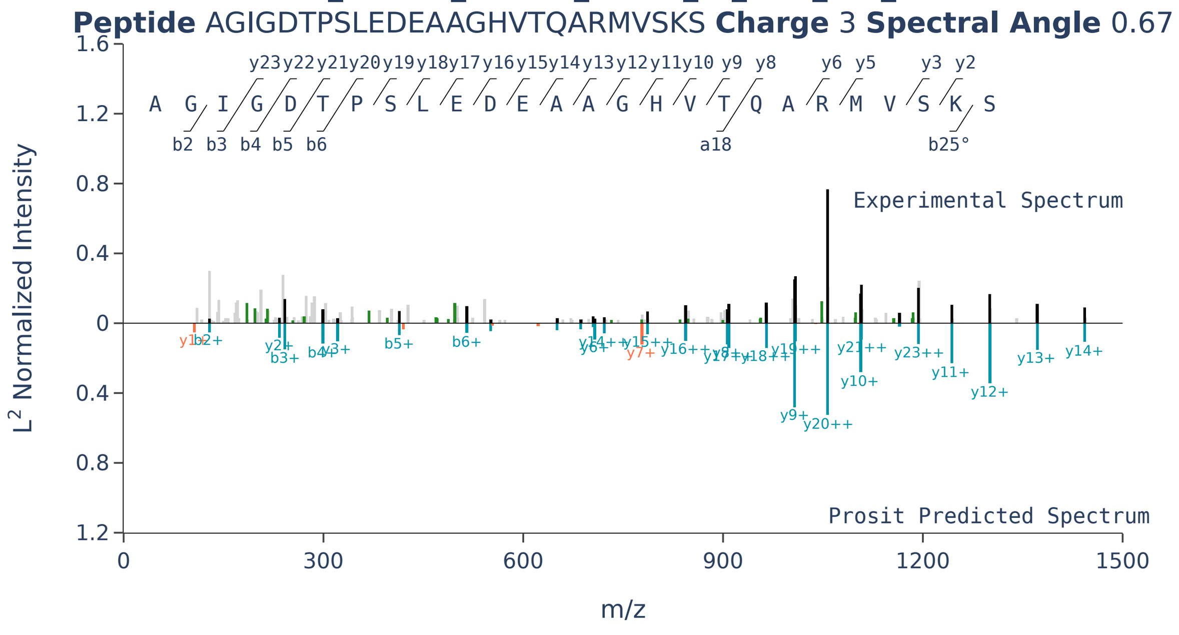


**Source** WSoh_221121_011221_HFGoe_L1_hTau_24h_R1 **Scan** 9488
**Peptide** NATRIPAKTPPAPKTPPSSGEPPKSGDR **Charge** 4 **Spectral Angle** 0.74

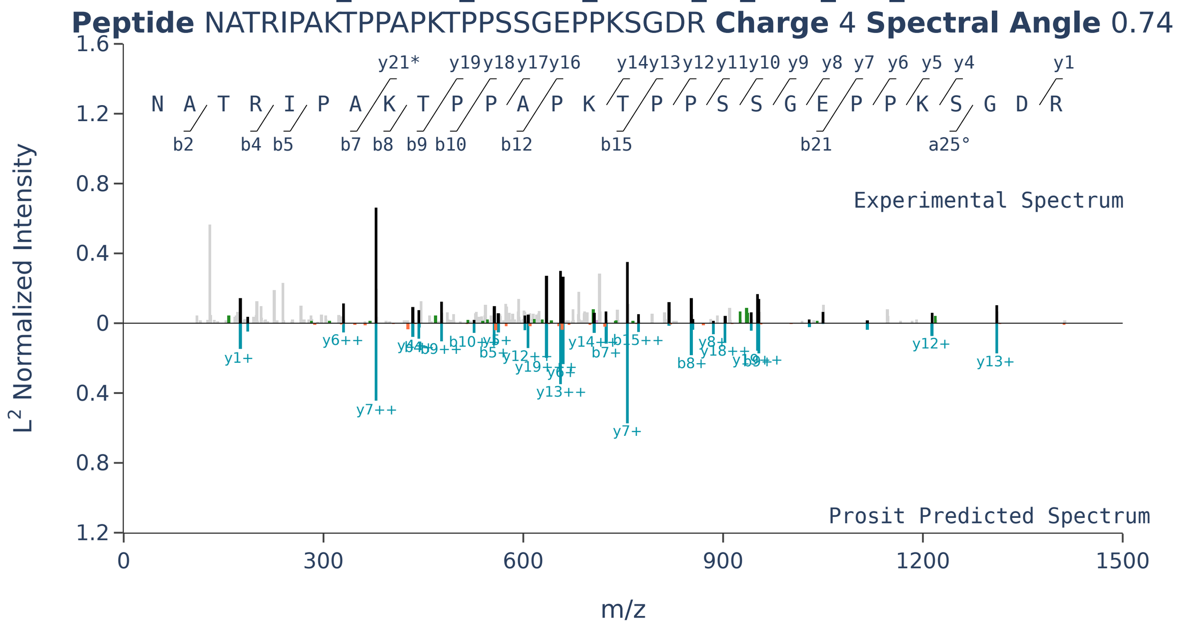


**Source** WSoh_221121_011221_HFGoe_L1_hTau_24h_R1 **Scan** 9522
**Peptide** GSLGNIHHKPGGGQVEVKSEKLD **Charge** 3 **Spectral Angle** 0.7

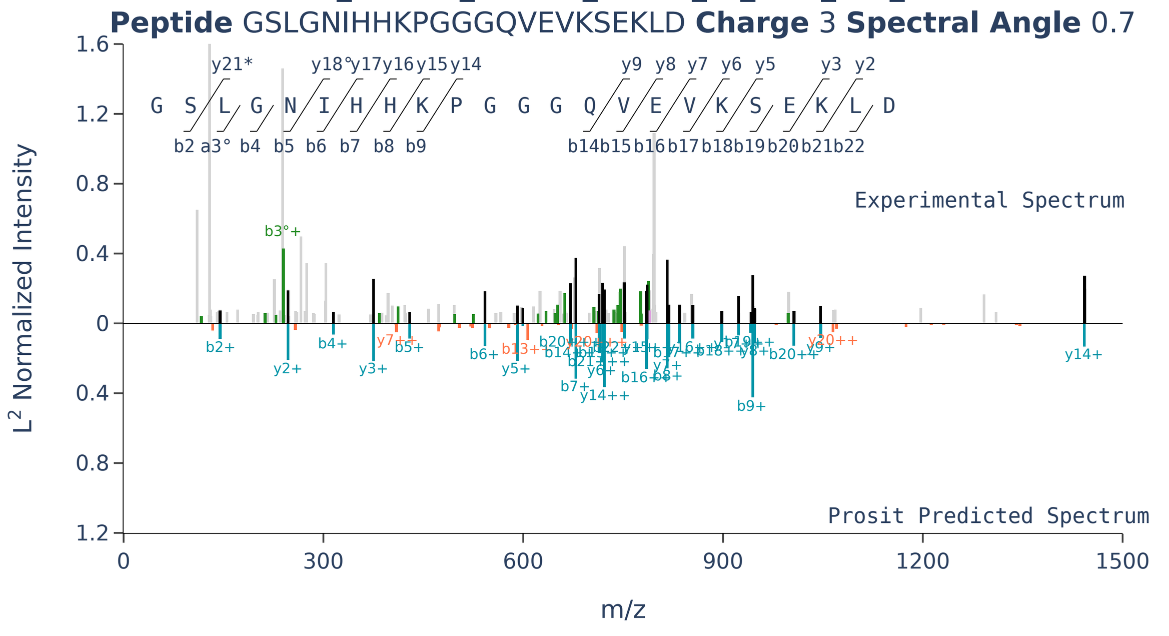


**Source** WSoh_221121_011221_HFGoe_L2_hTau_4h_R2 **Scan** 16096
**Peptide** HGAEIVYKSPVVSGDTSPRHLSNVS **Charge** 3 **Spectral Angle** 0.72

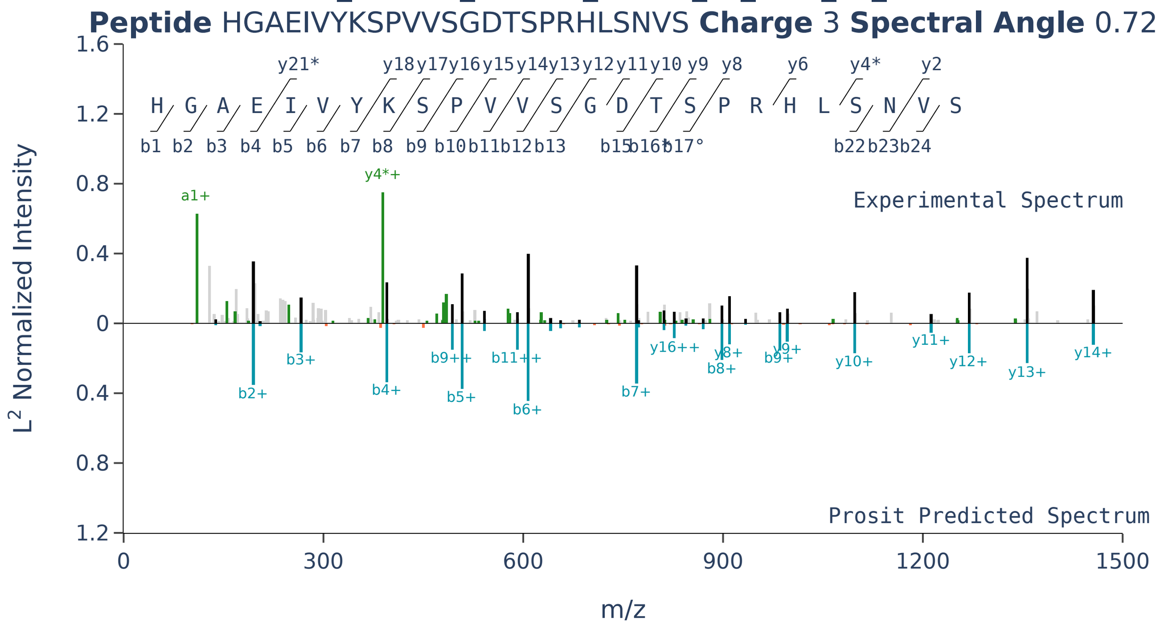


**Source** WSoh_221121_011221_HFGoe_L1_hTau_4h_R1 **Scan** 15414
**Peptide** LGSTENLKHQPGGGKVQII **Charge** 3 **Spectral Angle** 0.76

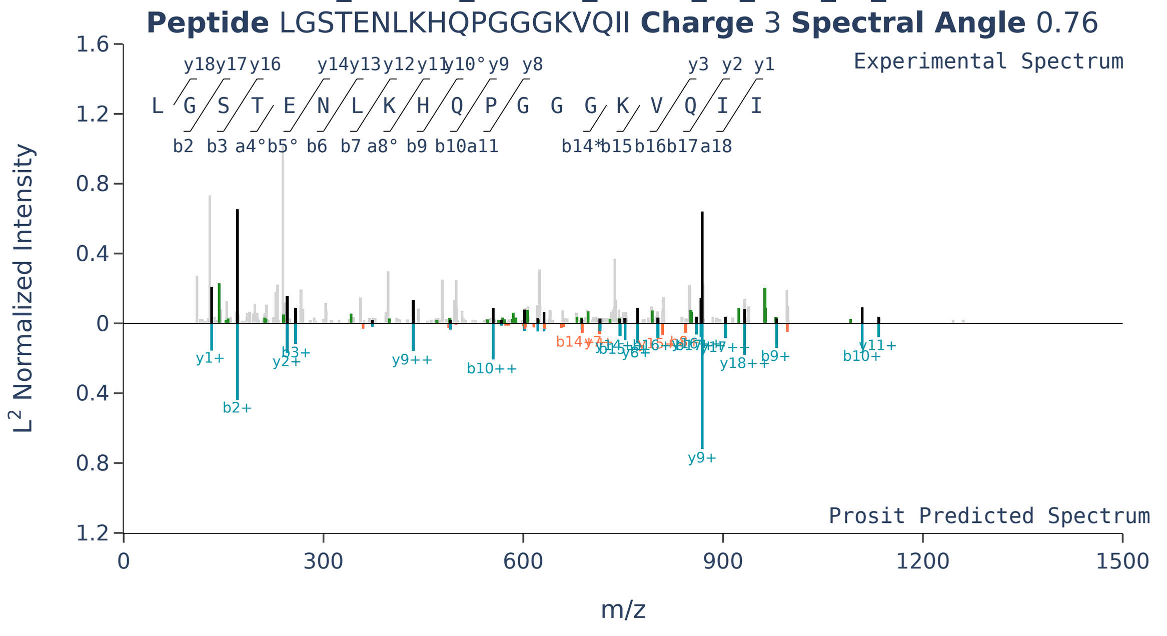


**Source** WSoh_221121_011221_HFGoe_L2_hTau_24h_R1 **Scan** 15287
**Peptide** GSLDNITHVPGGGNKKLE **Charge** 4 **Spectral Angle** 0.8

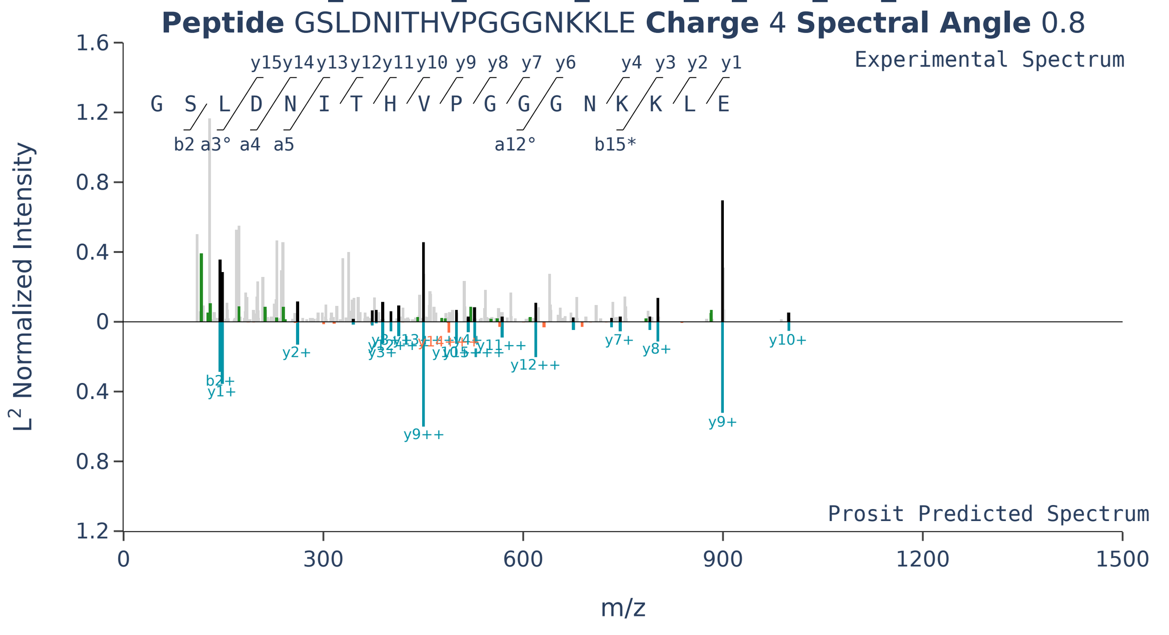


**Source** WSoh_221121_011221_HFGoe_L1_hTau_4h_R1 **Scan** 10901
**Peptide** QPHTEIPEGTT **Charge** 2 **Spectral Angle** 0.91

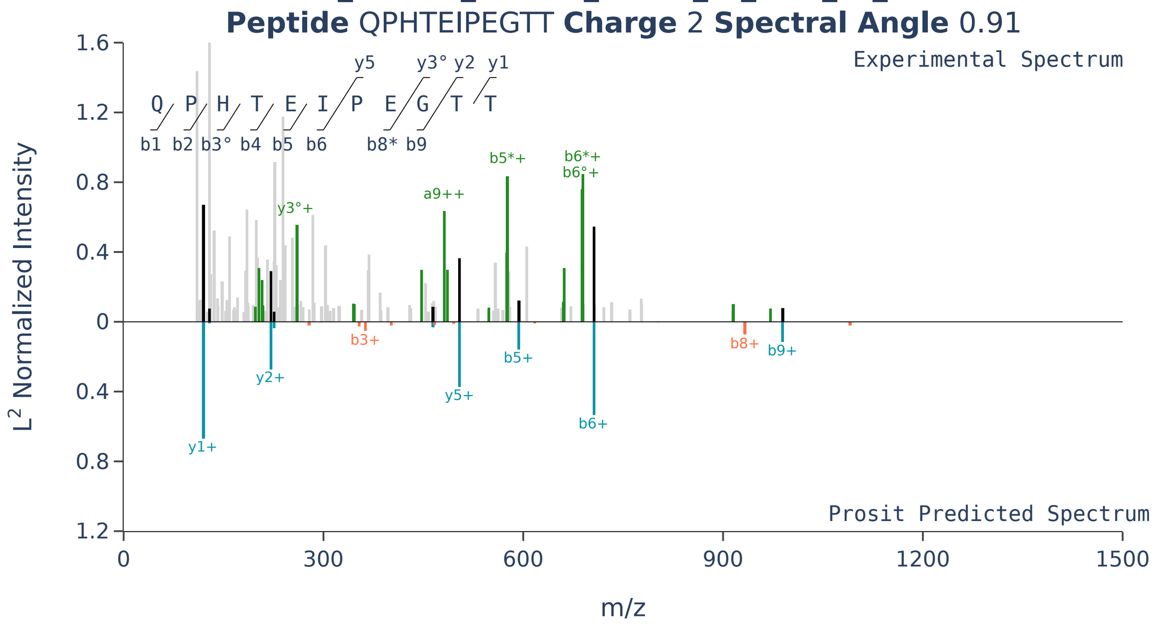


**Source** WSoh_221121_011221_HFGoe_L1_hTau_2h_R2 **Scan** 18994
**Peptide** KSTPTAEDVTAPLVDEGAPGKQA **Charge** 3 **Spectral Angle** 0.87

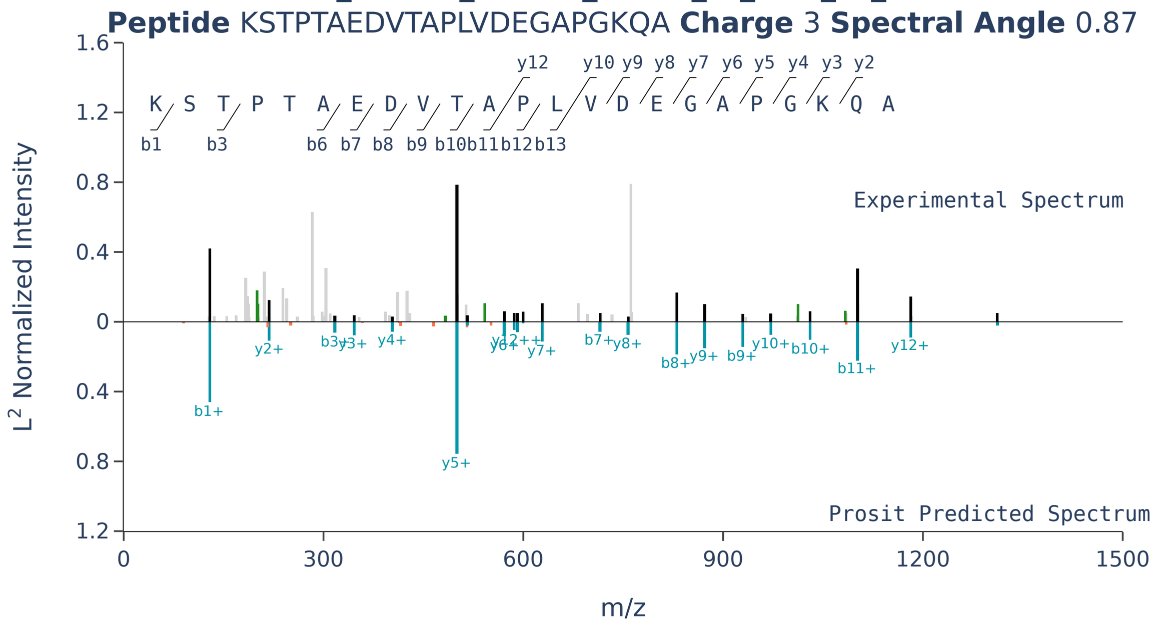


**Source** WSoh_221121_011221_HFGoe_L1_hTau_24h_R2 **Scan** 22299
**Peptide** TPPKSPSSAKSRLQTAPVPMPDL **Charge** 4 **Spectral Angle** 0.88

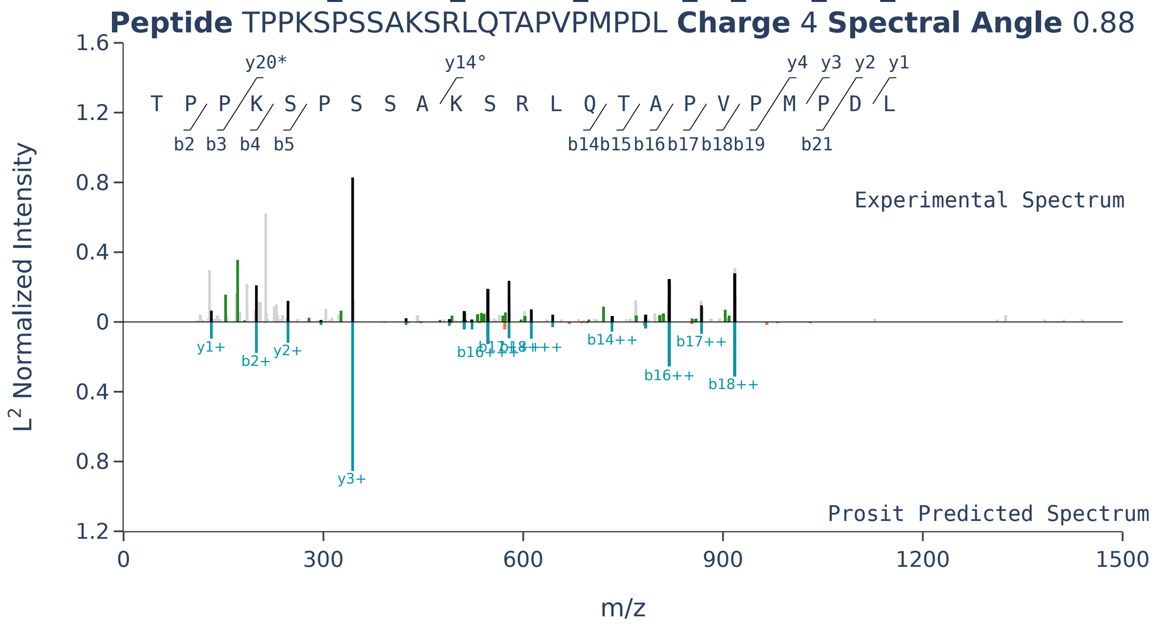


**Source** WSoh_221121_011221_HFGoe_L2_hTau_24h_R2 **Scan** 27537
**Peptide** SPQLATLADEVSA **Charge** 1 **Spectral Angle** 0.77

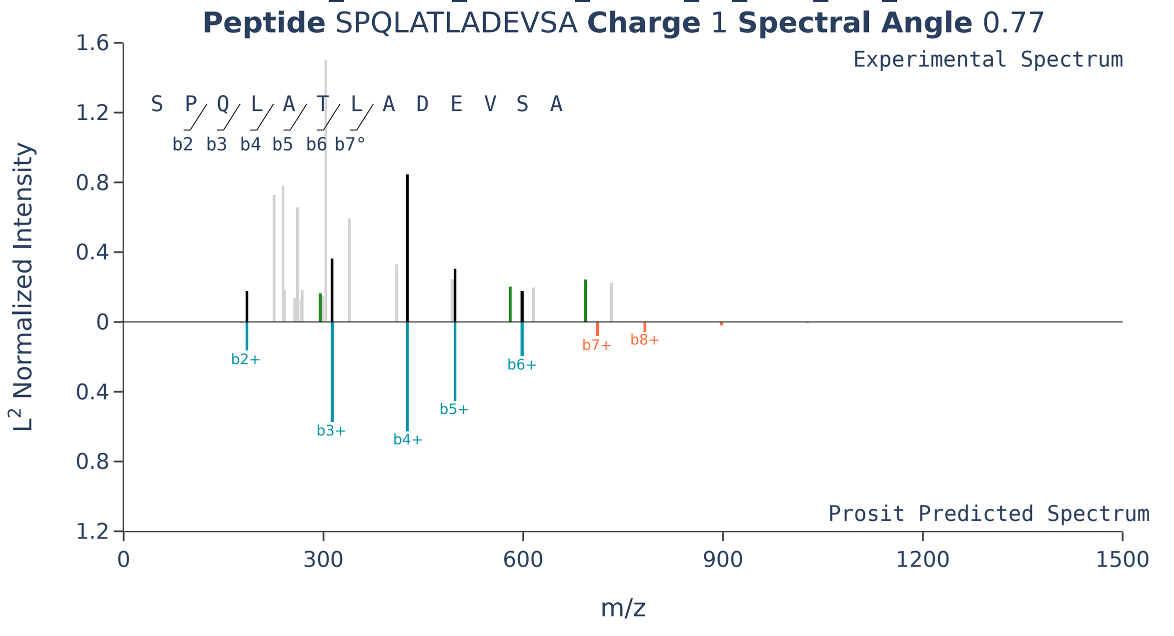


**Source** WSoh_221121_011221_HFGoe_L1_hTau_24h_R2 **Scan** 14134
**Peptide** LGSTENL **Charge** 1 **Spectral Angle** 0.82

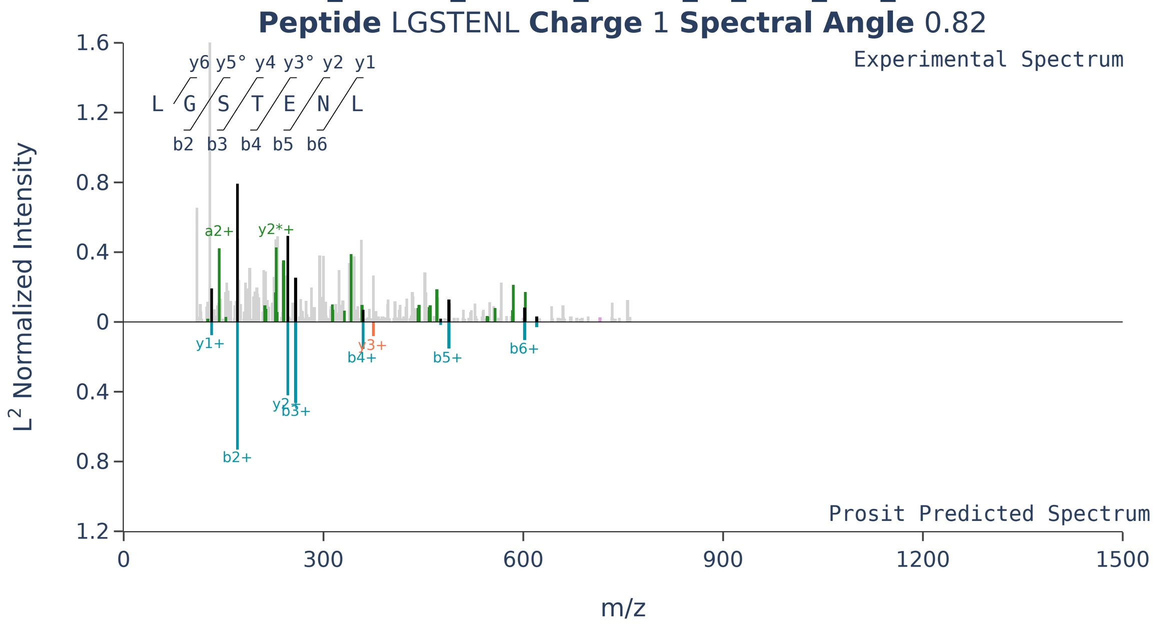

**Source** WSoh_221121_011221_HFGoe_L1_hTau_24h_R1 **Scan** 16678
**Peptide** PMPDLKNVKS **Charge** 2 **Spectral Angle** 0.82

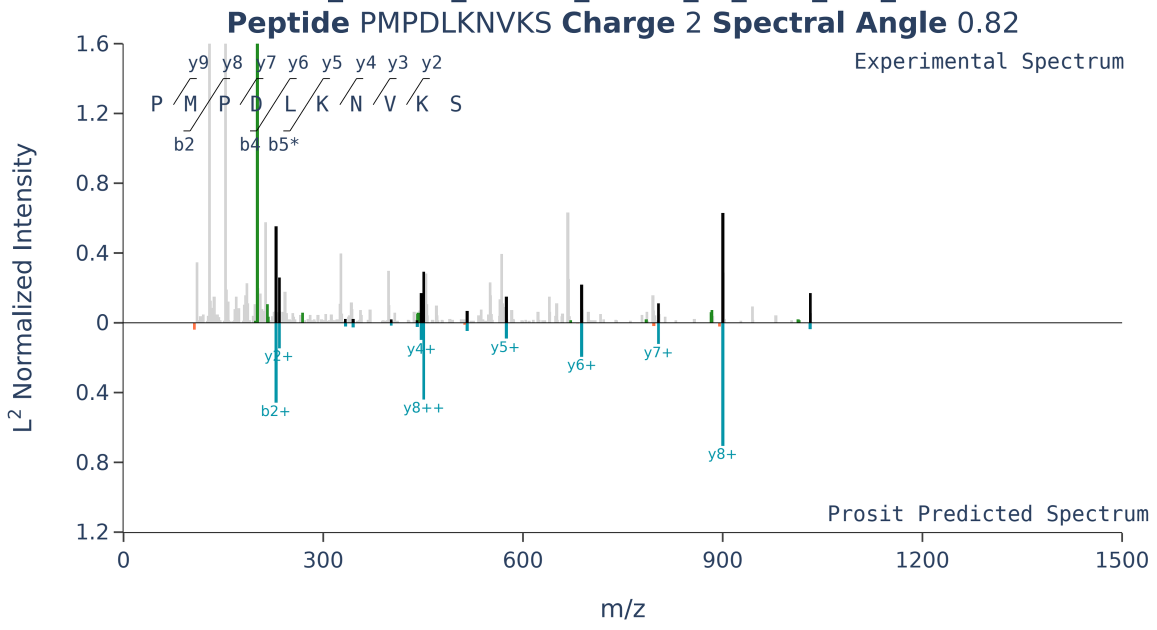


**Source** WSoh_221121_011221_HFGoe_L1_hTau_24h_R2 **Scan** 8802
**Peptide** PPTREPKKVAV **Charge** 3 **Spectral Angle** 0.84

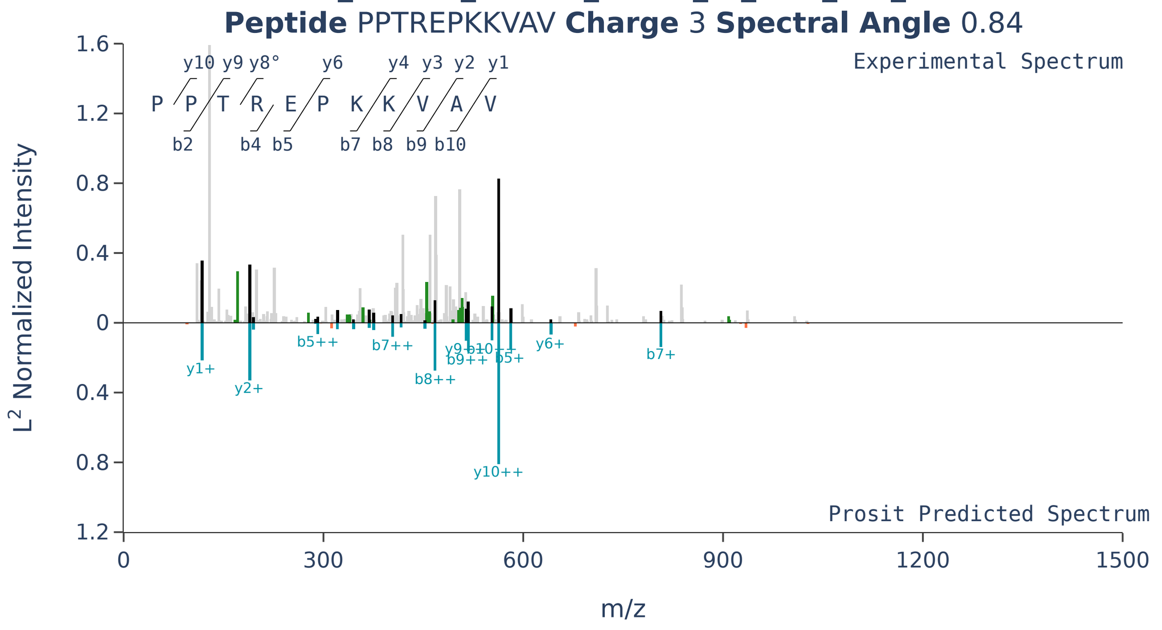


**Source** WSoh_221121_011221_HFGoe_L2_hTau_24h_R1 **Scan** 12756
**Peptide** SLGNIHHKPGGGQVEVKSEKLDFKDRVQS **Charge** 3 **Spectral Angle** 0.71

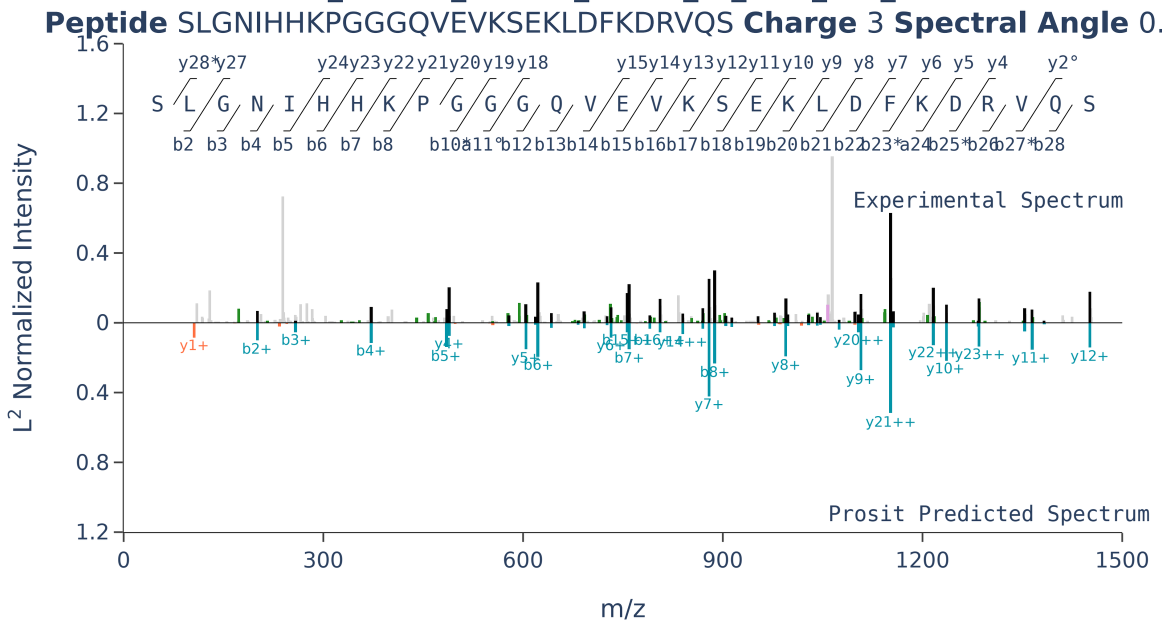


**Source** WSoh_221121_011221_HFGoe_L1_hTau_24h_R2 **Scan** 10608
**Peptide** NLTHVPGGGNKKIETHKLTFRENAKA **Charge** 5 **Spectral Angle** 0.81

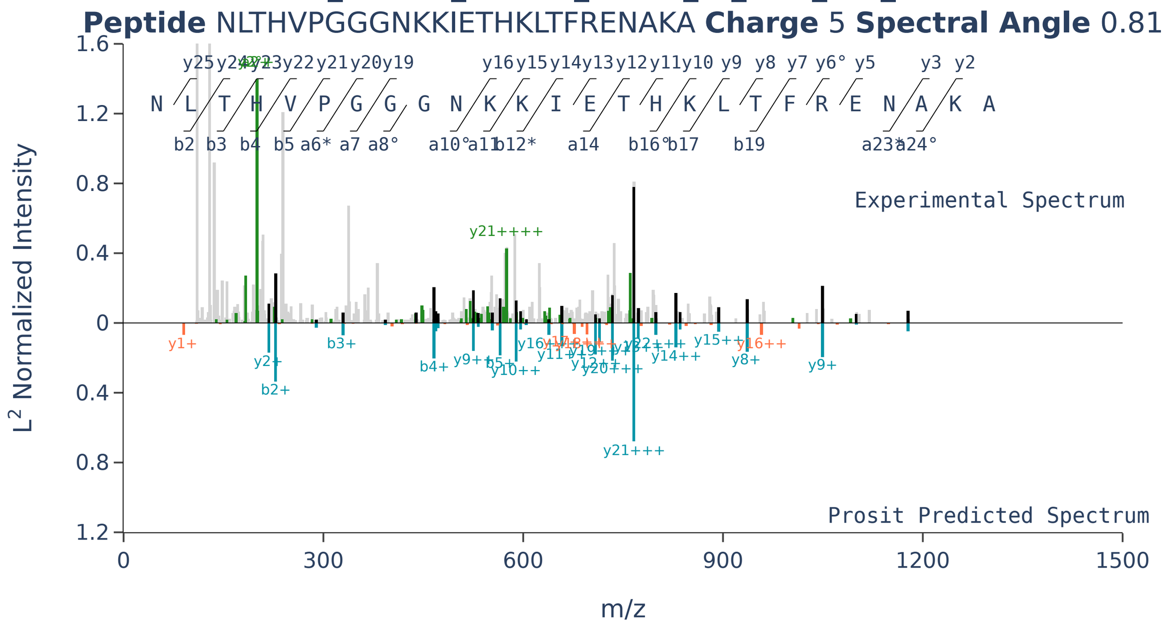


**Source** WSoh_221121_011221_HFGoe_L1_hTau_24h_R1 **Scan** 11062
**Peptide** IGSTENLKHQPGGGKVQIINK **Charge** 5 **Spectral Angle** 0.78

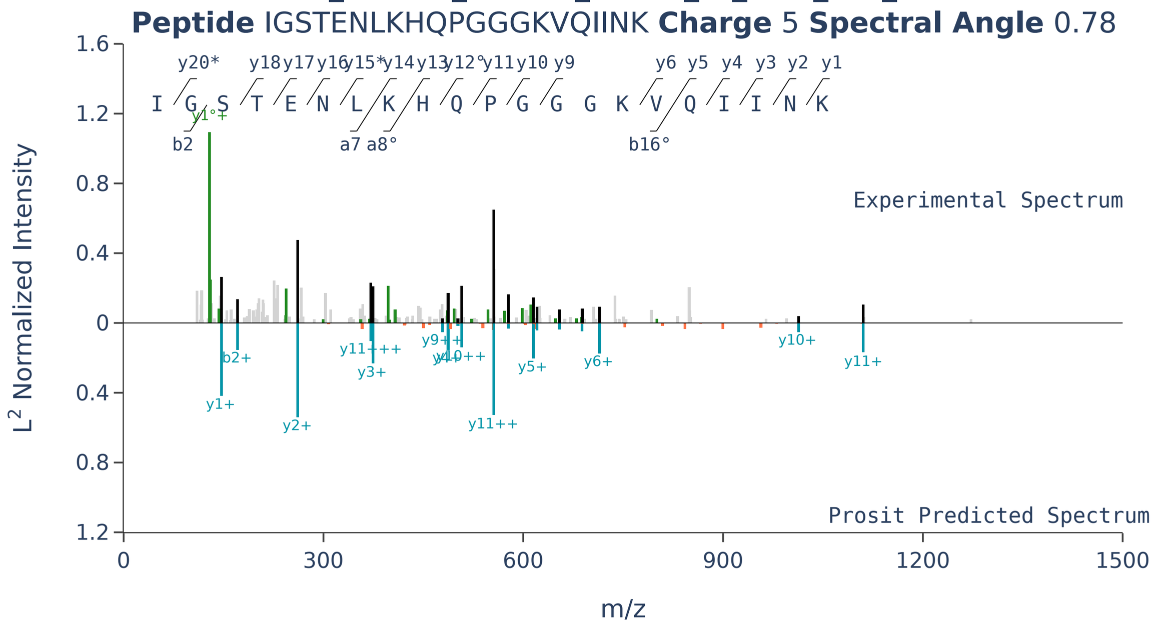


**Source** WSoh_221121_011221_HFGoe_L1_hTau_24h_R2 **Scan** 13811
**Peptide** TFRENAKAKTDHGAEIVYKSPVVSGDTS **Charge** 4 **Spectral Angle** 0.73

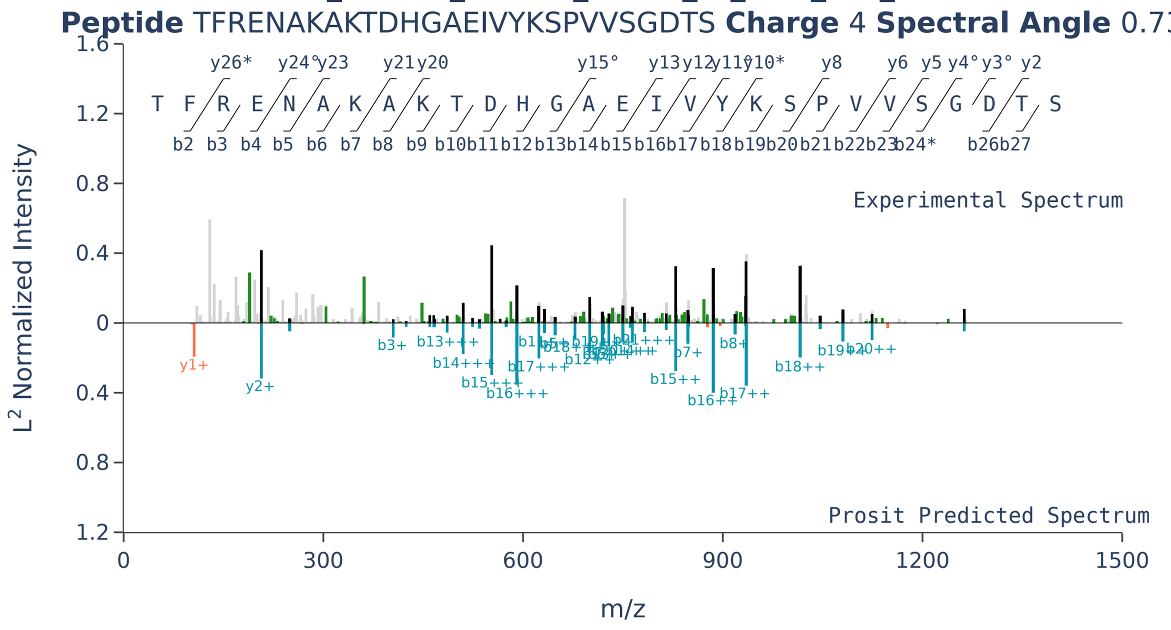


**Source** WSoh_221121_011221_HFGoe_L1_hTau_24h_R2 **Scan** 20805
**Peptide** DFKDRVQSKIGSLDNITH **Charge** 4 **Spectral Angle** 0.89

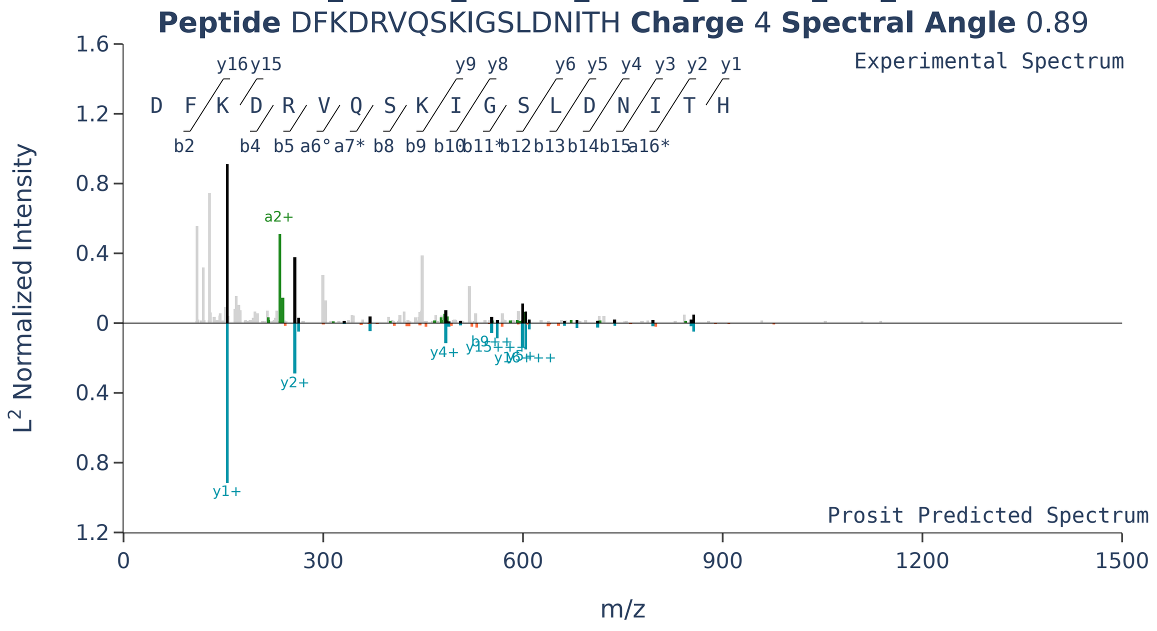


**Source** WSoh_221121_011221_HFGoe_L1_hTau_4h_R2 **Scan** 24519
**Peptide** RLQTAPVPMPDLKNV **Charge** 3 **Spectral Angle** 0.89

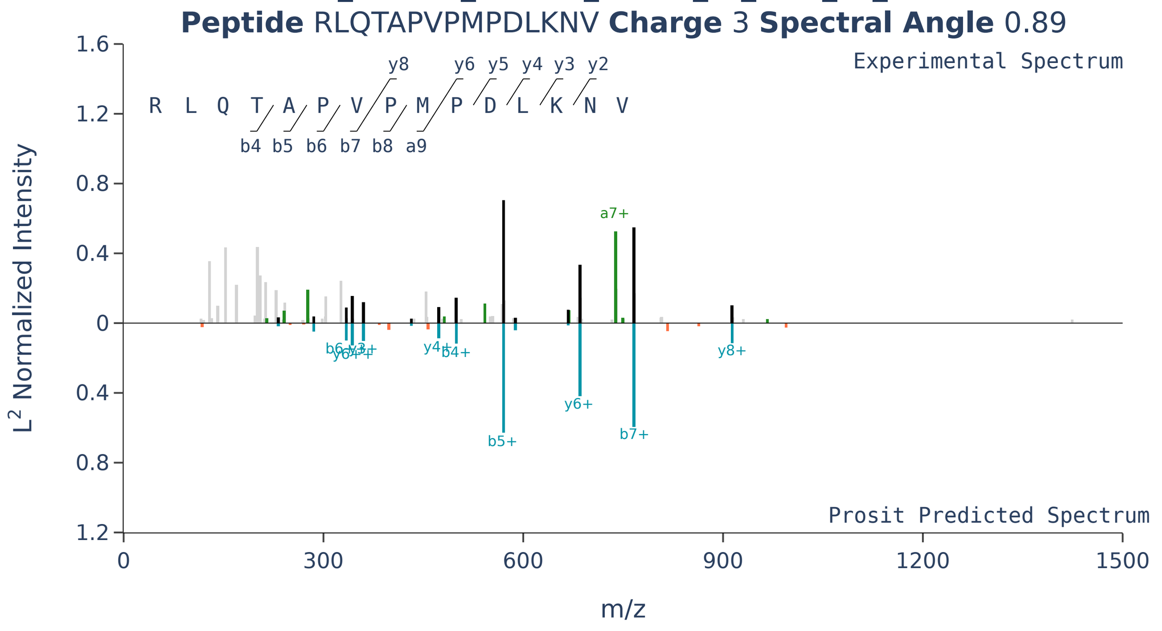


**Source** WSoh_221121_011221_HFGoe_L2_hTau_24h_R2 **Scan** 20363
**Peptide** DQEGDTDAGLKESPLQTPTE **Charge** 3 **Spectral Angle** 0.93

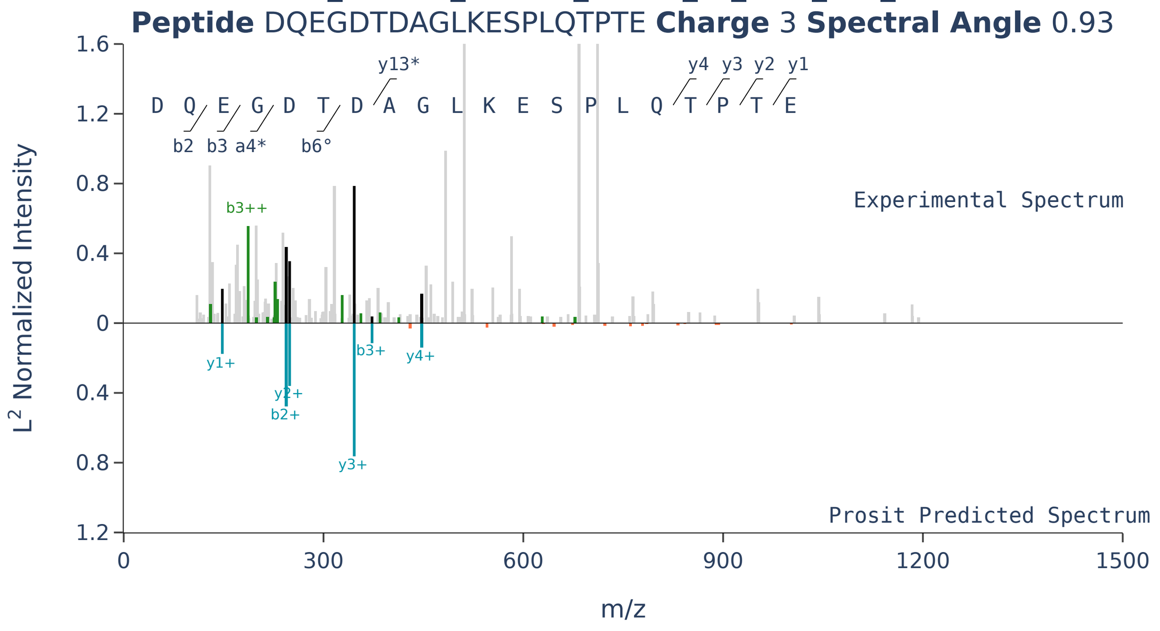


**Source** WSoh_221121_011221_HFGoe_L2_hTau_24h_R2 **Scan** 18238
**Peptide** AGIGDTPSLEDEAAGHVTQARMVSKSK **Charge** 5 **Spectral Angle** 0.76

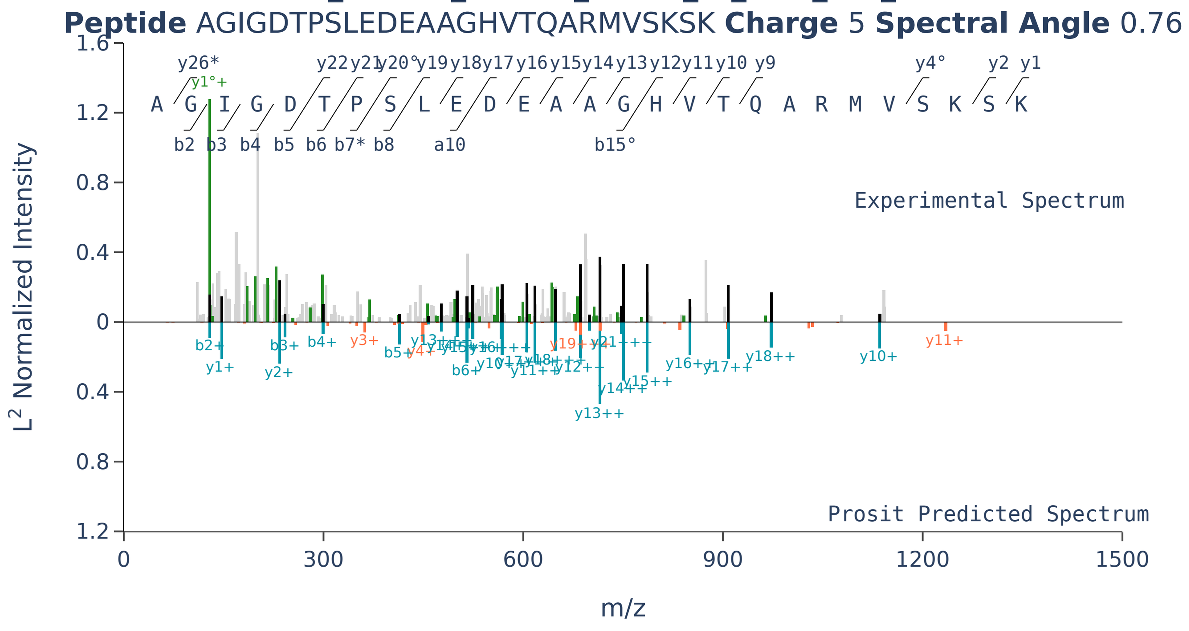


**Source** WSoh_221121_011221_HFGoe_L1_hTau_24h_R2 **Scan** 18787
**Peptide** LDLSNVQSKCGSKDNIKHVPGGGSVQI **Charge** 4 **Spectral Angle** 0.78

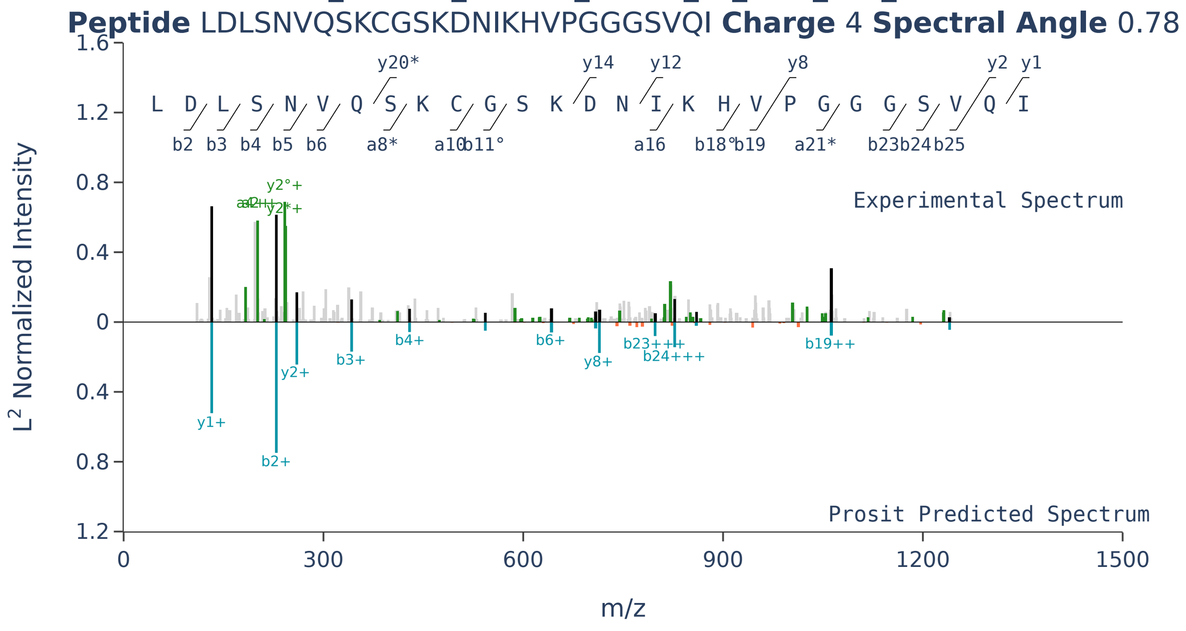


**Source** WSoh_221121_011221_HFGoe_L2_hTau_4h_R2 **Scan** 13675
**Peptide** GSTENLKHQPGGGKVQIL **Charge** 3 **Spectral Angle** 0.79

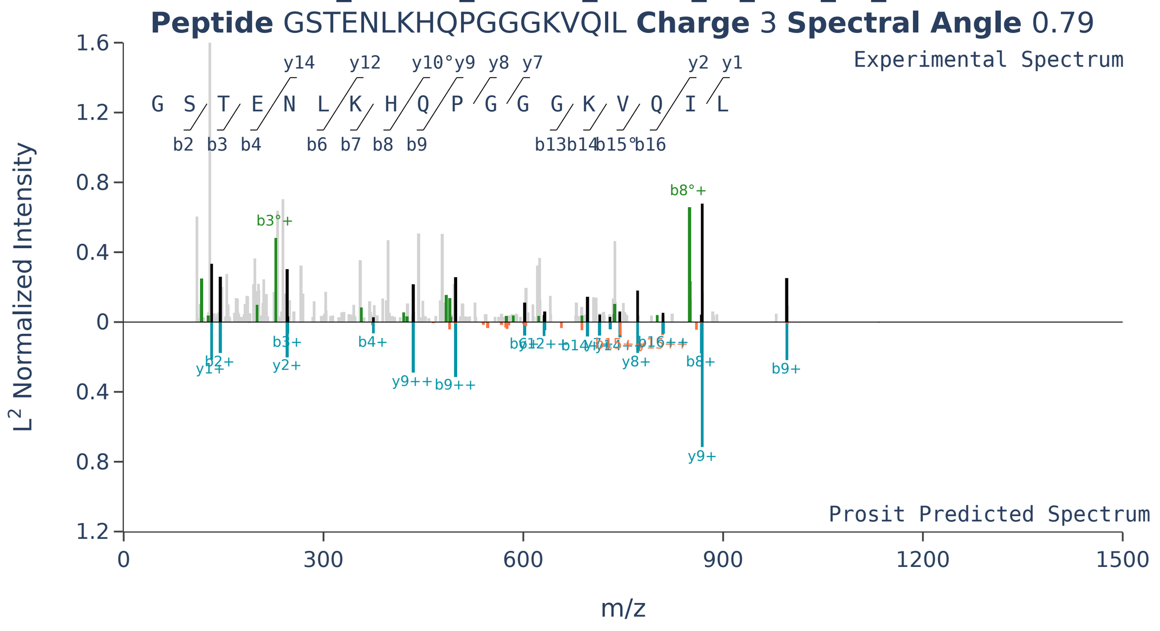


**Source** WSoh_221121_011221_HFGoe_L2_hTau_24h_R2 **Scan** 9293
**Peptide** KNVKSKIGSTENLKHQPGGGKVQL **Charge** 3 **Spectral Angle** 0.84

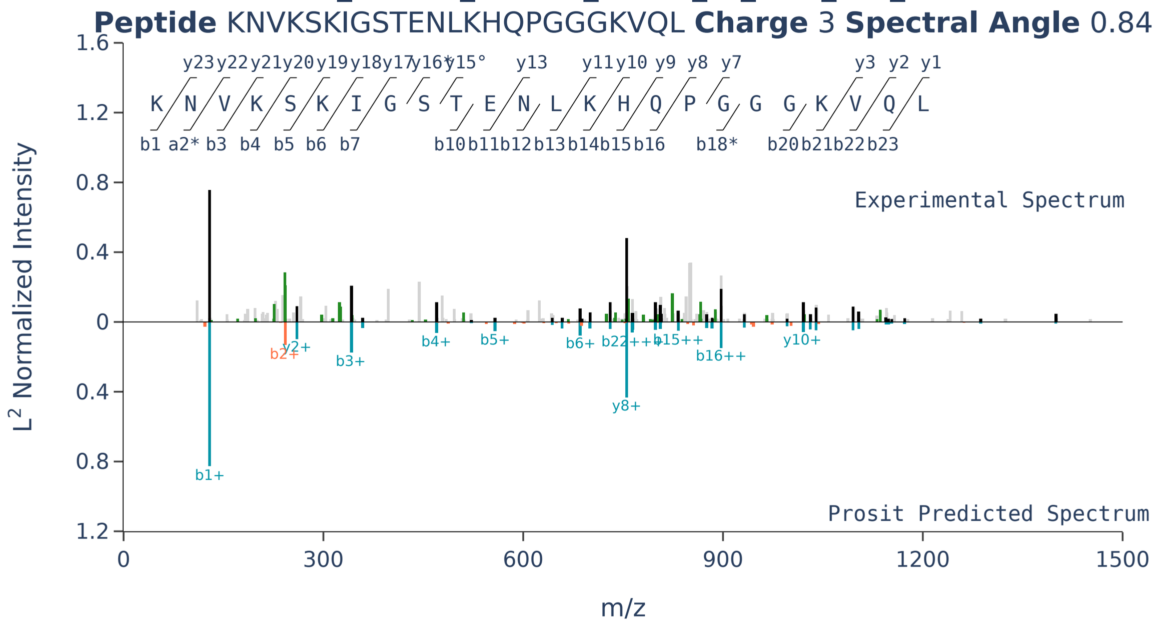


**Source** WSoh_221121_011221_HFGoe_L1_hTau_24h_R1 **Scan** 17307
**Peptide** AVVRTPPKSPSSAKSRLQTAPVPMPDL **Charge** 4 **Spectral Angle** 0.7

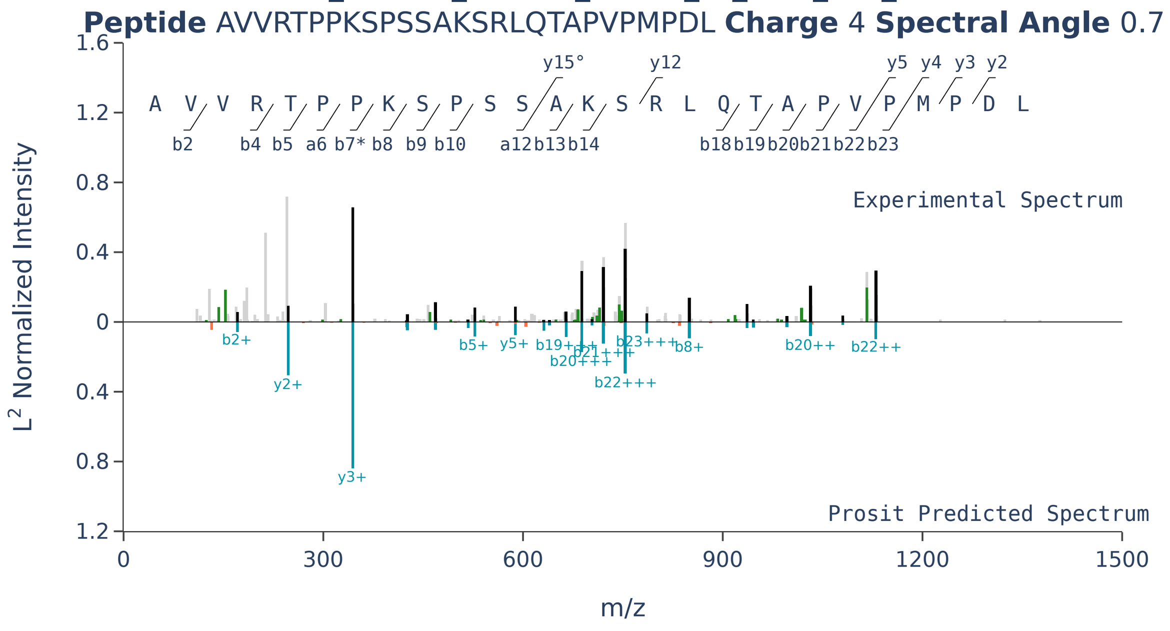


**Source** WSoh_221121_011221_HFGoe_L2_hTau_24h_R2 **Scan** 11966
**Peptide** LNKKLDL **Charge** 1 **Spectral Angle** 0.77

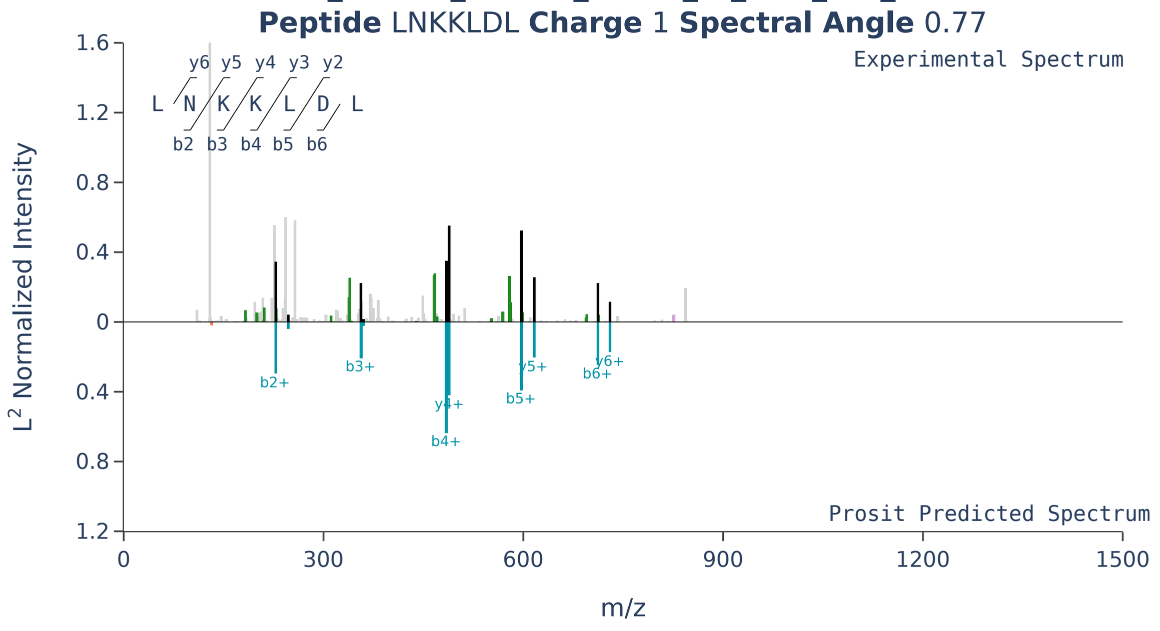

**Source** WSoh_221121_011221_HFGoe_L1_hTau_24h_R1 **Scan** 13928
**Peptide** EKLDFKDRVQSKIGS **Charge** 4 **Spectral Angle** 0.75

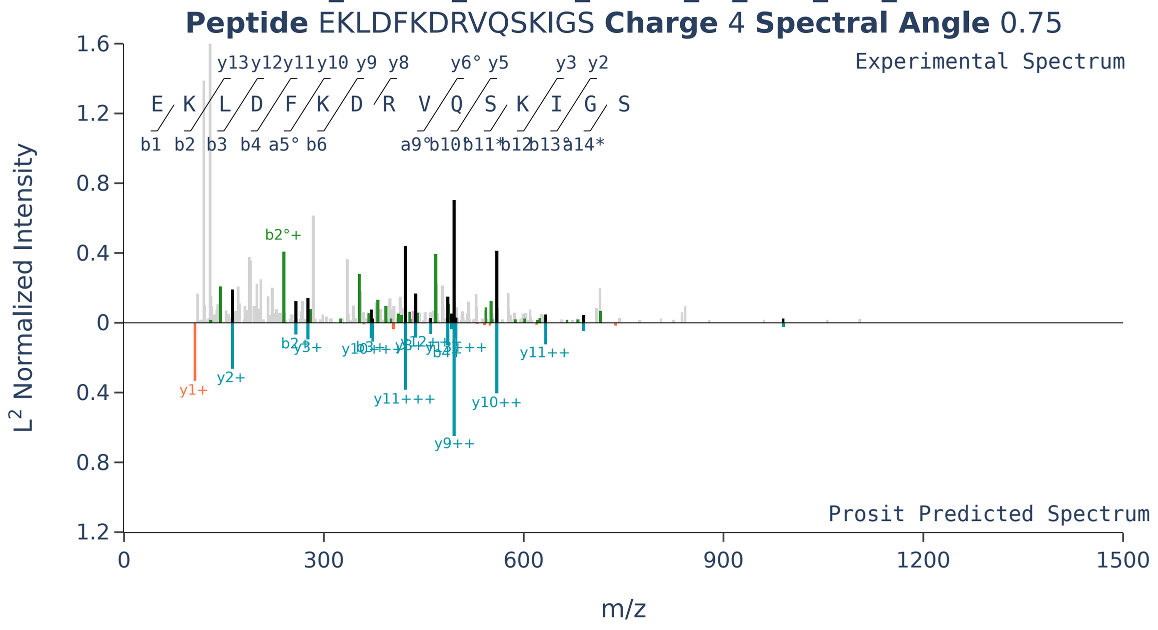


**Source** WSoh_221121_011221_HFGoe_L1_hTau_4h_R1 **Scan** 6530
**Peptide** ETHKLTFRENAKAKTD **Charge** 3 **Spectral Angle** 0.78

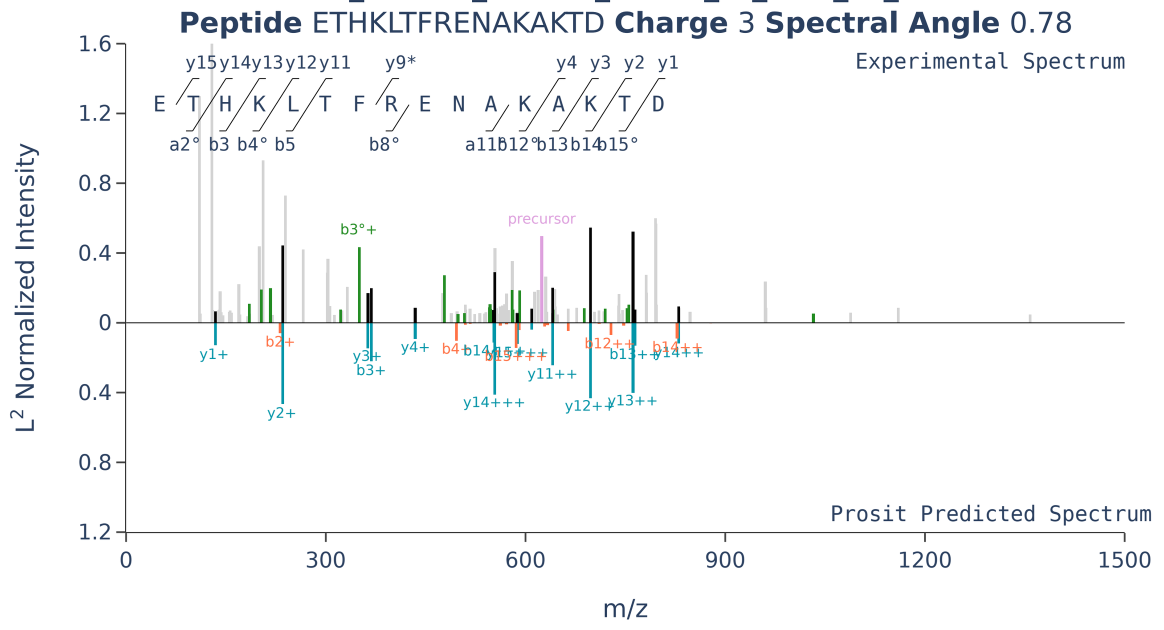


**Source** WSoh_221121_011221_HFGoe_L1_hTau_4h_R2 **Scan** 17983
**Peptide** KSPVVSGDTSPRHLSNVSSTGSIDMVD **Charge** 4 **Spectral Angle** 0.69

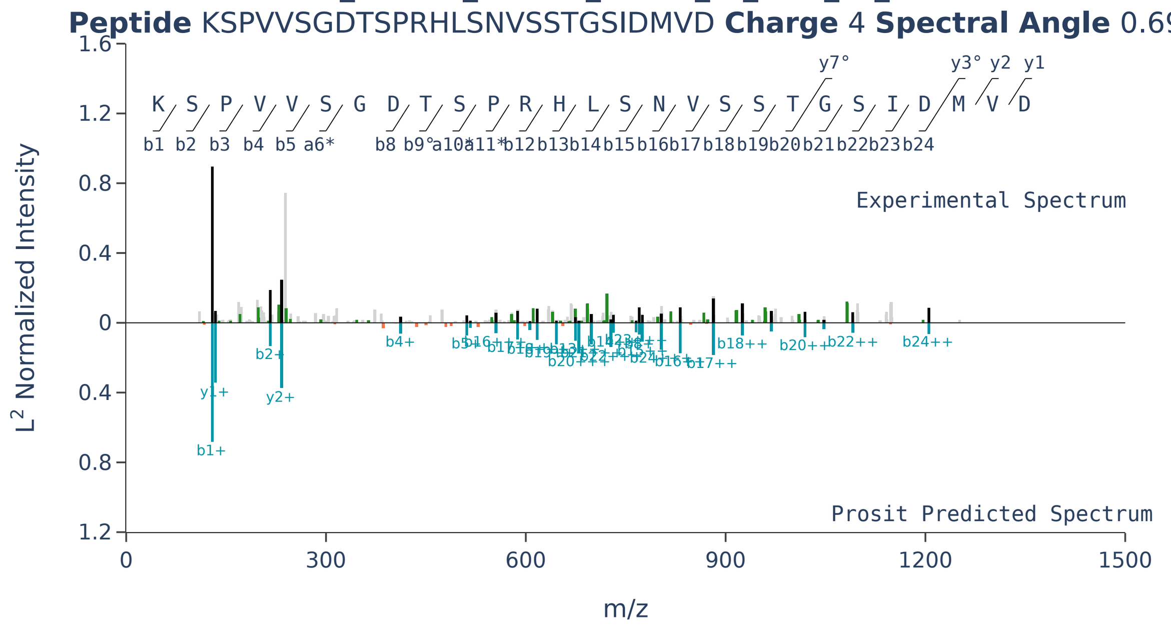


**Source** WSoh_221121_011221_HFGoe_L2_hTau_24h_R1 **Scan** 16214
**Peptide** IGSLDNITHVPGGGNKKIETHKLT **Charge** 5 **Spectral Angle** 0.85

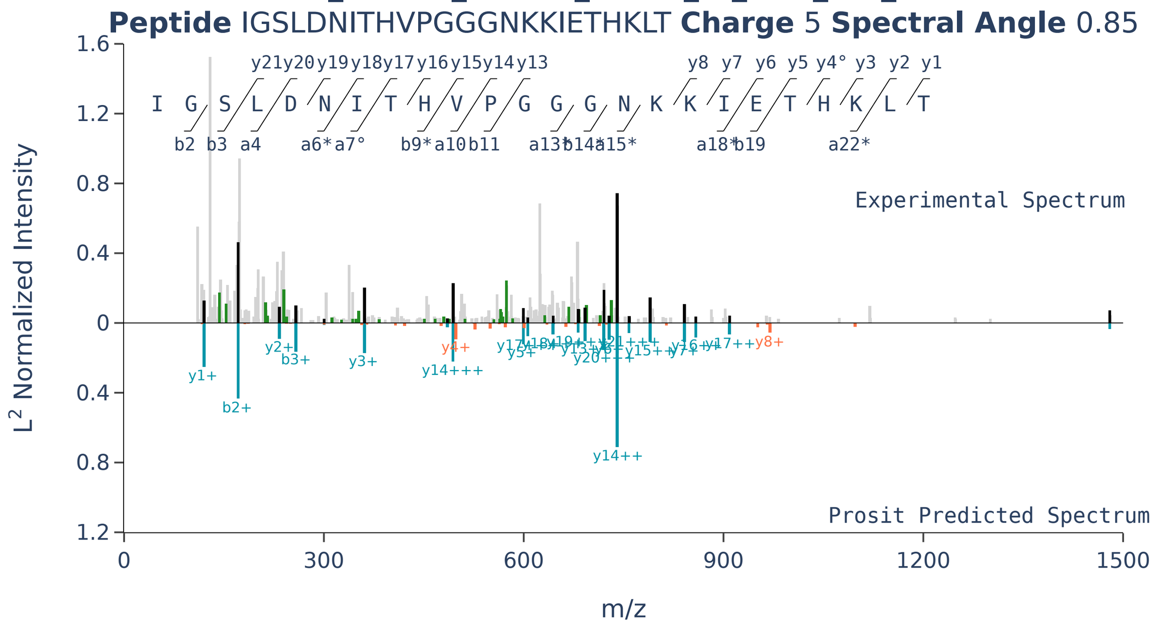


**Source** WSoh_221121_011221_HFGoe_L1_hTau_24h_R2 **Scan** 16902
**Peptide** QSKIGSLDNITHVPGGGNKKL **Charge** 3 **Spectral Angle** 0.9

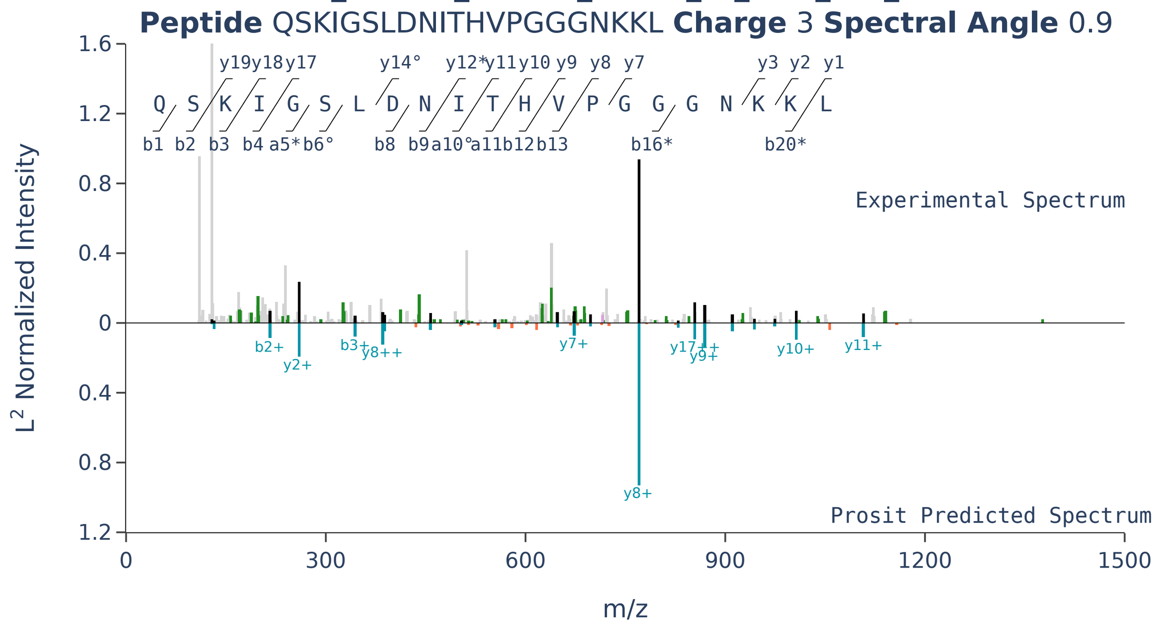


**Source** WSoh_221121_011221_HFGoe_L2_hTau_24h_R1 **Scan** 10272
**Peptide** KSPVVSGDTSPRHLSNVS **Charge** 2 **Spectral Angle** 0.8

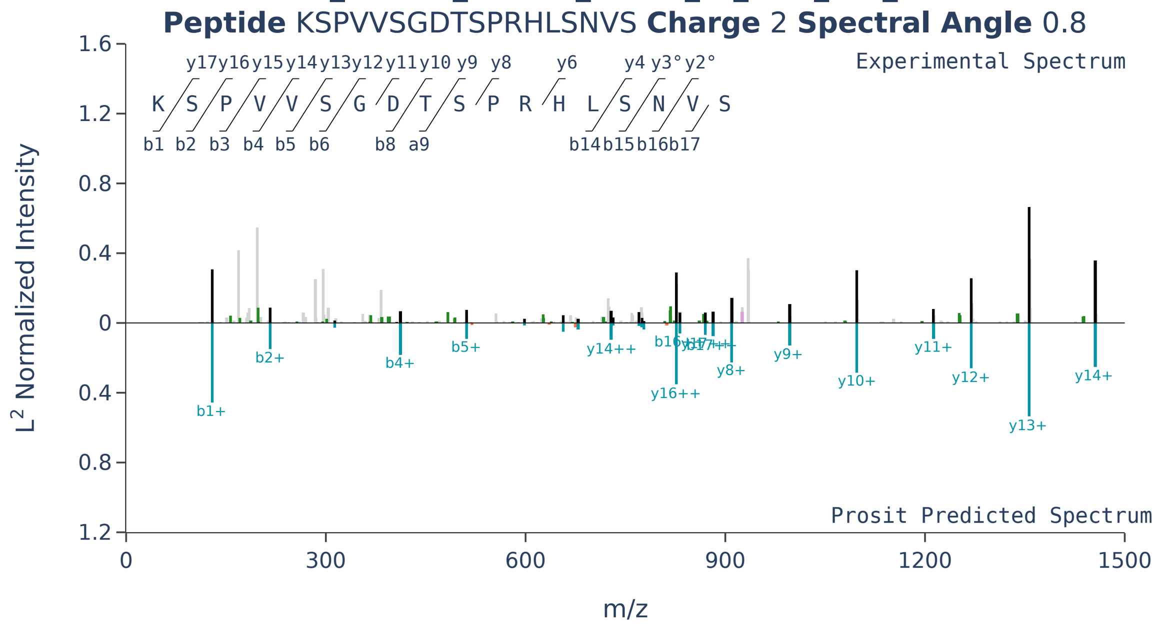


**Source** WSoh_221121_011221_HFGoe_L1_hTau_2h_R2 **Scan** 6863
**Peptide** GADGKTKIATPRGA **Charge** 3 **Spectral Angle** 0.84

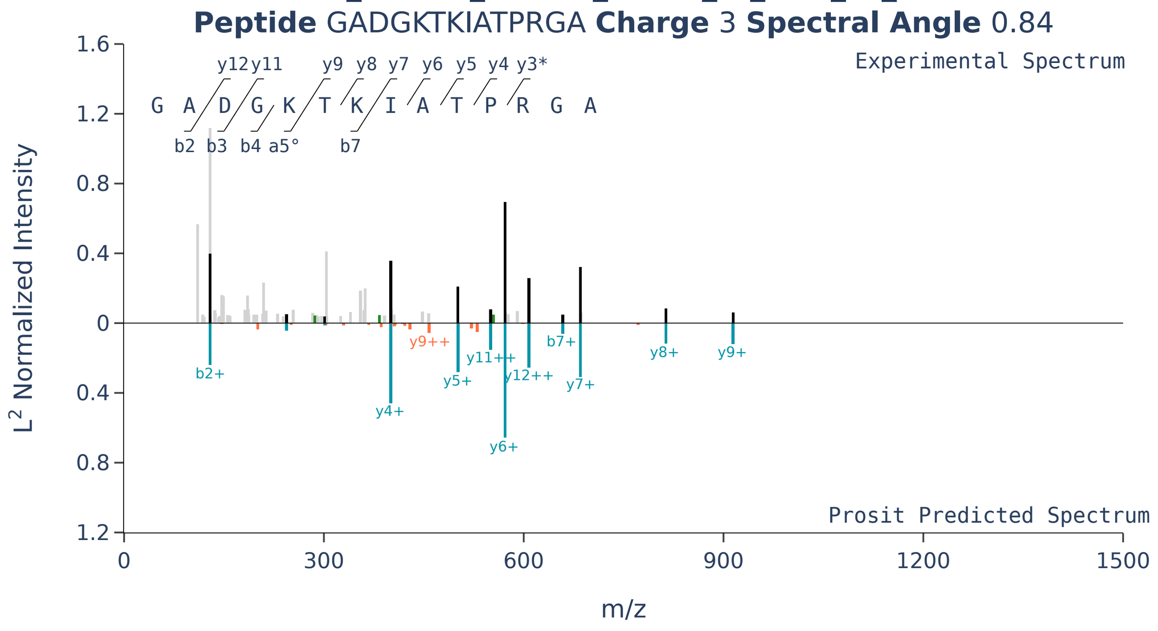


**Source** WSoh_221121_011221_HFGoe_L2_hTau_24h_R1 **Scan** 8327
**Peptide** DEAAGHVTQARMVSKS **Charge** 3 **Spectral Angle** 0.79

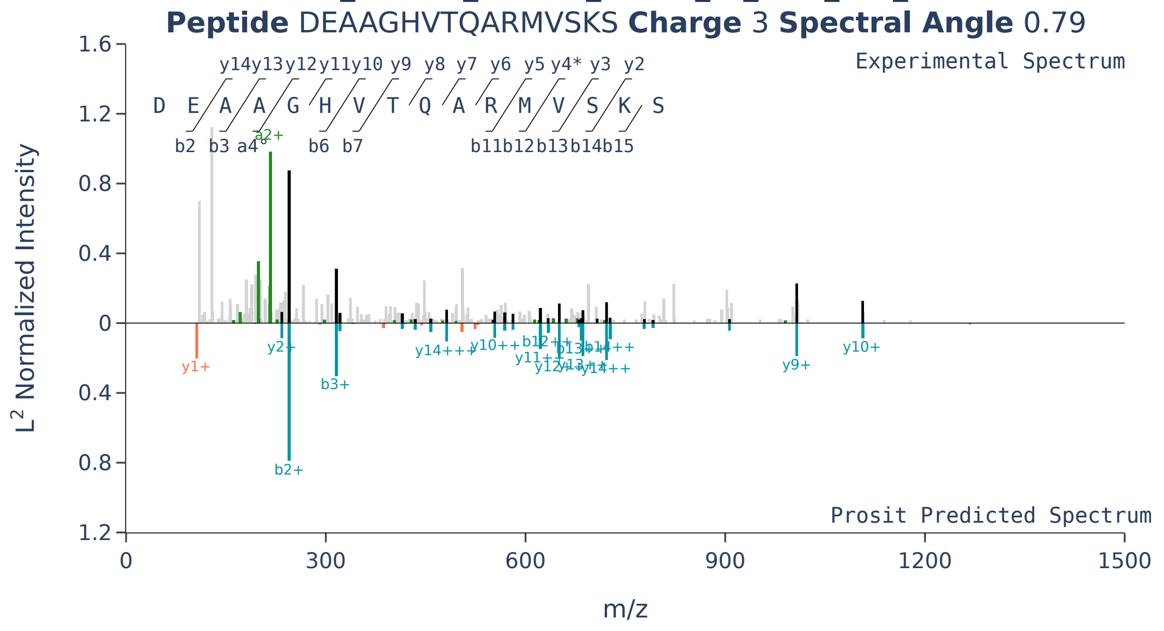


**Source** WSoh_221121_011221_HFGoe_L2_hTau_24h_R2 **Scan** 22348
**Peptide** IGDTPSLEDEAAGHVTQARMV **Charge** 3 **Spectral Angle** 0.74

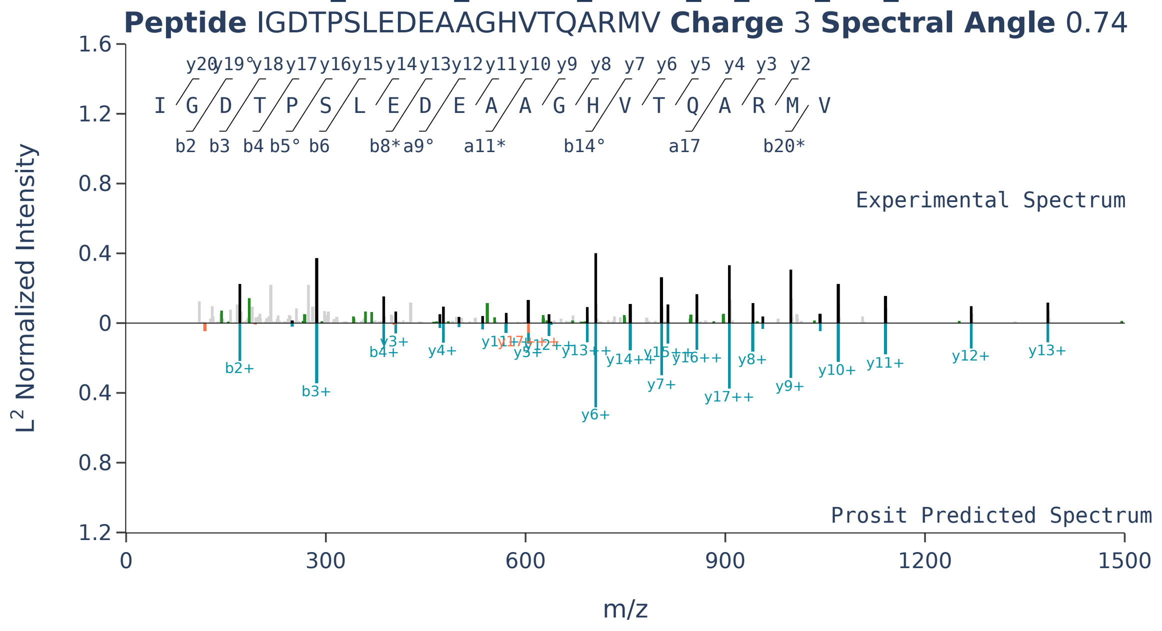


**Source** WSoh_221121_011221_HFGoe_L1_hTau_24h_R2 **Scan** 17877
**Peptide** AEIVYKSPVVSGDTSPRHLSNVSSTG **Charge** 3 **Spectral Angle** 0.86

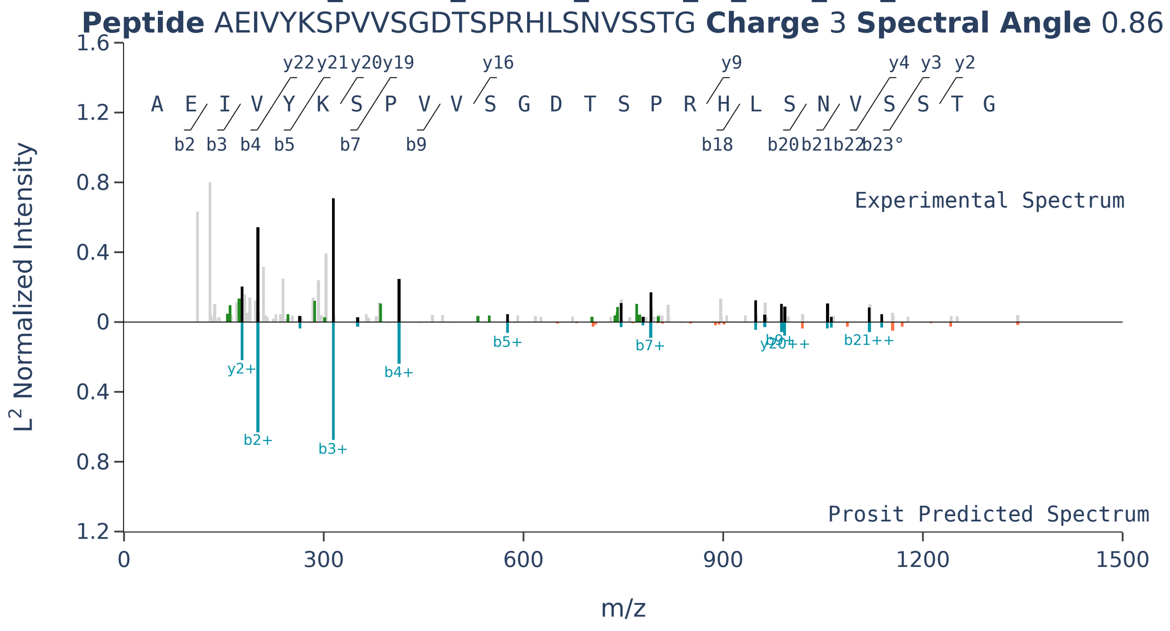


**Source** WSoh_221121_011221_HFGoe_L1_hTau_4h_R2 **Scan** 22405
**Peptide** PEGTTAEEAGIGDTPSLEDEAAGHVTQA **Charge** 3 **Spectral Angle** 0.72

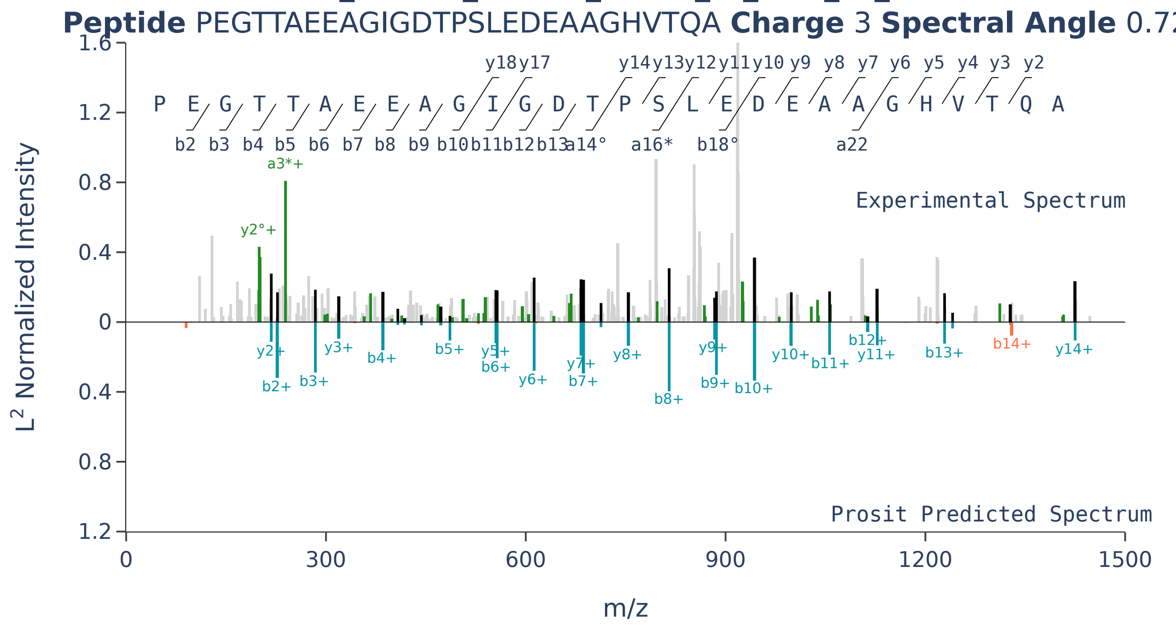


**Source** WSoh_221121_011221_HFGoe_L1_hTau_4h_R1 **Scan** 6473
**Peptide** GSTENLKHQ **Charge** 2 **Spectral Angle** 0.79

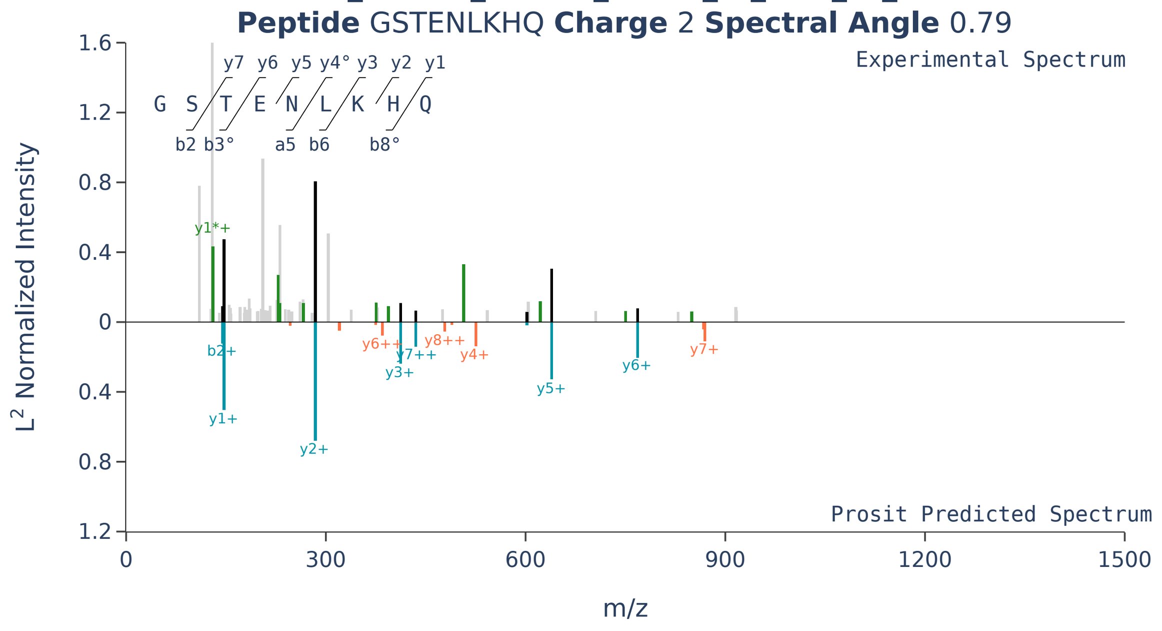


**Source** WSoh_221121_011221_HFGoe_L1_hTau_24h_R2 **Scan** 13870
**Peptide** IGSLDNITHVPGGGNKKIETHK **Charge** 5 **Spectral Angle** 0.84

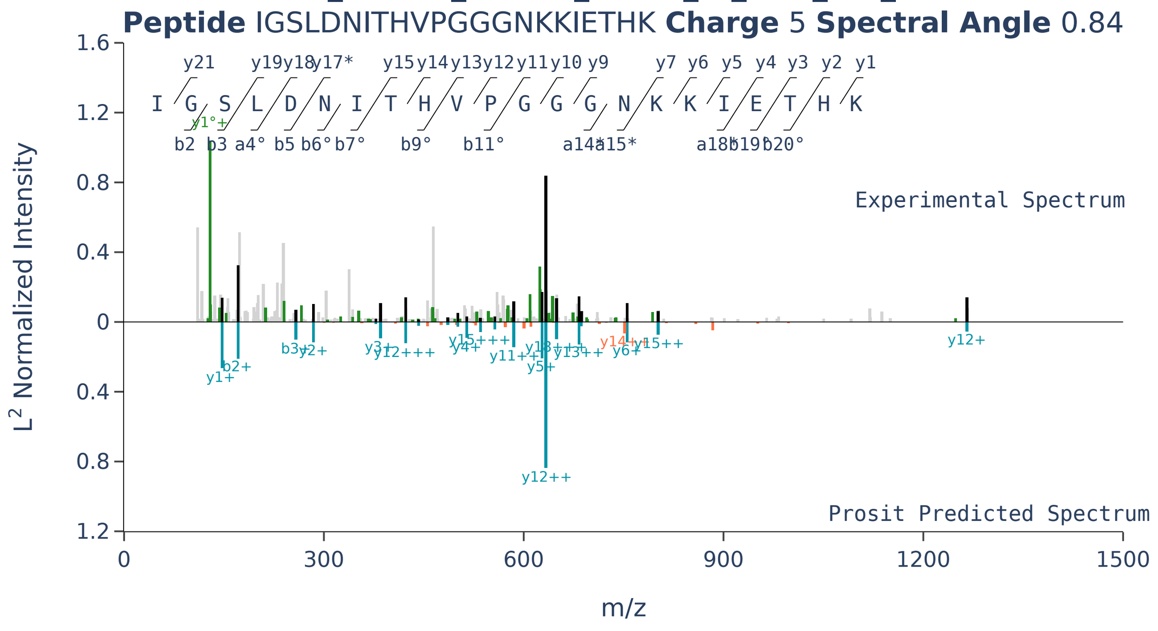


**Source** WSoh_221121_011221_HFGoe_L2_hTau_24h_R2 **Scan** 19917
**Peptide** QIINKKLDLSNV **Charge** 2 **Spectral Angle** 0.73

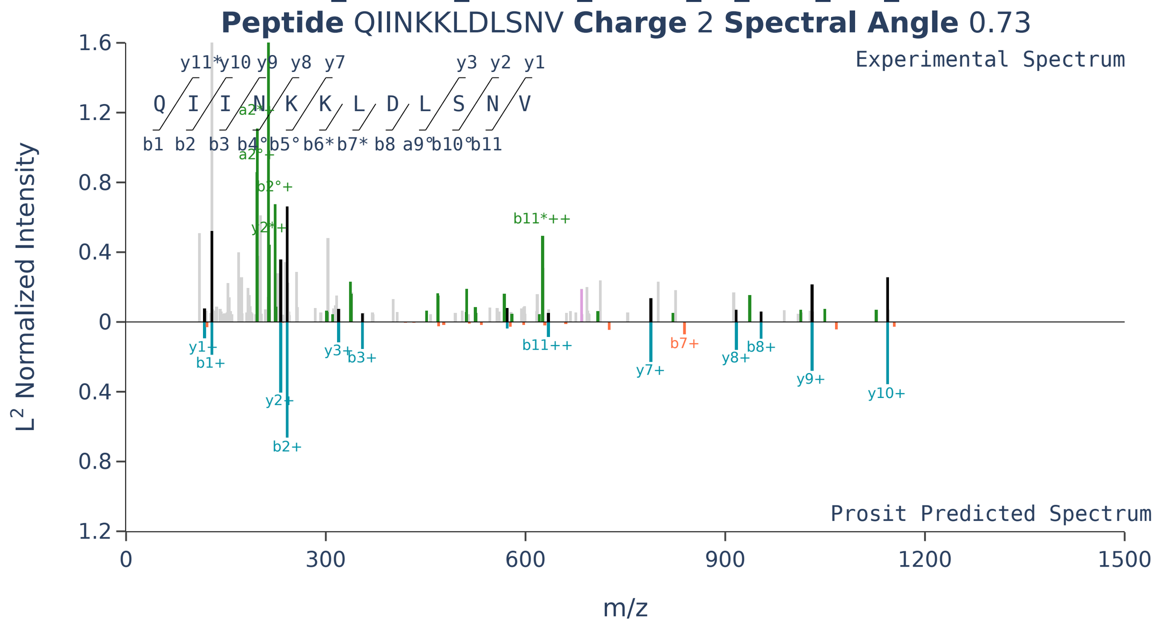


**Source** WSoh_221121_011221_HFGoe_L1_hTau_24h_R1 **Scan** 10644
**Peptide** AKAKTDHGAEIVYKSPVVSGDTSPRH **Charge** 3 **Spectral Angle** 0.72

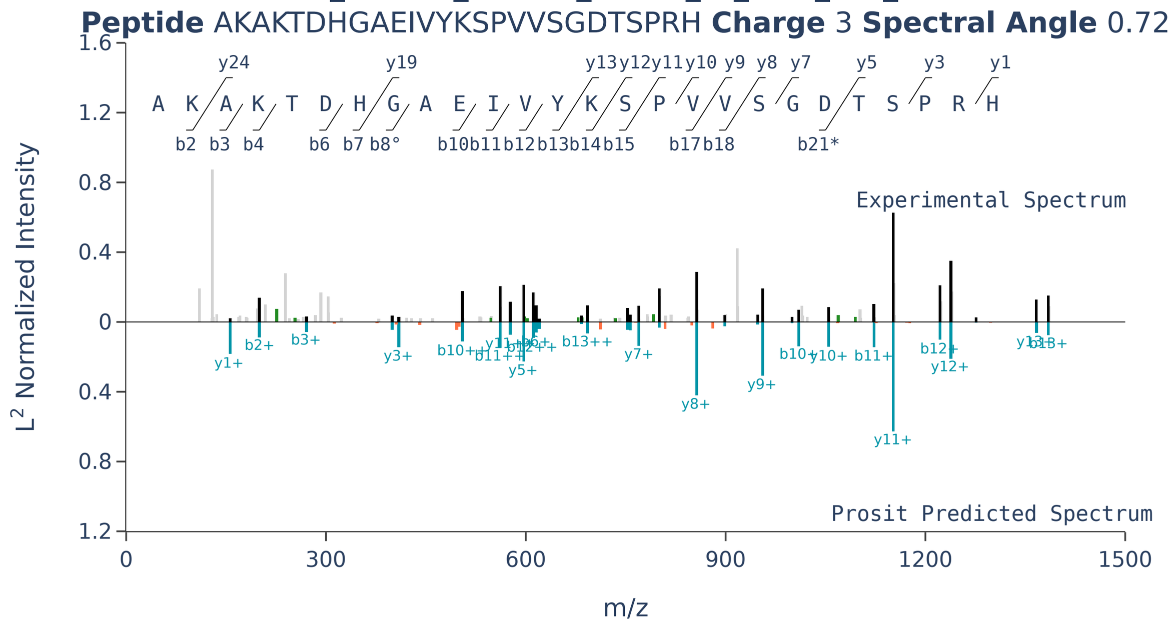

**Source** WSoh_221121_011221_HFGoe_L1_hTau_24h_R1 **Scan** 6615
**Peptide** RENAKAKTDHGAELV **Charge** 3 **Spectral Angle** 0.79

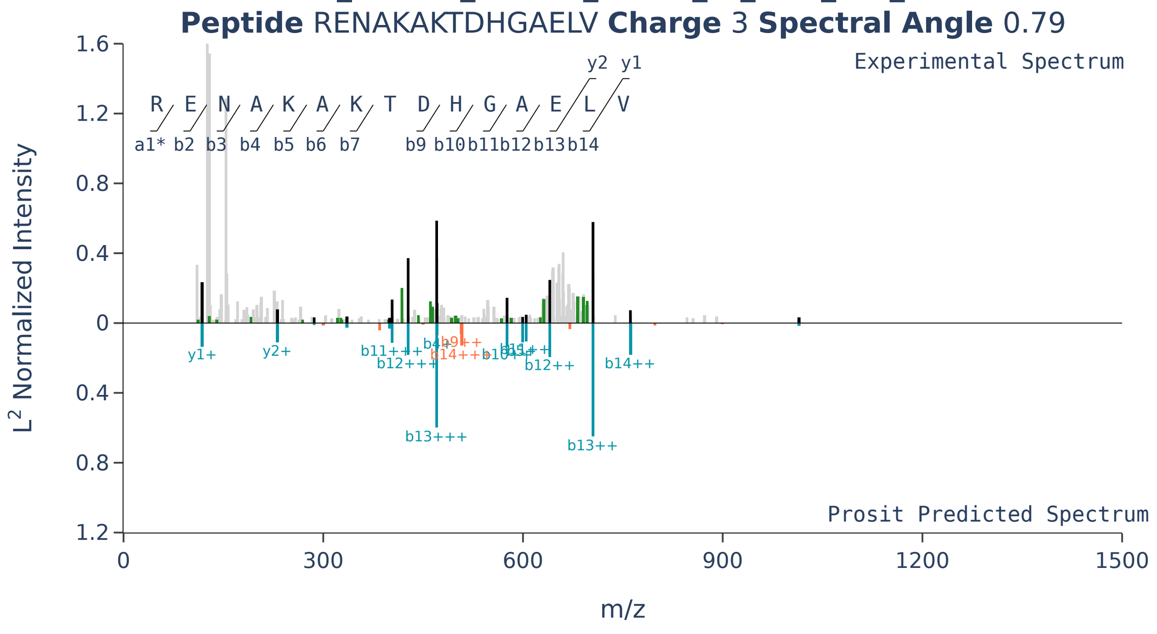


**Source** WSoh_221121_011221_HFGoe_L1_hTau_24h_R1 **Scan** 7179
**Peptide** PAKTPPAPKTPPSSGEPPKSGDRS **Charge** 4 **Spectral Angle** 0.83

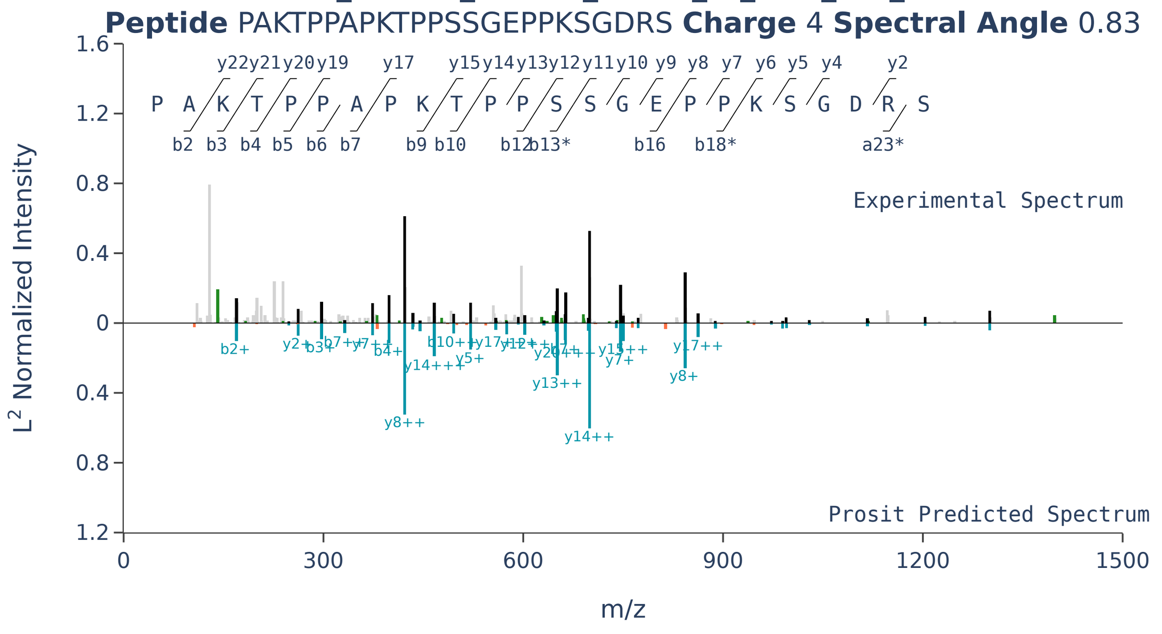


**Source** WSoh_221121_011221_HFGoe_L1_hTau_24h_R2 **Scan** 6781
**Peptide** KLGSTENLKHQPGG **Charge** 3 **Spectral Angle** 0.77

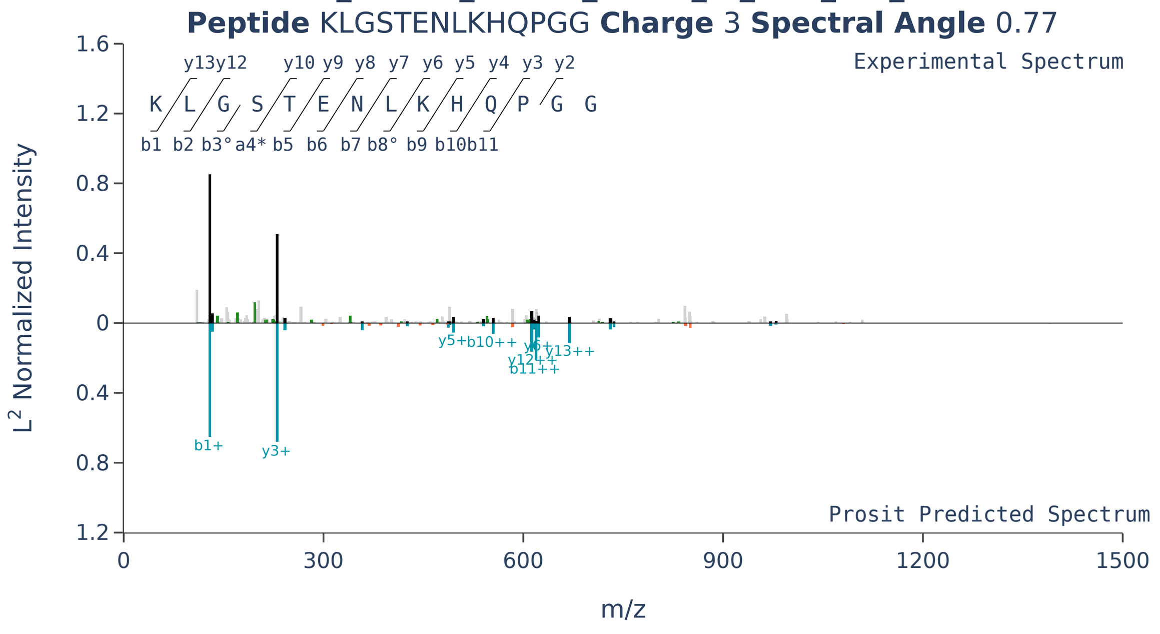


**Source** WSoh_221121_011221_HFGoe_L1_hTau_4h_R2 **Scan** 9342
**Peptide** VSGDTSPRHL **Charge** 3 **Spectral Angle** 0.67

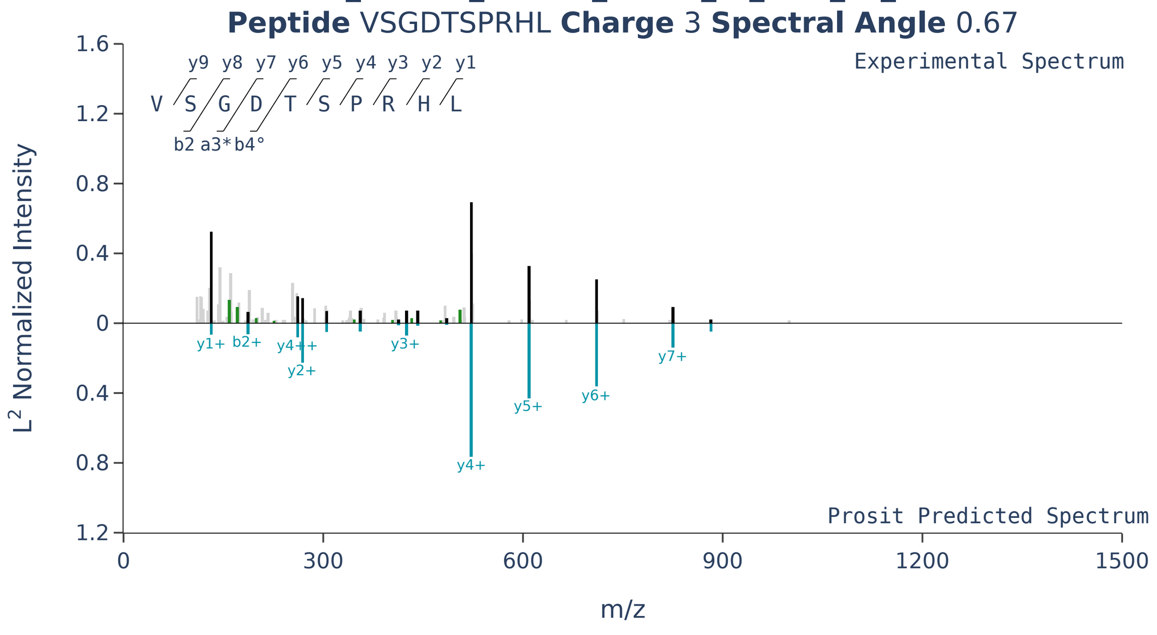


**Source** WSoh_221121_011221_HFGoe_L1_hTau_4h_R2 **Scan** 17462
**Peptide** IGSLDNITHVPGGGNKKIETHKLTFREN **Charge** 3 **Spectral Angle** 0.74

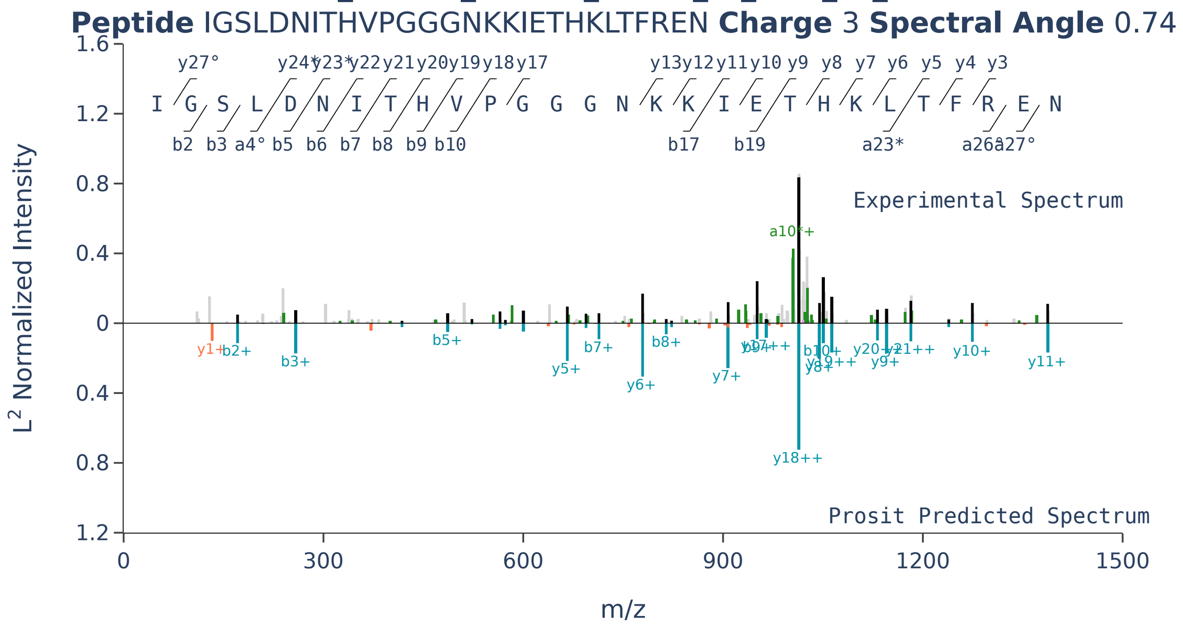


**Source** WSoh_221121_011221_HFGoe_L1_hTau_4h_R1 **Scan** 19956
**Peptide** RLQTAPVPMPDLKNVKSKIGSTENLKH **Charge** 5 **Spectral Angle** 0.87

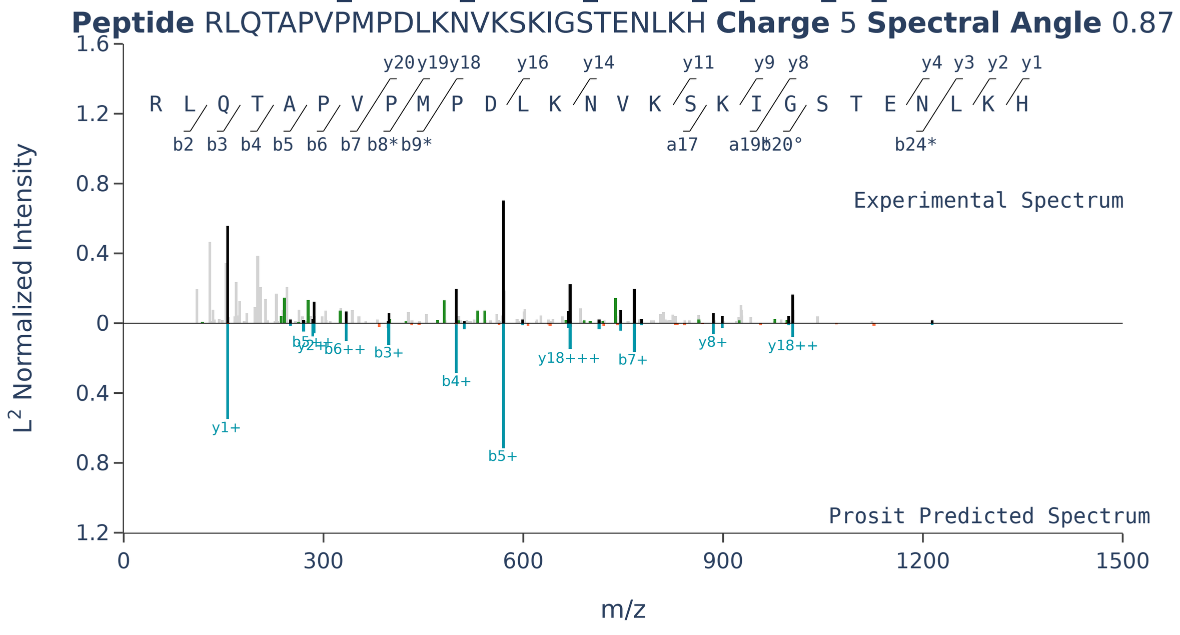


**Source** WSoh_221121_011221_HFGoe_L1_hTau_24h_R1 **Scan** 12558
**Peptide** NAKAKTDHGAEIVYKSPVVS **Charge** 3 **Spectral Angle** 0.74

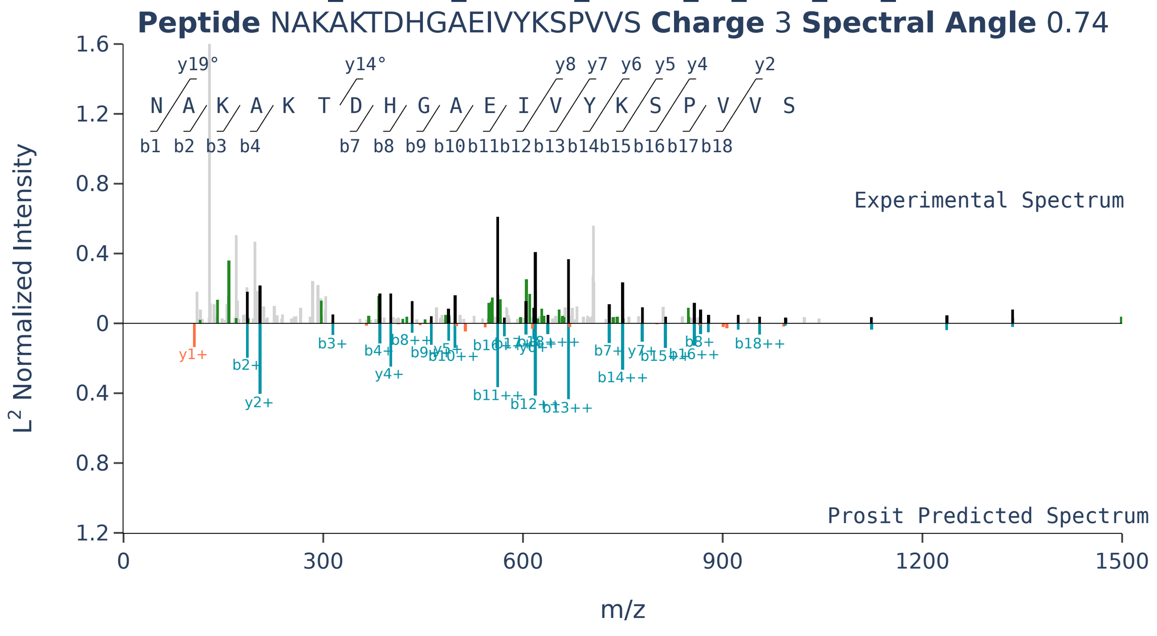


**Source** WSoh_221121_011221_HFGoe_L1_hTau_4h_R2 **Scan** 14731
**Peptide** KTDHGAEIVYKSPVVSG **Charge** 3 **Spectral Angle** 0.7

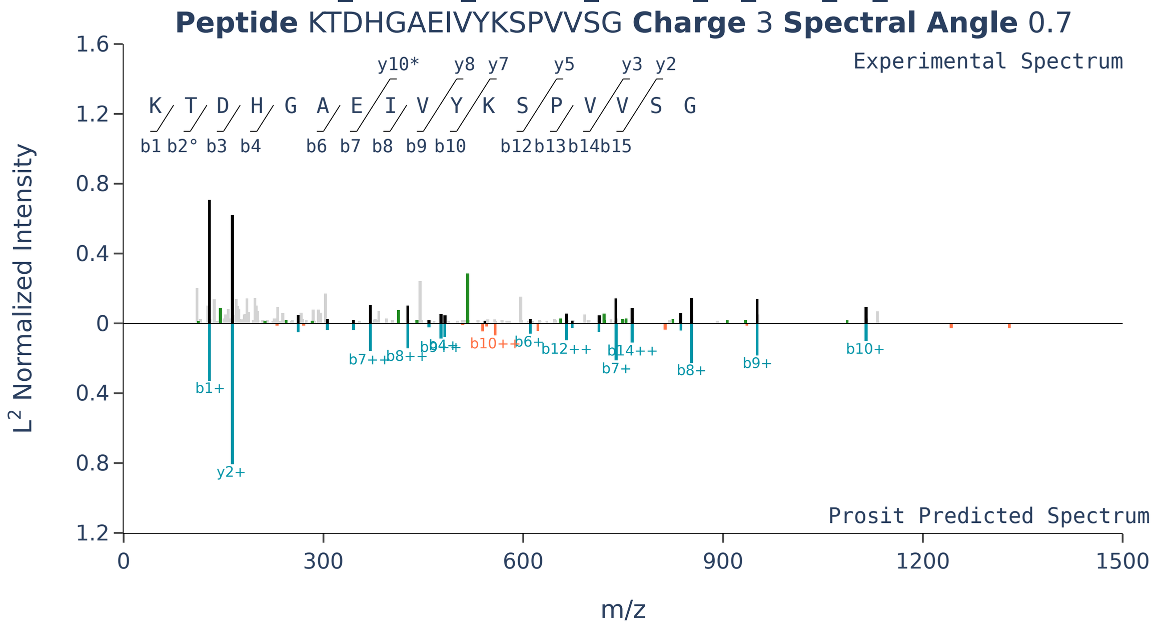


**Source** WSoh_221121_011221_HFGoe_L1_hTau_24h_R1 **Scan** 6636
**Peptide** KIGSTENLKHQPGG **Charge** 2 **Spectral Angle** 0.73

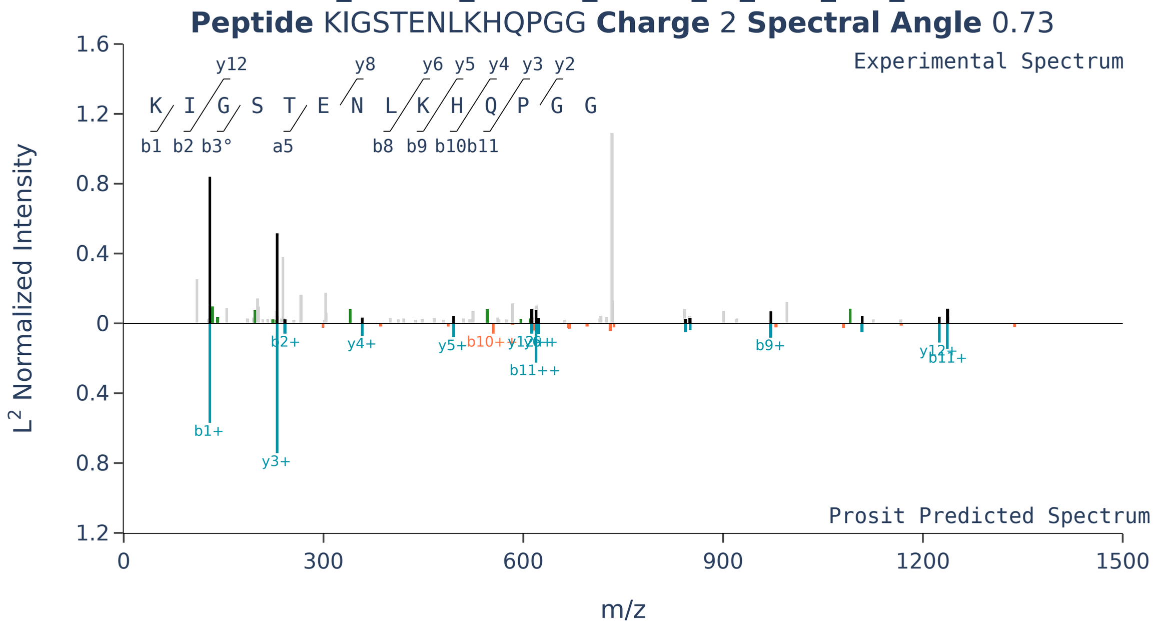


**Source** WSoh_221121_011221_HFGoe_L1_hTau_24h_R1 **Scan** 10023
**Peptide** RVQSKIGSLD **Charge** 3 **Spectral Angle** 0.8

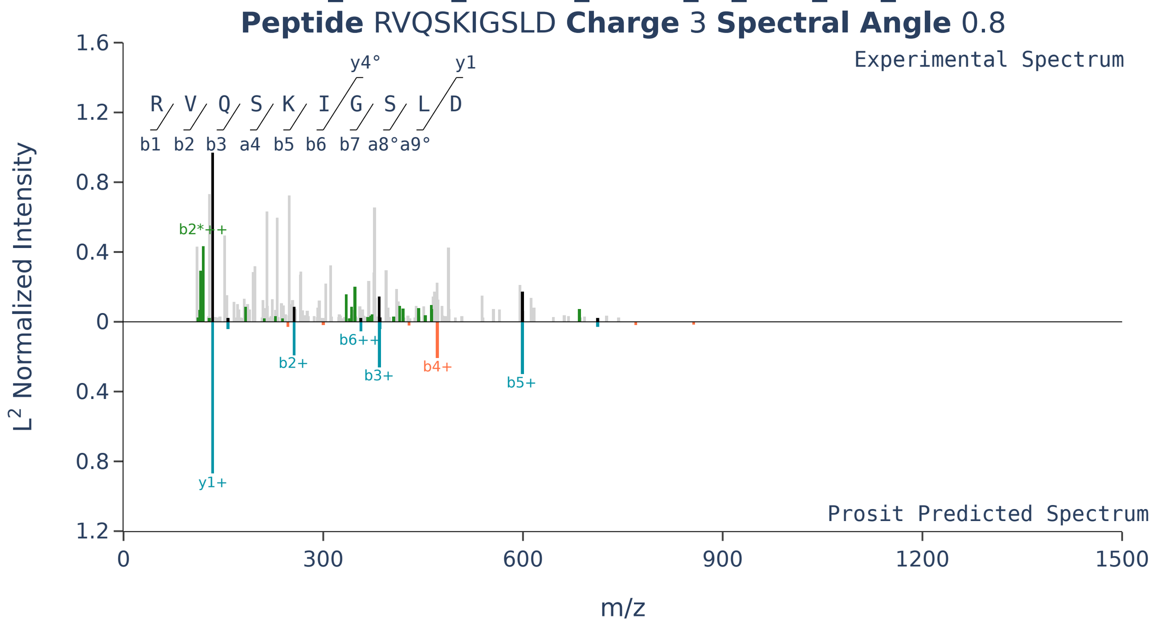


**Source** WSoh_221121_011221_HFGoe_L2_hTau_24h_R1 **Scan** 8762
**Peptide** GKTKIATPRGAAPPGQKGQANATRL **Charge** 4 **Spectral Angle** 0.8

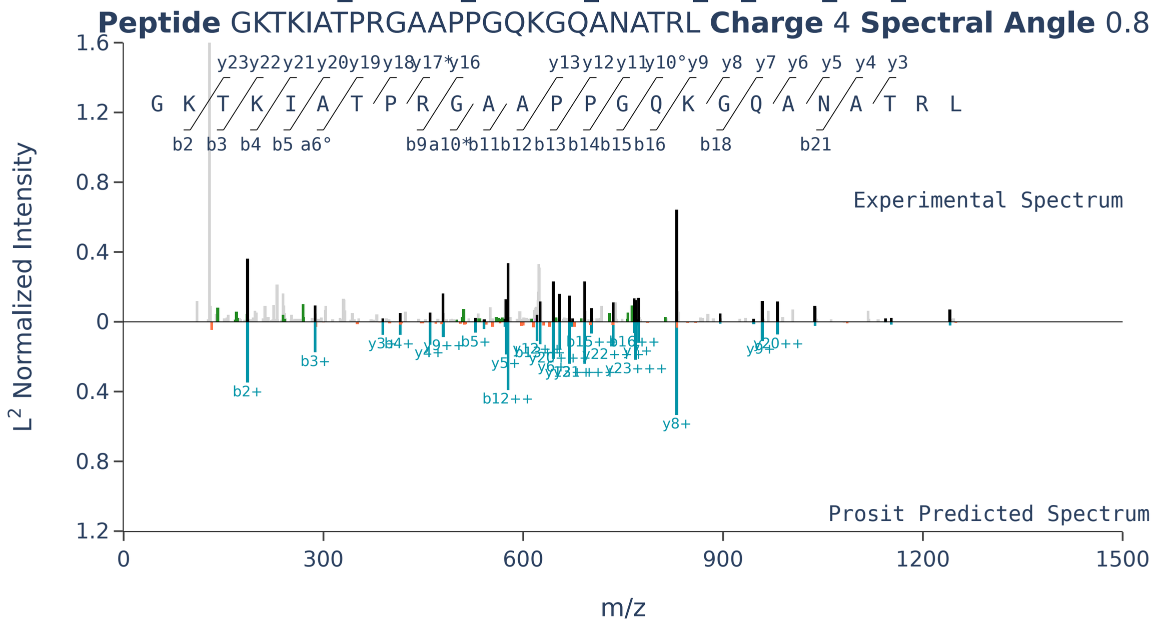


**Source** WSoh_221121_011221_HFGoe_L1_hTau_24h_R1 **Scan** 23283
**Peptide** DGSEEPGSETSDAKSTPTAEDVTAPLVD **Charge** 2 **Spectral Angle** 0.85

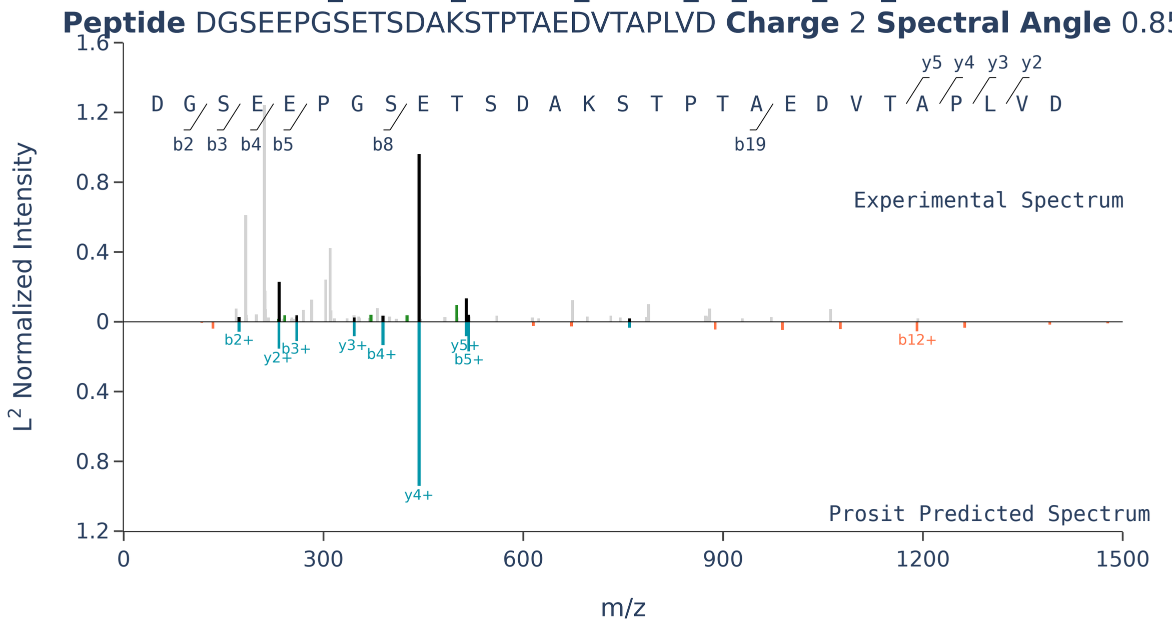


**Source** WSoh_221121_011221_HFGoe_L2_hTau_24h_R1 **Scan** 22298
**Peptide** VPGGGSVQIVYKPVD **Charge** 2 **Spectral Angle** 0.65

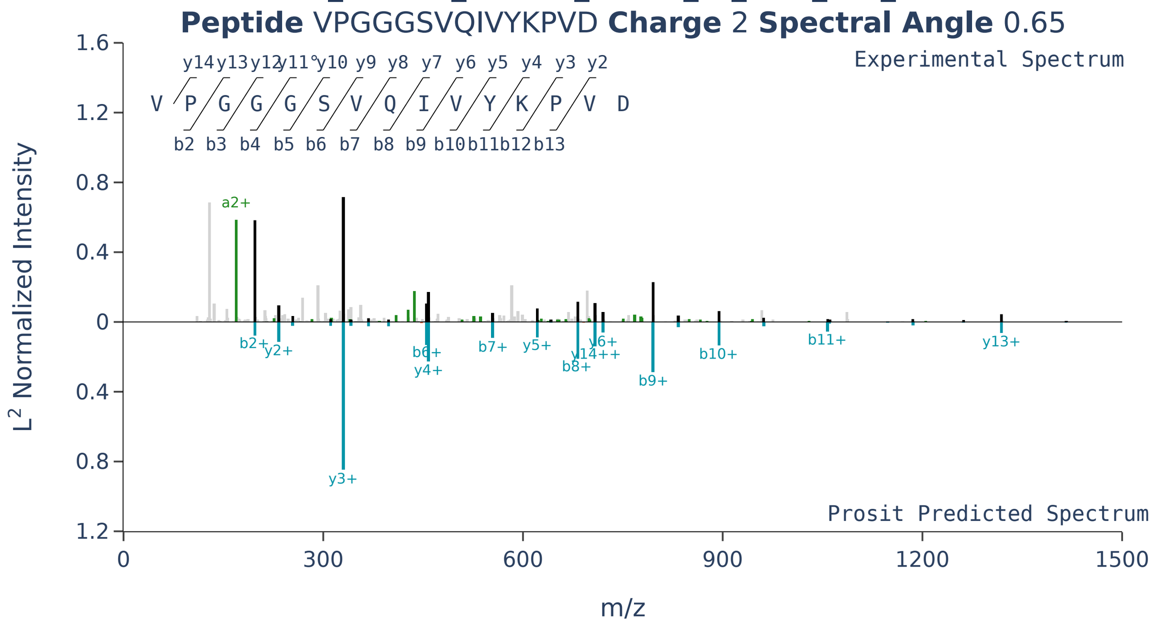


**Source** WSoh_221121_011221_HFGoe_L1_hTau_4h_R2 **Scan** 14026
**Peptide** KESPLQTPTEDGSEEPGSETSDAKSTPTA **Charge** 3 **Spectral Angle** 0.68

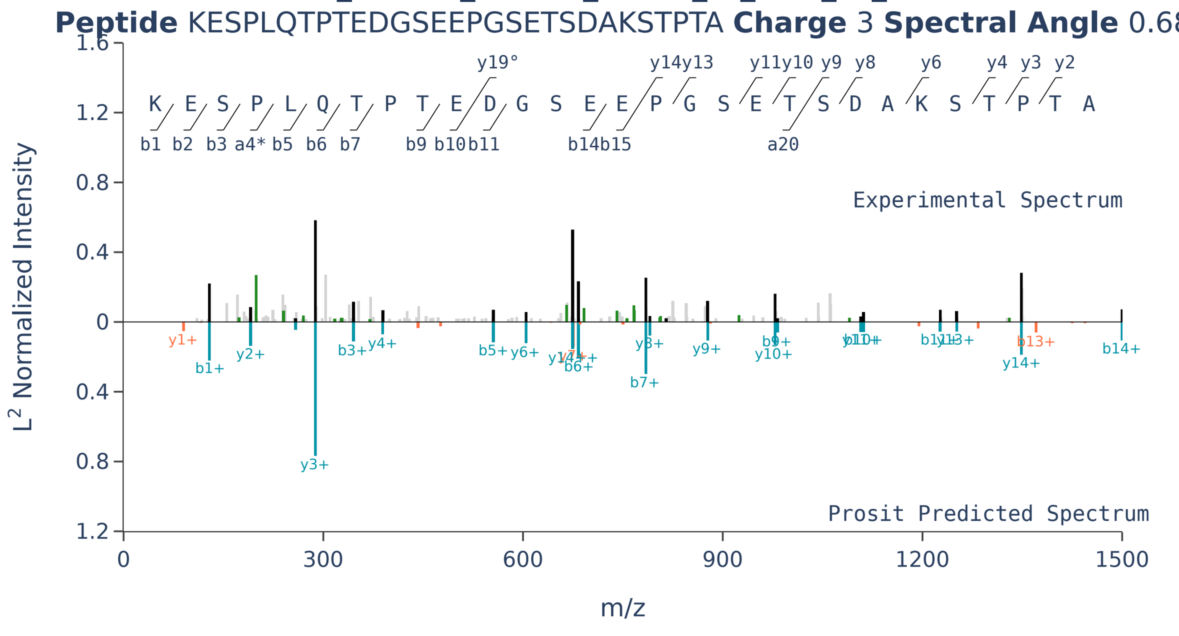


**Source** WSoh_221121_011221_HFGoe_L2_hTau_2h_R2 **Scan** 17423
**Peptide** AEPRQEFEVMEDHAGT **Charge** 3 **Spectral Angle** 0.77

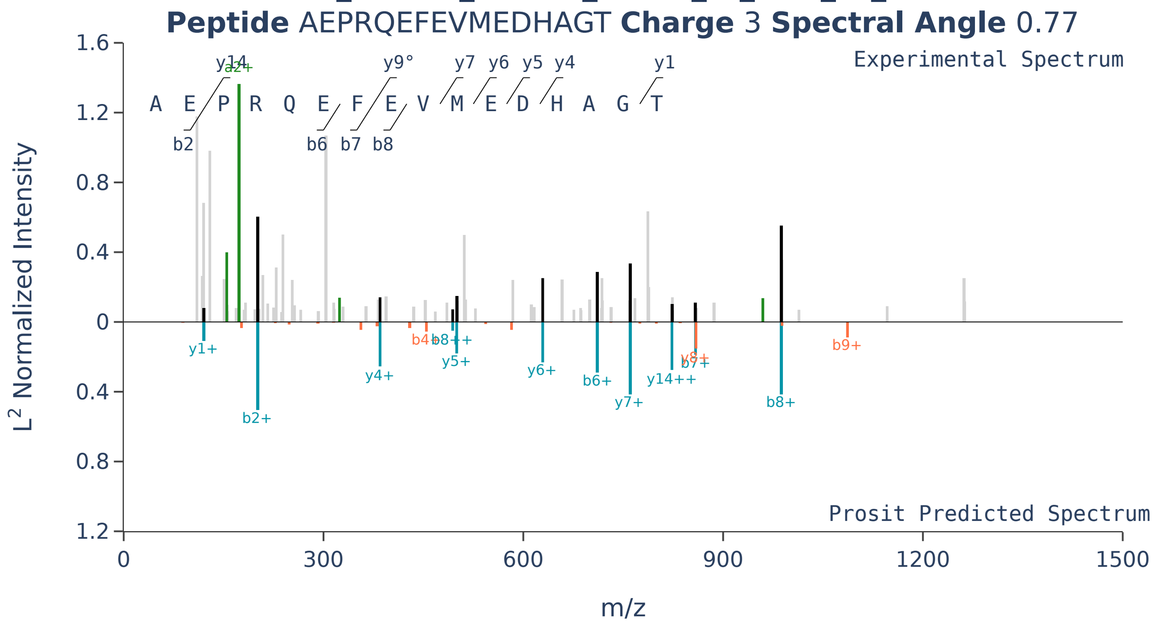

**Source** WSoh_221121_011221_HFGoe_L1_hTau_24h_R2 **Scan** 14928
**Peptide** KLGSLDNITHVPGGGNKKIETHKLT **Charge** 4 **Spectral Angle** 0.87

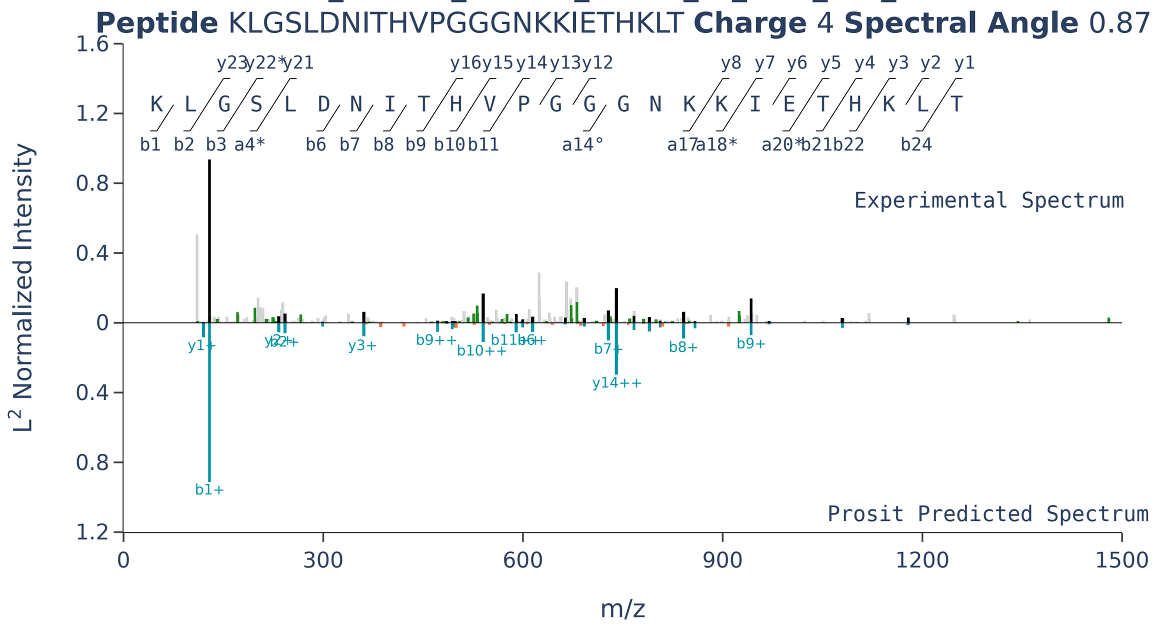


**Source** WSoh_221121_011221_HFGoe_L1_hTau_4h_R2 **Scan** 6856
**Peptide** KGADGKTKIATPRGAAPPGQKGQANA **Charge** 4 **Spectral Angle** 0.83

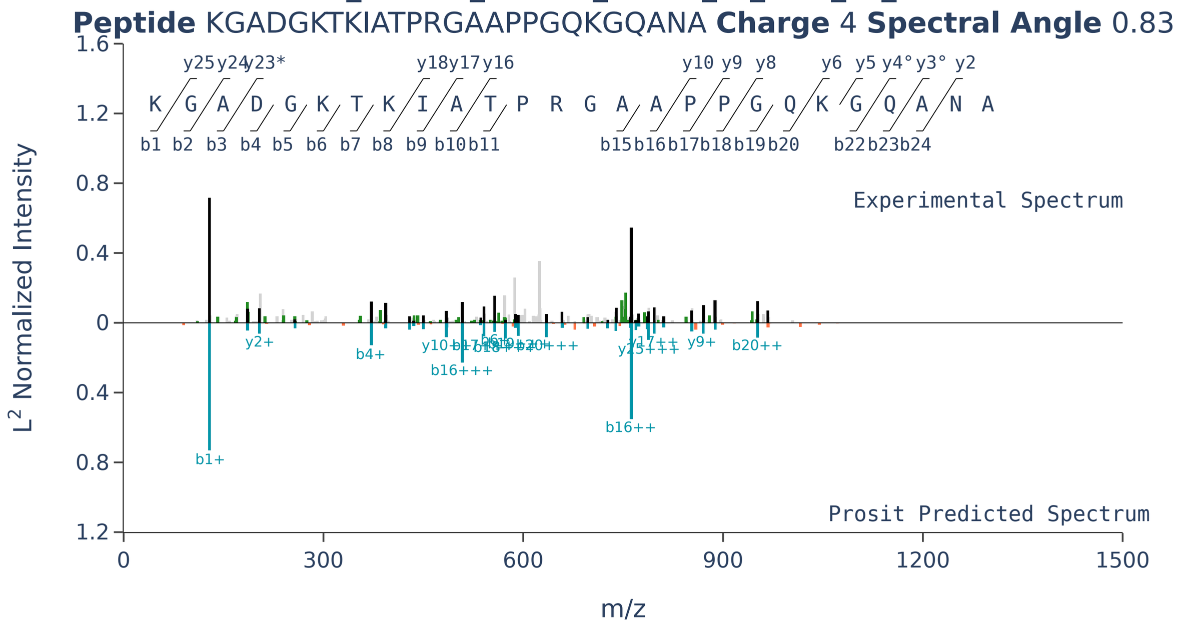


**Source** WSoh_221121_011221_HFGoe_L2_hTau_24h_R1 **Scan** 20632
**Peptide** PVPMPDLKNVKSKIGS **Charge** 4 **Spectral Angle** 0.77

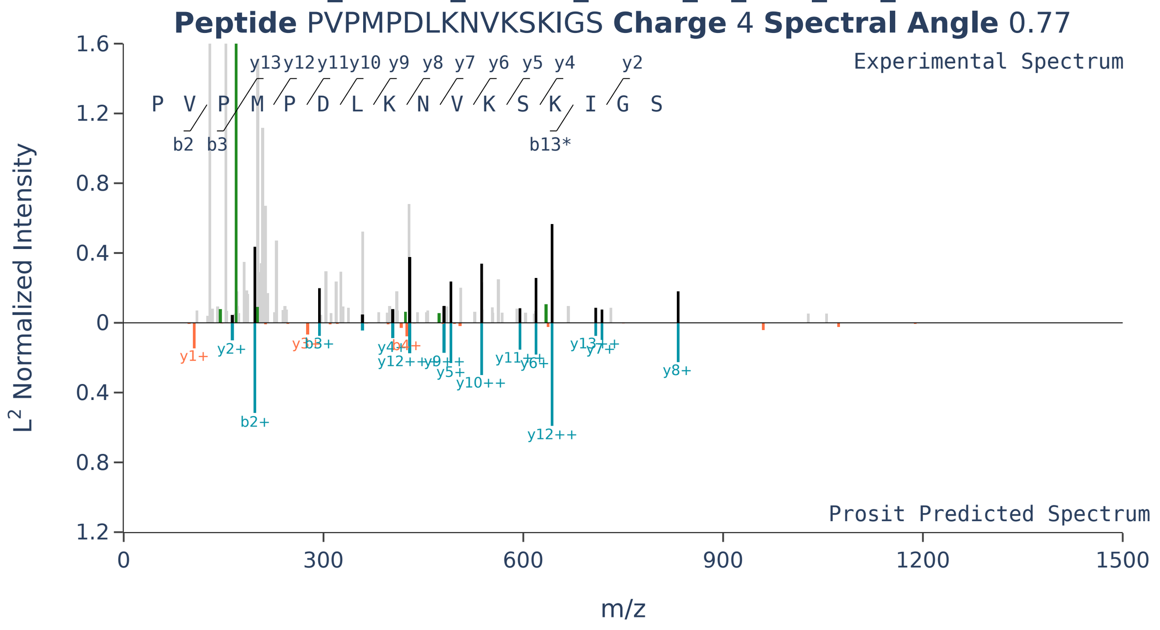


**Source** WSoh_221121_011221_HFGoe_L1_hTau_4h_R1 **Scan** 19027
**Peptide** KCGSKDNIKHVPGGGSVQIVYKPVDL **Charge** 4 **Spectral Angle** 0.73

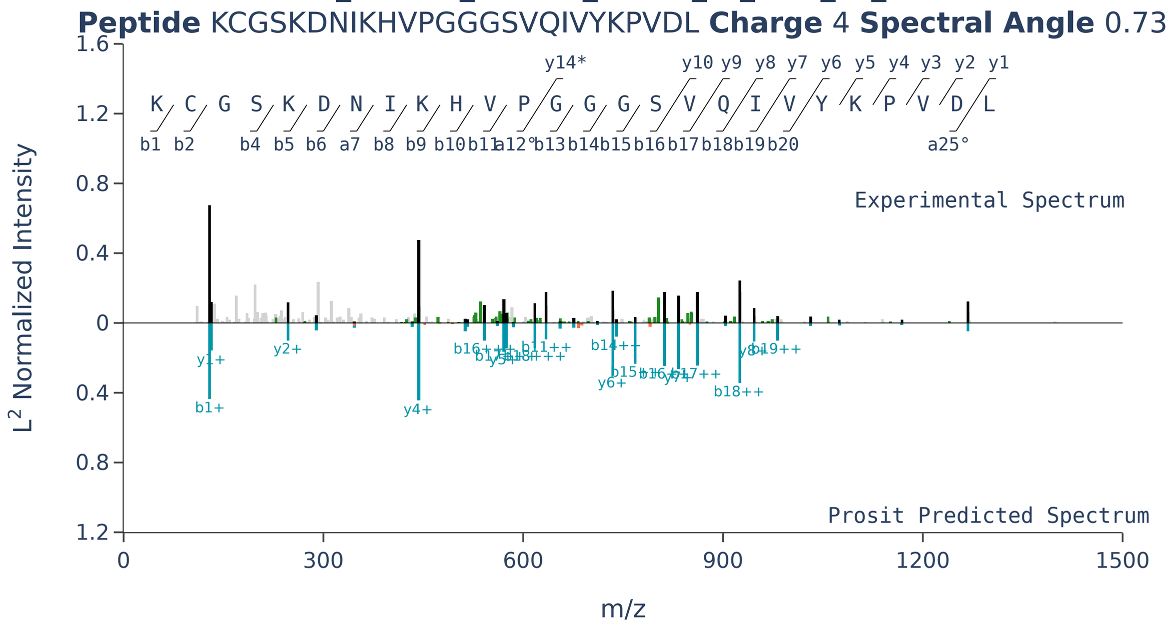


**Source** WSoh_221121_011221_HFGoe_L1_hTau_4h_R2 **Scan** 17784
**Peptide** PRQEFEVMEDHAGTYGLGDRKD **Charge** 4 **Spectral Angle** 0.79

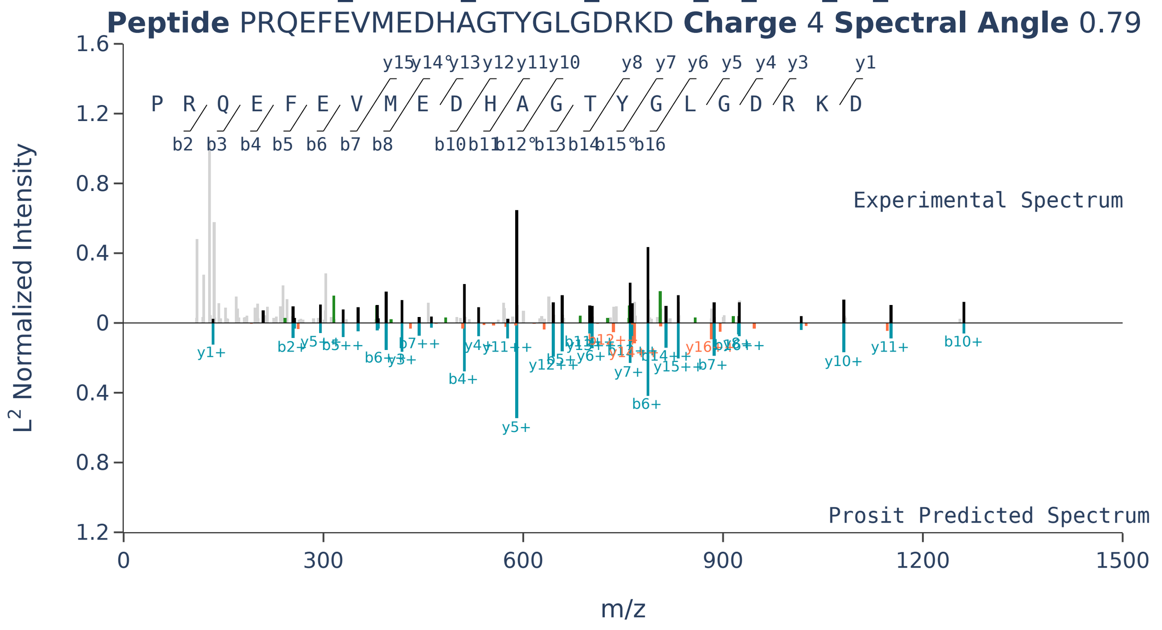


**Source** WSoh_221121_011221_HFGoe_L1_hTau_24h_R1 **Scan** 16389
**Peptide** VMEDHAGTYGLGDRKDQGGYTM **Charge** 3 **Spectral Angle** 0.8

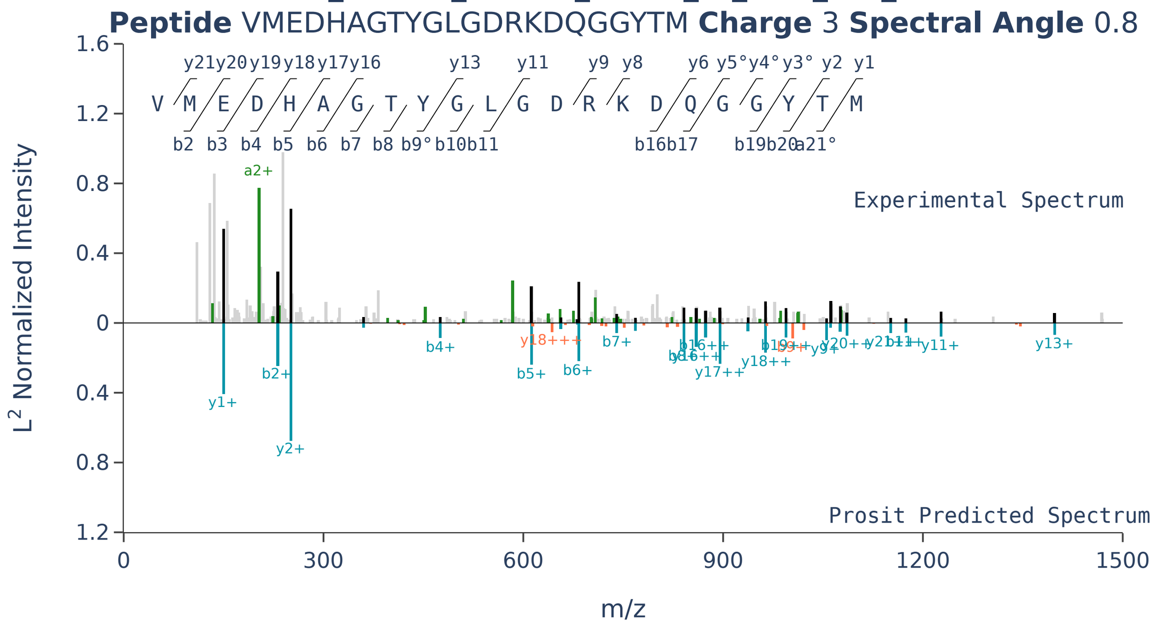


**Source** WSoh_221121_011221_HFGoe_L1_hTau_24h_R1 **Scan** 15543
**Peptide** PPKSPSSAKSRLQTAPVPMPDLKNVKSK **Charge** 6 **Spectral Angle** 0.69

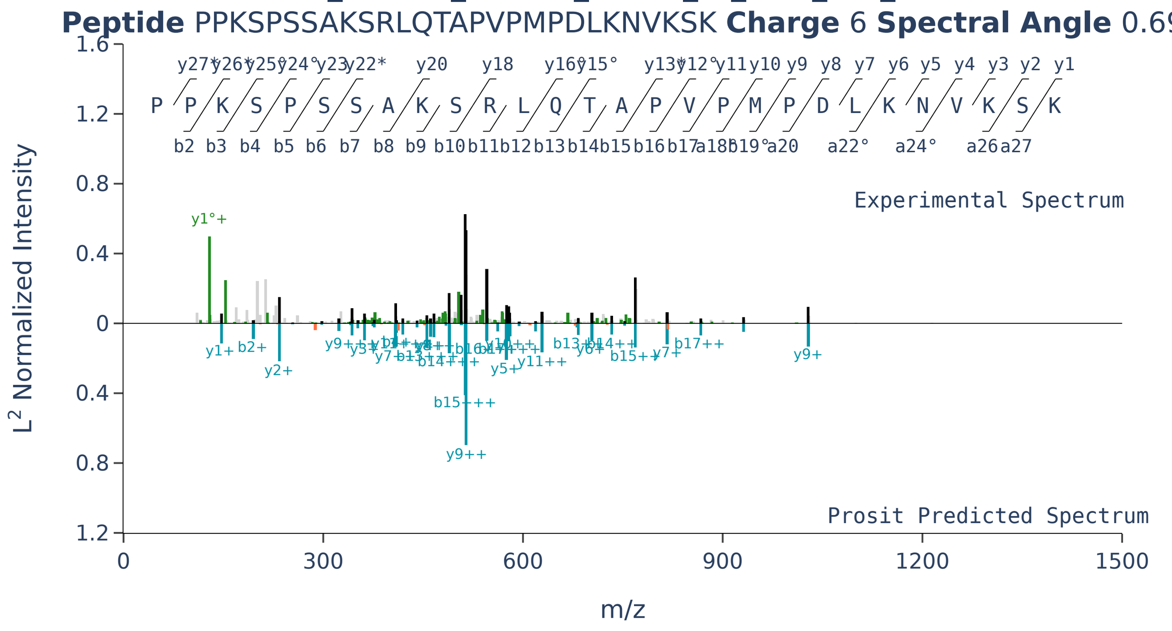


**Source** WSoh_221121_011221_HFGoe_L1_hTau_2h_R1 **Scan** 18364
**Peptide** PGGGSVQIV **Charge** 1 **Spectral Angle** 0.72

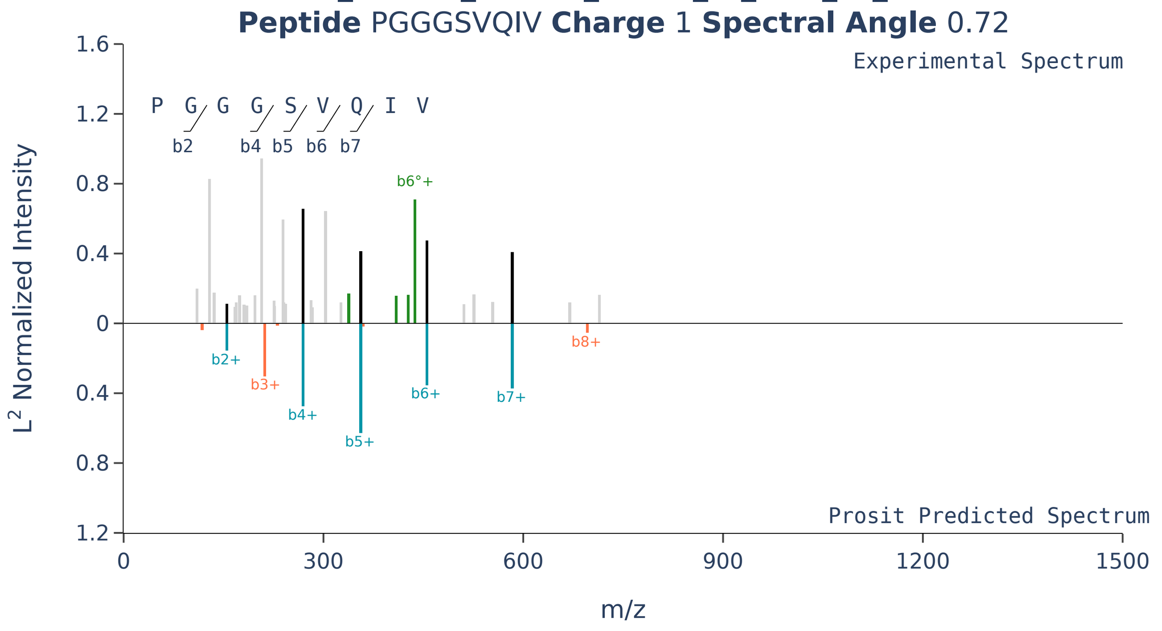


**Source** WSoh_221121_011221_HFGoe_L1_hTau_4h_R2 **Scan** 15658
**Peptide** IGSTENLKHQPGGGKVQII **Charge** 3 **Spectral Angle** 0.71

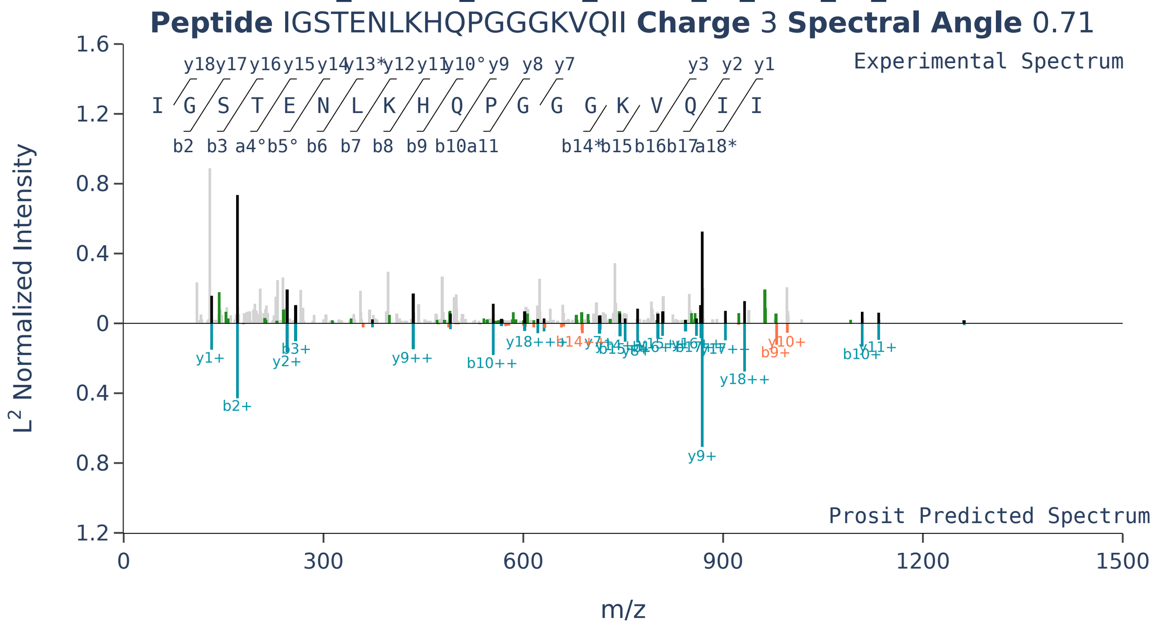


**Source** WSoh_221121_011221_HFGoe_L2_hTau_4h_R2 **Scan** 13467
**Peptide** GSLGNIHHKPGGGQVEVKSEKLDFKDRV **Charge** 3 **Spectral Angle** 0.71

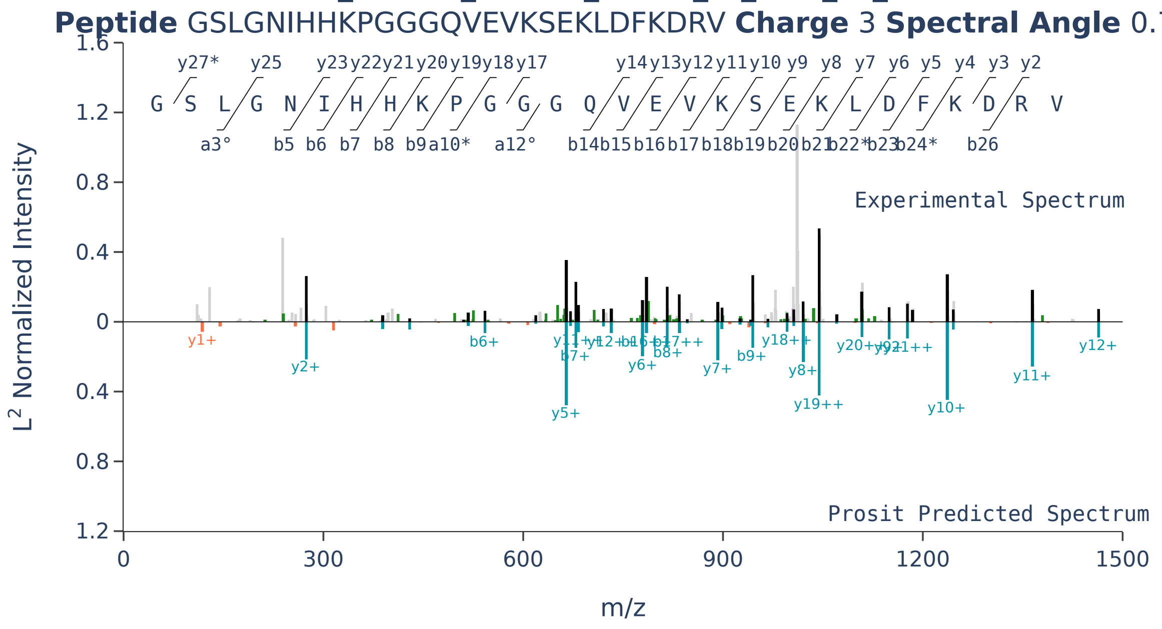


**Source** WSoh_221121_011221_HFGoe_L1_hTau_4h_R1 **Scan** 16842
**Peptide** PPKSPSSAKSRLQTAPVPMPDLKNVKS **Charge** 4 **Spectral Angle** 0.8

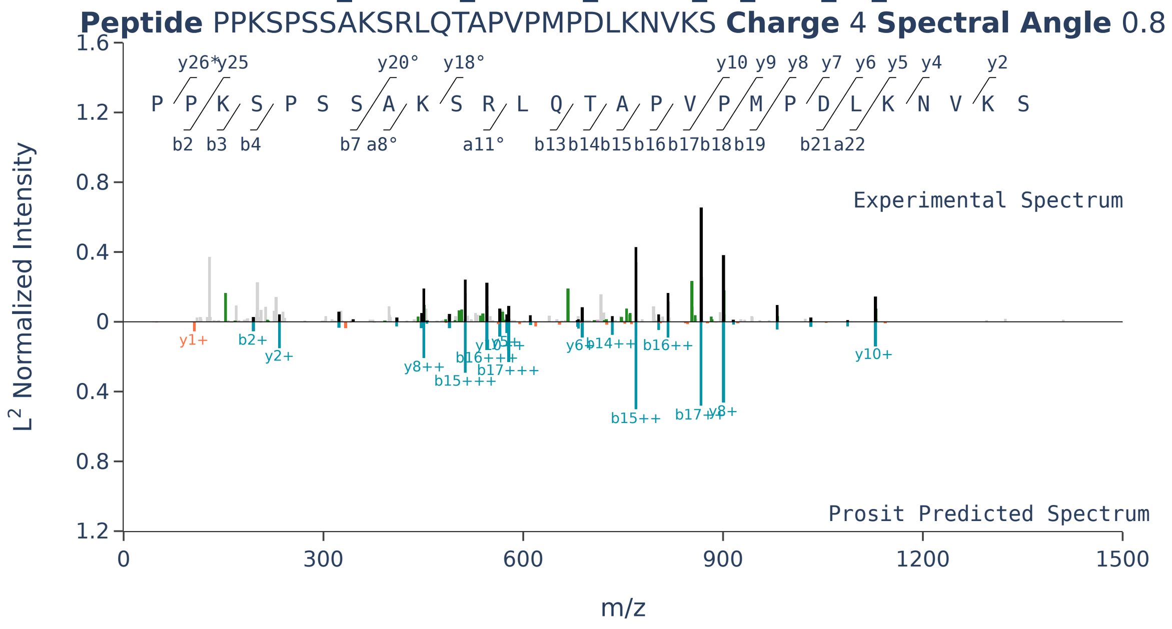


**Source** WSoh_221121_011221_HFGoe_L2_hTau_4h_R2 **Scan** 10389
**Peptide** INKKLDLSNVQSKCGSKDNIKHV **Charge** 5 **Spectral Angle** 0.88

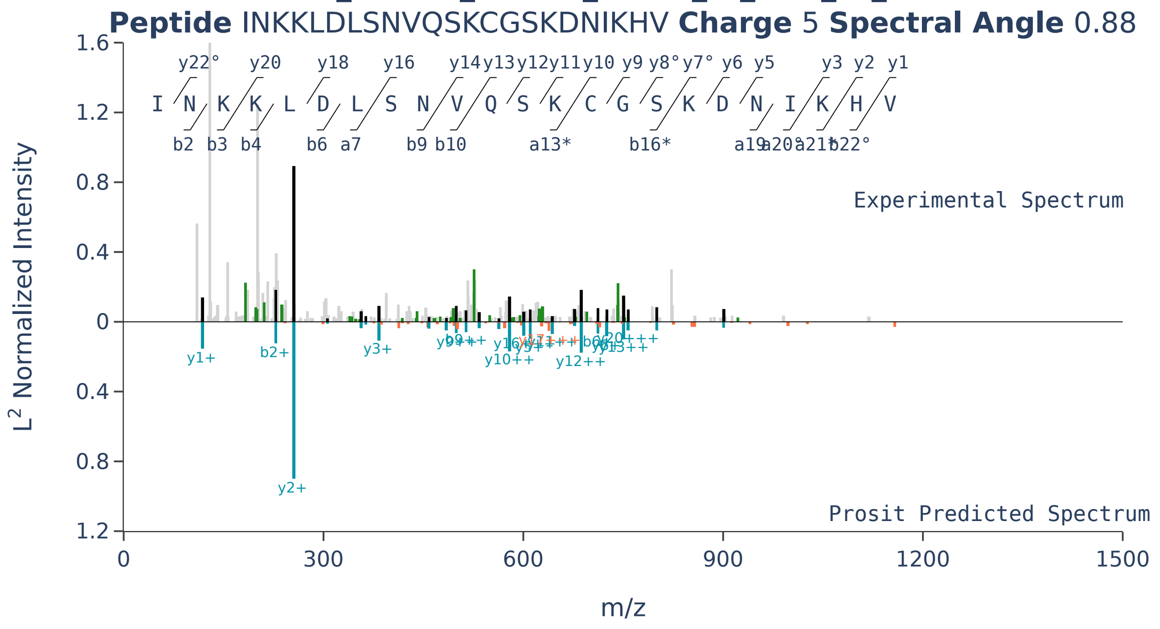


**Source** WSoh_221121_011221_HFGoe_L2_hTau_24h_R2 **Scan** 20081
**Peptide** KSRLQTAPVPMPDLKNVKSKIGSTE **Charge** 4 **Spectral Angle** 0.71

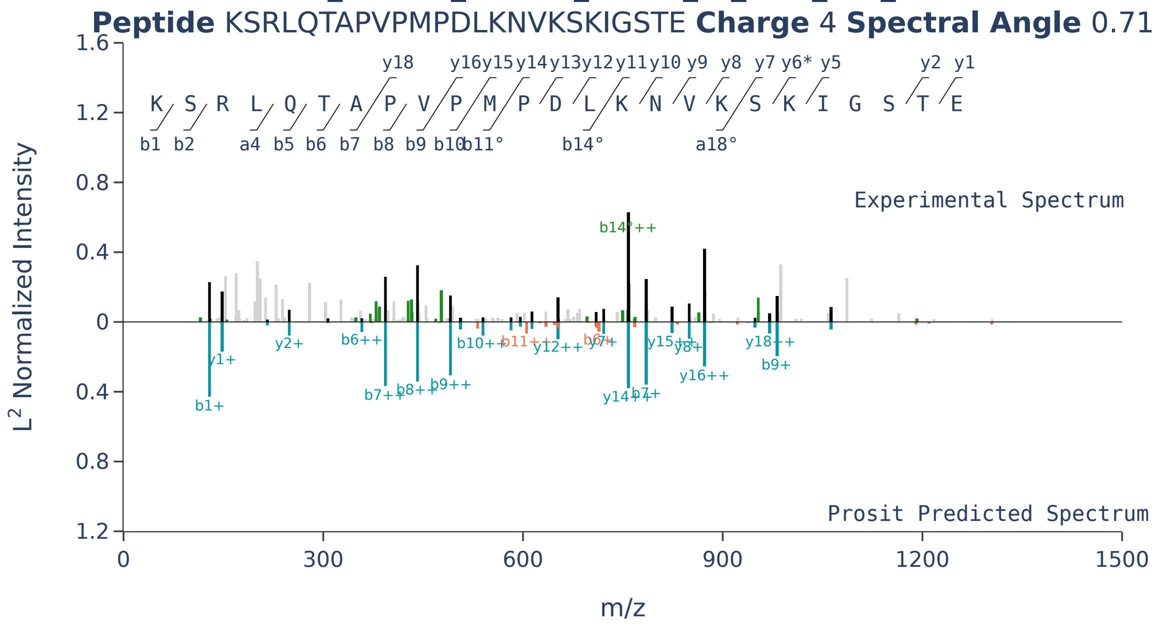


**Source** WSoh_221121_011221_HFGoe_L2_hTau_4h_R1 **Scan** 7174
**Peptide** PRGAAPPGQKGQANATRIPAKT **Charge** 3 **Spectral Angle** 0.72

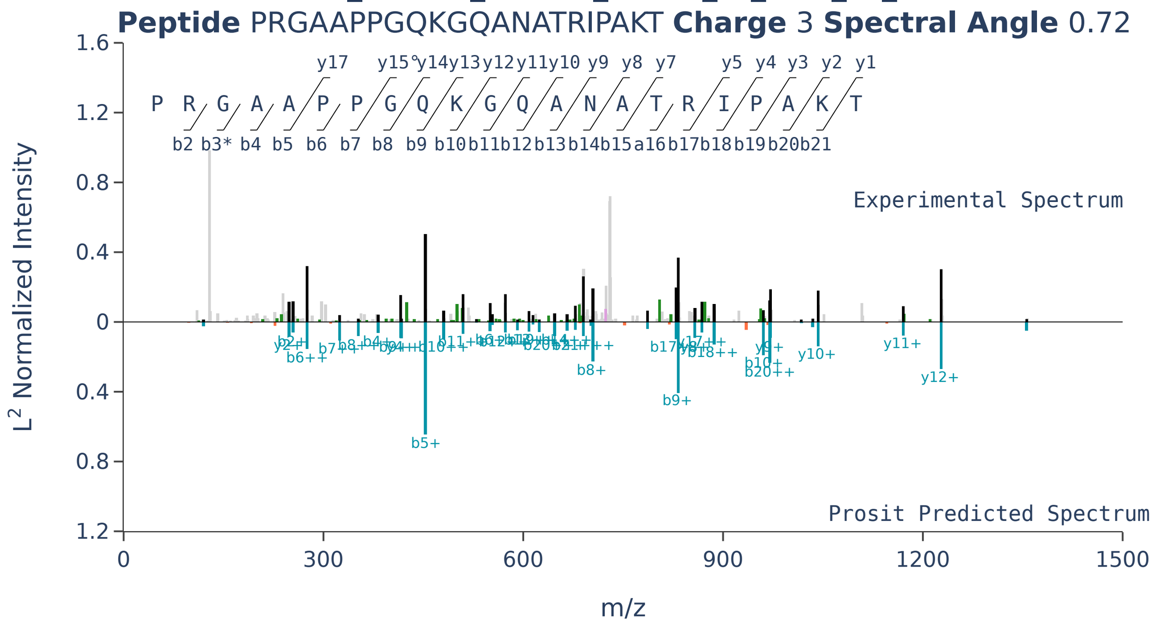


**Source** WSoh_221121_011221_HFGoe_L1_hTau_24h_R1 **Scan** 26471
**Peptide** SPQLATLADEVS **Charge** 2 **Spectral Angle** 0.73

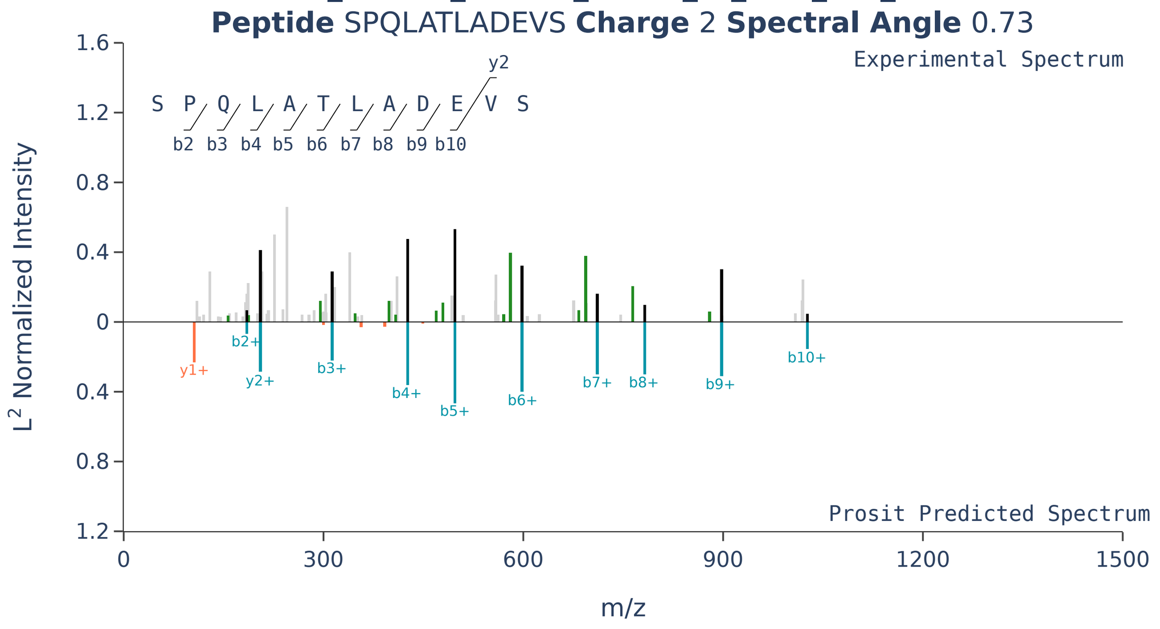

**Source** WSoh_221121_011221_HFGoe_L1_hTau_24h_R2 **Scan** 6282
**Peptide** RTPPKSPSSAKSRLQTA **Charge** 3 **Spectral Angle** 0.83

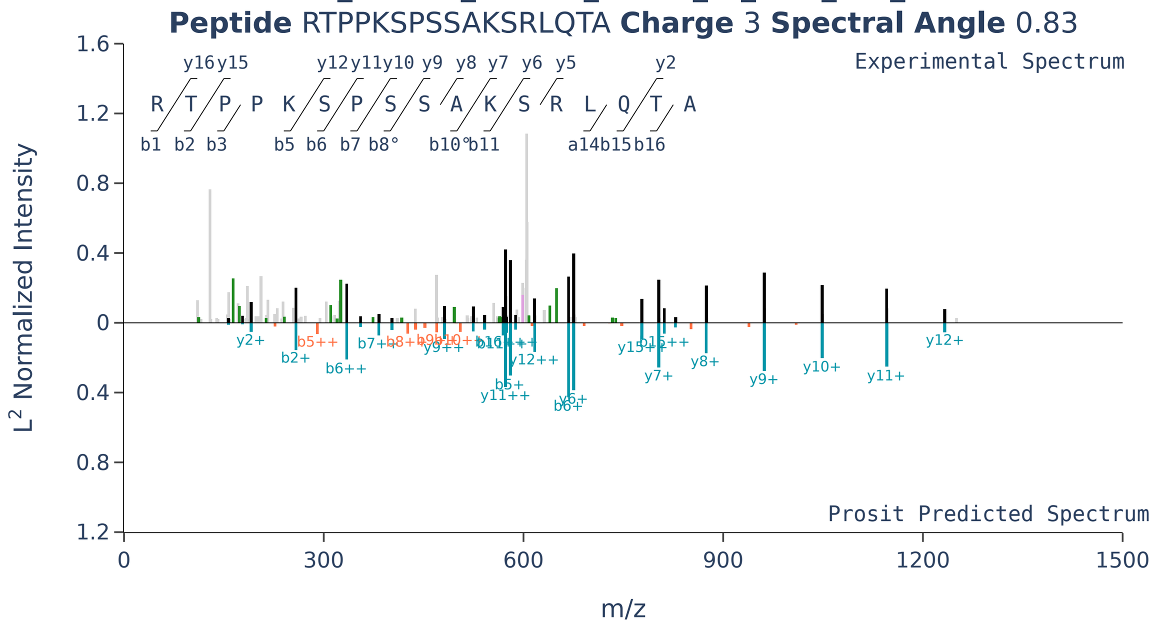


**Source** WSoh_221121_011221_HFGoe_L2_hTau_24h_R1 **Scan** 21306
**Peptide** AGIGDTPSLEDEAAG **Charge** 2 **Spectral Angle** 0.75

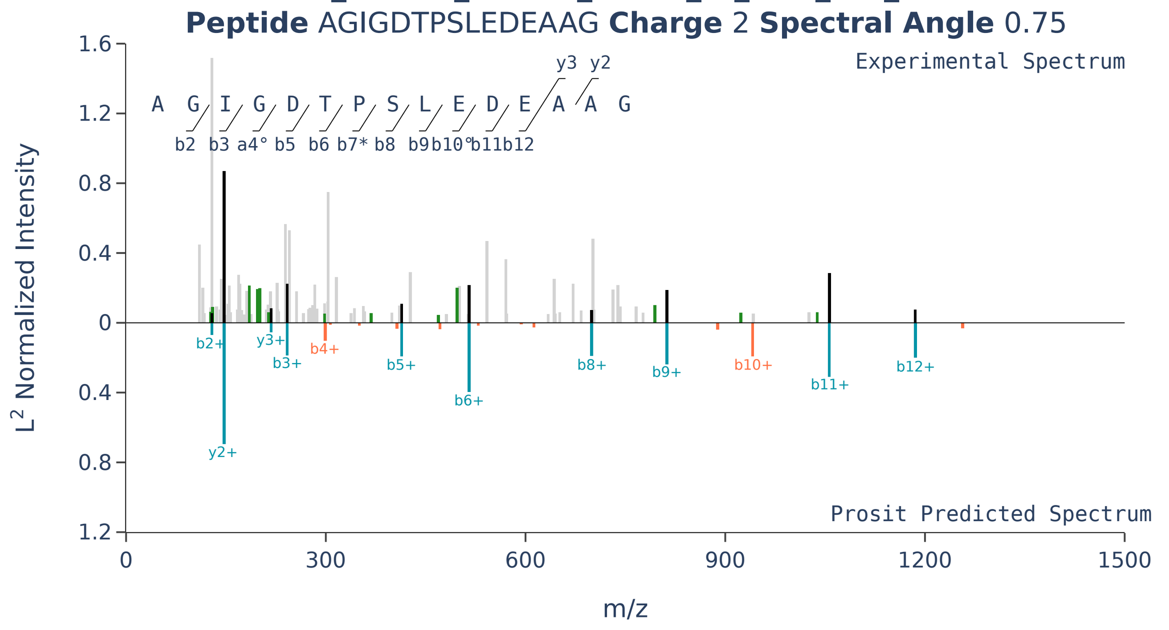


**Source** WSoh_221121_011221_HFGoe_L2_hTau_4h_R1 **Scan** 9368
**Peptide** HQDQEGDTDAGL **Charge** 2 **Spectral Angle** 0.78

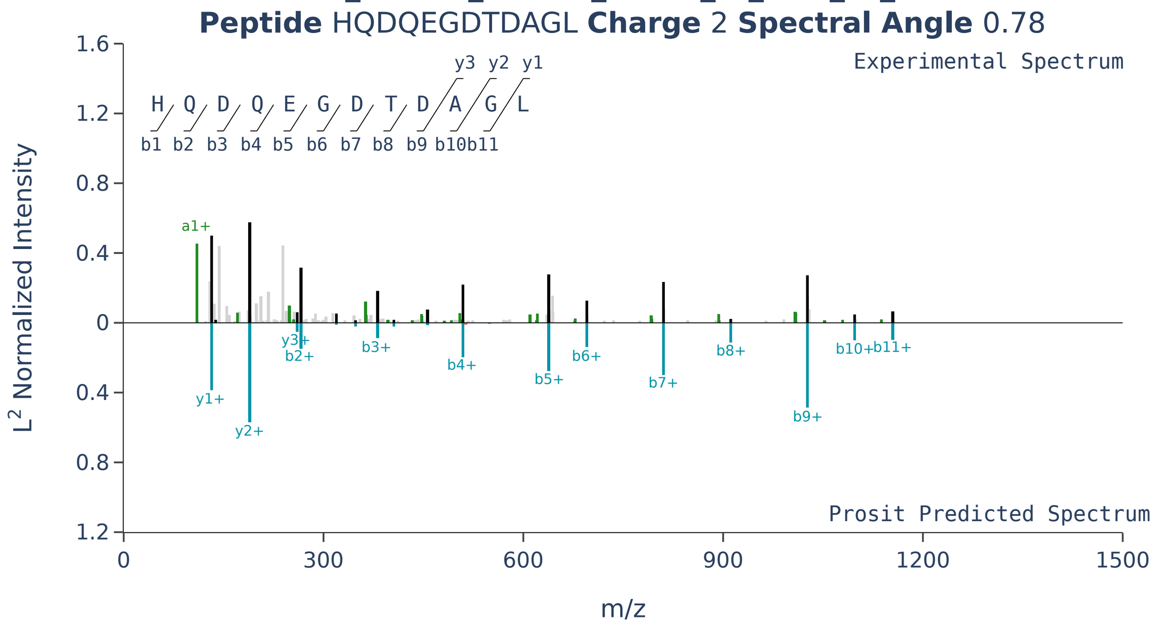


**Source** WSoh_221121_011221_HFGoe_L1_hTau_4h_R2 **Scan** 5616
**Peptide** LHHKPGGGQVEVKSE **Charge** 3 **Spectral Angle** 0.84

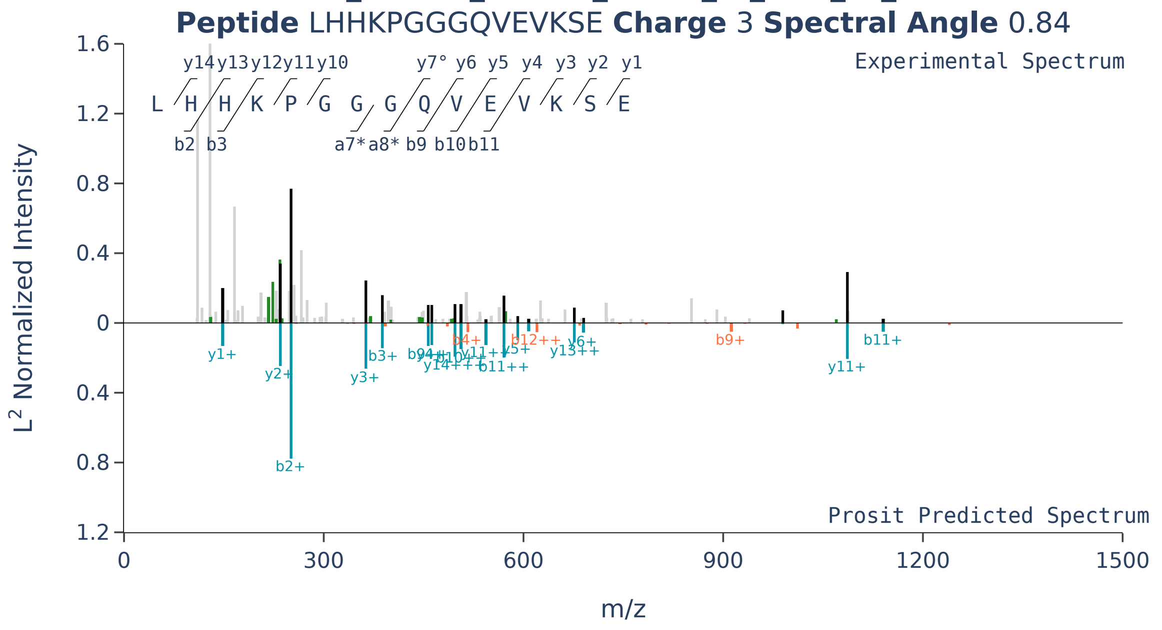


**Source** WSoh_221121_011221_HFGoe_L1_hTau_4h_R2 **Scan** 9714
**Peptide** EGAPGKQAAAQPHTEL **Charge** 3 **Spectral Angle** 0.84

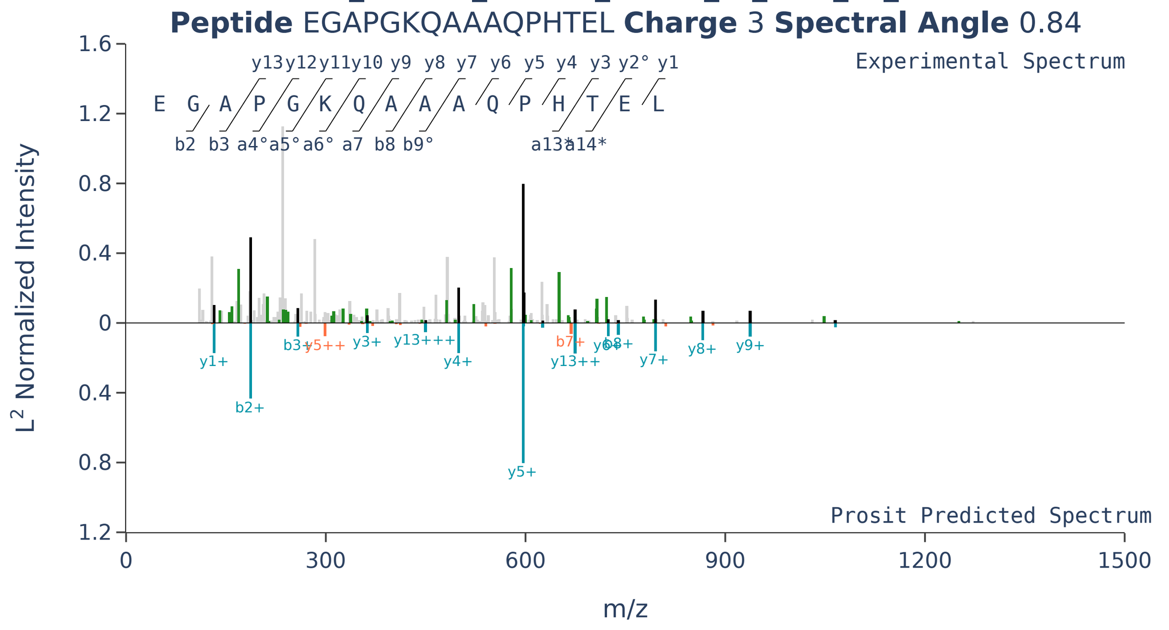


**Source** WSoh_221121_011221_HFGoe_L1_hTau_24h_R2 **Scan** 10394
**Peptide** HHKPGGGQVEVKSEKLDFKDRVQS **Charge** 4 **Spectral Angle** 0.8

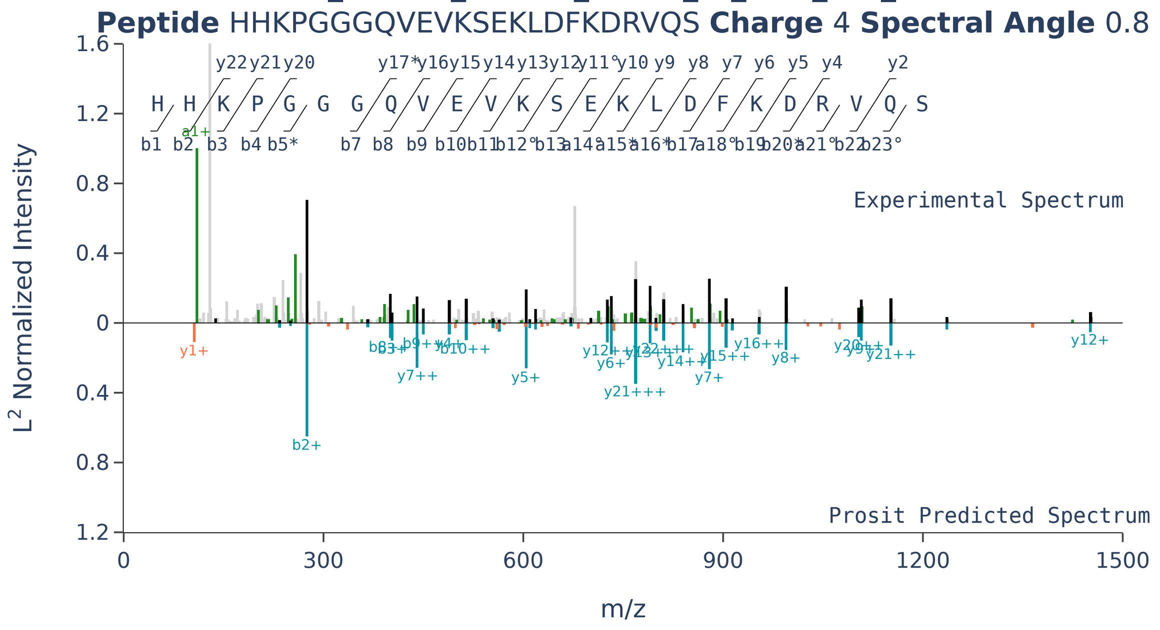


**Source** WSoh_221121_011221_HFGoe_L1_hTau_24h_R2 **Scan** 14244
**Peptide** LDNITHVPGGGNKKIETHKLTFREN **Charge** 6 **Spectral Angle** 0.76

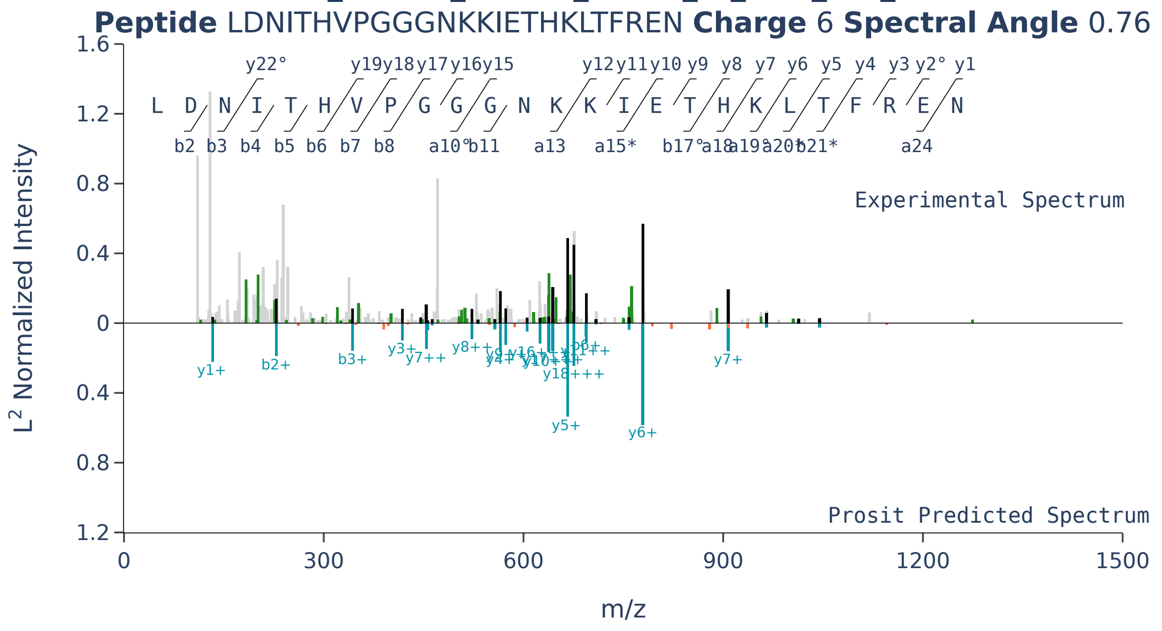


**Source** WSoh_221121_011221_HFGoe_L1_hTau_4h_R1 **Scan** 17259
**Peptide** GDTSPRHLSNVSSTGSIDMVDS **Charge** 3 **Spectral Angle** 0.72

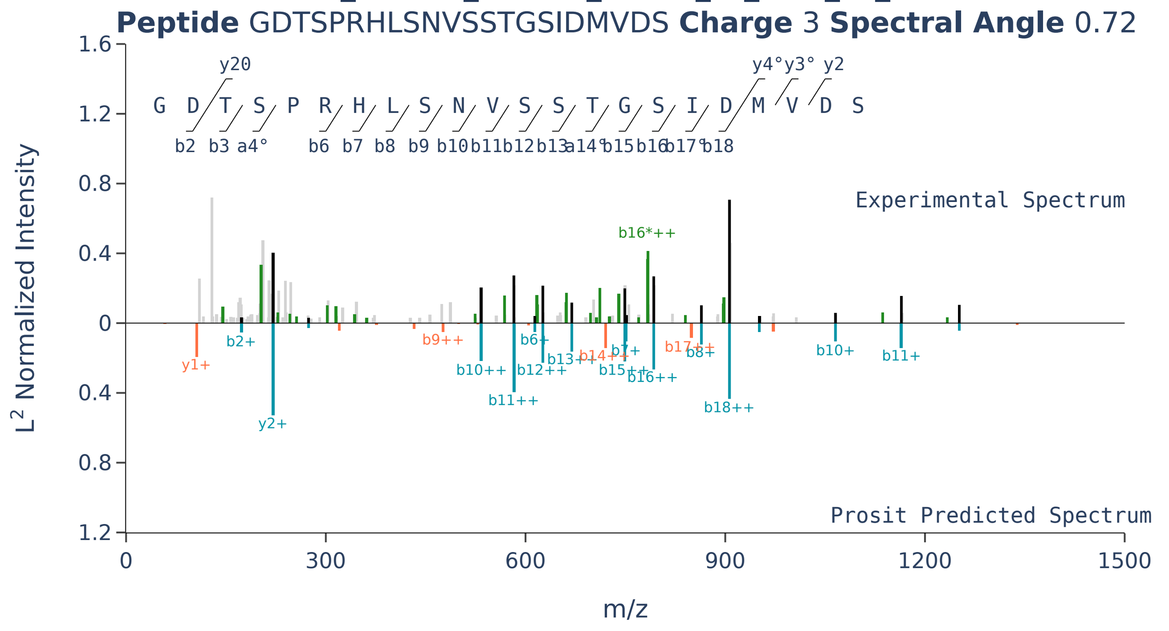


**Source** WSoh_221121_011221_HFGoe_L2_hTau_4h_R2 **Scan** 21898
**Peptide** PRHLSNVSSTGSIDMVDSPQL **Charge** 3 **Spectral Angle** 0.8

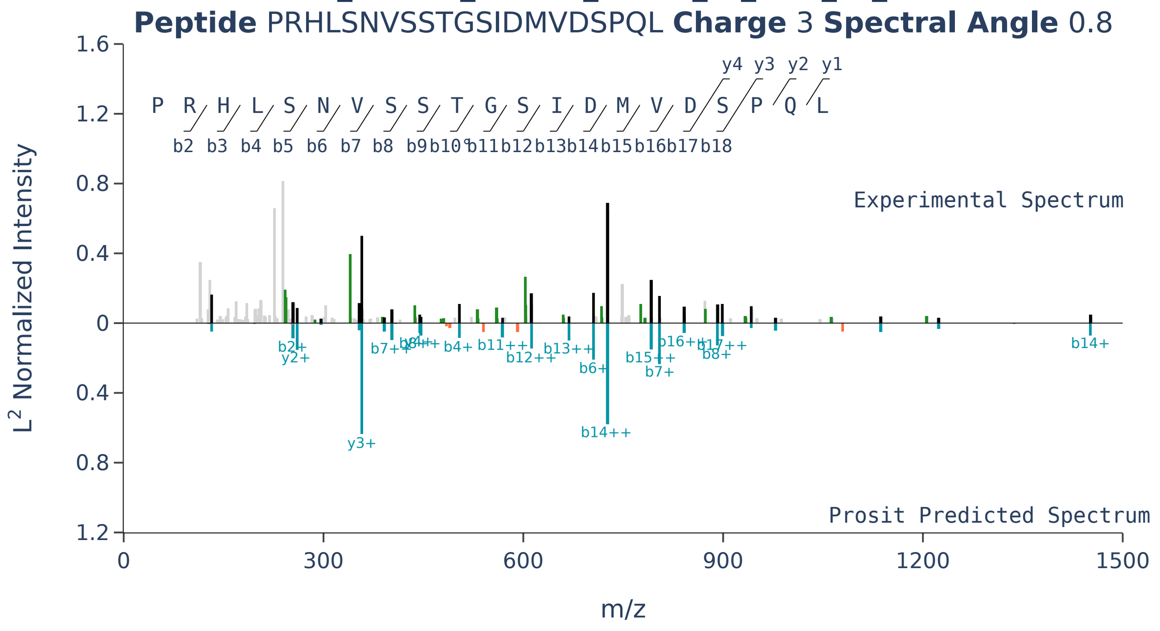


**Source** WSoh_221121_011221_HFGoe_L2_hTau_24h_R1 **Scan** 17561
**Peptide** PSLPTPPTREPKKVAVV **Charge** 3 **Spectral Angle** 0.72

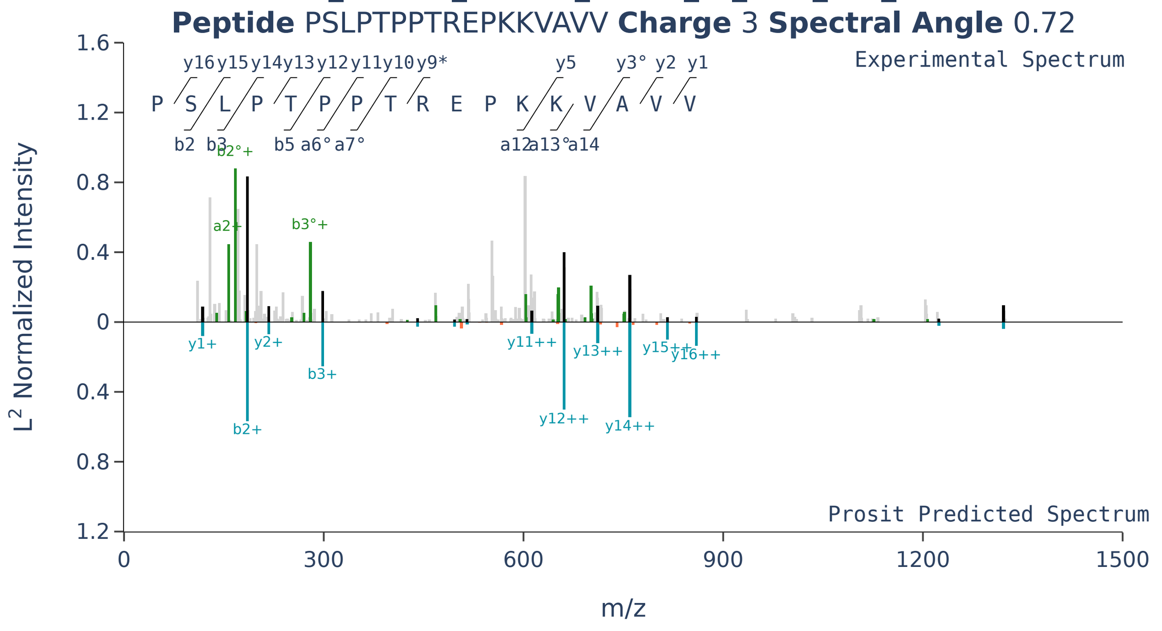


**Source** WSoh_221121_011221_HFGoe_L2_hTau_2h_R2 **Scan** 8276
**Peptide** RIPAKTPPAPKTPPSSGEPPKSGDRS **Charge** 5 **Spectral Angle** 0.79

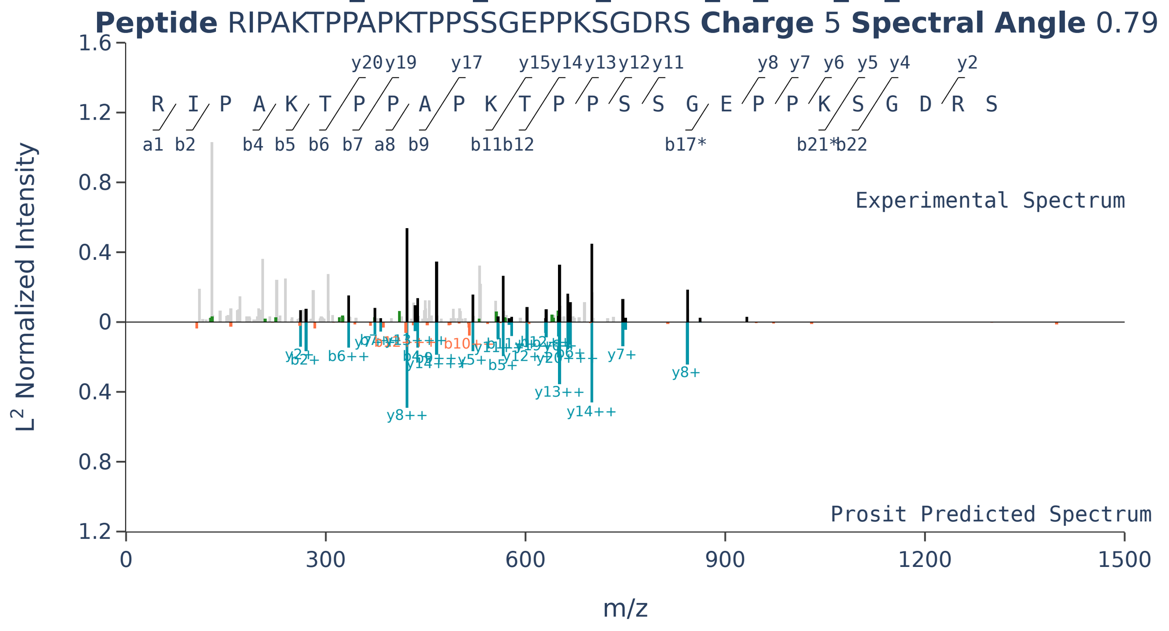


**Source** WSoh_221121_011221_HFGoe_L2_hTau_4h_R1 **Scan** 14794
**Peptide** QSKCGSKDNIKHVPGGGSVQIVY **Charge** 4 **Spectral Angle** 0.8

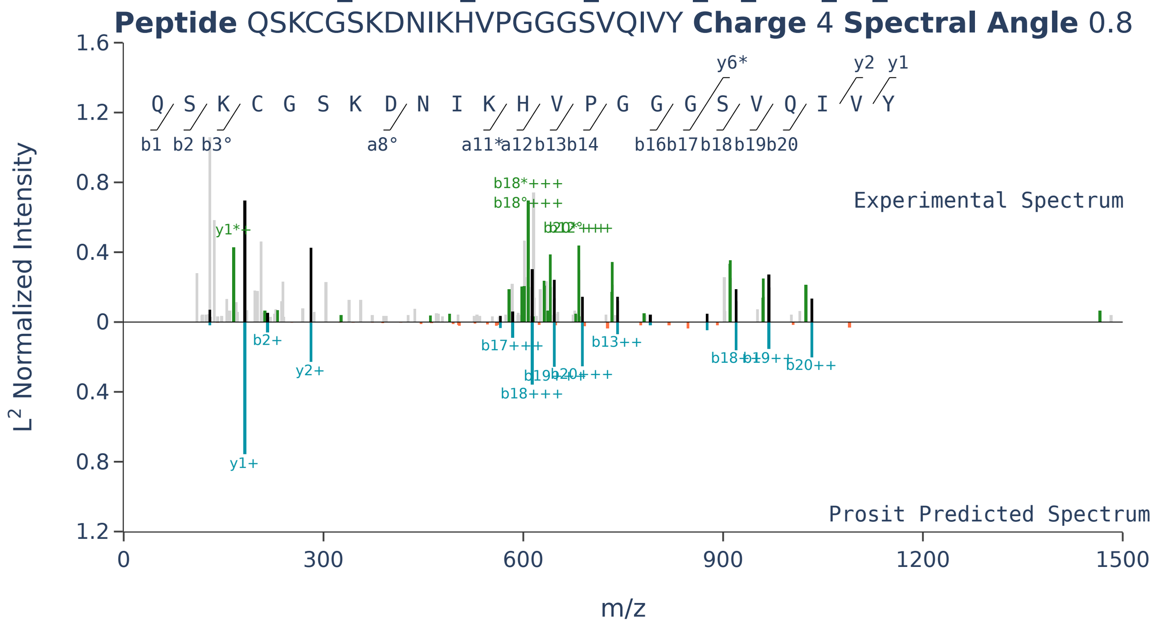


**Source** WSoh_221121_011221_HFGoe_L2_hTau_4h_R2 **Scan** 15562
**Peptide** KLGSTENLKHQPGGGKVQIINKKLDL **Charge** 6 **Spectral Angle** 0.9

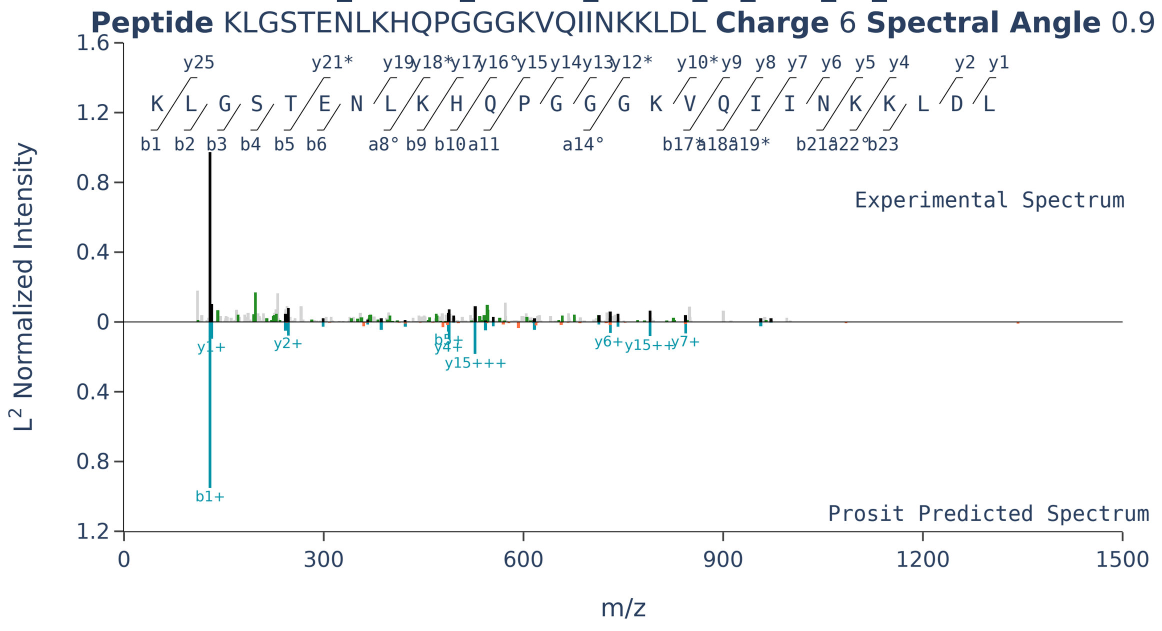


**Source** WSoh_221121_011221_HFGoe_L2_hTau_4h_R1 **Scan** 17632
**Peptide** DFKDRVQSKIGSLD **Charge** 3 **Spectral Angle** 0.72

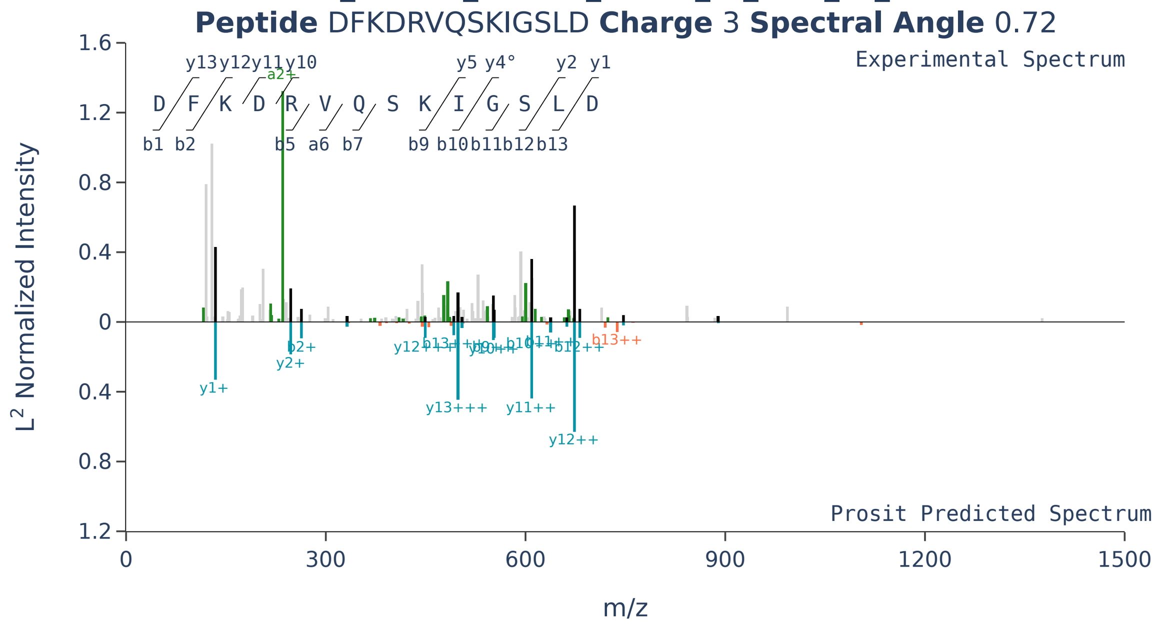


**Source** WSoh_221121_011221_HFGoe_L2_hTau_24h_R1 **Scan** 9912
**Peptide** DNITHVPGGGNKKIETHKLT **Charge** 4 **Spectral Angle** 0.82

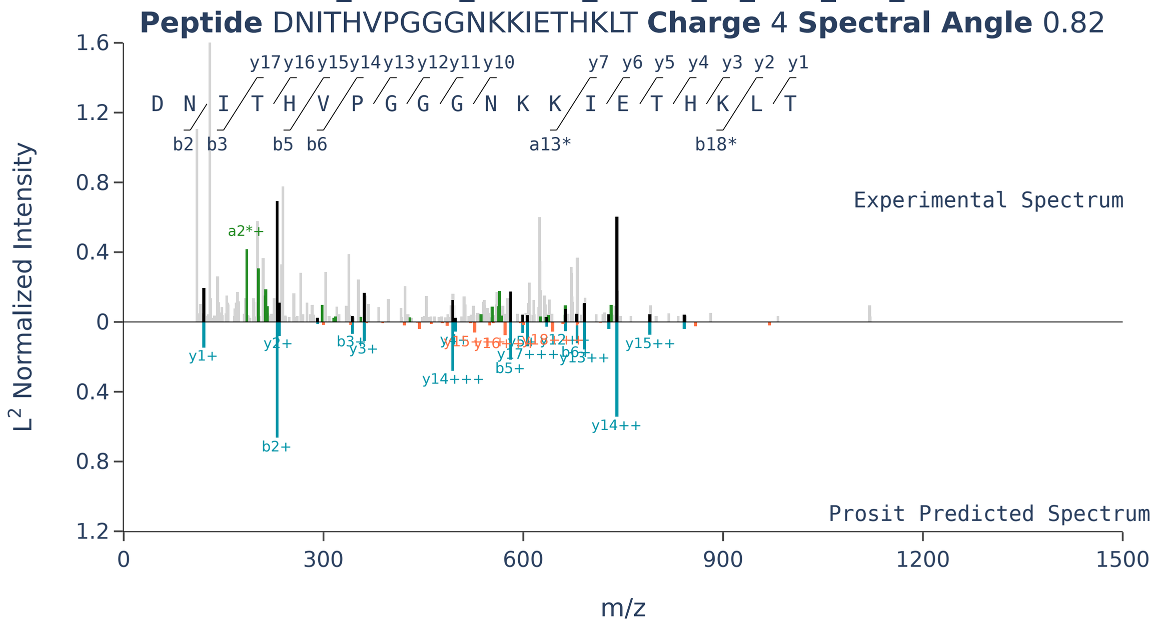

**Source** WSoh_221121_011221_HFGoe_L2_hTau_24h_R1 **Scan** 9726
**Peptide** QLINKKL **Charge** 2 **Spectral Angle** 0.76

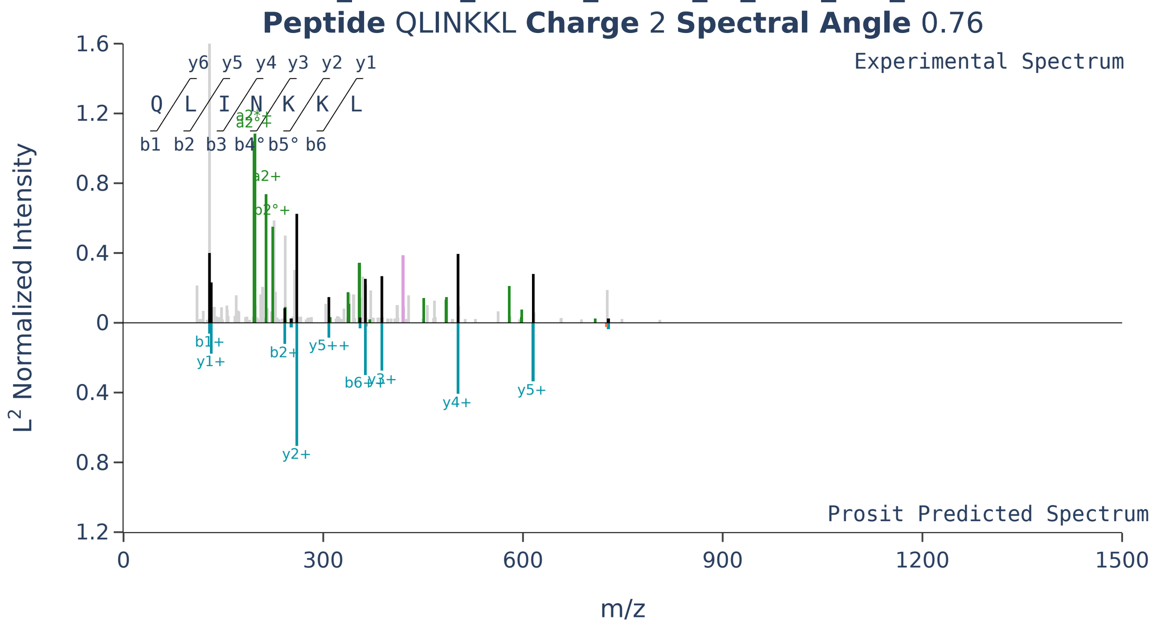

**Source** WSoh_221121_011221_HFGoe_L2_hTau_24h_R1 **Scan** 9582
**Peptide** ADGKTKIATPRGAAPPGQKGQANATRI **Charge** 4 **Spectral Angle** 0.8

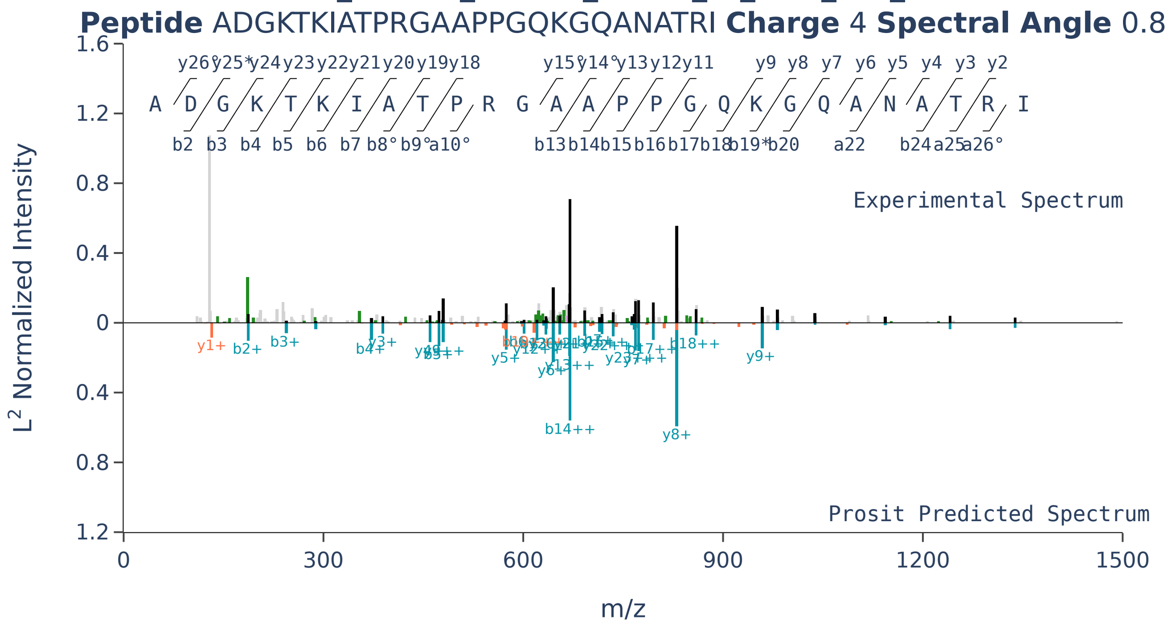

**Source** WSoh_221121_011221_HFGoe_L2_hTau_24h_R1 **Scan** 11664
**Peptide** VYKSPVVS **Charge** 1 **Spectral Angle** 0.88

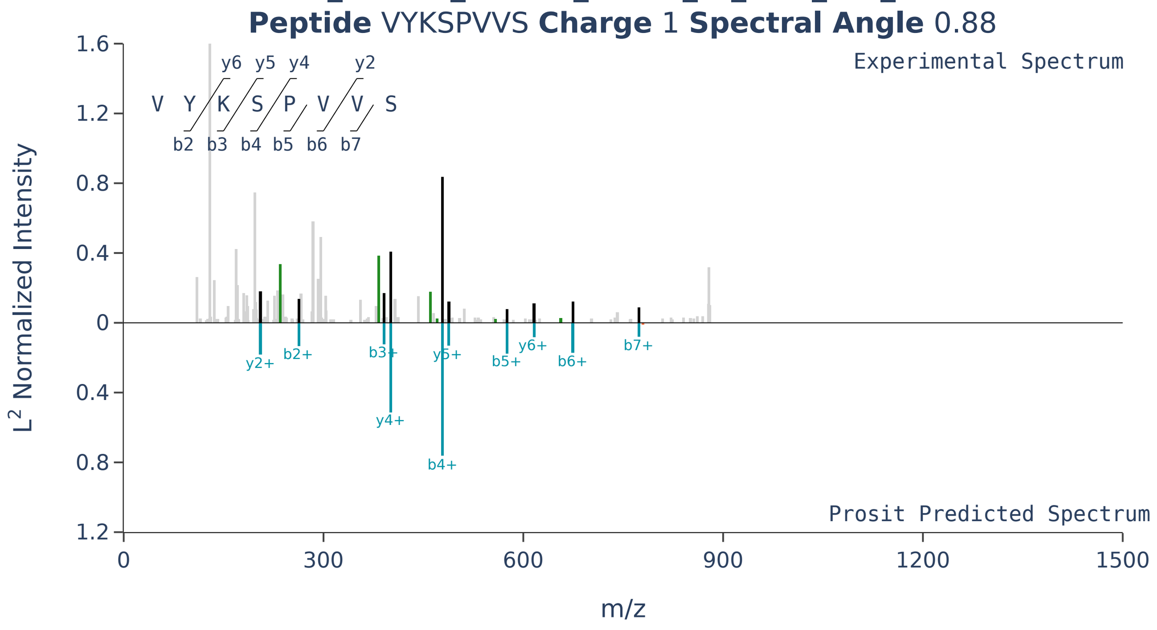

**Source** WSoh_221121_011221_HFGoe_L2_hTau_4h_R2 **Scan** 16729
**Peptide** RVQSKIGSLDNI **Charge** 2 **Spectral Angle** 0.82

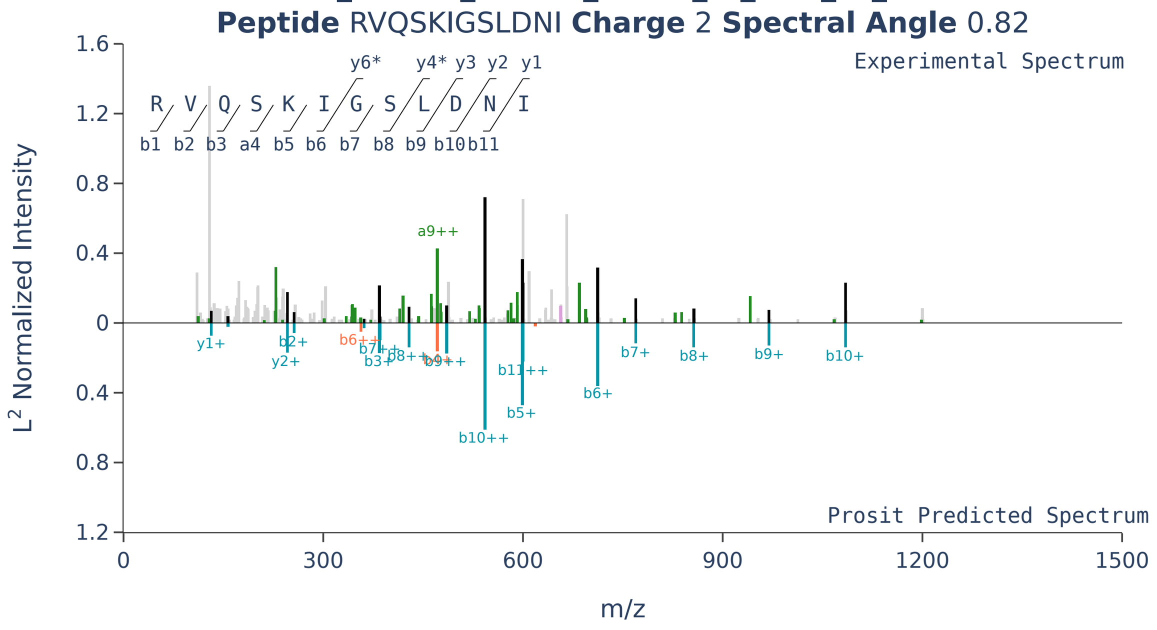

**Source** WSoh_221121_011221_HFGoe_L2_hTau_4h_R1 **Scan** 21603
**Peptide** KLDFKDRVQSKIGSLDNL **Charge** 4 **Spectral Angle** 0.82

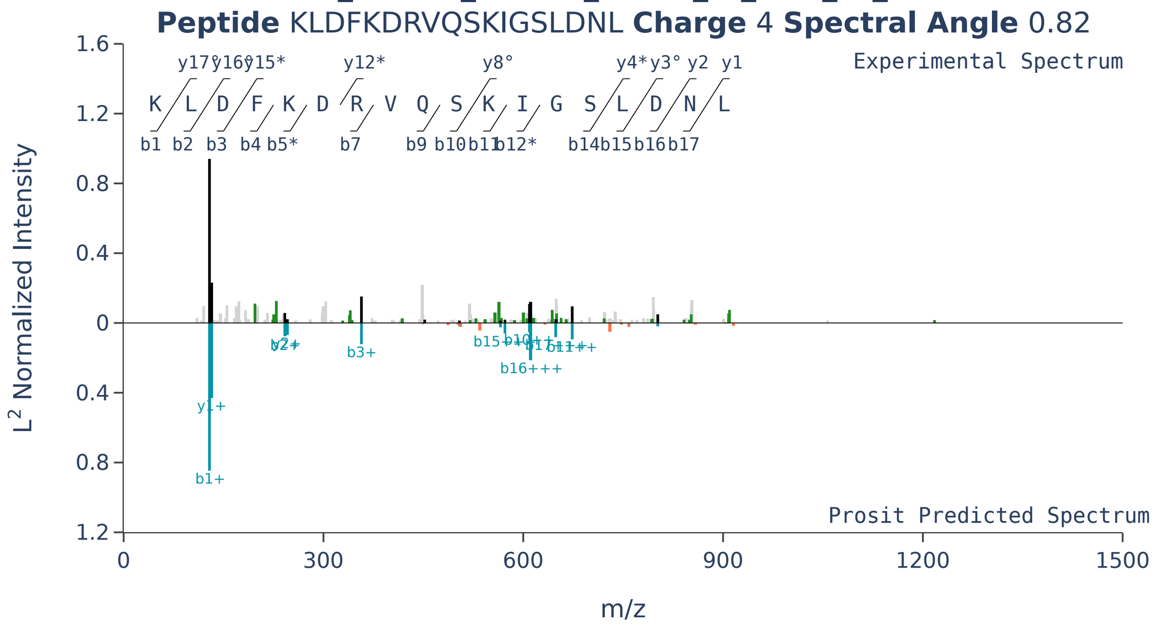

**Source** WSoh_221121_011221_HFGoe_L2_hTau_24h_R1 **Scan** 9127
**Peptide** KLDLSNVQSKCGSKDNLKH **Charge** 3 **Spectral Angle** 0.9

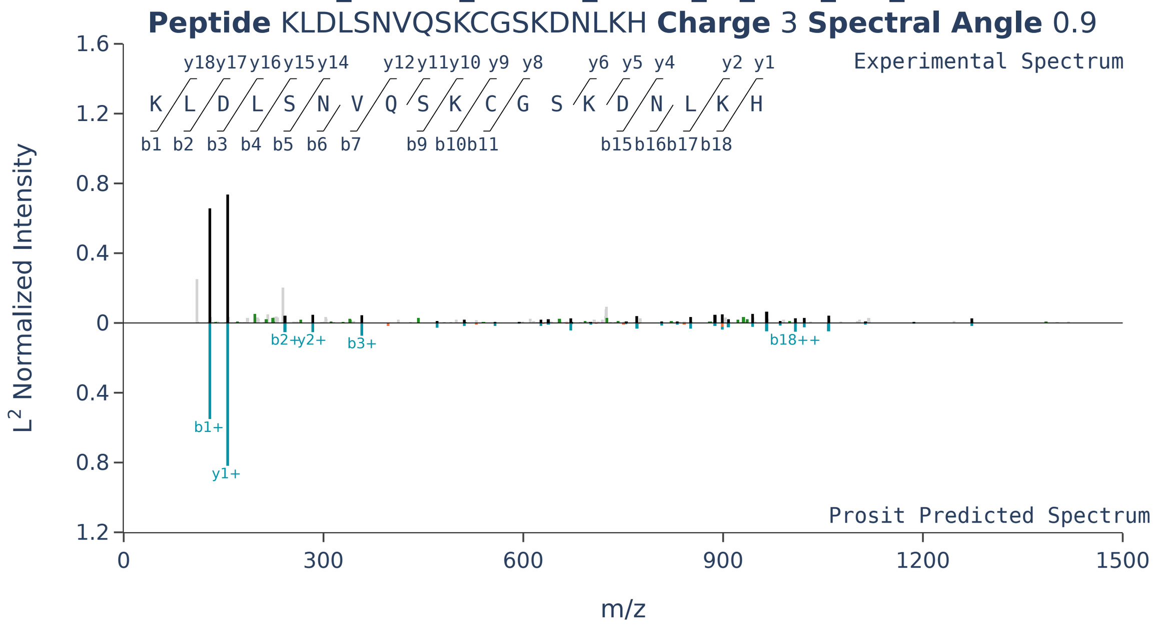

**Source** WSoh_221121_011221_HFGoe_L2_hTau_24h_R2 **Scan** 5769
**Peptide** GTGSDDKKAKGADGKTKIATPRGA **Charge** 3 **Spectral Angle** 0.82

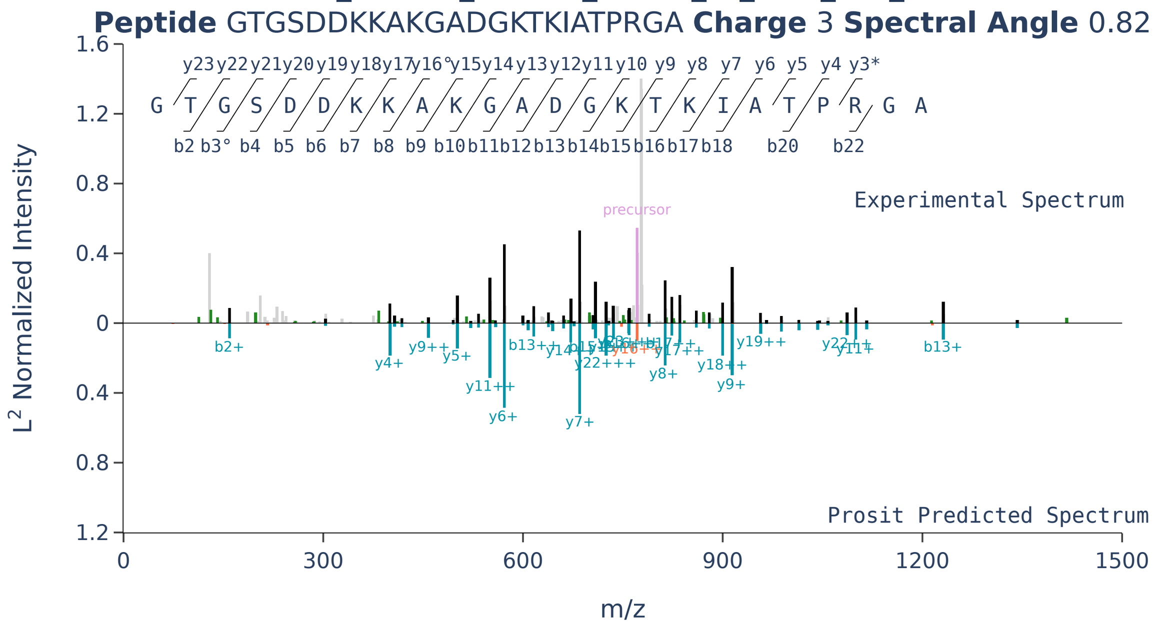

**Source** WSoh_221121_011221_HFGoe_L1_hTau_24h_R1 **Scan** 20057
**Peptide** SSTGSIDMV **Charge** 1 **Spectral Angle** 0.73

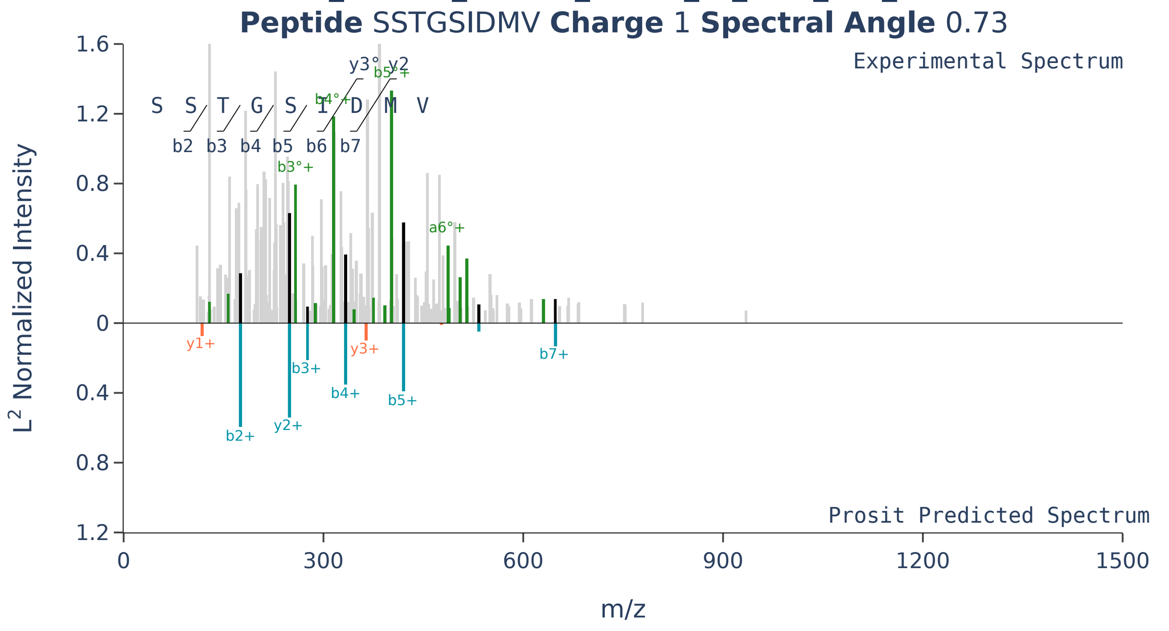

**Source** WSoh_221121_011221_HFGoe_L1_hTau_24h_R1 **Scan** 10510
**Peptide** NIHHKPGGGQVEVKSEKLDFKDRVQS **Charge** 5 **Spectral Angle** 0.75

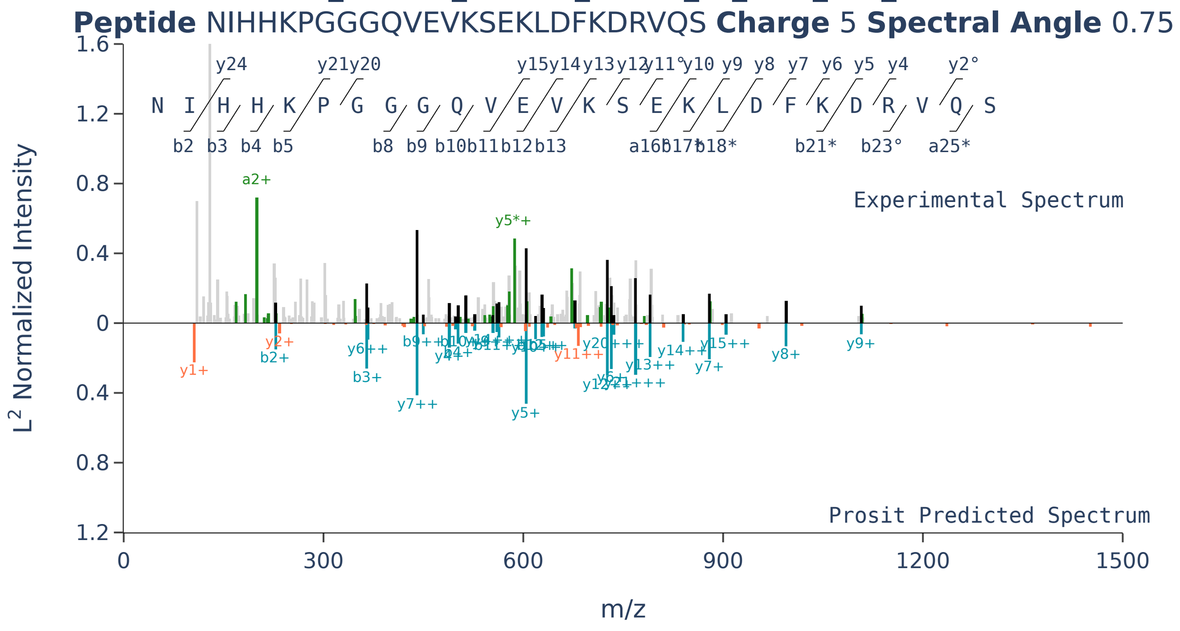

**Source** WSoh_221121_011221_HFGoe_L2_hTau_24h_R2 **Scan** 10943
**Peptide** APPGQKGQANATRIPAKTPPAPKTPPSS **Charge** 3 **Spectral Angle** 0.88

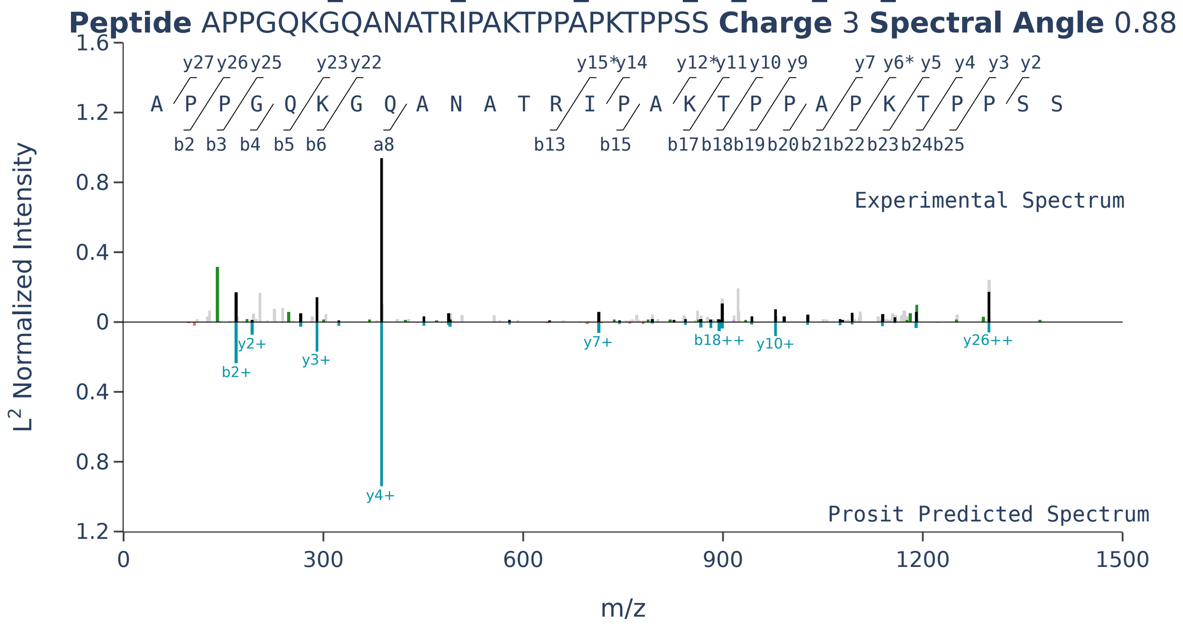

**Source** WSoh_221121_011221_HFGoe_L1_hTau_4h_R2 **Scan** 18891
**Peptide** SNVSSTGSIDMVDS **Charge** 2 **Spectral Angle** 0.88

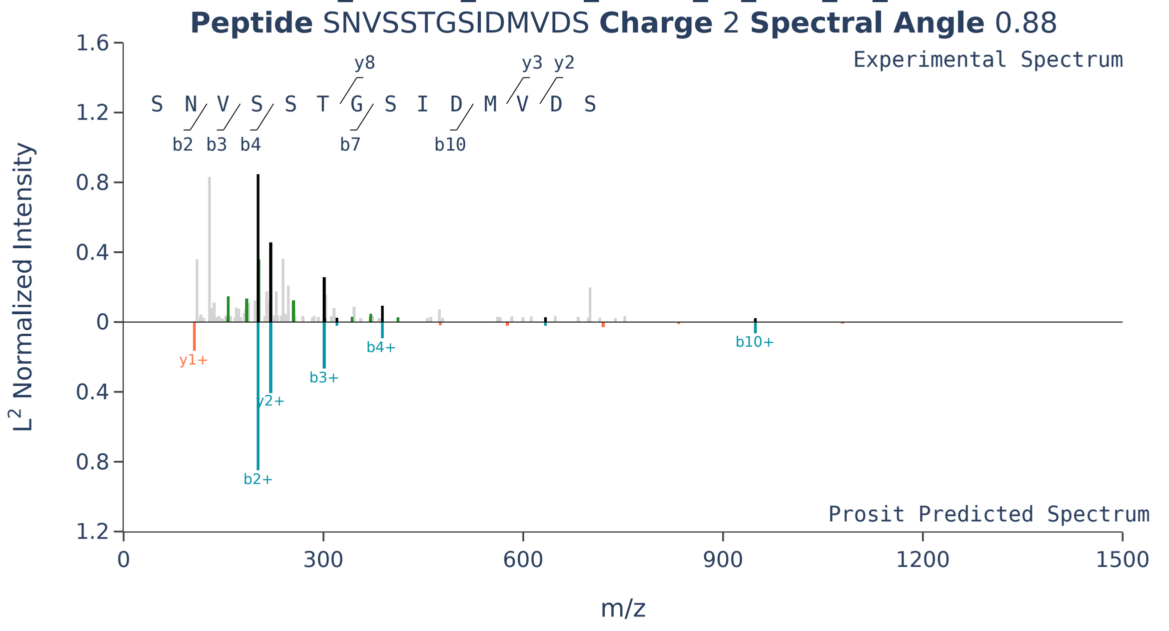

**Source** WSoh_221121_011221_HFGoe_L2_hTau_24h_R2 **Scan** 10423
**Peptide** CGSLGNIHHKPGGGQVEVKSEKL **Charge** 4 **Spectral Angle** 0.79

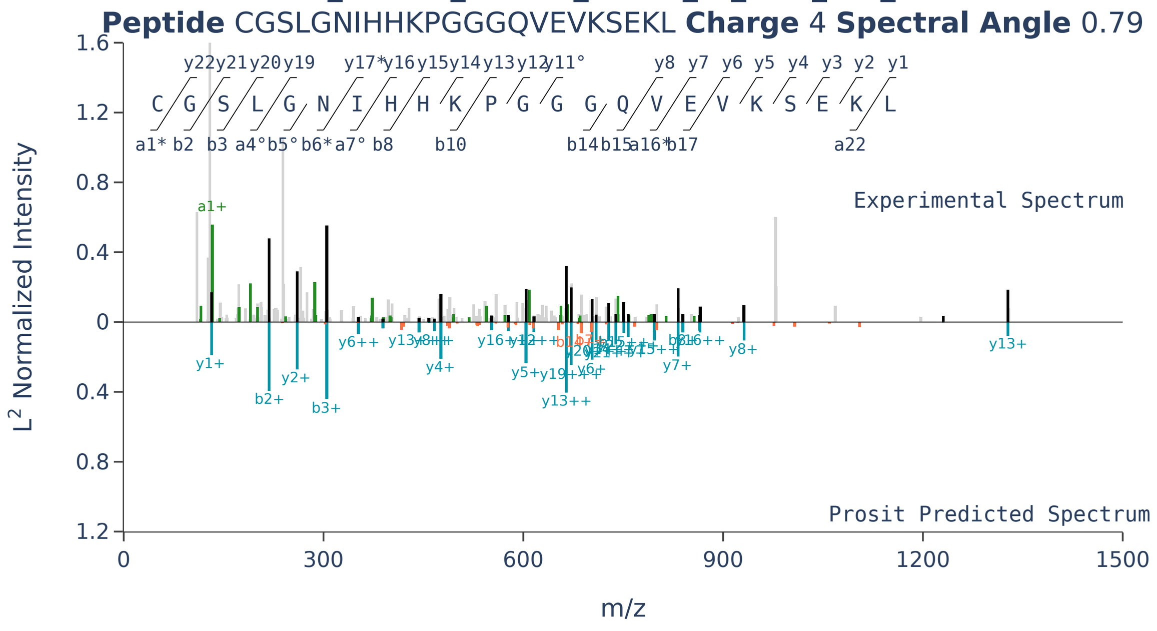

**Source** WSoh_221121_011221_HFGoe_L2_hTau_24h_R2 **Scan** 26814
**Peptide** PQLATLADEVSA **Charge** 2 **Spectral Angle** 0.77

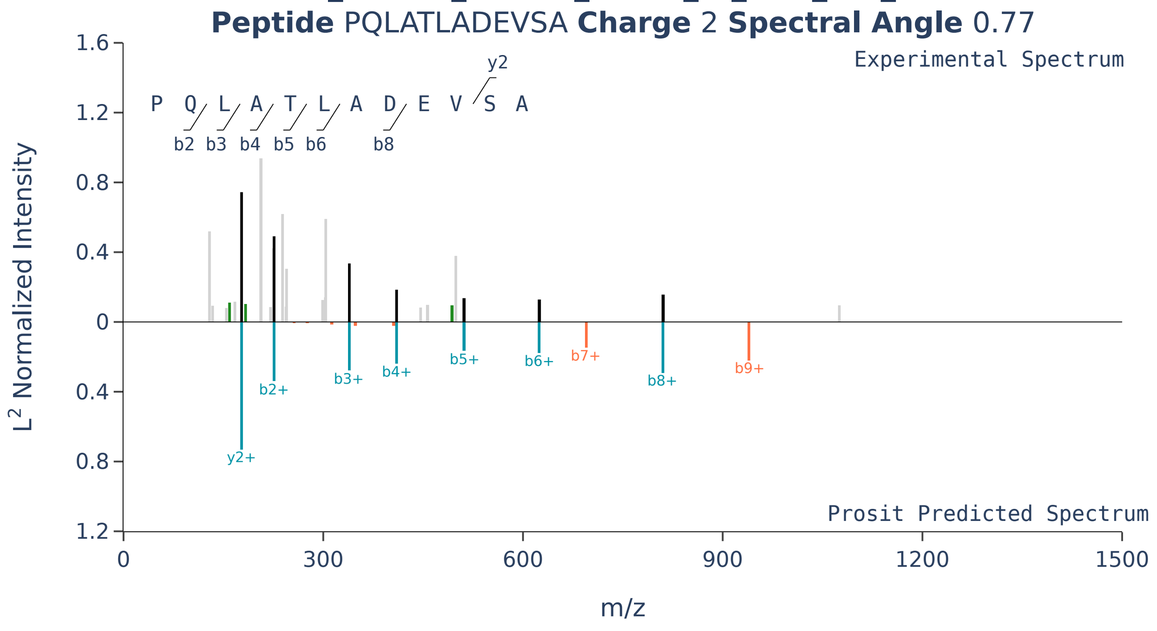

**Source** WSoh_221121_011221_HFGoe_L2_hTau_24h_R1 **Scan** 15884
**Peptide** STENLKHQPGGGKVQIINKKLDL **Charge** 4 **Spectral Angle** 0.81

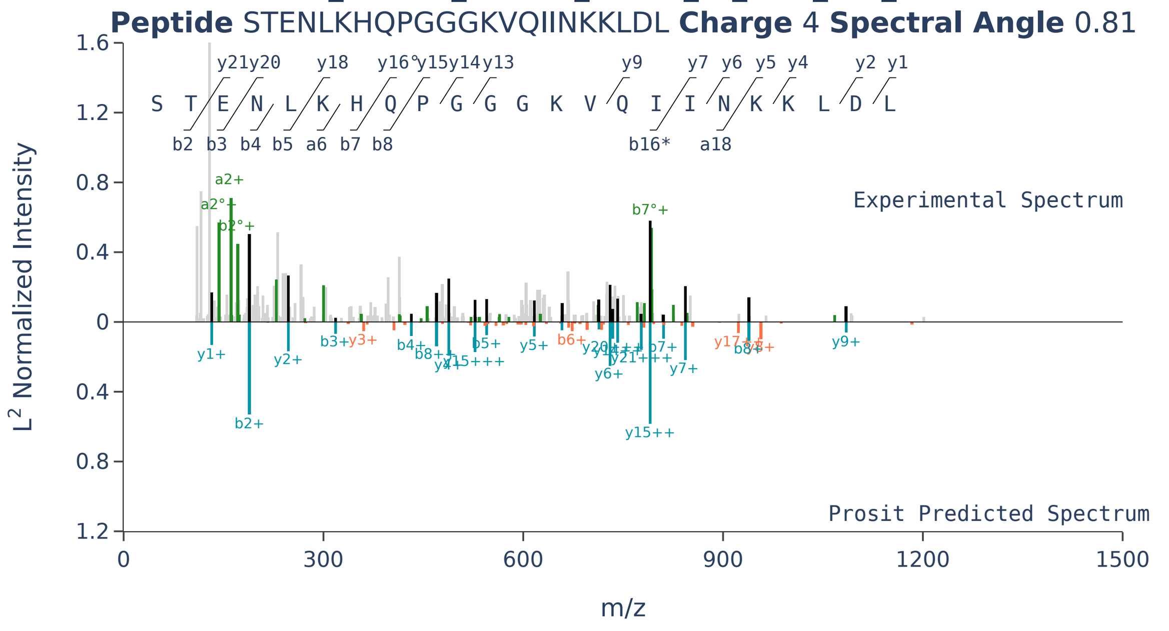

**Source** WSoh_221121_011221_HFGoe_L2_hTau_24h_R1 **Scan** 21692
**Peptide** QLVYKPVDLSKVTS **Charge** 2 **Spectral Angle** 0.84

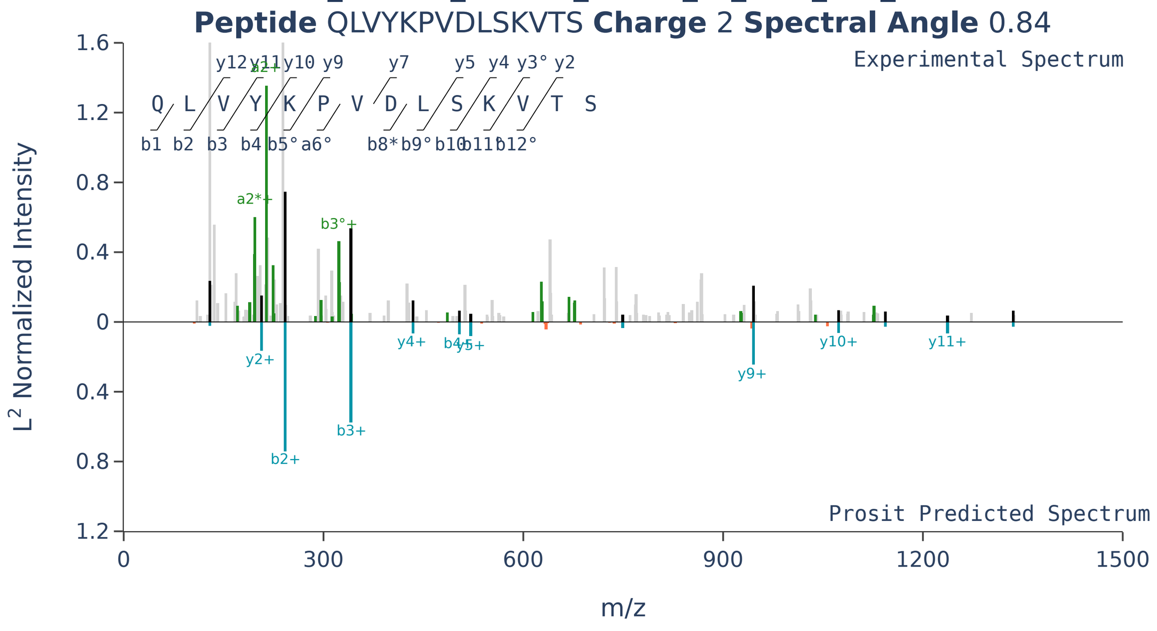

**Source** WSoh_221121_011221_HFGoe_L2_hTau_4h_R1 **Scan** 16411
**Peptide** QSKIGSLDNITHVPGGGNKKI **Charge** 3 **Spectral Angle** 0.89

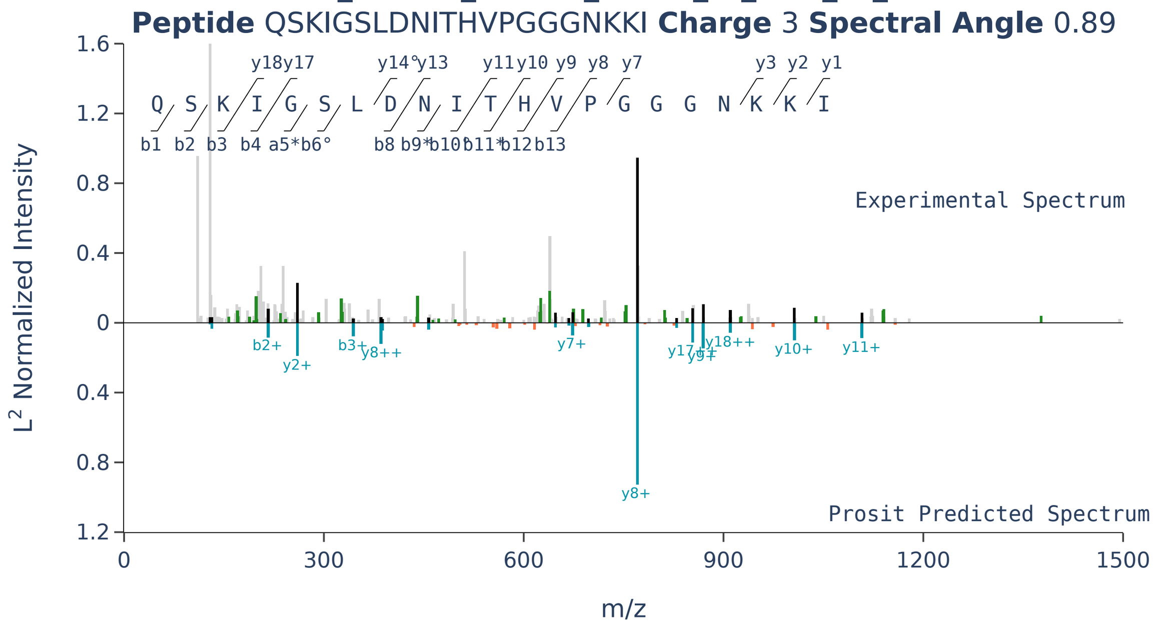


**Source** WSoh_221121_011221_HFGoe_L2_hTau_24h_R1 **Scan** 10531
**Peptide** TRIPAKTPPAPKTPPSSGEPPKSGDRSGY **Charge** 5 **Spectral Angle** 0.79

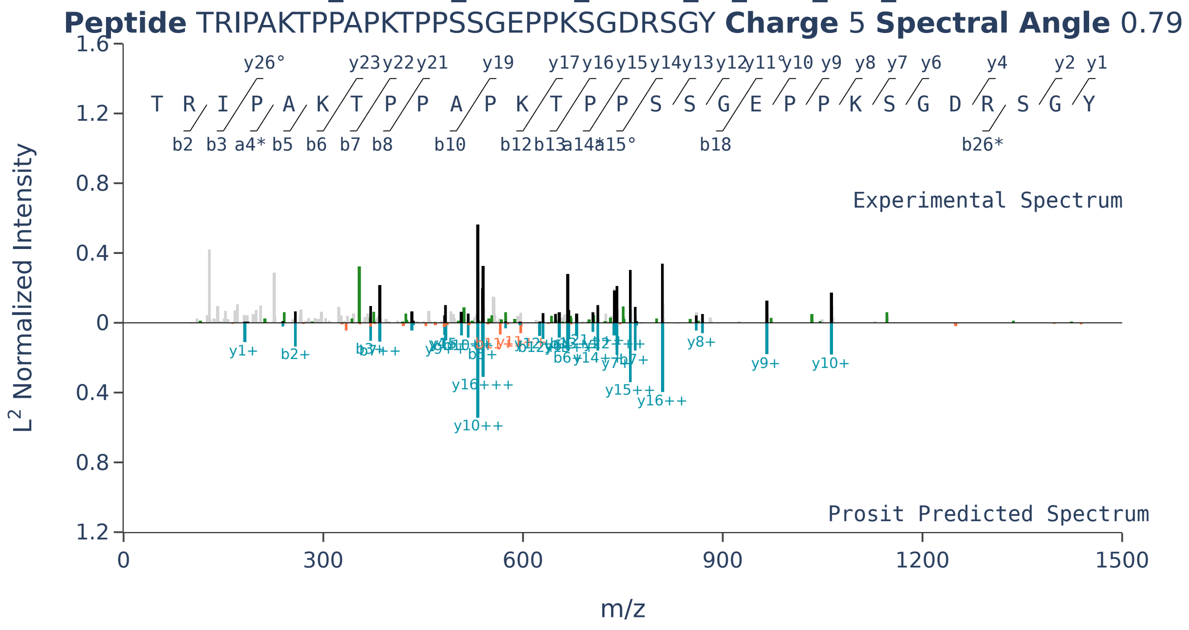


**Source** WSoh_221121_011221_HFGoe_L1_hTau_4h_R1 **Scan** 5974
**Peptide** SKVTSKCGSLGNIHHKPGG **Charge** 3 **Spectral Angle** 0.85

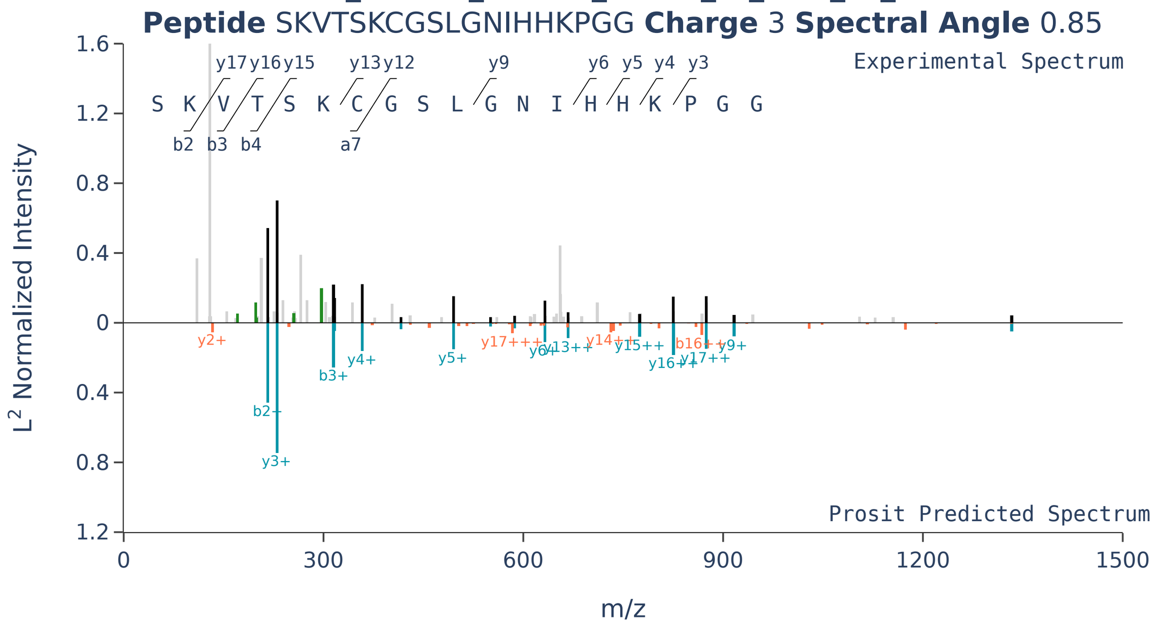


**Source** WSoh_221121_011221_HFGoe_L1_hTau_4h_R2 **Scan** 16335
**Peptide** PLVDEGAPGKQAAAQPHTEI **Charge** 3 **Spectral Angle** 0.83

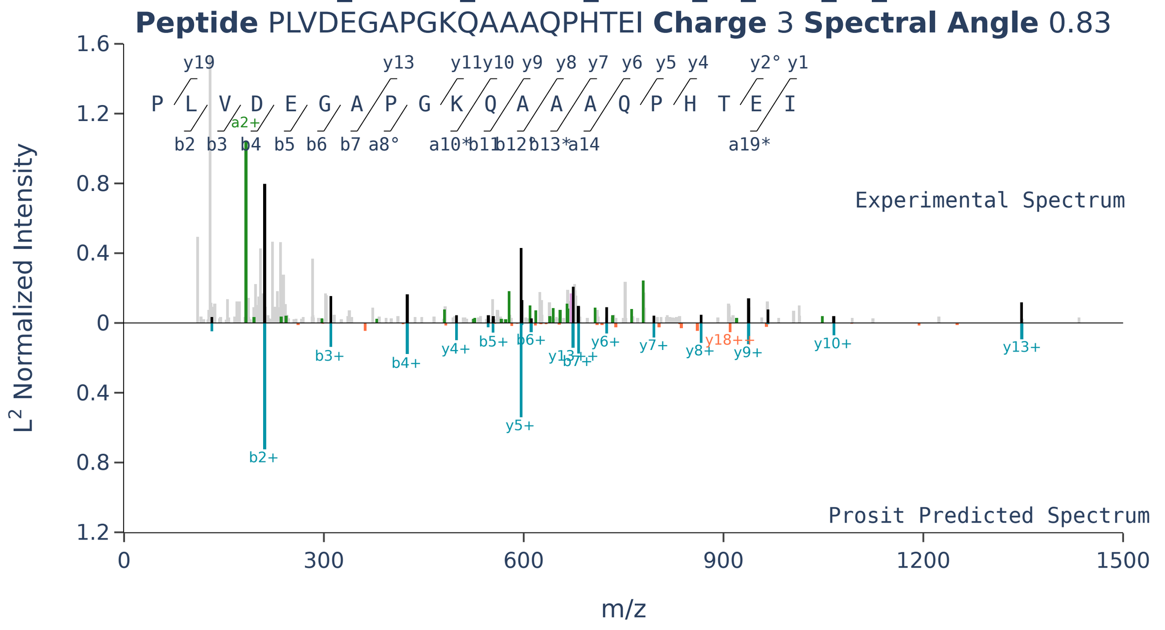


**Source** WSoh_221121_011221_HFGoe_L2_hTau_24h_R1 **Scan** 6226
**Peptide** ENAKAKTDHGAEI **Charge** 2 **Spectral Angle** 0.66

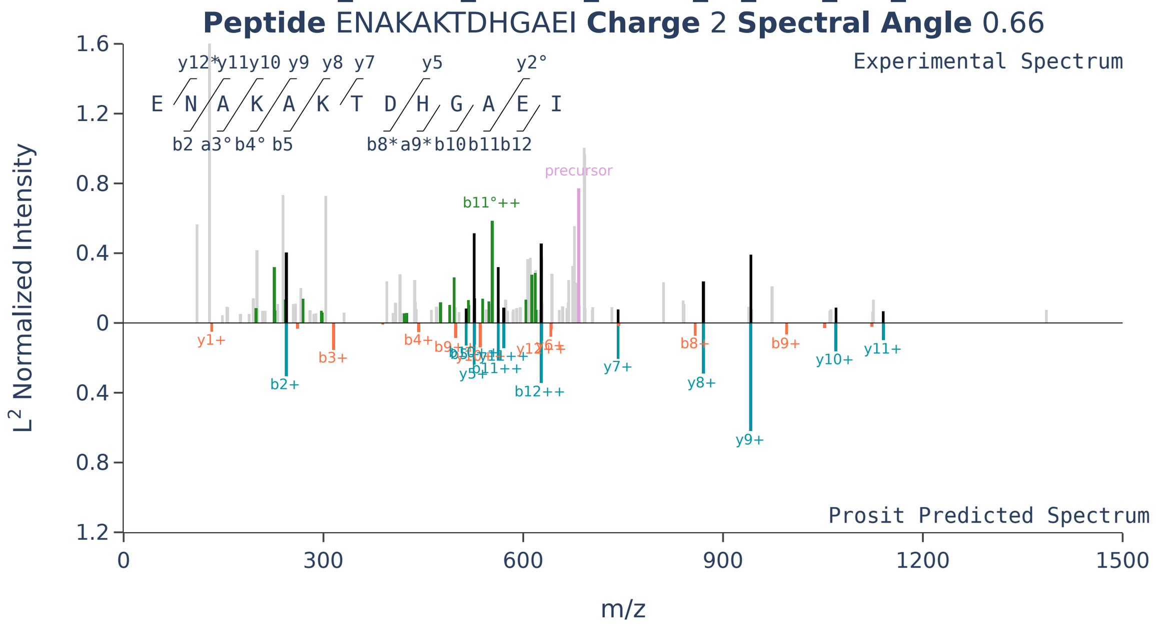


**Source** WSoh_221121_011221_HFGoe_L1_hTau_4h_R1 **Scan** 15497
**Peptide** VTAPLVDEGAPGKQAAAQPHTE **Charge** 3 **Spectral Angle** 0.86

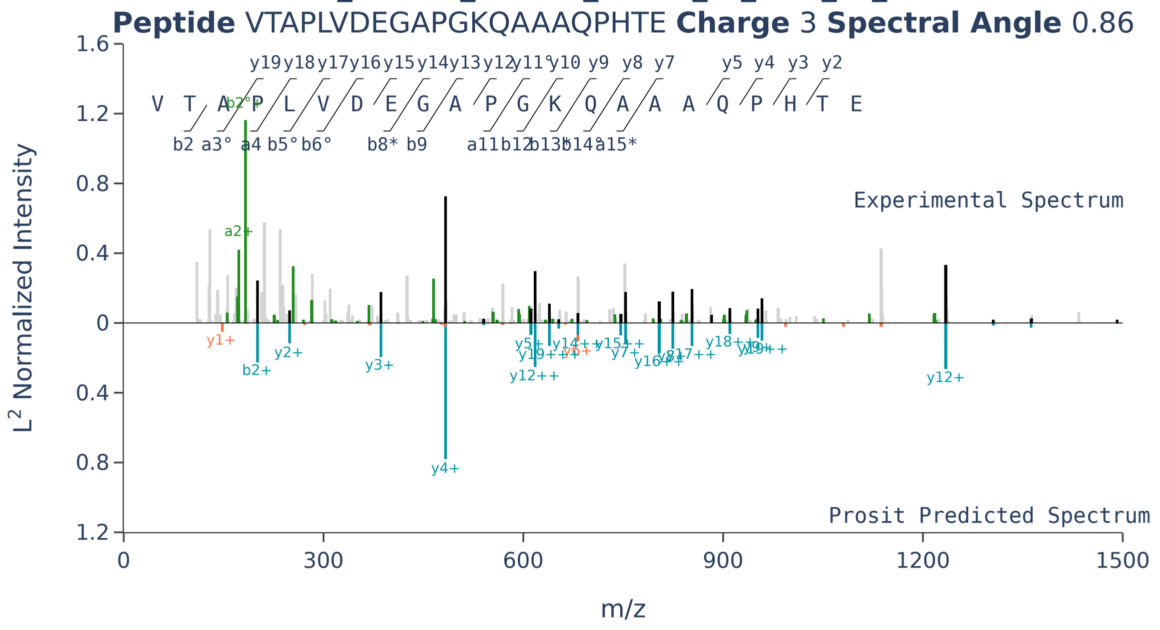


**Source** WSoh_221121_011221_HFGoe_L2_hTau_24h_R1 **Scan** 15019
**Peptide** QSKCGSKDNIKHVPGGGSVQIVYKPVD **Charge** 4 **Spectral Angle** 0.84

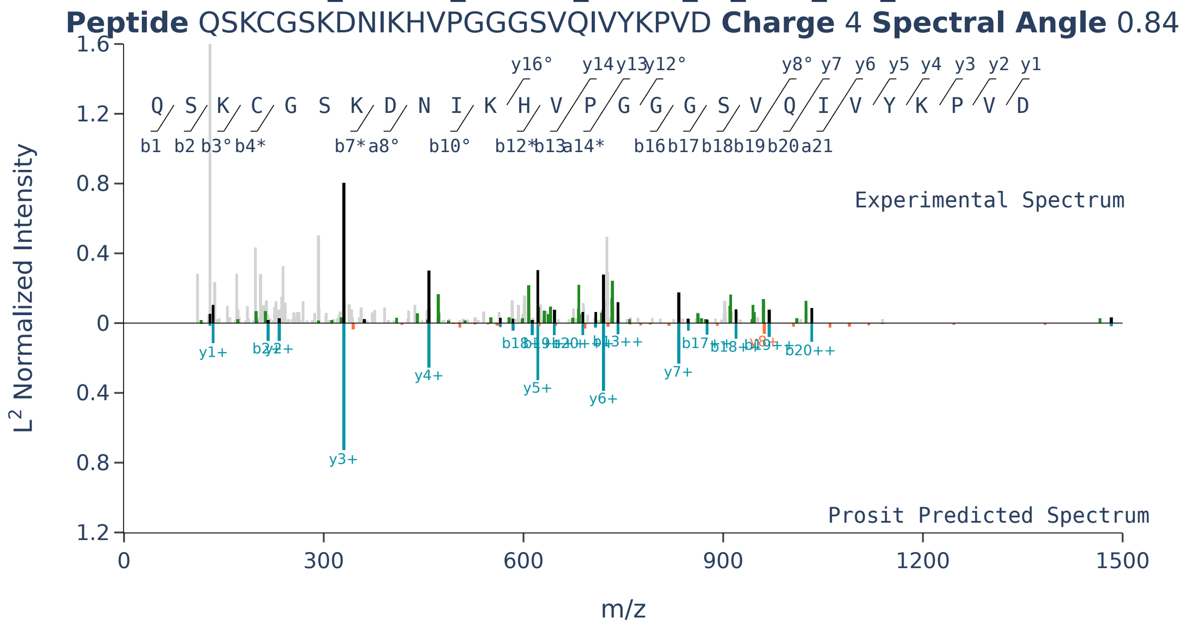


**Source** WSoh_221121_011221_HFGoe_L2_hTau_4h_R2 **Scan** 10679
**Peptide** GNIHHKPGGGQVEVKSEKLDFKDRVQ **Charge** 4 **Spectral Angle** 0.67

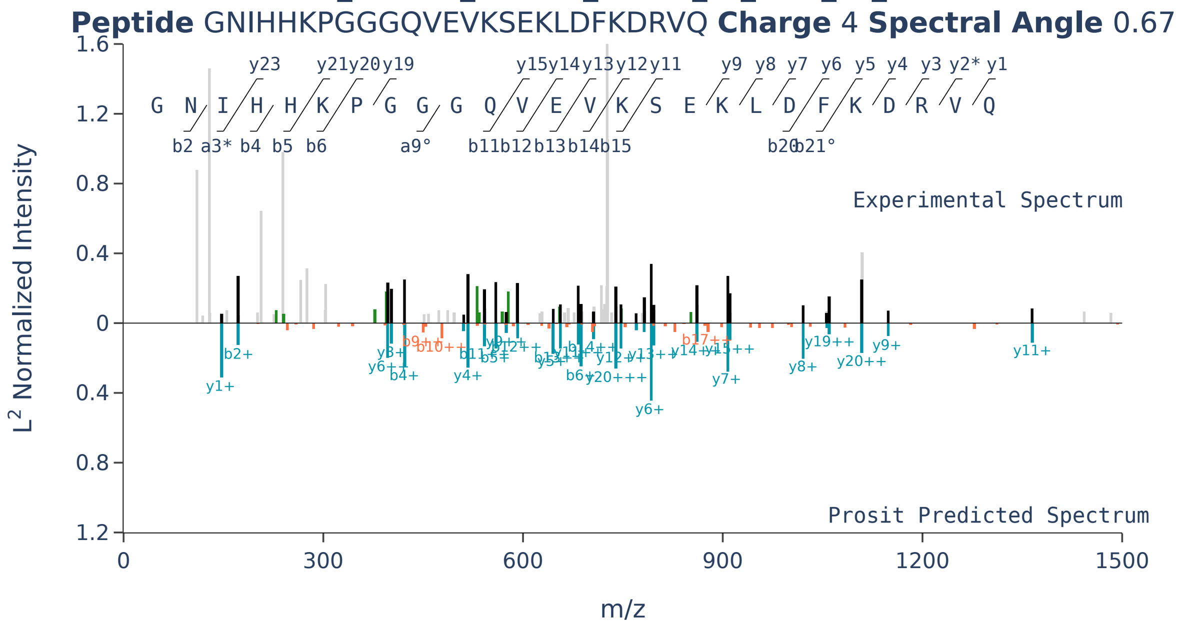


**Source** WSoh_221121_011221_HFGoe_L2_hTau_24h_R2 **Scan** 17121
**Peptide** PGGGKVQIINKKLDLS **Charge** 3 **Spectral Angle** 0.67

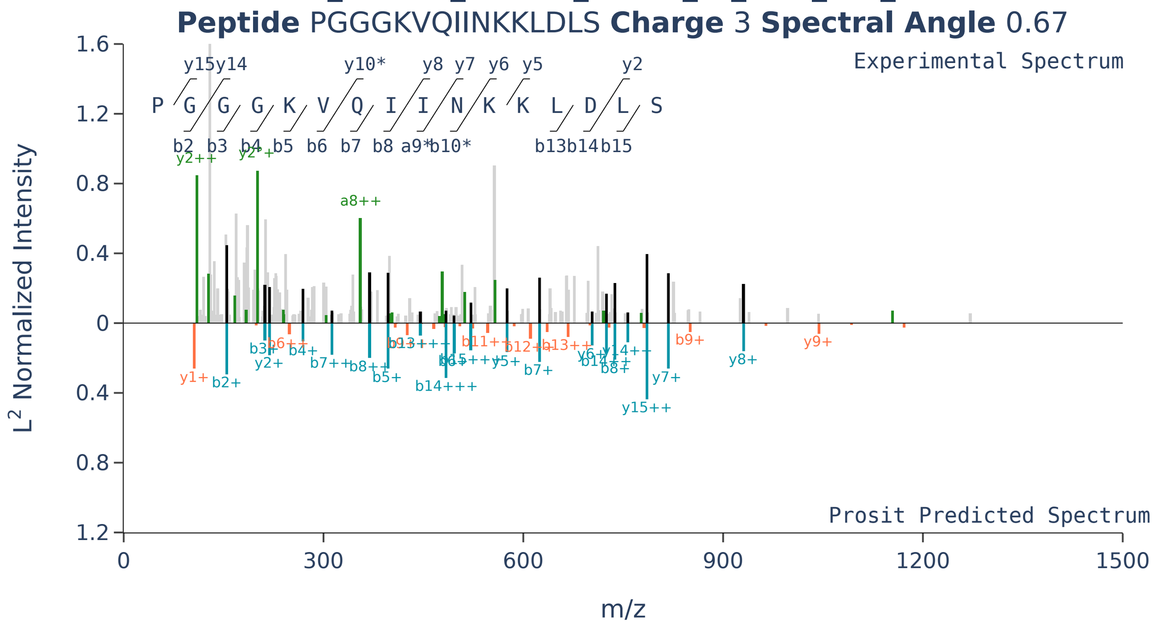


**Source** WSoh_221121_011221_HFGoe_L2_hTau_24h_R2 **Scan** 23081
**Peptide** AAQPHTEIPEGTTAEEAGIGDTPSLED **Charge** 3 **Spectral Angle** 0.84

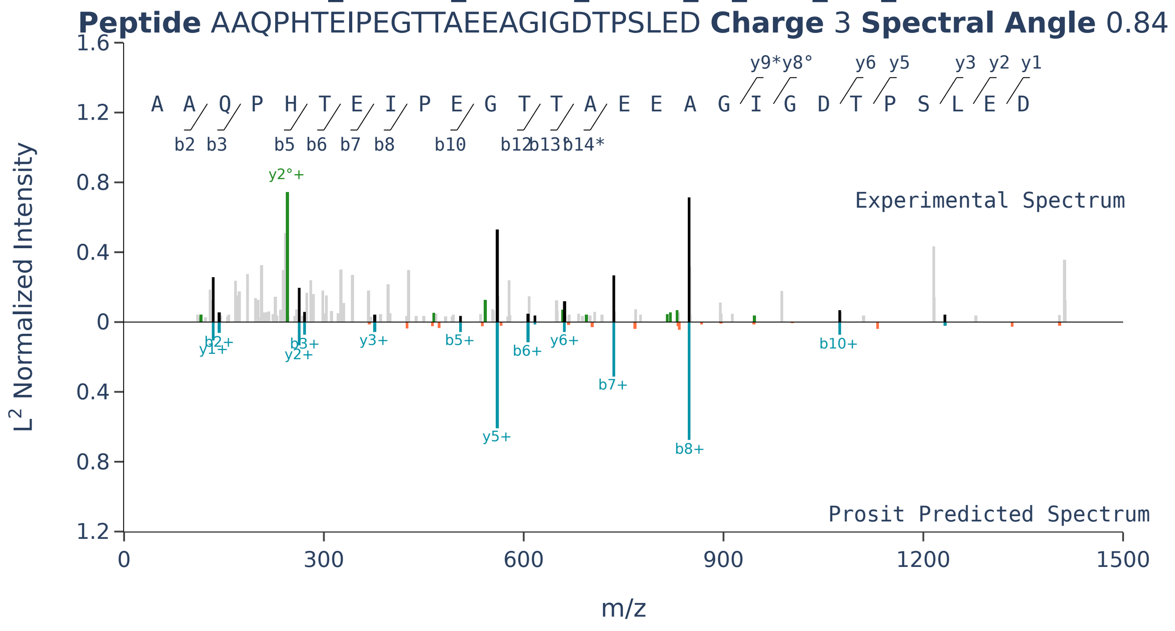


**Source** WSoh_221121_011221_HFGoe_L2_hTau_1h_R1 **Scan** 8777
**Peptide** PGGGQVEVK **Charge** 1 **Spectral Angle** 0.79

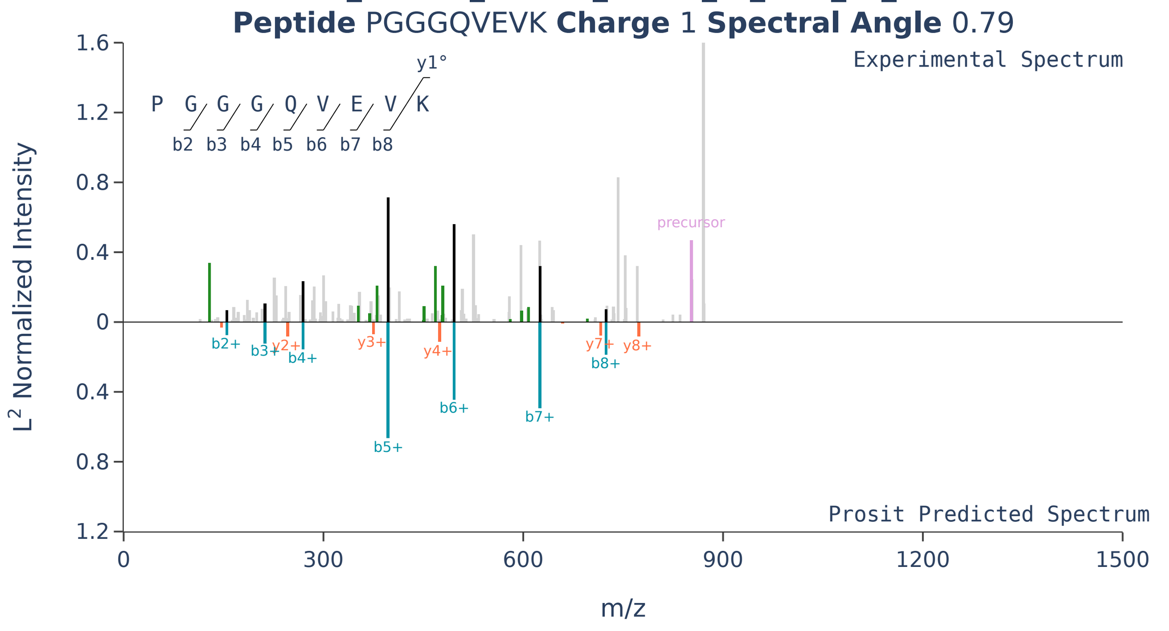


**Source** WSoh_221121_011221_HFGoe_L2_hTau_24h_R1 **Scan** 28780
**Peptide** TAPVPMPDL **Charge** 2 **Spectral Angle** 0.76

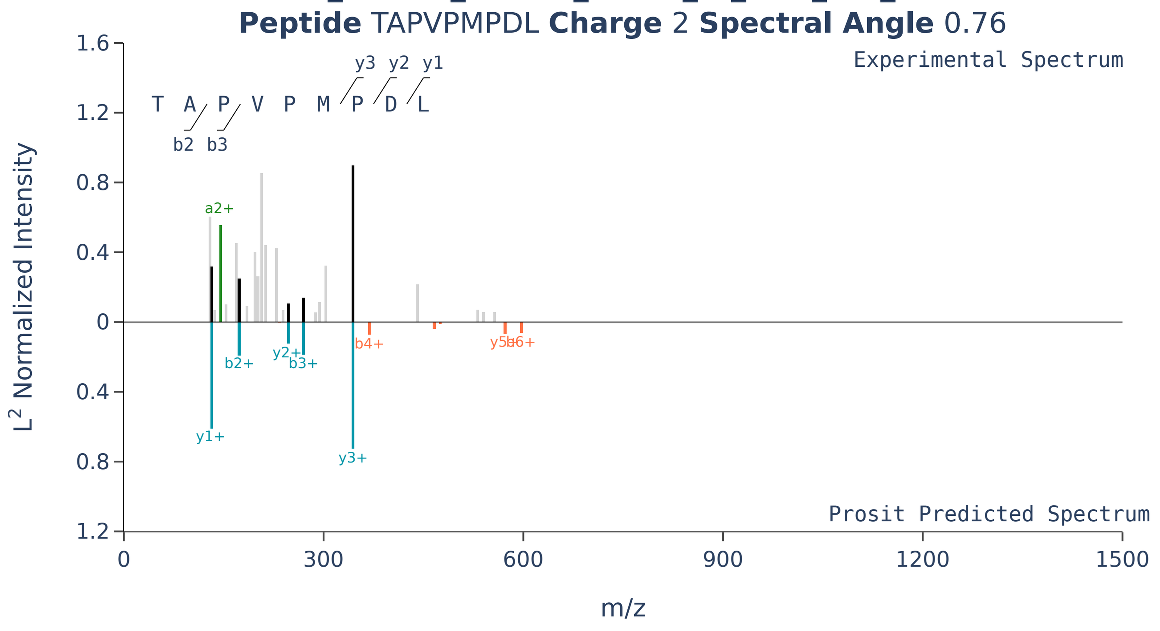


**Source** WSoh_221121_011221_HFGoe_L1_hTau_24h_R2 **Scan** 7309
**Peptide** VVSGDTSPRHLSN **Charge** 2 **Spectral Angle** 0.83

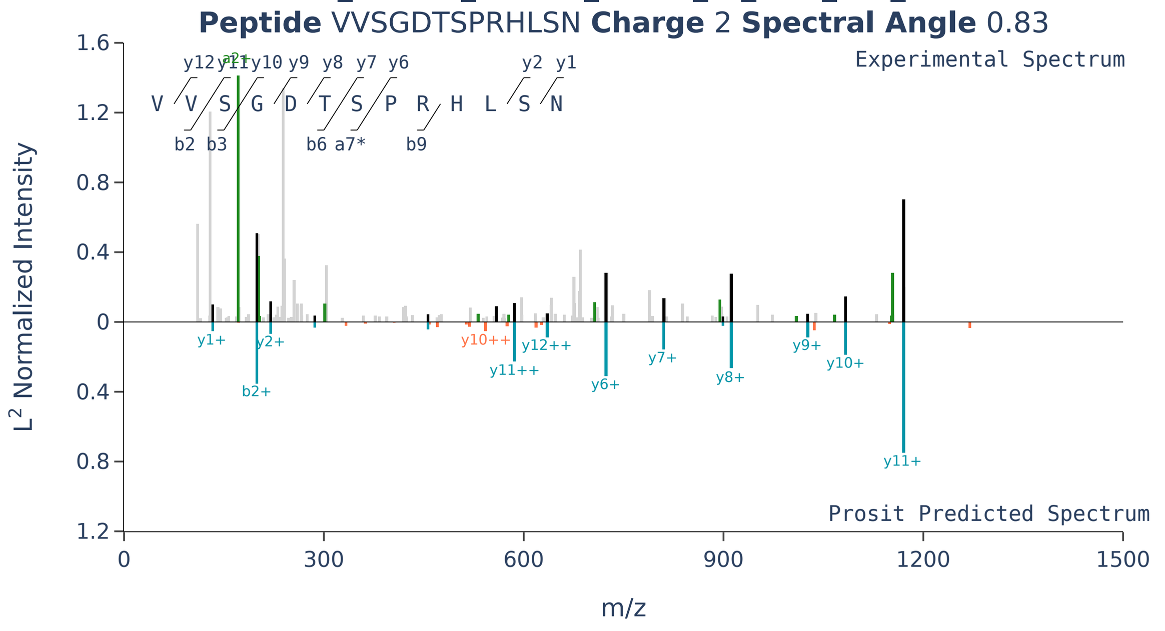


**Source** WSoh_221121_011221_HFGoe_L1_hTau_24h_R2 **Scan** 20629
**Peptide** IGSLDNITHVPGGGNKKIETHKLTF **Charge** 5 **Spectral Angle** 0.73

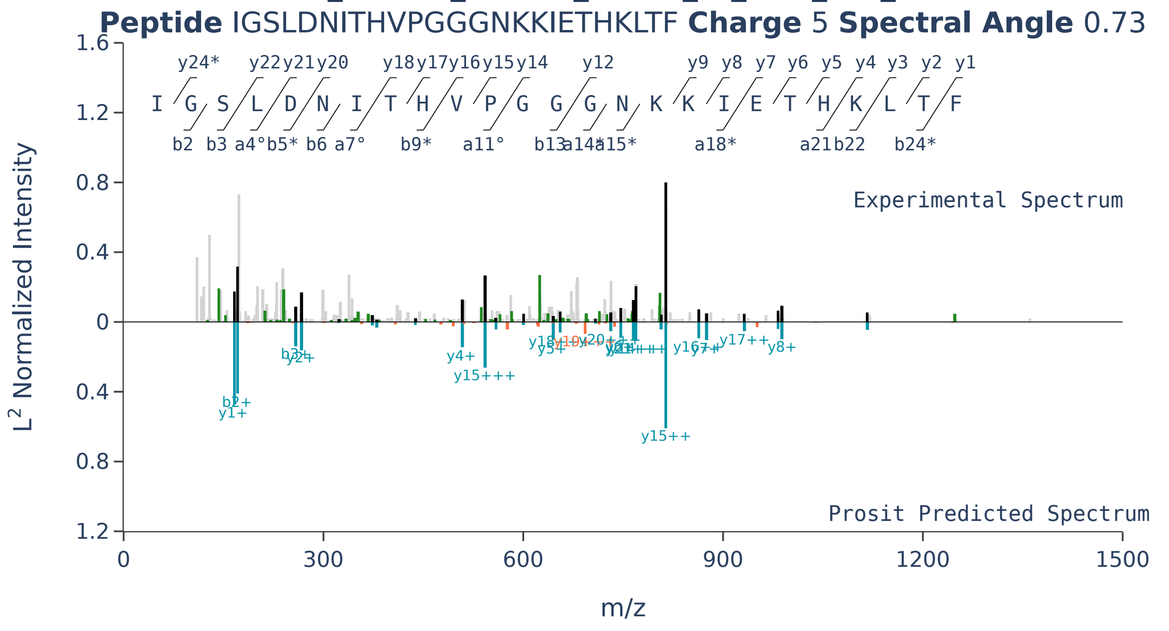


**Source** WSoh_221121_011221_HFGoe_L2_hTau_24h_R1 **Scan** 23838
**Peptide** AEEAGIGDTPSLEDEAAGHVTQARMV **Charge** 3 **Spectral Angle** 0.63

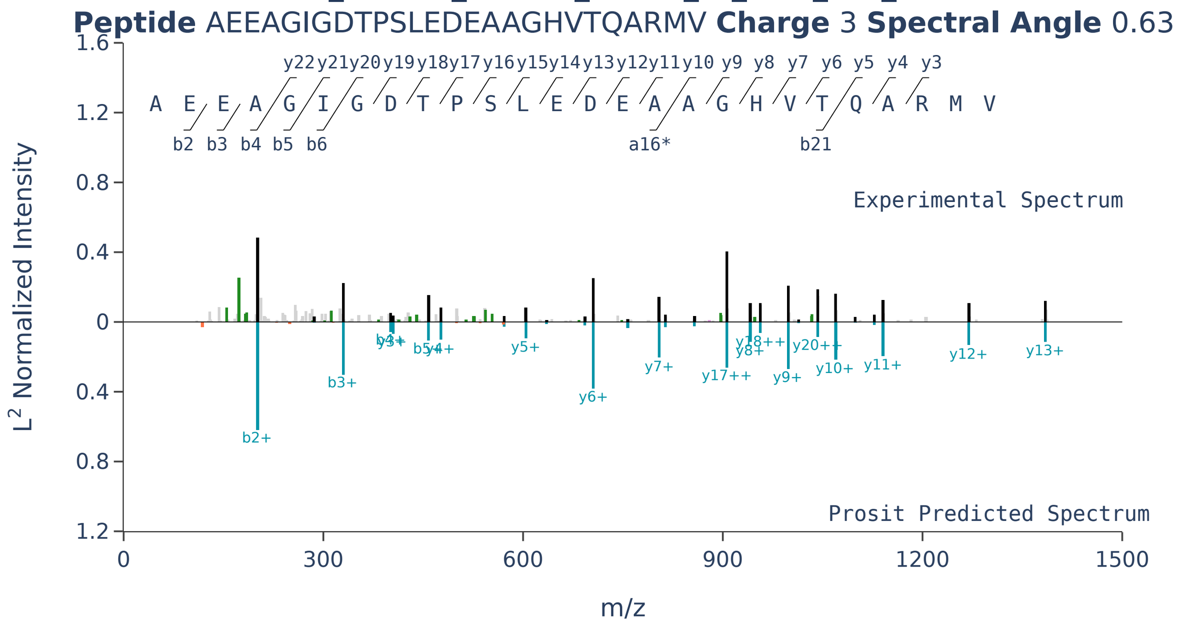

**Source** WSoh_221121_011221_HFGoe_L2_hTau_4h_R1 **Scan** 10016
**Peptide** GNIHHKPGGGQVEVKSEKLDFKD **Charge** 5 **Spectral Angle** 0.72

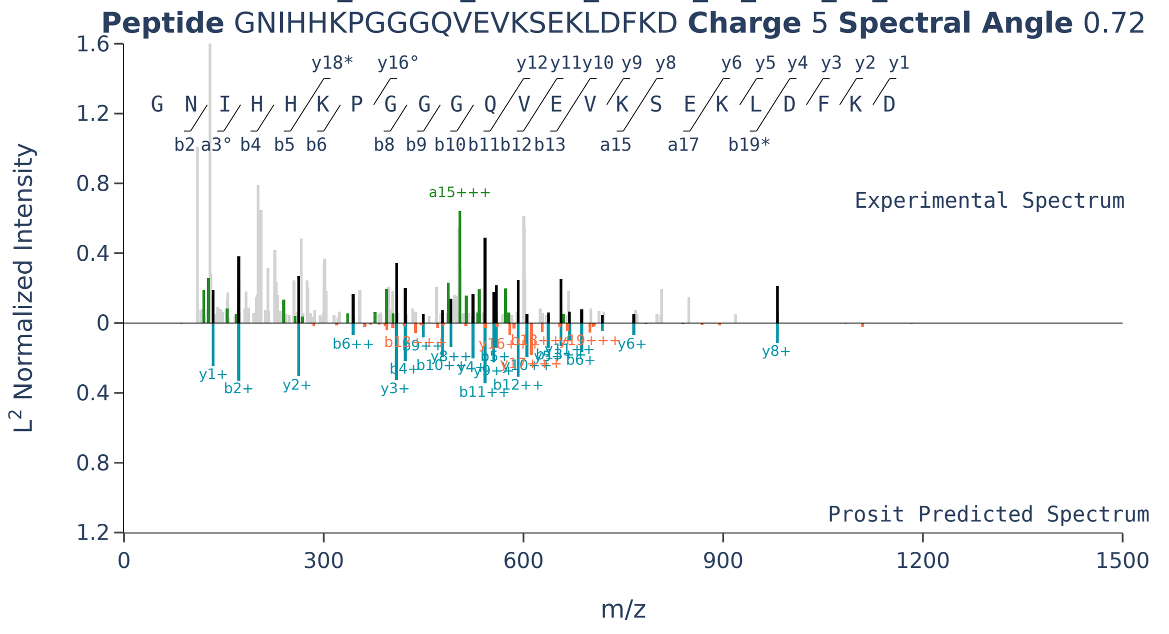


**Source** WSoh_221121_011221_HFGoe_L1_hTau_24h_R2 **Scan** 9318
**Peptide** IPAKTPPAPKTPPSSGEPPKSGDR **Charge** 4 **Spectral Angle** 0.81

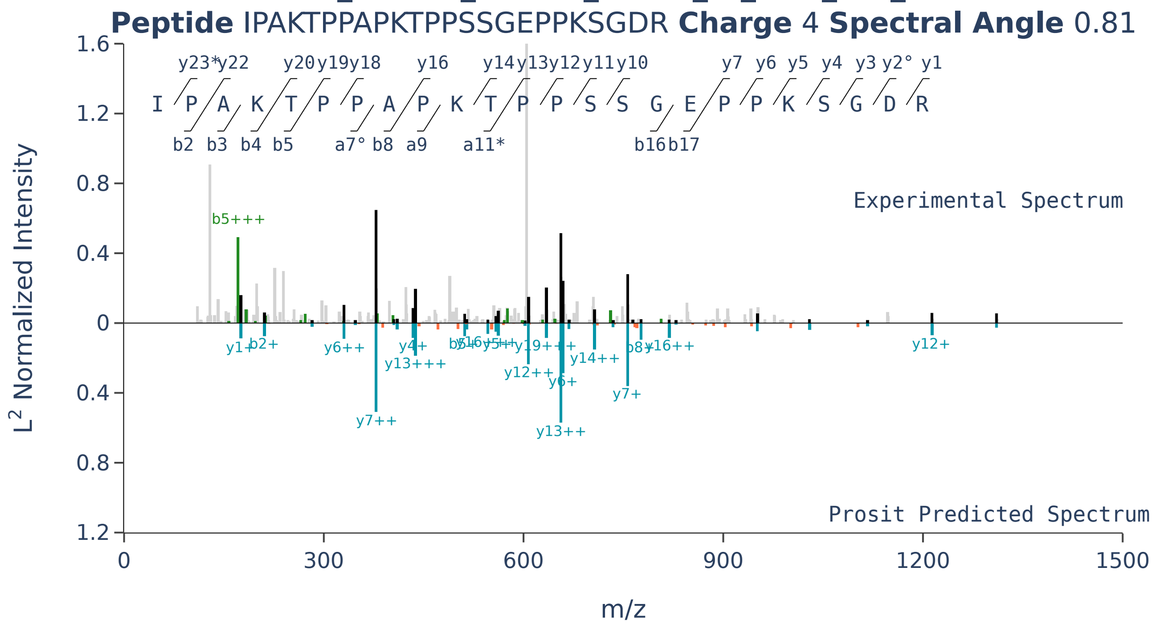


**Source** WSoh_221121_011221_HFGoe_L1_hTau_4h_R2 **Scan** 12804
**Peptide** GAPGKQAAAQPHTEIPEGTTAEEA **Charge** 3 **Spectral Angle** 0.71

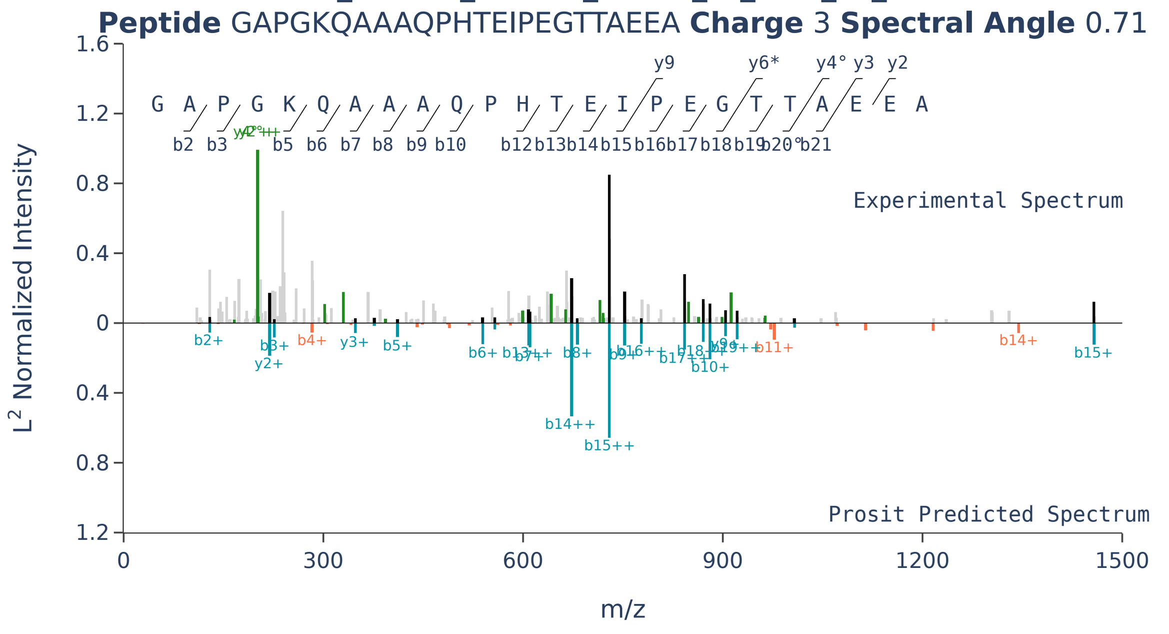


**Source** WSoh_221121_011221_HFGoe_L1_hTau_1h_R1 **Scan** 13925
**Peptide** EVKSEKLDF **Charge** 2 **Spectral Angle** 0.77

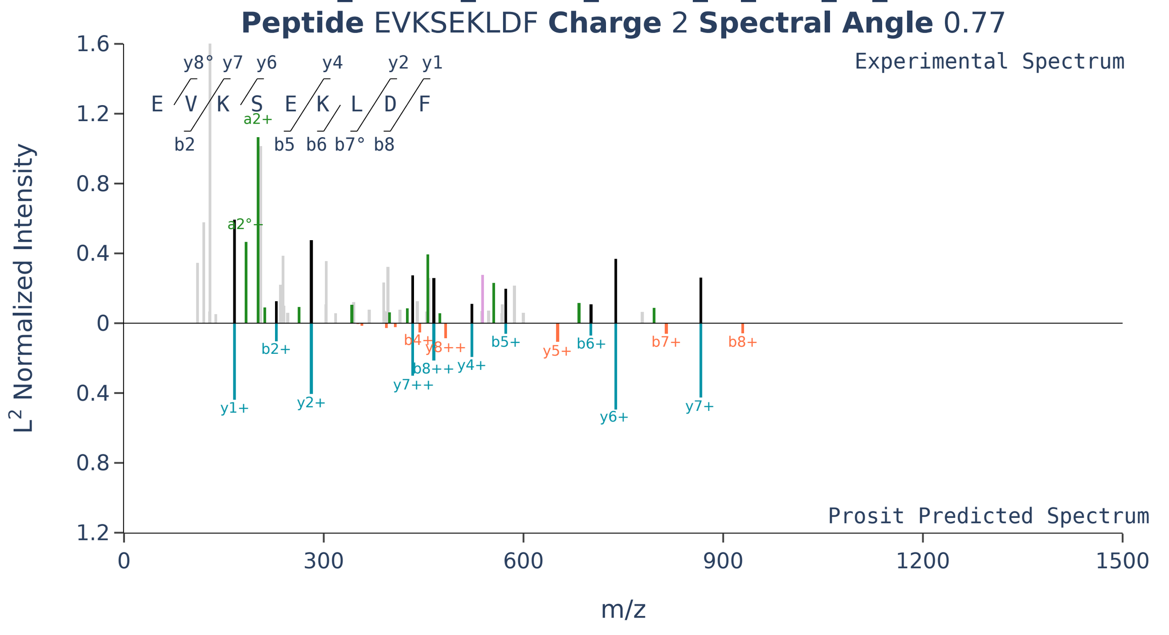


**Source** WSoh_221121_011221_HFGoe_L2_hTau_24h_R1 **Scan** 16087
**Peptide** GQVEVKSEKLDFKDRVQSKI **Charge** 4 **Spectral Angle** 0.79

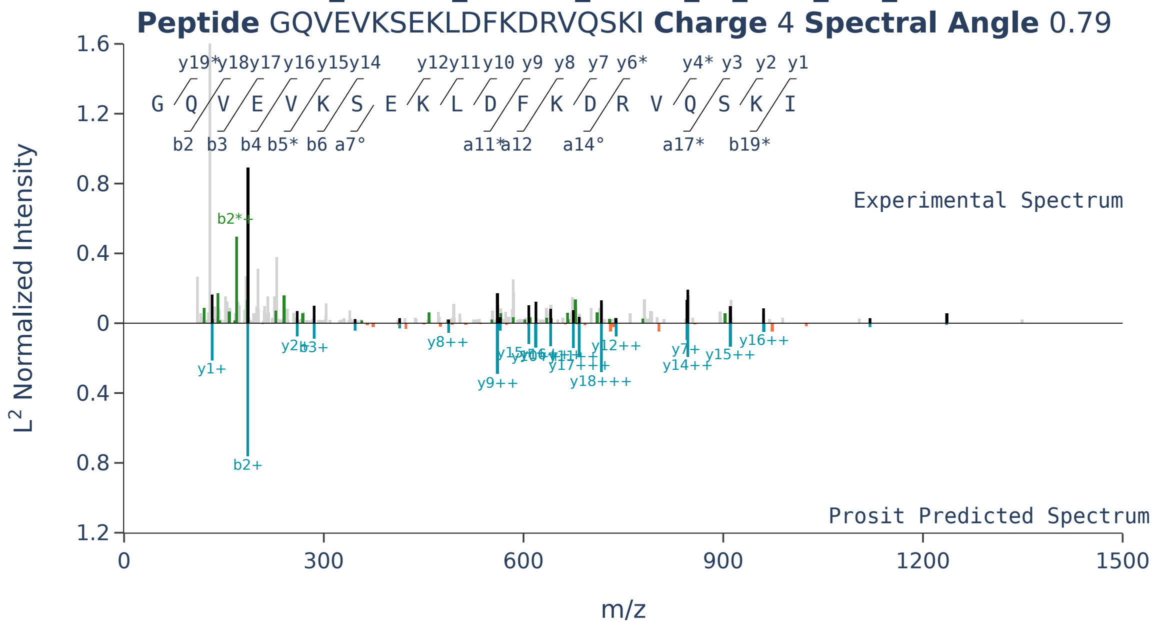


**Source** WSoh_221121_011221_HFGoe_L1_hTau_24h_R1 **Scan** 18732
**Peptide** PSSAKSRLQTAPVPMPDLKNVKS **Charge** 5 **Spectral Angle** 0.71

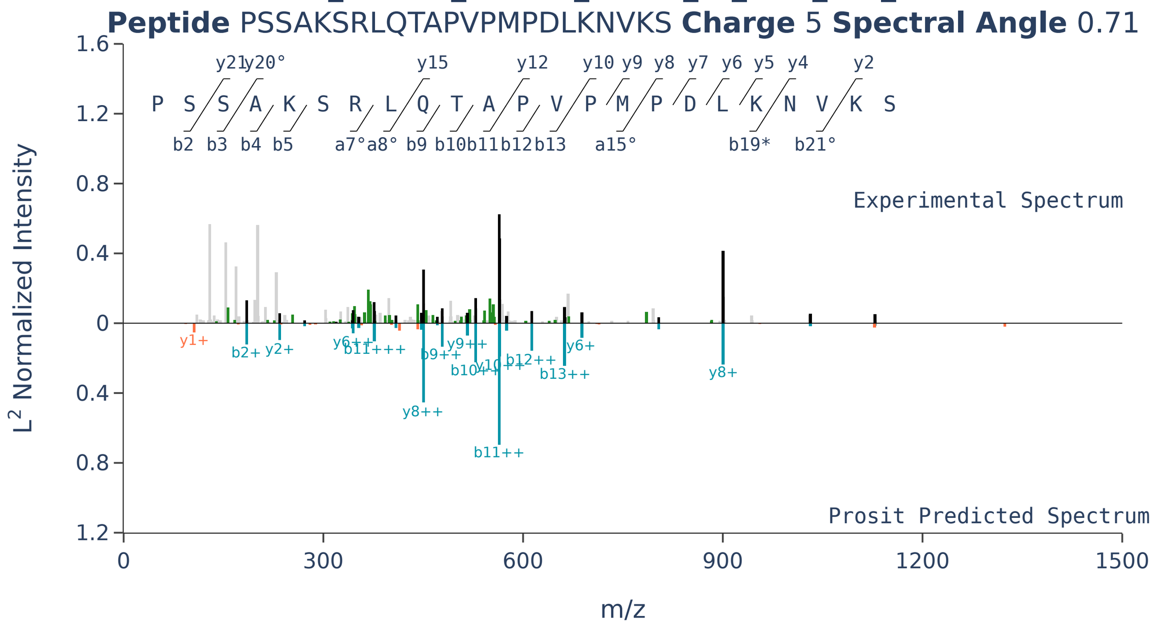


**Source** WSoh_221121_011221_HFGoe_L1_hTau_24h_R1 **Scan** 17865
**Peptide** TDHGAEIVYKSPVVSGD **Charge** 3 **Spectral Angle** 0.79

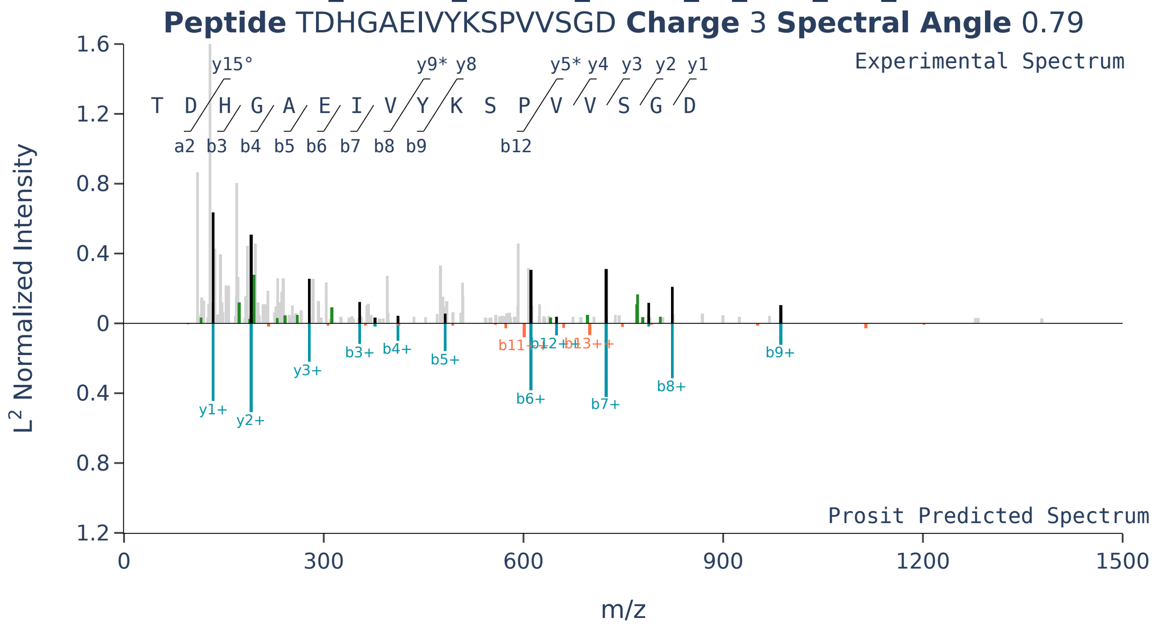


**Source** WSoh_221121_011221_HFGoe_L1_hTau_24h_R2 **Scan** 22559
**Peptide** EPGSETSDAKSTPTAEDVTAPLVD **Charge** 3 **Spectral Angle** 0.84

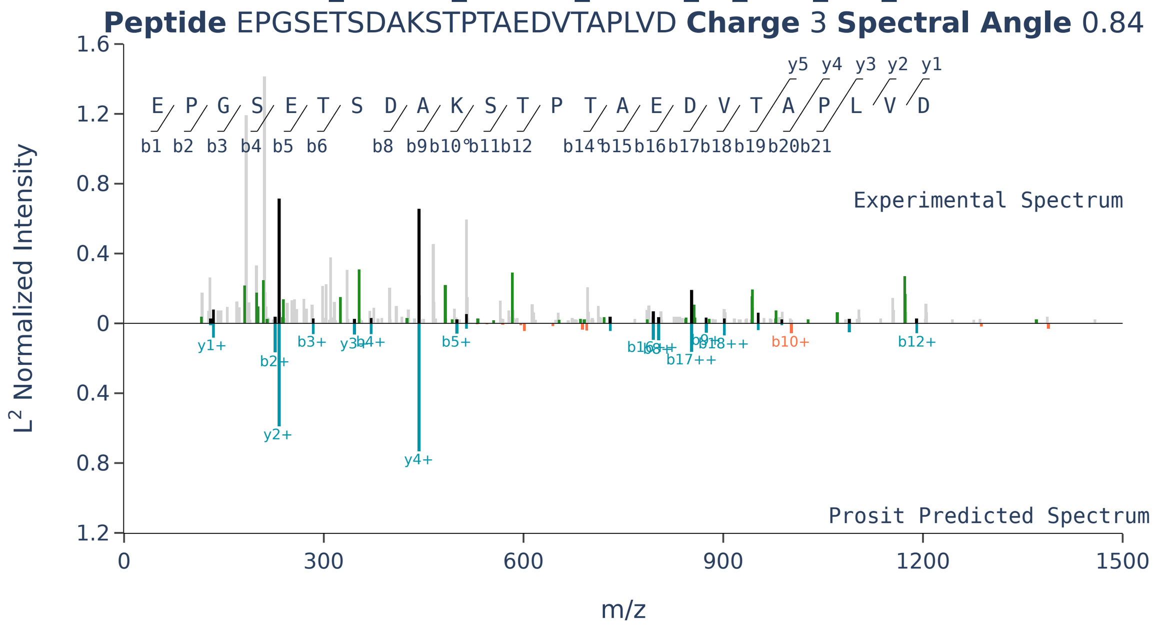


**Source** WSoh_221121_011221_HFGoe_L1_hTau_4h_R1 **Scan** 12138
**Peptide** NLKHQPGGGKVQII **Charge** 3 **Spectral Angle** 0.71

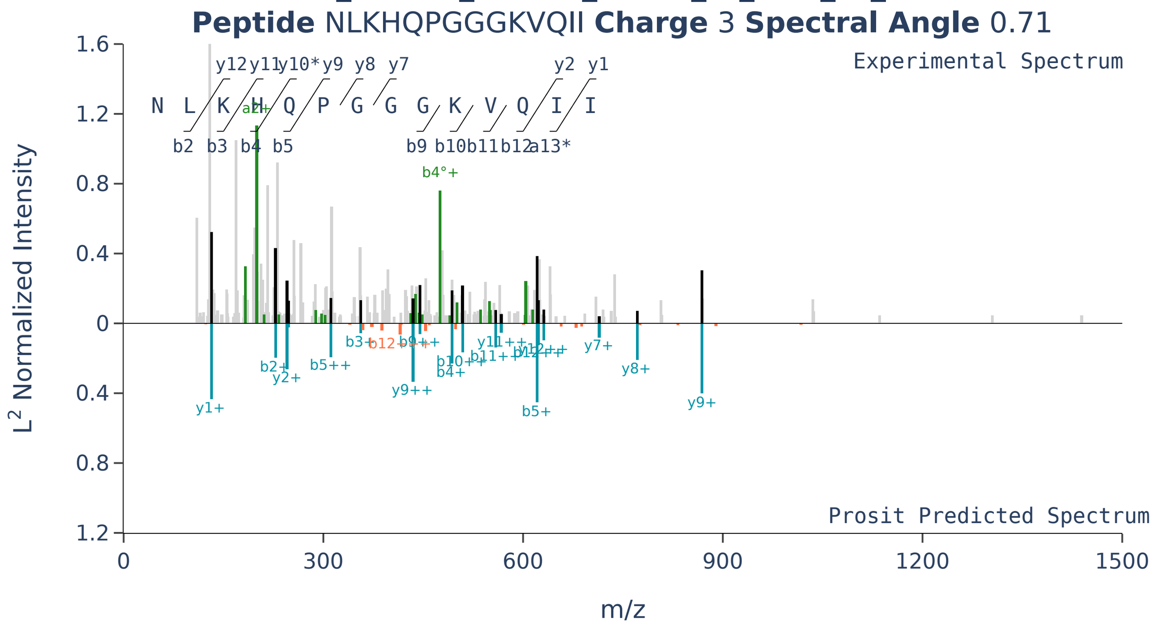


**Source** WSoh_221121_011221_HFGoe_L1_hTau_24h_R1 **Scan** 21745
**Peptide** RLQTAPVPMPDLKNVKSKIGST **Charge** 3 **Spectral Angle** 0.82

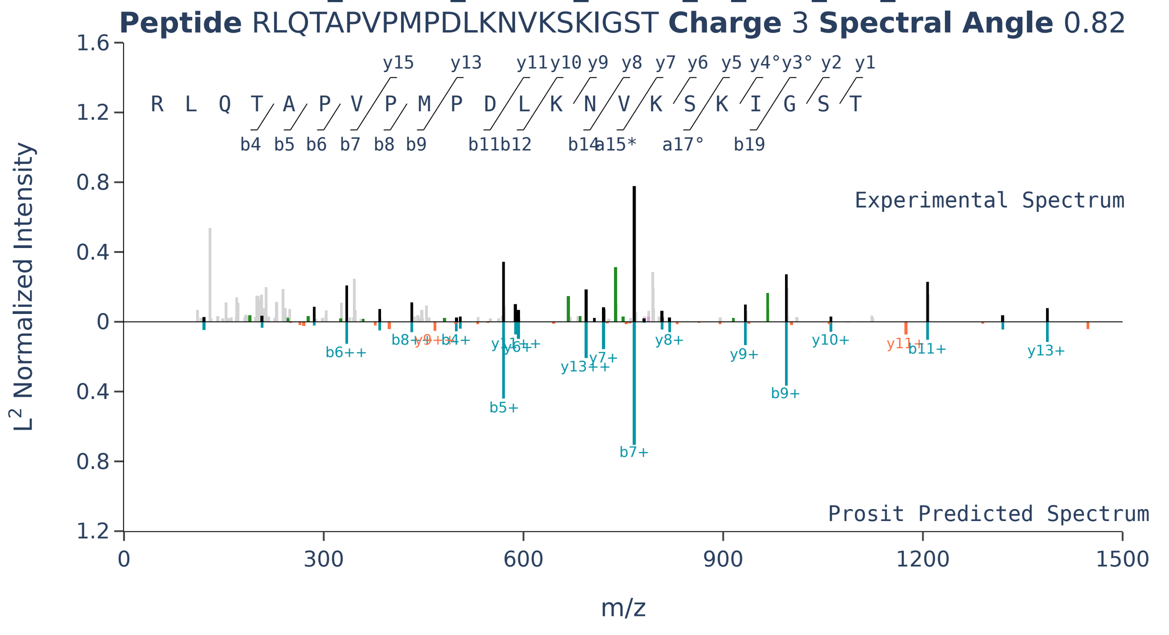


**Source** WSoh_221121_011221_HFGoe_L2_hTau_24h_R2 **Scan** 9379
**Peptide** LPAKTPPAPKTPPSSGEPPKSGDR **Charge** 4 **Spectral Angle** 0.81

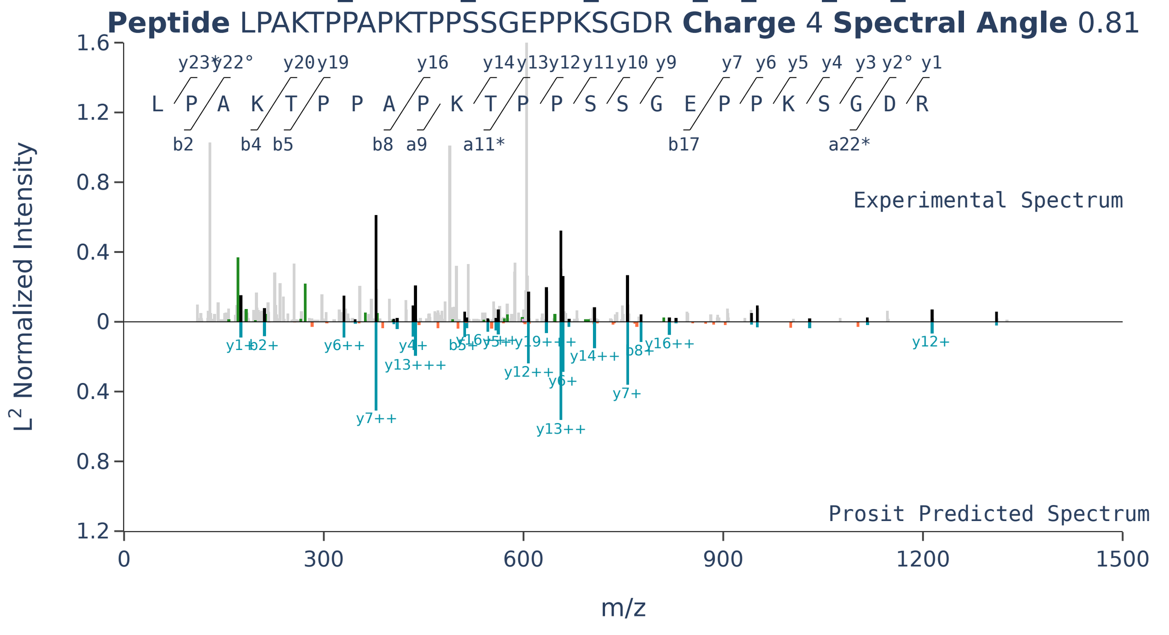


**Source** WSoh_221121_011221_HFGoe_L1_hTau_4h_R1 **Scan** 6993
**Peptide** KIATPRGAAPPGQKG **Charge** 3 **Spectral Angle** 0.81

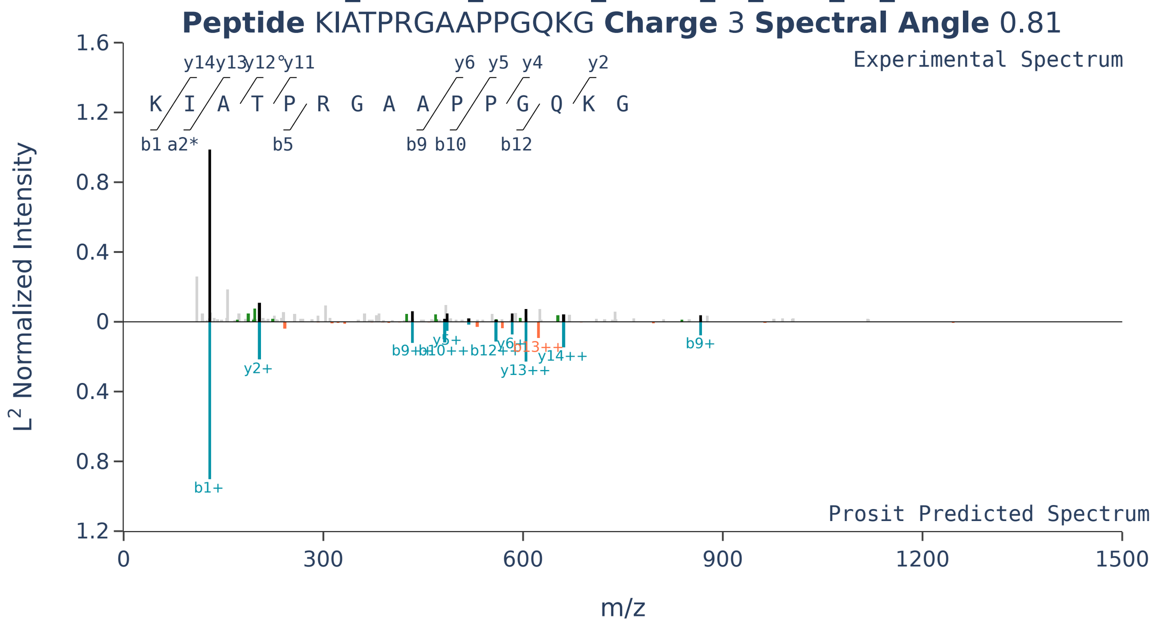


**Source** WSoh_221121_011221_HFGoe_L2_hTau_24h_R1 **Scan** 11258
**Peptide** PGSRSRTPSLPTPPTREPKKV **Charge** 5 **Spectral Angle** 0.87

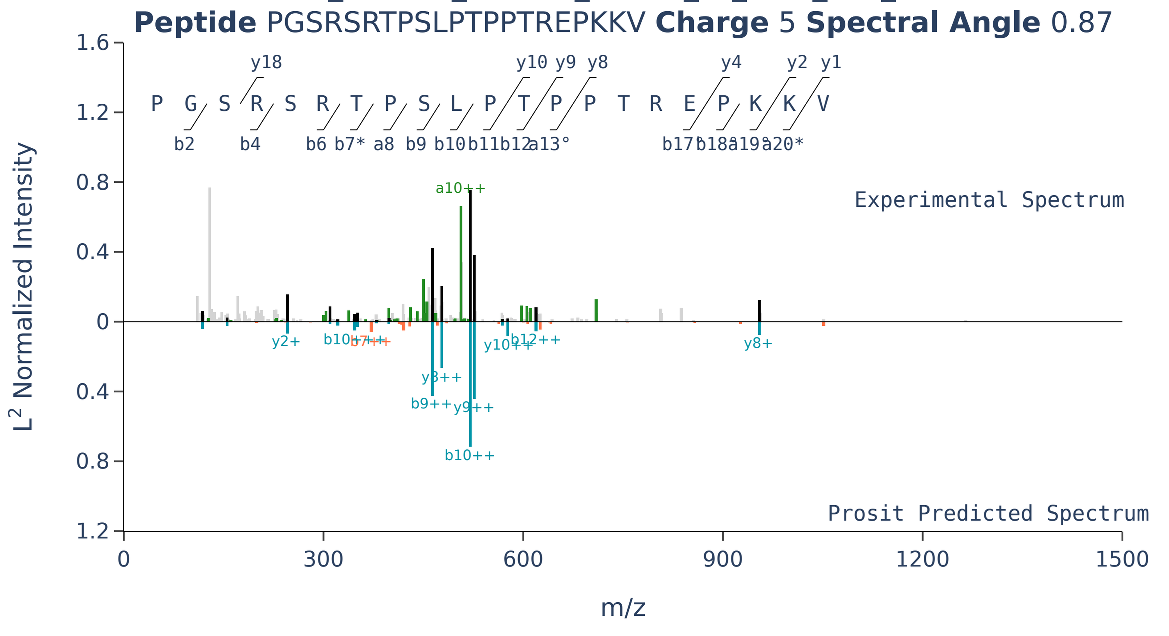


**Source** WSoh_221121_011221_HFGoe_L1_hTau_24h_R2 **Scan** 21010
**Peptide** RLQTAPVPMPDLKNVK **Charge** 3 **Spectral Angle** 0.86

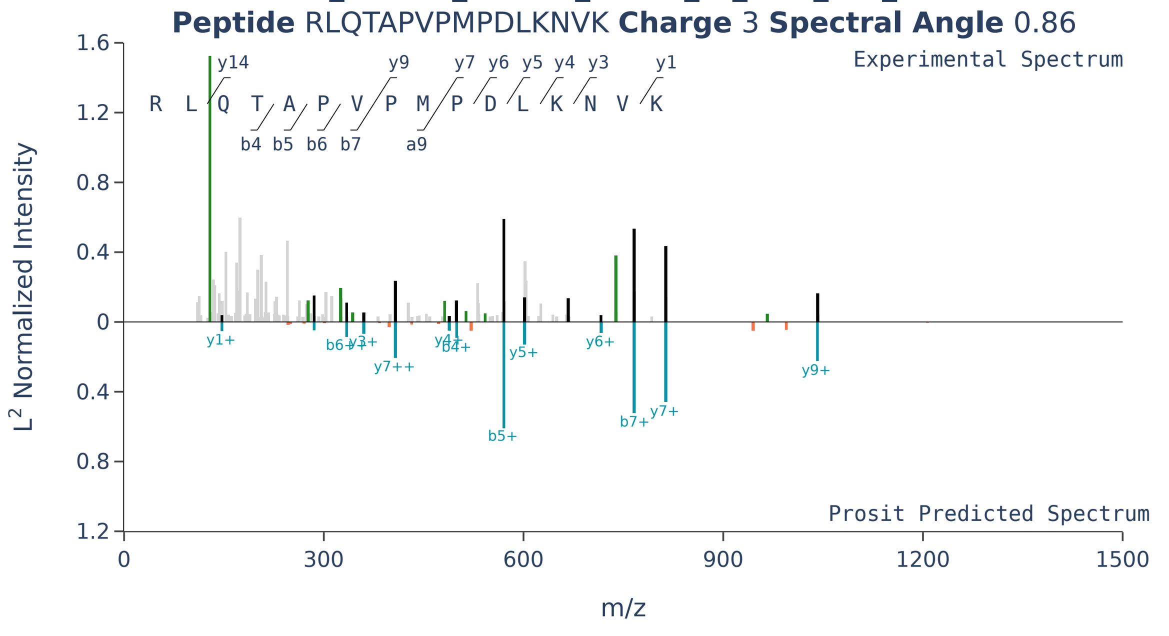


**Source** WSoh_221121_011221_HFGoe_L1_hTau_24h_R1 **Scan** 5615
**Peptide** KDGTGSDDKKAKGADGKTKIATPRGA **Charge** 4 **Spectral Angle** 0.85

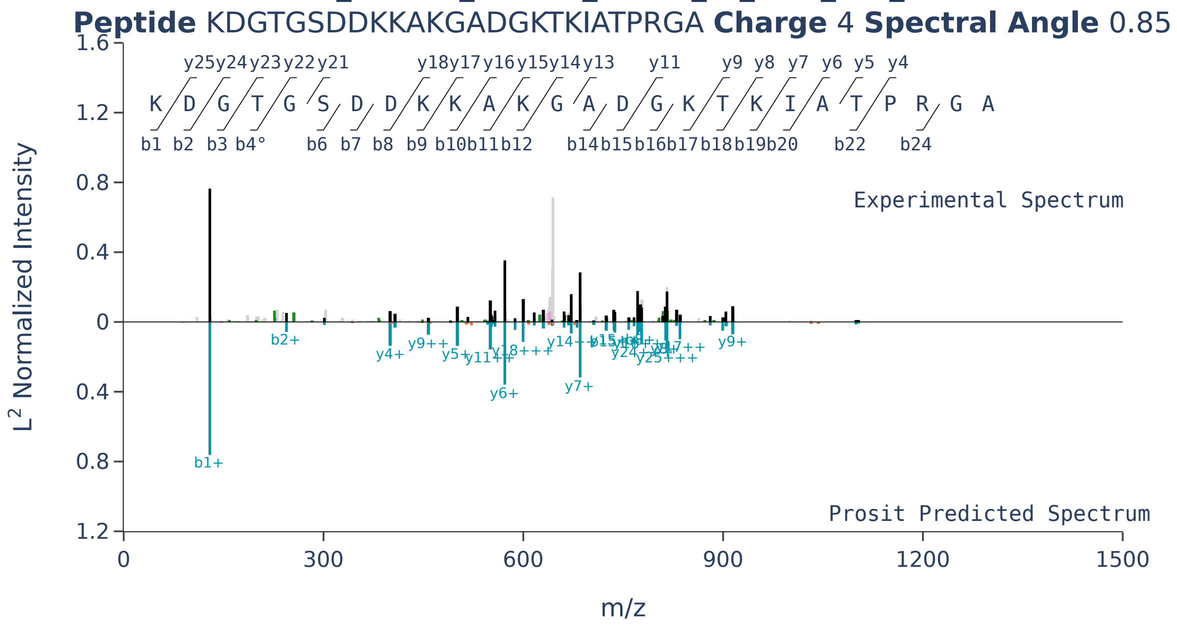

**Source** WSoh_221121_011221_HFGoe_L2_hTau_24h_R2 **Scan** 7574
**Peptide** KIATPRGAAPPGQKGQA **Charge** 3 **Spectral Angle** 0.76

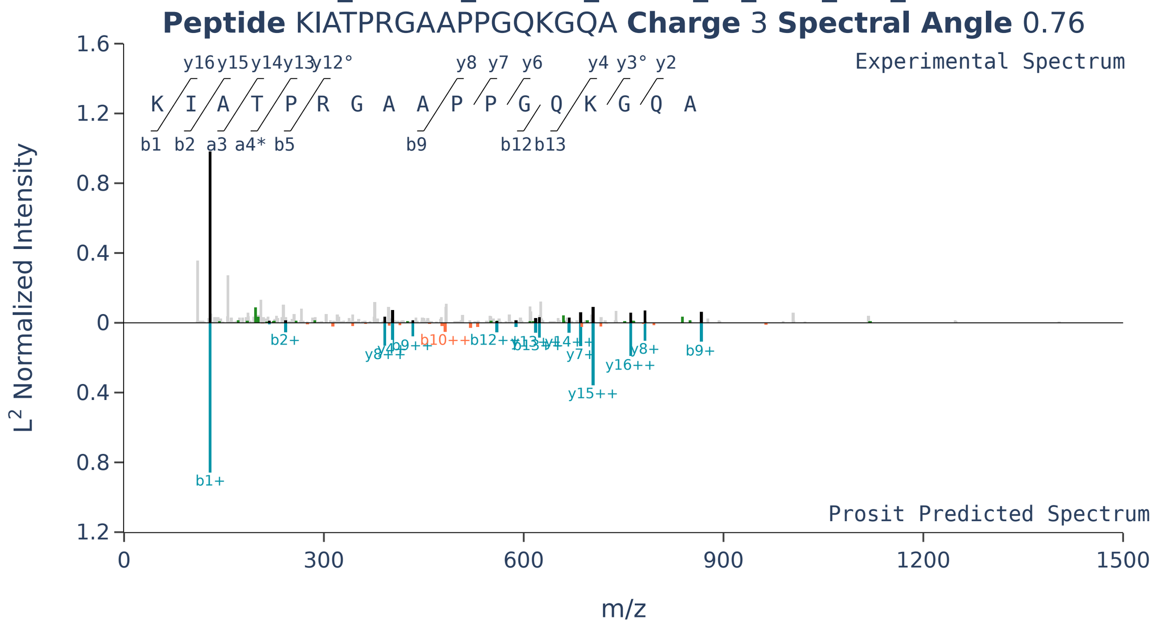


**Source** WSoh_221121_011221_HFGoe_L1_hTau_2h_R1 **Scan** 12963
**Peptide** DEGAPGKQAAAQPHTEIPEGTTA **Charge** 3 **Spectral Angle** 0.85

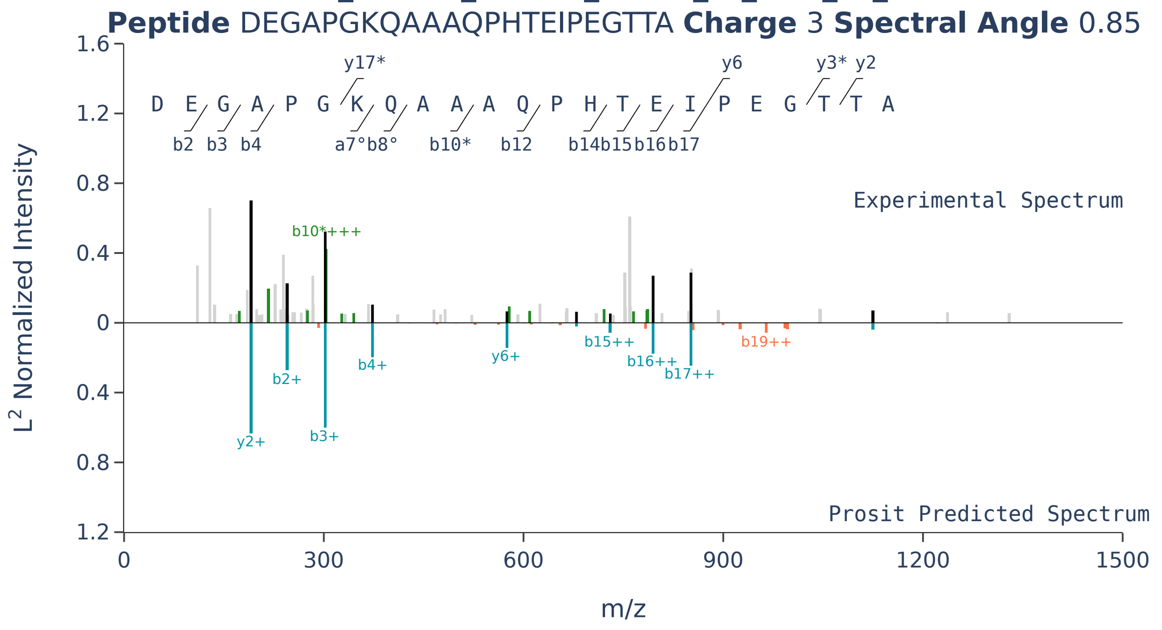


**Source** WSoh_221121_011221_HFGoe_L1_hTau_4h_R2 **Scan** 23603
**Peptide** MVDSPQLATLAD **Charge** 2 **Spectral Angle** 0.74

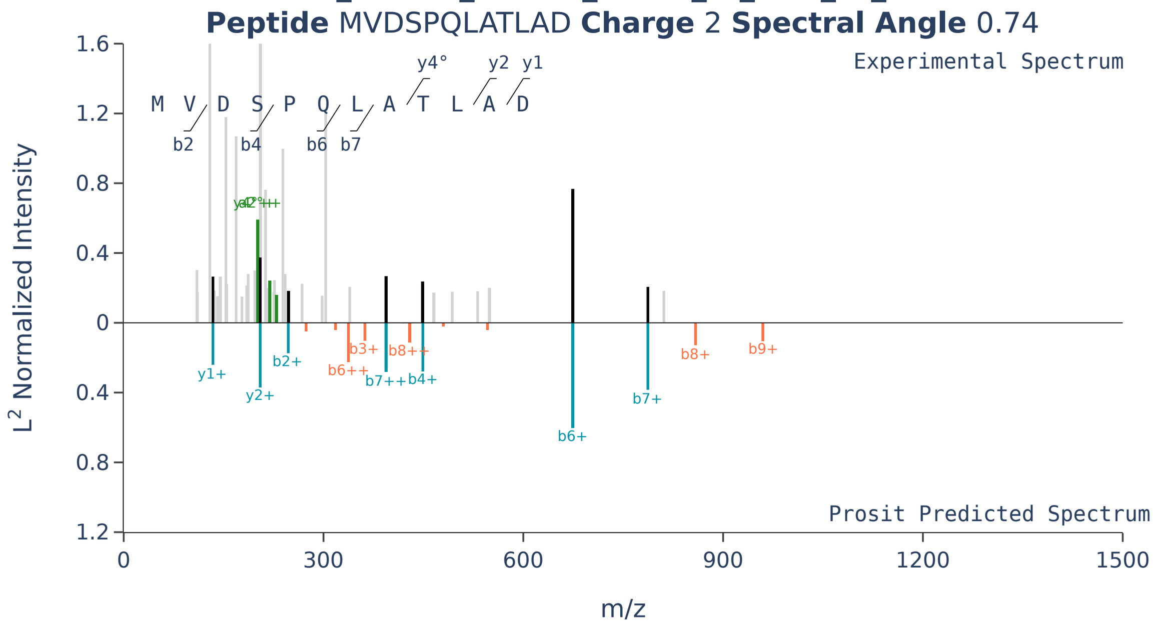


**Source** WSoh_221121_011221_HFGoe_L2_hTau_24h_R2 **Scan** 16608
**Peptide** VYKSPVVSGDTSPRHLSNVSSTGSID **Charge** 4 **Spectral Angle** 0.74

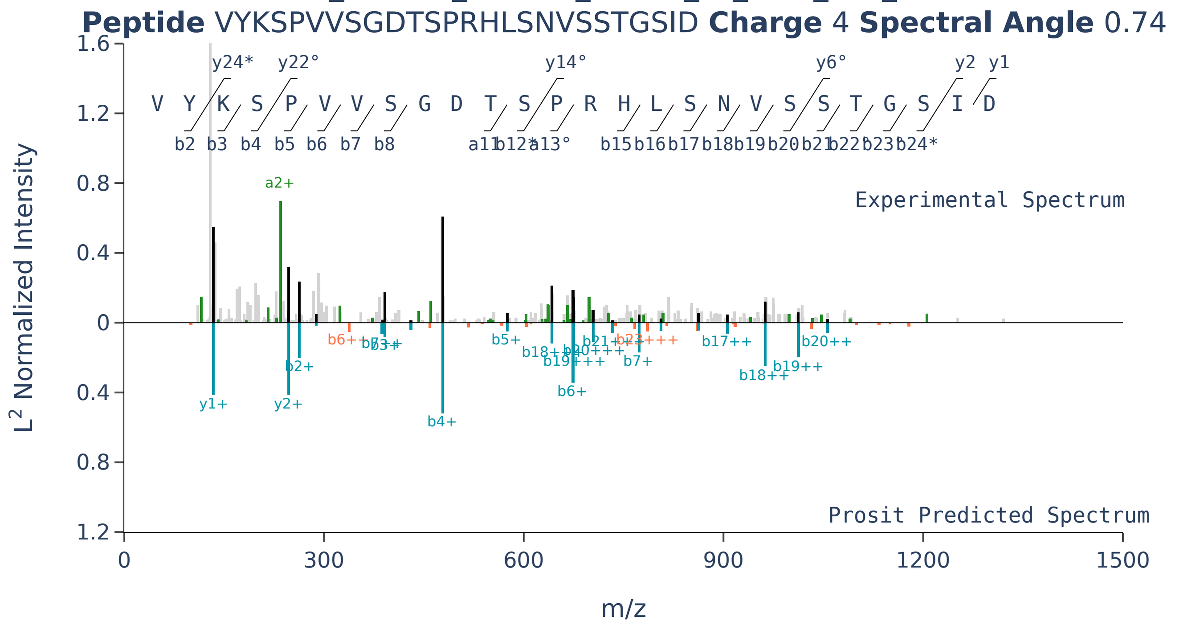


**Source** WSoh_221121_011221_HFGoe_L2_hTau_2h_R2 **Scan** 11945
**Peptide** RENAKAKTDHGAEIVYKSPVVSGDTS **Charge** 4 **Spectral Angle** 0.73

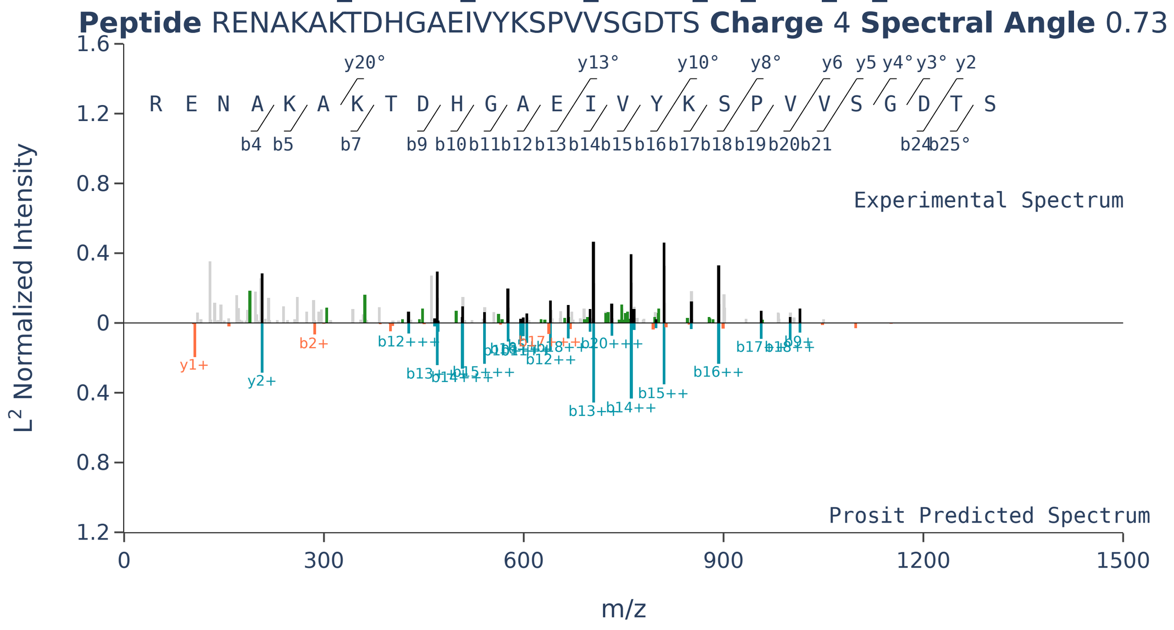


**Source** WSoh_221121_011221_HFGoe_L1_hTau_24h_R2 **Scan** 6834
**Peptide** IGSTENLKH **Charge** 1 **Spectral Angle** 0.83

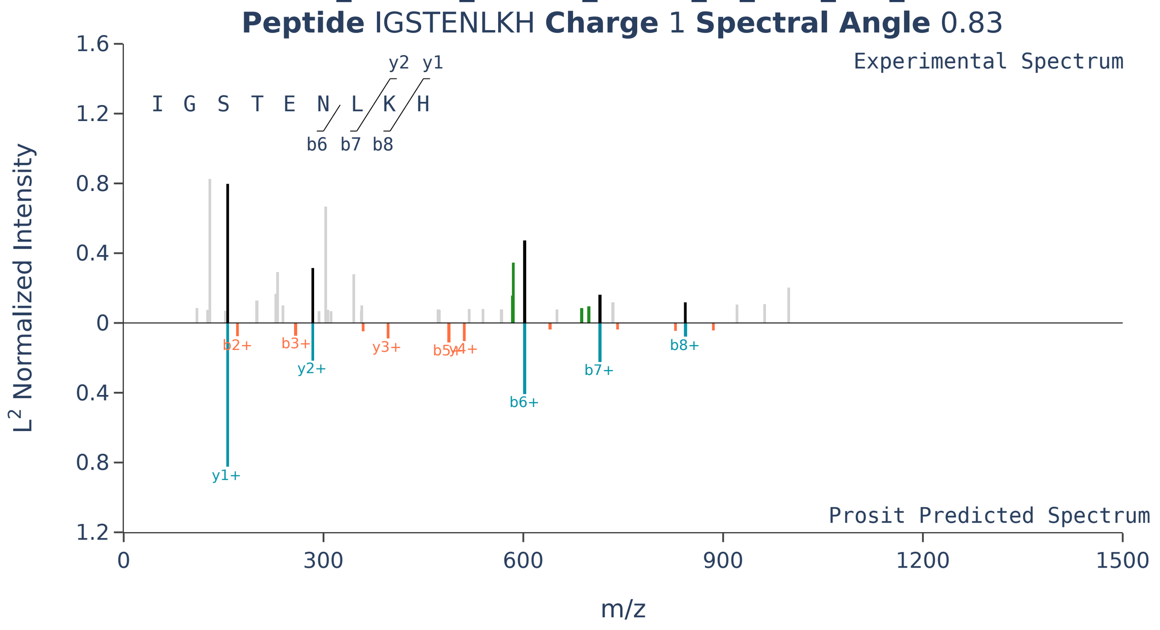


**Source** WSoh_221121_011221_HFGoe_L2_hTau_2h_R2 **Scan** 7397
**Peptide** EPPKSGDRSGYSSPGSPGTPGSR **Charge** 3 **Spectral Angle** 0.76

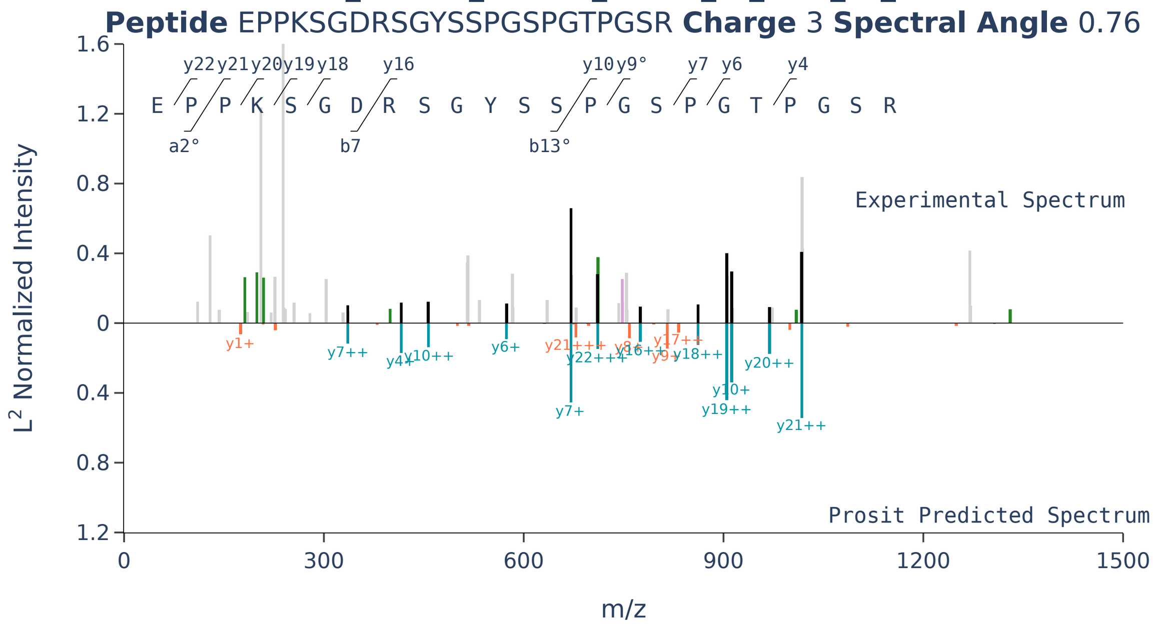


**Source** WSoh_221121_011221_HFGoe_L2_hTau_4h_R2 **Scan** 6924
**Peptide** KGADGKTKIATPRGAAPPGQKGQANAT **Charge** 4 **Spectral Angle** 0.76

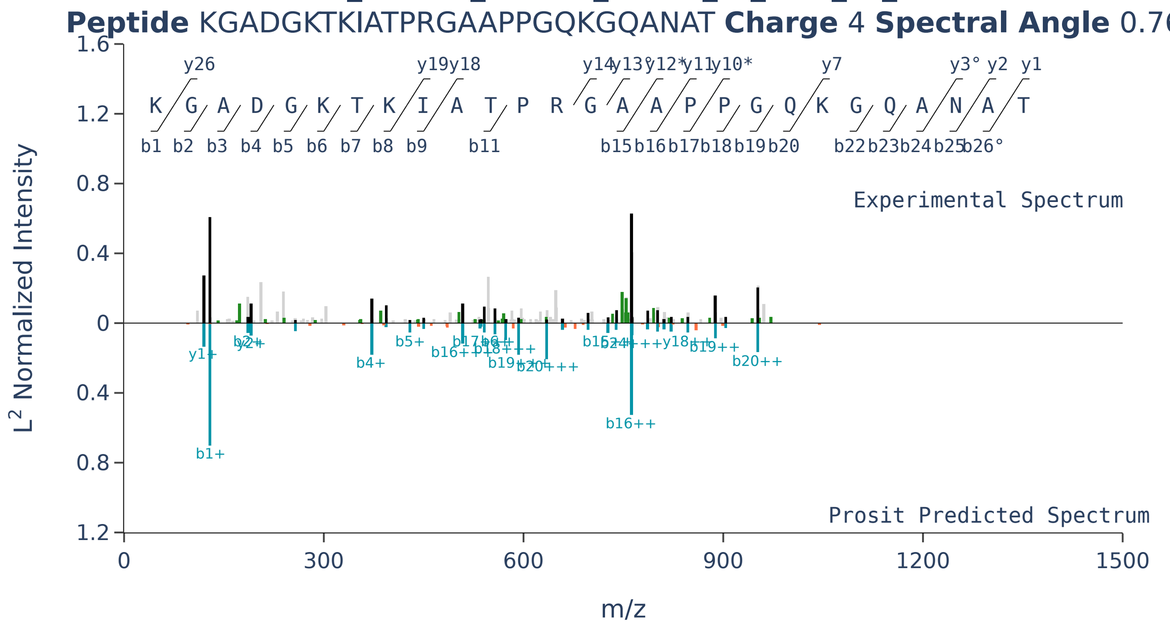


**Source** WSoh_221121_011221_HFGoe_L2_hTau_2h_R2 **Scan** 15414
**Peptide** KIGSTENLKHQPGGGKVQIINKKLDL **Charge** 6 **Spectral Angle** 0.92

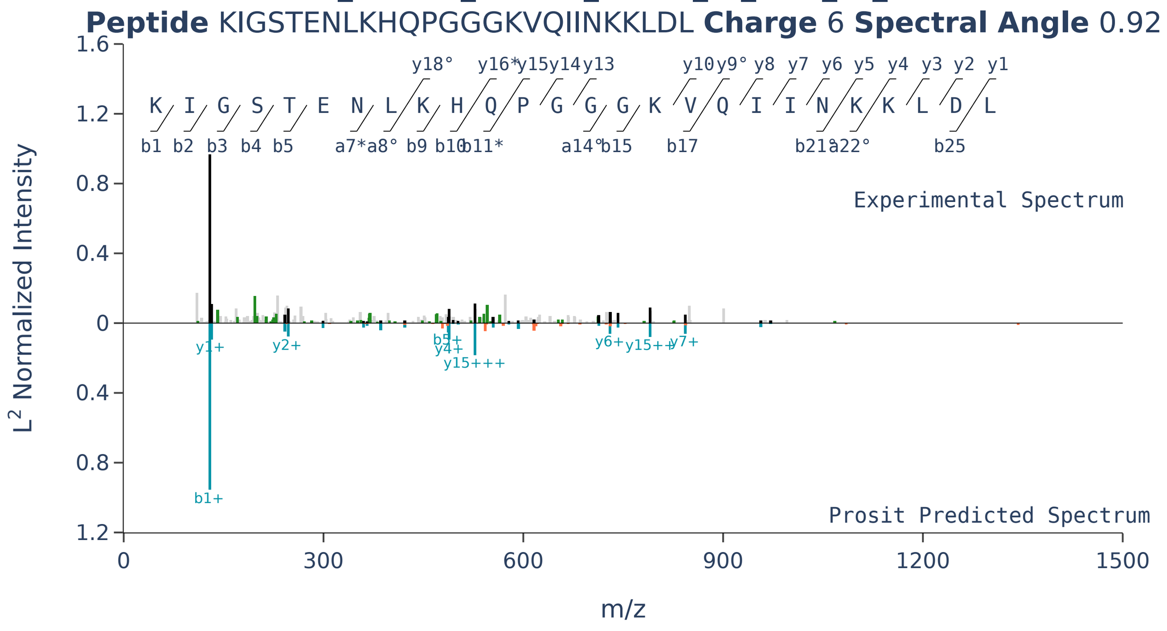


**Source** WSoh_221121_011221_HFGoe_L1_hTau_24h_R1 **Scan** 14330
**Peptide** KPGGGQVEVKSEKLDFKDRVQSKIG **Charge** 4 **Spectral Angle** 0.89

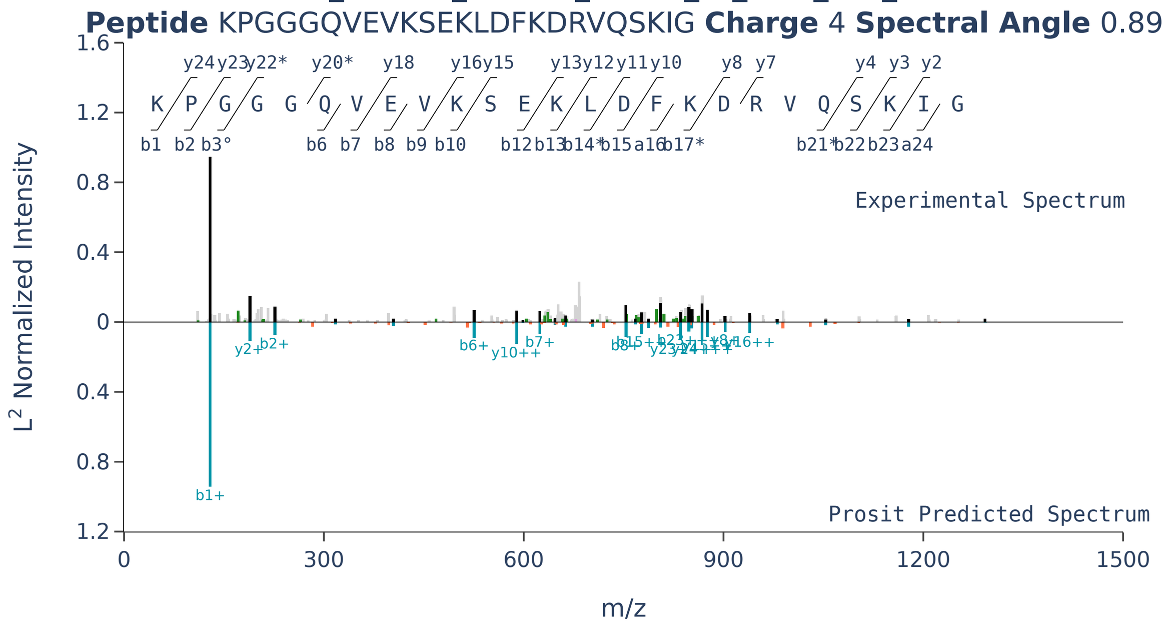


**Source** WSoh_221121_011221_HFGoe_L1_hTau_4h_R1 **Scan** 10497
**Peptide** PHTEIPEGTT **Charge** 2 **Spectral Angle** 0.75

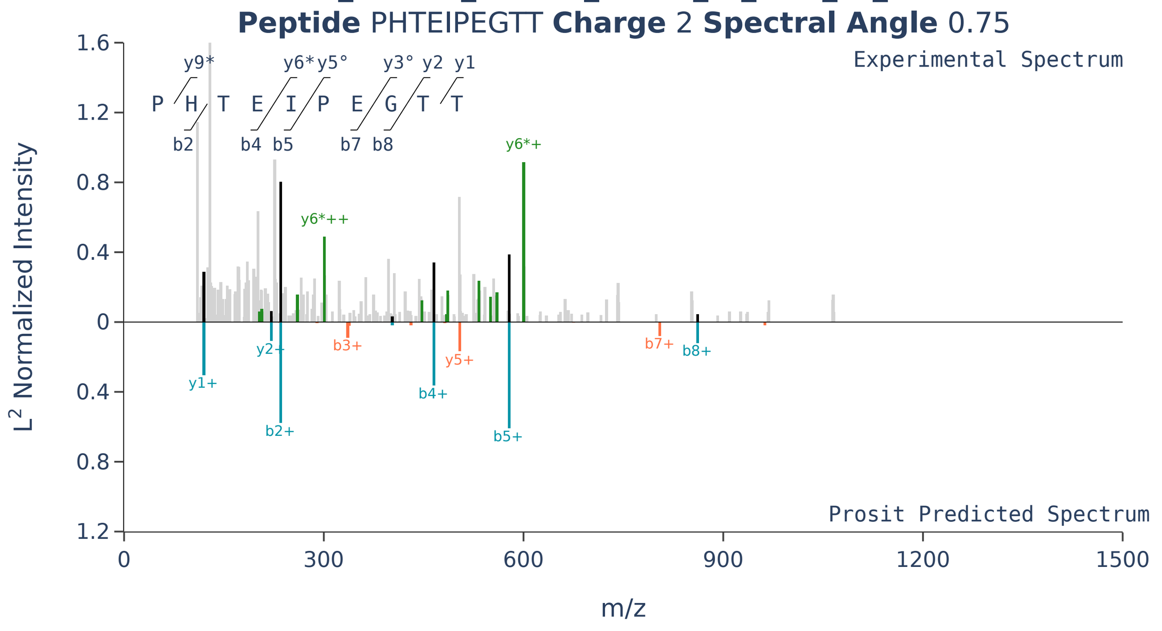


**Source** WSoh_221121_011221_HFGoe_L1_hTau_24h_R1 **Scan** 22498
**Peptide** SSTGSIDMVDSPQL **Charge** 2 **Spectral Angle** 0.9

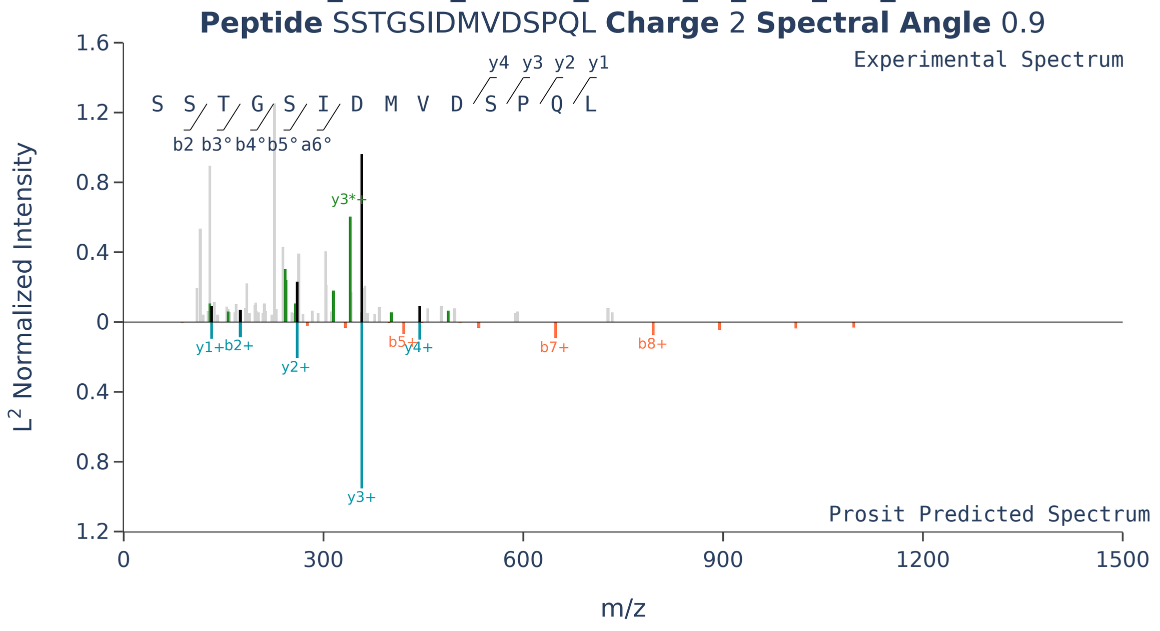


**Source** WSoh_221121_011221_HFGoe_L1_hTau_2h_R2 **Scan** 19836
**Peptide** SDAKSTPTAEDVTAPLVDEGAPGKQA **Charge** 3 **Spectral Angle** 0.81

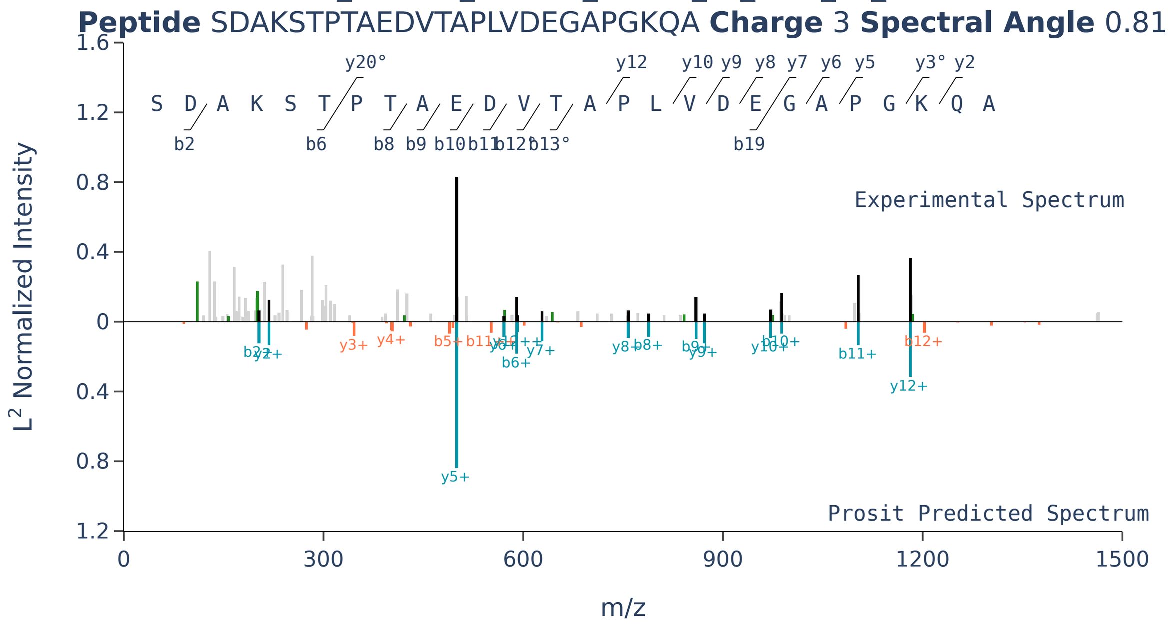


**Source** WSoh_221121_011221_HFGoe_L1_hTau_2h_R1 **Scan** 13401
**Peptide** KLDLSNVQSKCGSKDNI **Charge** 3 **Spectral Angle** 0.88

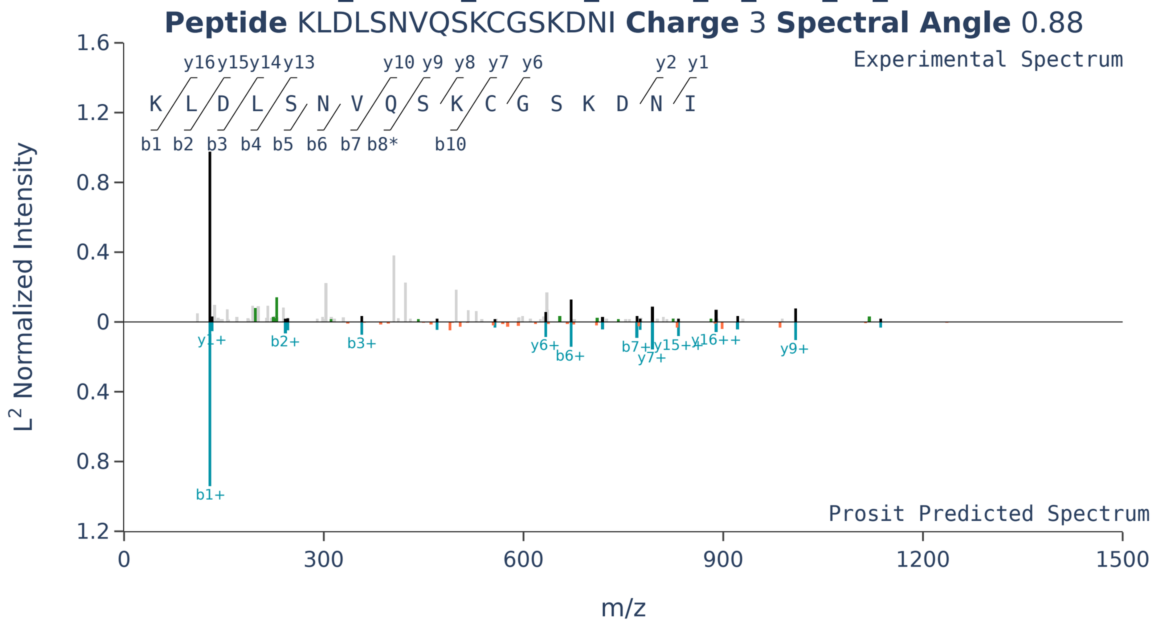


**Source** WSoh_221121_011221_HFGoe_L2_hTau_24h_R1 **Scan** 8238
**Peptide** LKHQPGGGKVQL **Charge** 2 **Spectral Angle** 0.84

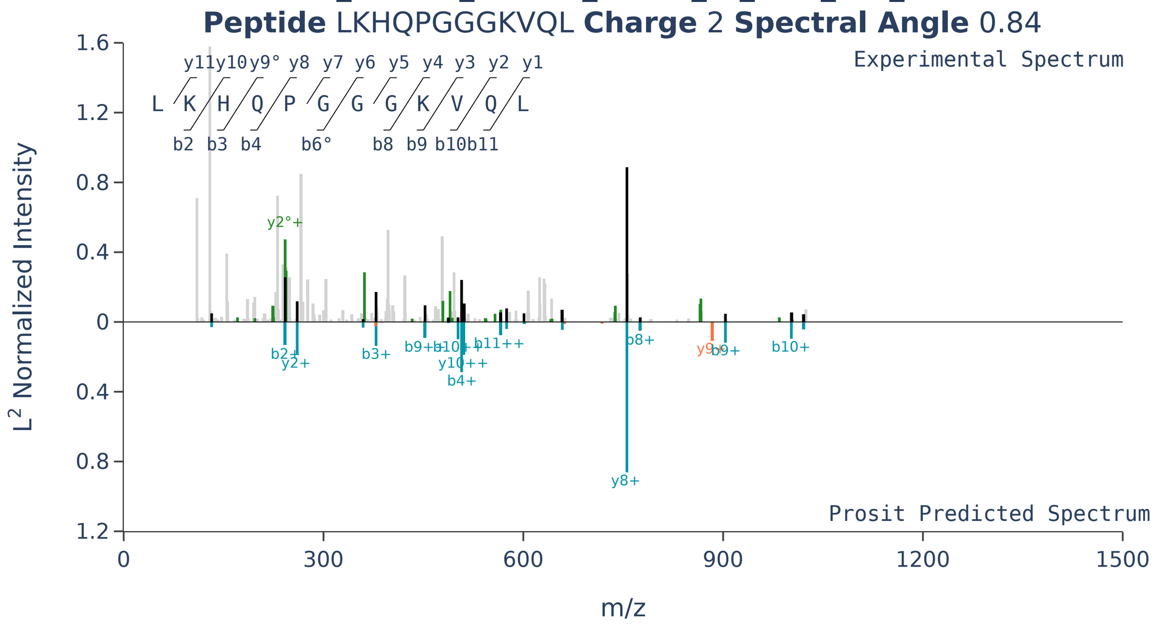

**Source** WSoh_221121_011221_HFGoe_L1_hTau_2h_R2 **Scan** 9847
**Peptide** QPGGGKVQIINK **Charge** 3 **Spectral Angle** 0.72

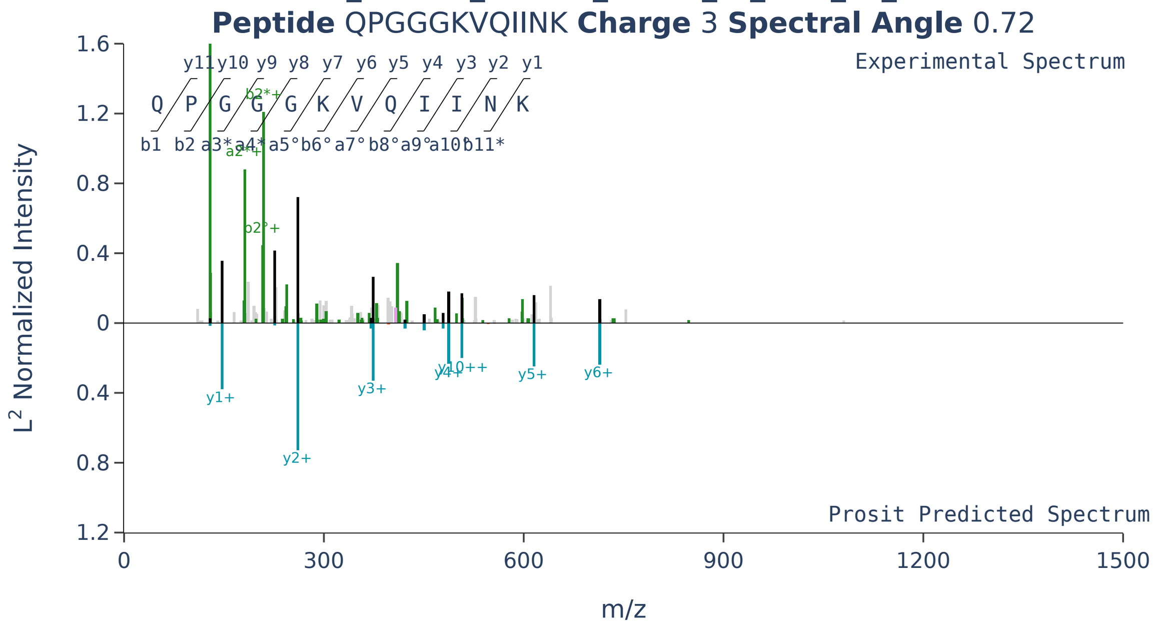


**Source** WSoh_221121_011221_HFGoe_L1_hTau_4h_R2 **Scan** 20259
**Peptide** KSEKLDFKDRVQSKIGSLDNI **Charge** 3 **Spectral Angle** 0.8

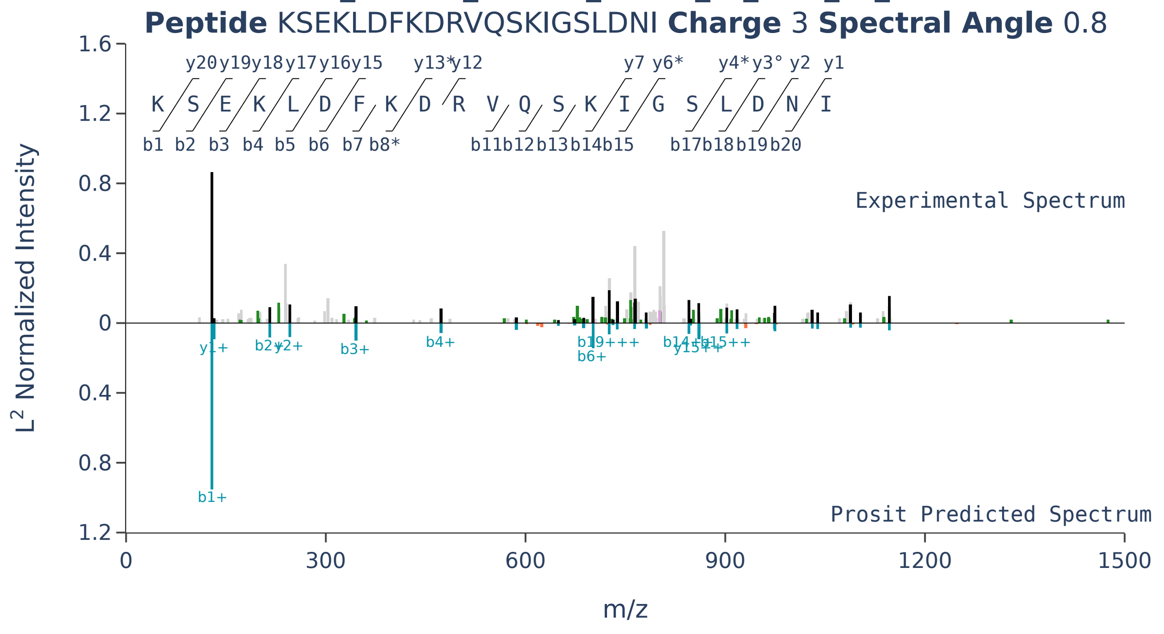


**Source** WSoh_221121_011221_HFGoe_L1_hTau_24h_R2 **Scan** 5783
**Peptide** NLHHKPGGGQVEVKS **Charge** 4 **Spectral Angle** 0.76

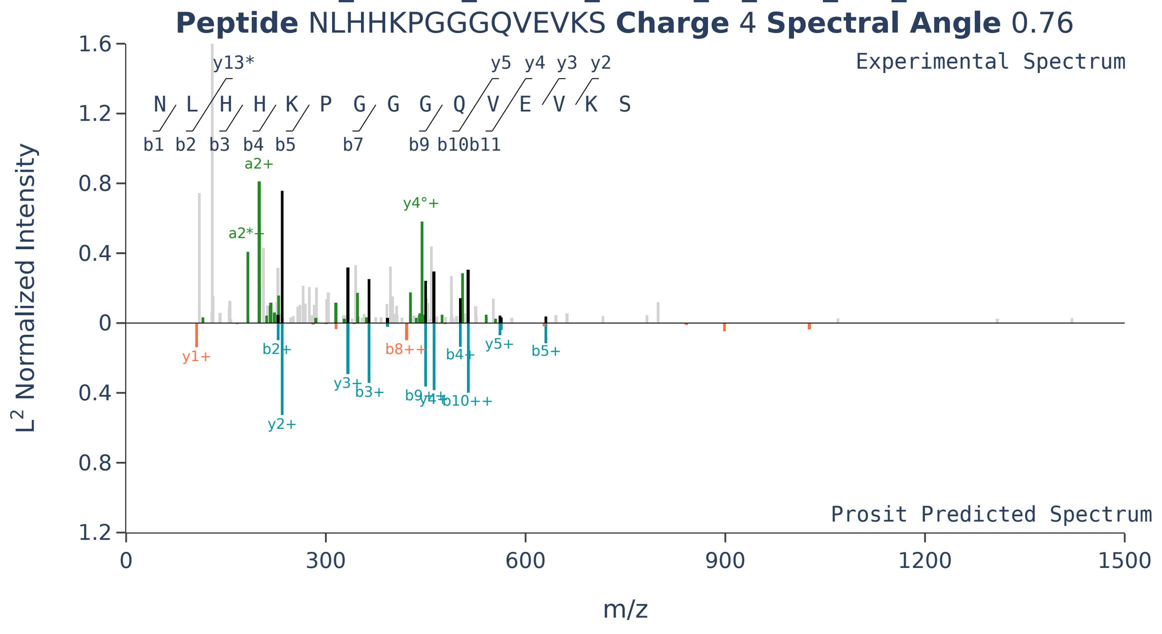


**Source** WSoh_221121_011221_HFGoe_L1_hTau_2h_R2 **Scan** 11375
**Peptide** DLSNVQSKCGSKDNIKHVPGGGSV **Charge** 3 **Spectral Angle** 0.91

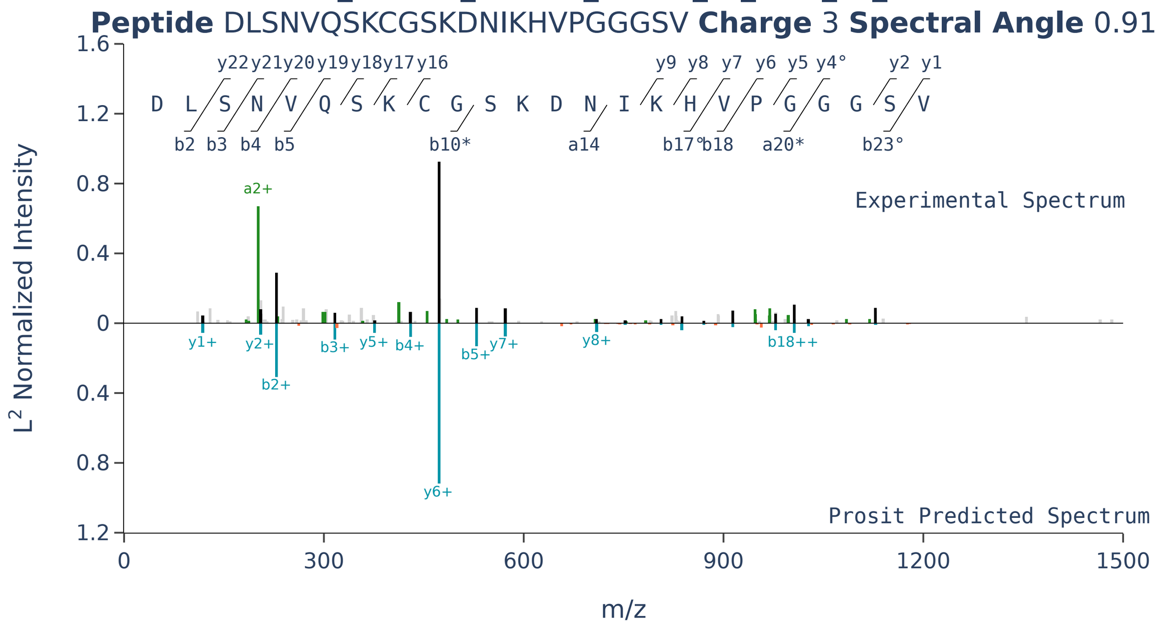


**Source** WSoh_221121_011221_HFGoe_L2_hTau_24h_R2 **Scan** 12170
**Peptide** IINKKLDLSNVQSKCGSKDNIKHV **Charge** 6 **Spectral Angle** 0.86

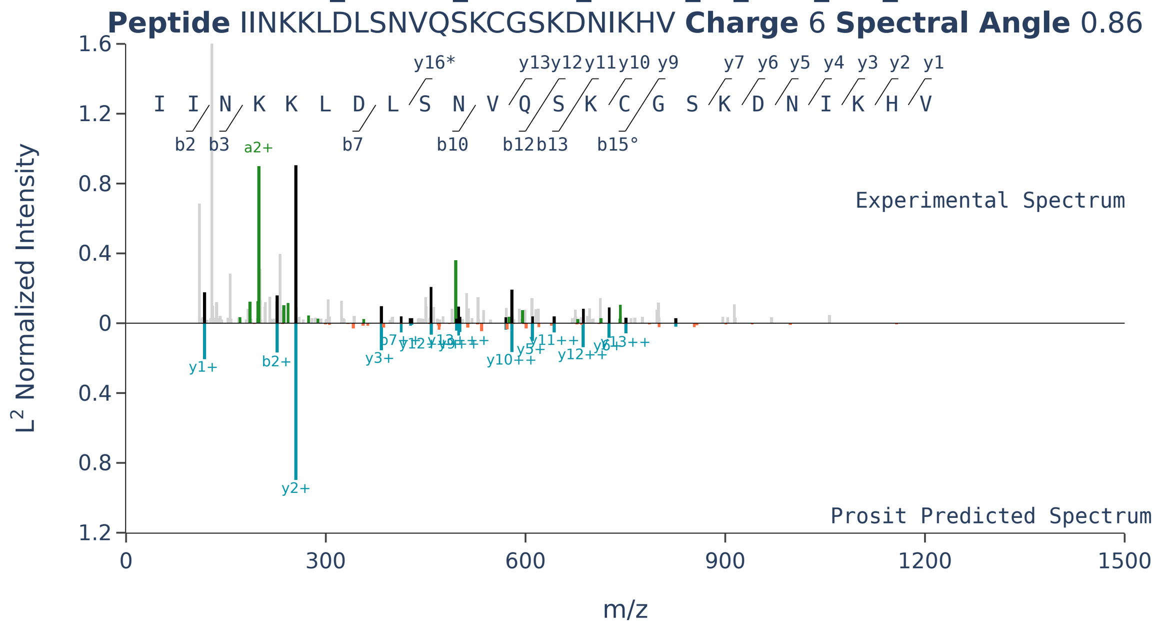


**Source** WSoh_221121_011221_HFGoe_L1_hTau_24h_R1 **Scan** 20535
**Peptide** PVVSGDTSPRHLSNVSSTGSIDMVD **Charge** 3 **Spectral Angle** 0.75

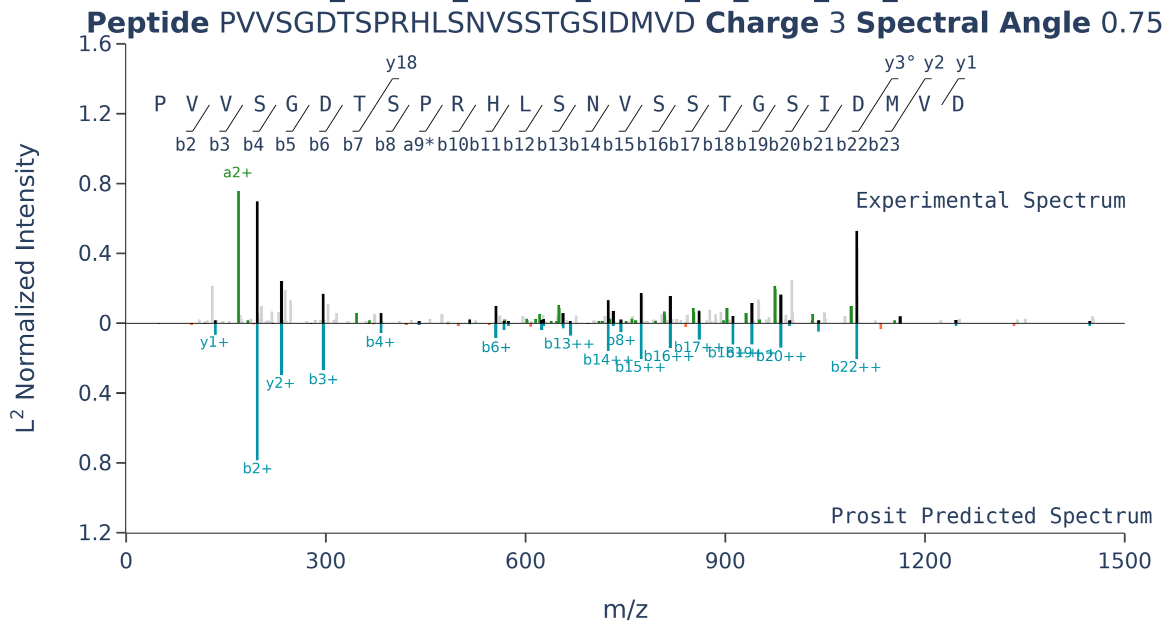


**Source** WSoh_221121_011221_HFGoe_L1_hTau_24h_R1 **Scan** 14654
**Peptide** TPSLPTPPTREPKKVAVVRTPPKSPSS **Charge** 4 **Spectral Angle** 0.85

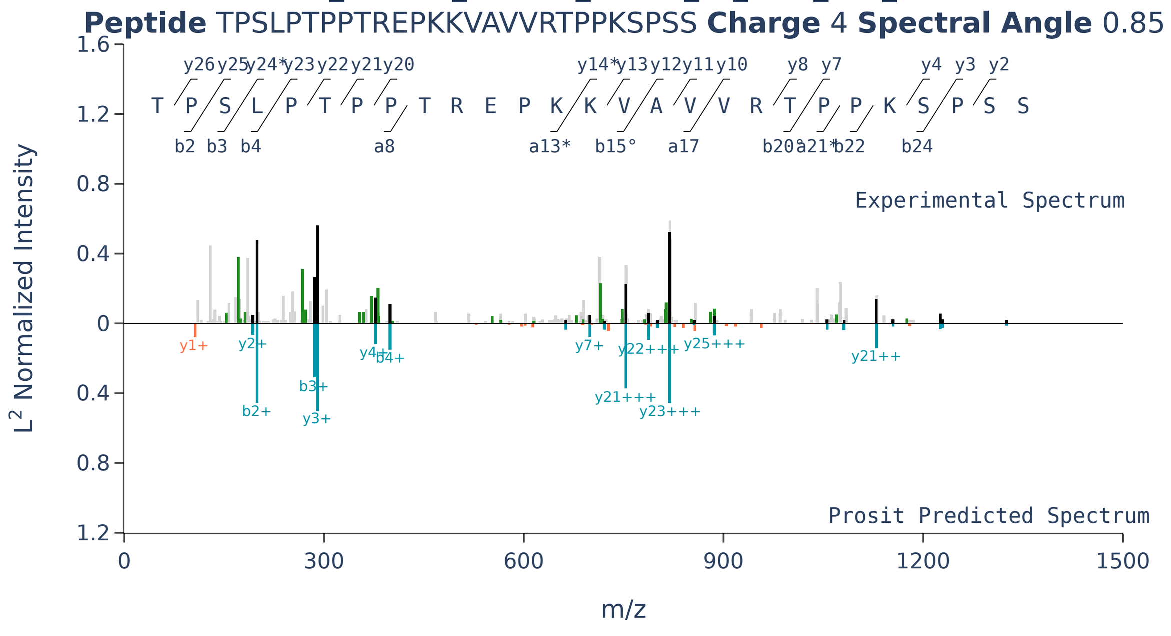


**Source** WSoh_221121_011221_HFGoe_L2_hTau_24h_R1 **Scan** 5975
**Peptide** TPRGAAPPGQKGQANA **Charge** 3 **Spectral Angle** 0.88

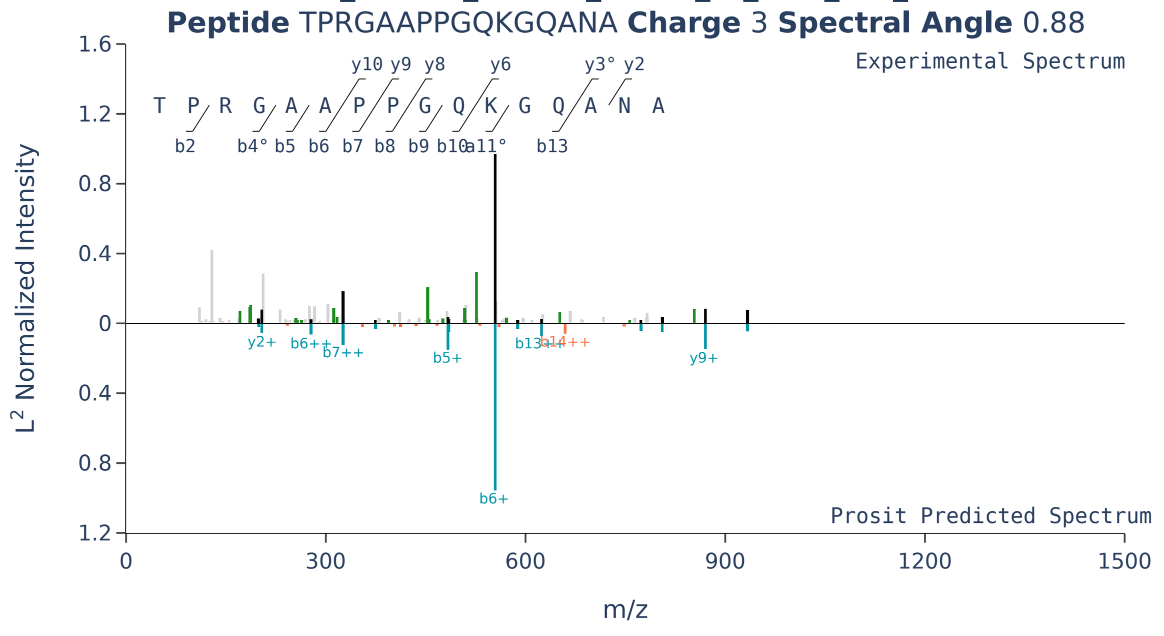


**Source** WSoh_221121_011221_HFGoe_L2_hTau_24h_R1 **Scan** 10666
**Peptide** NATRIPAKTPPAPKTPPSSGEPPKS **Charge** 4 **Spectral Angle** 0.78

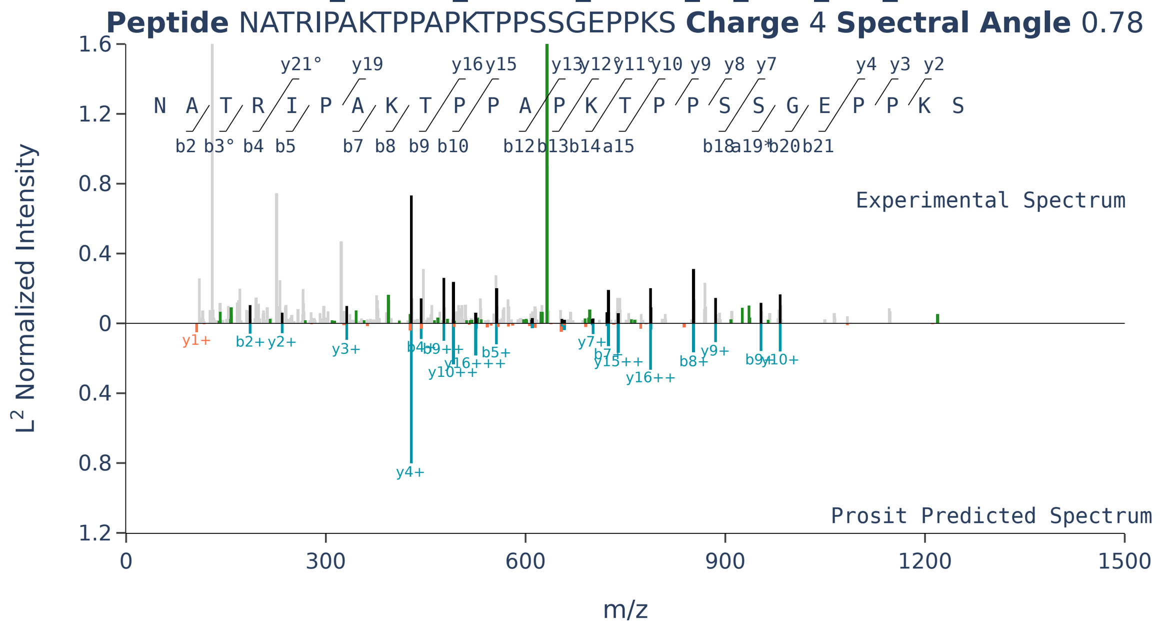


**Source** WSoh_221121_011221_HFGoe_L2_hTau_24h_R2 **Scan** 18318
**Peptide** DVTAPLVDEGAPGKQAAAQPHTE **Charge** 3 **Spectral Angle** 0.87

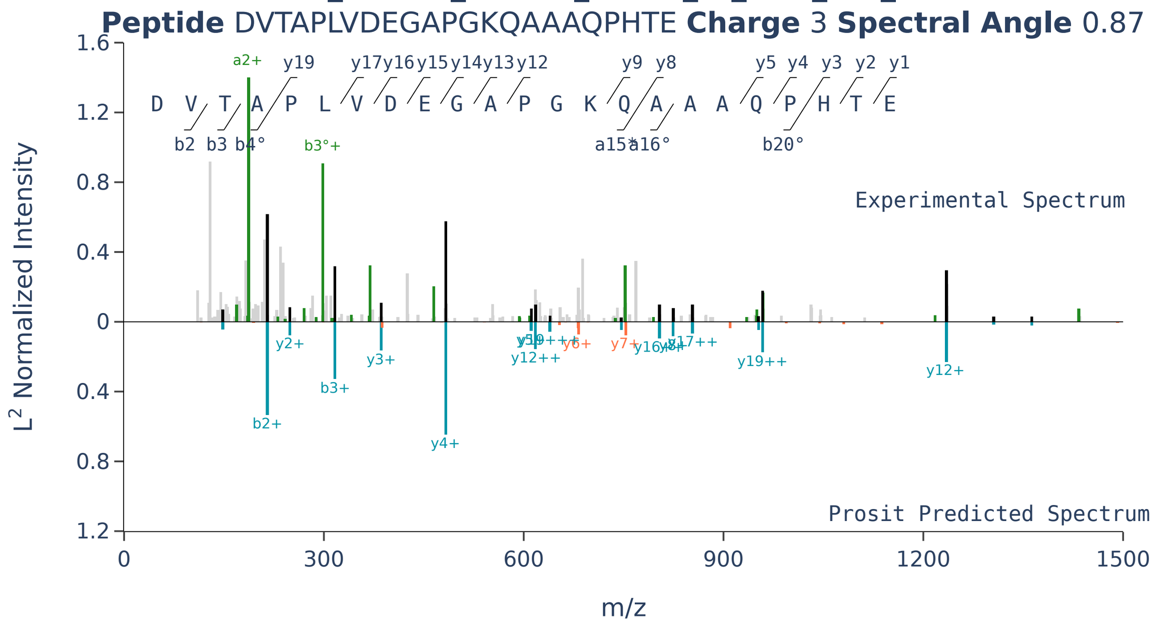


**Source** WSoh_221121_011221_HFGoe_L1_hTau_24h_R1 **Scan** 16871
**Peptide** KLDLSNVQSKCGSKDNIKHVPGGGSVQL **Charge** 4 **Spectral Angle** 0.81

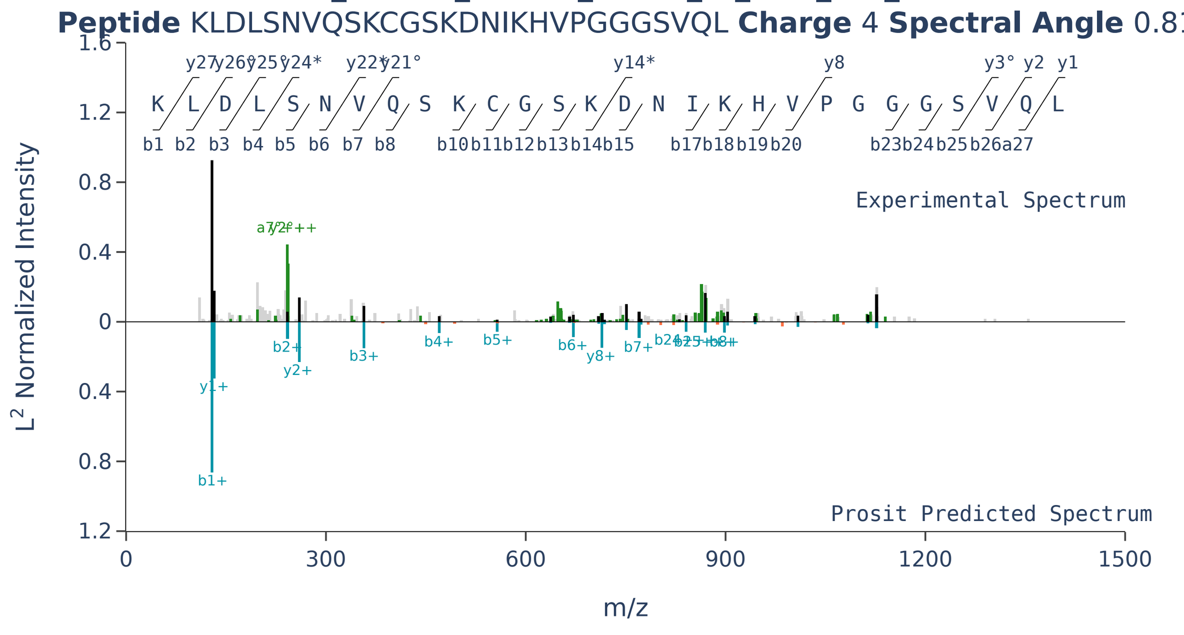


**Source** WSoh_221121_011221_HFGoe_L2_hTau_24h_R1 **Scan** 23212
**Peptide** ETSDAKSTPTAEDVTAPLV **Charge** 2 **Spectral Angle** 0.9

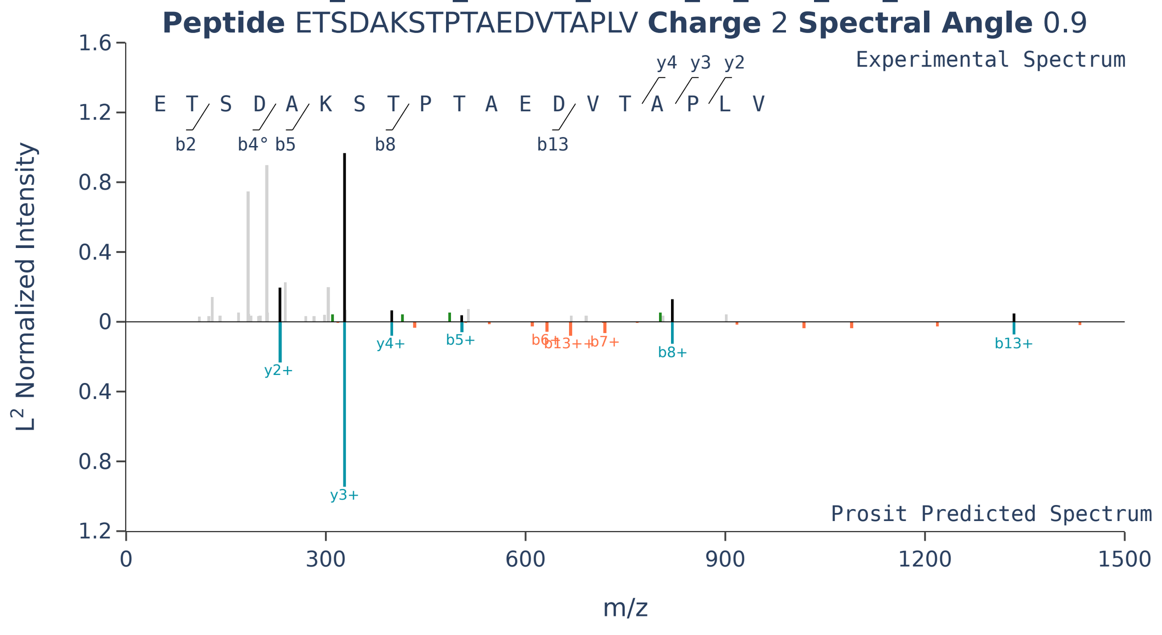


**Source** WSoh_221121_011221_HFGoe_L2_hTau_4h_R2 **Scan** 11810
**Peptide** NVQSKCGSKDNIKHVPGGGSVQI **Charge** 4 **Spectral Angle** 0.76

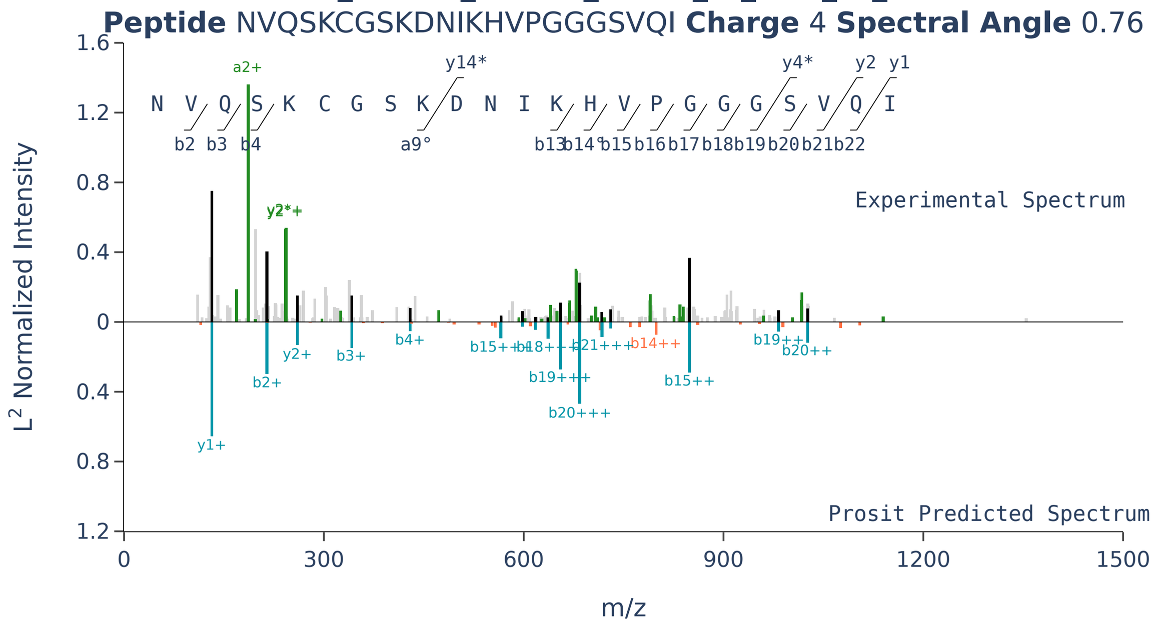


**Source** WSoh_221121_011221_HFGoe_L1_hTau_24h_R1 **Scan** 7584
**Peptide** GDTSPRHLSNVSSTGS **Charge** 3 **Spectral Angle** 0.72

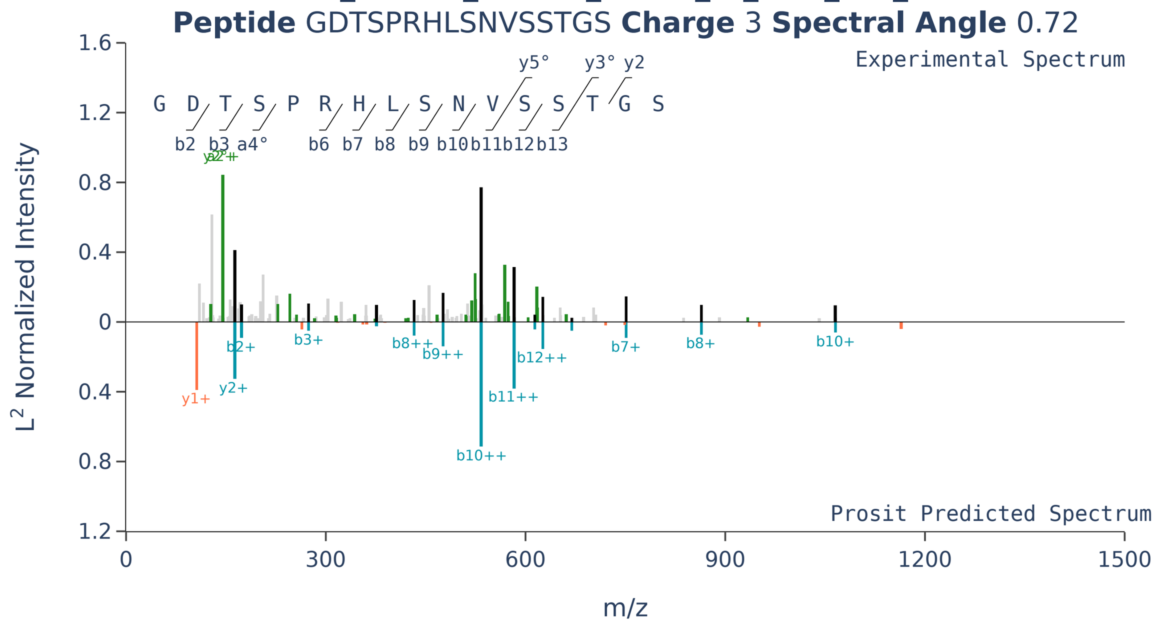


**Source** WSoh_221121_011221_HFGoe_L1_hTau_24h_R2 **Scan** 16145
**Peptide** GQVEVKSEKLDFKDRVQSKL **Charge** 4 **Spectral Angle** 0.79

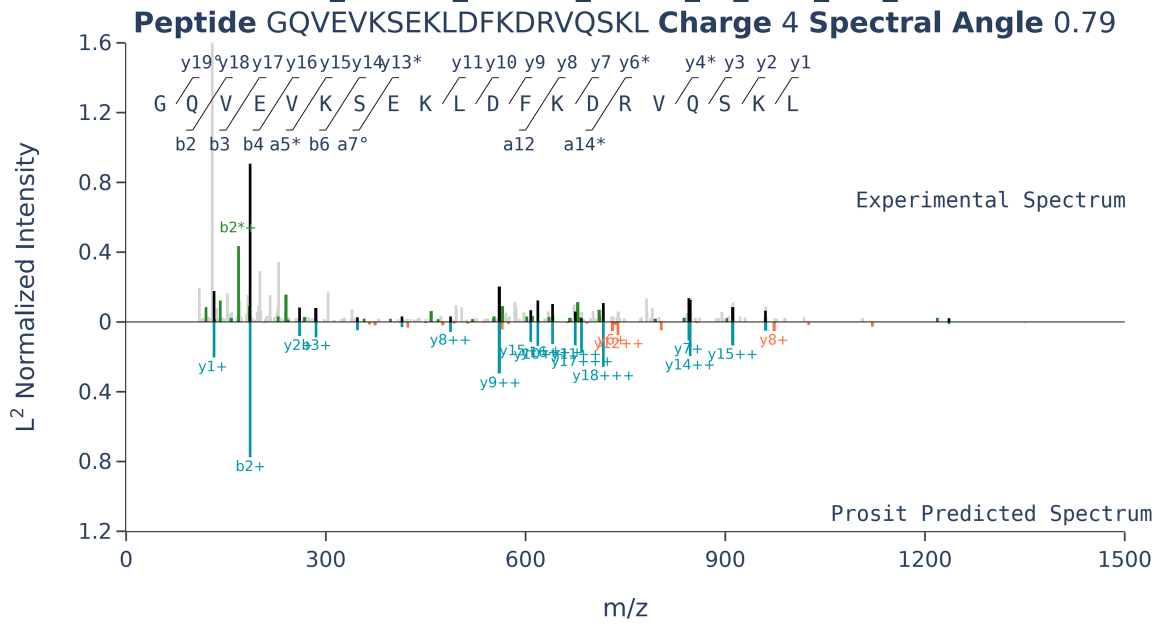

**Source** WSoh_221121_011221_HFGoe_L2_hTau_4h_R2 **Scan** 9026
**Peptide** SKVTSKCGSLGNIHHKPGGGQVEVKSEKL **Charge** 6 **Spectral Angle** 0.75

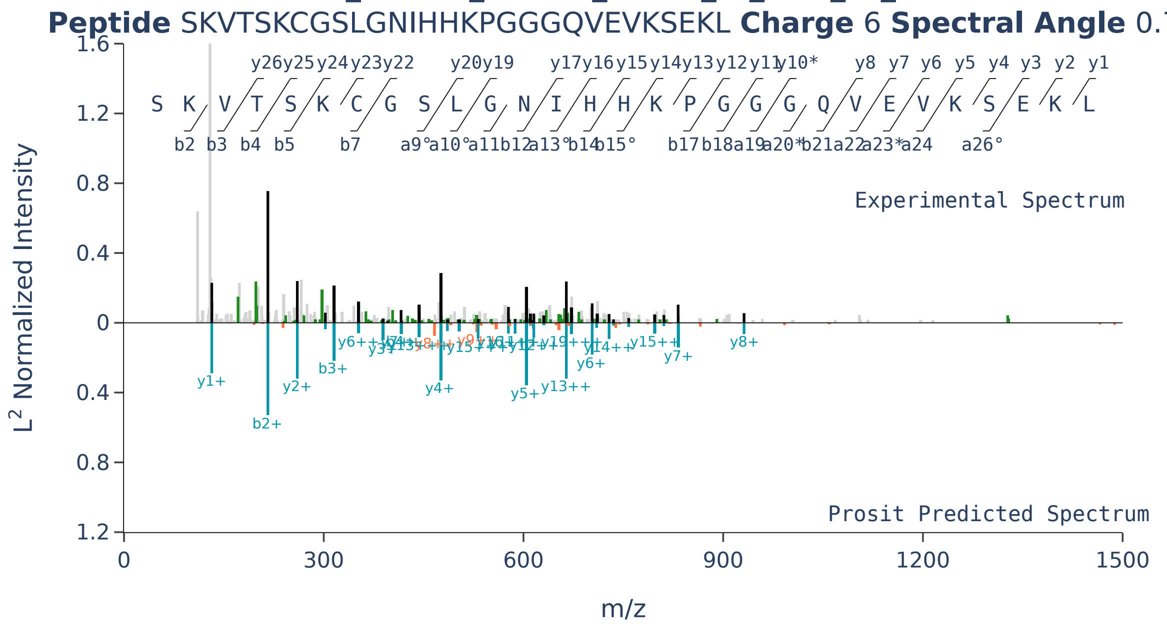


**Source** WSoh_221121_011221_HFGoe_L2_hTau_4h_R2 **Scan** 16025
**Peptide** HGAEIVYKSPVVSGDTSPRHLSNVSSTG **Charge** 4 **Spectral Angle** 0.72

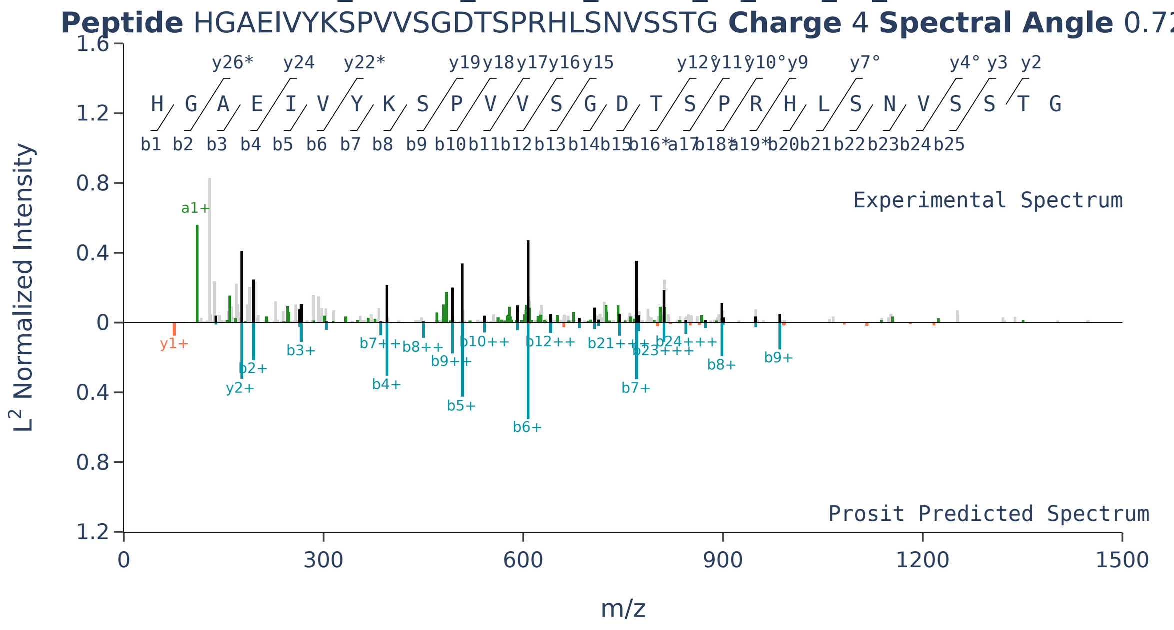


**Source** WSoh_221121_011221_HFGoe_L2_hTau_4h_R1 **Scan** 19131
**Peptide** DFKDRVQSKIGSLDNITHVPGGGNKKIETH **Charge** 5 **Spectral Angle** 0.79

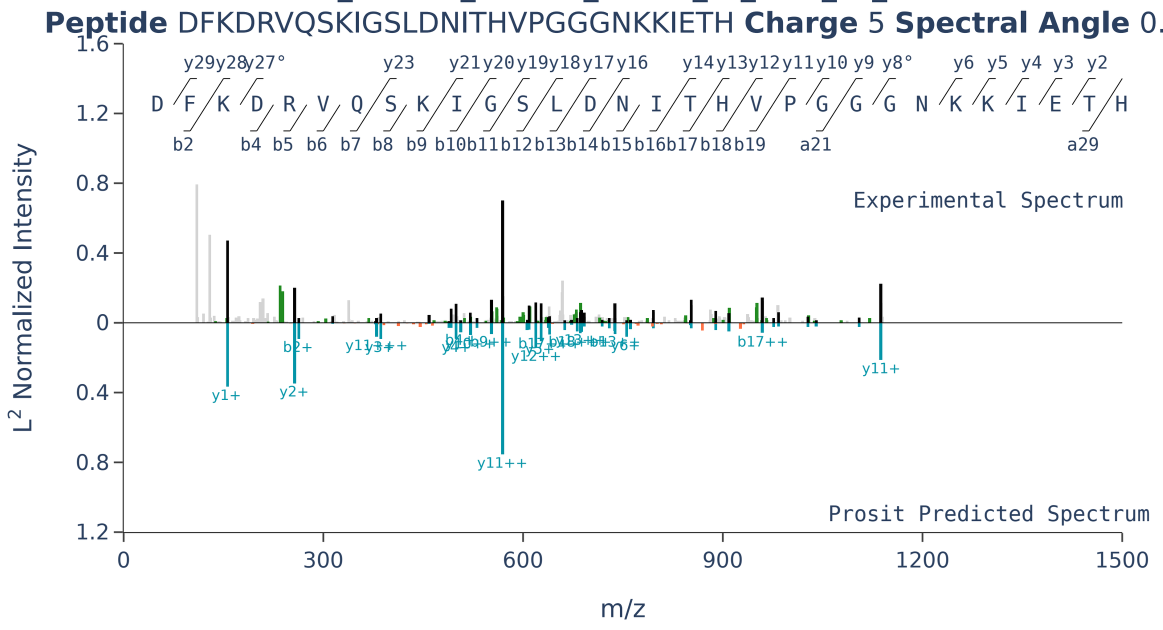


**Source** WSoh_221121_011221_HFGoe_L1_hTau_24h_R2 **Scan** 21976
**Peptide** KSEKLDFKDRVQSKIGSLDNITHV **Charge** 4 **Spectral Angle** 0.84

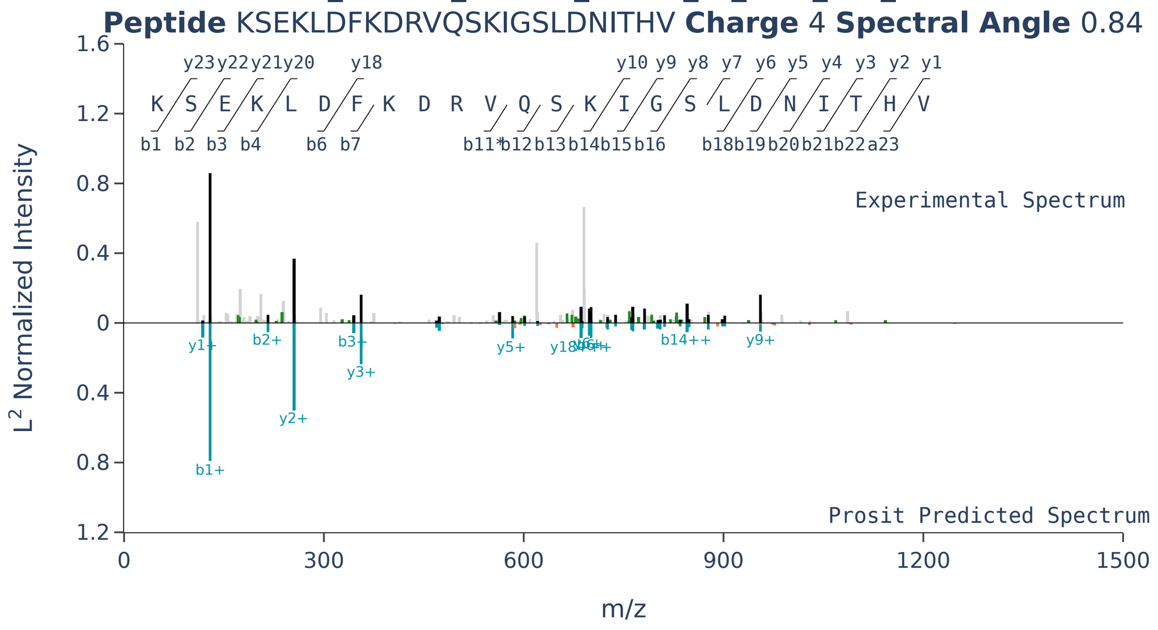


**Source** WSoh_221121_011221_HFGoe_L2_hTau_24h_R1 **Scan** 8431
**Peptide** LATPRGAAPPGQKGQAN **Charge** 3 **Spectral Angle** 0.81

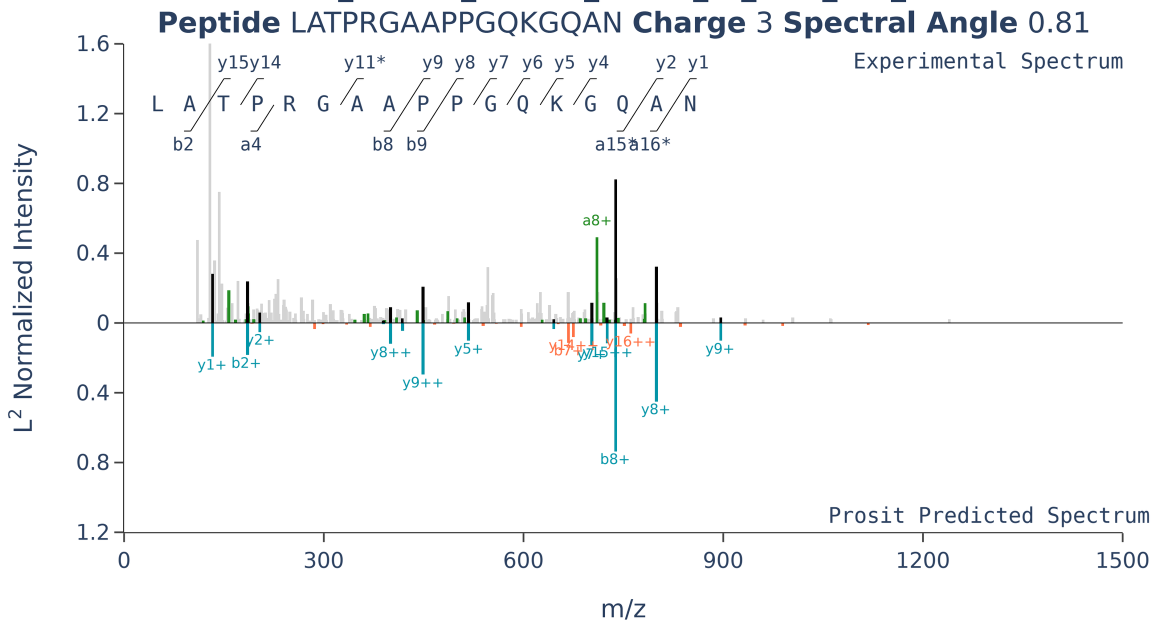


**Source** WSoh_221121_011221_HFGoe_L2_hTau_24h_R1 **Scan** 14808
**Peptide** PLQTPTEDGSEEPGSETSDAKSTPTAE **Charge** 3 **Spectral Angle** 0.66

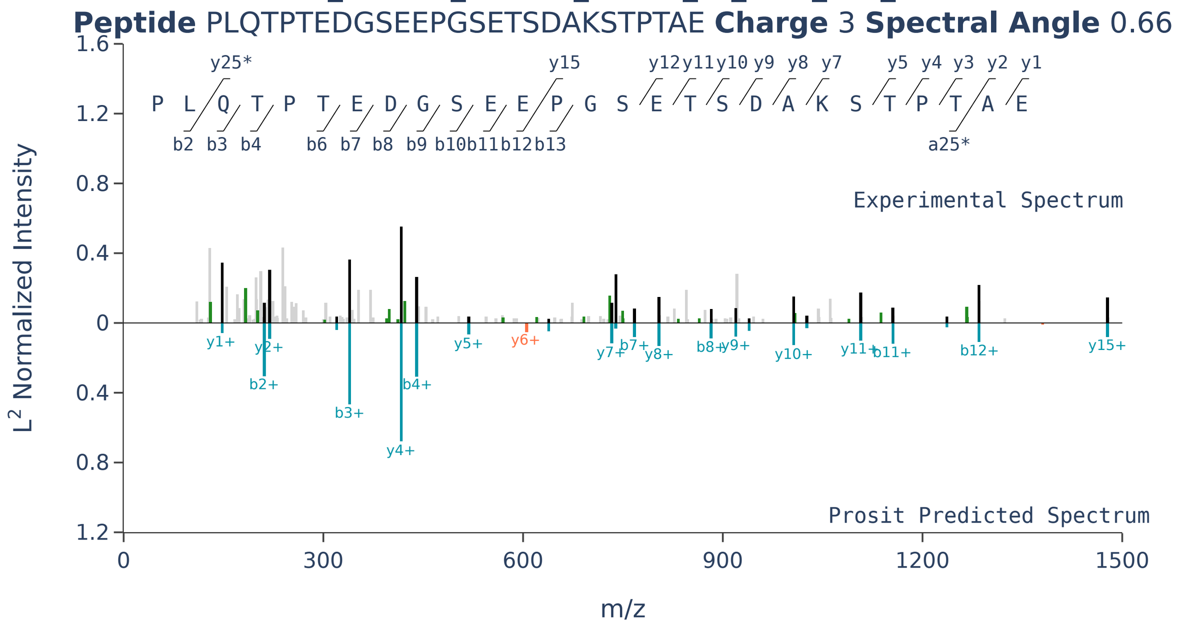


**Source** WSoh_221121_011221_HFGoe_L2_hTau_4h_R2 **Scan** 10934
**Peptide** DLSNVQSKCGSKDNI **Charge** 2 **Spectral Angle** 0.83

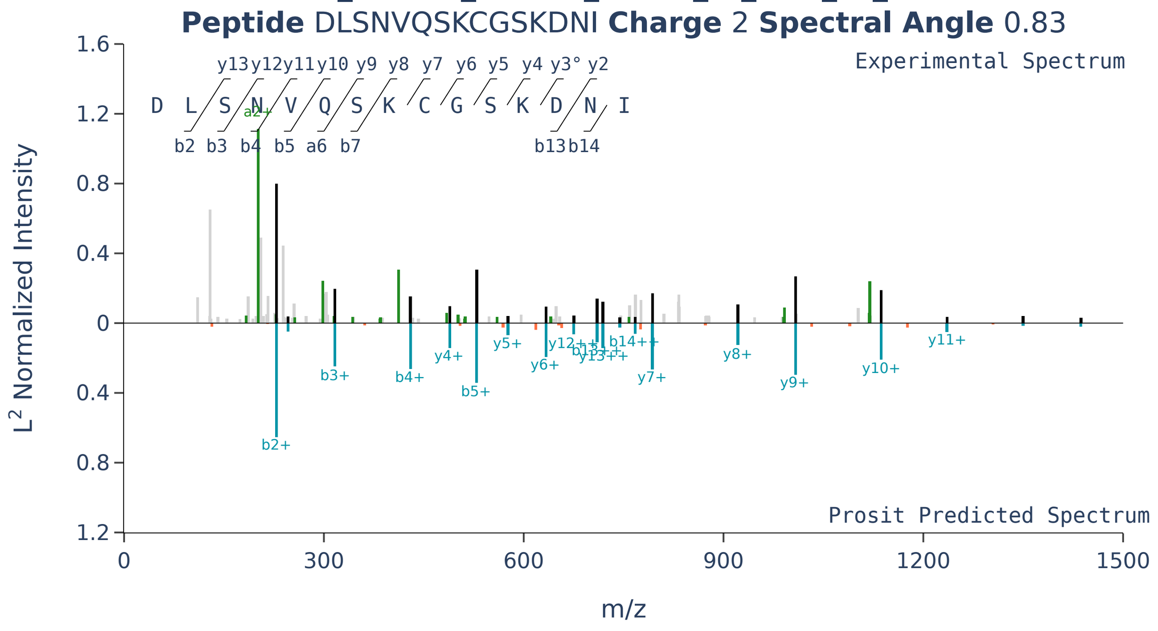


**Source** WSoh_221121_011221_HFGoe_L1_hTau_24h_R1 **Scan** 14524
**Peptide** KLGSLDNITHVPGGGNKKIETH **Charge** 5 **Spectral Angle** 0.73

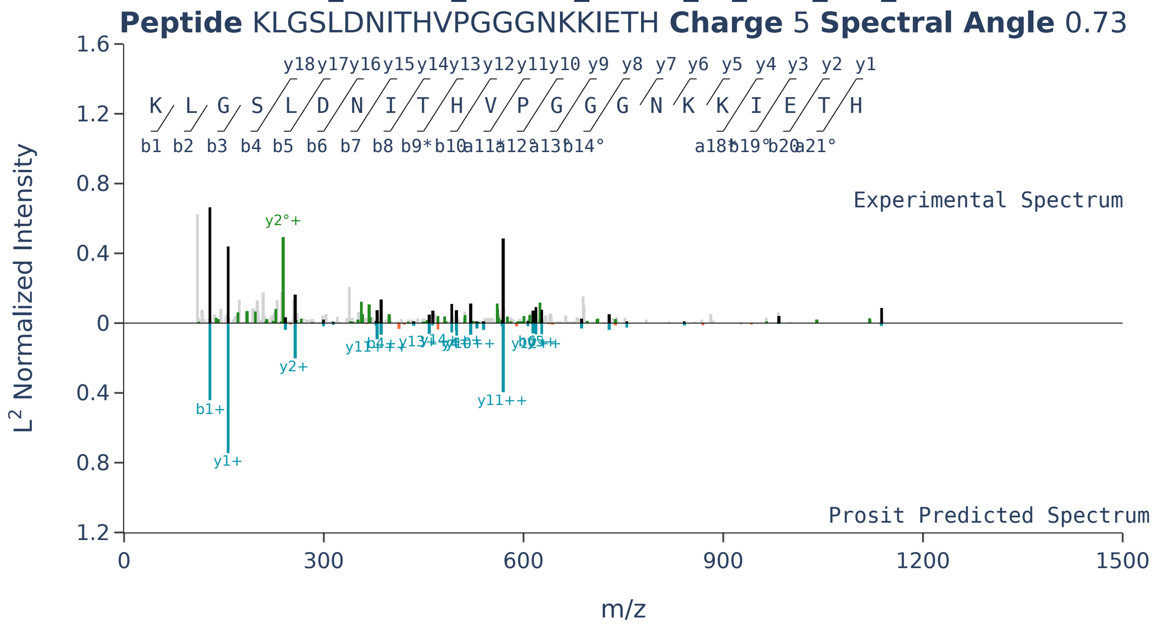


**Source** WSoh_221121_011221_HFGoe_L2_hTau_4h_R1 **Scan** 24350
**Peptide** PVPMPDLKNV **Charge** 2 **Spectral Angle** 0.81

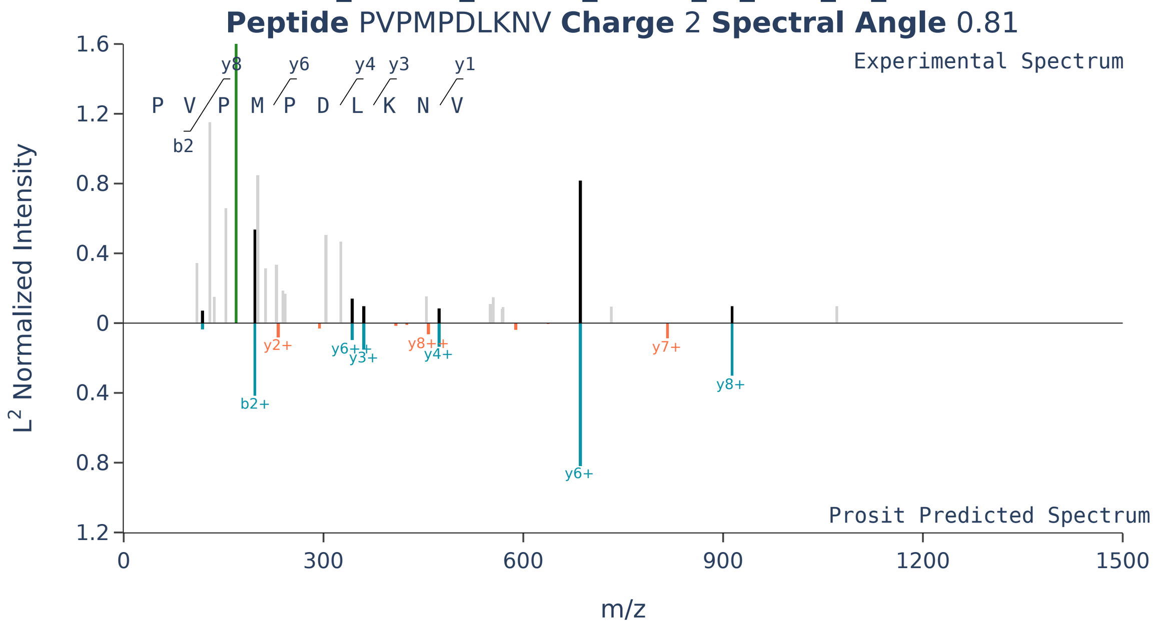


**Source** WSoh_221121_011221_HFGoe_L1_hTau_24h_R2 **Scan** 14826
**Peptide** RVQSKIGSLDNITHVPGGGNKKIETHKL **Charge** 5 **Spectral Angle** 0.82

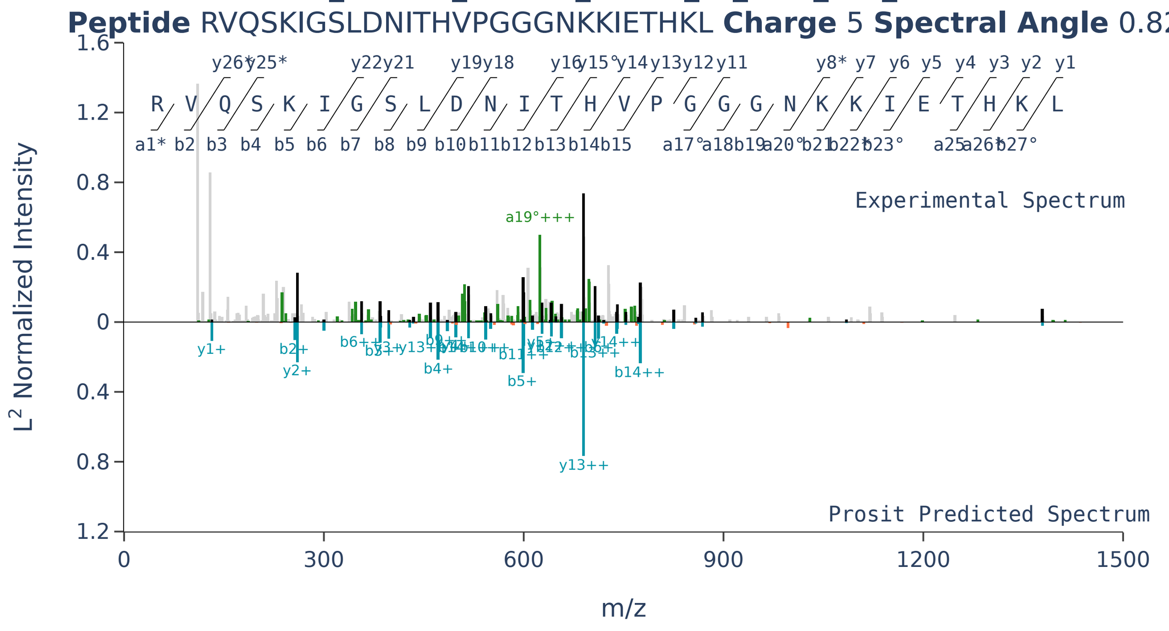


**Source** WSoh_221121_011221_HFGoe_L1_hTau_2h_R2 **Scan** 5382
**Peptide** TFRENAKAKTDHG **Charge** 3 **Spectral Angle** 0.76

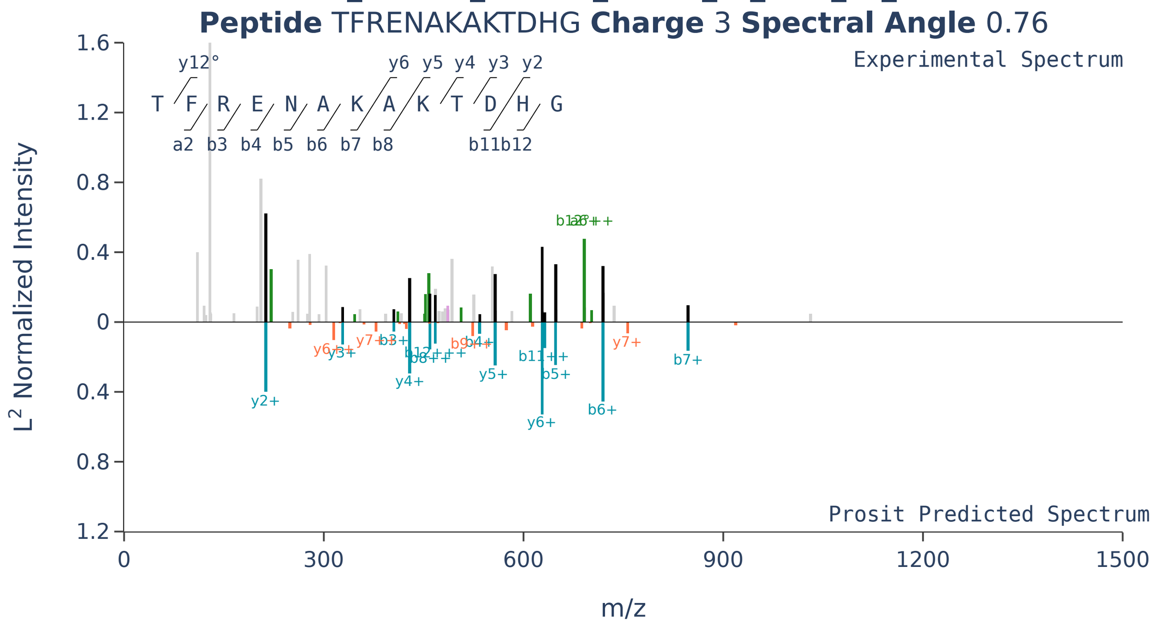


**Source** WSoh_221121_011221_HFGoe_L1_hTau_2h_R1 **Scan** 12520
**Peptide** AAAQPHTEIPEGTTA **Charge** 2 **Spectral Angle** 0.83

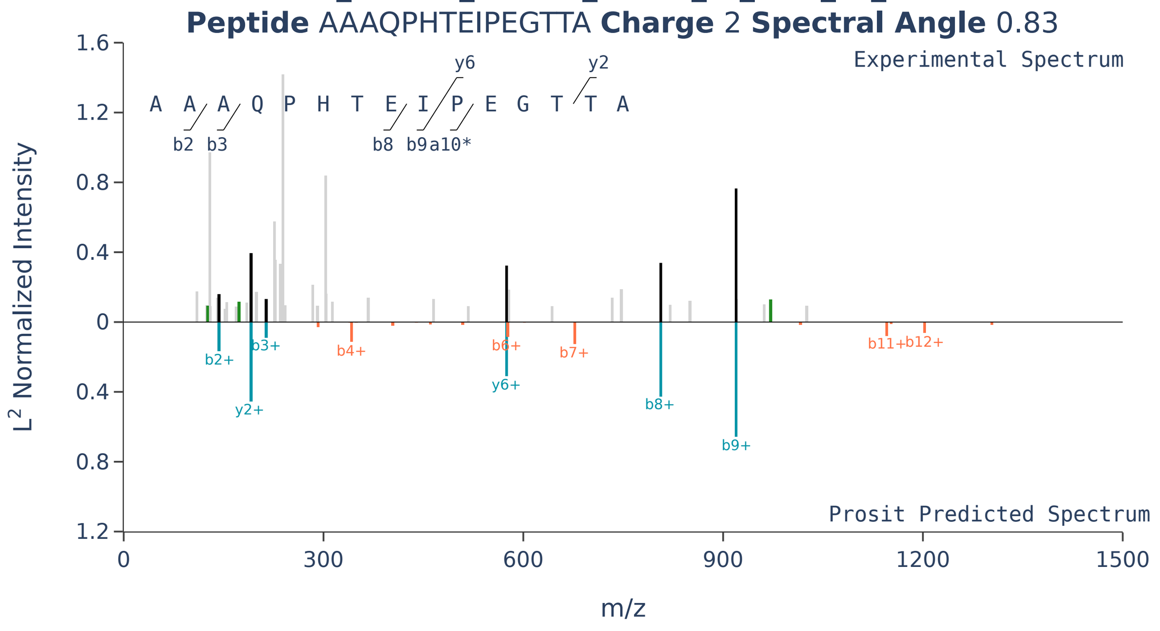


**Source** WSoh_221121_011221_HFGoe_L1_hTau_24h_R1 **Scan** 18178
**Peptide** YKPVDLSKVT **Charge** 2 **Spectral Angle** 0.83

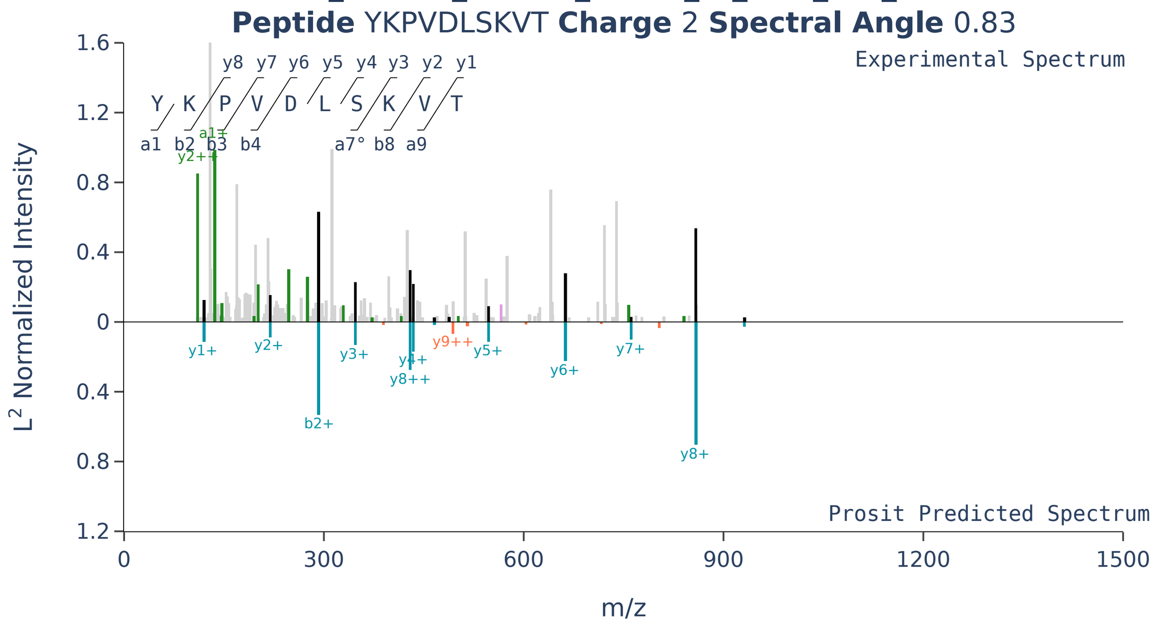


**Source** WSoh_221121_011221_HFGoe_L2_hTau_24h_R2 **Scan** 12420
**Peptide** PGSPGTPGSRSRTPSLPTPPTREPKKV **Charge** 6 **Spectral Angle** 0.73

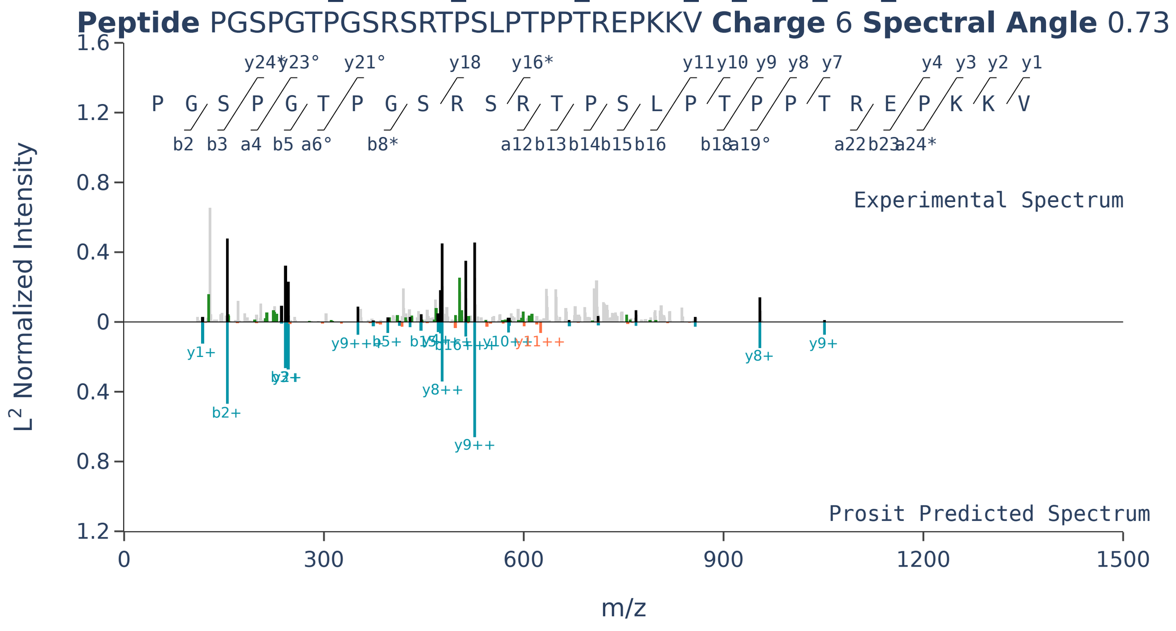


**Source** WSoh_221121_011221_HFGoe_L2_hTau_2h_R2 **Scan** 8861
**Peptide** TENLKHQPGGGKVQIINK **Charge** 4 **Spectral Angle** 0.77

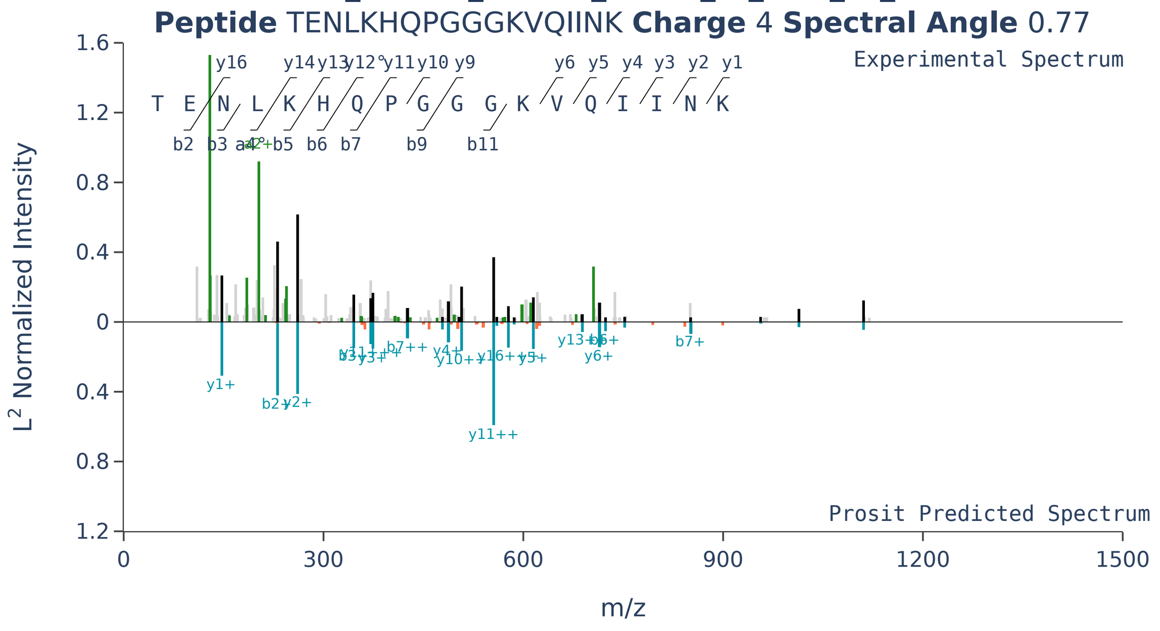

**Source** WSoh_221121_011221_HFGoe_L2_hTau_4h_R2 **Scan** 15534
**Peptide** VYKPVDLSKVTSKCGSLGNIHHKPGGG **Charge** 4 **Spectral Angle** 0.77

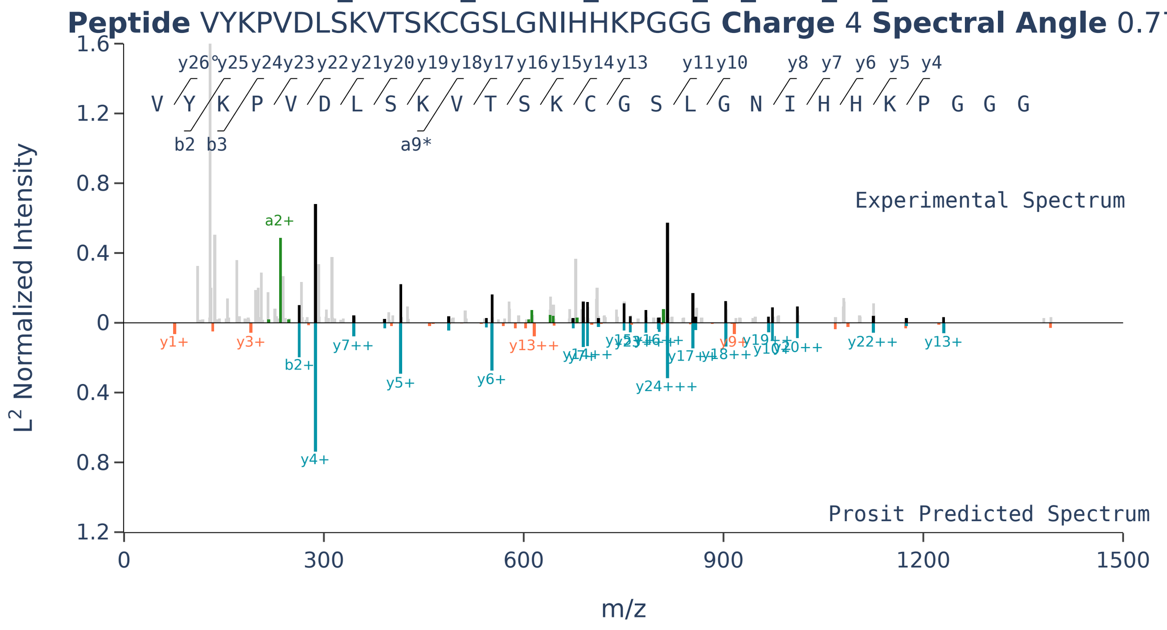


**Source** WSoh_221121_011221_HFGoe_L2_hTau_24h_R1 **Scan** 25674
**Peptide** PQLATLADEVS **Charge** 2 **Spectral Angle** 0.61

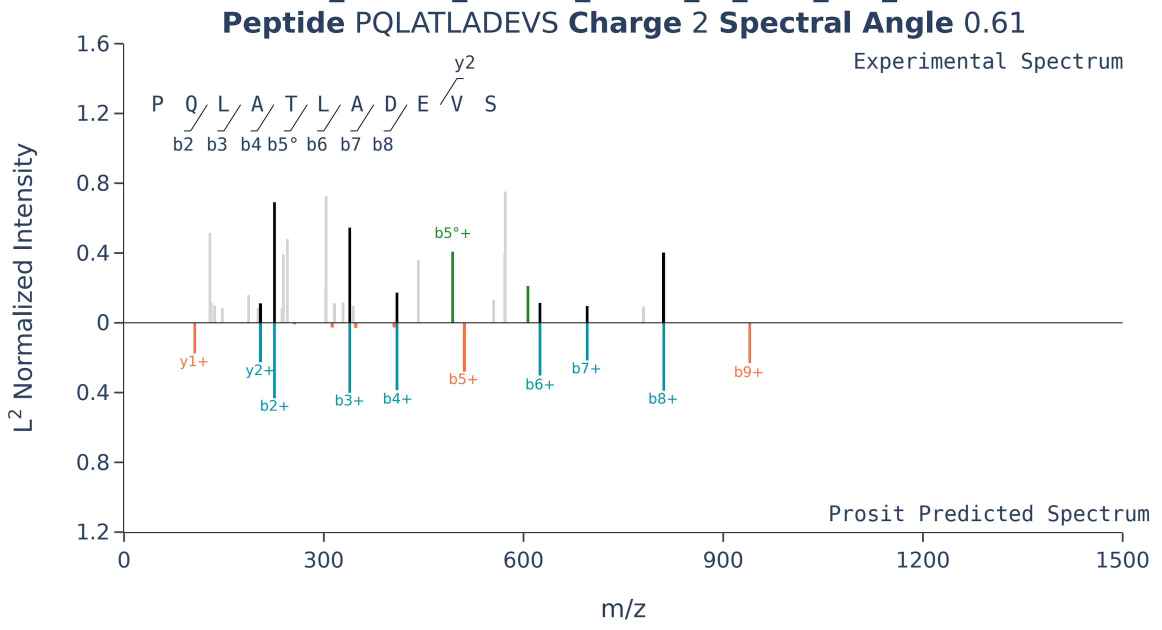


**Source** WSoh_221121_011221_HFGoe_L1_hTau_24h_R1 **Scan** 13856
**Peptide** LGNIHHKPGGGQVEVKSEKLDF **Charge** 4 **Spectral Angle** 0.72

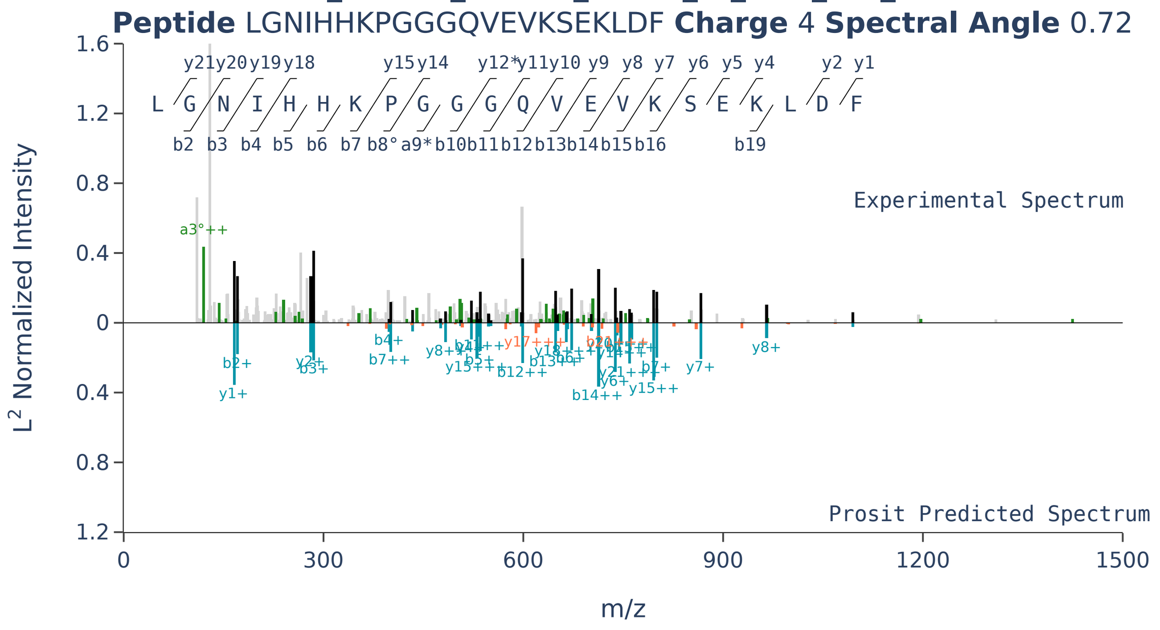


**Source** WSoh_221121_011221_HFGoe_L2_hTau_24h_R2 **Scan** 6987
**Peptide** GSLGNIHHKPGGGQVEVKSEK **Charge** 3 **Spectral Angle** 0.73

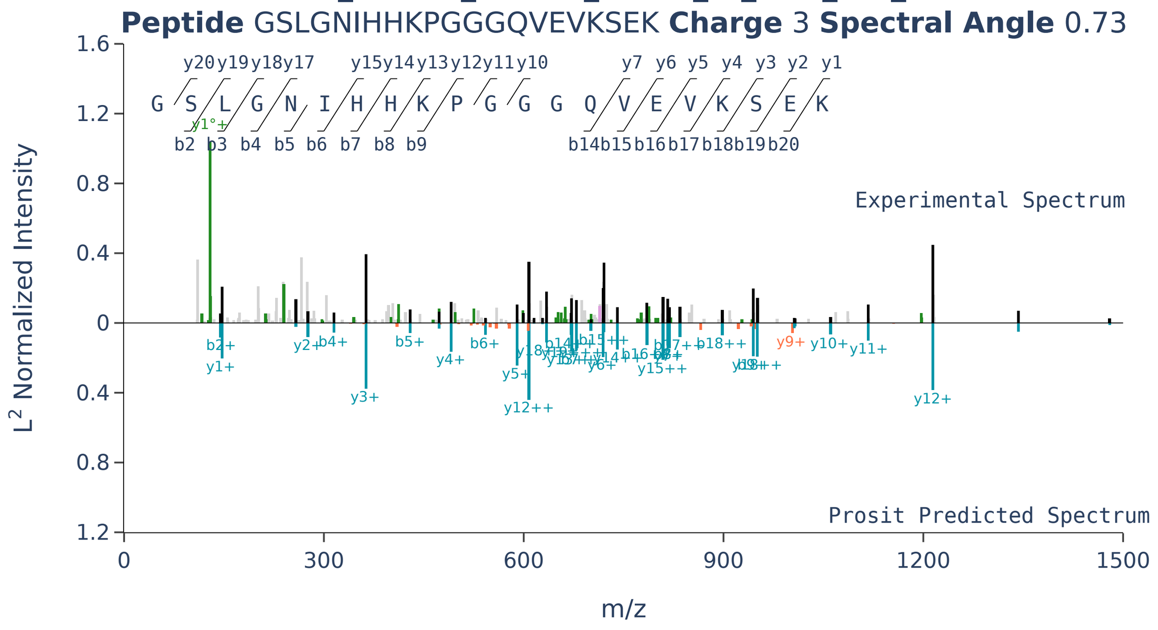


**Source** WSoh_221121_011221_HFGoe_L2_hTau_24h_R1 **Scan** 10879
**Peptide** RSRTPSLPTPPTREPKKV **Charge** 4 **Spectral Angle** 0.74

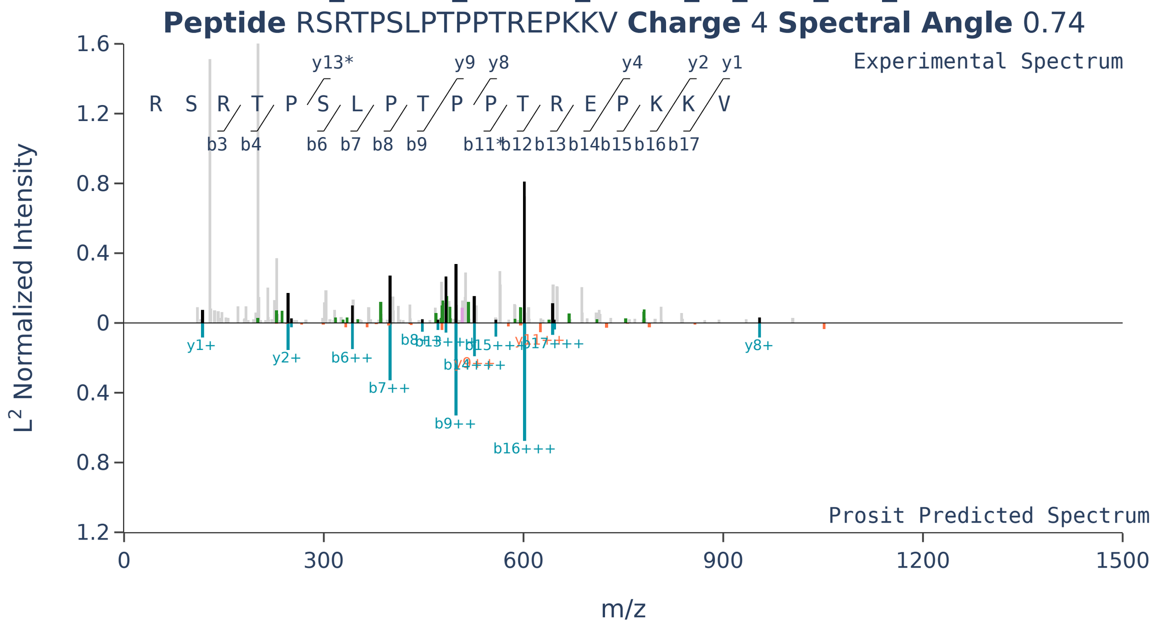


**Source** WSoh_221121_011221_HFGoe_L2_hTau_24h_R2 **Scan** 16916
**Peptide** KIGSLDNITHVPGGGNKKIETHKLTFR **Charge** 5 **Spectral Angle** 0.8

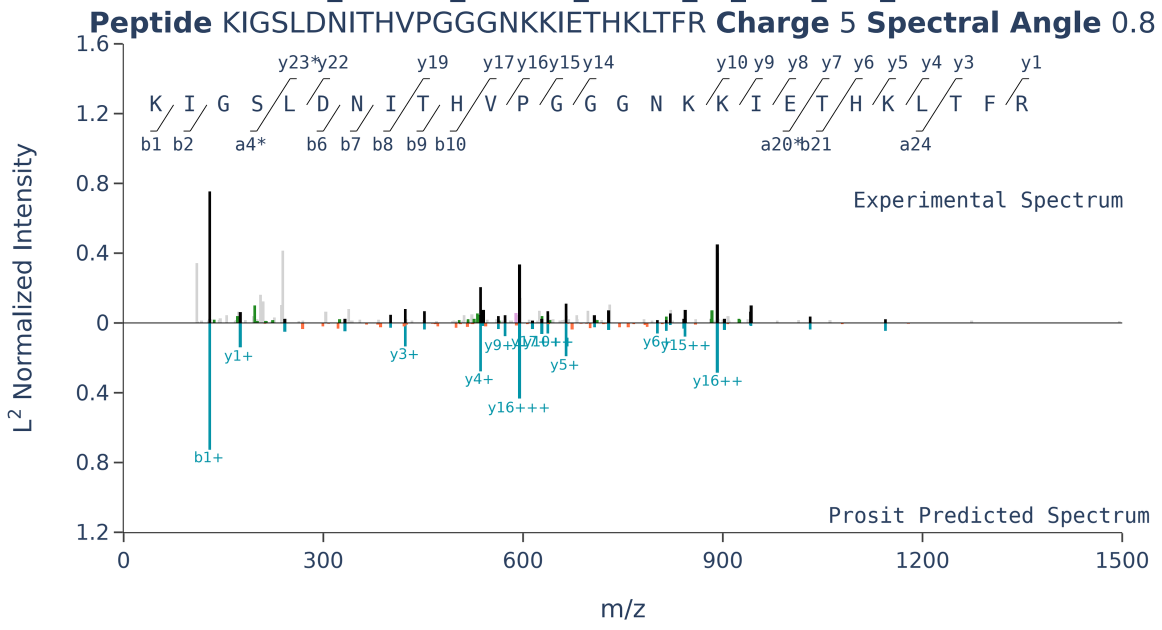


**Source** WSoh_221121_011221_HFGoe_L1_hTau_24h_R2 **Scan** 5509
**Peptide** KDGTGSDDKKAKGADGKTKIATPRG **Charge** 4 **Spectral Angle** 0.84

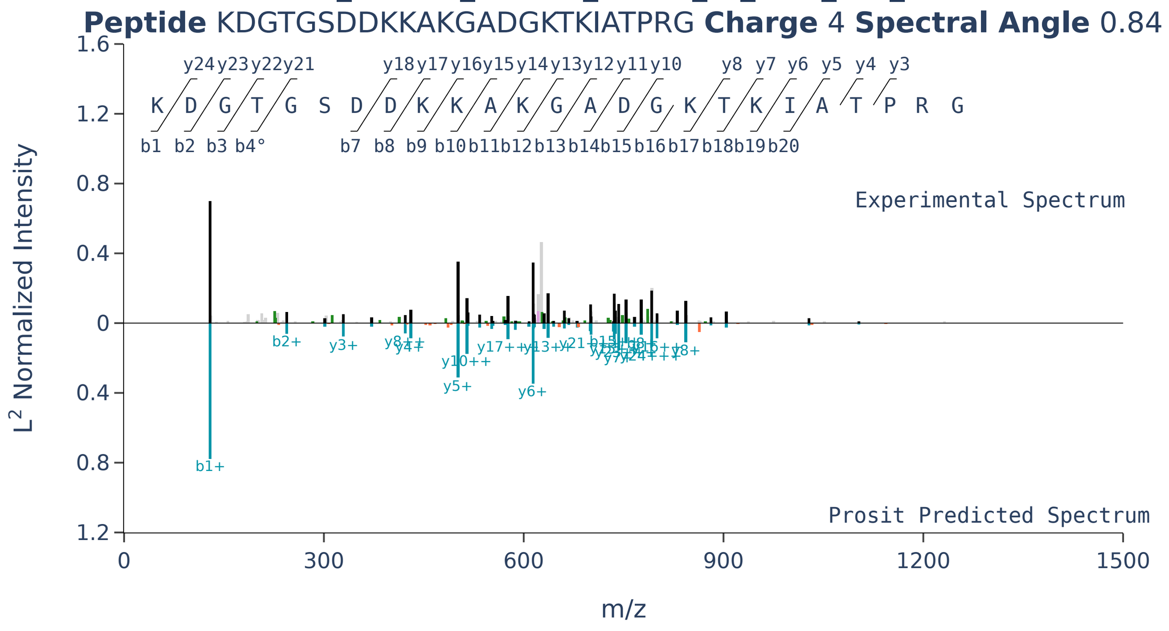


**Source** WSoh_221121_011221_HFGoe_L1_hTau_4h_R2 **Scan** 10565
**Peptide** ITHVPGGGNKKIETHKLTFREN **Charge** 4 **Spectral Angle** 0.81

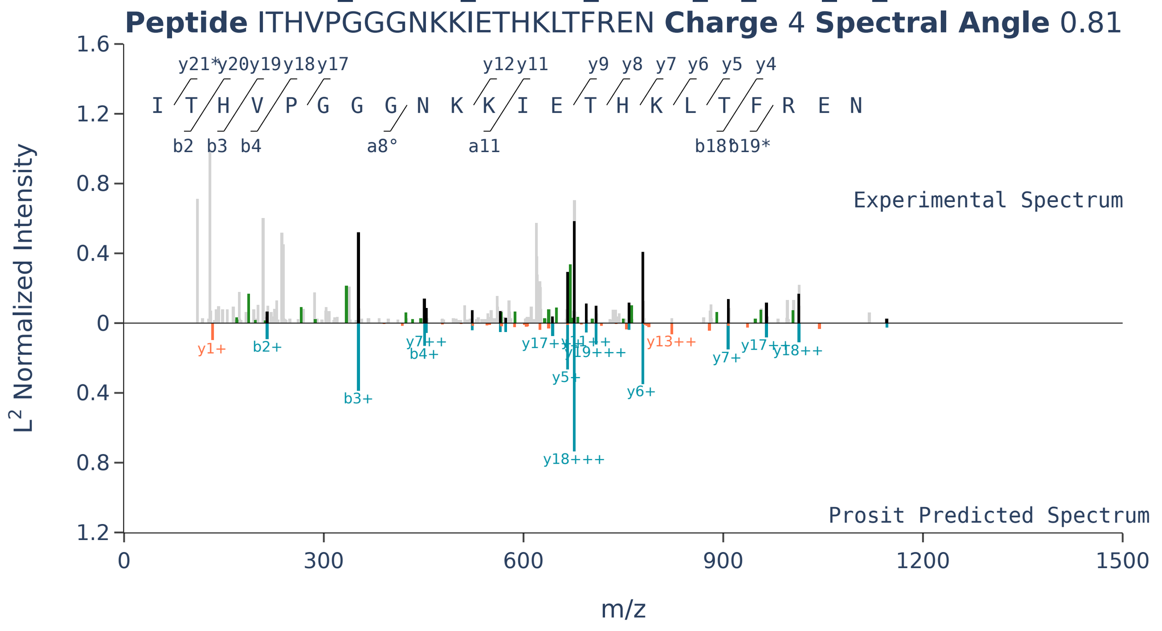


**Source** WSoh_221121_011221_HFGoe_L2_hTau_24h_R2 **Scan** 12305
**Peptide** LDFKDRV **Charge** 2 **Spectral Angle** 0.89

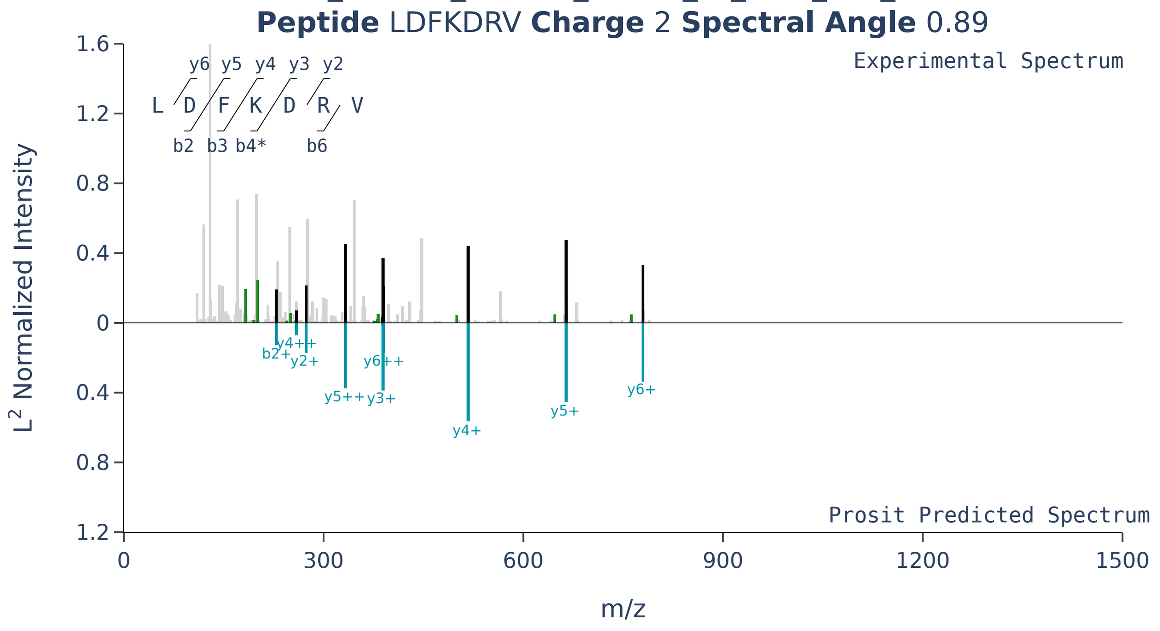


**Source** WSoh_221121_011221_HFGoe_L1_hTau_24h_R1 **Scan** 13016
**Peptide** AKAKTDHGAEIVYKSPVVSGDTS **Charge** 2 **Spectral Angle** 0.74

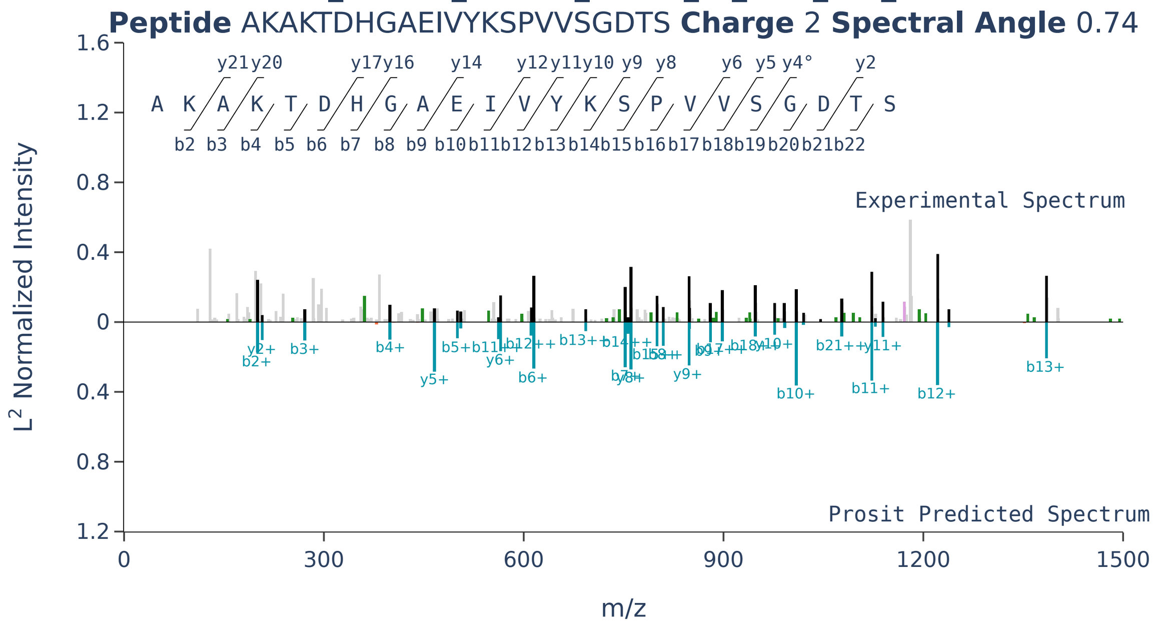


**Source** WSoh_221121_011221_HFGoe_L2_hTau_24h_R2 **Scan** 6235
**Peptide** KNVKSKIGSTENLKHQPGGGKVQ **Charge** 4 **Spectral Angle** 0.89

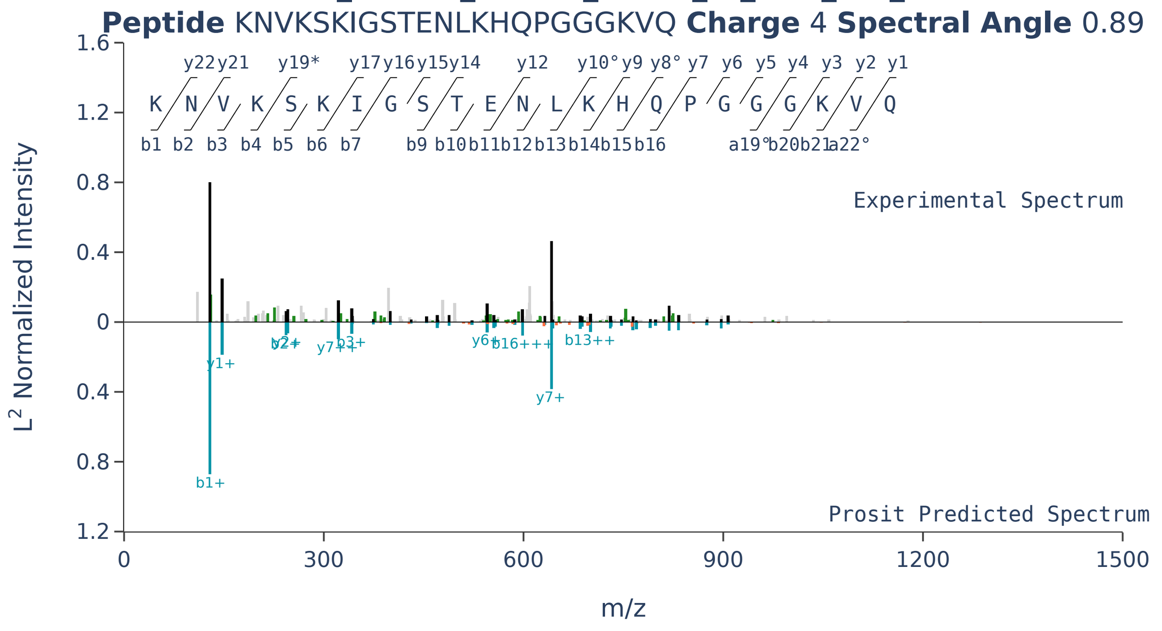


**Source** WSoh_221121_011221_HFGoe_L1_hTau_24h_R1 **Scan** 20444
**Peptide** EDVTAPLVDEGAPGKQAAA **Charge** 2 **Spectral Angle** 0.84

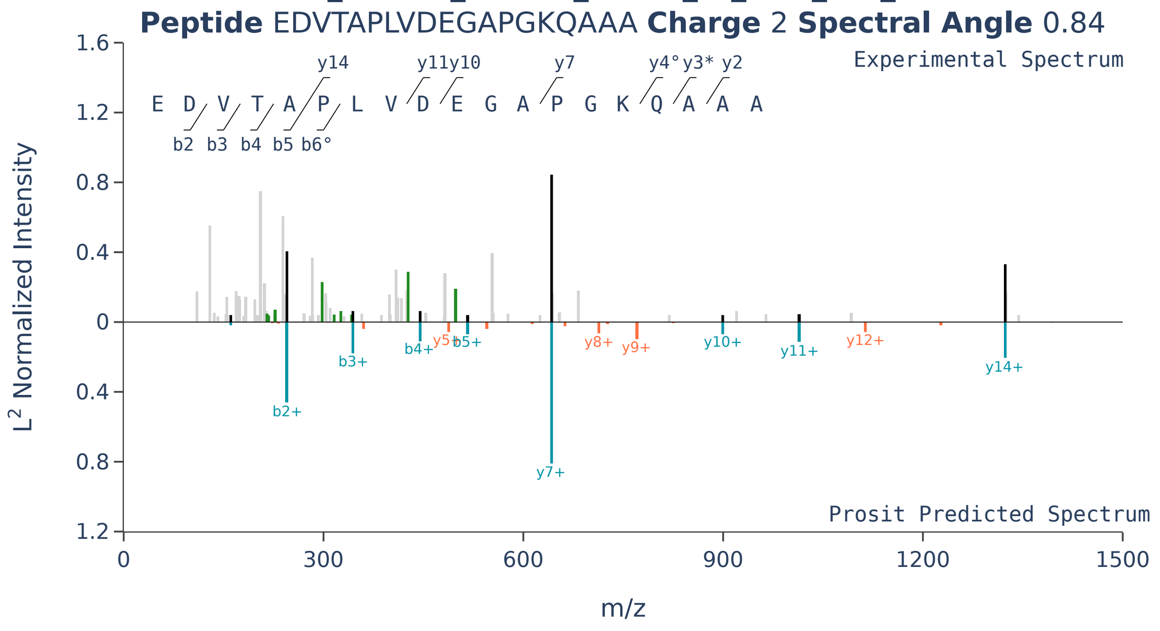


**Source** WSoh_221121_011221_HFGoe_L2_hTau_24h_R2 **Scan** 9752
**Peptide** KLETHKLTFREN **Charge** 4 **Spectral Angle** 0.61

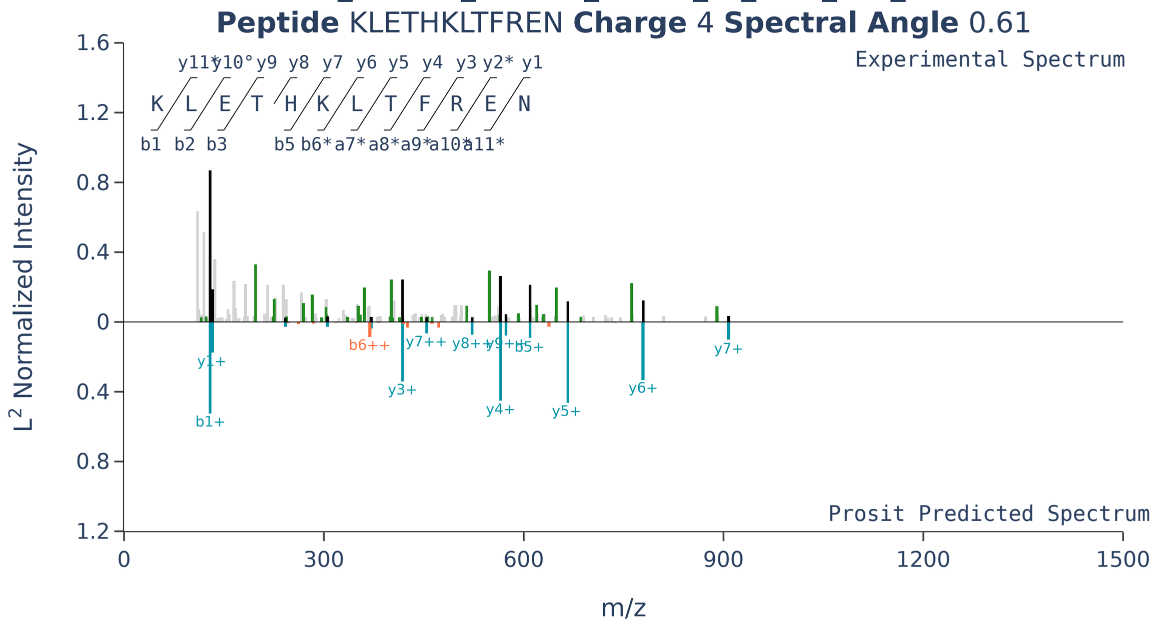


**Source** WSoh_221121_011221_HFGoe_L2_hTau_4h_R1 **Scan** 11037
**Peptide** TMHQDQEGDTDAGL **Charge** 2 **Spectral Angle** 0.73

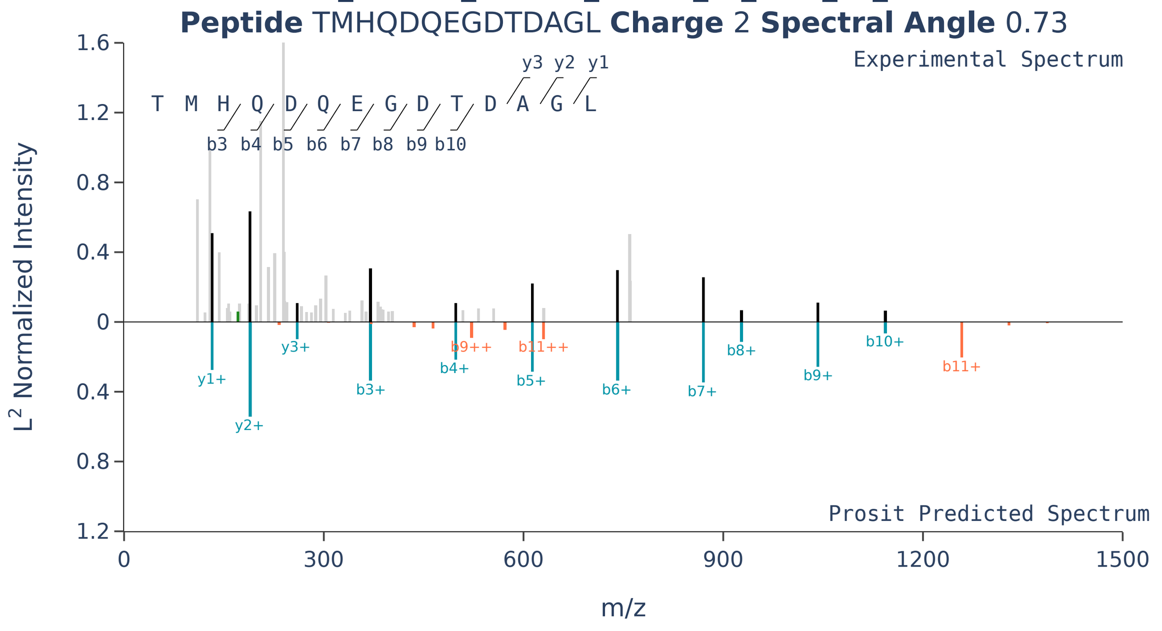


**Source** WSoh_221121_011221_HFGoe_L2_hTau_4h_R2 **Scan** 6620
**Peptide** KGADGKTKIATPRGAAPPGQKG **Charge** 3 **Spectral Angle** 0.88

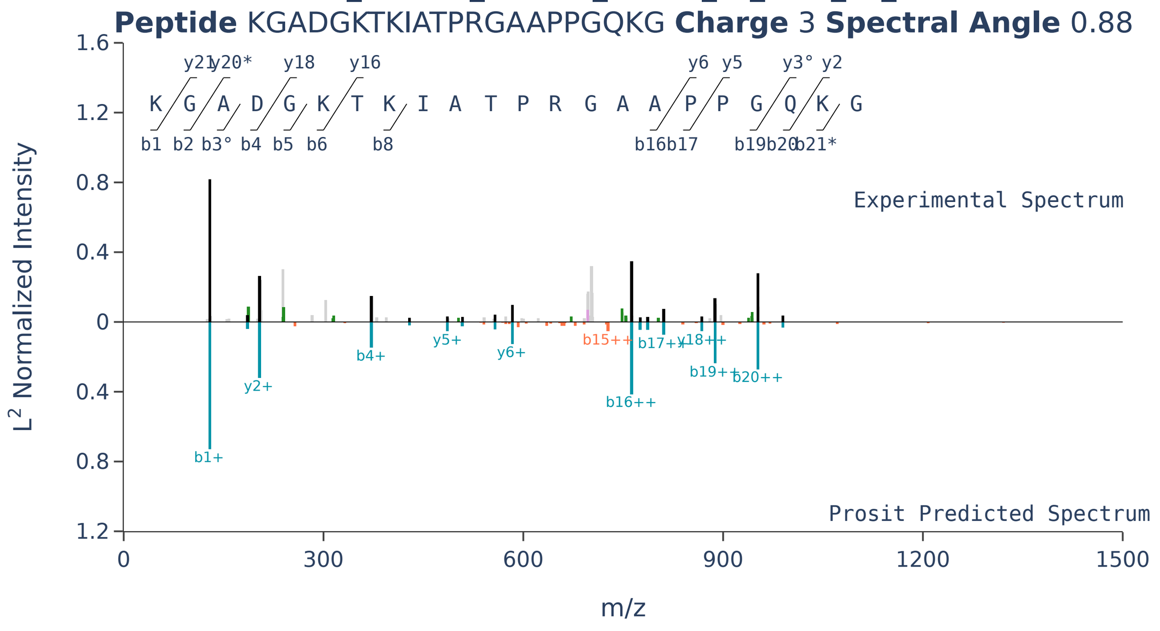

**Source** WSoh_221121_011221_HFGoe_L2_hTau_24h_R1 **Scan** 23345
**Peptide** AQPHTEIPEGTTAEEAGIGDTPSLE **Charge** 3 **Spectral Angle** 0.82

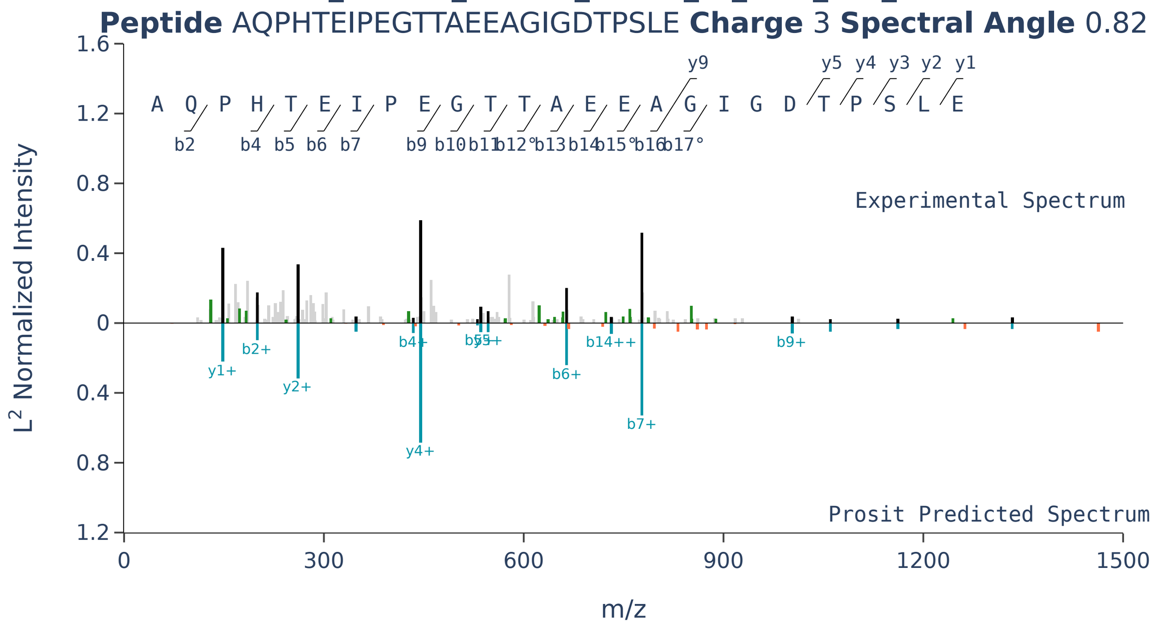


**Source** WSoh_221121_011221_HFGoe_L1_hTau_4h_R1 **Scan** 16114
**Peptide** SKIGSLDNITHVPGGGNKKI **Charge** 4 **Spectral Angle** 0.83

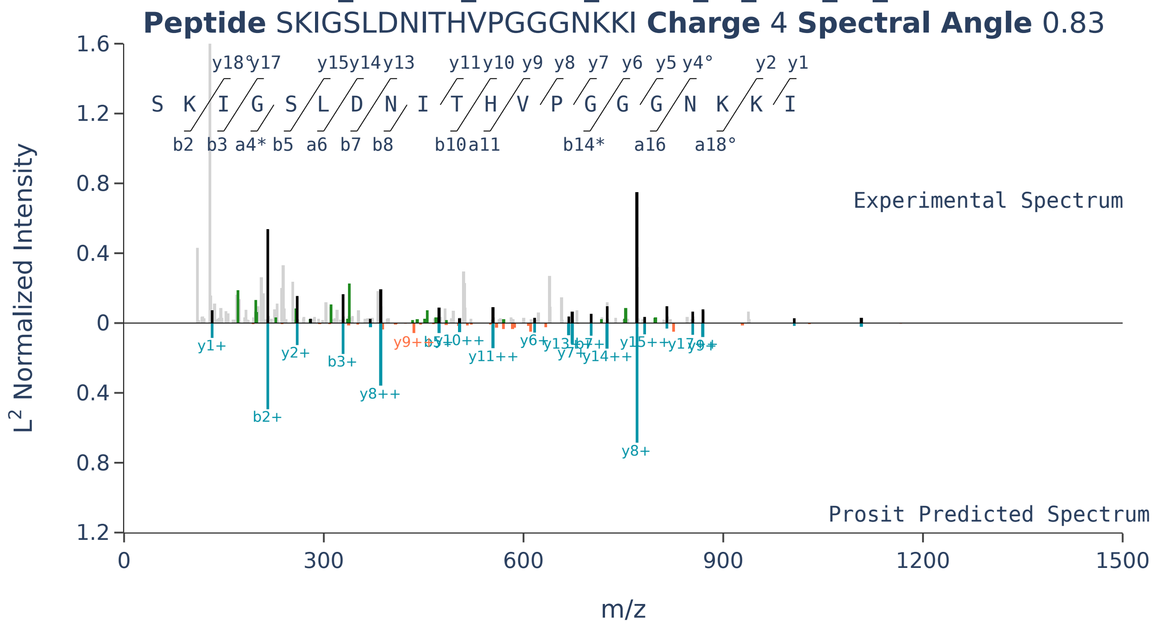


**Source** WSoh_221121_011221_HFGoe_L2_hTau_24h_R1 **Scan** 11356
**Peptide** QSKCGSKDNIKHVPGGGSVQL **Charge** 2 **Spectral Angle** 0.9

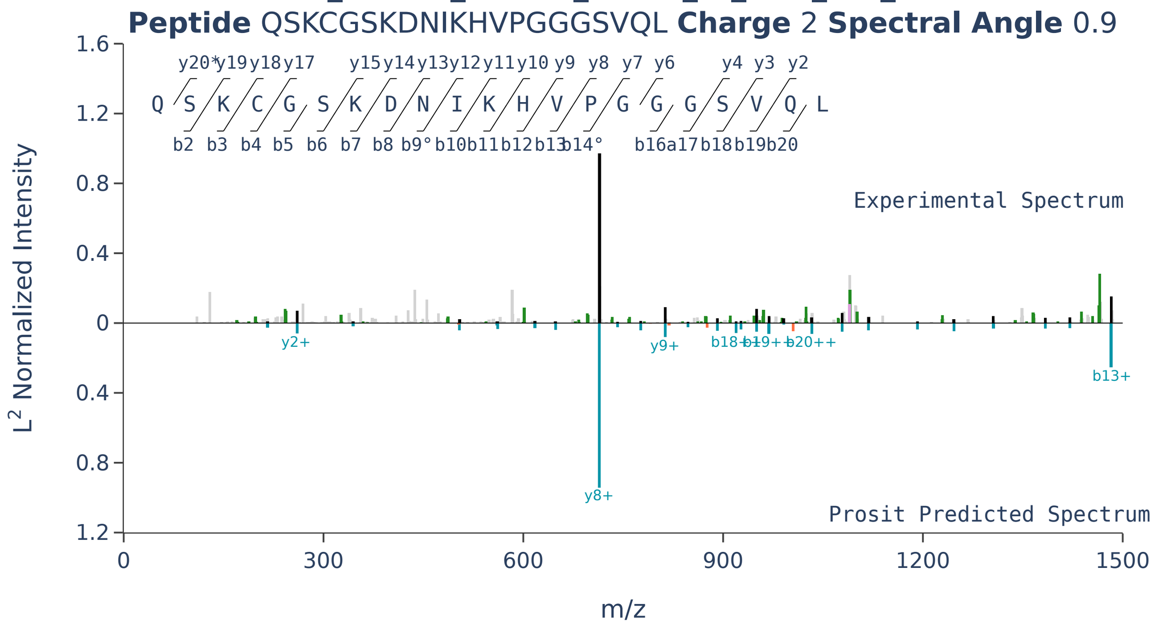


**Source** WSoh_221121_011221_HFGoe_L2_hTau_24h_R1 **Scan** 24443
**Peptide** PHTEIPEGTTAEEAGIGDTPSL **Charge** 3 **Spectral Angle** 0.89

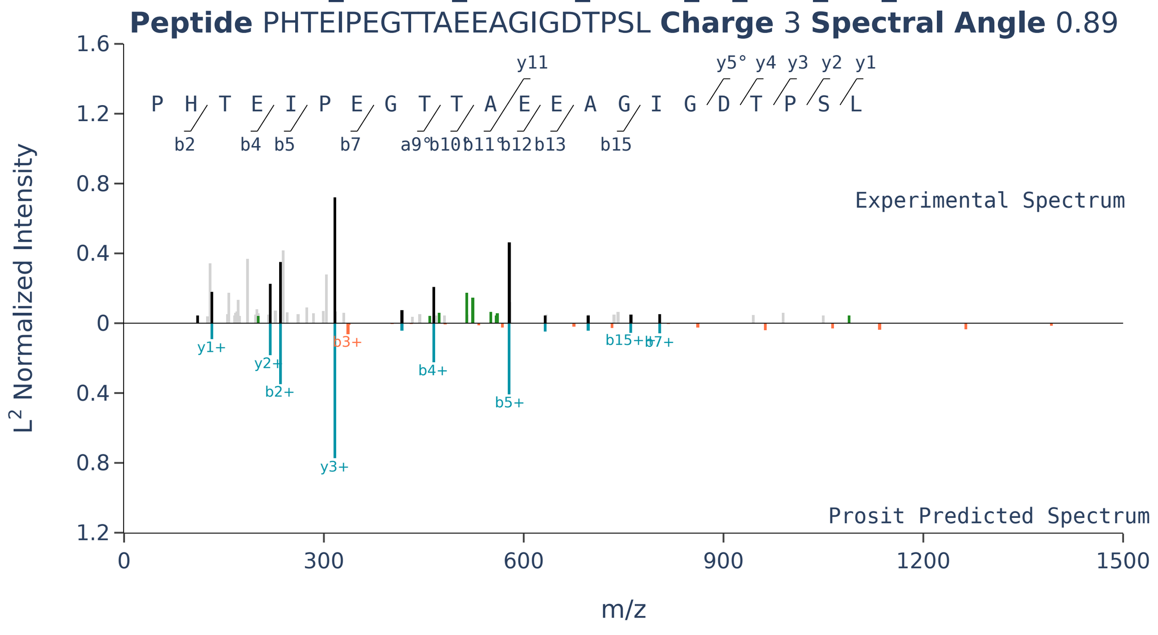


**Source** WSoh_221121_011221_HFGoe_L2_hTau_24h_R1 **Scan** 8439
**Peptide** PAKTPPAPKTPPSSGEPPKSGDRSGYSS **Charge** 4 **Spectral Angle** 0.81

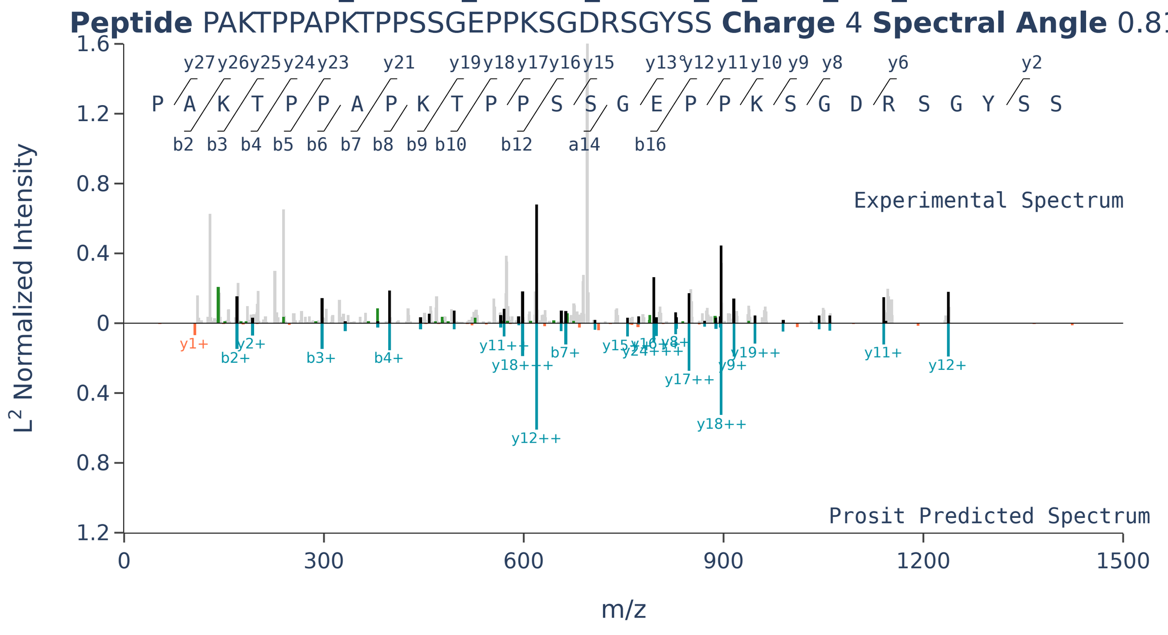


**Source** WSoh_221121_011221_HFGoe_L2_hTau_24h_R2 **Scan** 15003
**Peptide** SPLQTPTEDGSEE **Charge** 2 **Spectral Angle** 0.81

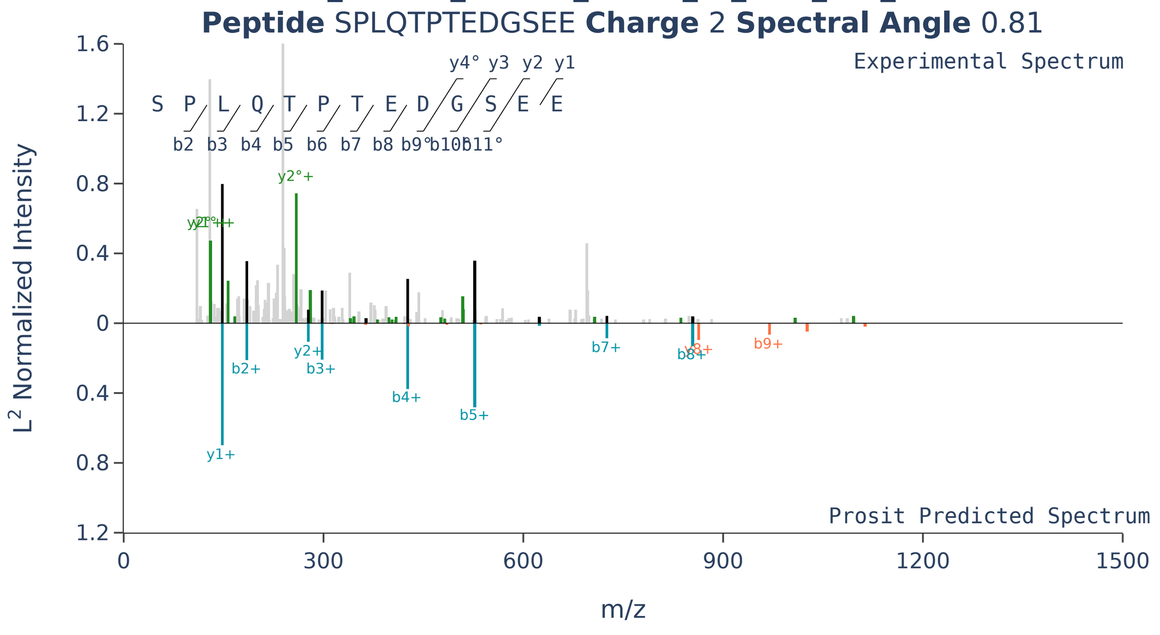


**Source** WSoh_221121_011221_HFGoe_L2_hTau_24h_R2 **Scan** 17828
**Peptide** LGSLDNITHVPGGGNKKIE **Charge** 3 **Spectral Angle** 0.85

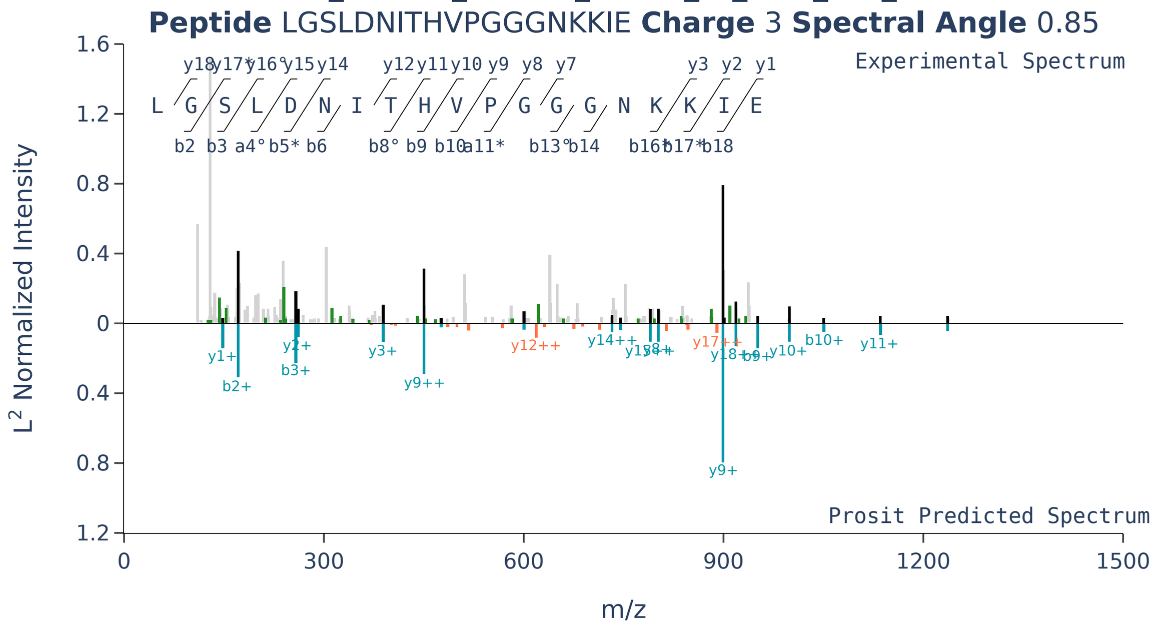


**Source** WSoh_221121_011221_HFGoe_L2_hTau_24h_R2 **Scan** 15661
**Peptide** NVQSKCGSKDNIKHVPGGGSVQIVY **Charge** 4 **Spectral Angle** 0.78

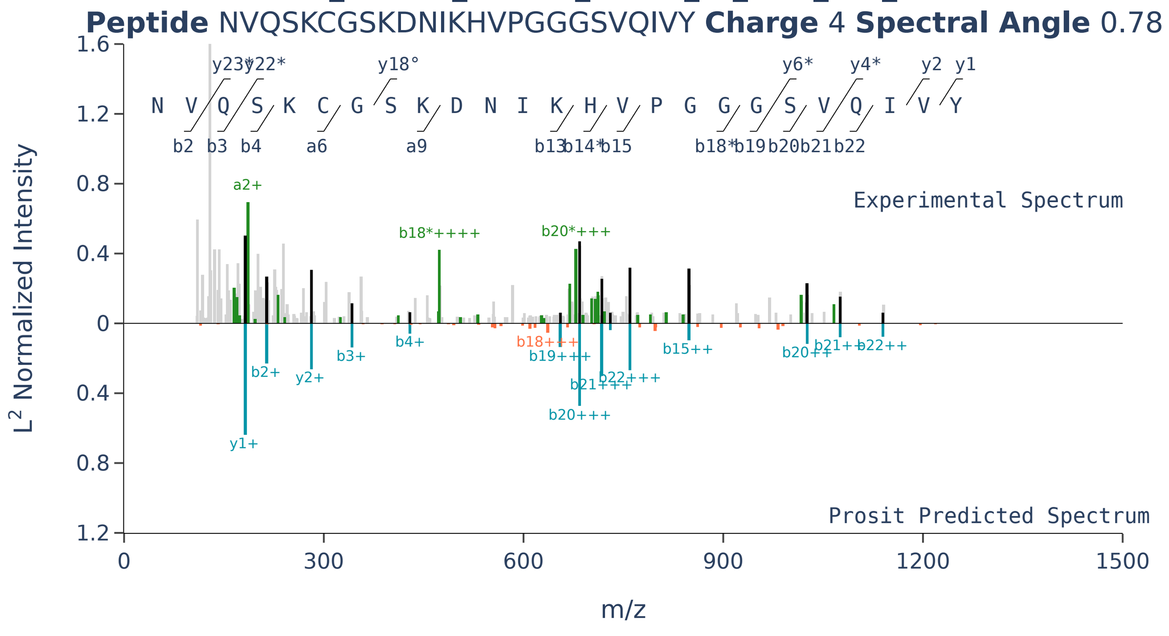


**Source** WSoh_221121_011221_HFGoe_L1_hTau_4h_R2 **Scan** 19796
**Peptide** NIKHVPGGGSVQIVYKPVDLSKVTSK **Charge** 4 **Spectral Angle** 0.78

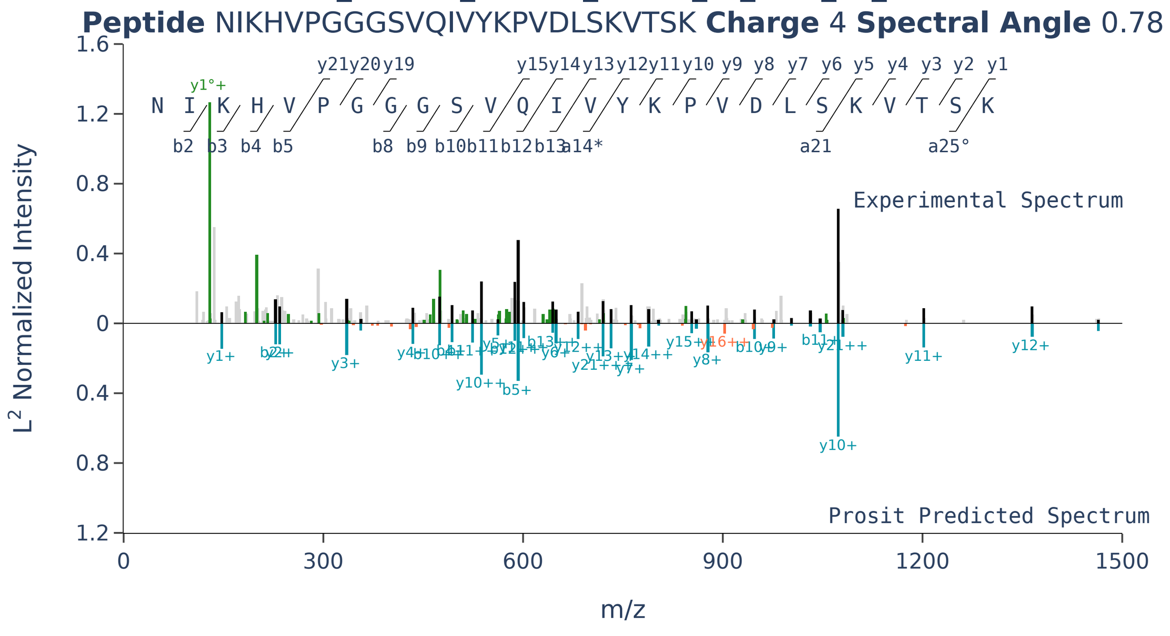


**Source** WSoh_221121_011221_HFGoe_L2_hTau_24h_R1 **Scan** 24532
**Peptide** AAAQPHTEIPEGTTAEEAGIGDTPSL **Charge** 3 **Spectral Angle** 0.83

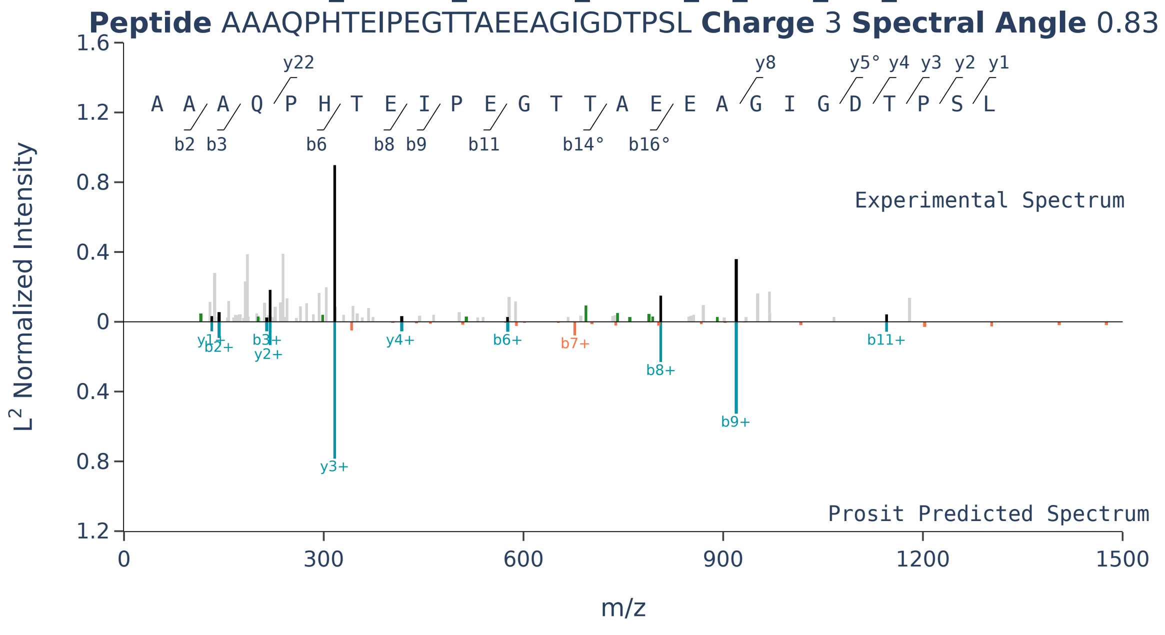


**Source** WSoh_221121_011221_HFGoe_L2_hTau_4h_R1 **Scan** 11798
**Peptide** LINKKLDLSNVQSKCGSKDNIKHV **Charge** 4 **Spectral Angle** 0.86

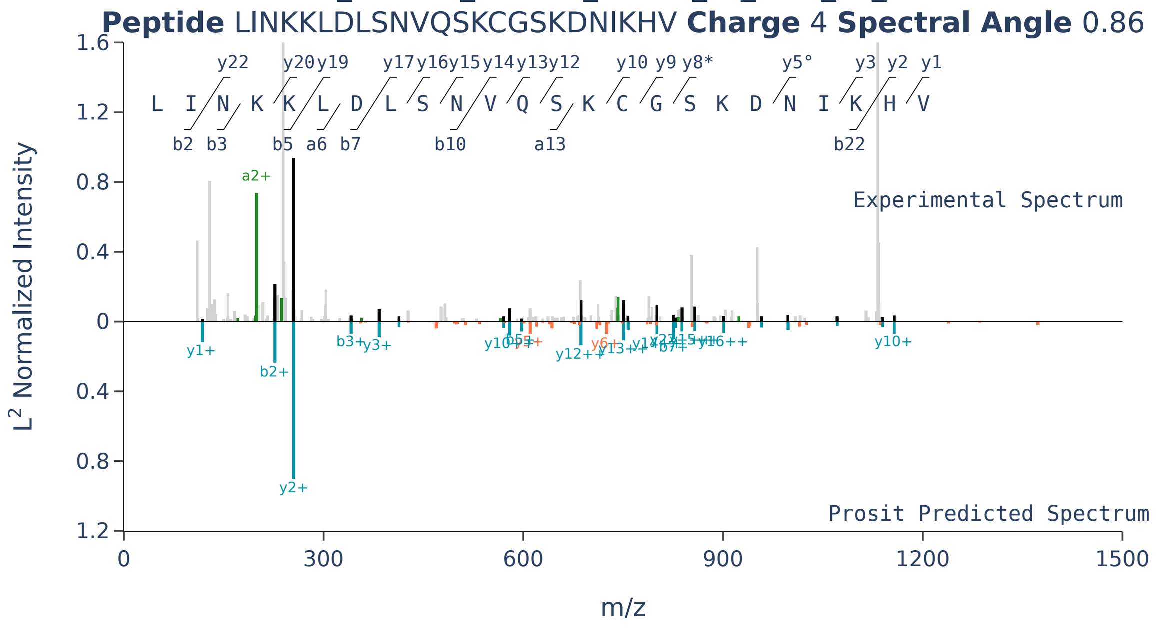


**Source** WSoh_221121_011221_HFGoe_L1_hTau_24h_R2 **Scan** 14986
**Peptide** TPSLPTPPTREPKKVAVVRTPPKSPSSA **Charge** 4 **Spectral Angle** 0.79

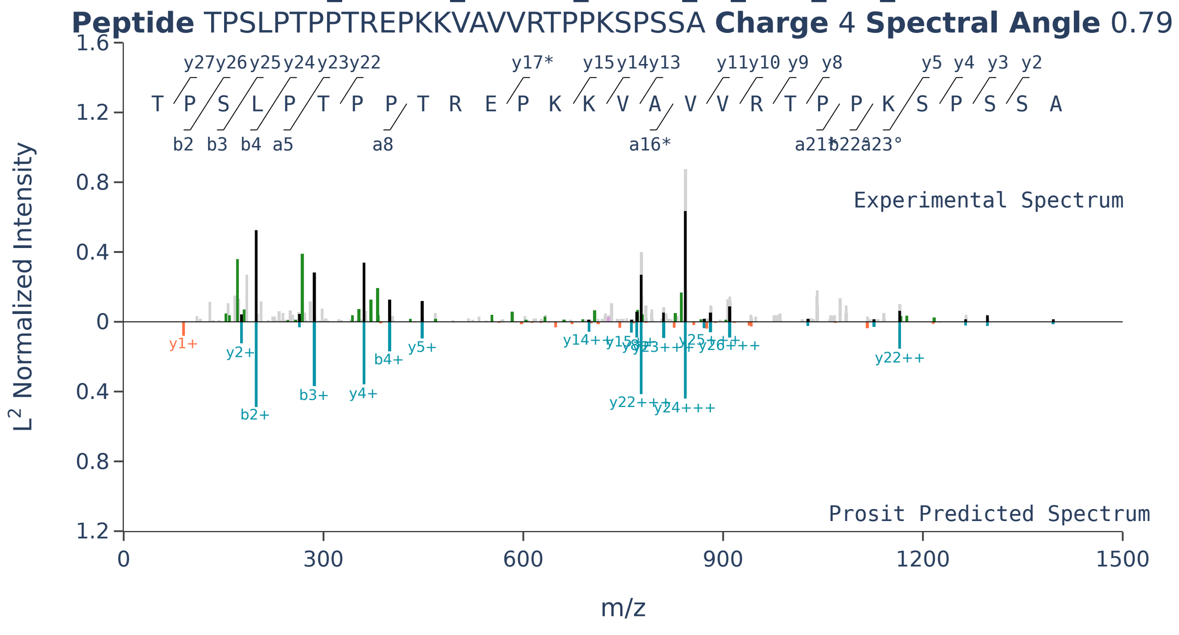


**Source** WSoh_221121_011221_HFGoe_L1_hTau_24h_R2 **Scan** 16336
**Peptide** LVYKSPVVS **Charge** 2 **Spectral Angle** 0.68

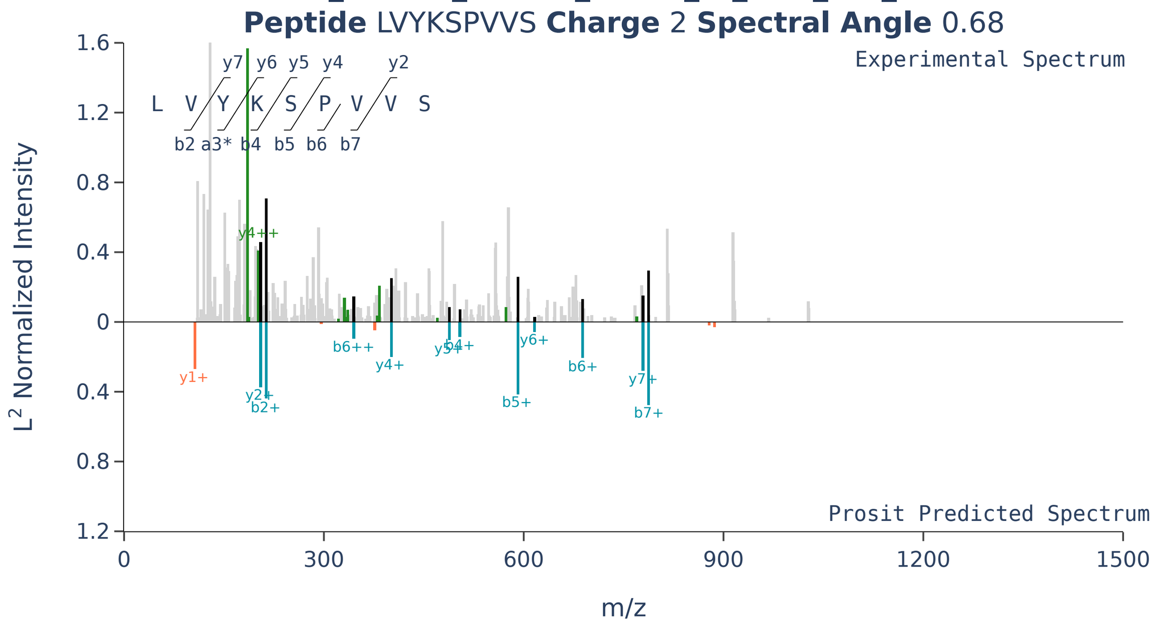


**Source** WSoh_221121_011221_HFGoe_L2_hTau_4h_R2 **Scan** 6358
**Peptide** RSGYSSPGSPGTPGSRSRT **Charge** 4 **Spectral Angle** 0.76

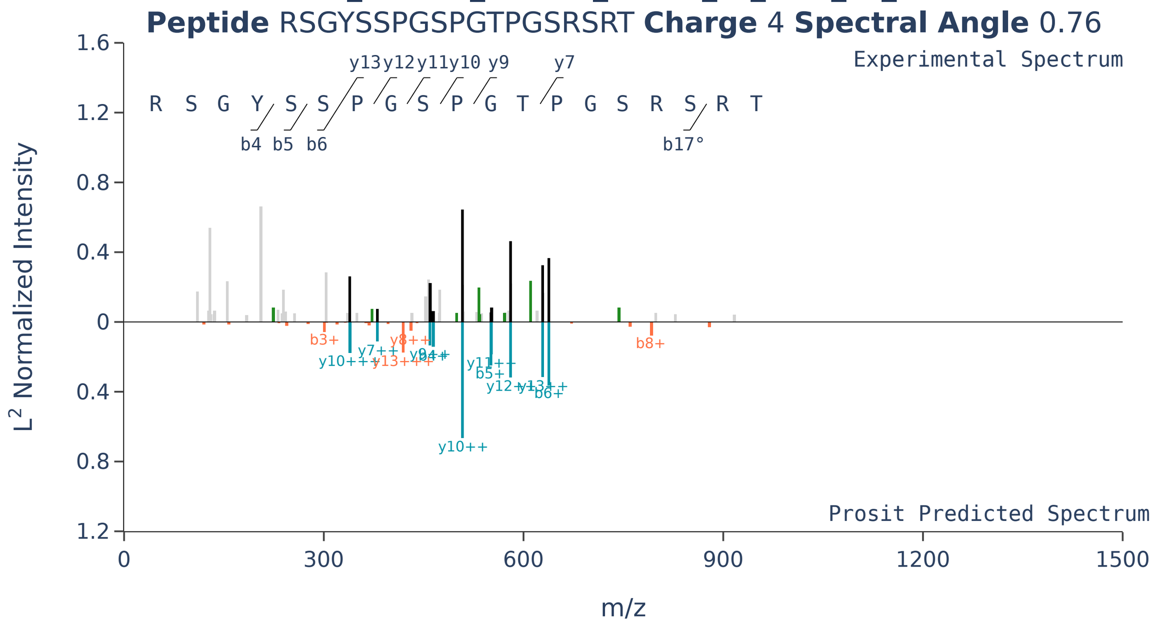


**Source** WSoh_221121_011221_HFGoe_L1_hTau_24h_R1 **Scan** 24599
**Peptide** SPSSAKSRLQTAPVPMPDL **Charge** 3 **Spectral Angle** 0.84

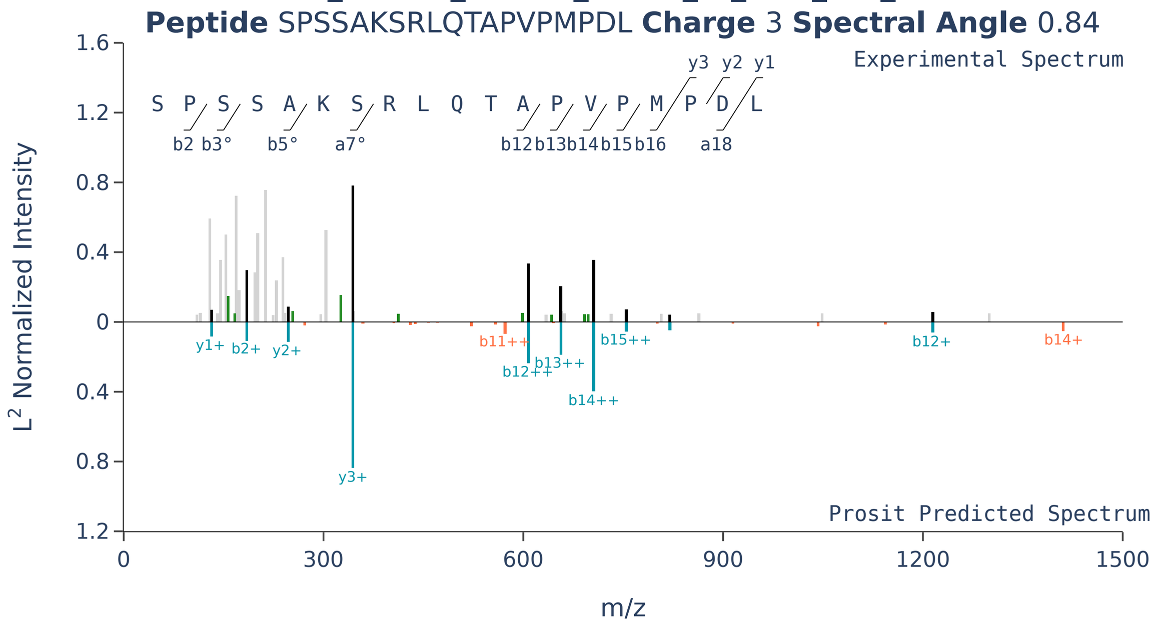

**Source** WSoh_221121_011221_HFGoe_L2_hTau_24h_R2 **Scan** 16723
**Peptide** KLGSLDNITHVPGGGNKKIETHKLTFREN **Charge** 5 **Spectral Angle** 0.81

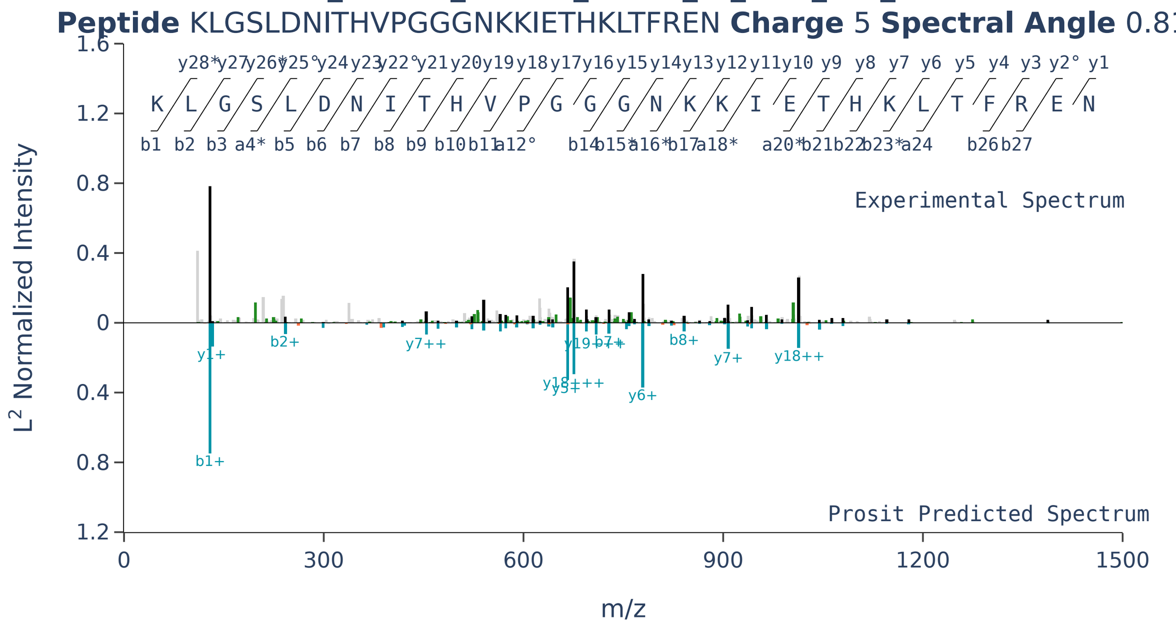


**Source** WSoh_221121_011221_HFGoe_L1_hTau_24h_R1 **Scan** 10406
**Peptide** IPAKTPPAPKTPPSSGEPPKSGD **Charge** 4 **Spectral Angle** 0.9

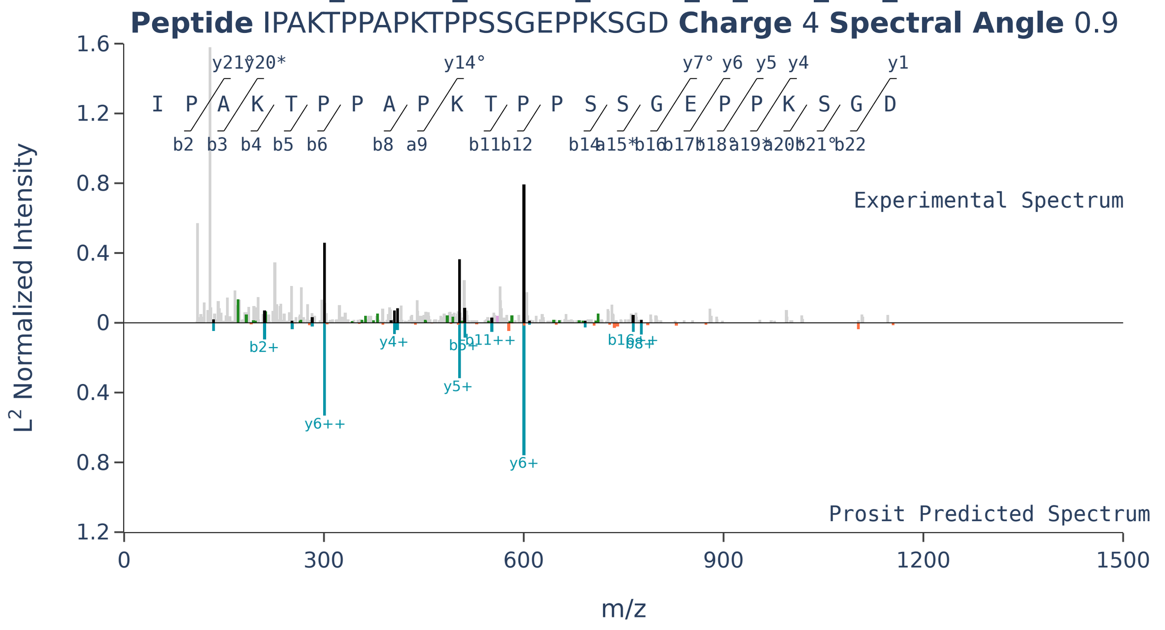


**Source** WSoh_221121_011221_HFGoe_L1_hTau_4h_R1 **Scan** 9814
**Peptide** INKKLDLS **Charge** 2 **Spectral Angle** 0.72

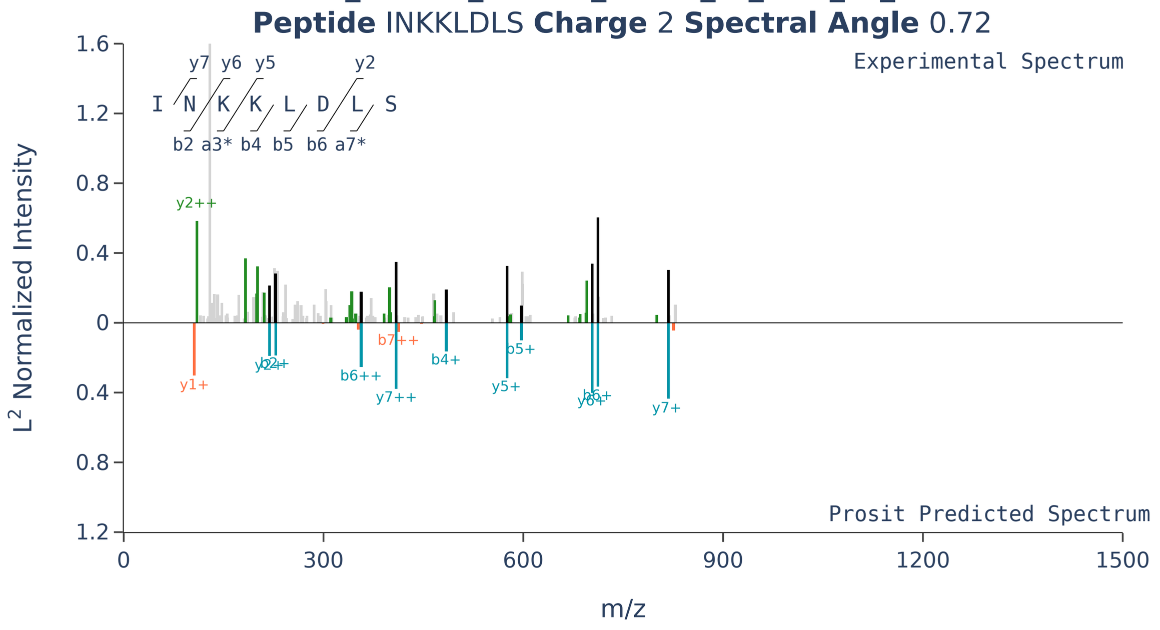


**Source** WSoh_221121_011221_HFGoe_L1_hTau_2h_R2 **Scan** 14157
**Peptide** VDSPQLA **Charge** 1 **Spectral Angle** 0.74

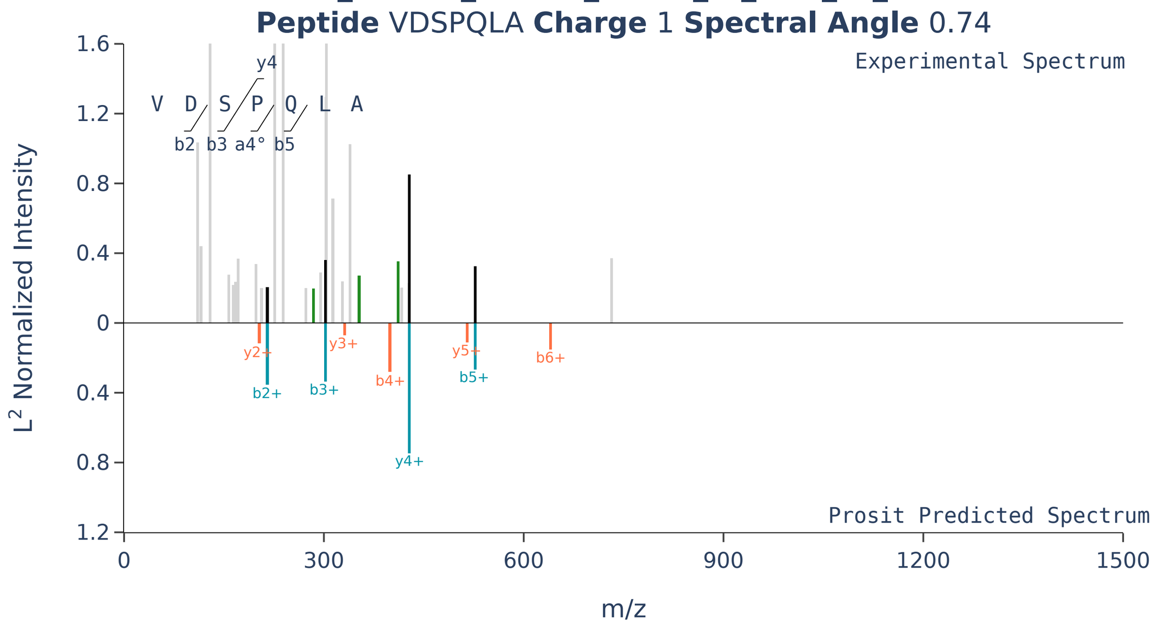


**Source** WSoh_221121_011221_HFGoe_L1_hTau_24h_R1 **Scan** 13067
**Peptide** QLINKKLDLSNVQSKCGSKDNIKHV **Charge** 4 **Spectral Angle** 0.86

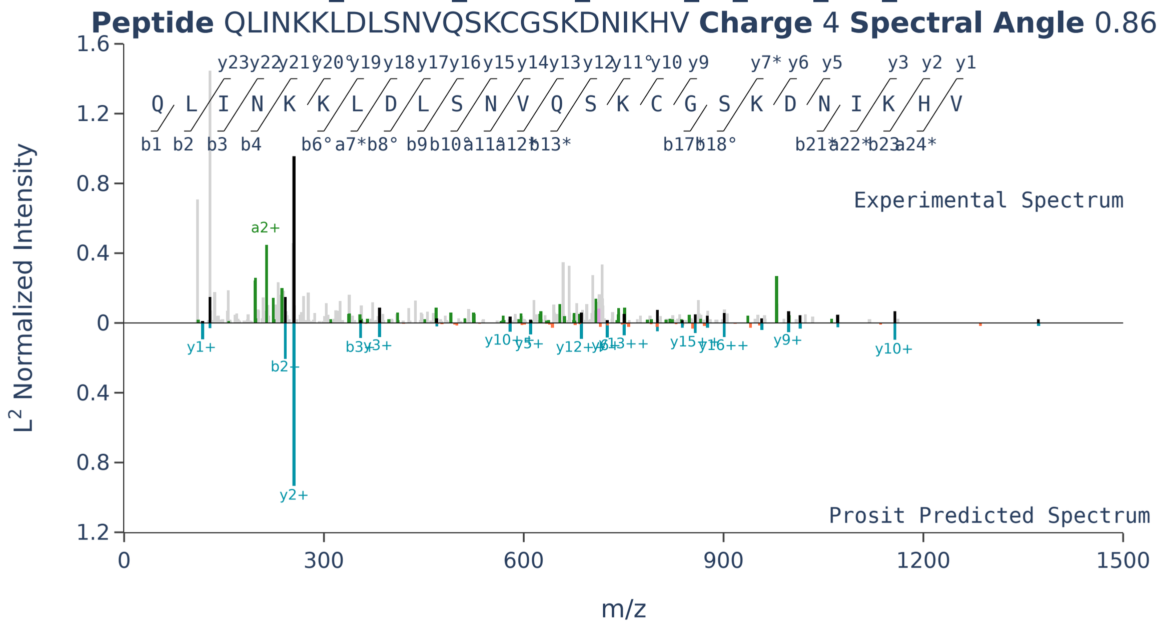


**Source** WSoh_221121_011221_HFGoe_L1_hTau_24h_R1 **Scan** 17616
**Peptide** GGGKVQIINKKLDL **Charge** 3 **Spectral Angle** 0.78

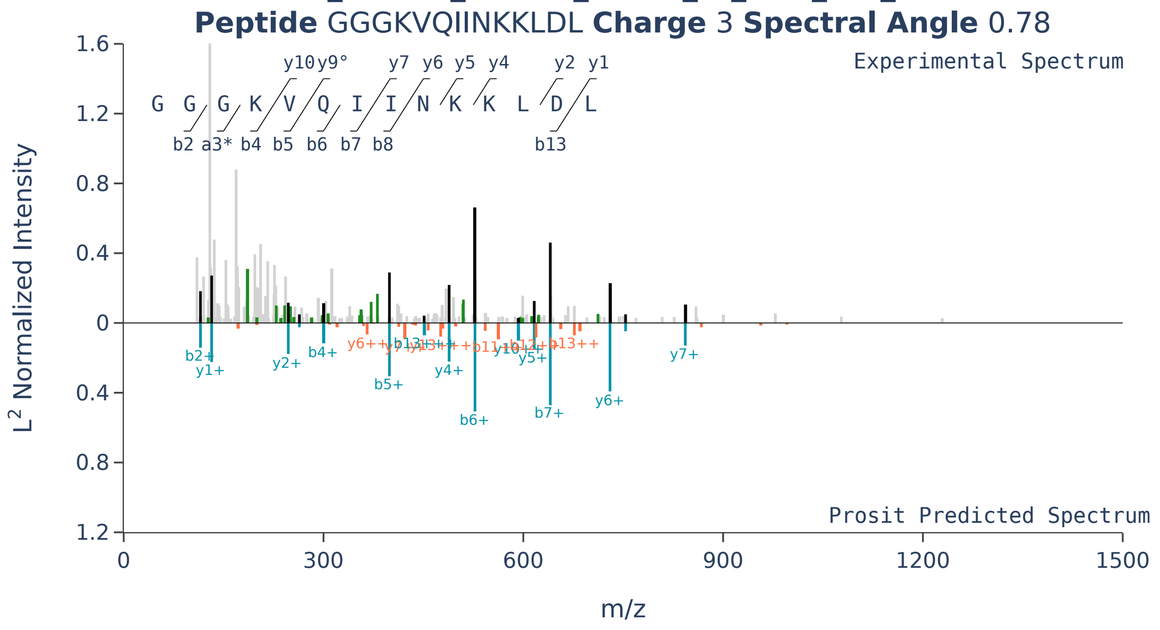


**Source** WSoh_221121_011221_HFGoe_L2_hTau_24h_R1 **Scan** 11684
**Peptide** EDHAGTYGLGDRKDQGGYTMHQDQEGD **Charge** 5 **Spectral Angle** 0.8

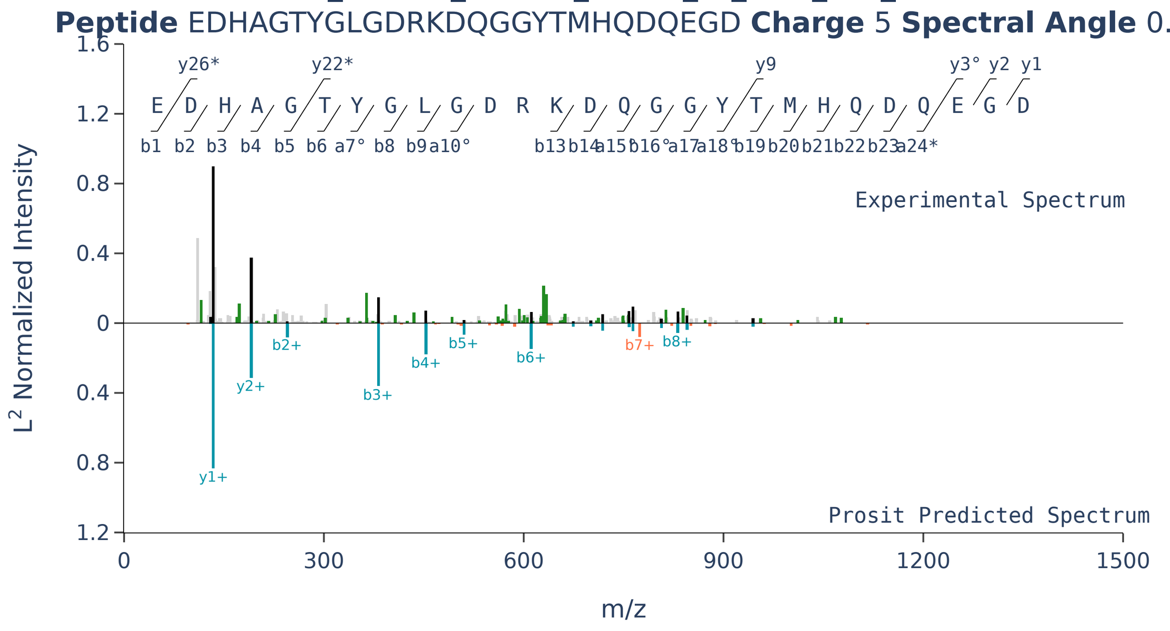


**Source** WSoh_221121_011221_HFGoe_L2_hTau_24h_R1 **Scan** 16301
**Peptide** PGGGKVQIINKKLDLSNVQSK **Charge** 5 **Spectral Angle** 0.73

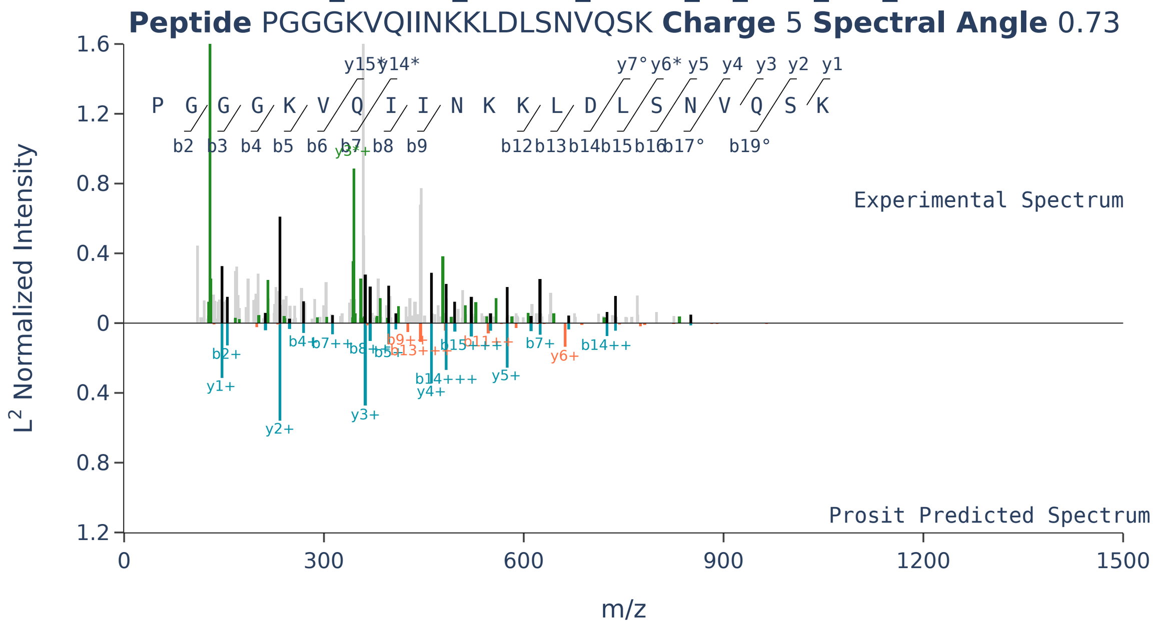


**Source** WSoh_221121_011221_HFGoe_L2_hTau_24h_R1 **Scan** 13829
**Peptide** EGAPGKQAAAQPHTEIPEGTTAEEA **Charge** 3 **Spectral Angle** 0.75

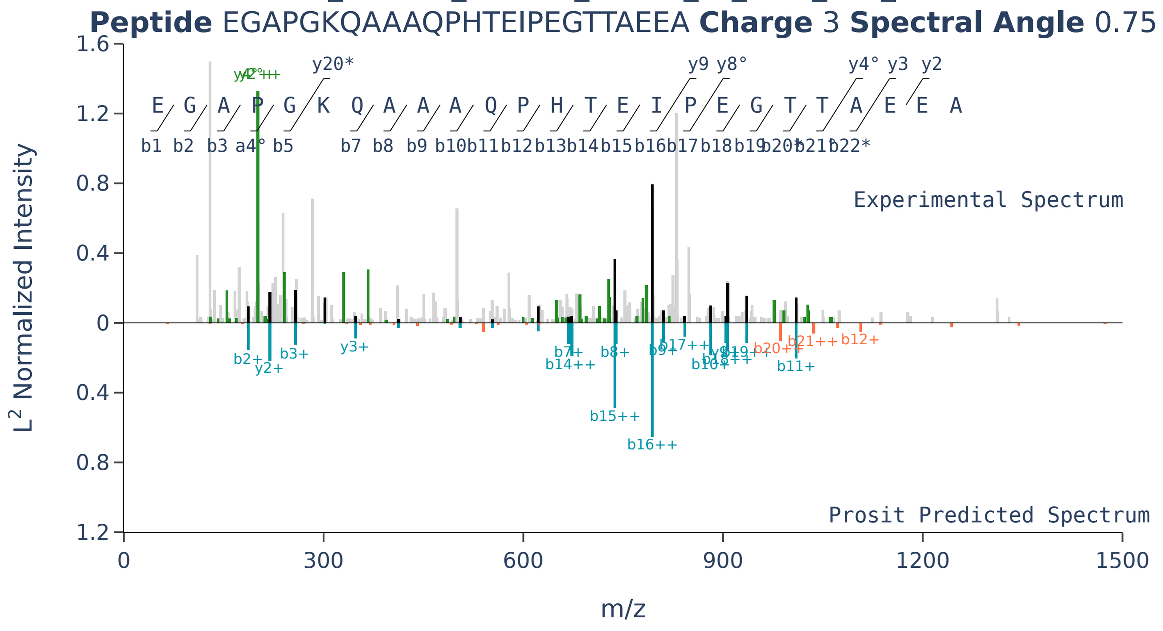


**Source** WSoh_221121_011221_HFGoe_L1_hTau_24h_R2 **Scan** 7027
**Peptide** KGADGKTKIATPRGAAPPGQKGQA **Charge** 4 **Spectral Angle** 0.84

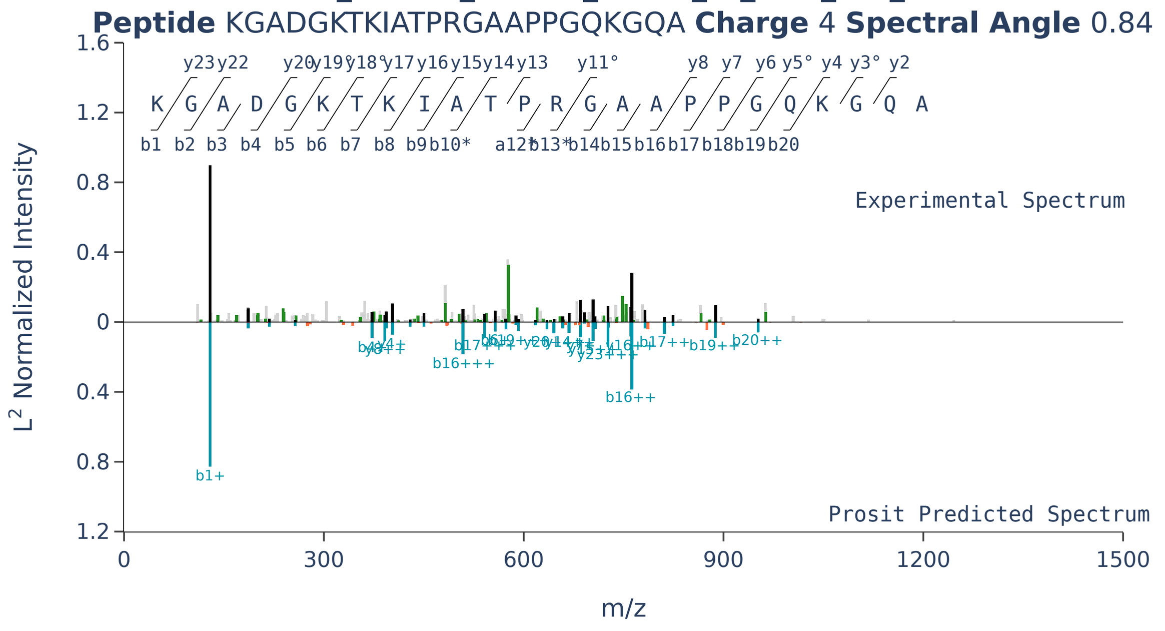


**Source** WSoh_221121_011221_HFGoe_L1_hTau_24h_R2 **Scan** 17583
**Peptide** IGDTPSLEDEAAGHVTQAR **Charge** 3 **Spectral Angle** 0.75

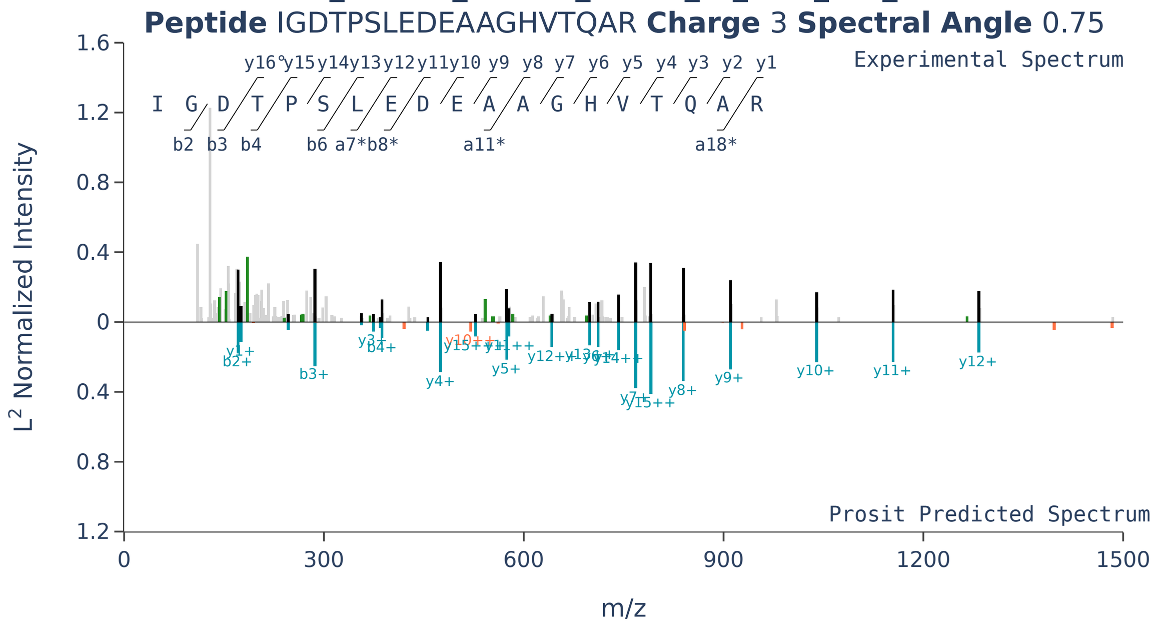


**Source** WSoh_221121_011221_HFGoe_L1_hTau_24h_R2 **Scan** 9732
**Peptide** KLATPRGAAPPGQKGQANATRI **Charge** 4 **Spectral Angle** 0.7

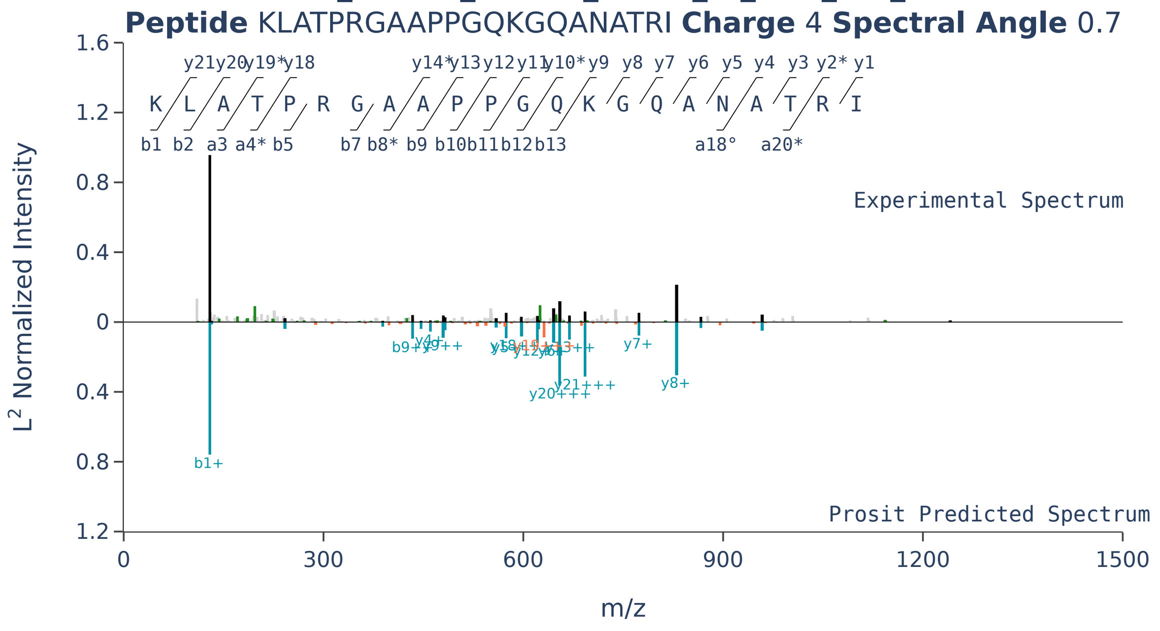


**Source** WSoh_221121_011221_HFGoe_L2_hTau_2h_R1 **Scan** 16874
**Peptide** AEPRQEFEVMEDHAG **Charge** 3 **Spectral Angle** 0.73

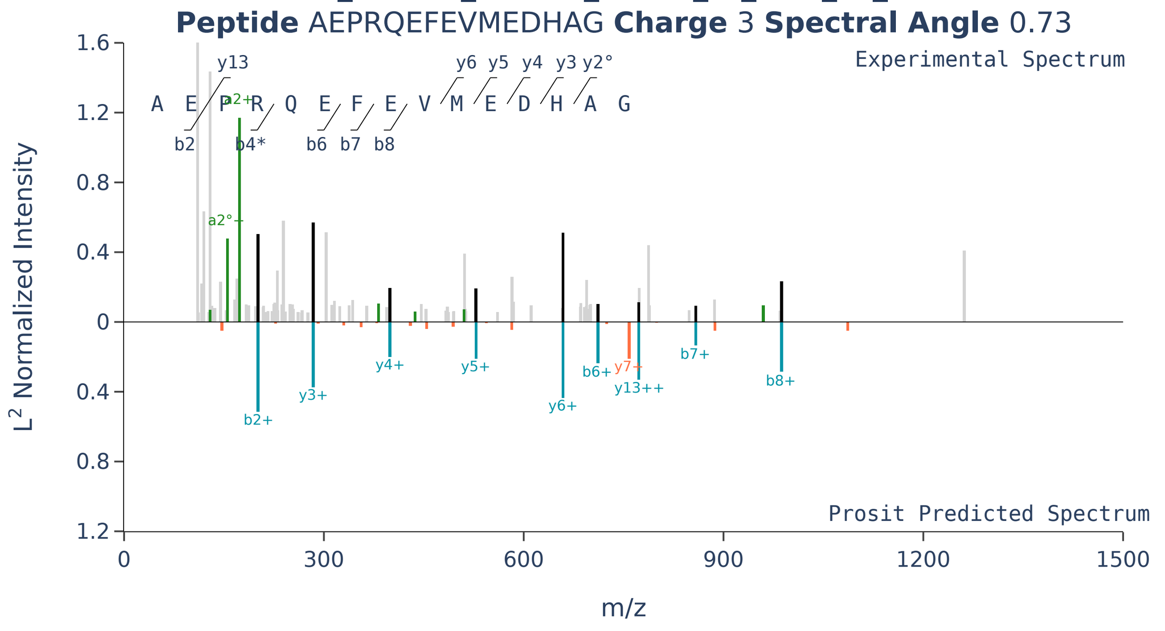


**Source** WSoh_221121_011221_HFGoe_L2_hTau_24h_R2 **Scan** 7056
**Peptide** GKTKIATPRGAAPPGQKGQANA **Charge** 4 **Spectral Angle** 0.89

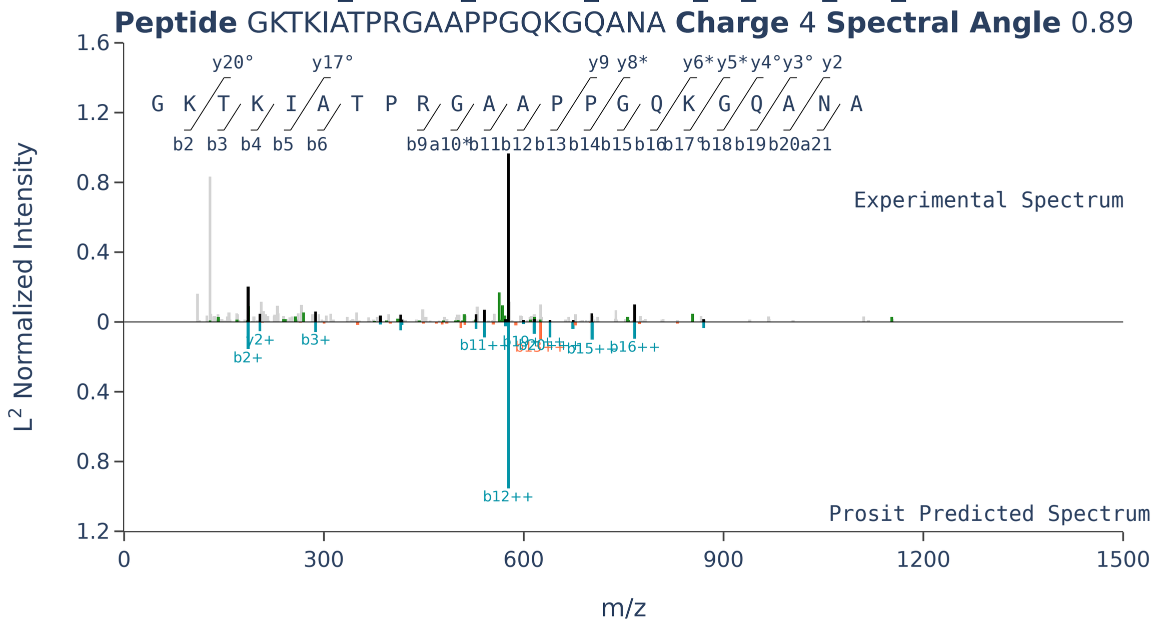


**Source** WSoh_221121_011221_HFGoe_L1_hTau_24h_R2 **Scan** 19723
**Peptide** QPHTEIPEGTTAEEAGI **Charge** 2 **Spectral Angle** 0.77

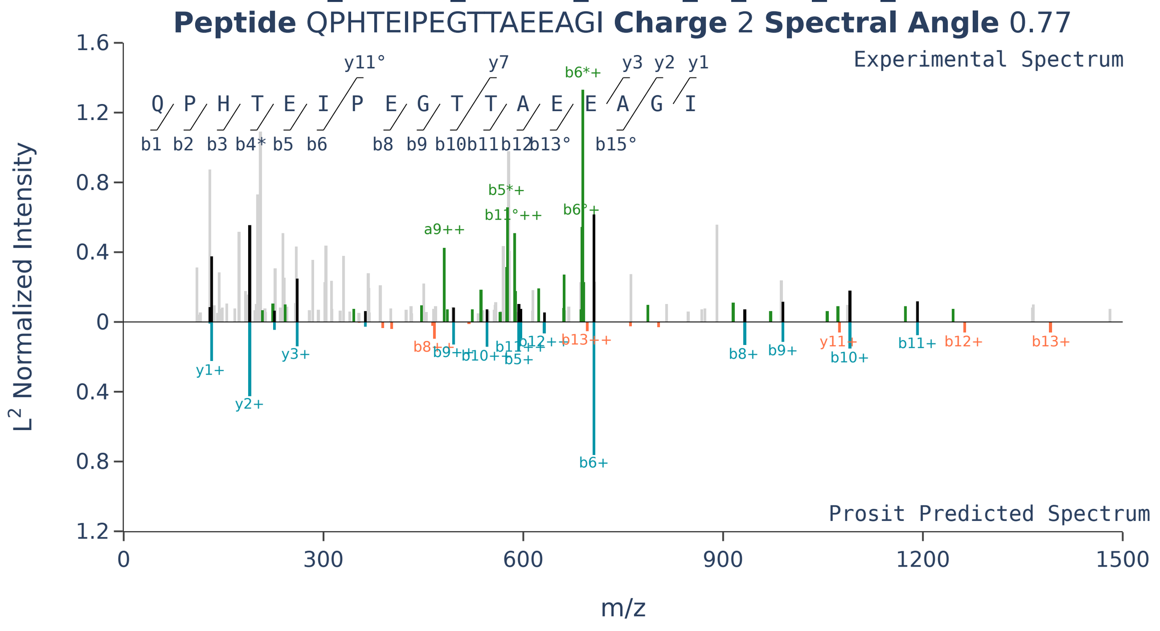

**Source** WSoh_221121_011221_HFGoe_L1_hTau_24h_R1 **Scan** 13327
**Peptide** HQPGGGKVQIL **Charge** 2 **Spectral Angle** 0.87

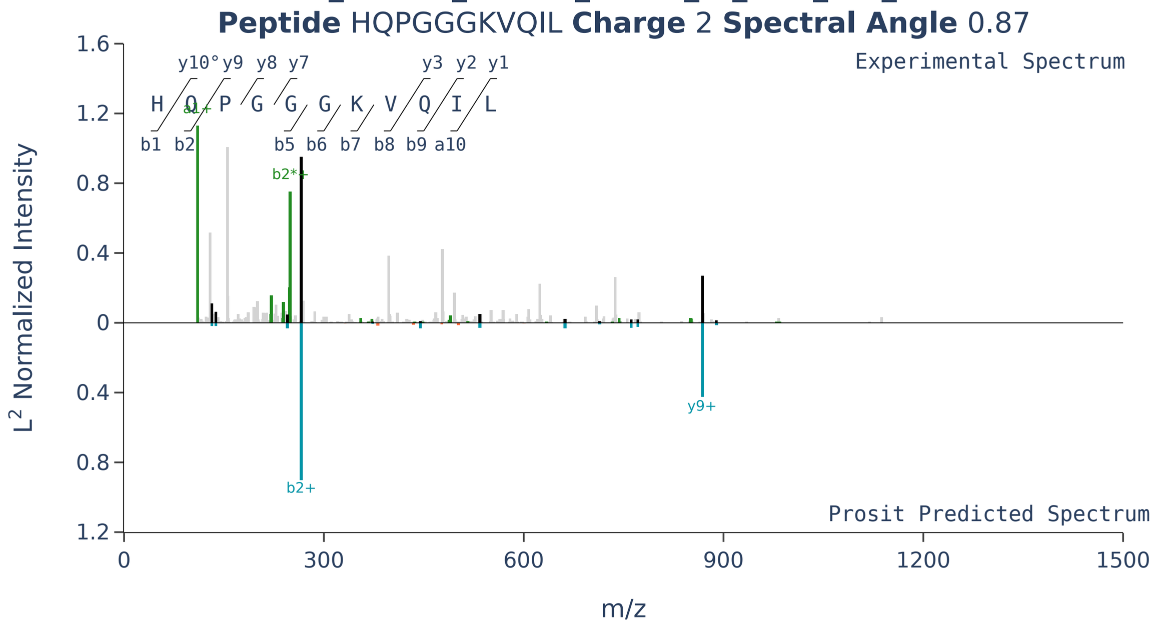


**Source** WSoh_221121_011221_HFGoe_L1_hTau_24h_R2 **Scan** 11562
**Peptide** DHAGTYGLGDRKDQGGYTMHQ **Charge** 4 **Spectral Angle** 0.88

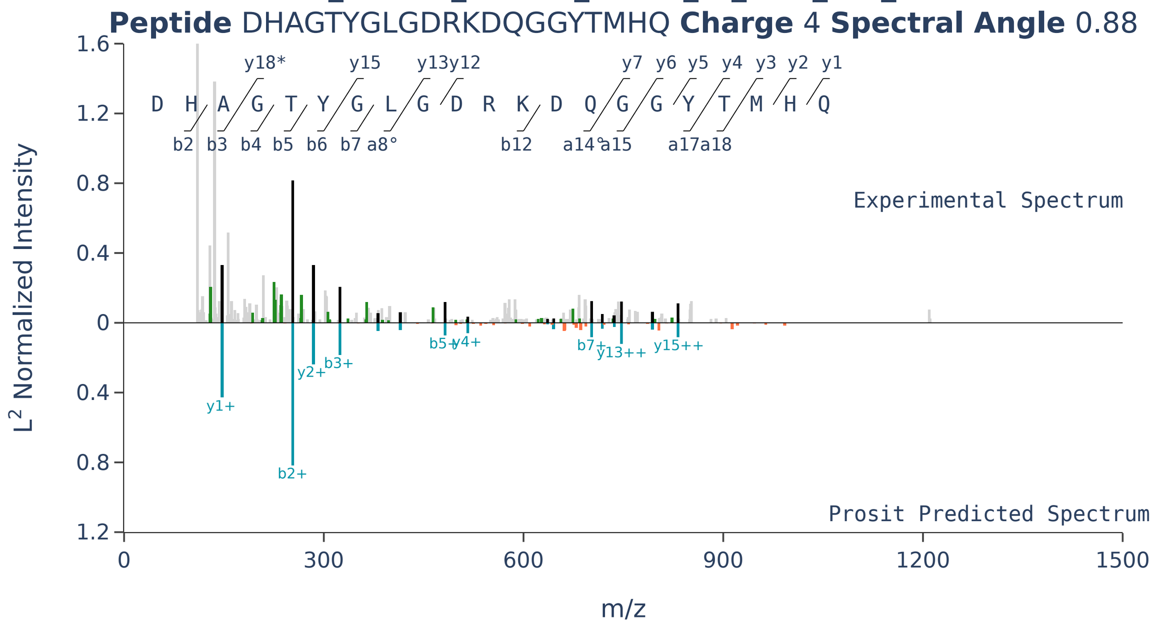


**Source** WSoh_221121_011221_HFGoe_L1_hTau_24h_R1 **Scan** 11914
**Peptide** LGNIHHKPGGGQVEVKSEKLDFKDRVQS **Charge** 5 **Spectral Angle** 0.73

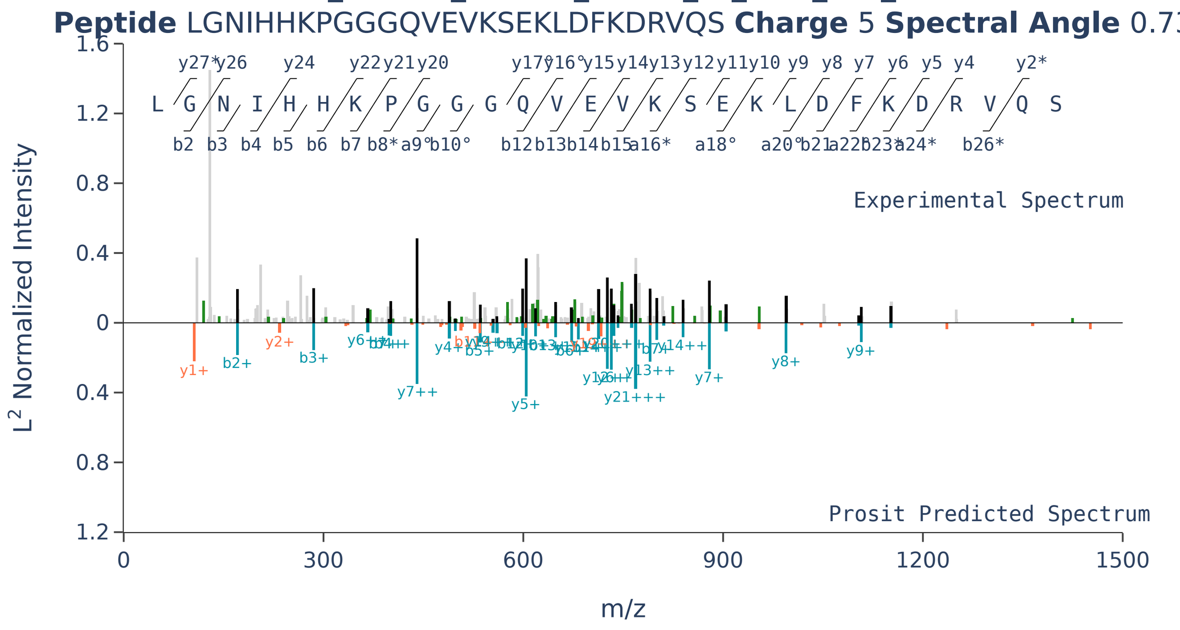


**Source** WSoh_221121_011221_HFGoe_L1_hTau_24h_R2 **Scan** 14118
**Peptide** RTPSLPTPPTREPKKVAVVRT **Charge** 5 **Spectral Angle** 0.79

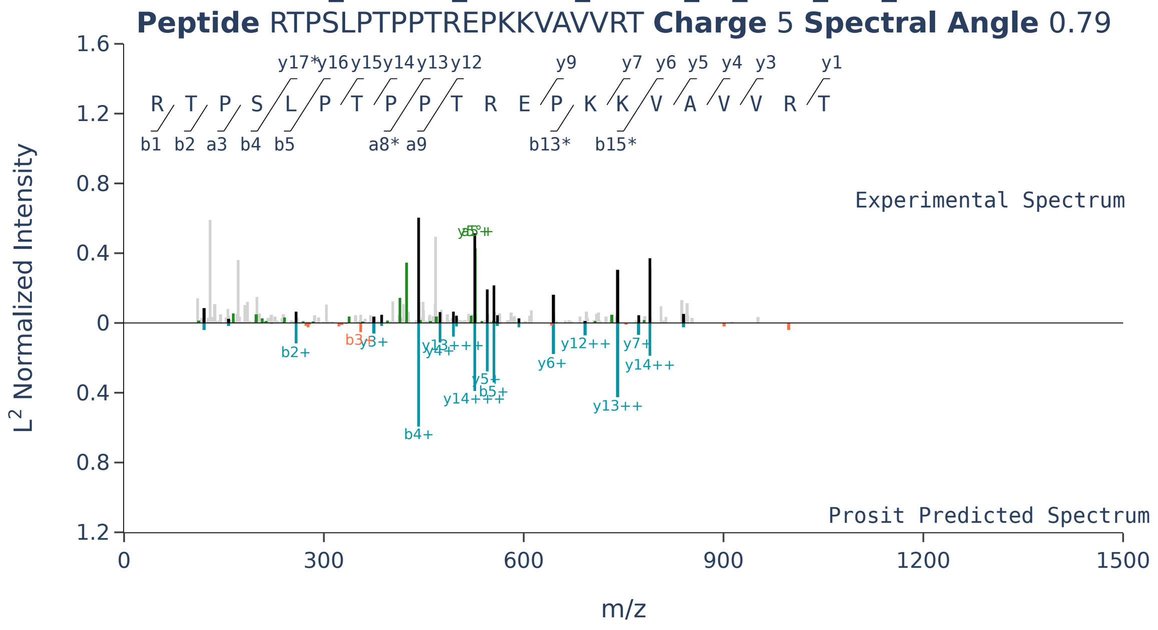


**Source** WSoh_221121_011221_HFGoe_L2_hTau_24h_R2 **Scan** 18656
**Peptide** EAGIGDTPSLEDEAAGHVTQARMVSKSKD **Charge** 4 **Spectral Angle** 0.77

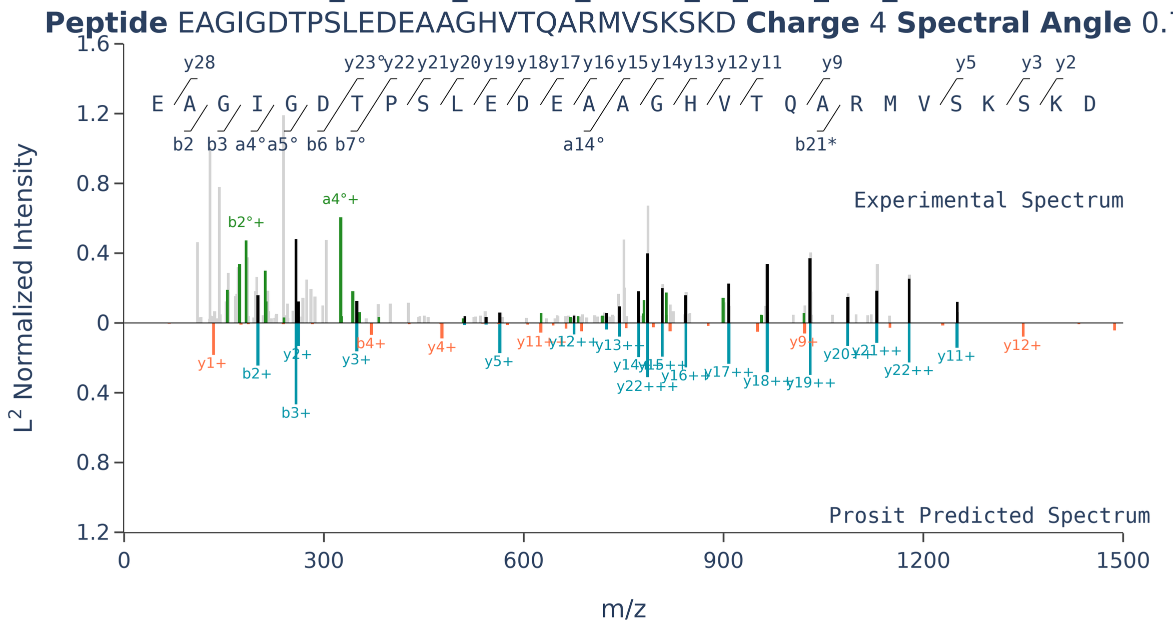


**Source** WSoh_221121_011221_HFGoe_L1_hTau_4h_R1 **Scan** 14407
**Peptide** KIGSLDNITHVPGGGNKKIETHKLT **Charge** 4 **Spectral Angle** 0.87

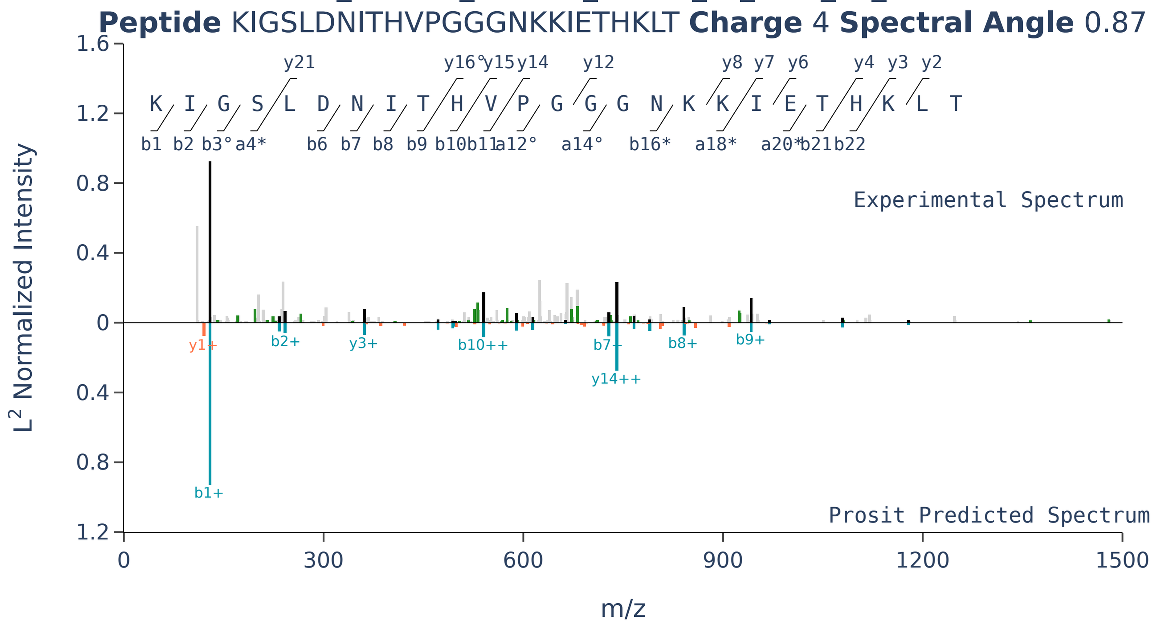


**Source** WSoh_221121_011221_HFGoe_L2_hTau_2h_R2 **Scan** 11160
**Peptide** QSKCGSKDNIKHVPGGGSVQI **Charge** 2 **Spectral Angle** 0.86

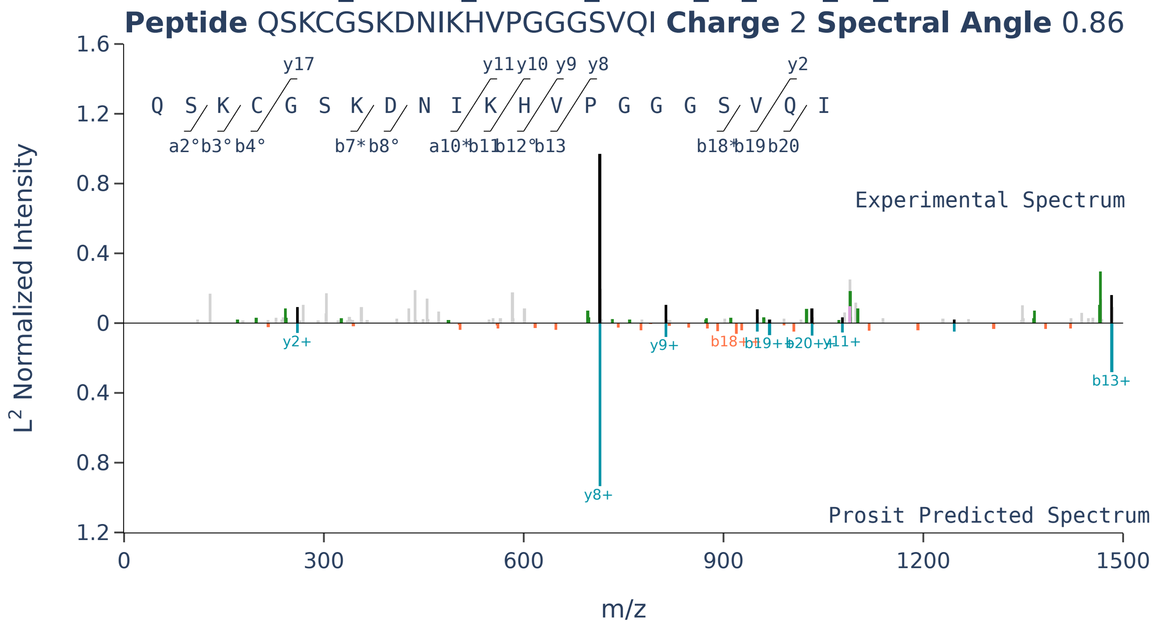


**Source** WSoh_221121_011221_HFGoe_L1_hTau_1h_R1 **Scan** 11976
**Peptide** DEGAPGKQAAAQPHTEIPEG **Charge** 3 **Spectral Angle** 0.83

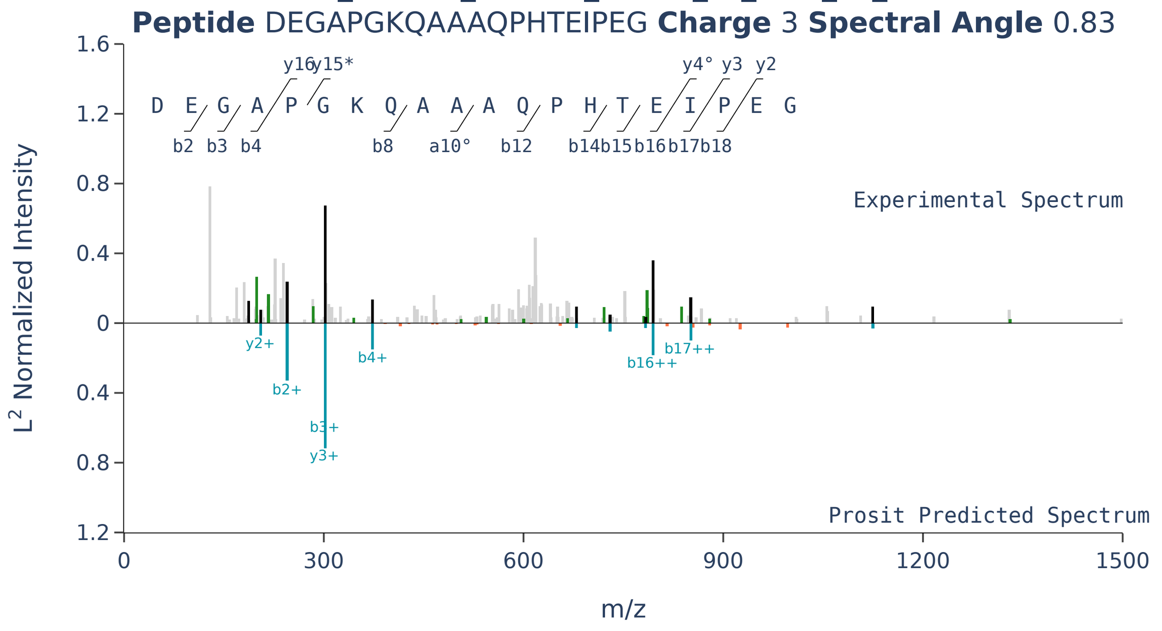


**Source** WSoh_221121_011221_HFGoe_L2_hTau_4h_R1 **Scan** 21458
**Peptide** DVTAPLVDEGAPGKQAAAQPHTEI **Charge** 3 **Spectral Angle** 0.81

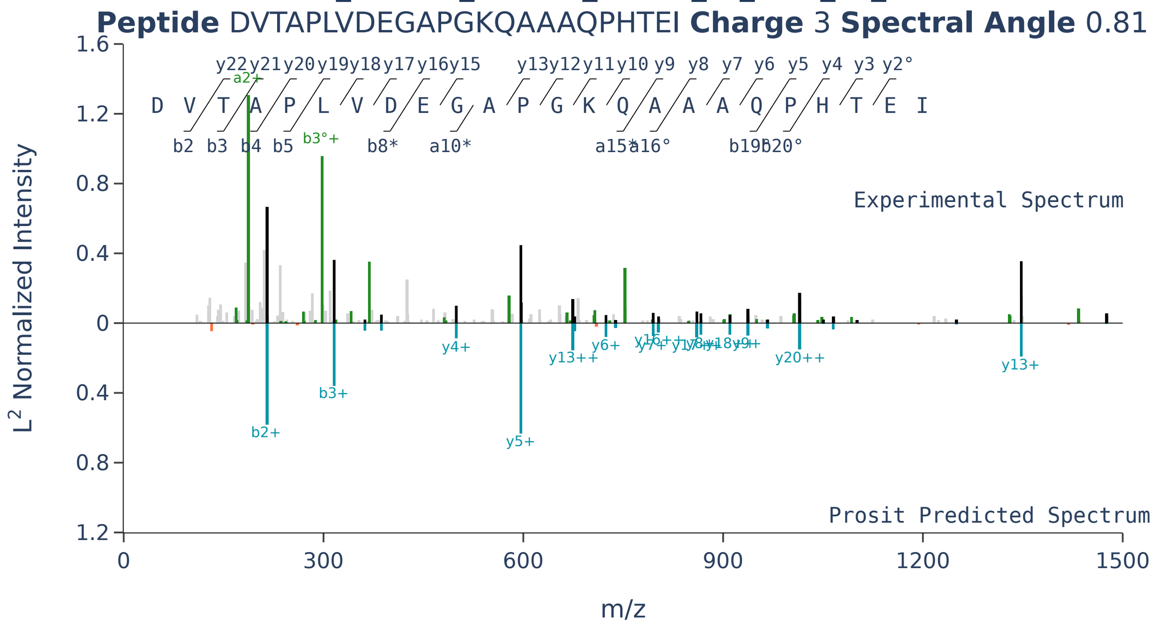


**Source** WSoh_221121_011221_HFGoe_L1_hTau_24h_R1 **Scan** 5988
**Peptide** DGTGSDDKKAKGADGKTKIATPRGA **Charge** 3 **Spectral Angle** 0.8

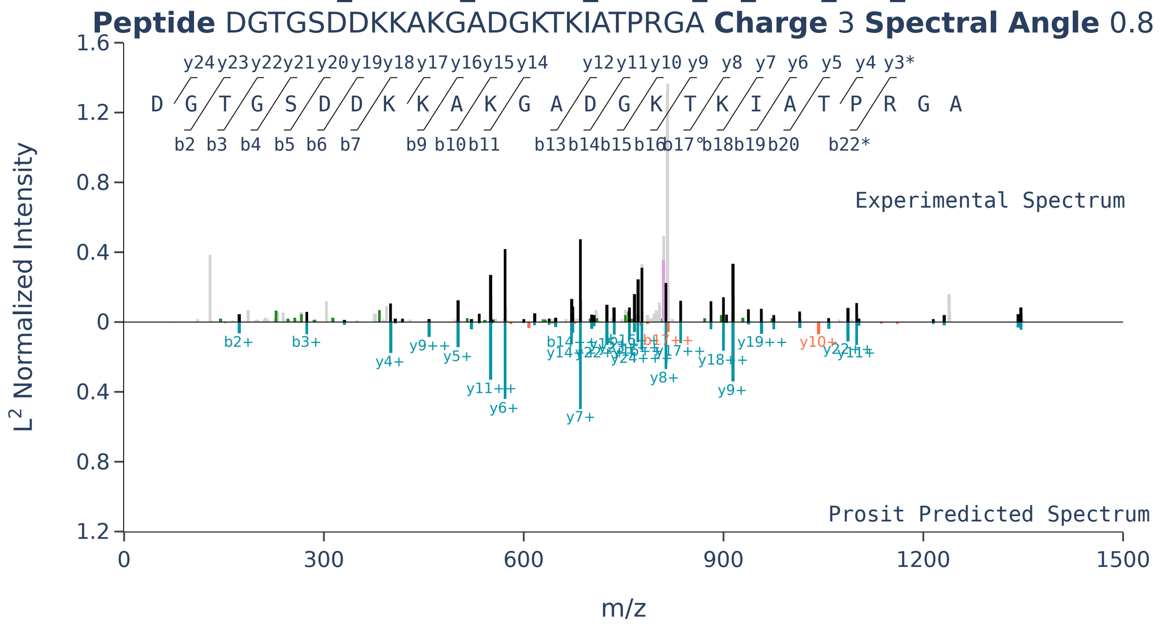


**Source** WSoh_221121_011221_HFGoe_L2_hTau_4h_R2 **Scan** 11973
**Peptide** HHKPGGGQVEVKSEKLDF **Charge** 4 **Spectral Angle** 0.78

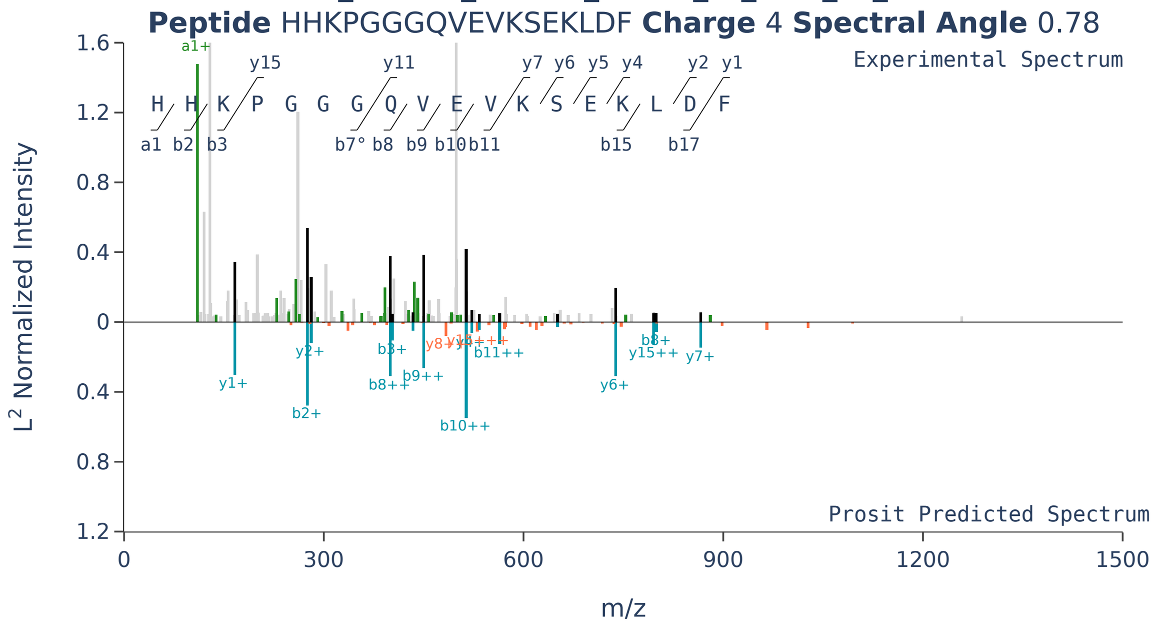


**Source** WSoh_221121_011221_HFGoe_L2_hTau_24h_R1 **Scan** 9718
**Peptide** VAVVRTPPKSPSSAKSRL **Charge** 4 **Spectral Angle** 0.74

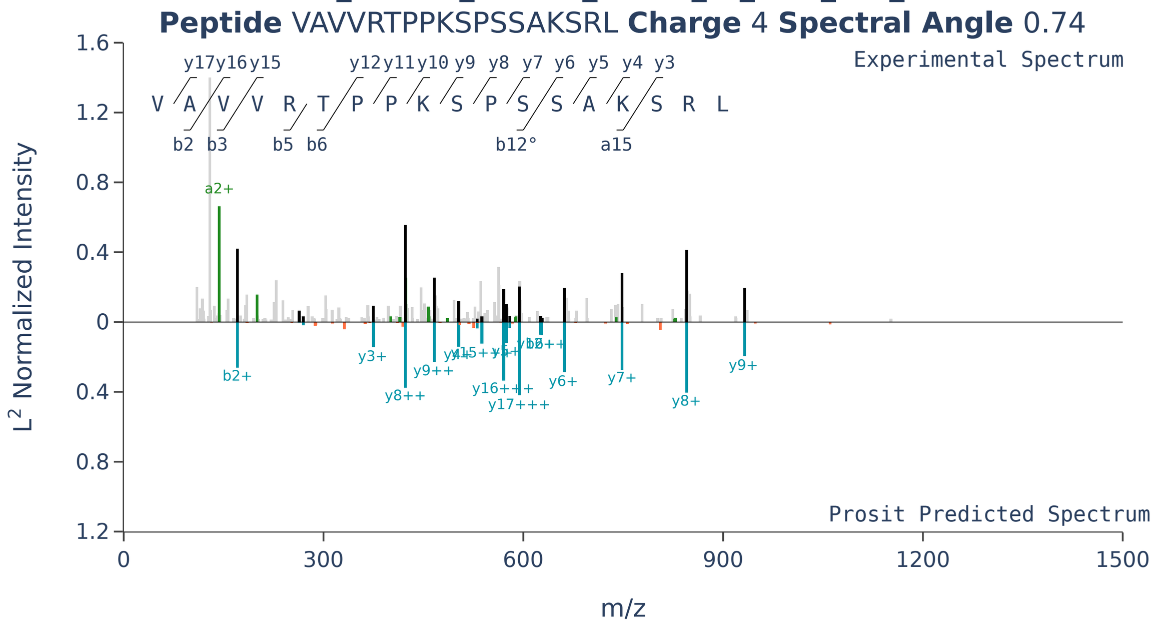


**Source** WSoh_221121_011221_HFGoe_L1_hTau_24h_R1 **Scan** 16562
**Peptide** VVRTPPKSPSSAKSRLQTAPVPMPDLKN **Charge** 5 **Spectral Angle** 0.77

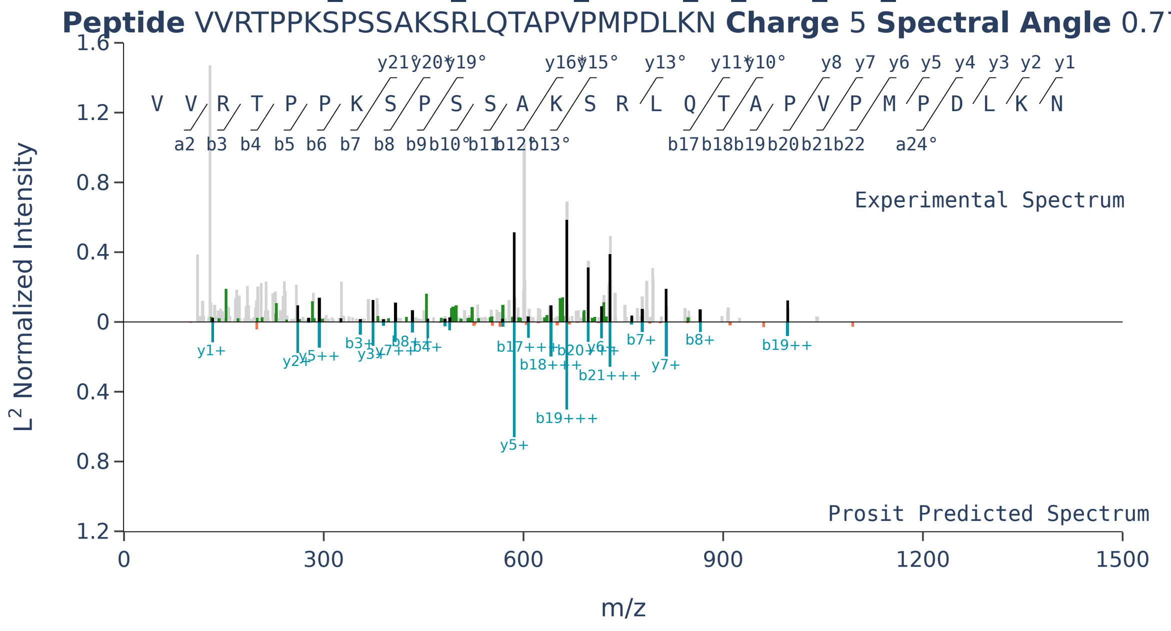


**Source** WSoh_221121_011221_HFGoe_L2_hTau_4h_R2 **Scan** 13606
**Peptide** VYKSPVVSGDTSPRHLSNVSSTG **Charge** 2 **Spectral Angle** 0.66

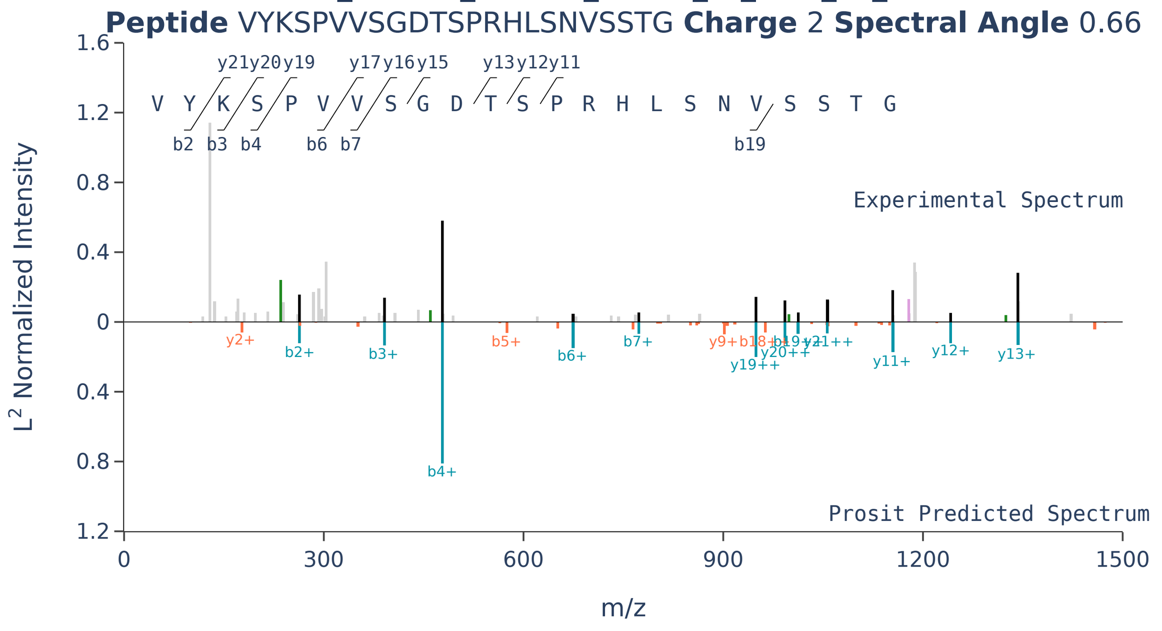


**Source** WSoh_221121_011221_HFGoe_L1_hTau_24h_R2 **Scan** 10948
**Peptide** KPGGGQVEVKSEKLDFKDR **Charge** 4 **Spectral Angle** 0.69

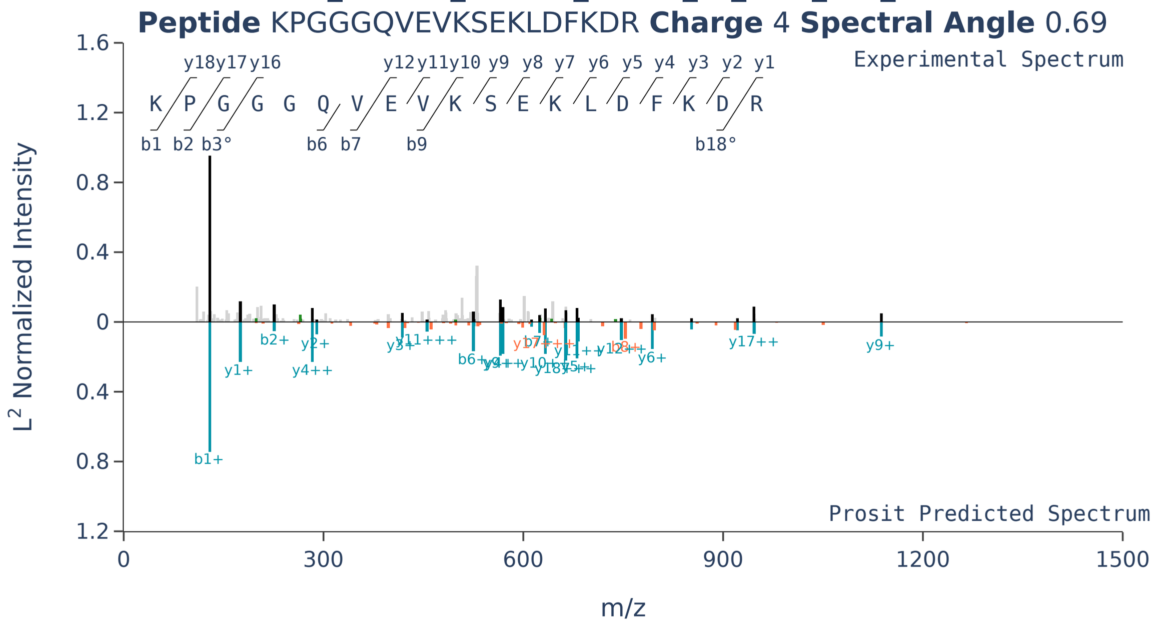

**Source** WSoh_221121_011221_HFGoe_L1_hTau_24h_R2 **Scan** 6154
**Peptide** AGHVTQARMVSK **Charge** 3 **Spectral Angle** 0.8

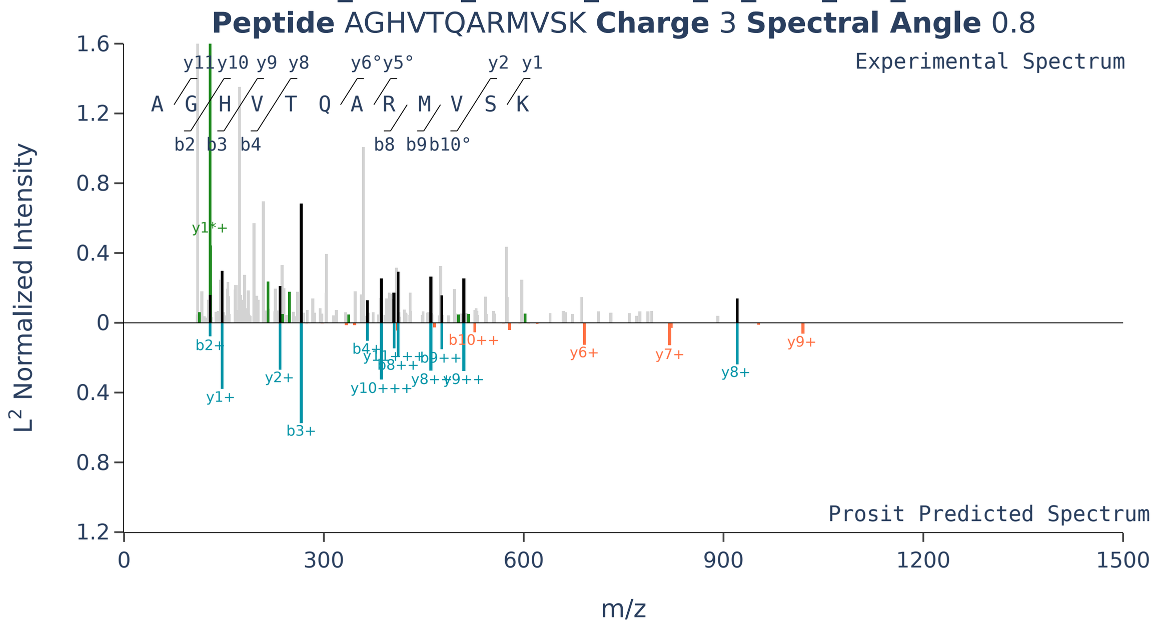


**Source** WSoh_221121_011221_HFGoe_L2_hTau_24h_R1 **Scan** 11614
**Peptide** YGLGDRKDQGGYTMHQDQEGD **Charge** 4 **Spectral Angle** 0.81

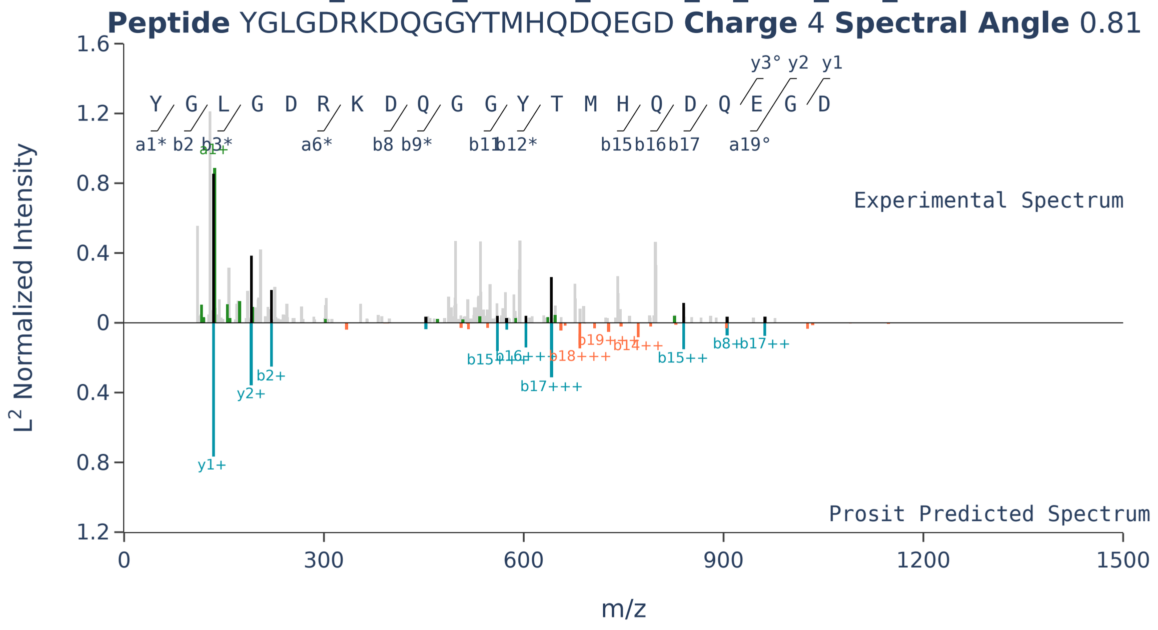


**Source** WSoh_221121_011221_HFGoe_L1_hTau_4h_R1 **Scan** 23423
**Peptide** PRQEFEVMEDHAGTYGL **Charge** 3 **Spectral Angle** 0.77

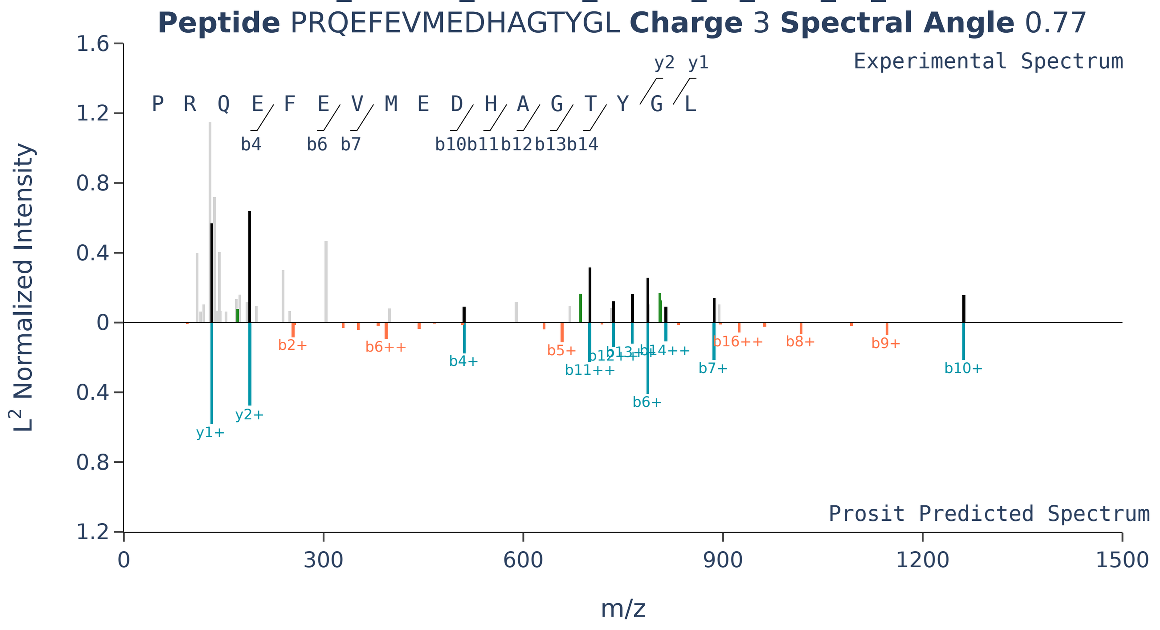


**Source** WSoh_221121_011221_HFGoe_L1_hTau_24h_R1 **Scan** 9173
**Peptide** PTPPTREPKKVAVVRTPPKSPSSA **Charge** 3 **Spectral Angle** 0.73

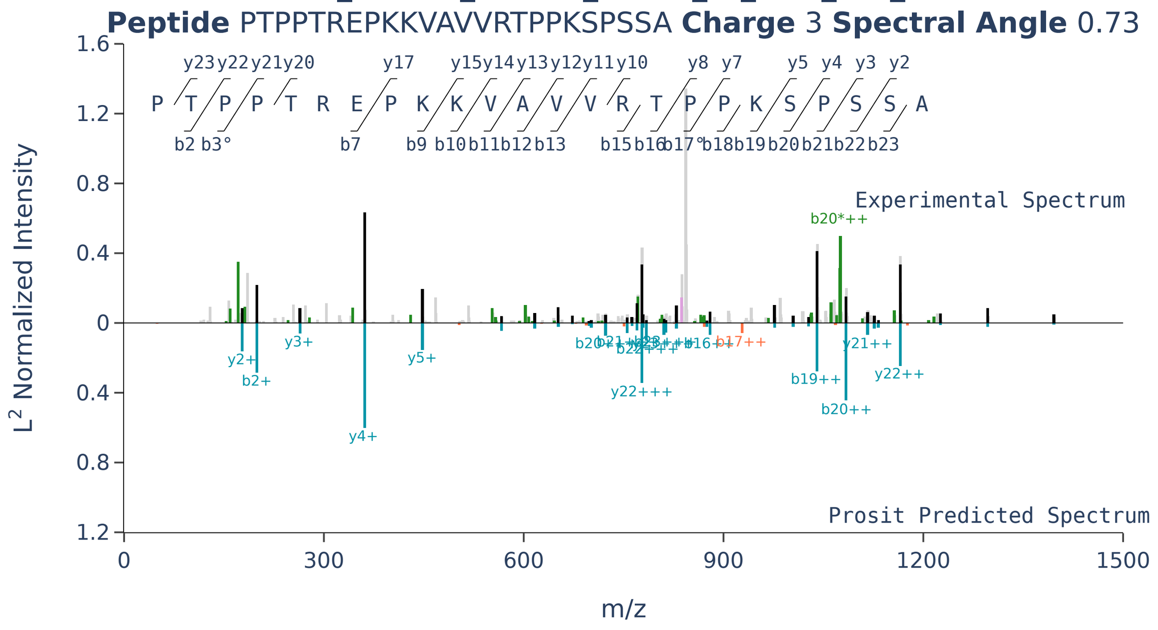


**Source** WSoh_221121_011221_HFGoe_L2_hTau_4h_R1 **Scan** 18911
**Peptide** SPVVSGDTSPRHLSNVSSTGSIDM **Charge** 3 **Spectral Angle** 0.93

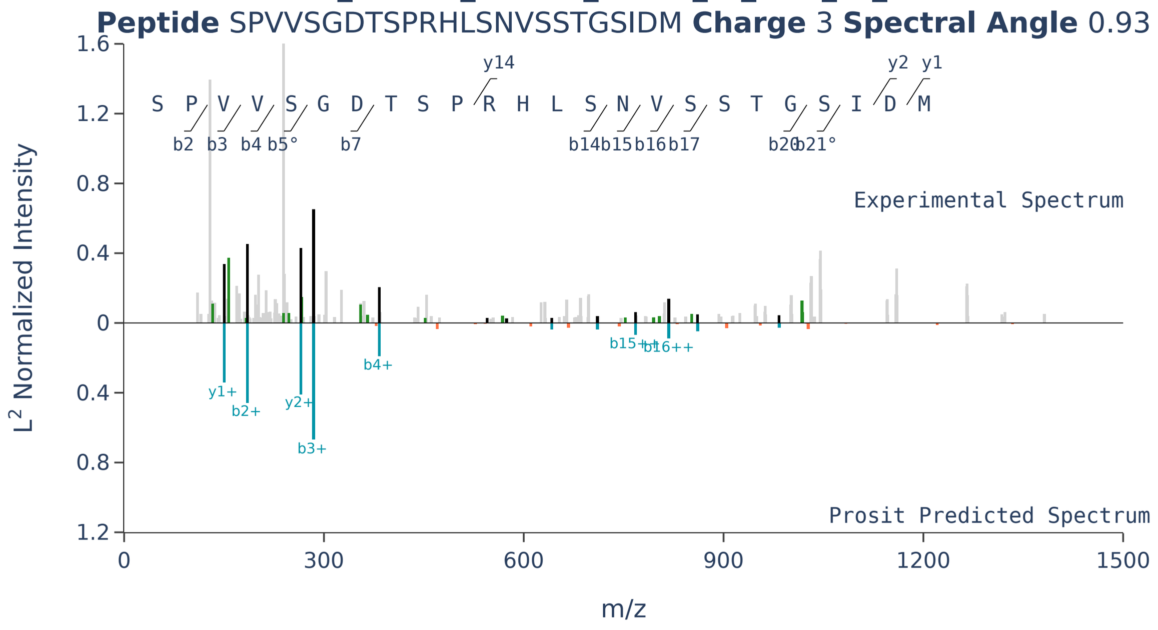


**Source** WSoh_221121_011221_HFGoe_L1_hTau_24h_R2 **Scan** 14079
**Peptide** LSNVSSTGSLD **Charge** 2 **Spectral Angle** 0.76

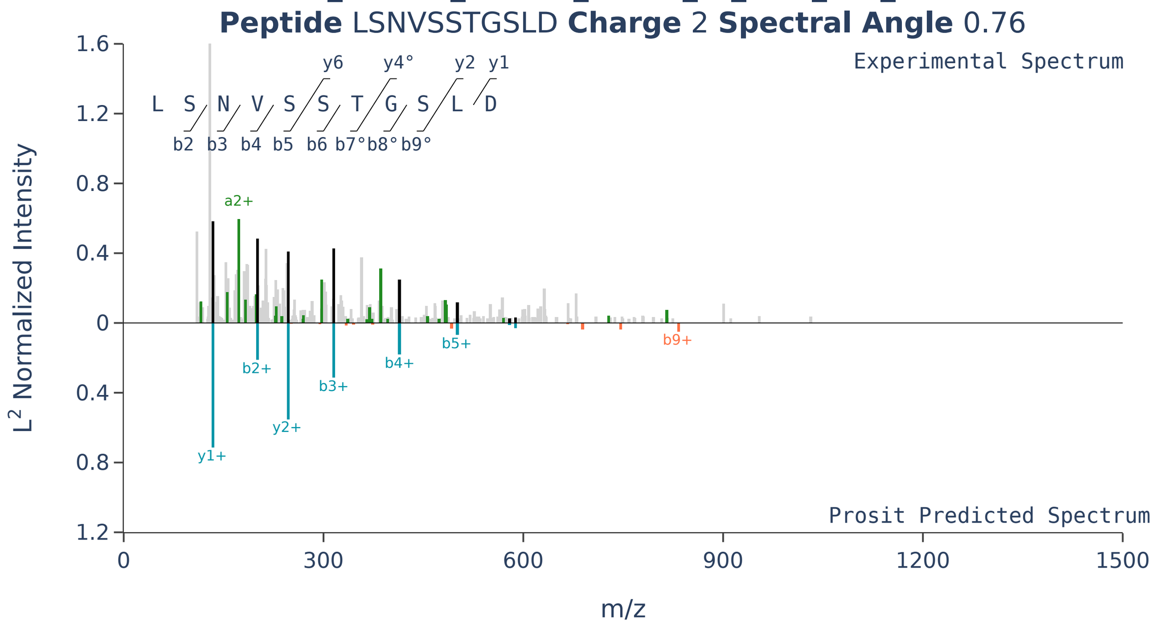


**Source** WSoh_221121_011221_HFGoe_L2_hTau_2h_R2 **Scan** 14597
**Peptide** KHQPGGGKVQIINKKLDLSNVQ **Charge** 4 **Spectral Angle** 0.91

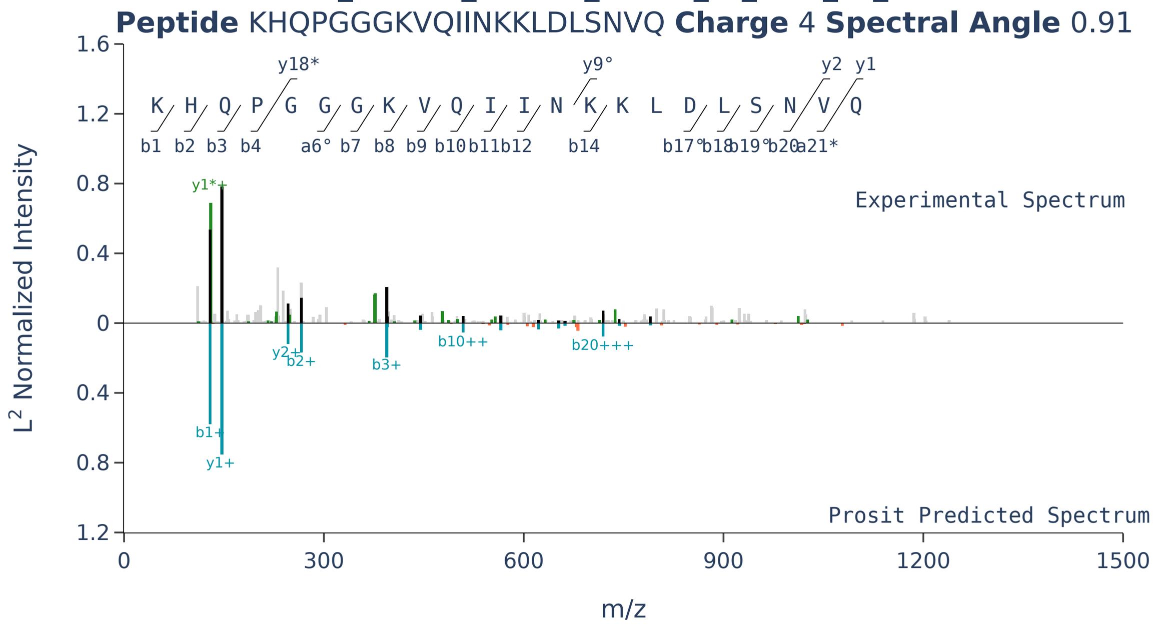


**Source** WSoh_221121_011221_HFGoe_L2_hTau_24h_R1 **Scan** 15044
**Peptide** QVEVKSEKLDFKDRV **Charge** 4 **Spectral Angle** 0.78

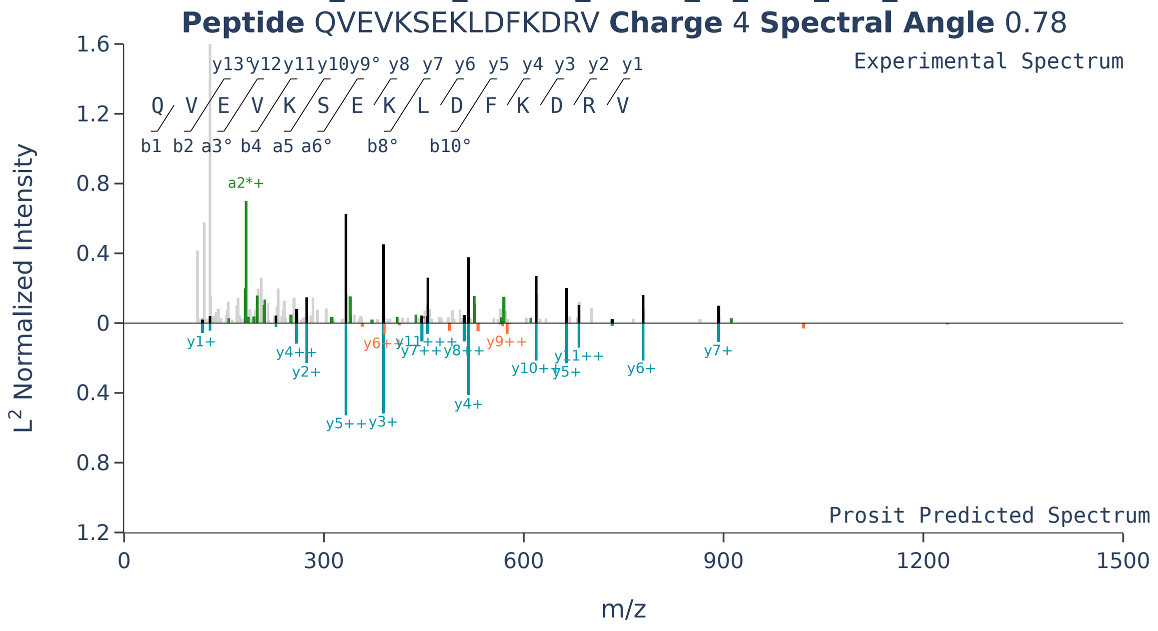


**Source** WSoh_221121_011221_HFGoe_L2_hTau_24h_R1 **Scan** 12560
**Peptide** AKAKTDHGAEIVYKSPVVSGDTSPRHLSN **Charge** 4 **Spectral Angle** 0.62

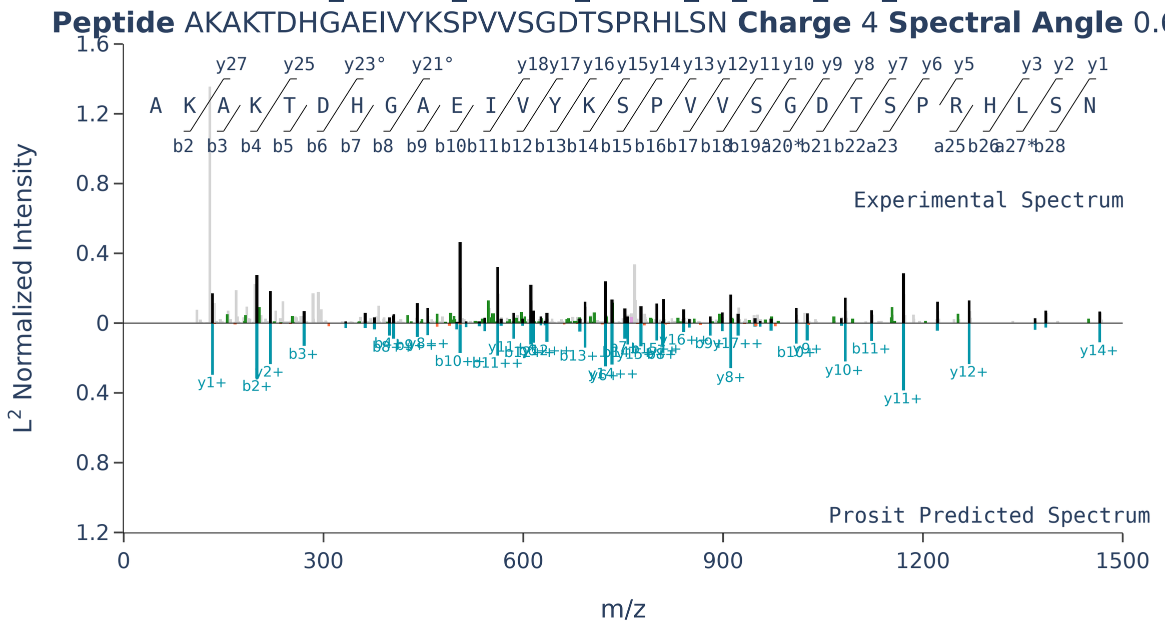


**Source** WSoh_221121_011221_HFGoe_L2_hTau_24h_R1 **Scan** 5740
**Peptide** DGTGSDDKKAKGADGKTKIATPRG **Charge** 4 **Spectral Angle** 0.79

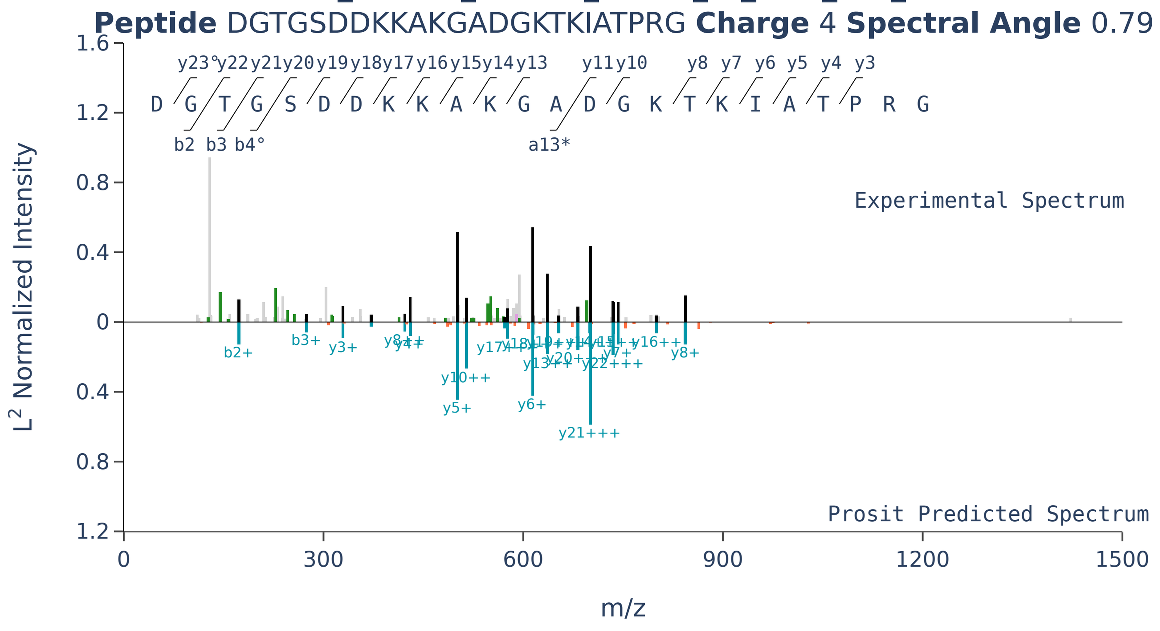


**Source** WSoh_221121_011221_HFGoe_L2_hTau_4h_R1 **Scan** 19840
**Peptide** AGIGDTPSLEDE **Charge** 2 **Spectral Angle** 0.75

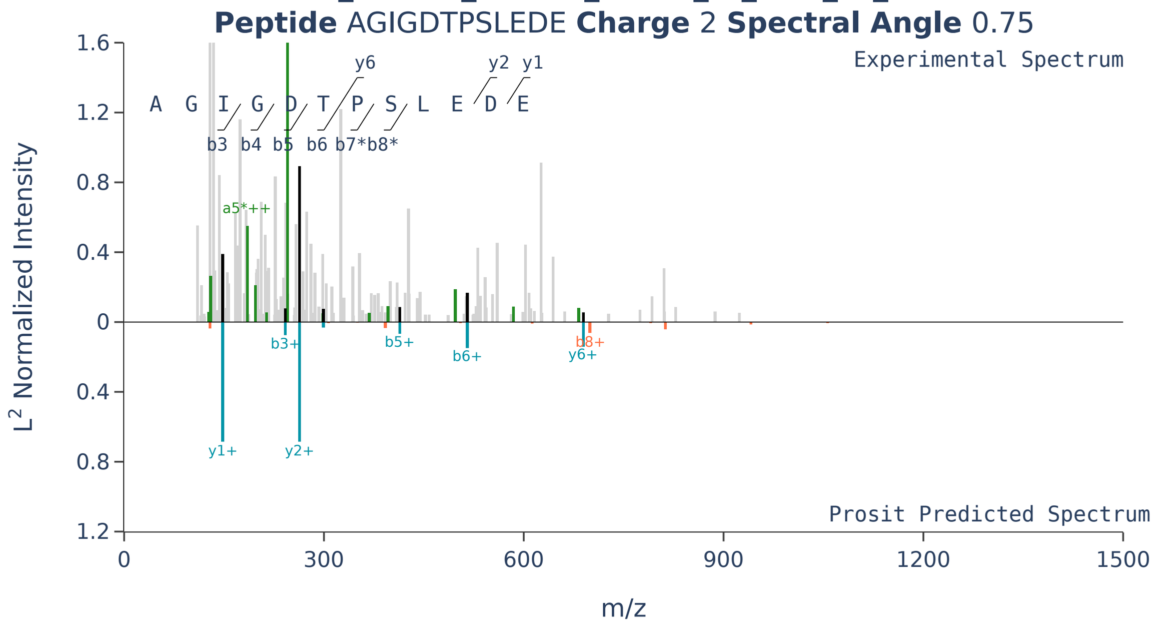


**Source** WSoh_221121_011221_HFGoe_L2_hTau_24h_R2 **Scan** 15119
**Peptide** KAKTDHGAEIVYKSPVVSGDTSPRHLSNV **Charge** 4 **Spectral Angle** 0.79

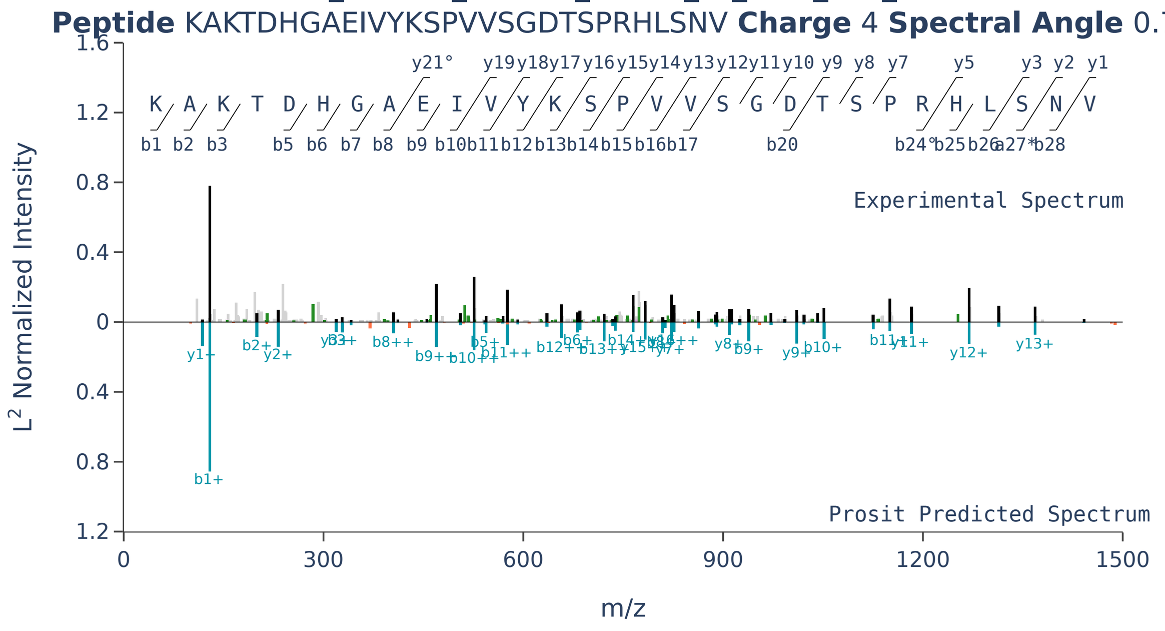


**Source** WSoh_221121_011221_HFGoe_L2_hTau_24h_R1 **Scan** 6145
**Peptide** SLGNIHHKPG **Charge** 3 **Spectral Angle** 0.85

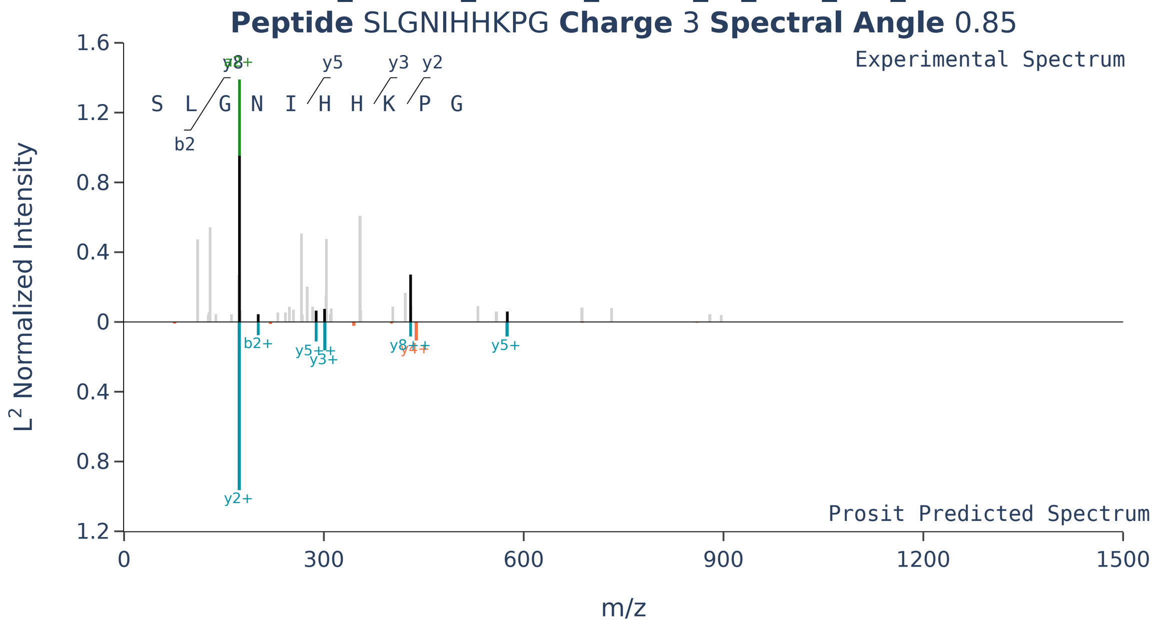


**Source** WSoh_221121_011221_HFGoe_L1_hTau_4h_R2 **Scan** 16763
**Peptide** IGSTENLKHQPGGGKVQIINKKLDL **Charge** 4 **Spectral Angle** 0.78

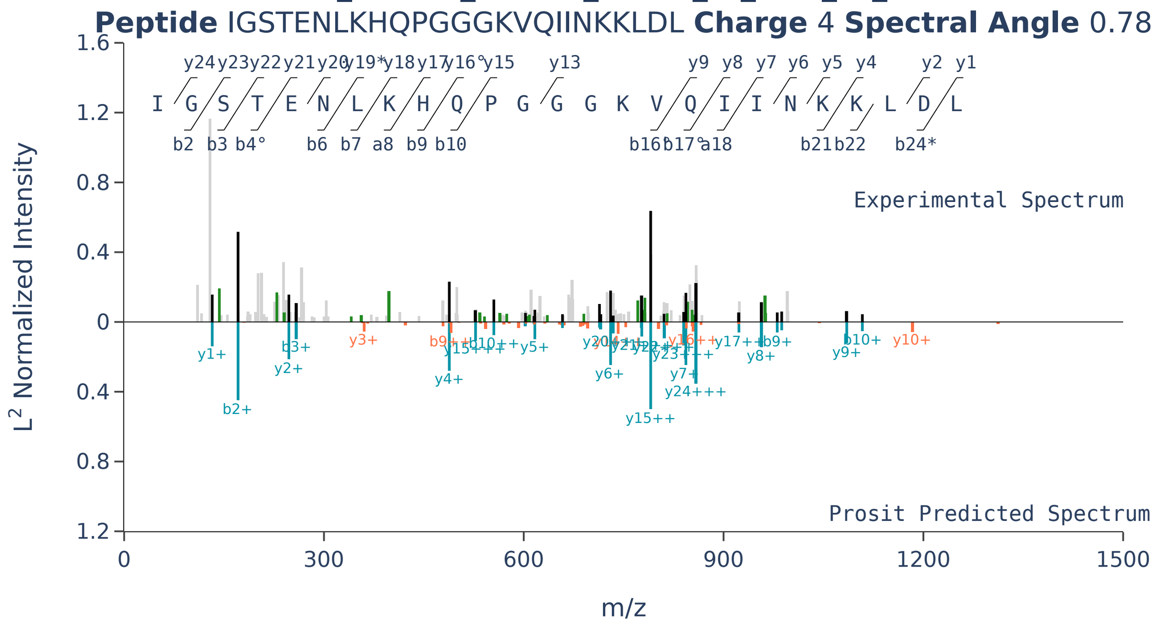


**Source** WSoh_221121_011221_HFGoe_L1_hTau_24h_R2 **Scan** 12885
**Peptide** GSLGNIHHKPGGGQVEVKSEKLDFKD **Charge** 3 **Spectral Angle** 0.7

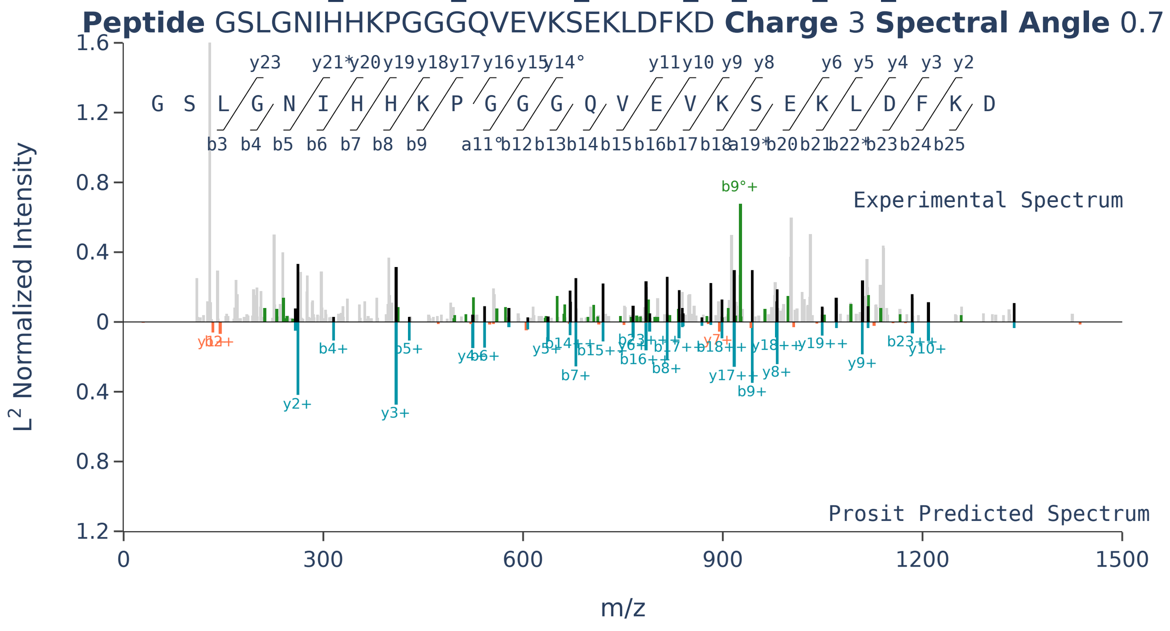

**Source** WSoh_221121_011221_HFGoe_L1_hTau_24h_R1 **Scan** 6607
**Peptide** KLGSTENLKHQPGGG **Charge** 3 **Spectral Angle** 0.69

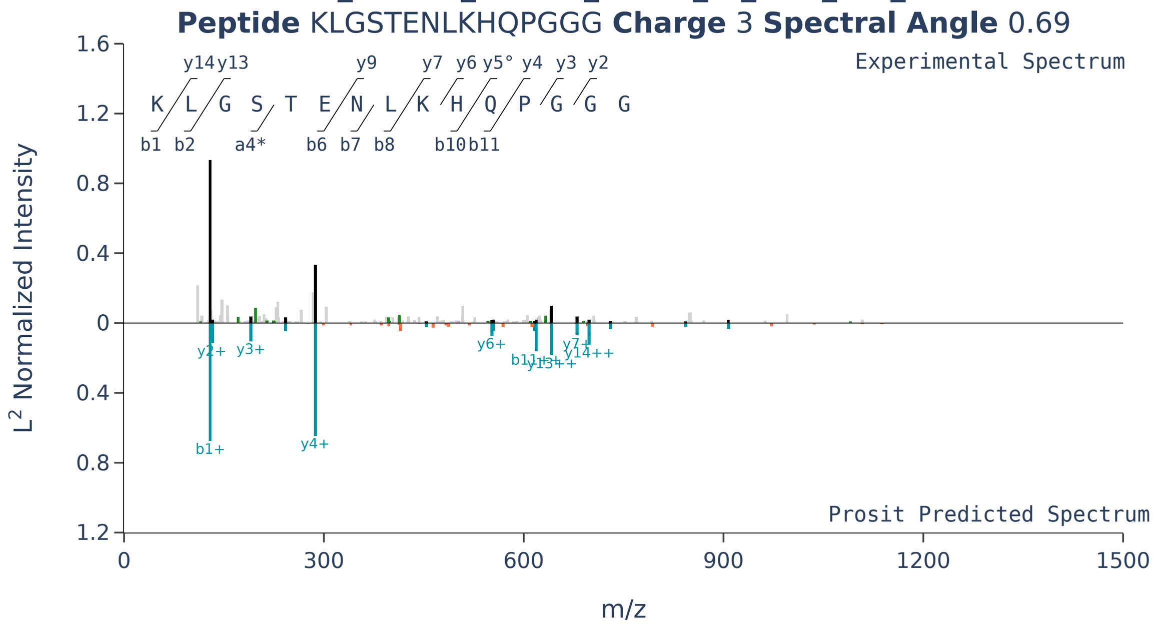


**Source** WSoh_221121_011221_HFGoe_L1_hTau_4h_R1 **Scan** 5436
**Peptide** HHKPGGGQVEVKSE **Charge** 2 **Spectral Angle** 0.83

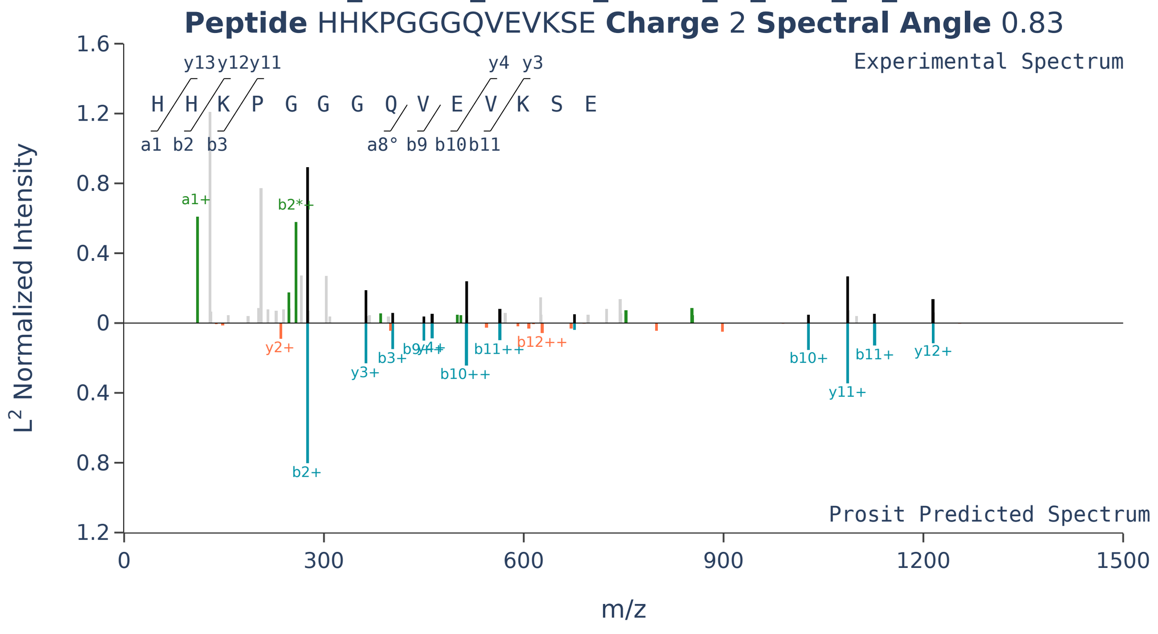


**Source** WSoh_221121_011221_HFGoe_L1_hTau_24h_R2 **Scan** 15435
**Peptide** GSEEPGSETSDAKSTPTAEDVTAP **Charge** 2 **Spectral Angle** 0.74

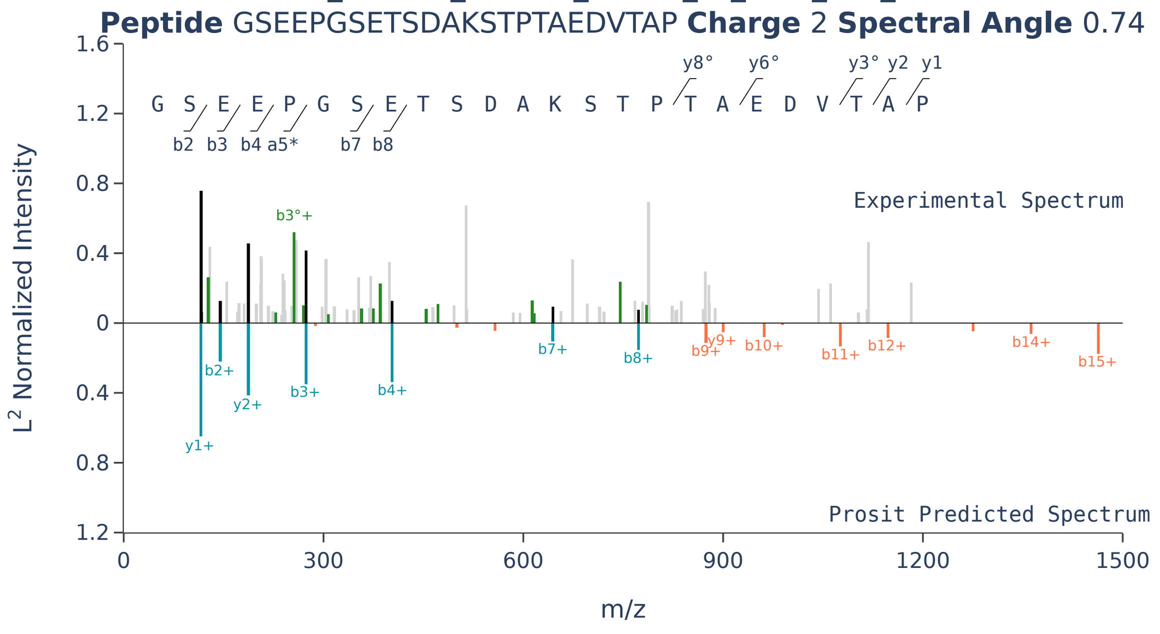


**Source** WSoh_221121_011221_HFGoe_L1_hTau_24h_R1 **Scan** 8004
**Peptide** HKLTFRENAKAKTDHGAEL **Charge** 3 **Spectral Angle** 0.72

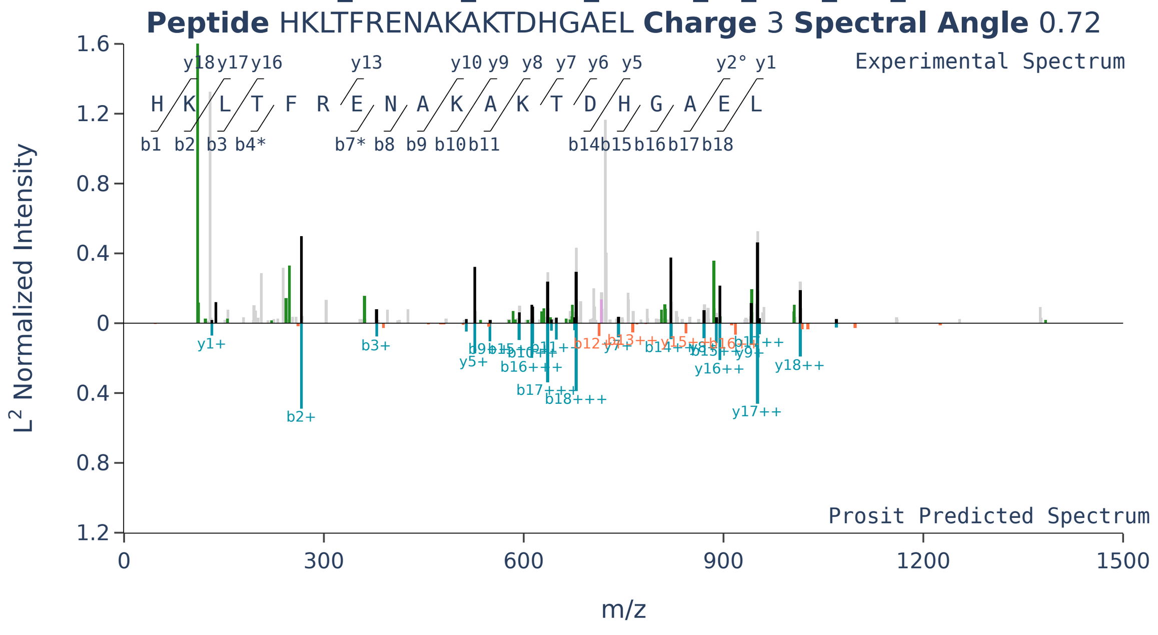


**Source** WSoh_221121_011221_HFGoe_L2_hTau_4h_R2 **Scan** 13179
**Peptide** PVDLSKVTSKCGS **Charge** 3 **Spectral Angle** 0.74

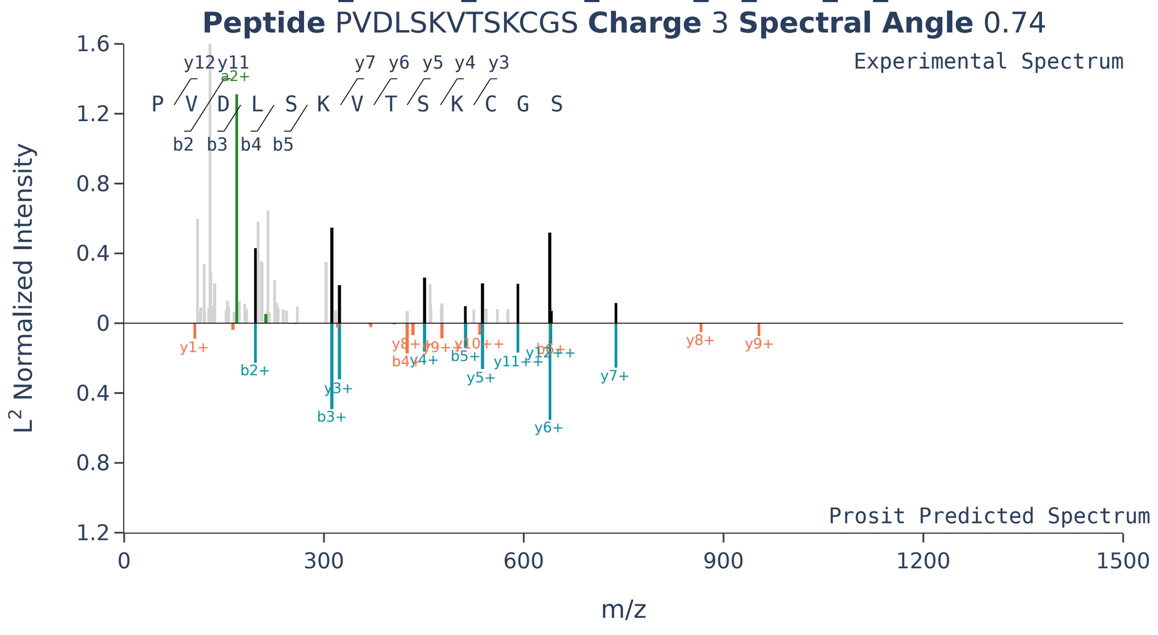


**Source** WSoh_221121_011221_HFGoe_L1_hTau_24h_R2 **Scan** 10987
**Peptide** LGDRKDQGGYTMHQDQEGDTDAGLKE **Charge** 3 **Spectral Angle** 0.78

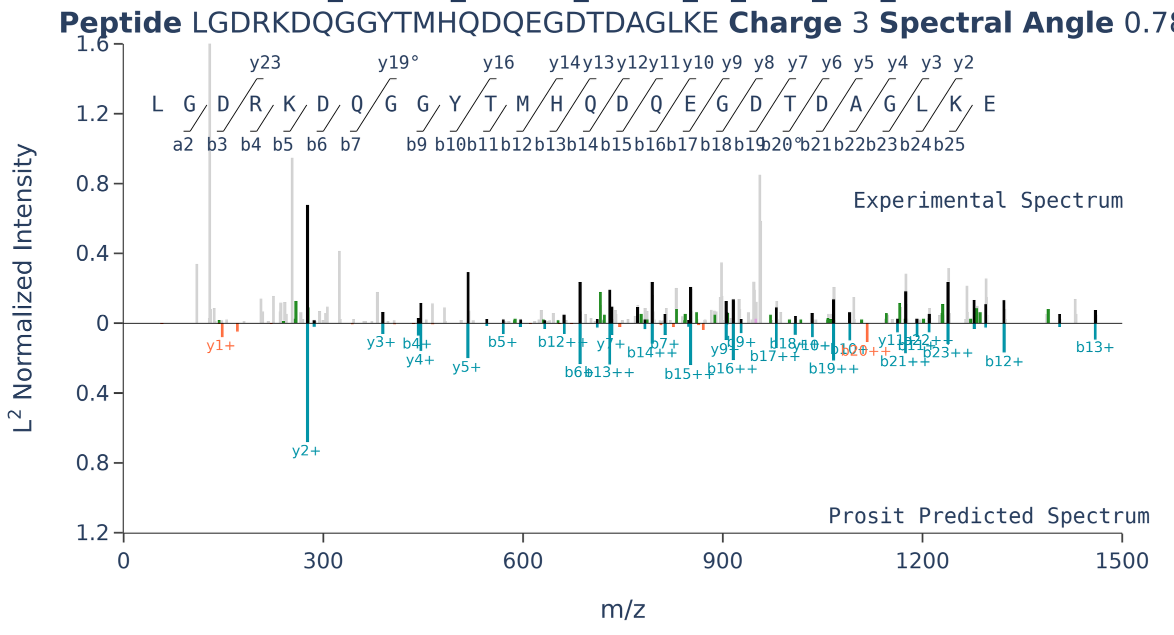


**Source** WSoh_221121_011221_HFGoe_L2_hTau_4h_R1 **Scan** 9752
**Peptide** DGKTKIATPRGAAPPGQKGQANATRI **Charge** 5 **Spectral Angle** 0.82

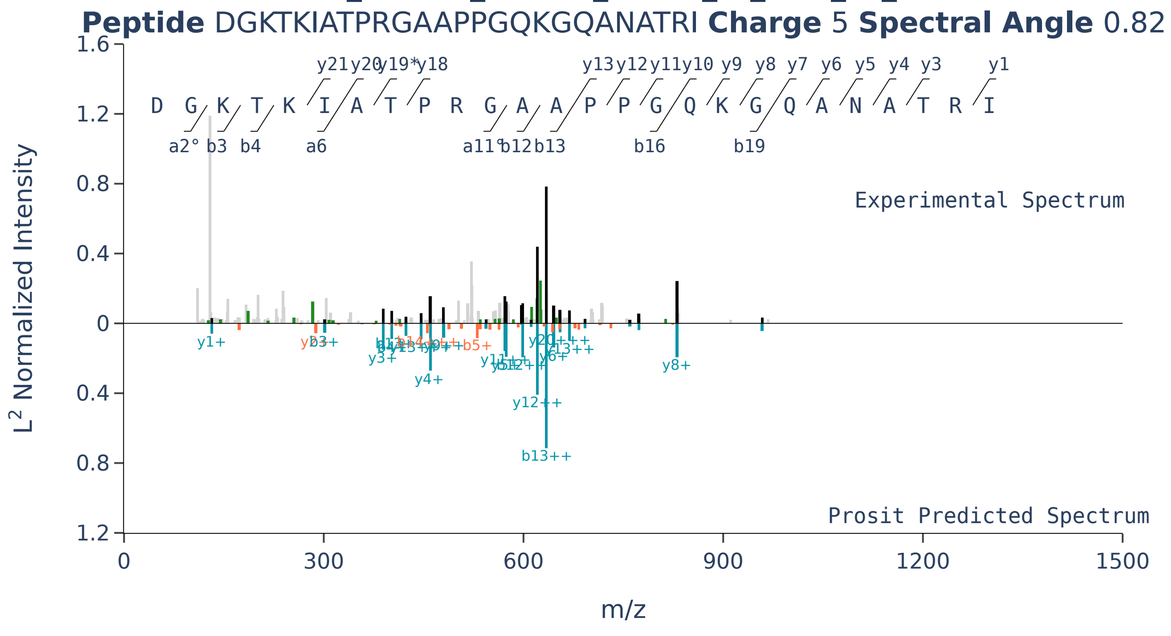


**Source** WSoh_221121_011221_HFGoe_L2_hTau_24h_R1 **Scan** 16483
**Peptide** VYKSPVVSGDTSPRHLSNVSSTGSLD **Charge** 4 **Spectral Angle** 0.73

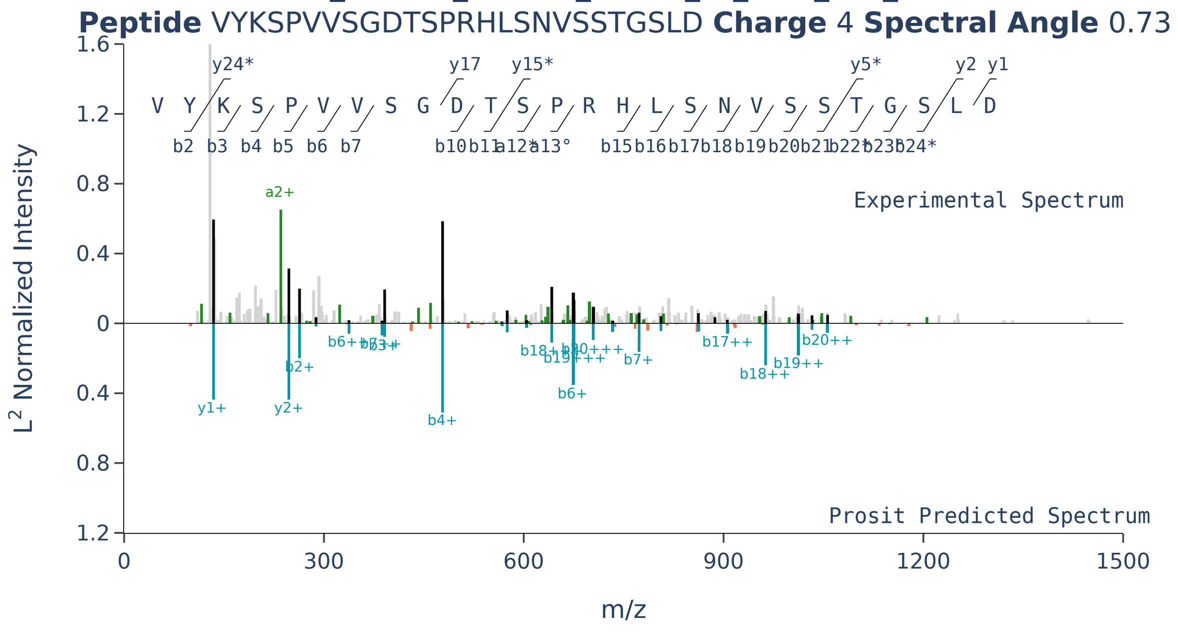


**Source** WSoh_221121_011221_HFGoe_L1_hTau_24h_R1 **Scan** 17797
**Peptide** LGSLDNITHVPGGGNKKIETHKLTFREN **Charge** 3 **Spectral Angle** 0.74

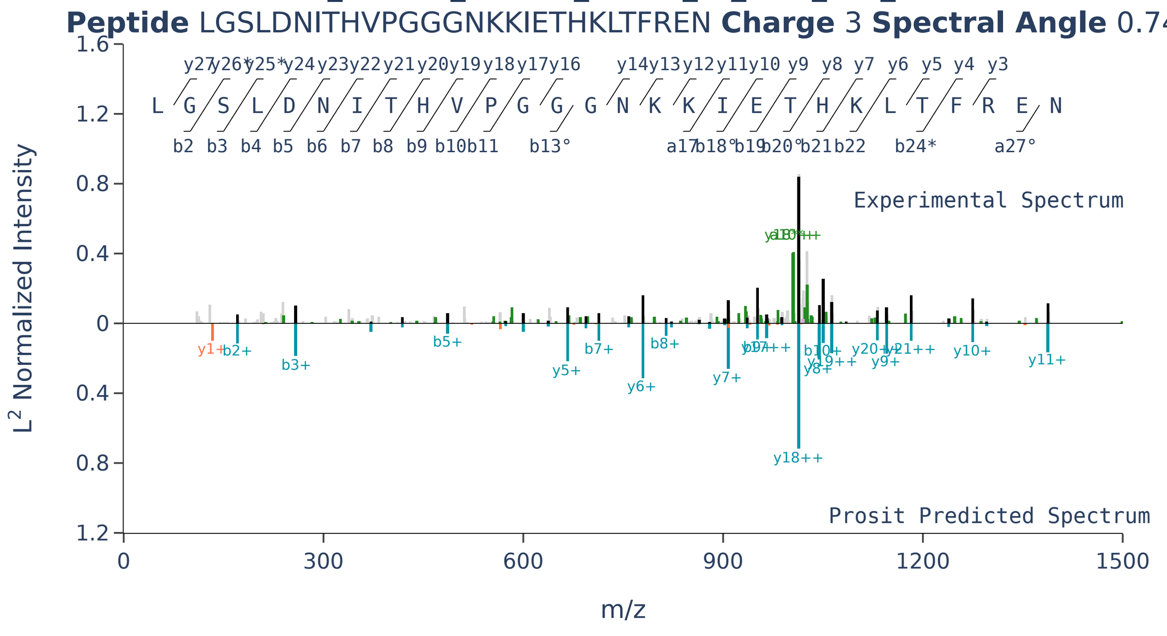


**Source** WSoh_221121_011221_HFGoe_L1_hTau_4h_R2 **Scan** 14394
**Peptide** DEGAPGKQAAAQPHTEIPEGTTAEEA **Charge** 3 **Spectral Angle** 0.7

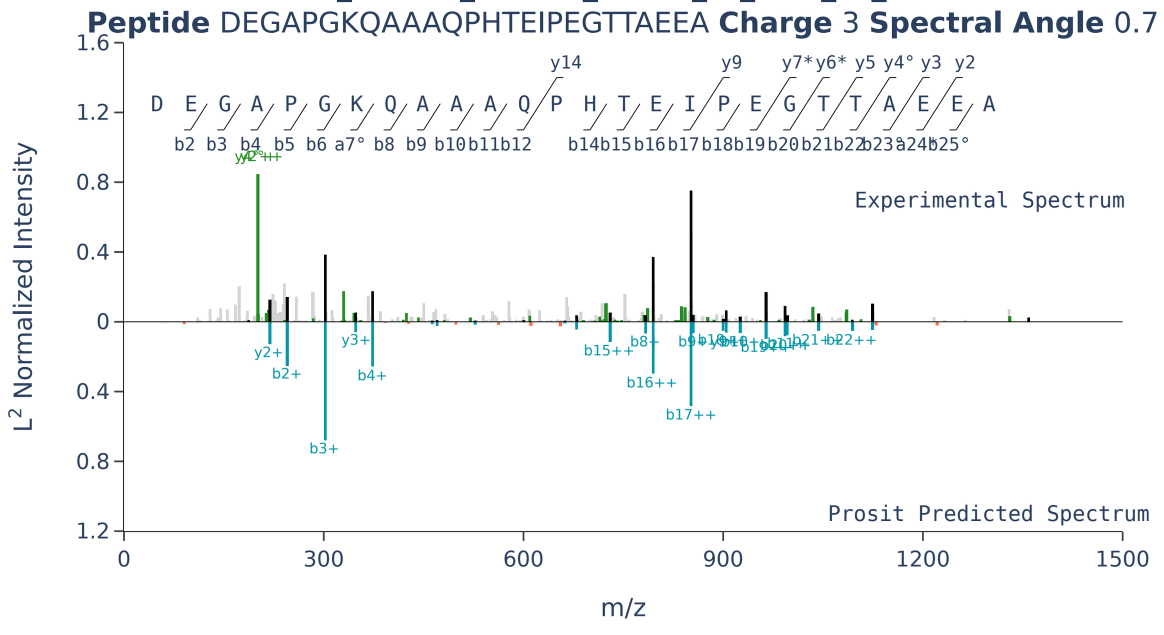


**Source** WSoh_221121_011221_HFGoe_L1_hTau_24h_R1 **Scan** 21565
**Peptide** TAPVPMPDLKNVKSKIGSTEN **Charge** 4 **Spectral Angle** 0.66

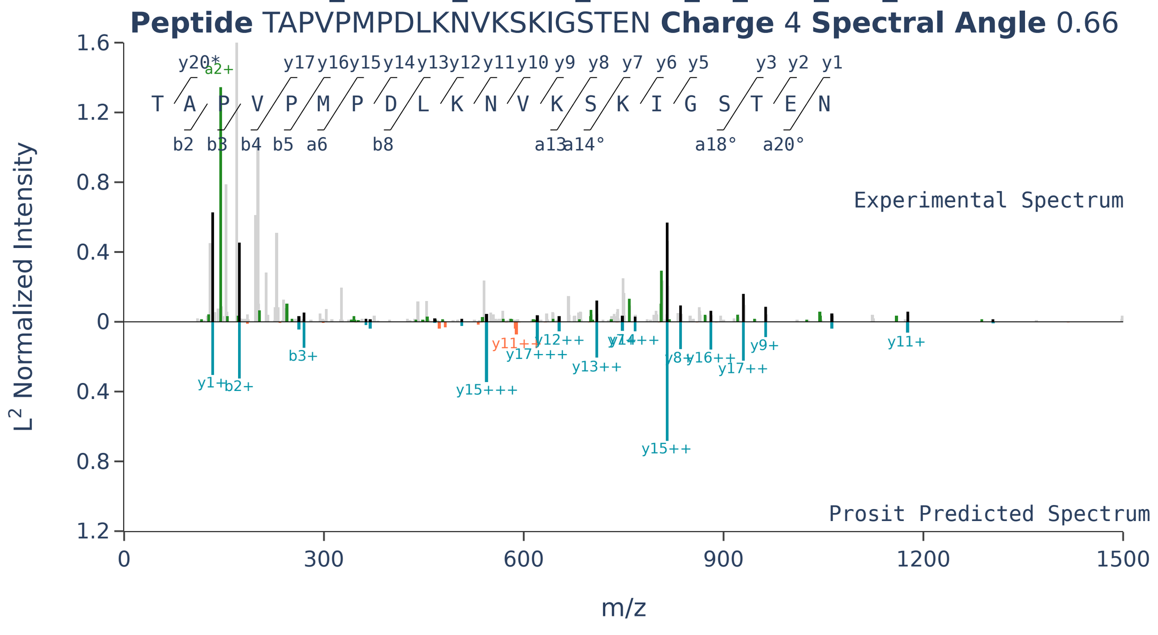


**Source** WSoh_221121_011221_HFGoe_L1_hTau_24h_R1 **Scan** 21417
**Peptide** RLQTAPVPMPDLKNVKSKIGS **Charge** 4 **Spectral Angle** 0.8

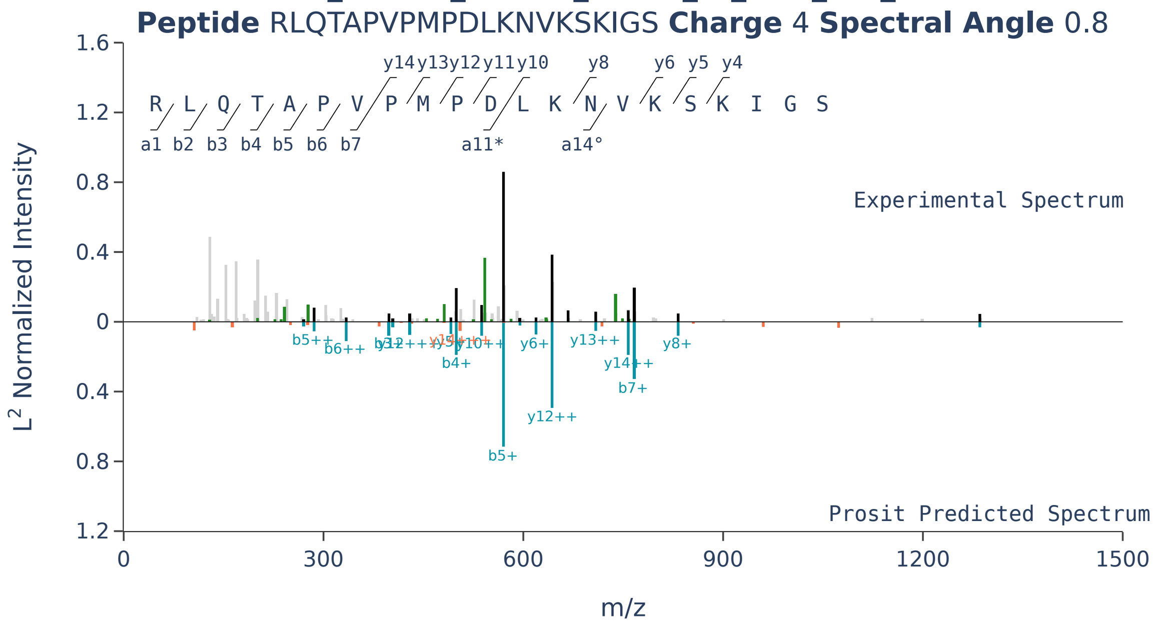


**Source** WSoh_221121_011221_HFGoe_L1_hTau_24h_R2 **Scan** 12443
**Peptide** VPGGGNKKIETHKLTF **Charge** 4 **Spectral Angle** 0.77

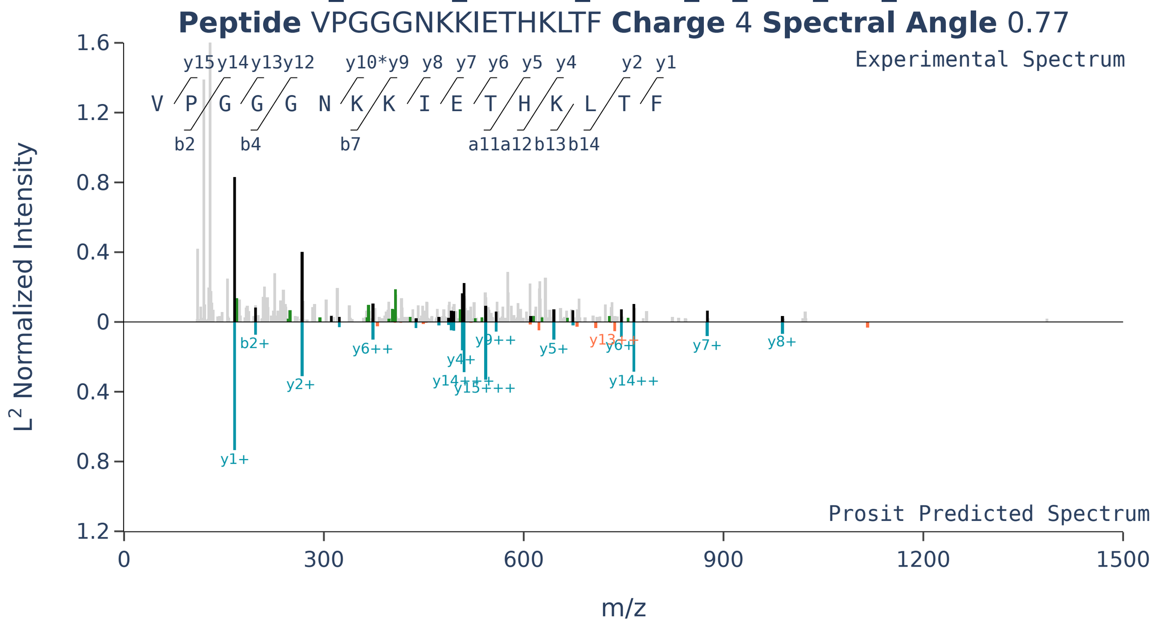


**Source** WSoh_221121_011221_HFGoe_L1_hTau_4h_R2 **Scan** 17173
**Peptide** MHQDQEGDTDAGLKESPLQTPTE **Charge** 3 **Spectral Angle** 0.86

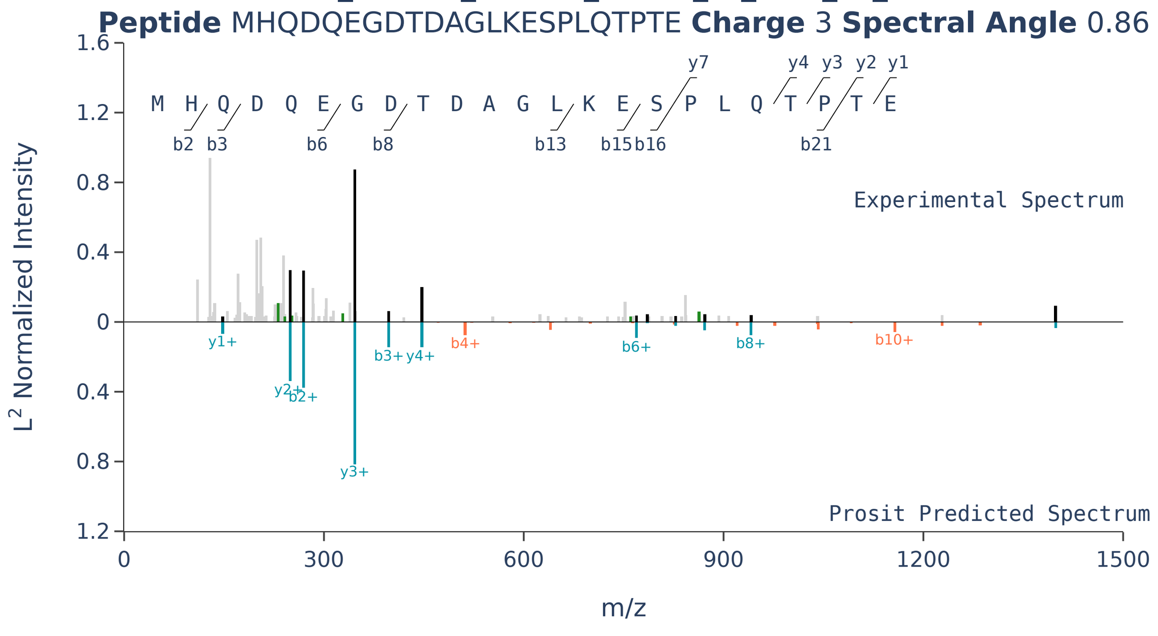


**Source** WSoh_221121_011221_HFGoe_L2_hTau_4h_R1 **Scan** 16354
**Peptide** QSKIGSLDNITHVPGGGNKKIET **Charge** 5 **Spectral Angle** 0.76

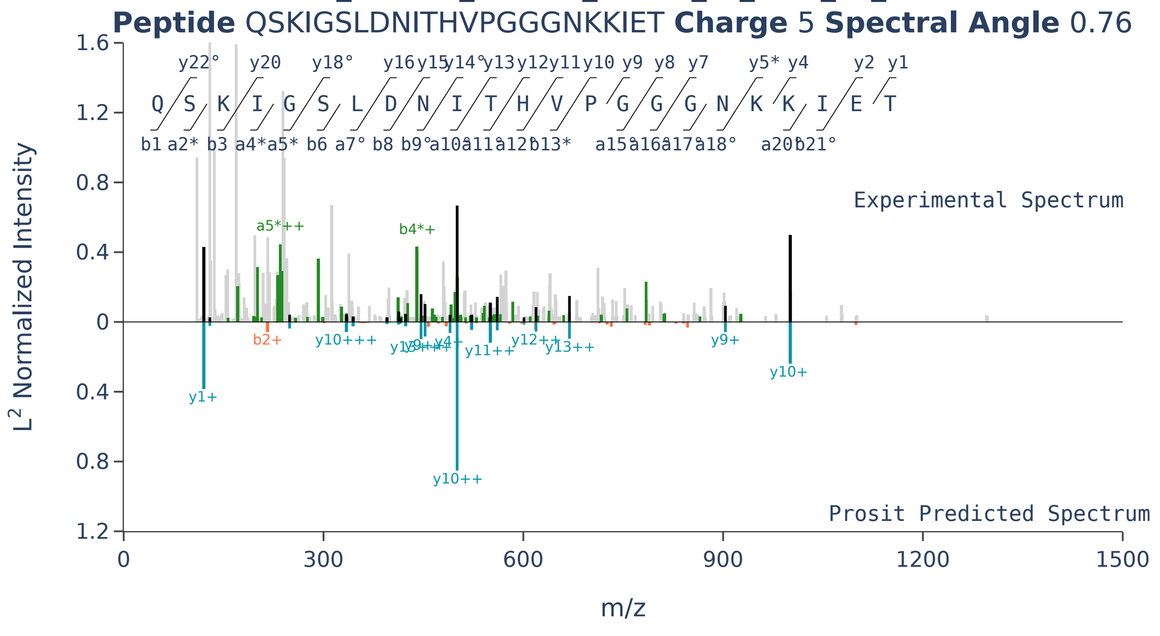

**Source** WSoh_221121_011221_HFGoe_L2_hTau_24h_R1 **Scan** 7029
**Peptide** QSKCGSKDNIKHVPGGGSV **Charge** 3 **Spectral Angle** 0.8

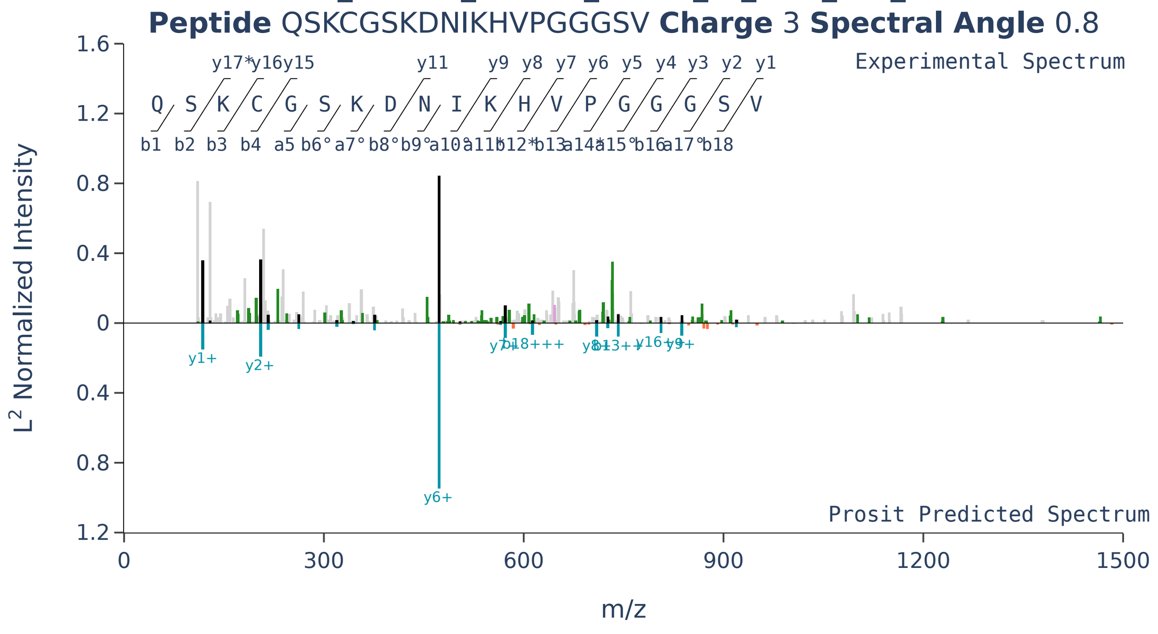


**Source** WSoh_221121_011221_HFGoe_L1_hTau_4h_R1 **Scan** 10698
**Peptide** DLSNVQSKCGSKDNL **Charge** 3 **Spectral Angle** 0.76

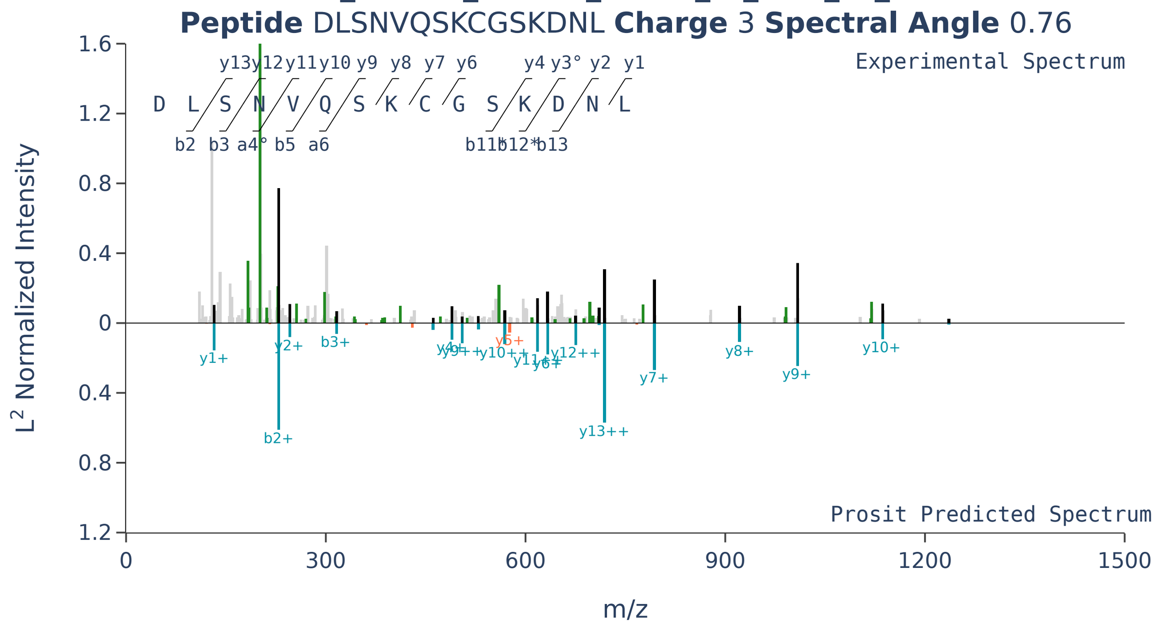


**Source** WSoh_221121_011221_HFGoe_L1_hTau_24h_R1 **Scan** 17649
**Peptide** IGSLDNITHVPGGGNKKIE **Charge** 3 **Spectral Angle** 0.8

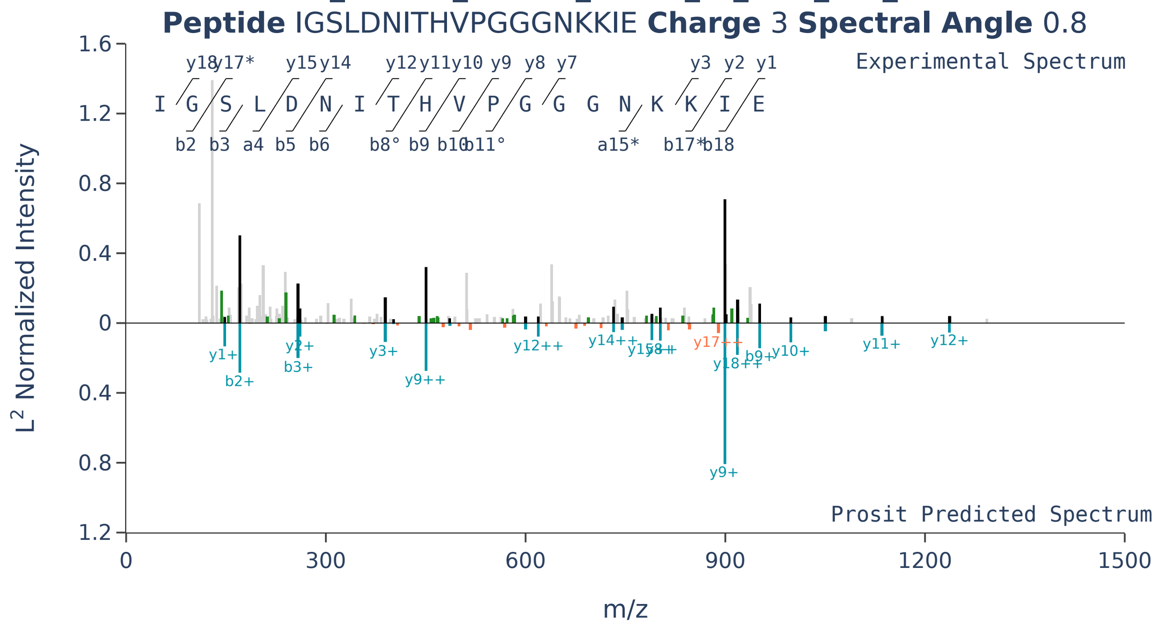


**Source** WSoh_221121_011221_HFGoe_L1_hTau_24h_R1 **Scan** 5726
**Peptide** KNVKSKIGSTENLKHQ **Charge** 4 **Spectral Angle** 0.91

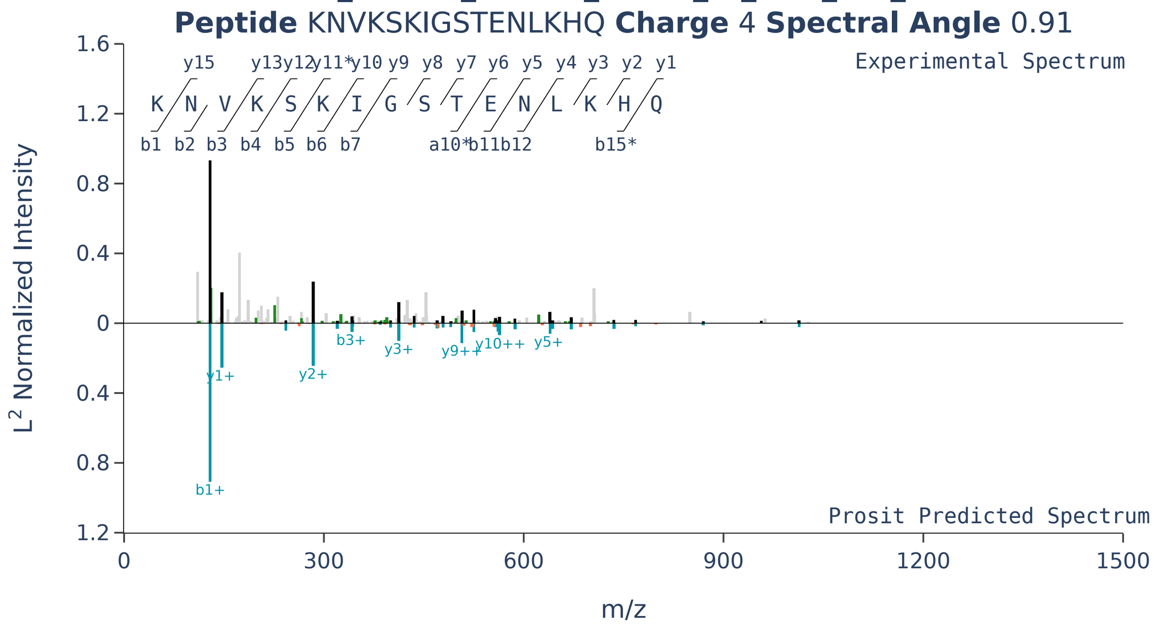


**Source** WSoh_221121_011221_HFGoe_L2_hTau_24h_R1 **Scan** 15138
**Peptide** KLGSTENLKHQPGGGKVQIINKKLDLS **Charge** 4 **Spectral Angle** 0.9

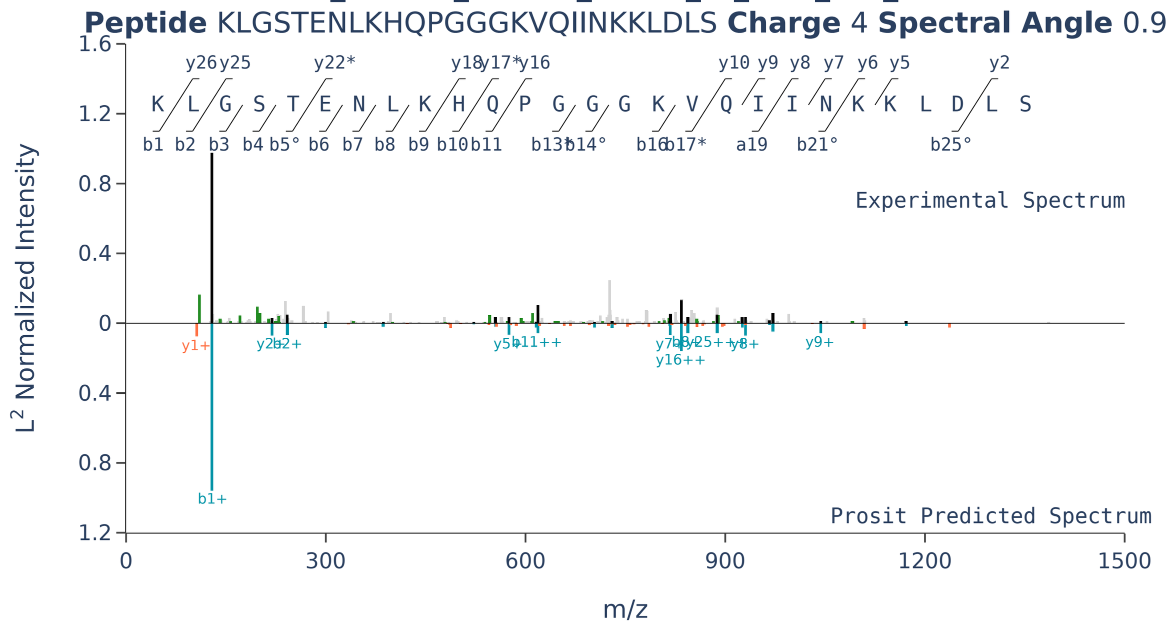


**Source** WSoh_221121_011221_HFGoe_L2_hTau_1h_R1 **Scan** 5372
**Peptide** HVPGGGNKKIETH **Charge** 3 **Spectral Angle** 0.92

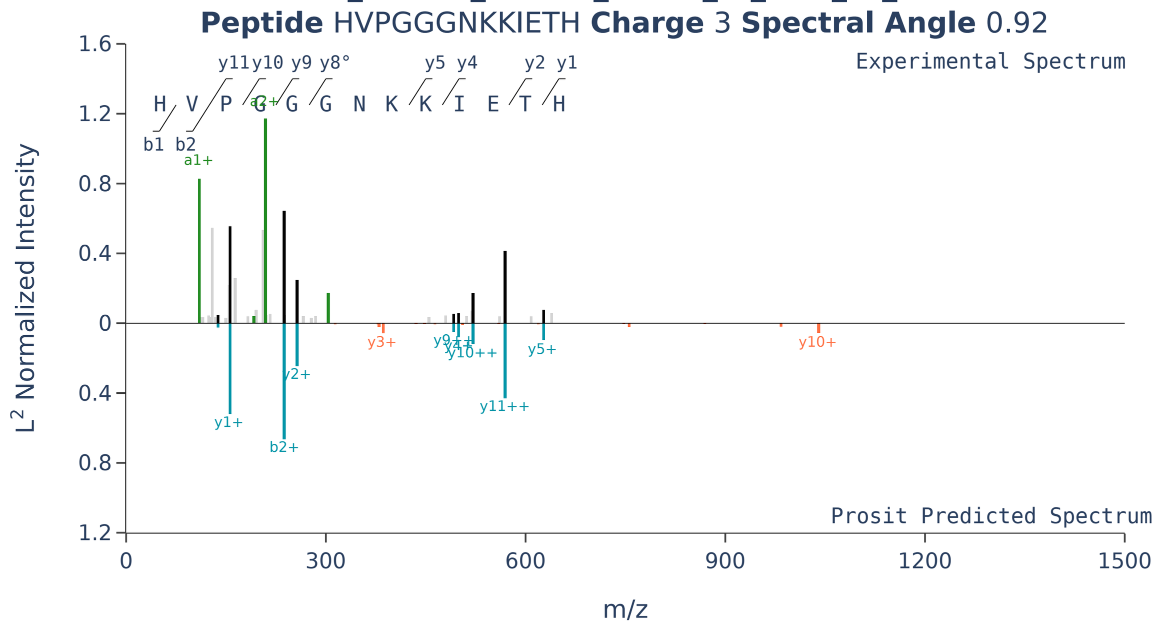


**Source** WSoh_221121_011221_HFGoe_L2_hTau_24h_R1 **Scan** 9677
**Peptide** KIATPRGAAPPGQKGQANATRI **Charge** 4 **Spectral Angle** 0.7

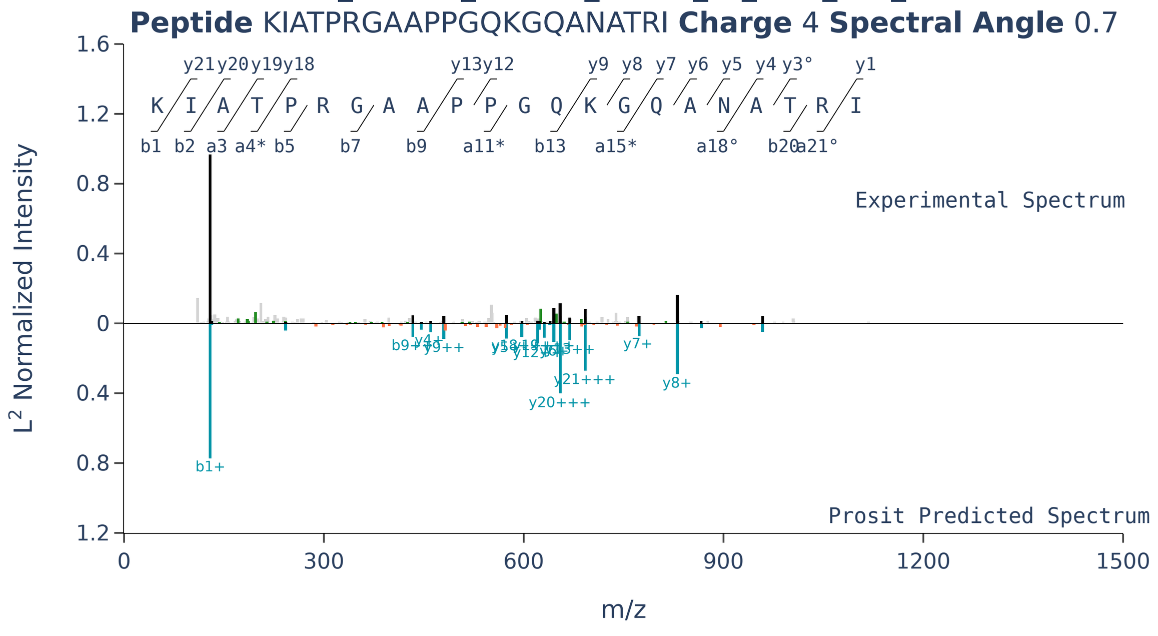


**Source** WSoh_221121_011221_HFGoe_L1_hTau_24h_R1 **Scan** 17572
**Peptide** DHGAEIVYKSPVVSGDTSPRHLSNVSSTG **Charge** 4 **Spectral Angle** 0.85

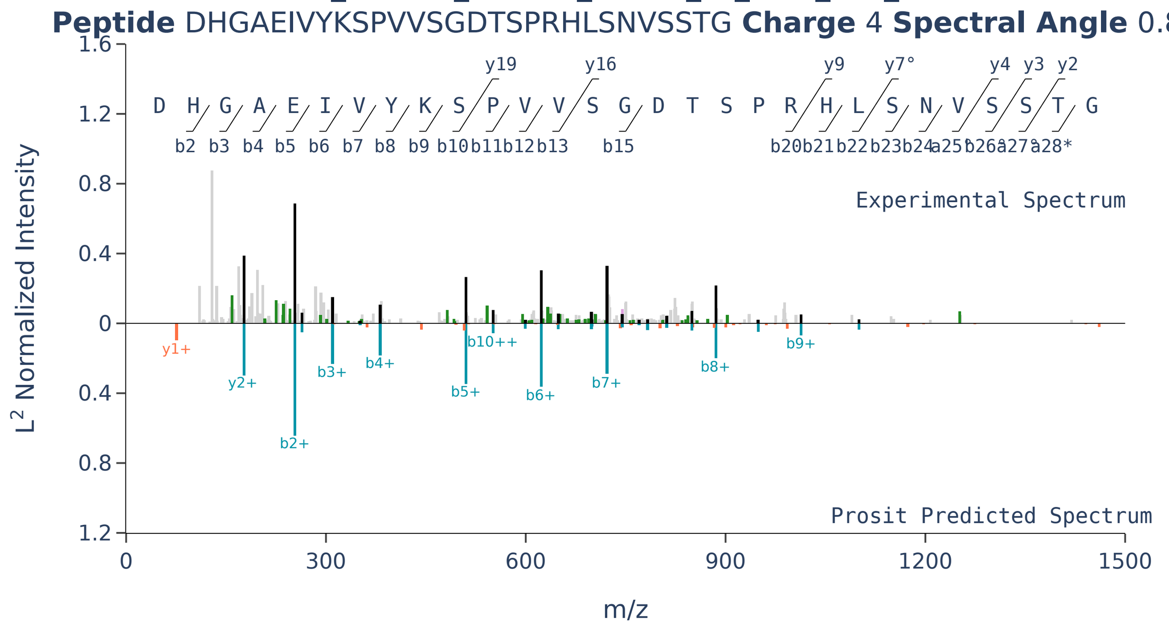


**Source** WSoh_221121_011221_HFGoe_L1_hTau_4h_R2 **Scan** 12380
**Peptide** QPGGGKVQIINKKL **Charge** 3 **Spectral Angle** 0.66

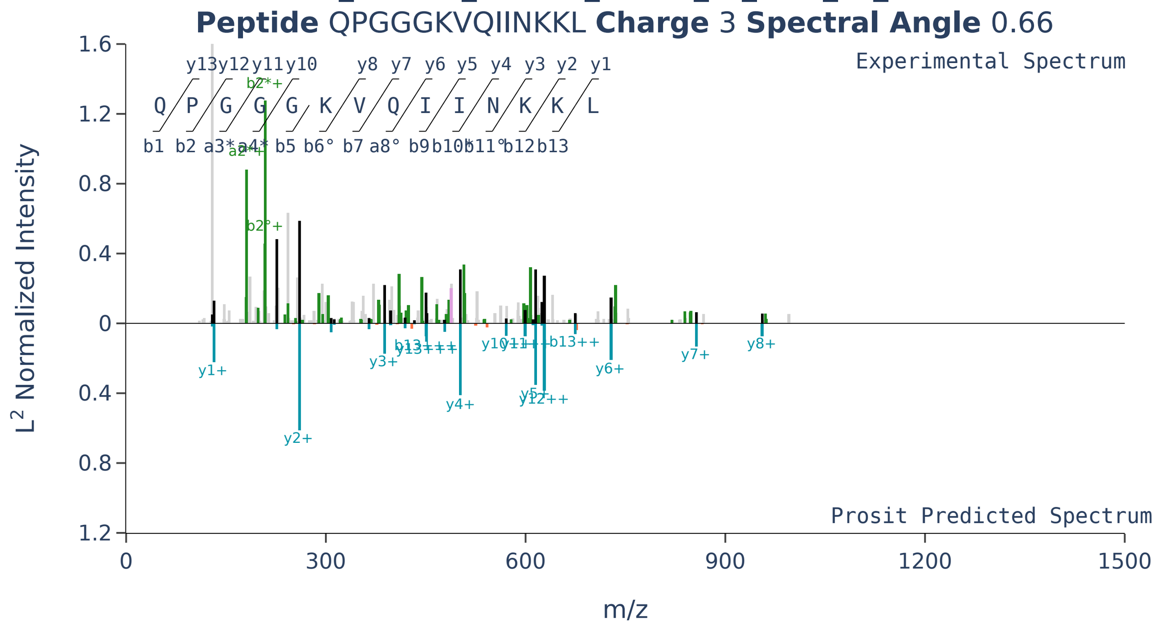


**Source** WSoh_221121_011221_HFGoe_L2_hTau_4h_R1 **Scan** 18866
**Peptide** KHVPGGGSVQIVYKPVDLSKVTSKC **Charge** 4 **Spectral Angle** 0.85

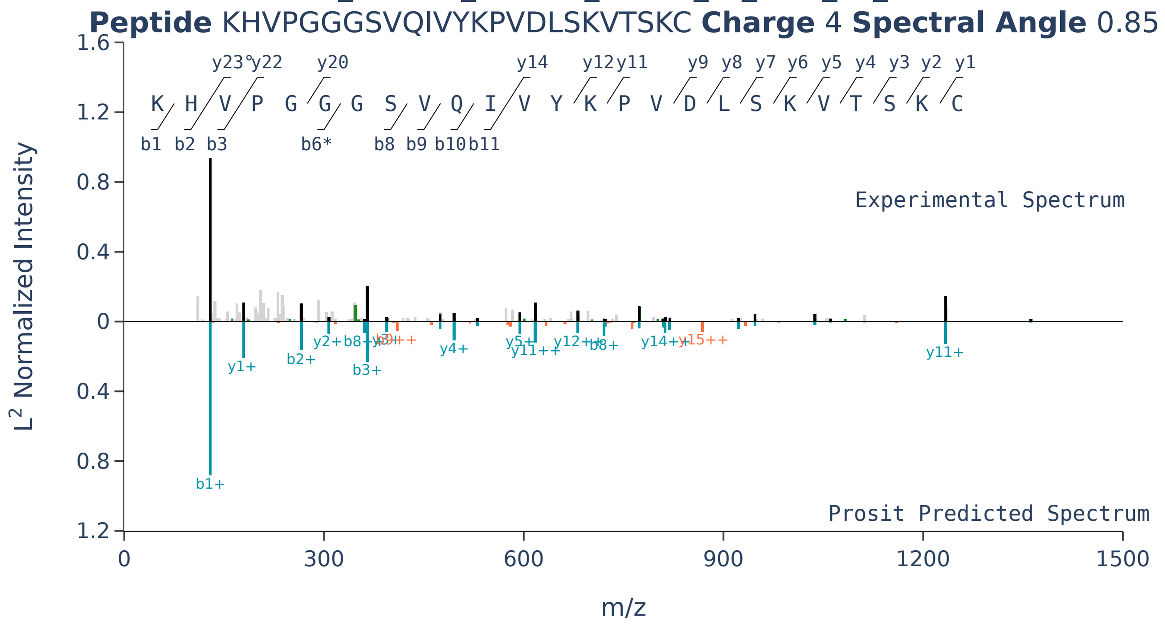


**Source** WSoh_221121_011221_HFGoe_L1_hTau_24h_R2 **Scan** 20940
**Peptide** KSEKLDFKDRVQSKIGSLDNL **Charge** 3 **Spectral Angle** 0.8

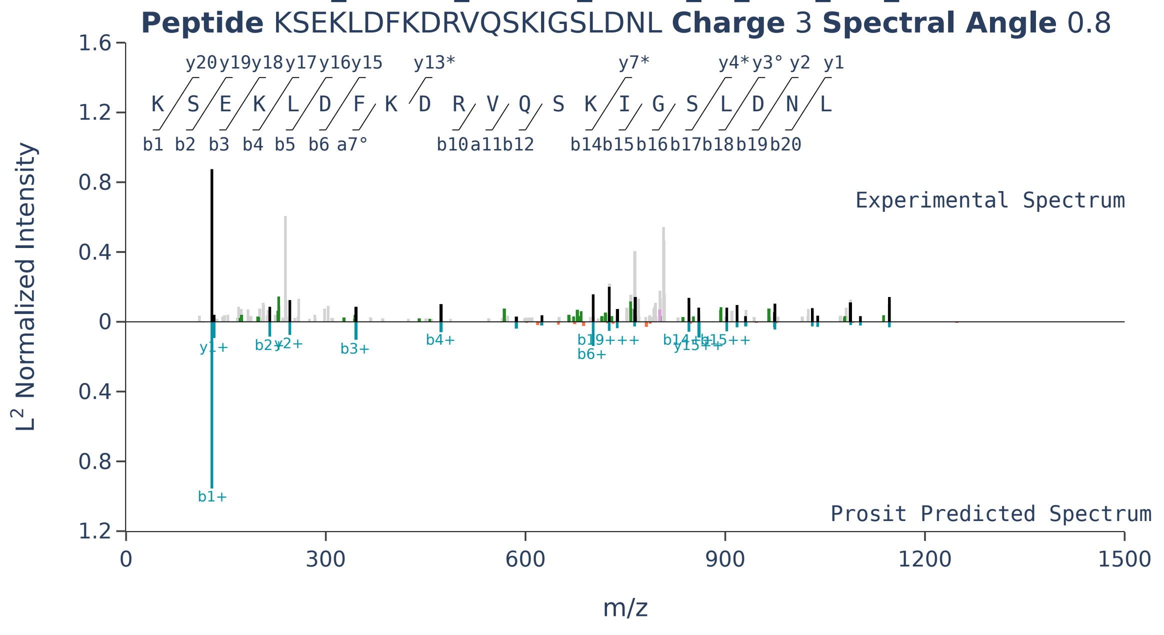


**Source** WSoh_221121_011221_HFGoe_L2_hTau_24h_R1 **Scan** 22945
**Peptide** GSEEPGSETSDAKSTPTAEDVTAPLVD **Charge** 2 **Spectral Angle** 0.85

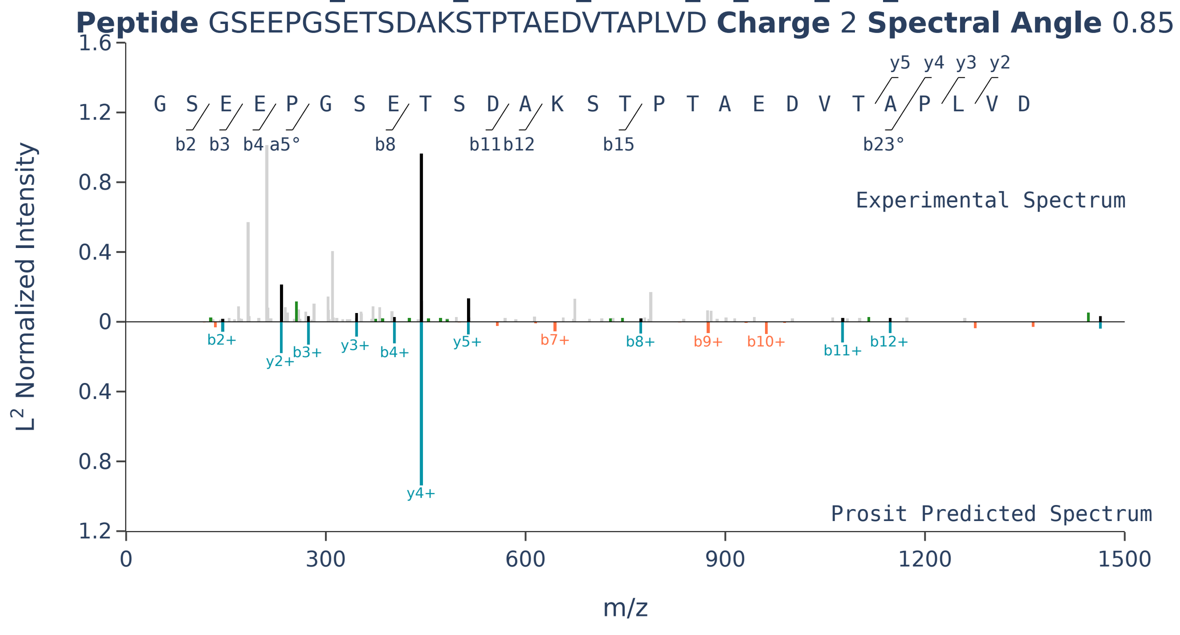


**Source** WSoh_221121_011221_HFGoe_L1_hTau_24h_R1 **Scan** 9327
**Peptide** ATRIPAKTPPAPKTPPSSGEPPKSGDR **Charge** 4 **Spectral Angle** 0.77

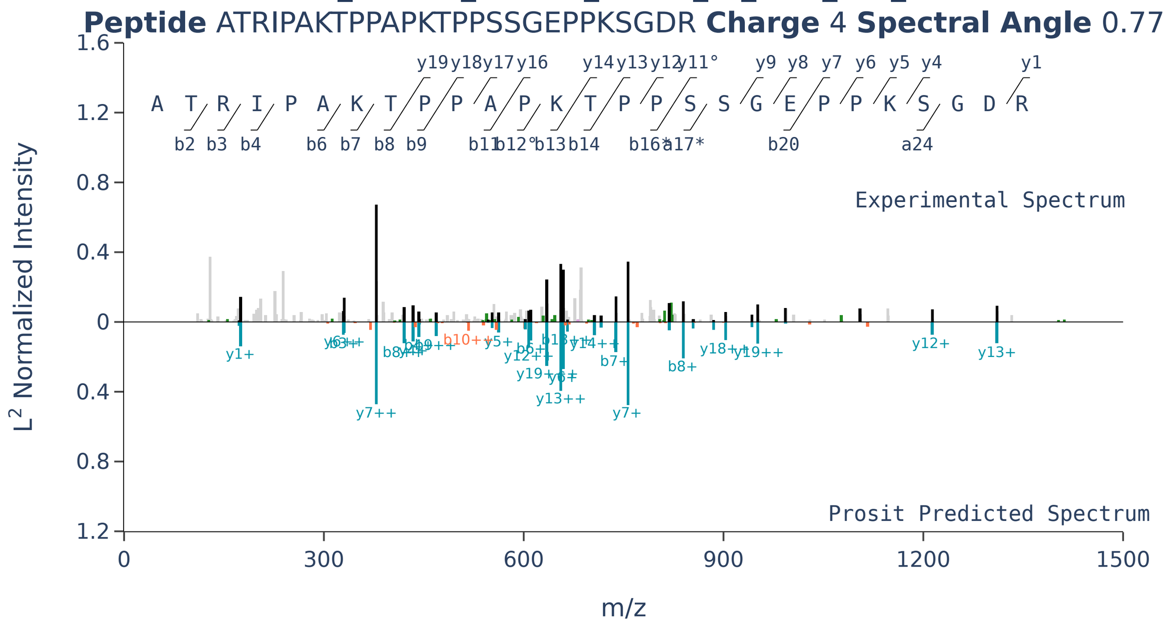


**Source** WSoh_221121_011221_HFGoe_L1_hTau_24h_R2 **Scan** 10439
**Peptide** LATPRGAAPPGQKGQANATRIPAKT **Charge** 4 **Spectral Angle** 0.76

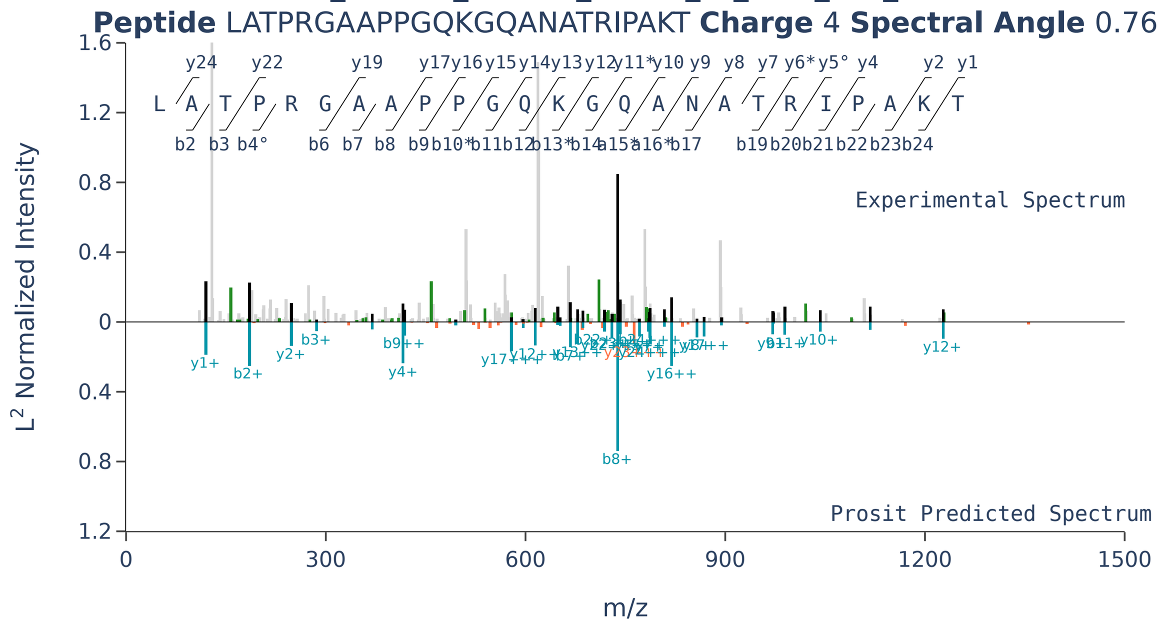


**Source** WSoh_221121_011221_HFGoe_L2_hTau_24h_R2 **Scan** 21840
**Peptide** EDVTAPLVDEGAPGKQAAAQPHTEI **Charge** 3 **Spectral Angle** 0.83

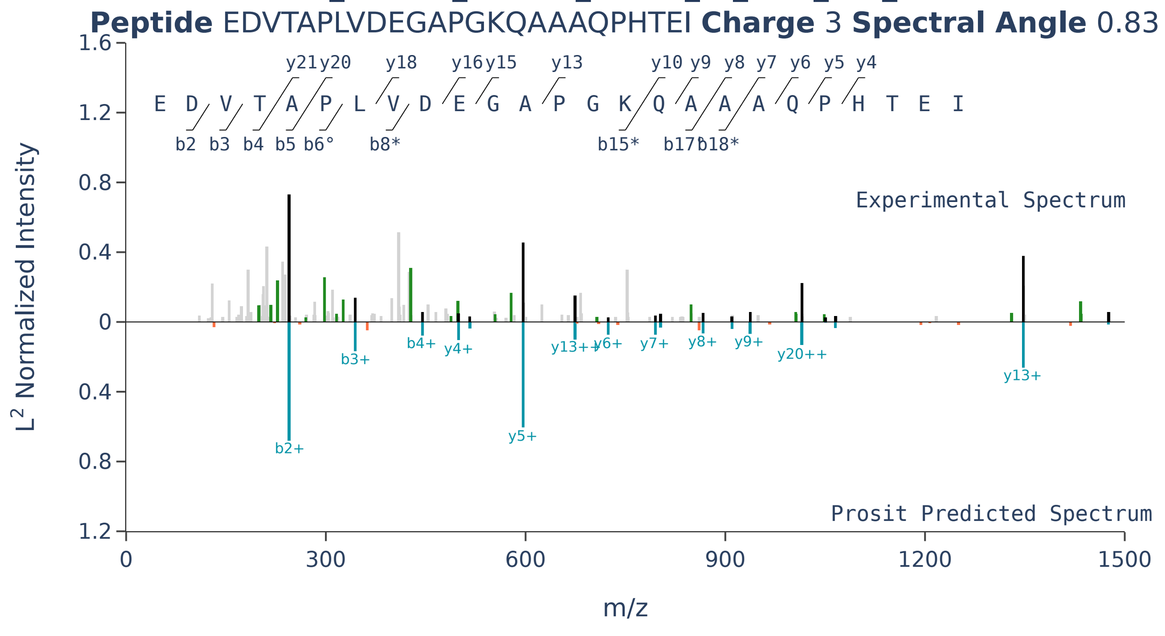

**Source** WSoh_221121_011221_HFGoe_L2_hTau_24h_R1 **Scan** 19147
**Peptide** KIGSLDNITHVPGGGN **Charge** 3 **Spectral Angle** 0.52

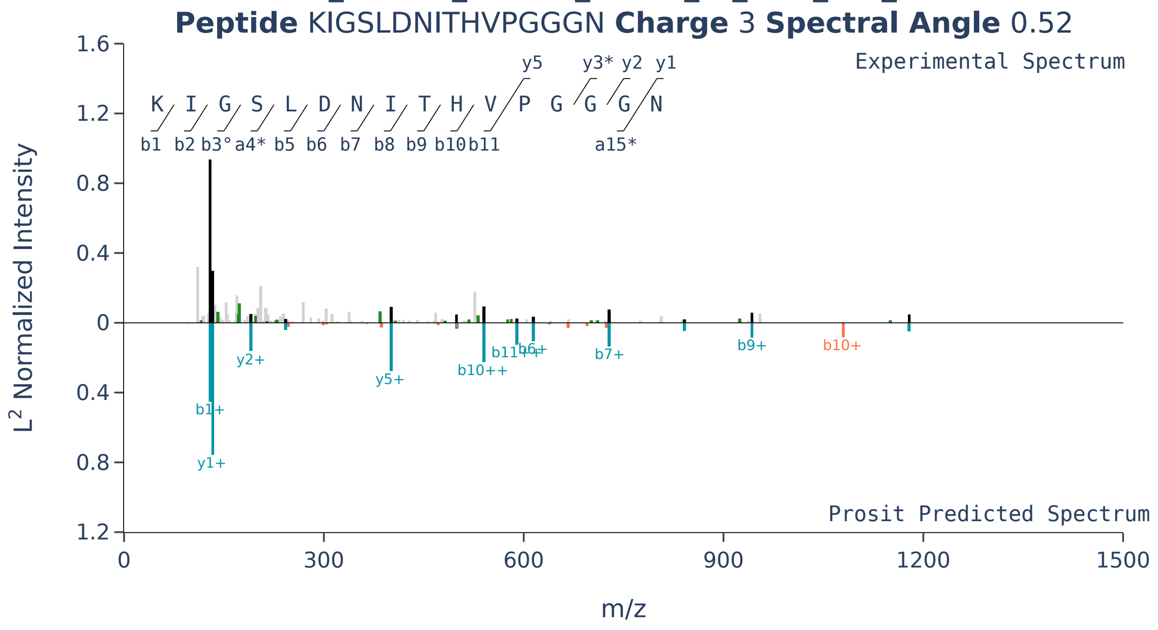


**Source** WSoh_221121_011221_HFGoe_L2_hTau_24h_R2 **Scan** 6576
**Peptide** KNVKSKIGSTENLKHQPGGGKV **Charge** 3 **Spectral Angle** 0.89

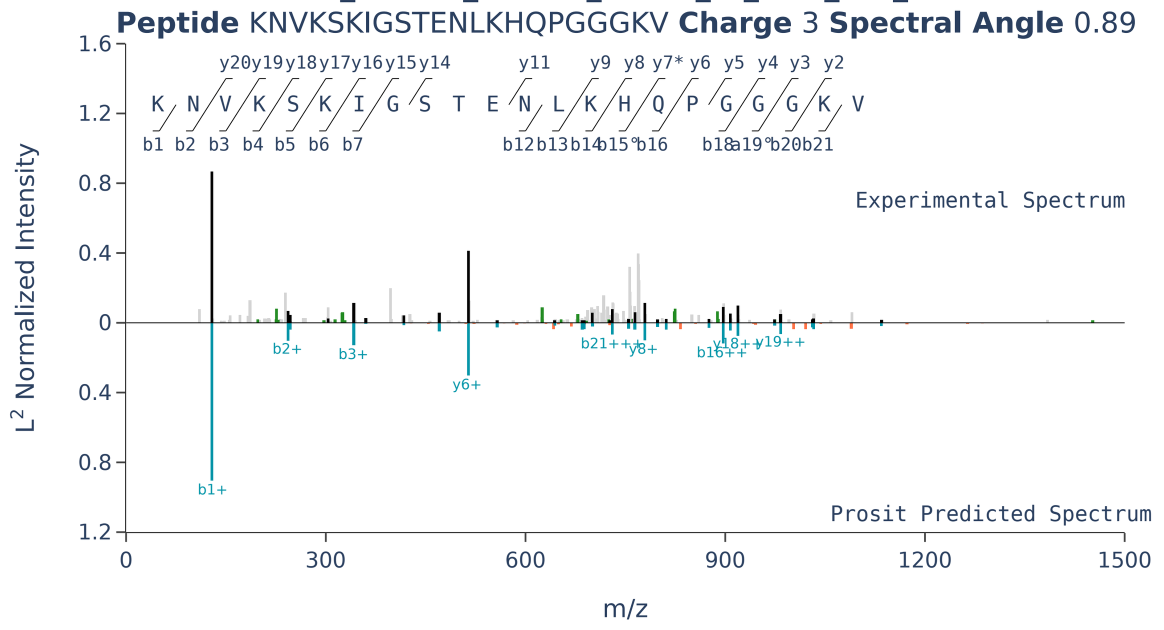


**Source** WSoh_221121_011221_HFGoe_L1_hTau_24h_R1 **Scan** 8903
**Peptide** VMEDHAGTYG **Charge** 2 **Spectral Angle** 0.54

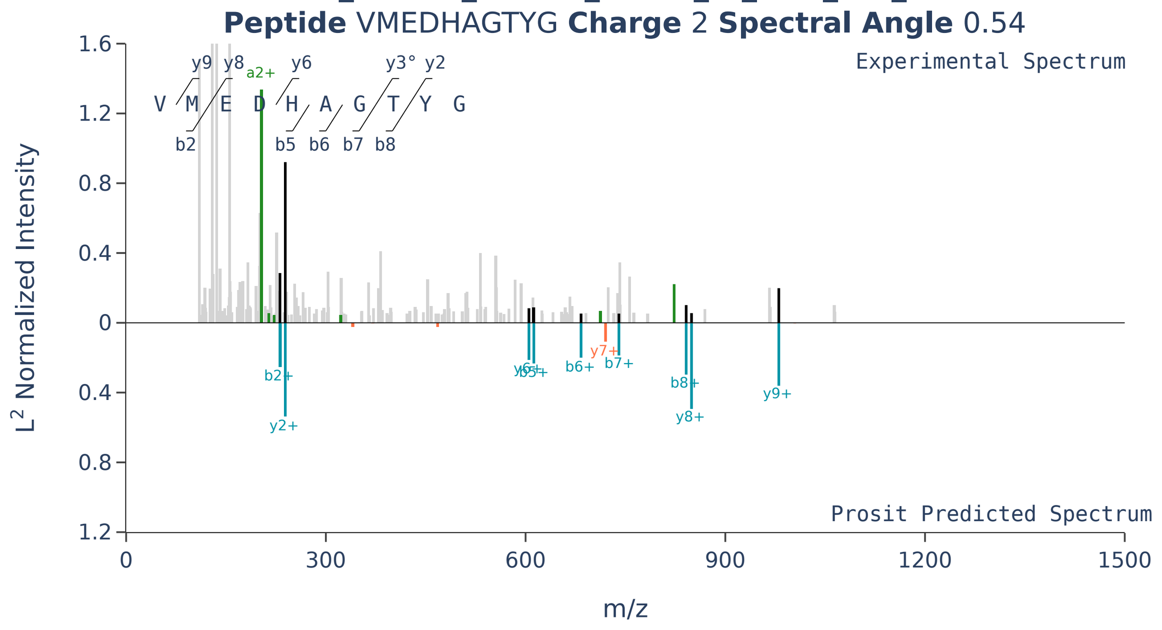


**Source** WSoh_221121_011221_HFGoe_L1_hTau_2h_R2 **Scan** 17595
**Peptide** EGAPGKQAAAQPHTEIPEGTTAEEAGL **Charge** 3 **Spectral Angle** 0.77

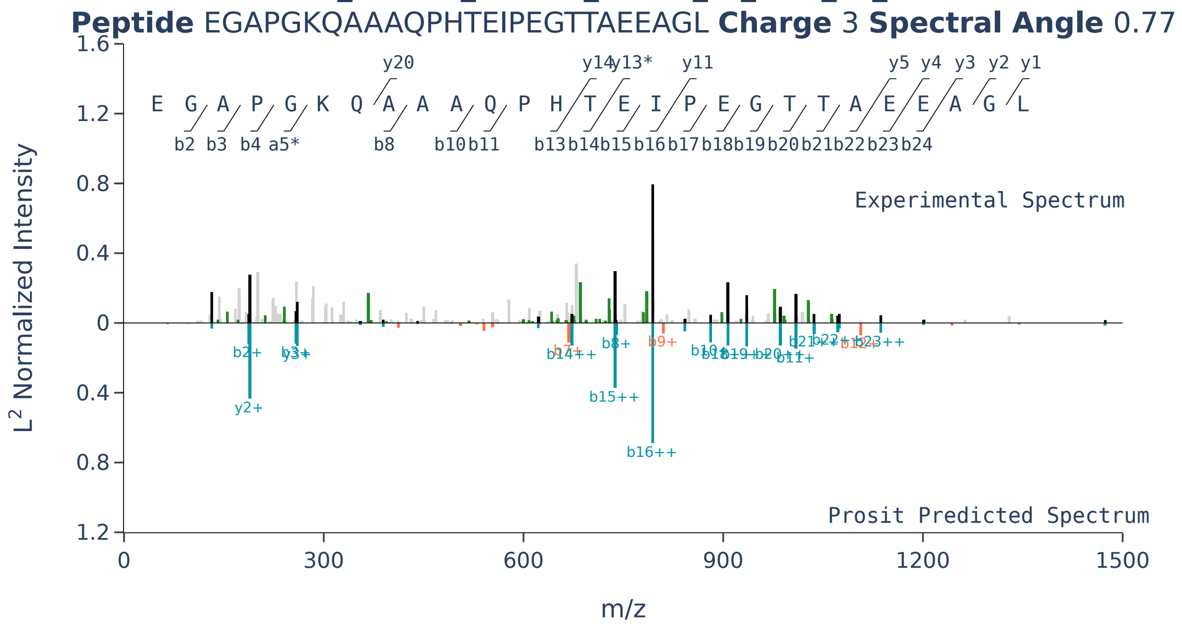


**Source** WSoh_221121_011221_HFGoe_L1_hTau_24h_R1 **Scan** 20842
**Peptide** QLVYKPVDLSKVTSKC **Charge** 3 **Spectral Angle** 0.62

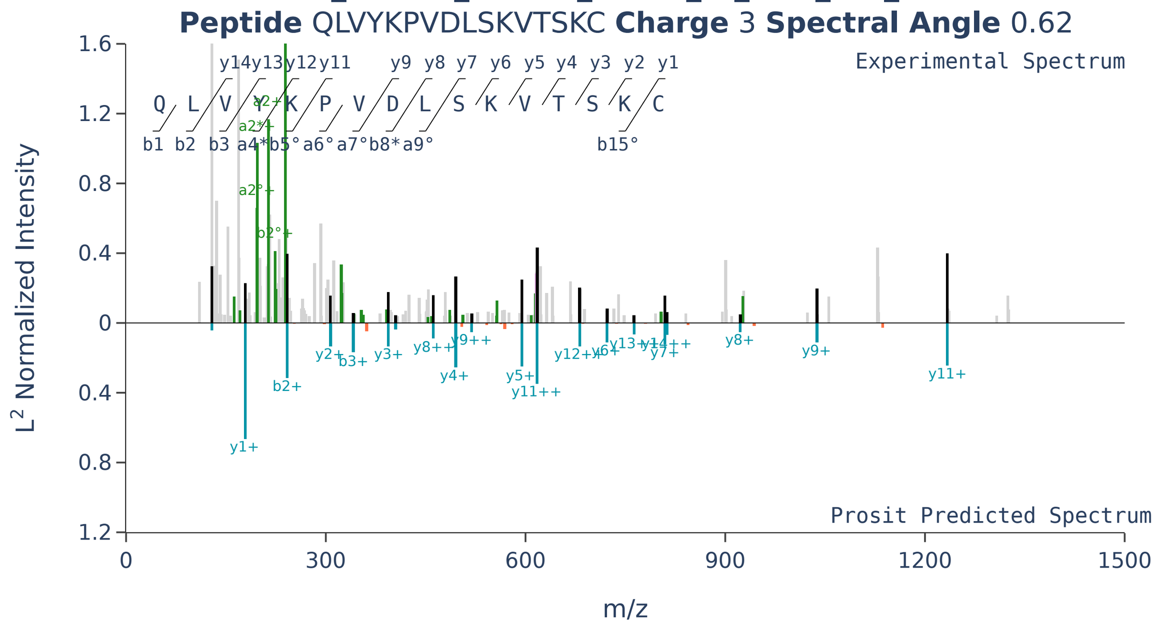


**Source** WSoh_221121_011221_HFGoe_L1_hTau_4h_R1 **Scan** 6758
**Peptide** PGGGNKKIETHKLTFRENAKAKTD **Charge** 4 **Spectral Angle** 0.72

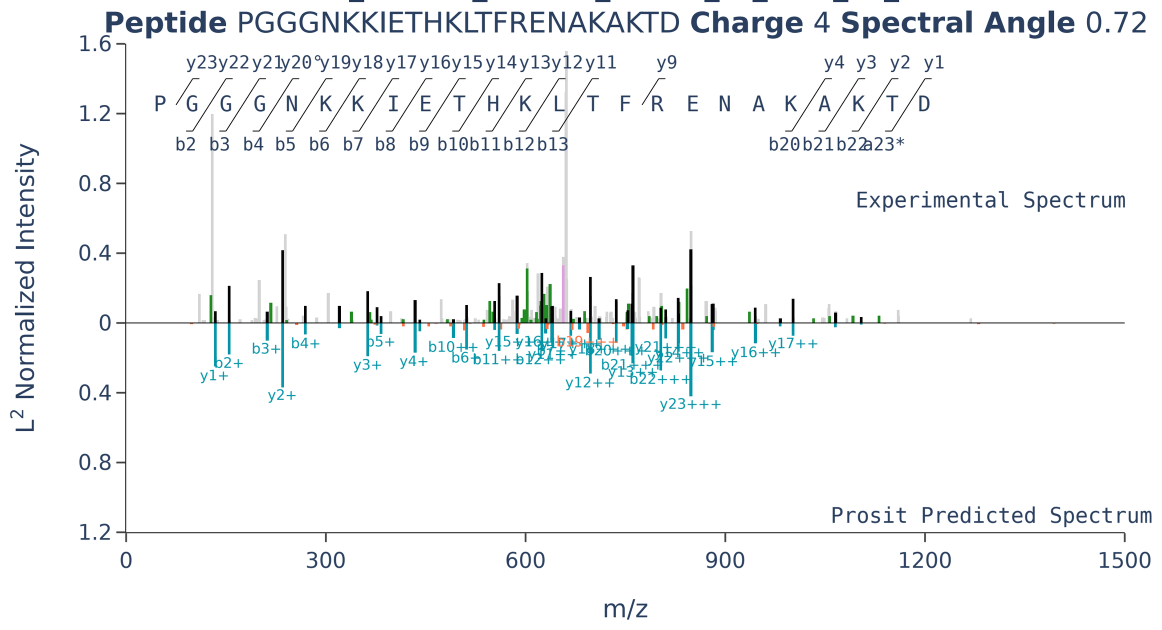


**Source** WSoh_221121_011221_HFGoe_L2_hTau_2h_R2 **Scan** 13529
**Peptide** GSTENLKHQPGGGKVQII **Charge** 3 **Spectral Angle** 0.8

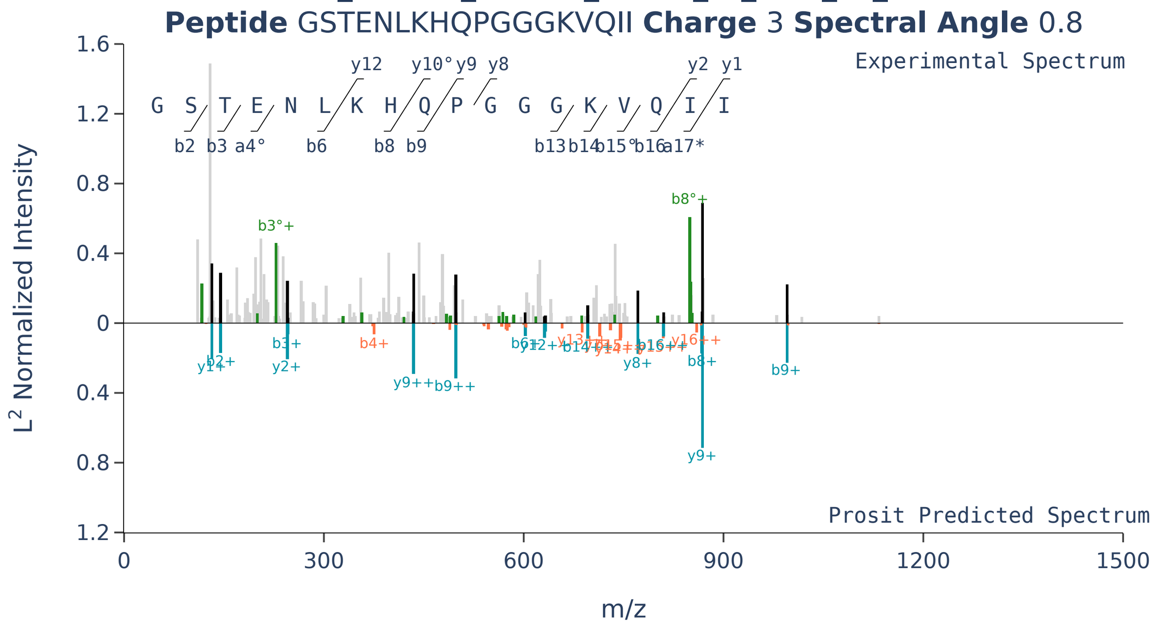


**Source** WSoh_221121_011221_HFGoe_L2_hTau_4h_R1 **Scan** 24298
**Peptide** VDSPQLATL **Charge** 2 **Spectral Angle** 0.78

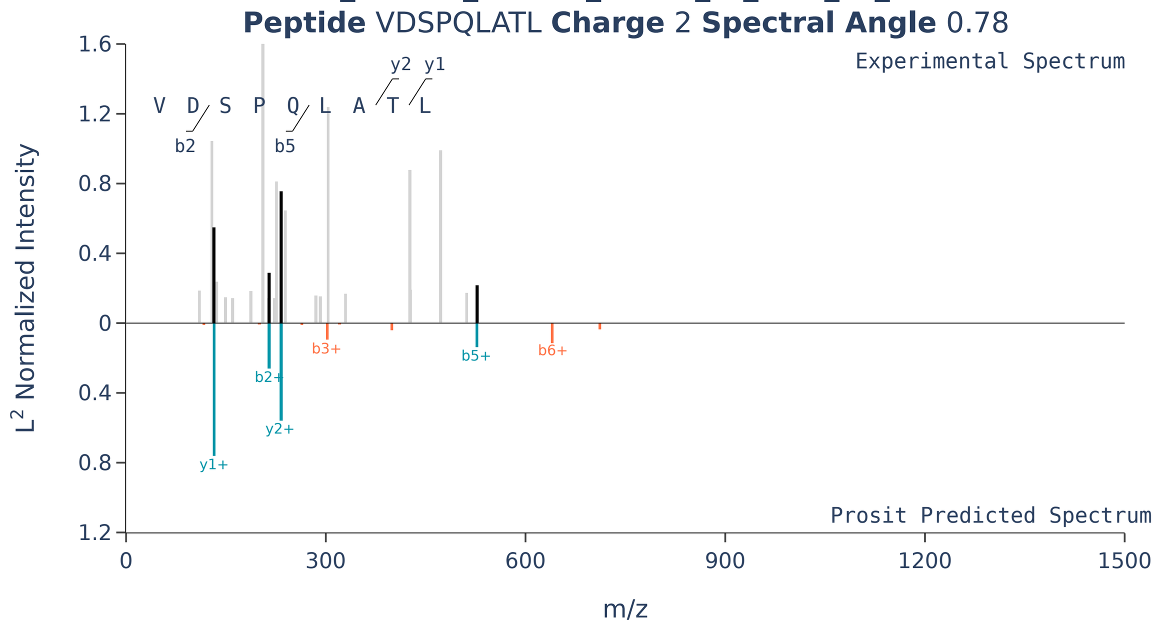


**Source** WSoh_221121_011221_HFGoe_L1_hTau_4h_R1 **Scan** 6555
**Peptide** SKCGSLGNIHHKPGGGQVE **Charge** 3 **Spectral Angle** 0.77

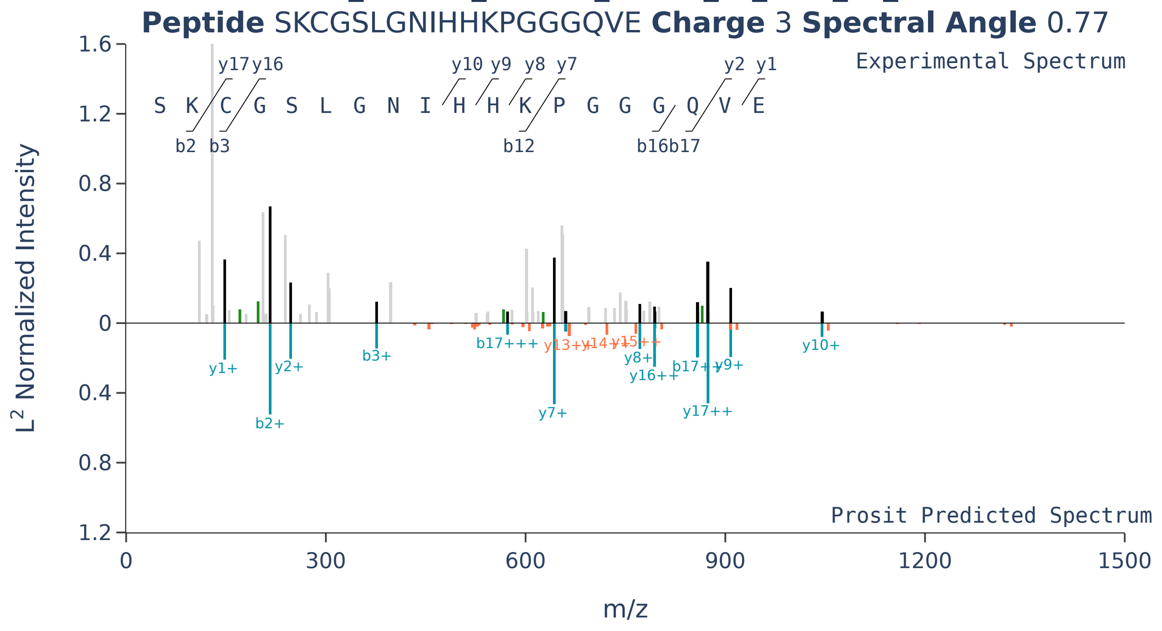


**Source** WSoh_221121_011221_HFGoe_L1_hTau_4h_R1 **Scan** 11564
**Peptide** NVQSKCGSKDNIKHVPGGGSVQL **Charge** 4 **Spectral Angle** 0.81

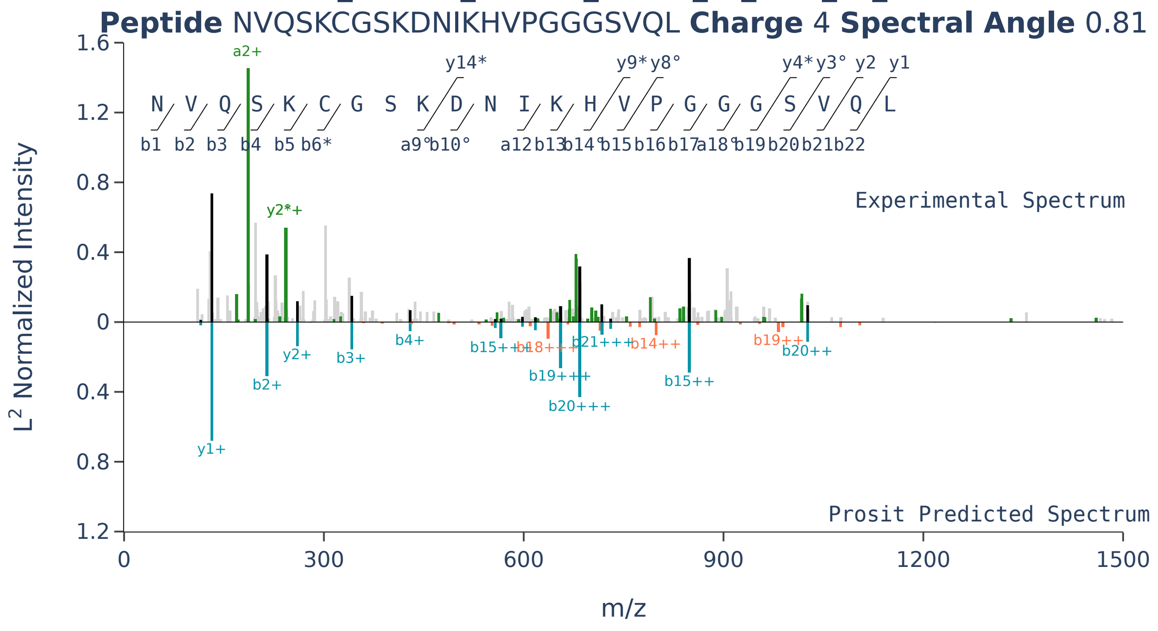


**Source** WSoh_221121_011221_HFGoe_L2_hTau_24h_R2 **Scan** 15195
**Peptide** SEKLDFKDRVQSKL **Charge** 4 **Spectral Angle** 0.74

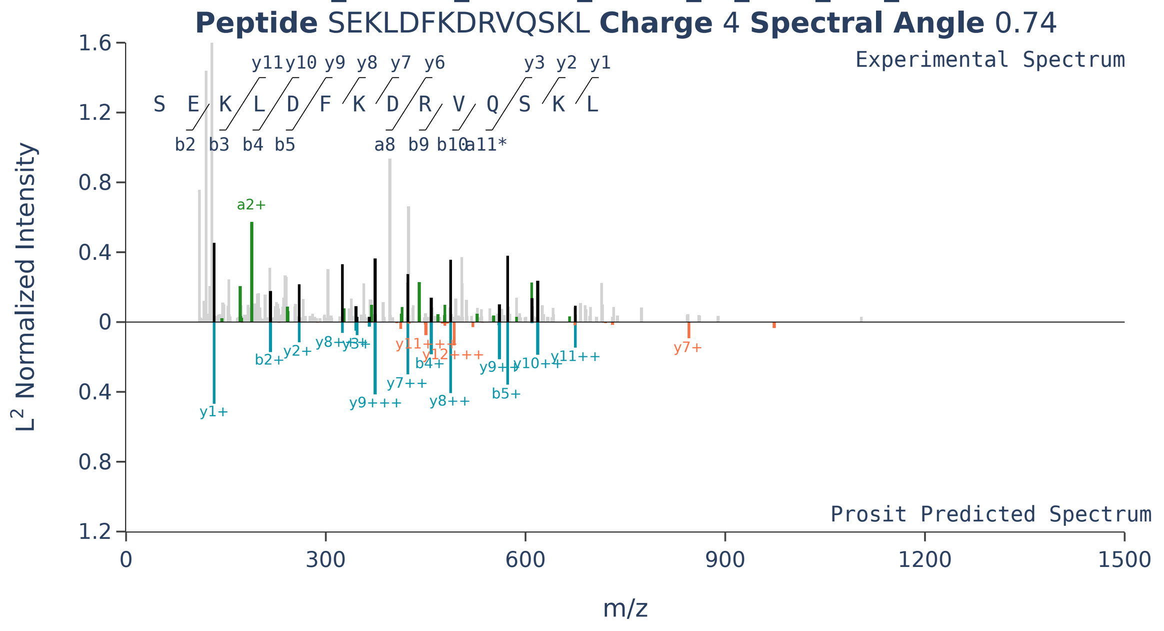


**Source** WSoh_221121_011221_HFGoe_L1_hTau_4h_R2 **Scan** 15097
**Peptide** QSKIGSLDNITHVPGGGNKKIETHKL **Charge** 5 **Spectral Angle** 0.84

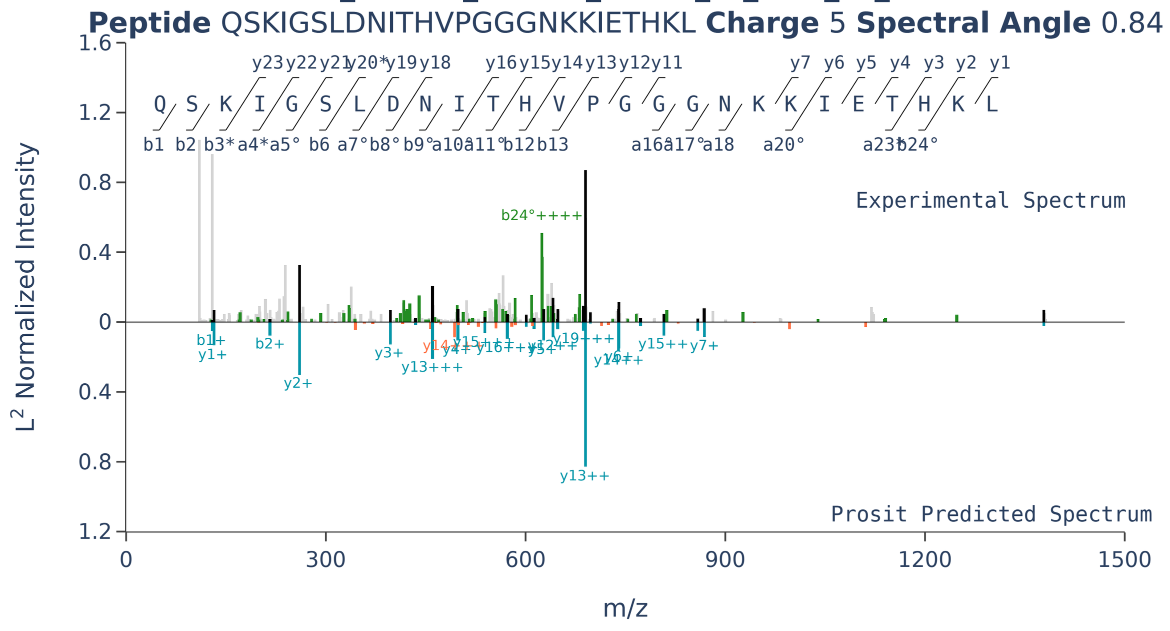


**Source** WSoh_221121_011221_HFGoe_L1_hTau_24h_R1 **Scan** 18202
**Peptide** SLDNITHVPGGGNKKIETHKLTF **Charge** 4 **Spectral Angle** 0.78

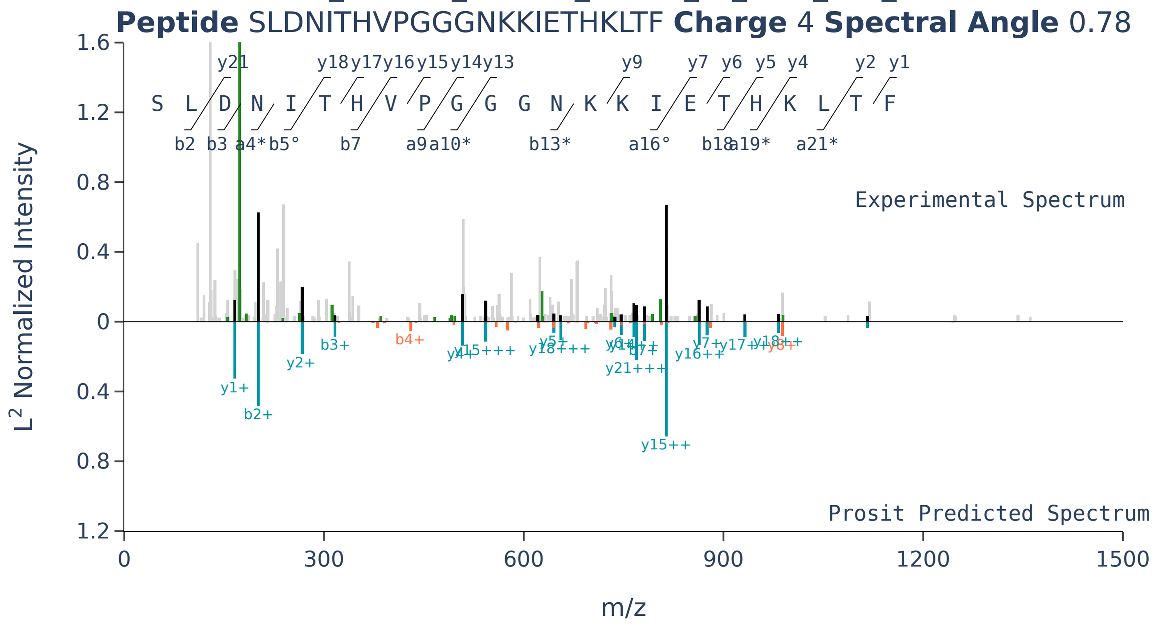


**Source** WSoh_221121_011221_HFGoe_L2_hTau_2h_R2 **Scan** 7358
**Peptide** SKVTSKCGSLGNIHHKPGGGQVEVKSE **Charge** 4 **Spectral Angle** 0.78

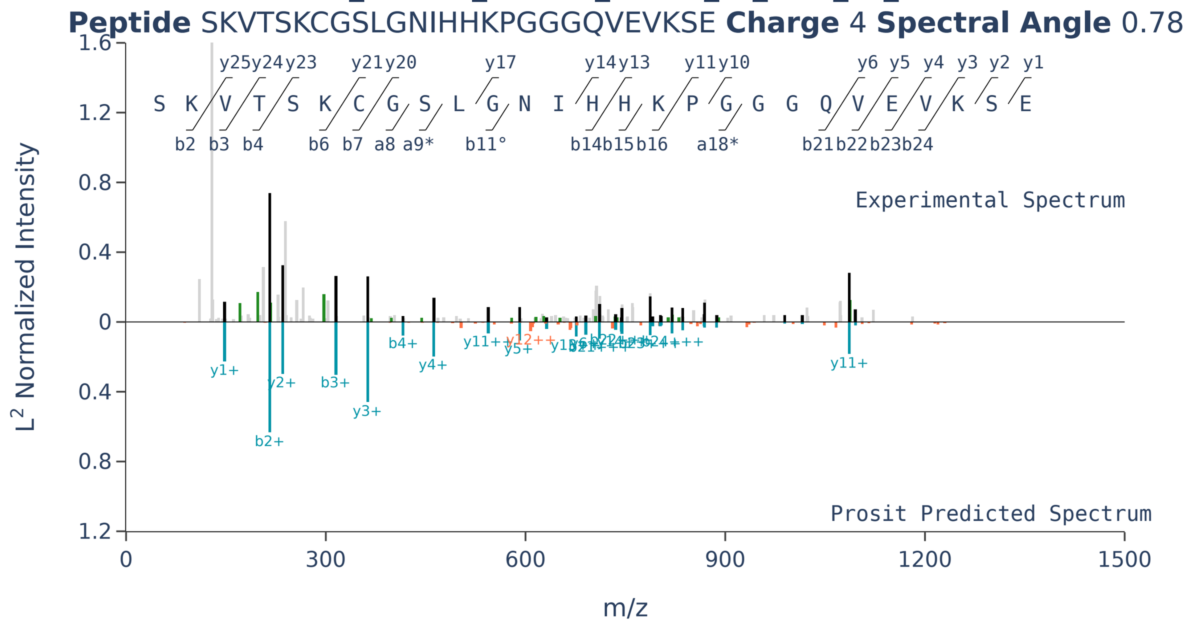


**Source** WSoh_221121_011221_HFGoe_L2_hTau_24h_R2 **Scan** 5707
**Peptide** RENAKAKTDHGAEL **Charge** 2 **Spectral Angle** 0.63

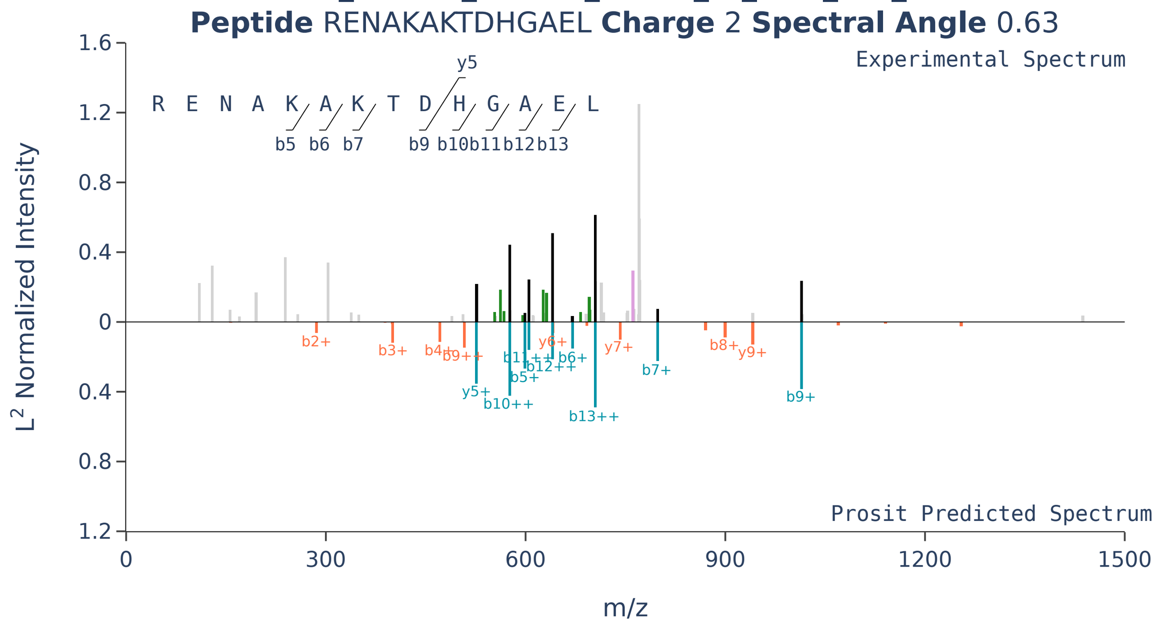

**Source** WSoh_221121_011221_HFGoe_L2_hTau_24h_R1 **Scan** 9198
**Peptide** KNVKSKIGSTENLKHQPGGGKVQI **Charge** 3 **Spectral Angle** 0.82

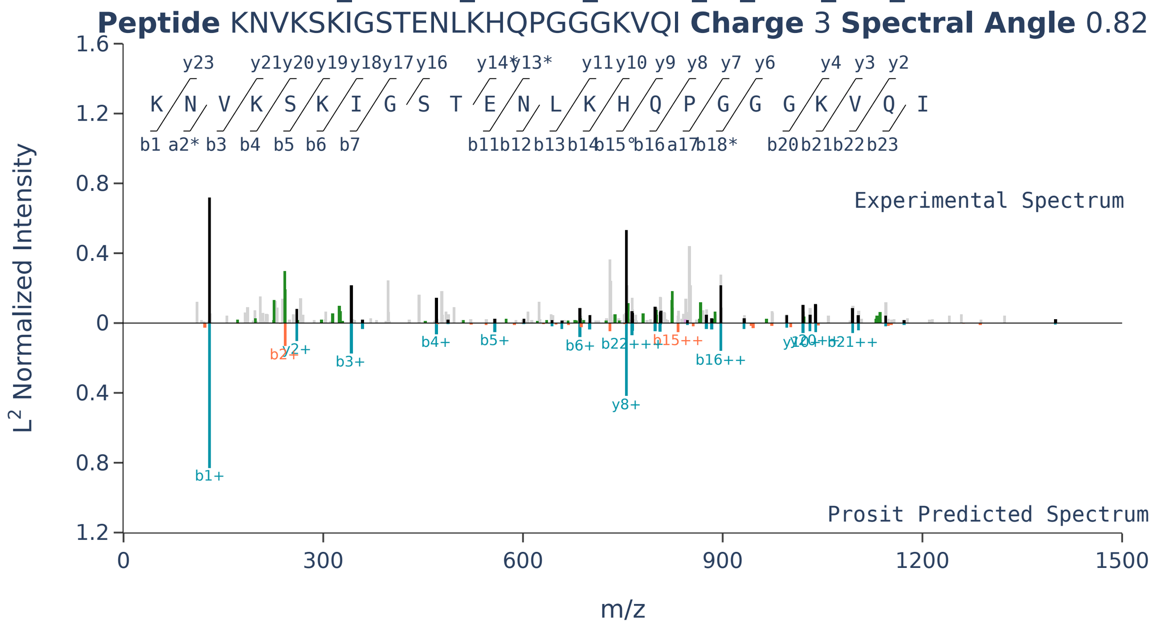


**Source** WSoh_221121_011221_HFGoe_L2_hTau_24h_R1 **Scan** 13656
**Peptide** STENLKHQPGGGKVQIL **Charge** 3 **Spectral Angle** 0.79

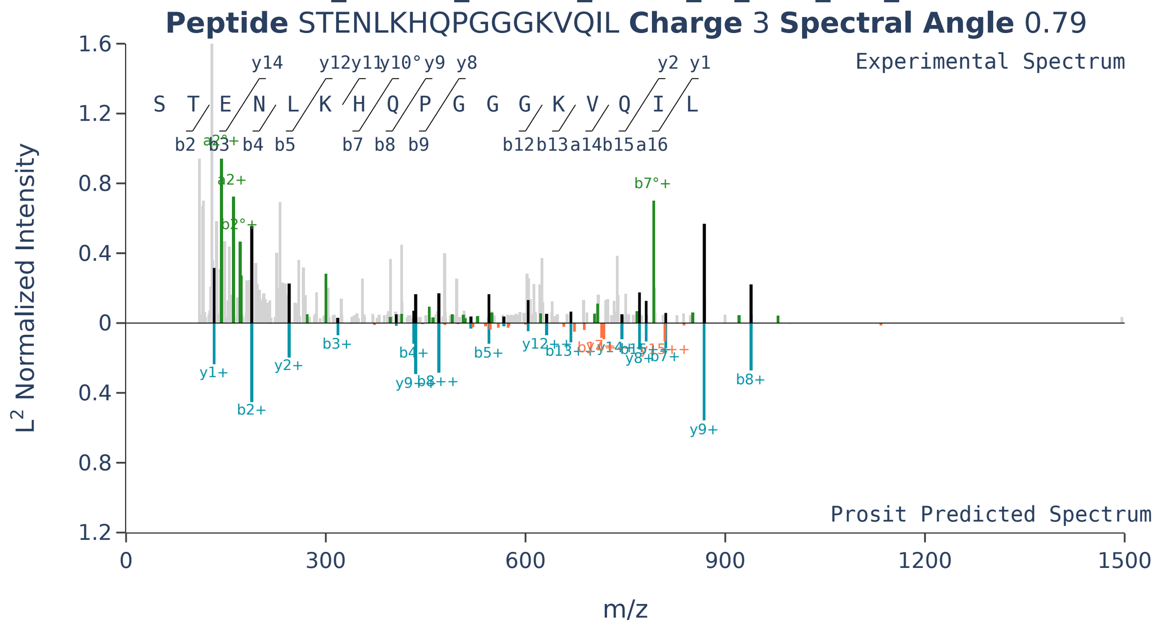


**Source** WSoh_221121_011221_HFGoe_L1_hTau_24h_R1 **Scan** 20312
**Peptide** NLKHVPGGGSVQIVYKPVDLSKVTSK **Charge** 4 **Spectral Angle** 0.75

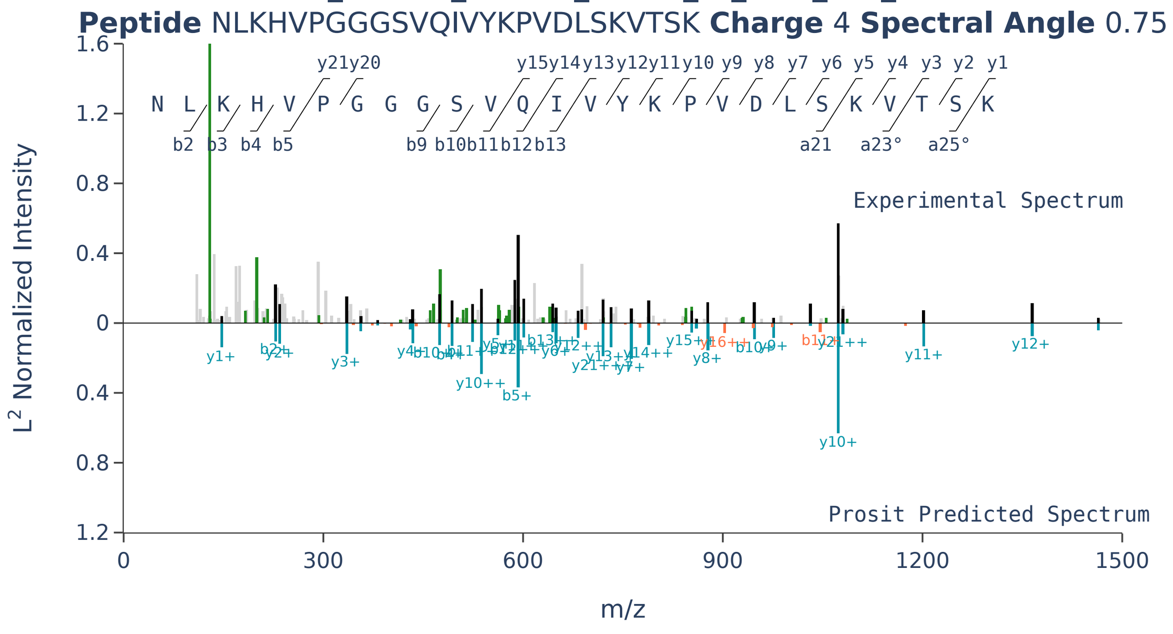


**Source** WSoh_221121_011221_HFGoe_L2_hTau_4h_R2 **Scan** 12171
**Peptide** KIGSTENLKHQPGGGKVQIIN **Charge** 3 **Spectral Angle** 0.88

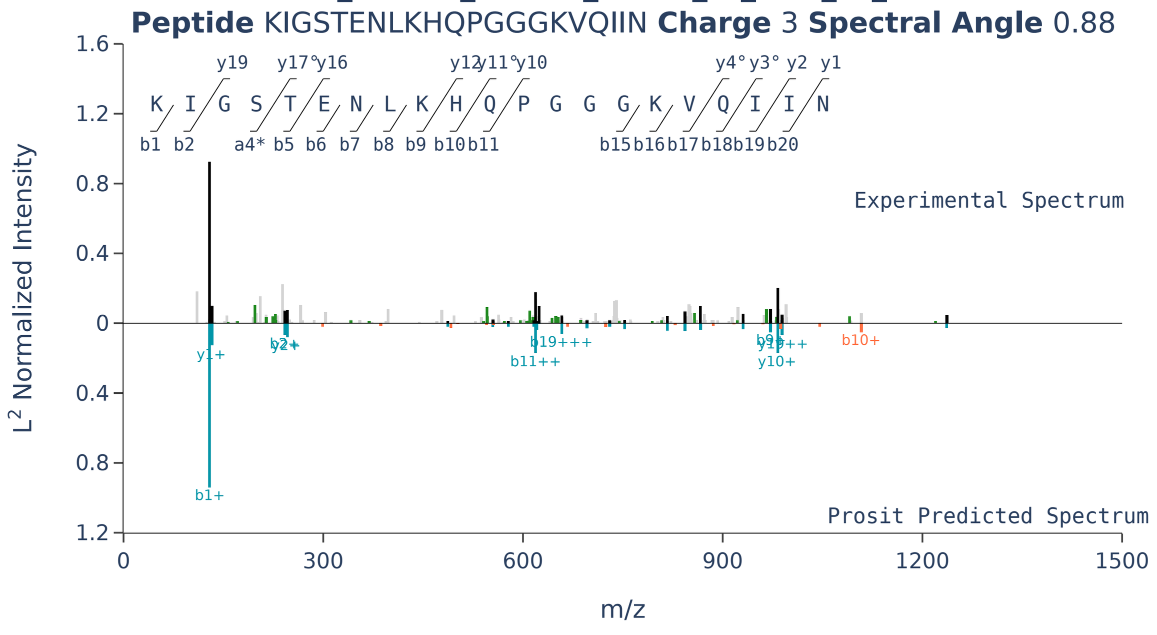


**Source** WSoh_221121_011221_HFGoe_L1_hTau_4h_R2 **Scan** 20369
**Peptide** IVYKPVDLSKVTS **Charge** 3 **Spectral Angle** 0.74

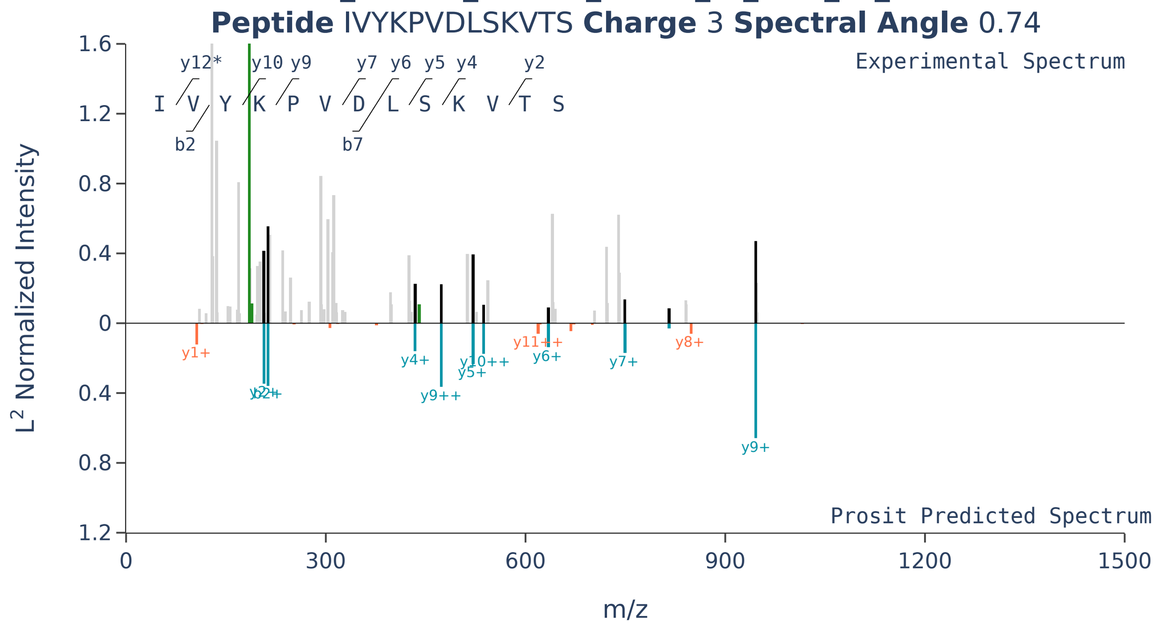


**Source** WSoh_221121_011221_HFGoe_L1_hTau_24h_R1 **Scan** 10335
**Peptide** NITHVPGGGNKKIETHKLTFRENAKA **Charge** 5 **Spectral Angle** 0.8

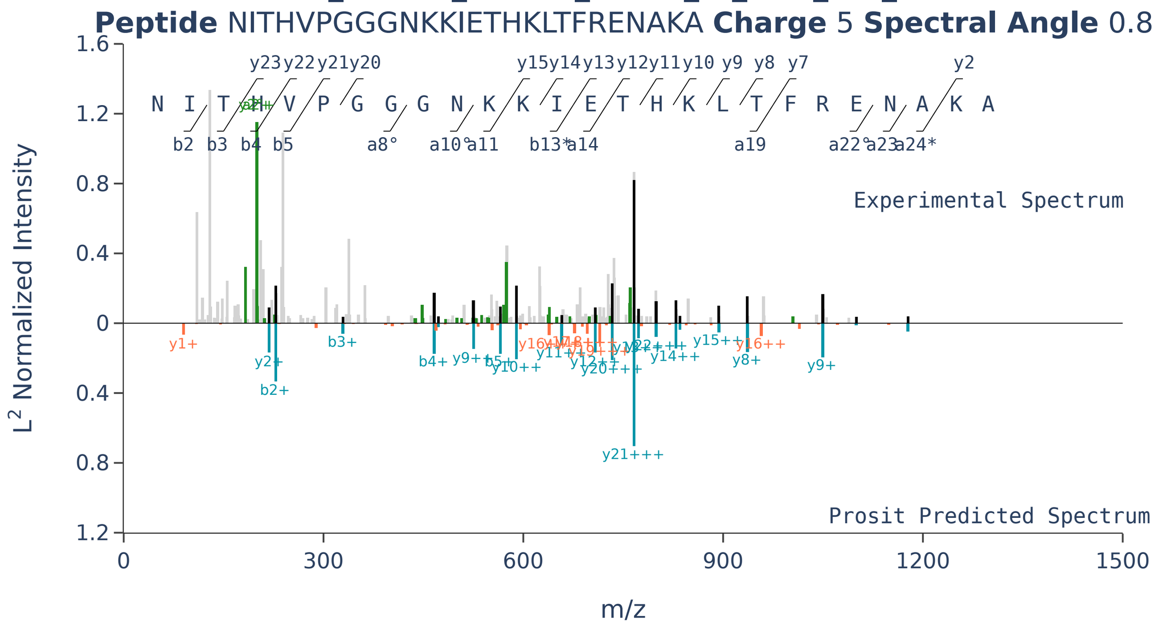


**Source** WSoh_221121_011221_HFGoe_L1_hTau_2h_R1 **Scan** 12836
**Peptide** VYKSPVVSGDTSPRHLSNVSS **Charge** 3 **Spectral Angle** 0.76

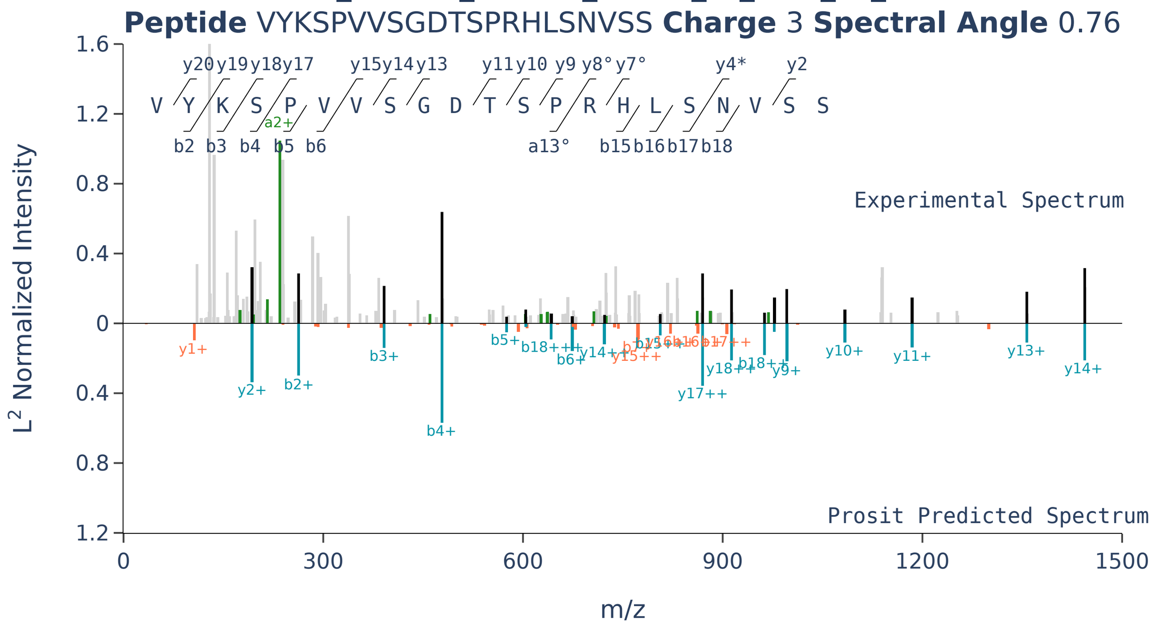


**Source** WSoh_221121_011221_HFGoe_L1_hTau_24h_R1 **Scan** 23136
**Peptide** PGSETSDAKSTPTAEDVTAPLV **Charge** 3 **Spectral Angle** 0.74

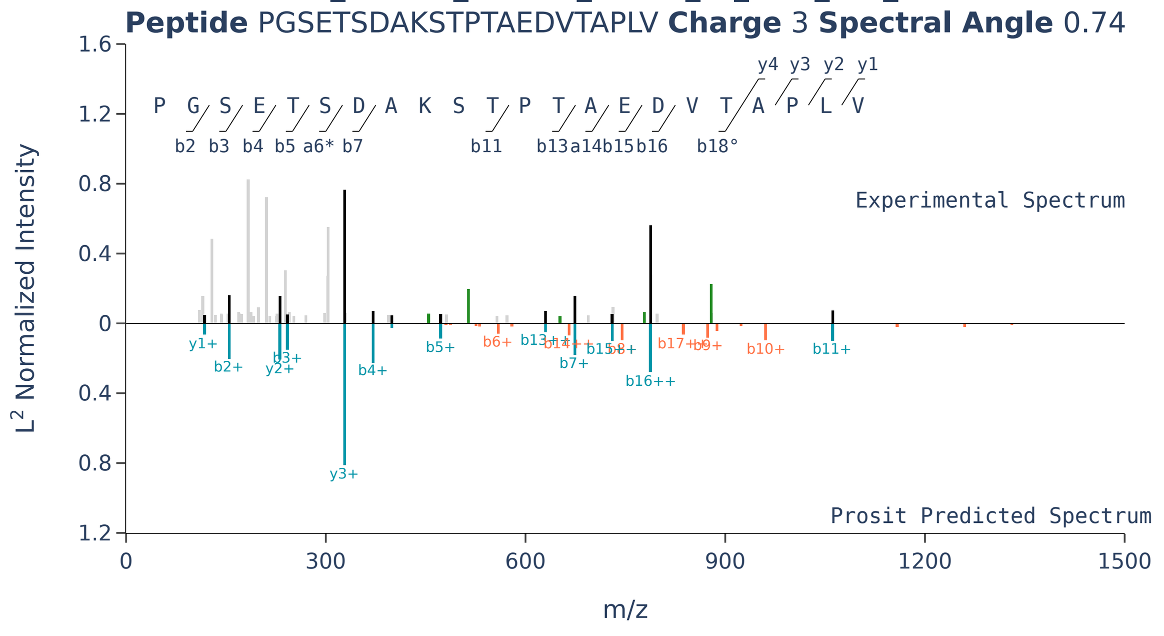


**Source** WSoh_221121_011221_HFGoe_L2_hTau_2h_R1 **Scan** 12542
**Peptide** AKAKTDHGAEIVYKSPVVSGDTSPRHLS **Charge** 5 **Spectral Angle** 0.68

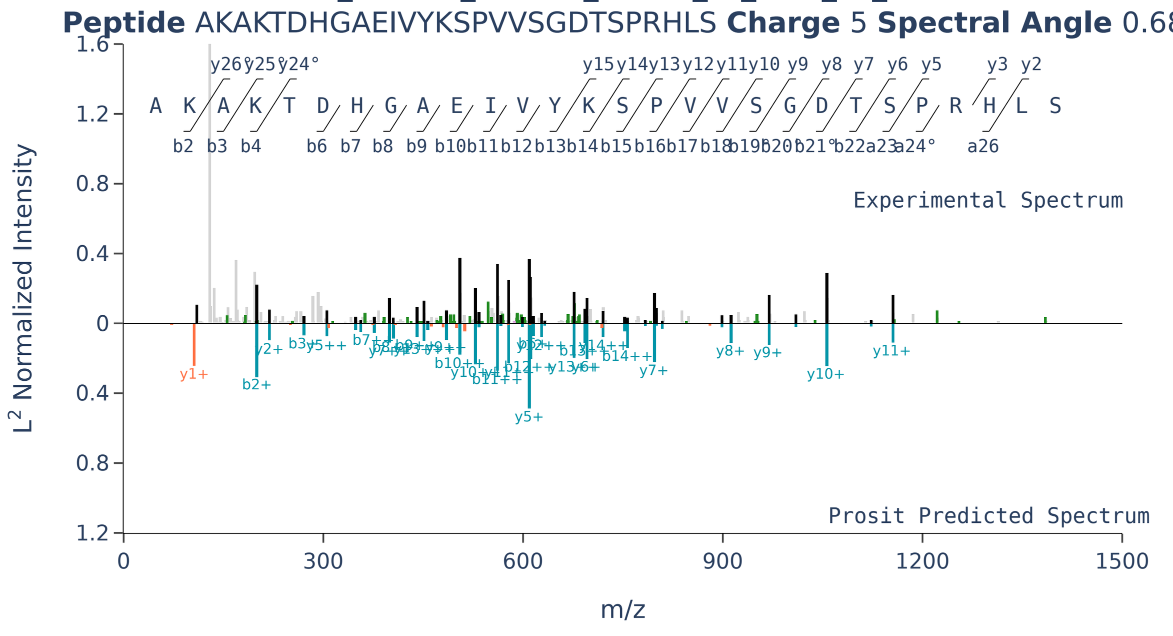


**Source** WSoh_221121_011221_HFGoe_L2_hTau_24h_R1 **Scan** 10210
**Peptide** TRIPAKTPPAPKTPPSSGEPPKSG **Charge** 3 **Spectral Angle** 0.74

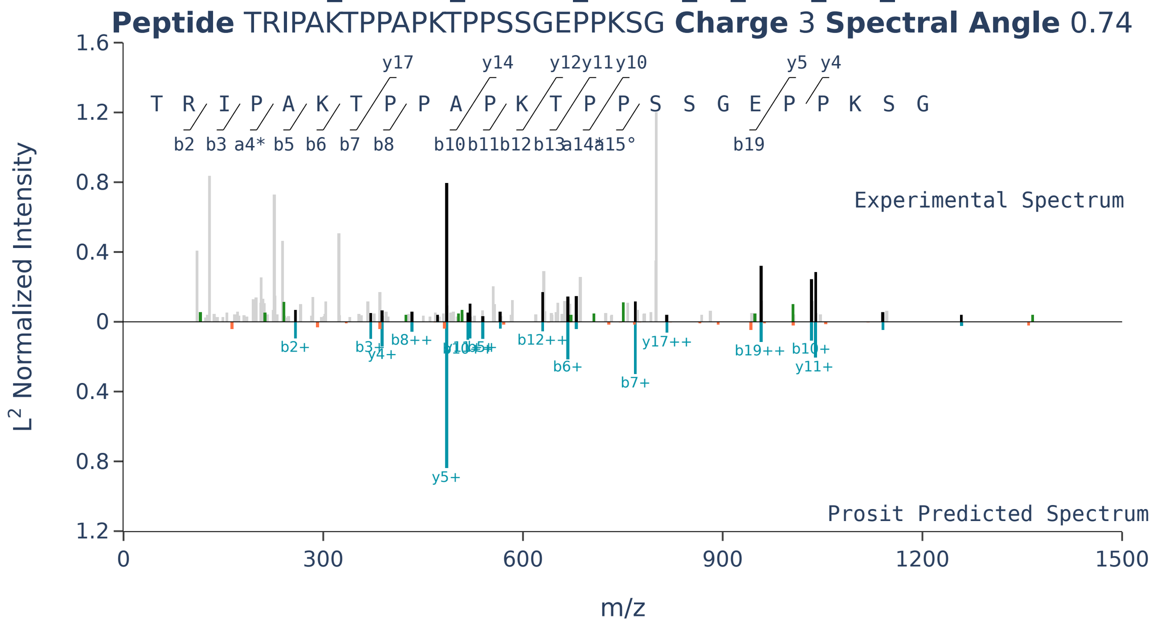


**Source** WSoh_221121_011221_HFGoe_L2_hTau_24h_R1 **Scan** 20194
**Peptide** LVYKPVDLSKVTSKC **Charge** 3 **Spectral Angle** 0.7

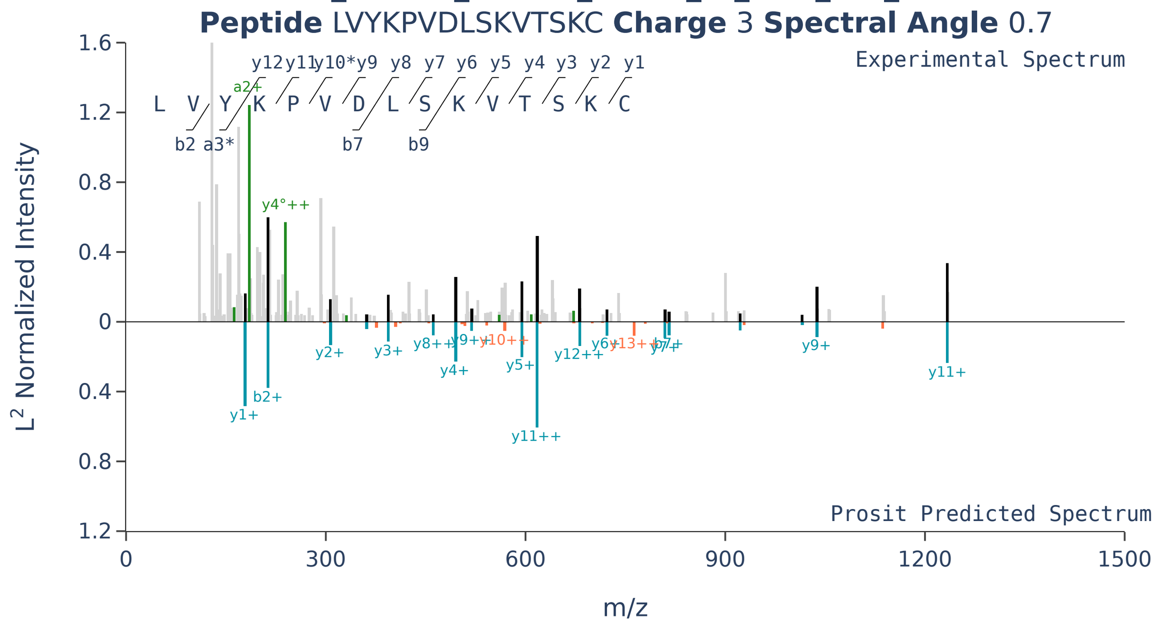


**Source** WSoh_221121_011221_HFGoe_L1_hTau_24h_R2 **Scan** 10978
**Peptide** LPAKTPPAPKTPPSSGEPPKSGDRSGY **Charge** 5 **Spectral Angle** 0.75

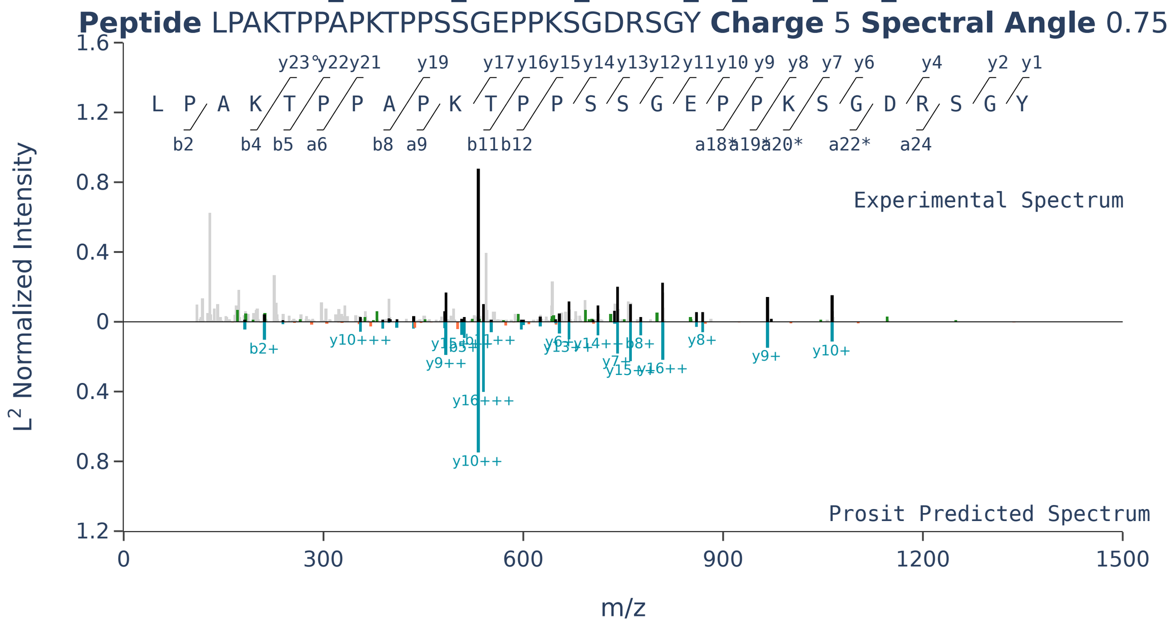


**Source** WSoh_221121_011221_HFGoe_L1_hTau_24h_R2 **Scan** 12557
**Peptide** NLKHQPGGGKVQIL **Charge** 3 **Spectral Angle** 0.72

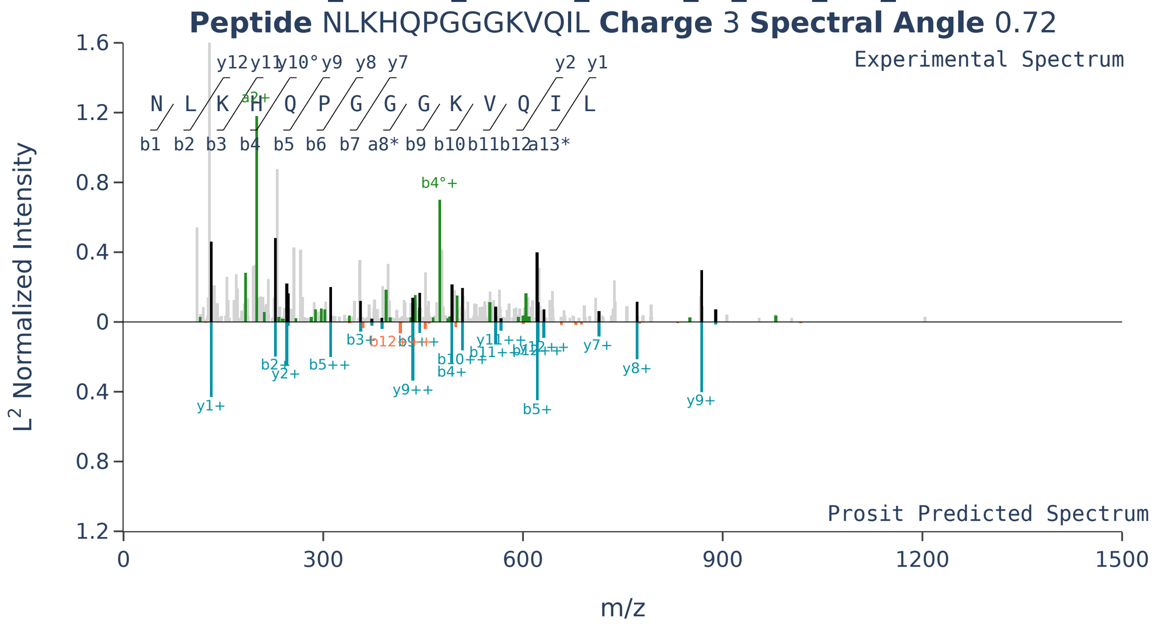


**Source** WSoh_221121_011221_HFGoe_L2_hTau_24h_R1 **Scan** 22041
**Peptide** DVTAPLVDEGAPGKQAAAQPHTEIPEGT **Charge** 3 **Spectral Angle** 0.84

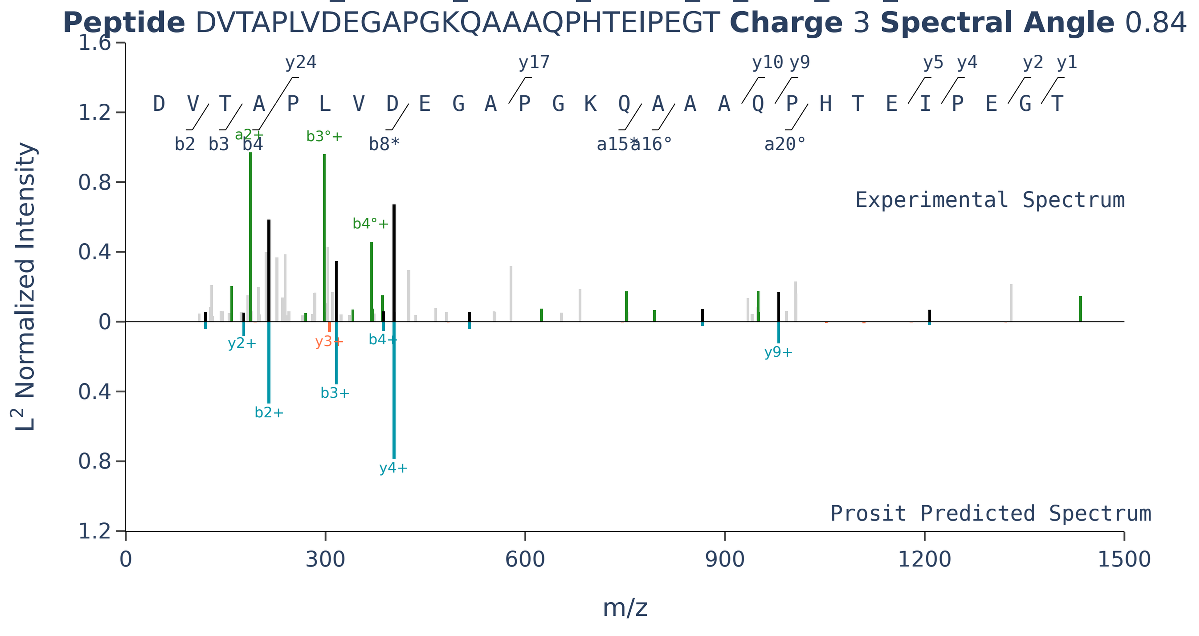


**Source** WSoh_221121_011221_HFGoe_L2_hTau_24h_R2 **Scan** 19431
**Peptide** DEGAPGKQAAAQPHTEIPEGTTAEEAGI **Charge** 3 **Spectral Angle** 0.65

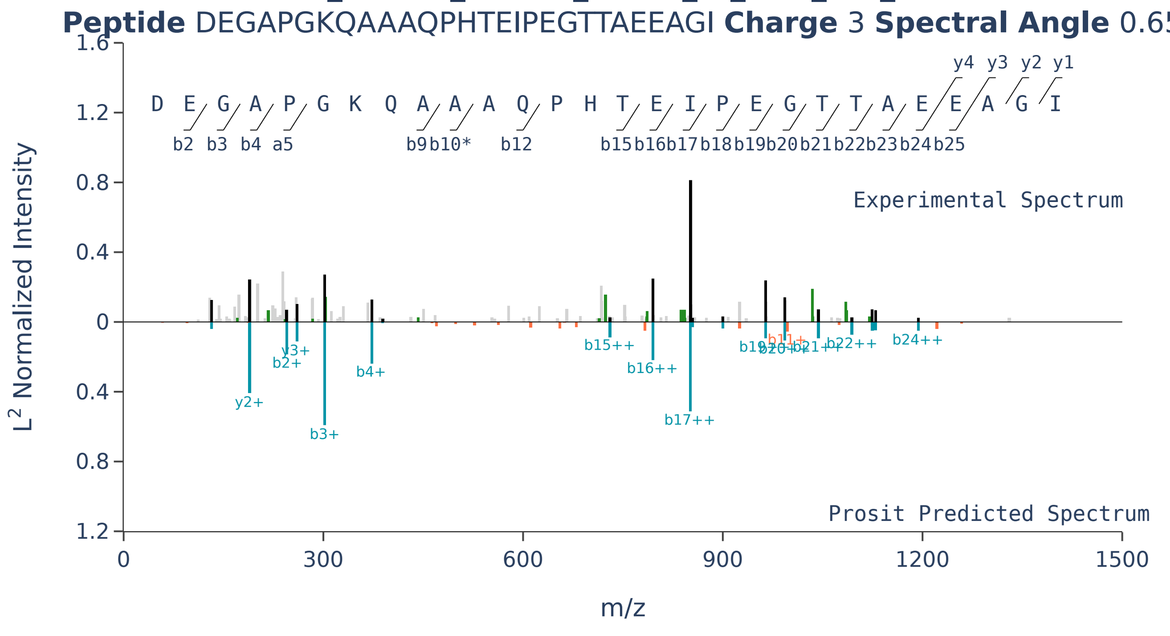

**Source** WSoh_221121_011221_HFGoe_L1_hTau_4h_R1 **Scan** 13265
**Peptide** STENLKHQPGGGKVQII **Charge** 3 **Spectral Angle** 0.78

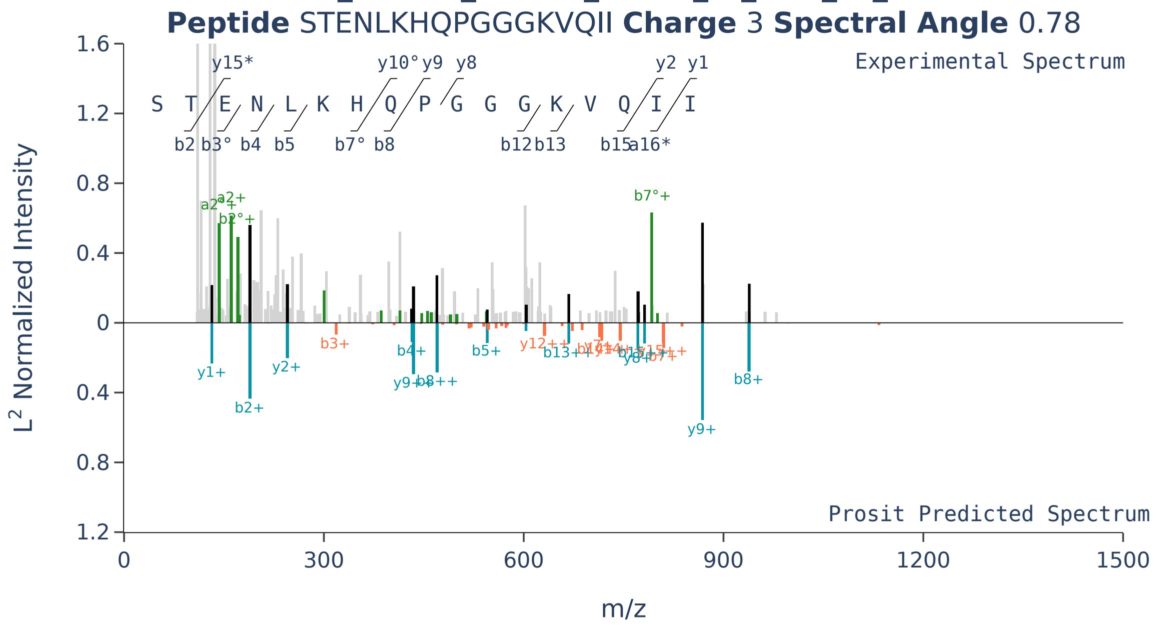


**Source** WSoh_221121_011221_HFGoe_L1_hTau_4h_R1 **Scan** 5537
**Peptide** SKDGTGSDDKKAKGADGKTKIATPRGA **Charge** 4 **Spectral Angle** 0.8

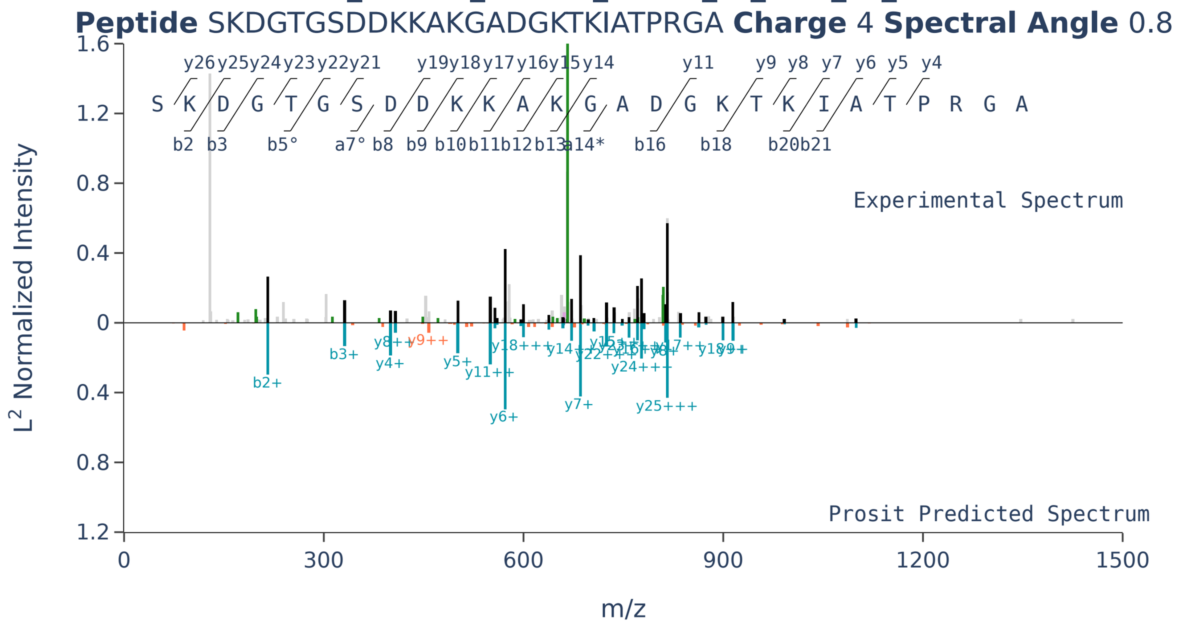


**Source** WSoh_221121_011221_HFGoe_L2_hTau_24h_R2 **Scan** 19180
**Peptide** RTPPKSPSSAKSRLQTAPVPMPDLKNV **Charge** 5 **Spectral Angle** 0.75

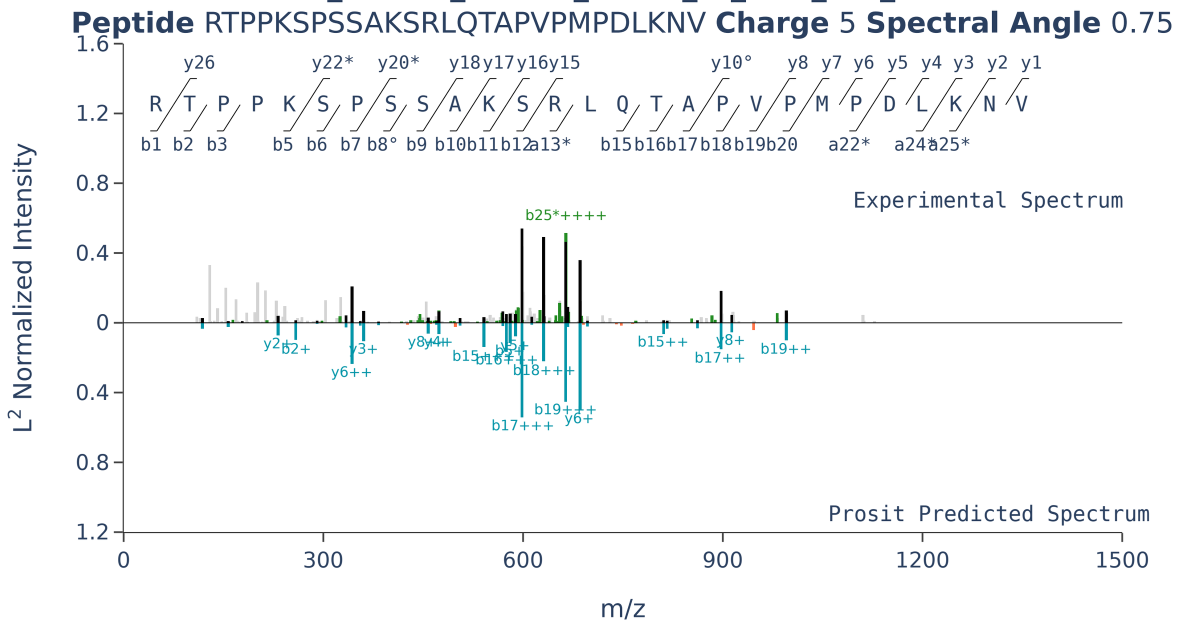


**Source** WSoh_221121_011221_HFGoe_L1_hTau_24h_R2 **Scan** 6868
**Peptide** KGADGKTKIATPRGAAPPGQKGQAN **Charge** 3 **Spectral Angle** 0.8

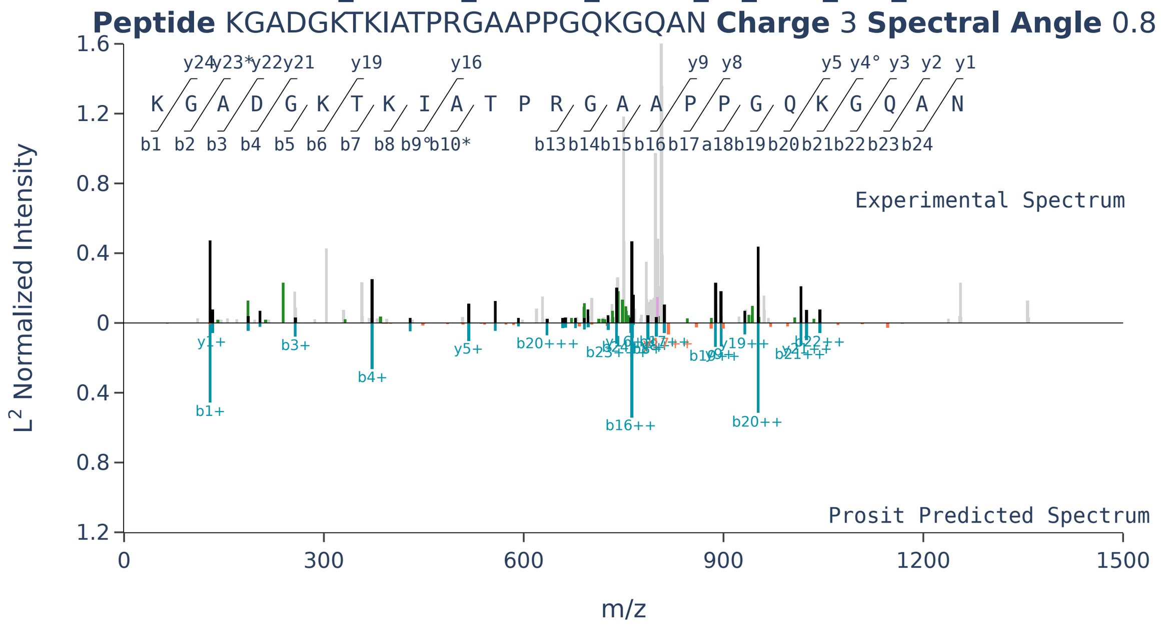


**Source** WSoh_221121_011221_HFGoe_L1_hTau_24h_R1 **Scan** 22024
**Peptide** AEEAGIGDTPSLEDEAAGHVTQA **Charge** 2 **Spectral Angle** 0.63

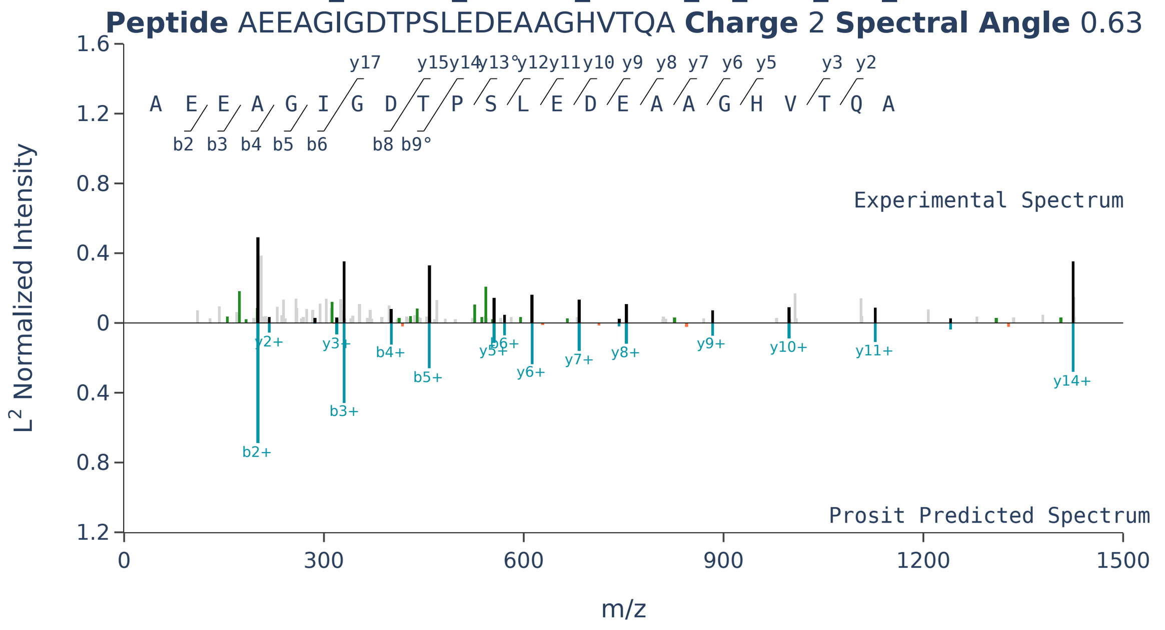


**Source** WSoh_221121_011221_HFGoe_L1_hTau_24h_R2 **Scan** 12698
**Peptide** KHQPGGGKVQIINKKLDLSN **Charge** 3 **Spectral Angle** 0.88

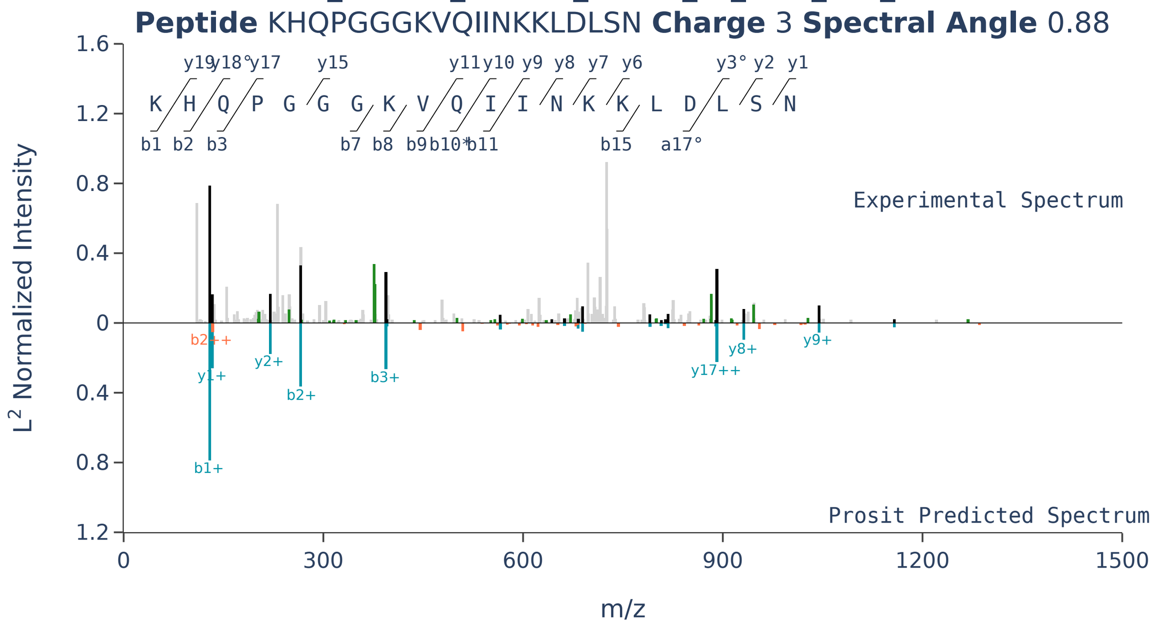


**Source** WSoh_221121_011221_HFGoe_L1_hTau_24h_R2 **Scan** 15213
**Peptide** KIGSTENLKHQPGGGKVQIINKKLDLS **Charge** 4 **Spectral Angle** 0.91

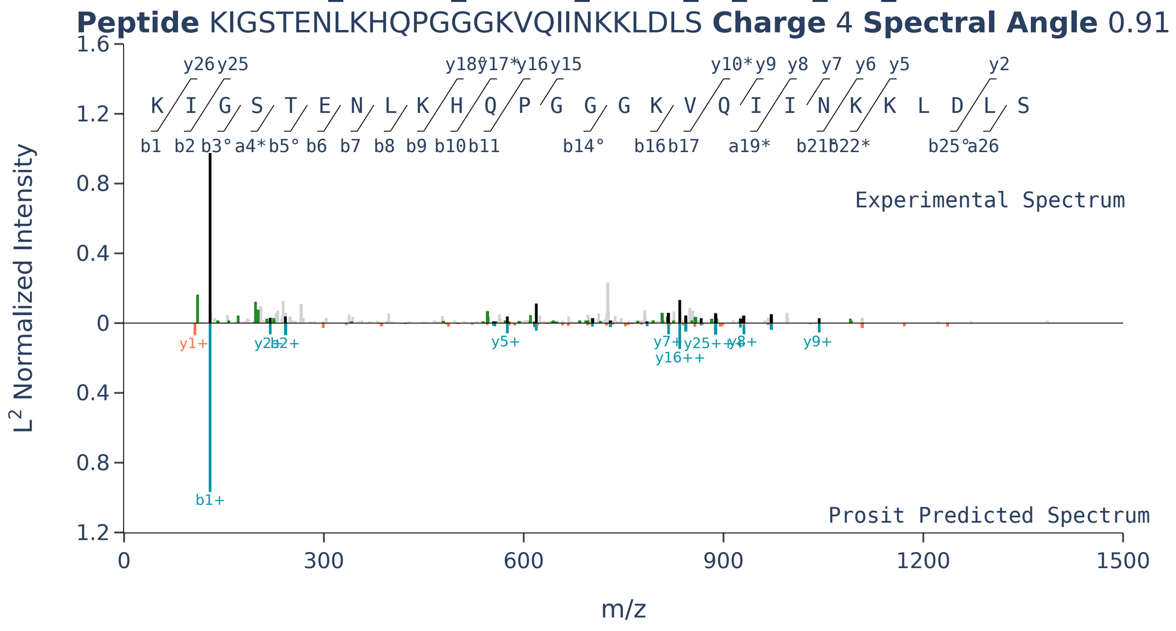


**Source** WSoh_221121_011221_HFGoe_L1_hTau_2h_R2 **Scan** 13953
**Peptide** PRHLSNVSSTGSIDM **Charge** 3 **Spectral Angle** 0.78

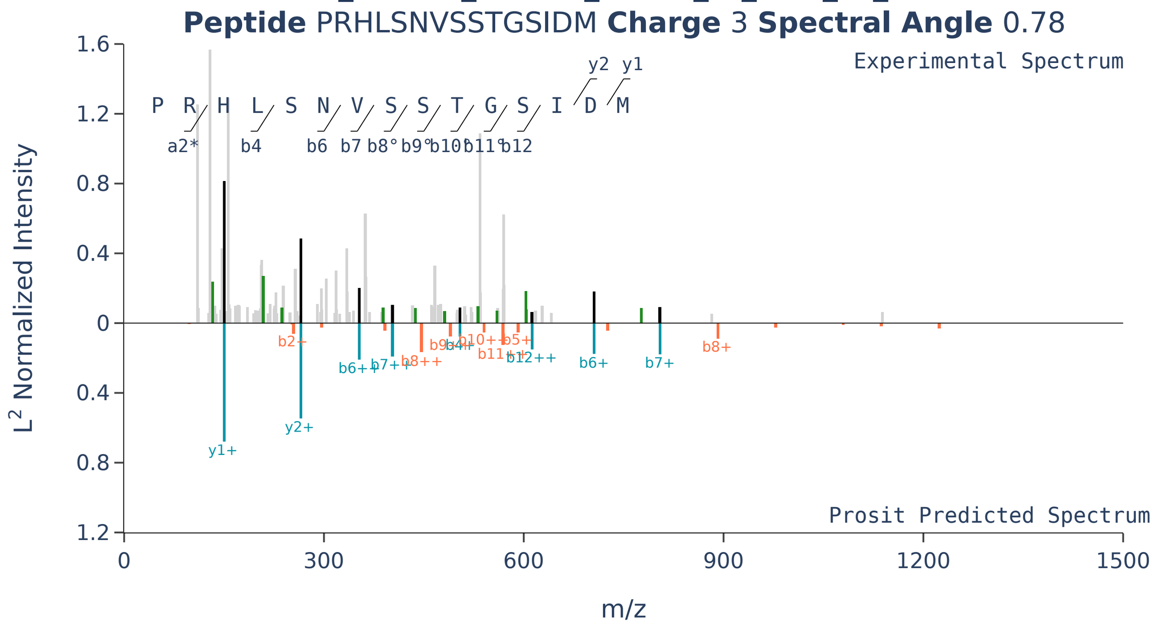


**Source** WSoh_221121_011221_HFGoe_L2_hTau_24h_R2 **Scan** 17246
**Peptide** TDHGAEIVYKSPVVSGDTSPRHLSNVSS **Charge** 4 **Spectral Angle** 0.81

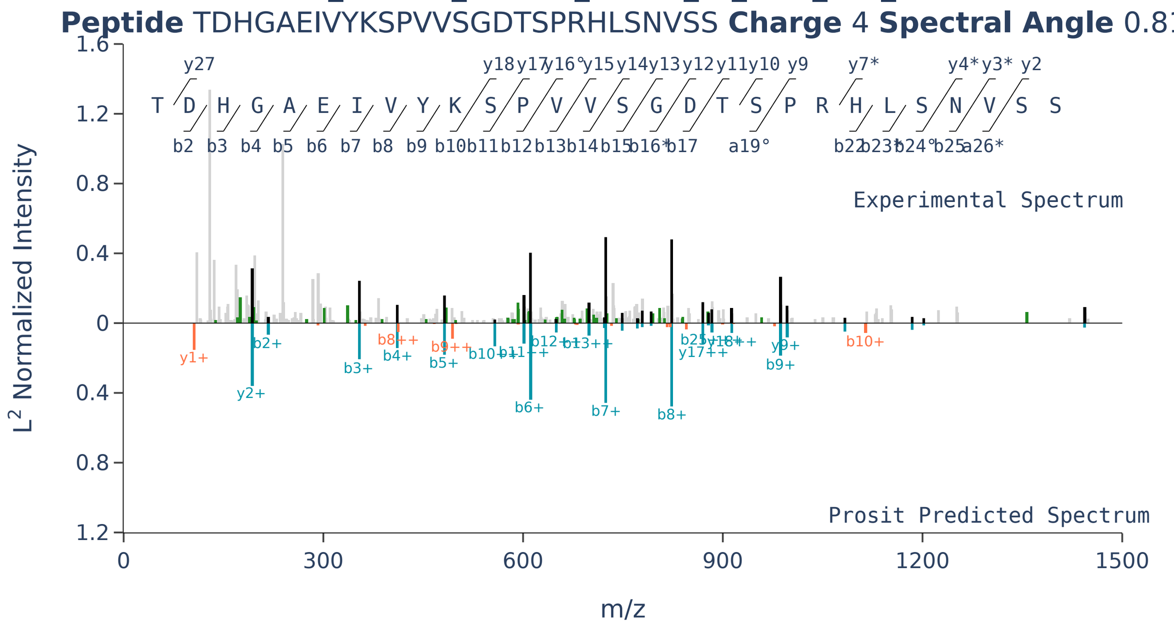


**Source** WSoh_221121_011221_HFGoe_L2_hTau_4h_R2 **Scan** 23459
**Peptide** TTAEEAGIGDTPSLEDEAAGHVTQARMV **Charge** 3 **Spectral Angle** 0.68

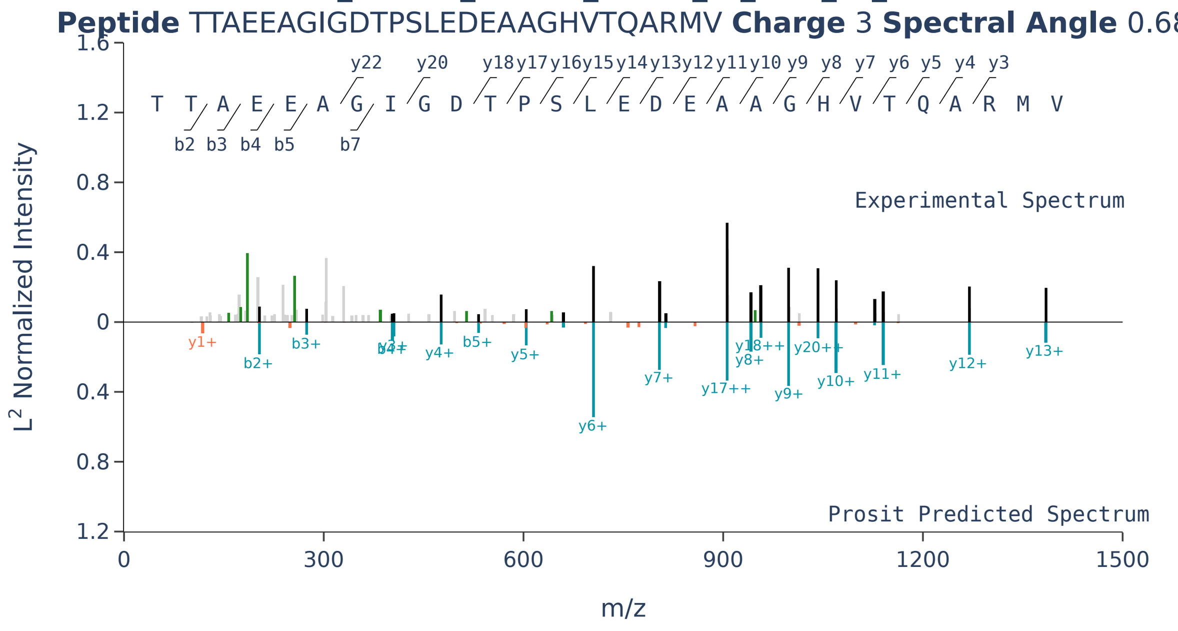


**Source** WSoh_221121_011221_HFGoe_L1_hTau_24h_R1 **Scan** 16272
**Peptide** TDHGAEIVYKSPVVSGDTSPRHLS **Charge** 4 **Spectral Angle** 0.66

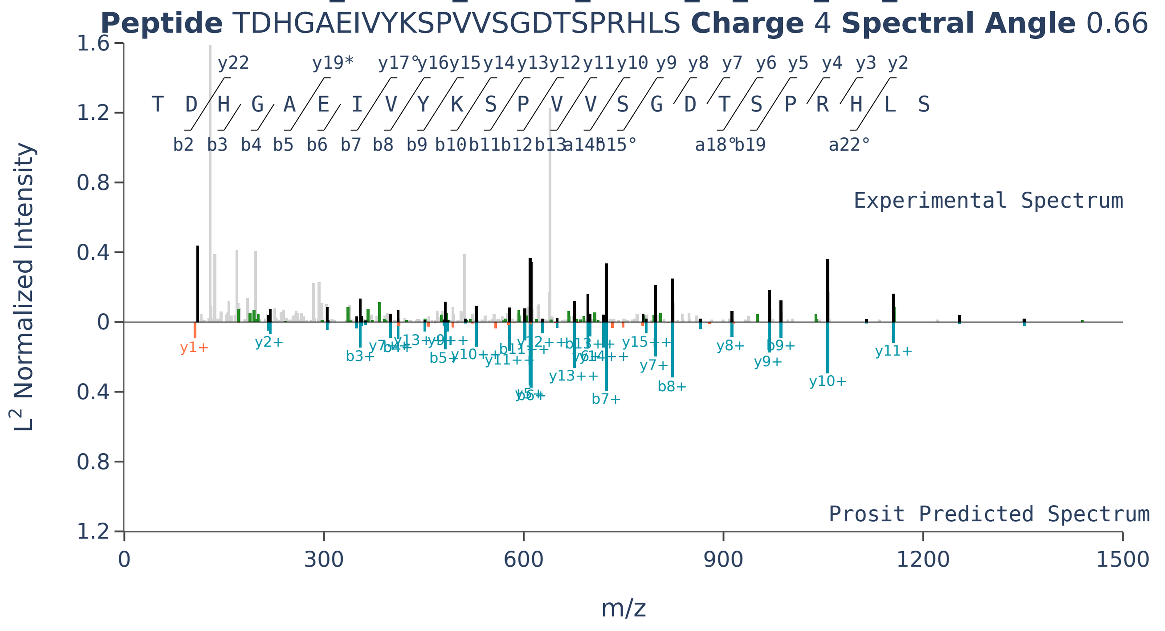


**Source** WSoh_221121_011221_HFGoe_L2_hTau_24h_R1 **Scan** 6231
**Peptide** GNIHHKPGGGQ **Charge** 2 **Spectral Angle** 0.85

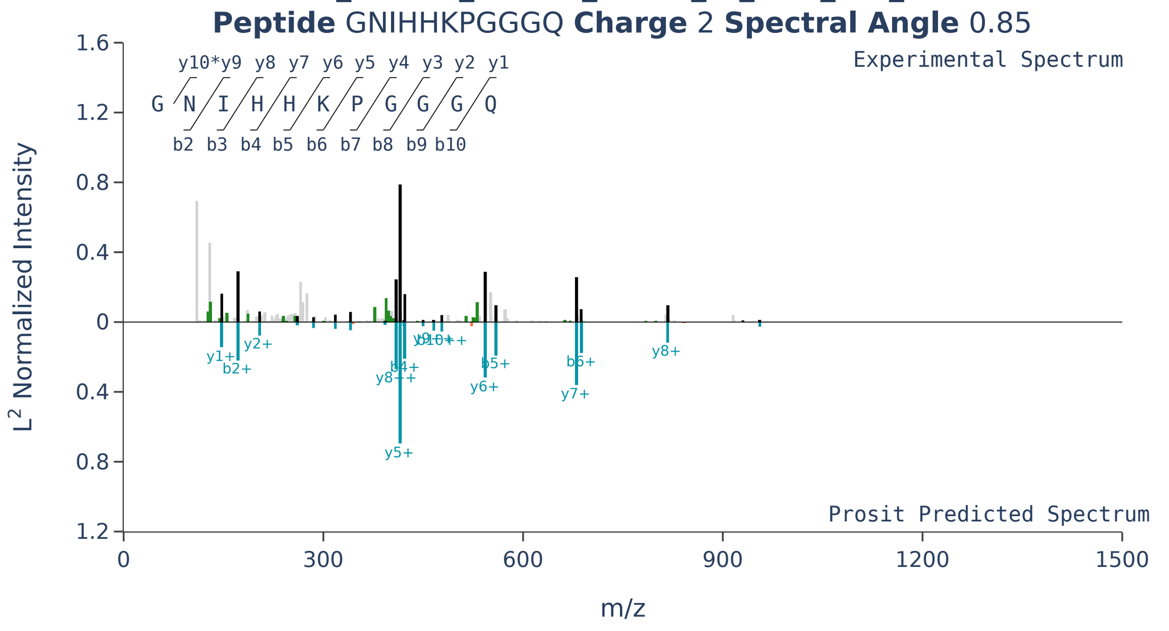


**Source** WSoh_221121_011221_HFGoe_L2_hTau_24h_R1 **Scan** 20025
**Peptide** IGDTPSLEDEAAGHVTQA **Charge** 3 **Spectral Angle** 0.65

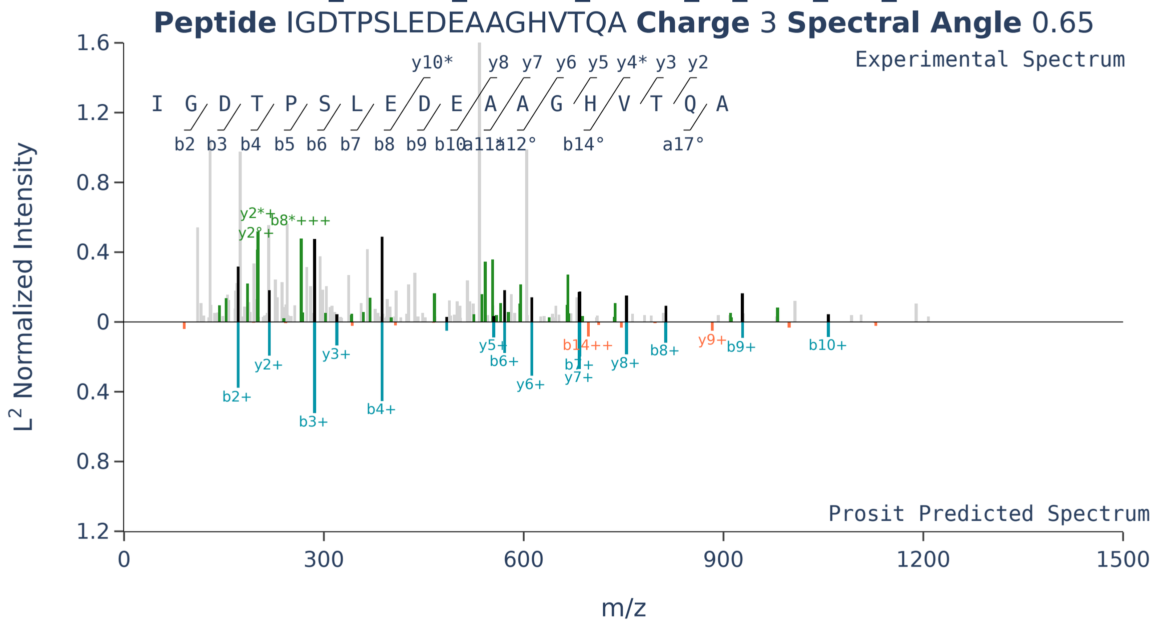


**Source** WSoh_221121_011221_HFGoe_L1_hTau_24h_R2 **Scan** 17967
**Peptide** KLGSLDNIT **Charge** 2 **Spectral Angle** 0.6

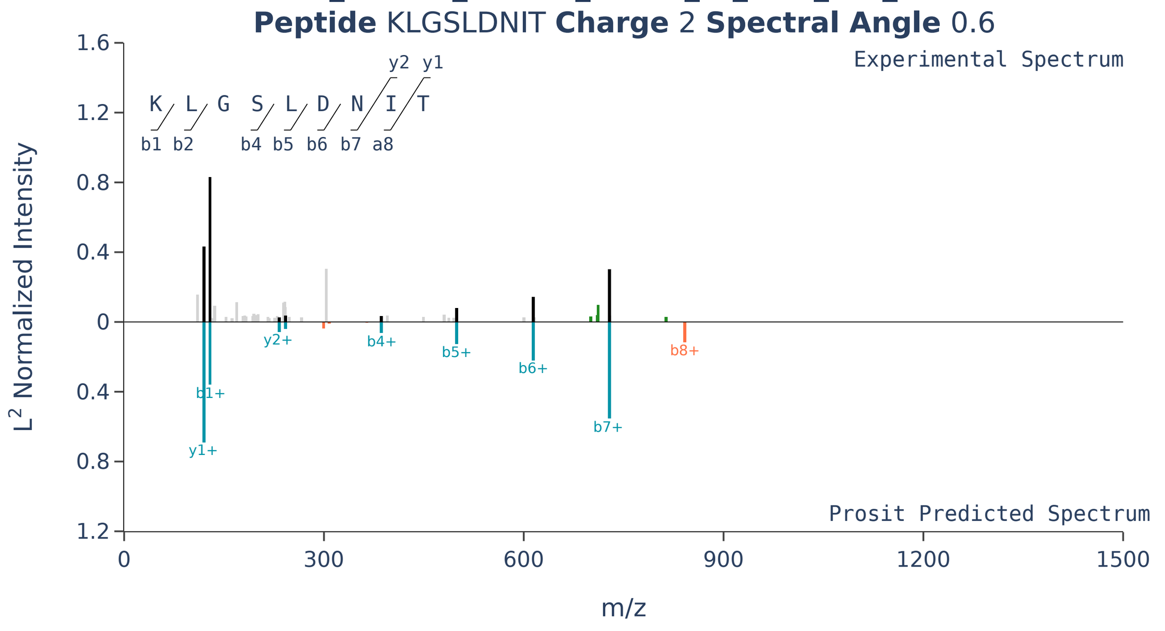


**Source** WSoh_221121_011221_HFGoe_L2_hTau_24h_R2 **Scan** 16010
**Peptide** SLDNITHVPGGGNKKIETHKLTFR **Charge** 6 **Spectral Angle** 0.67

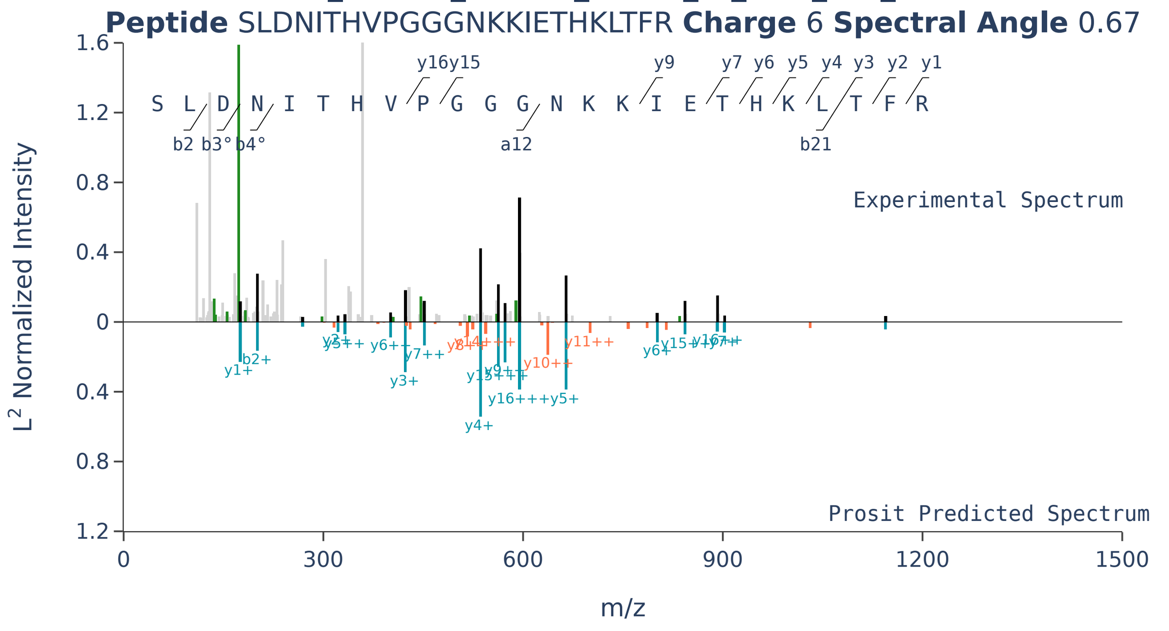

**Source** WSoh_221121_011221_HFGoe_L2_hTau_24h_R1 **Scan** 17430
**Peptide** PLVDEGAPGKQAAAQPHTEIPEGT **Charge** 3 **Spectral Angle** 0.9

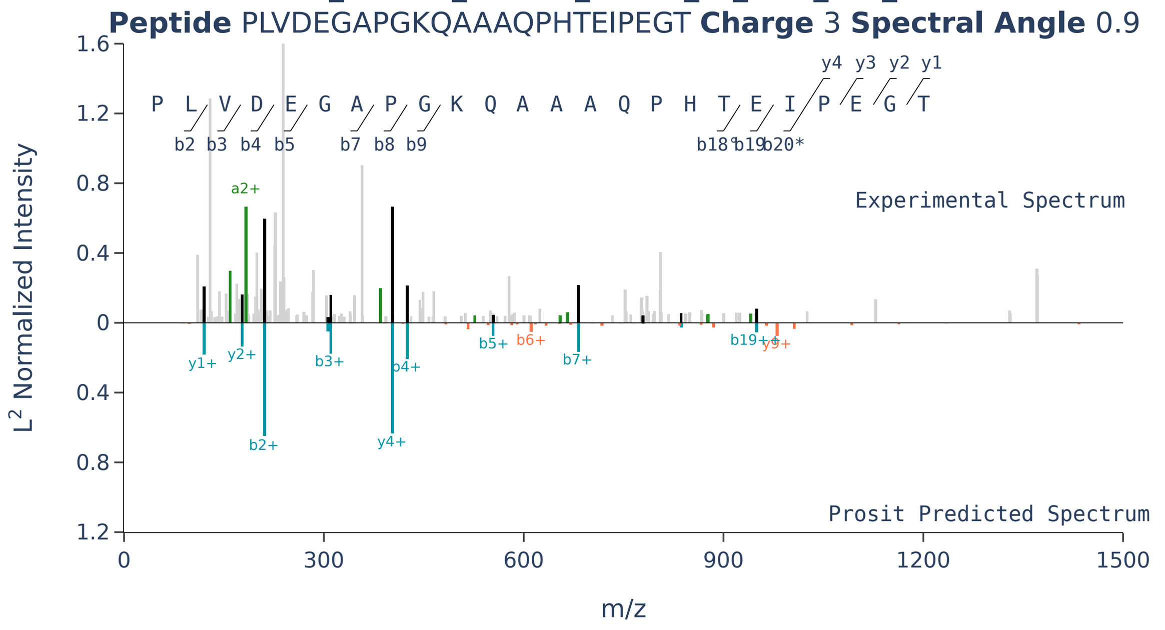


**Source** WSoh_221121_011221_HFGoe_L2_hTau_24h_R2 **Scan** 10075
**Peptide** TENLKHQPGGGKVQL **Charge** 2 **Spectral Angle** 0.66

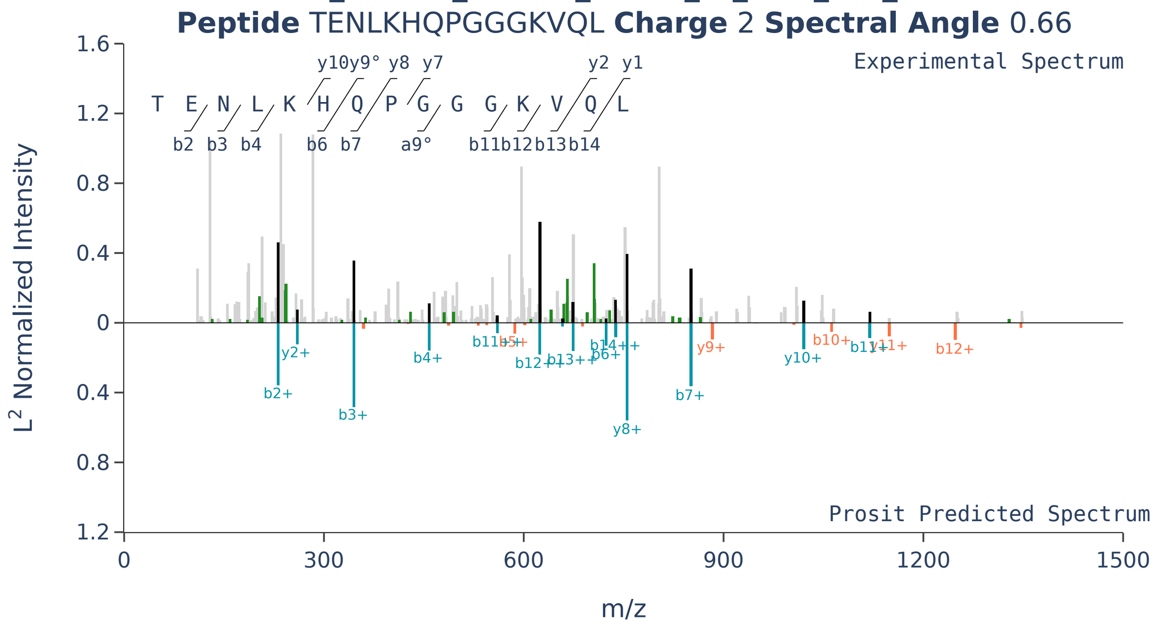


**Source** WSoh_221121_011221_HFGoe_L2_hTau_24h_R2 **Scan** 7321
**Peptide** KLATPRGAAPPGQKG **Charge** 3 **Spectral Angle** 0.79

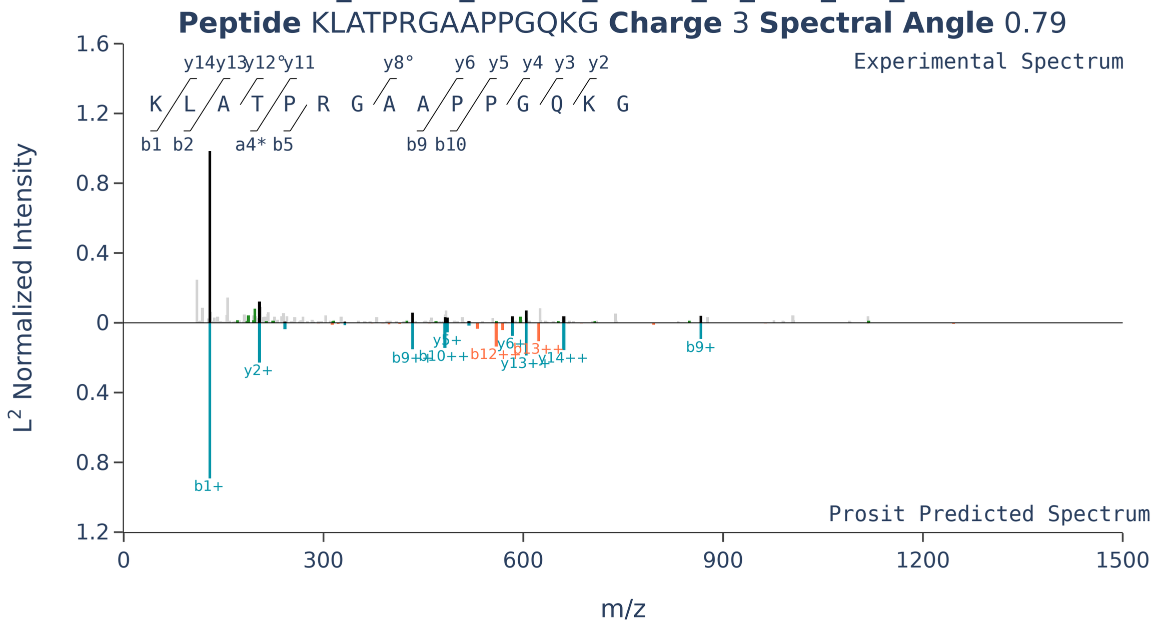


**Source** WSoh_221121_011221_HFGoe_L2_hTau_2h_R2 **Scan** 12357
**Peptide** FKDRVQSKIGSLD **Charge** 3 **Spectral Angle** 0.83

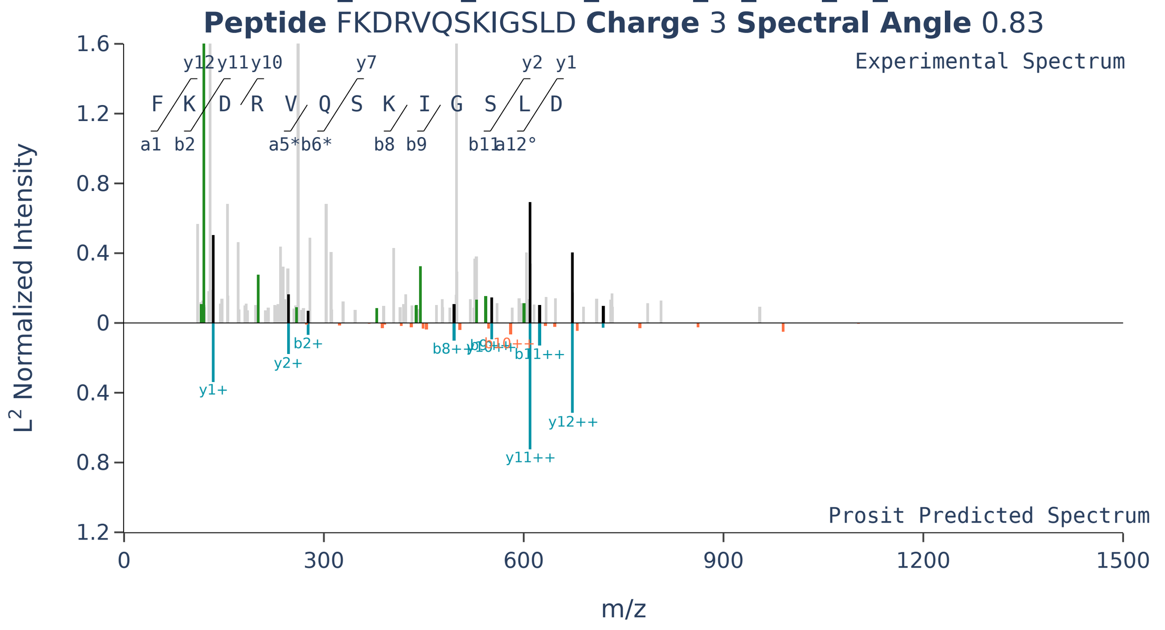


**Source** WSoh_221121_011221_HFGoe_L2_hTau_4h_R2 **Scan** 12594
**Peptide** SPVVSGDTSPRHLSNVSST **Charge** 3 **Spectral Angle** 0.87

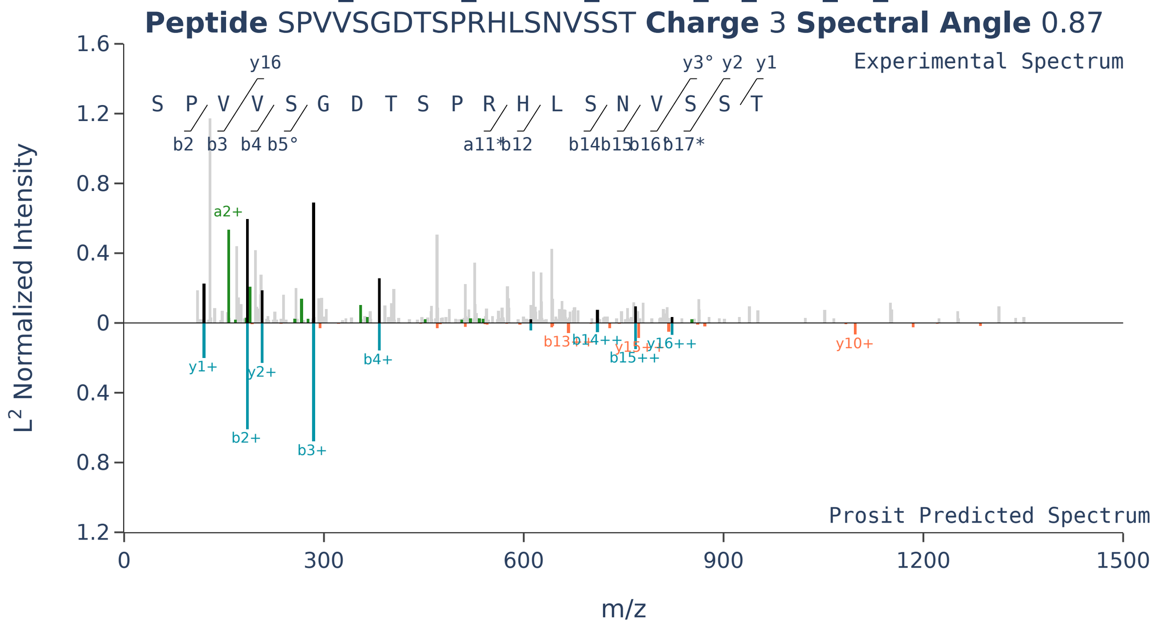


**Source** WSoh_221121_011221_HFGoe_L1_hTau_24h_R1 **Scan** 9653
**Peptide** LSKVTSK **Charge** 2 **Spectral Angle** 0.79

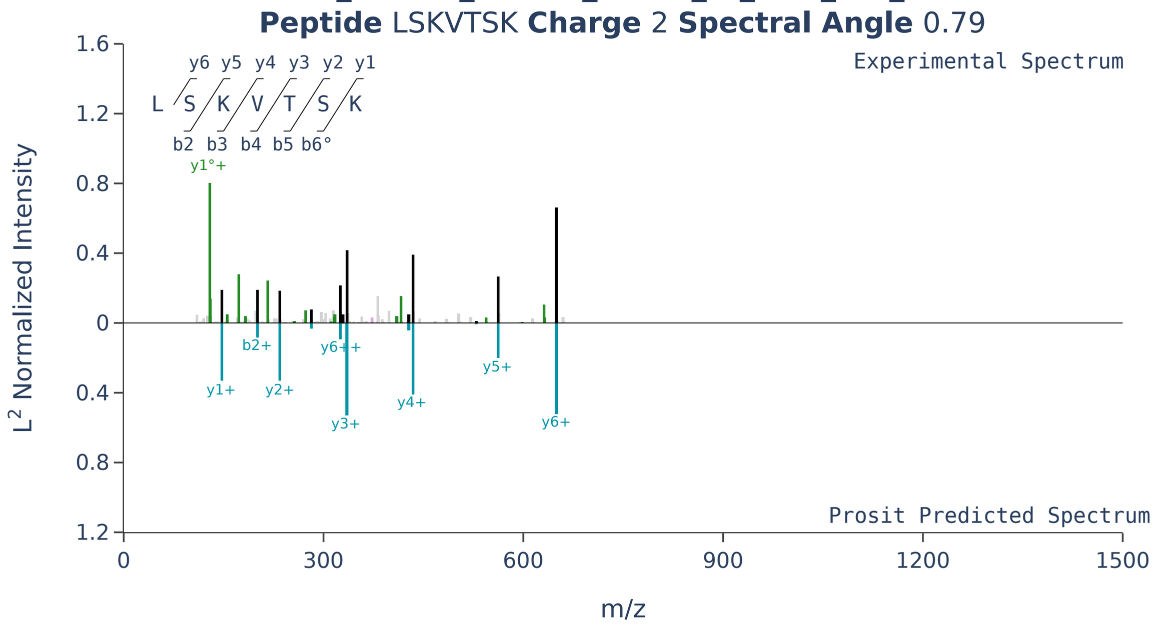


**Source** WSoh_221121_011221_HFGoe_L1_hTau_4h_R2 **Scan** 9654
**Peptide** DNITHVPGGGNKKIE **Charge** 2 **Spectral Angle** 0.81

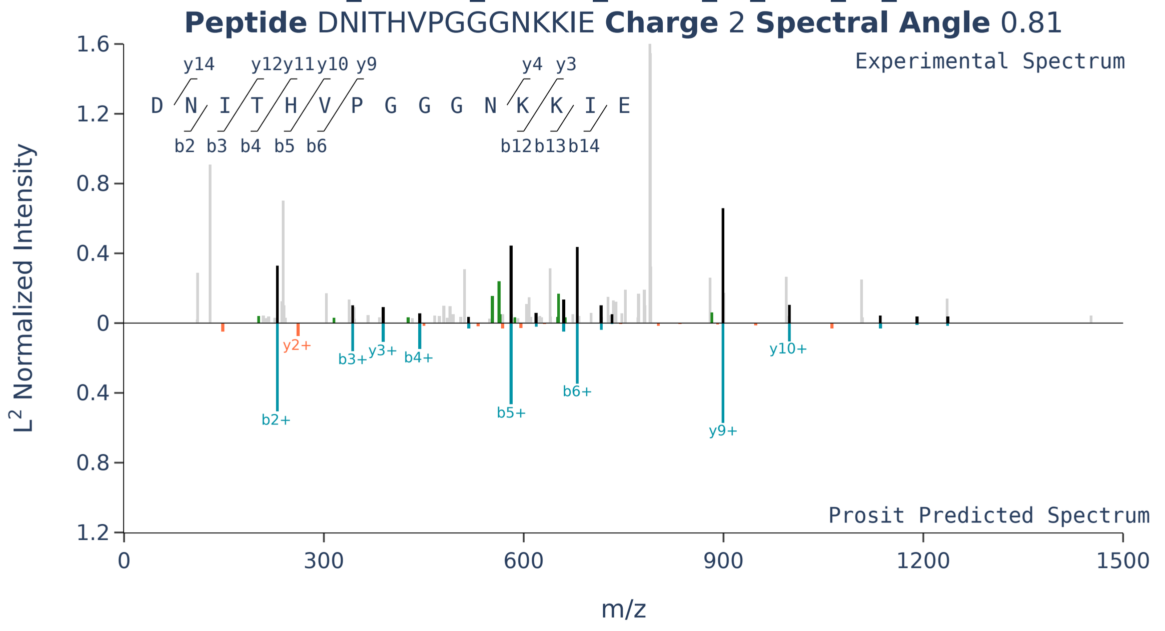


**Source** WSoh_221121_011221_HFGoe_L1_hTau_24h_R1 **Scan** 17898
**Peptide** IGDTPSLEDE **Charge** 1 **Spectral Angle** 0.55

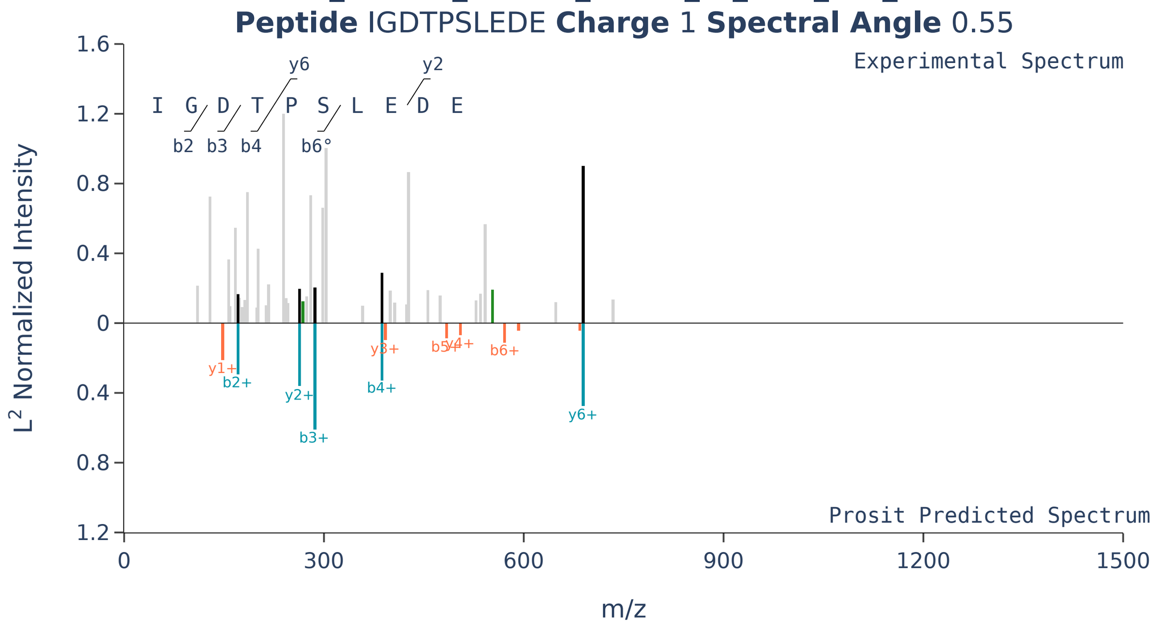


**Source** WSoh_221121_011221_HFGoe_L1_hTau_4h_R2 **Scan** 19324
**Peptide** AEPRQEFEVMEDHAGTYGLGDRKDQ **Charge** 4 **Spectral Angle** 0.73

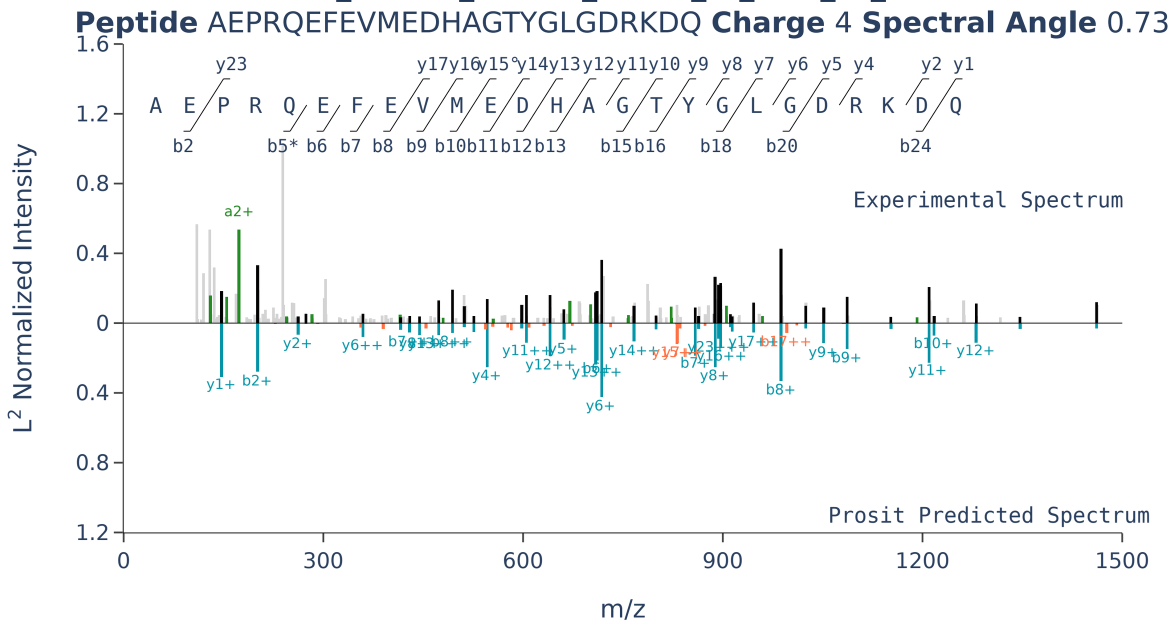


**Source** WSoh_221121_011221_HFGoe_L1_hTau_24h_R1 **Scan** 6059
**Peptide** KSKIGSTENLKHQPGGGKVQ **Charge** 3 **Spectral Angle** 0.83

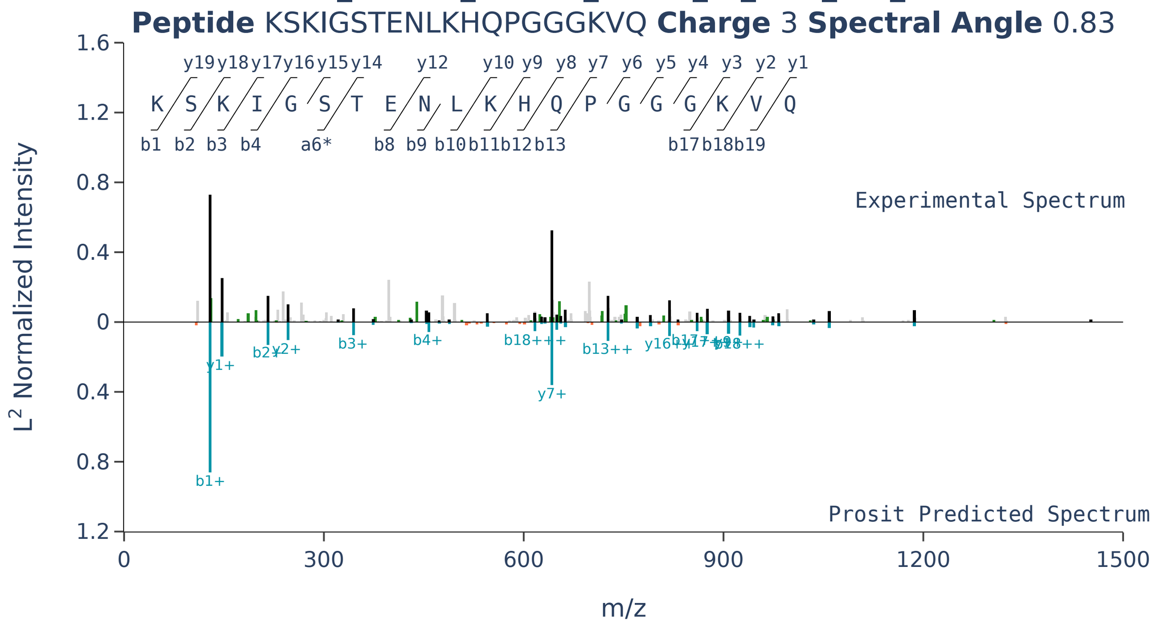


**Source** WSoh_221121_011221_HFGoe_L2_hTau_4h_R2 **Scan** 6713
**Peptide** LSKVTSKCGSLGNIHHK **Charge** 3 **Spectral Angle** 0.77

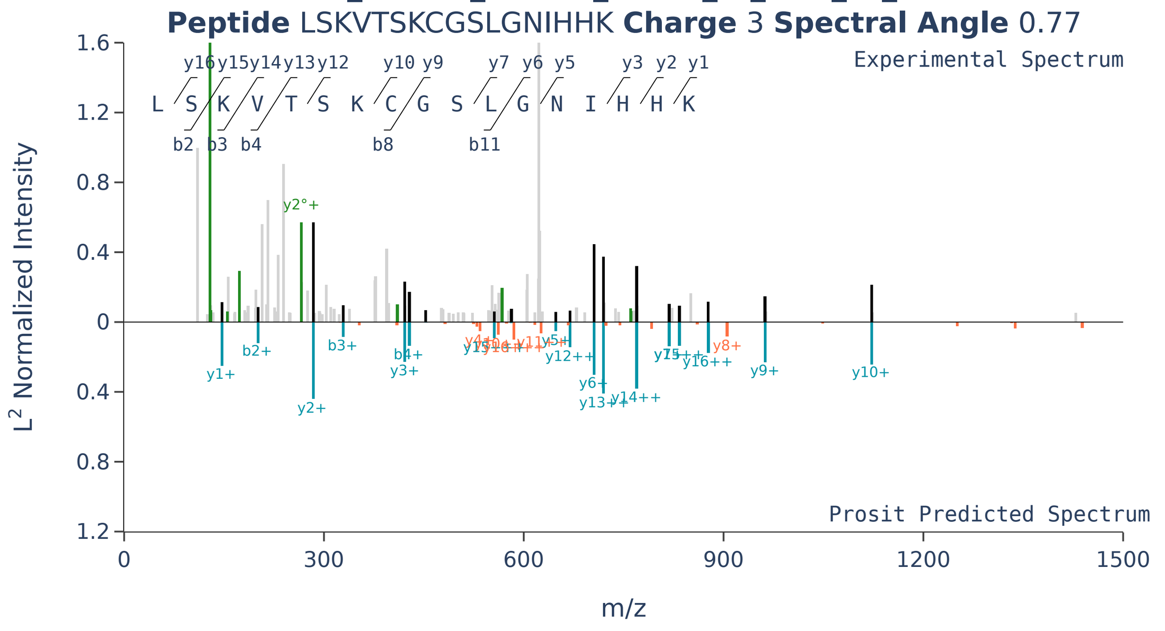


**Source** WSoh_221121_011221_HFGoe_L2_hTau_24h_R1 **Scan** 8748
**Peptide** KNVKSKIGSTENLKHQPGGGKVQIINK **Charge** 5 **Spectral Angle** 0.92

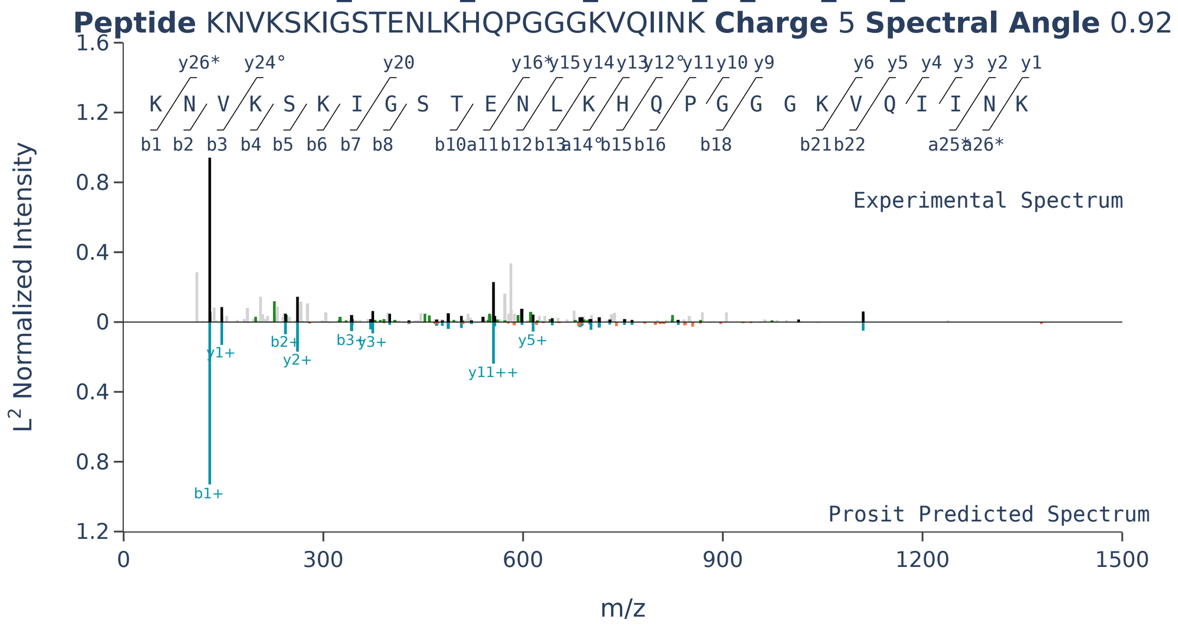


**Source** WSoh_221121_011221_HFGoe_L1_hTau_24h_R1 **Scan** 22552
**Peptide** VQIINKKLDLSNV **Charge** 3 **Spectral Angle** 0.94

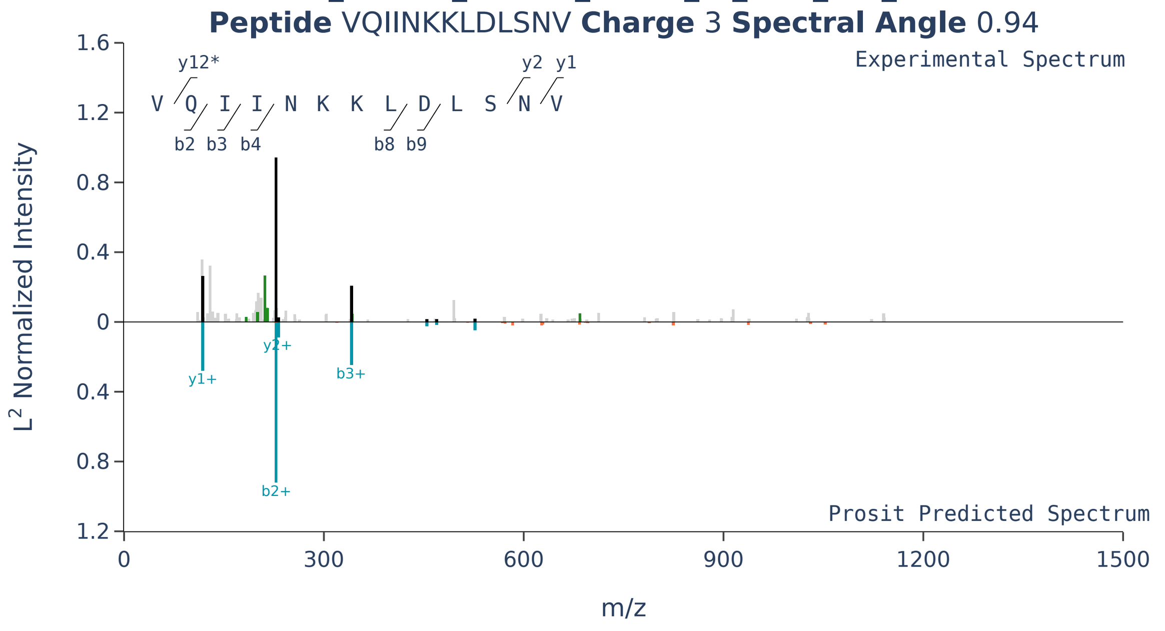


**Source** WSoh_221121_011221_HFGoe_L2_hTau_24h_R1 **Scan** 15613
**Peptide** KPVDLSKVTSKCGSLGNIHHKPGGGQVEV **Charge** 5 **Spectral Angle** 0.87

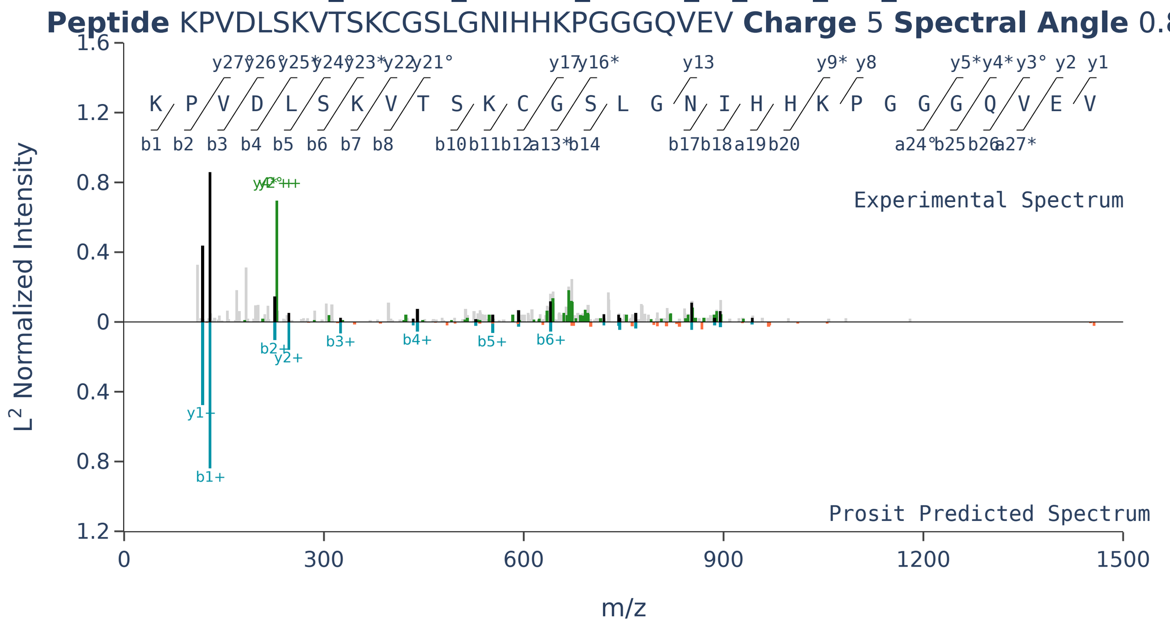


**Source** WSoh_221121_011221_HFGoe_L1_hTau_24h_R2 **Scan** 8818
**Peptide** GKTKIATPRGAAPPGQKGQANATRI **Charge** 4 **Spectral Angle** 0.76

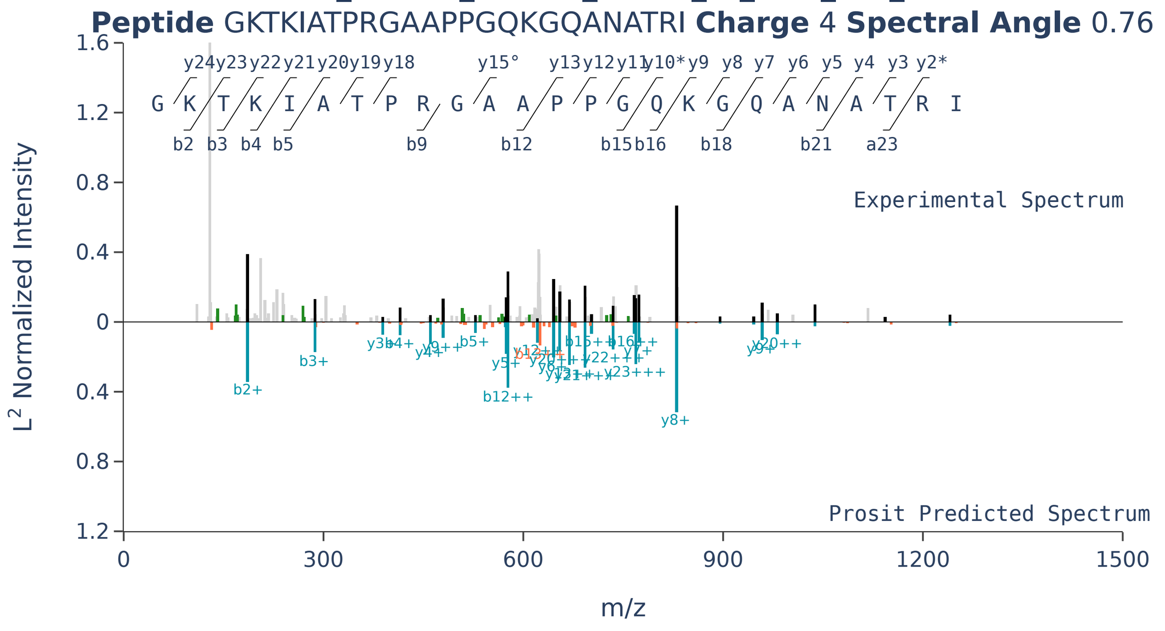

**Source** WSoh_221121_011221_HFGoe_L1_hTau_4h_R2 **Scan** 7094
**Peptide** SLGNIHH **Charge** 2 **Spectral Angle** 0.85

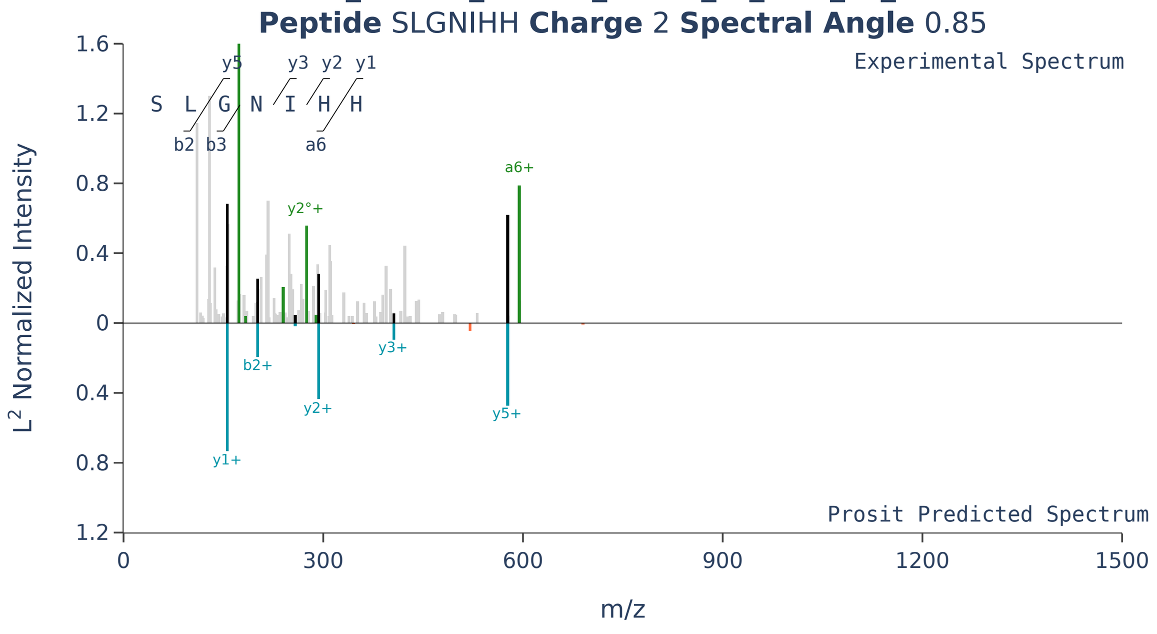


**Source** WSoh_221121_011221_HFGoe_L2_hTau_2h_R2 **Scan** 21554
**Peptide** QIVYKPVDLSKV **Charge** 3 **Spectral Angle** 0.79

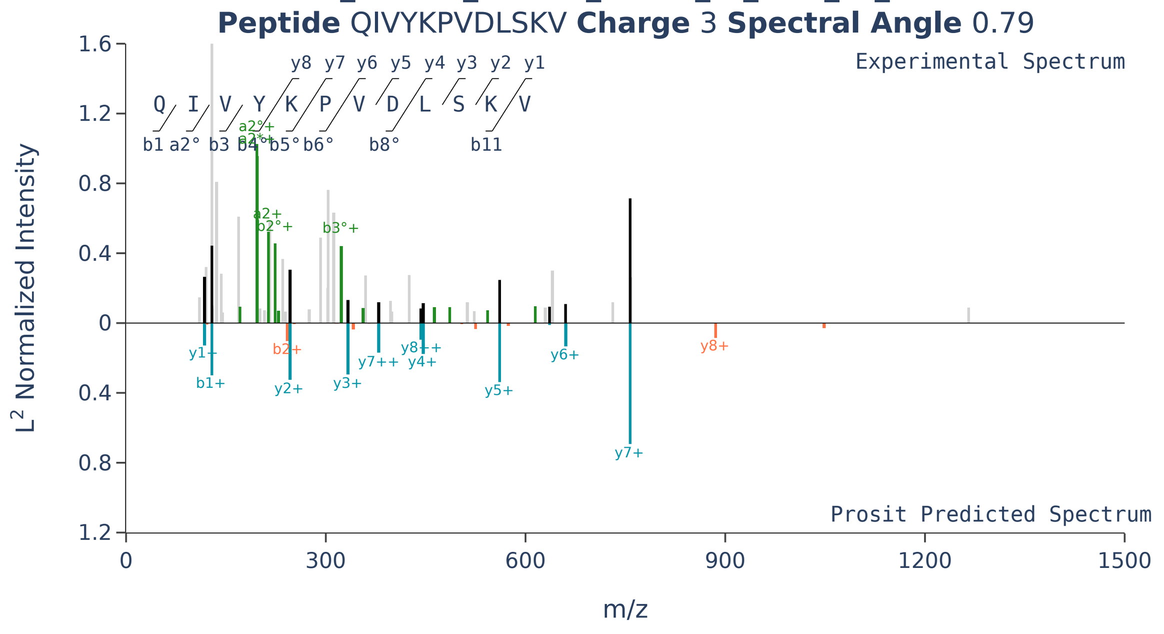


**Source** WSoh_221121_011221_HFGoe_L1_hTau_4h_R1 **Scan** 14312
**Peptide** PGGGQVEVKSEKLDFKD **Charge** 4 **Spectral Angle** 0.77

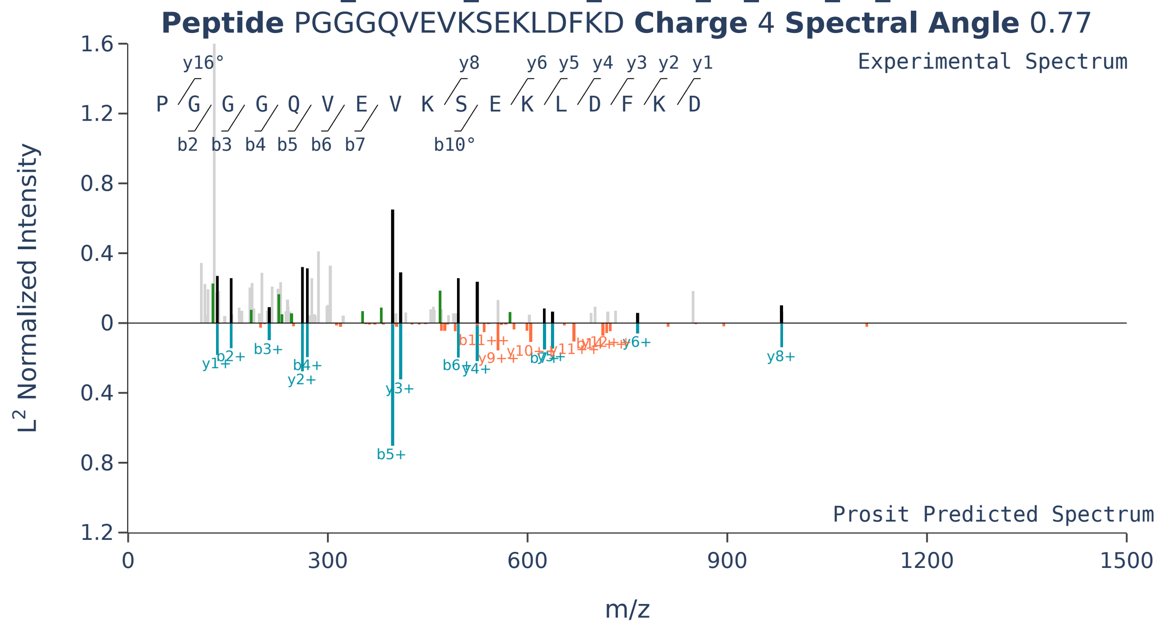


**Source** WSoh_221121_011221_HFGoe_L1_hTau_24h_R2 **Scan** 7560
**Peptide** KLATPRGAAPPGQKGQA **Charge** 3 **Spectral Angle** 0.75

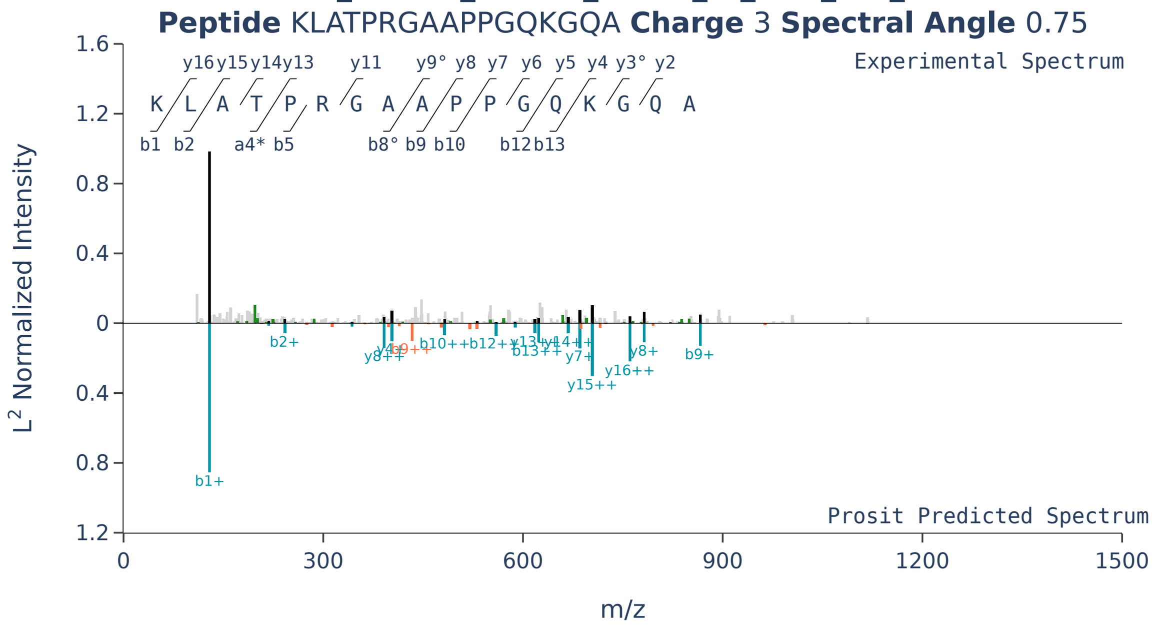


**Source** WSoh_221121_011221_HFGoe_L2_hTau_4h_R1 **Scan** 13824
**Peptide** KESPLQTPTEDGSEEPGSETSDAKSTPTAE **Charge** 3 **Spectral Angle** 0.66

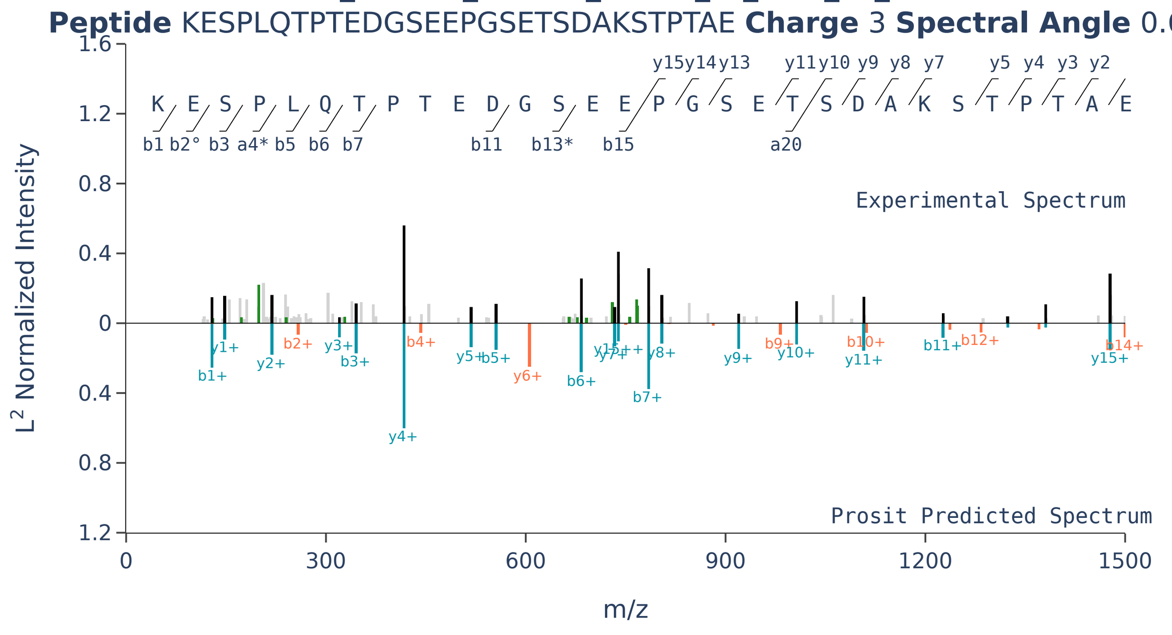


**Source** WSoh_221121_011221_HFGoe_L1_hTau_24h_R1 **Scan** 12027
**Peptide** LHHKPGGGQVEVKSEKLDF **Charge** 4 **Spectral Angle** 0.8

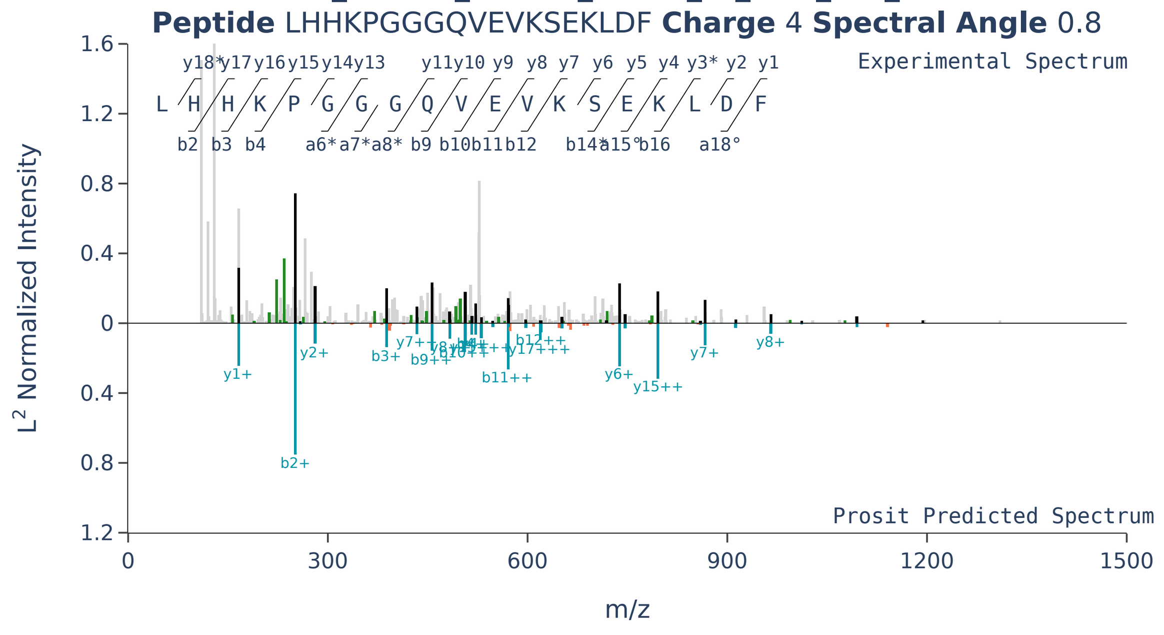


**Source** WSoh_221121_011221_HFGoe_L1_hTau_24h_R2 **Scan** 6275
**Peptide** HKLTFRENAKAKT **Charge** 3 **Spectral Angle** 0.68

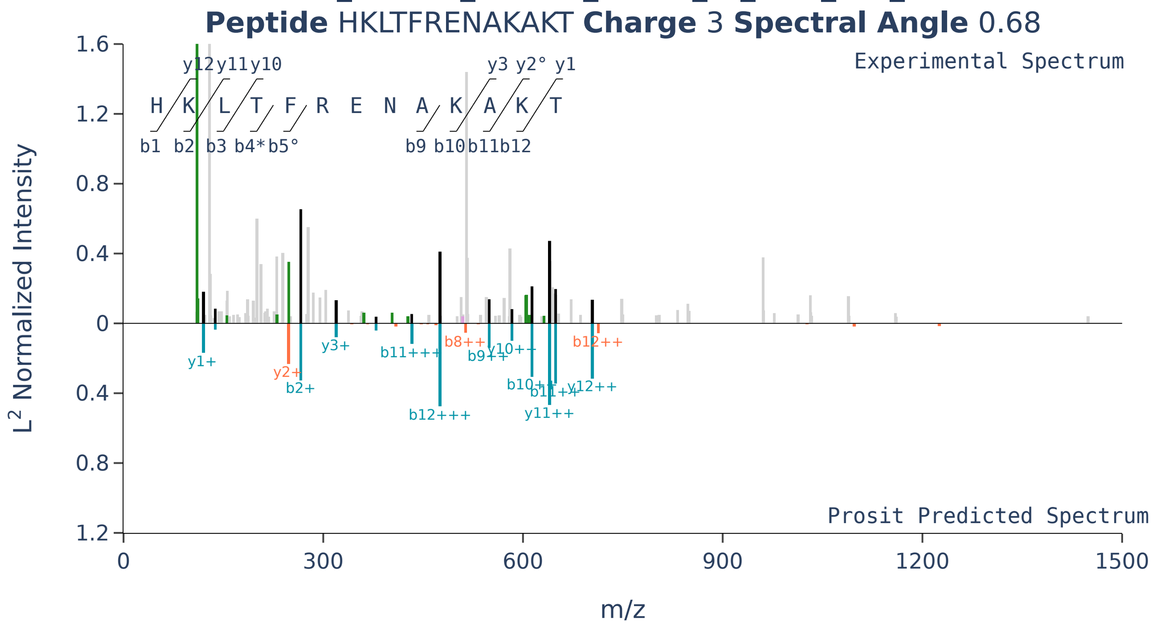


**Source** WSoh_221121_011221_HFGoe_L2_hTau_4h_R1 **Scan** 20423
**Peptide** DFKDRVQSKIGSLDNITHVPGGGNKKIET **Charge** 4 **Spectral Angle** 0.75

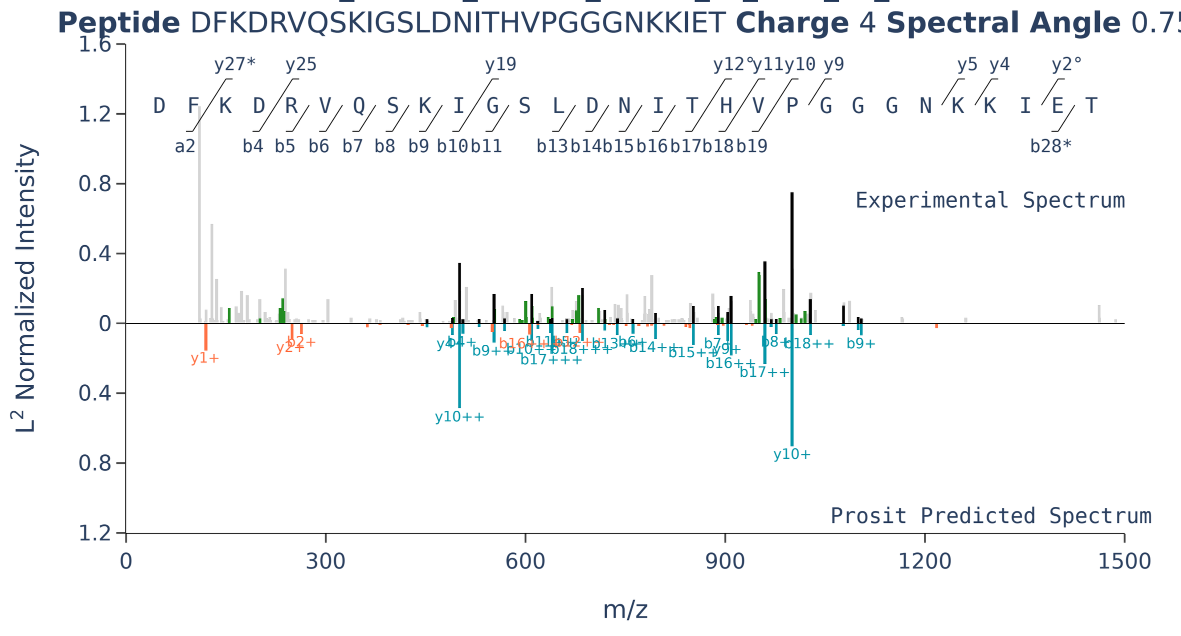


**Source** WSoh_221121_011221_HFGoe_L2_hTau_24h_R2 **Scan** 19380
**Peptide** QGGYTMHQDQEGDTDAGLKESPLQTPTE **Charge** 3 **Spectral Angle** 0.92

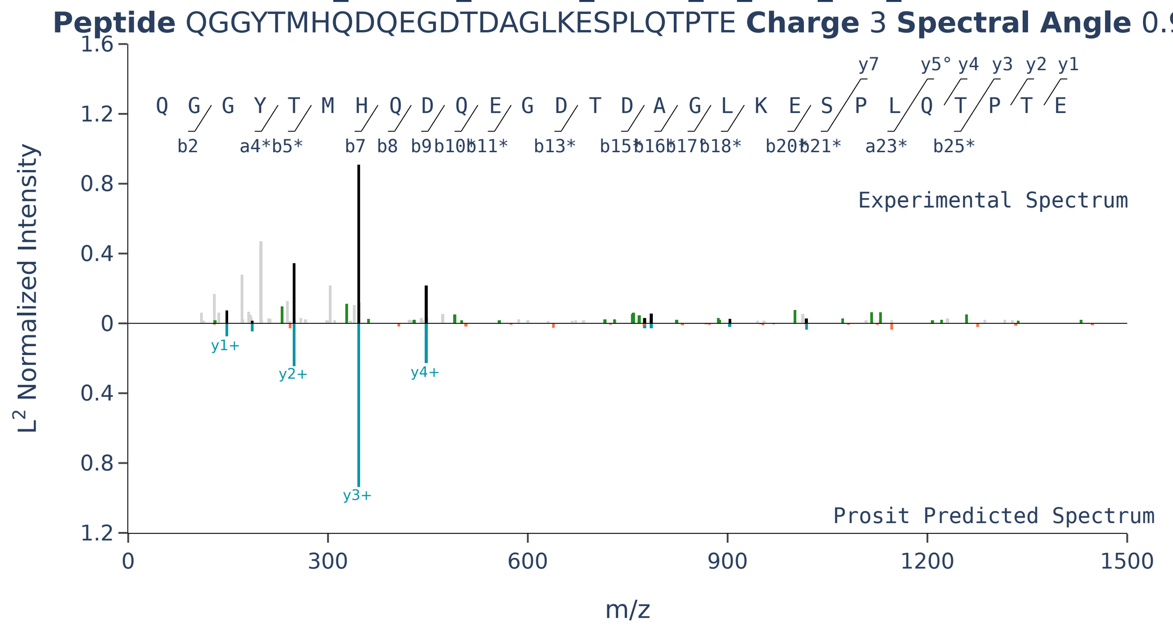


**Source** WSoh_221121_011221_HFGoe_L2_hTau_2h_R1 **Scan** 6259
**Peptide** PRHLSNV **Charge** 2 **Spectral Angle** 0.84

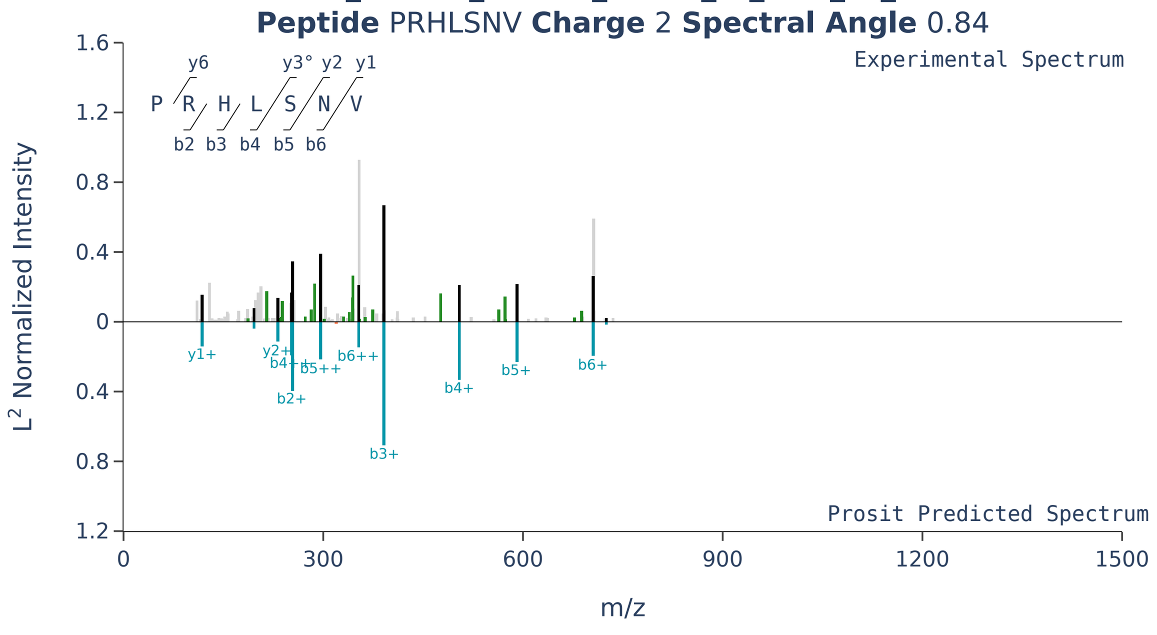


**Source** WSoh_221121_011221_HFGoe_L2_hTau_4h_R1 **Scan** 5961
**Peptide** VPGGGNKKIE **Charge** 2 **Spectral Angle** 0.88

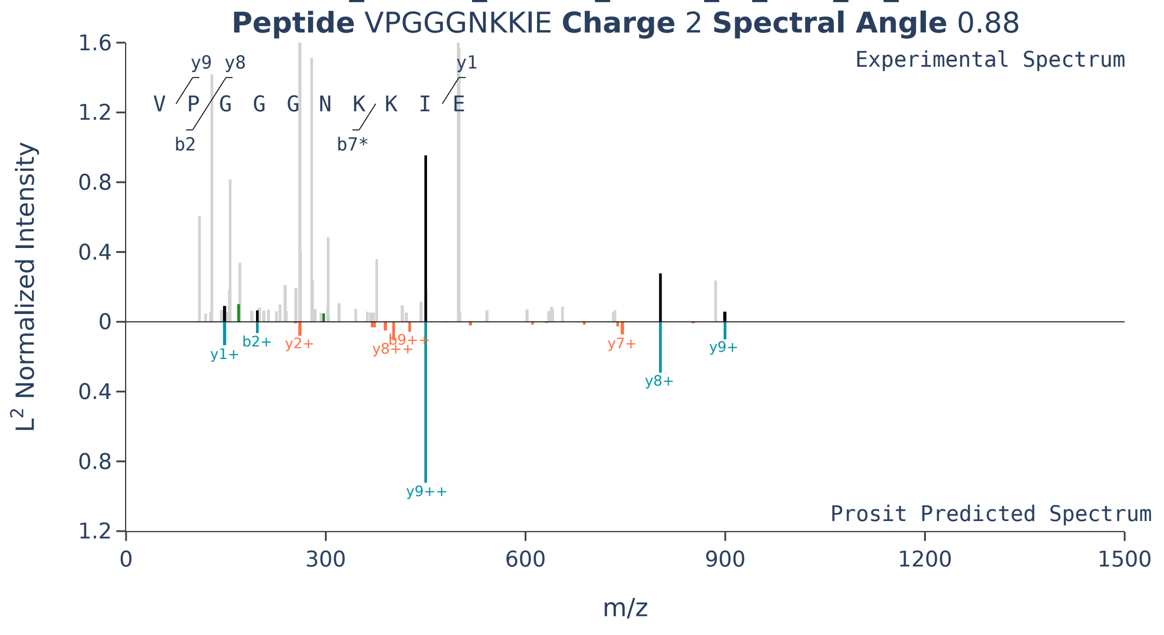


**Source** WSoh_221121_011221_HFGoe_L2_hTau_24h_R1 **Scan** 5658
**Peptide** GNIHHKPGGGQVEVK **Charge** 3 **Spectral Angle** 0.73

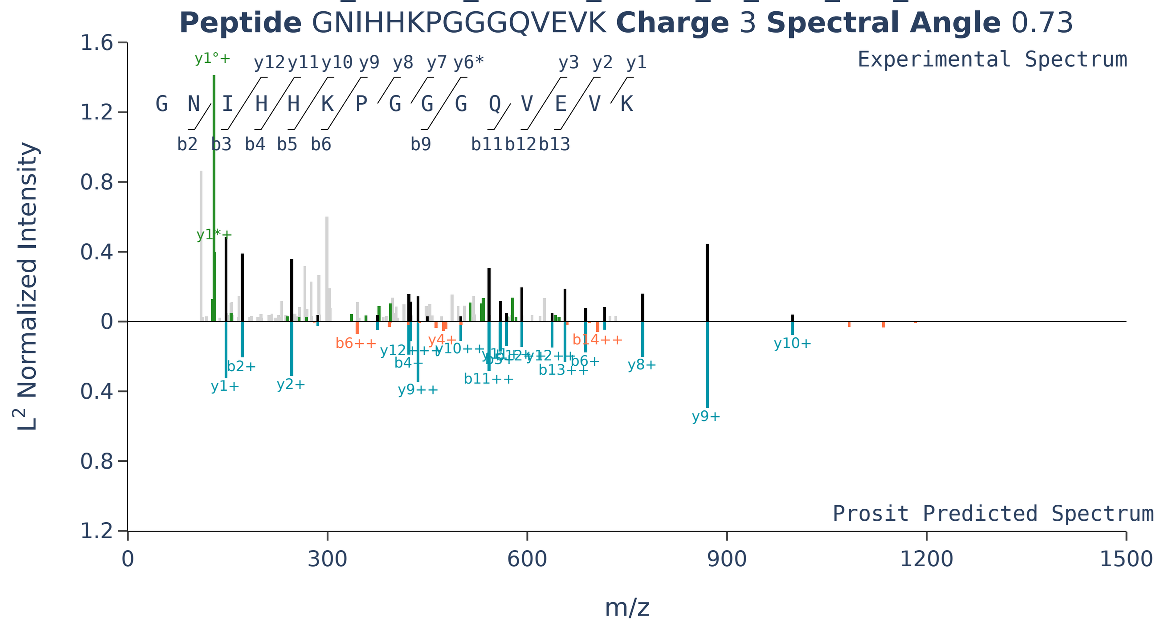


**Source** WSoh_221121_011221_HFGoe_L1_hTau_4h_R2 **Scan** 13466
**Peptide** RVQSKIGSLDNITH **Charge** 3 **Spectral Angle** 0.82

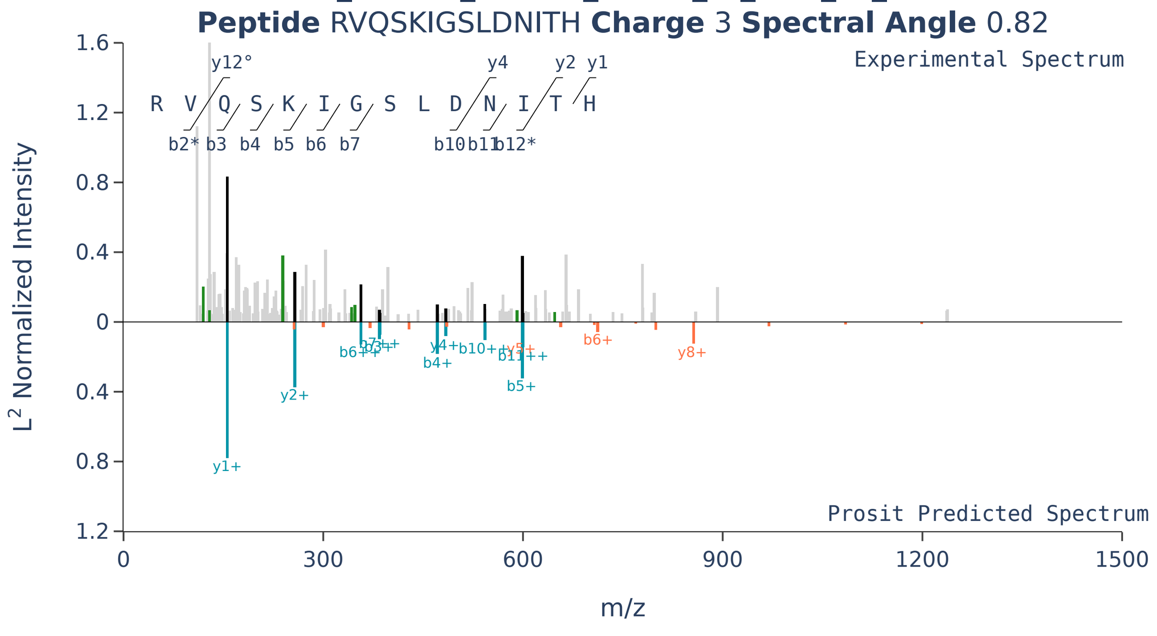


**Source** WSoh_221121_011221_HFGoe_L1_hTau_24h_R2 **Scan** 10384
**Peptide** GSTENLKHQPGGGKVQI **Charge** 2 **Spectral Angle** 0.85

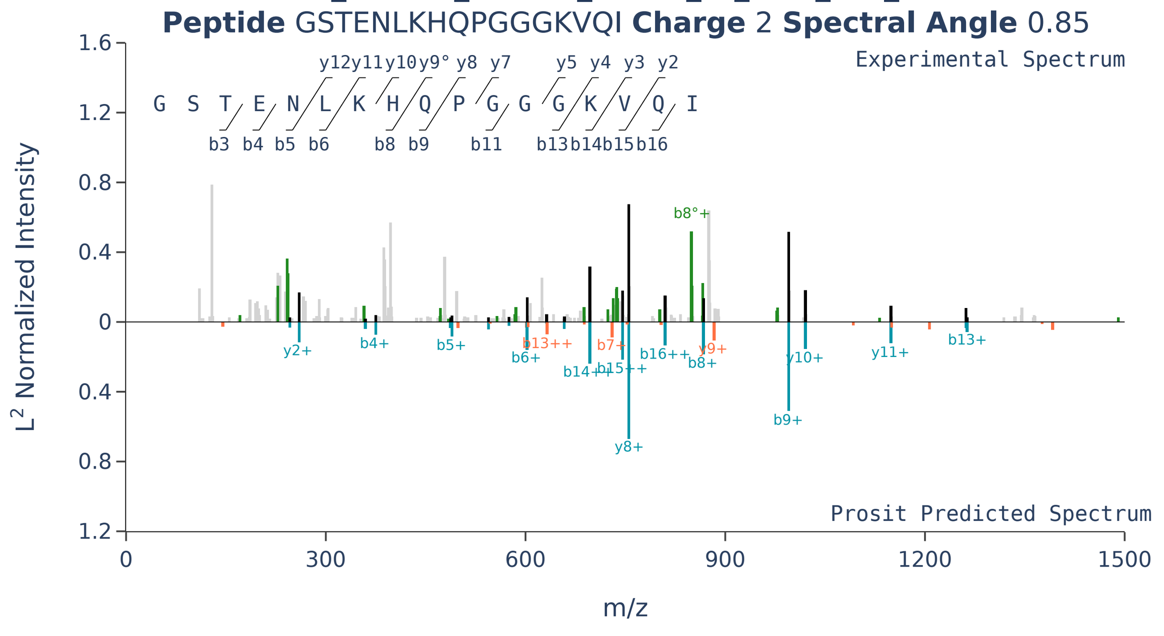


**Source** WSoh_221121_011221_HFGoe_L2_hTau_4h_R2 **Scan** 7617
**Peptide** GDRSGYSSPGSPGTPGSR **Charge** 2 **Spectral Angle** 0.71

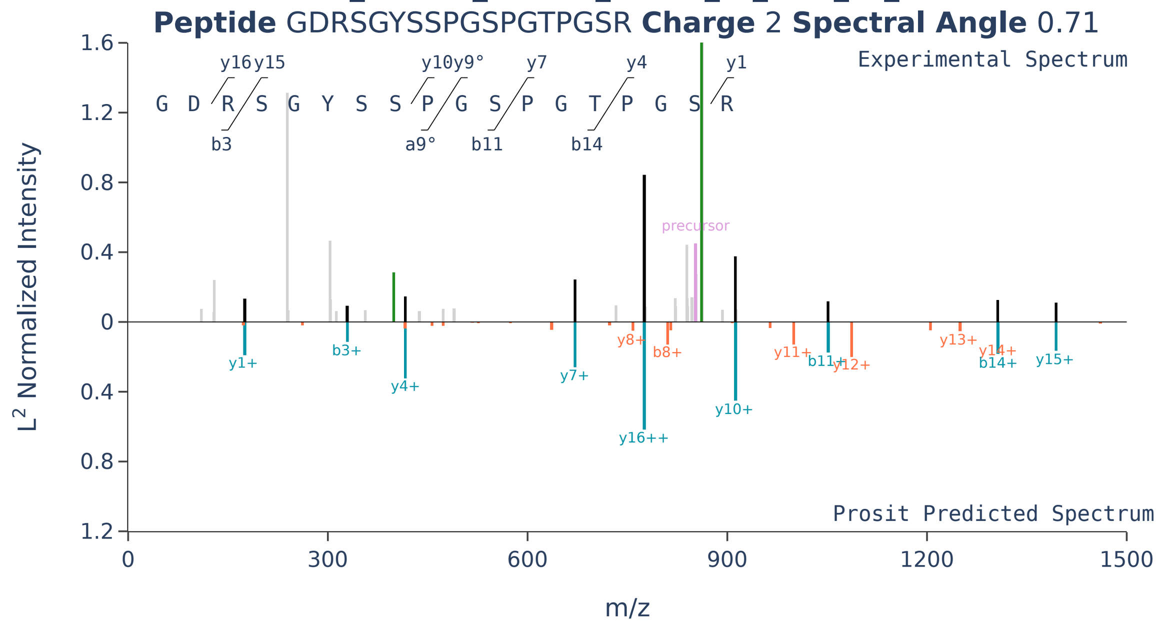

**Source** WSoh_221121_011221_HFGoe_L1_hTau_24h_R2 **Scan** 13388
**Peptide** VKSEKLDFKDRVQSKI **Charge** 5 **Spectral Angle** 0.64

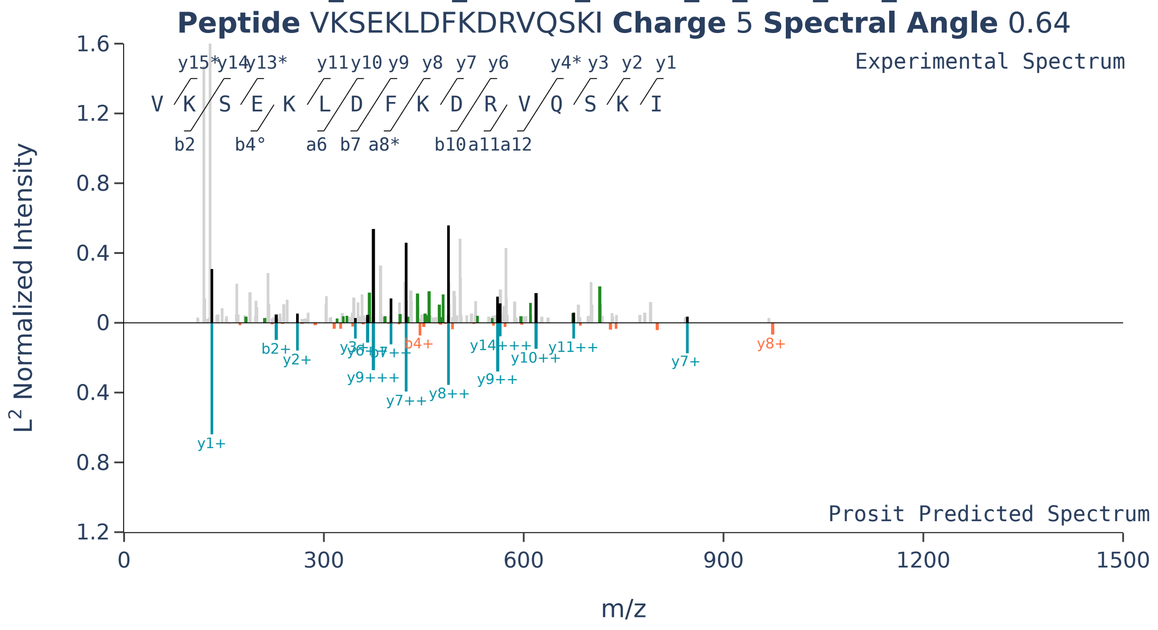


**Source** WSoh_221121_011221_HFGoe_L2_hTau_24h_R2 **Scan** 9949
**Peptide** AAPPGQKGQANATRIPAKTPPAPKT **Charge** 4 **Spectral Angle** 0.79

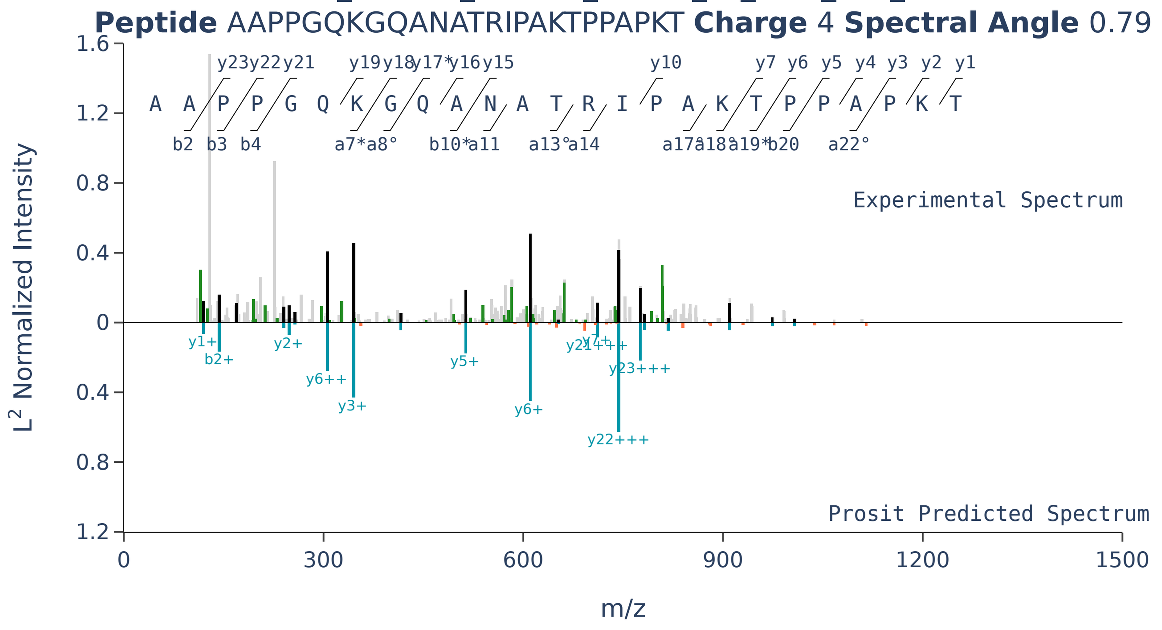


**Source** WSoh_221121_011221_HFGoe_L2_hTau_4h_R1 **Scan** 20908
**Peptide** CGSKDNIKHVPGGGSVQIVYKPVDL **Charge** 4 **Spectral Angle** 0.75

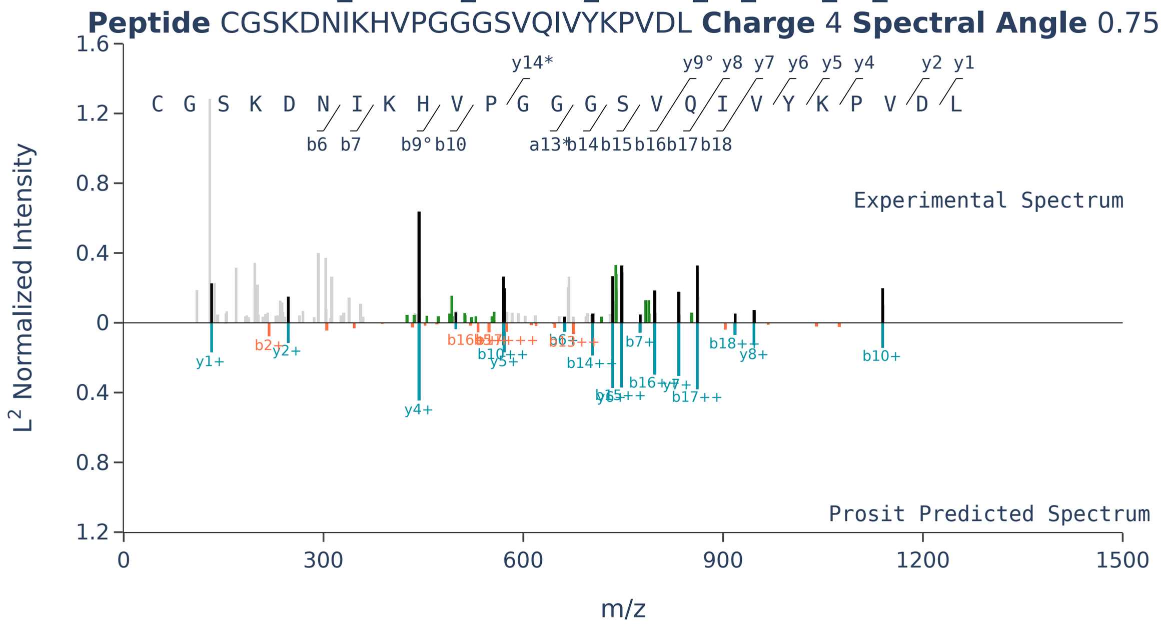


**Source** WSoh_221121_011221_HFGoe_L1_hTau_24h_R1 **Scan** 20096
**Peptide** AKSTPTAEDVTAPLVDEGAPGKQAA **Charge** 3 **Spectral Angle** 0.82

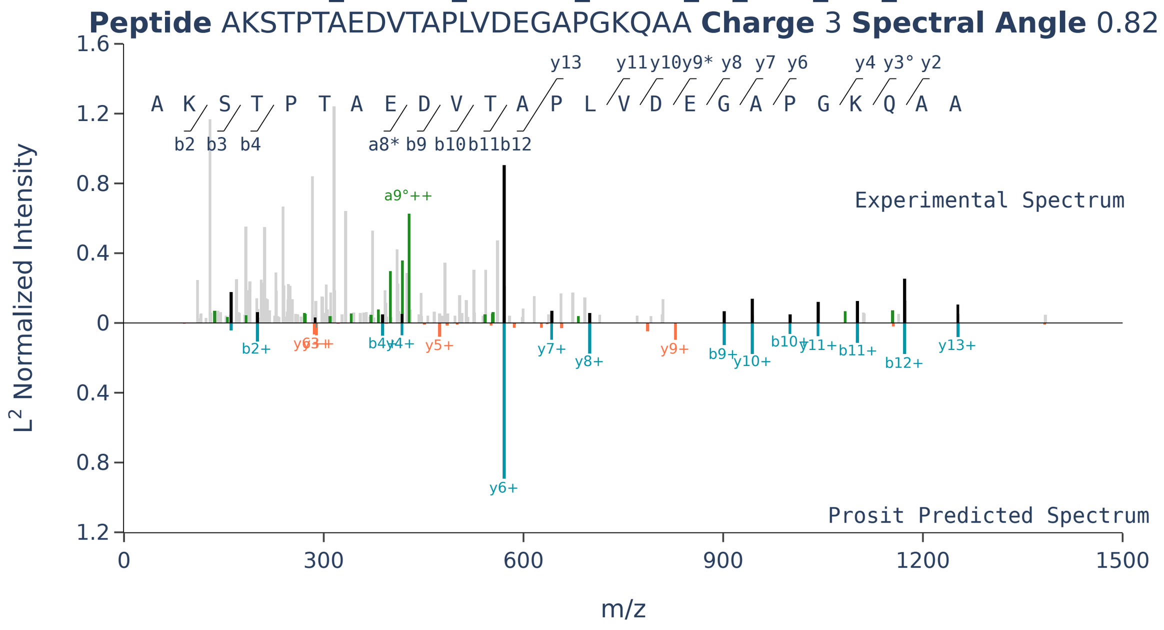


**Source** WSoh_221121_011221_HFGoe_L1_hTau_24h_R1 **Scan** 16459
**Peptide** VSSTGSIDM **Charge** 1 **Spectral Angle** 0.67

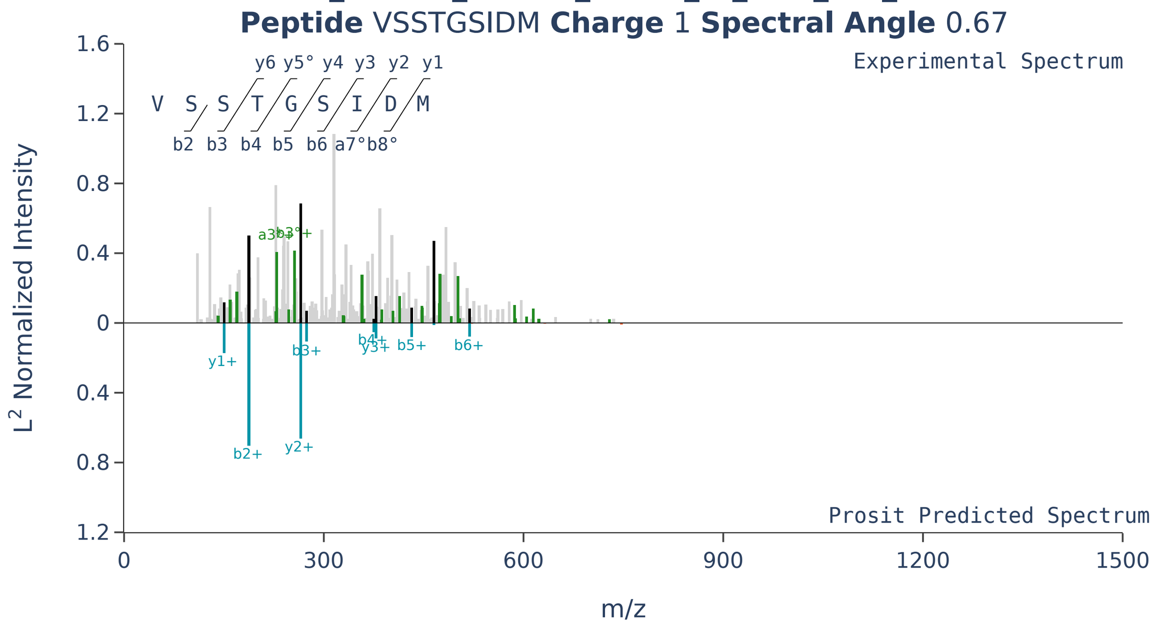


**Source** WSoh_221121_011221_HFGoe_L2_hTau_4h_R1 **Scan** 12814
**Peptide** DNITHVPGGGNKKIETHKLTFRE **Charge** 5 **Spectral Angle** 0.78

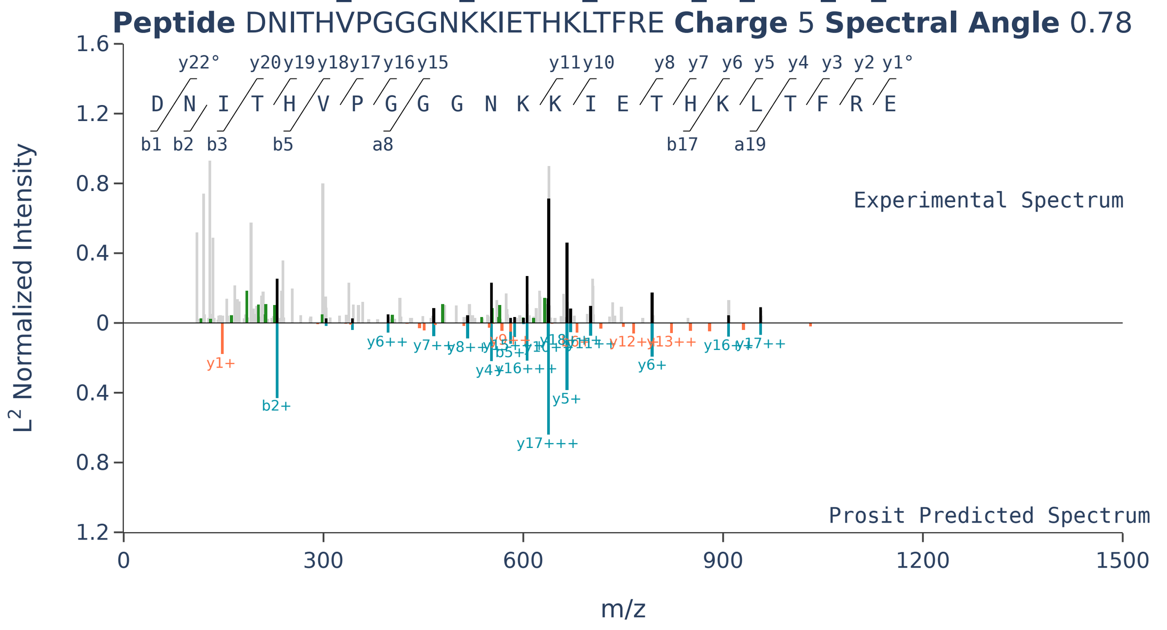


**Source** WSoh_221121_011221_HFGoe_L2_hTau_24h_R2 **Scan** 8751
**Peptide** LDNITHVPGGGNKKIETHK **Charge** 5 **Spectral Angle** 0.73

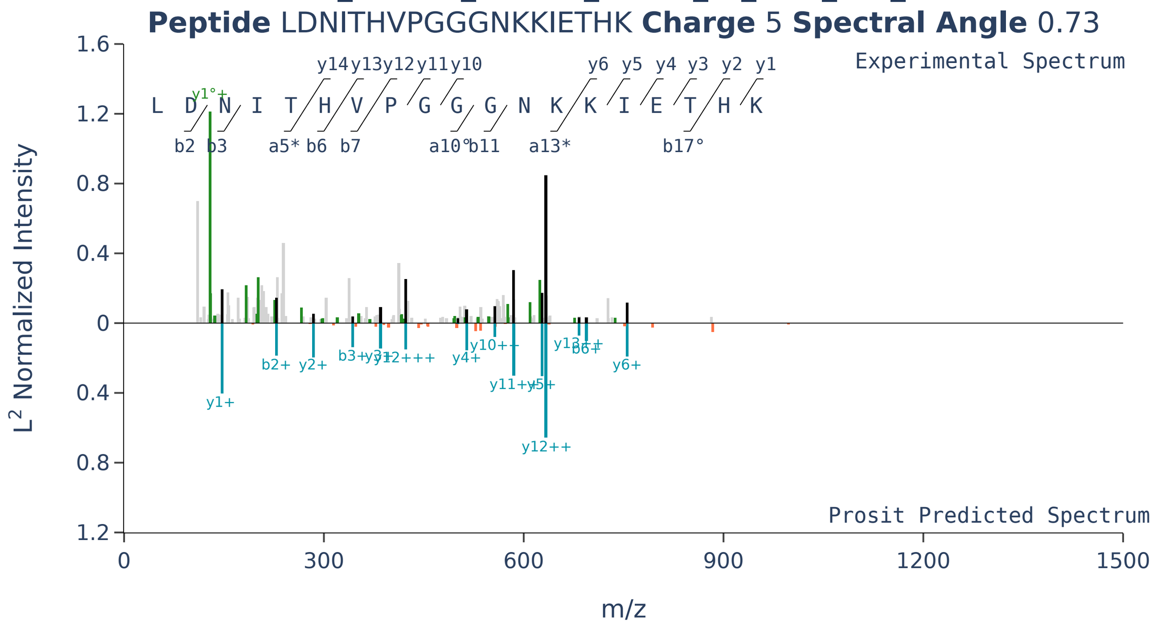


**Source** WSoh_221121_011221_HFGoe_L1_hTau_24h_R2 **Scan** 21053
**Peptide** AEEAGIGDTPSLED **Charge** 1 **Spectral Angle** 0.68

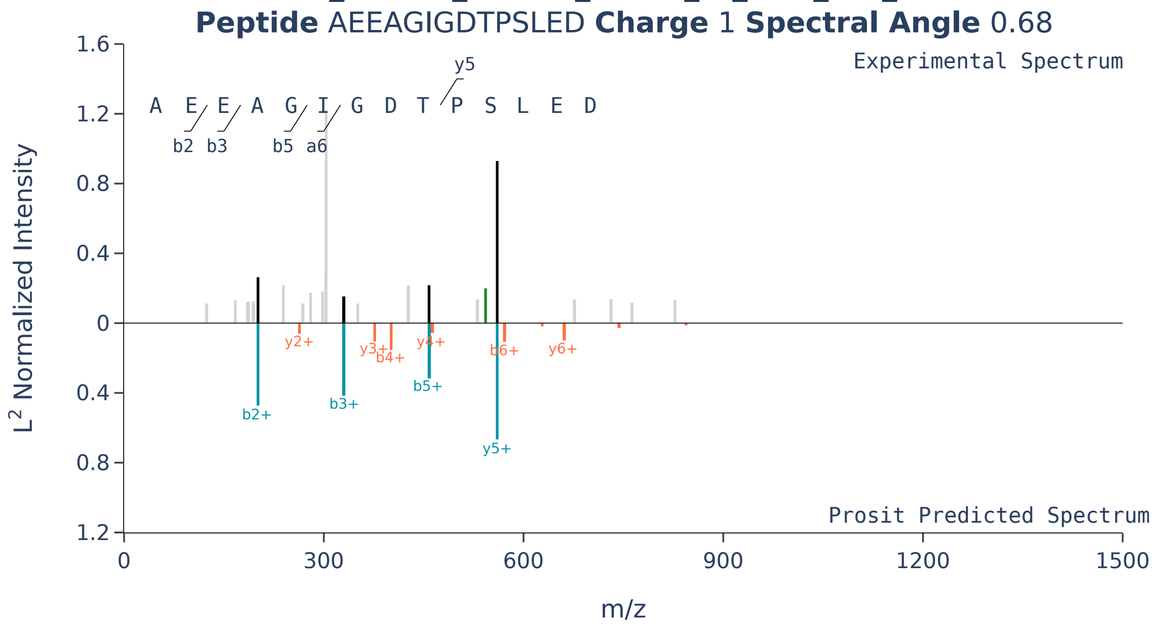


**Source** WSoh_221121_011221_HFGoe_L2_hTau_4h_R1 **Scan** 14564
**Peptide** PLQTPTEDGSEEPGSETSDAKSTPTA **Charge** 3 **Spectral Angle** 0.74

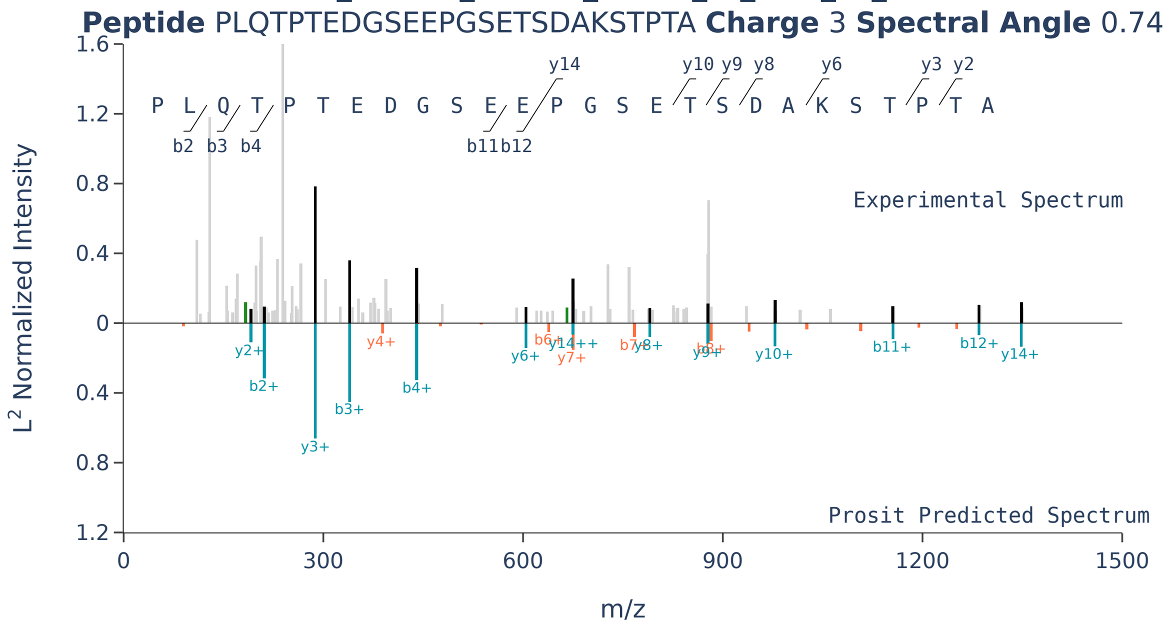


**Source** WSoh_221121_011221_HFGoe_L2_hTau_2h_R1 **Scan** 12464
**Peptide** SGDTSPRHLSNVSSTGSI **Charge** 3 **Spectral Angle** 0.82

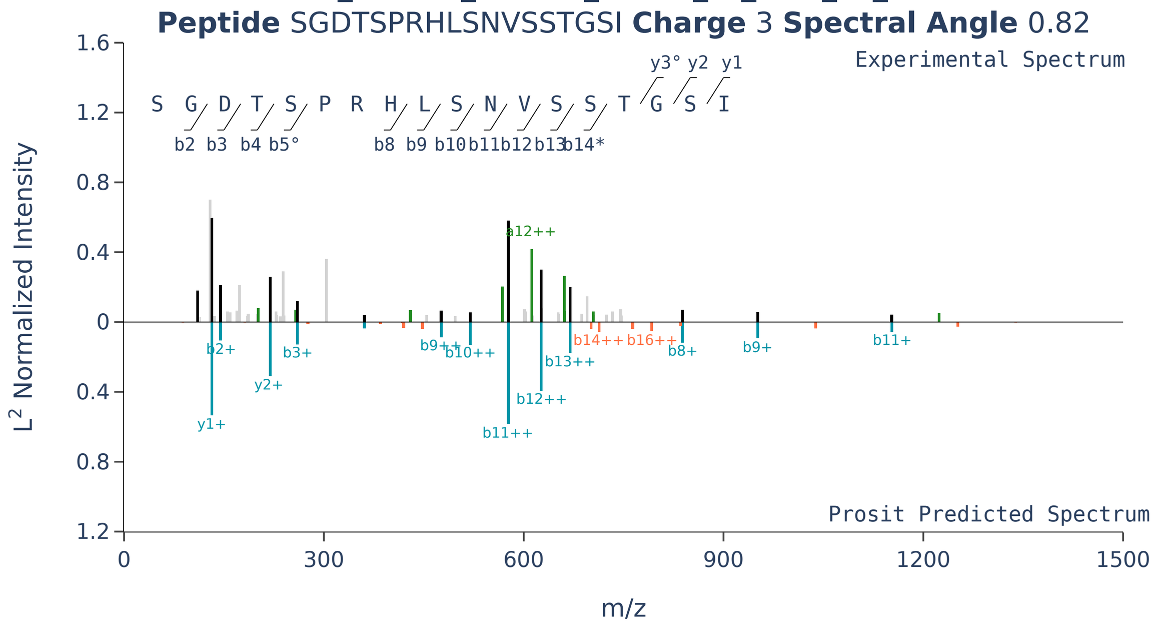


**Source** WSoh_221121_011221_HFGoe_L1_hTau_24h_R1 **Scan** 8938
**Peptide** SKCGSLGNIHHKPGGGQVEVKSEKL **Charge** 3 **Spectral Angle** 0.75

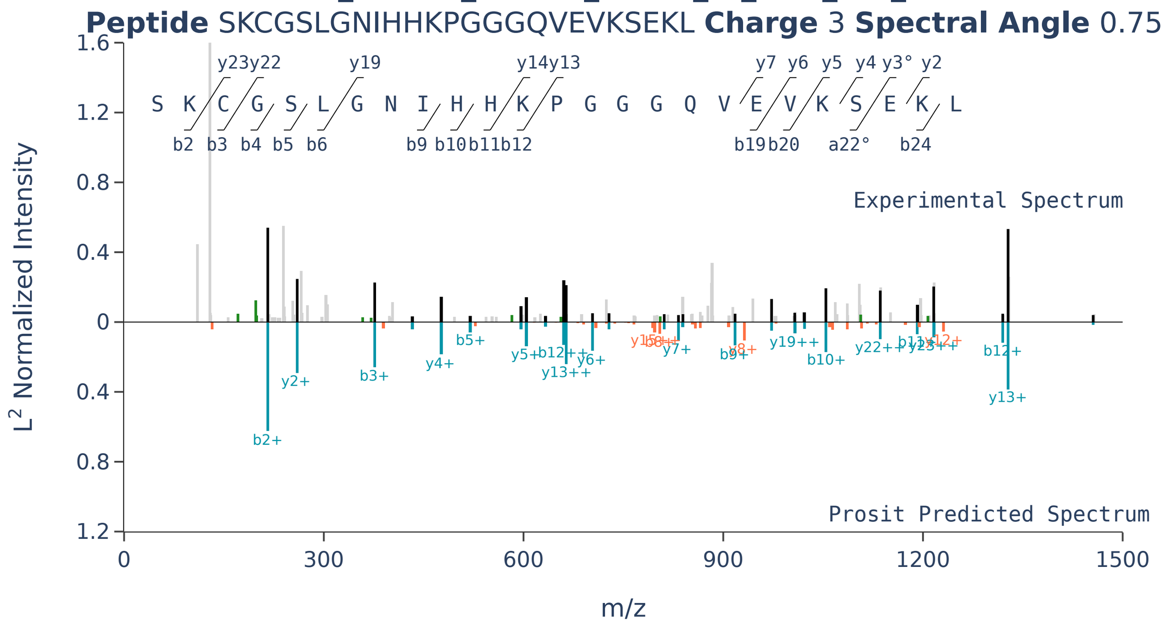


**Source** WSoh_221121_011221_HFGoe_L1_hTau_4h_R1 **Scan** 21833
**Peptide** TSPRHLSNVSSTGSIDMVDSPQL **Charge** 3 **Spectral Angle** 0.75

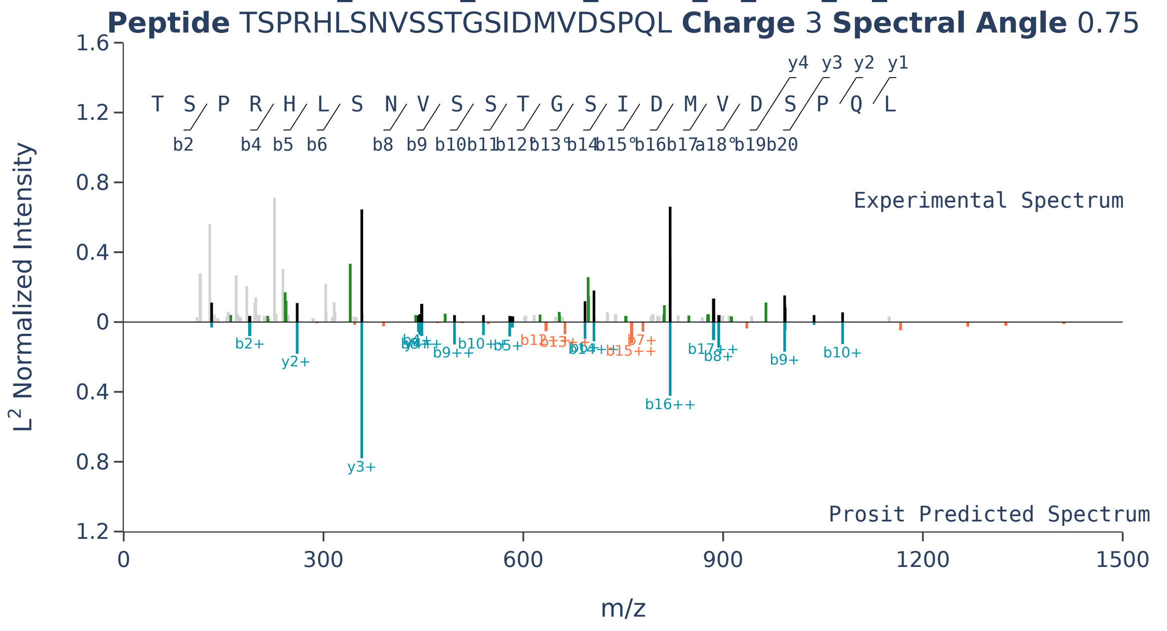


**Source** WSoh_221121_011221_HFGoe_L1_hTau_24h_R1 **Scan** 15827
**Peptide** SLDNITHVPGGGNKKIETHKLTFRENA **Charge** 5 **Spectral Angle** 0.8

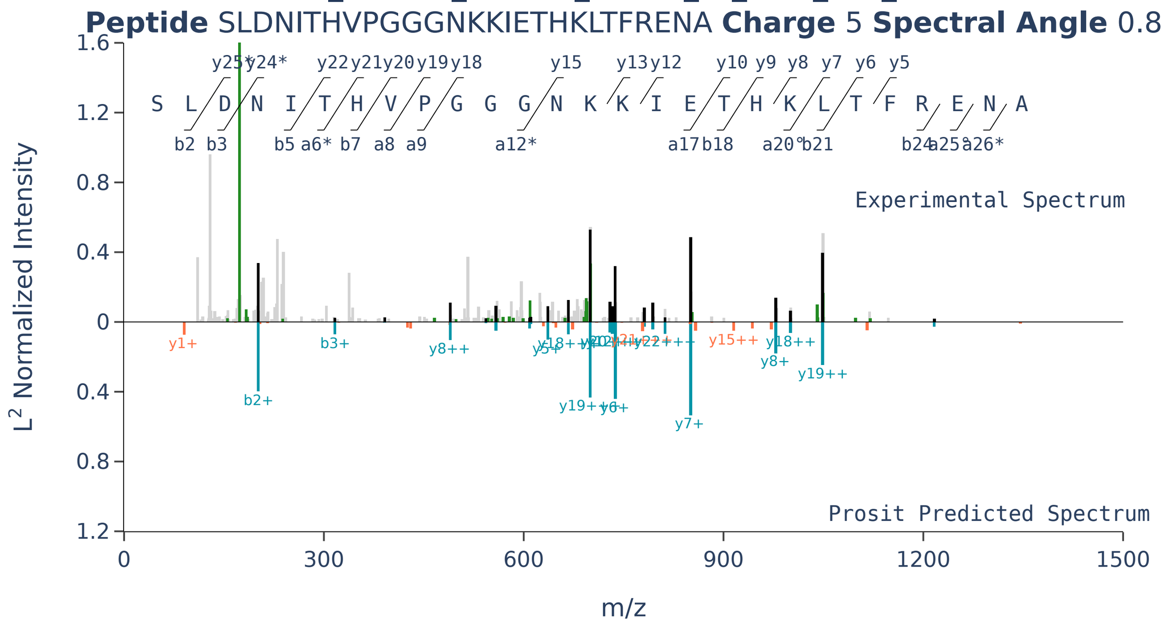


**Source** WSoh_221121_011221_HFGoe_L2_hTau_2h_R2 **Scan** 19428
**Peptide** EDVTAPLVDEGAPGKQAA **Charge** 2 **Spectral Angle** 0.84

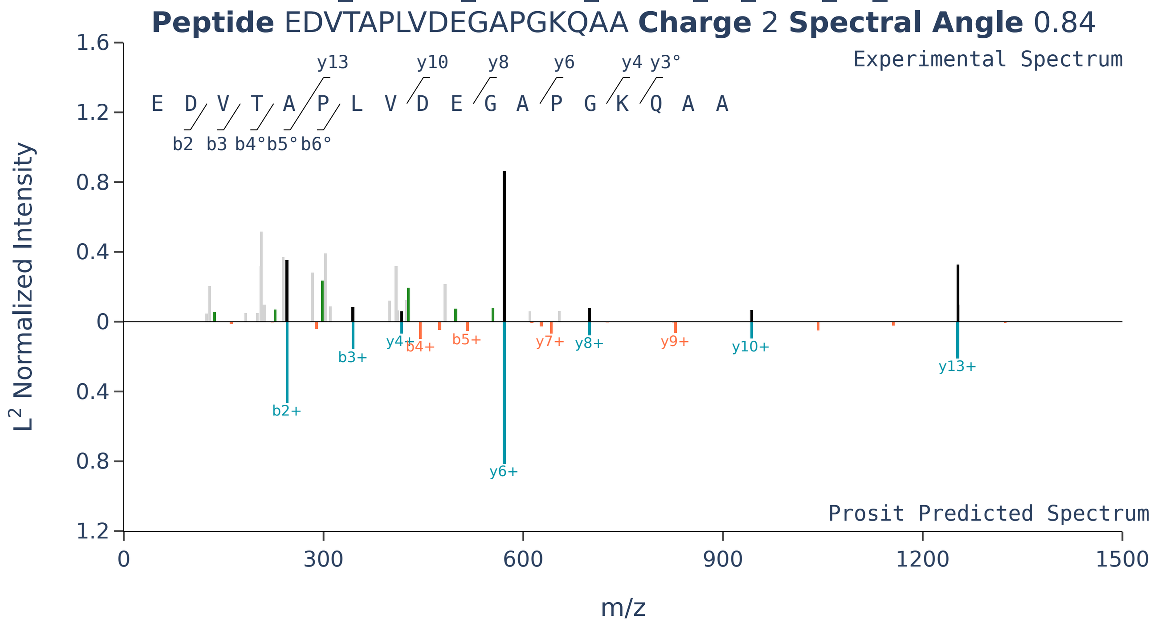


**Source** WSoh_221121_011221_HFGoe_L1_hTau_24h_R2 **Scan** 25413
**Peptide** MVDSPQLATL **Charge** 2 **Spectral Angle** 0.73

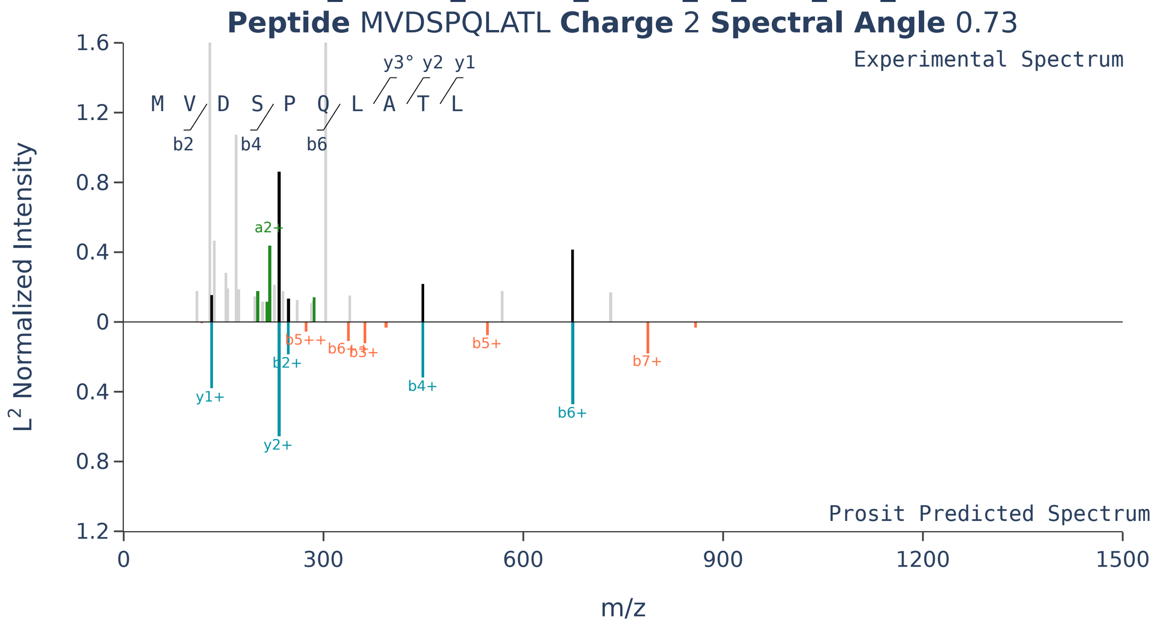

**Source** WSoh_221121_011221_HFGoe_L2_hTau_4h_R1 **Scan** 5992
**Peptide** GNIHHKPGGG **Charge** 2 **Spectral Angle** 0.84

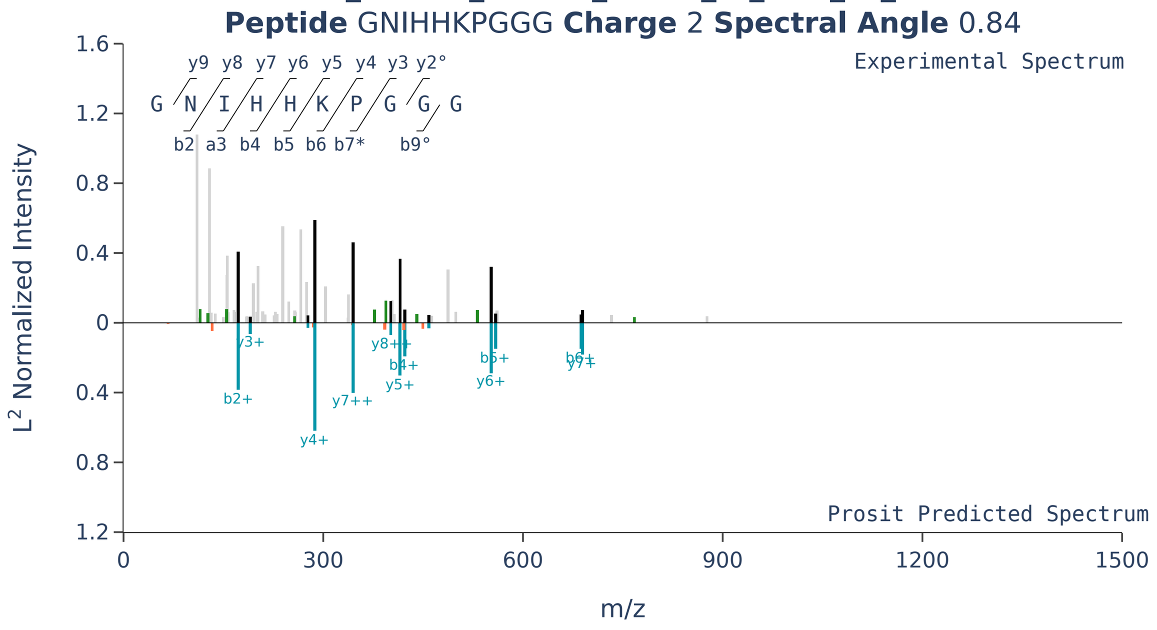


**Source** WSoh_221121_011221_HFGoe_L1_hTau_24h_R1 **Scan** 17218
**Peptide** TDHGAEIVYKSPVVSGDTSPRHLSNVSSTG **Charge** 4 **Spectral Angle** 0.75

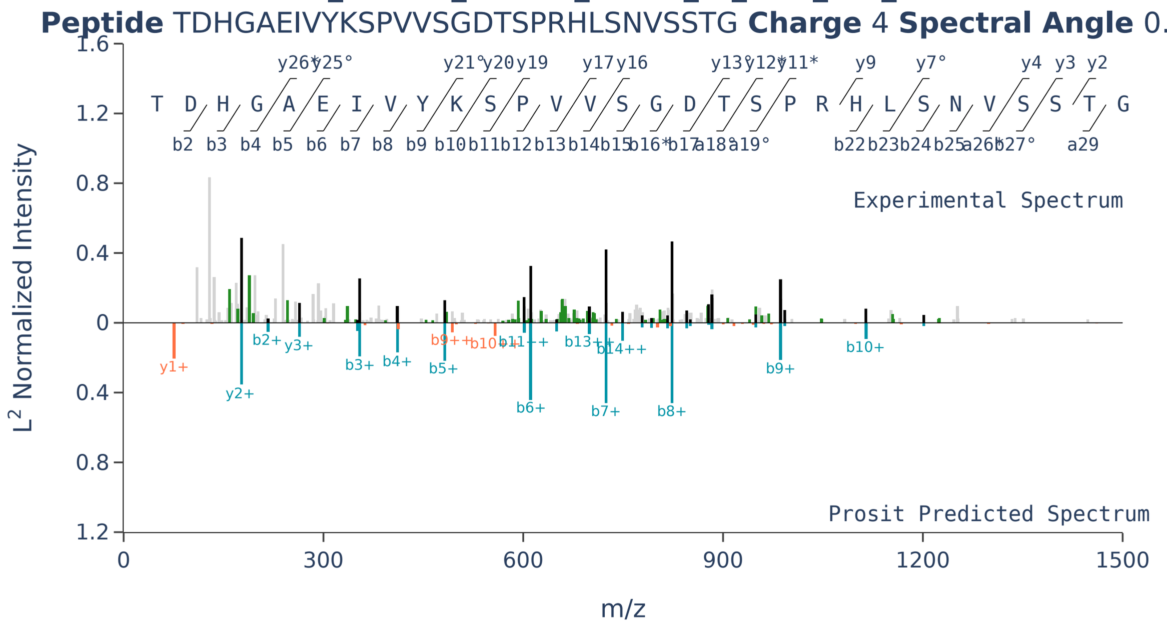


**Source** WSoh_221121_011221_HFGoe_L1_hTau_24h_R1 **Scan** 21471
**Peptide** TAEEAGIGDTPSLEDEAAGHVTQ **Charge** 2 **Spectral Angle** 0.67

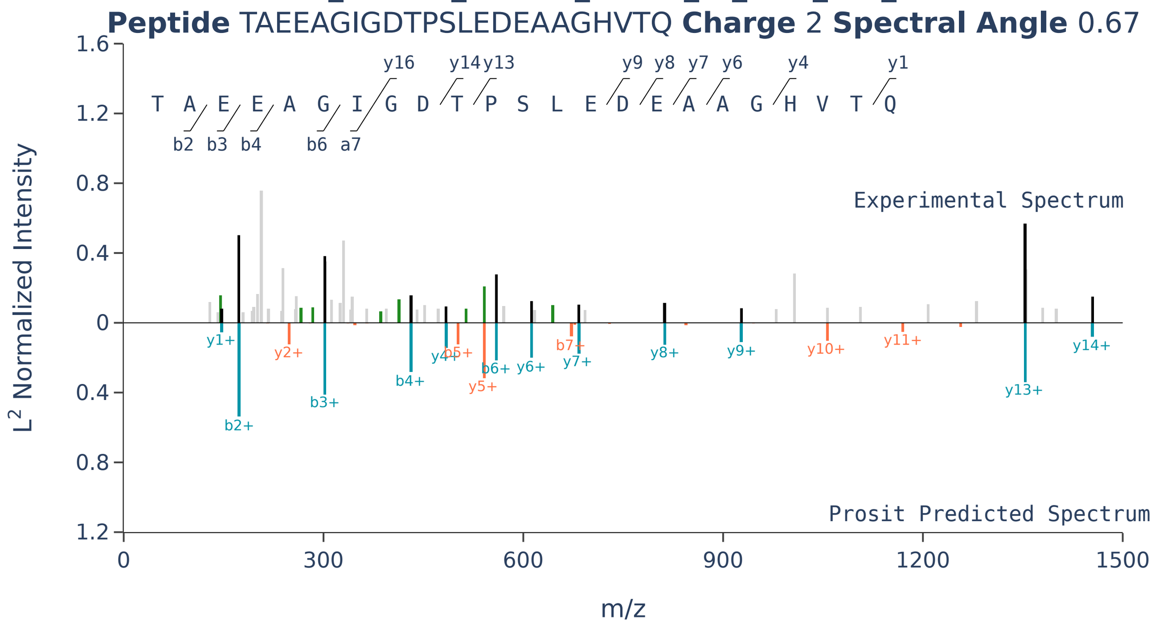


**Source** WSoh_221121_011221_HFGoe_L2_hTau_24h_R2 **Scan** 18717
**Peptide** LDLSNVQSKCGSKDNIKHVPGGGSVQL **Charge** 4 **Spectral Angle** 0.73

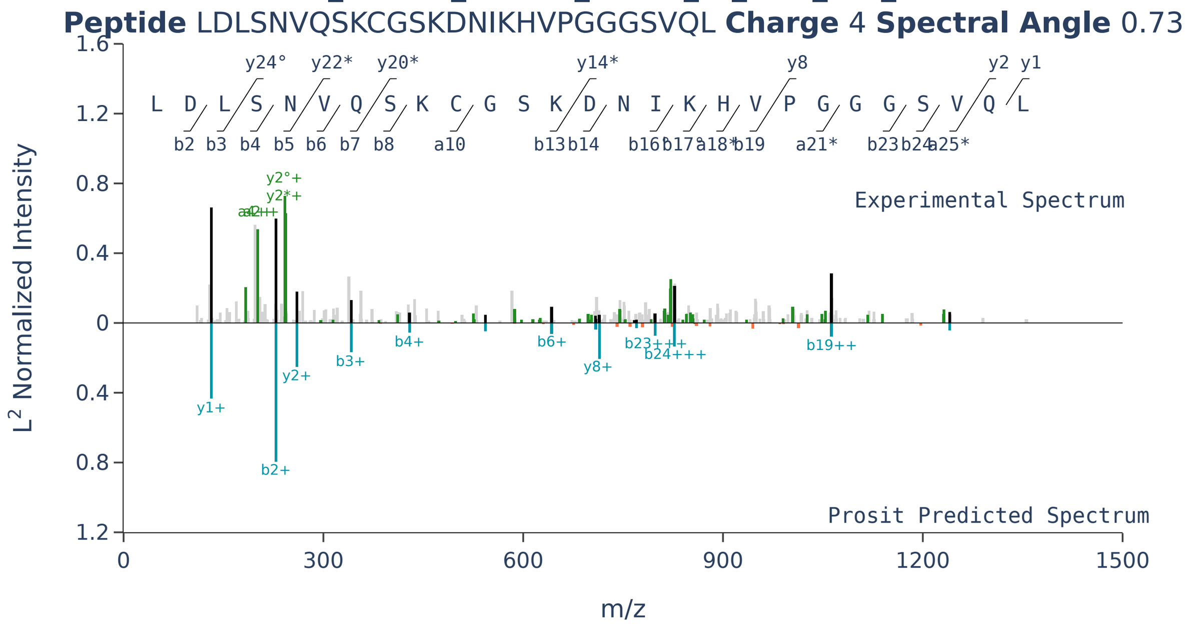


**Source** WSoh_221121_011221_HFGoe_L2_hTau_24h_R2 **Scan** 11033
**Peptide** IPAKTPPAPKTPPSSGEPPKSGDRSGY **Charge** 5 **Spectral Angle** 0.74

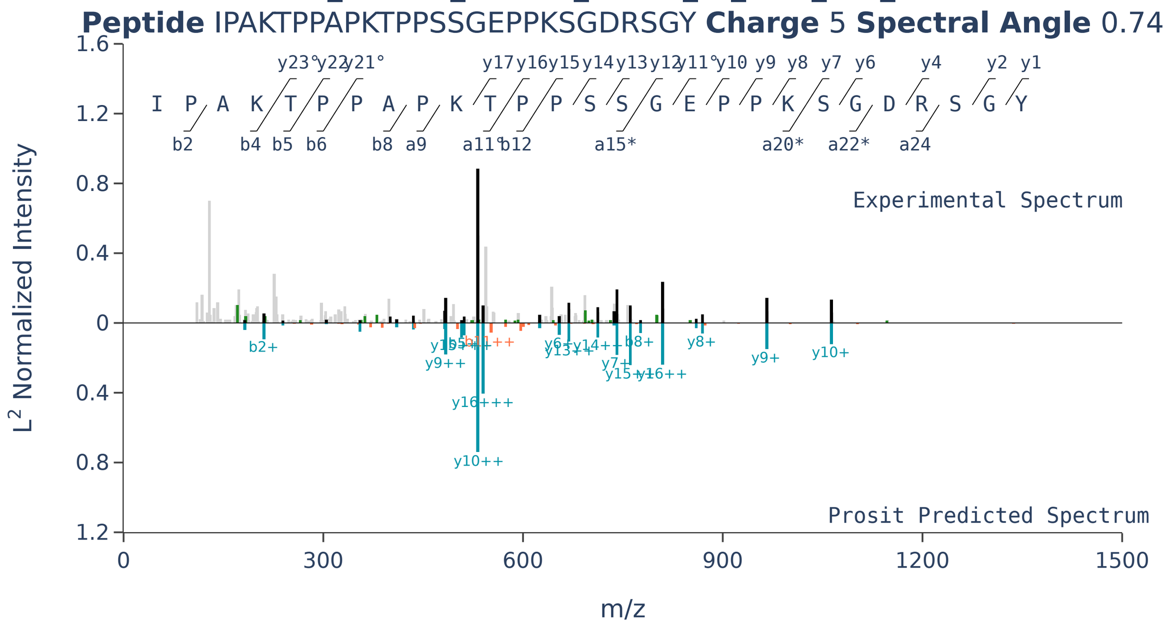


**Source** WSoh_221121_011221_HFGoe_L2_hTau_24h_R2 **Scan** 17201
**Peptide** AVVRTPPKSPSSAKSRLQTAPVPMPDLKN **Charge** 5 **Spectral Angle** 0.73

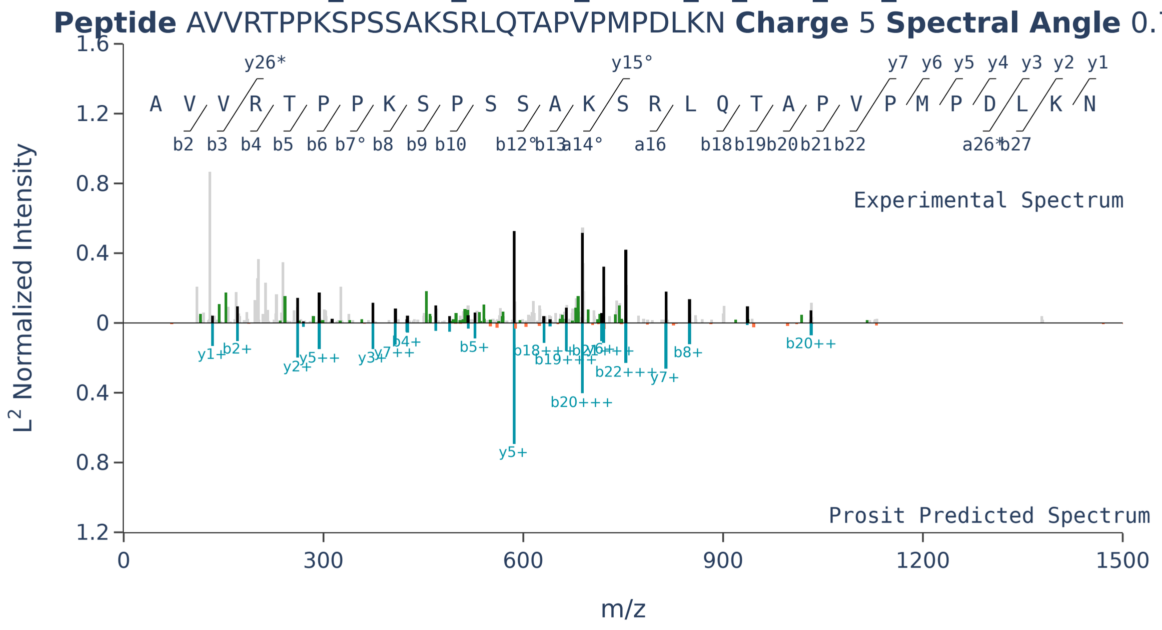


**Source** WSoh_221121_011221_HFGoe_L1_hTau_4h_R2 **Scan** 8632
**Peptide** NLTHVPGGGNKKIETHKLT **Charge** 5 **Spectral Angle** 0.62

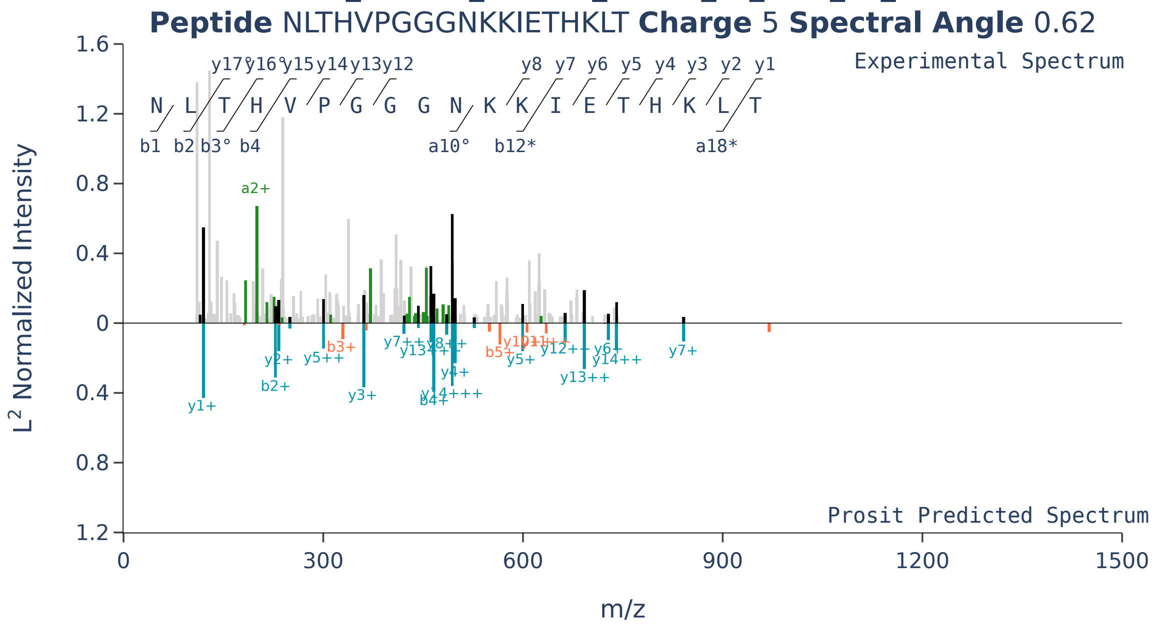


**Source** WSoh_221121_011221_HFGoe_L2_hTau_24h_R2 **Scan** 15252
**Peptide** LDLSNVQSKCG **Charge** 2 **Spectral Angle** 0.74

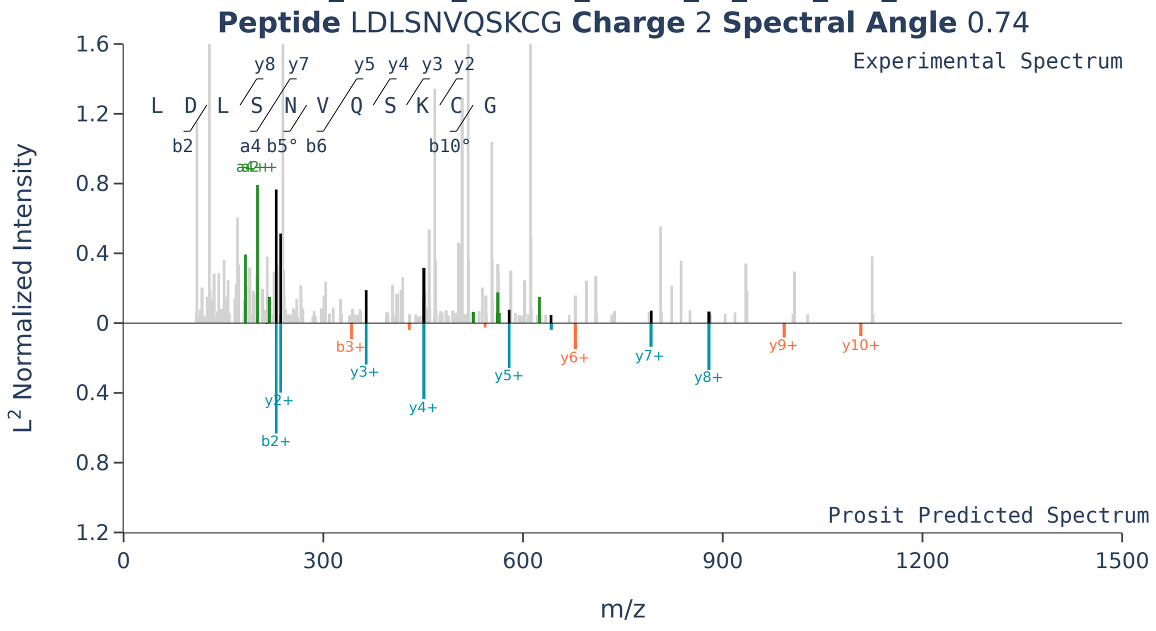


**Source** WSoh_221121_011221_HFGoe_L1_hTau_4h_R2 **Scan** 15995
**Peptide** DHGAEIVYKSPVVSGDTSPRHLSN **Charge** 4 **Spectral Angle** 0.77

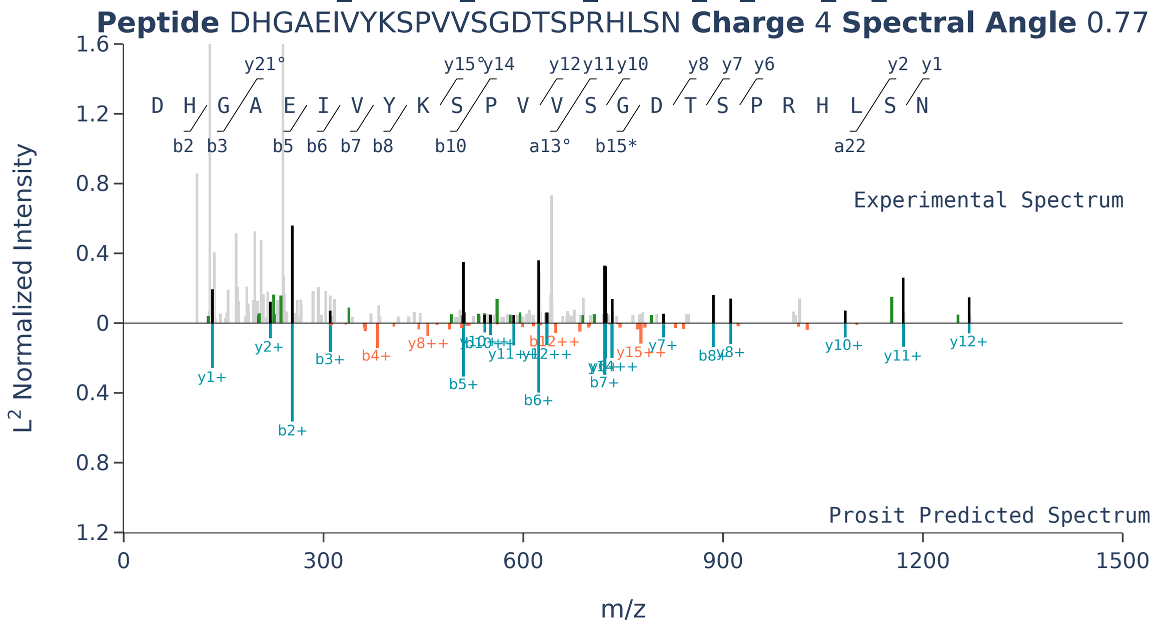


**Source** WSoh_221121_011221_HFGoe_L2_hTau_4h_R1 **Scan** 18486
**Peptide** QVEVKSEKLDFKDRVQSKIGSLD **Charge** 4 **Spectral Angle** 0.69

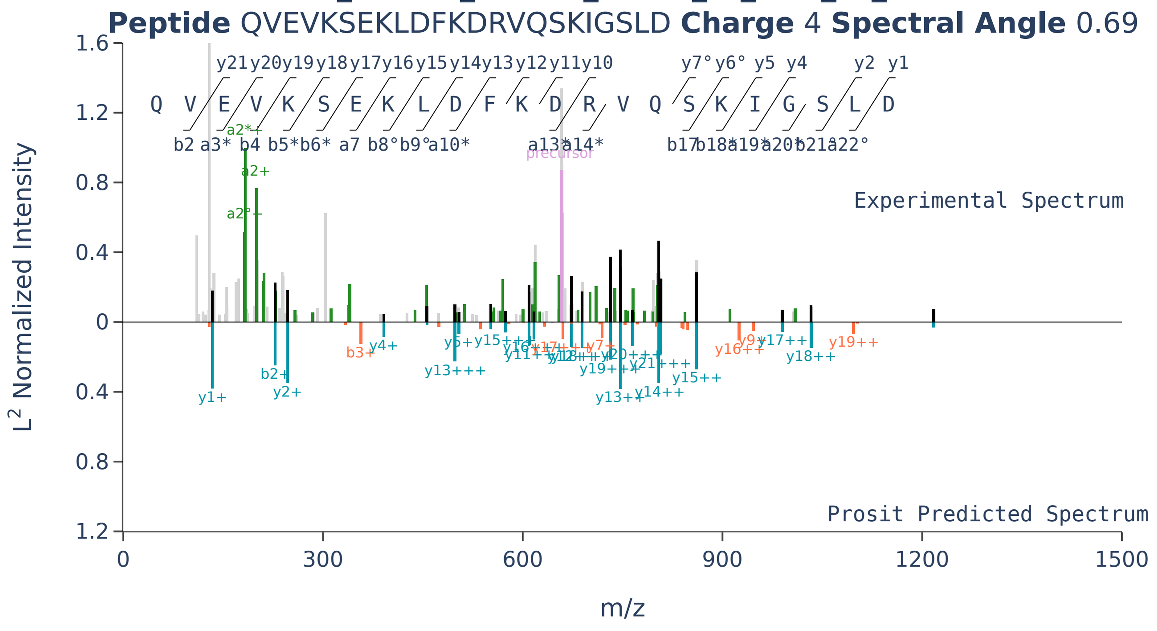


**Source** WSoh_221121_011221_HFGoe_L2_hTau_24h_R1 **Scan** 19341
**Peptide** QGGYTMHQDQEGDTDAGLKESPLQTPTED **Charge** 3 **Spectral Angle** 0.91

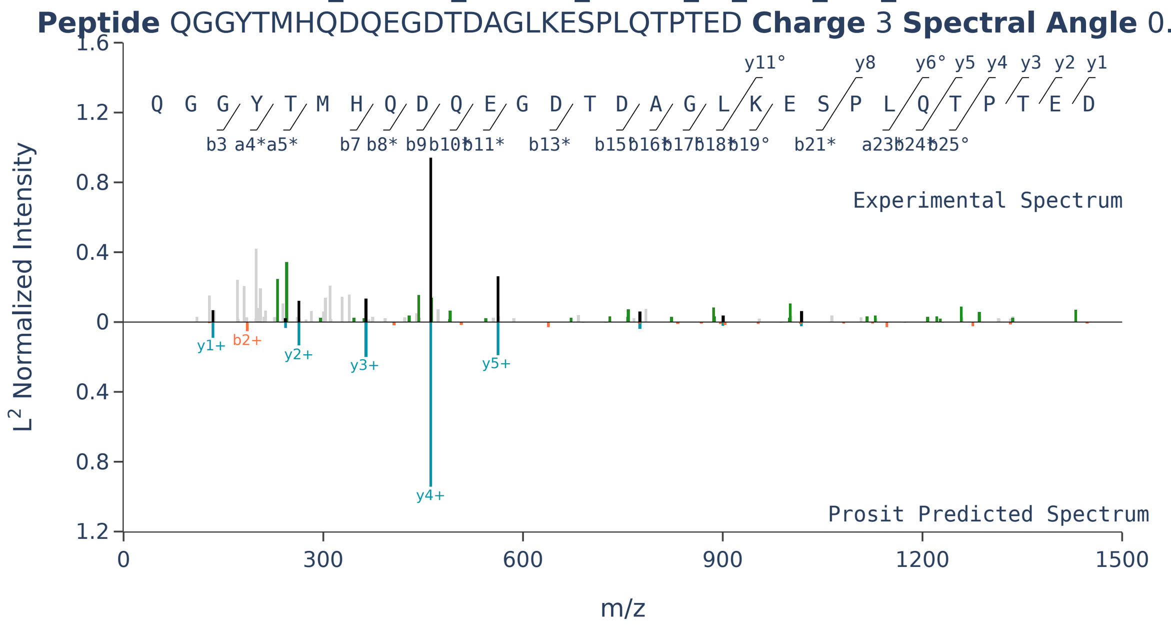


**Source** WSoh_221121_011221_HFGoe_L1_hTau_24h_R1 **Scan** 14125
**Peptide** SLDNITHVPGGGNKKLE **Charge** 4 **Spectral Angle** 0.78

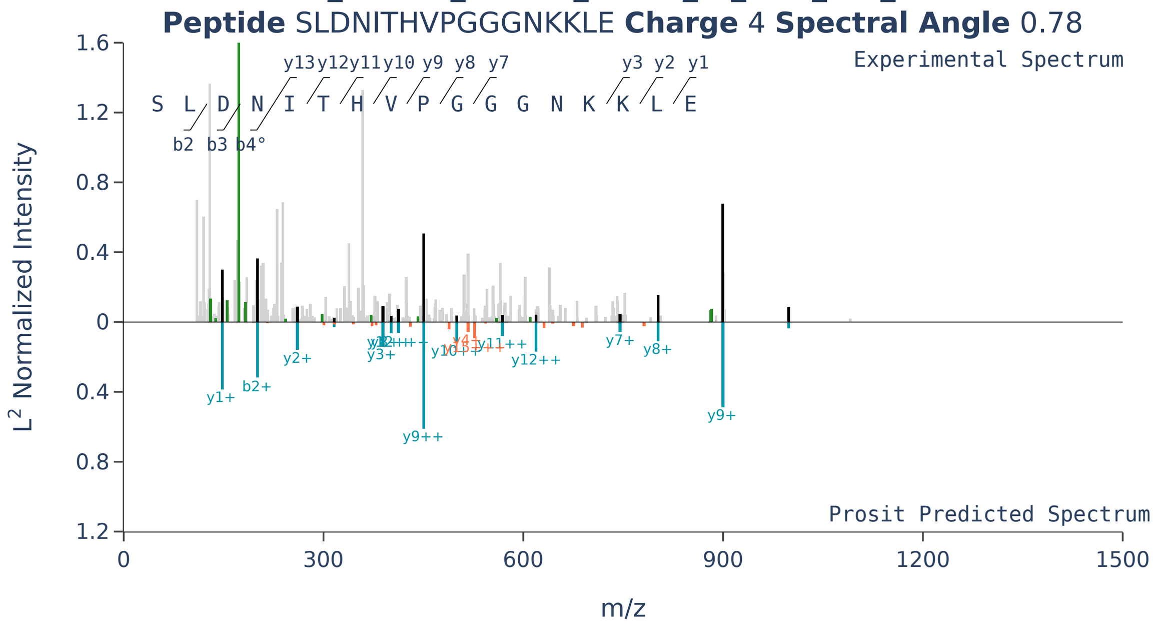


**Source** WSoh_221121_011221_HFGoe_L2_hTau_4h_R2 **Scan** 18983
**Peptide** GSKDNIKHVPGGGSVQIVYKPVDLSKVTSK **Charge** 4 **Spectral Angle** 0.74

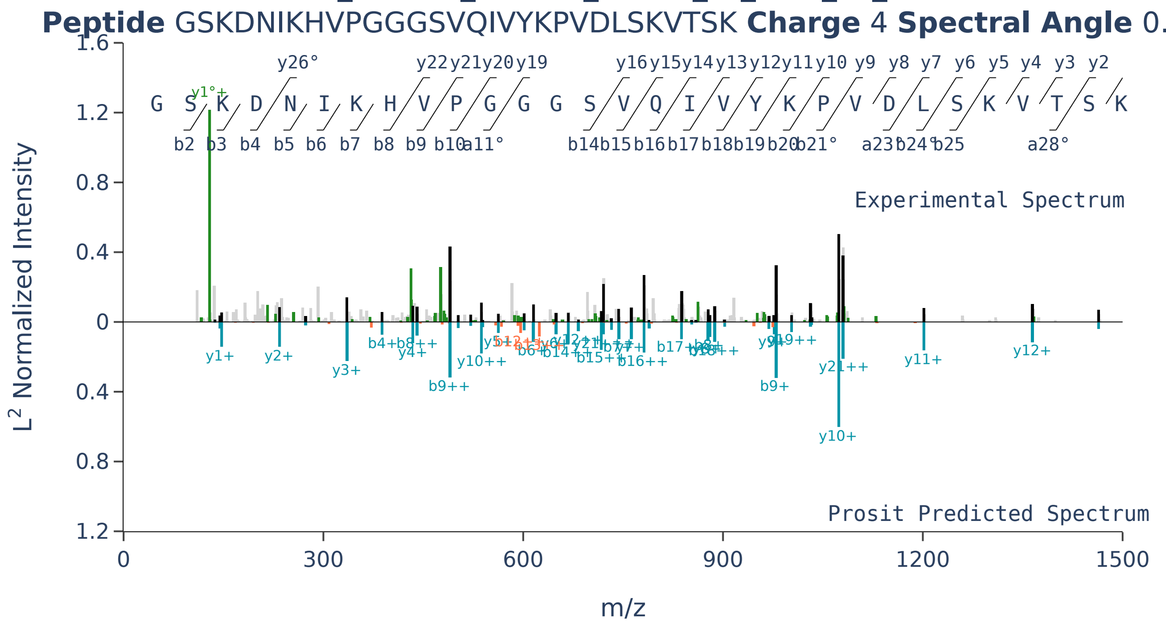


**Source** WSoh_221121_011221_HFGoe_L1_hTau_4h_R2 **Scan** 12785
**Peptide** GSLGNIHHKPGGGQVEVKSEKLDFKDRVQ **Charge** 6 **Spectral Angle** 0.73

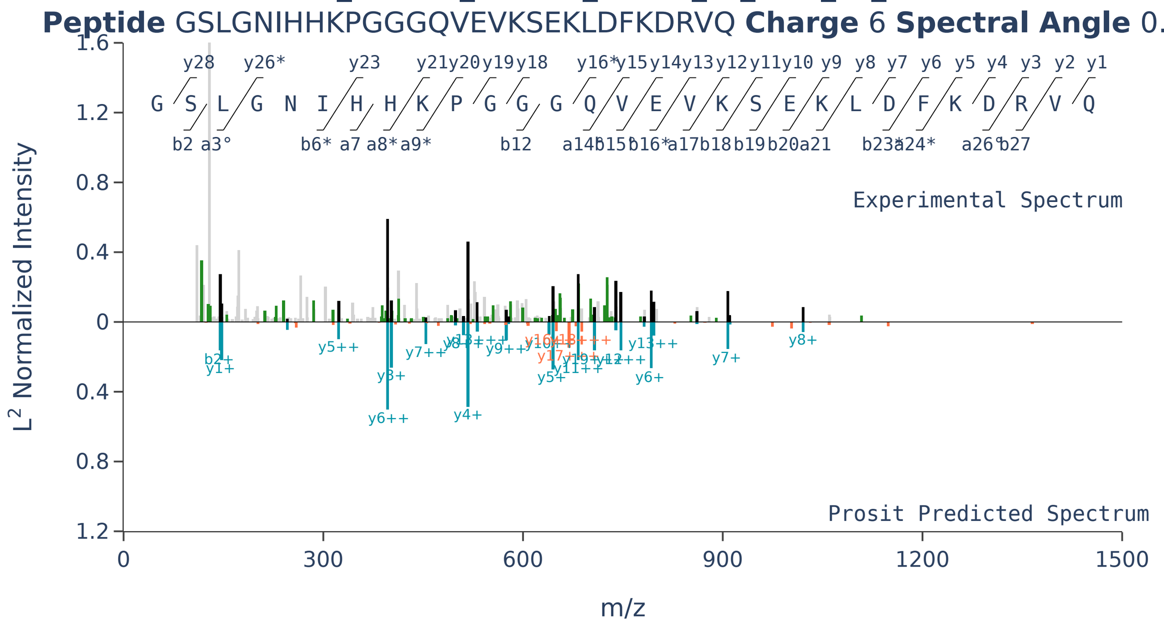


**Source** WSoh_221121_011221_HFGoe_L1_hTau_24h_R1 **Scan** 10871
**Peptide** ANATRIPAKTPPAPKTPPSSGEPPKSGD **Charge** 4 **Spectral Angle** 0.83

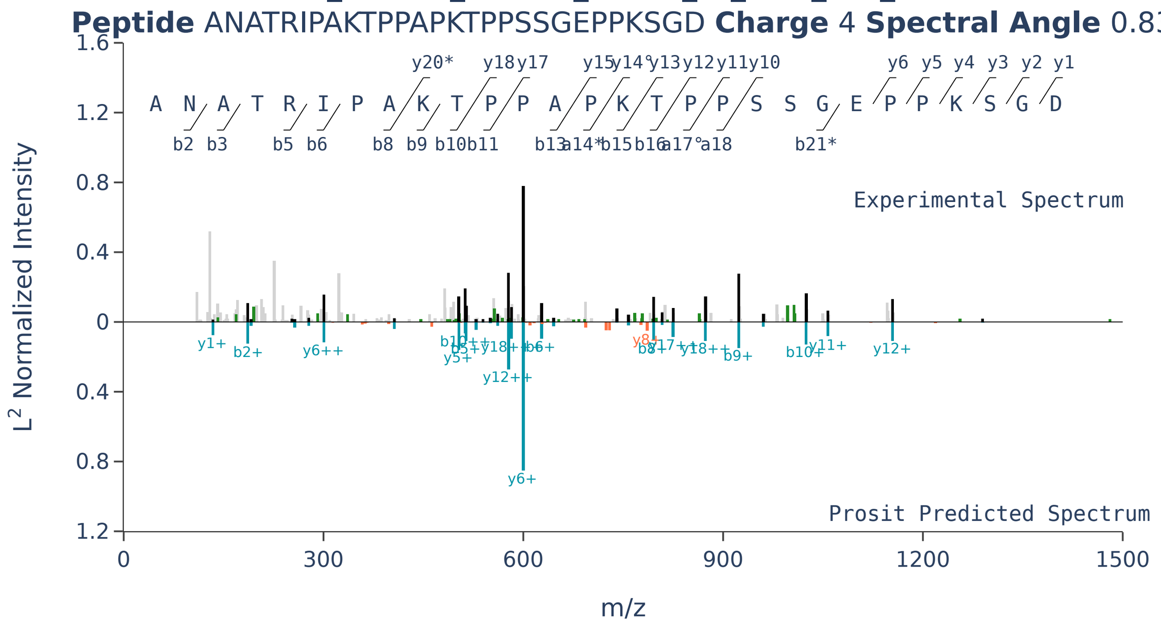

**Source** WSoh_221121_011221_HFGoe_L1_hTau_24h_R2 **Scan** 12139
**Peptide** RENAKAKTDHGAEIVYKSPVVSGD **Charge** 3 **Spectral Angle** 0.67

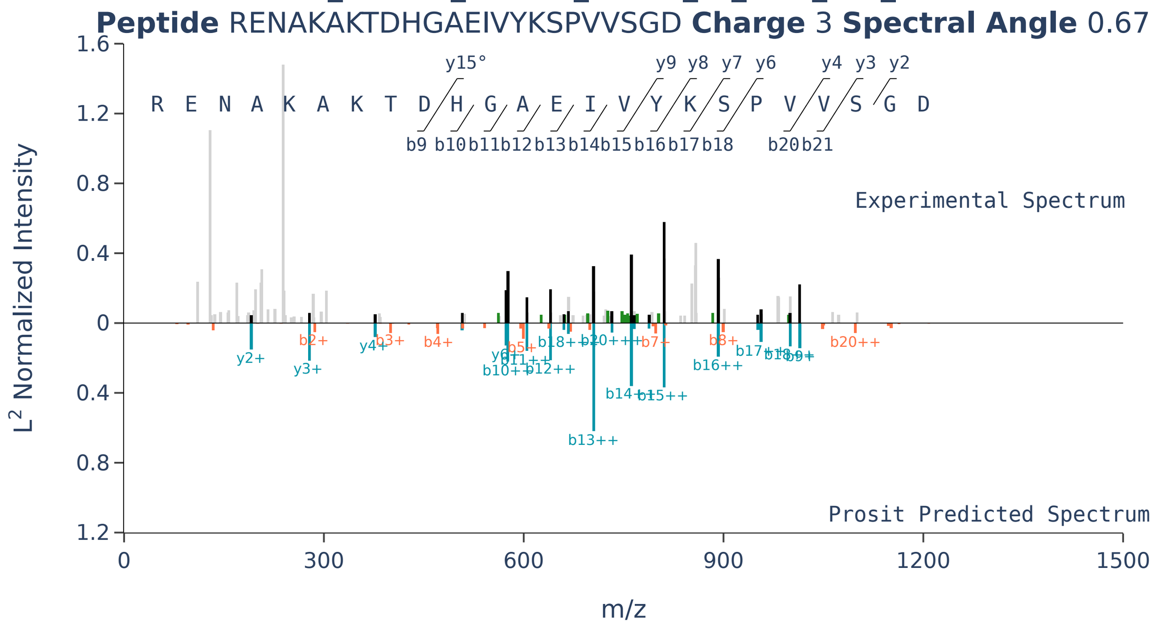


**Source** WSoh_221121_011221_HFGoe_L1_hTau_24h_R1 **Scan** 15623
**Peptide** VYKPVDLSKVTSKCGS **Charge** 3 **Spectral Angle** 0.74

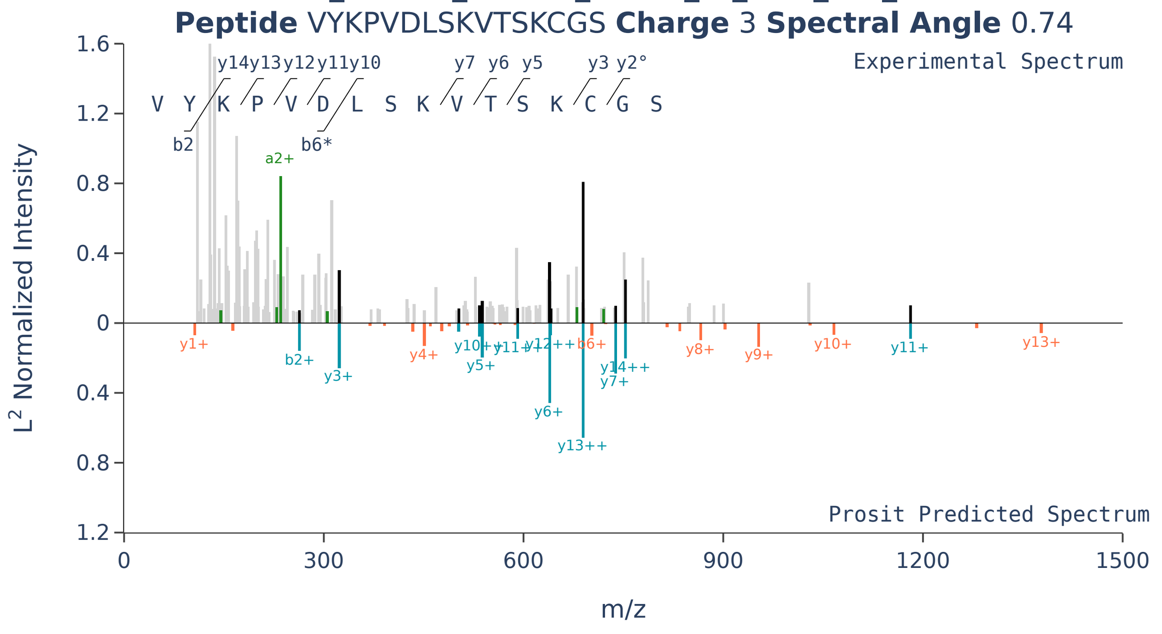


**Source** WSoh_221121_011221_HFGoe_L1_hTau_4h_R1 **Scan** 14422
**Peptide** YKPVDLSKVTSKCGSLGNIHHKPGGGQ **Charge** 4 **Spectral Angle** 0.81

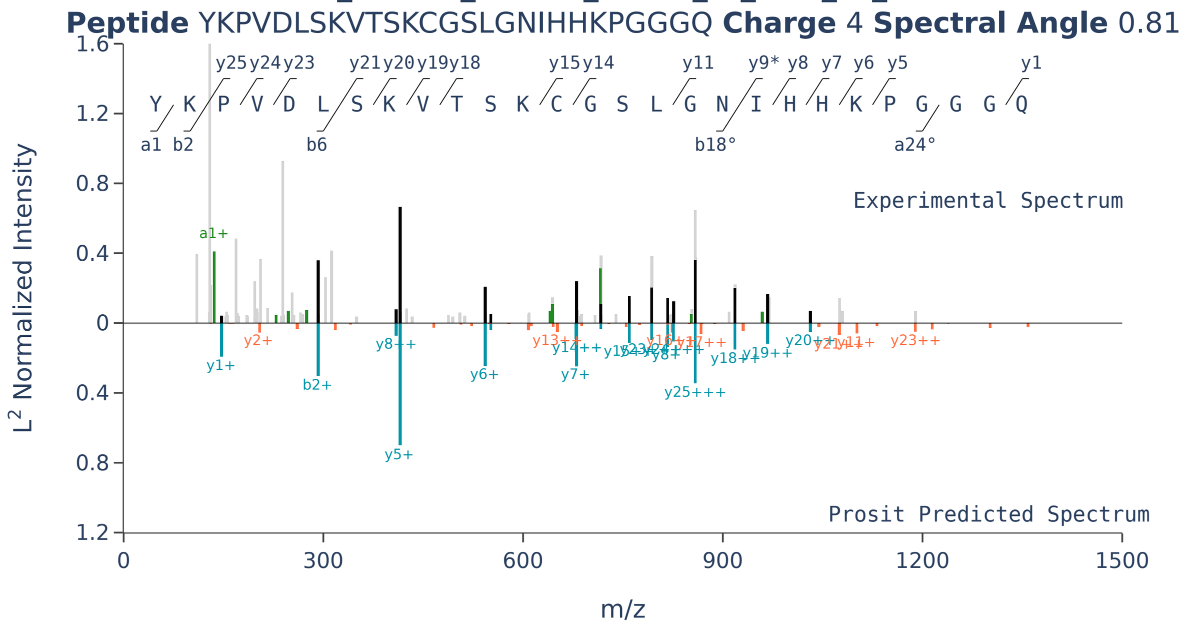


**Source** WSoh_221121_011221_HFGoe_L1_hTau_4h_R2 **Scan** 9133
**Peptide** PTPPTREPKKVAVVRTPPKSPSSAKSRL **Charge** 5 **Spectral Angle** 0.73

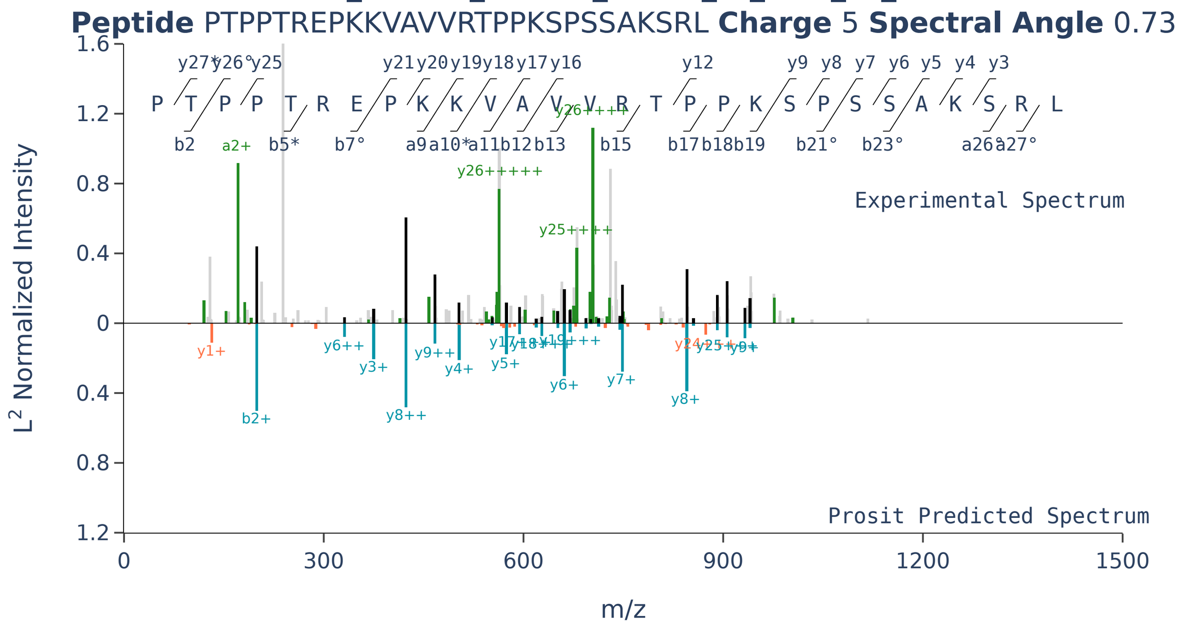


**Source** WSoh_221121_011221_HFGoe_L1_hTau_1h_R2 **Scan** 6291
**Peptide** DEGAPGKQAAAQPHT **Charge** 3 **Spectral Angle** 0.88

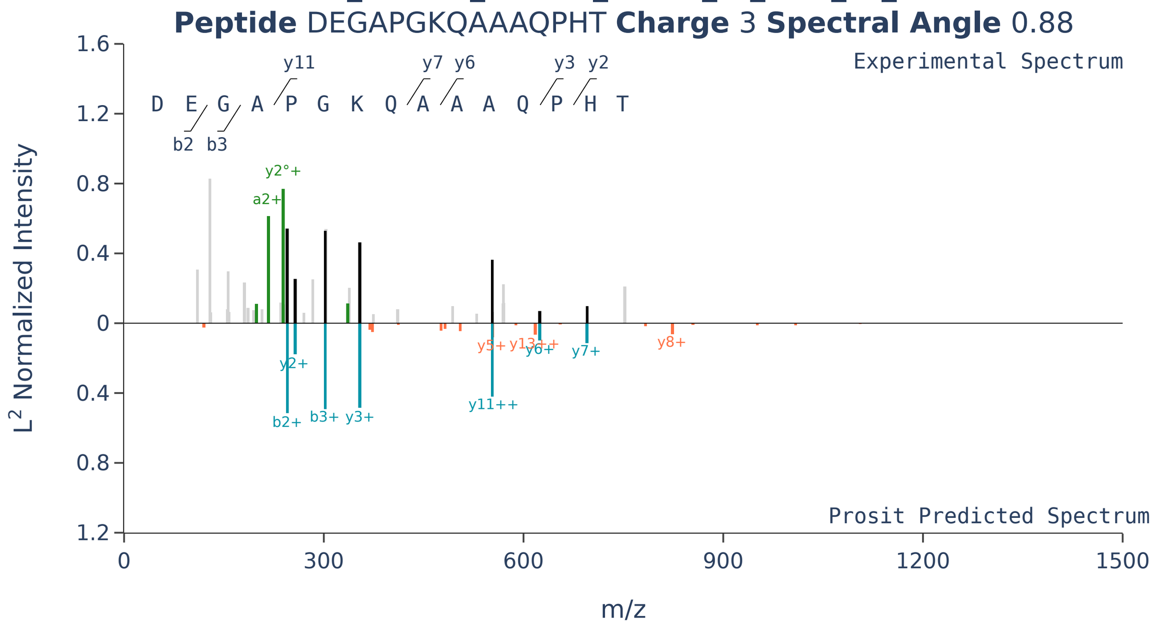


**Source** WSoh_221121_011221_HFGoe_L2_hTau_24h_R2 **Scan** 22166
**Peptide** KSTPTAEDVTAPLV **Charge** 2 **Spectral Angle** 0.57

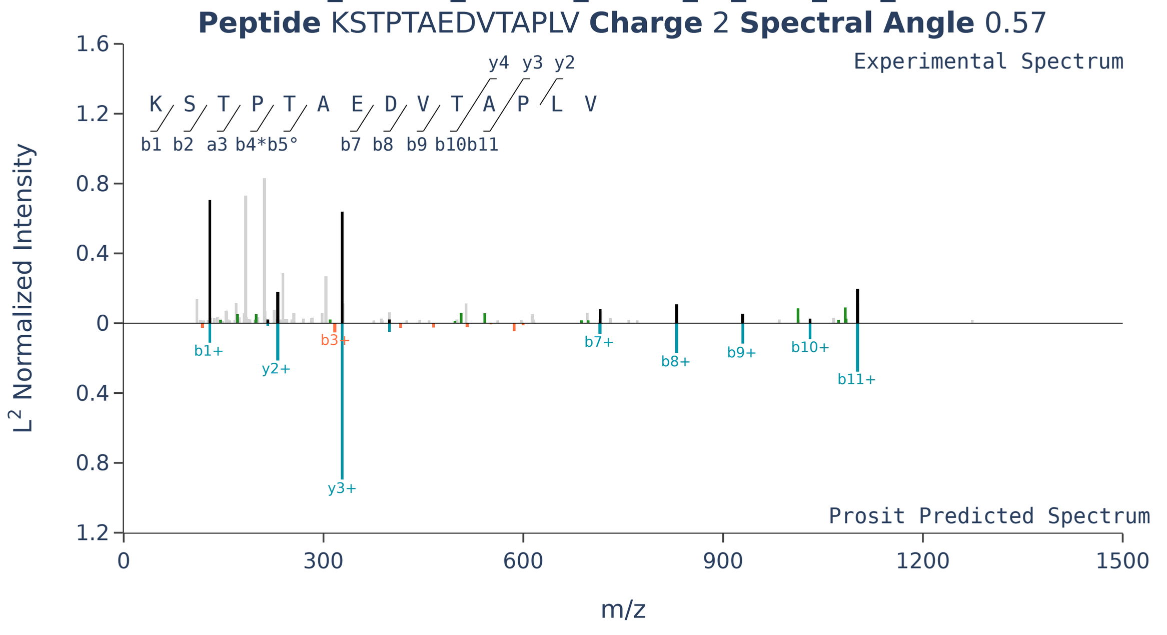


**Source** WSoh_221121_011221_HFGoe_L2_hTau_24h_R1 **Scan** 9581
**Peptide** KSKIGSTENLKHQPGGGKVQL **Charge** 3 **Spectral Angle** 0.88

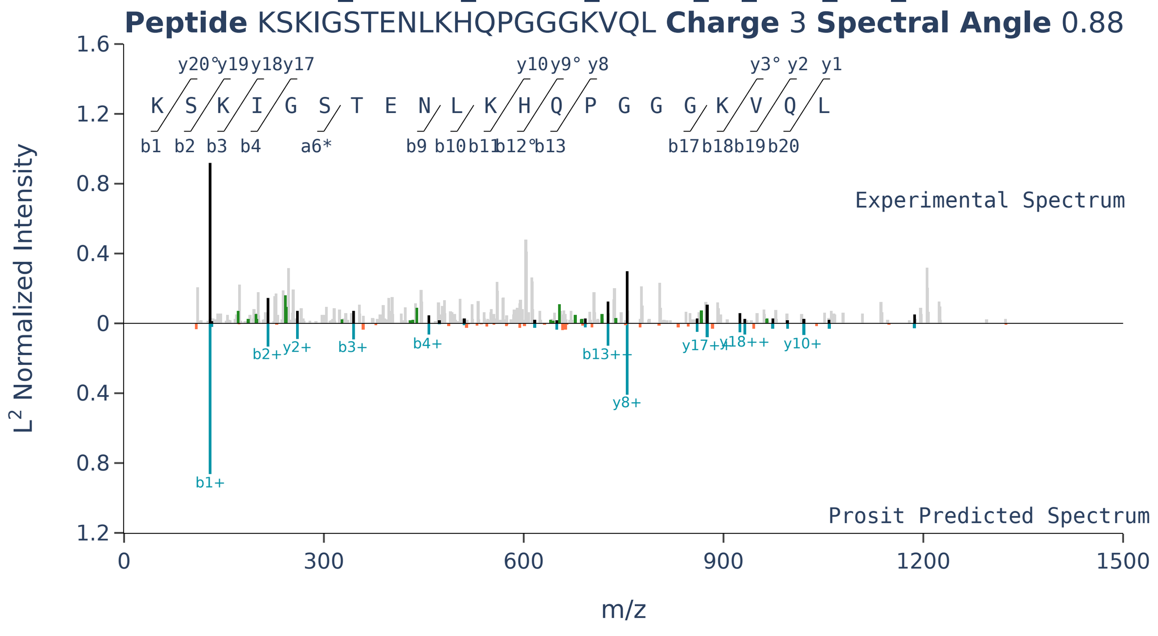


**Source** WSoh_221121_011221_HFGoe_L1_hTau_4h_R2 **Scan** 20883
**Peptide** GSKDNIKHVPGGGSVQIVYKPVDLSKV **Charge** 4 **Spectral Angle** 0.76

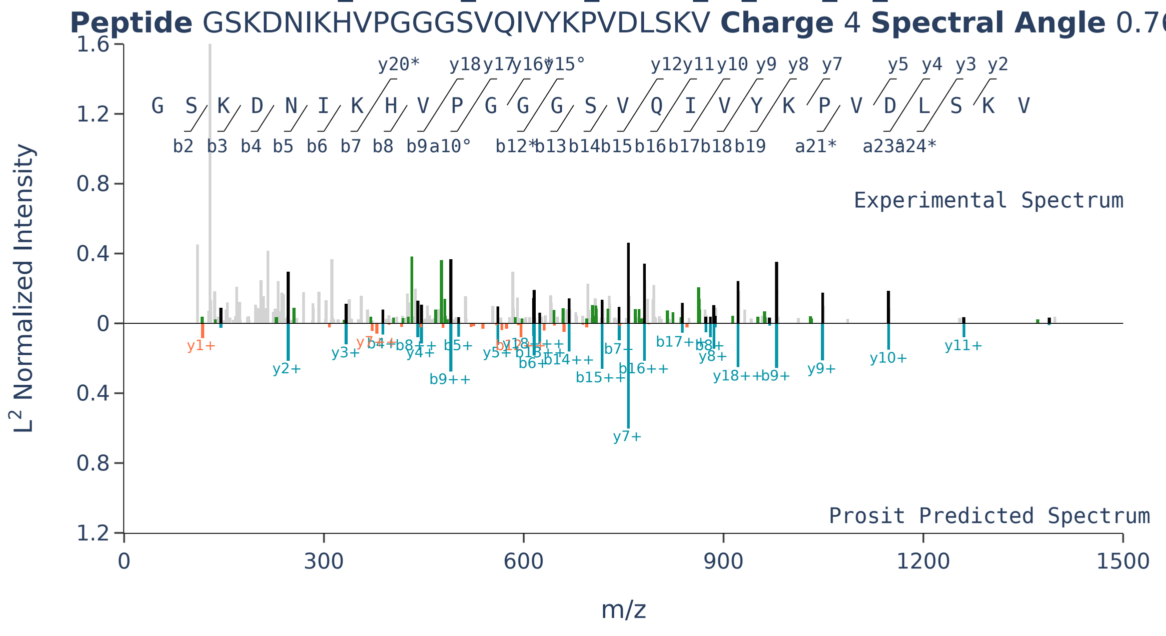


**Source** WSoh_221121_011221_HFGoe_L1_hTau_24h_R1 **Scan** 19073
**Peptide** KSEKLDFKDRVQSKIGSLDNITH **Charge** 4 **Spectral Angle** 0.74

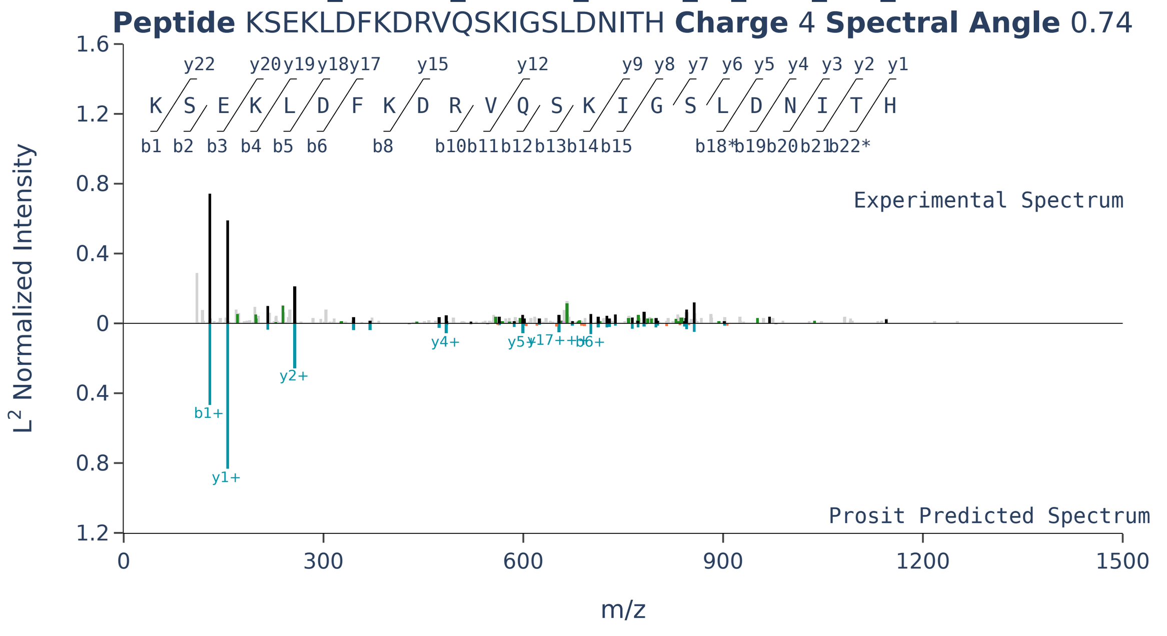


**Source** WSoh_221121_011221_HFGoe_L2_hTau_24h_R2 **Scan** 19404
**Peptide** VRTPPKSPSSAKSRLQTAPVPMPDLKNV **Charge** 4 **Spectral Angle** 0.75

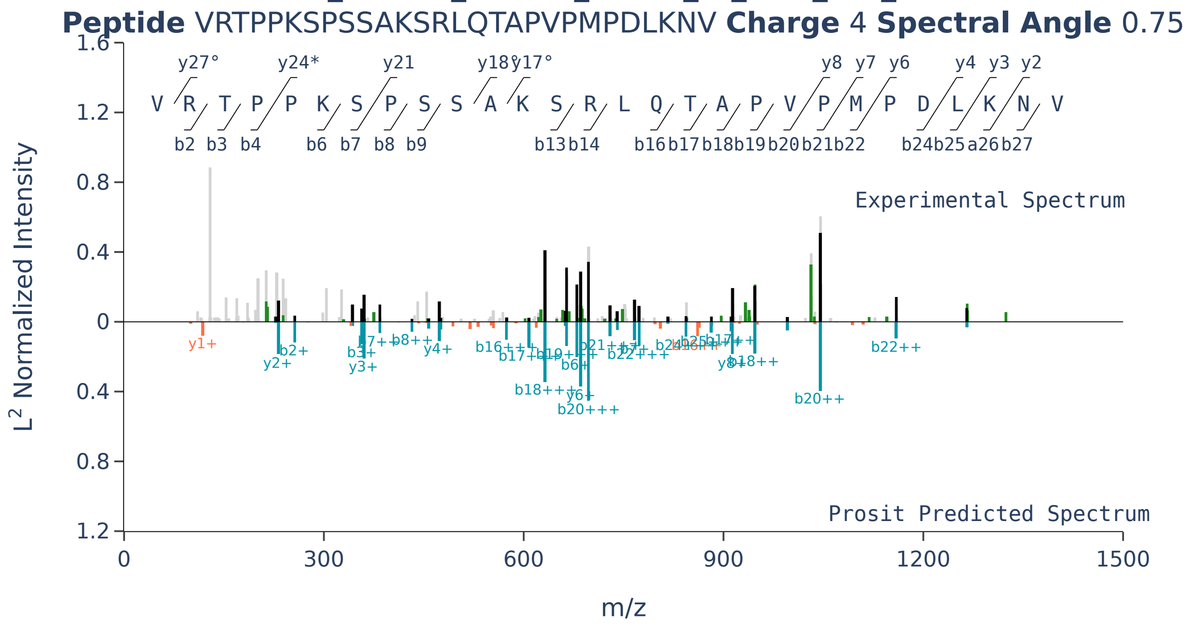


**Source** WSoh_221121_011221_HFGoe_L1_hTau_24h_R2 **Scan** 13397
**Peptide** TPSLPTPPTREPKKVAVVRTPPKSPSSAK **Charge** 5 **Spectral Angle** 0.73

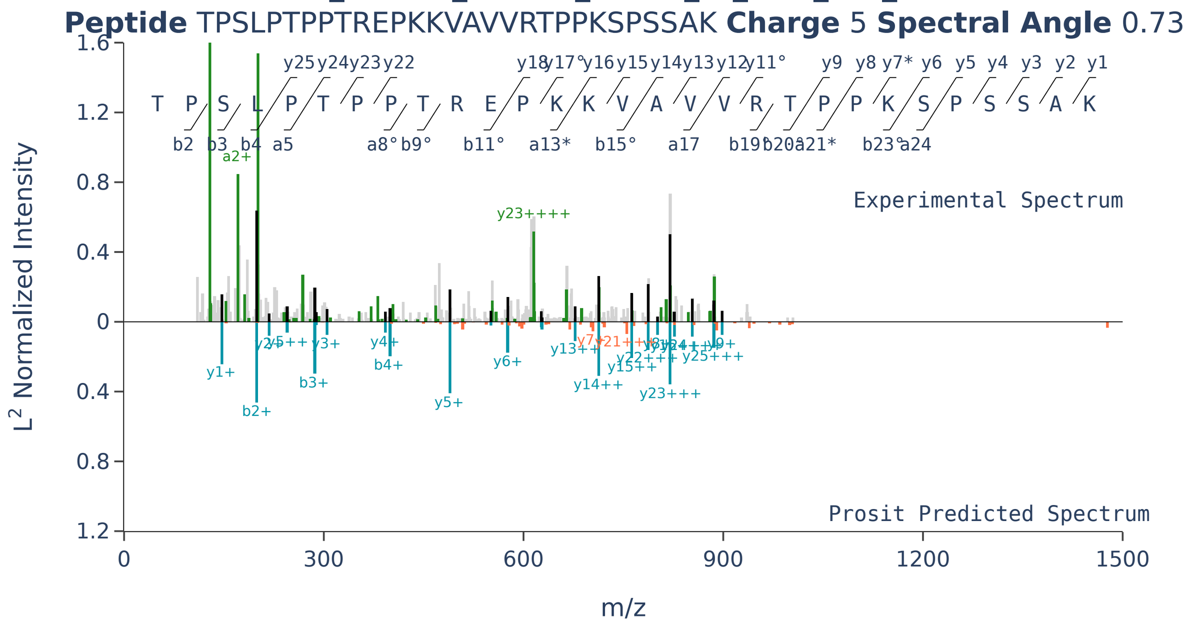


**Source** WSoh_221121_011221_HFGoe_L1_hTau_24h_R1 **Scan** 26009
**Peptide** IPEGTTAEEAGIGDTPSL **Charge** 2 **Spectral Angle** 0.9

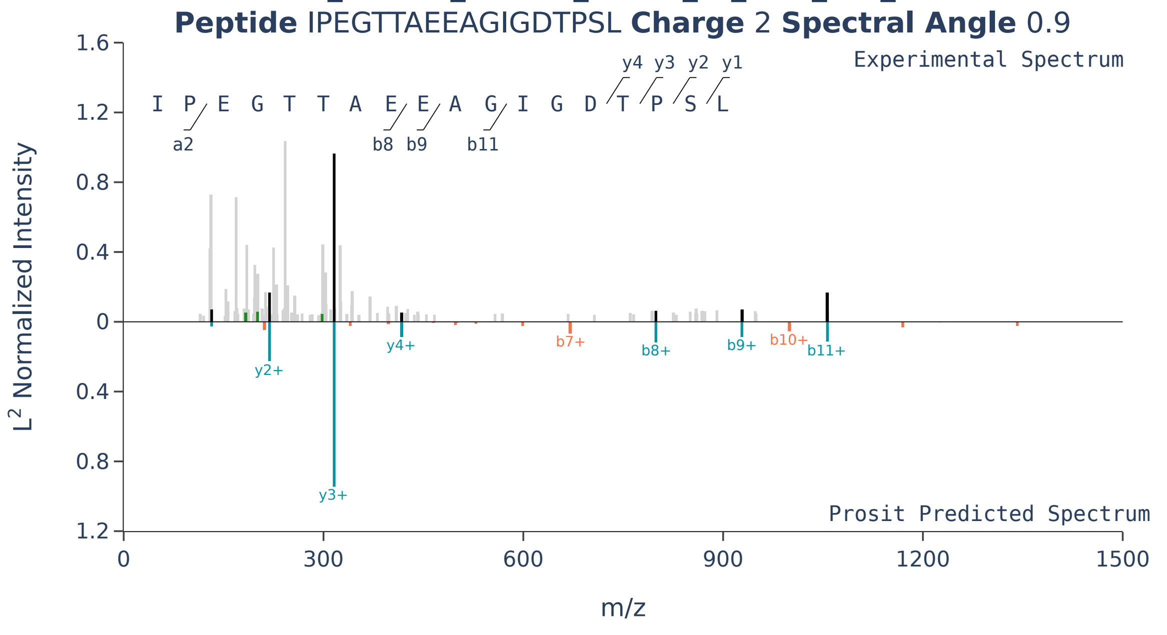


**Source** WSoh_221121_011221_HFGoe_L1_hTau_24h_R2 **Scan** 10560
**Peptide** IPAKTPPAPKTPPSSG **Charge** 3 **Spectral Angle** 0.67

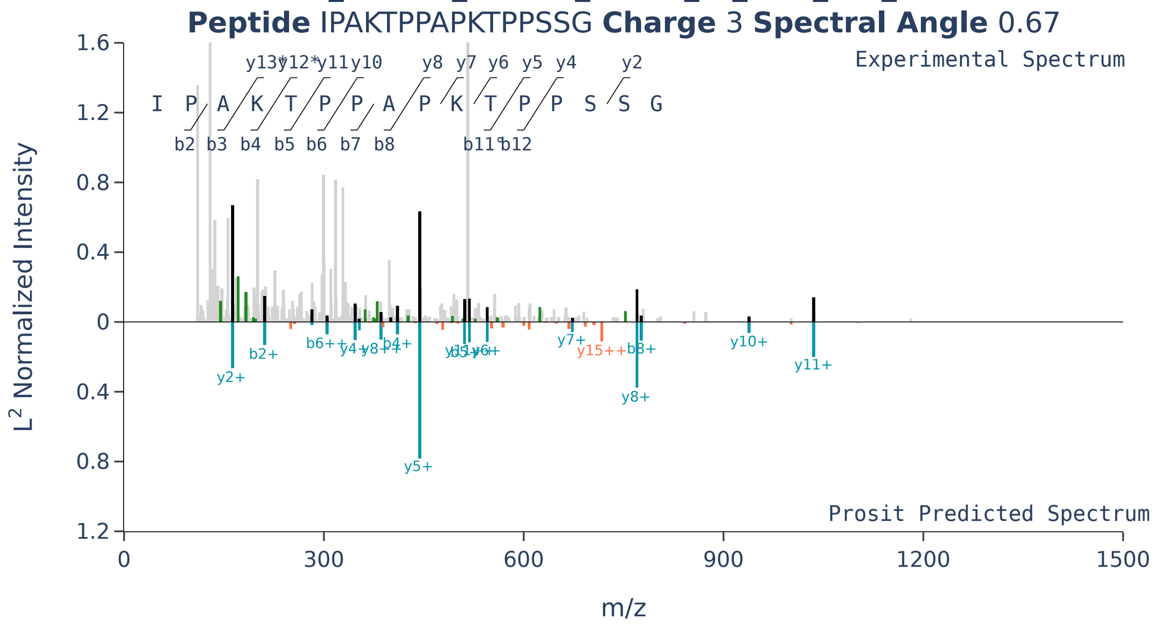


**Source** WSoh_221121_011221_HFGoe_L1_hTau_24h_R1 **Scan** 17008
**Peptide** EGAPGKQAAAQPHTEIPEGTTAEEAGIGD **Charge** 3 **Spectral Angle** 0.55

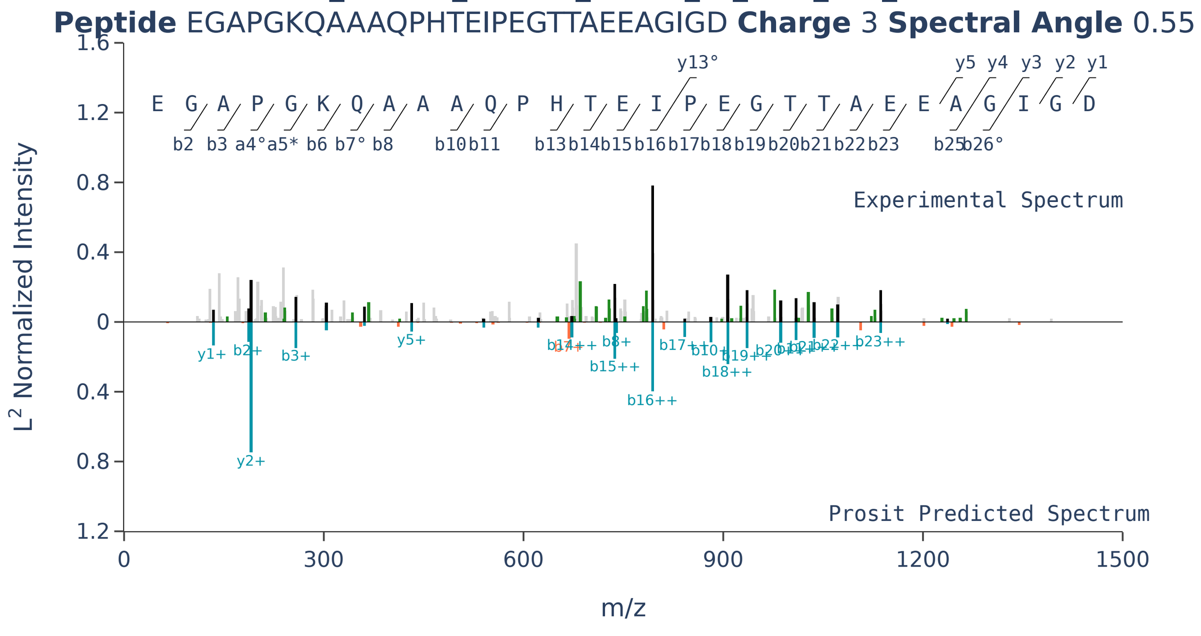


**Source** WSoh_221121_011221_HFGoe_L2_hTau_4h_R1 **Scan** 15852
**Peptide** HQPGGGKVQIINKKLDL **Charge** 4 **Spectral Angle** 0.88

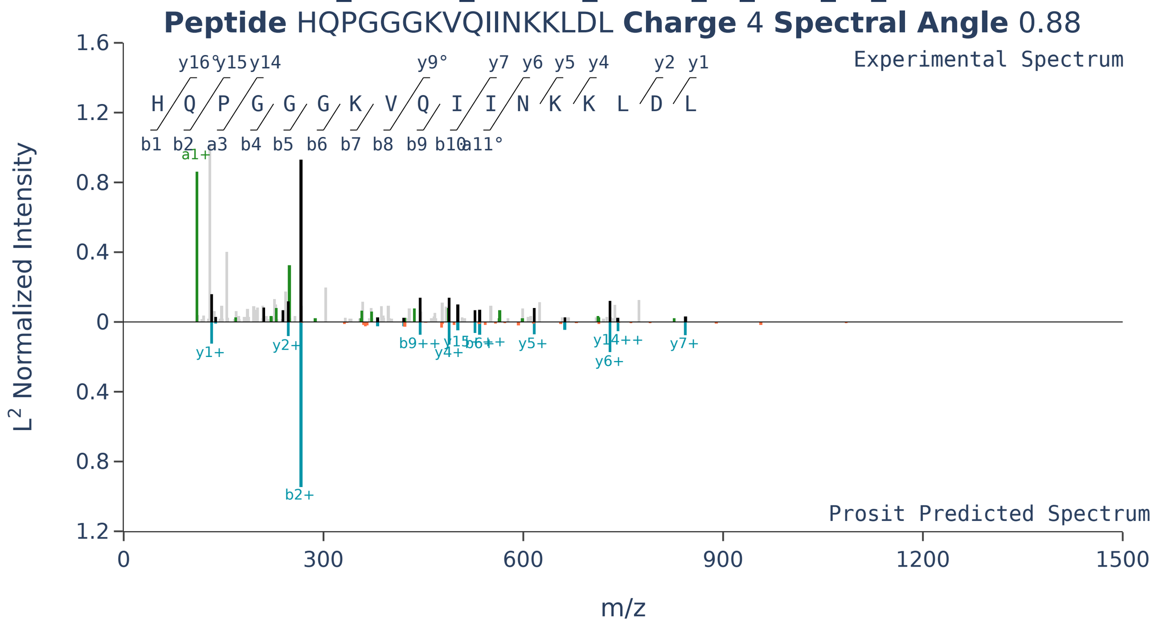

**Source** WSoh_221121_011221_HFGoe_L1_hTau_24h_R1 **Scan** 16343
**Peptide** QSKIGSLDNITHVPGGGNKKLE **Charge** 4 **Spectral Angle** 0.82

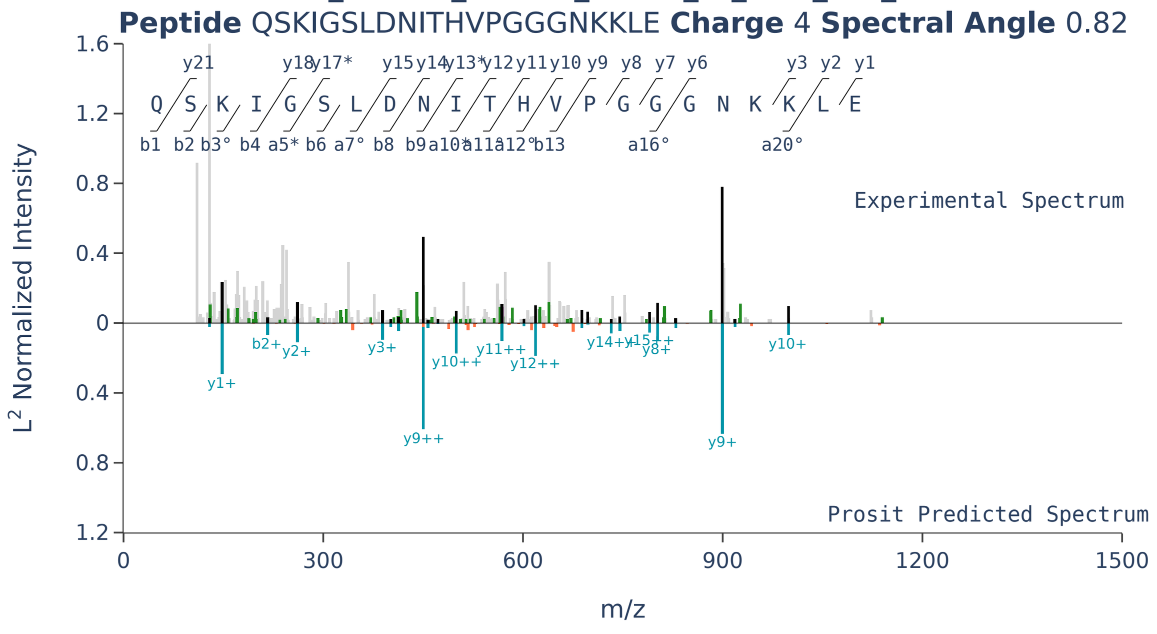


**Source** WSoh_221121_011221_HFGoe_L1_hTau_24h_R2 **Scan** 9597
**Peptide** GNIHHKPGGGQVEVKSEKLDFKDRVQSK **Charge** 5 **Spectral Angle** 0.68

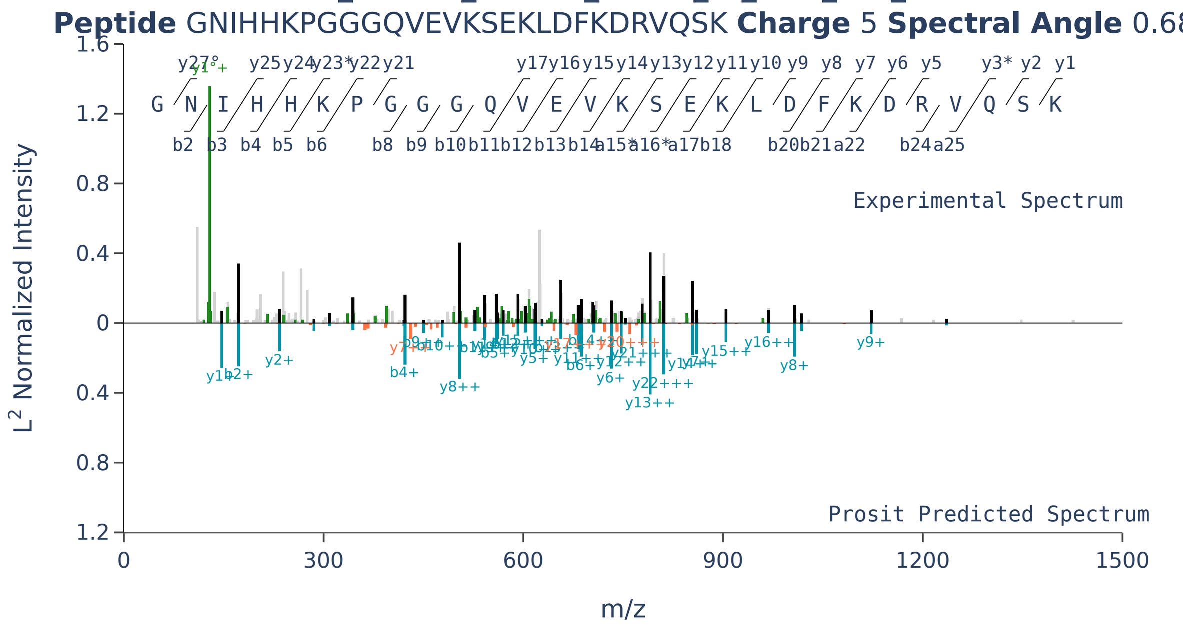


**Source** WSoh_221121_011221_HFGoe_L2_hTau_4h_R2 **Scan** 12881
**Peptide** FKDRVQS **Charge** 2 **Spectral Angle** 0.94

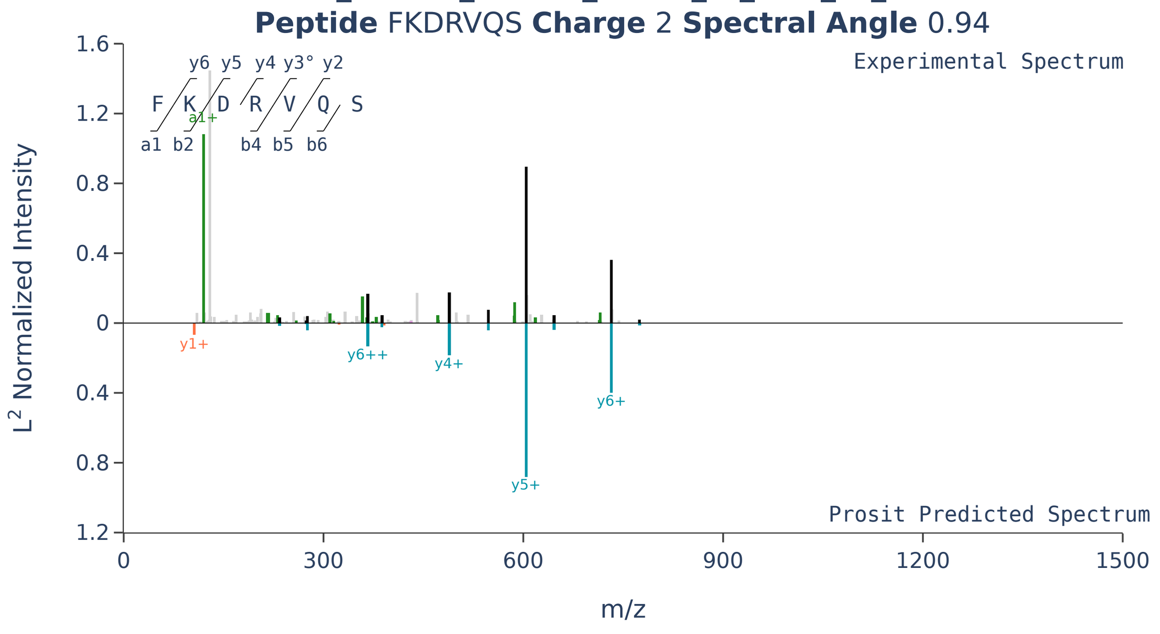


**Source** WSoh_221121_011221_HFGoe_L1_hTau_24h_R2 **Scan** 10995
**Peptide** ANATRIPAKTPPAPKTPPSSGEPPKS **Charge** 4 **Spectral Angle** 0.75

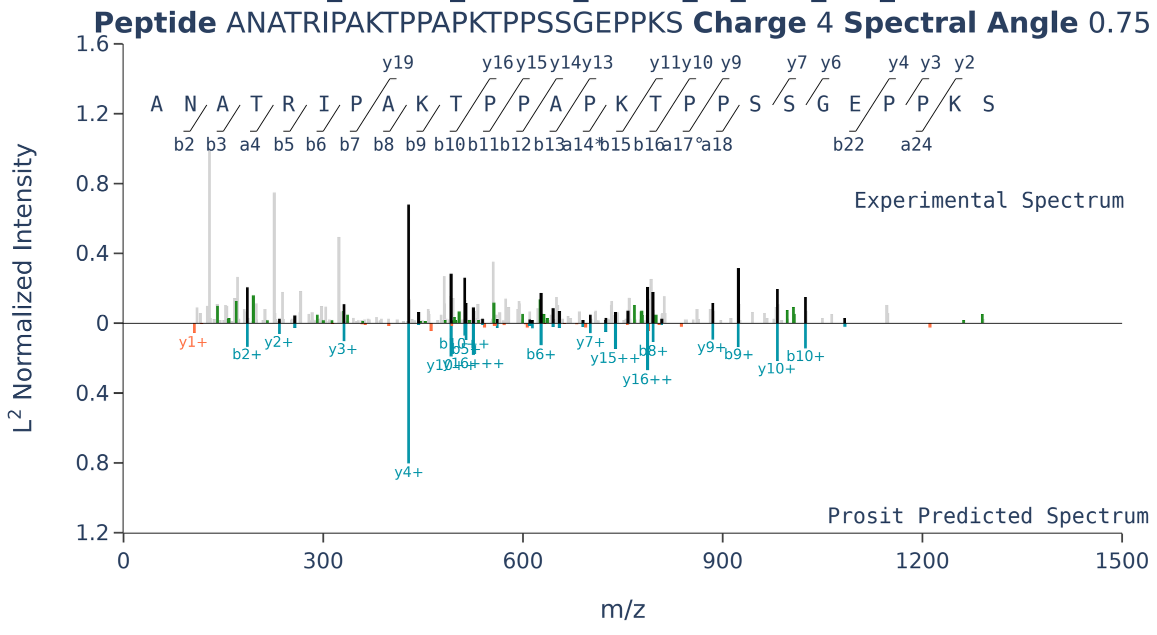


**Source** WSoh_221121_011221_HFGoe_L1_hTau_24h_R2 **Scan** 9921
**Peptide** TFRENAKAKTDHGAEIVYKSP **Charge** 4 **Spectral Angle** 0.82

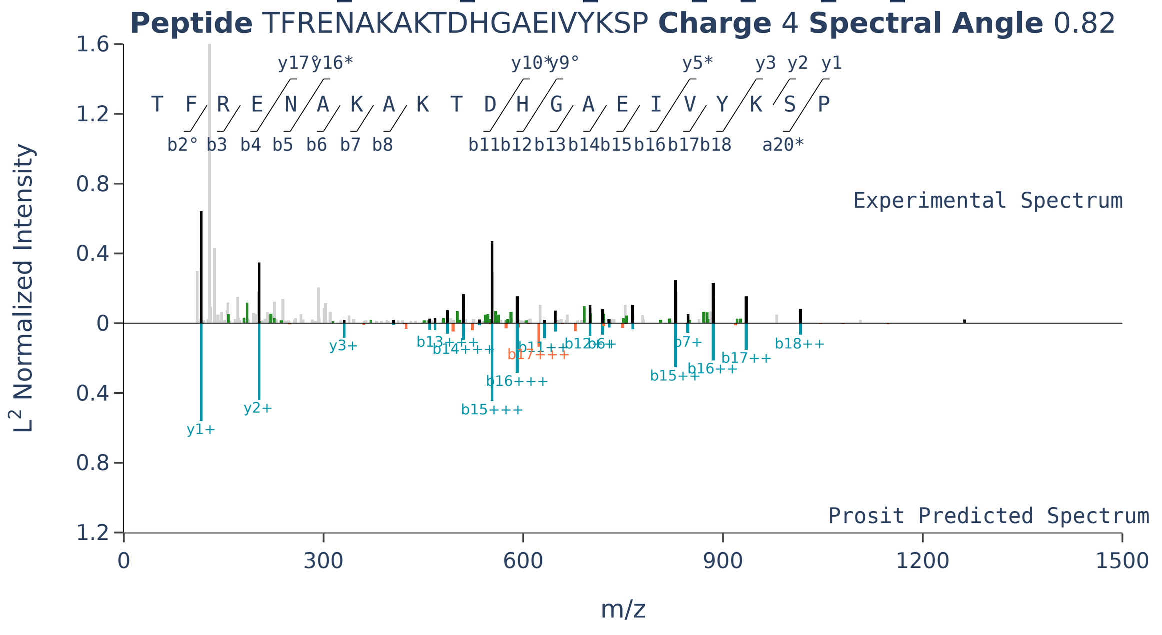


**Source** WSoh_221121_011221_HFGoe_L2_hTau_2h_R2 **Scan** 19597
**Peptide** HVPGGGSVQIVYKPVDLSK **Charge** 3 **Spectral Angle** 0.85

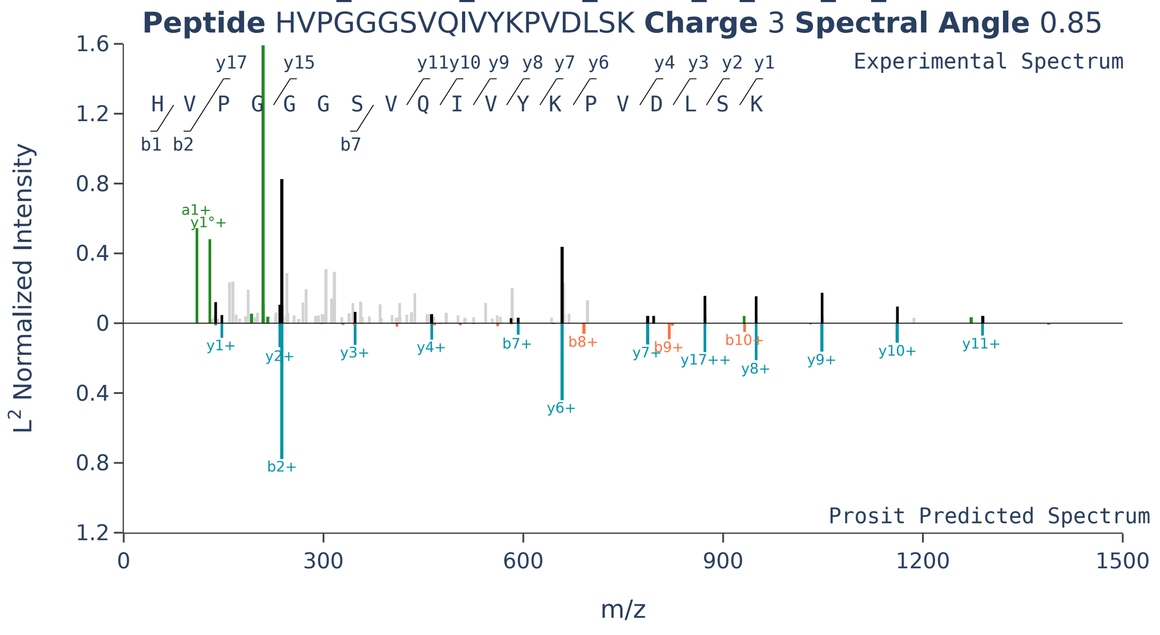


**Source** WSoh_221121_011221_HFGoe_L1_hTau_24h_R2 **Scan** 6441
**Peptide** HVPGGGNKKIETHKL **Charge** 3 **Spectral Angle** 0.87

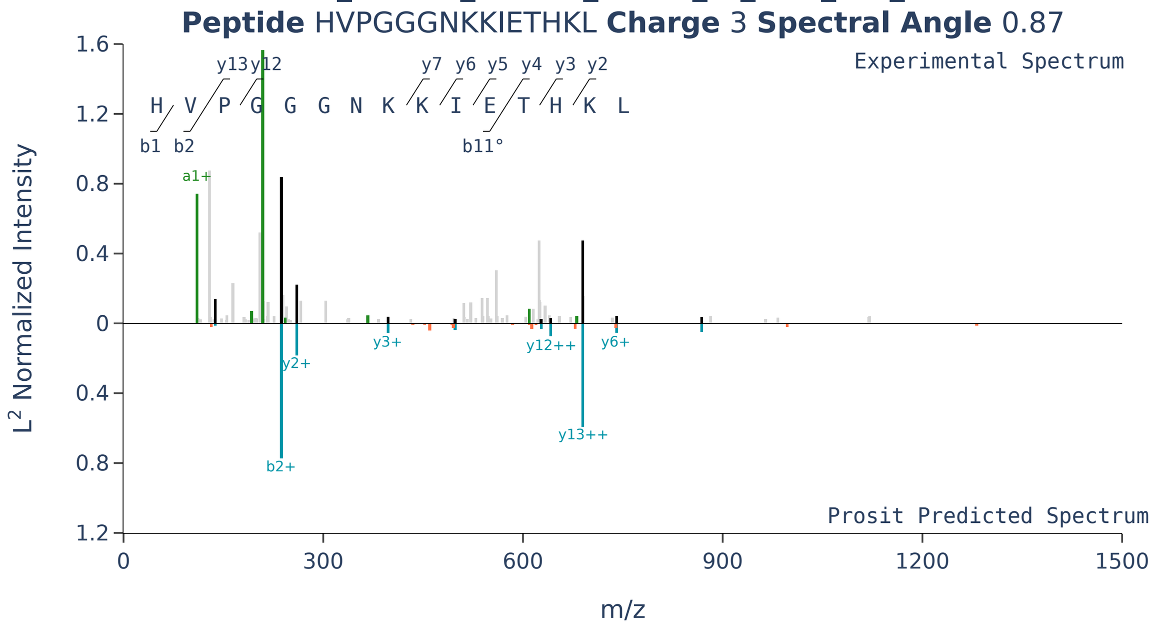


**Source** WSoh_221121_011221_HFGoe_L2_hTau_4h_R2 **Scan** 13203
**Peptide** KDNIKHVPGGGSVQIVYK **Charge** 4 **Spectral Angle** 0.6

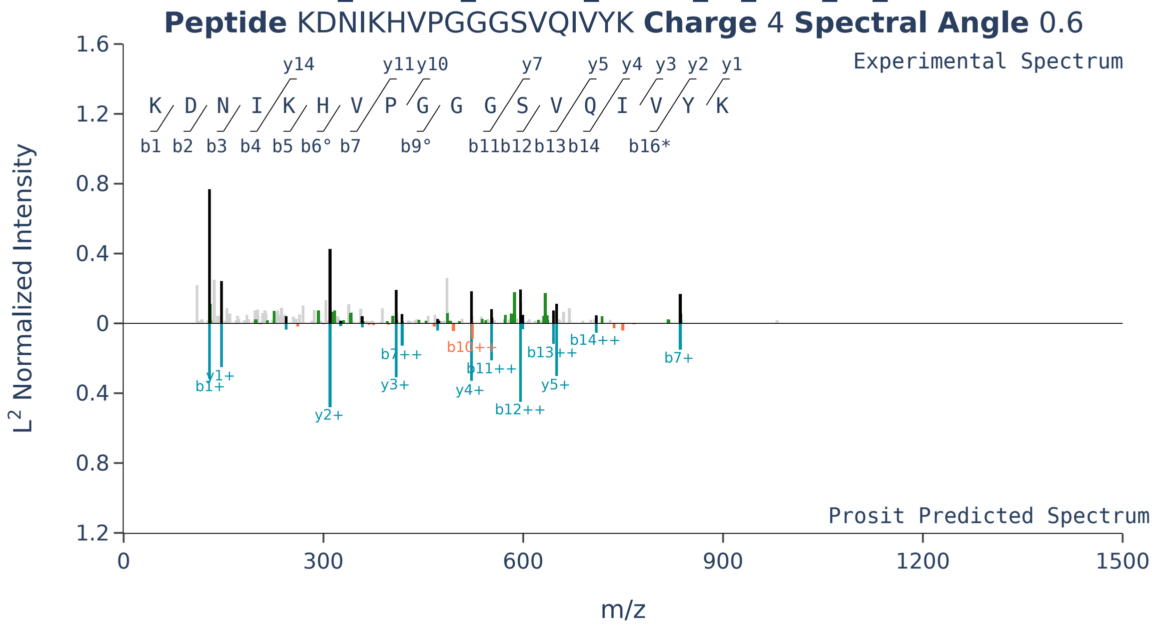


**Source** WSoh_221121_011221_HFGoe_L1_hTau_1h_R1 **Scan** 12004
**Peptide** AKAKTDHGAEIVYKSPVVS **Charge** 3 **Spectral Angle** 0.68

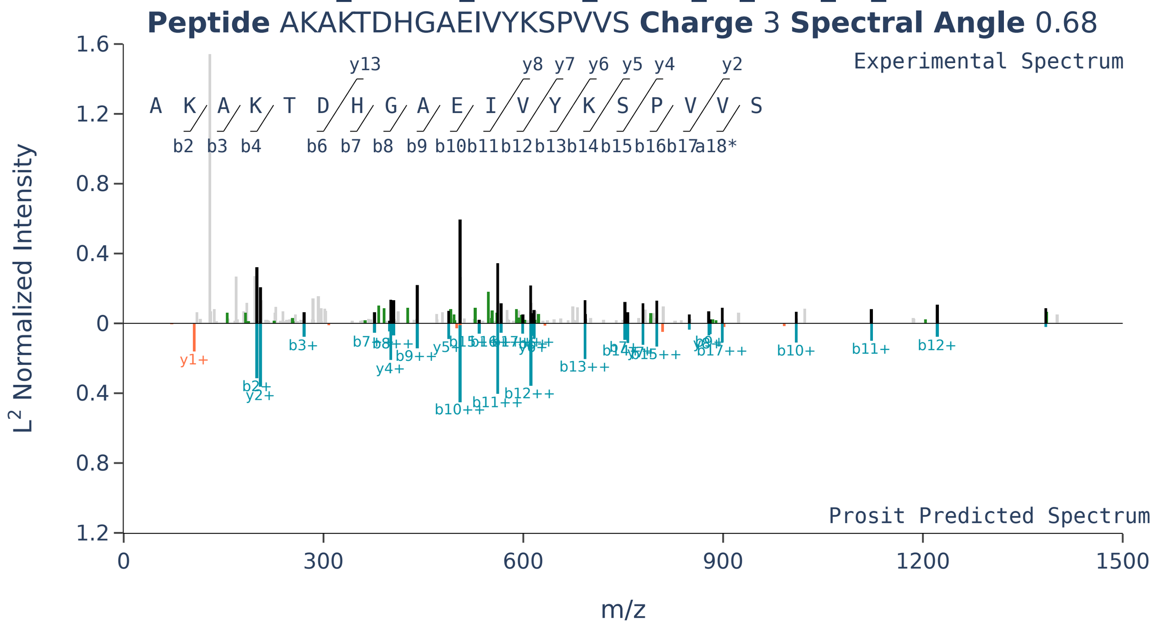


**Source** WSoh_221121_011221_HFGoe_L2_hTau_24h_R2 **Scan** 20908
**Peptide** ETSDAKSTPTAEDVTAPLVDEGAPGKQA **Charge** 3 **Spectral Angle** 0.85

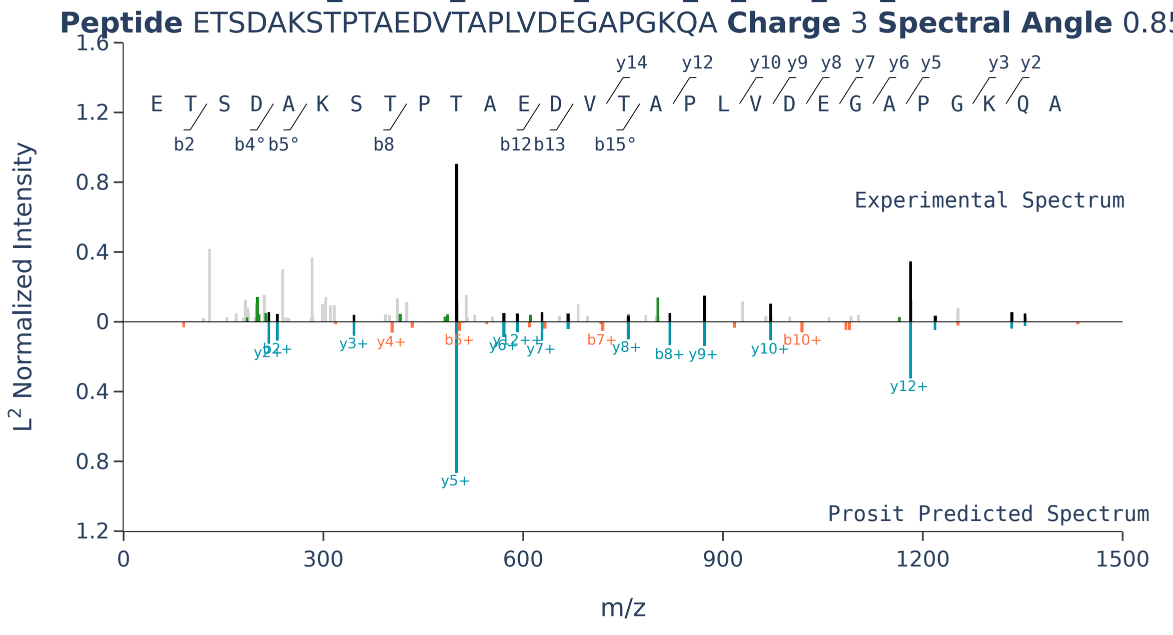


**Source** WSoh_221121_011221_HFGoe_L1_hTau_2h_R1 **Scan** 14701
**Peptide** LKESPLQTPTED **Charge** 2 **Spectral Angle** 0.75

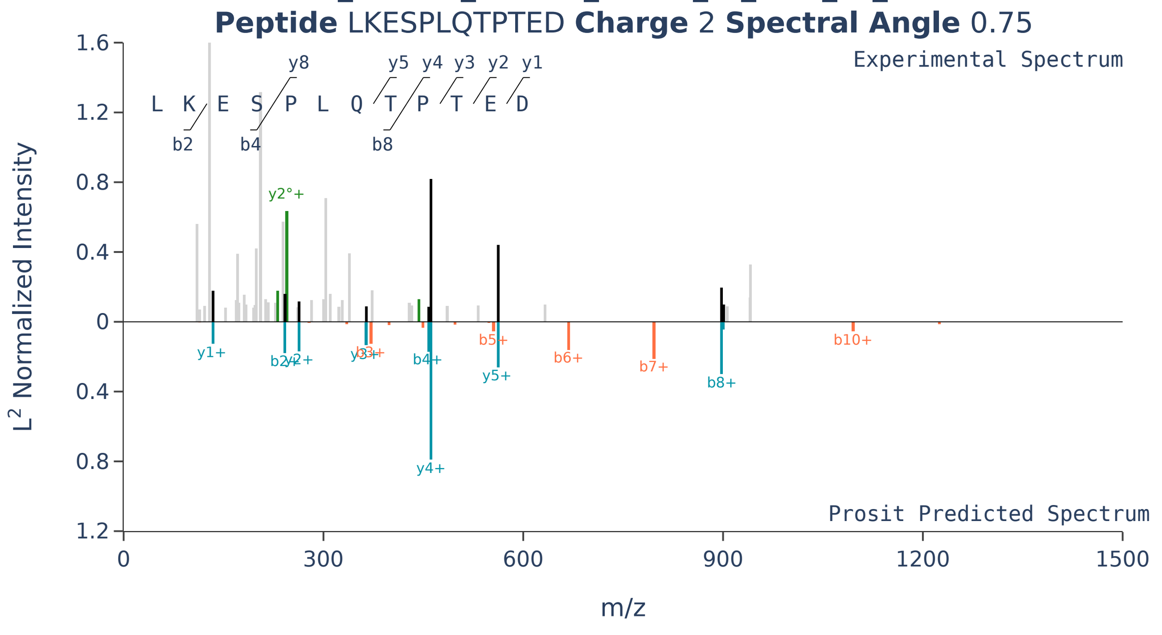


**Source** WSoh_221121_011221_HFGoe_L1_hTau_4h_R1 **Scan** 14807
**Peptide** CGSLGNIHHKPGGGQVEVKSEKLDF **Charge** 3 **Spectral Angle** 0.76

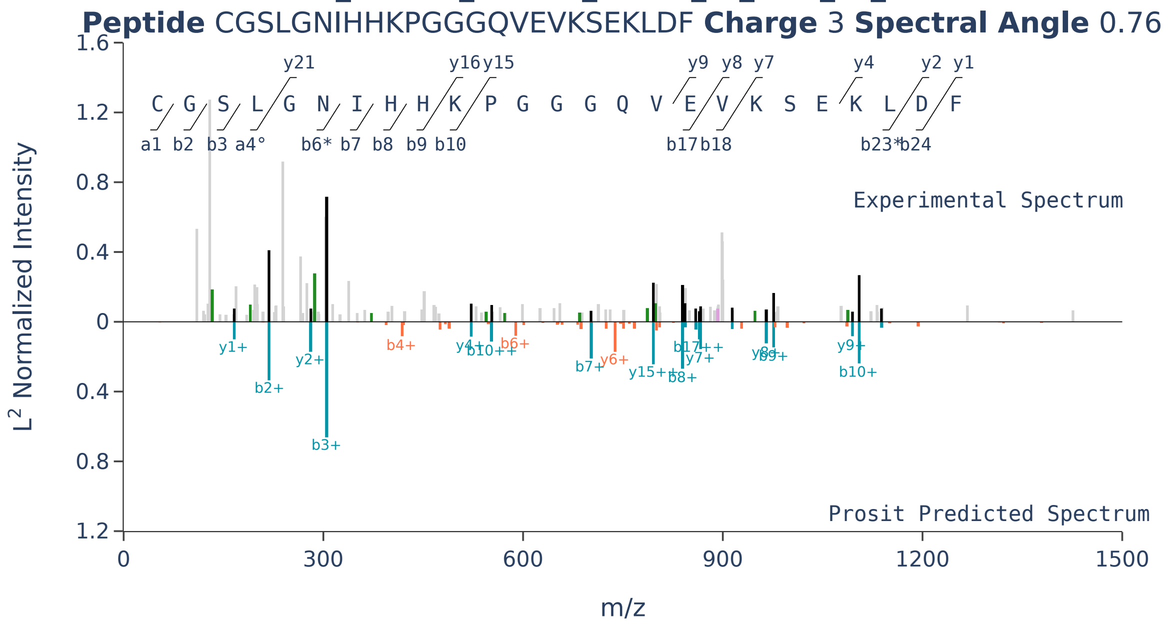


**Source** WSoh_221121_011221_HFGoe_L1_hTau_4h_R1 **Scan** 19454
**Peptide** VTAPLVDEGAPGKQAAAQPHTEIPEGT **Charge** 3 **Spectral Angle** 0.92

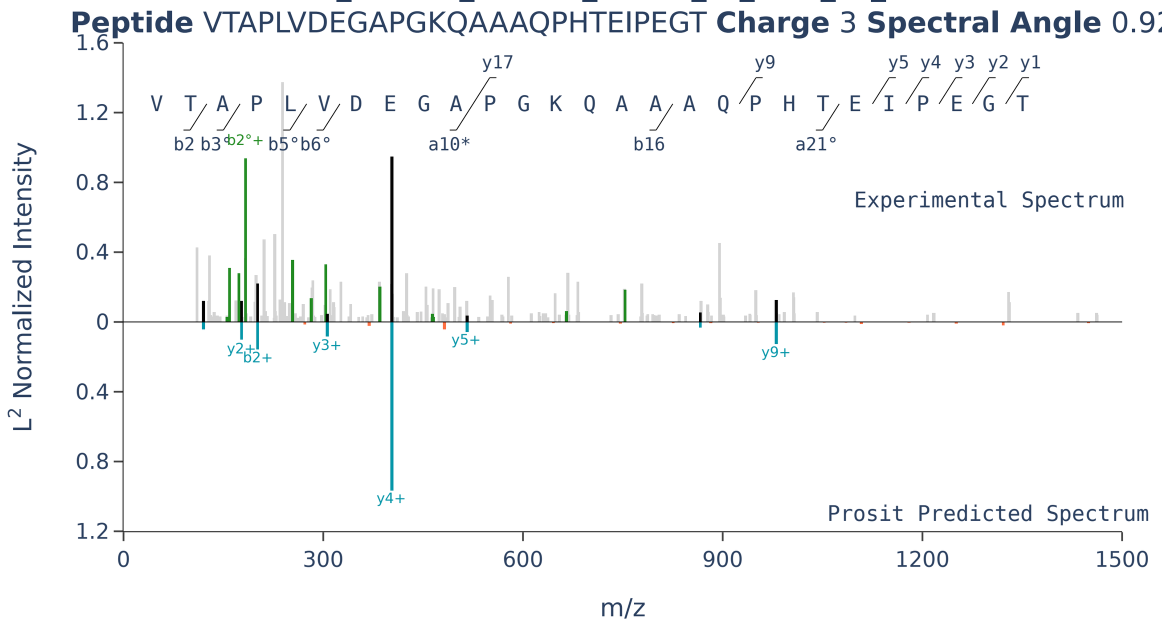


**Source** WSoh_221121_011221_HFGoe_L1_hTau_24h_R2 **Scan** 14573
**Peptide** DEGAPGKQAAAQPHTEIPEGTTAEEAG **Charge** 3 **Spectral Angle** 0.77

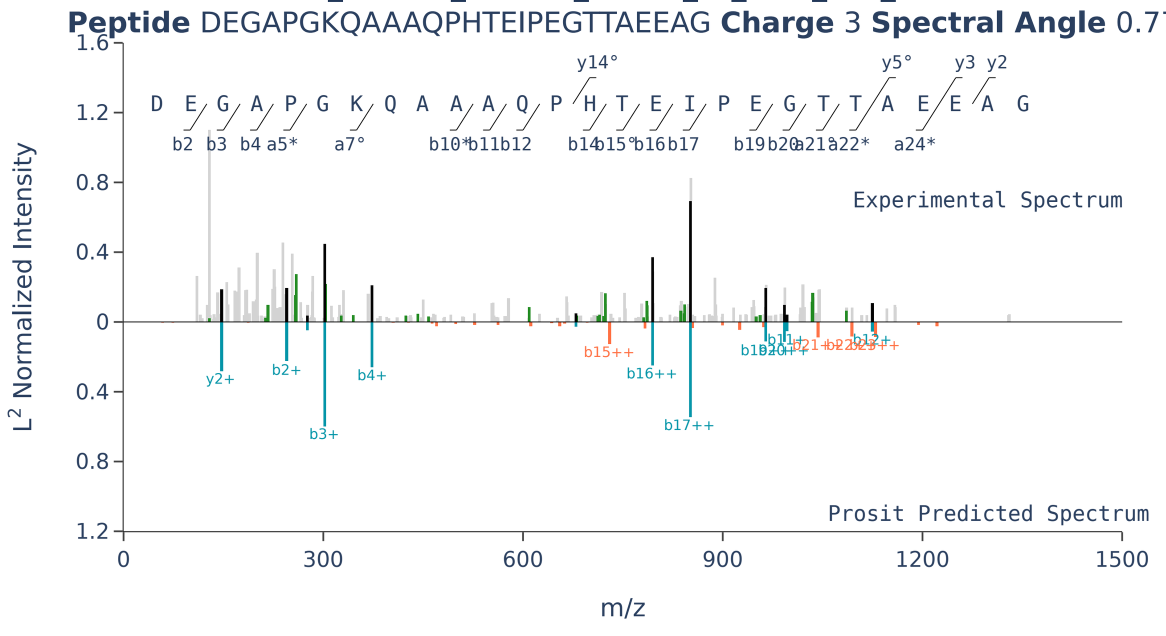


**Source** WSoh_221121_011221_HFGoe_L1_hTau_2h_R2 **Scan** 6086
**Peptide** KPGGGQVEVKS **Charge** 3 **Spectral Angle** 0.95

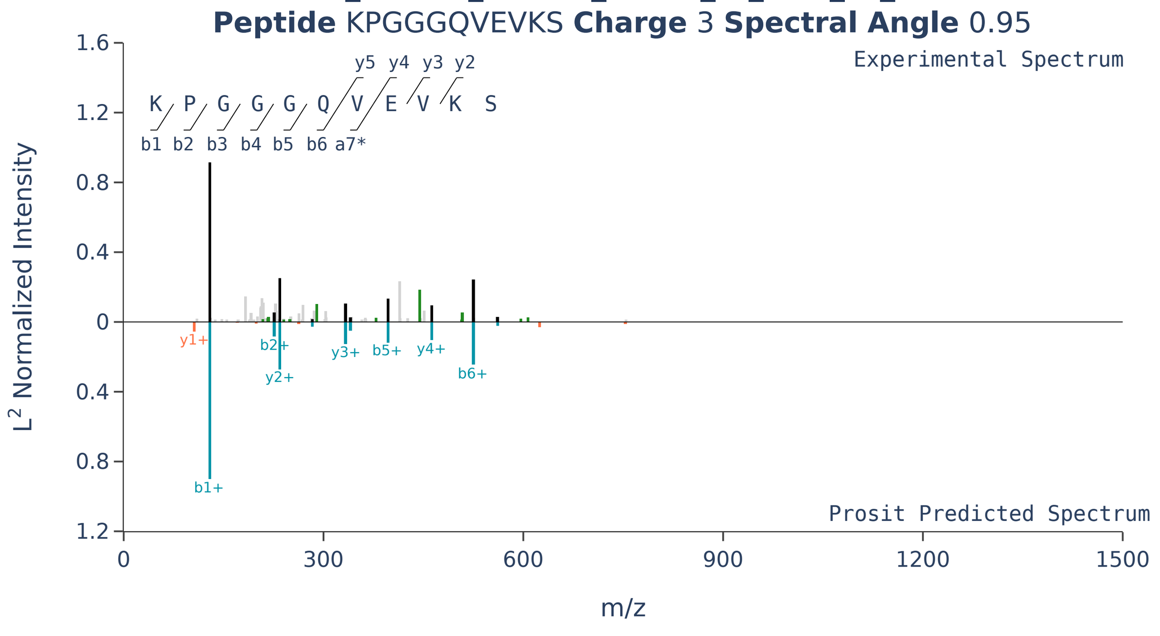

**Source** WSoh_221121_011221_HFGoe_L1_hTau_24h_R2 **Scan** 19918
**Peptide** AEIVYKSPVVSGDTSPRHLSNVSSTGSLD **Charge** 4 **Spectral Angle** 0.87

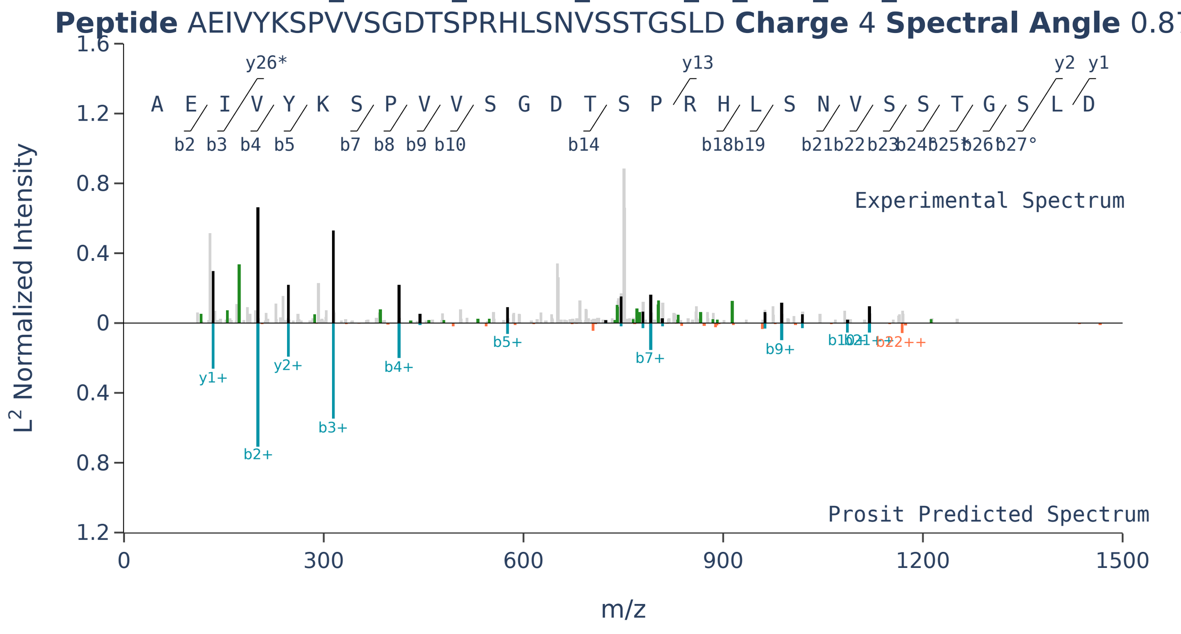


**Source** WSoh_221121_011221_HFGoe_L1_hTau_24h_R1 **Scan** 17354
**Peptide** TPPKSPSSAKSRLQTAPVPMPDLKNVKS **Charge** 4 **Spectral Angle** 0.75

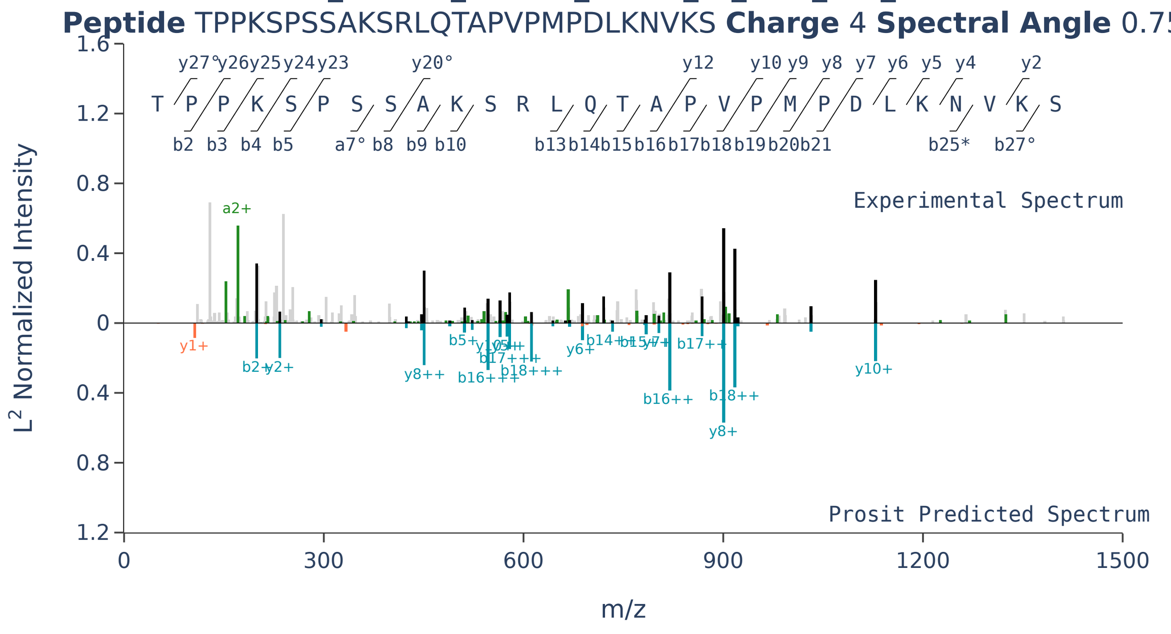


**Source** WSoh_221121_011221_HFGoe_L1_hTau_24h_R2 **Scan** 12493
**Peptide** NIHHKPGGGQVEVKSEKLDF **Charge** 5 **Spectral Angle** 0.72

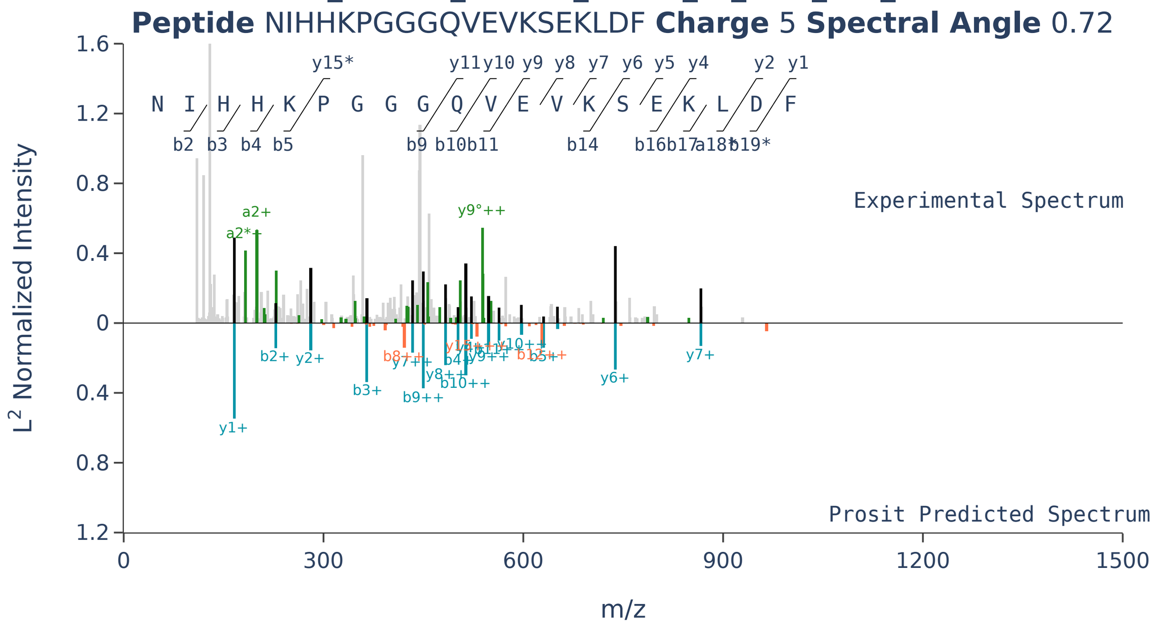


**Source** WSoh_221121_011221_HFGoe_L2_hTau_24h_R1 **Scan** 11656
**Peptide** DNITHVPGGGNKKIETHKLTFRENAKA **Charge** 5 **Spectral Angle** 0.76

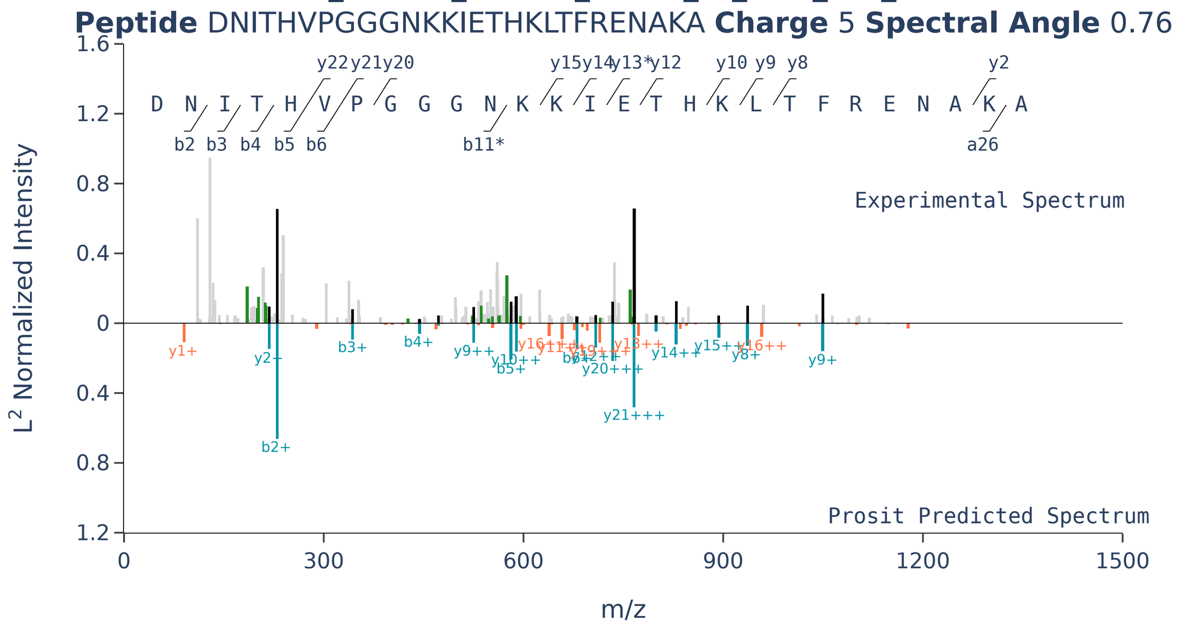


**Source** WSoh_221121_011221_HFGoe_L2_hTau_4h_R2 **Scan** 10712
**Peptide** NLHHKPGGGQVEVKSEKLDFKDRVQS **Charge** 4 **Spectral Angle** 0.64

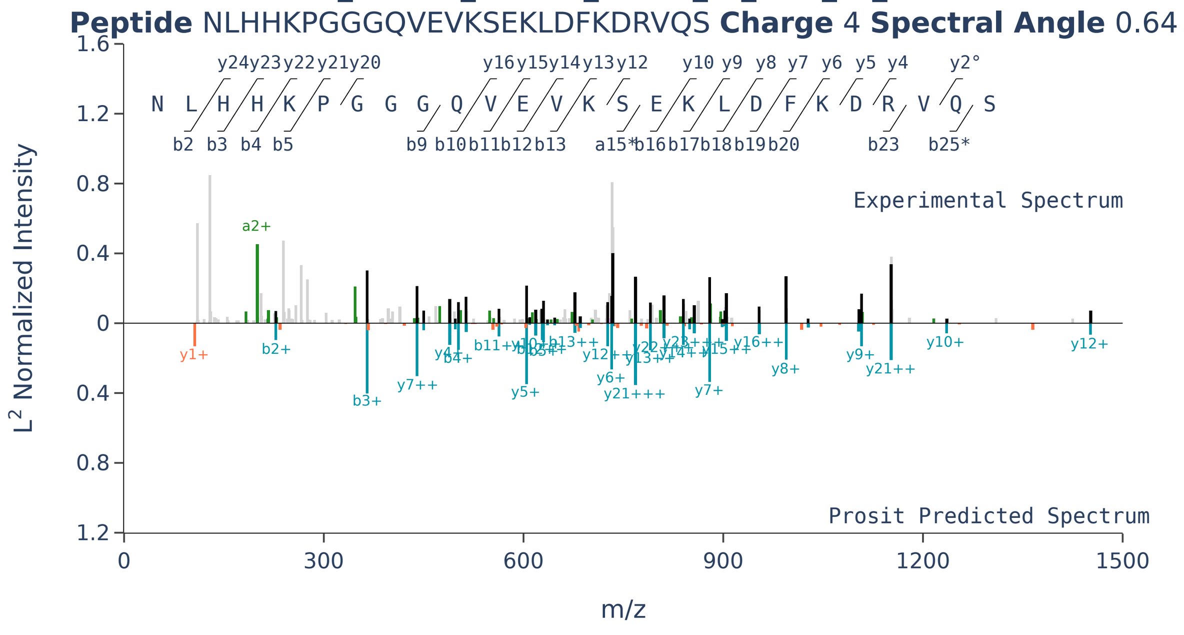


**Source** WSoh_221121_011221_HFGoe_L2_hTau_24h_R1 **Scan** 26497
**Peptide** SIDMVDSPQL **Charge** 2 **Spectral Angle** 0.82

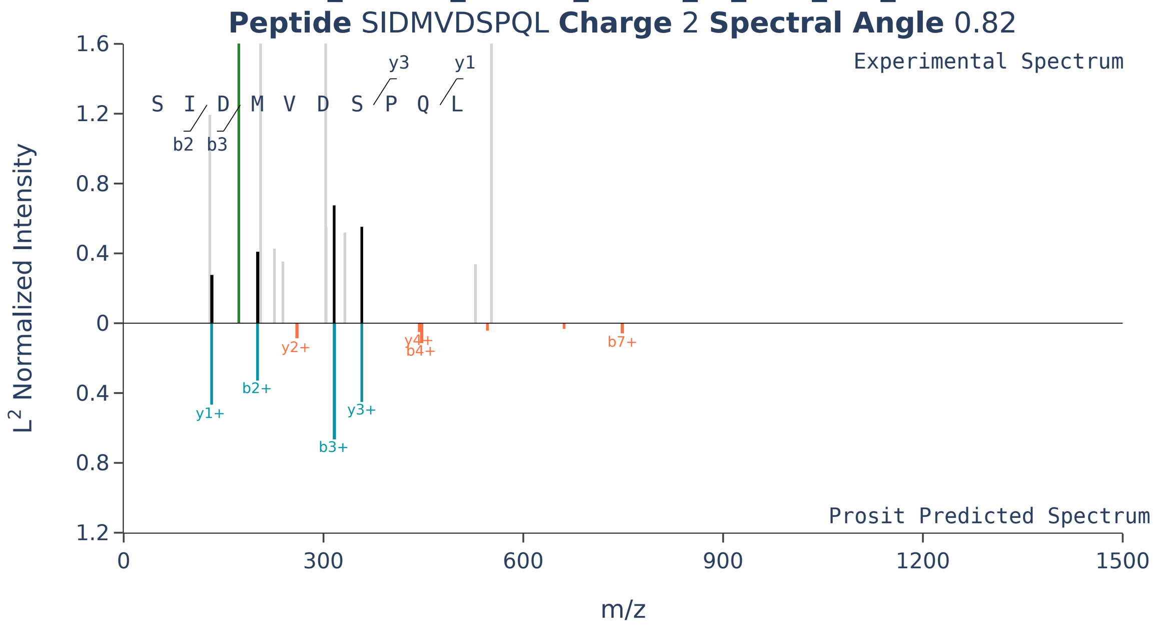


**Source** WSoh_221121_011221_HFGoe_L1_hTau_24h_R1 **Scan** 9341
**Peptide** GDRKDQGGYTMHQDQEGDTDAGLKE **Charge** 3 **Spectral Angle** 0.75

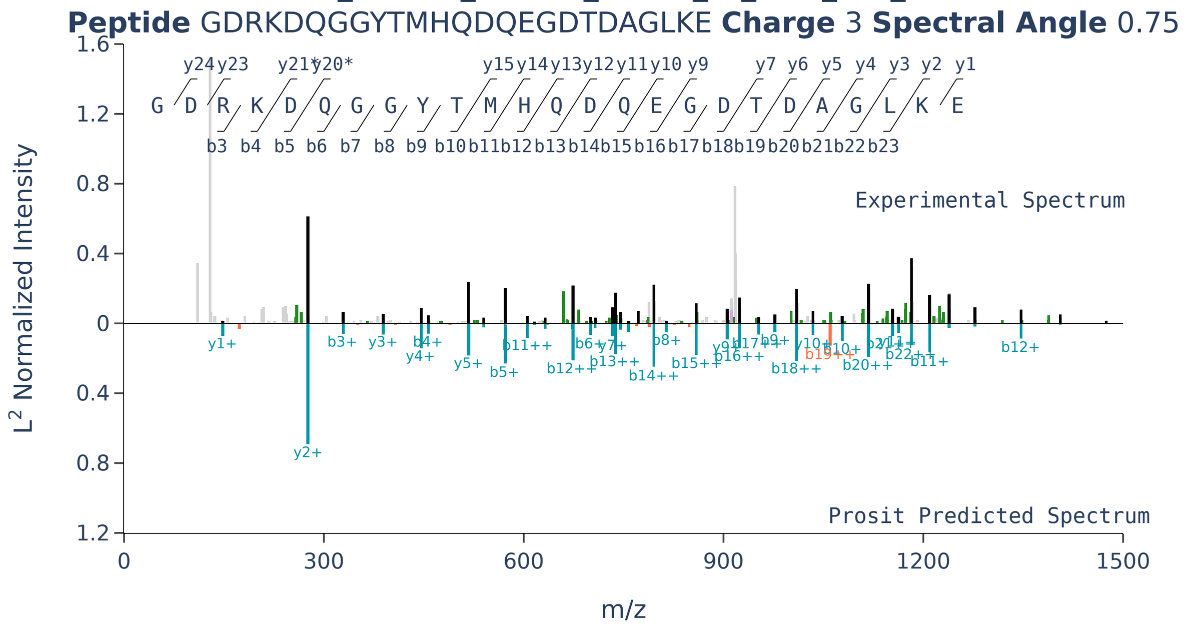


**Source** WSoh_221121_011221_HFGoe_L2_hTau_24h_R2 **Scan** 8993
**Peptide** HQPGGGKVQL **Charge** 2 **Spectral Angle** 0.91

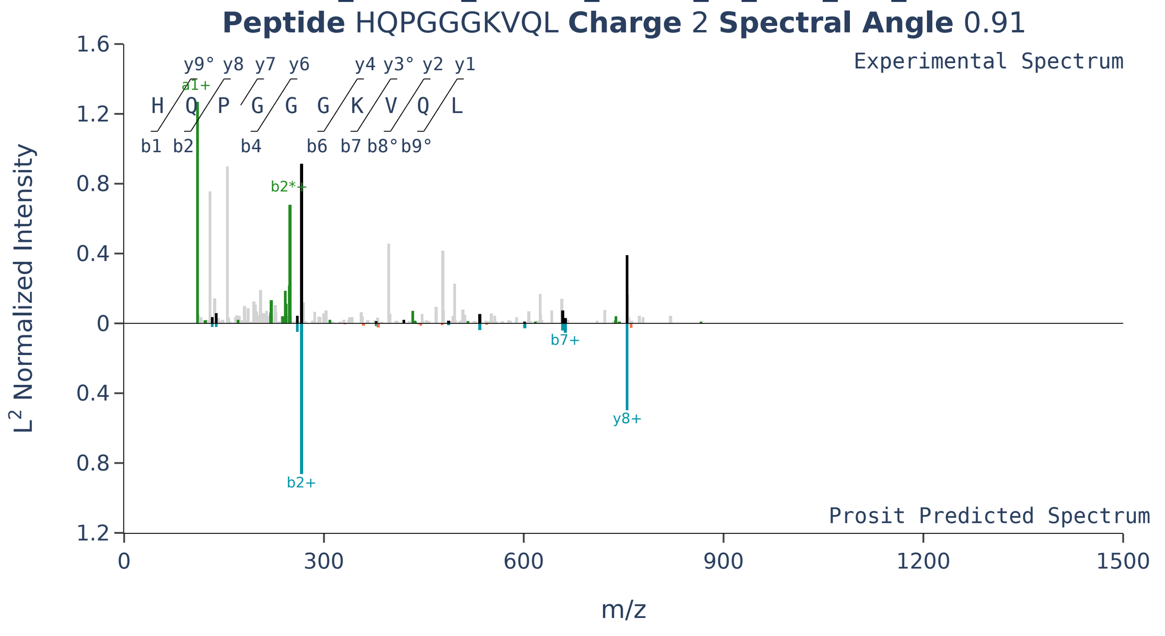


**Source** WSoh_221121_011221_HFGoe_L1_hTau_4h_R2 **Scan** 24713
**Peptide** PGGGSVQIVYKPVDLSKV **Charge** 3 **Spectral Angle** 0.81

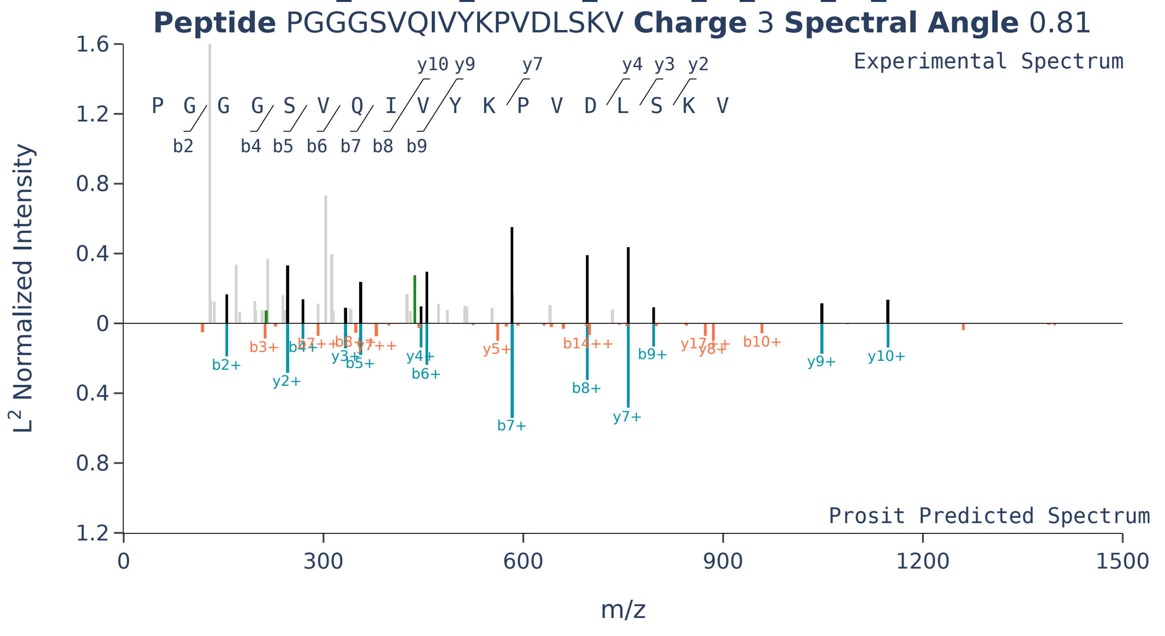


**Source** WSoh_221121_011221_HFGoe_L2_hTau_24h_R2 **Scan** 7432
**Peptide** SKVTSKCGSLGNIHHKPGGGQVEVKS **Charge** 3 **Spectral Angle** 0.79

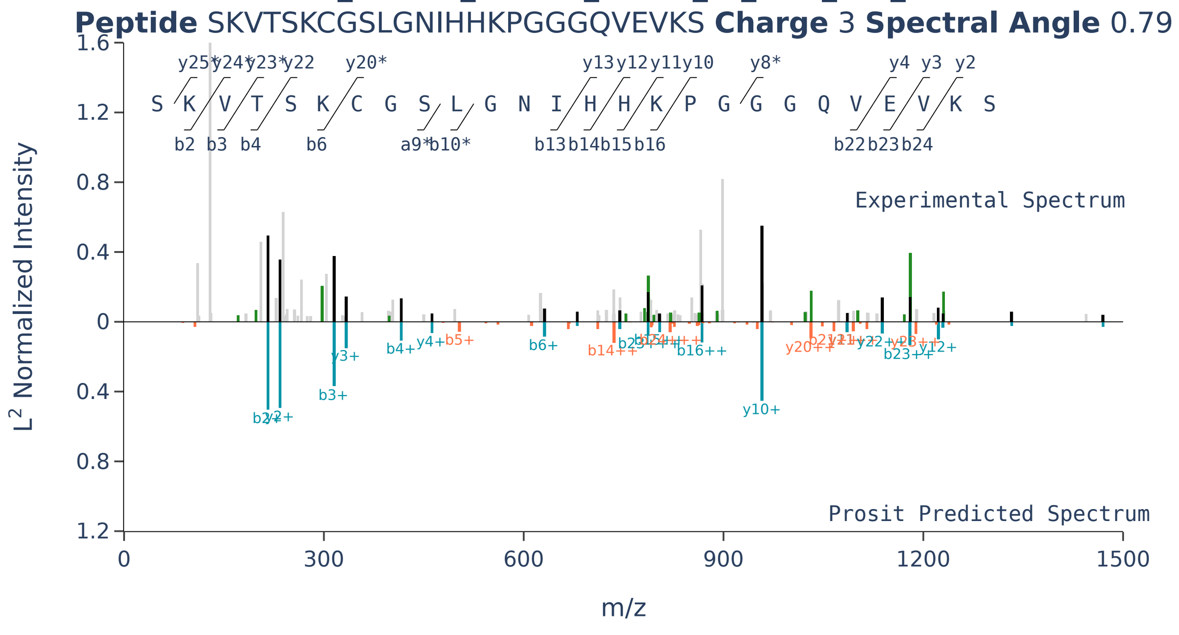


**Source** WSoh_221121_011221_HFGoe_L2_hTau_24h_R1 **Scan** 25658
**Peptide** SIDMVDSPQLA **Charge** 2 **Spectral Angle** 0.8

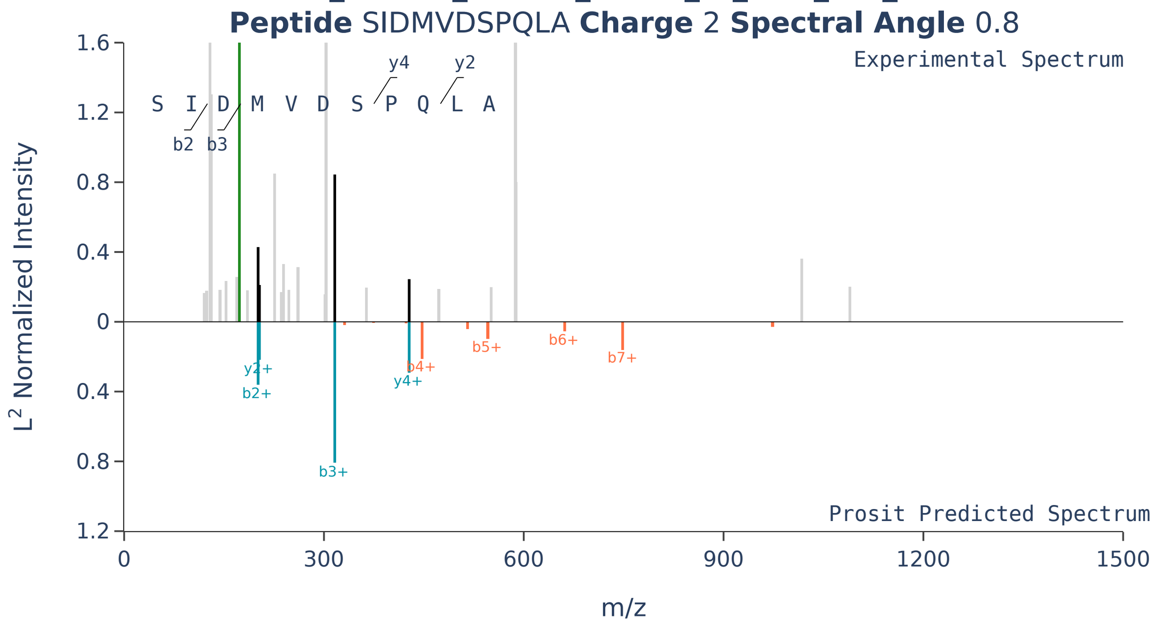


**Source** WSoh_221121_011221_HFGoe_L2_hTau_24h_R1 **Scan** 5476
**Peptide** SKDGTGSDDKKAKGADGKTKIATPRG **Charge** 4 **Spectral Angle** 0.84

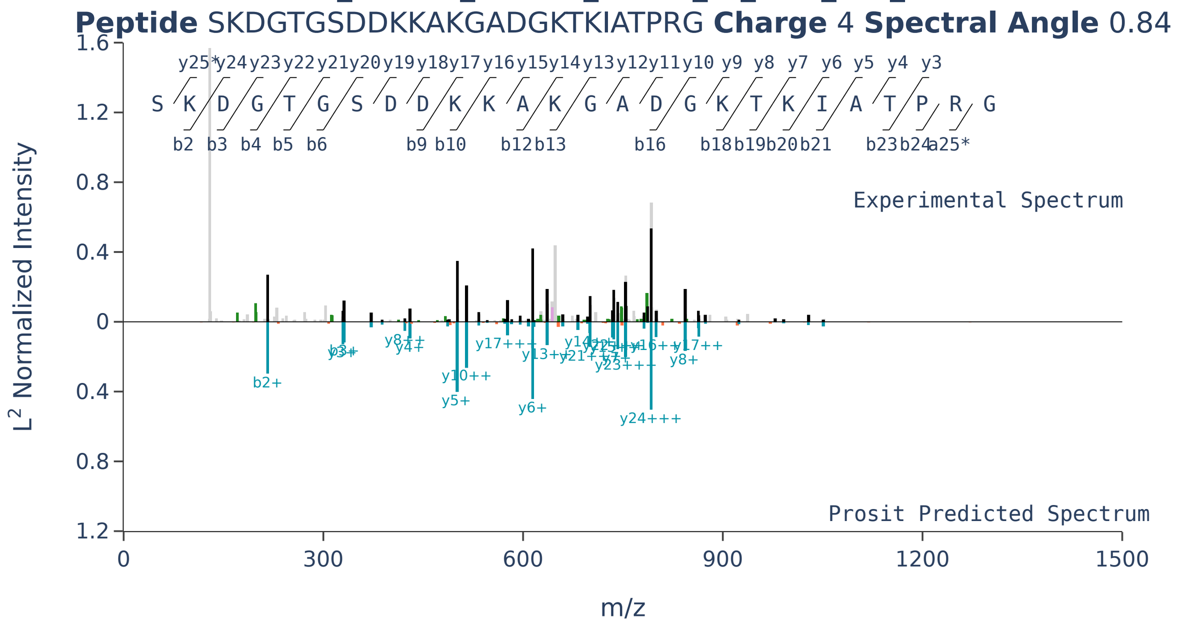


**Source** WSoh_221121_011221_HFGoe_L1_hTau_24h_R1 **Scan** 9959
**Peptide** STENLKHQPGGGKVQI **Charge** 3 **Spectral Angle** 0.8

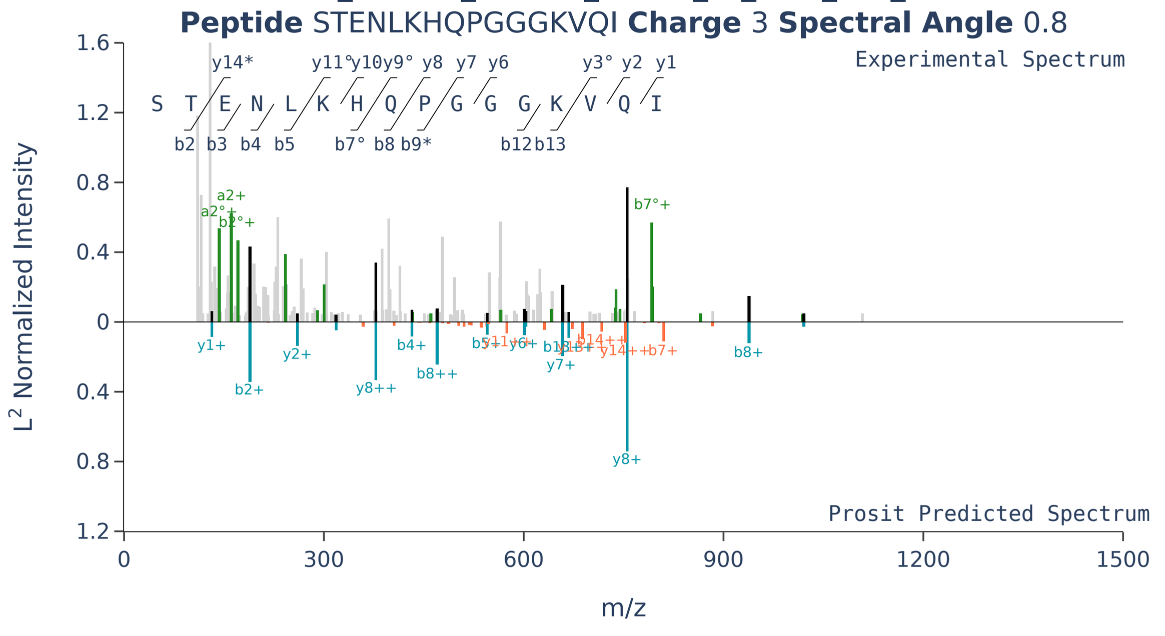


**Source** WSoh_221121_011221_HFGoe_L1_hTau_24h_R2 **Scan** 18208
**Peptide** EVKSEKLDFKDRVQSKIGSLD **Charge** 4 **Spectral Angle** 0.7

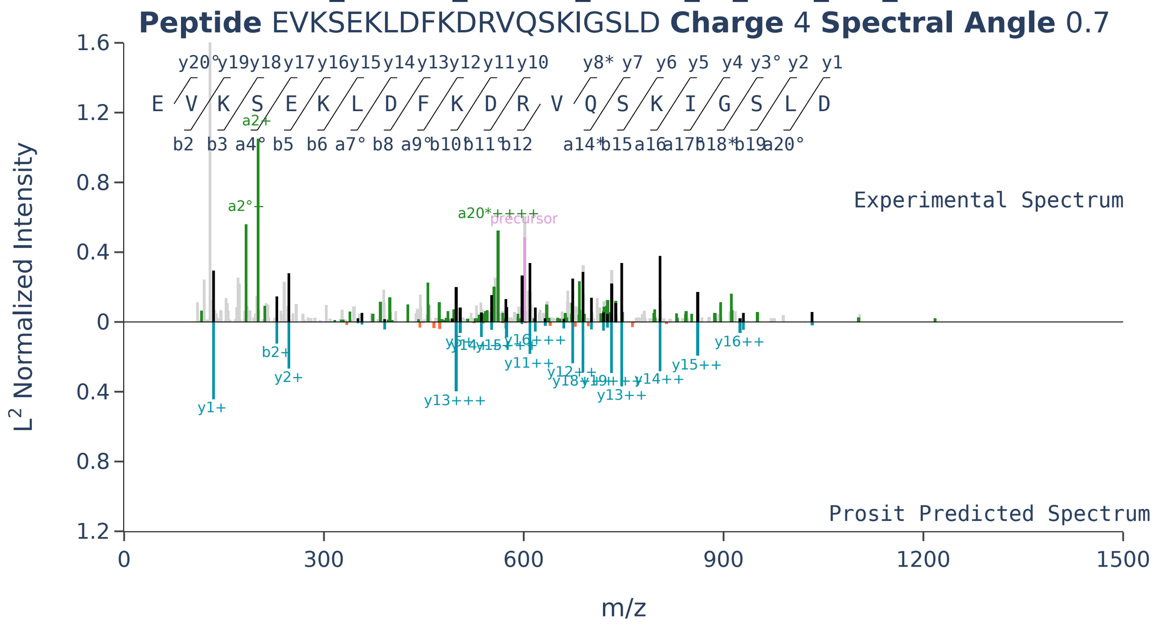


**Source** WSoh_221121_011221_HFGoe_L1_hTau_4h_R2 **Scan** 10784
**Peptide** APPGQKGQANATRIPAKTPPAPKTPPSSG **Charge** 4 **Spectral Angle** 0.8

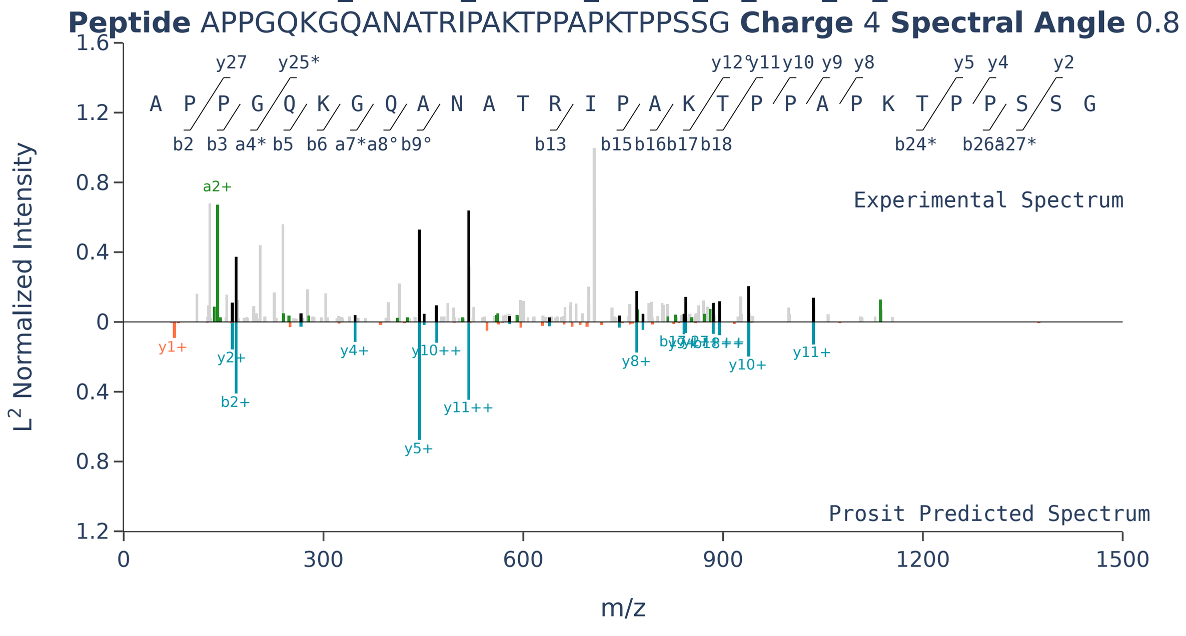

**Source** WSoh_221121_011221_HFGoe_L2_hTau_4h_R1 **Scan** 8831
**Peptide** KVTSKCGSLGNIHHKPGGGQVEV **Charge** 3 **Spectral Angle** 0.85

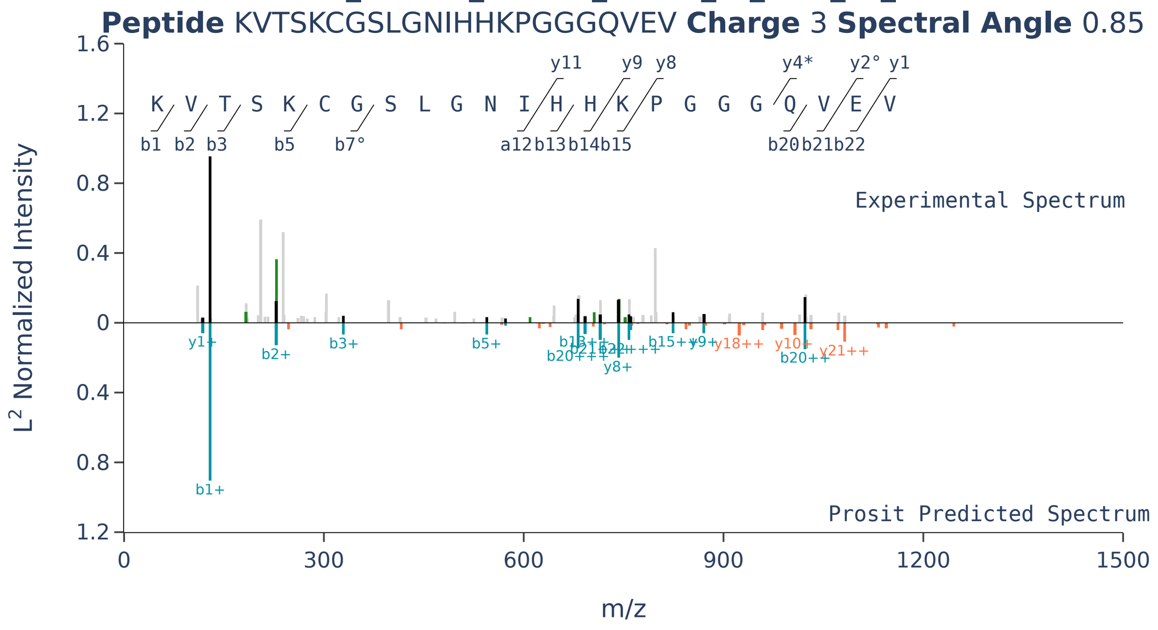


**Source** WSoh_221121_011221_HFGoe_L2_hTau_24h_R2 **Scan** 13565
**Peptide** KCGSLGNIHHKPGGGQVEVKSEKLDF **Charge** 4 **Spectral Angle** 0.85

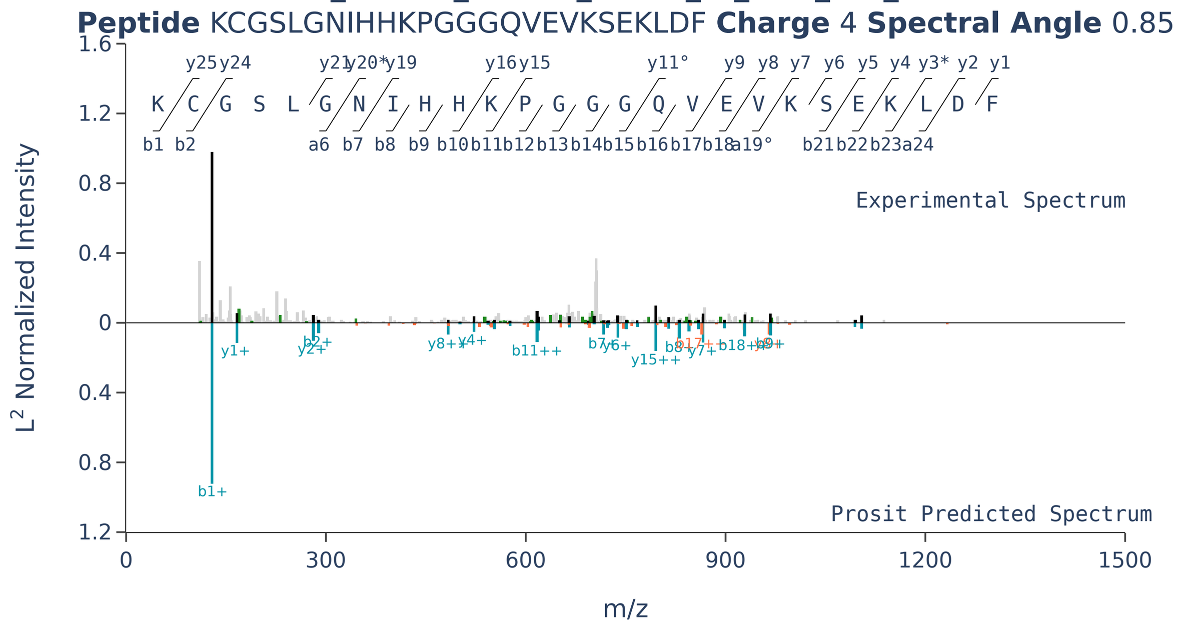


**Source** WSoh_221121_011221_HFGoe_L1_hTau_4h_R2 **Scan** 15882
**Peptide** HGAEIVYKSPVVSGDTSPRHLSNVSS **Charge** 3 **Spectral Angle** 0.7

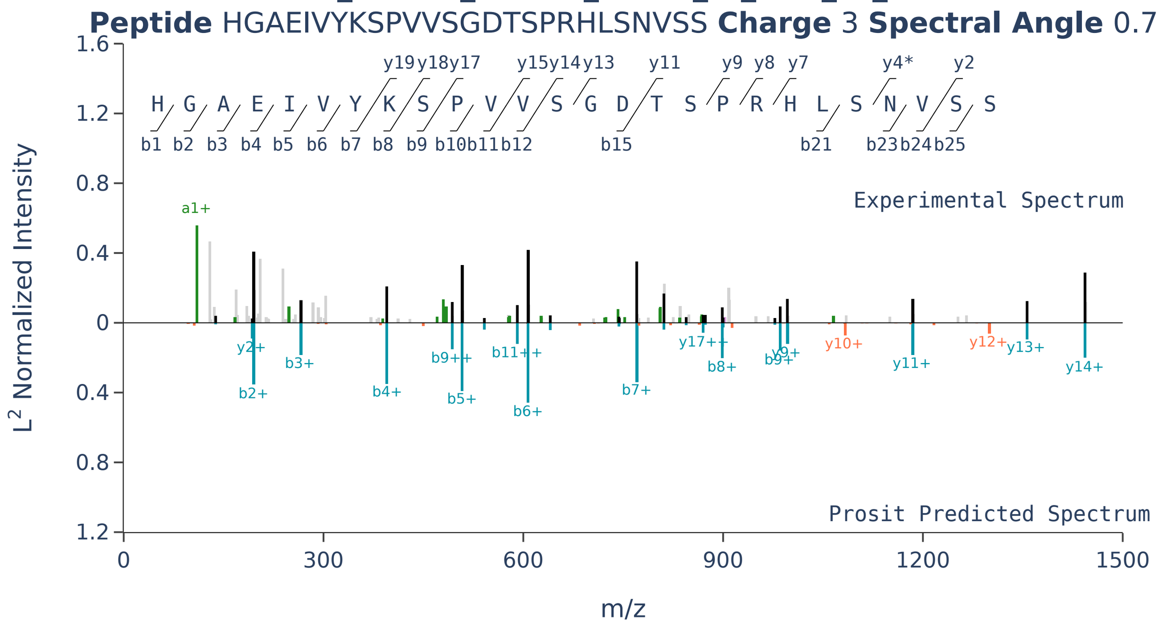


**Source** WSoh_221121_011221_HFGoe_L2_hTau_24h_R2 **Scan** 24779
**Peptide** PGGGSVQIVYKPVDLSKVTS **Charge** 2 **Spectral Angle** 0.74

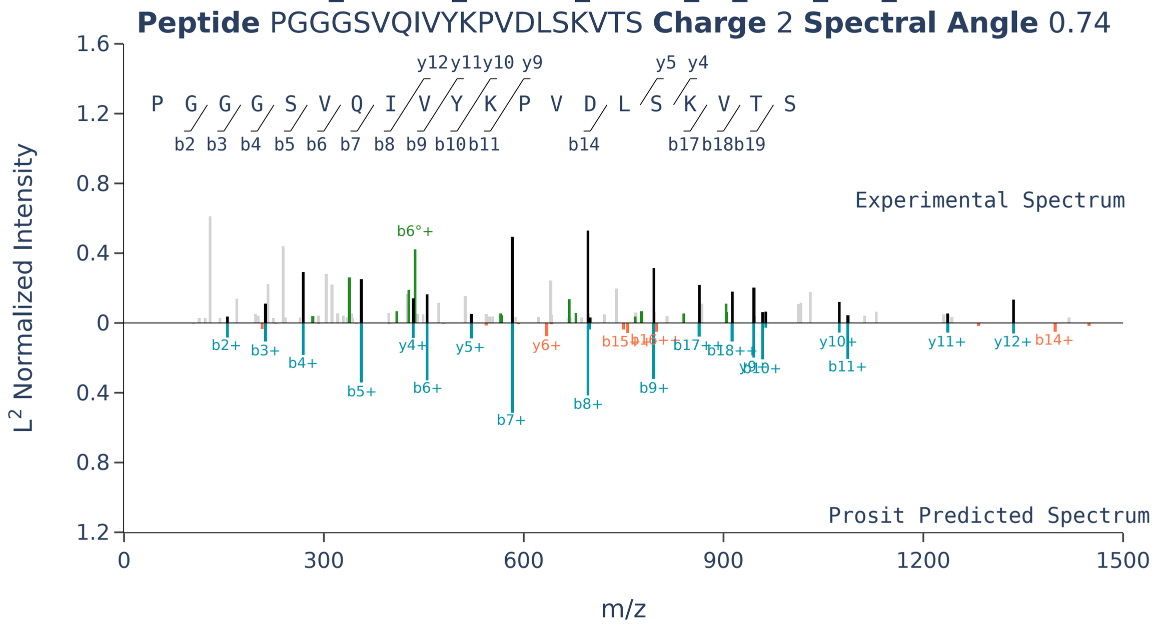


**Source** WSoh_221121_011221_HFGoe_L2_hTau_4h_R1 **Scan** 19608
**Peptide** VTAPLVDEGAPGKQAAAQPHTEIPEG **Charge** 3 **Spectral Angle** 0.93

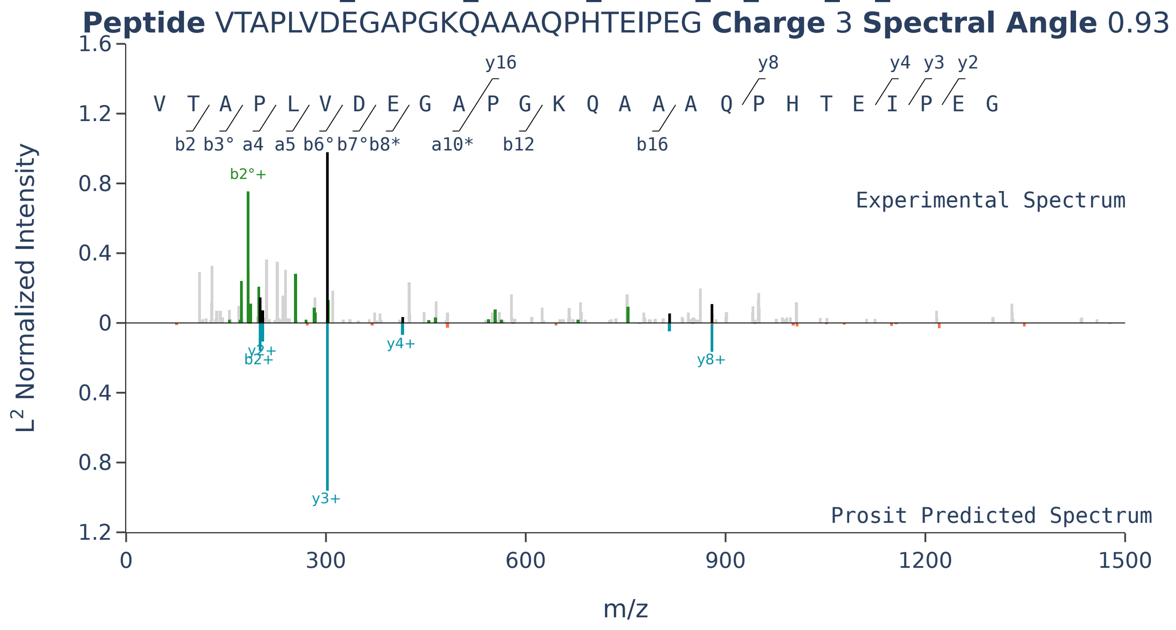


**Source** WSoh_221121_011221_HFGoe_L1_hTau_24h_R1 **Scan** 6648
**Peptide** KGADGKTKIATPRGAAPPGQKGQ **Charge** 3 **Spectral Angle** 0.85

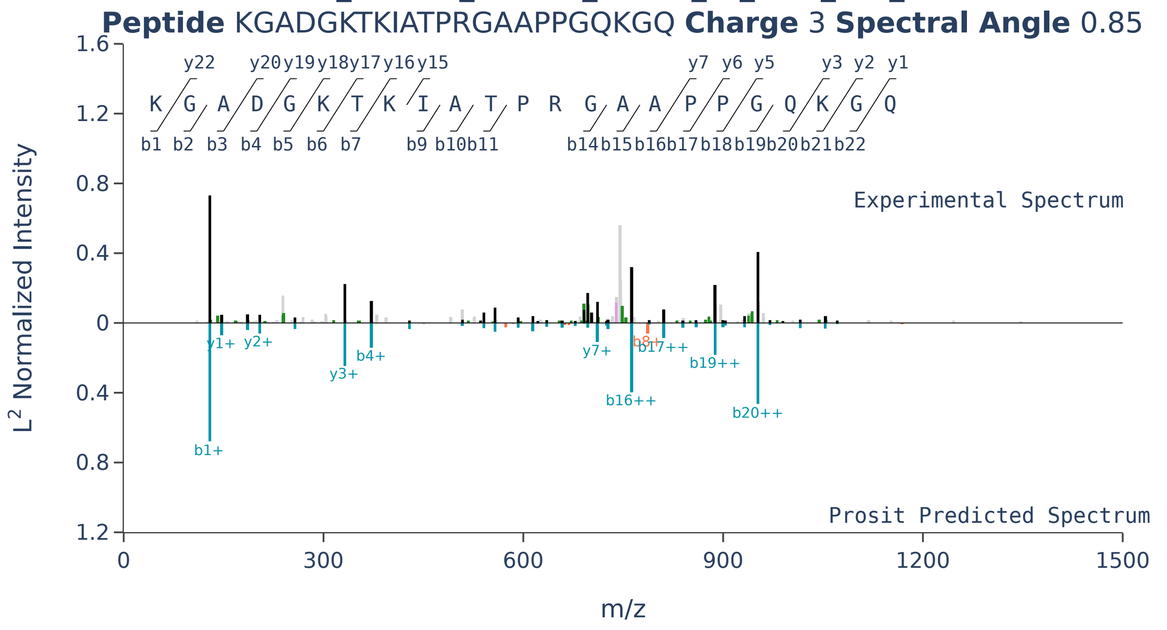


**Source** WSoh_221121_011221_HFGoe_L1_hTau_4h_R1 **Scan** 10204
**Peptide** LPAKTPPAPKTPPSSG **Charge** 3 **Spectral Angle** 0.67

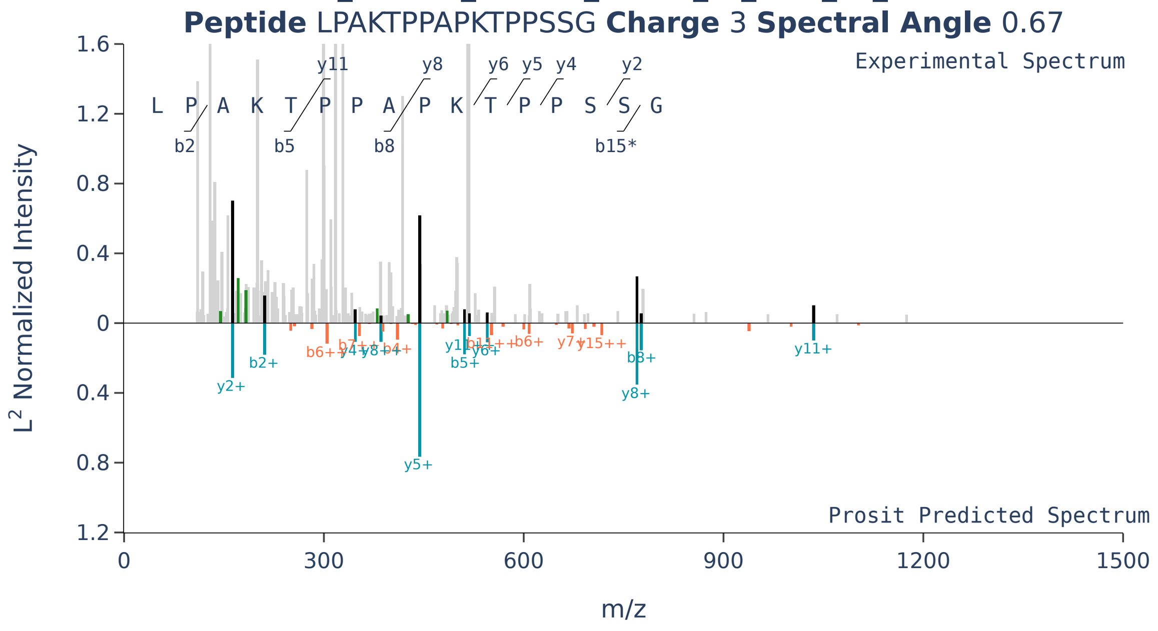


**Source** WSoh_221121_011221_HFGoe_L2_hTau_24h_R1 **Scan** 16371
**Peptide** TENLKHQPGGGKVQIINKKLDLSNVQS **Charge** 4 **Spectral Angle** 0.72

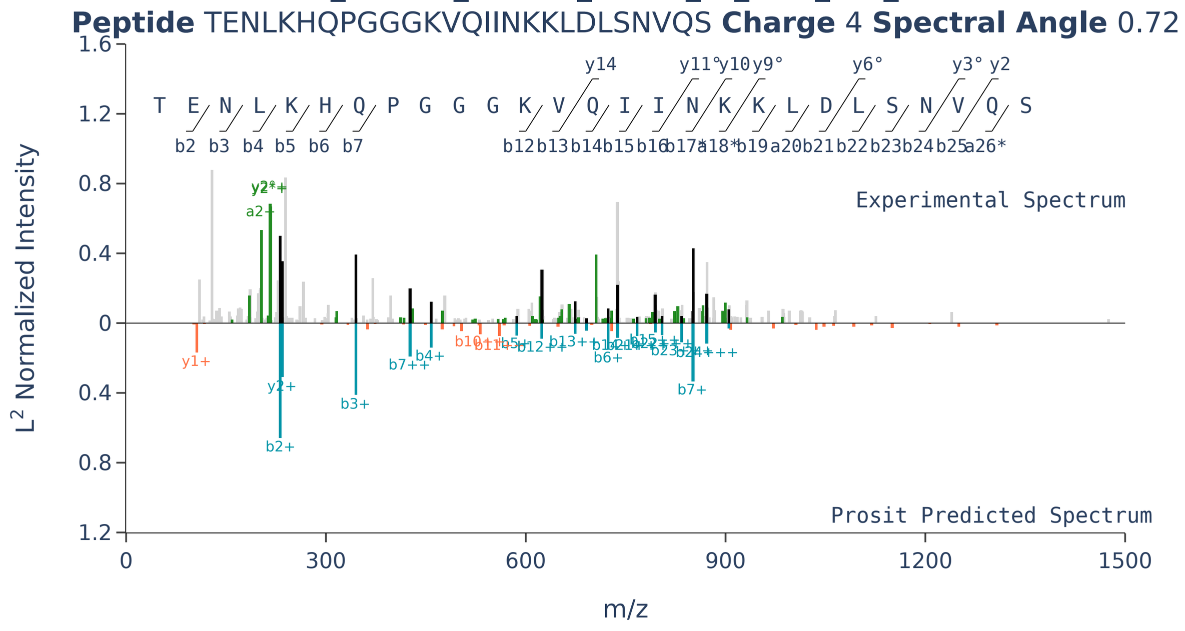


**Source** WSoh_221121_011221_HFGoe_L1_hTau_24h_R2 **Scan** 8008
**Peptide** PTPPTREPKKVAVVRTPPKSPSSAKS **Charge** 5 **Spectral Angle** 0.73

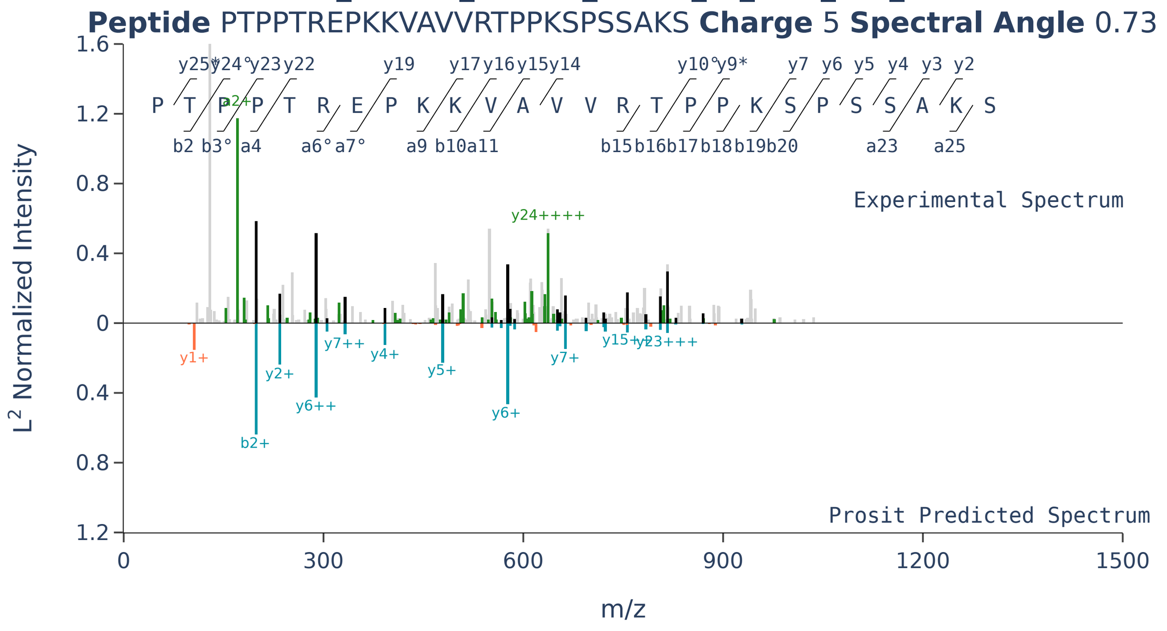


**Source** WSoh_221121_011221_HFGoe_L2_hTau_24h_R2 **Scan** 14647
**Peptide** KSKIGSTENLKHQPGGGKVQIINKKLDL **Charge** 4 **Spectral Angle** 0.87

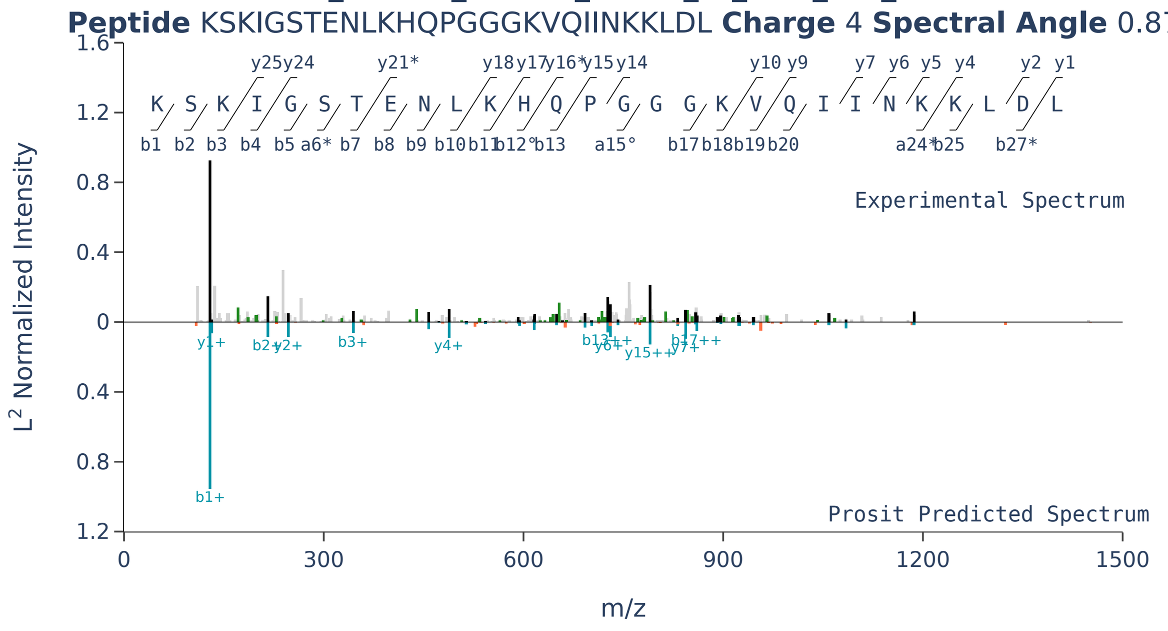


**Source** WSoh_221121_011221_HFGoe_L2_hTau_2h_R1 **Scan** 13206
**Peptide** KLDLSNVQSKCGSKDNIKHVPGGGSV **Charge** 5 **Spectral Angle** 0.86

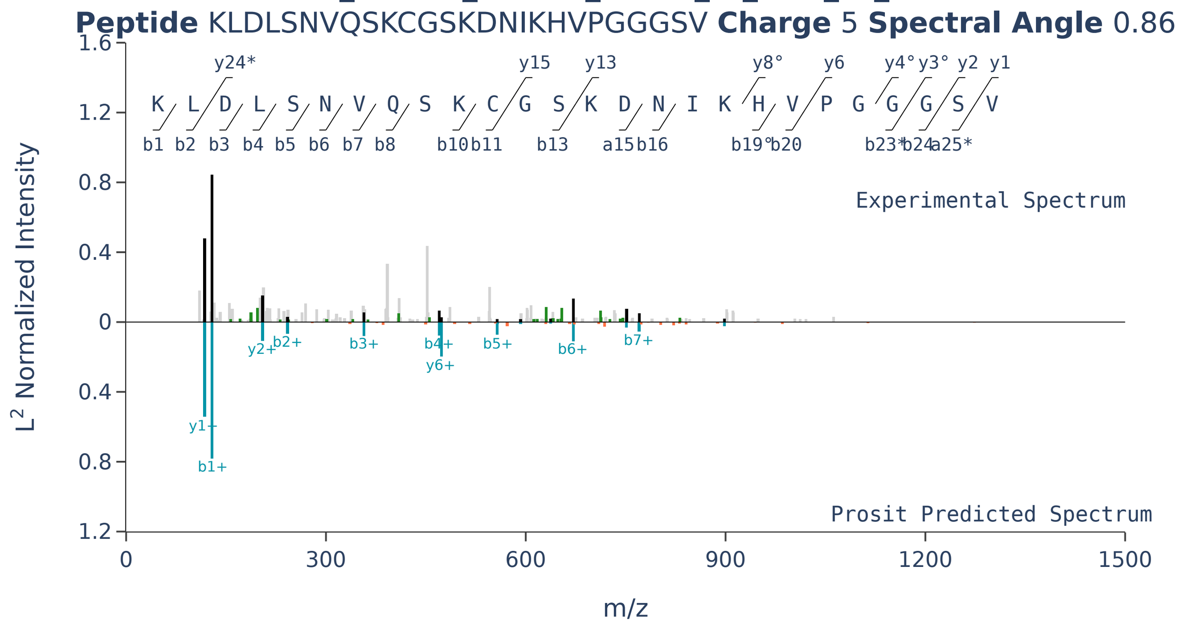


**Source** WSoh_221121_011221_HFGoe_L2_hTau_24h_R1 **Scan** 12819
**Peptide** SLGNIHHKPGGGQVEVKSEKLDFKDRVQ **Charge** 5 **Spectral Angle** 0.74

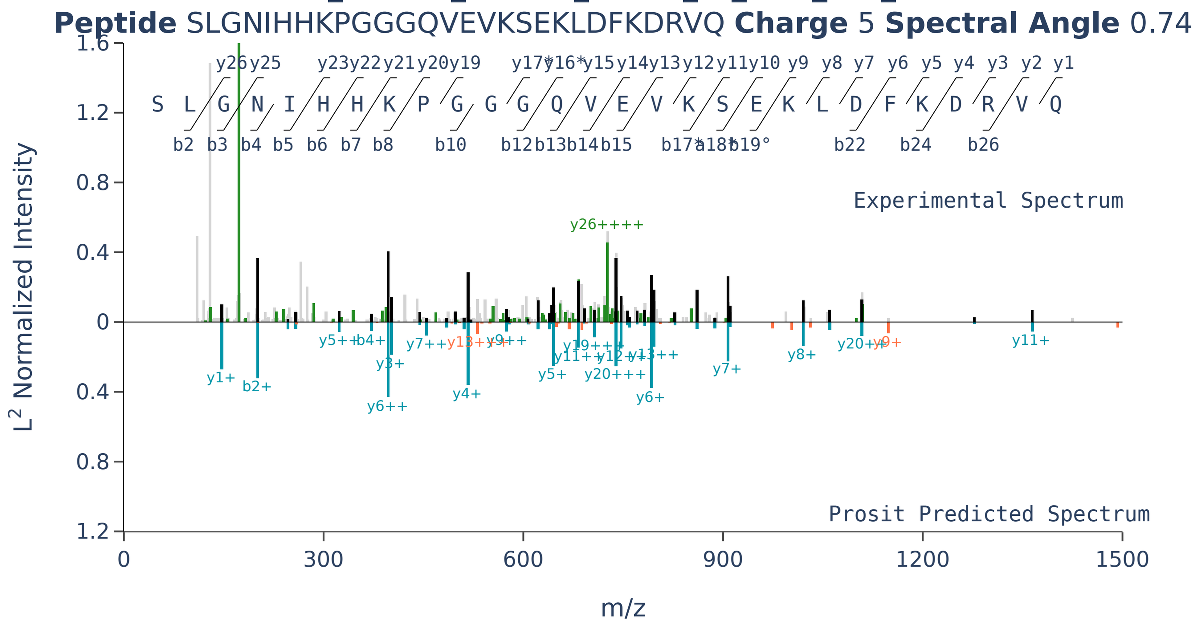


**Source** WSoh_221121_011221_HFGoe_L1_hTau_24h_R1 **Scan** 12873
**Peptide** KLDFKDRVQSKIGS **Charge** 3 **Spectral Angle** 0.84

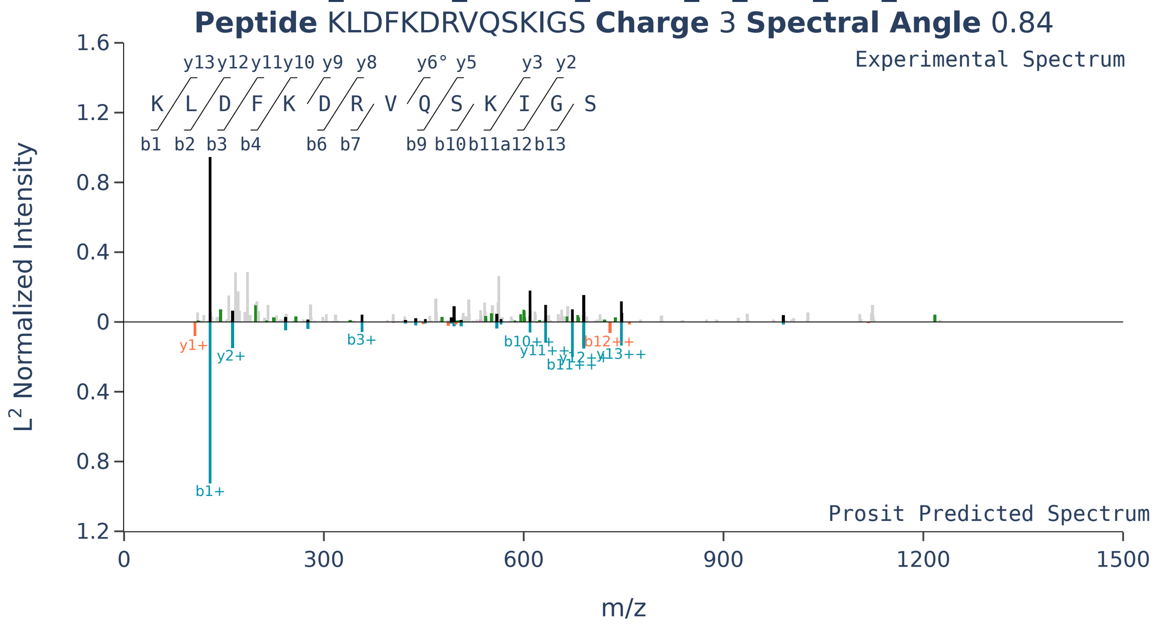


**Source** WSoh_221121_011221_HFGoe_L2_hTau_24h_R1 **Scan** 9252
**Peptide** NLTHVPGGGNKKIETHKLTFRENAKAK **Charge** 4 **Spectral Angle** 0.75

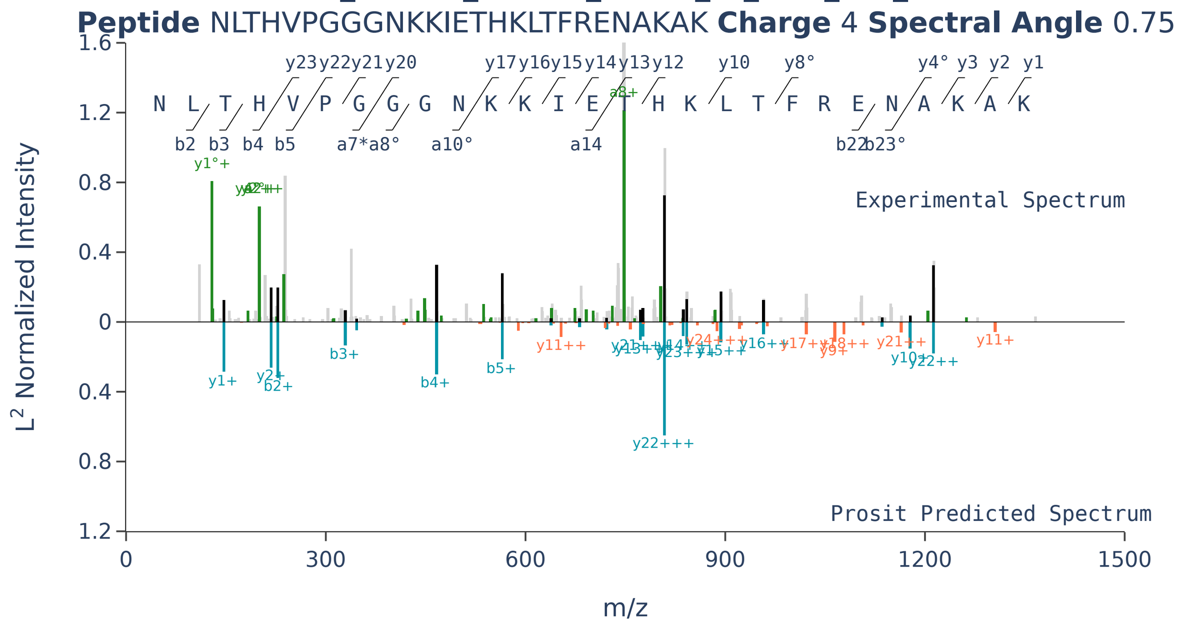


**Source** WSoh_221121_011221_HFGoe_L1_hTau_24h_R2 **Scan** 22394
**Peptide** PMPDLKNVKSKIGSTE **Charge** 3 **Spectral Angle** 0.93

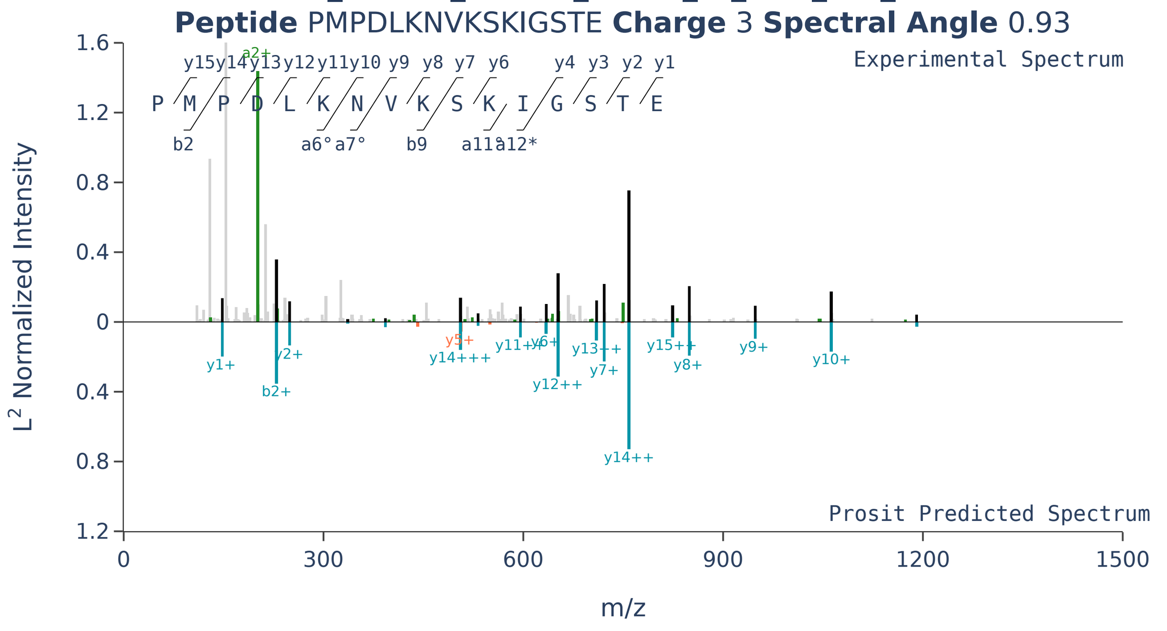

**Source** WSoh_221121_011221_HFGoe_L1_hTau_24h_R1 **Scan** 9502
**Peptide** KIETHKLTFREN **Charge** 4 **Spectral Angle** 0.55

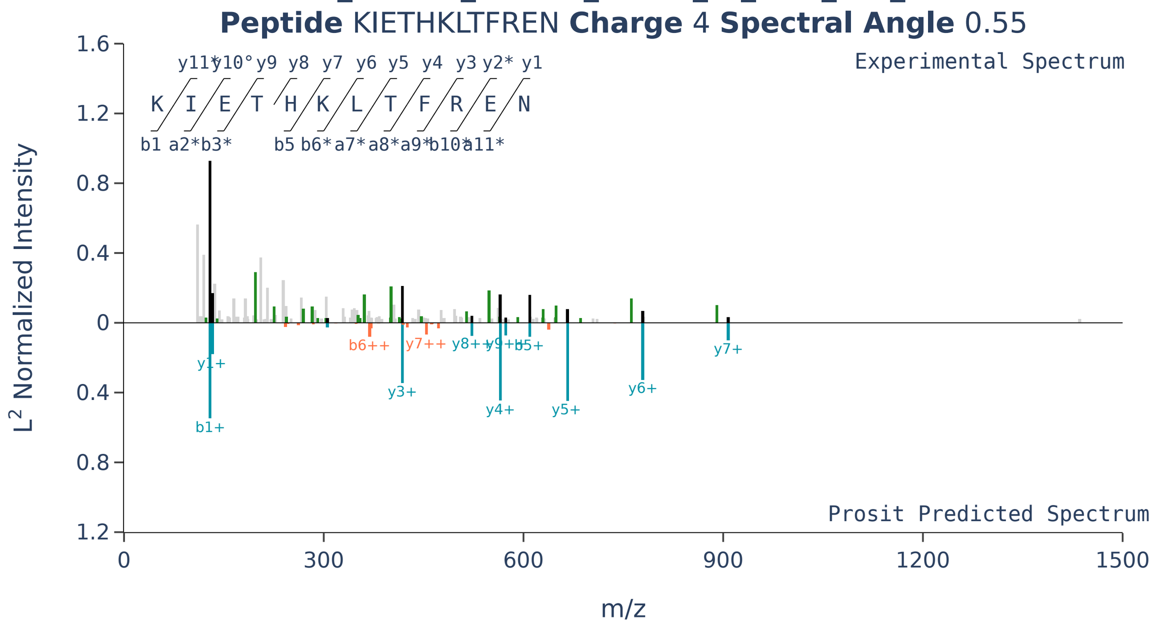


**Source** WSoh_221121_011221_HFGoe_L2_hTau_24h_R2 **Scan** 10662
**Peptide** LDLSNVQSKCGSKDNIKH **Charge** 4 **Spectral Angle** 0.88

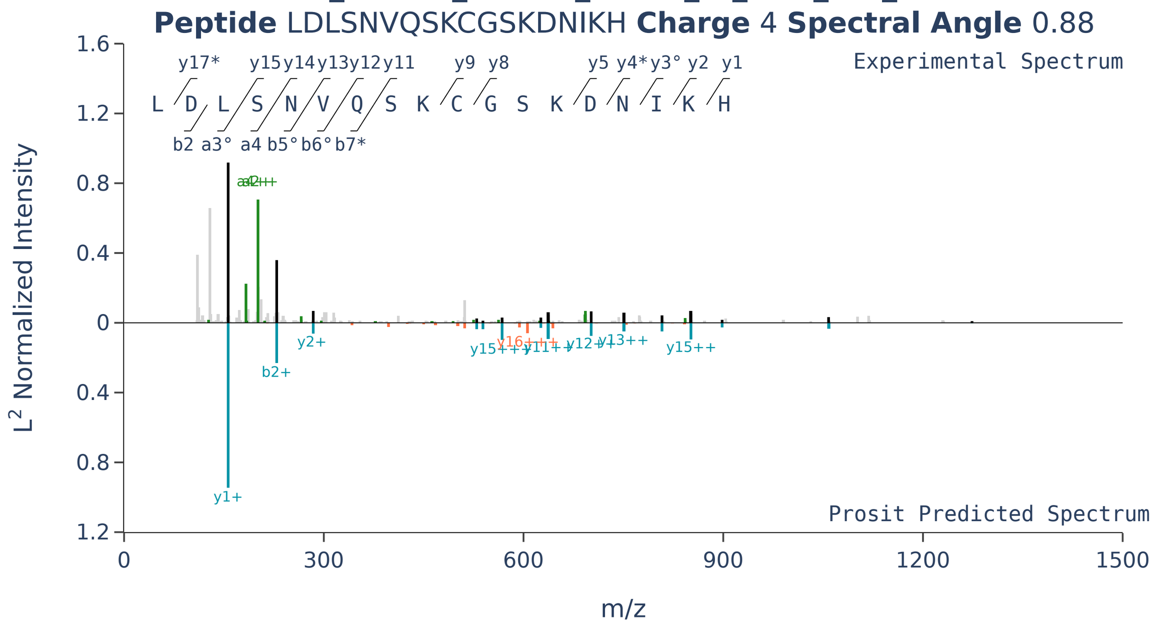


**Source** WSoh_221121_011221_HFGoe_L1_hTau_24h_R1 **Scan** 17987
**Peptide** LGSLDNITHVPGGGNKKIET **Charge** 4 **Spectral Angle** 0.8

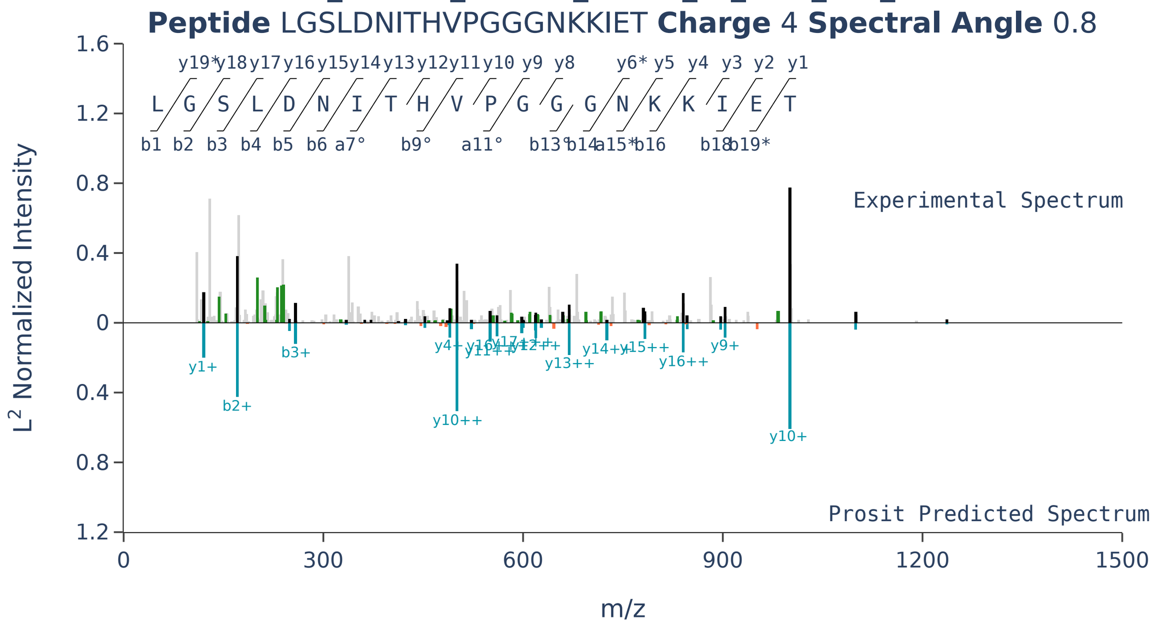


**Source** WSoh_221121_011221_HFGoe_L2_hTau_24h_R2 **Scan** 23349
**Peptide** PHTEIPEGTTAEEAGIGDTPSLE **Charge** 3 **Spectral Angle** 0.82

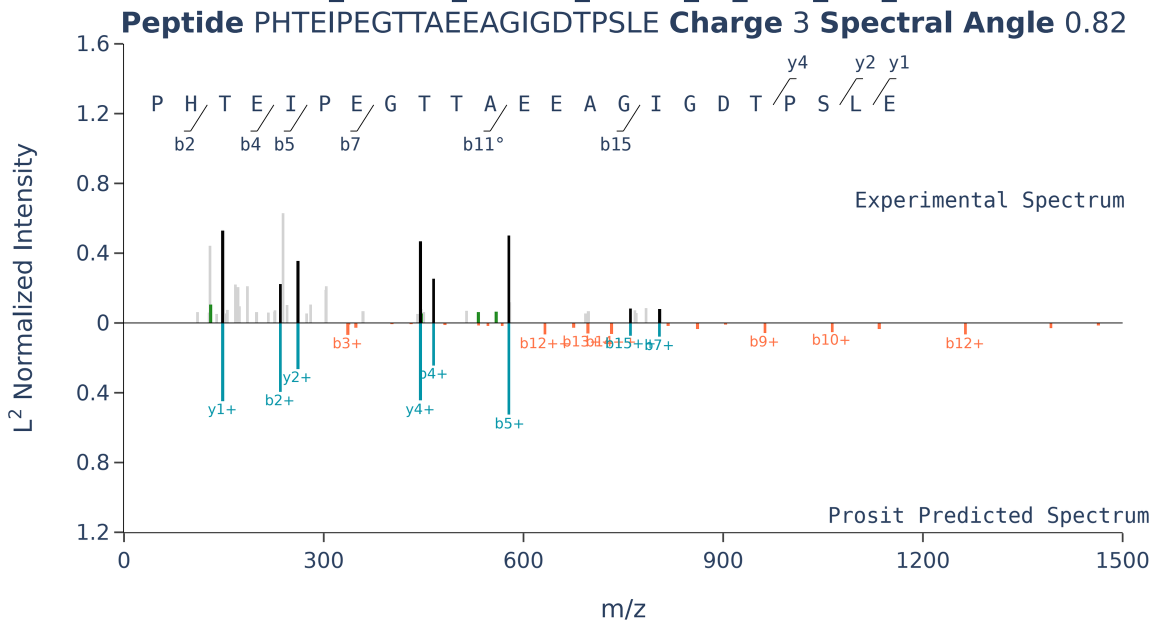


**Source** WSoh_221121_011221_HFGoe_L2_hTau_24h_R2 **Scan** 14352
**Peptide** PSLPTPPTREPKKVAVVRTPPKSPSSA **Charge** 4 **Spectral Angle** 0.76

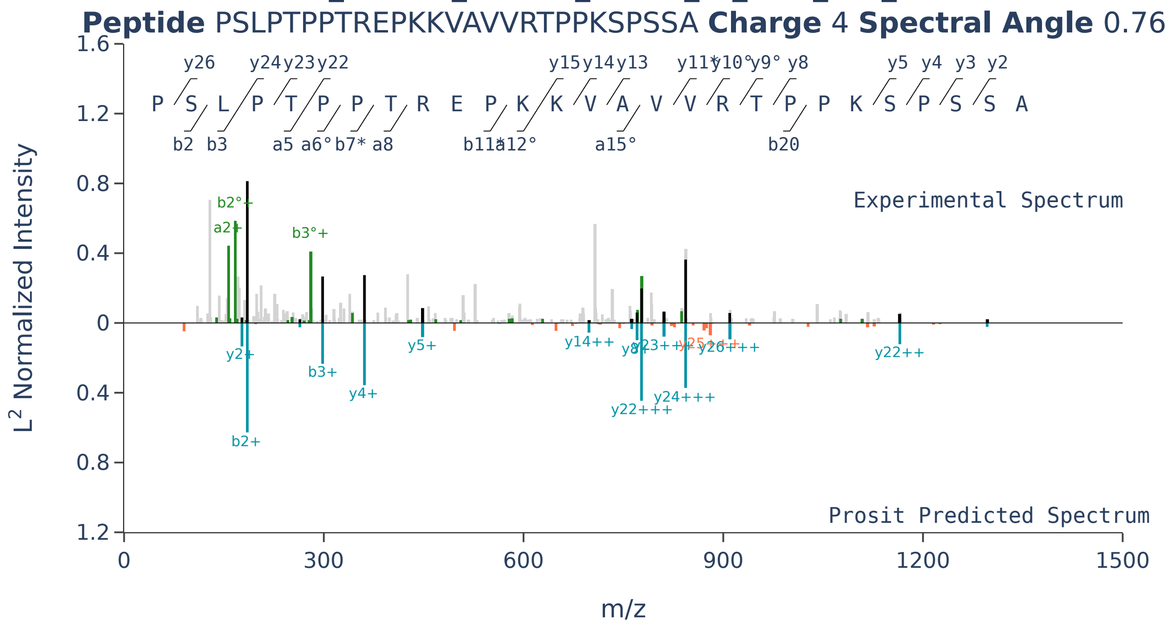


**Source** WSoh_221121_011221_HFGoe_L1_hTau_24h_R2 **Scan** 16964
**Peptide** PDLKNVKS **Charge** 2 **Spectral Angle** 0.82

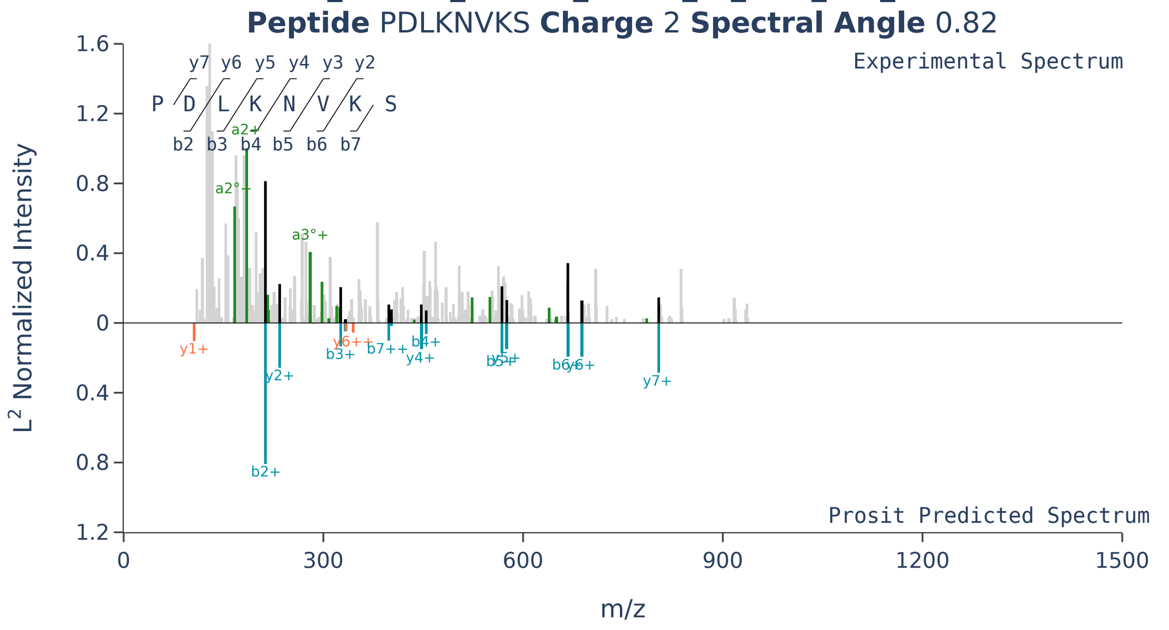


**Source** WSoh_221121_011221_HFGoe_L2_hTau_24h_R1 **Scan** 16996
**Peptide** PMPDLKNVKSK **Charge** 3 **Spectral Angle** 0.89

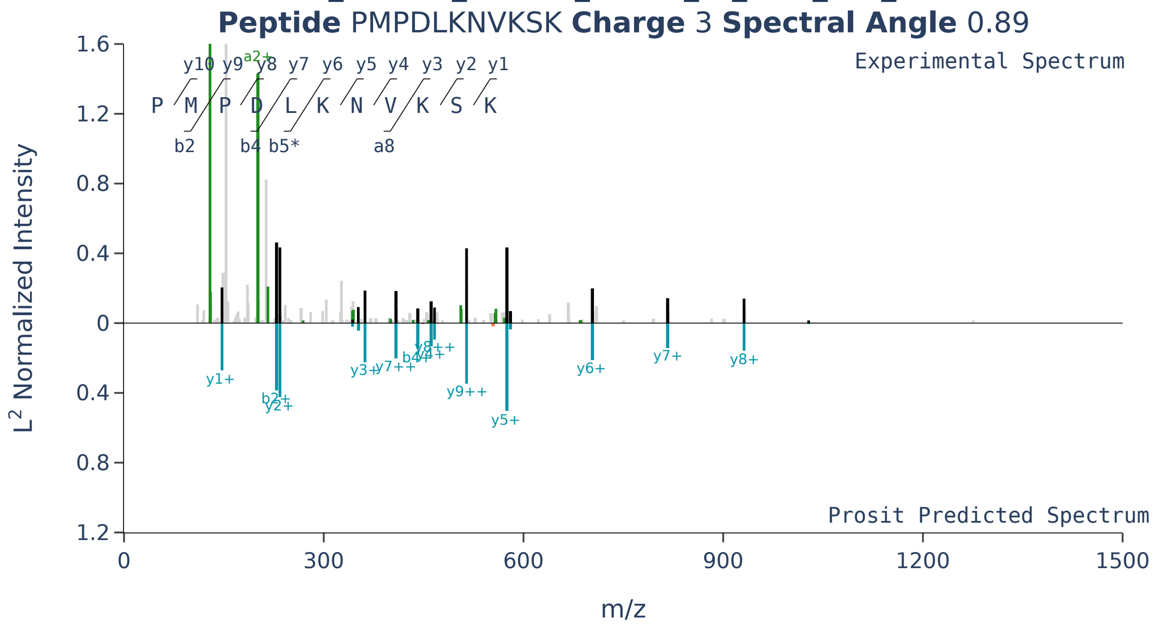


**Source** WSoh_221121_011221_HFGoe_L2_hTau_4h_R1 **Scan** 11747
**Peptide** QSKIGSL **Charge** 2 **Spectral Angle** 0.77

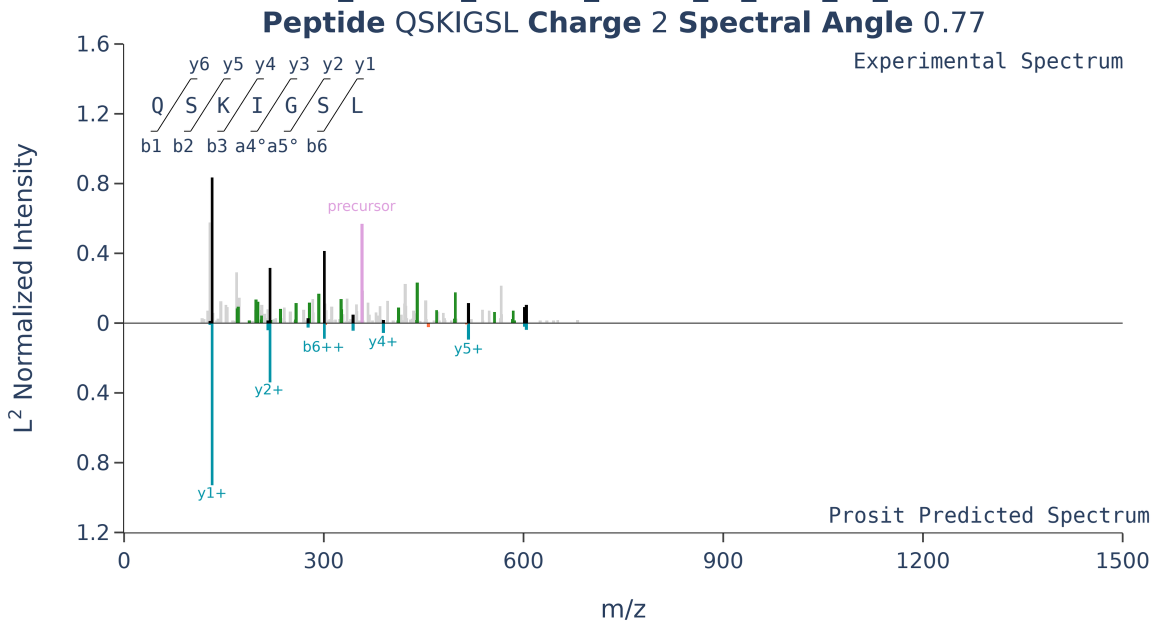


**Source** WSoh_221121_011221_HFGoe_L1_hTau_24h_R1 **Scan** 6431
**Peptide** AVVRTPPKS **Charge** 2 **Spectral Angle** 0.75

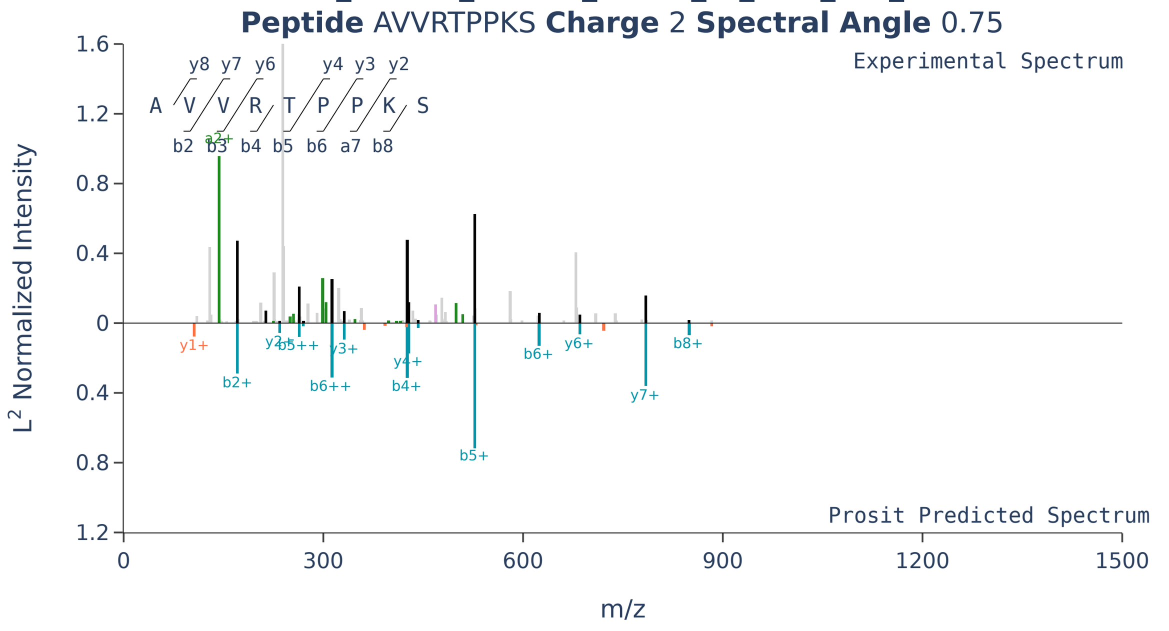


**Source** WSoh_221121_011221_HFGoe_L1_hTau_24h_R1 **Scan** 6653
**Peptide** GDRSGYSSPGSPGTPGSRSRT **Charge** 3 **Spectral Angle** 0.7

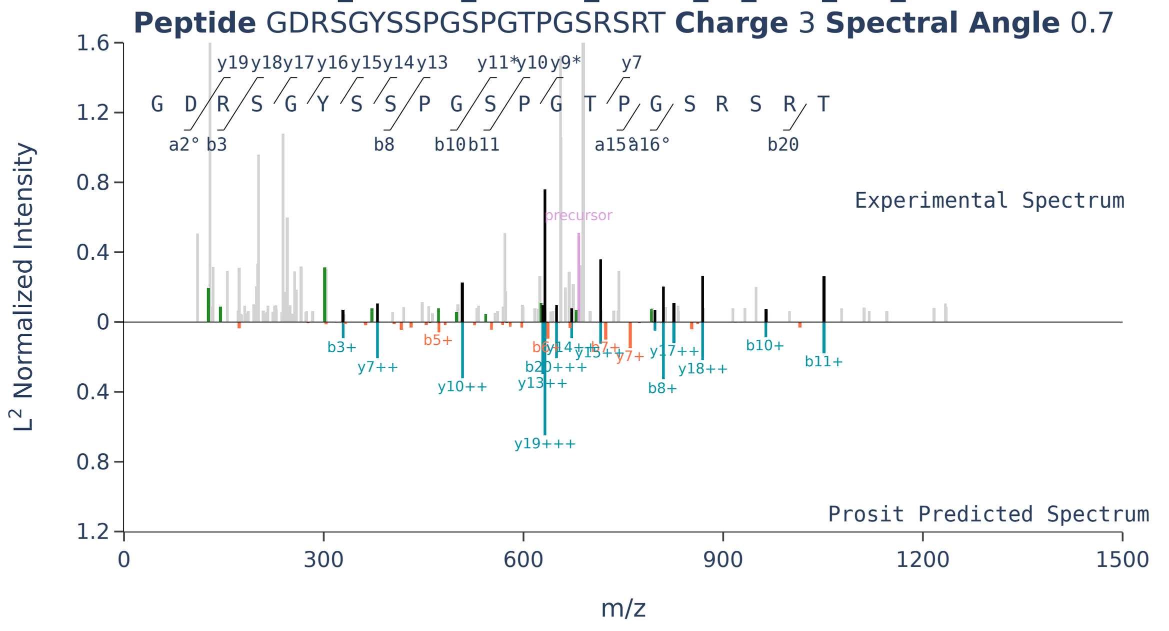


**Source** WSoh_221121_011221_HFGoe_L2_hTau_4h_R1 **Scan** 11072
**Peptide** NKKLDLSNVQSKCGSKDNI **Charge** 3 **Spectral Angle** 0.73

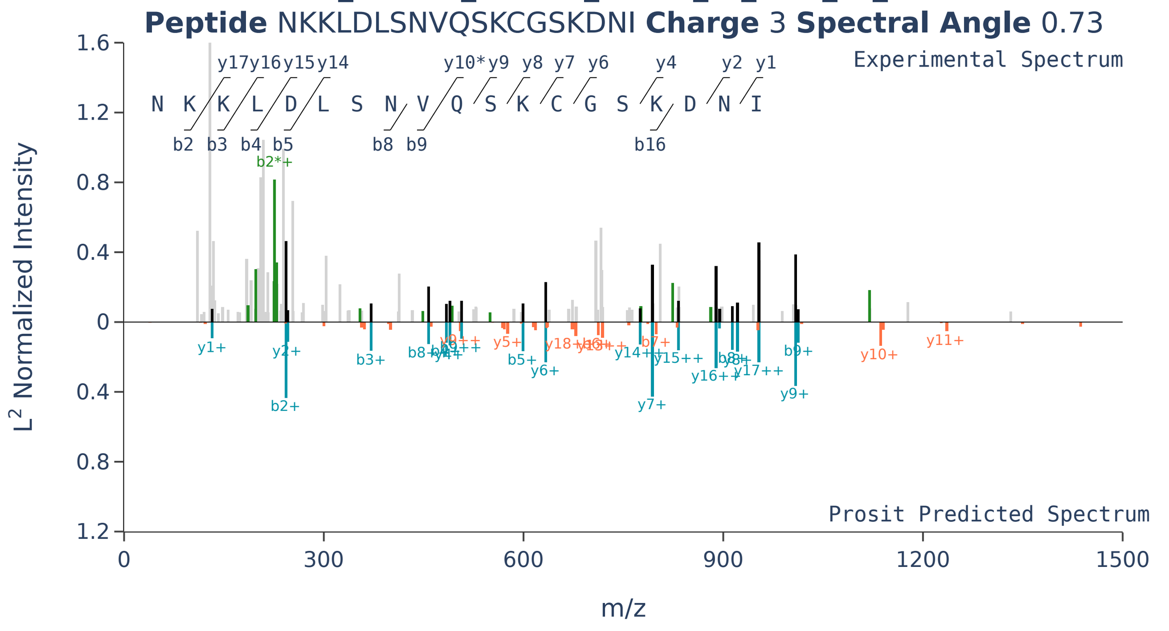


**Source** WSoh_221121_011221_HFGoe_L1_hTau_4h_R2 **Scan** 16290
**Peptide** KIGSLDNITHVPGGGNKKIETHKLTFREN **Charge** 4 **Spectral Angle** 0.68

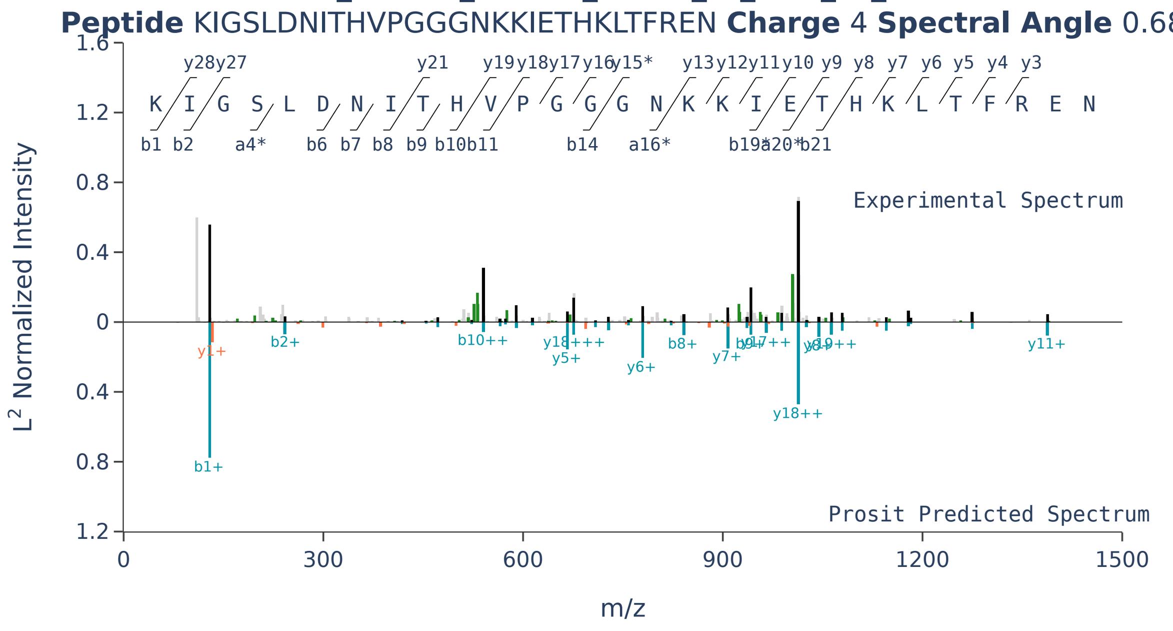


**Source** WSoh_221121_011221_HFGoe_L1_hTau_24h_R2 **Scan** 24459
**Peptide** IPEGTTAEEAGIGDTPSLEDEAAGHVTQA **Charge** 3 **Spectral Angle** 0.64

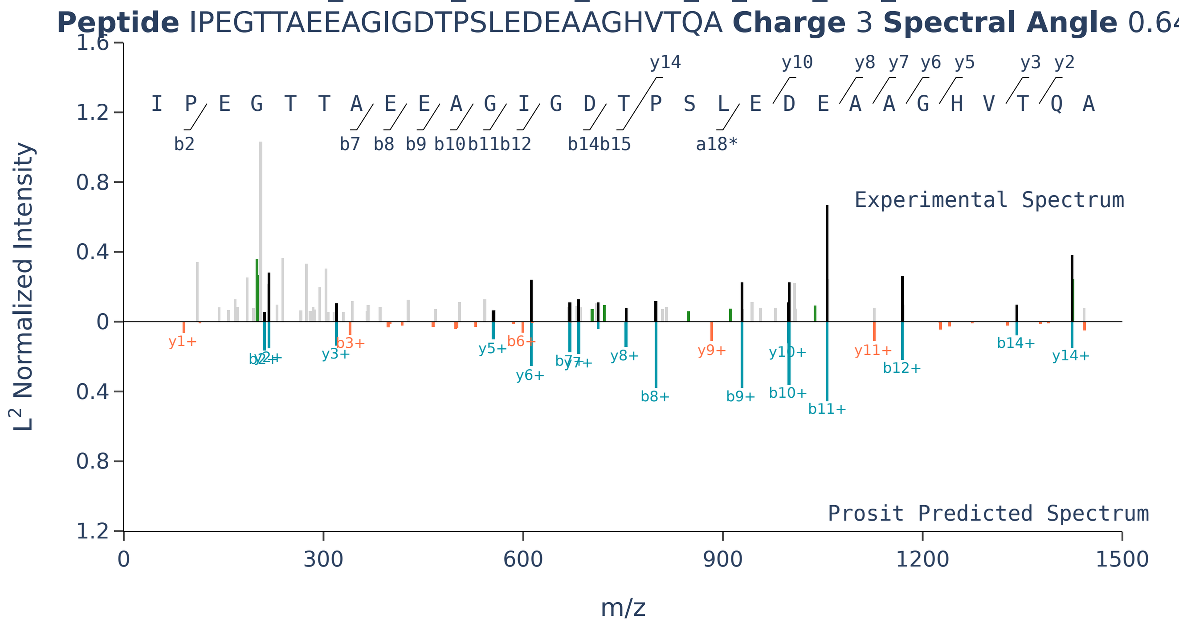


**Source** WSoh_221121_011221_HFGoe_L1_hTau_1h_R2 **Scan** 5341
**Peptide** GNIHHKPGGGQVE **Charge** 3 **Spectral Angle** 0.9

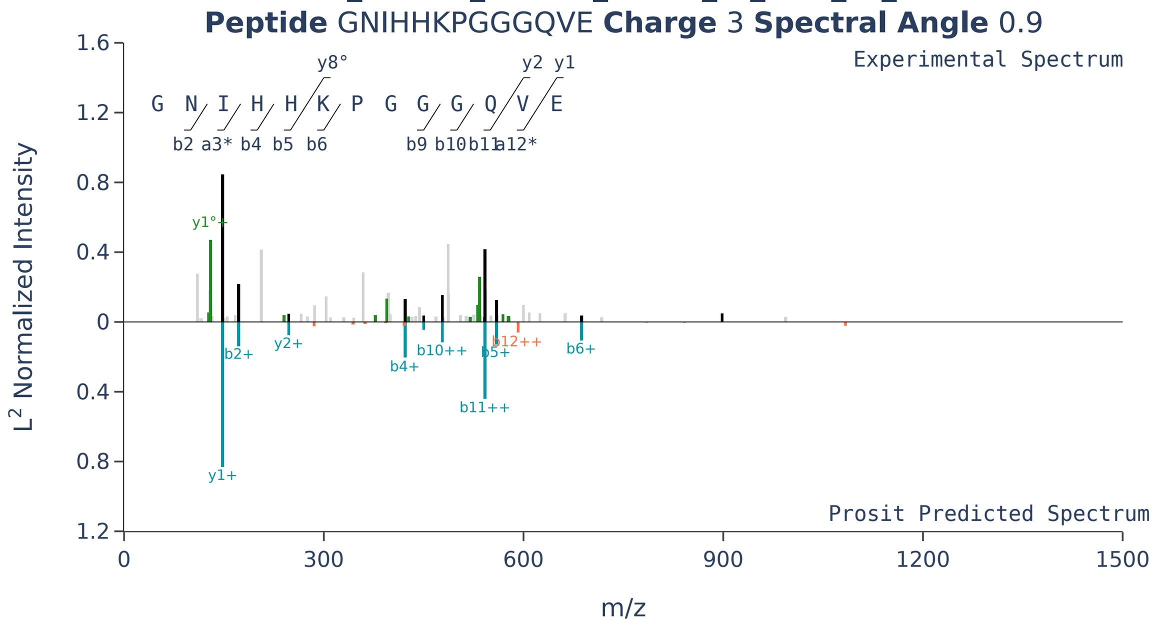


**Source** WSoh_221121_011221_HFGoe_L1_hTau_4h_R2 **Scan** 16253
**Peptide** GSLDNITHVPGGGNKKIETHKLTFRE **Charge** 4 **Spectral Angle** 0.72

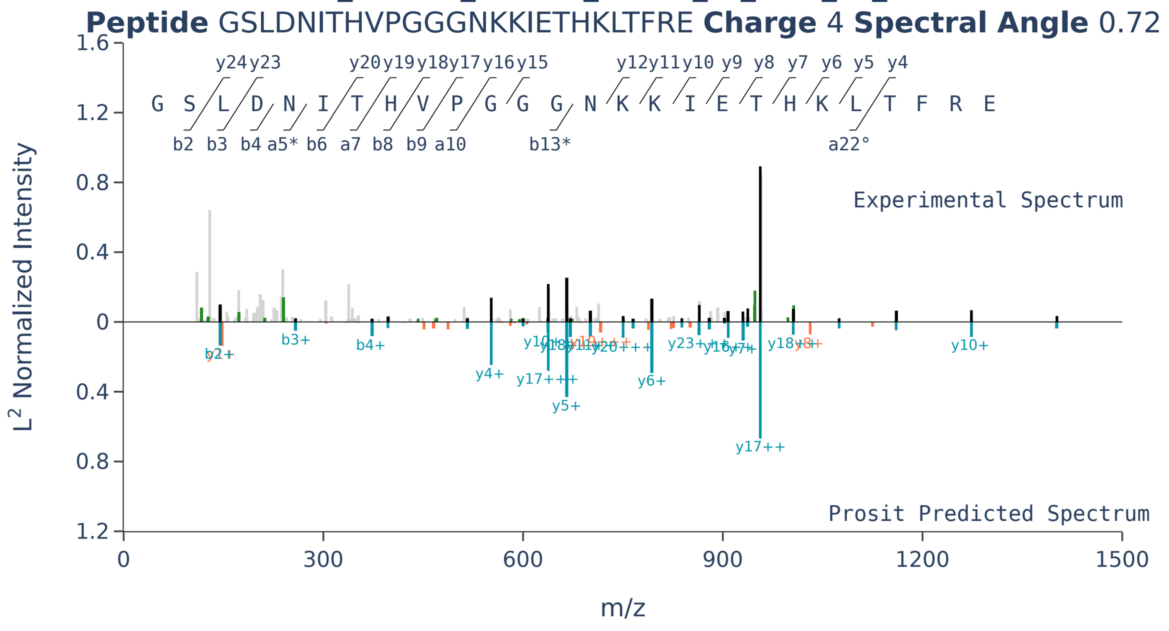

**Source** WSoh_221121_011221_HFGoe_L2_hTau_24h_R1 **Scan** 6685
**Peptide** SKVTSKCGSL **Charge** 3 **Spectral Angle** 0.94

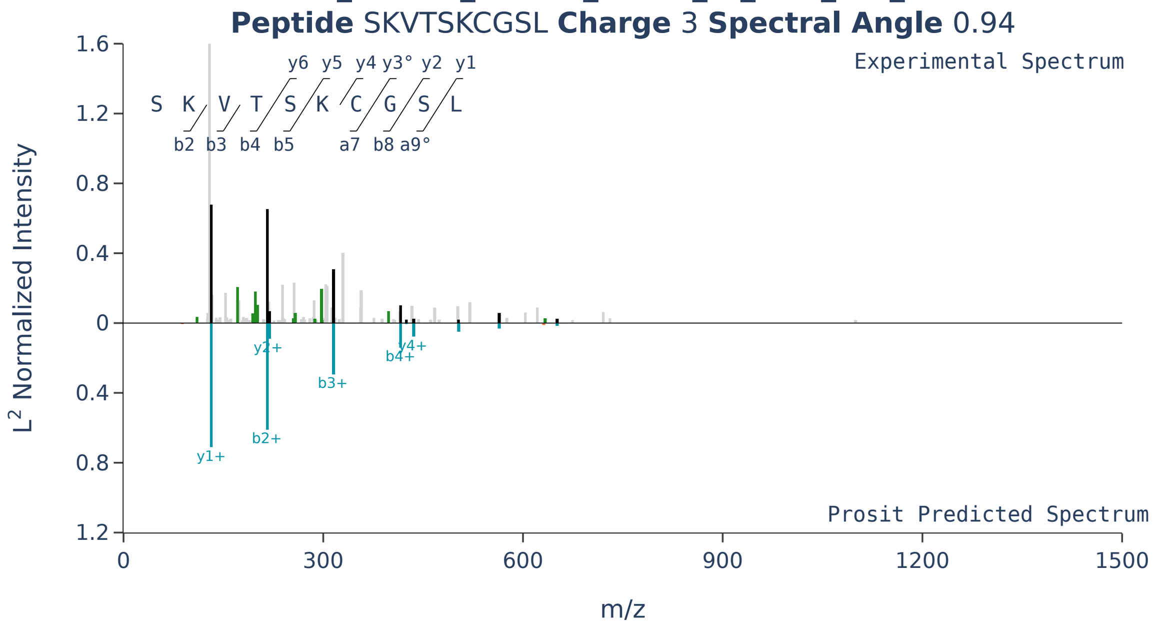


**Source** WSoh_221121_011221_HFGoe_L1_hTau_24h_R1 **Scan** 20285
**Peptide** DQEGDTDAGLKESPLQTPTED **Charge** 3 **Spectral Angle** 0.86

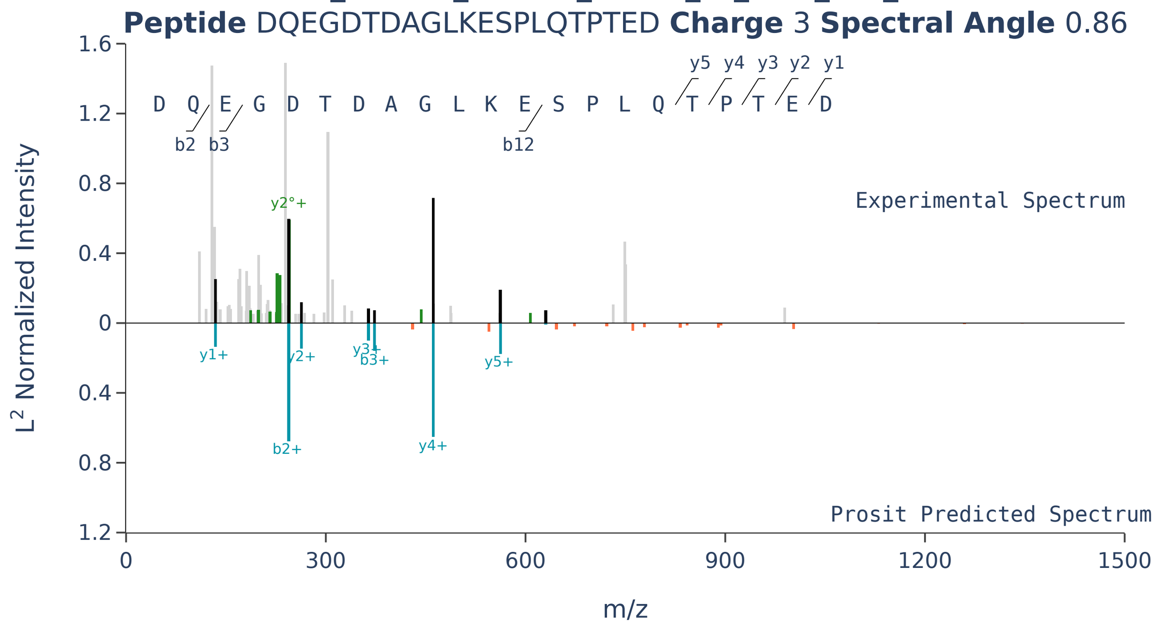


**Source** WSoh_221121_011221_HFGoe_L1_hTau_24h_R1 **Scan** 15989
**Peptide** VQSKIGSLDNITHVPGGGNKKIETHKL **Charge** 5 **Spectral Angle** 0.82

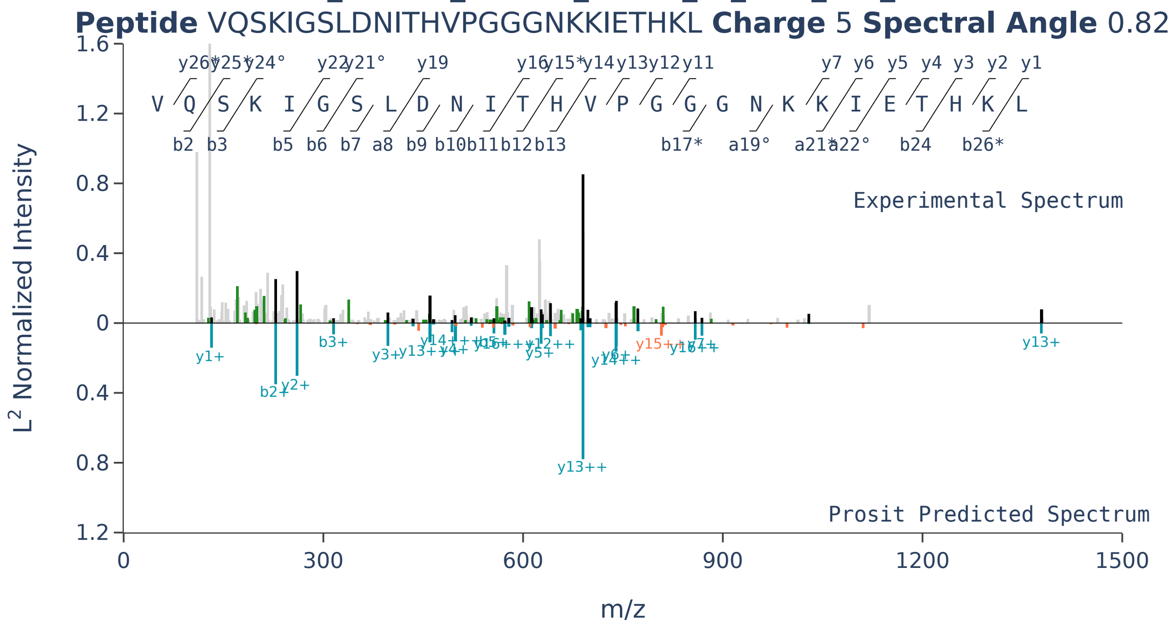


**Source** WSoh_221121_011221_HFGoe_L1_hTau_24h_R2 **Scan** 19455
**Peptide** SKDNIKHVPGGGSVQIVYKPVDLSKVTSK **Charge** 4 **Spectral Angle** 0.72

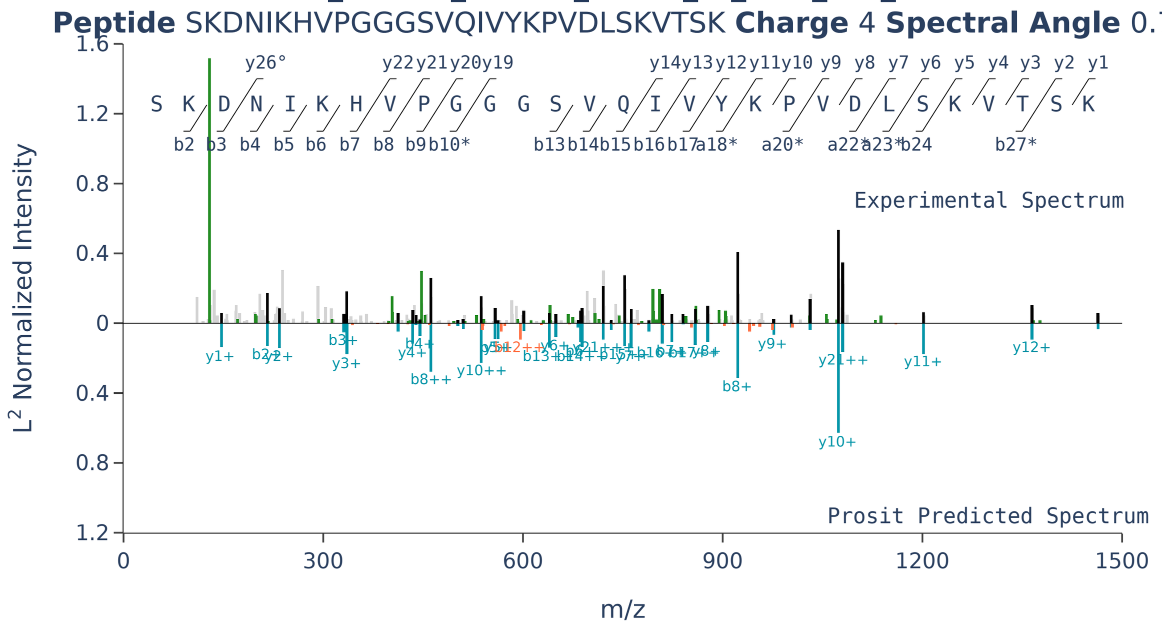


**Source** WSoh_221121_011221_HFGoe_L1_hTau_4h_R2 **Scan** 9589
**Peptide** KSKIGSTENLKHQPGGGKVQI **Charge** 5 **Spectral Angle** 0.85

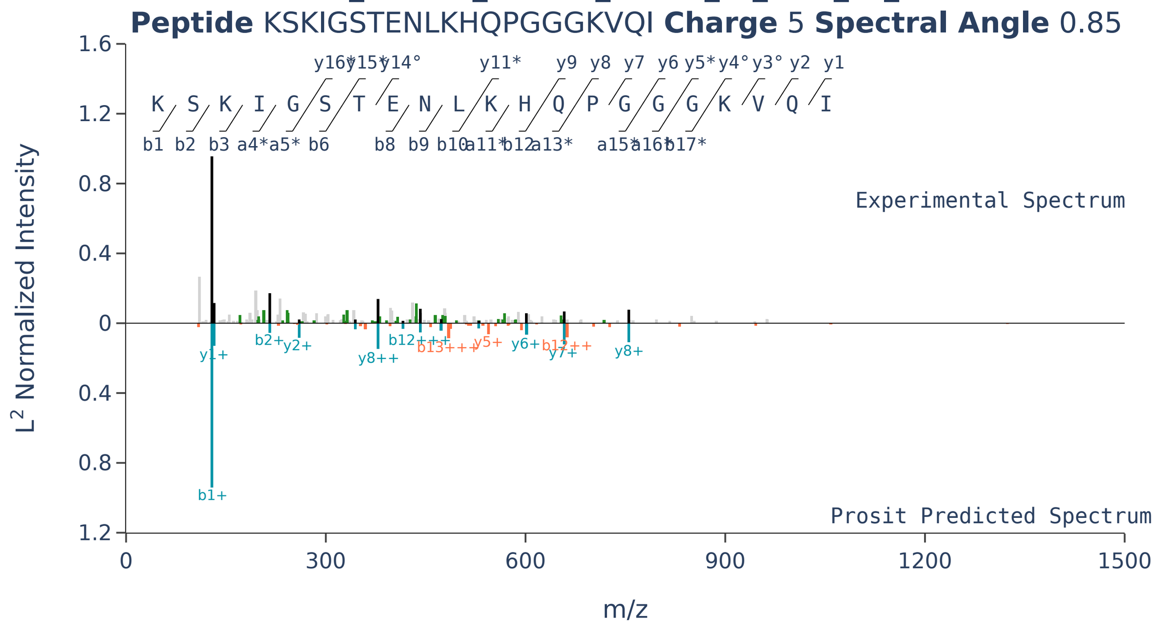


**Source** WSoh_221121_011221_HFGoe_L1_hTau_4h_R2 **Scan** 11759
**Peptide** DHAGTYGLGDRKDQGGYTMHQDQEGDTD **Charge** 4 **Spectral Angle** 0.89

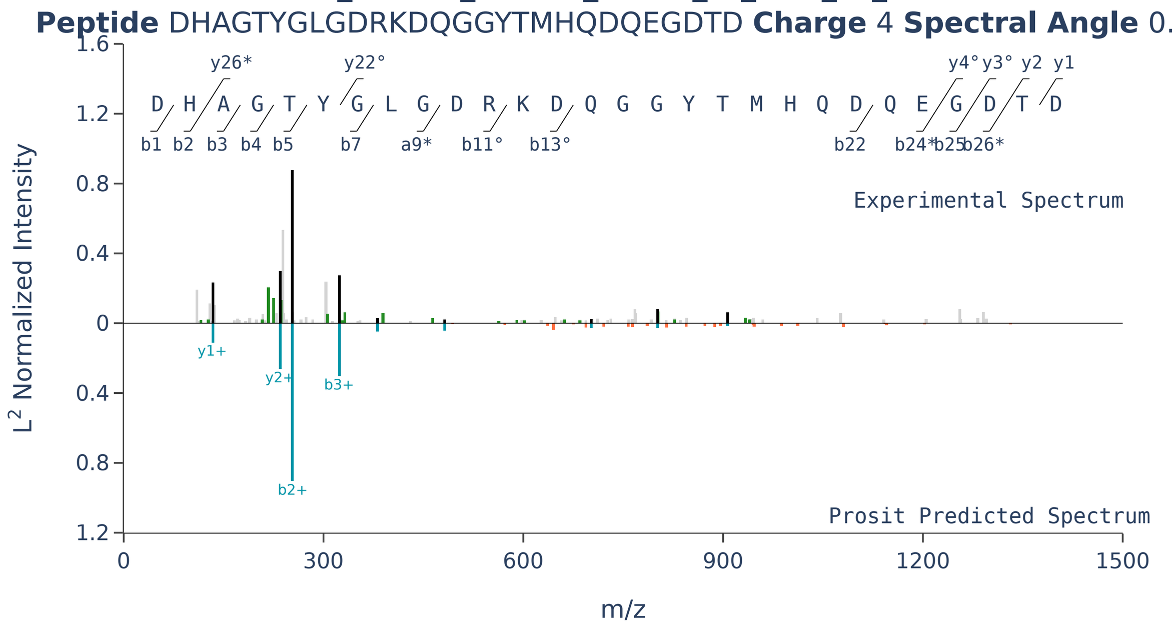


**Source** WSoh_221121_011221_HFGoe_L2_hTau_4h_R2 **Scan** 13193
**Peptide** KPVDLSKVTSKCGSLGNIHHKPGGGQVE **Charge** 4 **Spectral Angle** 0.83

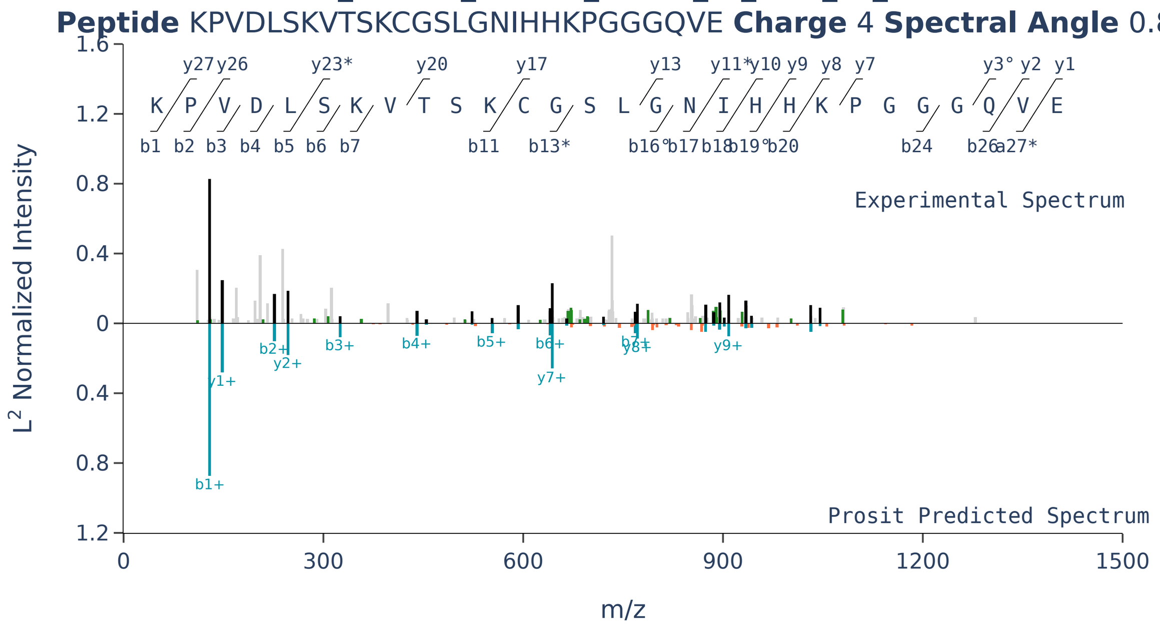


**Source** WSoh_221121_011221_HFGoe_L2_hTau_24h_R1 **Scan** 15225
**Peptide** PSLPTPPTREPKKVAVVR **Charge** 4 **Spectral Angle** 0.64

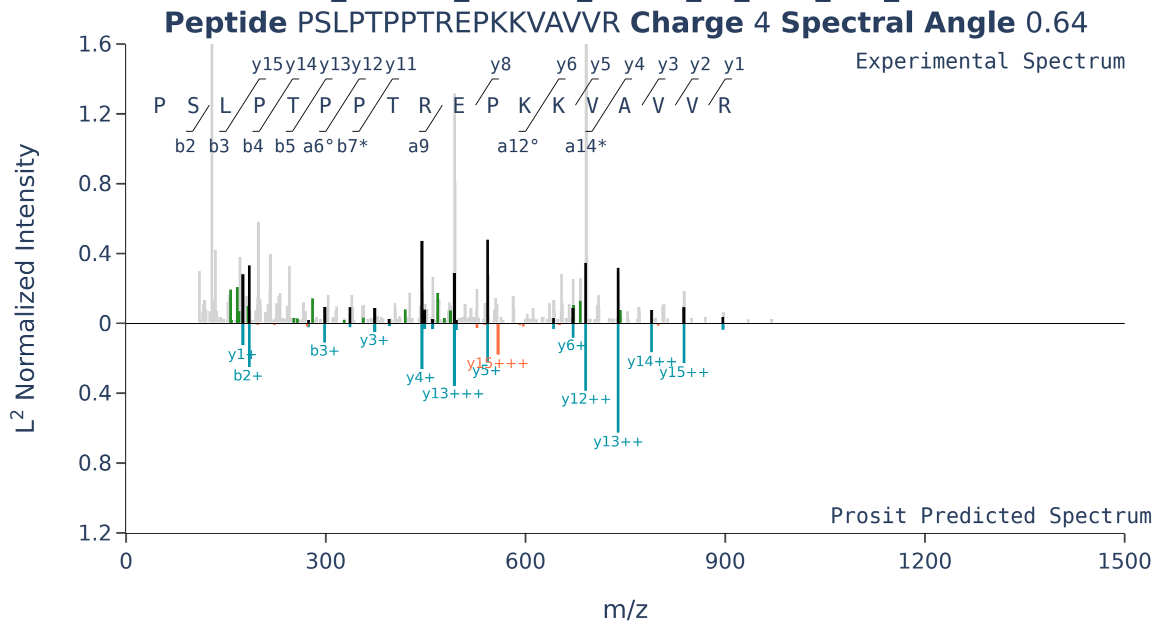


**Source** WSoh_221121_011221_HFGoe_L2_hTau_4h_R1 **Scan** 14076
**Peptide** SLDNITHVPGGGNKKIE **Charge** 4 **Spectral Angle** 0.77

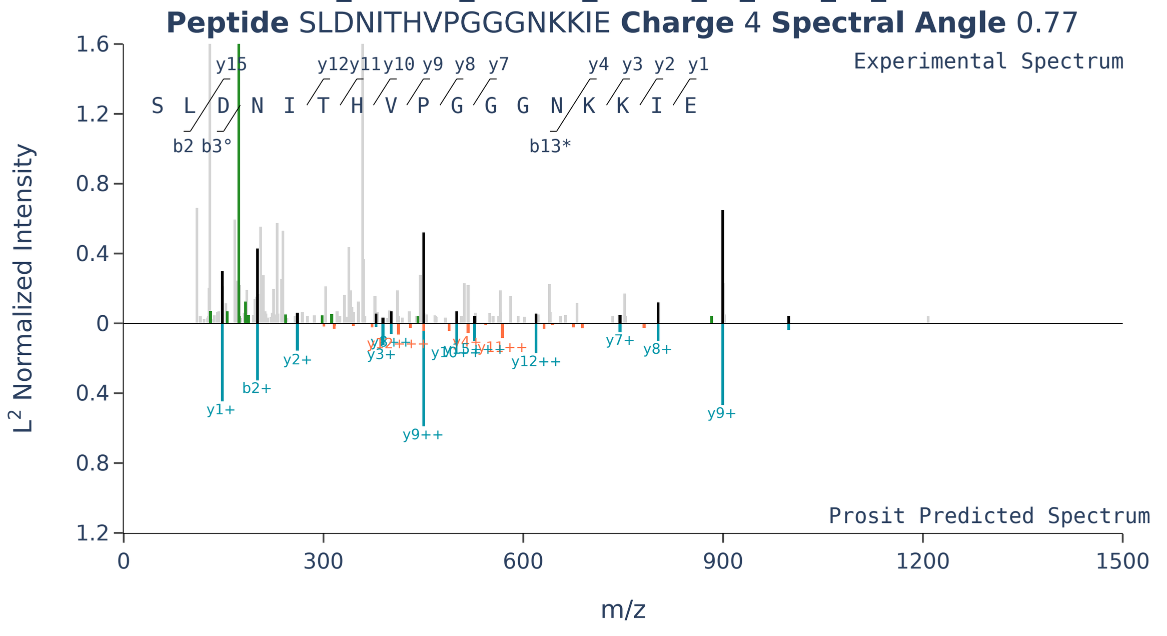


**Source** WSoh_221121_011221_HFGoe_L1_hTau_24h_R1 **Scan** 20844
**Peptide** TPPKSPSSAKSRLQTAPVPMPDLKNV **Charge** 4 **Spectral Angle** 0.76

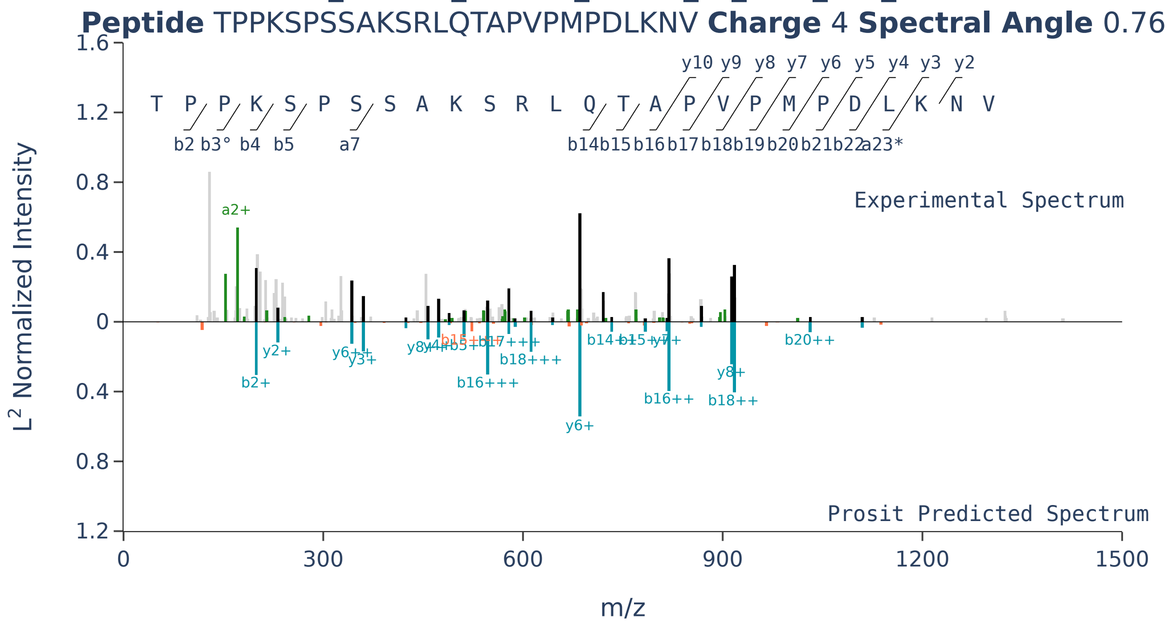


**Source** WSoh_221121_011221_HFGoe_L2_hTau_4h_R1 **Scan** 22266
**Peptide** EKLDFKDRVQSKIGSLDNI **Charge** 3 **Spectral Angle** 0.72

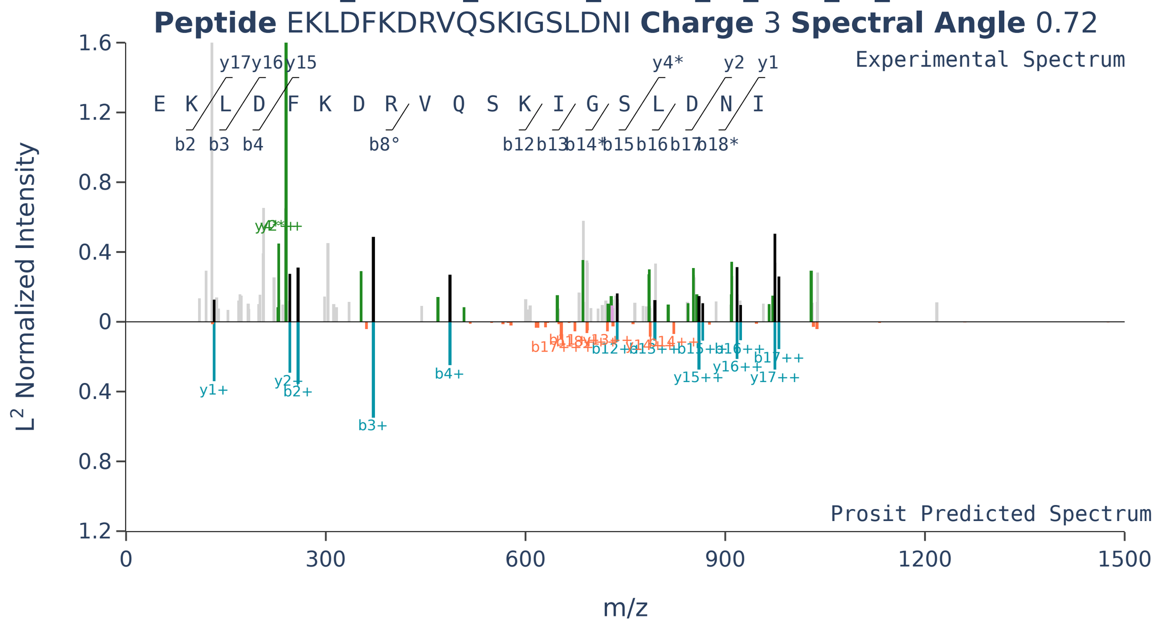


**Source** WSoh_221121_011221_HFGoe_L1_hTau_4h_R2 **Scan** 7709
**Peptide** PPGQKGQANATRIPAKT **Charge** 4 **Spectral Angle** 0.75

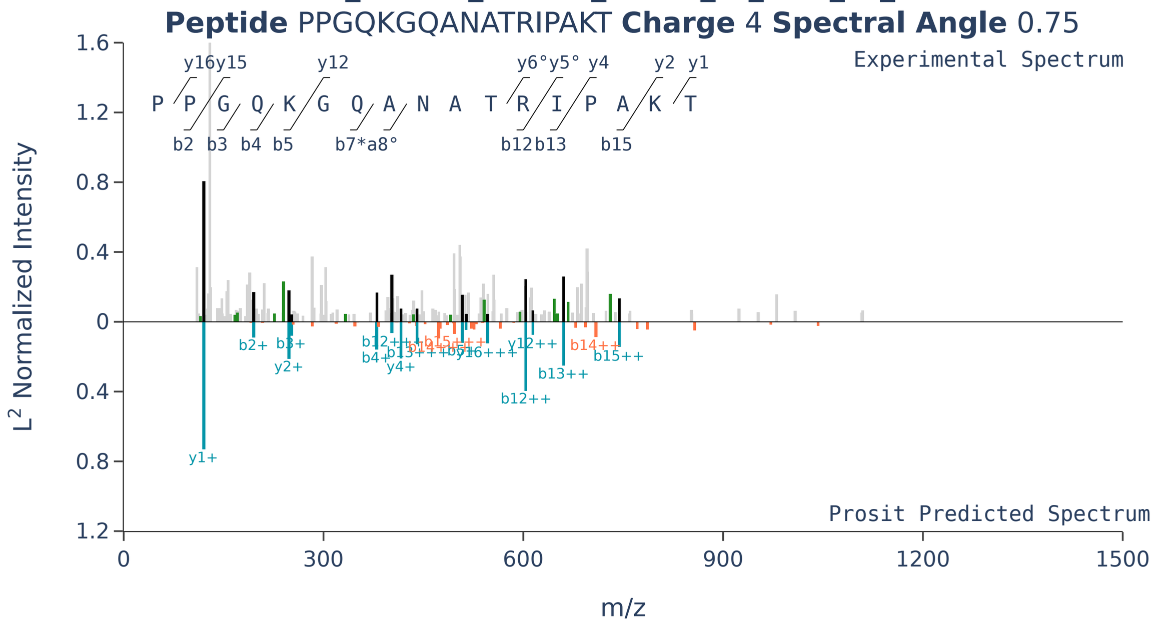


**Source** WSoh_221121_011221_HFGoe_L2_hTau_4h_R1 **Scan** 7940
**Peptide** SEEPGSETSDAKSTPTAED **Charge** 2 **Spectral Angle** 0.78

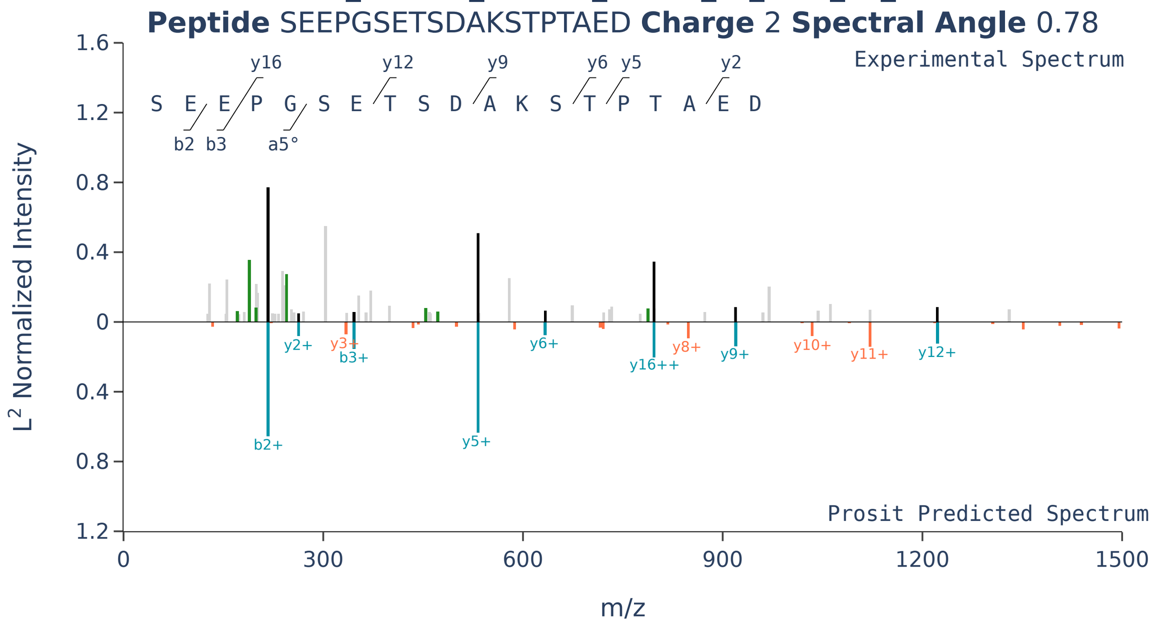


**Source** WSoh_221121_011221_HFGoe_L2_hTau_24h_R2 **Scan** 11739
**Peptide** KIGSTENLKHQPGGGKVQIINKKLD **Charge** 5 **Spectral Angle** 0.87

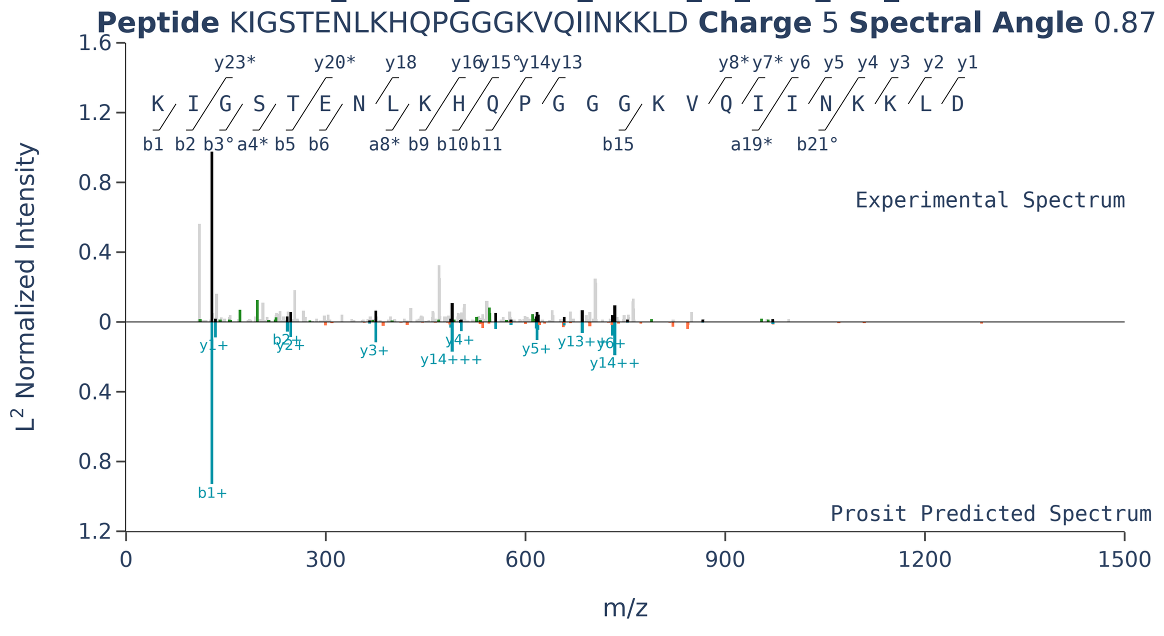


**Source** WSoh_221121_011221_HFGoe_L2_hTau_24h_R2 **Scan** 22983
**Peptide** GSEEPGSETSDAKSTPTAEDVTAPLVDE **Charge** 3 **Spectral Angle** 0.8

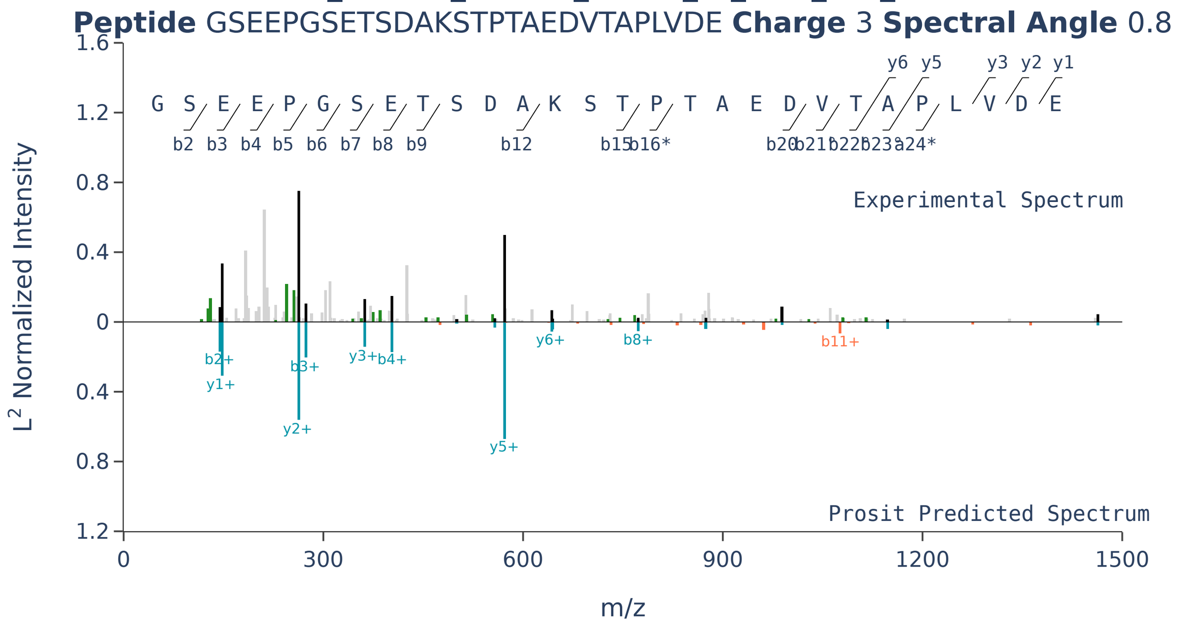

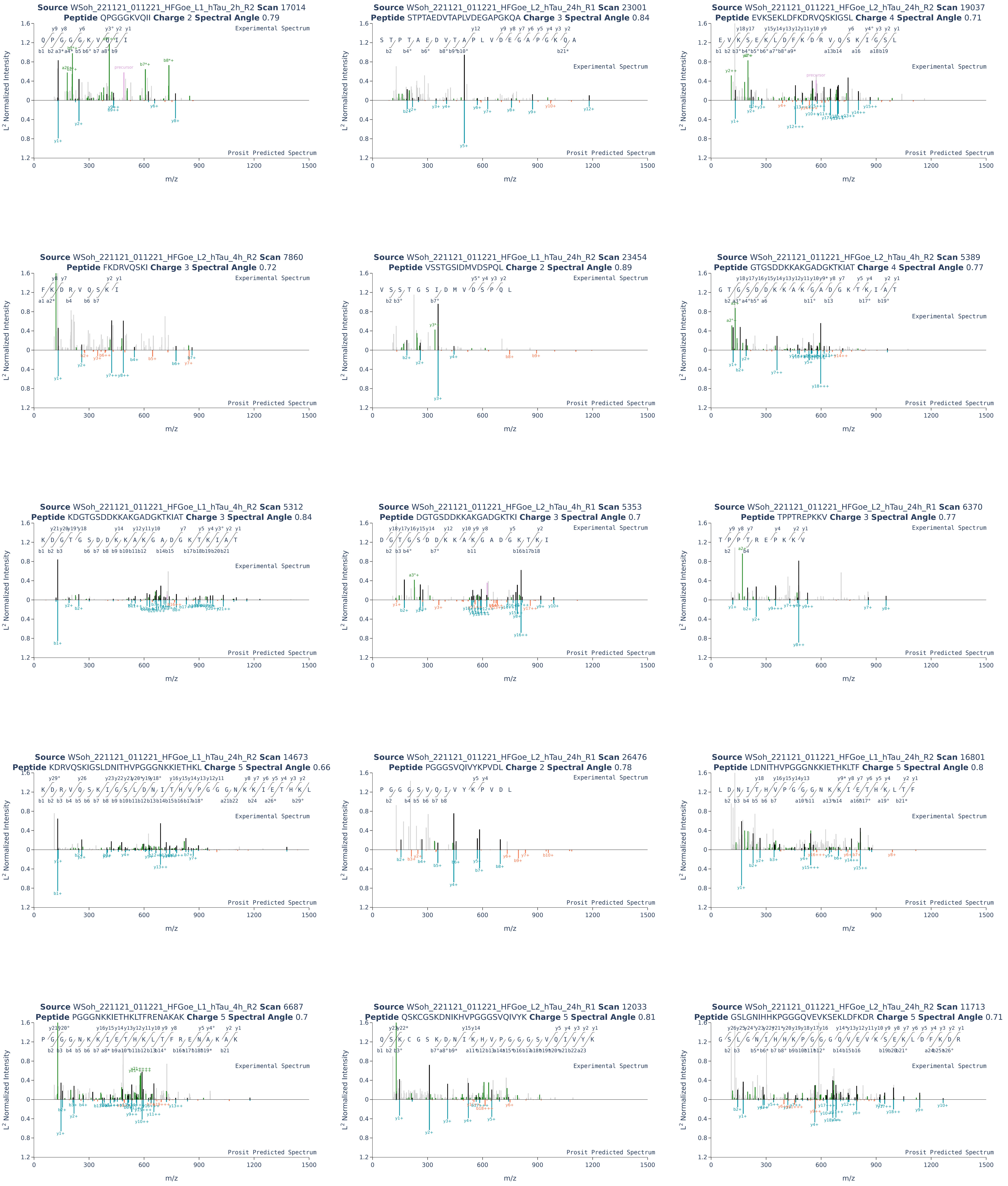
Source WSoh_221121_011221_HFGoe_L1_hTau_2h_R2 Scan 17014
Peptide QPGGGKVQII Charge 2 Spectral Angle 0.79
Source WSoh_221121_011221_HFGoe_L2_hTau_24h_R1 Scan 23001
Peptide STPTAEDVTAPLVDEGAPGKQA Charge 3 Spectral Angle 0.84
Source WSoh_221121_011221_HFGoe_L2_hTau_24h_R2 Scan 19037
Peptide EVKSEKLDFKDRVQSKIGSL Charge 4 Spectral Angle 0.71
Source WSoh_221121_011221_HFGoe_L2_hTau_4h_R2 Scan 7860
Peptide FKDRVQSKI Charge 3 Spectral Angle 0.72
Source WSoh_221121_011221_HFGoe_L1_hTau_24h_R2 Scan 23454
Peptide VSSTGSIDMVDSPQL Charge 2 Spectral Angle 0.89
Source WSoh_221121_011221_HFGoe_L2_hTau_4h_R2 Scan 5389
Peptide GTGSDDKKAKGADGKTKIAT Charge 4 Spectral Angle 0.77
Source WSoh_221121_011221_HFGoe_L1_hTau_4h_R2 Scan 5312
Peptide KDGTGSDDKKAKGADGKTKIAT Charge 3 Spectral Angle 0.84
Source WSoh_221121_011221_HFGoe_L2_hTau_4h_R1 Scan 5353
Peptide DGTGSDDKKAKGADGKTKI Charge 3 Spectral Angle 0.7
Source WSoh_221121_011221_HFGoe_L2_hTau_24h_R1 Scan 6370
Peptide TPPTREPKKV Charge 3 Spectral Angle 0.77
Source WSoh_221121_011221_HFGoe_L1_hTau_24h_R2 Scan 14673
Peptide KDRVQSKIGSLDNITHVPGGGNKKIETHKL Charge 5 Spectral Angle 0.66
Source WSoh_221121_011221_HFGoe_L2_hTau_24h_R1 Scan 26476
Peptide PGGGSVQIVYKPVDL Charge 2 Spectral Angle 0.78
Source WSoh_221121_011221_HFGoe_L1_hTau_24h_R2 Scan 16801
Peptide LDNITHVPGGGNKKIETHKLTF Charge 5 Spectral Angle 0.8
Source WSoh_221121_011221_HFGoe_L1_hTau_4h_R2 Scan 6687
Peptide PGGGNKKIETHKLTFRENAKAK Charge 5 Spectral Angle 0.7
Source WSoh_221121_011221_HFGoe_L2_hTau_24h_R1 Scan 12033
Peptide QSKCGSKDNIKHVPGGGSVQIVYK Charge 5 Spectral Angle 0.81
Source WSoh_221121_011221_HFGoe_L2_hTau_24h_R2 Scan 11713
Peptide GSLGNIHHKPGGGQVEVKSEKLDFKDR Charge 5 Spectral Angle 0.71
Experimental Spectrum
Prosit Predicted Spectrum
L² Normalized Intensity
m/z

**Source** WSoh_221121_011221_HFGoe_L2_hTau_24h_R1 **Scan** 23618
**Peptide** QTAPVPMPDL **Charge** 2 **Spectral Angle** 0.75

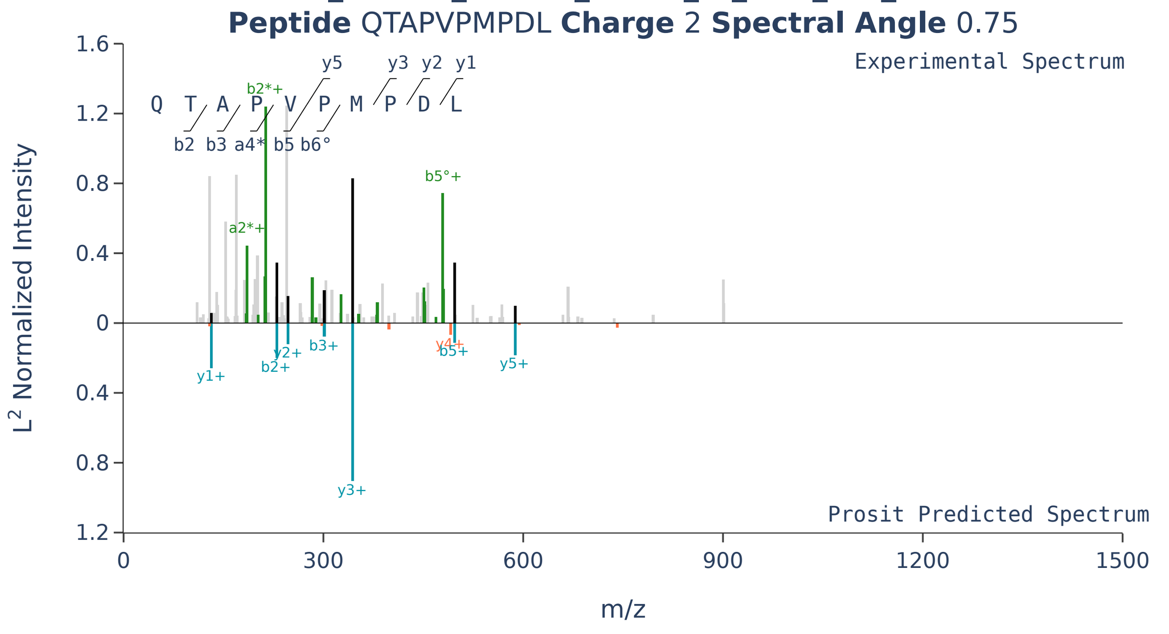


**Source** WSoh_221121_011221_HFGoe_L2_hTau_24h_R2 **Scan** 18796
**Peptide** LGDTPSLEDEAAGHVTQARMVSK **Charge** 3 **Spectral Angle** 0.68

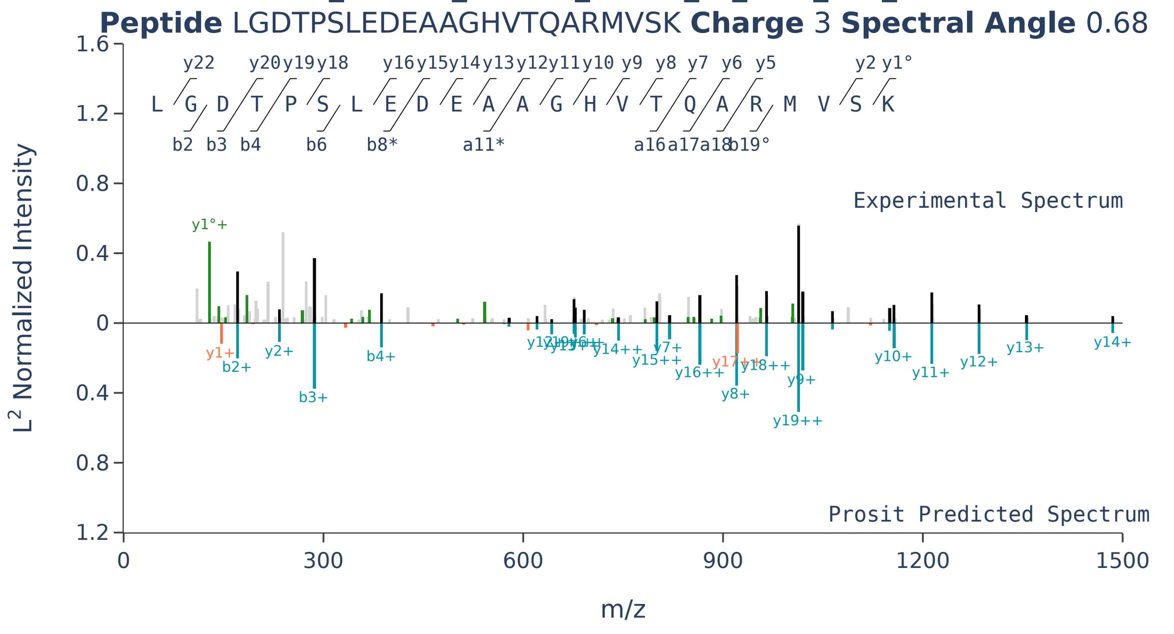


**Source** WSoh_221121_011221_HFGoe_L1_hTau_1h_R2 **Scan** 6320
**Peptide** CGSLGNIHHKPGGG **Charge** 3 **Spectral Angle** 0.68

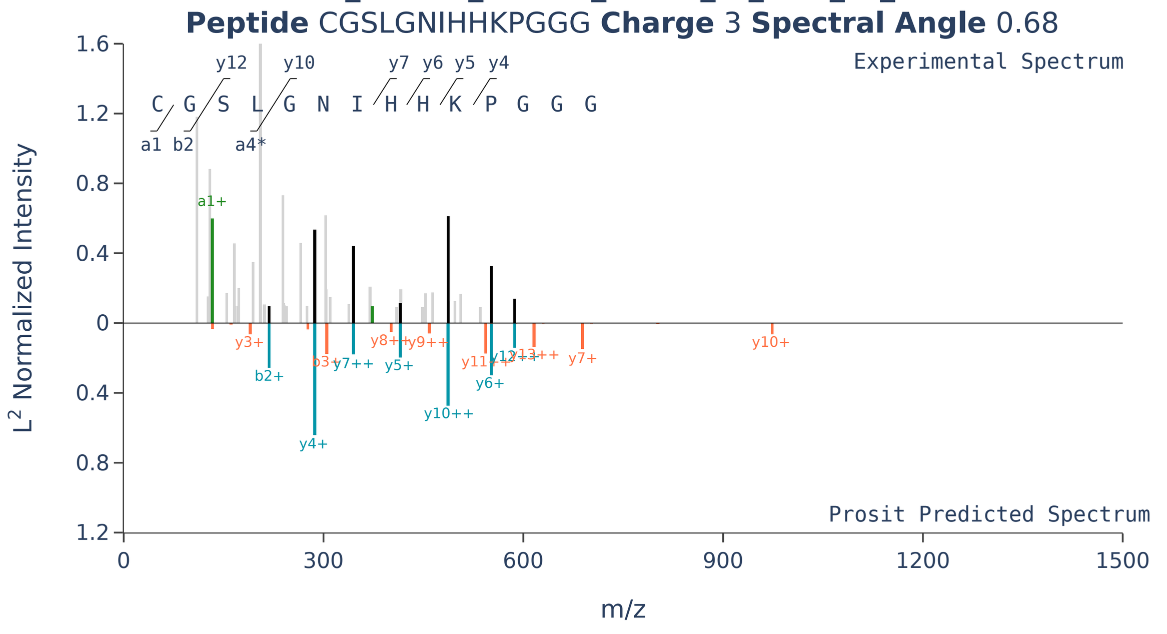


**Source** WSoh_221121_011221_HFGoe_L2_hTau_4h_R2 **Scan** 21854
**Peptide** KSRLQTAPVPMPDLKNVKSKIGSTENL **Charge** 5 **Spectral Angle** 0.76

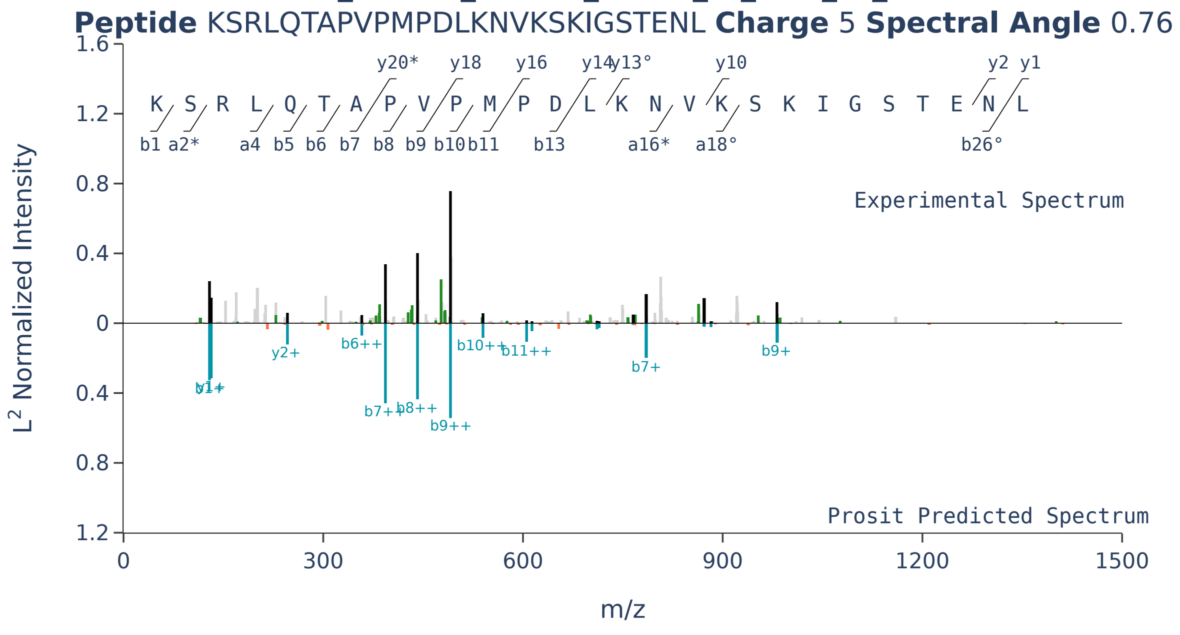


**Source** WSoh_221121_011221_HFGoe_L2_hTau_24h_R2 **Scan** 8225
**Peptide** HKLTFRENAKAKTDHGAEI **Charge** 3 **Spectral Angle** 0.69

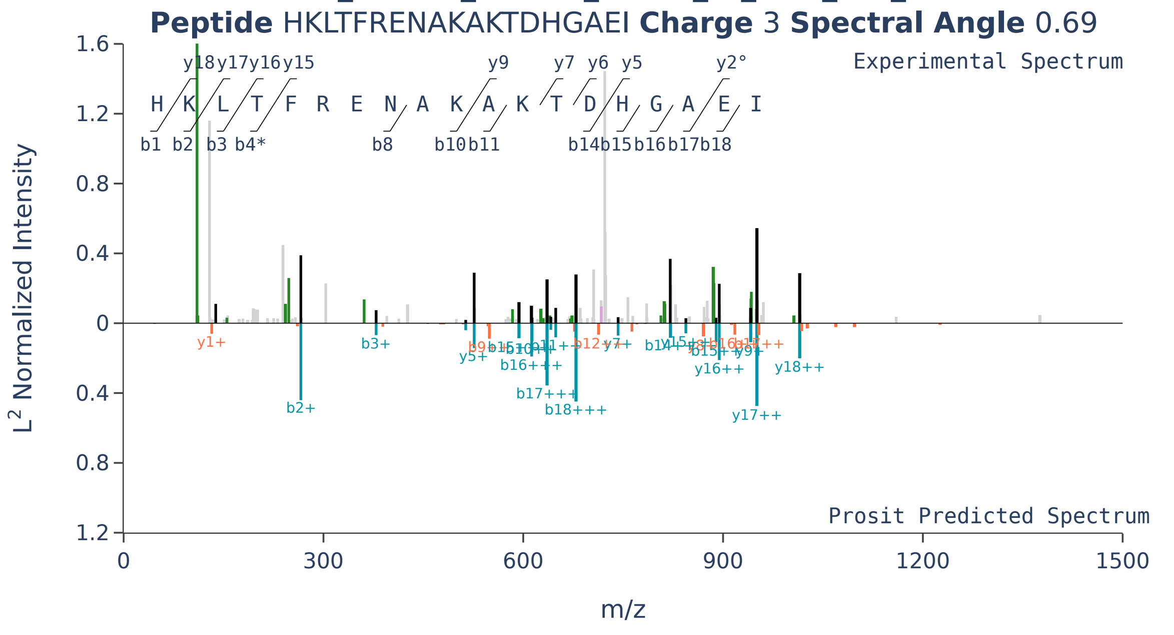


**Source** WSoh_221121_011221_HFGoe_L1_hTau_24h_R1 **Scan** 19183
**Peptide** AAQPHTEIPEGTTAEEAGIGDTPS **Charge** 2 **Spectral Angle** 0.9

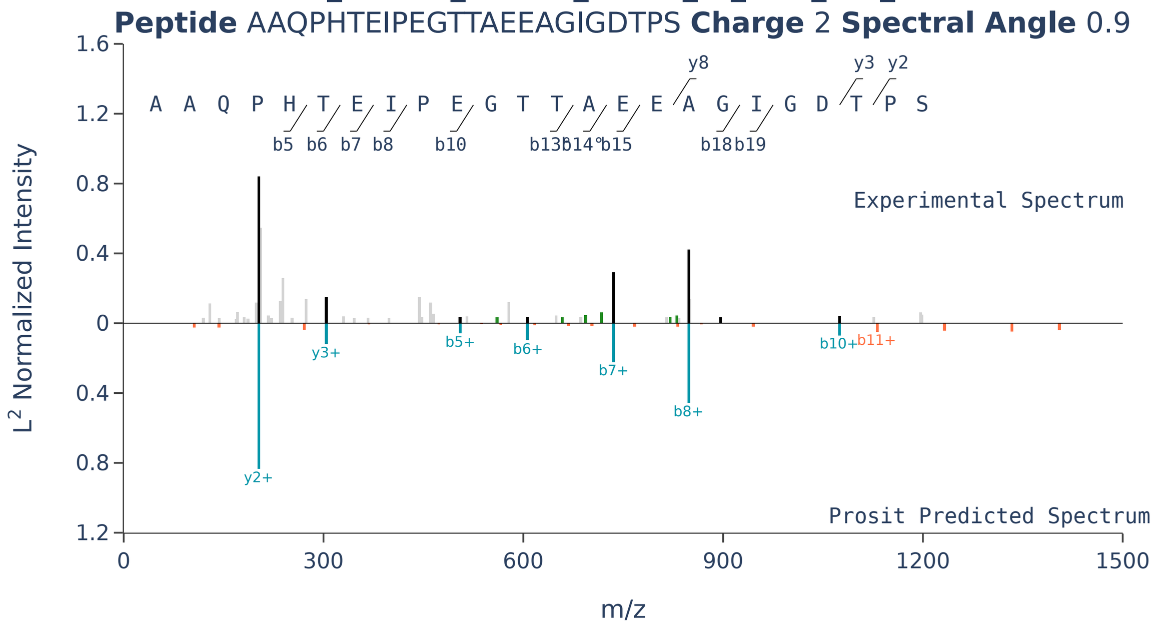


**Source** WSoh_221121_011221_HFGoe_L1_hTau_24h_R1 **Scan** 9456
**Peptide** APPGQKGQANATRIPAKTPPAPKT **Charge** 3 **Spectral Angle** 0.69

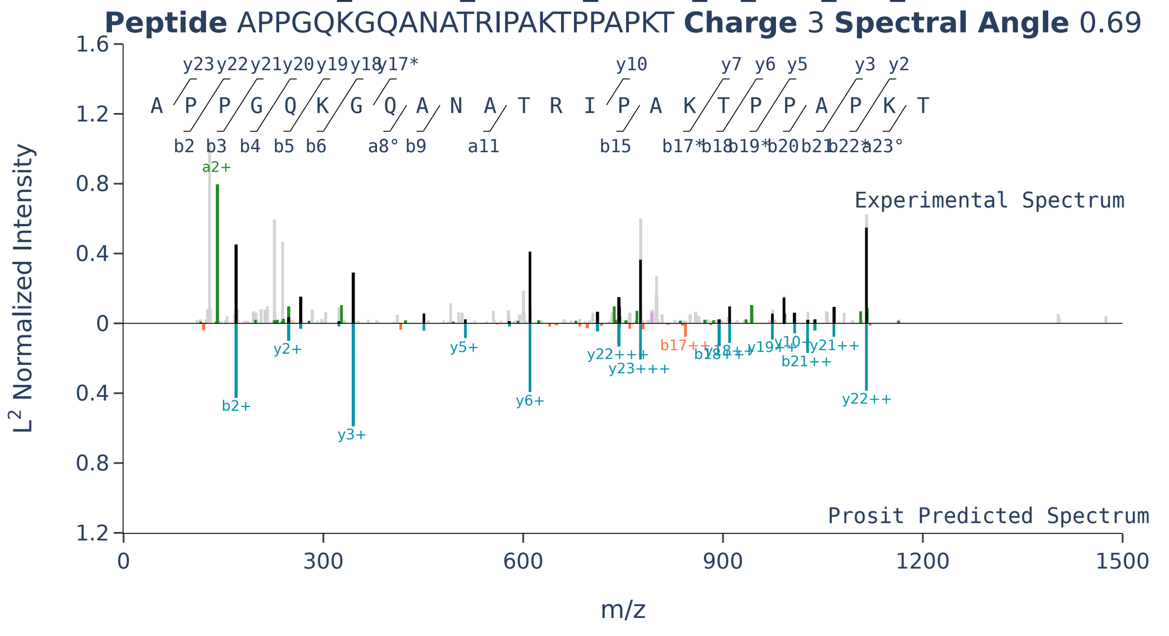


**Source** WSoh_221121_011221_HFGoe_L1_hTau_24h_R2 **Scan** 13203
**Peptide** RENAKAKTDHGAEIVYKSPVVSGDTSPRHL **Charge** 4 **Spectral Angle** 0.66

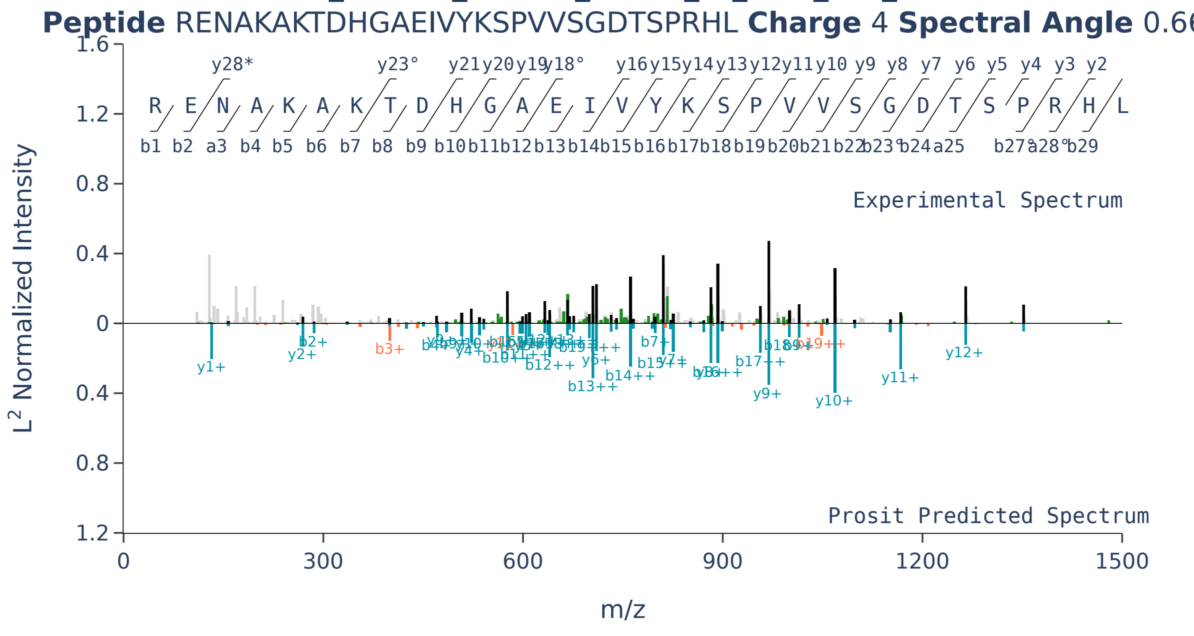


**Source** WSoh_221121_011221_HFGoe_L2_hTau_24h_R2 **Scan** 12137
**Peptide** KHQPGGGKVQIINKKLDLSNVQSKCGSKD **Charge** 6 **Spectral Angle** 0.83

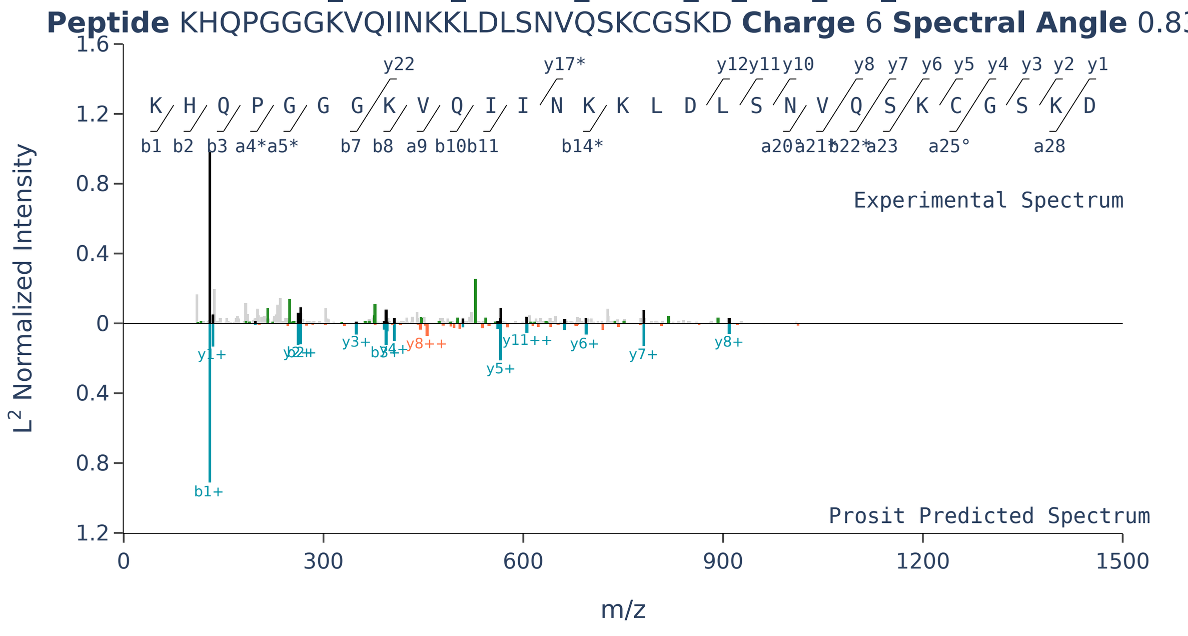


**Source** WSoh_221121_011221_HFGoe_L1_hTau_24h_R2 **Scan** 6071
**Peptide** EAAGHVTQA **Charge** 2 **Spectral Angle** 0.57

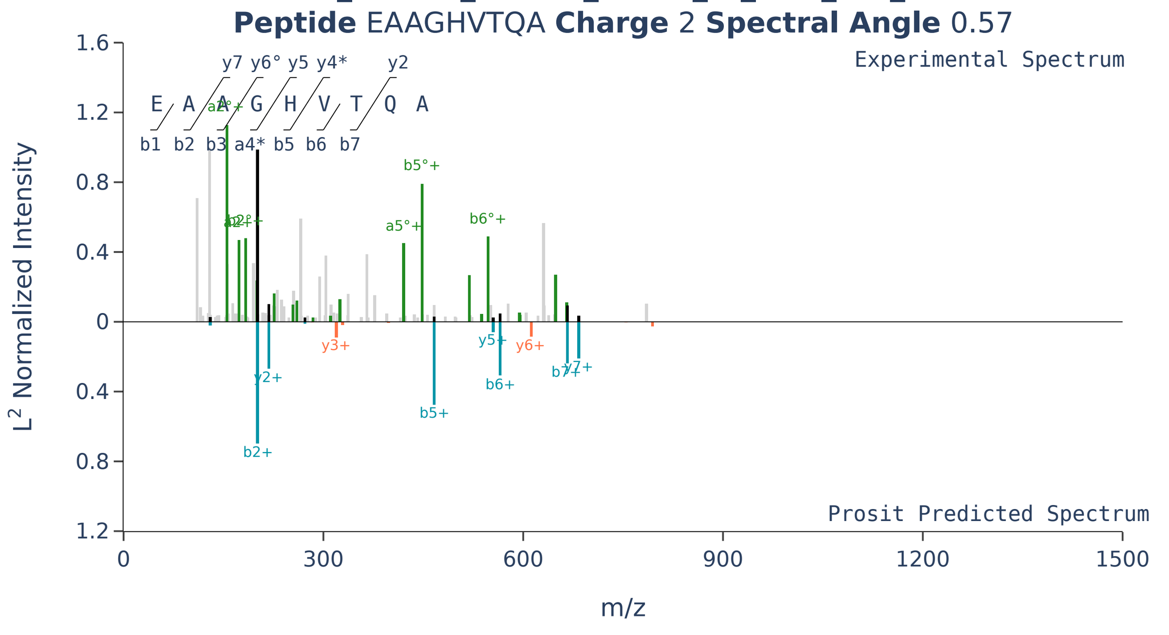


**Source** WSoh_221121_011221_HFGoe_L1_hTau_24h_R1 **Scan** 16351
**Peptide** HGAEIVYKSPVVSGDTSPRHLSNVSST **Charge** 4 **Spectral Angle** 0.67

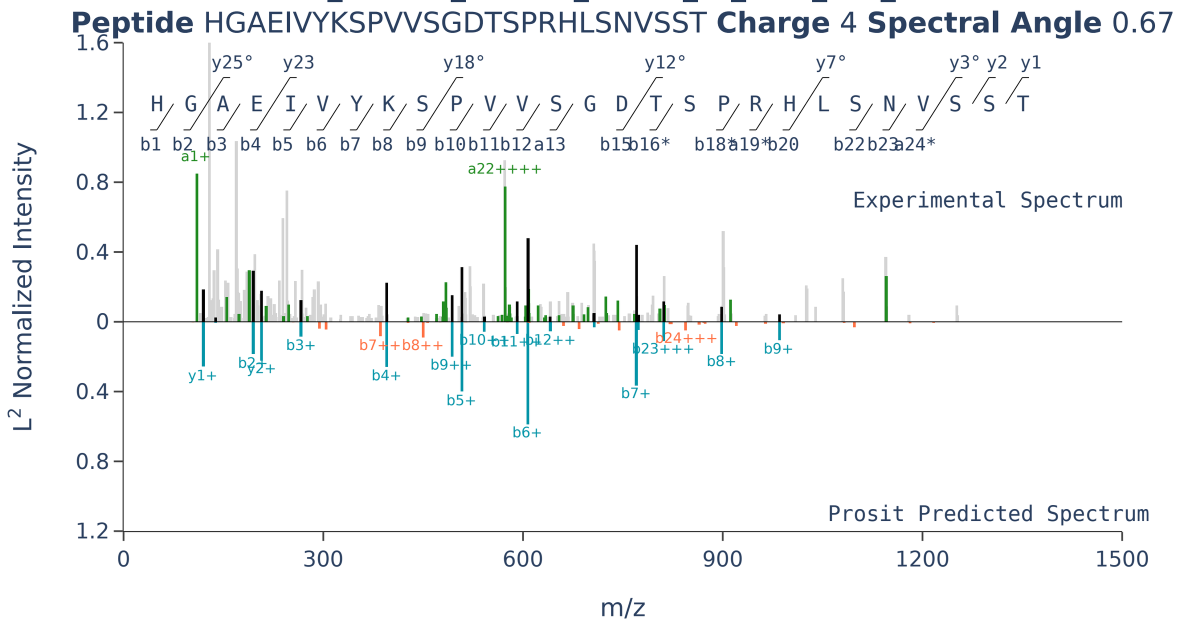


**Source** WSoh_221121_011221_HFGoe_L2_hTau_24h_R1 **Scan** 13787
**Peptide** LGSLDNITHVPGGGNKKIETHK **Charge** 4 **Spectral Angle** 0.68

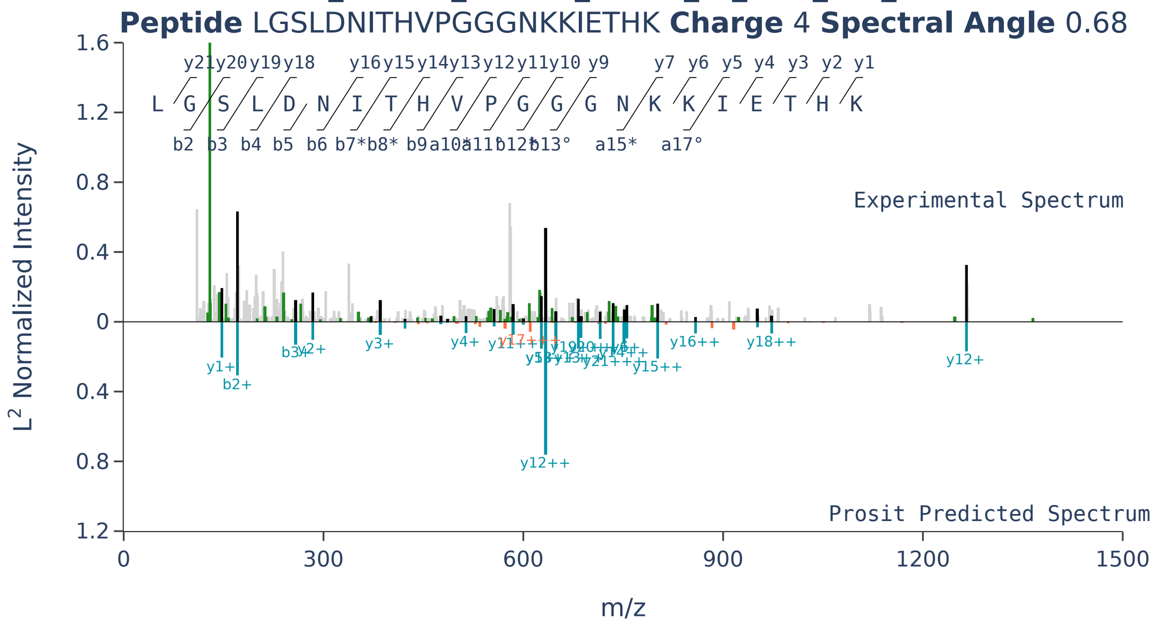


**Source** WSoh_221121_011221_HFGoe_L2_hTau_24h_R1 **Scan** 10546
**Peptide** IPAKTPPAPKTPPSSGEPPKS **Charge** 4 **Spectral Angle** 0.85

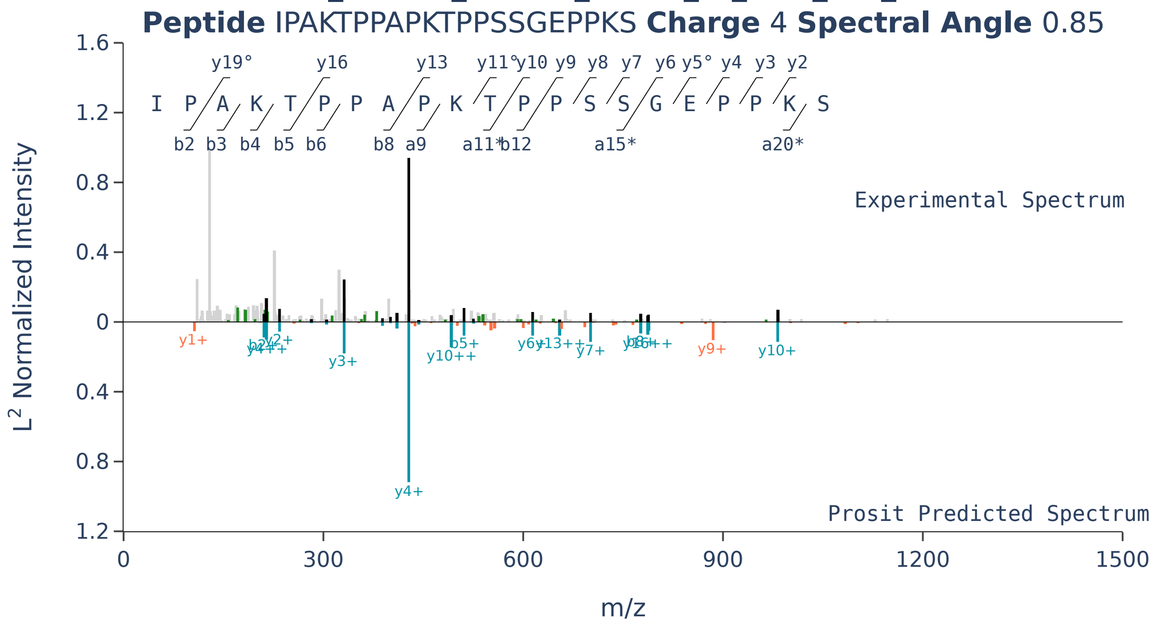


**Source** WSoh_221121_011221_HFGoe_L1_hTau_24h_R2 **Scan** 21837
**Peptide** RLQTAPVPMPDLKNVKSKLG **Charge** 3 **Spectral Angle** 0.81

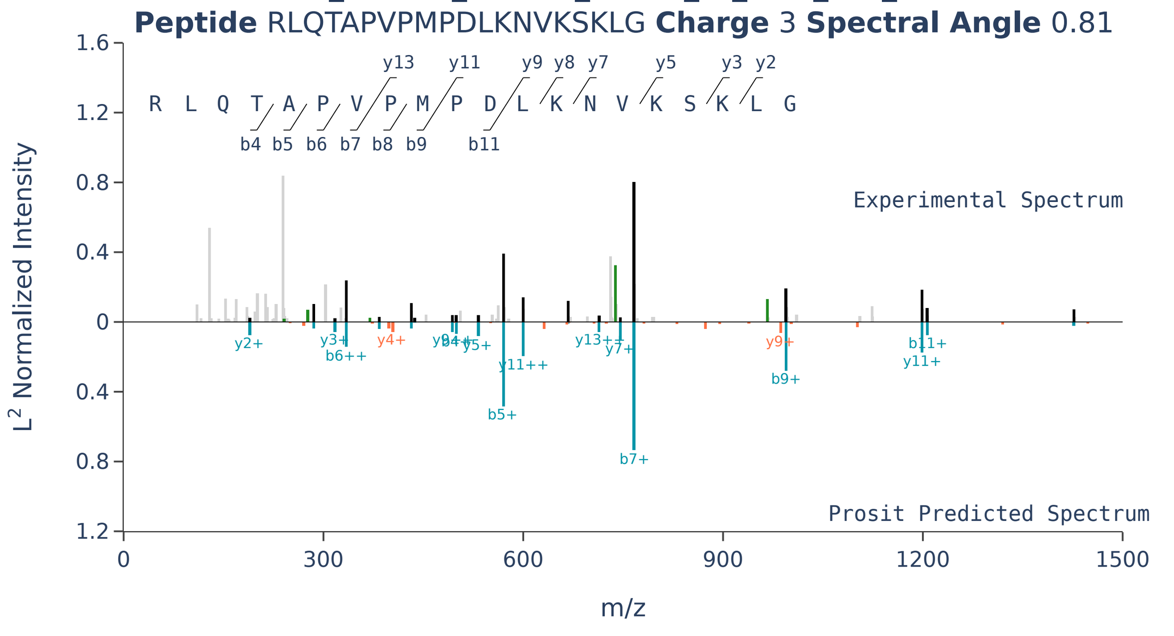


**Source** WSoh_221121_011221_HFGoe_L1_hTau_4h_R2 **Scan** 12926
**Peptide** GSEEPGSETSDAKSTPTAEDVTA **Charge** 2 **Spectral Angle** 0.76

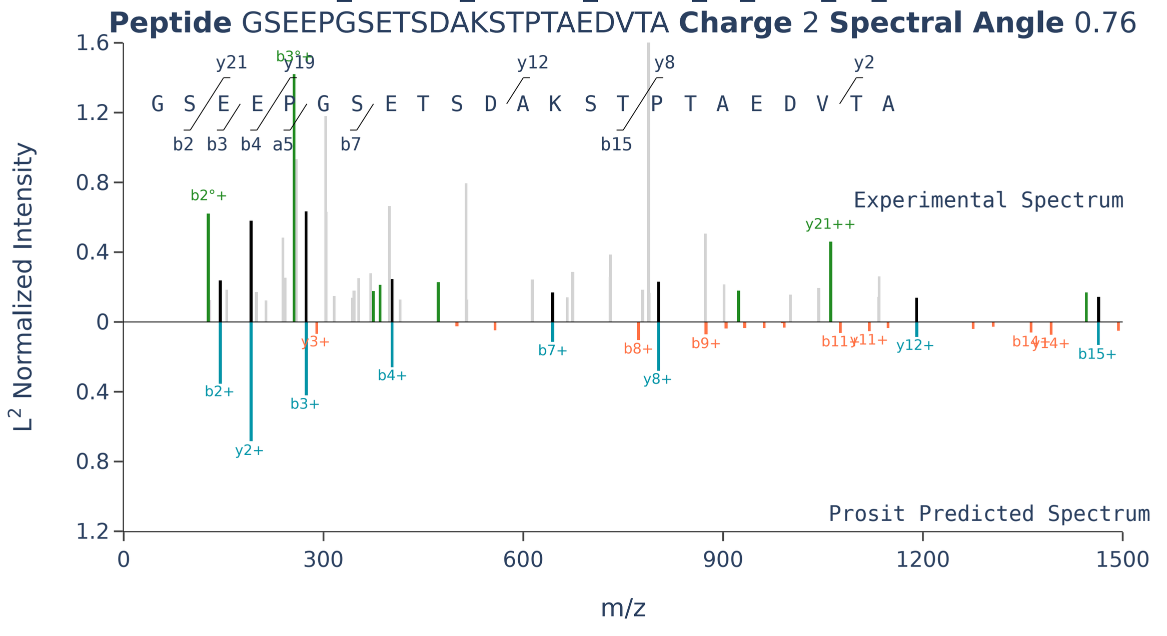

**Source** WSoh_221121_011221_HFGoe_L2_hTau_4h_R1 **Scan** 16971
**Peptide** KLGSTENLKHQPGGGKVQIINKKLDLSNV **Charge** 4 **Spectral Angle** 0.87

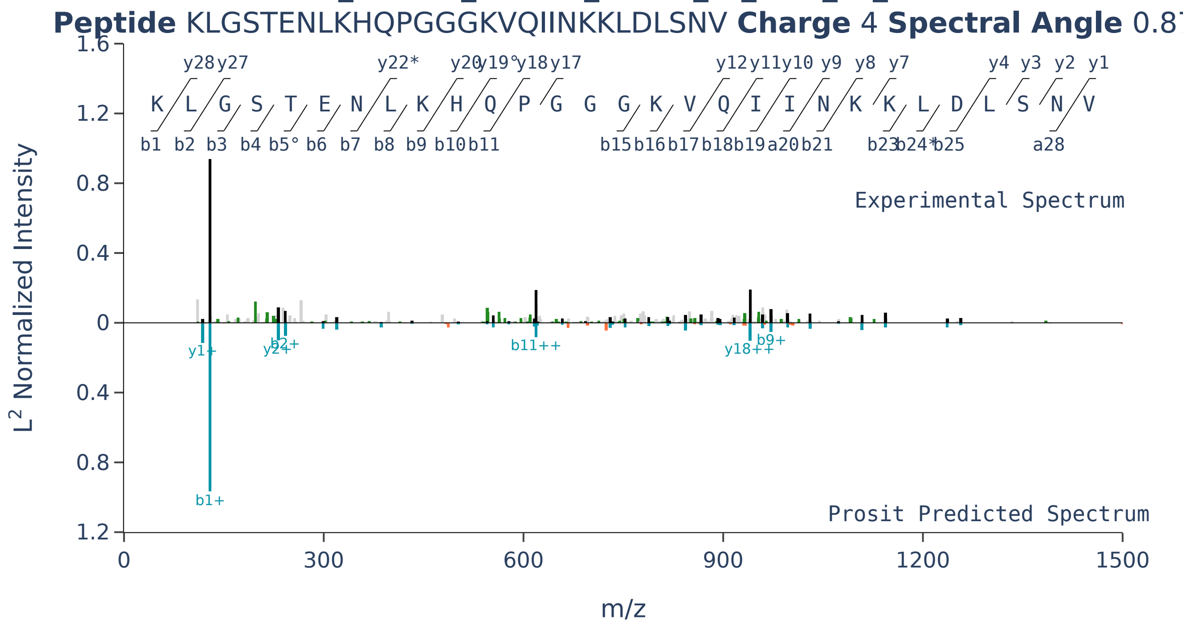


**Source** WSoh_221121_011221_HFGoe_L1_hTau_4h_R2 **Scan** 12743
**Peptide** GNIHHKPGGGQVEVKSEKLDFKDRVQSKL **Charge** 5 **Spectral Angle** 0.66

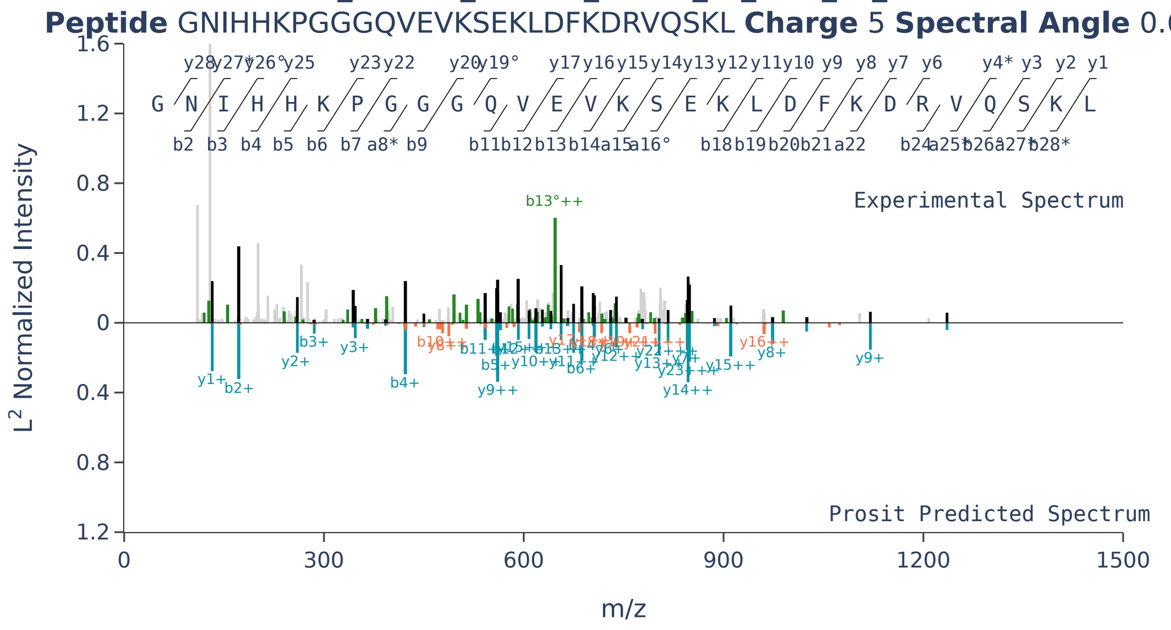


**Source** WSoh_221121_011221_HFGoe_L1_hTau_24h_R1 **Scan** 19721
**Peptide** AEIVYKSPVVSGDTSPRHLSNVSSTGSID **Charge** 4 **Spectral Angle** 0.85

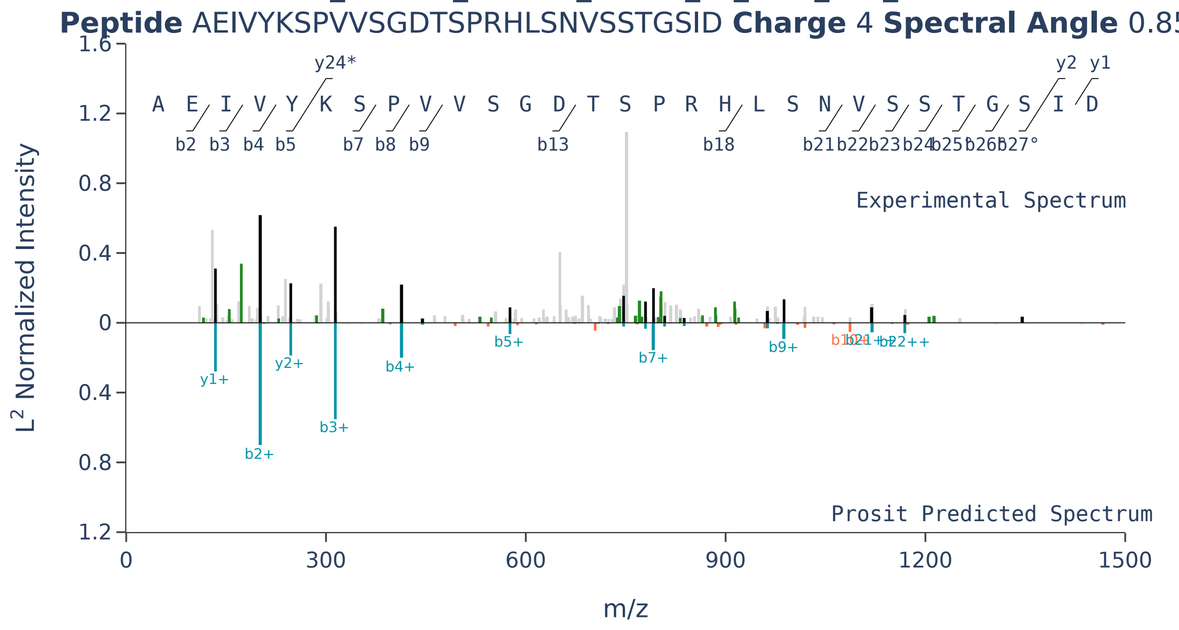


**Source** WSoh_221121_011221_HFGoe_L2_hTau_24h_R2 **Scan** 17065
**Peptide** KLGSLDNITHVPGGGNKKIETHKLTFRE **Charge** 5 **Spectral Angle** 0.72

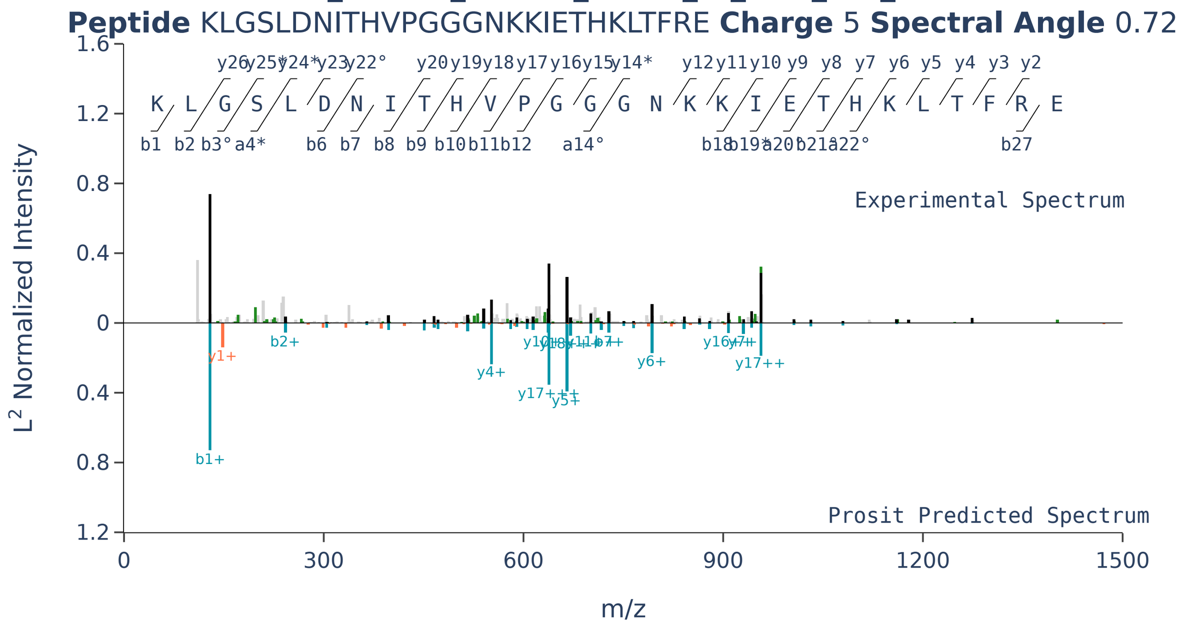


**Source** WSoh_221121_011221_HFGoe_L2_hTau_24h_R2 **Scan** 23015
**Peptide** VPMPDLKNVKSKIGSTENL **Charge** 3 **Spectral Angle** 0.79

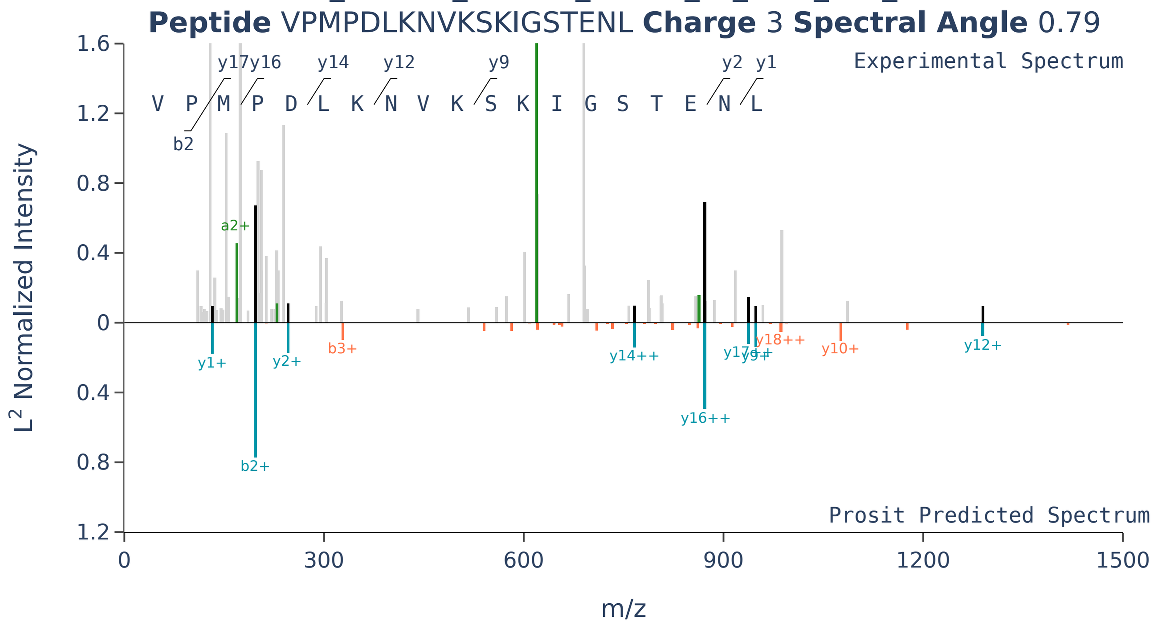


**Source** WSoh_221121_011221_HFGoe_L2_hTau_4h_R2 **Scan** 11832
**Peptide** PGKQAAAQPHTEIPEGTTAEE **Charge** 3 **Spectral Angle** 0.62

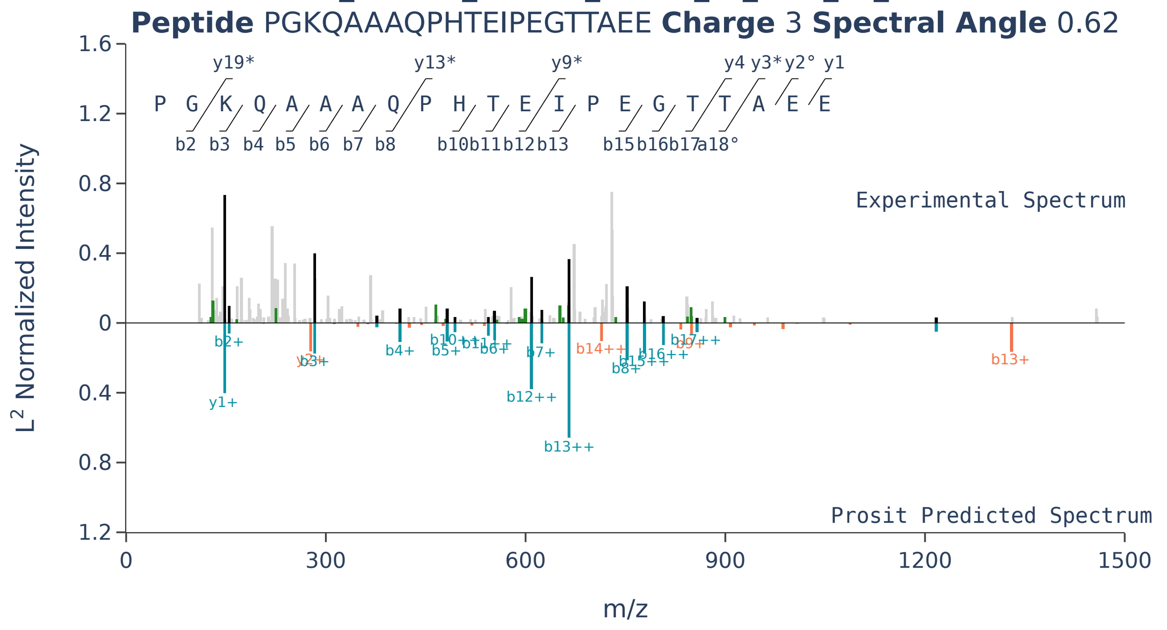


**Source** WSoh_221121_011221_HFGoe_L2_hTau_24h_R2 **Scan** 21416
**Peptide** EEAGIGDTPSLEDEAAGHVTQ **Charge** 2 **Spectral Angle** 0.68

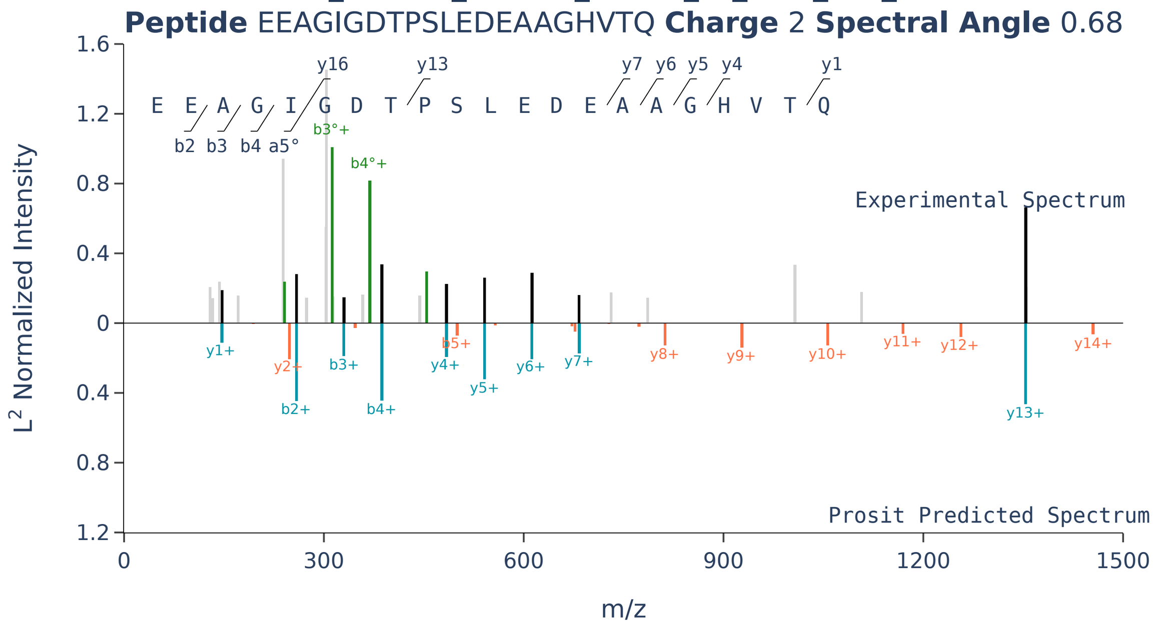


**Source** WSoh_221121_011221_HFGoe_L1_hTau_24h_R1 **Scan** 21350
**Peptide** IGDTPSLEDEAAGHVTQARMVS **Charge** 3 **Spectral Angle** 0.67

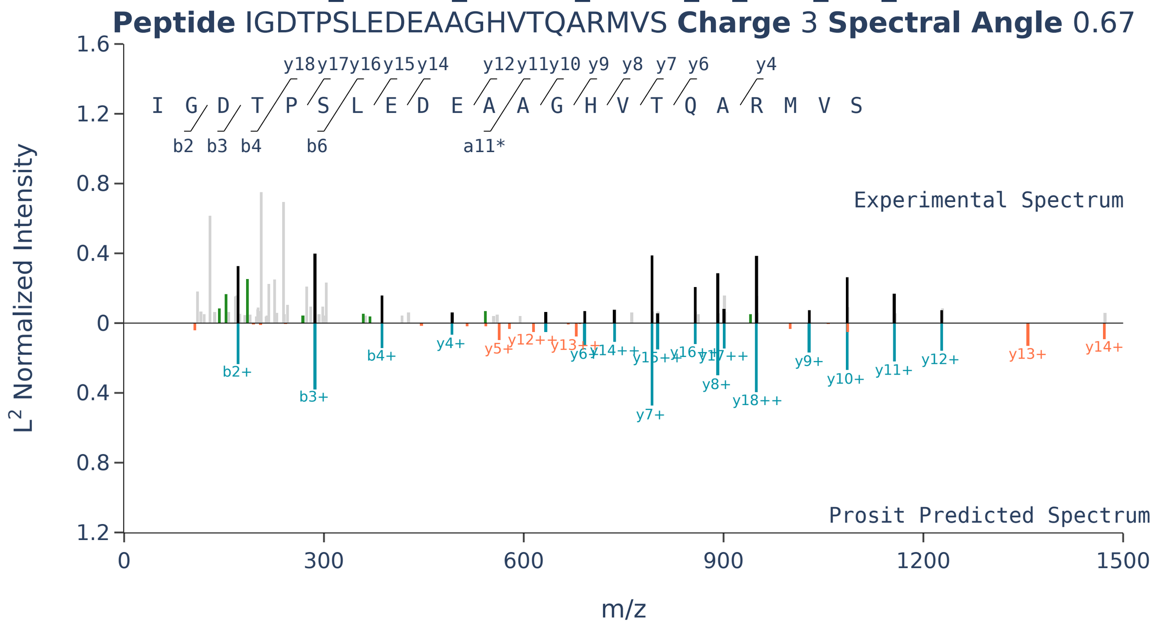


**Source** WSoh_221121_011221_HFGoe_L1_hTau_24h_R1 **Scan** 25170
**Peptide** PMPDLKNV **Charge** 2 **Spectral Angle** 0.82

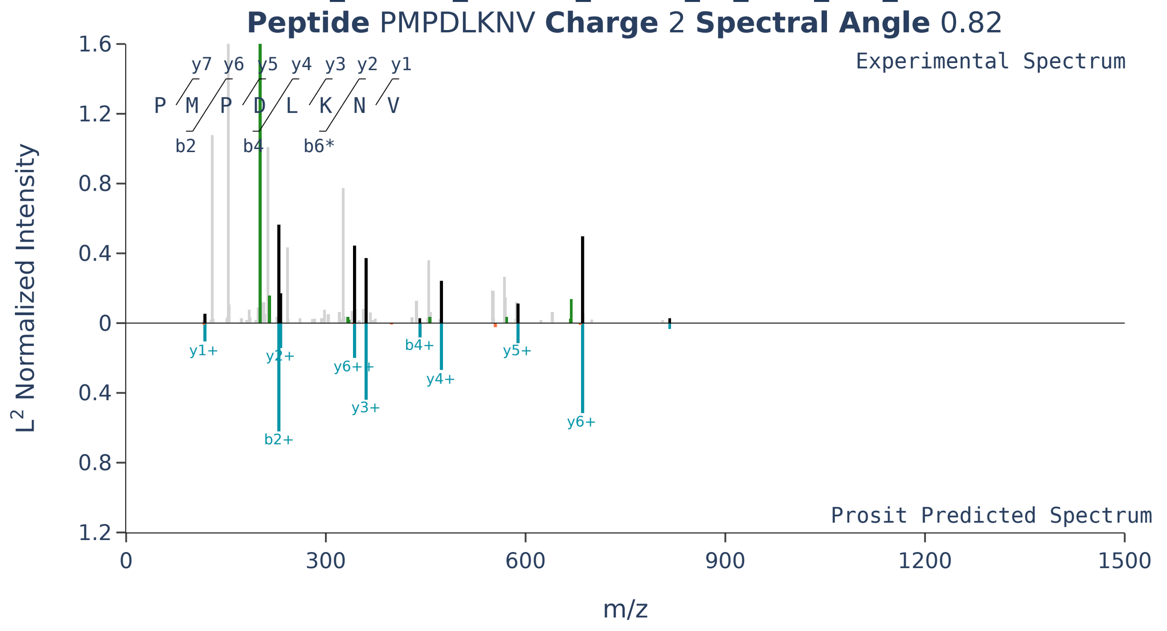


**Source** WSoh_221121_011221_HFGoe_L2_hTau_24h_R1 **Scan** 10197
**Peptide** LKNVKSKIGSTENL **Charge** 3 **Spectral Angle** 0.79

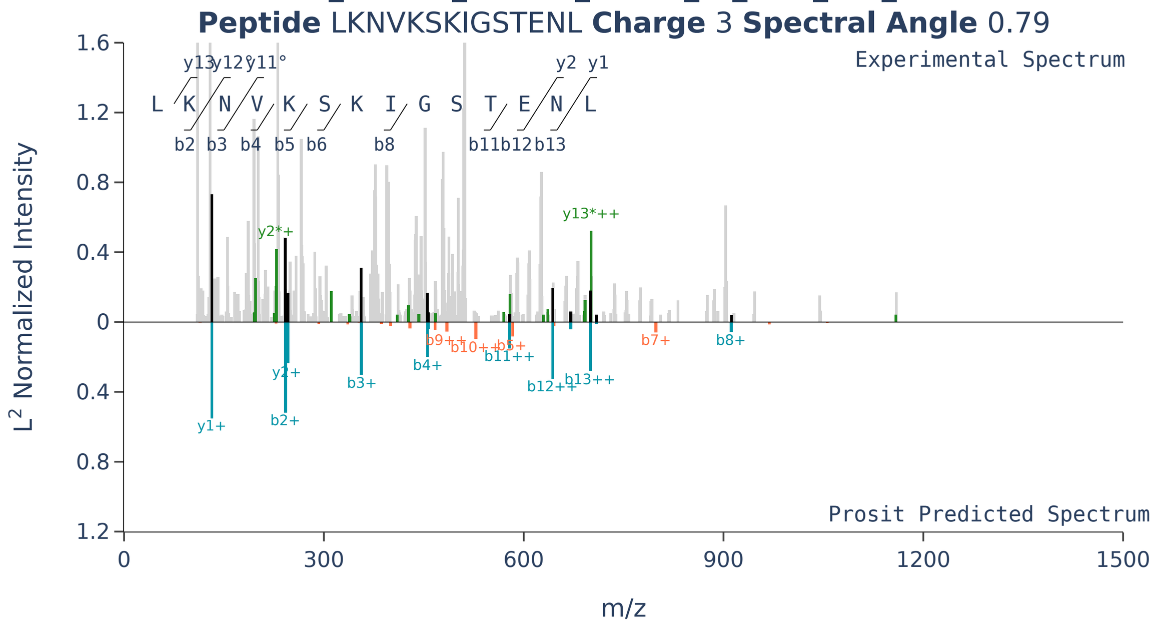


**Source** WSoh_221121_011221_HFGoe_L2_hTau_24h_R2 **Scan** 20258
**Peptide** TTAEEAGIGDTPSLEDEAAGHVTQAR **Charge** 3 **Spectral Angle** 0.69

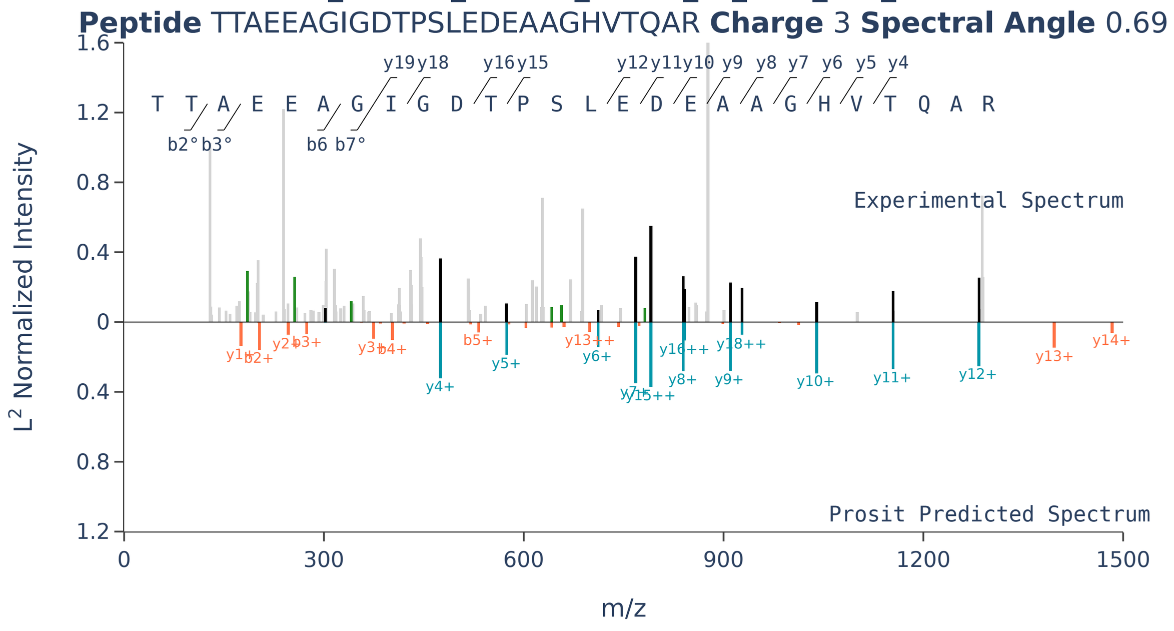


**Source** WSoh_221121_011221_HFGoe_L2_hTau_2h_R2 **Scan** 11544
**Peptide** NKKLDLSNVQSKCGSKDNIKHVPGGGSV **Charge** 4 **Spectral Angle** 0.86

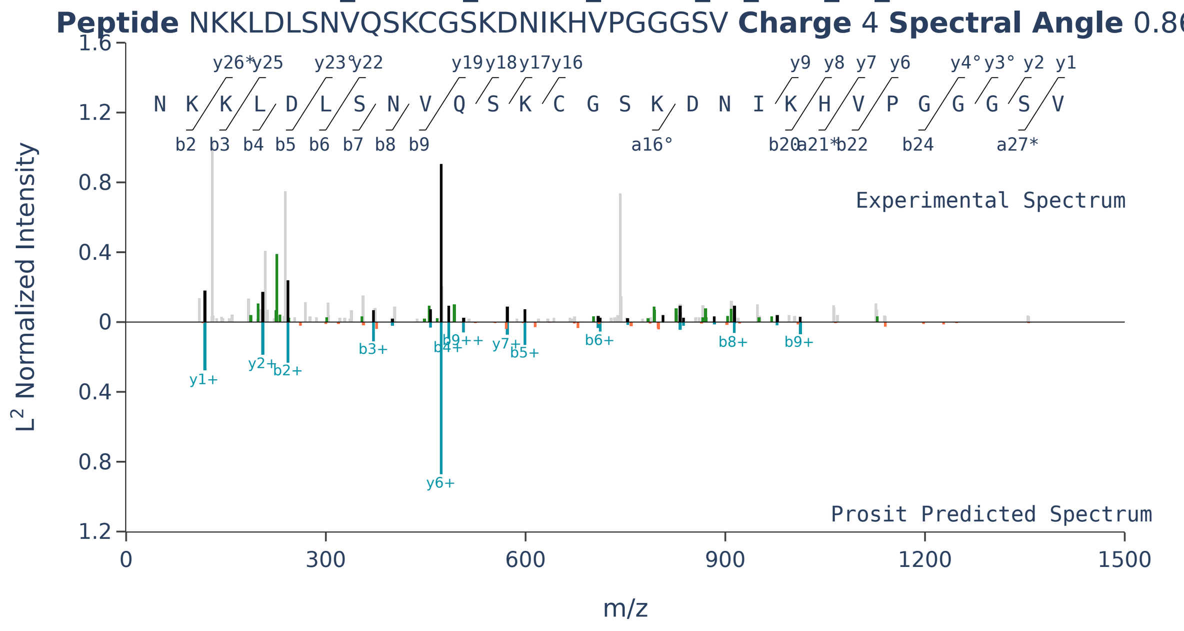


**Source** WSoh_221121_011221_HFGoe_L2_hTau_4h_R1 **Scan** 12502
**Peptide** SLGNIHHKPGGGQVEVKSEKLDFKD **Charge** 4 **Spectral Angle** 0.73

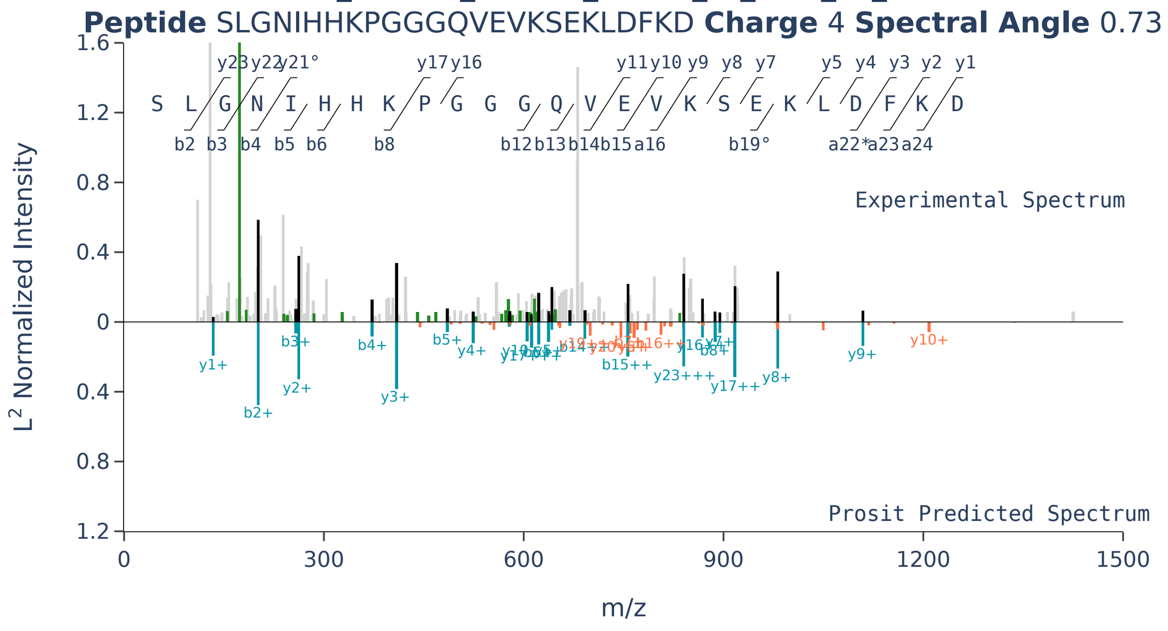


**Source** WSoh_221121_011221_HFGoe_L2_hTau_24h_R1 **Scan** 12817
**Peptide** GSLGNIHHKPGGGQVEVKSEKLDFKDRVQS **Charge** 3 **Spectral Angle** 0.69

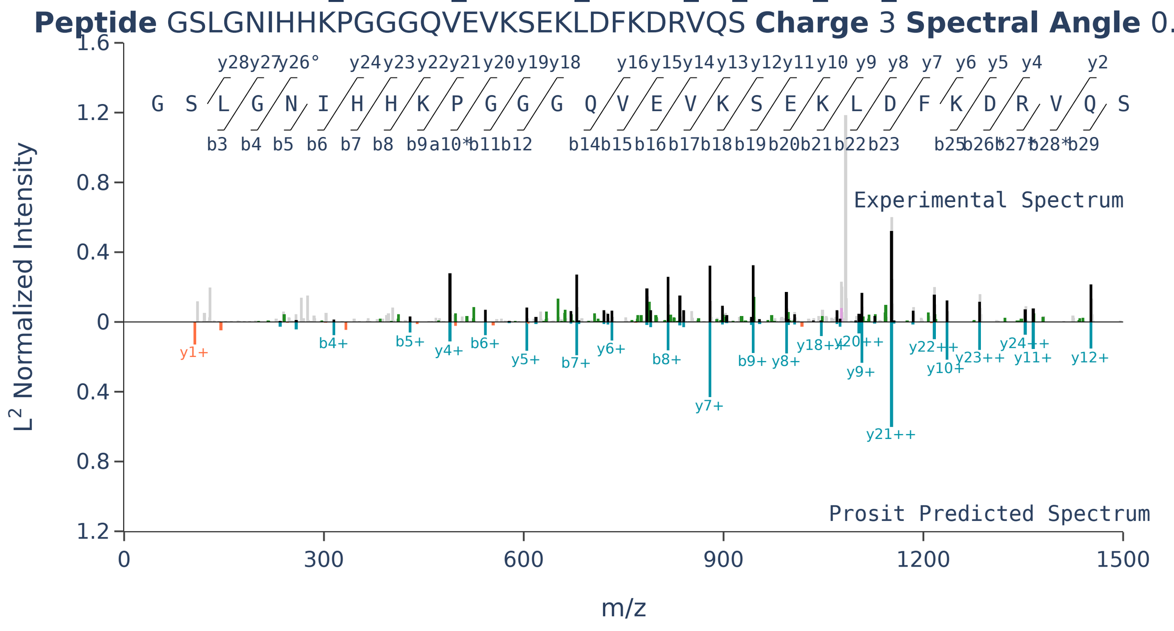


**Source** WSoh_221121_011221_HFGoe_L1_hTau_4h_R1 **Scan** 13037
**Peptide** HKLTFRENAKAKTDHGAEIVYKSPVVS **Charge** 4 **Spectral Angle** 0.65

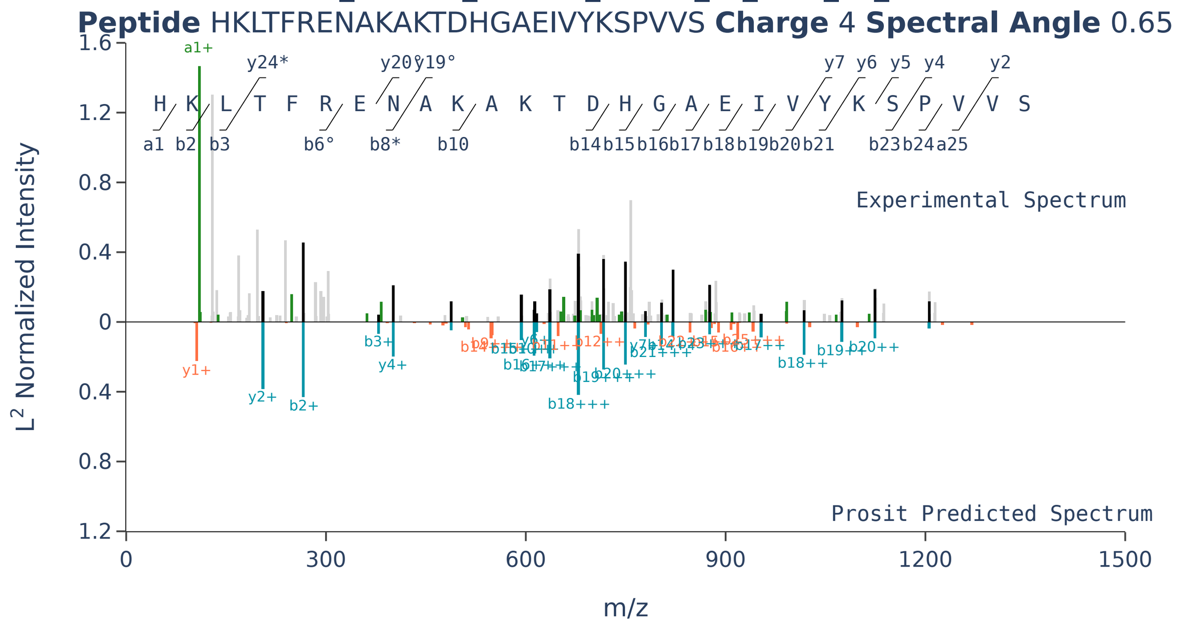

**Source** WSoh_221121_011221_HFGoe_L2_hTau_24h_R2 **Scan** 10069
**Peptide** ANATRIPAKTPPAPKTPPSSGEPPKSGDR **Charge** 4 **Spectral Angle** 0.7

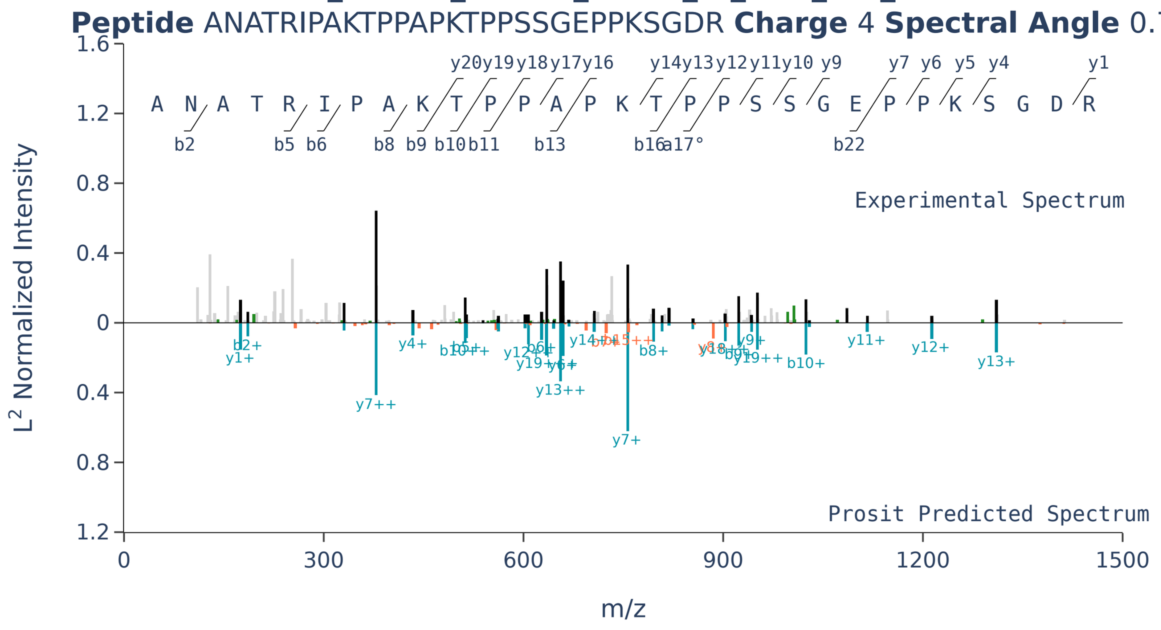


**Source** WSoh_221121_011221_HFGoe_L2_hTau_4h_R1 **Scan** 19488
**Peptide** LGDTPSLEDEAAGHVTQA **Charge** 3 **Spectral Angle** 0.65

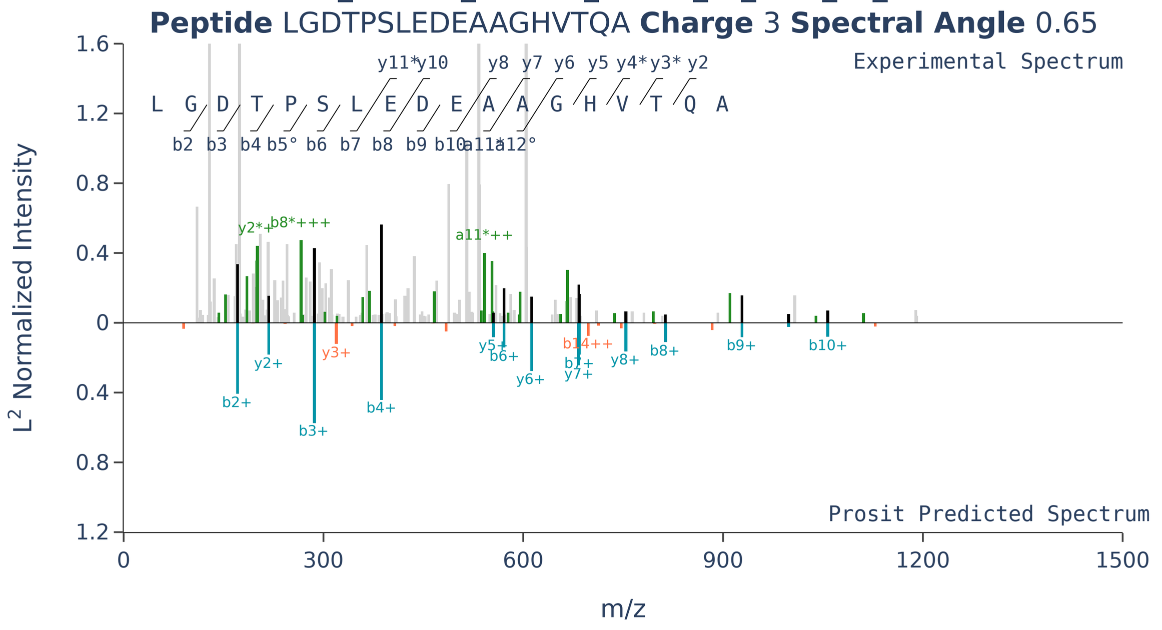


**Source** WSoh_221121_011221_HFGoe_L2_hTau_2h_R1 **Scan** 18948
**Peptide** YKPVDLSKVTSKCGSL **Charge** 3 **Spectral Angle** 0.78

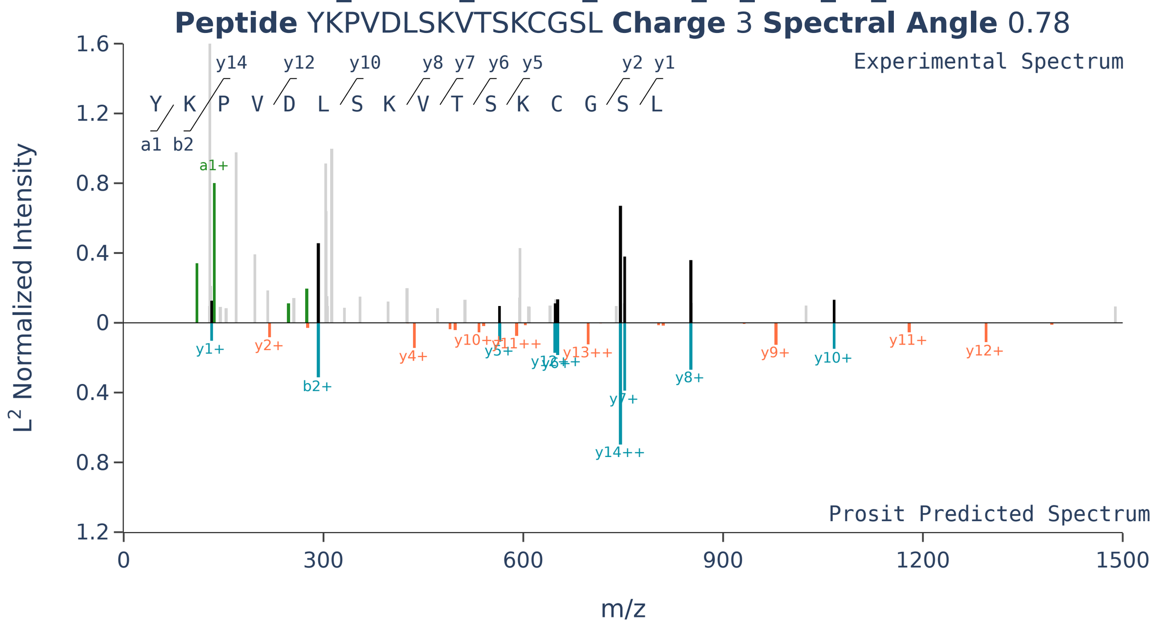


**Source** WSoh_221121_011221_HFGoe_L1_hTau_24h_R2 **Scan** 8701
**Peptide** LSKVTSKCGSLGNIHHKPGGGQVEVKSE **Charge** 4 **Spectral Angle** 0.74

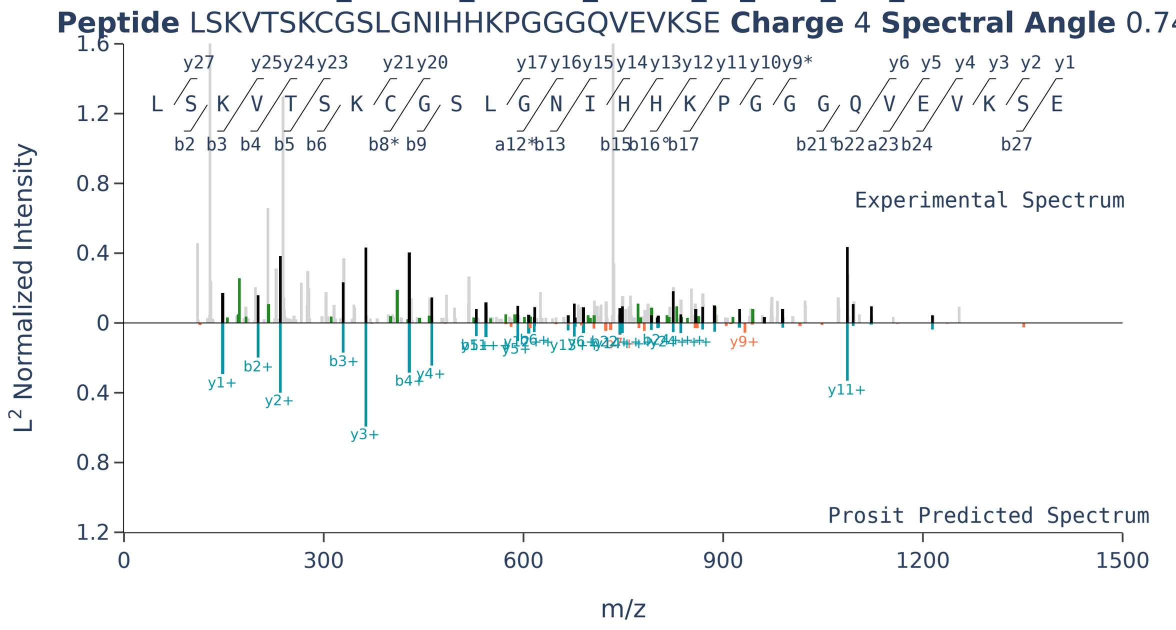


**Source** WSoh_221121_011221_HFGoe_L2_hTau_1h_R1 **Scan** 11794
**Peptide** AAAQPHTEIPEGTT **Charge** 2 **Spectral Angle** 0.7

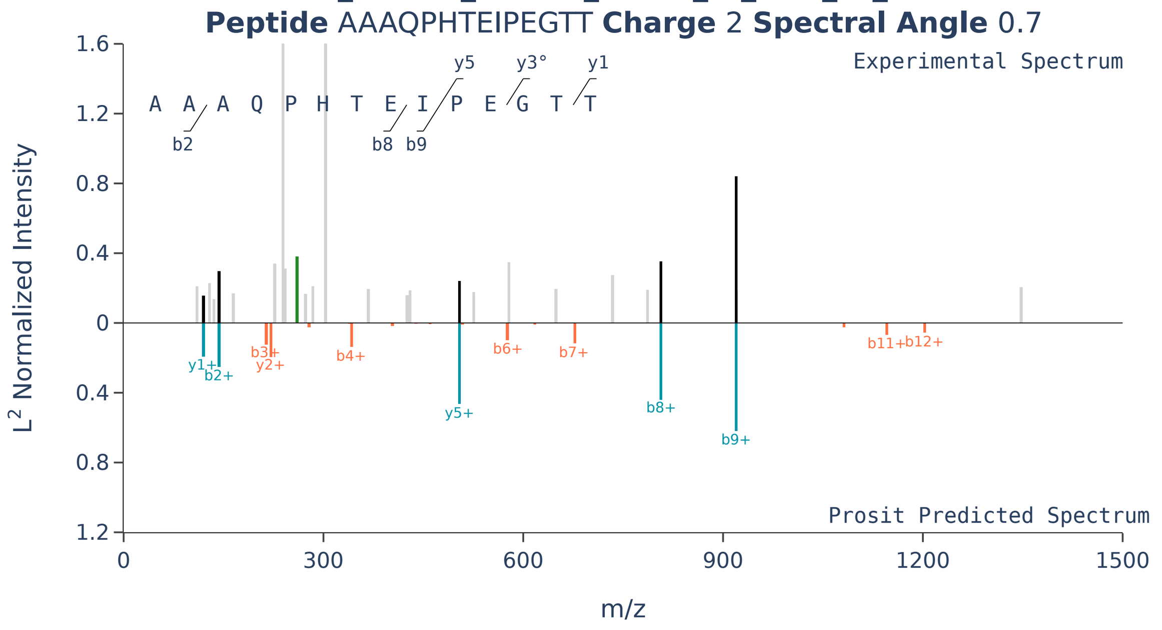


**Source** WSoh_221121_011221_HFGoe_L1_hTau_24h_R2 **Scan** 19504
**Peptide** PPKSPSSAKSRLQTAPVPMPDLKNVKSKI **Charge** 5 **Spectral Angle** 0.65

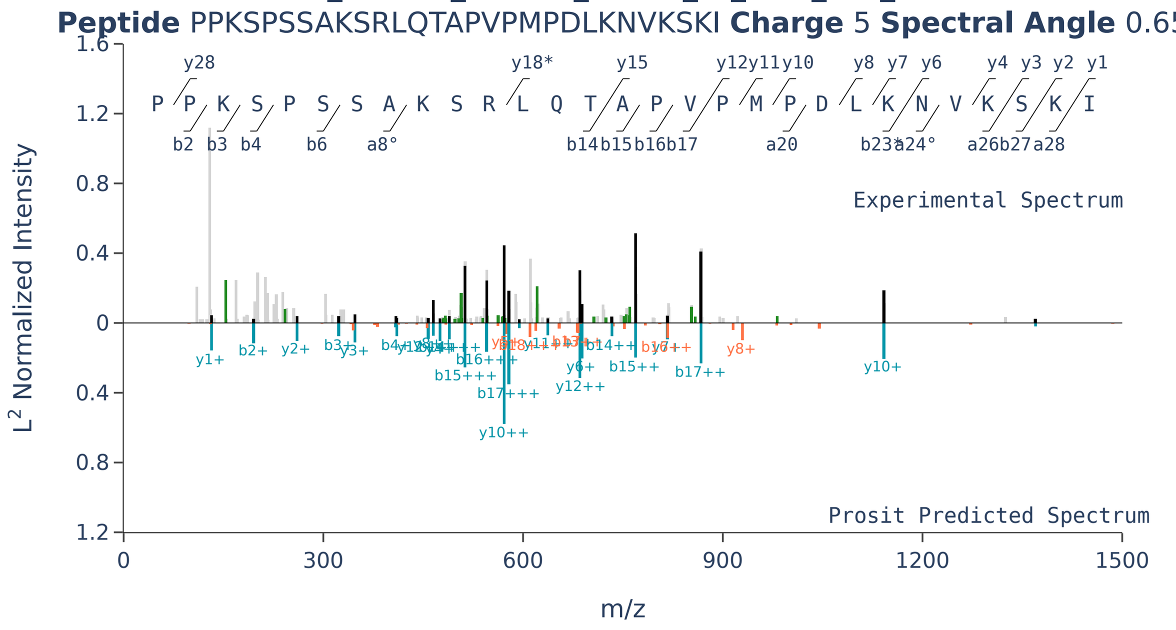


**Source** WSoh_221121_011221_HFGoe_L1_hTau_24h_R2 **Scan** 20417
**Peptide** GAEIVYKSPVVSGDTSPRHLSNVSSTGSID **Charge** 4 **Spectral Angle** 0.89

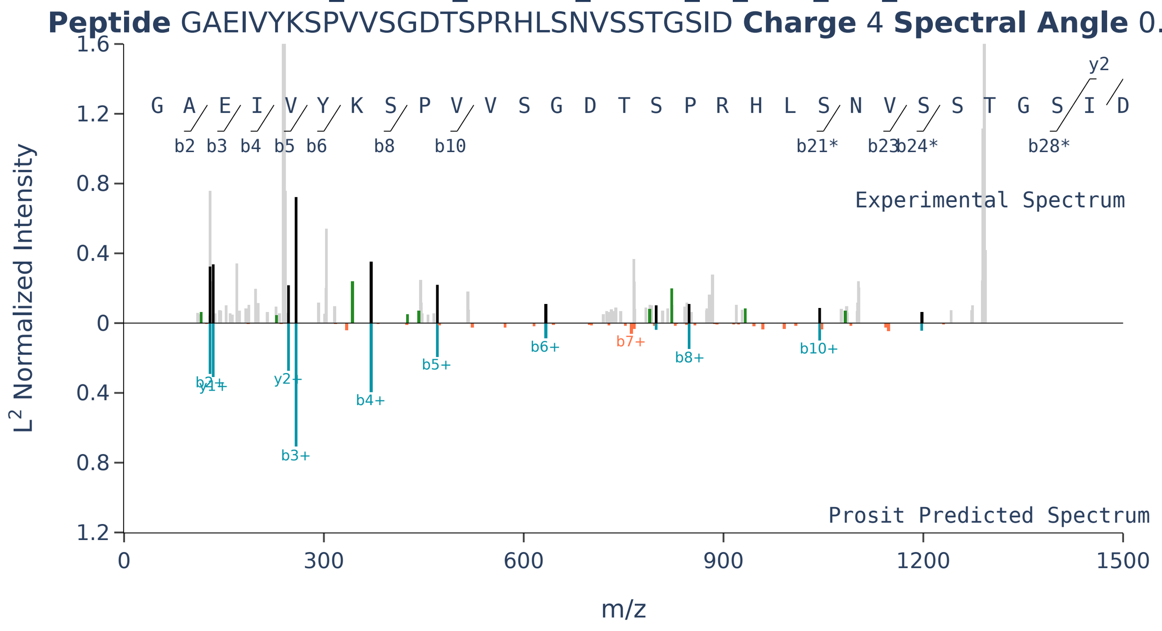


**Source** WSoh_221121_011221_HFGoe_L1_hTau_24h_R2 **Scan** 13508
**Peptide** VMEDHAGTYGLGDRKDQGGYTMHQDQEGD **Charge** 4 **Spectral Angle** 0.73

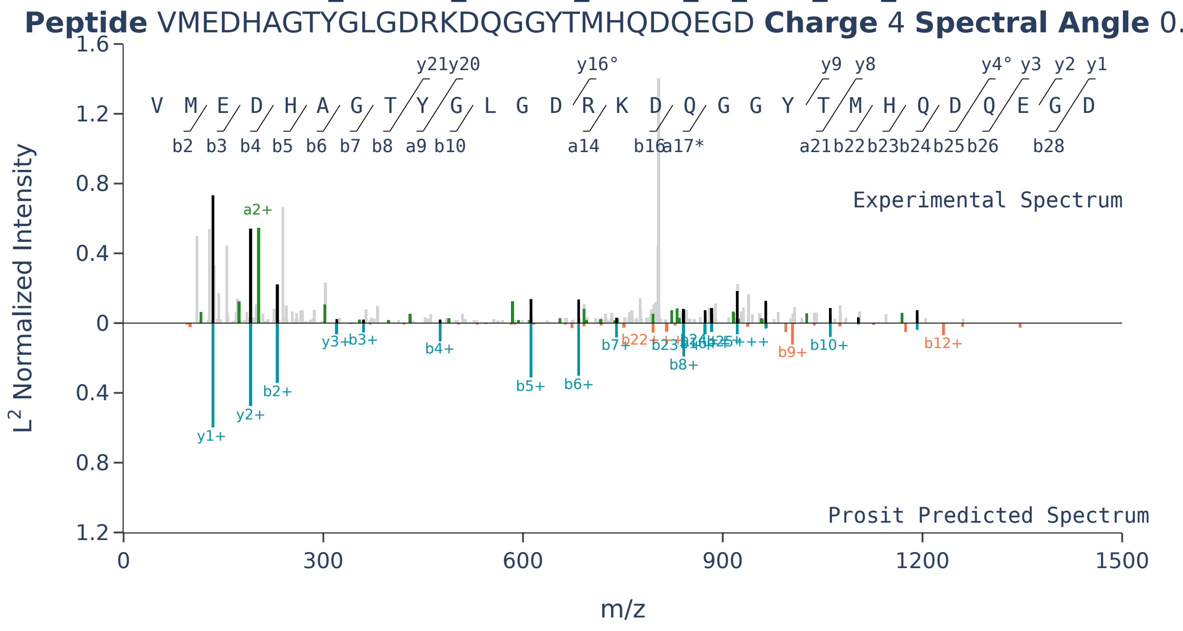


**Source** WSoh_221121_011221_HFGoe_L2_hTau_24h_R1 **Scan** 10588
**Peptide** LDLSNVQSKCGSKDNLKH **Charge** 4 **Spectral Angle** 0.92

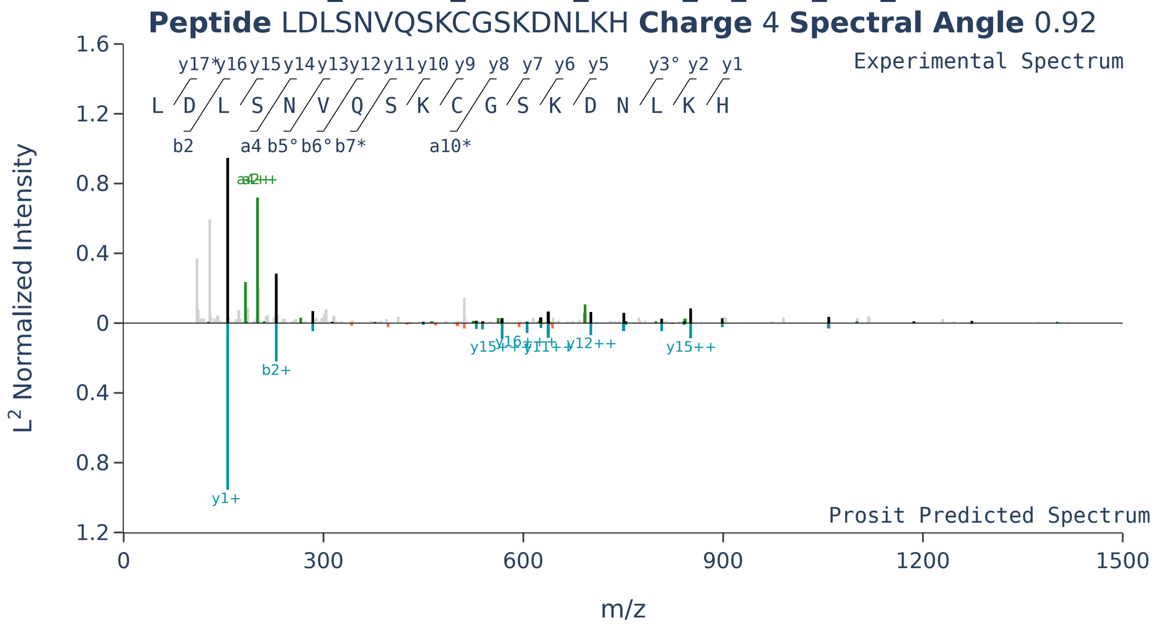


**Source** WSoh_221121_011221_HFGoe_L2_hTau_1h_R2 **Scan** 9895
**Peptide** DNIKHVPGGGSV **Charge** 2 **Spectral Angle** 0.81

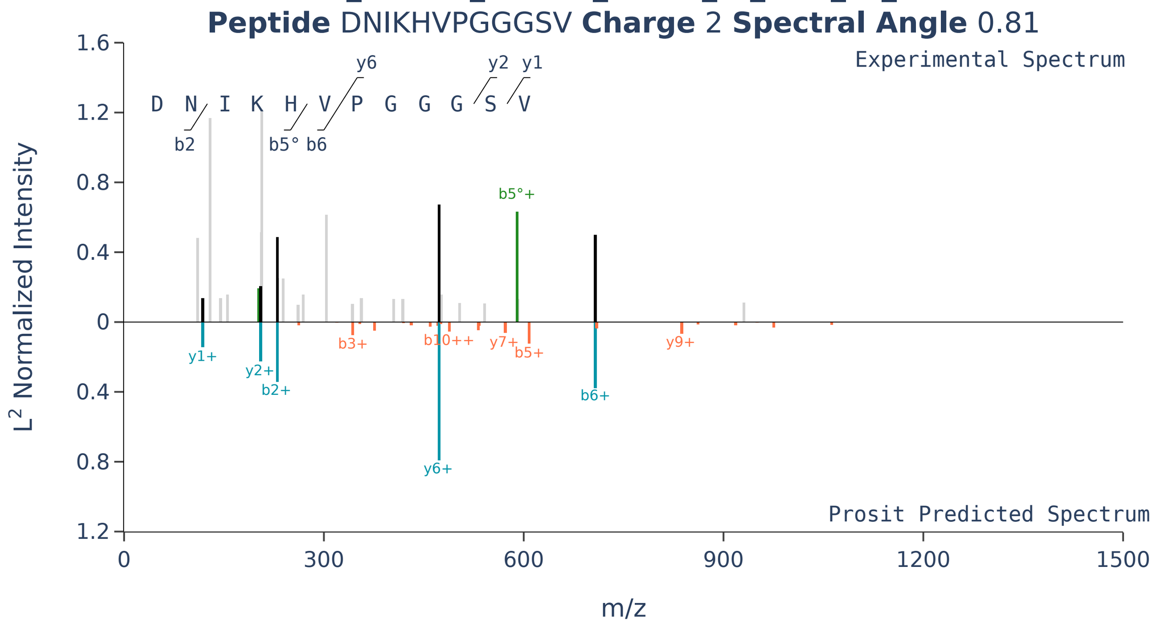


**Source** WSoh_221121_011221_HFGoe_L1_hTau_4h_R2 **Scan** 20936
**Peptide** RLQTAPVPMPDLKNVKSKIGSTEN **Charge** 4 **Spectral Angle** 0.85

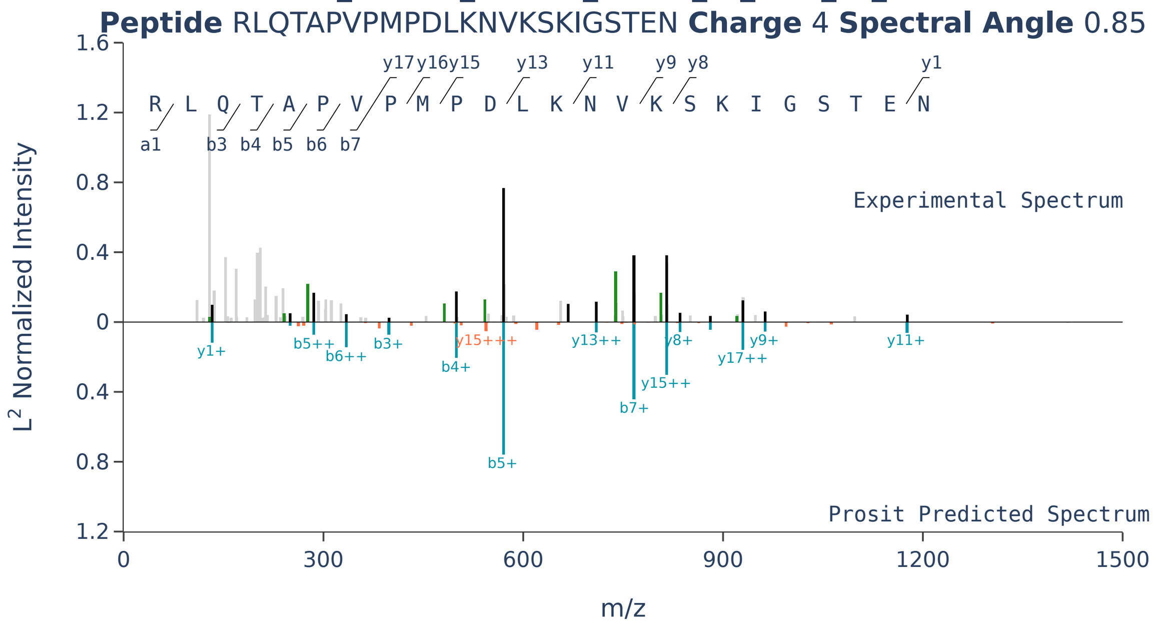


**Source** WSoh_221121_011221_HFGoe_L1_hTau_24h_R2 **Scan** 21325
**Peptide** DFKDRVQSKIGSLDNITHVPGGGNKKI **Charge** 4 **Spectral Angle** 0.72

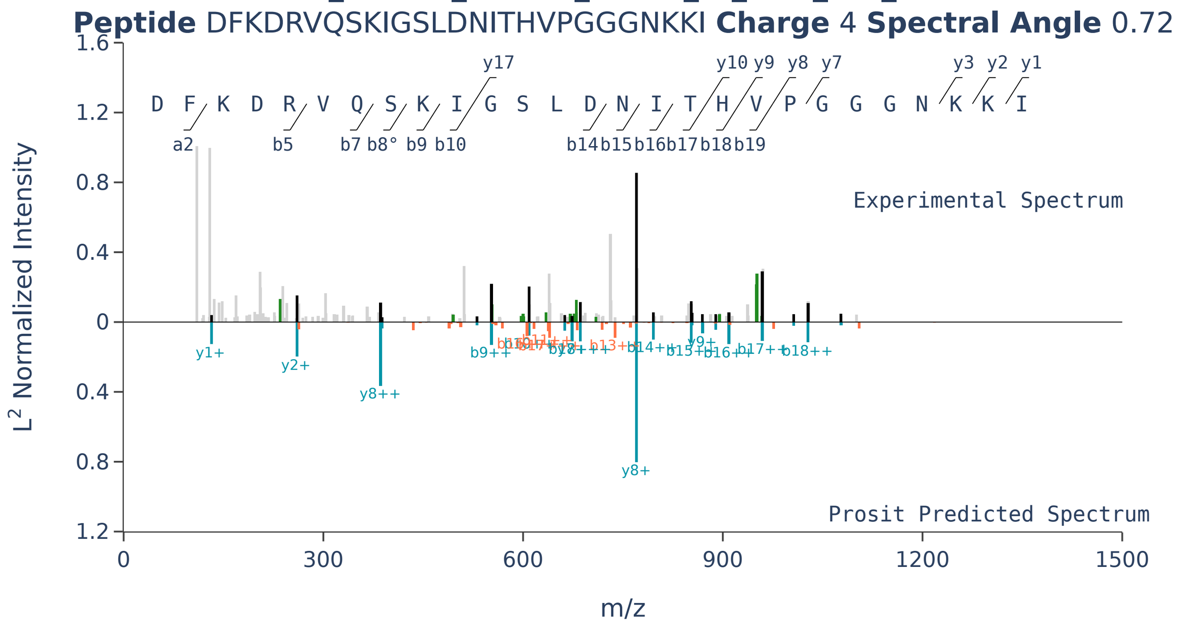


**Source** WSoh_221121_011221_HFGoe_L1_hTau_24h_R2 **Scan** 8798
**Peptide** TPPTREPKKVAV **Charge** 3 **Spectral Angle** 0.74

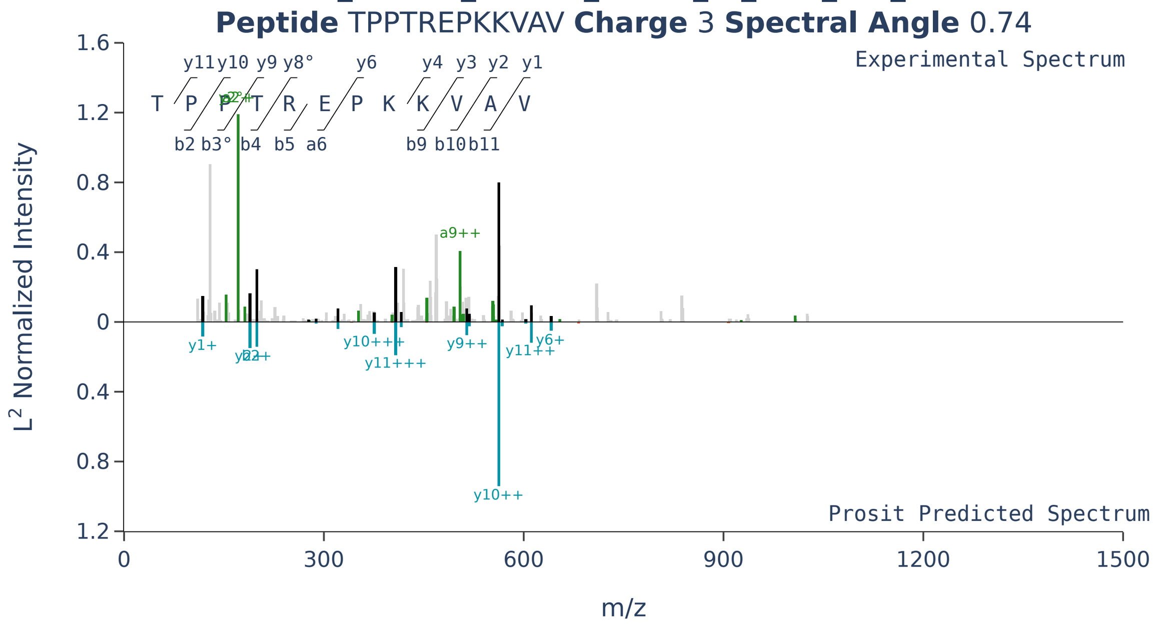


**Source** WSoh_221121_011221_HFGoe_L2_hTau_24h_R1 **Scan** 17387
**Peptide** KIGSTENLKHQPGGGKVQIINKKLDLSNV **Charge** 4 **Spectral Angle** 0.86

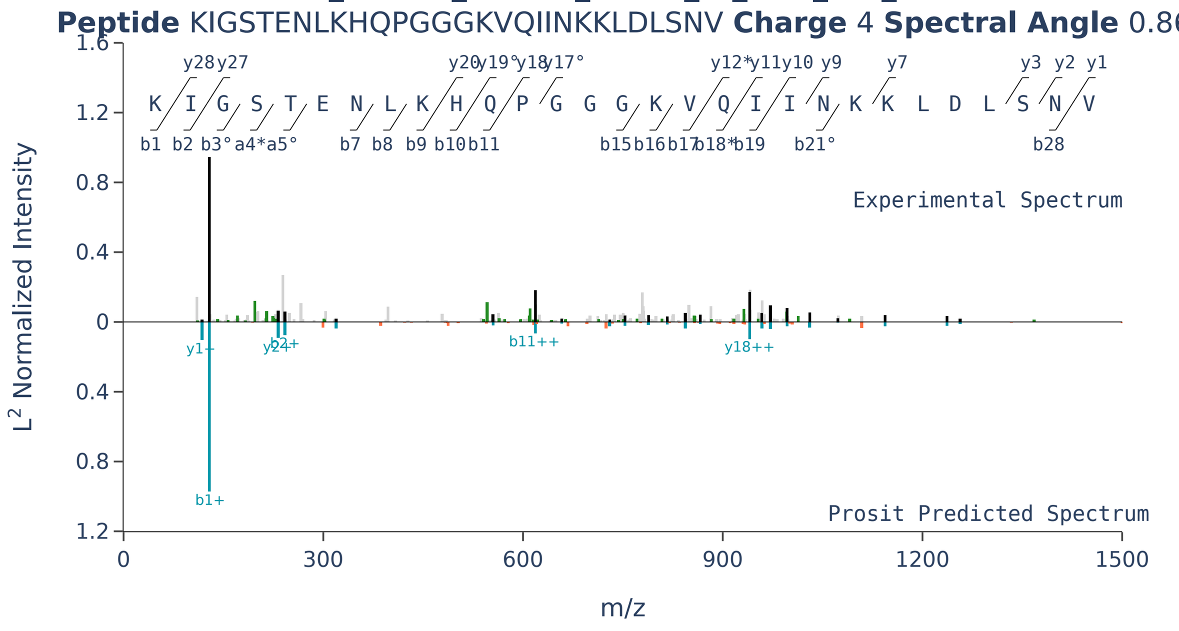


**Source** WSoh_221121_011221_HFGoe_L2_hTau_24h_R1 **Scan** 19363
**Peptide** APVPMPDLKNVKSKIGSTENLKHQPGGGKV **Charge** 4 **Spectral Angle** 0.73

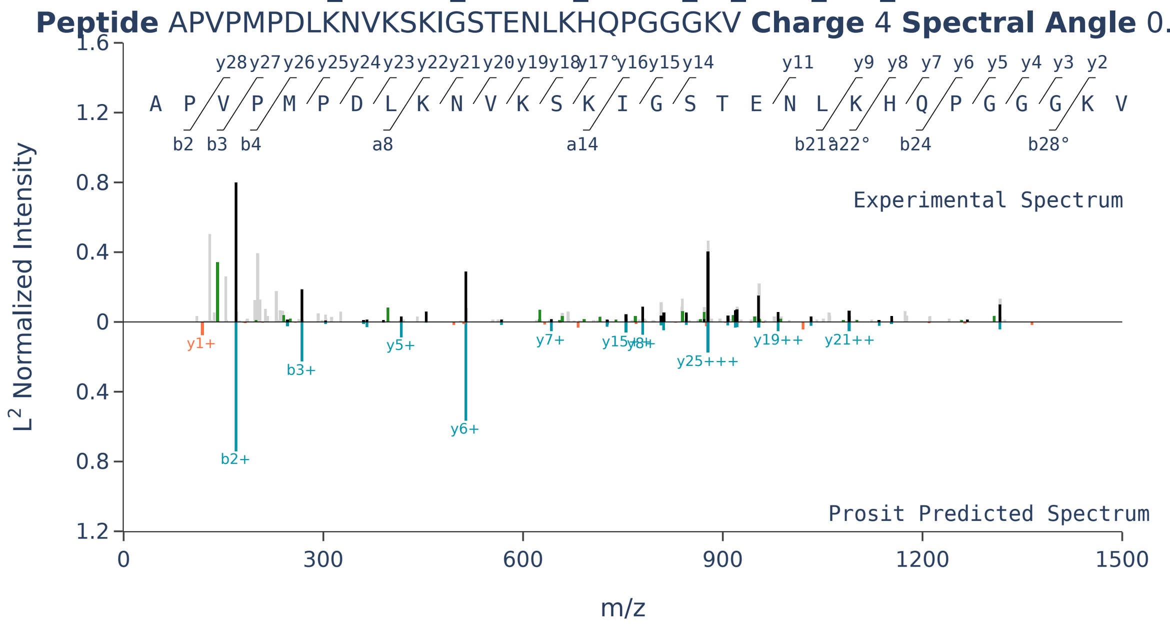

**Source** WSoh_221121_011221_HFGoe_L1_hTau_2h_R2 **Scan** 7496
**Peptide** GNIHHKPGGGQV **Charge** 2 **Spectral Angle** 0.85

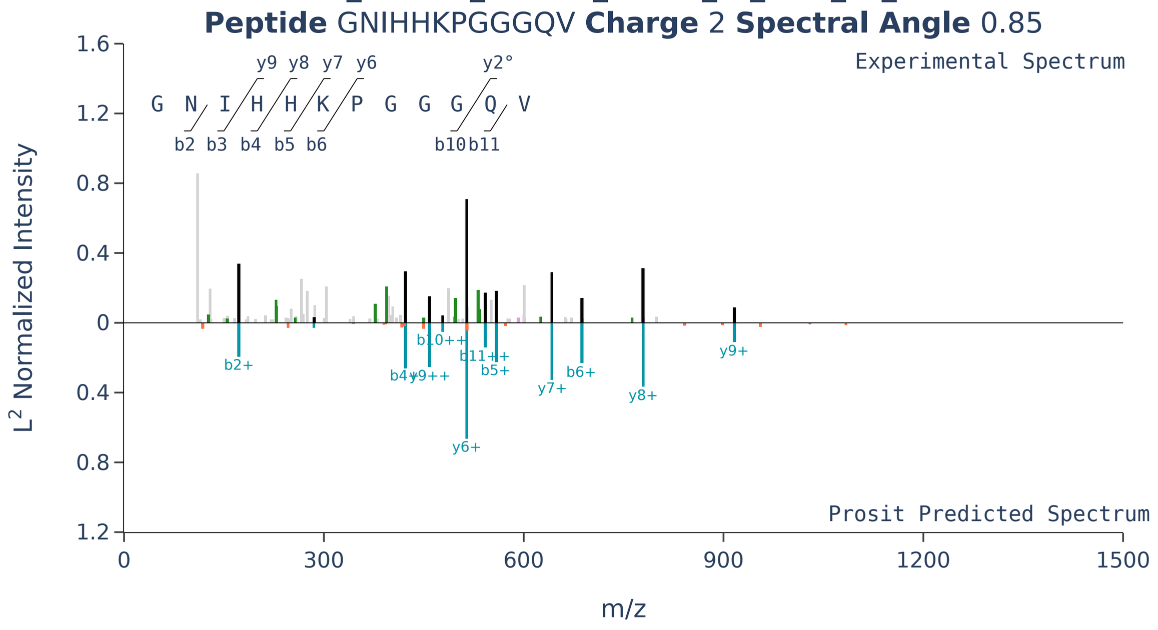


**Source** WSoh_221121_011221_HFGoe_L2_hTau_4h_R2 **Scan** 9483
**Peptide** TSDAKSTPTAEDV **Charge** 2 **Spectral Angle** 0.68

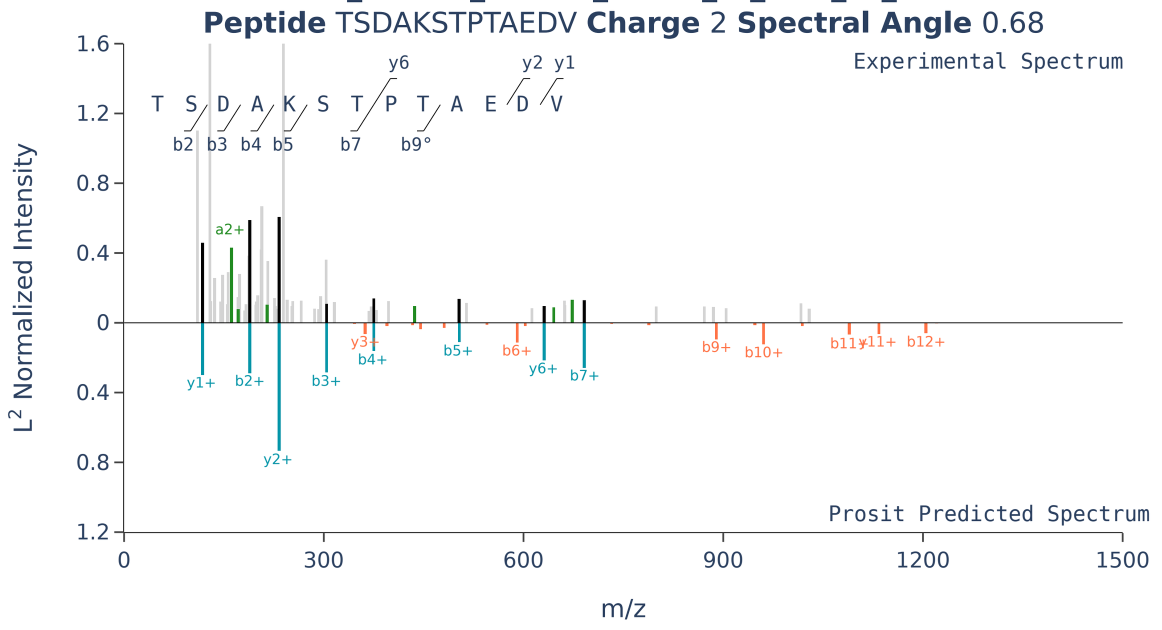


**Source** WSoh_221121_011221_HFGoe_L1_hTau_4h_R2 **Scan** 19226
**Peptide** QEGDTDAGLKESPLQTPTEDGSEEPGSE **Charge** 3 **Spectral Angle** 0.91

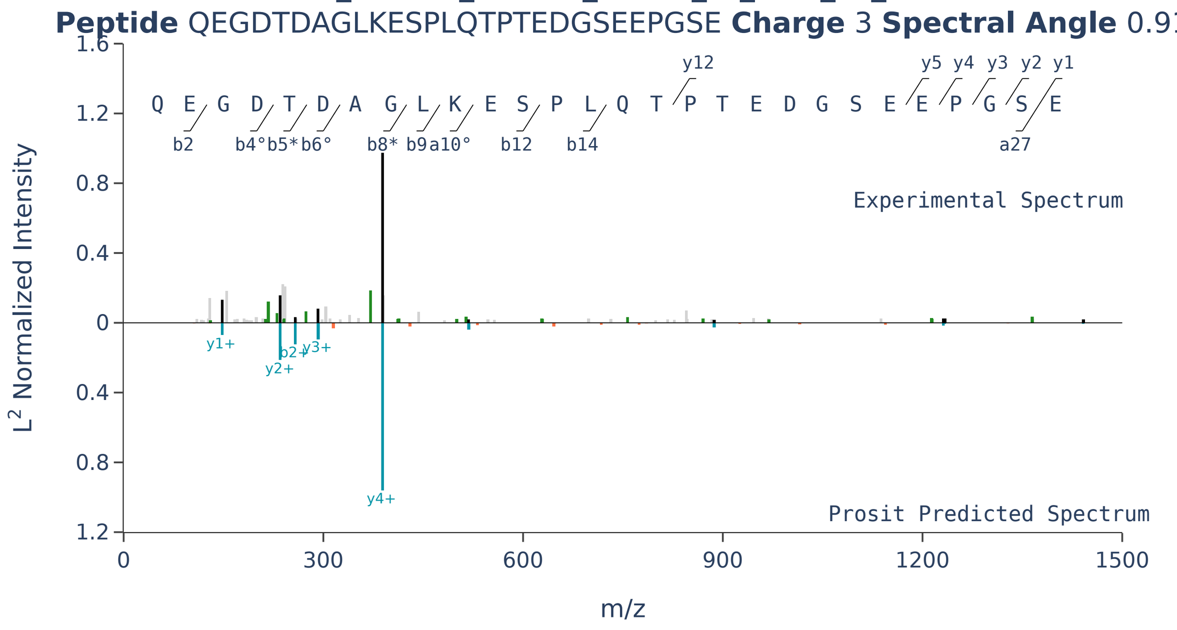


**Source** WSoh_221121_011221_HFGoe_L2_hTau_24h_R2 **Scan** 17041
**Peptide** SKIGSLDNITHVPGGGNKKIETHKLTFREN **Charge** 3 **Spectral Angle** 0.7

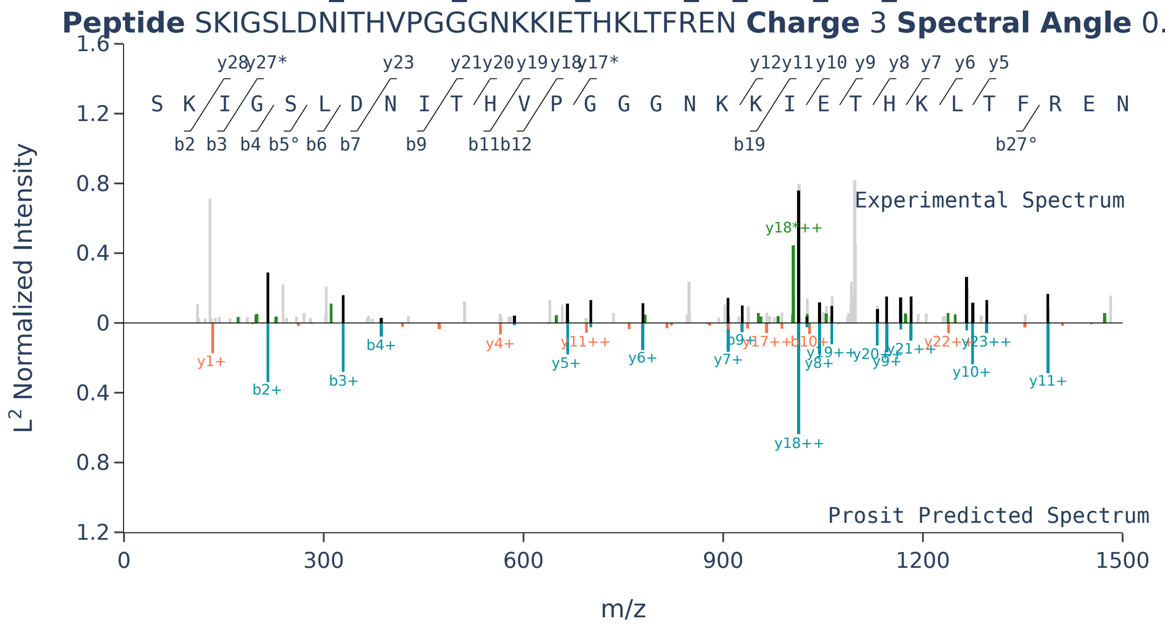


**Source** WSoh_221121_011221_HFGoe_L2_hTau_2h_R2 **Scan** 8791
**Peptide** HQPGGGKVQI **Charge** 2 **Spectral Angle** 0.89

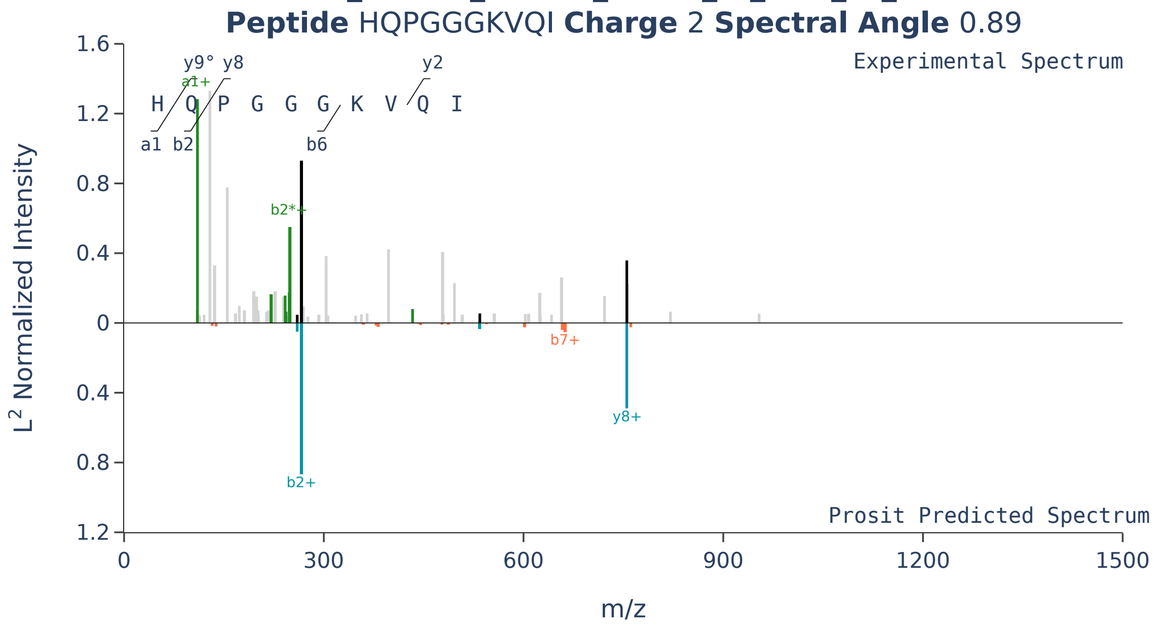


**Source** WSoh_221121_011221_HFGoe_L1_hTau_24h_R2 **Scan** 11694
**Peptide** TYGLGDRKDQGGY **Charge** 3 **Spectral Angle** 0.27

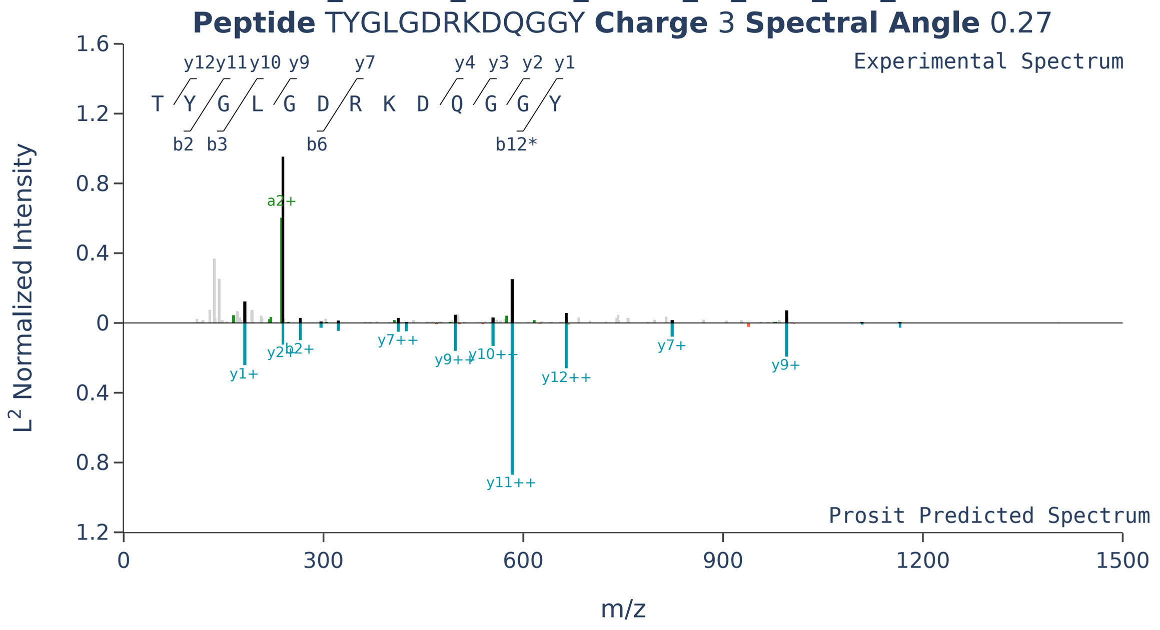


**Source** WSoh_221121_011221_HFGoe_L2_hTau_24h_R1 **Scan** 23095
**Peptide** DVTAPLVDE **Charge** 2 **Spectral Angle** 0.71

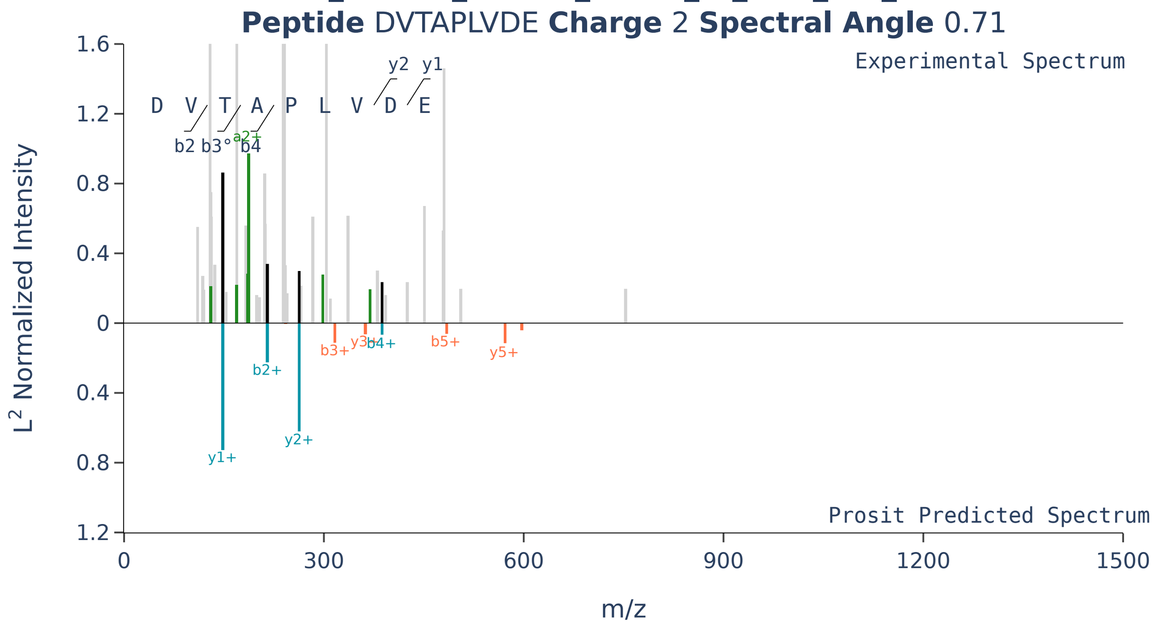


**Source** WSoh_221121_011221_HFGoe_L2_hTau_24h_R2 **Scan** 11758
**Peptide** SLGNIHHKPGGGQVEVKSEKLDFKDRVQSK **Charge** 4 **Spectral Angle** 0.63

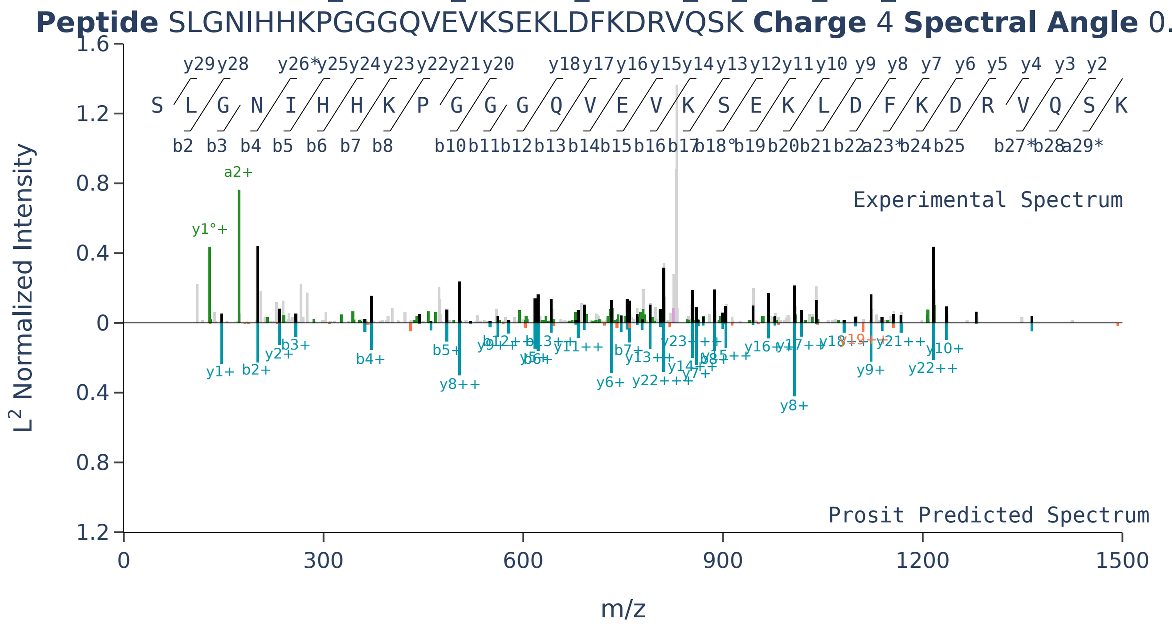


**Source** WSoh_221121_011221_HFGoe_L1_hTau_24h_R2 **Scan** 10045
**Peptide** TENLKHQPGGGKVQI **Charge** 3 **Spectral Angle** 0.74

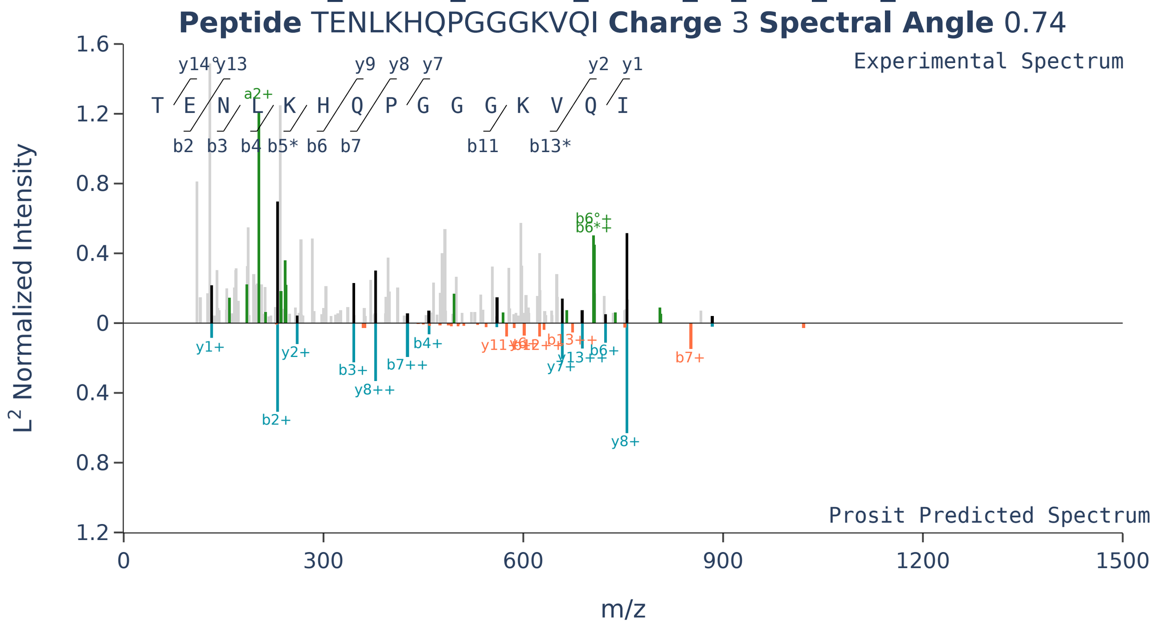


**Source** WSoh_221121_011221_HFGoe_L2_hTau_4h_R2 **Scan** 23757
**Peptide** DTSPRHLSNVSSTGSIDMVDSPQL **Charge** 3 **Spectral Angle** 0.72

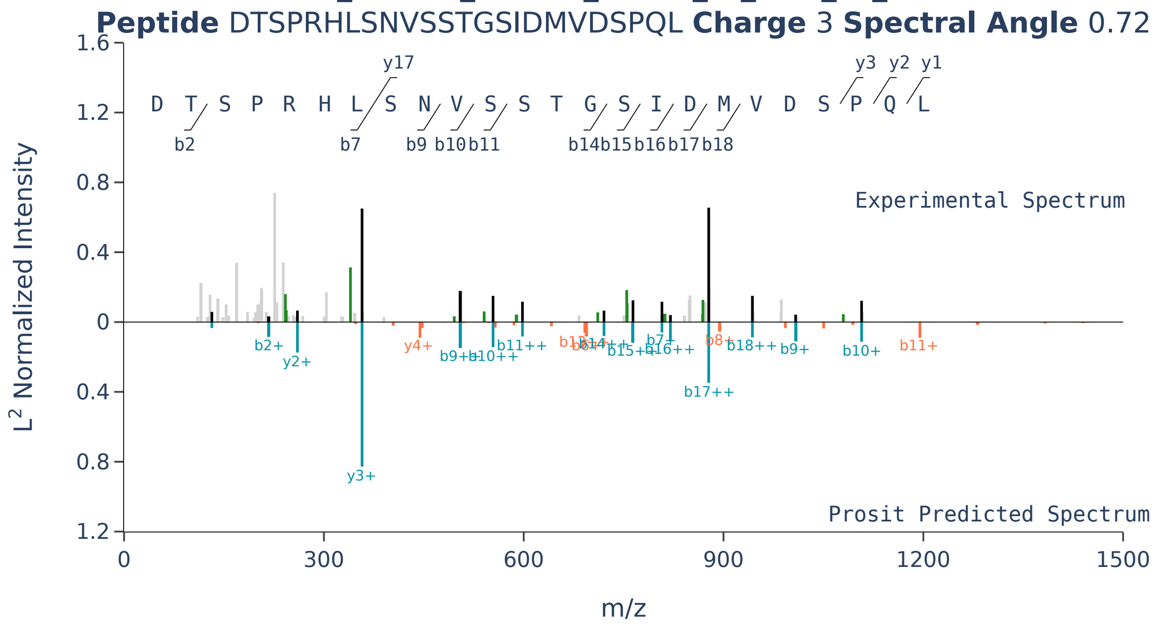


**Source** WSoh_221121_011221_HFGoe_L2_hTau_24h_R2 **Scan** 13358
**Peptide** RSRTPSLPTPPTREPKKVAV **Charge** 5 **Spectral Angle** 0.74

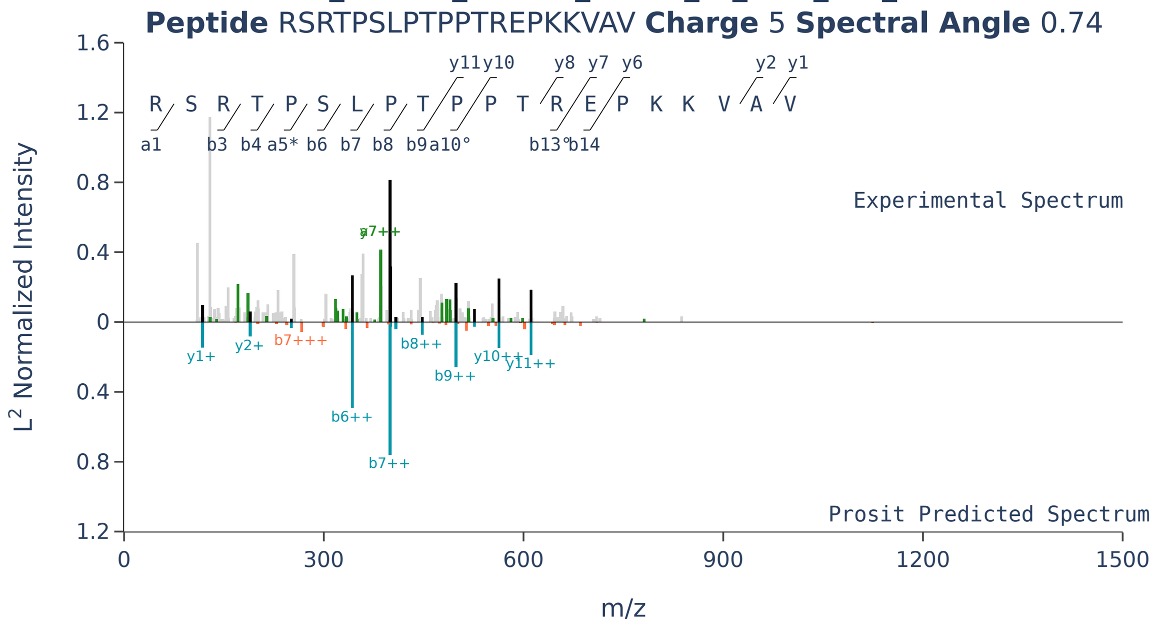


**Source** WSoh_221121_011221_HFGoe_L2_hTau_24h_R1 **Scan** 10518
**Peptide** DNITHVPGGGNKKIETHKLTFRENAKAKTD **Charge** 4 **Spectral Angle** 0.63

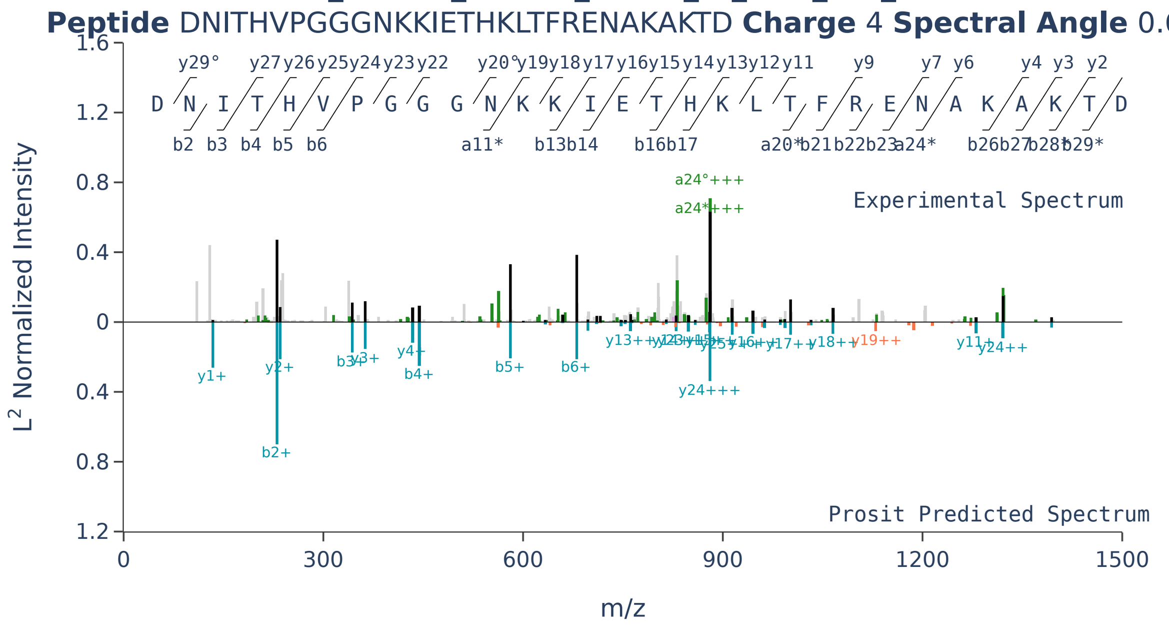


**Source** WSoh_221121_011221_HFGoe_L2_hTau_24h_R2 **Scan** 7164
**Peptide** GKTKIATPRGAAPPGQKGQANAT **Charge** 3 **Spectral Angle** 0.77

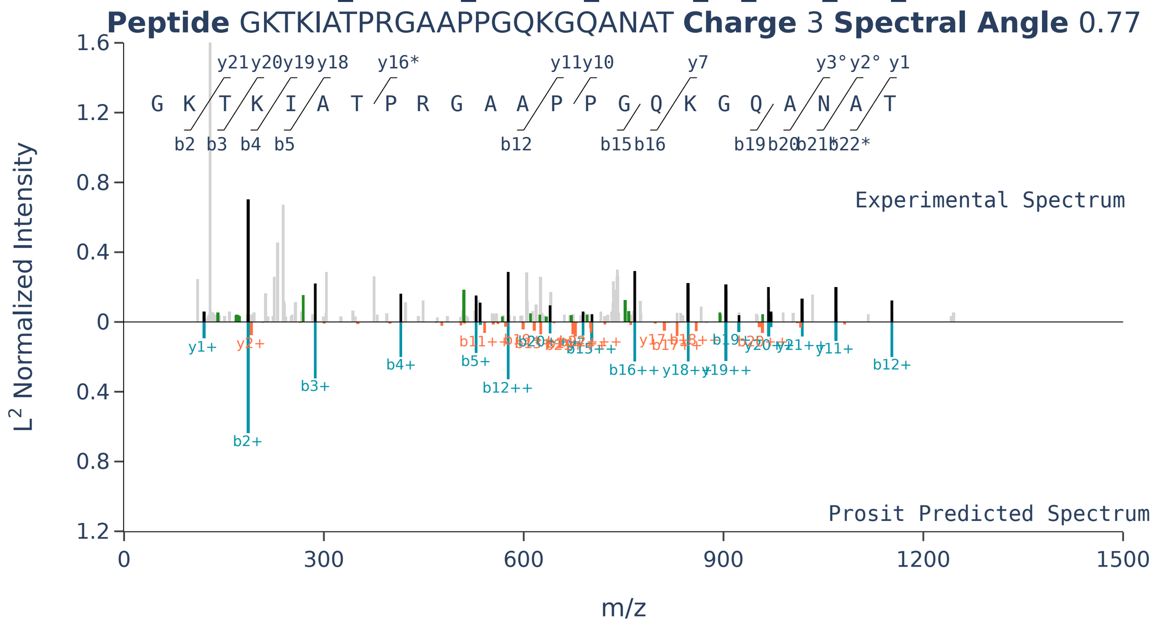


**Source** WSoh_221121_011221_HFGoe_L2_hTau_24h_R2 **Scan** 5817
**Peptide** STENLKHQPGGGKVQ **Charge** 2 **Spectral Angle** 0.74

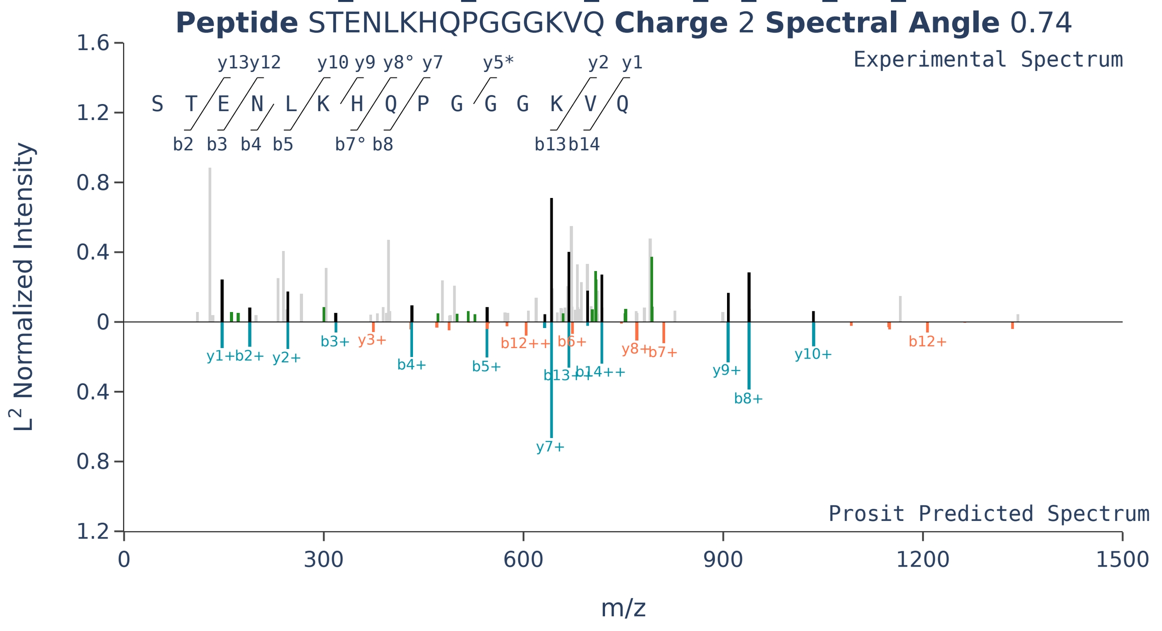


**Source** WSoh_221121_011221_HFGoe_L2_hTau_24h_R2 **Scan** 18876
**Peptide** IVYKPVDLSKVTSKC **Charge** 4 **Spectral Angle** 0.73

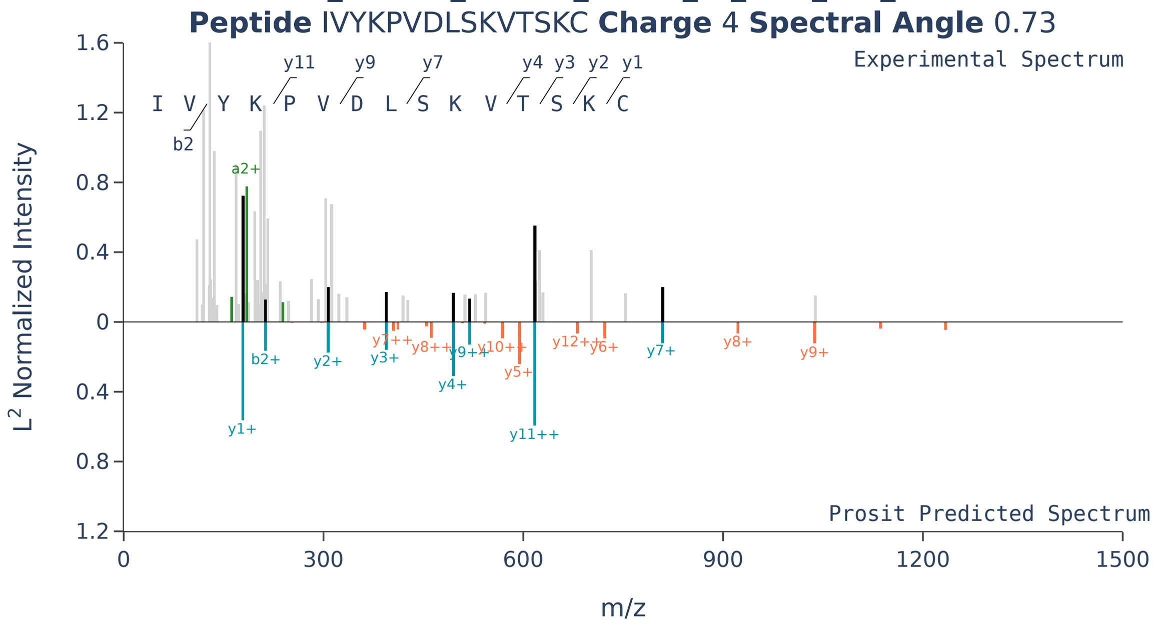

**Source** WSoh_221121_011221_HFGoe_L1_hTau_24h_R2 **Scan** 12514
**Peptide** SPGSPGTPGSRSRTPSLPTPPTREPKKV **Charge** 6 **Spectral Angle** 0.68

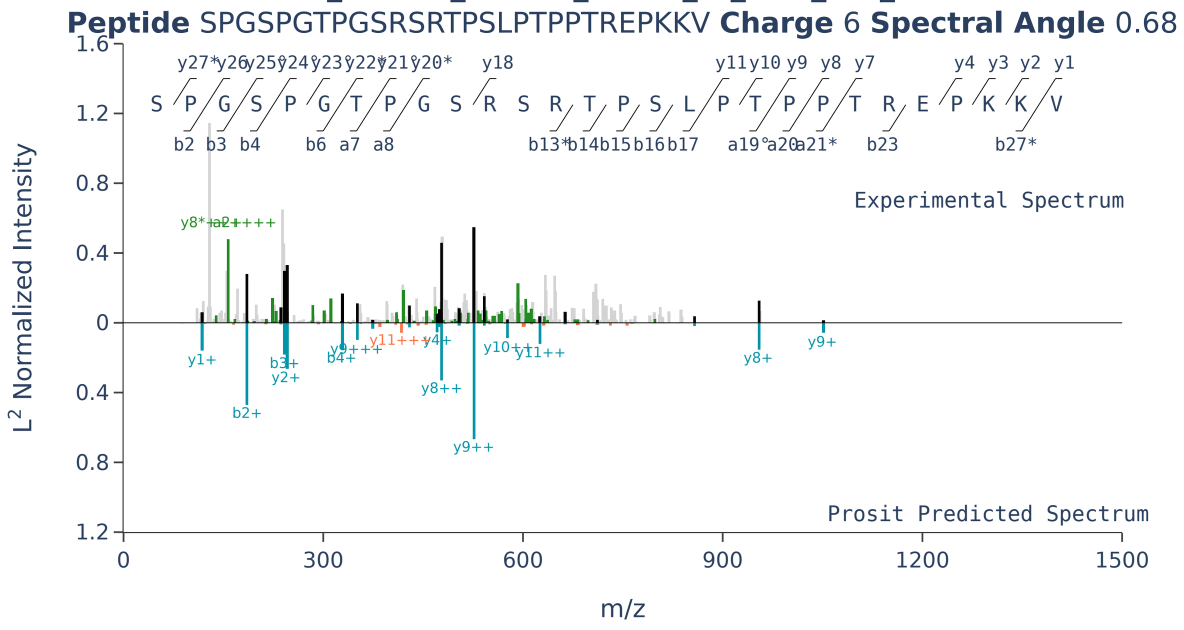


**Source** WSoh_221121_011221_HFGoe_L2_hTau_24h_R1 **Scan** 7433
**Peptide** PPSSGEPPKSGDRSGYSSPGSPGTPGSRSR **Charge** 4 **Spectral Angle** 0.76

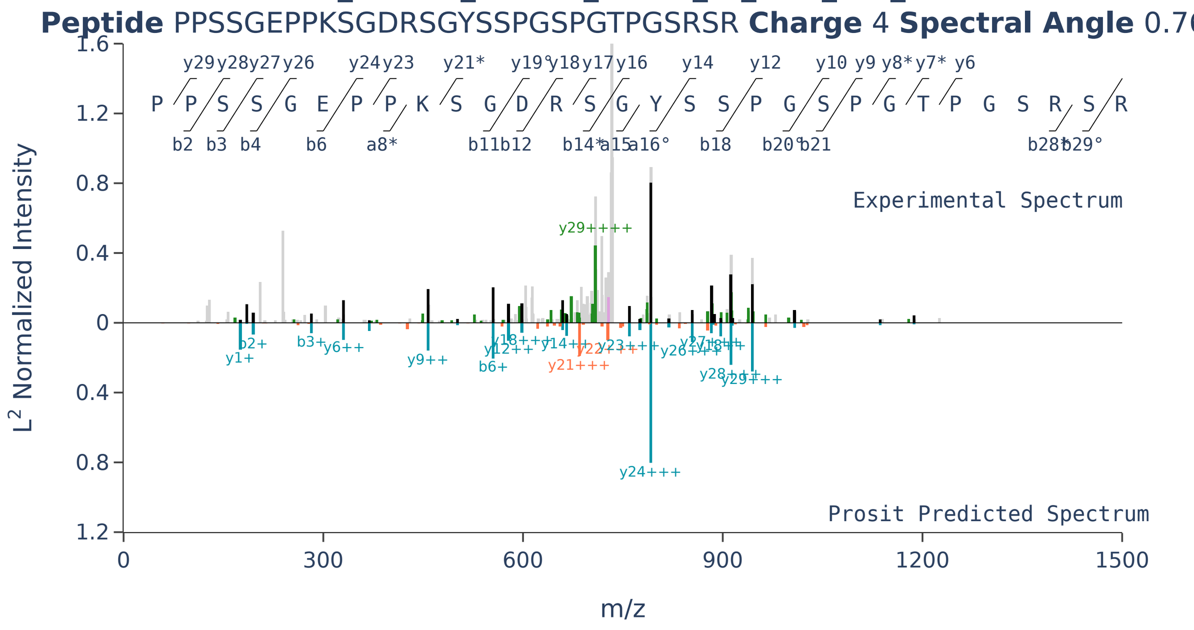


**Source** WSoh_221121_011221_HFGoe_L2_hTau_4h_R1 **Scan** 14894
**Peptide** PVDLSKVTSKCGSLGNIHHKPGGGQVE **Charge** 4 **Spectral Angle** 0.82

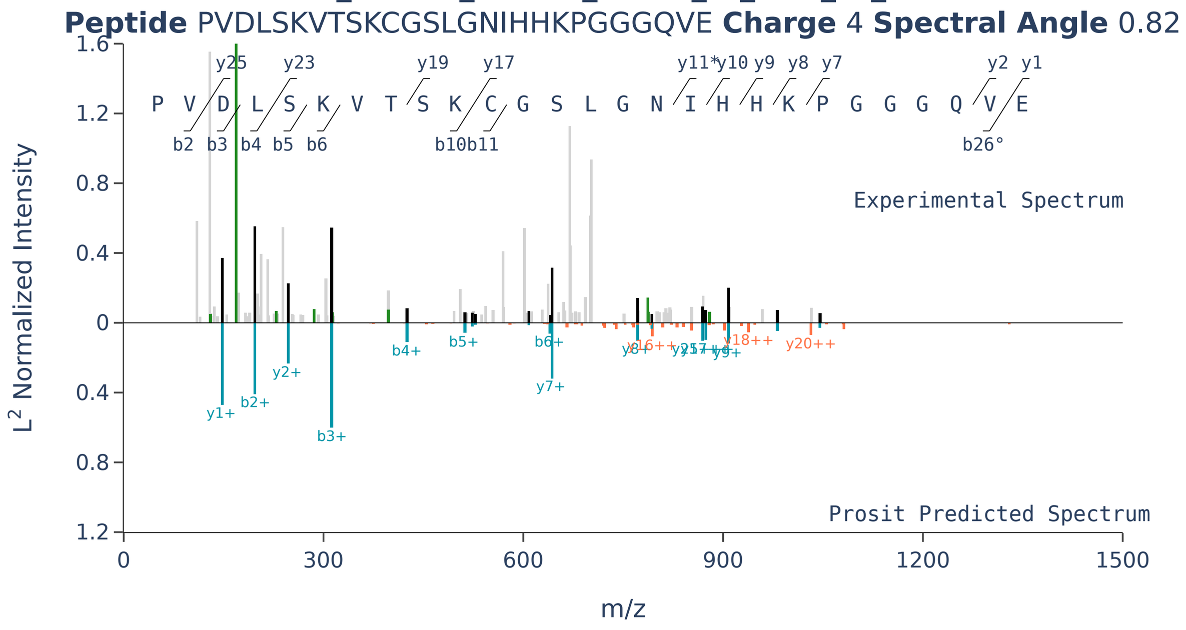


**Source** WSoh_221121_011221_HFGoe_L2_hTau_4h_R2 **Scan** 12357
**Peptide** ETHKLTFRENAKAKTDHGAEIVY **Charge** 4 **Spectral Angle** 0.88

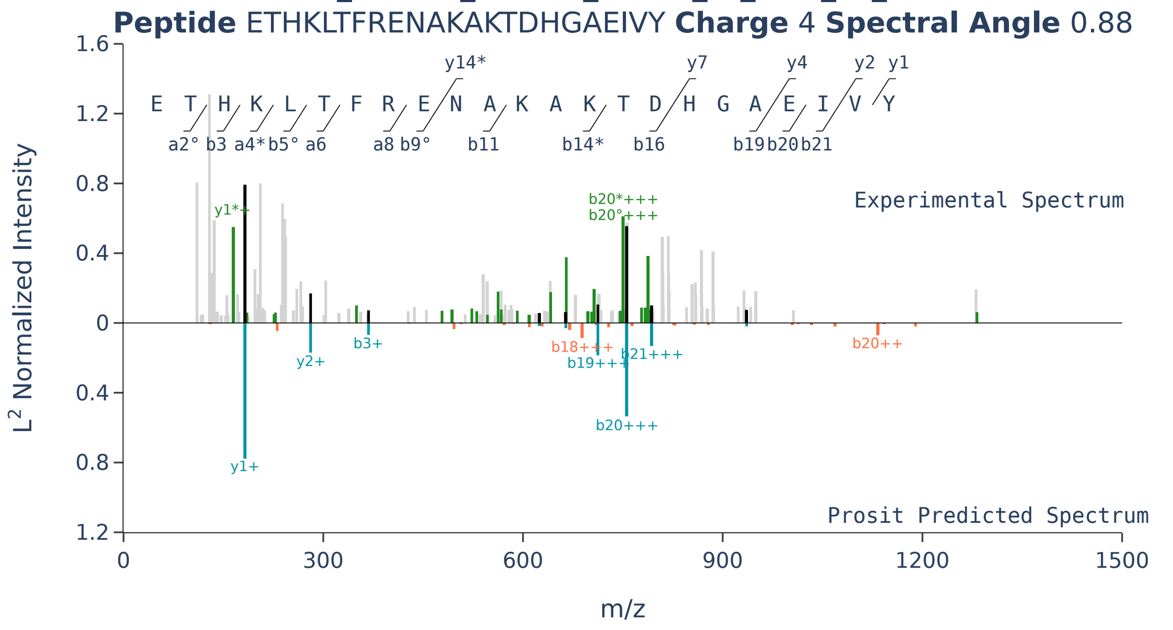


**Source** WSoh_221121_011221_HFGoe_L1_hTau_4h_R1 **Scan** 13758
**Peptide** AKTDHGAEIVYKSPVVSGDTSPRHLSN **Charge** 4 **Spectral Angle** 0.67

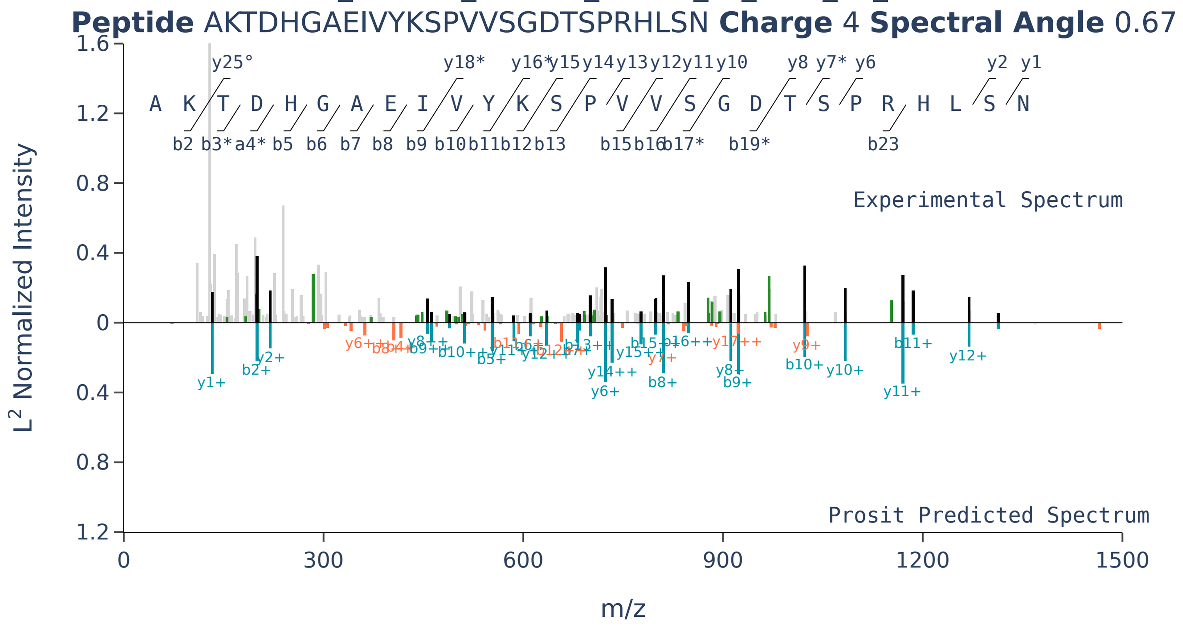


**Source** WSoh_221121_011221_HFGoe_L1_hTau_4h_R2 **Scan** 21823
**Peptide** TSDAKSTPTAEDVTAPLVDE **Charge** 2 **Spectral Angle** 0.75

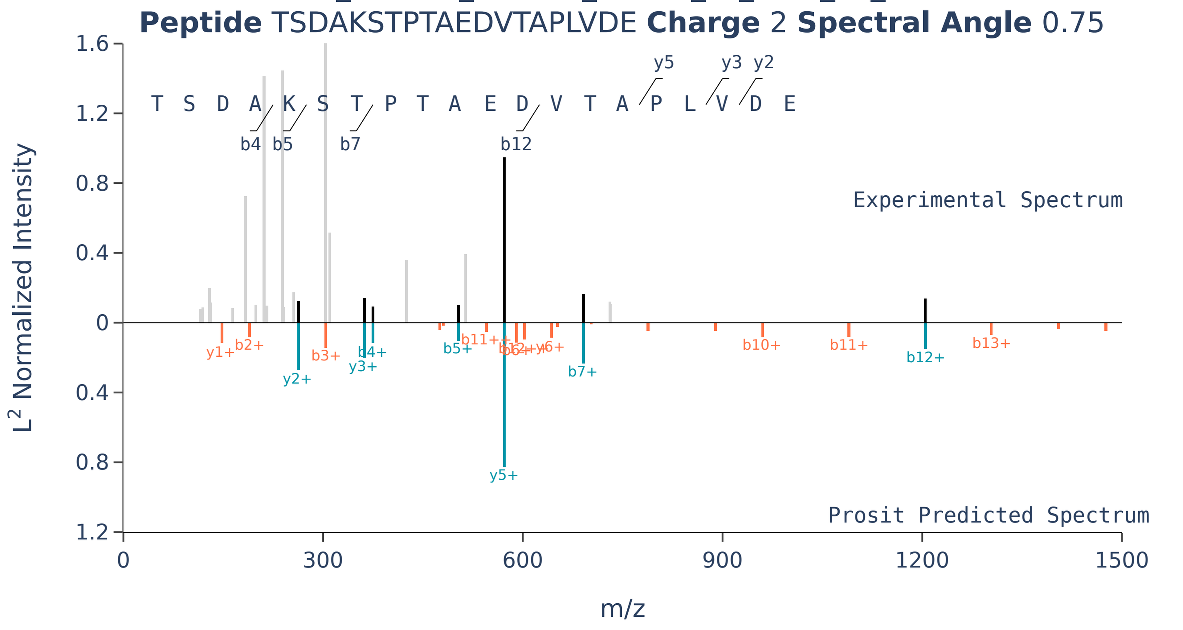


**Source** WSoh_221121_011221_HFGoe_L1_hTau_4h_R2 **Scan** 19176
**Peptide** VVRTPPKSPSSAKSRLQTAPVPMPDLKNV **Charge** 5 **Spectral Angle** 0.66

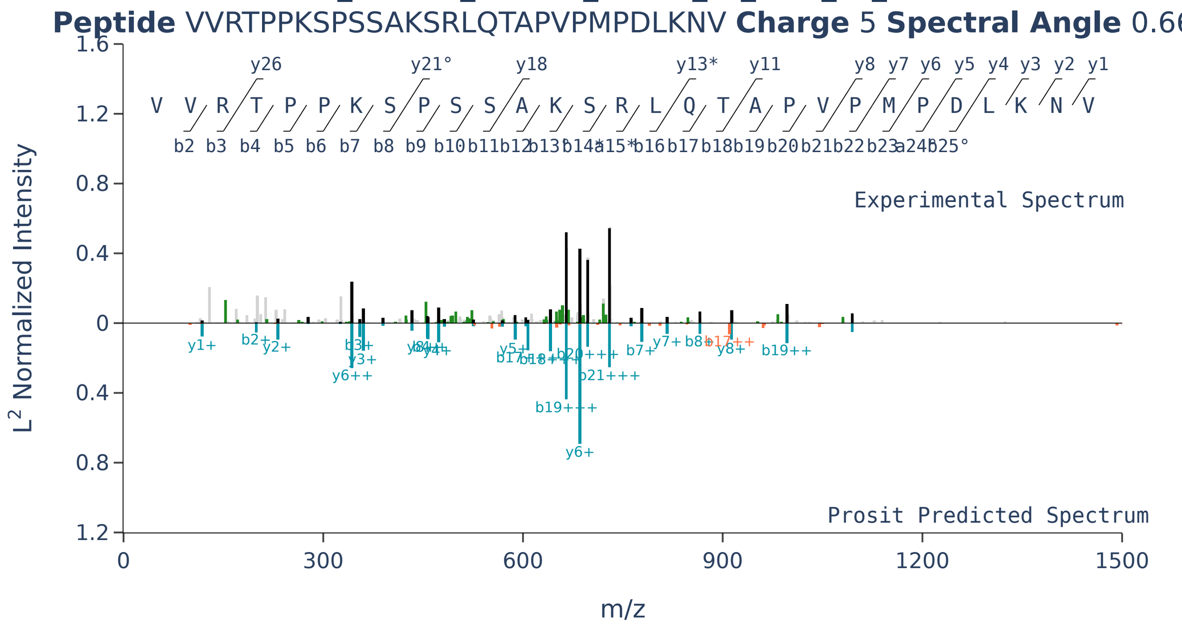


**Source** WSoh_221121_011221_HFGoe_L2_hTau_24h_R2 **Scan** 8298
**Peptide** PTPPTREPKKVAVVRTPPKS **Charge** 4 **Spectral Angle** 0.72

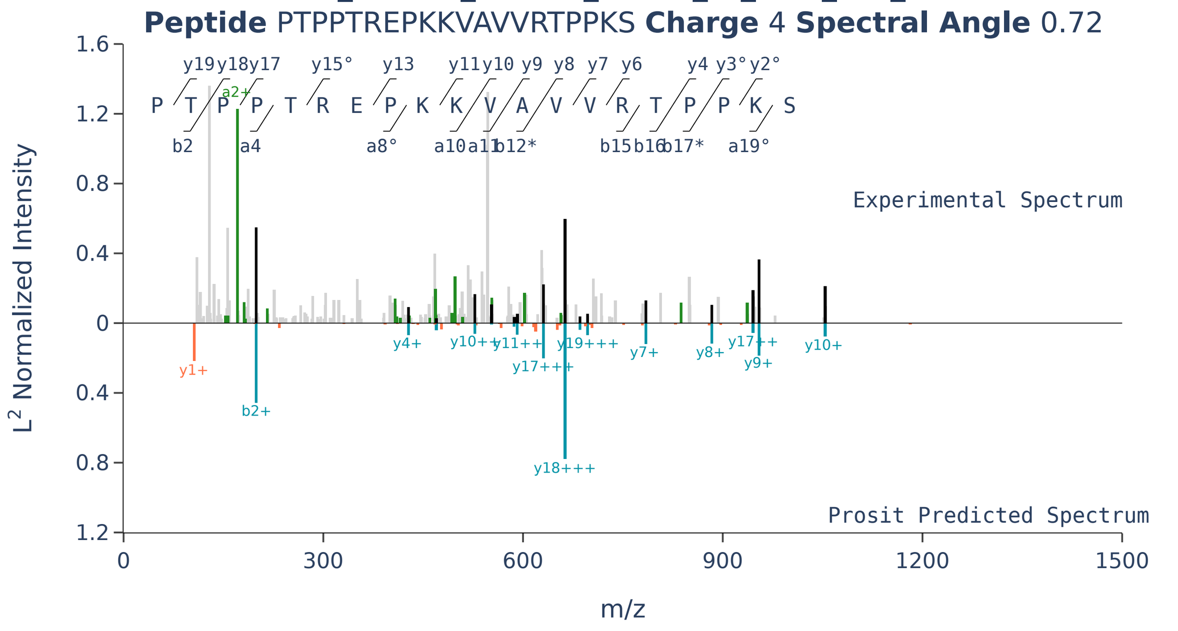


**Source** WSoh_221121_011221_HFGoe_L1_hTau_24h_R1 **Scan** 24350
**Peptide** QPHTEIPEGTTAEEAGIGDTPSL **Charge** 3 **Spectral Angle** 0.92

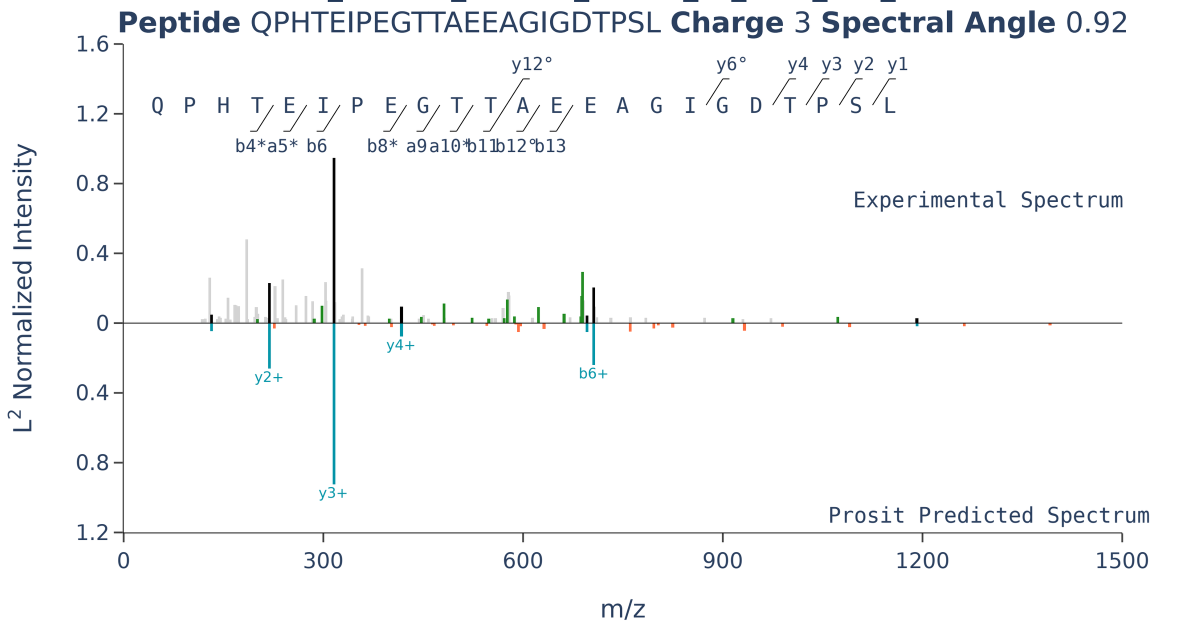


**Source** WSoh_221121_011221_HFGoe_L1_hTau_24h_R2 **Scan** 9806
**Peptide** VVSGDTSPRHLSNVS **Charge** 2 **Spectral Angle** 0.78

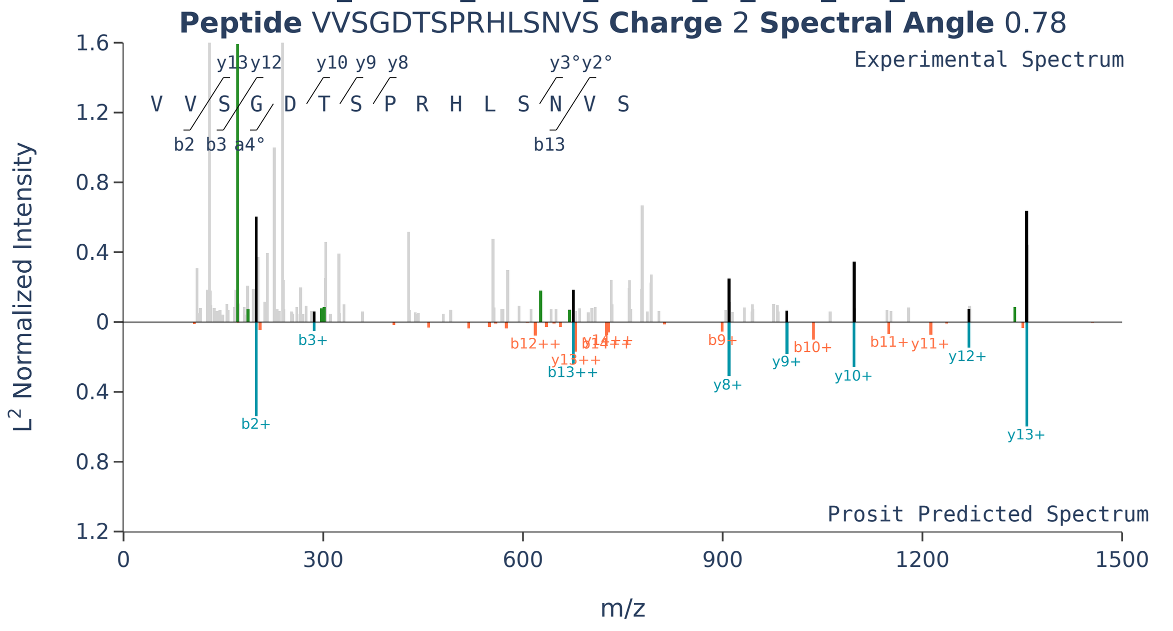


**Source** WSoh_221121_011221_HFGoe_L2_hTau_24h_R2 **Scan** 5329
**Peptide** SKDGTGSDDKKAKGADGKTKIAT **Charge** 3 **Spectral Angle** 0.73

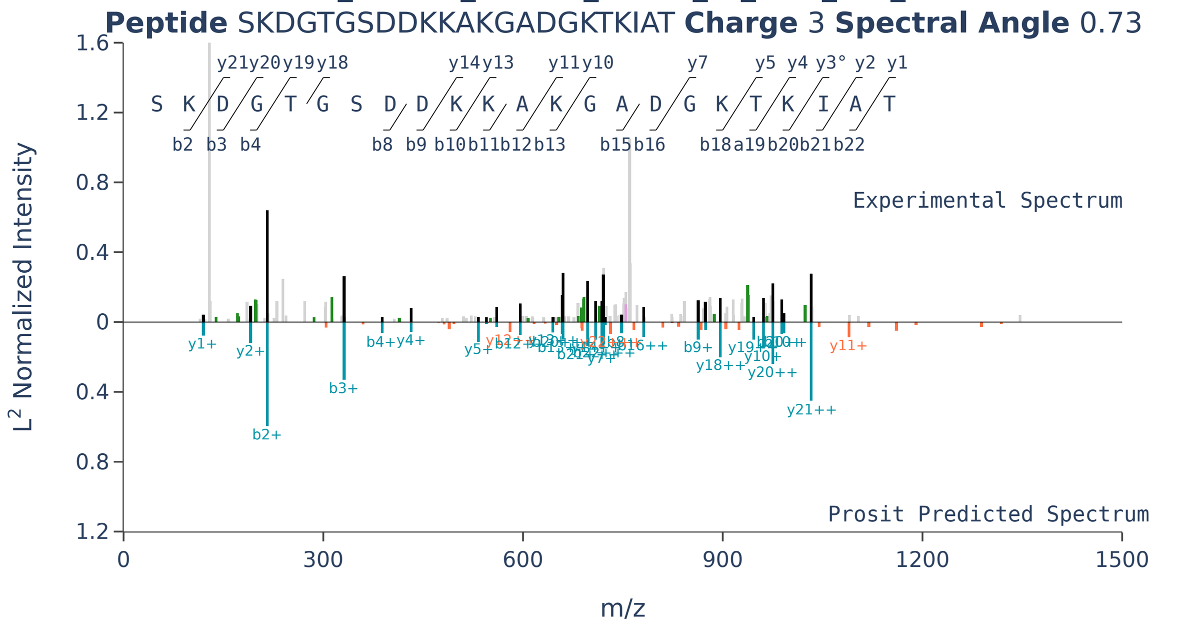


**Source** WSoh_221121_011221_HFGoe_L1_hTau_24h_R1 **Scan** 7658
**Peptide** KGADGKTKIATPRGAAPPGQ **Charge** 3 **Spectral Angle** 0.59

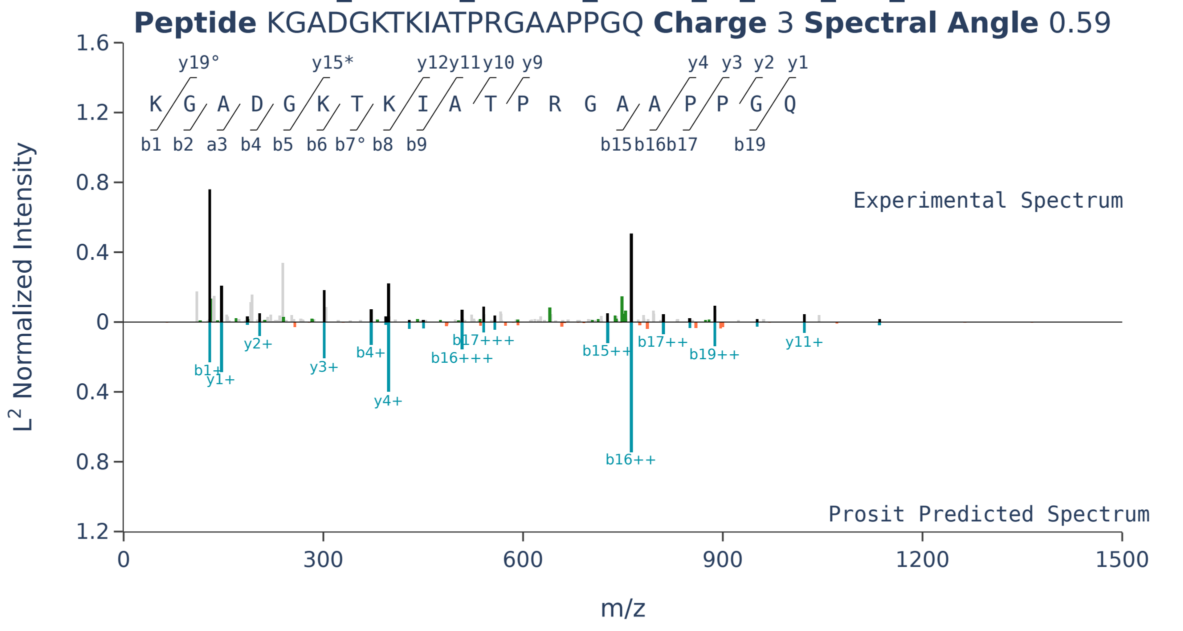


**Source** WSoh_221121_011221_HFGoe_L1_hTau_24h_R1 **Scan** 10935
**Peptide** KLTFRENAKAKTDHGAELV **Charge** 4 **Spectral Angle** 0.64

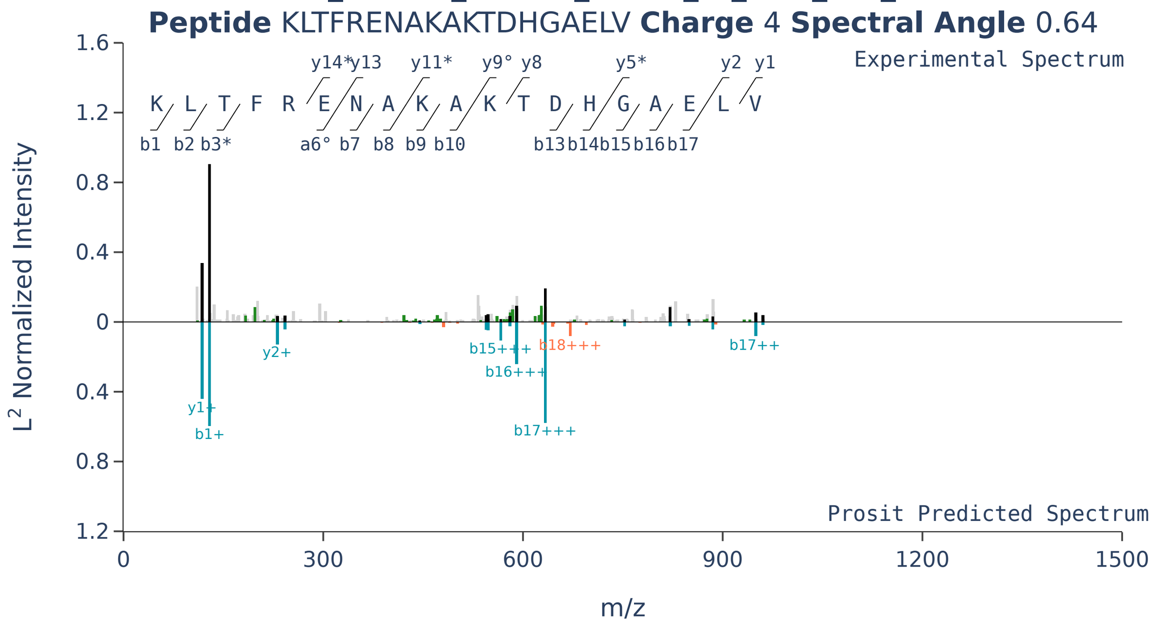


**Source** WSoh_221121_011221_HFGoe_L2_hTau_24h_R1 **Scan** 20344
**Peptide** AKSTPTAEDVTAPLVDEGAPGKQAAA **Charge** 3 **Spectral Angle** 0.8

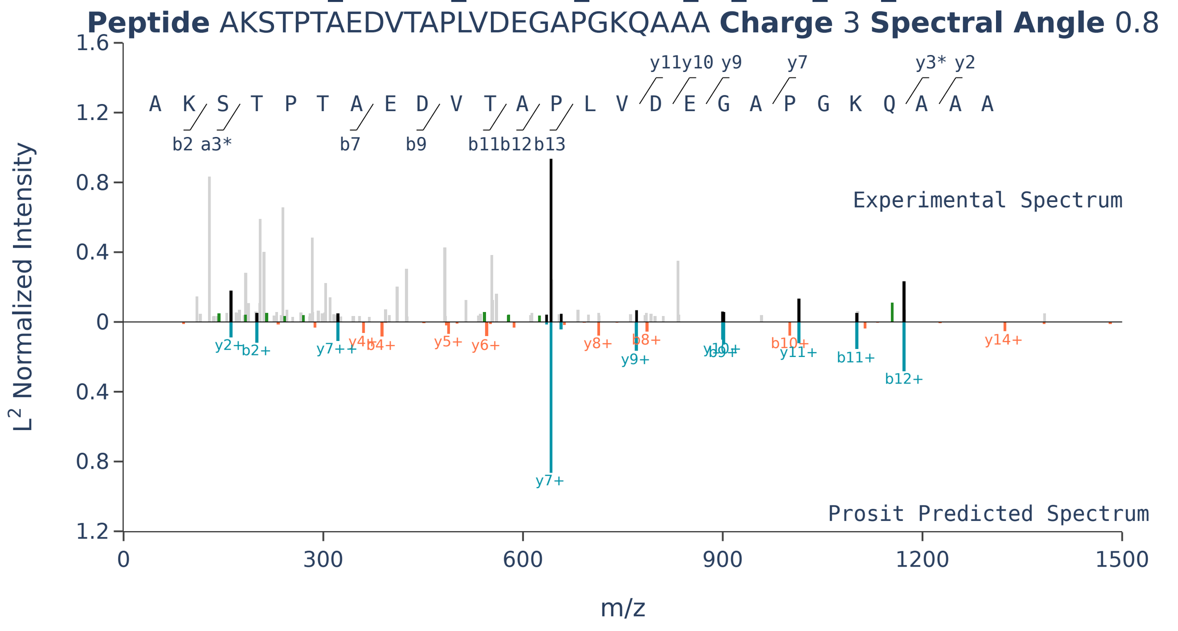


**Source** WSoh_221121_011221_HFGoe_L2_hTau_4h_R2 **Scan** 16188
**Peptide** VYKPVDLSKVTSKCGSLGNIHHKPGGGQV **Charge** 4 **Spectral Angle** 0.66

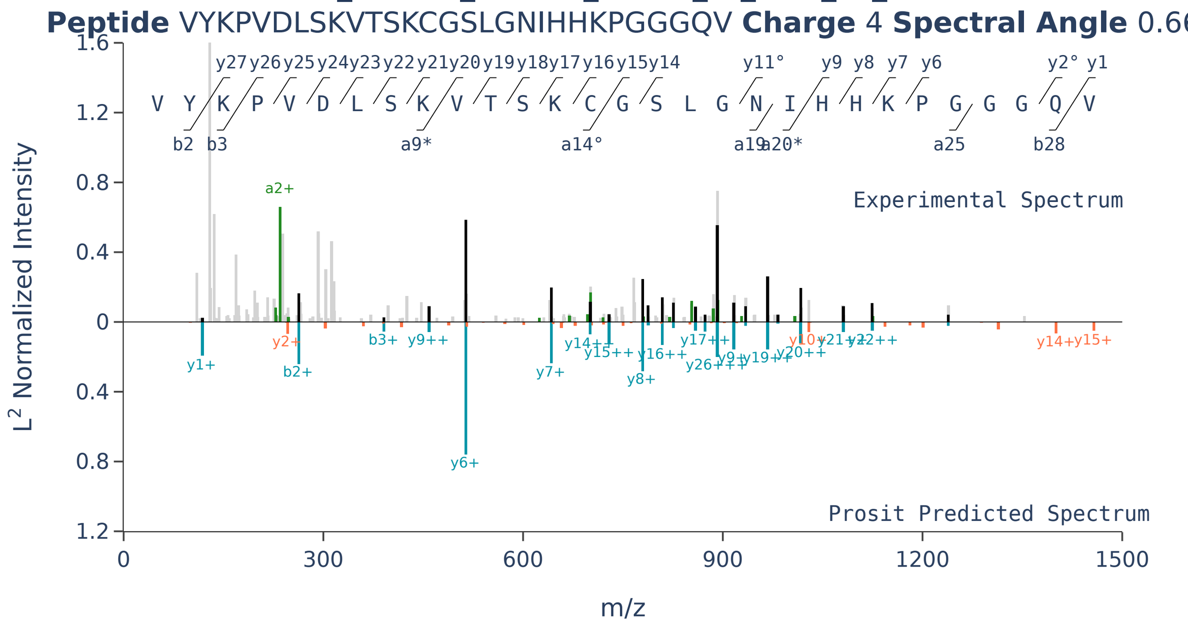

**Source** WSoh_221121_011221_HFGoe_L2_hTau_24h_R1 **Scan** 12451
**Peptide** SSPGSPGTPGSRSRTPSLPTPPTREPKKV **Charge** 4 **Spectral Angle** 0.65

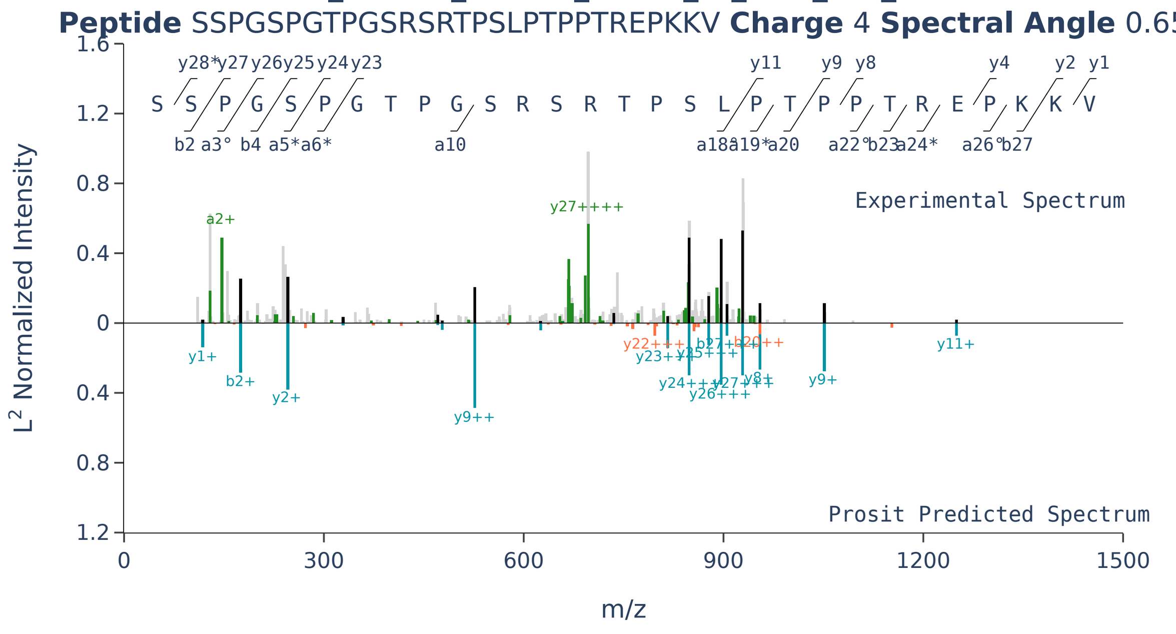


**Source** WSoh_221121_011221_HFGoe_L1_hTau_24h_R1 **Scan** 25642
**Peptide** TGSIDMVDSPQLA **Charge** 2 **Spectral Angle** 0.62

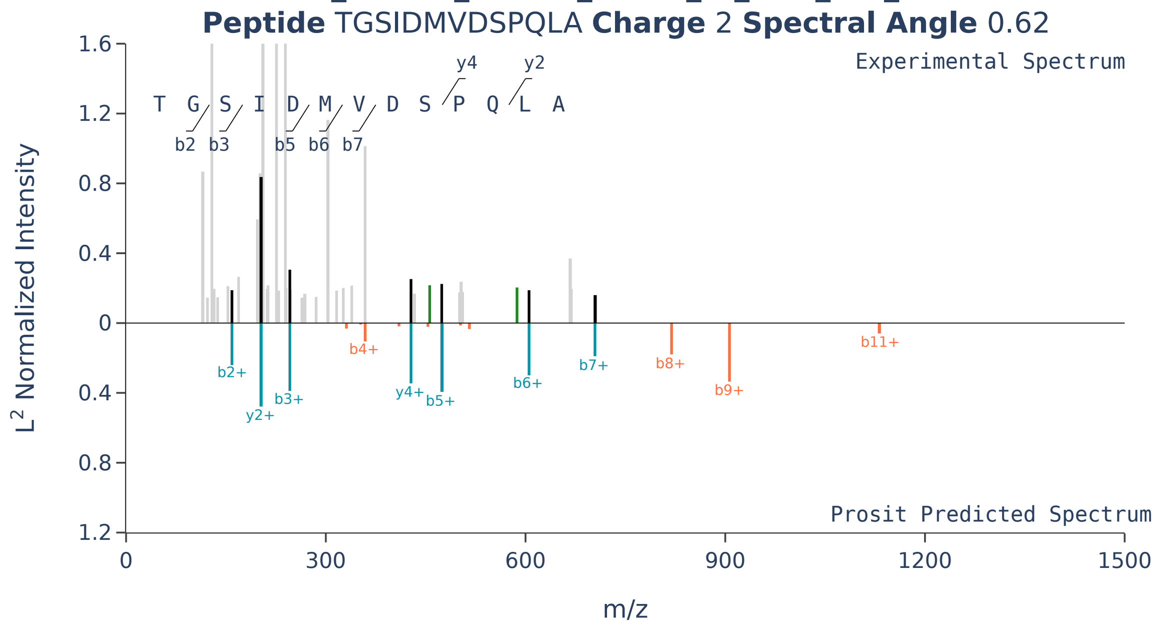


**Source** WSoh_221121_011221_HFGoe_L2_hTau_24h_R2 **Scan** 22972
**Peptide** AEEAGIGDTPSL **Charge** 2 **Spectral Angle** 0.74

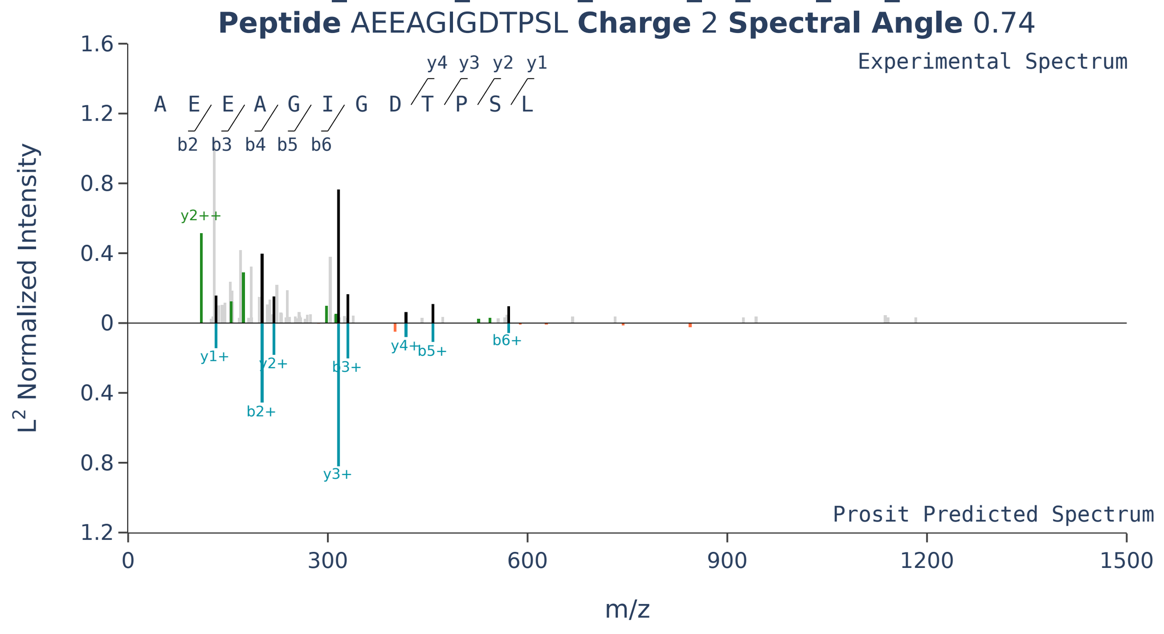


**Source** WSoh_221121_011221_HFGoe_L2_hTau_24h_R2 **Scan** 17879
**Peptide** GAPGKQAAAQPHTEIPEGTTAEEAGI **Charge** 3 **Spectral Angle** 0.72

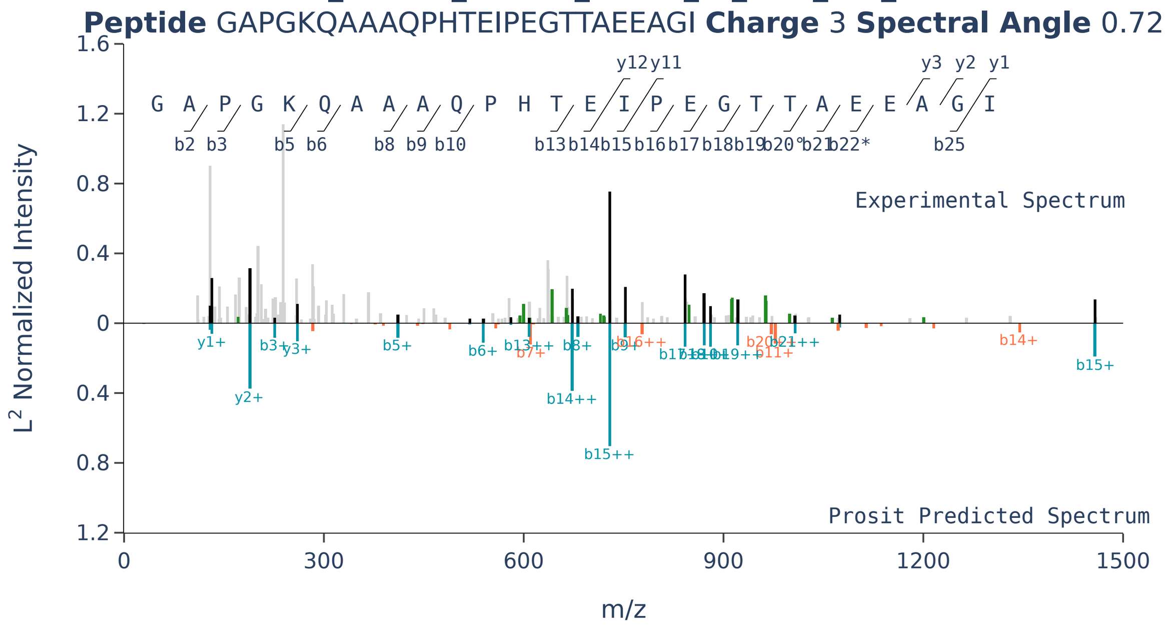


**Source** WSoh_221121_011221_HFGoe_L1_hTau_24h_R1 **Scan** 13954
**Peptide** PTREPKKV **Charge** 2 **Spectral Angle** 0.74

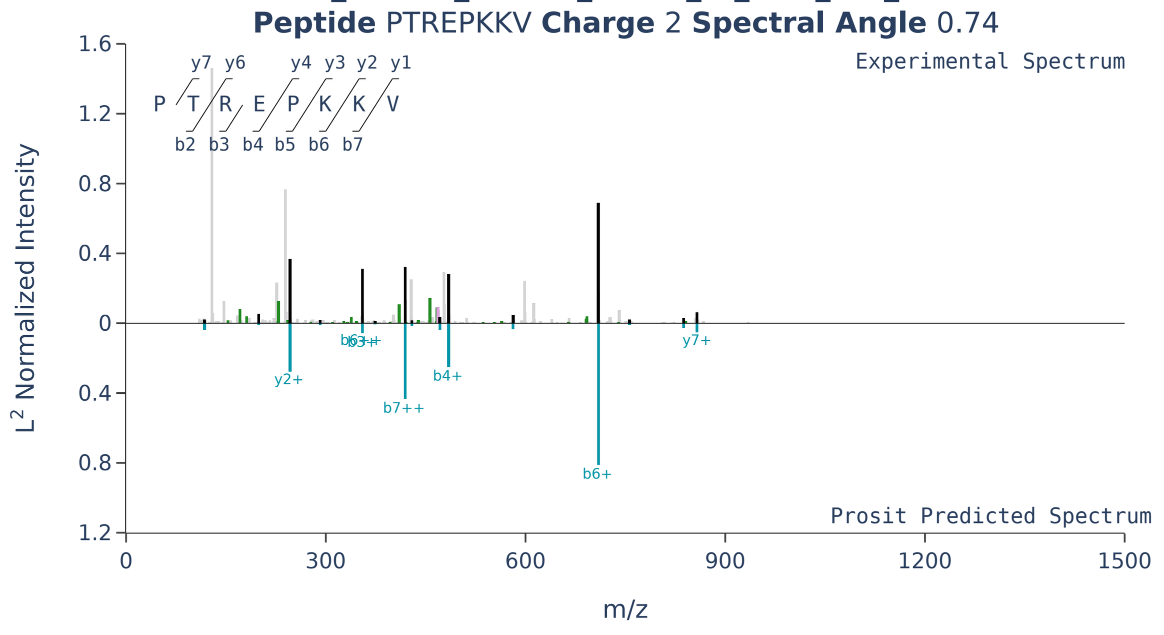


**Source** WSoh_221121_011221_HFGoe_L1_hTau_2h_R1 **Scan** 11043
**Peptide** SKVTSKCGSLGNI **Charge** 3 **Spectral Angle** 0.87

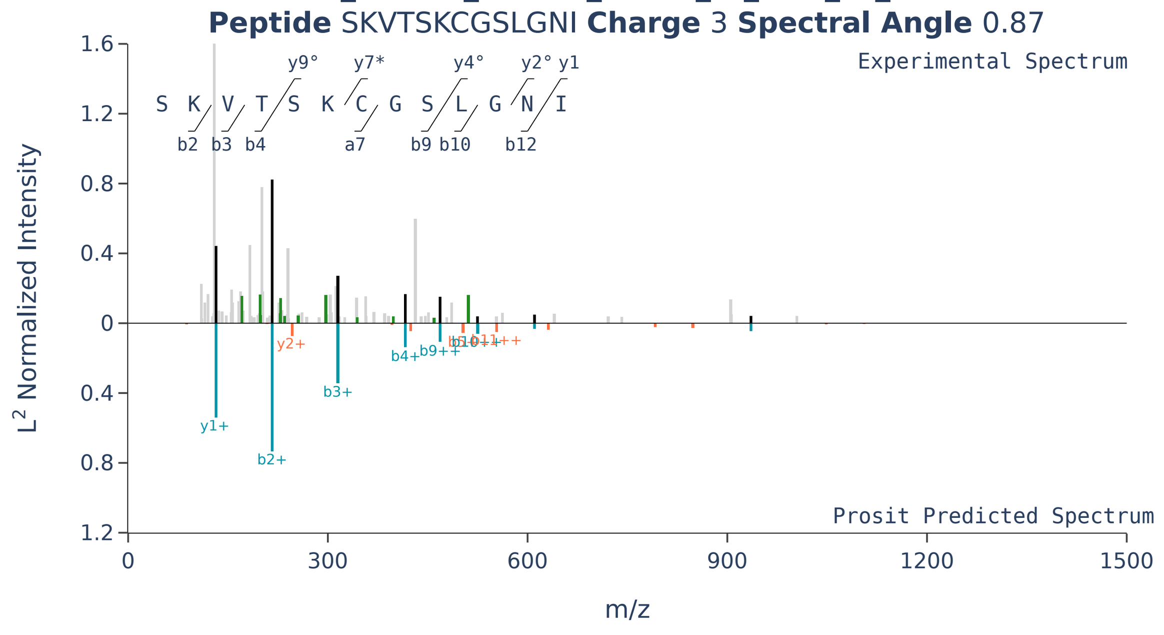


**Source** WSoh_221121_011221_HFGoe_L2_hTau_4h_R2 **Scan** 13045
**Peptide** CGSLGNIHHKPGGGQVEVKSEKLDFKDRVQ **Charge** 5 **Spectral Angle** 0.69

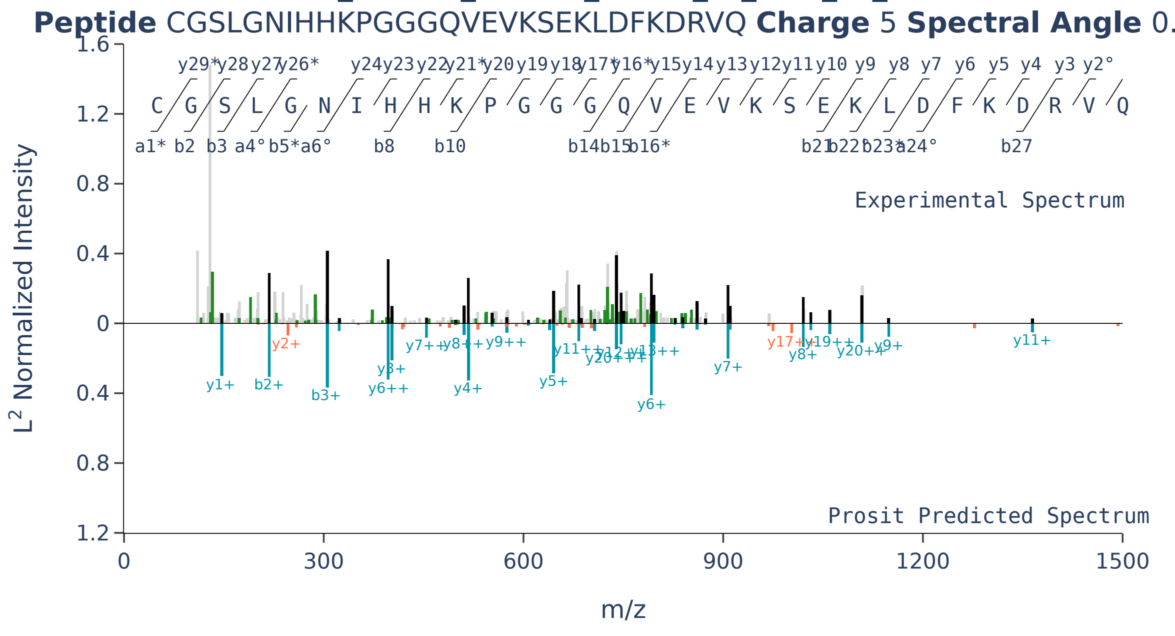


**Source** WSoh_221121_011221_HFGoe_L2_hTau_24h_R2 **Scan** 13337
**Peptide** TPSLPTPPTREPKKVAVVRTPPKSPSSAKS **Charge** 4 **Spectral Angle** 0.63

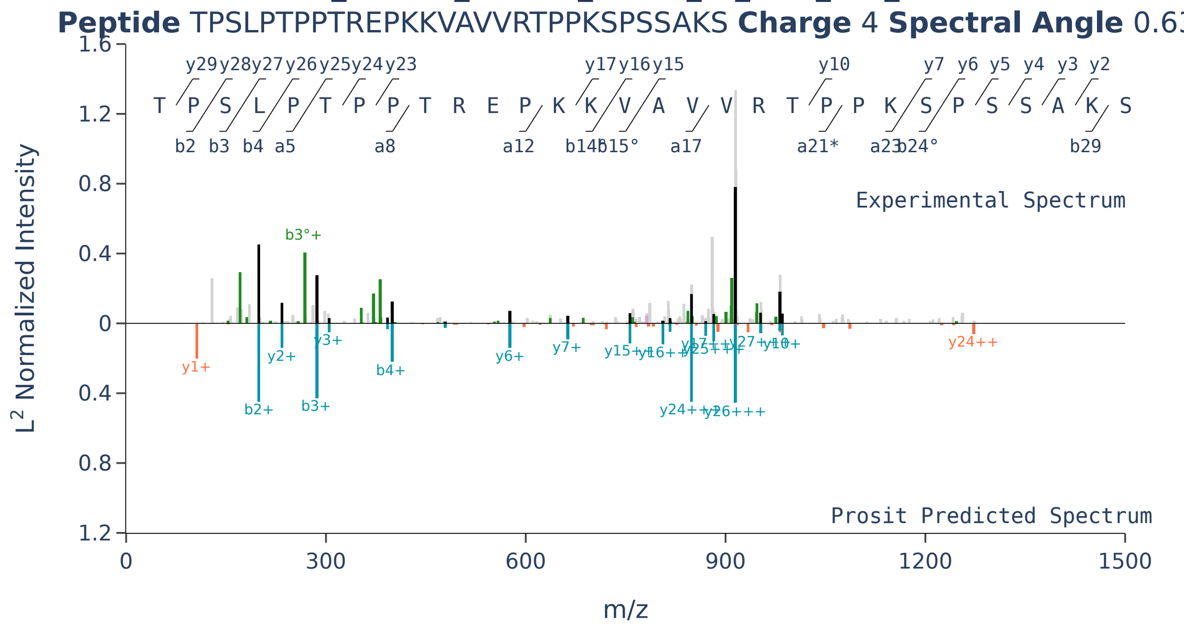


**Source** WSoh_221121_011221_HFGoe_L2_hTau_4h_R1 **Scan** 15284
**Peptide** QIINKKLDLS **Charge** 2 **Spectral Angle** 0.62

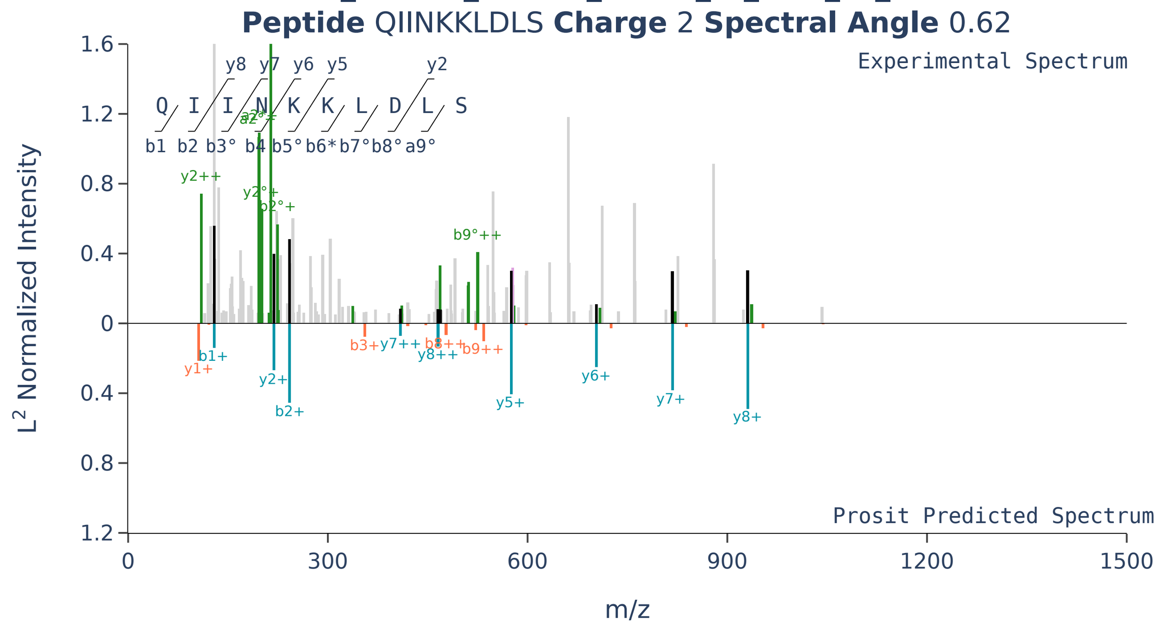


**Source** WSoh_221121_011221_HFGoe_L2_hTau_4h_R2 **Scan** 8255
**Peptide** LSKVTSKCGSLGNIHHKPGGGQV **Charge** 3 **Spectral Angle** 0.78

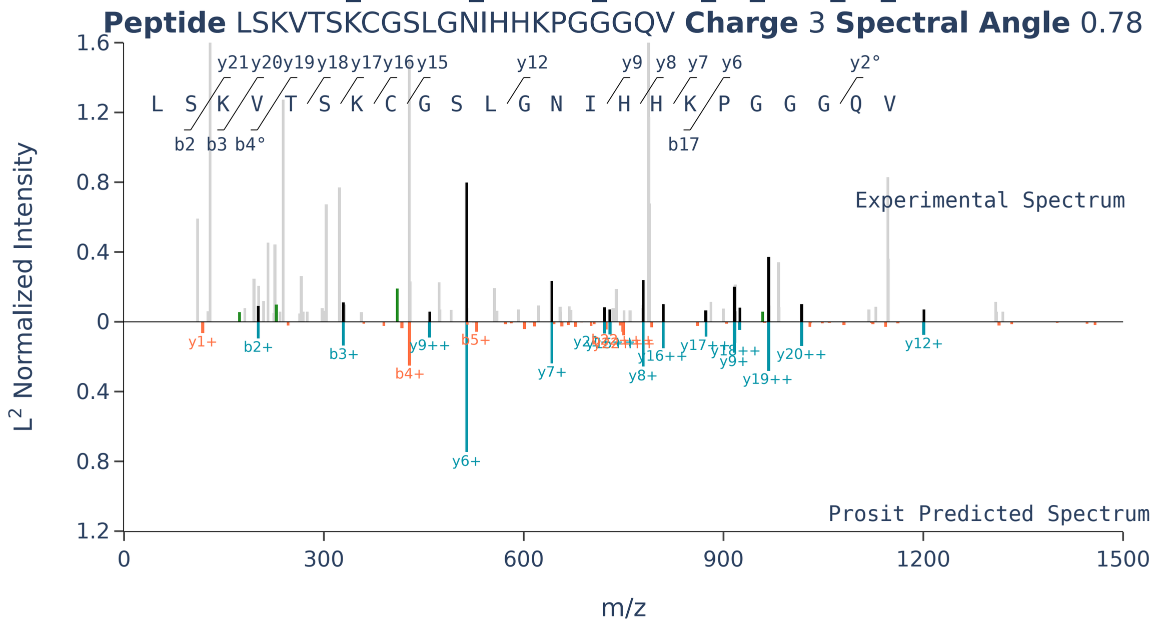


**Source** WSoh_221121_011221_HFGoe_L2_hTau_24h_R1 **Scan** 7070
**Peptide** SNVQSKCGSKDNIKHVPGGGSVQ **Charge** 4 **Spectral Angle** 0.9

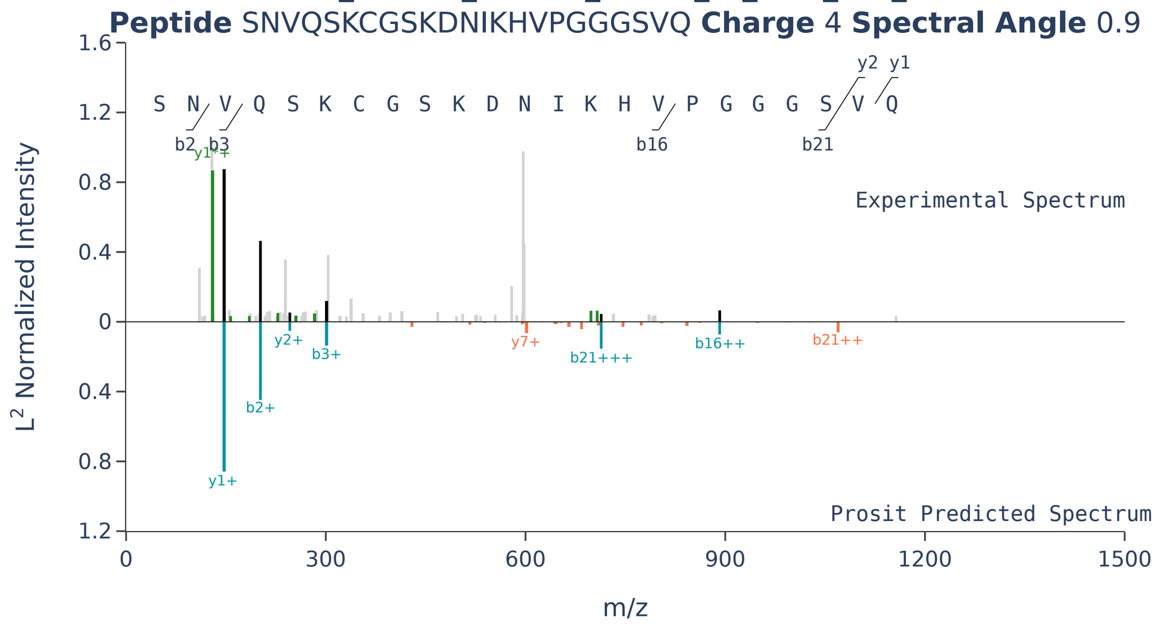


**Source** WSoh_221121_011221_HFGoe_L1_hTau_24h_R1 **Scan** 13523
**Peptide** RTPSLPTPPTREPKKVAVVRTPPKSPSSA **Charge** 5 **Spectral Angle** 0.84

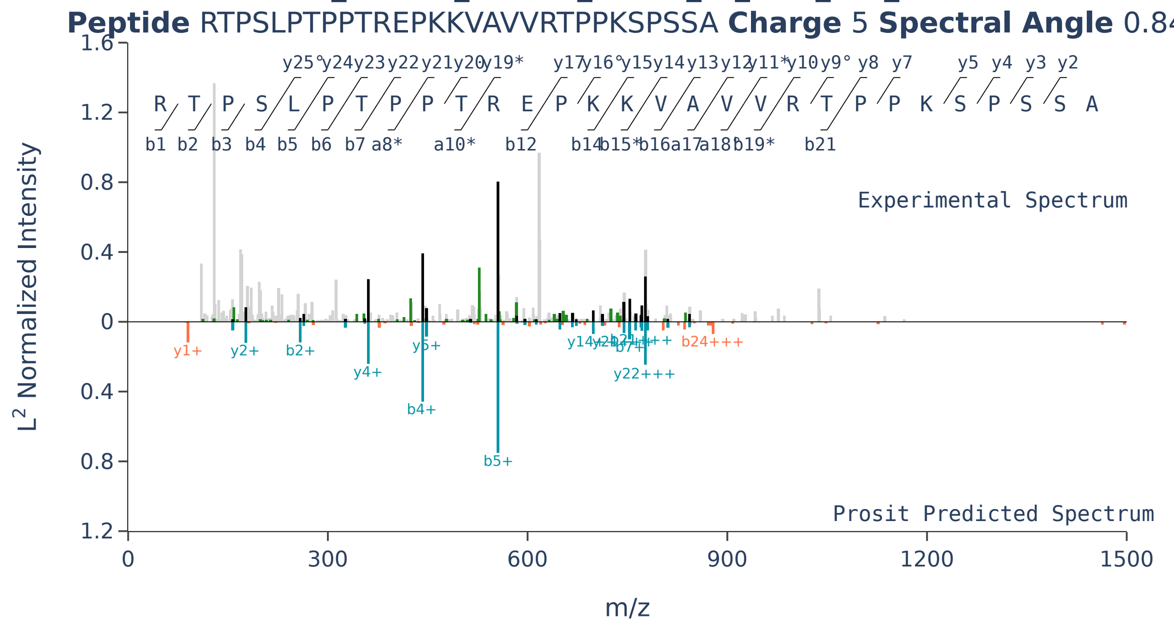


**Source** WSoh_221121_011221_HFGoe_L1_hTau_24h_R2 **Scan** 30406
**Peptide** SIDMVDSPQLATL **Charge** 2 **Spectral Angle** 0.87

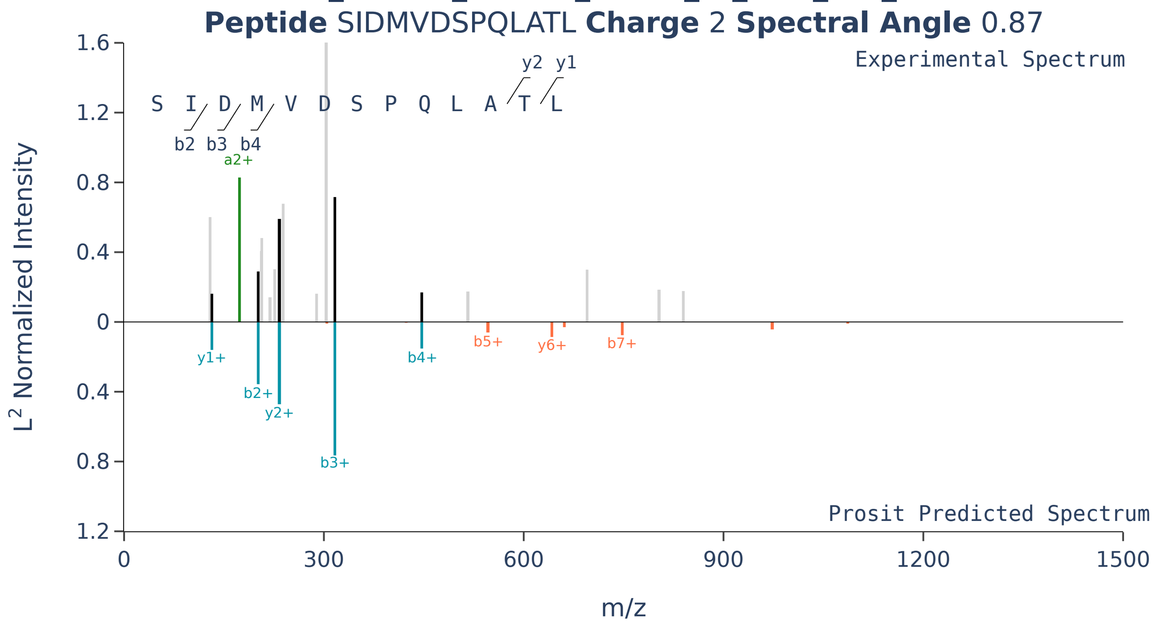


**Source** WSoh_221121_011221_HFGoe_L2_hTau_24h_R1 **Scan** 7873
**Peptide** KSEKLDFK **Charge** 2 **Spectral Angle** 0.75

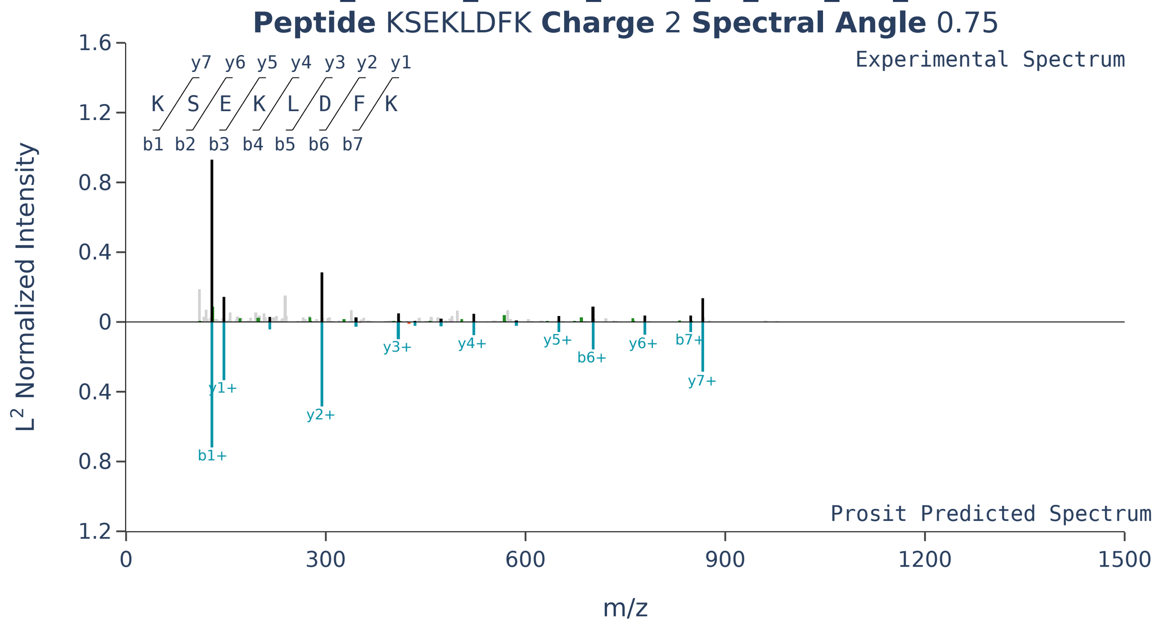


**Source** WSoh_221121_011221_HFGoe_L1_hTau_24h_R2 **Scan** 24463
**Peptide** PMPDLKNVKSKIGSTENL **Charge** 3 **Spectral Angle** 0.84

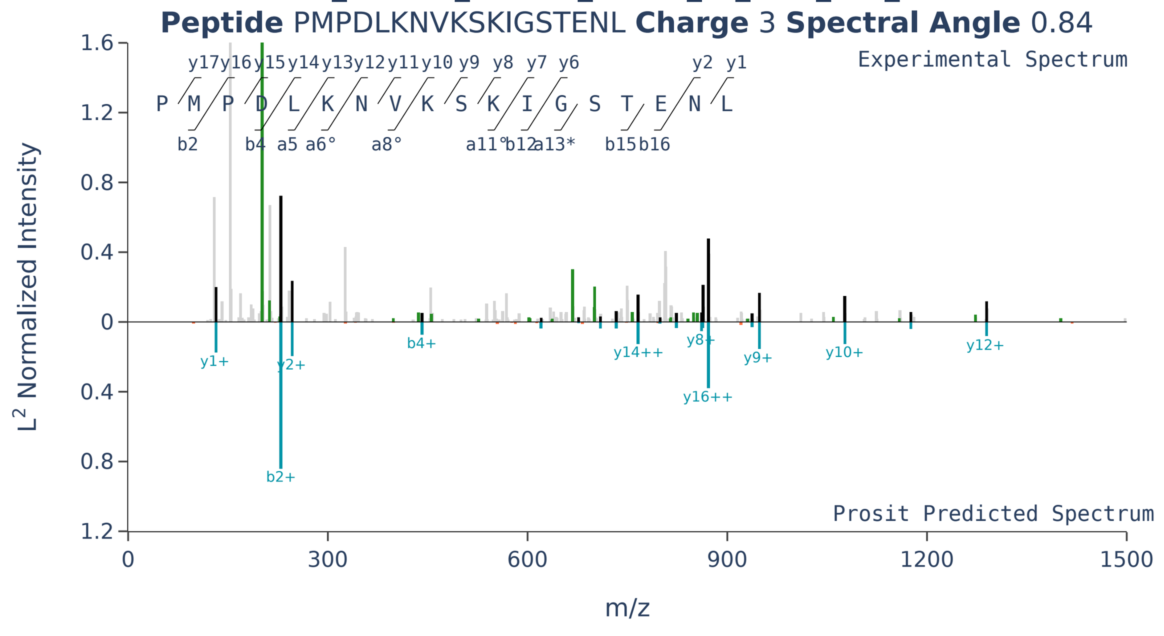

**Source** WSoh_221121_011221_HFGoe_L1_hTau_4h_R1 **Scan** 7226
**Peptide** ENAKAKTDHGAEIVYKS **Charge** 3 **Spectral Angle** 0.79

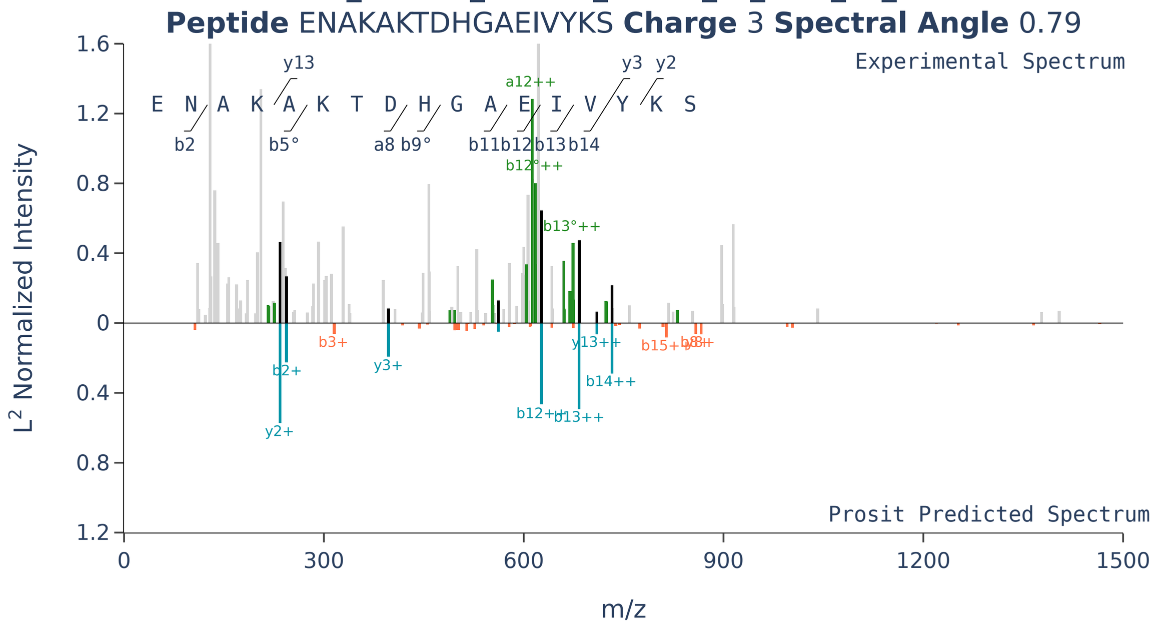


**Source** WSoh_221121_011221_HFGoe_L2_hTau_24h_R2 **Scan** 23268
**Peptide** AAAQPHTEIPEGTTAEEAGIGDTPSLED **Charge** 3 **Spectral Angle** 0.82

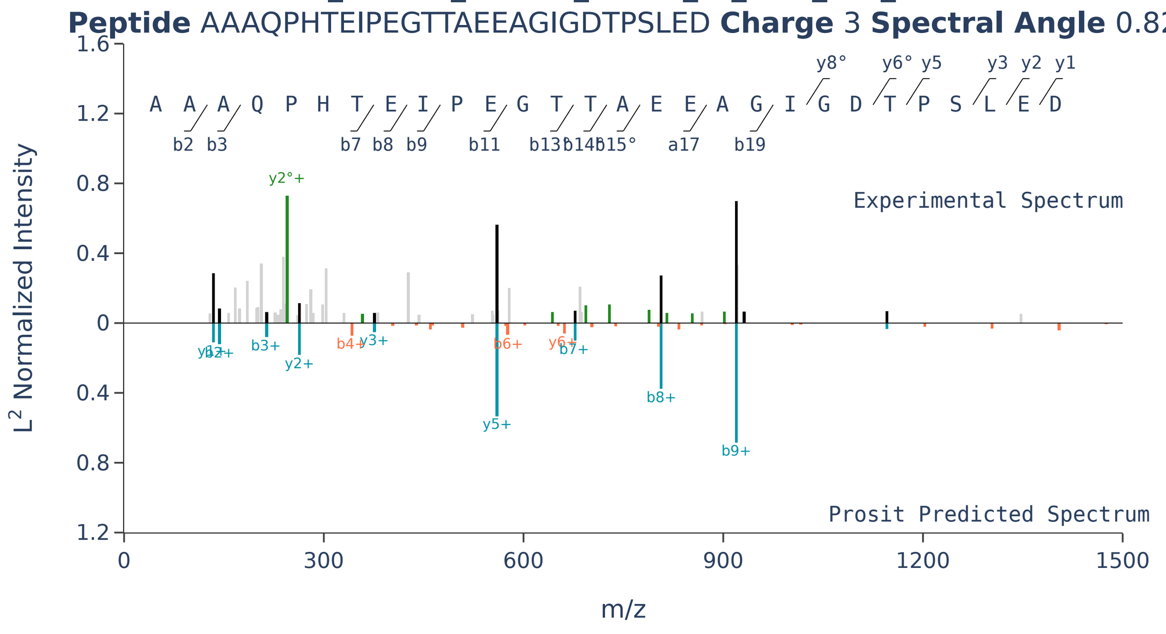


**Source** WSoh_221121_011221_HFGoe_L1_hTau_24h_R2 **Scan** 11752
**Peptide** PSLPTPPTREPKKVAVVRTPPKSPSSAKSR **Charge** 6 **Spectral Angle** 0.75

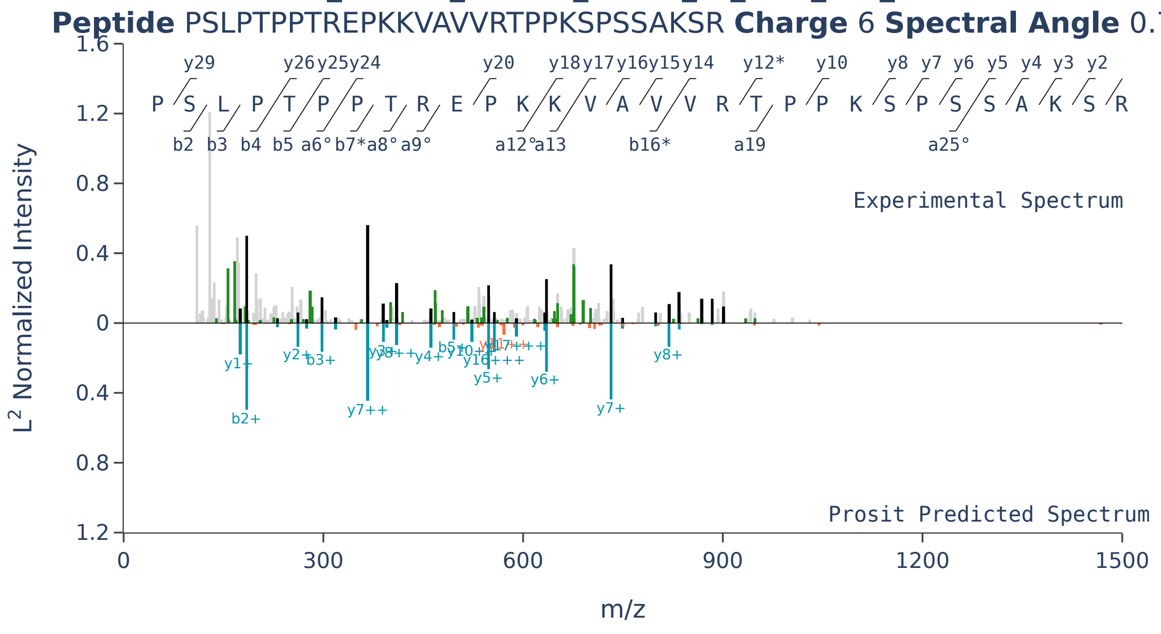


**Source** WSoh_221121_011221_HFGoe_L1_hTau_24h_R2 **Scan** 13876
**Peptide** FRENAKAKTDHGAEIVYKSPVV **Charge** 3 **Spectral Angle** 0.72

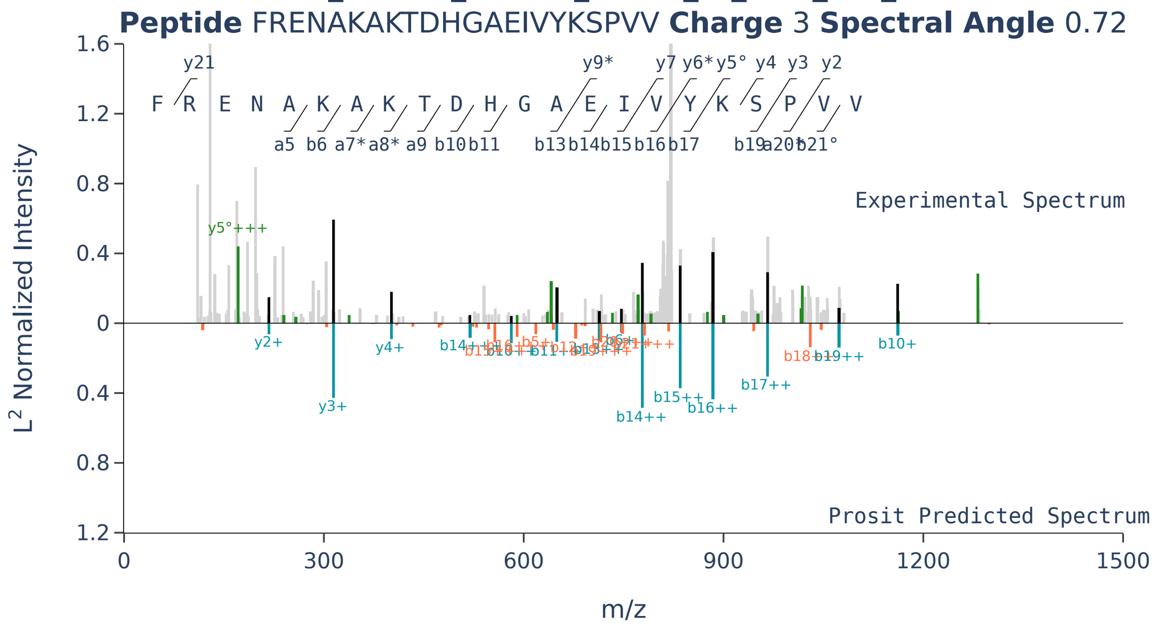


**Source** WSoh_221121_011221_HFGoe_L1_hTau_24h_R1 **Scan** 14127
**Peptide** KAKTDHGAEIVYKSPVVSGDTSPRHLSNVS **Charge** 5 **Spectral Angle** 0.79

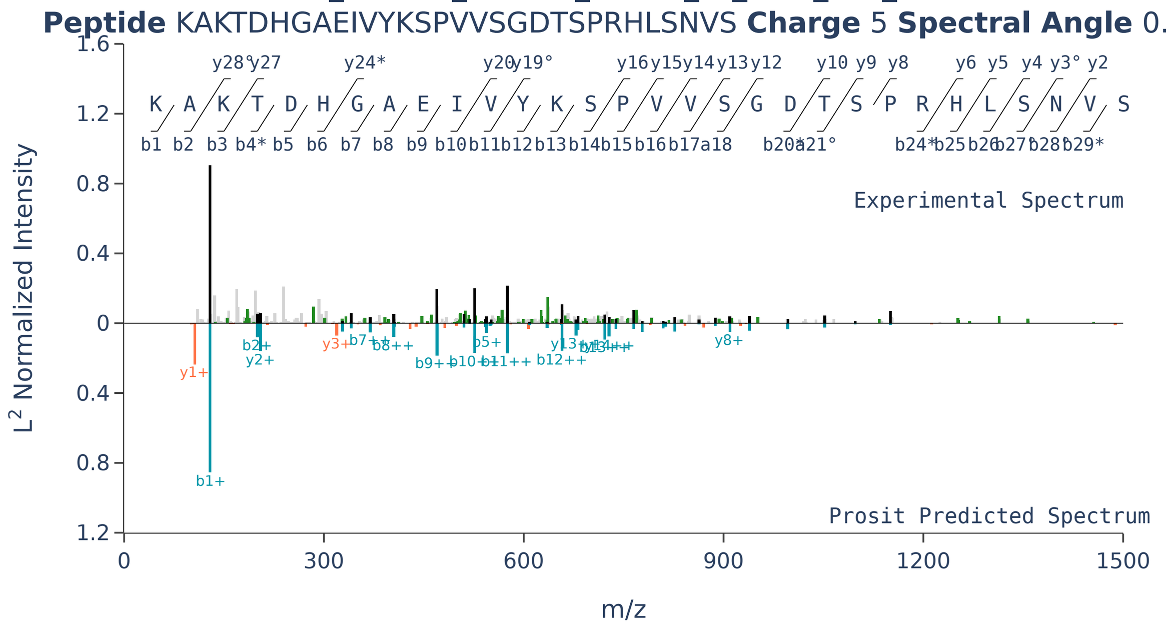


**Source** WSoh_221121_011221_HFGoe_L1_hTau_24h_R1 **Scan** 15070
**Peptide** KTDHGAEIVYKSPVVSGD **Charge** 3 **Spectral Angle** 0.55

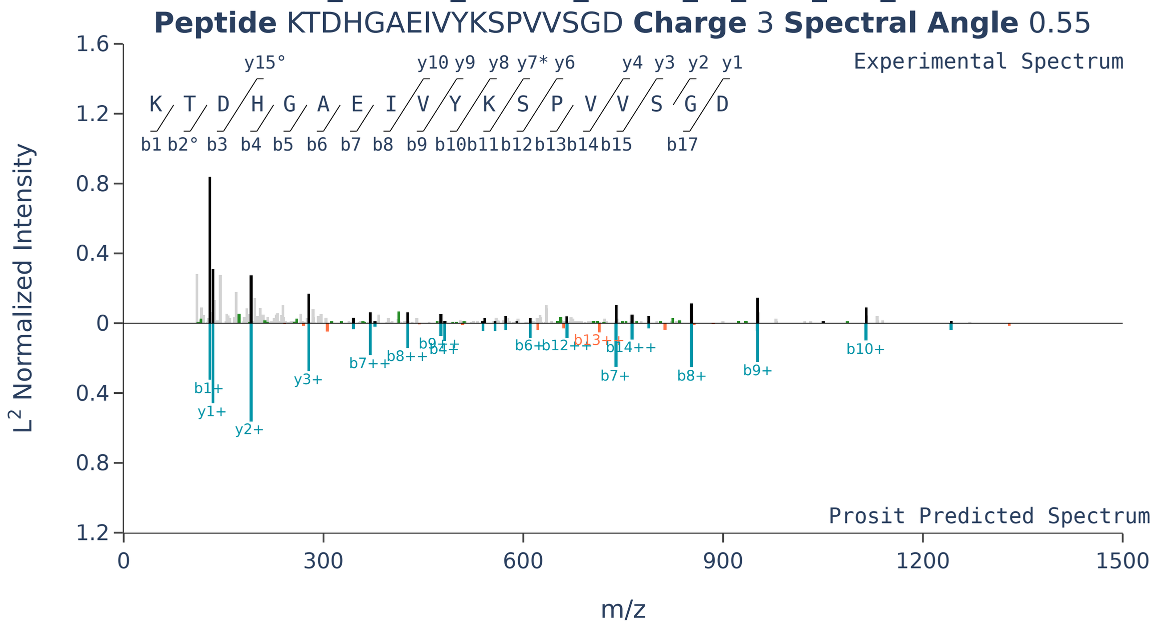


**Source** WSoh_221121_011221_HFGoe_L1_hTau_4h_R1 **Scan** 7896
**Peptide** CGSLGNIHHKPGGGQVEVK **Charge** 3 **Spectral Angle** 0.69

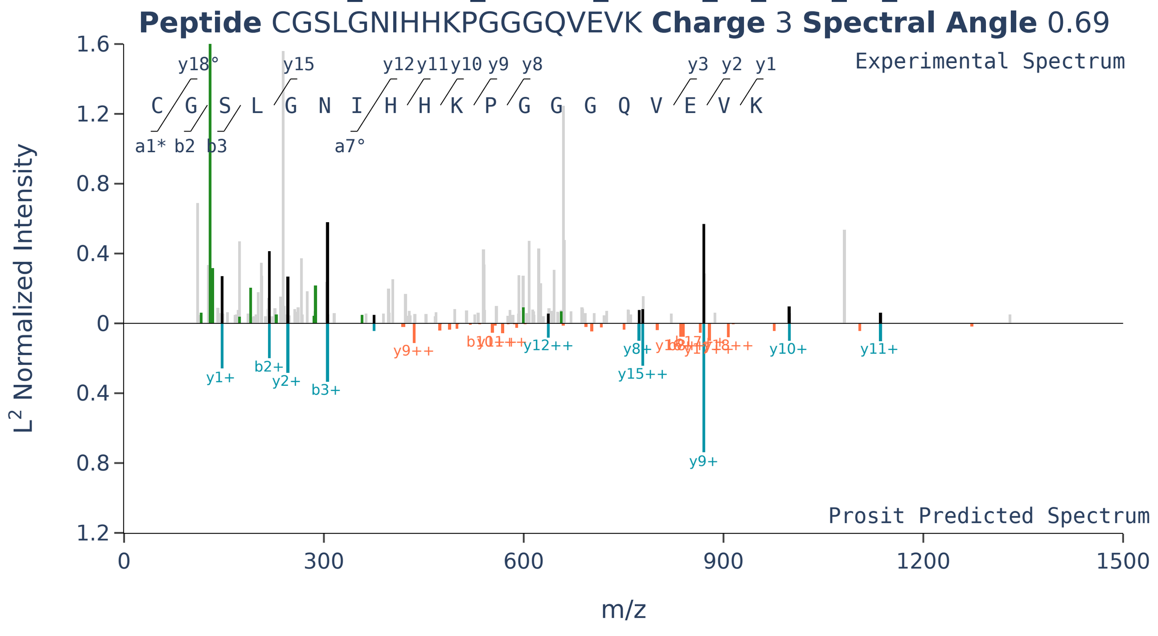


**Source** WSoh_221121_011221_HFGoe_L1_hTau_4h_R1 **Scan** 12460
**Peptide** GNIHHKPGGGQVEVKSEKLDFKDRVQSKI **Charge** 5 **Spectral Angle** 0.64

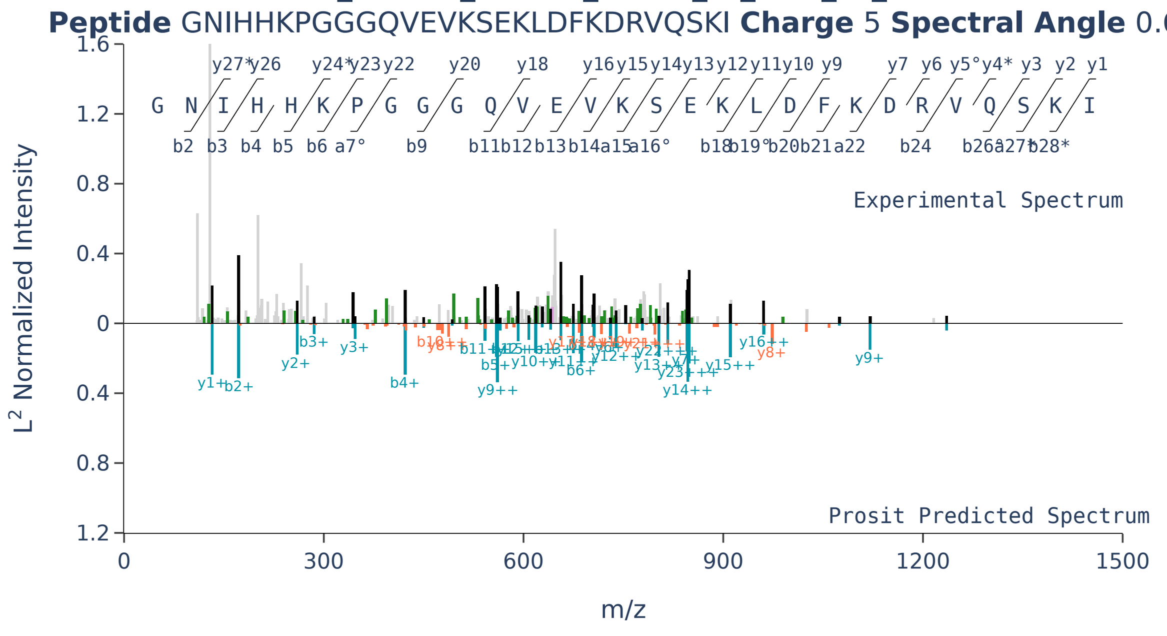


**Source** WSoh_221121_011221_HFGoe_L2_hTau_24h_R2 **Scan** 17812
**Peptide** QLINKKLDLSNVQS **Charge** 2 **Spectral Angle** 0.69

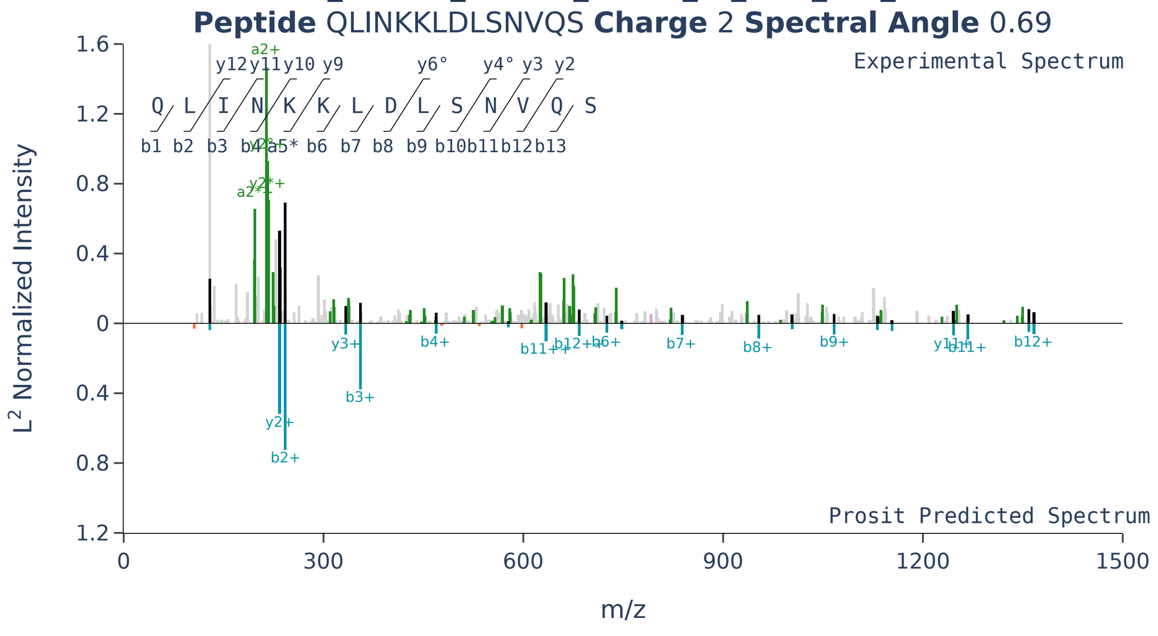


**Source** WSoh_221121_011221_HFGoe_L2_hTau_4h_R1 **Scan** 21465
**Peptide** VKSEKLDFKDRVQSKIGSLDNITHV **Charge** 4 **Spectral Angle** 0.82

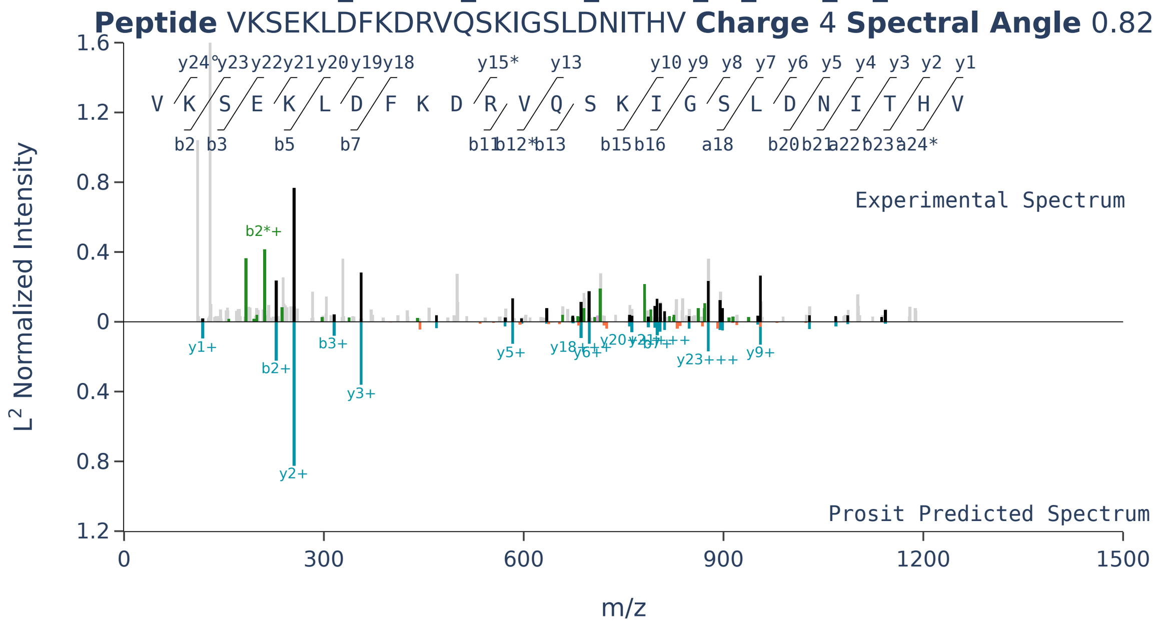


**Source** WSoh_221121_011221_HFGoe_L1_hTau_24h_R1 **Scan** 17596
**Peptide** PLVDEGAPGKQAAAQPHTEIPEGTTAE **Charge** 3 **Spectral Angle** 0.71

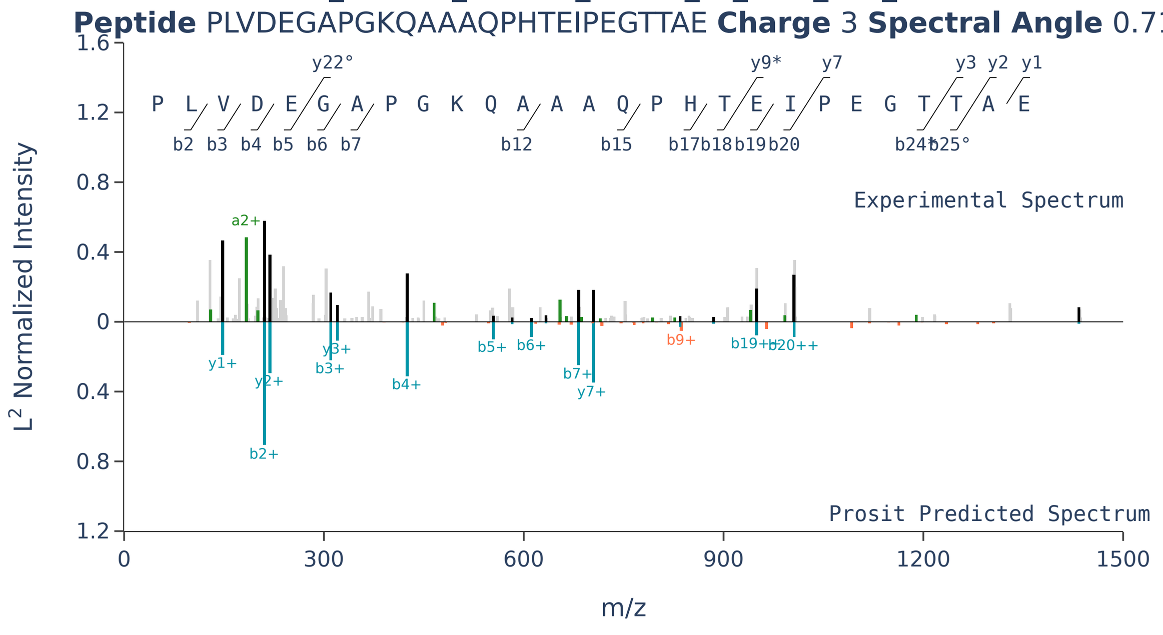


**Source** WSoh_221121_011221_HFGoe_L2_hTau_24h_R1 **Scan** 17314
**Peptide** KDNIKHVPGGGSVQIVY **Charge** 3 **Spectral Angle** 0.39

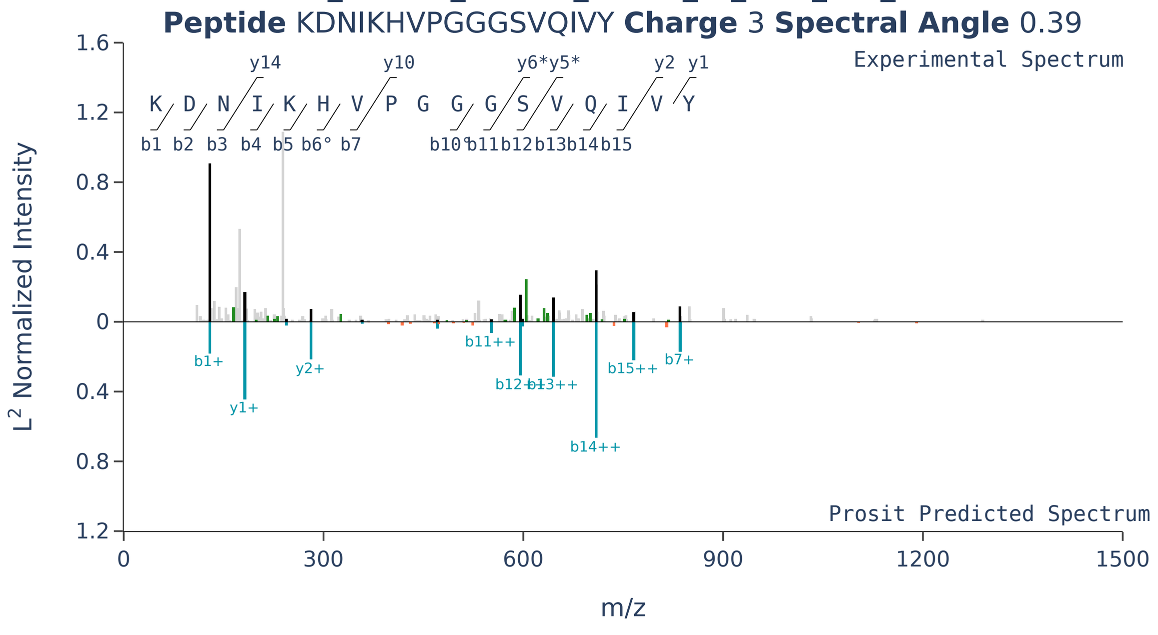


**Source** WSoh_221121_011221_HFGoe_L1_hTau_24h_R1 **Scan** 23259
**Peptide** AAQPHTEIPEGTTAEEAGIGDTPSLEDE **Charge** 2 **Spectral Angle** 0.83

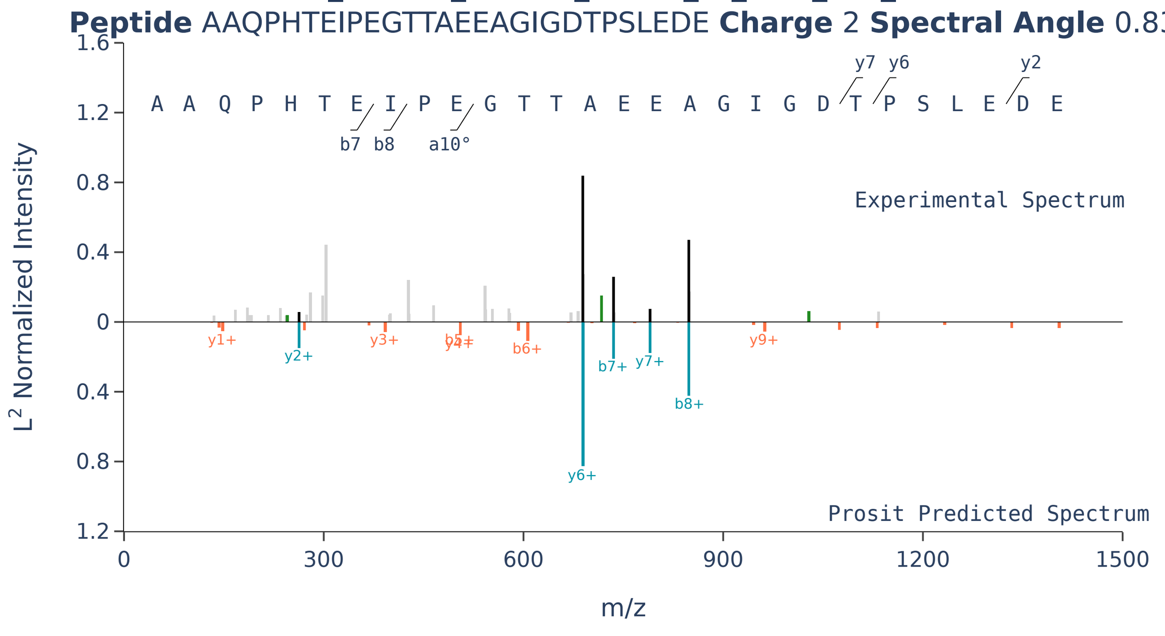


**Source** WSoh_221121_011221_HFGoe_L1_hTau_4h_R2 **Scan** 24435
**Peptide** PGGGSVQIVYKPVDLS **Charge** 2 **Spectral Angle** 0.74

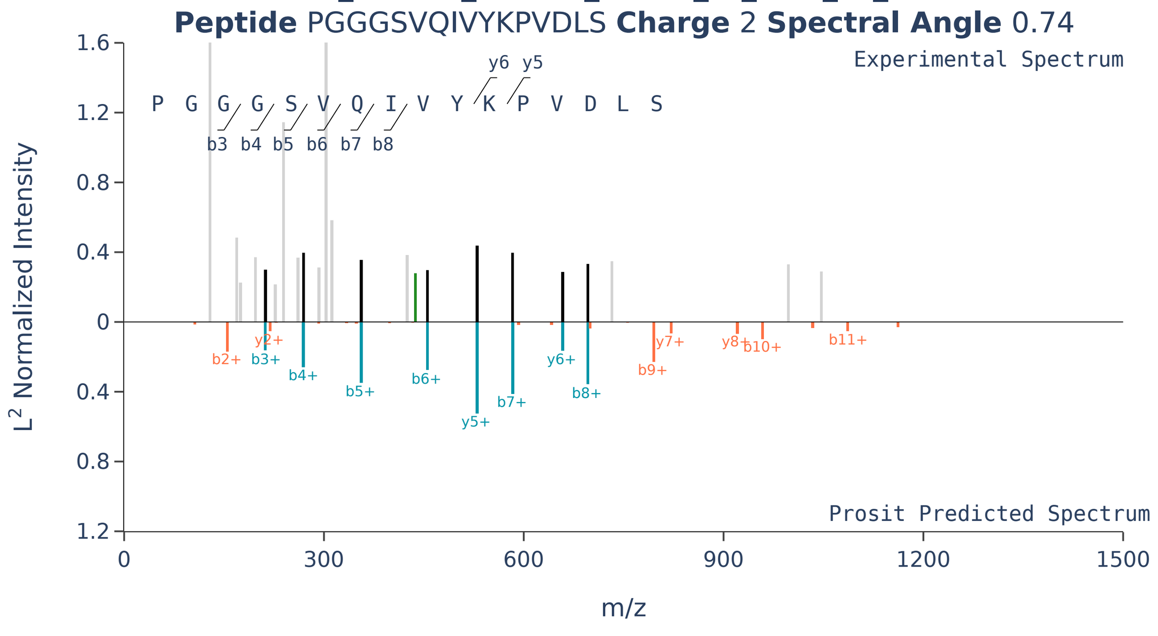


**Source** WSoh_221121_011221_HFGoe_L2_hTau_24h_R2 **Scan** 11296
**Peptide** QANATRIPAKTPPAPKTPPSSGEPPKS **Charge** 5 **Spectral Angle** 0.86

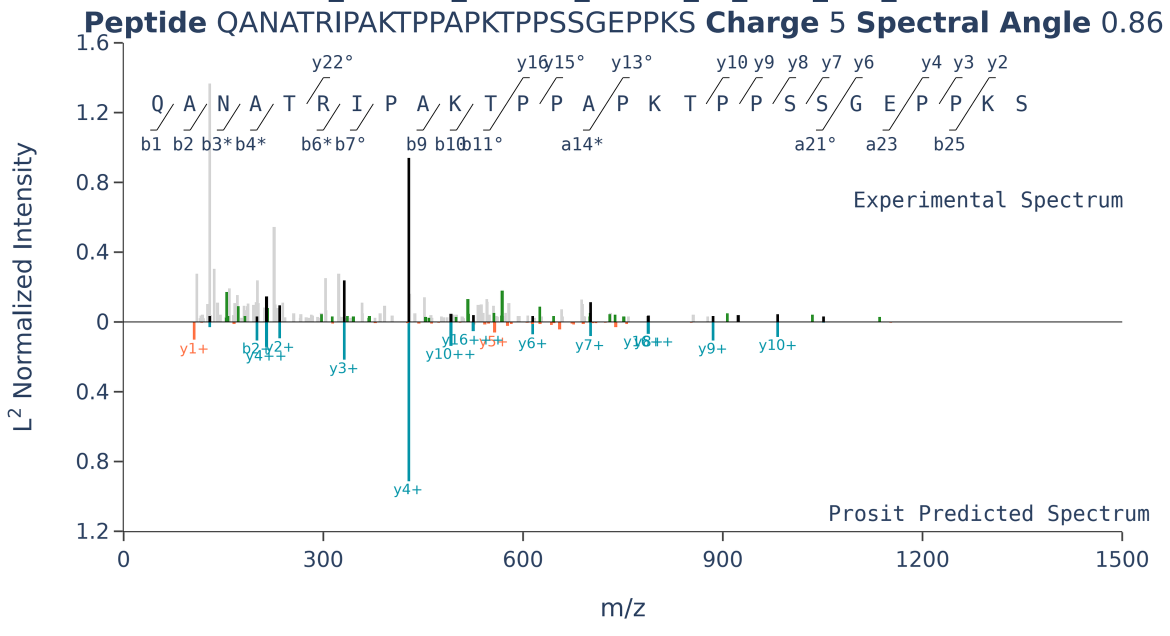

**Source** WSoh_221121_011221_HFGoe_L1_hTau_24h_R1 **Scan** 24111
**Peptide** SRLQTAPVPMPDLKNVKSKIGSTENL **Charge** 4 **Spectral Angle** 0.7

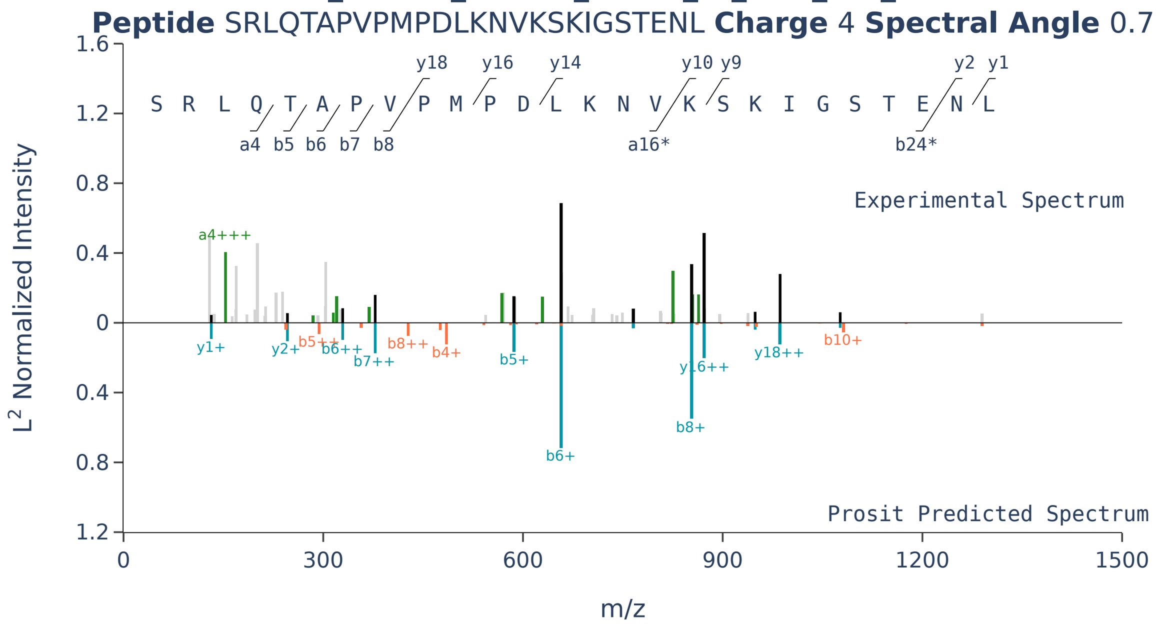


**Source** WSoh_221121_011221_HFGoe_L1_hTau_4h_R2 **Scan** 13414
**Peptide** KPVDLSKVTSKCGSLGNIHHKPGGGQV **Charge** 5 **Spectral Angle** 0.83

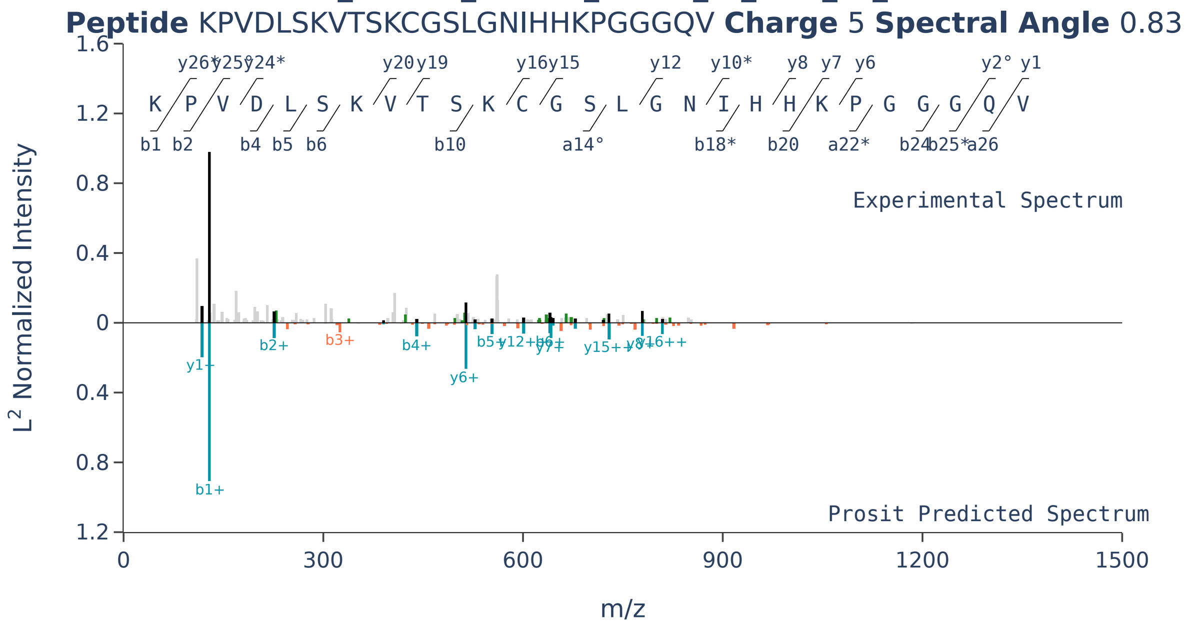


**Source** WSoh_221121_011221_HFGoe_L1_hTau_4h_R2 **Scan** 21360
**Peptide** TSPRHLSNVSSTGSIDMVDSPQLA **Charge** 3 **Spectral Angle** 0.7

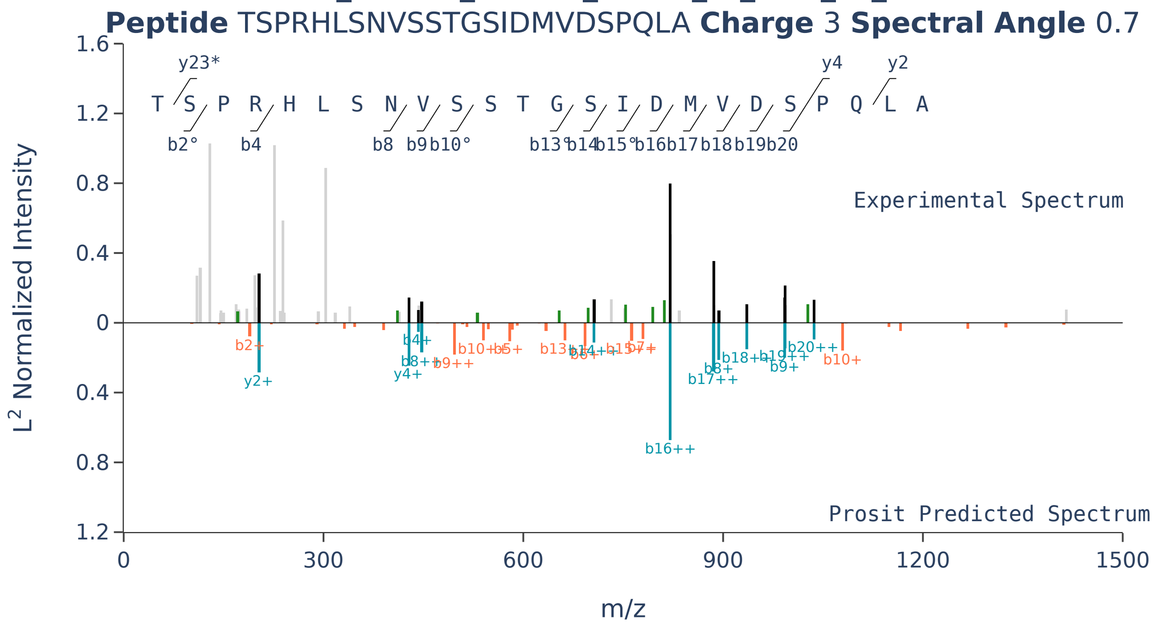


**Source** WSoh_221121_011221_HFGoe_L2_hTau_24h_R2 **Scan** 17187
**Peptide** EGAPGKQAAAQPHTEIPEGTTAEEAGLGD **Charge** 3 **Spectral Angle** 0.59

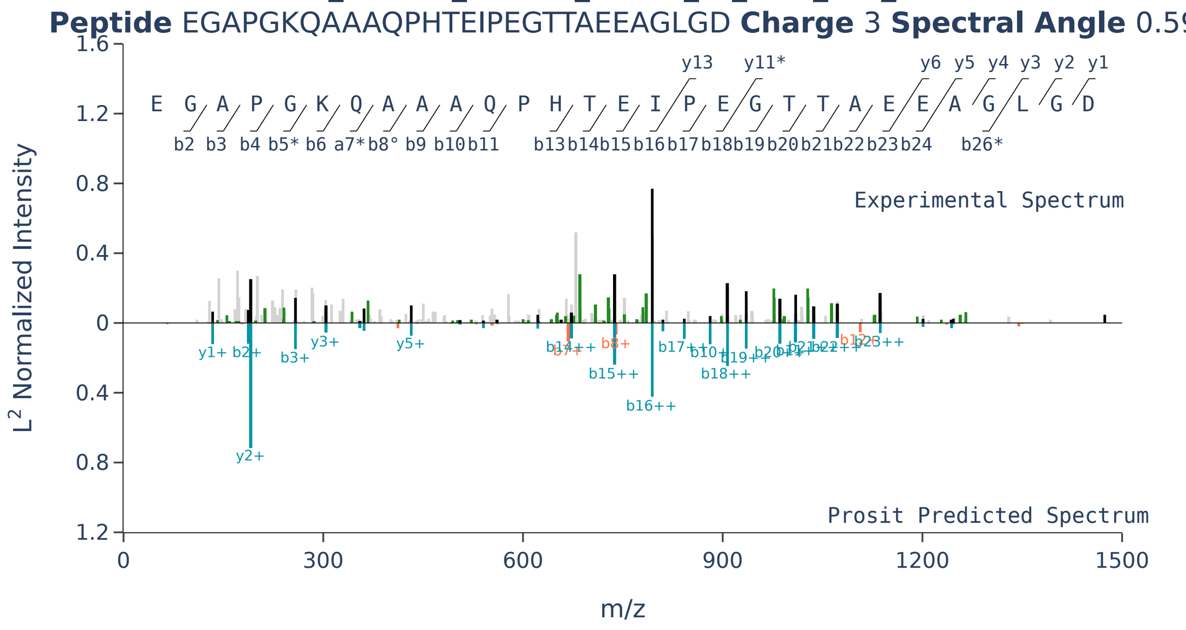


**Source** WSoh_221121_011221_HFGoe_L1_hTau_24h_R1 **Scan** 21943
**Peptide** SVQIVYKPVDLSKVTSK **Charge** 4 **Spectral Angle** 0.71

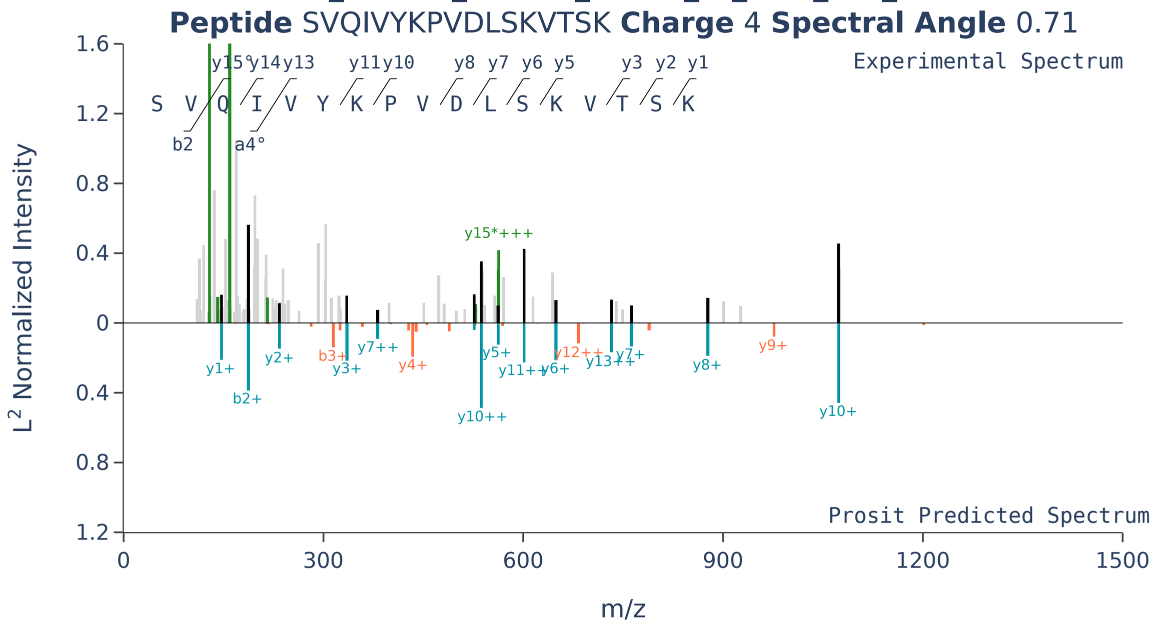


**Source** WSoh_221121_011221_HFGoe_L1_hTau_24h_R1 **Scan** 15803
**Peptide** LINKKLDLSNVQS **Charge** 3 **Spectral Angle** 0.53

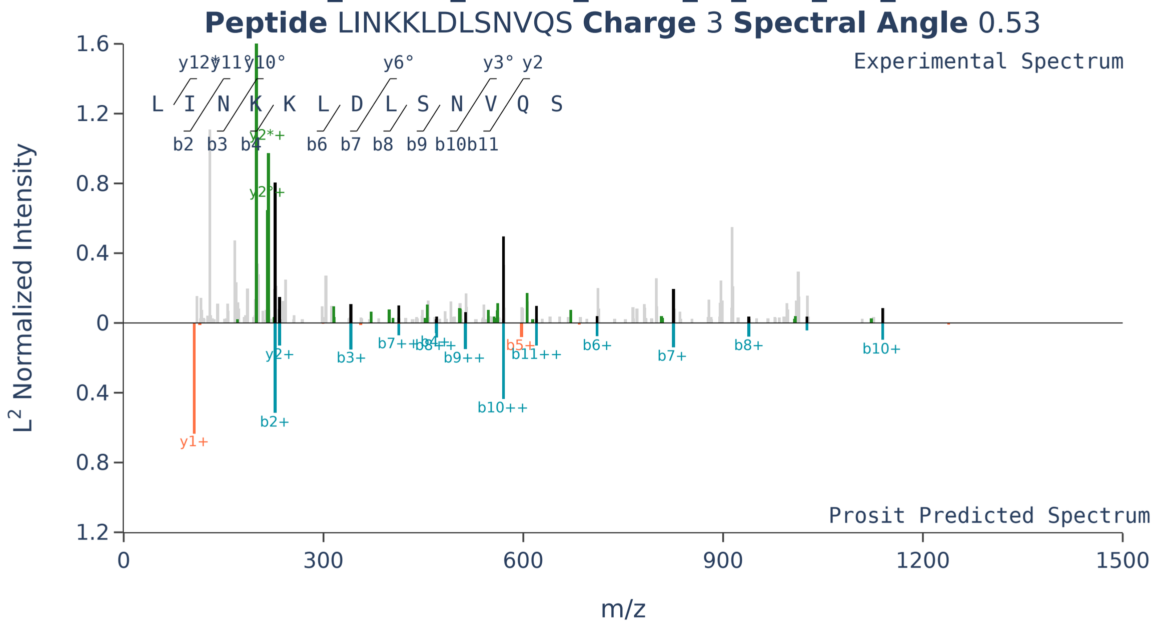


**Source** WSoh_221121_011221_HFGoe_L1_hTau_4h_R2 **Scan** 7167
**Peptide** KIATPRGAAPPGQKGQAN **Charge** 3 **Spectral Angle** 0.69

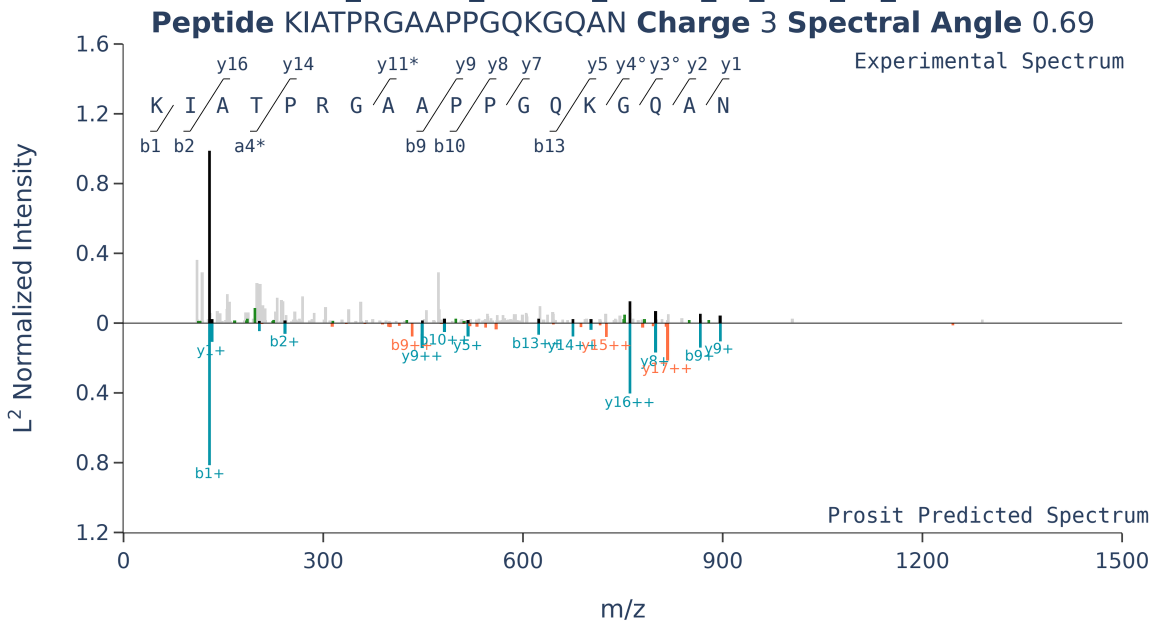


**Source** WSoh_221121_011221_HFGoe_L2_hTau_4h_R2 **Scan** 14542
**Peptide** KLTFRENAKAKTDHGAEIVYKSPVVSG **Charge** 4 **Spectral Angle** 0.74

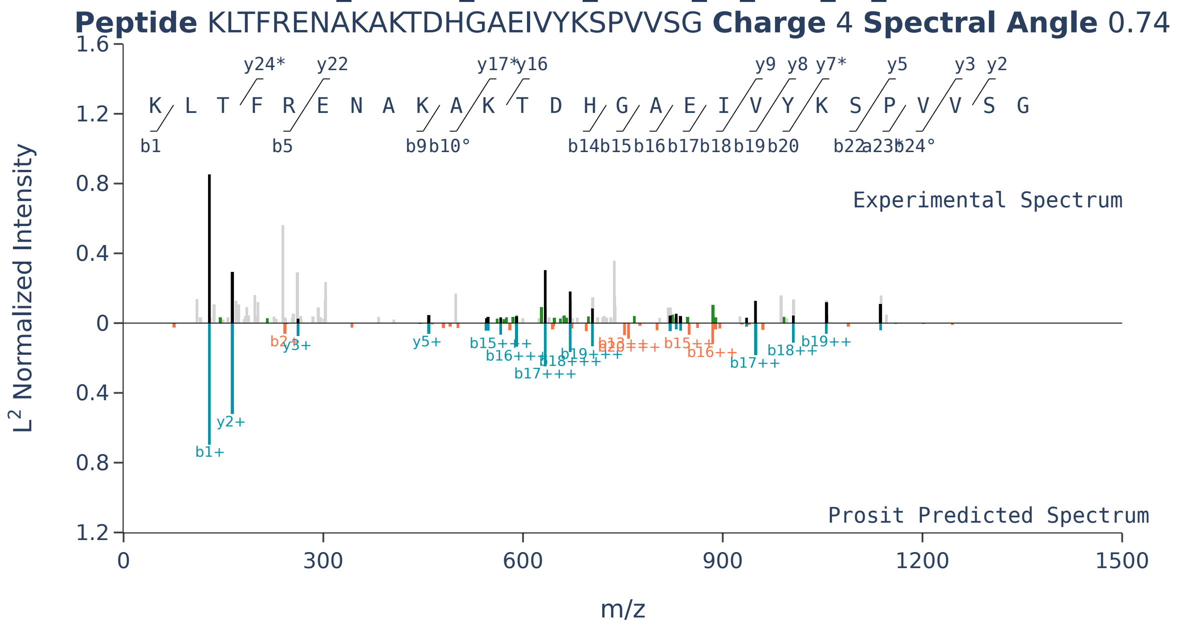


**Source** WSoh_221121_011221_HFGoe_L1_hTau_24h_R1 **Scan** 23627
**Peptide** NVSSTGSIDMVDSPQL **Charge** 2 **Spectral Angle** 0.86

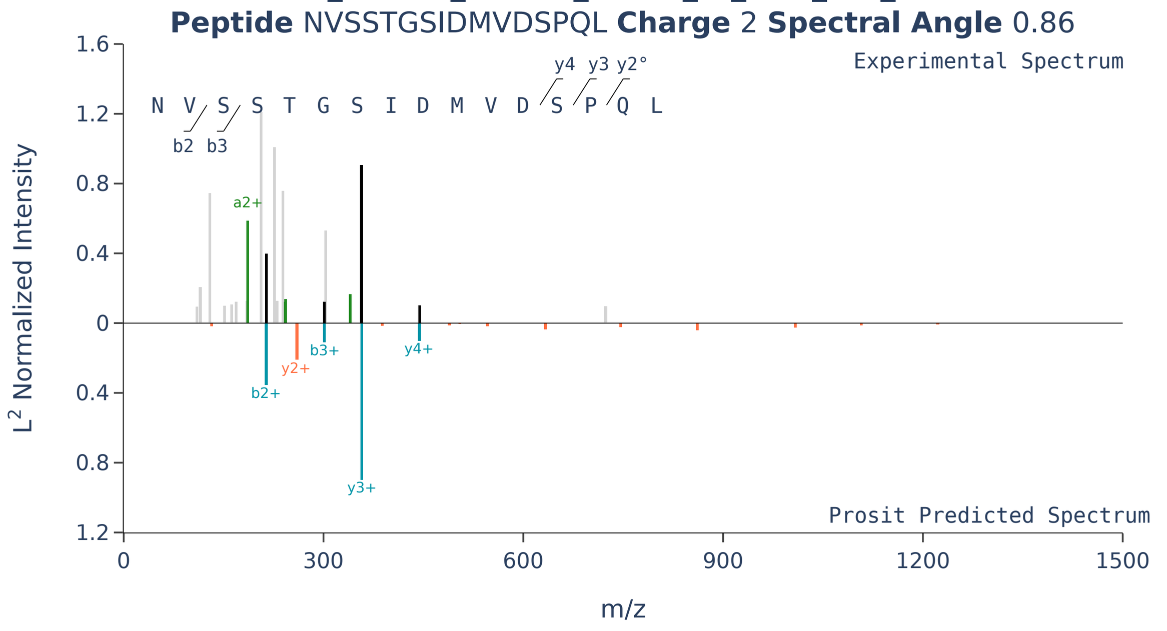


**Source** WSoh_221121_011221_HFGoe_L1_hTau_24h_R2 **Scan** 6726
**Peptide** PGGGNKKIETHKLTFRENAKAKTDH **Charge** 4 **Spectral Angle** 0.78

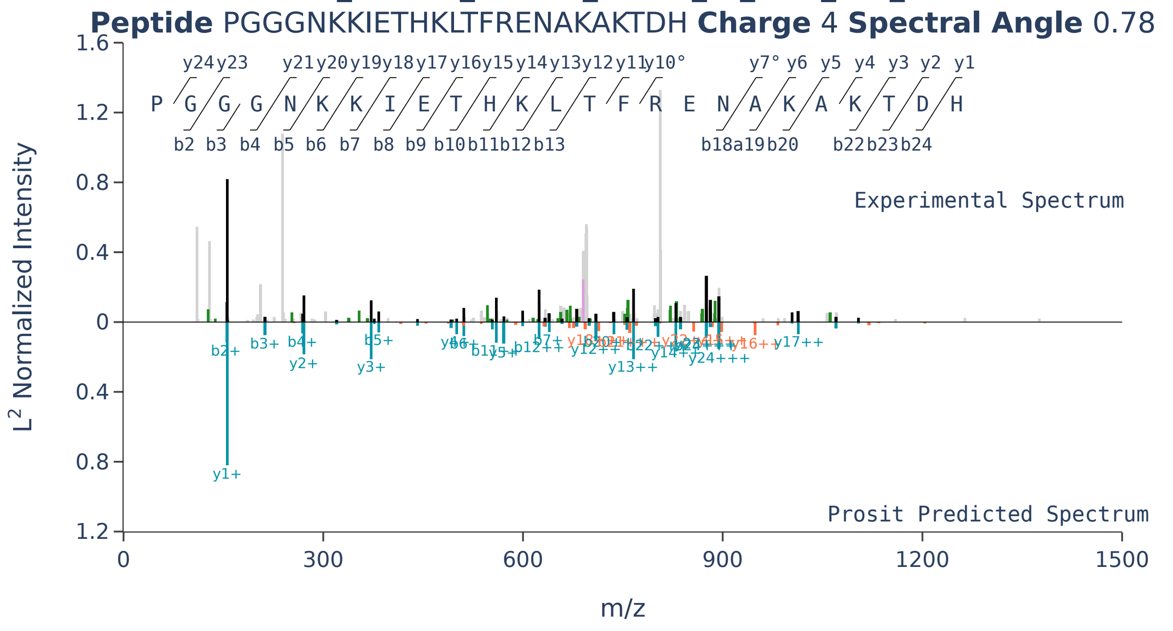


**Source** WSoh_221121_011221_HFGoe_L2_hTau_24h_R1 **Scan** 12449
**Peptide** PGKQAAAQPHTEIPEGTTAEEAG **Charge** 2 **Spectral Angle** 0.49

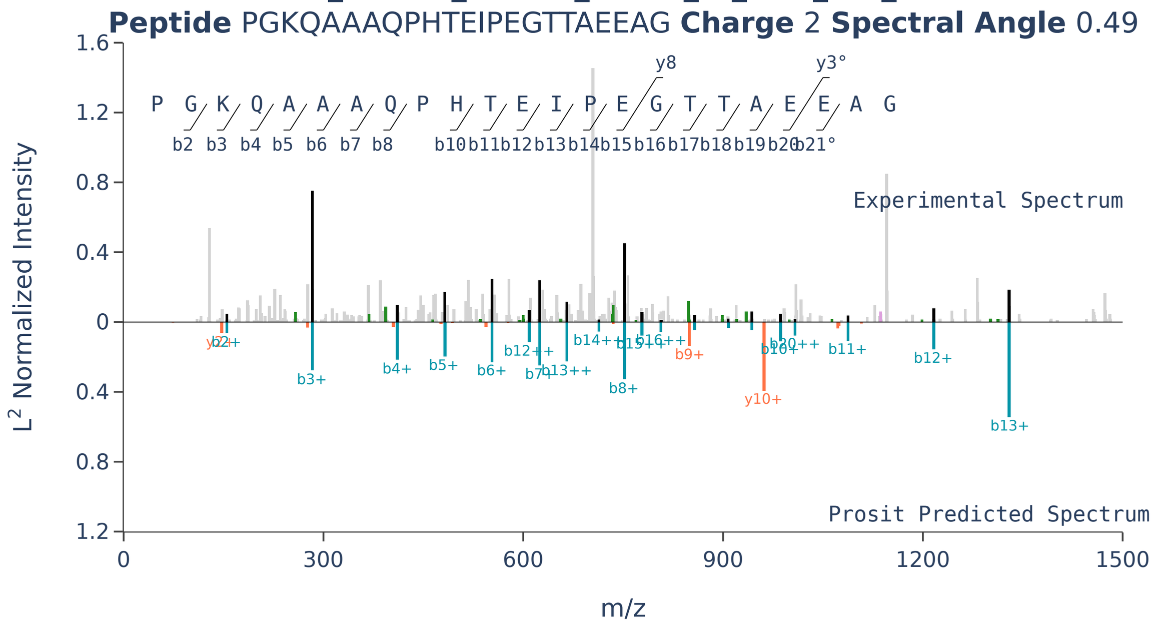


**Source** WSoh_221121_011221_HFGoe_L2_hTau_4h_R2 **Scan** 11586
**Peptide** VYKSPVVSG **Charge** 2 **Spectral Angle** 0.83

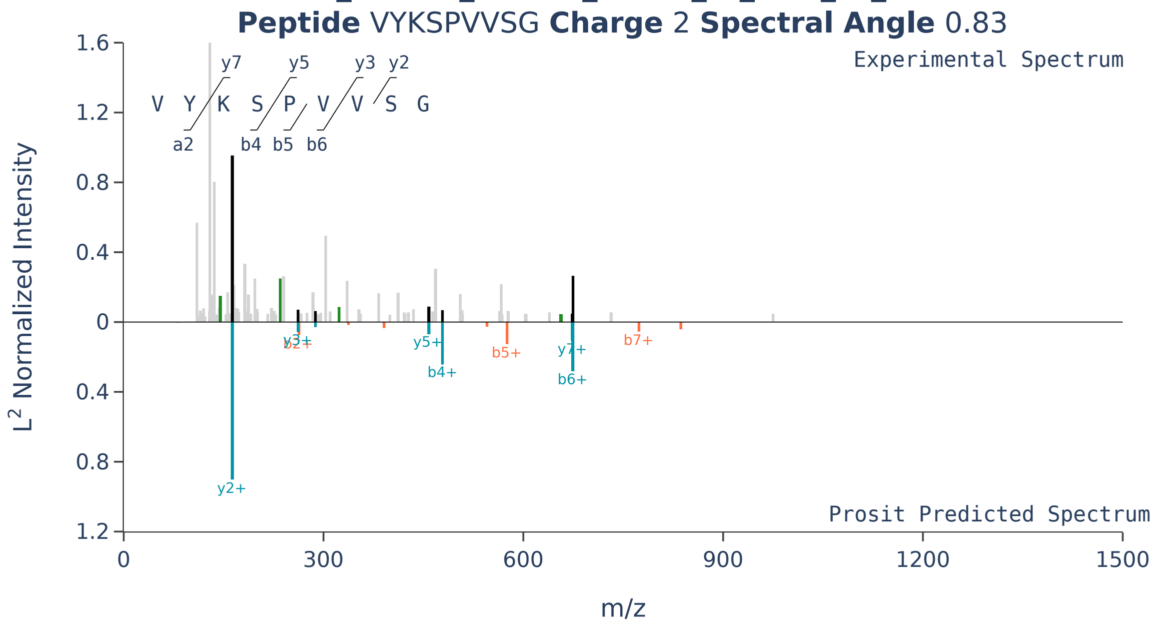


**Source** WSoh_221121_011221_HFGoe_L1_hTau_24h_R1 **Scan** 17533
**Peptide** KVQIINKKLDLSNVQS **Charge** 3 **Spectral Angle** 0.62

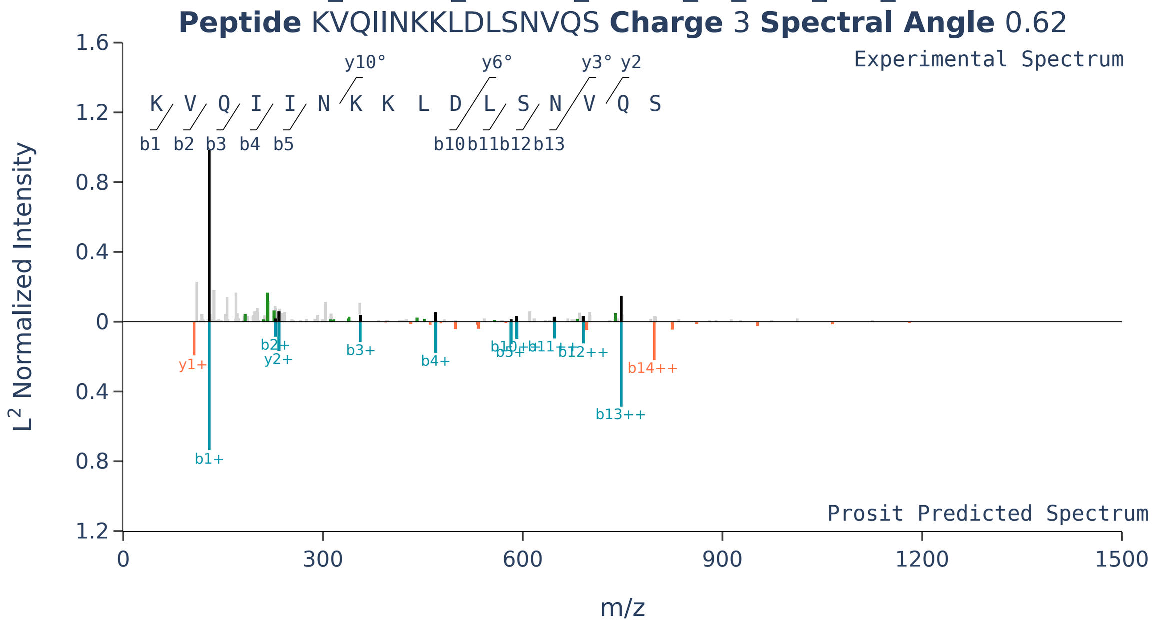


**Source** WSoh_221121_011221_HFGoe_L1_hTau_4h_R1 **Scan** 20194
**Peptide** KHVPGGGSVQIVYKPVDLSKVTS **Charge** 3 **Spectral Angle** 0.71

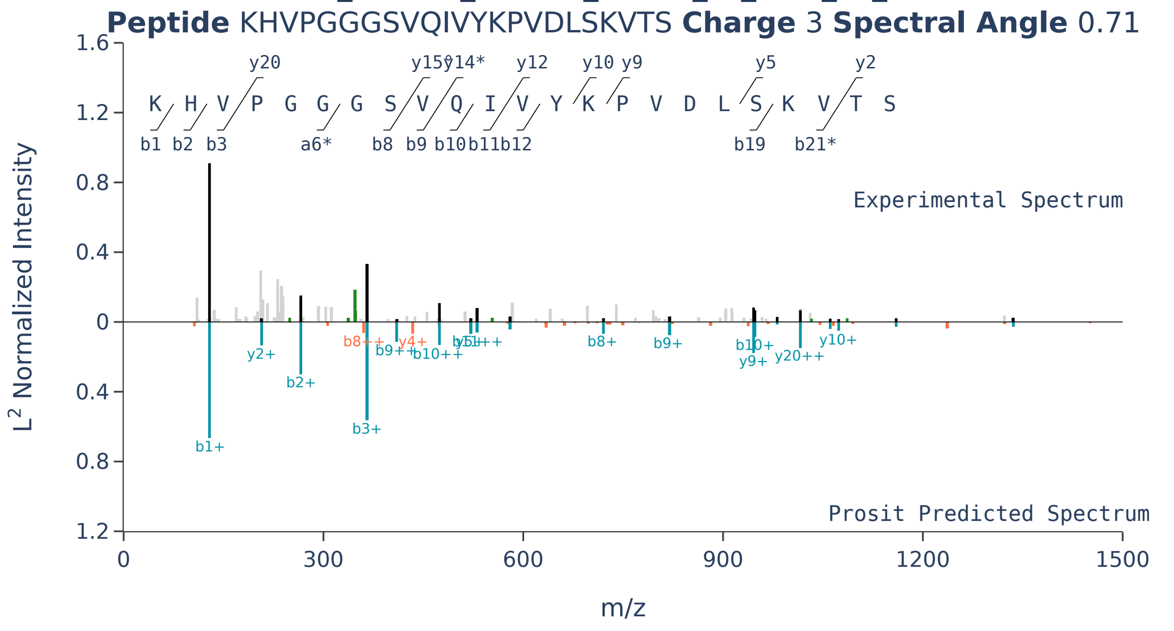


**Source** WSoh_221121_011221_HFGoe_L2_hTau_24h_R1 **Scan** 11136
**Peptide** AAPPGQKGQANATRIPAKTPPAPKTPPSS **Charge** 3 **Spectral Angle** 0.87

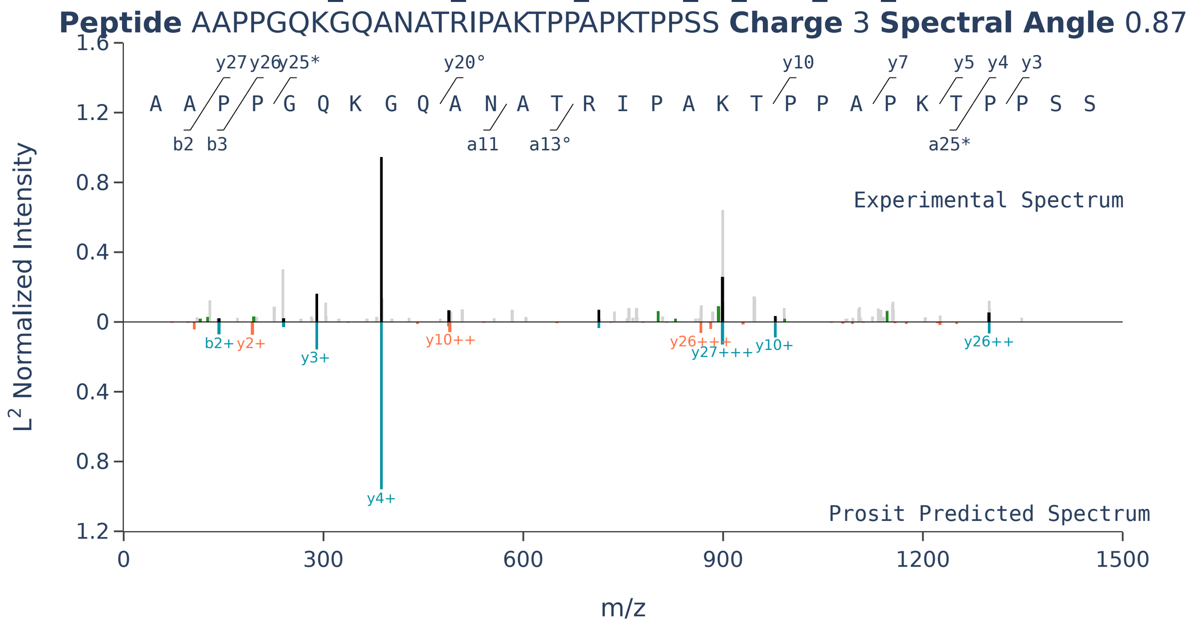

**Source** WSoh_221121_011221_HFGoe_L1_hTau_24h_R1 **Scan** 15457
**Peptide** QGGYTMHQDQEGDTDAGL **Charge** 2 **Spectral Angle** 0.71

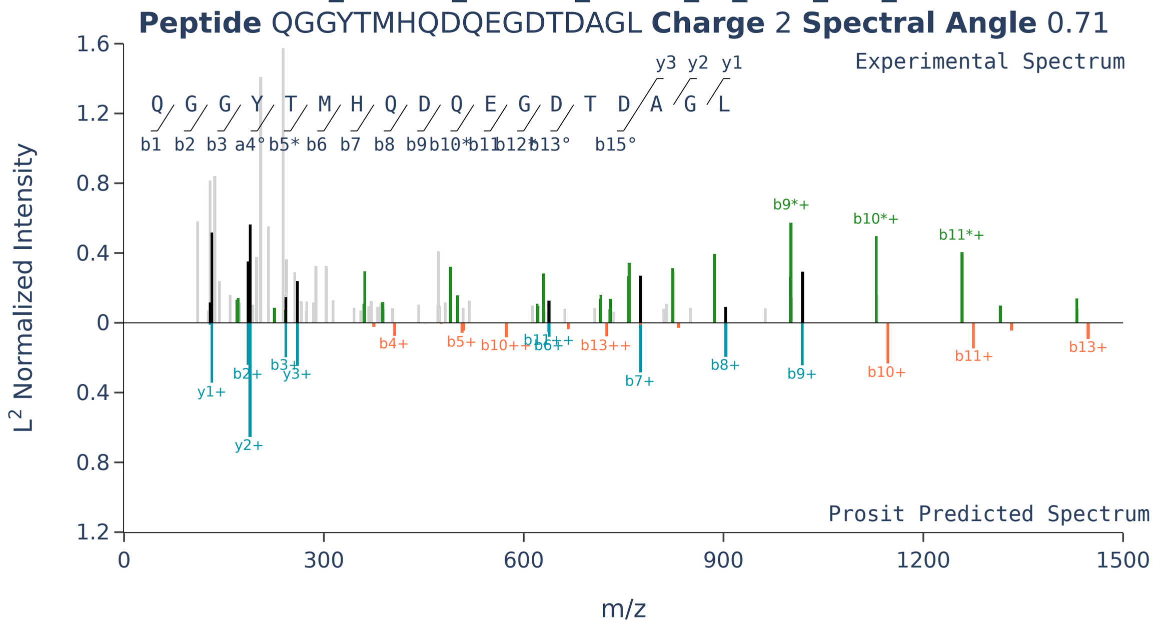


**Source** WSoh_221121_011221_HFGoe_L2_hTau_24h_R2 **Scan** 10780
**Peptide** PPKSGDRSGY **Charge** 2 **Spectral Angle** 0.9

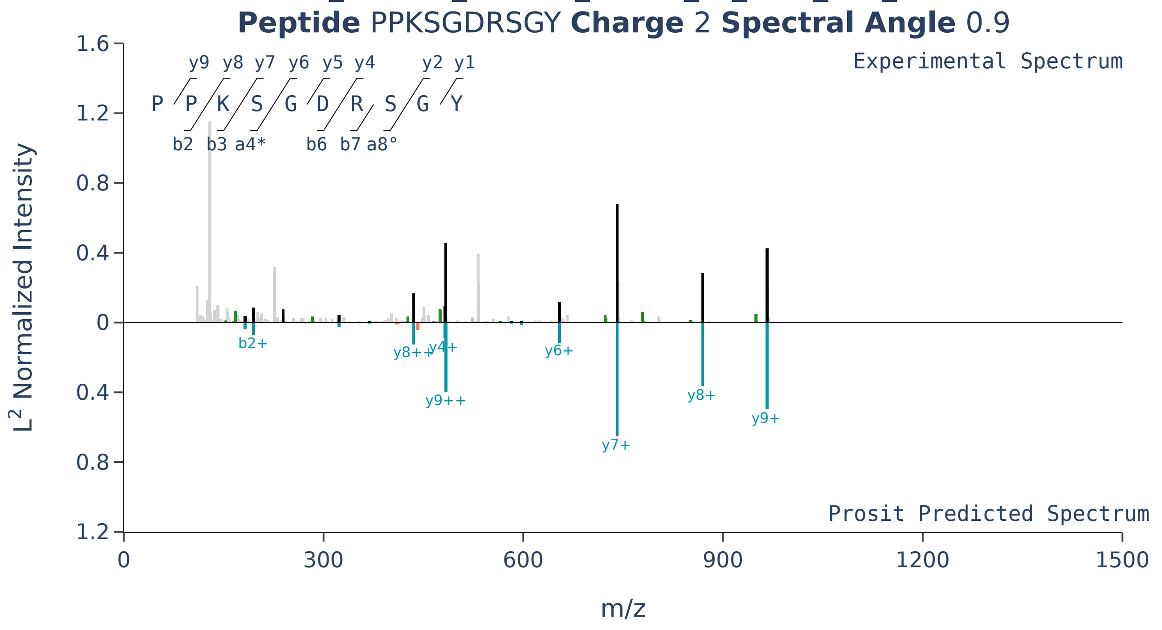


**Source** WSoh_221121_011221_HFGoe_L2_hTau_2h_R2 **Scan** 11151
**Peptide** SKCGSKDNIKHVPGGGSVQL **Charge** 4 **Spectral Angle** 0.74

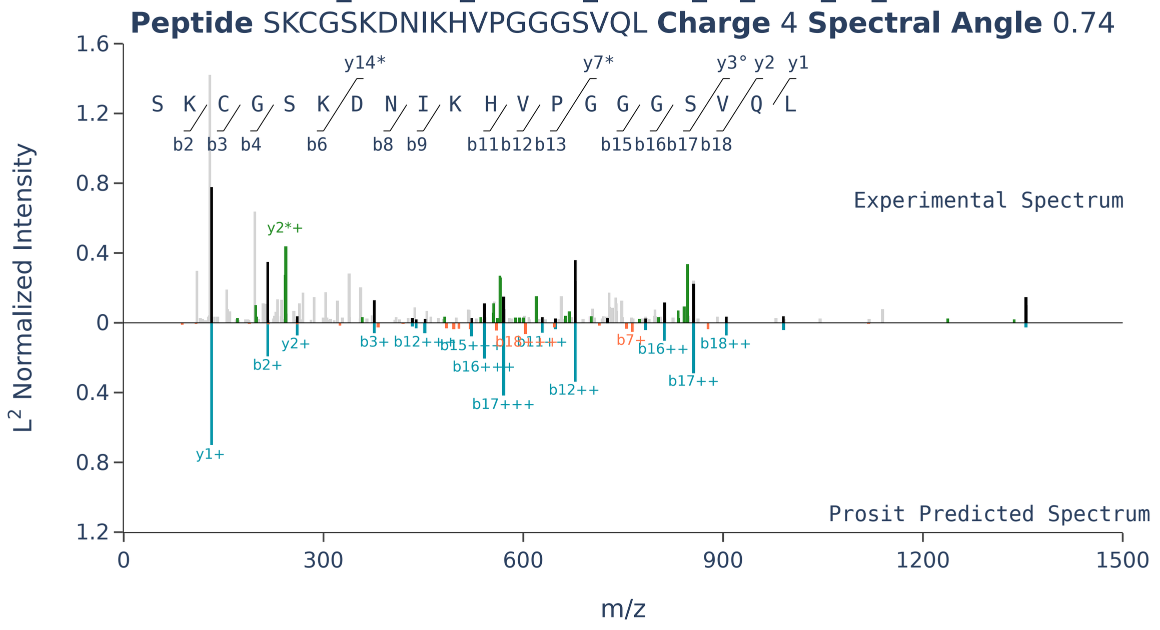


**Source** WSoh_221121_011221_HFGoe_L1_hTau_24h_R2 **Scan** 12457
**Peptide** PGSPGTPGSRSRTPSLPTPPTREPKKVA **Charge** 4 **Spectral Angle** 0.56

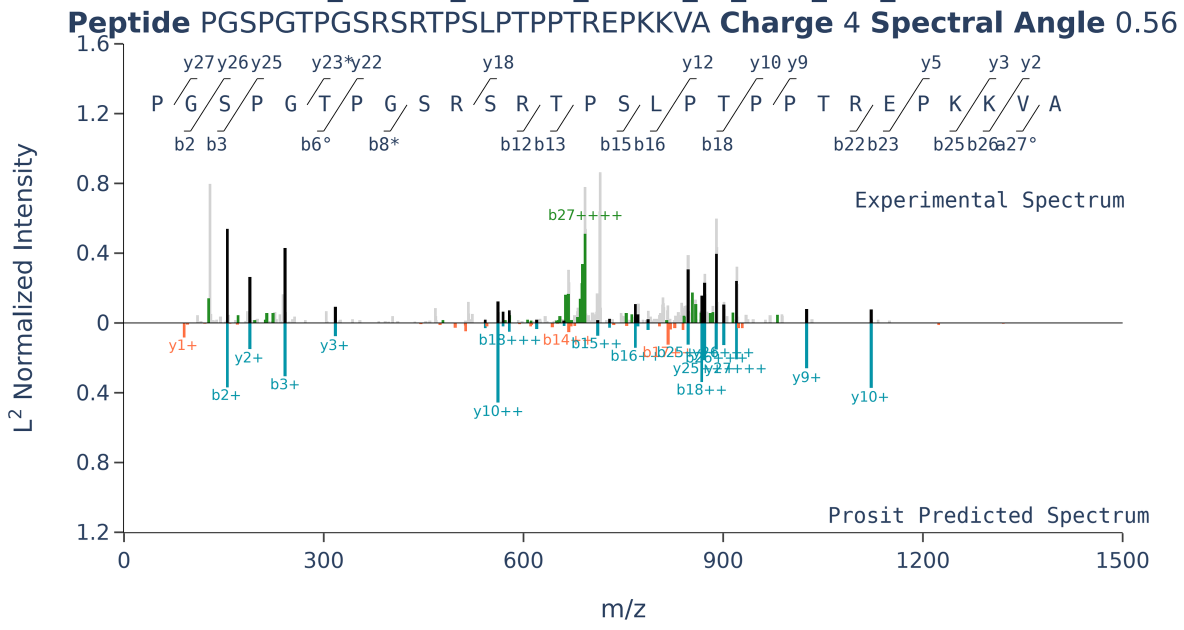


**Source** WSoh_221121_011221_HFGoe_L1_hTau_4h_R1 **Scan** 11180
**Peptide** EPPKSGDRSGYSSPGSPGTPGSRSRTPSL **Charge** 5 **Spectral Angle** 0.77

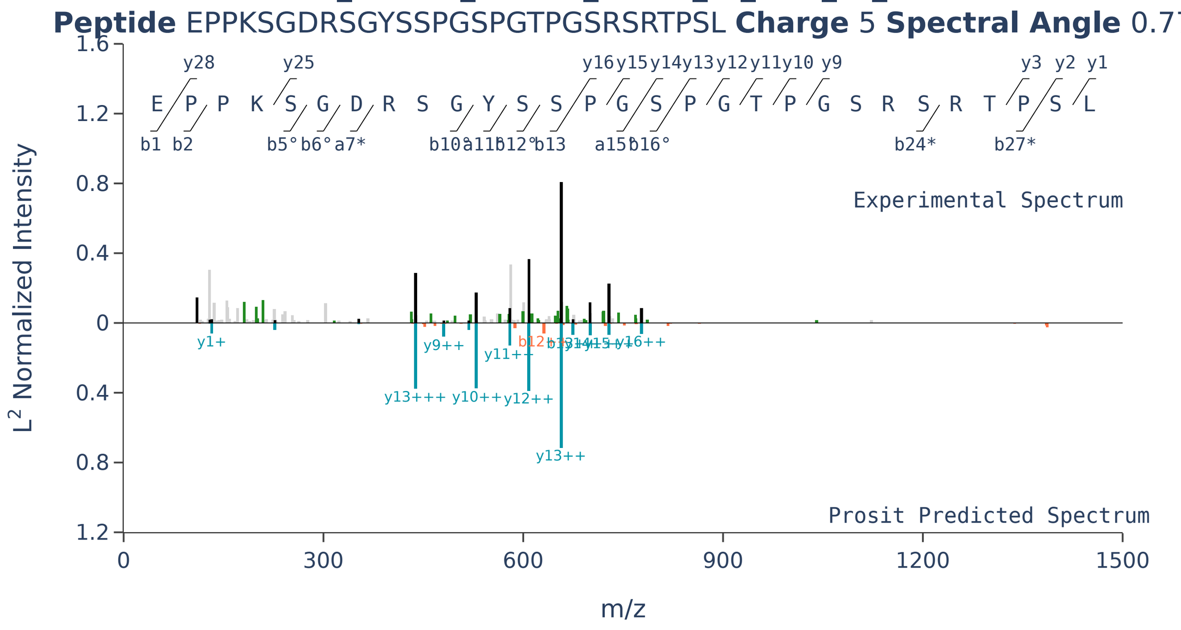


**Source** WSoh_221121_011221_HFGoe_L1_hTau_24h_R2 **Scan** 5520
**Peptide** DGTGSDDKKAKGADGKTKIAT **Charge** 3 **Spectral Angle** 0.7

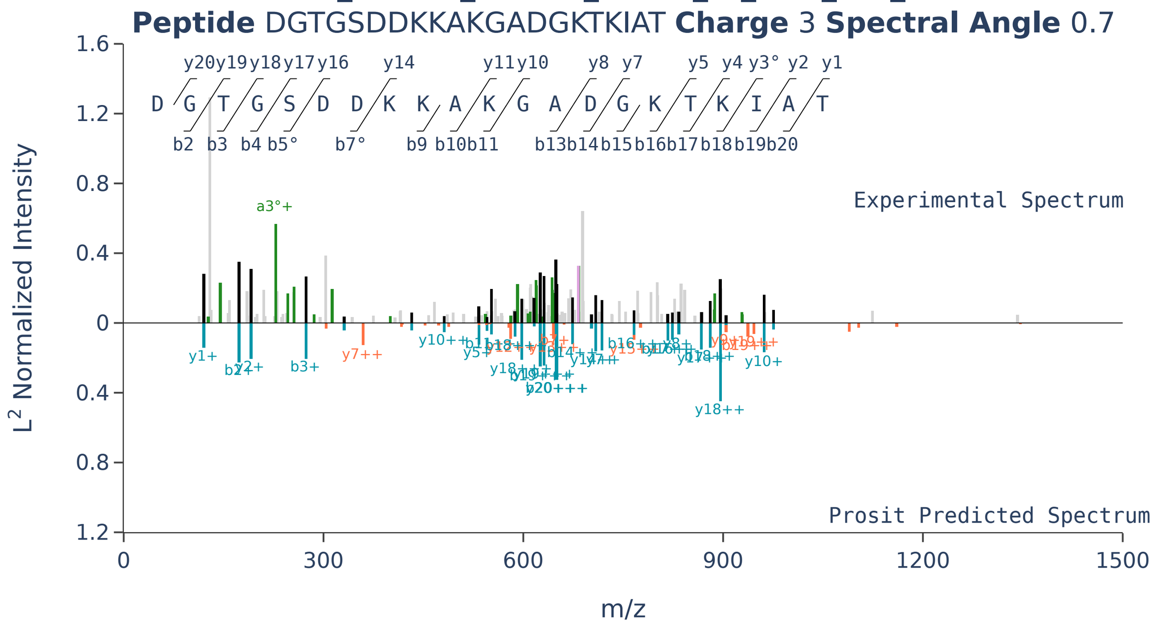


**Source** WSoh_221121_011221_HFGoe_L2_hTau_4h_R2 **Scan** 6533
**Peptide** PSSAKSRL **Charge** 2 **Spectral Angle** 0.89

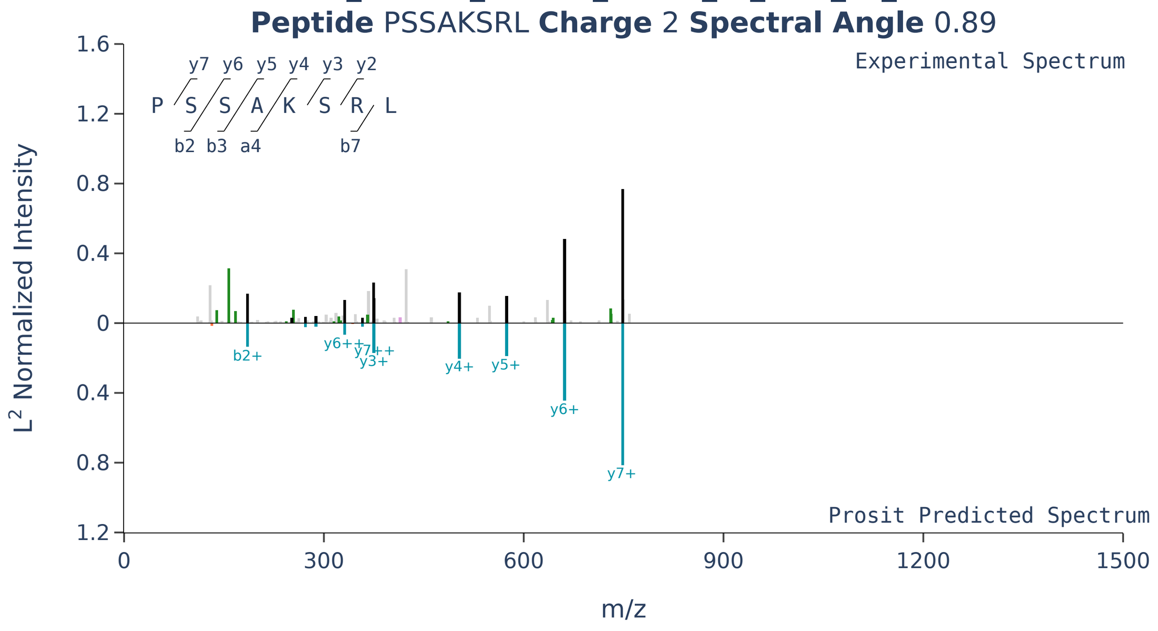


**Source** WSoh_221121_011221_HFGoe_L1_hTau_2h_R2 **Scan** 13012
**Peptide** ENLKHQPGGGKVQII **Charge** 2 **Spectral Angle** 0.68

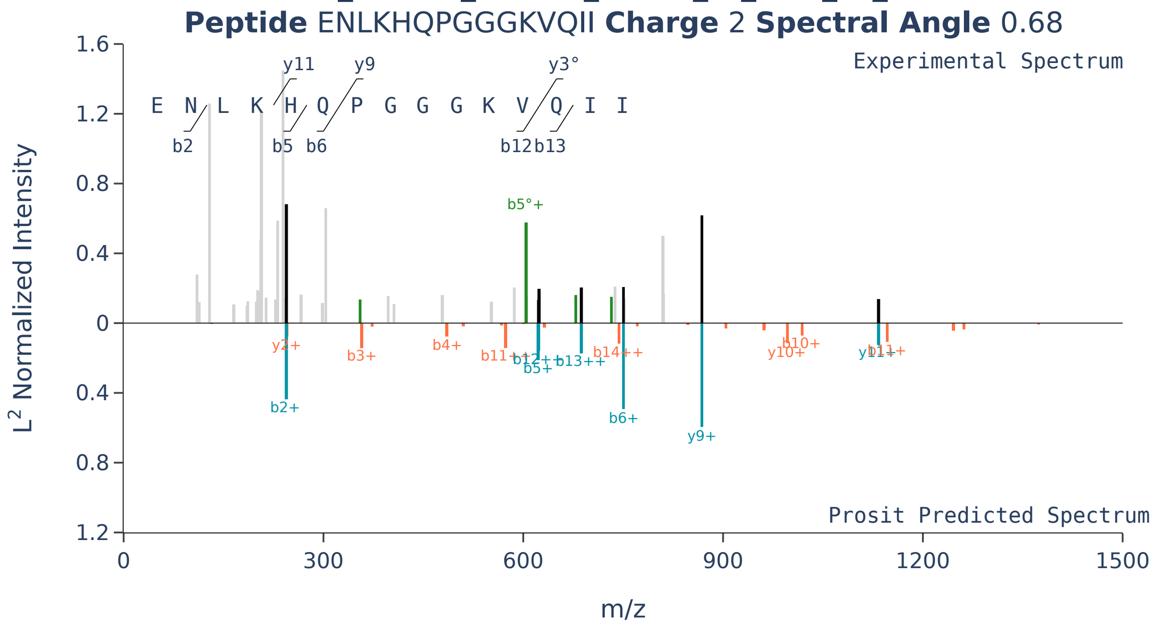


**Source** WSoh_221121_011221_HFGoe_L2_hTau_4h_R2 **Scan** 9290
**Peptide** NITHVPGGGNKKIETHKLTFRENAKAK **Charge** 5 **Spectral Angle** 0.69

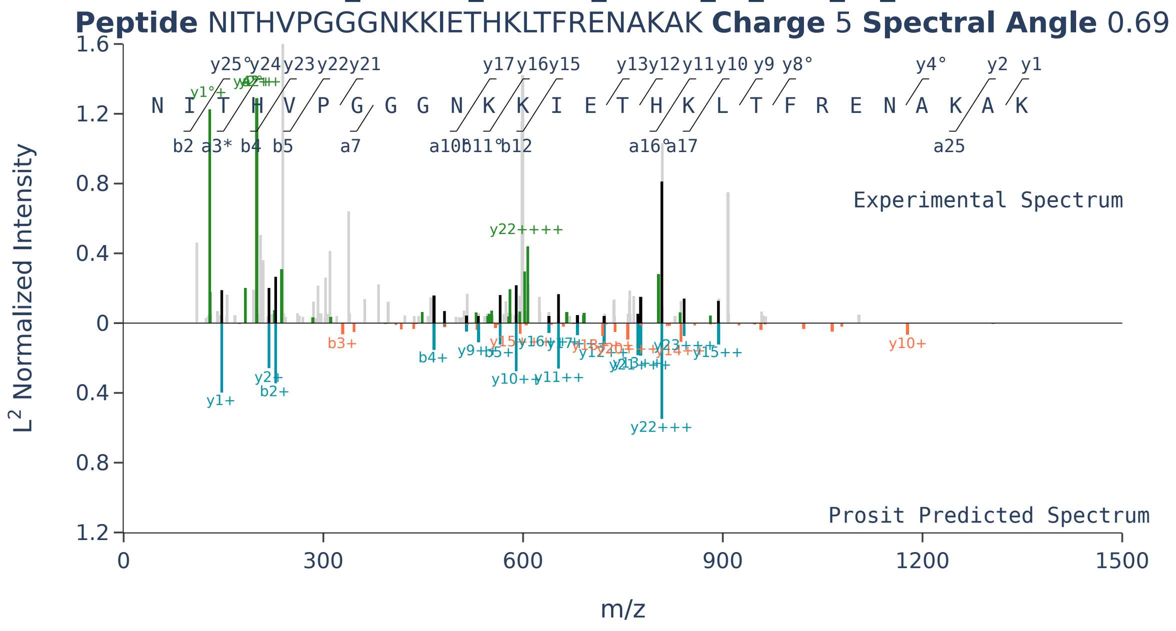


**Source** WSoh_221121_011221_HFGoe_L1_hTau_4h_R2 **Scan** 6166
**Peptide** SKVTSKCGSLG **Charge** 2 **Spectral Angle** 0.86

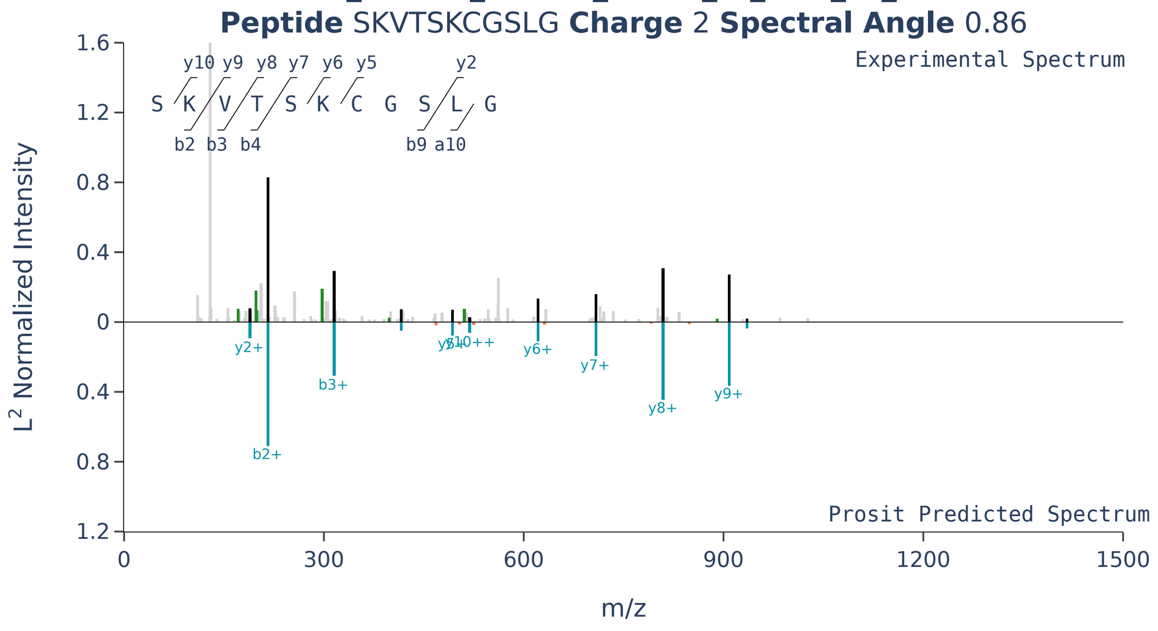


**Source** WSoh_221121_011221_HFGoe_L1_hTau_24h_R1 **Scan** 19839
**Peptide** DFKDRVQSKIGSLDNITHVPGGGNKK **Charge** 5 **Spectral Angle** 0.81

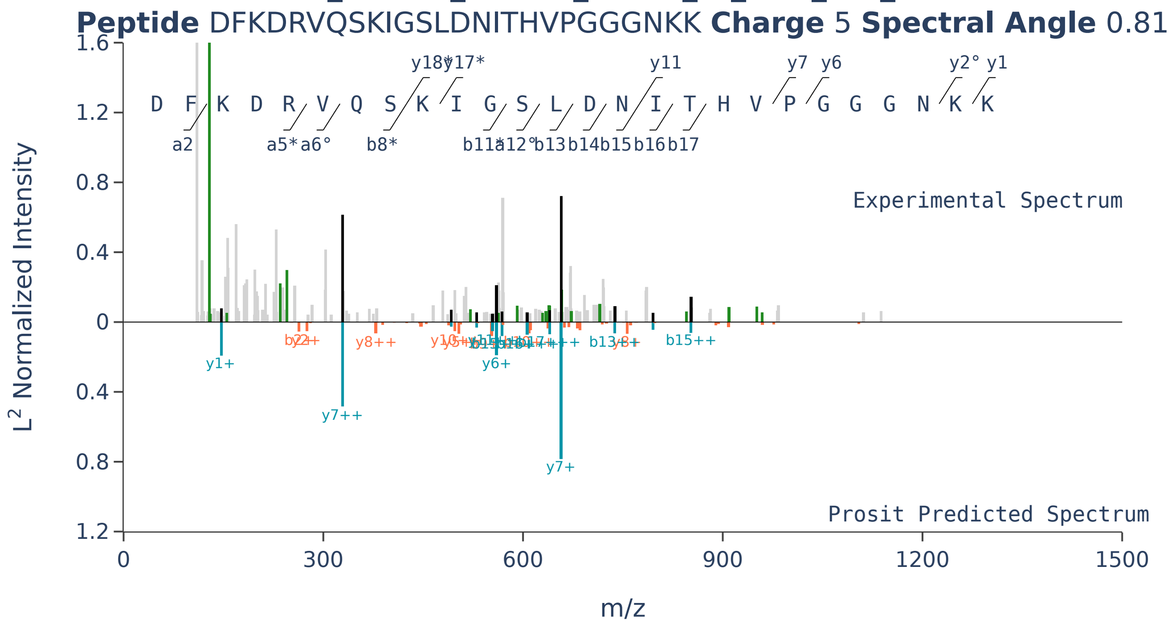


**Source** WSoh_221121_011221_HFGoe_L2_hTau_24h_R1 **Scan** 15520
**Peptide** KTDHGAEIVYKSPVVSGDTSPRHLSNVSS **Charge** 3 **Spectral Angle** 0.62

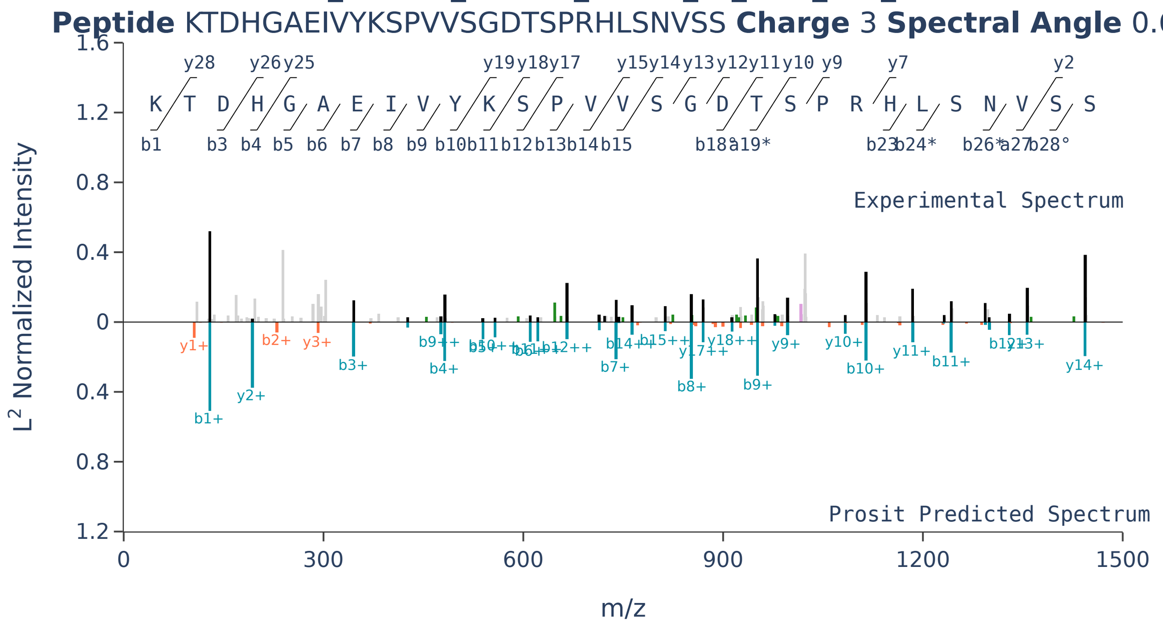


**Source** WSoh_221121_011221_HFGoe_L1_hTau_4h_R1 **Scan** 6673
**Peptide** TSKCGSLGNIHHKPGGGQVE **Charge** 3 **Spectral Angle** 0.71

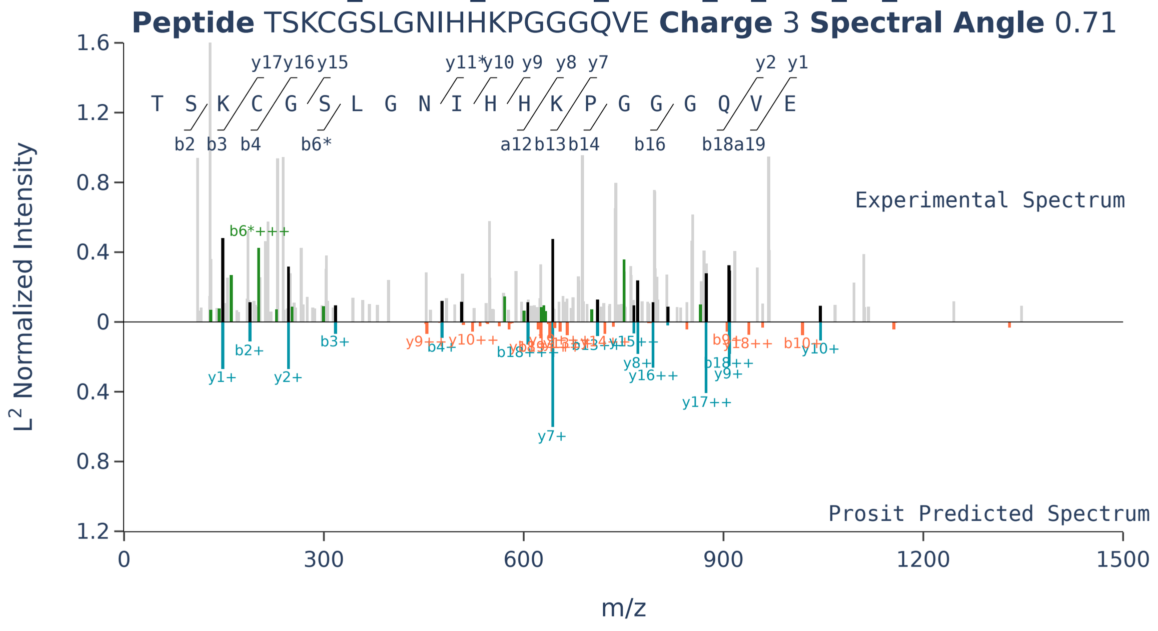


**Source** WSoh_221121_011221_HFGoe_L1_hTau_24h_R1 **Scan** 17916
**Peptide** PPKSPSSAKSRLQTAPVPMPDLK **Charge** 4 **Spectral Angle** 0.77

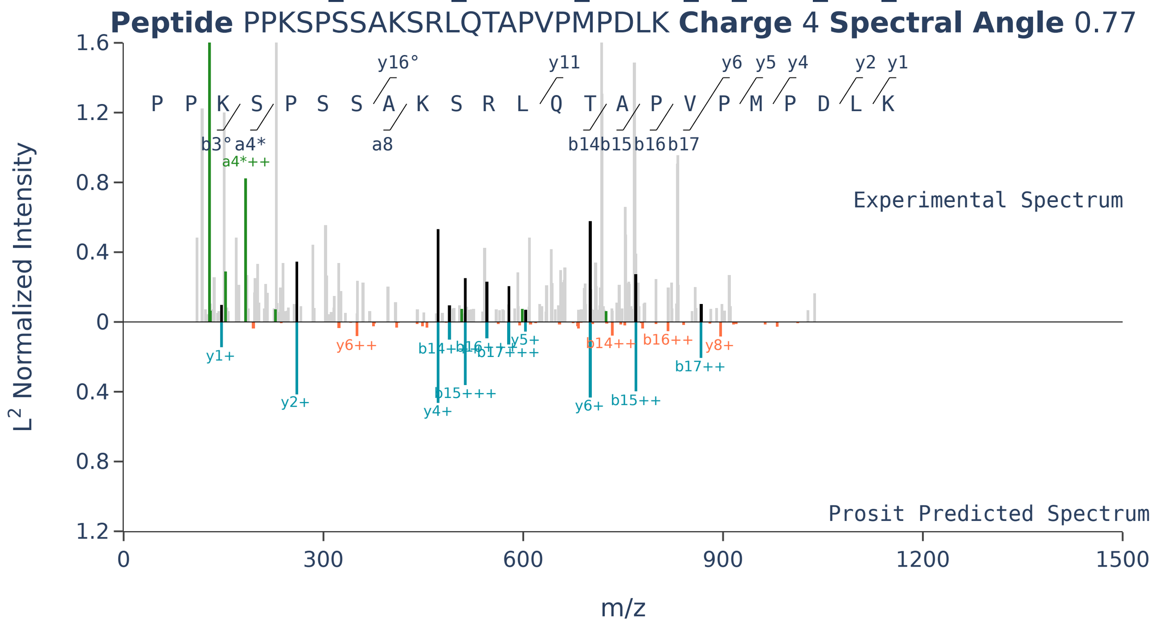


**Source** WSoh_221121_011221_HFGoe_L2_hTau_24h_R2 **Scan** 10290
**Peptide** DNITHVPGGGNKKIETHKLTFRENAKAK **Charge** 5 **Spectral Angle** 0.69

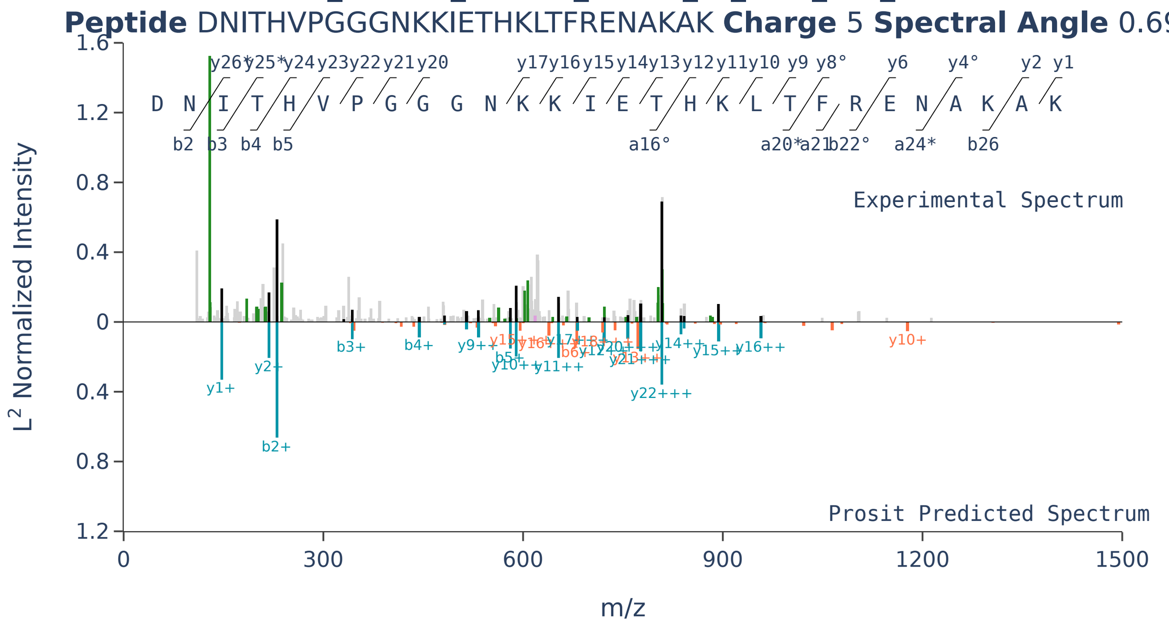

**Source** WSoh_221121_011221_HFGoe_L2_hTau_2h_R1 **Scan** 9364
**Peptide** NITHVPGGGNKKIETHKLTFRENAKAKTD **Charge** 4 **Spectral Angle** 0.63

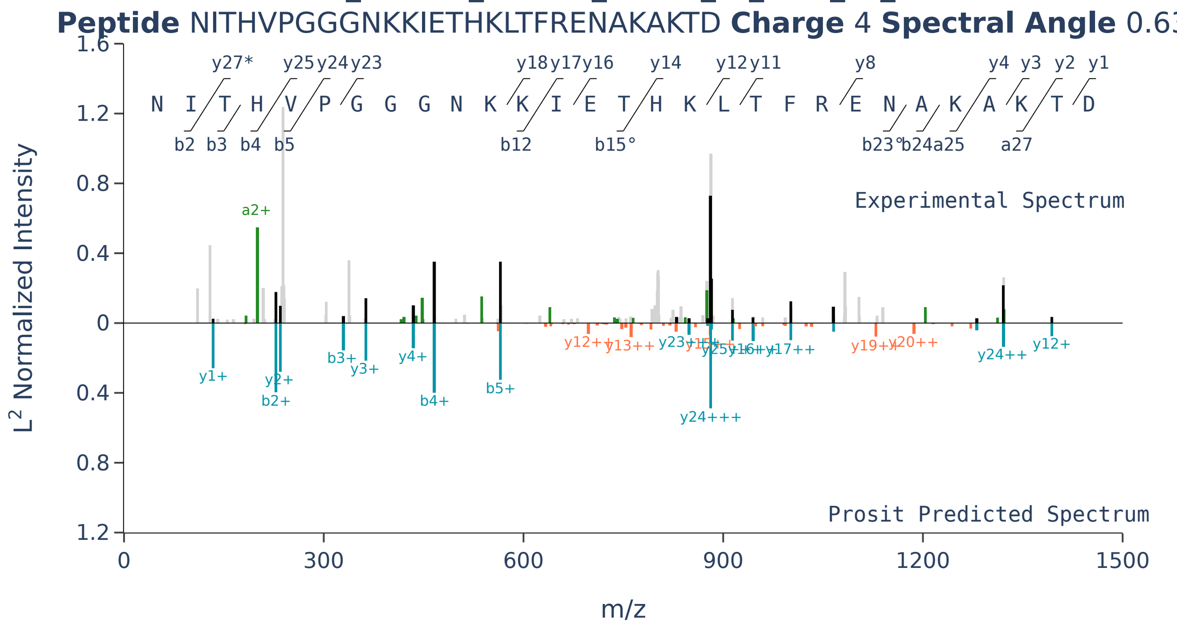


**Source** WSoh_221121_011221_HFGoe_L1_hTau_24h_R2 **Scan** 5556
**Peptide** KSKDGTGSDDKKAKGADGKTKIATPRGA **Charge** 4 **Spectral Angle** 0.79

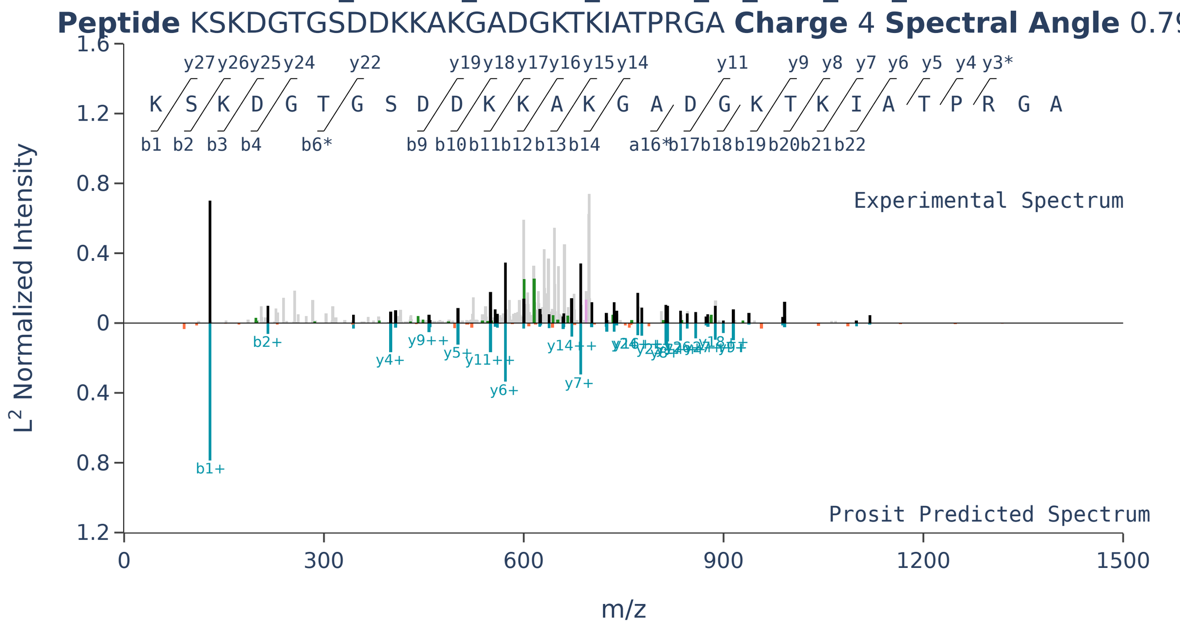


**Source** WSoh_221121_011221_HFGoe_L2_hTau_24h_R2 **Scan** 15413
**Peptide** LDLSNVQSKCGSKDNIKHVPGGGSV **Charge** 4 **Spectral Angle** 0.87

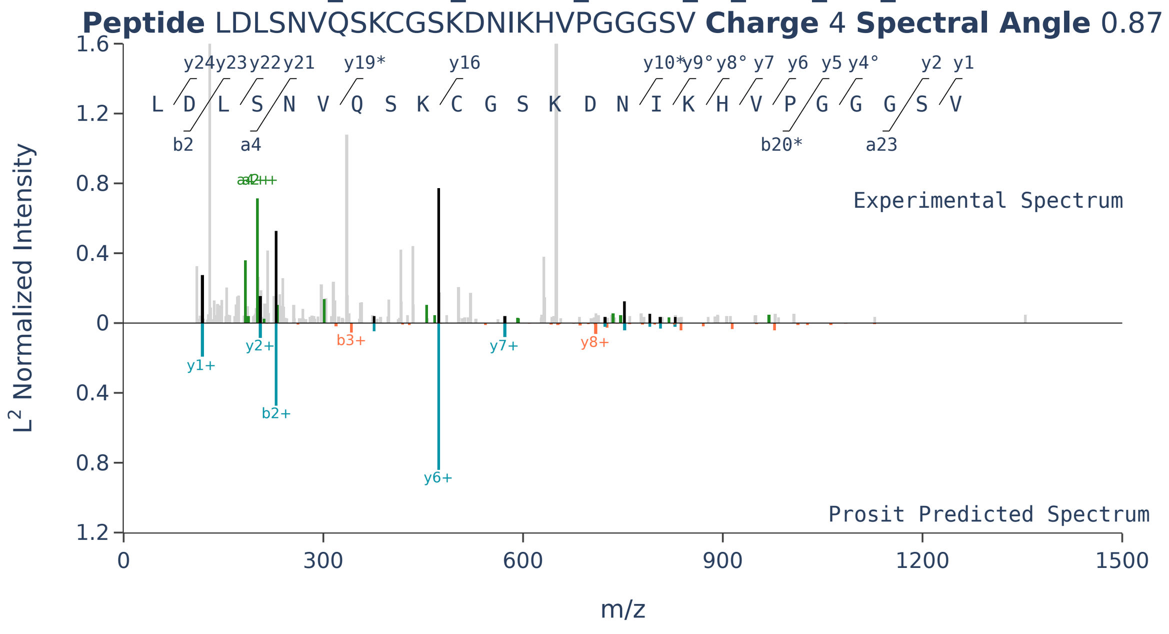


**Source** WSoh_221121_011221_HFGoe_L2_hTau_4h_R1 **Scan** 10973
**Peptide** KLTFRENAKAKTDHGAEIV **Charge** 4 **Spectral Angle** 0.56

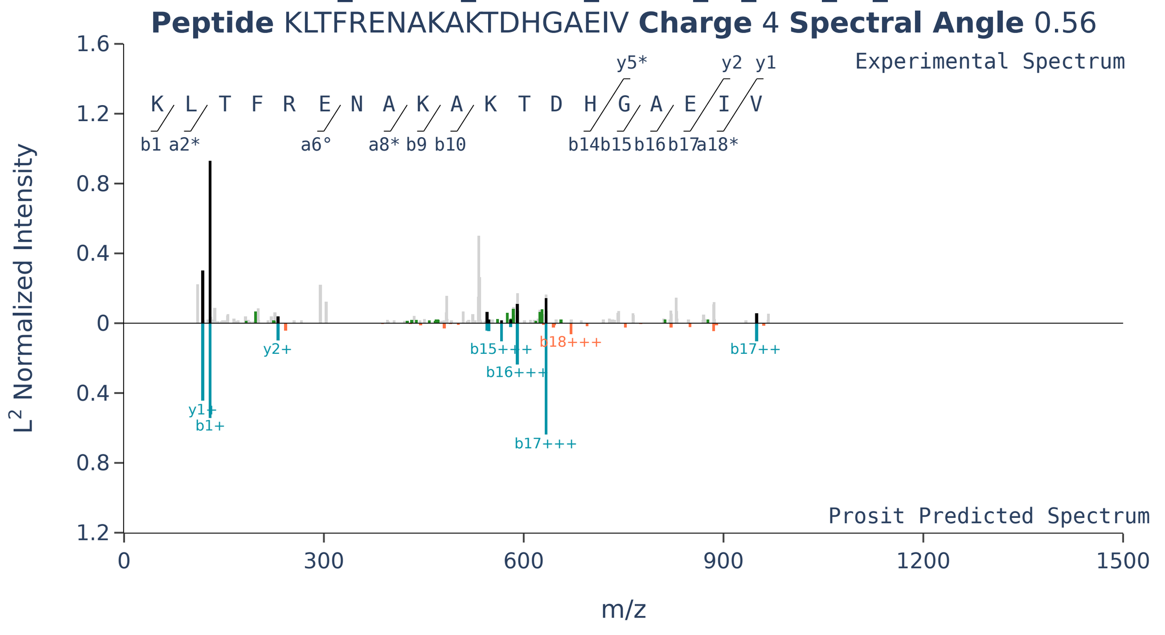


**Source** WSoh_221121_011221_HFGoe_L2_hTau_4h_R2 **Scan** 16633
**Peptide** KIGSLDNITHVPGGGNKKIETHKLTFRE **Charge** 5 **Spectral Angle** 0.7

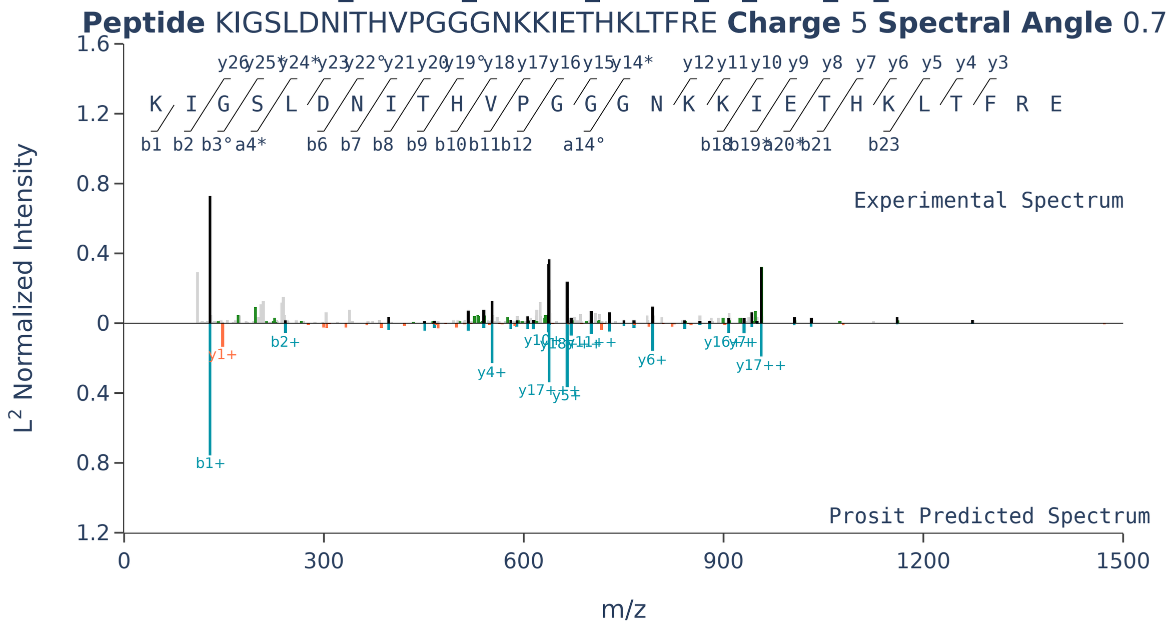


**Source** WSoh_221121_011221_HFGoe_L1_hTau_24h_R1 **Scan** 16199
**Peptide** SKIGSLDNITHVPGGGNKKLE **Charge** 4 **Spectral Angle** 0.81

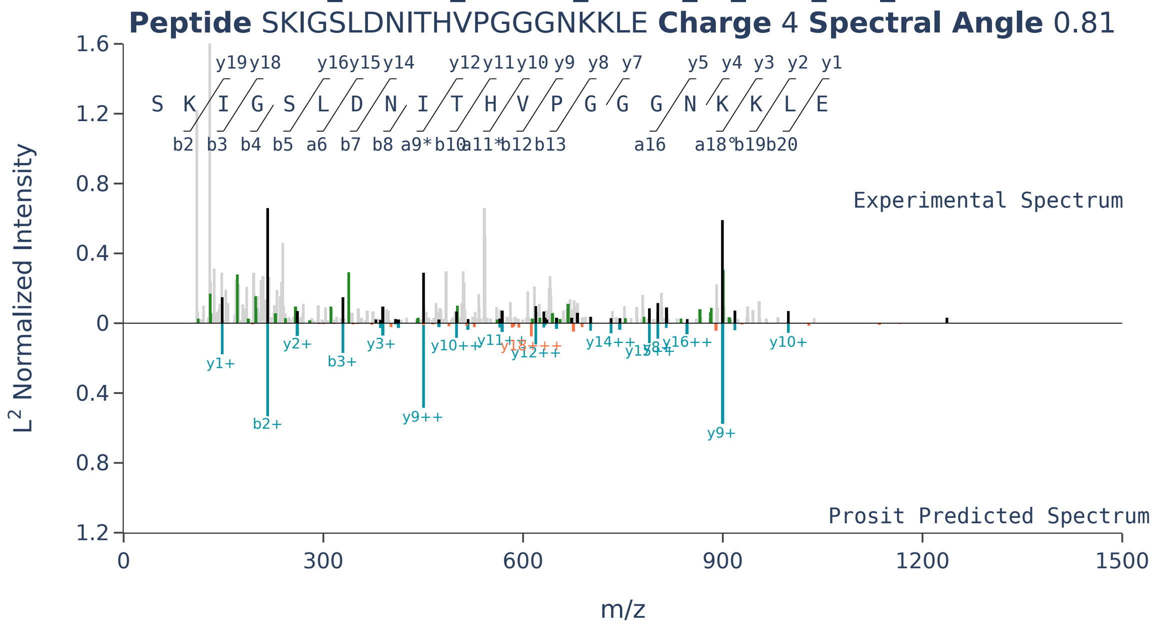


**Source** WSoh_221121_011221_HFGoe_L1_hTau_24h_R1 **Scan** 17702
**Peptide** PLVDEGAPGKQAAAQPHTEIPEGTTAEE **Charge** 3 **Spectral Angle** 0.69

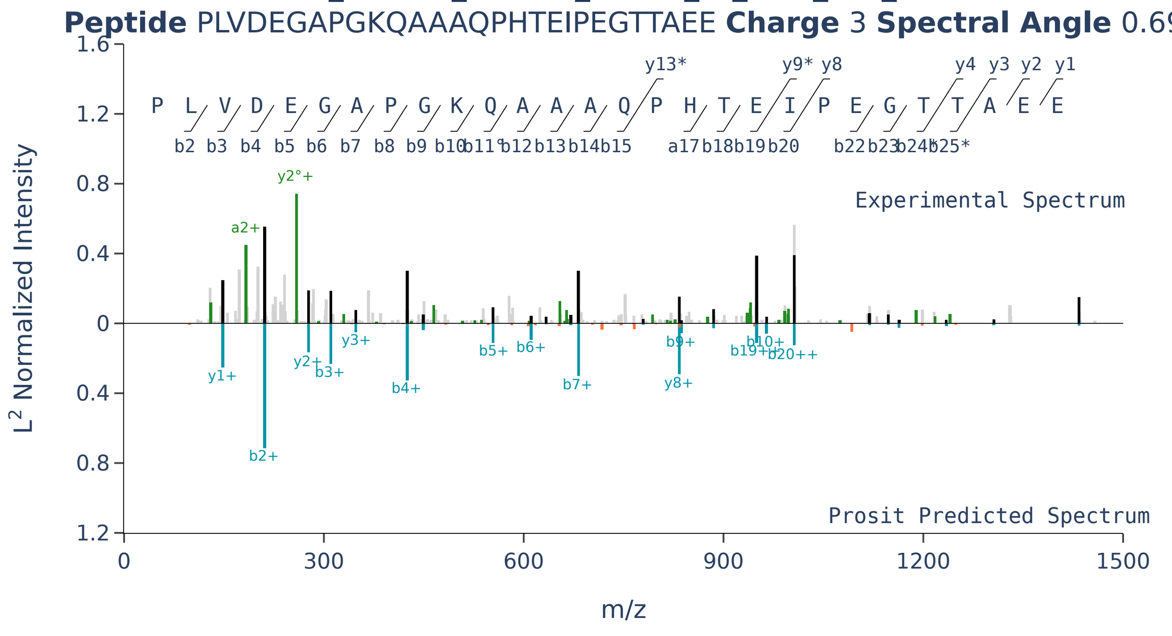


**Source** WSoh_221121_011221_HFGoe_L1_hTau_24h_R1 **Scan** 23050
**Peptide** PHTEIPEGTTAEEAGIGDTPSLED **Charge** 3 **Spectral Angle** 0.74

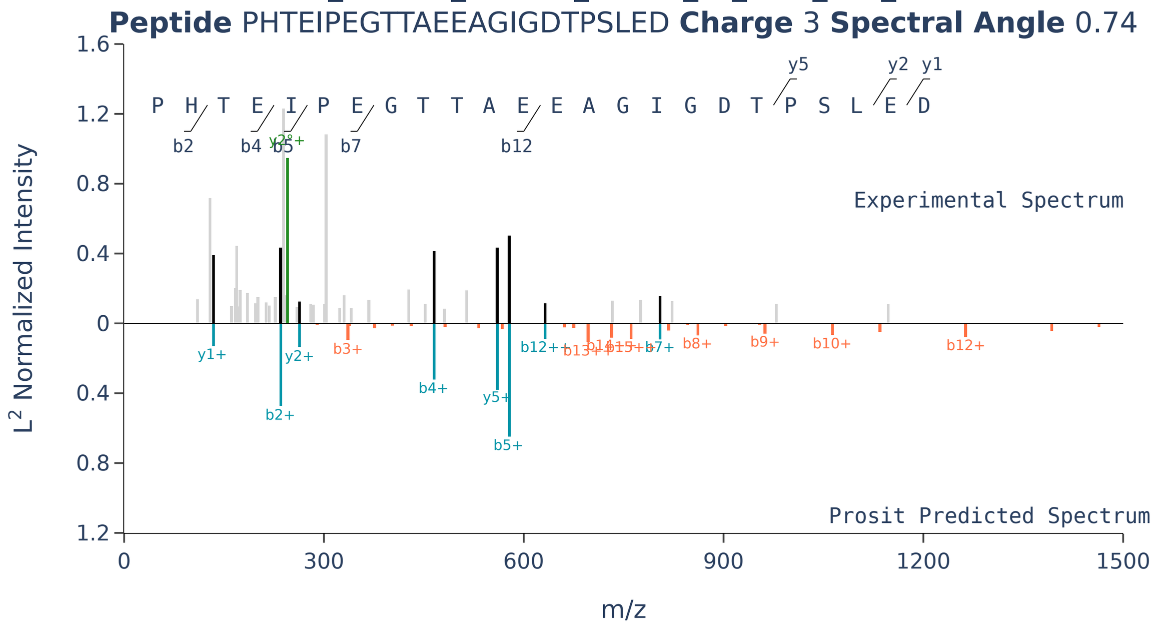


**Source** WSoh_221121_011221_HFGoe_L1_hTau_24h_R1 **Scan** 23024
**Peptide** TAEEAGIGDTPSLEDEAAGHVTQARMVS **Charge** 3 **Spectral Angle** 0.67

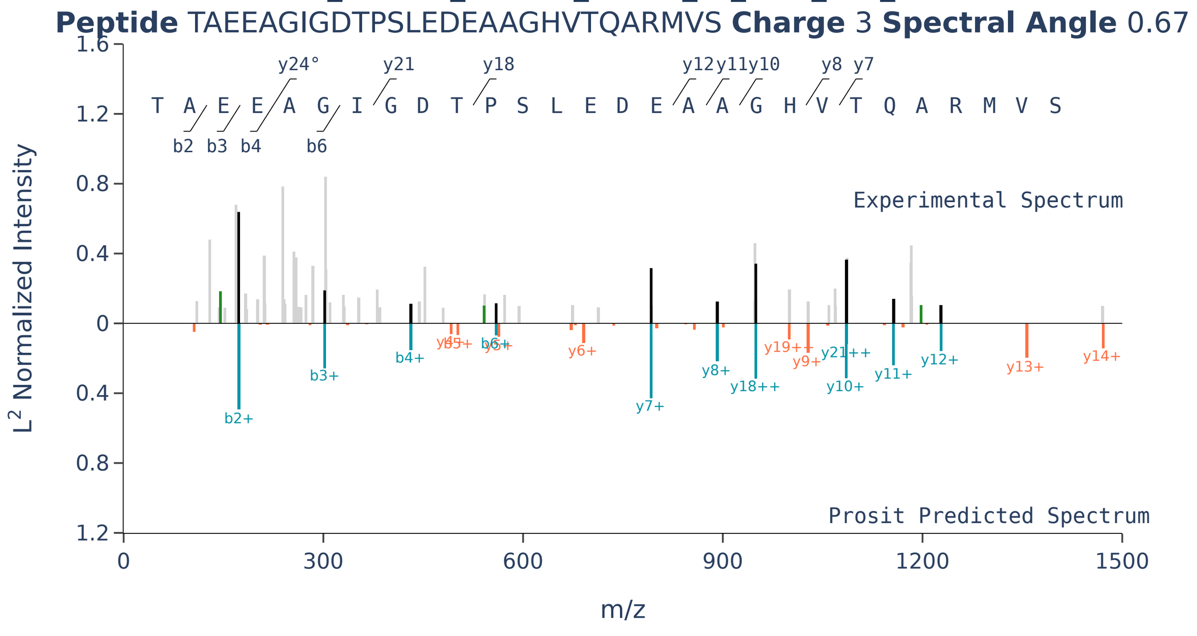


**Source** WSoh_221121_011221_HFGoe_L2_hTau_24h_R1 **Scan** 13750
**Peptide** TFRENAKAKTDHGAEIVYKSPVVSGD **Charge** 3 **Spectral Angle** 0.58

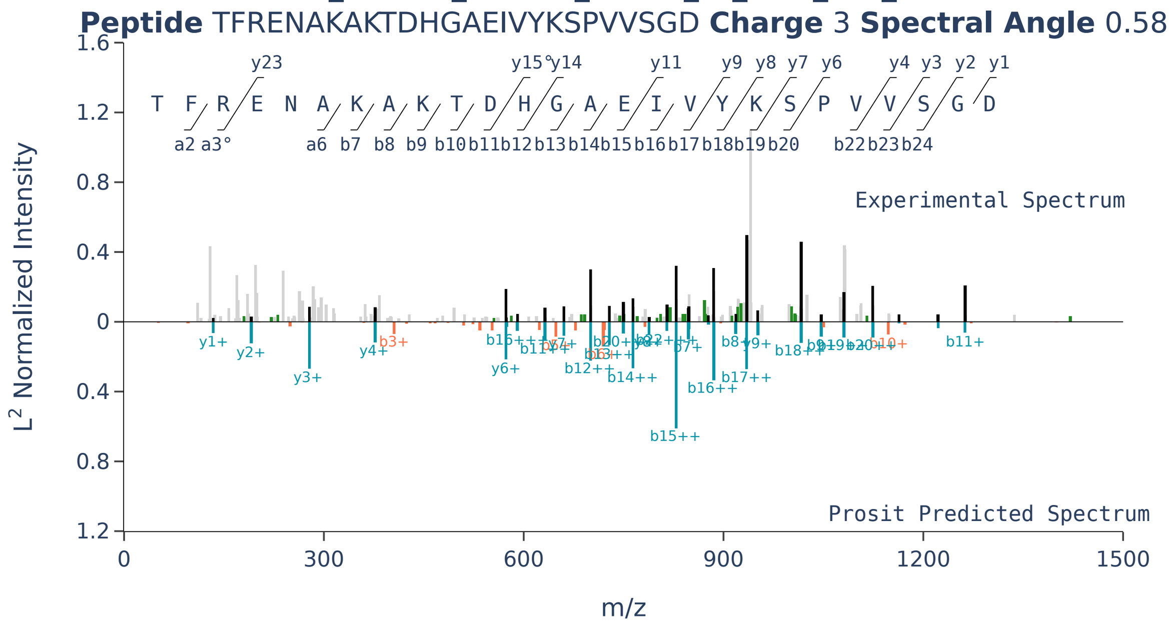


**Source** WSoh_221121_011221_HFGoe_L2_hTau_24h_R2 **Scan** 22465
**Peptide** SEEPGSETSDAKSTPTAEDVTAPLVD **Charge** 2 **Spectral Angle** 0.83

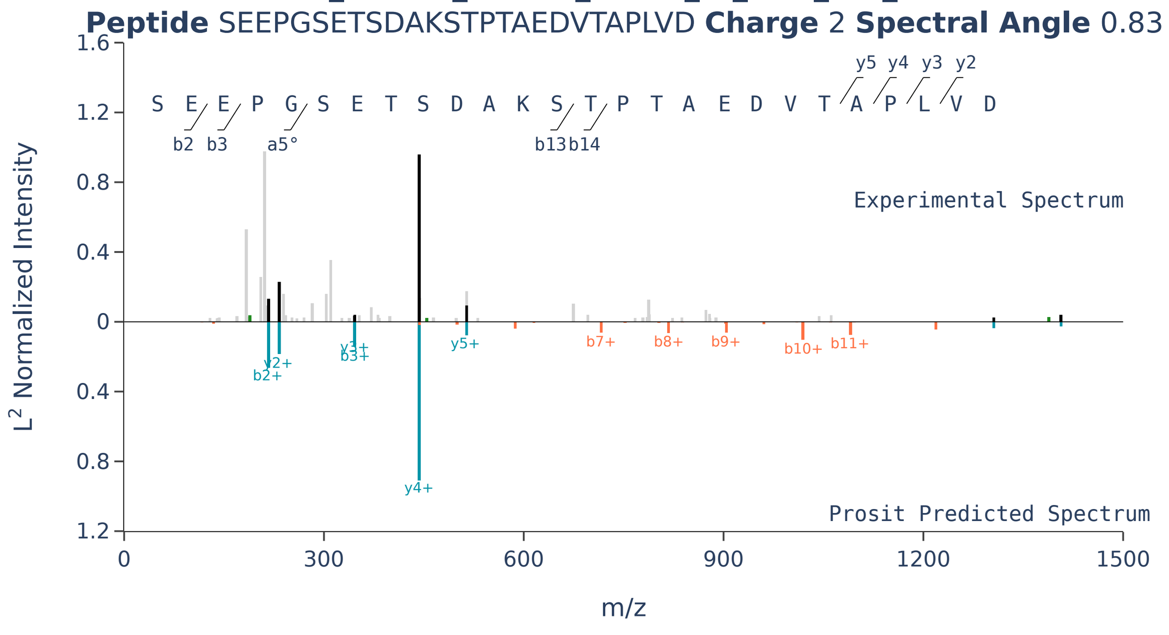


**Source** WSoh_221121_011221_HFGoe_L2_hTau_24h_R1 **Scan** 7601
**Peptide** GQANATRIPAKT **Charge** 2 **Spectral Angle** 0.52

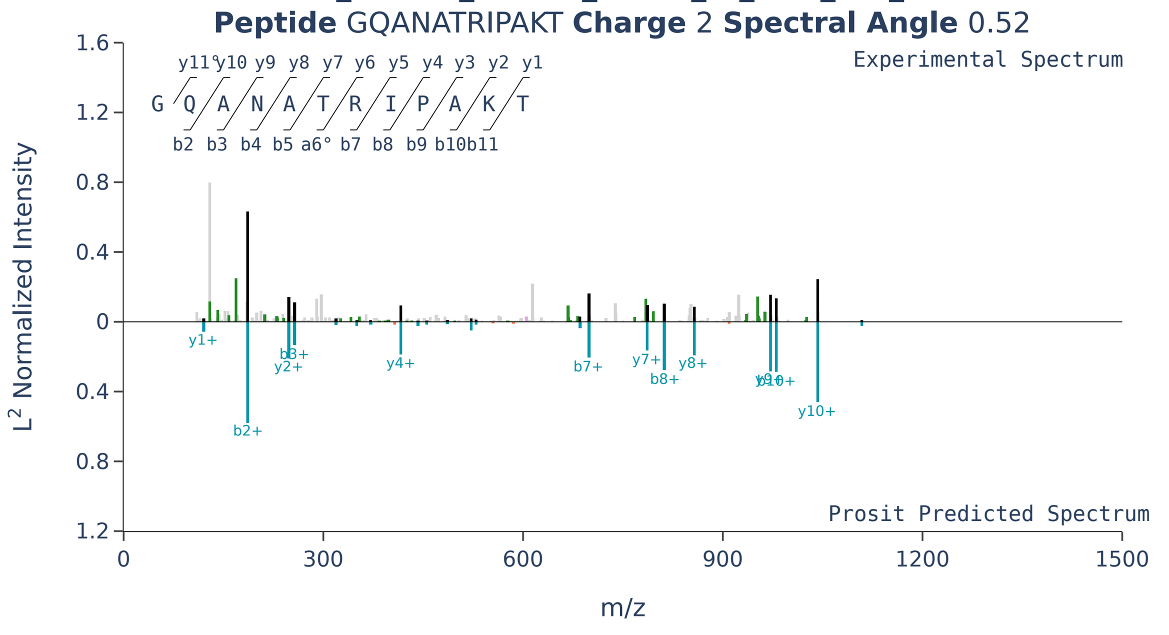


**Source** WSoh_221121_011221_HFGoe_L1_hTau_4h_R1 **Scan** 10071
**Peptide** KNVKSKIGSTENLKHQPGGGKVQIINKKL **Charge** 4 **Spectral Angle** 0.85

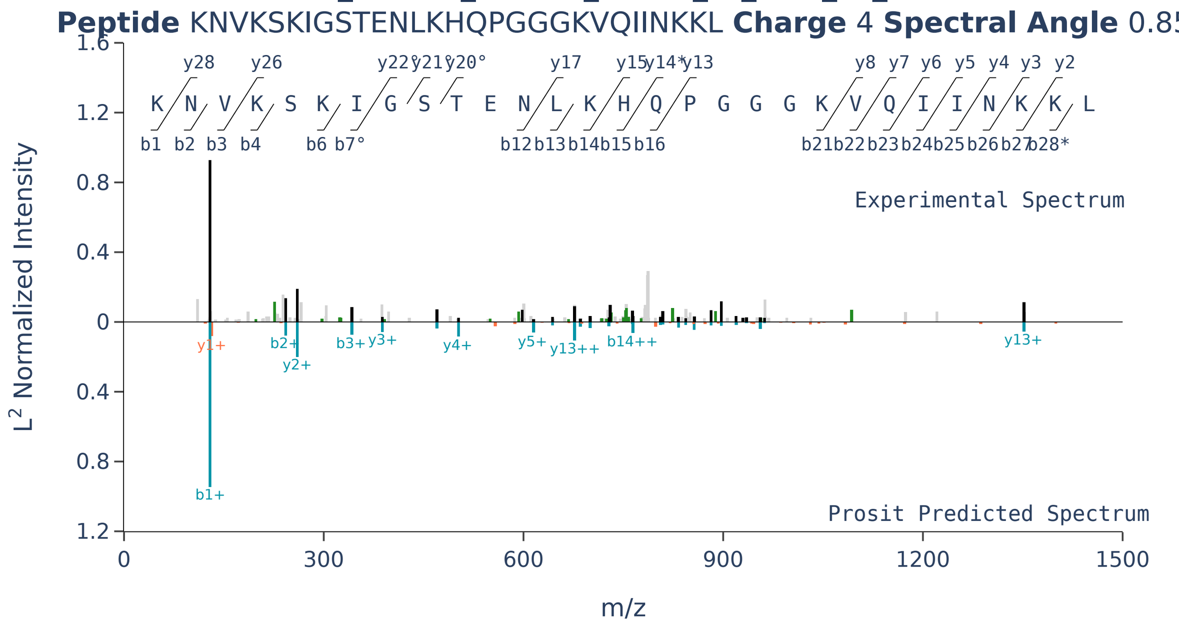


**Source** WSoh_221121_011221_HFGoe_L1_hTau_24h_R1 **Scan** 29845
**Peptide** SIDMVDSPQLATLAD **Charge** 2 **Spectral Angle** 0.84

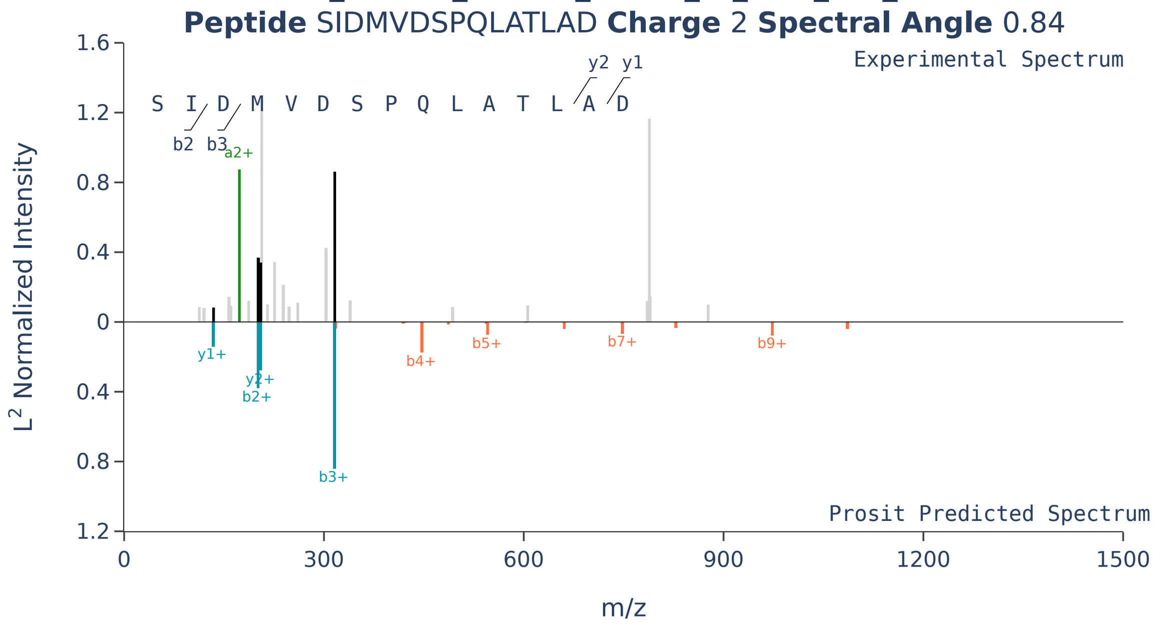


**Source** WSoh_221121_011221_HFGoe_L1_hTau_2h_R1 **Scan** 17317
**Peptide** DHGAEIVYKSPVVSGDTS **Charge** 3 **Spectral Angle** 0.76

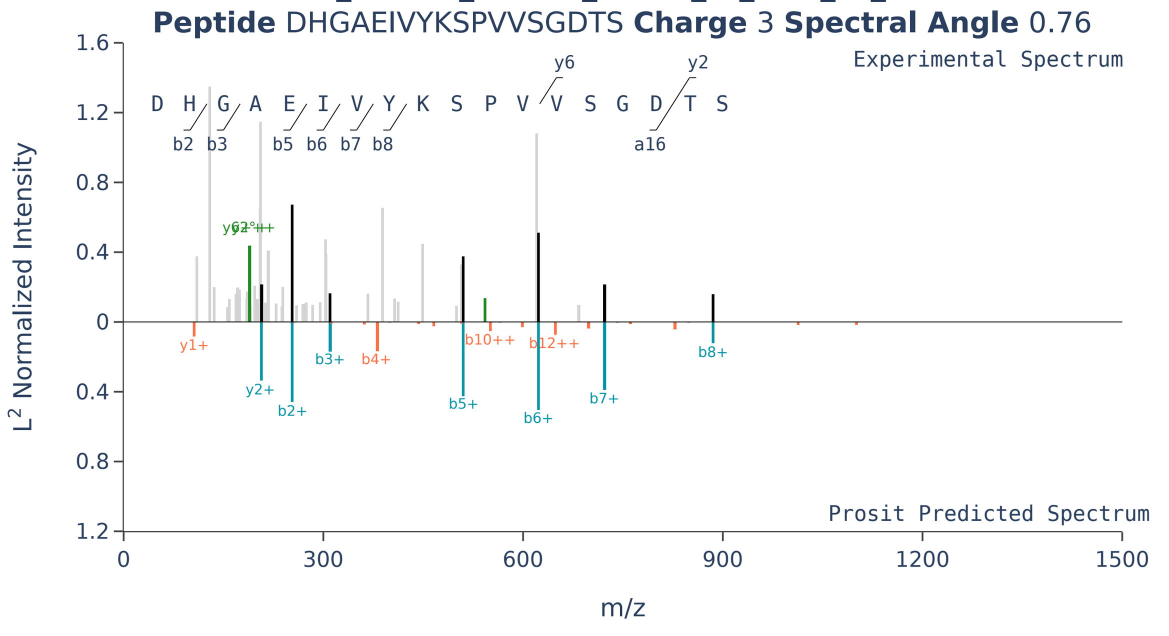

**Source** WSoh_221121_011221_HFGoe_L1_hTau_24h_R1 **Scan** 8787
**Peptide** PTPPTREPKKVAVVRTPPKSPSS **Charge** 4 **Spectral Angle** 0.79

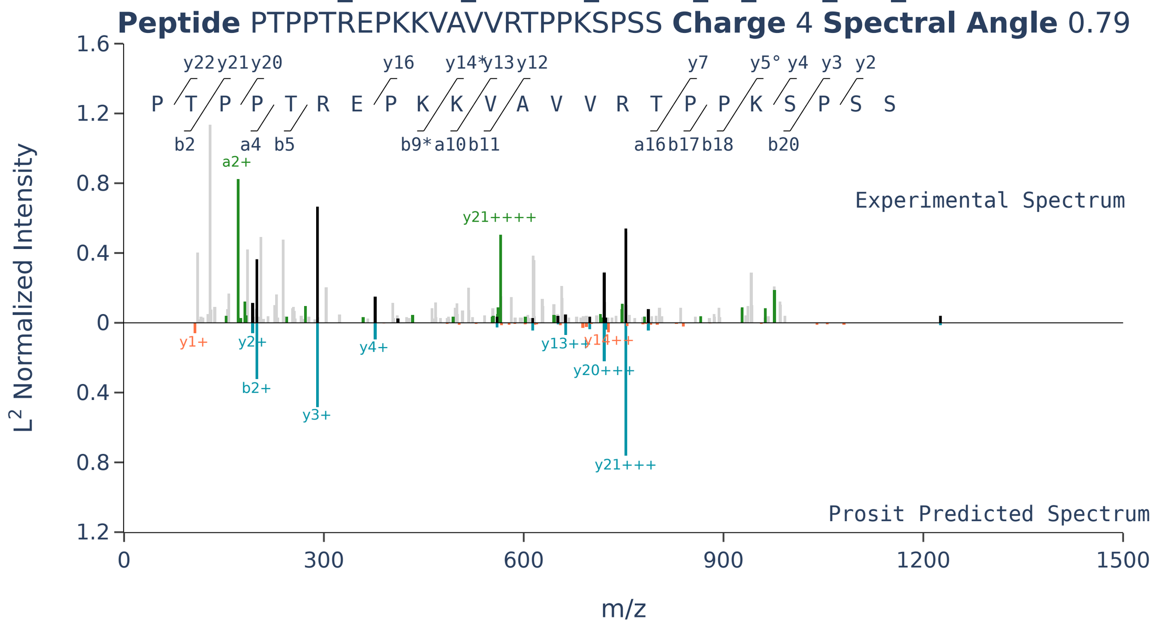


**Source** WSoh_221121_011221_HFGoe_L2_hTau_4h_R1 **Scan** 17706
**Peptide** QGGYTMHQDQEGDTDAGLKESPLQT **Charge** 3 **Spectral Angle** 0.6

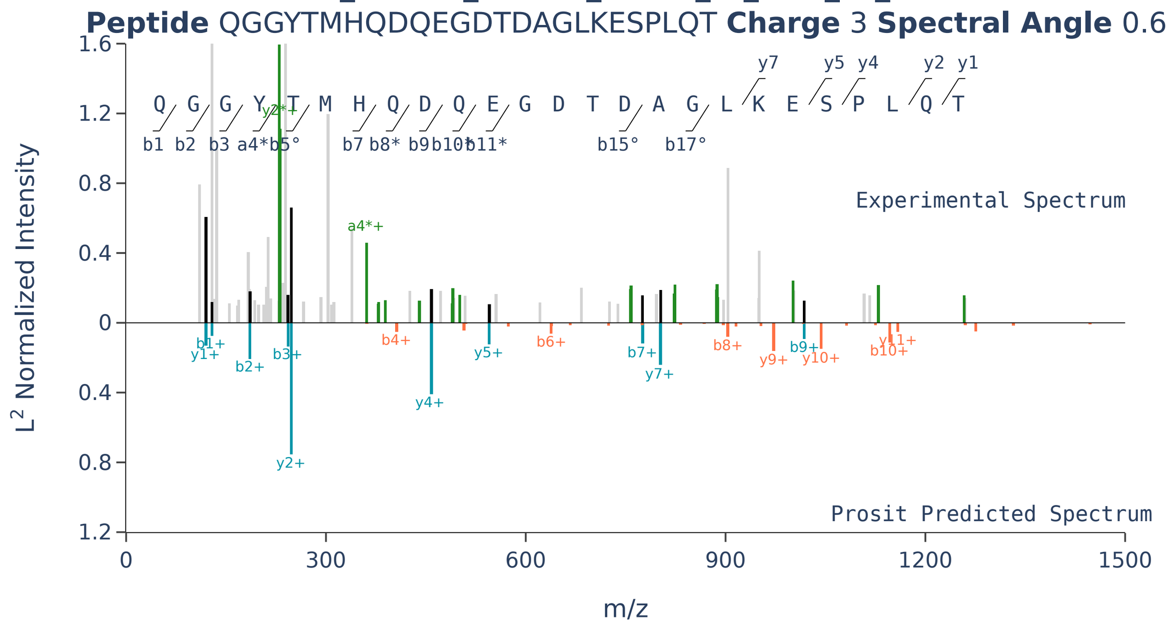


**Source** WSoh_221121_011221_HFGoe_L2_hTau_24h_R1 **Scan** 7964
**Peptide** QGGYTMHQDQEGD **Charge** 2 **Spectral Angle** 0.78

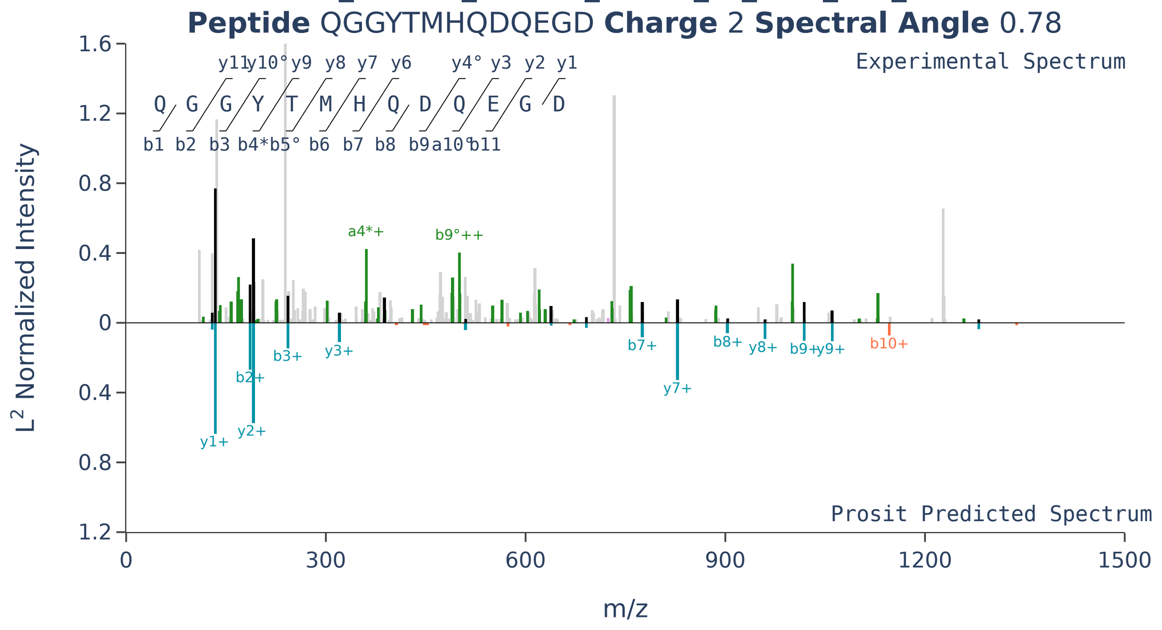


**Source** WSoh_221121_011221_HFGoe_L1_hTau_24h_R2 **Scan** 22014
**Peptide** GSETSDAKSTPTAEDVTAPLVD **Charge** 2 **Spectral Angle** 0.8

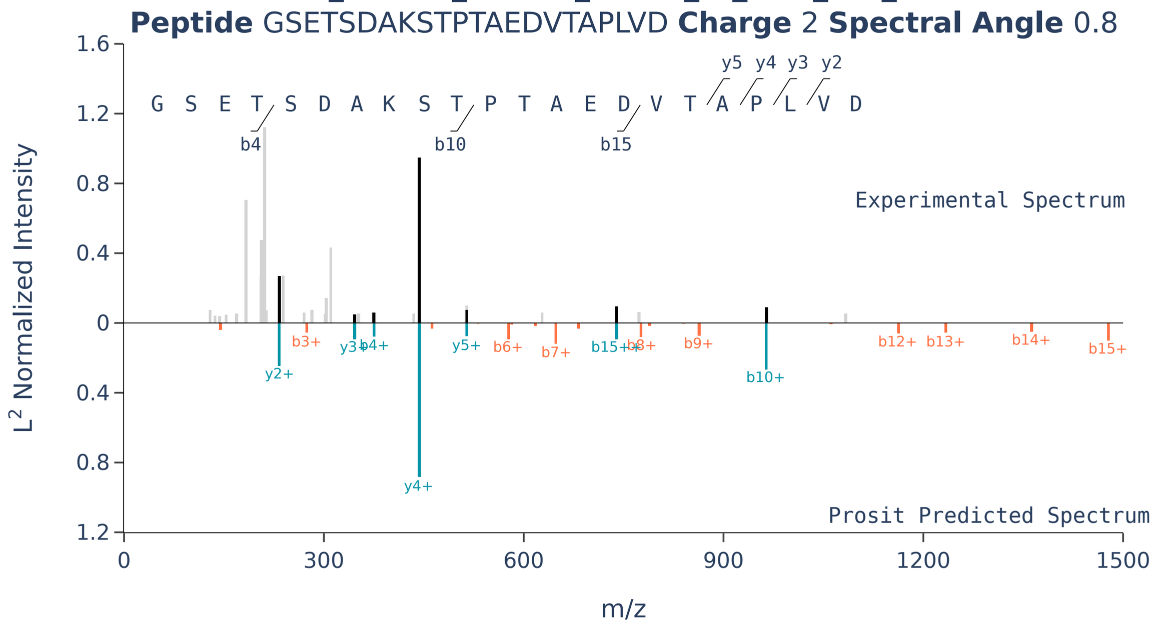


**Source** WSoh_221121_011221_HFGoe_L1_hTau_24h_R1 **Scan** 25041
**Peptide** PGGGSVQIVYKPVDLSKVT **Charge** 3 **Spectral Angle** 0.78

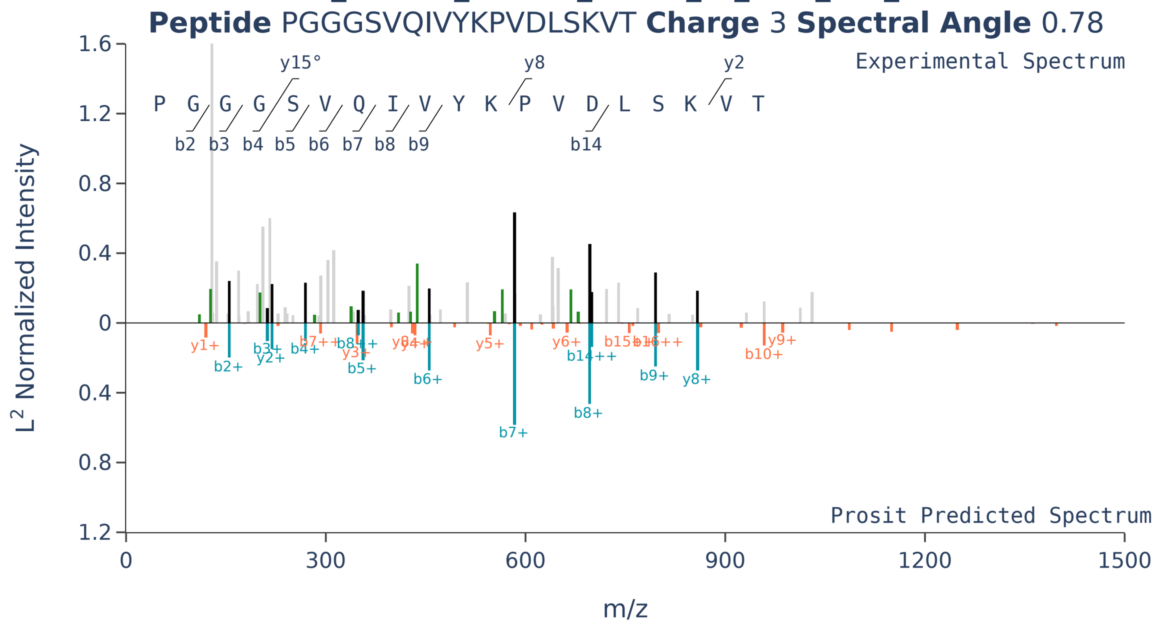


**Source** WSoh_221121_011221_HFGoe_L1_hTau_1h_R2 **Scan** 5338
**Peptide** NIHHKPGGGQVE **Charge** 3 **Spectral Angle** 0.81

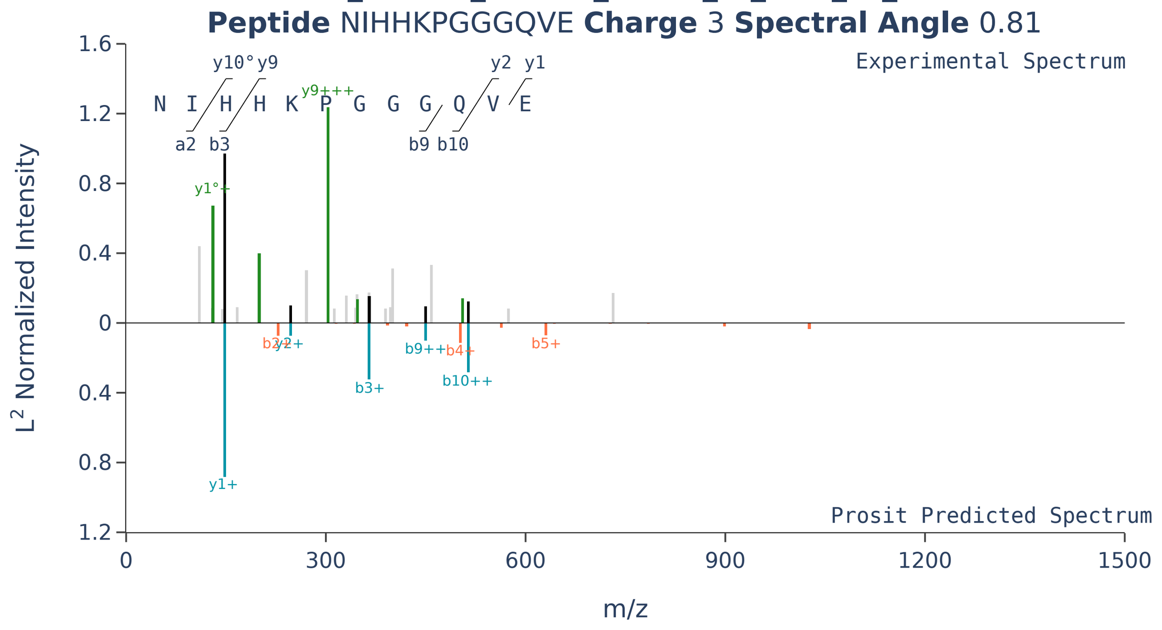


**Source** WSoh_221121_011221_HFGoe_L2_hTau_24h_R2 **Scan** 23748
**Peptide** LQTAPVPMPDLKNV **Charge** 2 **Spectral Angle** 0.76

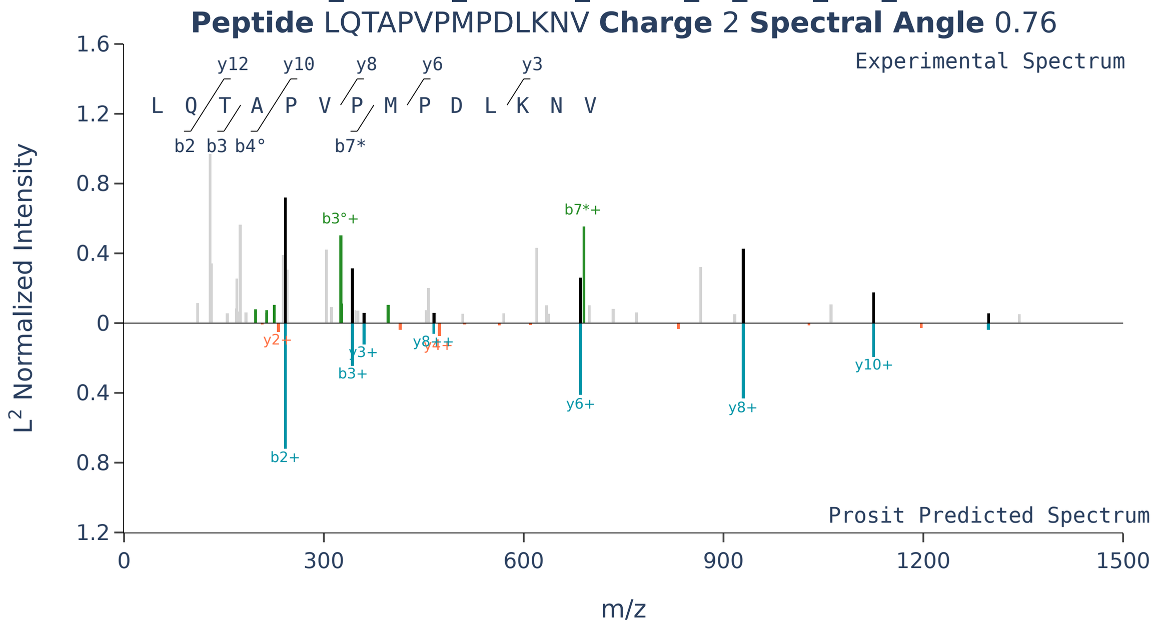


**Source** WSoh_221121_011221_HFGoe_L1_hTau_24h_R1 **Scan** 21467
**Peptide** LDFKDRVQSKIGSL **Charge** 3 **Spectral Angle** 0.67

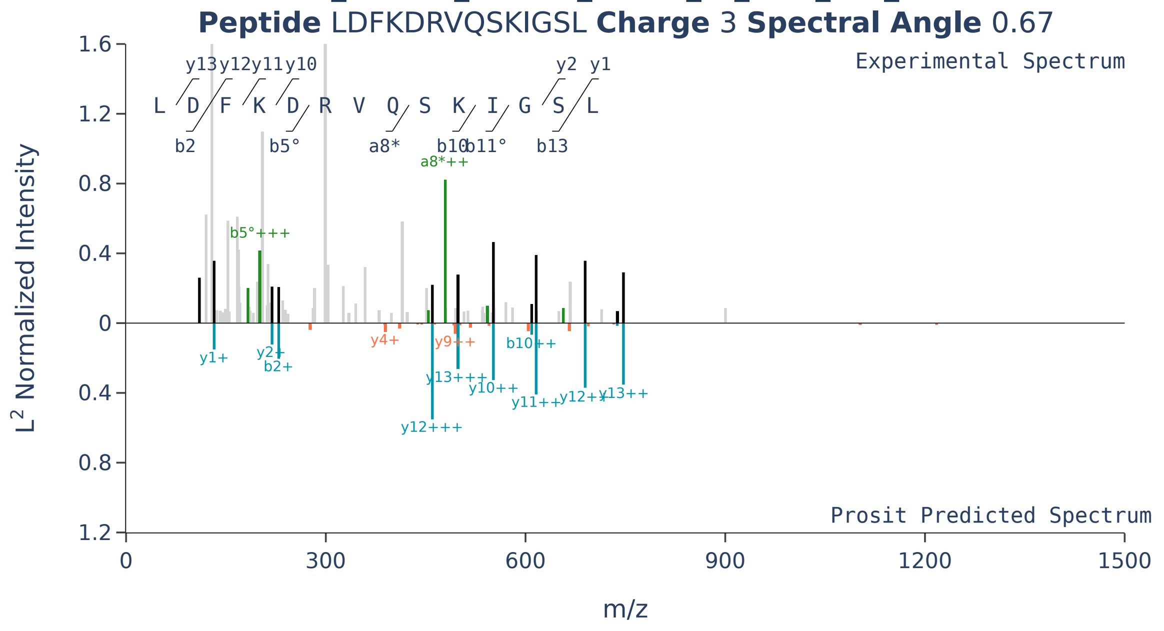


**Source** WSoh_221121_011221_HFGoe_L1_hTau_24h_R2 **Scan** 16561
**Peptide** QSKIGSLDNITHVPGGGNKKIE **Charge** 4 **Spectral Angle** 0.81

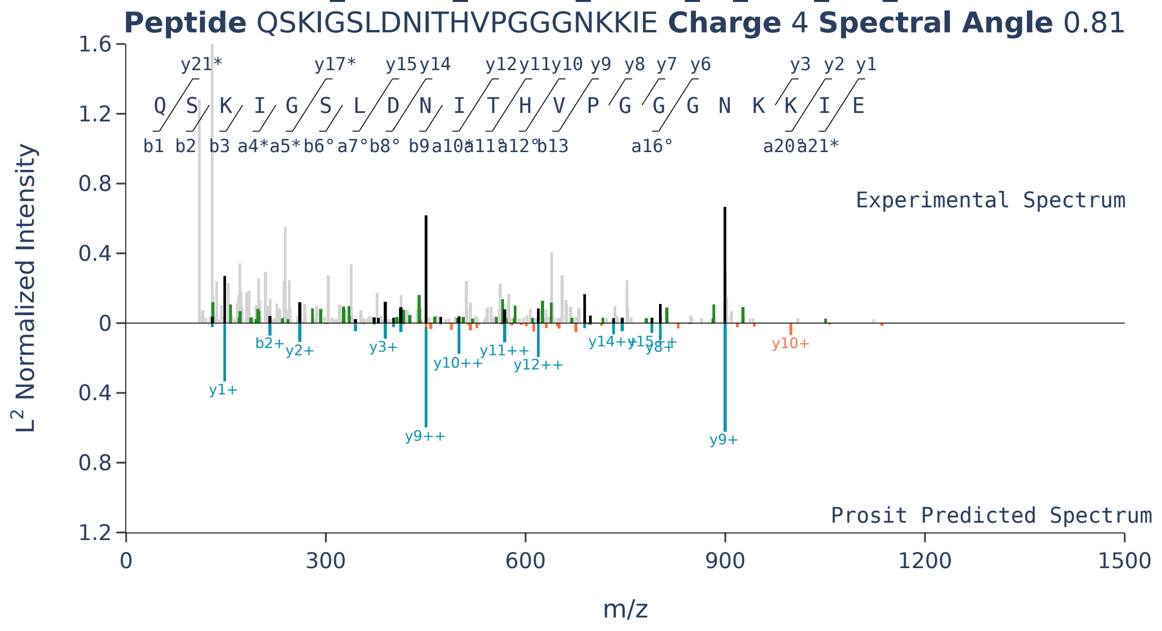


**Source** WSoh_221121_011221_HFGoe_L1_hTau_24h_R2 **Scan** 5734
**Peptide** EAAGHVTQARMVSKSKDGTGSDDKKA **Charge** 3 **Spectral Angle** 0.72

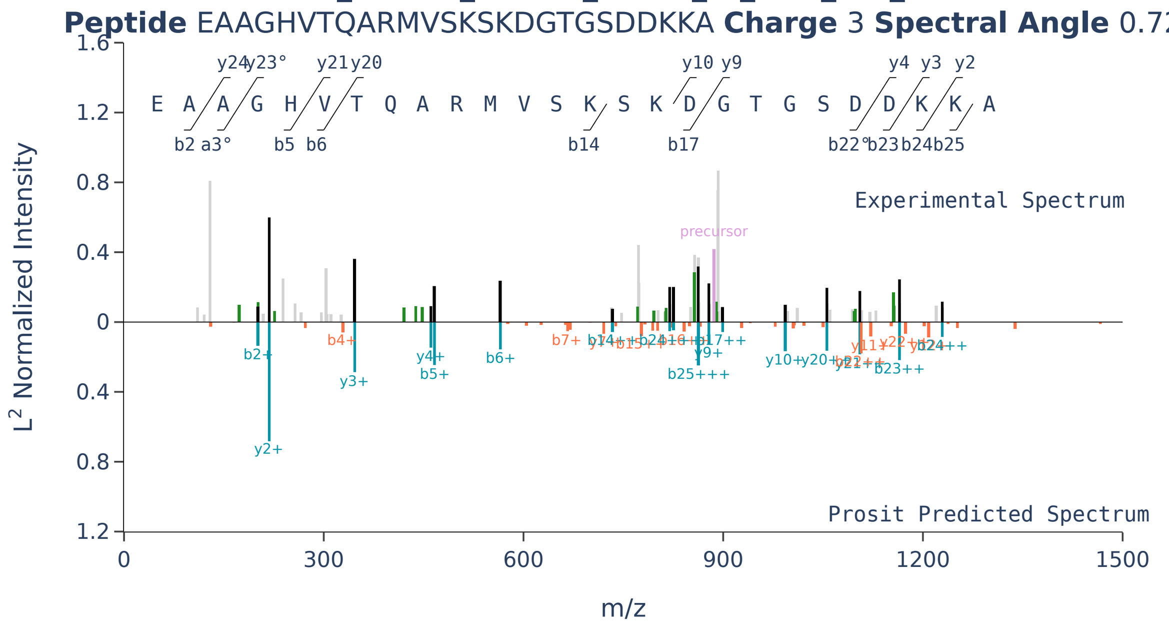


**Source** WSoh_221121_011221_HFGoe_L1_hTau_24h_R2 **Scan** 8545
**Peptide** LSKVTSKCGSLGNIHHKPGGGQVEVKS **Charge** 4 **Spectral Angle** 0.73

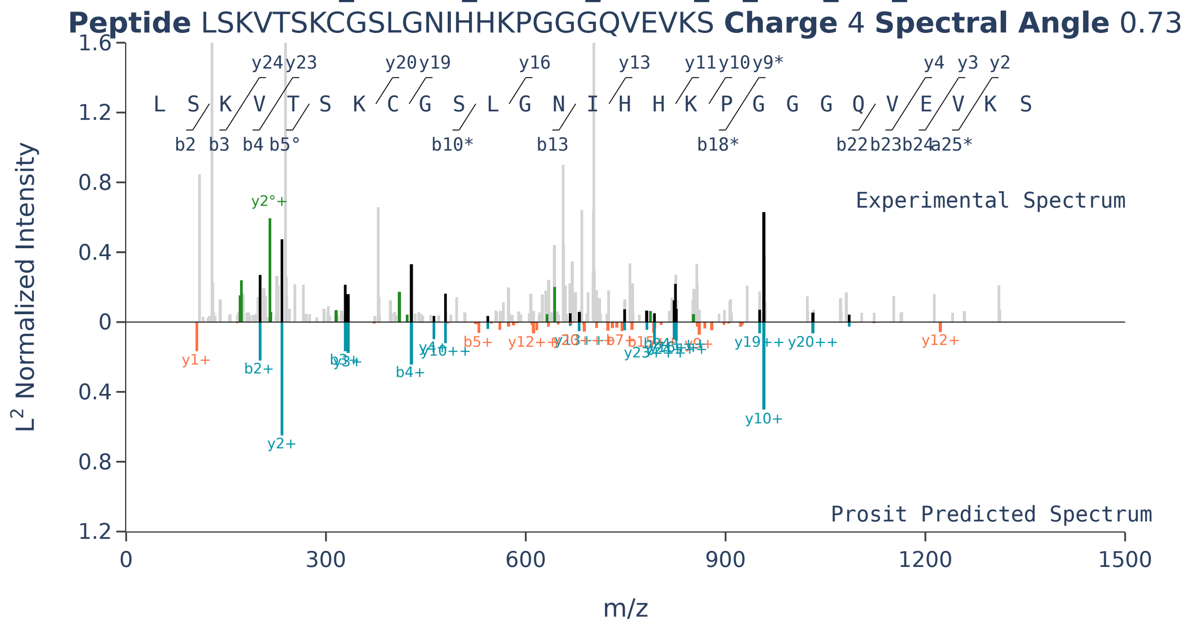


**Source** WSoh_221121_011221_HFGoe_L2_hTau_24h_R1 **Scan** 7958
**Peptide** FKDRVQSKL **Charge** 3 **Spectral Angle** 0.76

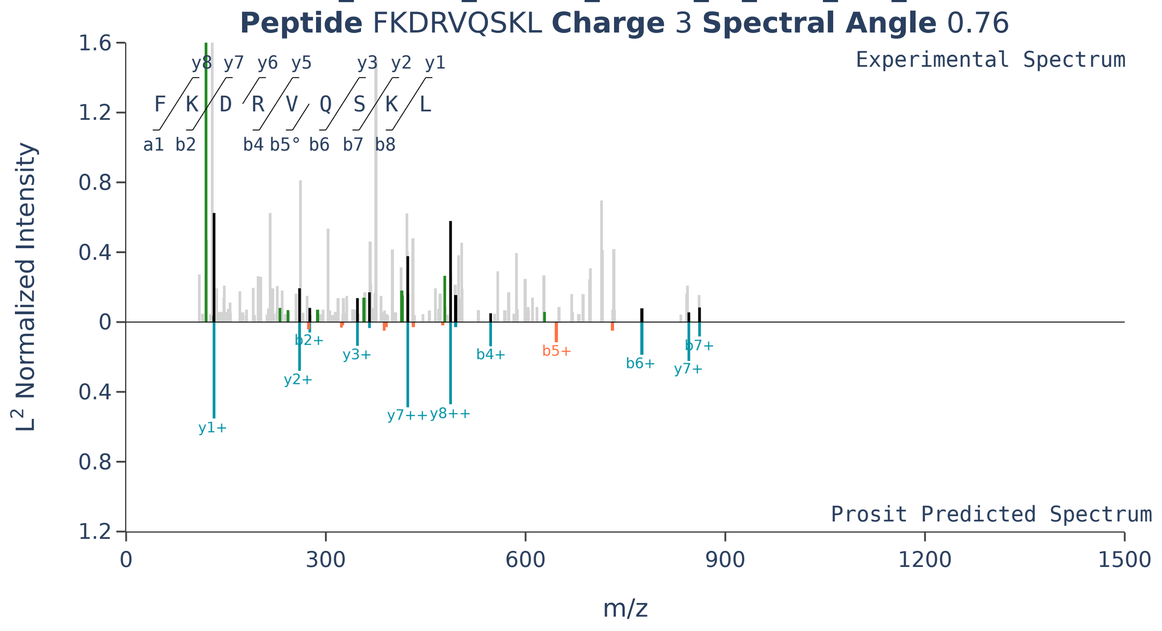


**Source** WSoh_221121_011221_HFGoe_L1_hTau_24h_R1 **Scan** 21868
**Peptide** SRLQTAPVPMPDLKNVKSKIGSTE **Charge** 4 **Spectral Angle** 0.78

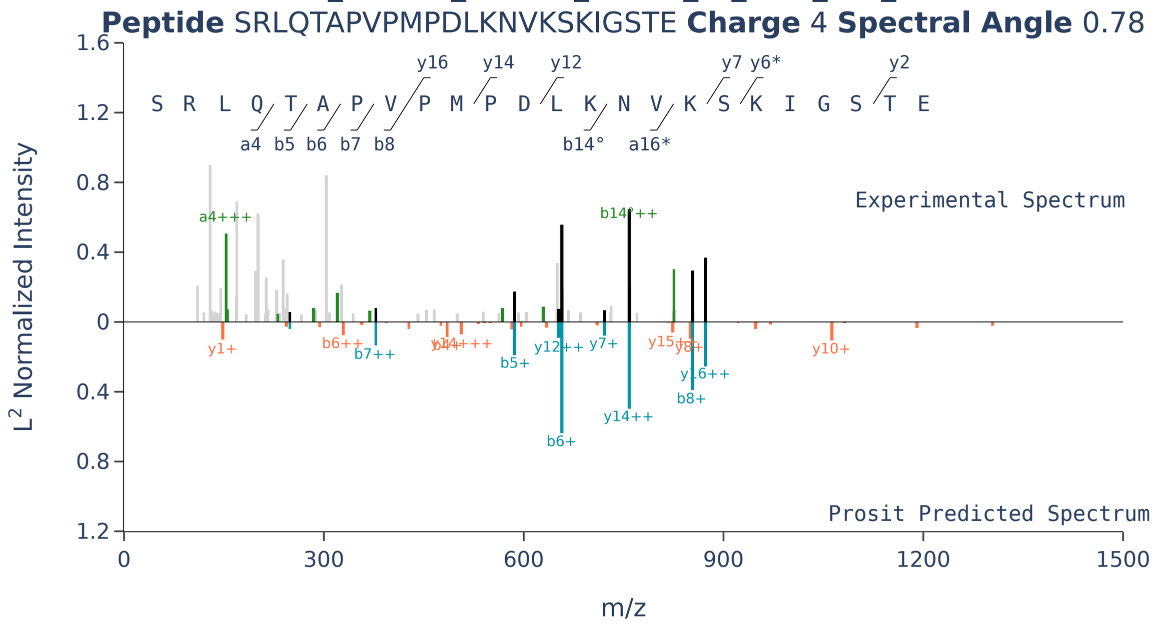


**Source** WSoh_221121_011221_HFGoe_L1_hTau_4h_R2 **Scan** 14322
**Peptide** GLGDRKDQGGYTMHQDQEGDTDAGL **Charge** 4 **Spectral Angle** 0.76

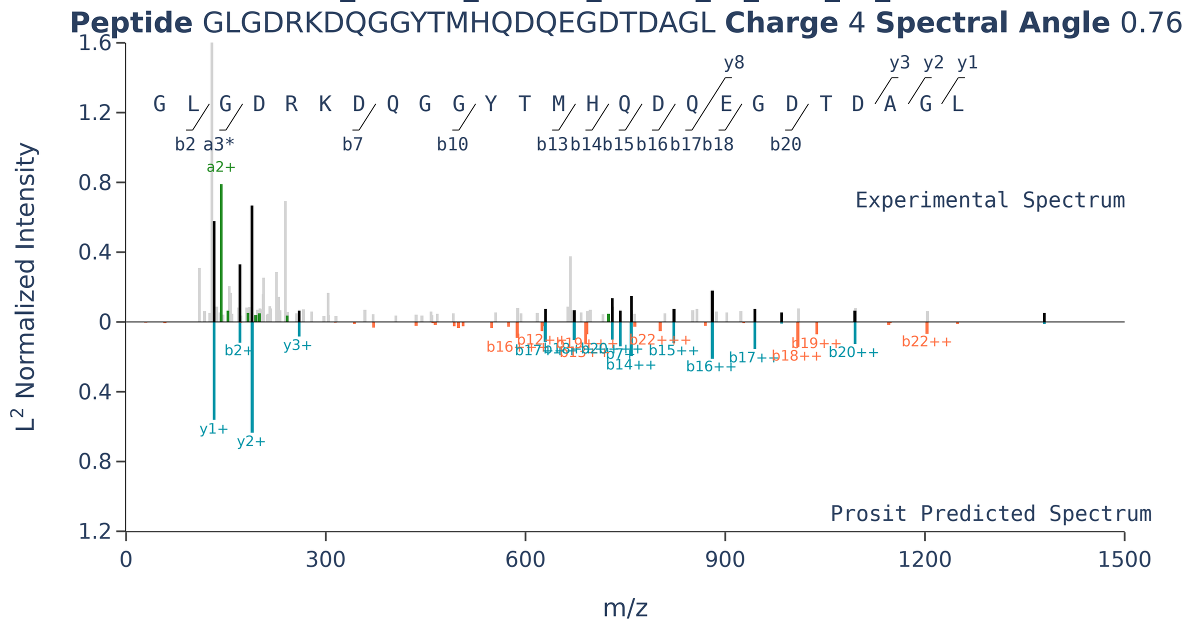


**Source** WSoh_221121_011221_HFGoe_L1_hTau_24h_R2 **Scan** 9581
**Peptide** NLTHVPGGGNKKIETHKLTFRENAKATD **Charge** 5 **Spectral Angle** 0.57

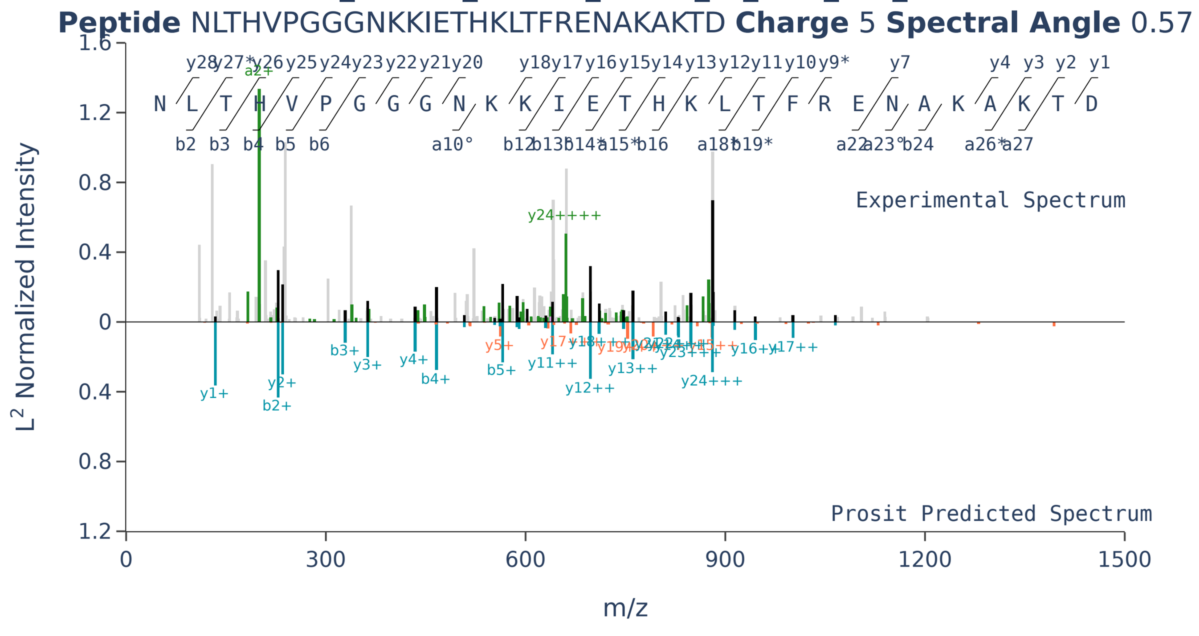

**Source** WSoh_221121_011221_HFGoe_L1_hTau_24h_R1 **Scan** 12427
**Peptide** PSLPTPPTREPKKVAVVRTPPKSPSSAKS **Charge** 4 **Spectral Angle** 0.66

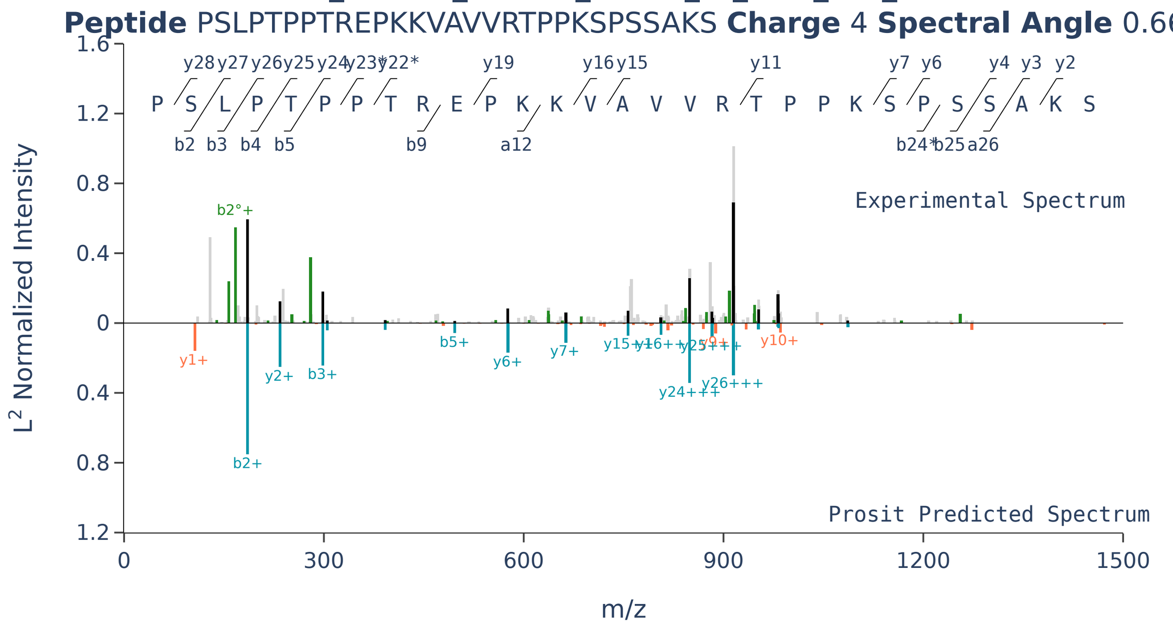


**Source** WSoh_221121_011221_HFGoe_L1_hTau_4h_R2 **Scan** 5425
**Peptide** HVTQARMVSKSKD **Charge** 3 **Spectral Angle** 0.67

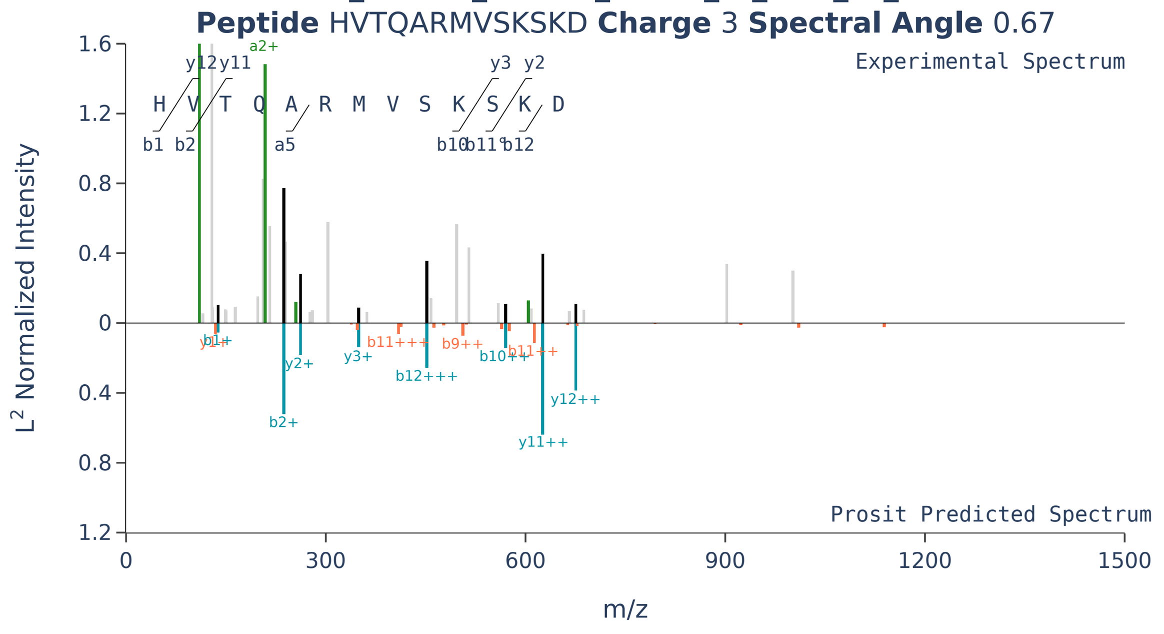


**Source** WSoh_221121_011221_HFGoe_L1_hTau_24h_R2 **Scan** 16150
**Peptide** RTPPKSPSSAKSRLQTAPVPMPDLKNVKS **Charge** 4 **Spectral Angle** 0.71

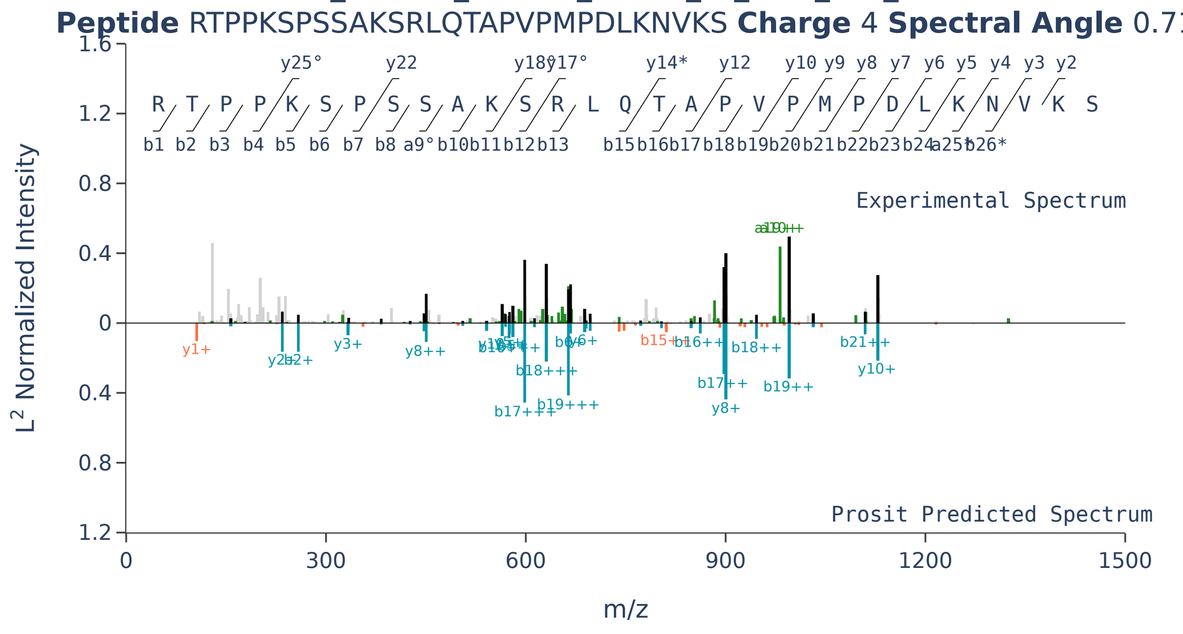


**Source** WSoh_221121_011221_HFGoe_L2_hTau_4h_R2 **Scan** 6109
**Peptide** PRHLSNVSST **Charge** 2 **Spectral Angle** 0.83

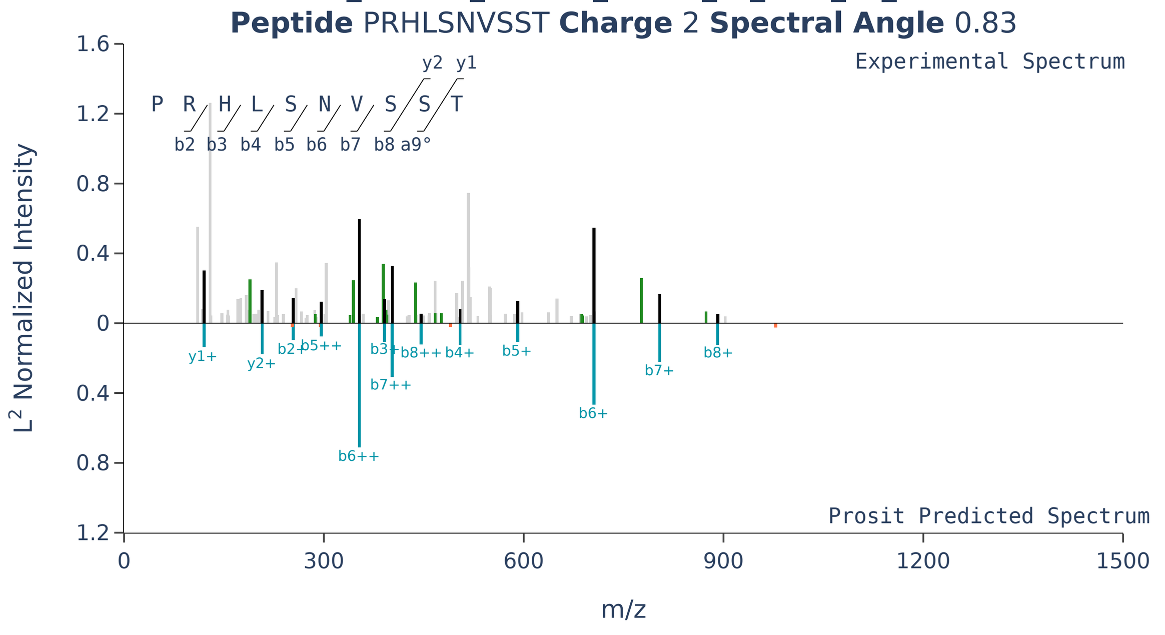


**Source** WSoh_221121_011221_HFGoe_L1_hTau_4h_R1 **Scan** 6600
**Peptide** GGNKKIETHKL **Charge** 3 **Spectral Angle** 0.65

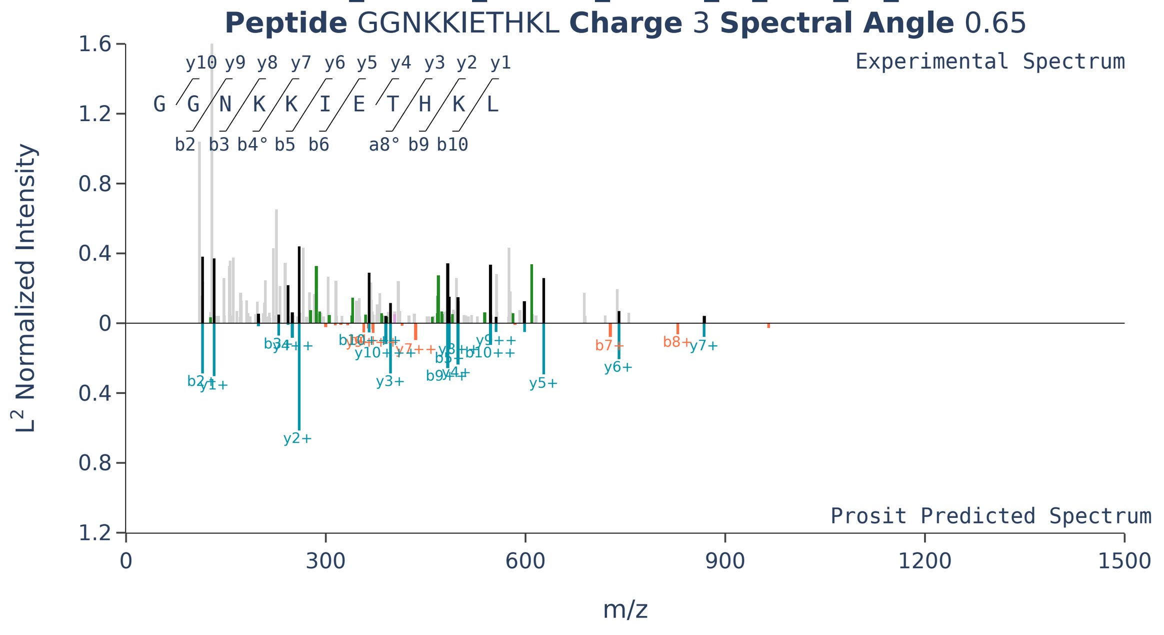


**Source** WSoh_221121_011221_HFGoe_L2_hTau_4h_R2 **Scan** 15095
**Peptide** NVQSKCGSKDNIKHVPGGGSVQIVYKPVD **Charge** 4 **Spectral Angle** 0.73

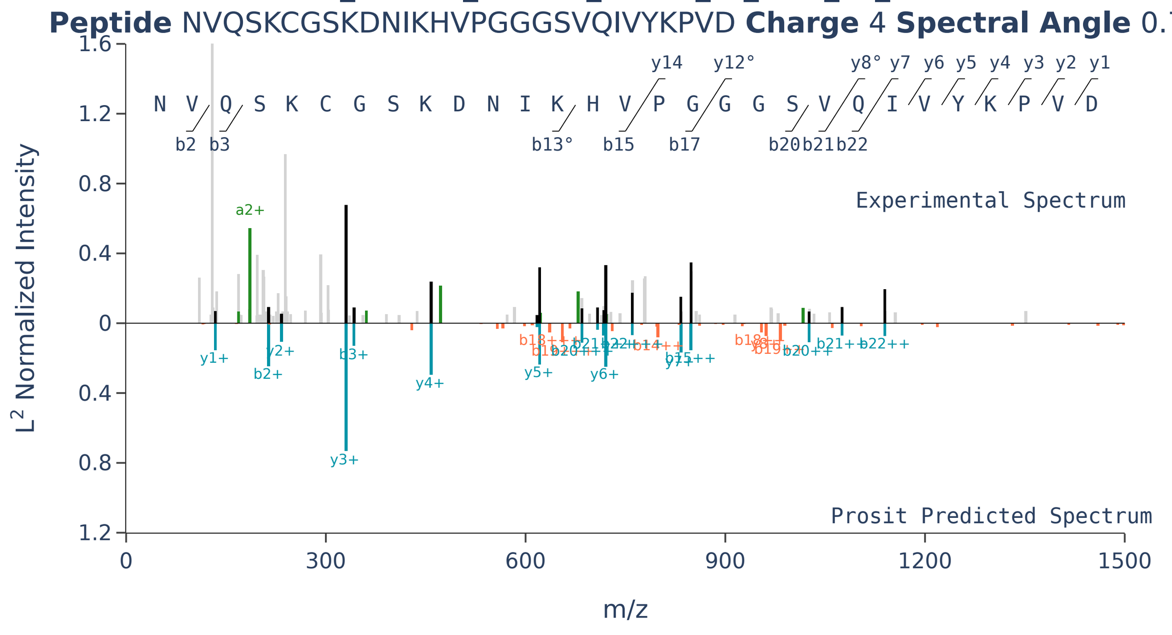


**Source** WSoh_221121_011221_HFGoe_L2_hTau_24h_R2 **Scan** 12519
**Peptide** NLHHKPGGGQVEVKSEKLDF **Charge** 5 **Spectral Angle** 0.67

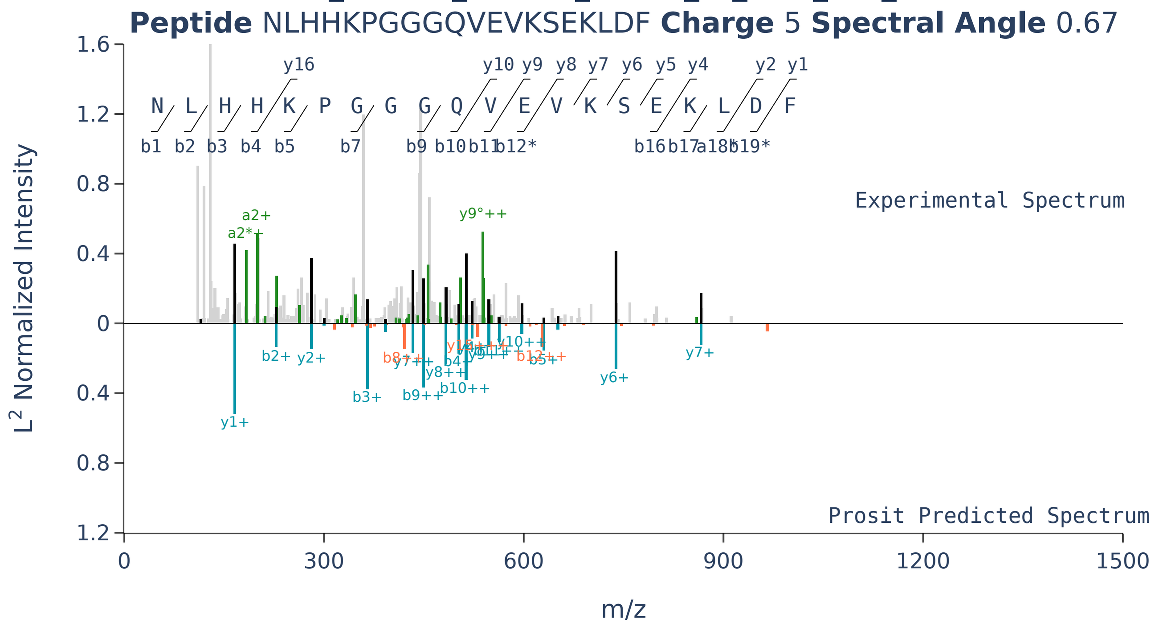


**Source** WSoh_221121_011221_HFGoe_L1_hTau_24h_R1 **Scan** 26076
**Peptide** SNVSSTGSIDMVDSPQLA **Charge** 2 **Spectral Angle** 0.81

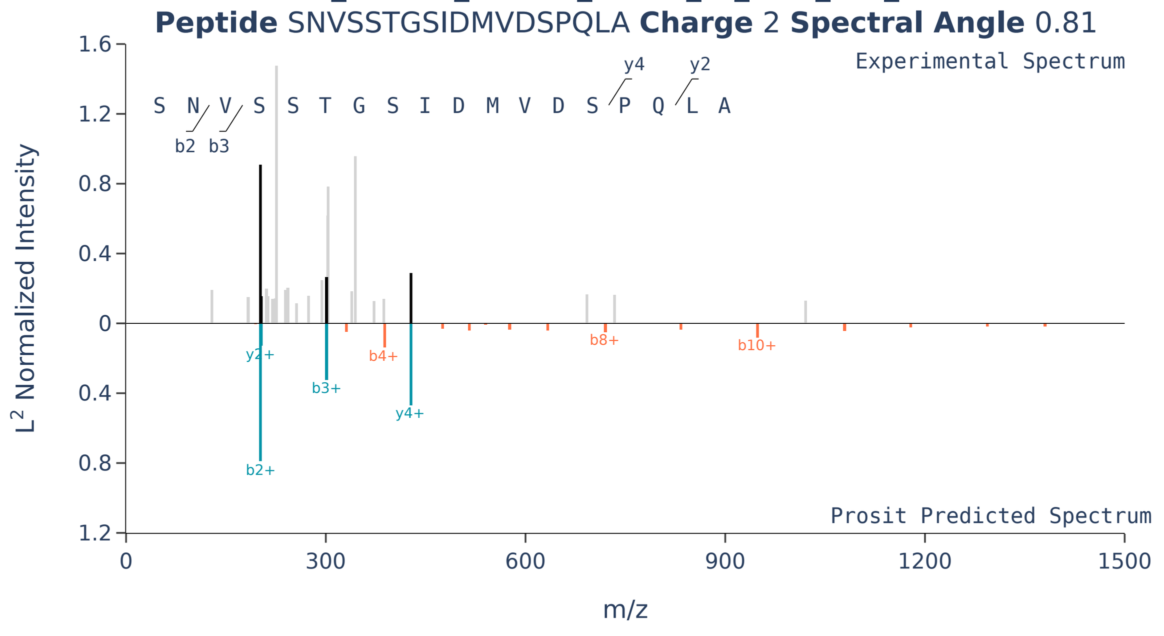


**Source** WSoh_221121_011221_HFGoe_L2_hTau_4h_R2 **Scan** 5573
**Peptide** CGSKDNIKHV **Charge** 2 **Spectral Angle** 0.8

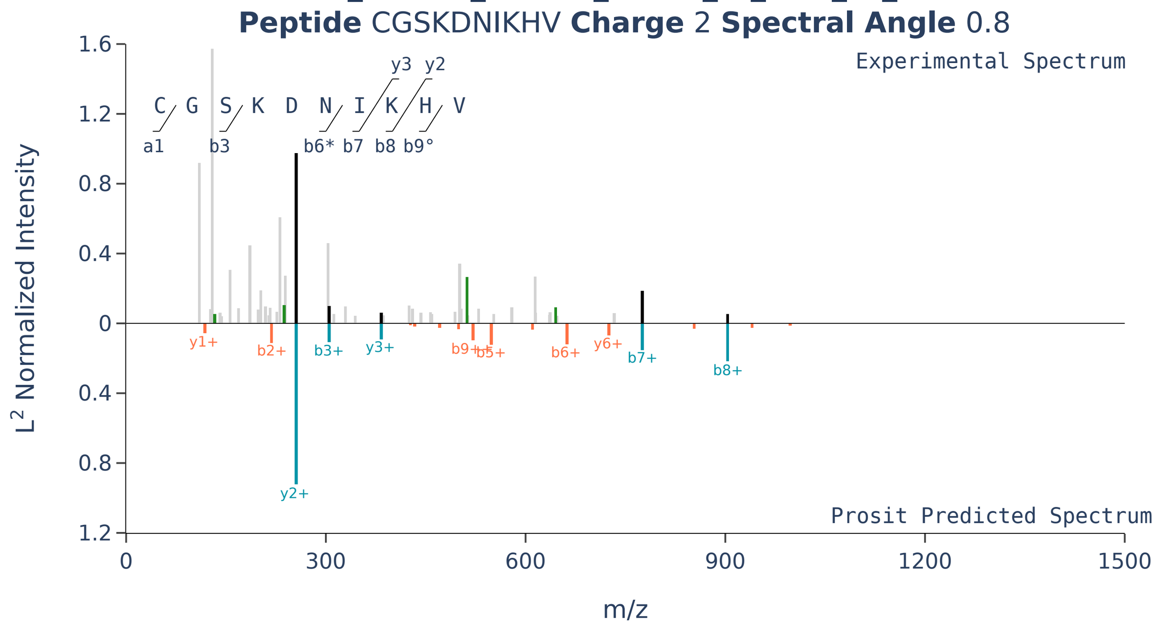


**Source** WSoh_221121_011221_HFGoe_L1_hTau_4h_R2 **Scan** 7410
**Peptide** TSKCGSLGNIHHKPGGGQVEVKSE **Charge** 4 **Spectral Angle** 0.66

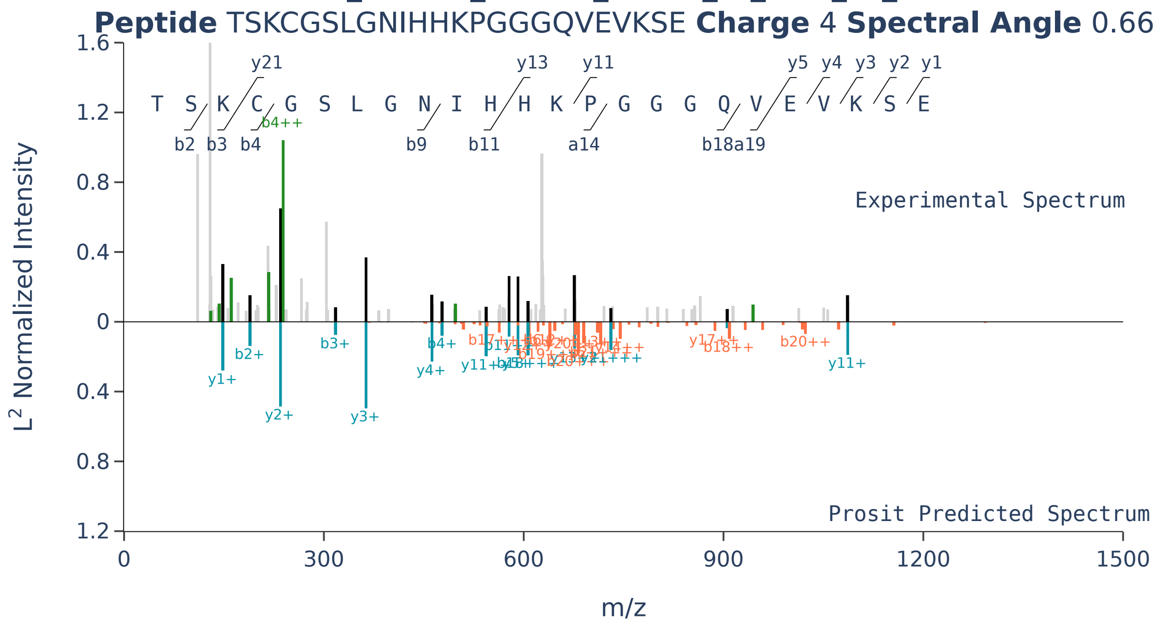


**Source** WSoh_221121_011221_HFGoe_L1_hTau_24h_R1 **Scan** 25137
**Peptide** SRLQTAPVPMPDLKNV **Charge** 3 **Spectral Angle** 0.8

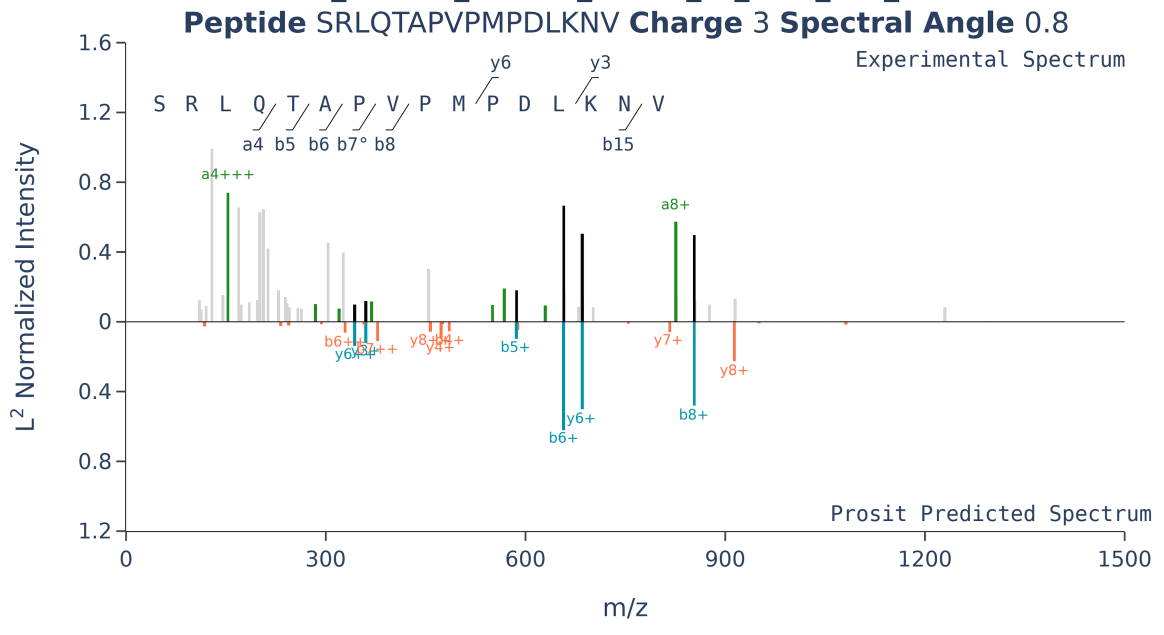


**Source** WSoh_221121_011221_HFGoe_L2_hTau_24h_R1 **Scan** 5306
**Peptide** GTGSDDKKAKGADGKTKI **Charge** 3 **Spectral Angle** 0.68

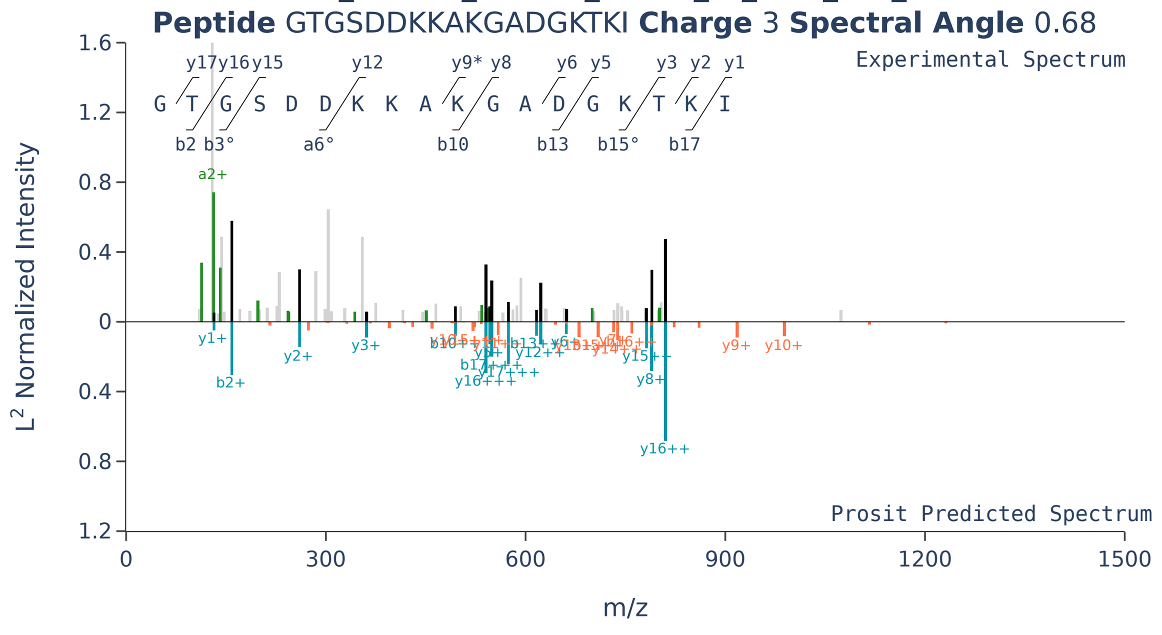


**Source** WSoh_221121_011221_HFGoe_L1_hTau_24h_R1 **Scan** 17482
**Peptide** ENLKHQPGGGKVQIINKKLDLSNV **Charge** 4 **Spectral Angle** 0.76

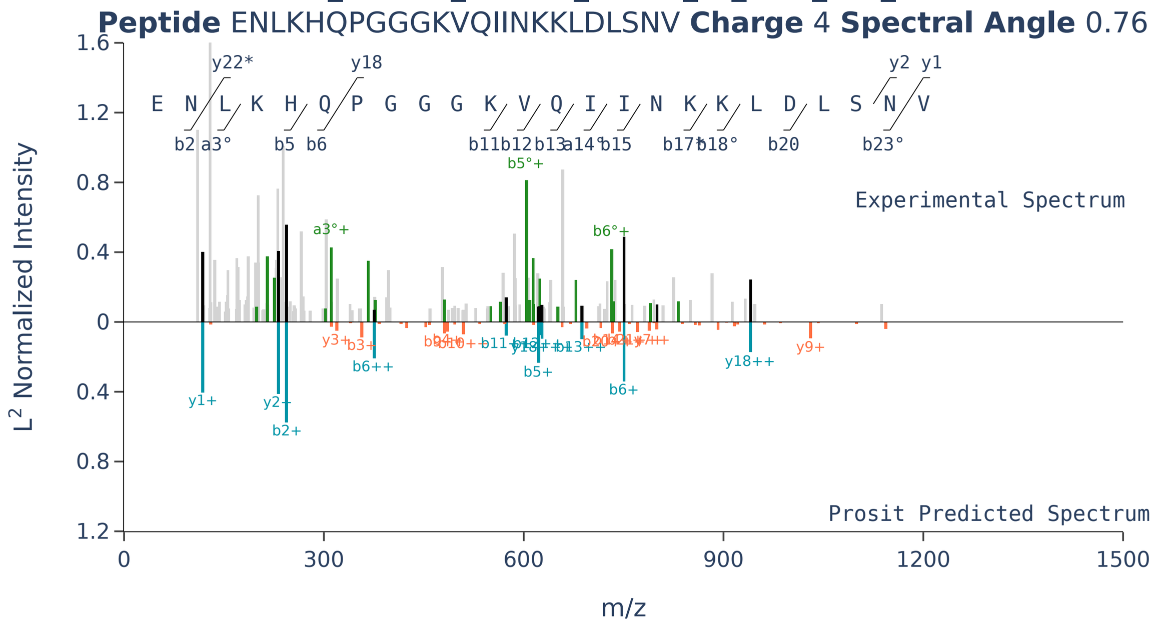


**Source** WSoh_221121_011221_HFGoe_L1_hTau_4h_R2 **Scan** 25621
**Peptide** PRHLSNVSSTGSIDMVDSPQLATL **Charge** 3 **Spectral Angle** 0.68

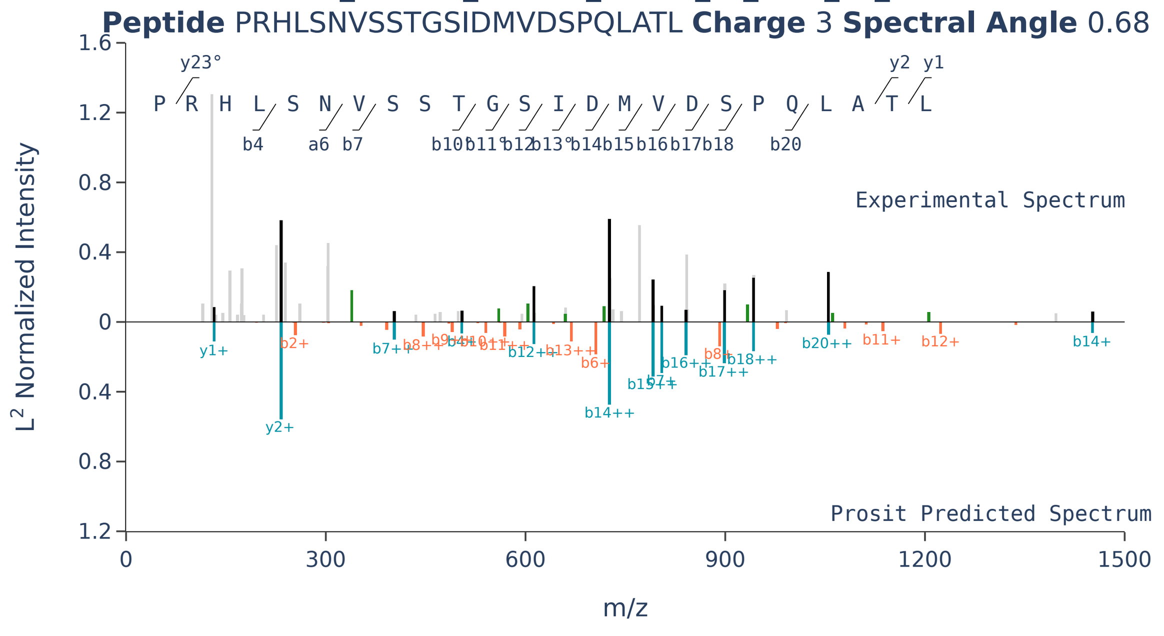


**Source** WSoh_221121_011221_HFGoe_L1_hTau_4h_R1 **Scan** 11854
**Peptide** EIPEGTTAEE **Charge** 2 **Spectral Angle** 0.71

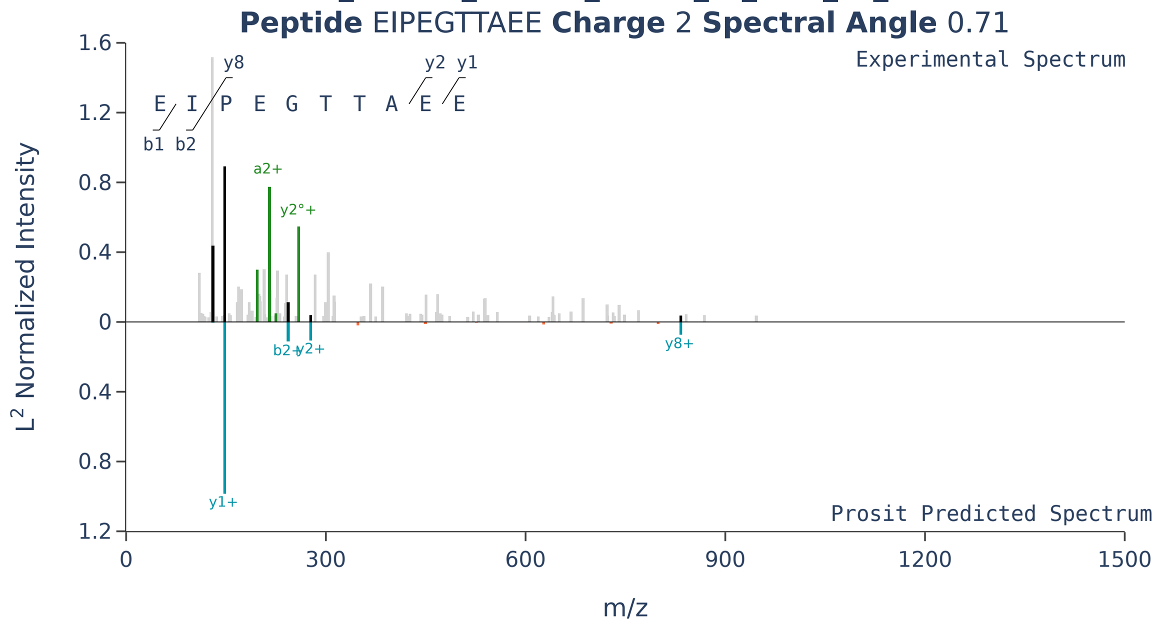

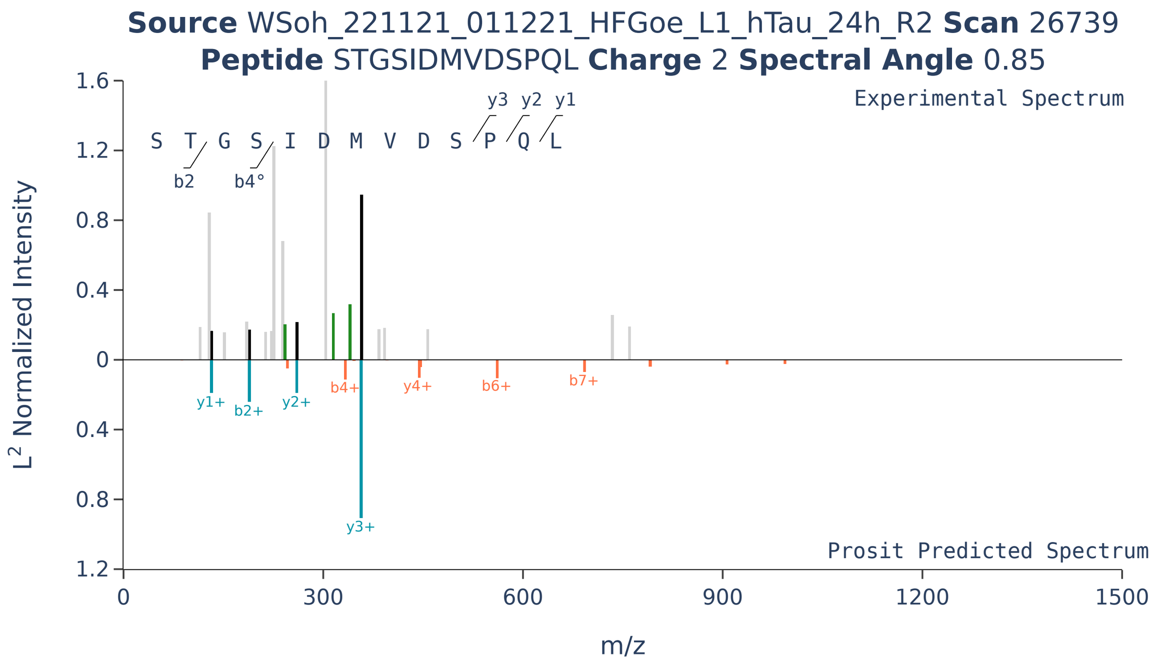
Source WSoh_221121_011221_HFGoe_L1_hTau_24h_R2 Scan 26739
Peptide STGSIDMVDSPQL Charge 2 Spectral Angle 0.85

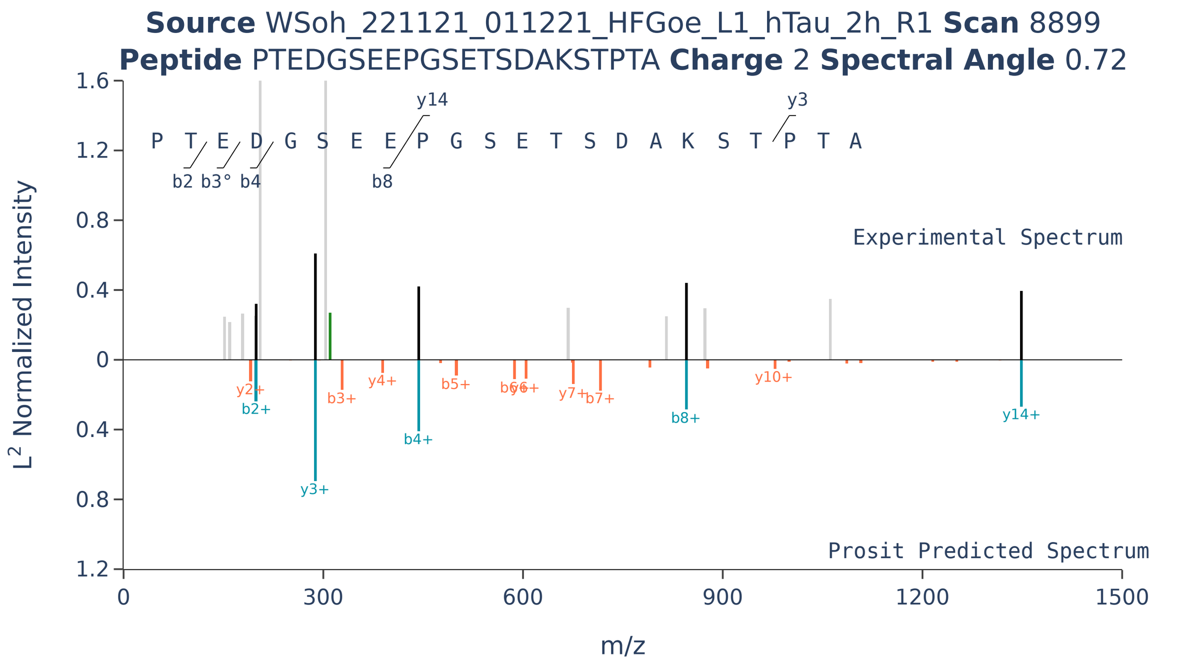
Source WSoh_221121_011221_HFGoe_L1_hTau_2h_R1 Scan 8899
Peptide PTEDGSEEPGSETSDAKSTPTA Charge 2 Spectral Angle 0.72

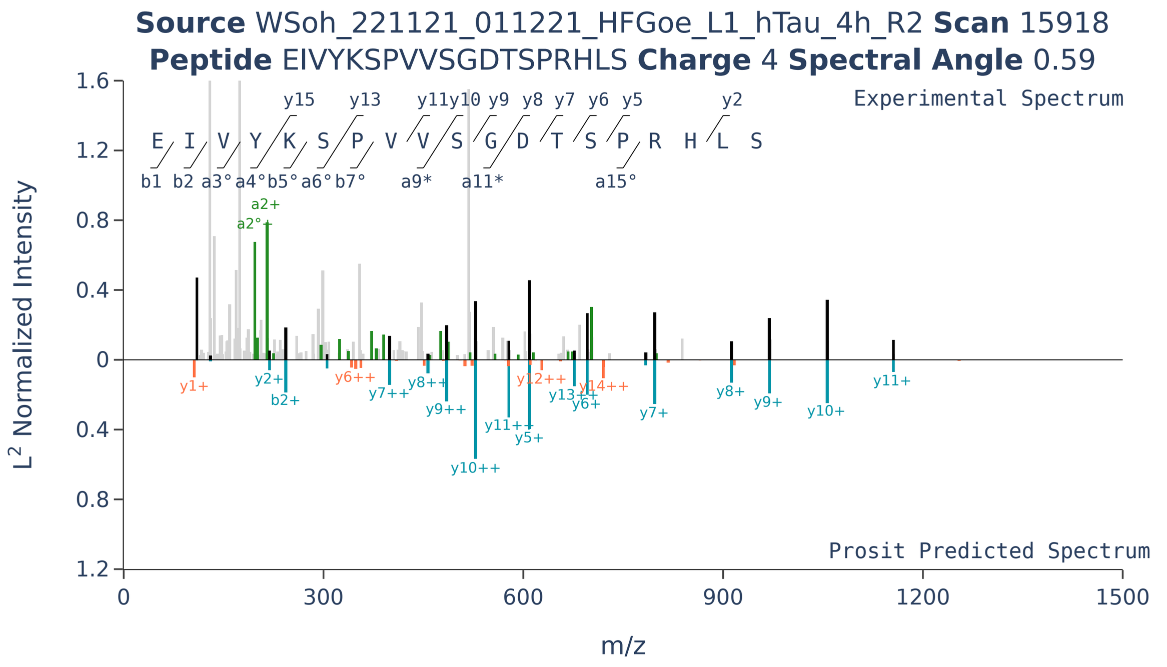
Source WSoh_221121_011221_HFGoe_L1_hTau_4h_R2 Scan 15918
Peptide EIVYKSPVVSGDTSPRHLS Charge 4 Spectral Angle 0.59

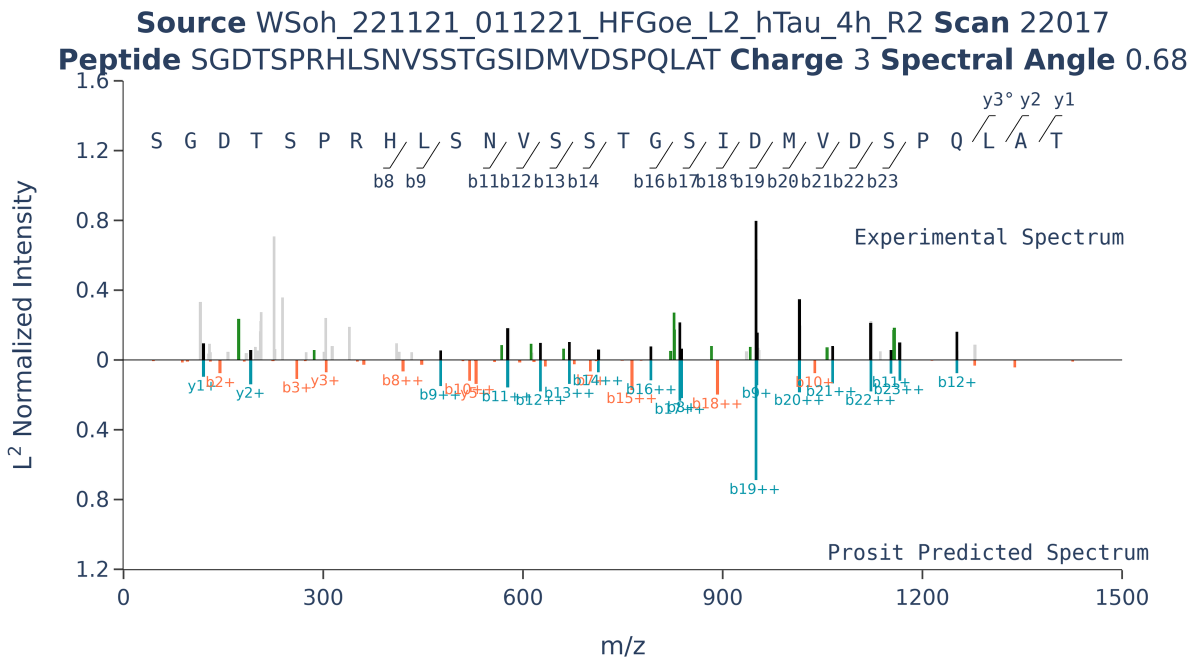
Source WSoh_221121_011221_HFGoe_L2_hTau_4h_R2 Scan 22017
Peptide SGDTSPRHLSNVSSTGSIDMVDSPQLAT Charge 3 Spectral Angle 0.68

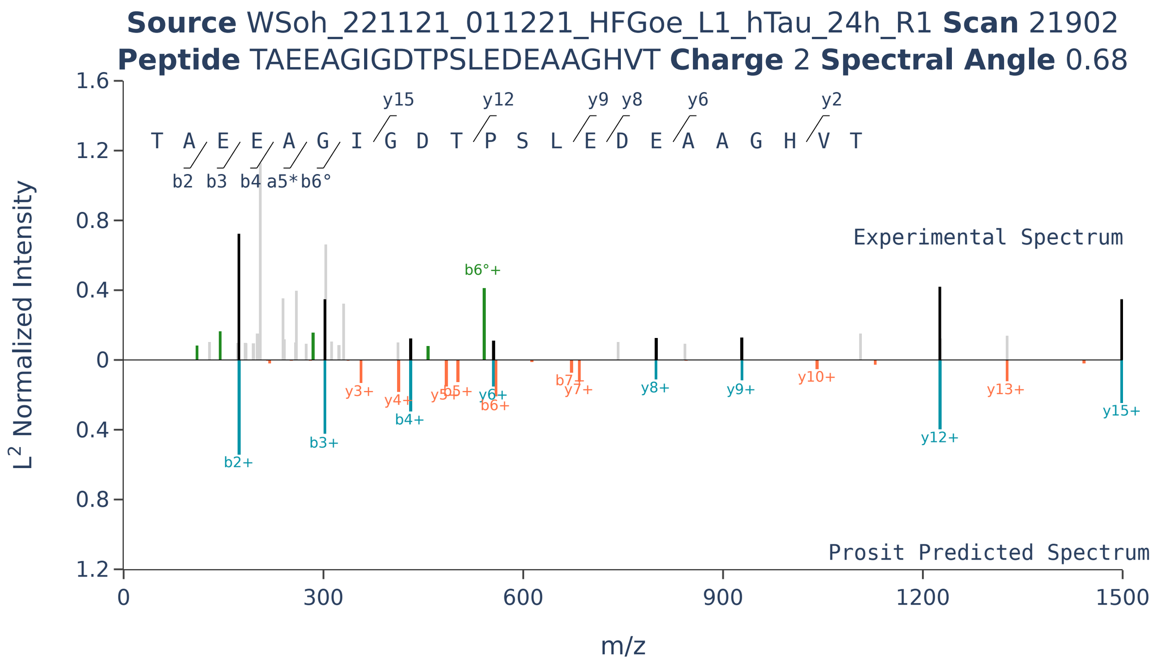
Source WSoh_221121_011221_HFGoe_L1_hTau_24h_R1 Scan 21902
Peptide TAEEAGIGDTPSLEDEAAGHVT Charge 2 Spectral Angle 0.68

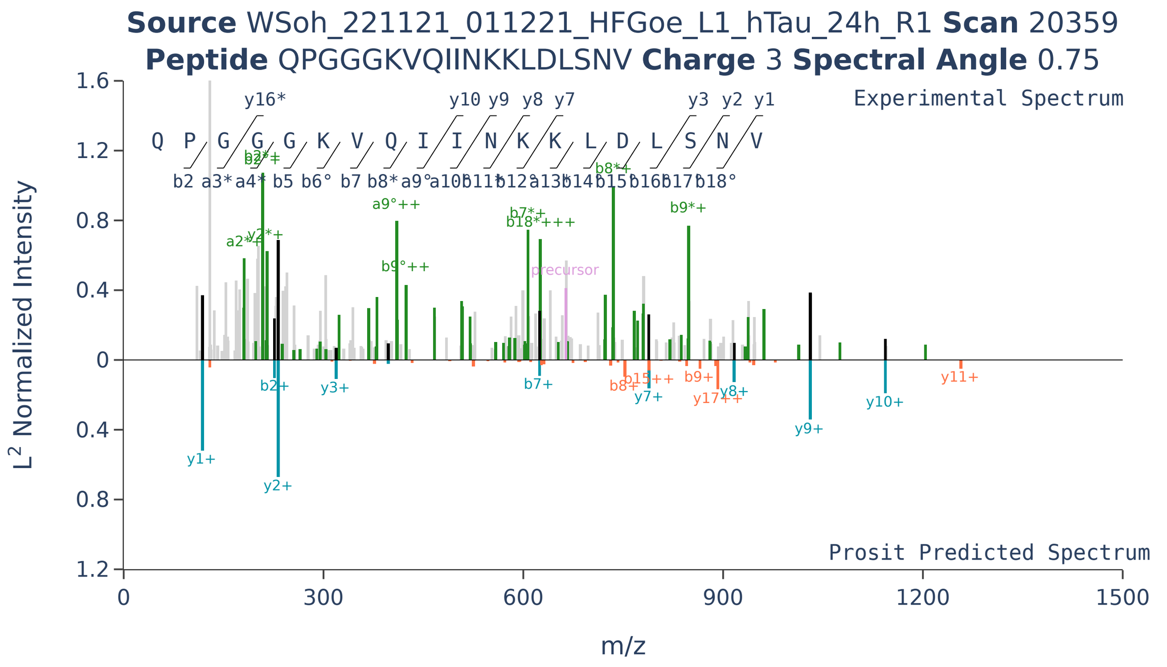
Source WSoh_221121_011221_HFGoe_L1_hTau_24h_R1 Scan 20359
Peptide QPGGGKVQIINKKLDLSNV Charge 3 Spectral Angle 0.75

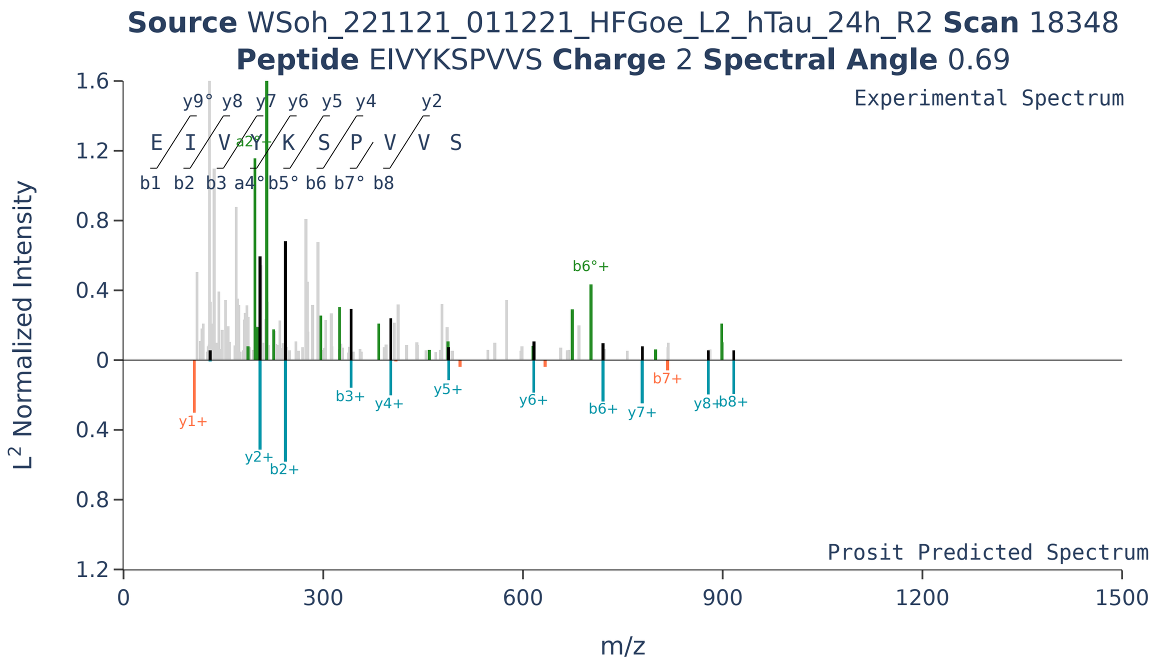
Source WSoh_221121_011221_HFGoe_L2_hTau_24h_R2 Scan 18348
Peptide EIVYKSPVVS Charge 2 Spectral Angle 0.69

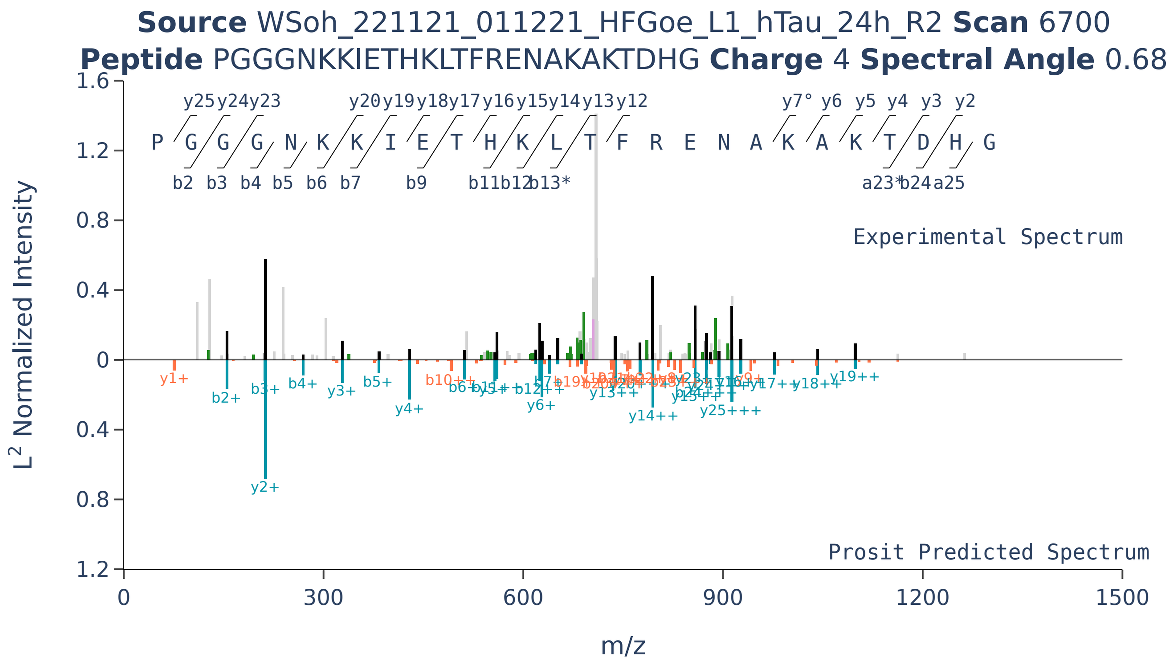
Source WSoh_221121_011221_HFGoe_L1_hTau_24h_R2 Scan 6700
Peptide PGGGNKKIETHKLTFRENAKAKTDHG Charge 4 Spectral Angle 0.68

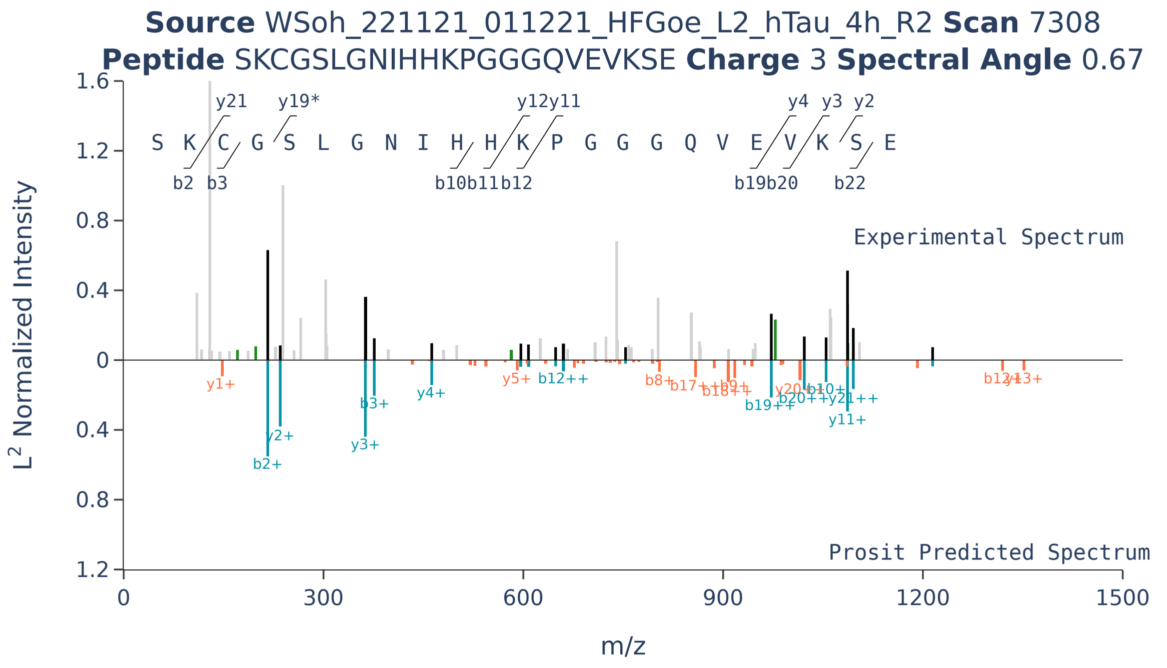
Source WSoh_221121_011221_HFGoe_L2_hTau_4h_R2 Scan 7308
Peptide SKCGSLGNIHHKPGGGQVEVKSE Charge 3 Spectral Angle 0.67

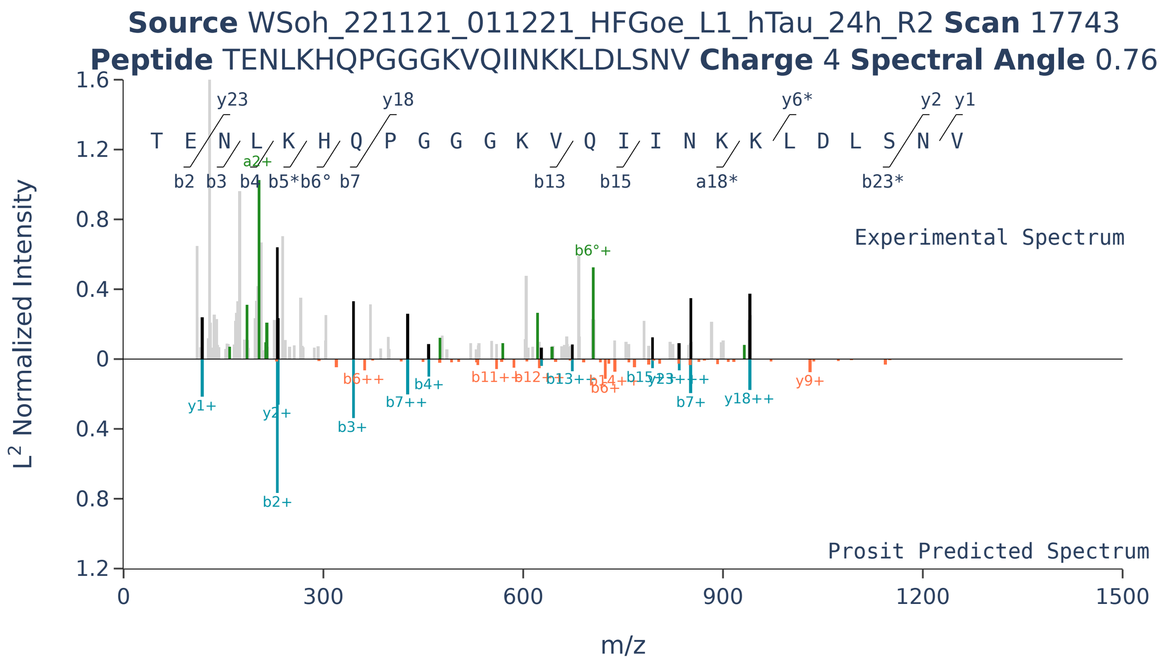
Source WSoh_221121_011221_HFGoe_L1_hTau_24h_R2 Scan 17743
Peptide TENLKHQPGGGKVQIINKKLDLSNV Charge 4 Spectral Angle 0.76

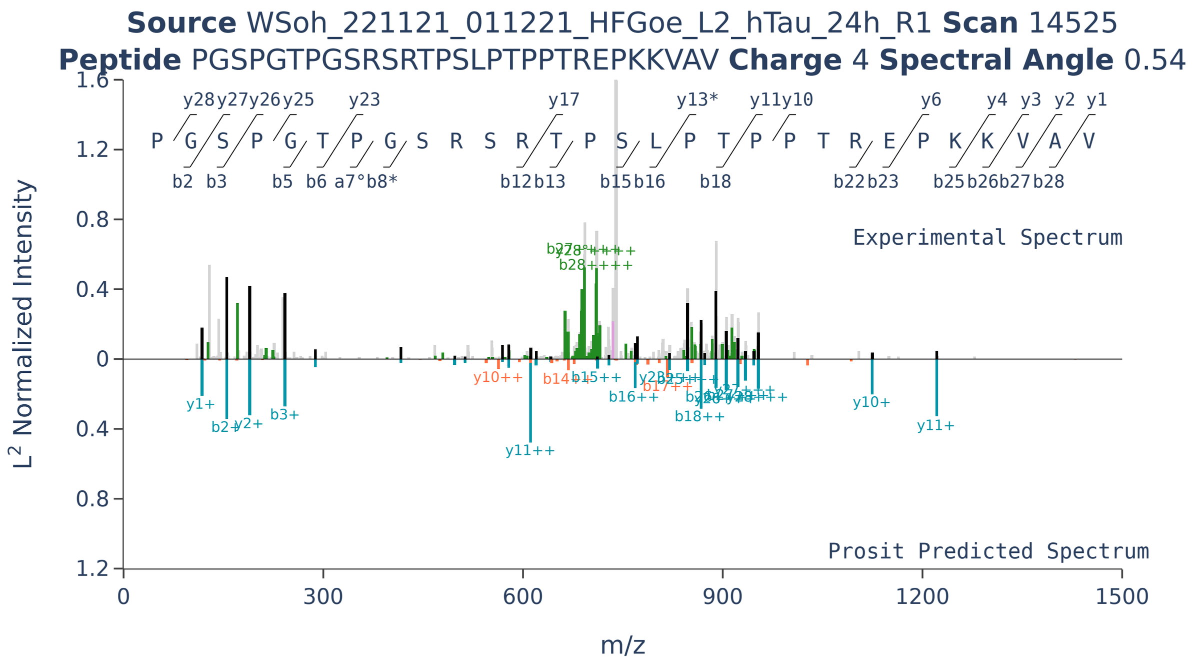
Source WSoh_221121_011221_HFGoe_L2_hTau_24h_R1 Scan 14525
Peptide PGSPGTPGSRSRTPSLPTPPTREPKKVAV Charge 4 Spectral Angle 0.54

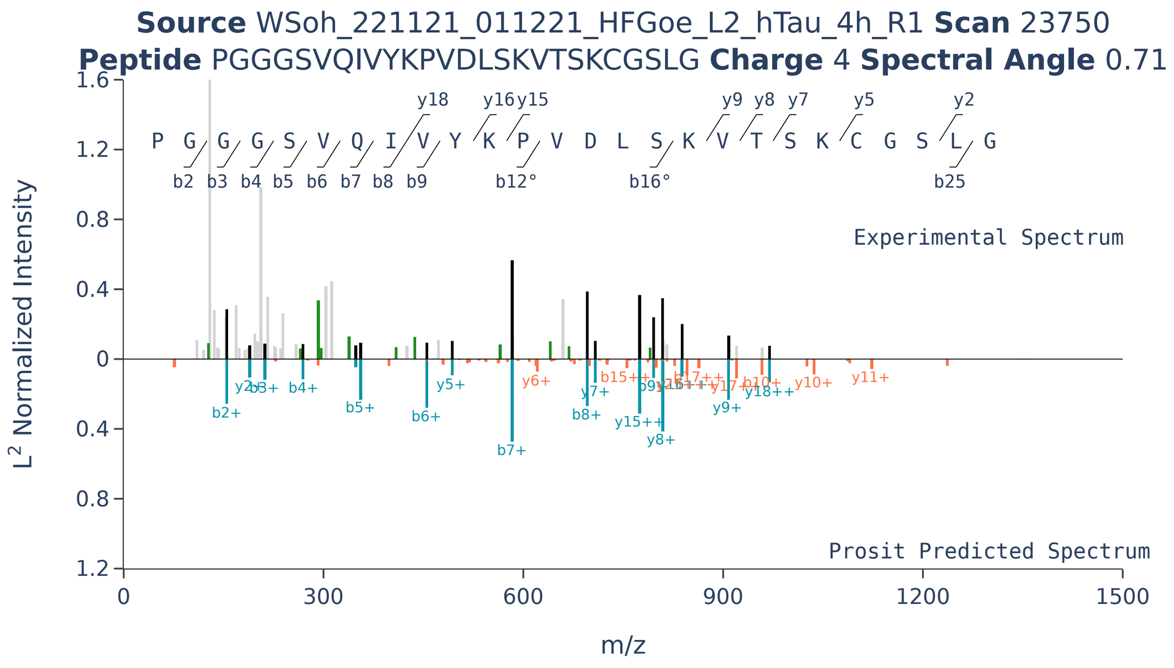
Source WSoh_221121_011221_HFGoe_L2_hTau_4h_R1 Scan 23750
Peptide PGGGSVQIVYKPVDLSKVTSKCGSLG Charge 4 Spectral Angle 0.71

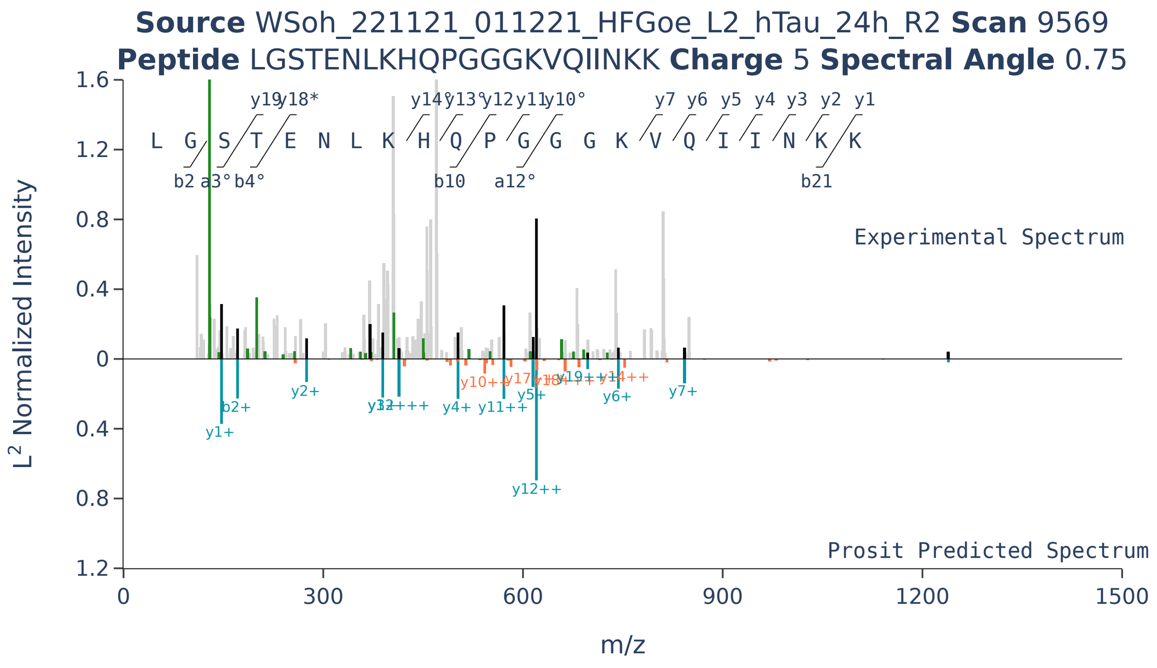
Source WSoh_221121_011221_HFGoe_L2_hTau_24h_R2 Scan 9569
Peptide LGSTENLKHQPGGGKVQIINKK Charge 5 Spectral Angle 0.75

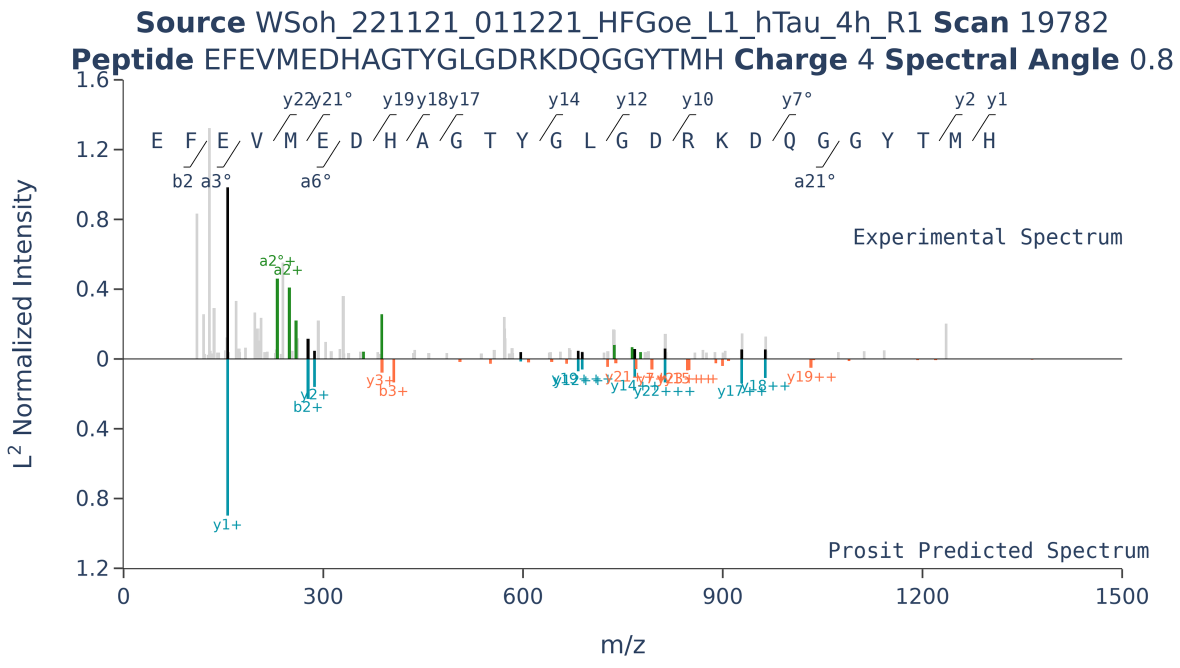
Source WSoh_221121_011221_HFGoe_L1_hTau_4h_R1 Scan 19782
Peptide EFEVMEDHAGTYGLGDRKDQGGYTMH Charge 4 Spectral Angle 0.8

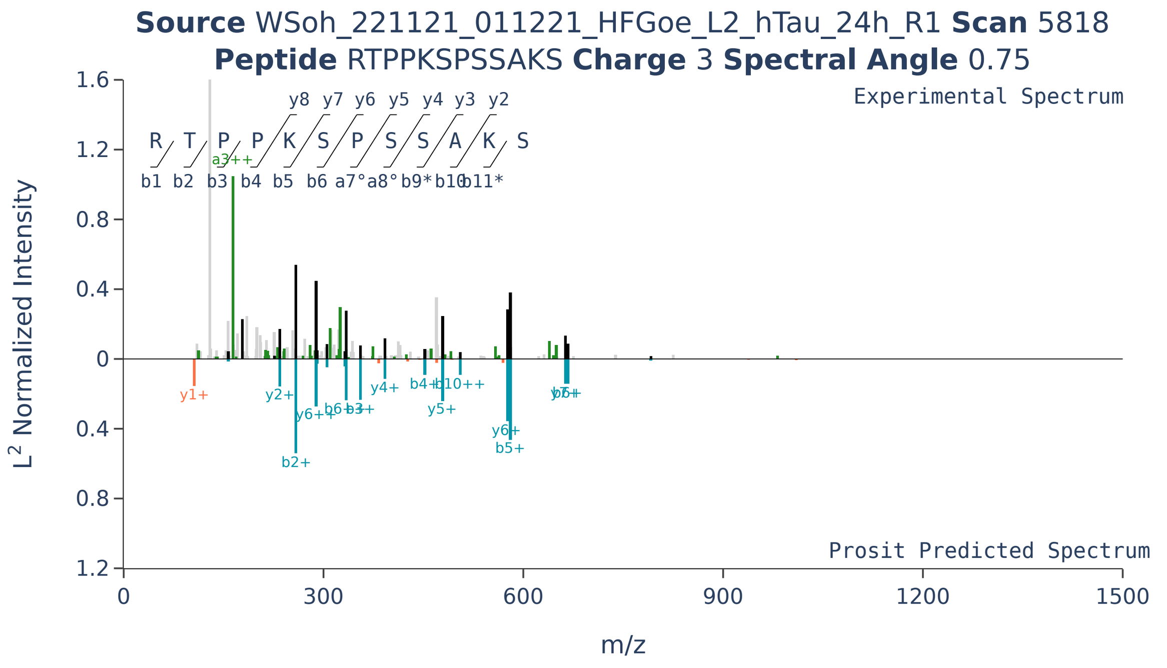
Source WSoh_221121_011221_HFGoe_L2_hTau_24h_R1 Scan 5818
Peptide RTPPKSPSSAKS Charge 3 Spectral Angle 0.75

**Source** WSoh_221121_011221_HFGoe_L2_hTau_24h_R1 **Scan** 19034
**Peptide** IGDTPSLEDEAAGHVTQ **Charge** 2 **Spectral Angle** 0.64

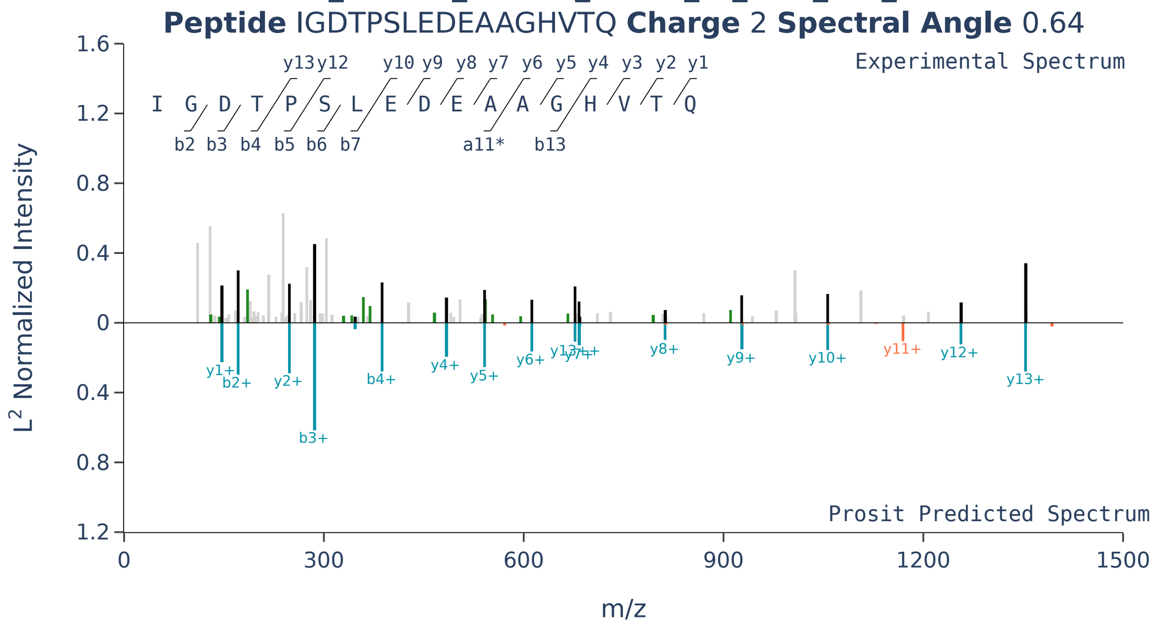


**Source** WSoh_221121_011221_HFGoe_L1_hTau_4h_R2 **Scan** 5394
**Peptide** KSKDGTGSDDKKAKGADGKTKIATPRG **Charge** 4 **Spectral Angle** 0.79

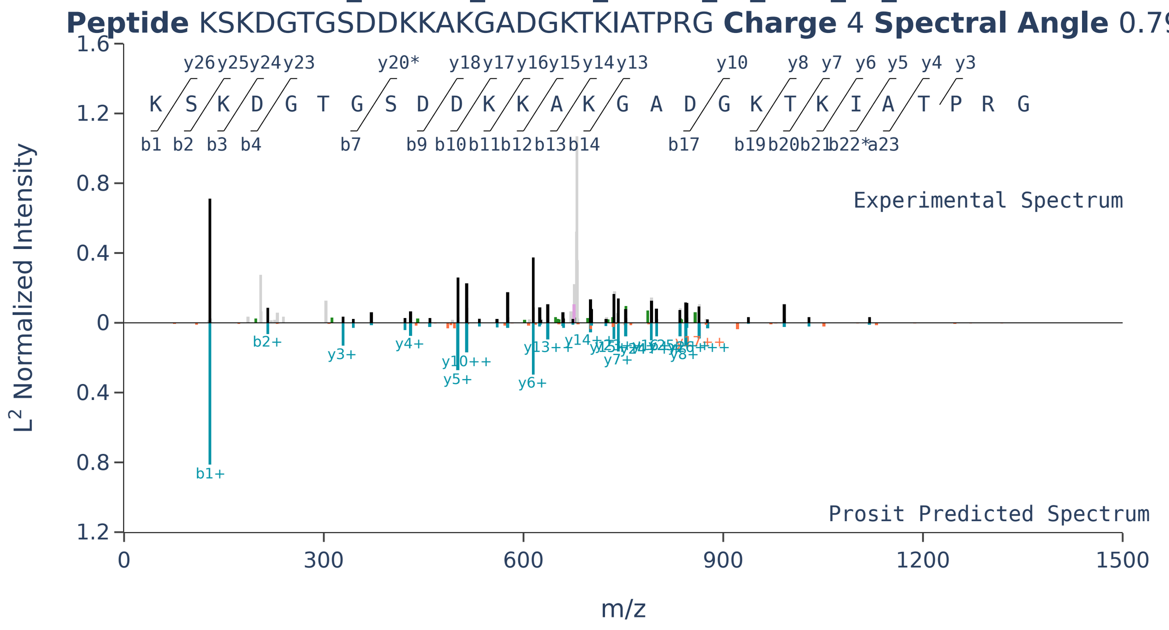


**Source** WSoh_221121_011221_HFGoe_L1_hTau_24h_R2 **Scan** 8530
**Peptide** GKTKIATPRGAAPPGQKGQANATRIPAKT **Charge** 4 **Spectral Angle** 0.67

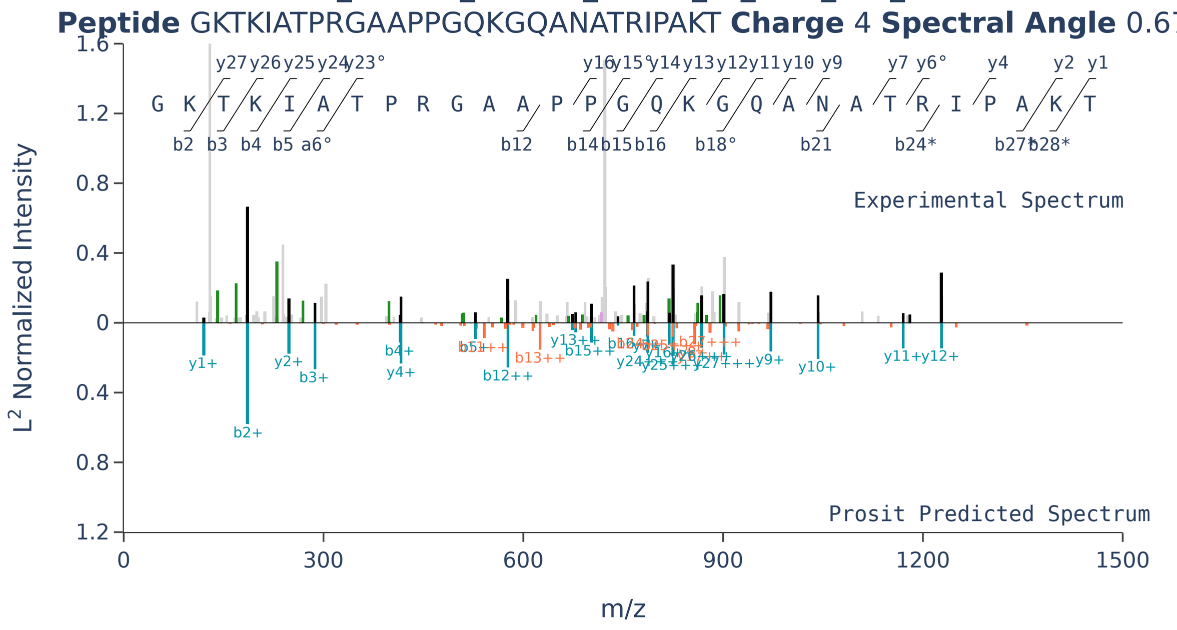


**Source** WSoh_221121_011221_HFGoe_L1_hTau_4h_R2 **Scan** 19849
**Peptide** VTAPLVDEGAPGKQAAAQPHTEIPEGTTA **Charge** 3 **Spectral Angle** 0.79

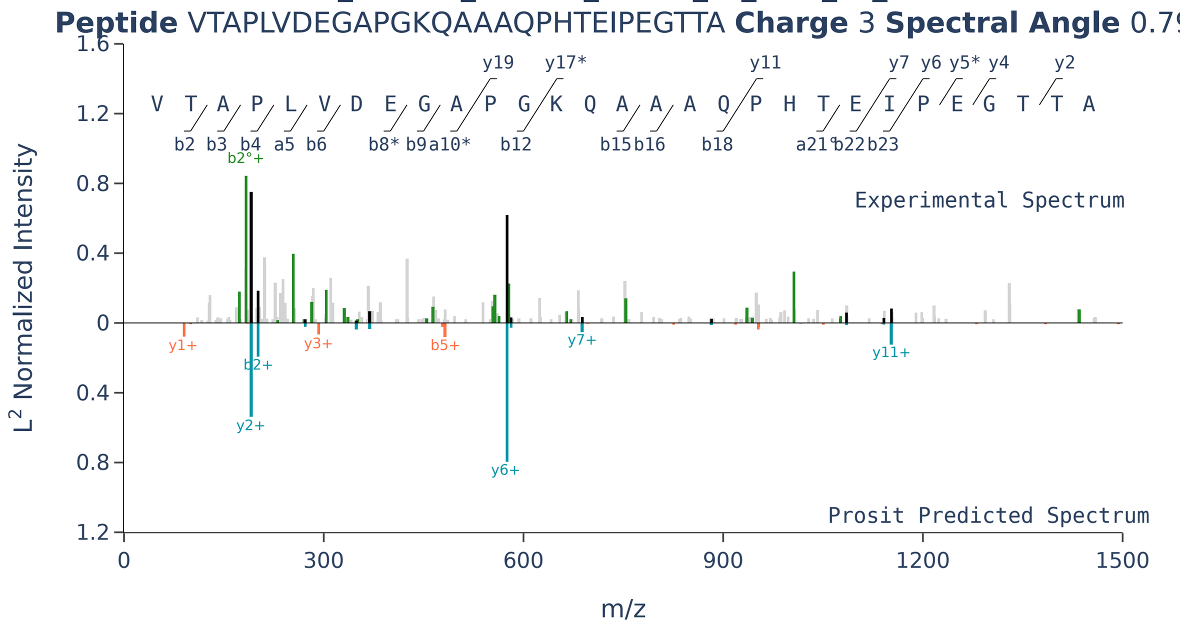


**Source** WSoh_221121_011221_HFGoe_L2_hTau_1h_R2 **Scan** 12176
**Peptide** PLVDEGAPGKQAA **Charge** 2 **Spectral Angle** 0.84

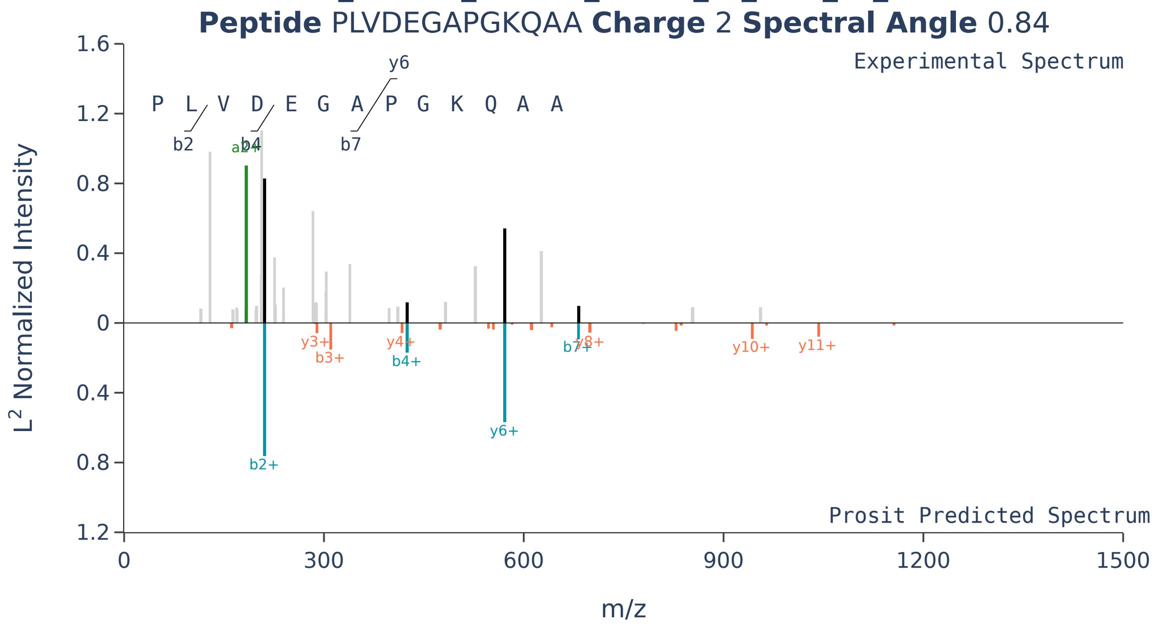


**Source** WSoh_221121_011221_HFGoe_L1_hTau_4h_R1 **Scan** 10884
**Peptide** QANATRIPAKTPPAPKTPPSSGEPPKSGD **Charge** 4 **Spectral Angle** 0.73

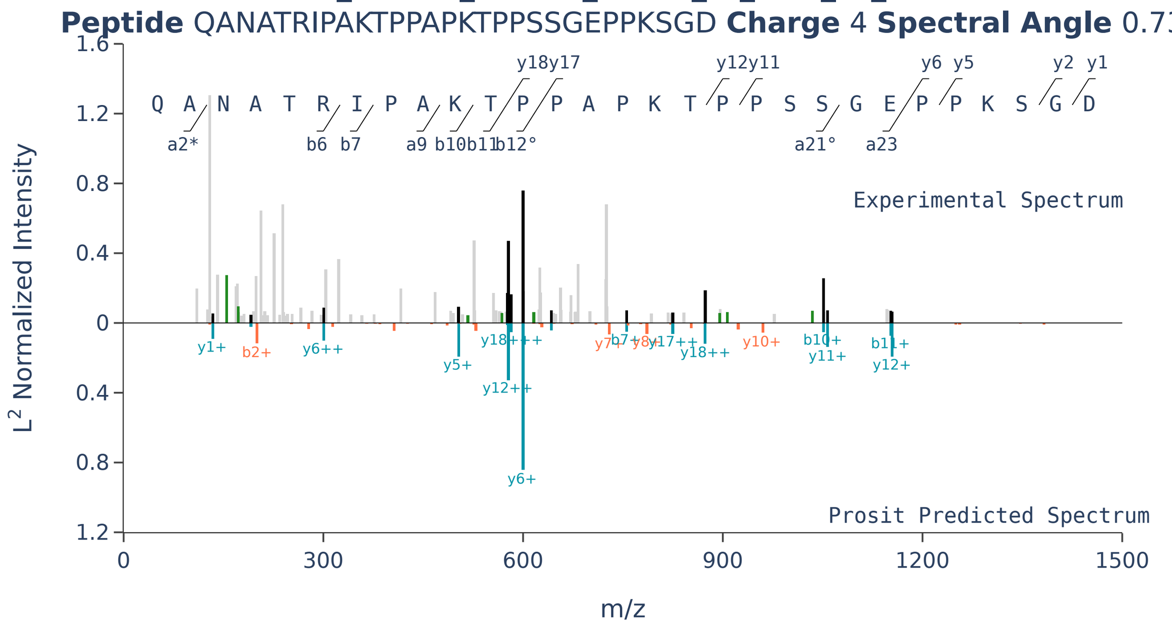


**Source** WSoh_221121_011221_HFGoe_L1_hTau_4h_R1 **Scan** 11927
**Peptide** IHHKPGGGQVEVKSEKLDF **Charge** 3 **Spectral Angle** 0.82

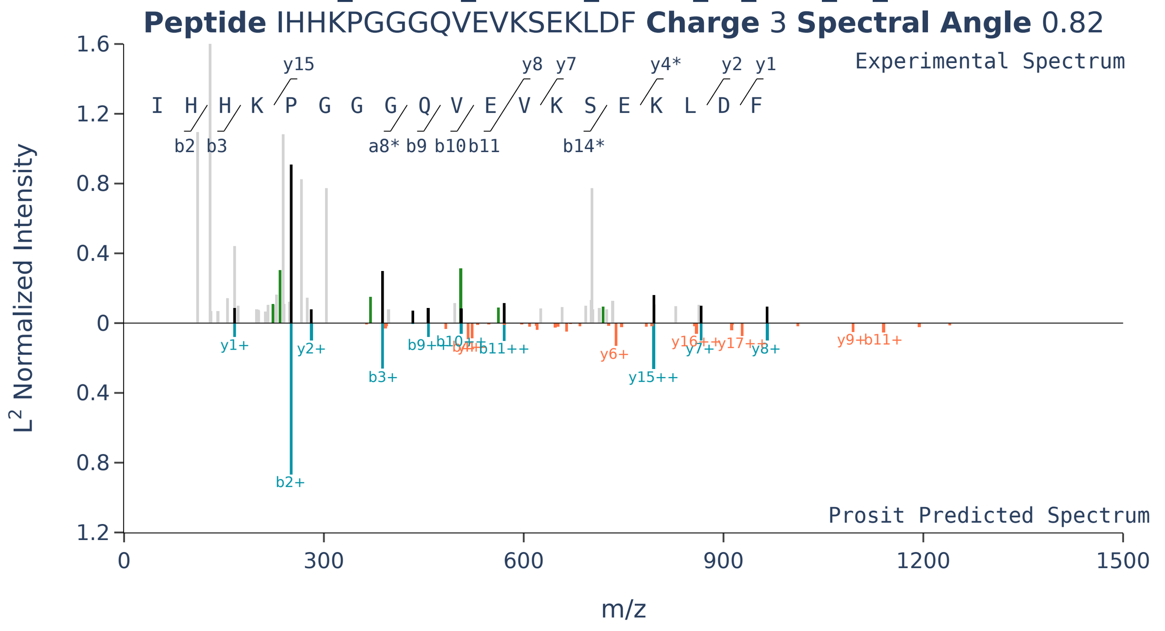


**Source** WSoh_221121_011221_HFGoe_L2_hTau_4h_R1 **Scan** 14422
**Peptide** ETHKLTFRENAKAKTDHGAEIVYKSPVV **Charge** 5 **Spectral Angle** 0.65

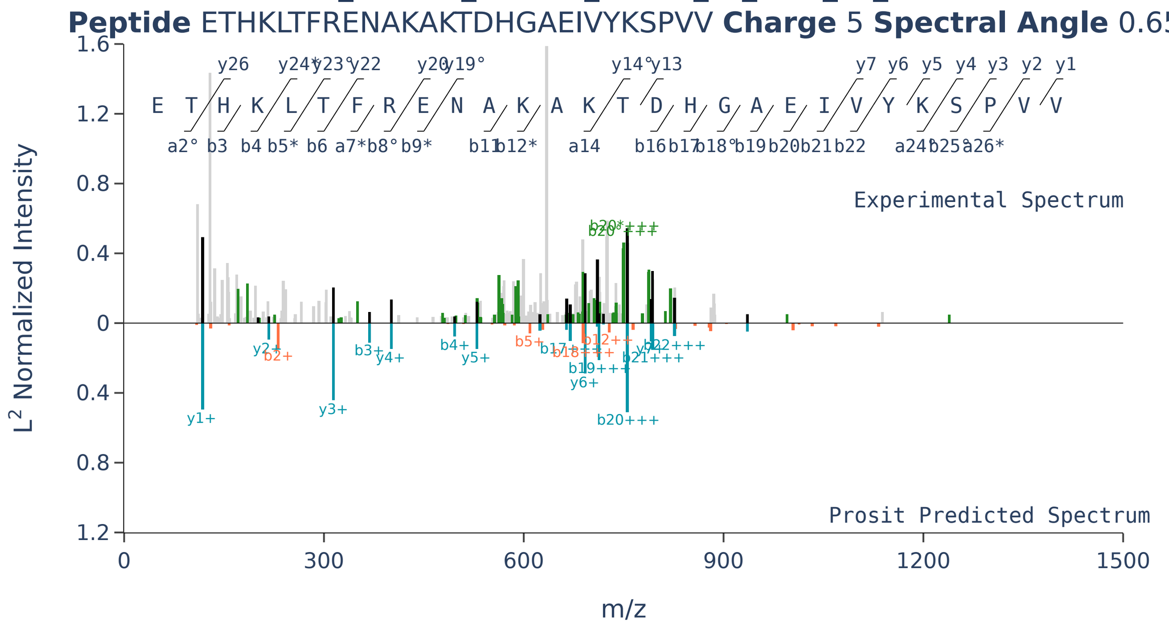


**Source** WSoh_221121_011221_HFGoe_L2_hTau_4h_R2 **Scan** 24403
**Peptide** PGGGSVQIVYKPVDLSKVTSKCGSL **Charge** 4 **Spectral Angle** 0.72

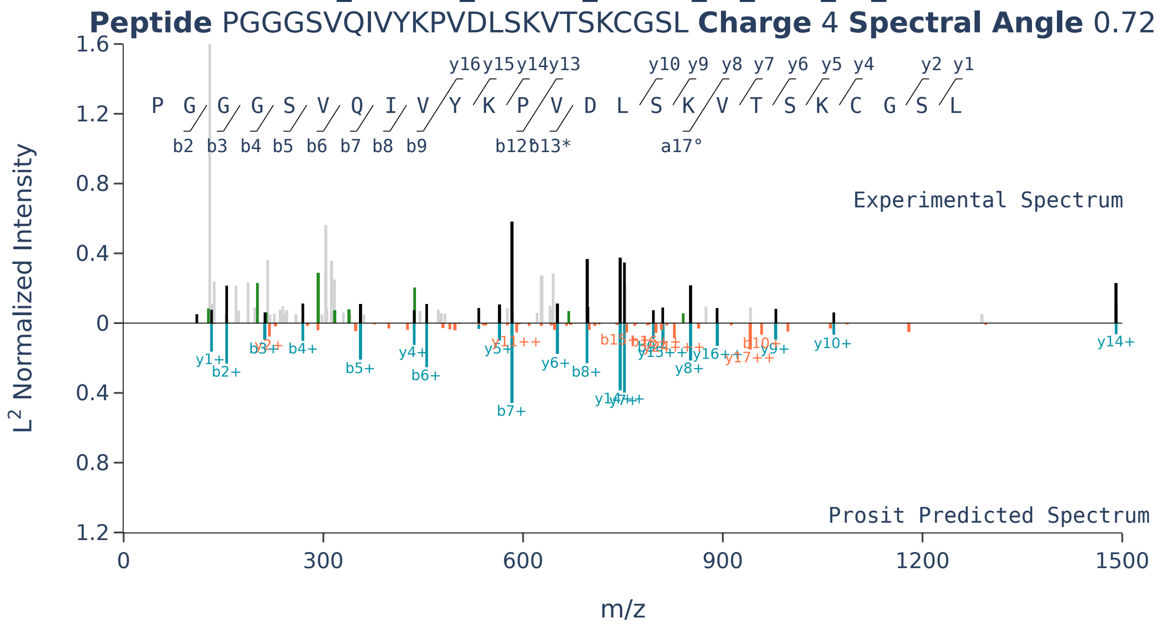


**Source** WSoh_221121_011221_HFGoe_L2_hTau_24h_R2 **Scan** 20006
**Peptide** KSRLQTAPVPMPDLKNVKSKIGST **Charge** 4 **Spectral Angle** 0.66

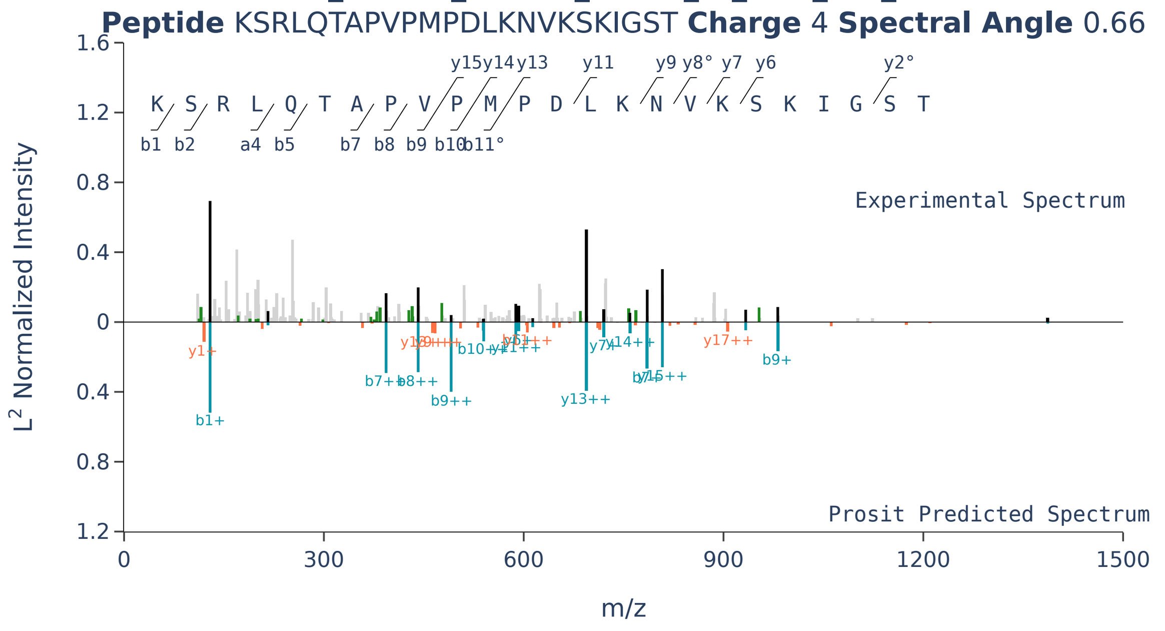


**Source** WSoh_221121_011221_HFGoe_L1_hTau_24h_R1 **Scan** 17847
**Peptide** IGSLDNITHVPGGGNKKIETHKLTFRENA **Charge** 6 **Spectral Angle** 0.63

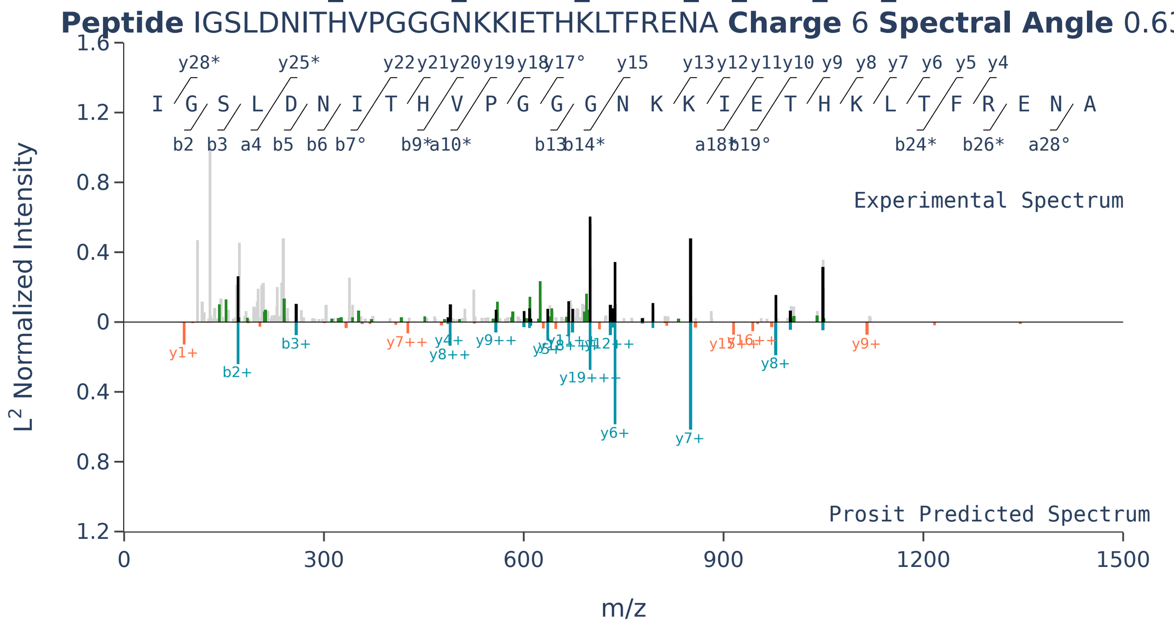


**Source** WSoh_221121_011221_HFGoe_L2_hTau_4h_R2 **Scan** 12899
**Peptide** PGTPGSRSRTPSLPTPPTREPKKV **Charge** 5 **Spectral Angle** 0.58

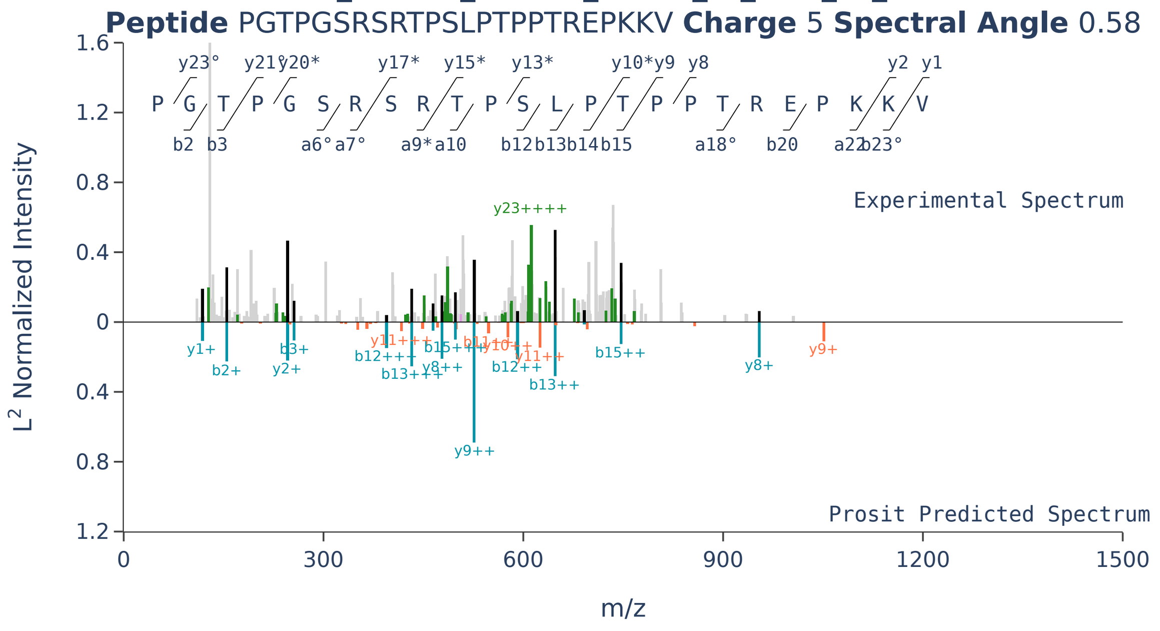


**Source** WSoh_221121_011221_HFGoe_L2_hTau_24h_R1 **Scan** 21785
**Peptide** TTAEEAGIGDTPSLEDE **Charge** 2 **Spectral Angle** 0.68

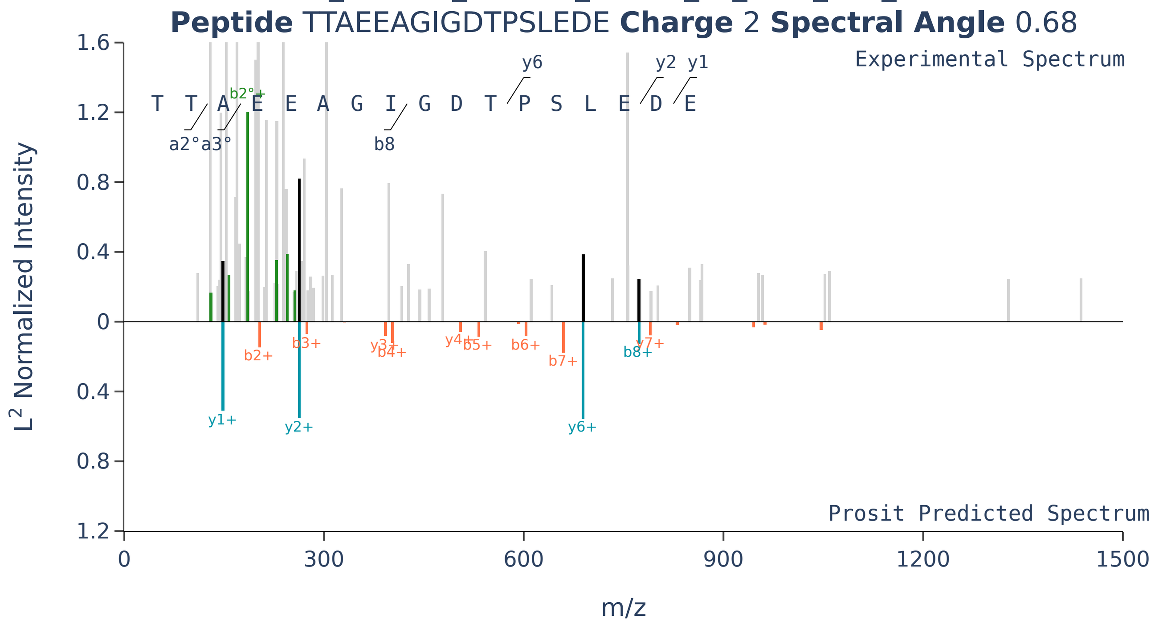


**Source** WSoh_221121_011221_HFGoe_L2_hTau_1h_R1 **Scan** 6241
**Peptide** PGSETSDAKSTPTA **Charge** 2 **Spectral Angle** 0.73

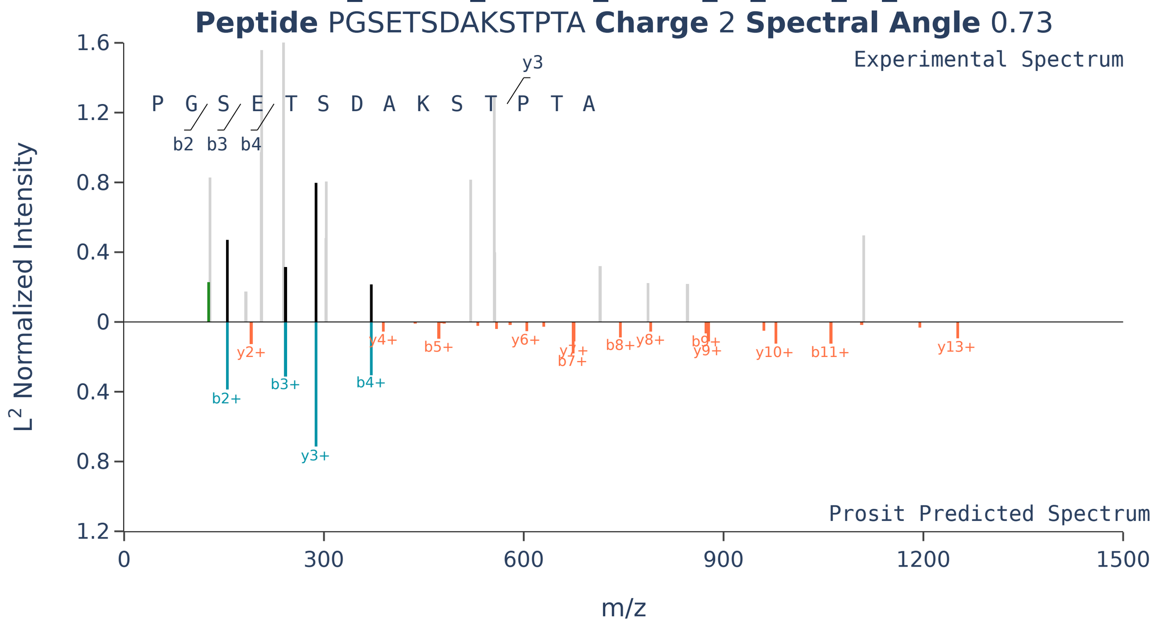


**Source** WSoh_221121_011221_HFGoe_L1_hTau_24h_R1 **Scan** 11007
**Peptide** GEPPKSGDRSGYSSPGSPGTPGSRSRTPSL **Charge** 4 **Spectral Angle** 0.8

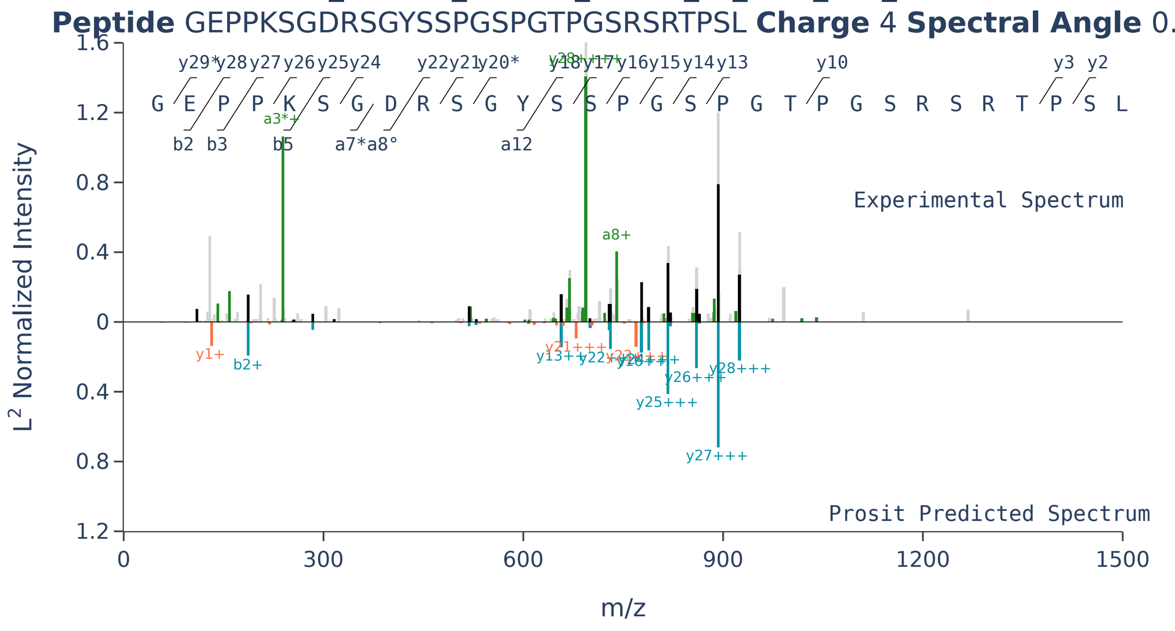

**Source** WSoh_221121_011221_HFGoe_L1_hTau_24h_R1 **Scan** 18495
**Peptide** GSLDNITHVPGGGN **Charge** 2 **Spectral Angle** 0.76

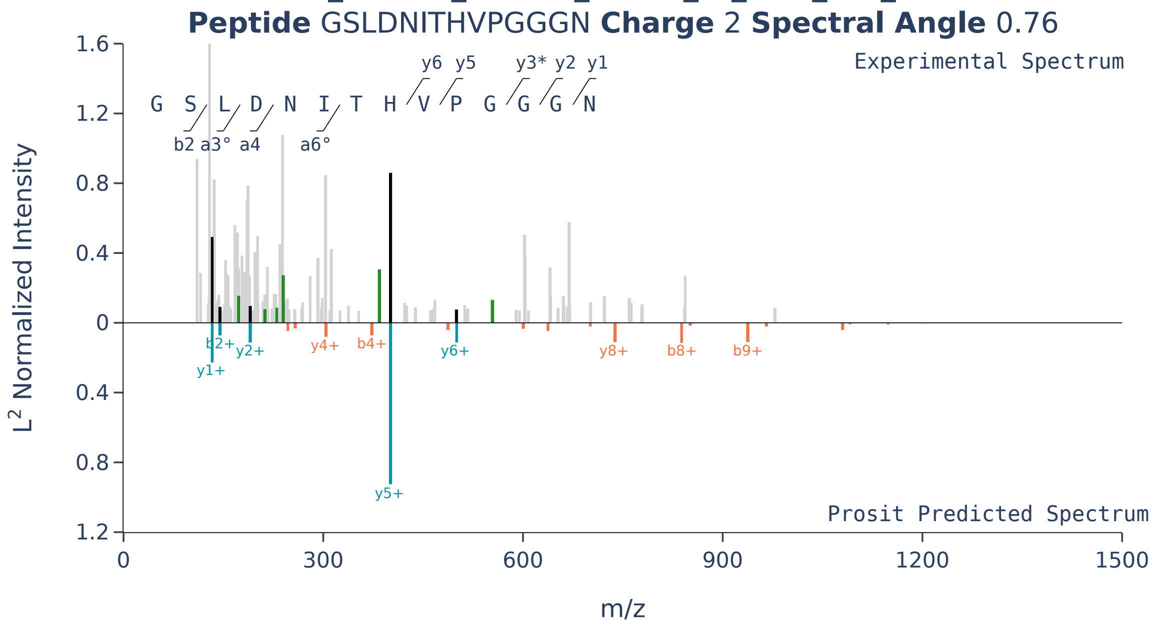


**Source** WSoh_221121_011221_HFGoe_L1_hTau_24h_R1 **Scan** 12327
**Peptide** MEDHAGTYGLGDRKDQGGYTMHQDQEGD **Charge** 4 **Spectral Angle** 0.68

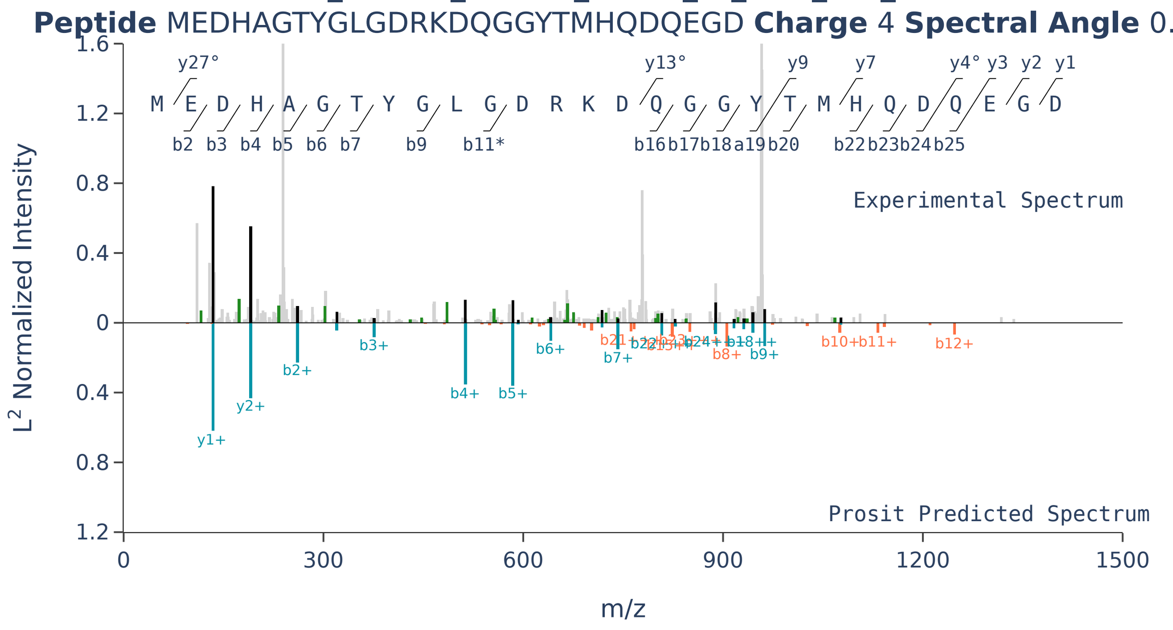


**Source** WSoh_221121_011221_HFGoe_L1_hTau_24h_R1 **Scan** 17644
**Peptide** DHGAEIVYKSPVVSGDTSPRHLSNVS **Charge** 4 **Spectral Angle** 0.82

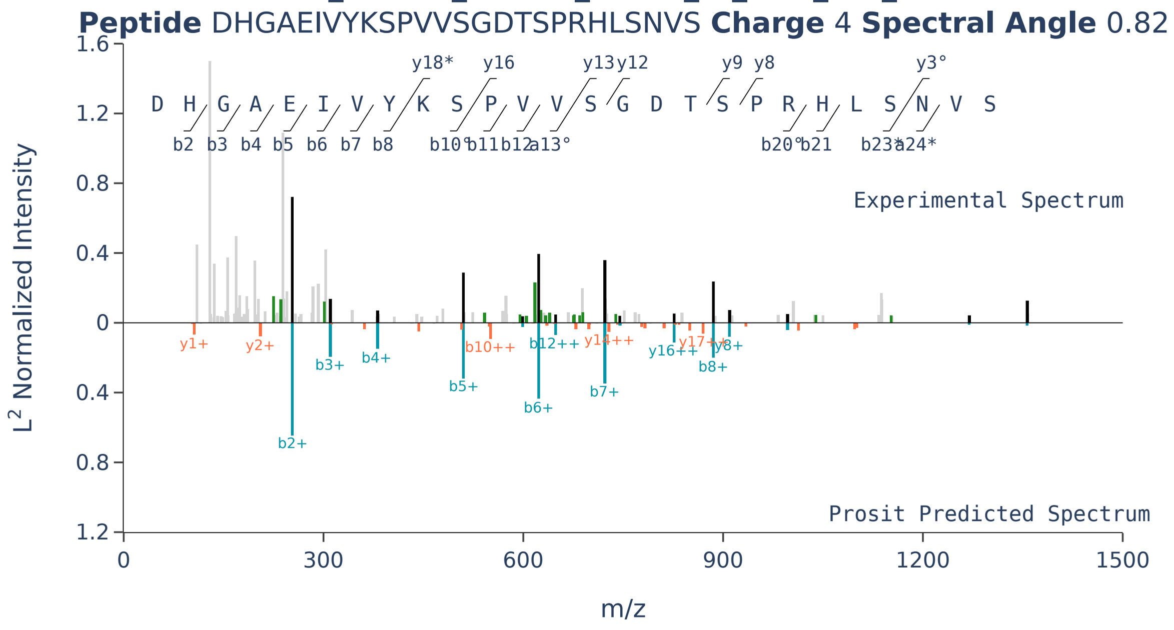


**Source** WSoh_221121_011221_HFGoe_L2_hTau_4h_R2 **Scan** 15338
**Peptide** LKHQPGGGKVQIINKKLDLSNVQS **Charge** 4 **Spectral Angle** 0.71

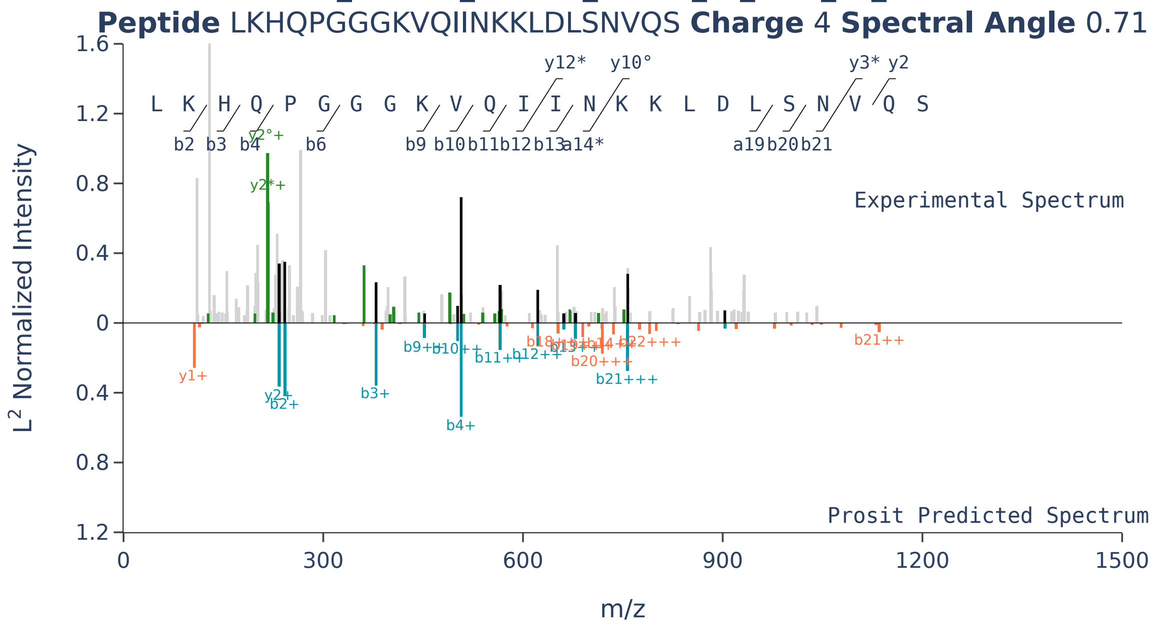


**Source** WSoh_221121_011221_HFGoe_L1_hTau_4h_R1 **Scan** 22864
**Peptide** EKLDFKDRVQSKIGSLDNITHV **Charge** 4 **Spectral Angle** 0.81

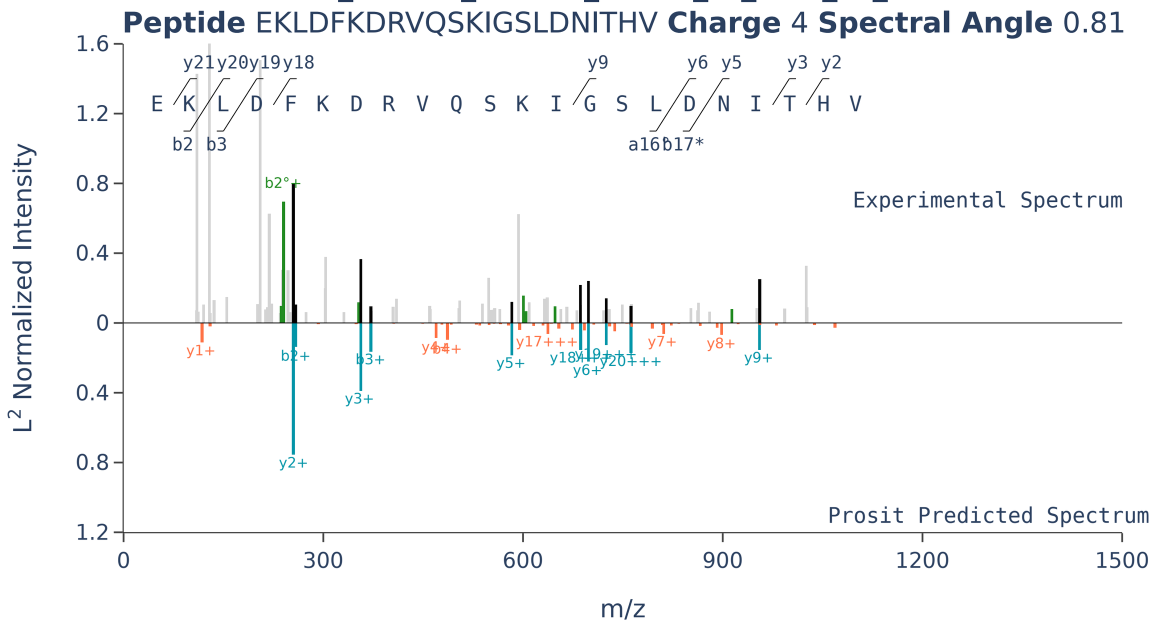


**Source** WSoh_221121_011221_HFGoe_L1_hTau_4h_R2 **Scan** 21295
**Peptide** EEAGIGDTPSLEDEAAGHVT **Charge** 2 **Spectral Angle** 0.6

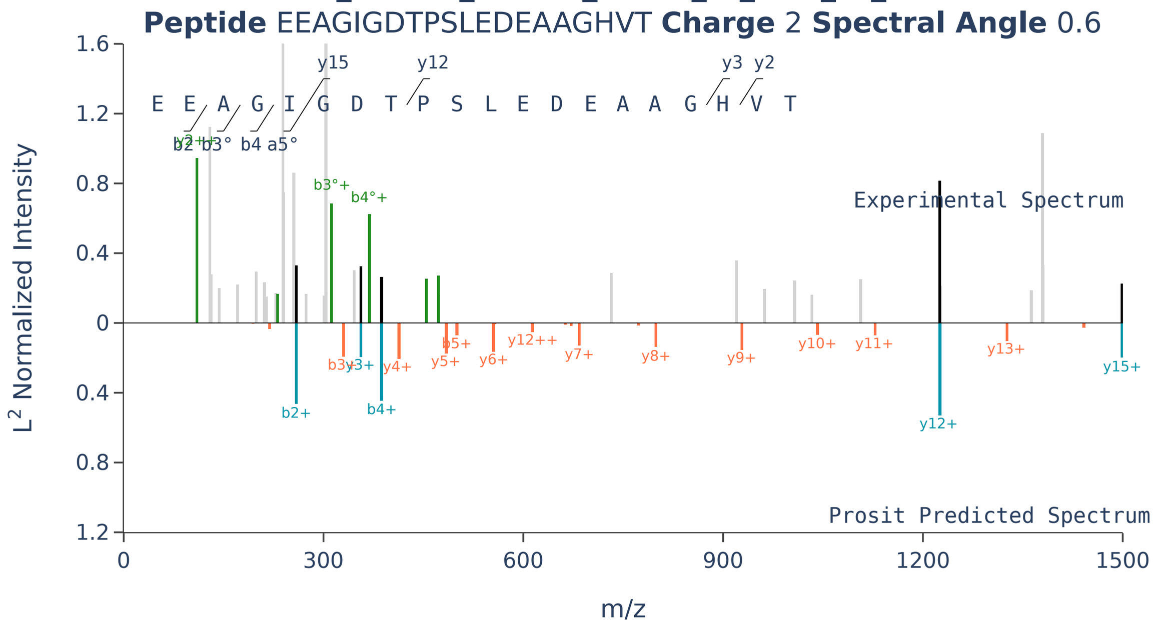


**Source** WSoh_221121_011221_HFGoe_L1_hTau_24h_R2 **Scan** 6793
**Peptide** KTKIATPRGAAPPGQKGQ **Charge** 3 **Spectral Angle** 0.81

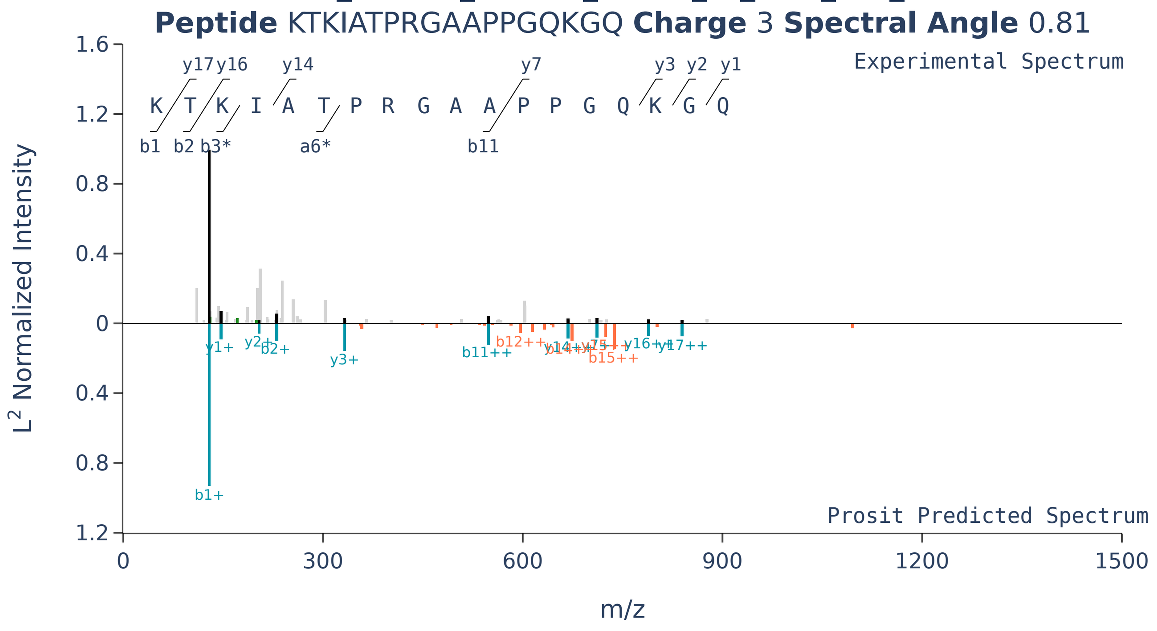


**Source** WSoh_221121_011221_HFGoe_L1_hTau_4h_R1 **Scan** 19619
**Peptide** GAEIVYKSPVVSGDTSPRHLSNVSSTGSLD **Charge** 4 **Spectral Angle** 0.81

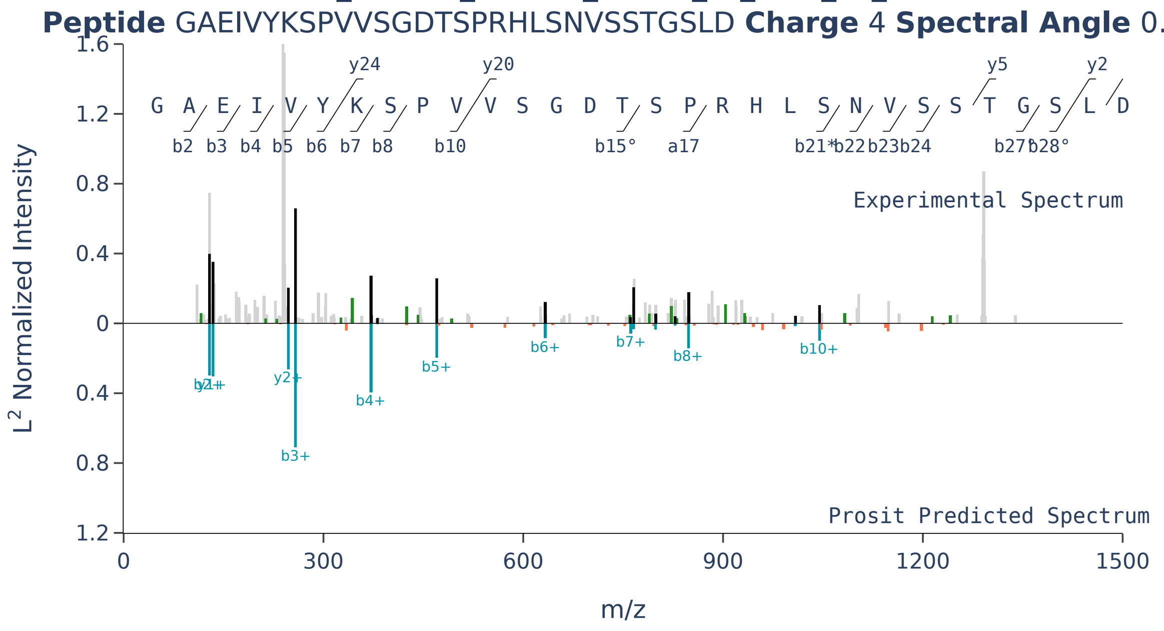


**Source** WSoh_221121_011221_HFGoe_L2_hTau_24h_R2 **Scan** 7443
**Peptide** LGDRKDQGGYTMHQDQ **Charge** 3 **Spectral Angle** 0.65

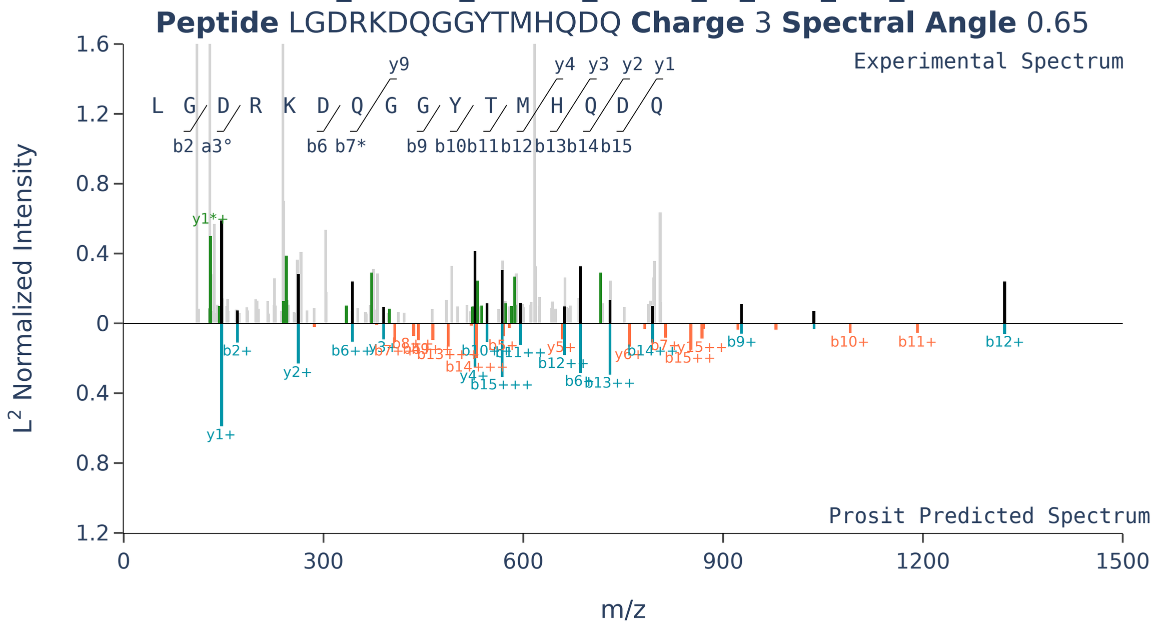


**Source** WSoh_221121_011221_HFGoe_L2_hTau_4h_R2 **Scan** 27156
**Peptide** SPQLATLADEV **Charge** 2 **Spectral Angle** 0.7

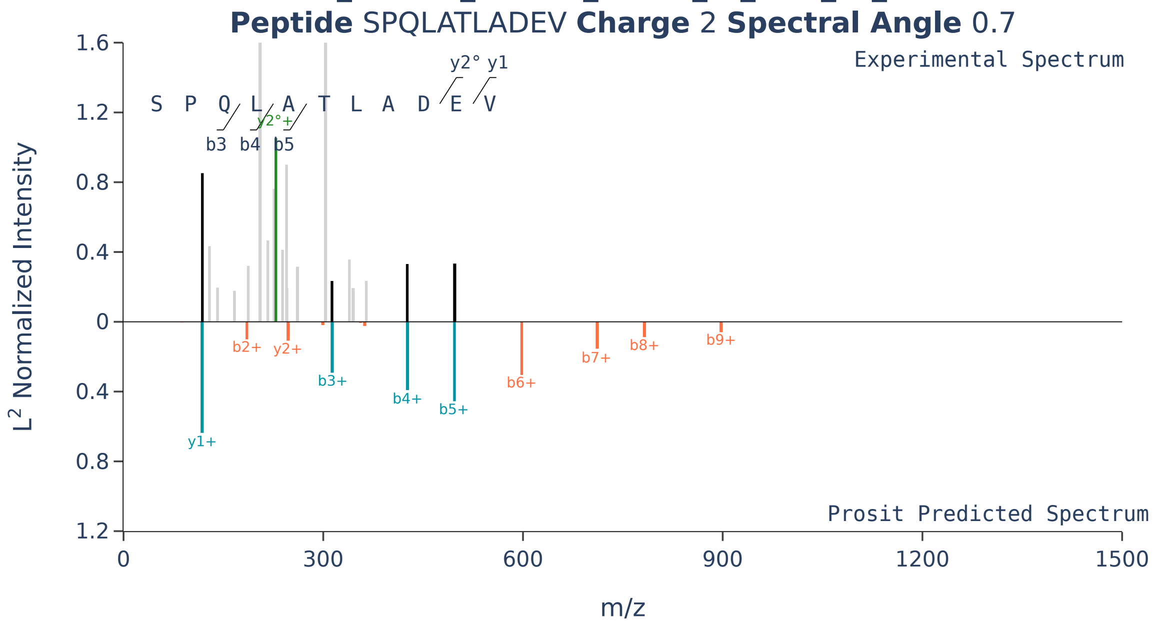


**Source** WSoh_221121_011221_HFGoe_L1_hTau_24h_R1 **Scan** 20476
**Peptide** AEEAGIGDTPSLEDEAAGHVTQARMVSK **Charge** 4 **Spectral Angle** 0.58

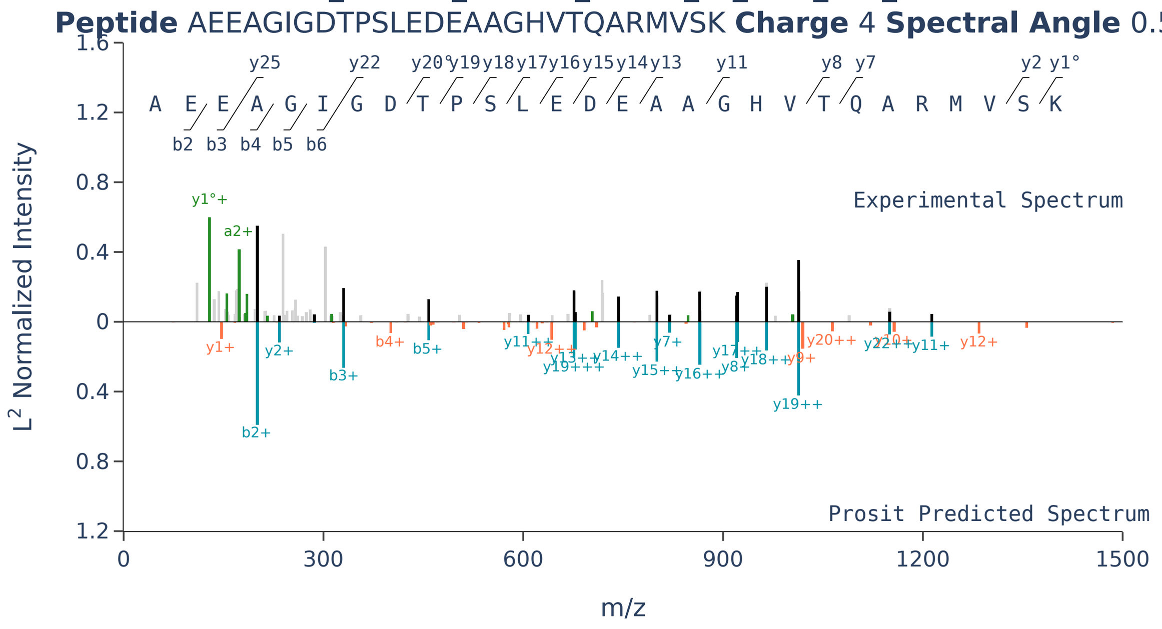


**Source** WSoh_221121_011221_HFGoe_L1_hTau_24h_R1 **Scan** 10260
**Peptide** KDQGGYTMHQDQEGDTDAGLKE **Charge** 4 **Spectral Angle** 0.62

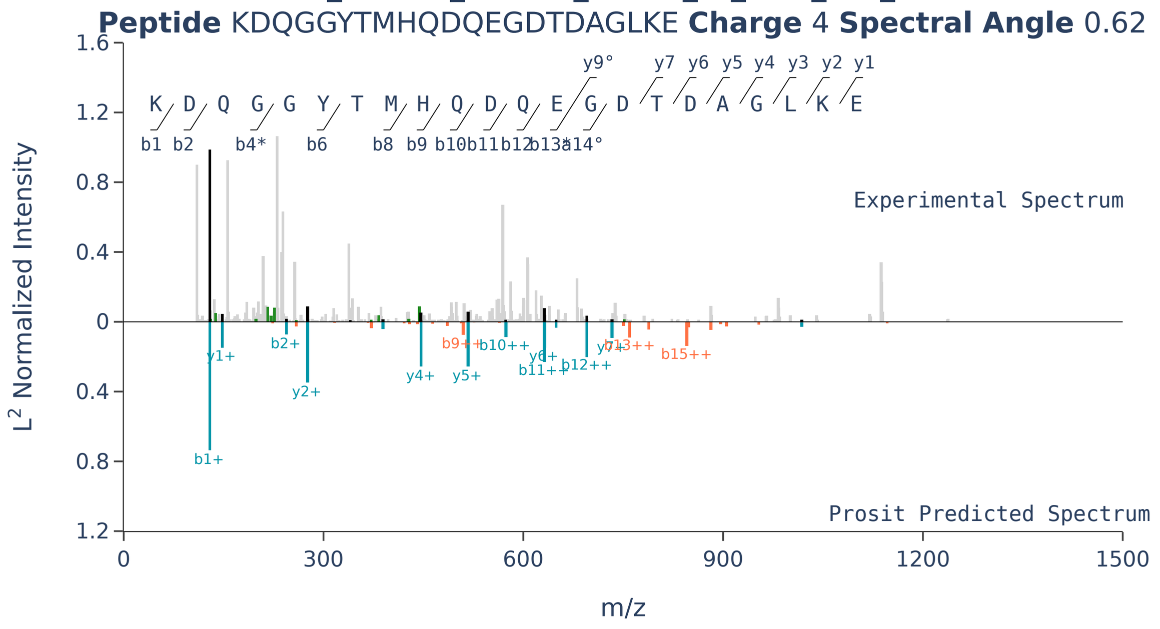


**Source** WSoh_221121_011221_HFGoe_L2_hTau_4h_R1 **Scan** 10070
**Peptide** LSKVTSKCGSLGNIHHKPGGGQVEVKSEKL **Charge** 4 **Spectral Angle** 0.52

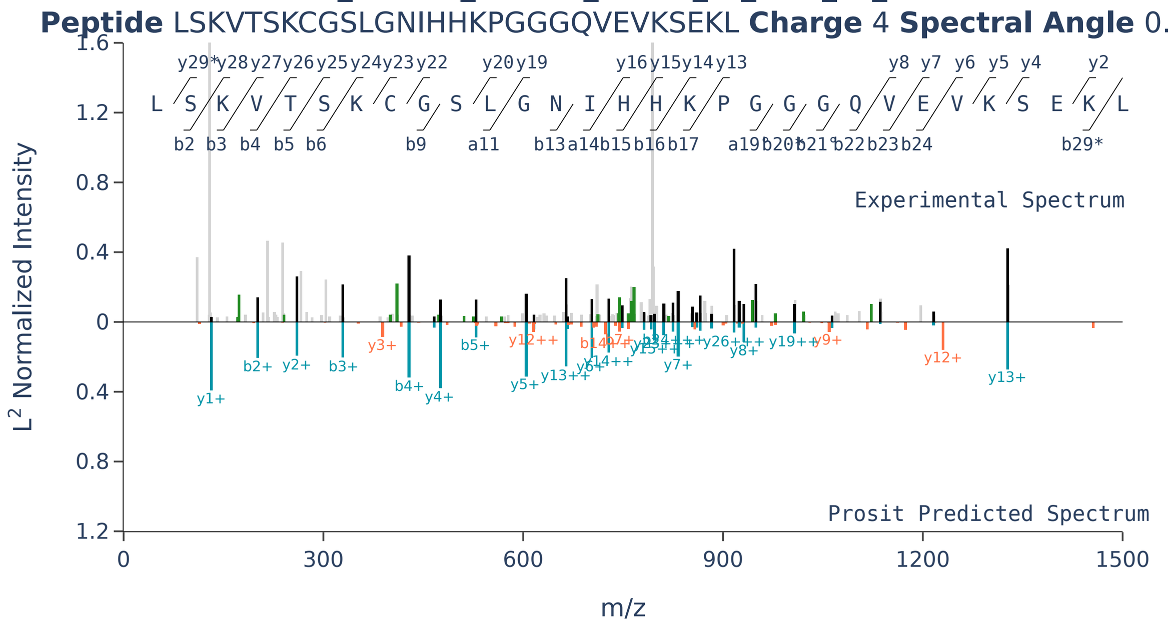


**Source** WSoh_221121_011221_HFGoe_L1_hTau_24h_R1 **Scan** 16911
**Peptide** AVVRTPPKSPSSAKSRLQTAPVPMPDLKNV **Charge** 5 **Spectral Angle** 0.59

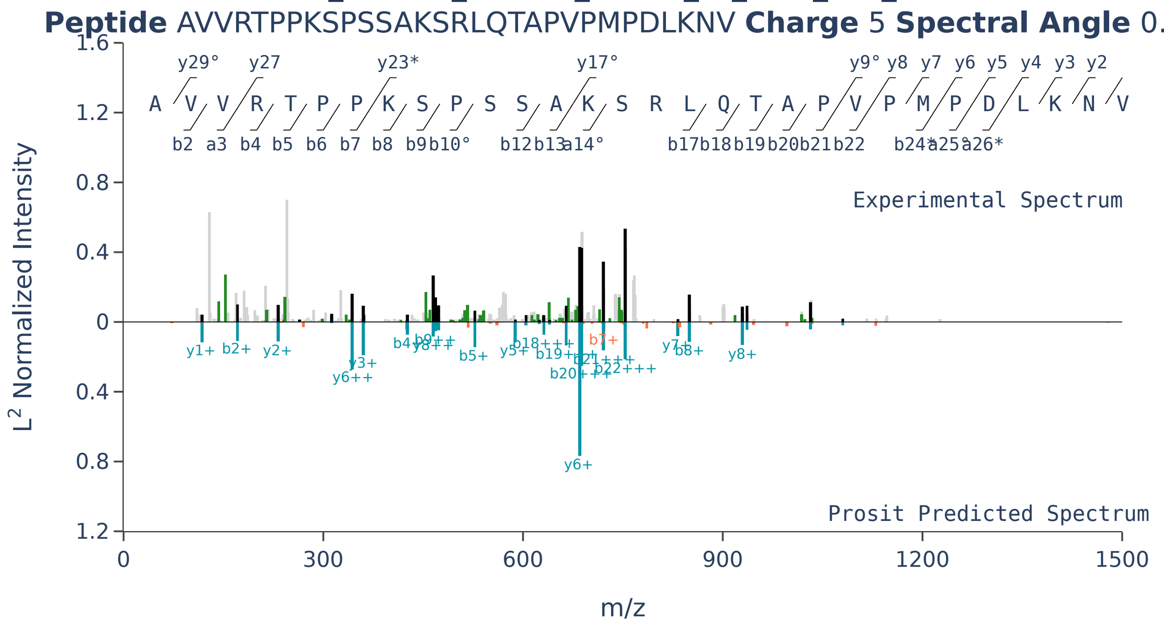


**Source** WSoh_221121_011221_HFGoe_L2_hTau_24h_R1 **Scan** 11257
**Peptide** GAPGKQAAAQPHTEIPEGTT **Charge** 3 **Spectral Angle** 0.63

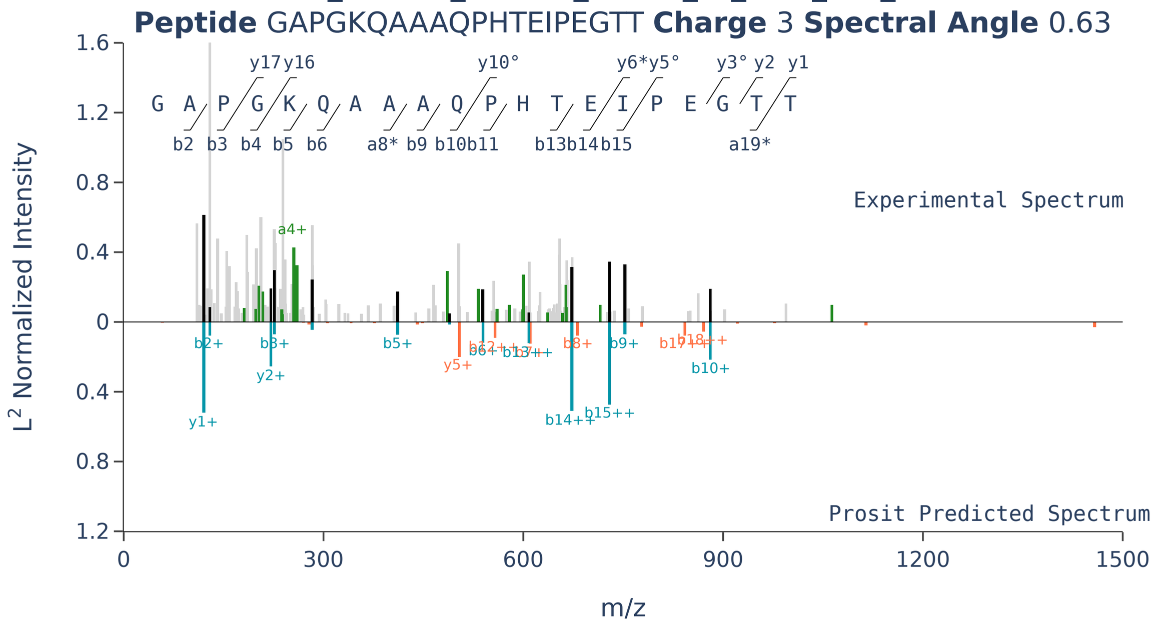

**Source** WSoh_221121_011221_HFGoe_L2_hTau_4h_R2 **Scan** 19383
**Peptide** KCGSKDNIKHVPGGGSVQIVYKPVDLSKV **Charge** 4 **Spectral Angle** 0.73

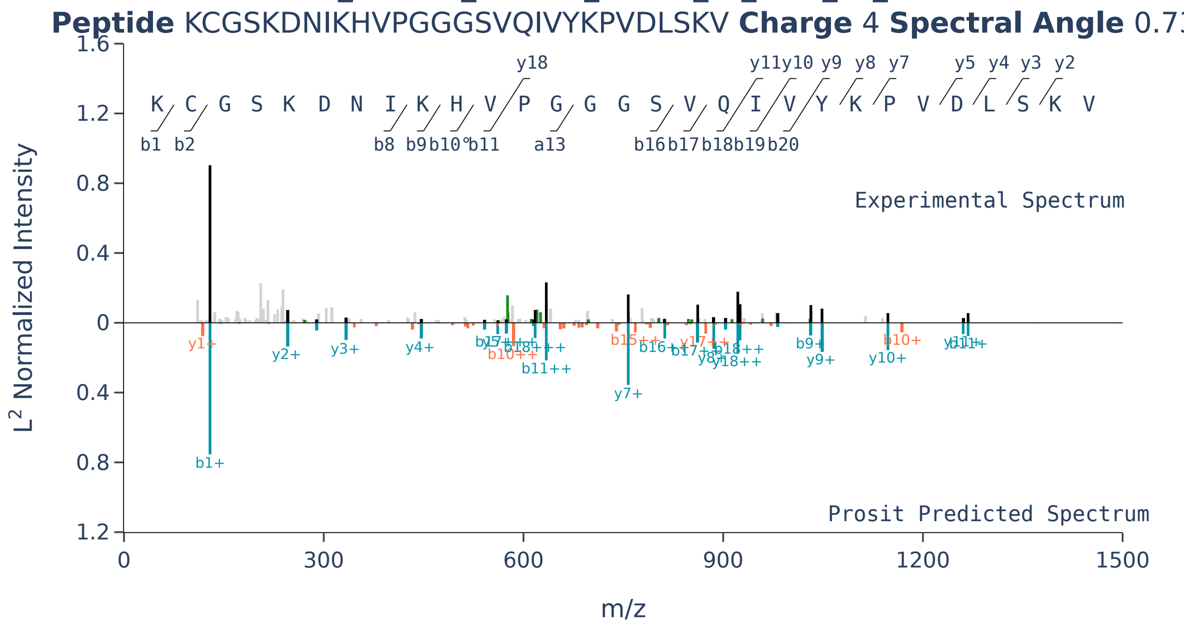


**Source** WSoh_221121_011221_HFGoe_L2_hTau_24h_R2 **Scan** 16448
**Peptide** ENLKHQPGGGKVQIINKKLDLSNVQS **Charge** 4 **Spectral Angle** 0.69

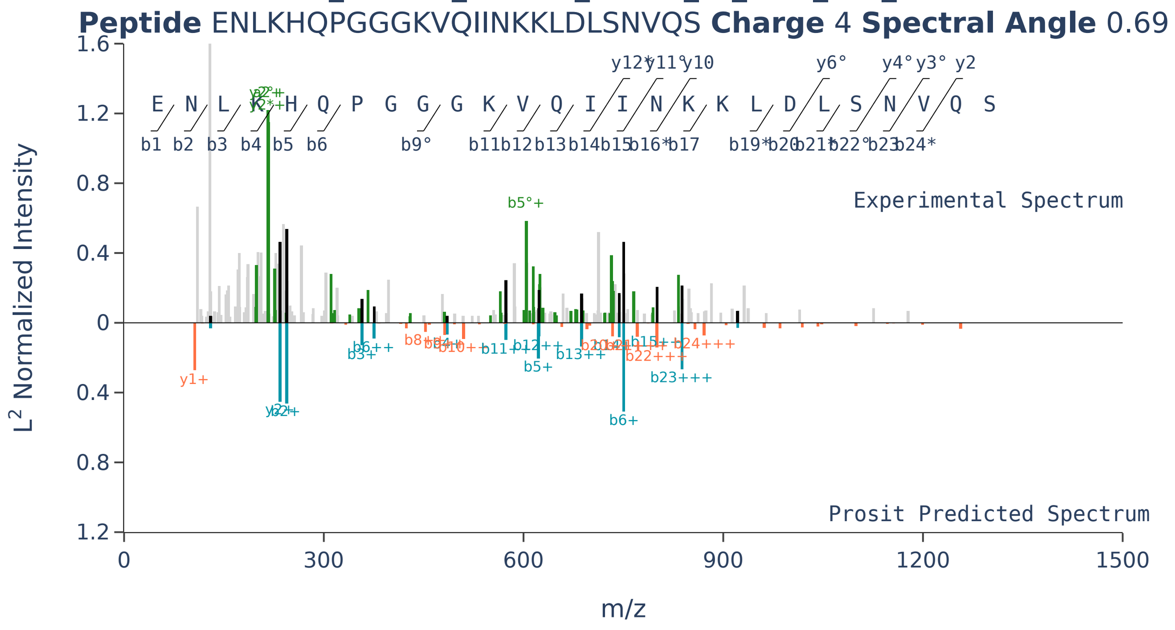


**Source** WSoh_221121_011221_HFGoe_L2_hTau_24h_R1 **Scan** 20913
**Peptide** DFKDRVQSKIGSLDNITHVPGGGNKKIE **Charge** 4 **Spectral Angle** 0.71

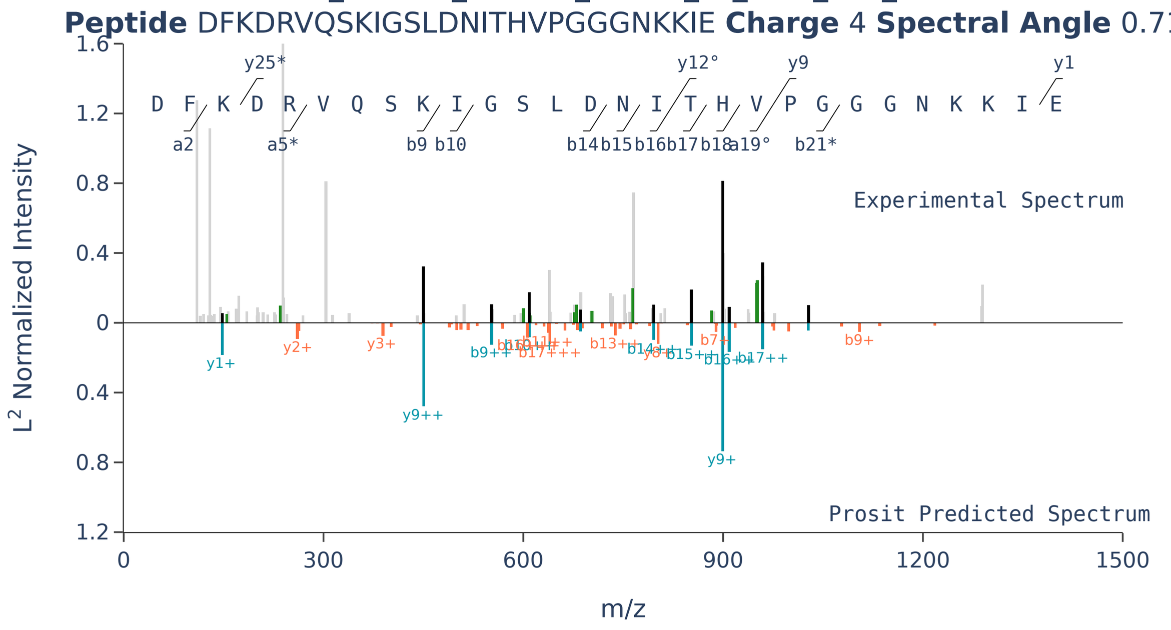


**Source** WSoh_221121_011221_HFGoe_L2_hTau_4h_R2 **Scan** 21325
**Peptide** EFEVMEDHAGTY **Charge** 2 **Spectral Angle** 0.62

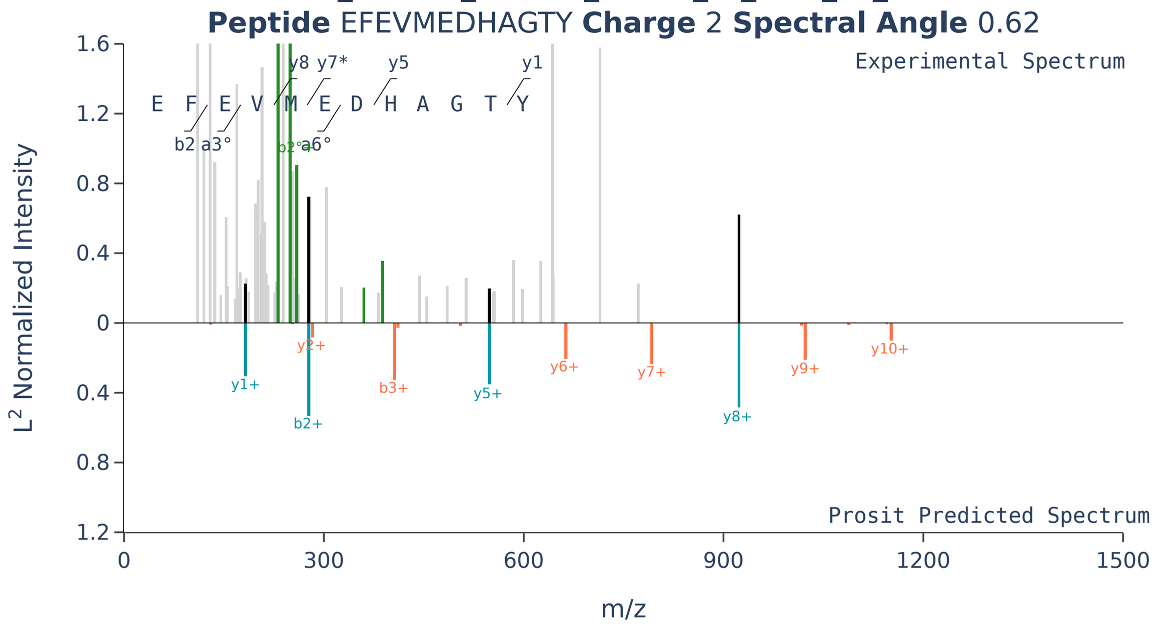


**Source** WSoh_221121_011221_HFGoe_L1_hTau_24h_R1 **Scan** 10197
**Peptide** VKSEKLDFKDR **Charge** 1 **Spectral Angle** 0.76

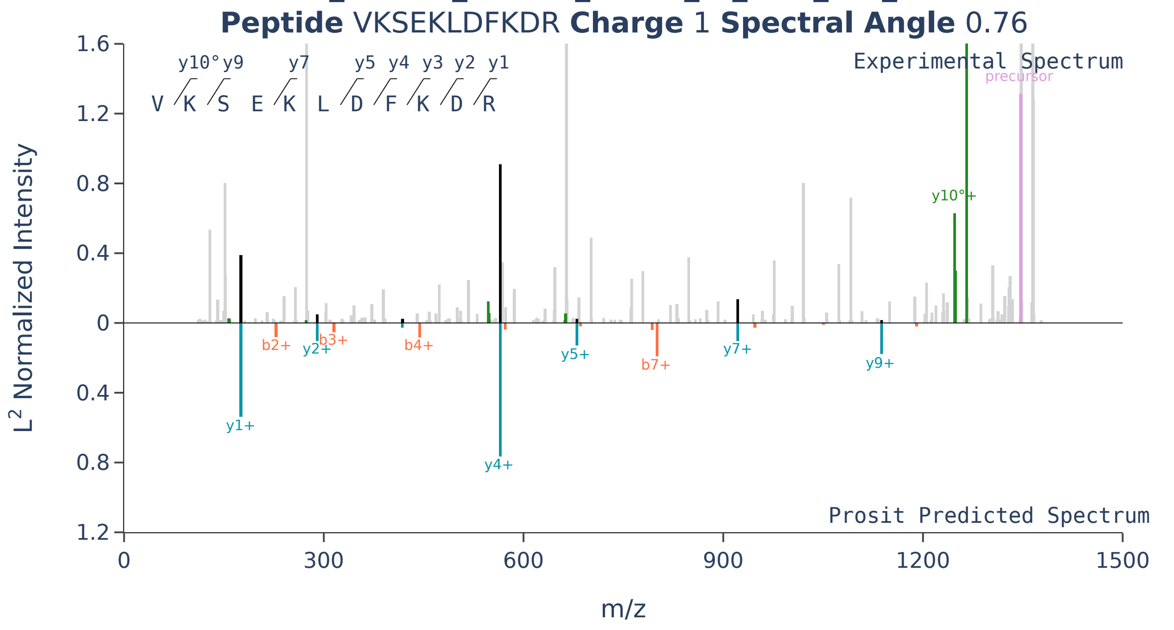


**Source** WSoh_221121_011221_HFGoe_L1_hTau_4h_R1 **Scan** 12026
**Peptide** MEDHAGTYGLGDRKDQGGYTMHQD **Charge** 4 **Spectral Angle** 0.66

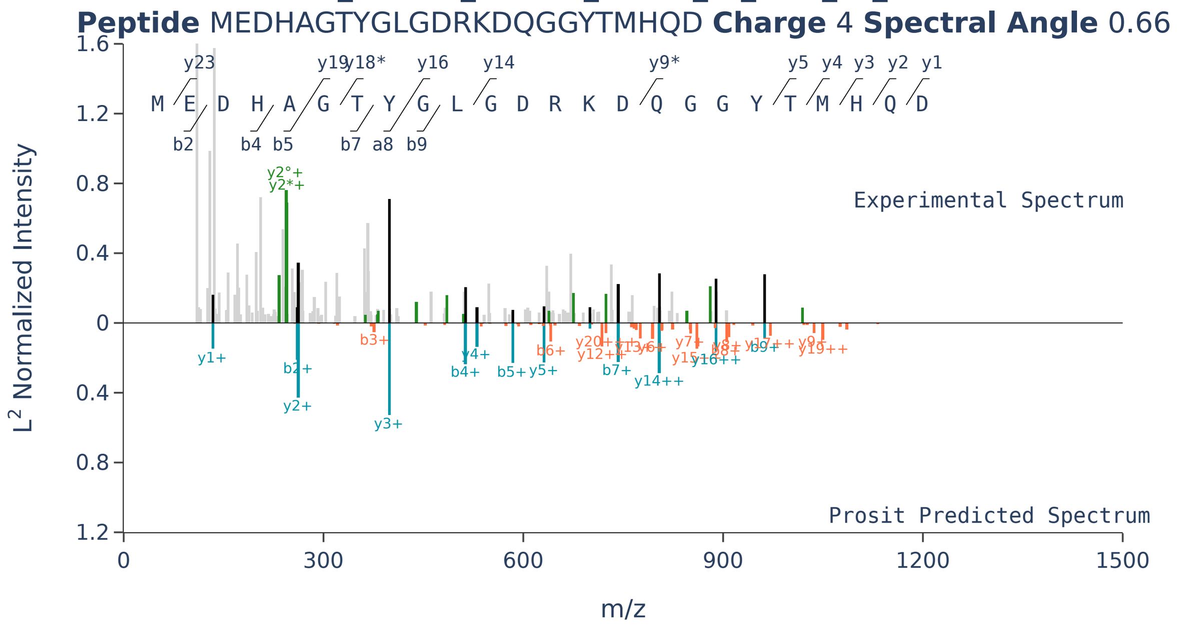


**Source** WSoh_221121_011221_HFGoe_L1_hTau_4h_R2 **Scan** 6699
**Peptide** SLGNIHHKPGGGQVEVKSEK **Charge** 3 **Spectral Angle** 0.61

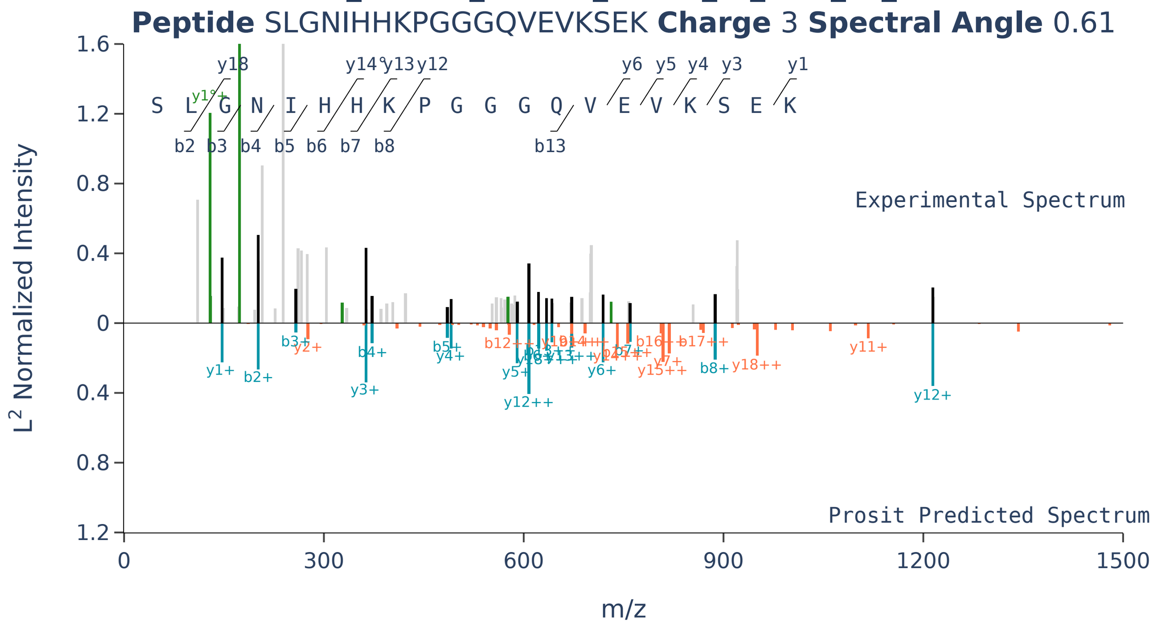


**Source** WSoh_221121_011221_HFGoe_L1_hTau_4h_R2 **Scan** 9787
**Peptide** VSGDTSPRHLS **Charge** 2 **Spectral Angle** 0.74

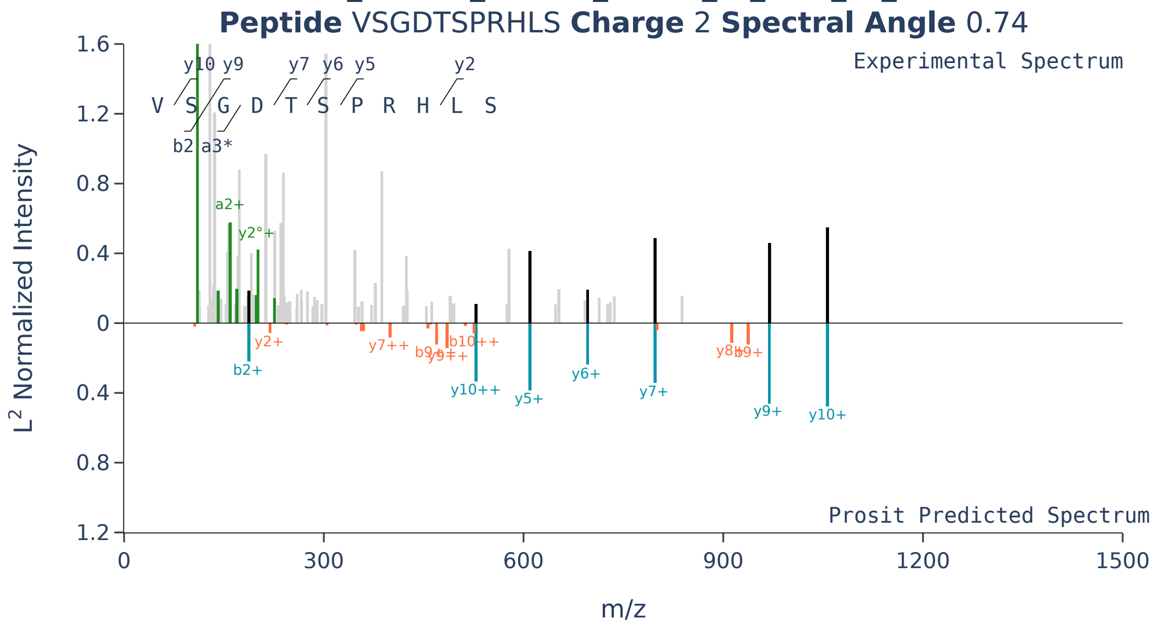


**Source** WSoh_221121_011221_HFGoe_L2_hTau_4h_R2 **Scan** 11235
**Peptide** NKKLDLSNVQSKCGSKDNL **Charge** 3 **Spectral Angle** 0.64

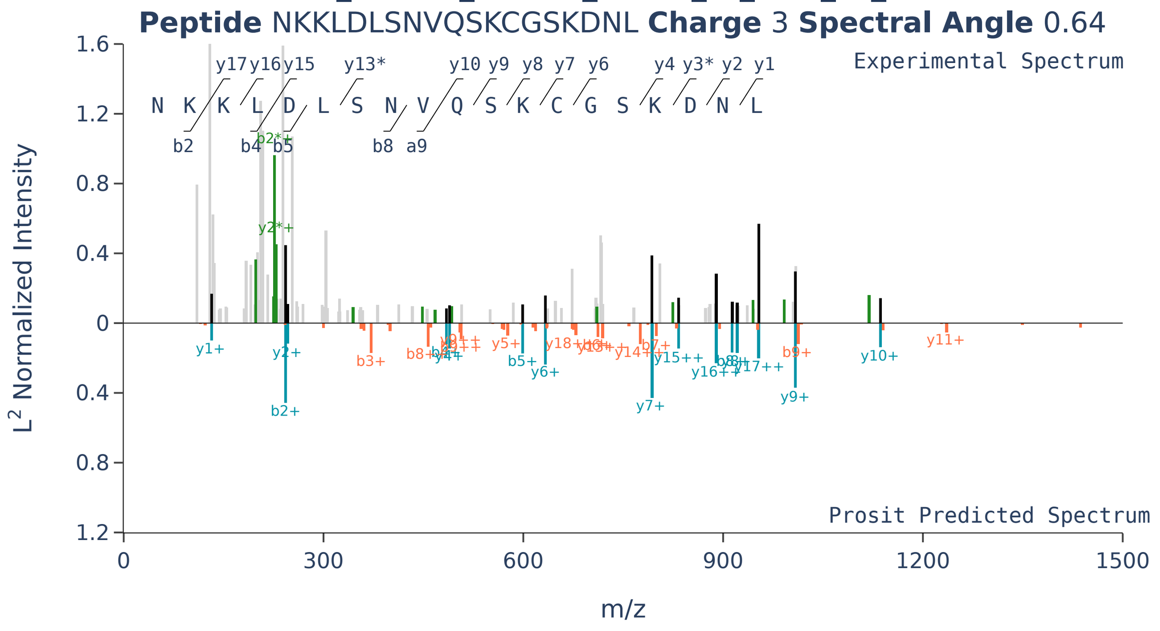


**Source** WSoh_221121_011221_HFGoe_L1_hTau_24h_R1 **Scan** 17869
**Peptide** LGSLDNITHVPGGGNKKIETHKLTFRENA **Charge** 4 **Spectral Angle** 0.58

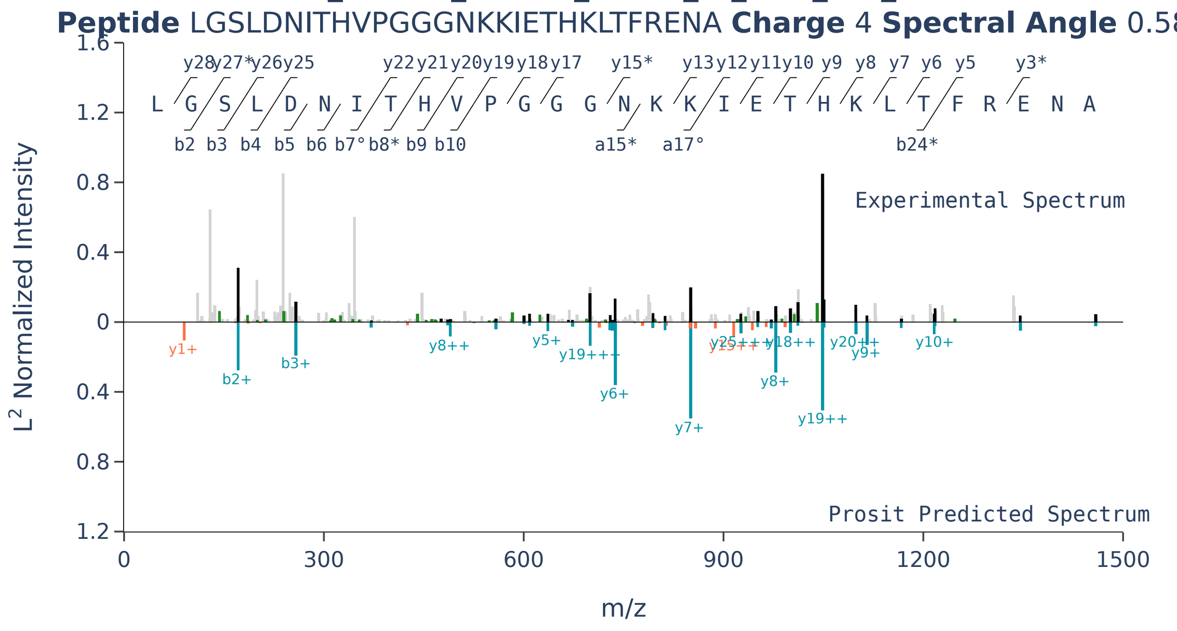


**Source** WSoh_221121_011221_HFGoe_L1_hTau_24h_R2 **Scan** 11497
**Peptide** DHAGTYGLGDRKDQGGYTMHQDQEG **Charge** 4 **Spectral Angle** 0.83

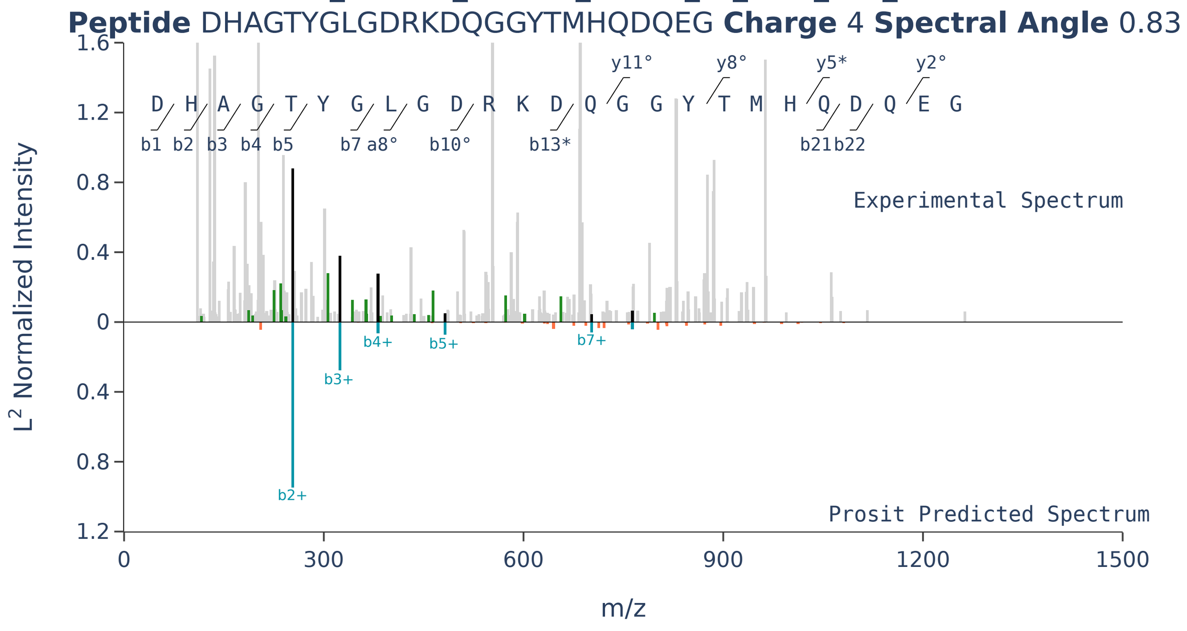


**Source** WSoh_221121_011221_HFGoe_L2_hTau_24h_R1 **Scan** 18681
**Peptide** DLSNVQSKCGSKDNIKHVPGGGSVQIVY **Charge** 3 **Spectral Angle** 0.55

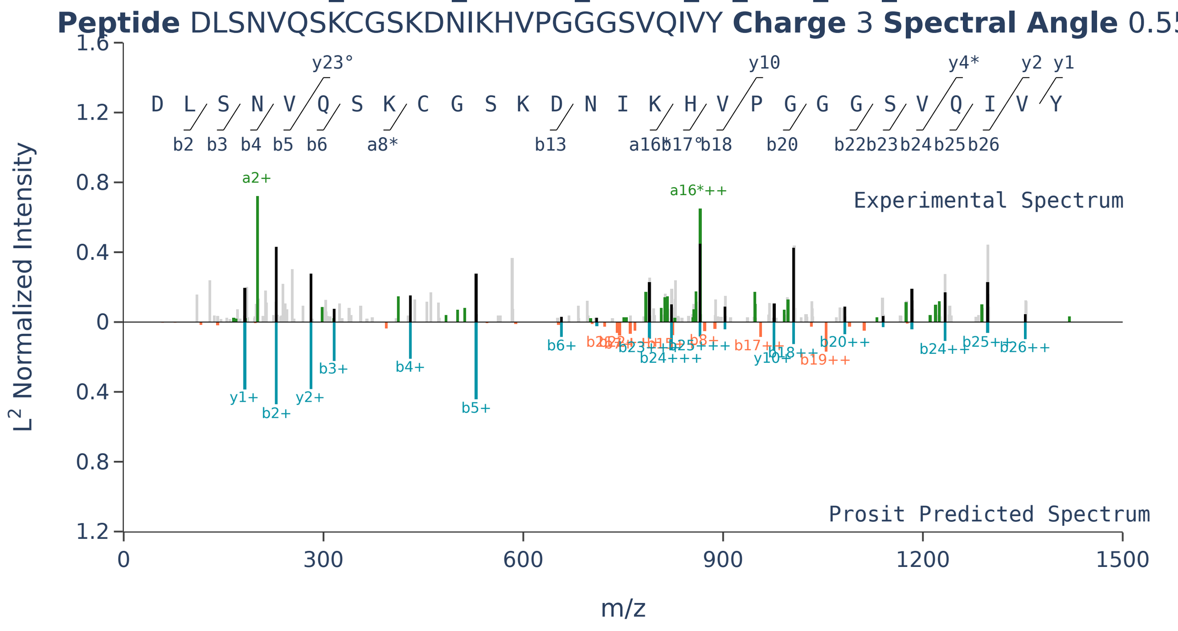


**Source** WSoh_221121_011221_HFGoe_L2_hTau_4h_R1 **Scan** 17120
**Peptide** DHGAEIVYKSPVVSGDTSPRHLSNVSS **Charge** 4 **Spectral Angle** 0.73

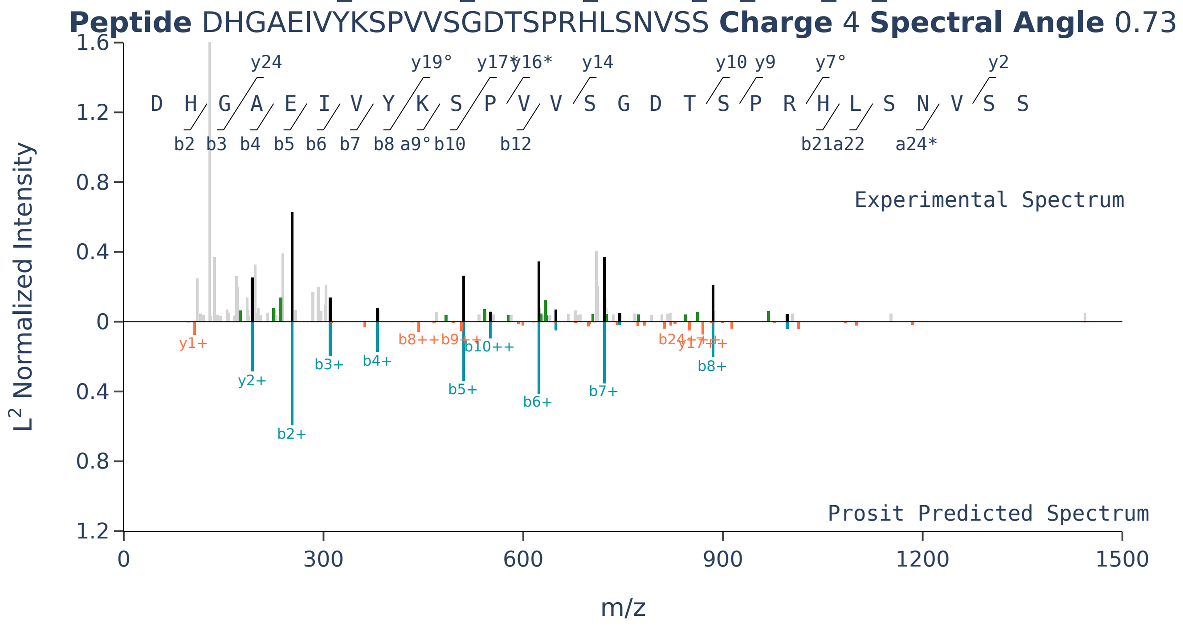


**Source** WSoh_221121_011221_HFGoe_L1_hTau_24h_R1 **Scan** 10046
**Peptide** HAGTYGLGDRKDQGGYTMHQ **Charge** 4 **Spectral Angle** 0.76

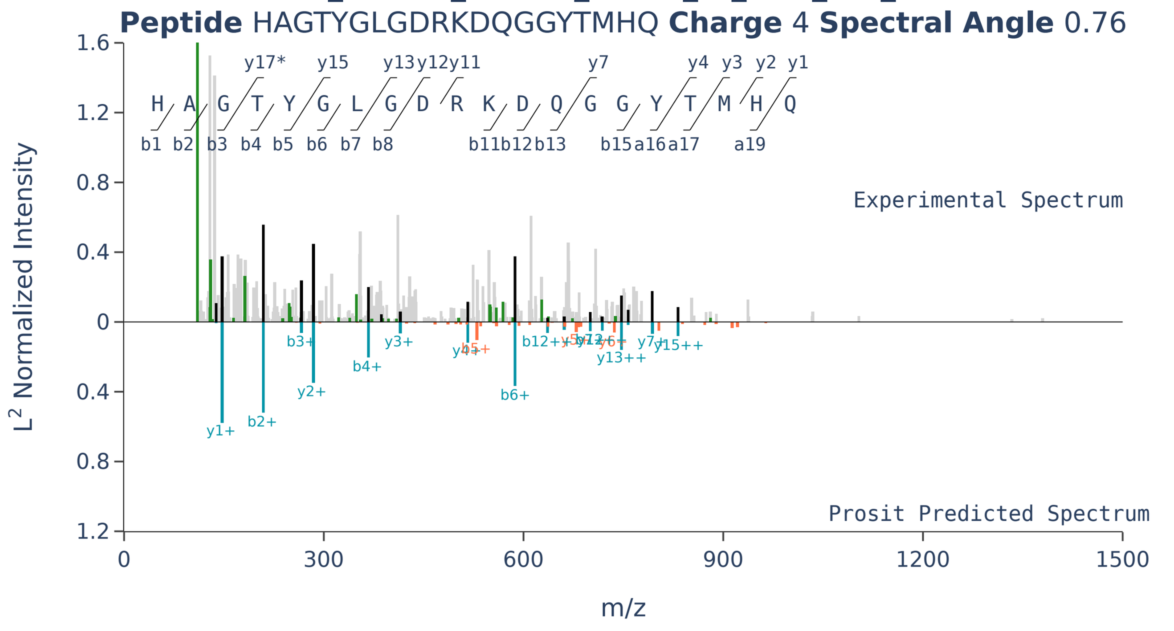


**Source** WSoh_221121_011221_HFGoe_L2_hTau_2h_R2 **Scan** 15381
**Peptide** RVQSKIGSLDNITHVPGGGNKKI **Charge** 4 **Spectral Angle** 0.69

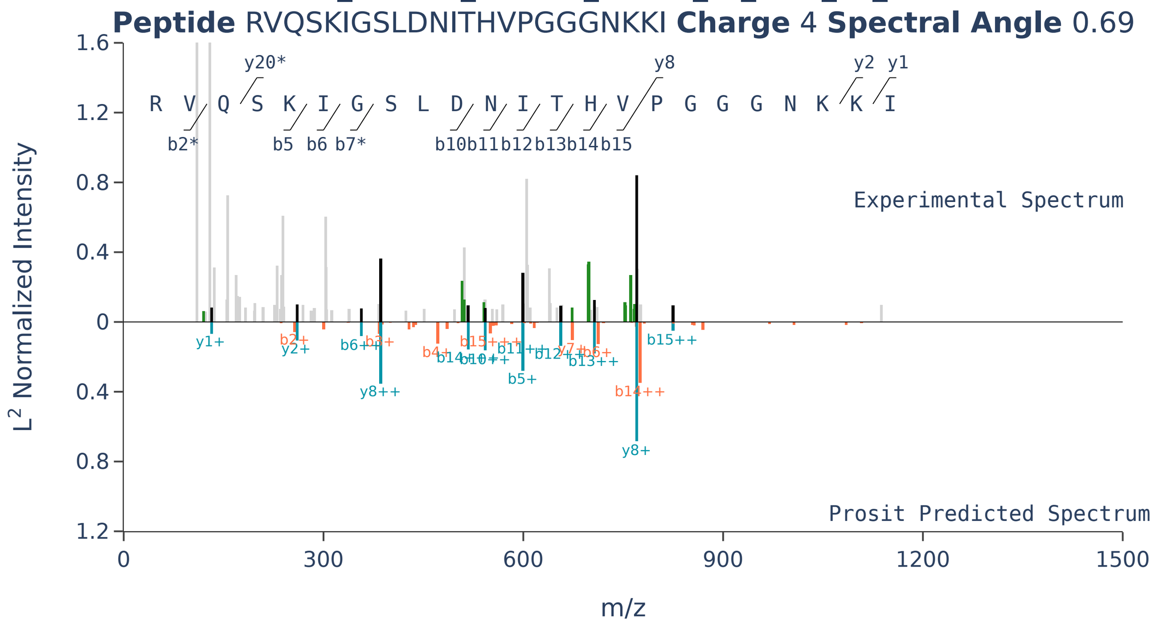

**Source** WSoh_221121_011221_HFGoe_L2_hTau_1h_R2 **Scan** 13988
**Peptide** SKIGSLDNITH **Charge** 2 **Spectral Angle** 0.68

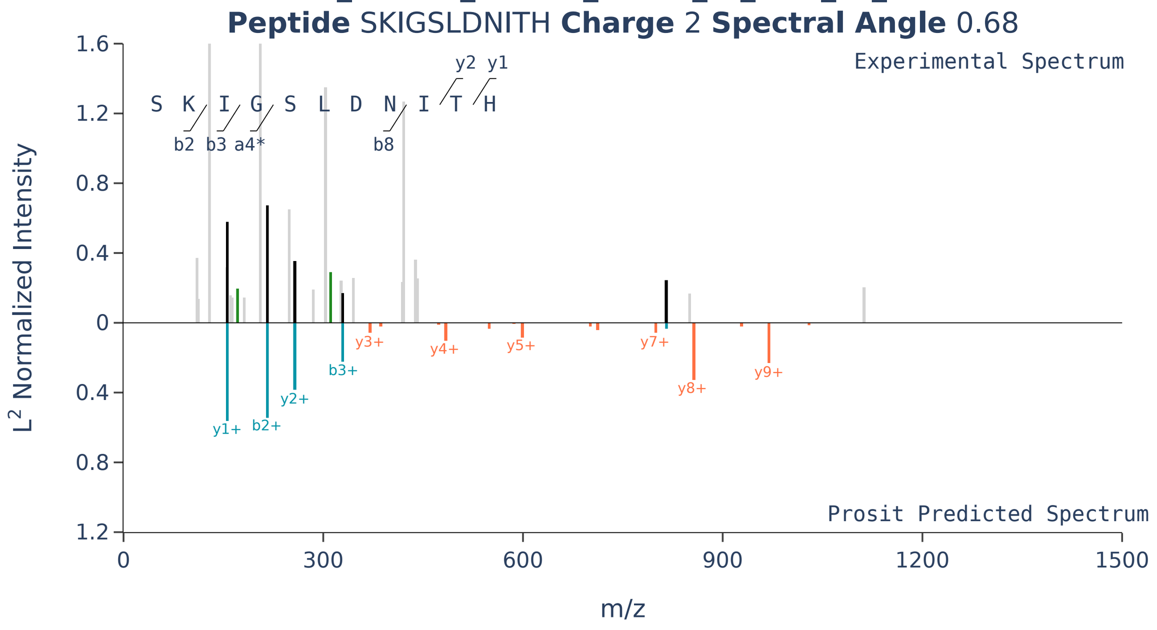


**Source** WSoh_221121_011221_HFGoe_L1_hTau_24h_R1 **Scan** 8601
**Peptide** PTPPTREPKKVAVVRTPPKSPSSAKSRLQ **Charge** 5 **Spectral Angle** 0.69

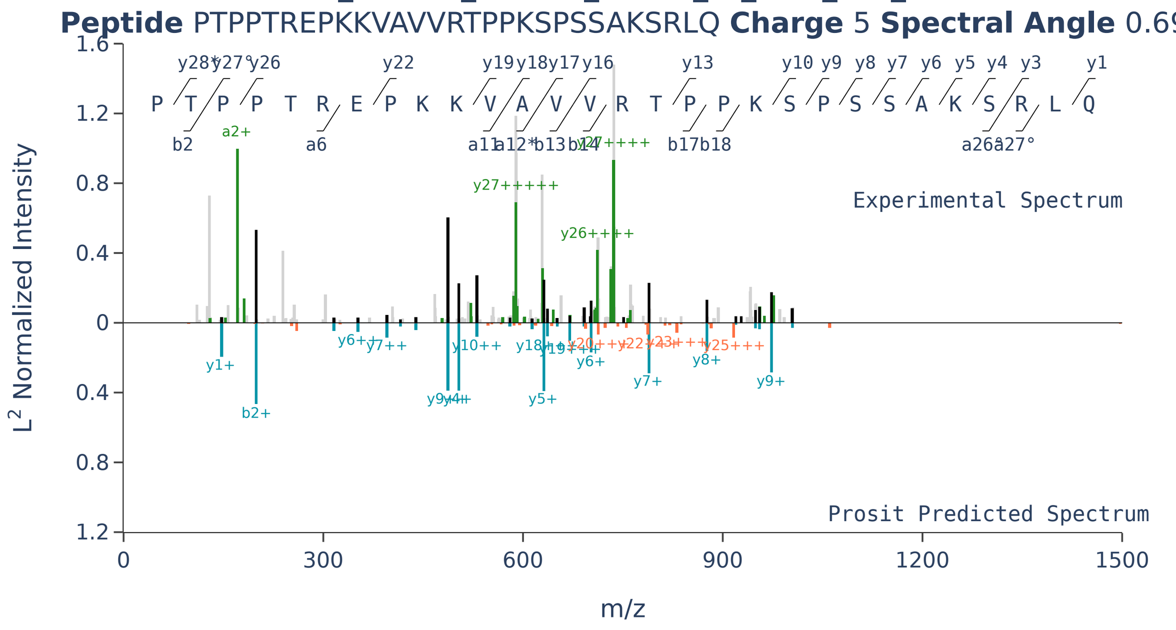


**Source** WSoh_221121_011221_HFGoe_L1_hTau_24h_R1 **Scan** 18188
**Peptide** EDVTAPLVDEGAPGKQAAAQPHTE **Charge** 3 **Spectral Angle** 0.76

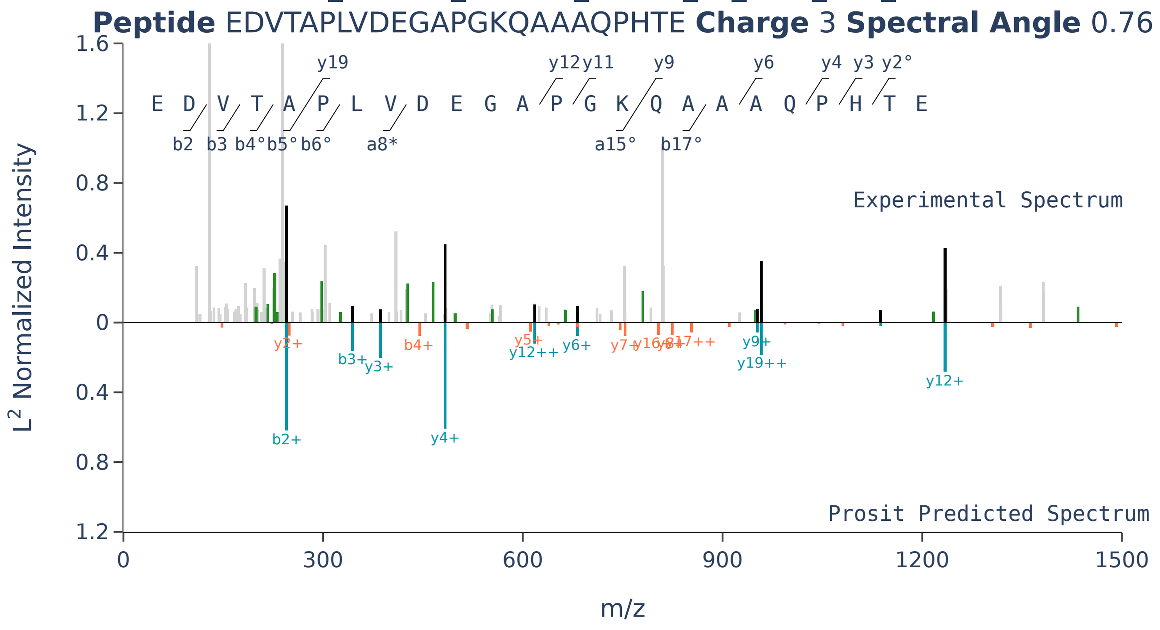


**Source** WSoh_221121_011221_HFGoe_L1_hTau_24h_R1 **Scan** 20866
**Peptide** DFKDRVQSKIGSLDNITHVPGGGNKKLE **Charge** 5 **Spectral Angle** 0.7

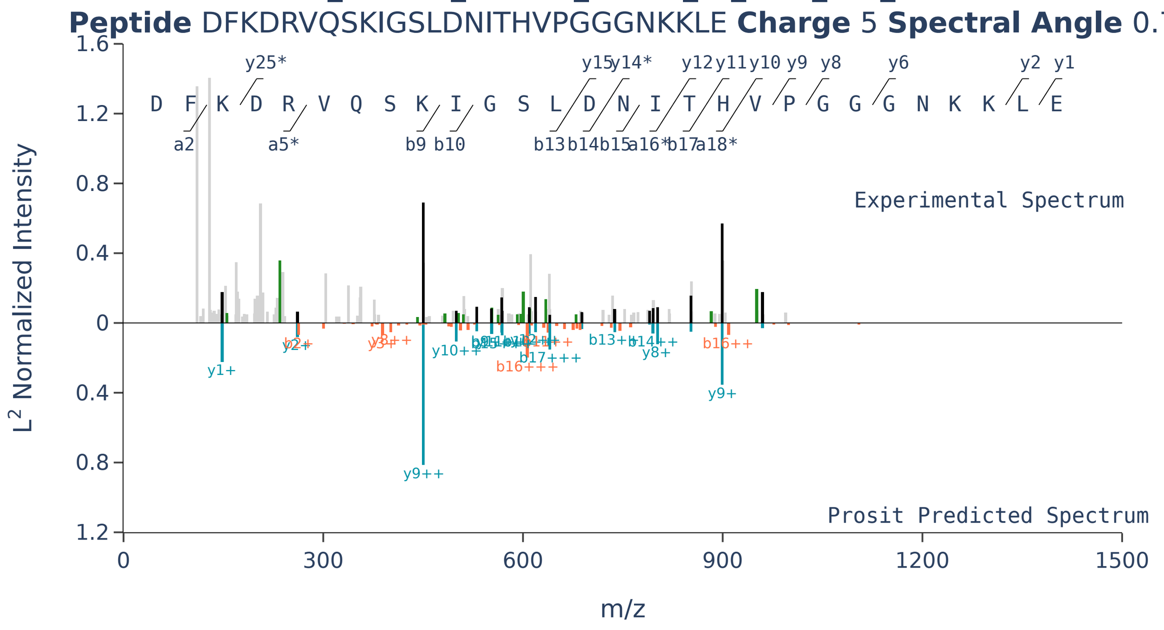


**Source** WSoh_221121_011221_HFGoe_L1_hTau_24h_R2 **Scan** 17284
**Peptide** NLKHQPGGGKVQIINKKLDLSNV **Charge** 4 **Spectral Angle** 0.73

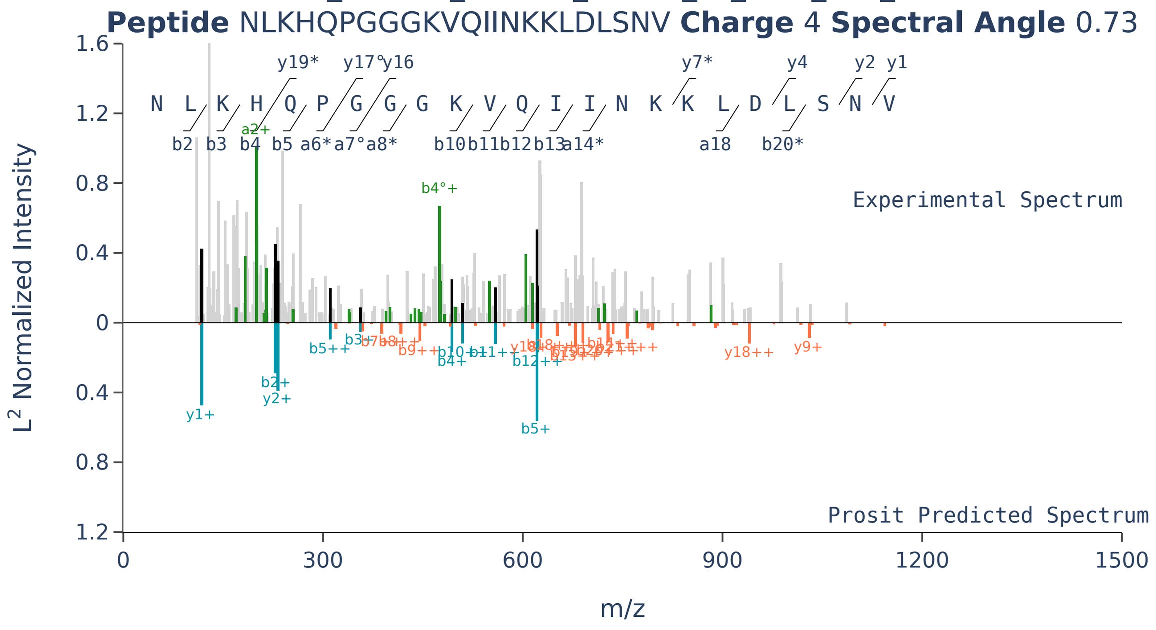


**Source** WSoh_221121_011221_HFGoe_L2_hTau_24h_R2 **Scan** 25356
**Peptide** STGSIDMVDSPQLAT **Charge** 2 **Spectral Angle** 0.66

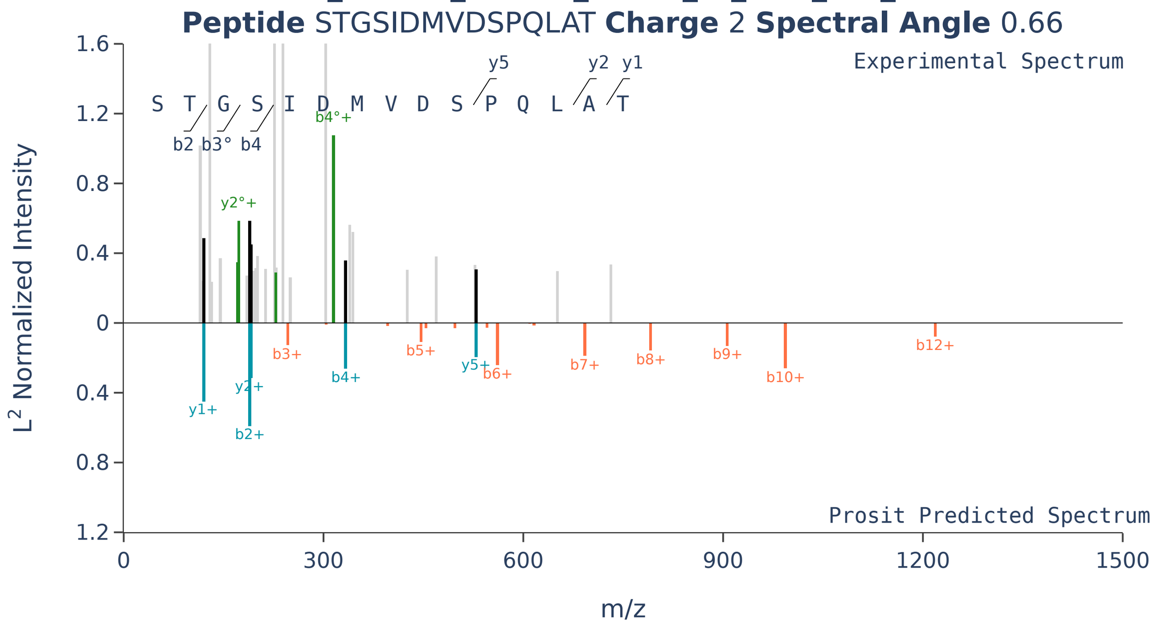


**Source** WSoh_221121_011221_HFGoe_L2_hTau_24h_R2 **Scan** 16109
**Peptide** GGSVQIV **Charge** 1 **Spectral Angle** 0.75

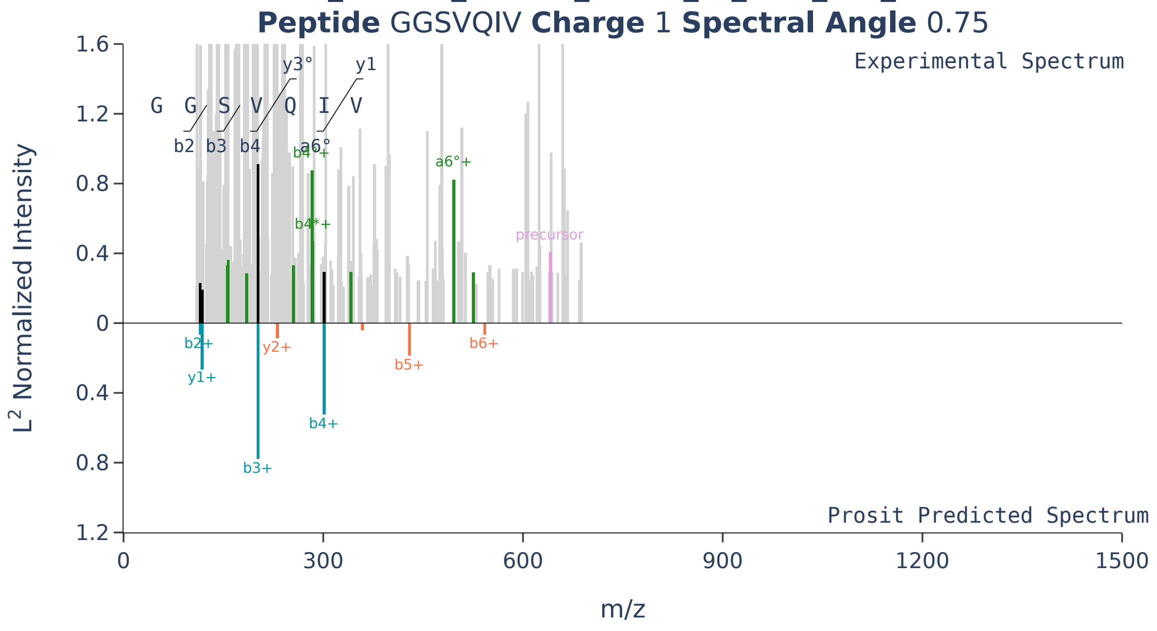


**Source** WSoh_221121_011221_HFGoe_L2_hTau_24h_R1 **Scan** 14646
**Peptide** SPGSPGTPGSRSRTPSLPTPPTREPKKVAV **Charge** 4 **Spectral Angle** 0.64

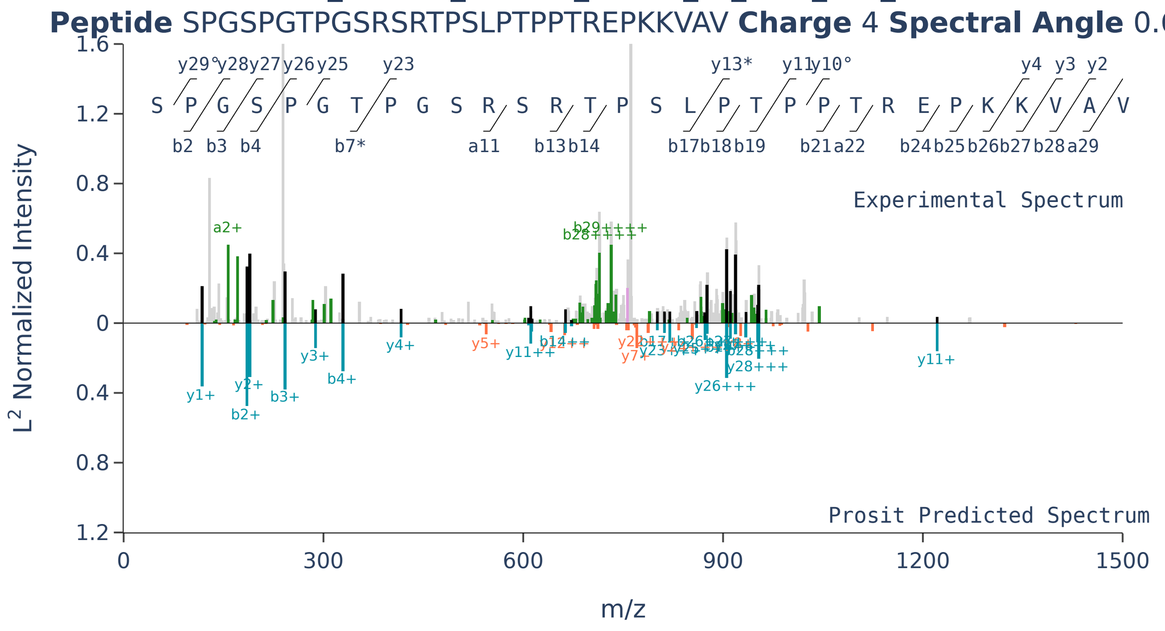


**Source** WSoh_221121_011221_HFGoe_L1_hTau_24h_R2 **Scan** 19104
**Peptide** QPGGGKVQIINKKLDLSNVQ **Charge** 4 **Spectral Angle** 0.9

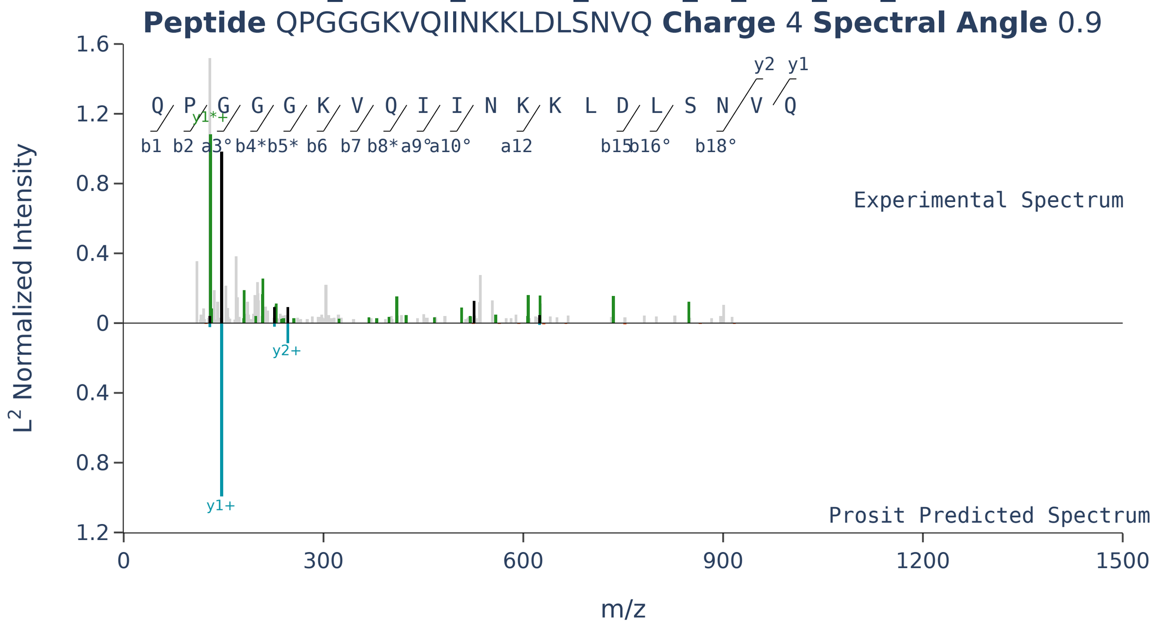


**Source** WSoh_221121_011221_HFGoe_L2_hTau_4h_R2 **Scan** 20562
**Peptide** AEPRQEFEVMEDHAGTYGLGDRKDQGGY **Charge** 5 **Spectral Angle** 0.66

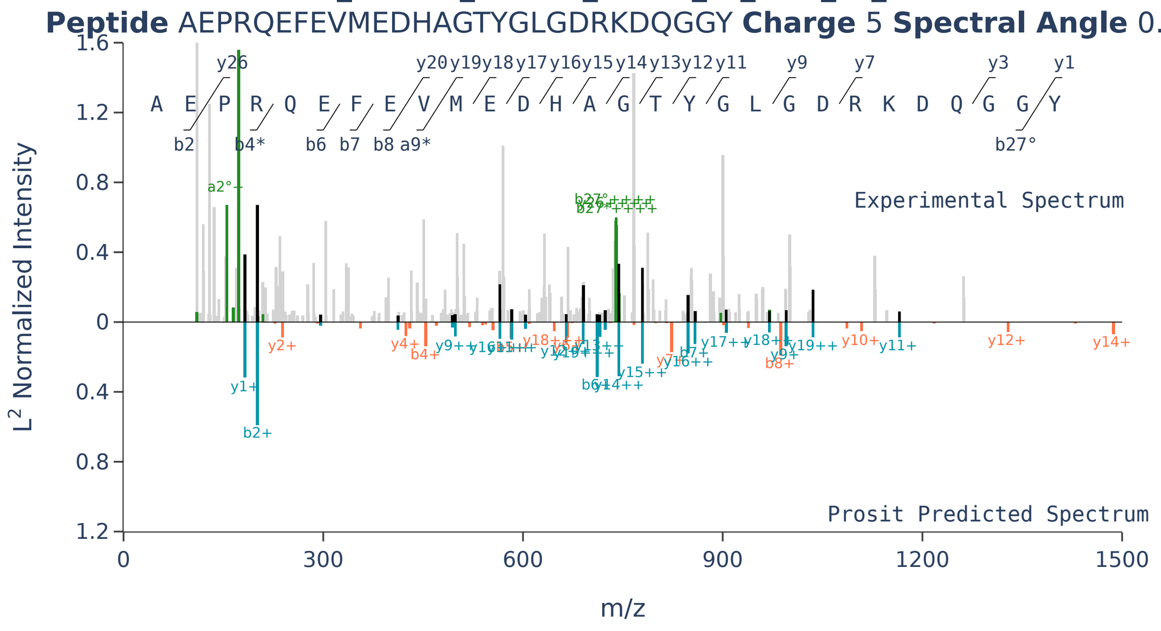


**Source** WSoh_221121_011221_HFGoe_L1_hTau_4h_R2 **Scan** 21566
**Peptide** QIVYKPVDLSKVT **Charge** 3 **Spectral Angle** 0.64

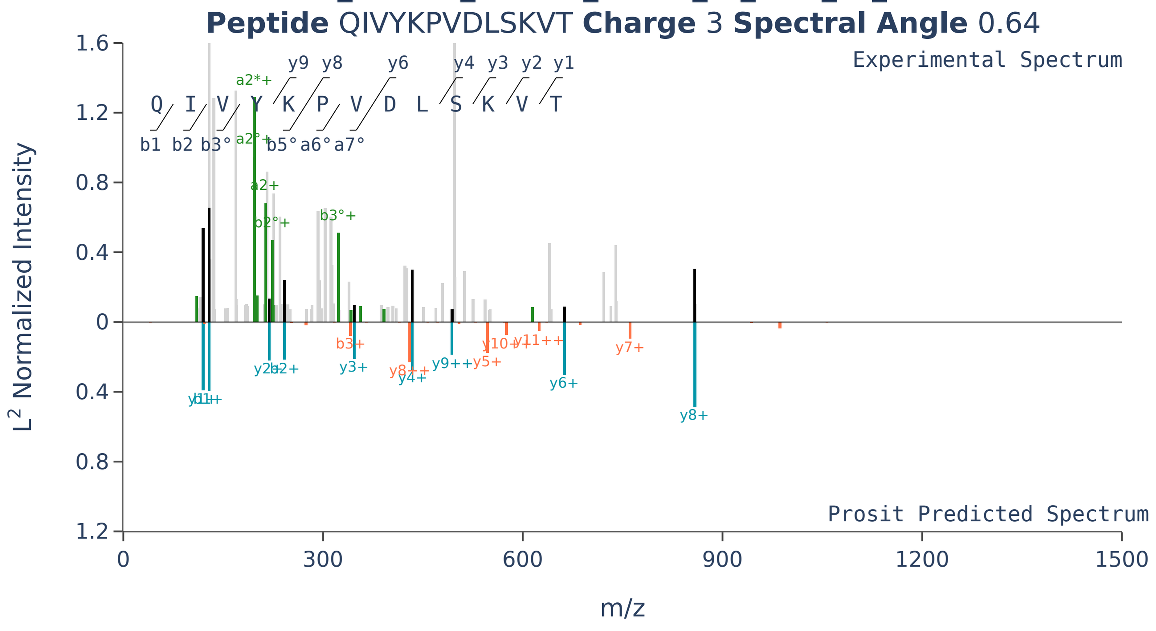


**Source** WSoh_221121_011221_HFGoe_L2_hTau_2h_R1 **Scan** 12480
**Peptide** YKSPVVSGDTSPRHLSNVS **Charge** 4 **Spectral Angle** 0.67

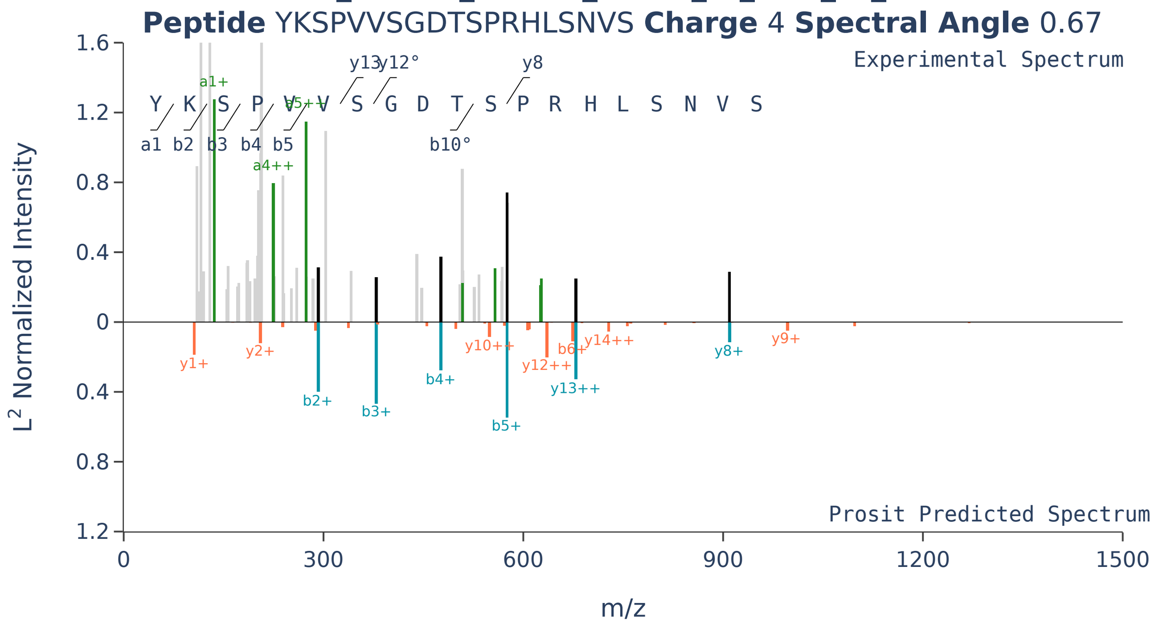


**Source** WSoh_221121_011221_HFGoe_L1_hTau_4h_R1 **Scan** 10670
**Peptide** AAPPGQKGQANATRIPAKTPPAPKTPPSSG **Charge** 4 **Spectral Angle** 0.73

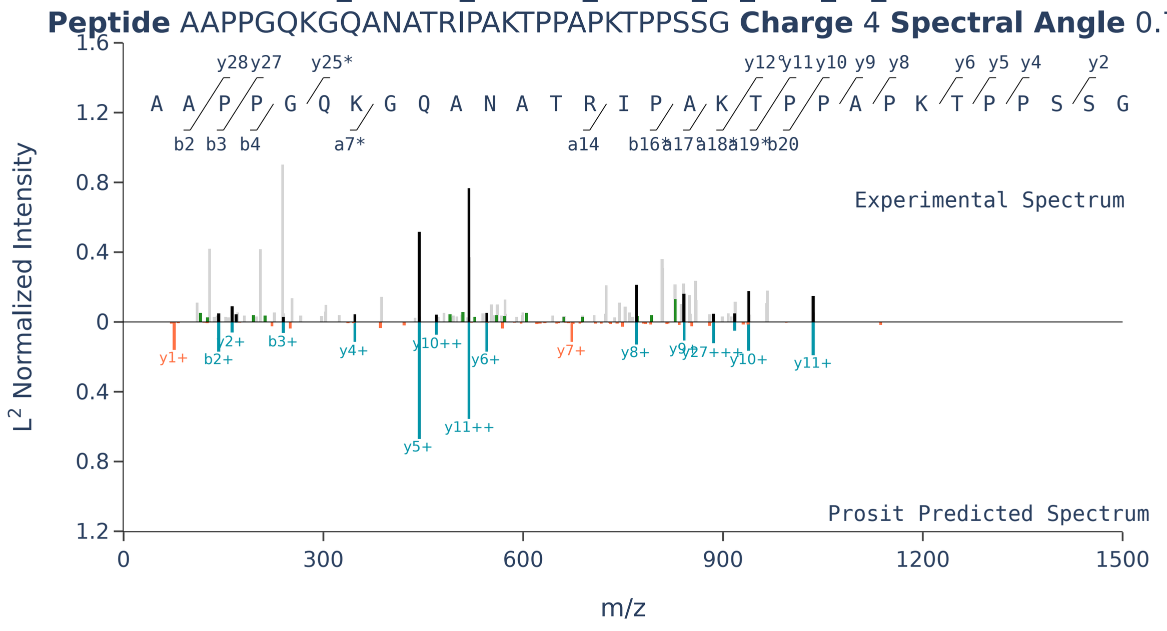


**Source** WSoh_221121_011221_HFGoe_L2_hTau_24h_R1 **Scan** 15275
**Peptide** SNVQSKCGSKDNIKHVPGGGSVQIVYKPVD **Charge** 4 **Spectral Angle** 0.53

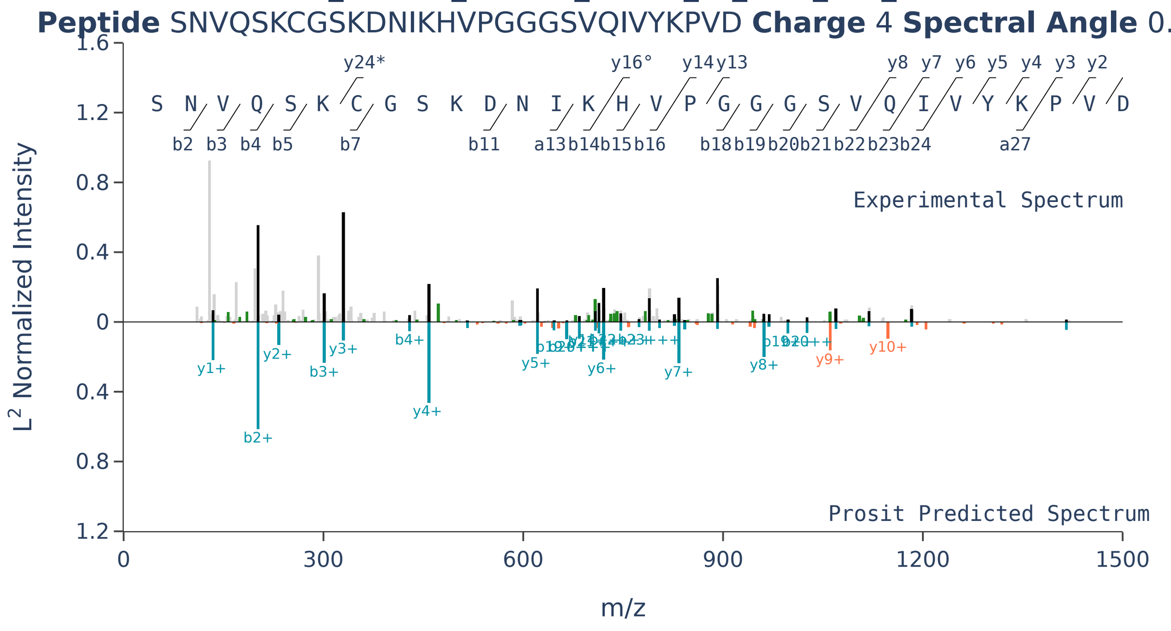


**Source** WSoh_221121_011221_HFGoe_L2_hTau_4h_R2 **Scan** 21234
**Peptide** QTAPVPMPDLKNVKSKIGSTEN **Charge** 3 **Spectral Angle** 0.76

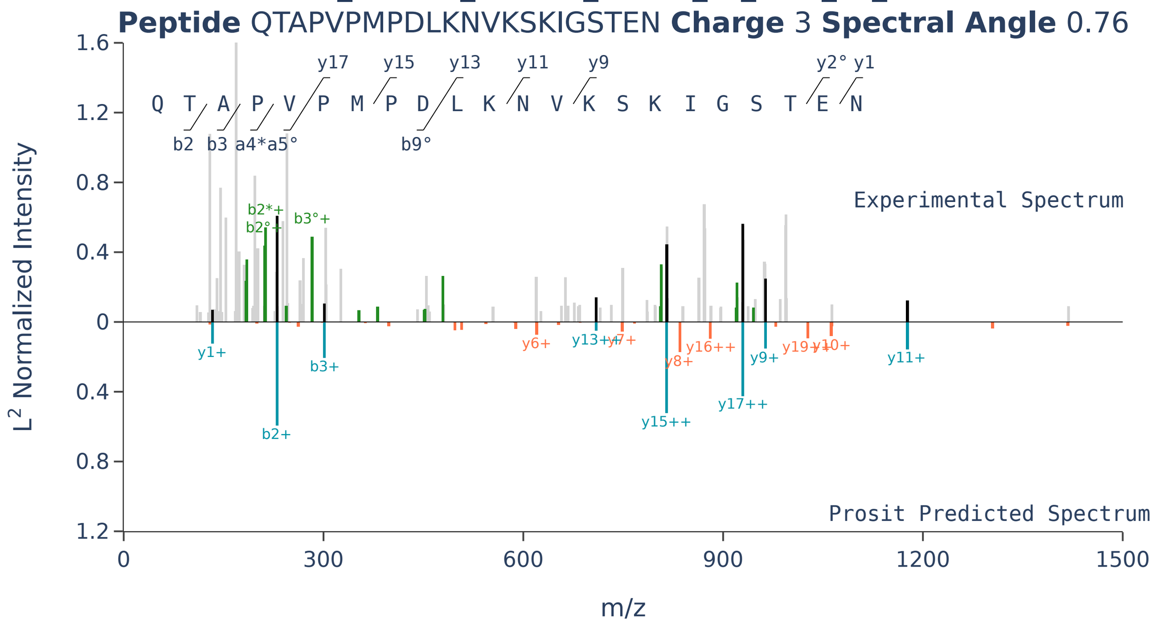

**Source** WSoh_221121_011221_HFGoe_L1_hTau_1h_R2 **Scan** 10359
**Peptide** EVKSEKLDFKD **Charge** 3 **Spectral Angle** 0.68

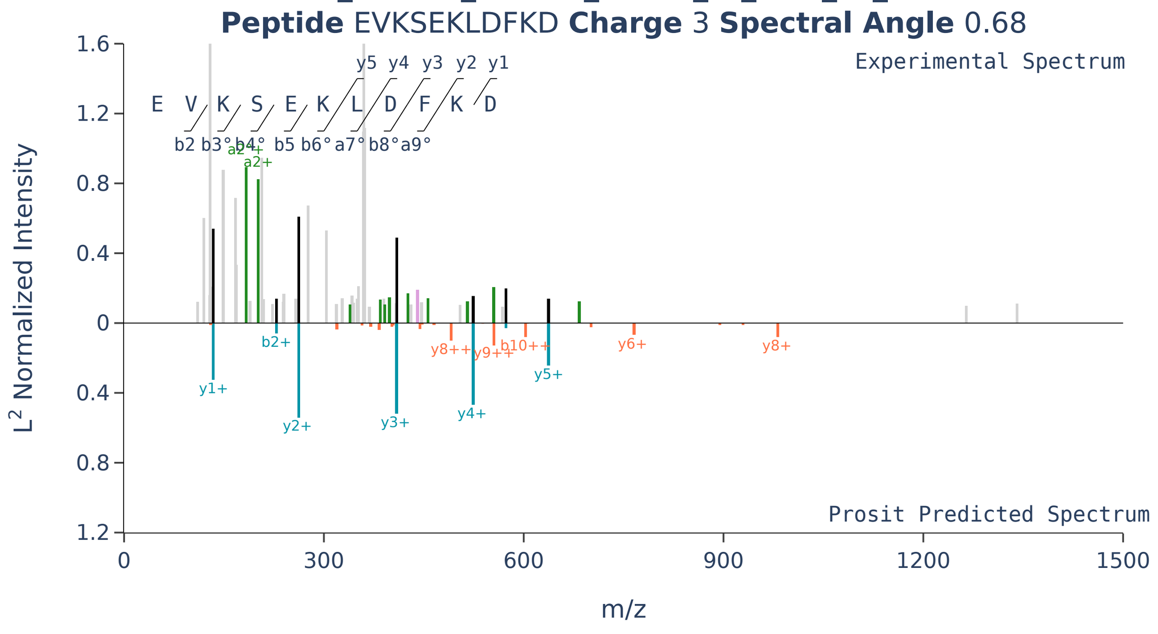


**Source** WSoh_221121_011221_HFGoe_L1_hTau_24h_R2 **Scan** 10173
**Peptide** LTFRENAKA **Charge** 3 **Spectral Angle** 0.63

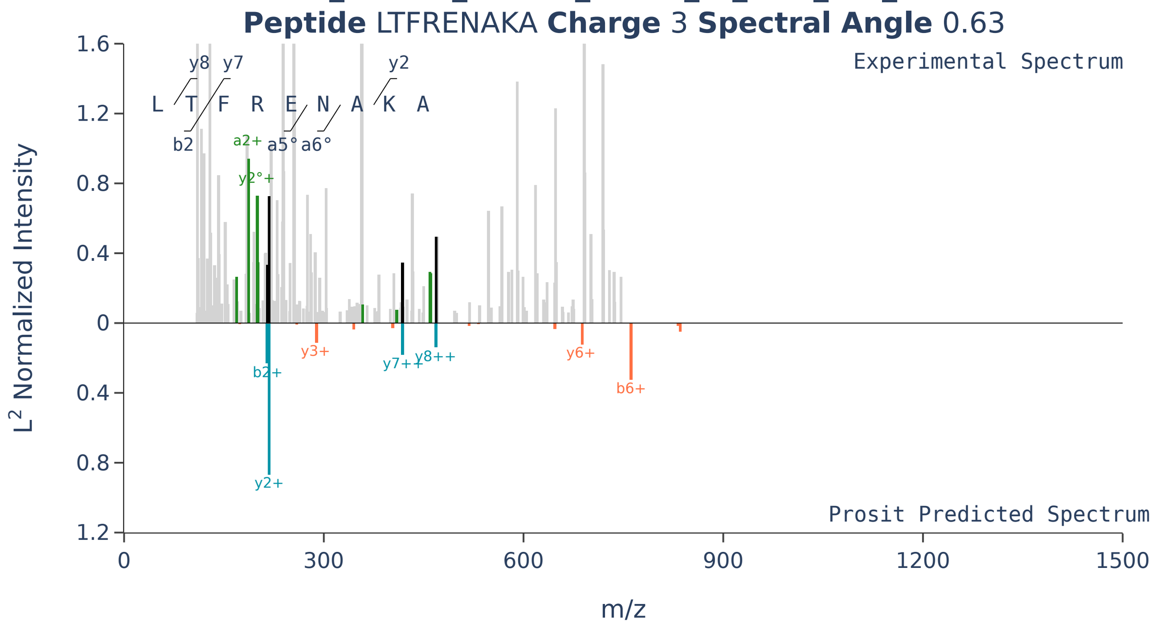


**Source** WSoh_221121_011221_HFGoe_L2_hTau_24h_R1 **Scan** 18245
**Peptide** PLVDEGAPGKQAAAQPHTEIPEGTTAEEA **Charge** 3 **Spectral Angle** 0.59

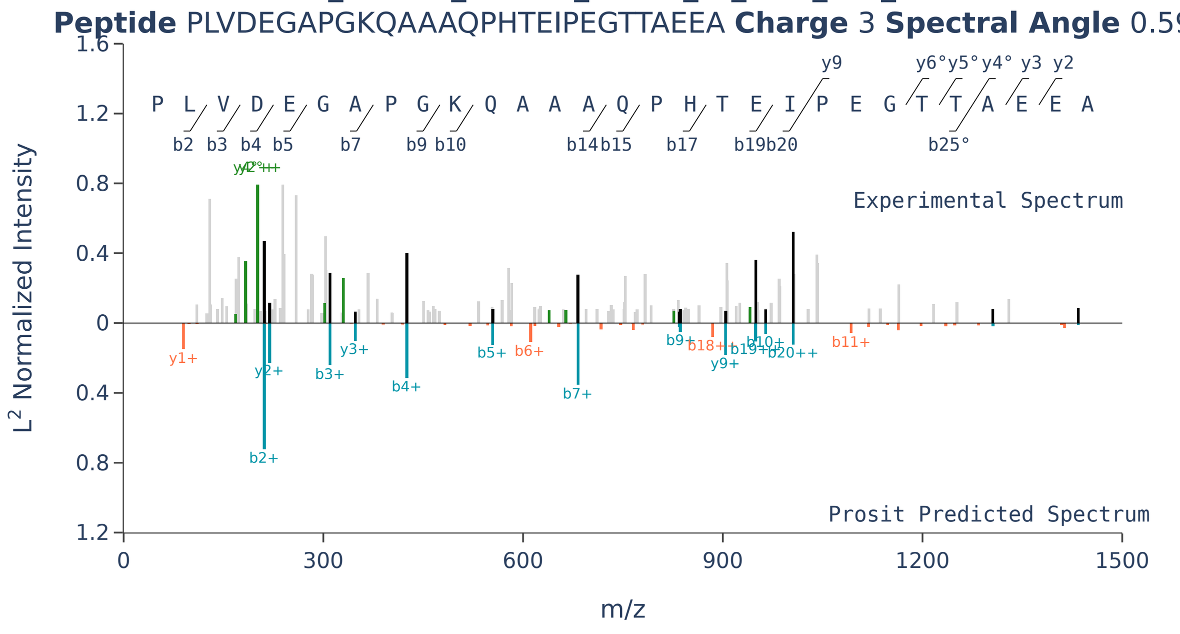


**Source** WSoh_221121_011221_HFGoe_L1_hTau_24h_R1 **Scan** 5986
**Peptide** EDEAAGHVTQARMVSKSKDGTGSDDKKAKG **Charge** 4 **Spectral Angle** 0.65

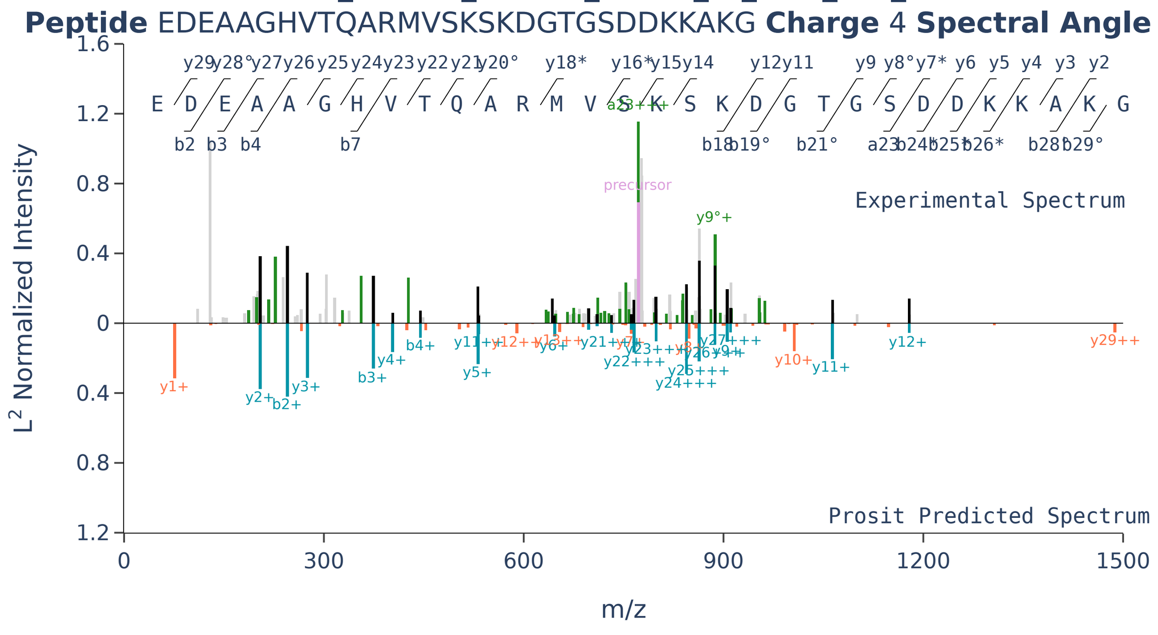


**Source** WSoh_221121_011221_HFGoe_L2_hTau_1h_R2 **Scan** 11269
**Peptide** EVKSEKLDFKDRVQ **Charge** 4 **Spectral Angle** 0.61

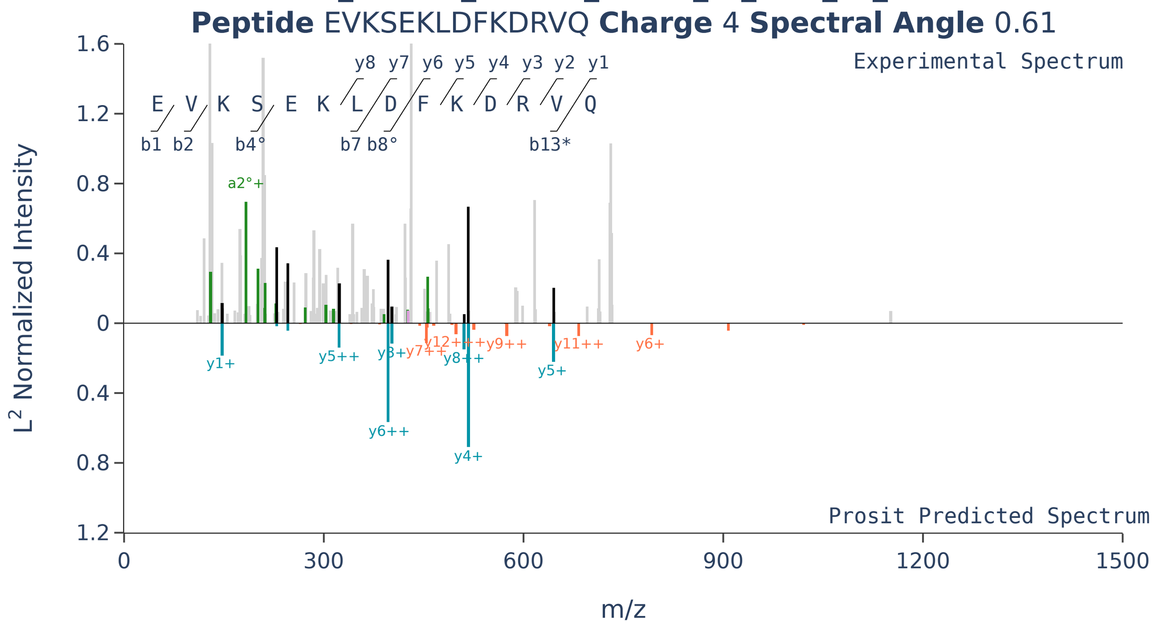


**Source** WSoh_221121_011221_HFGoe_L1_hTau_24h_R1 **Scan** 16901
**Peptide** LGDTPSLEDEAAGHVTQARMVSKSKD **Charge** 3 **Spectral Angle** 0.59

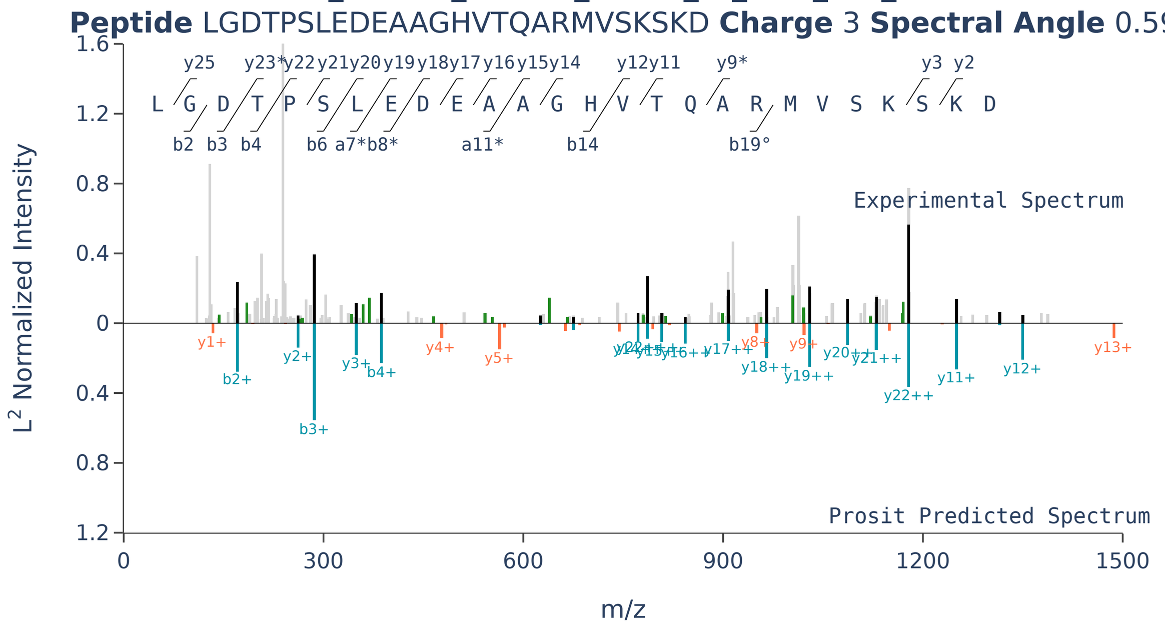


**Source** WSoh_221121_011221_HFGoe_L2_hTau_24h_R1 **Scan** 16828
**Peptide** KLGSLDNITHVPGGGNKKIETHKLTFRENA **Charge** 4 **Spectral Angle** 0.35

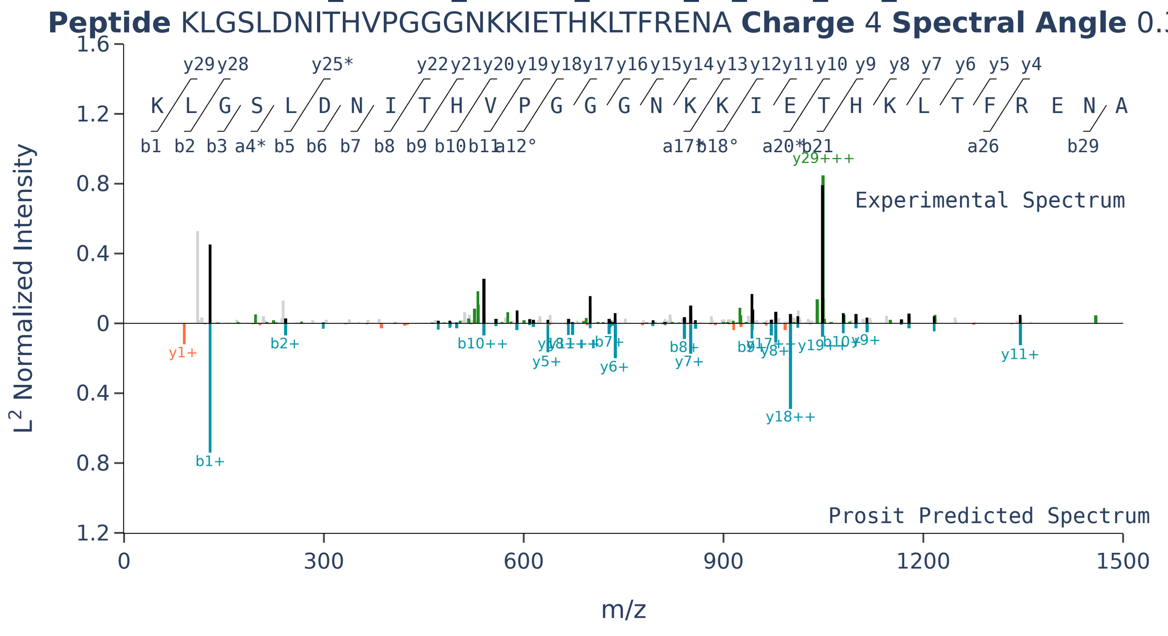


**Source** WSoh_221121_011221_HFGoe_L1_hTau_4h_R2 **Scan** 12823
**Peptide** FRENAKAKTDHGAEIVYKSPVVS **Charge** 4 **Spectral Angle** 0.66

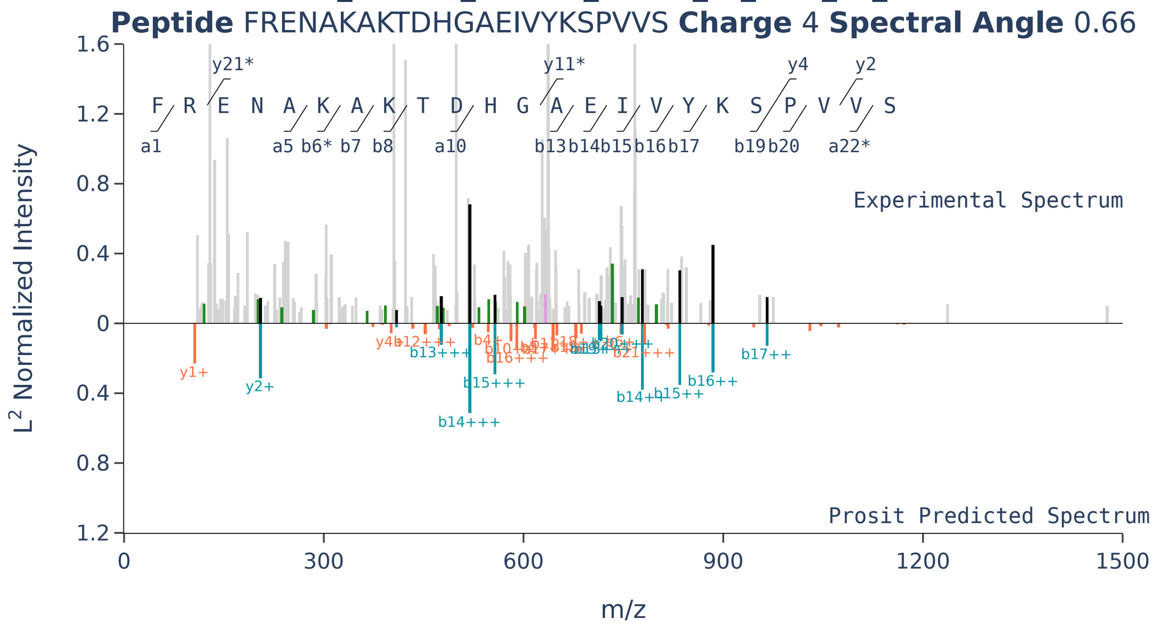


**Source** WSoh_221121_011221_HFGoe_L2_hTau_24h_R1 **Scan** 6504
**Peptide** KLTFRENAKAKTDHGAE **Charge** 2 **Spectral Angle** 0.59

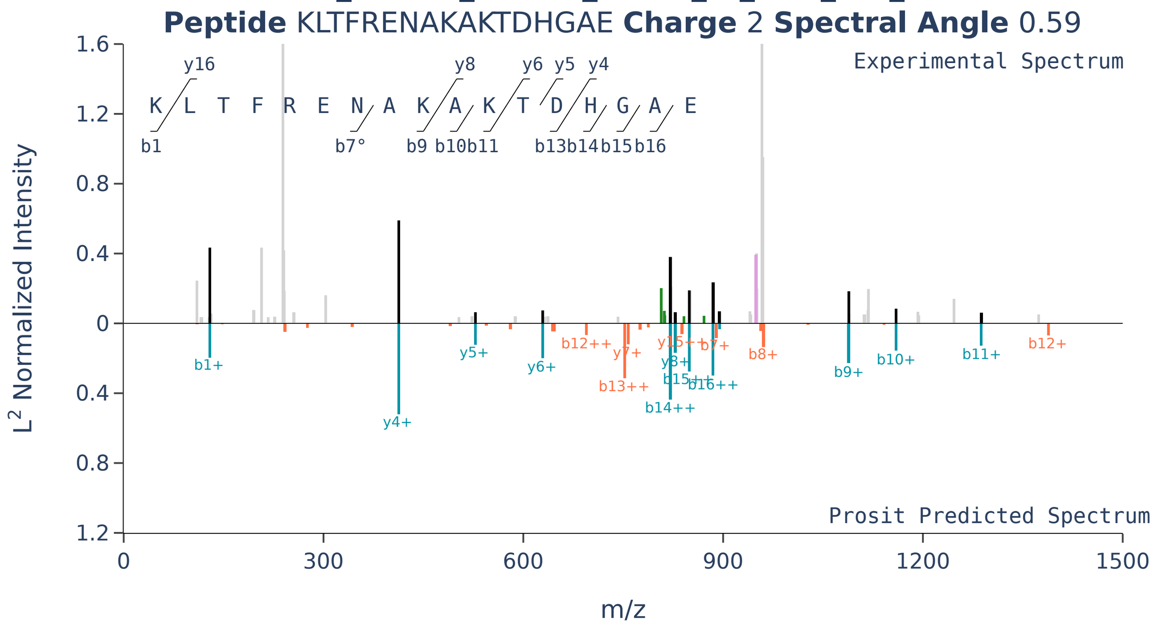


**Source** WSoh_221121_011221_HFGoe_L2_hTau_24h_R1 **Scan** 18836
**Peptide** SPLQTPTEDGSEEPGSETSDAKSTPTAEDV **Charge** 3 **Spectral Angle** 0.66

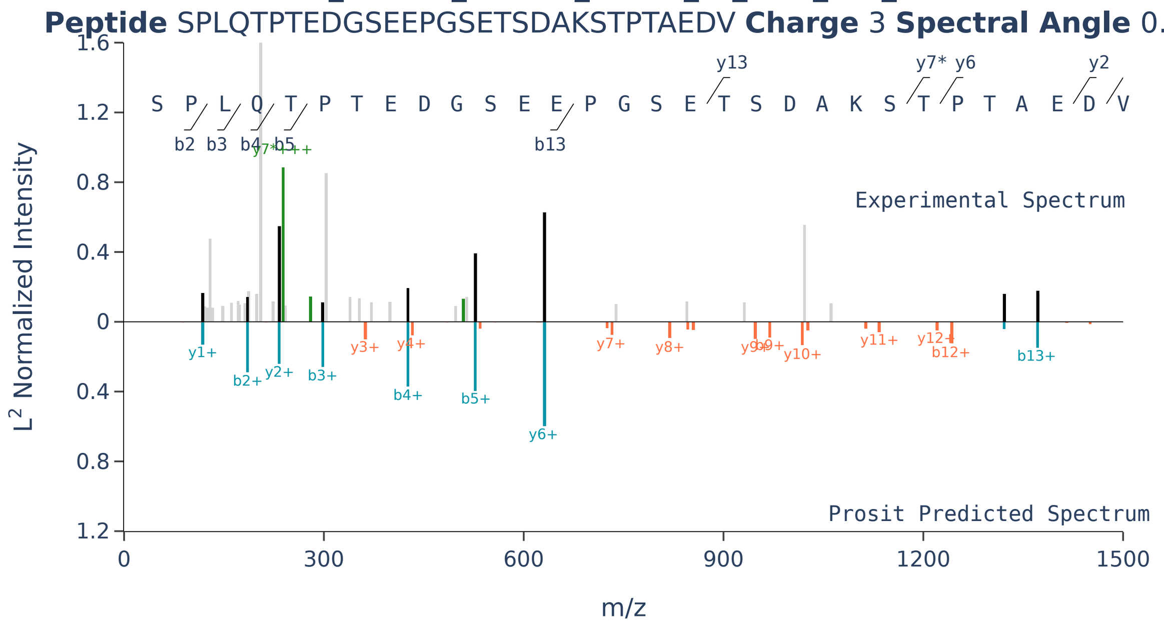


**Source** WSoh_221121_011221_HFGoe_L2_hTau_4h_R2 **Scan** 20992
**Peptide** KDNIKHVPGGGSVQIVYKPVDL **Charge** 4 **Spectral Angle** 0.61

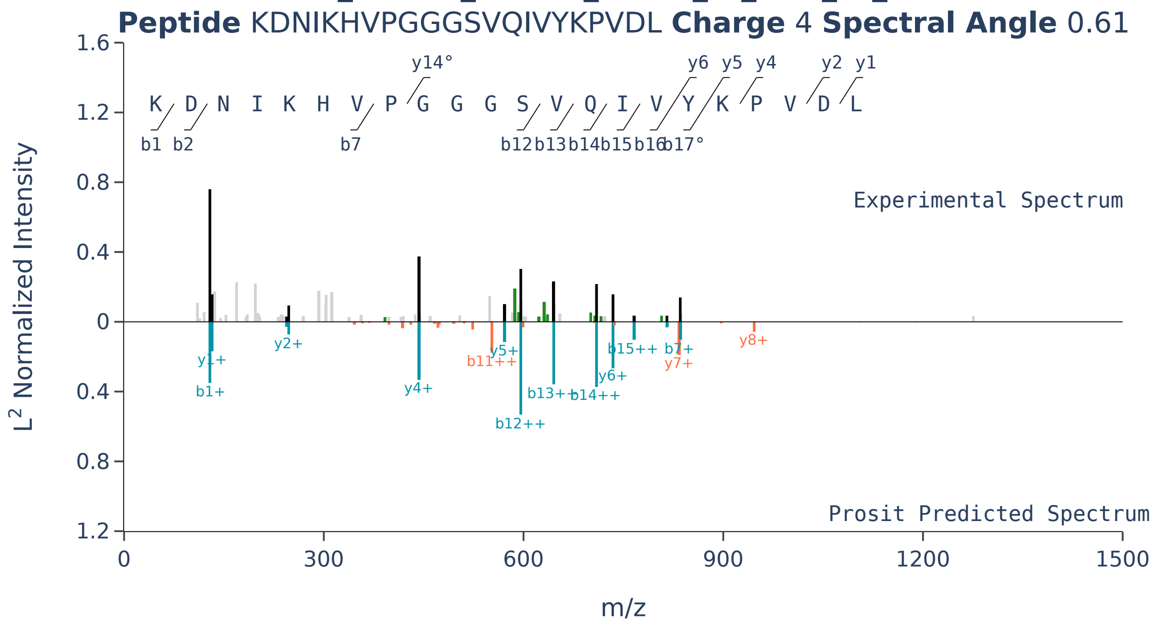


**Source** WSoh_221121_011221_HFGoe_L2_hTau_1h_R2 **Scan** 12458
**Peptide** PEGTTAEE **Charge** 1 **Spectral Angle** 0.93

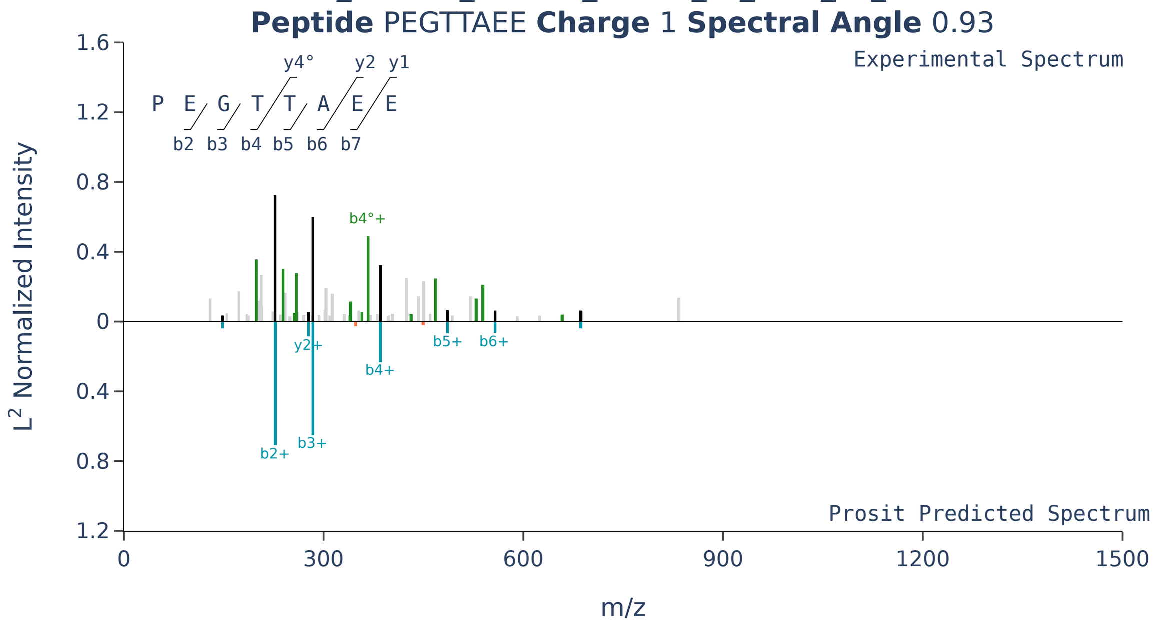


**Source** WSoh_221121_011221_HFGoe_L1_hTau_24h_R2 **Scan** 20181
**Peptide** AEDVTAPLVDEGAPGKQA **Charge** 2 **Spectral Angle** 0.86

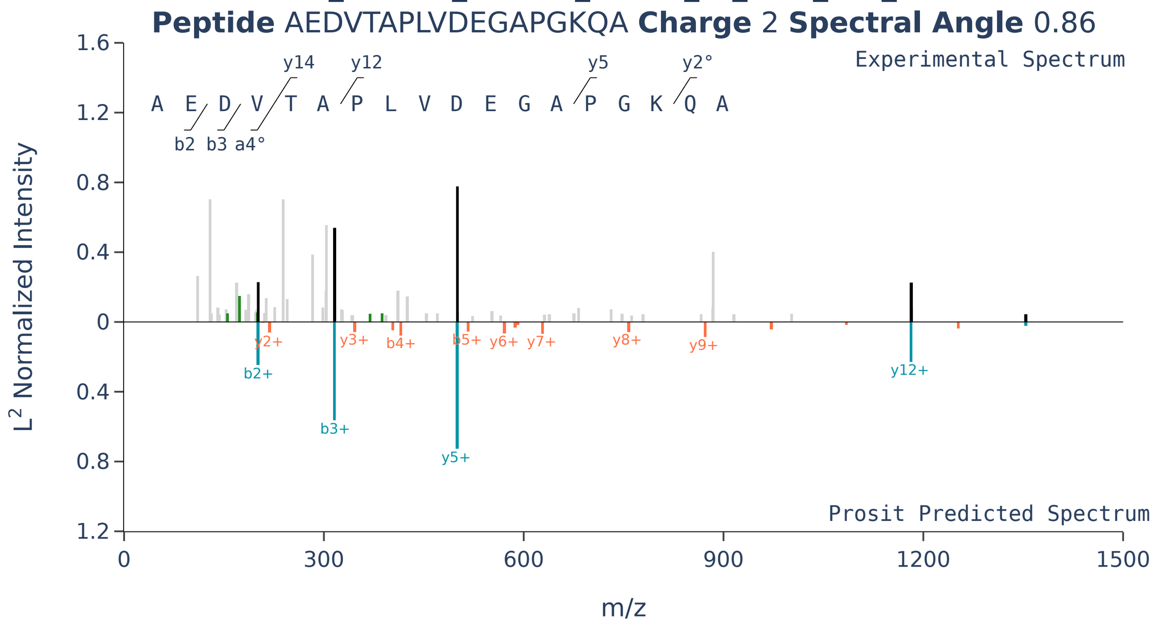


**Source** WSoh_221121_011221_HFGoe_L1_hTau_2h_R2 **Scan** 13047
**Peptide** SNVQSKCGSKDNIKHVPGGGSVQIV **Charge** 4 **Spectral Angle** 0.74

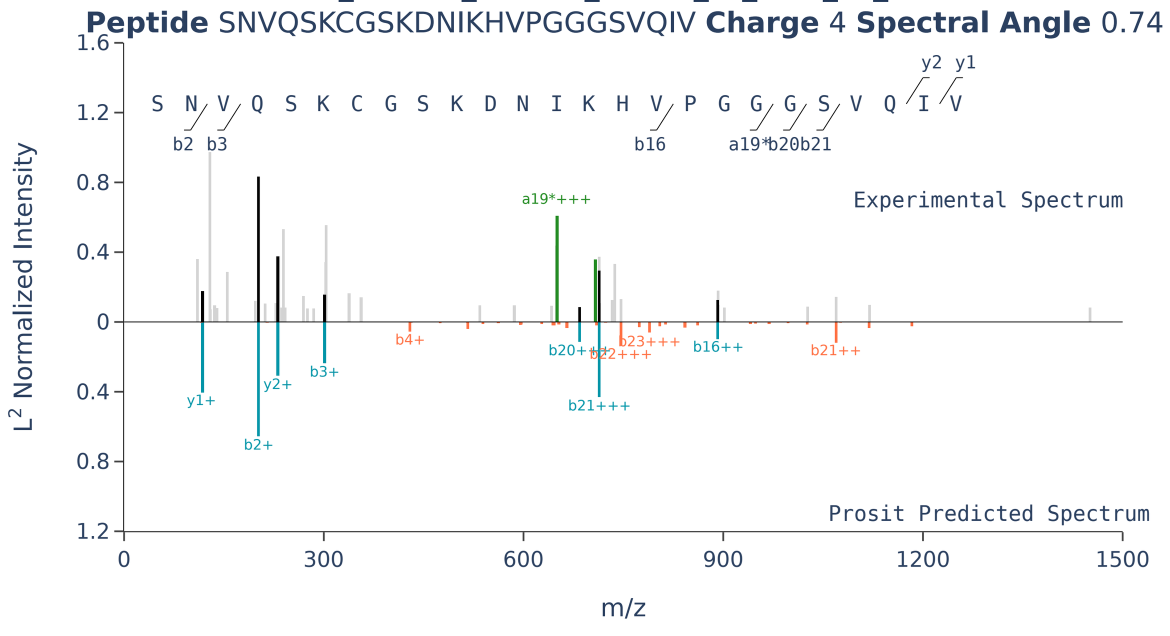


**Source** WSoh_221121_011221_HFGoe_L2_hTau_4h_R1 **Scan** 20479
**Peptide** GSKDNIKHVPGGGSVQIVYKPVDLSKVTS **Charge** 3 **Spectral Angle** 0.58

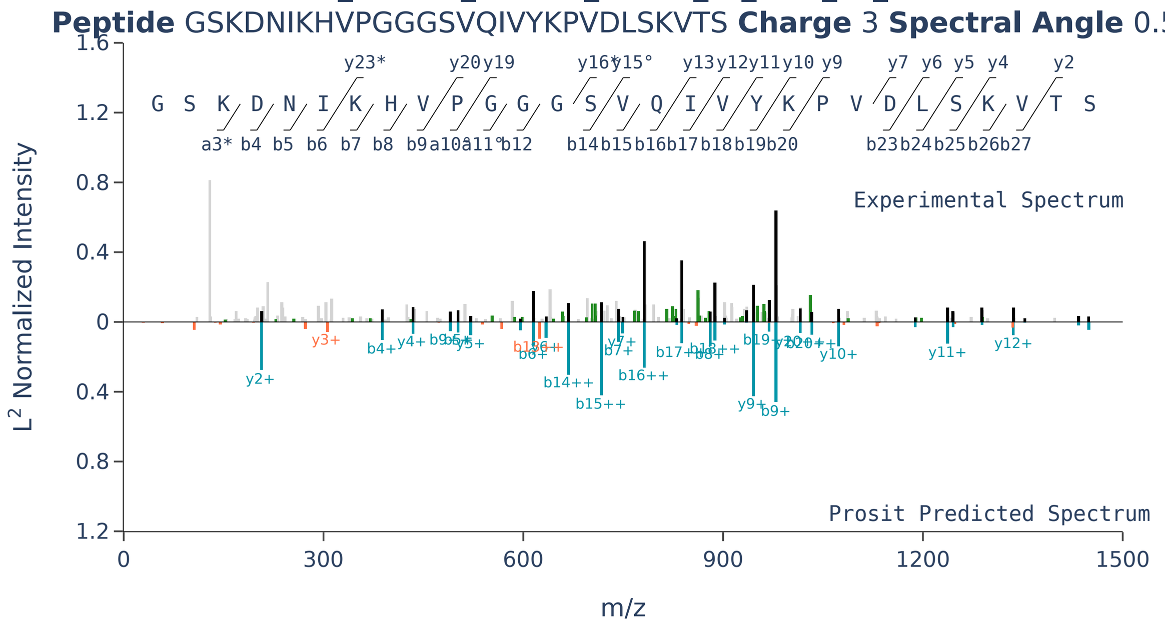

**Source** WSoh_221121_011221_HFGoe_L1_hTau_4h_R1 **Scan** 23191
**Peptide** DGSEEPGSETSDAKSTPTAEDVTAPLVDE **Charge** 3 **Spectral Angle** 0.8

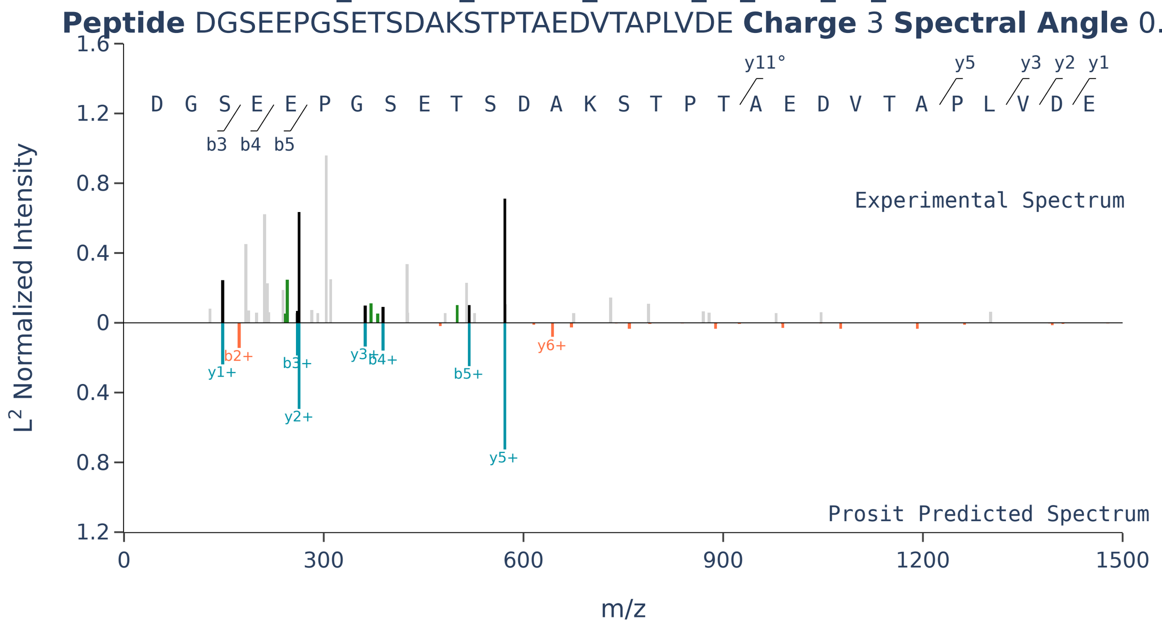


**Source** WSoh_221121_011221_HFGoe_L2_hTau_4h_R2 **Scan** 20439
**Peptide** SKDNIKHVPGGGSVQIVYKPVDLSKVTS **Charge** 4 **Spectral Angle** 0.62

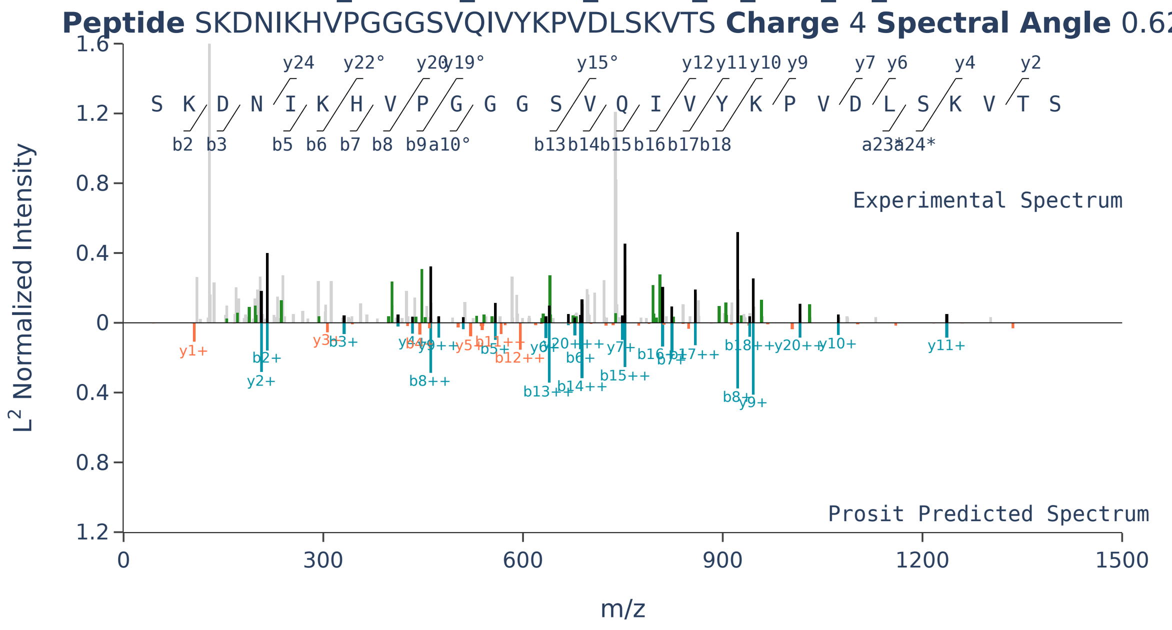


**Source** WSoh_221121_011221_HFGoe_L1_hTau_24h_R2 **Scan** 20134
**Peptide** PVPMPDLKNVKSKIGSTENLKHQ **Charge** 5 **Spectral Angle** 0.75

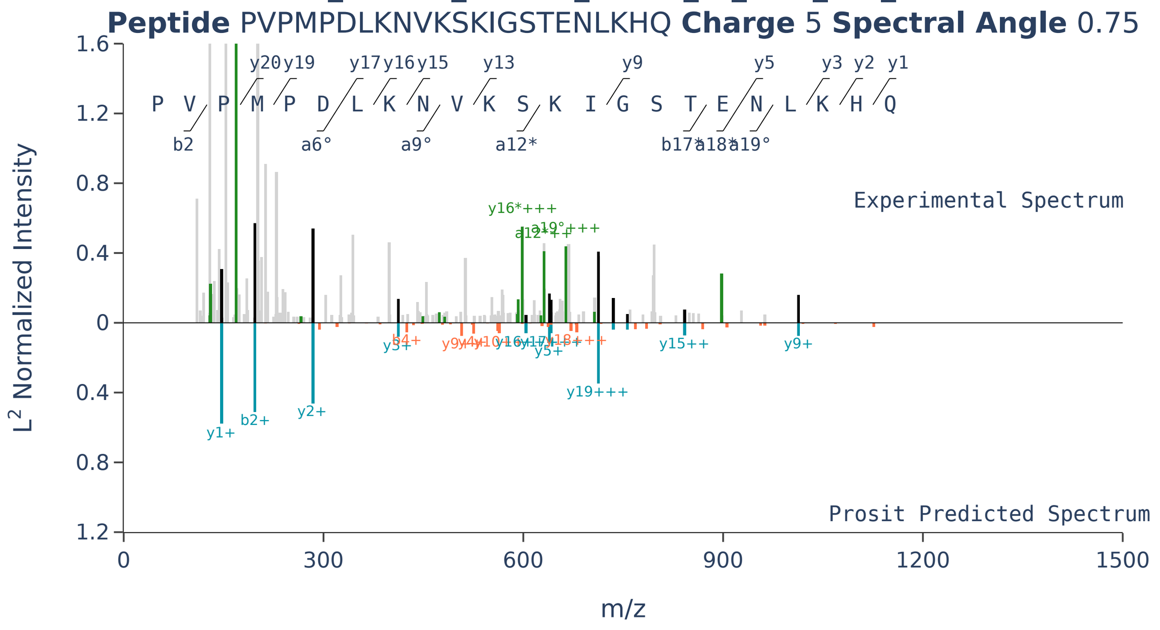


**Source** WSoh_221121_011221_HFGoe_L1_hTau_24h_R2 **Scan** 18654
**Peptide** PVPMPDLKNVKSKIGSTENLKHQPGGGKVQ **Charge** 6 **Spectral Angle** 0.79

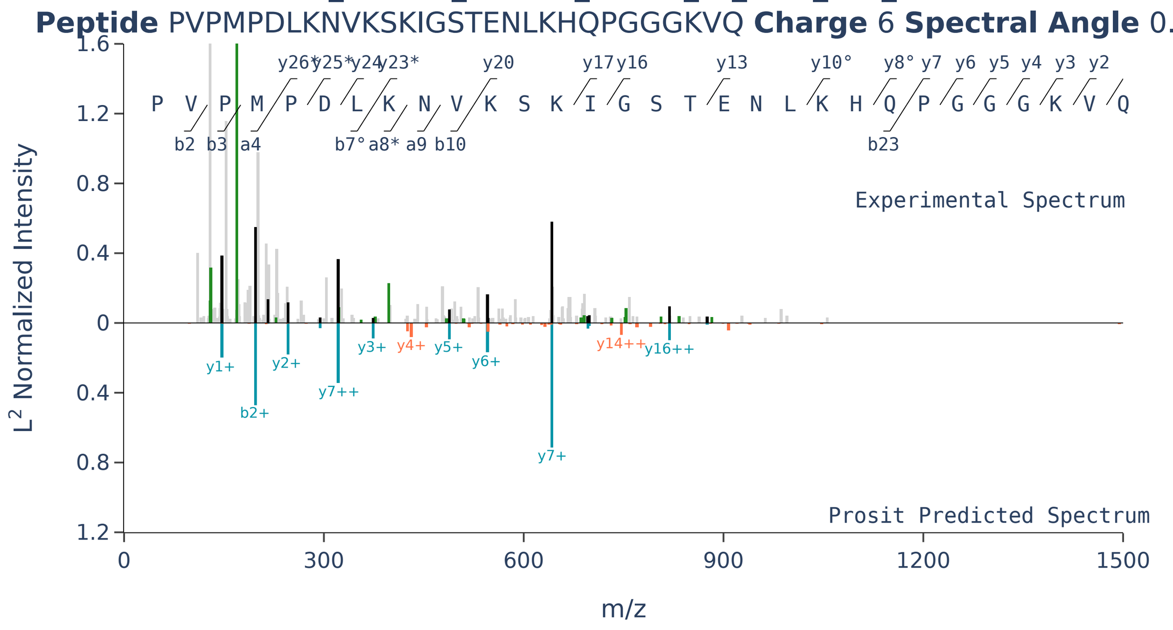


**Source** WSoh_221121_011221_HFGoe_L2_hTau_24h_R1 **Scan** 19820
**Peptide** KSRLQTAPVPMPDLKNVKSKIG **Charge** 5 **Spectral Angle** 0.75

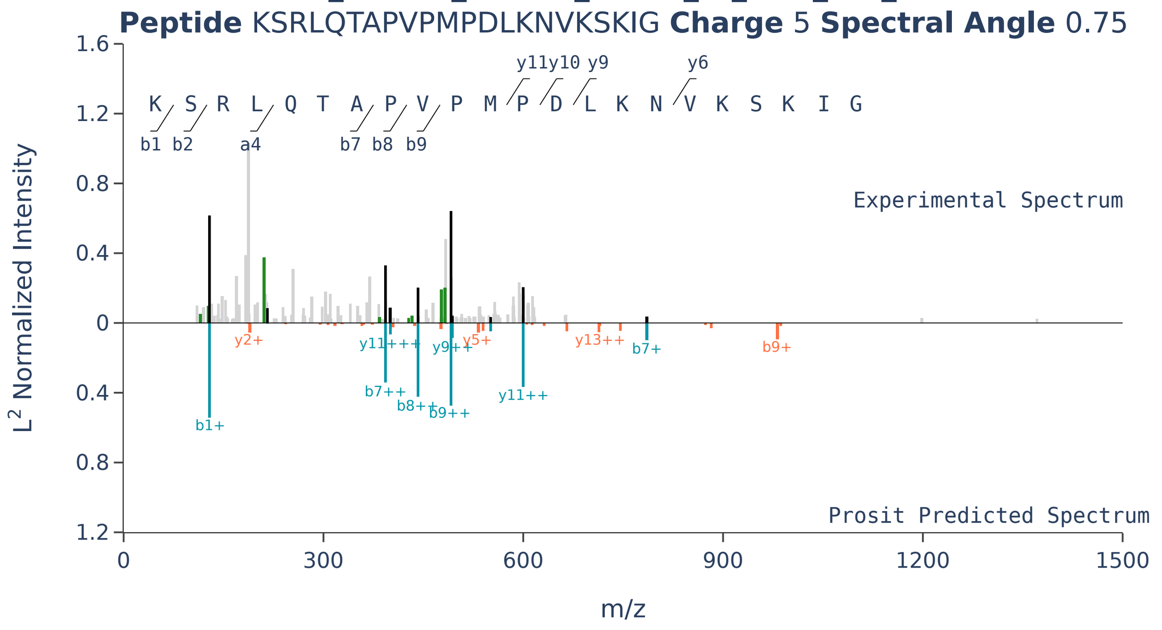


**Source** WSoh_221121_011221_HFGoe_L2_hTau_24h_R1 **Scan** 20154
**Peptide** VTAPLVDEGAPGKQAAAQPHTEIPEGTT **Charge** 3 **Spectral Angle** 0.82

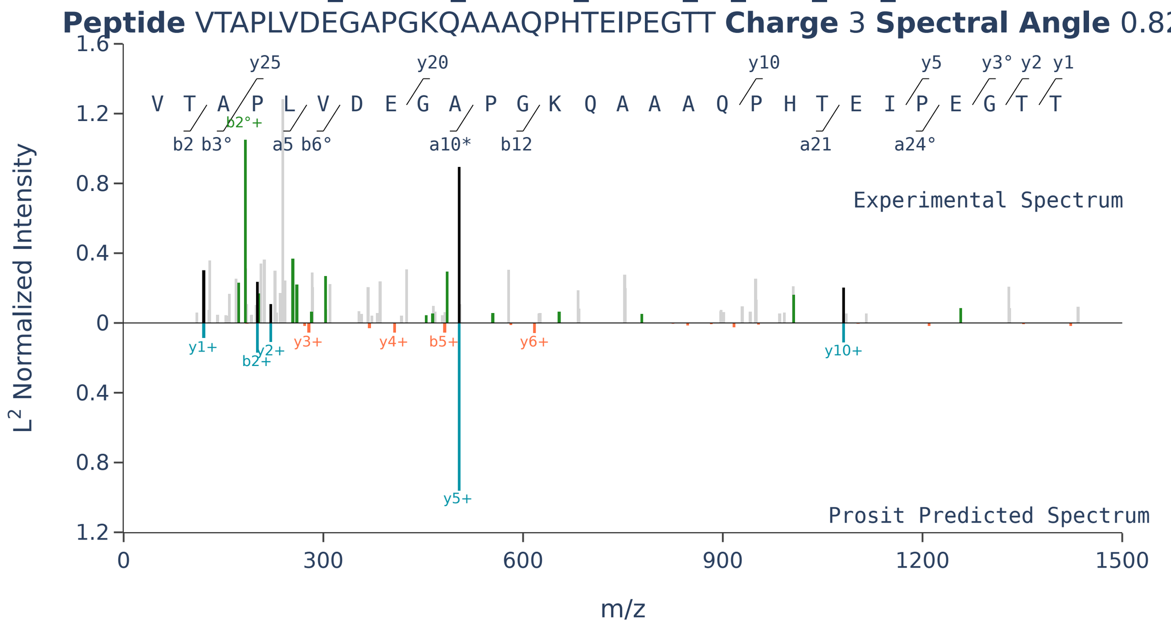


**Source** WSoh_221121_011221_HFGoe_L1_hTau_1h_R2 **Scan** 12298
**Peptide** KCGSLGNI **Charge** 2 **Spectral Angle** 0.45

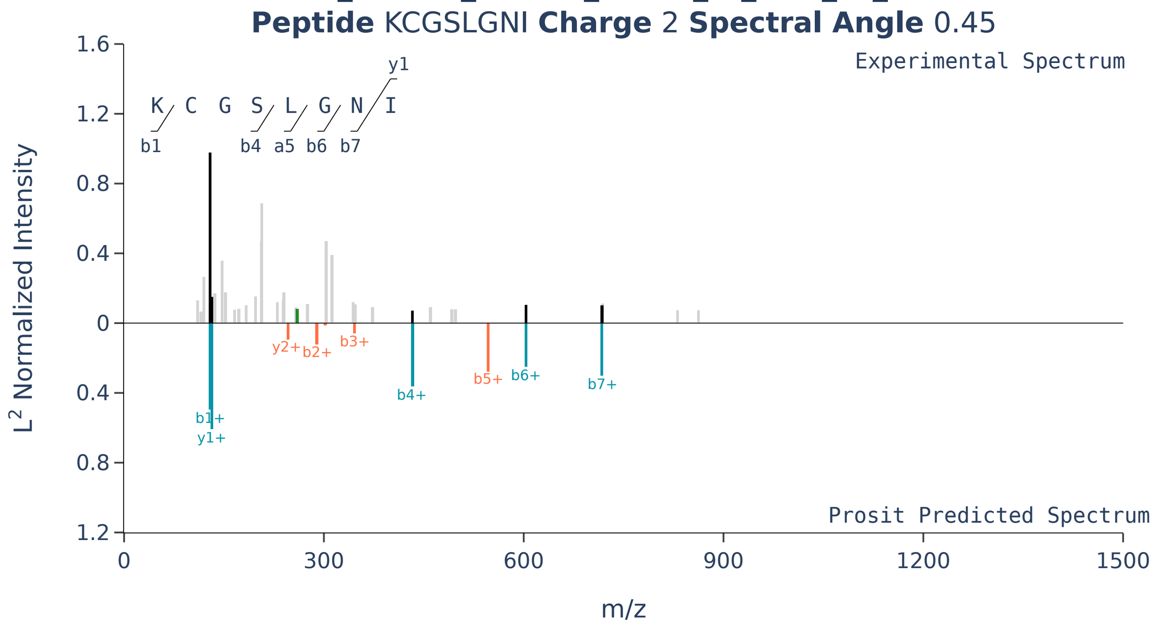


**Source** WSoh_221121_011221_HFGoe_L1_hTau_4h_R2 **Scan** 7146
**Peptide** LSKVTSKCGSLGNIHHKPGG **Charge** 3 **Spectral Angle** 0.76

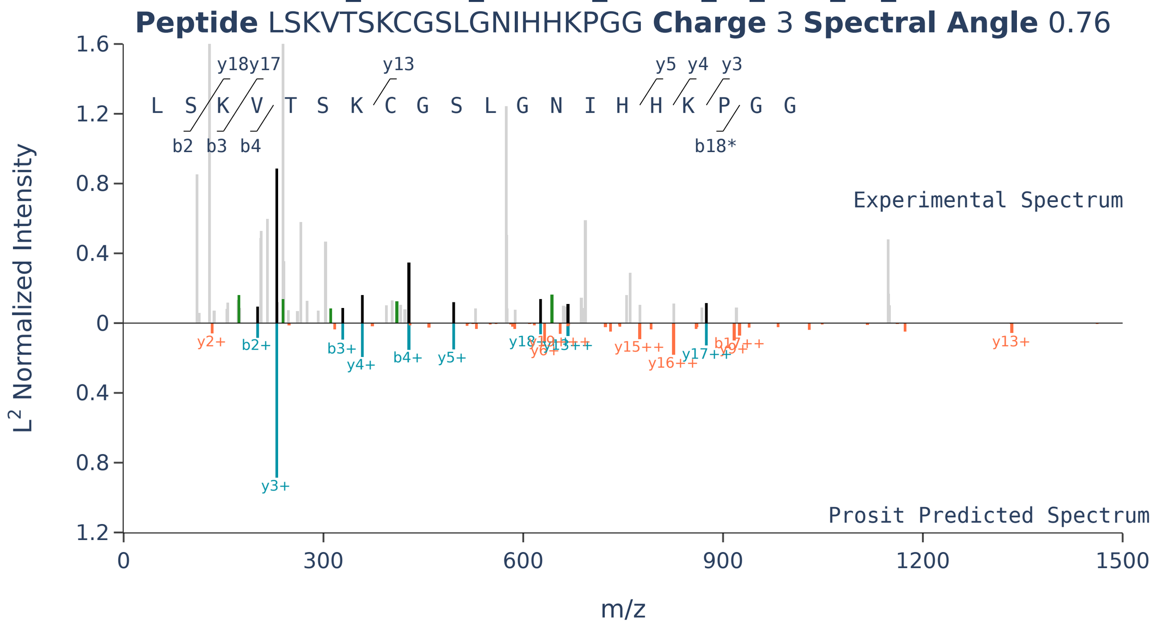


**Source** WSoh_221121_011221_HFGoe_L1_hTau_24h_R2 **Scan** 15154
**Peptide** GSLDNITHVPGGGNKKIETHKLTFRENAKA **Charge** 6 **Spectral Angle** 0.58

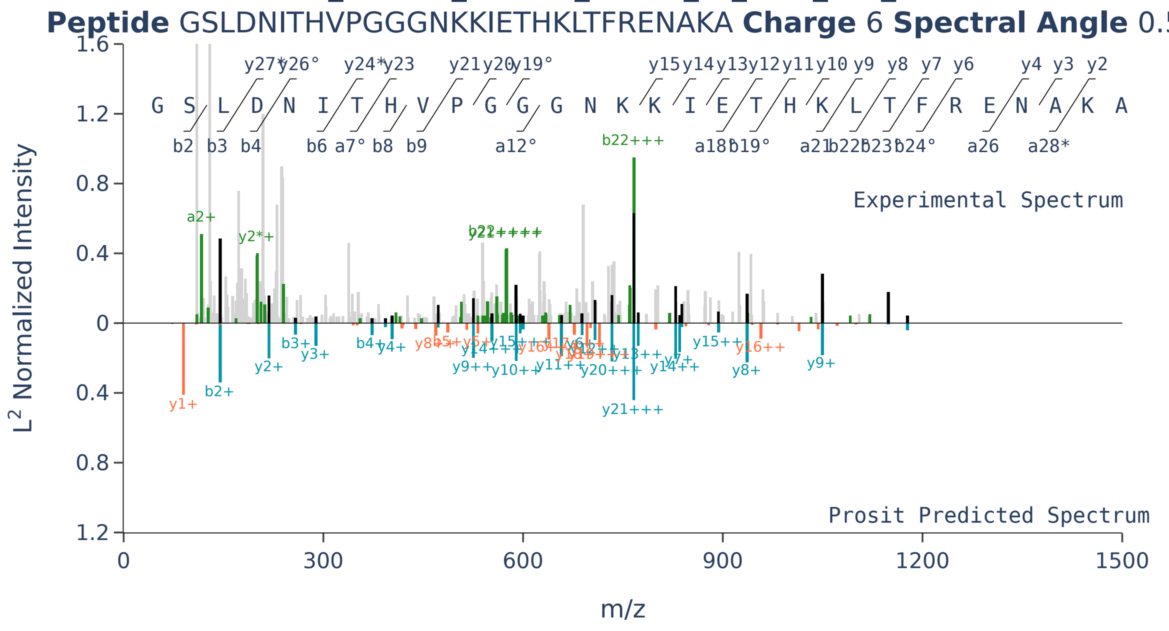


**Source** WSoh_221121_011221_HFGoe_L2_hTau_24h_R1 **Scan** 15054
**Peptide** NKKLDLSNVQSKCGSKDNIKHVPGGGSVQL **Charge** 4 **Spectral Angle** 0.65

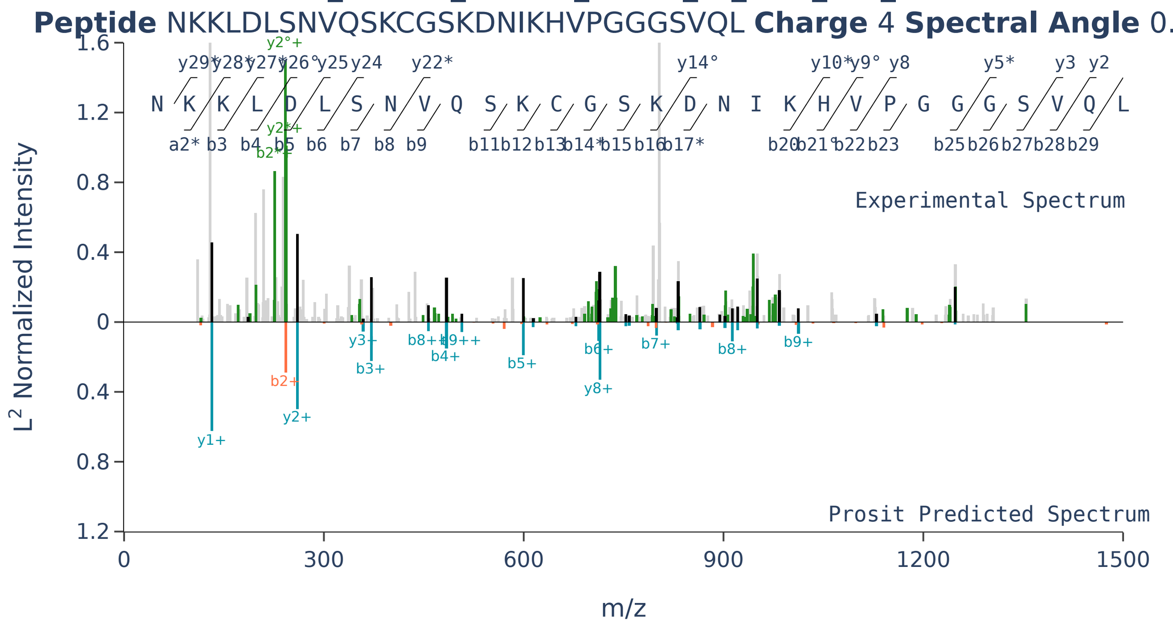


**Source** WSoh_221121_011221_HFGoe_L1_hTau_24h_R1 **Scan** 6896
**Peptide** TDAGLKE **Charge** 2 **Spectral Angle** 0.89

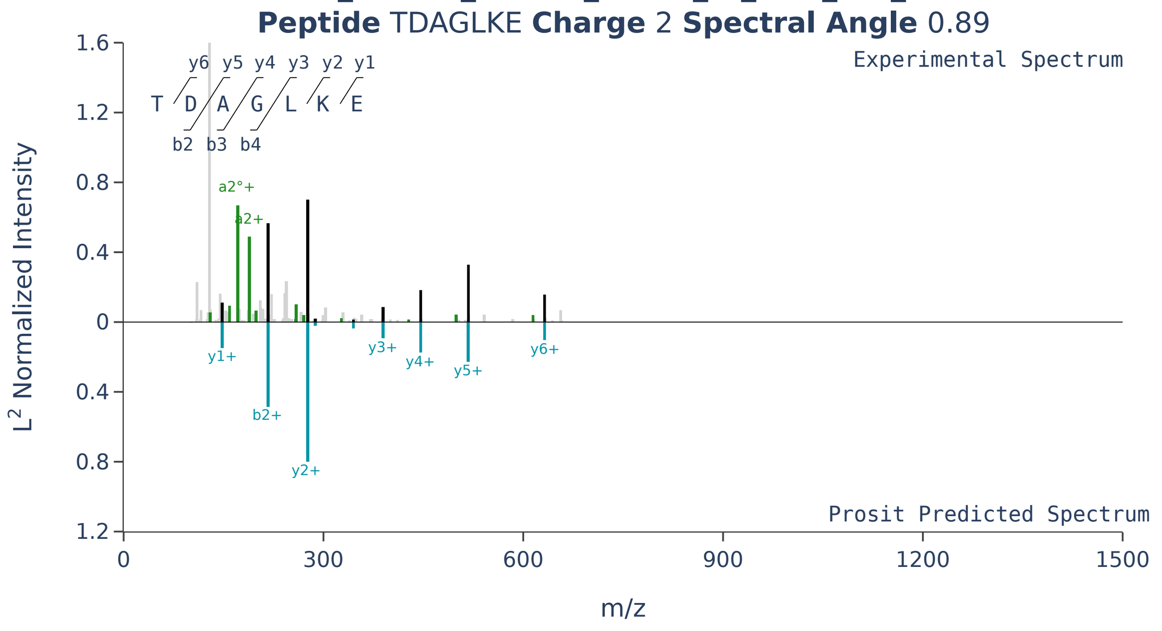


**Source** WSoh_221121_011221_HFGoe_L2_hTau_4h_R1 **Scan** 21429
**Peptide** NIKHVPGGGSVQIVYKPVDLSKVTS **Charge** 4 **Spectral Angle** 0.66

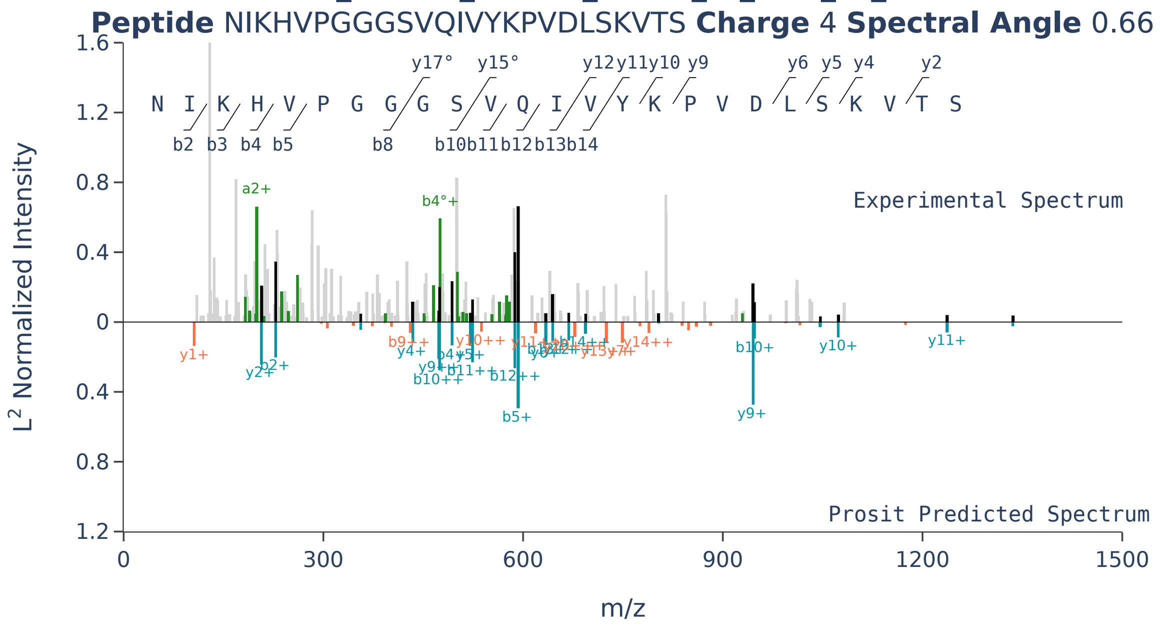


**Source** WSoh_221121_011221_HFGoe_L1_hTau_24h_R1 **Scan** 20748
**Peptide** PEGTTAEEAGIGDTPSLEDEAAGHVTQAR **Charge** 3 **Spectral Angle** 0.58

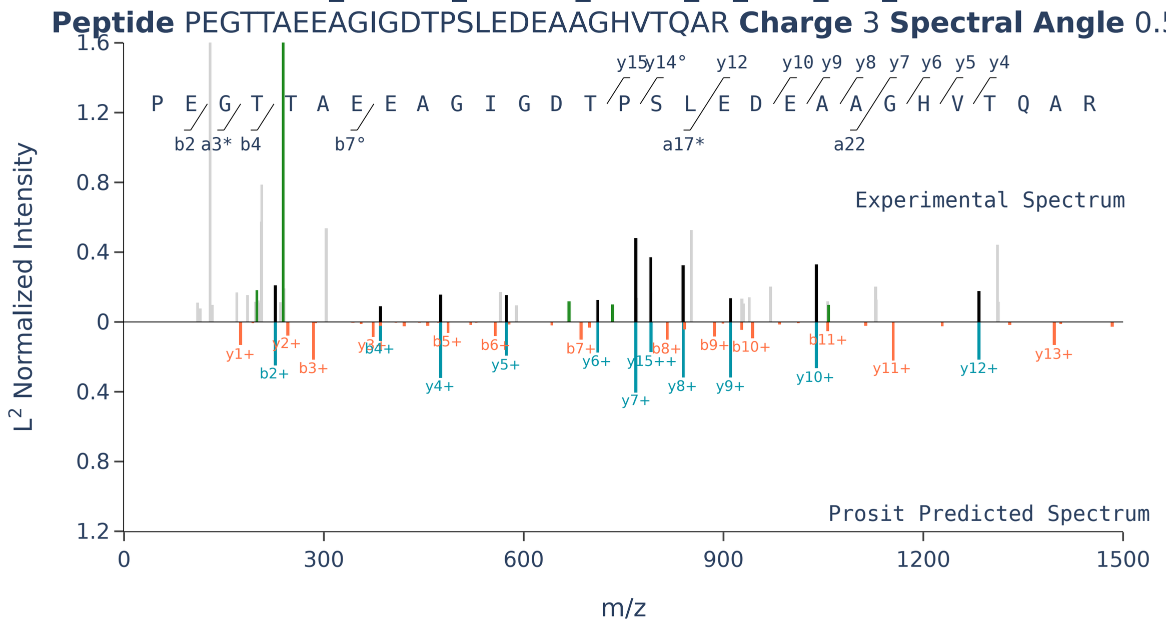


**Source** WSoh_221121_011221_HFGoe_L2_hTau_24h_R1 **Scan** 23699
**Peptide** AAQPHTEIPEGTTAEEAGIGDTPSLEDEA **Charge** 3 **Spectral Angle** 0.75

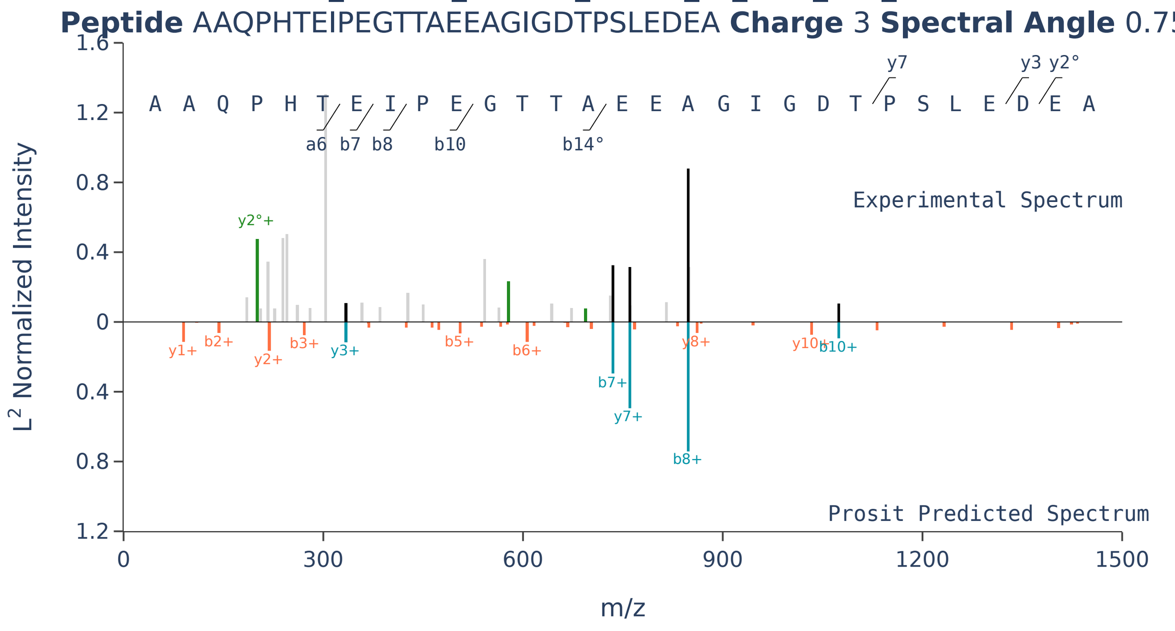


**Source** WSoh_221121_011221_HFGoe_L2_hTau_2h_R2 **Scan** 13669
**Peptide** QPGGGKVQIIN **Charge** 2 **Spectral Angle** 0.84

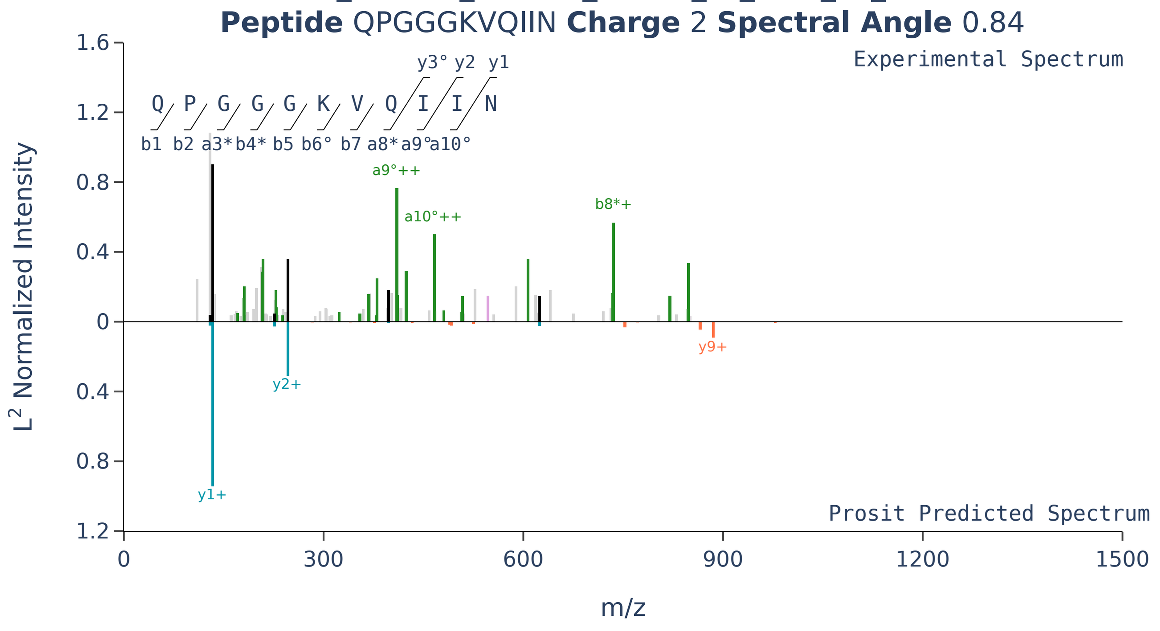

**Source** WSoh_221121_011221_HFGoe_L2_hTau_24h_R1 **Scan** 25423
**Peptide** SIDMVDSPQLAT **Charge** 2 **Spectral Angle** 0.69

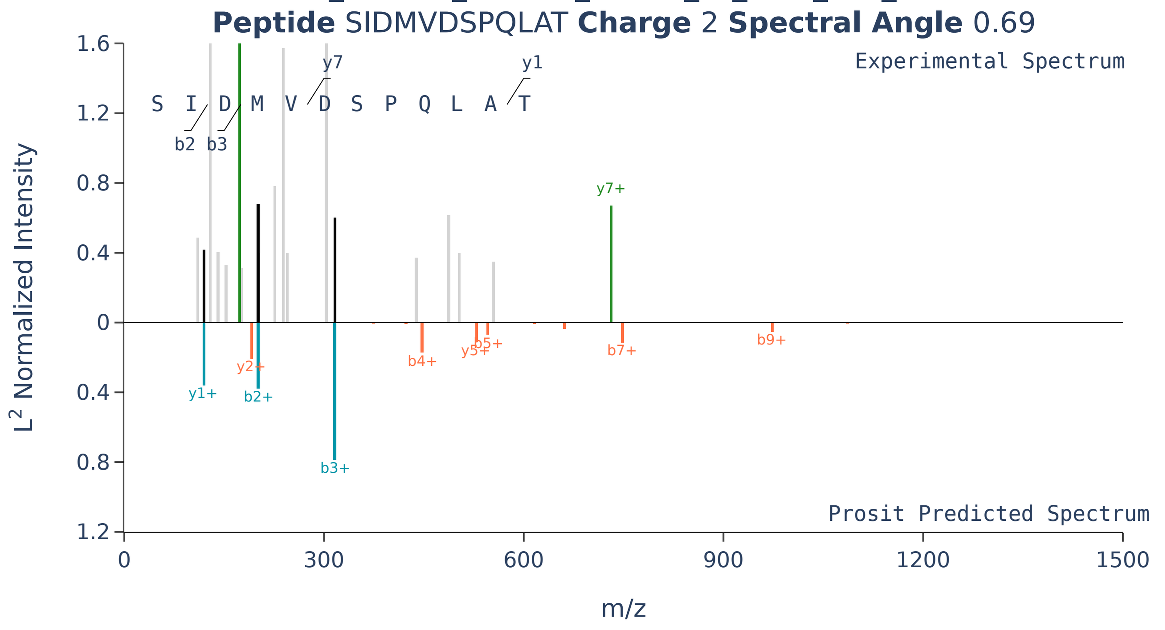


**Source** WSoh_221121_011221_HFGoe_L1_hTau_24h_R1 **Scan** 25048
**Peptide** STPTAEDVTAPLVD **Charge** 2 **Spectral Angle** 0.84

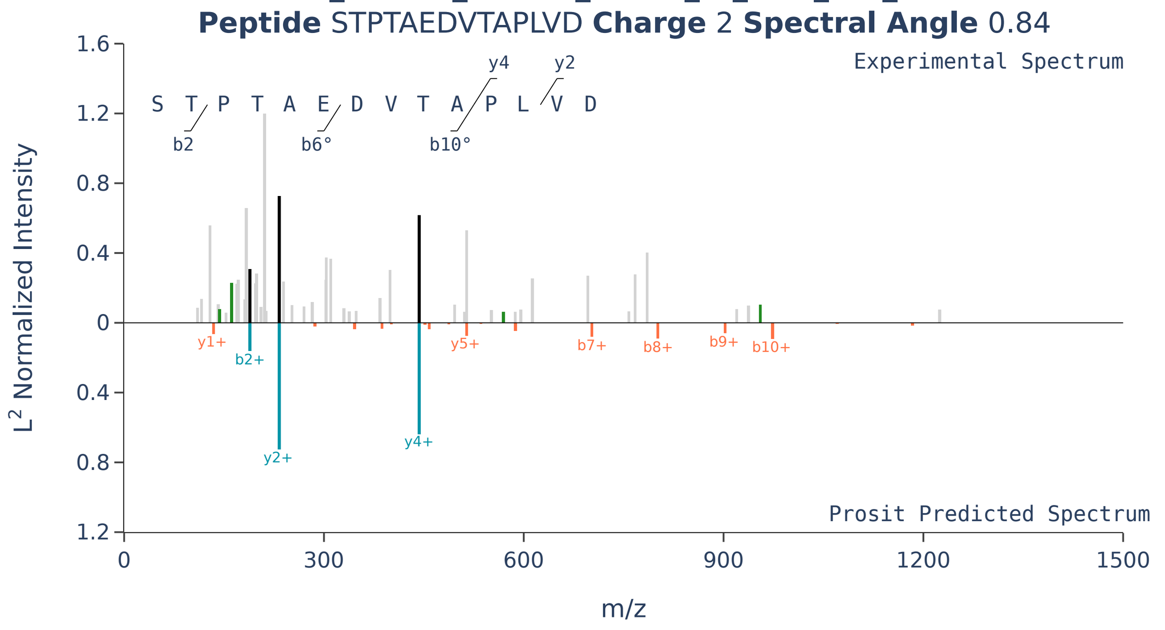


**Source** WSoh_221121_011221_HFGoe_L2_hTau_24h_R1 **Scan** 12453
**Peptide** GNIHHKPGGGQVEVKSEKLDFKDRVQSKIG **Charge** 4 **Spectral Angle** 0.58

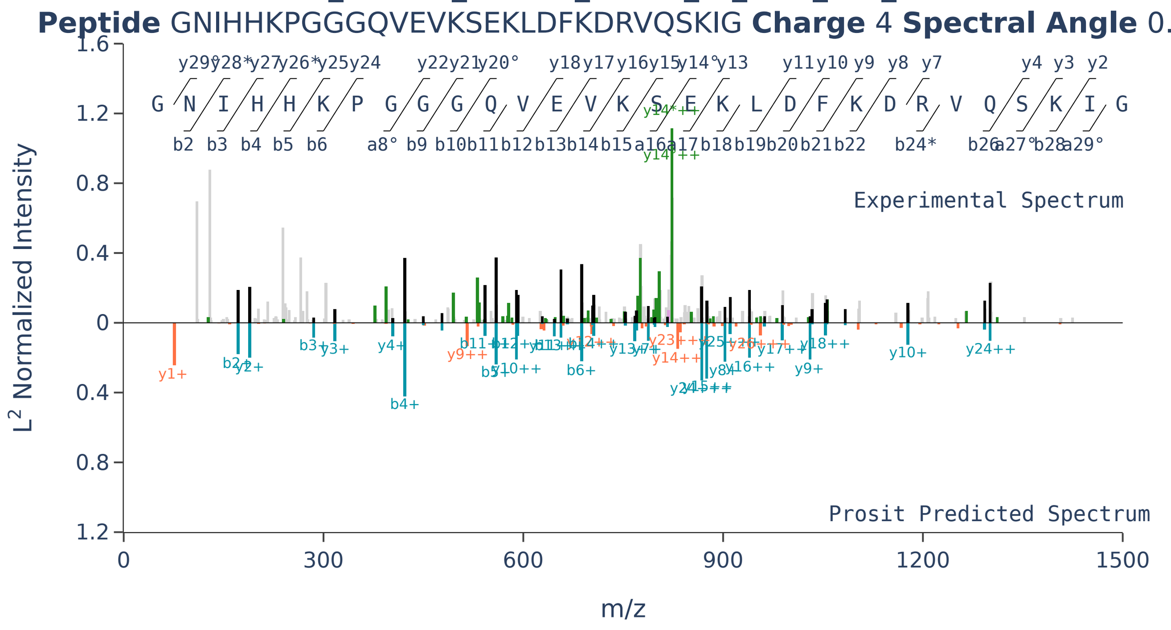


**Source** WSoh_221121_011221_HFGoe_L1_hTau_4h_R2 **Scan** 9923
**Peptide** EVKSEKL **Charge** 2 **Spectral Angle** 0.77

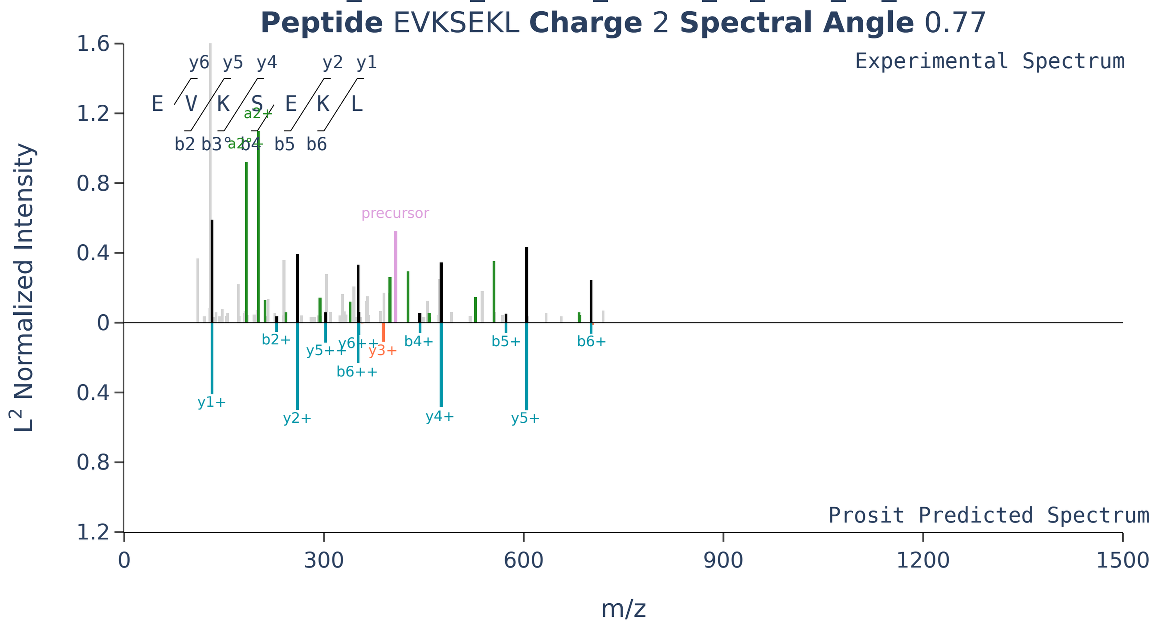


**Source** WSoh_221121_011221_HFGoe_L2_hTau_24h_R1 **Scan** 12265
**Peptide** KLGSTENLKHQPGGGKVQIIN **Charge** 4 **Spectral Angle** 0.69

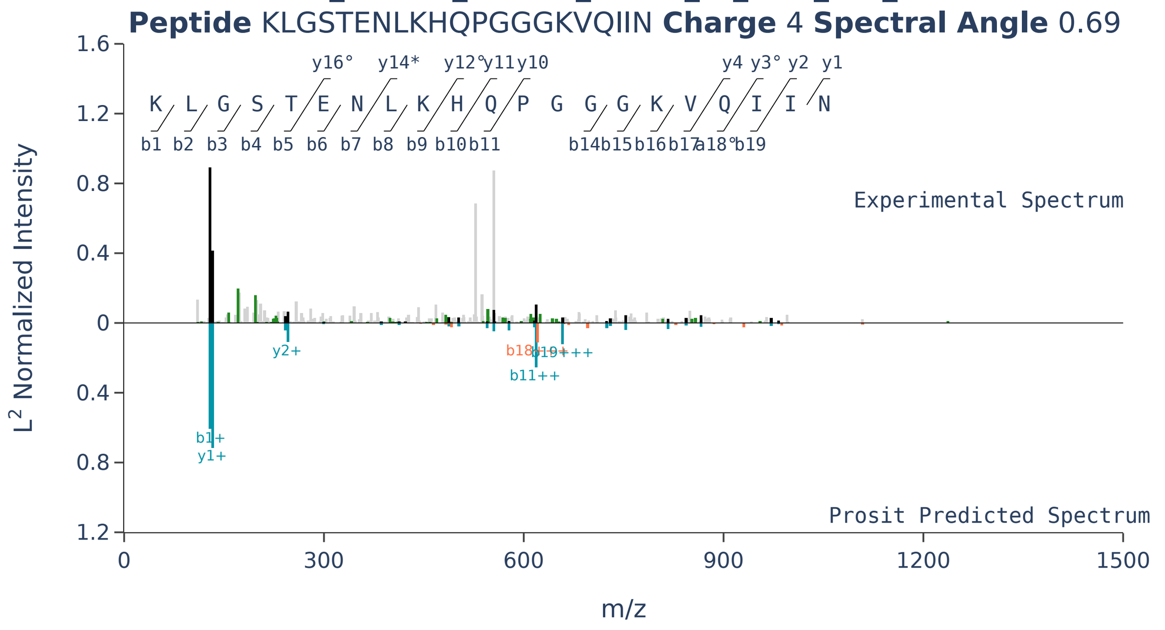


**Source** WSoh_221121_011221_HFGoe_L2_hTau_24h_R1 **Scan** 5334
**Peptide** RMVSKSKDGTGSDDKKAKGADGKTKLAT **Charge** 6 **Spectral Angle** 0.63

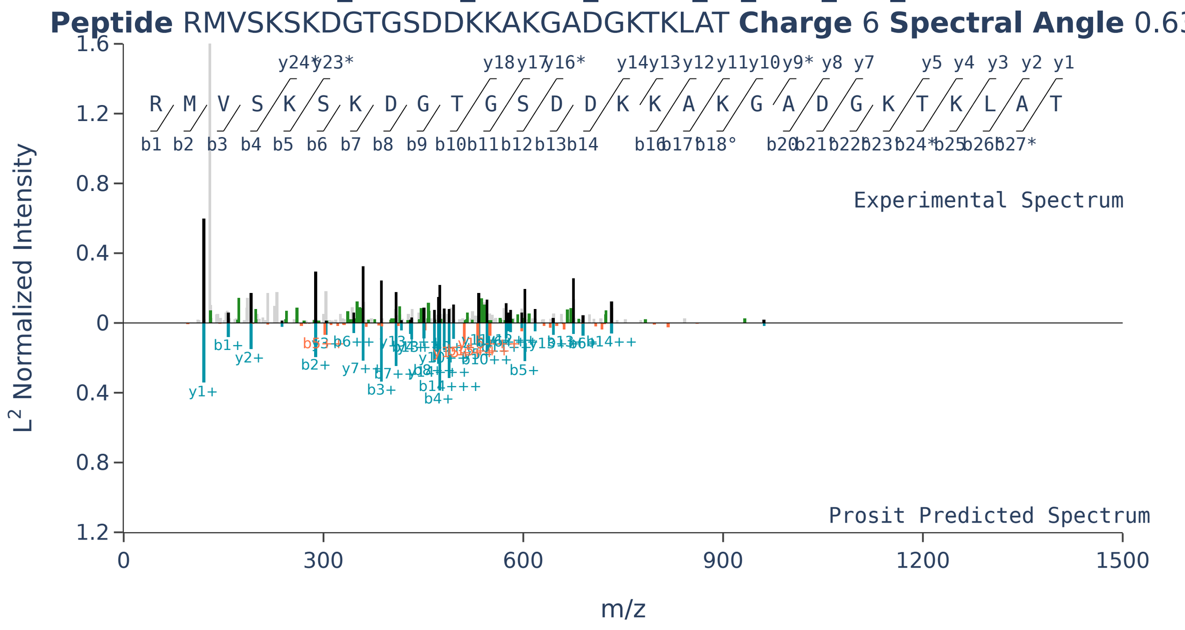


**Source** WSoh_221121_011221_HFGoe_L1_hTau_4h_R1 **Scan** 7683
**Peptide** RSRTPSL **Charge** 2 **Spectral Angle** 0.7

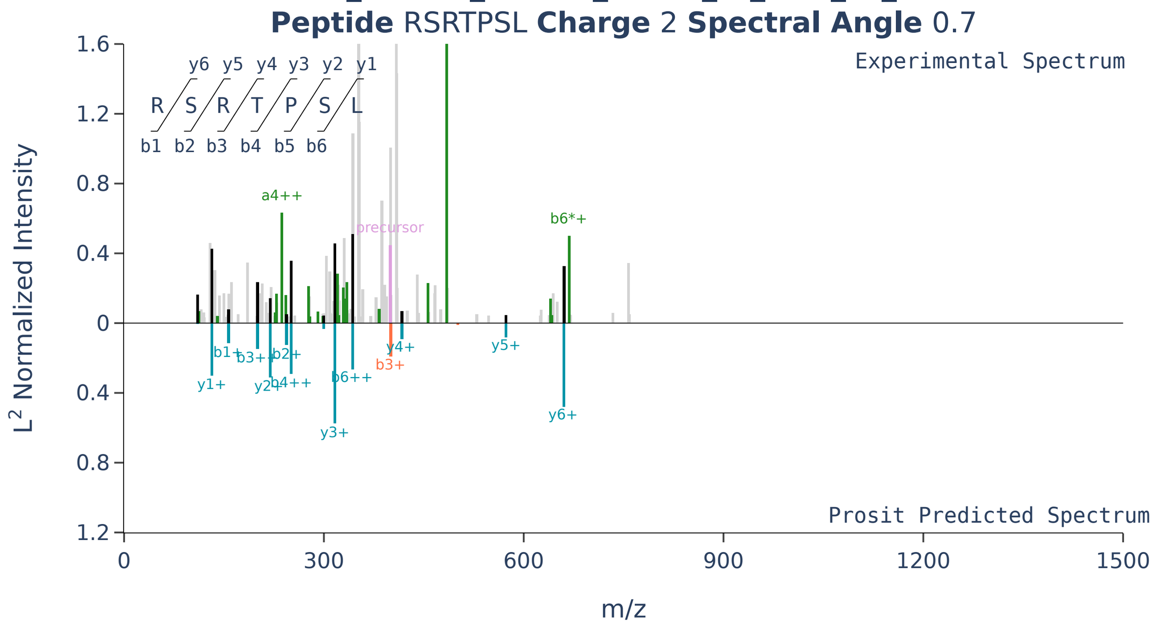


**Source** WSoh_221121_011221_HFGoe_L1_hTau_4h_R1 **Scan** 19366
**Peptide** EFEVMEDHAGTYGLGDRKDQGGYTMHQD **Charge** 4 **Spectral Angle** 0.67

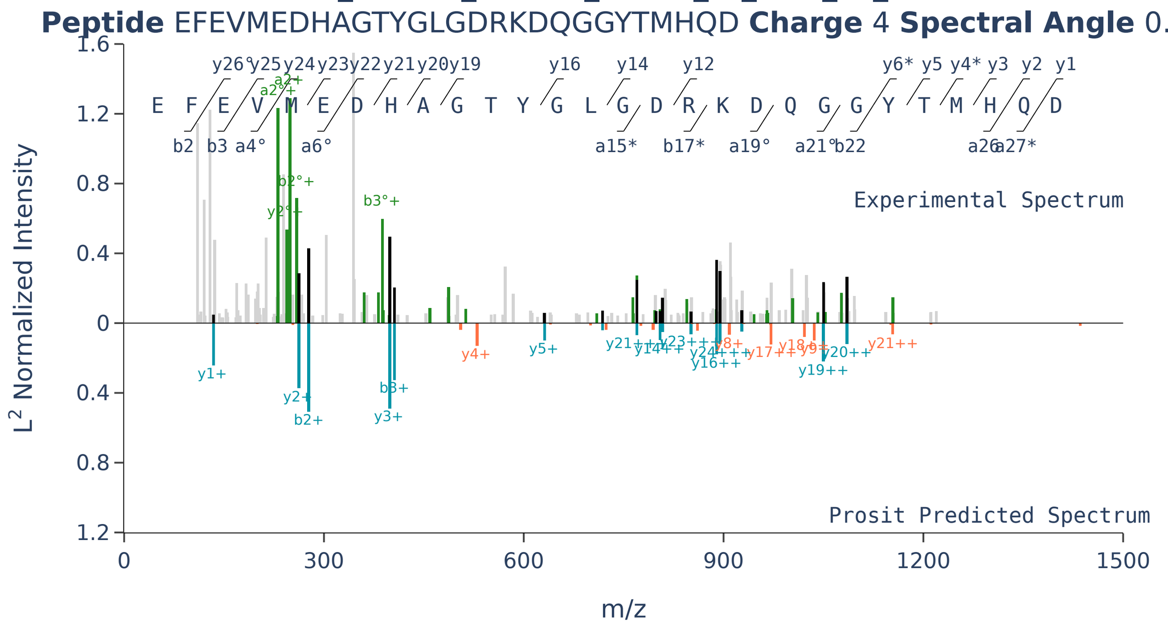


**Source** WSoh_221121_011221_HFGoe_L1_hTau_4h_R1 **Scan** 19601
**Peptide** VTAPLVDEGAPGKQAAAQPHTEIPEGTTAE **Charge** 3 **Spectral Angle** 0.59

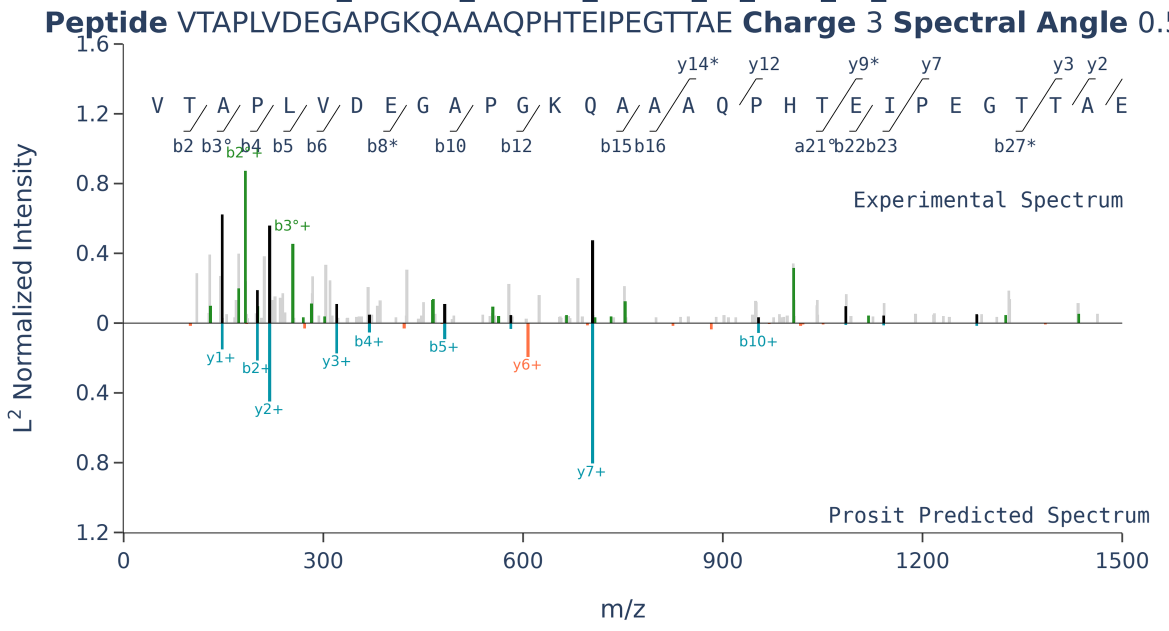


**Source** WSoh_221121_011221_HFGoe_L1_hTau_24h_R2 **Scan** 6885
**Peptide** GKTKIATPRGAAPPGQKGQ **Charge** 3 **Spectral Angle** 0.58

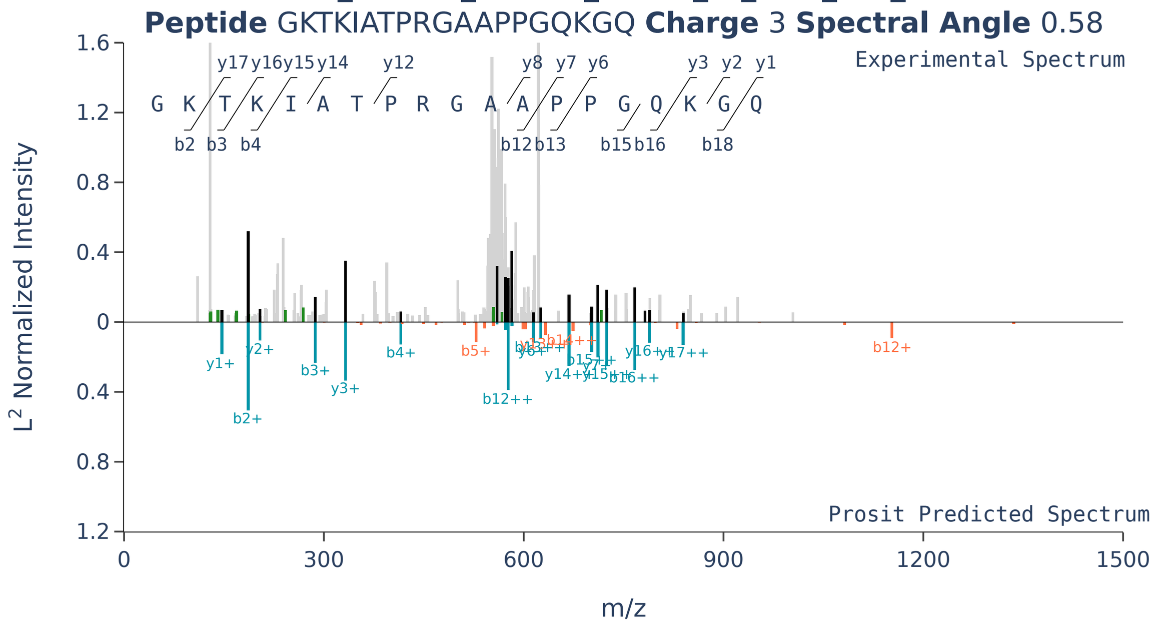


**Source** WSoh_221121_011221_HFGoe_L1_hTau_24h_R1 **Scan** 15943
**Peptide** QVEVKSEKLDFKDRVQSKI **Charge** 5 **Spectral Angle** 0.56

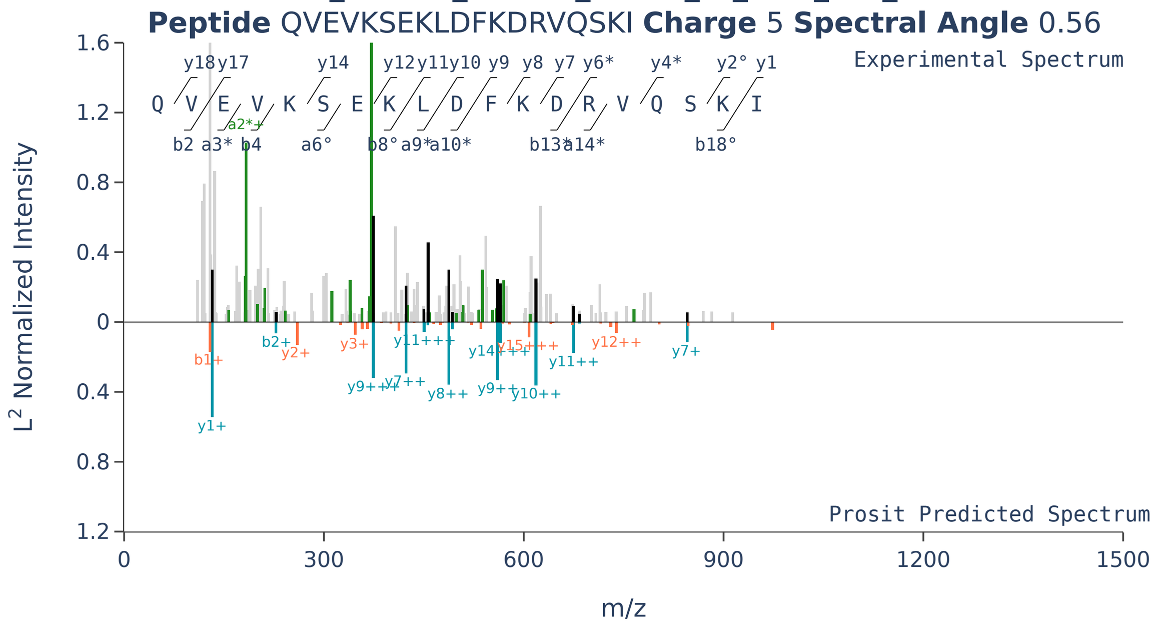


**Source** WSoh_221121_011221_HFGoe_L2_hTau_1h_R1 **Scan** 6716
**Peptide** KSPVVSGDTS **Charge** 2 **Spectral Angle** 0.59

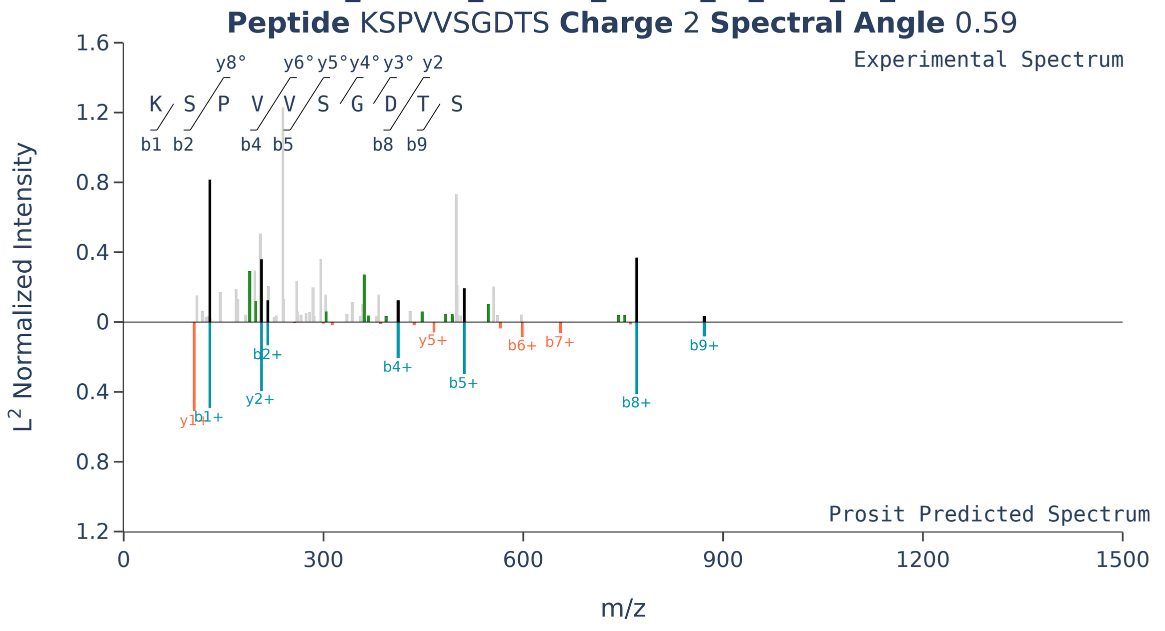


**Source** WSoh_221121_011221_HFGoe_L2_hTau_4h_R1 **Scan** 18063
**Peptide** KPGGGQVEVKSEKLDFKDRVQSKIGSL **Charge** 4 **Spectral Angle** 0.72

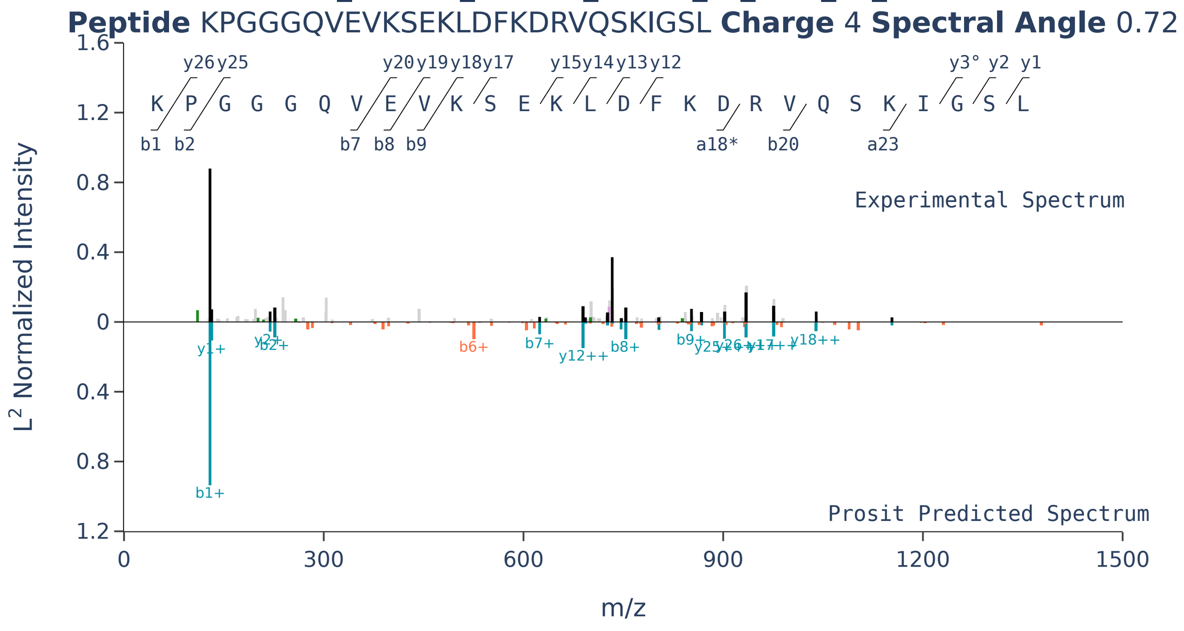


**Source** WSoh_221121_011221_HFGoe_L1_hTau_24h_R2 **Scan** 12085
**Peptide** GSEEPGSETSDAKSTPTAEDVT **Charge** 2 **Spectral Angle** 0.68

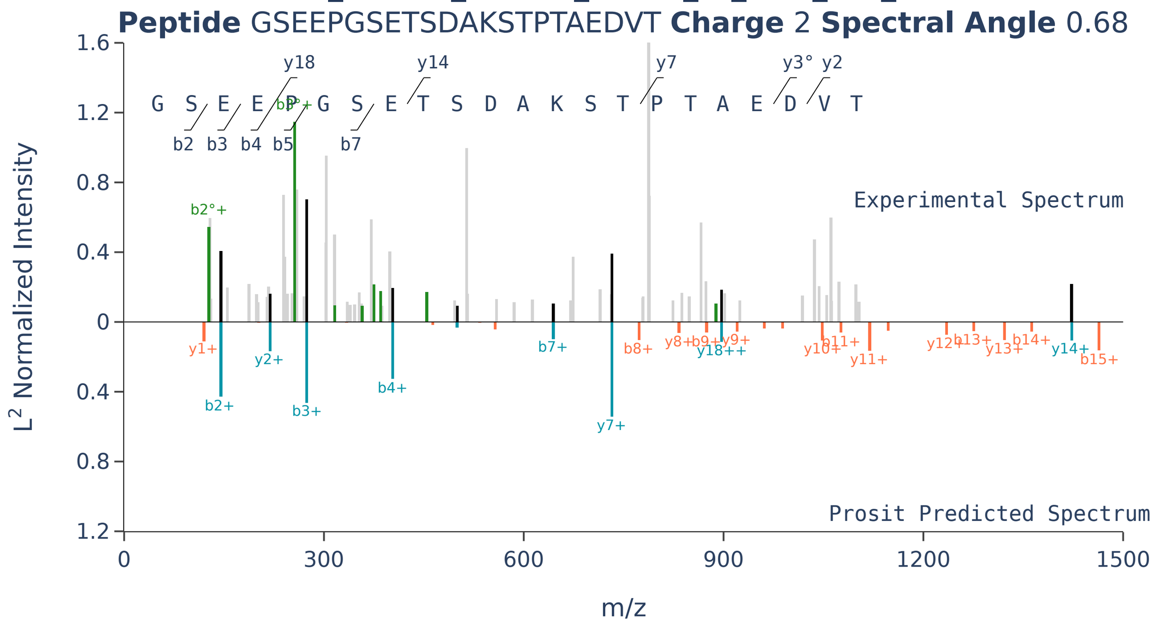


**Source** WSoh_221121_011221_HFGoe_L1_hTau_24h_R1 **Scan** 18115
**Peptide** APLVDEGAPGKQAAAQPHTEIPEGTTAE **Charge** 3 **Spectral Angle** 0.54

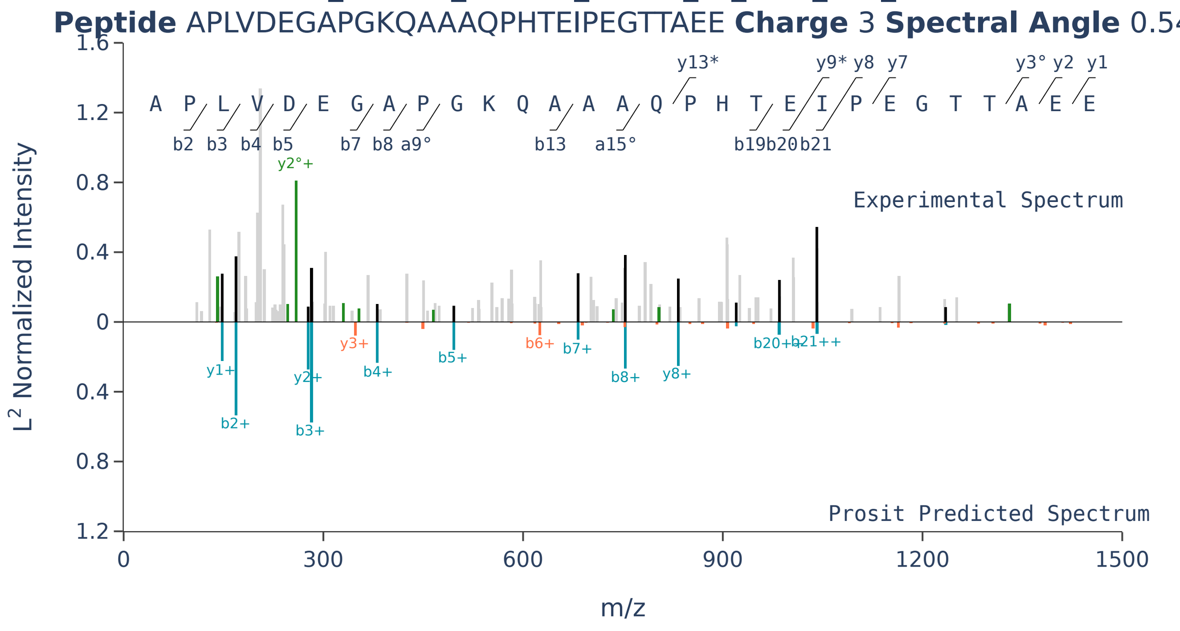

**Source** WSoh_221121_011221_HFGoe_L2_hTau_24h_R1 **Scan** 7374
**Peptide** PTPPTREPKKVAVVRTPPKSPSSAKSR **Charge** 5 **Spectral Angle** 0.64

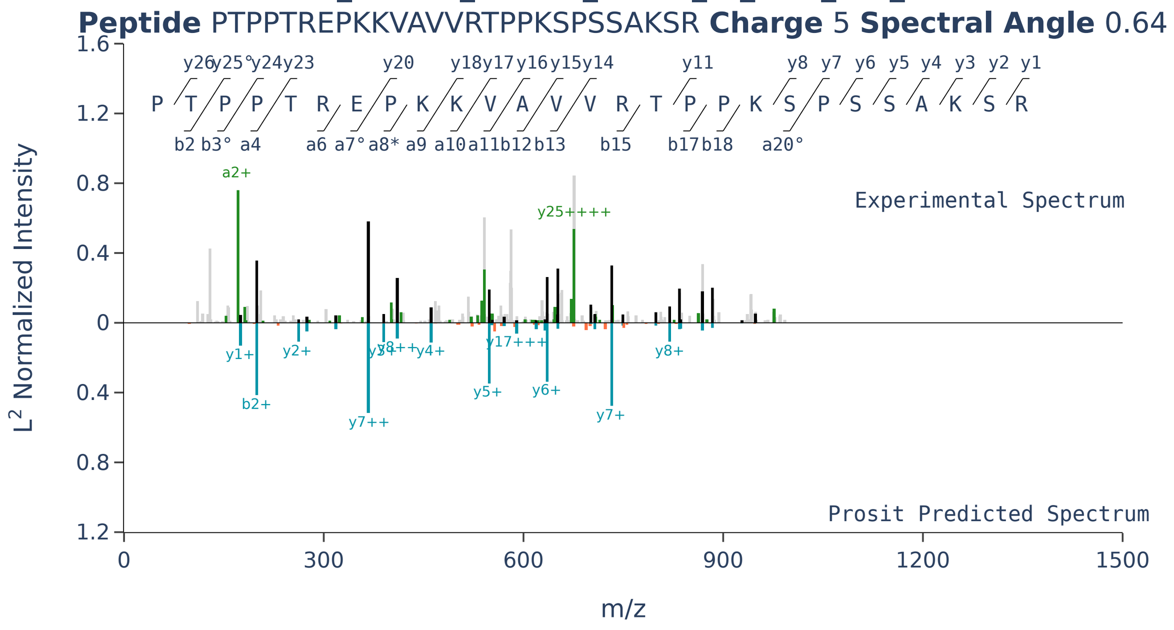


**Source** WSoh_221121_011221_HFGoe_L1_hTau_4h_R2 **Scan** 18280
**Peptide** QPGGGKVQIINKKLDLSNVQS **Charge** 3 **Spectral Angle** 0.81

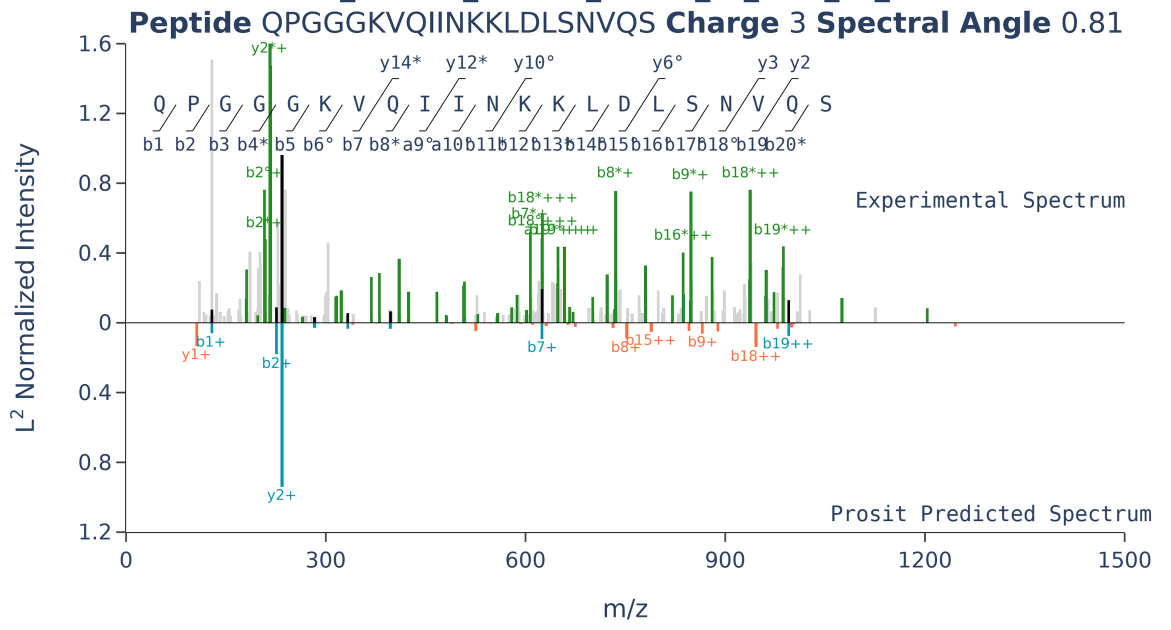


**Source** WSoh_221121_011221_HFGoe_L2_hTau_24h_R2 **Scan** 19133
**Peptide** KSRLQTAPVPMPDLKNVKSKIGSTENLKHQ **Charge** 6 **Spectral Angle** 0.67

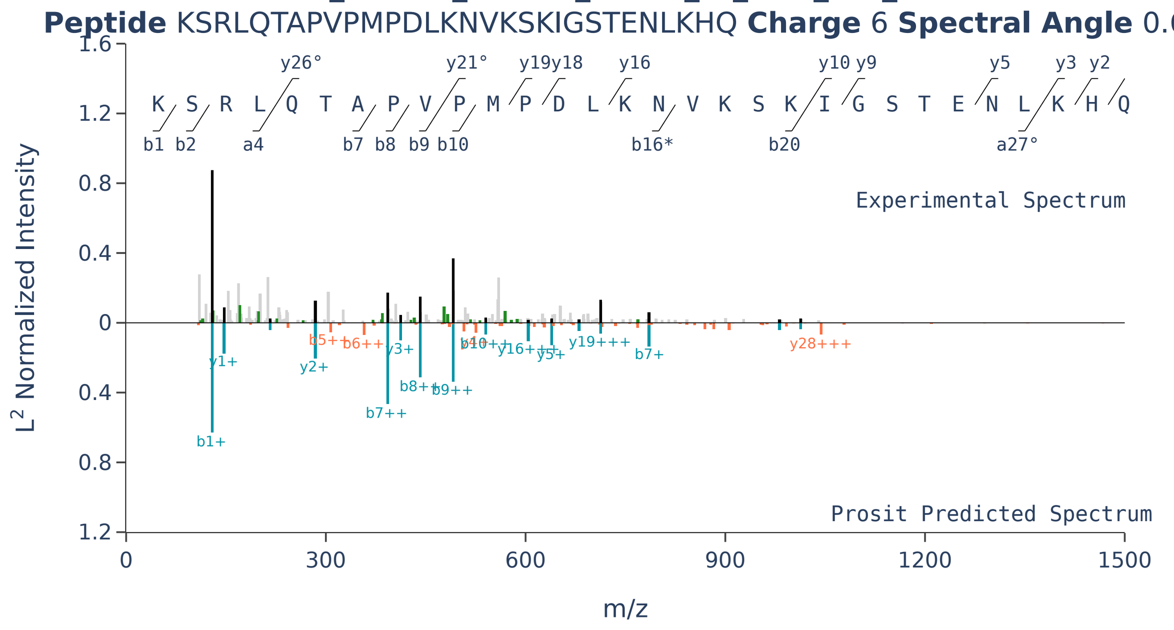


**Source** WSoh_221121_011221_HFGoe_L1_hTau_24h_R2 **Scan** 22009
**Peptide** EDVTAPLVDEGAPGKQAAAQPHTEIPEGT **Charge** 3 **Spectral Angle** 0.82

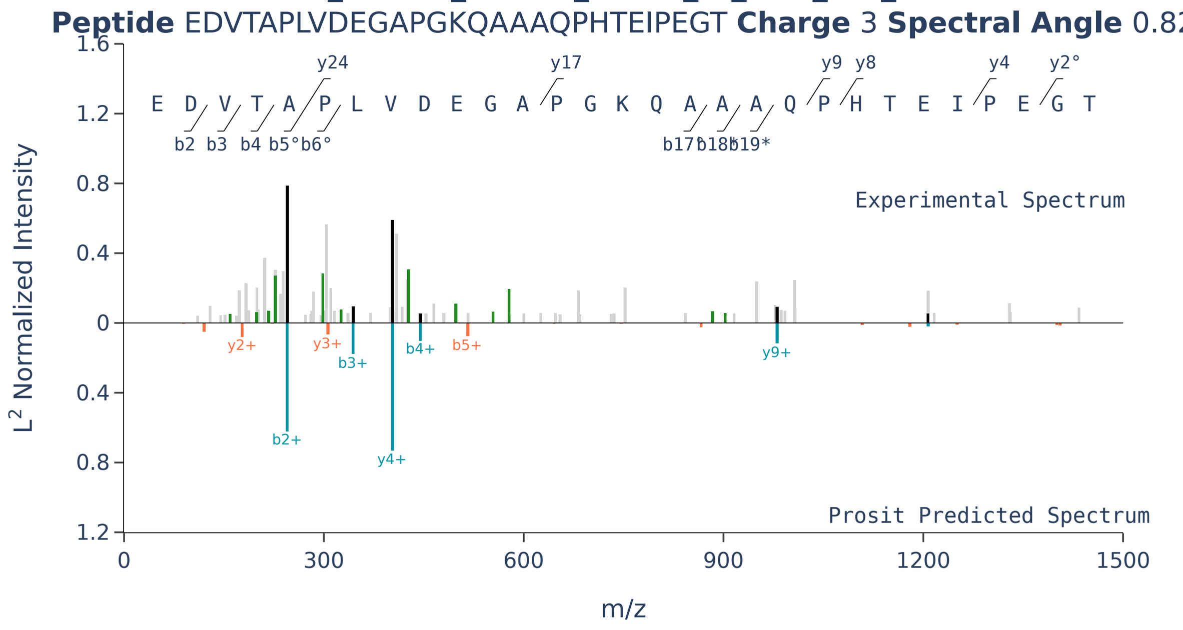


**Source** WSoh_221121_011221_HFGoe_L2_hTau_4h_R1 **Scan** 6812
**Peptide** KTKIATPRGAAPPGQKGQANAT **Charge** 3 **Spectral Angle** 0.82

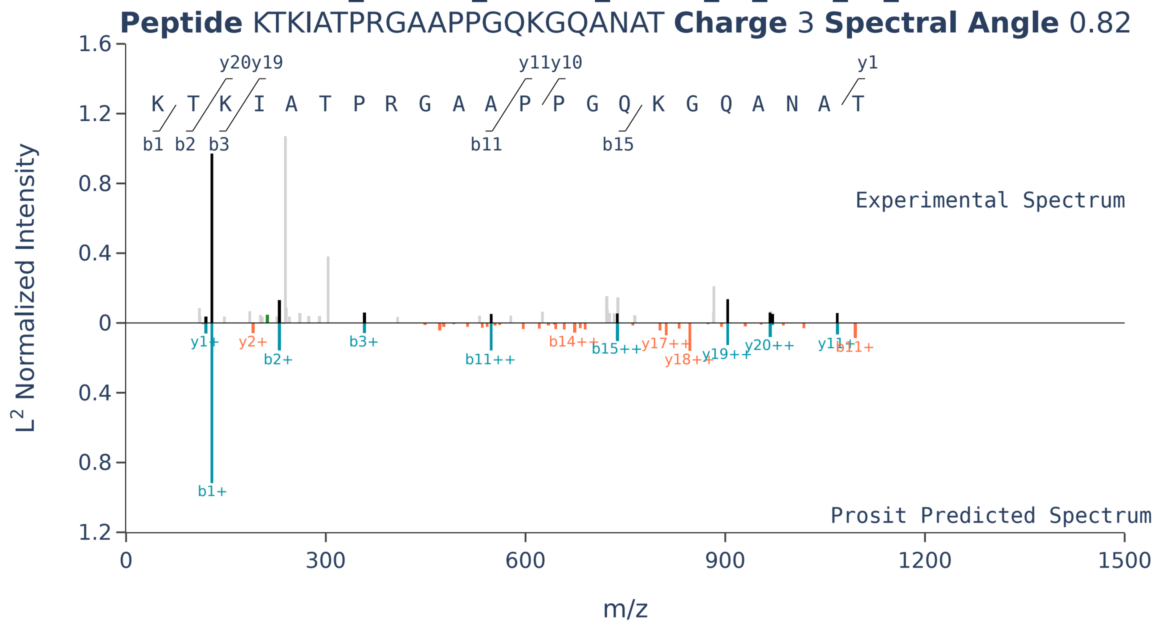


**Source** WSoh_221121_011221_HFGoe_L2_hTau_4h_R1 **Scan** 16342
**Peptide** DHGAEIVYKSPVVSGDTSPRHLS **Charge** 4 **Spectral Angle** 0.6

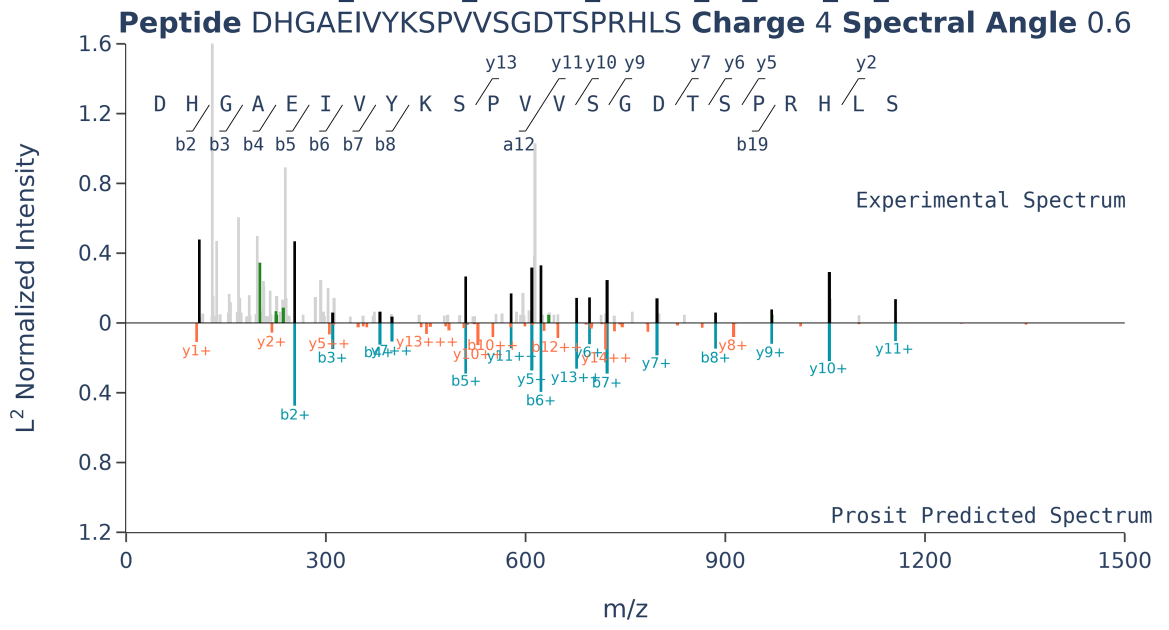


**Source** WSoh_221121_011221_HFGoe_L1_hTau_24h_R2 **Scan** 17161
**Peptide** PDLKNVKSK **Charge** 2 **Spectral Angle** 0.87

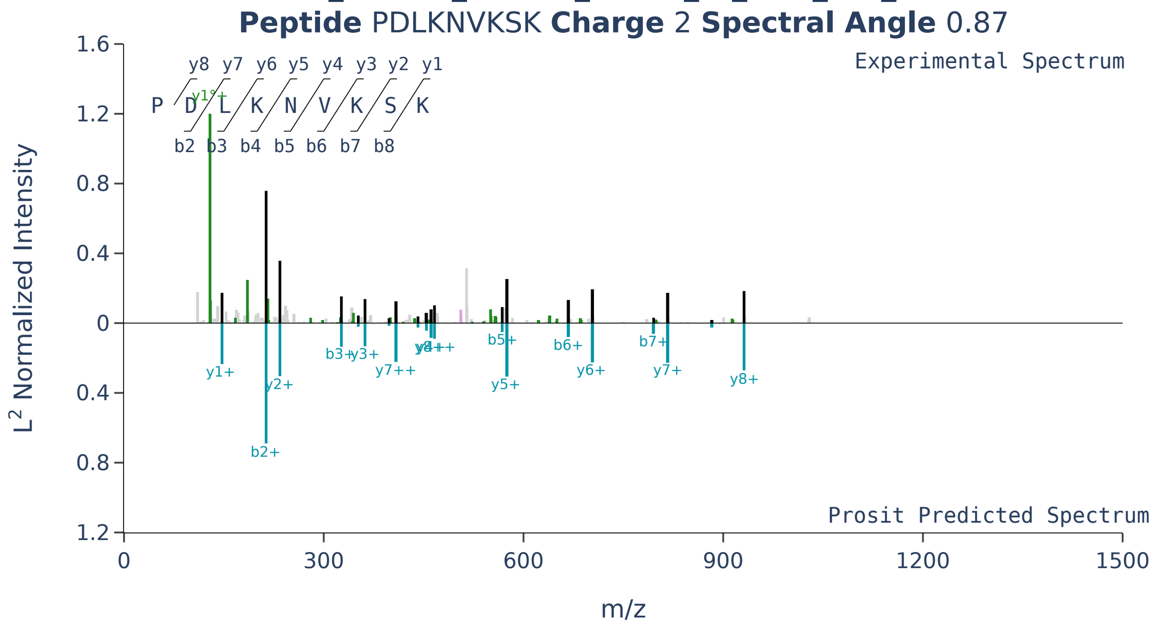


**Source** WSoh_221121_011221_HFGoe_L2_hTau_4h_R2 **Scan** 21324
**Peptide** EDVTAPLVDEGAPGKQAAAQPHTEIPEG **Charge** 3 **Spectral Angle** 0.87

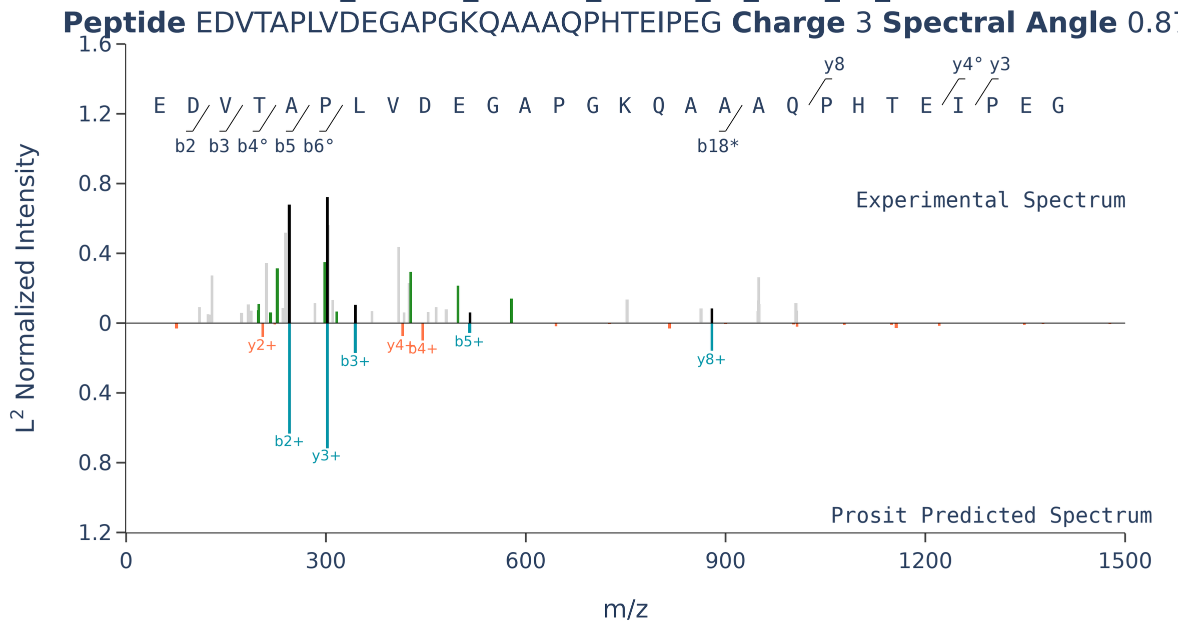


**Source** WSoh_221121_011221_HFGoe_L1_hTau_24h_R1 **Scan** 24611
**Peptide** IPEGTTAEEAGIGDTPSLE **Charge** 2 **Spectral Angle** 0.72

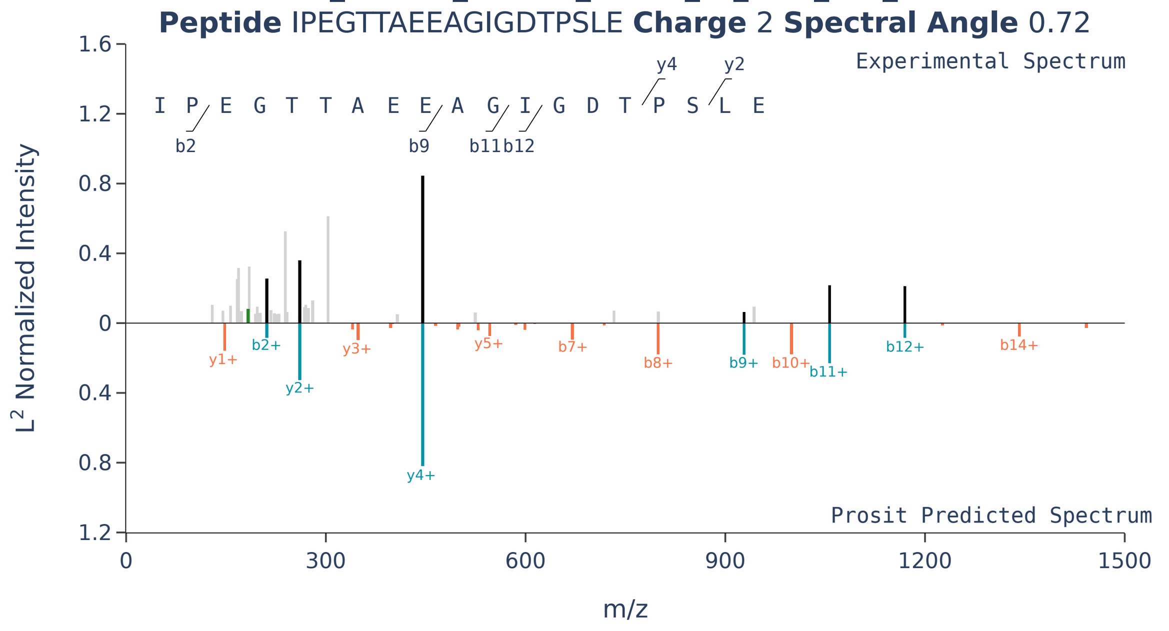


**Source** WSoh_221121_011221_HFGoe_L2_hTau_24h_R1 **Scan** 21378
**Peptide** AGIGDTPSLEDEAAGHVT **Charge** 2 **Spectral Angle** 0.63

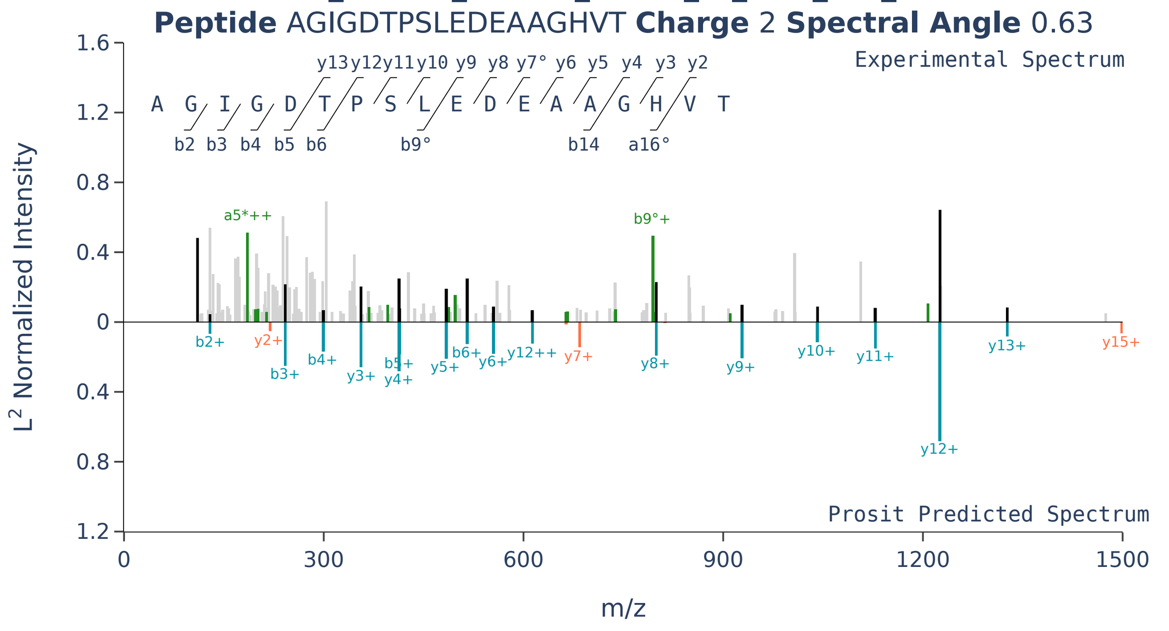


**Source** WSoh_221121_011221_HFGoe_L2_hTau_24h_R1 **Scan** 24219
**Peptide** IPEGTTAEEAGIGDTPSLED **Charge** 2 **Spectral Angle** 0.69

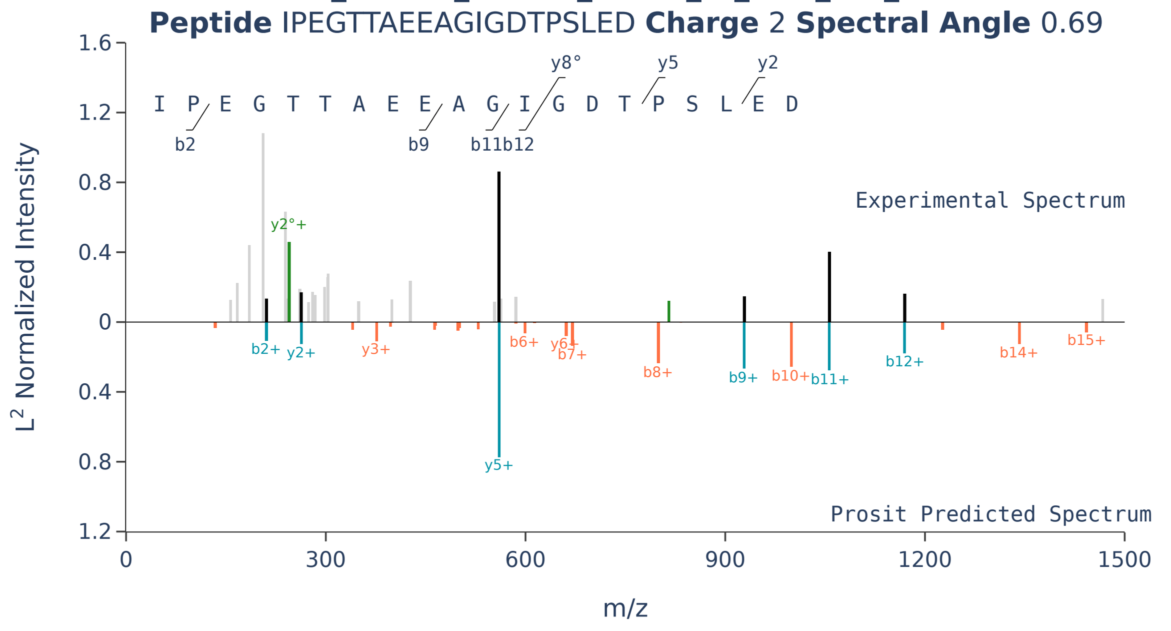


**Source** WSoh_221121_011221_HFGoe_L2_hTau_24h_R1 **Scan** 9279
**Peptide** TSKCGSLGNIHHKPGGGQVEVKSEKL **Charge** 4 **Spectral Angle** 0.6

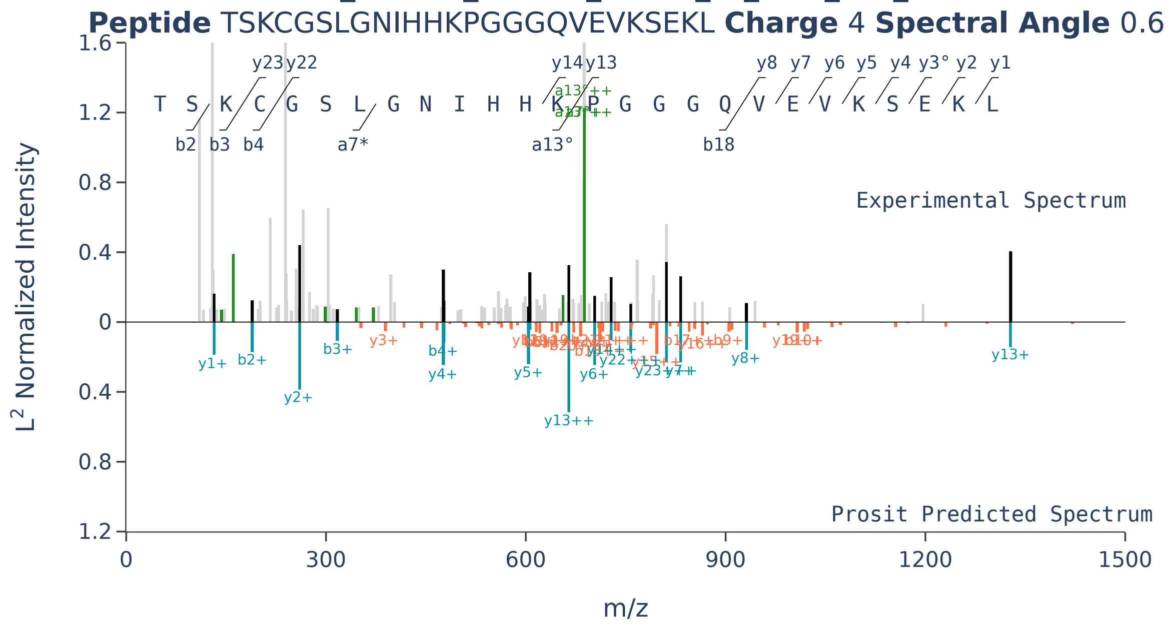


**Source** WSoh_221121_011221_HFGoe_L2_hTau_24h_R2 **Scan** 13064
**Peptide** NLHHKPGGGQVEVKSEKLDFKDRVQSKI **Charge** 5 **Spectral Angle** 0.56

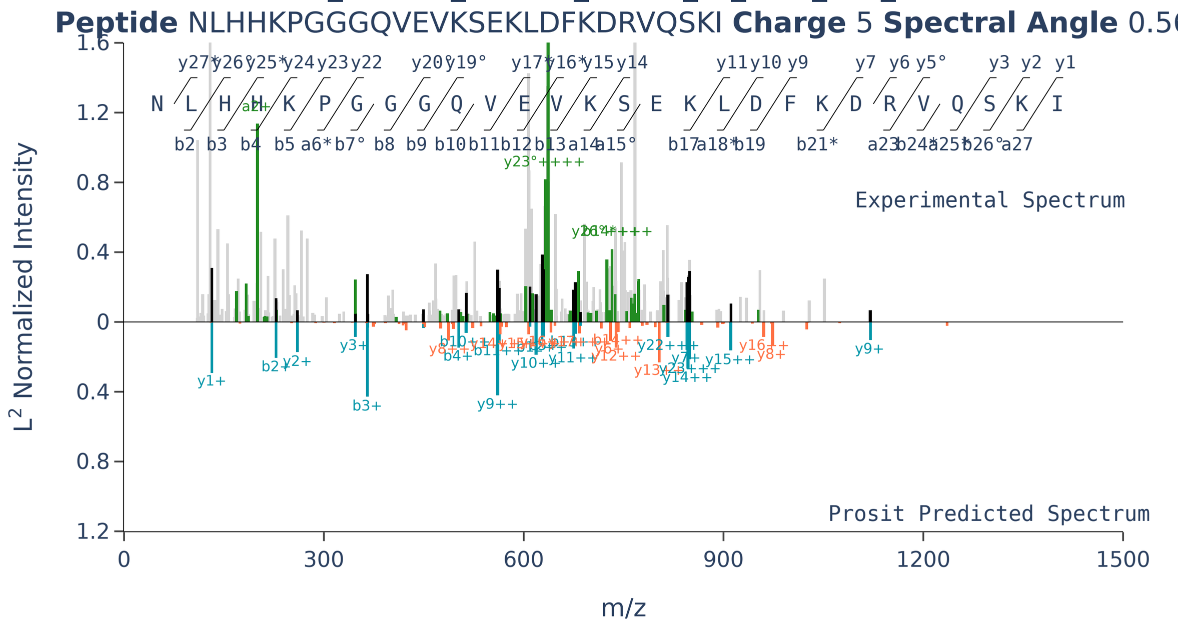


**Source** WSoh_221121_011221_HFGoe_L2_hTau_2h_R2 **Scan** 21708
**Peptide** QTAPVPMPDLKNVKSKL **Charge** 3 **Spectral Angle** 0.74

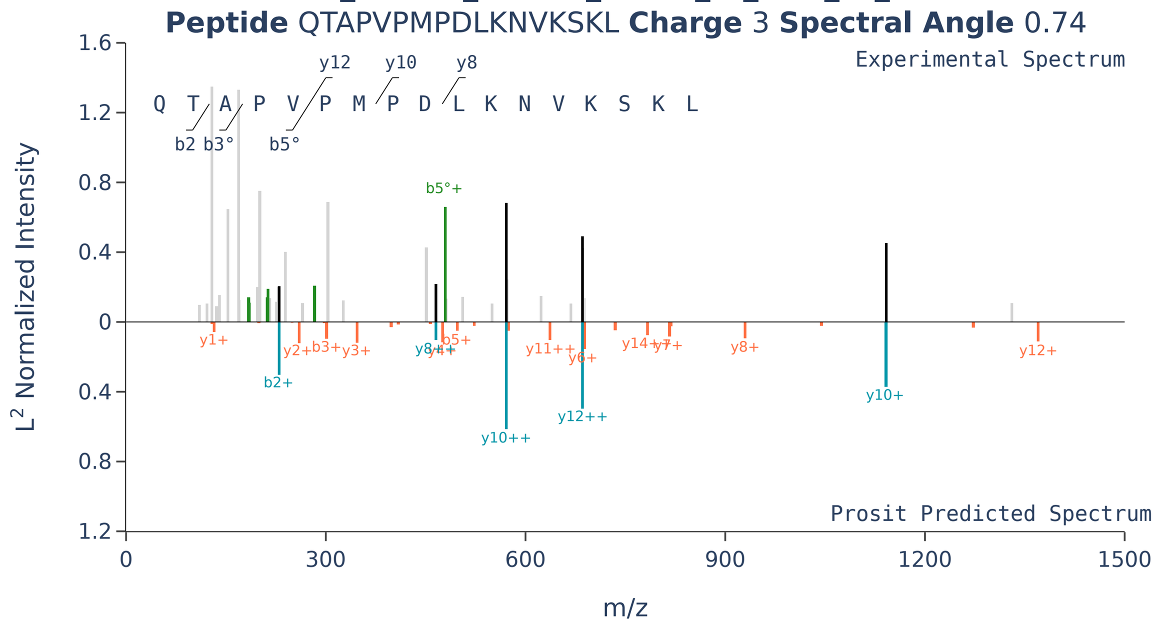


**Source** WSoh_221121_011221_HFGoe_L1_hTau_4h_R1 **Scan** 23788
**Peptide** GDTSPRHLSNVSSTGSIDMVDSPQLATL **Charge** 3 **Spectral Angle** 0.7

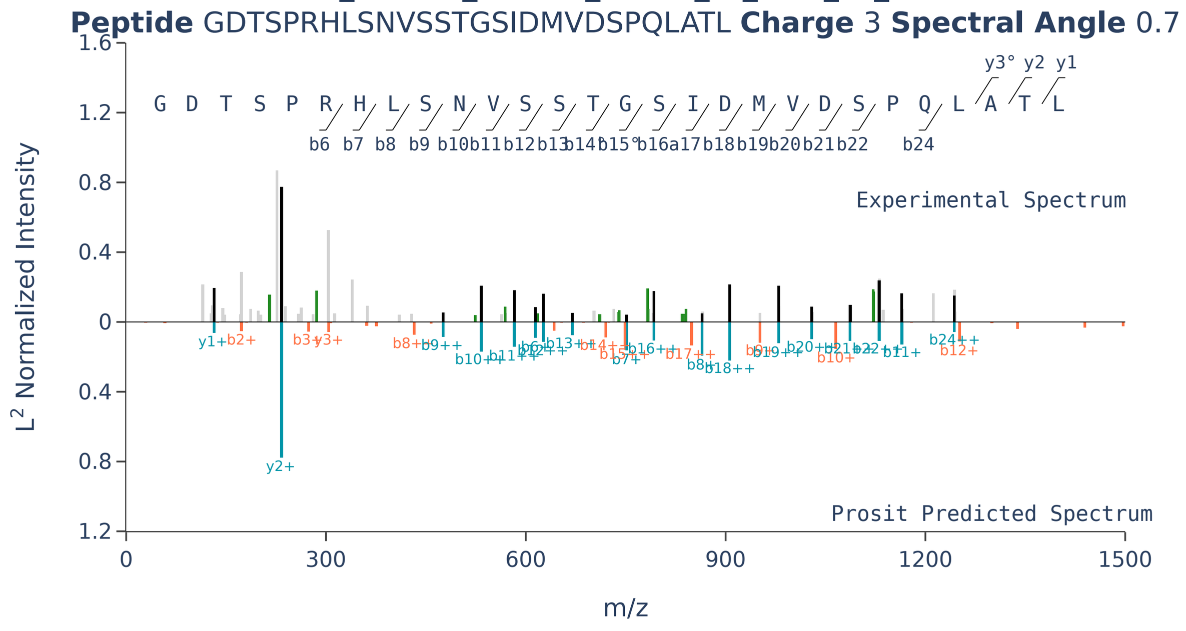

**Source** WSoh_221121_011221_HFGoe_L1_hTau_4h_R1 **Scan** 14040
**Peptide** KESPLQTPTEDGSEEPGSETSD **Charge** 2 **Spectral Angle** 0.79

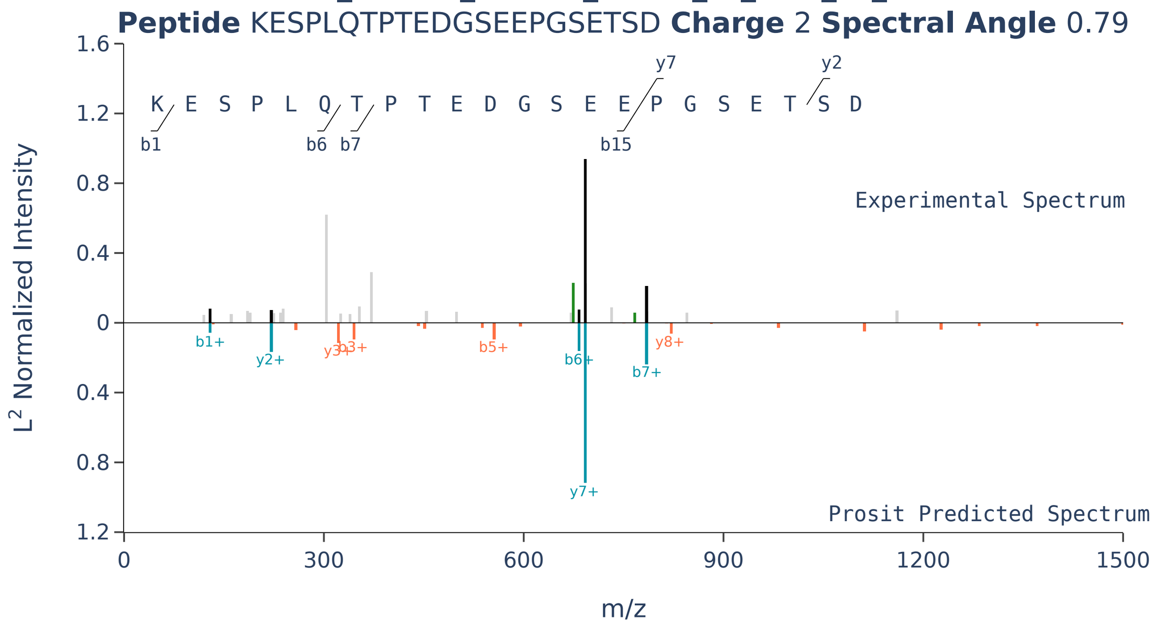


**Source** WSoh_221121_011221_HFGoe_L1_hTau_4h_R1 **Scan** 6722
**Peptide** GTGSDDKKAKGADGKTKIATPRGAAPPGQ **Charge** 4 **Spectral Angle** 0.73

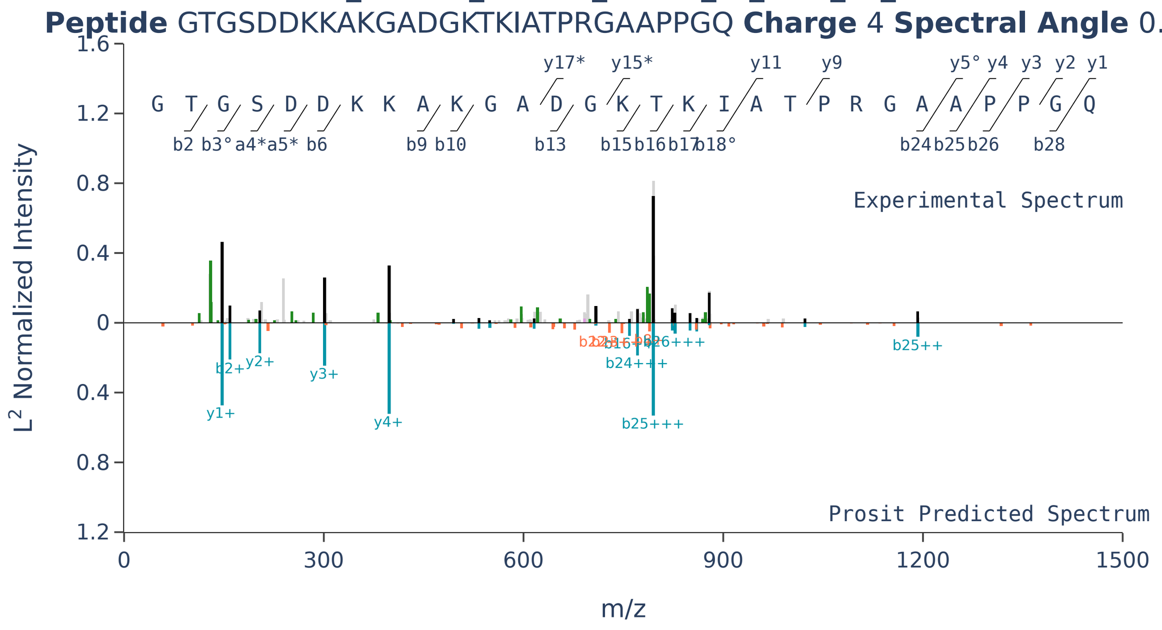


**Source** WSoh_221121_011221_HFGoe_L2_hTau_4h_R2 **Scan** 23655
**Peptide** FEVMEDHAGTYGLGD **Charge** 2 **Spectral Angle** 0.8

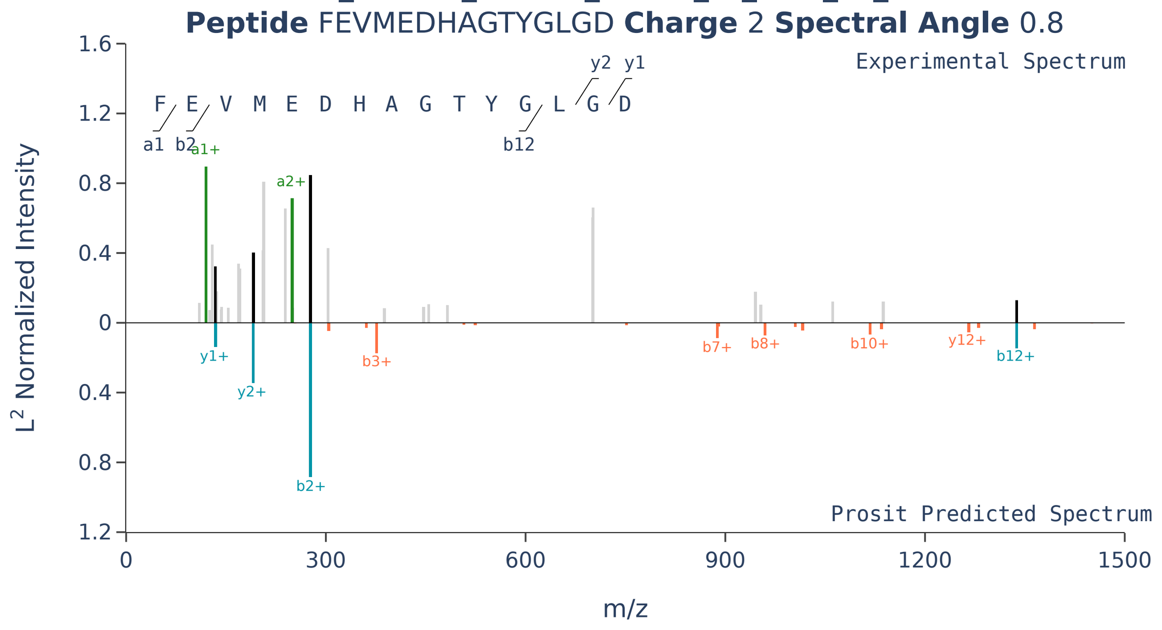


**Source** WSoh_221121_011221_HFGoe_L1_hTau_24h_R1 **Scan** 18742
**Peptide** GYTMHQDQEGDTDAGLKESPLQTPTE **Charge** 3 **Spectral Angle** 0.83

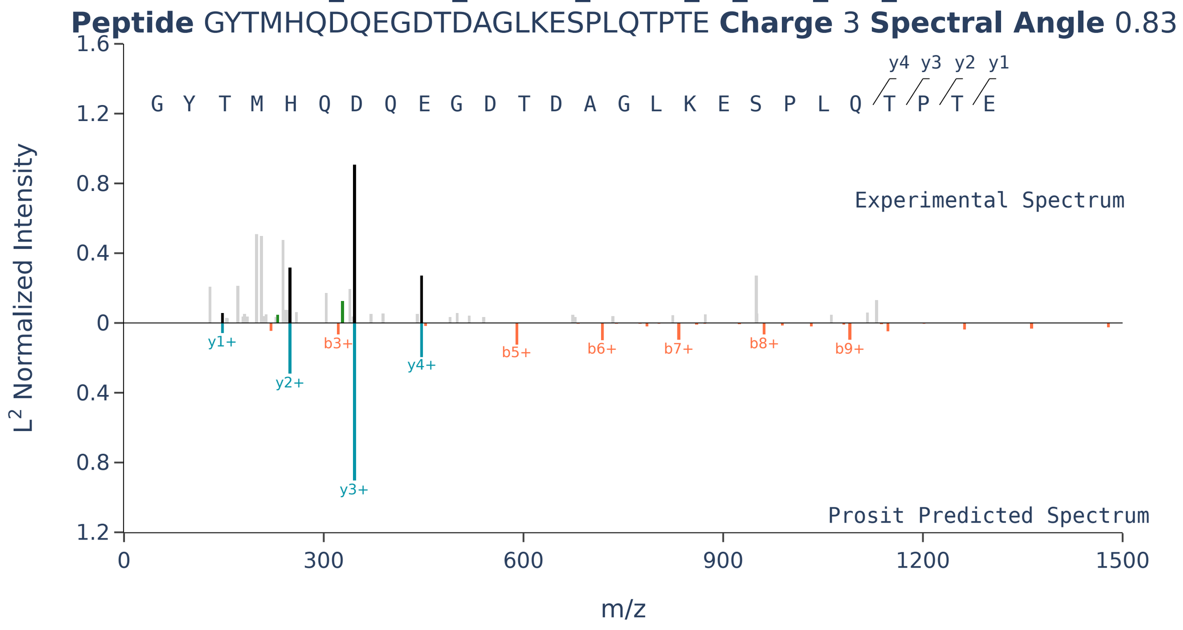


**Source** WSoh_221121_011221_HFGoe_L1_hTau_4h_R1 **Scan** 12316
**Peptide** SPGSPGTPGSRSRTPSLPTPPTREPKKVA **Charge** 6 **Spectral Angle** 0.67

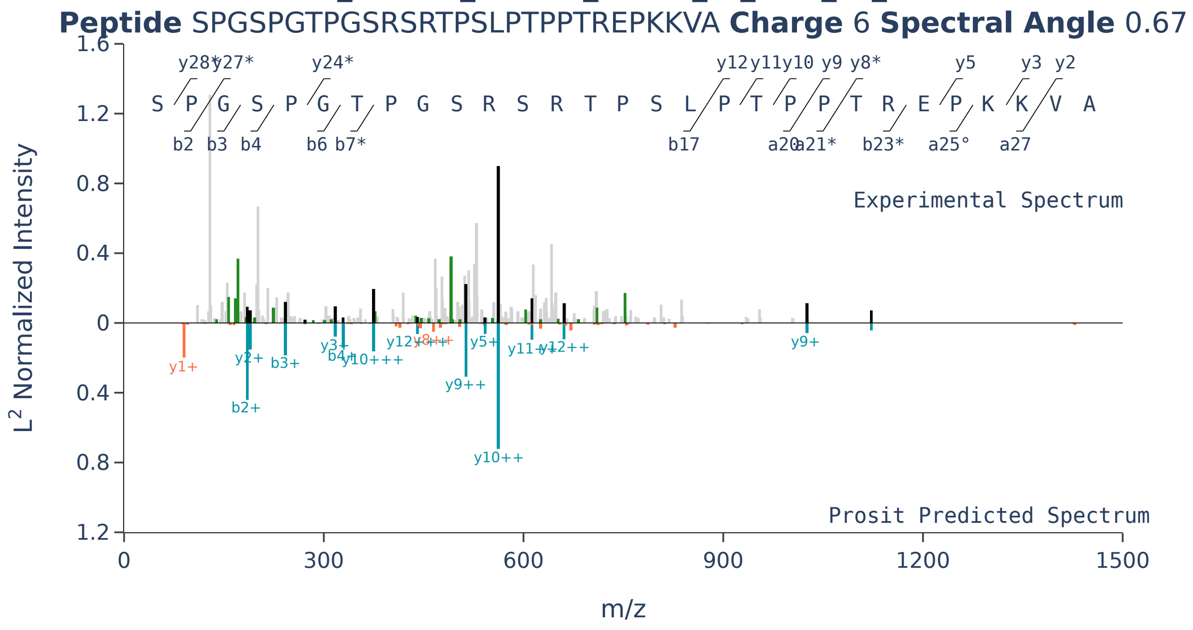


**Source** WSoh_221121_011221_HFGoe_L1_hTau_2h_R2 **Scan** 16335
**Peptide** QPGGGKVQIINKKLDLSNVQSKC **Charge** 4 **Spectral Angle** 0.74

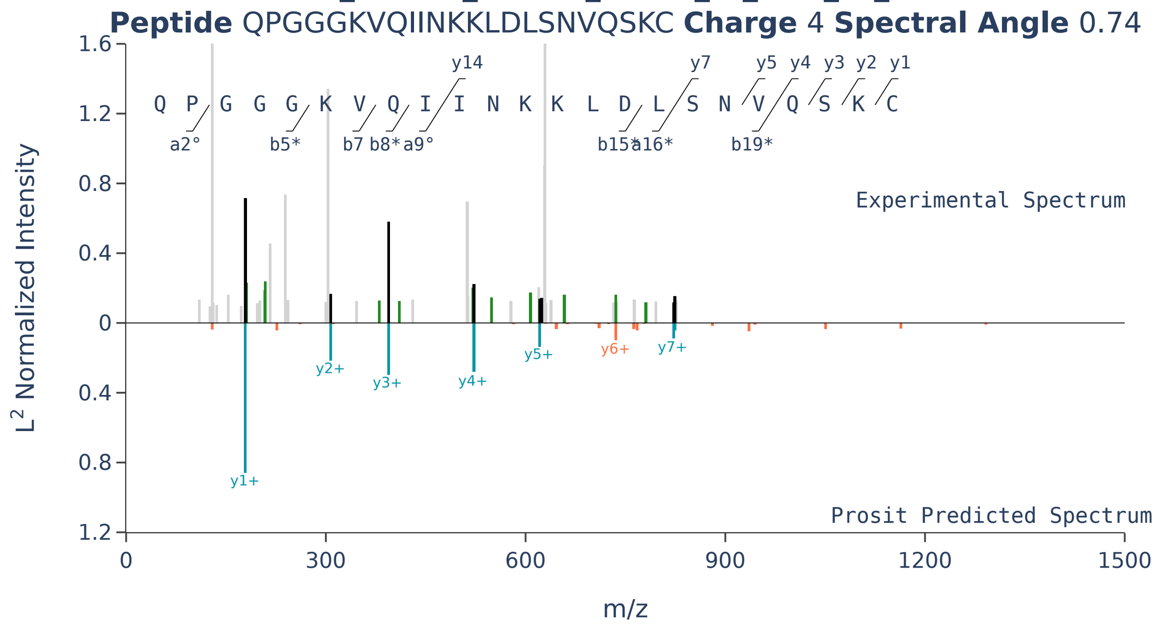


**Source** WSoh_221121_011221_HFGoe_L2_hTau_24h_R1 **Scan** 5166
**Peptide** RTPPKSPSSAKSR **Charge** 3 **Spectral Angle** 0.67

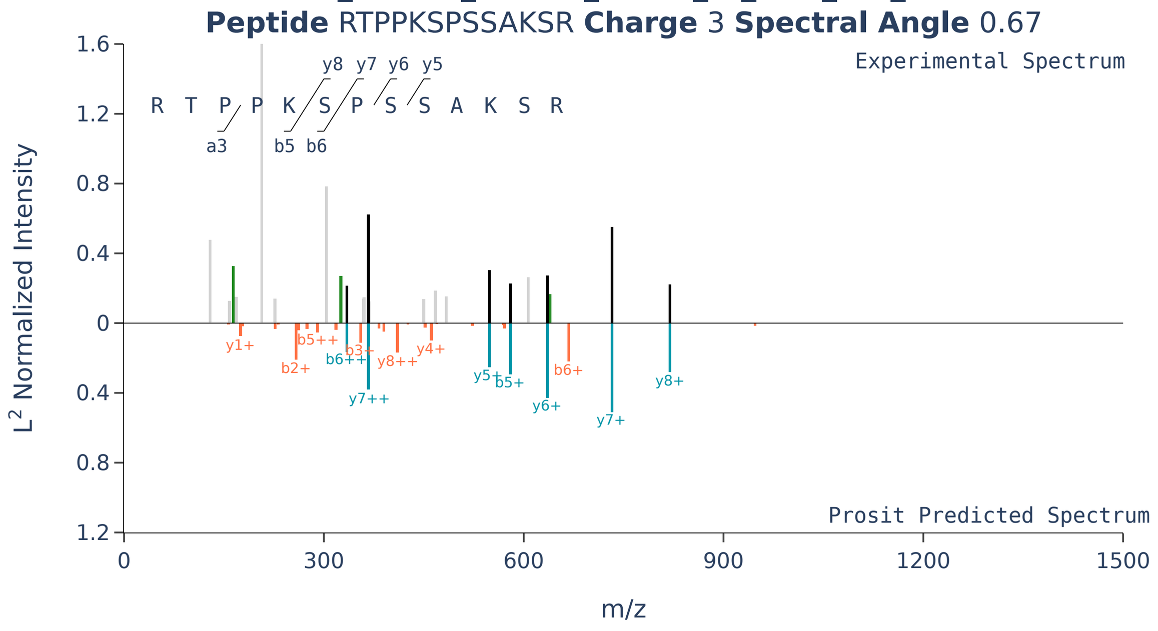


**Source** WSoh_221121_011221_HFGoe_L2_hTau_24h_R2 **Scan** 7037
**Peptide** PGGGNKKIETHKLTFRENAKAKT **Charge** 4 **Spectral Angle** 0.64

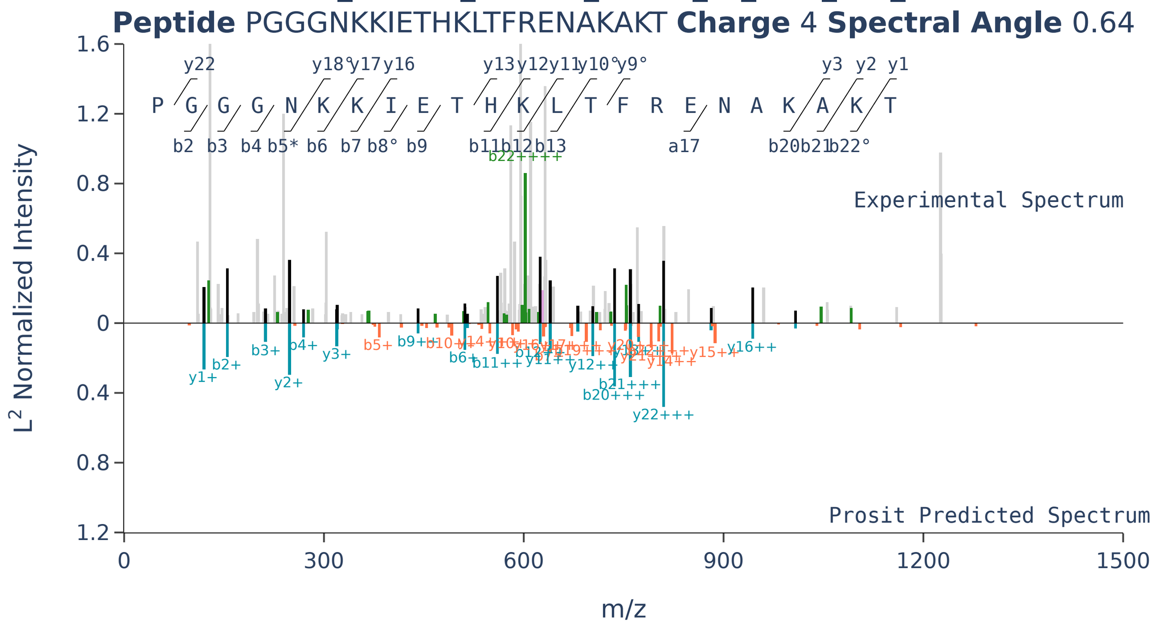


**Source** WSoh_221121_011221_HFGoe_L2_hTau_4h_R1 **Scan** 5317
**Peptide** RMVSKSKDGTGSDDKKAKGADGKTKIAT **Charge** 6 **Spectral Angle** 0.61

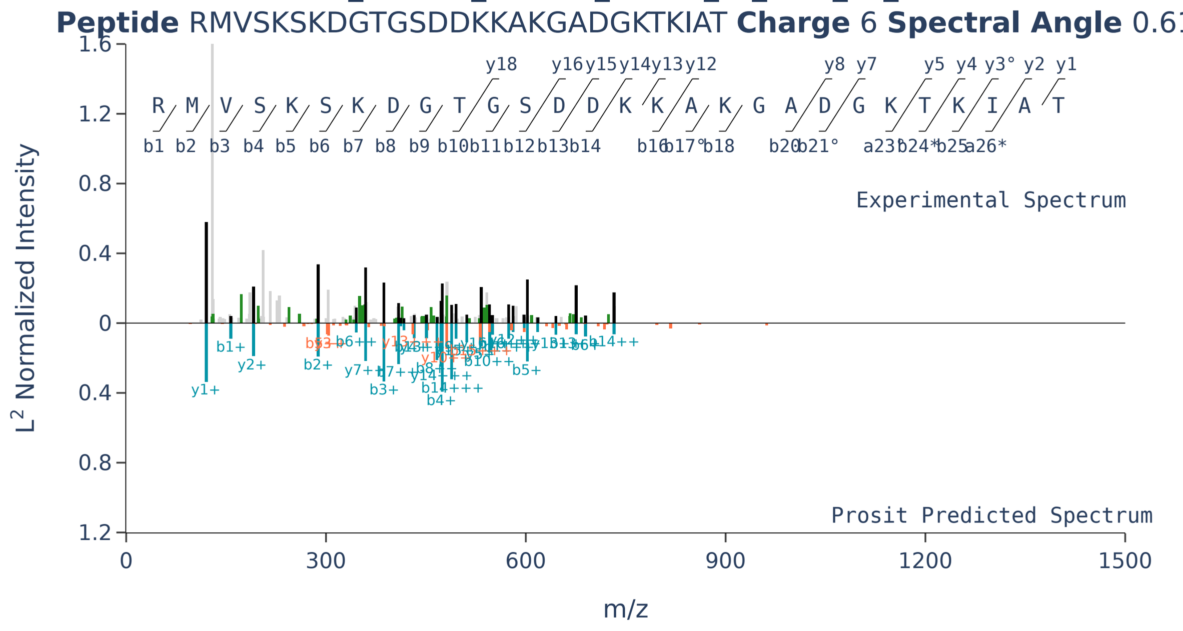


**Source** WSoh_221121_011221_HFGoe_L1_hTau_4h_R1 **Scan** 11826
**Peptide** PLVDEGAPGKQAAAQPHT **Charge** 3 **Spectral Angle** 0.75

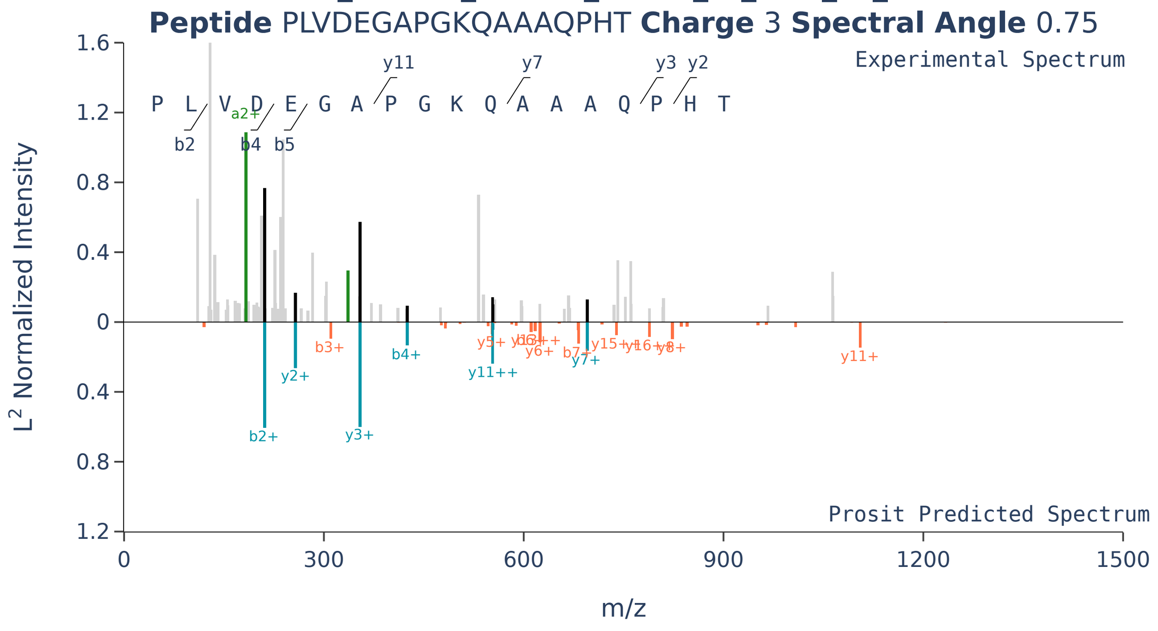


**Source** WSoh_221121_011221_HFGoe_L1_hTau_24h_R1 **Scan** 19136
**Peptide** KSRLQTAPVPMPDLKNVKSKIGSTENLKH **Charge** 5 **Spectral Angle** 0.66

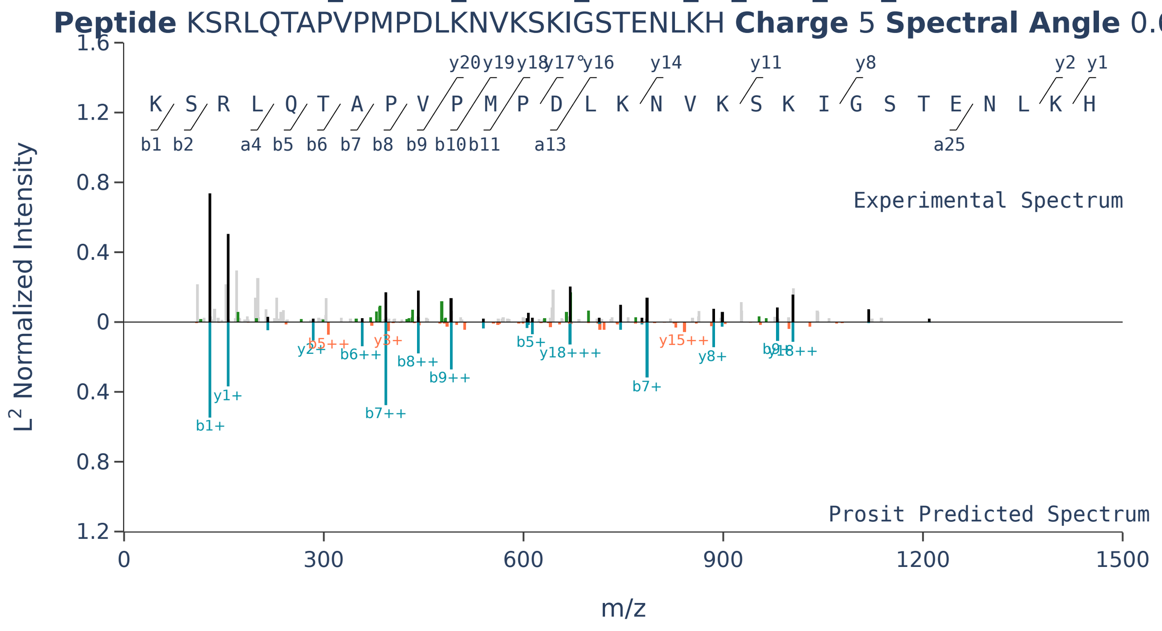


**Source** WSoh_221121_011221_HFGoe_L2_hTau_4h_R1 **Scan** 19903
**Peptide** KSEKLDFKDRVQSKIGSLDNIT **Charge** 4 **Spectral Angle** 0.63

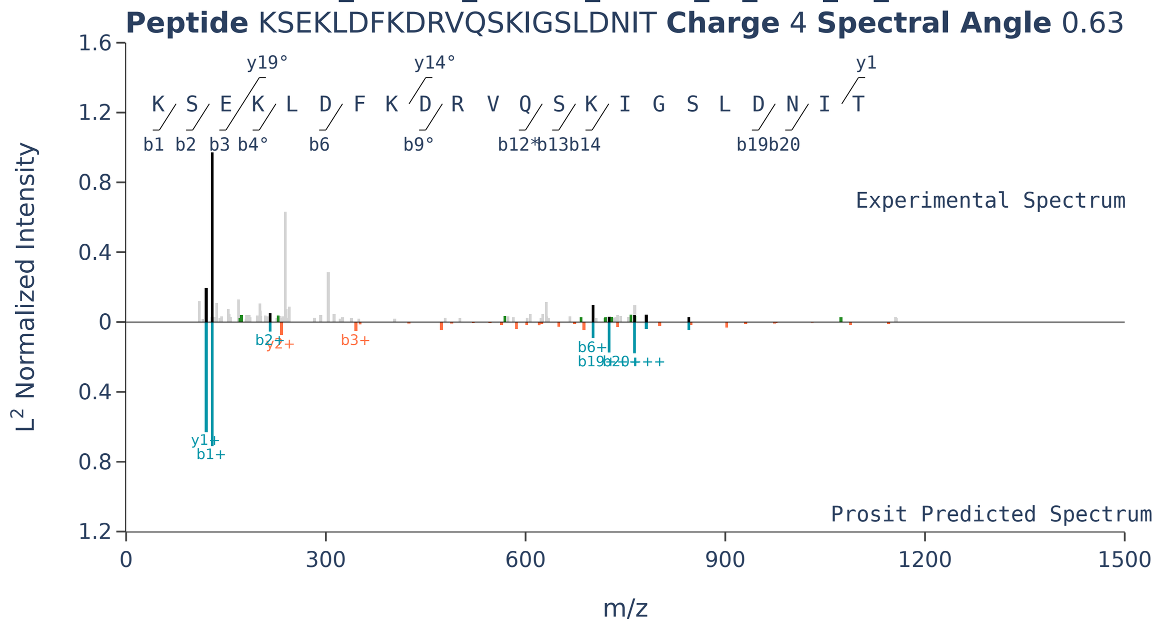


**Source** WSoh_221121_011221_HFGoe_L1_hTau_24h_R2 **Scan** 22414
**Peptide** DVTAPLVDEGAPGKQAAAQPHTEIPEGTTA **Charge** 3 **Spectral Angle** 0.7

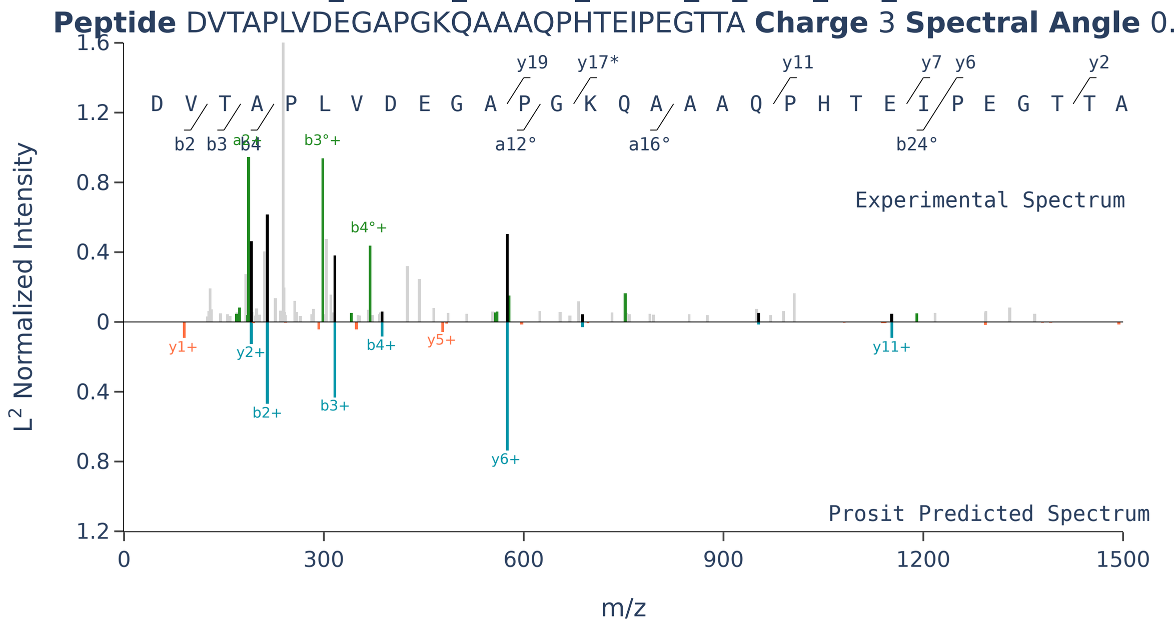


**Source** WSoh_221121_011221_HFGoe_L1_hTau_2h_R2 **Scan** 12617
**Peptide** KCGSKDNIKHVPGGGSVQIV **Charge** 4 **Spectral Angle** 0.59

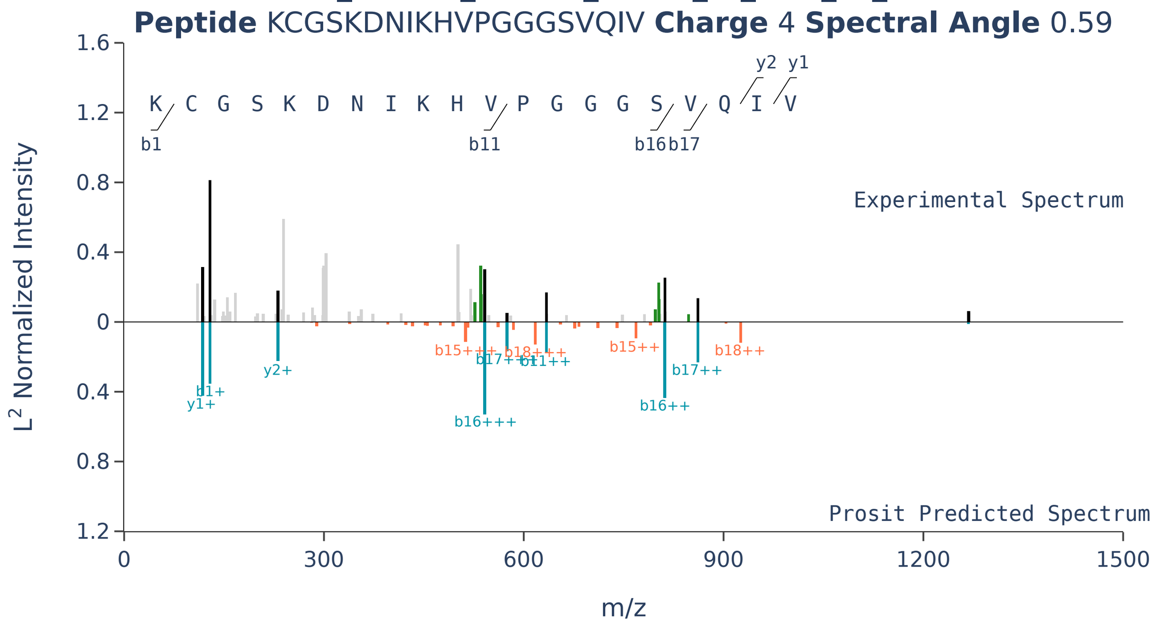


**Source** WSoh_221121_011221_HFGoe_L1_hTau_24h_R2 **Scan** 19377
**Peptide** SKIGSLDNITHVPGGGNKKIETHKLTF **Charge** 6 **Spectral Angle** 0.65

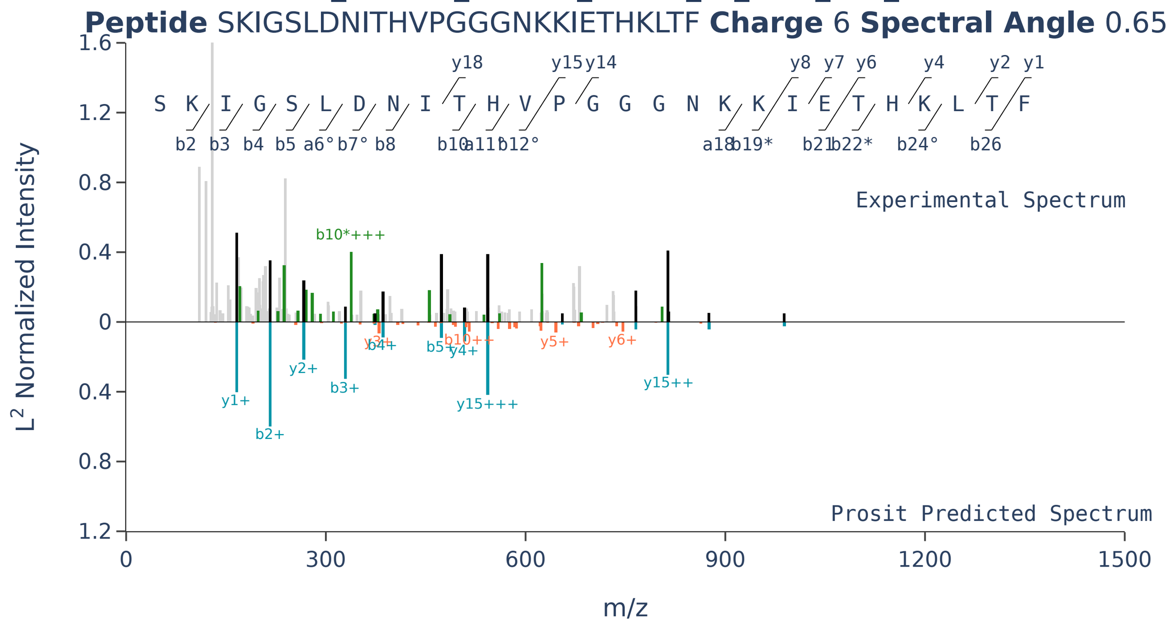

**Source** WSoh_221121_011221_HFGoe_L1_hTau_24h_R1 **Scan** 13333
**Peptide** VMEDHAGTYGLGDRKDQGGY **Charge** 3 **Spectral Angle** 0.21

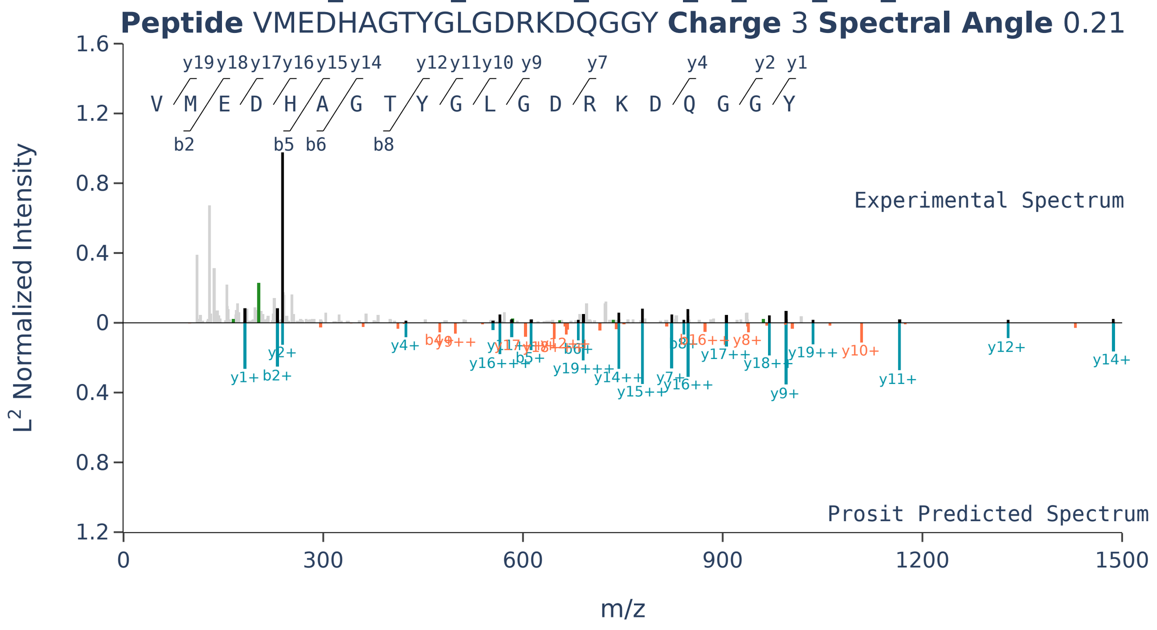


**Source** WSoh_221121_011221_HFGoe_L2_hTau_4h_R2 **Scan** 5375
**Peptide** DGTGSDDKKAKGADGKTKIA **Charge** 3 **Spectral Angle** 0.55

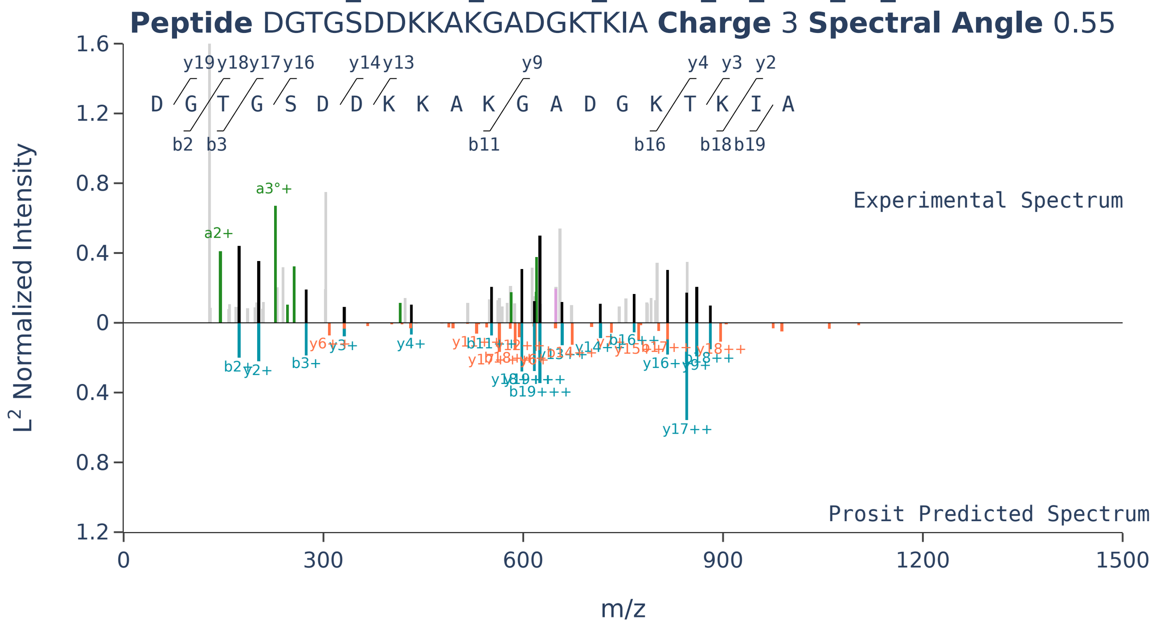


**Source** WSoh_221121_011221_HFGoe_L1_hTau_24h_R2 **Scan** 14363
**Peptide** GSLDNITHVPGGGNKKIETHKLT **Charge** 4 **Spectral Angle** 0.72

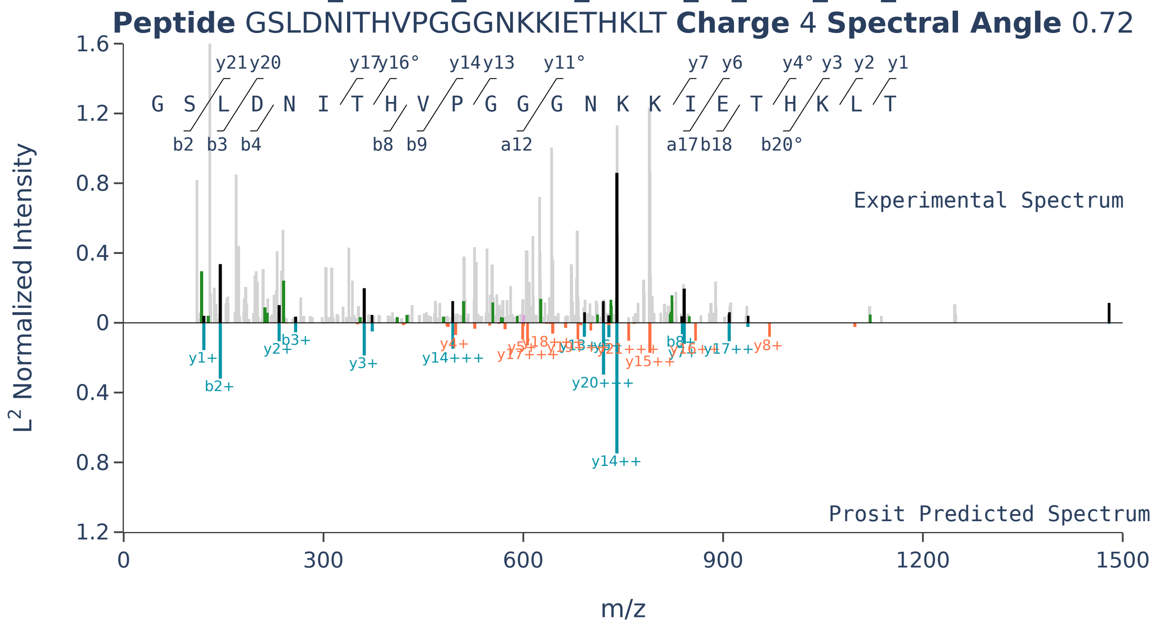


**Source** WSoh_221121_011221_HFGoe_L1_hTau_24h_R2 **Scan** 5533
**Peptide** SKSKDGTGSDDKKAKGADGKTKIATPRGA **Charge** 5 **Spectral Angle** 0.72

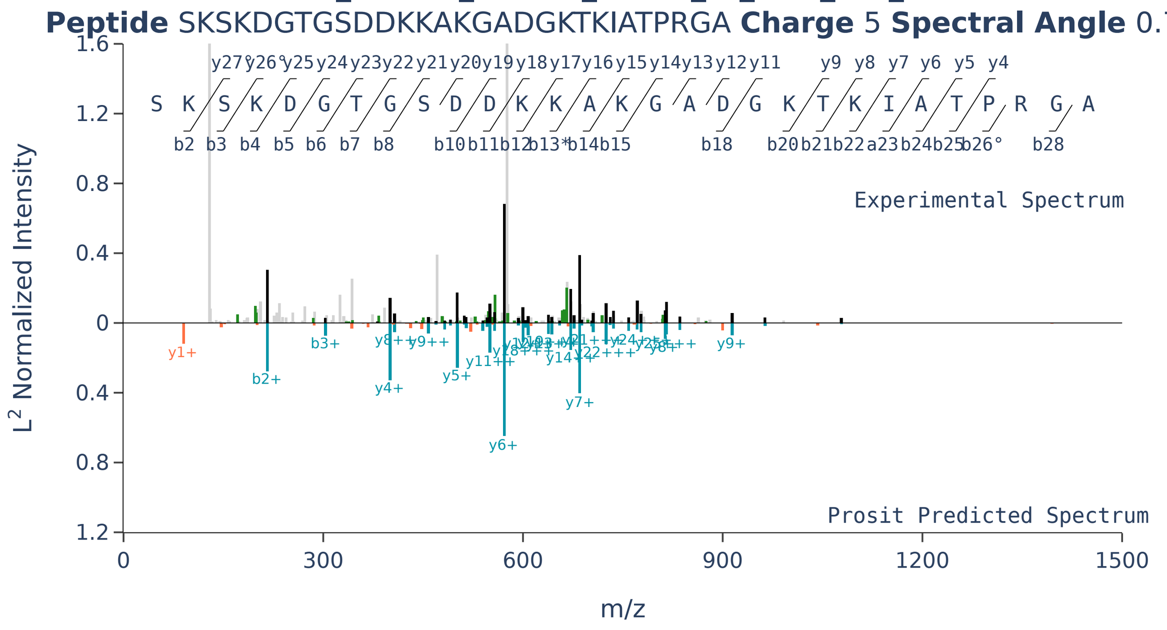


**Source** WSoh_221121_011221_HFGoe_L1_hTau_4h_R1 **Scan** 17538
**Peptide** LDLSNVQSKCGSKDNIKHVPGGGSVQIVYK **Charge** 5 **Spectral Angle** 0.66

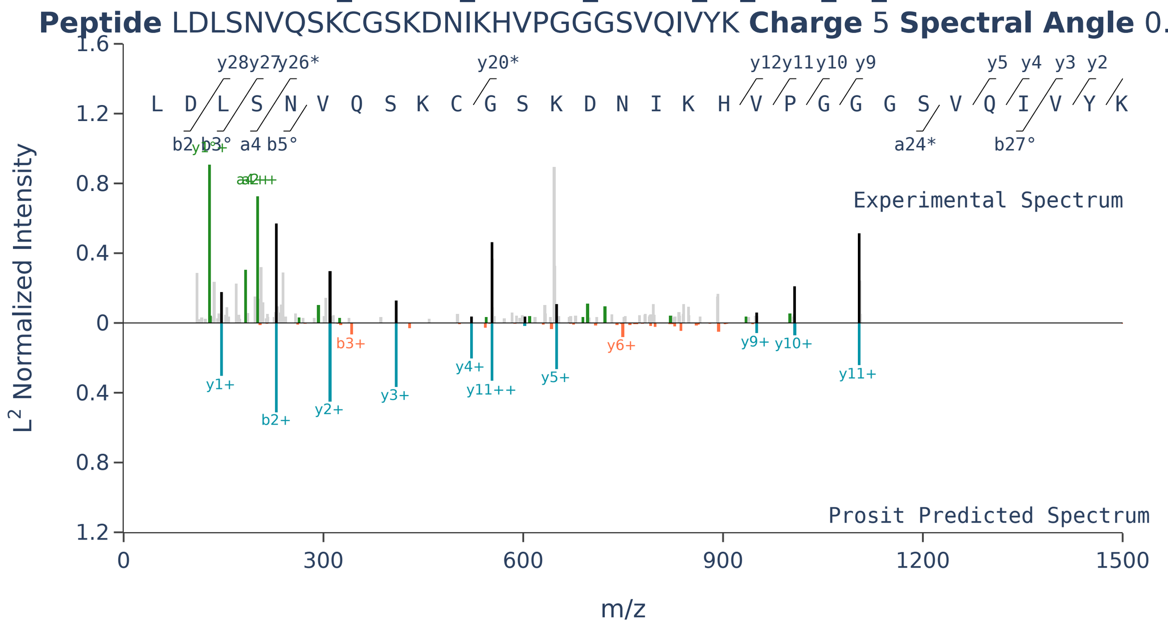


**Source** WSoh_221121_011221_HFGoe_L2_hTau_1h_R2 **Scan** 13061
**Peptide** PGGGNKKIETH **Charge** 2 **Spectral Angle** 0.88

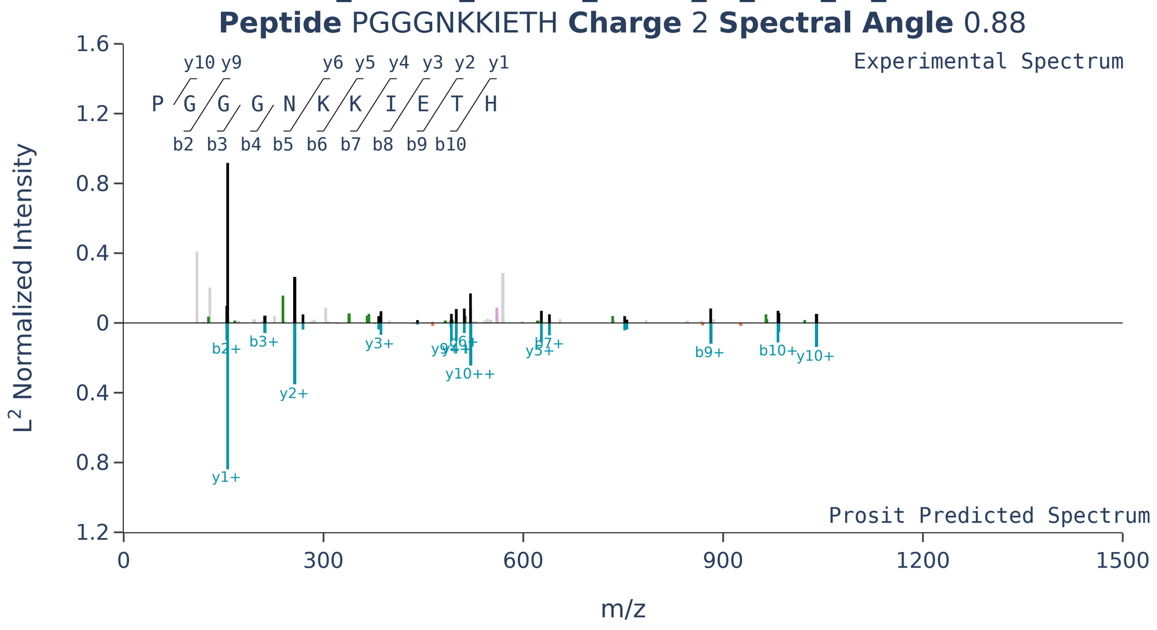


**Source** WSoh_221121_011221_HFGoe_L2_hTau_24h_R2 **Scan** 7988
**Peptide** GLGDRKD **Charge** 2 **Spectral Angle** 0.92

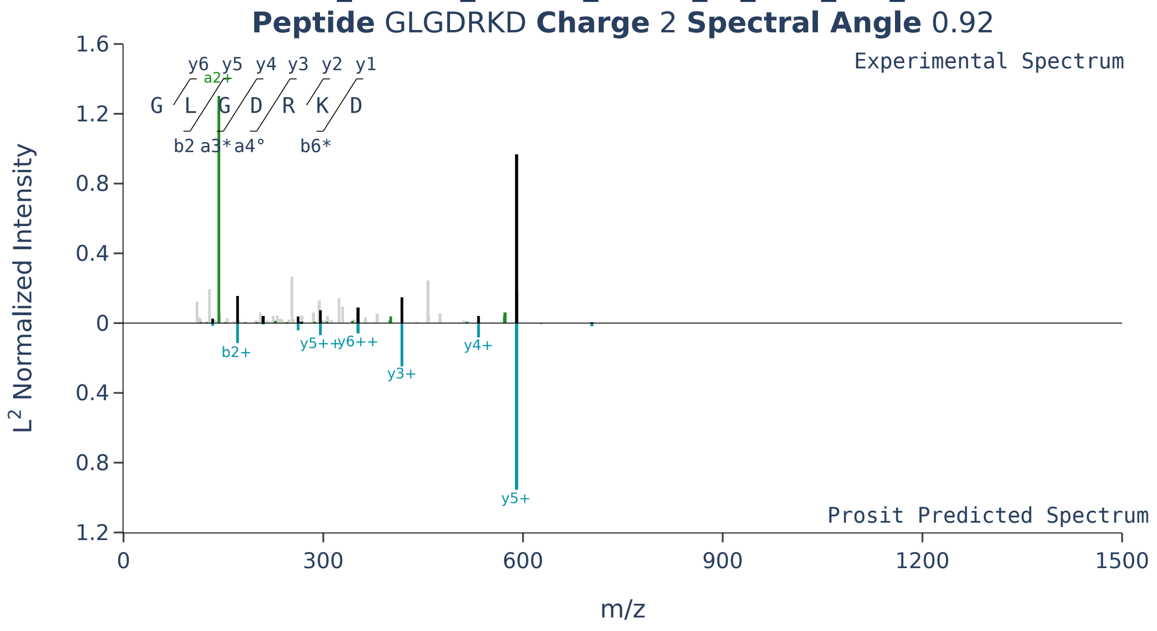


**Source** WSoh_221121_011221_HFGoe_L2_hTau_24h_R1 **Scan** 15867
**Peptide** NLKHQPGGGKVQIINKKLDLSNVQS **Charge** 4 **Spectral Angle** 0.66

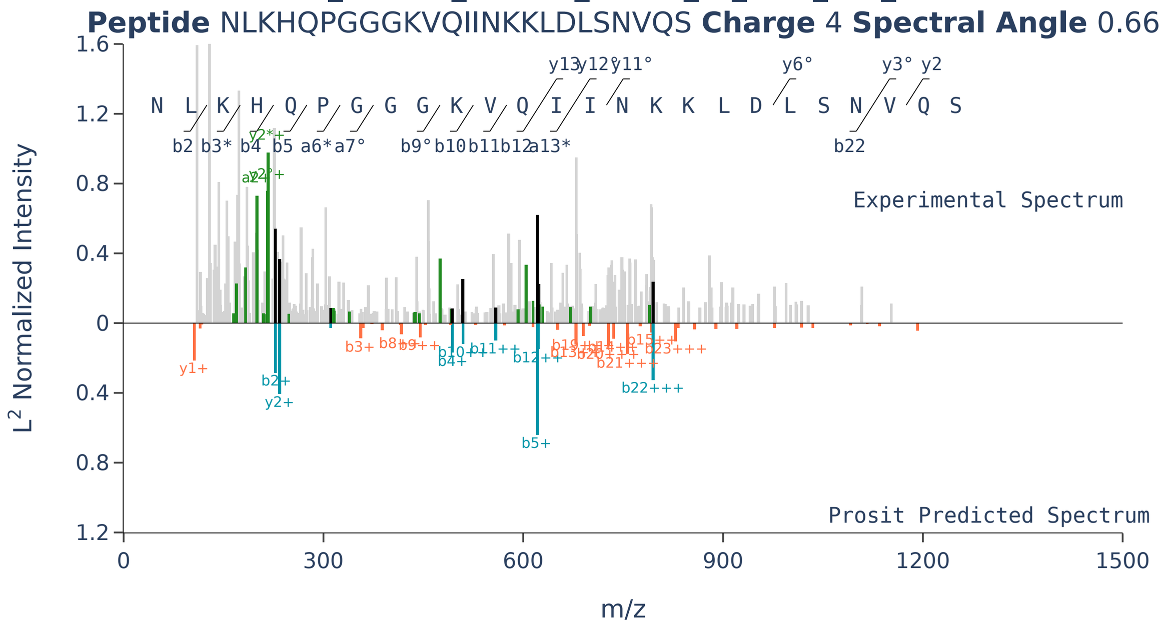


**Source** WSoh_221121_011221_HFGoe_L1_hTau_24h_R2 **Scan** 11512
**Peptide** KKLDLSNVQSKCG **Charge** 4 **Spectral Angle** 0.74

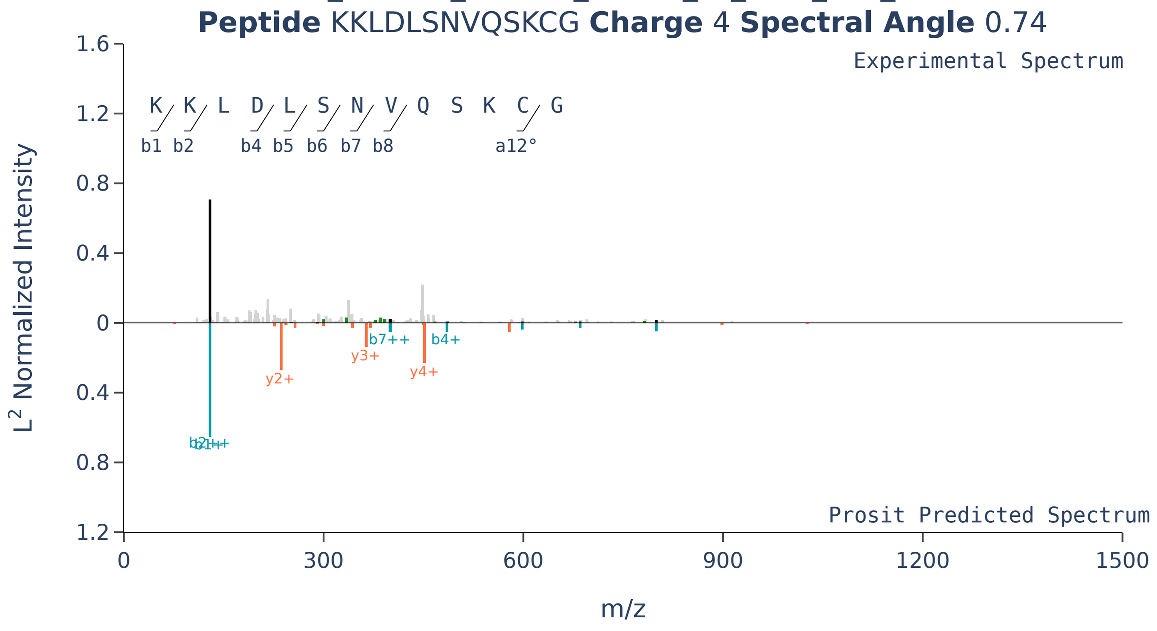


**Source** WSoh_221121_011221_HFGoe_L1_hTau_24h_R1 **Scan** 6323
**Peptide** PKKVAVVRTPPKSPSSAKS **Charge** 3 **Spectral Angle** 0.8

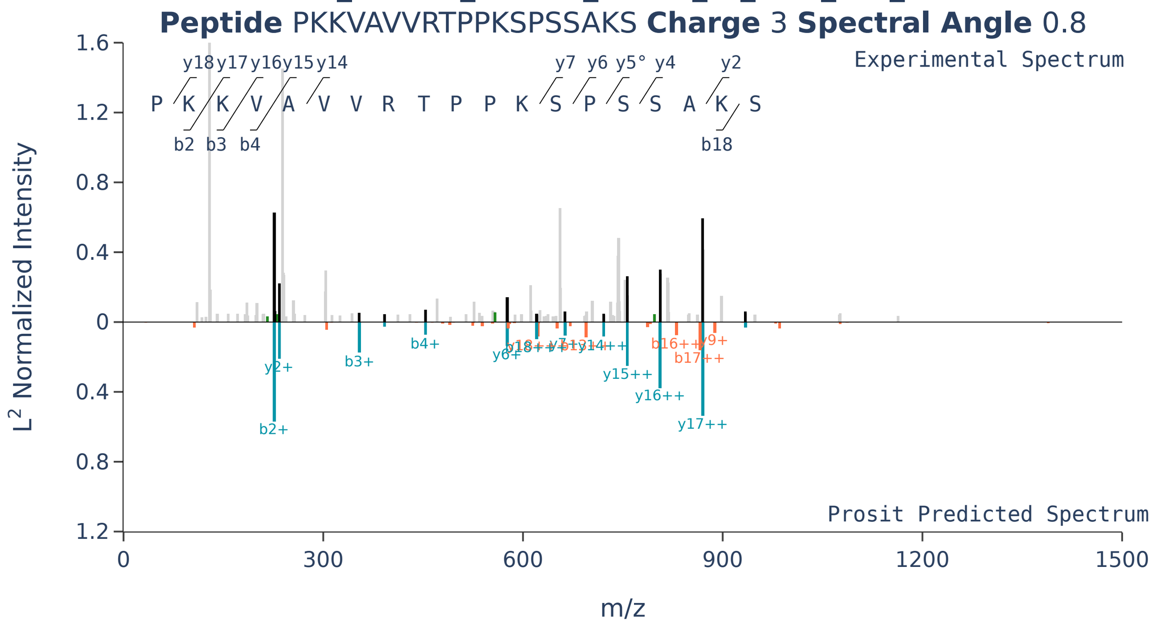


**Source** WSoh_221121_011221_HFGoe_L1_hTau_4h_R2 **Scan** 18960
**Peptide** GGYTMHQDQEGDTDAGLKESPLQTPTE **Charge** 3 **Spectral Angle** 0.84

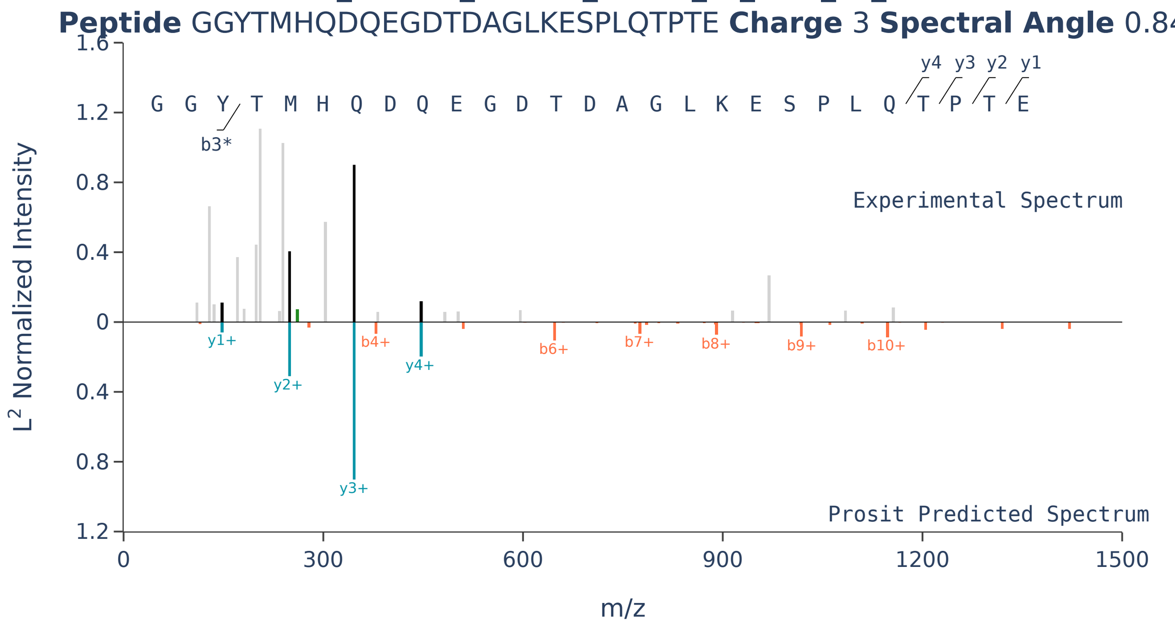


**Source** WSoh_221121_011221_HFGoe_L2_hTau_4h_R2 **Scan** 13940
**Peptide** TYGLGDRKDQGGYTMHQDQEGDTDAGLKE **Charge** 4 **Spectral Angle** 0.67

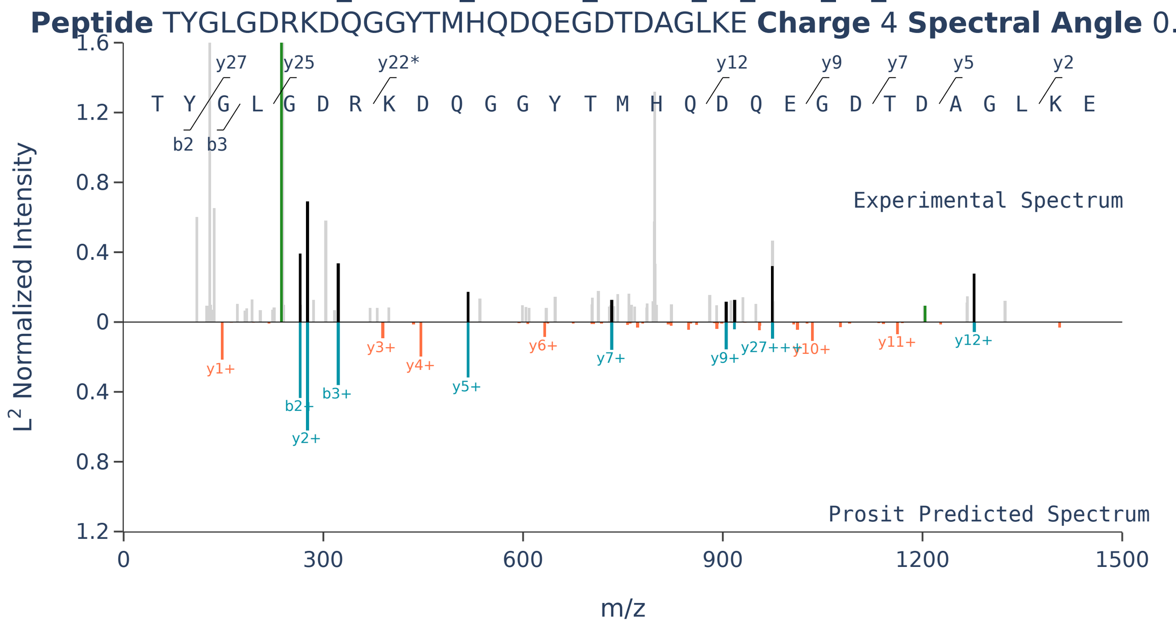


**Source** WSoh_221121_011221_HFGoe_L2_hTau_4h_R1 **Scan** 22685
**Peptide** QPHTEIPEGTTAEEAGIGDTPSLE **Charge** 3 **Spectral Angle** 0.79

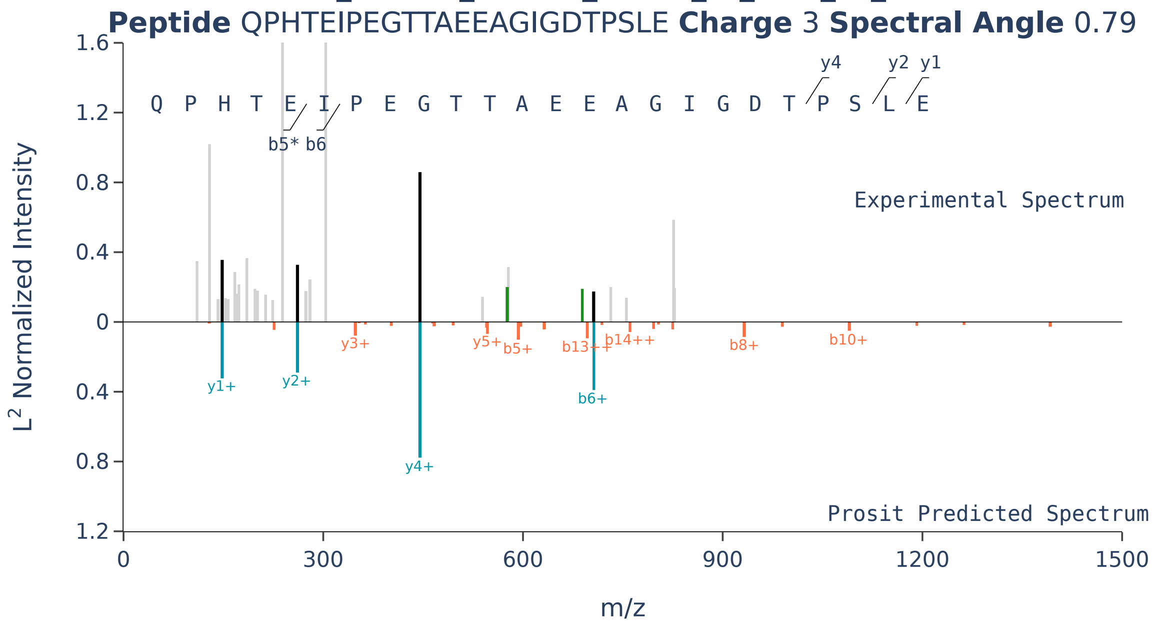


**Source** WSoh_221121_011221_HFGoe_L1_hTau_4h_R2 **Scan** 16943
**Peptide** QPGGGKVQIINKKLDLS **Charge** 3 **Spectral Angle** 0.54

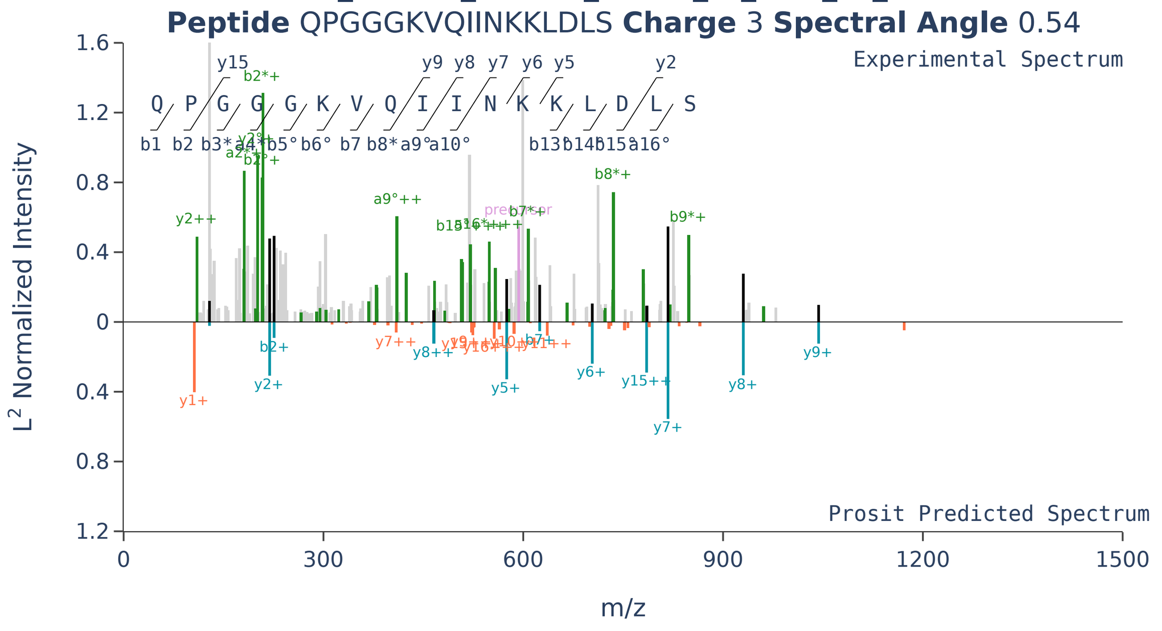


**Source** WSoh_221121_011221_HFGoe_L2_hTau_24h_R2 **Scan** 13887
**Peptide** SRTPSLPTPPTREPKKVAVVRTPPKSPSSA **Charge** 5 **Spectral Angle** 0.76

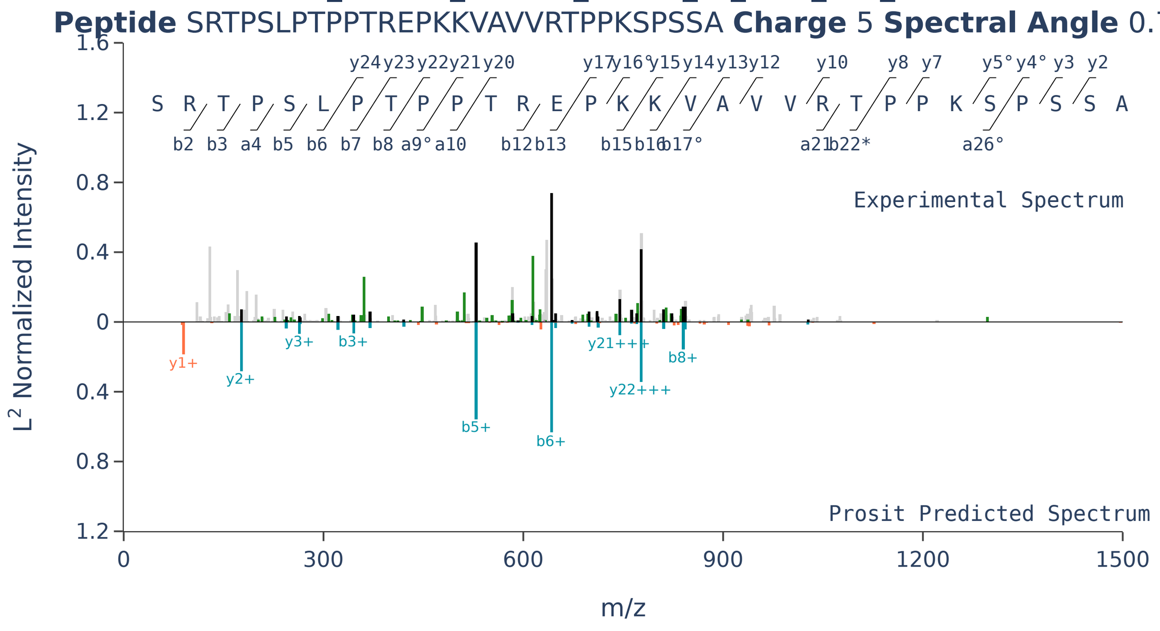

**Source** WSoh_221121_011221_HFGoe_L2_hTau_24h_R2 **Scan** 19021
**Peptide** PVPMPDLKNVKSKIGSTENLKHQPGGGKV **Charge** 5 **Spectral Angle** 0.71

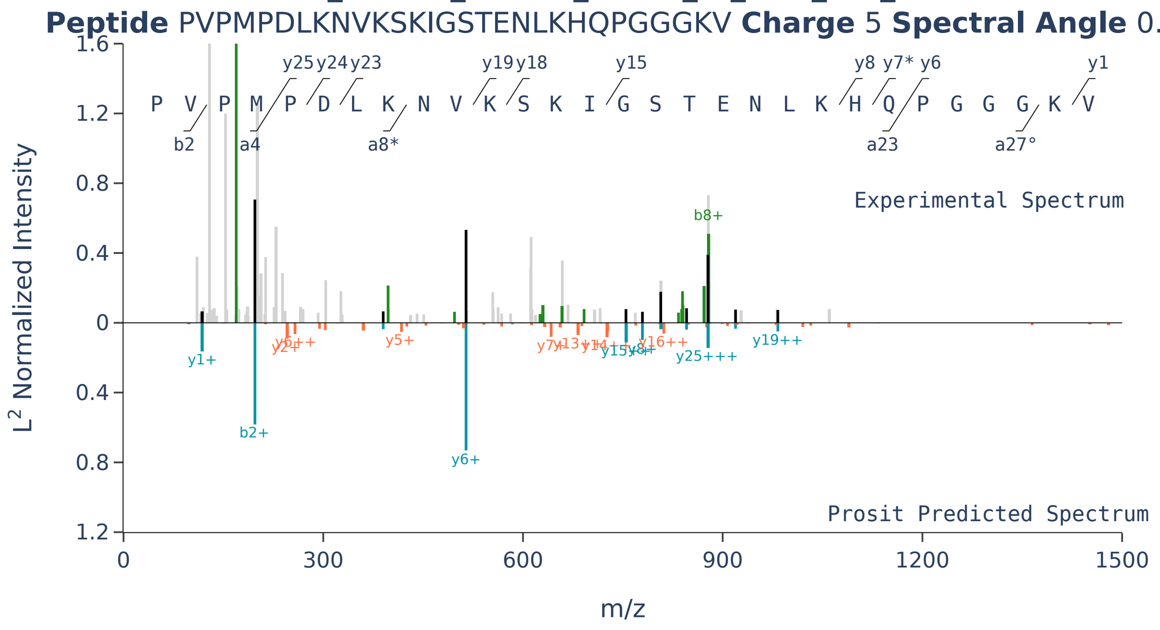

**Source** WSoh_221121_011221_HFGoe_L2_hTau_4h_R2 **Scan** 28829
**Peptide** STGSIDMVDSPQLATLAD **Charge** 2 **Spectral Angle** 0.57

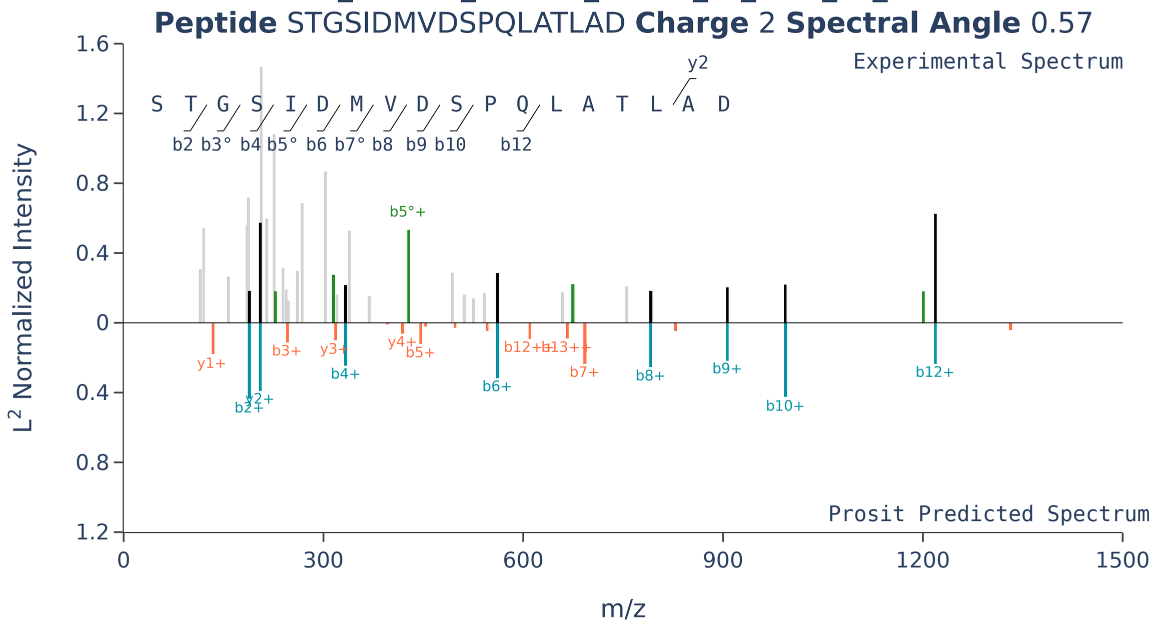

**Source** WSoh_221121_011221_HFGoe_L1_hTau_1h_R1 **Scan** 20130
**Peptide** APVPMPDLKNVKSKL **Charge** 4 **Spectral Angle** 0.64

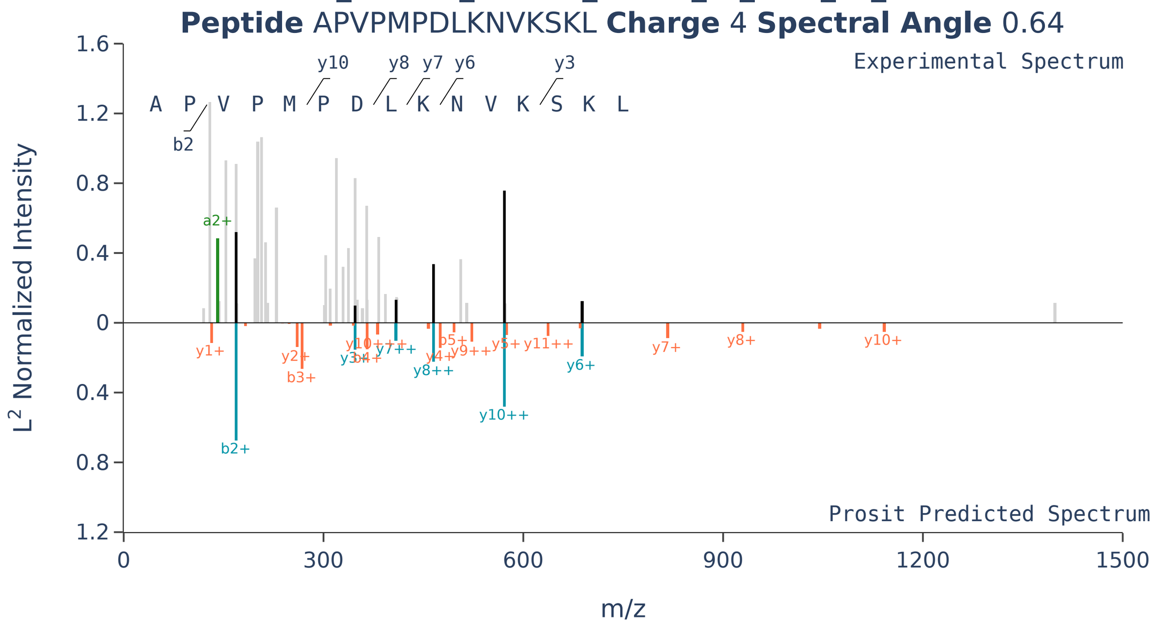

**Source** WSoh_221121_011221_HFGoe_L1_hTau_24h_R1 **Scan** 5472
**Peptide** EAAGHVTQARMVSKSKDGTGSDDKKAKG **Charge** 4 **Spectral Angle** 0.69

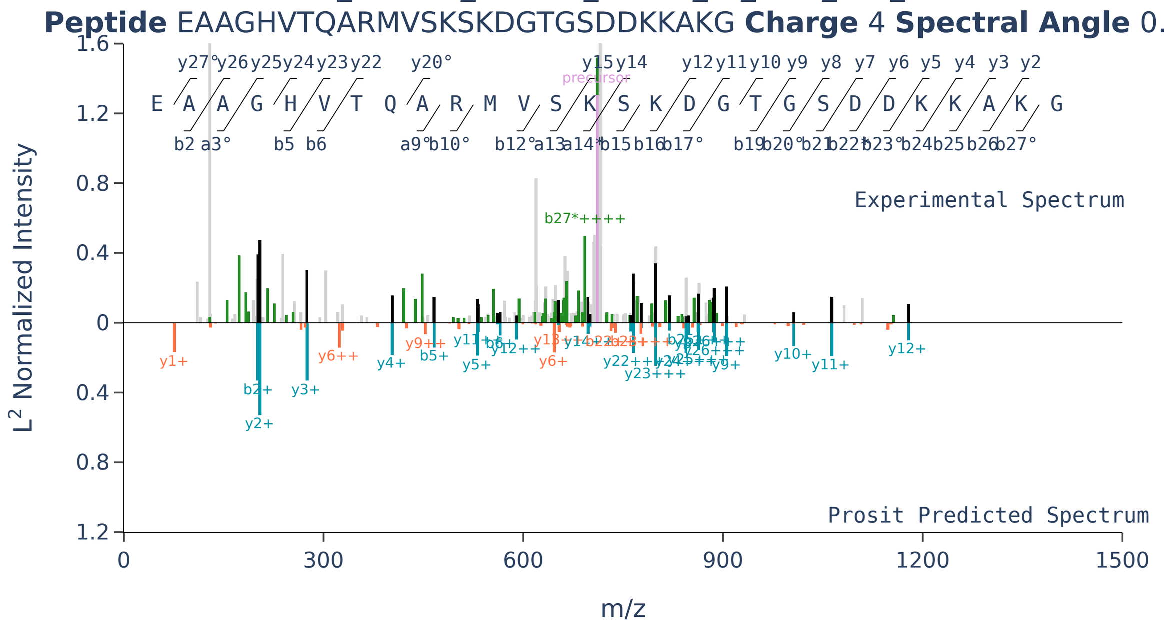

**Source** WSoh_221121_011221_HFGoe_L2_hTau_2h_R2 **Scan** 17145
**Peptide** QIINKKLDLSNVQS **Charge** 2 **Spectral Angle** 0.74

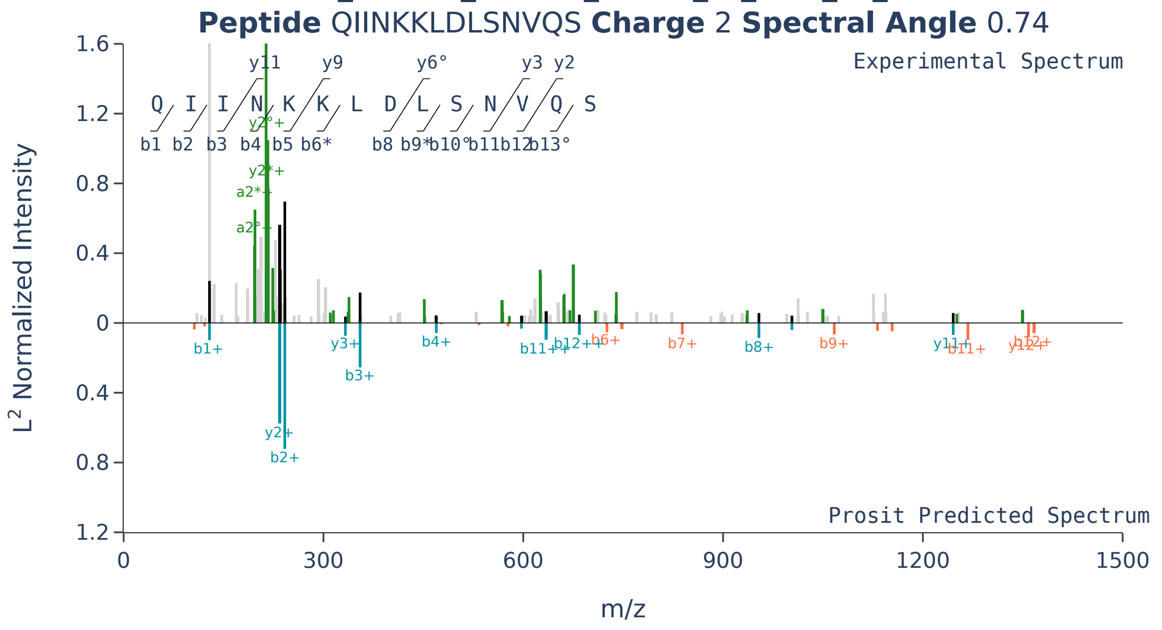

**Source** WSoh_221121_011221_HFGoe_L1_hTau_4h_R1 **Scan** 14760
**Peptide** KDRVQSKIGSLDNITHVPGGGNKKIE **Charge** 5 **Spectral Angle** 0.65

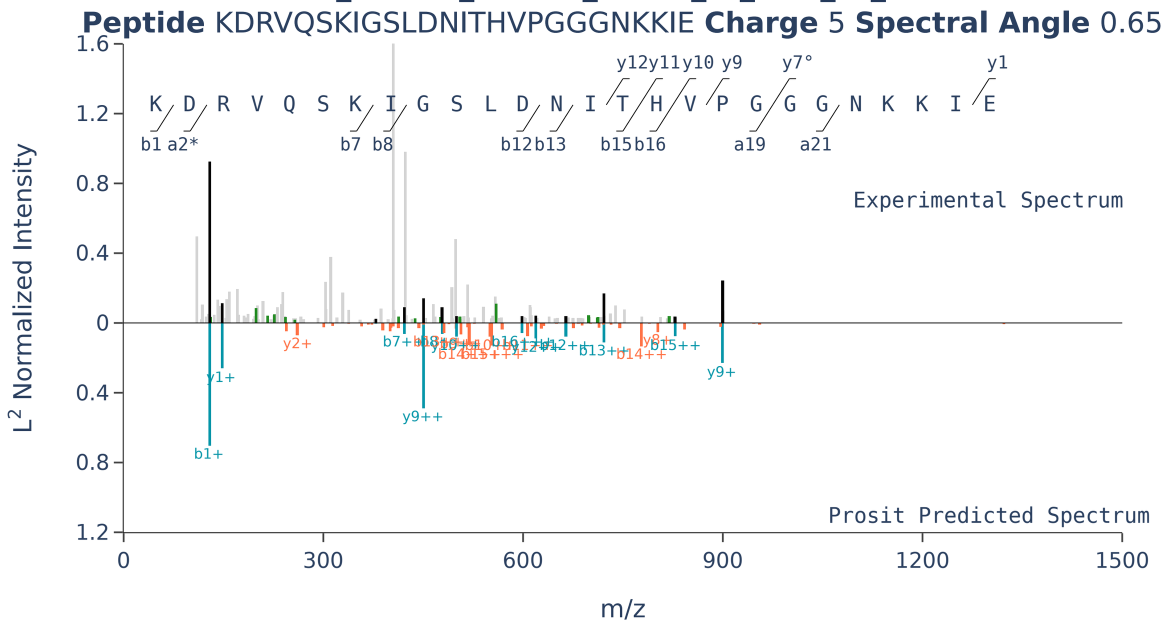

**Source** WSoh_221121_011221_HFGoe_L1_hTau_4h_R1 **Scan** 21972
**Peptide** EVKSEKLDFKDRVQSKIGSLDNITHV **Charge** 4 **Spectral Angle** 0.73

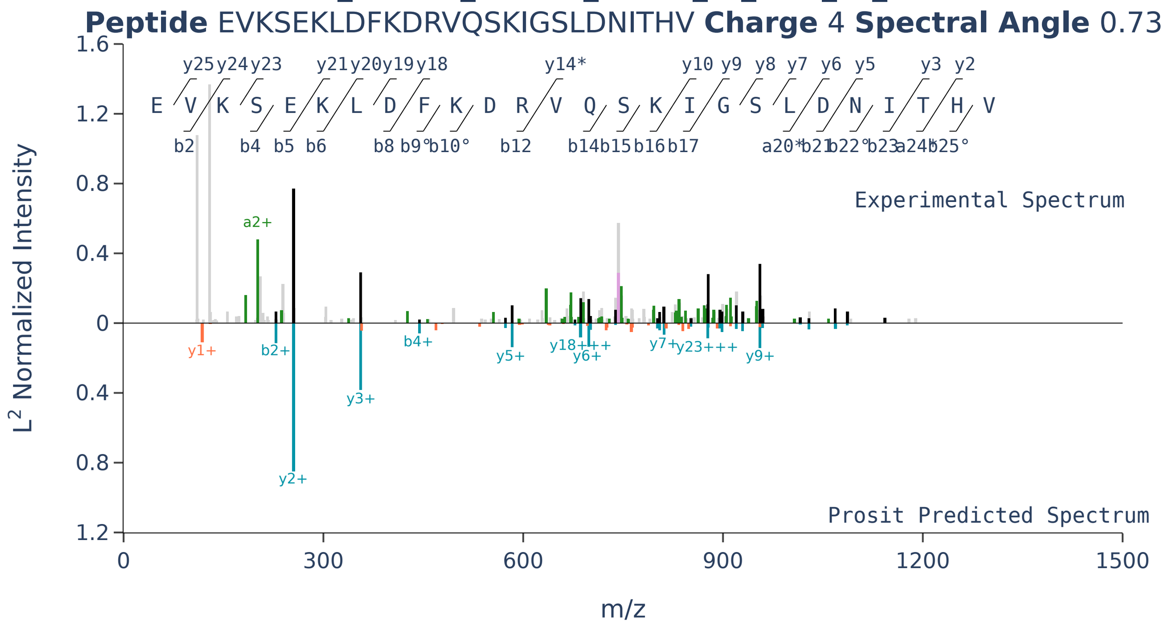

**Source** WSoh_221121_011221_HFGoe_L2_hTau_4h_R2 **Scan** 7585
**Peptide** THVPGGGNKKIETHKLTFRENAKAK **Charge** 5 **Spectral Angle** 0.84

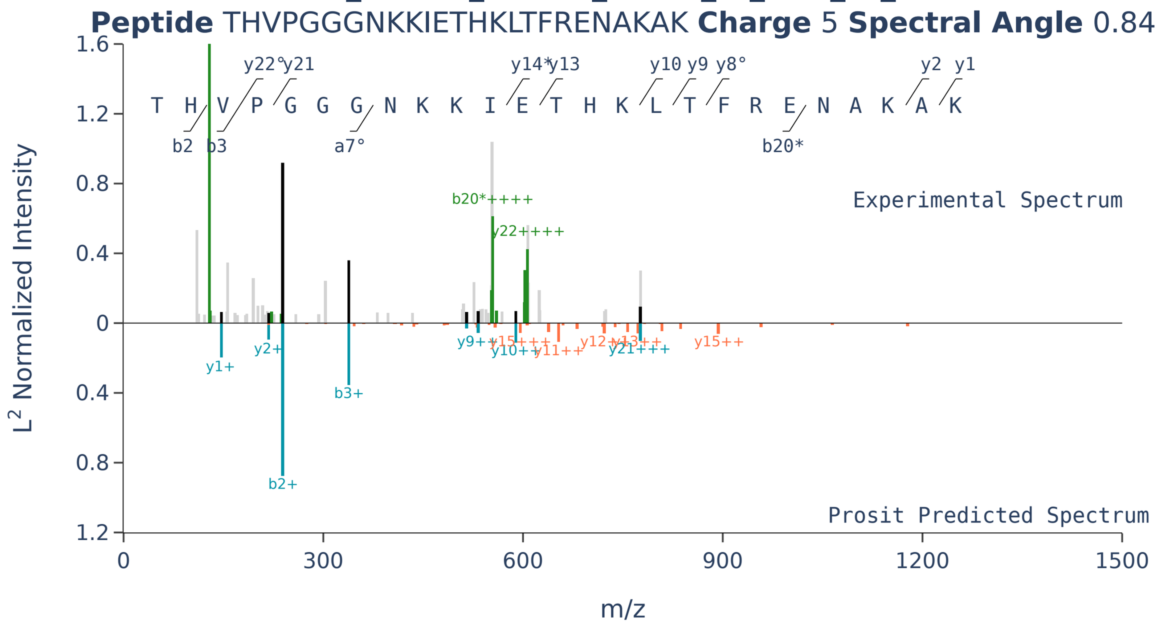

**Source** WSoh_221121_011221_HFGoe_L1_hTau_24h_R2 **Scan** 23397
**Peptide** PTAEDVTAPLVDEGAPGKQAAAQPHTEI **Charge** 3 **Spectral Angle** 0.78

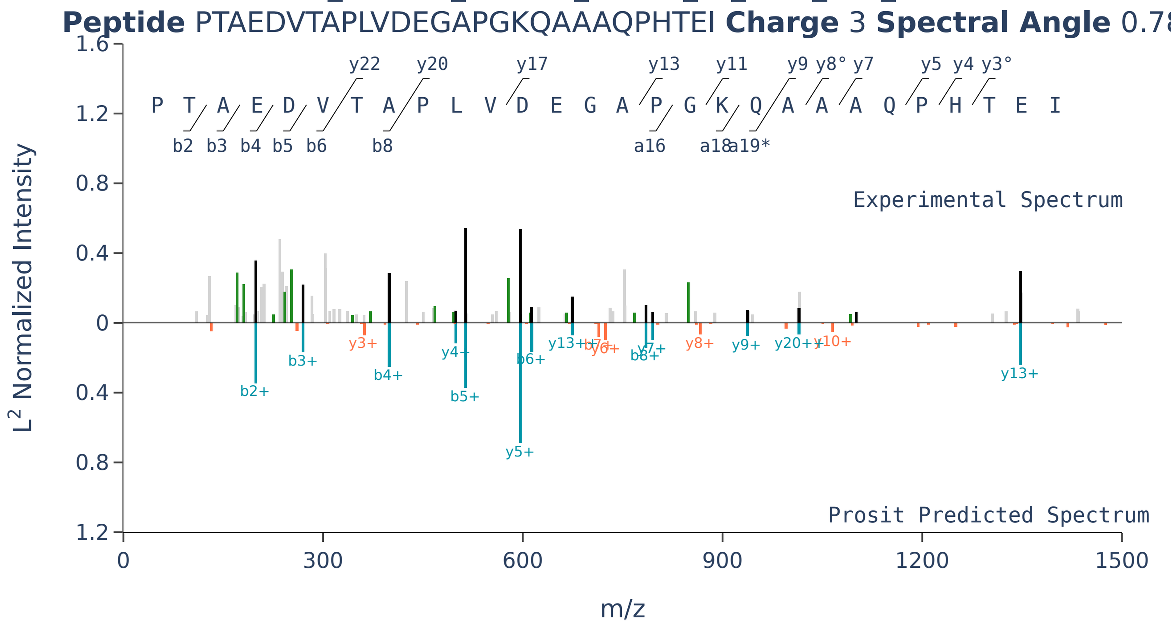

**Source** WSoh_221121_011221_HFGoe_L2_hTau_4h_R2 **Scan** 15374
**Peptide** QLINKKLDLS **Charge** 2 **Spectral Angle** 0.55

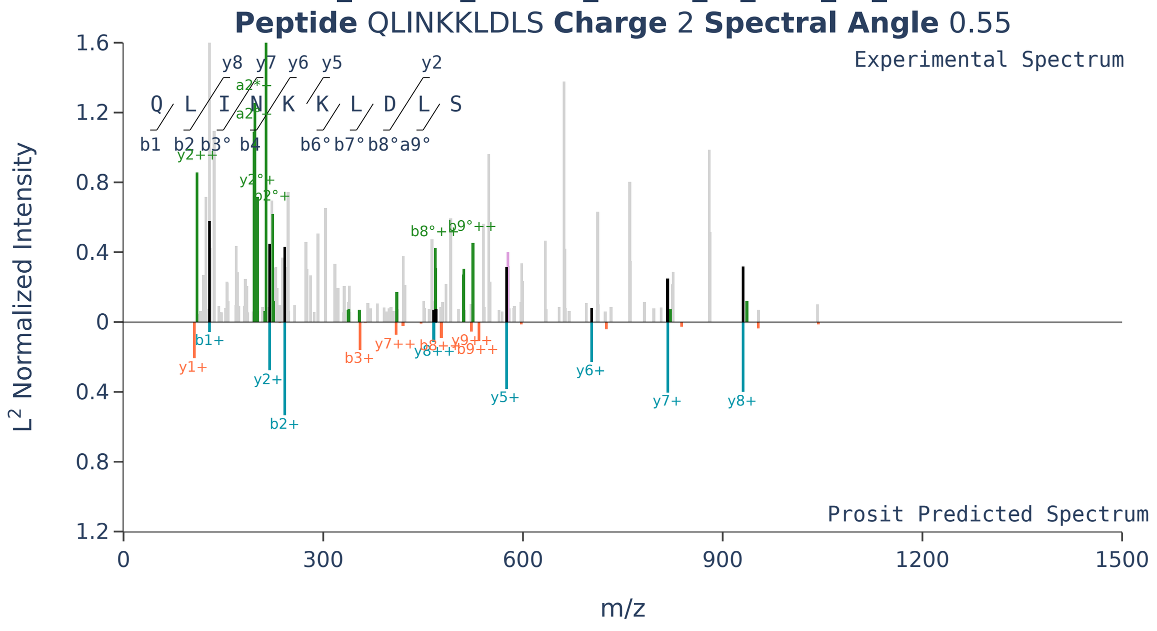

**Source** WSoh_221121_011221_HFGoe_L2_hTau_1h_R2 **Scan** 8750
**Peptide** LATPRGAAPPGQKGQANA **Charge** 2 **Spectral Angle** 0.74

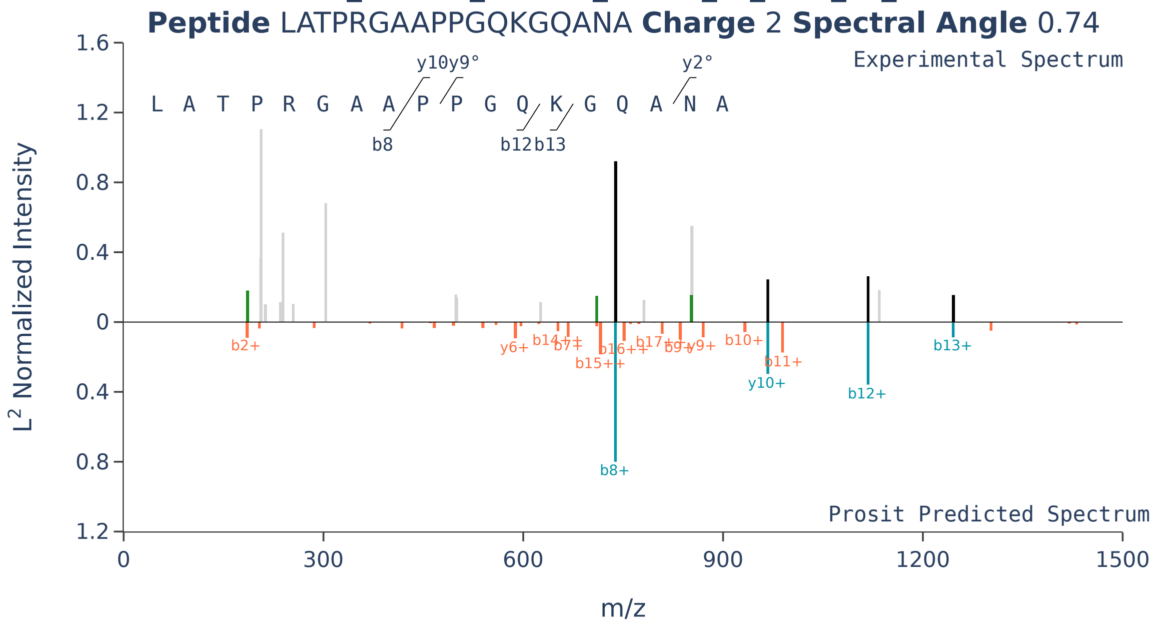

**Source** WSoh_221121_011221_HFGoe_L1_hTau_24h_R1 **Scan** 16654
**Peptide** KIGSLDNITHVPGGGNKKIETHKLTFRENA **Charge** 5 **Spectral Angle** 0.59

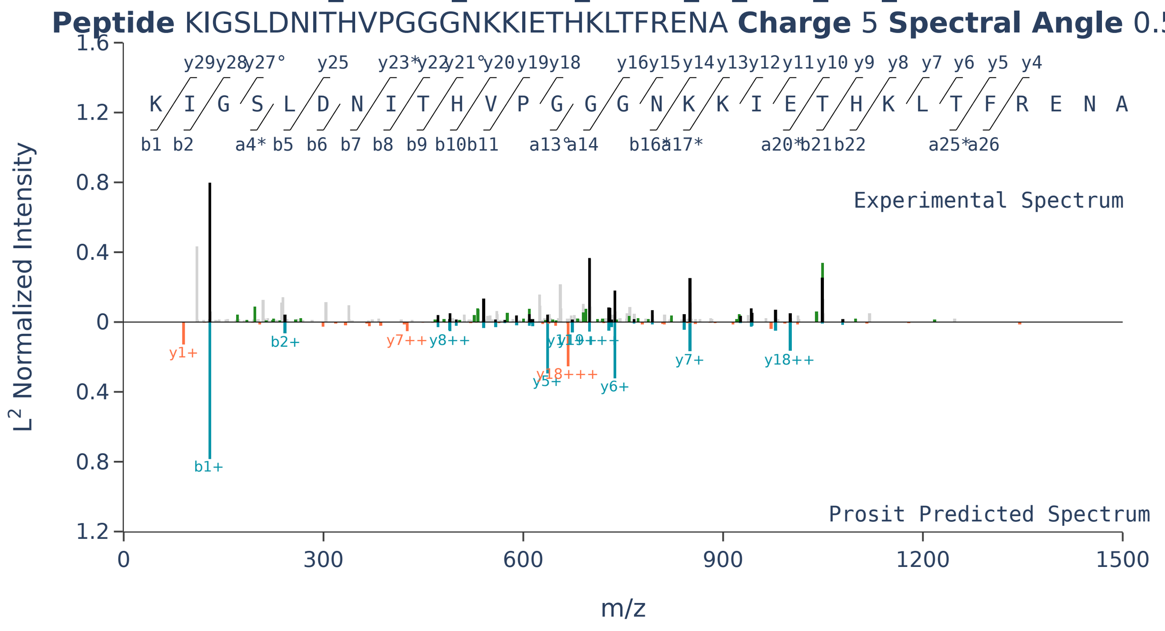

**Source** WSoh_221121_011221_HFGoe_L1_hTau_24h_R1 **Scan** 25376
**Peptide** TGSIDMVDSPQLAT **Charge** 2 **Spectral Angle** 0.54

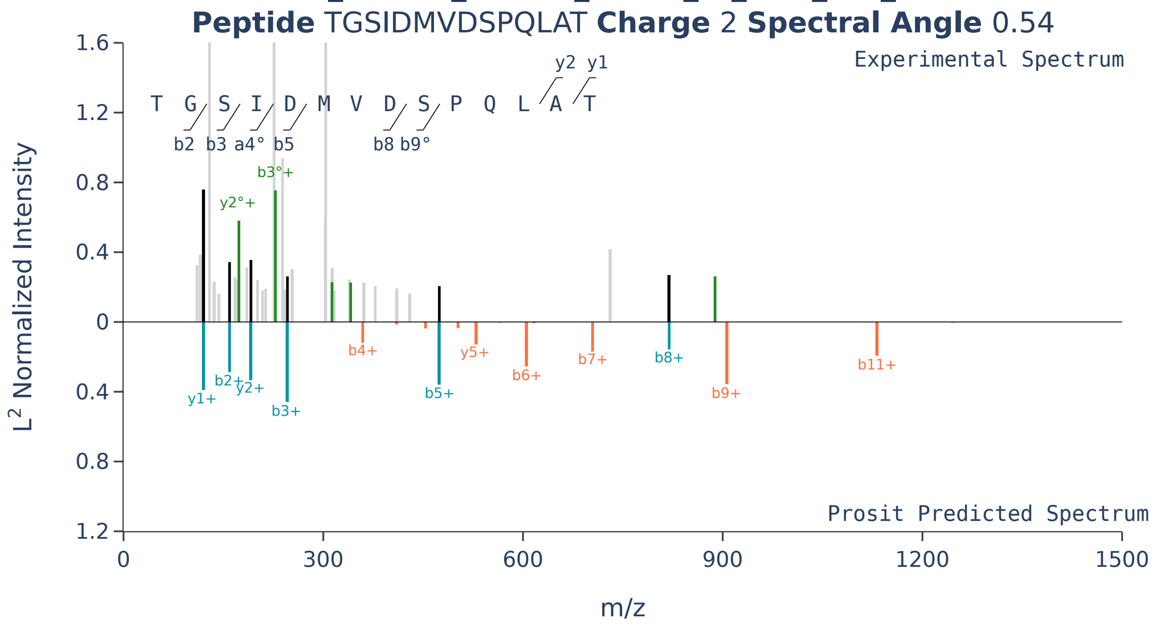

**Source** WSoh_221121_011221_HFGoe_L2_hTau_24h_R1 **Scan** 19291
**Peptide** QSKIGSLDNITHVPGGGNKKIETHKLTF **Charge** 5 **Spectral Angle** 0.69

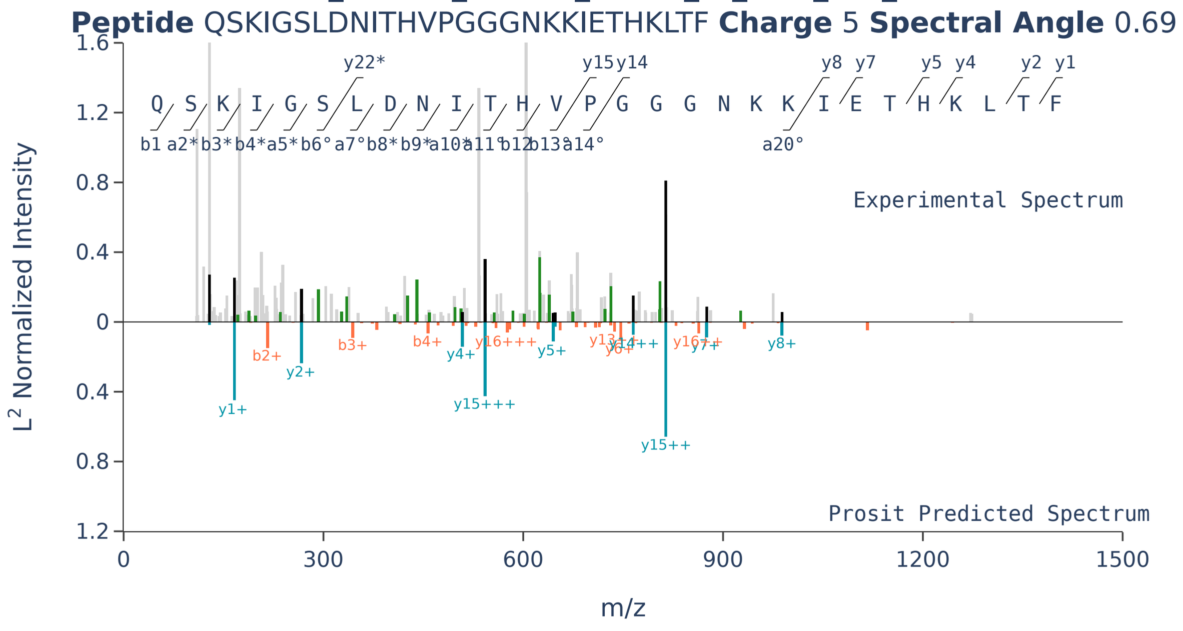

**Source** WSoh_221121_011221_HFGoe_L1_hTau_4h_R2 **Scan** 15784
**Peptide** IVYKSPVVSGDTSPRHLSNVSSTG **Charge** 3 **Spectral Angle** 0.72

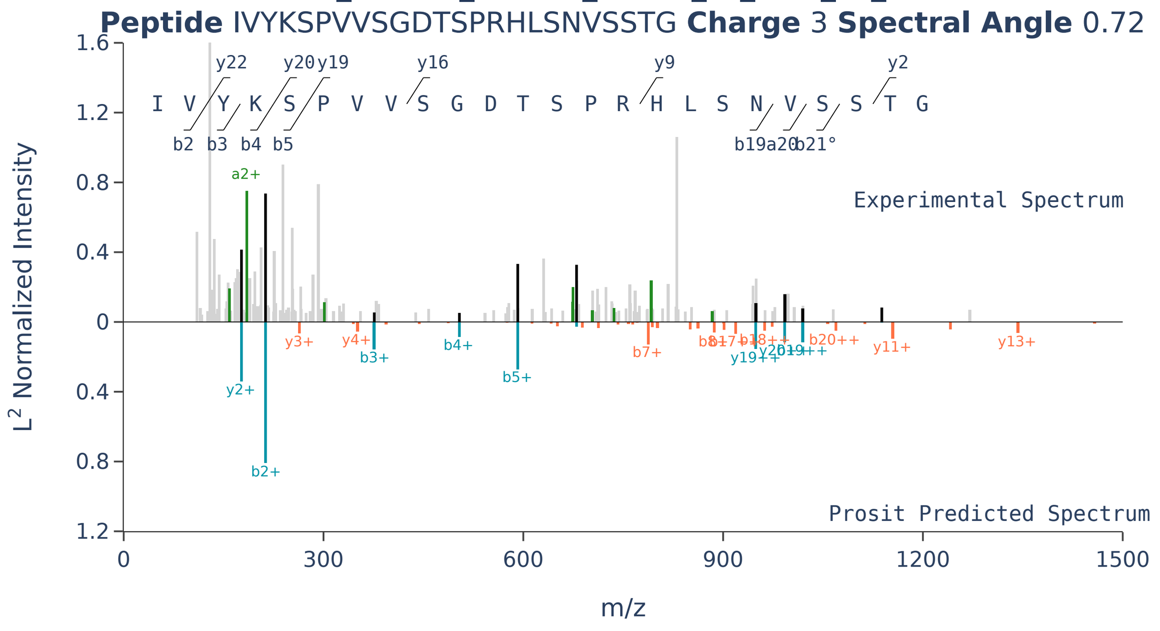

**Source** WSoh_221121_011221_HFGoe_L2_hTau_24h_R1 **Scan** 15071
**Peptide** NKKLDLSNVQSKCGSKDNIKHVPGGGSVQI **Charge** 3 **Spectral Angle** 0.65

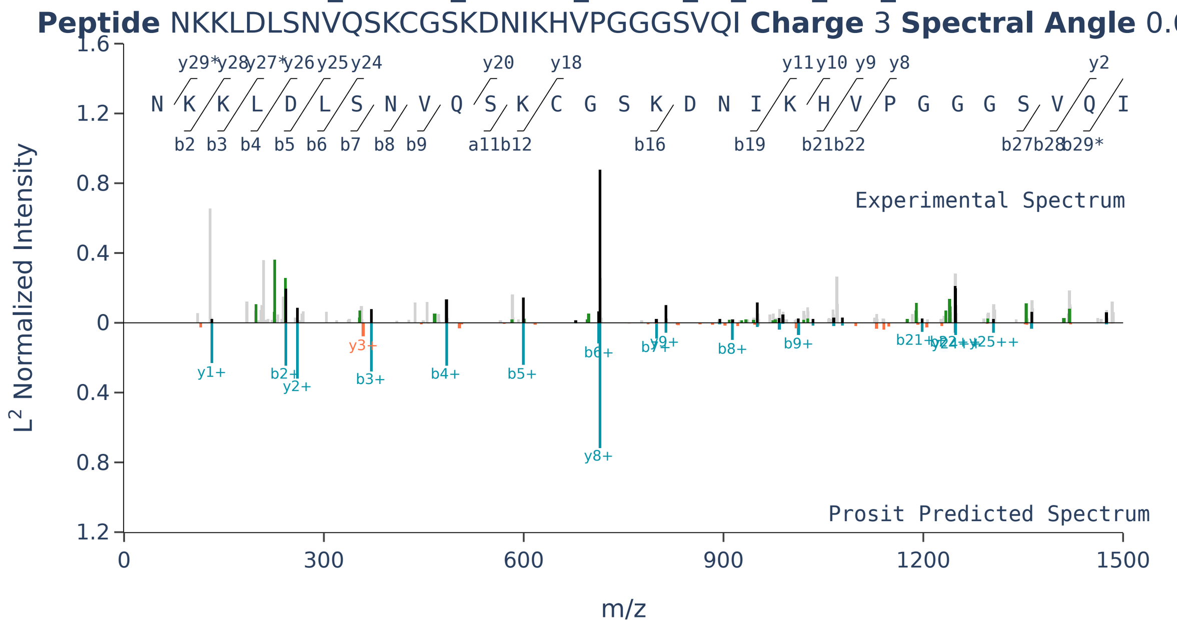


**Source** WSoh_221121_011221_HFGoe_L1_hTau_2h_R2 **Scan** 16422
**Peptide** DLSNVQSKCGSKDNIKHVPGGGSVQIV **Charge** 4 **Spectral Angle** 0.56

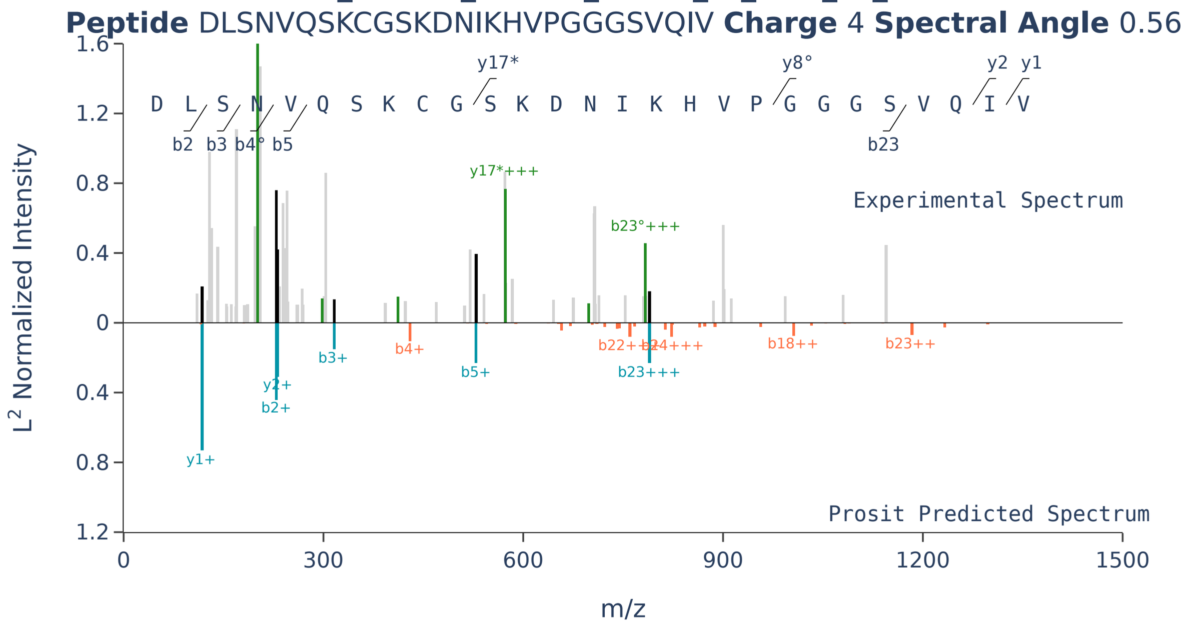


**Source** WSoh_221121_011221_HFGoe_L2_hTau_24h_R2 **Scan** 14160
**Peptide** GTYGLGDRKDQGGYTMHQDQEGDTDAGLKE **Charge** 3 **Spectral Angle** 0.5

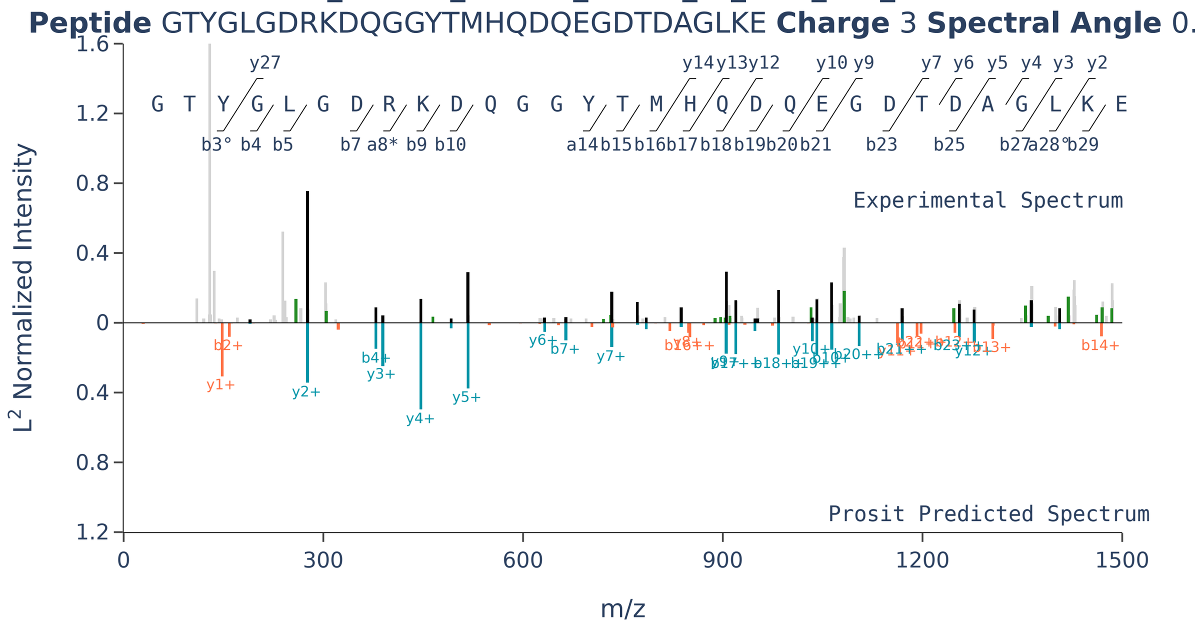


**Source** WSoh_221121_011221_HFGoe_L2_hTau_2h_R1 **Scan** 9302
**Peptide** QSKIGSLDN **Charge** 2 **Spectral Angle** 0.89

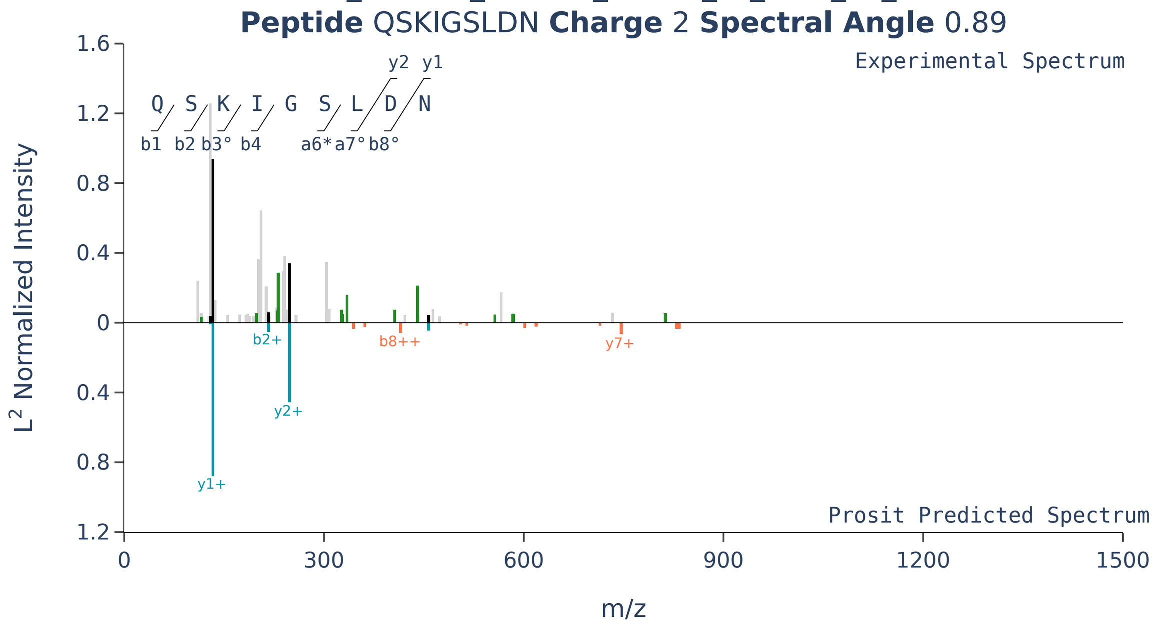


**Source** WSoh_221121_011221_HFGoe_L1_hTau_24h_R2 **Scan** 8933
**Peptide** PGGGNKKIETHKLTFRENAKAKTDHGAEI **Charge** 3 **Spectral Angle** 0.5

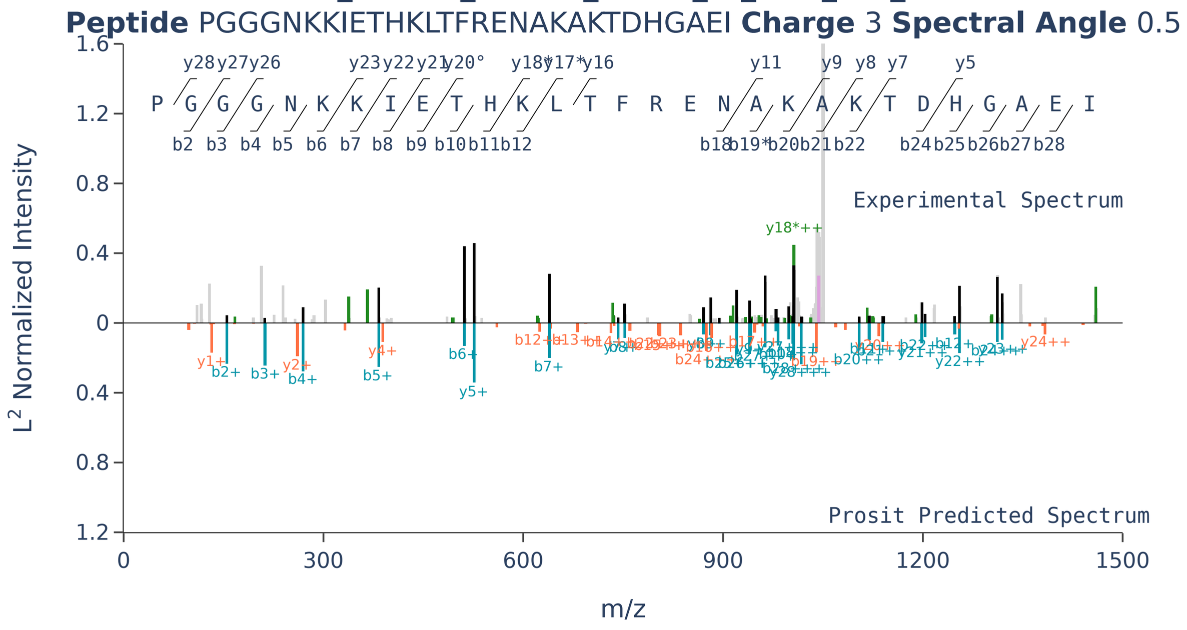


**Source** WSoh_221121_011221_HFGoe_L2_hTau_24h_R1 **Scan** 5198
**Peptide** RMVSKSKDGTGSDDKKAKGADGKTKI **Charge** 6 **Spectral Angle** 0.53

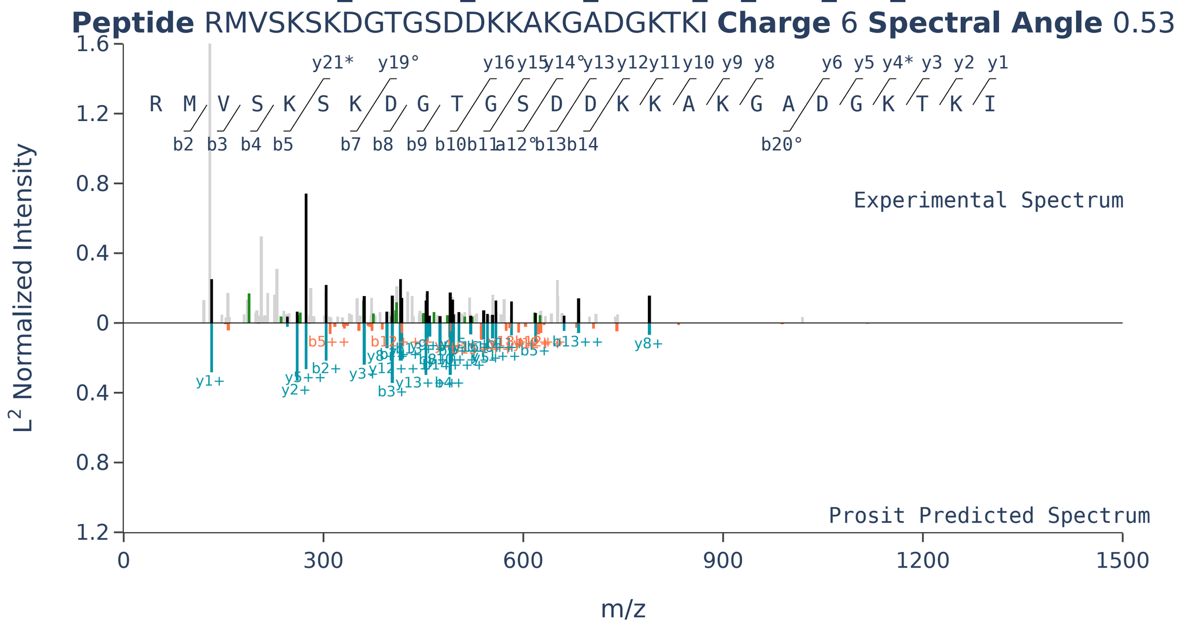


**Source** WSoh_221121_011221_HFGoe_L1_hTau_24h_R2 **Scan** 16395
**Peptide** VYKPVDLSKVTSKCGSLGNIHHKPGGGQVE **Charge** 4 **Spectral Angle** 0.46

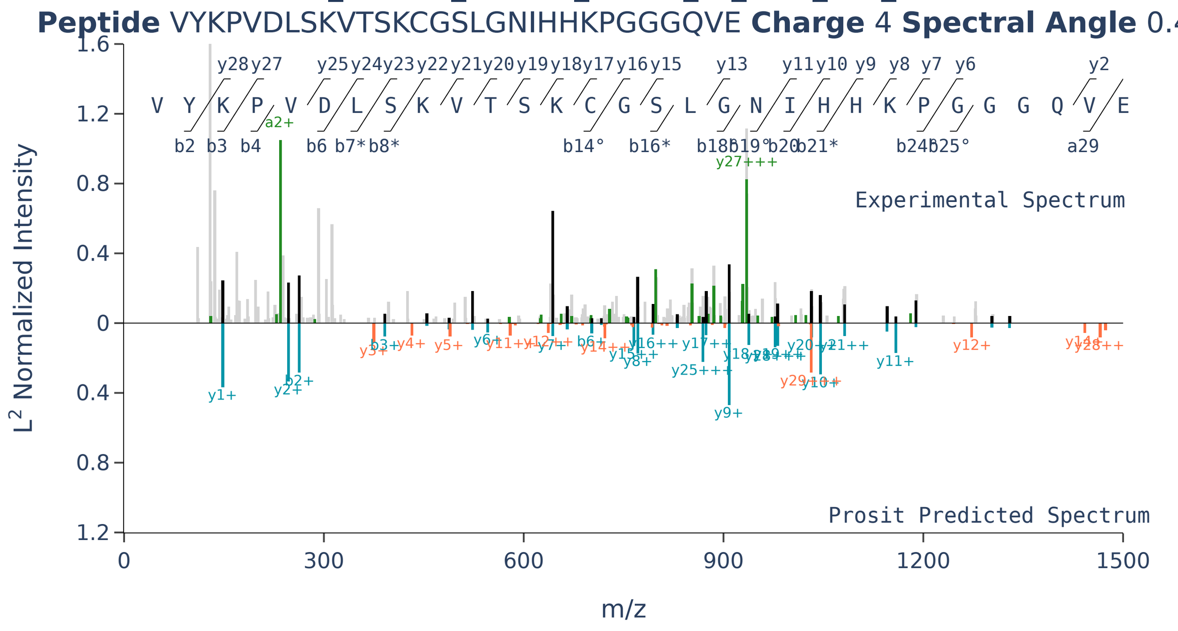


**Source** WSoh_221121_011221_HFGoe_L2_hTau_4h_R2 **Scan** 11603
**Peptide** VQSKCGSKDNIKHVPGGGSVQI **Charge** 3 **Spectral Angle** 0.63

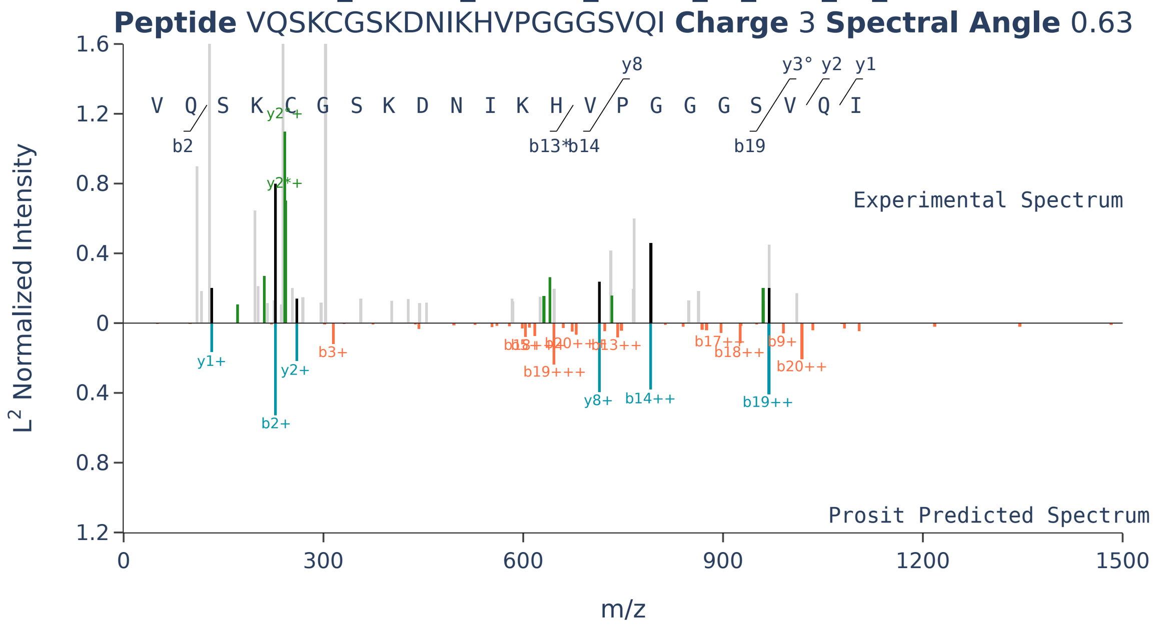


**Source** WSoh_221121_011221_HFGoe_L1_hTau_2h_R1 **Scan** 12225
**Peptide** INKKLDLSNVQSKCGSKDNIKHVPGGGSV **Charge** 4 **Spectral Angle** 0.79

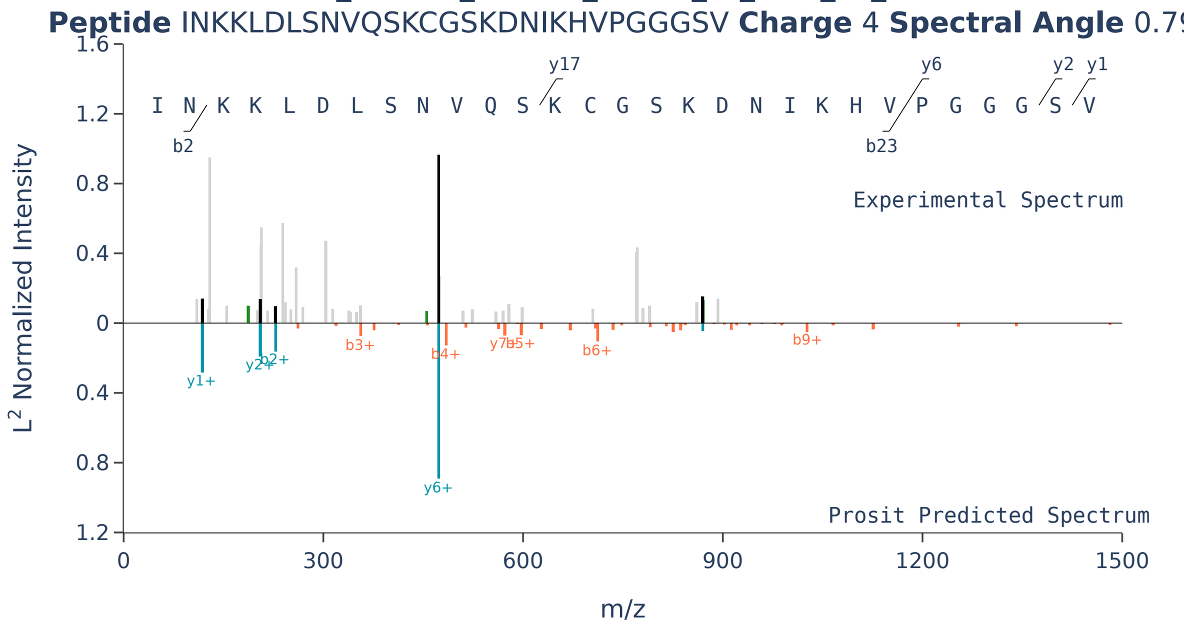


**Source** WSoh_221121_011221_HFGoe_L1_hTau_24h_R1 **Scan** 16338
**Peptide** GSTENLKHQPGGGKVQIINKKLDLSNVQS **Charge** 4 **Spectral Angle** 0.59

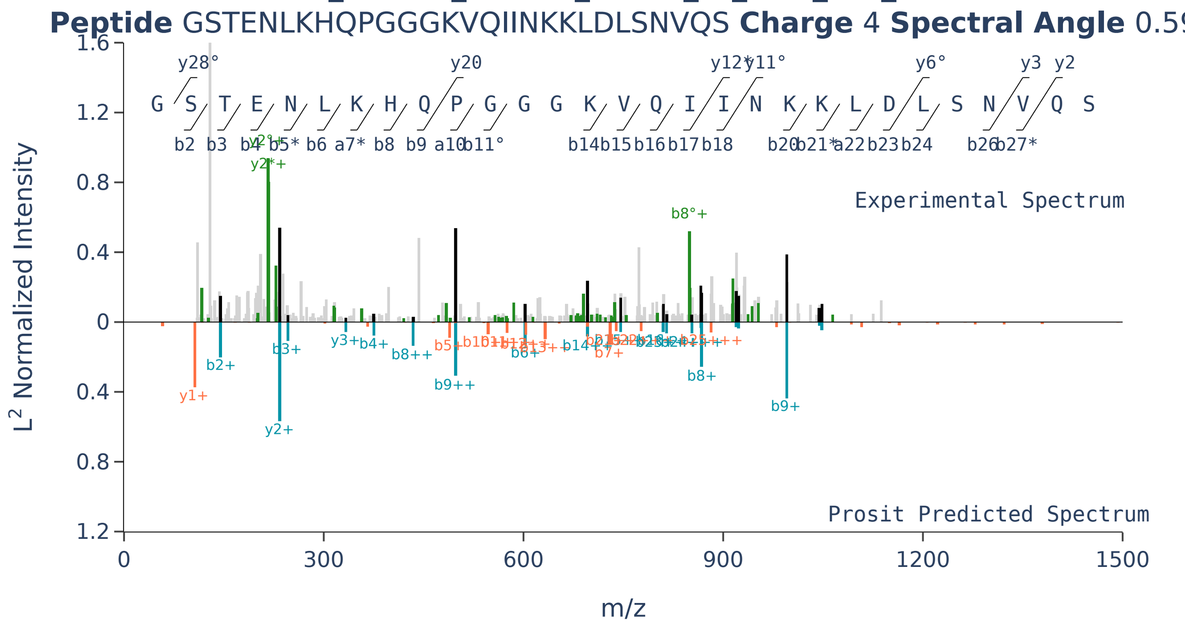


**Source** WSoh_221121_011221_HFGoe_L1_hTau_24h_R2 **Scan** 9475
**Peptide** RIPAKTPPAPKTPPSSGEPPKSGDRSGYSS **Charge** 5 **Spectral Angle** 0.54

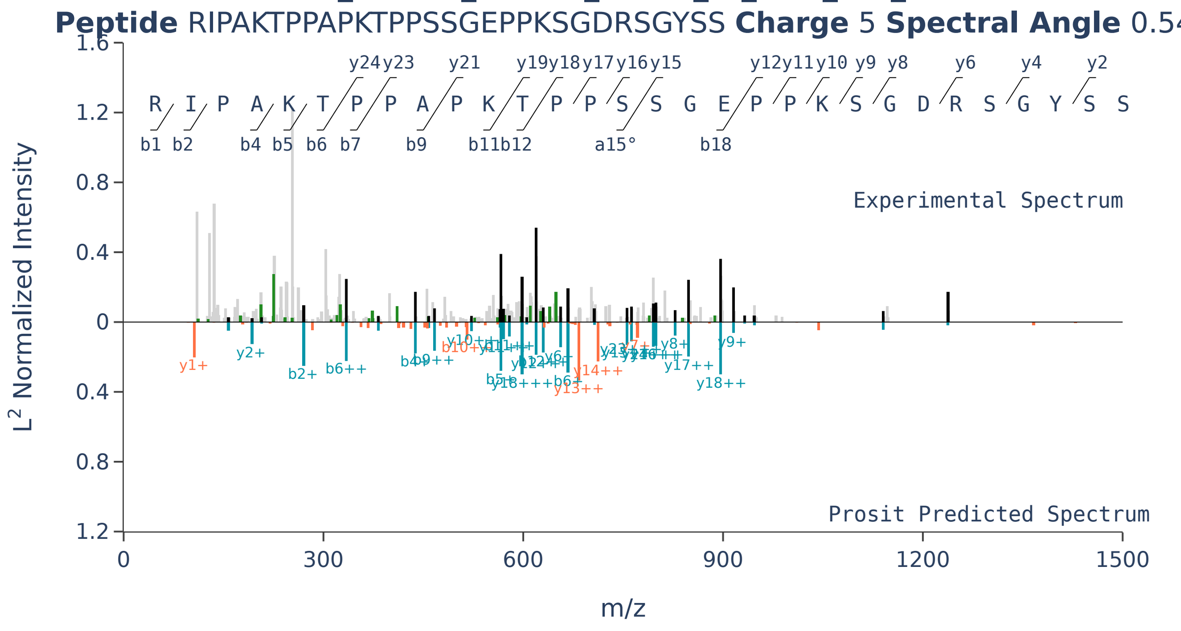


**Source** WSoh_221121_011221_HFGoe_L1_hTau_2h_R1 **Scan** 10639
**Peptide** KKLDLSNVQ **Charge** 2 **Spectral Angle** 0.28

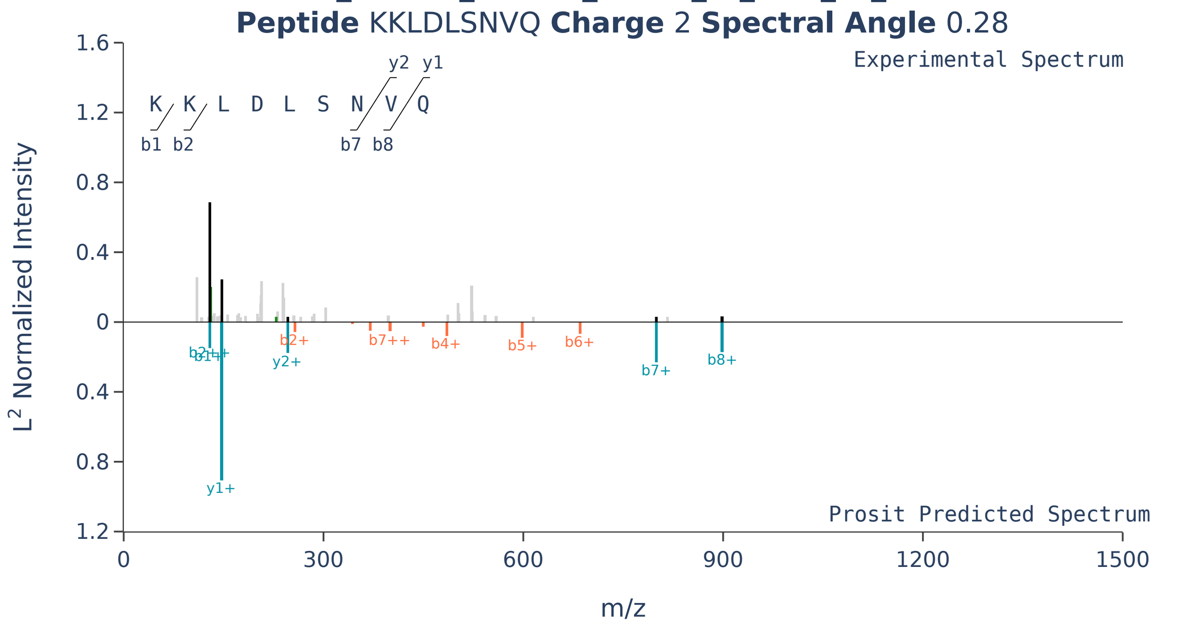


**Source** WSoh_221121_011221_HFGoe_L1_hTau_24h_R2 **Scan** 9239
**Peptide** PTPPTREPKKVAVVRTPPKSPSSAKSRLQT **Charge** 6 **Spectral Angle** 0.56

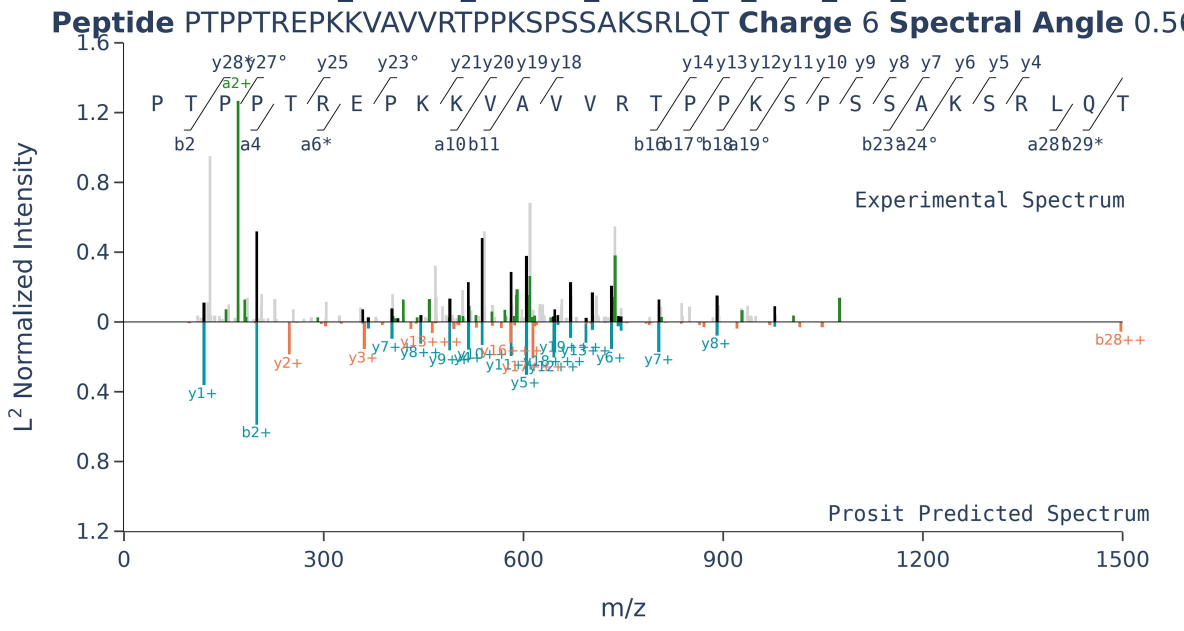


**Source** WSoh_221121_011221_HFGoe_L1_hTau_24h_R2 **Scan** 15043
**Peptide** AKAKTDHGAEIVYKSPVVSGDTSPRHLSNV **Charge** 6 **Spectral Angle** 0.48

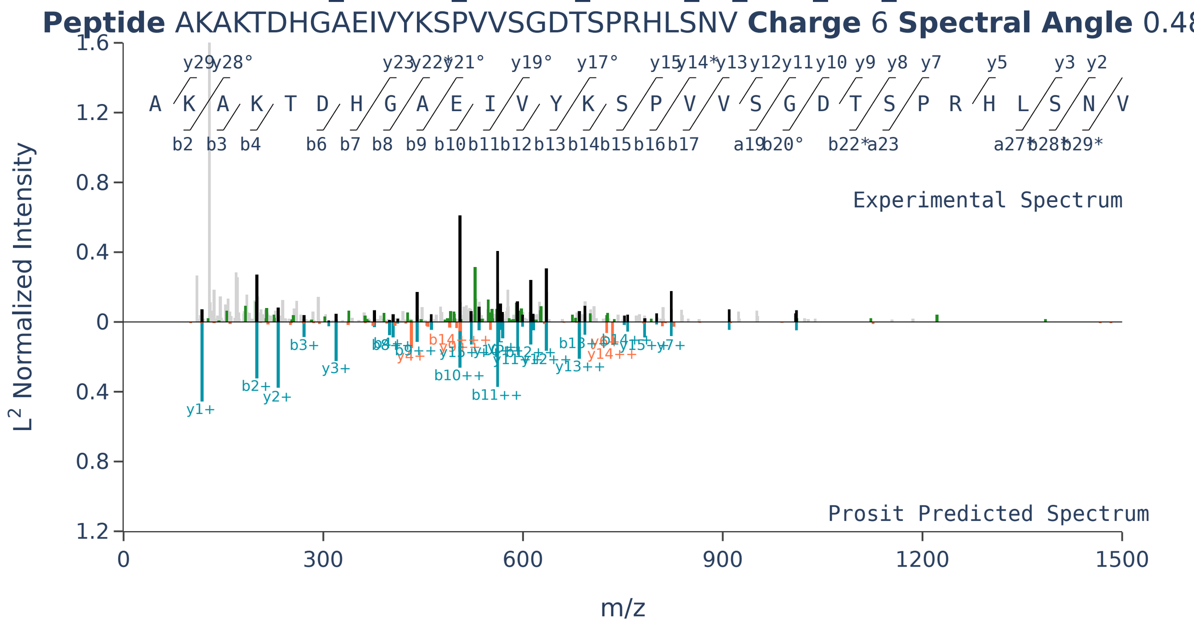


**Source** WSoh_221121_011221_HFGoe_L2_hTau_4h_R1 **Scan** 6755
**Peptide** GKTKIATPRGAAPPGQKGQA **Charge** 3 **Spectral Angle** 0.62

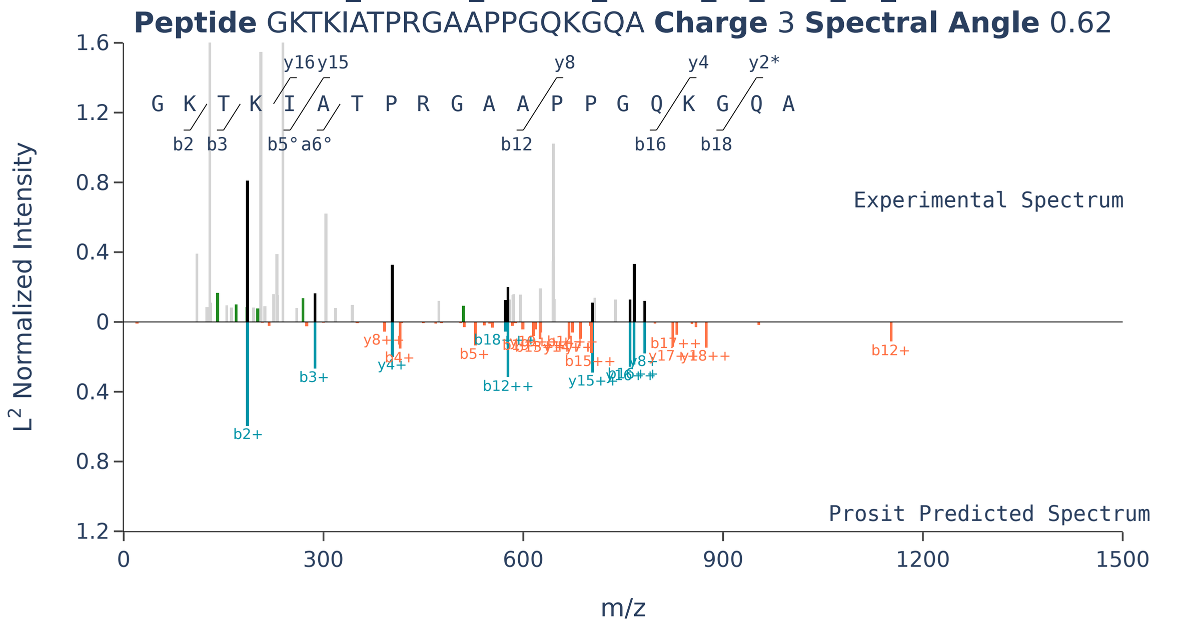

**Source** WSoh_221121_011221_HFGoe_L2_hTau_1h_R1 **Scan** 17025
**Peptide** IVYKPVDLSKVTSK **Charge** 3 **Spectral Angle** 0.73

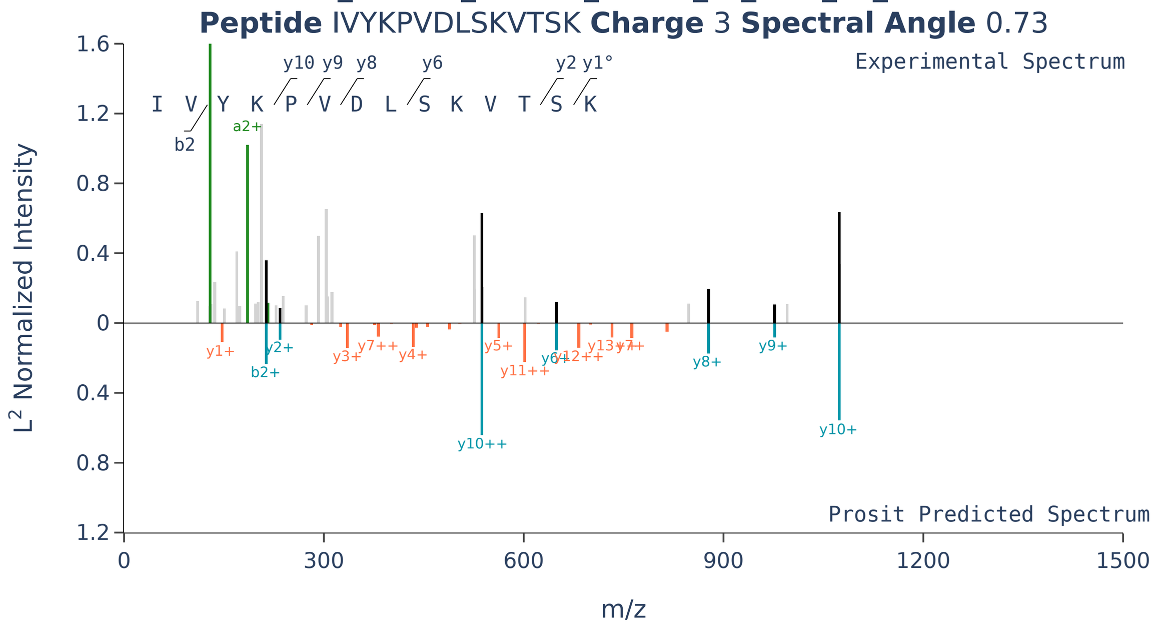

**Source** WSoh_221121_011221_HFGoe_L2_hTau_24h_R1 **Scan** 11260
**Peptide** ANATRIPAKTPPAPKTPPSSG **Charge** 4 **Spectral Angle** 0.55

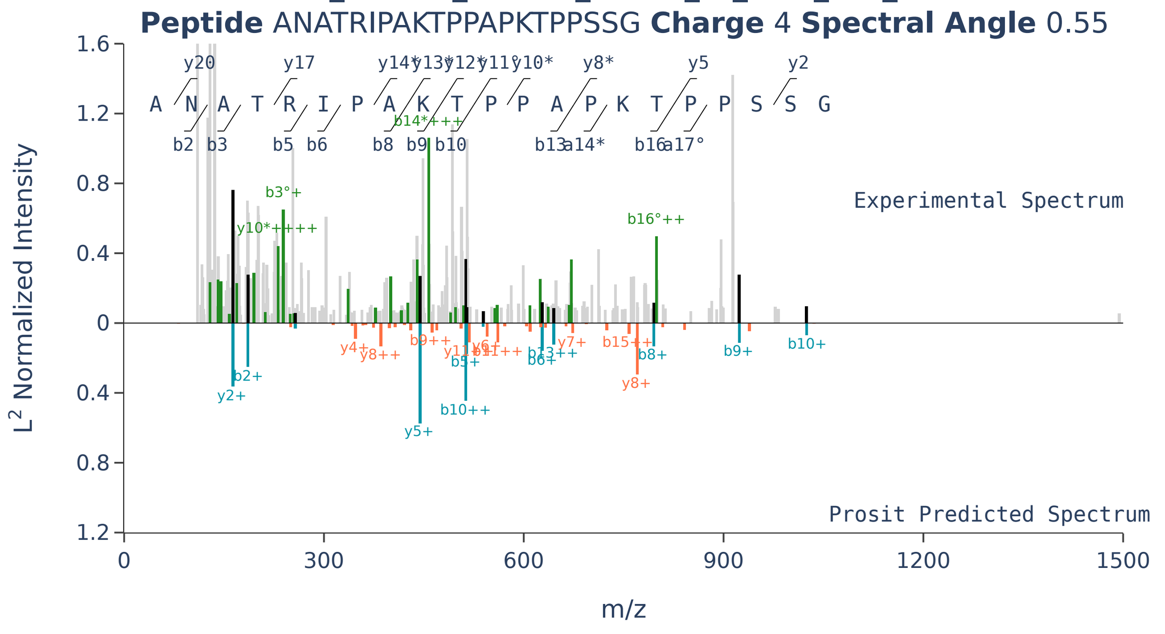

**Source** WSoh_221121_011221_HFGoe_L1_hTau_4h_R2 **Scan** 6632
**Peptide** GKTKIATPRGAAPPGQKGQAN **Charge** 3 **Spectral Angle** 0.63

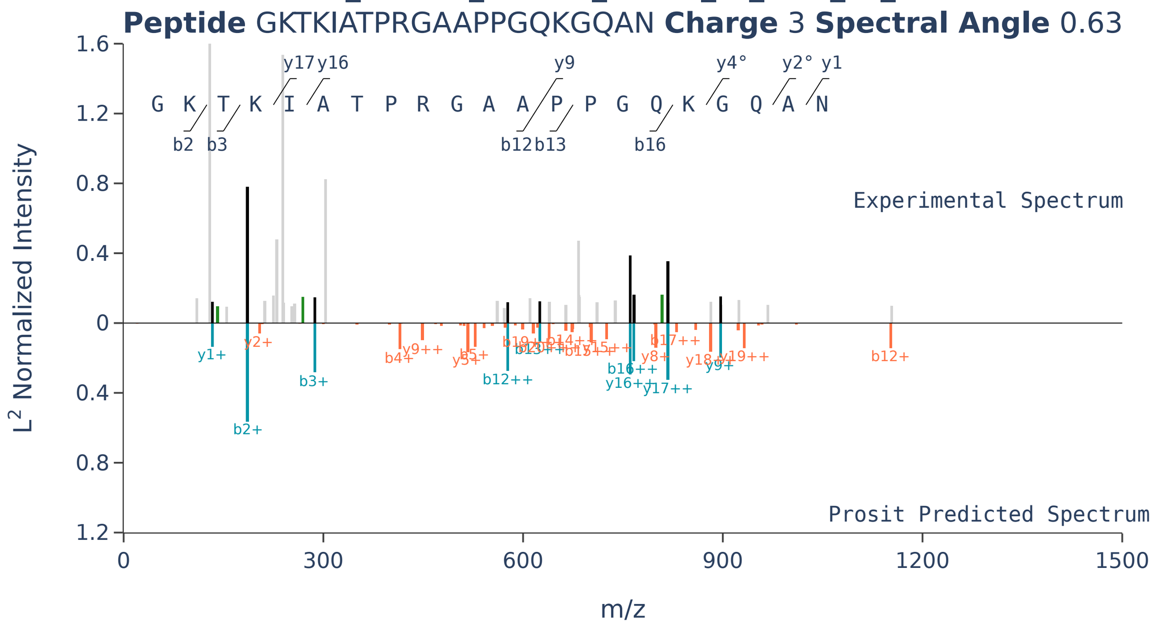

**Source** WSoh_221121_011221_HFGoe_L2_hTau_4h_R2 **Scan** 5390
**Peptide** SKSKDGTGSDDKKAKGADGKTKIATPRG **Charge** 3 **Spectral Angle** 0.61

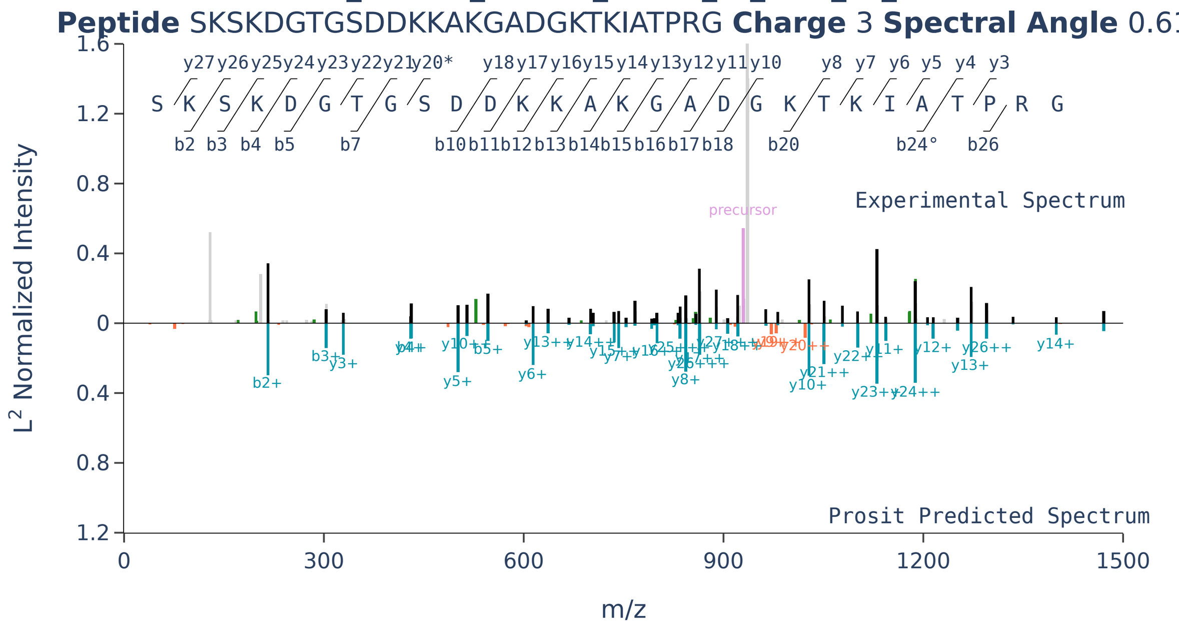

**Source** WSoh_221121_011221_HFGoe_L2_hTau_24h_R2 **Scan** 16018
**Peptide** EVMEDHAGTYGL **Charge** 2 **Spectral Angle** 0.58

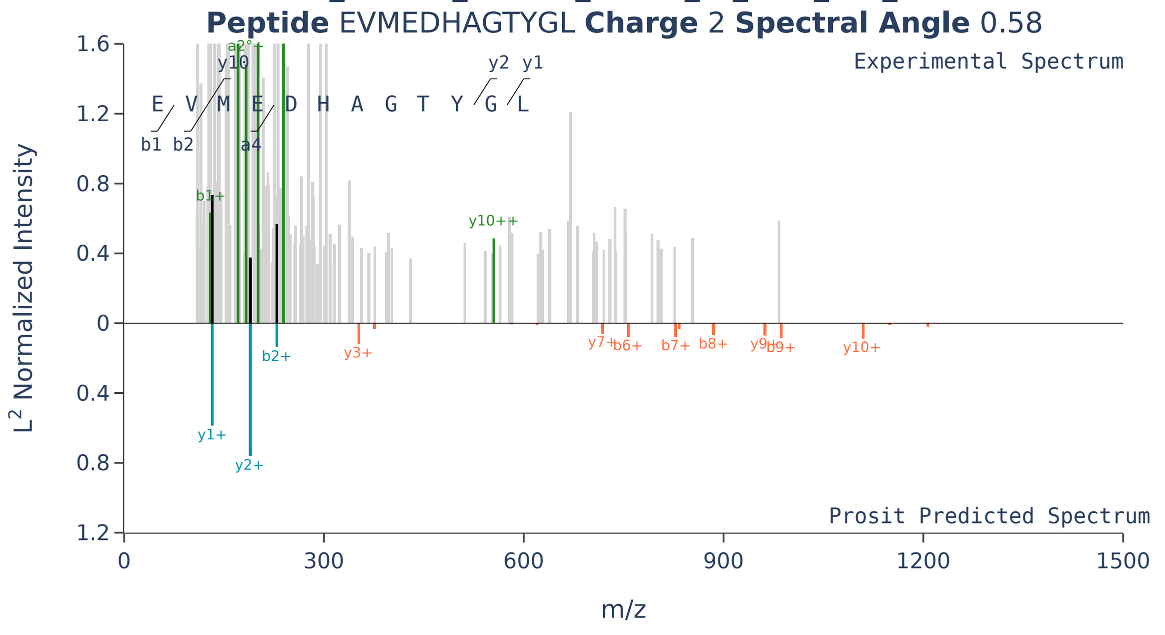

**Source** WSoh_221121_011221_HFGoe_L2_hTau_4h_R1 **Scan** 7068
**Peptide** SKCGSLGNIHHKPGGGQVEVKS **Charge** 3 **Spectral Angle** 0.68

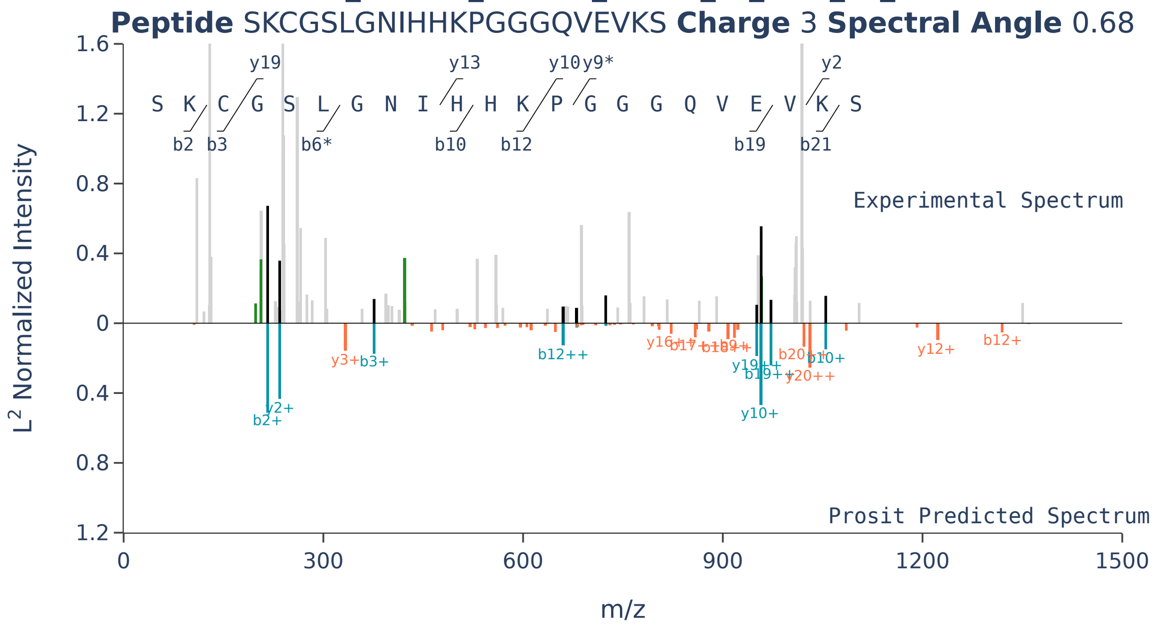

**Source** WSoh_221121_011221_HFGoe_L2_hTau_4h_R2 **Scan** 20400
**Peptide** GIGDTPSLEDEA **Charge** 2 **Spectral Angle** 0.58

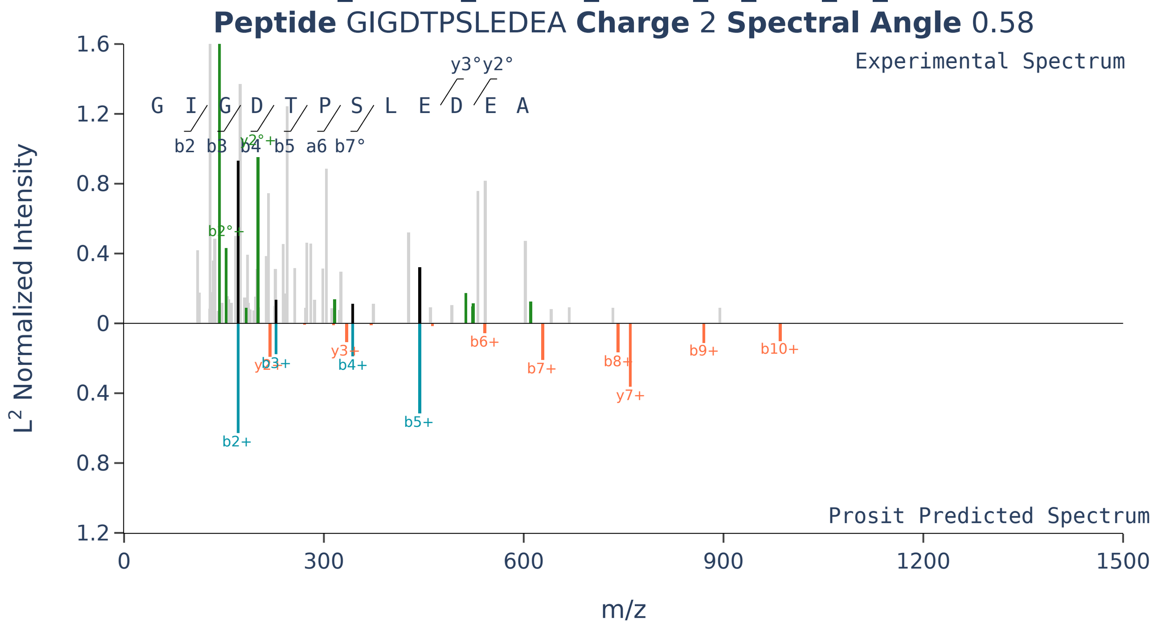

**Source** WSoh_221121_011221_HFGoe_L1_hTau_24h_R1 **Scan** 5407
**Peptide** AGHVTQARMVSKSKDGTGSDDKKA **Charge** 3 **Spectral Angle** 0.7

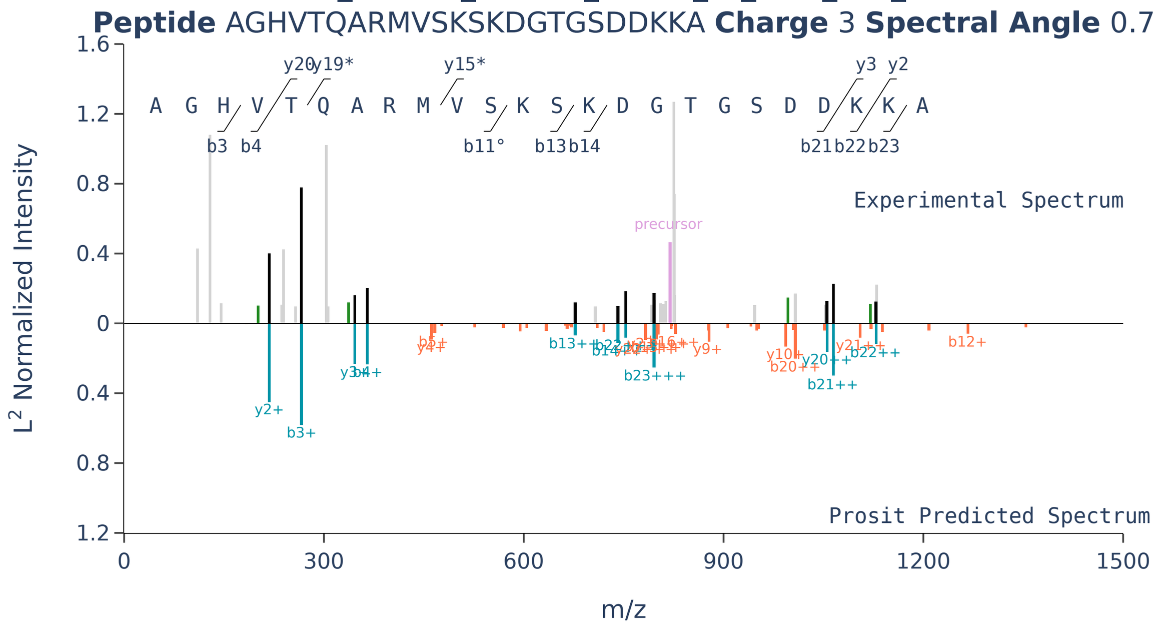

**Source** WSoh_221121_011221_HFGoe_L1_hTau_2h_R2 **Scan** 13441
**Peptide** IINKKLDLSNVQSKC **Charge** 3 **Spectral Angle** 0.67

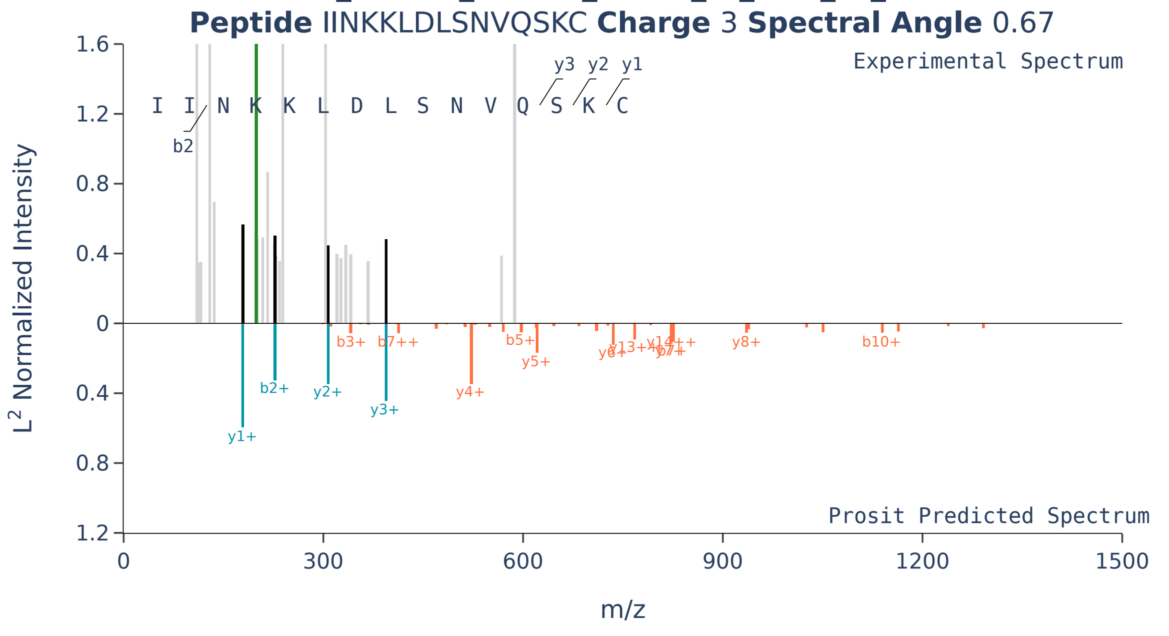

**Source** WSoh_221121_011221_HFGoe_L1_hTau_4h_R1 **Scan** 12493
**Peptide** KCGSKDNIKHVPGGGSVQLV **Charge** 4 **Spectral Angle** 0.44

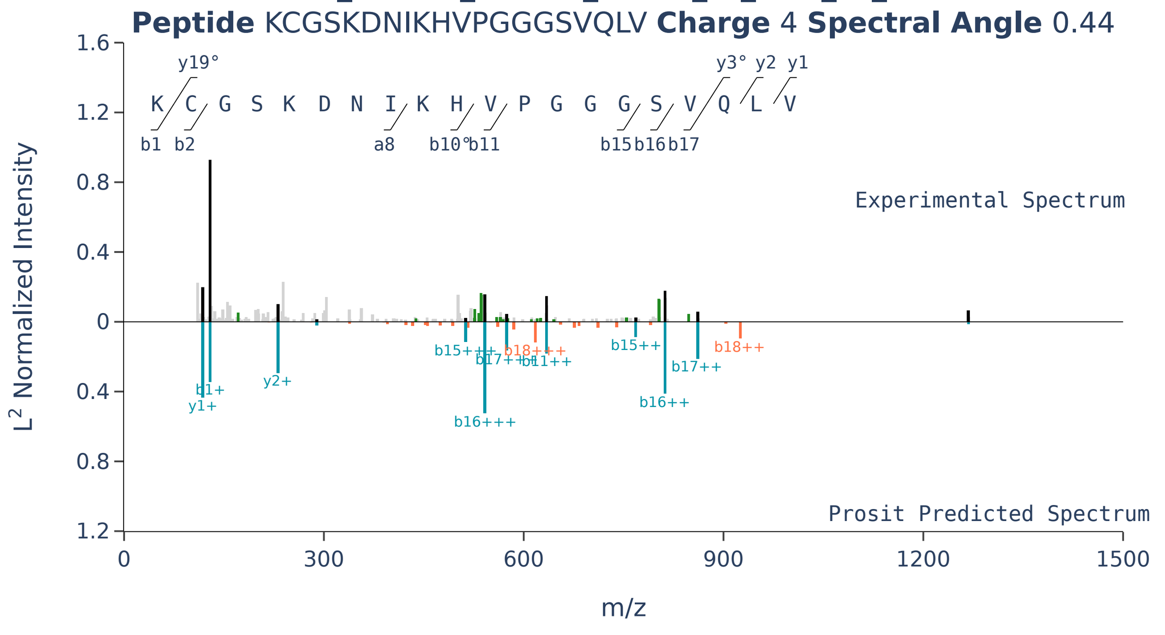

**Source** WSoh_221121_011221_HFGoe_L1_hTau_24h_R1 **Scan** 23507
**Peptide** LQTAPVPMPDLKNVKSKIGSTENLK **Charge** 4 **Spectral Angle** 0.73

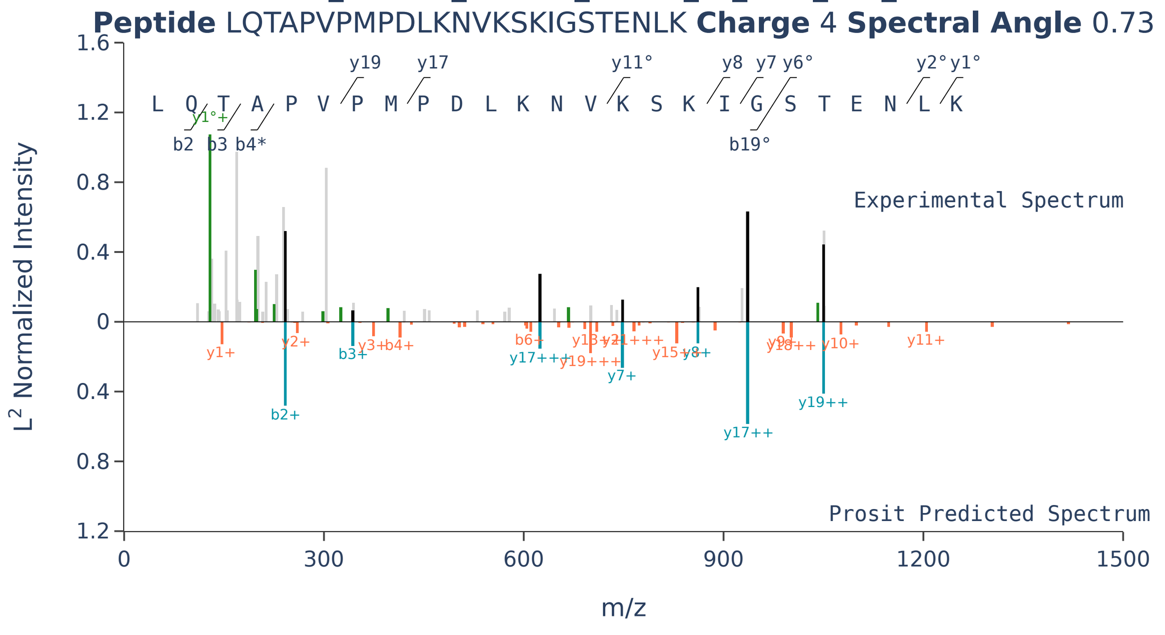

**Source** WSoh_221121_011221_HFGoe_L2_hTau_4h_R2 **Scan** 5354
**Peptide** PPKSPSSAKSR **Charge** 3 **Spectral Angle** 0.64

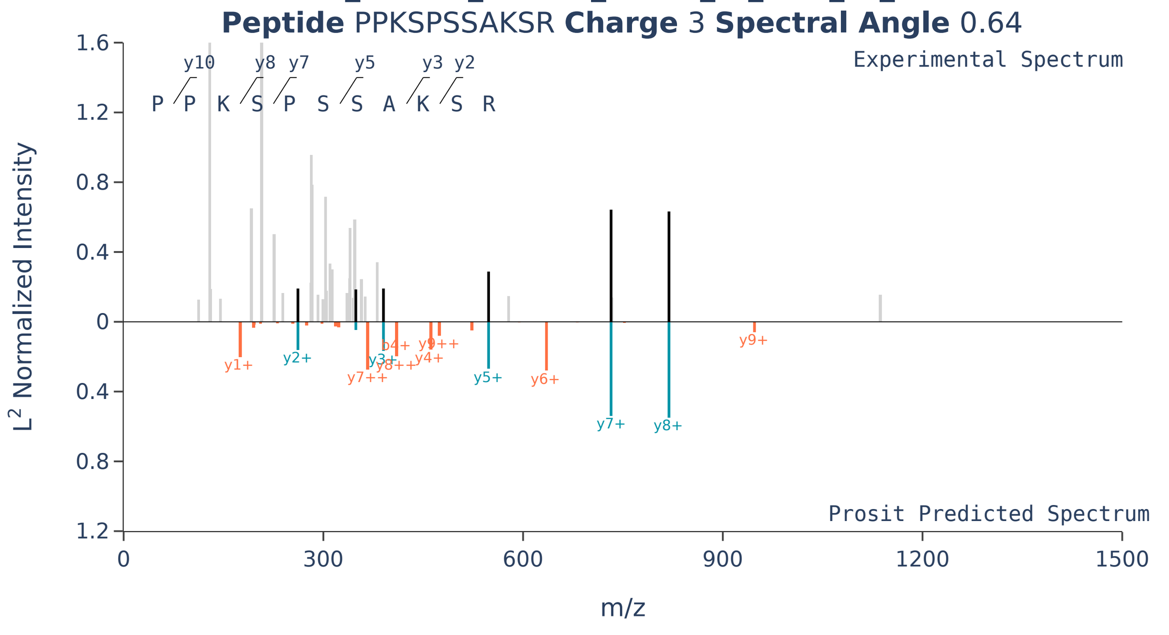

**Source** WSoh_221121_011221_HFGoe_L2_hTau_4h_R1 **Scan** 22167
**Peptide** VYKPVDLSKVTSKCGSLGNI **Charge** 3 **Spectral Angle** 0.62

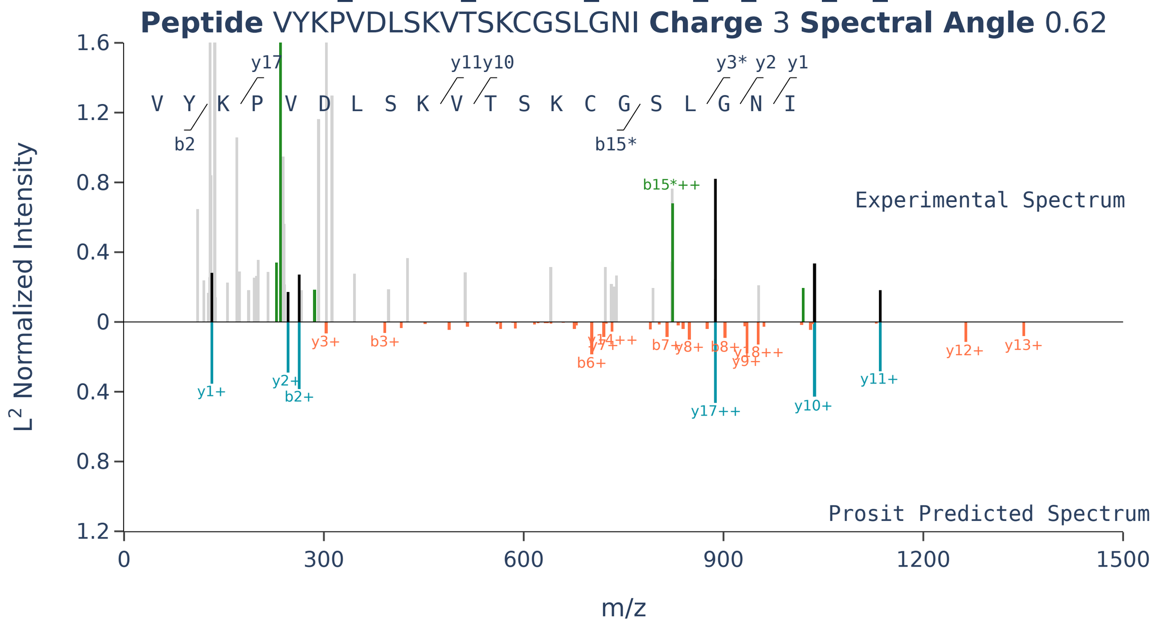

**Source** WSoh_221121_011221_HFGoe_L1_hTau_24h_R2 **Scan** 15821
**Peptide** AKTDHGAEIVYKSPVVSGDTSPRHLSNVS **Charge** 3 **Spectral Angle** 0.55

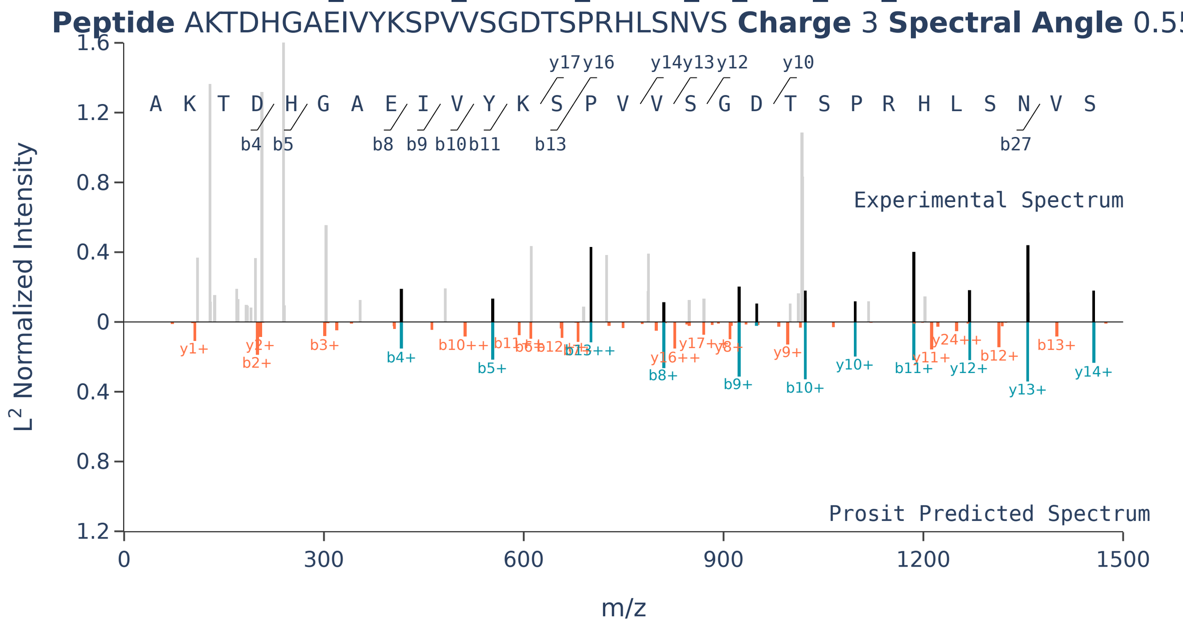

**Source** WSoh_221121_011221_HFGoe_L2_hTau_1h_R2 **Scan** 18343
**Peptide** AAQPHTEIPEGTTAEEAGL **Charge** 2 **Spectral Angle** 0.81

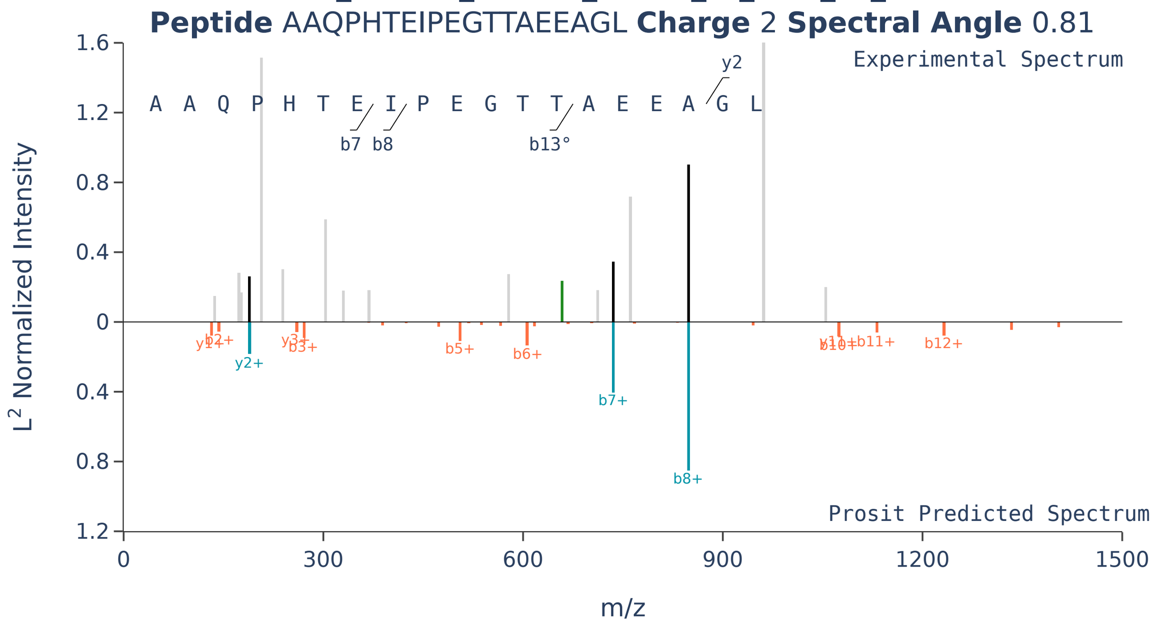

**Source** WSoh_221121_011221_HFGoe_L2_hTau_4h_R1 **Scan** 23194
**Peptide** VPGGGSVQIVYKPVDLSKVTSKC **Charge** 4 **Spectral Angle** 0.69

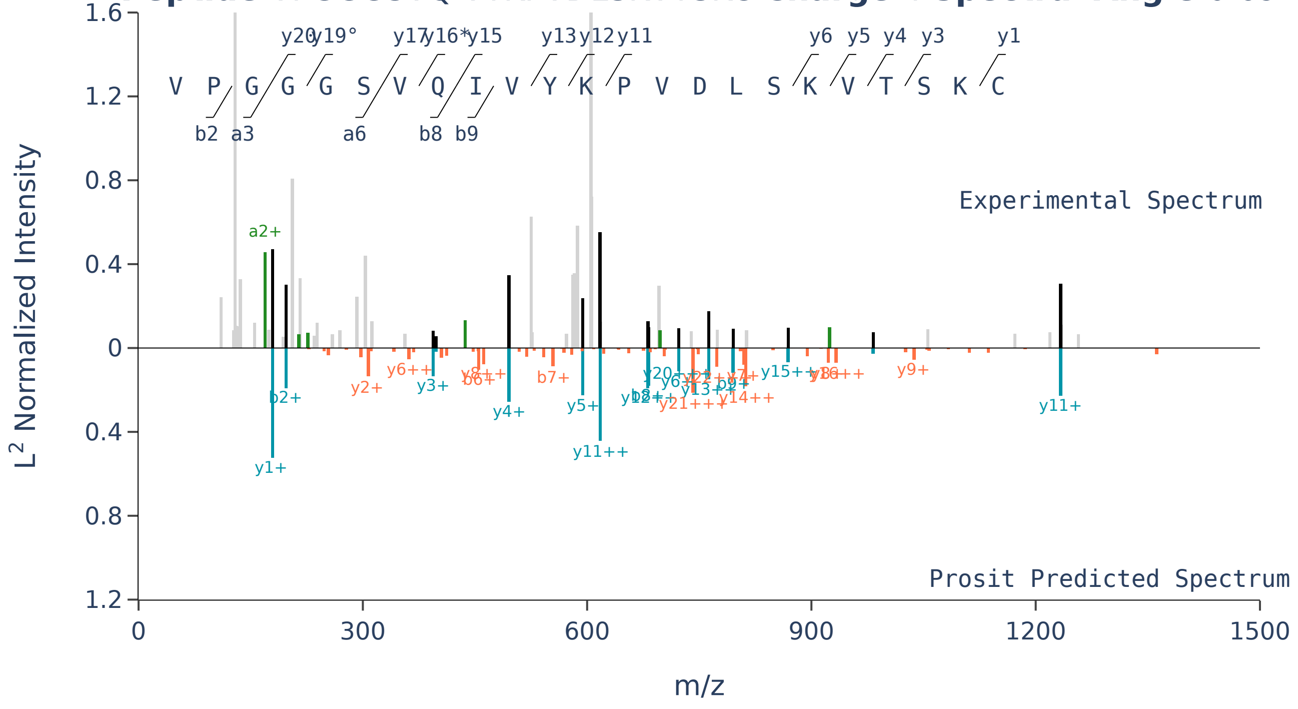


**Source** WSoh_221121_011221_HFGoe_L2_hTau_4h_R1 **Scan** 18774
**Peptide** KLDLSNVQSKCGSKDNIKHVPGGGSVQIVY **Charge** 5 **Spectral Angle** 0.64

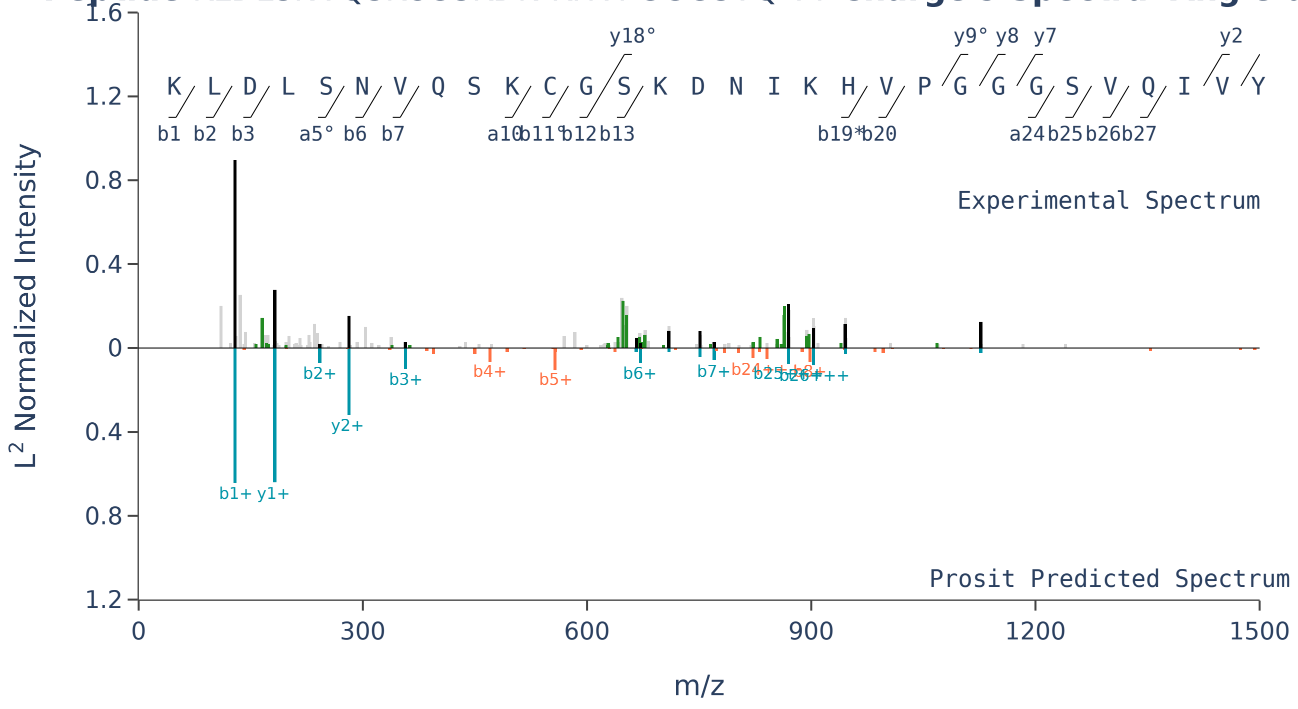


**Source** WSoh_221121_011221_HFGoe_L1_hTau_4h_R1 **Scan** 28624
**Peptide** TLADEVSASLAKQGL **Charge** 3 **Spectral Angle** 0.63

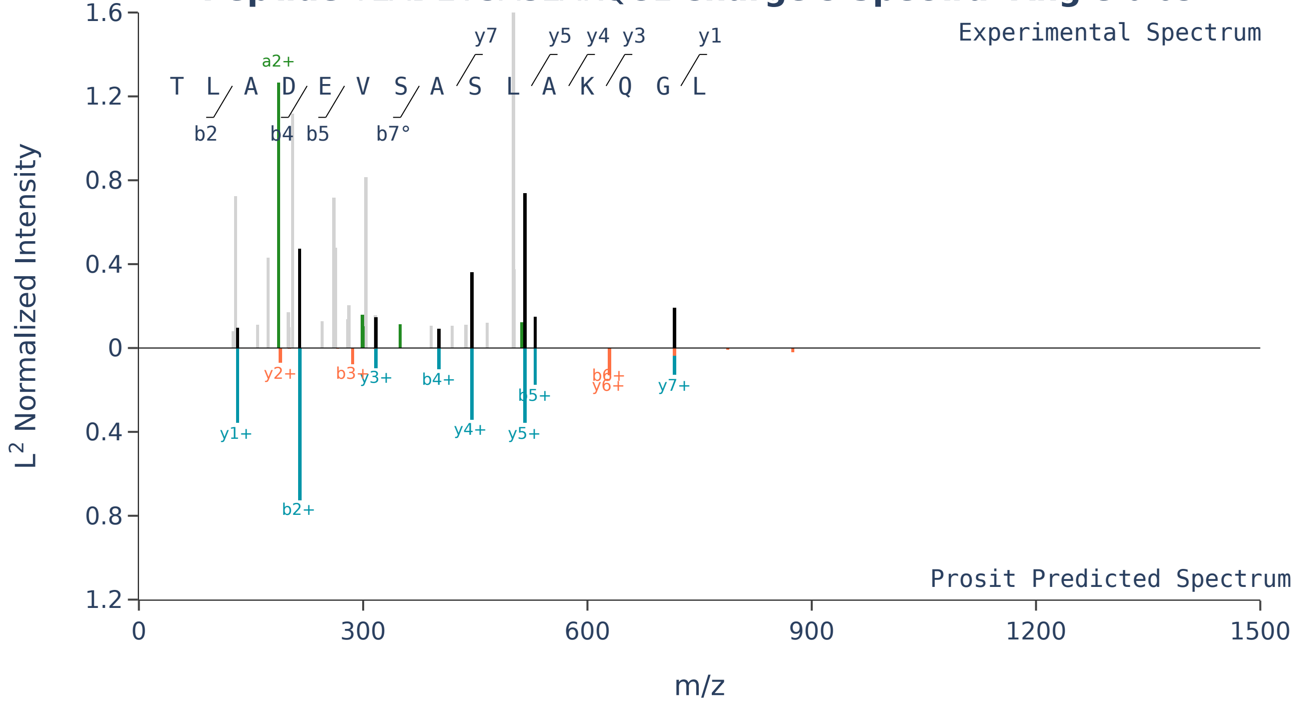


**Source** WSoh_221121_011221_HFGoe_L1_hTau_4h_R1 **Scan** 20306
**Peptide** KLDFKDRVQSKIGSLDNITHVPGGGNKKI **Charge** 5 **Spectral Angle** 0.82

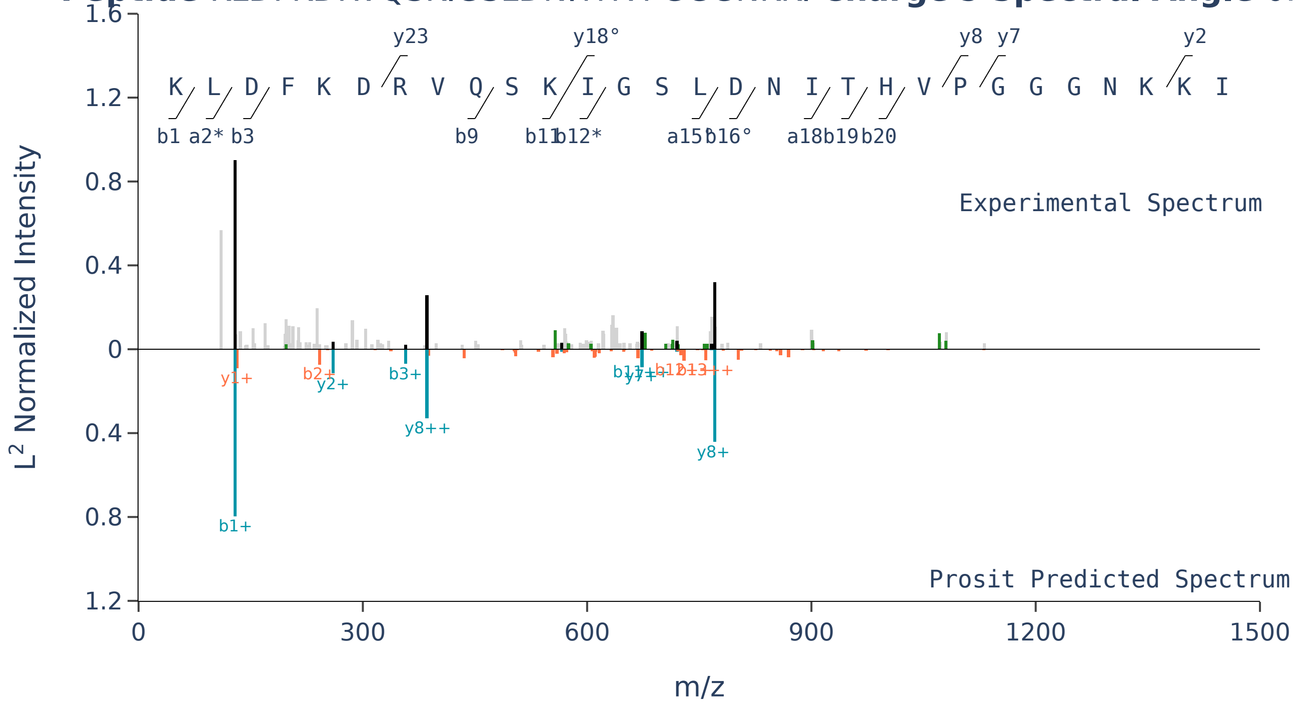


**Source** WSoh_221121_011221_HFGoe_L1_hTau_4h_R2 **Scan** 19767
**Peptide** VYKSPVVSGDTSPRHLSNVSSTGSIDMVD **Charge** 4 **Spectral Angle** 0.54

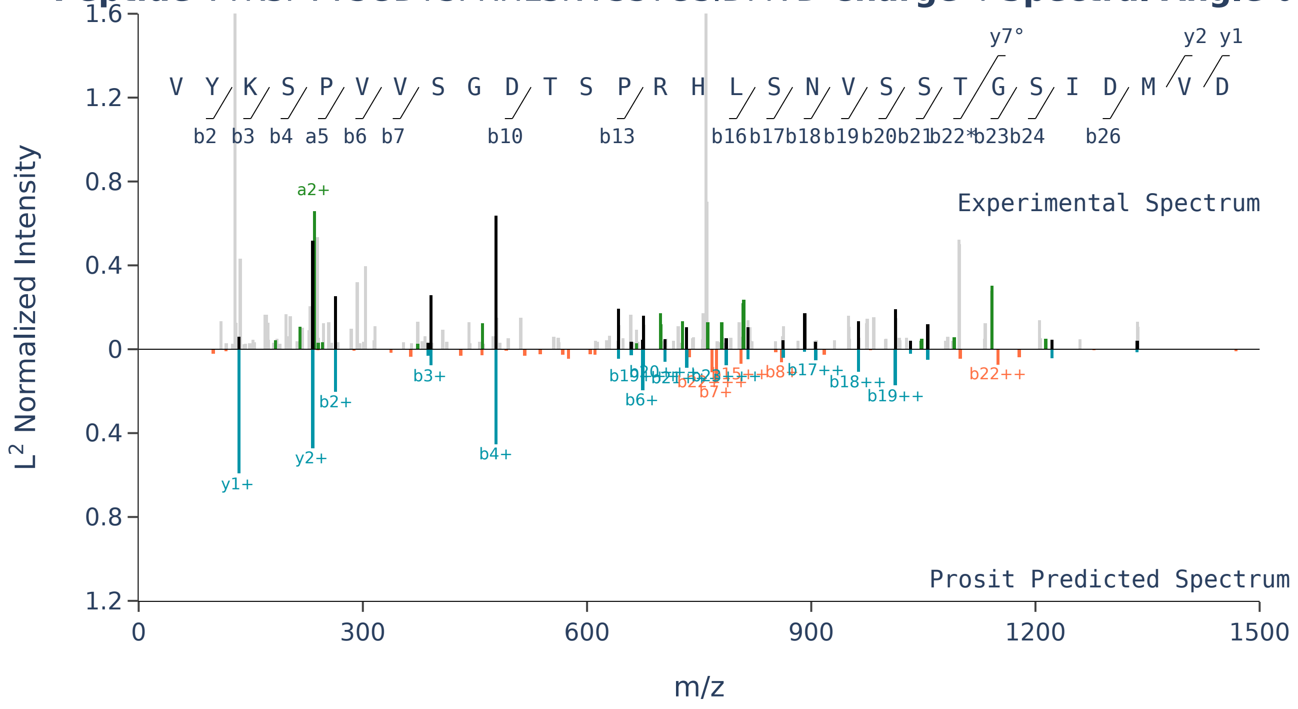


**Source** WSoh_221121_011221_HFGoe_L2_hTau_24h_R1 **Scan** 23436
**Peptide** PHTEIPEGTTAEEAGIGDTPSLEDEA **Charge** 3 **Spectral Angle** 0.7

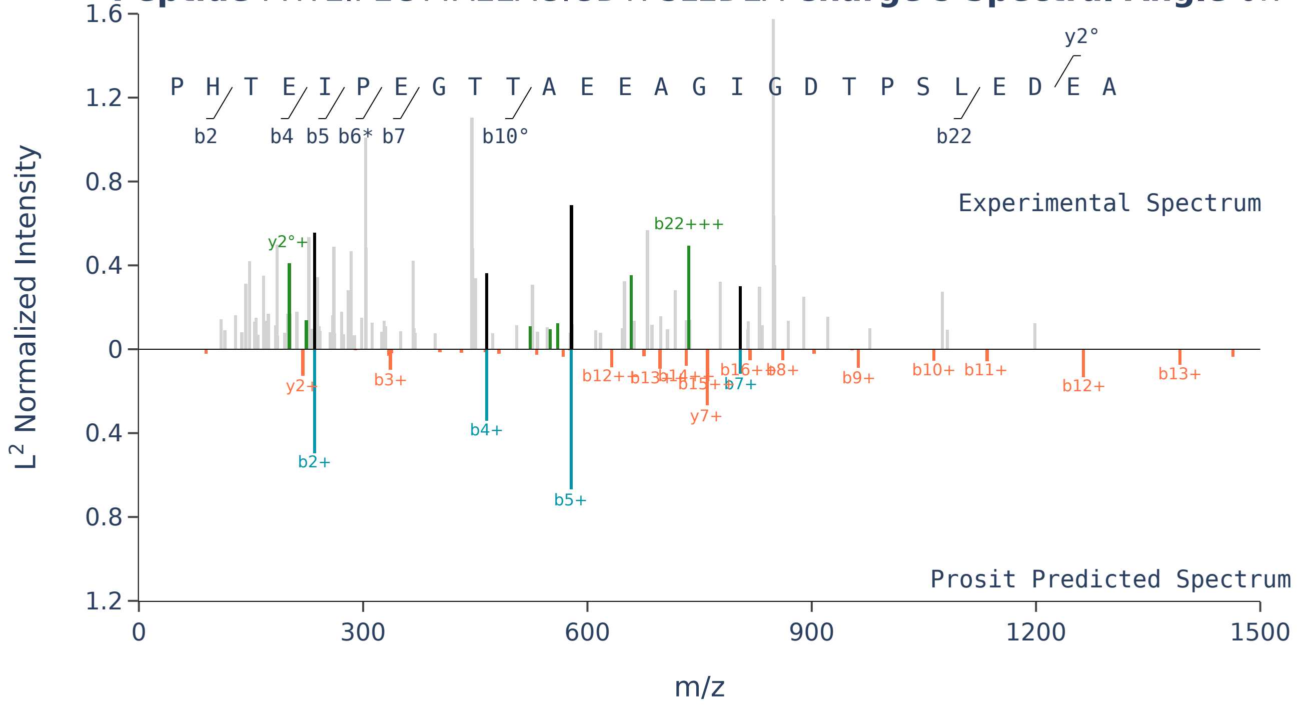


**Source** WSoh_221121_011221_HFGoe_L1_hTau_4h_R1 **Scan** 20347
**Peptide** VYKSPVVSGDTSPRHLSNVSSTGSIDMV **Charge** 4 **Spectral Angle** 0.67

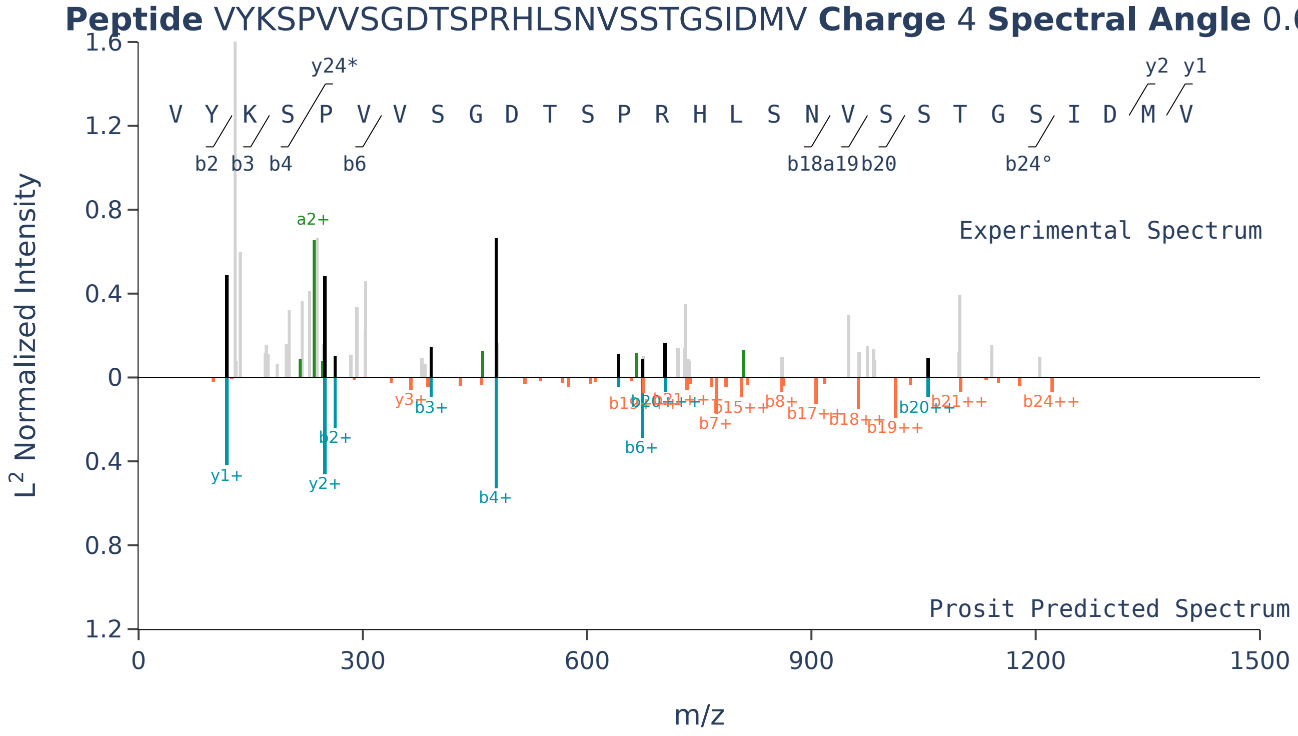

**Source** HRoetschke_211222_160123_HFGoe_IF2_b1R1_24h **Scan** 9814
**Peptide** TVLVREG **Charge** 2 **Spectral Angle** 0.94

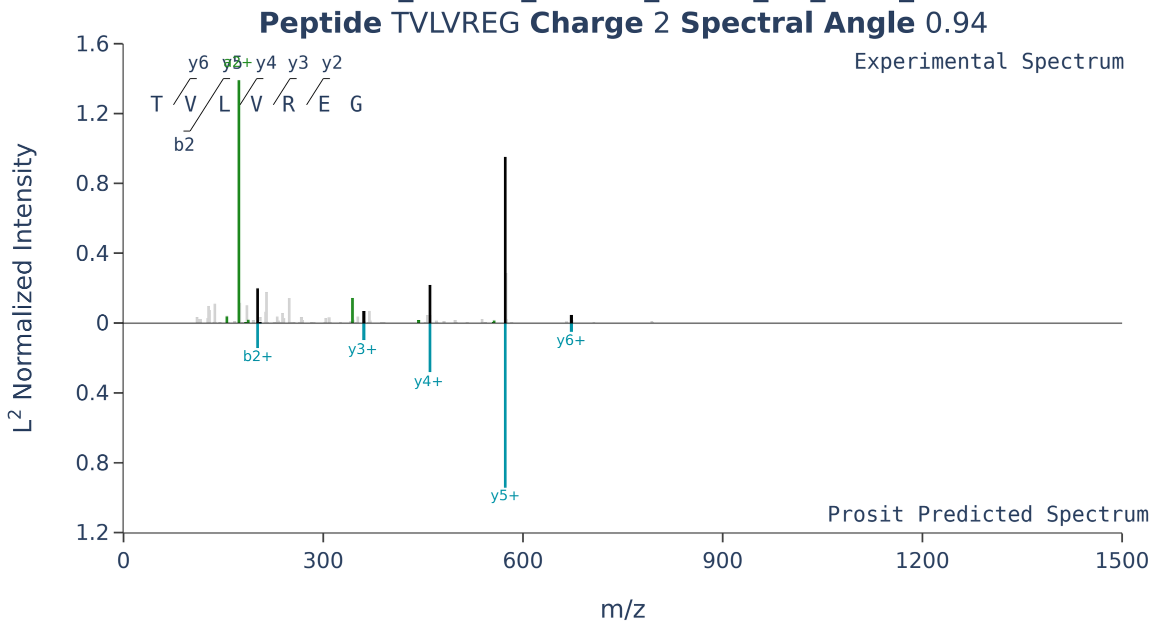


**Source** HRoetschke_211222_160123_HFGoe_IF2_b1R2_24h **Scan** 11717
**Peptide** MASGAVIE **Charge** 2 **Spectral Angle** 0.95

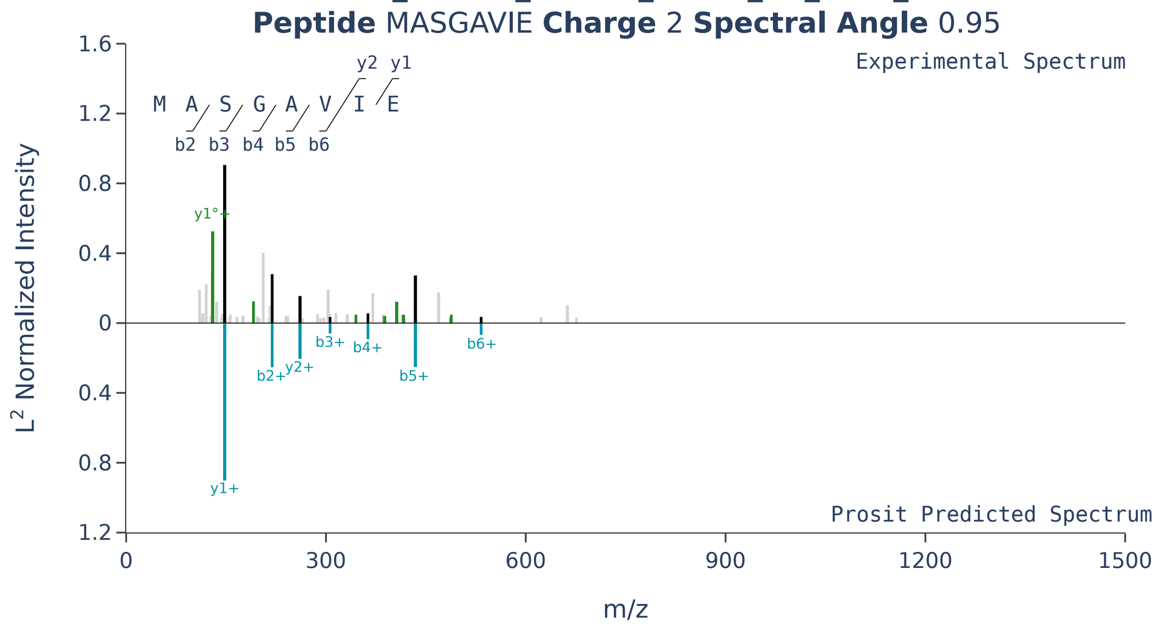


**Source** HRoetschke_211222_160123_HFGoe_IF2_b2R1_1h **Scan** 6476
**Peptide** GFQKPAQ **Charge** 2 **Spectral Angle** 0.92

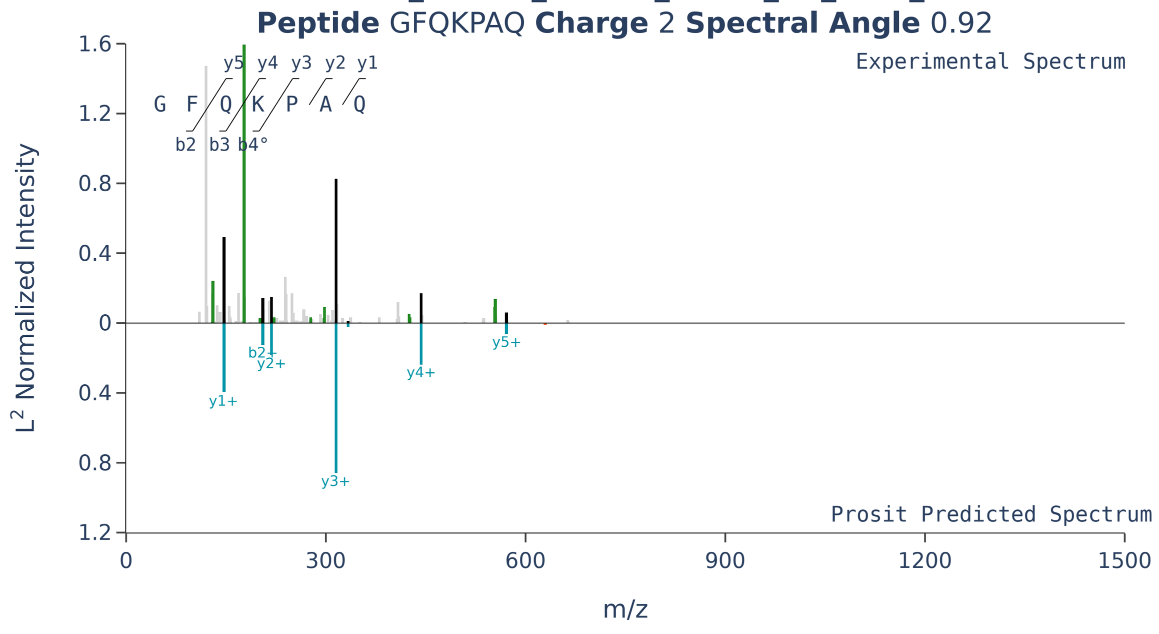


**Source** HRoetschke_211222_160123_HFGoe_IF2_b1R1_2h **Scan** 10463
**Peptide** NQVIDQETA **Charge** 1 **Spectral Angle** 0.93

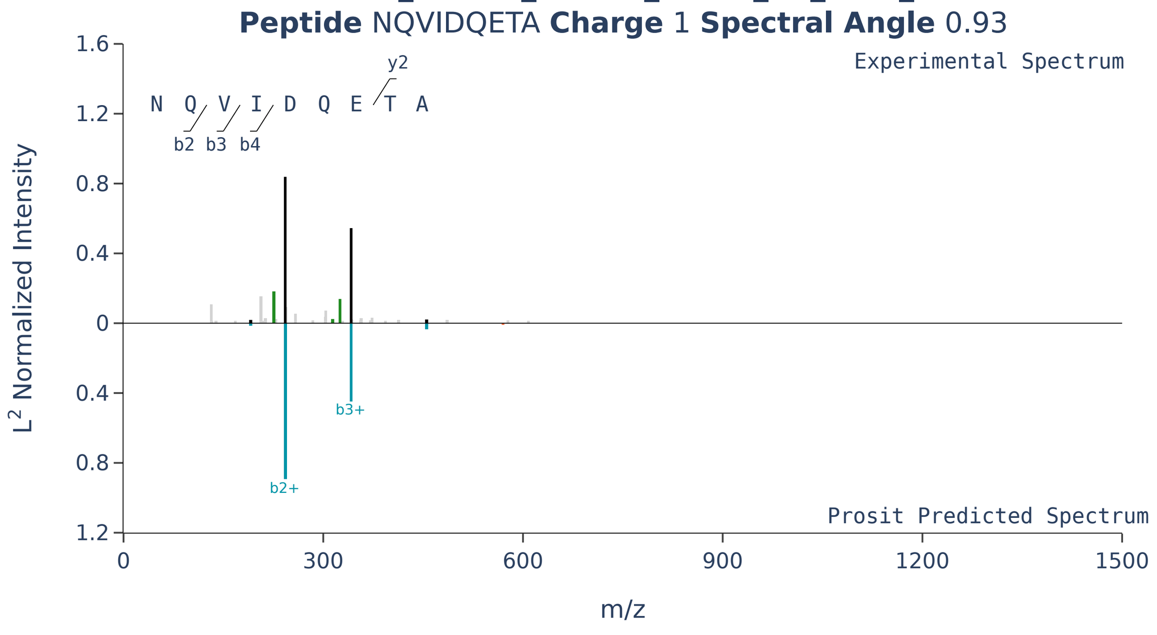


**Source** HRoetschke_211222_160123_HFGoe_IF2_b2R1_4h **Scan** 9863
**Peptide** DEVTVVR **Charge** 2 **Spectral Angle** 0.96

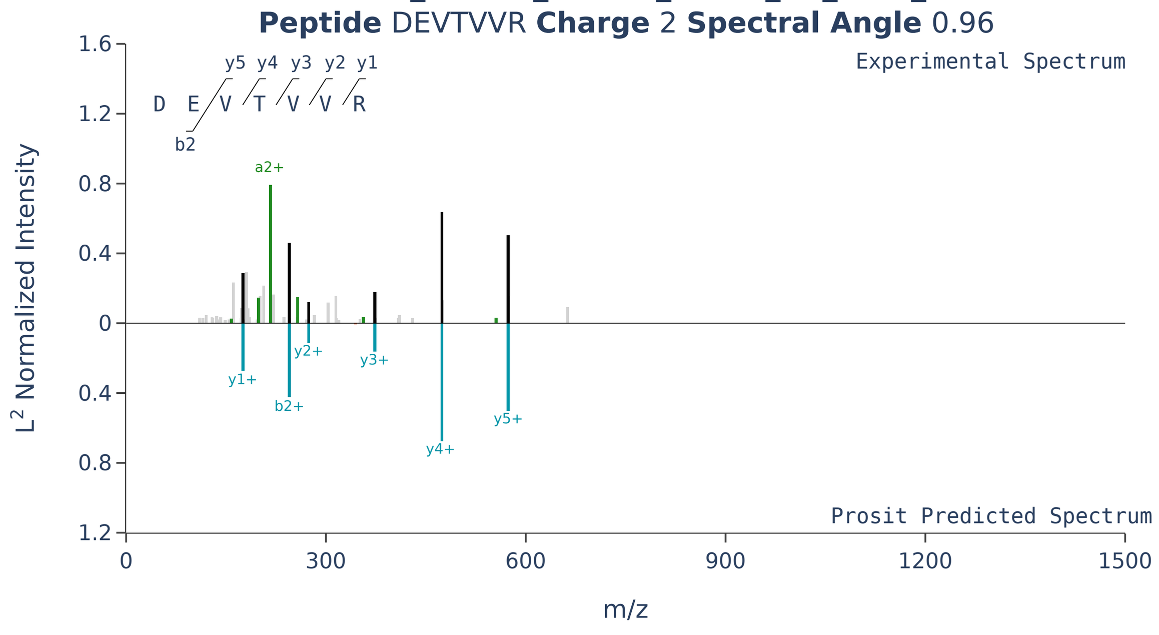


**Source** HRoetschke_211222_160123_HFGoe_IF2_b1R1_24h **Scan** 14110
**Peptide** GGESQFV **Charge** 2 **Spectral Angle** 0.91

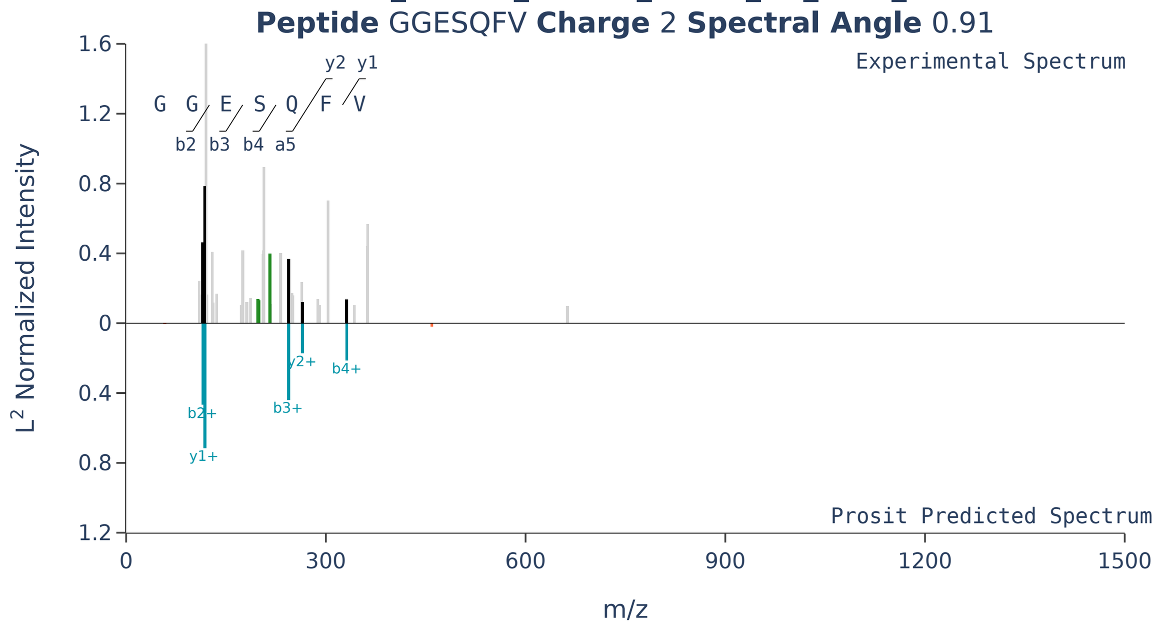


**Source** HRoetschke_211222_160123_HFGoe_IF2_b1R1_4h **Scan** 6728
**Peptide** EMGHKVILRR **Charge** 3 **Spectral Angle** 0.95

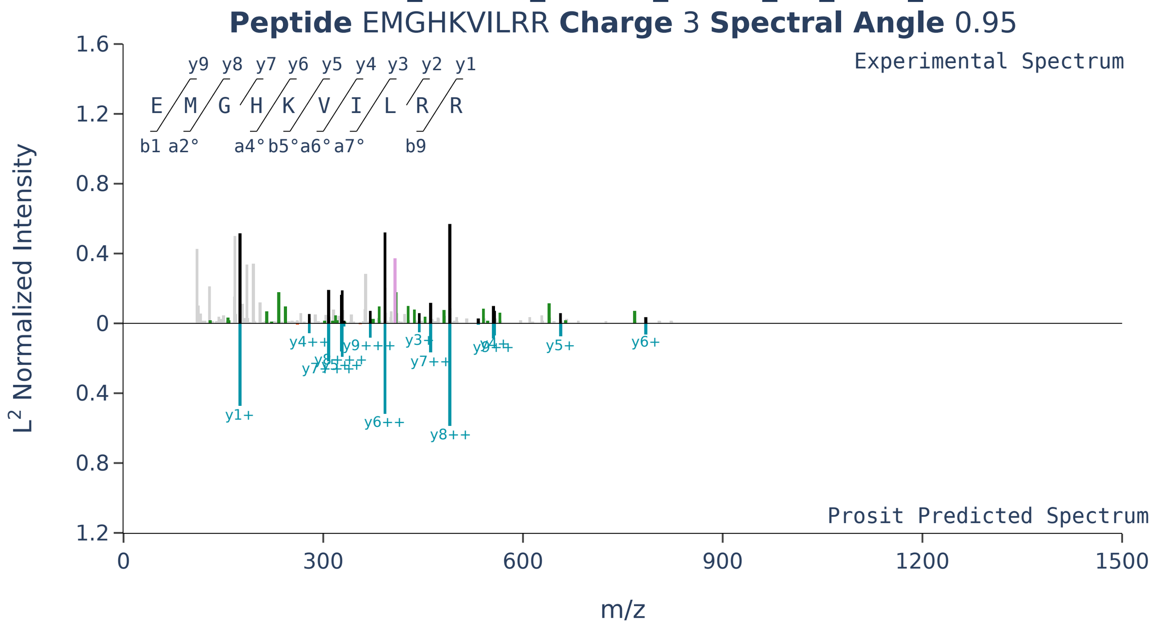


**Source** HRoetschke_211222_160123_HFGoe_IF2_b1R1_4h **Scan** 11659
**Peptide** NRDVVIGE **Charge** 2 **Spectral Angle** 0.9

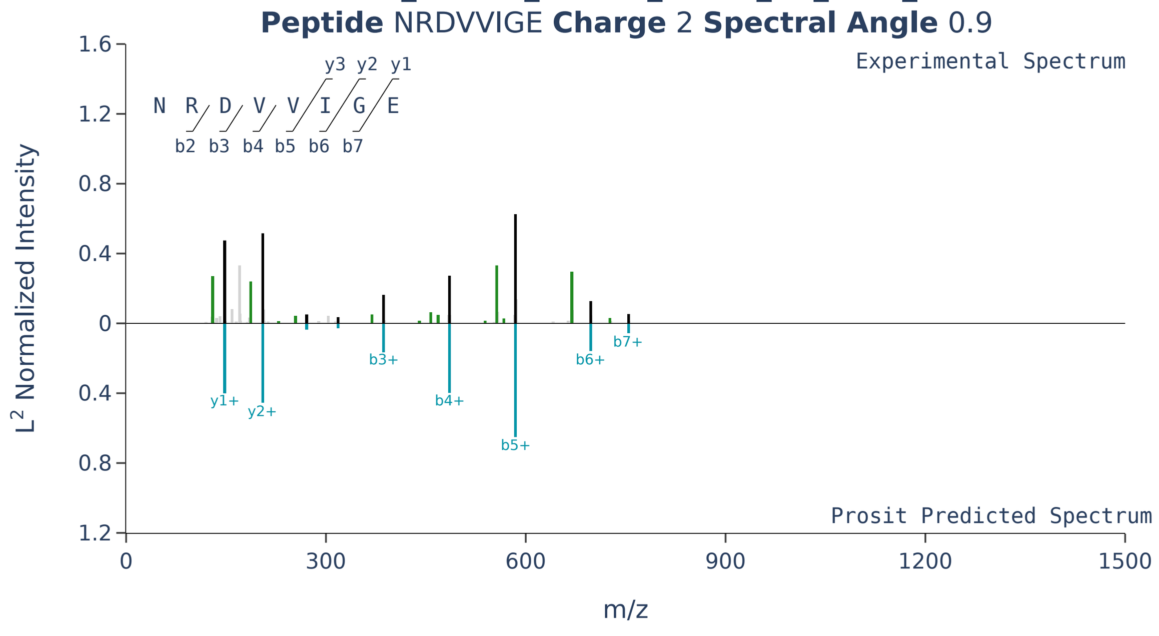


**Source** HRoetschke_211222_160123_HFGoe_IF2_b1R2_24h **Scan** 17507
**Peptide** DVTIKTL **Charge** 2 **Spectral Angle** 0.92

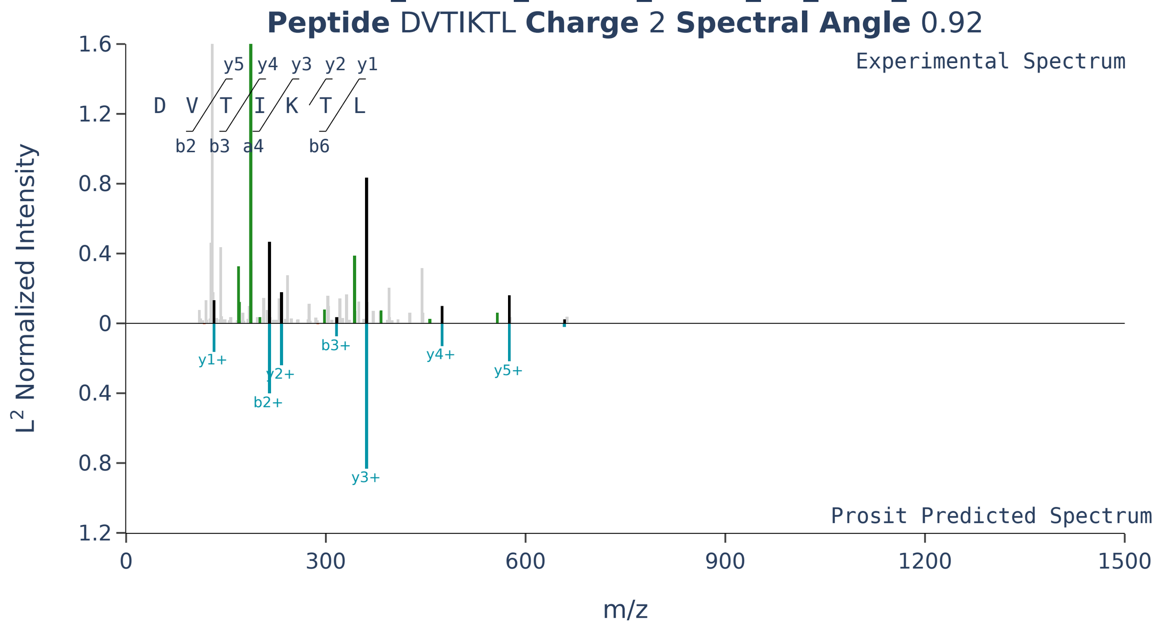


**Source** HRoetschke_211222_160123_HFGoe_IF2_b1R1_4h **Scan** 14231
**Peptide** QETAQLVAE **Charge** 2 **Spectral Angle** 0.92

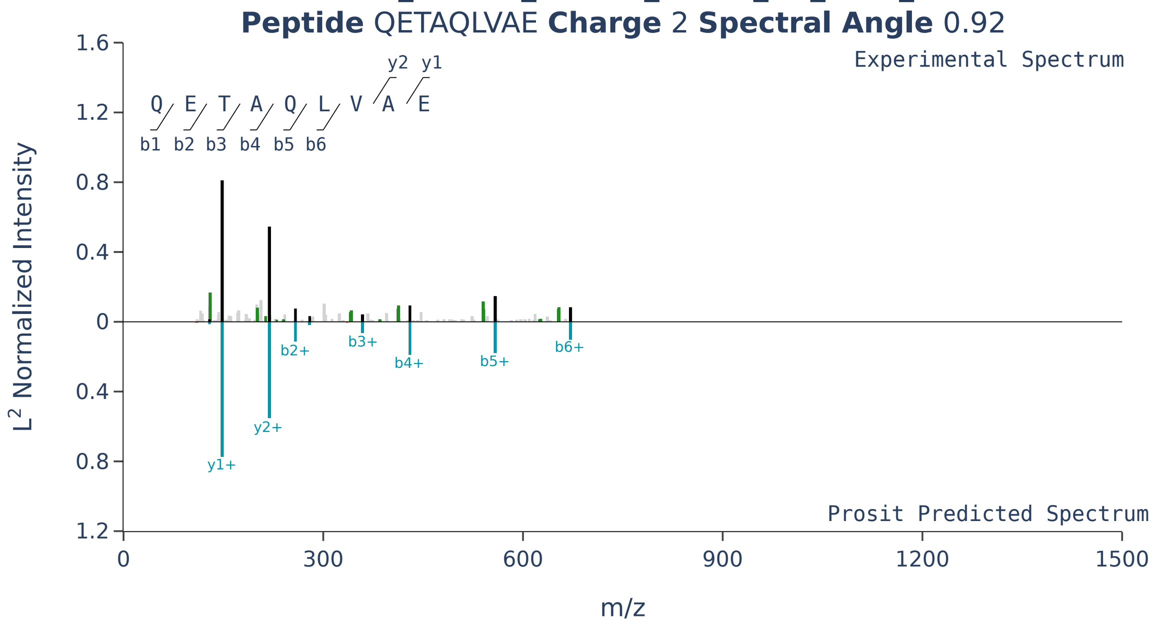


**Source** HRoetschke_211222_160123_HFGoe_IF2_b1R1_24h **Scan** 15829
**Peptide** AEVRDVF **Charge** 2 **Spectral Angle** 0.92

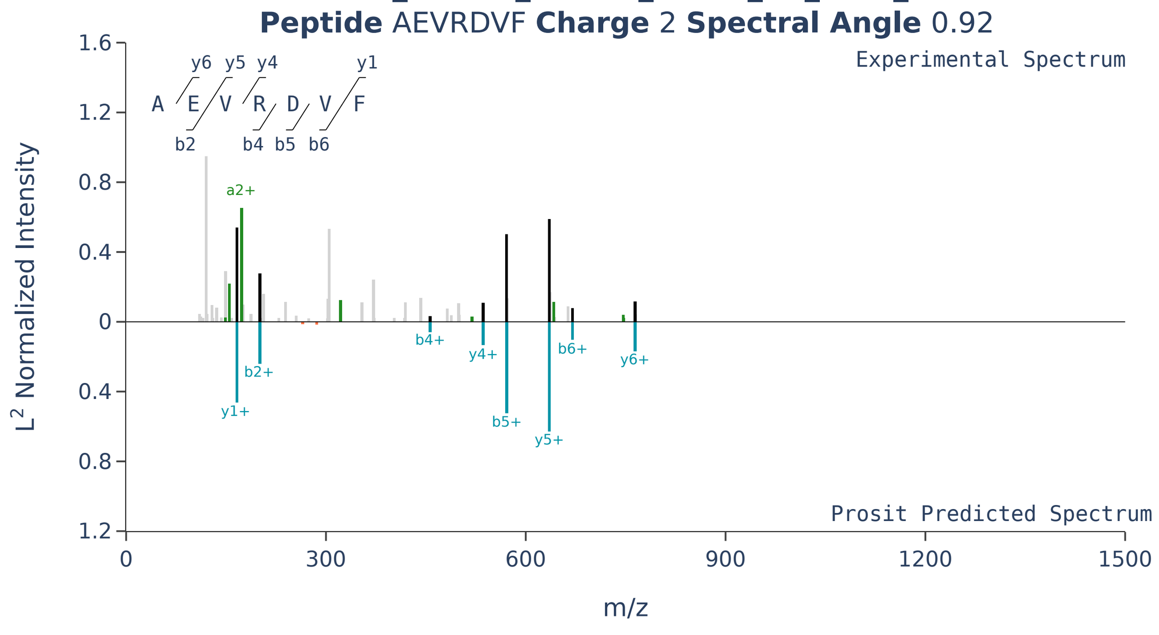


**Source** HRoetschke_211222_160123_HFGoe_IF2_b1R1_24h **Scan** 10097
**Peptide** NKWTDNAEPTE **Charge** 2 **Spectral Angle** 0.95

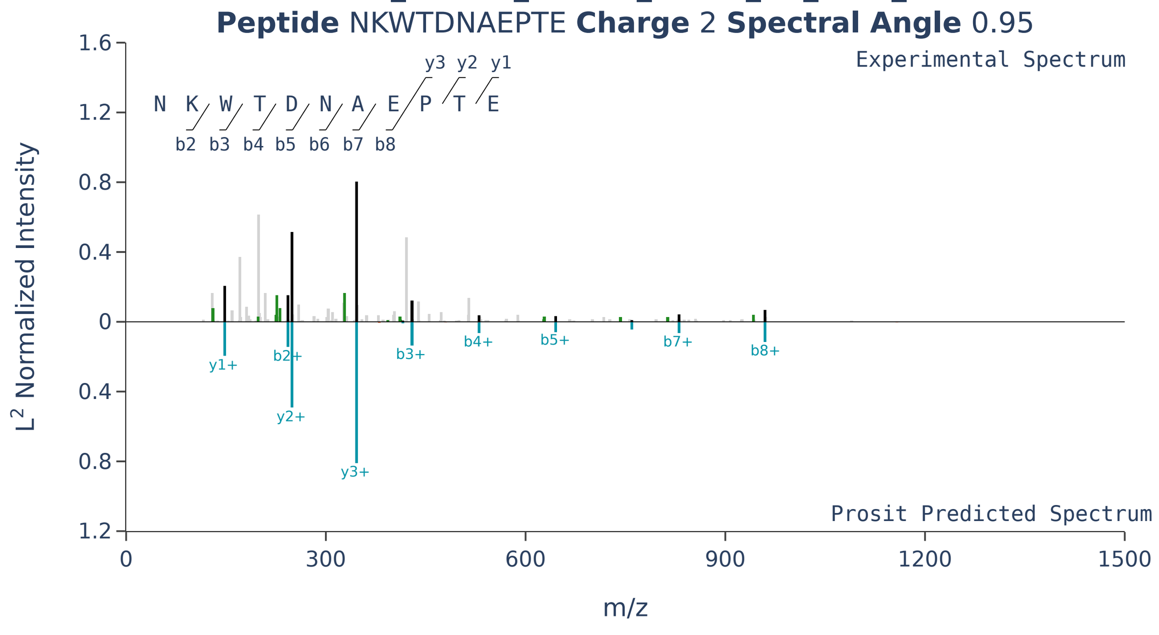


**Source** HRoetschke_211222_160123_HFGoe_IF2_b1R2_2h **Scan** 10155
**Peptide** VLELKAVRK **Charge** 3 **Spectral Angle** 0.93

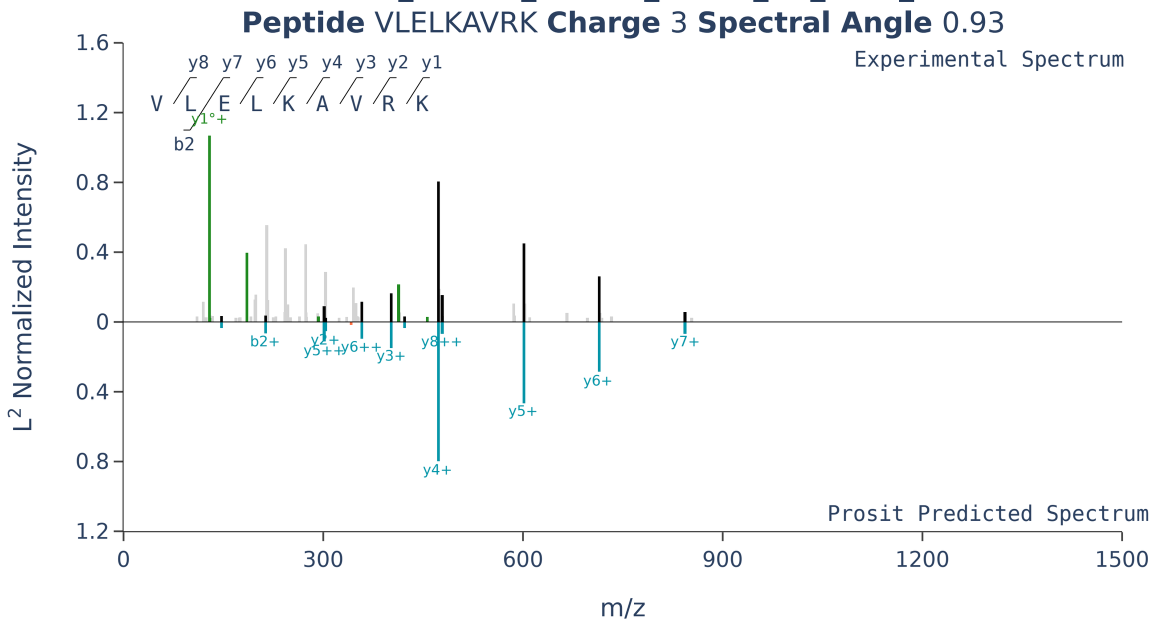


**Source** HRoetschke_211222_160123_HFGoe_IF2_b2R2_4h **Scan** 9811
**Peptide** VLELKAVRKG **Charge** 3 **Spectral Angle** 0.91

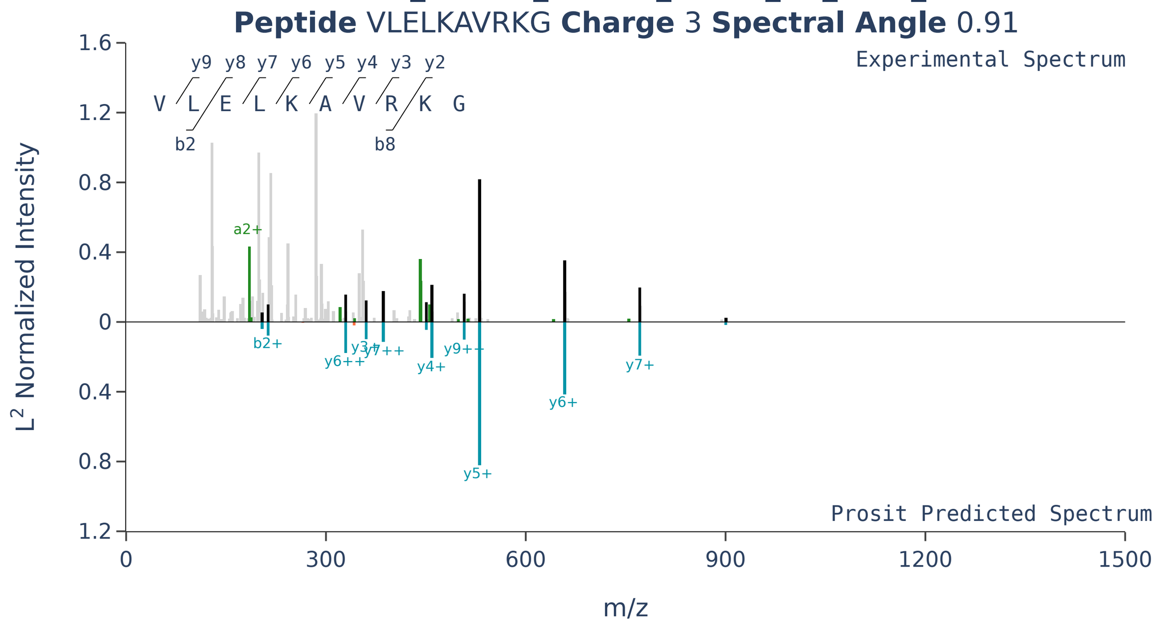


**Source** HRoetschke_211222_160123_HFGoe_IF2_b2R1_2h **Scan** 5950
**Peptide** RLAAEEQAQRE **Charge** 3 **Spectral Angle** 0.92

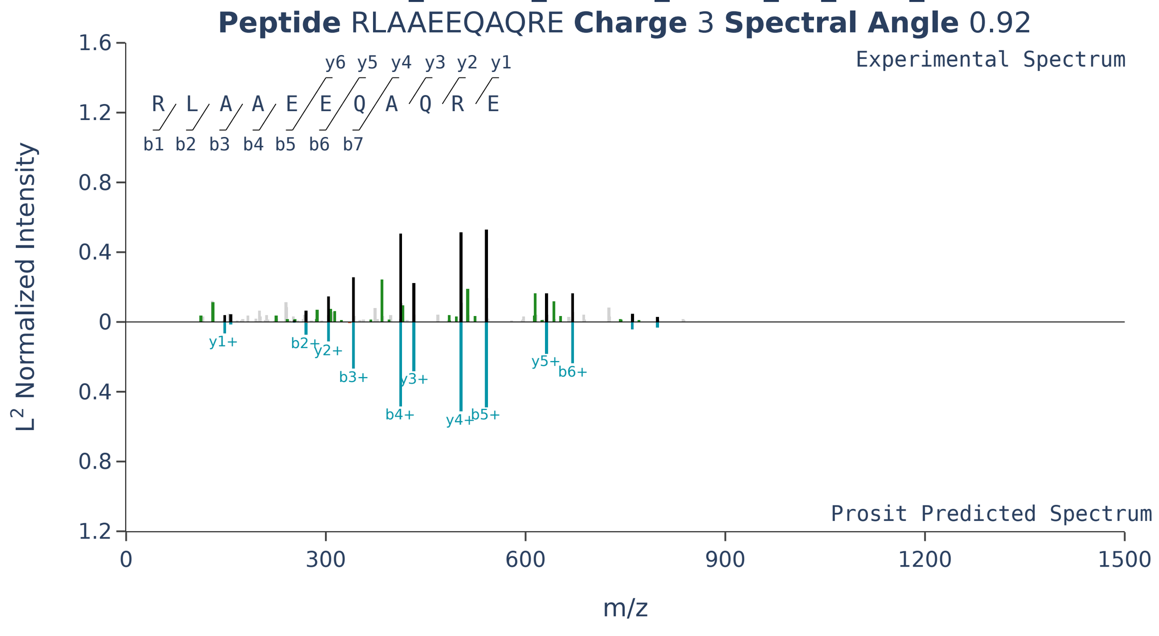

**Source** HRoetschke_211222_160123_HFGoe_IF2_b1R1_4h **Scan** 13298
**Peptide** VTIKTLAAER **Charge** 3 **Spectral Angle** 0.94

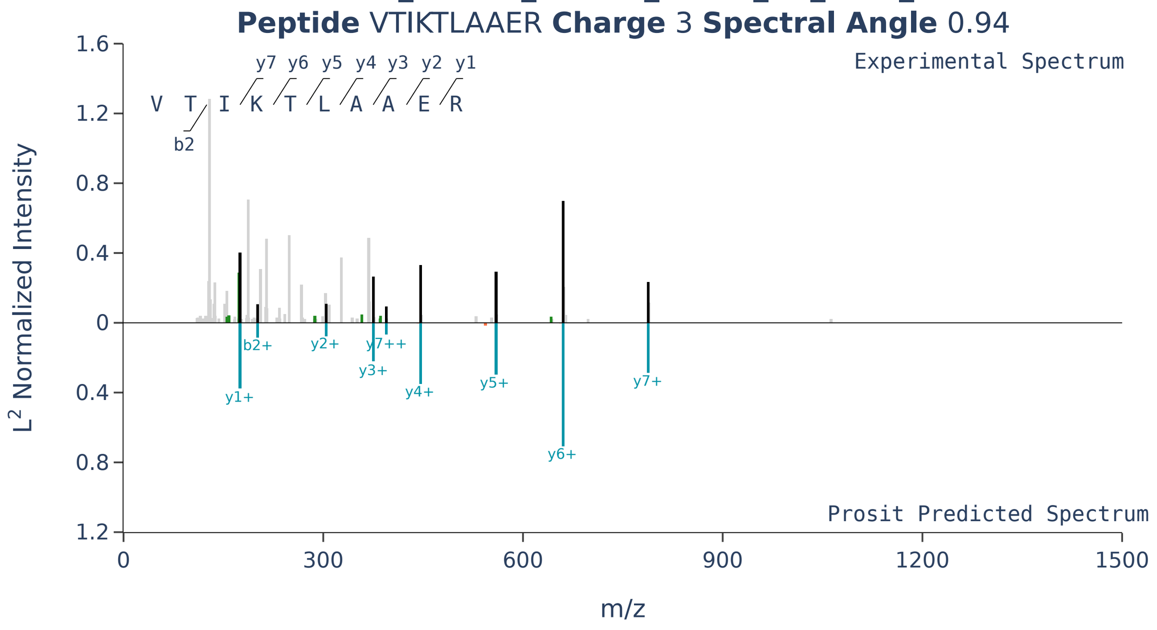


**Source** HRoetschke_211222_160123_HFGoe_IF2_b1R1_24h **Scan** 13325
**Peptide** GEVHEVNI **Charge** 2 **Spectral Angle** 0.85

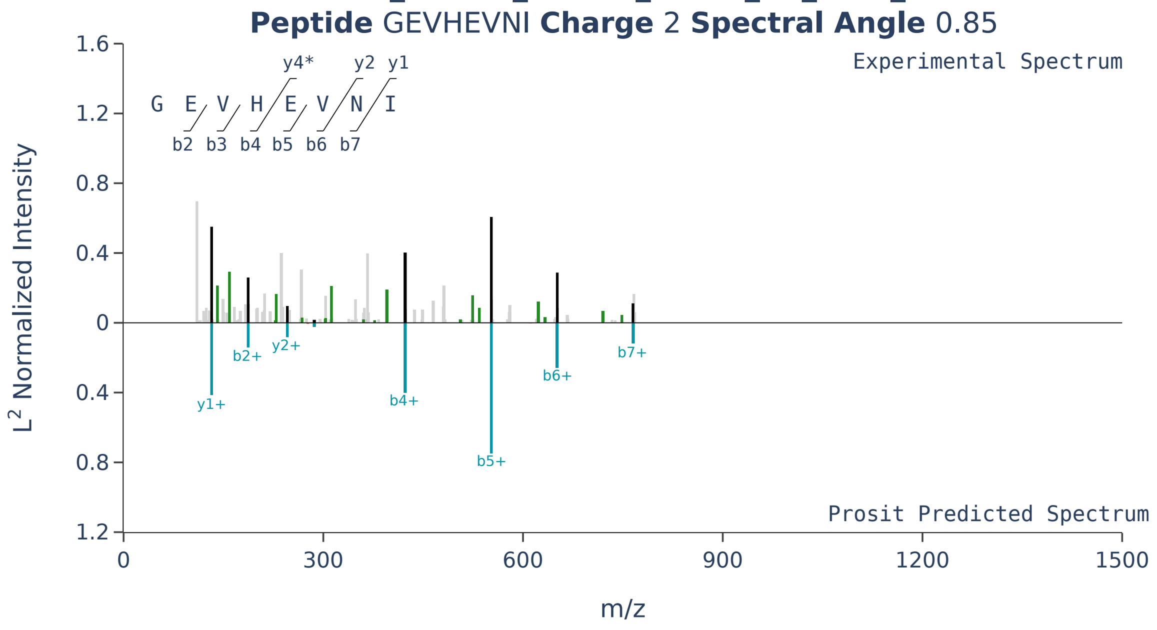


**Source** HRoetschke_211222_160123_HFGoe_IF2_b1R1_1h **Scan** 10581
**Peptide** GFNVRAD **Charge** 2 **Spectral Angle** 0.93

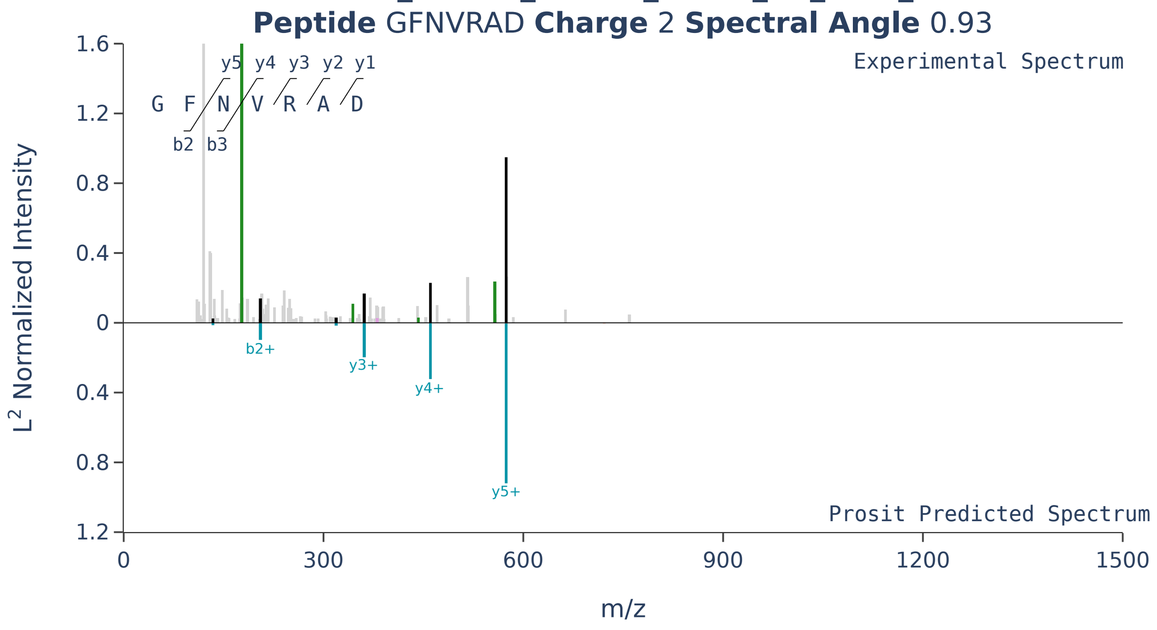


**Source** HRoetschke_211222_160123_HFGoe_IF2_b1R1_4h **Scan** 16319
**Peptide** DQETAQLV **Charge** 1 **Spectral Angle** 0.94

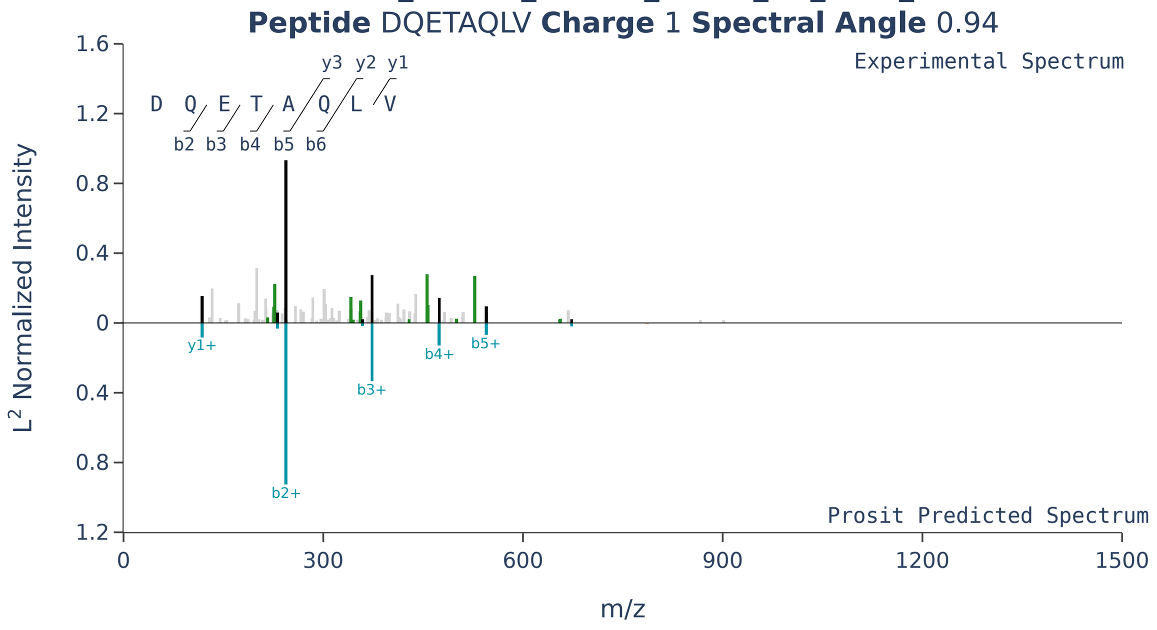


**Source** HRoetschke_211222_160123_HFGoe_IF2_b1R2_2h **Scan** 12782
**Peptide** SVQIEVR **Charge** 2 **Spectral Angle** 0.91

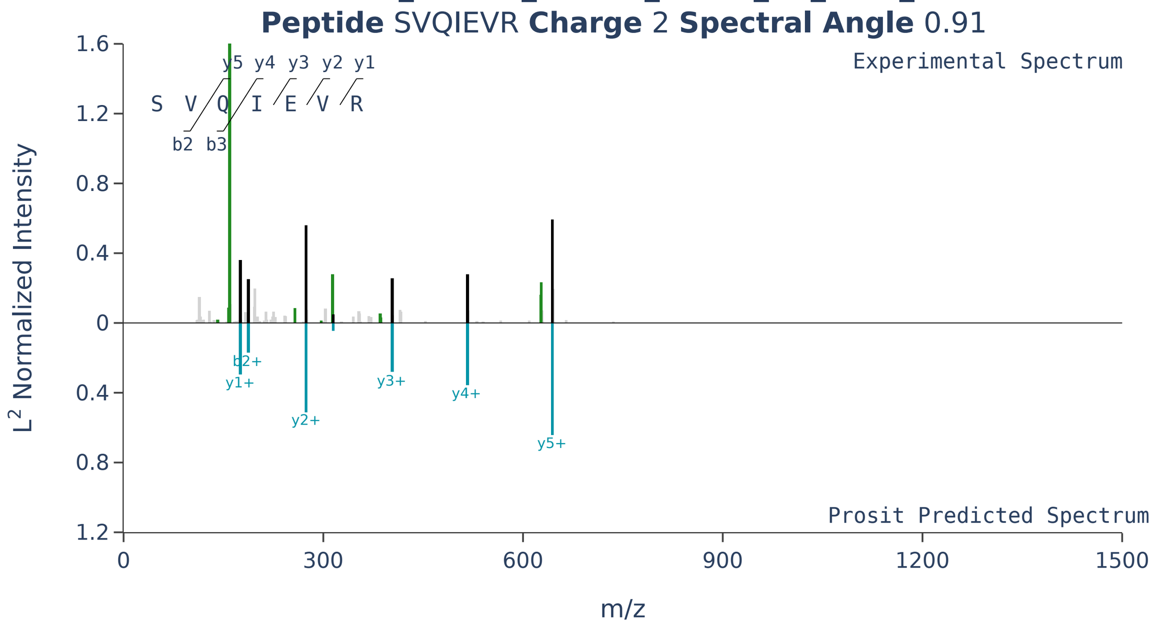


**Source** HRoetschke_211222_160123_HFGoe_IF2_b1R2_2h **Scan** 9374
**Peptide** EVTVVRDE **Charge** 2 **Spectral Angle** 0.91

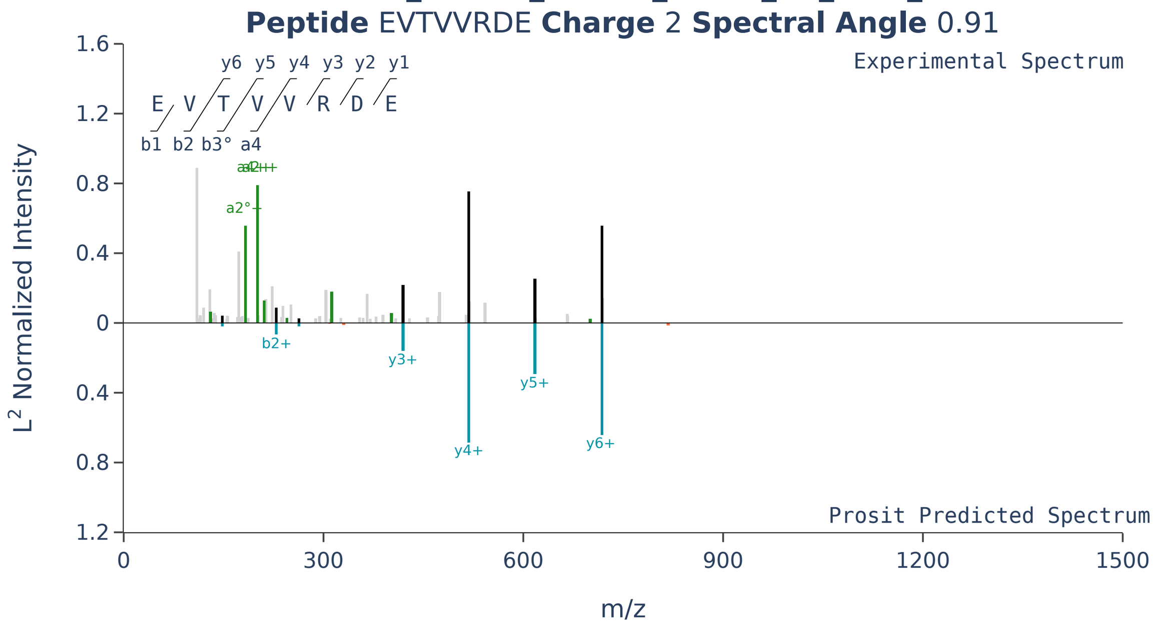


**Source** HRoetschke_211222_160123_HFGoe_IF2_b2R2_2h **Scan** 7609
**Peptide** GFQKPAQAVNRD **Charge** 3 **Spectral Angle** 0.91

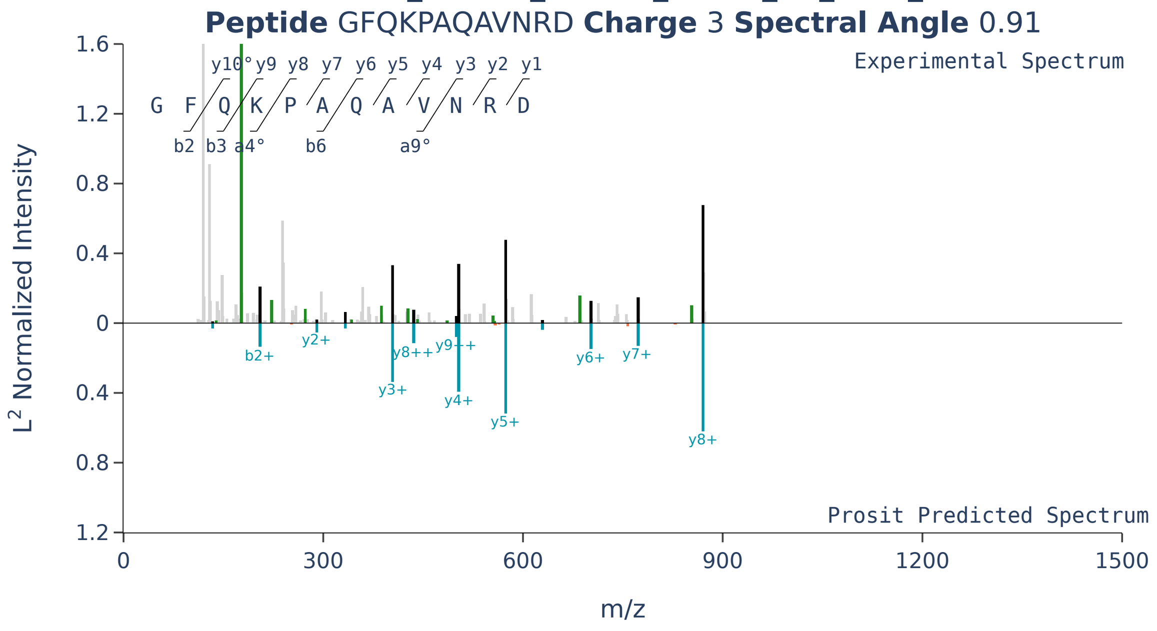


**Source** HRoetschke_211222_160123_HFGoe_IF2_b1R1_4h **Scan** 7001
**Peptide** EMGHKVILR **Charge** 3 **Spectral Angle** 0.92

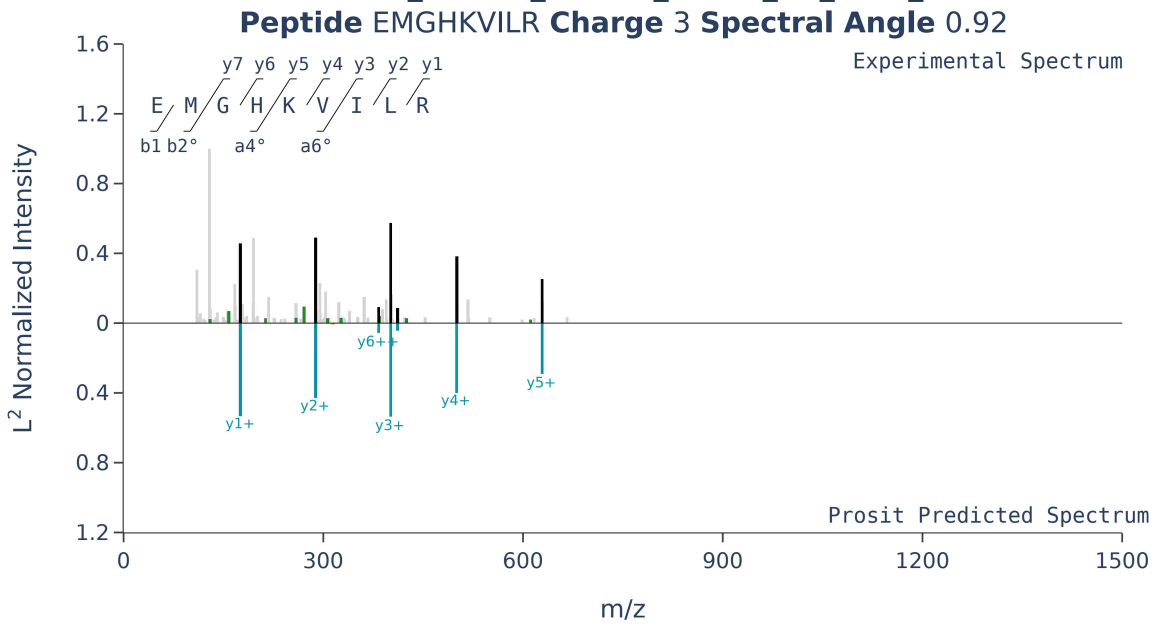


**Source** HRoetschke_211222_160123_HFGoe_IF2_b2R2_2h **Scan** 14633
**Peptide** DQETAQLVAE **Charge** 2 **Spectral Angle** 0.96

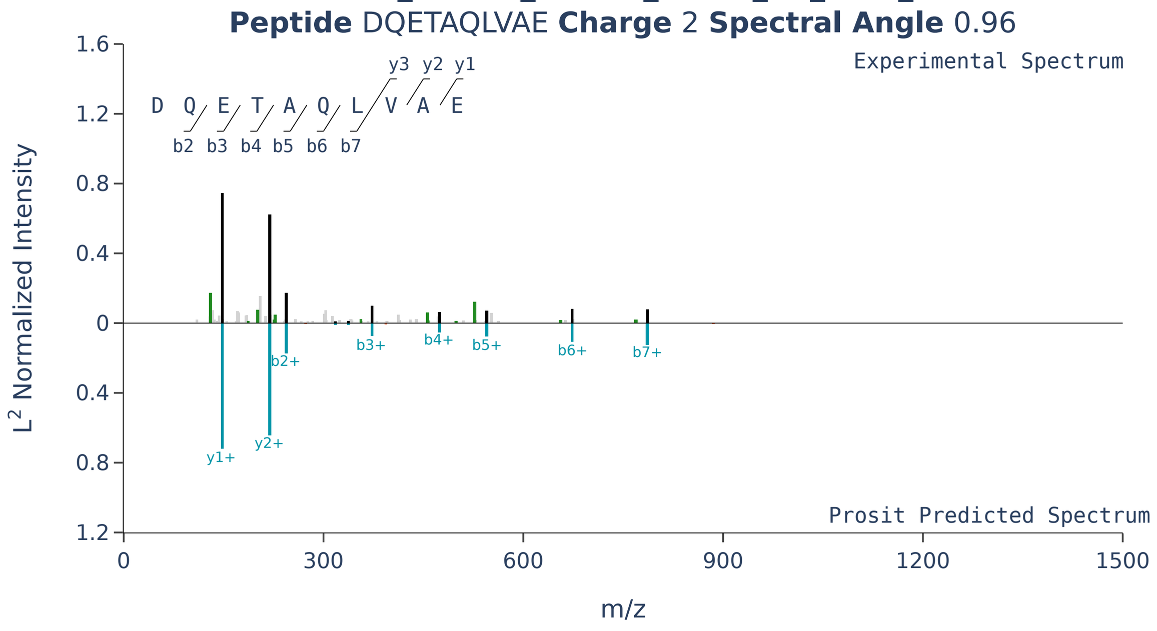


**Source** HRoetschke_211222_160123_HFGoe_IF2_b2R2_4h **Scan** 12747
**Peptide** QAEVLELKAVRKG **Charge** 4 **Spectral Angle** 0.92

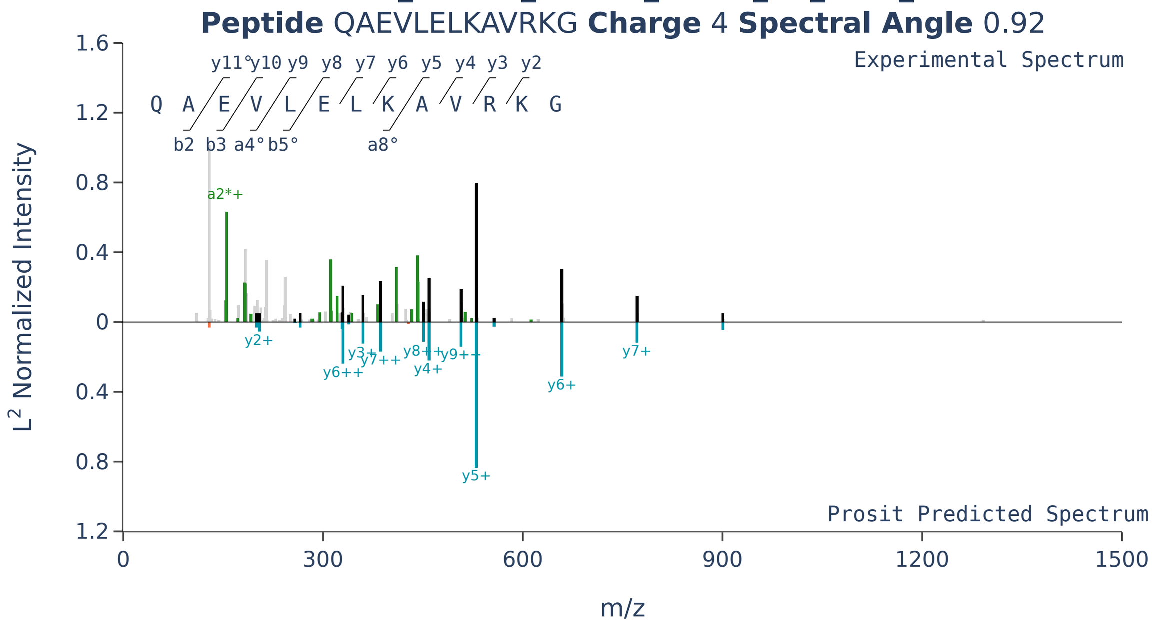


**Source** HRoetschke_211222_160123_HFGoe_IF2_b2R2_4h **Scan** 17132
**Peptide** KQQIIGL **Charge** 2 **Spectral Angle** 0.92

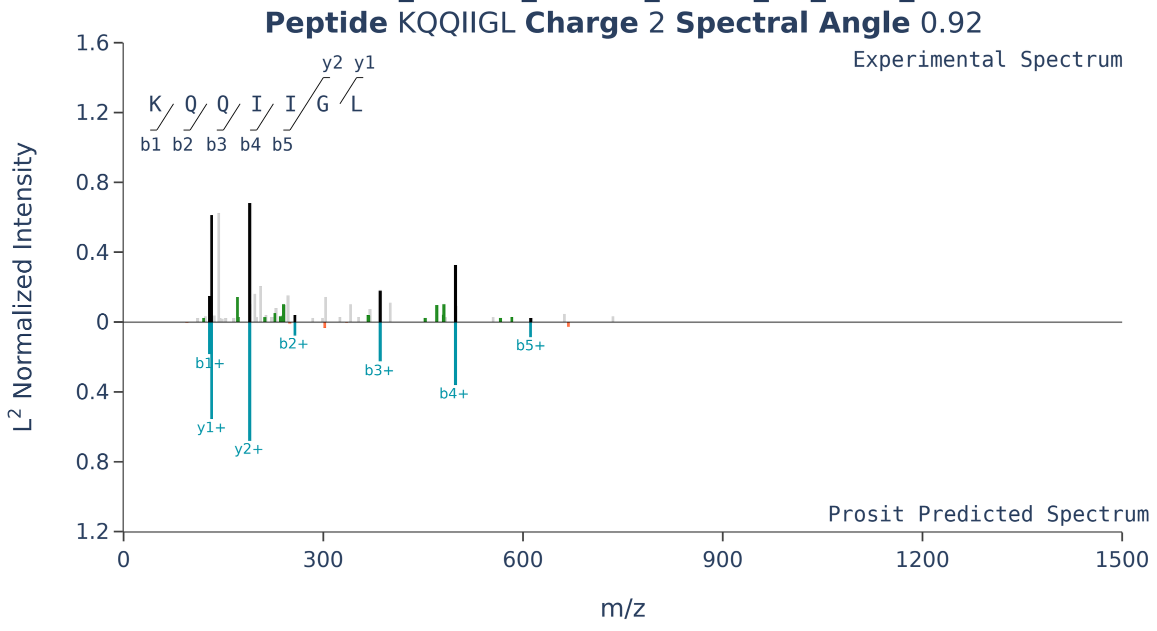


**Source** HRoetschke_211222_160123_HFGoe_IF2_b2R2_2h **Scan** 8809
**Peptide** EVTVVRD **Charge** 2 **Spectral Angle** 0.93

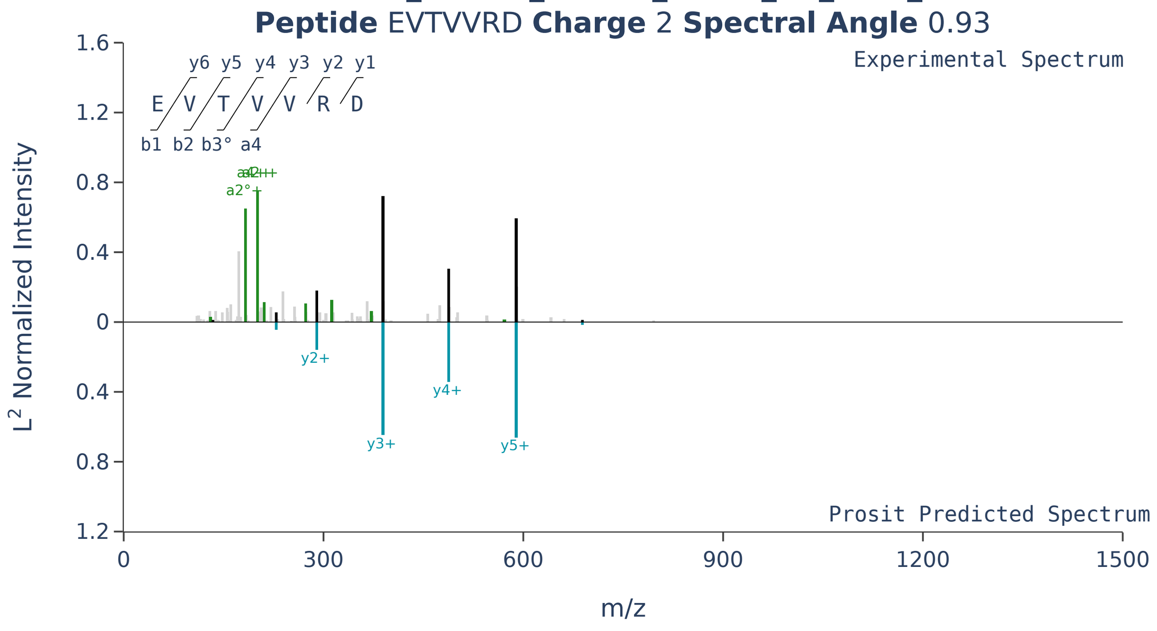


**Source** HRoetschke_211222_160123_HFGoe_IF2_b1R1_1h **Scan** 7408
**Peptide** GFQKPAQAVNR **Charge** 3 **Spectral Angle** 0.9

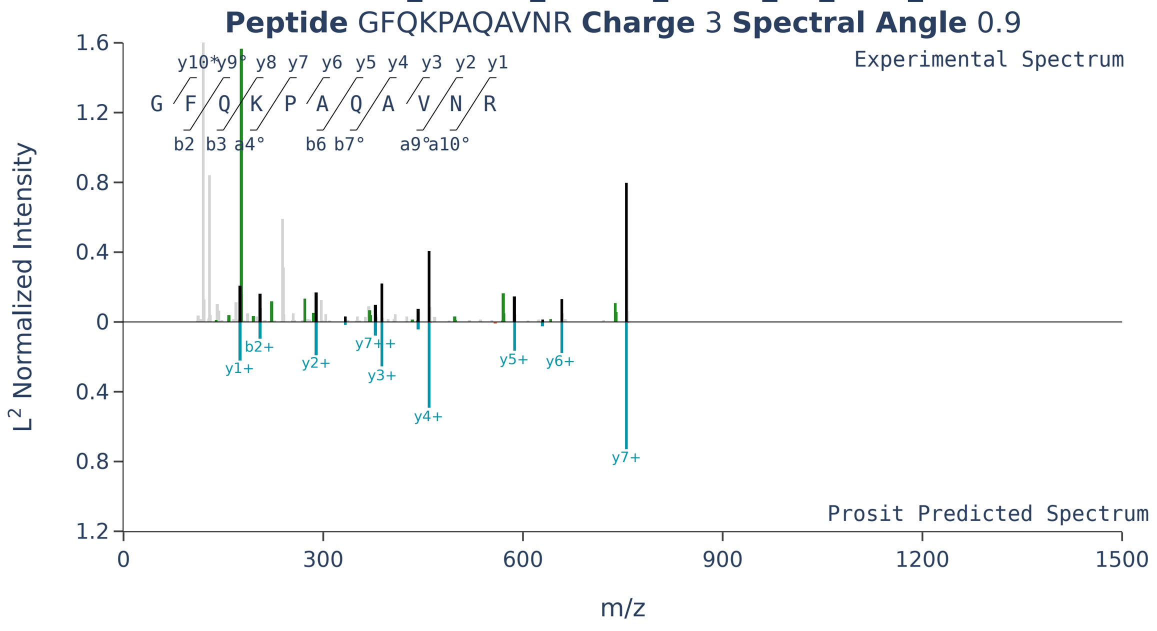


**Source** HRoetschke_211222_160123_HFGoe_IF2_b1R1_4h **Scan** 6074
**Peptide** AIQHAKAAQV **Charge** 1 **Spectral Angle** 0.83

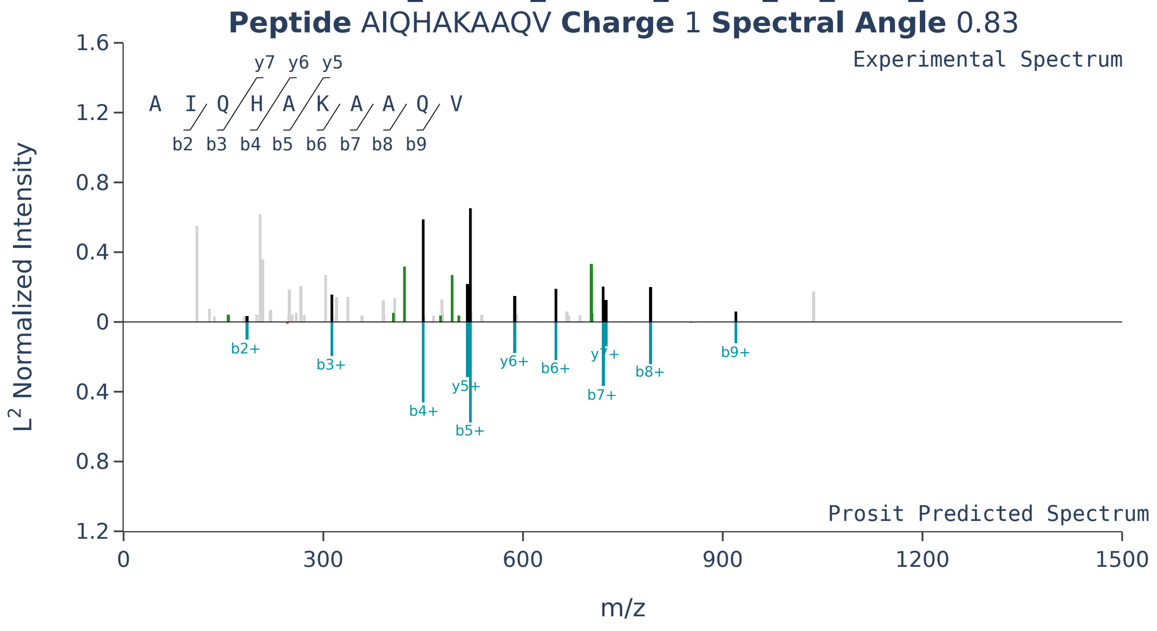


**Source** HRoetschke_211222_160123_HFGoe_IF2_b1R1_4h **Scan** 11662
**Peptide** RDVVIGE **Charge** 2 **Spectral Angle** 0.9

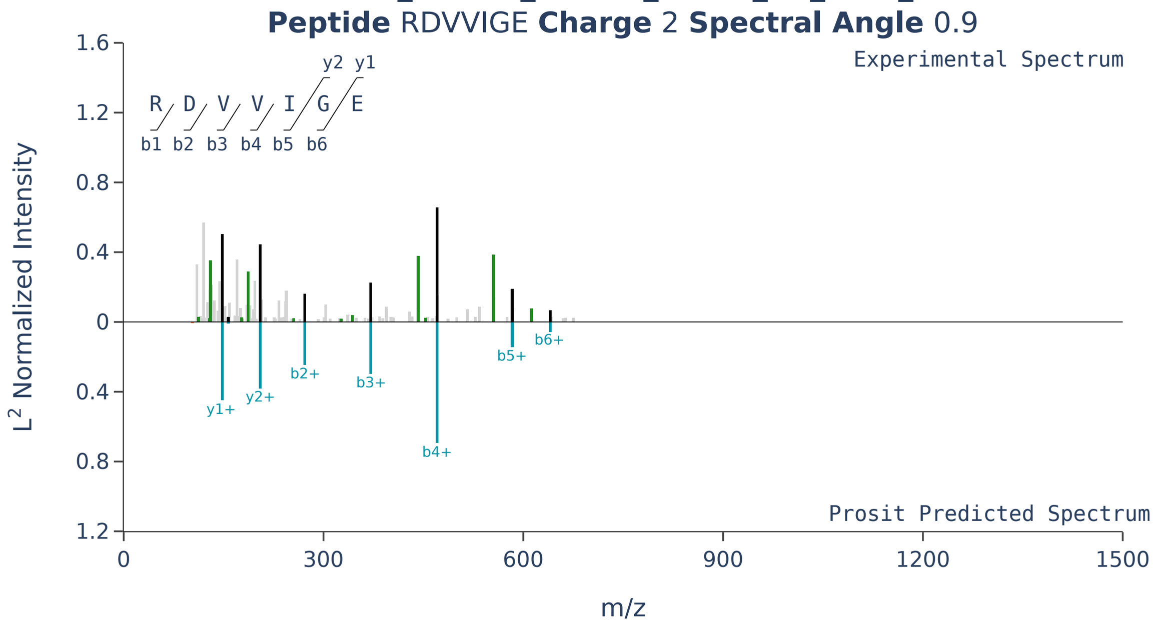

**Source** HRoetschke_211222_160123_HFGoe_IF2_b1R1_2h **Scan** 7964
**Peptide** QGFQKPAQAVNR **Charge** 2 **Spectral Angle** 0.93

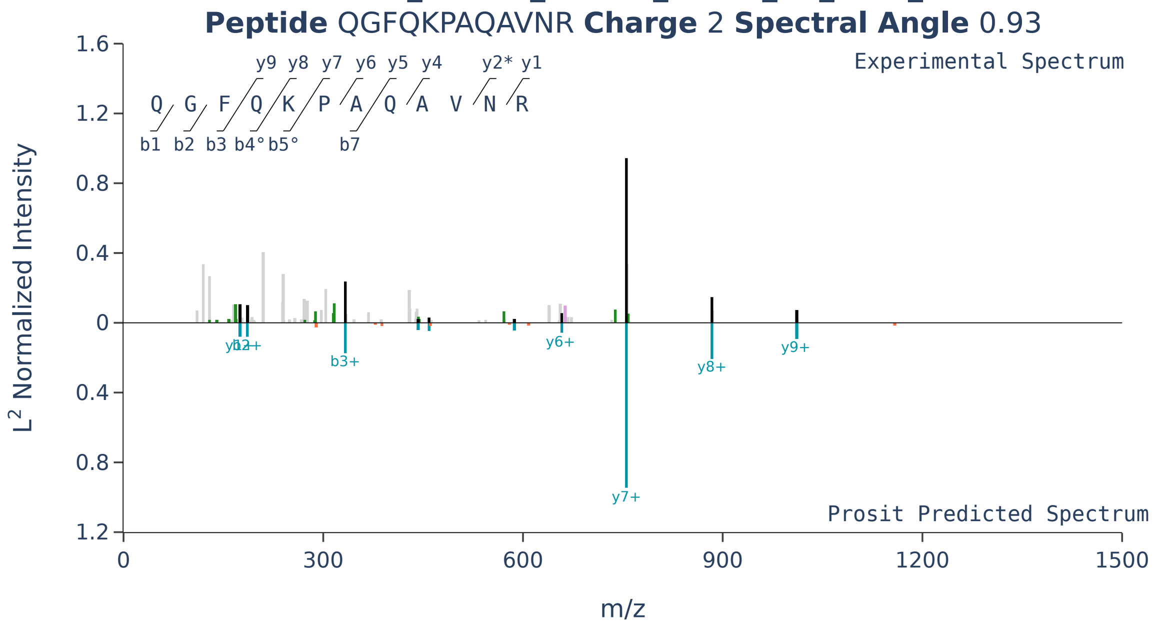


**Source** HRoetschke_211222_160123_HFGoe_IF2_b1R2_24h **Scan** 7470
**Peptide** LDTPGHAA **Charge** 2 **Spectral Angle** 0.91

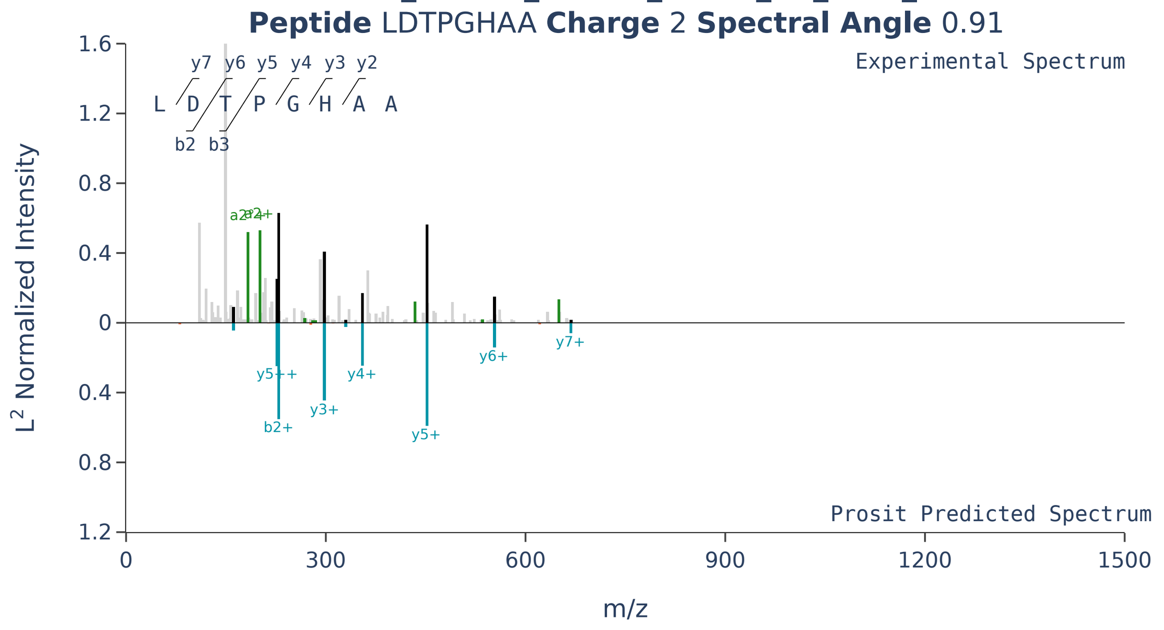


**Source** HRoetschke_211222_160123_HFGoe_IF2_b2R1_24h **Scan** 9132
**Peptide** IEAIQHA **Charge** 2 **Spectral Angle** 0.94

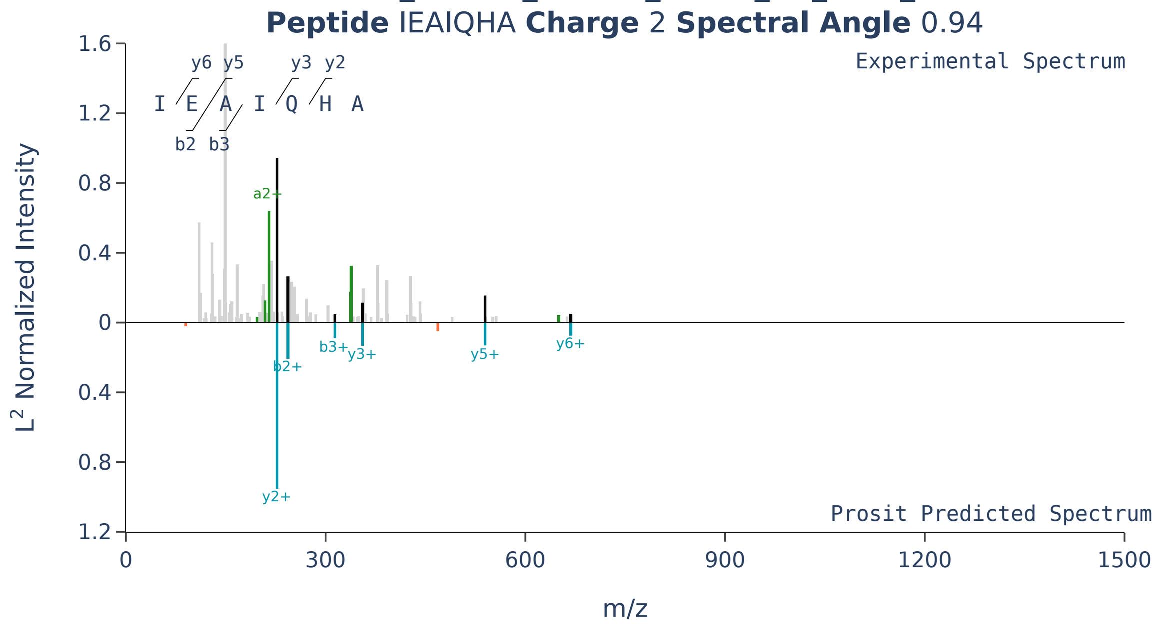


**Source** HRoetschke_211222_160123_HFGoe_IF2_b1R2_1h **Scan** 6123
**Peptide** VNKIDKPEAD **Charge** 3 **Spectral Angle** 0.93

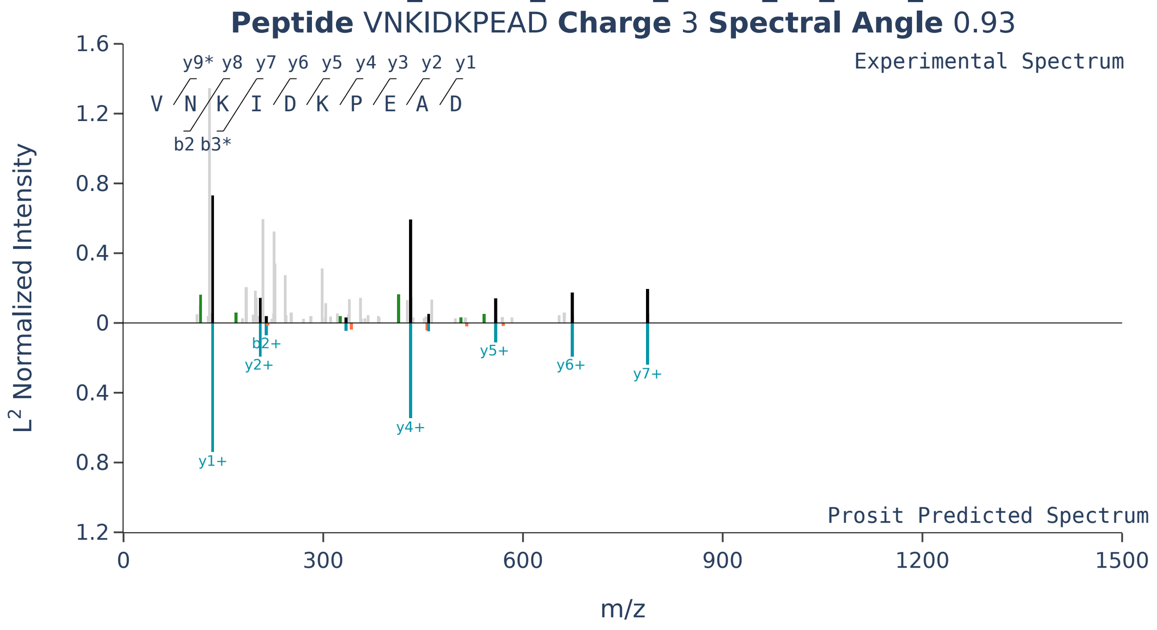


**Source** HRoetschke_211222_160123_HFGoe_IF2_b1R1_1h **Scan** 10230
**Peptide** DEVTVVRD **Charge** 2 **Spectral Angle** 0.86

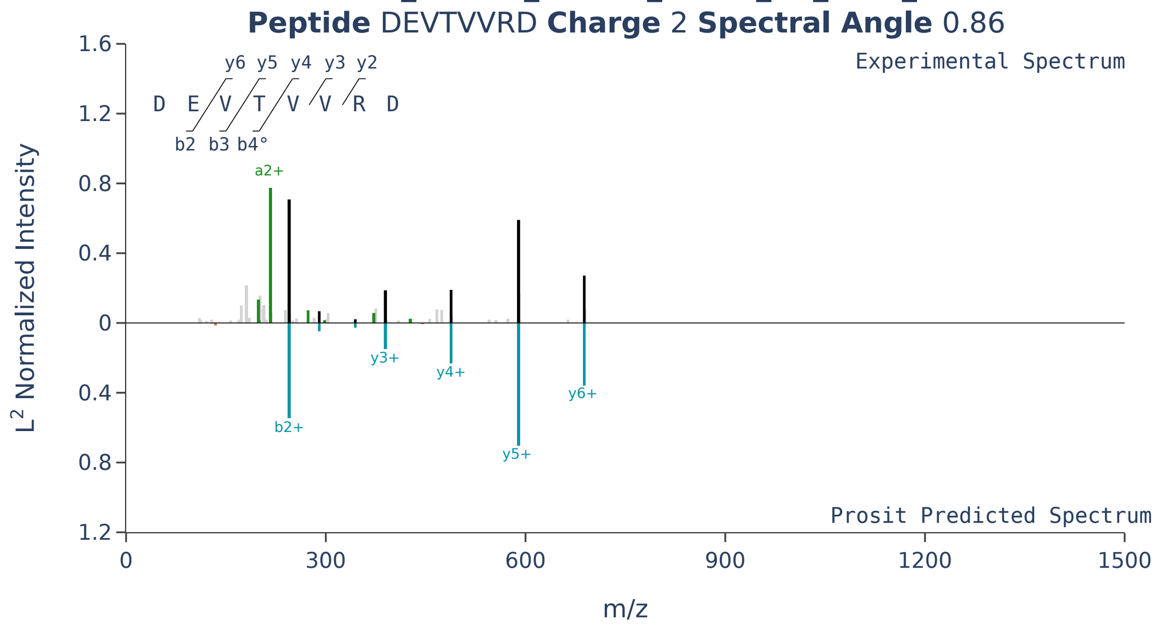


**Source** HRoetschke_211222_160123_HFGoe_IF2_b1R2_1h **Scan** 16697
**Peptide** RNELGQEVL **Charge** 2 **Spectral Angle** 0.88

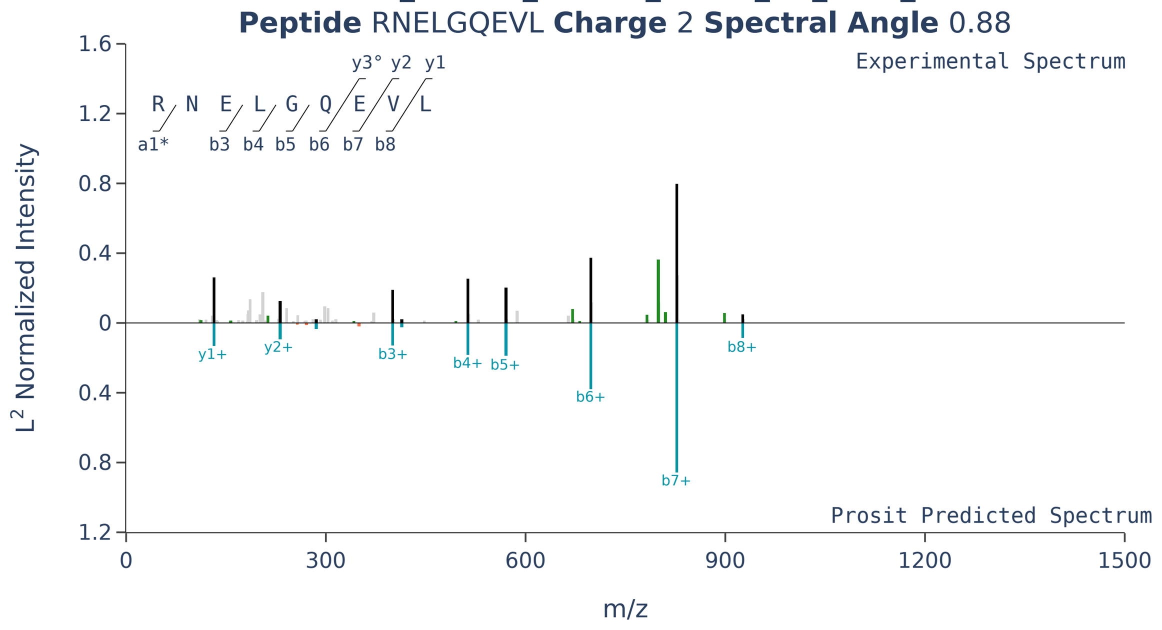


**Source** HRoetschke_211222_160123_HFGoe_IF2_b2R2_4h **Scan** 8809
**Peptide** GGESQFVH **Charge** 2 **Spectral Angle** 0.89

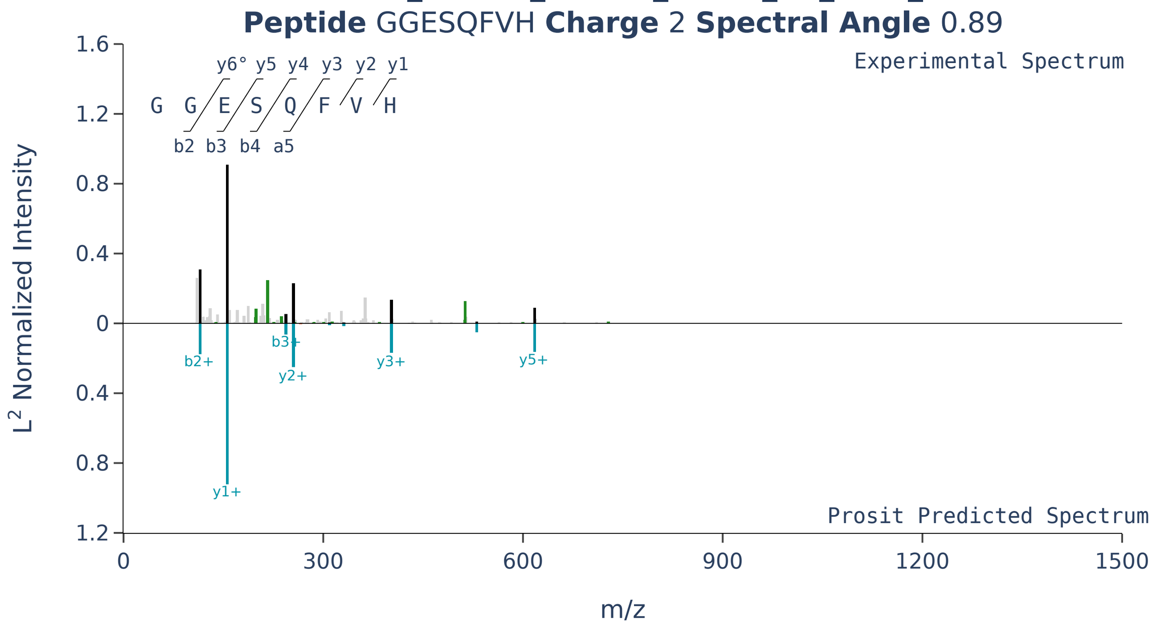


**Source** HRoetschke_211222_160123_HFGoe_IF2_b1R2_4h **Scan** 13203
**Peptide** HNPIRVL **Charge** 2 **Spectral Angle** 0.9

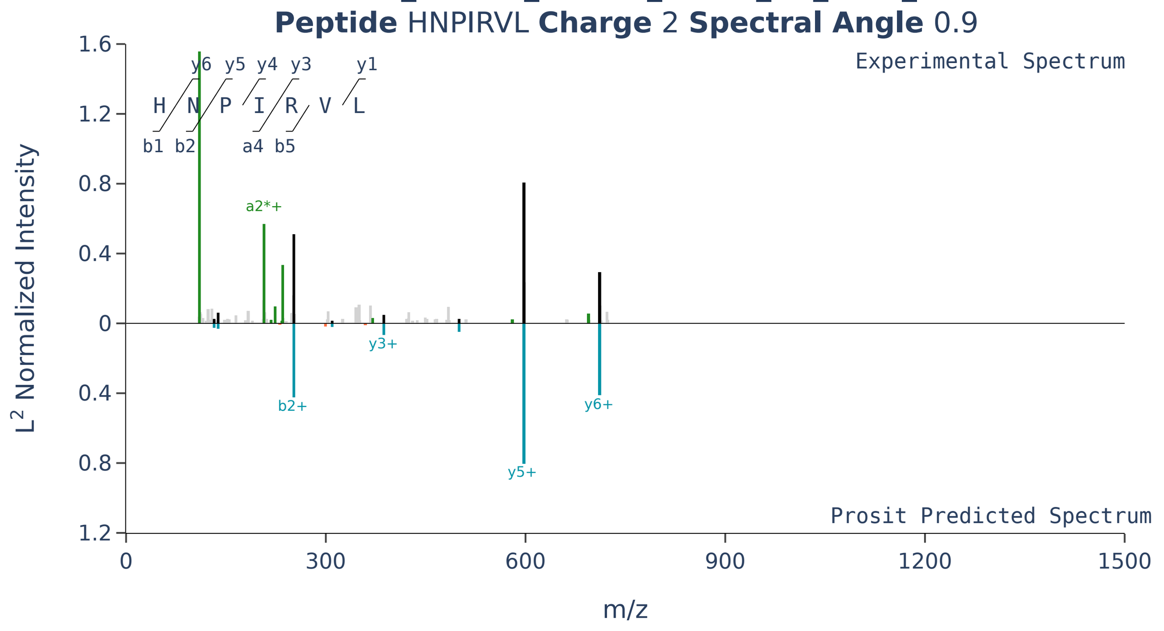


**Source** HRoetschke_211222_160123_HFGoe_IF2_b1R2_24h **Scan** 13959
**Peptide** GLSGVPAAGD **Charge** 2 **Spectral Angle** 0.89

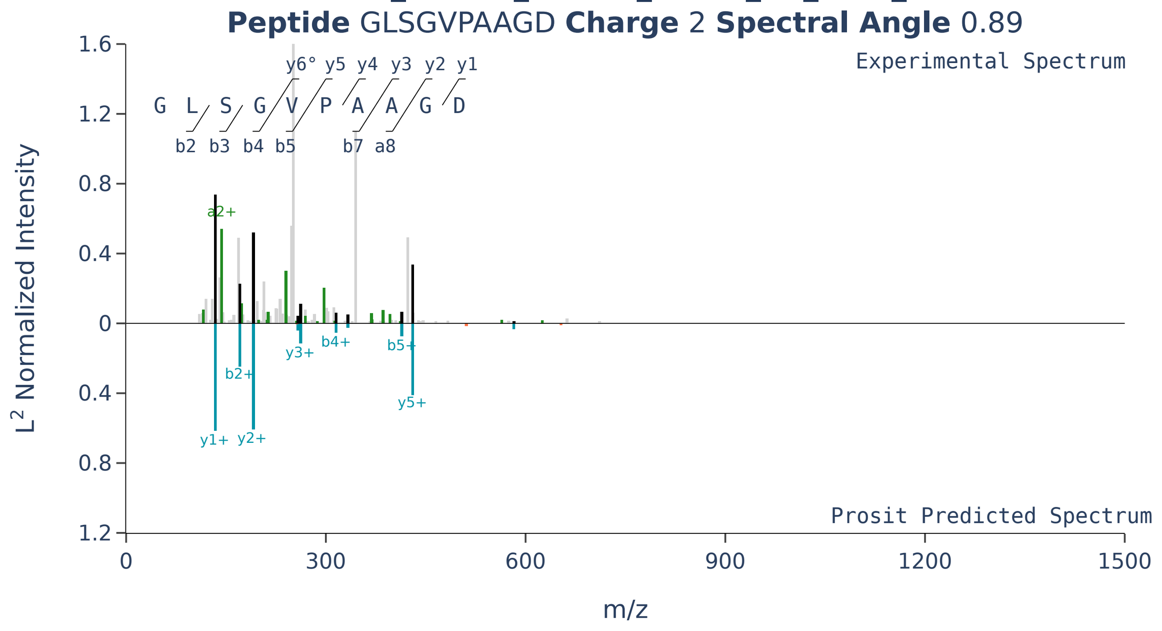


**Source** HRoetschke_211222_160123_HFGoe_IF2_b1R1_1h **Scan** 6488
**Peptide** KIDKPEAD **Charge** 2 **Spectral Angle** 0.91

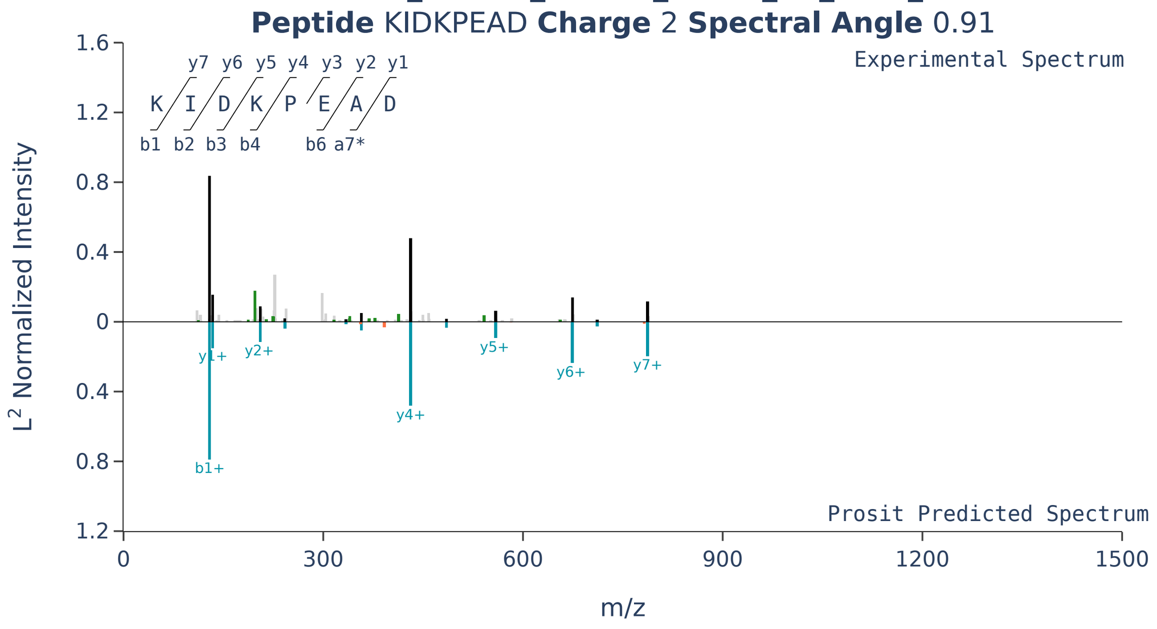


**Source** HRoetschke_211222_160123_HFGoe_IF2_b1R1_2h **Scan** 9953
**Peptide** DVNEVRNGME **Charge** 2 **Spectral Angle** 0.89

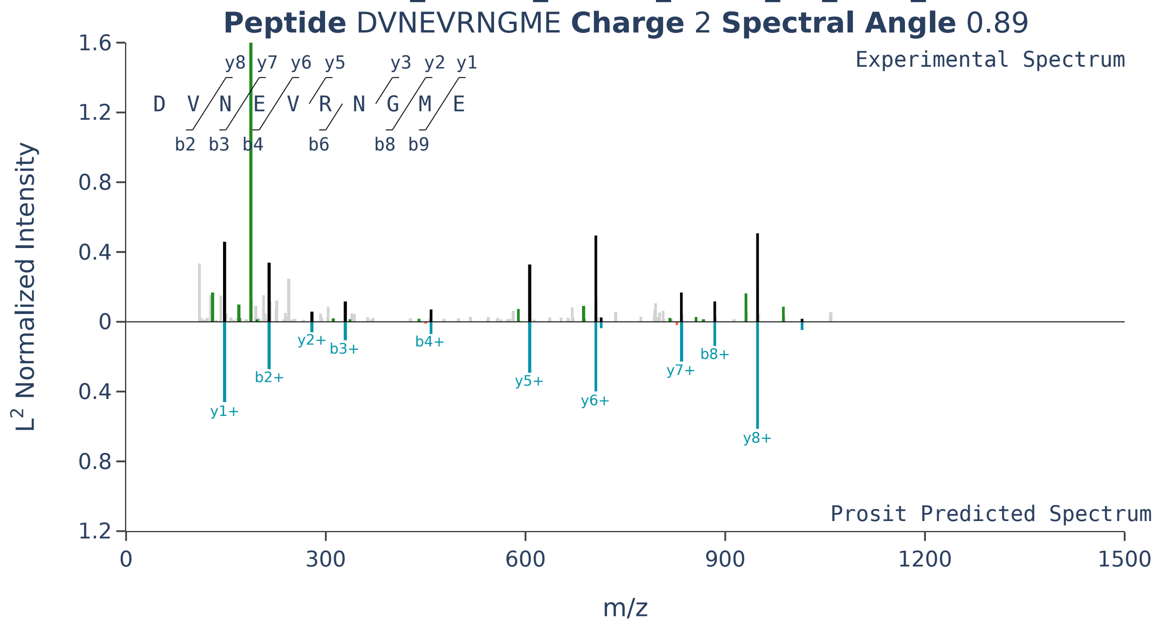


**Source** HRoetschke_211222_160123_HFGoe_IF2_b1R2_4h **Scan** 11002
**Peptide** SVERLVQ **Charge** 2 **Spectral Angle** 0.85

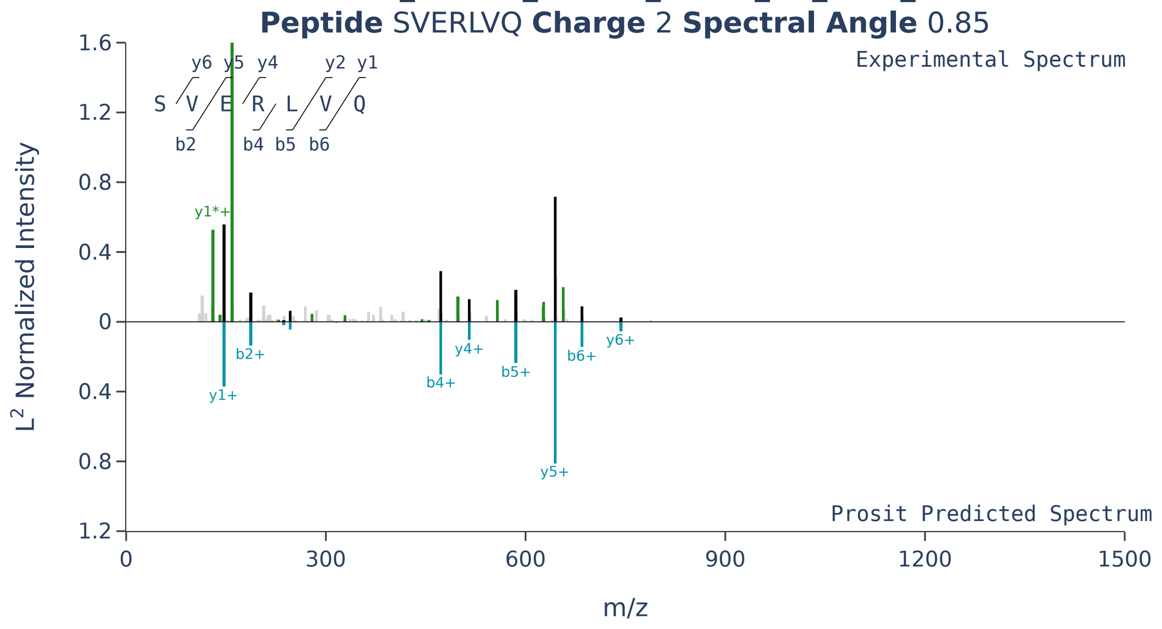


**Source** HRoetschke_211222_160123_HFGoe_IF2_b1R1_2h **Scan** 6453
**Peptide** DTGAAAEPRA **Charge** 2 **Spectral Angle** 0.85

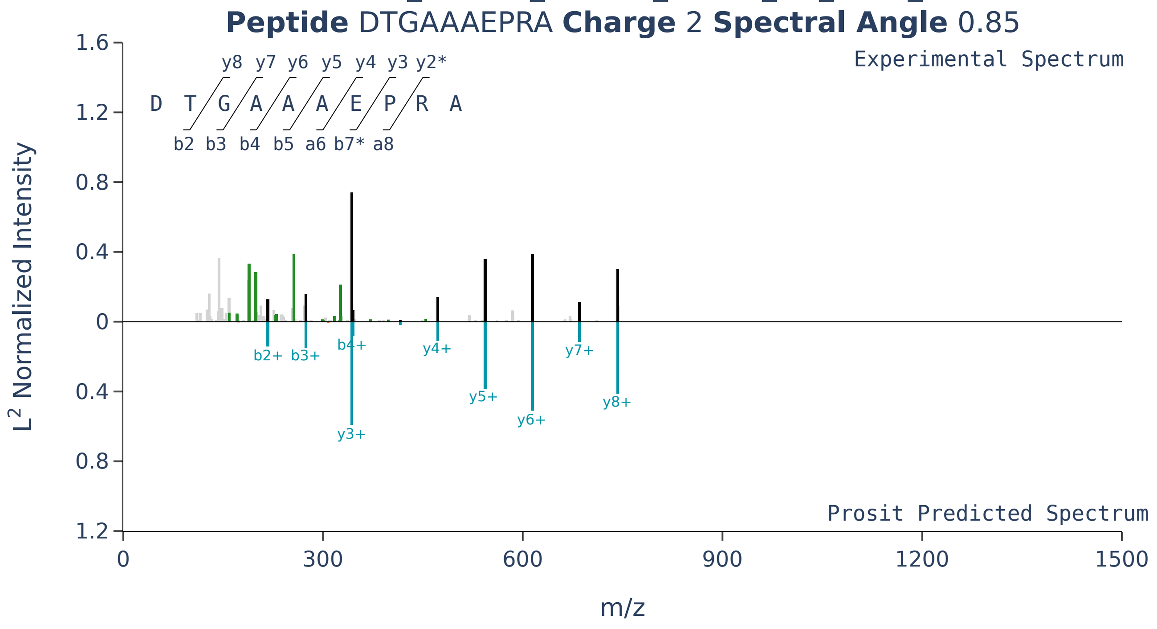


**Source** HRoetschke_211222_160123_HFGoe_IF2_b2R2_2h **Scan** 7388
**Peptide** AERLAAEE **Charge** 2 **Spectral Angle** 0.89

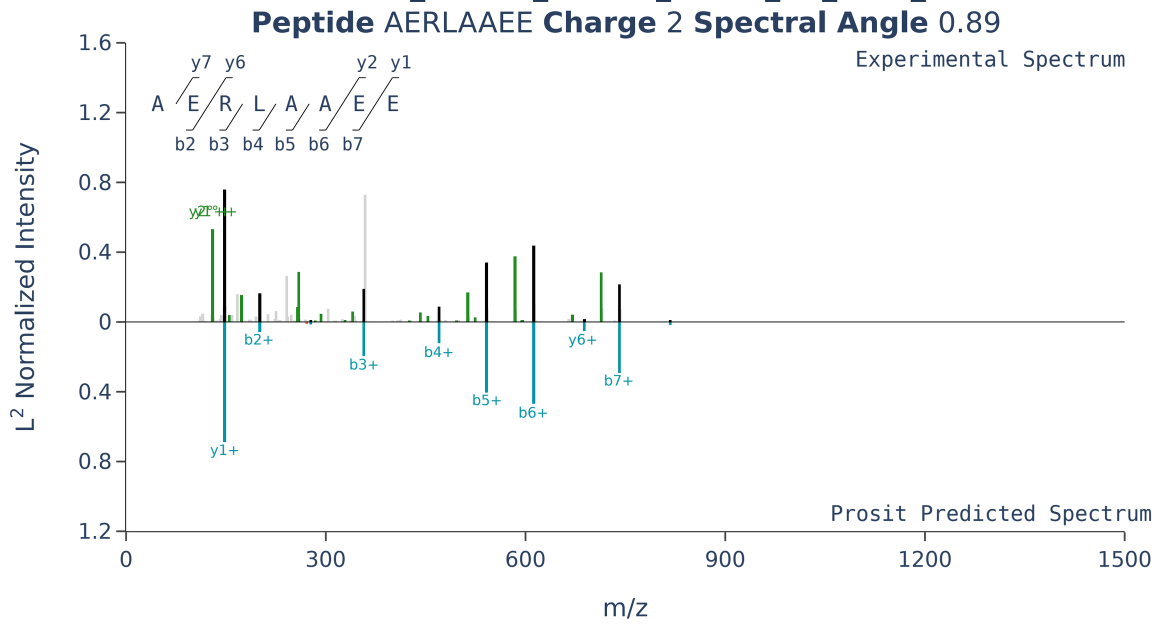


**Source** HRoetschke_211222_160123_HFGoe_IF2_b2R2_24h **Scan** 12096
**Peptide** EVLELKAVRKG **Charge** 3 **Spectral Angle** 0.94

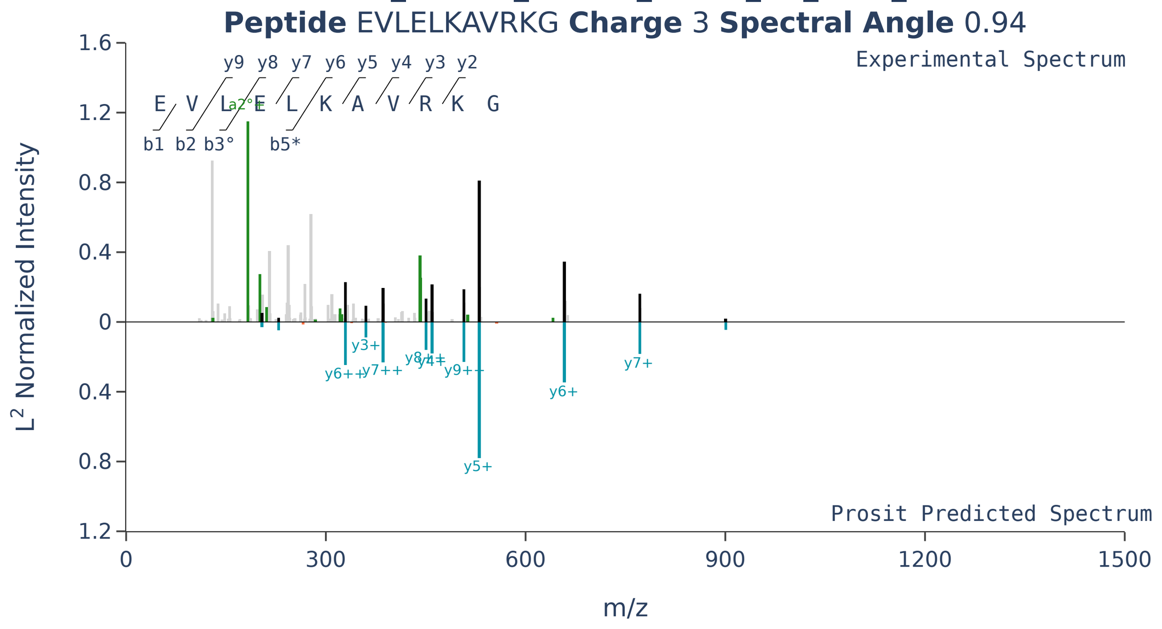

**Source** HRoetschke_211222_160123_HFGoe_IF2_b1R1_24h **Scan** 10290
**Peptide** TDVTIKT **Charge** 1 **Spectral Angle** 0.92

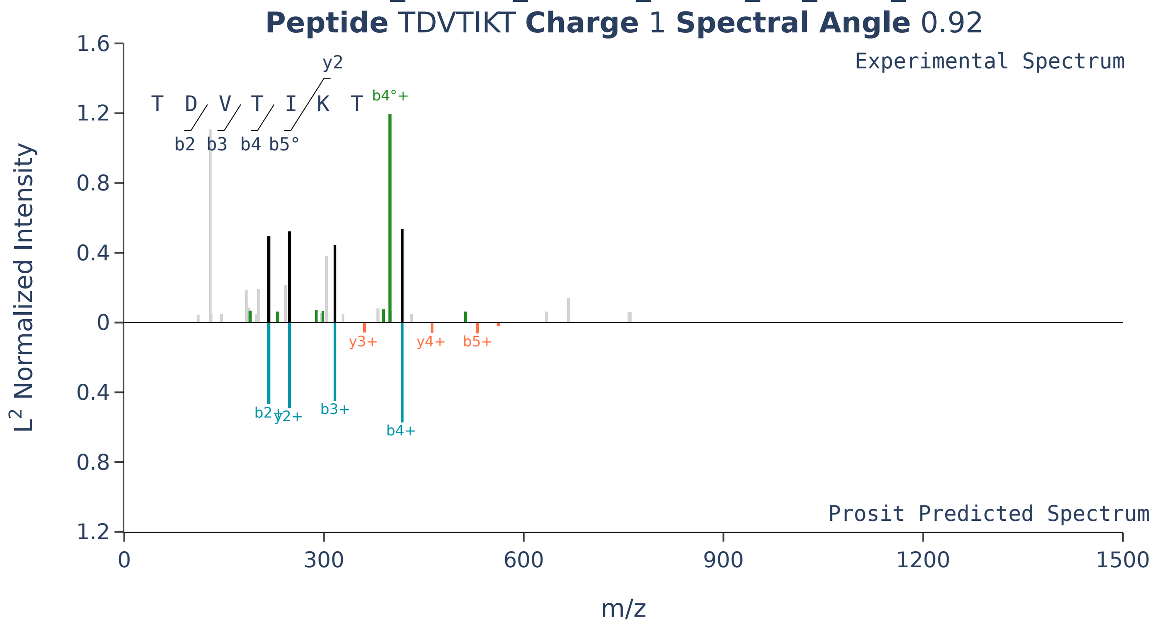


**Source** HRoetschke_211222_160123_HFGoe_IF2_b2R2_24h **Scan** 11248
**Peptide** GIGVKNYNDVRT **Charge** 3 **Spectral Angle** 0.9

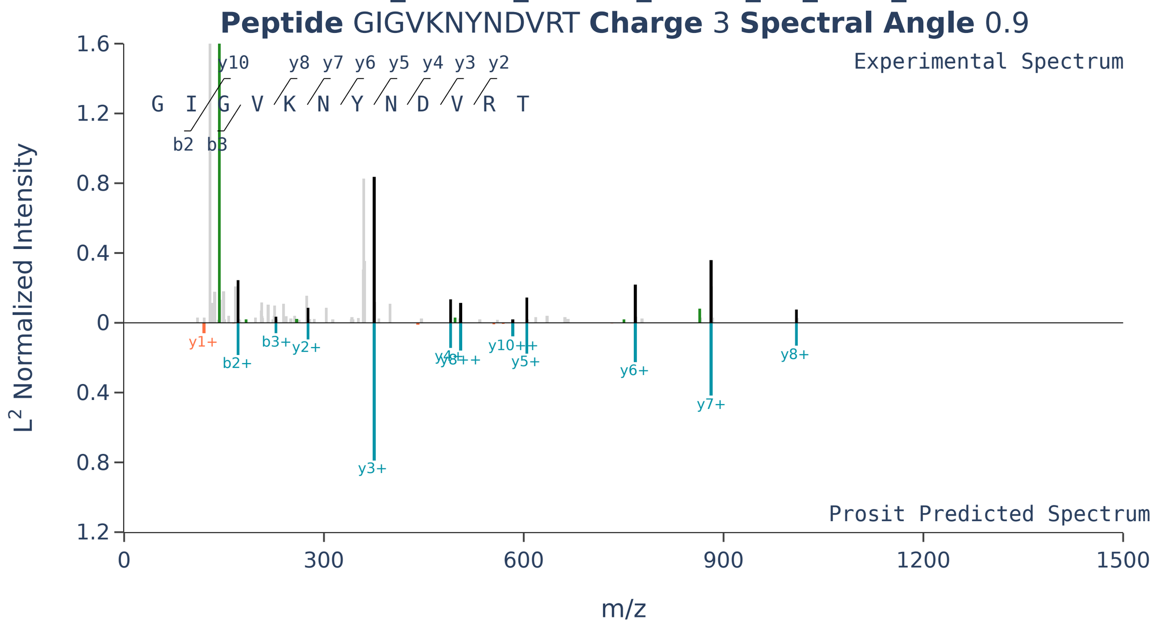


**Source** HRoetschke_211222_160123_HFGoe_IF2_b2R2_4h **Scan** 9987
**Peptide** ALYRQGKFREV **Charge** 3 **Spectral Angle** 0.91

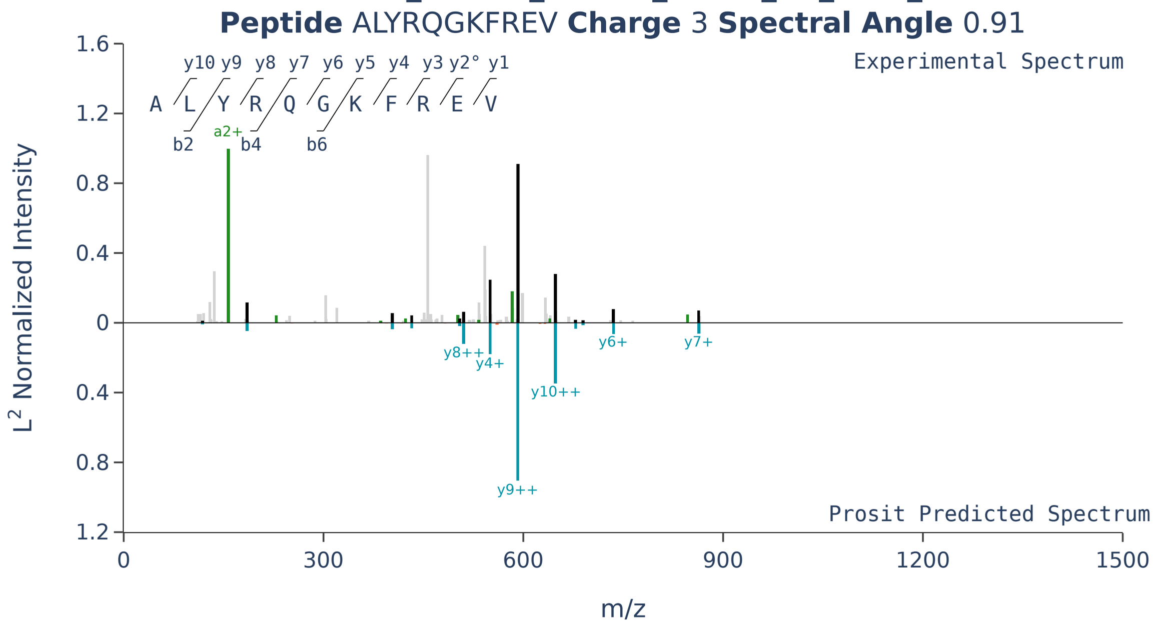


**Source** HRoetschke_211222_160123_HFGoe_IF2_b1R2_24h **Scan** 15571
**Peptide** SSLQQGF **Charge** 2 **Spectral Angle** 0.9

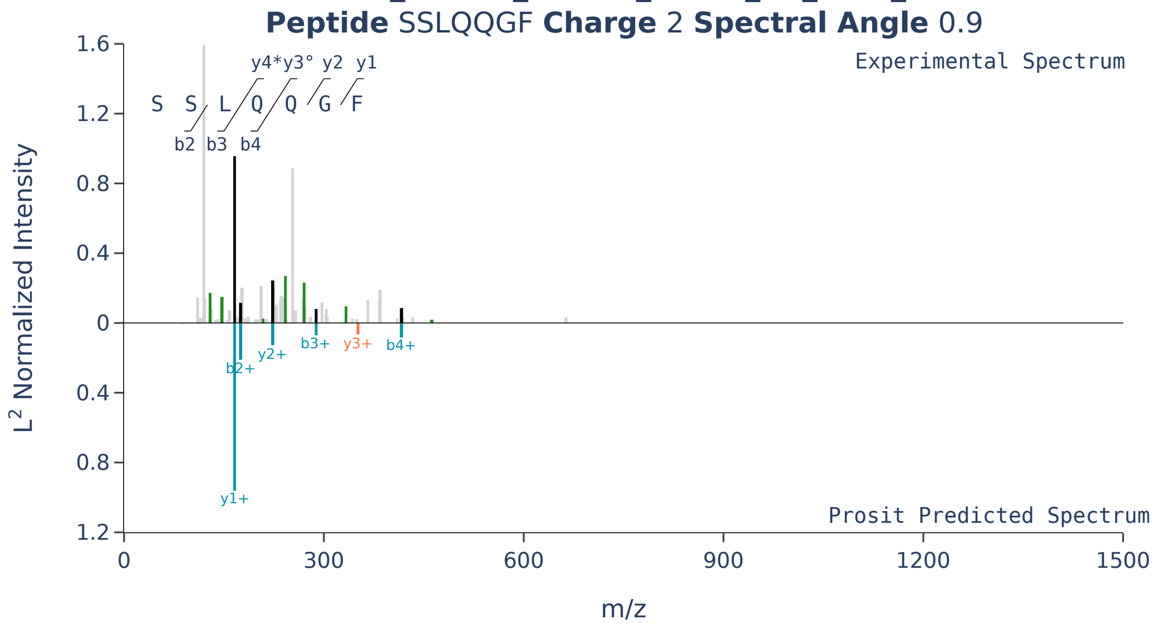


**Source** HRoetschke_211222_160123_HFGoe_IF2_b2R1_24h **Scan** 6781
**Peptide** AVNKIDKPEA **Charge** 3 **Spectral Angle** 0.93

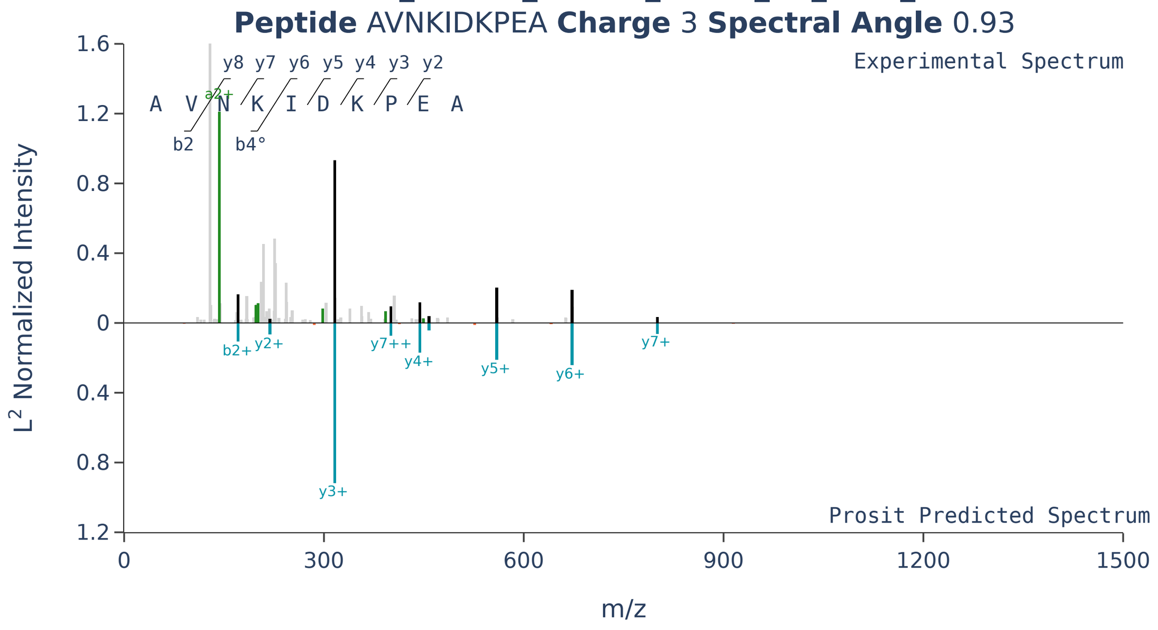


**Source** HRoetschke_211222_160123_HFGoe_IF2_b1R1_4h **Scan** 11280
**Peptide** PGHAAFTSMRA **Charge** 3 **Spectral Angle** 0.94

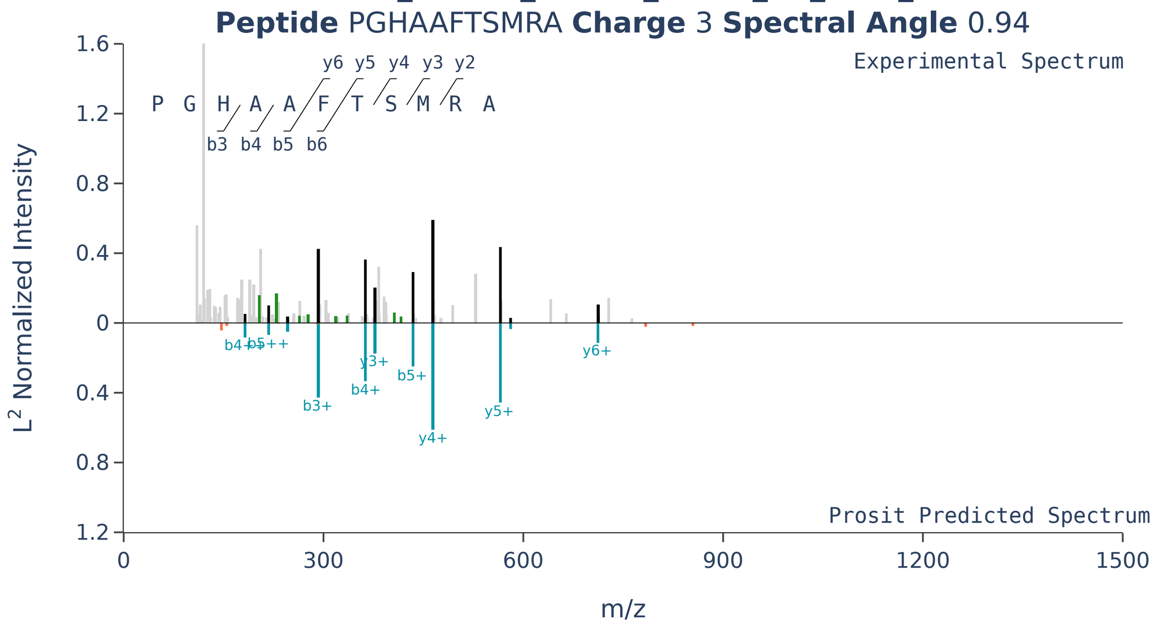


**Source** HRoetschke_211222_160123_HFGoe_IF2_b2R1_2h **Scan** 7118
**Peptide** SGVPAAGD **Charge** 1 **Spectral Angle** 0.93

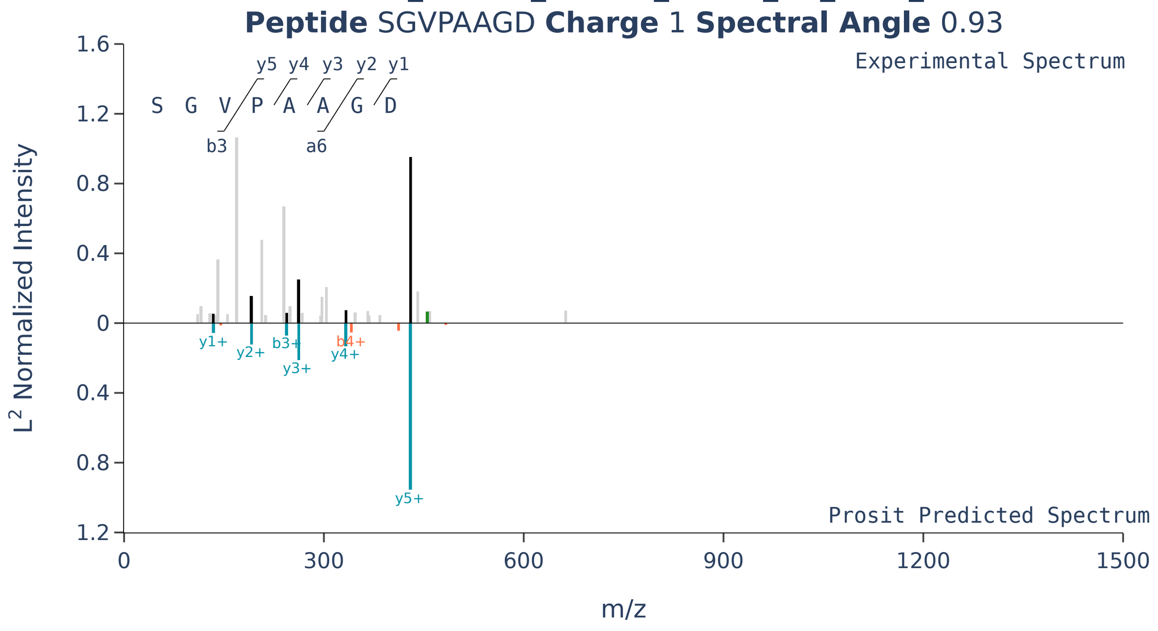


**Source** HRoetschke_211222_160123_HFGoe_IF2_b2R1_1h **Scan** 11914
**Peptide** AEVLELKAVRKG **Charge** 3 **Spectral Angle** 0.88

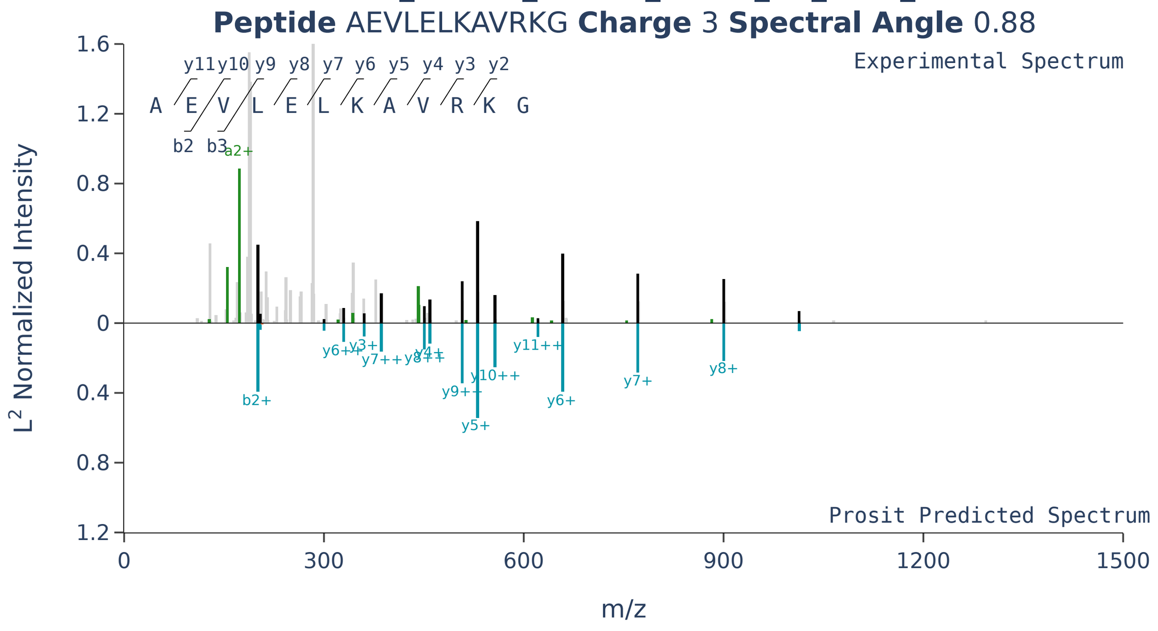


**Source** HRoetschke_211222_160123_HFGoe_IF2_b1R1_24h **Scan** 7222
**Peptide** LDTPGHA **Charge** 2 **Spectral Angle** 0.9

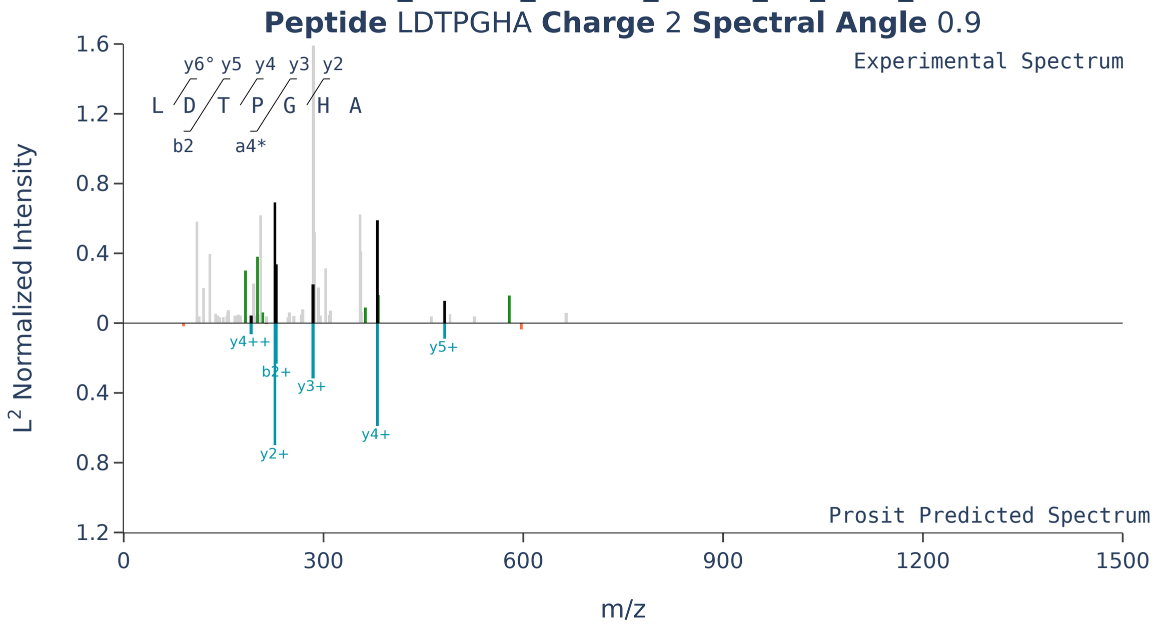


**Source** HRoetschke_211222_160123_HFGoe_IF2_b2R1_4h **Scan** 8866
**Peptide** IDKPEADPDRV **Charge** 3 **Spectral Angle** 0.88

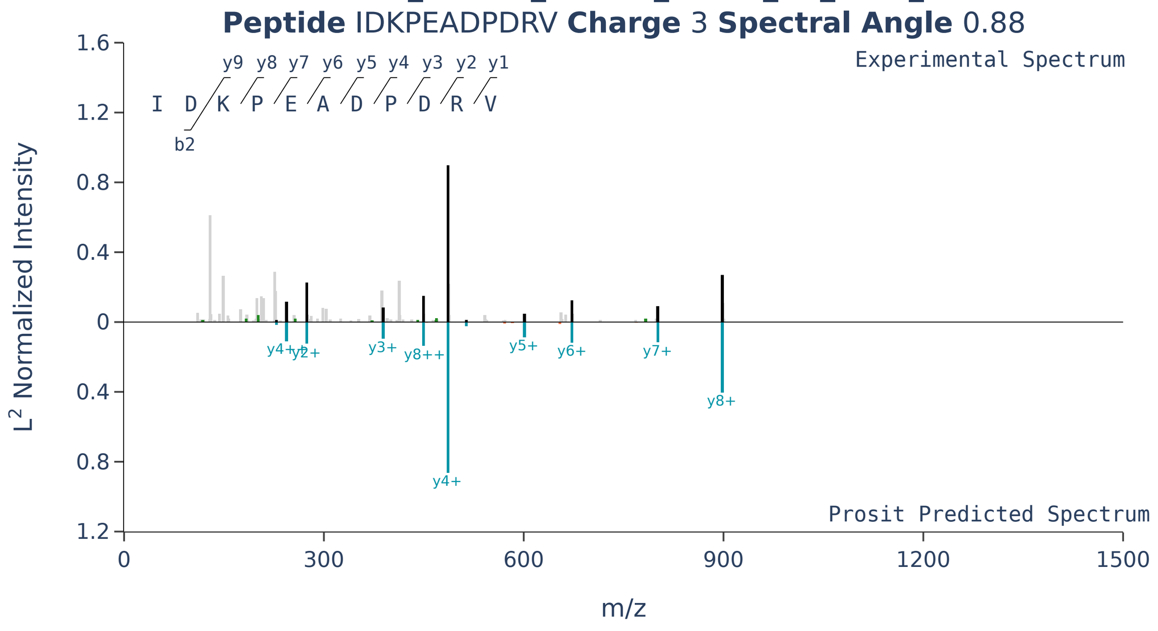


**Source** HRoetschke_211222_160123_HFGoe_IF2_b2R1_24h **Scan** 10890
**Peptide** GGESQFVHVSAK **Charge** 3 **Spectral Angle** 0.82

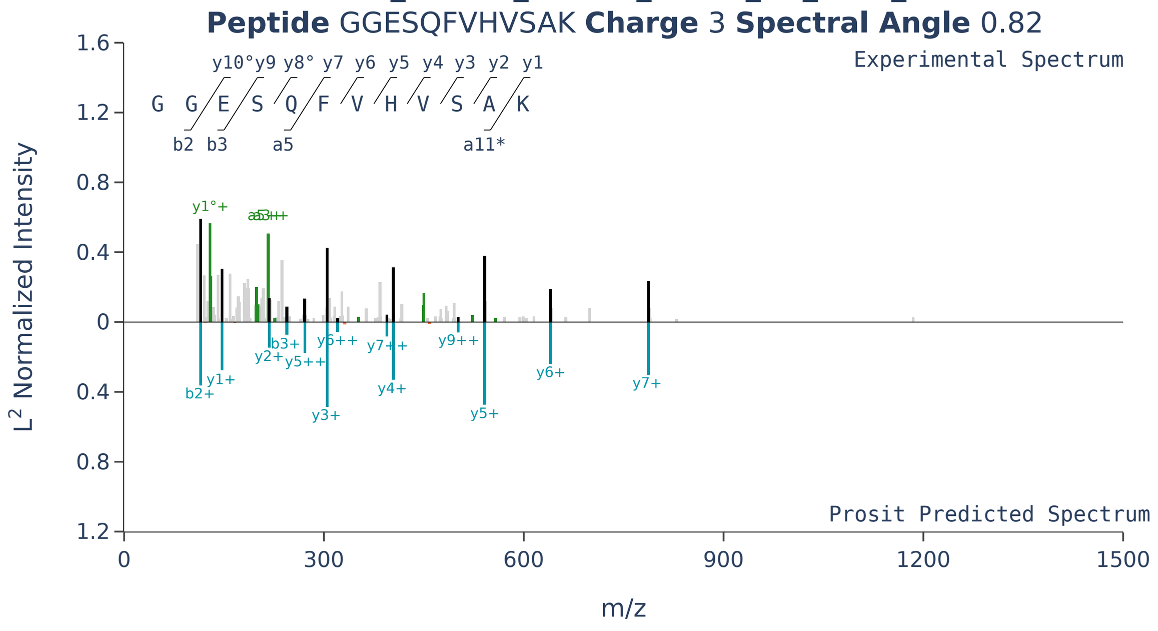


**Source** HRoetschke_211222_160123_HFGoe_IF2_b1R1_24h **Scan** 15661
**Peptide** RNELGQEVLE **Charge** 2 **Spectral Angle** 0.85

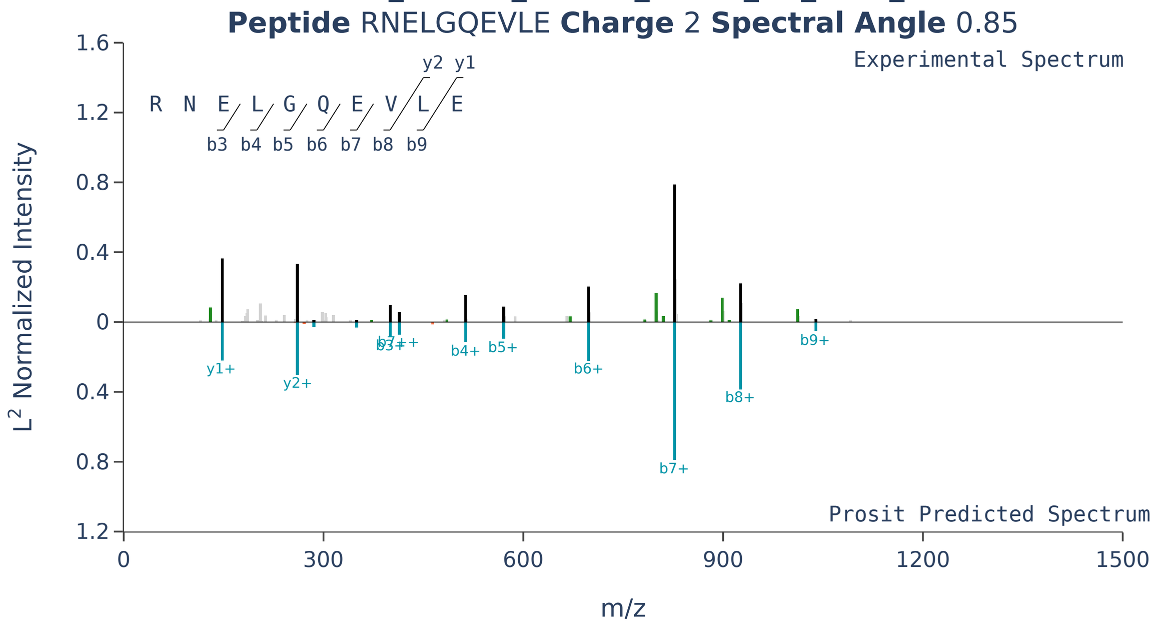


**Source** HRoetschke_211222_160123_HFGoe_IF2_b2R2_4h **Scan** 13233
**Peptide** LEEAVMSD **Charge** 1 **Spectral Angle** 0.88

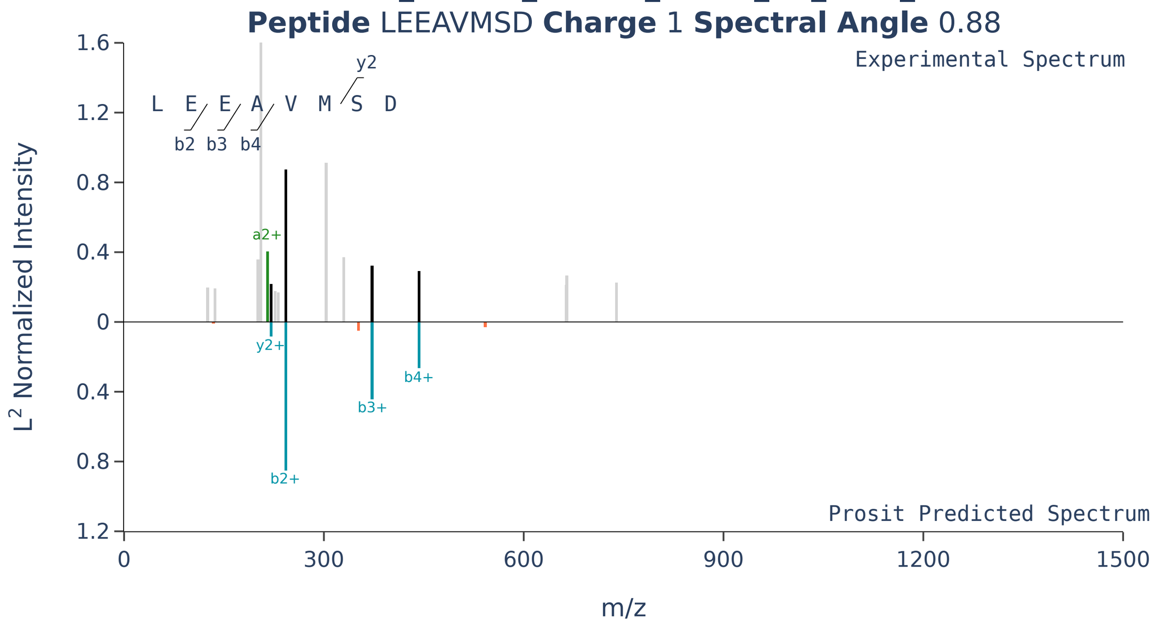


**Source** HRoetschke_211222_160123_HFGoe_IF2_b1R1_24h **Scan** 11925
**Peptide** EVLELKAVRK **Charge** 3 **Spectral Angle** 0.9

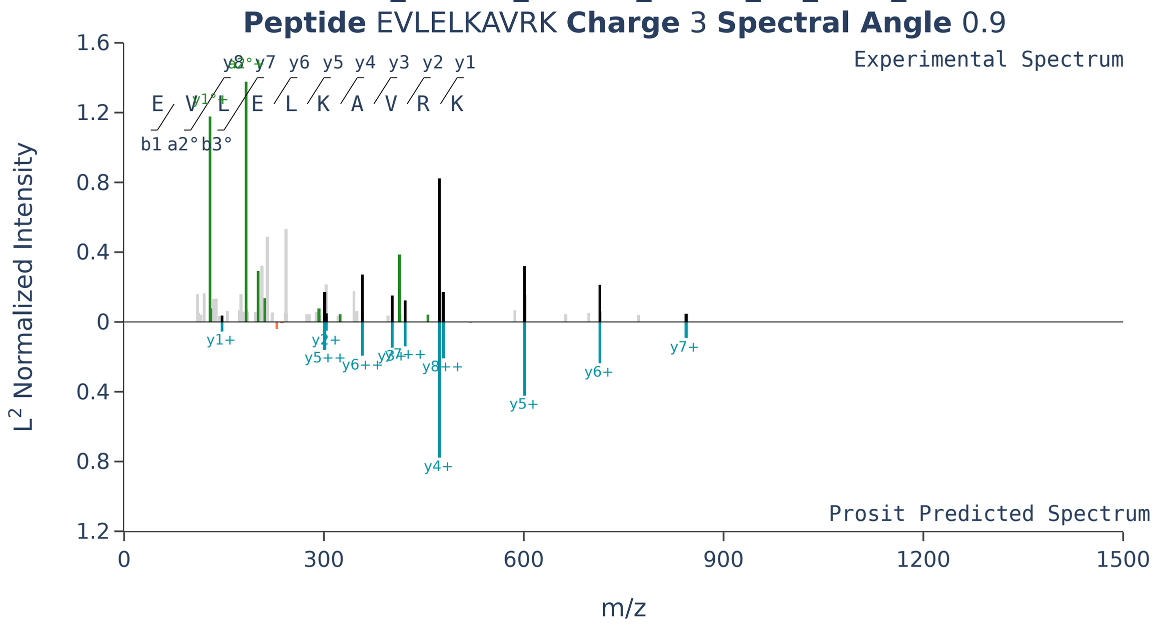


**Source** HRoetschke_211222_160123_HFGoe_IF2_b2R2_24h **Scan** 6964
**Peptide** KVASGEAGGITQH **Charge** 3 **Spectral Angle** 0.88

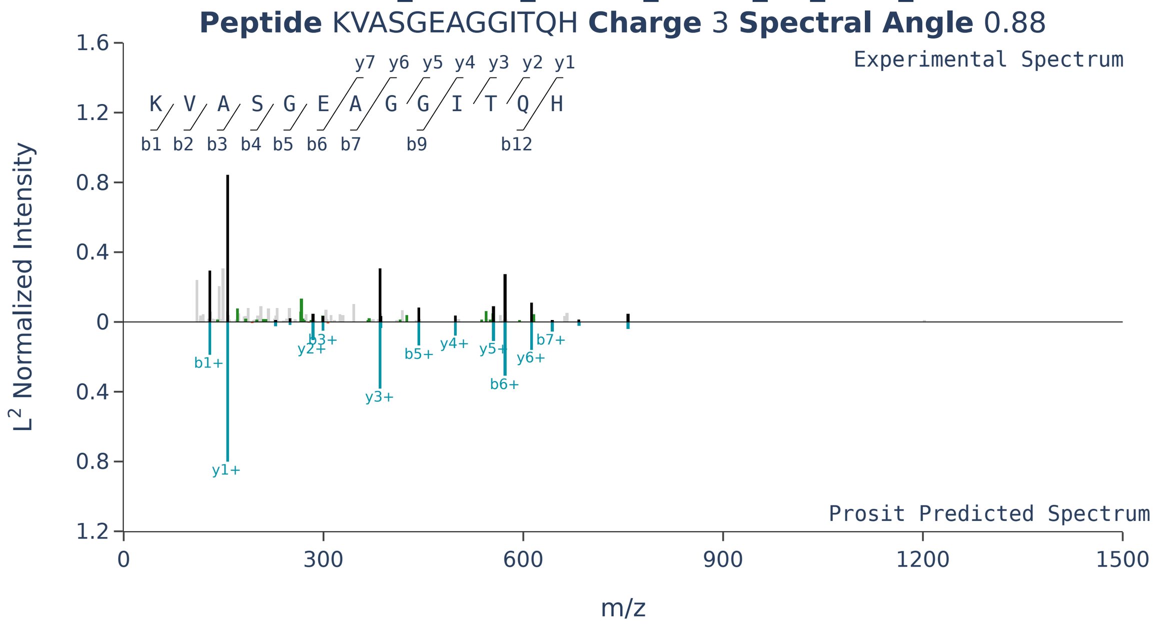

**Source** HRoetschke_211222_160123_HFGoe_IF2_b1R2_1h **Scan** 10223
**Peptide** GESQFVHVSAK **Charge** 3 **Spectral Angle** 0.86

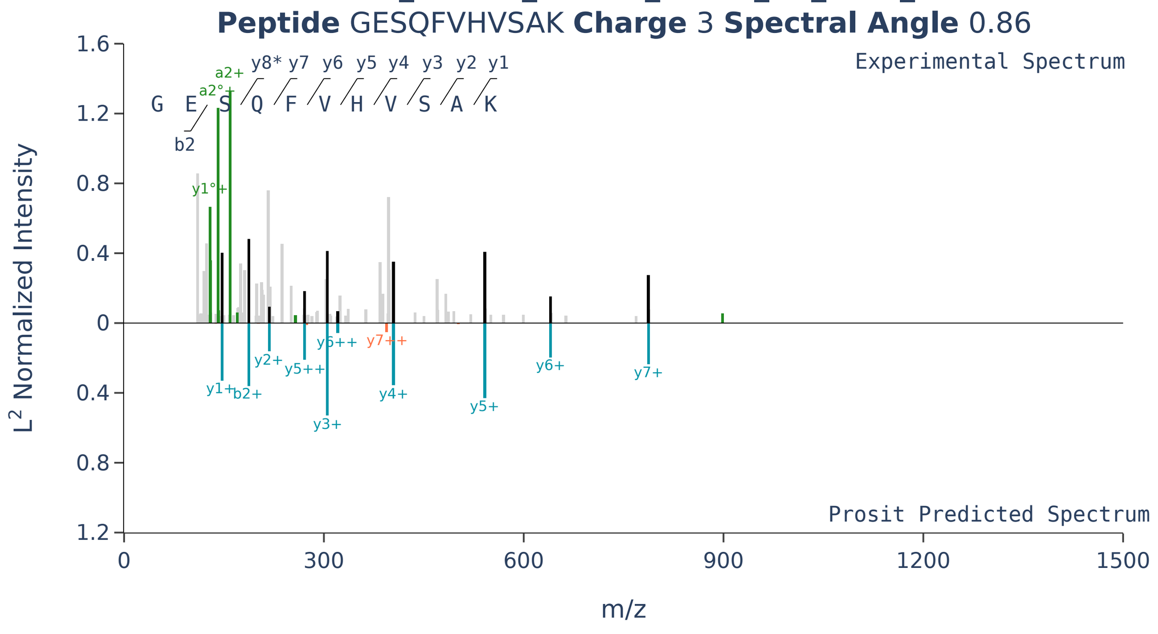


**Source** HRoetschke_211222_160123_HFGoe_IF2_b1R1_24h **Scan** 9148
**Peptide** SFLDKGRG **Charge** 2 **Spectral Angle** 0.94

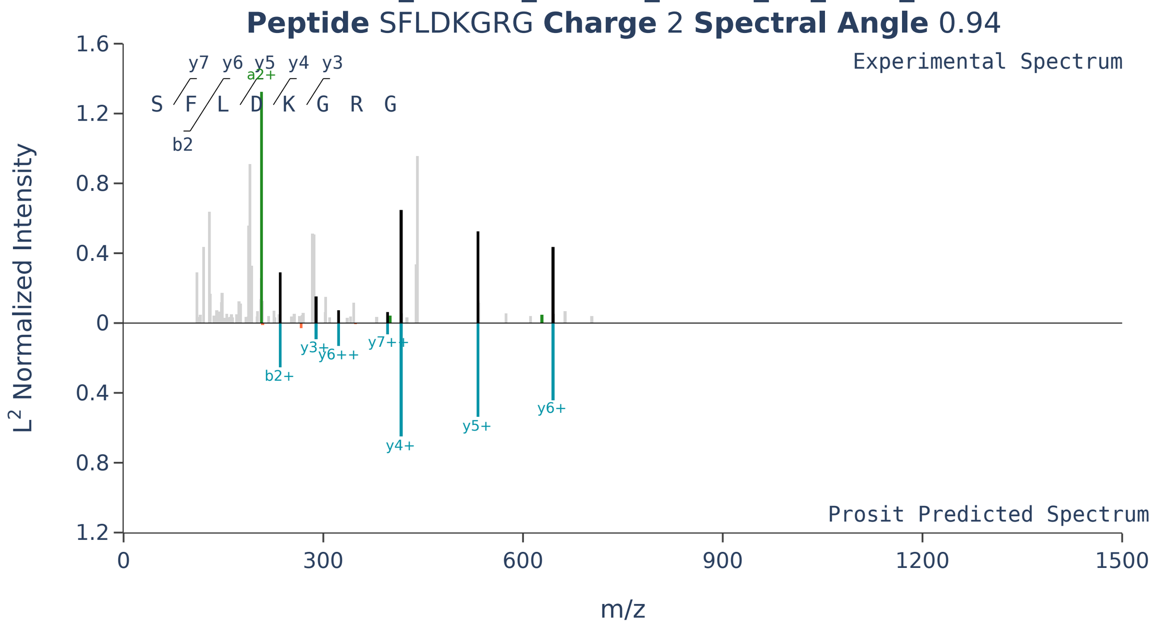


**Source** HRoetschke_211222_160123_HFGoe_IF2_b1R2_24h **Scan** 12565
**Peptide** LDYIRST **Charge** 2 **Spectral Angle** 0.89

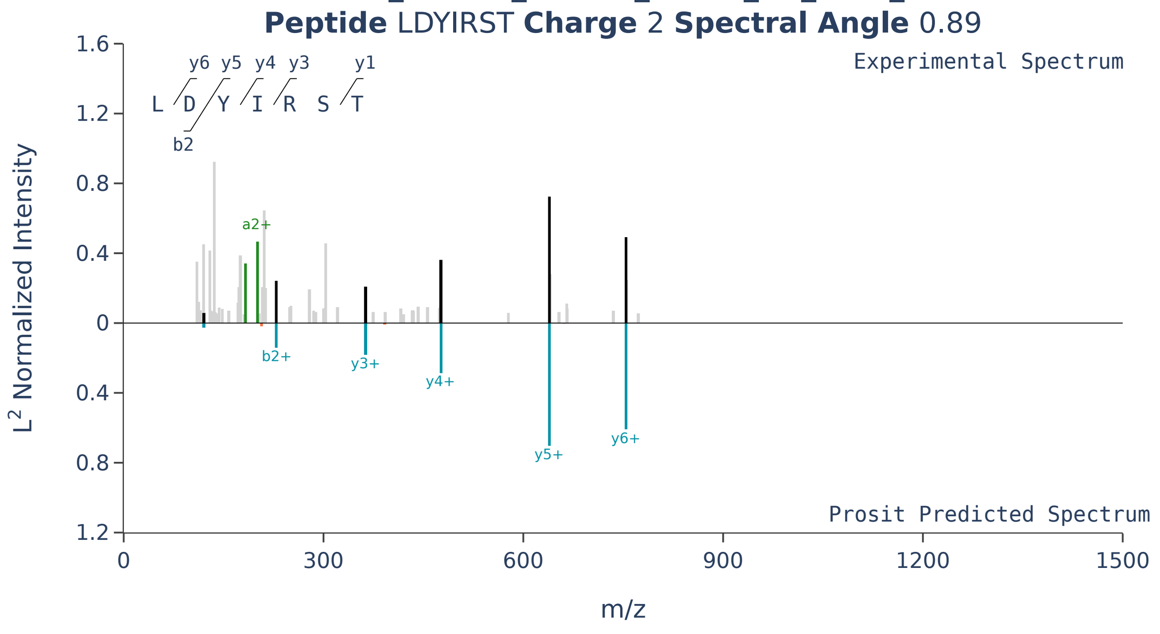


**Source** HRoetschke_211222_160123_HFGoe_IF2_b1R2_24h **Scan** 6216
**Peptide** SSDYHVTTSQH **Charge** 2 **Spectral Angle** 0.86

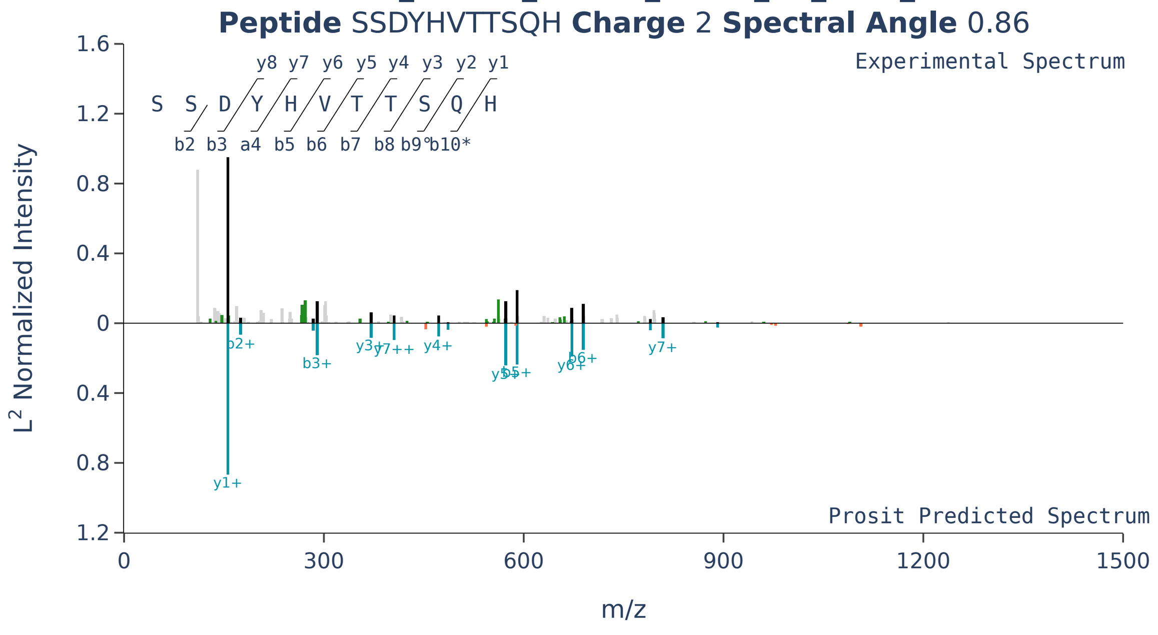


**Source** HRoetschke_211222_160123_HFGoe_IF2_b2R1_2h **Scan** 8454
**Peptide** GELANKM **Charge** 2 **Spectral Angle** 0.89

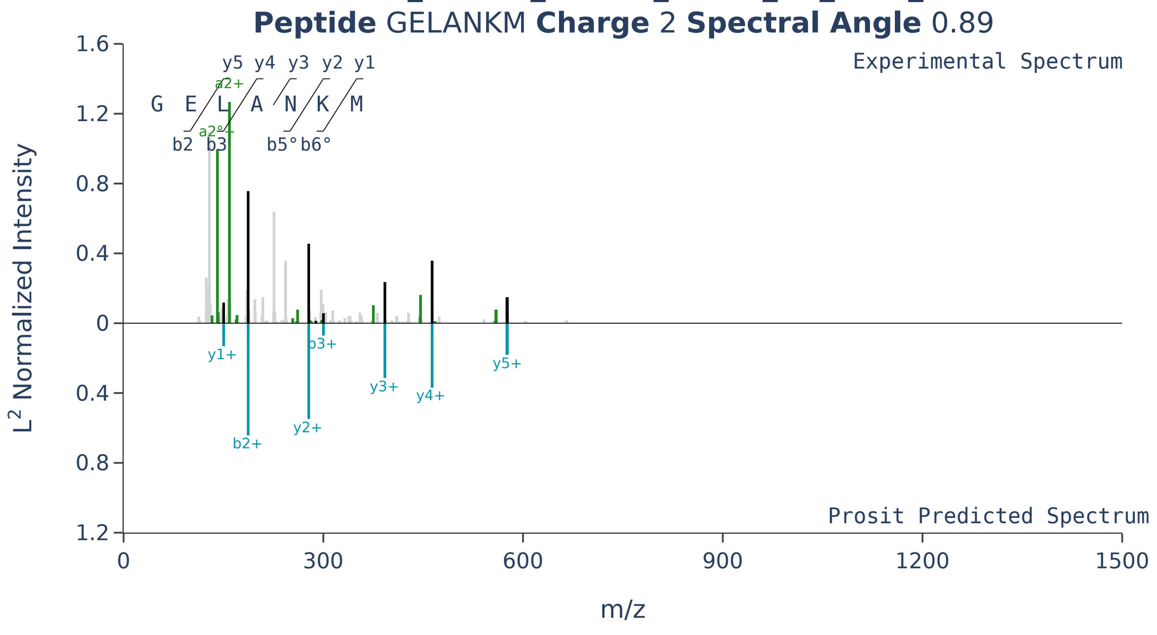


**Source** HRoetschke_211222_160123_HFGoe_IF2_b2R1_4h **Scan** 7002
**Peptide** AEEMGHKVILR **Charge** 3 **Spectral Angle** 0.87

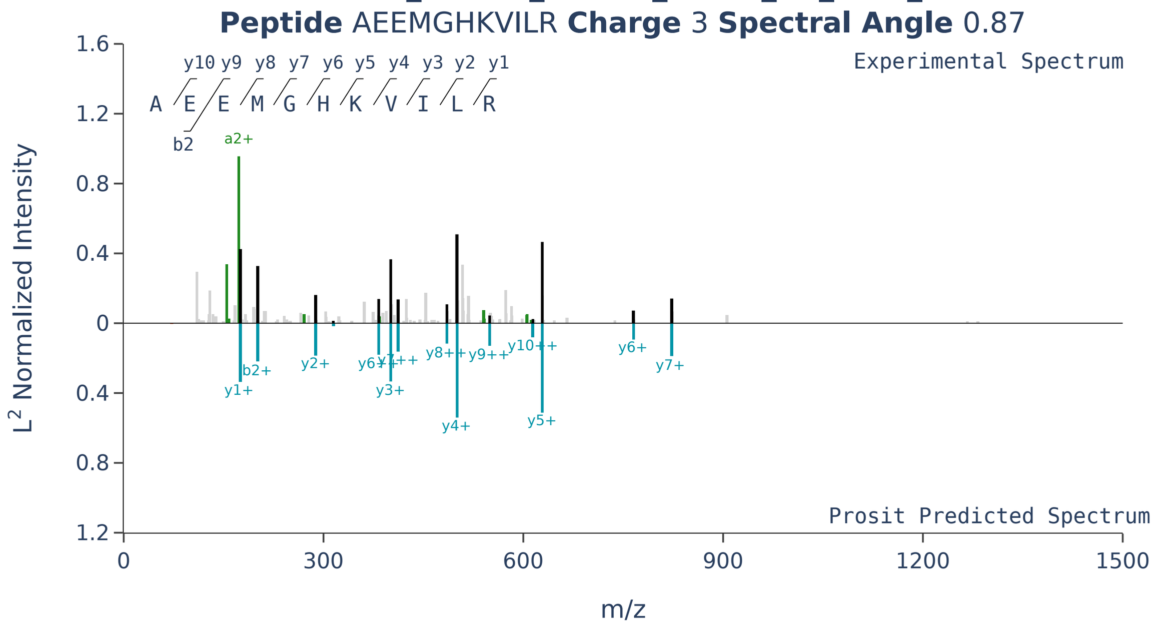


**Source** HRoetschke_211222_160123_HFGoe_IF2_b2R2_24h **Scan** 12165
**Peptide** SGVGGITE **Charge** 1 **Spectral Angle** 0.92

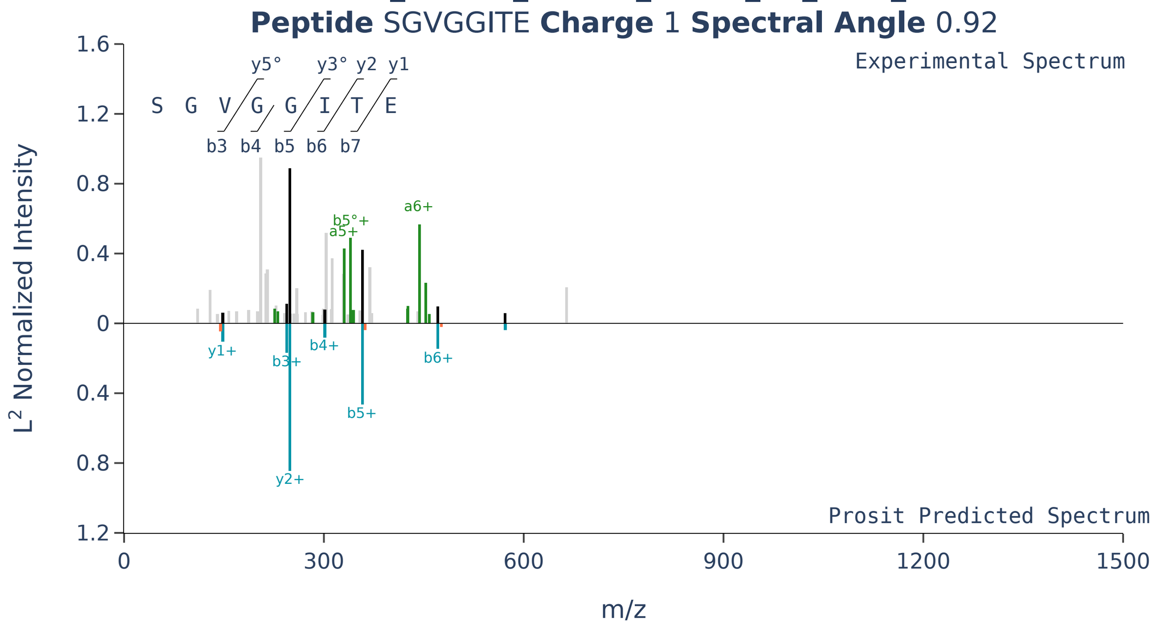


**Source** HRoetschke_211222_160123_HFGoe_IF2_b1R1_24h **Scan** 14818
**Peptide** QETAQLV **Charge** 2 **Spectral Angle** 0.88

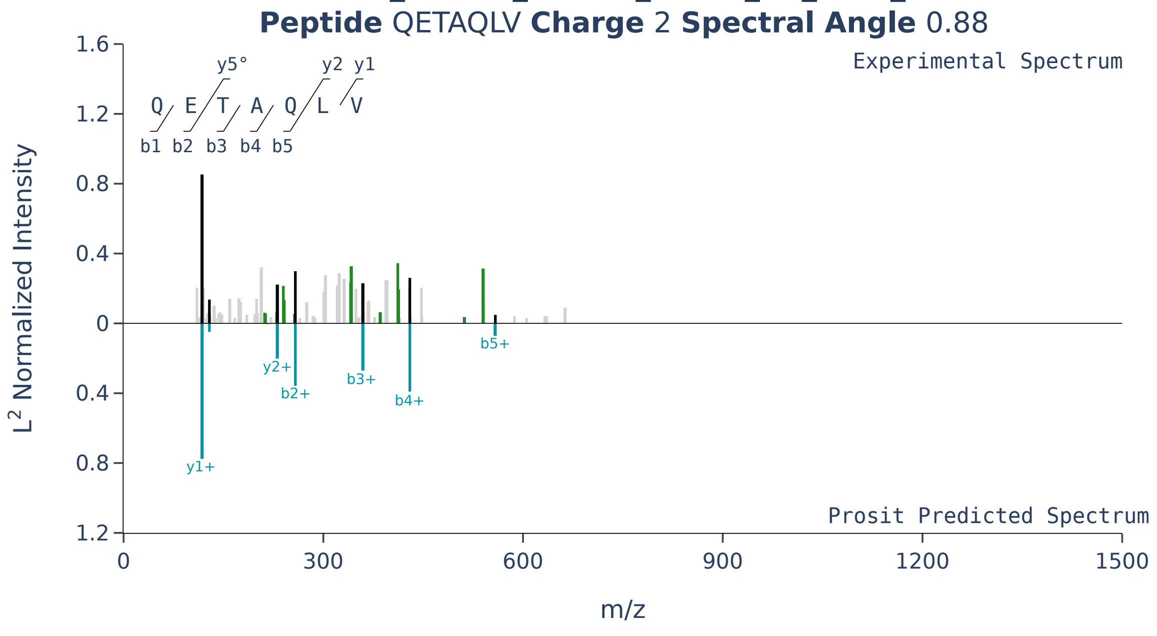


**Source** HRoetschke_211222_160123_HFGoe_IF2_b1R1_4h **Scan** 6703
**Peptide** SGPDKLTLQRKTR **Charge** 4 **Spectral Angle** 0.85

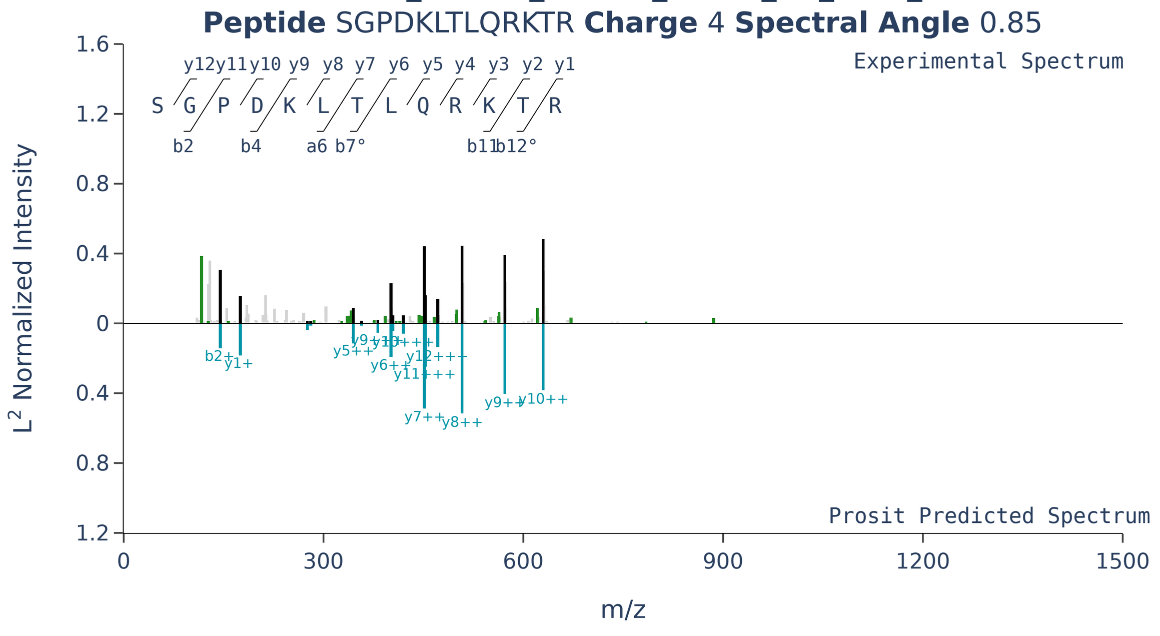


**Source** HRoetschke_211222_160123_HFGoe_IF2_b2R2_4h **Scan** 14980
**Peptide** DQETAQLVAEE **Charge** 2 **Spectral Angle** 0.9

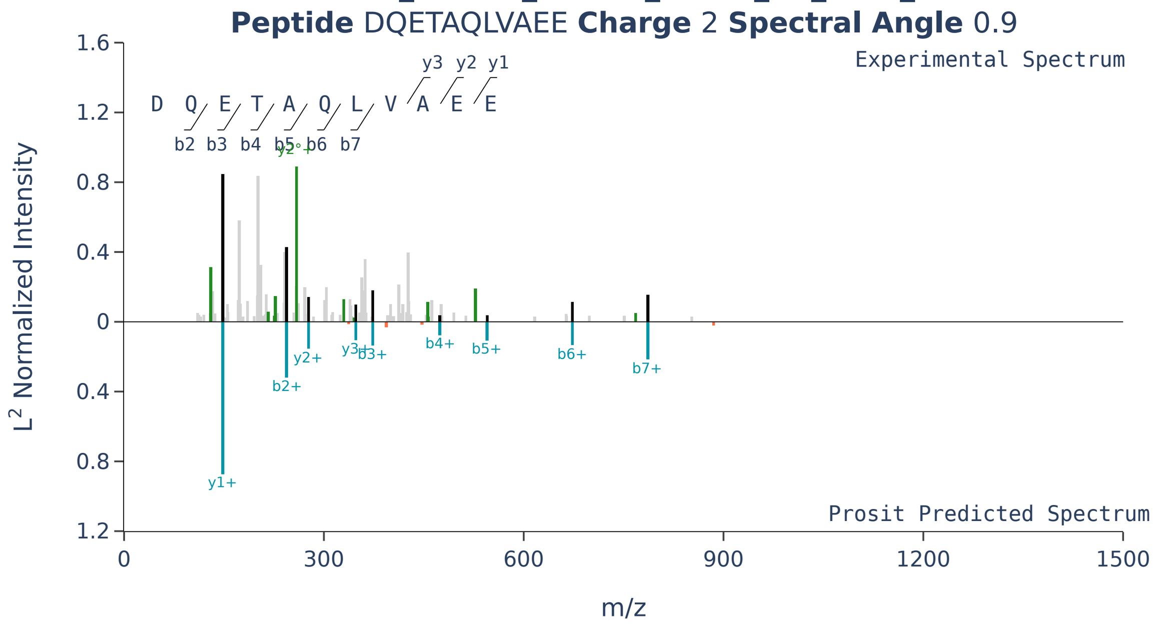


**Source** HRoetschke_211222_160123_HFGoe_IF2_b1R2_24h **Scan** 7054
**Peptide** EVTVVRDEK **Charge** 2 **Spectral Angle** 0.9

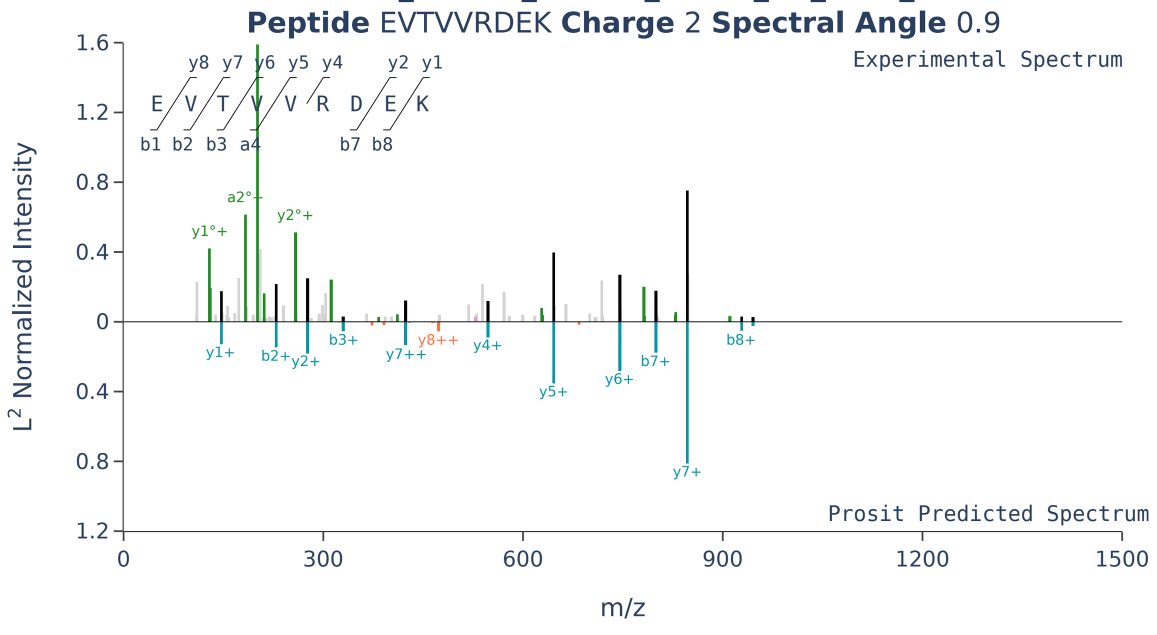


**Source** HRoetschke_211222_160123_HFGoe_IF2_b1R2_1h **Scan** 8432
**Peptide** ERQTSVERL **Charge** 3 **Spectral Angle** 0.92

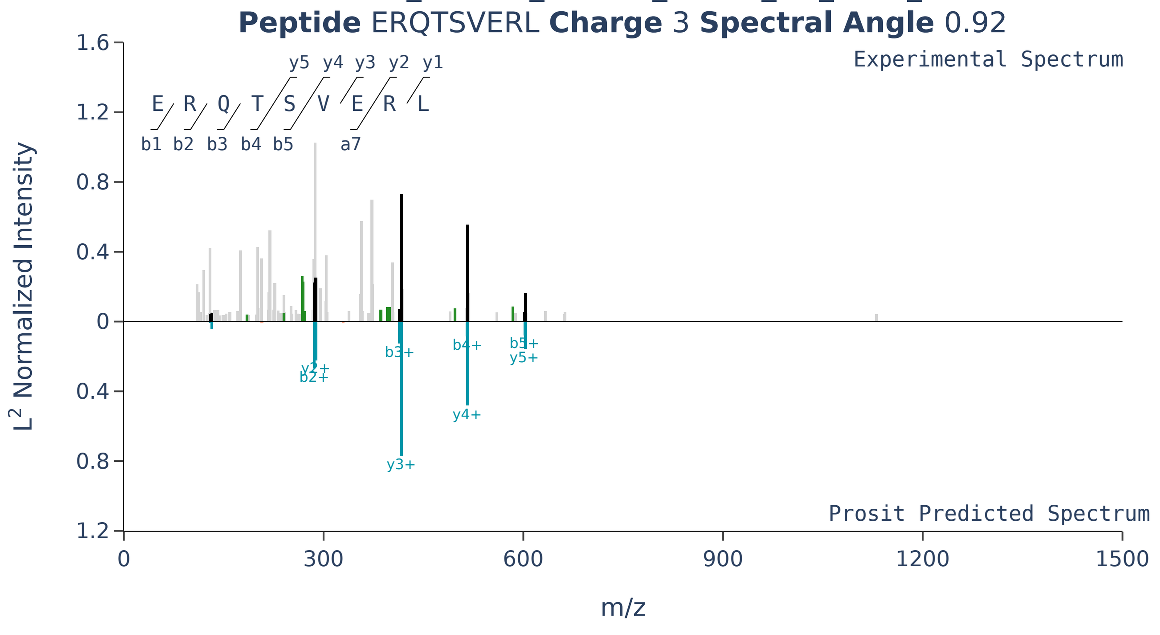


**Source** HRoetschke_211222_160123_HFGoe_IF2_b2R1_2h **Scan** 10106
**Peptide** DPQEAERL **Charge** 2 **Spectral Angle** 0.86

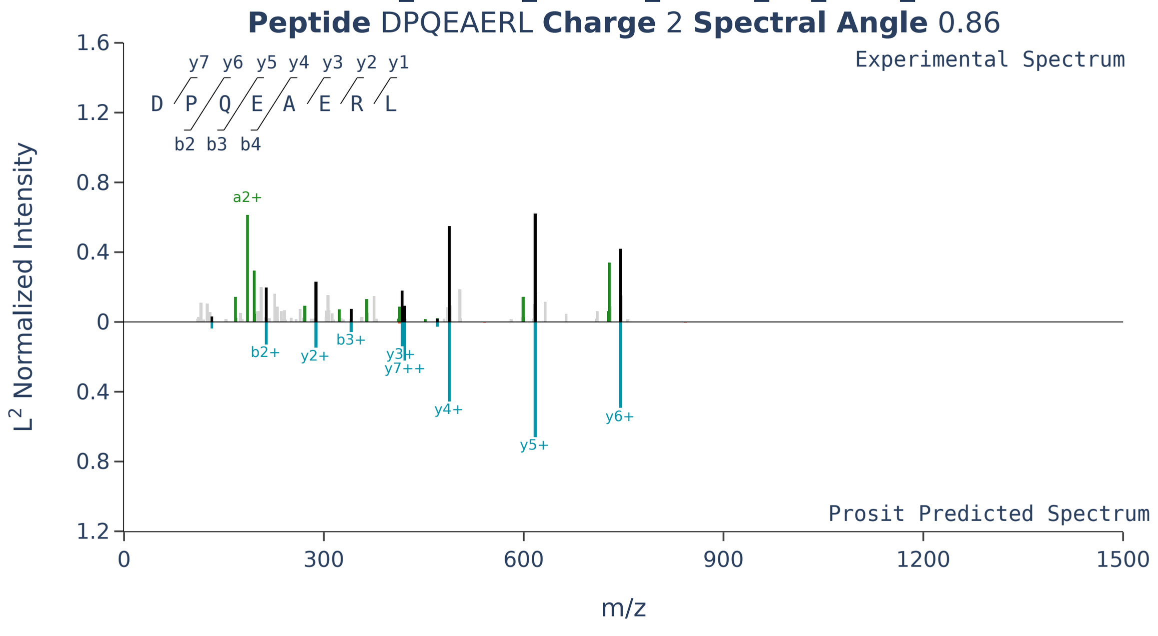


**Source** HRoetschke_211222_160123_HFGoe_IF2_b2R2_24h **Scan** 9860
**Peptide** TPGHAAF **Charge** 2 **Spectral Angle** 0.94

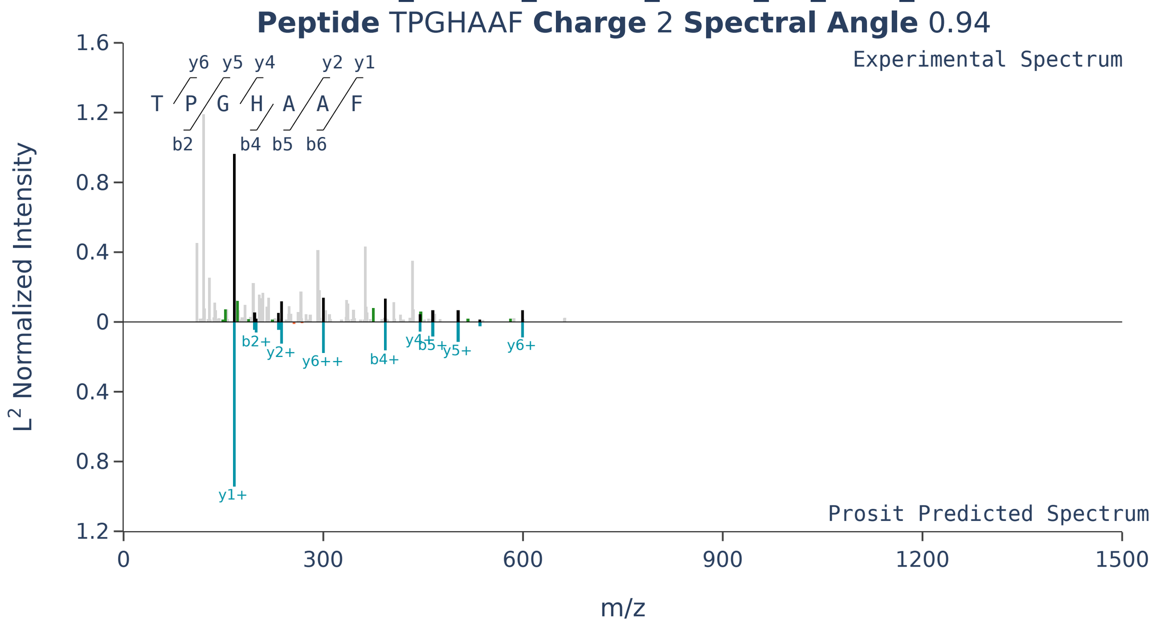


**Source** HRoetschke_211222_160123_HFGoe_IF2_b1R2_24h **Scan** 8113
**Peptide** LELKAVRKG **Charge** 2 **Spectral Angle** 0.89

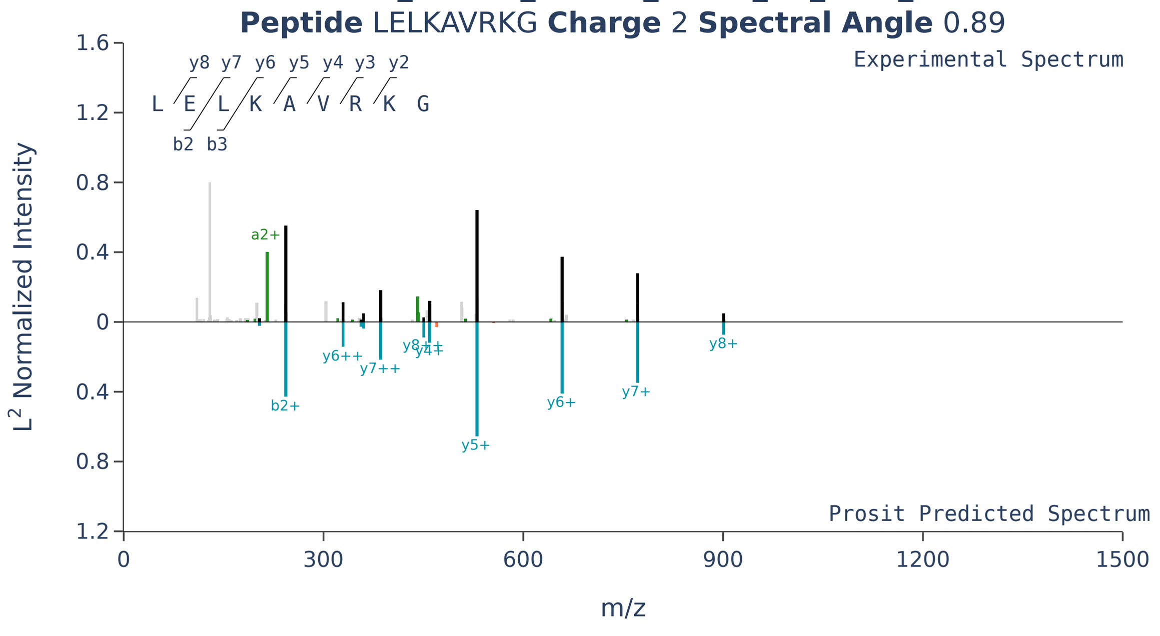

**Source** HRoetschke_211222_160123_HFGoe_IF2_b1R2_24h **Scan** 12994
**Peptide** VTIKTLAAERQT **Charge** 3 **Spectral Angle** 0.91

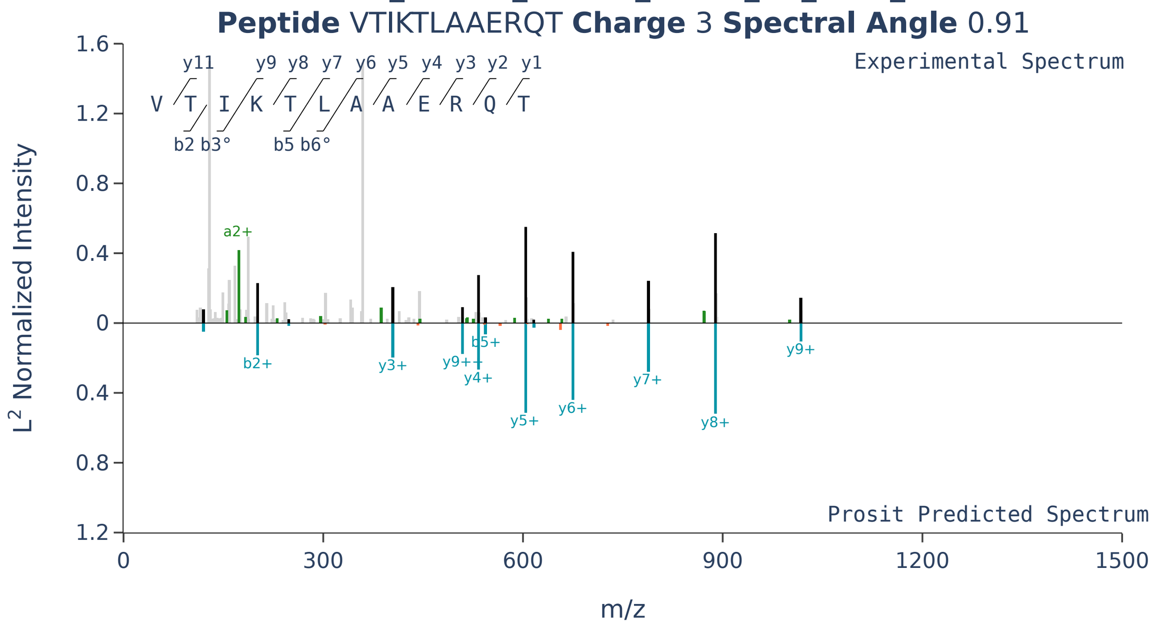


**Source** HRoetschke_211222_160123_HFGoe_IF2_b1R2_24h **Scan** 8727
**Peptide** GESQFVH **Charge** 2 **Spectral Angle** 0.92

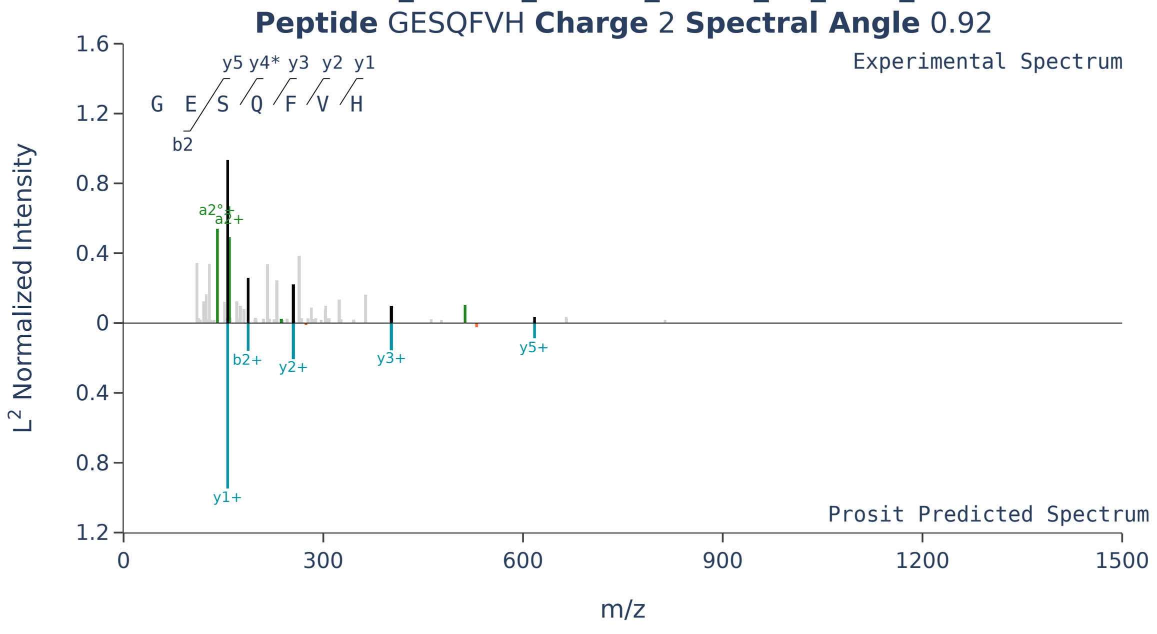


**Source** HRoetschke_211222_160123_HFGoe_IF2_b2R2_24h **Scan** 12939
**Peptide** AERQTSVERLV **Charge** 3 **Spectral Angle** 0.91

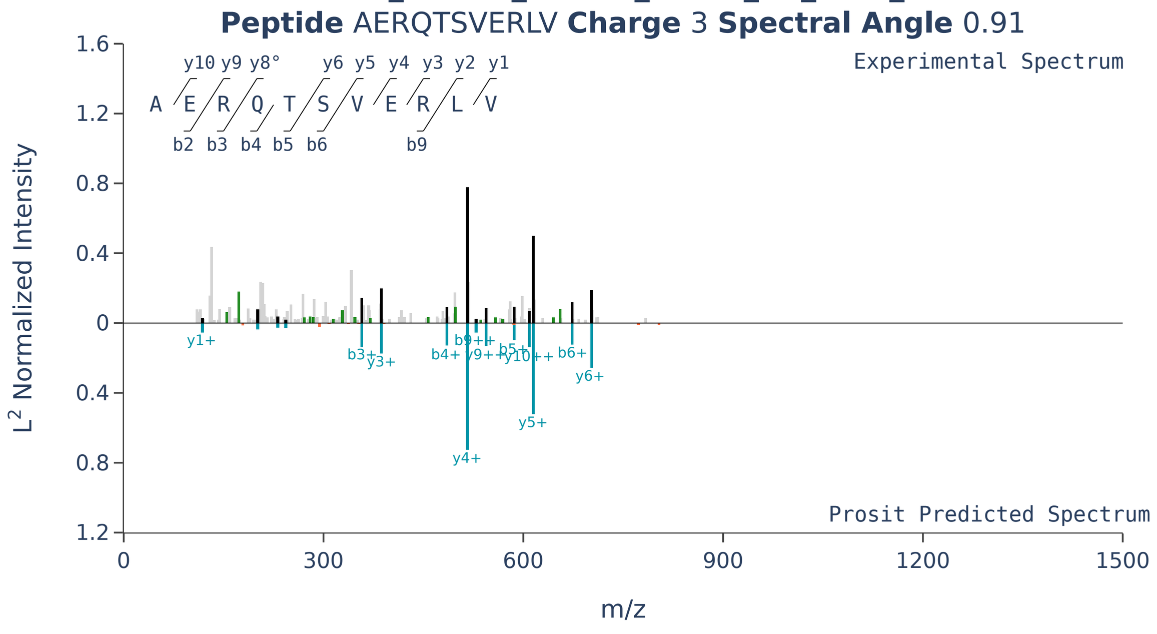


**Source** HRoetschke_211222_160123_HFGoe_IF2_b1R1_2h **Scan** 5693
**Peptide** SDRDTGAAAEPRA **Charge** 3 **Spectral Angle** 0.89

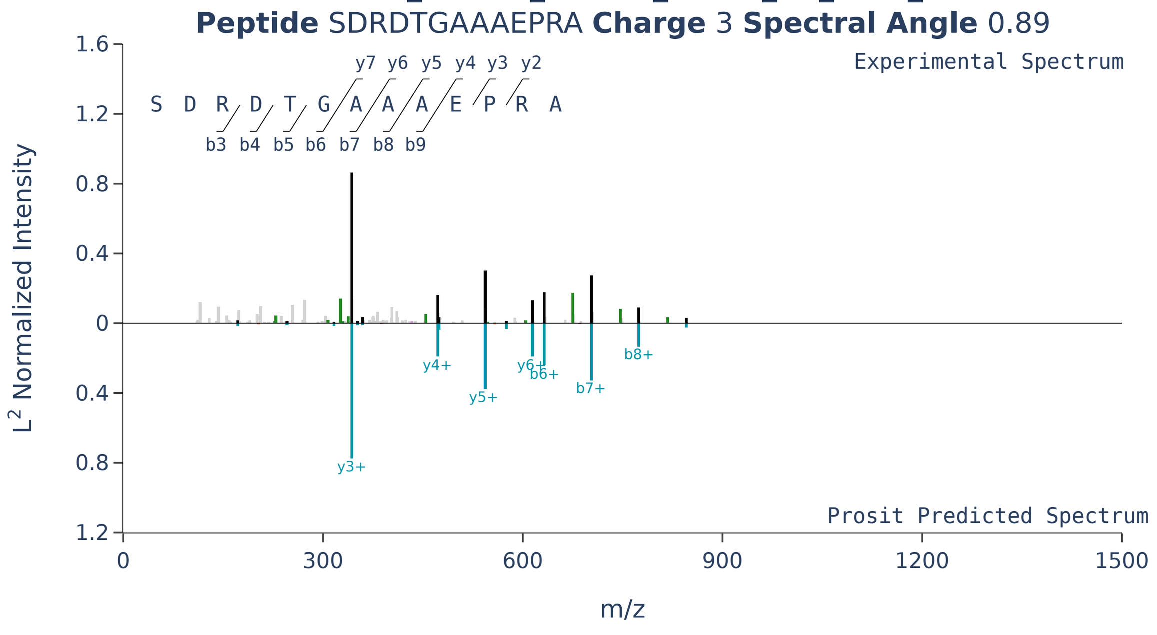


**Source** HRoetschke_211222_160123_HFGoe_IF2_b1R2_24h **Scan** 17986
**Peptide** LLDYIRST **Charge** 2 **Spectral Angle** 0.91

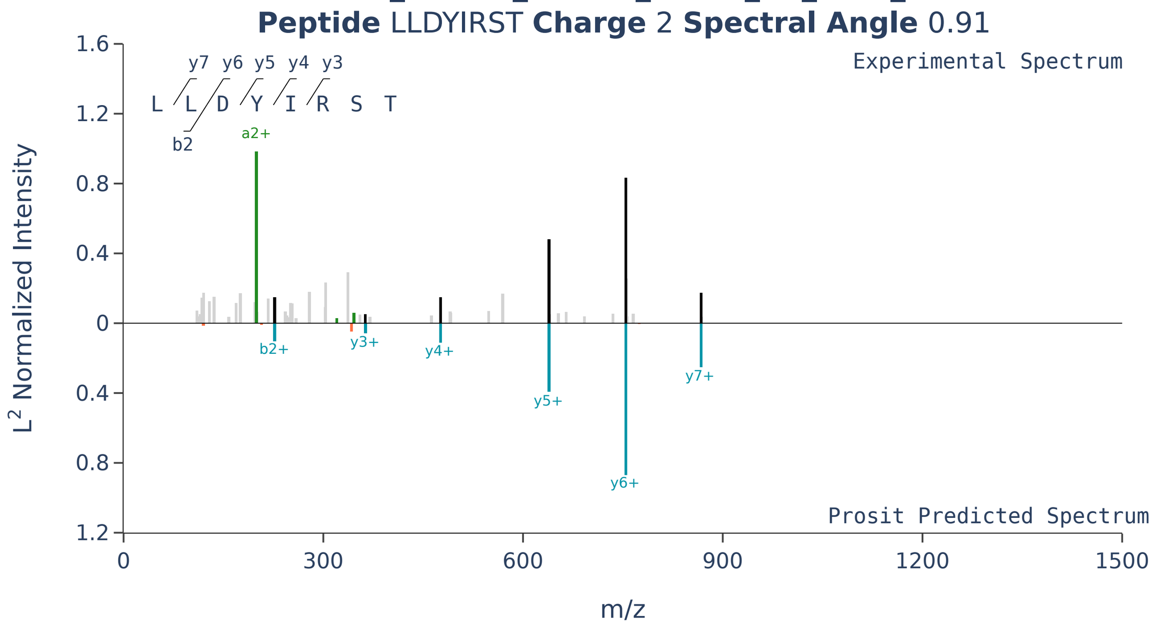


**Source** HRoetschke_211222_160123_HFGoe_IF2_b1R1_2h **Scan** 6773
**Peptide** SDYHVTTSQH **Charge** 2 **Spectral Angle** 0.83

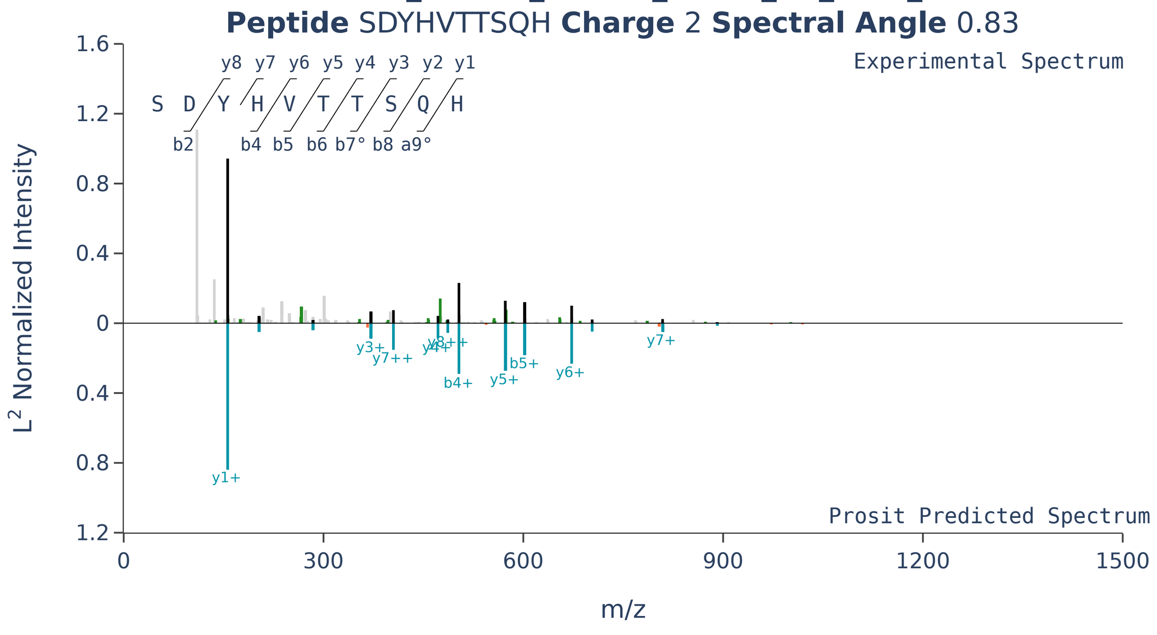


**Source** HRoetschke_211222_160123_HFGoe_IF2_b1R1_4h **Scan** 8203
**Peptide** AMRNELGQ **Charge** 2 **Spectral Angle** 0.93

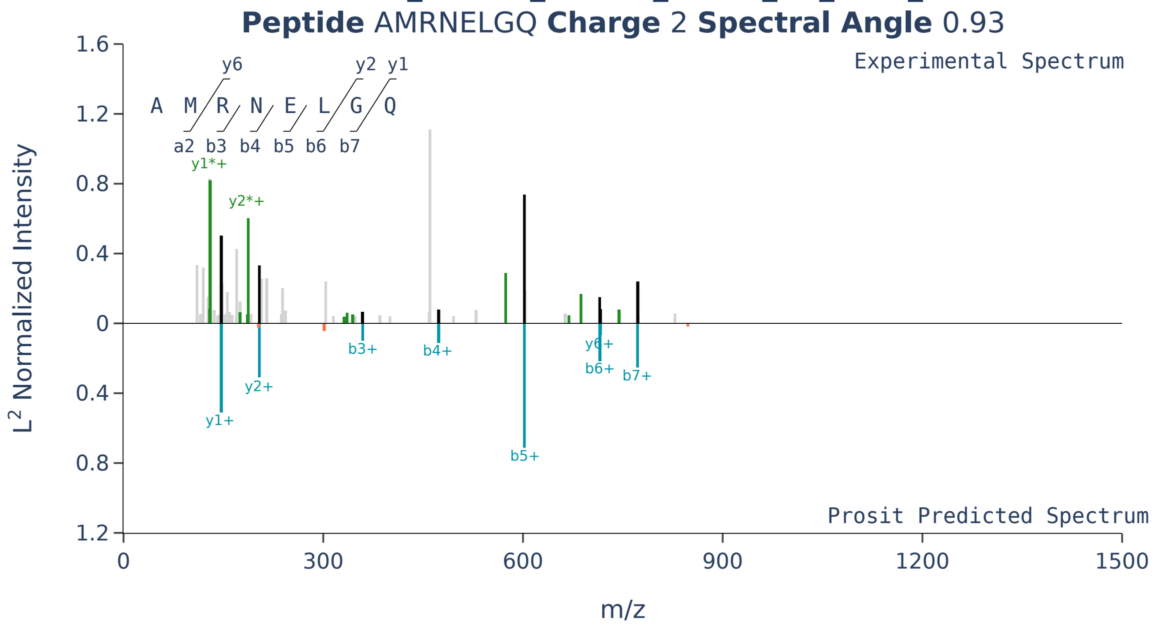


**Source** HRoetschke_211222_160123_HFGoe_IF2_b1R2_24h **Scan** 17620
**Peptide** GKTSLLDY **Charge** 2 **Spectral Angle** 0.87

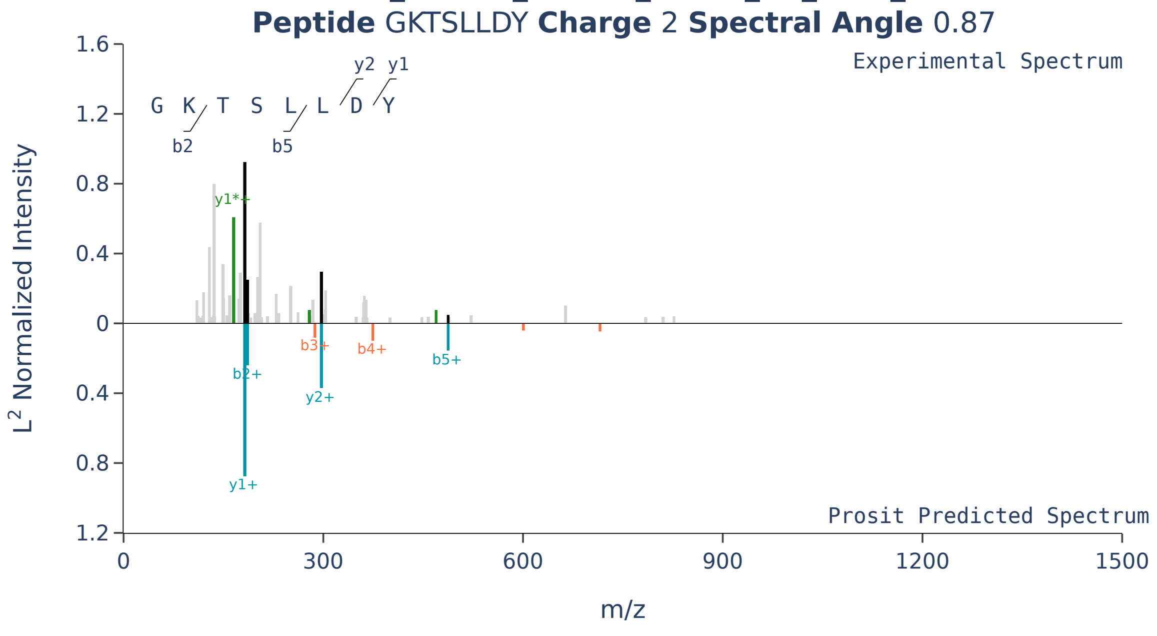


**Source** HRoetschke_211222_160123_HFGoe_IF2_b2R2_24h **Scan** 12385
**Peptide** VNRDVVIG **Charge** 2 **Spectral Angle** 0.92

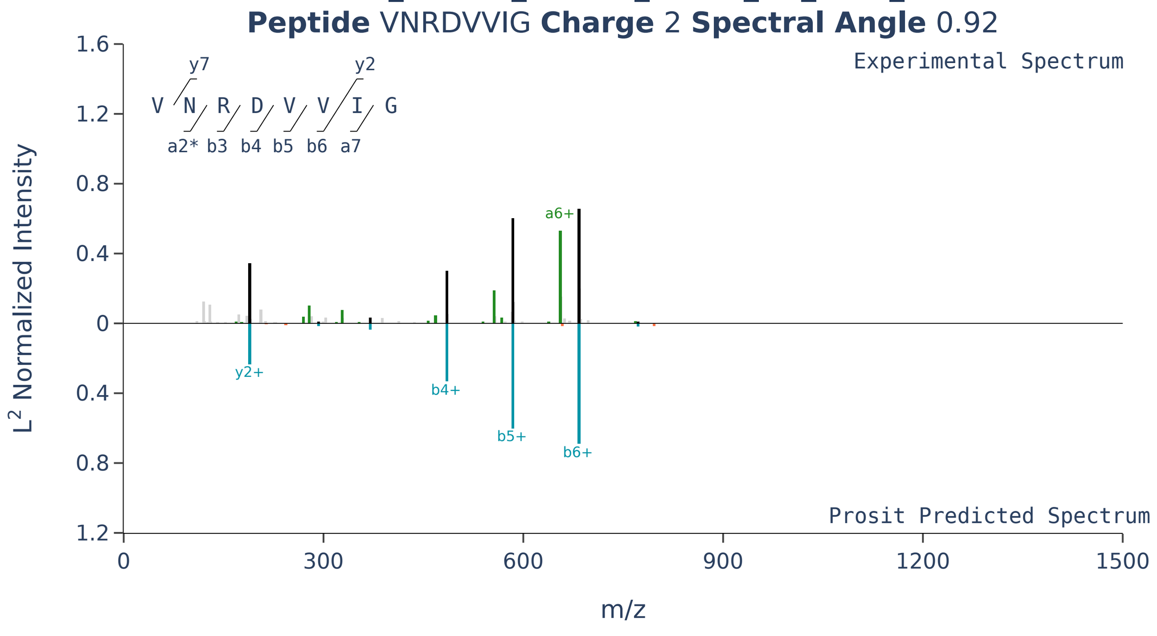


**Source** HRoetschke_211222_160123_HFGoe_IF2_b1R2_24h **Scan** 17409
**Peptide** RNELGQEVLEA **Charge** 2 **Spectral Angle** 0.9

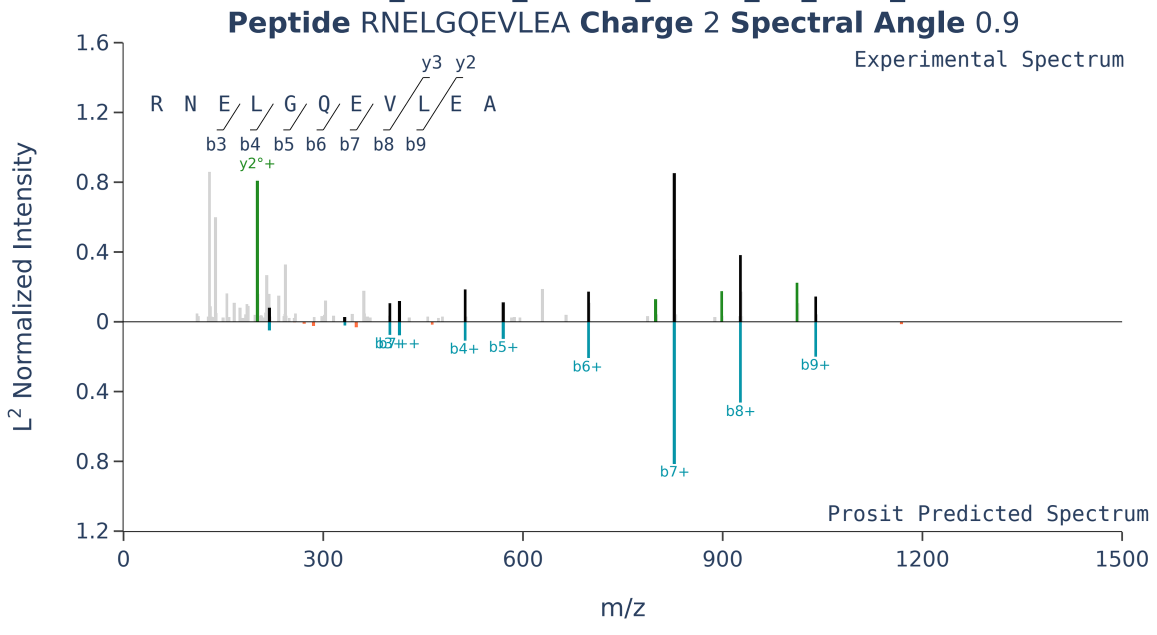


**Source** HRoetschke_211222_160123_HFGoe_IF2_b1R2_24h **Scan** 16361
**Peptide** AQEKQTLIDHL **Charge** 3 **Spectral Angle** 0.9

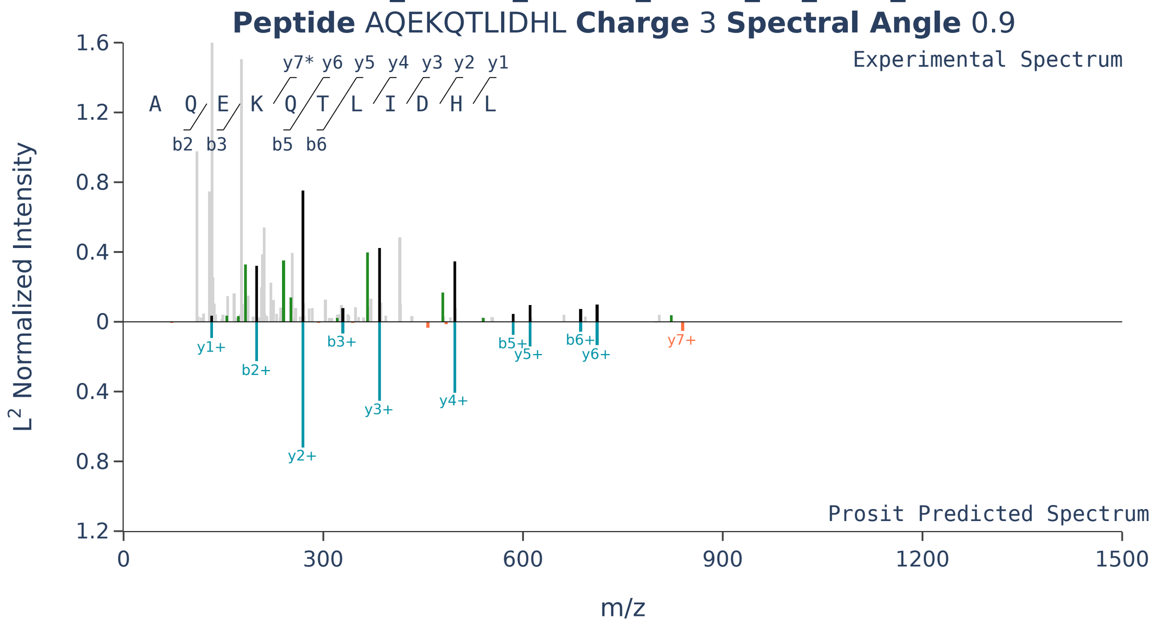


**Source** HRoetschke_211222_160123_HFGoe_IF2_b1R1_24h **Scan** 13636
**Peptide** GSSLQQGFQ **Charge** 2 **Spectral Angle** 0.97

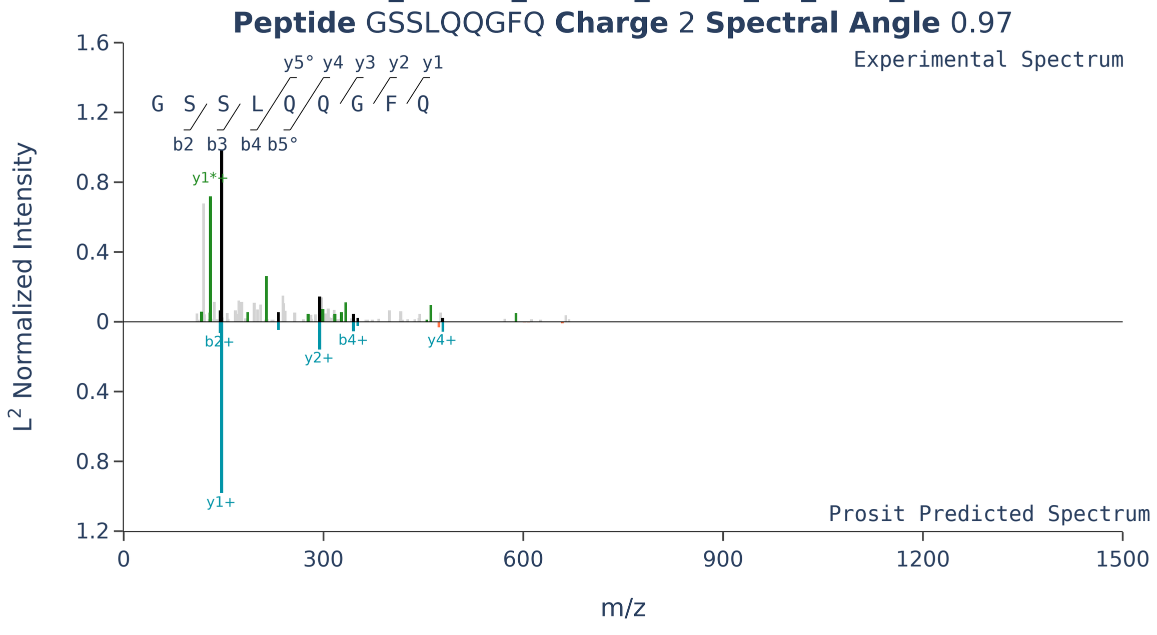


**Source** HRoetschke_211222_160123_HFGoe_IF2_b2R2_2h **Scan** 17509
**Peptide** NELGQEVLE **Charge** 2 **Spectral Angle** 0.92

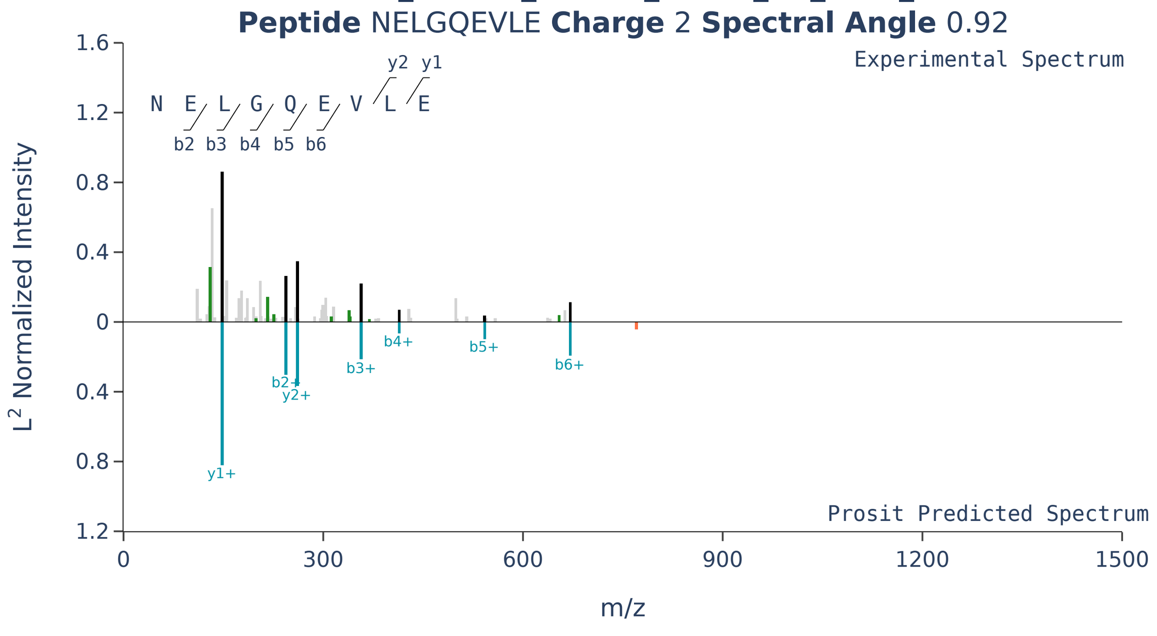


**Source** HRoetschke_211222_160123_HFGoe_IF2_b1R2_24h **Scan** 9041
**Peptide** REVALYRQG **Charge** 3 **Spectral Angle** 0.85

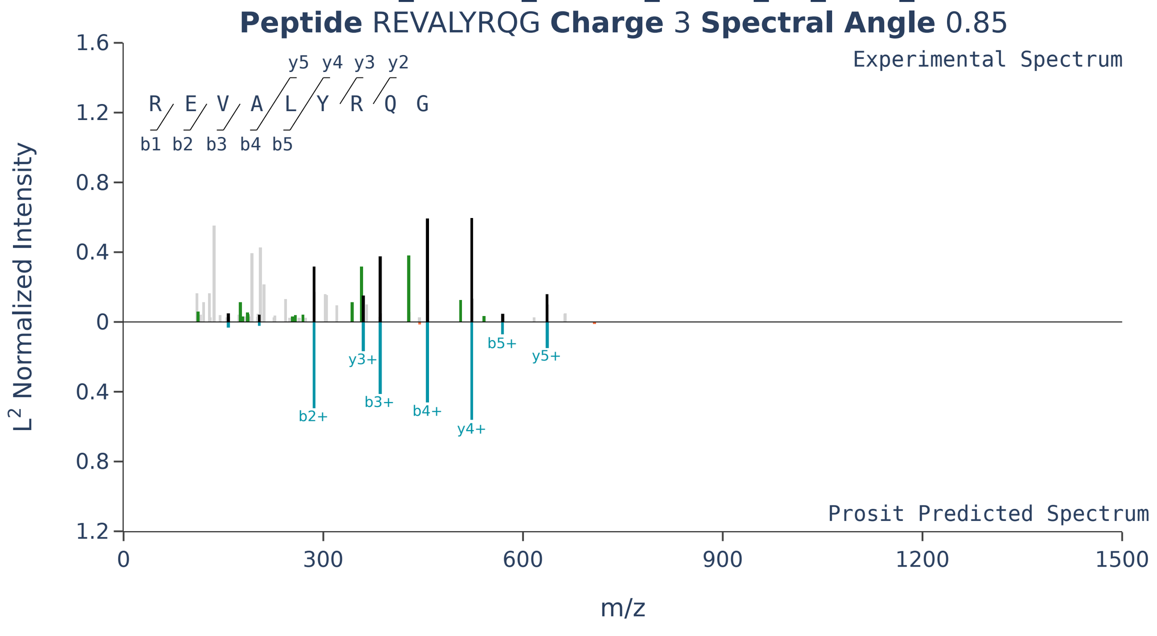


**Source** HRoetschke_211222_160123_HFGoe_IF2_b1R1_2h **Scan** 10957
**Peptide** GIGVKNY **Charge** 1 **Spectral Angle** 0.86

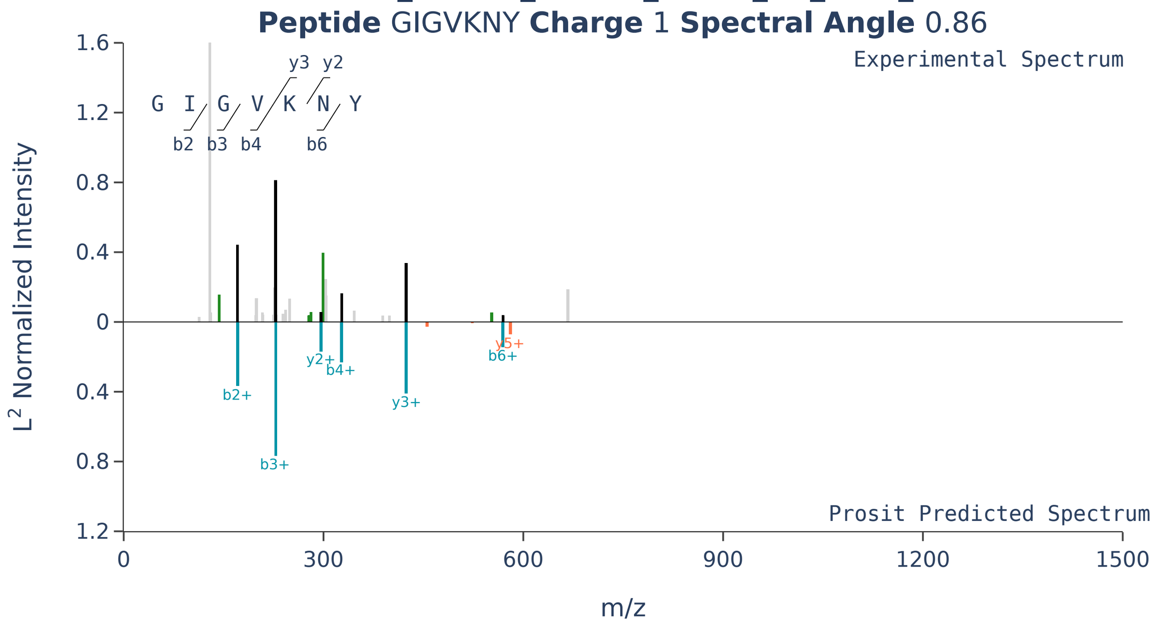

**Source** HRoetschke_211222_160123_HFGoe_IF2_b2R2_24h **Scan** 11785
**Peptide** TDATLAAA **Charge** 1 **Spectral Angle** 0.88

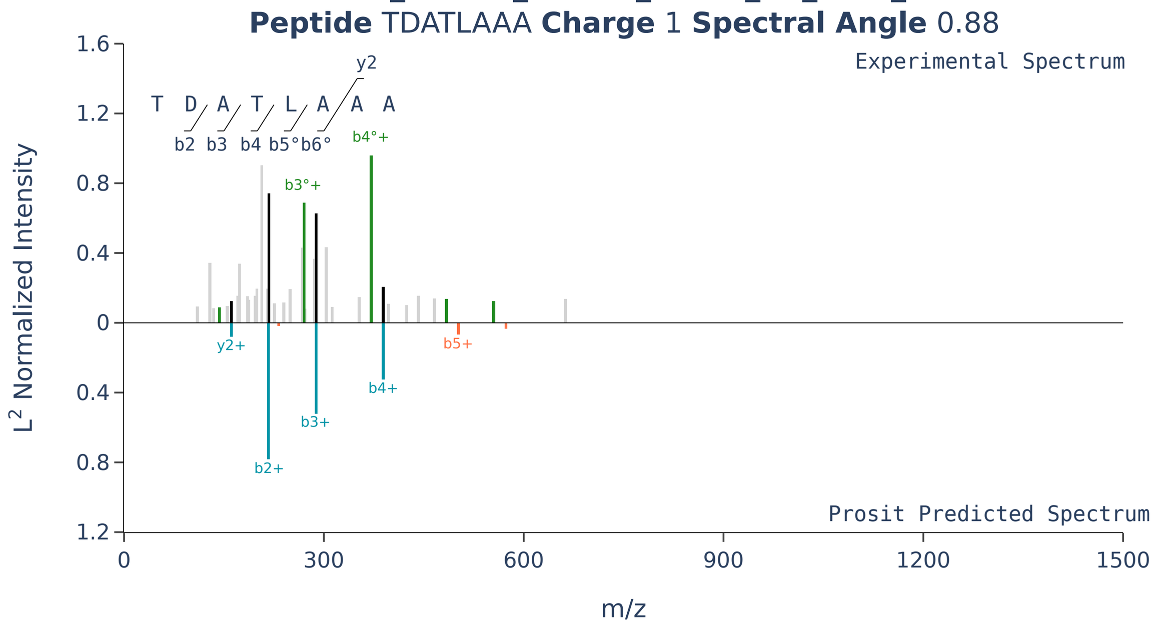


**Source** HRoetschke_211222_160123_HFGoe_IF2_b1R1_1h **Scan** 10612
**Peptide** AQEKQTLID **Charge** 1 **Spectral Angle** 0.86

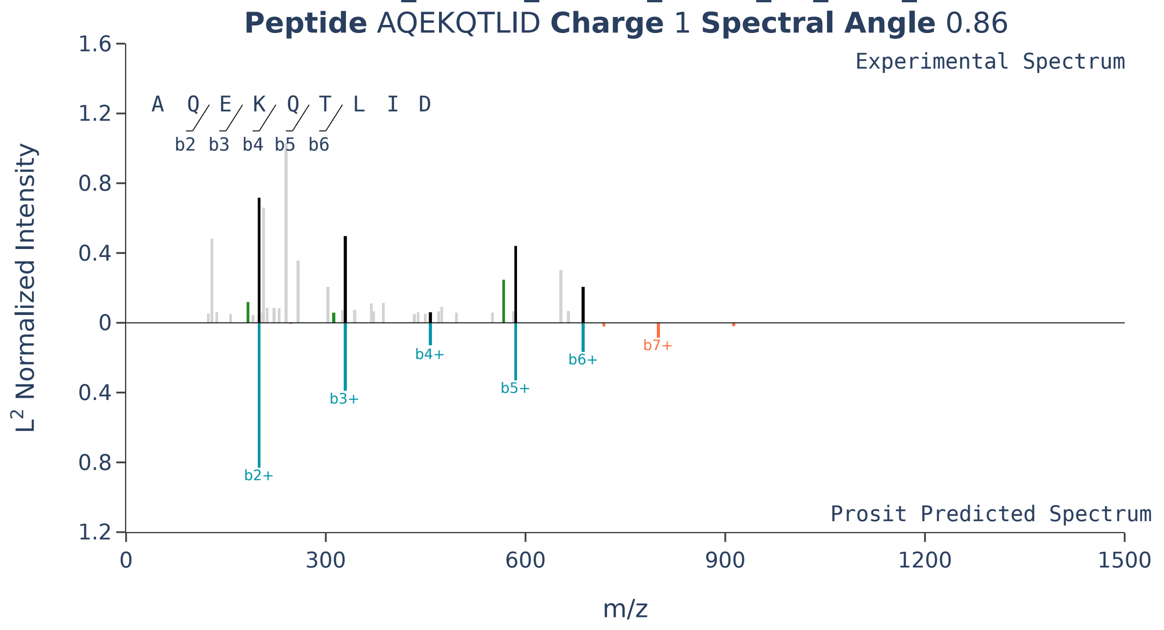


**Source** HRoetschke_211222_160123_HFGoe_IF2_b2R1_24h **Scan** 8073
**Peptide** VAEEMGHKVILR **Charge** 4 **Spectral Angle** 0.92

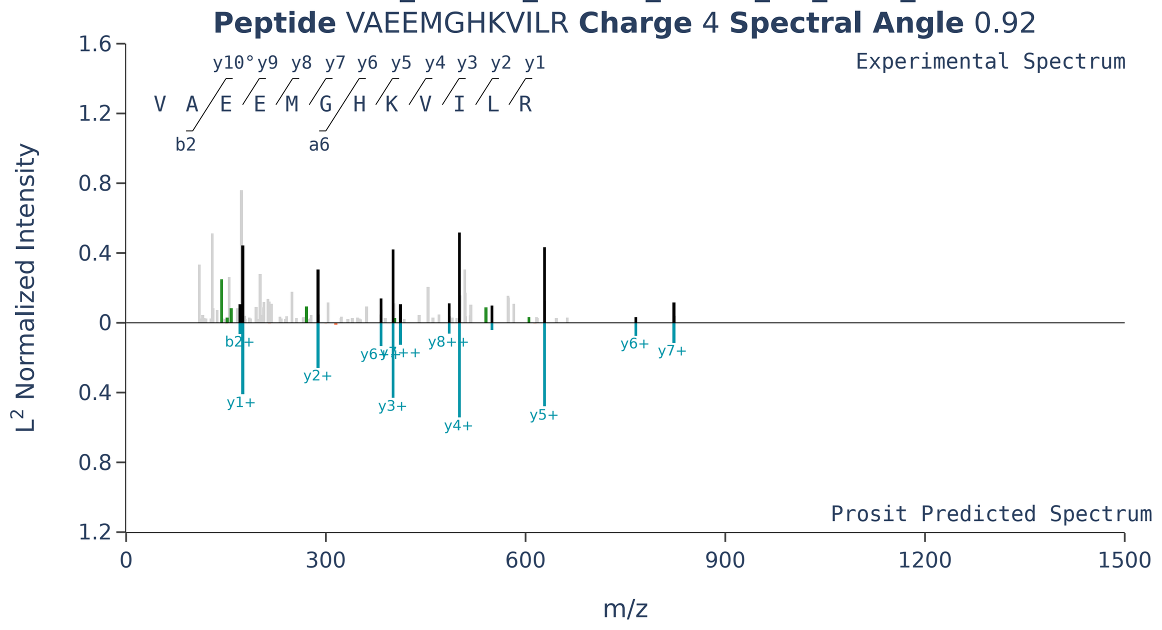


**Source** HRoetschke_211222_160123_HFGoe_IF2_b2R2_4h **Scan** 12258
**Peptide** EAGGITQHI **Charge** 2 **Spectral Angle** 0.91

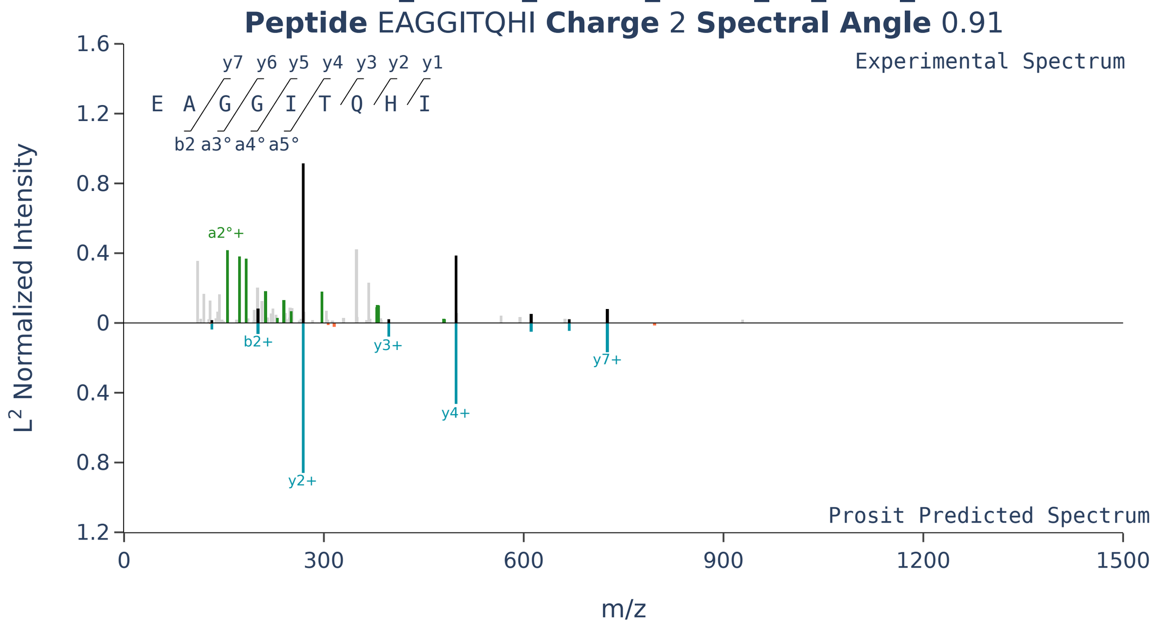


**Source** HRoetschke_211222_160123_HFGoe_IF2_b1R2_24h **Scan** 11618
**Peptide** GFEYGRVRAM **Charge** 3 **Spectral Angle** 0.91

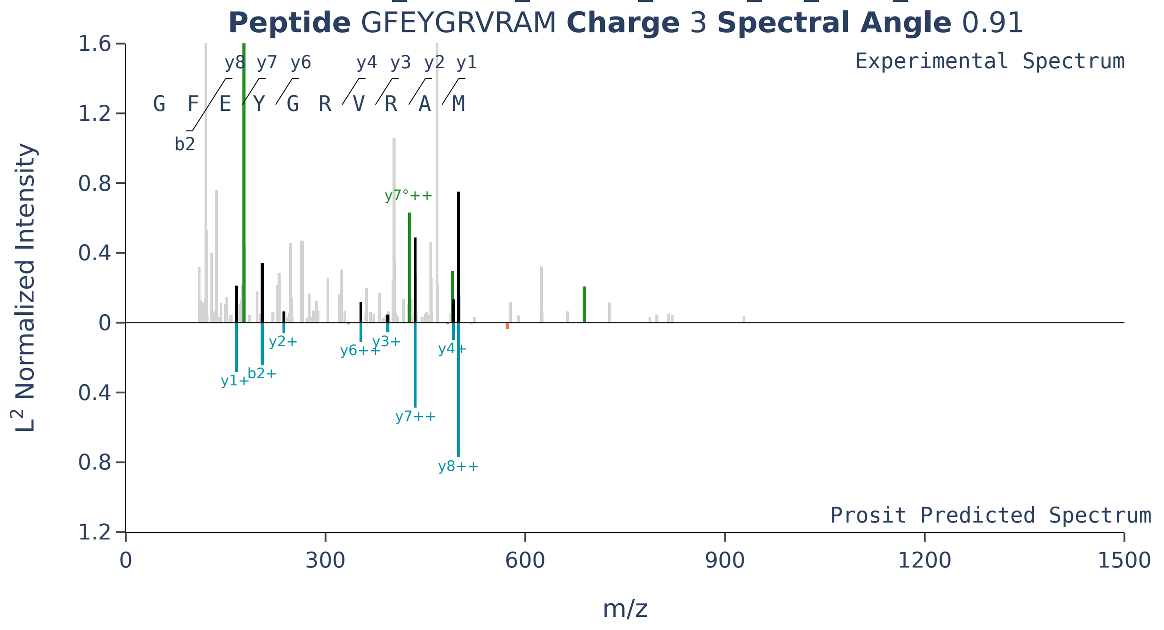


**Source** HRoetschke_211222_160123_HFGoe_IF2_b2R2_4h **Scan** 12431
**Peptide** VNRDVVIGE **Charge** 2 **Spectral Angle** 0.87

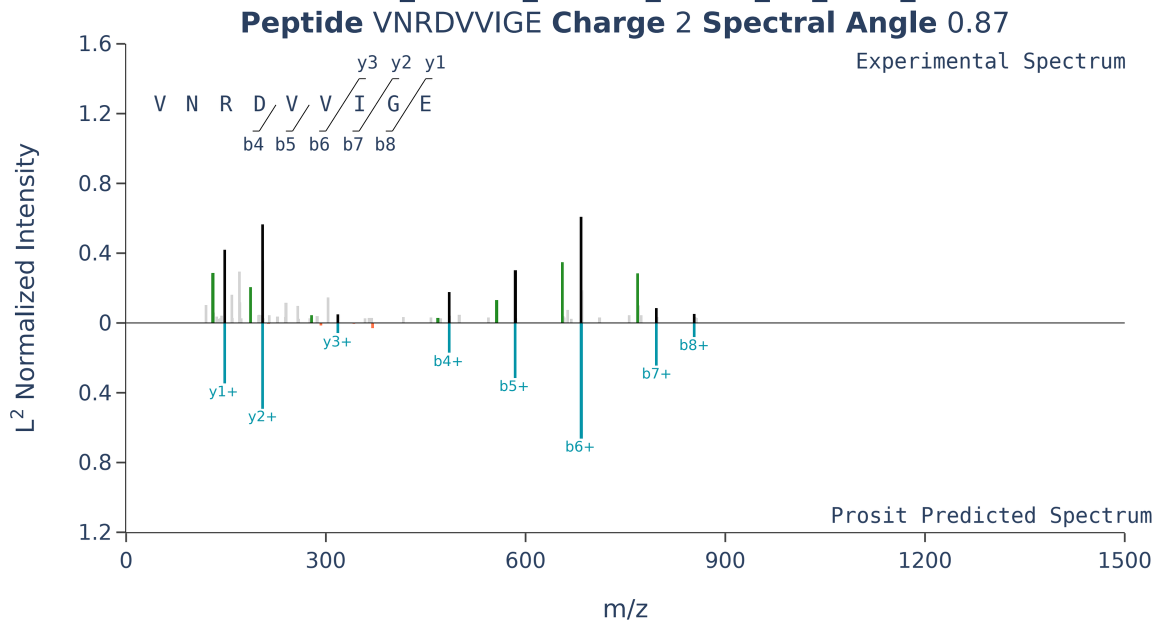


**Source** HRoetschke_211222_160123_HFGoe_IF2_b1R2_24h **Scan** 8417
**Peptide** MRNELGQE **Charge** 2 **Spectral Angle** 0.91

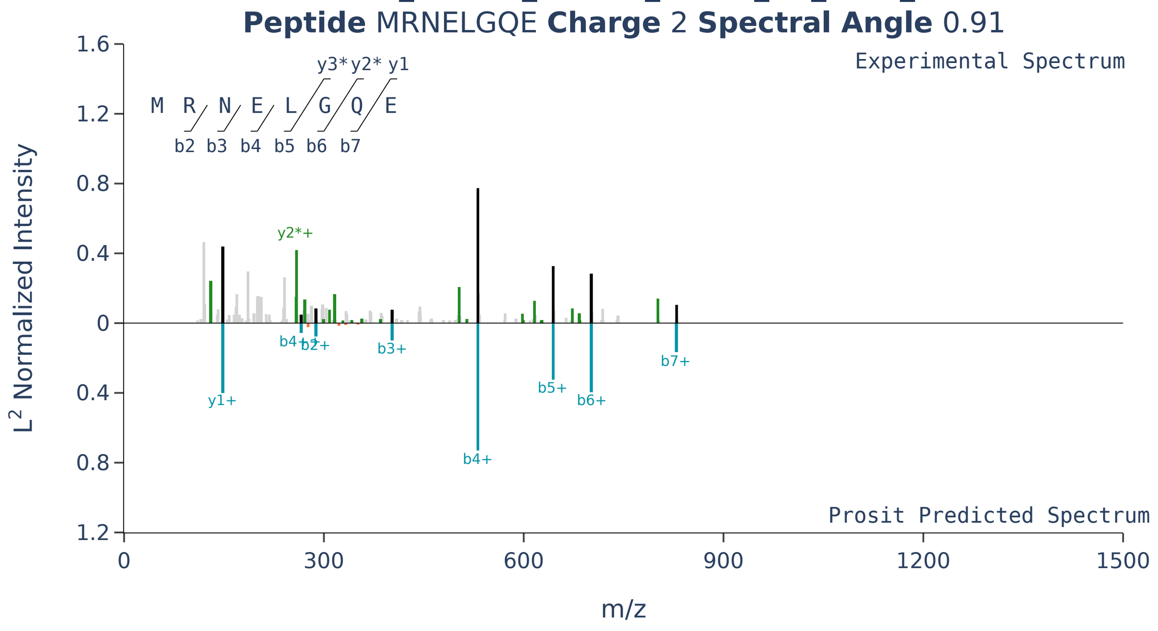


**Source** HRoetschke_211222_160123_HFGoe_IF2_b2R1_4h **Scan** 9398
**Peptide** TDATLAAASN **Charge** 2 **Spectral Angle** 0.94

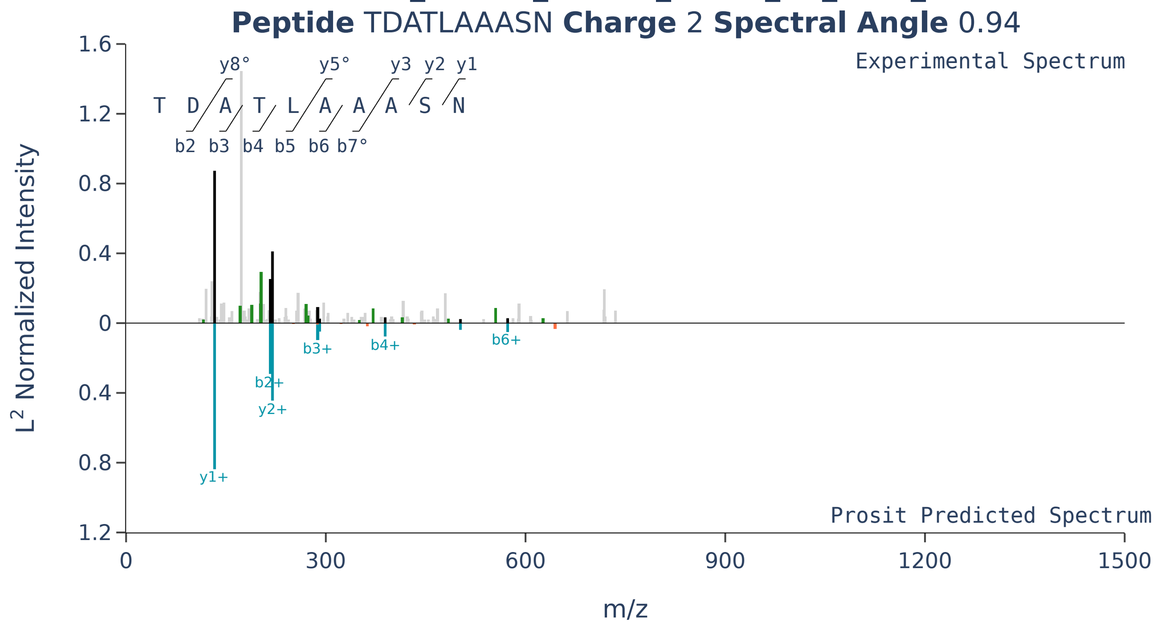


**Source** HRoetschke_211222_160123_HFGoe_IF2_b2R1_1h **Scan** 6903
**Peptide** AMRNELGQE **Charge** 2 **Spectral Angle** 0.85

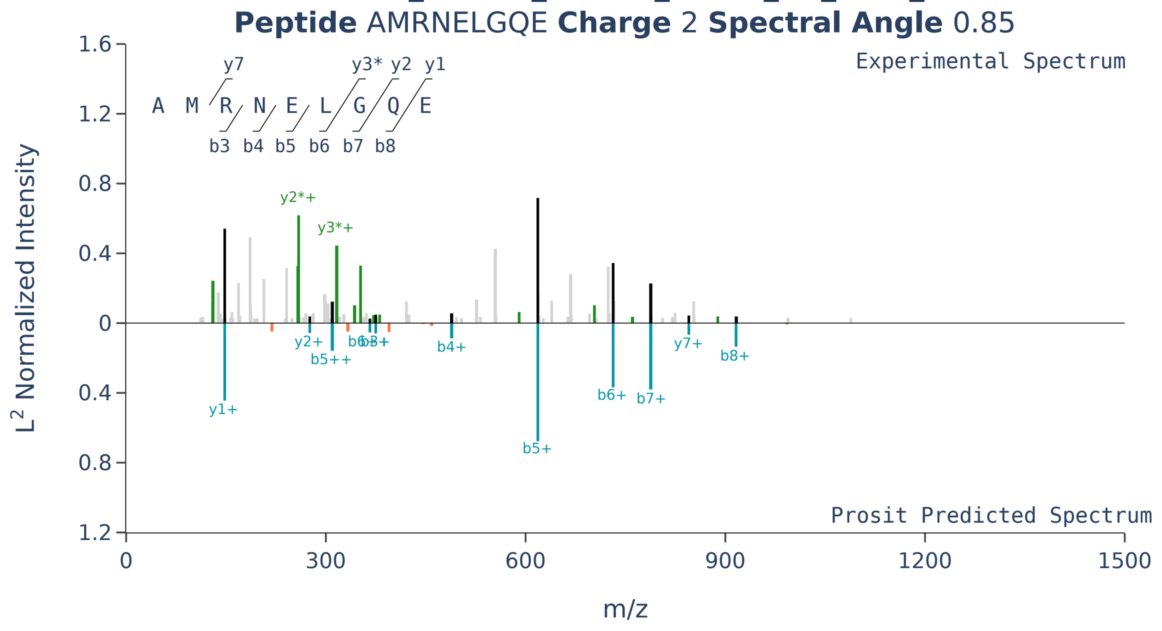


**Source** HRoetschke_211222_160123_HFGoe_IF2_b1R1_2h **Scan** 8245
**Peptide** QGFQKPAQAVNRD **Charge** 2 **Spectral Angle** 0.93

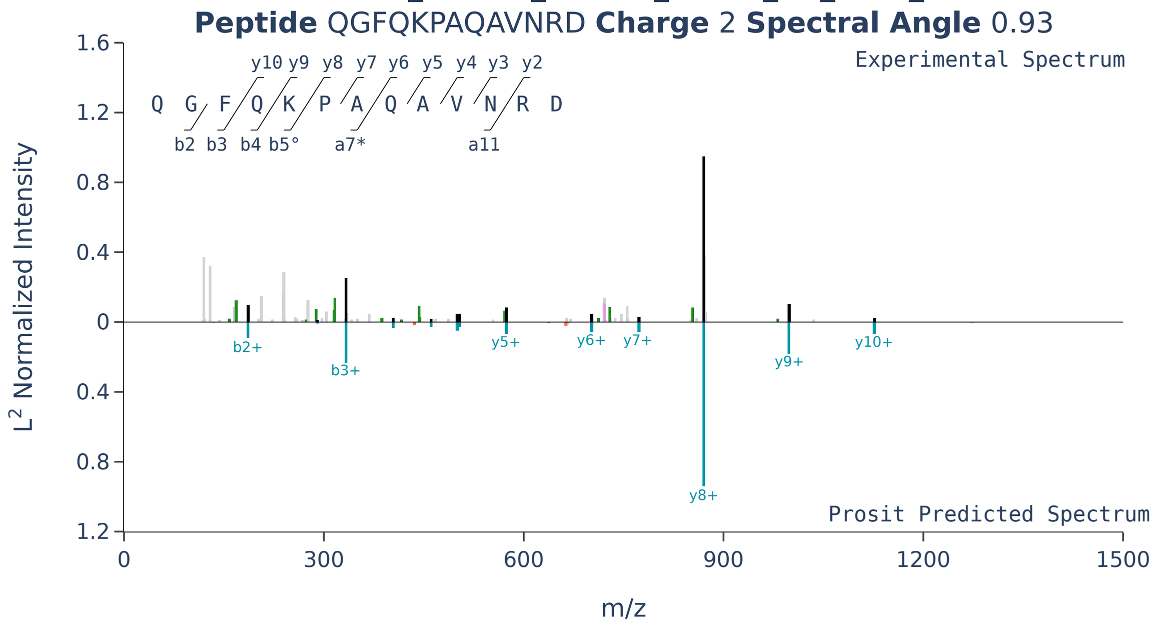


**Source** HRoetschke_211222_160123_HFGoe_IF2_b1R2_24h **Scan** 5870
**Peptide** KVILRRENE **Charge** 3 **Spectral Angle** 0.94

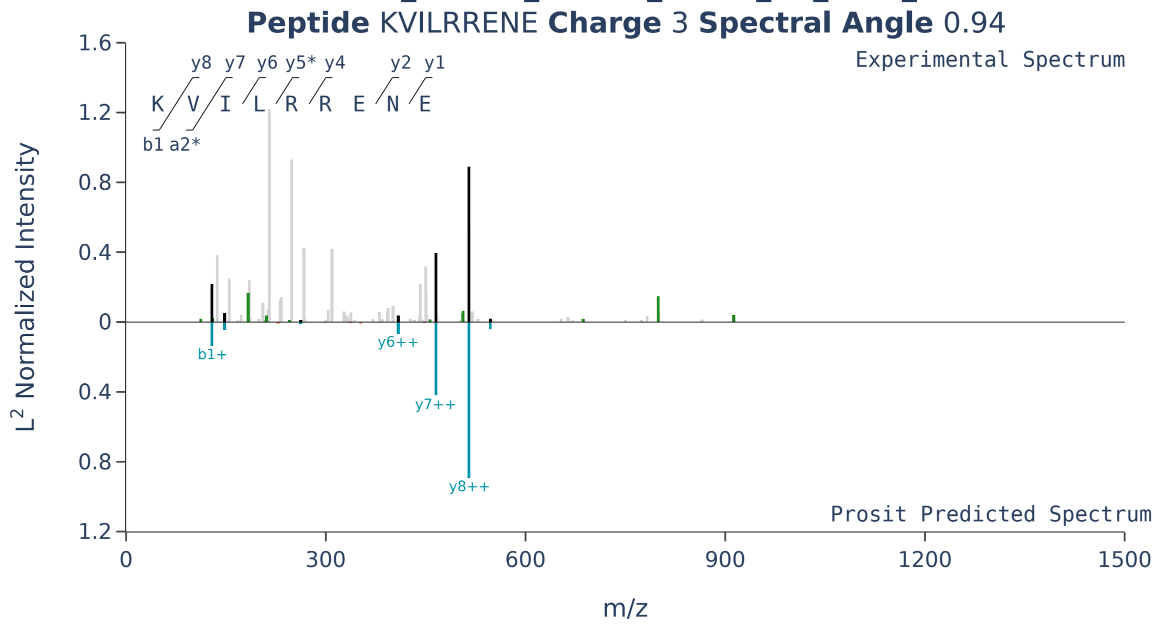


**Source** HRoetschke_211222_160123_HFGoe_IF2_b1R2_24h **Scan** 7068
**Peptide** NIPGTGGKSKSV **Charge** 2 **Spectral Angle** 0.91

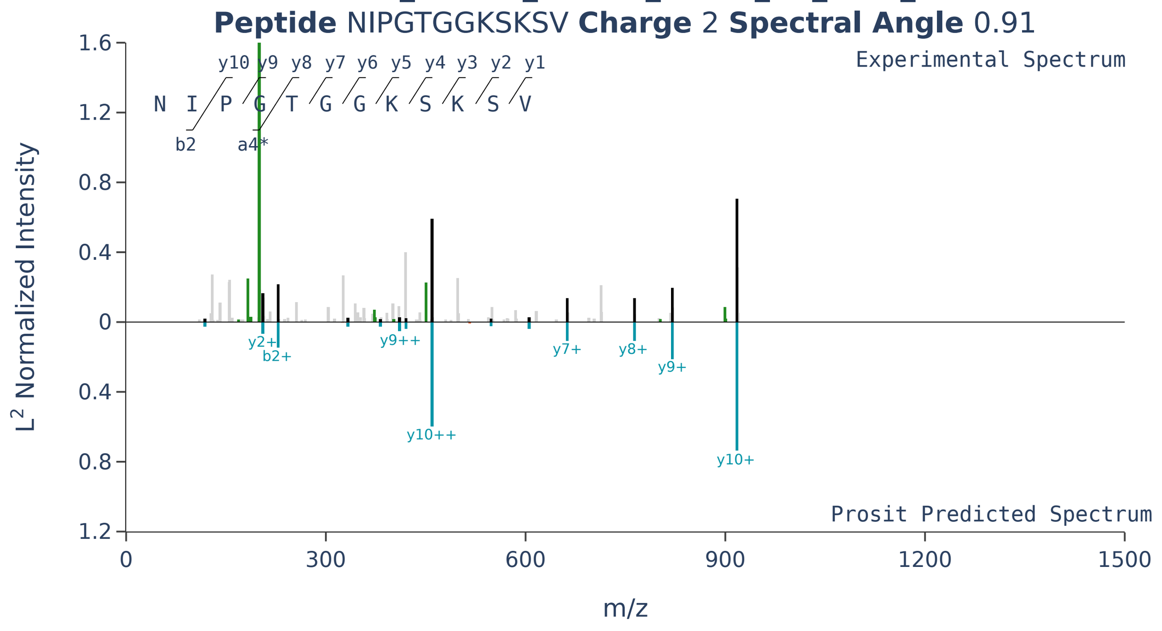


**Source** HRoetschke_211222_160123_HFGoe_IF2_b2R2_4h **Scan** 8910
**Peptide** KGMASGAVIE **Charge** 2 **Spectral Angle** 0.84

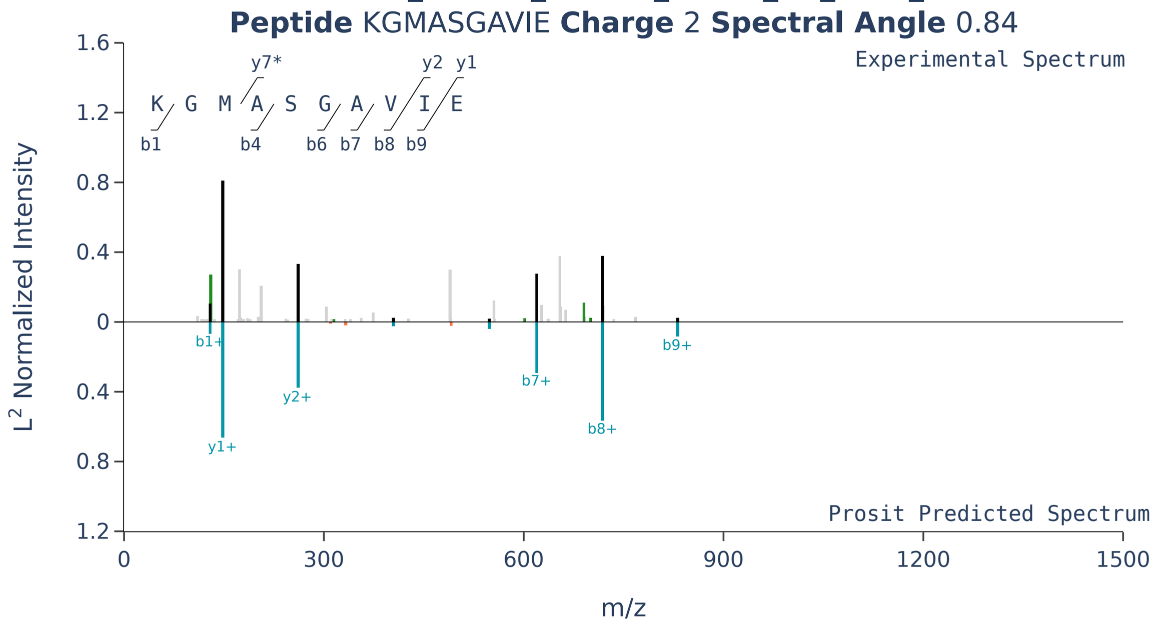


**Source** HRoetschke_211222_160123_HFGoe_IF2_b1R1_1h **Scan** 11122
**Peptide** DQETAQL **Charge** 2 **Spectral Angle** 0.89

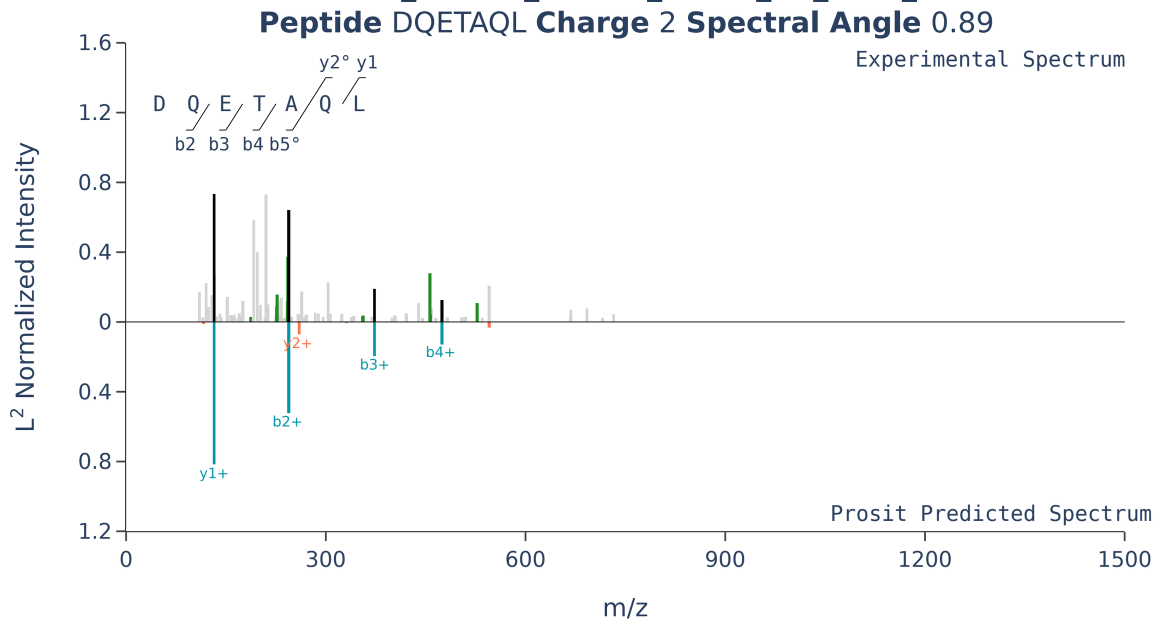


**Source** HRoetschke_211222_160123_HFGoe_IF2_b1R1_4h **Scan** 7124
**Peptide** AYHVETENG **Charge** 2 **Spectral Angle** 0.86

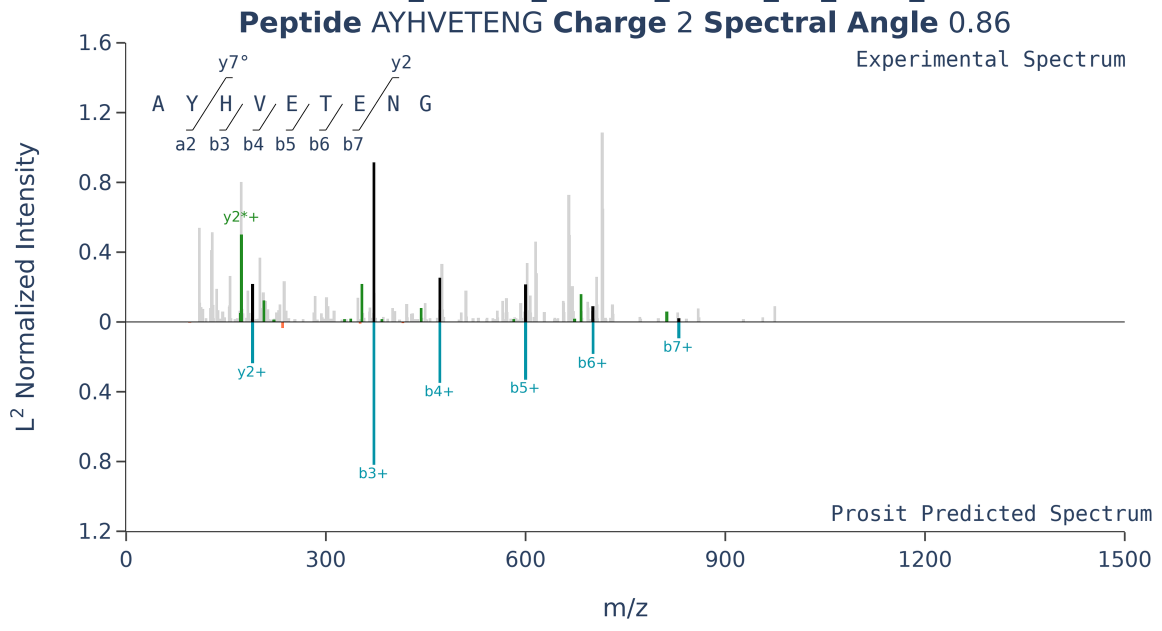

**Source** HRoetschke_211222_160123_HFGoe_IF2_b1R2_4h **Scan** 11878
**Peptide** GKTSLLD **Charge** 2 **Spectral Angle** 0.88

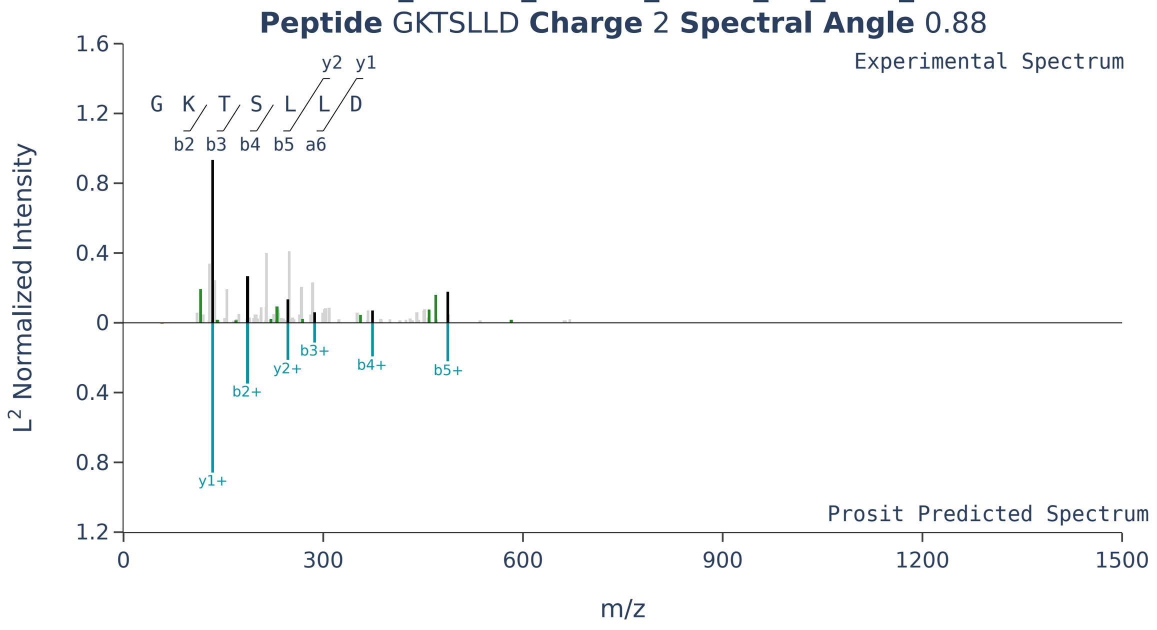


**Source** HRoetschke_211222_160123_HFGoe_IF2_b1R1_4h **Scan** 13625
**Peptide** AAFTSMRA **Charge** 2 **Spectral Angle** 0.9

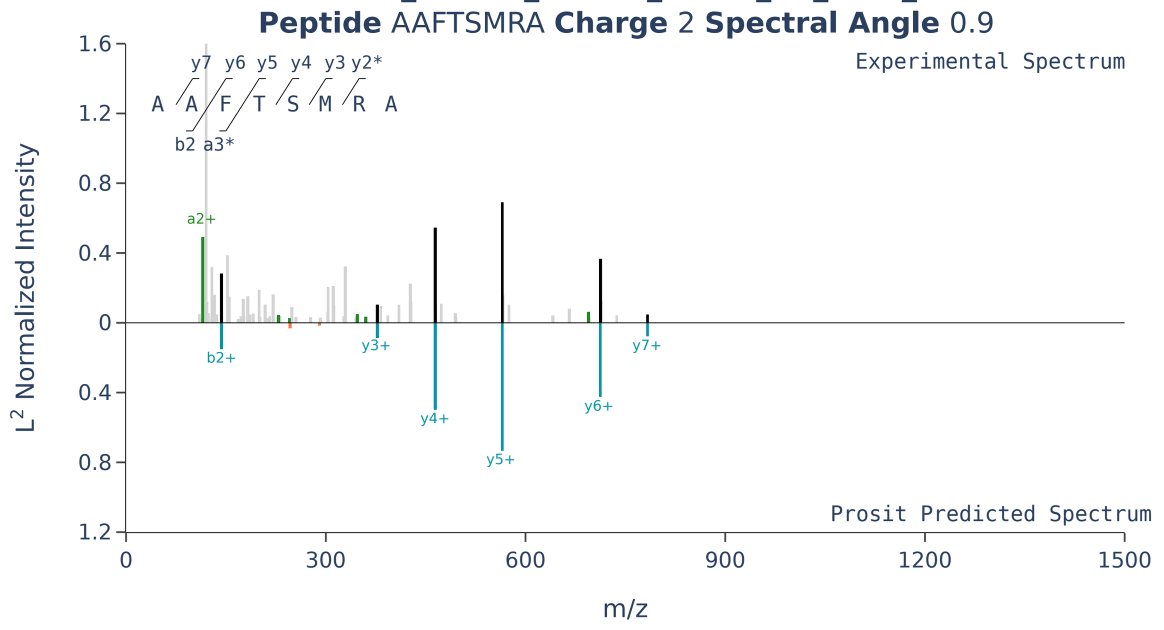


**Source** HRoetschke_211222_160123_HFGoe_IF2_b1R2_24h **Scan** 14449
**Peptide** VNRDVVI **Charge** 2 **Spectral Angle** 0.92

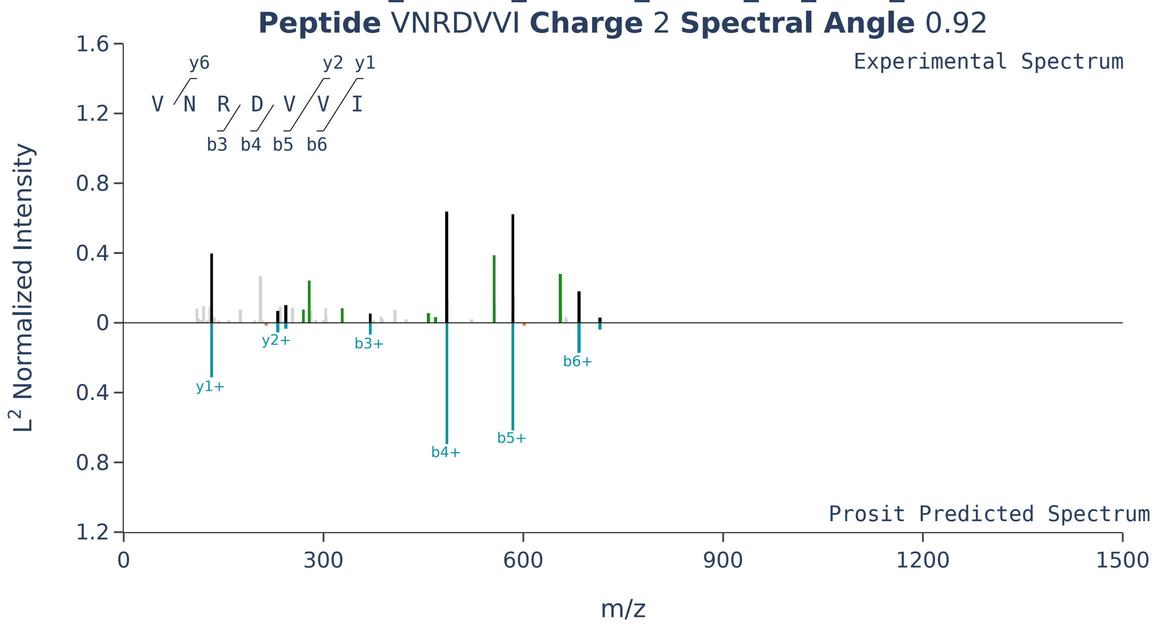


**Source** HRoetschke_211222_160123_HFGoe_IF2_b1R1_4h **Scan** 14184
**Peptide** SQFVHVSA **Charge** 1 **Spectral Angle** 0.89

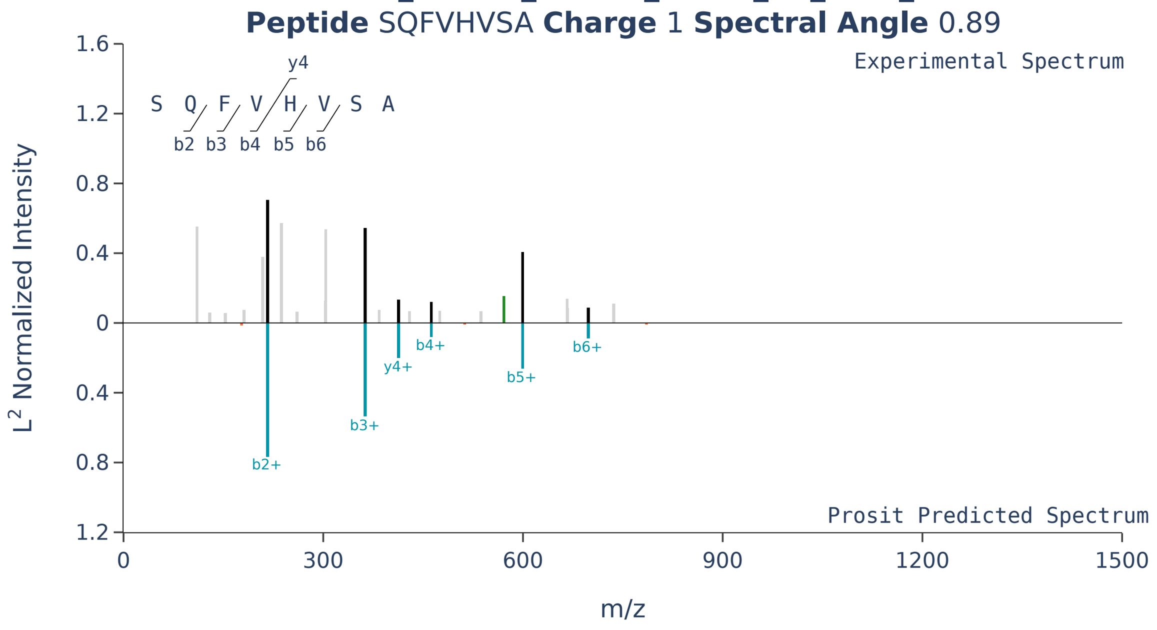


**Source** HRoetschke_211222_160123_HFGoe_IF2_b1R2_24h **Scan** 9676
**Peptide** RSTLNIPGTGGKSK **Charge** 3 **Spectral Angle** 0.87

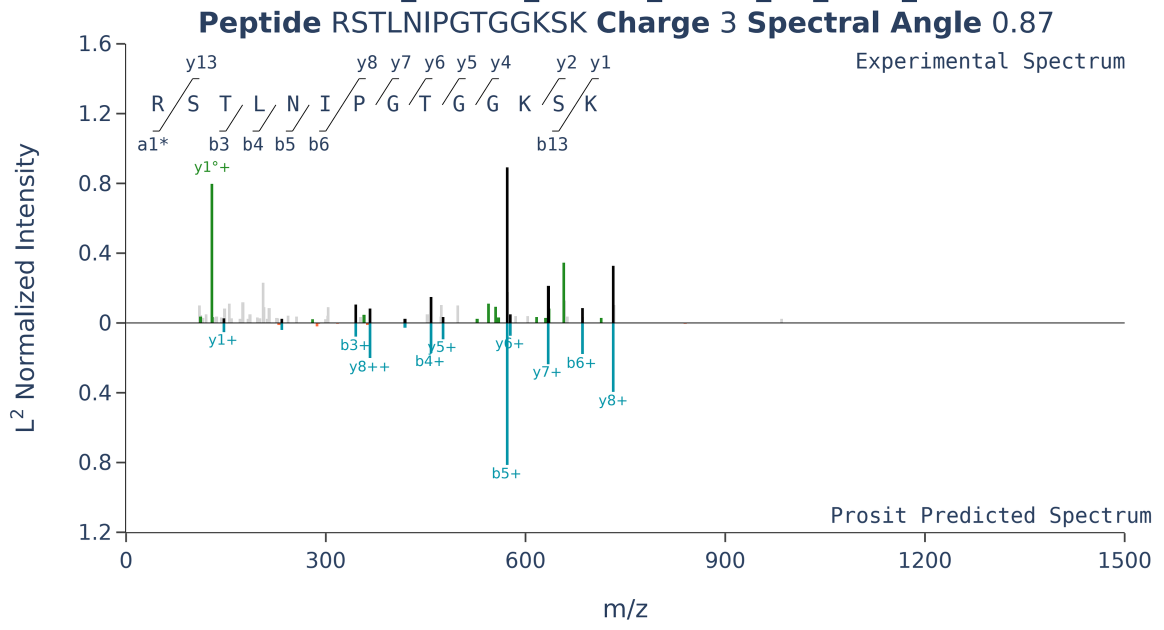


**Source** HRoetschke_211222_160123_HFGoe_IF2_b1R1_24h **Scan** 6746
**Peptide** AVNKIDKPEAD **Charge** 3 **Spectral Angle** 0.9

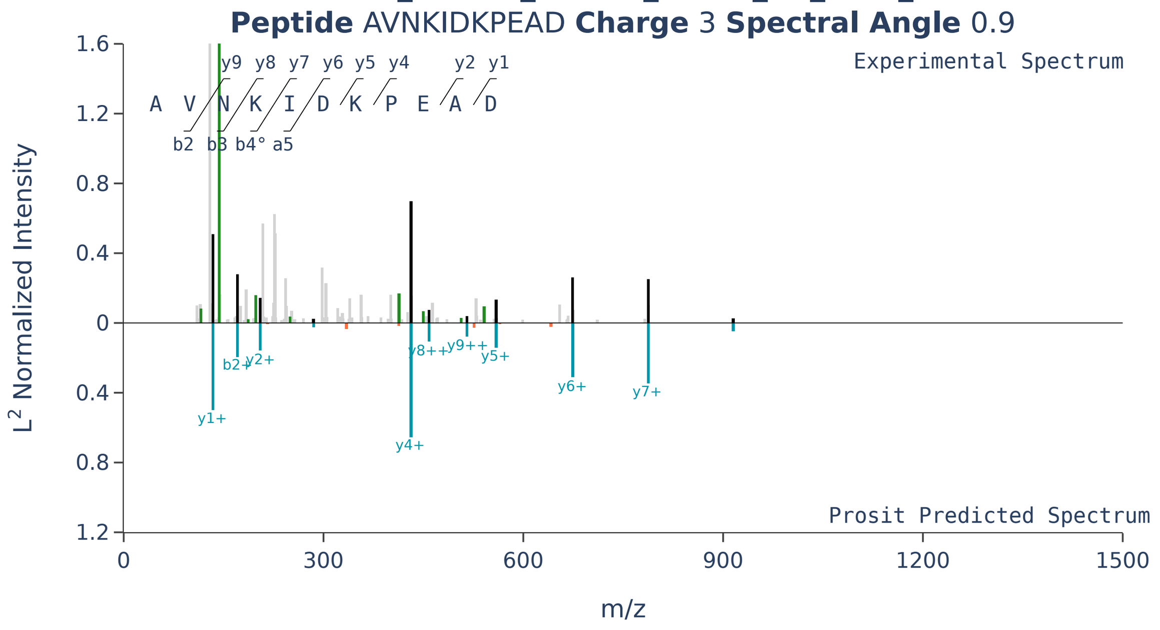


**Source** HRoetschke_211222_160123_HFGoe_IF2_b1R2_1h **Scan** 10033
**Peptide** VQQFADAGIRKS **Charge** 3 **Spectral Angle** 0.86

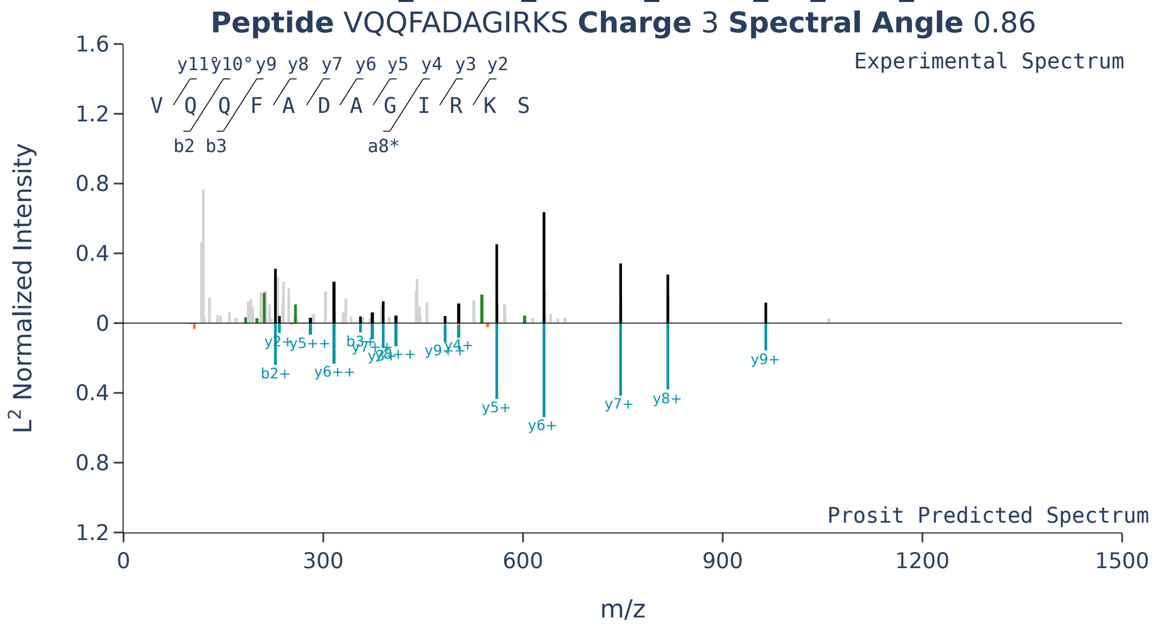


**Source** HRoetschke_211222_160123_HFGoe_IF2_b2R2_4h **Scan** 11141
**Peptide** AEENKWTDNAEPTE **Charge** 2 **Spectral Angle** 0.94

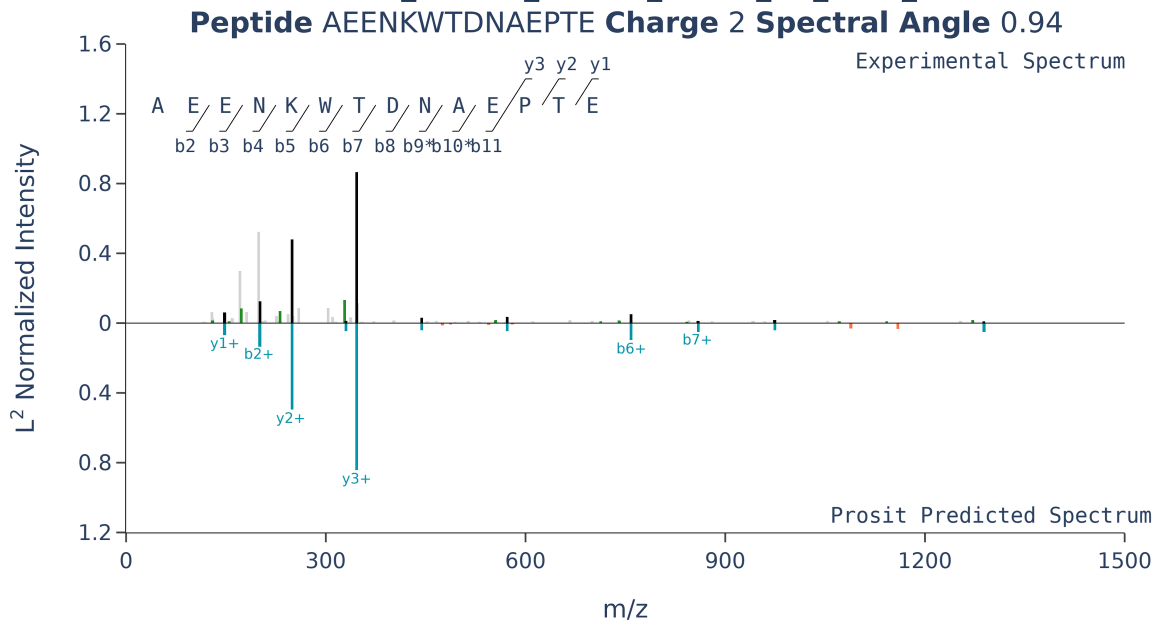


**Source** HRoetschke_211222_160123_HFGoe_IF2_b1R1_4h **Scan** 7740
**Peptide** FADAGIRK **Charge** 2 **Spectral Angle** 0.91

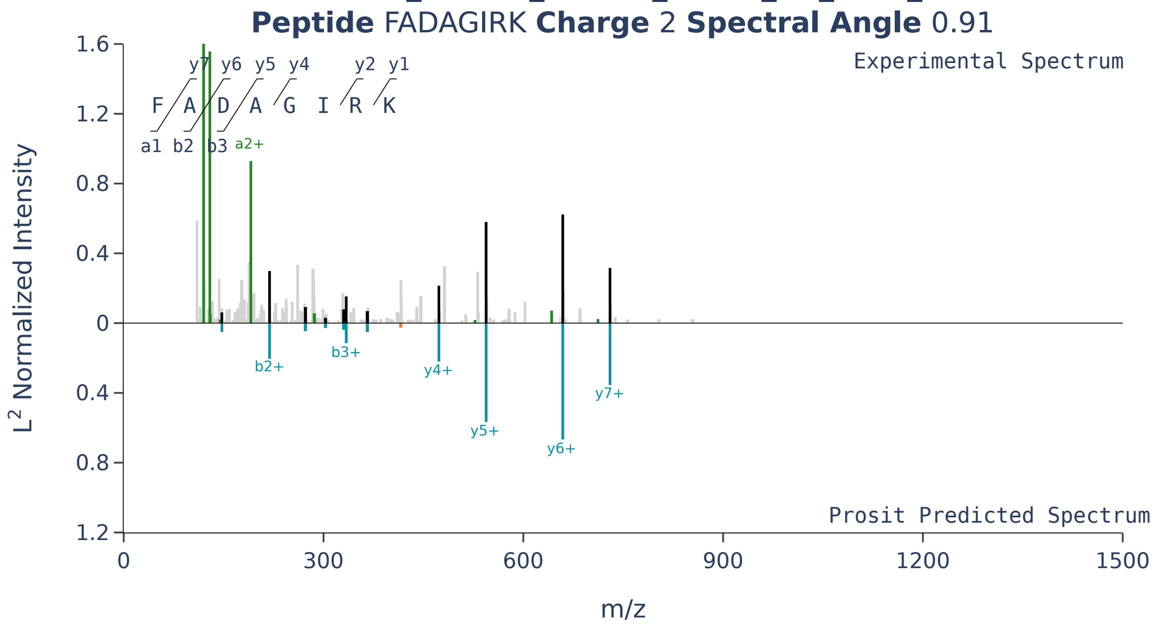


**Source** HRoetschke_211222_160123_HFGoe_IF2_b1R1_4h **Scan** 9644
**Peptide** PGHAAFTSMR **Charge** 3 **Spectral Angle** 0.91

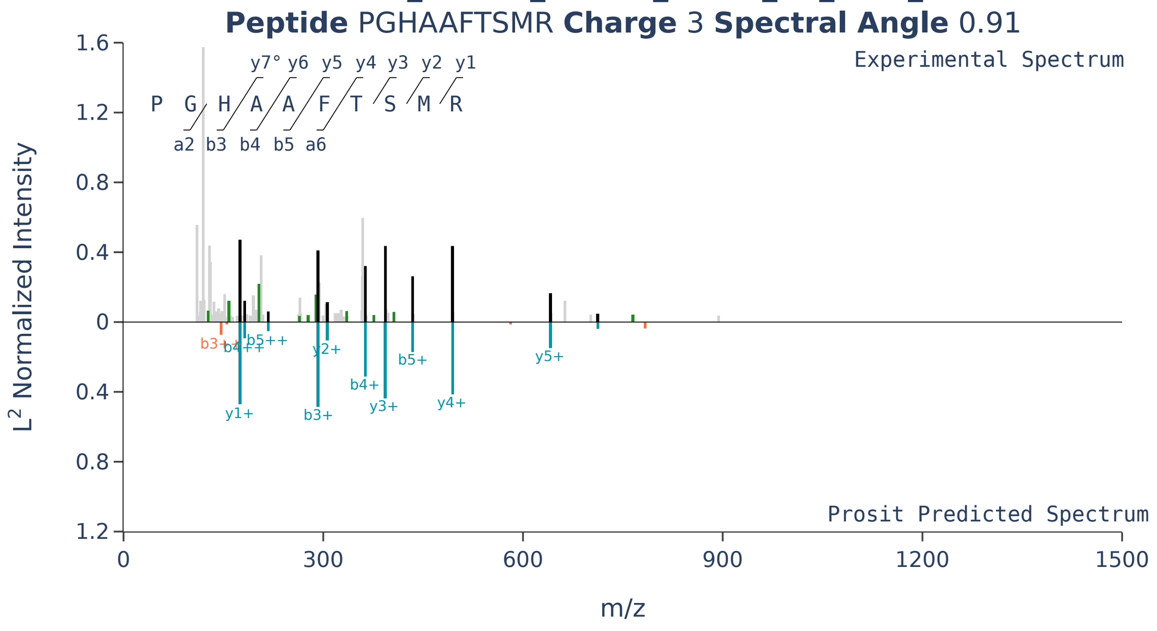


**Source** HRoetschke_211222_160123_HFGoe_IF2_b1R1_1h **Scan** 8960
**Peptide** TLAAERQTSVE **Charge** 2 **Spectral Angle** 0.86

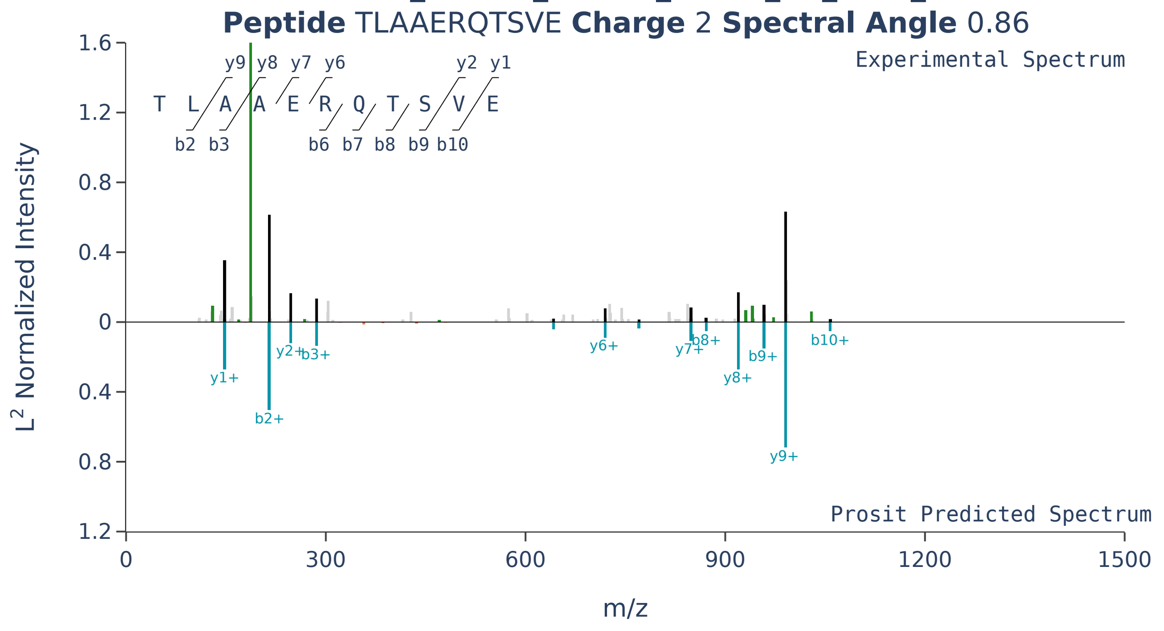


**Source** HRoetschke_211222_160123_HFGoe_IF2_b2R2_24h **Scan** 7694
**Peptide** GFQKPAQA **Charge** 2 **Spectral Angle** 0.86

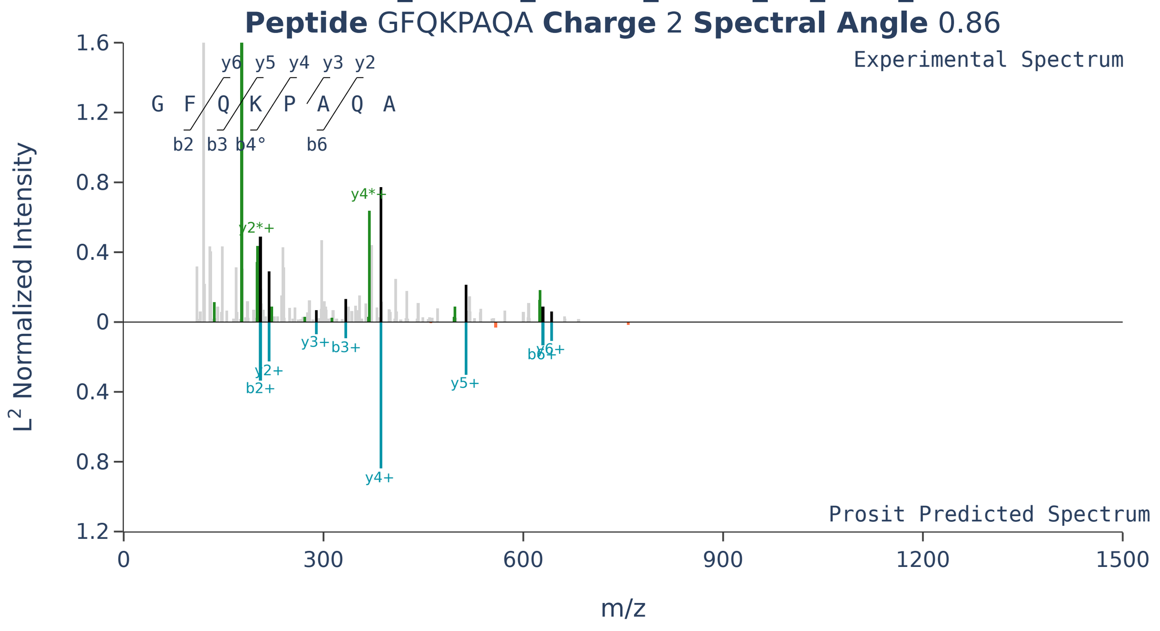


**Source** HRoetschke_211222_160123_HFGoe_IF2_b1R2_4h **Scan** 13501
**Peptide** TLHKGDIVLC **Charge** 3 **Spectral Angle** 0.94

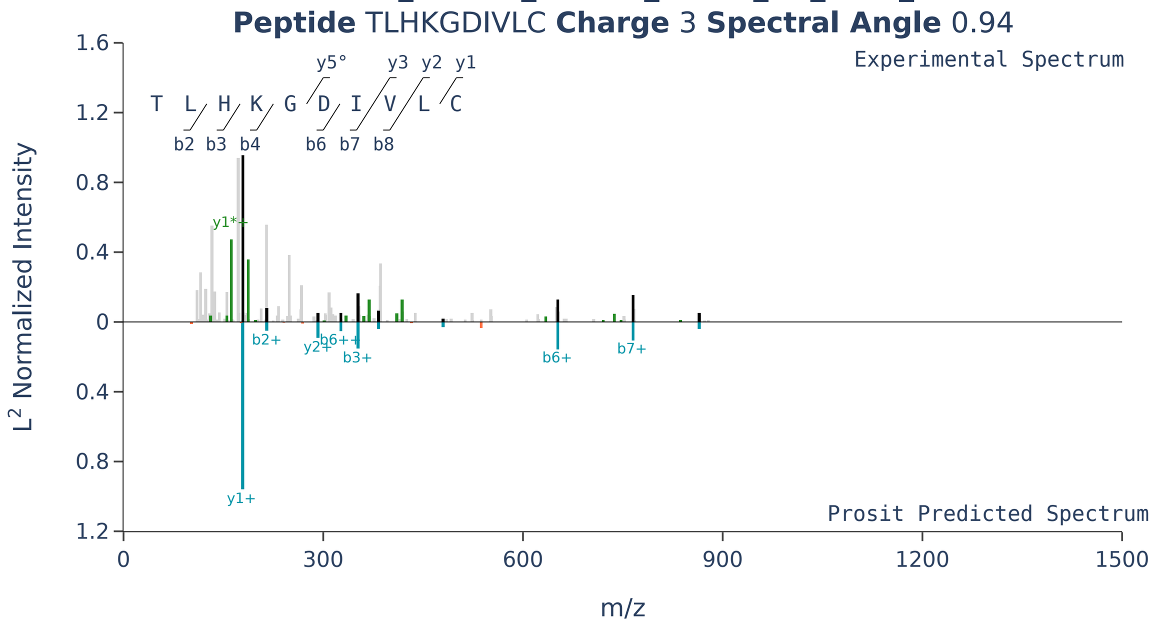


**Source** HRoetschke_211222_160123_HFGoe_IF2_b2R2_24h **Scan** 12675
**Peptide** HKGDIVL **Charge** 1 **Spectral Angle** 0.9

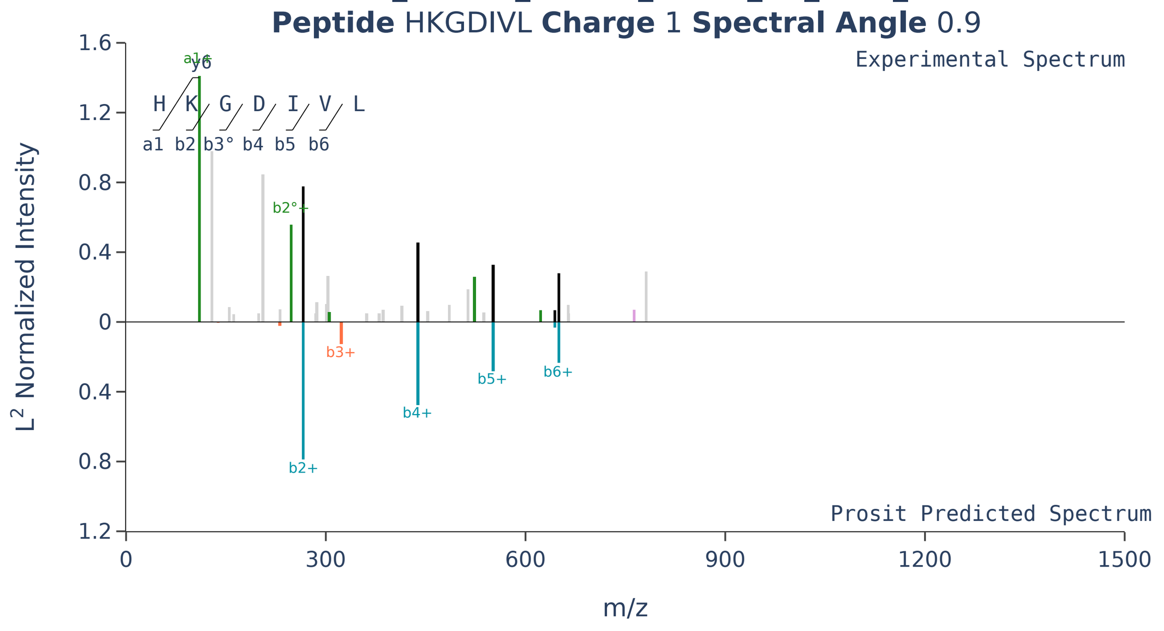


**Source** HRoetschke_211222_160123_HFGoe_IF2_b1R2_24h **Scan** 8221
**Peptide** PGHAAFTSM **Charge** 2 **Spectral Angle** 0.92

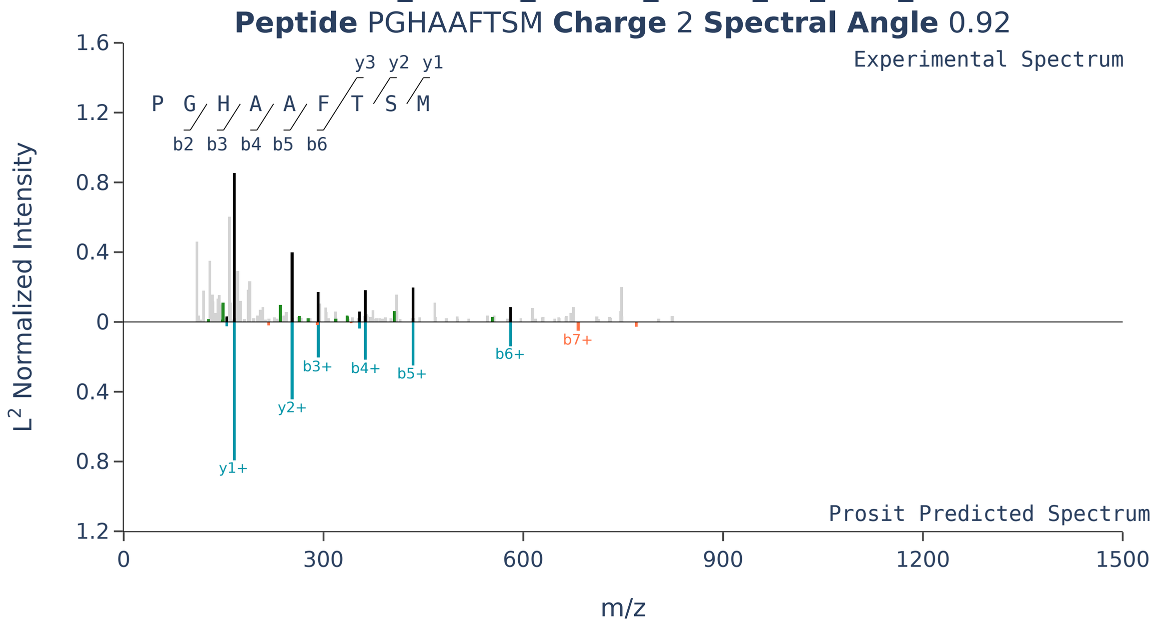

**Source** HRoetschke_211222_160123_HFGoe_IF2_b2R1_2h **Scan** 6569
**Peptide** PDRVKNELSQ **Charge** 2 **Spectral Angle** 0.84

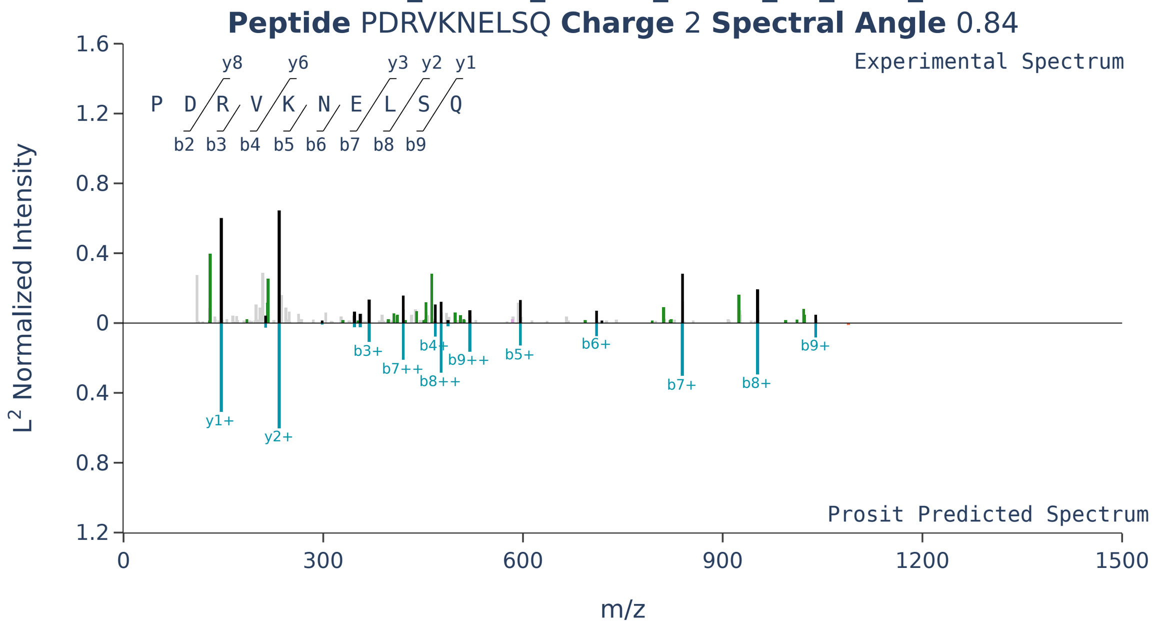


**Source** HRoetschke_211222_160123_HFGoe_IF2_b1R1_24h **Scan** 9851
**Peptide** VRKGMASGAVIE **Charge** 3 **Spectral Angle** 0.87

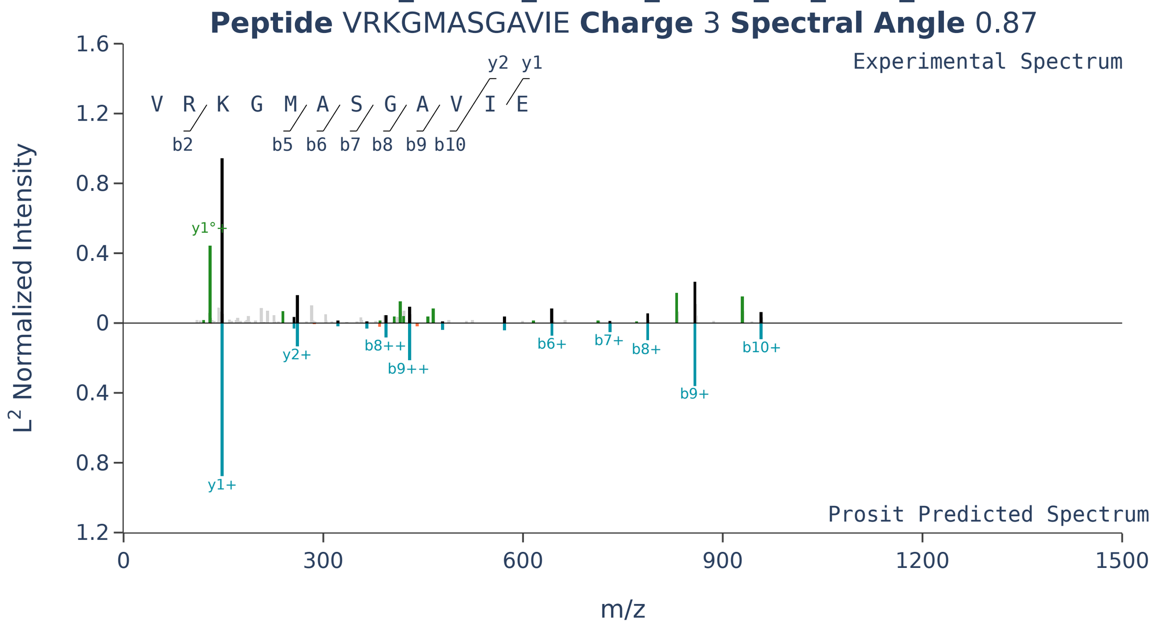


**Source** HRoetschke_211222_160123_HFGoe_IF2_b1R1_2h **Scan** 8284
**Peptide** SGVPAAGDE **Charge** 1 **Spectral Angle** 0.85

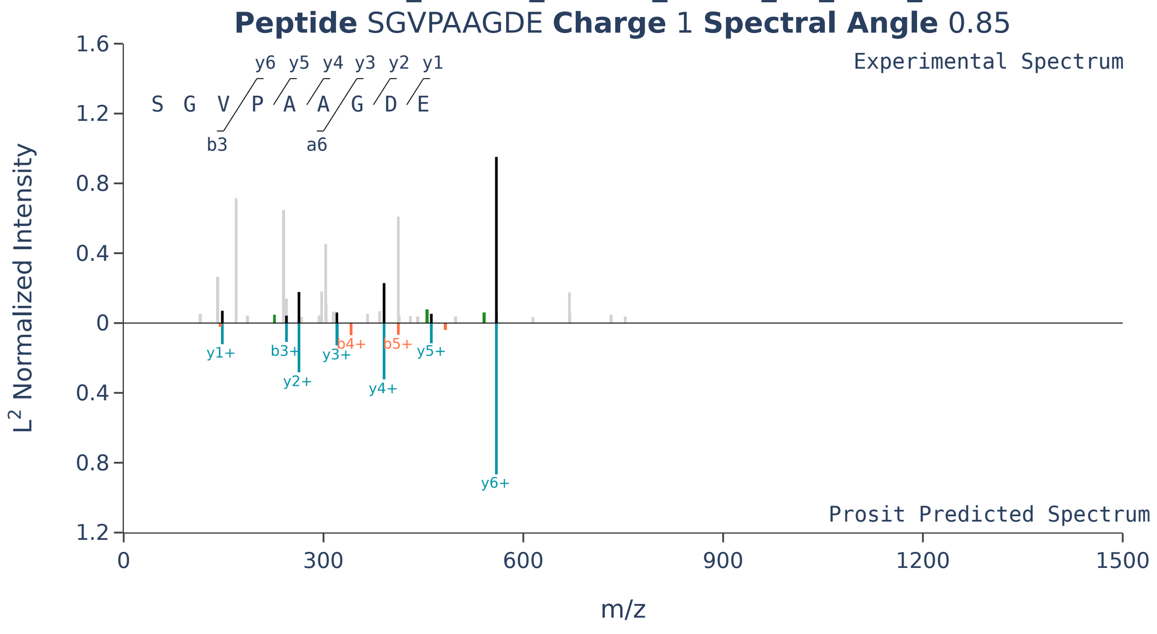


**Source** HRoetschke_211222_160123_HFGoe_IF2_b1R2_1h **Scan** 14248
**Peptide** DSVSAQEKQTLID **Charge** 2 **Spectral Angle** 0.87

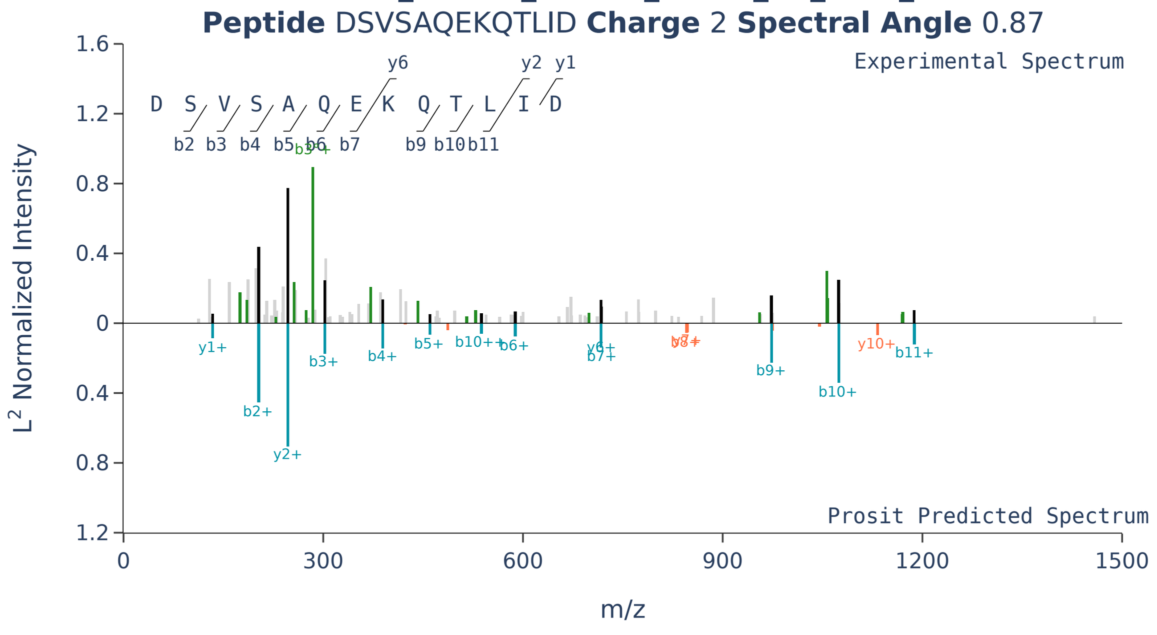


**Source** HRoetschke_211222_160123_HFGoe_IF2_b2R2_1h **Scan** 9234
**Peptide** SLQQGFQK **Charge** 2 **Spectral Angle** 0.84

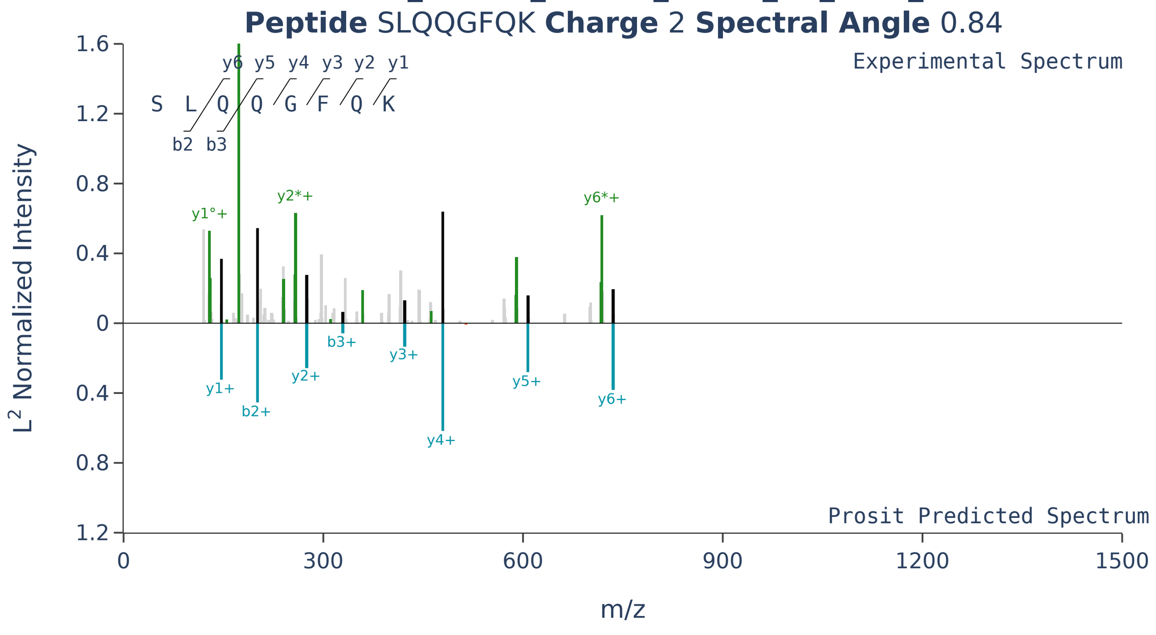


**Source** HRoetschke_211222_160123_HFGoe_IF2_b1R1_4h **Scan** 12743
**Peptide** LSGVPAAGD **Charge** 1 **Spectral Angle** 0.92

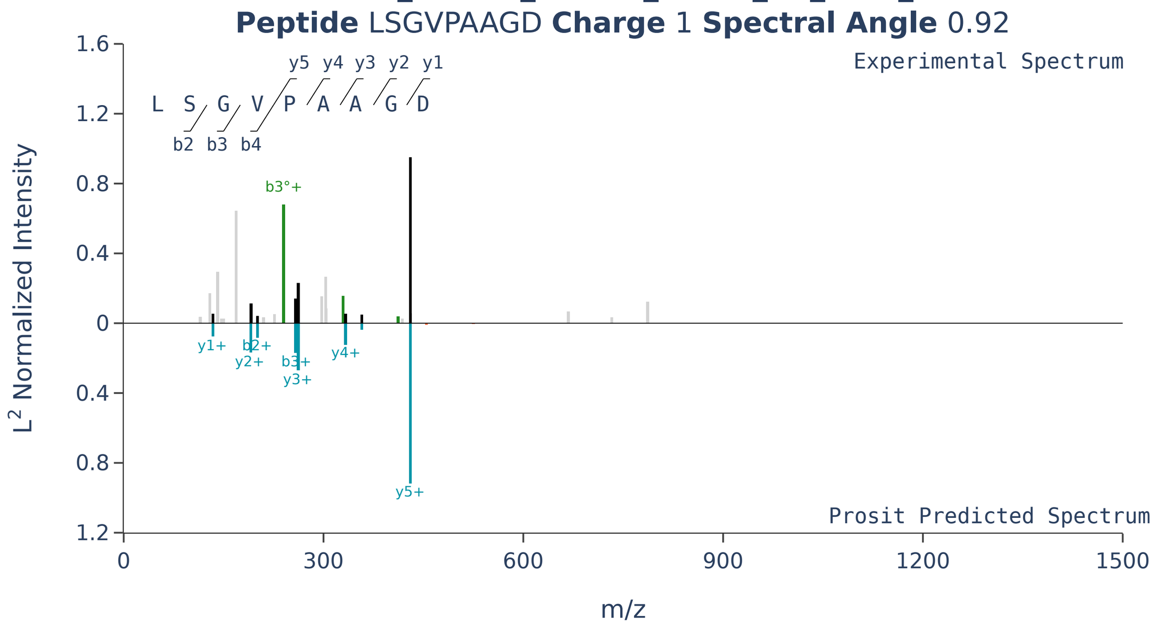


**Source** HRoetschke_211222_160123_HFGoe_IF2_b1R2_2h **Scan** 12050
**Peptide** GMASGAVIE **Charge** 1 **Spectral Angle** 0.91

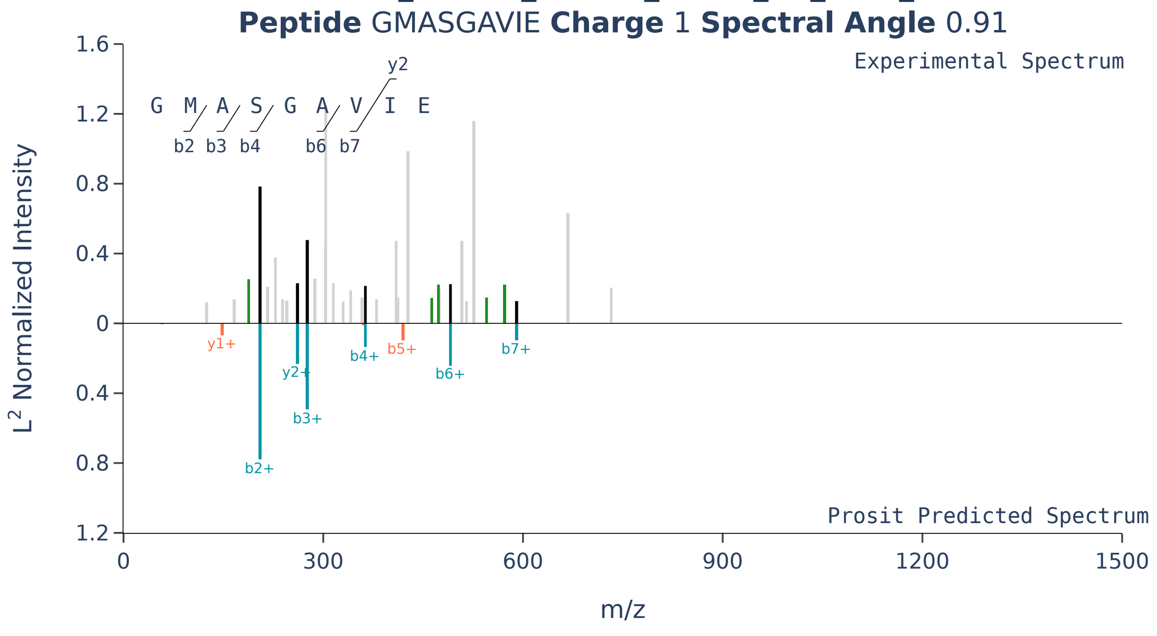


**Source** HRoetschke_211222_160123_HFGoe_IF2_b1R1_4h **Scan** 9413
**Peptide** FTSMRAR **Charge** 2 **Spectral Angle** 0.89

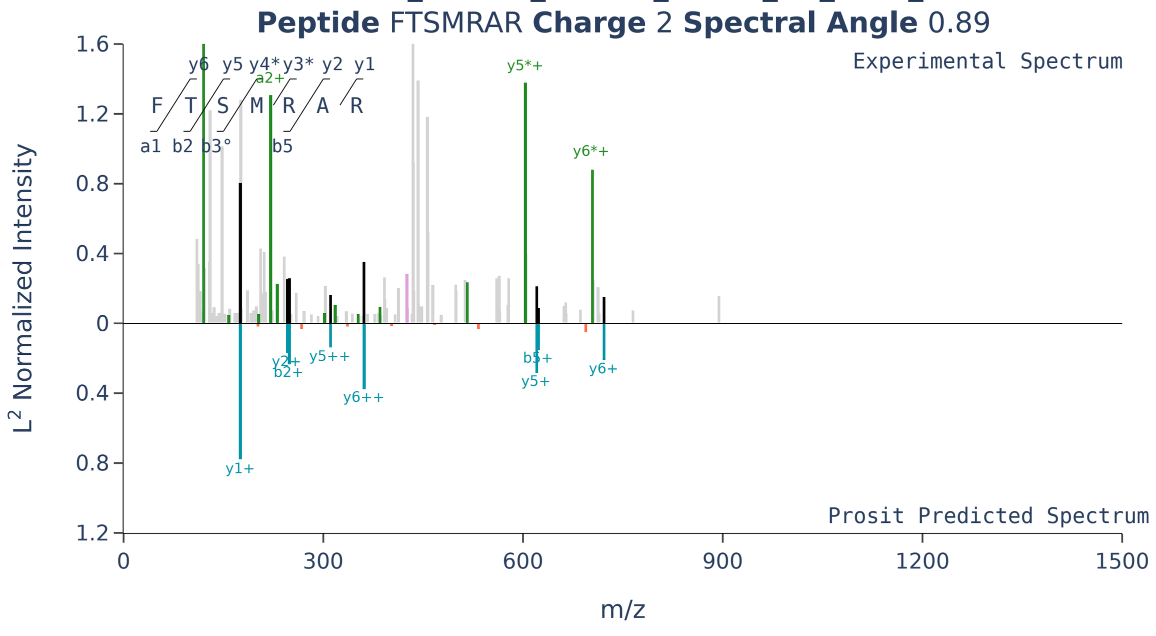


**Source** HRoetschke_211222_160123_HFGoe_IF2_b1R1_4h **Scan** 16251
**Peptide** DVTIKTLAAER **Charge** 3 **Spectral Angle** 0.9

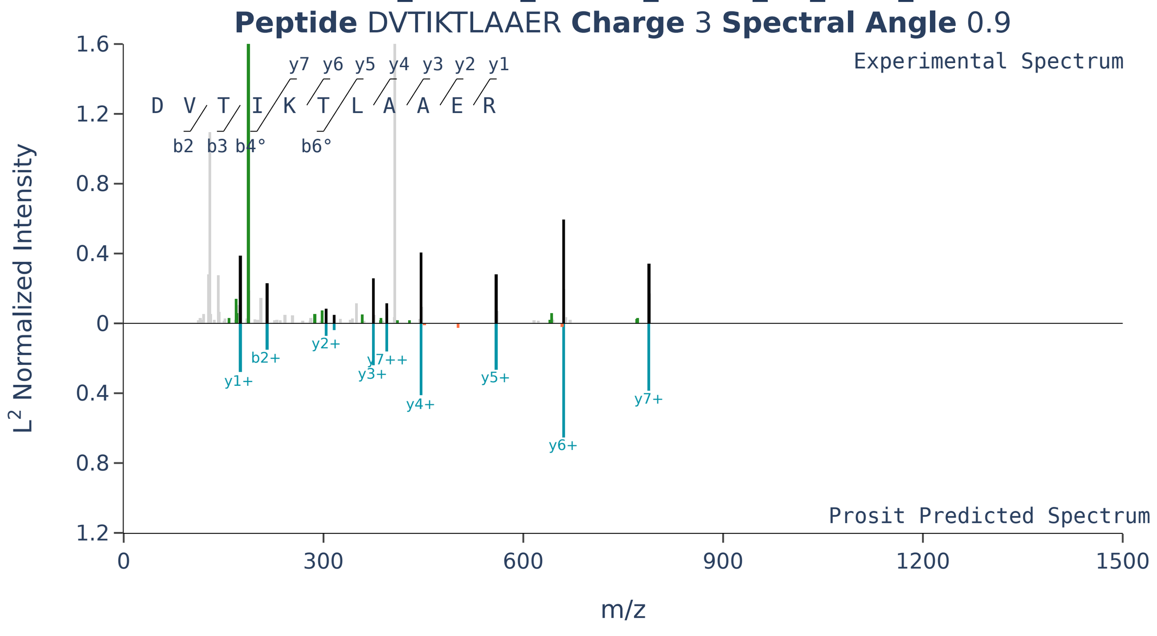


**Source** HRoetschke_211222_160123_HFGoe_IF2_b1R1_24h **Scan** 6231
**Peptide** LDKGRGPVA **Charge** 2 **Spectral Angle** 0.89

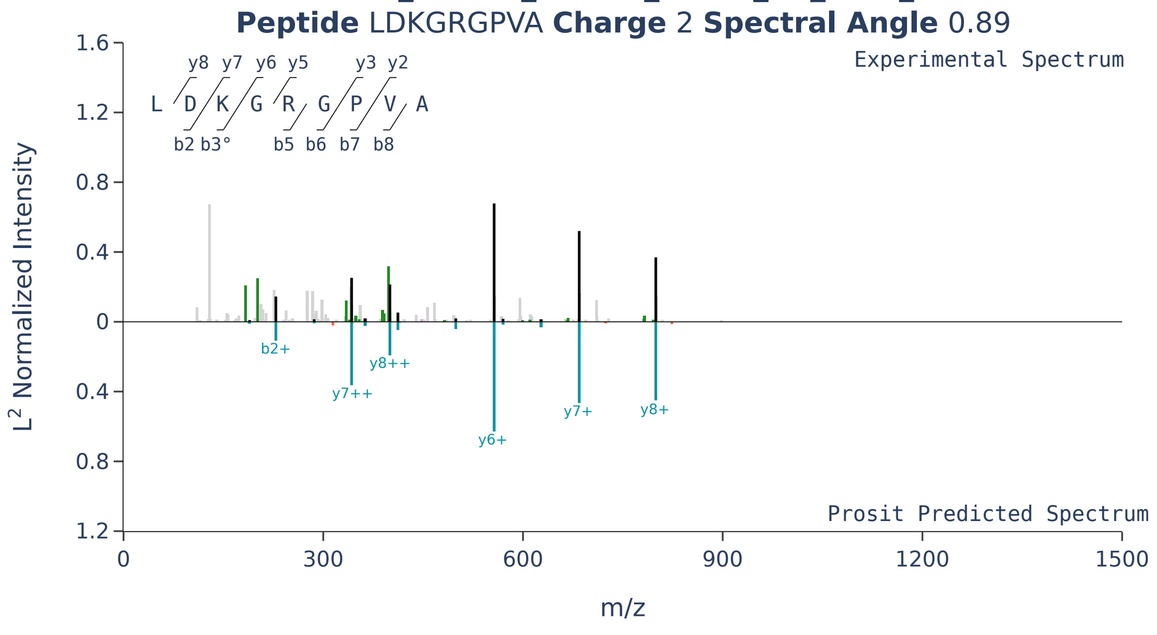


**Source** HRoetschke_211222_160123_HFGoe_IF2_b2R2_4h **Scan** 12662
**Peptide** DPQEAERLAAEE **Charge** 2 **Spectral Angle** 0.82

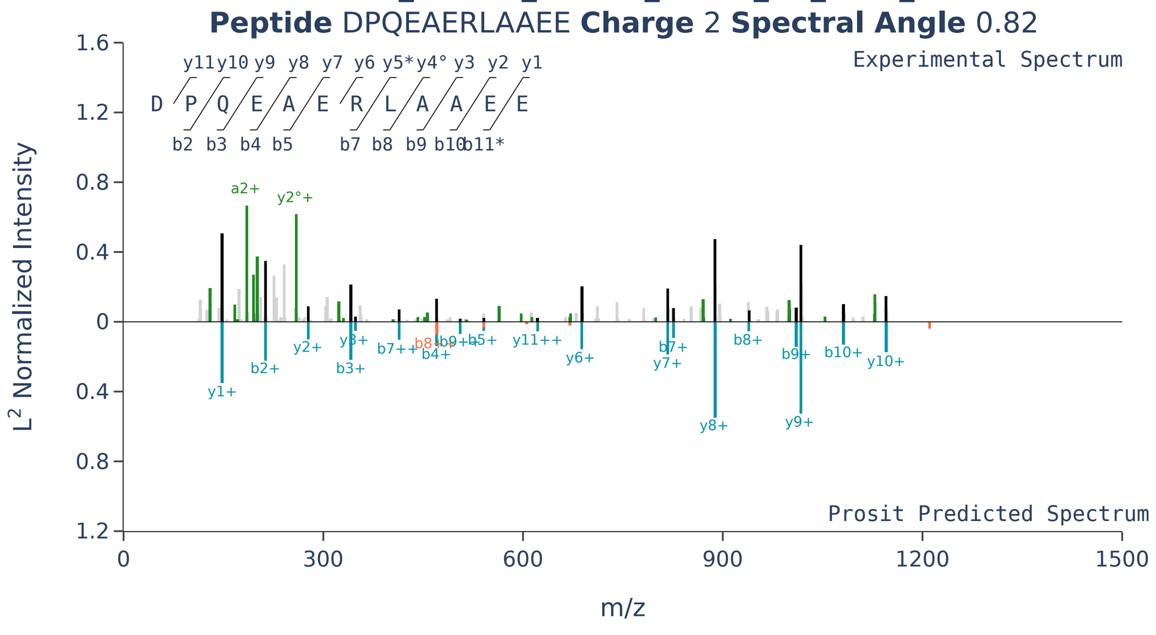


**Source** HRoetschke_211222_160123_HFGoe_IF2_b1R1_4h **Scan** 16572
**Peptide** LGQEVLE **Charge** 1 **Spectral Angle** 0.92

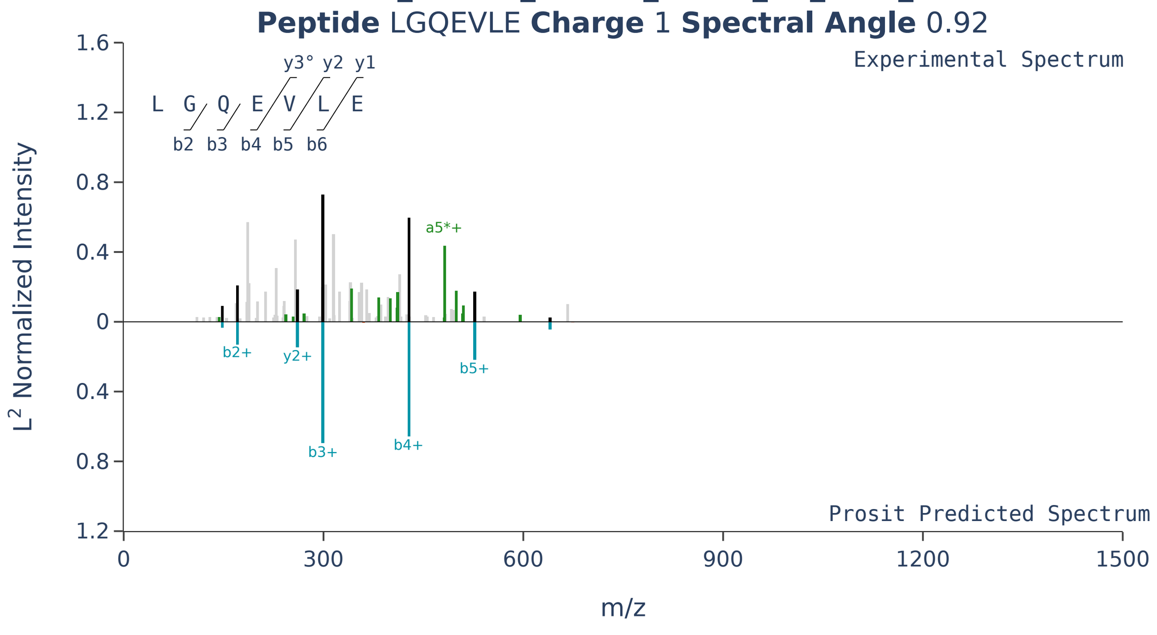


**Source** HRoetschke_211222_160123_HFGoe_IF2_b1R1_2h **Scan** 13879
**Peptide** KGDIVLC **Charge** 2 **Spectral Angle** 0.95

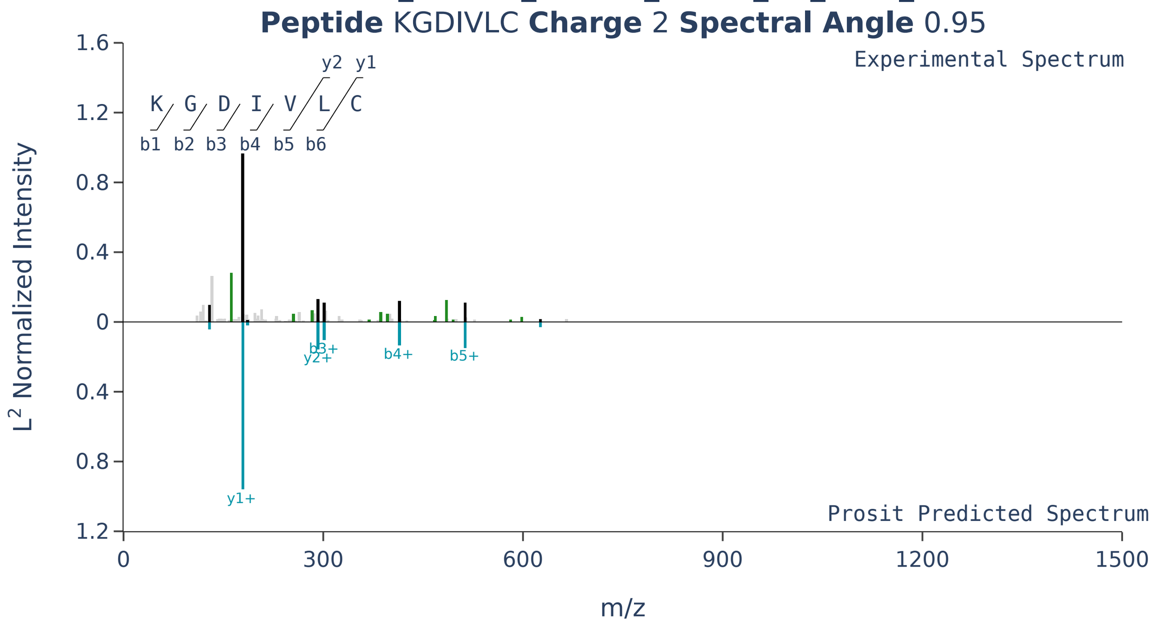


**Source** HRoetschke_211222_160123_HFGoe_IF2_b1R1_4h **Scan** 10597
**Peptide** FEYGRVR **Charge** 2 **Spectral Angle** 0.88

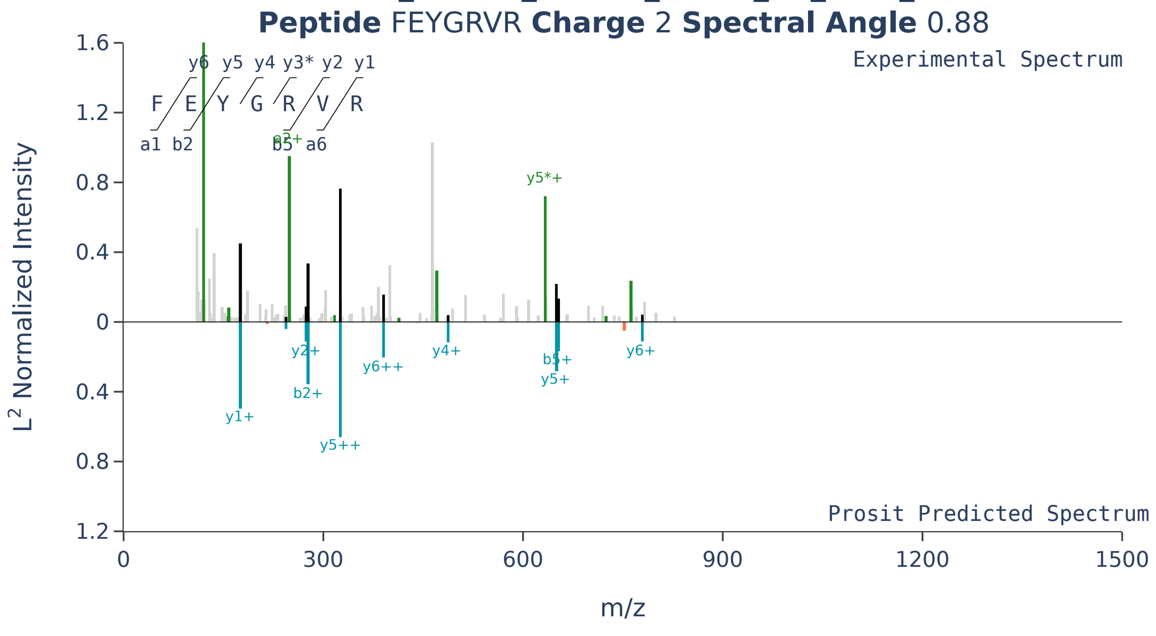


**Source** HRoetschke_211222_160123_HFGoe_IF2_b2R2_1h **Scan** 10895
**Peptide** CGFEYGRVR **Charge** 3 **Spectral Angle** 0.82

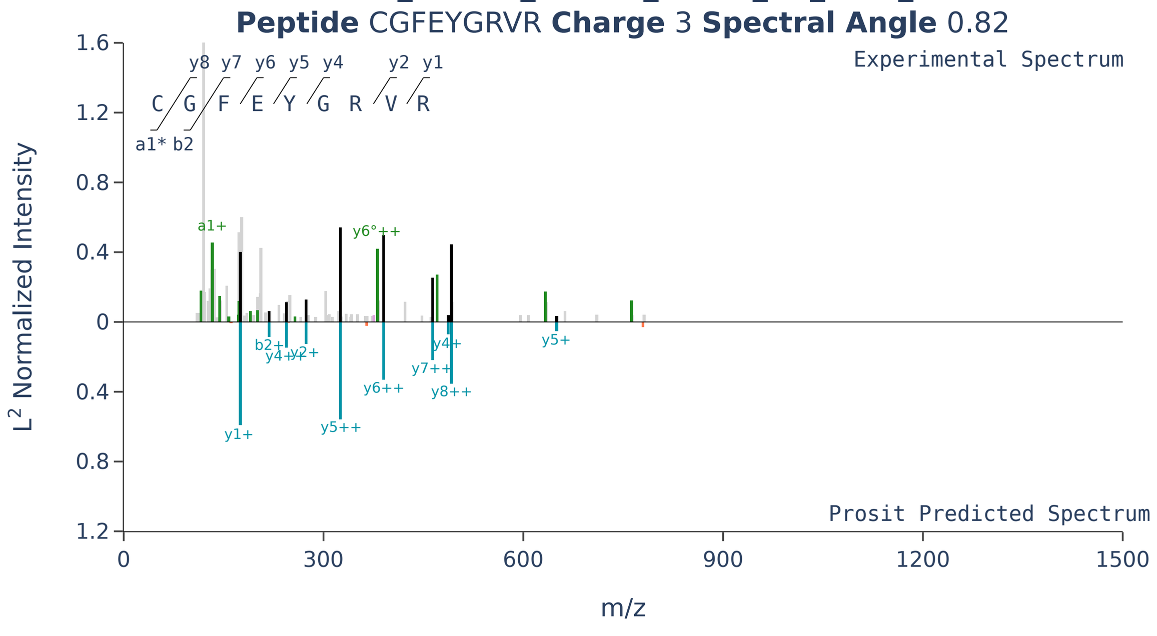

**Source** HRoetschke_211222_160123_HFGoe_IF2_b1R2_2h **Scan** 11294
**Peptide** RSTLNIPGTGGKSKSV **Charge** 3 **Spectral Angle** 0.88

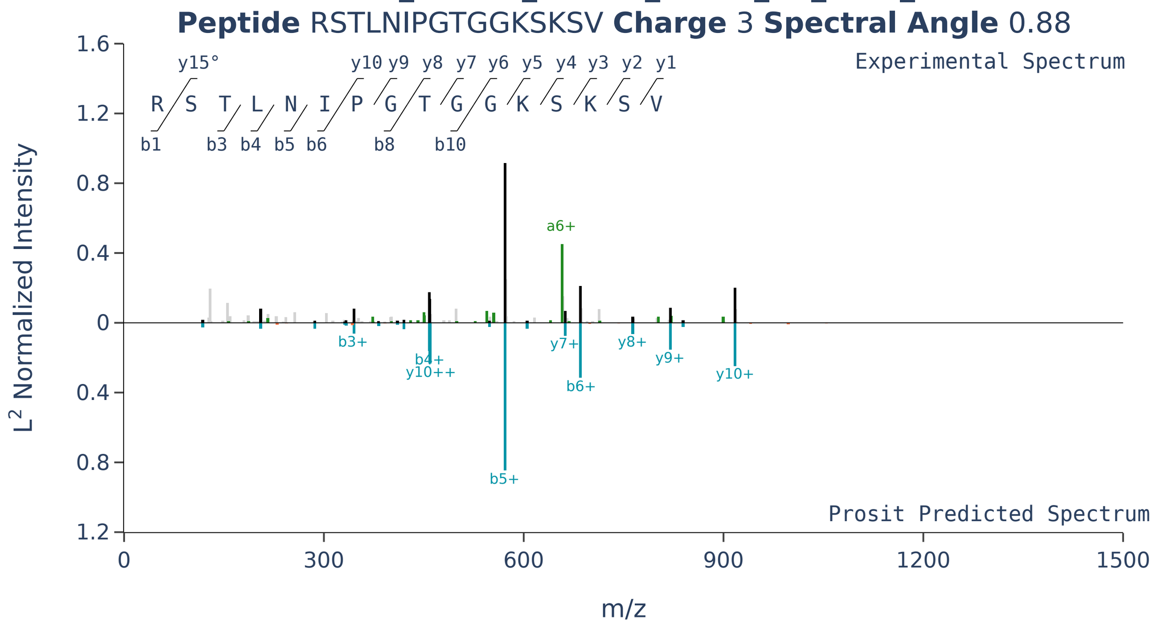


**Source** HRoetschke_211222_160123_HFGoe_IF2_b1R1_4h **Scan** 17457
**Peptide** TDVTIKTL **Charge** 1 **Spectral Angle** 0.91

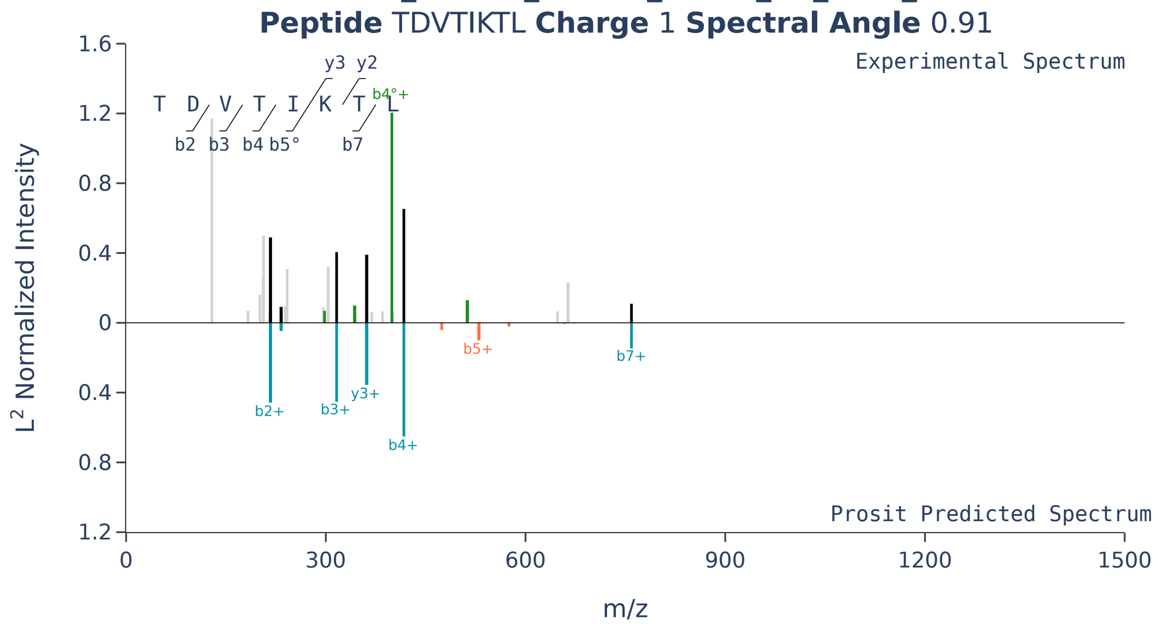


**Source** HRoetschke_211222_160123_HFGoe_IF2_b1R2_24h **Scan** 6921
**Peptide** YRQGKFREV **Charge** 3 **Spectral Angle** 0.95

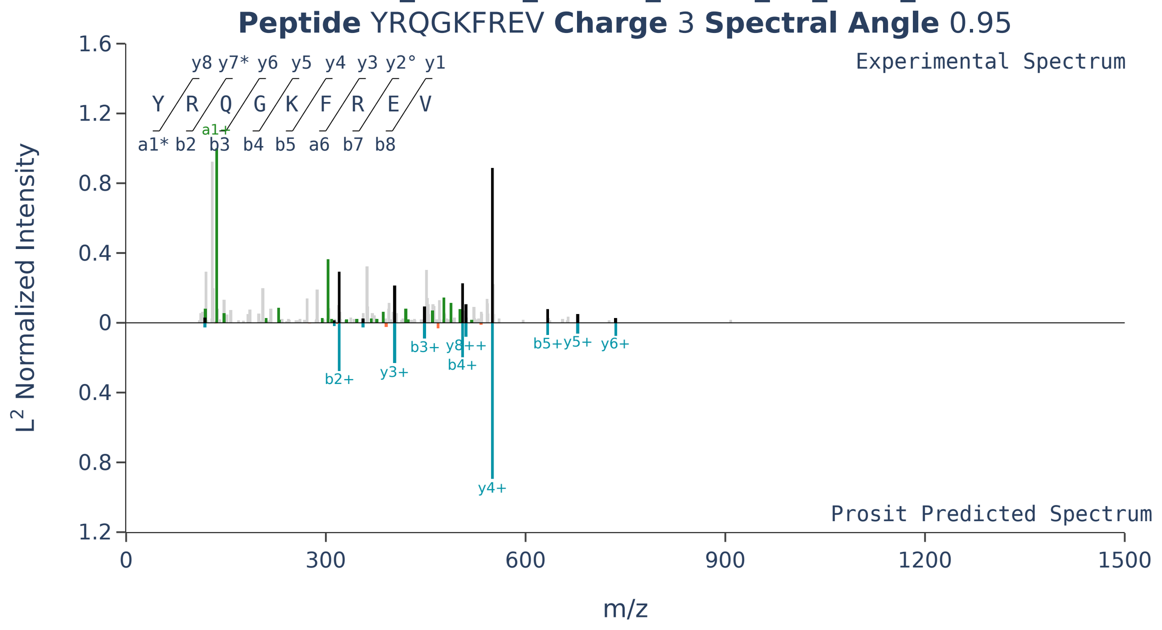


**Source** HRoetschke_211222_160123_HFGoe_IF2_b1R1_2h **Scan** 11384
**Peptide** SAQEKQTLID **Charge** 2 **Spectral Angle** 0.84

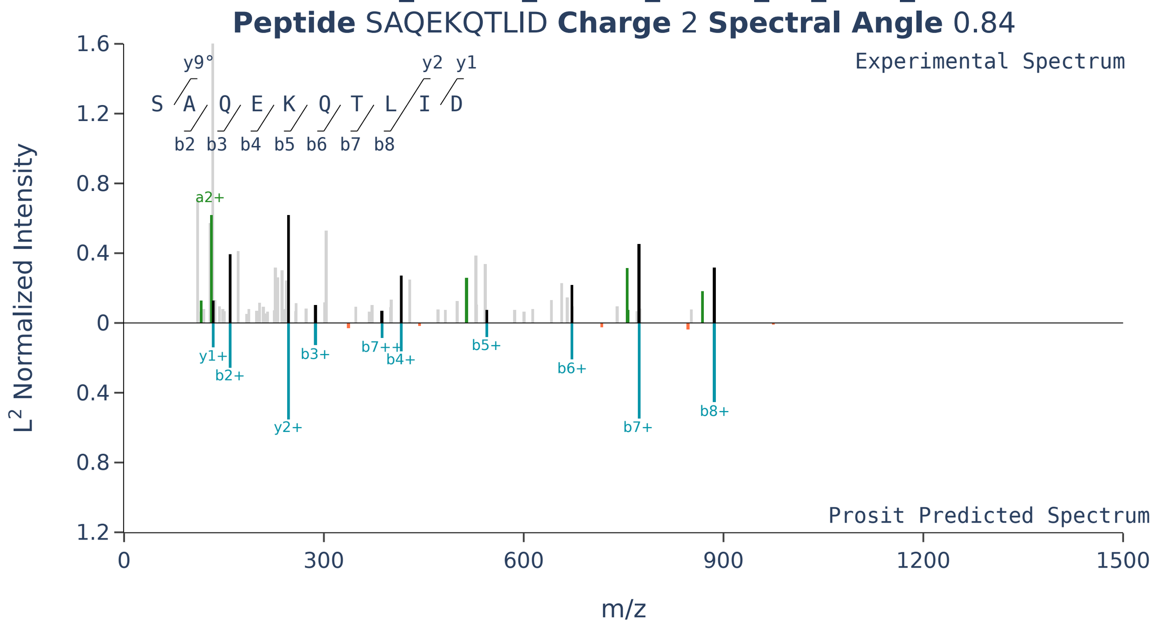


**Source** HRoetschke_211222_160123_HFGoe_IF2_b2R1_24h **Scan** 10219
**Peptide** VALYRQG **Charge** 2 **Spectral Angle** 0.85

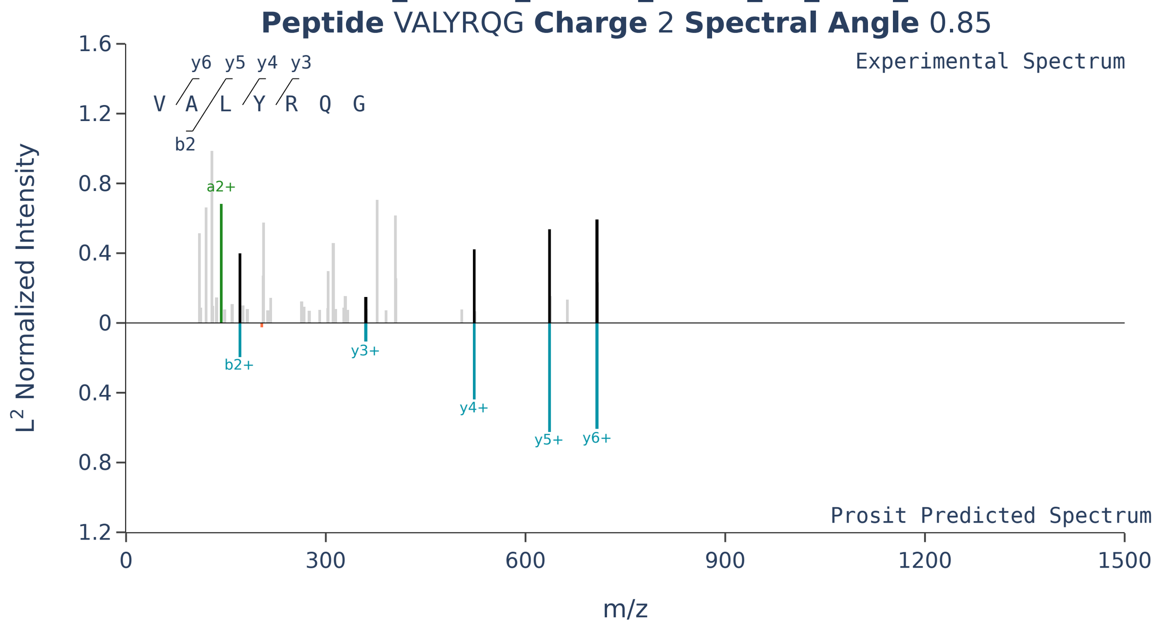


**Source** HRoetschke_211222_160123_HFGoe_IF2_b1R2_4h **Scan** 11985
**Peptide** DPQEAERLAAE **Charge** 2 **Spectral Angle** 0.89

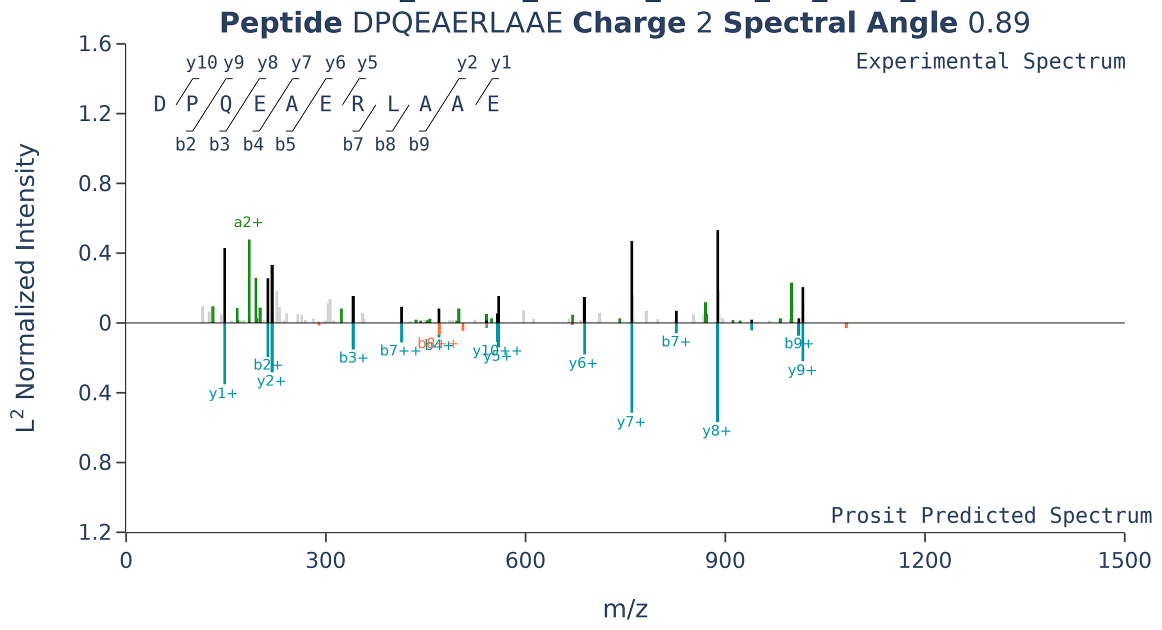


**Source** HRoetschke_211222_160123_HFGoe_IF2_b1R1_4h **Scan** 9716
**Peptide** DYIRSTKVA **Charge** 2 **Spectral Angle** 0.88

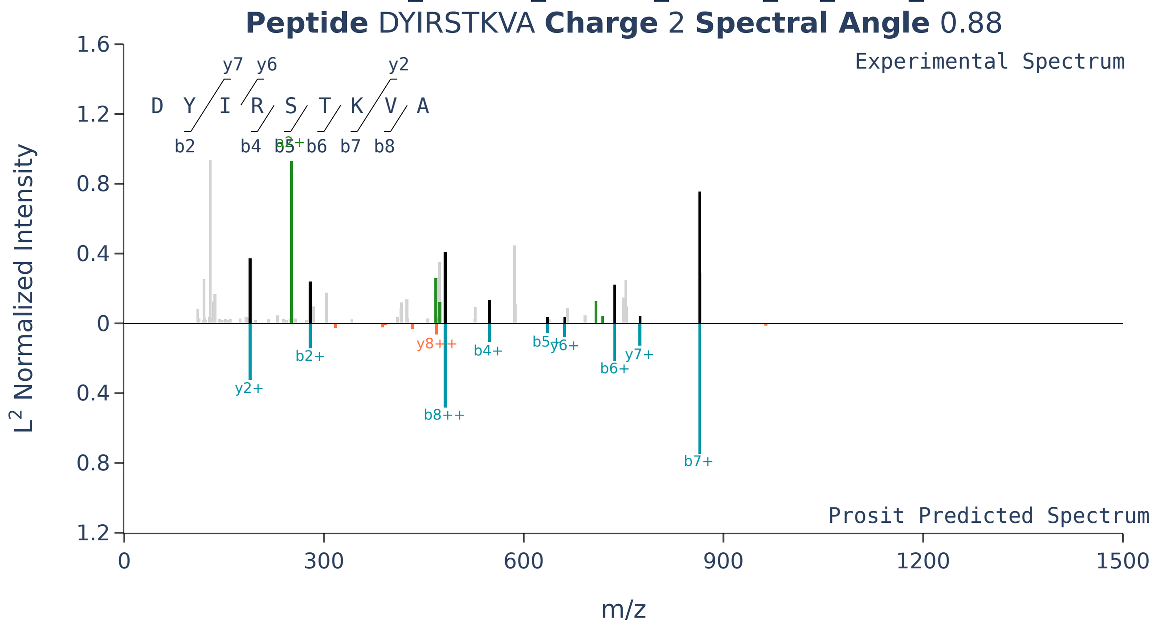


**Source** HRoetschke_211222_160123_HFGoe_IF2_b1R1_24h **Scan** 7479
**Peptide** PEADPDRV **Charge** 2 **Spectral Angle** 0.91

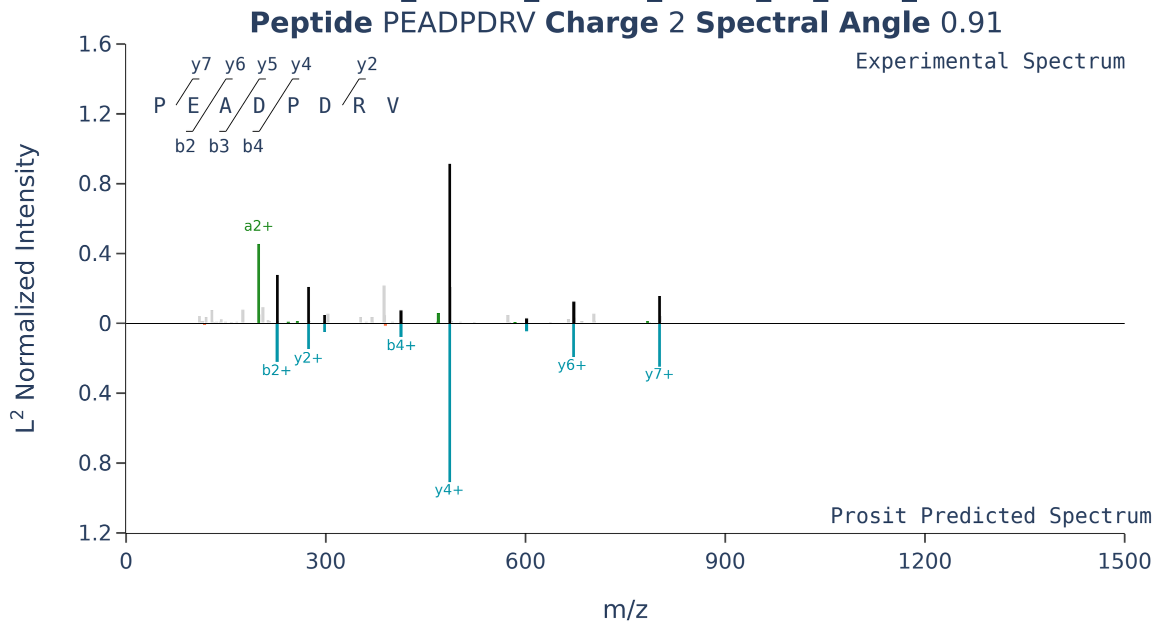


**Source** HRoetschke_211222_160123_HFGoe_IF2_b1R2_2h **Scan** 8814
**Peptide** TIKTLAAERQT **Charge** 3 **Spectral Angle** 0.88

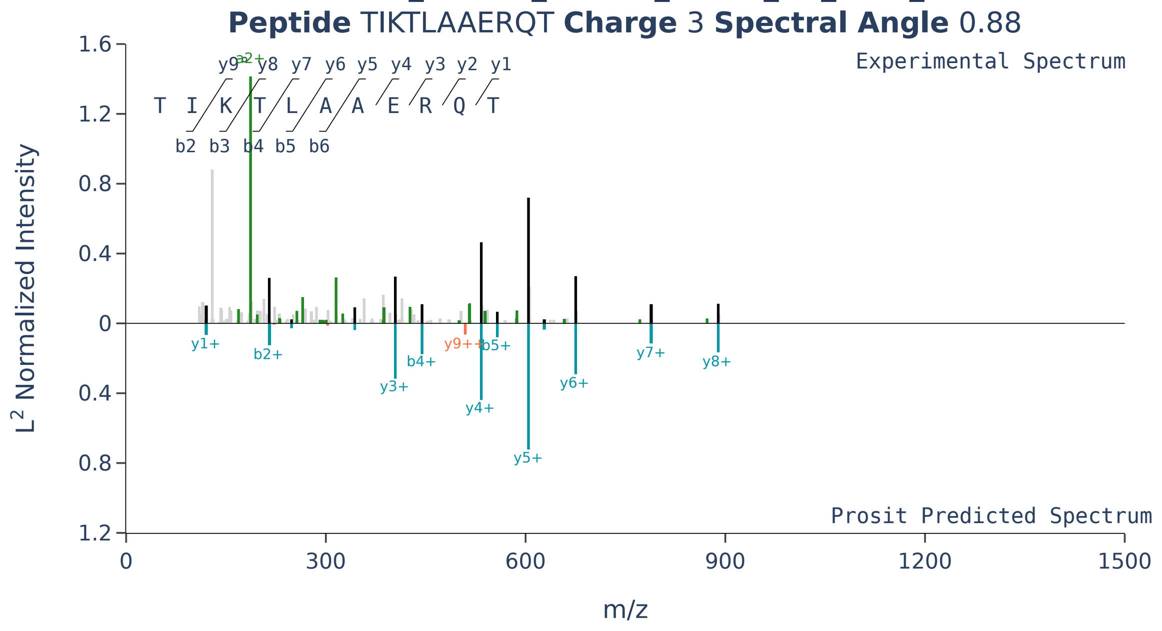


**Source** HRoetschke_211222_160123_HFGoe_IF2_b1R2_24h **Scan** 16022
**Peptide** AMRNELGQEVLE **Charge** 2 **Spectral Angle** 0.82

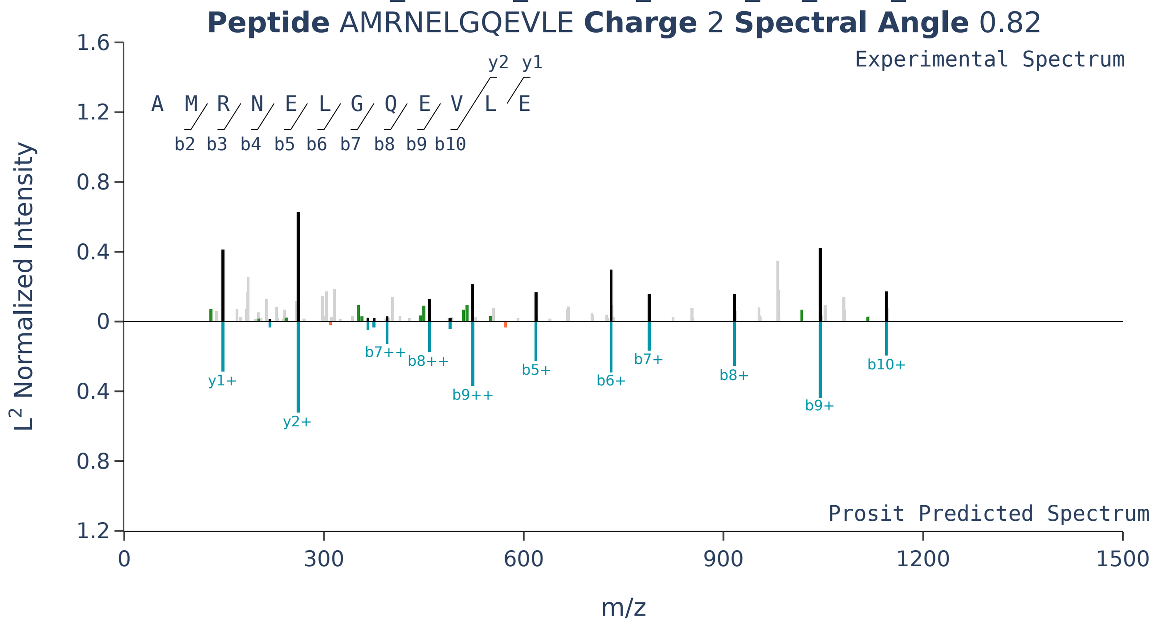


**Source** HRoetschke_211222_160123_HFGoe_IF2_b1R1_4h **Scan** 11808
**Peptide** AGGITQHI **Charge** 2 **Spectral Angle** 0.9

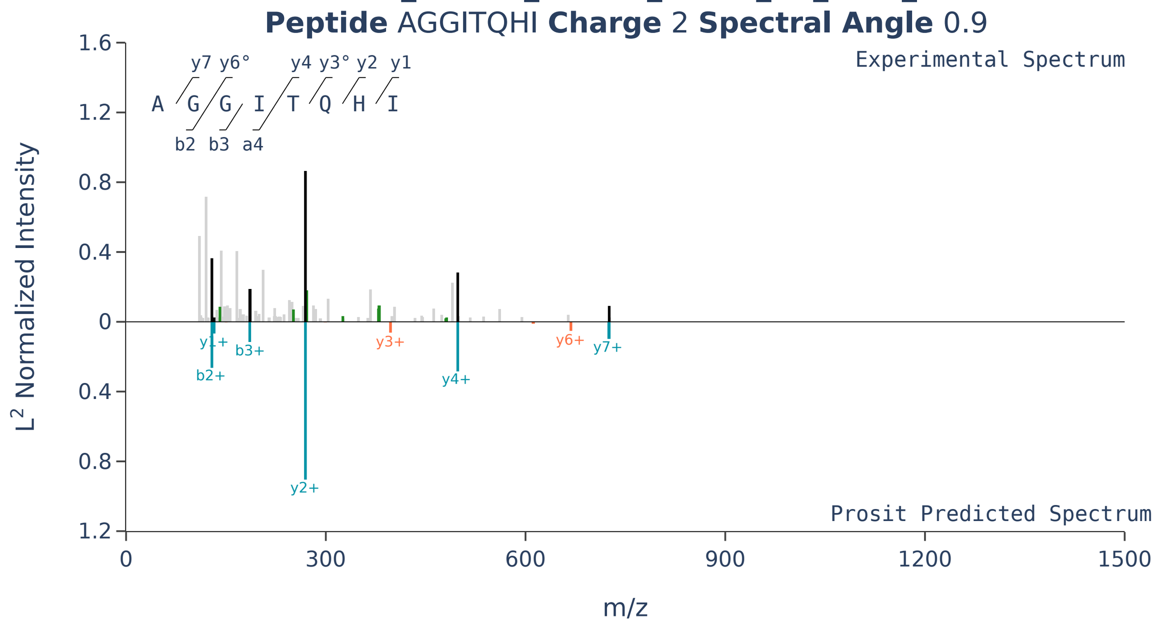


**Source** HRoetschke_211222_160123_HFGoe_IF2_b1R2_24h **Scan** 7033
**Peptide** AYHVETEN **Charge** 2 **Spectral Angle** 0.87

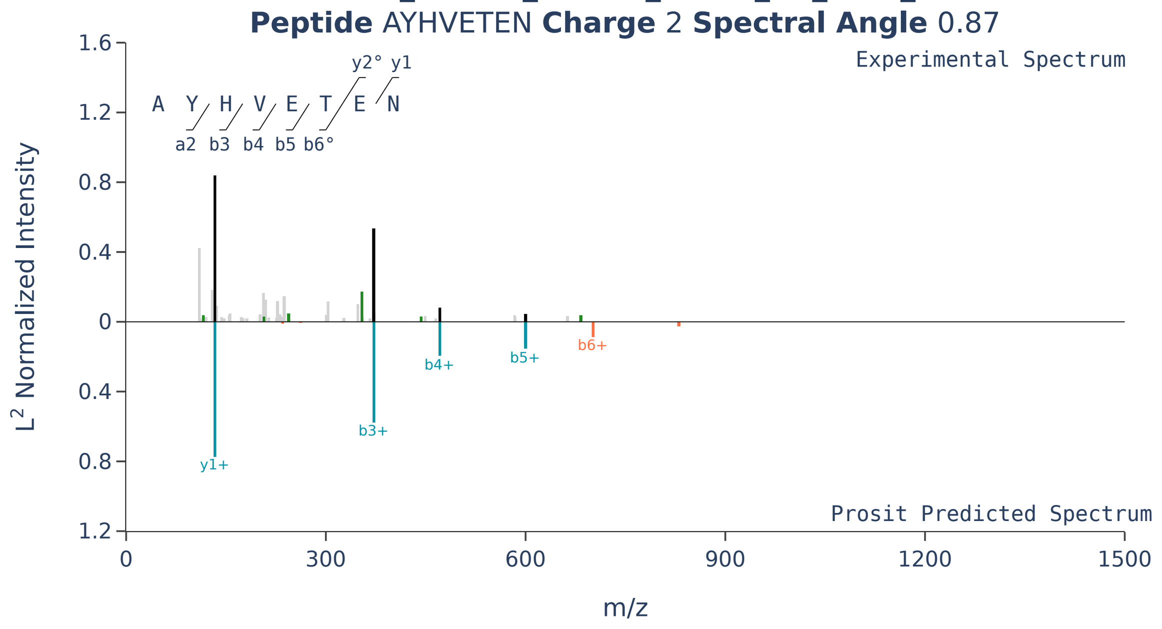


**Source** HRoetschke_211222_160123_HFGoe_IF2_b2R2_2h **Scan** 7124
**Peptide** AERLAAEEQ **Charge** 2 **Spectral Angle** 0.84

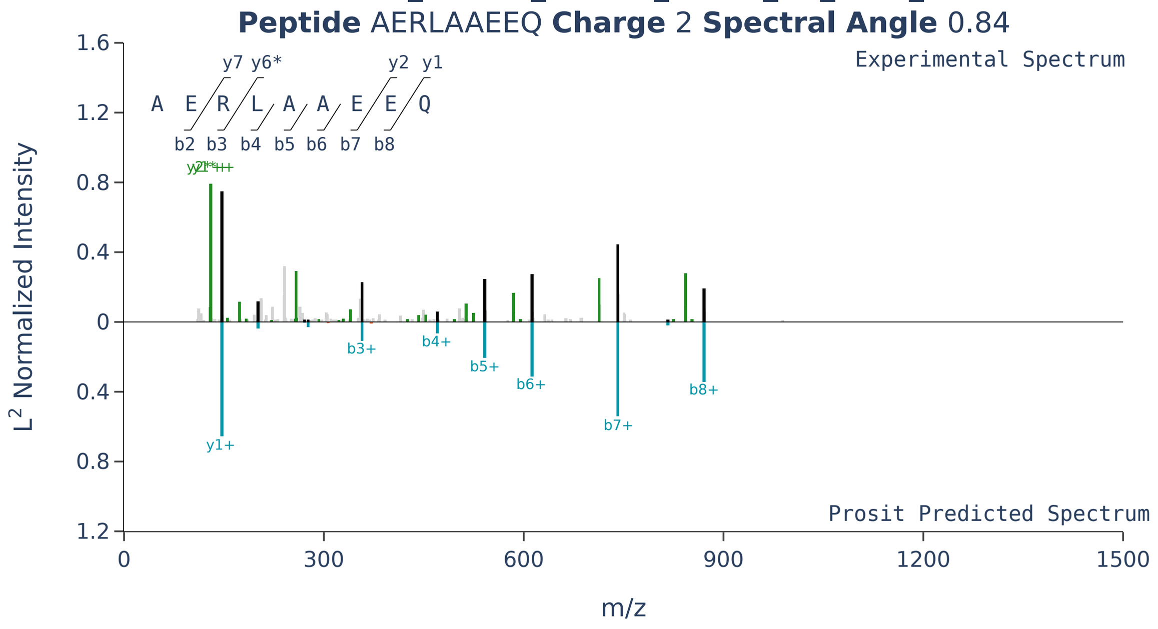


**Source** HRoetschke_211222_160123_HFGoe_IF2_b1R1_4h **Scan** 11726
**Peptide** DPQEAERLAA **Charge** 2 **Spectral Angle** 0.86

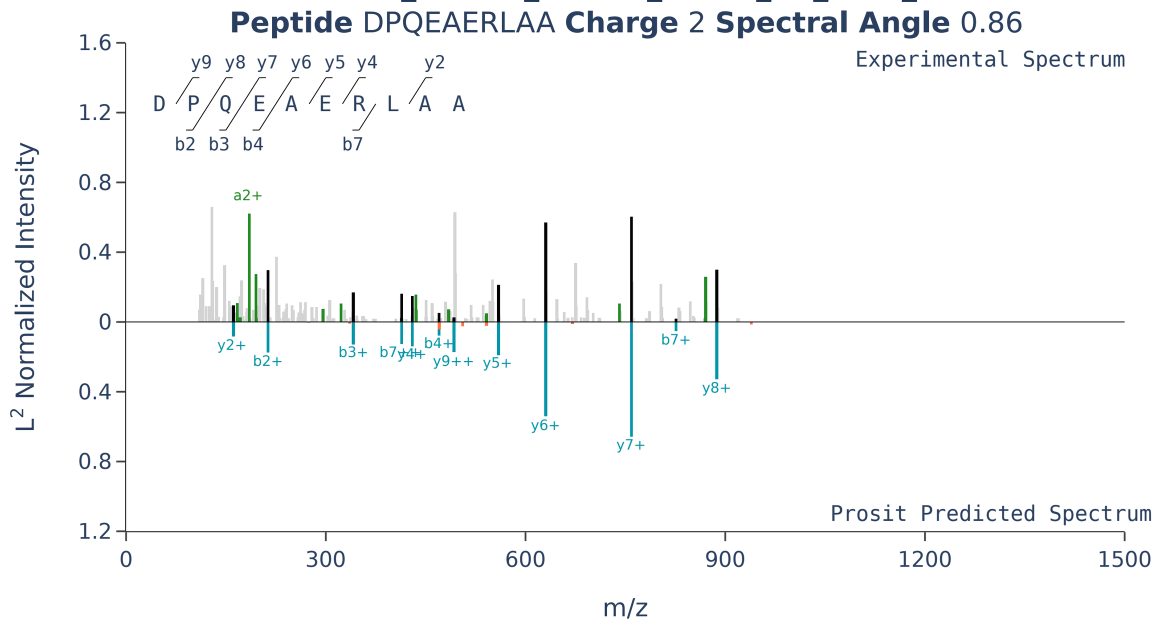


**Source** HRoetschke_211222_160123_HFGoe_IF2_b1R2_2h **Scan** 8367
**Peptide** FADAGIRKSAD **Charge** 3 **Spectral Angle** 0.86

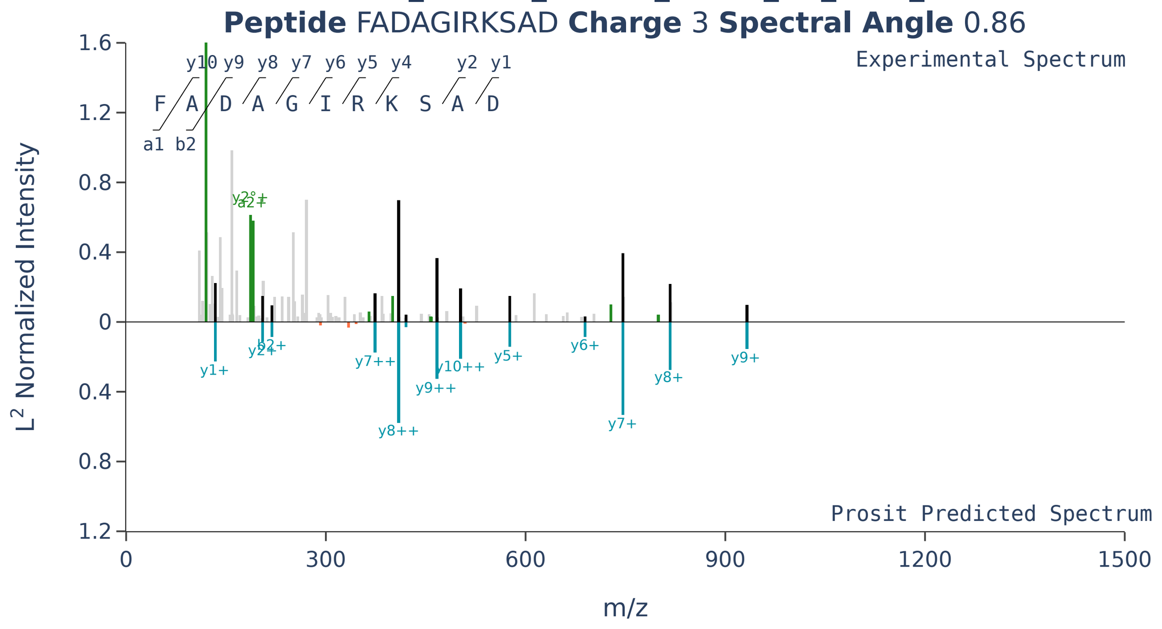

**Source** HRoetschke_211222_160123_HFGoe_IF2_b2R2_4h **Scan** 14427
**Peptide** TINQVID **Charge** 1 **Spectral Angle** 0.85

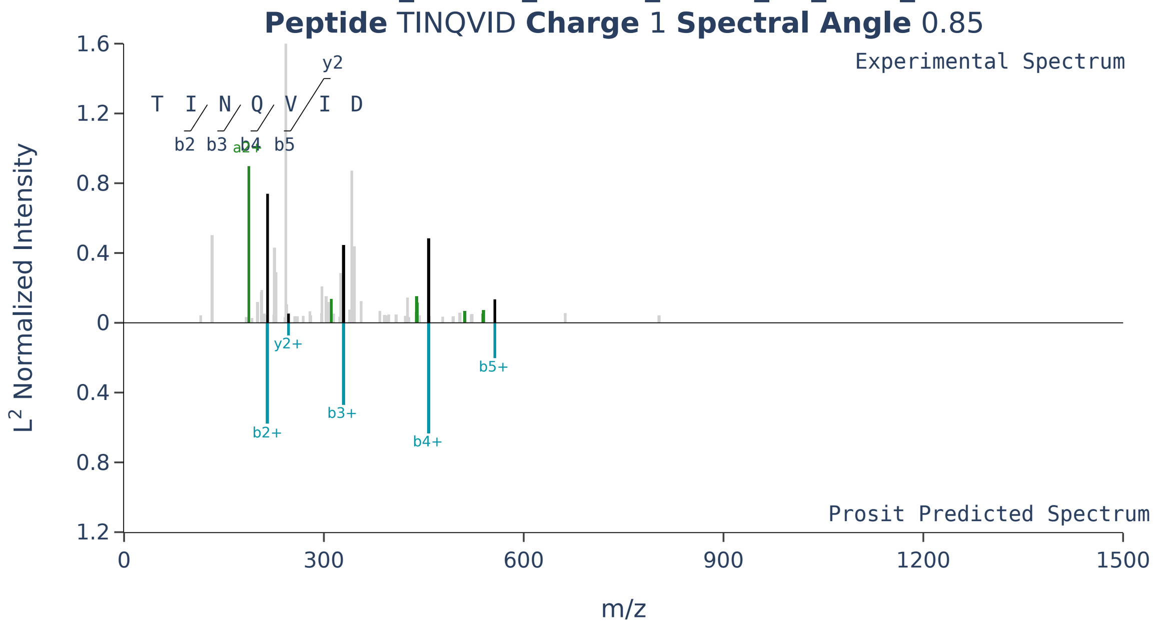


**Source** HRoetschke_211222_160123_HFGoe_IF2_b1R2_24h **Scan** 17120
**Peptide** EVLELKA **Charge** 2 **Spectral Angle** 0.96

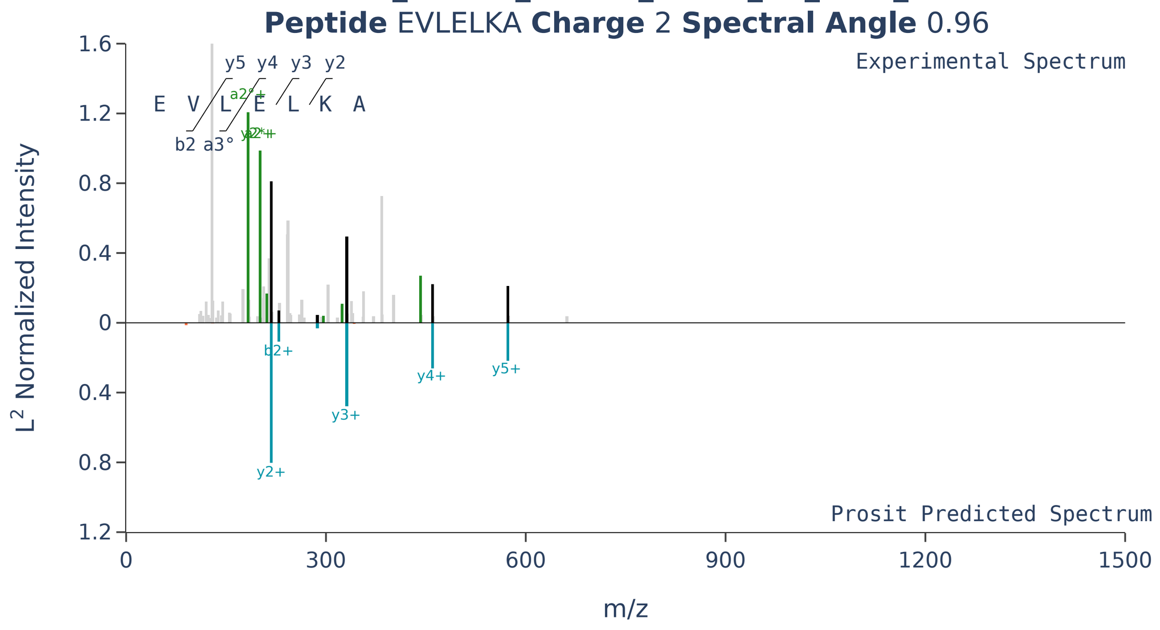

**Source** HRoetschke_211222_160123_HFGoe_IF2_b2R1_24h **Scan** 12368
**Peptide** RSTLNIPGTGGKS **Charge** 3 **Spectral Angle** 0.91

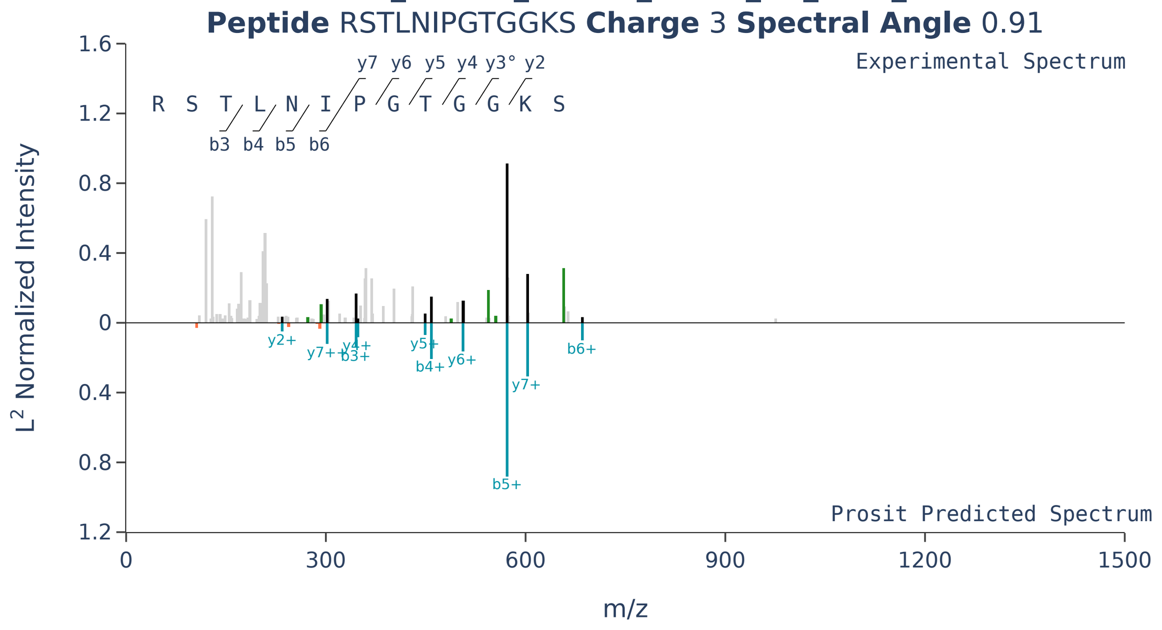

**Source** HRoetschke_211222_160123_HFGoe_IF2_b1R1_4h **Scan** 10715
**Peptide** DPQEAERLA **Charge** 2 **Spectral Angle** 0.85

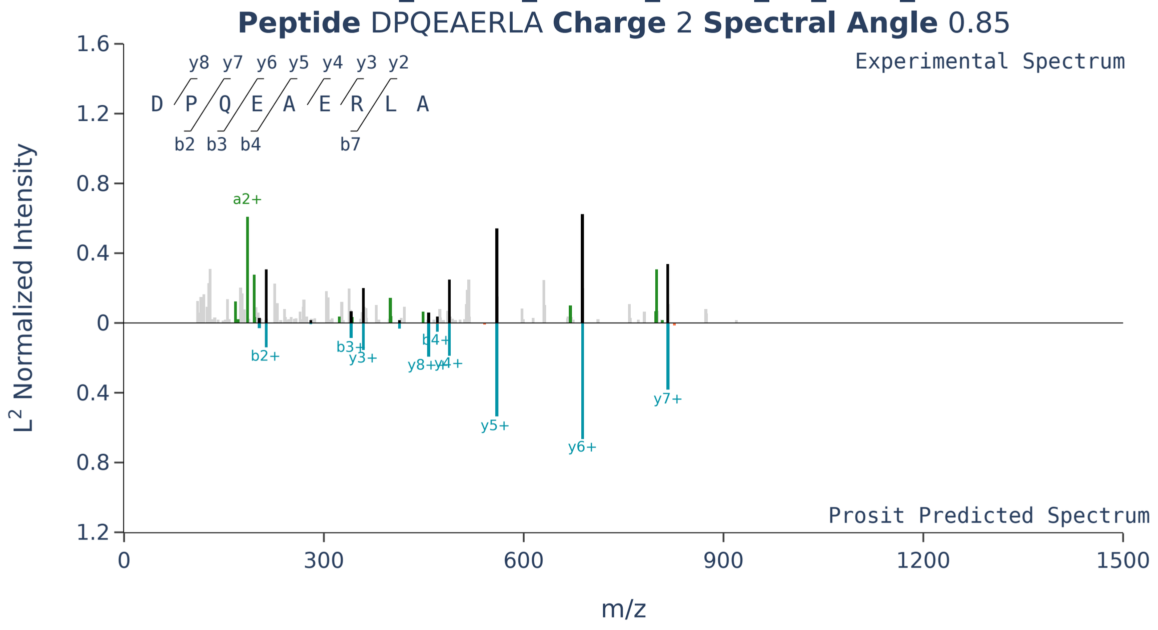

**Source** HRoetschke_211222_160123_HFGoe_IF2_b1R2_1h **Scan** 12573
**Peptide** AEVLELKAVRK **Charge** 3 **Spectral Angle** 0.84

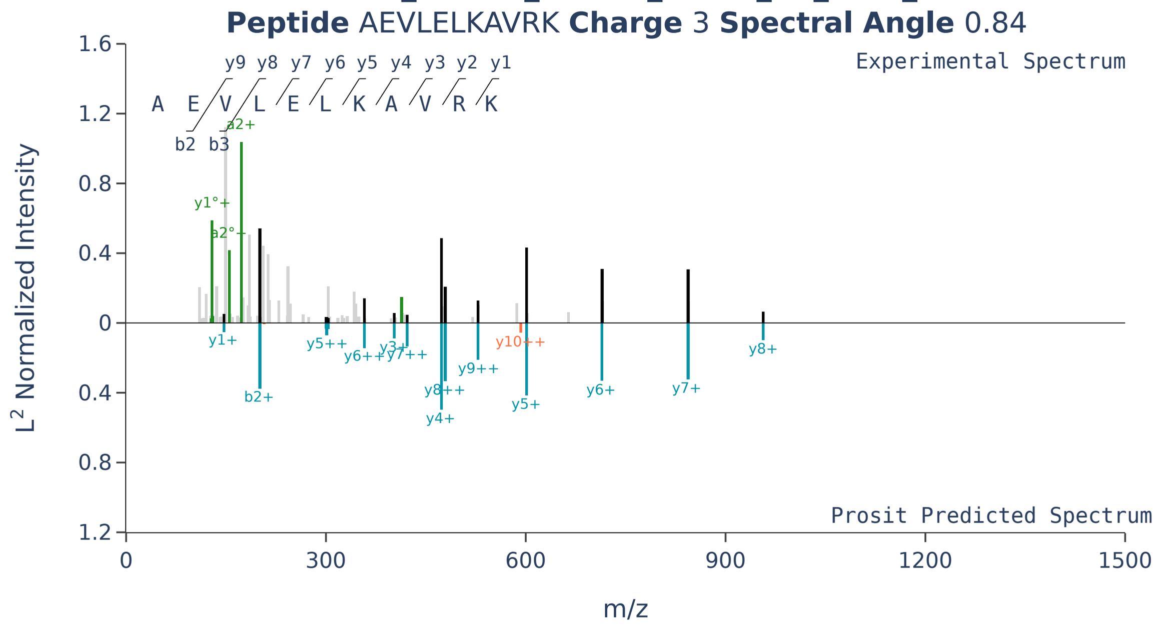

**Source** HRoetschke_211222_160123_HFGoe_IF2_b1R1_4h **Scan** 11742
**Peptide** MASGAVLE **Charge** 2 **Spectral Angle** 0.91

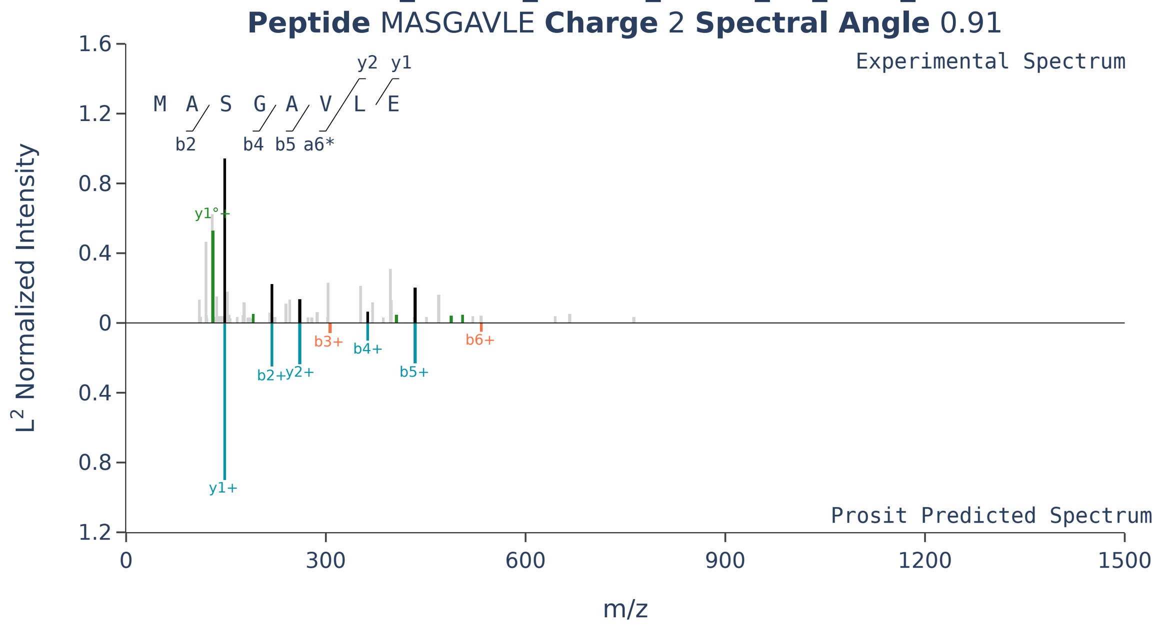

**Source** HRoetschke_211222_160123_HFGoe_IF2_b1R2_24h **Scan** 7080
**Peptide** LRRENELEE **Charge** 3 **Spectral Angle** 0.86

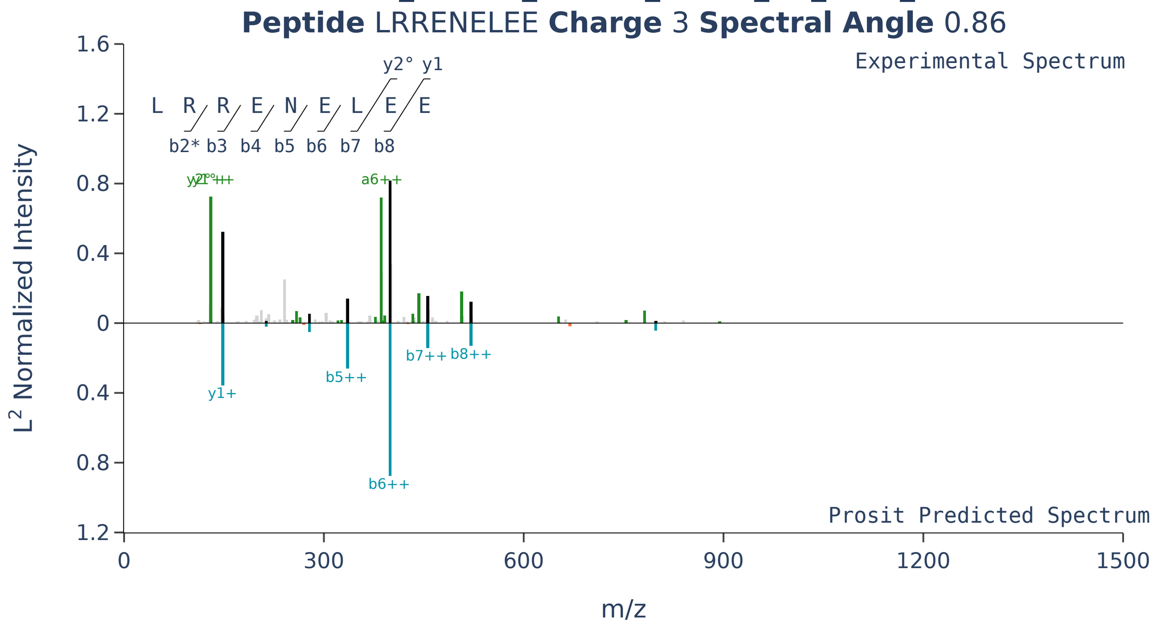

**Source** HRoetschke_211222_160123_HFGoe_IF2_b1R1_1h **Scan** 6354
**Peptide** AGIRKSADDSVSA **Charge** 3 **Spectral Angle** 0.86

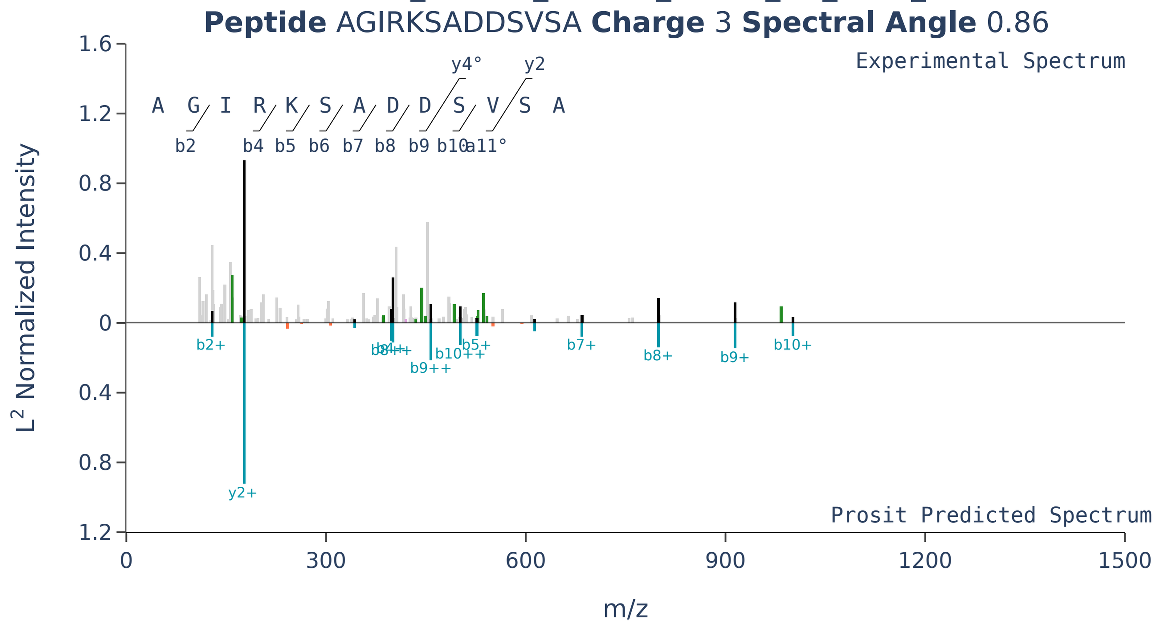

**Source** HRoetschke_211222_160123_HFGoe_IF2_b1R2_2h **Scan** 7137
**Peptide** QGFQKPAQ **Charge** 1 **Spectral Angle** 0.89

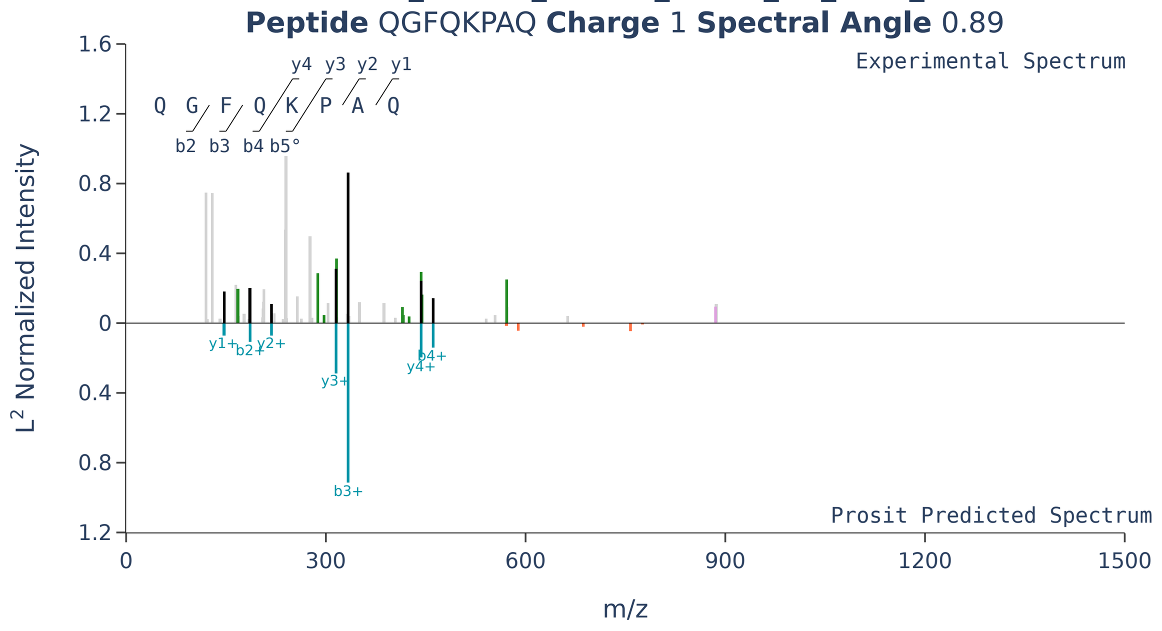

**Source** HRoetschke_211222_160123_HFGoe_IF2_b1R1_4h **Scan** 12392
**Peptide** STLNIPGTGGKSK **Charge** 3 **Spectral Angle** 0.87

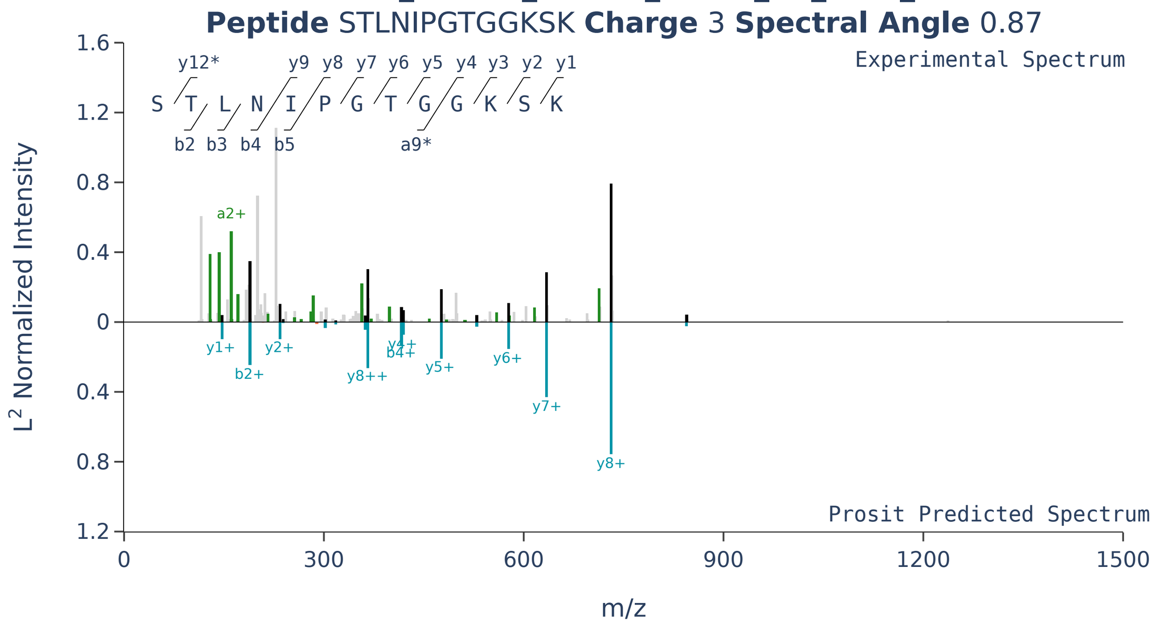

**Source** HRoetschke_211222_160123_HFGoe_IF2_b2R2_2h **Scan** 7410
**Peptide** LAAERQTSVE **Charge** 2 **Spectral Angle** 0.84

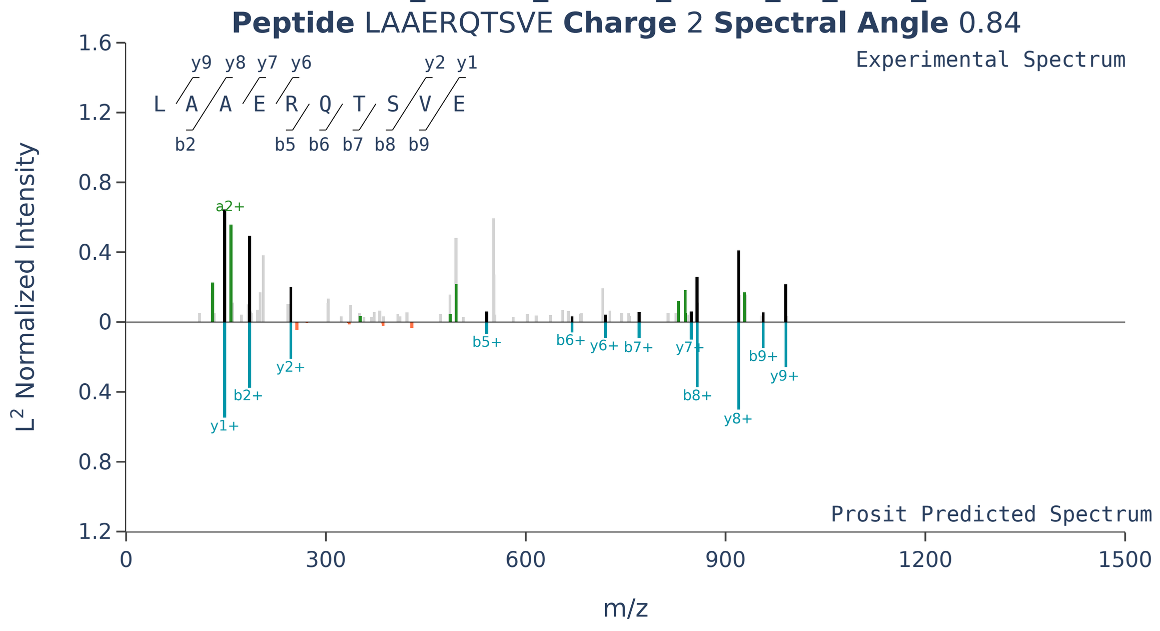

**Source** HRoetschke_211222_160123_HFGoe_IF2_b1R2_24h **Scan** 7598
**Peptide** FADAGIRKS **Charge** 2 **Spectral Angle** 0.86

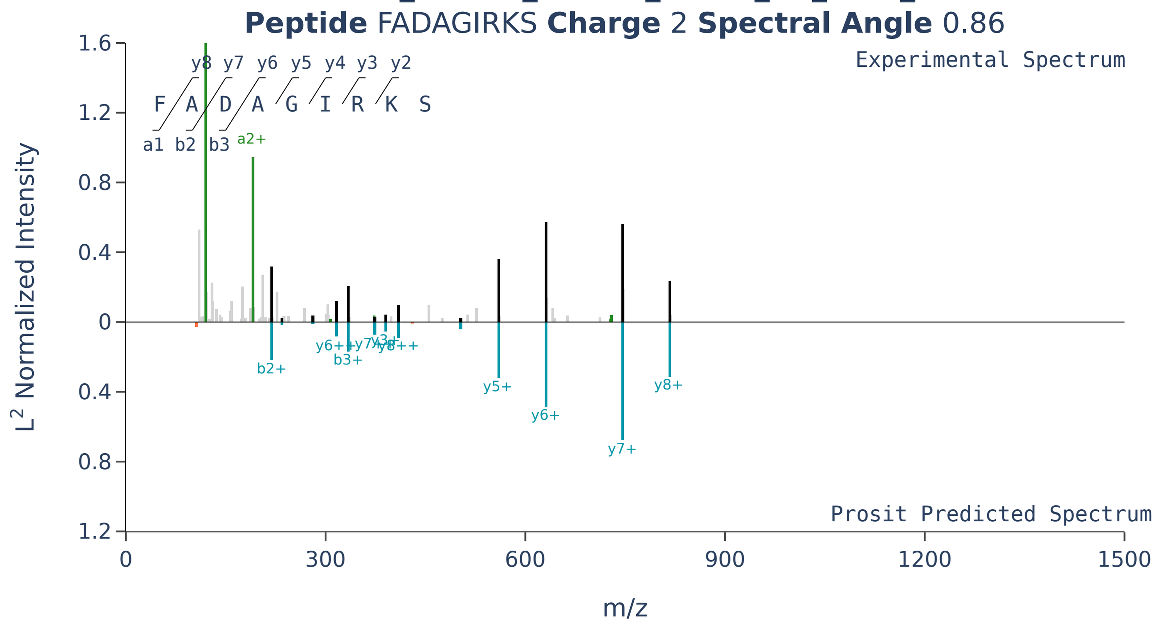

**Source** HRoetschke_211222_160123_HFGoe_IF2_b1R1_4h **Scan** 9281
**Peptide** REVALYR **Charge** 2 **Spectral Angle** 0.83

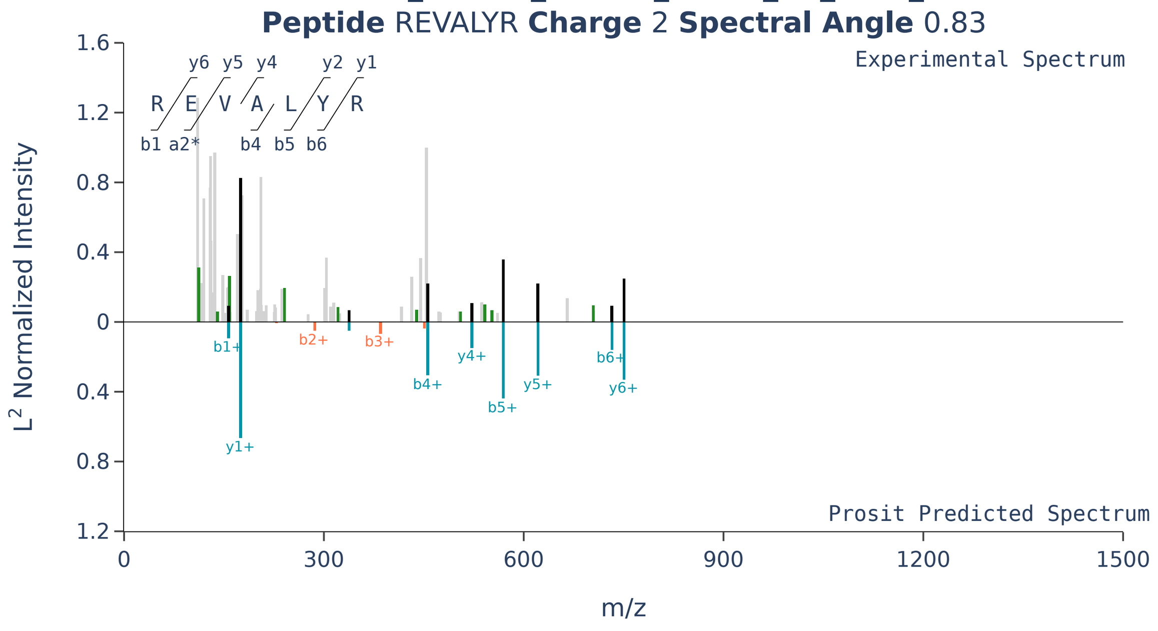

**Source** HRoetschke_211222_160123_HFGoe_IF2_b1R2_24h **Scan** 15016
**Peptide** AVNRDVVI **Charge** 2 **Spectral Angle** 0.89

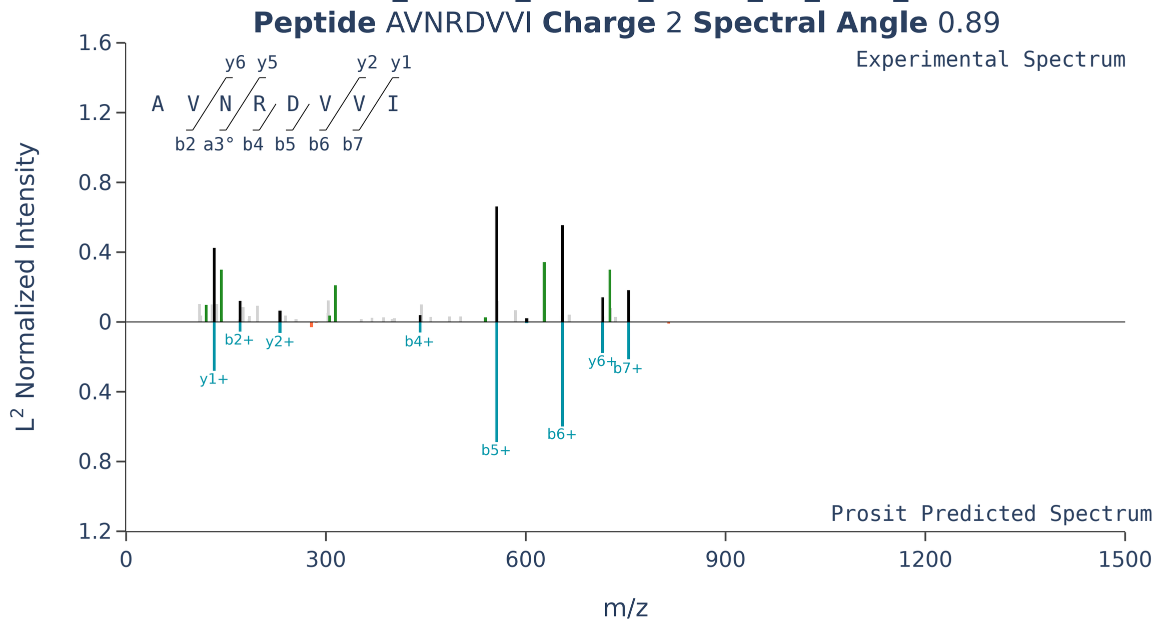

**Source** HRoetschke_211222_160123_HFGoe_IF2_b2R1_4h **Scan** 7381
**Peptide** AYHVETE **Charge** 2 **Spectral Angle** 0.89

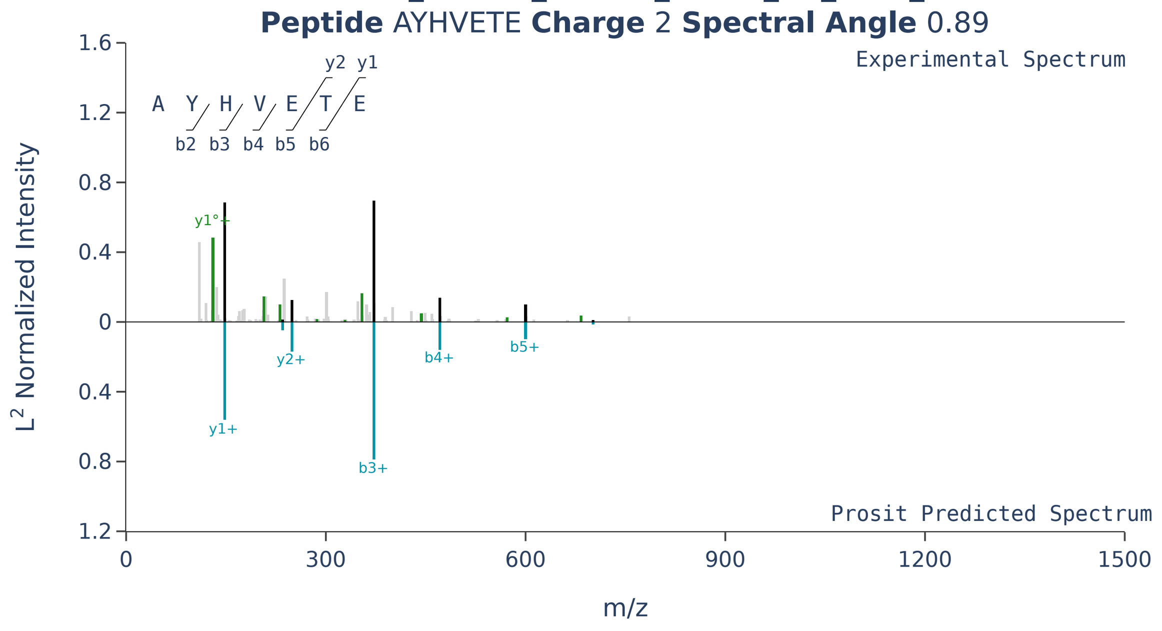

**Source** HRoetschke_211222_160123_HFGoe_IF2_b1R1_4h **Scan** 16689
**Peptide** SGPDKLTL **Charge** 1 **Spectral Angle** 0.88

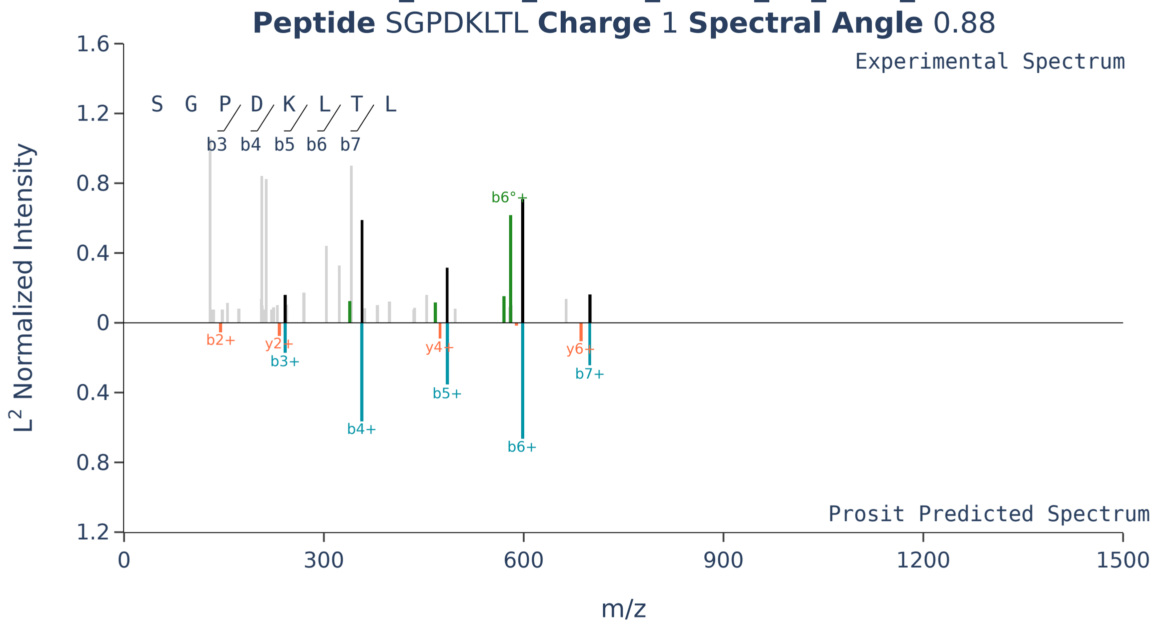


**Source** HRoetschke_211222_160123_HFGoe_IF2_b1R1_4h **Scan** 6645
**Peptide** YHVETENG **Charge** 2 **Spectral Angle** 0.91

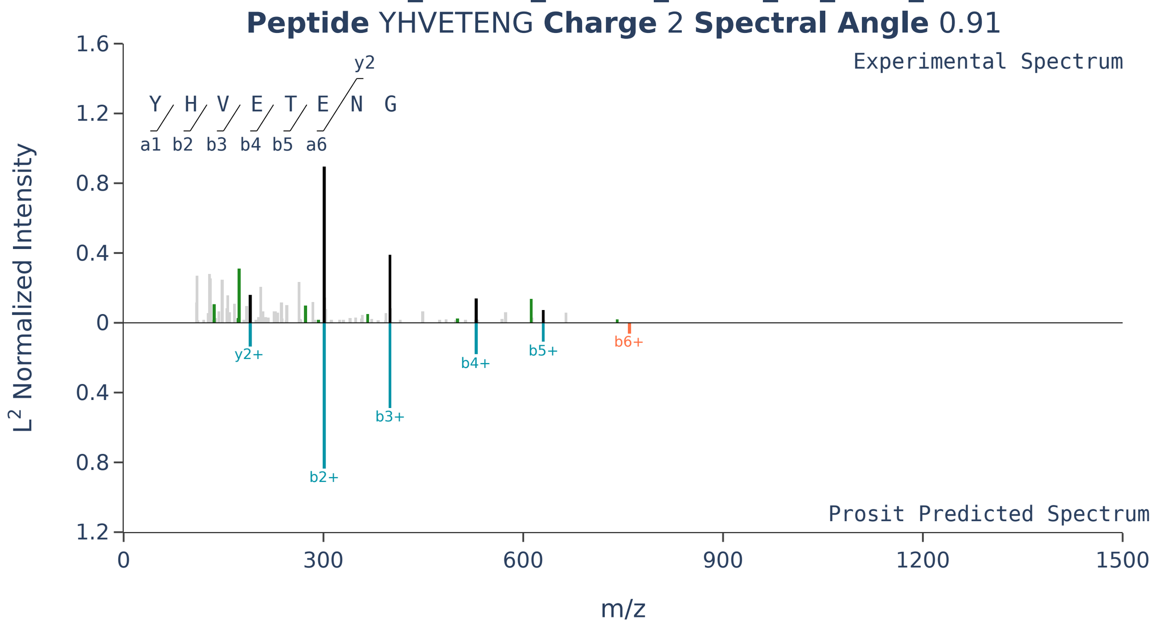


**Source** HRoetschke_211222_160123_HFGoe_IF2_b2R2_4h **Scan** 10204
**Peptide** DTPGHAAFTSM **Charge** 2 **Spectral Angle** 0.89

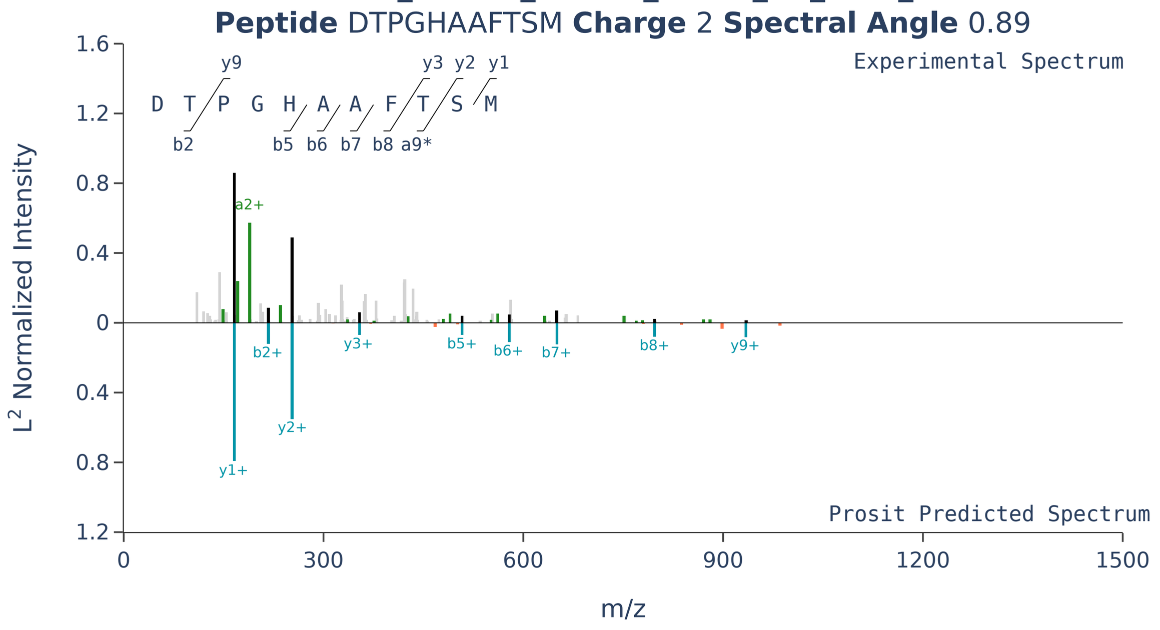


**Source** HRoetschke_211222_160123_HFGoe_IF2_b1R1_4h **Scan** 15870
**Peptide** EVLELKAVR **Charge** 3 **Spectral Angle** 0.89

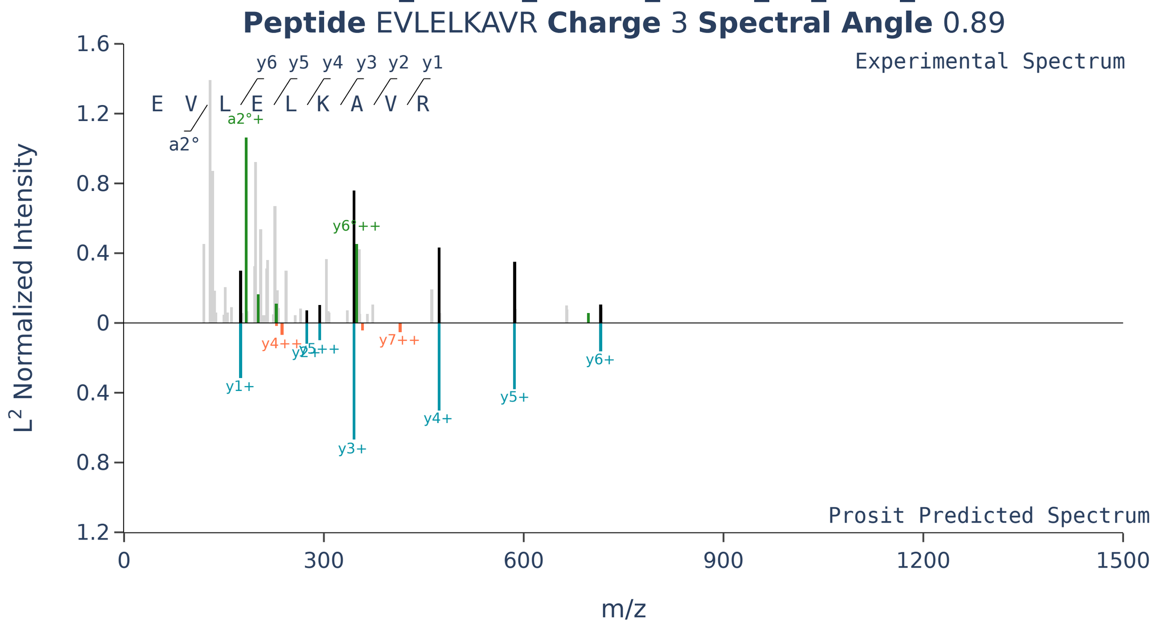


**Source** HRoetschke_211222_160123_HFGoe_IF2_b1R2_24h **Scan** 17122
**Peptide** IDQETAQLVAE **Charge** 2 **Spectral Angle** 0.9

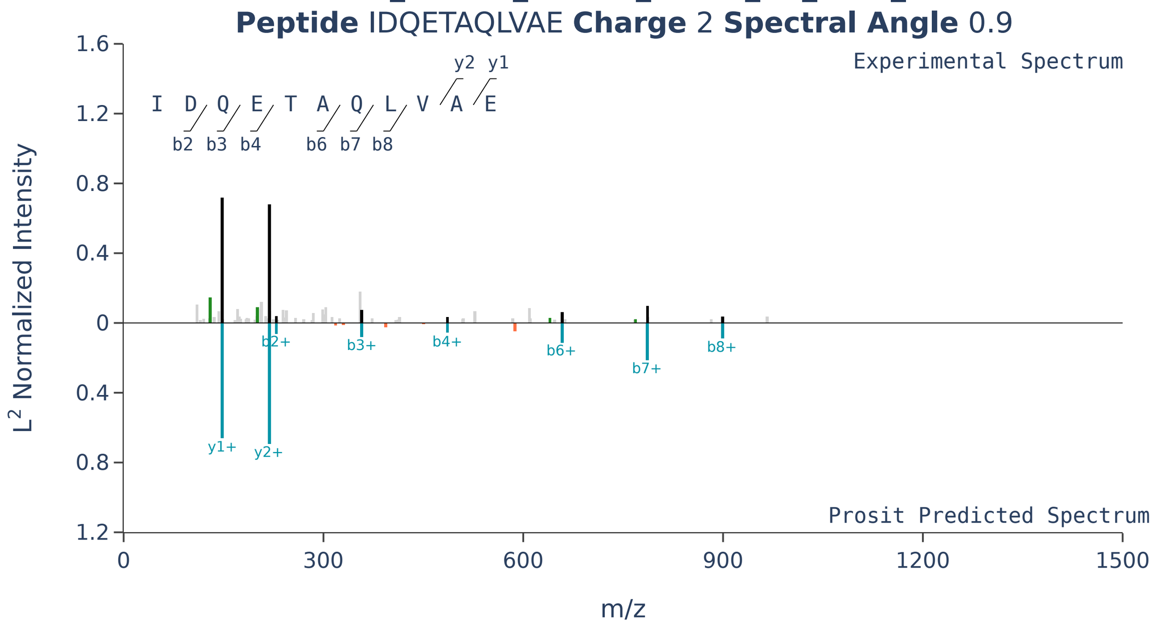


**Source** HRoetschke_211222_160123_HFGoe_IF2_b2R1_1h **Scan** 6724
**Peptide** KTLAAERQTSVE **Charge** 2 **Spectral Angle** 0.82

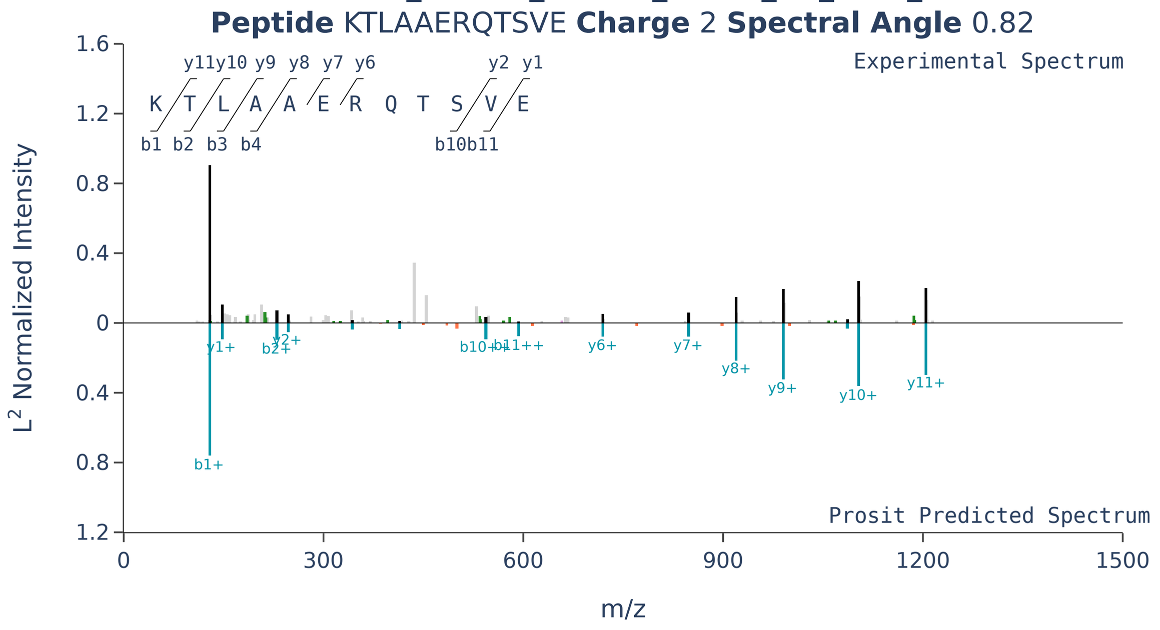


**Source** HRoetschke_211222_160123_HFGoe_IF2_b1R1_2h **Scan** 8979
**Peptide** TPGHAAFTSMRAR **Charge** 4 **Spectral Angle** 0.87

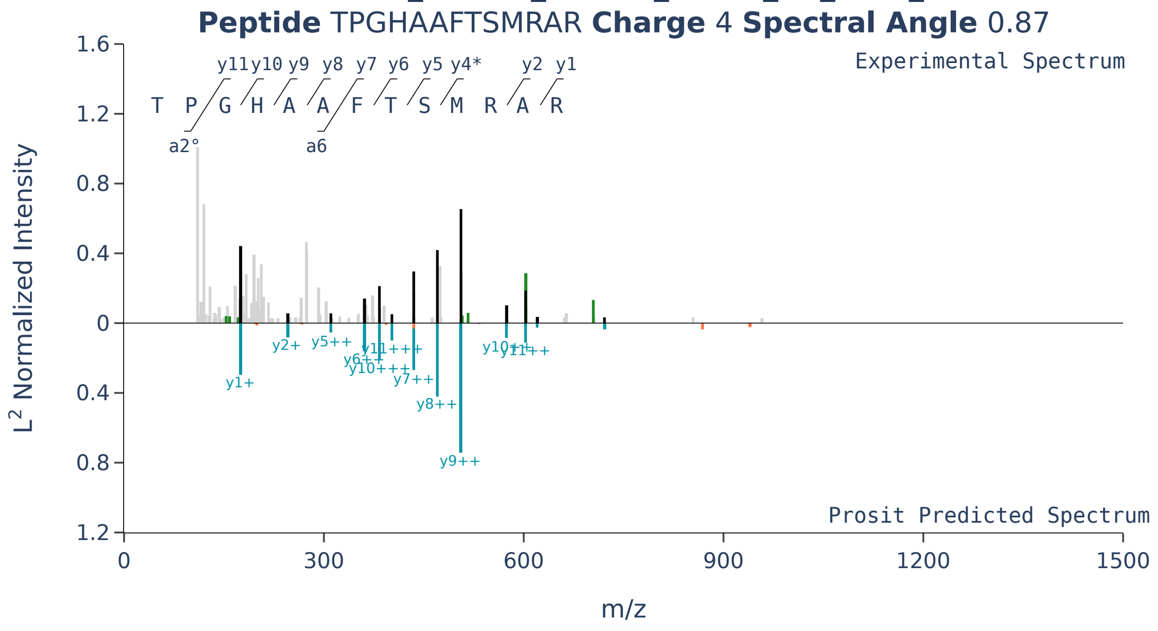


**Source** HRoetschke_211222_160123_HFGoe_IF2_b2R2_1h **Scan** 9831
**Peptide** AMATINQ **Charge** 1 **Spectral Angle** 0.75

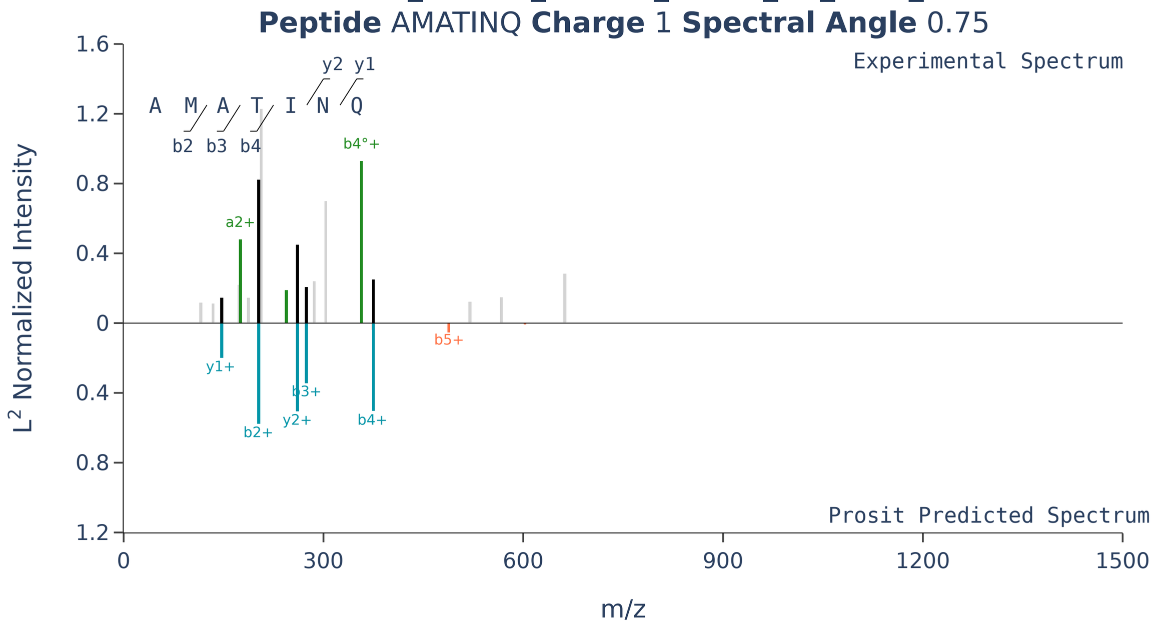


**Source** HRoetschke_211222_160123_HFGoe_IF2_b1R1_4h **Scan** 6965
**Peptide** AVMSDRDTGA **Charge** 2 **Spectral Angle** 0.83

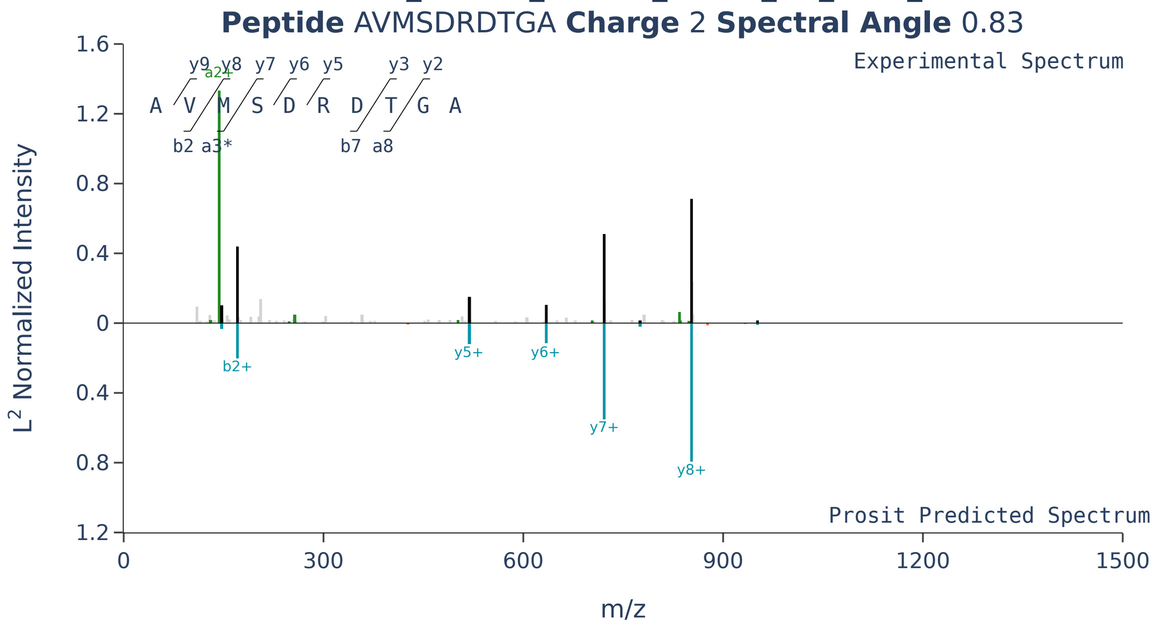


**Source** HRoetschke_211222_160123_HFGoe_IF2_b2R2_4h **Scan** 15463
**Peptide** VIDQETAQL **Charge** 2 **Spectral Angle** 0.9

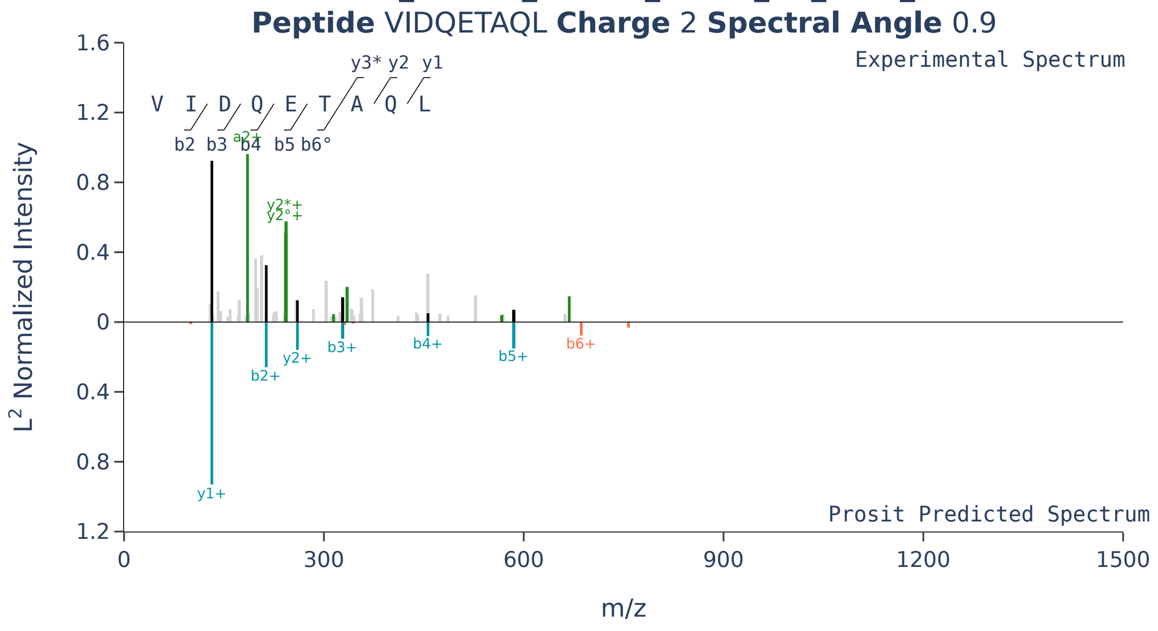


**Source** HRoetschke_211222_160123_HFGoe_IF2_b2R2_2h **Scan** 6996
**Peptide** AERLAAEEQAQRE **Charge** 3 **Spectral Angle** 0.89

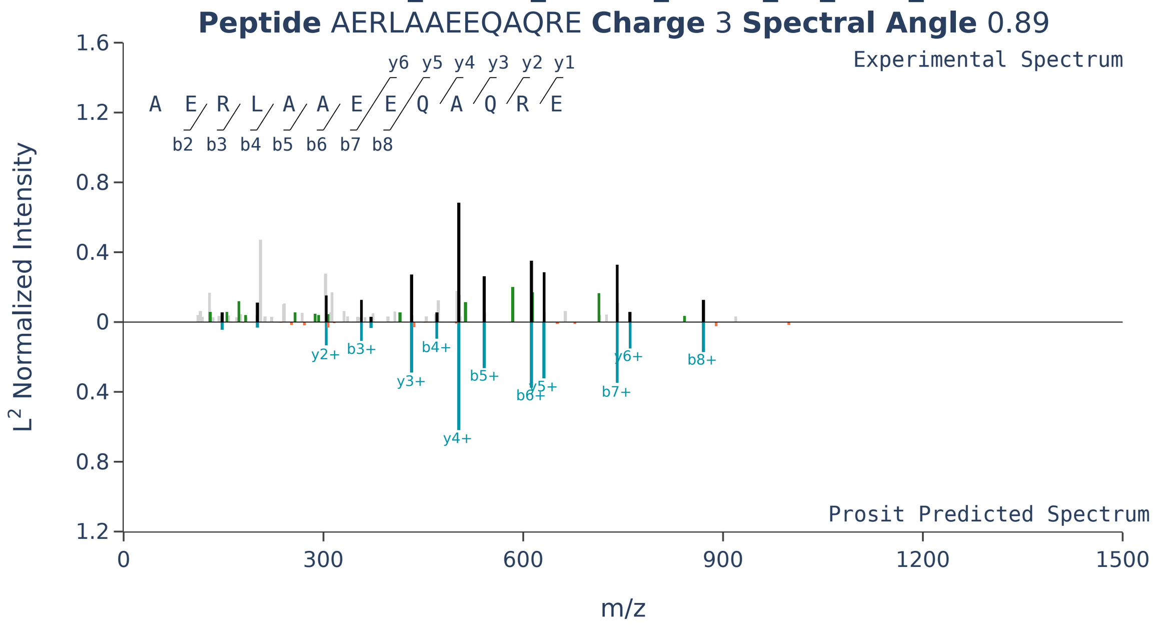


**Source** HRoetschke_211222_160123_HFGoe_IF2_b1R1_4h **Scan** 5852
**Peptide** YHVTTSQHA **Charge** 2 **Spectral Angle** 0.83

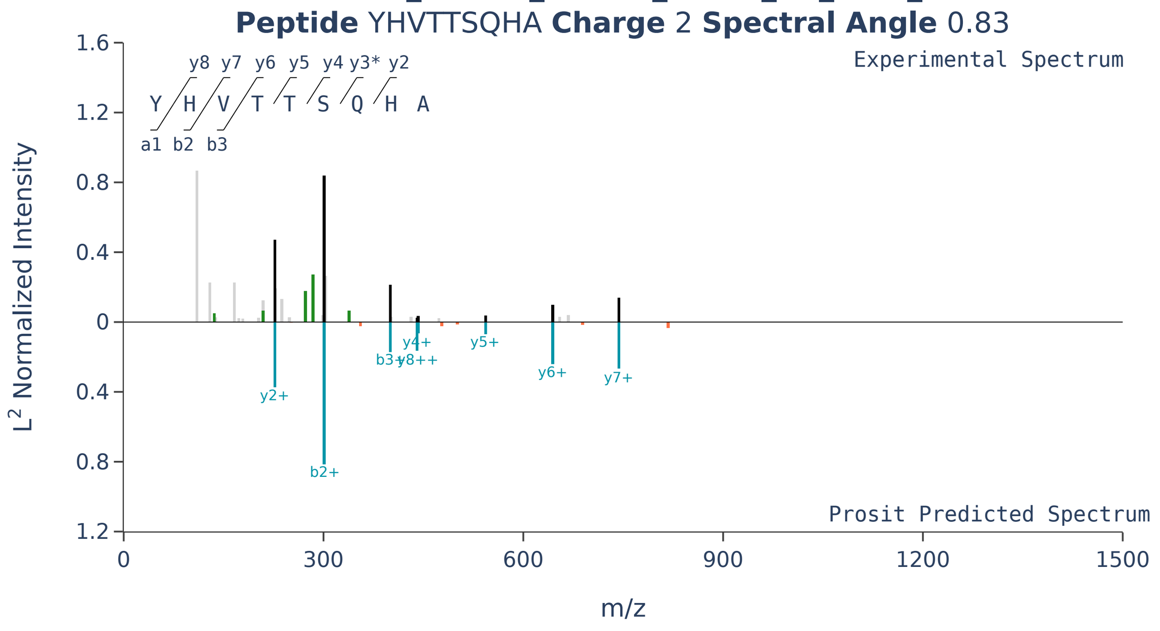


**Source** HRoetschke_211222_160123_HFGoe_IF2_b2R2_4h **Scan** 8066
**Peptide** ANMTEGEVHE **Charge** 2 **Spectral Angle** 0.82

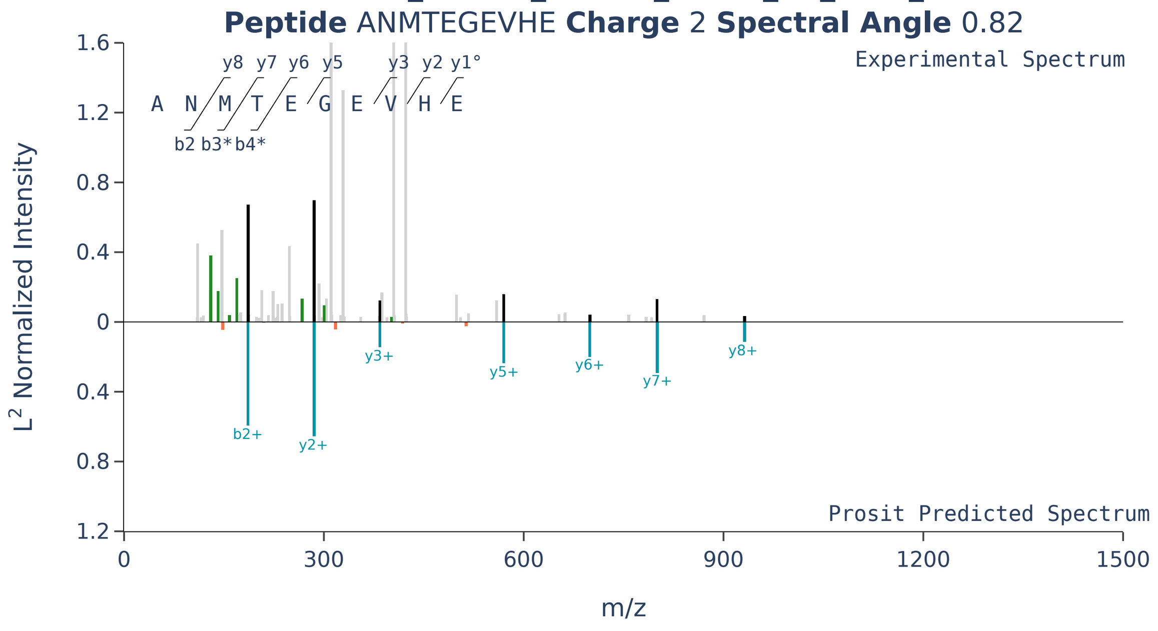


**Source** HRoetschke_211222_160123_HFGoe_IF2_b1R1_4h **Scan** 7386
**Peptide** SGEAGGITQ **Charge** 1 **Spectral Angle** 0.93

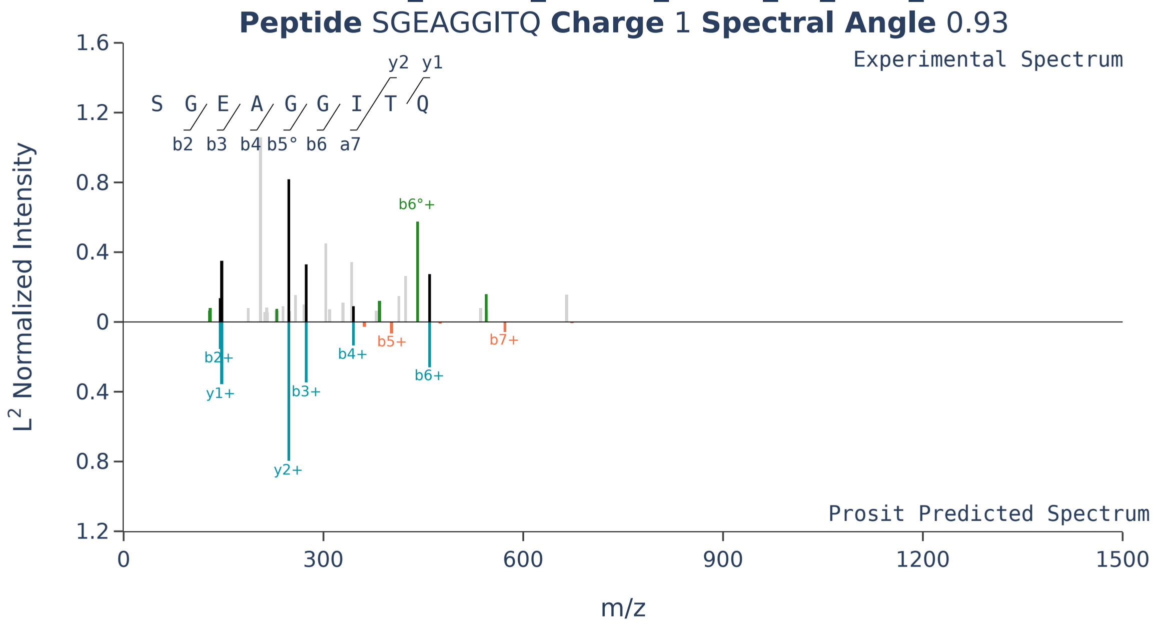


**Source** HRoetschke_211222_160123_HFGoe_IF2_b2R2_24h **Scan** 17952
**Peptide** LLDYIRS **Charge** 2 **Spectral Angle** 0.94

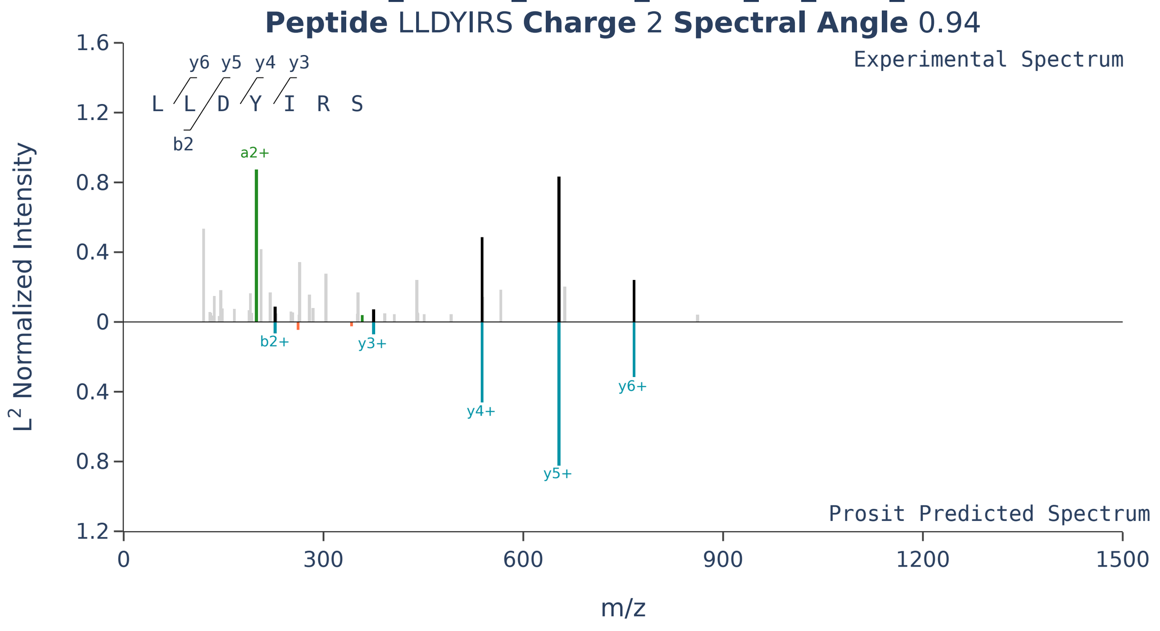

**Source** HRoetschke_211222_160123_HFGoe_IF2_b1R1_2h **Scan** 6579
**Peptide** KLDKPEAD **Charge** 2 **Spectral Angle** 0.89

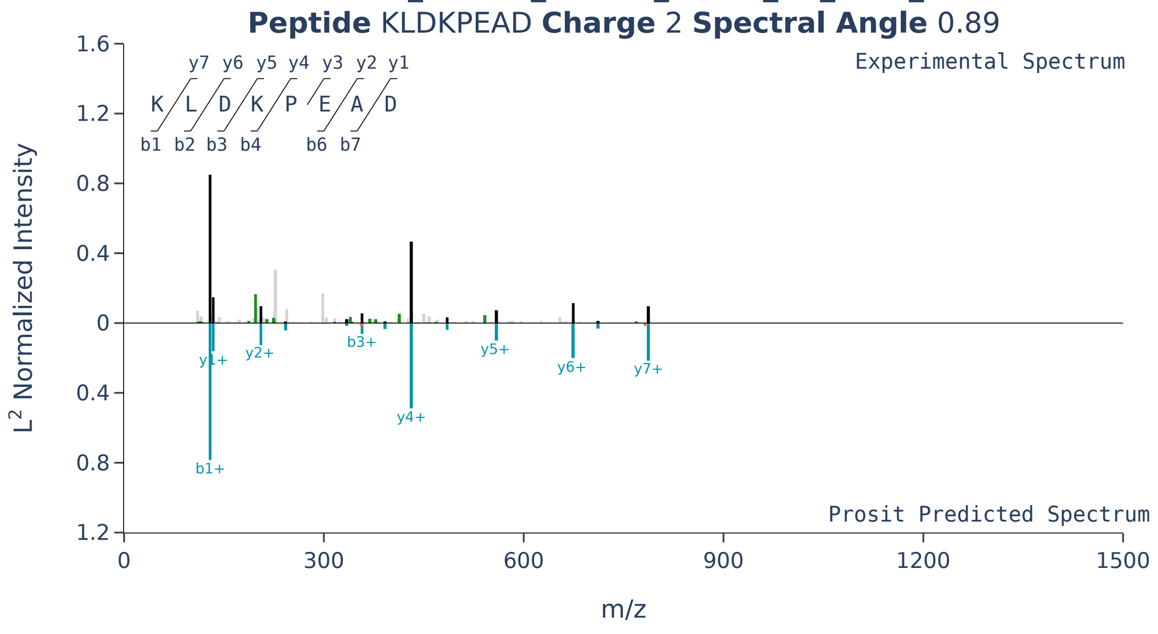


**Source** HRoetschke_211222_160123_HFGoe_IF2_b1R1_24h **Scan** 13339
**Peptide** SGEAGGITQHL **Charge** 2 **Spectral Angle** 0.78

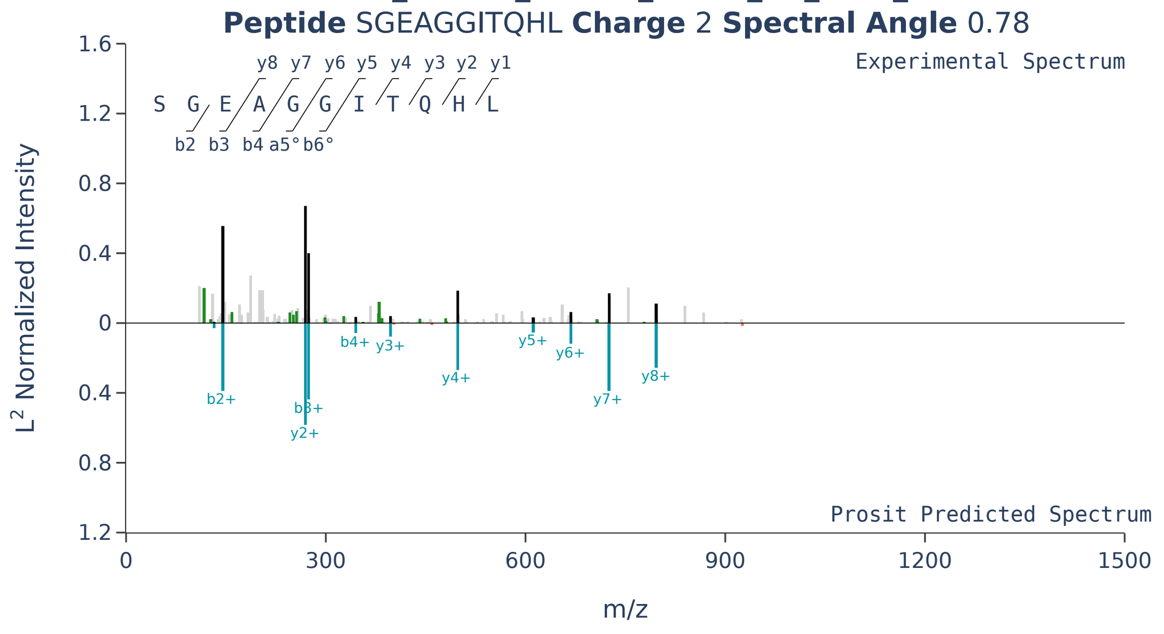


**Source** HRoetschke_211222_160123_HFGoe_IF2_b1R1_4h **Scan** 11107
**Peptide** GFQKPAQAV **Charge** 2 **Spectral Angle** 0.83

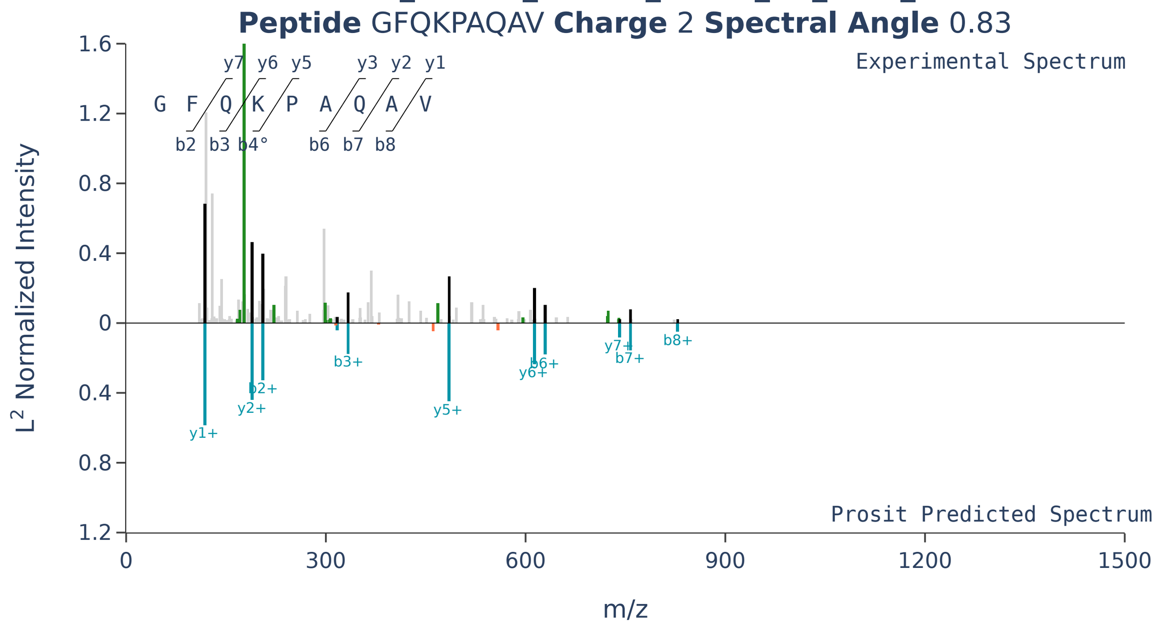


**Source** HRoetschke_211222_160123_HFGoe_IF2_b1R1_4h **Scan** 8105
**Peptide** LELKAVRK **Charge** 2 **Spectral Angle** 0.88

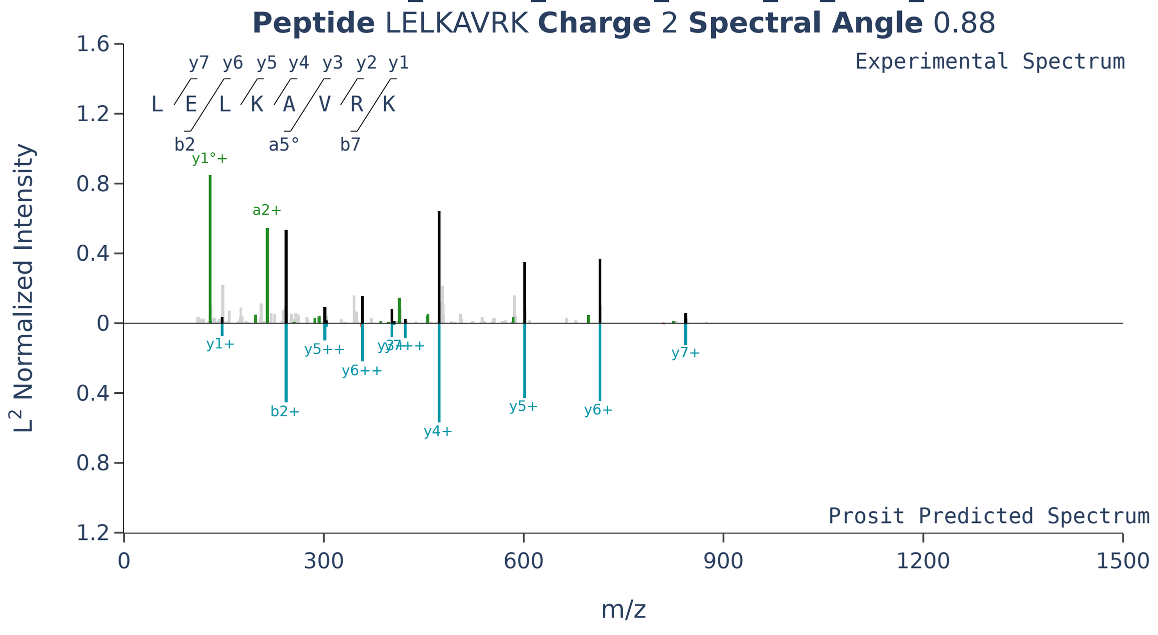


**Source** HRoetschke_211222_160123_HFGoe_IF2_b1R1_4h **Scan** 12900
**Peptide** GSSLQQGFQKPAQA **Charge** 2 **Spectral Angle** 0.88

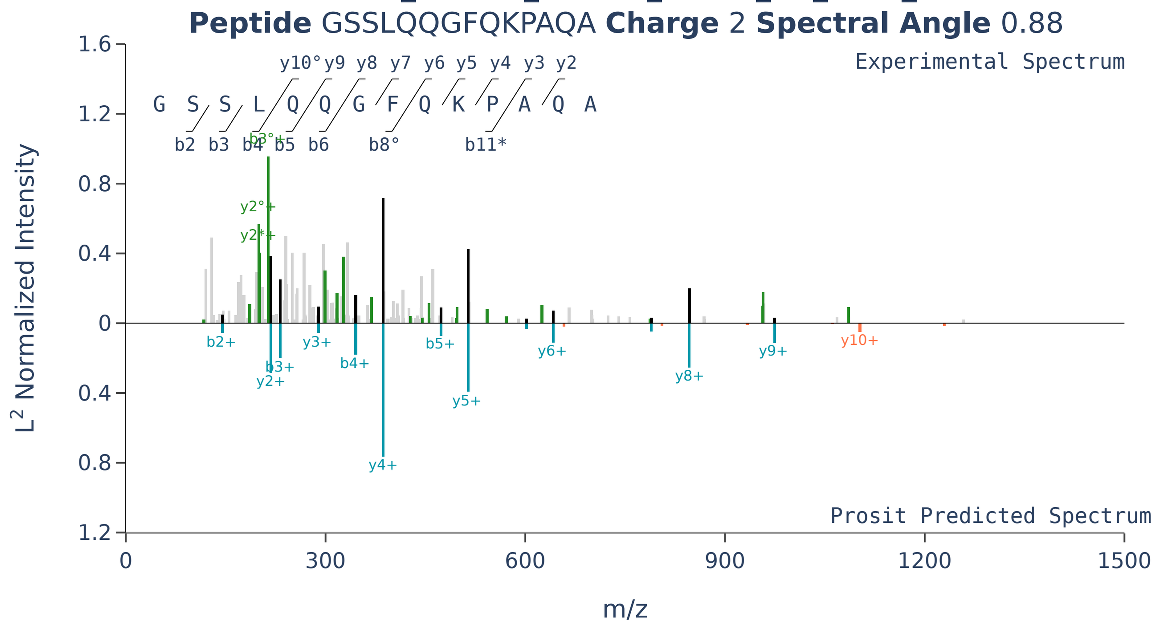


**Source** HRoetschke_211222_160123_HFGoe_IF2_b1R2_2h **Scan** 9207
**Peptide** GLAEVRD **Charge** 2 **Spectral Angle** 0.84

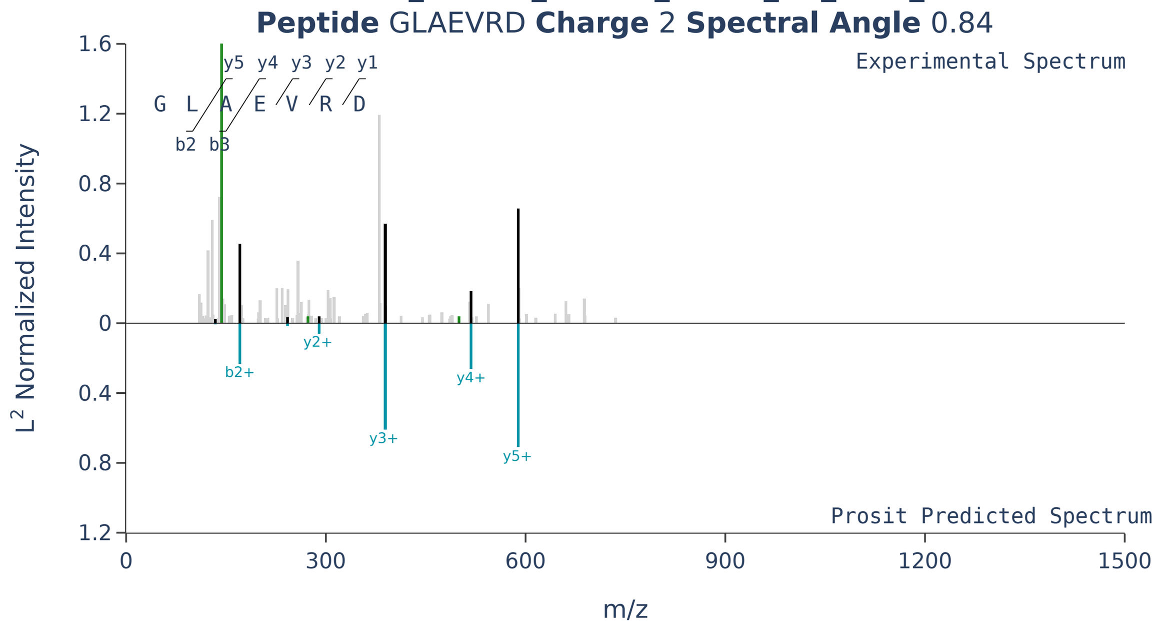


**Source** HRoetschke_211222_160123_HFGoe_IF2_b1R1_2h **Scan** 8494
**Peptide** MVTEGVVKR **Charge** 2 **Spectral Angle** 0.85

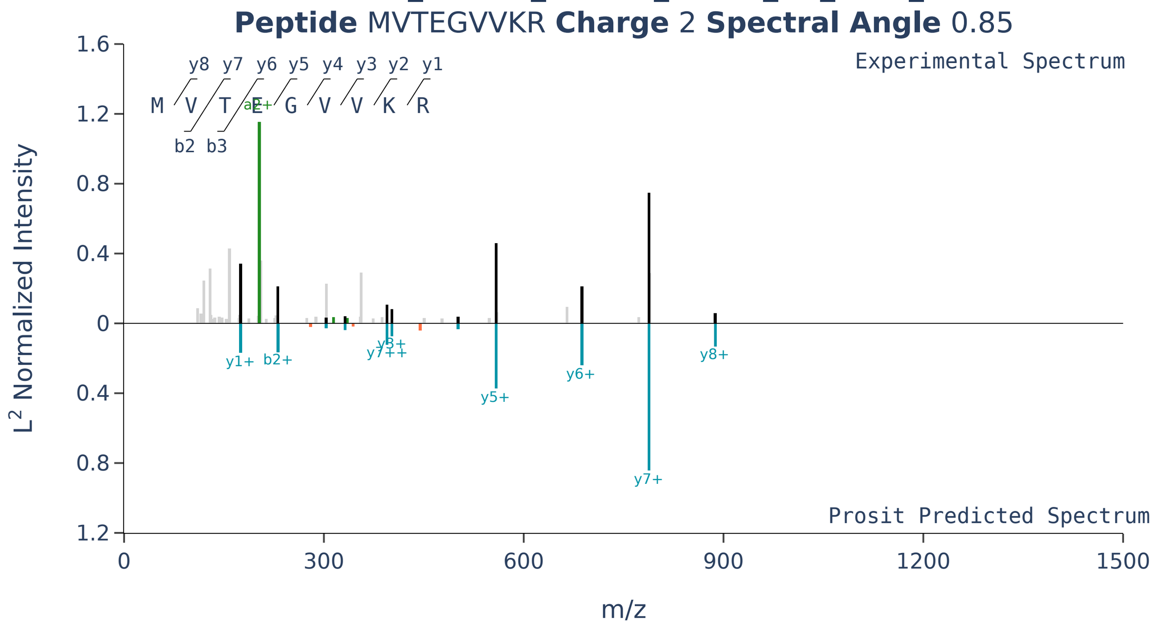


**Source** HRoetschke_211222_160123_HFGoe_IF2_b1R1_4h **Scan** 11527
**Peptide** LAAERQTSVERL **Charge** 3 **Spectral Angle** 0.89

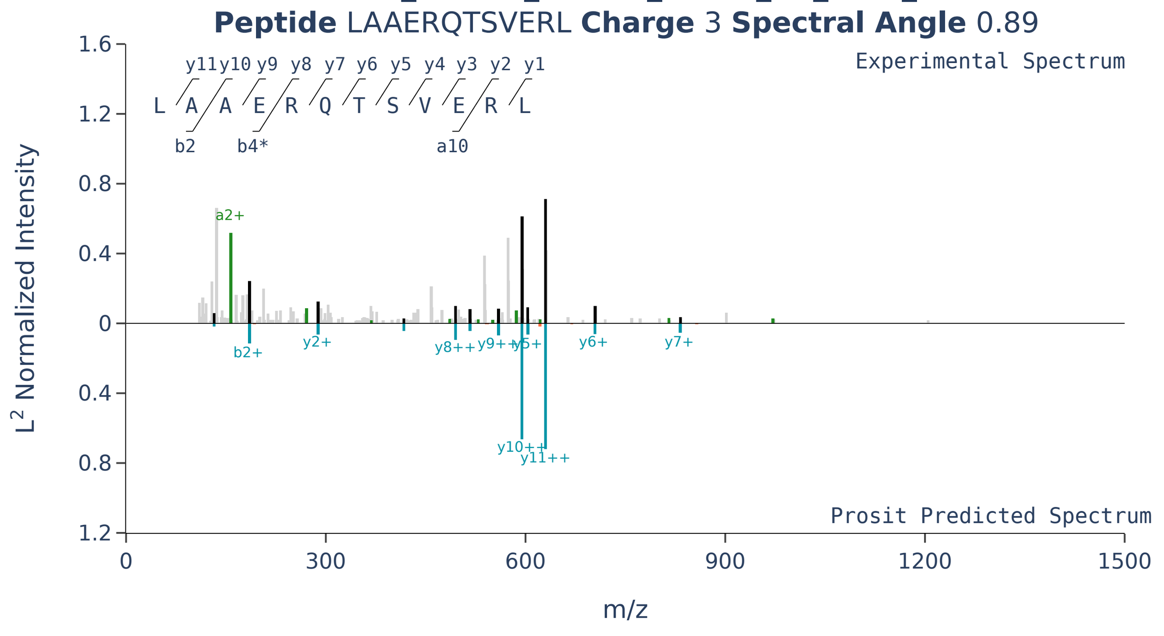


**Source** HRoetschke_211222_160123_HFGoe_IF2_b1R2_1h **Scan** 13520
**Peptide** RLVQQFAD **Charge** 2 **Spectral Angle** 0.86

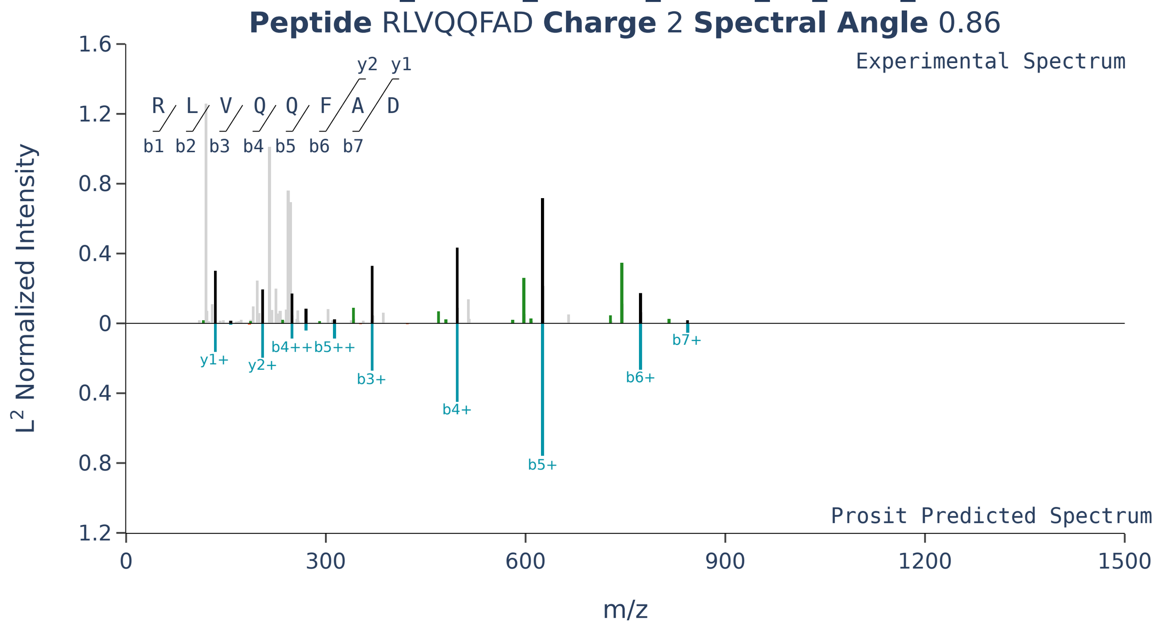


**Source** HRoetschke_211222_160123_HFGoe_IF2_b2R1_4h **Scan** 12970
**Peptide** SSLQQGFQ **Charge** 2 **Spectral Angle** 0.95

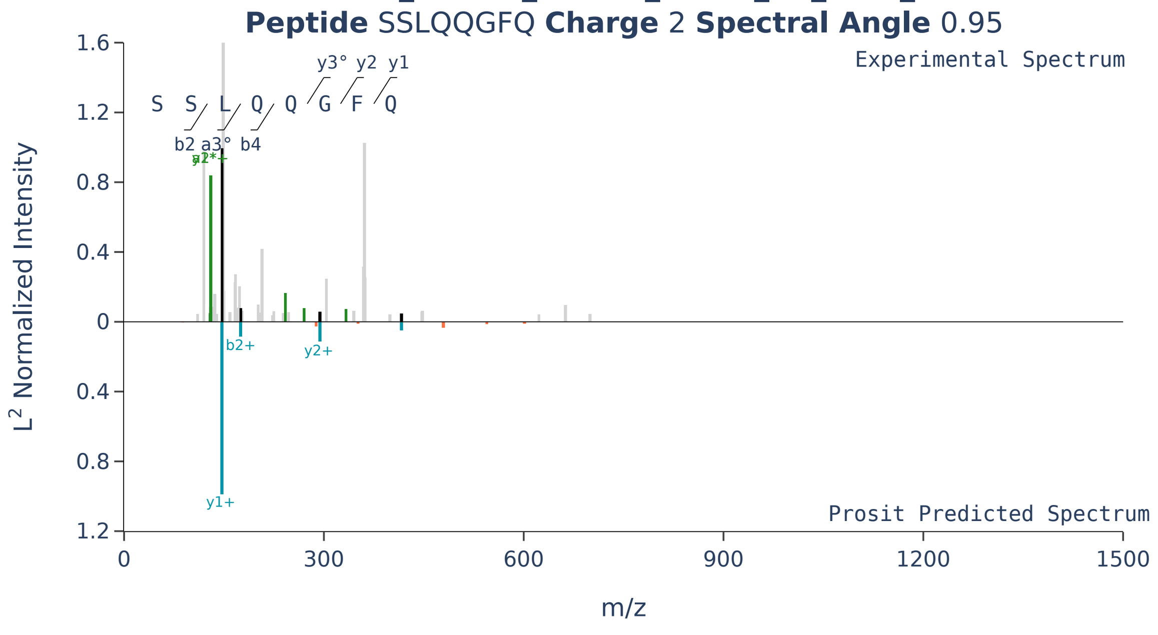


**Source** HRoetschke_211222_160123_HFGoe_IF2_b2R2_24h **Scan** 12816
**Peptide** TAQLVAE **Charge** 1 **Spectral Angle** 0.91

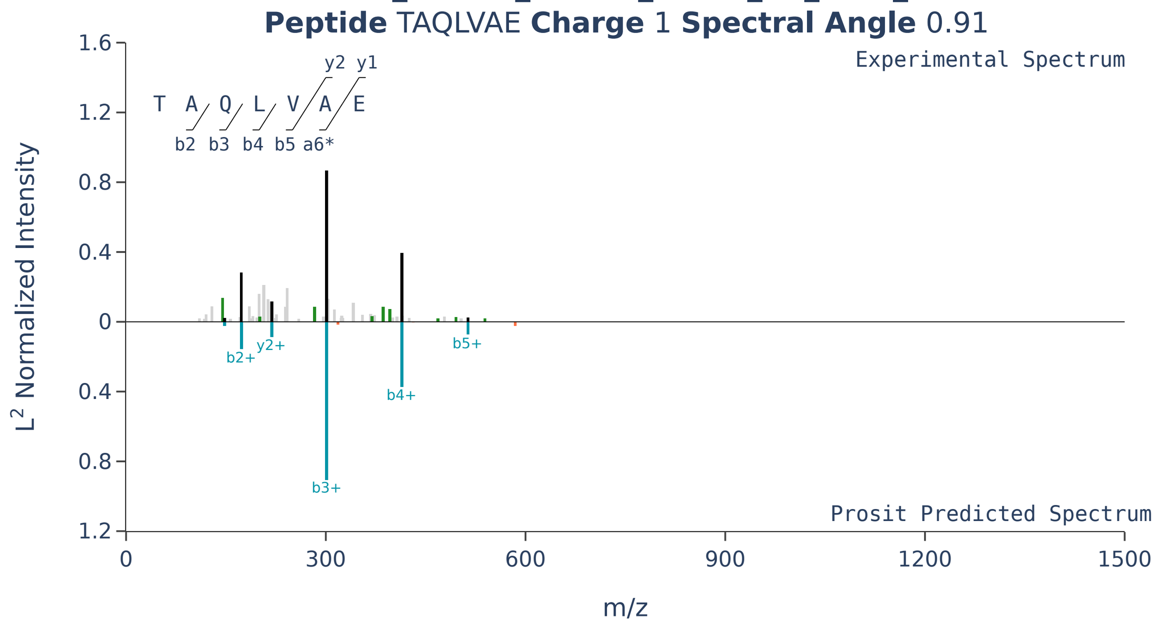


**Source** HRoetschke_211222_160123_HFGoe_IF2_b1R1_2h **Scan** 6891
**Peptide** EMGHKVILRRENE **Charge** 3 **Spectral Angle** 0.84

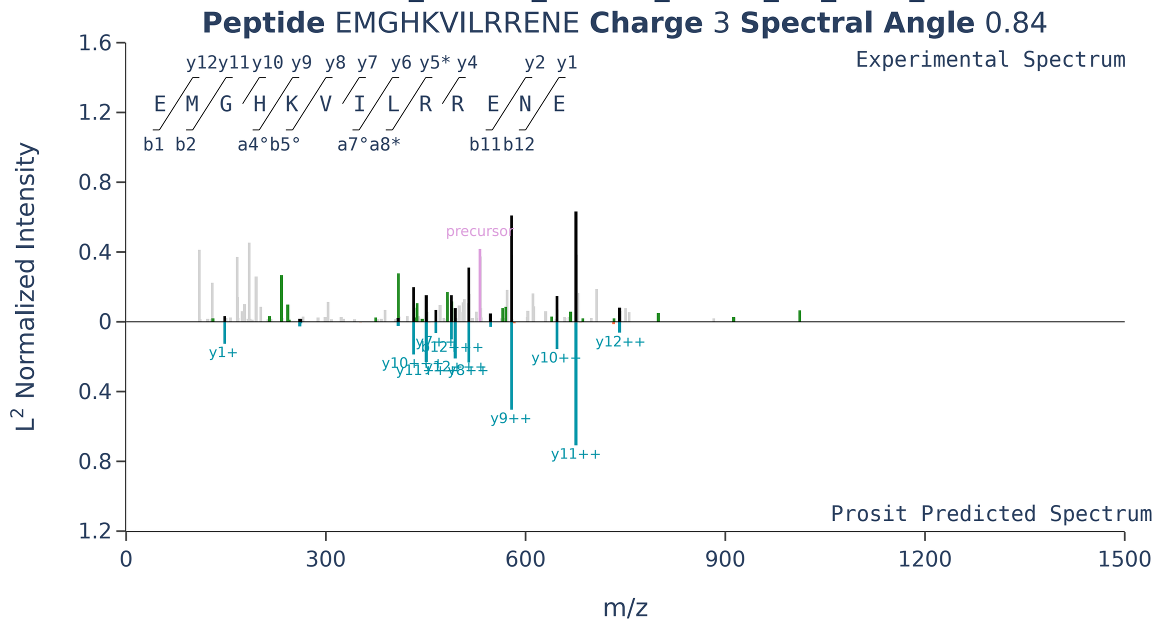


**Source** HRoetschke_211222_160123_HFGoe_IF2_b2R2_24h **Scan** 11872
**Peptide** GEAGGITQHIG **Charge** 2 **Spectral Angle** 0.86

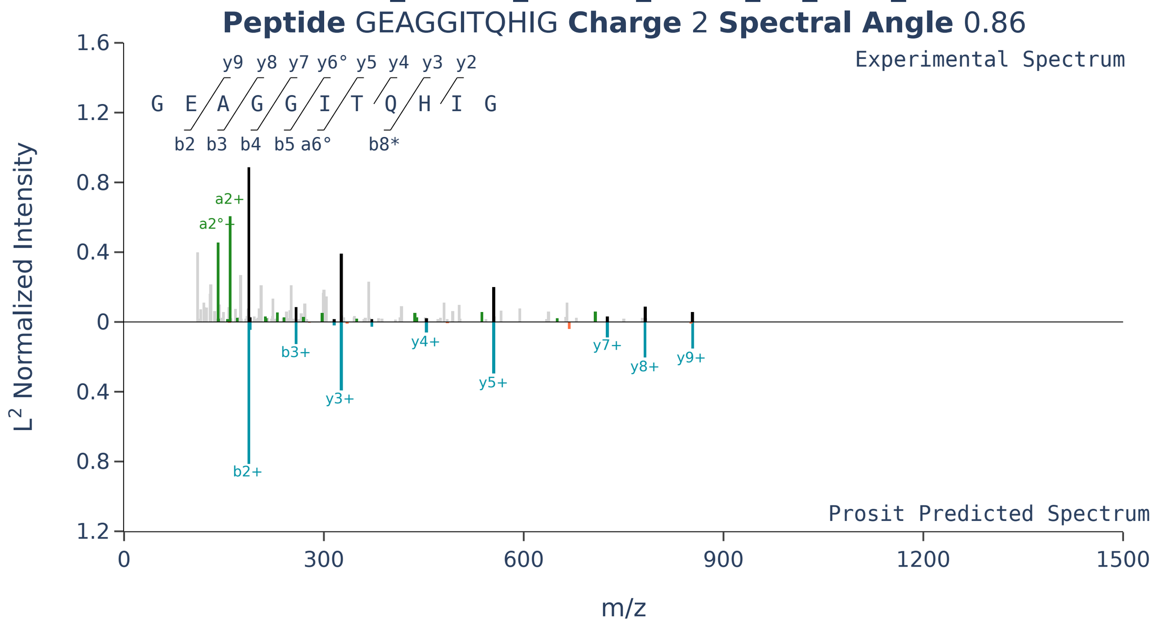


**Source** HRoetschke_211222_160123_HFGoe_IF2_b2R2_4h **Scan** 7367
**Peptide** PAQAVNRDV **Charge** 2 **Spectral Angle** 0.81

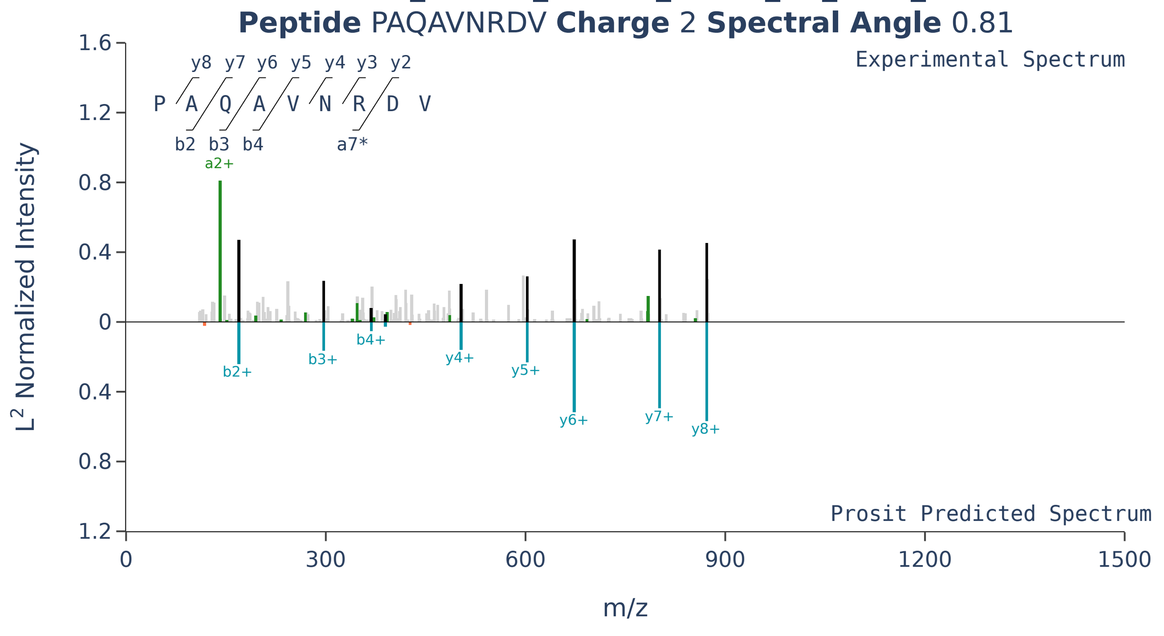


**Source** HRoetschke_211222_160123_HFGoe_IF2_b2R2_2h **Scan** 14814
**Peptide** GESQFVHV **Charge** 2 **Spectral Angle** 0.92

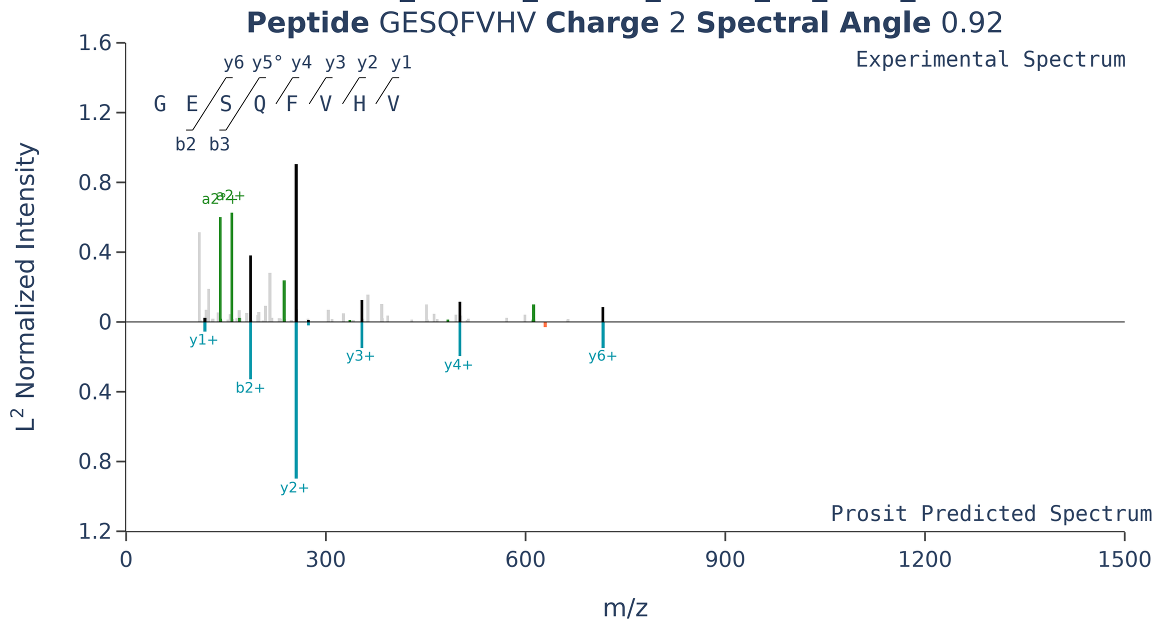

**Source** HRoetschke_211222_160123_HFGoe_IF2_b1R1_24h **Scan** 10139
**Peptide** AVKGSQVI **Charge** 1 **Spectral Angle** 0.77

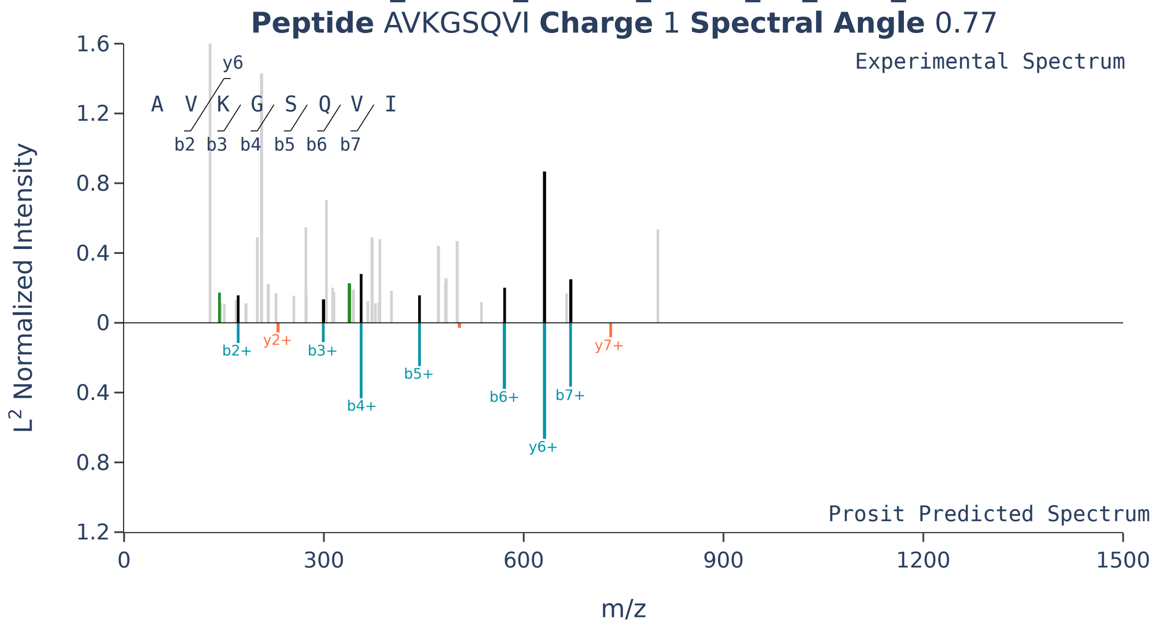


**Source** HRoetschke_211222_160123_HFGoe_IF2_b1R1_4h **Scan** 5699
**Peptide** SSDYHVTTSQHARQAE **Charge** 2 **Spectral Angle** 0.84

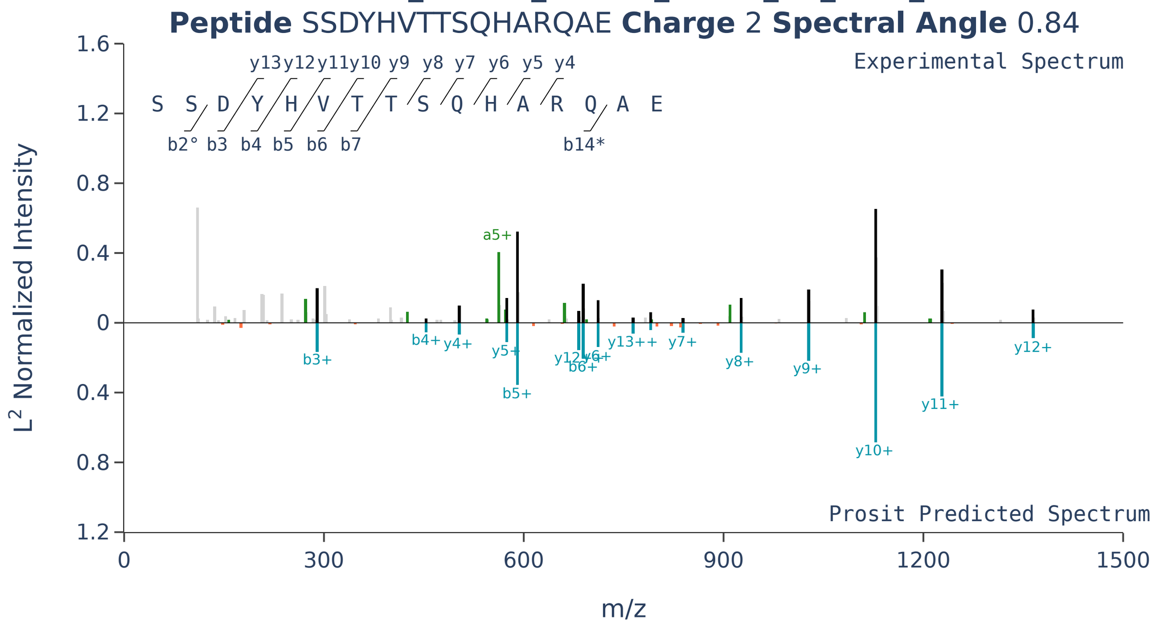


**Source** HRoetschke_211222_160123_HFGoe_IF2_b1R1_24h **Scan** 16438
**Peptide** QAEVLELKAVR **Charge** 3 **Spectral Angle** 0.89

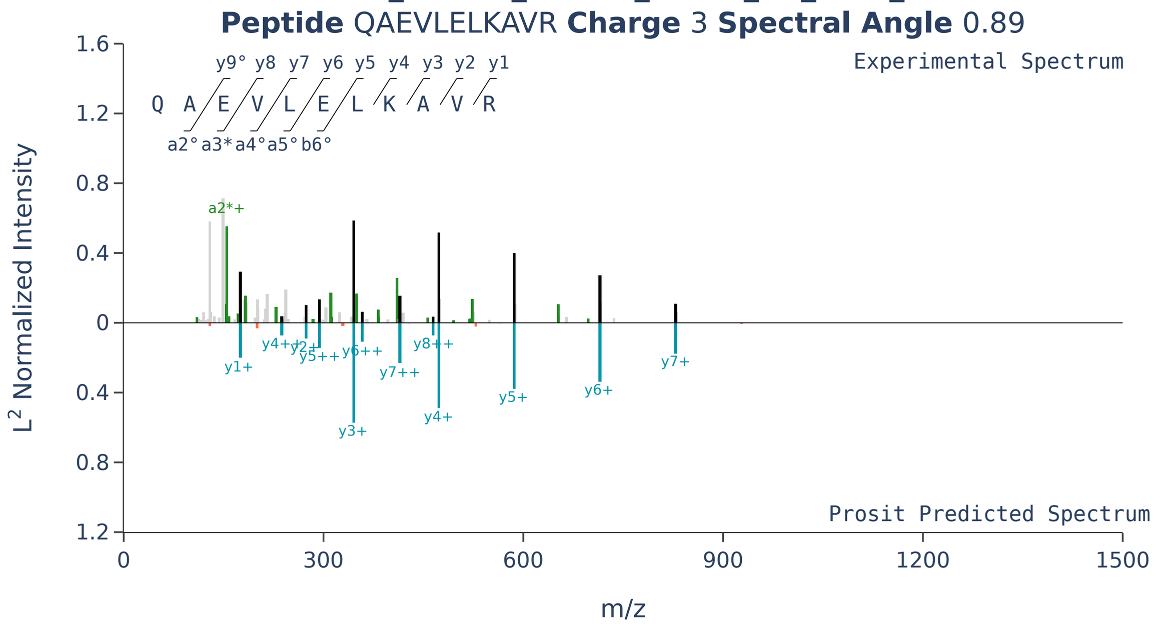


**Source** HRoetschke_211222_160123_HFGoe_IF2_b1R1_4h **Scan** 11411
**Peptide** FLDKGRGPVA **Charge** 2 **Spectral Angle** 0.91

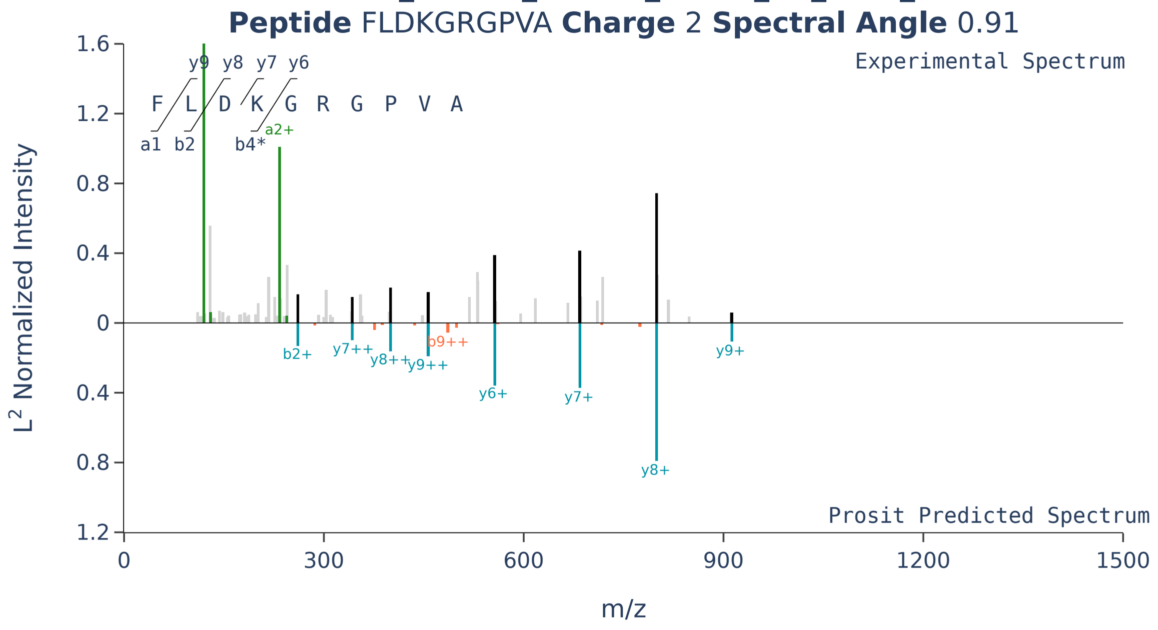


**Source** HRoetschke_211222_160123_HFGoe_IF2_b1R1_2h **Scan** 9328
**Peptide** EEAVMSD **Charge** 1 **Spectral Angle** 0.86

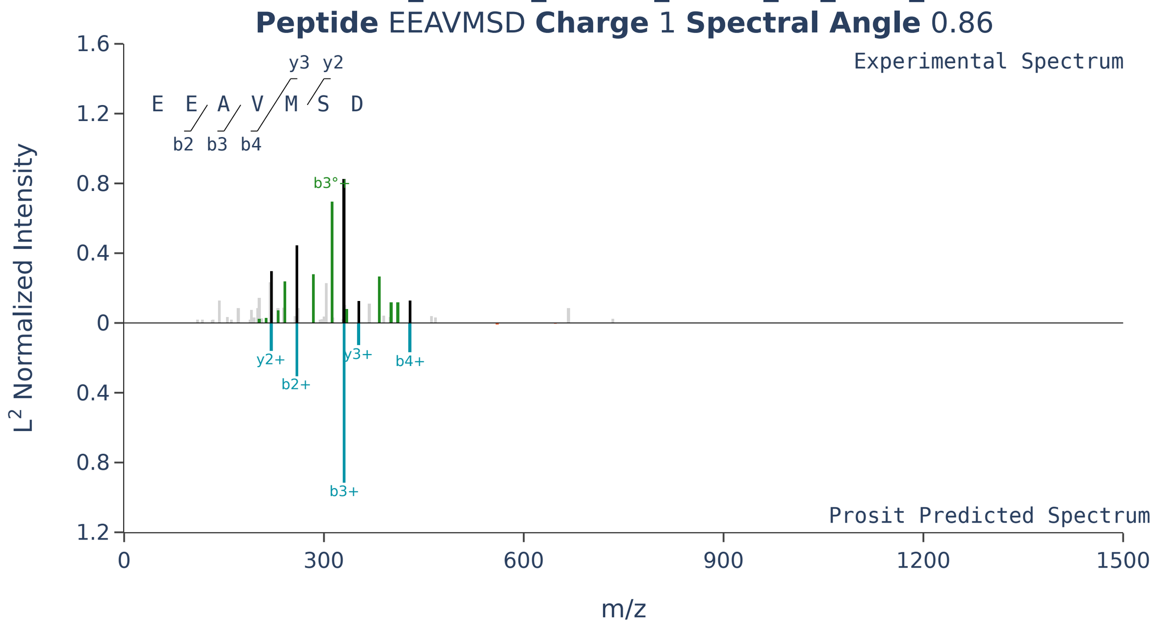


**Source** HRoetschke_211222_160123_HFGoe_IF2_b1R1_24h **Scan** 9046
**Peptide** SVQIEVRK **Charge** 2 **Spectral Angle** 0.84

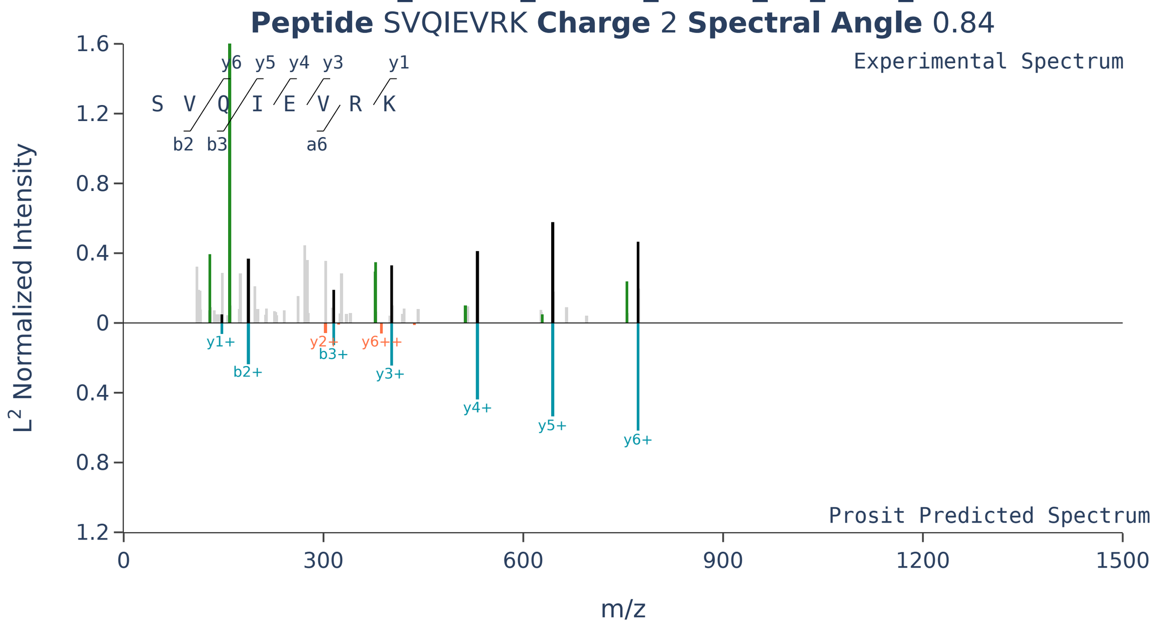


**Source** HRoetschke_211222_160123_HFGoe_IF2_b1R1_4h **Scan** 7201
**Peptide** MGHKVILR **Charge** 2 **Spectral Angle** 0.91

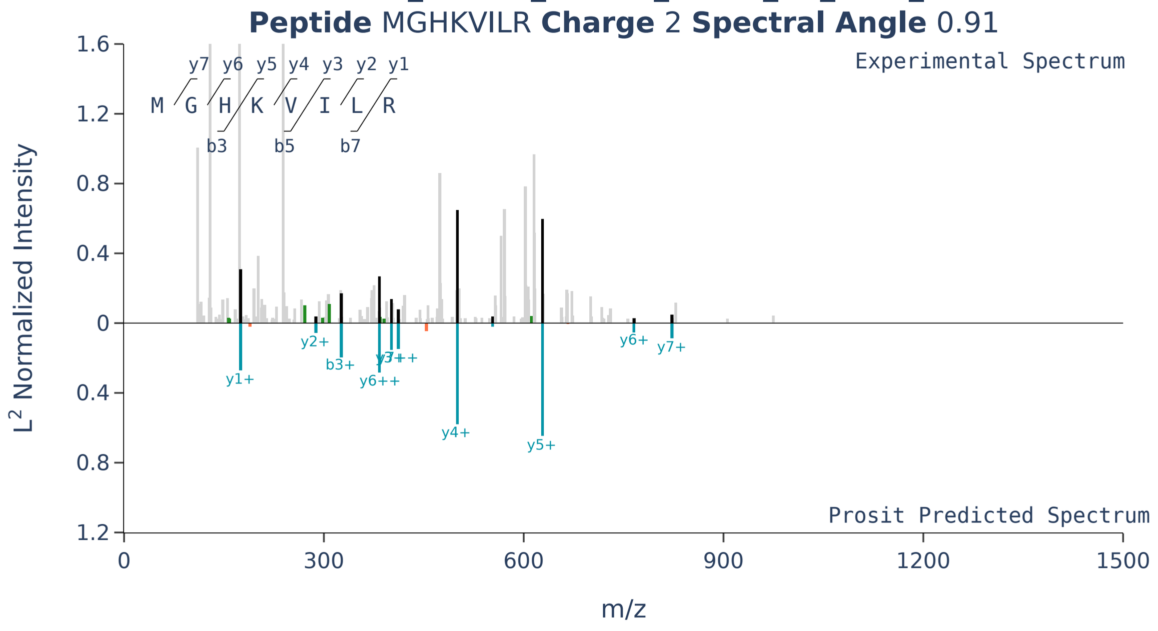


**Source** HRoetschke_211222_160123_HFGoe_IF2_b1R2_4h **Scan** 14186
**Peptide** NELEEAVM **Charge** 2 **Spectral Angle** 0.94

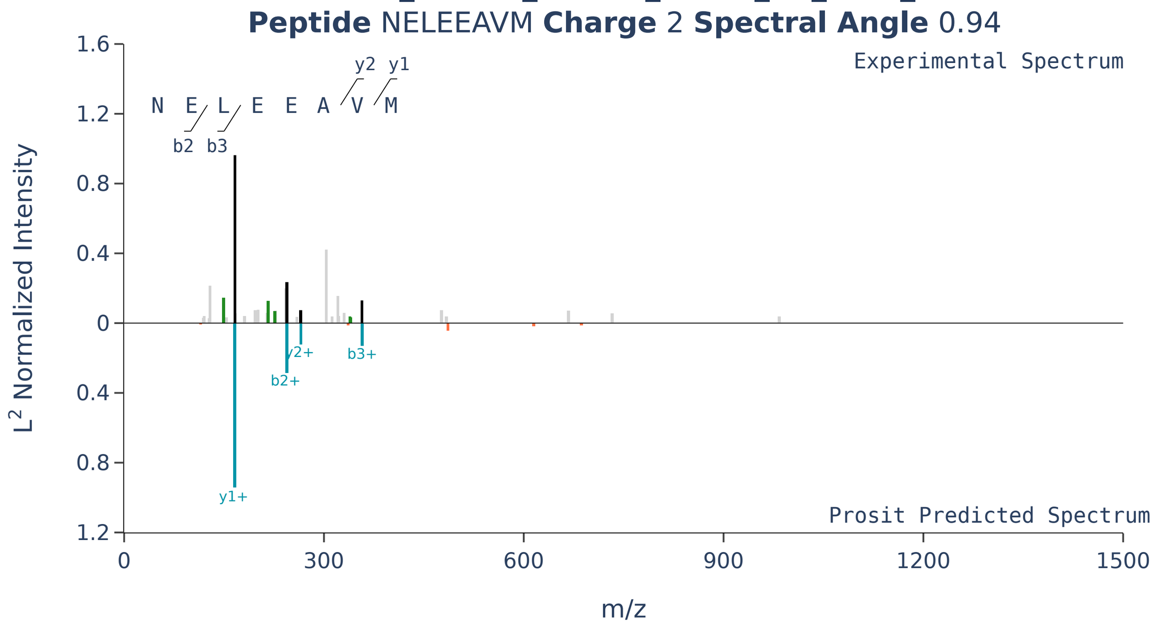


**Source** HRoetschke_211222_160123_HFGoe_IF2_b1R1_1h **Scan** 9066
**Peptide** DSSDYHVTT **Charge** 1 **Spectral Angle** 0.91

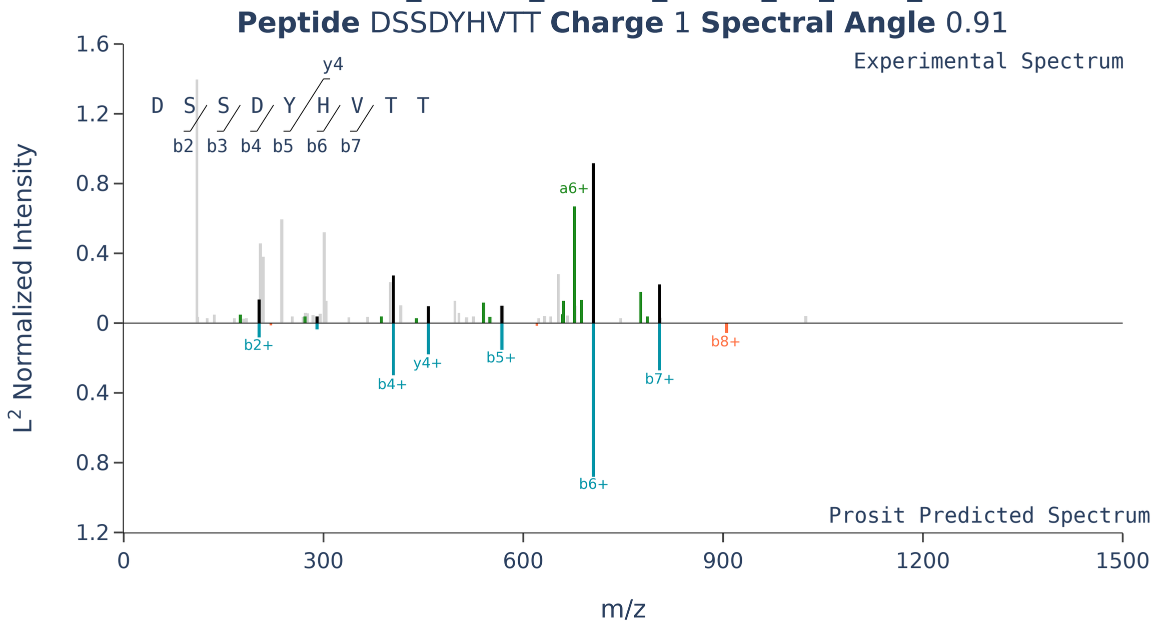


**Source** HRoetschke_211222_160123_HFGoe_IF2_b2R2_24h **Scan** 7878
**Peptide** NKWTDNAE **Charge** 2 **Spectral Angle** 0.87

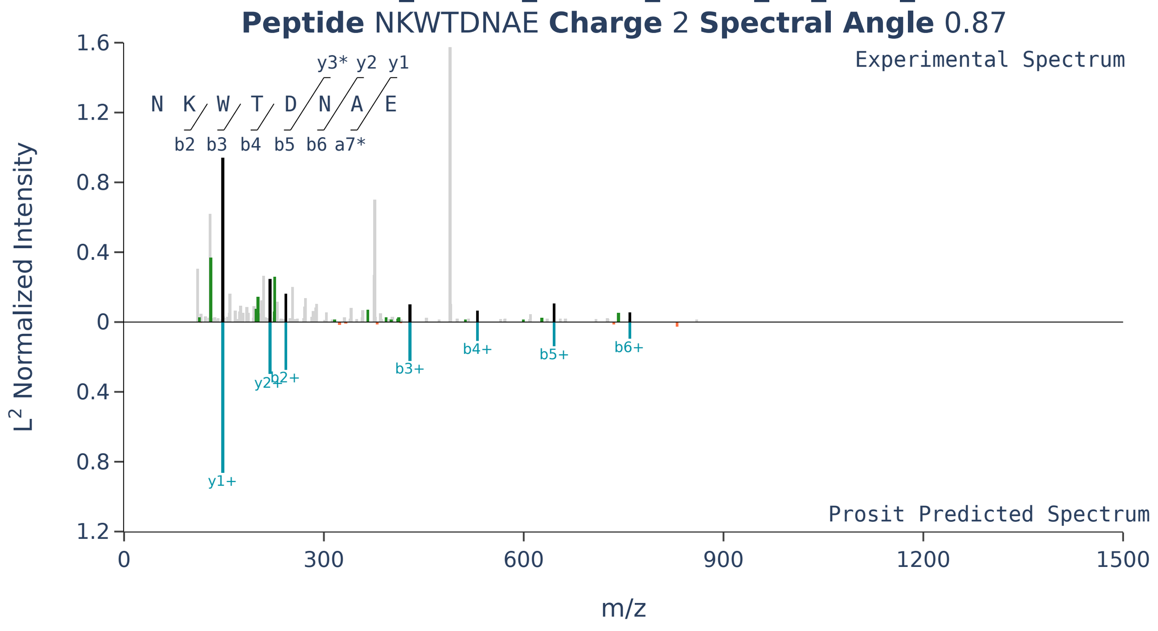


**Source** HRoetschke_211222_160123_HFGoe_IF2_b1R2_24h **Scan** 15828
**Peptide** ESQFVHV **Charge** 1 **Spectral Angle** 0.94

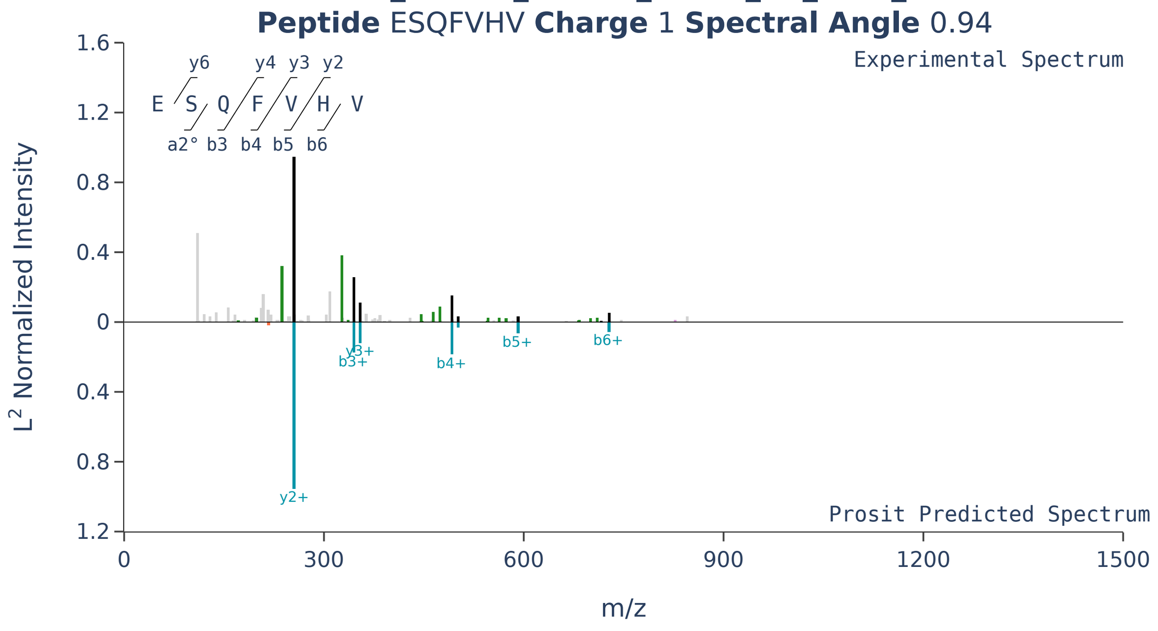


**Source** HRoetschke_211222_160123_HFGoe_IF2_b2R2_2h **Scan** 11757
**Peptide** SGEAGGITQHIG **Charge** 2 **Spectral Angle** 0.81

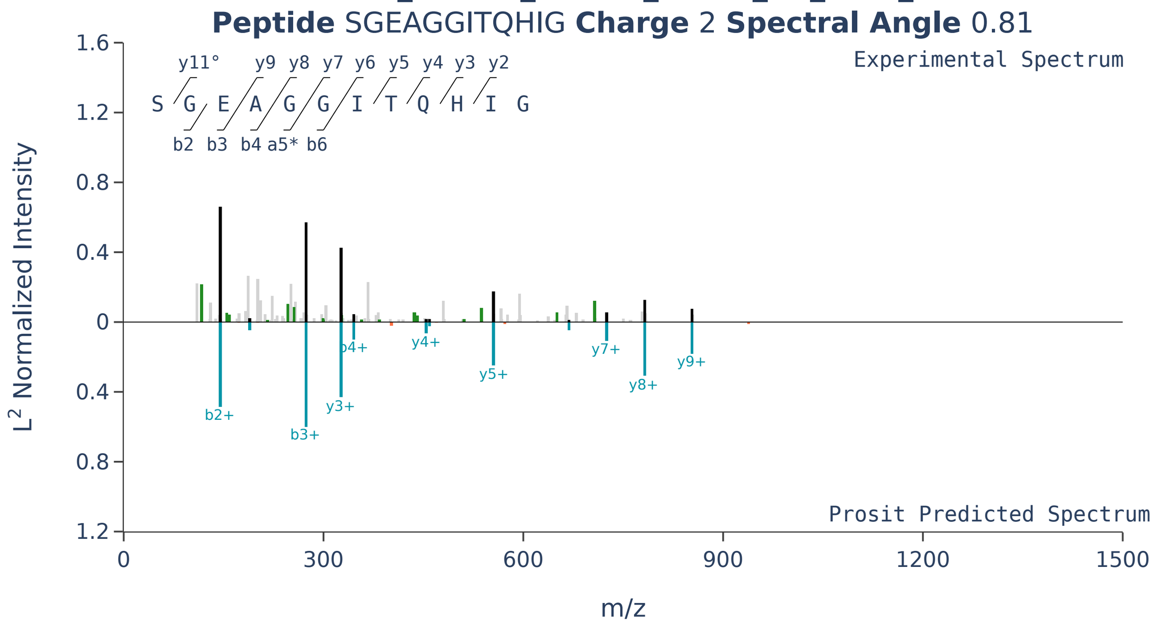


**Source** HRoetschke_211222_160123_HFGoe_IF2_b1R1_1h **Scan** 10181
**Peptide** RNELGQEV **Charge** 2 **Spectral Angle** 0.87

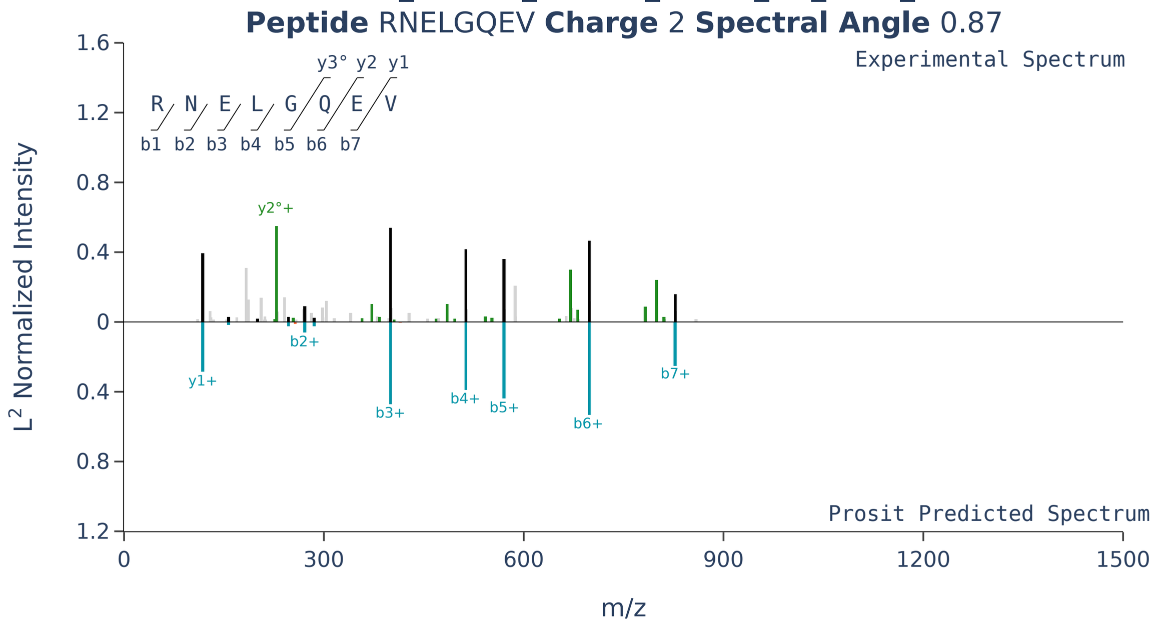


**Source** HRoetschke_211222_160123_HFGoe_IF2_b1R1_24h **Scan** 6445
**Peptide** LAAEEQAQRE **Charge** 2 **Spectral Angle** 0.83

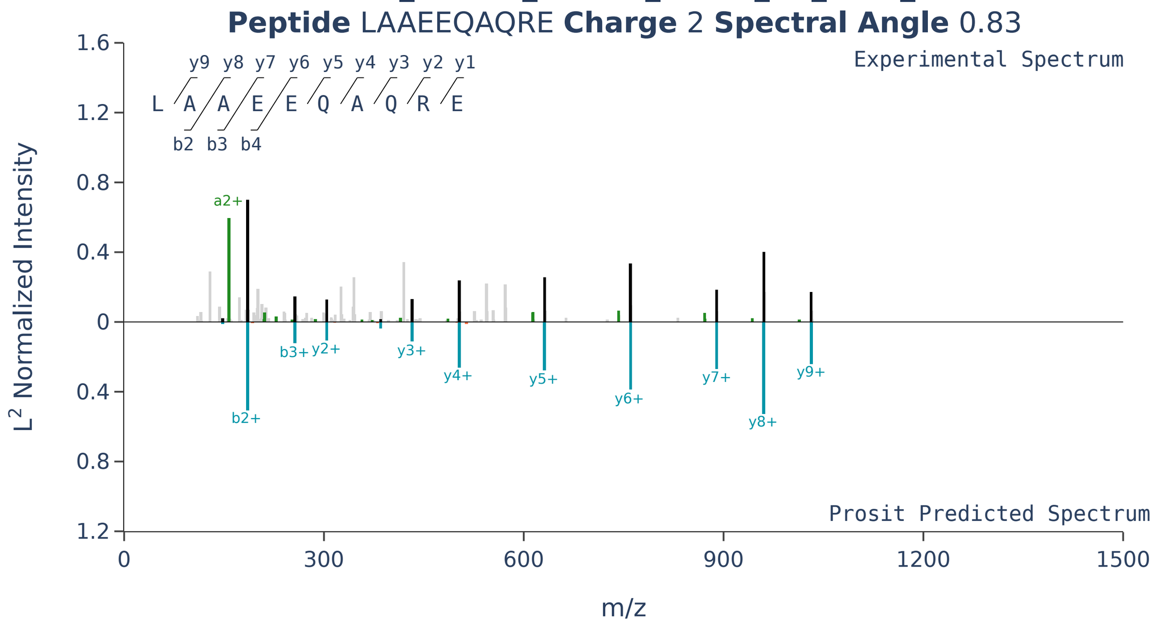


**Source** HRoetschke_211222_160123_HFGoe_IF2_b1R1_24h **Scan** 6644
**Peptide** DSSDYHVTTSQH **Charge** 2 **Spectral Angle** 0.91

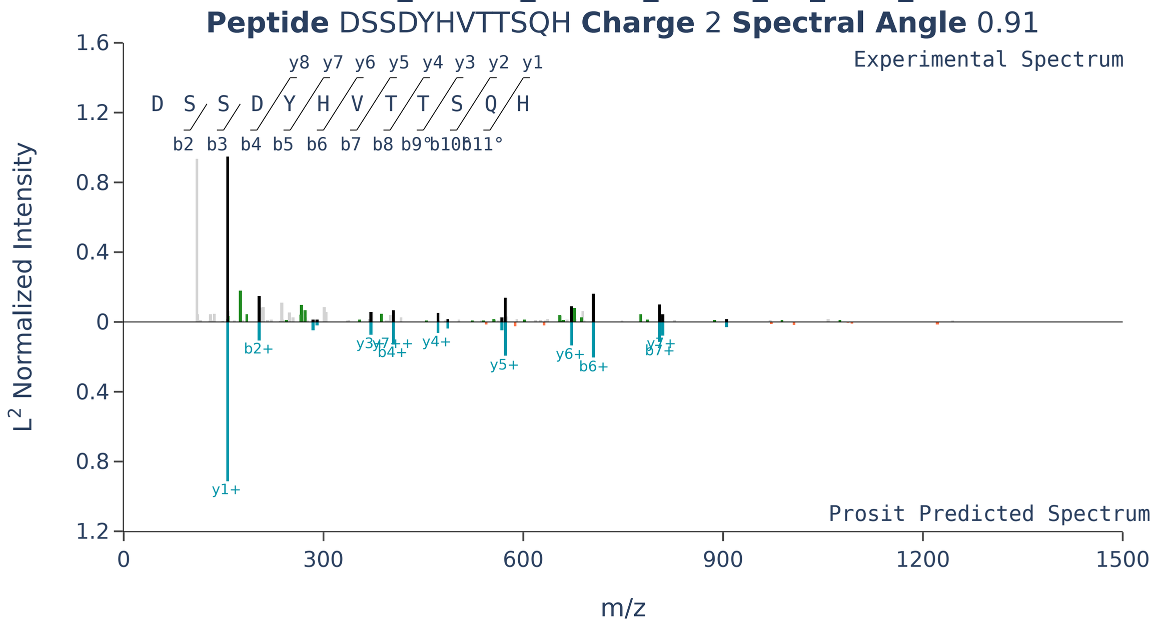

**Source** HRoetschke_211222_160123_HFGoe_IF2_b1R2_2h **Scan** 9145
**Peptide** LDKPEADPDRV **Charge** 3 **Spectral Angle** 0.9

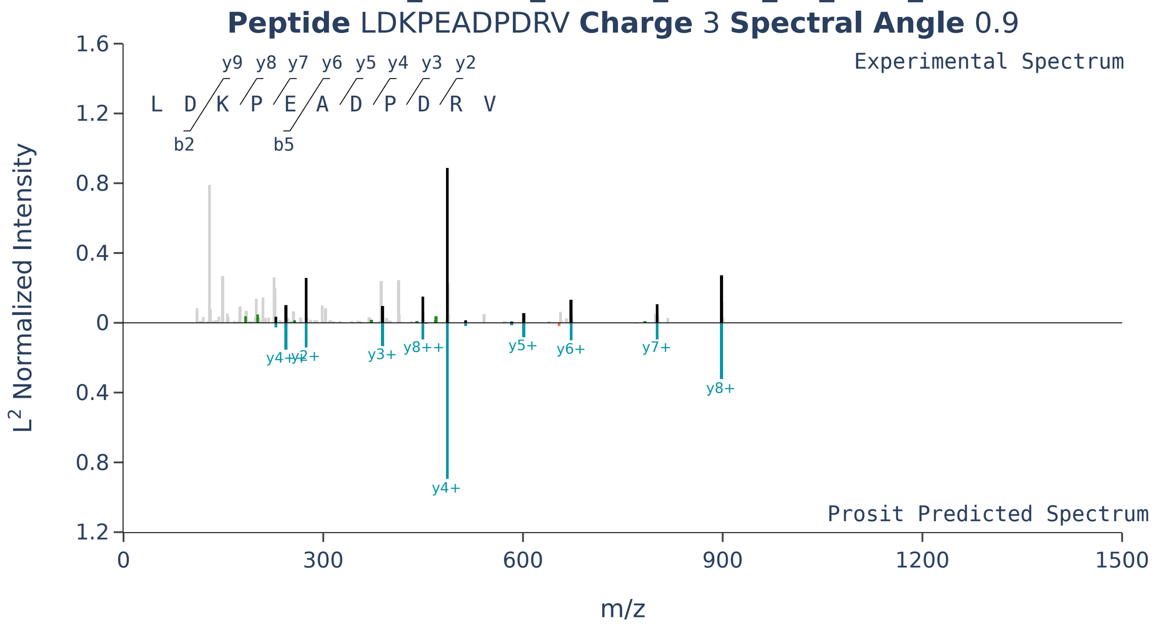


**Source** HRoetschke_211222_160123_HFGoe_IF2_b1R1_2h **Scan** 10256
**Peptide** HLGAYHVETE **Charge** 2 **Spectral Angle** 0.85

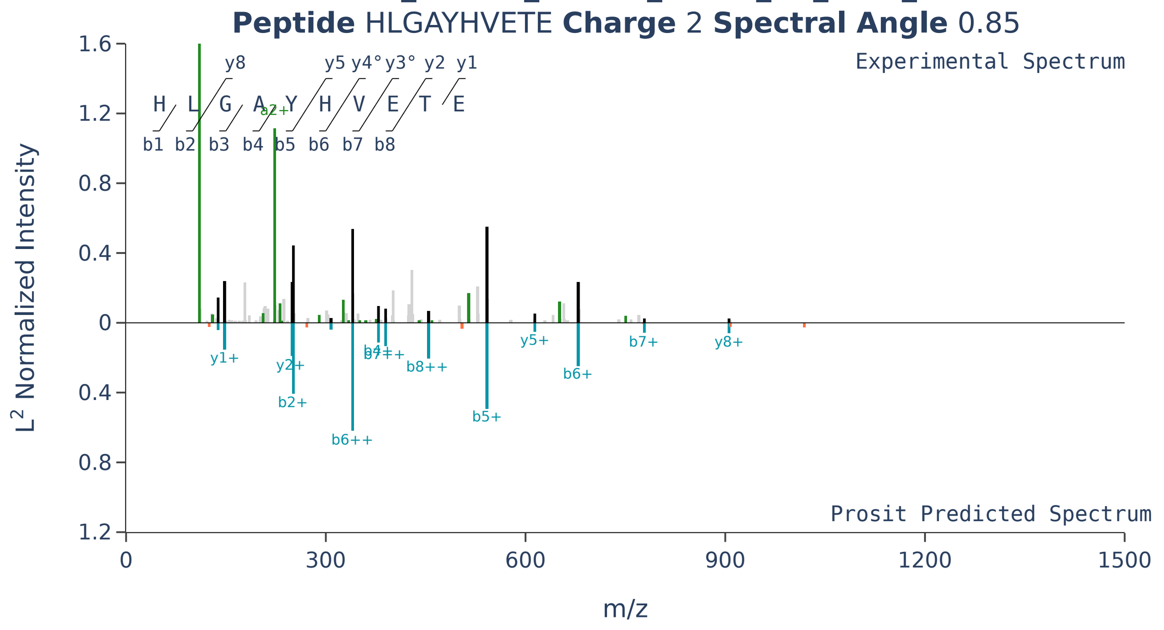


**Source** HRoetschke_211222_160123_HFGoe_IF2_b2R2_2h **Scan** 15004
**Peptide** DVQGSVEAISD **Charge** 2 **Spectral Angle** 0.93

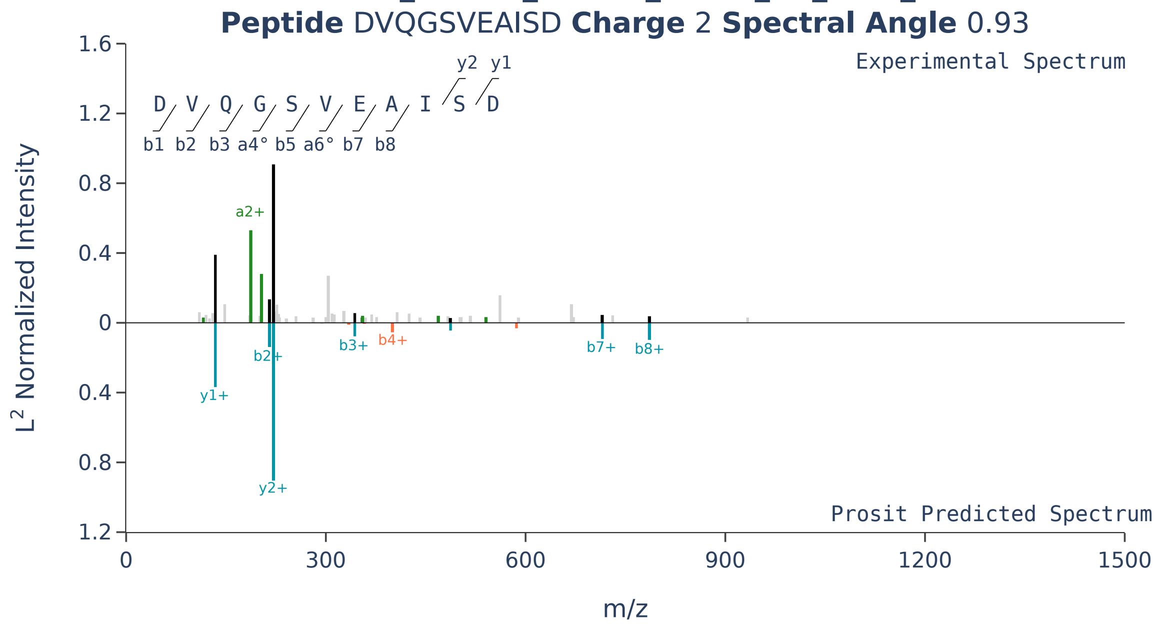


**Source** HRoetschke_211222_160123_HFGoe_IF2_b2R1_24h **Scan** 11647
**Peptide** SLQQGFQKPAQAVNR **Charge** 3 **Spectral Angle** 0.91

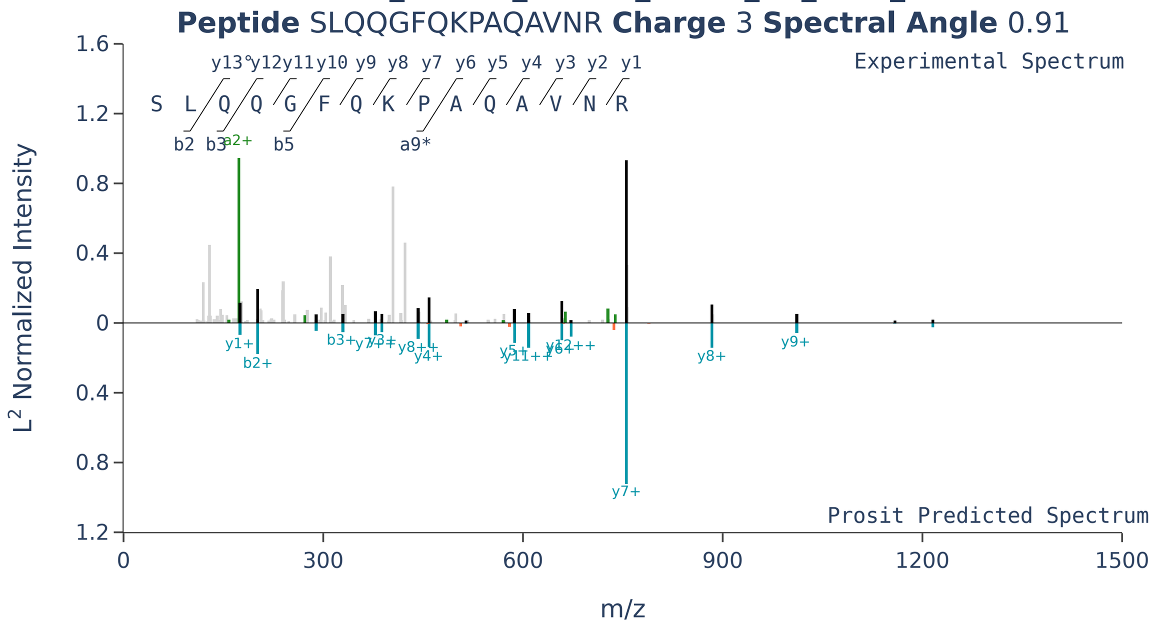


**Source** HRoetschke_211222_160123_HFGoe_IF2_b1R1_1h **Scan** 9010
**Peptide** TPGHAAFTSM **Charge** 2 **Spectral Angle** 0.88

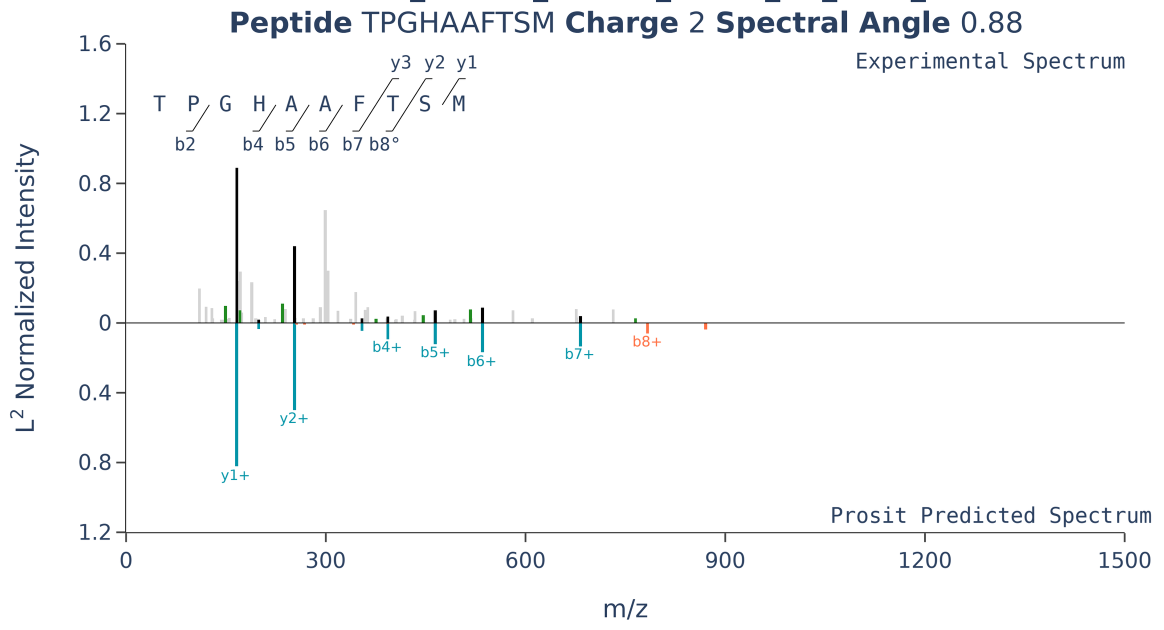


**Source** HRoetschke_211222_160123_HFGoe_IF2_b1R1_24h **Scan** 10737
**Peptide** EKQTLID **Charge** 1 **Spectral Angle** 0.78

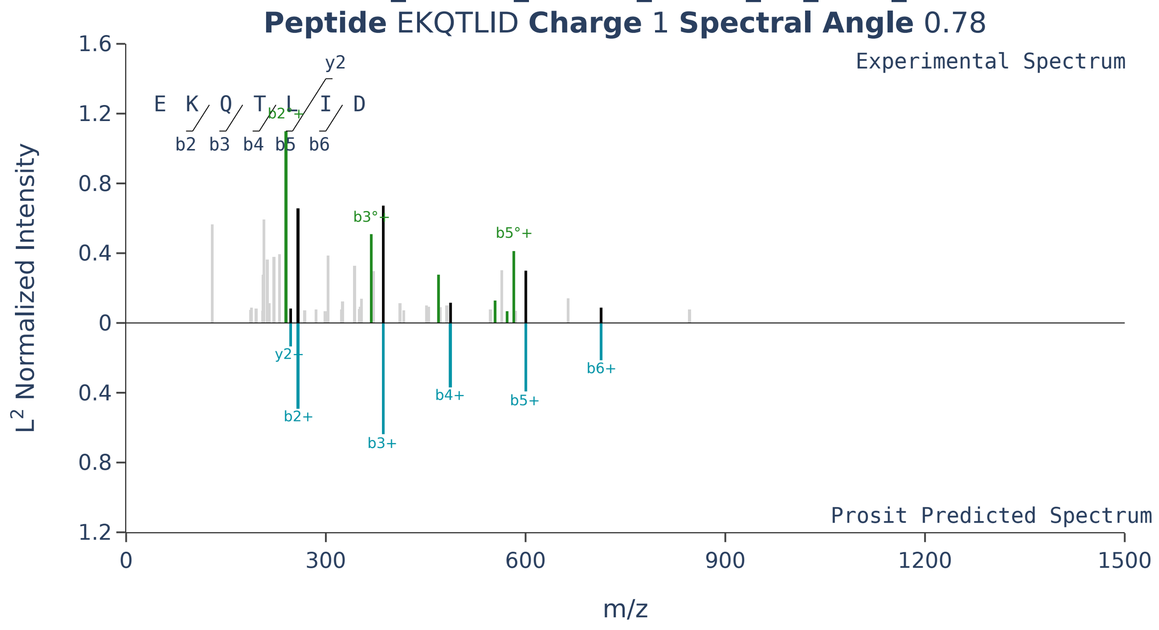


**Source** HRoetschke_211222_160123_HFGoe_IF2_b2R2_4h **Scan** 7699
**Peptide** RDPQEAERL **Charge** 3 **Spectral Angle** 0.93

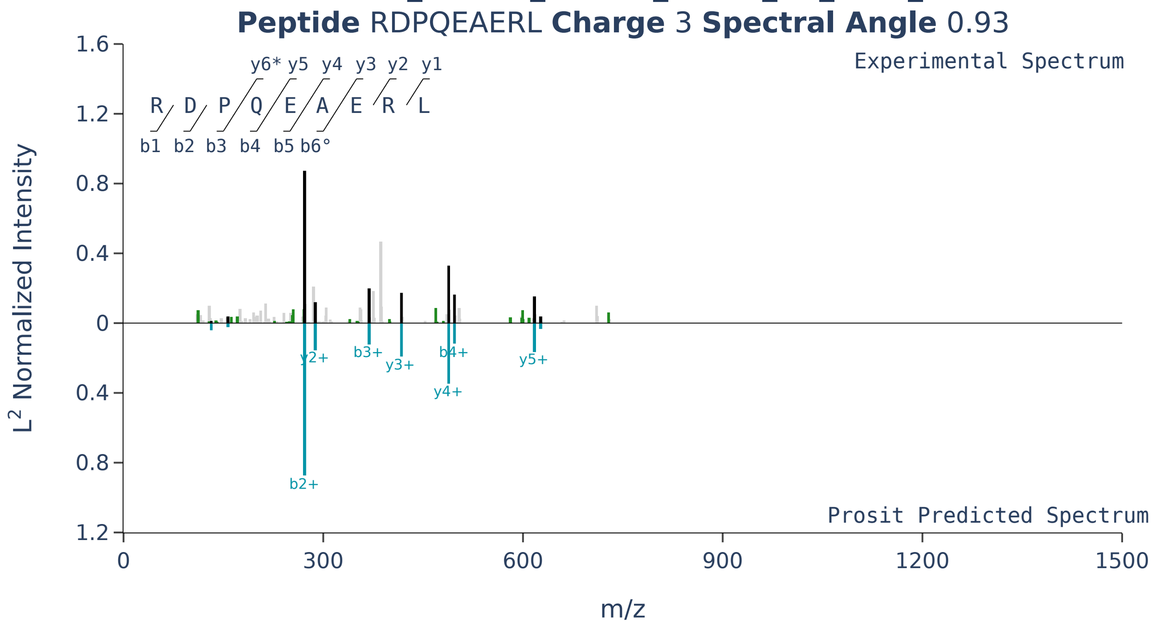


**Source** HRoetschke_211222_160123_HFGoe_IF2_b1R1_4h **Scan** 14580
**Peptide** RLVQQFA **Charge** 2 **Spectral Angle** 0.88

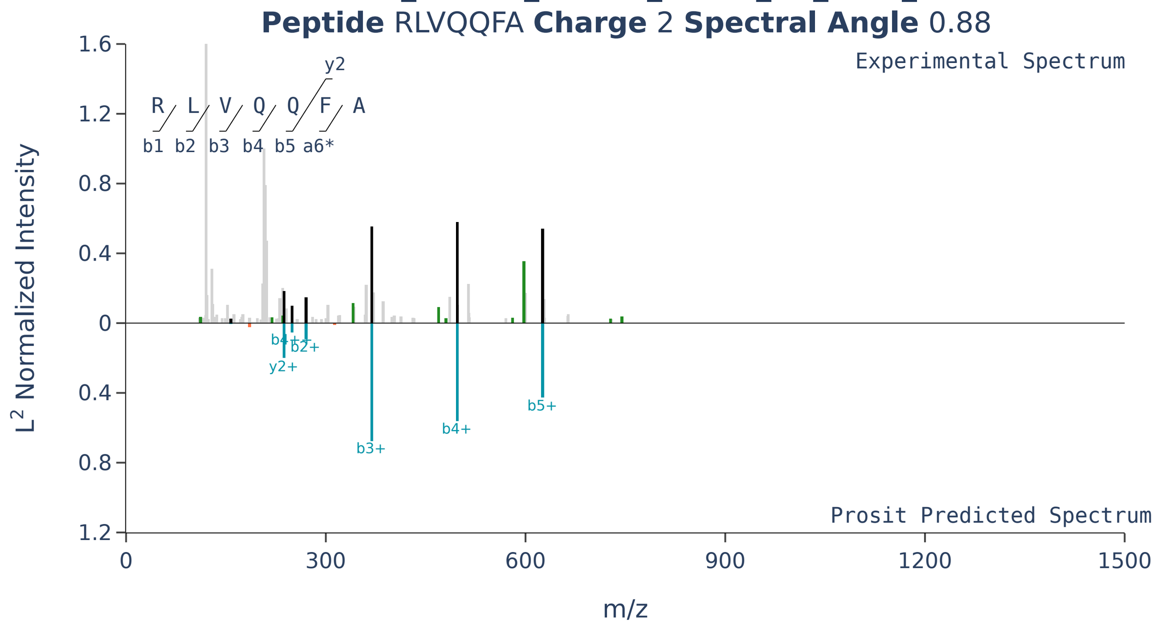


**Source** HRoetschke_211222_160123_HFGoe_IF2_b1R1_2h **Scan** 11376
**Peptide** HKGDIVLC **Charge** 2 **Spectral Angle** 0.93

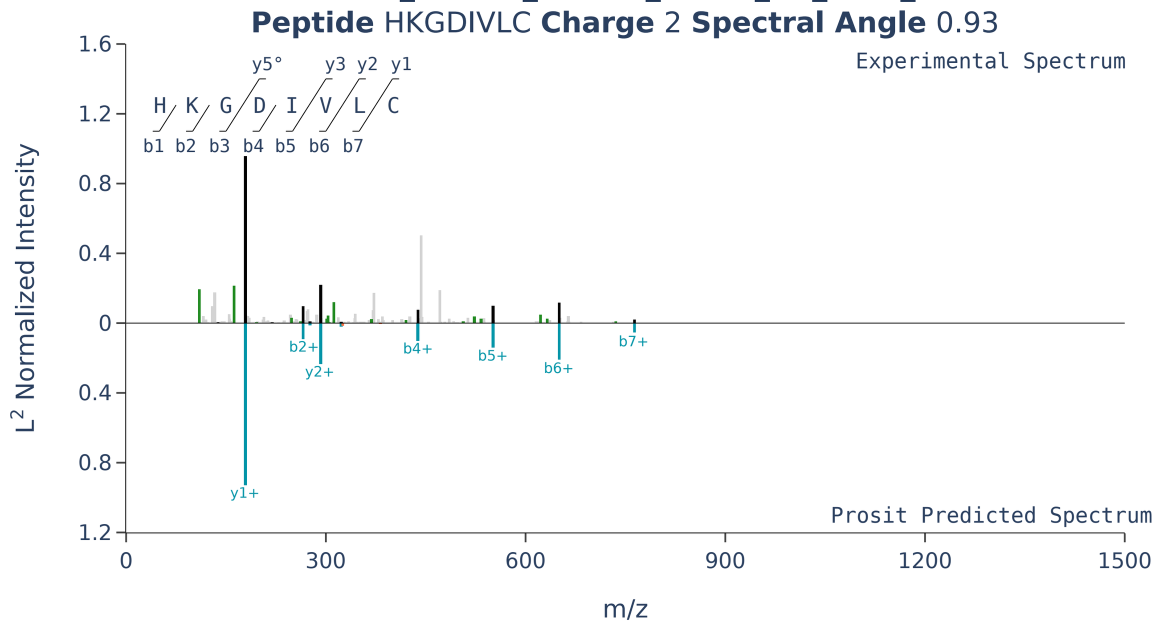


**Source** HRoetschke_211222_160123_HFGoe_IF2_b2R1_2h **Scan** 6442
**Peptide** SSDYHVTTSQHA **Charge** 3 **Spectral Angle** 0.85

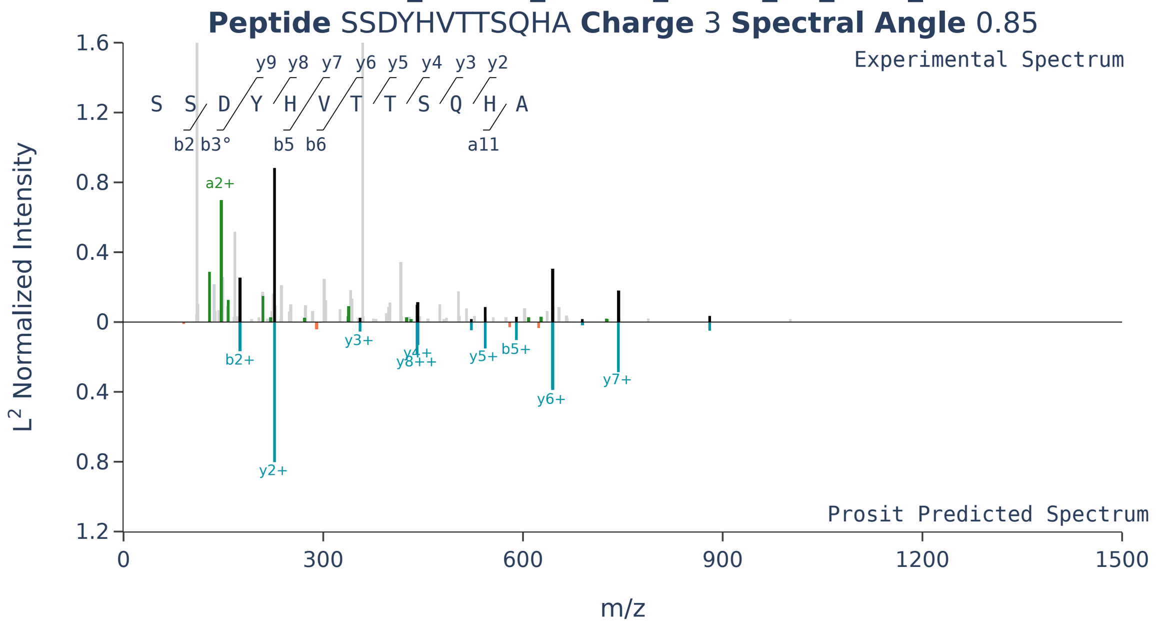


**Source** HRoetschke_211222_160123_HFGoe_IF2_b1R1_4h **Scan** 6936
**Peptide** EMGHKVILRRE **Charge** 3 **Spectral Angle** 0.87

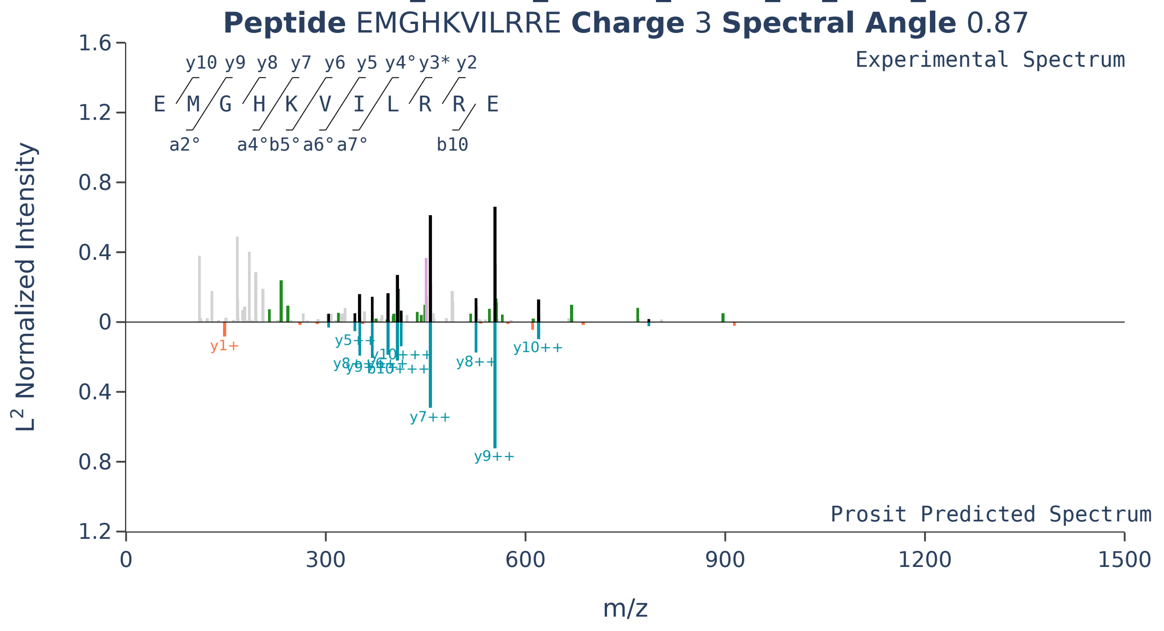


**Source** HRoetschke_211222_160123_HFGoe_IF2_b2R2_2h **Scan** 7890
**Peptide** RDPQEAERLA **Charge** 3 **Spectral Angle** 0.9

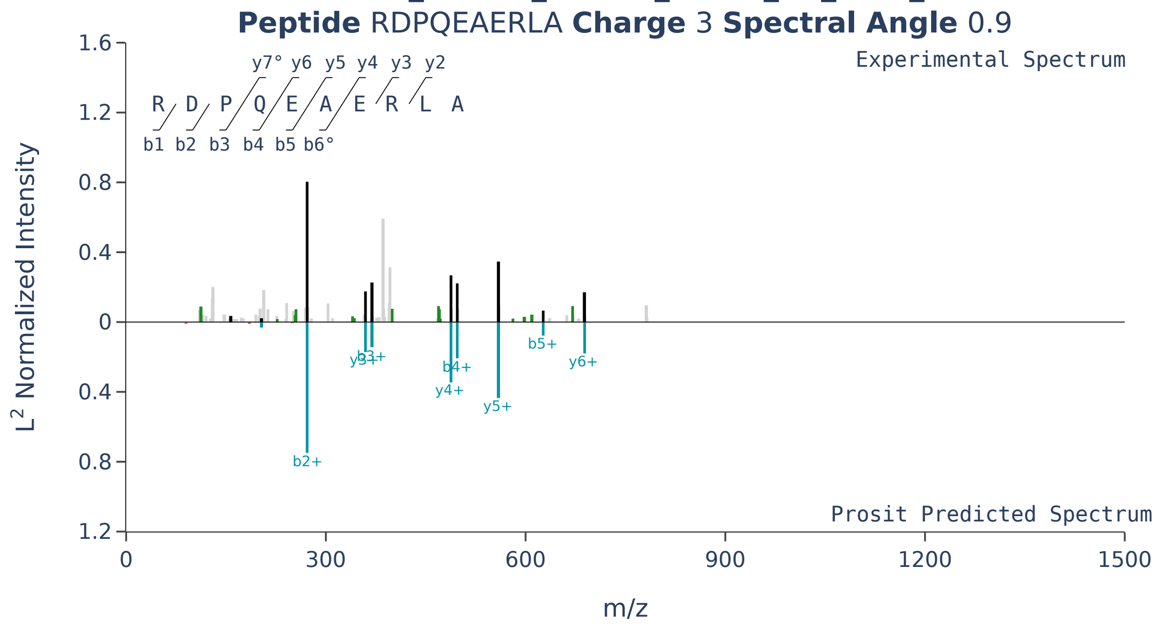


**Source** HRoetschke_211222_160123_HFGoe_IF2_b1R1_4h **Scan** 12207
**Peptide** TLNIPGTGGKSK **Charge** 3 **Spectral Angle** 0.85

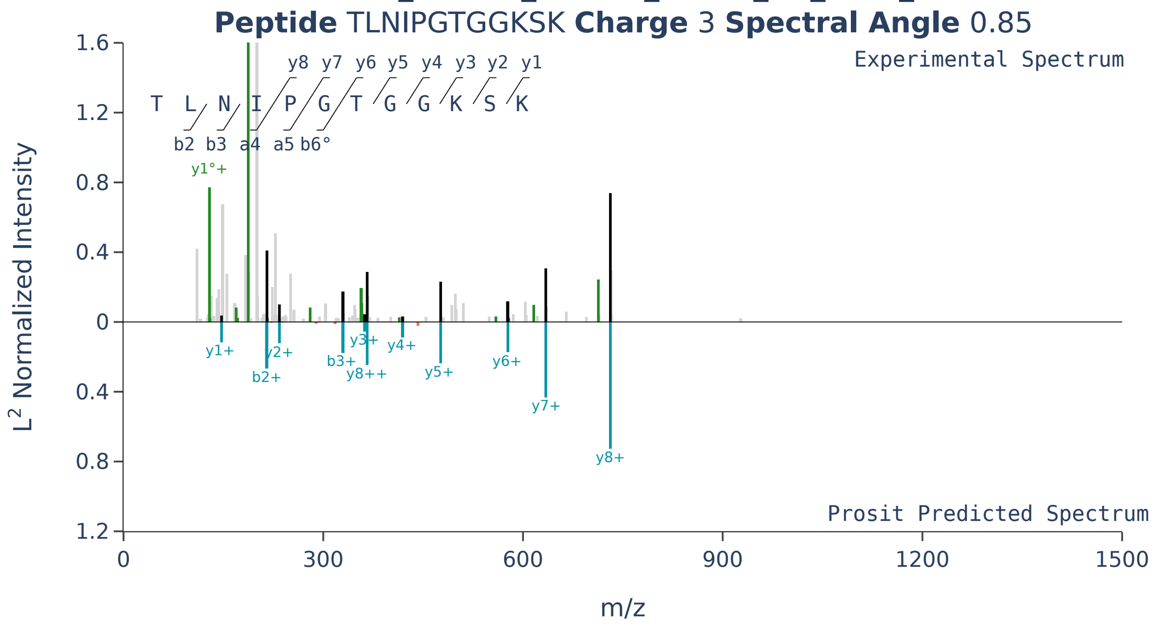


**Source** HRoetschke_211222_160123_HFGoe_IF2_b1R2_4h **Scan** 12952
**Peptide** INQVIDQET **Charge** 2 **Spectral Angle** 0.89

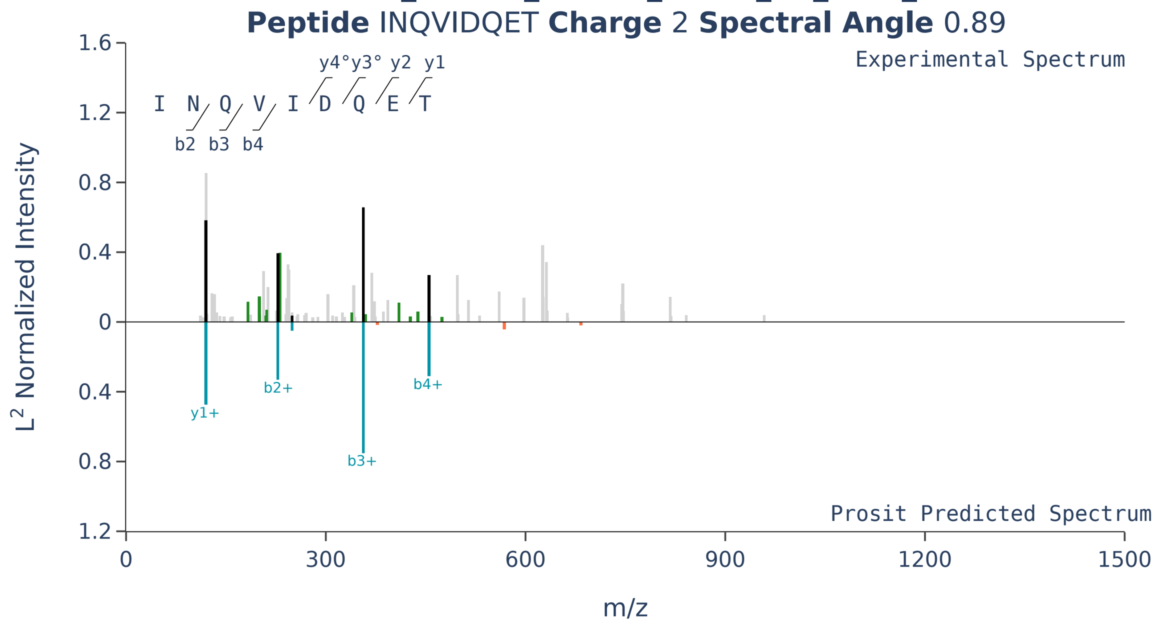


**Source** HRoetschke_211222_160123_HFGoe_IF2_b1R1_4h **Scan** 10536
**Peptide** AGGITQHIG **Charge** 2 **Spectral Angle** 0.87

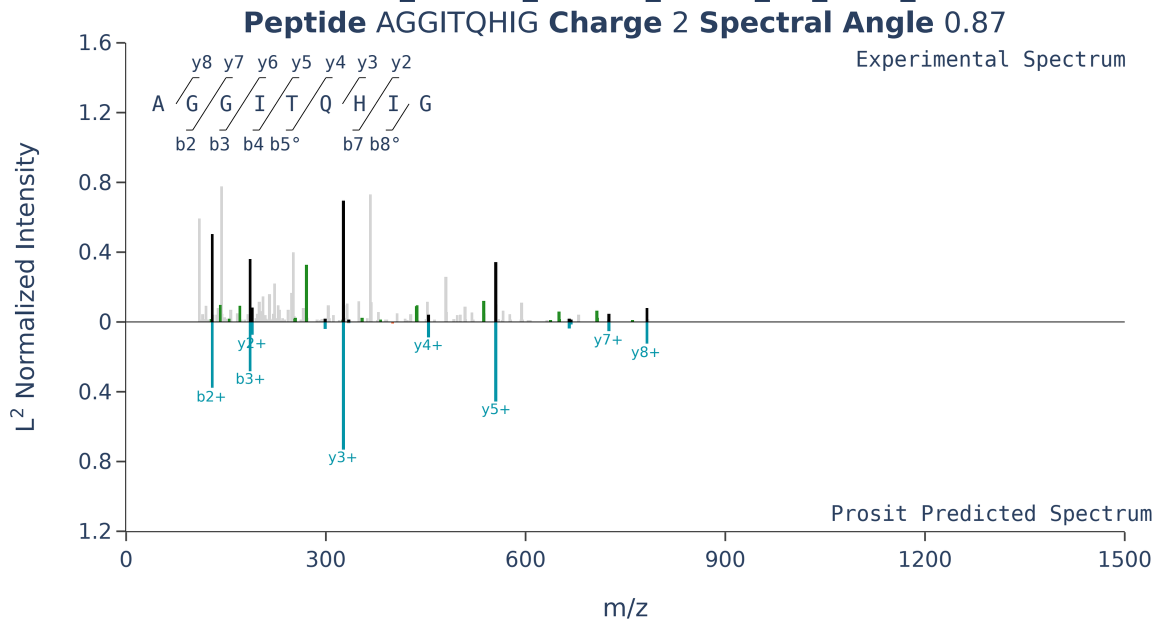

**Source** HRoetschke_211222_160123_HFGoe_IF2_b1R1_4h **Scan** 6959
**Peptide** MVTEGVVKRH **Charge** 3 **Spectral Angle** 0.85

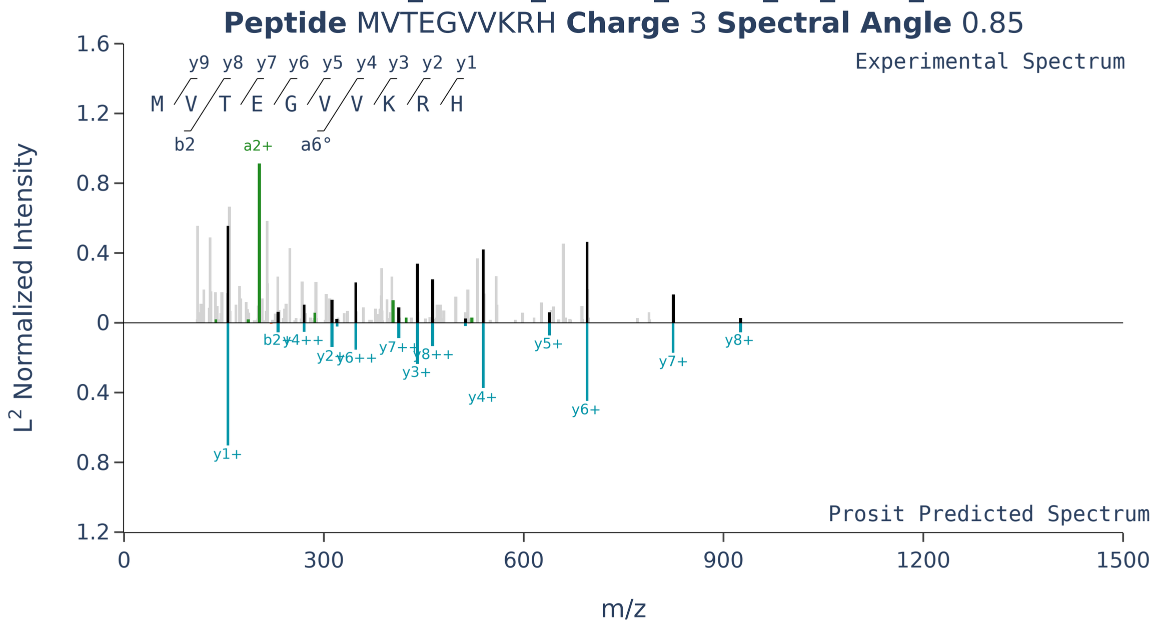


**Source** HRoetschke_211222_160123_HFGoe_IF2_b2R1_4h **Scan** 9627
**Peptide** KSKLENMF **Charge** 2 **Spectral Angle** 0.83

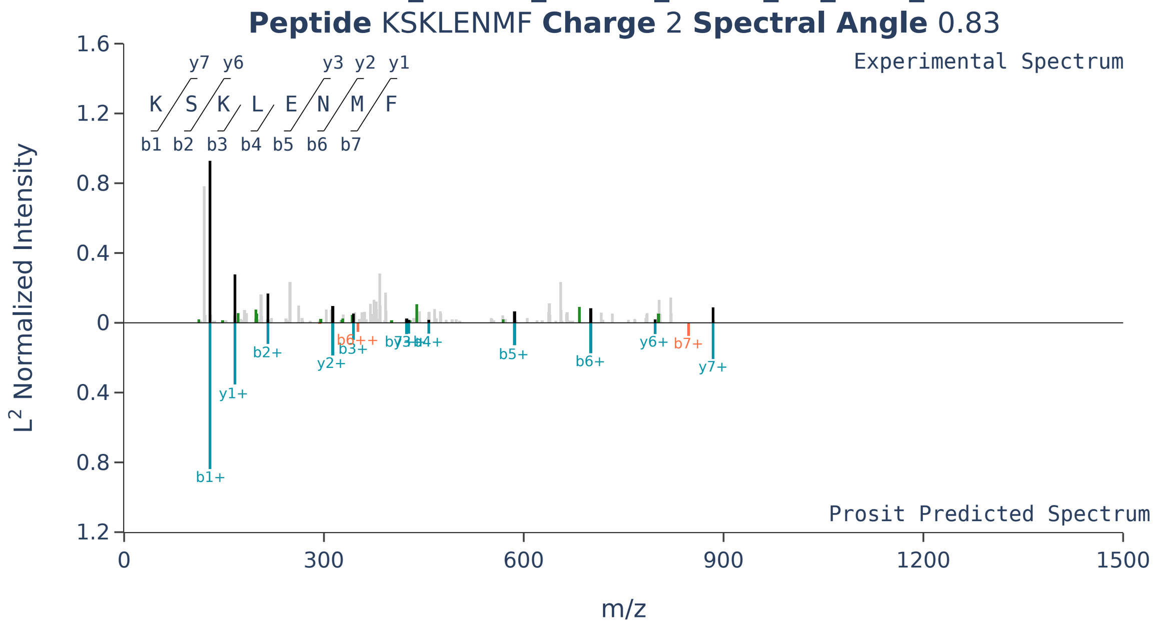


**Source** HRoetschke_211222_160123_HFGoe_IF2_b2R2_2h **Scan** 15437
**Peptide** TLTVGELANKM **Charge** 2 **Spectral Angle** 0.81

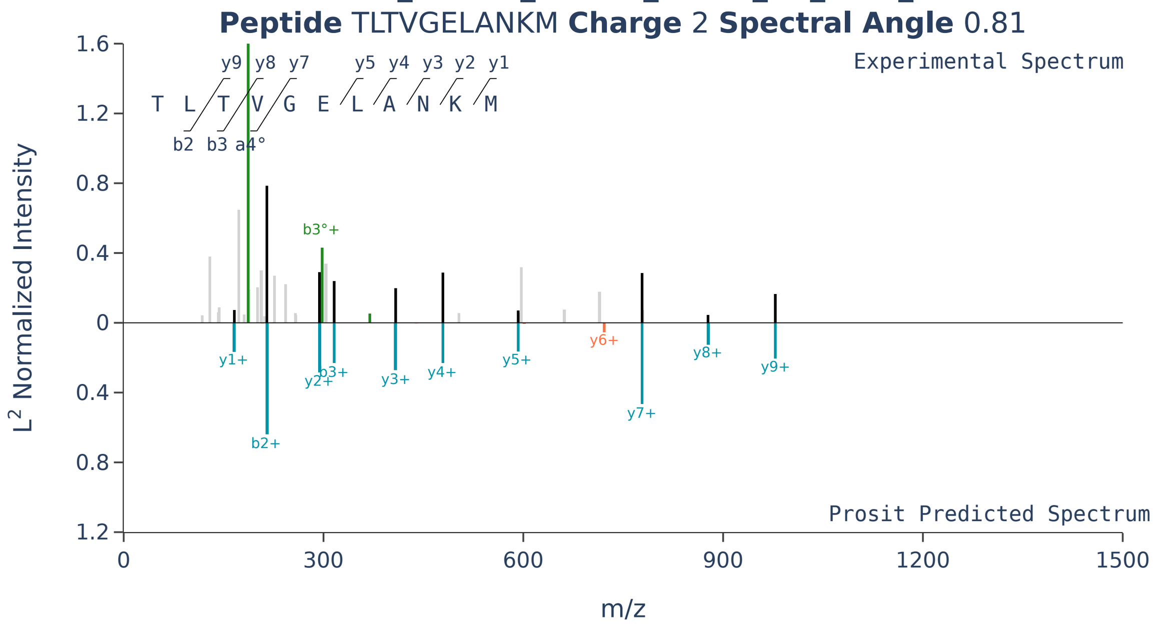


**Source** HRoetschke_211222_160123_HFGoe_IF2_b2R1_2h **Scan** 10295
**Peptide** VDHGKTSLLD **Charge** 3 **Spectral Angle** 0.91

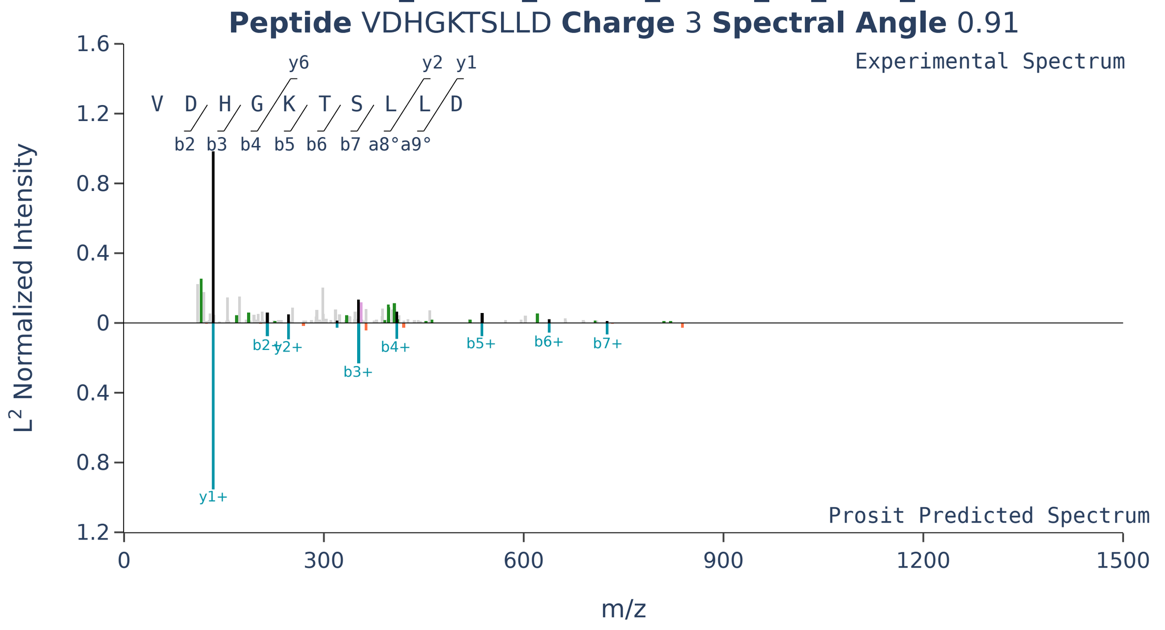


**Source** HRoetschke_211222_160123_HFGoe_IF2_b1R1_1h **Scan** 8897
**Peptide** GELANKMAVK **Charge** 3 **Spectral Angle** 0.83

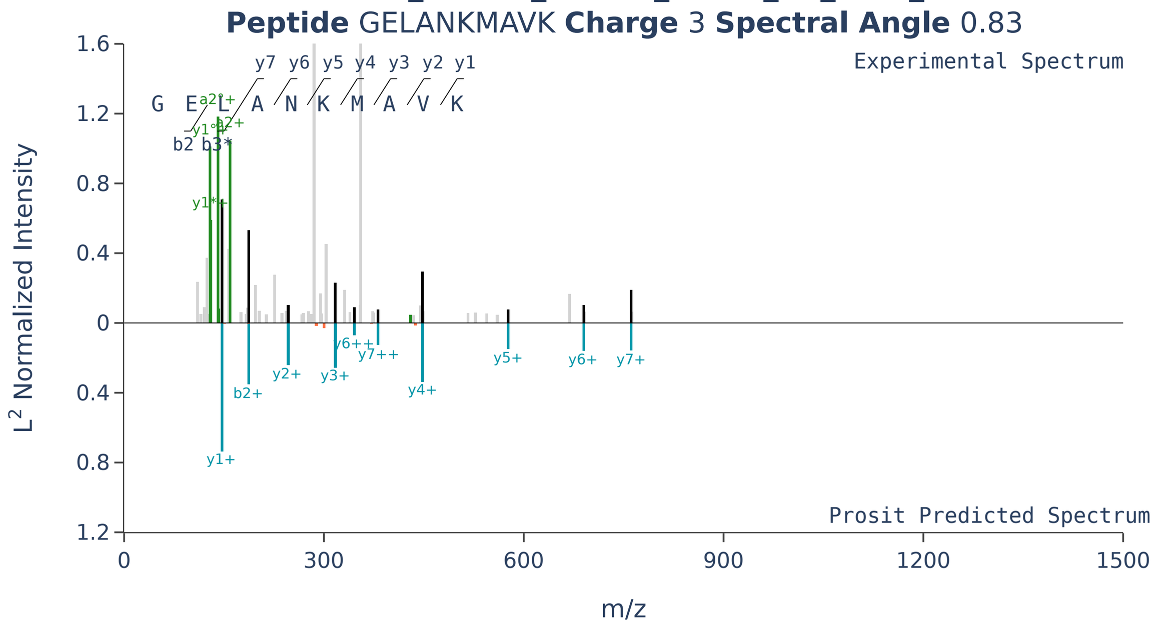


**Source** HRoetschke_211222_160123_HFGoe_IF2_b1R1_4h **Scan** 13058
**Peptide** ERQTSVERLV **Charge** 3 **Spectral Angle** 0.92

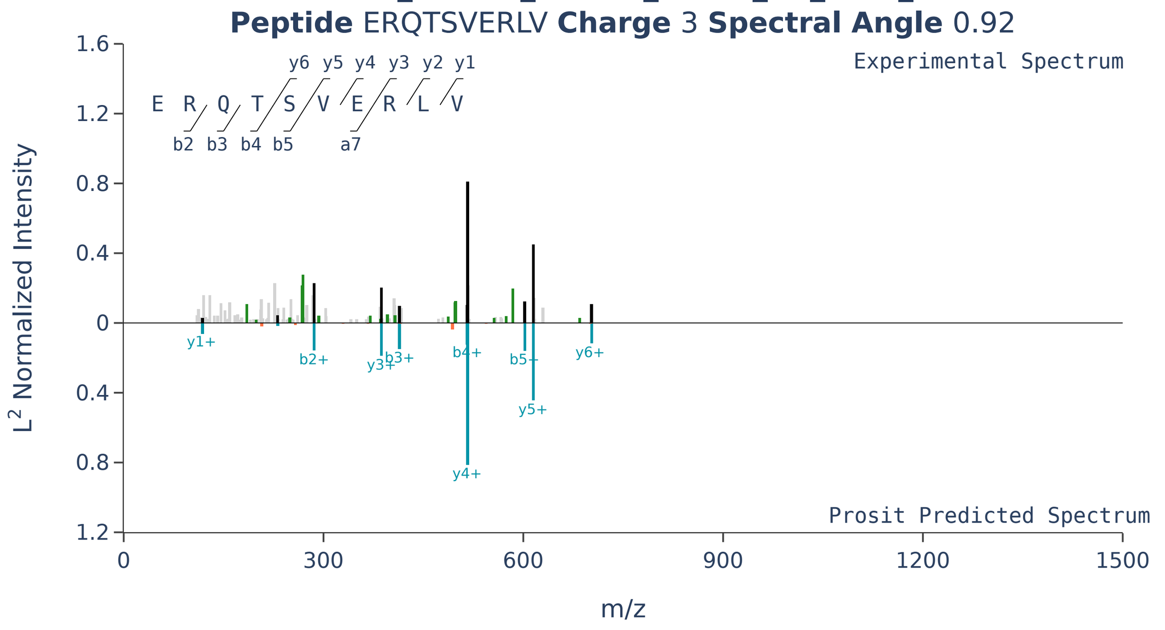


**Source** HRoetschke_211222_160123_HFGoe_IF2_b2R2_4h **Scan** 17330
**Peptide** RDVVIGETI **Charge** 2 **Spectral Angle** 0.84

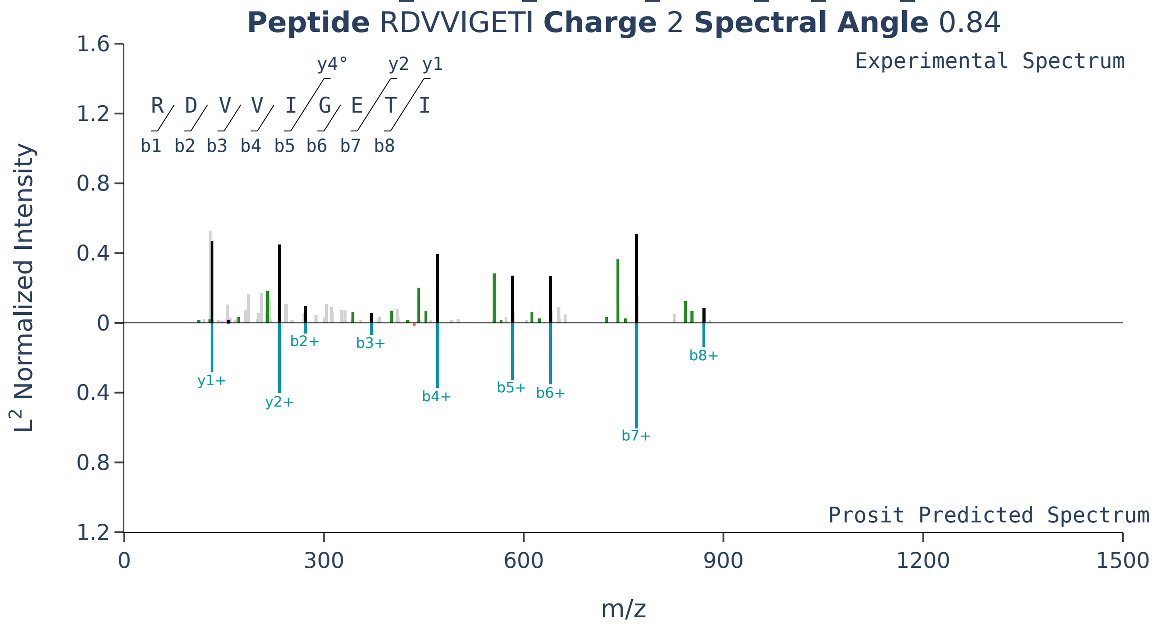


**Source** HRoetschke_211222_160123_HFGoe_IF2_b1R1_4h **Scan** 6800
**Peptide** NSGPDKLTLQRKTR **Charge** 4 **Spectral Angle** 0.84

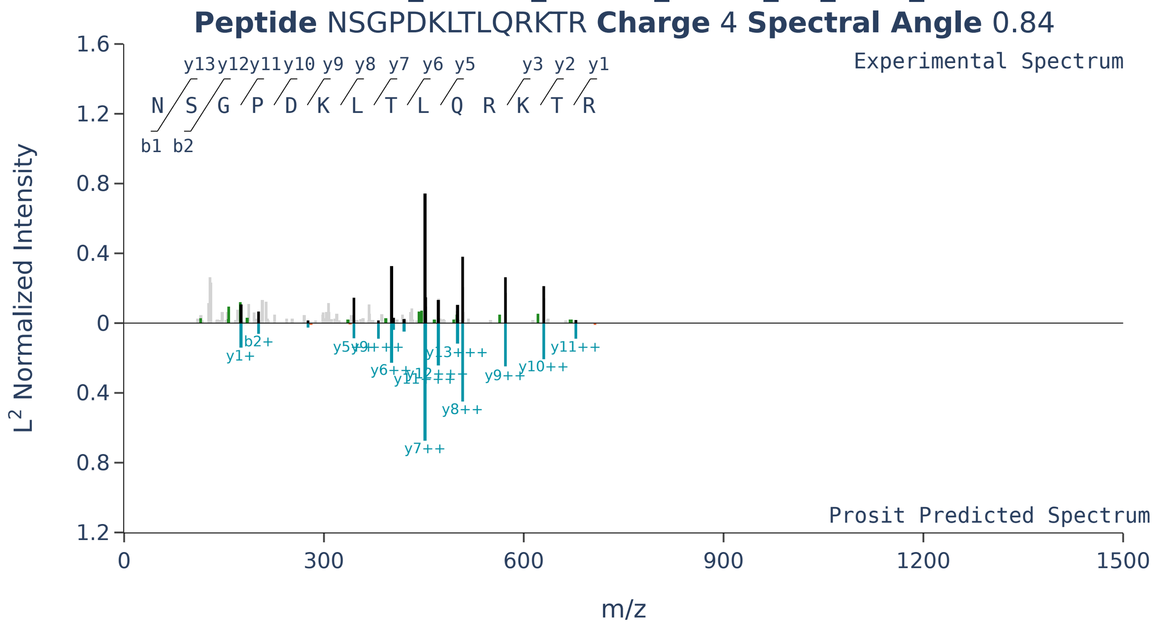


**Source** HRoetschke_211222_160123_HFGoe_IF2_b2R1_1h **Scan** 12170
**Peptide** AAERQTSVERLV **Charge** 3 **Spectral Angle** 0.84

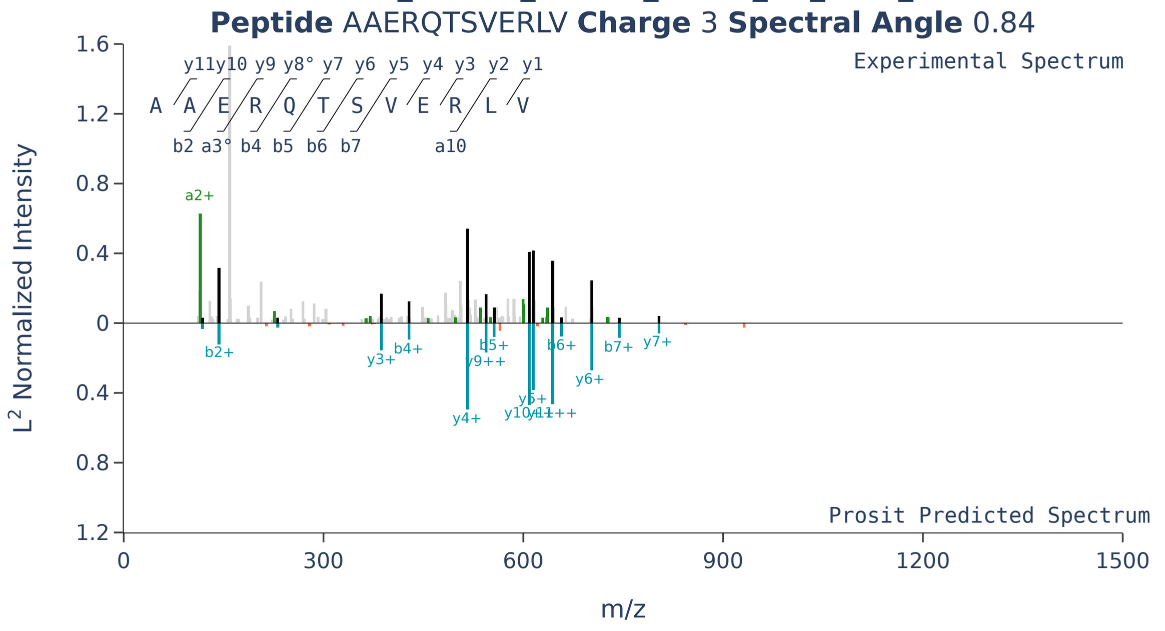


**Source** HRoetschke_211222_160123_HFGoe_IF2_b2R2_4h **Scan** 7634
**Peptide** VKGSQVLKA **Charge** 2 **Spectral Angle** 0.8

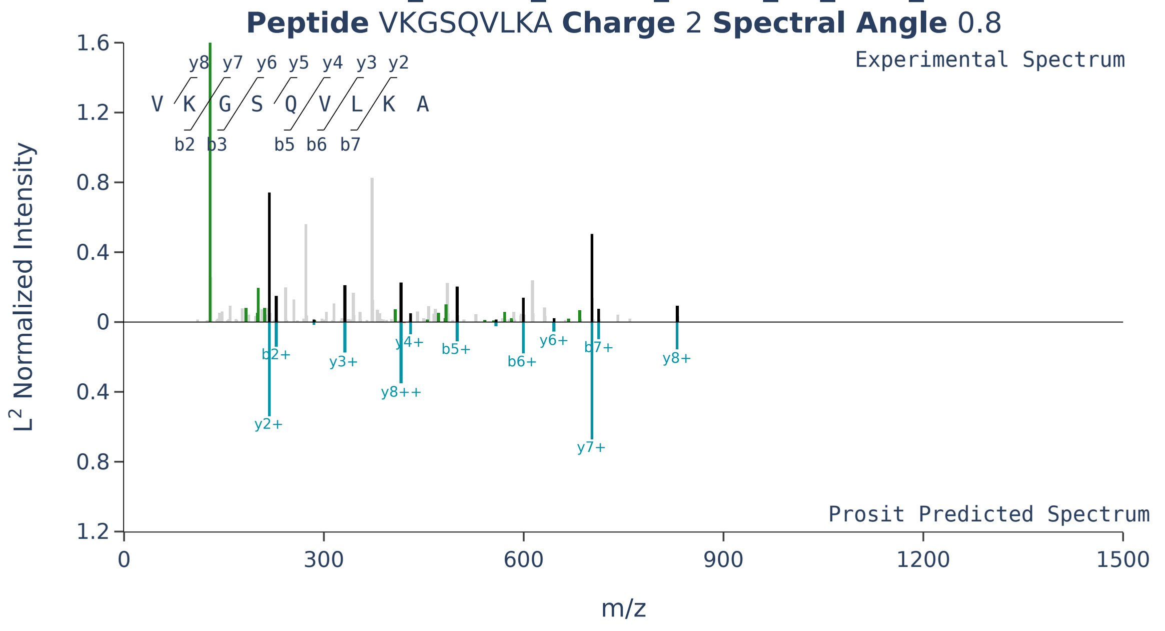


**Source** HRoetschke_211222_160123_HFGoe_IF2_b1R1_4h **Scan** 13286
**Peptide** KNSGPDKLTL **Charge** 2 **Spectral Angle** 0.84

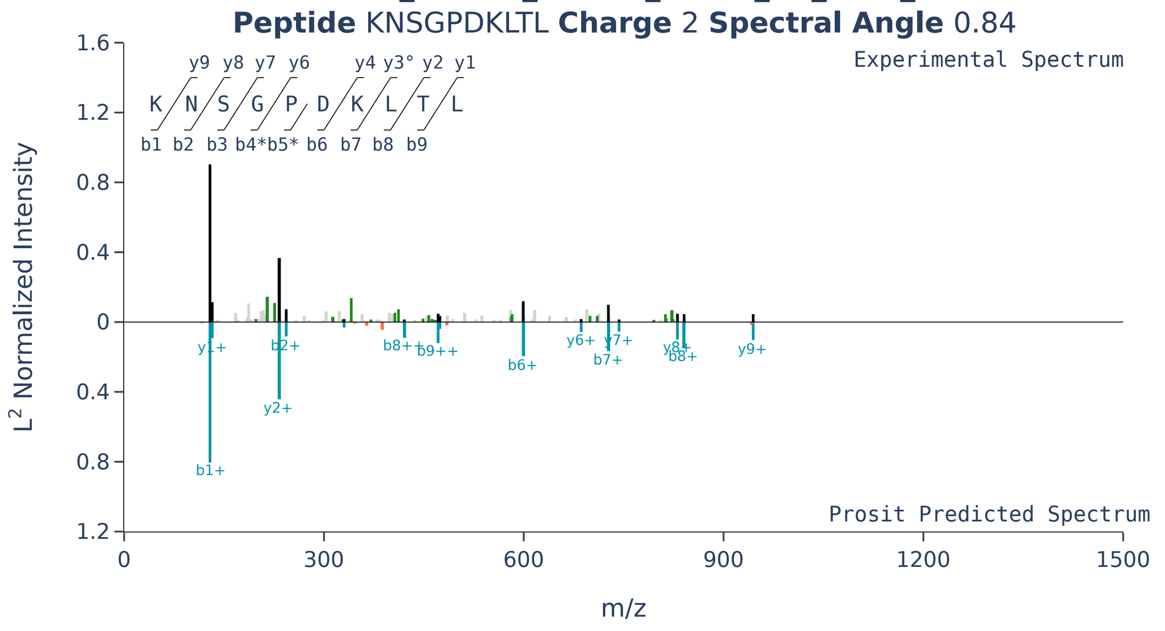


**Source** HRoetschke_211222_160123_HFGoe_IF2_b1R1_2h **Scan** 10626
**Peptide** GDEVTVVRD **Charge** 2 **Spectral Angle** 0.84

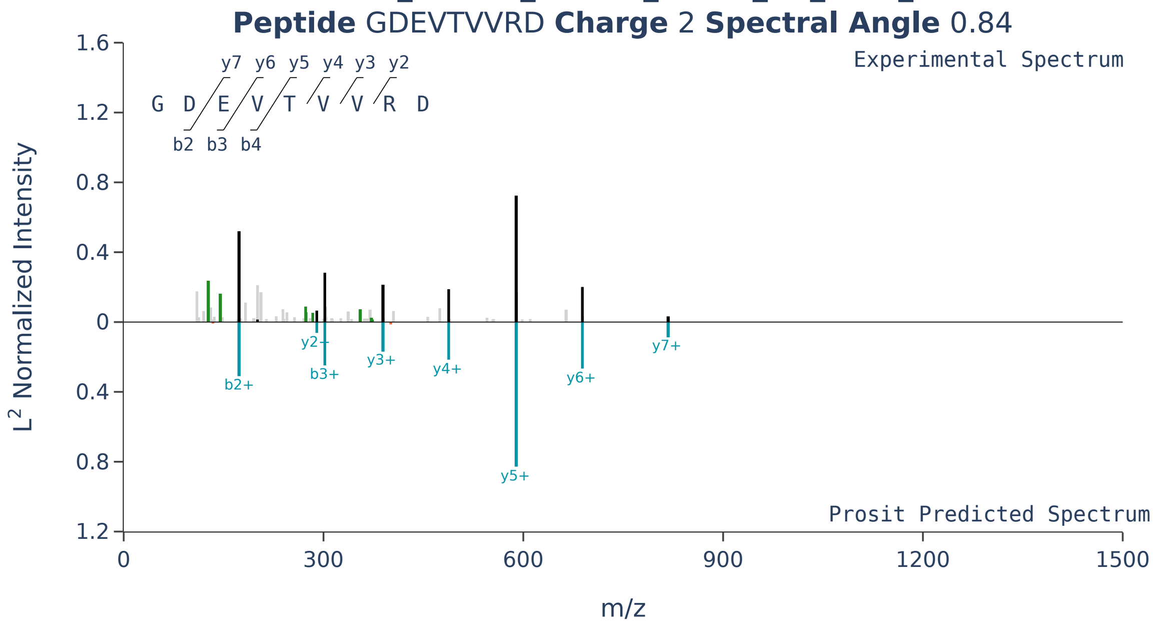


**Source** HRoetschke_211222_160123_HFGoe_IF2_b2R1_24h **Scan** 7216
**Peptide** AVKGSQVIKAM **Charge** 3 **Spectral Angle** 0.87

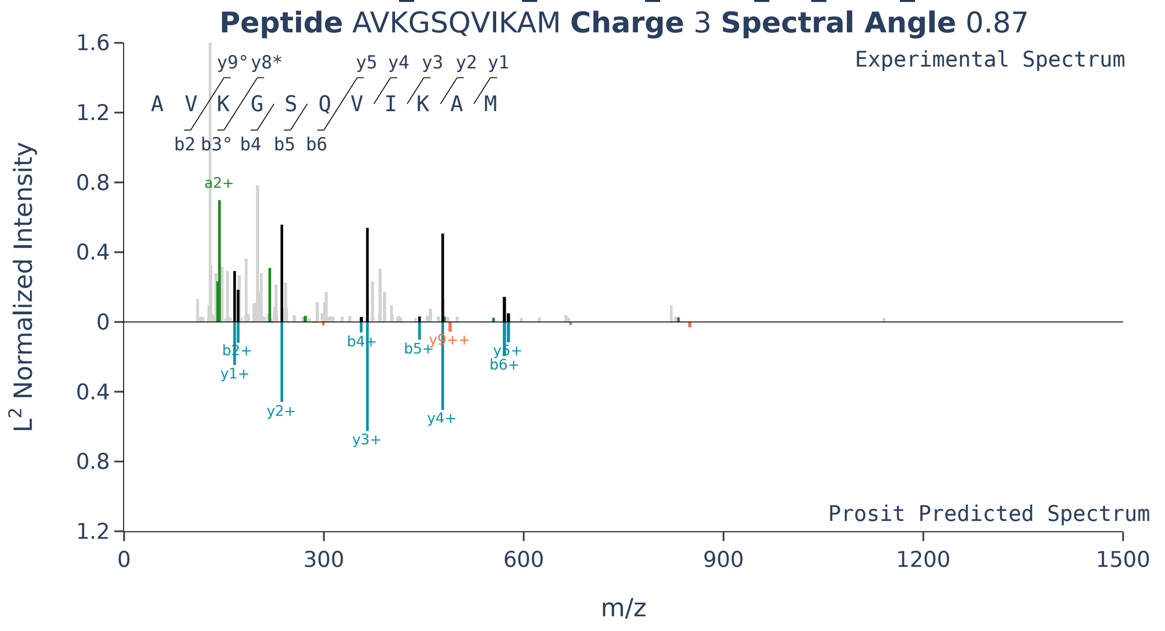


**Source** HRoetschke_211222_160123_HFGoe_IF2_b1R1_4h **Scan** 14724
**Peptide** SFLDKGRGPVA **Charge** 2 **Spectral Angle** 0.84

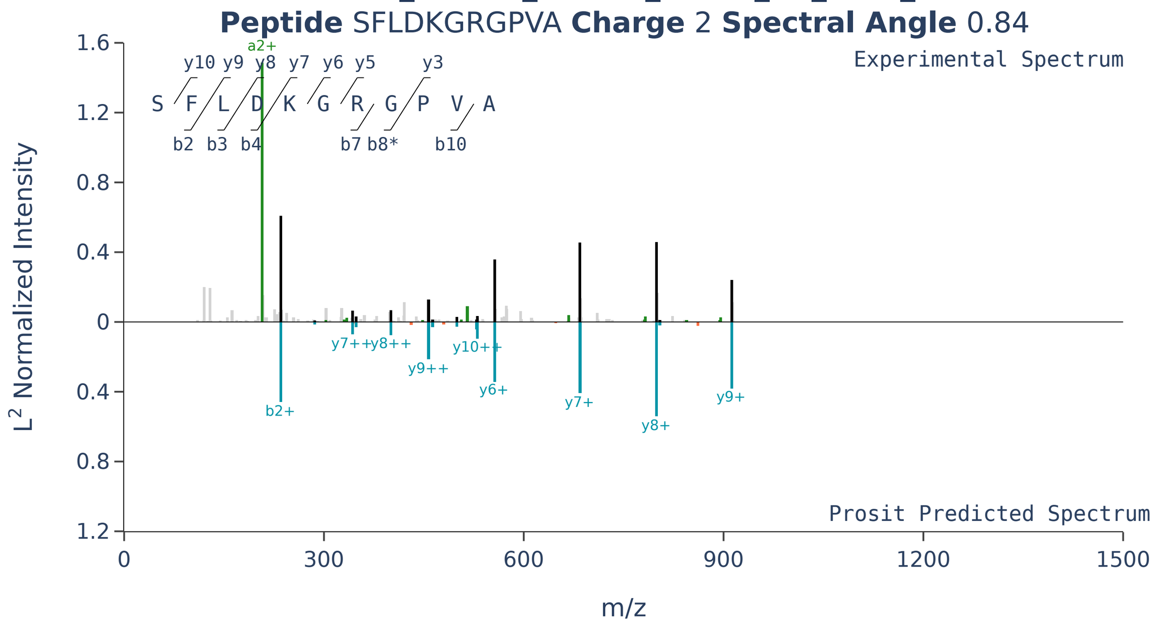


**Source** HRoetschke_211222_160123_HFGoe_IF2_b1R1_4h **Scan** 11858
**Peptide** PVVVAVNK **Charge** 2 **Spectral Angle** 0.86

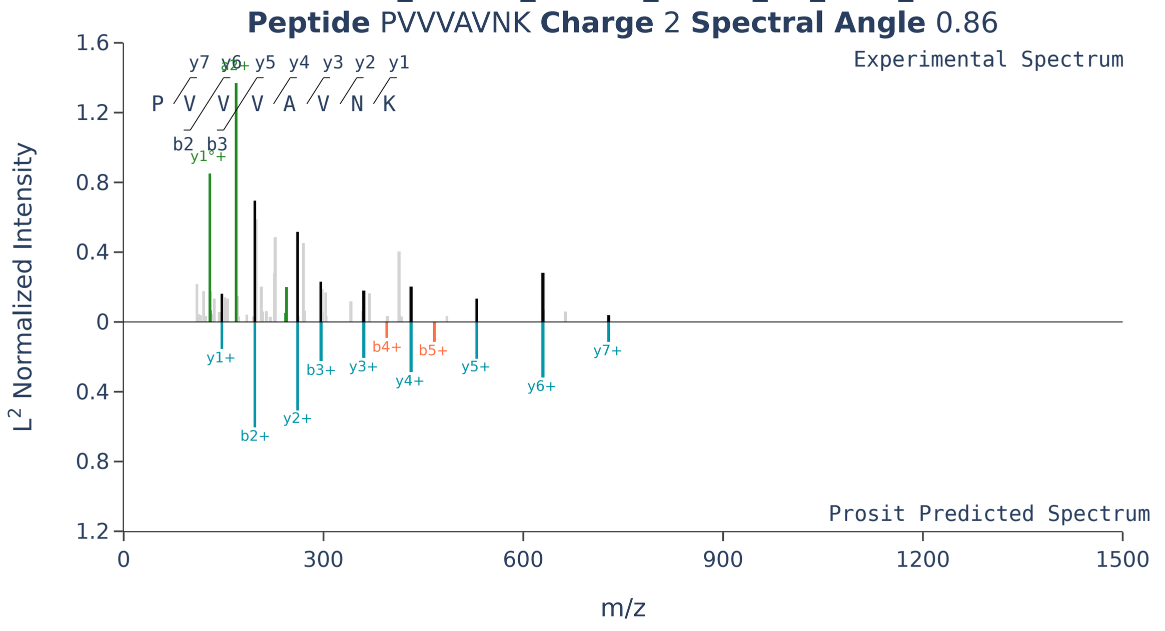

**Source** HRoetschke_211222_160123_HFGoe_IF2_b1R1_4h **Scan** 8902
**Peptide** ILRRENELEE **Charge** 2 **Spectral Angle** 0.9

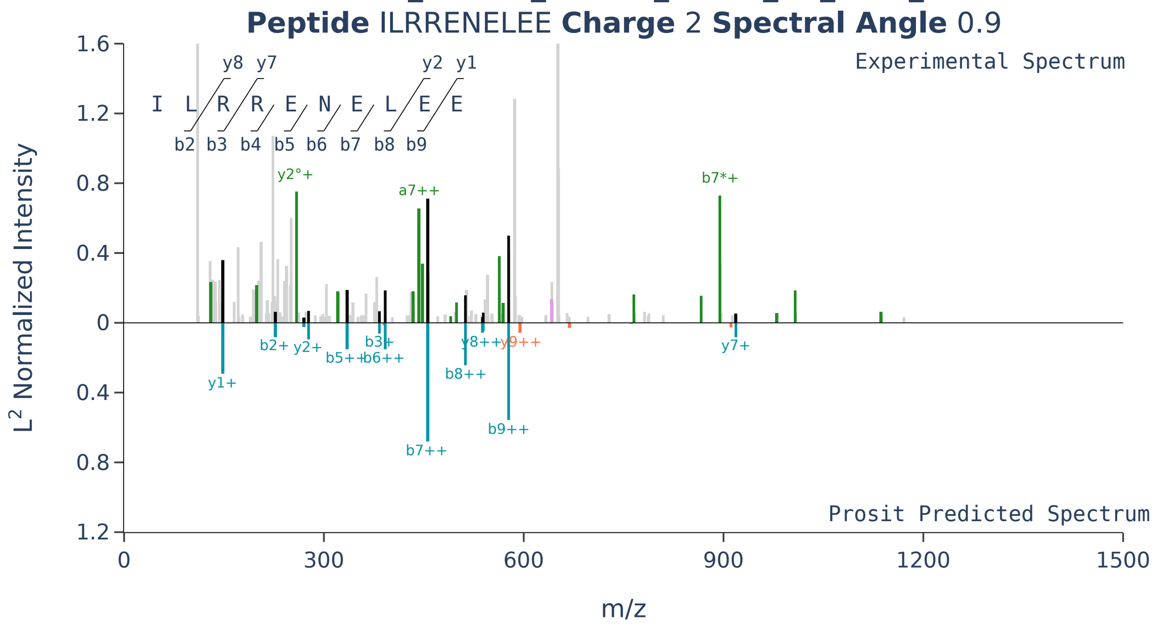

**Source** HRoetschke_211222_160123_HFGoe_IF2_b1R1_4h **Scan** 10600
**Peptide** SGPDKLTLQR **Charge** 2 **Spectral Angle** 0.85

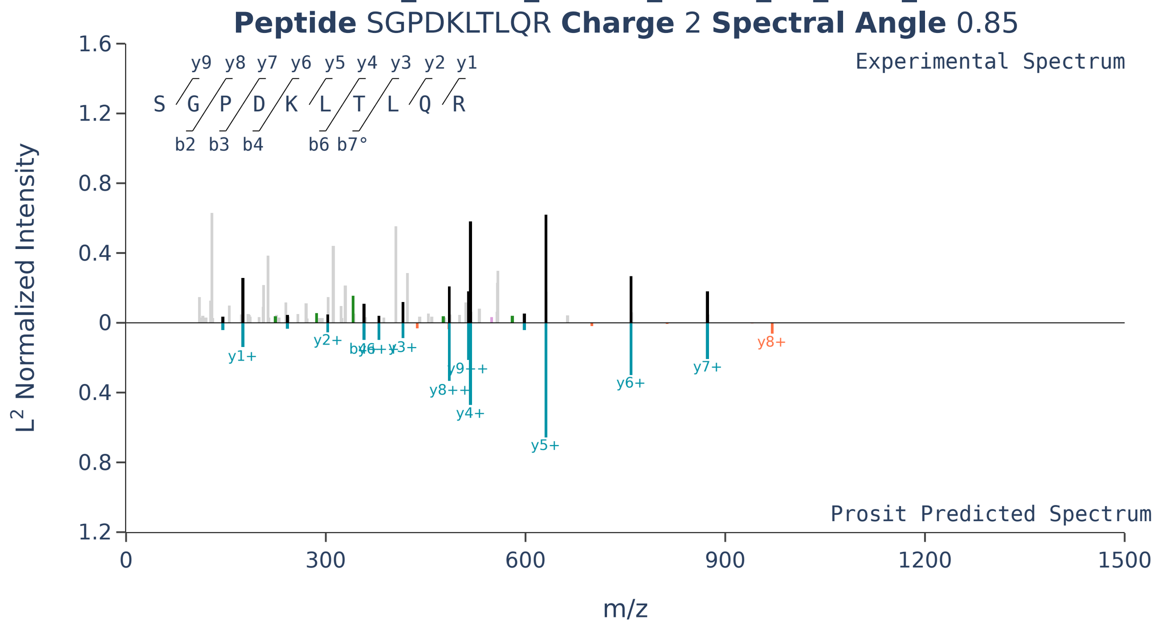

**Source** HRoetschke_211222_160123_HFGoe_IF2_b1R2_4h **Scan** 10863
**Peptide** SVERLVQQ **Charge** 2 **Spectral Angle** 0.83

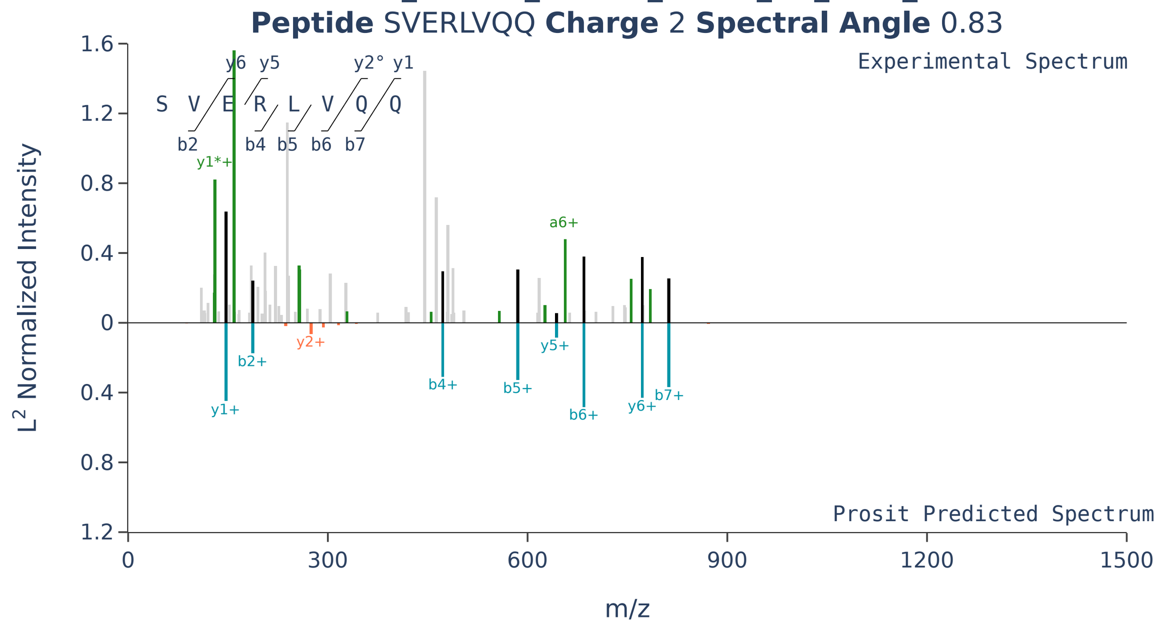

**Source** HRoetschke_211222_160123_HFGoe_IF2_b2R1_1h **Scan** 7200
**Peptide** PDRVKNEL **Charge** 2 **Spectral Angle** 0.85

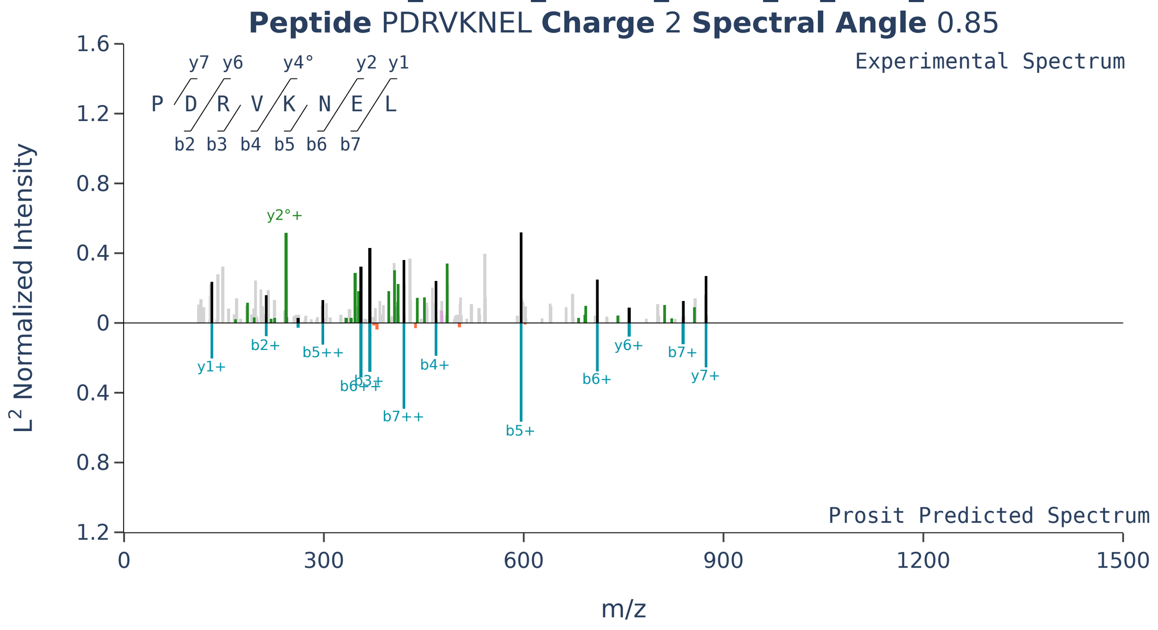

**Source** HRoetschke_211222_160123_HFGoe_IF2_b1R2_2h **Scan** 9970
**Peptide** DVNEVRNGM **Charge** 2 **Spectral Angle** 0.85

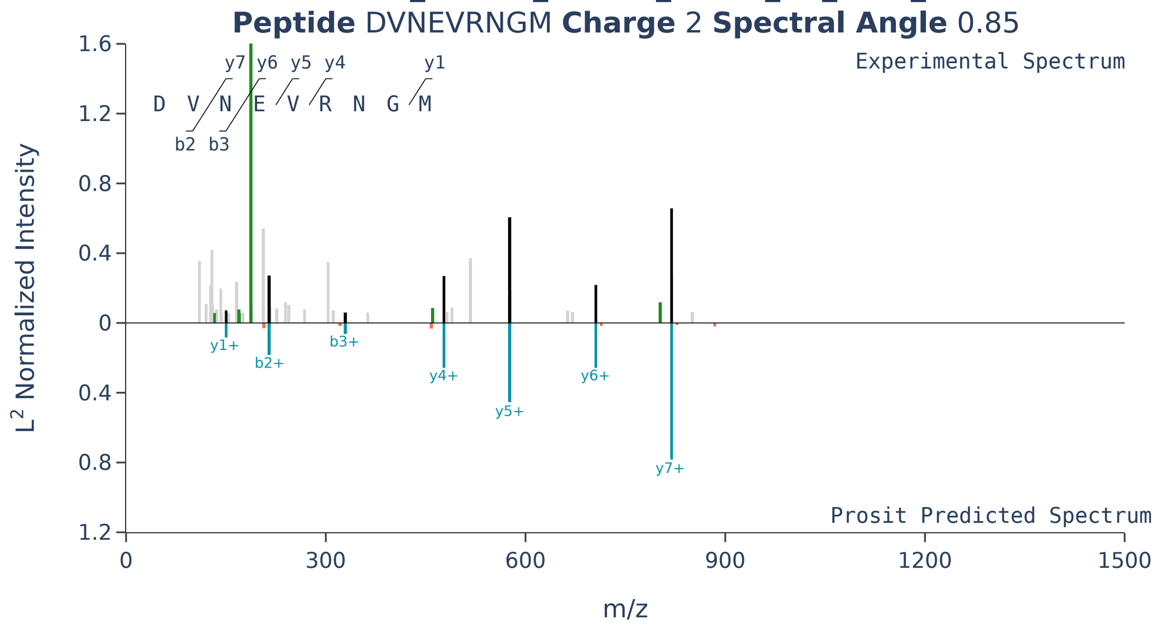

**Source** HRoetschke_211222_160123_HFGoe_IF2_b1R1_4h **Scan** 15759
**Peptide** TDVTIKTLAAER **Charge** 3 **Spectral Angle** 0.85

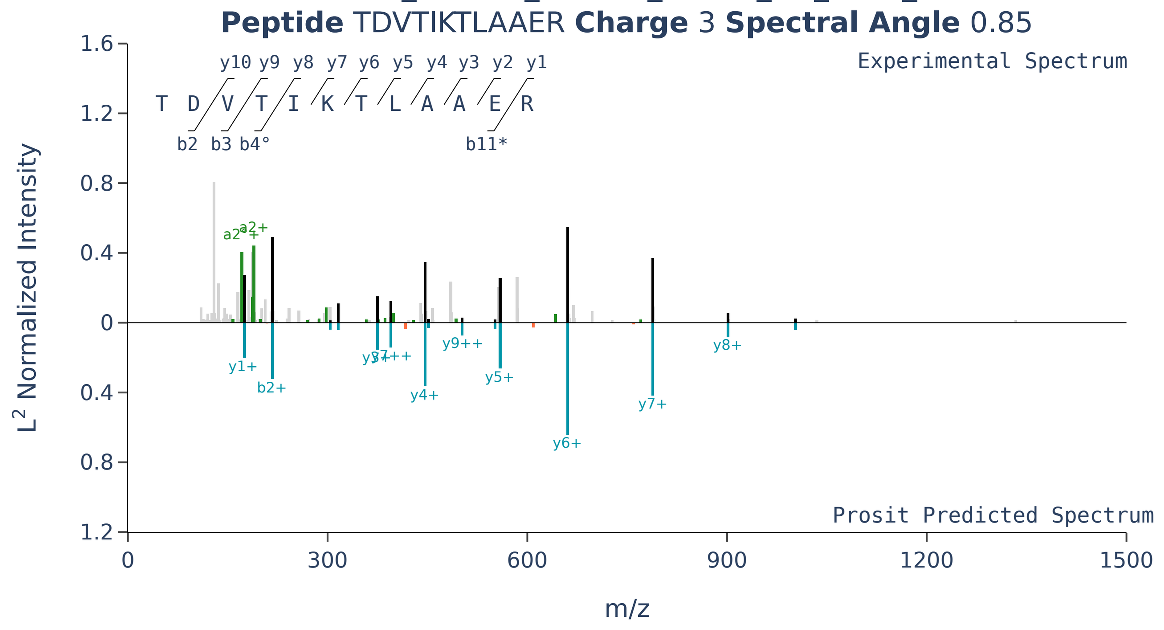

**Source** HRoetschke_211222_160123_HFGoe_IF2_b1R1_24h **Scan** 10688
**Peptide** ASGAVIE **Charge** 1 **Spectral Angle** 0.87

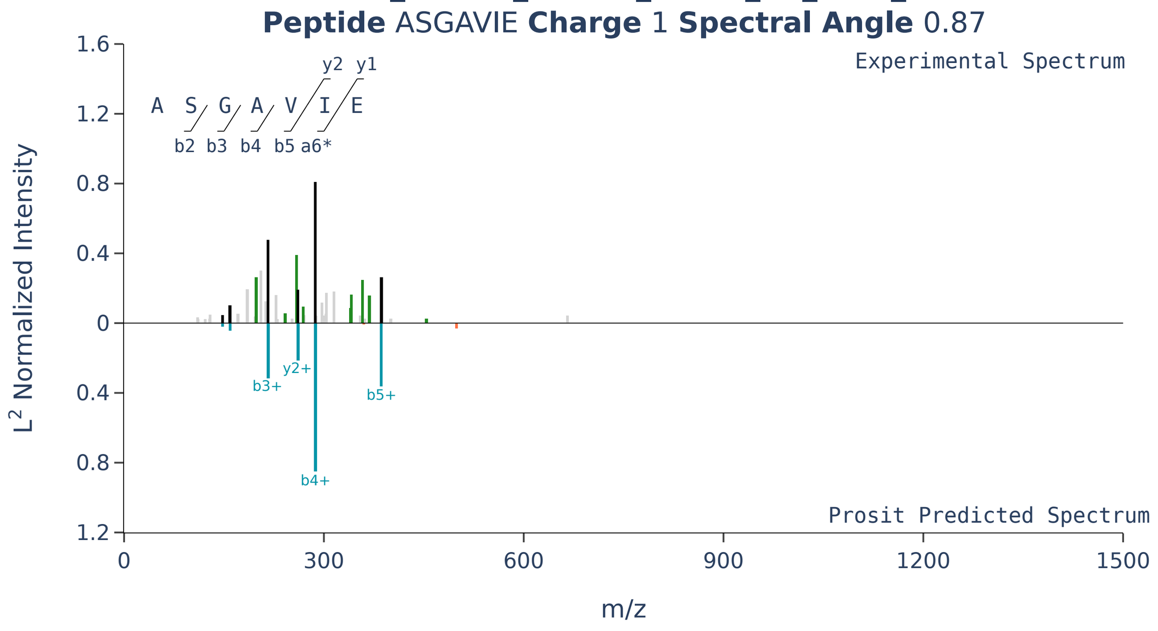

**Source** HRoetschke_211222_160123_HFGoe_IF2_b1R2_24h **Scan** 8234
**Peptide** TVGELANKM **Charge** 2 **Spectral Angle** 0.78

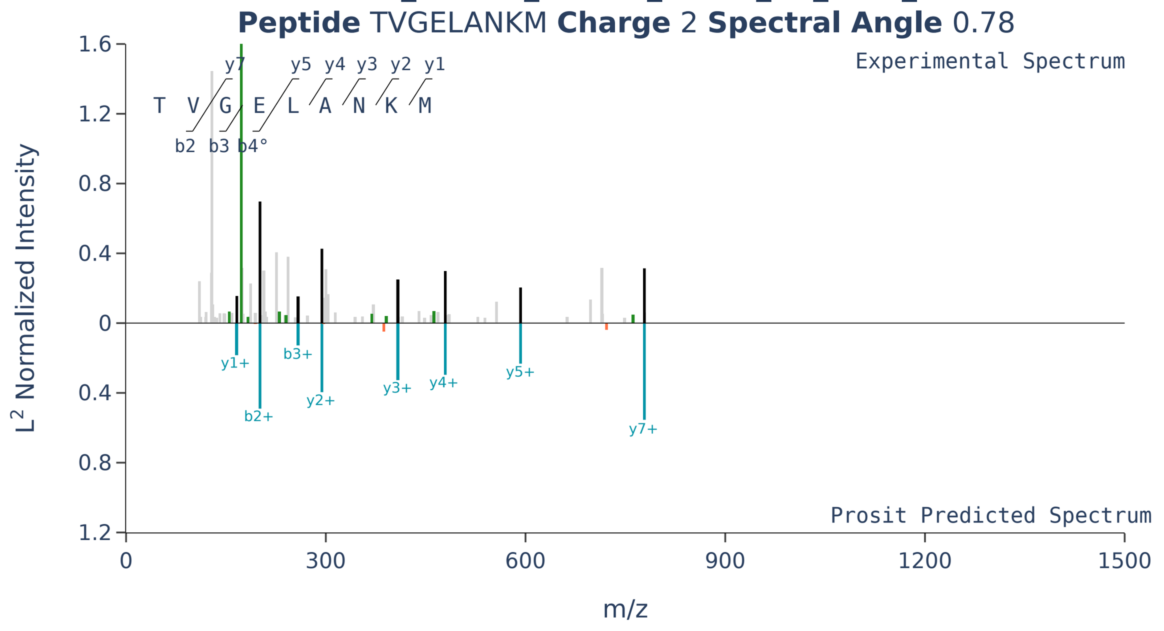

**Source** HRoetschke_211222_160123_HFGoe_IF2_b1R1_2h **Scan** 11392
**Peptide** DEVTVVRDE **Charge** 2 **Spectral Angle** 0.88

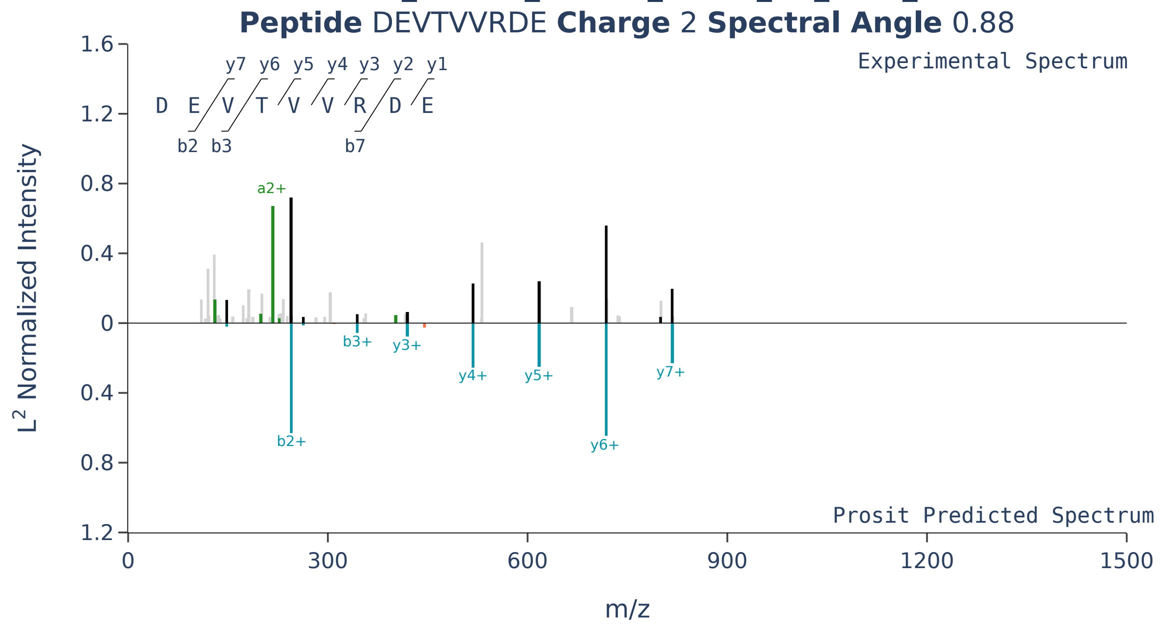

**Source** HRoetschke_211222_160123_HFGoe_IF2_b1R1_4h **Scan** 6366
**Peptide** YIRSTKVASGE **Charge** 3 **Spectral Angle** 0.89

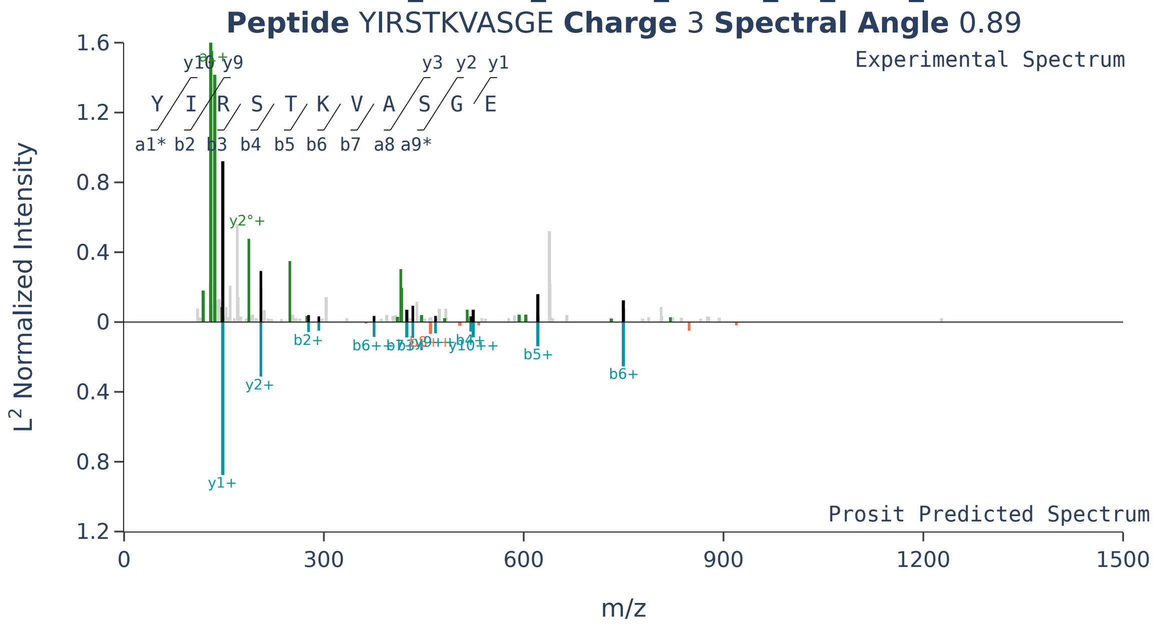

**Source** HRoetschke_211222_160123_HFGoe_IF2_b2R2_2h **Scan** 9051
**Peptide** AEENKWTDNAE **Charge** 2 **Spectral Angle** 0.87

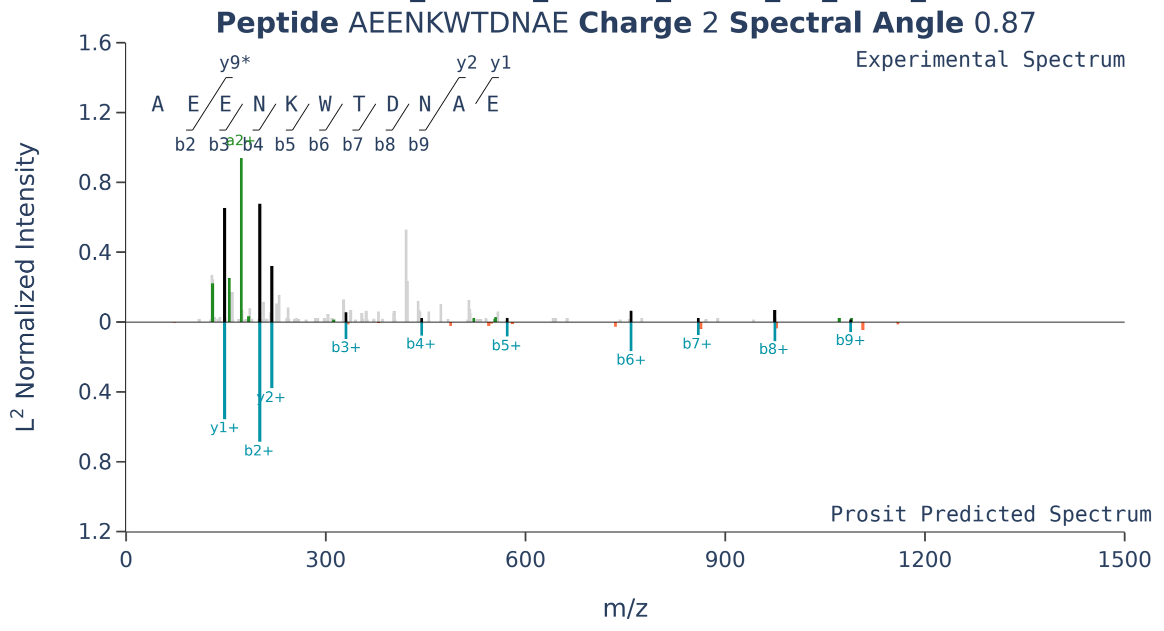

**Source** HRoetschke_211222_160123_HFGoe_IF2_b1R2_24h **Scan** 7362
**Peptide** RKGMASGAVIE **Charge** 3 **Spectral Angle** 0.9

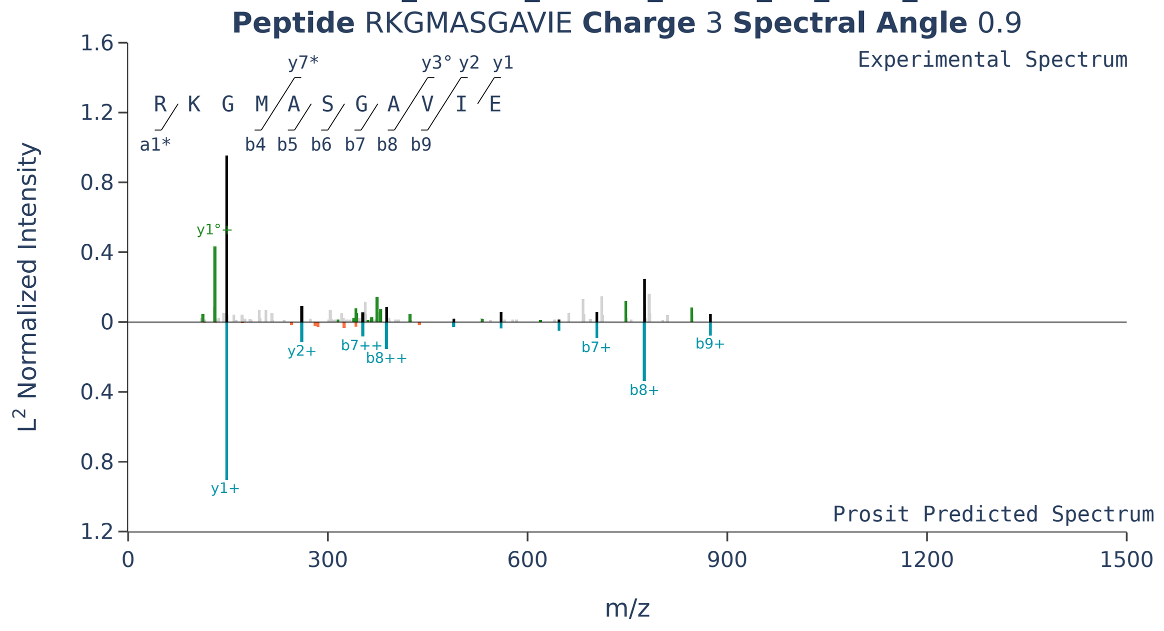

**Source** HRoetschke_211222_160123_HFGoe_IF2_b1R2_24h **Scan** 8349
**Peptide** FADAGIRKSADD **Charge** 3 **Spectral Angle** 0.84

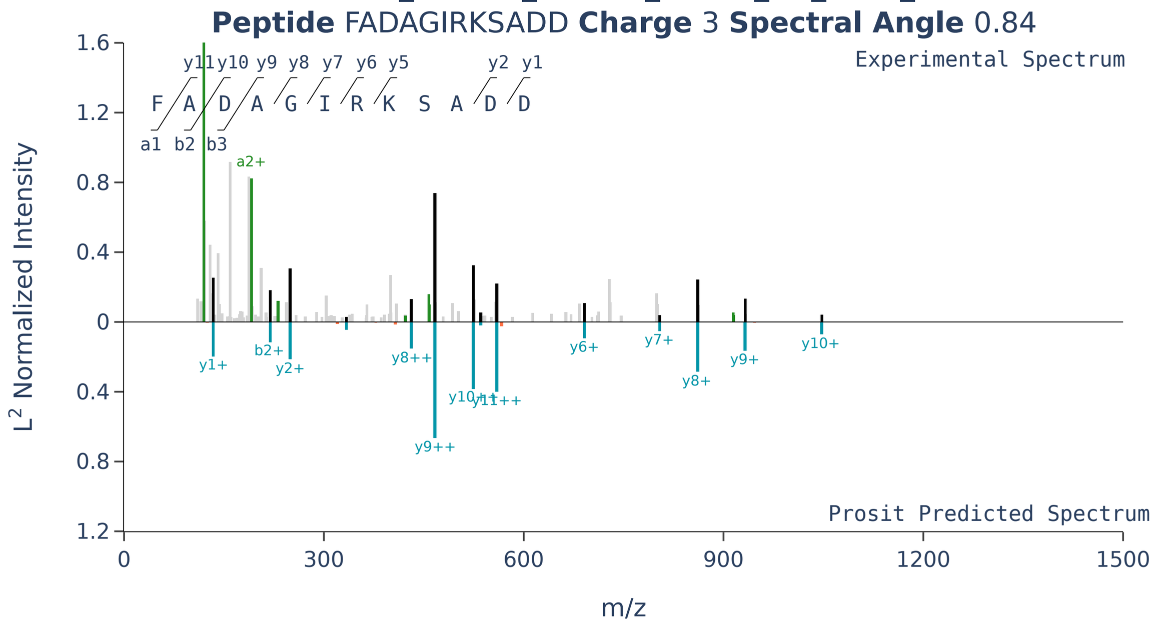

**Source** HRoetschke_211222_160123_HFGoe_IF2_b1R1_4h **Scan** 14371
**Peptide** IDQETAQL **Charge** 1 **Spectral Angle** 0.91

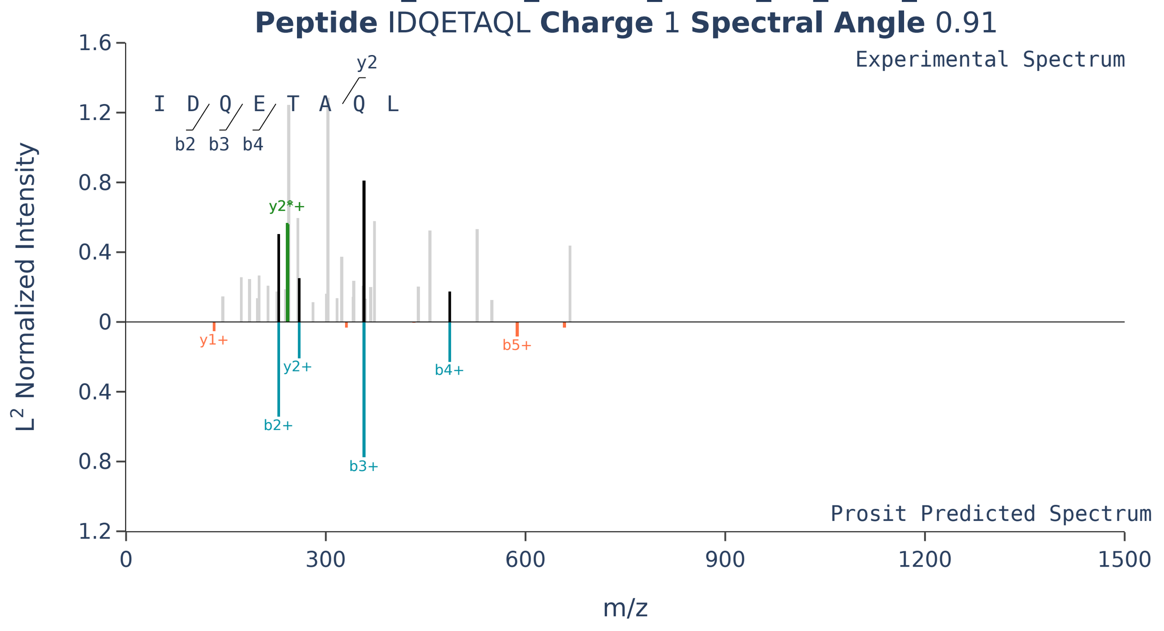

**Source** HRoetschke_211222_160123_HFGoe_IF2_b2R1_24h **Scan** 5493
**Peptide** EVTVVRDEKKARE **Charge** 4 **Spectral Angle** 0.88

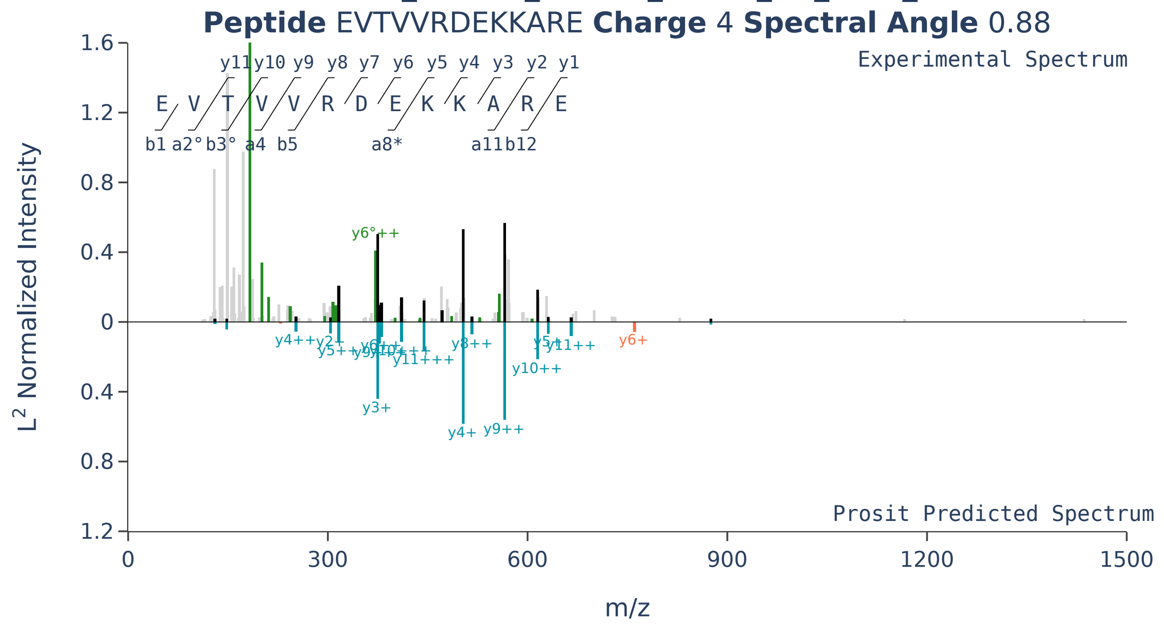

**Source** HRoetschke_211222_160123_HFGoe_IF2_b1R1_4h **Scan** 6230
**Peptide** ASGEAGGITQH **Charge** 1 **Spectral Angle** 0.83

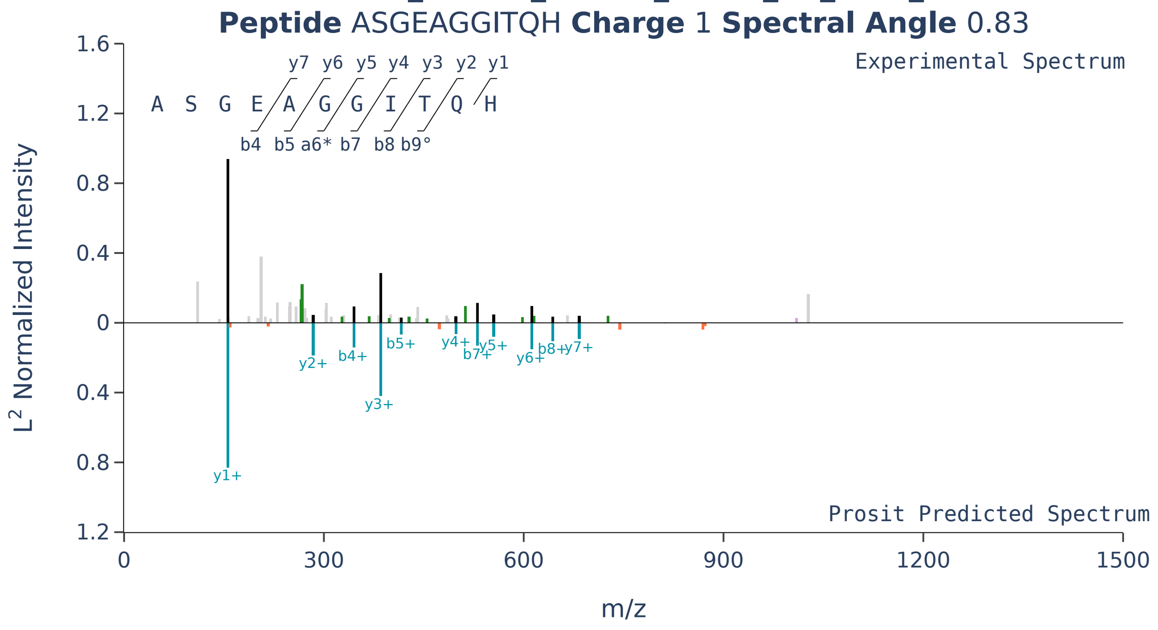


**Source** HRoetschke_211222_160123_HFGoe_IF2_b2R1_24h **Scan** 12477
**Peptide** GSGVGGITETD **Charge** 1 **Spectral Angle** 0.88

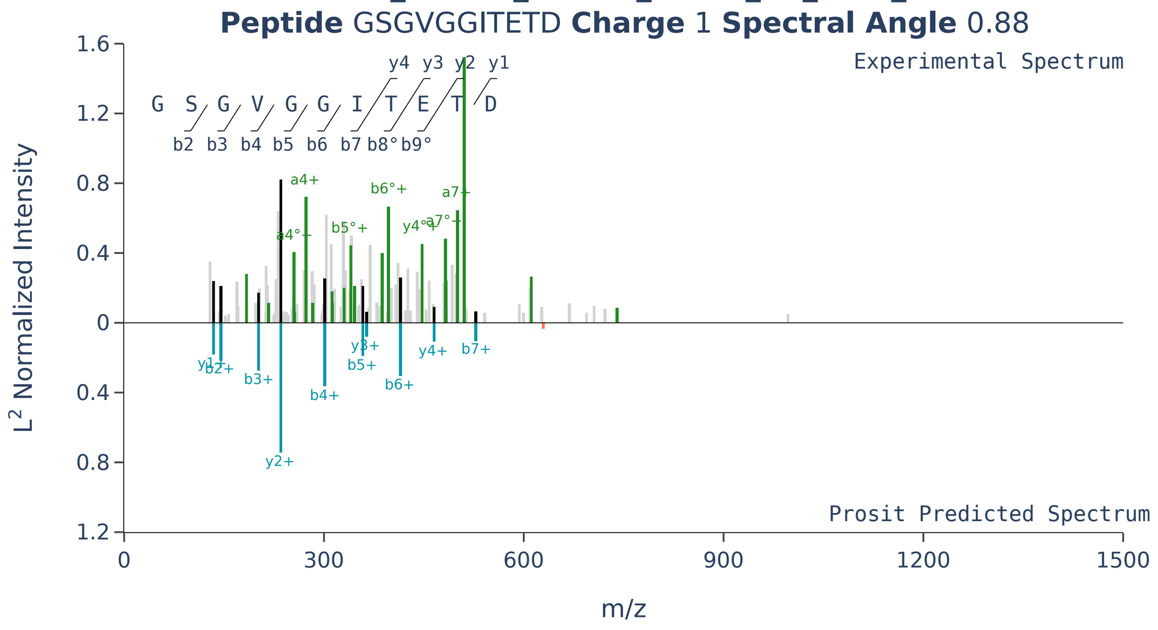


**Source** HRoetschke_211222_160123_HFGoe_IF2_b1R1_1h **Scan** 7612
**Peptide** ASGEAGGITQ **Charge** 2 **Spectral Angle** 0.95

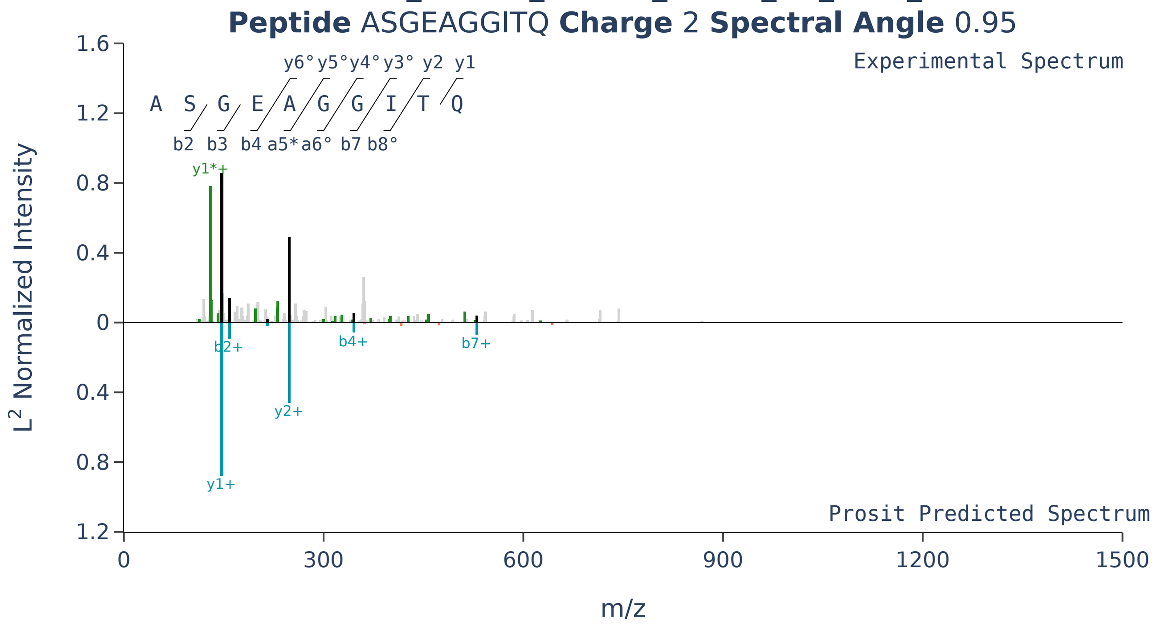


**Source** HRoetschke_211222_160123_HFGoe_IF2_b2R2_4h **Scan** 16087
**Peptide** DDGVMPQTIEAIQH **Charge** 2 **Spectral Angle** 0.86

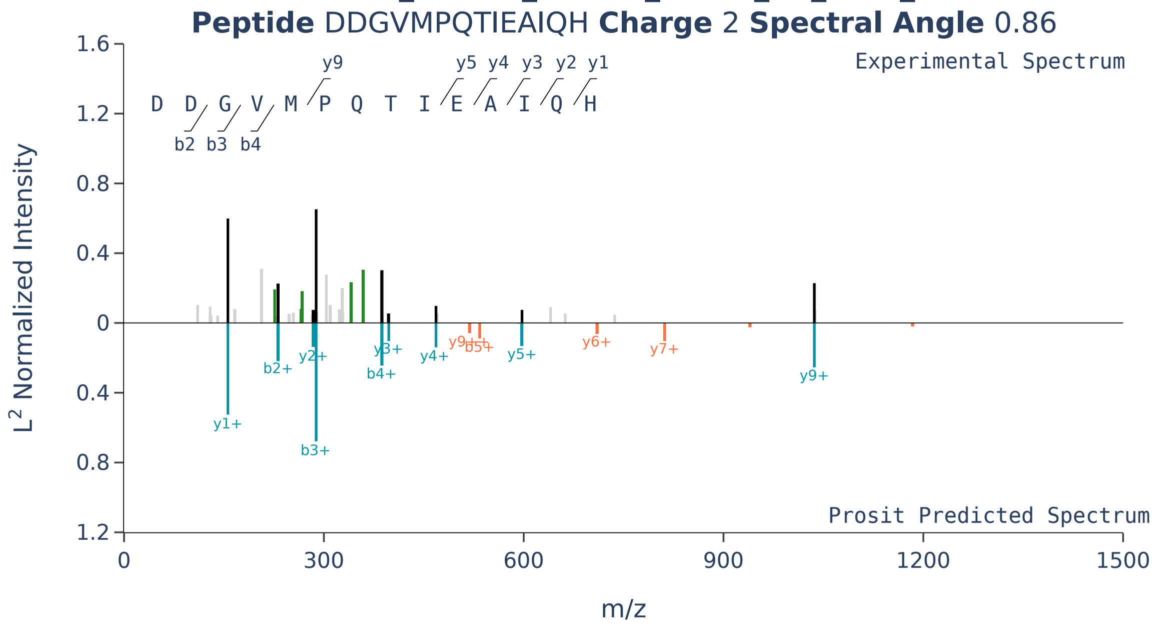


**Source** HRoetschke_211222_160123_HFGoe_IF2_b1R1_2h **Scan** 11931
**Peptide** CGIGVKNY **Charge** 2 **Spectral Angle** 0.85

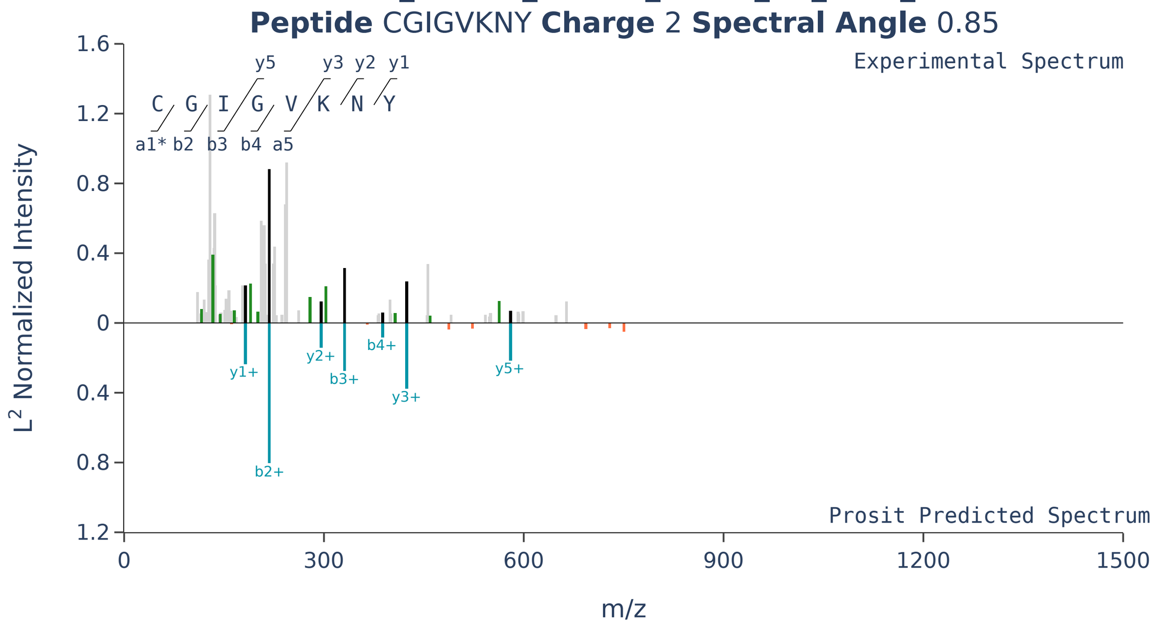


**Source** HRoetschke_211222_160123_HFGoe_IF2_b1R1_24h **Scan** 6513
**Peptide** TFVKRDPQE **Charge** 2 **Spectral Angle** 0.78

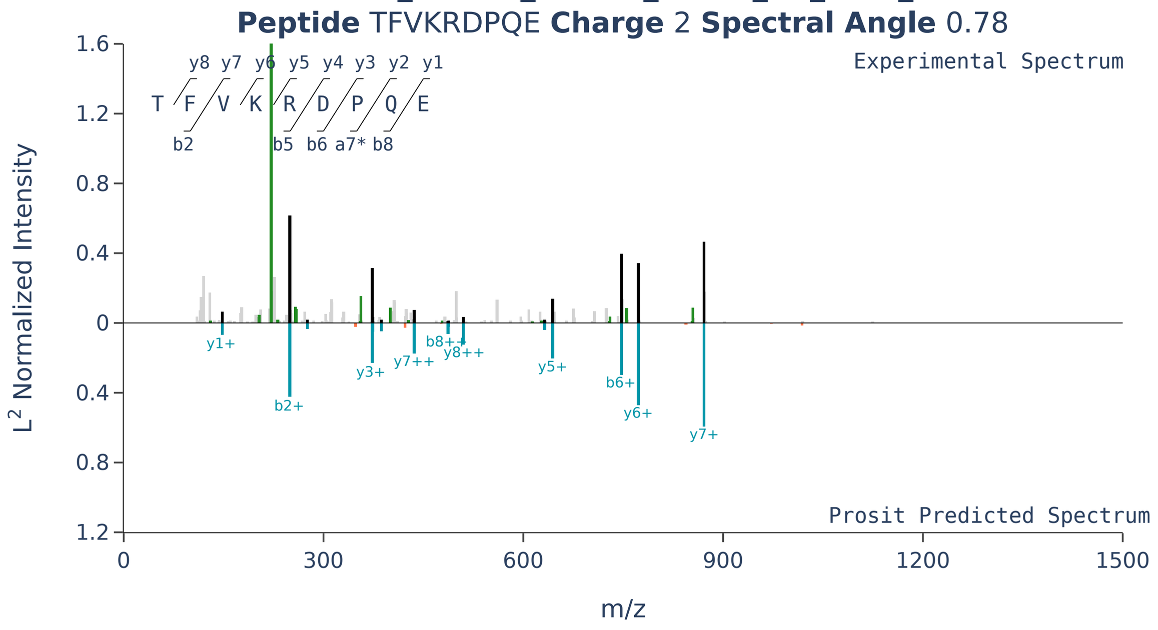


**Source** HRoetschke_211222_160123_HFGoe_IF2_b1R2_24h **Scan** 5716
**Peptide** PEADPDRVKNE **Charge** 2 **Spectral Angle** 0.84

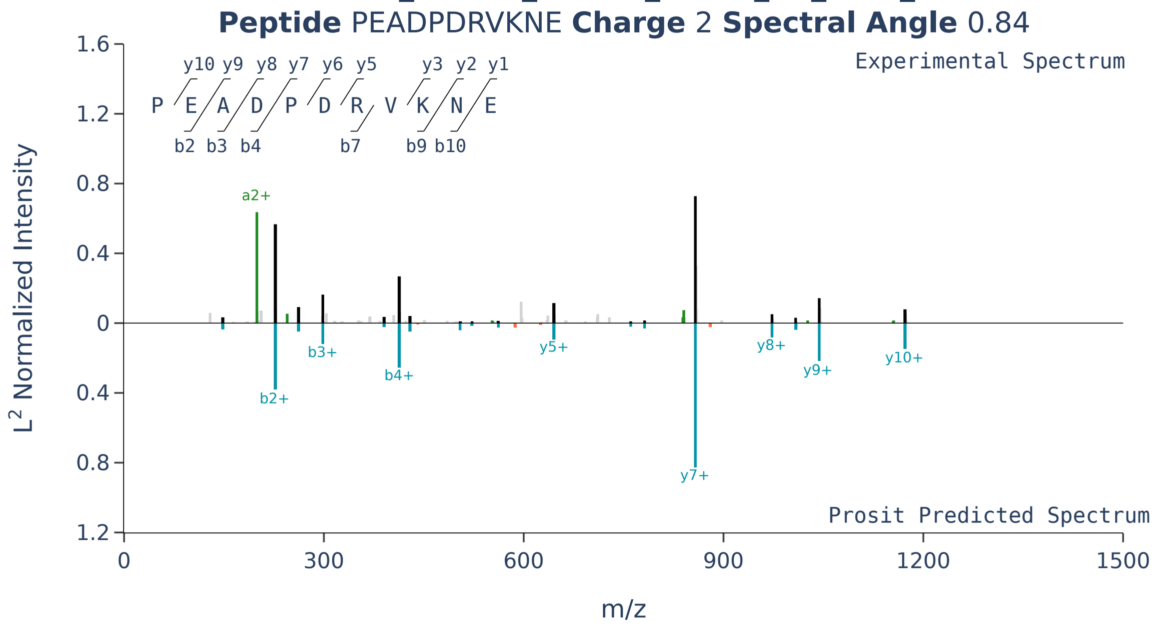


**Source** HRoetschke_211222_160123_HFGoe_IF2_b2R1_24h **Scan** 9836
**Peptide** HIGAYHVETE **Charge** 2 **Spectral Angle** 0.85

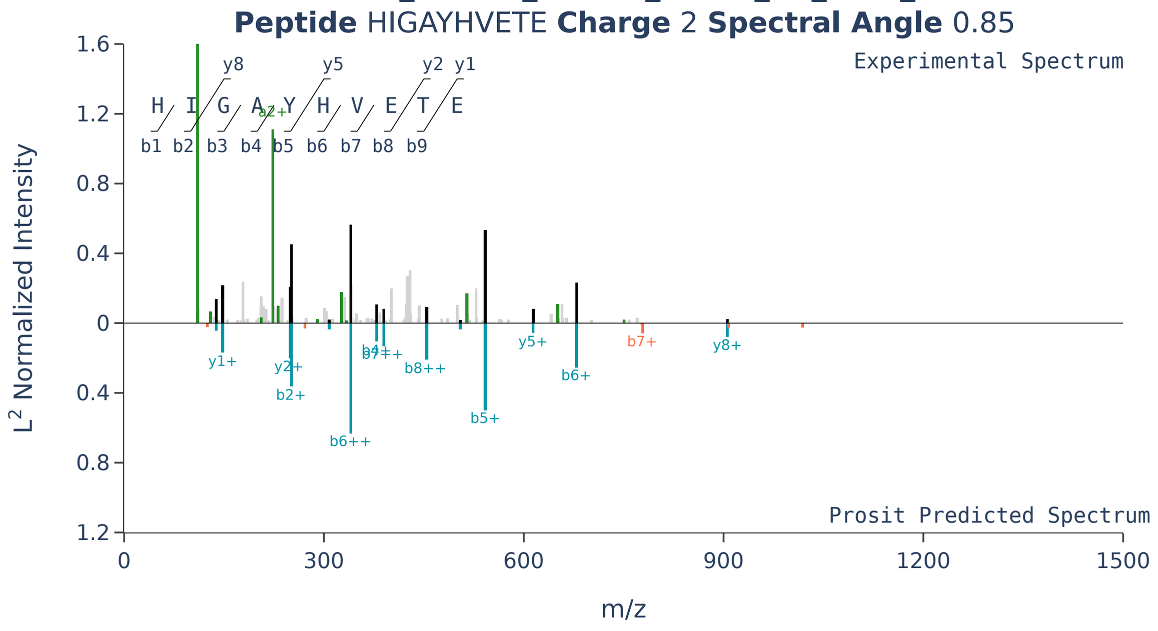


**Source** HRoetschke_211222_160123_HFGoe_IF2_b1R1_4h **Scan** 8802
**Peptide** SAQEKQTL **Charge** 1 **Spectral Angle** 0.87

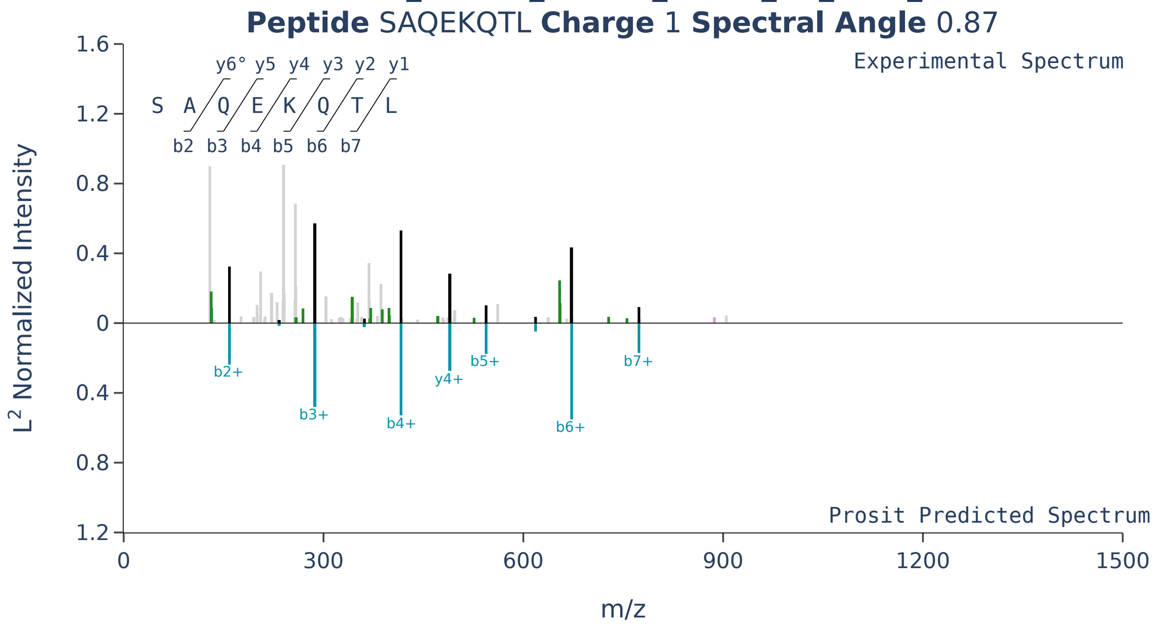


**Source** HRoetschke_211222_160123_HFGoe_IF2_b1R1_2h **Scan** 7554
**Peptide** QKPAQAVNR **Charge** 2 **Spectral Angle** 0.97

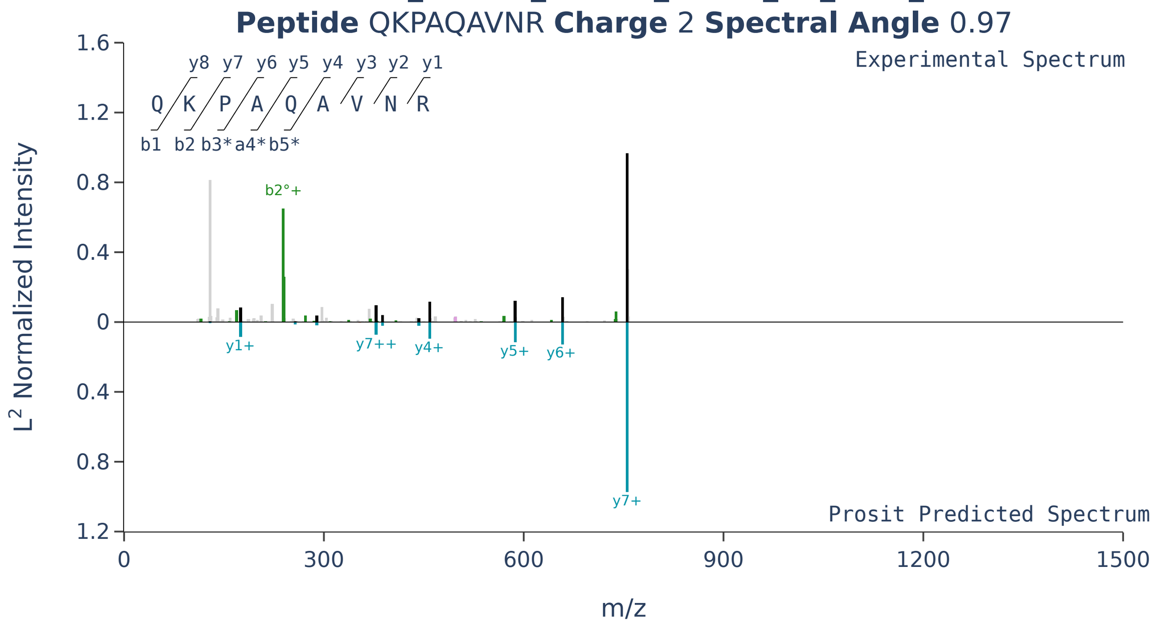


**Source** HRoetschke_211222_160123_HFGoe_IF2_b1R1_4h **Scan** 13095
**Peptide** QLVAEEMGH **Charge** 2 **Spectral Angle** 0.81

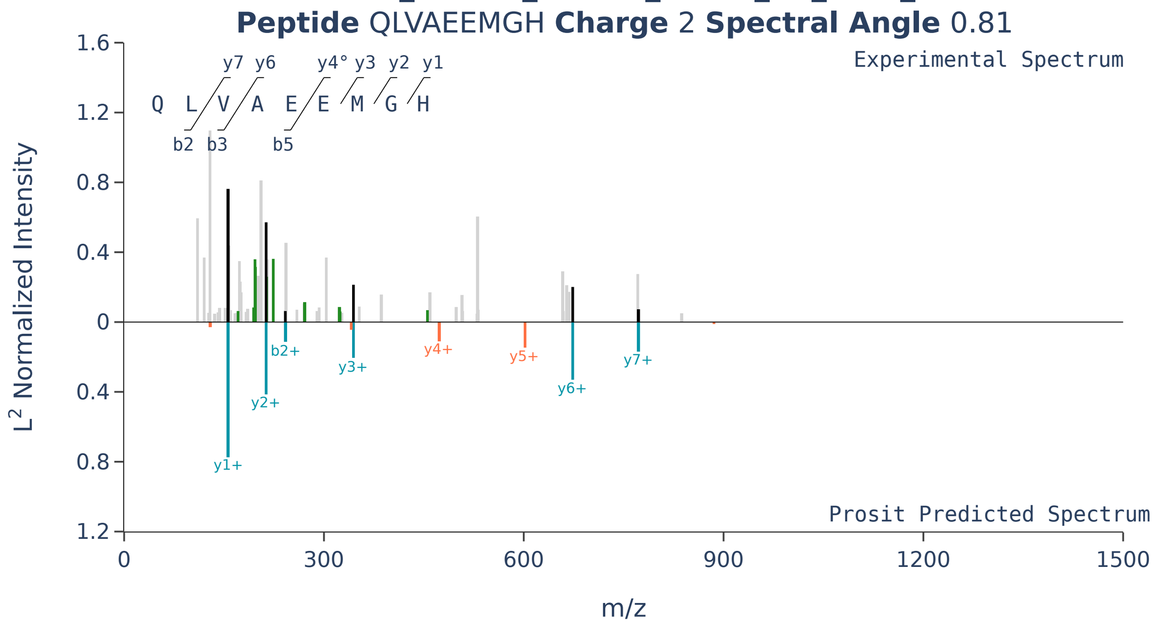


**Source** HRoetschke_211222_160123_HFGoe_IF2_b2R2_4h **Scan** 18986
**Peptide** AILLQAE **Charge** 1 **Spectral Angle** 0.94

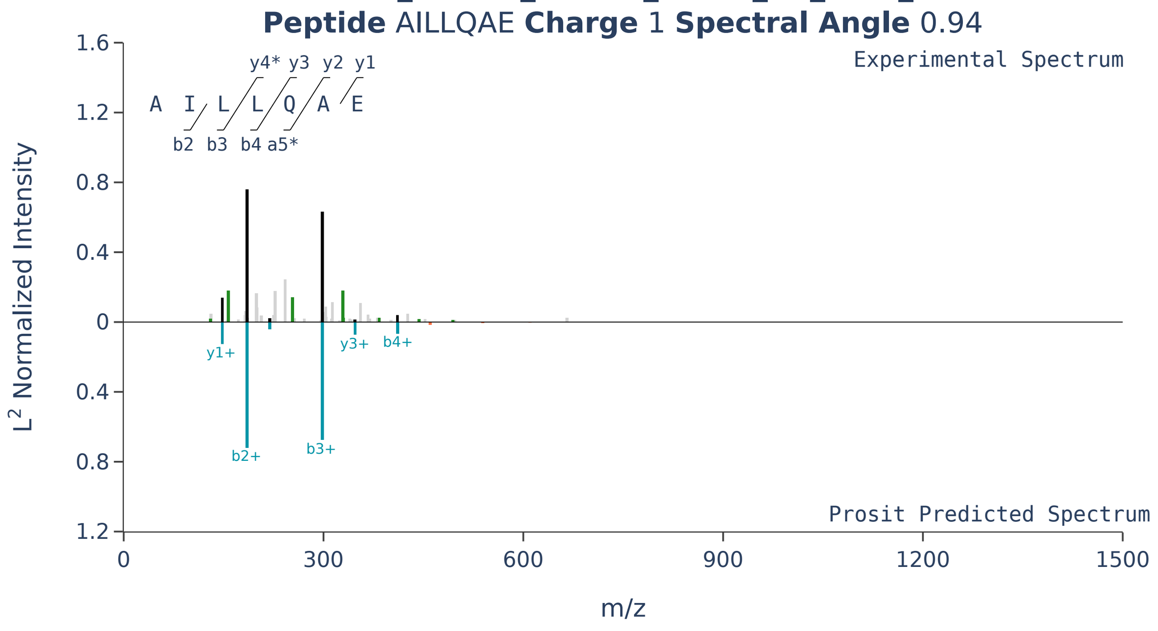


**Source** HRoetschke_211222_160123_HFGoe_IF2_b1R1_4h **Scan** 19547
**Peptide** TENGMITF **Charge** 2 **Spectral Angle** 0.94

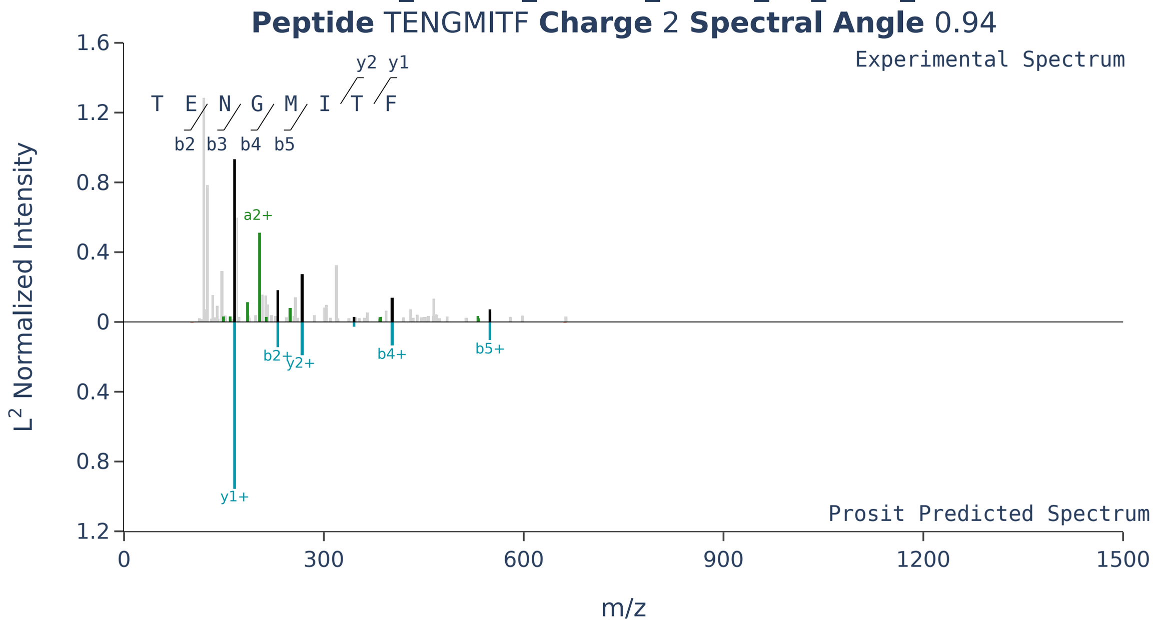


**Source** HRoetschke_211222_160123_HFGoe_IF2_b1R2_2h **Scan** 10813
**Peptide** GFEYGRVR **Charge** 2 **Spectral Angle** 0.83

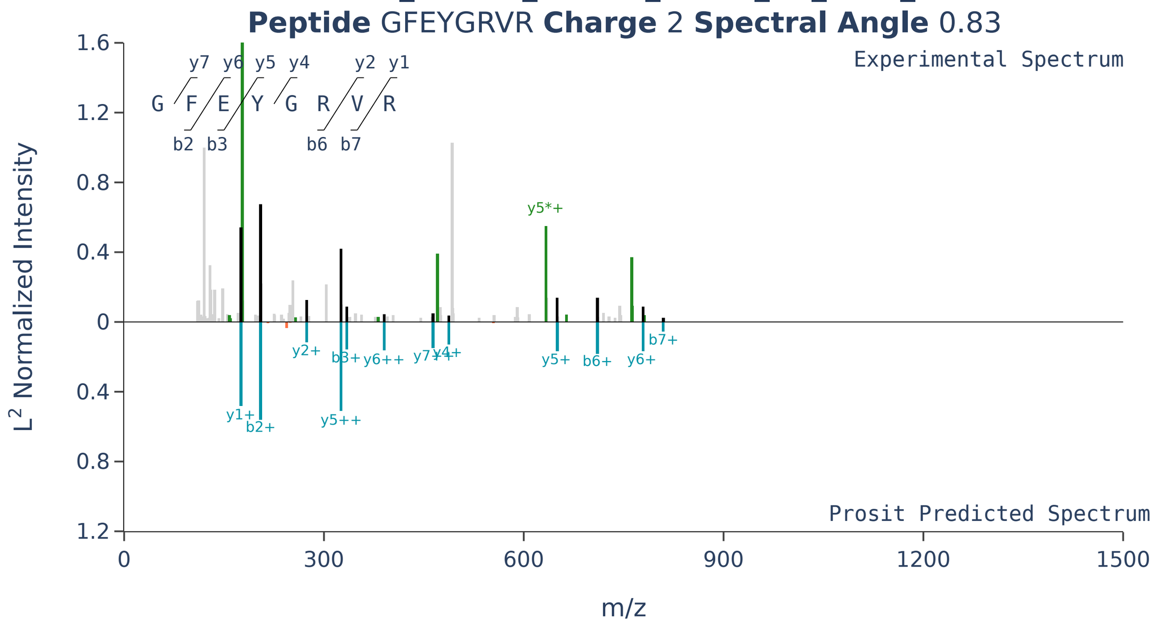


**Source** HRoetschke_211222_160123_HFGoe_IF2_b2R2_4h **Scan** 8516
**Peptide** QFADAGIRKS **Charge** 3 **Spectral Angle** 0.87

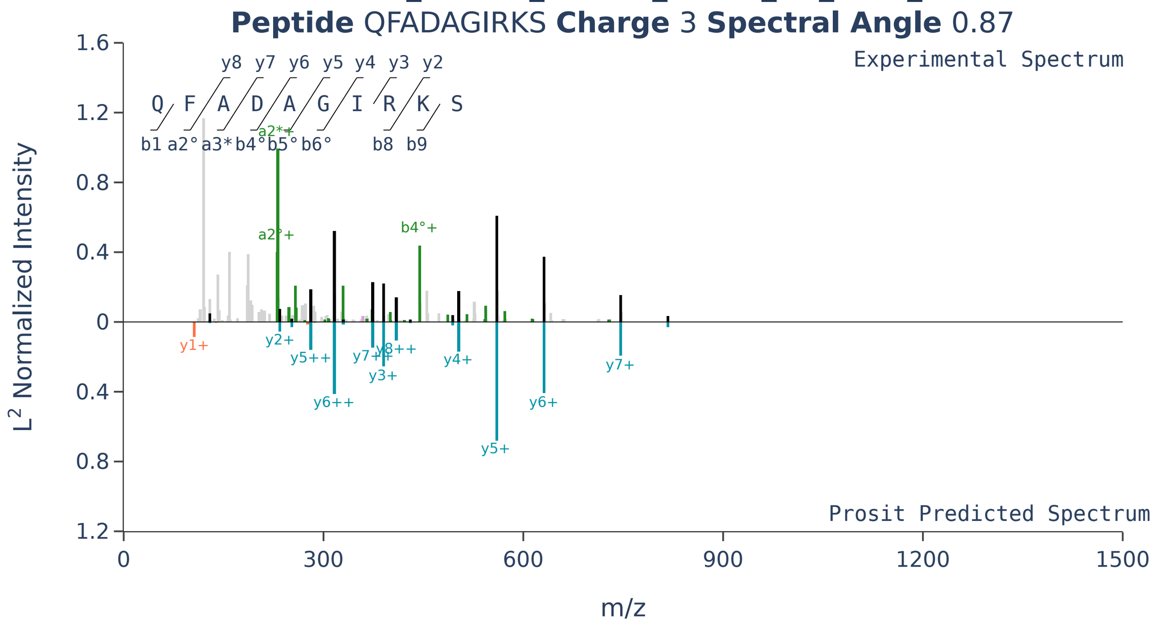

**Source** HRoetschke_211222_160123_HFGoe_IF2_b1R1_4h **Scan** 13537
**Peptide** AVNRDVVIGE **Charge** 2 **Spectral Angle** 0.82

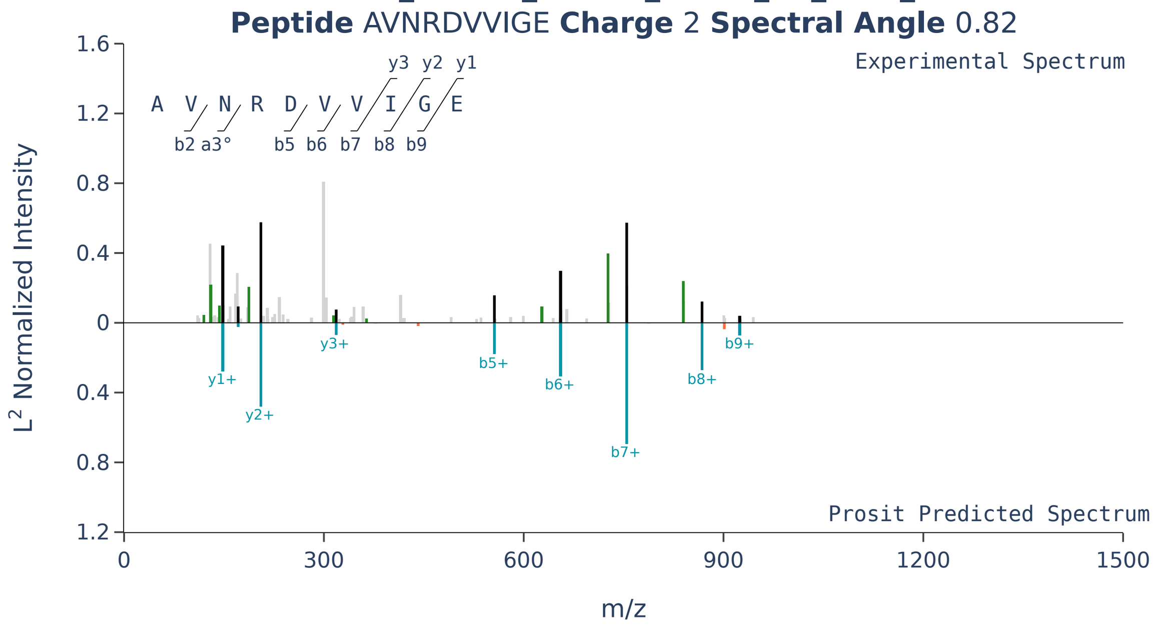


**Source** HRoetschke_211222_160123_HFGoe_IF2_b2R2_24h **Scan** 5302
**Peptide** ELKAVRKGMA **Charge** 2 **Spectral Angle** 0.89

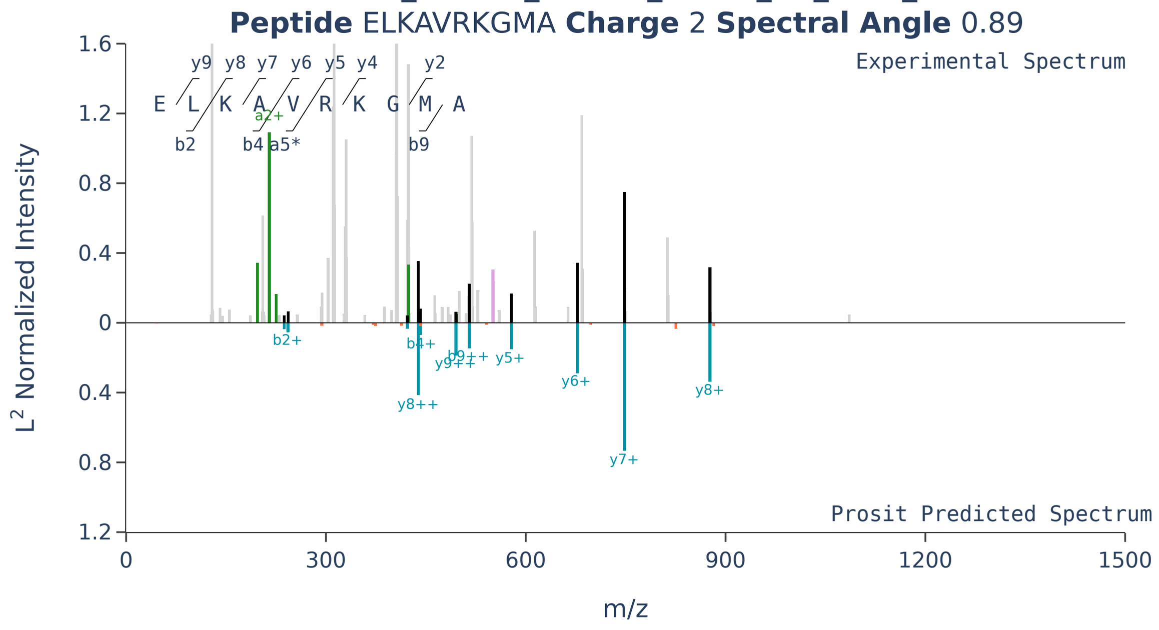


**Source** HRoetschke_211222_160123_HFGoe_IF2_b1R2_2h **Scan** 10128
**Peptide** GSSLQQGFQK **Charge** 2 **Spectral Angle** 0.76

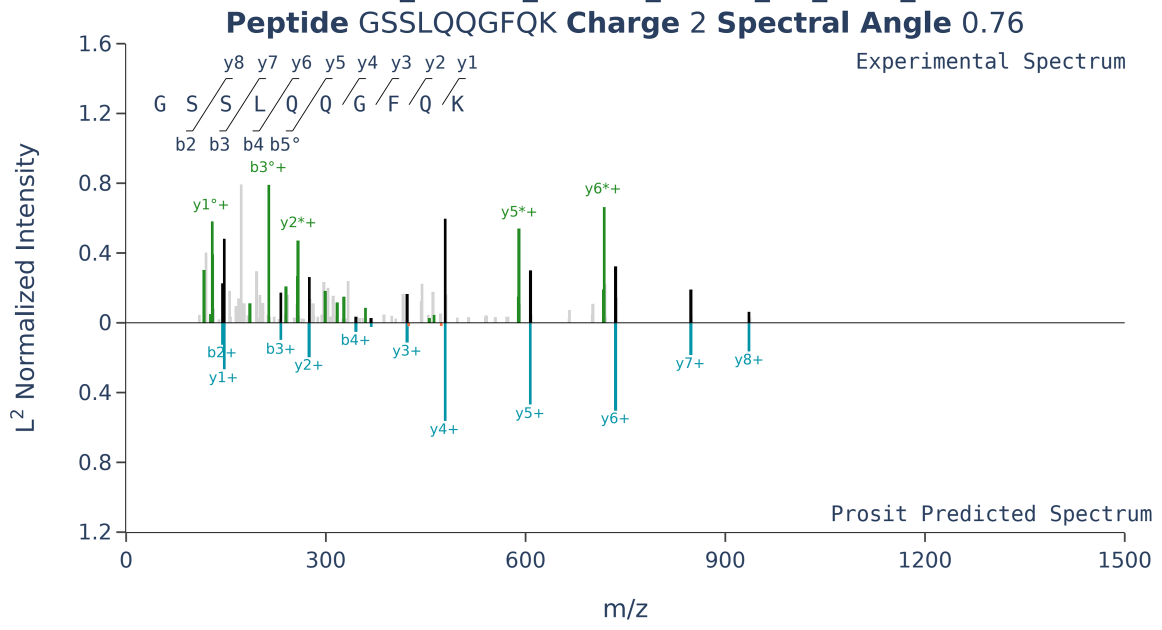


**Source** HRoetschke_211222_160123_HFGoe_IF2_b2R2_1h **Scan** 5934
**Peptide** RTFVKRDPQE **Charge** 3 **Spectral Angle** 0.83

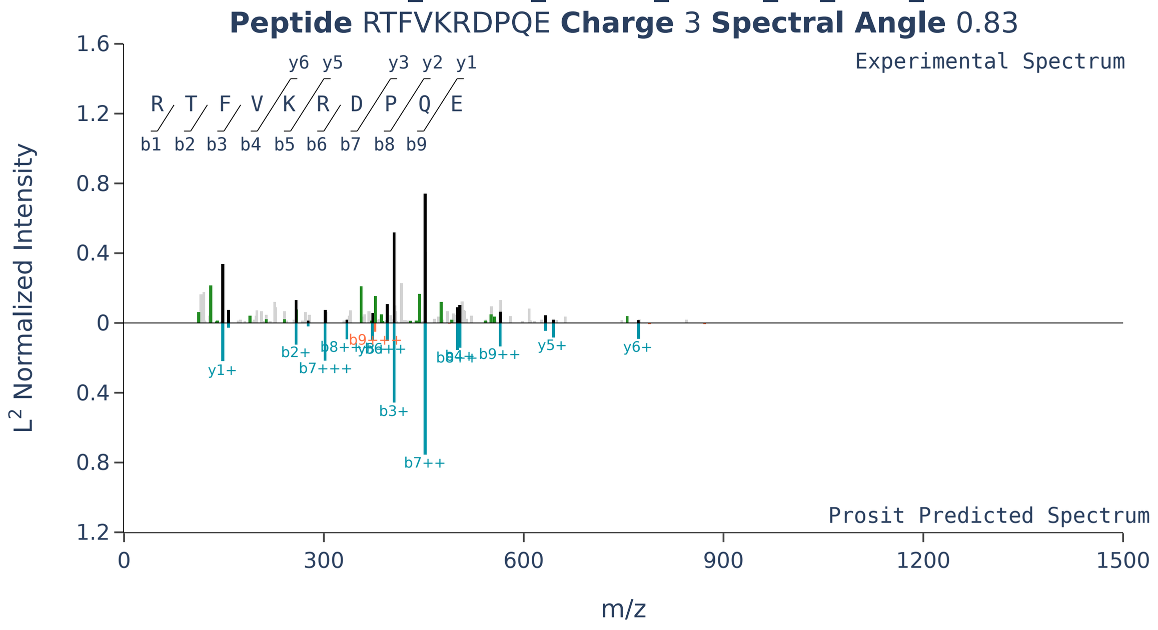


**Source** HRoetschke_211222_160123_HFGoe_IF2_b1R1_24h **Scan** 6977
**Peptide** RENELEE **Charge** 2 **Spectral Angle** 0.9

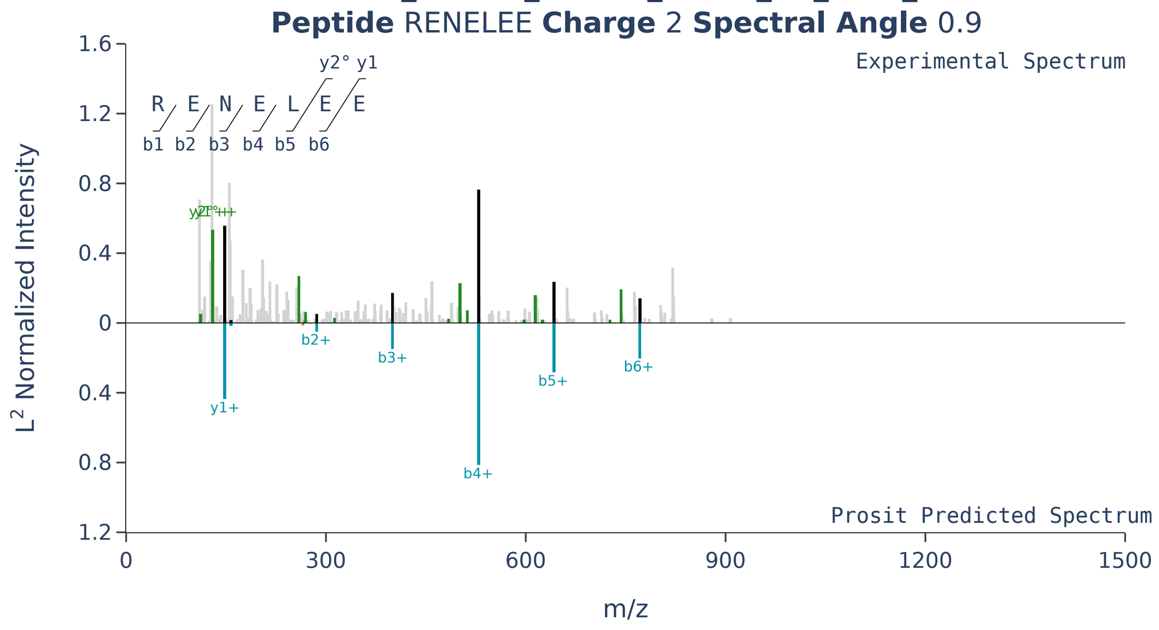


**Source** HRoetschke_211222_160123_HFGoe_IF2_b2R2_4h **Scan** 21267
**Peptide** DVVIGETI **Charge** 2 **Spectral Angle** 0.92

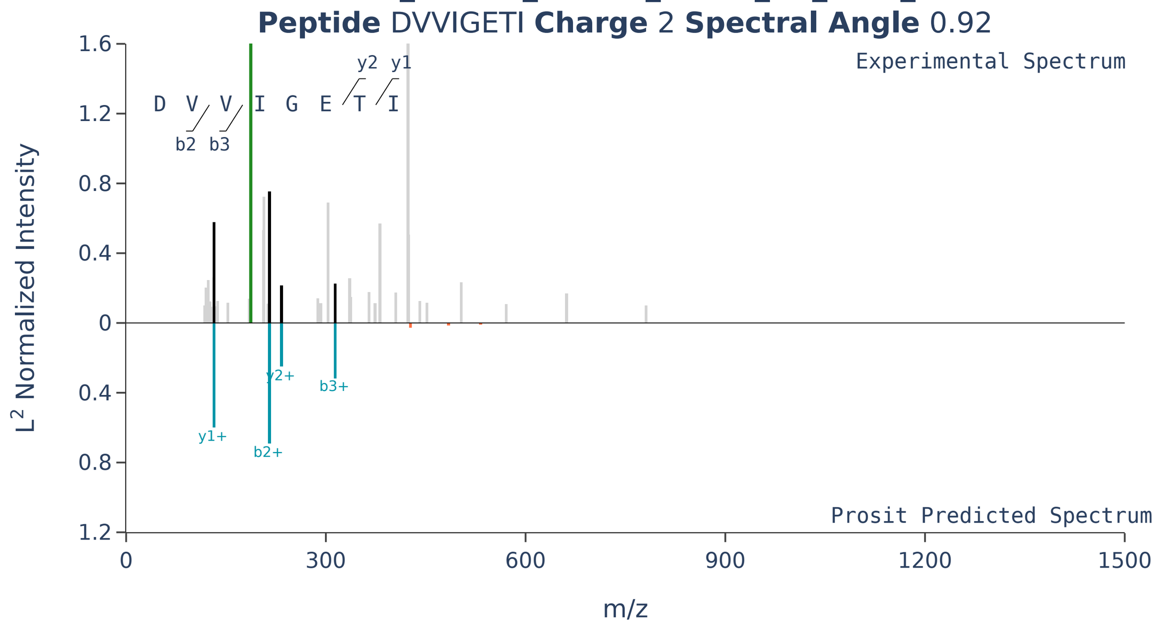


**Source** HRoetschke_211222_160123_HFGoe_IF2_b1R2_4h **Scan** 15174
**Peptide** TFLDTPGH **Charge** 2 **Spectral Angle** 0.91

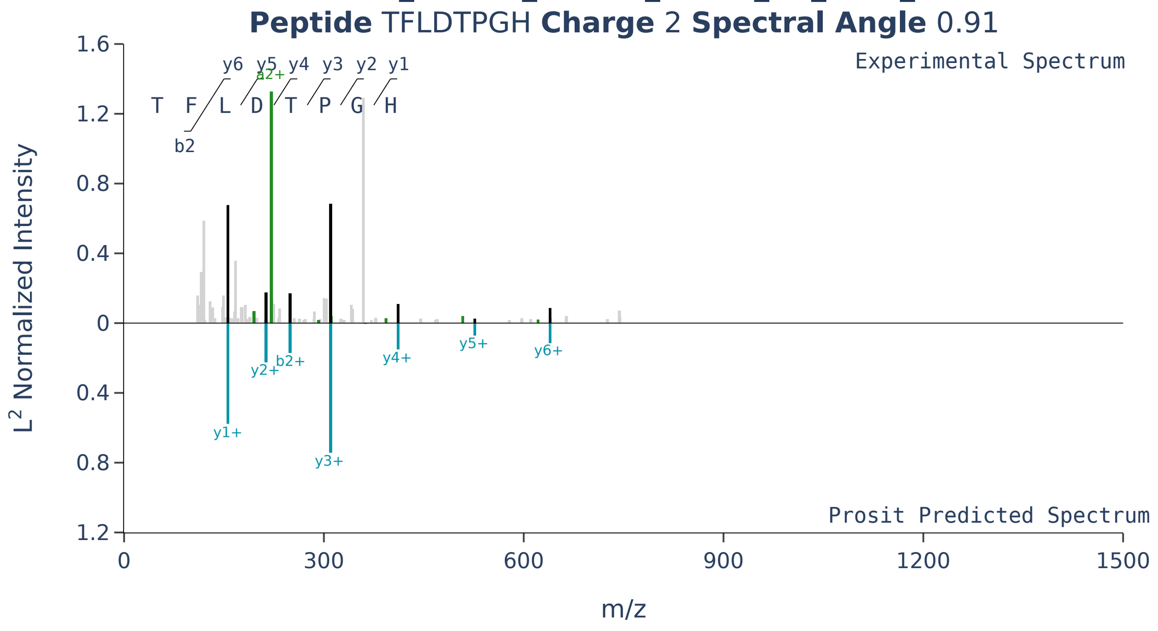


**Source** HRoetschke_211222_160123_HFGoe_IF2_b2R2_24h **Scan** 13455
**Peptide** RDVFKSPKF **Charge** 3 **Spectral Angle** 0.83

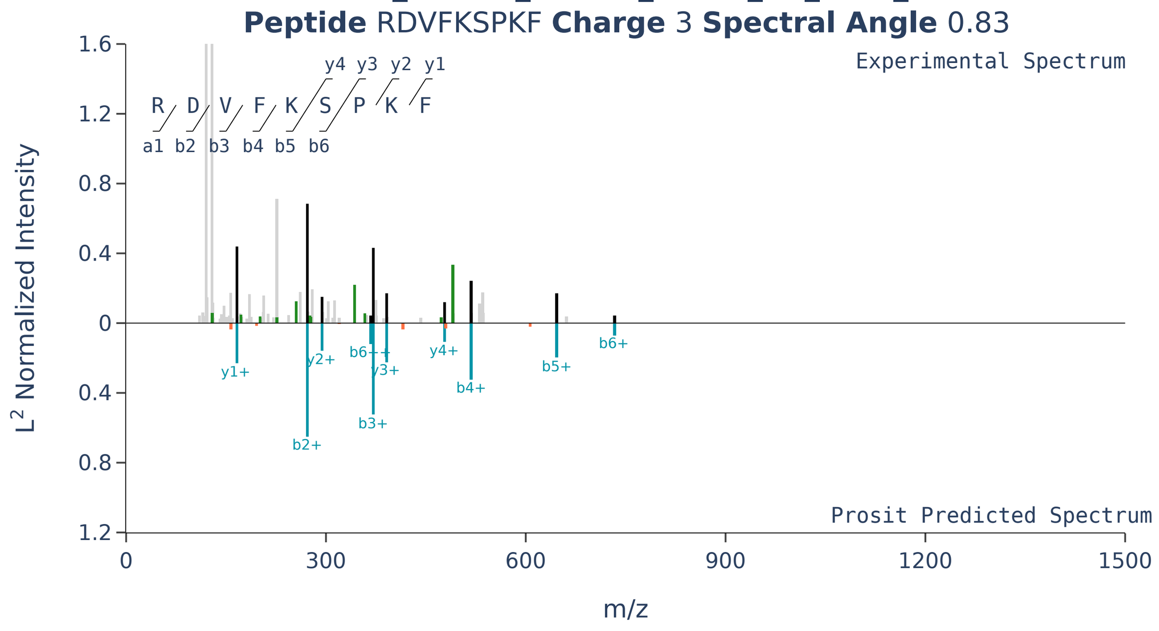


**Source** HRoetschke_211222_160123_HFGoe_IF2_b1R1_24h **Scan** 9083
**Peptide** EEMGHKVILR **Charge** 3 **Spectral Angle** 0.91

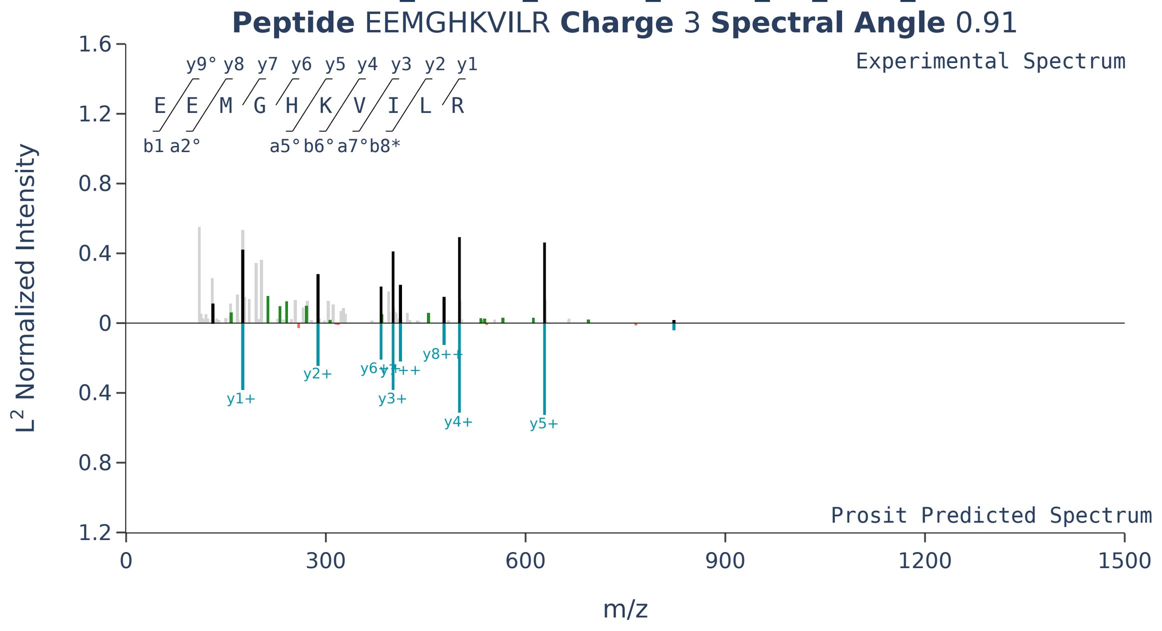


**Source** HRoetschke_211222_160123_HFGoe_IF2_b1R1_2h **Scan** 13454
**Peptide** SLQQGFQ **Charge** 2 **Spectral Angle** 0.97

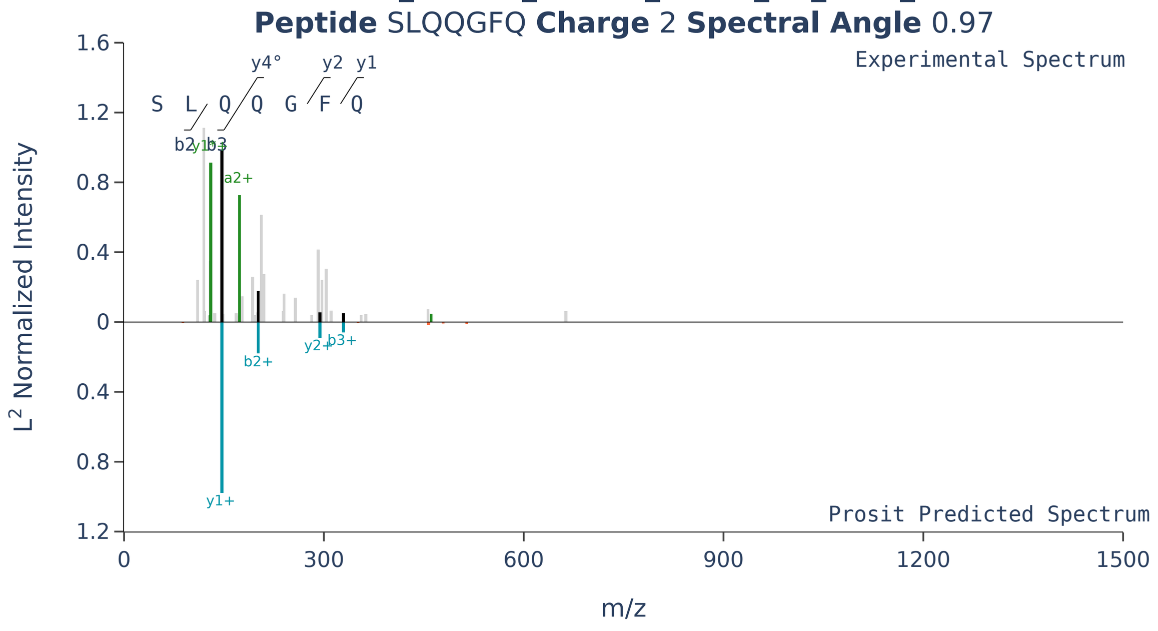


**Source** HRoetschke_211222_160123_HFGoe_IF2_b1R2_24h **Scan** 11113
**Peptide** DTPGHAAFT **Charge** 2 **Spectral Angle** 0.81

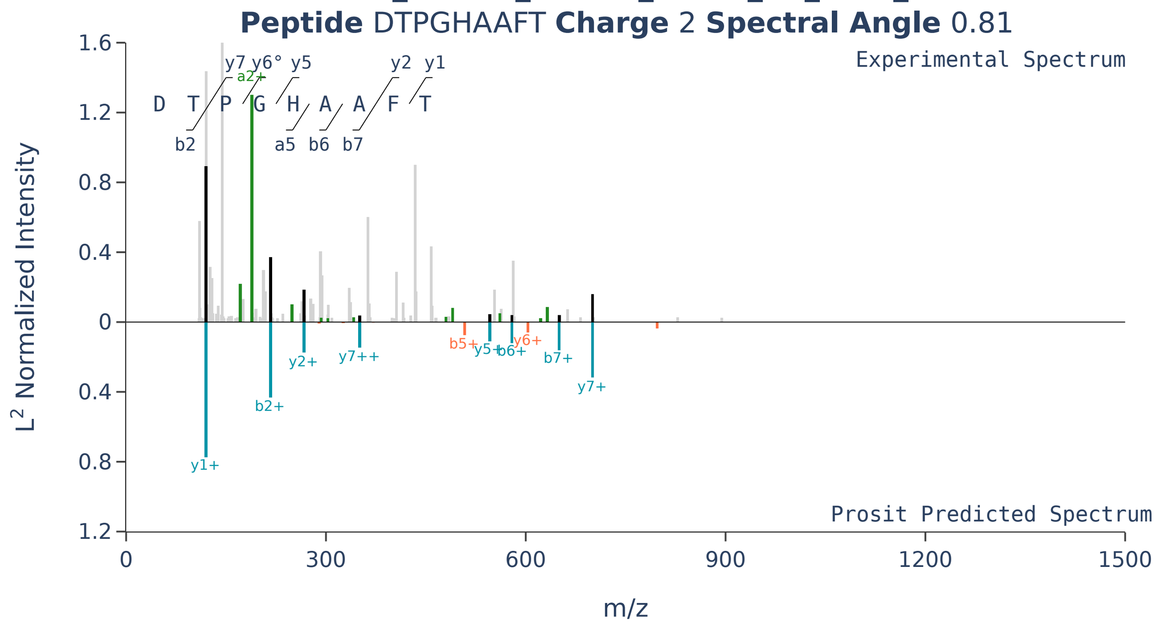


**Source** HRoetschke_211222_160123_HFGoe_IF2_b1R2_2h **Scan** 9341
**Peptide** GHVDHGKTSLL **Charge** 3 **Spectral Angle** 0.93

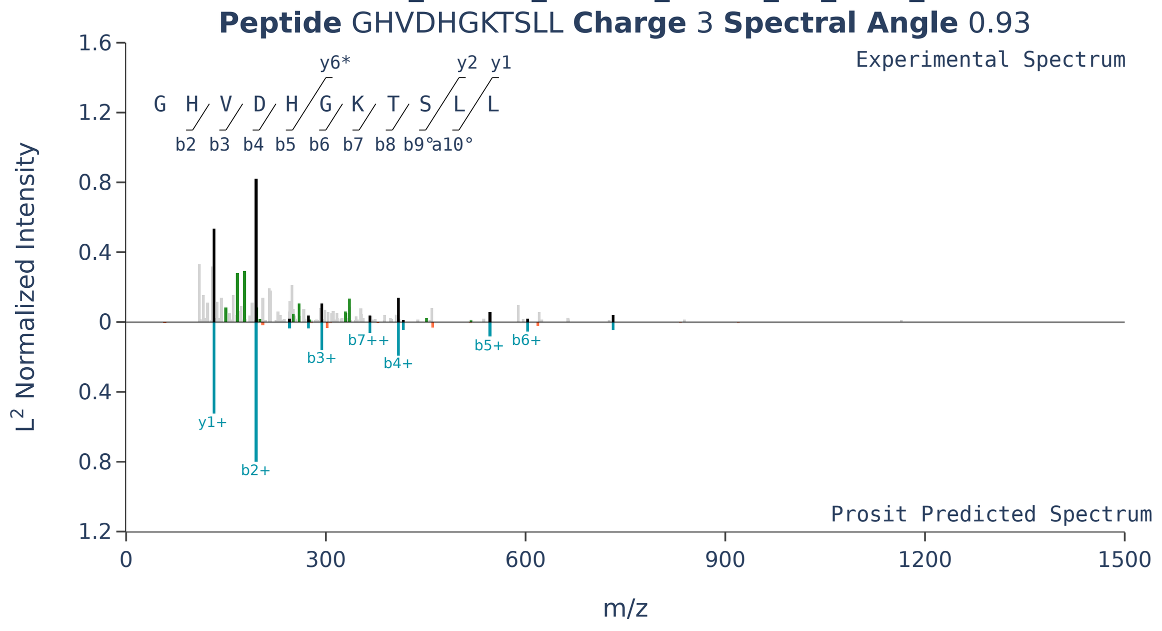


**Source** HRoetschke_211222_160123_HFGoe_IF2_b2R1_1h **Scan** 14944
**Peptide** TITVGELANKM **Charge** 2 **Spectral Angle** 0.83

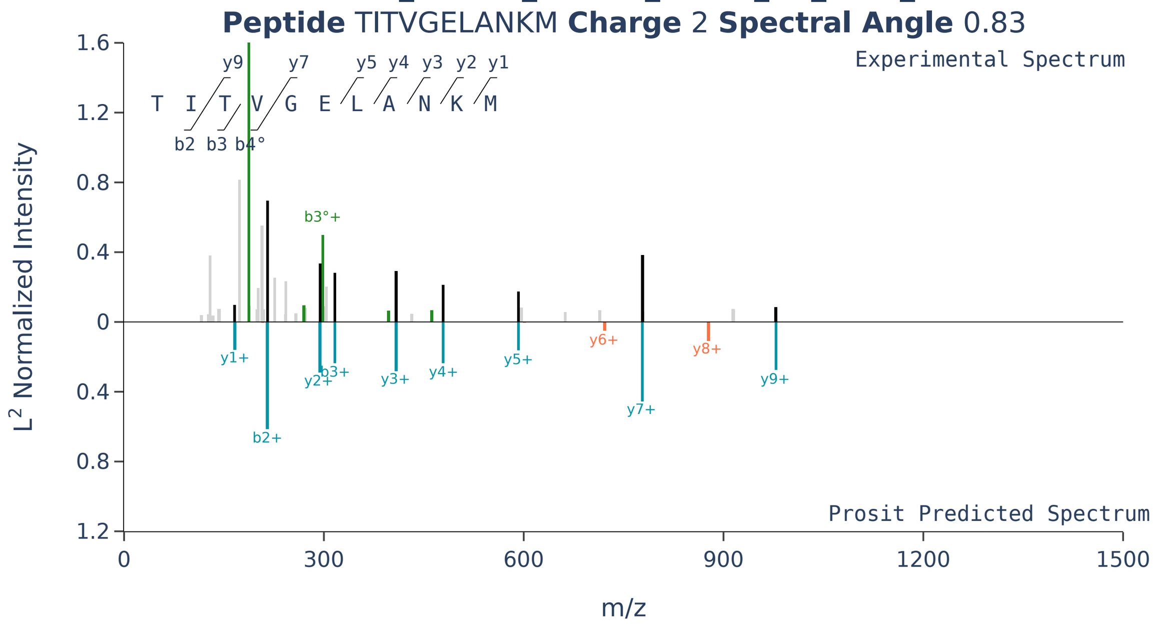


**Source** HRoetschke_211222_160123_HFGoe_IF2_b1R1_2h **Scan** 13881
**Peptide** GTLHKGDIVLC **Charge** 3 **Spectral Angle** 0.93

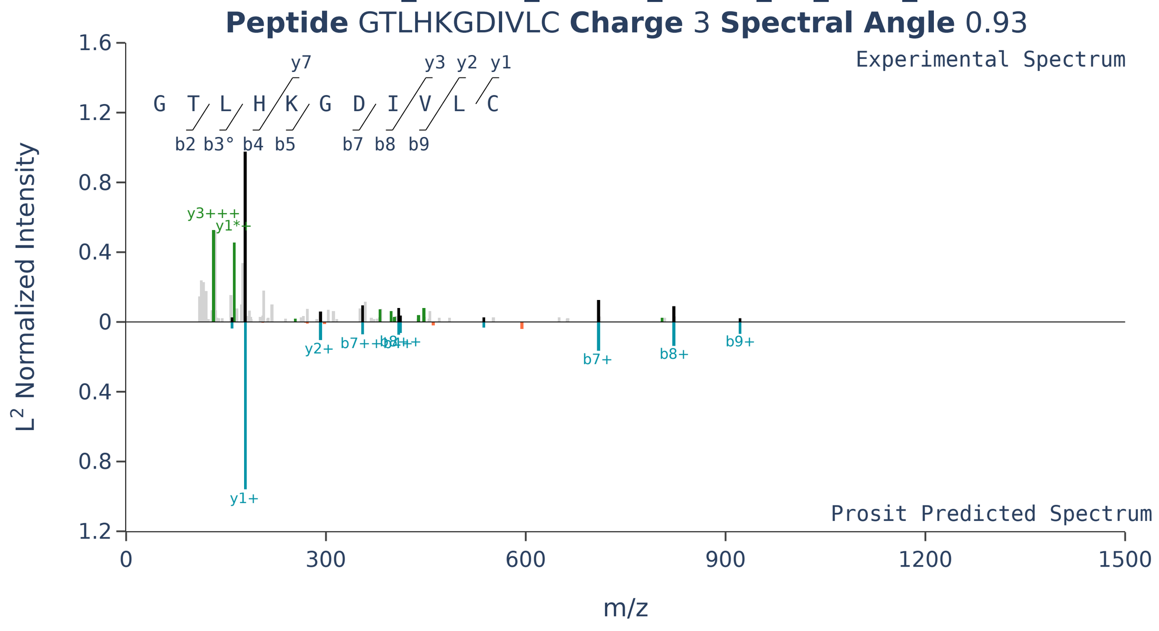


**Source** HRoetschke_211222_160123_HFGoe_IF2_b1R2_1h **Scan** 10247
**Peptide** NSGPDKLTLQR **Charge** 2 **Spectral Angle** 0.81

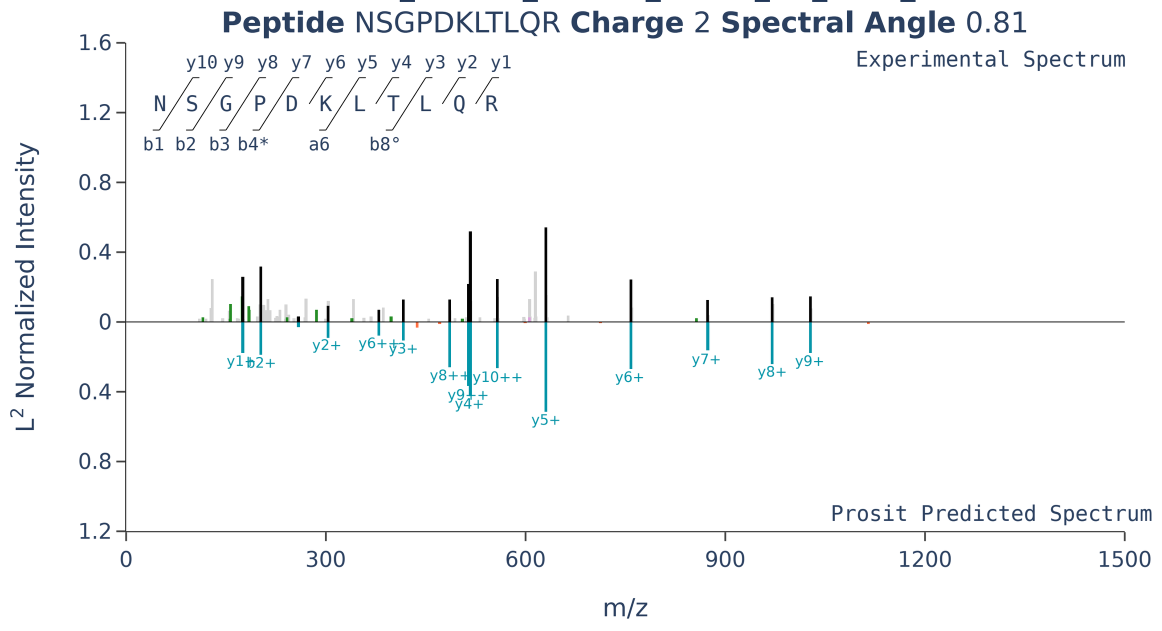

**Source** HRoetschke_211222_160123_HFGoe_IF2_b1R2_4h **Scan** 10034
**Peptide** KQTLIDHLNQKN **Charge** 4 **Spectral Angle** 0.86

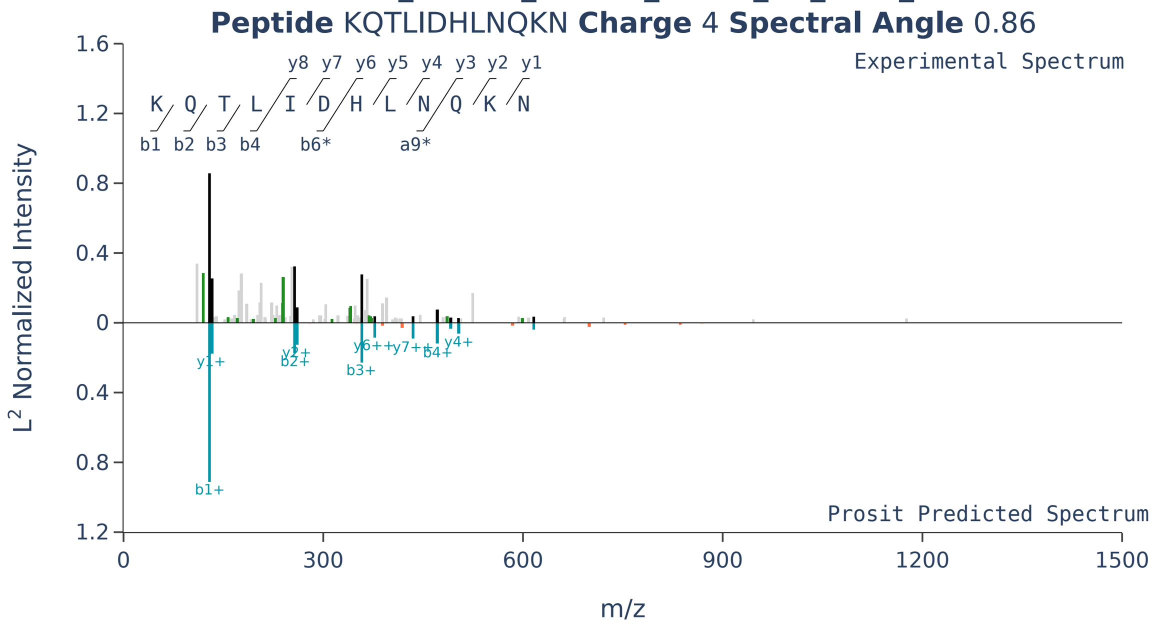


**Source** HRoetschke_211222_160123_HFGoe_IF2_b1R2_24h **Scan** 15423
**Peptide** RDNVVIY **Charge** 2 **Spectral Angle** 0.87

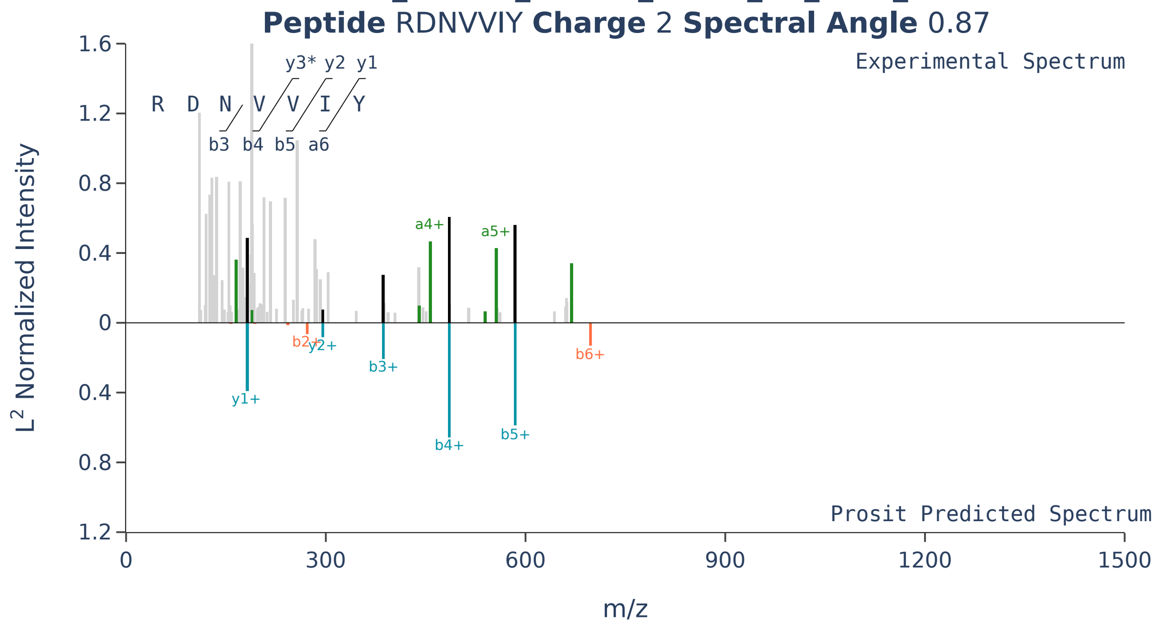


**Source** HRoetschke_211222_160123_HFGoe_IF2_b1R1_4h **Scan** 8817
**Peptide** EMGHKVILRRENEL **Charge** 3 **Spectral Angle** 0.88

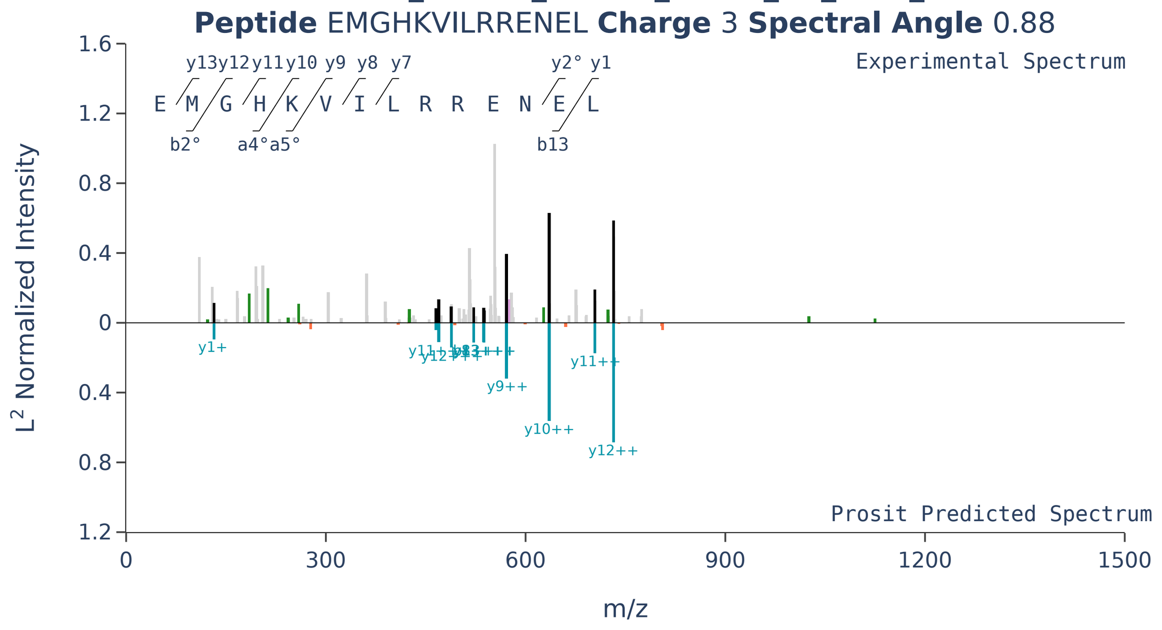


**Source** HRoetschke_211222_160123_HFGoe_IF2_b2R2_4h **Scan** 6857
**Peptide** TKVASGEAGGITQH **Charge** 3 **Spectral Angle** 0.8

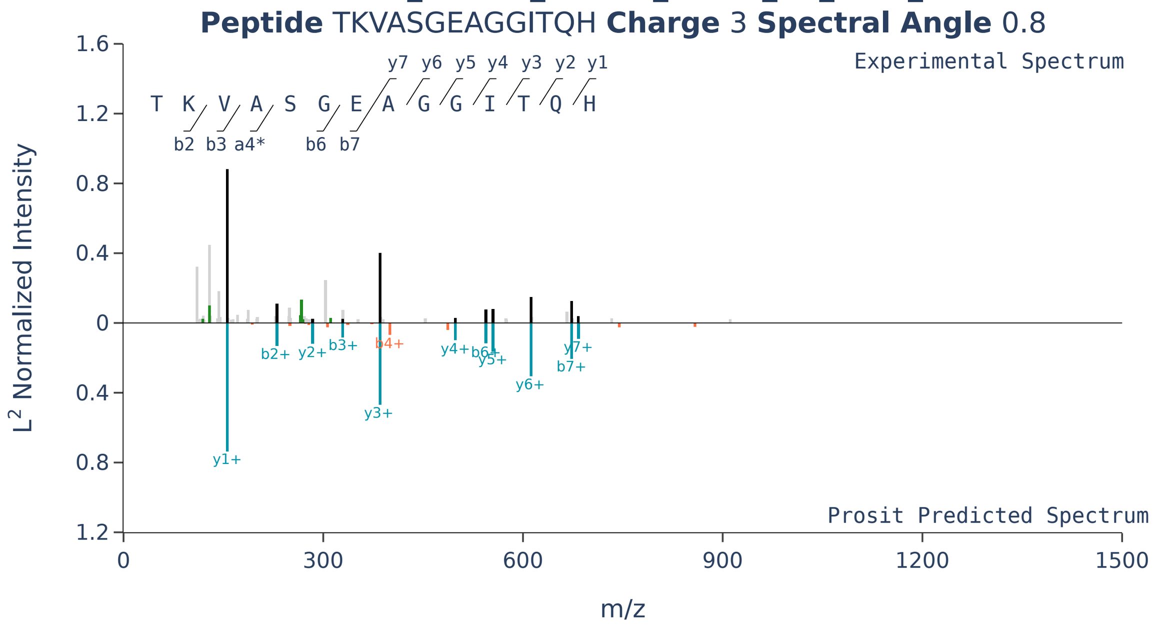


**Source** HRoetschke_211222_160123_HFGoe_IF2_b1R1_2h **Scan** 7000
**Peptide** VQIEVRKK **Charge** 2 **Spectral Angle** 0.85

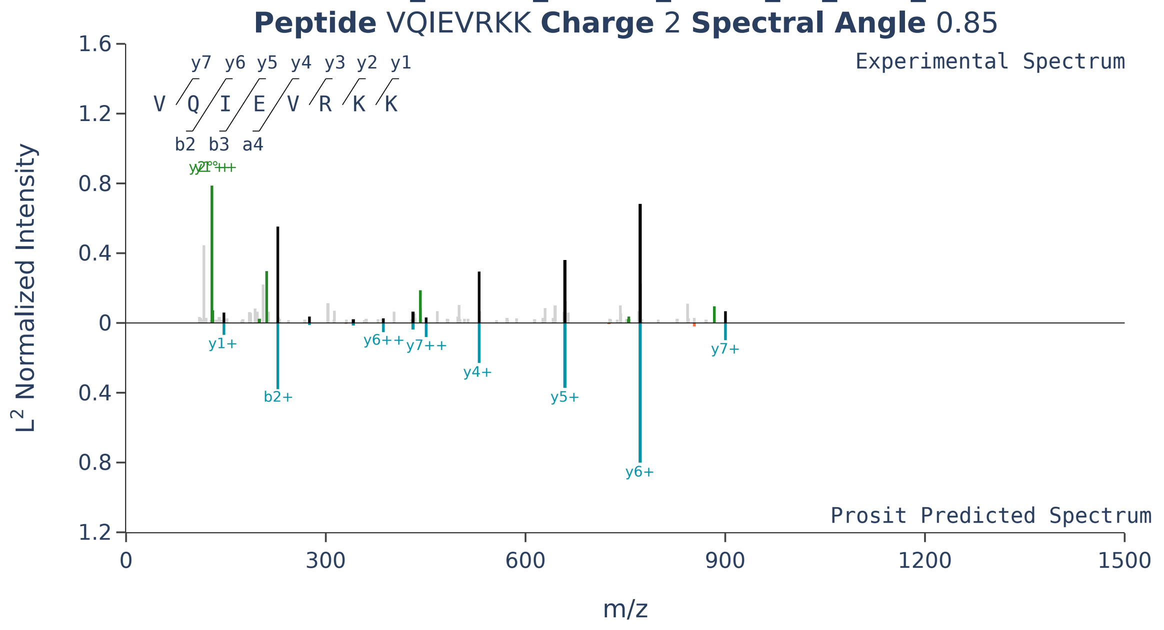


**Source** HRoetschke_211222_160123_HFGoe_IF2_b2R2_24h **Scan** 12125
**Peptide** SPELKQQI **Charge** 2 **Spectral Angle** 0.78

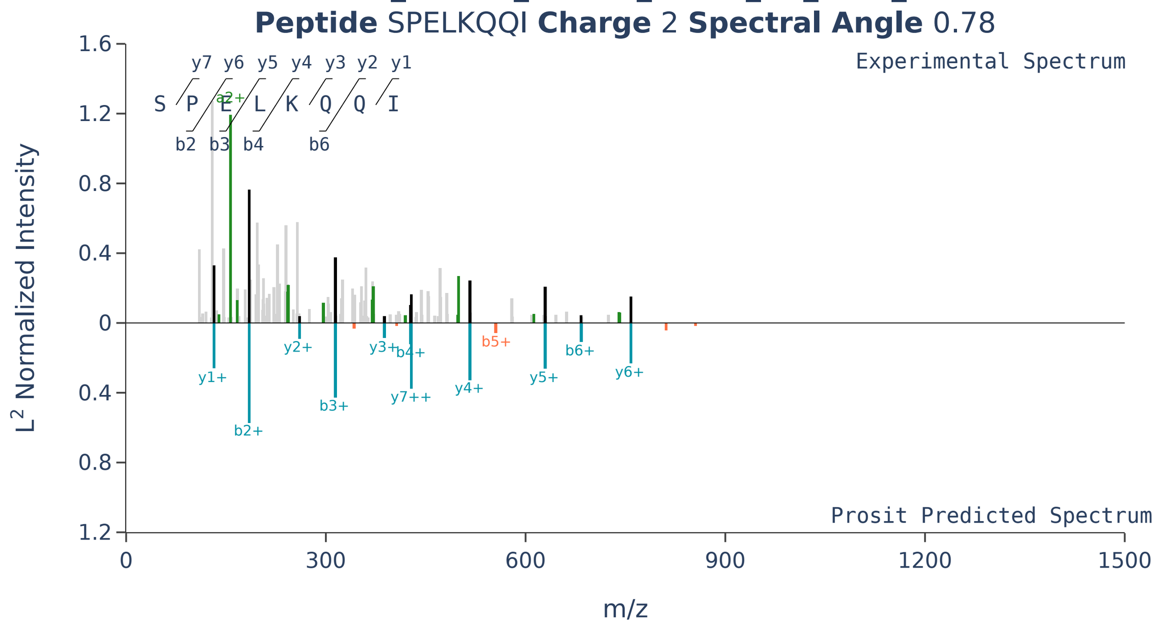


**Source** HRoetschke_211222_160123_HFGoe_IF2_b2R1_24h **Scan** 5952
**Peptide** FVKRDPQEAE **Charge** 3 **Spectral Angle** 0.9

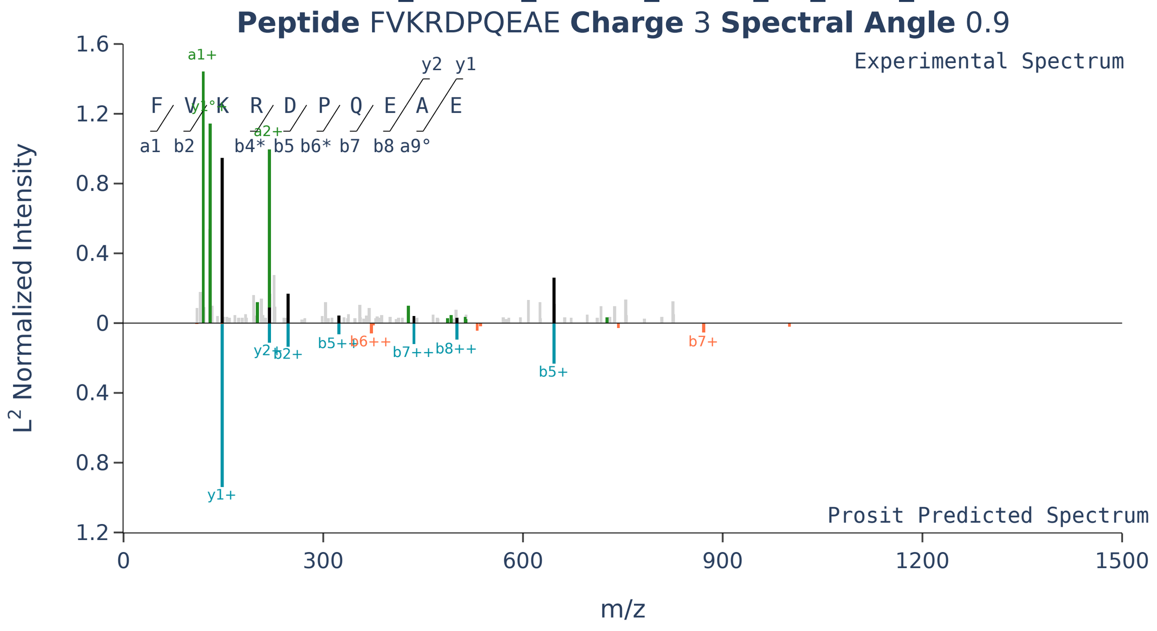


**Source** HRoetschke_211222_160123_HFGoe_IF2_b1R1_2h **Scan** 7618
**Peptide** AEEMGHKVILRR **Charge** 4 **Spectral Angle** 0.87

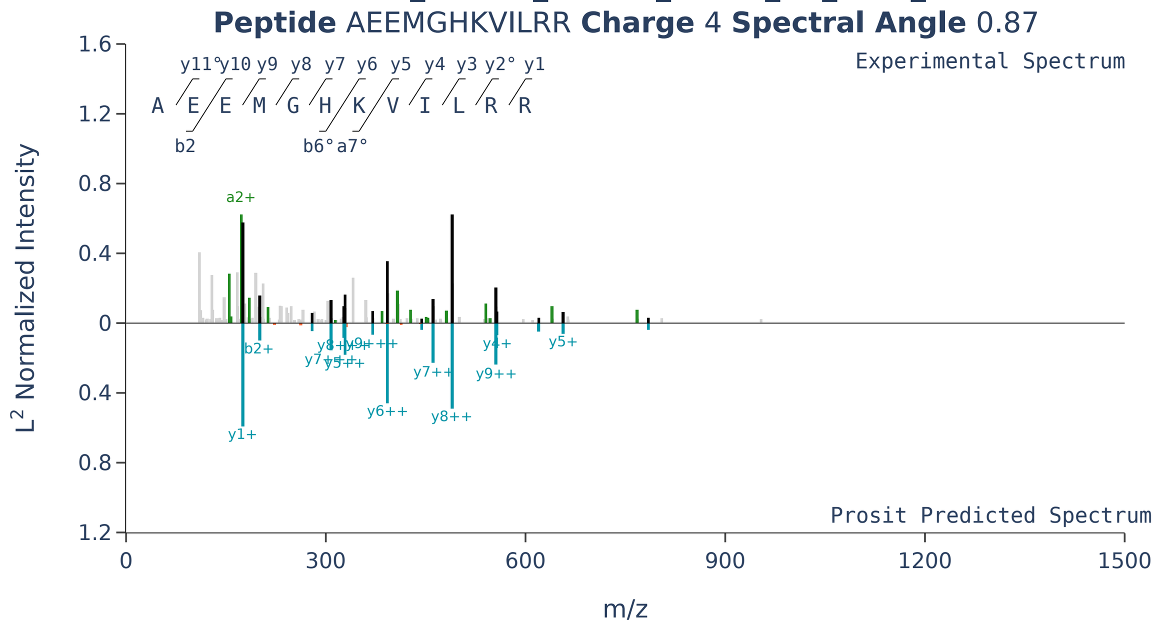


**Source** HRoetschke_211222_160123_HFGoe_IF2_b2R1_2h **Scan** 11069
**Peptide** NDVRTGDVIE **Charge** 2 **Spectral Angle** 0.84

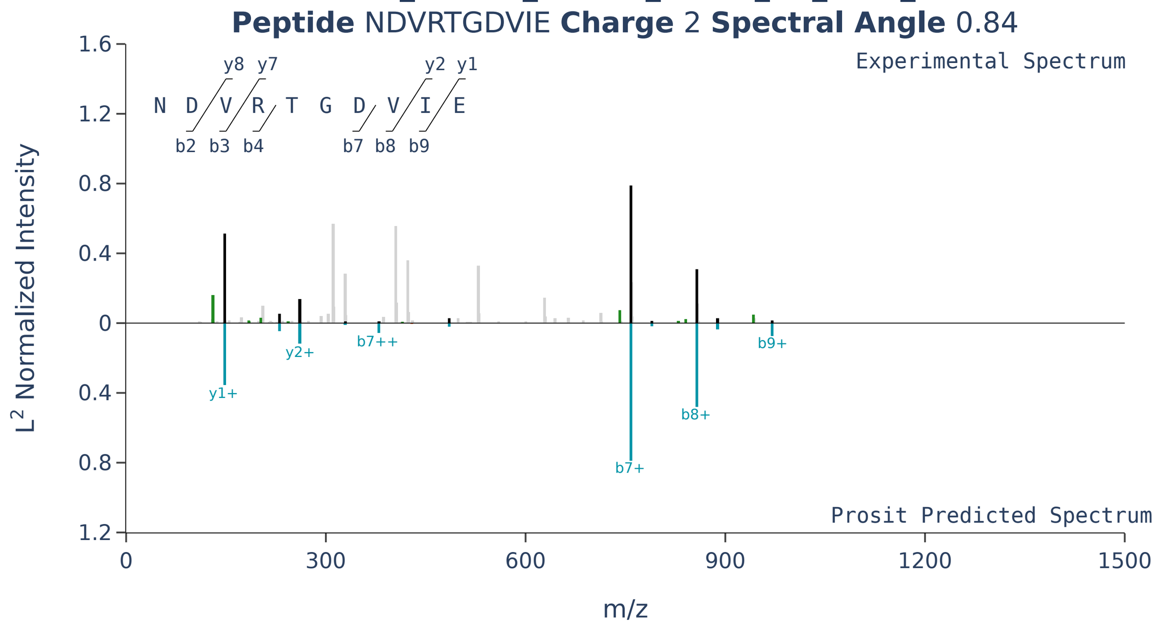


**Source** HRoetschke_211222_160123_HFGoe_IF2_b2R1_2h **Scan** 10544
**Peptide** RRENELEEAVM **Charge** 3 **Spectral Angle** 0.87

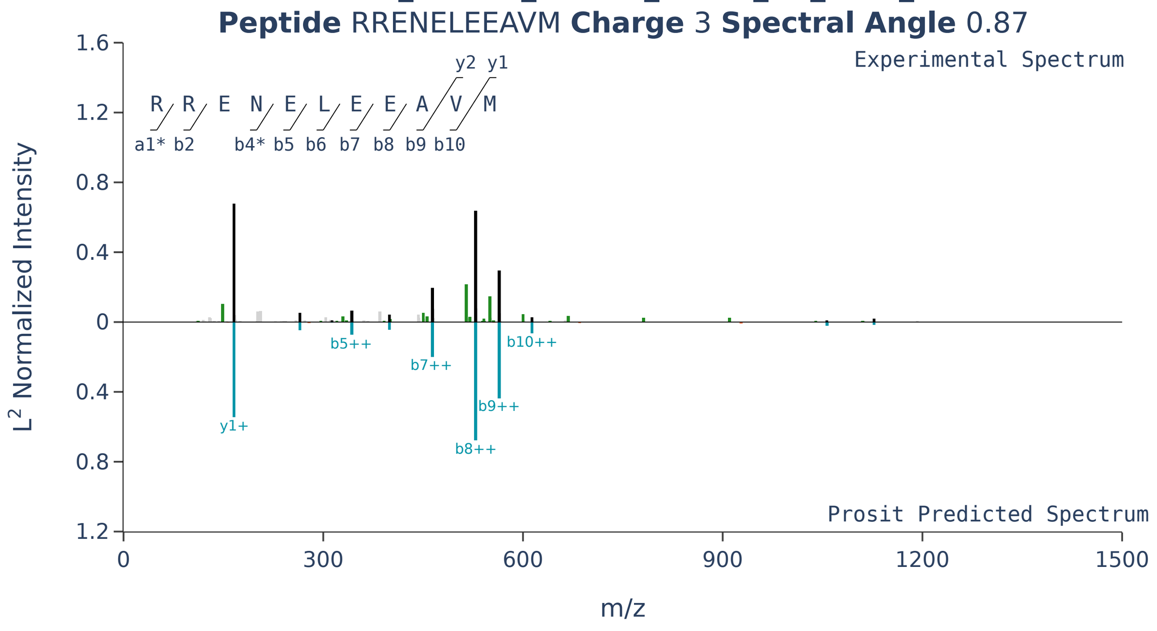


**Source** HRoetschke_211222_160123_HFGoe_IF2_b1R2_1h **Scan** 8293
**Peptide** AERQTSVERL **Charge** 3 **Spectral Angle** 0.89

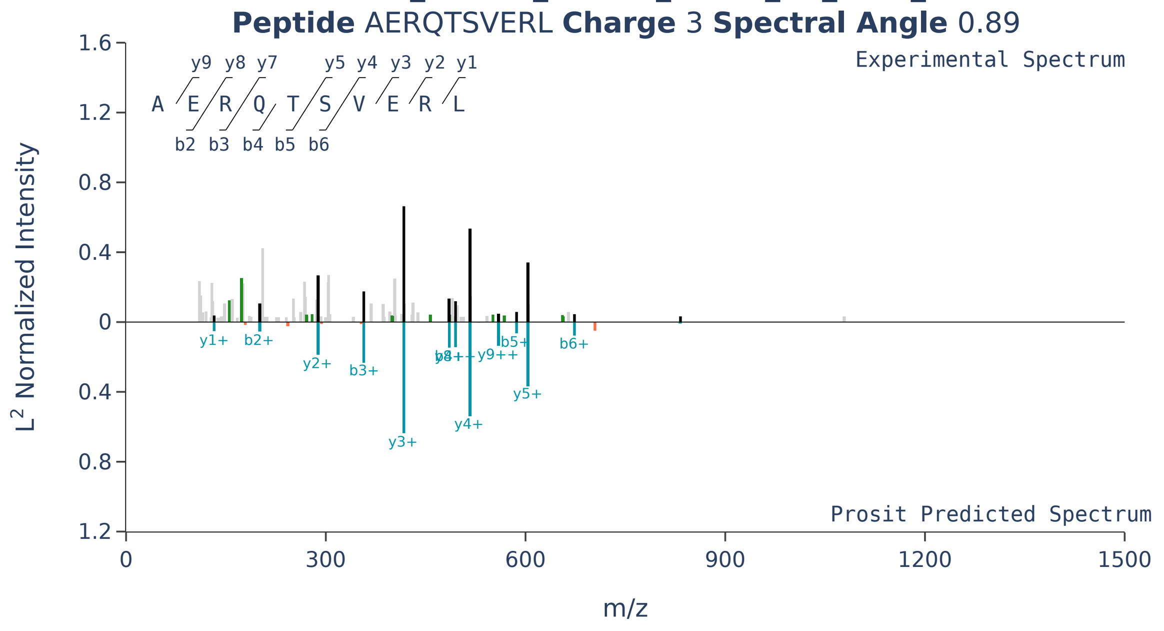


**Source** HRoetschke_211222_160123_HFGoe_IF2_b1R2_24h **Scan** 6281
**Peptide** AGIRKSADDSV **Charge** 2 **Spectral Angle** 0.78

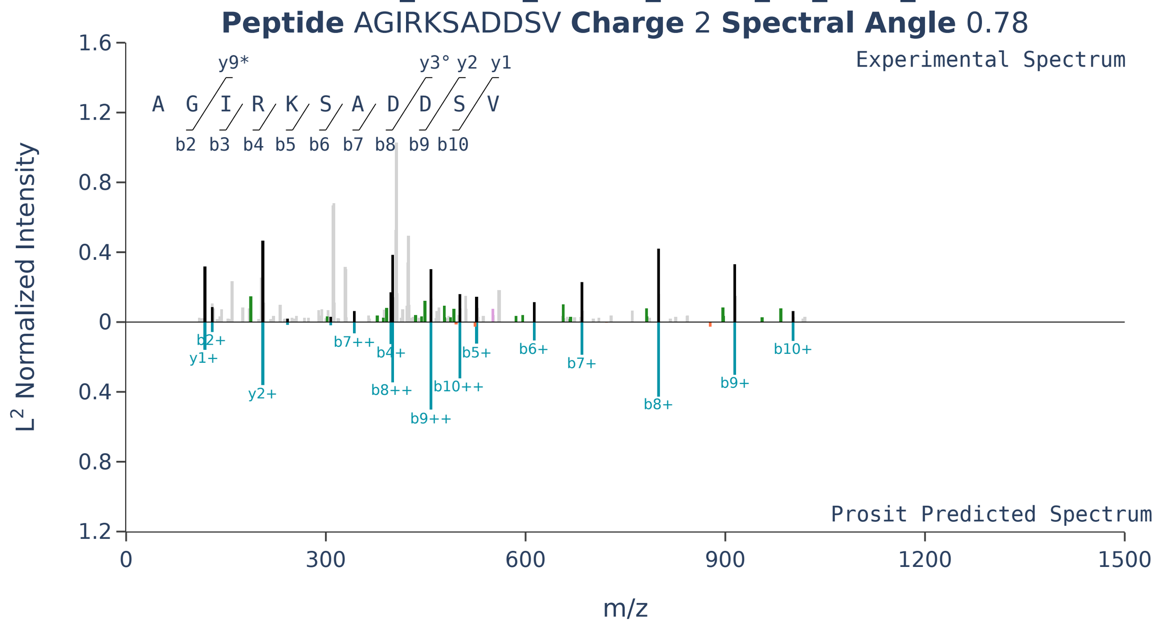


**Source** HRoetschke_211222_160123_HFGoe_IF2_b2R2_4h **Scan** 17755
**Peptide** VIDQETAQLVAE **Charge** 2 **Spectral Angle** 0.82

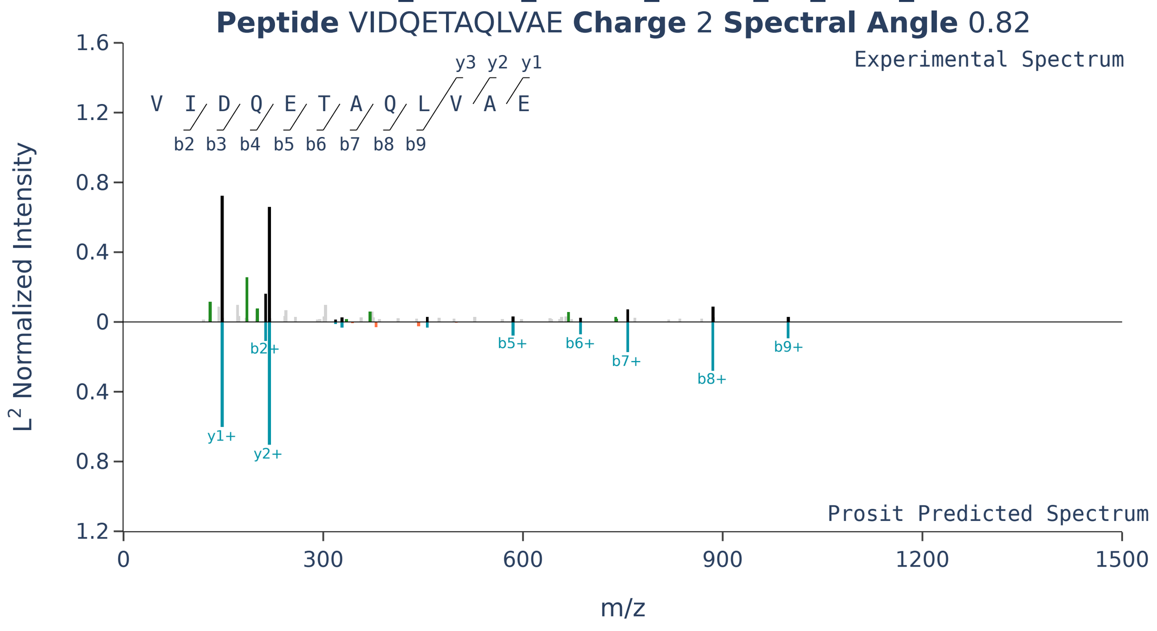


**Source** HRoetschke_211222_160123_HFGoe_IF2_b1R2_24h **Scan** 11087
**Peptide** LNIPGTGGKSK **Charge** 3 **Spectral Angle** 0.85

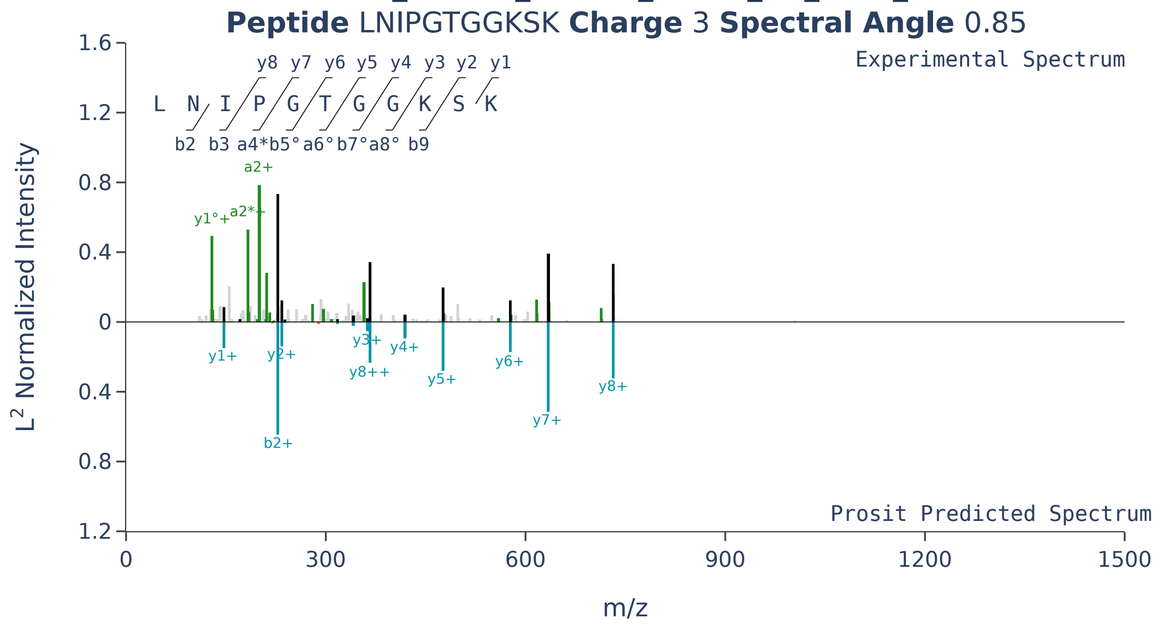


**Source** HRoetschke_211222_160123_HFGoe_IF2_b1R2_24h **Scan** 8214
**Peptide** ADAGIRKSAD **Charge** 2 **Spectral Angle** 0.8

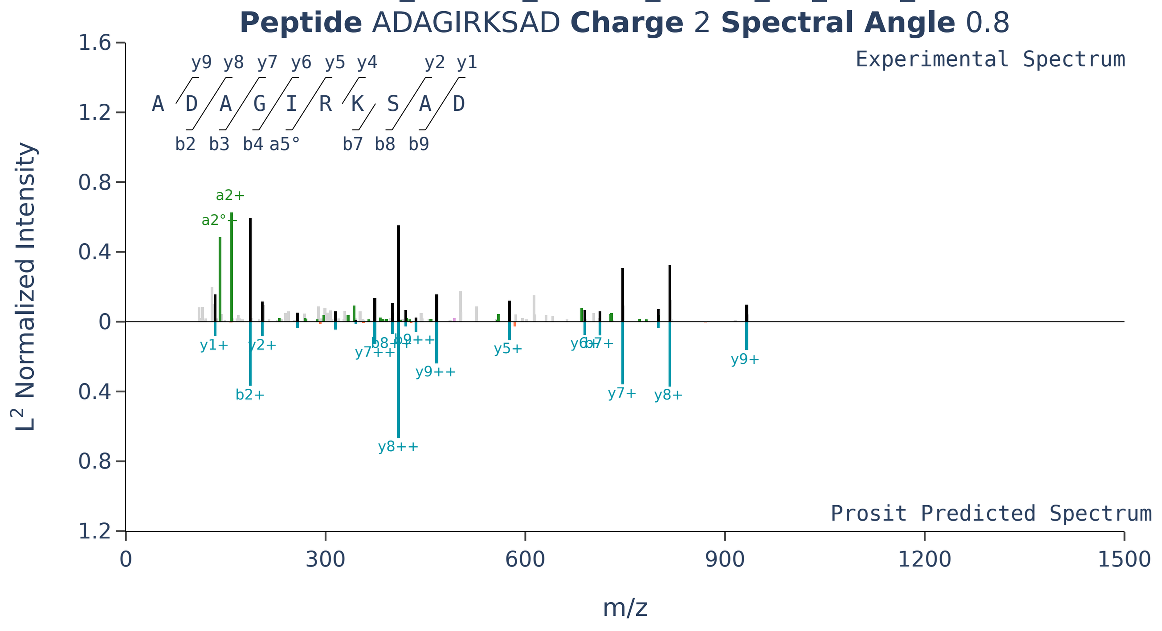

**Source** HRoetschke_211222_160123_HFGoe_IF2_b2R2_4h **Scan** 16244
**Peptide** ATINQVID **Charge** 1 **Spectral Angle** 0.86

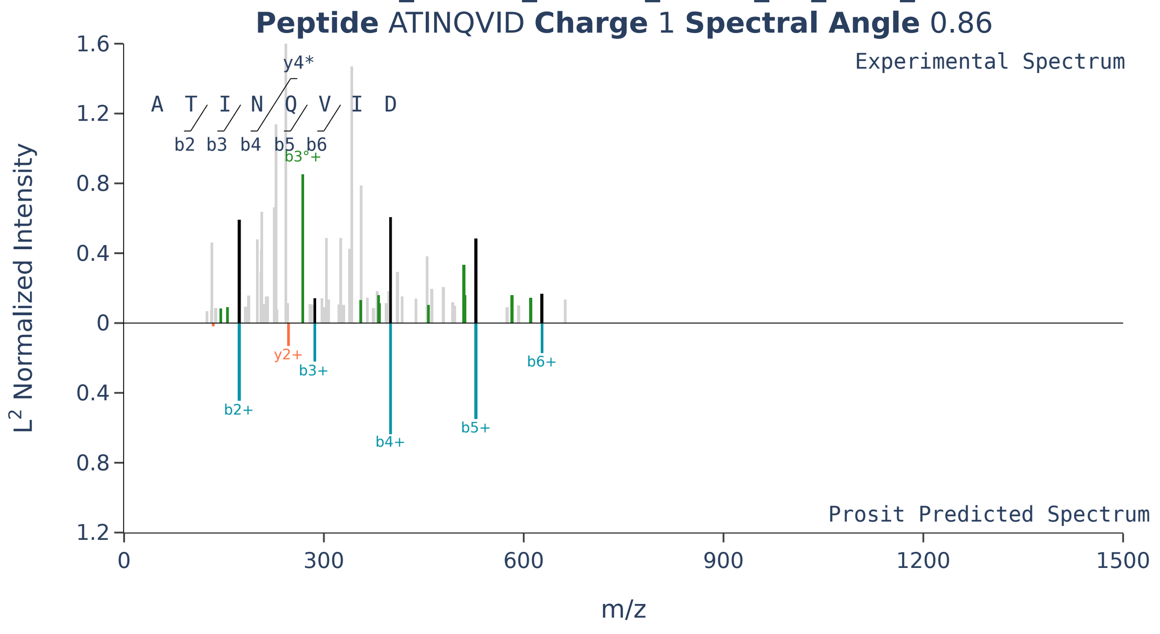


**Source** HRoetschke_211222_160123_HFGoe_IF2_b2R1_1h **Scan** 6828
**Peptide** RAMRNELGQE **Charge** 3 **Spectral Angle** 0.89

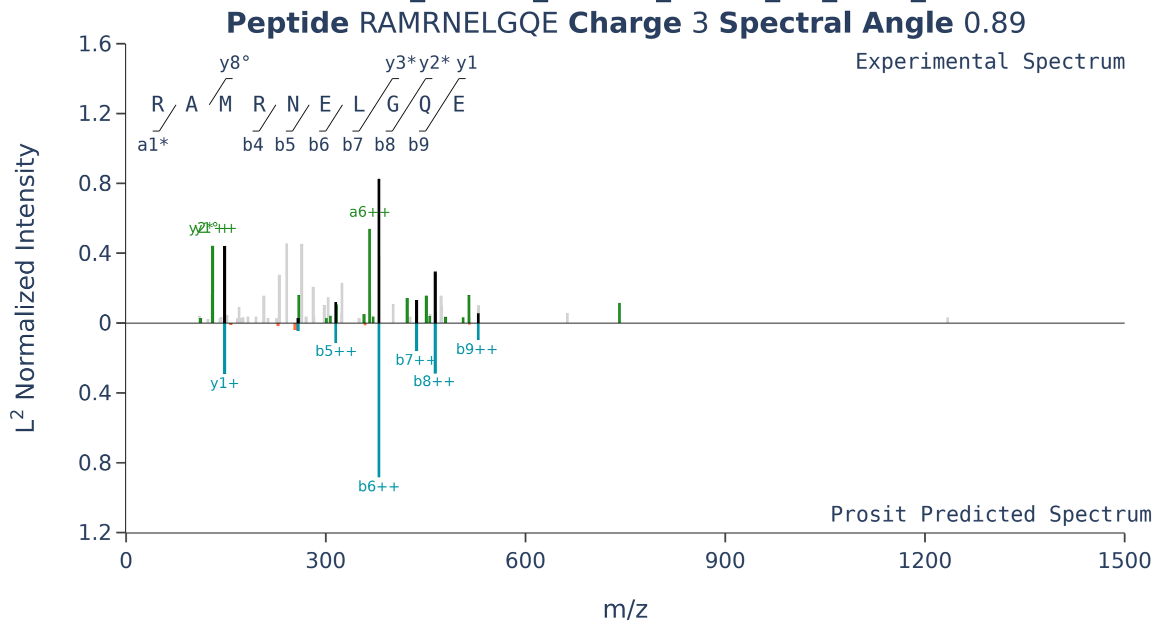


**Source** HRoetschke_211222_160123_HFGoe_IF2_b2R1_24h **Scan** 9397
**Peptide** KVILRRENEL **Charge** 2 **Spectral Angle** 0.9

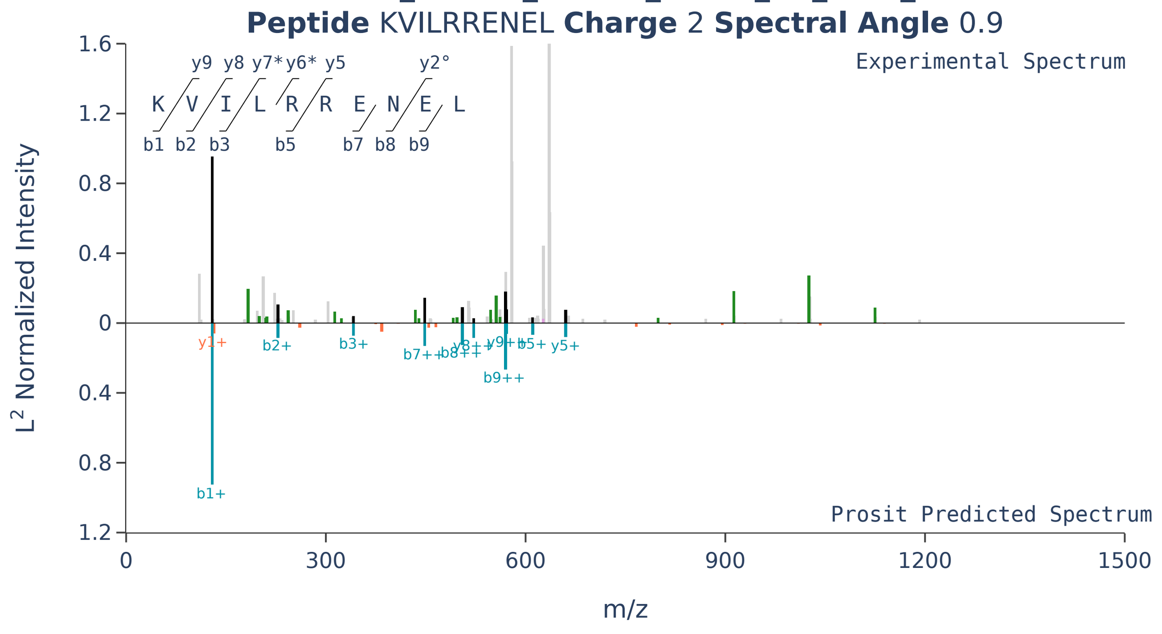


**Source** HRoetschke_211222_160123_HFGoe_IF2_b2R2_4h **Scan** 13633
**Peptide** LDTPGHAAFTSM **Charge** 2 **Spectral Angle** 0.91

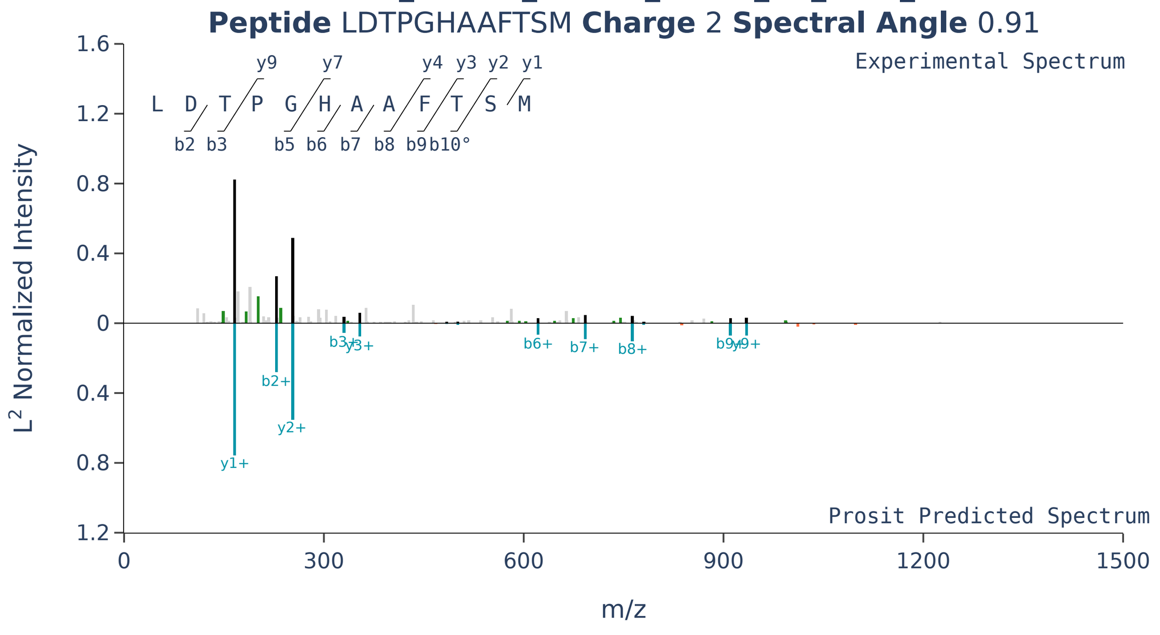


**Source** HRoetschke_211222_160123_HFGoe_IF2_b2R2_24h **Scan** 9842
**Peptide** KWTDNAEPTE **Charge** 2 **Spectral Angle** 0.88

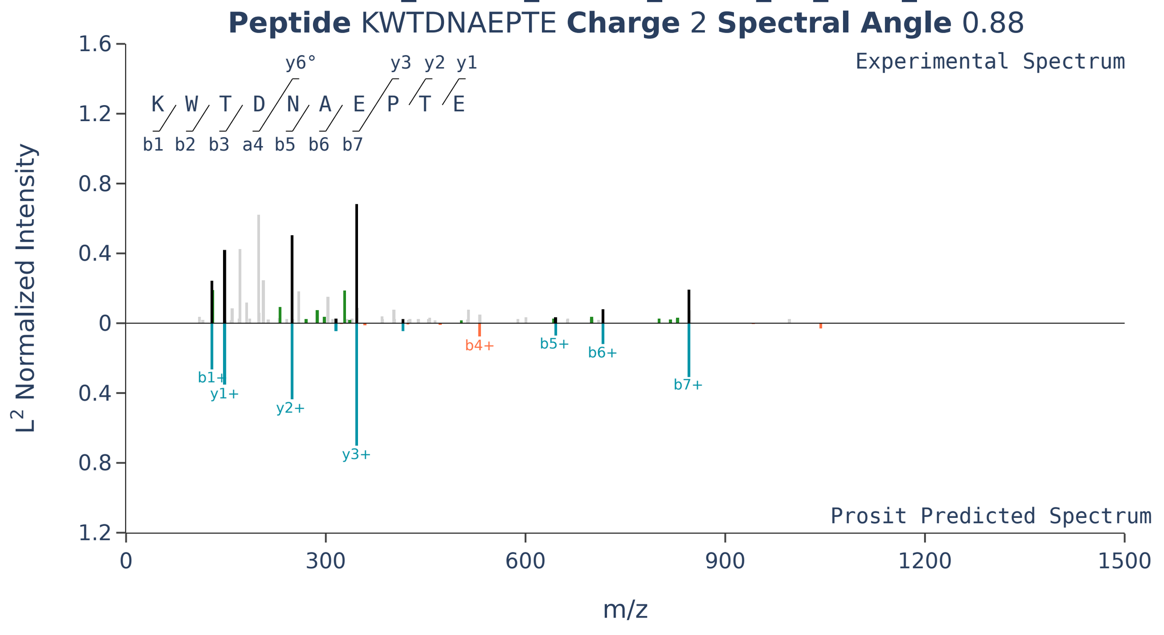


**Source** HRoetschke_211222_160123_HFGoe_IF2_b1R2_1h **Scan** 7849
**Peptide** ARGAQATDI **Charge** 2 **Spectral Angle** 0.85

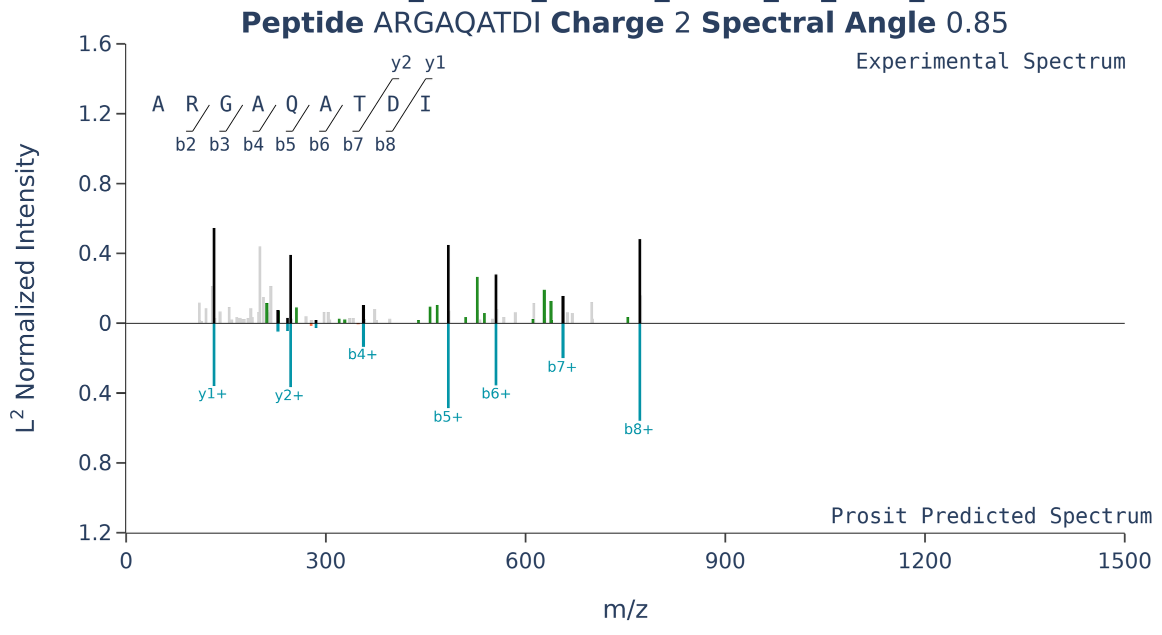


**Source** HRoetschke_211222_160123_HFGoe_IF2_b1R1_4h **Scan** 9304
**Peptide** VAVNKIDKPEA **Charge** 3 **Spectral Angle** 0.78

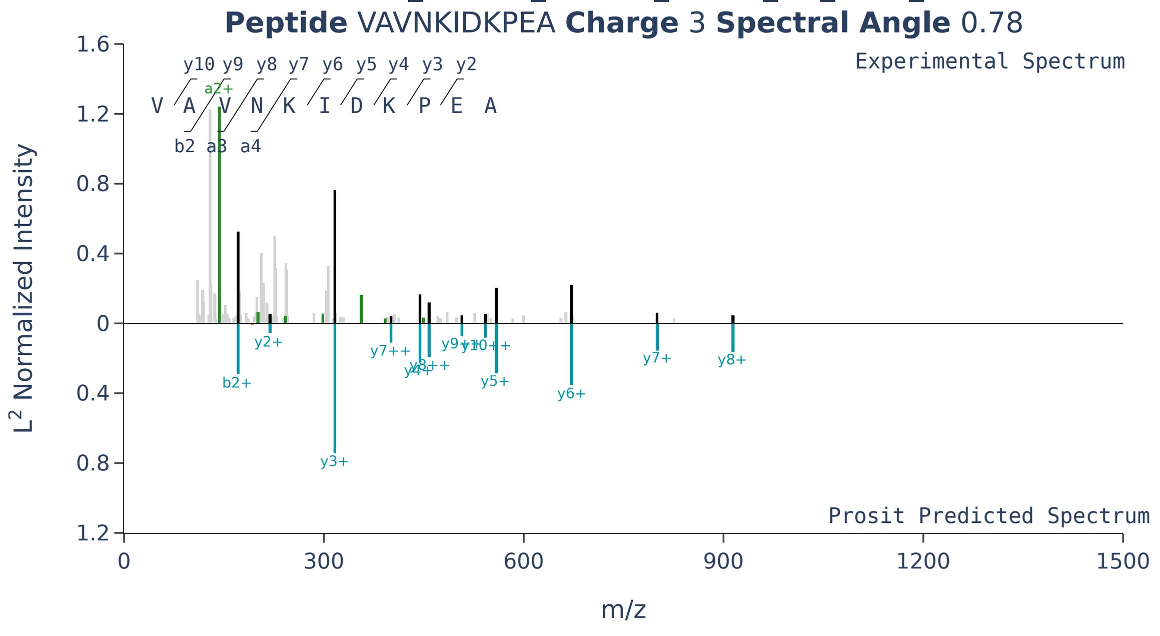


**Source** HRoetschke_211222_160123_HFGoe_IF2_b2R2_24h **Scan** 10760
**Peptide** GFQKPAQAVNRDV **Charge** 3 **Spectral Angle** 0.85

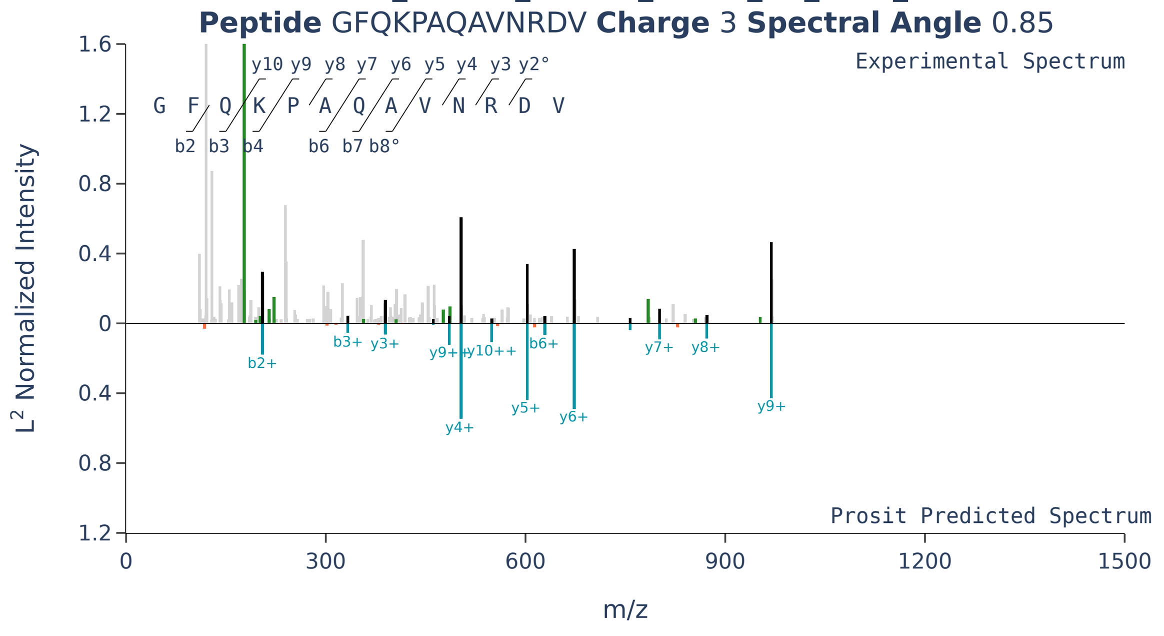


**Source** HRoetschke_211222_160123_HFGoe_IF2_b1R1_4h **Scan** 17466
**Peptide** DGVMPQTIEAIQH **Charge** 2 **Spectral Angle** 0.84

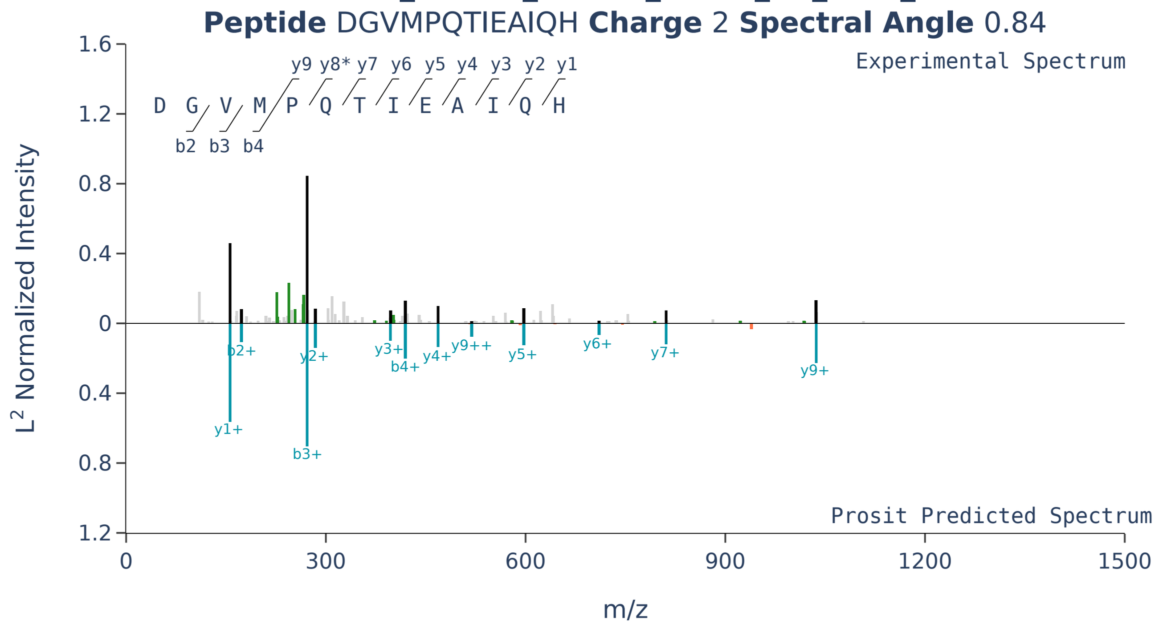


**Source** HRoetschke_211222_160123_HFGoe_IF2_b1R1_24h **Scan** 8814
**Peptide** LLRRENELEE **Charge** 2 **Spectral Angle** 0.93

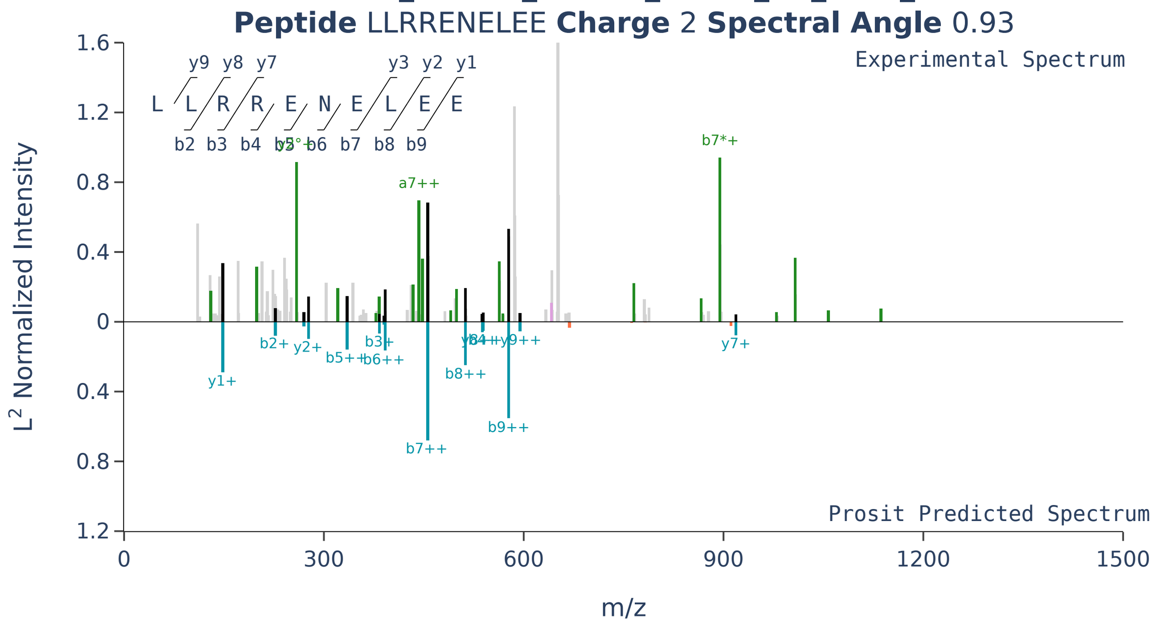


**Source** HRoetschke_211222_160123_HFGoe_IF2_b2R2_24h **Scan** 9748
**Peptide** PGTGGKSK **Charge** 2 **Spectral Angle** 0.84

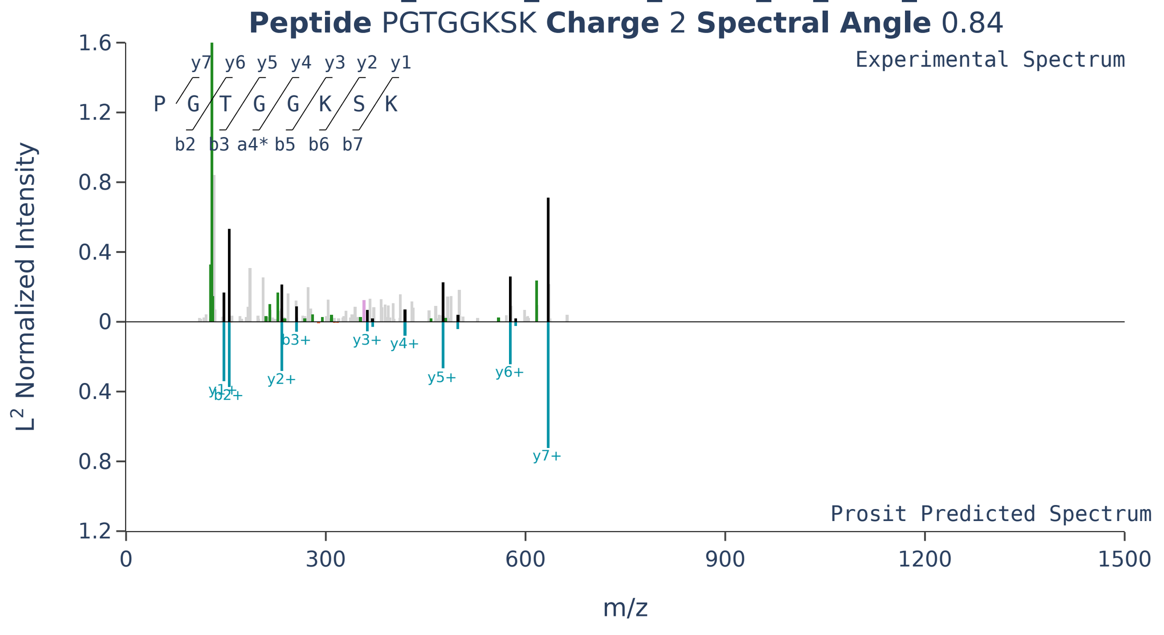


**Source** HRoetschke_211222_160123_HFGoe_IF2_b1R2_24h **Scan** 7184
**Peptide** DHLNQKNSGPDKL **Charge** 4 **Spectral Angle** 0.8

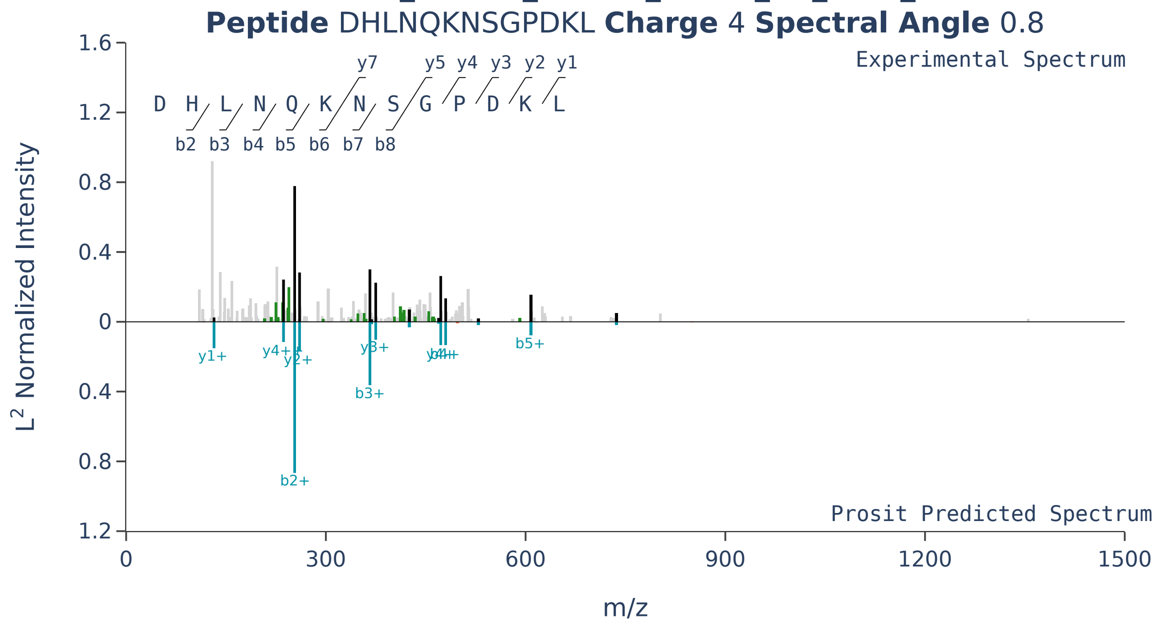


**Source** HRoetschke_211222_160123_HFGoe_IF2_b1R2_24h **Scan** 11272
**Peptide** ANKMAVKGSQVI **Charge** 3 **Spectral Angle** 0.88

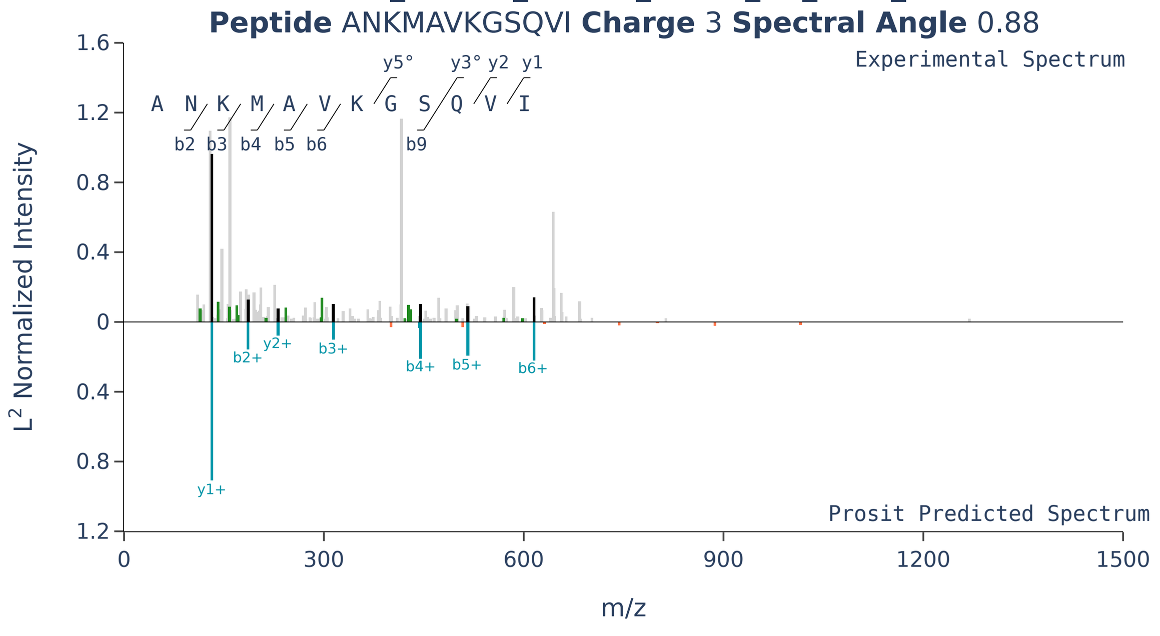


**Source** HRoetschke_211222_160123_HFGoe_IF2_b1R1_24h **Scan** 12868
**Peptide** LNIPGTGGKSKSV **Charge** 3 **Spectral Angle** 0.84

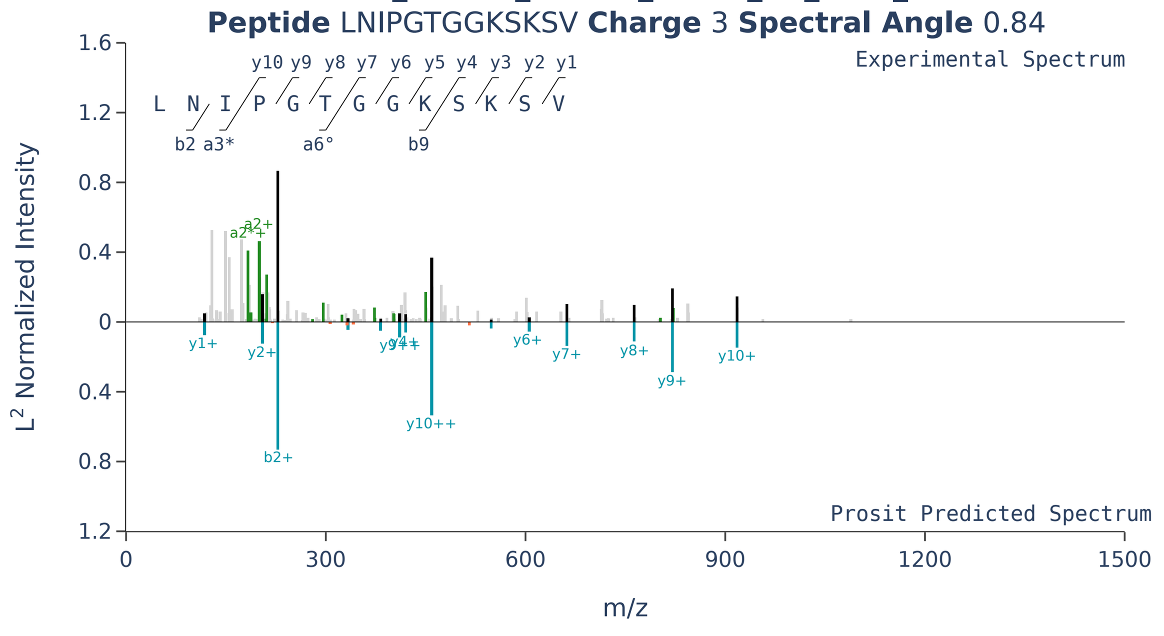


**Source** HRoetschke_211222_160123_HFGoe_IF2_b1R1_4h **Scan** 11556
**Peptide** SLQQGFQKPAQ **Charge** 2 **Spectral Angle** 0.87

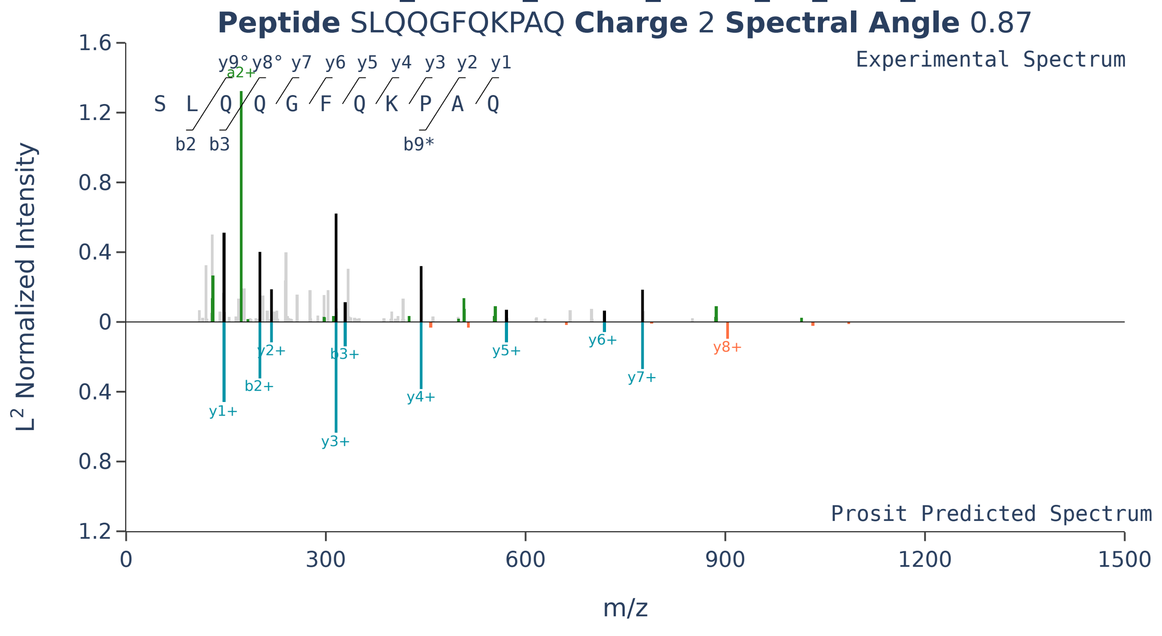

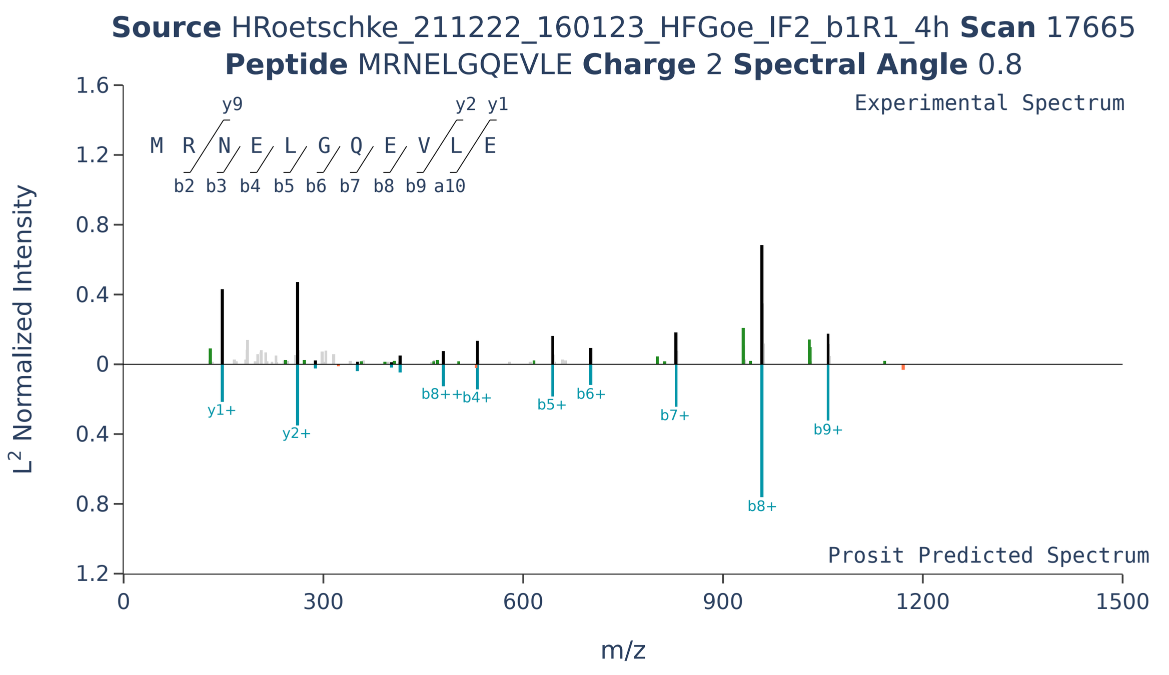
Source HRoetschke_211222_160123_HFGoe_IF2_b1R1_4h Scan 17665
Peptide MRNELGQEVLE Charge 2 Spectral Angle 0.8
Experimental Spectrum
Prosit Predicted Spectrum
L² Normalized Intensity
m/z

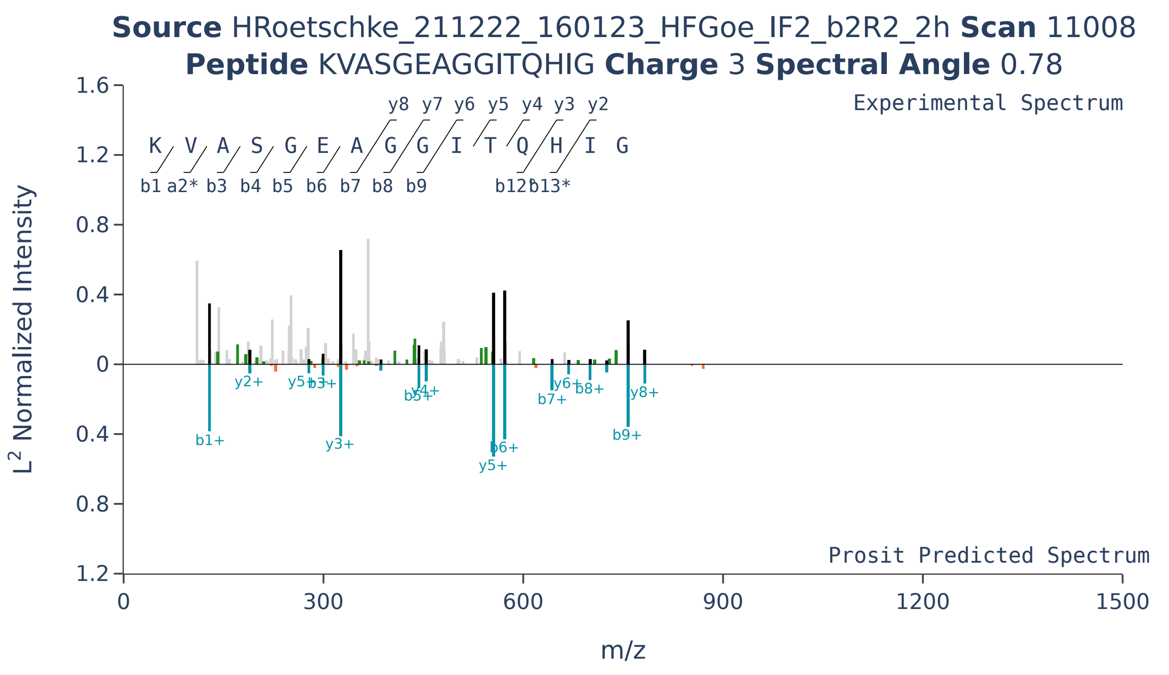
Source HRoetschke_211222_160123_HFGoe_IF2_b2R2_2h Scan 11008
Peptide KVASGEAGGITQHIG Charge 3 Spectral Angle 0.78
Experimental Spectrum
Prosit Predicted Spectrum
L² Normalized Intensity
m/z

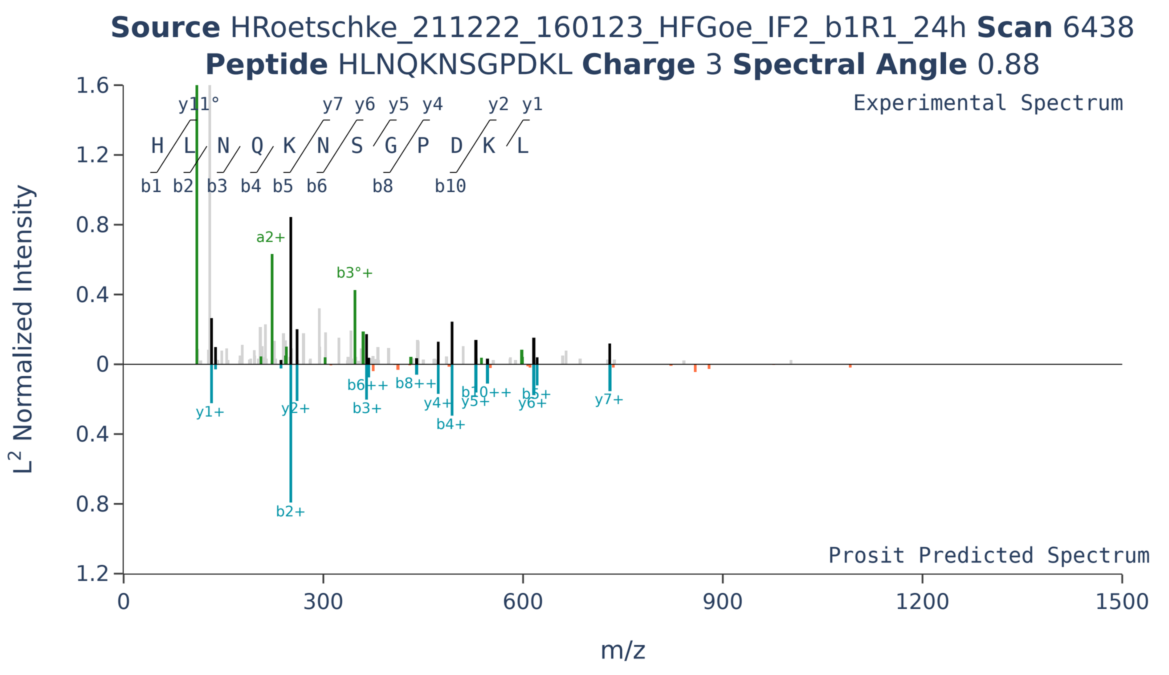
Source HRoetschke_211222_160123_HFGoe_IF2_b1R1_24h Scan 6438
Peptide HLNQKNSGPDKL Charge 3 Spectral Angle 0.88
Experimental Spectrum
Prosit Predicted Spectrum
L² Normalized Intensity
m/z

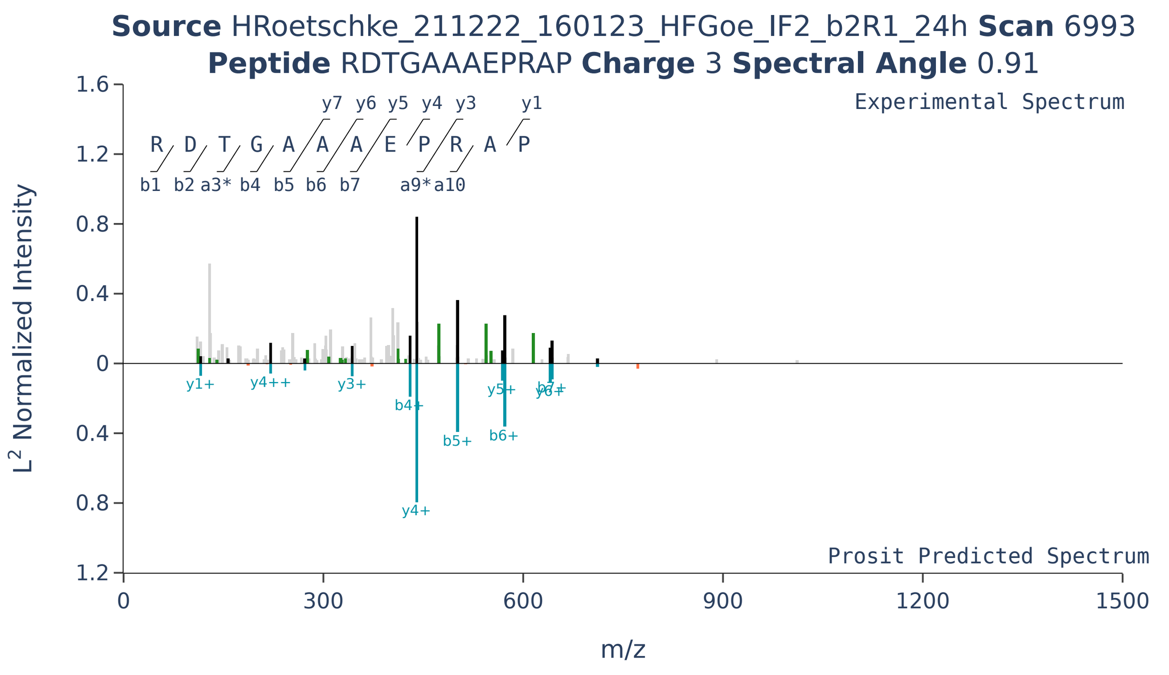
Source HRoetschke_211222_160123_HFGoe_IF2_b2R1_24h Scan 6993
Peptide RDTGAAAEPRAP Charge 3 Spectral Angle 0.91
Experimental Spectrum
Prosit Predicted Spectrum
L² Normalized Intensity
m/z

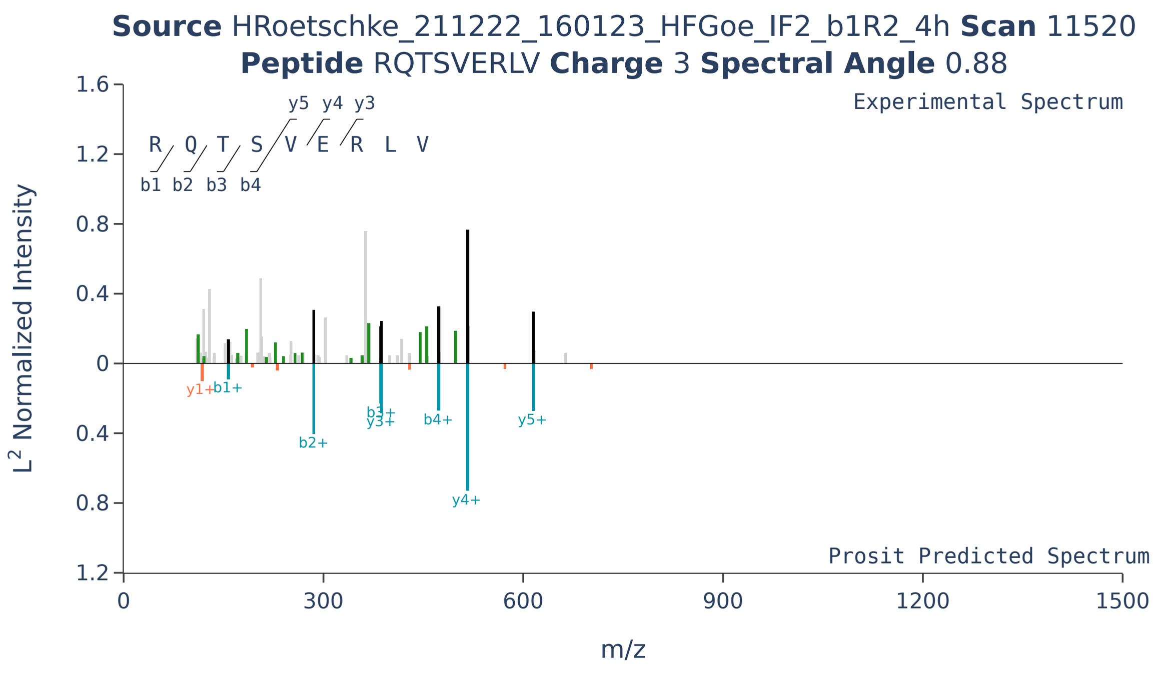
Source HRoetschke_211222_160123_HFGoe_IF2_b1R2_4h Scan 11520
Peptide RQTSVERLV Charge 3 Spectral Angle 0.88
Experimental Spectrum
Prosit Predicted Spectrum
L² Normalized Intensity
m/z

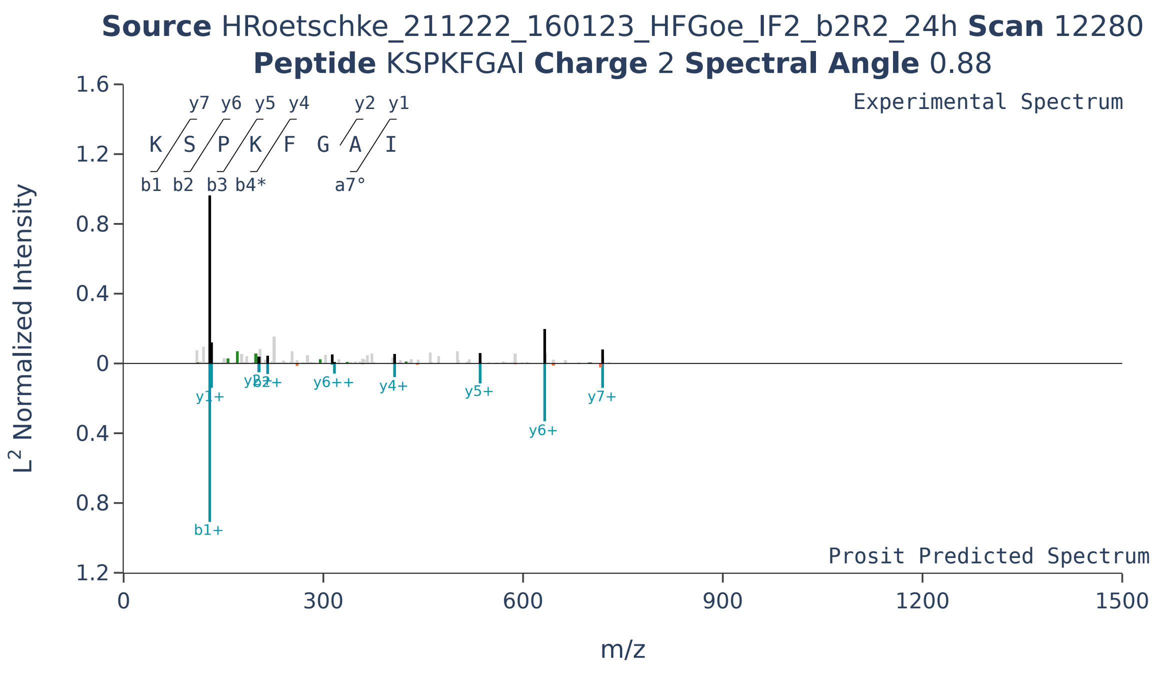
Source HRoetschke_211222_160123_HFGoe_IF2_b2R2_24h Scan 12280
Peptide KSPKFGAI Charge 2 Spectral Angle 0.88
Experimental Spectrum
Prosit Predicted Spectrum
L² Normalized Intensity
m/z

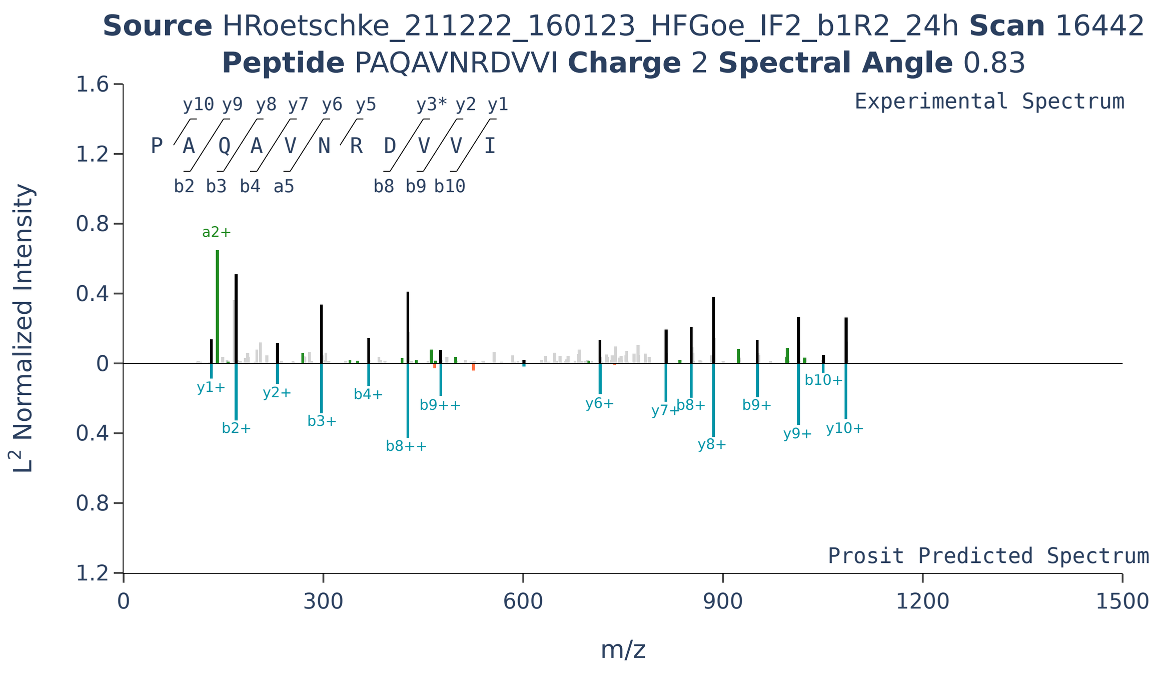
Source HRoetschke_211222_160123_HFGoe_IF2_b1R2_24h Scan 16442
Peptide PAQAVNRDVVI Charge 2 Spectral Angle 0.83
Experimental Spectrum
Prosit Predicted Spectrum
L² Normalized Intensity
m/z

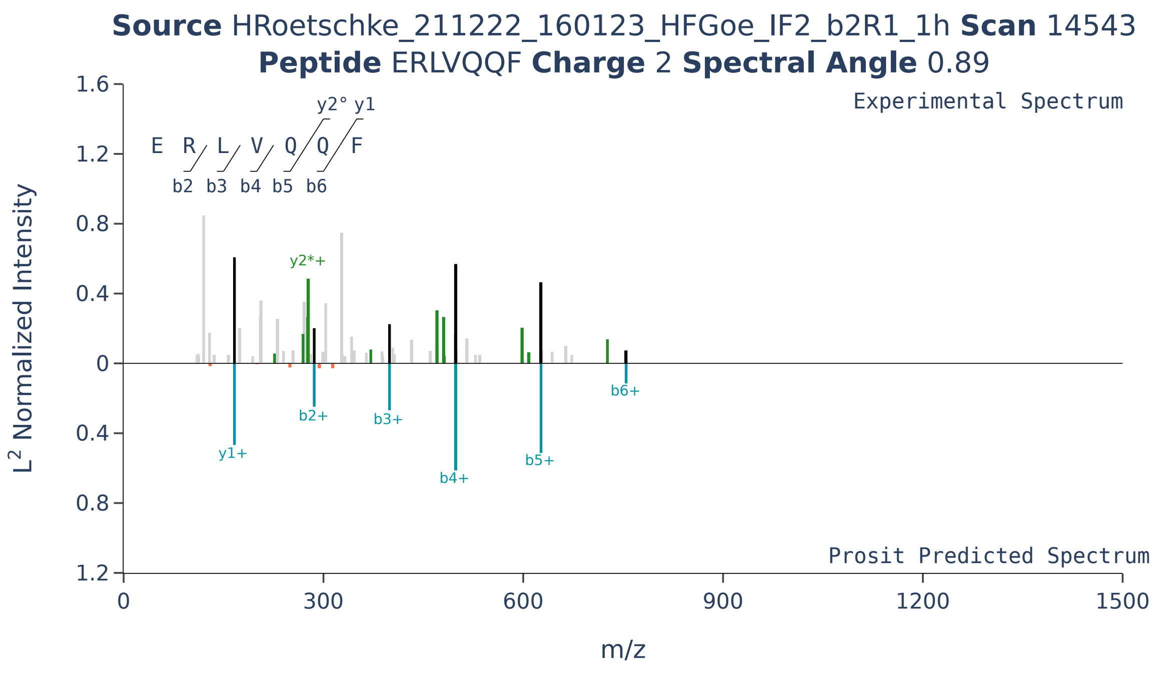
Source HRoetschke_211222_160123_HFGoe_IF2_b2R1_1h Scan 14543
Peptide ERLVQQF Charge 2 Spectral Angle 0.89
Experimental Spectrum
Prosit Predicted Spectrum
L² Normalized Intensity
m/z

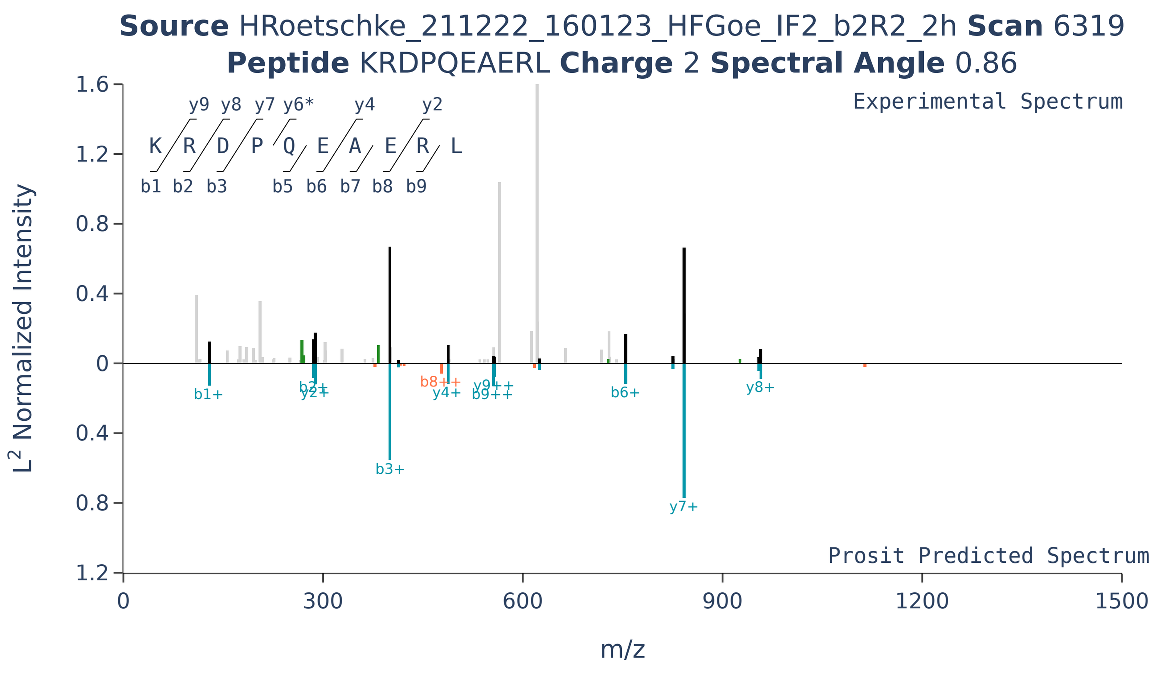
Source HRoetschke_211222_160123_HFGoe_IF2_b2R2_2h Scan 6319
Peptide KRDPQEAERL Charge 2 Spectral Angle 0.86
Experimental Spectrum
Prosit Predicted Spectrum
L² Normalized Intensity
m/z

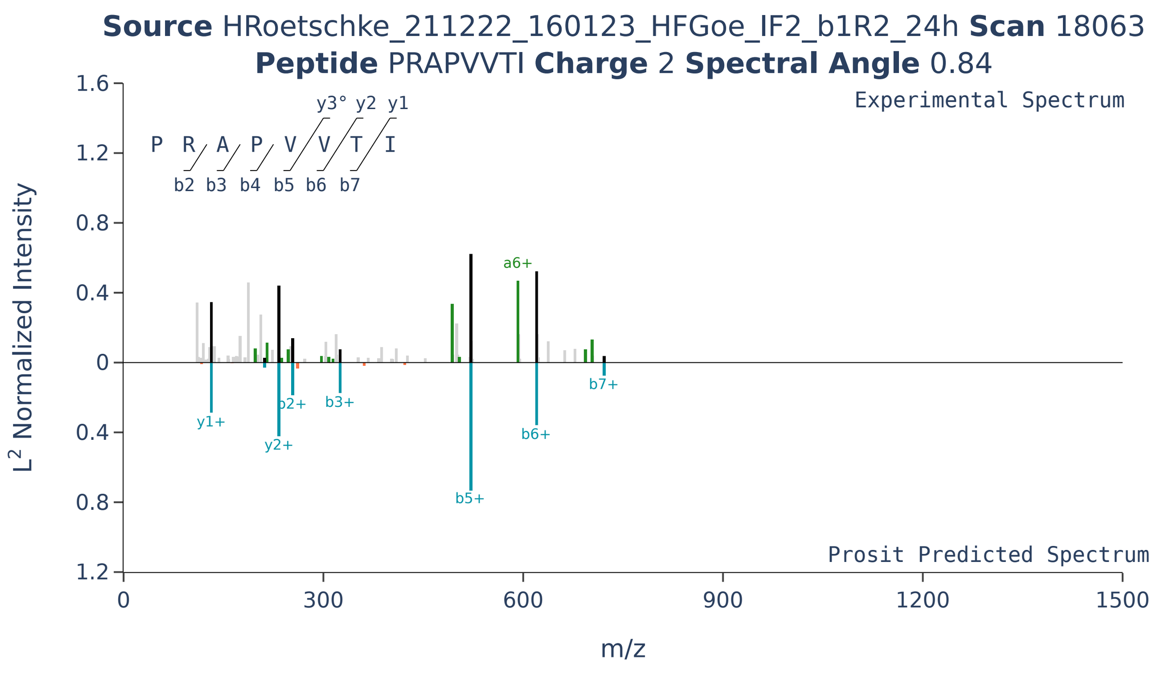
Source HRoetschke_211222_160123_HFGoe_IF2_b1R2_24h Scan 18063
Peptide PRAPVVTI Charge 2 Spectral Angle 0.84
Experimental Spectrum
Prosit Predicted Spectrum
L² Normalized Intensity
m/z

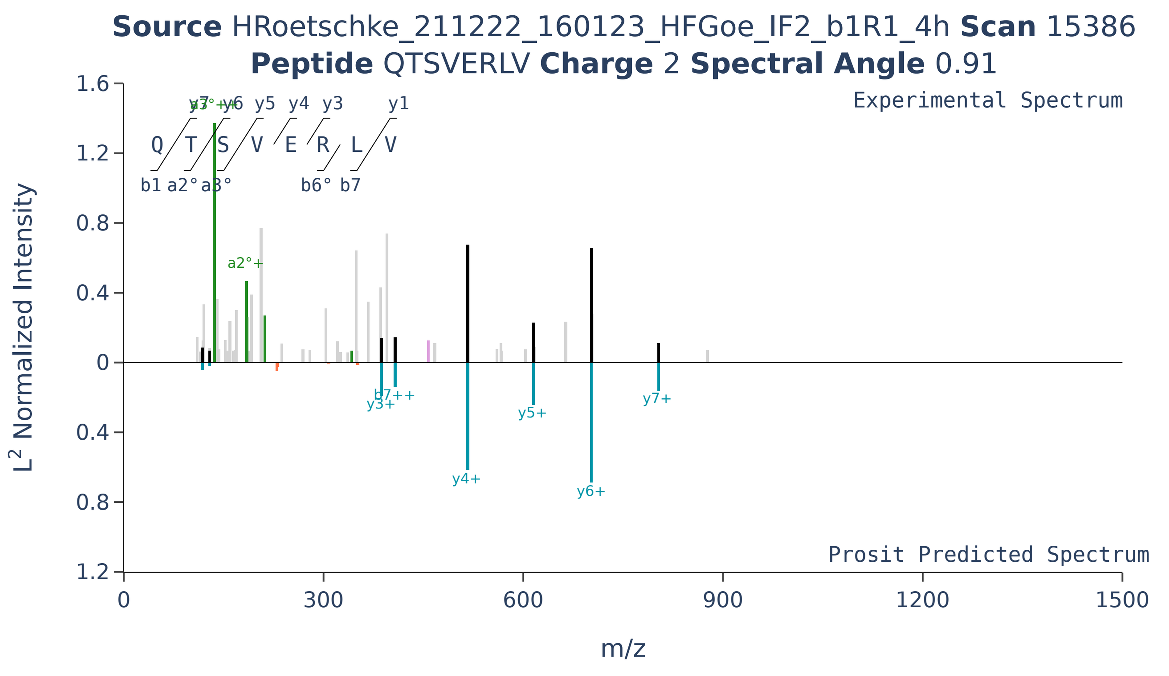
Source HRoetschke_211222_160123_HFGoe_IF2_b1R1_4h Scan 15386
Peptide QTSVERLV Charge 2 Spectral Angle 0.91
Experimental Spectrum
Prosit Predicted Spectrum
L² Normalized Intensity
m/z

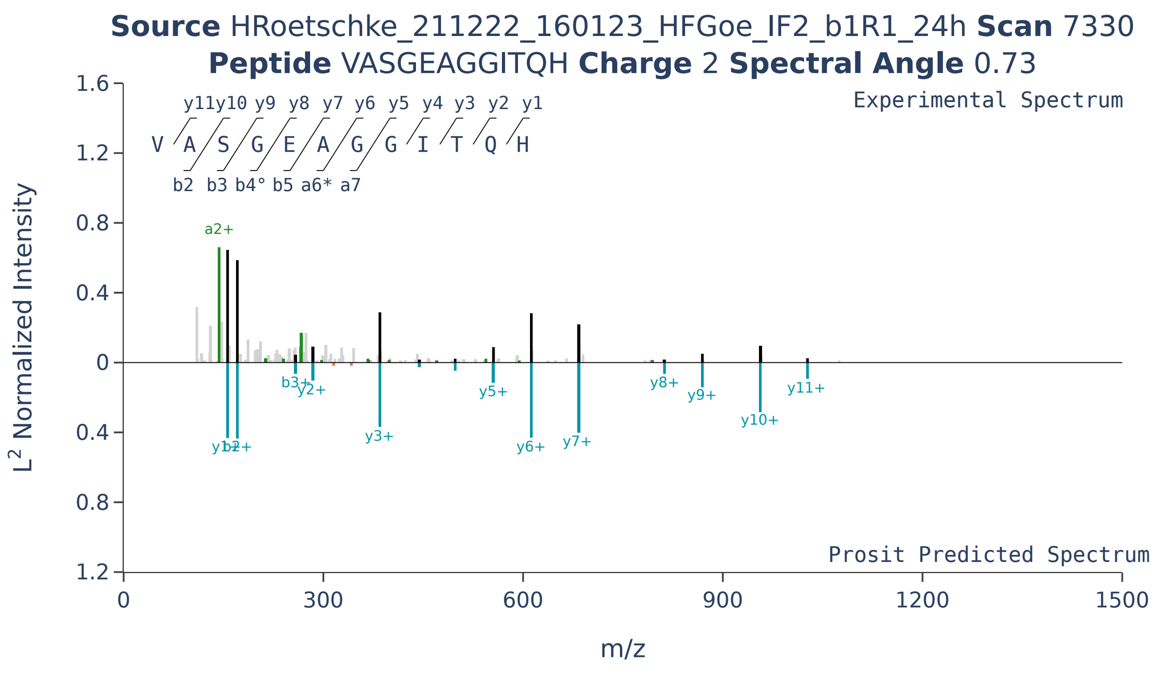
Source HRoetschke_211222_160123_HFGoe_IF2_b1R1_24h Scan 7330
Peptide VASGEAGGITQH Charge 2 Spectral Angle 0.73
Experimental Spectrum
Prosit Predicted Spectrum
L² Normalized Intensity
m/z

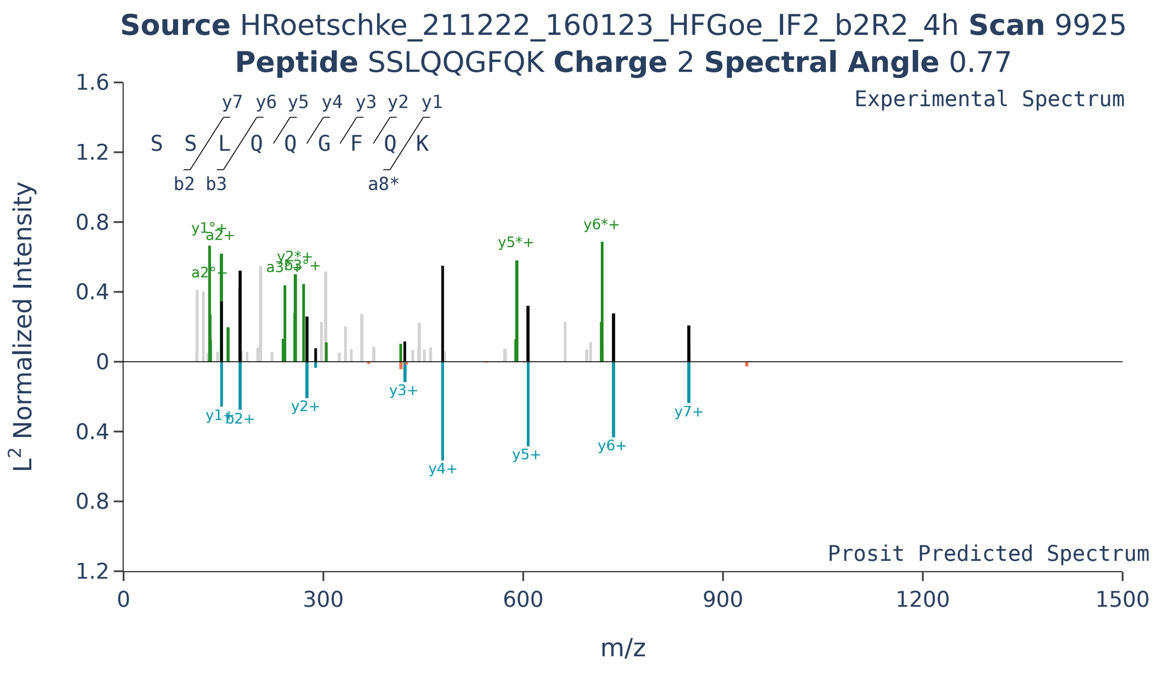
Source HRoetschke_211222_160123_HFGoe_IF2_b2R2_4h Scan 9925
Peptide SSLQQGFQK Charge 2 Spectral Angle 0.77
Experimental Spectrum
Prosit Predicted Spectrum
L² Normalized Intensity
m/z

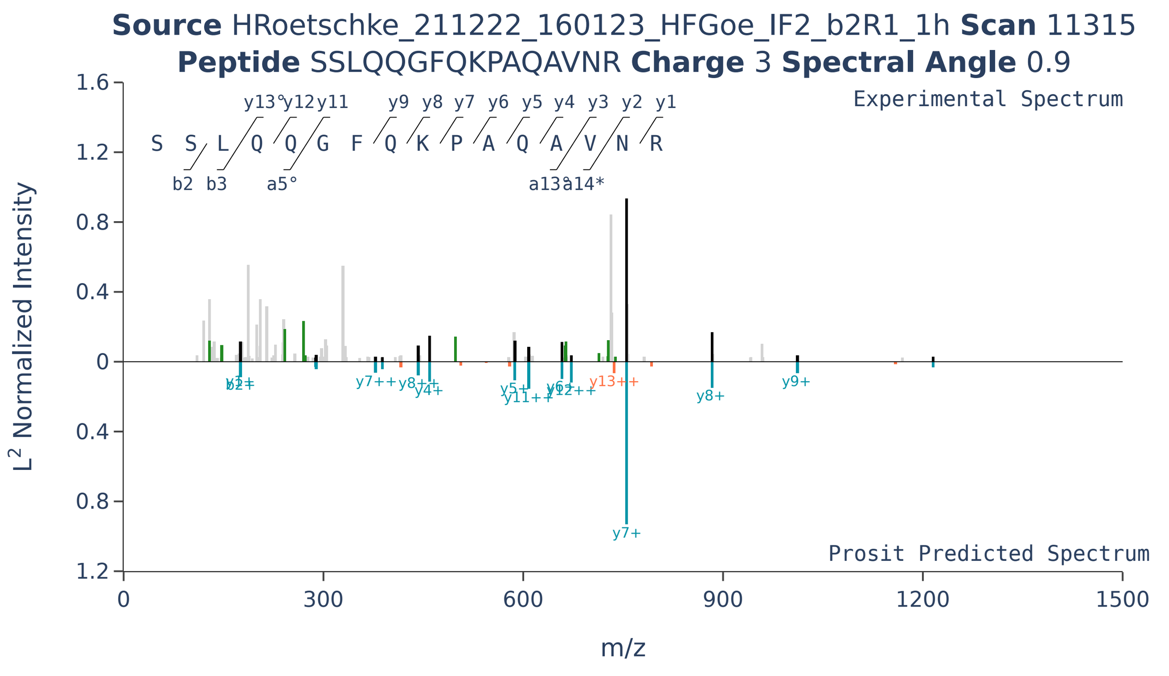
Source HRoetschke_211222_160123_HFGoe_IF2_b2R1_1h Scan 11315
Peptide SSLQQGFQKPAQAVNR Charge 3 Spectral Angle 0.9
Experimental Spectrum
Prosit Predicted Spectrum
L² Normalized Intensity
m/z

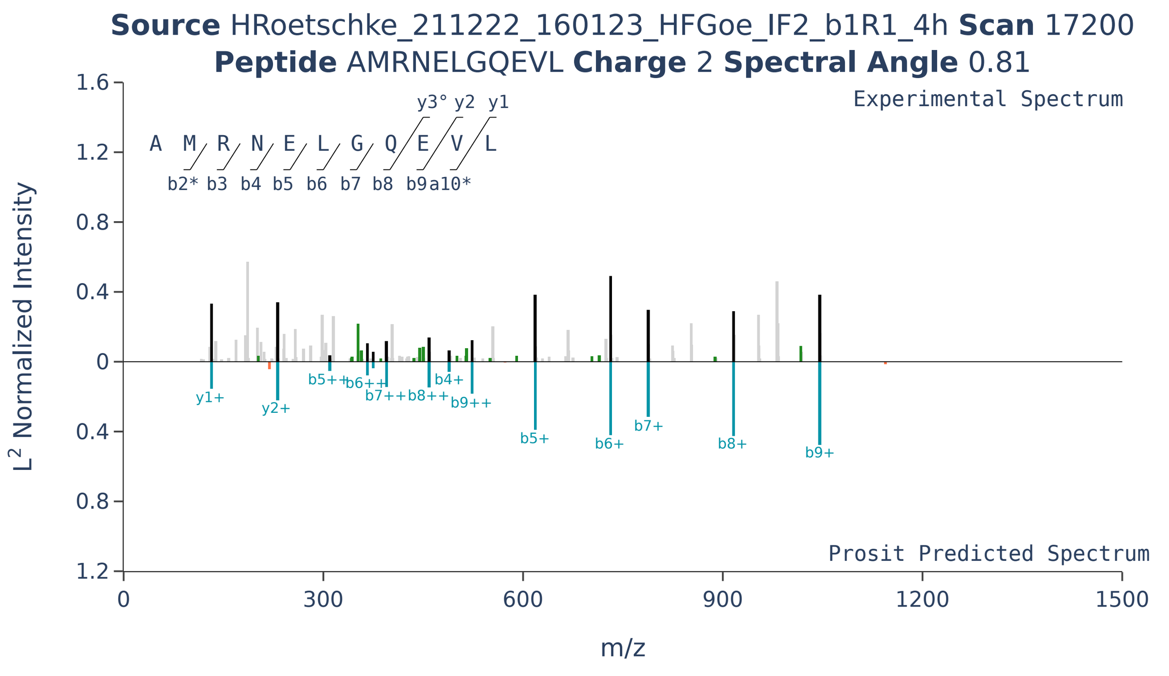
Source HRoetschke_211222_160123_HFGoe_IF2_b1R1_4h Scan 17200
Peptide AMRNELGQEVL Charge 2 Spectral Angle 0.81
Experimental Spectrum
Prosit Predicted Spectrum
L² Normalized Intensity
m/z

**Source** HRoetschke_211222_160123_HFGoe_IF2_b1R1_4h **Scan** 9143
**Peptide** RDPQEAERLAAE **Charge** 3 **Spectral Angle** 0.87

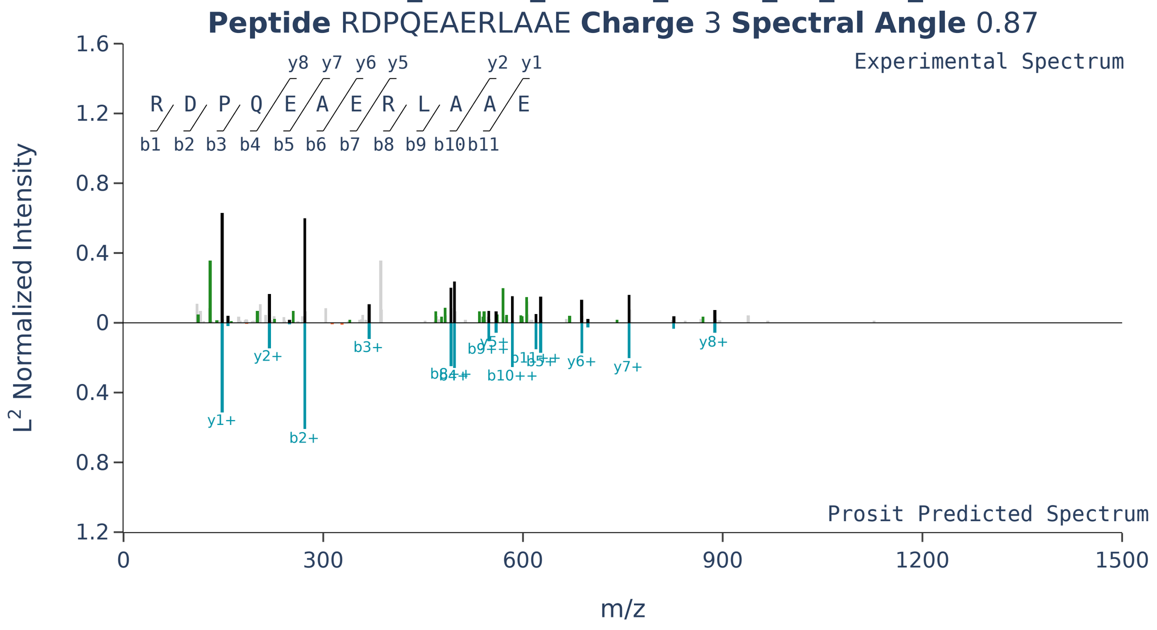


**Source** HRoetschke_211222_160123_HFGoe_IF2_b1R1_2h **Scan** 10916
**Peptide** IGAYHVE **Charge** 2 **Spectral Angle** 0.84

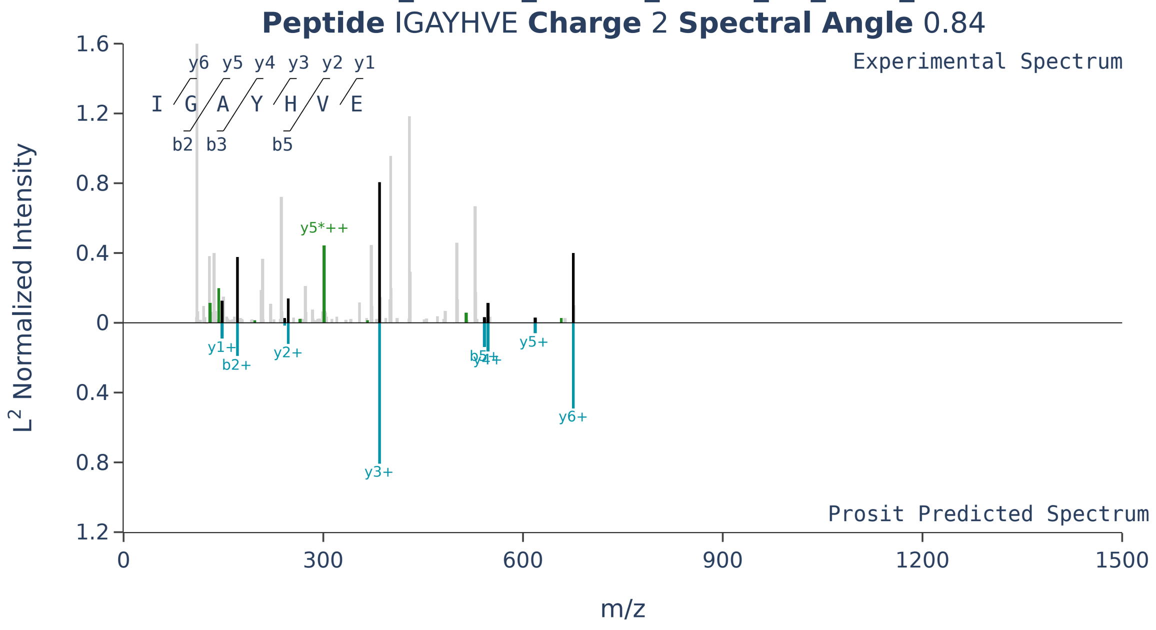


**Source** HRoetschke_211222_160123_HFGoe_IF2_b1R1_24h **Scan** 7287
**Peptide** LNQKNSGPDKL **Charge** 3 **Spectral Angle** 0.81

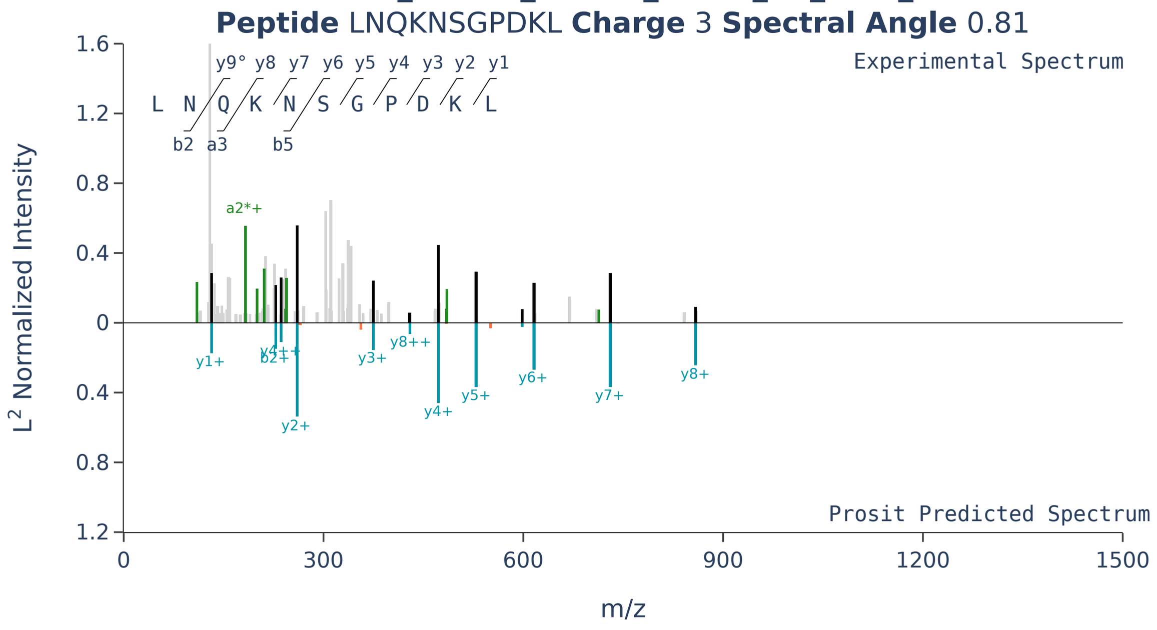


**Source** HRoetschke_211222_160123_HFGoe_IF2_b2R2_24h **Scan** 6098
**Peptide** VVRDEKKA **Charge** 2 **Spectral Angle** 0.8

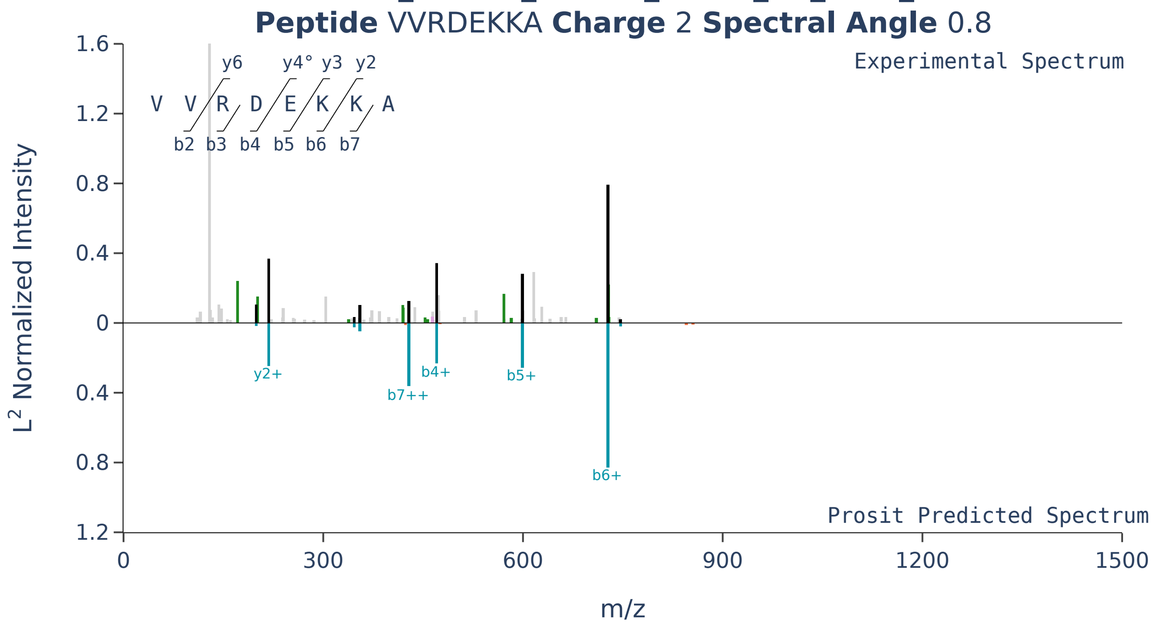


**Source** HRoetschke_211222_160123_HFGoe_IF2_b1R1_4h **Scan** 10375
**Peptide** VLVREGTLH **Charge** 2 **Spectral Angle** 0.86

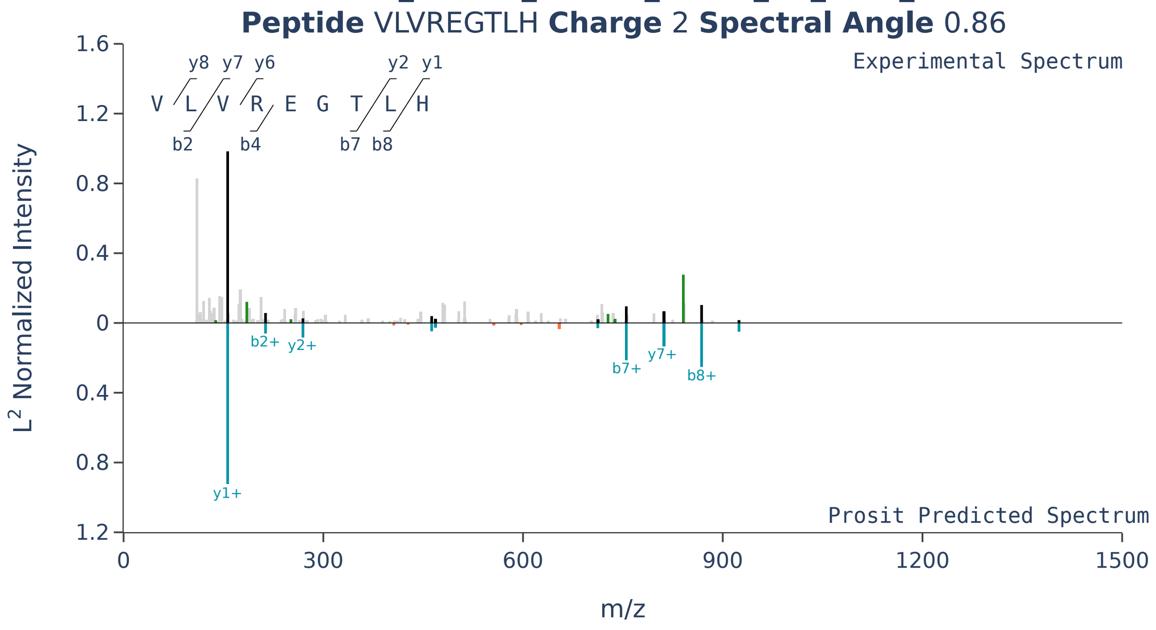


**Source** HRoetschke_211222_160123_HFGoe_IF2_b1R1_2h **Scan** 5855
**Peptide** DRDTGAAAEPRA **Charge** 3 **Spectral Angle** 0.86

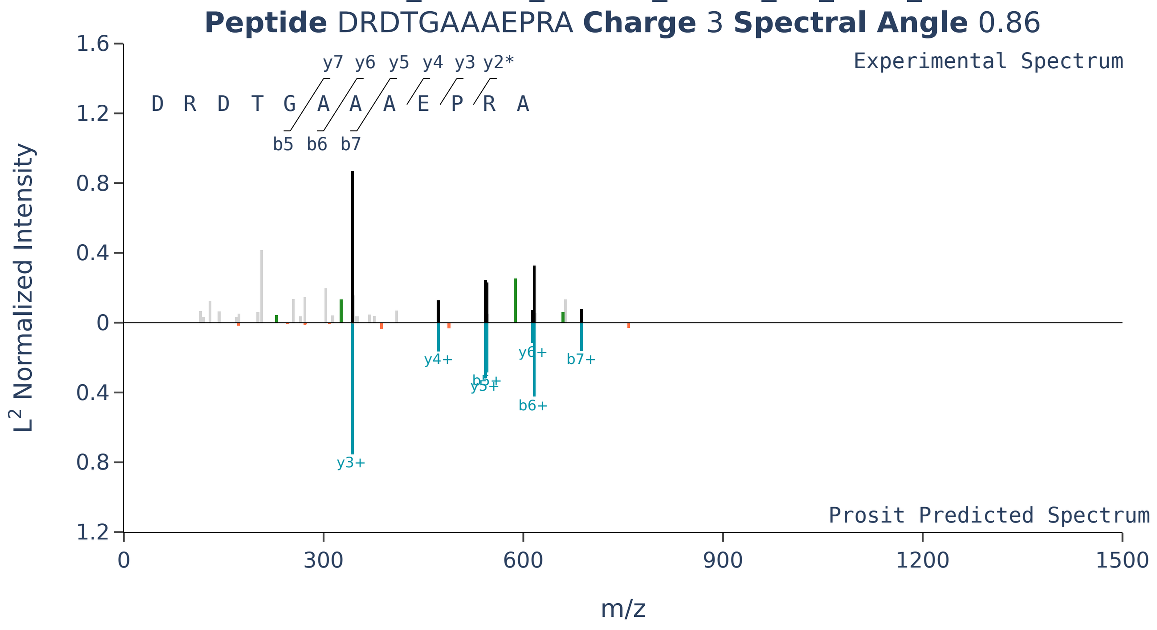


**Source** HRoetschke_211222_160123_HFGoe_IF2_b1R1_4h **Scan** 15622
**Peptide** ADVQGSVEAISD **Charge** 2 **Spectral Angle** 0.91

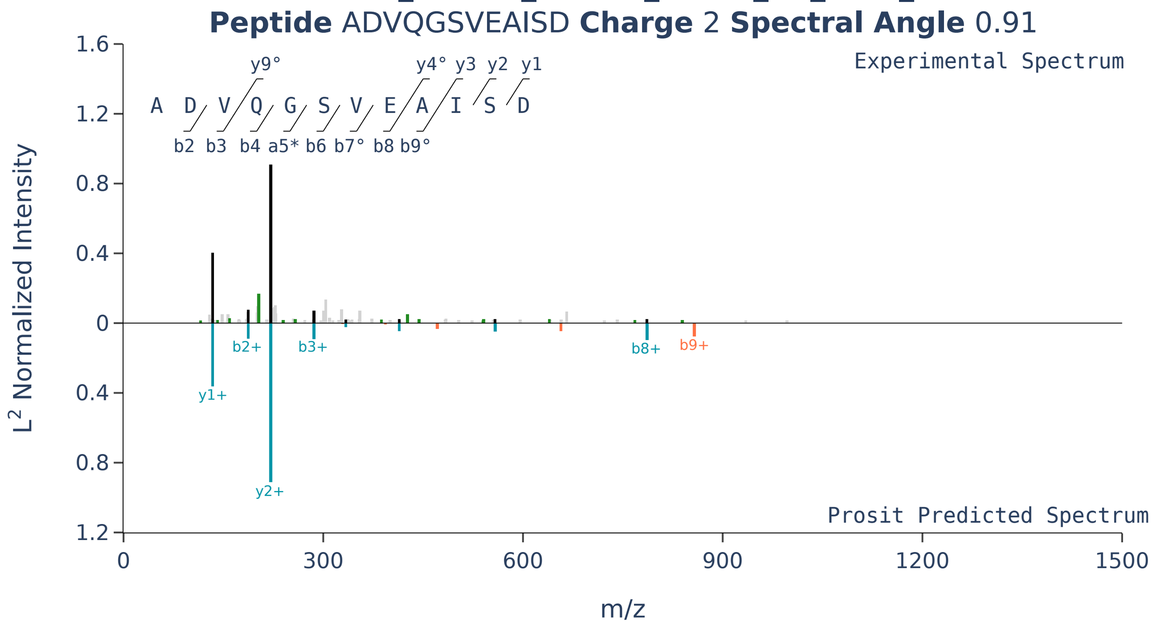


**Source** HRoetschke_211222_160123_HFGoe_IF2_b1R2_24h **Scan** 13224
**Peptide** REGTLHKGDIVL **Charge** 2 **Spectral Angle** 0.83

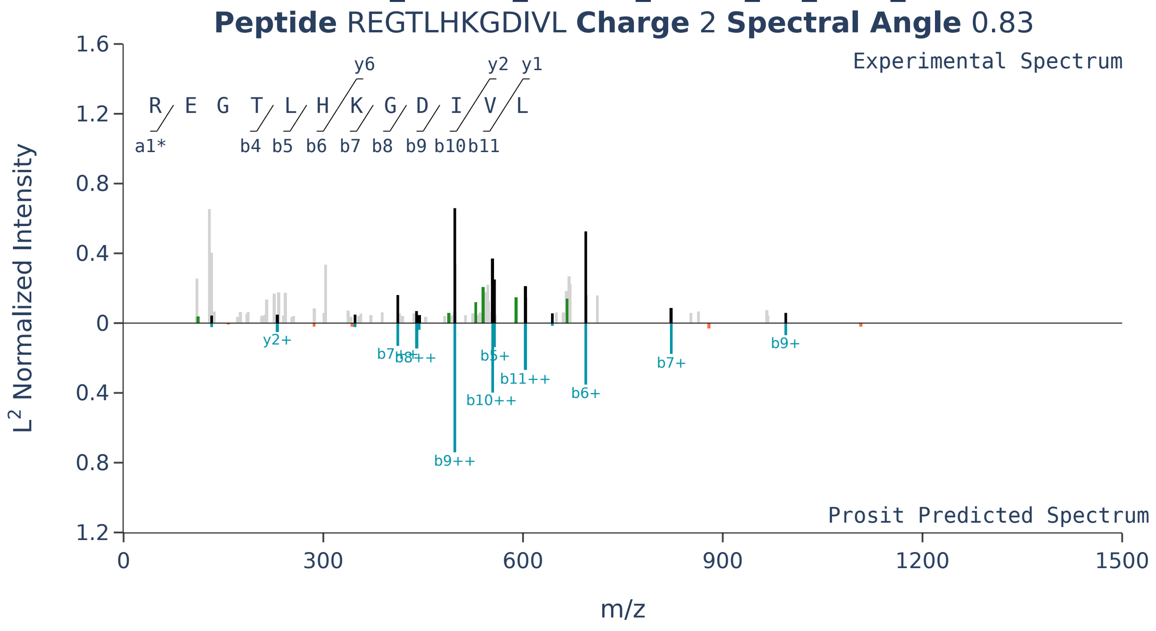


**Source** HRoetschke_211222_160123_HFGoe_IF2_b1R1_1h **Scan** 7556
**Peptide** DAGIRKSADDSVSA **Charge** 3 **Spectral Angle** 0.87

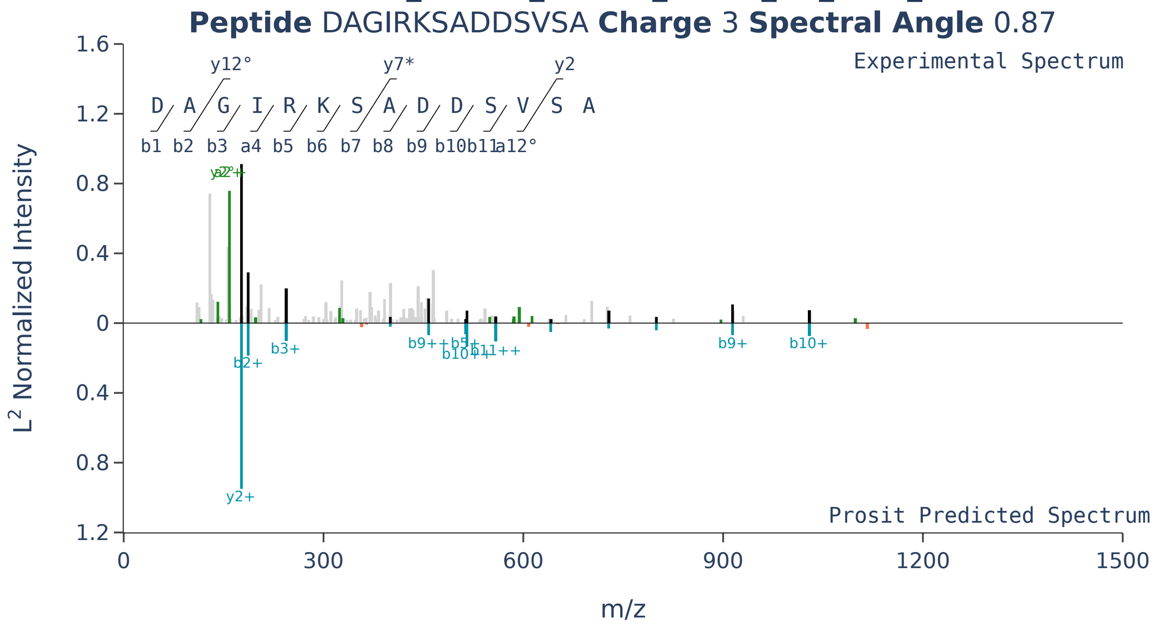


**Source** HRoetschke_211222_160123_HFGoe_IF2_b1R1_4h **Scan** 5863
**Peptide** IDKPEAD **Charge** 2 **Spectral Angle** 0.88

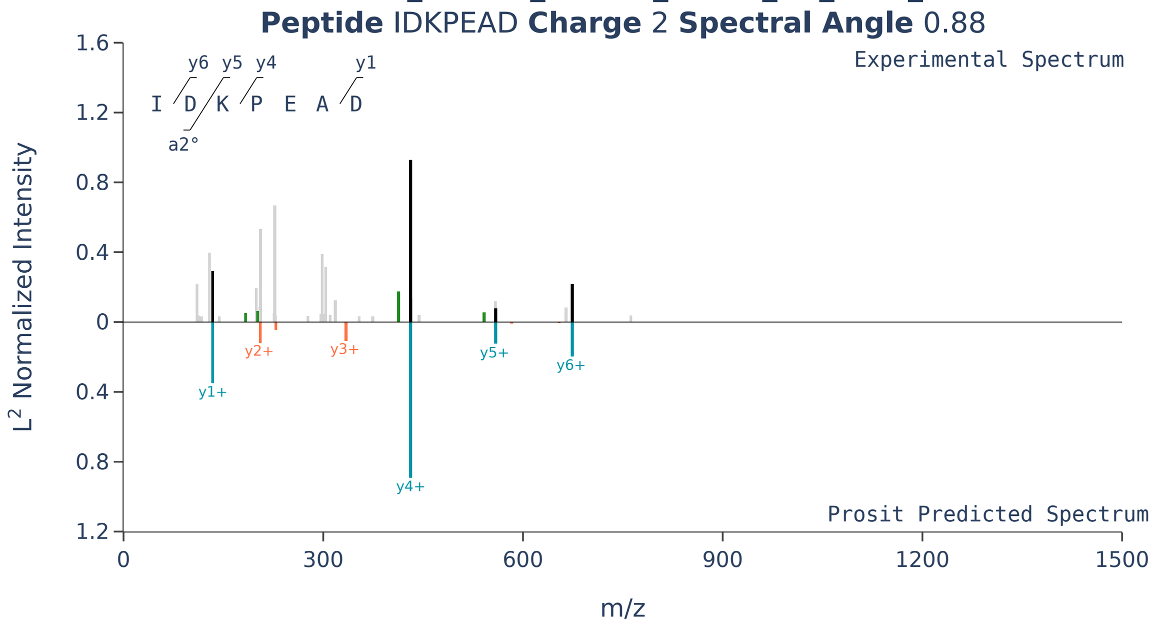


**Source** HRoetschke_211222_160123_HFGoe_IF2_b1R1_24h **Scan** 20259
**Peptide** SAKAGTGIDELLD **Charge** 2 **Spectral Angle** 0.84

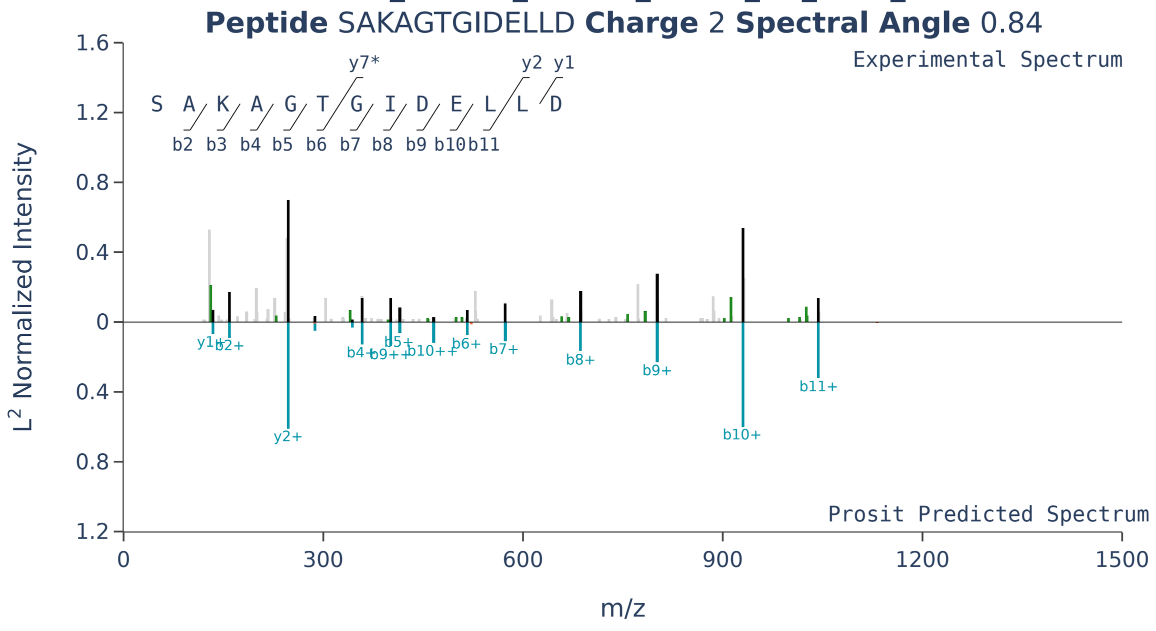


**Source** HRoetschke_211222_160123_HFGoe_IF2_b1R1_4h **Scan** 7237
**Peptide** KTLAAERQTSV **Charge** 3 **Spectral Angle** 0.89

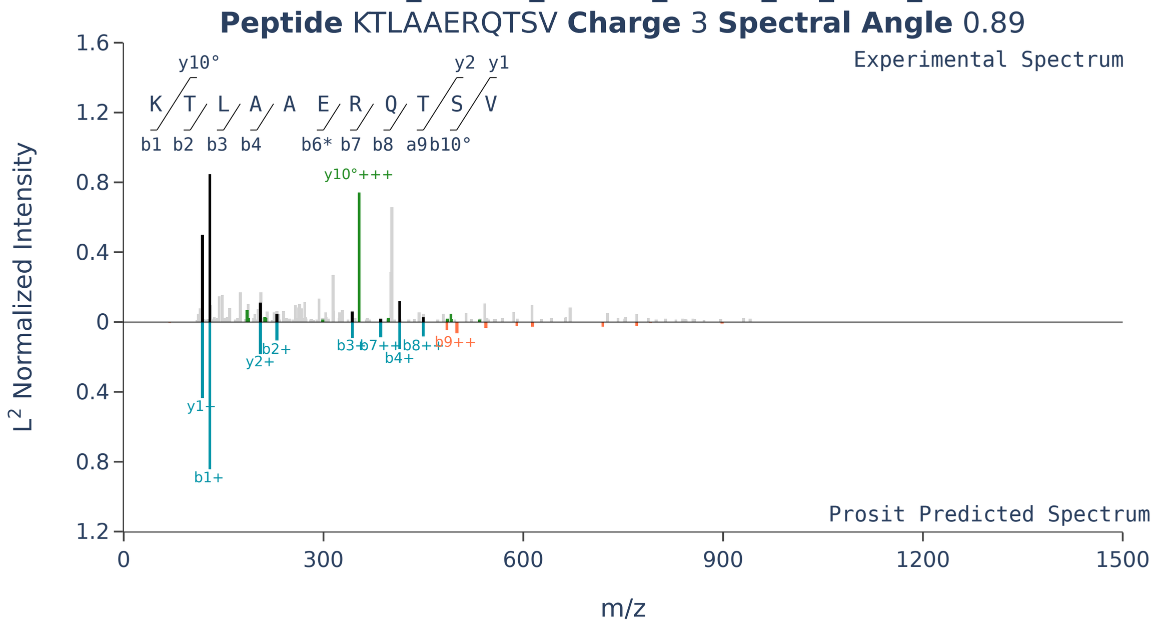


**Source** HRoetschke_211222_160123_HFGoe_IF2_b2R1_2h **Scan** 6111
**Peptide** NKIDKPEADPDRVKNE **Charge** 3 **Spectral Angle** 0.81

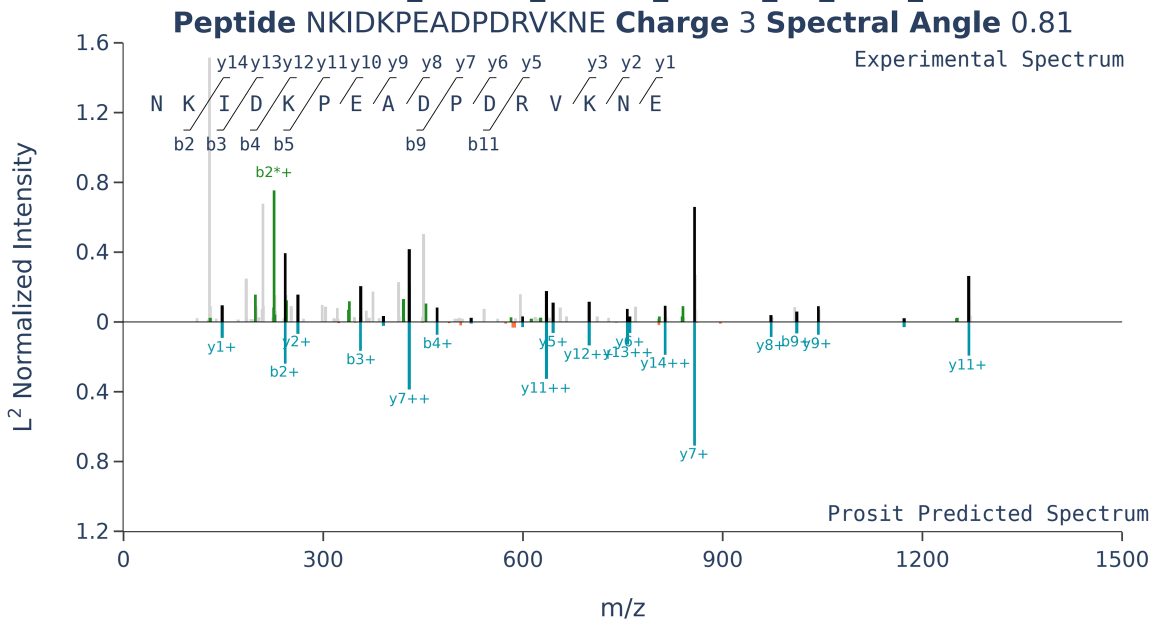


**Source** HRoetschke_211222_160123_HFGoe_IF2_b2R2_24h **Scan** 11309
**Peptide** ANKMAVKGSQVL **Charge** 3 **Spectral Angle** 0.89

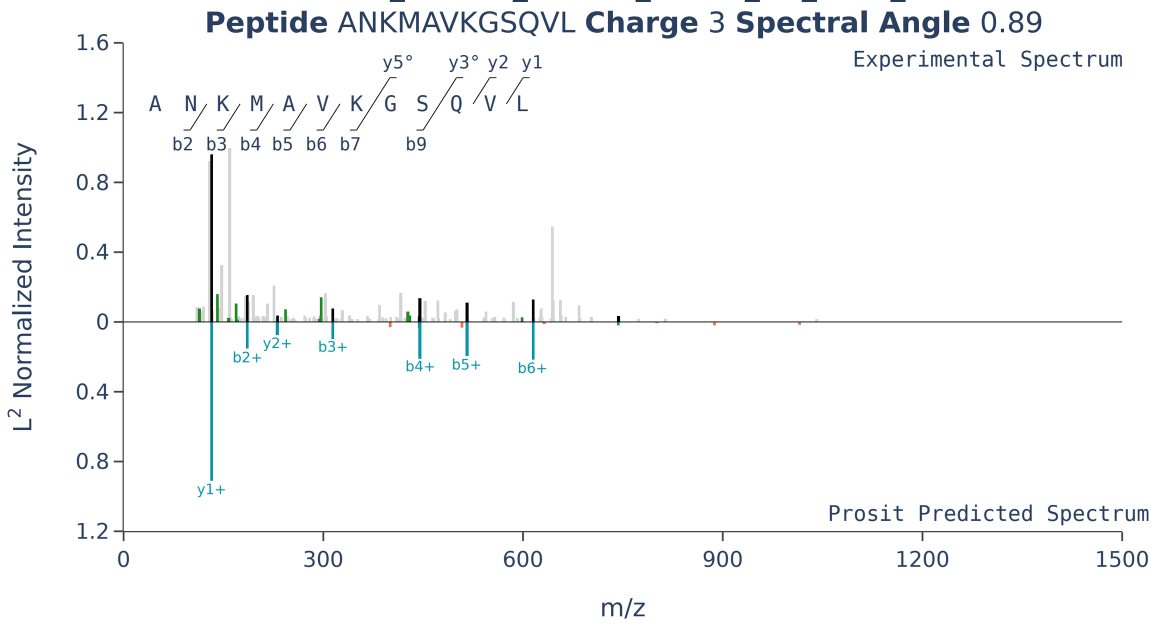


**Source** HRoetschke_211222_160123_HFGoe_IF2_b1R2_4h **Scan** 11957
**Peptide** KGMASGAVLE **Charge** 2 **Spectral Angle** 0.83

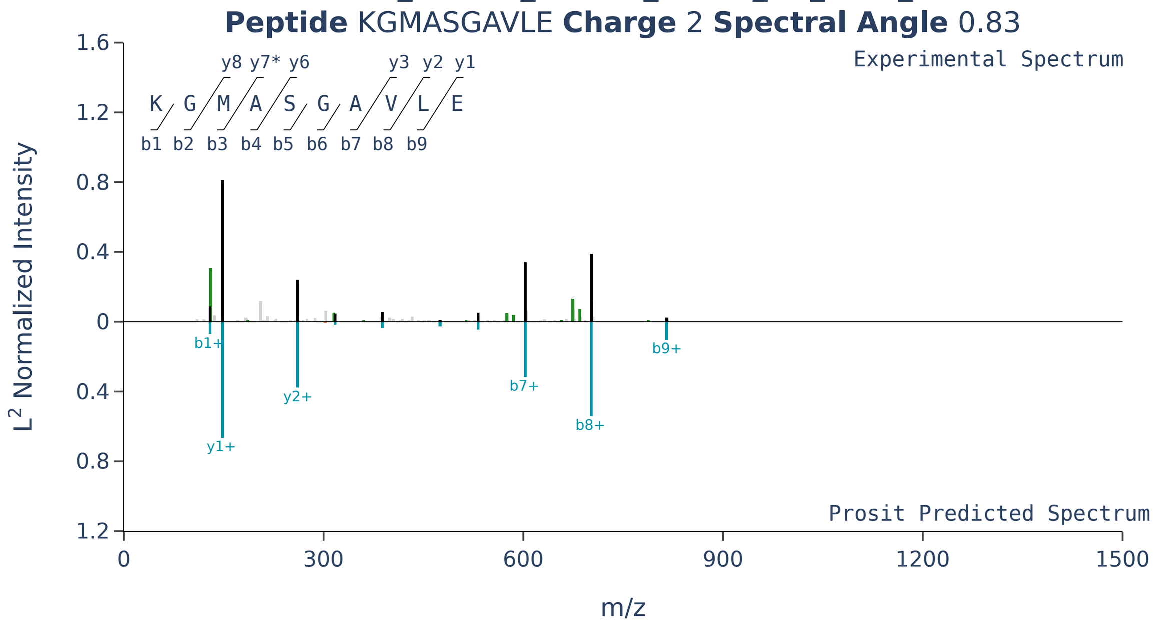

**Source** HRoetschke_211222_160123_HFGoe_IF2_b1R2_1h **Scan** 10444
**Peptide** IGAYHVETEN **Charge** 1 **Spectral Angle** 0.88

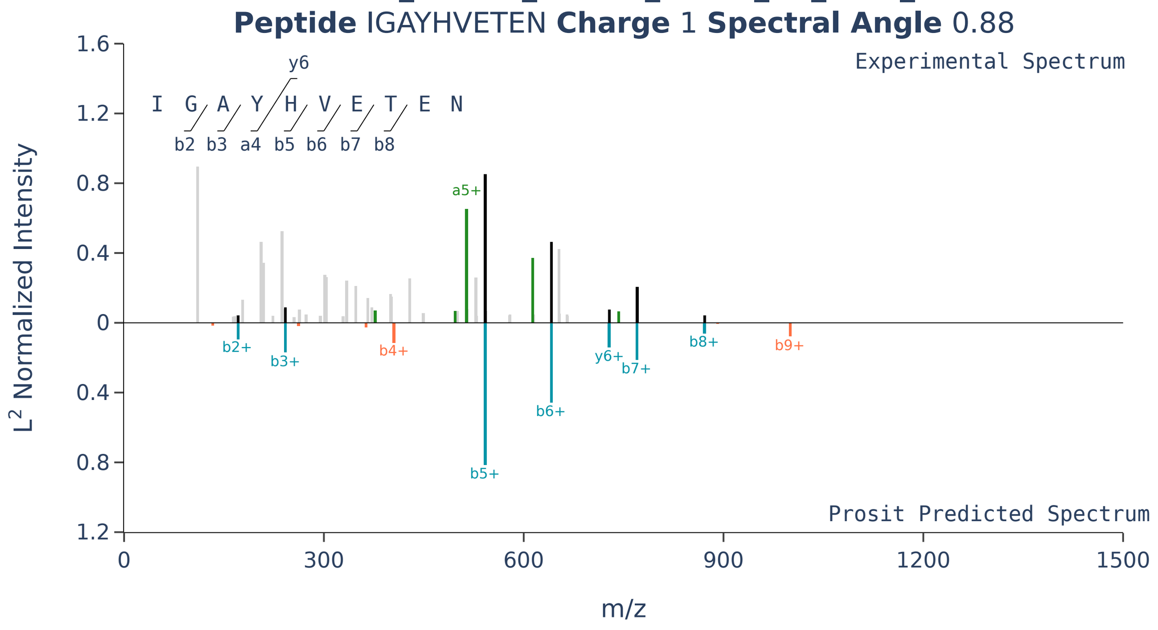


**Source** HRoetschke_211222_160123_HFGoe_IF2_b1R1_4h **Scan** 15797
**Peptide** VLVREGTL **Charge** 2 **Spectral Angle** 0.91

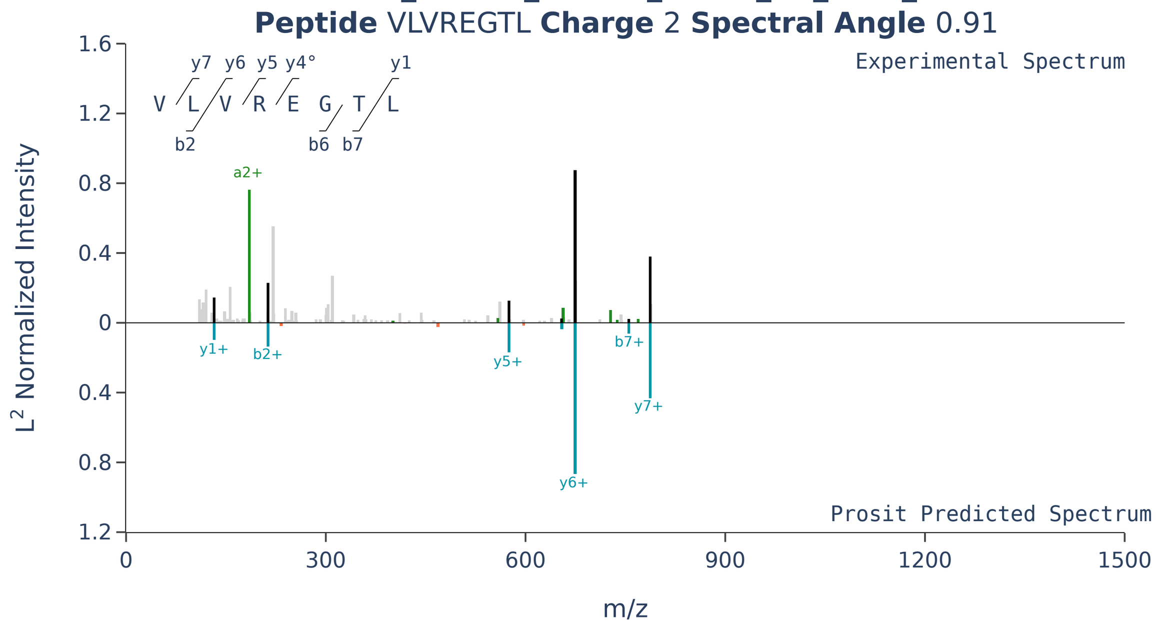


**Source** HRoetschke_211222_160123_HFGoe_IF2_b1R1_4h **Scan** 11446
**Peptide** GAMATINQ **Charge** 1 **Spectral Angle** 0.8

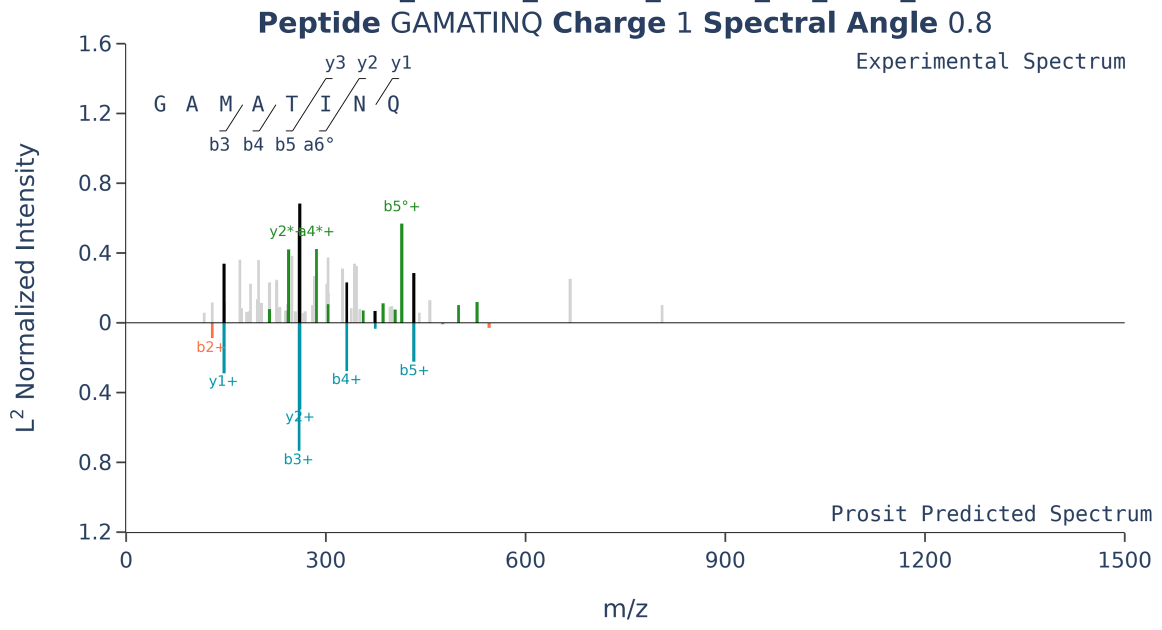


**Source** HRoetschke_211222_160123_HFGoe_IF2_b1R1_2h **Scan** 10950
**Peptide** KNSGPDKLTLQ **Charge** 3 **Spectral Angle** 0.89

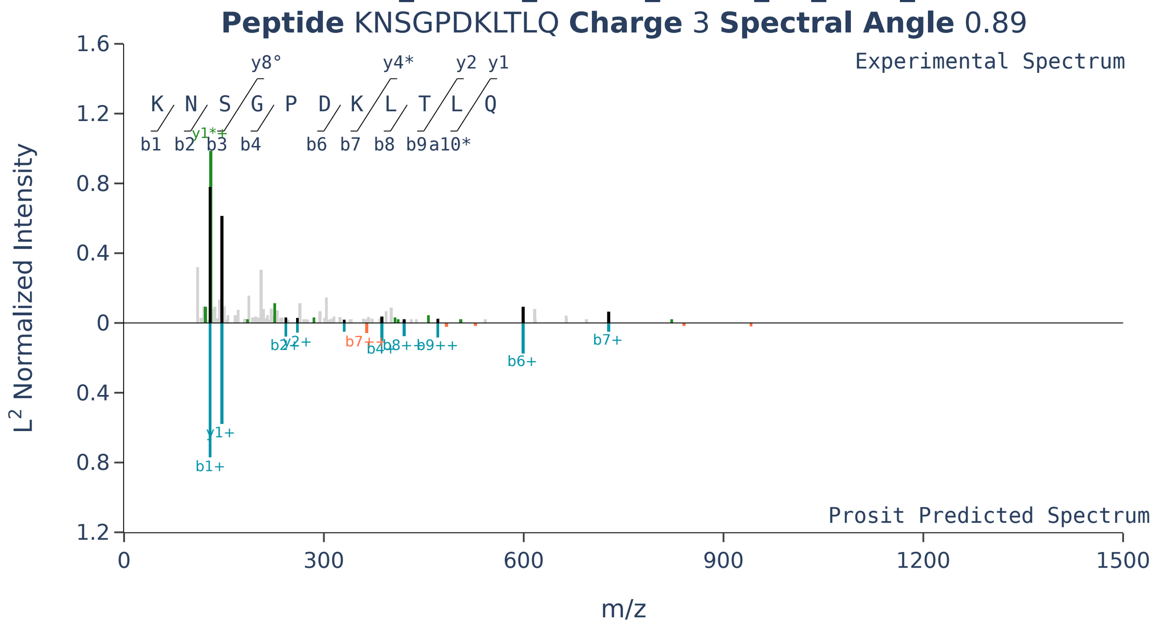


**Source** HRoetschke_211222_160123_HFGoe_IF2_b1R1_4h **Scan** 12796
**Peptide** SSLQQGFQKPAQA **Charge** 2 **Spectral Angle** 0.87

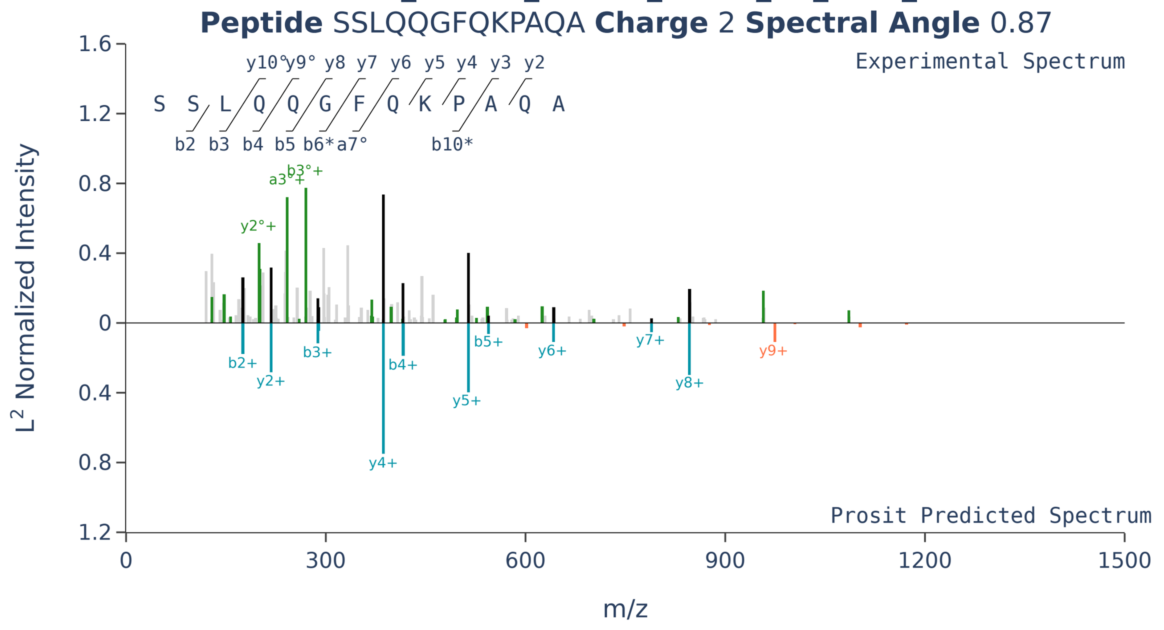


**Source** HRoetschke_211222_160123_HFGoe_IF2_b1R1_24h **Scan** 17467
**Peptide** IDQETAQLV **Charge** 2 **Spectral Angle** 0.83

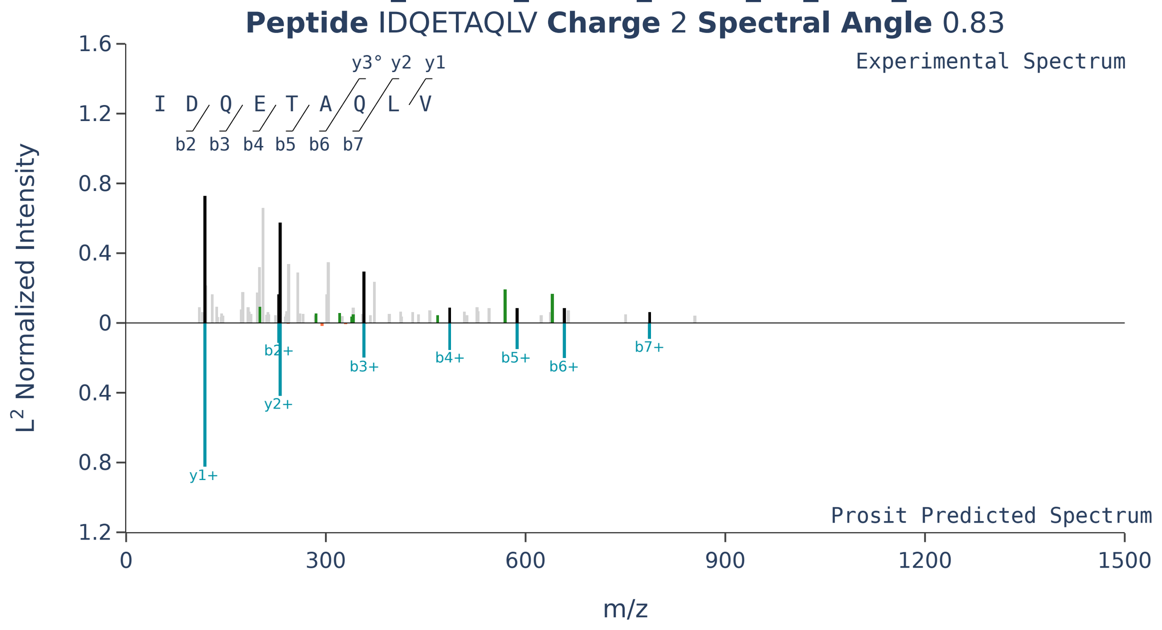


**Source** HRoetschke_211222_160123_HFGoe_IF2_b2R2_24h **Scan** 11804
**Peptide** VILRRENELEE **Charge** 3 **Spectral Angle** 0.81

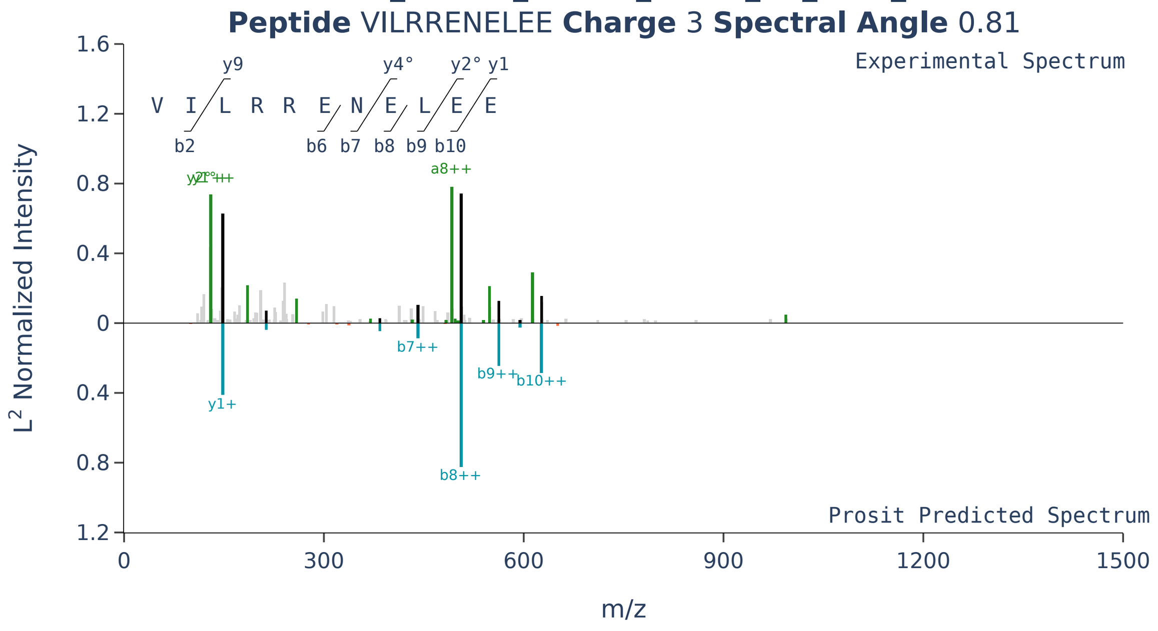


**Source** HRoetschke_211222_160123_HFGoe_IF2_b1R2_1h **Scan** 15273
**Peptide** ERLVQQFAD **Charge** 2 **Spectral Angle** 0.86

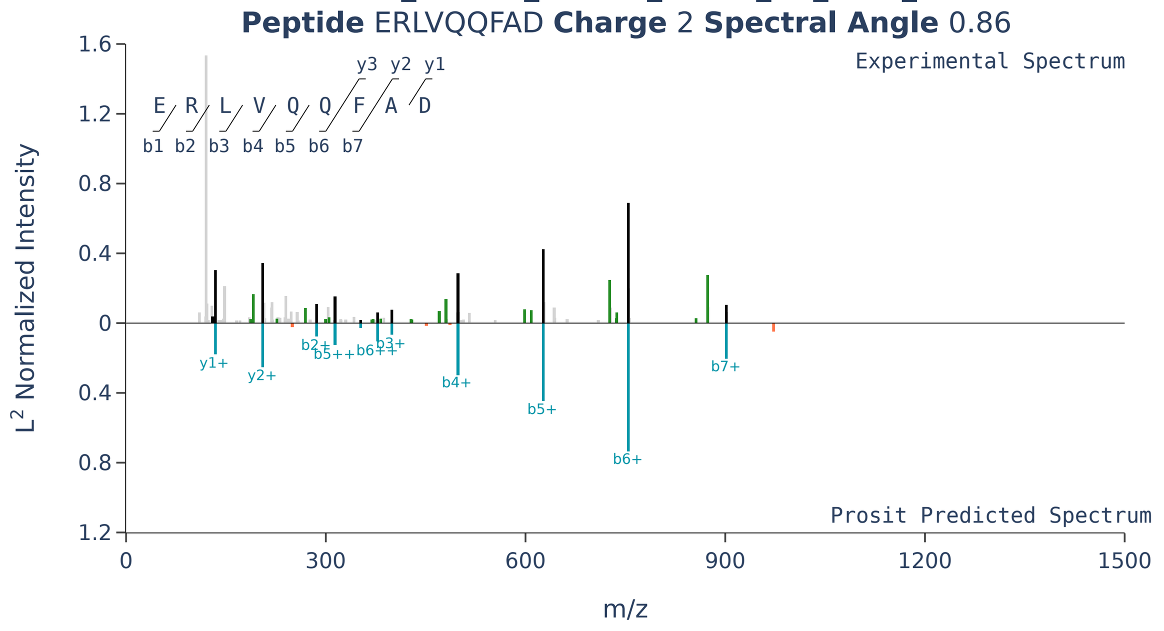


**Source** HRoetschke_211222_160123_HFGoe_IF2_b1R2_2h **Scan** 9368
**Peptide** PQEAERLAAE **Charge** 2 **Spectral Angle** 0.83

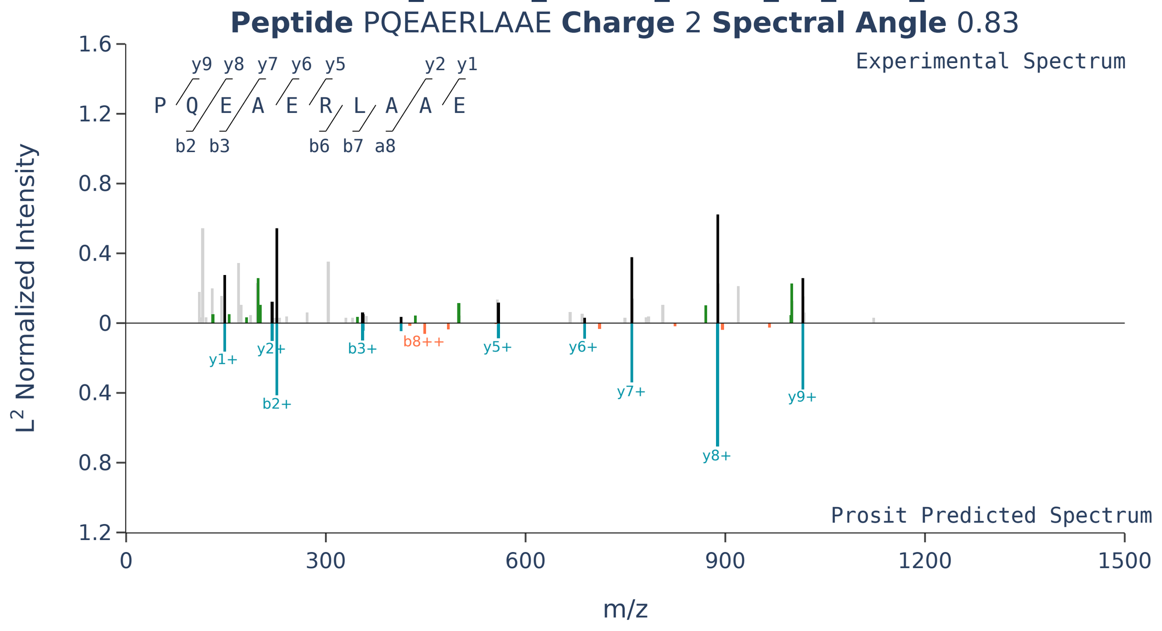


**Source** HRoetschke_211222_160123_HFGoe_IF2_b1R2_4h **Scan** 10820
**Peptide** QEKQTLID **Charge** 2 **Spectral Angle** 0.8

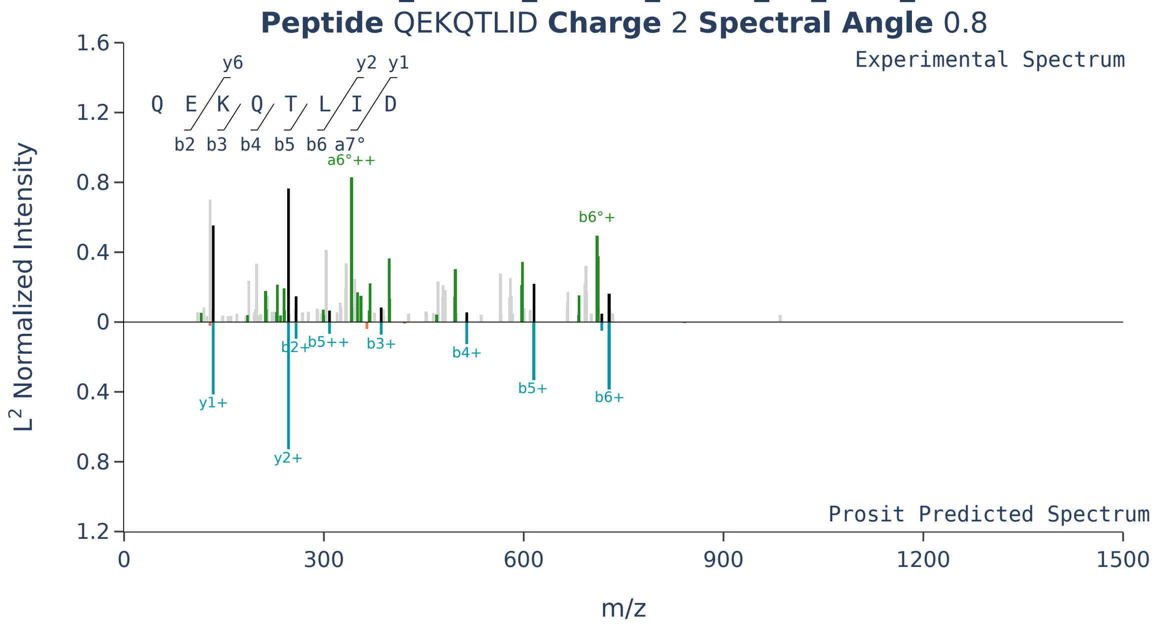


**Source** HRoetschke_211222_160123_HFGoe_IF2_b1R1_4h **Scan** 12811
**Peptide** VTIKTLAAERQ **Charge** 3 **Spectral Angle** 0.82

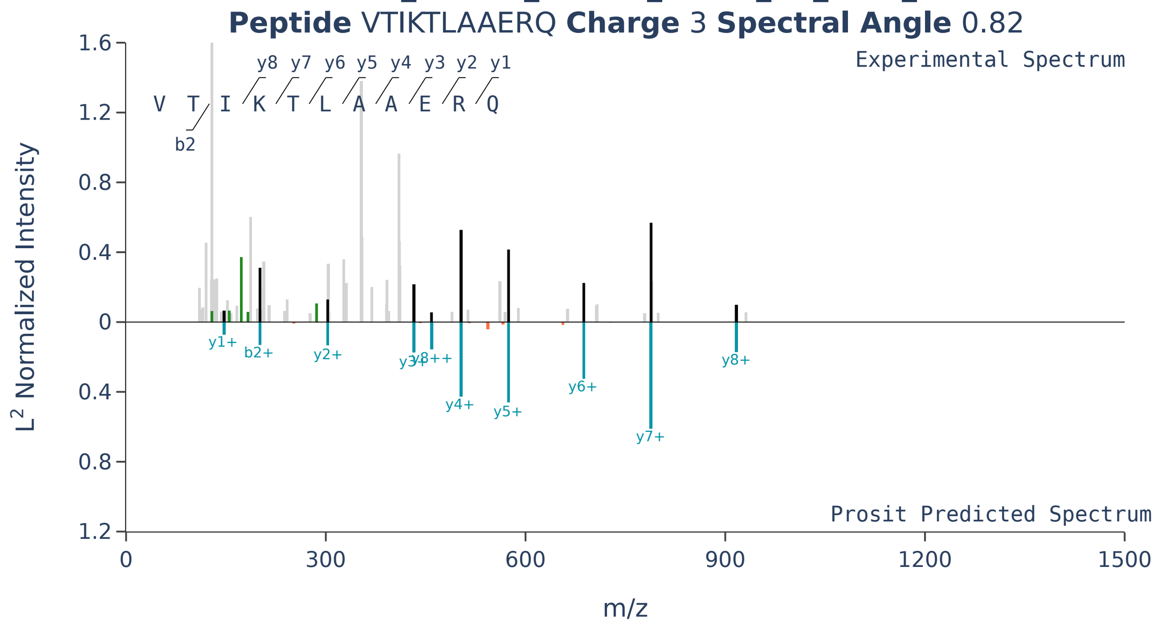


**Source** HRoetschke_211222_160123_HFGoe_IF2_b2R2_2h **Scan** 16496
**Peptide** RENELEEAVM **Charge** 2 **Spectral Angle** 0.8

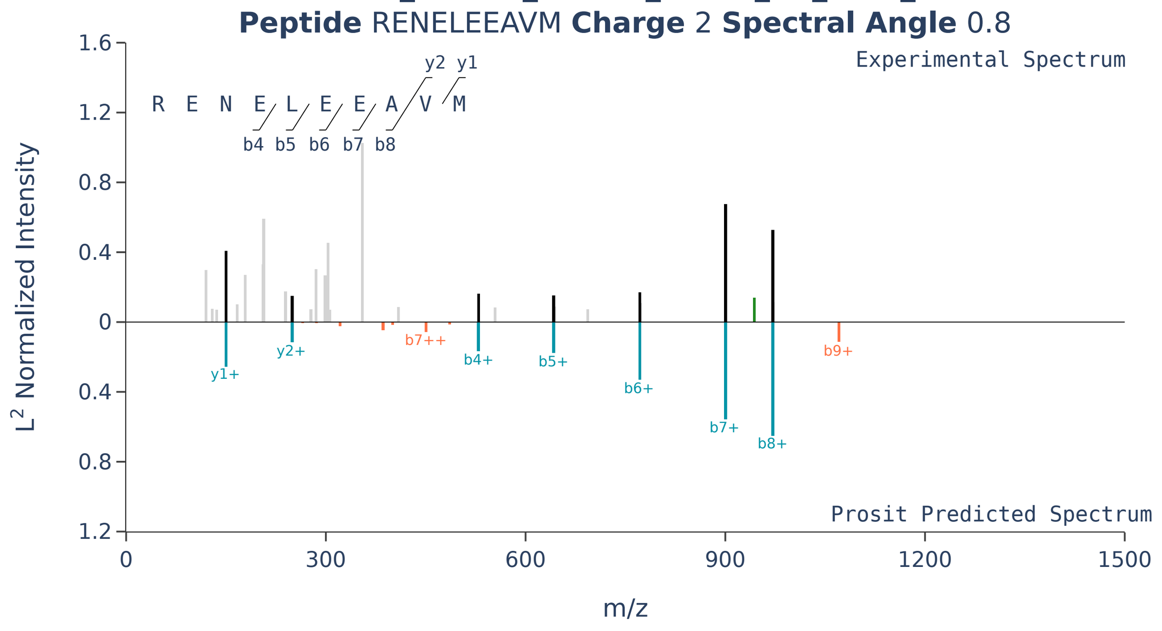


**Source** HRoetschke_211222_160123_HFGoe_IF2_b2R1_4h **Scan** 12809
**Peptide** SGEAGGITQHI **Charge** 2 **Spectral Angle** 0.77

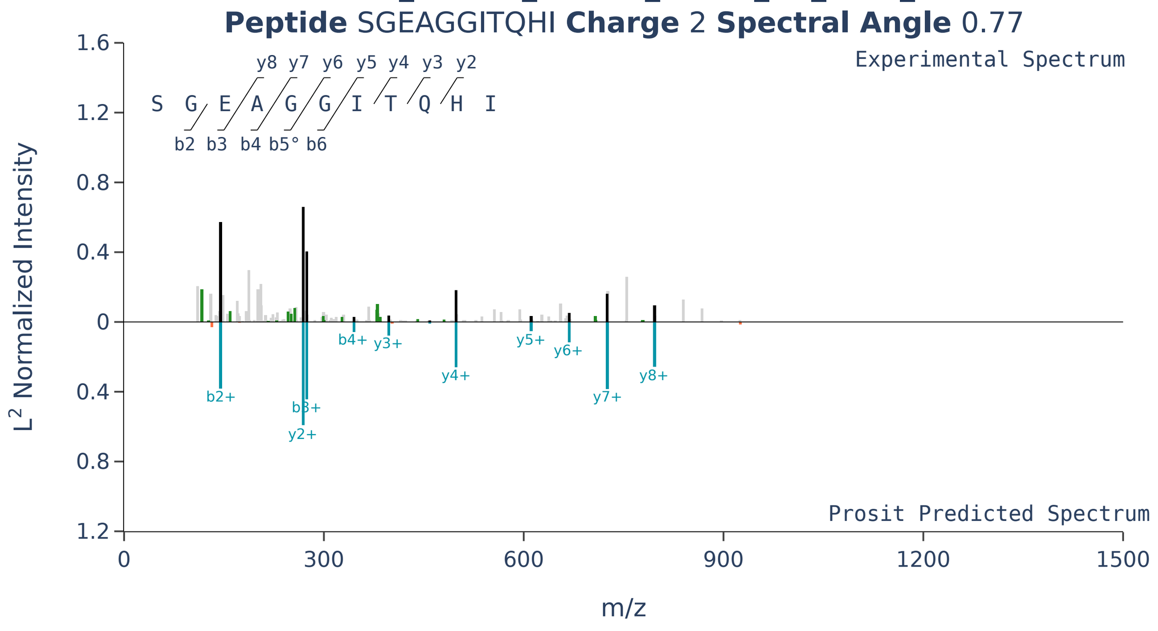


**Source** HRoetschke_211222_160123_HFGoe_IF2_b2R1_24h **Scan** 7014
**Peptide** VTVVRDEKKAREV **Charge** 3 **Spectral Angle** 0.88

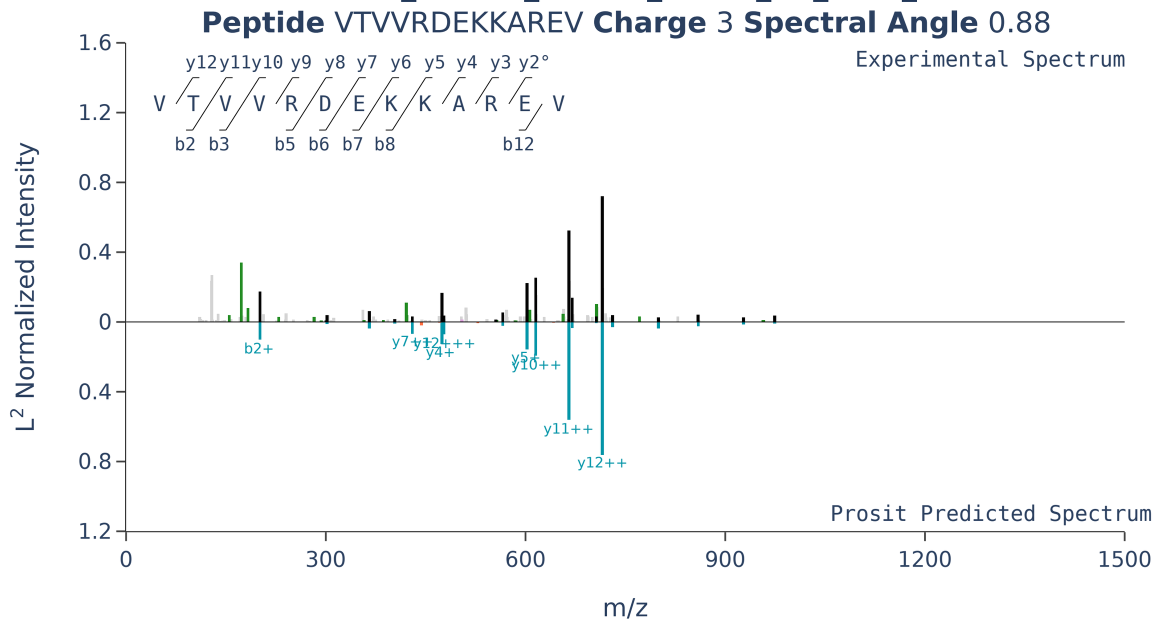


**Source** HRoetschke_211222_160123_HFGoe_IF2_b1R1_24h **Scan** 14664
**Peptide** SVSAQEKQTLL **Charge** 2 **Spectral Angle** 0.8

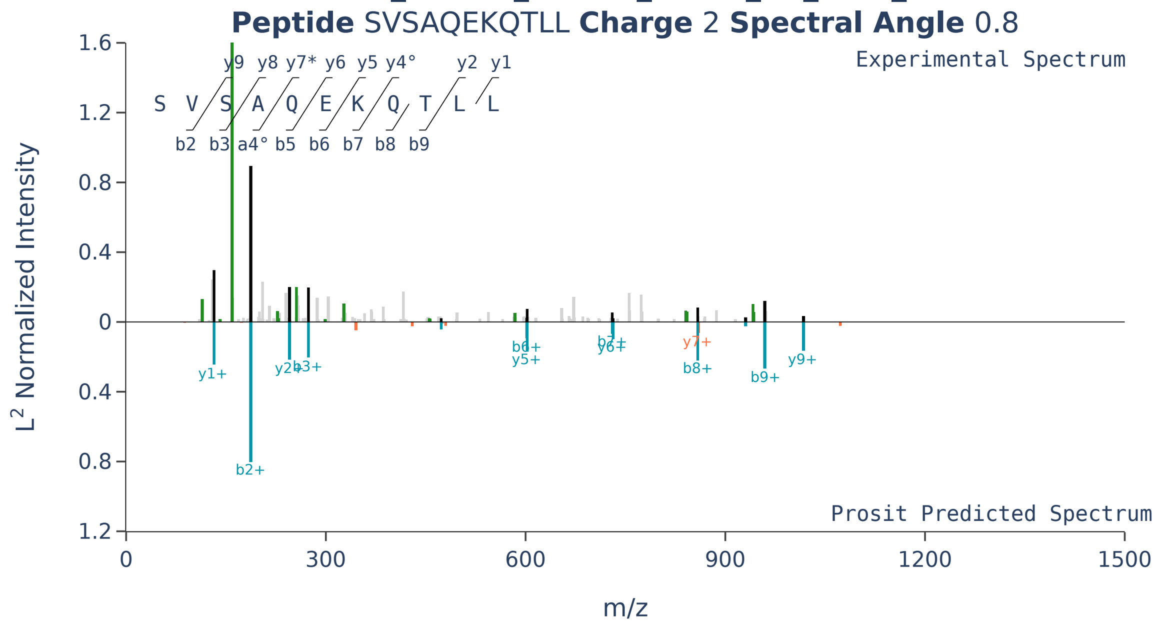

**Source** HRoetschke_211222_160123_HFGoe_IF2_b1R1_4h **Scan** 9089
**Peptide** AVNKIDKPEADPDRV **Charge** 4 **Spectral Angle** 0.91

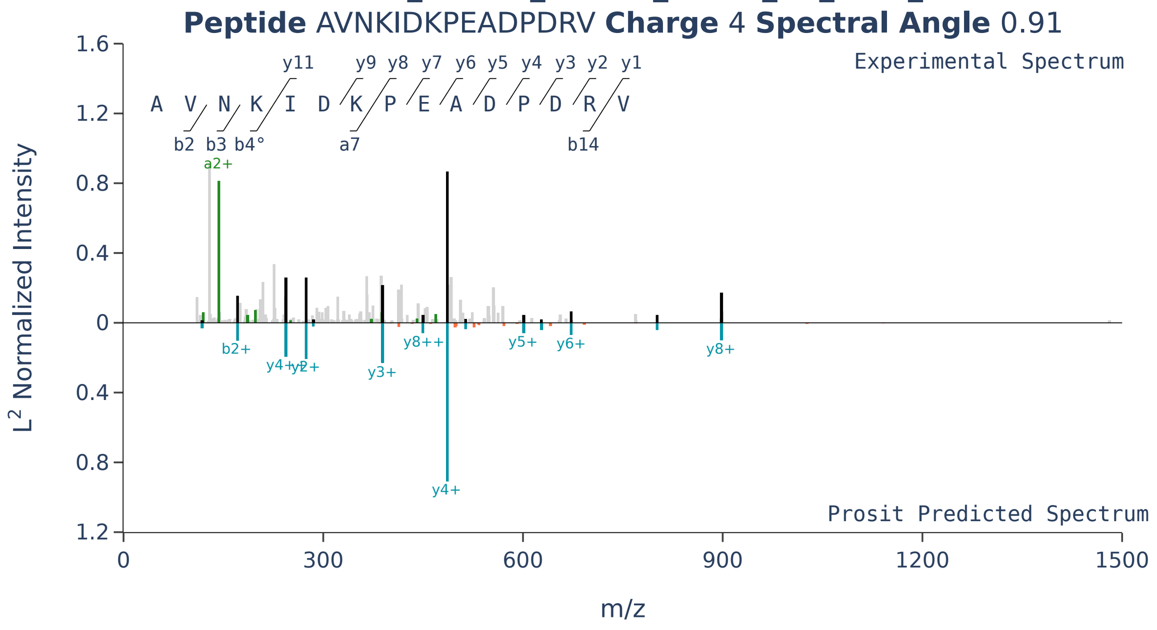


**Source** HRoetschke_211222_160123_HFGoe_IF2_b1R1_2h **Scan** 7907
**Peptide** DSSDYHVT **Charge** 1 **Spectral Angle** 0.87

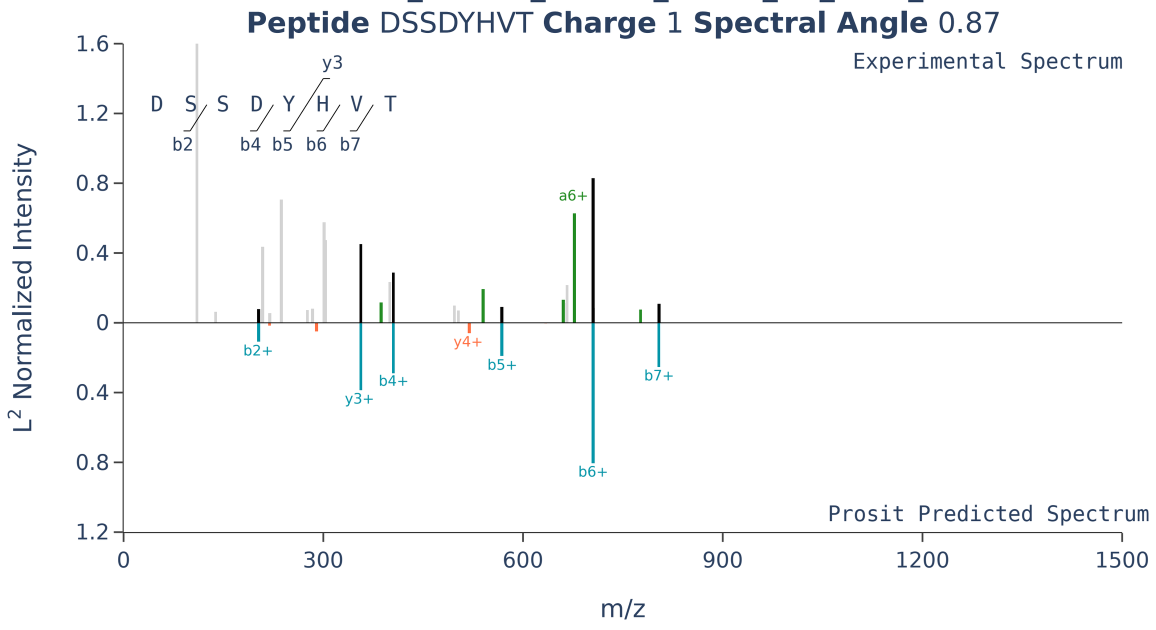


**Source** HRoetschke_211222_160123_HFGoe_IF2_b1R1_4h **Scan** 9116
**Peptide** DSSDYHV **Charge** 1 **Spectral Angle** 0.9

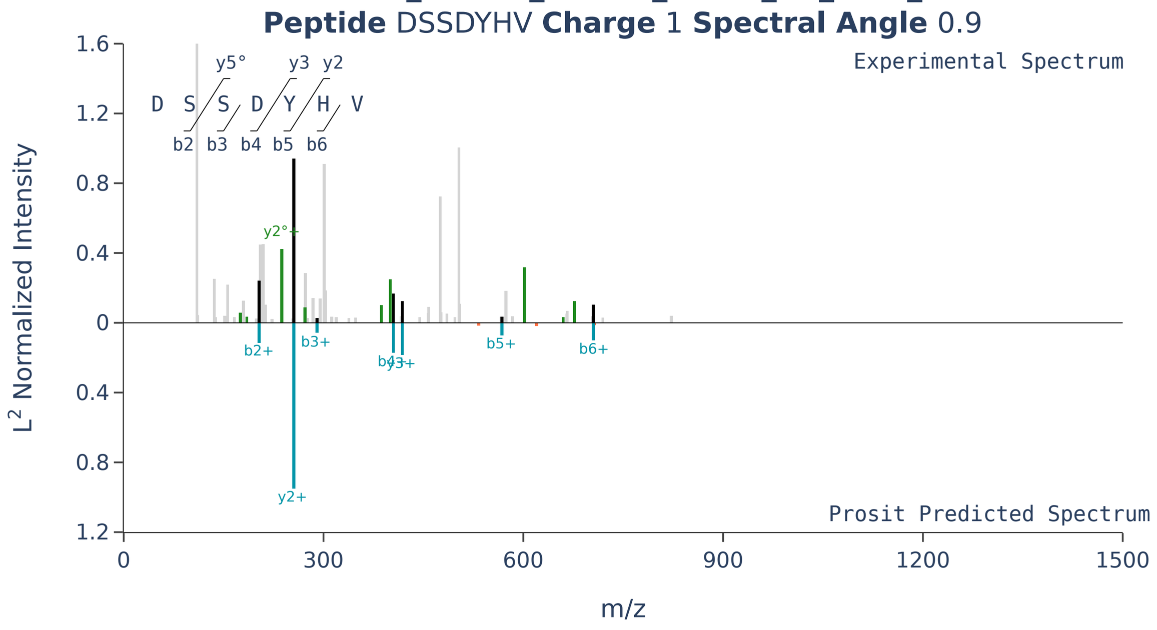


**Source** HRoetschke_211222_160123_HFGoe_IF2_b1R2_2h **Scan** 13428
**Peptide** AMRNELGQEV **Charge** 2 **Spectral Angle** 0.81

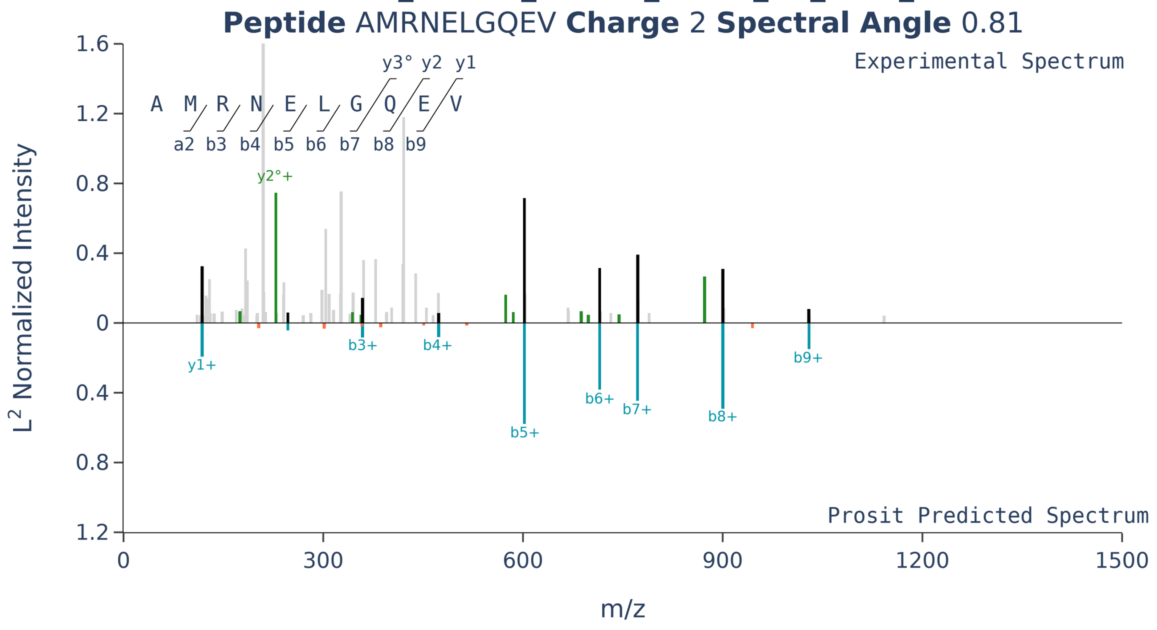


**Source** HRoetschke_211222_160123_HFGoe_IF2_b1R2_24h **Scan** 15925
**Peptide** GGESQFVHV **Charge** 2 **Spectral Angle** 0.85

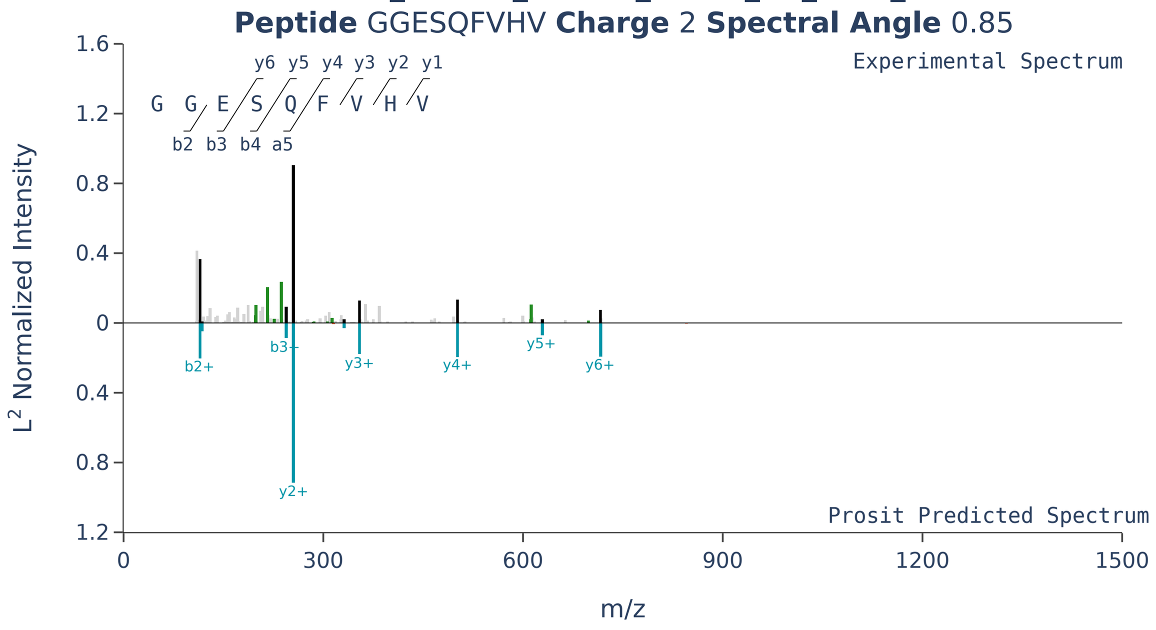


**Source** HRoetschke_211222_160123_HFGoe_IF2_b2R1_1h **Scan** 8138
**Peptide** ILRRENELE **Charge** 3 **Spectral Angle** 0.93

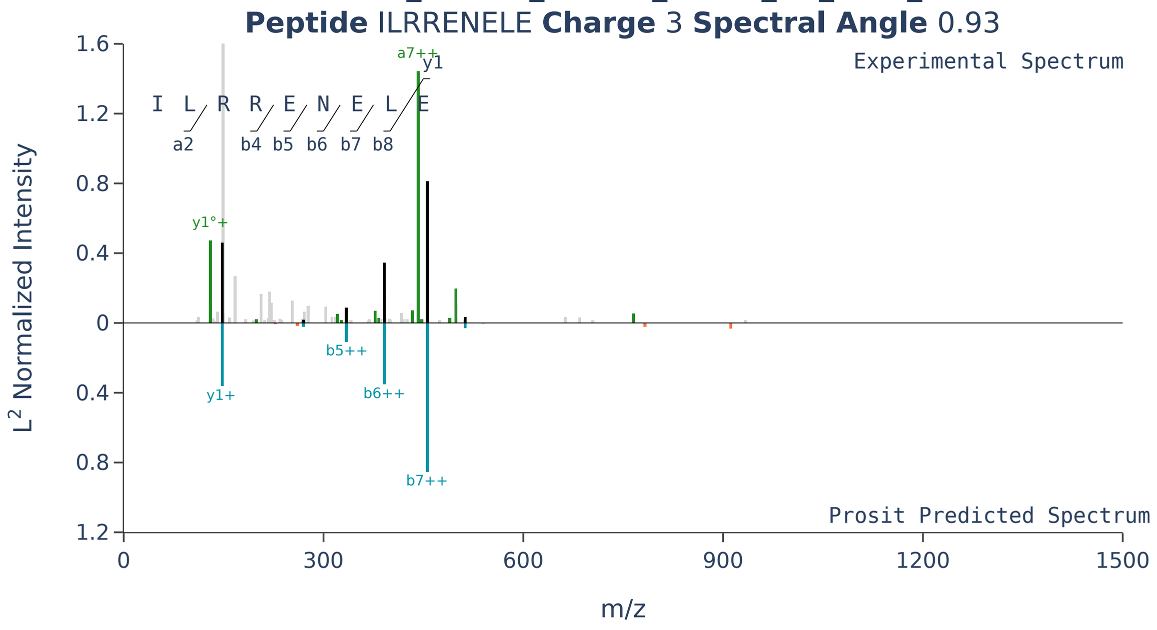


**Source** HRoetschke_211222_160123_HFGoe_IF2_b2R1_24h **Scan** 13138
**Peptide** GEAGGITQHI **Charge** 2 **Spectral Angle** 0.84

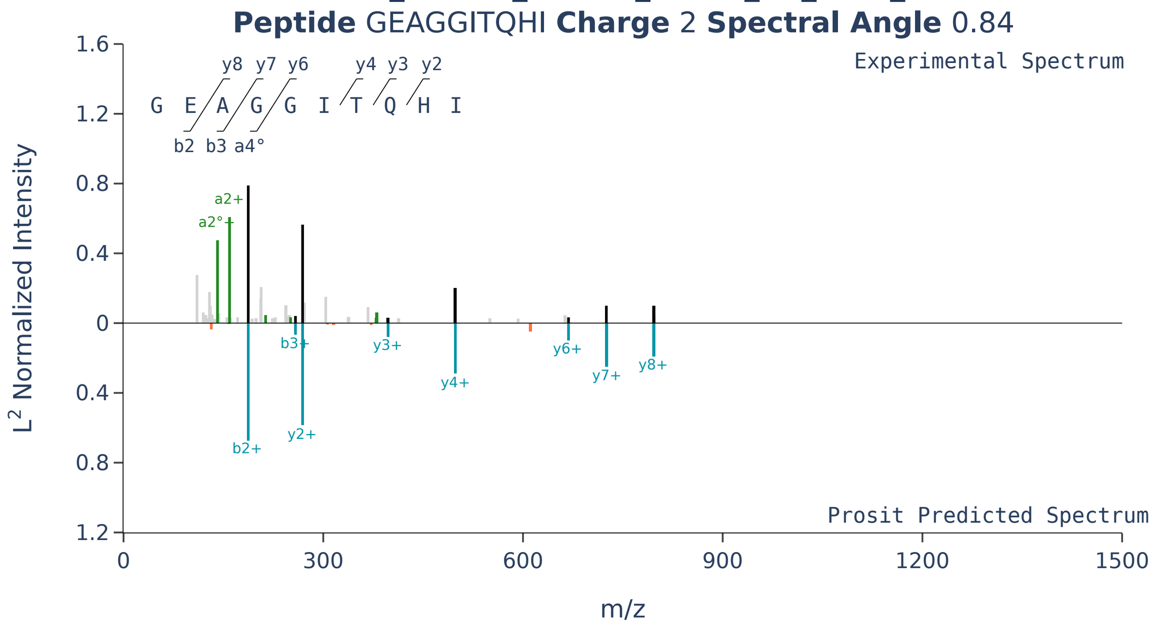


**Source** HRoetschke_211222_160123_HFGoe_IF2_b2R1_1h **Scan** 14078
**Peptide** TLNQVID **Charge** 2 **Spectral Angle** 0.75

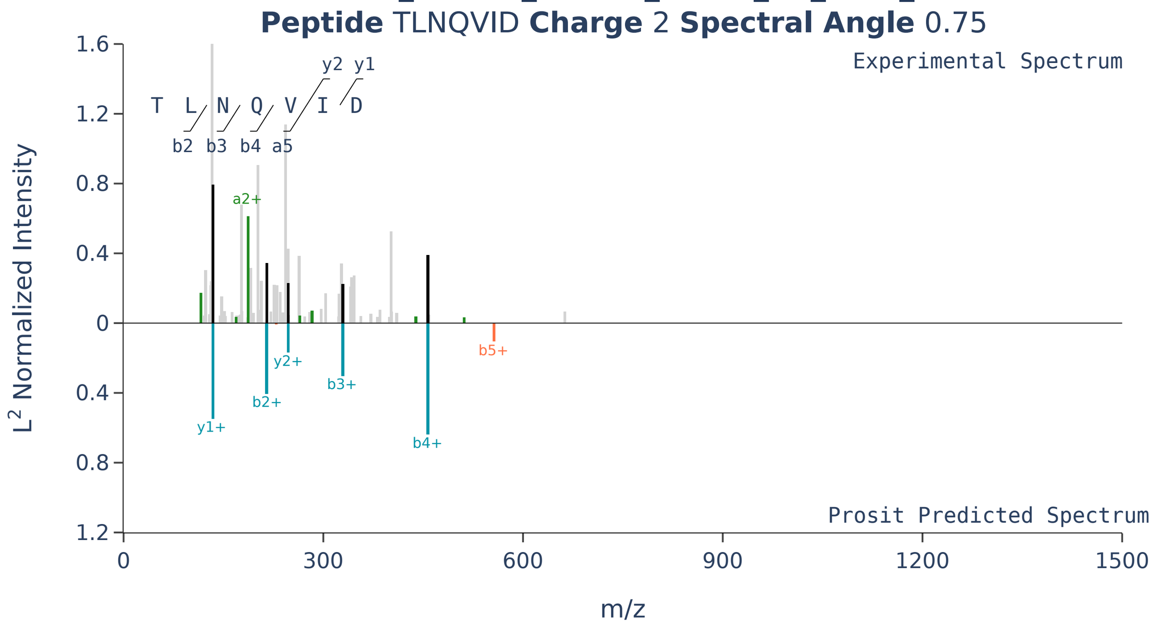


**Source** HRoetschke_211222_160123_HFGoe_IF2_b1R1_1h **Scan** 8915
**Peptide** GFQKPAQAVN **Charge** 2 **Spectral Angle** 0.77

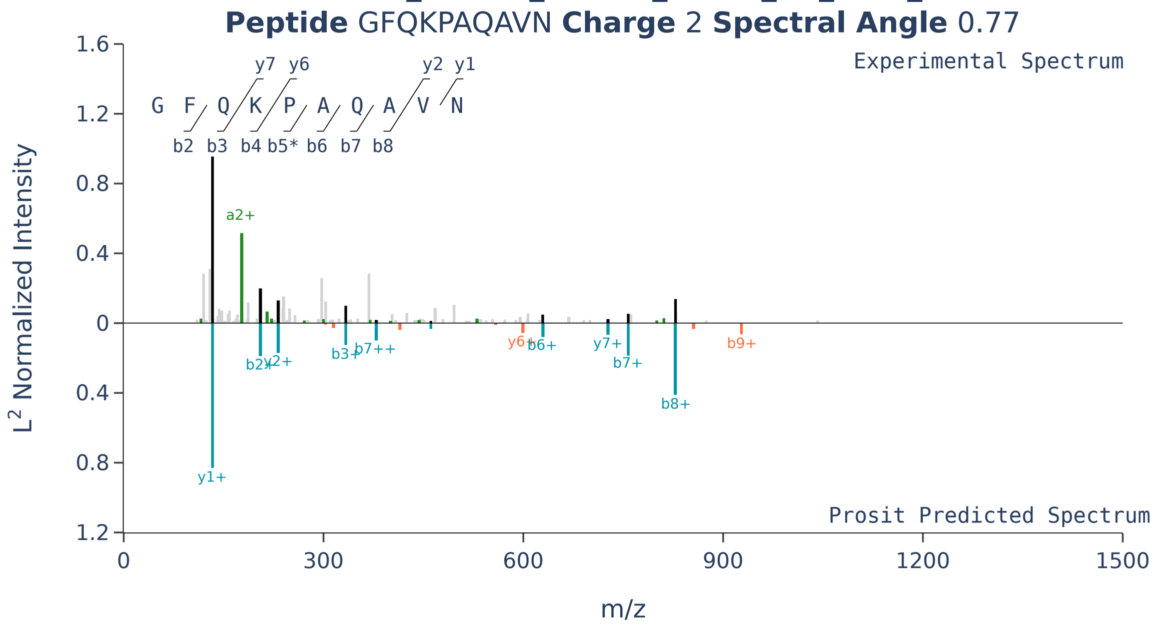


**Source** HRoetschke_211222_160123_HFGoe_IF2_b2R1_24h **Scan** 13275
**Peptide** AAEPRAPVV **Charge** 2 **Spectral Angle** 0.86

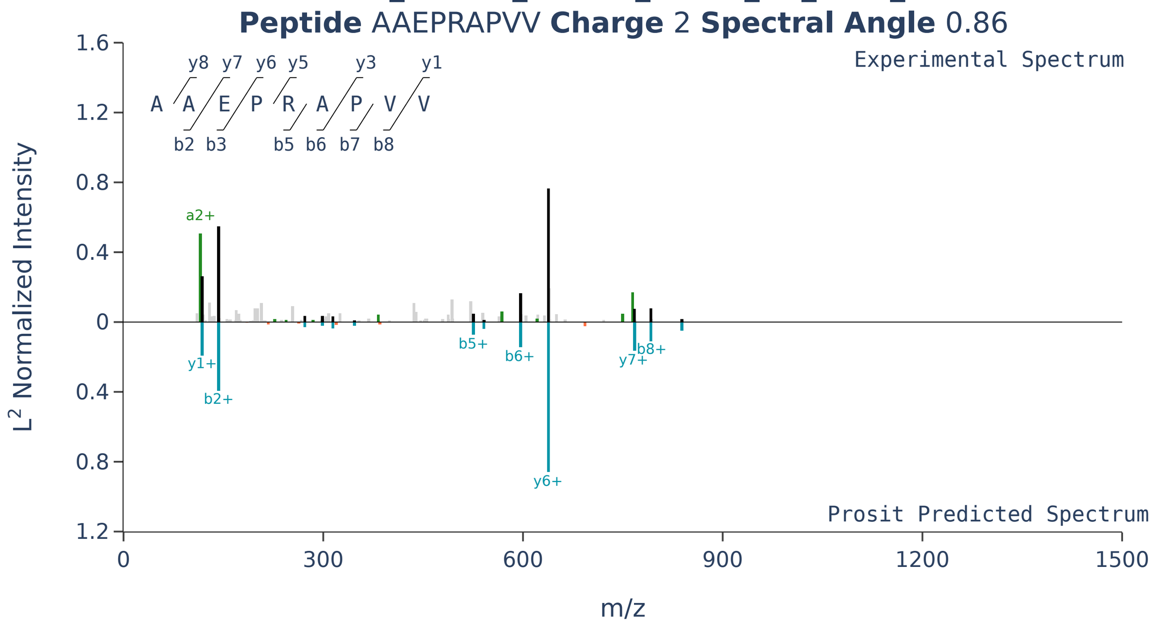


**Source** HRoetschke_211222_160123_HFGoe_IF2_b1R1_4h **Scan** 7938
**Peptide** MGHKVILRRENELE **Charge** 3 **Spectral Angle** 0.85

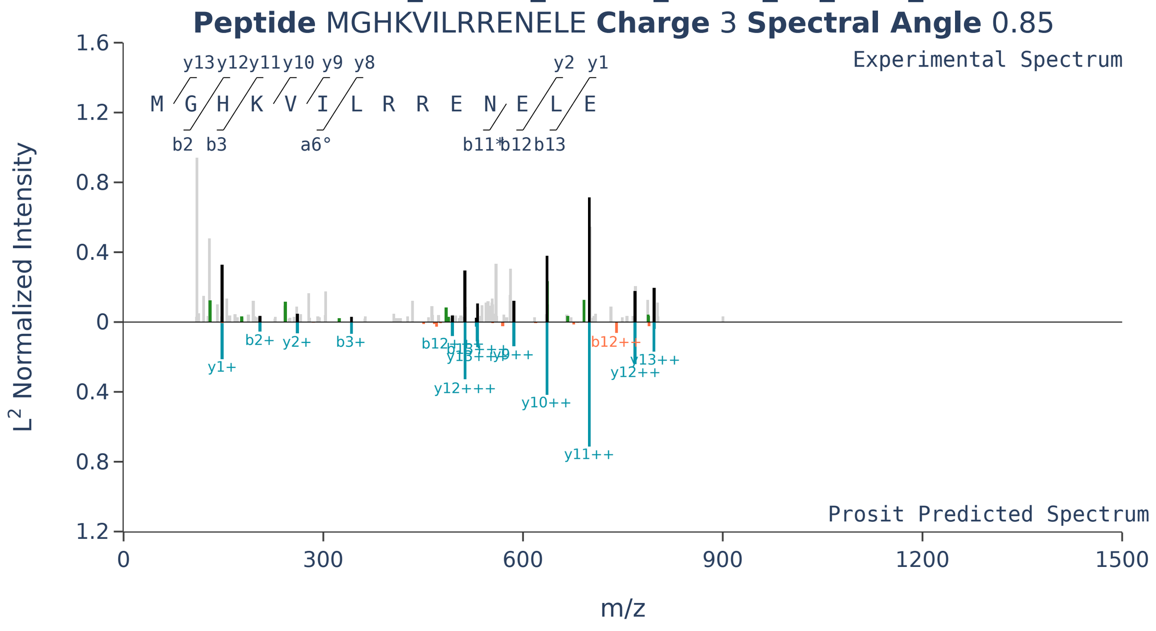


**Source** HRoetschke_211222_160123_HFGoe_IF2_b1R1_24h **Scan** 10314
**Peptide** TVGELAN **Charge** 1 **Spectral Angle** 0.8

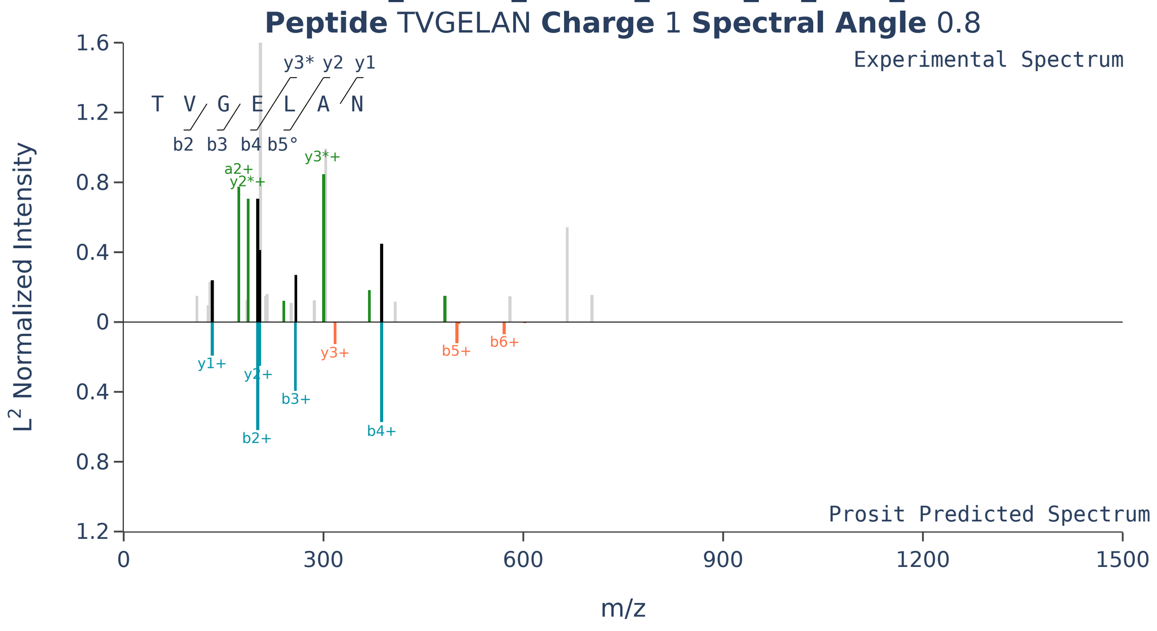


**Source** HRoetschke_211222_160123_HFGoe_IF2_b2R2_24h **Scan** 7252
**Peptide** TLAAERQTSVER **Charge** 3 **Spectral Angle** 0.87

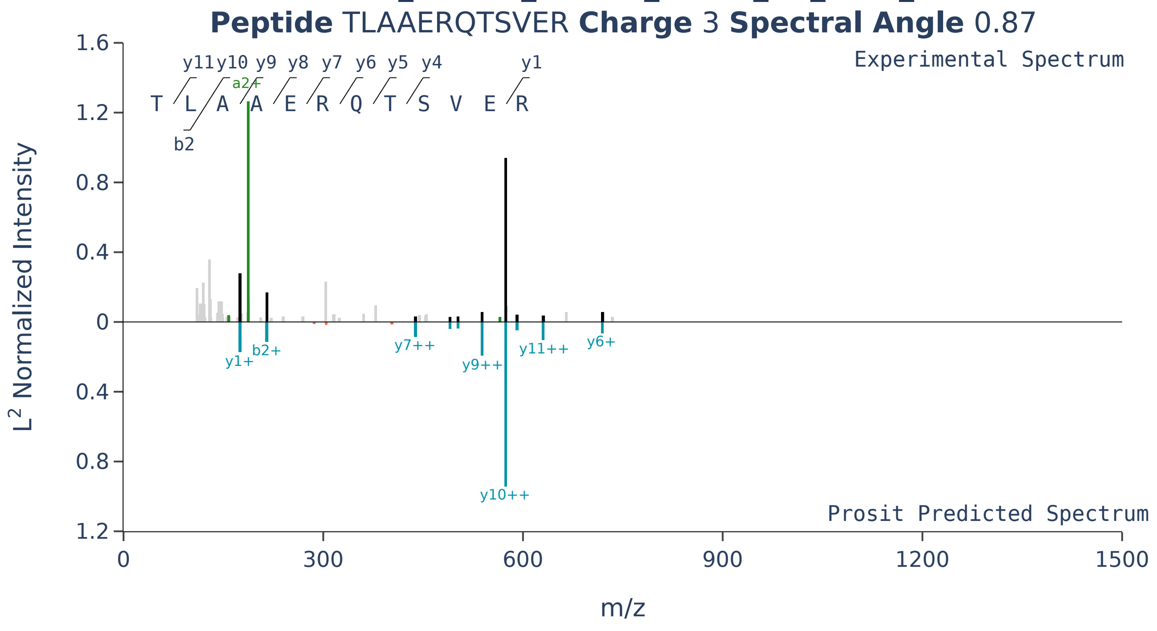


**Source** HRoetschke_211222_160123_HFGoe_IF2_b1R1_4h **Scan** 15203
**Peptide** LVQQFAD **Charge** 1 **Spectral Angle** 0.88

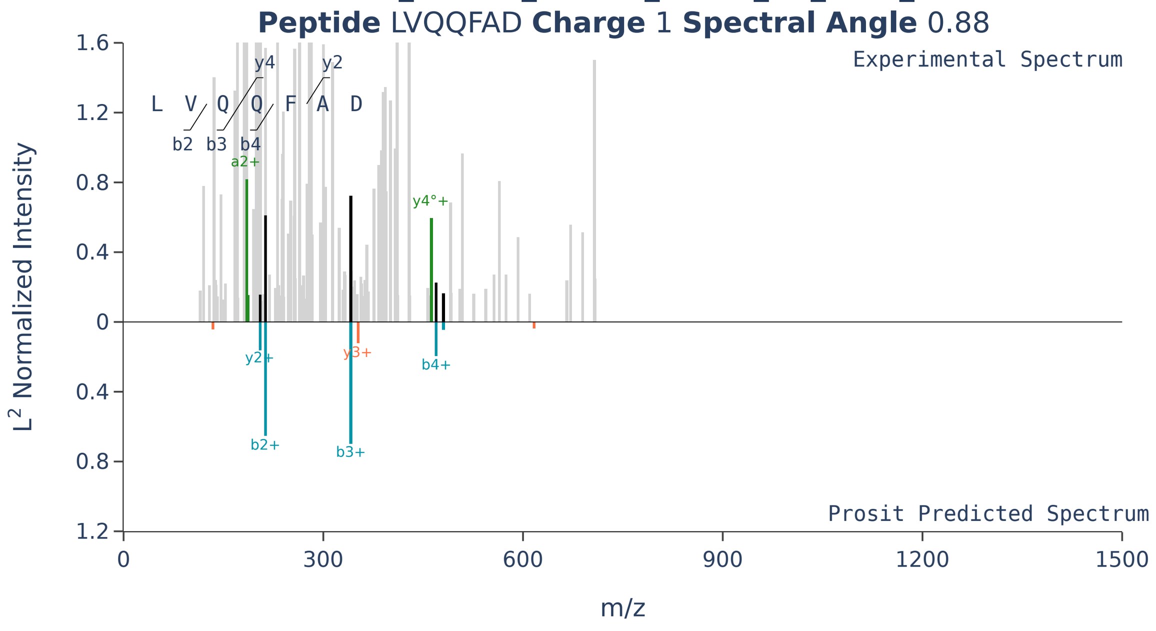


**Source** HRoetschke_211222_160123_HFGoe_IF2_b2R2_4h **Scan** 14418
**Peptide** TDVTIKTLAAERQT **Charge** 2 **Spectral Angle** 0.9

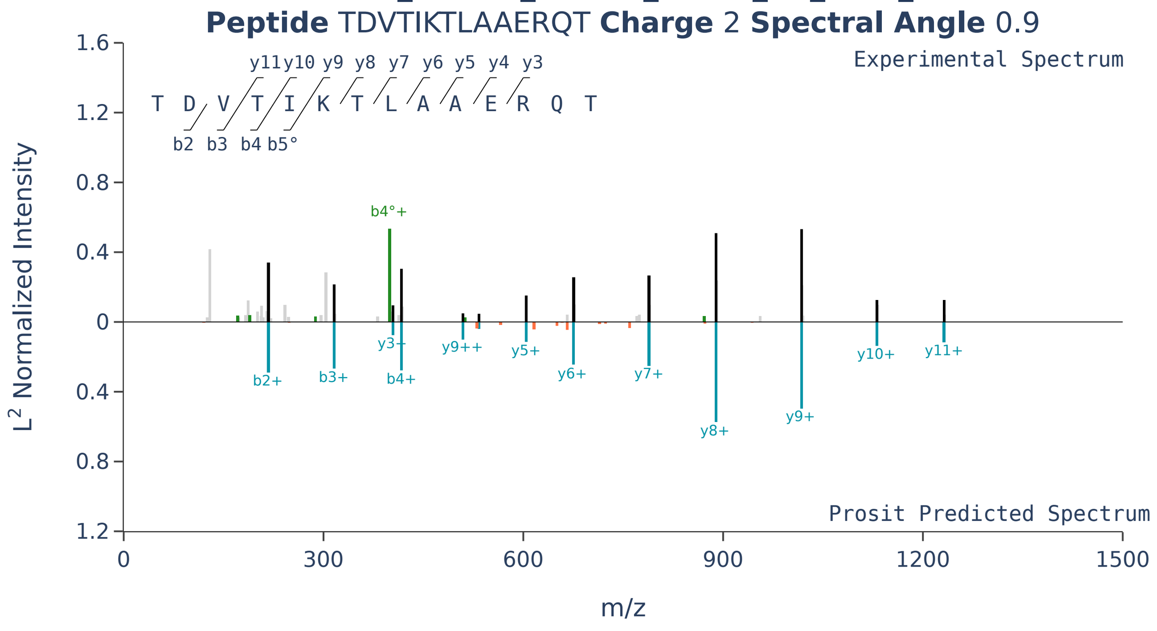

**Source** HRoetschke_211222_160123_HFGoe_IF2_b2R1_1h **Scan** 8457
**Peptide** VAVNKIDKPEAD **Charge** 3 **Spectral Angle** 0.73

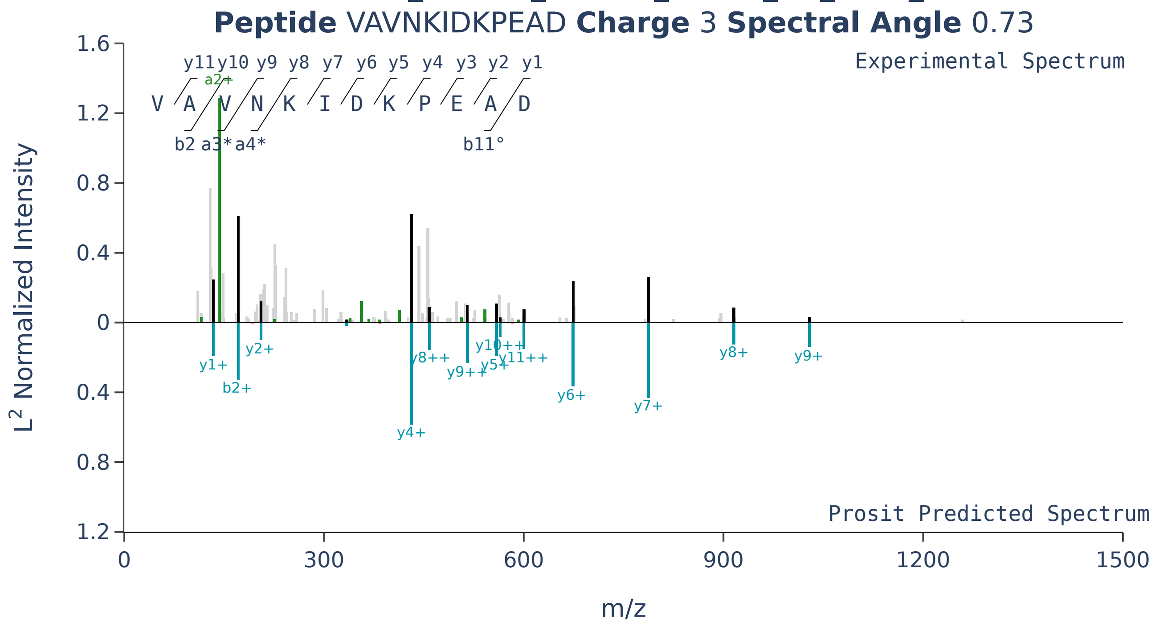


**Source** HRoetschke_211222_160123_HFGoe_IF2_b1R1_2h **Scan** 7345
**Peptide** IEAIQHAKAA **Charge** 3 **Spectral Angle** 0.85

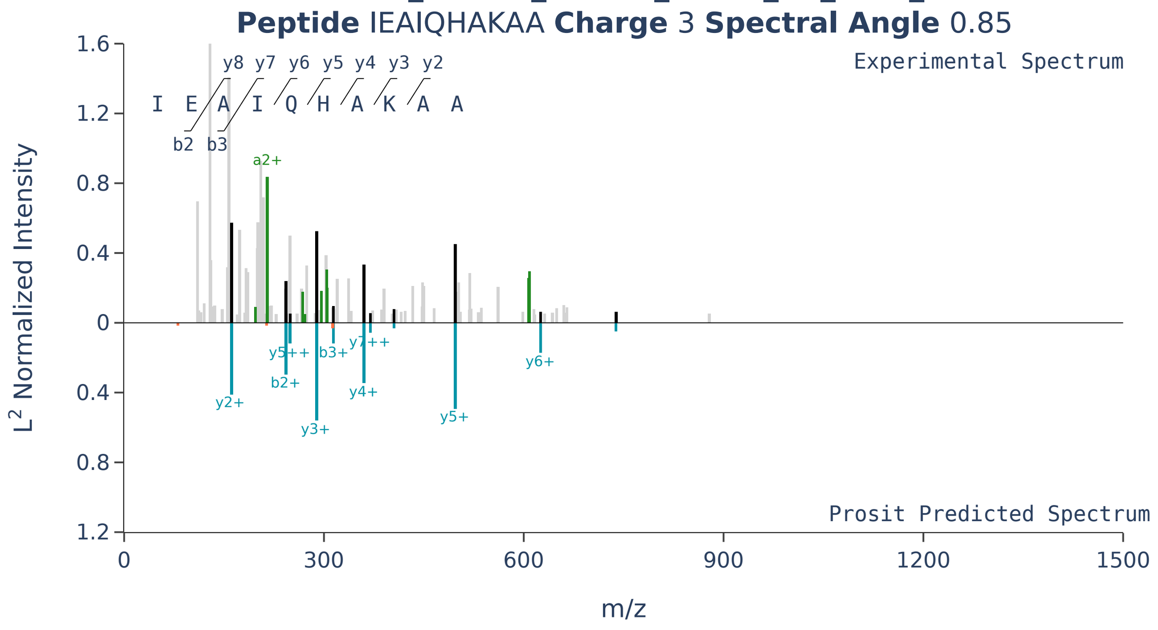


**Source** HRoetschke_211222_160123_HFGoe_IF2_b1R2_24h **Scan** 8916
**Peptide** TLKTLAAERQT **Charge** 3 **Spectral Angle** 0.86

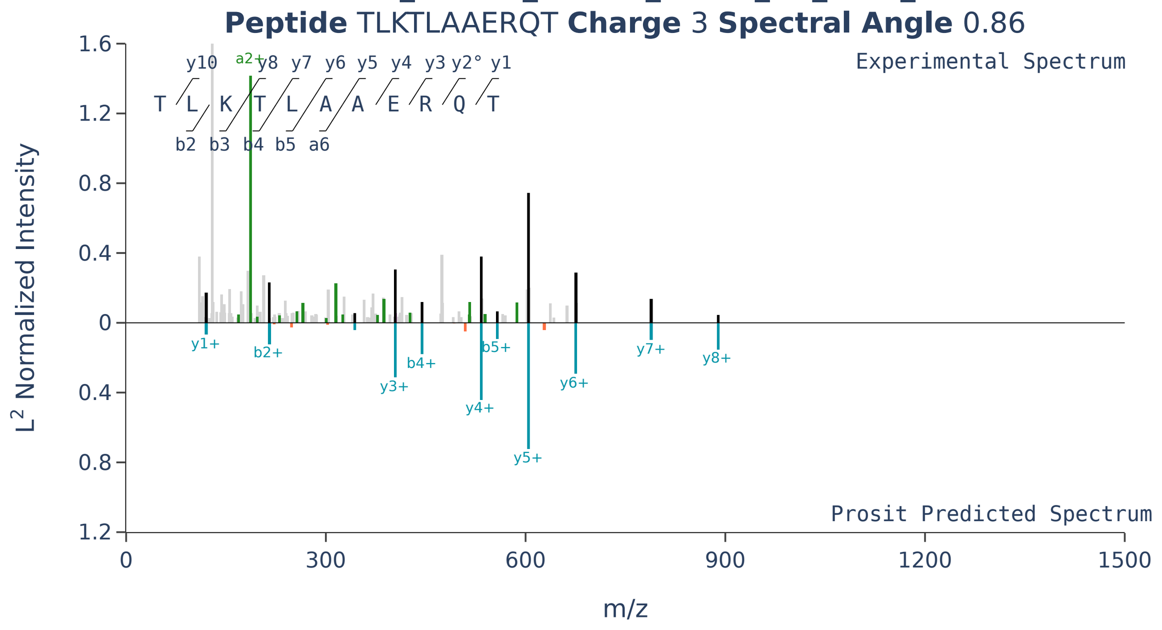


**Source** HRoetschke_211222_160123_HFGoe_IF2_b1R1_4h **Scan** 14394
**Peptide** SFLDKGRGPVAT **Charge** 3 **Spectral Angle** 0.93

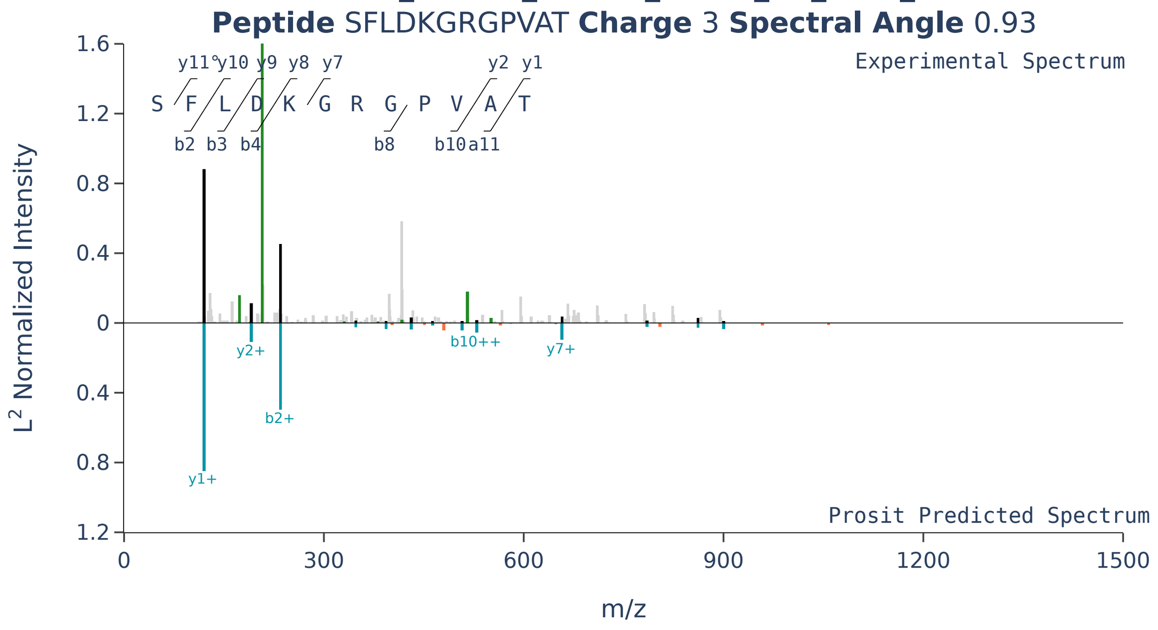


**Source** HRoetschke_211222_160123_HFGoe_IF2_b2R2_2h **Scan** 6962
**Peptide** NLPGTGGKSKSV **Charge** 2 **Spectral Angle** 0.89

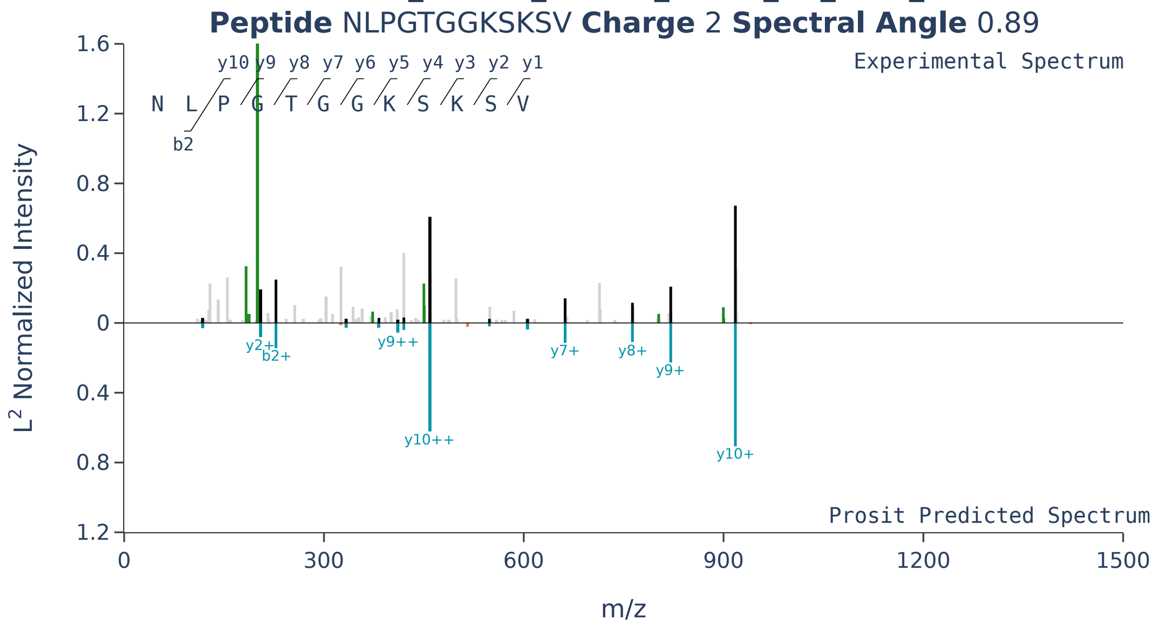


**Source** HRoetschke_211222_160123_HFGoe_IF2_b1R2_24h **Scan** 14006
**Peptide** INQVIDQETA **Charge** 2 **Spectral Angle** 0.88

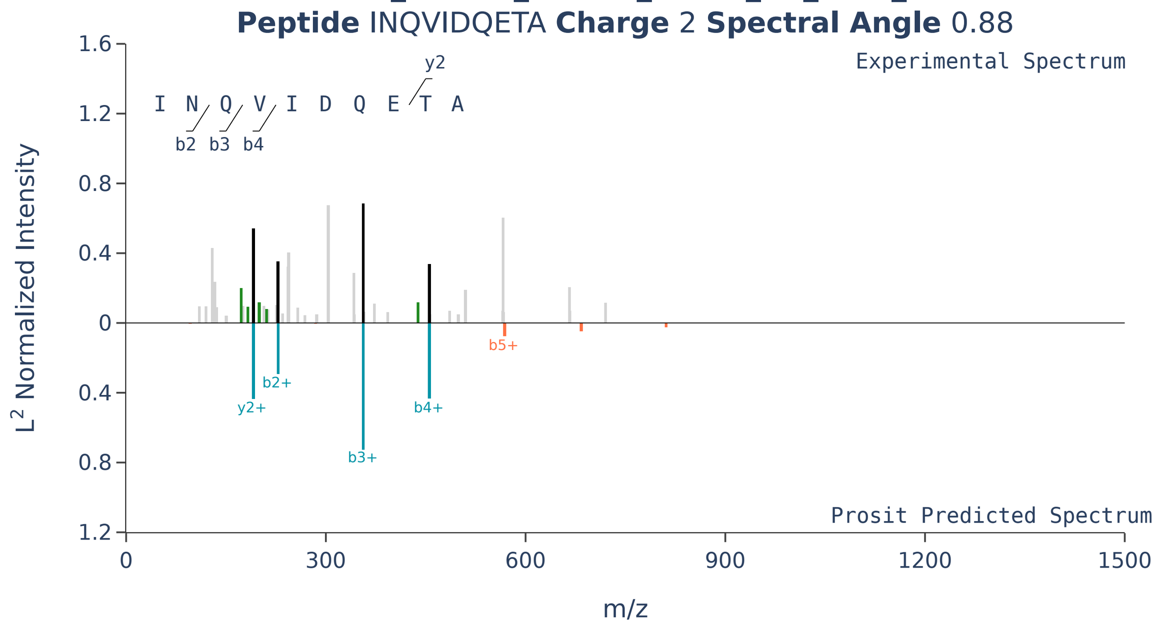


**Source** HRoetschke_211222_160123_HFGoe_IF2_b1R1_4h **Scan** 6090
**Peptide** MGHKVILRRE **Charge** 3 **Spectral Angle** 0.88

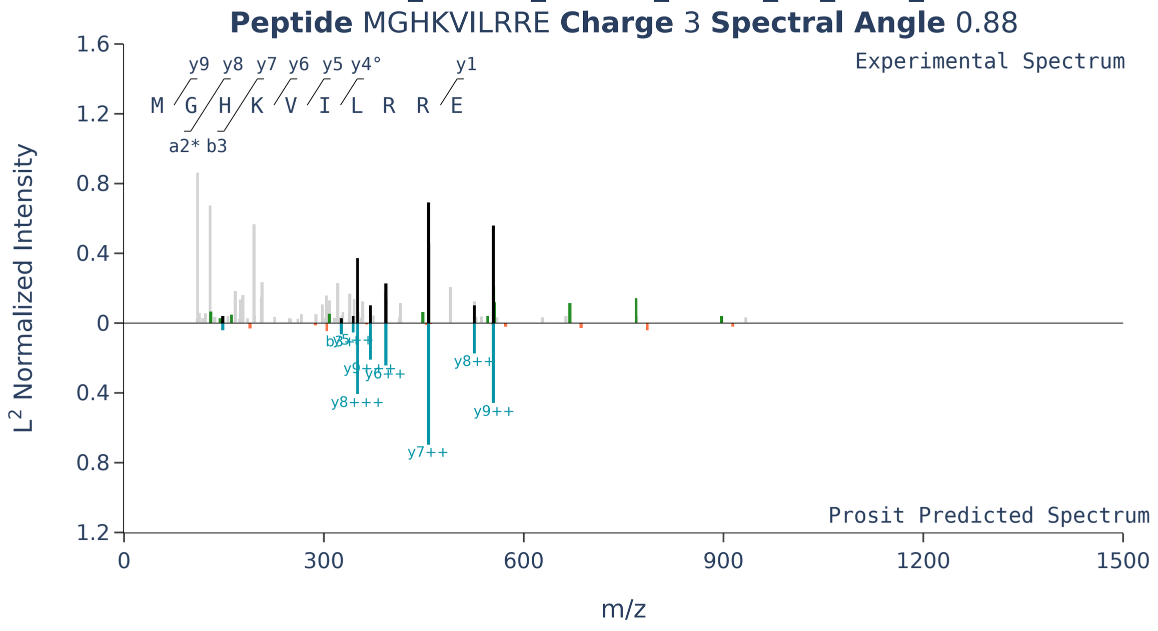


**Source** HRoetschke_211222_160123_HFGoe_IF2_b1R1_4h **Scan** 11816
**Peptide** GSSLQQGFQKPAQ **Charge** 2 **Spectral Angle** 0.84

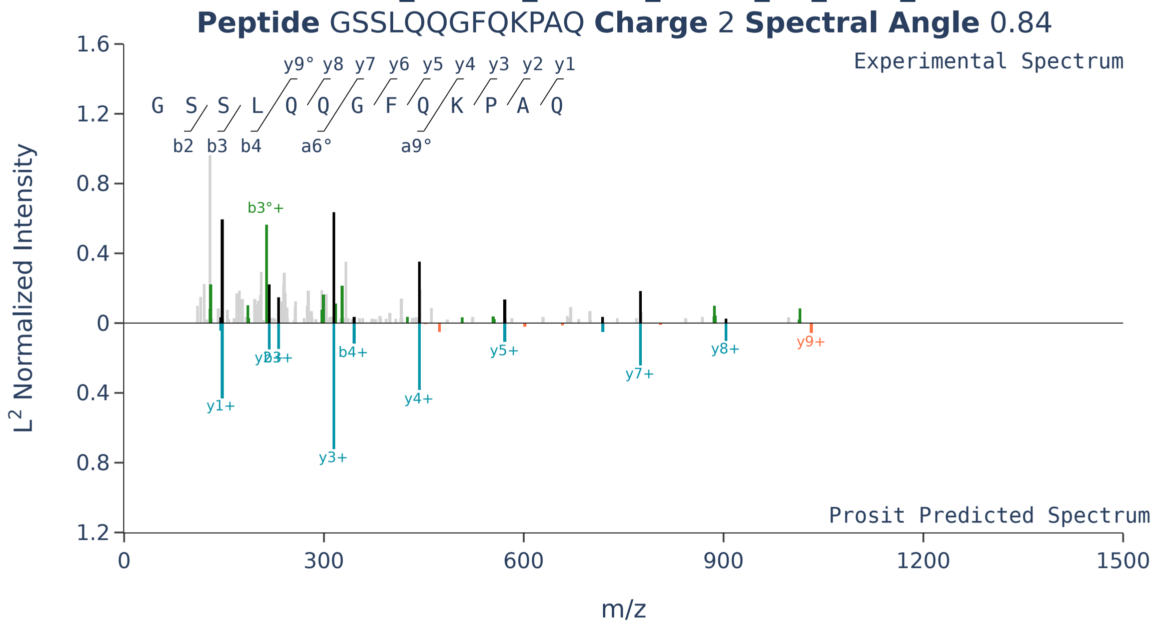


**Source** HRoetschke_211222_160123_HFGoe_IF2_b2R1_24h **Scan** 10105
**Peptide** ELKAVRKG **Charge** 2 **Spectral Angle** 0.9

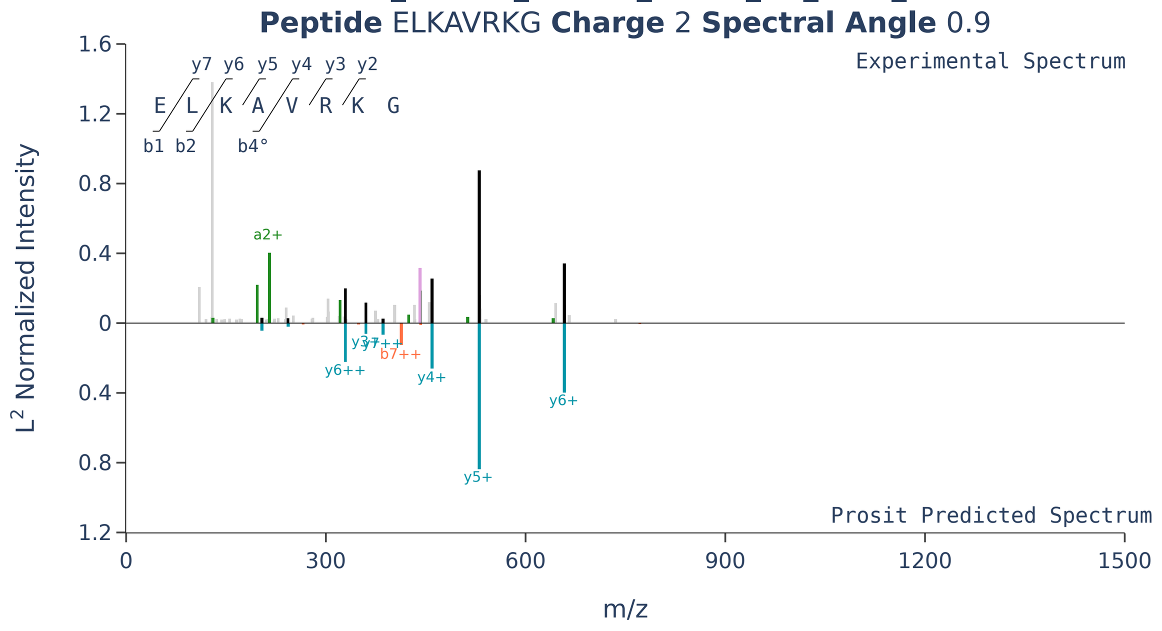


**Source** HRoetschke_211222_160123_HFGoe_IF2_b1R1_24h **Scan** 13693
**Peptide** GHVDHGKTSLLDY **Charge** 3 **Spectral Angle** 0.85

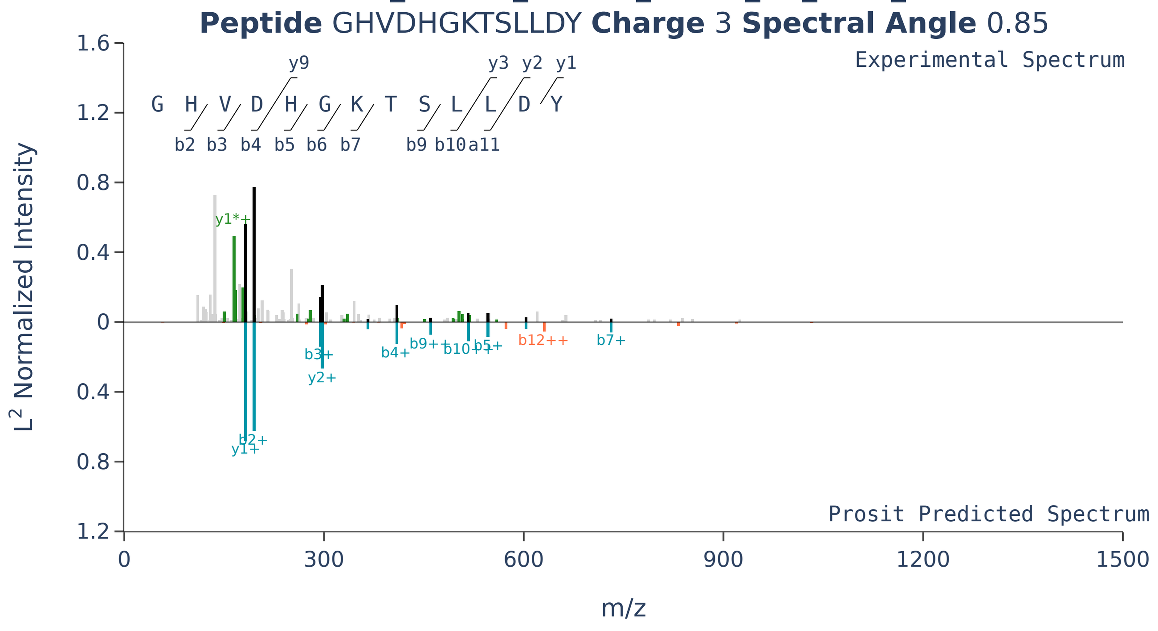


**Source** HRoetschke_211222_160123_HFGoe_IF2_b1R1_24h **Scan** 8311
**Peptide** SSDYHVTT **Charge** 2 **Spectral Angle** 0.8

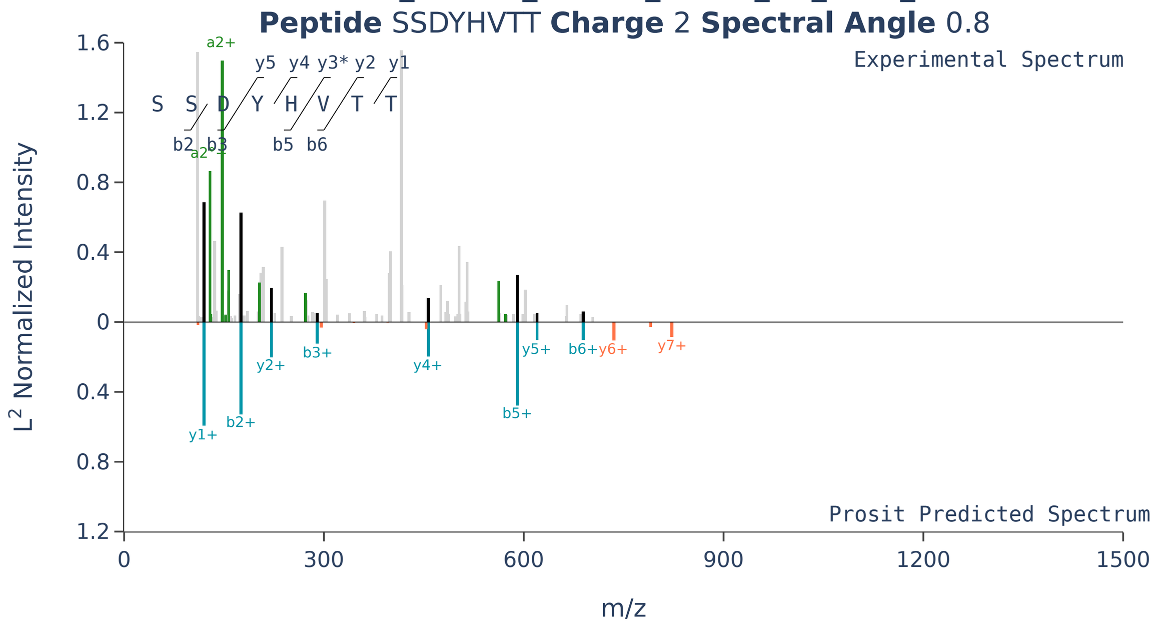


**Source** HRoetschke_211222_160123_HFGoe_IF2_b1R1_4h **Scan** 5850
**Peptide** KTLAAERQTSVER **Charge** 3 **Spectral Angle** 0.73

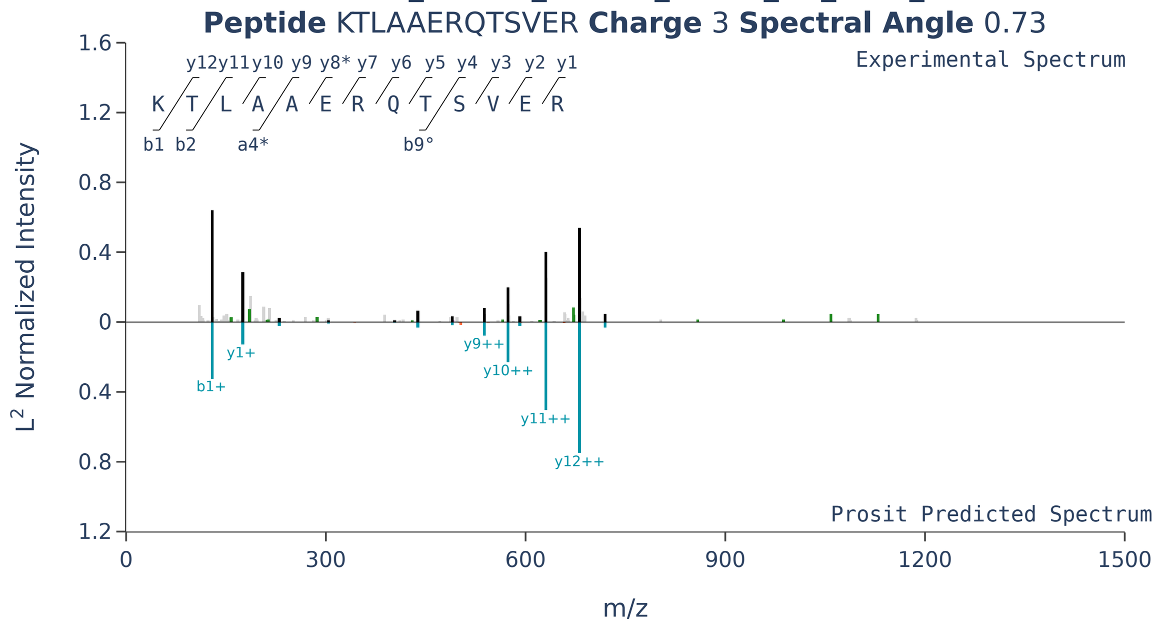


**Source** HRoetschke_211222_160123_HFGoe_IF2_b1R1_24h **Scan** 6088
**Peptide** SGEAGGITQH **Charge** 2 **Spectral Angle** 0.8

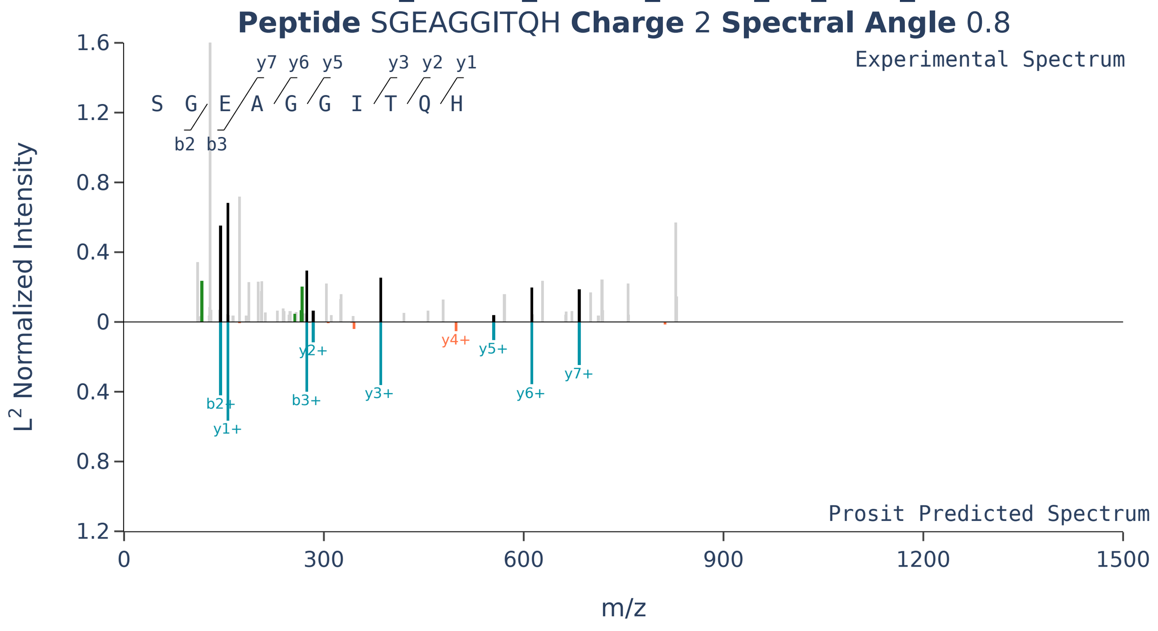


**Source** HRoetschke_211222_160123_HFGoe_IF2_b1R1_24h **Scan** 6961
**Peptide** DEVTVVRDEKK **Charge** 3 **Spectral Angle** 0.75

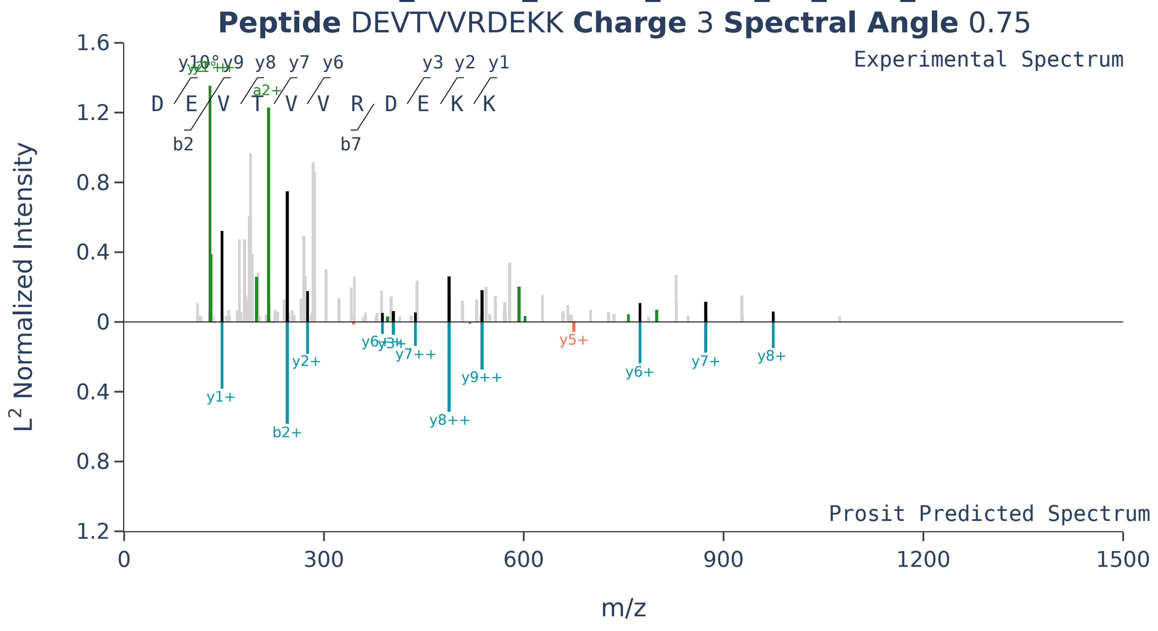


**Source** HRoetschke_211222_160123_HFGoe_IF2_b1R2_24h **Scan** 14592
**Peptide** LHKGDIVL **Charge** 2 **Spectral Angle** 0.86

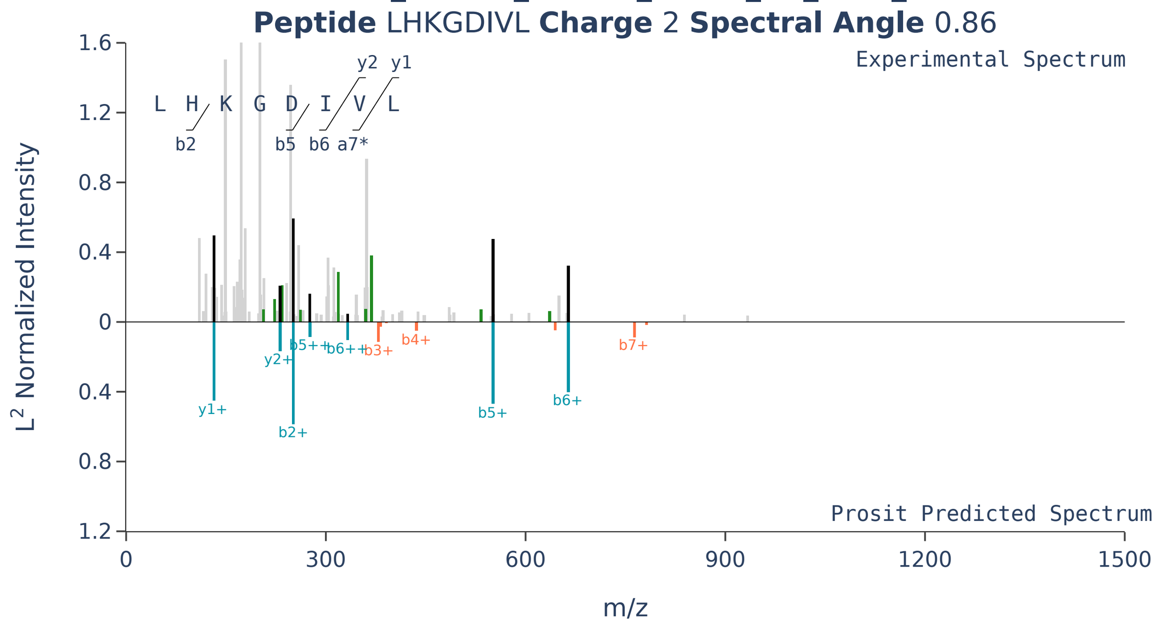

**Source** HRoetschke_211222_160123_HFGoe_IF2_b2R2_2h **Scan** 7368
**Peptide** KVASGEAGGITQ **Charge** 2 **Spectral Angle** 0.86

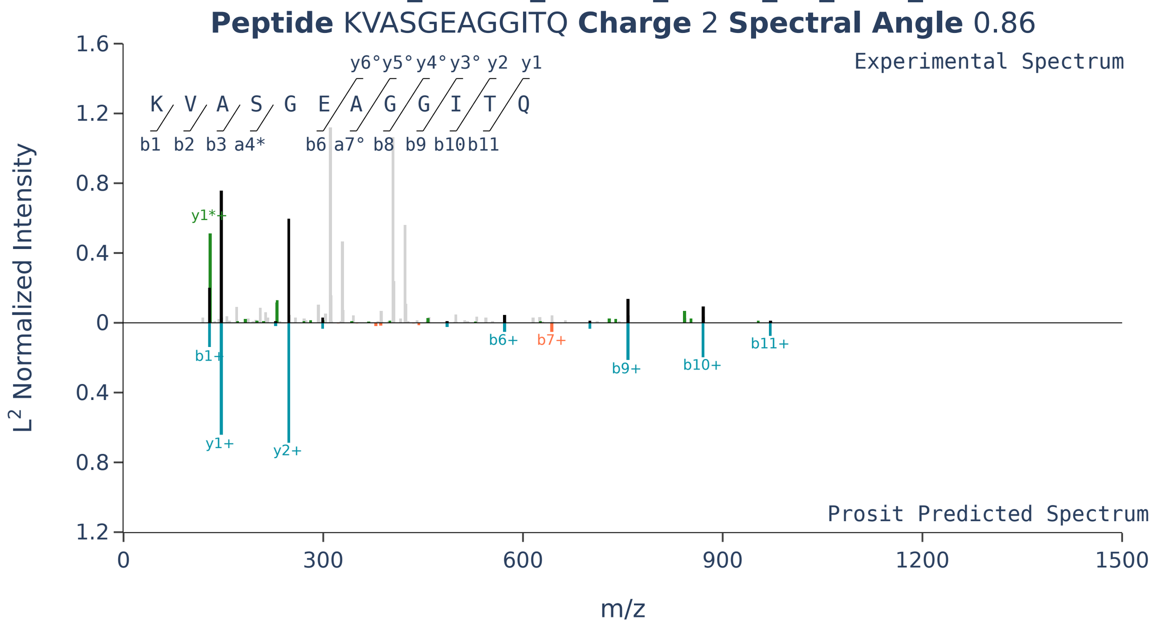


**Source** HRoetschke_211222_160123_HFGoe_IF2_b1R2_1h **Scan** 8834
**Peptide** QQFADAGIRK **Charge** 3 **Spectral Angle** 0.8

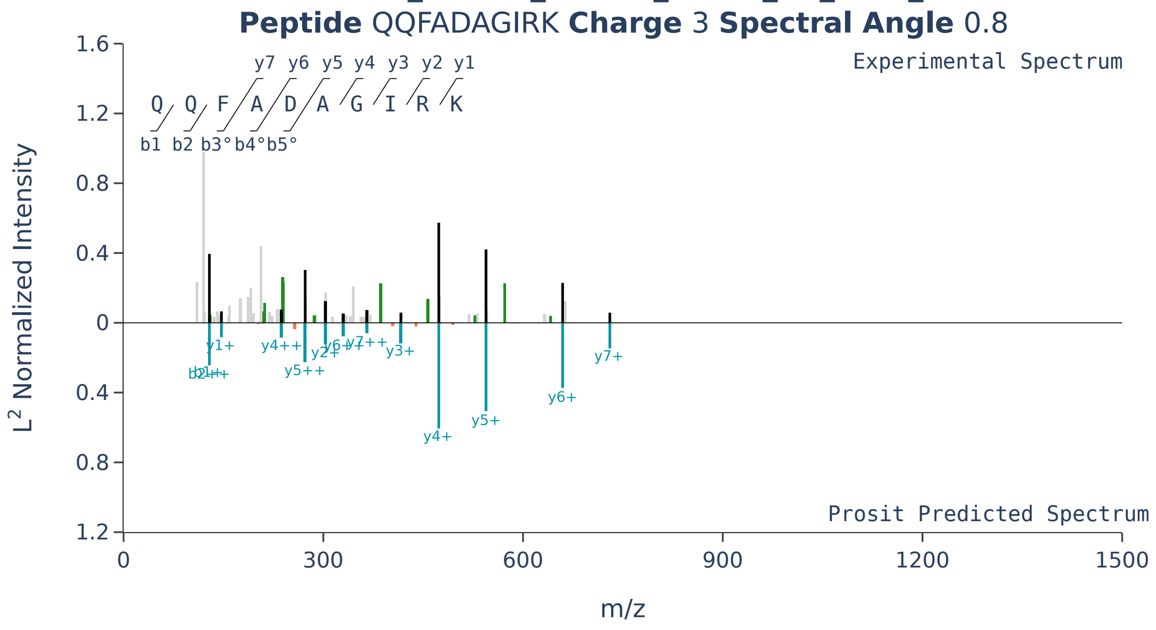


**Source** HRoetschke_211222_160123_HFGoe_IF2_b1R1_24h **Scan** 10519
**Peptide** MGHVDHGKTSLLD **Charge** 3 **Spectral Angle** 0.87

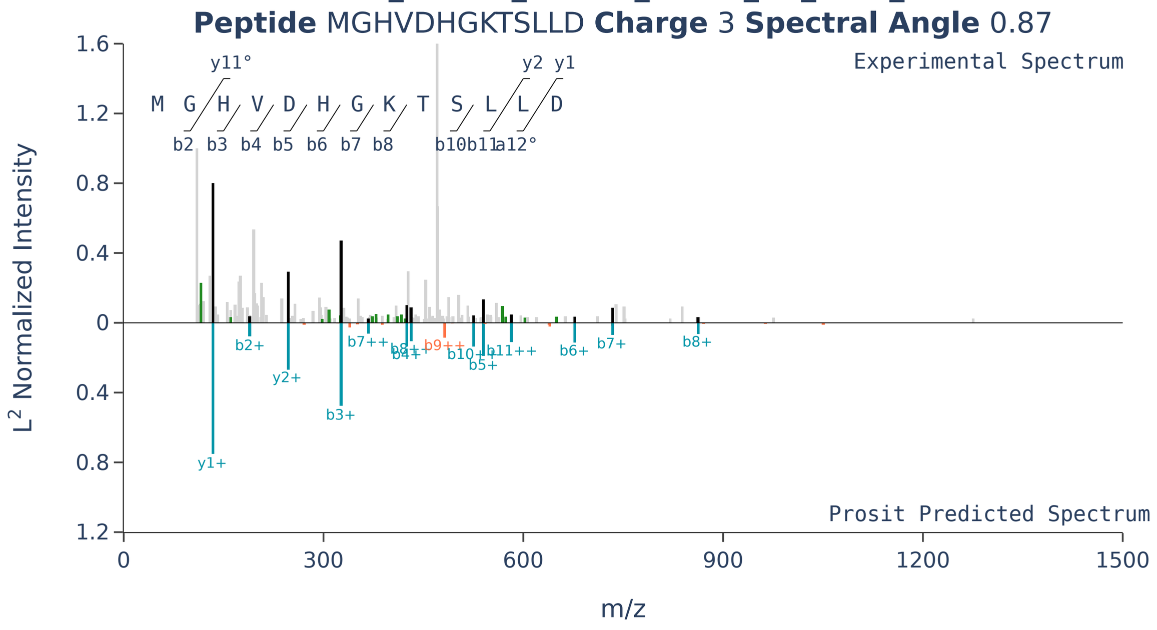


**Source** HRoetschke_211222_160123_HFGoe_IF2_b1R1_4h **Scan** 15704
**Peptide** GRGPVATVLVRE **Charge** 3 **Spectral Angle** 0.83

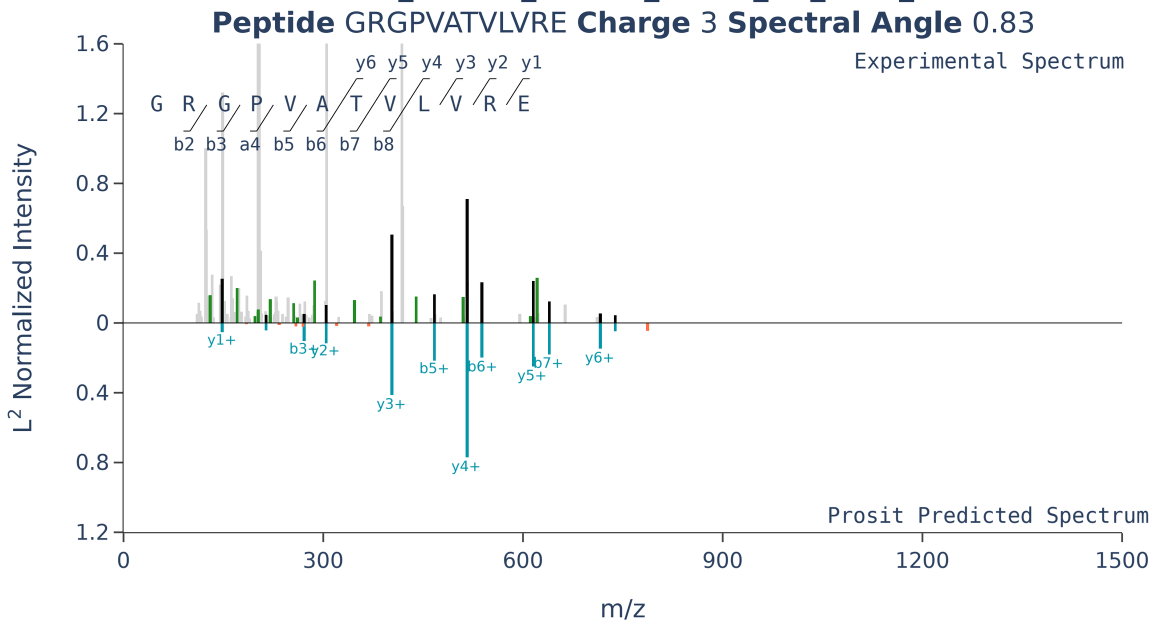


**Source** HRoetschke_211222_160123_HFGoe_IF2_b1R2_1h **Scan** 11443
**Peptide** LGAYHVETE **Charge** 2 **Spectral Angle** 0.78

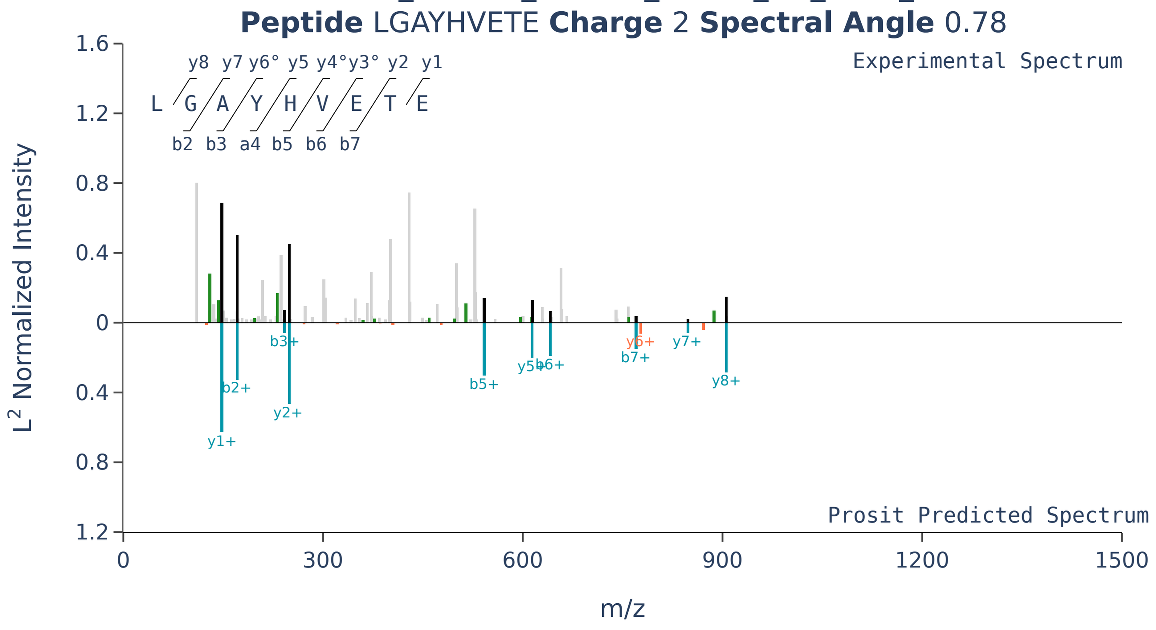


**Source** HRoetschke_211222_160123_HFGoe_IF2_b1R1_4h **Scan** 7700
**Peptide** AVKGSQVIKA **Charge** 1 **Spectral Angle** 0.83

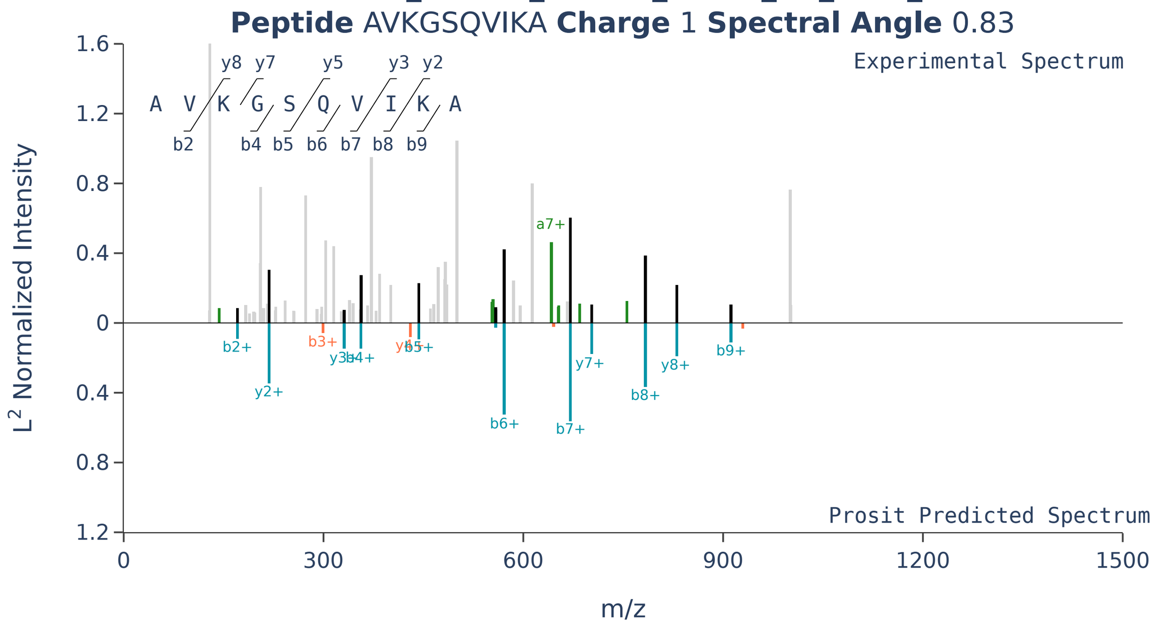


**Source** HRoetschke_211222_160123_HFGoe_IF2_b2R1_1h **Scan** 7412
**Peptide** KGSQVIKA **Charge** 2 **Spectral Angle** 0.96

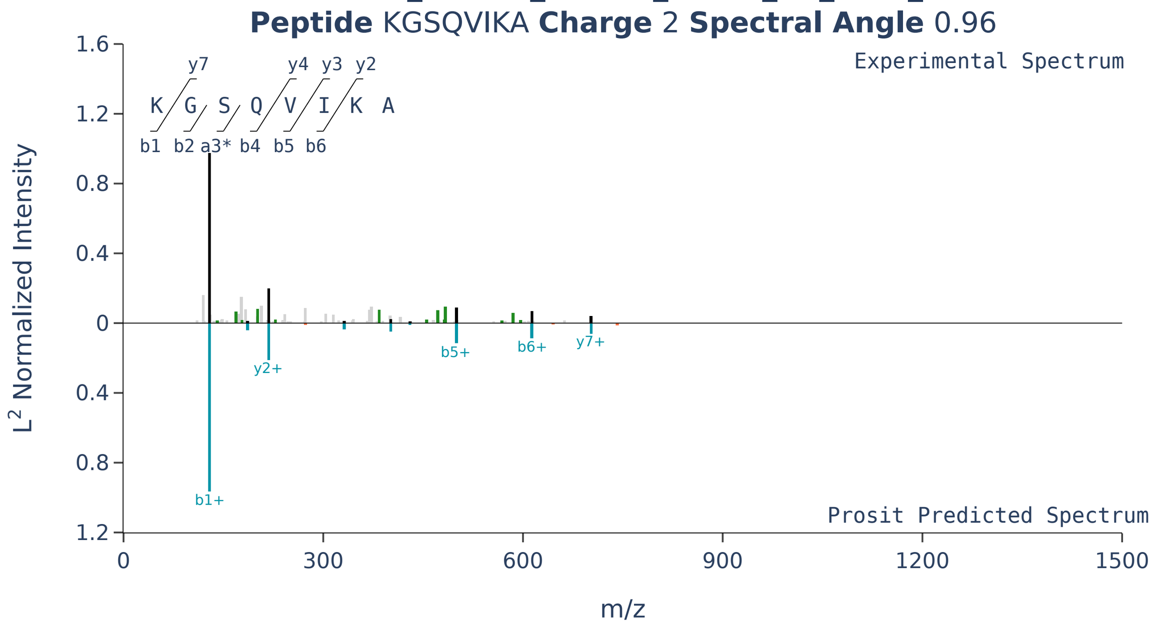


**Source** HRoetschke_211222_160123_HFGoe_IF2_b1R1_2h **Scan** 13054
**Peptide** TSVERLVQQ **Charge** 2 **Spectral Angle** 0.79

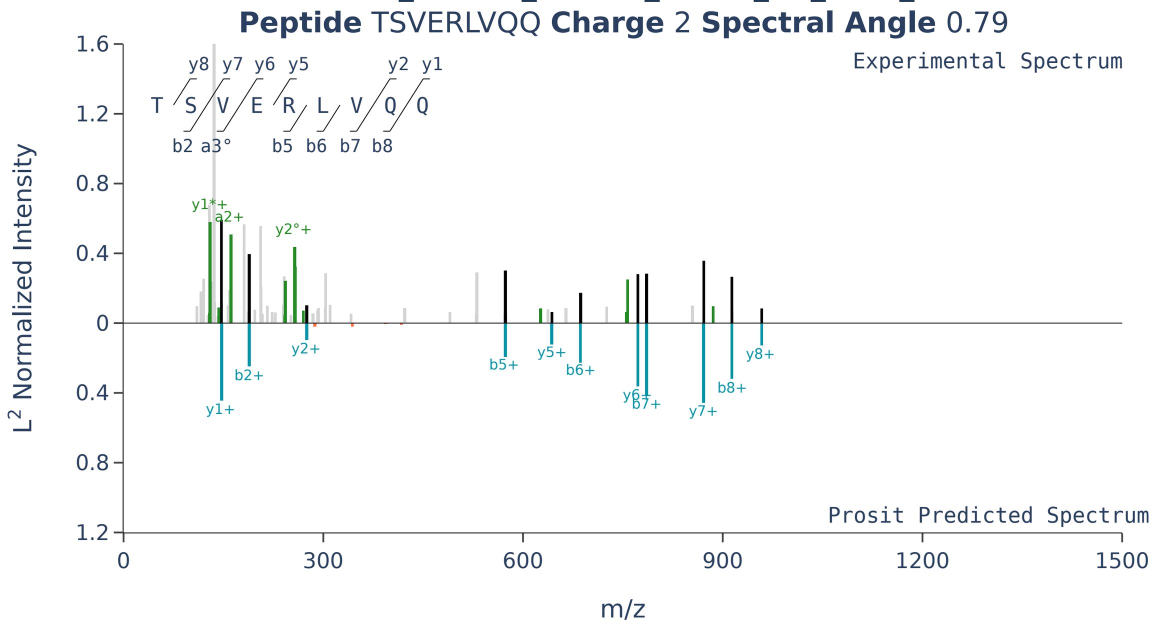


**Source** HRoetschke_211222_160123_HFGoe_IF2_b1R1_2h **Scan** 8493
**Peptide** PGHAAFTSMRARGA **Charge** 4 **Spectral Angle** 0.77

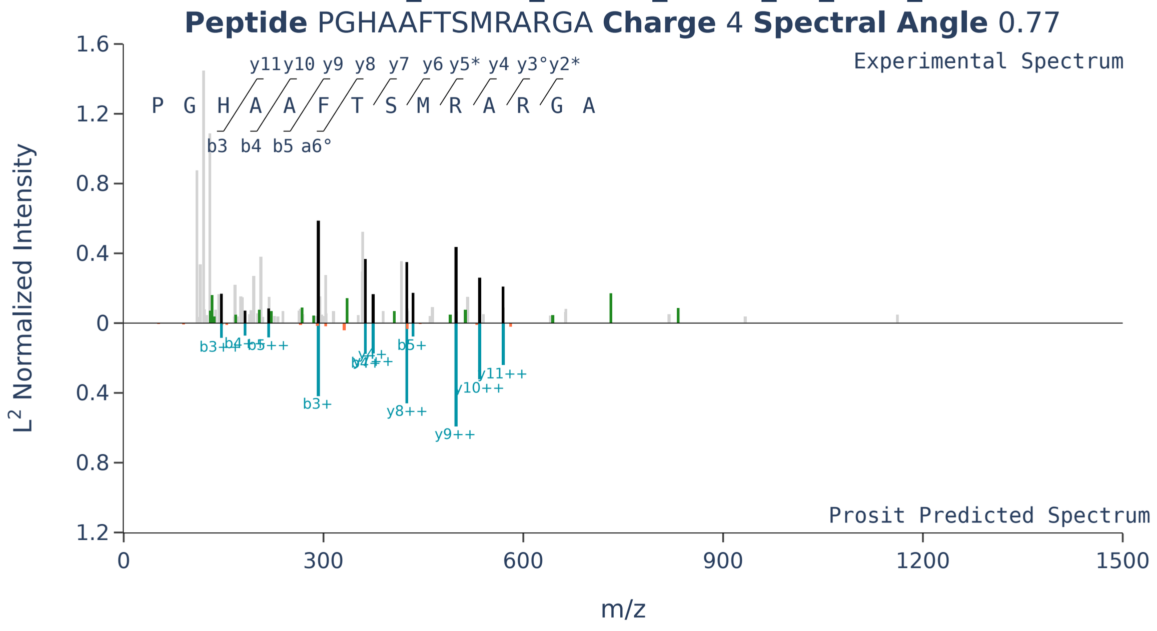


**Source** HRoetschke_211222_160123_HFGoe_IF2_b1R1_24h **Scan** 12070
**Peptide** KSPKFGAL **Charge** 2 **Spectral Angle** 0.89

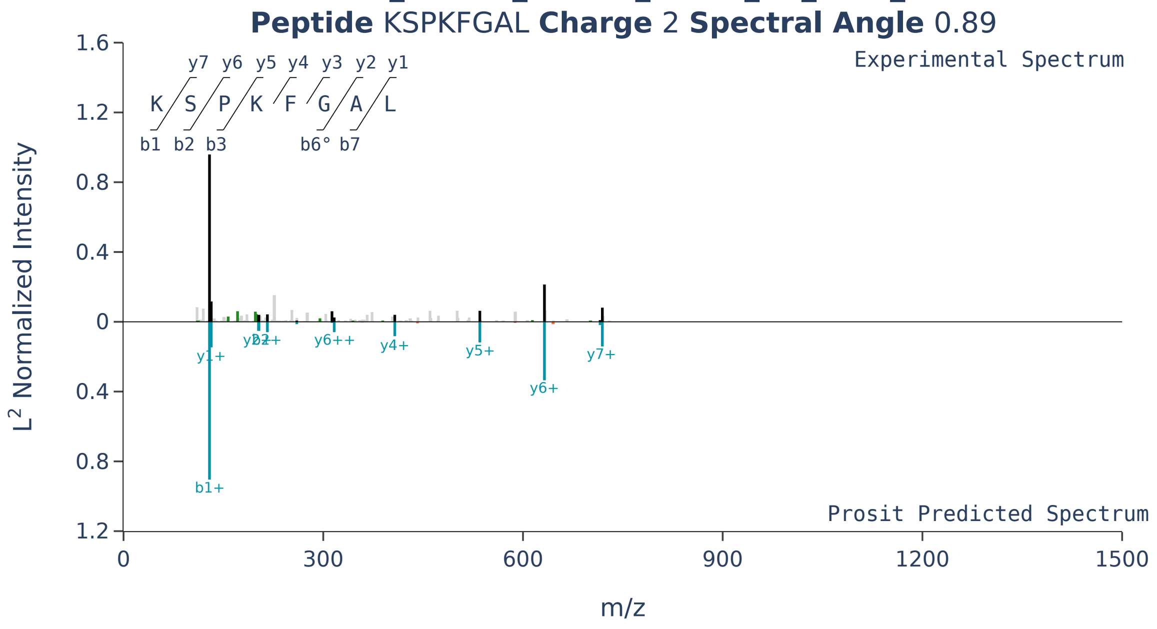


**Source** HRoetschke_211222_160123_HFGoe_IF2_b1R1_24h **Scan** 7595
**Peptide** EVTVVRDEKKAREV **Charge** 3 **Spectral Angle** 0.88

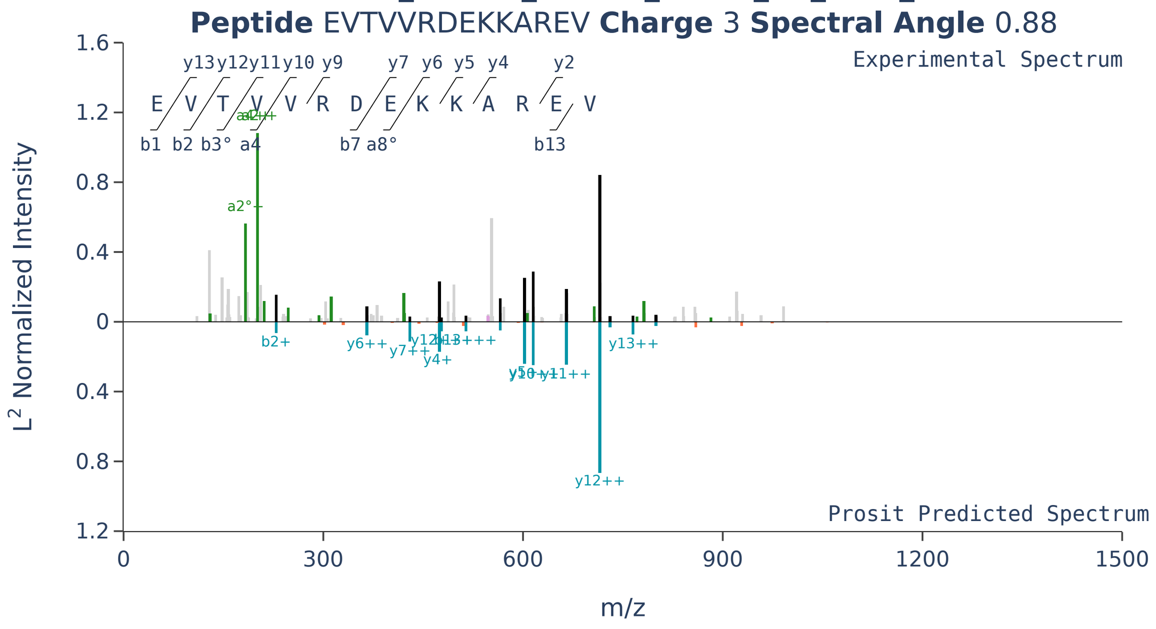


**Source** HRoetschke_211222_160123_HFGoe_IF2_b1R1_4h **Scan** 13072
**Peptide** DTPGHAAFTSMR **Charge** 2 **Spectral Angle** 0.83

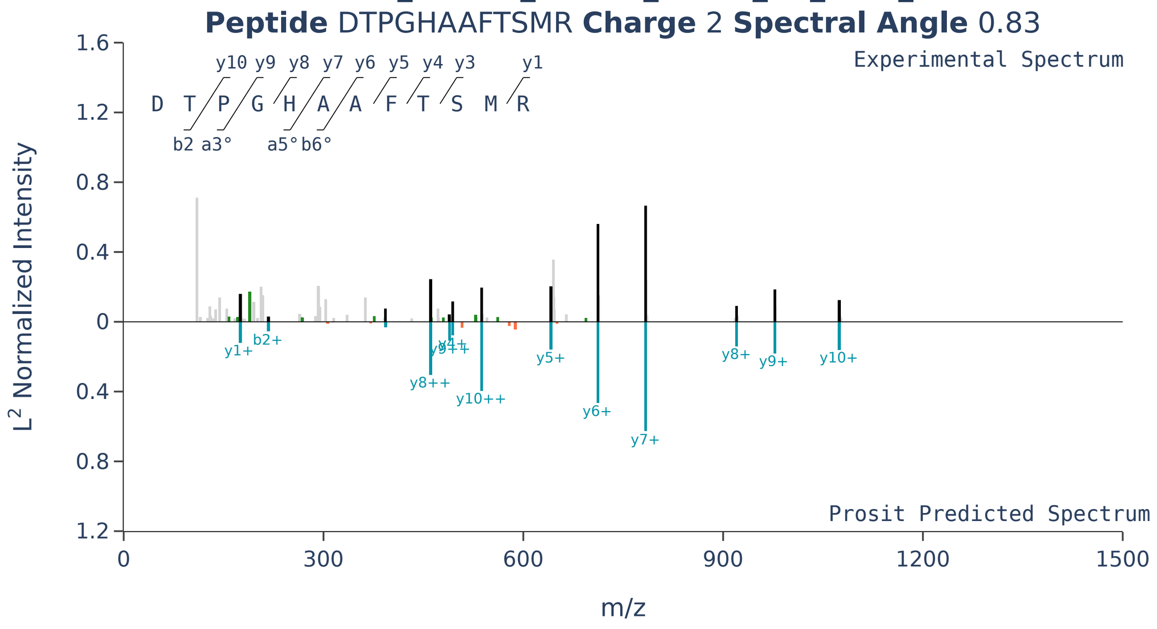


**Source** HRoetschke_211222_160123_HFGoe_IF2_b1R1_4h **Scan** 13450
**Peptide** RAMRNELGQEVLE **Charge** 3 **Spectral Angle** 0.78

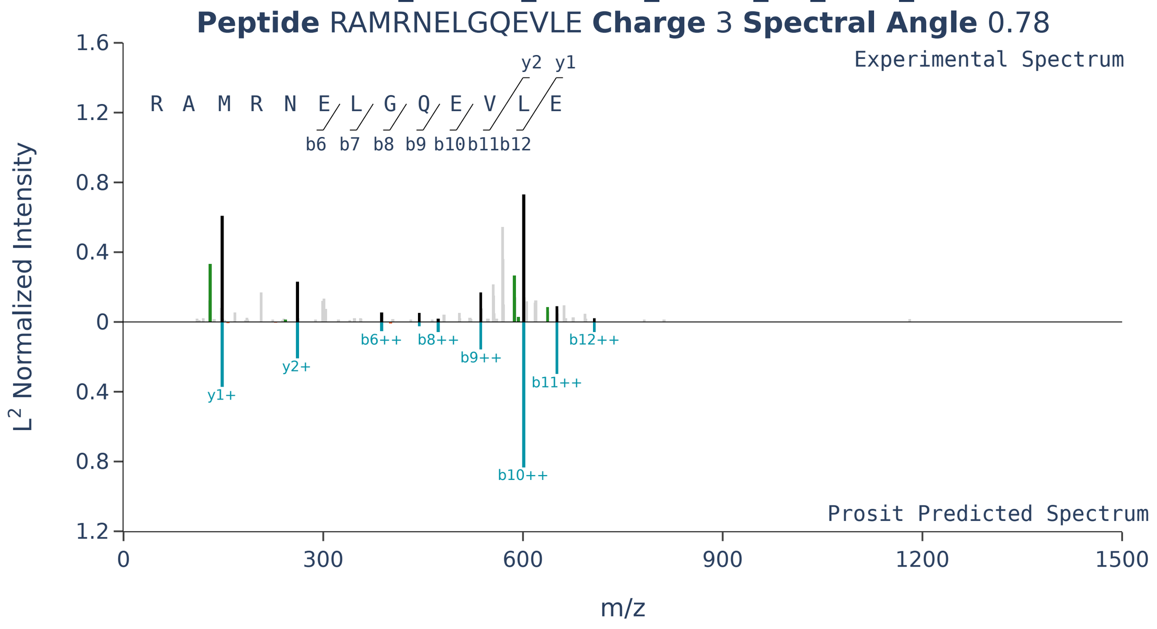


**Source** HRoetschke_211222_160123_HFGoe_IF2_b1R1_24h **Scan** 6229
**Peptide** RADASARKVIE **Charge** 2 **Spectral Angle** 0.76

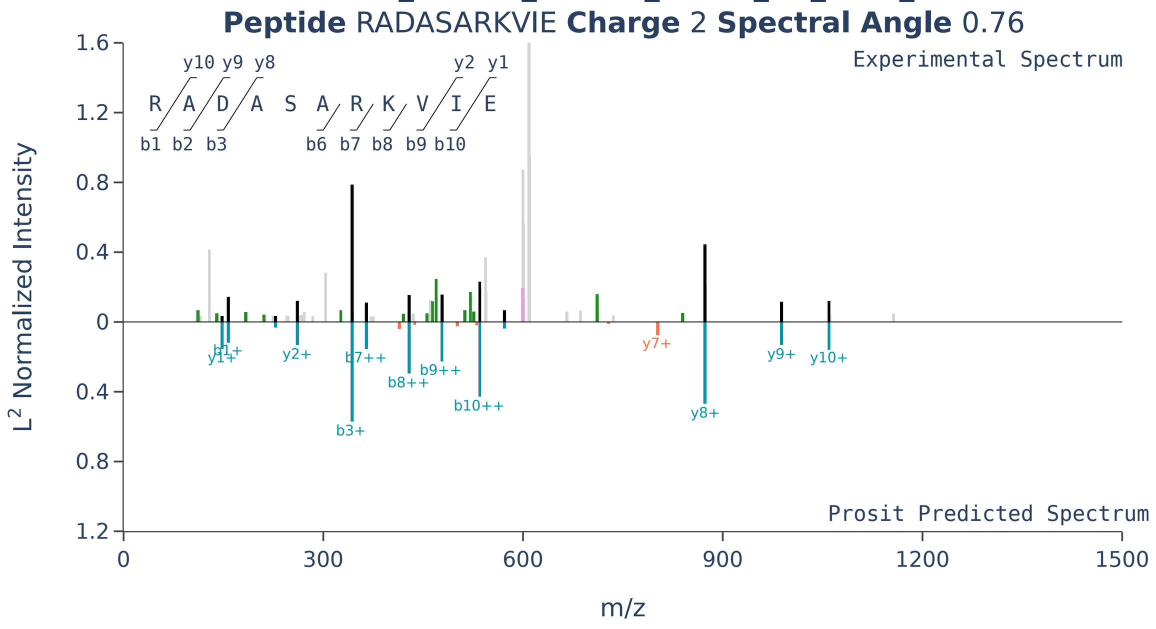


**Source** HRoetschke_211222_160123_HFGoe_IF2_b1R1_2h **Scan** 8285
**Peptide** QGFQKPAQA **Charge** 1 **Spectral Angle** 0.93

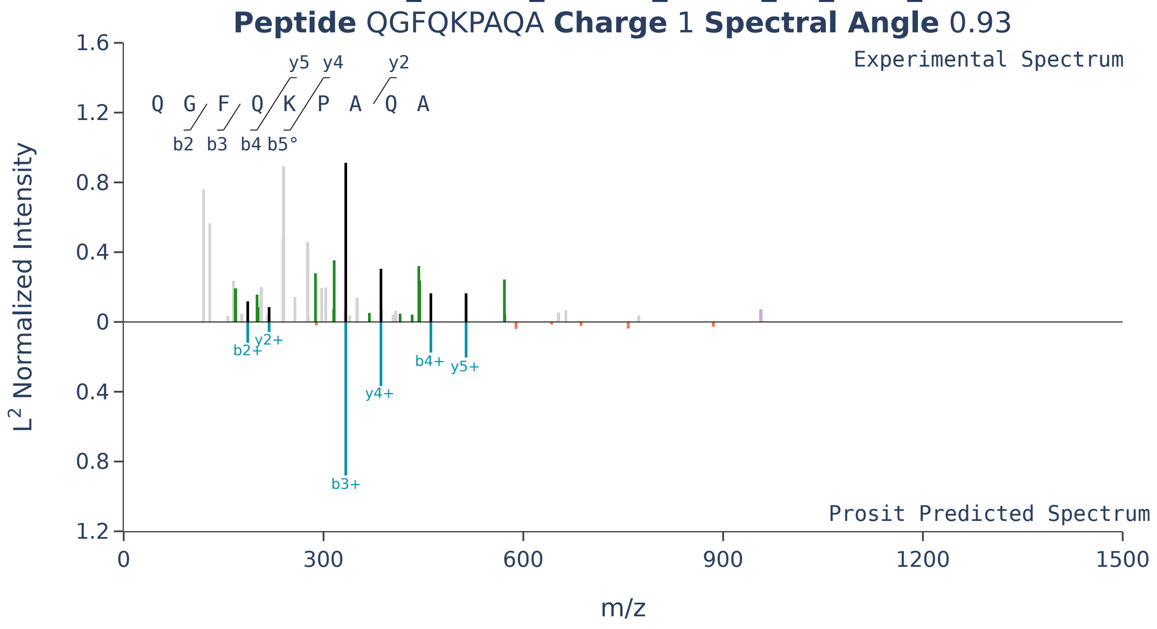

**Source** HRoetschke_211222_160123_HFGoe_IF2_b1R1_4h **Scan** 8998
**Peptide** DEVKAAM **Charge** 2 **Spectral Angle** 0.91

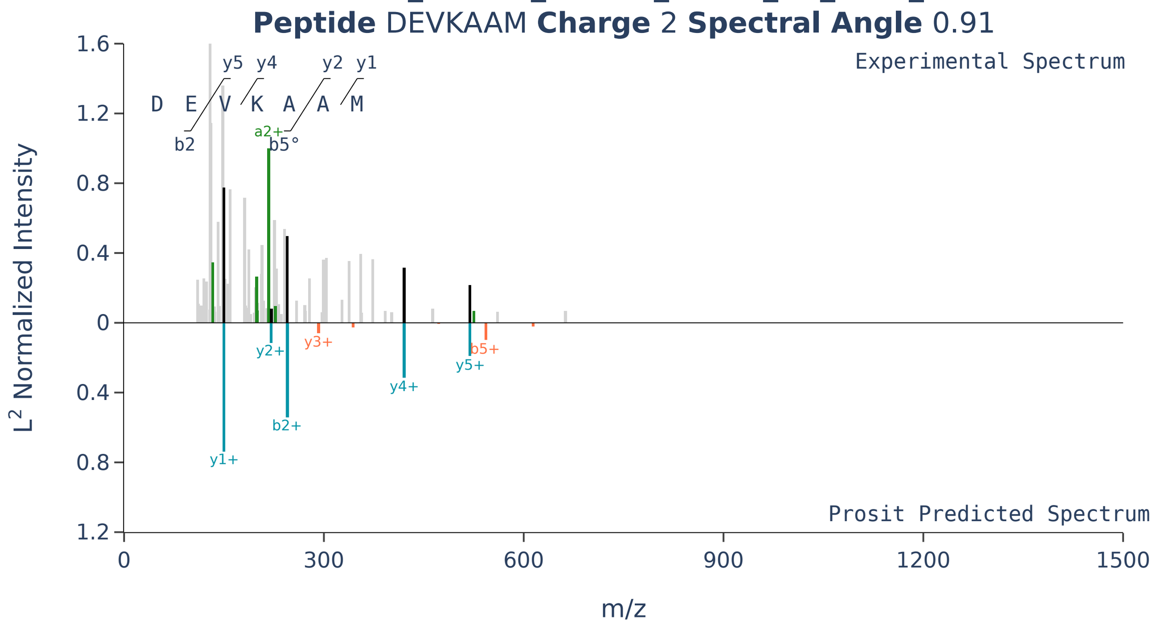


**Source** HRoetschke_211222_160123_HFGoe_IF2_b1R1_4h **Scan** 5675
**Peptide** IQHAKAAQV **Charge** 2 **Spectral Angle** 0.78

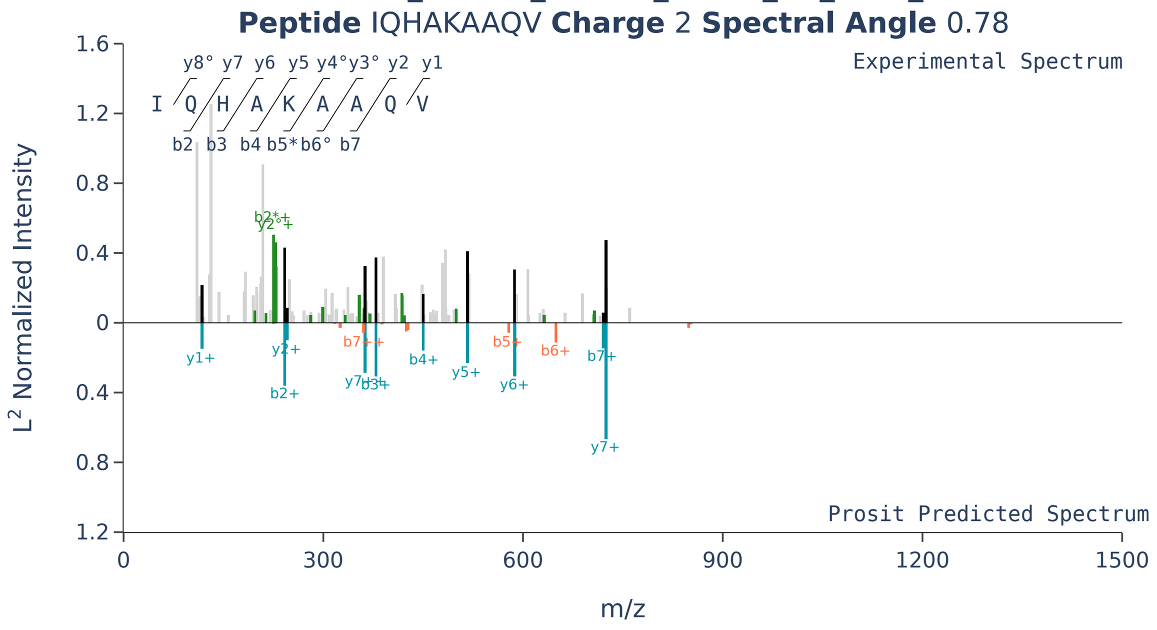


**Source** HRoetschke_211222_160123_HFGoe_IF2_b2R1_1h **Scan** 7362
**Peptide** AERLAAEEQAQ **Charge** 2 **Spectral Angle** 0.8

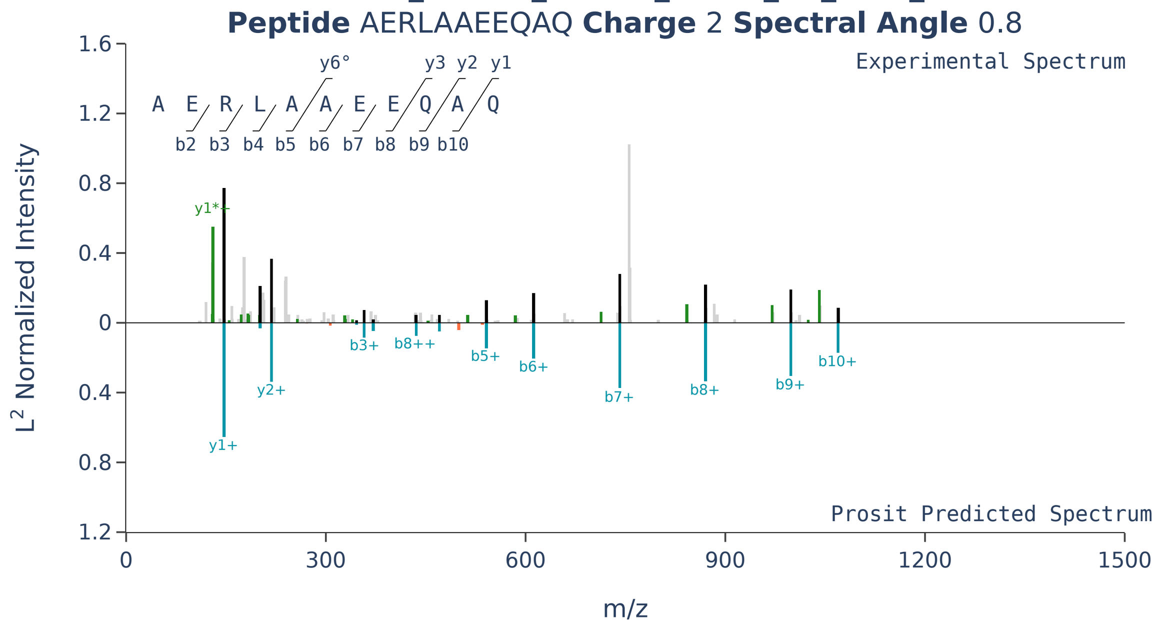


**Source** HRoetschke_211222_160123_HFGoe_IF2_b1R2_1h **Scan** 12894
**Peptide** TAQLVAEE **Charge** 1 **Spectral Angle** 0.94

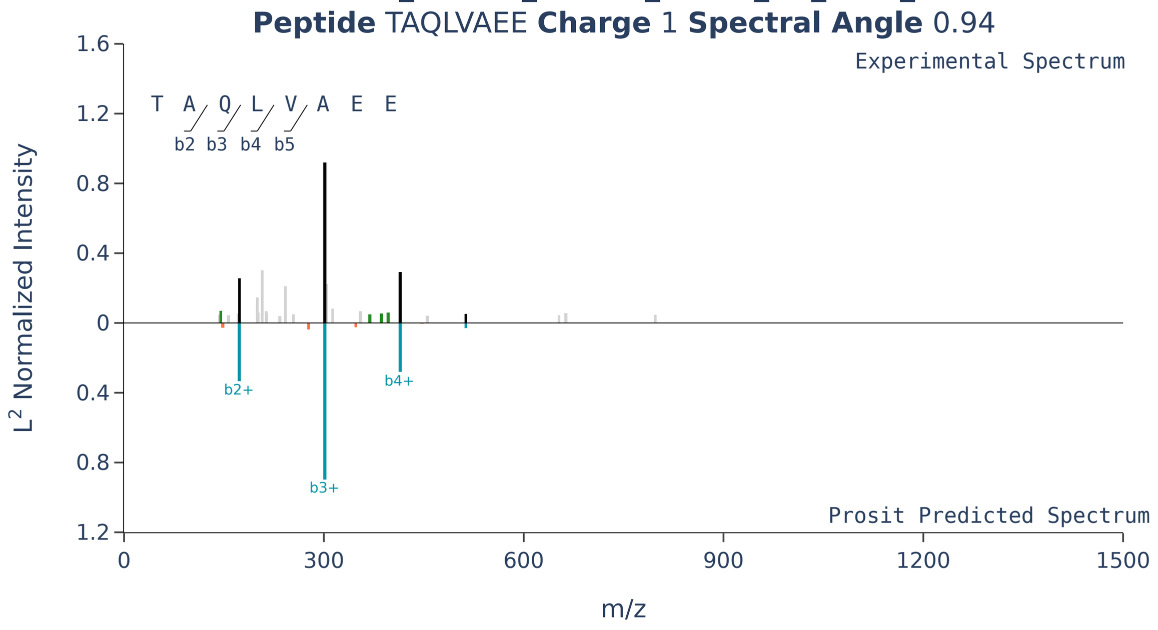


**Source** HRoetschke_211222_160123_HFGoe_IF2_b2R1_24h **Scan** 11586
**Peptide** VDHGKTSLL **Charge** 2 **Spectral Angle** 0.71

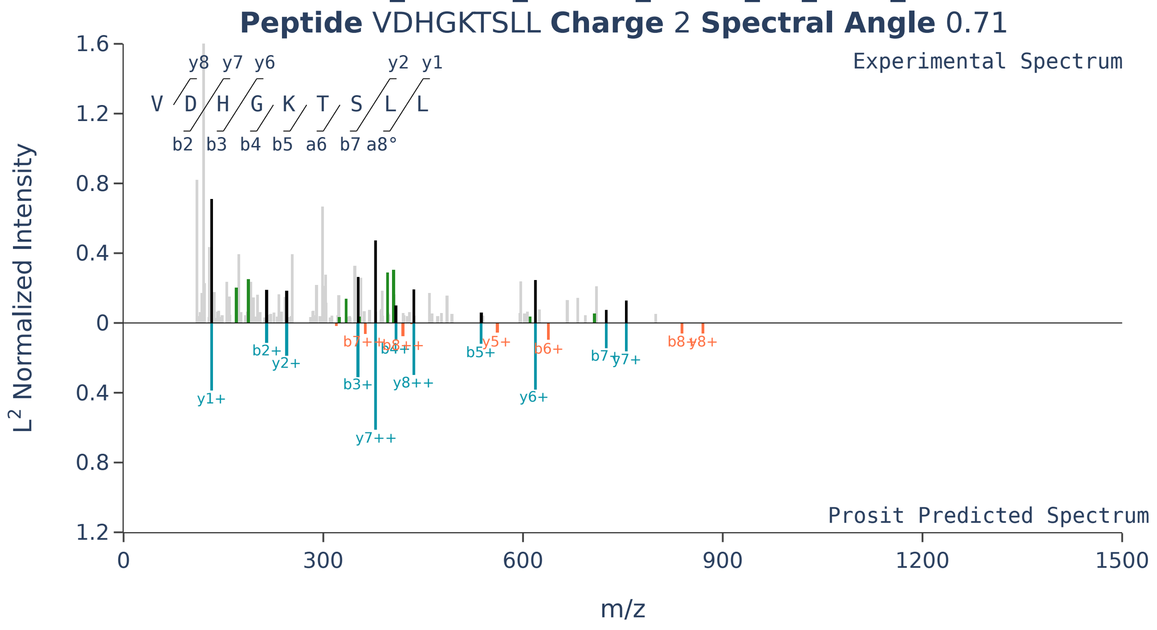


**Source** HRoetschke_211222_160123_HFGoe_IF2_b1R1_1h **Scan** 9143
**Peptide** TPGHAAFT **Charge** 2 **Spectral Angle** 0.79

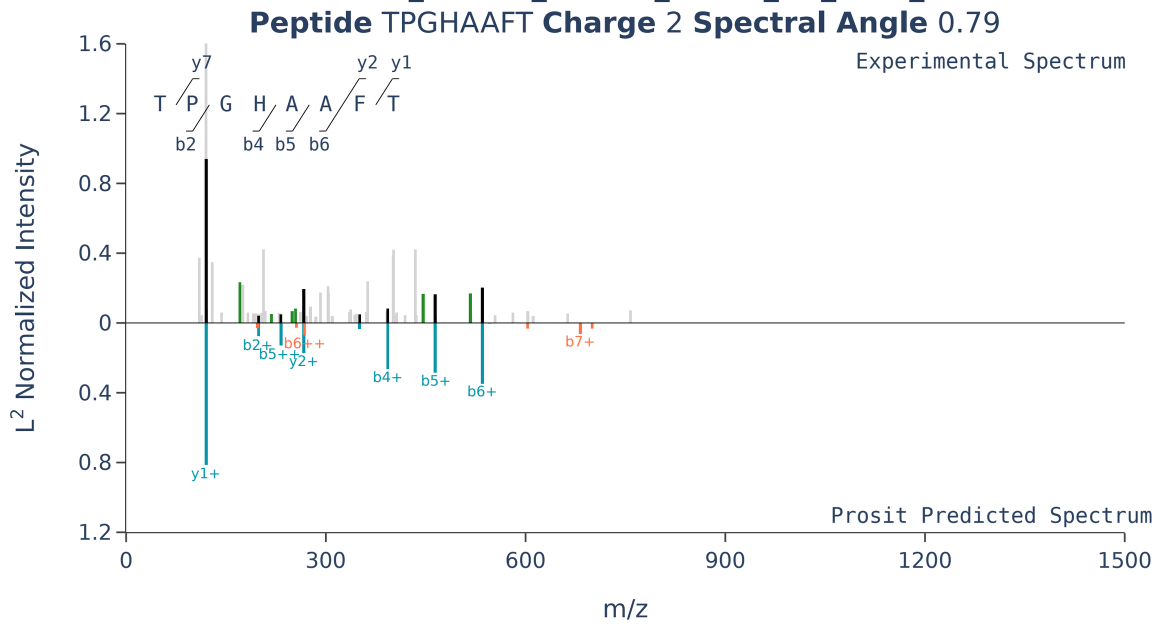


**Source** HRoetschke_211222_160123_HFGoe_IF2_b1R1_4h **Scan** 17996
**Peptide** MATINQVLD **Charge** 2 **Spectral Angle** 0.77

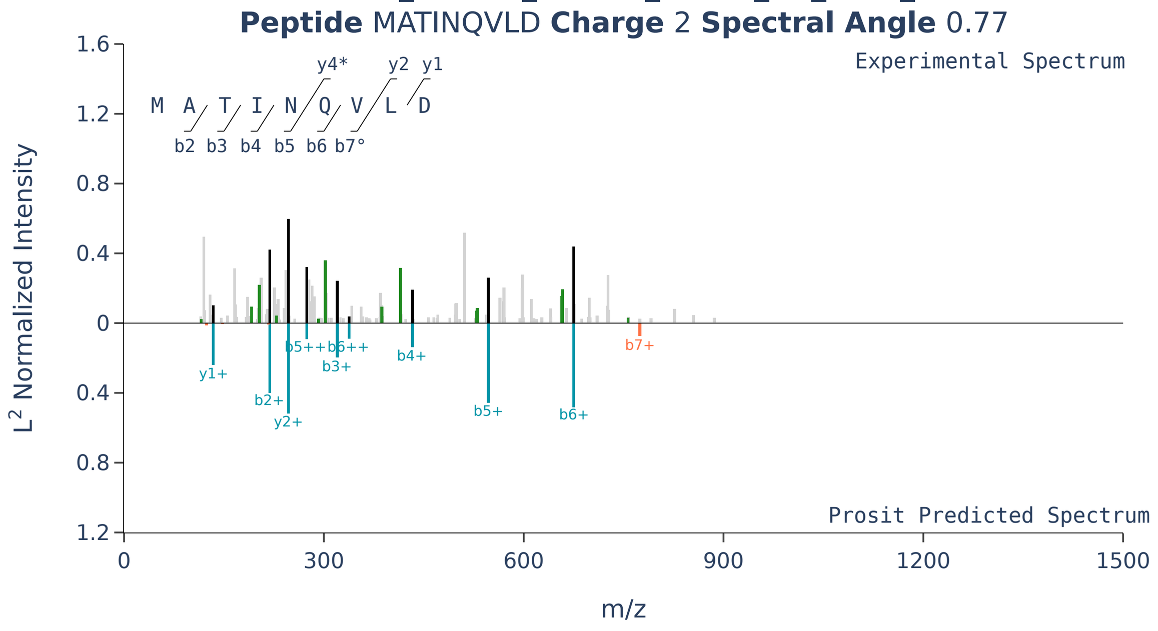


**Source** HRoetschke_211222_160123_HFGoe_IF2_b2R1_24h **Scan** 11929
**Peptide** TVLVREGTLH **Charge** 2 **Spectral Angle** 0.81

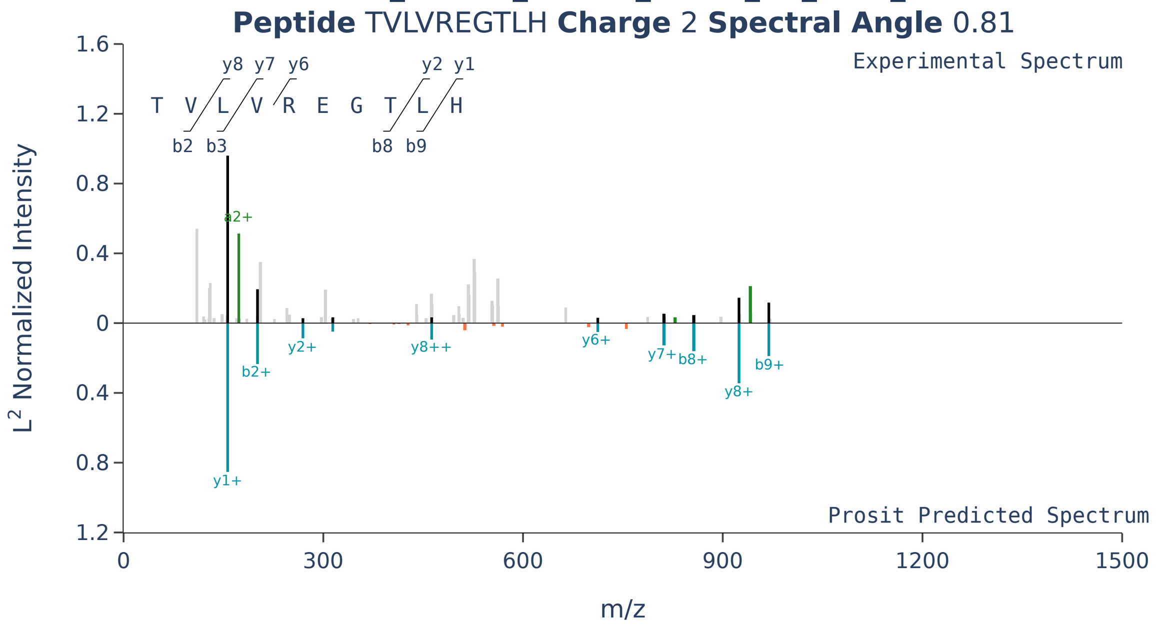


**Source** HRoetschke_211222_160123_HFGoe_IF2_b1R1_4h **Scan** 11457
**Peptide** DTPGHAAFTSMRAR **Charge** 4 **Spectral Angle** 0.87

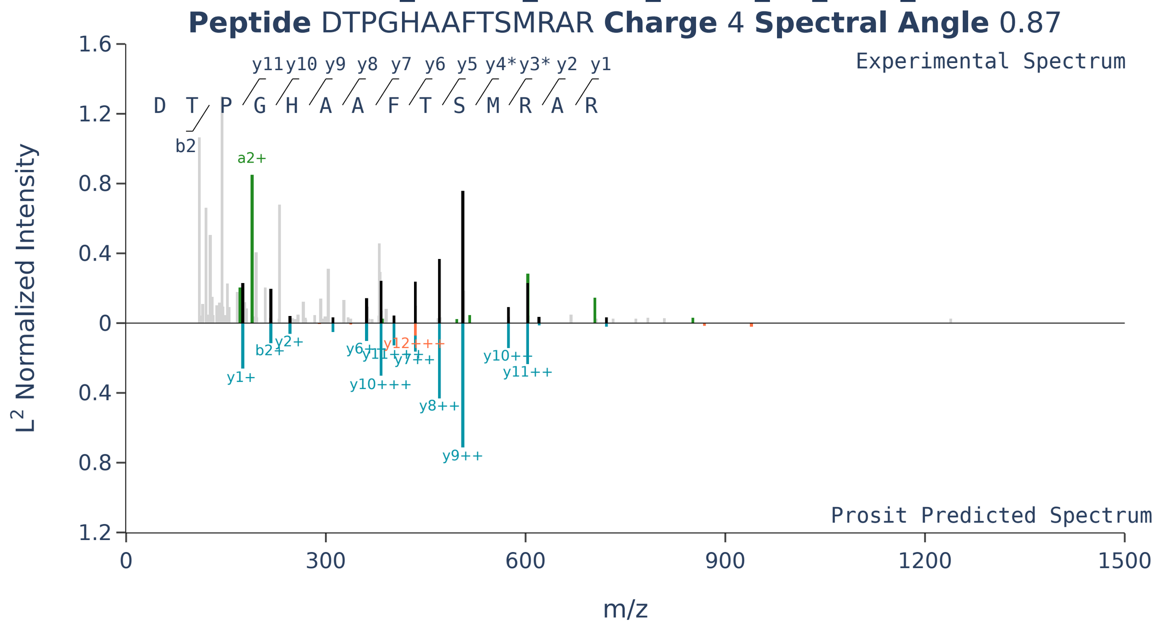


**Source** HRoetschke_211222_160123_HFGoe_IF2_b1R1_24h **Scan** 13461
**Peptide** ASGEAGGITQHI **Charge** 2 **Spectral Angle** 0.77

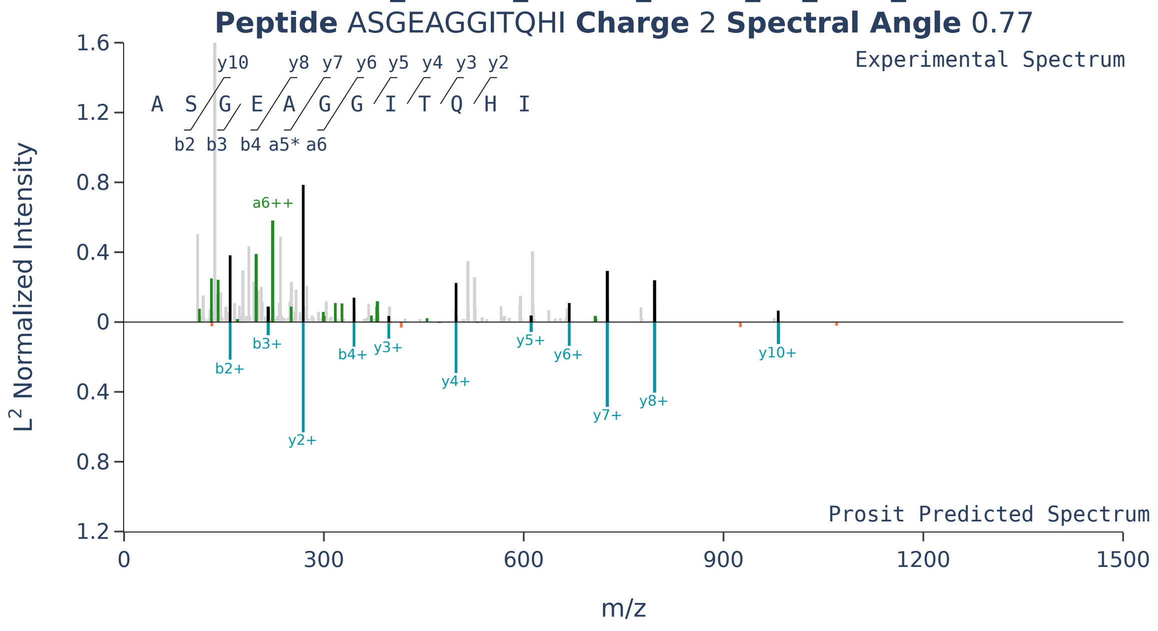


**Source** HRoetschke_211222_160123_HFGoe_IF2_b1R2_1h **Scan** 15265
**Peptide** DTGAAAEPRAPVV **Charge** 2 **Spectral Angle** 0.87

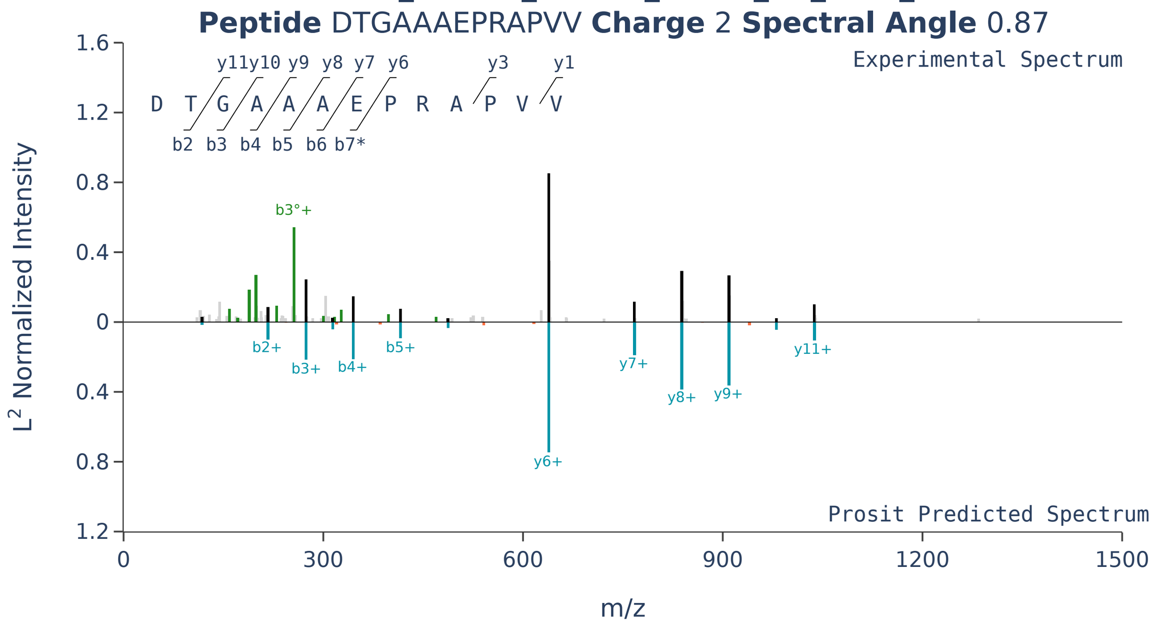


**Source** HRoetschke_211222_160123_HFGoe_IF2_b1R1_24h **Scan** 7763
**Peptide** GAYHVETE **Charge** 2 **Spectral Angle** 0.88

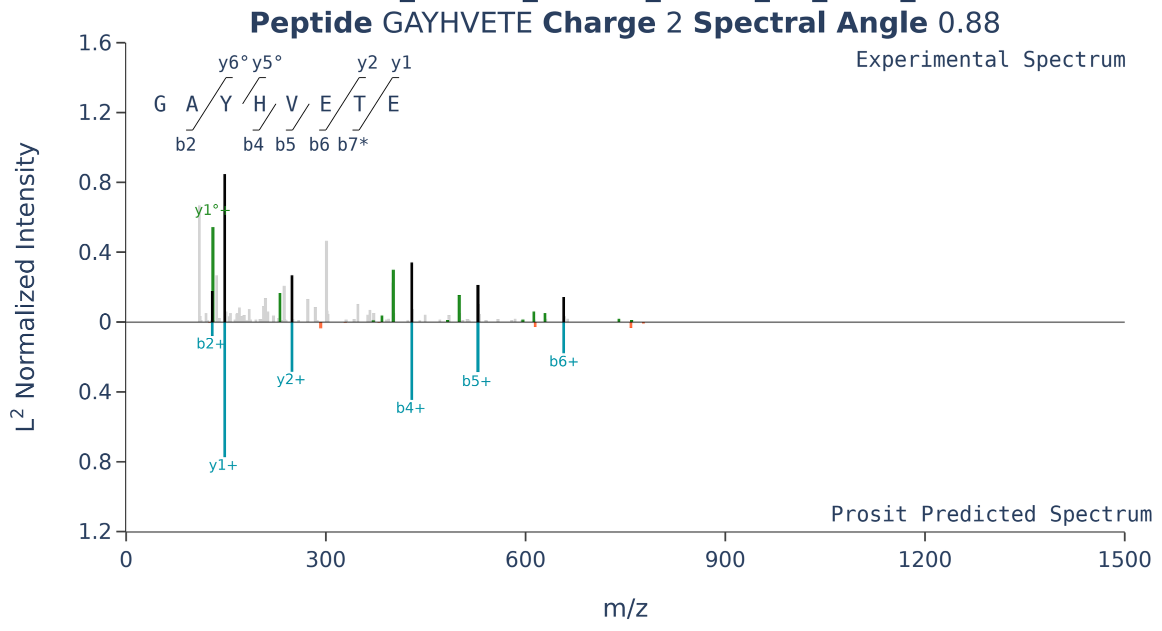


**Source** HRoetschke_211222_160123_HFGoe_IF2_b1R2_2h **Scan** 10715
**Peptide** ADDSVSAQEKQTL **Charge** 3 **Spectral Angle** 0.91

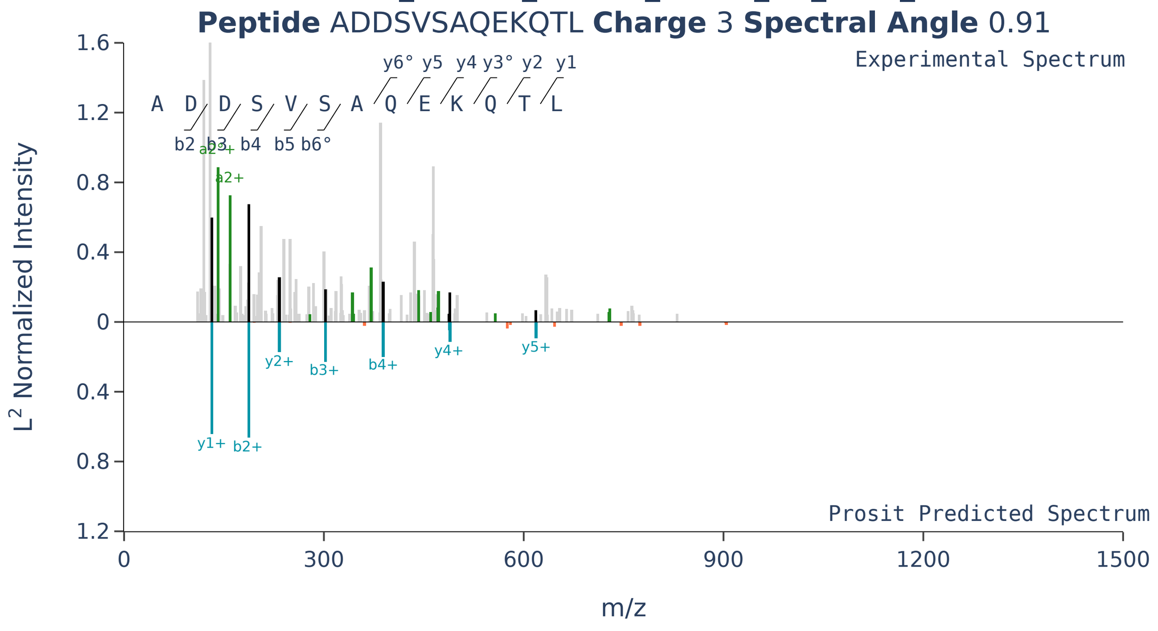


**Source** HRoetschke_211222_160123_HFGoe_IF2_b1R2_2h **Scan** 10169
**Peptide** QTSVERL **Charge** 2 **Spectral Angle** 0.82

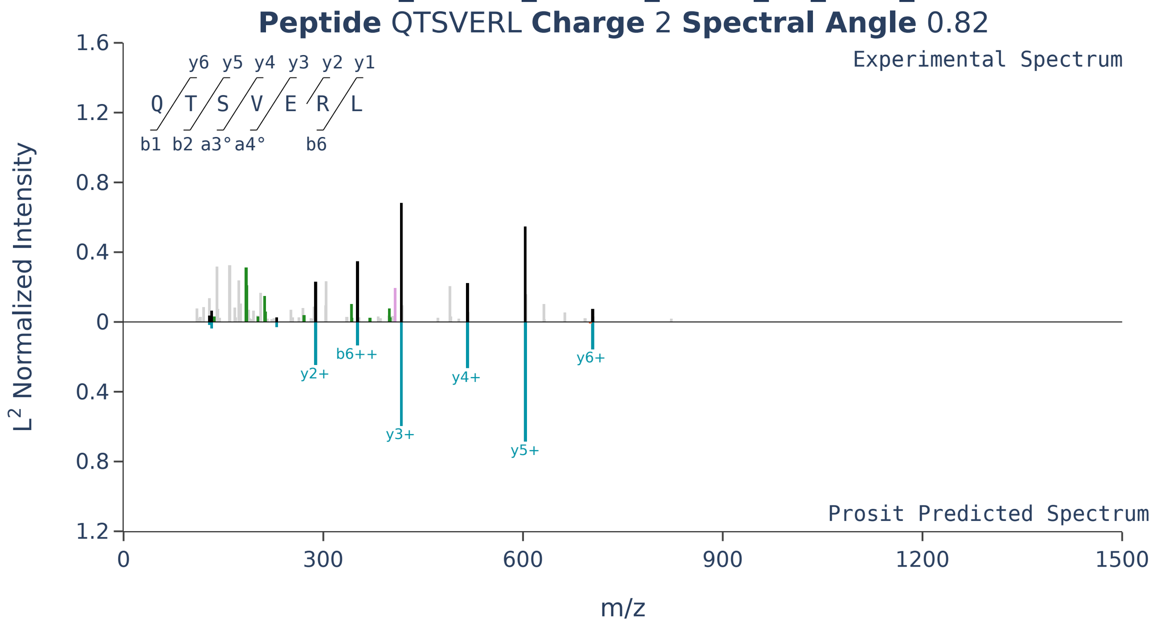


**Source** HRoetschke_211222_160123_HFGoe_IF2_b1R1_2h **Scan** 5695
**Peptide** SSDYHVTTSQHARQA **Charge** 2 **Spectral Angle** 0.89

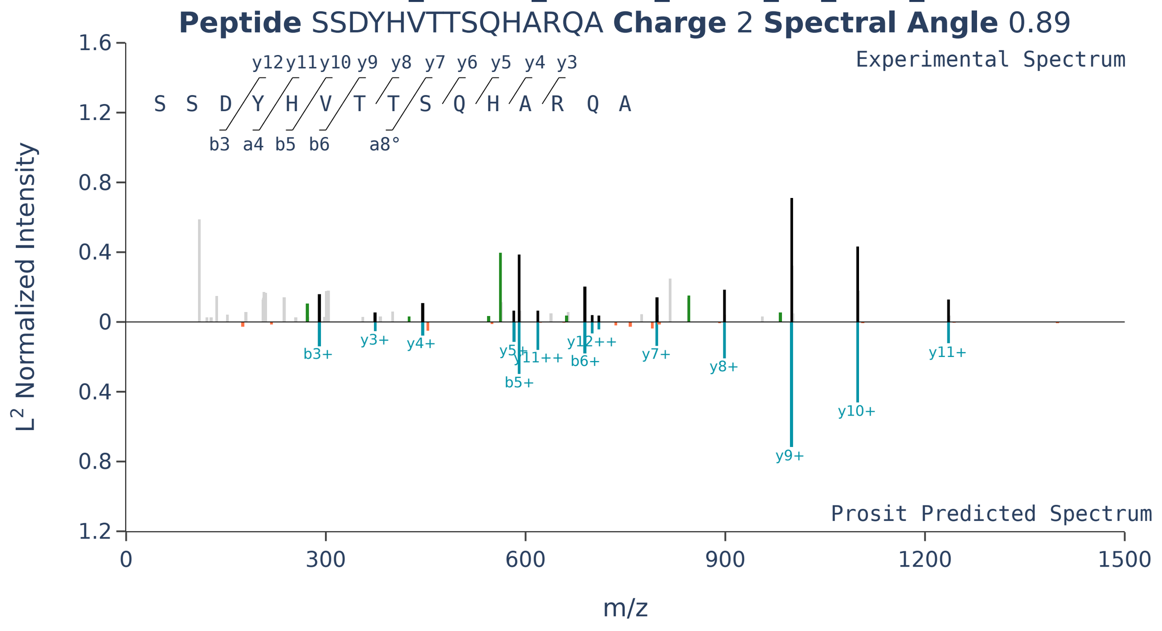

**Source** HRoetschke_211222_160123_HFGoe_IF2_b2R1_2h **Scan** 6883
**Peptide** ADASARKVIE **Charge** 2 **Spectral Angle** 0.77

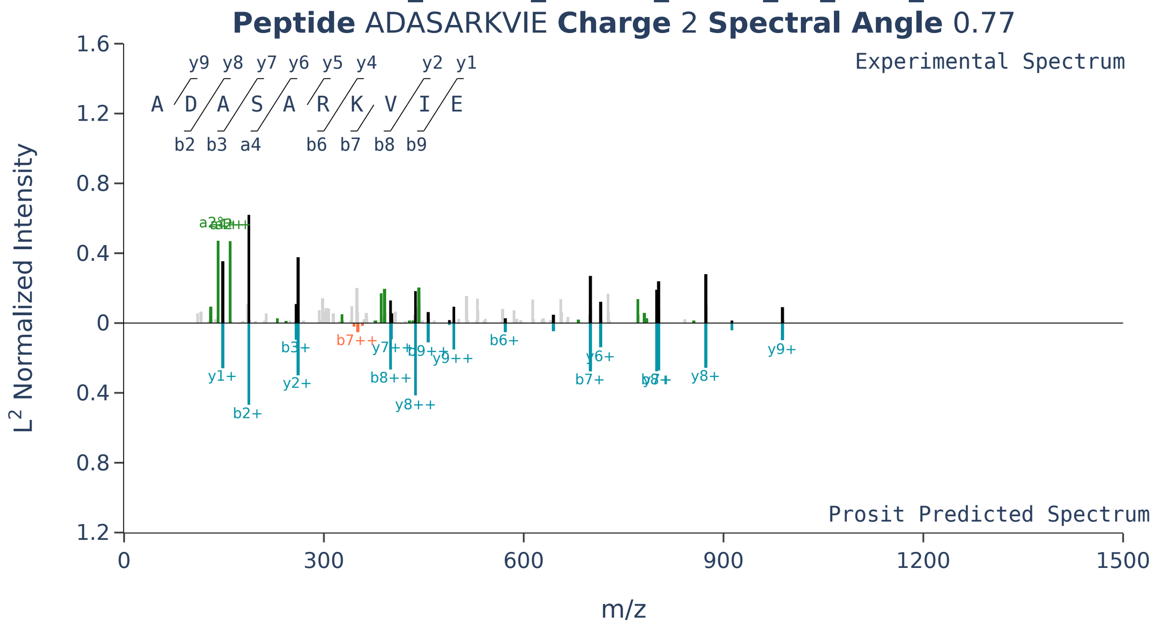


**Source** HRoetschke_211222_160123_HFGoe_IF2_b1R2_24h **Scan** 15249
**Peptide** DGVMPQTI **Charge** 2 **Spectral Angle** 0.82

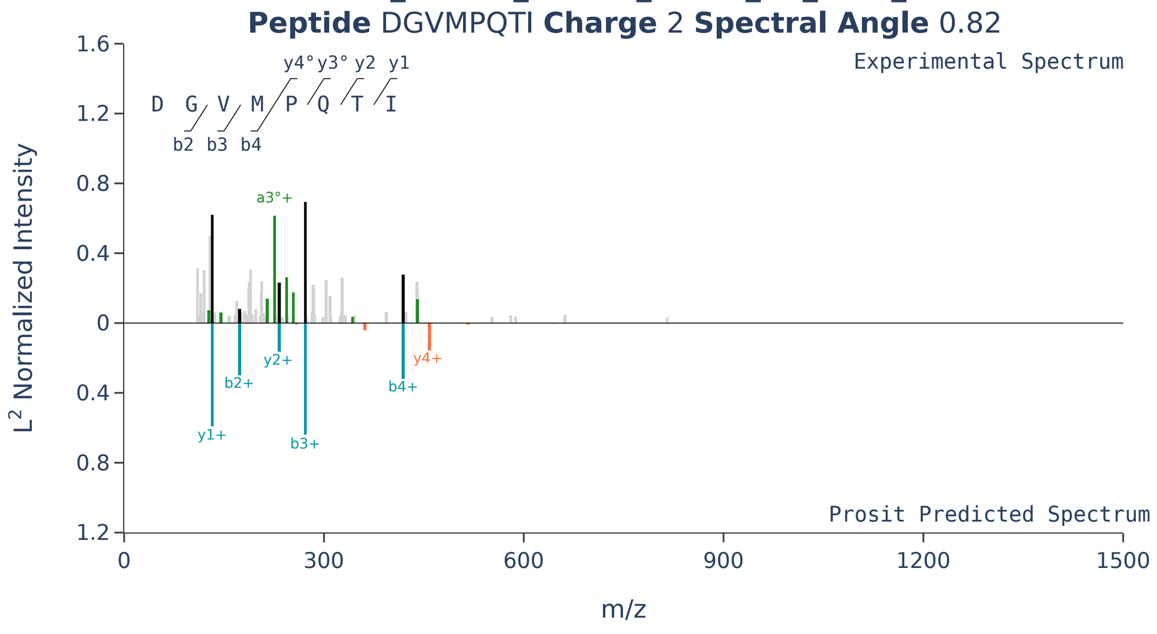


**Source** HRoetschke_211222_160123_HFGoe_IF2_b1R1_2h **Scan** 11363
**Peptide** FKSPKFG **Charge** 2 **Spectral Angle** 0.84

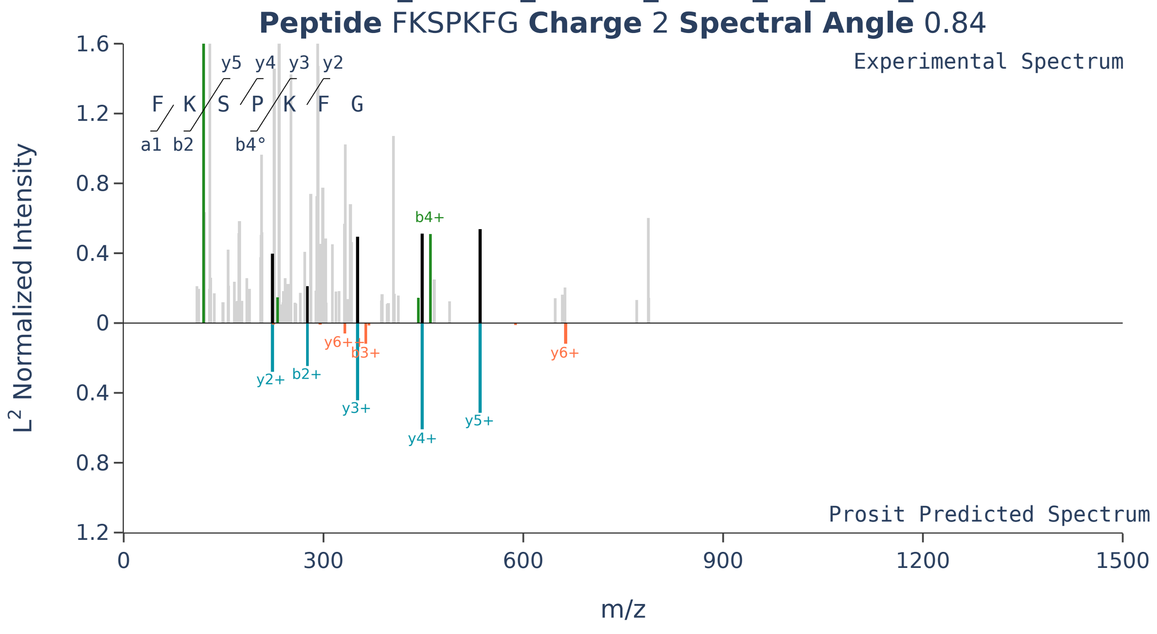


**Source** HRoetschke_211222_160123_HFGoe_IF2_b1R2_1h **Scan** 15168
**Peptide** GTLHKGDIVL **Charge** 3 **Spectral Angle** 0.86

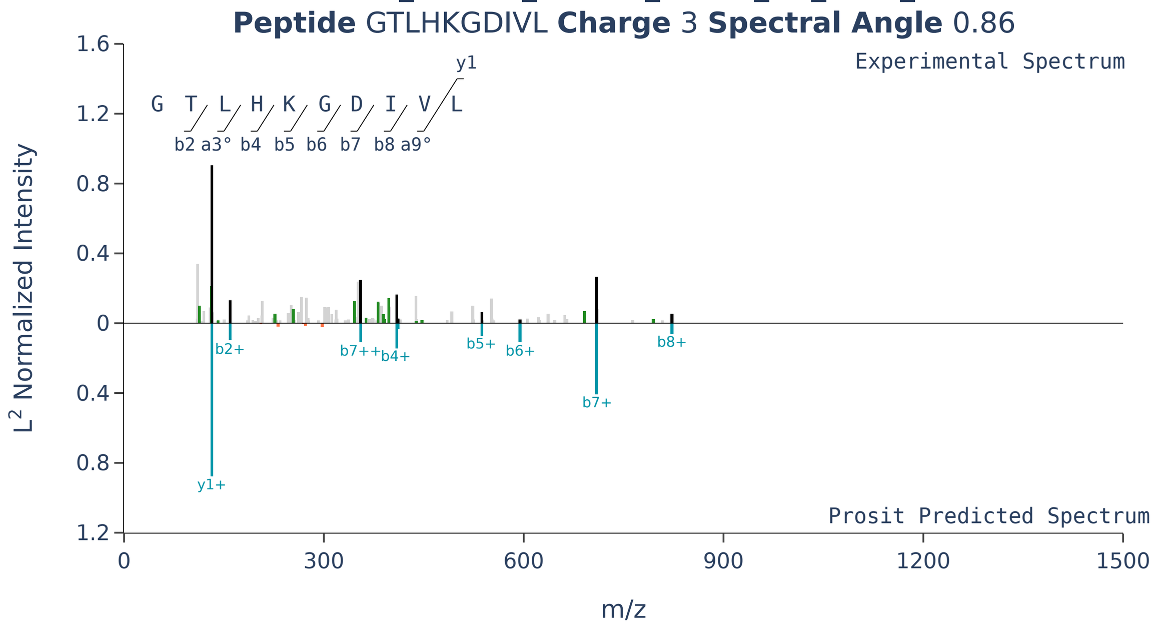


**Source** HRoetschke_211222_160123_HFGoe_IF2_b2R1_24h **Scan** 8263
**Peptide** KDDVNEVRNGME **Charge** 3 **Spectral Angle** 0.89

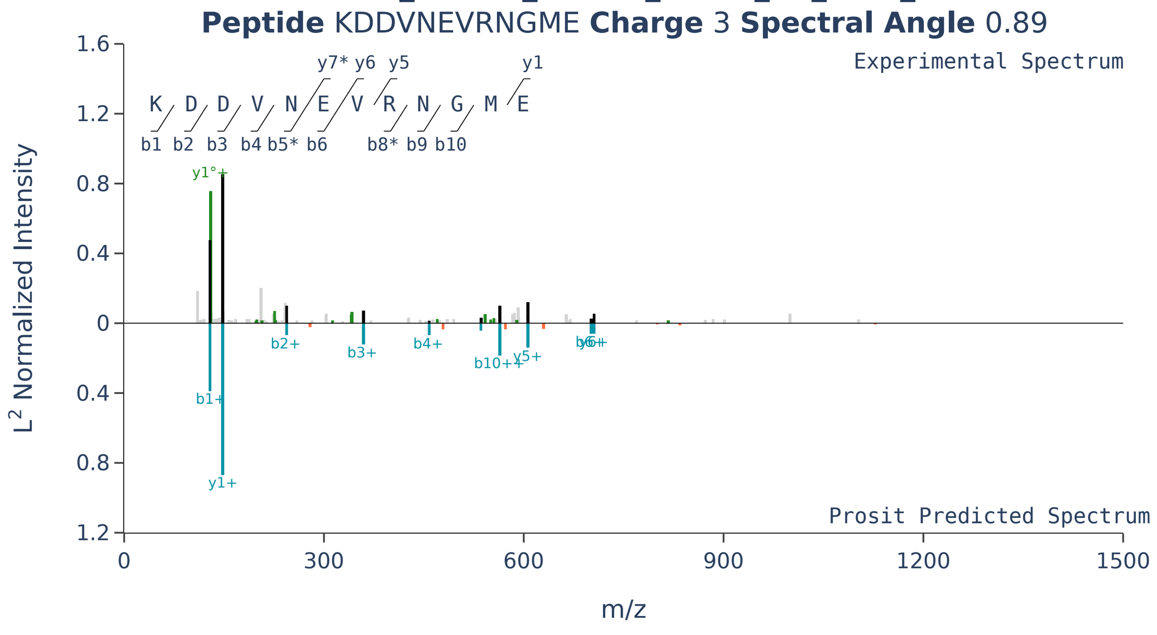


**Source** HRoetschke_211222_160123_HFGoe_IF2_b1R1_4h **Scan** 12496
**Peptide** SSLQQGFQKPAQAVNRD **Charge** 3 **Spectral Angle** 0.88

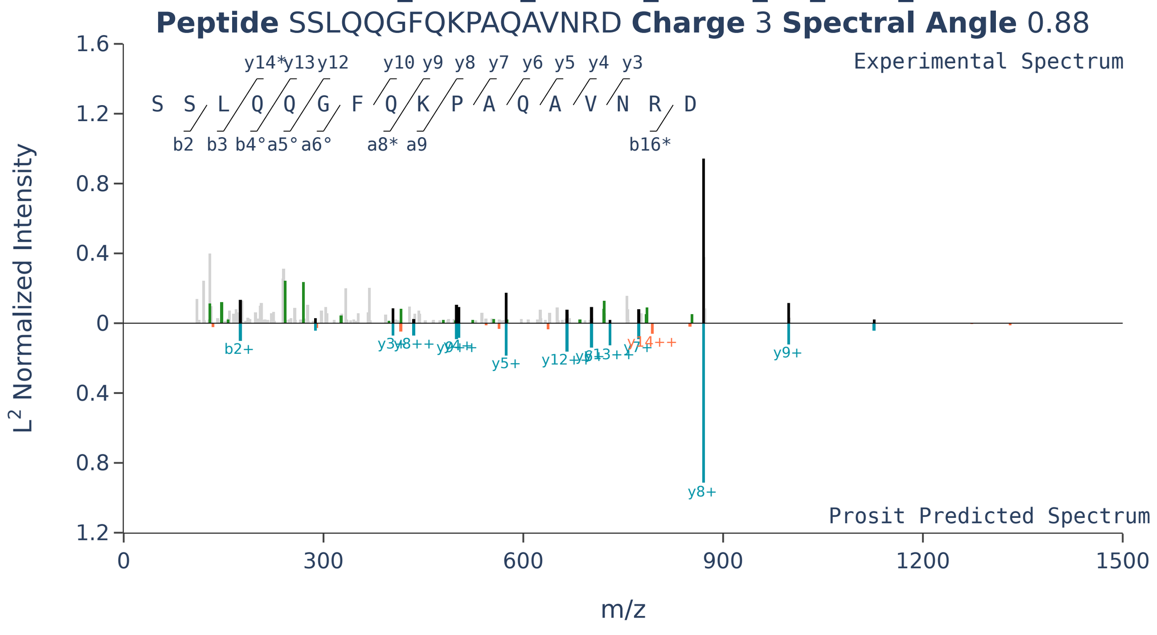


**Source** HRoetschke_211222_160123_HFGoe_IF2_b2R2_4h **Scan** 14978
**Peptide** SGVPAAGDEVTVVRD **Charge** 2 **Spectral Angle** 0.82

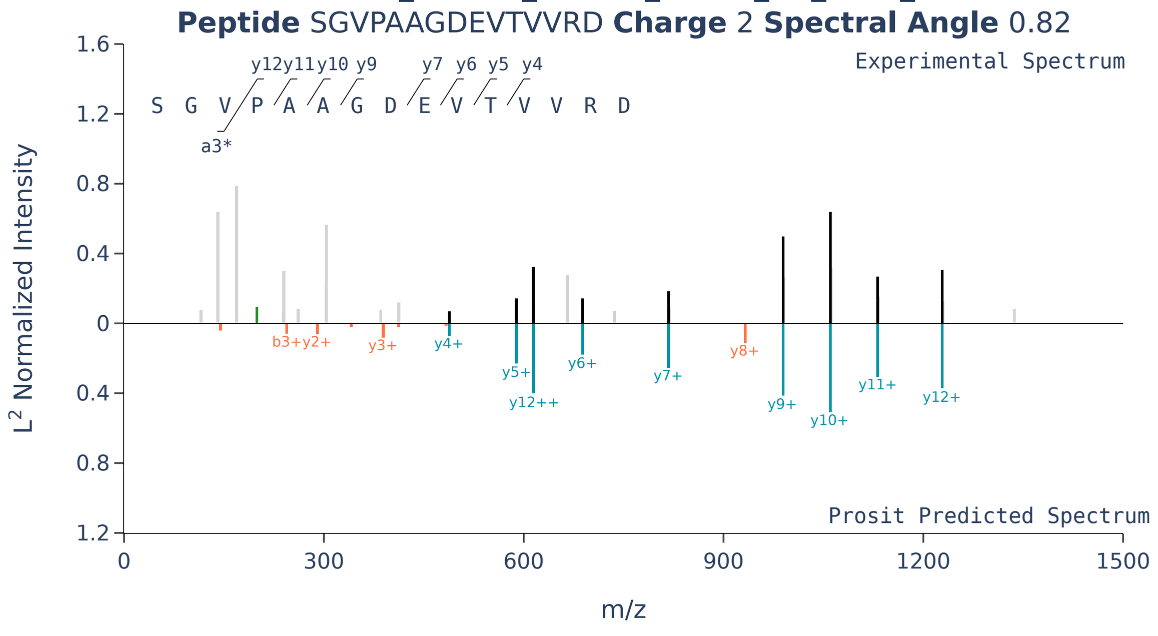


**Source** HRoetschke_211222_160123_HFGoe_IF2_b2R1_4h **Scan** 15212
**Peptide** PVVVAVN **Charge** 1 **Spectral Angle** 0.88

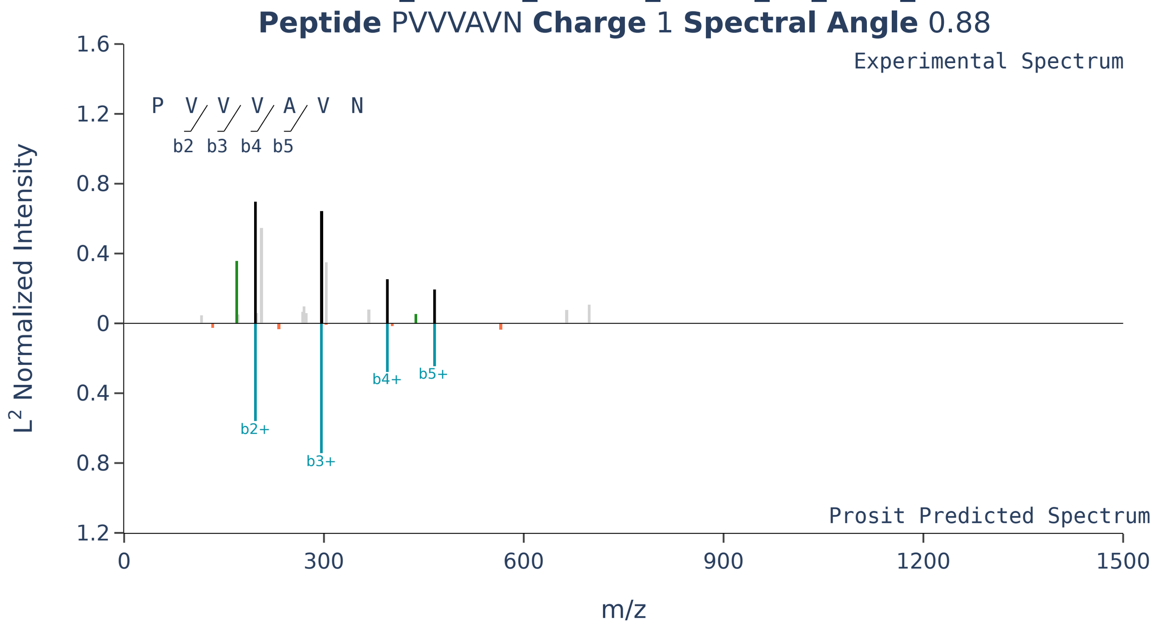


**Source** HRoetschke_211222_160123_HFGoe_IF2_b2R1_2h **Scan** 6703
**Peptide** RDEKKAREVAL **Charge** 3 **Spectral Angle** 0.7

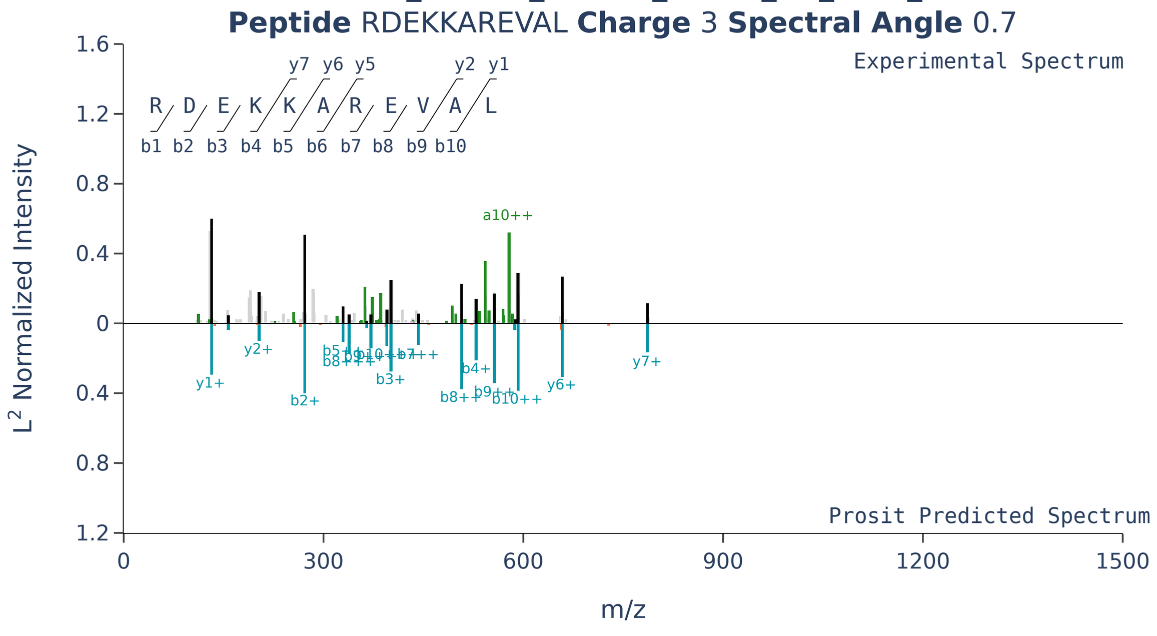


**Source** HRoetschke_211222_160123_HFGoe_IF2_b1R1_24h **Scan** 12062
**Peptide** PEADPDRVKNEL **Charge** 2 **Spectral Angle** 0.79

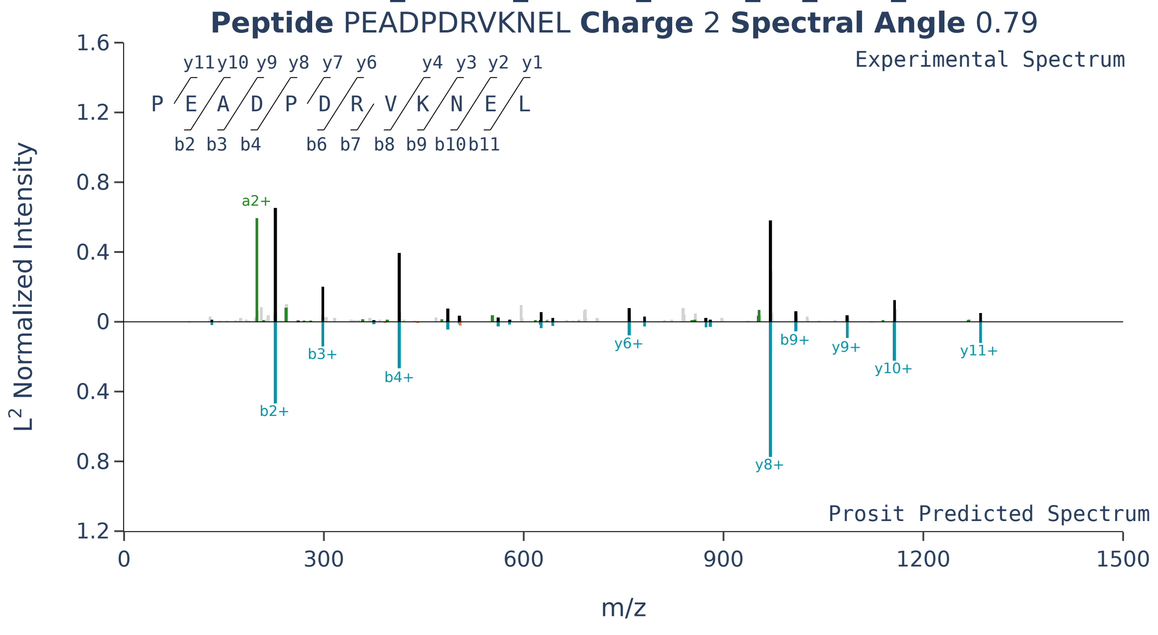


**Source** HRoetschke_211222_160123_HFGoe_IF2_b1R1_4h **Scan** 9688
**Peptide** KAMMKLG **Charge** 2 **Spectral Angle** 0.95

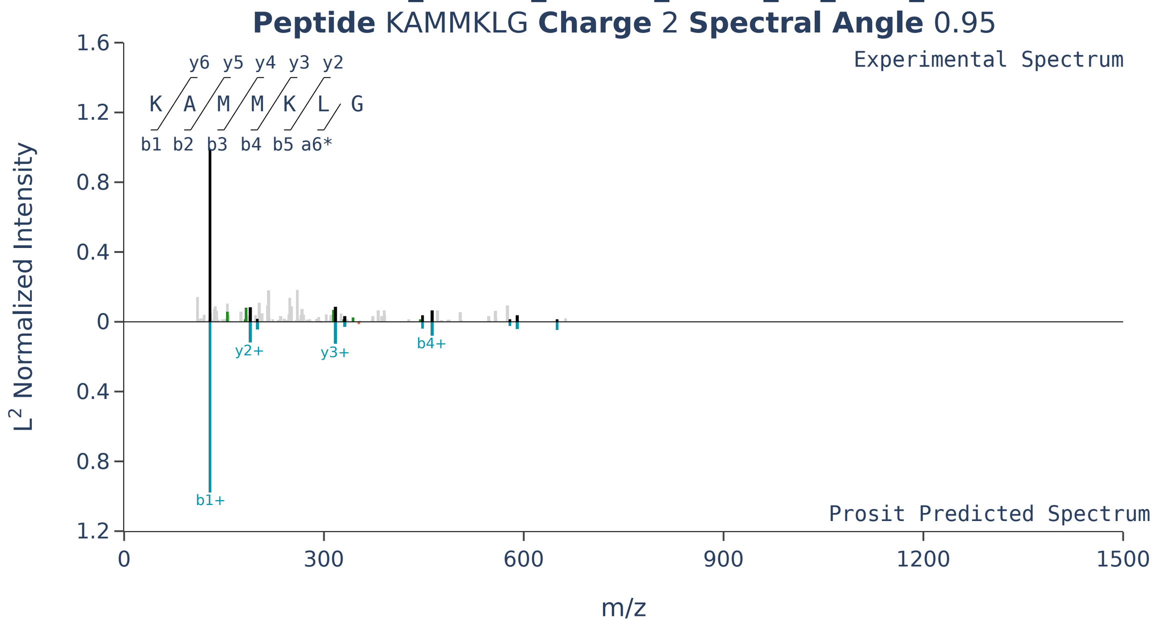


**Source** HRoetschke_211222_160123_HFGoe_IF2_b1R2_24h **Scan** 13338
**Peptide** TGAAAEPRAPVVT **Charge** 2 **Spectral Angle** 0.82

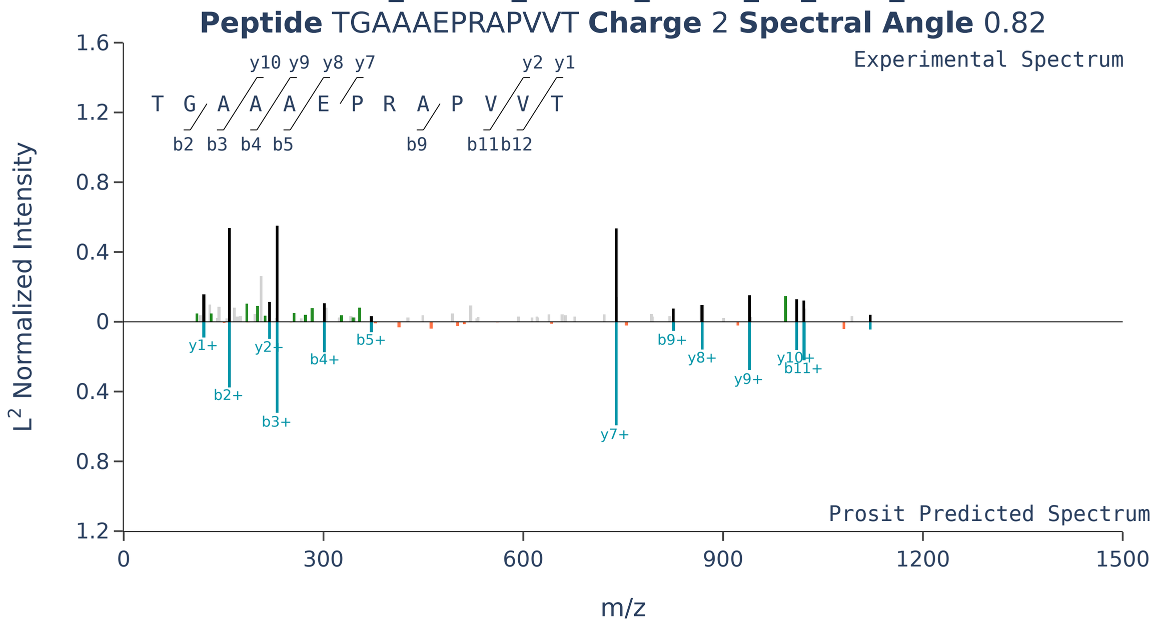


**Source** HRoetschke_211222_160123_HFGoe_IF2_b1R1_24h **Scan** 7791
**Peptide** RGAQATDI **Charge** 2 **Spectral Angle** 0.88

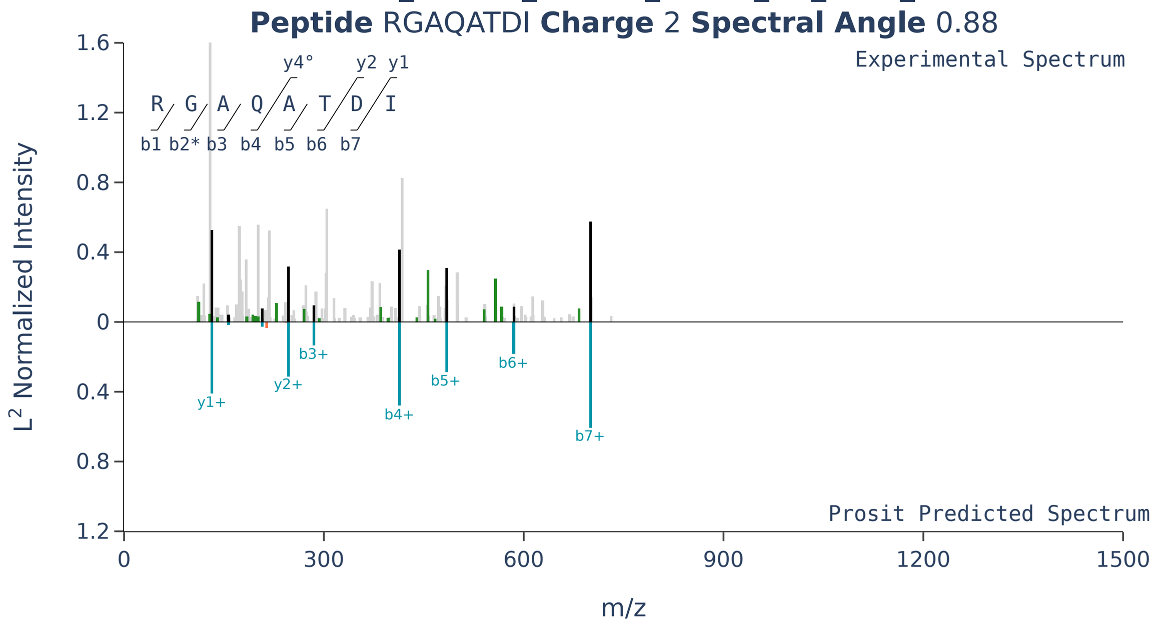


**Source** HRoetschke_211222_160123_HFGoe_IF2_b2R1_24h **Scan** 9080
**Peptide** KGSQVIKAM **Charge** 2 **Spectral Angle** 0.83

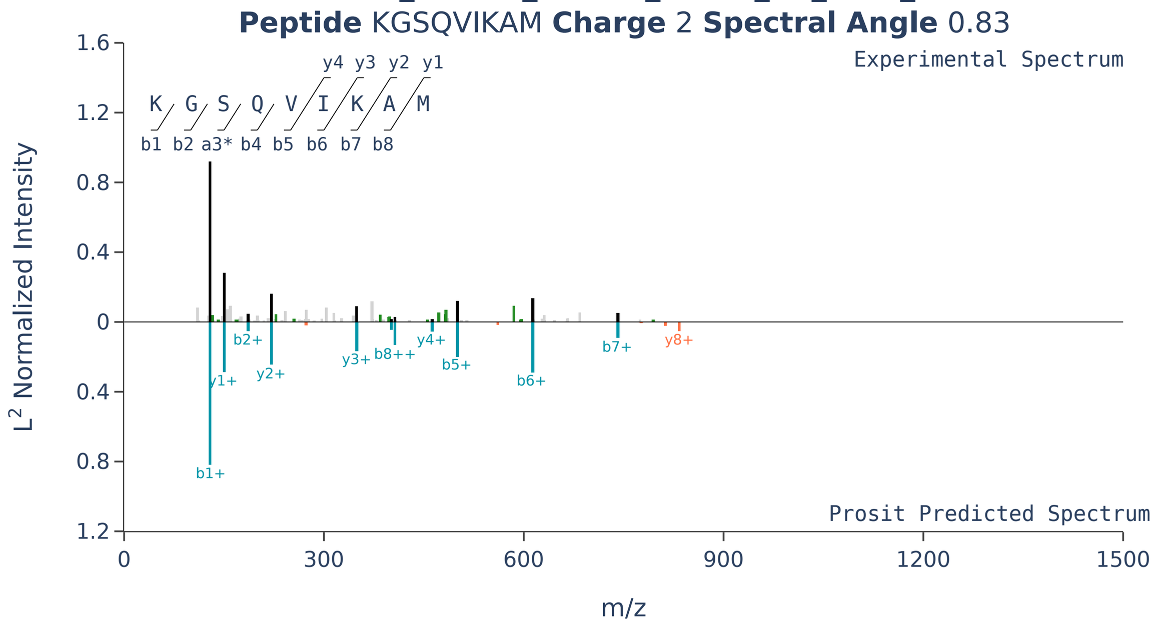


**Source** HRoetschke_211222_160123_HFGoe_IF2_b1R2_24h **Scan** 10165
**Peptide** KSKLENMFA **Charge** 2 **Spectral Angle** 0.79

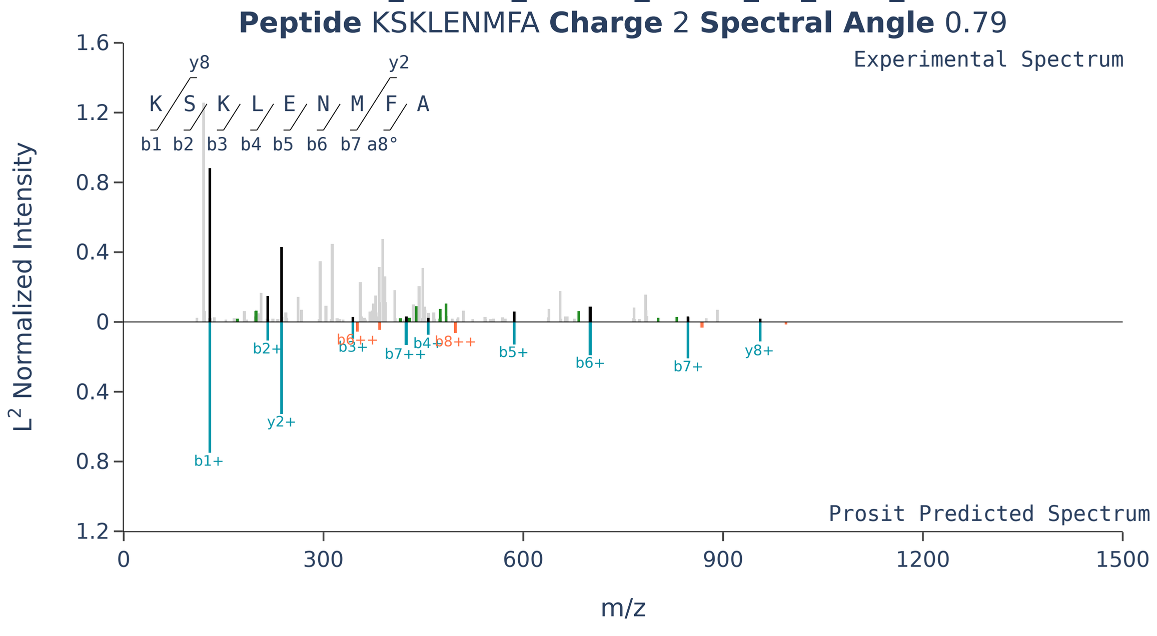

**Source** HRoetschke_211222_160123_HFGoe_IF2_b1R1_4h **Scan** 14923
**Peptide** ADDSVSAQEKQTLID **Charge** 2 **Spectral Angle** 0.86

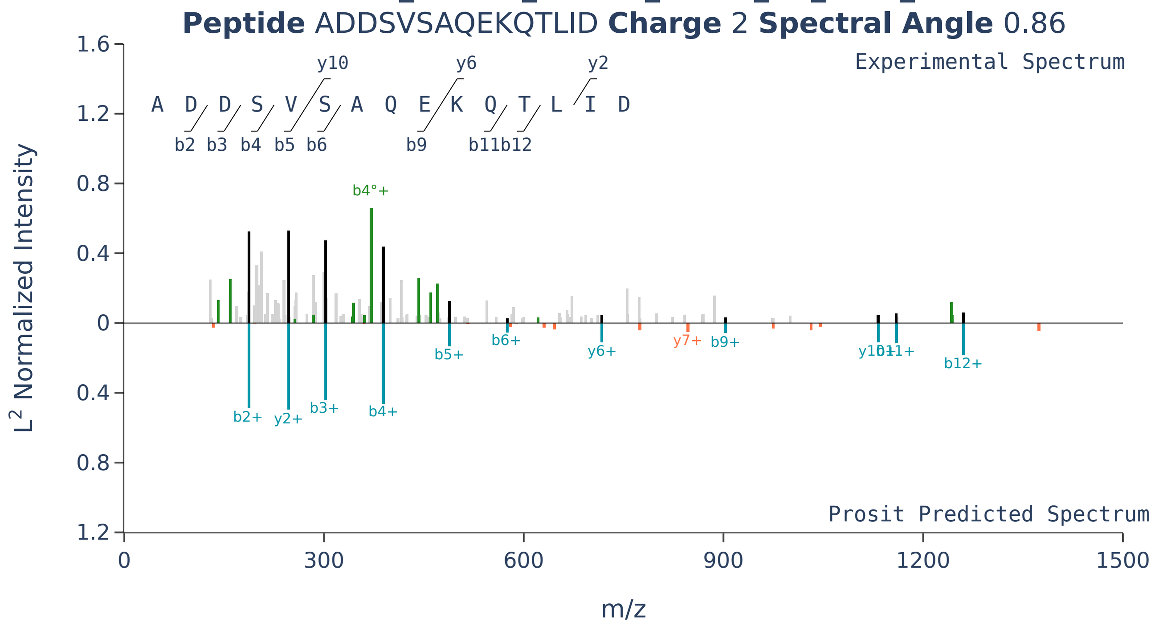


**Source** HRoetschke_211222_160123_HFGoe_IF2_b2R2_2h **Scan** 11423
**Peptide** IGAYHVETE **Charge** 2 **Spectral Angle** 0.75

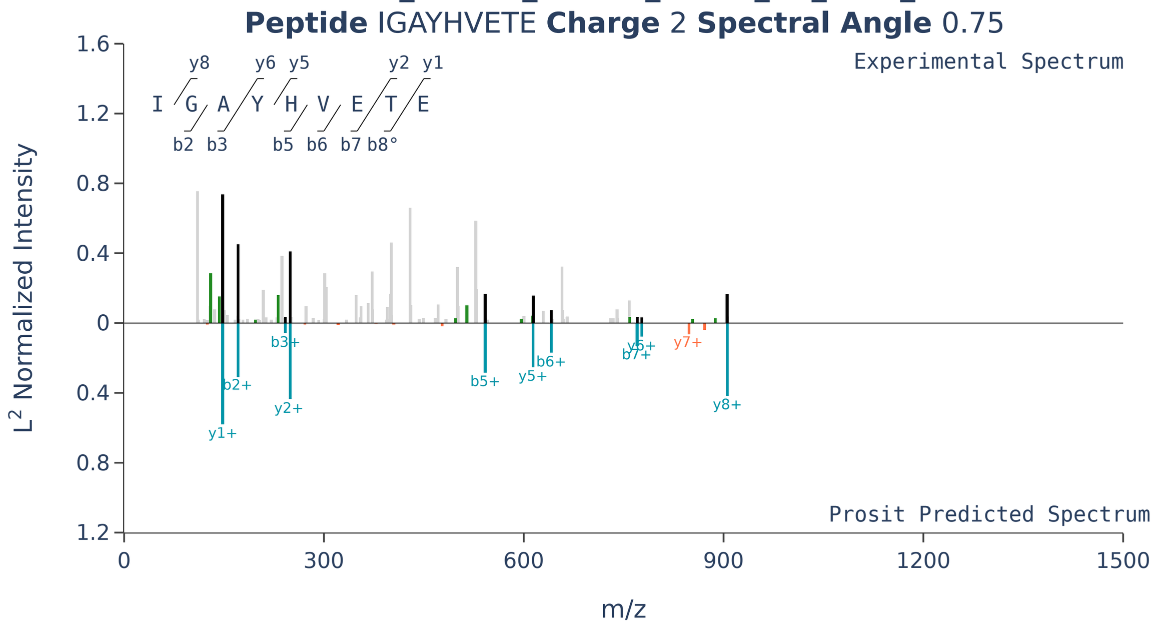


**Source** HRoetschke_211222_160123_HFGoe_IF2_b1R1_4h **Scan** 9207
**Peptide** VAEEMGHKVILRRENE **Charge** 4 **Spectral Angle** 0.81

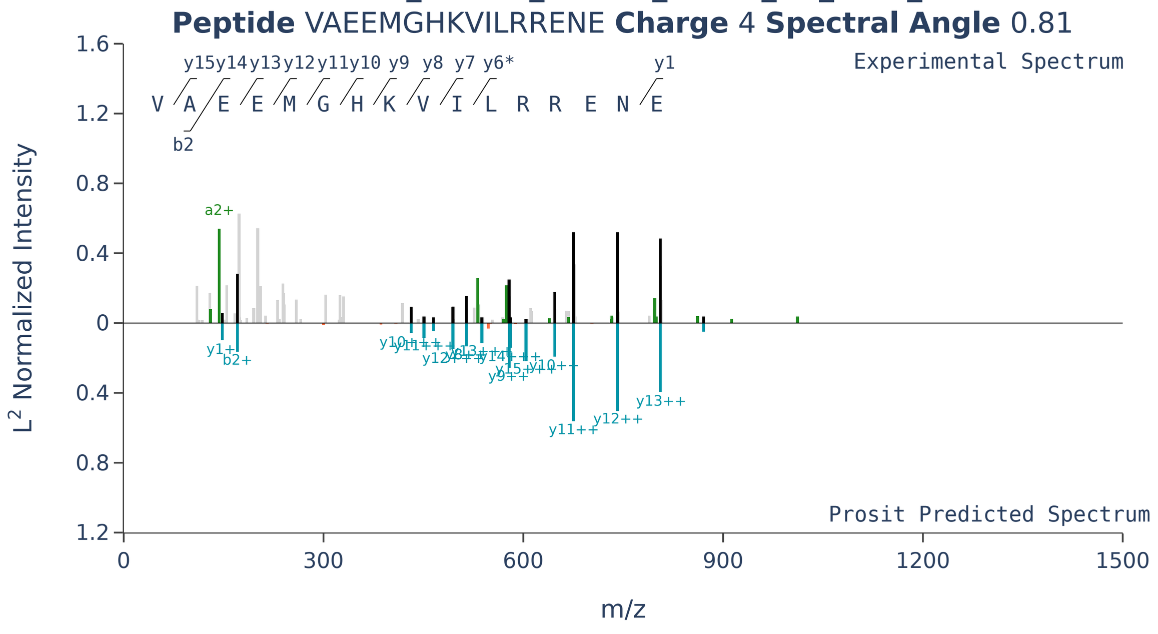


**Source** HRoetschke_211222_160123_HFGoe_IF2_b2R1_24h **Scan** 11522
**Peptide** SSLQQGFQKPAQ **Charge** 2 **Spectral Angle** 0.82

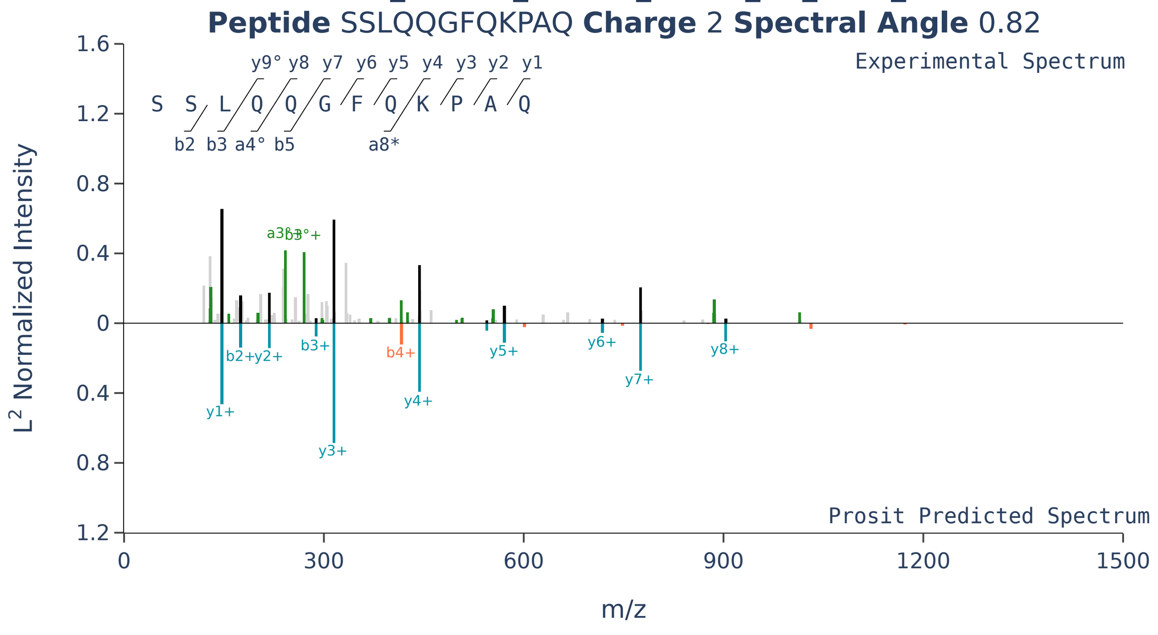


**Source** HRoetschke_211222_160123_HFGoe_IF2_b2R1_24h **Scan** 10813
**Peptide** AEVRDVFKSPK **Charge** 3 **Spectral Angle** 0.79

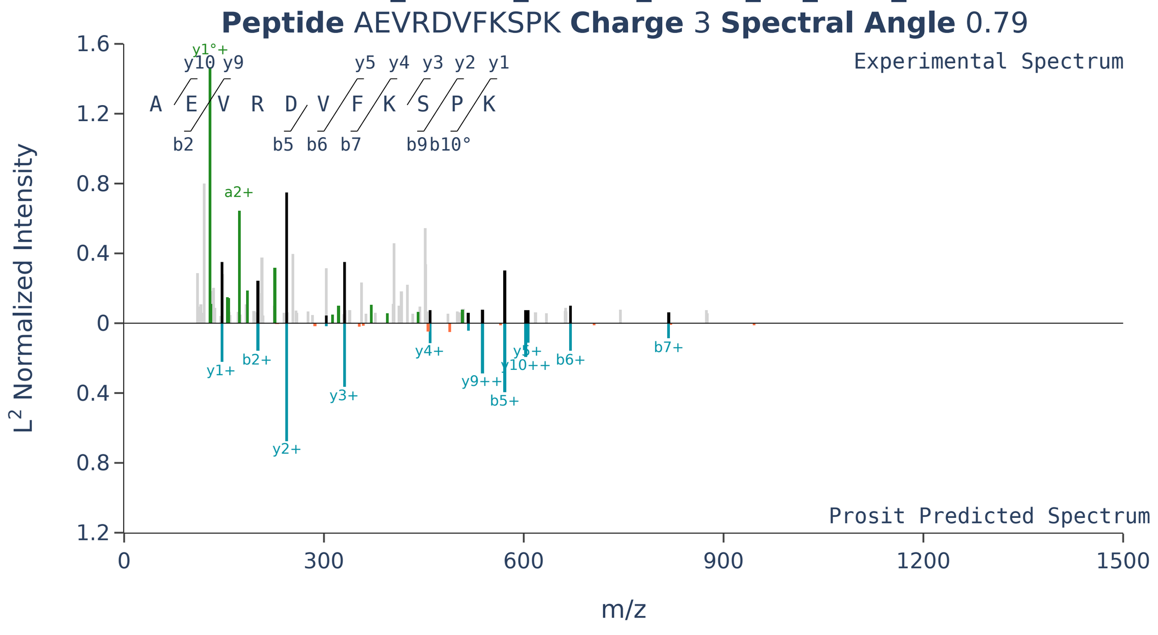


**Source** HRoetschke_211222_160123_HFGoe_IF2_b1R2_24h **Scan** 22866
**Peptide** QAEVLEL **Charge** 2 **Spectral Angle** 0.93

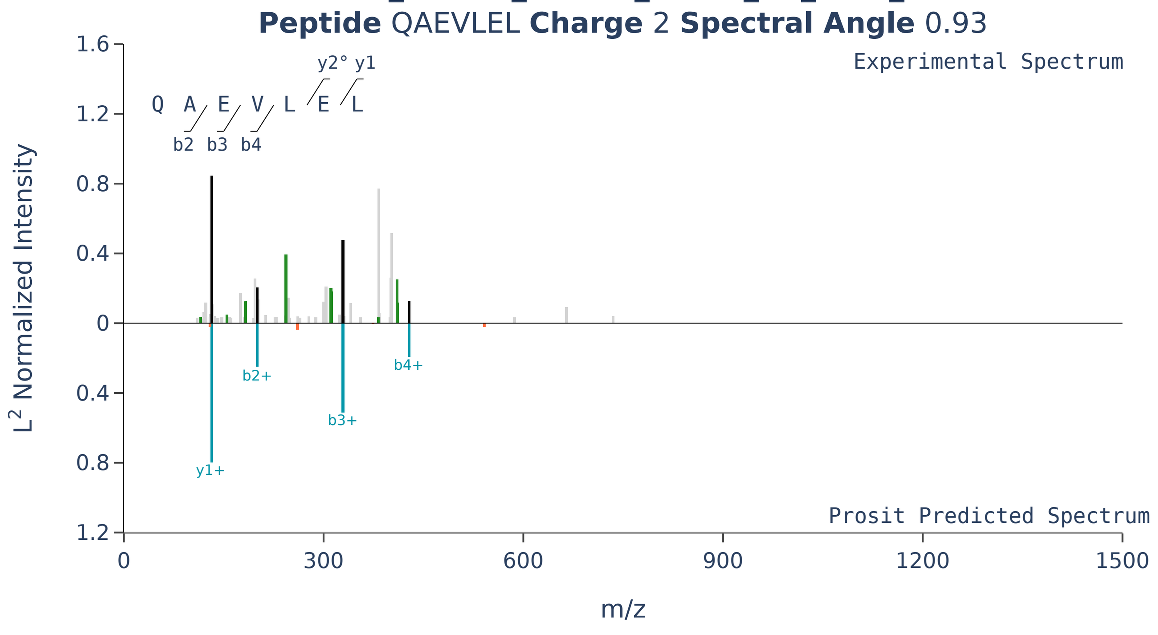


**Source** HRoetschke_211222_160123_HFGoe_IF2_b1R1_24h **Scan** 8754
**Peptide** GHVDHGKTSLLD **Charge** 3 **Spectral Angle** 0.85

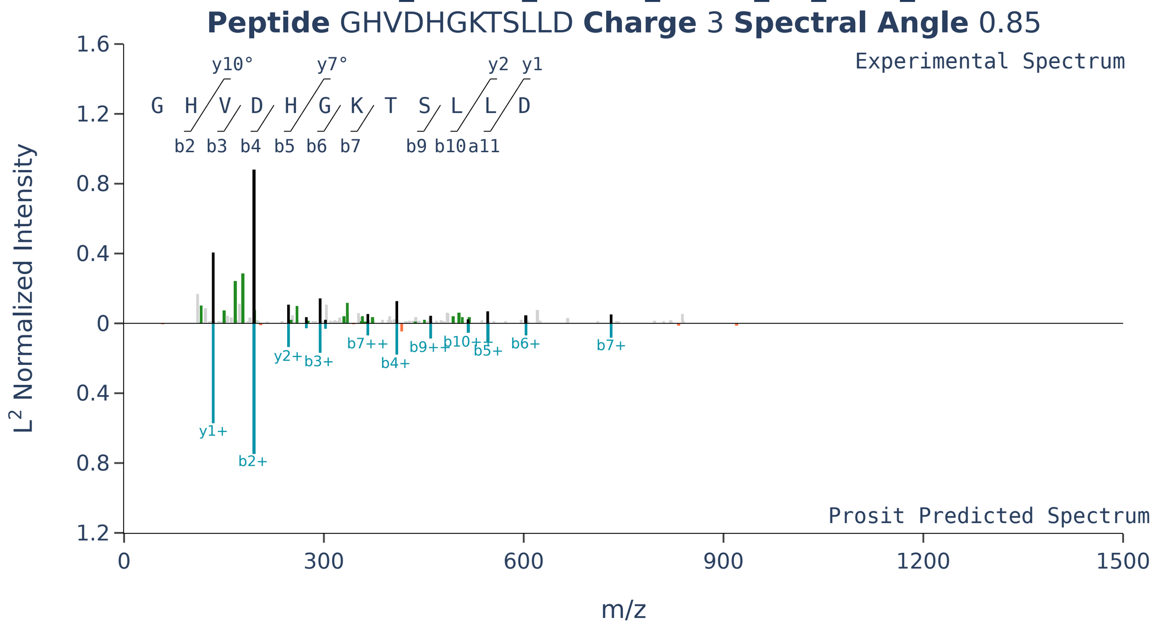


**Source** HRoetschke_211222_160123_HFGoe_IF2_b1R1_4h **Scan** 12602
**Peptide** YHVETENGMI **Charge** 2 **Spectral Angle** 0.86

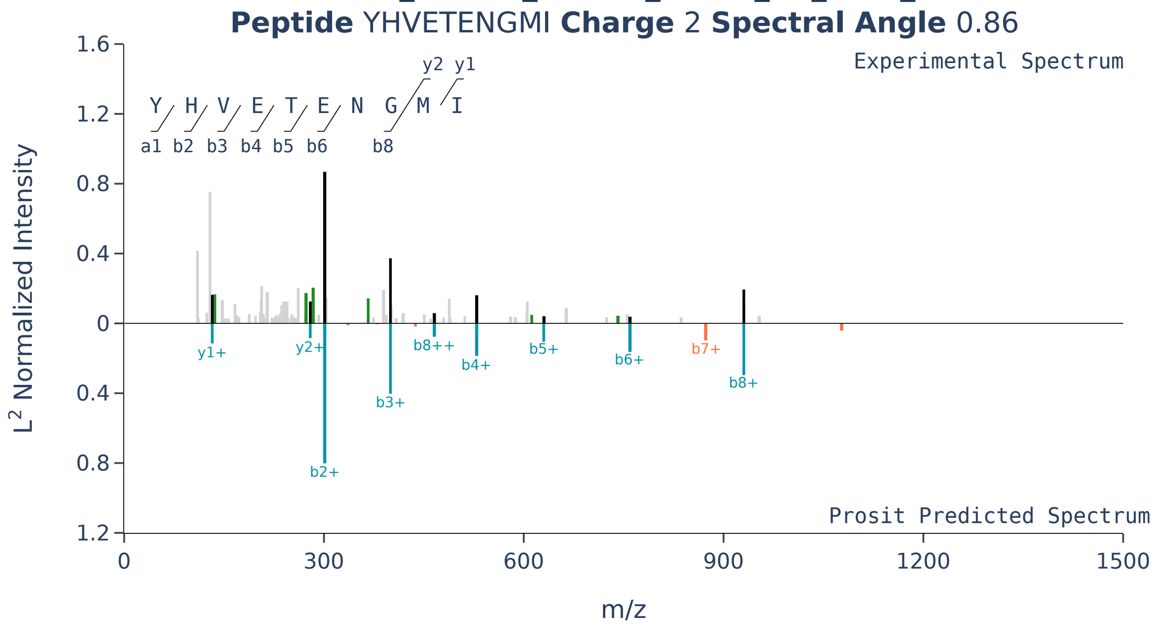


**Source** HRoetschke_211222_160123_HFGoe_IF2_b2R2_4h **Scan** 7517
**Peptide** ADASARKV **Charge** 2 **Spectral Angle** 0.78

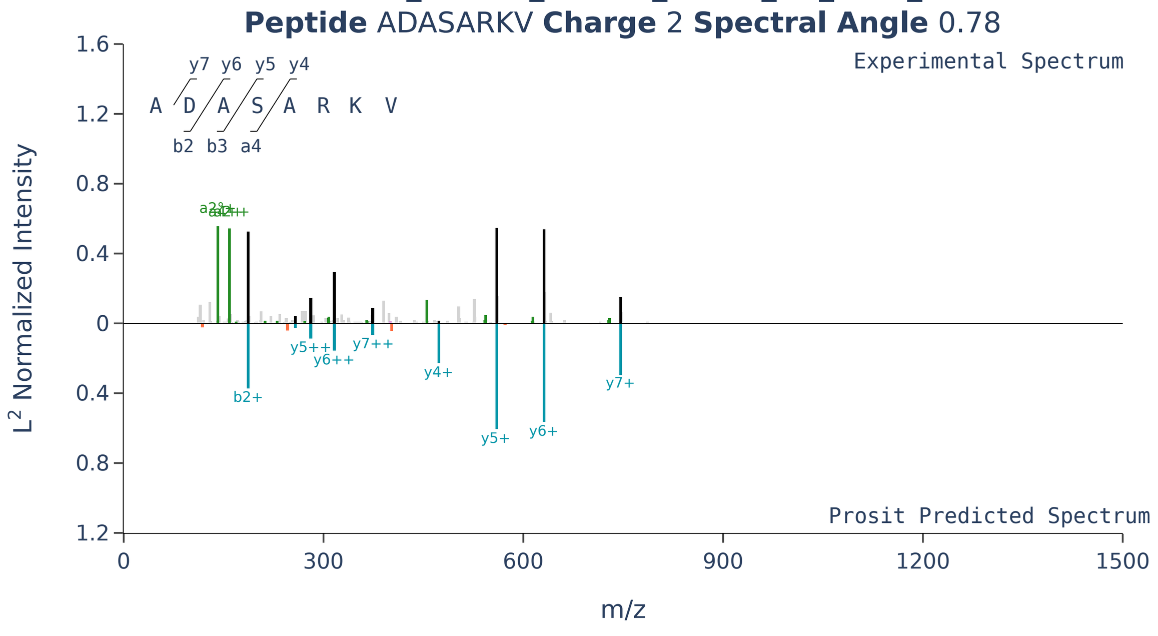


**Source** HRoetschke_211222_160123_HFGoe_IF2_b2R2_24h **Scan** 13042
**Peptide** LVVAADD **Charge** 1 **Spectral Angle** 0.88

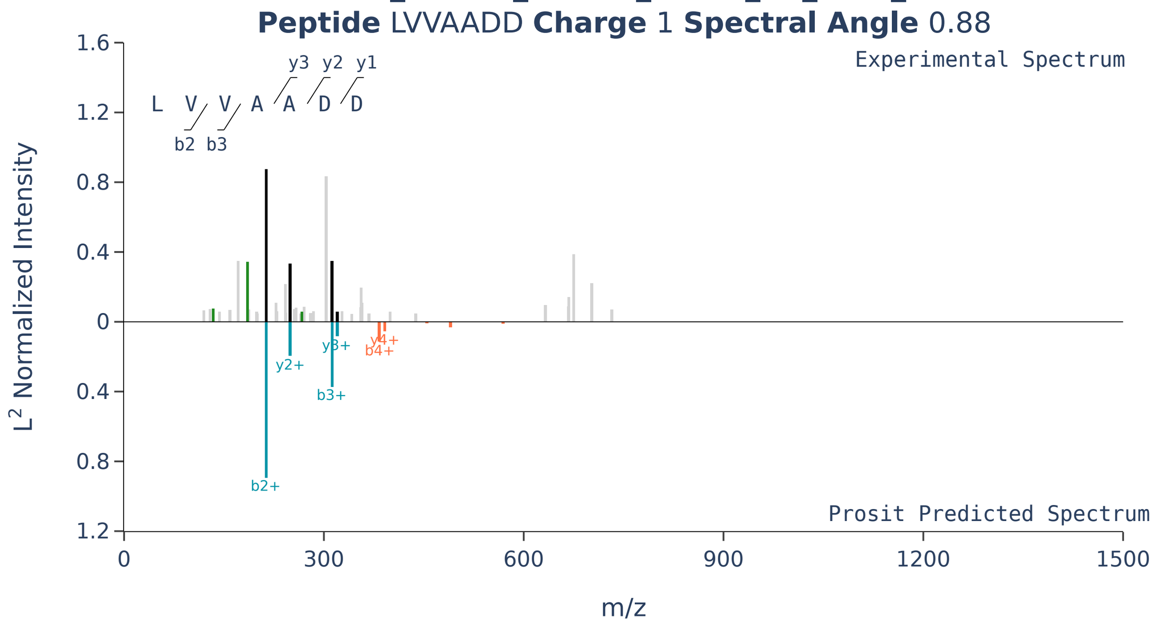


**Source** HRoetschke_211222_160123_HFGoe_IF2_b1R2_24h **Scan** 17169
**Peptide** TINQVIDQETAQ **Charge** 2 **Spectral Angle** 0.9

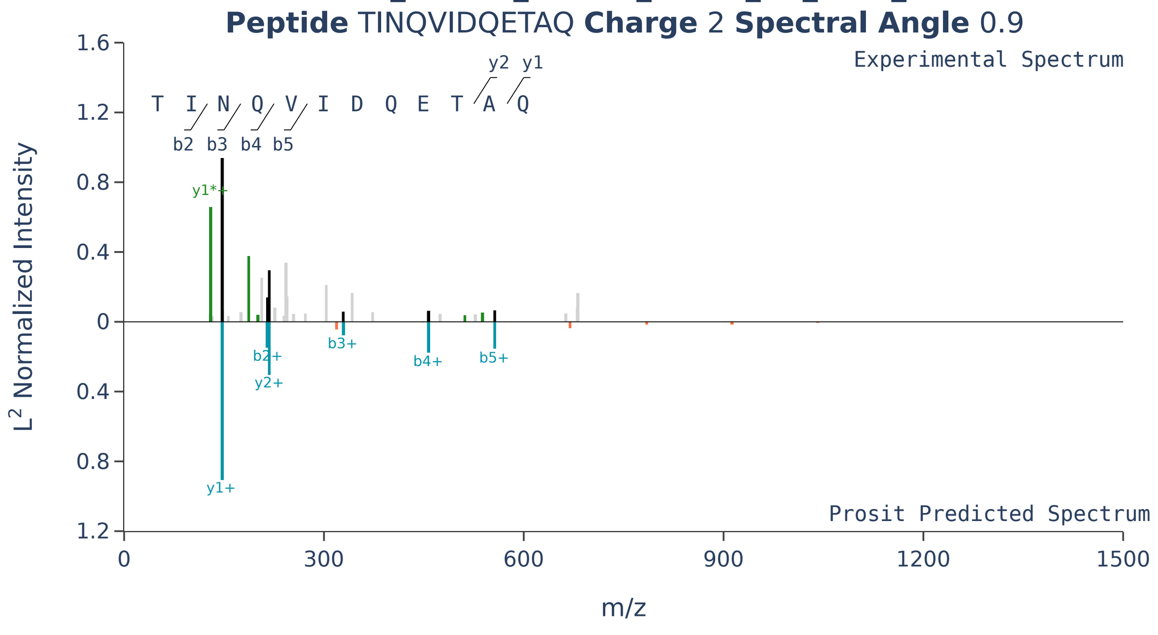


**Source** HRoetschke_211222_160123_HFGoe_IF2_b1R2_24h **Scan** 11829
**Peptide** DTPGHAAF **Charge** 2 **Spectral Angle** 0.75

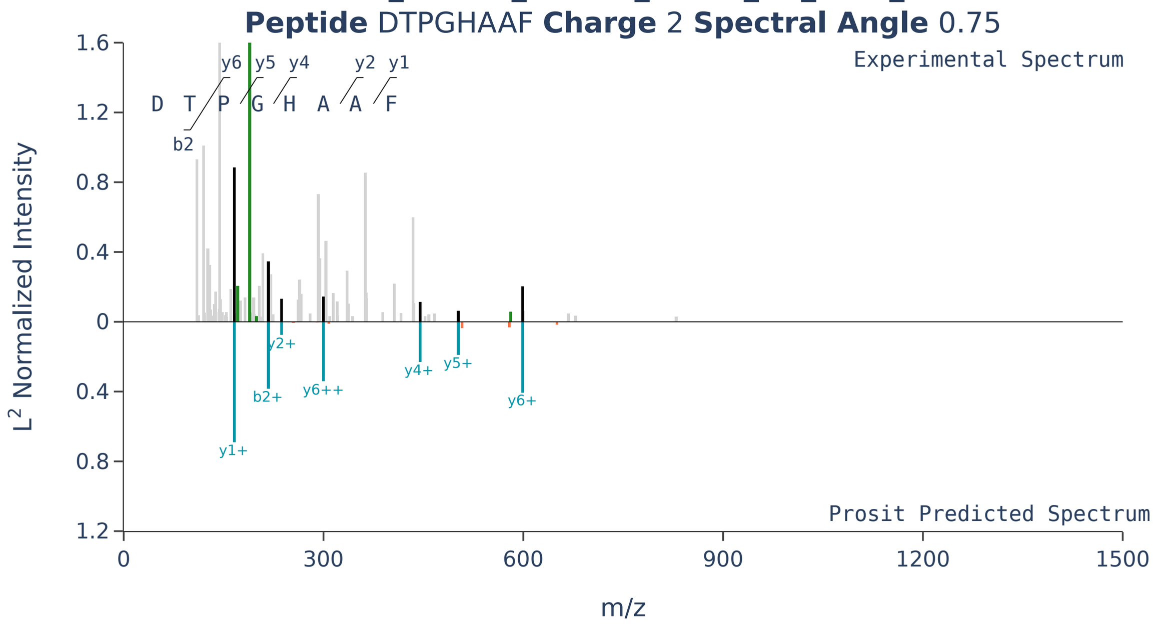


**Source** HRoetschke_211222_160123_HFGoe_IF2_b1R1_4h **Scan** 10180
**Peptide** KPEADPDRVKNEL **Charge** 2 **Spectral Angle** 0.87

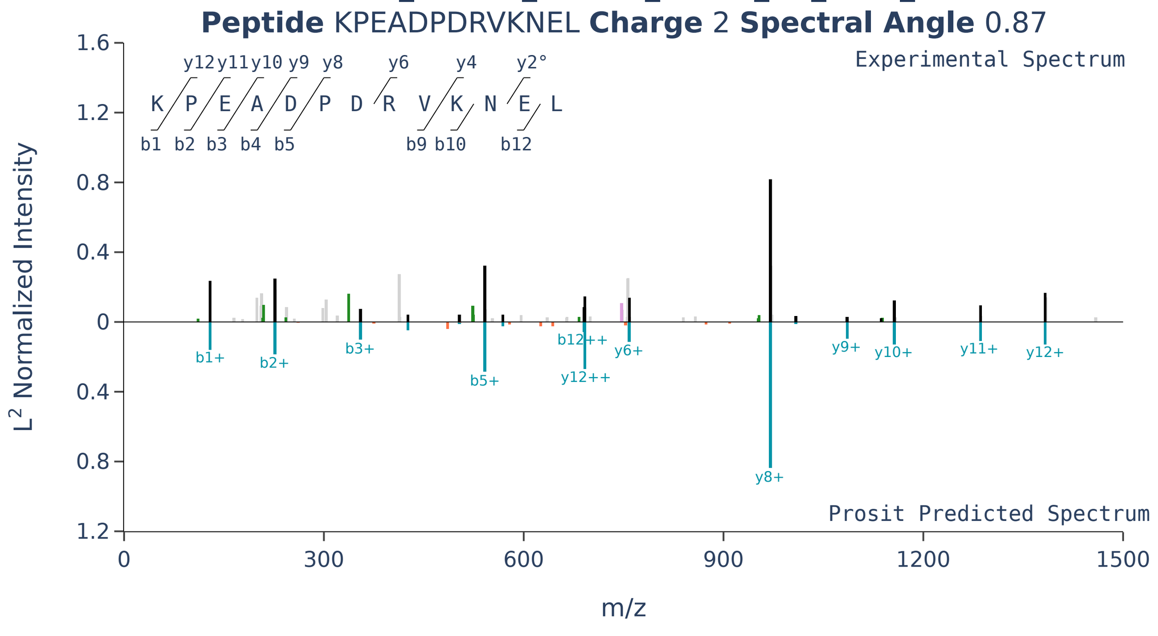


**Source** HRoetschke_211222_160123_HFGoe_IF2_b1R1_24h **Scan** 7635
**Peptide** KLSTDEVKVK **Charge** 3 **Spectral Angle** 0.88

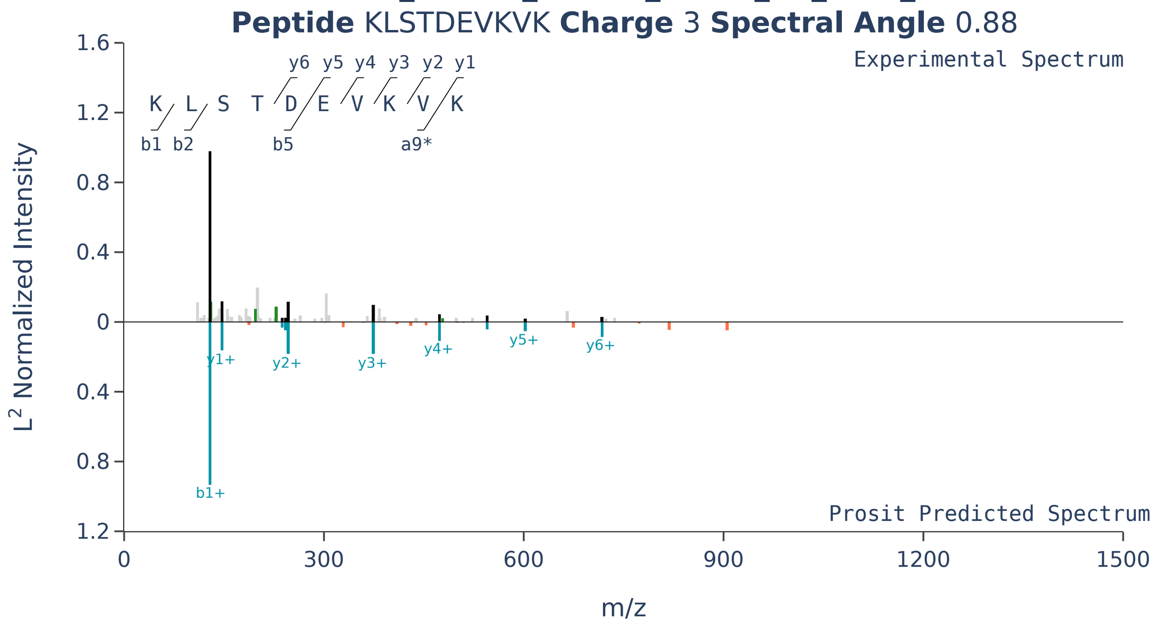


**Source** HRoetschke_211222_160123_HFGoe_IF2_b1R1_4h **Scan** 8744
**Peptide** TIKTLAAER **Charge** 2 **Spectral Angle** 0.82

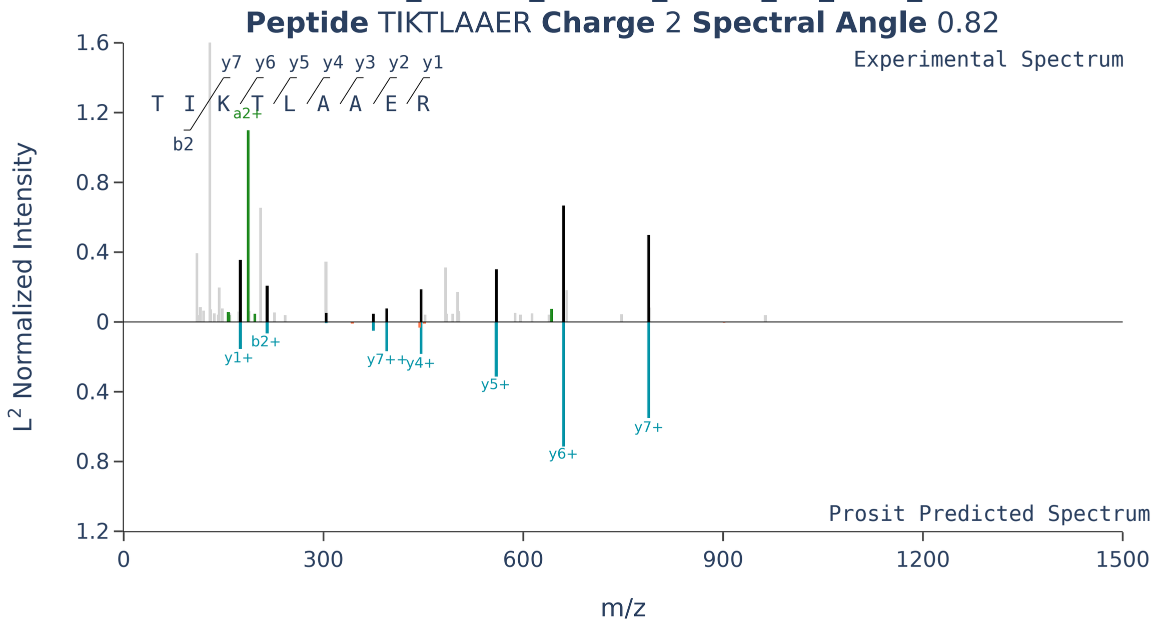

**Source** HRoetschke_211222_160123_HFGoe_IF2_b1R1_4h **Scan** 15683
**Peptide** DVTIKTLAAERQ **Charge** 3 **Spectral Angle** 0.85

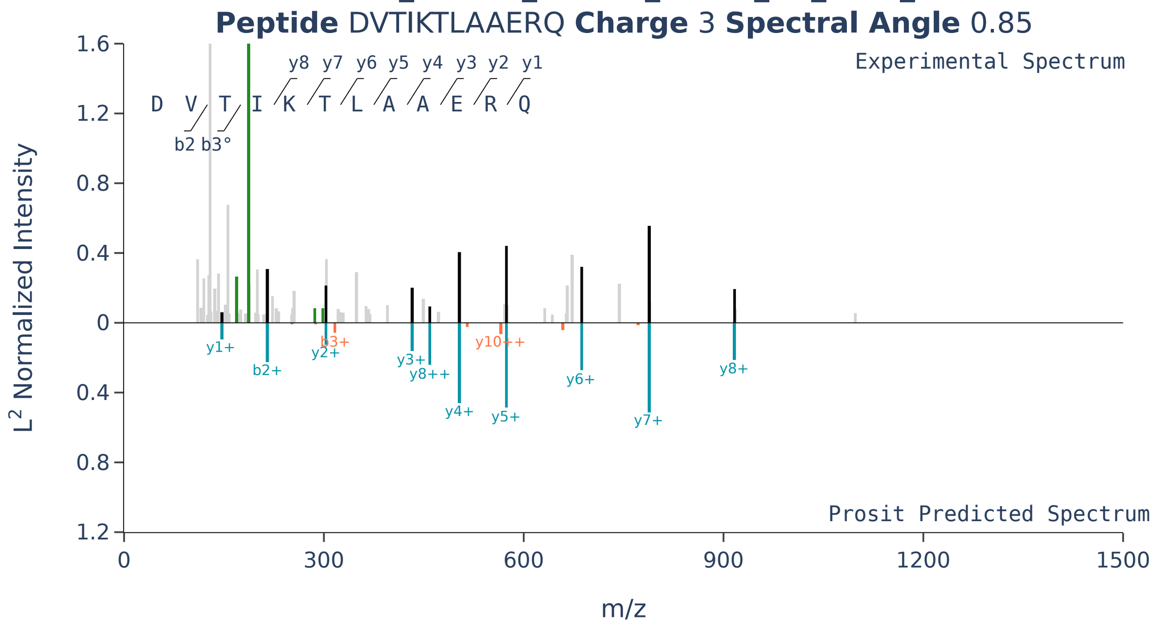


**Source** HRoetschke_211222_160123_HFGoe_IF2_b1R2_24h **Scan** 7206
**Peptide** IDKPEADPDRVKNE **Charge** 2 **Spectral Angle** 0.88

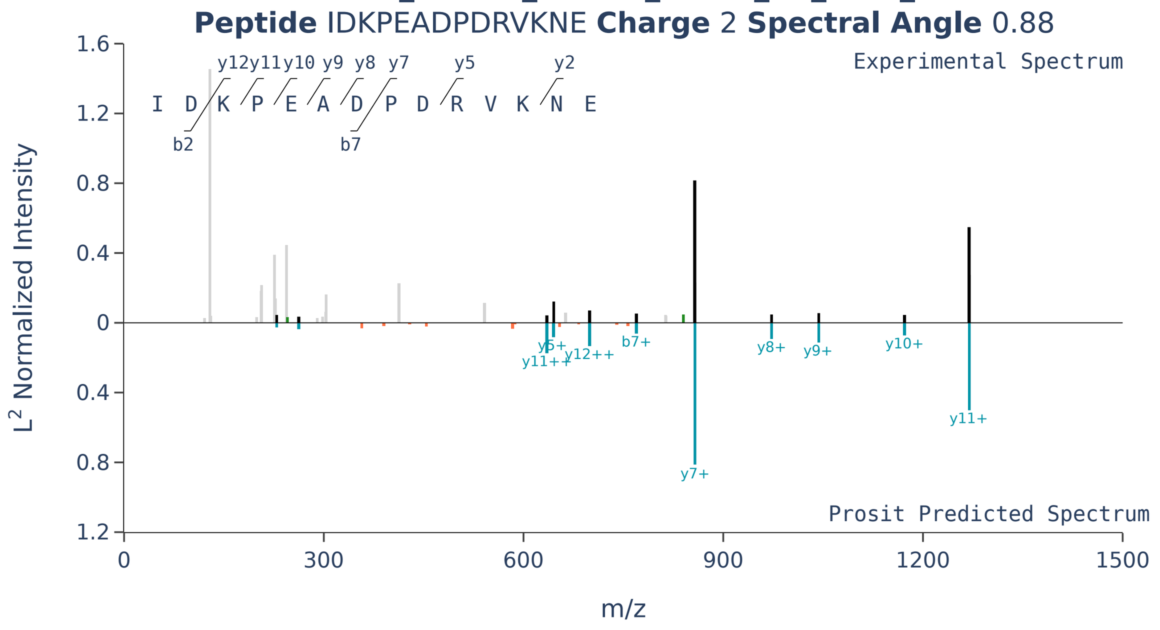


**Source** HRoetschke_211222_160123_HFGoe_IF2_b1R1_24h **Scan** 9118
**Peptide** STDEVKV **Charge** 2 **Spectral Angle** 0.9

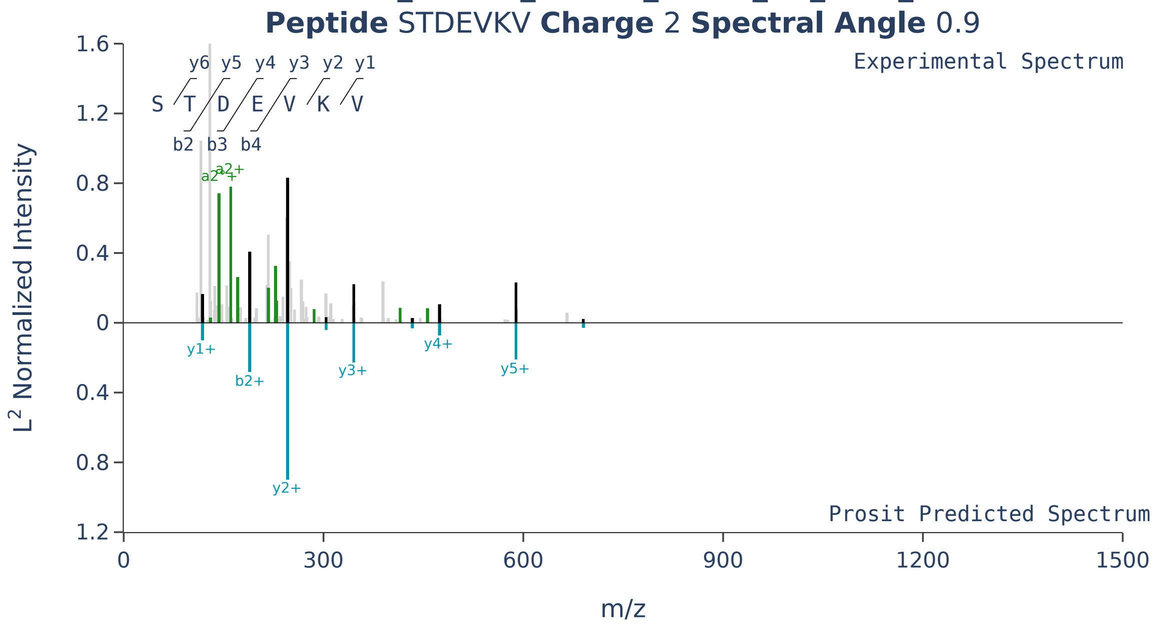


**Source** HRoetschke_211222_160123_HFGoe_IF2_b1R2_24h **Scan** 16232
**Peptide** GPSIPVE **Charge** 1 **Spectral Angle** 0.89

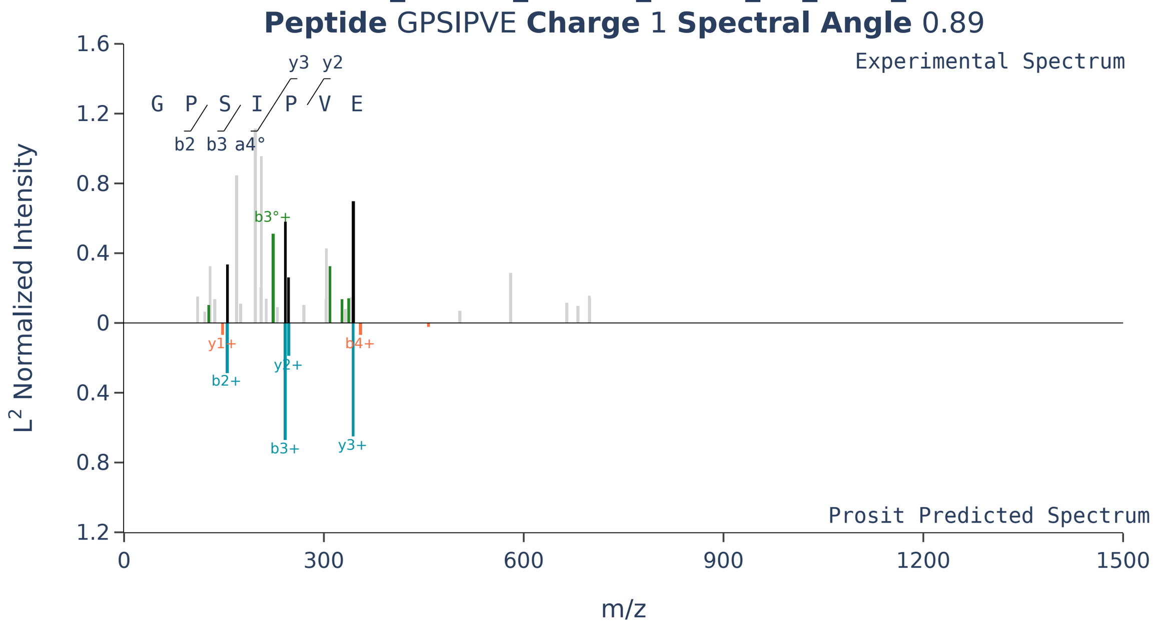


**Source** HRoetschke_211222_160123_HFGoe_IF2_b2R1_1h **Scan** 7491
**Peptide** NMTEGEVHE **Charge** 2 **Spectral Angle** 0.76

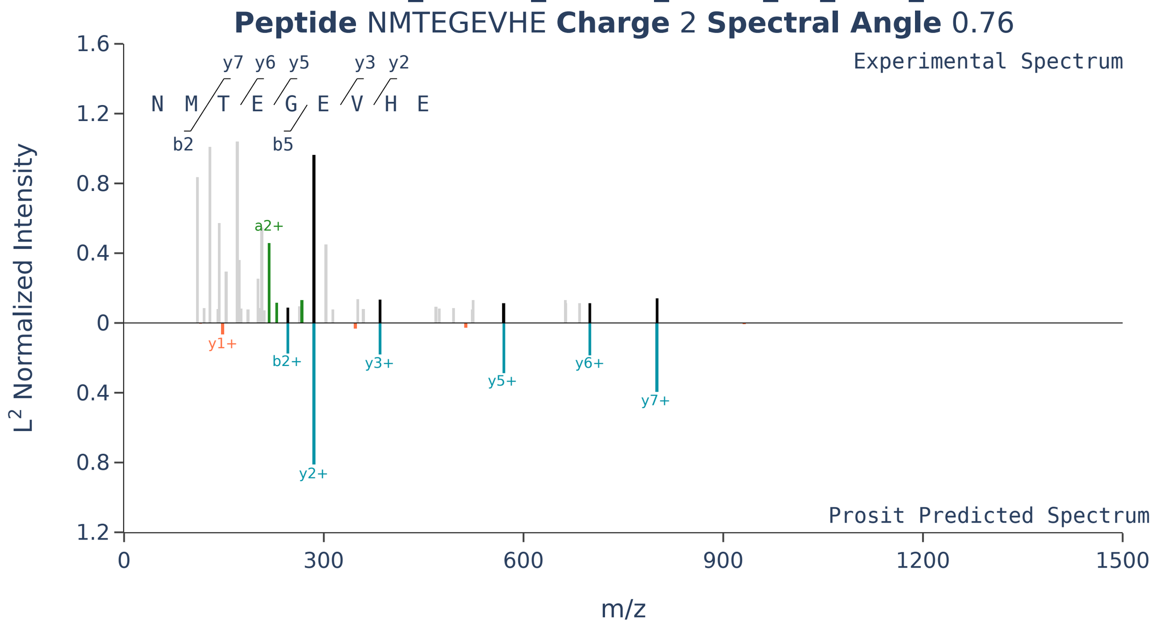


**Source** HRoetschke_211222_160123_HFGoe_IF2_b1R1_24h **Scan** 15169
**Peptide** TLHKGDIVL **Charge** 2 **Spectral Angle** 0.77

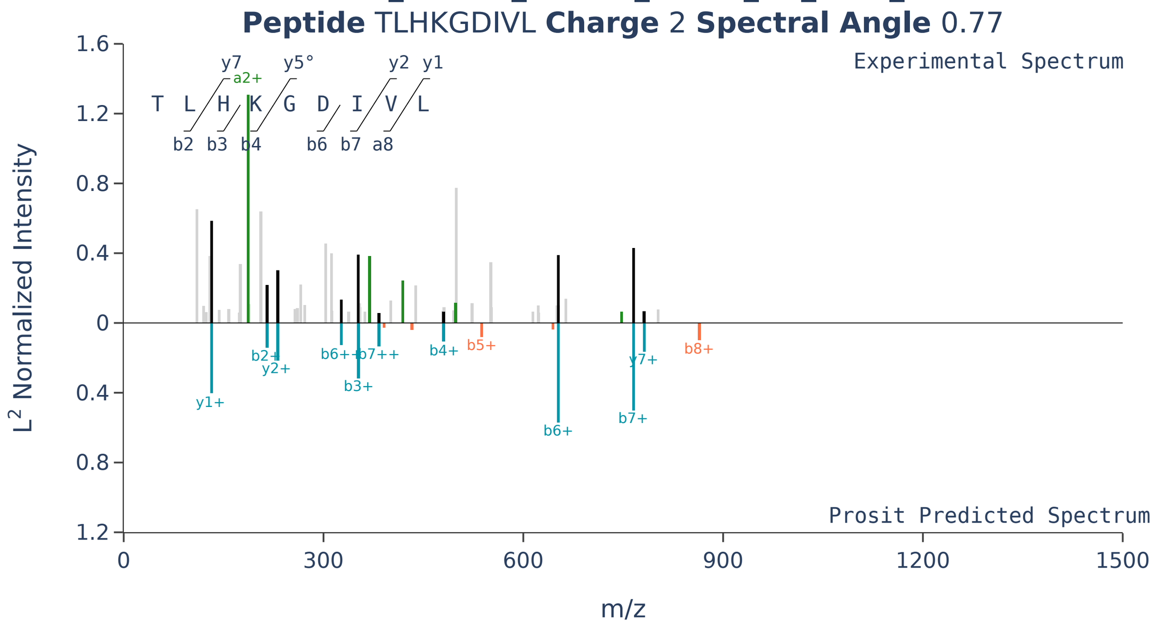


**Source** HRoetschke_211222_160123_HFGoe_IF2_b1R2_1h **Scan** 6525
**Peptide** GVKNYNDVRTGD **Charge** 3 **Spectral Angle** 0.88

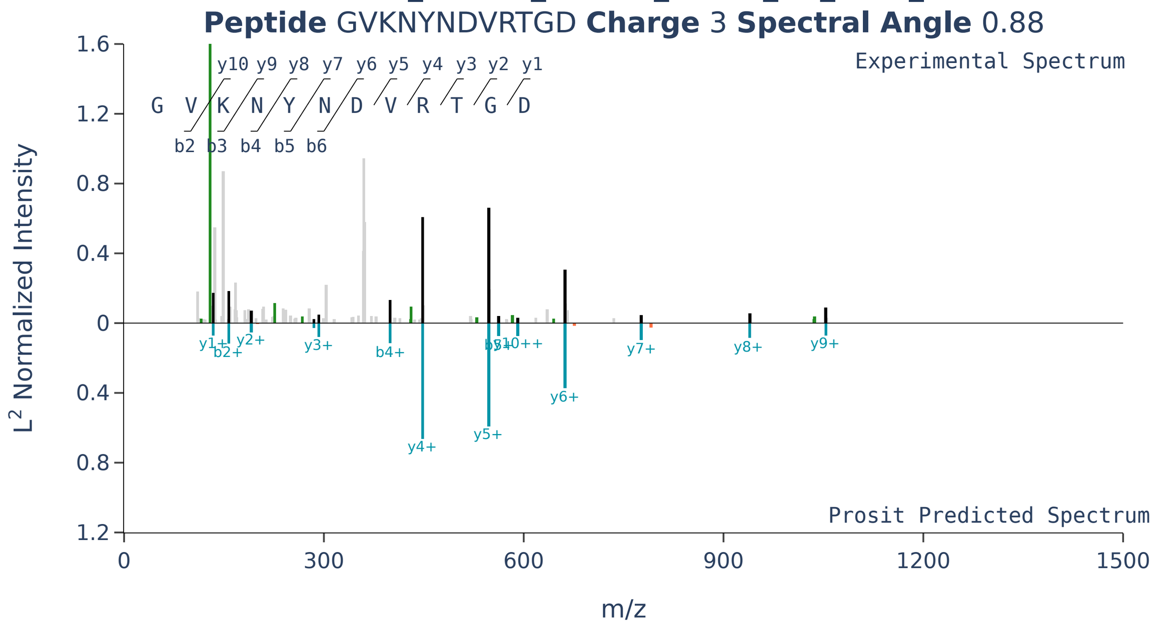


**Source** HRoetschke_211222_160123_HFGoe_IF2_b2R2_24h **Scan** 10932
**Peptide** PGHAAFT **Charge** 1 **Spectral Angle** 0.84

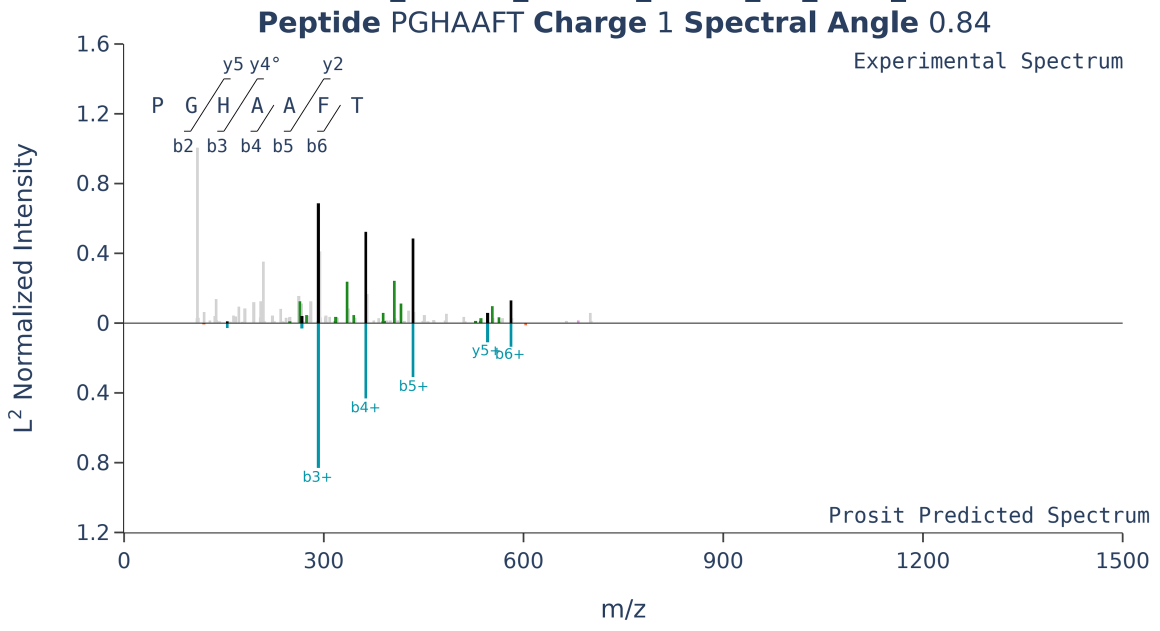


**Source** HRoetschke_211222_160123_HFGoe_IF2_b1R2_4h **Scan** 7652
**Peptide** QKPAQAVNRD **Charge** 2 **Spectral Angle** 0.97

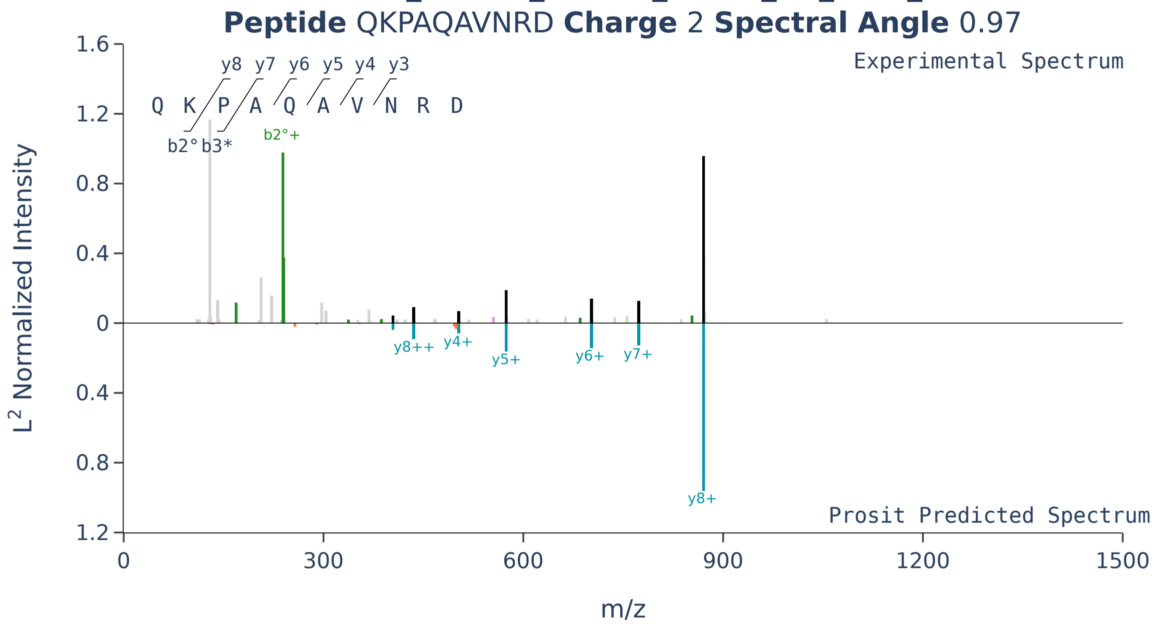


**Source** HRoetschke_211222_160123_HFGoe_IF2_b1R1_4h **Scan** 12479
**Peptide** ASGEAGGITQHIG **Charge** 1 **Spectral Angle** 0.83

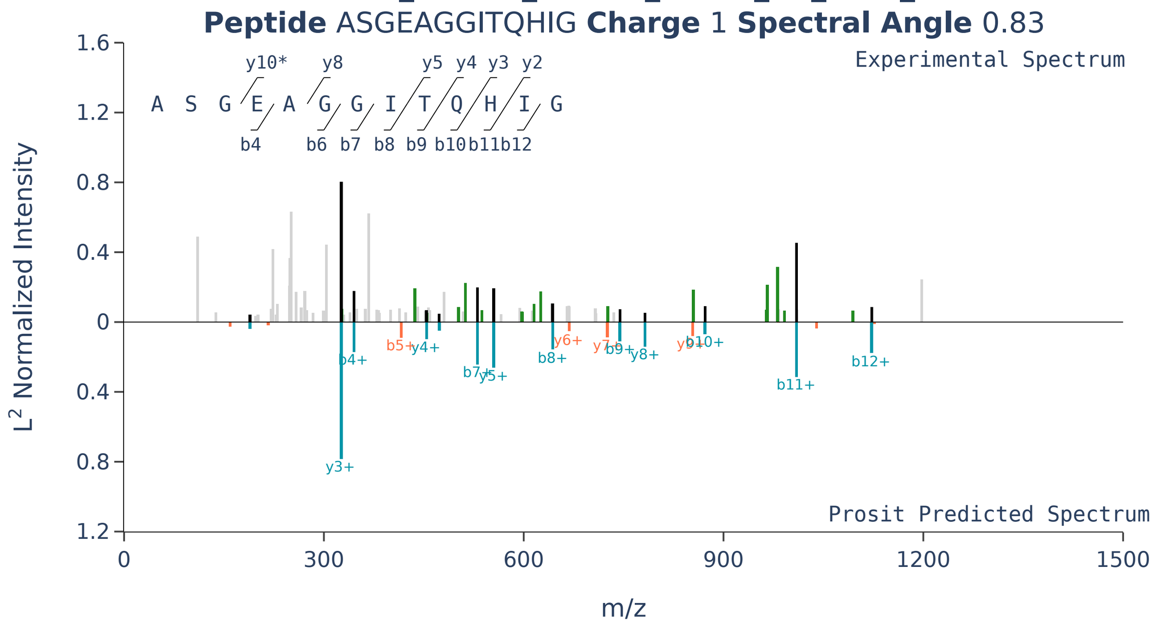


**Source** HRoetschke_211222_160123_HFGoe_IF2_b1R2_2h **Scan** 10063
**Peptide** RDPQEAERLAAEE **Charge** 3 **Spectral Angle** 0.71

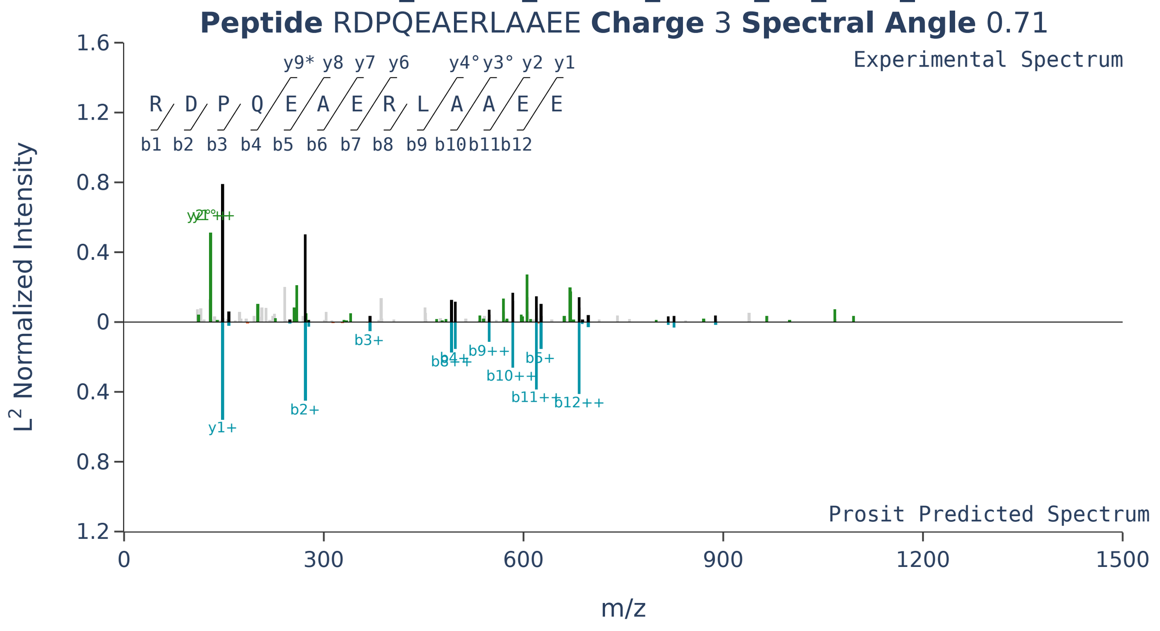


**Source** HRoetschke_211222_160123_HFGoe_IF2_b1R1_2h **Scan** 7727
**Peptide** ADAGIRK **Charge** 2 **Spectral Angle** 0.83

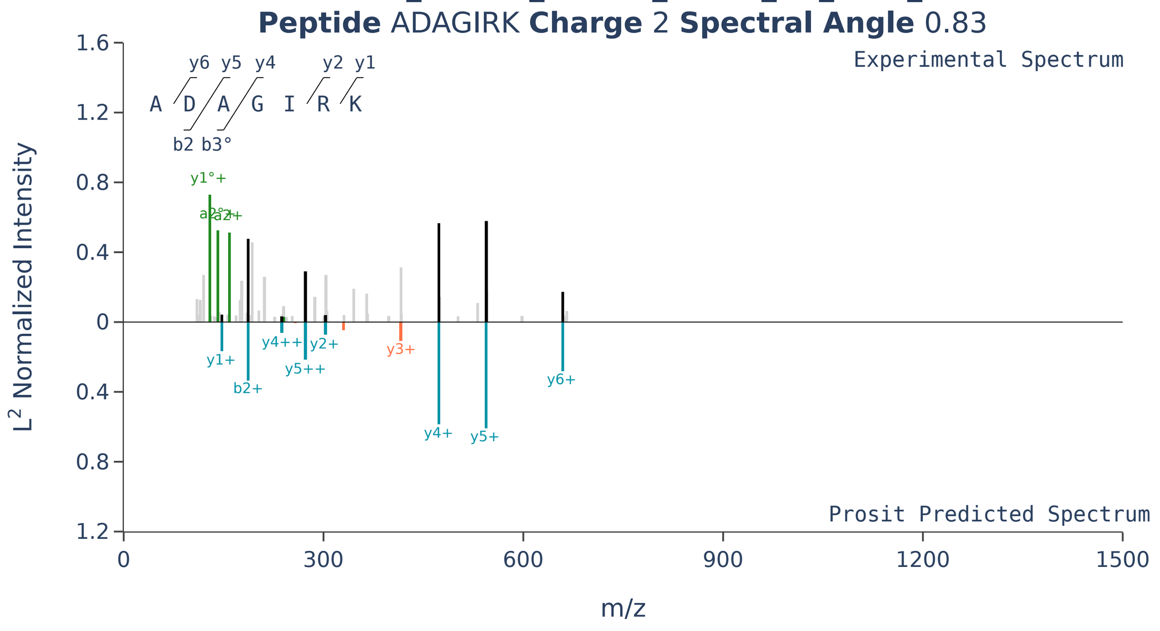


**Source** HRoetschke_211222_160123_HFGoe_IF2_b1R1_24h **Scan** 7931
**Peptide** VIDQETA **Charge** 2 **Spectral Angle** 0.74

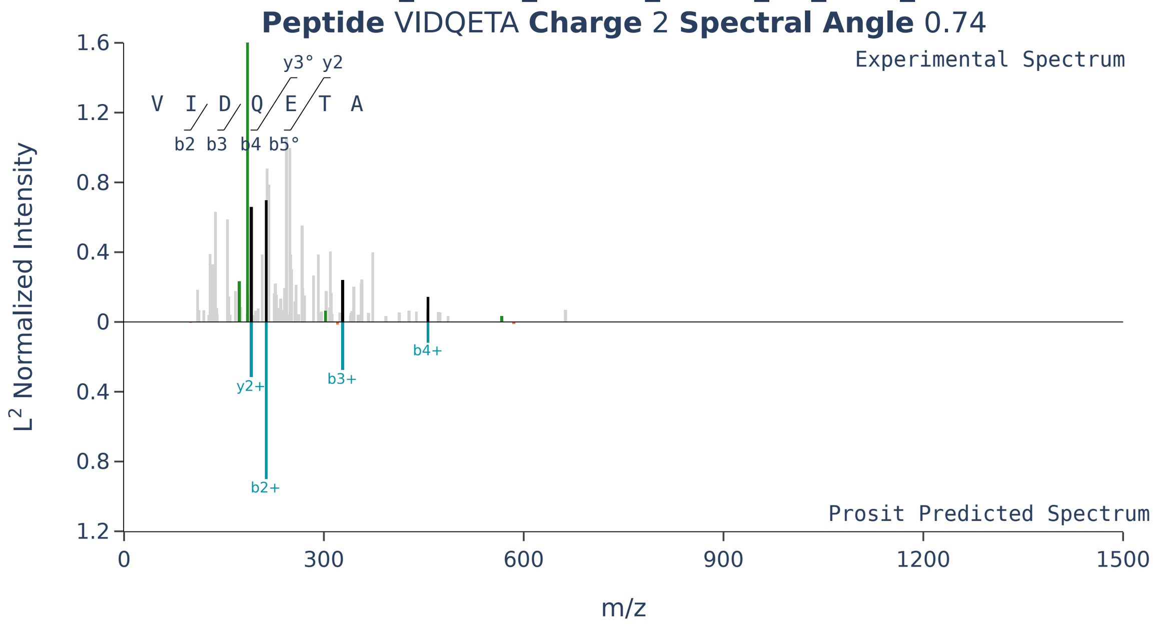


**Source** HRoetschke_211222_160123_HFGoe_IF2_b1R1_2h **Scan** 10300
**Peptide** PQEAERLAAEE **Charge** 2 **Spectral Angle** 0.78

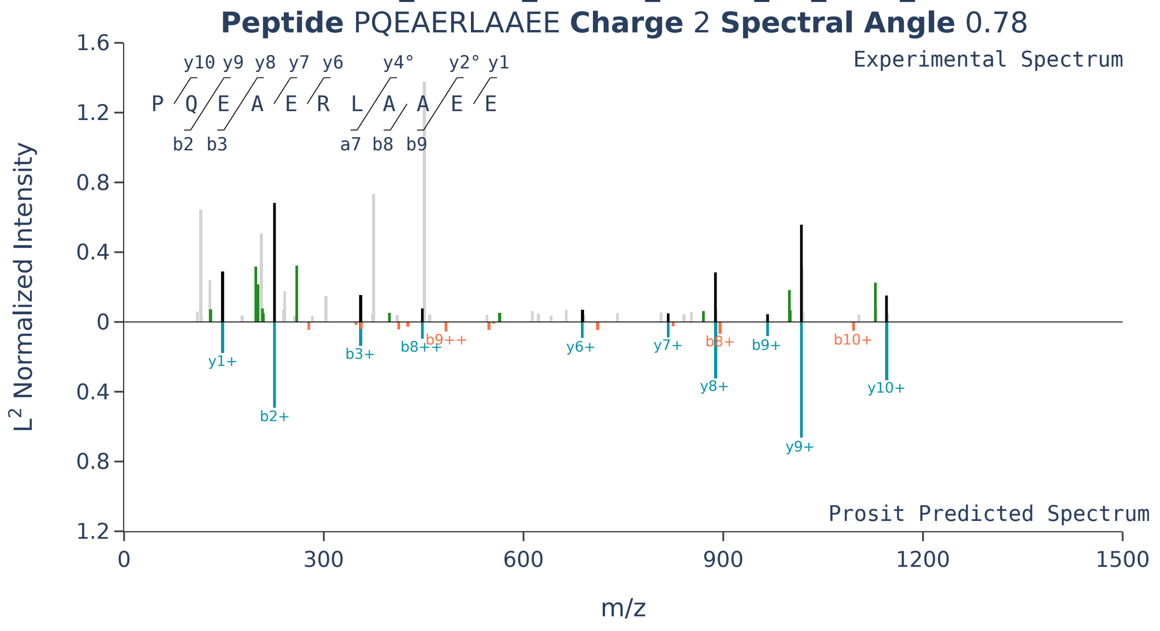


**Source** HRoetschke_211222_160123_HFGoe_IF2_b1R2_24h **Scan** 6974
**Peptide** VNKIDKPEADPDRVKNE **Charge** 3 **Spectral Angle** 0.83

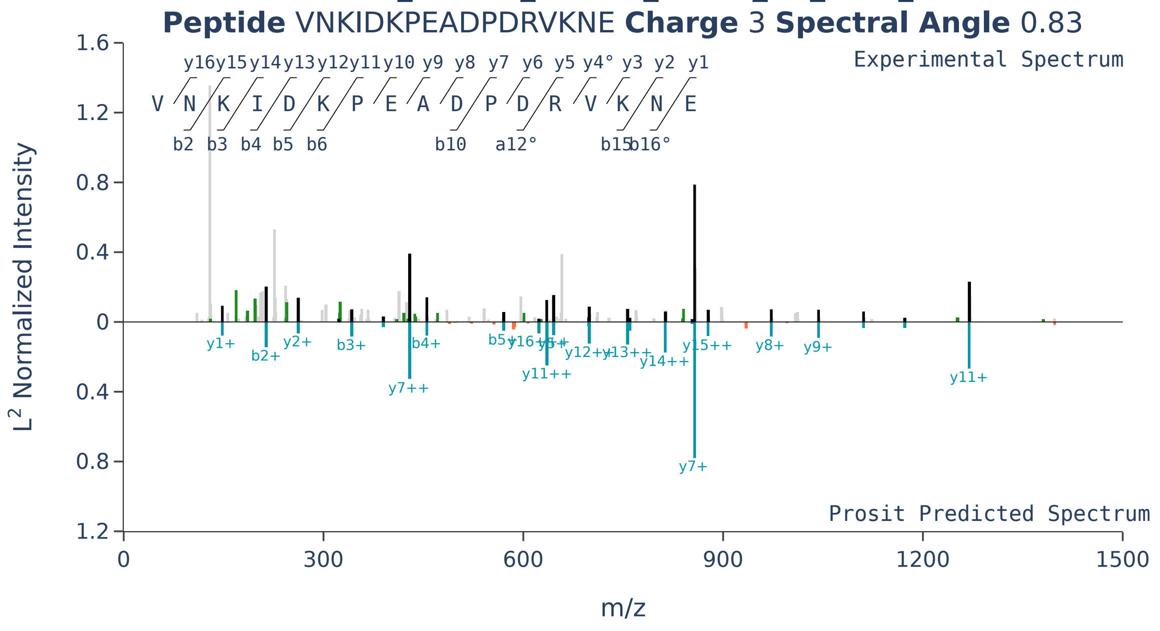

**Source** HRoetschke_211222_160123_HFGoe_IF2_b1R1_4h **Scan** 18572
**Peptide** KQQILGL **Charge** 1 **Spectral Angle** 0.78

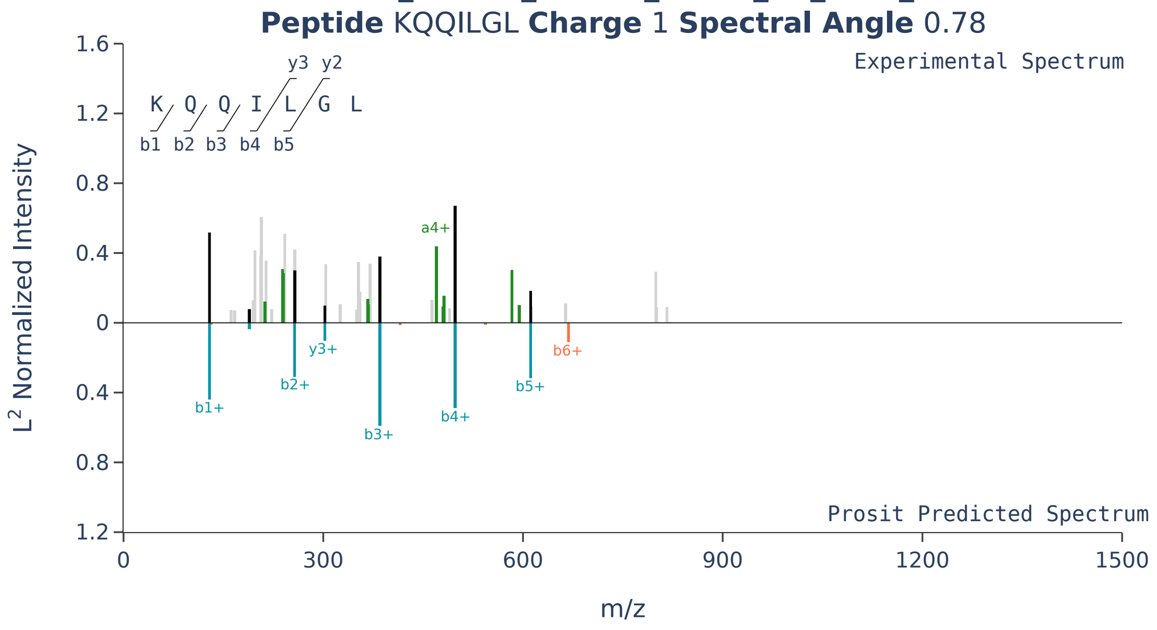


**Source** HRoetschke_211222_160123_HFGoe_IF2_b1R1_4h **Scan** 12612
**Peptide** KQTLIDHLNQ **Charge** 2 **Spectral Angle** 0.88

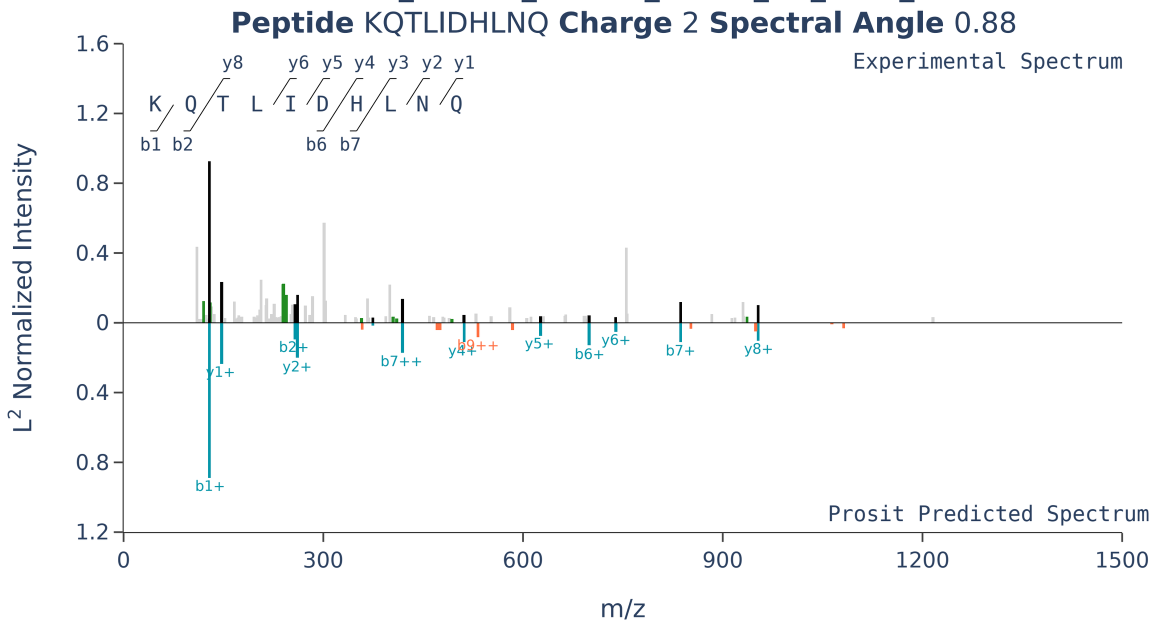


**Source** HRoetschke_211222_160123_HFGoe_IF2_b1R1_24h **Scan** 7891
**Peptide** AVKGSQVLKA **Charge** 2 **Spectral Angle** 0.74

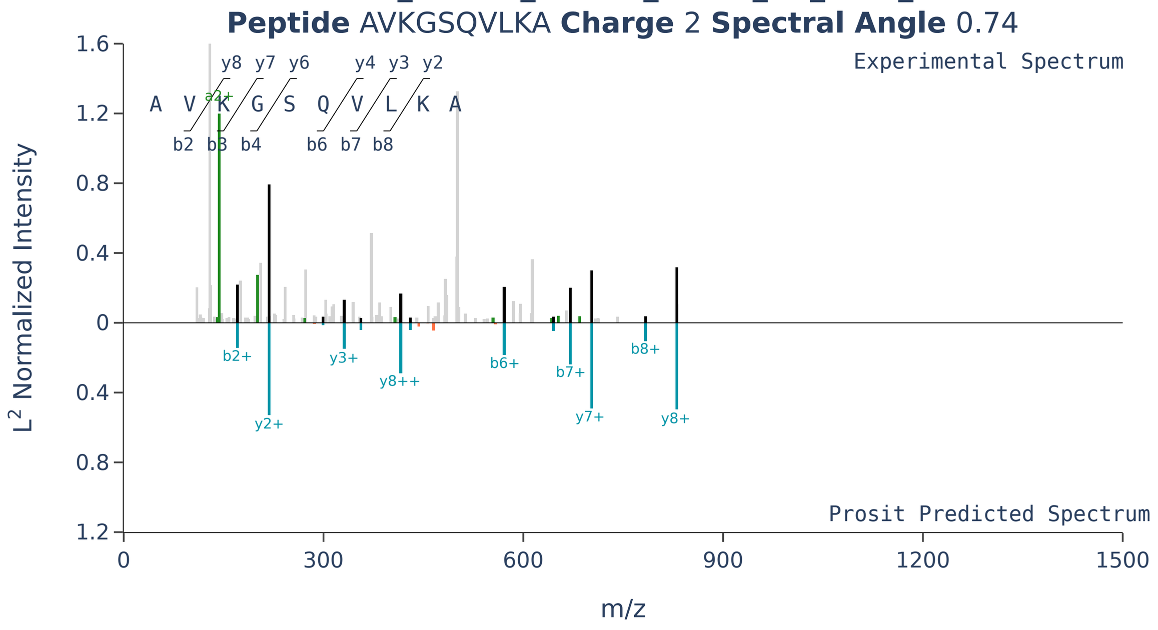


**Source** HRoetschke_211222_160123_HFGoe_IF2_b1R1_24h **Scan** 5994
**Peptide** EVTVVRDEKKA **Charge** 2 **Spectral Angle** 0.74

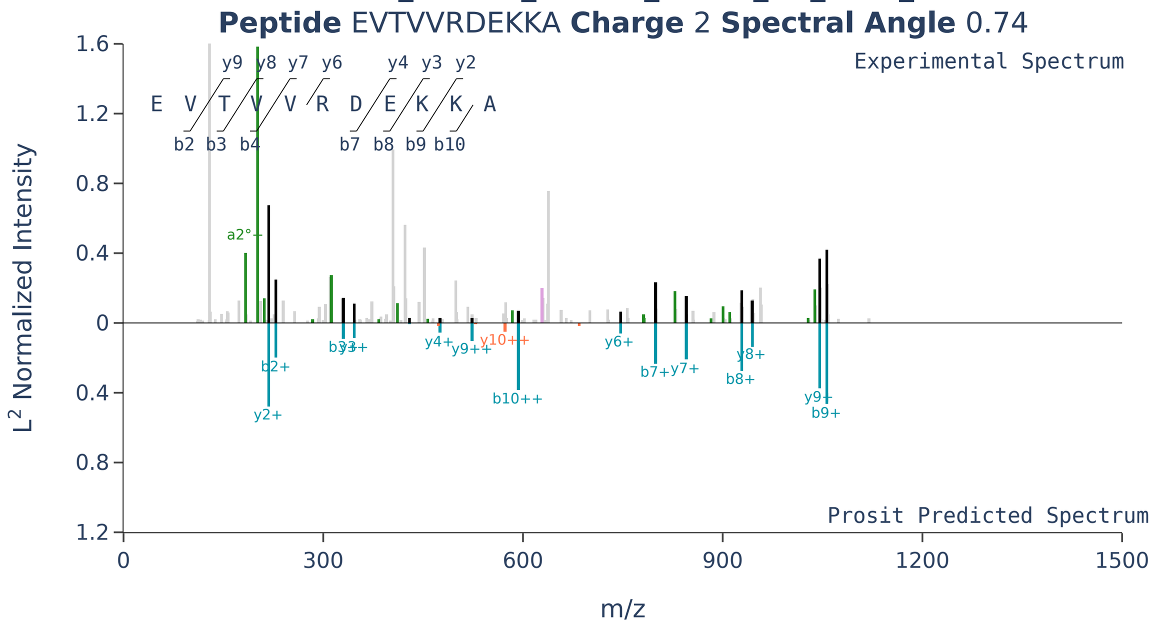


**Source** HRoetschke_211222_160123_HFGoe_IF2_b1R1_24h **Scan** 10321
**Peptide** KFREVKL **Charge** 2 **Spectral Angle** 0.79

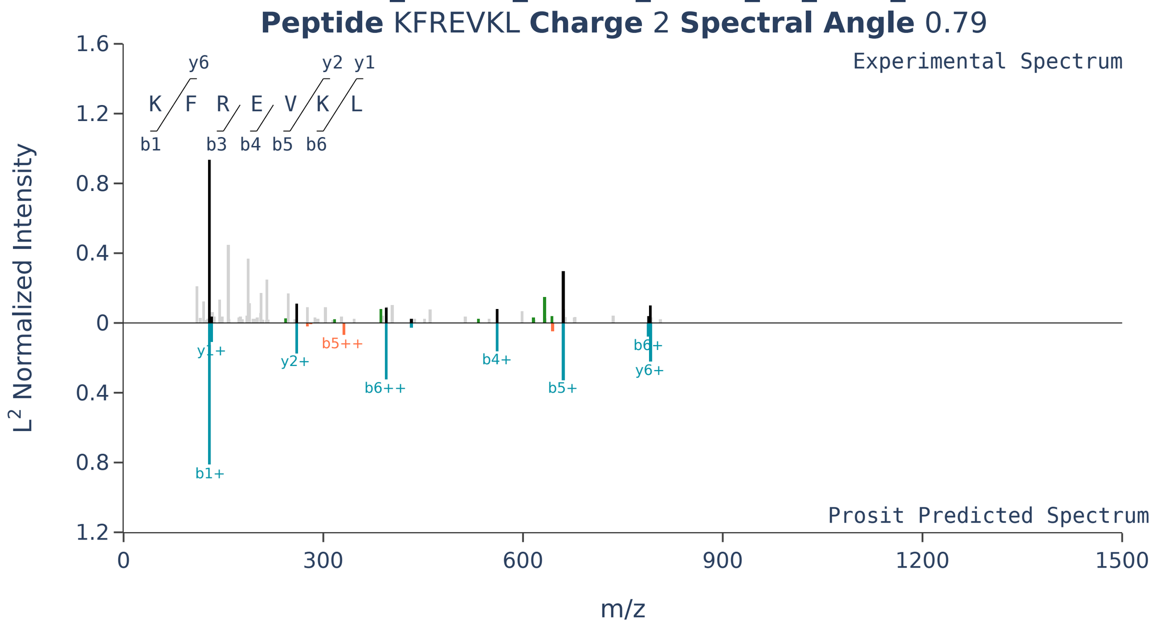


**Source** HRoetschke_211222_160123_HFGoe_IF2_b1R1_4h **Scan** 6499
**Peptide** LDKGRGPVAT **Charge** 2 **Spectral Angle** 0.74

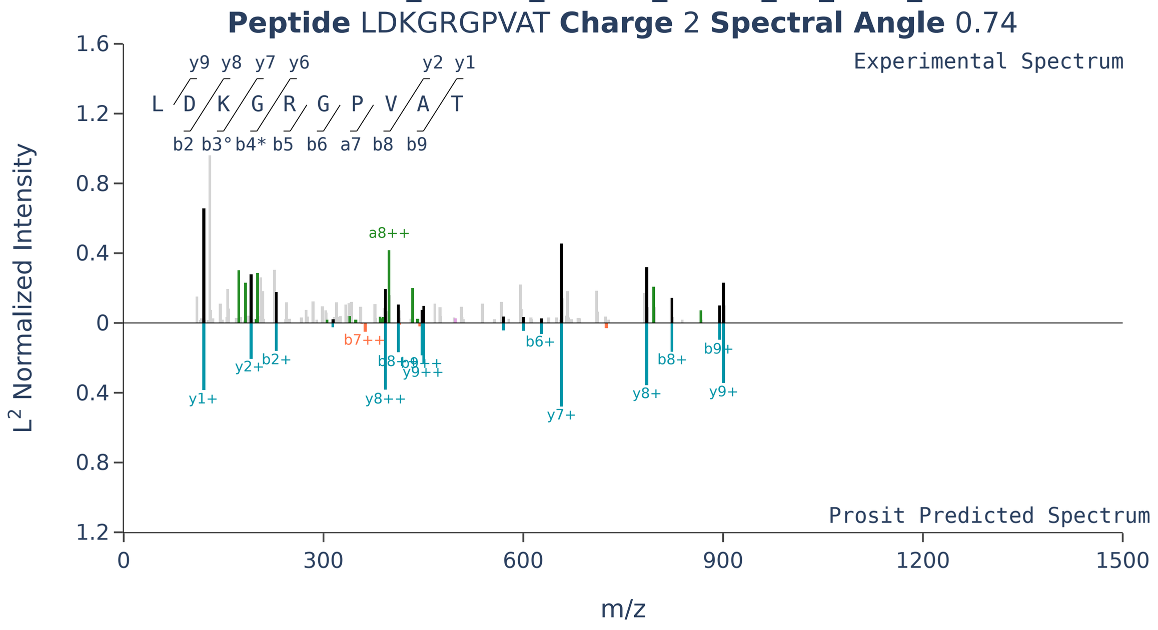


**Source** HRoetschke_211222_160123_HFGoe_IF2_b1R2_2h **Scan** 12517
**Peptide** GSSLQQGFQKPAQAVNRD **Charge** 3 **Spectral Angle** 0.87

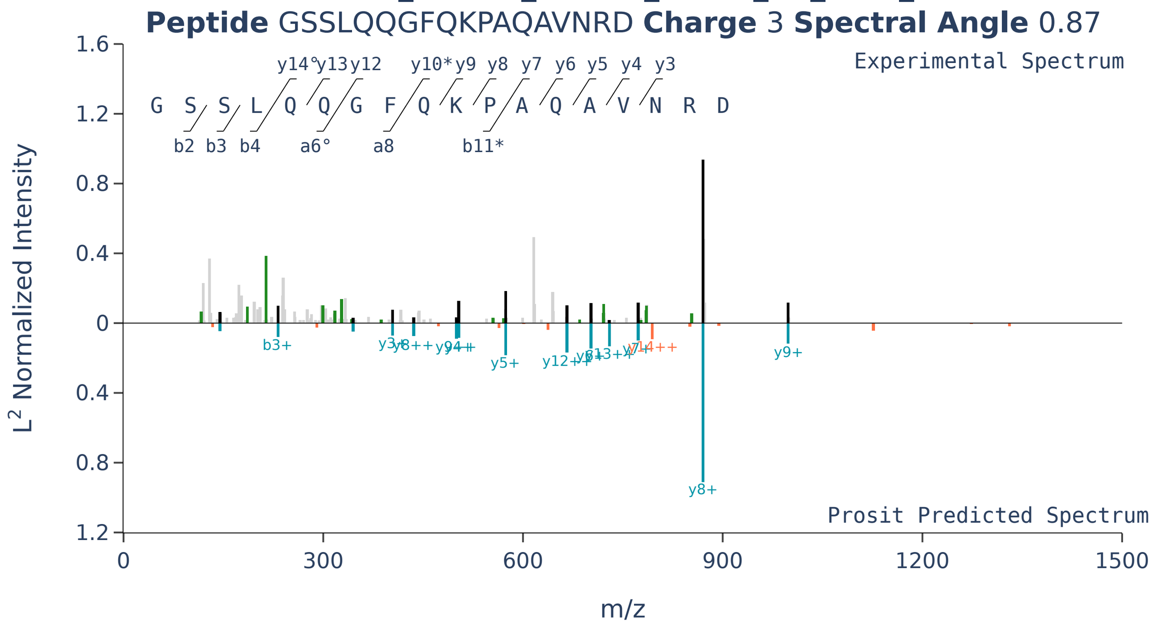


**Source** HRoetschke_211222_160123_HFGoe_IF2_b1R1_4h **Scan** 8103
**Peptide** MGHKVILRRENEL **Charge** 3 **Spectral Angle** 0.91

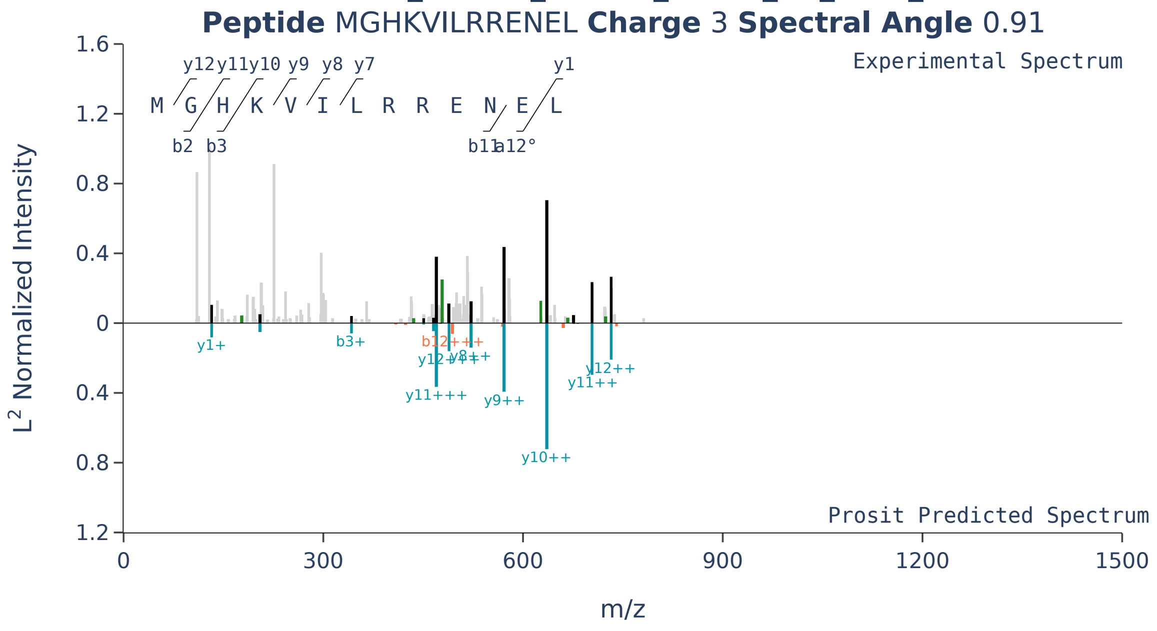


**Source** HRoetschke_211222_160123_HFGoe_IF2_b1R2_24h **Scan** 13364
**Peptide** DPQEAERLAAEEQAQRE **Charge** 3 **Spectral Angle** 0.75

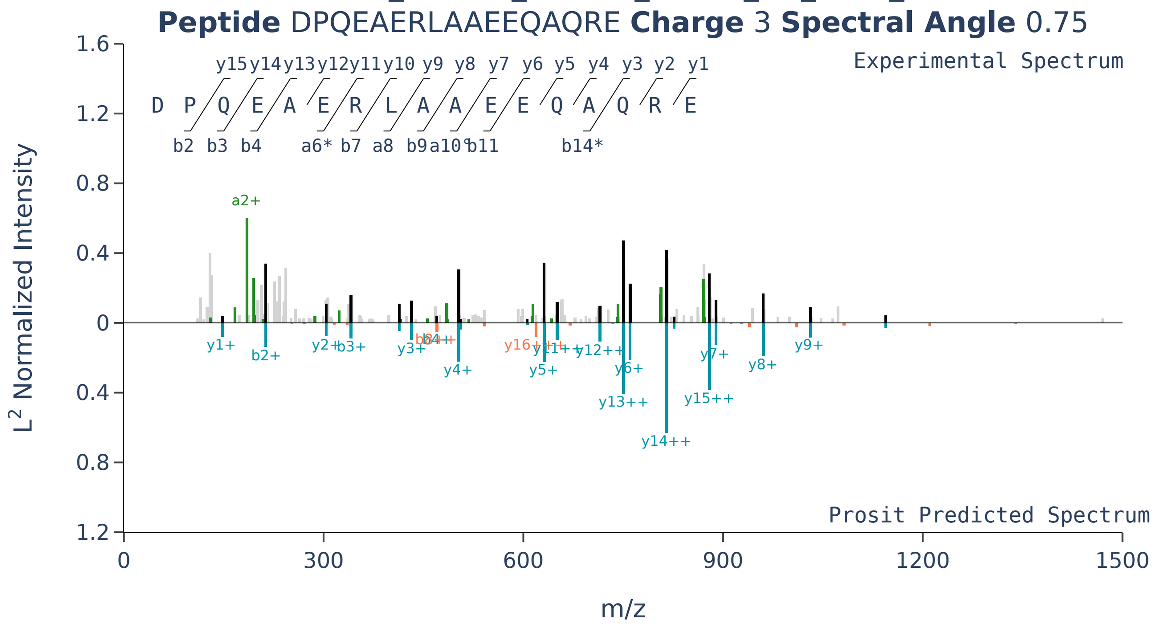


**Source** HRoetschke_211222_160123_HFGoe_IF2_b1R2_2h **Scan** 5536
**Peptide** TVVRDEKKARE **Charge** 3 **Spectral Angle** 0.89

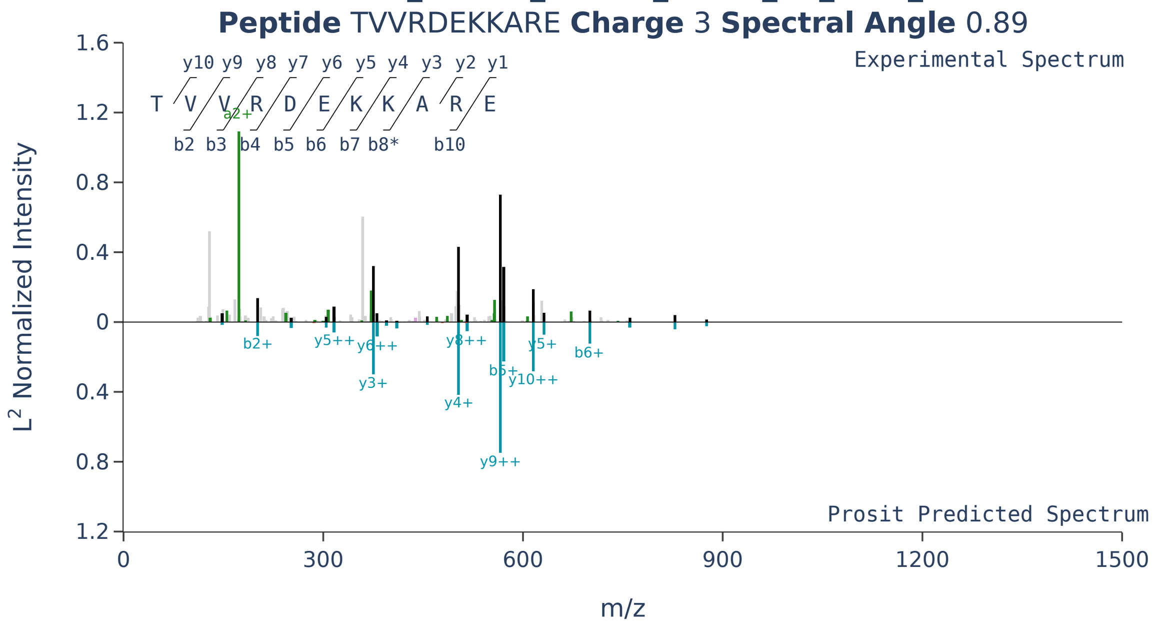


**Source** HRoetschke_211222_160123_HFGoe_IF2_b1R1_4h **Scan** 8654
**Peptide** NQKNSGPDKLTLQR **Charge** 4 **Spectral Angle** 0.82

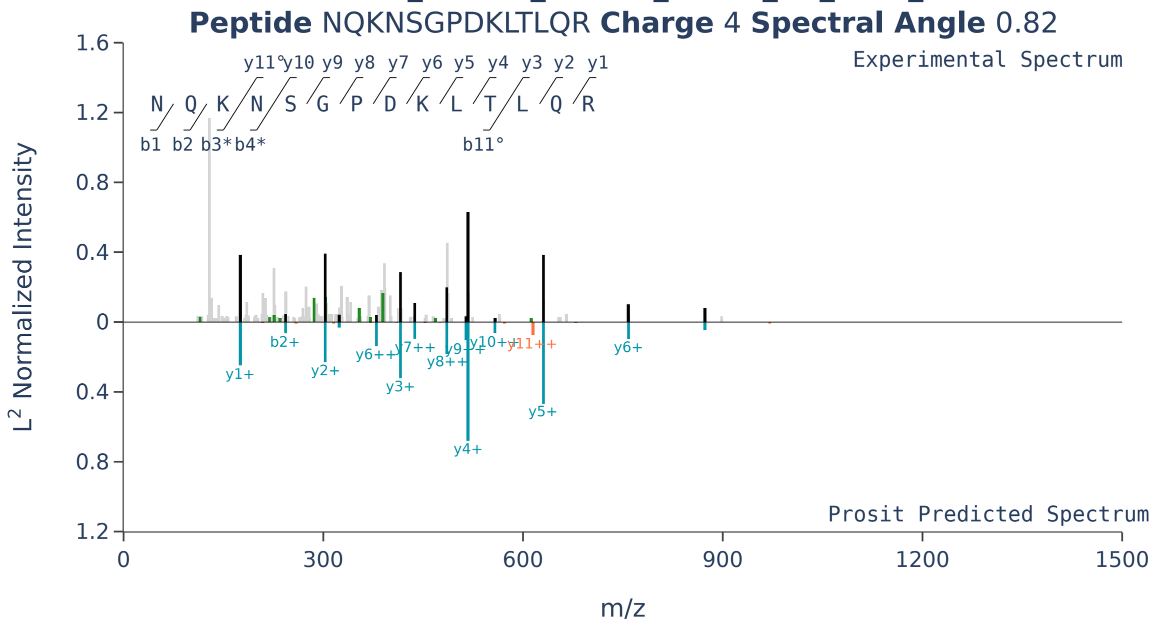


**Source** HRoetschke_211222_160123_HFGoe_IF2_b2R2_24h **Scan** 14485
**Peptide** QETAQLVA **Charge** 2 **Spectral Angle** 0.84

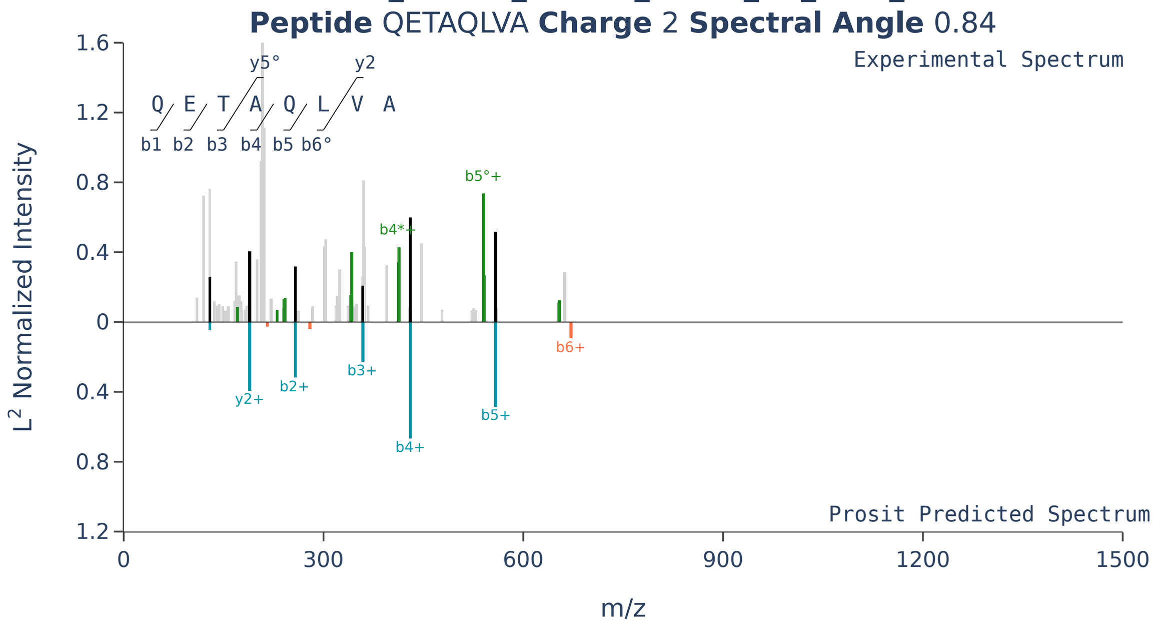


**Source** HRoetschke_211222_160123_HFGoe_IF2_b1R2_4h **Scan** 11375
**Peptide** GVGGITE **Charge** 1 **Spectral Angle** 0.88

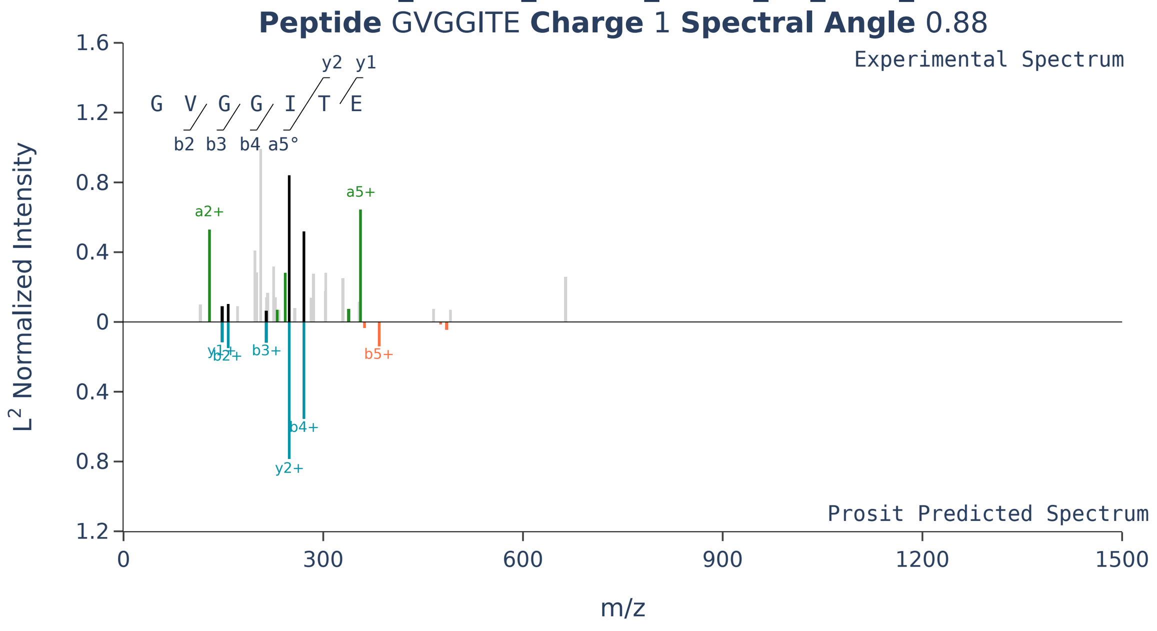


**Source** HRoetschke_211222_160123_HFGoe_IF2_b1R2_24h **Scan** 7205
**Peptide** LEEEARRVAE **Charge** 3 **Spectral Angle** 0.78

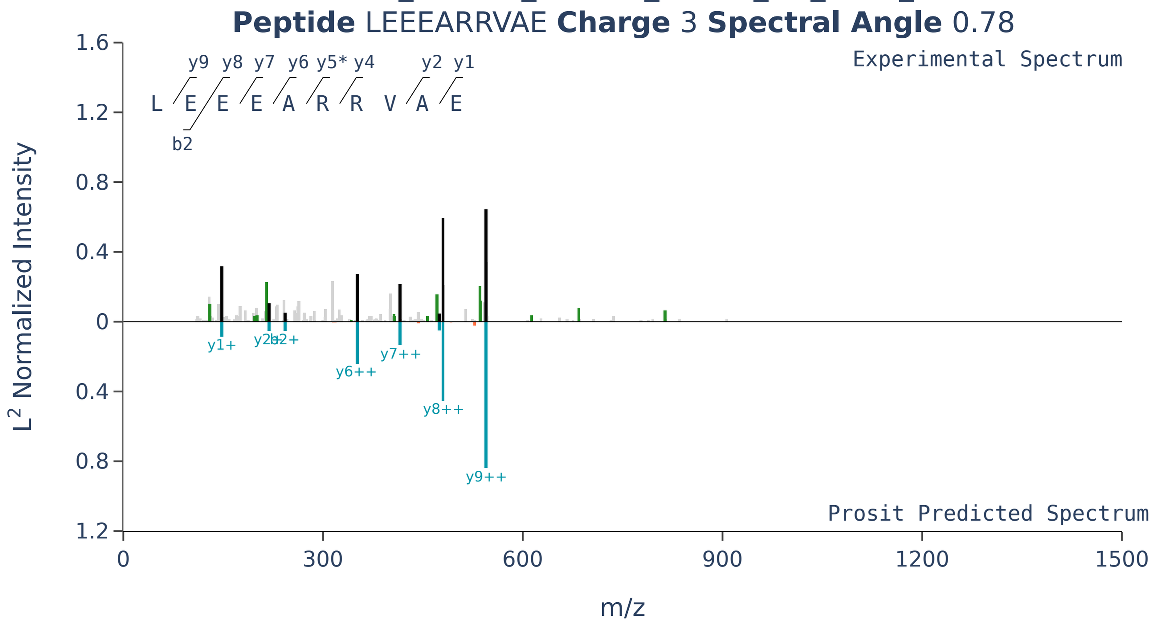


**Source** HRoetschke_211222_160123_HFGoe_IF2_b1R1_24h **Scan** 7625
**Peptide** RREQEAAEL **Charge** 3 **Spectral Angle** 0.84

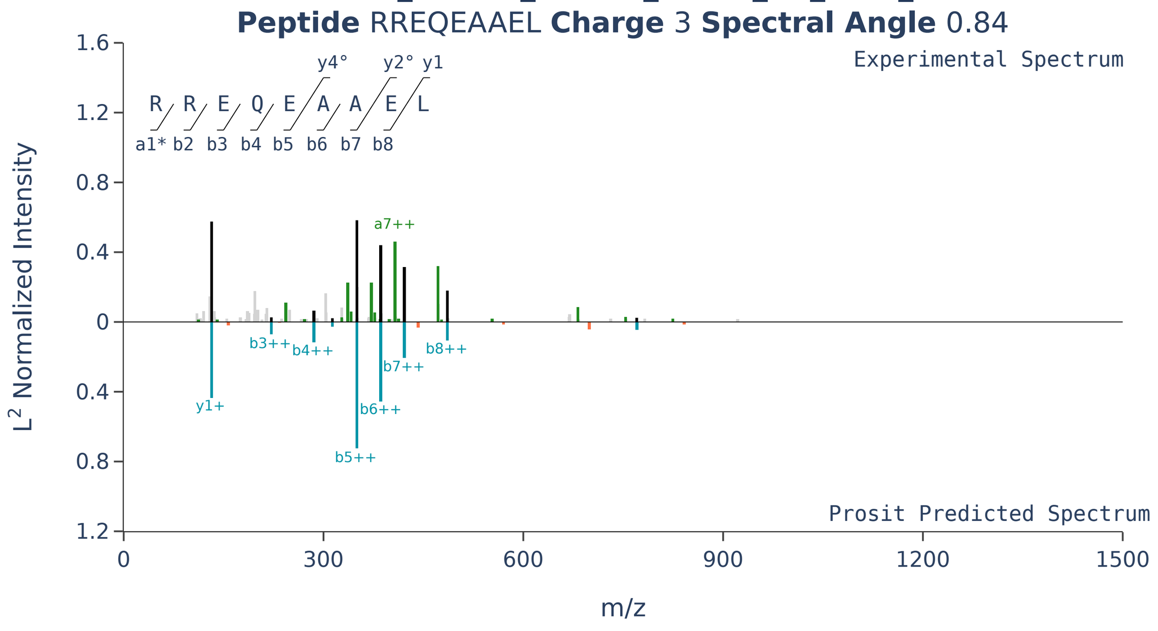

**Source** HRoetschke_211222_160123_HFGoe_IF2_b1R1_4h **Scan** 7326
**Peptide** VIDQETAQ **Charge** 2 **Spectral Angle** 0.92

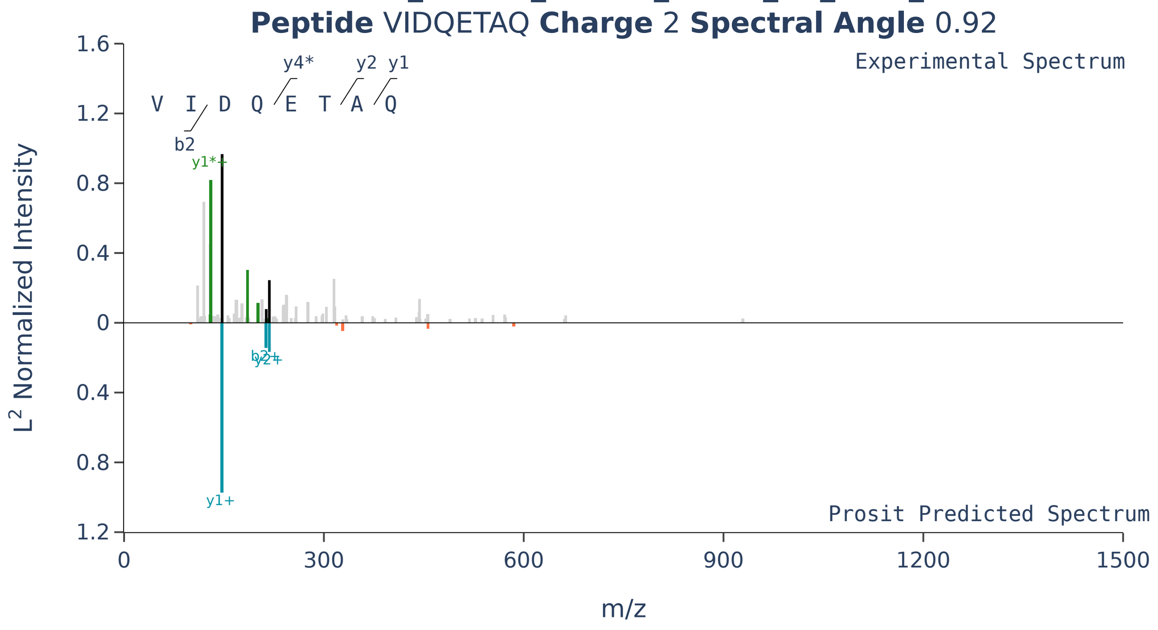


**Source** HRoetschke_211222_160123_HFGoe_IF2_b2R2_2h **Scan** 6678
**Peptide** DEVTVVRDEKKARE **Charge** 4 **Spectral Angle** 0.85

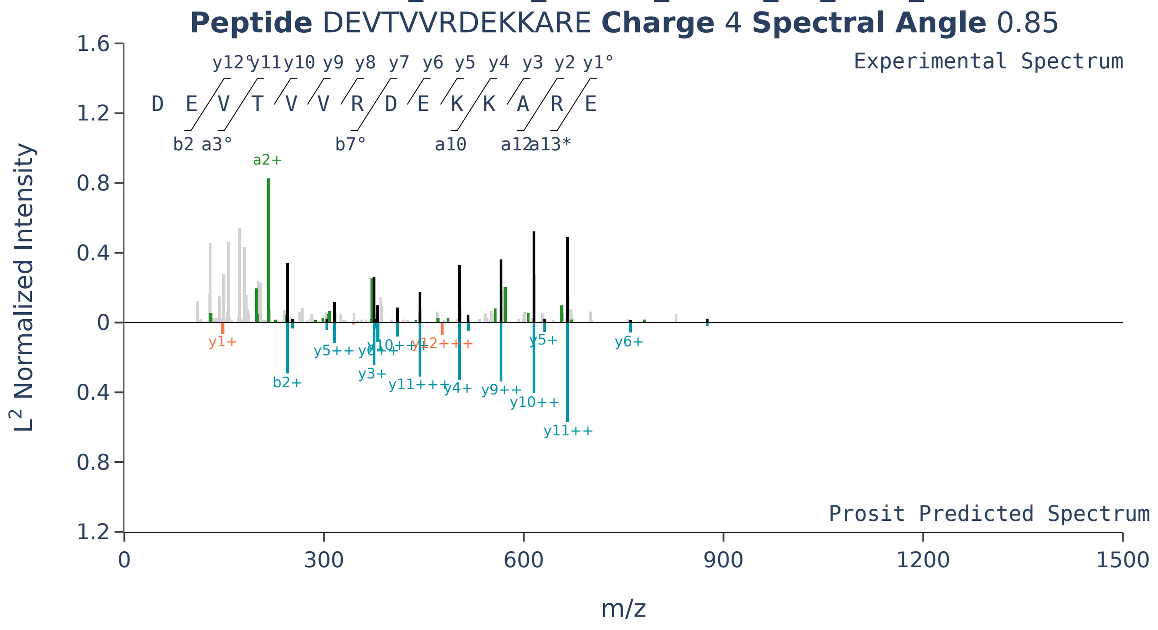


**Source** HRoetschke_211222_160123_HFGoe_IF2_b1R2_1h **Scan** 11409
**Peptide** PEADPDRVKNELSQ **Charge** 2 **Spectral Angle** 0.82

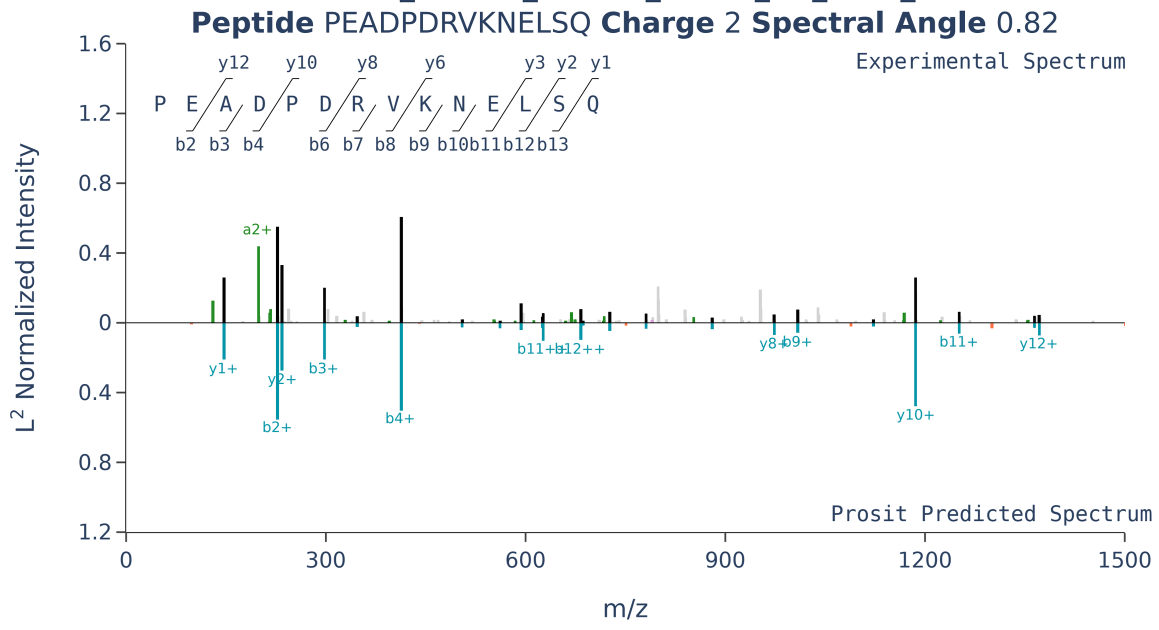


**Source** HRoetschke_211222_160123_HFGoe_IF2_b2R2_2h **Scan** 8962
**Peptide** RKGMASGAVLE **Charge** 3 **Spectral Angle** 0.91

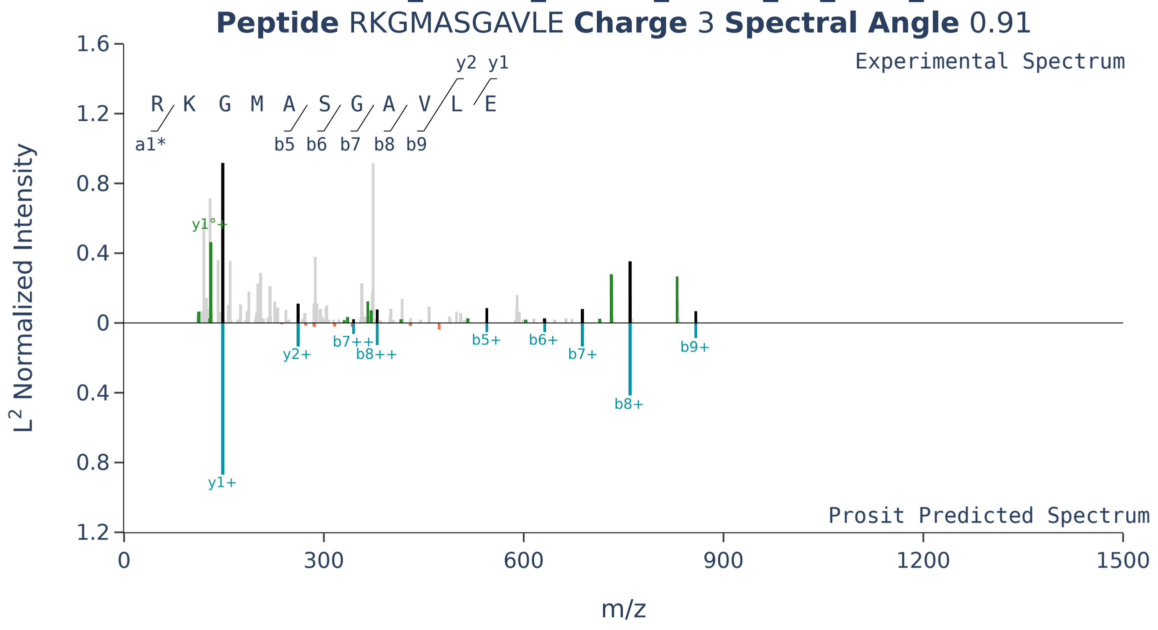


**Source** HRoetschke_211222_160123_HFGoe_IF2_b2R2_24h **Scan** 14036
**Peptide** KADVQGSVEAI **Charge** 2 **Spectral Angle** 0.71

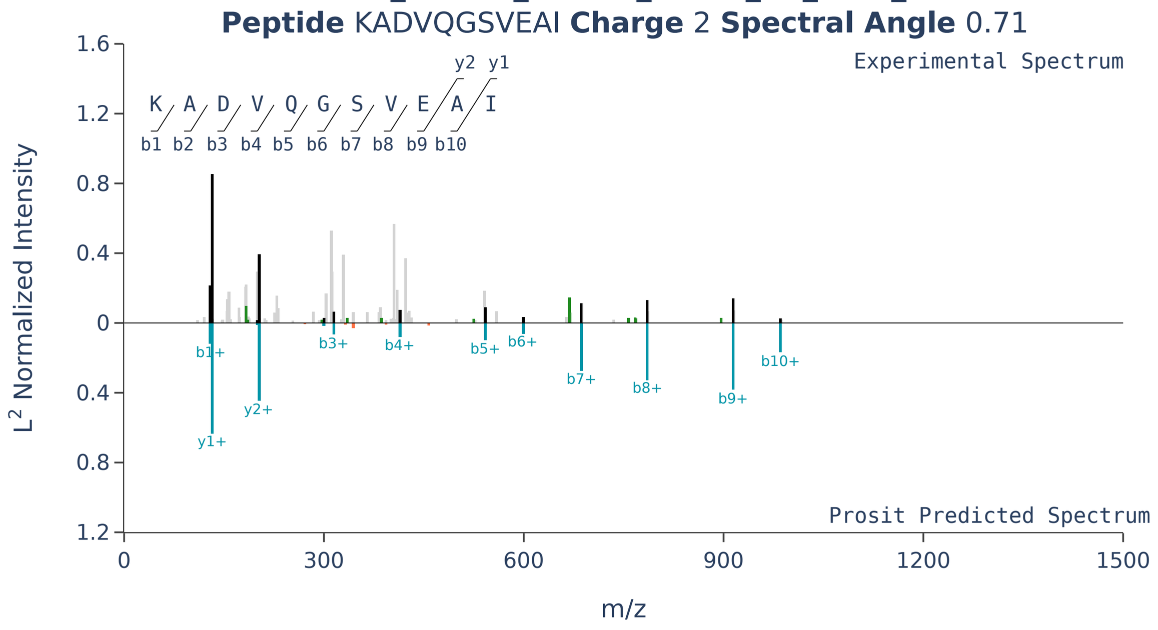


**Source** HRoetschke_211222_160123_HFGoe_IF2_b1R1_4h **Scan** 14226
**Peptide** DDGVMPQTIE **Charge** 2 **Spectral Angle** 0.83

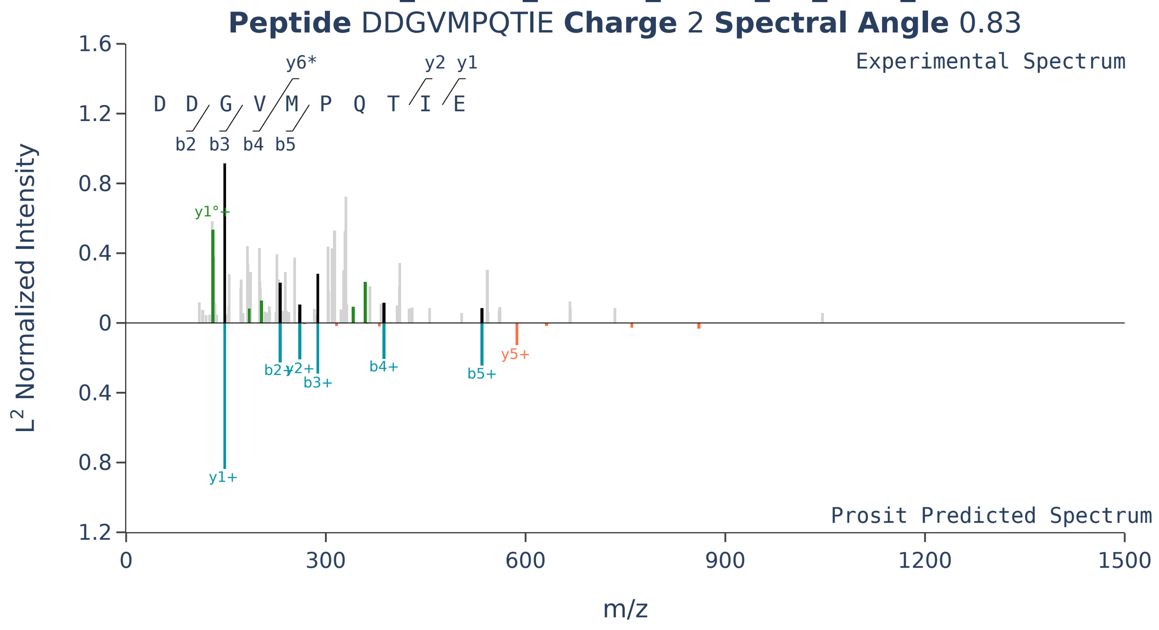


**Source** HRoetschke_211222_160123_HFGoe_IF2_b2R2_4h **Scan** 17644
**Peptide** IIEIQRTIA **Charge** 2 **Spectral Angle** 0.89

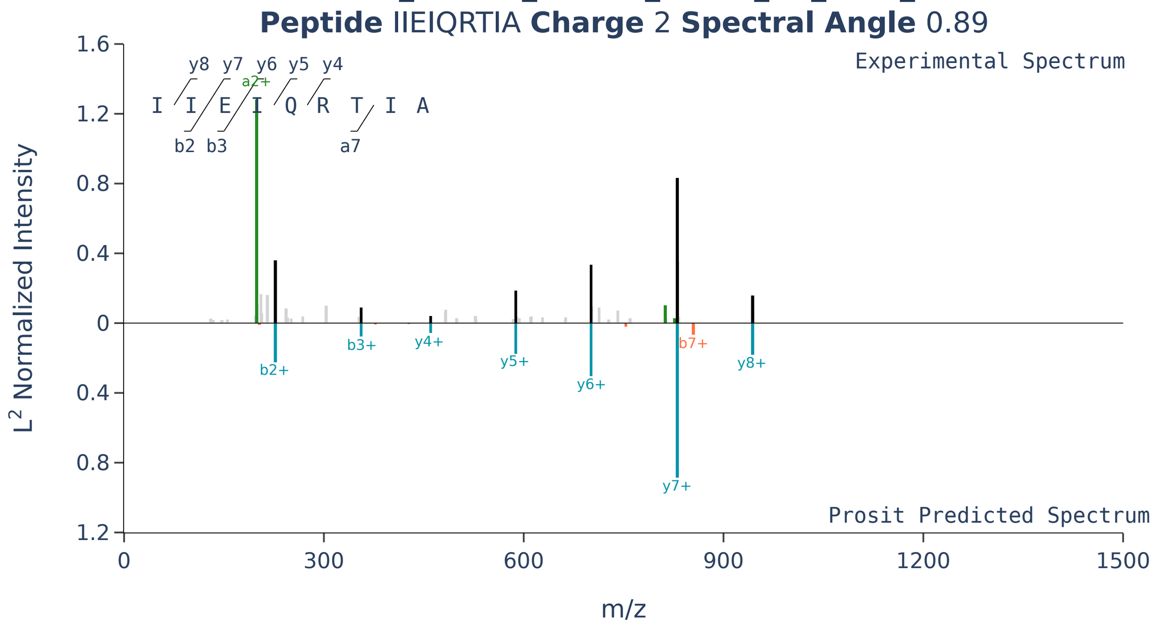


**Source** HRoetschke_211222_160123_HFGoe_IF2_b1R2_1h **Scan** 17547
**Peptide** TDVTIKTLAAE **Charge** 2 **Spectral Angle** 0.85

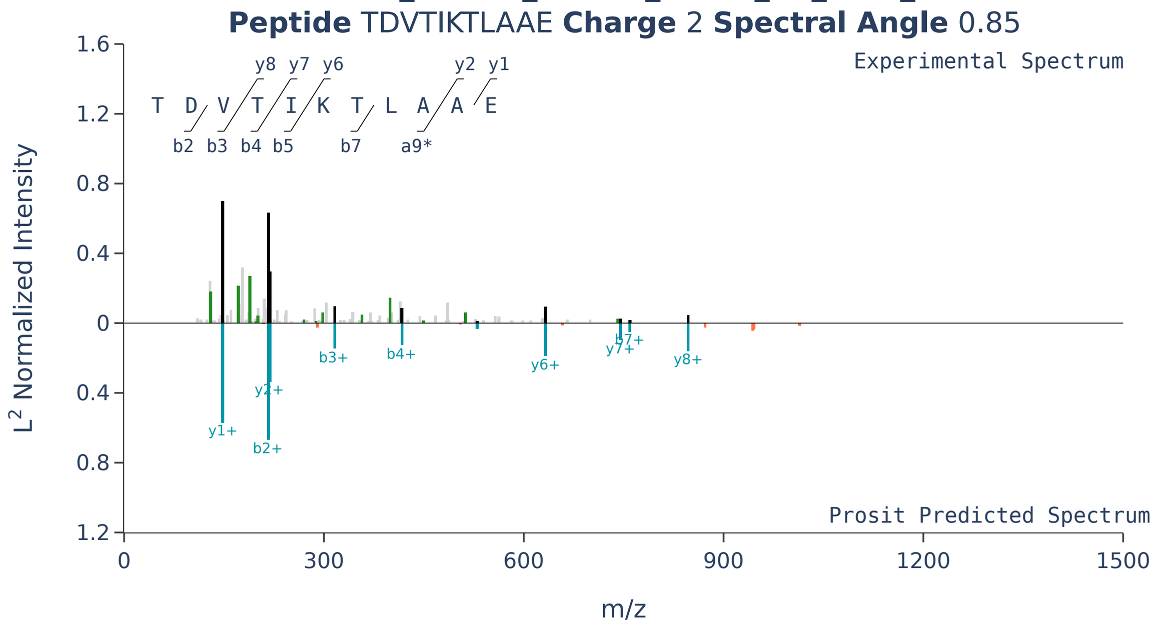


**Source** HRoetschke_211222_160123_HFGoe_IF2_b1R1_2h **Scan** 9831
**Peptide** HNPIRVLRD **Charge** 3 **Spectral Angle** 0.85

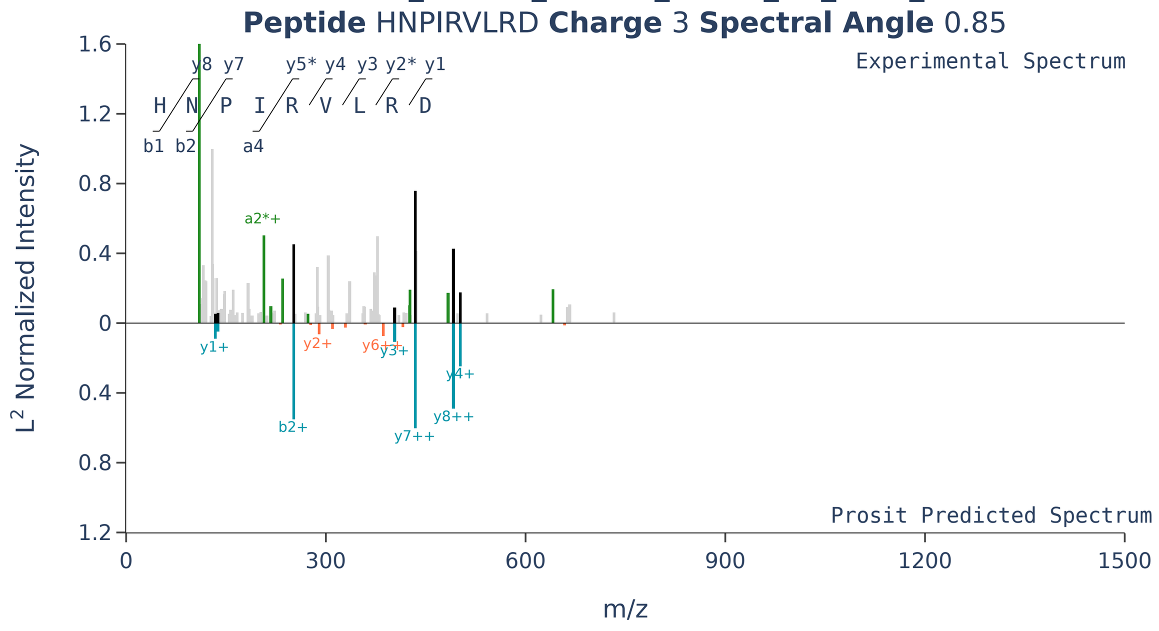


**Source** HRoetschke_211222_160123_HFGoe_IF2_b2R1_1h **Scan** 16518
**Peptide** NQVIDQETAQL **Charge** 2 **Spectral Angle** 0.9

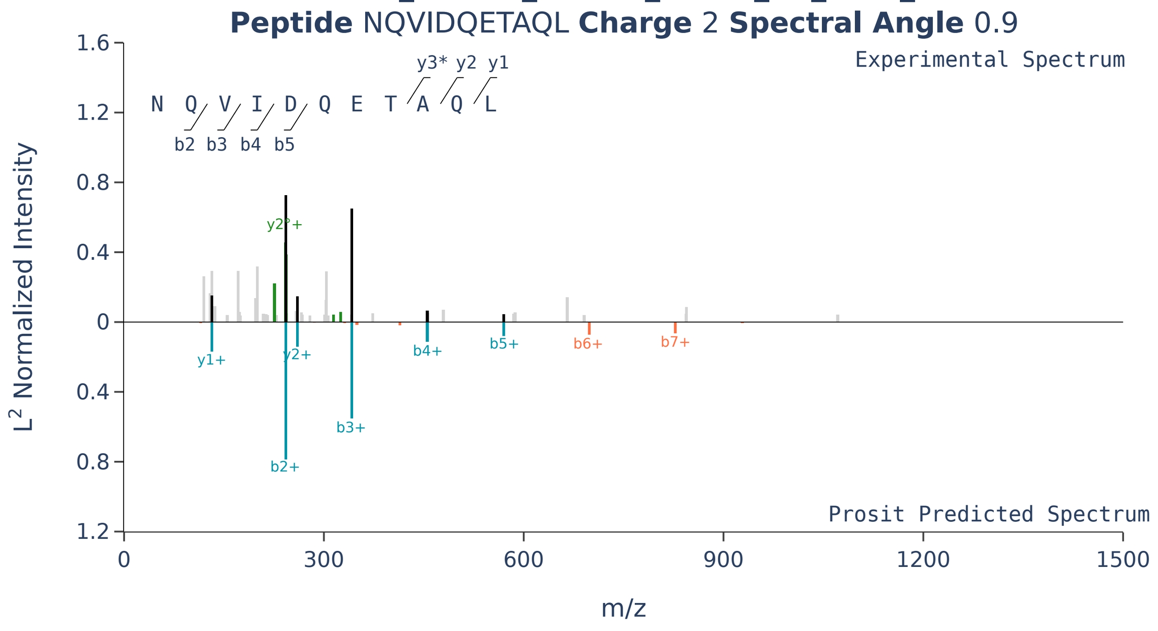


**Source** HRoetschke_211222_160123_HFGoe_IF2_b2R1_4h **Scan** 13977
**Peptide** RDNVVIYEGE **Charge** 2 **Spectral Angle** 0.83

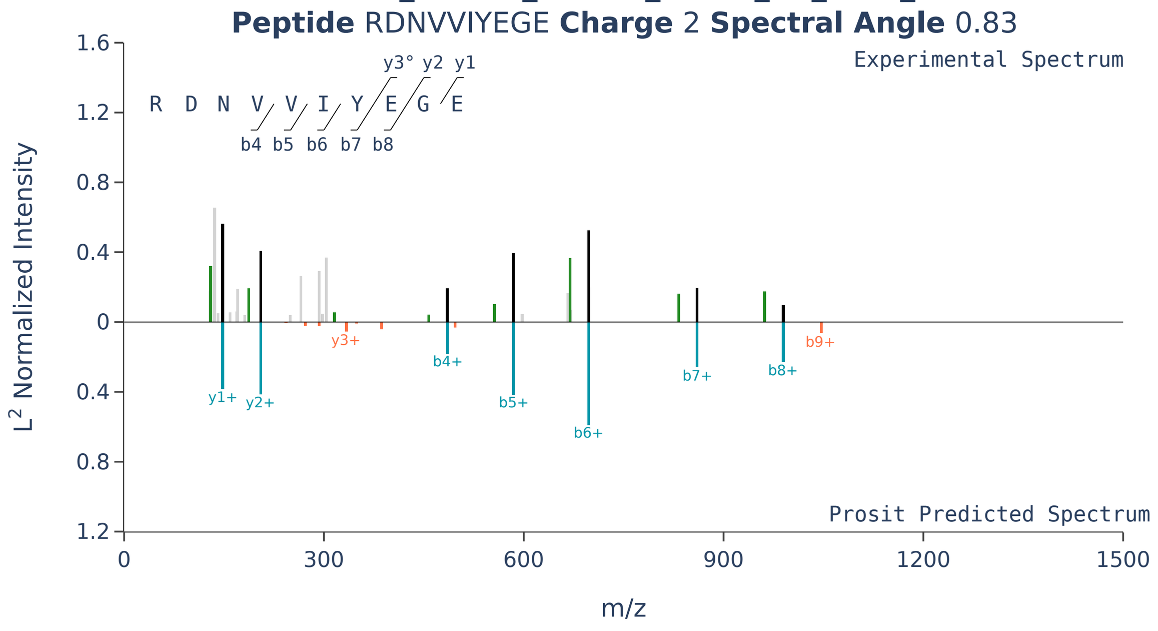


**Source** HRoetschke_211222_160123_HFGoe_IF2_b1R1_4h **Scan** 12119
**Peptide** DYIRSTKVASGEAGGITQH **Charge** 3 **Spectral Angle** 0.9

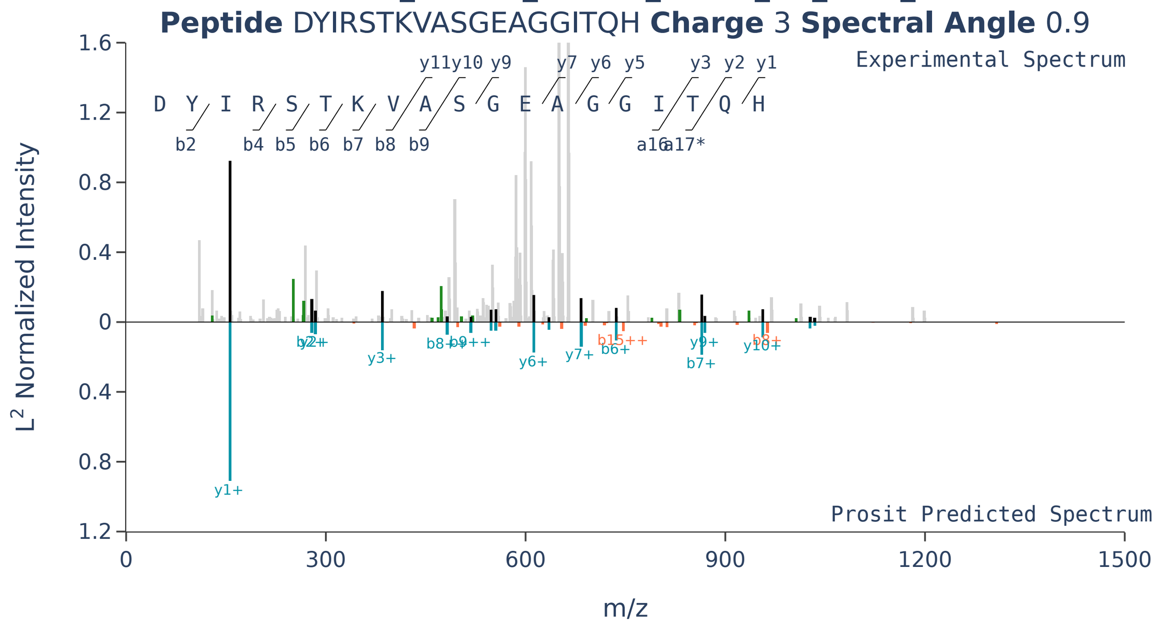


**Source** HRoetschke_211222_160123_HFGoe_IF2_b1R1_4h **Scan** 10563
**Peptide** RQTSVERLVQ **Charge** 3 **Spectral Angle** 0.82

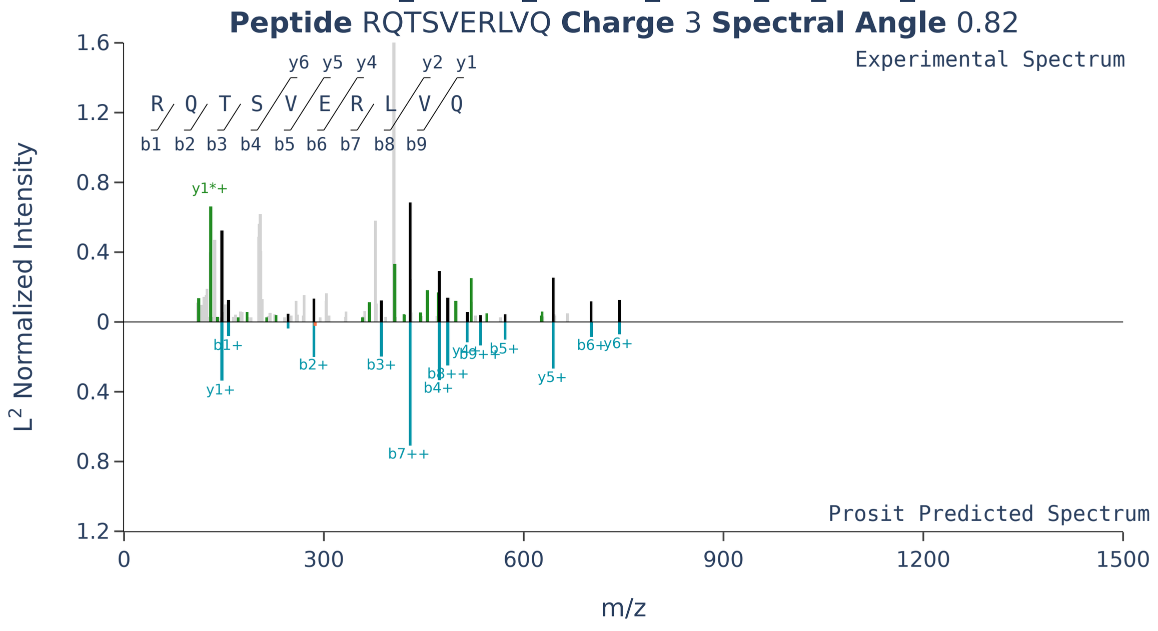


**Source** HRoetschke_211222_160123_HFGoe_IF2_b1R2_24h **Scan** 10337
**Peptide** KQTLIDHLNQK **Charge** 3 **Spectral Angle** 0.79

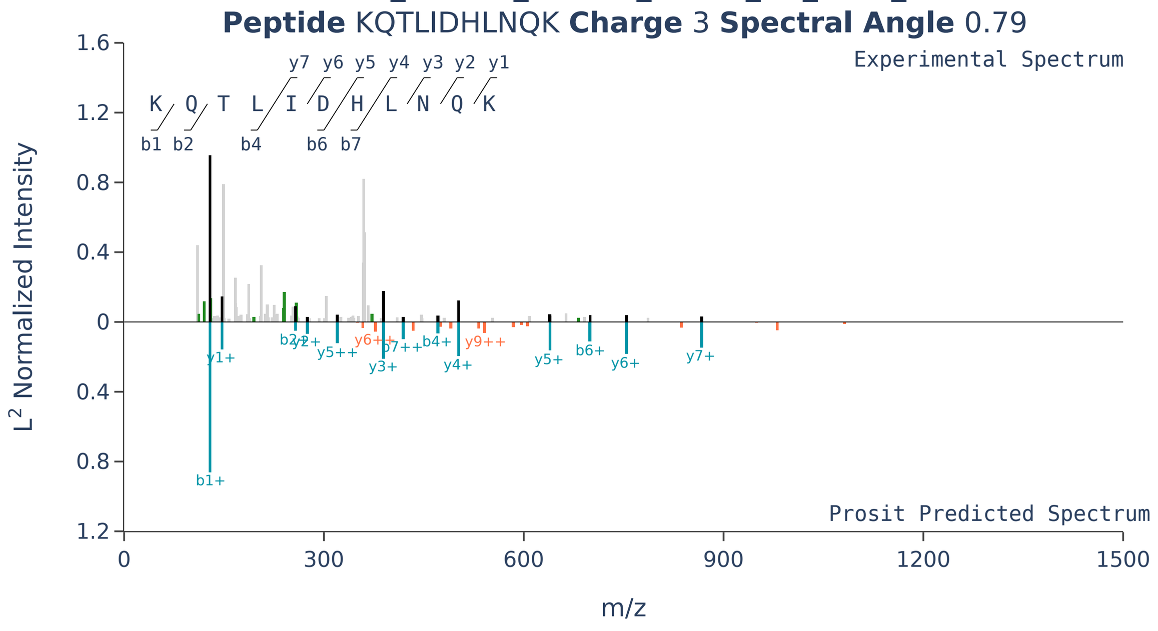


**Source** HRoetschke_211222_160123_HFGoe_IF2_b2R1_24h **Scan** 12360
**Peptide** SGVGGITETD **Charge** 2 **Spectral Angle** 0.91

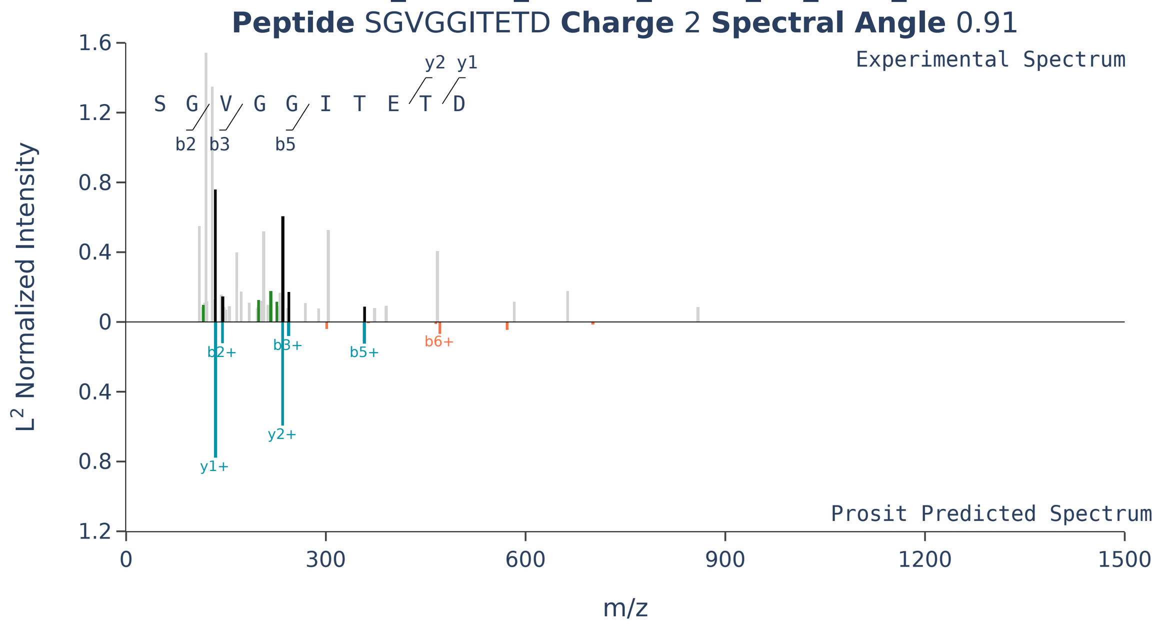

**Source** HRoetschke_211222_160123_HFGoe_IF2_b1R1_4h **Scan** 7955
**Peptide** KGSSLQQGFQK **Charge** 2 **Spectral Angle** 0.78

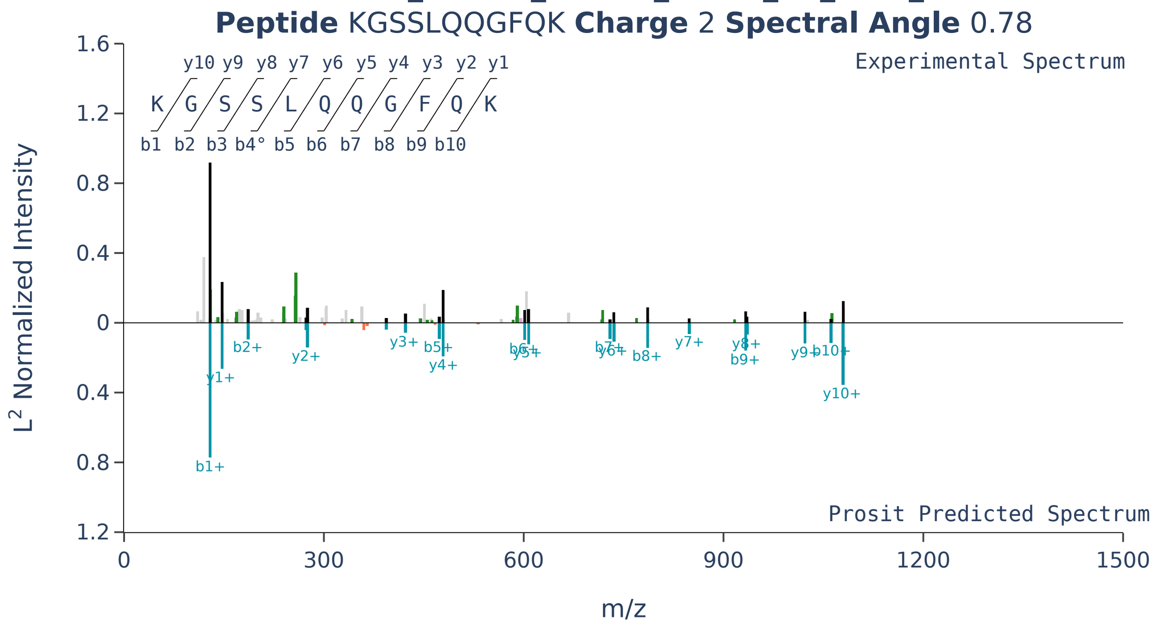


**Source** HRoetschke_211222_160123_HFGoe_IF2_b1R2_2h **Scan** 9362
**Peptide** KVILRRENELEE **Charge** 3 **Spectral Angle** 0.78

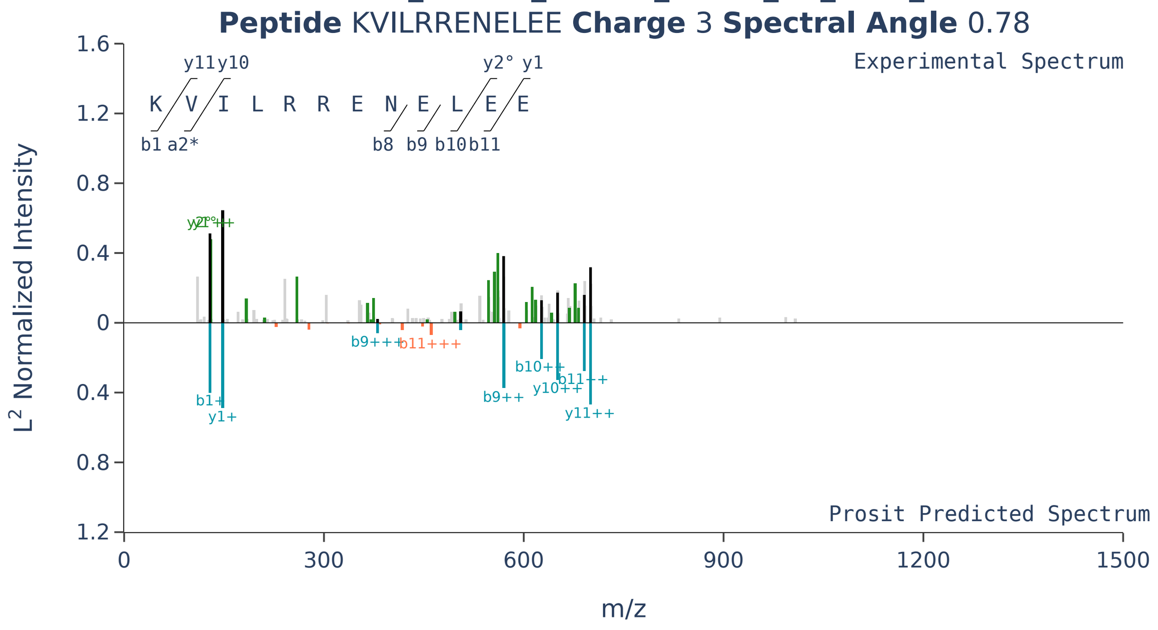


**Source** HRoetschke_211222_160123_HFGoe_IF2_b1R1_4h **Scan** 17408
**Peptide** TVLVREGTL **Charge** 2 **Spectral Angle** 0.9

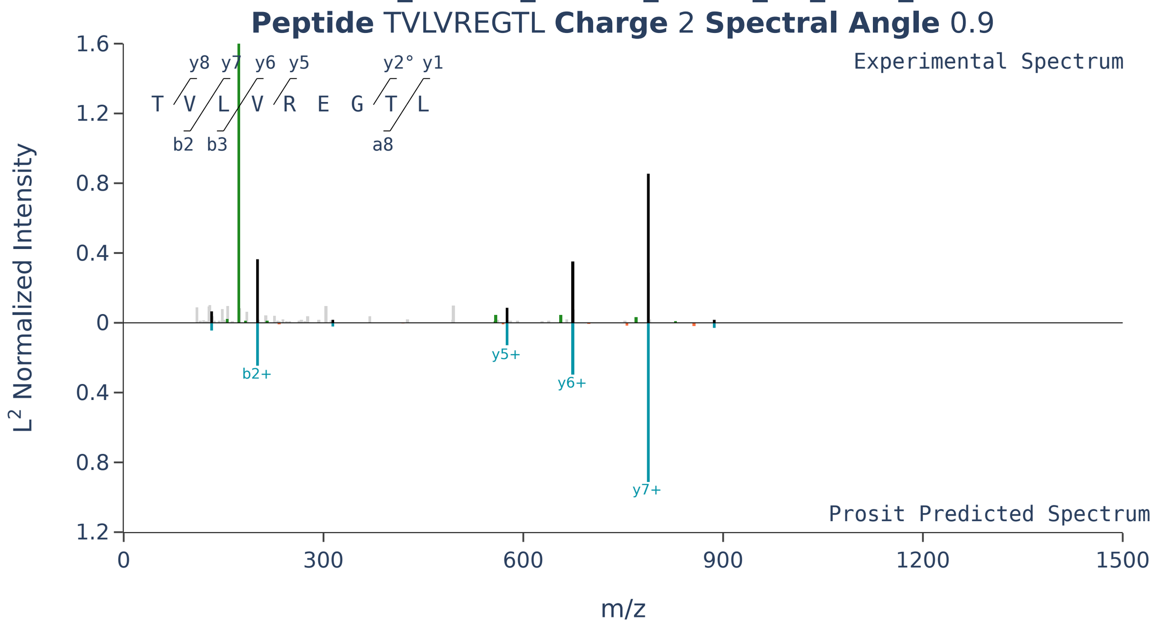


**Source** HRoetschke_211222_160123_HFGoe_IF2_b1R1_2h **Scan** 7547
**Peptide** EEMGHKVILRRE **Charge** 3 **Spectral Angle** 0.87

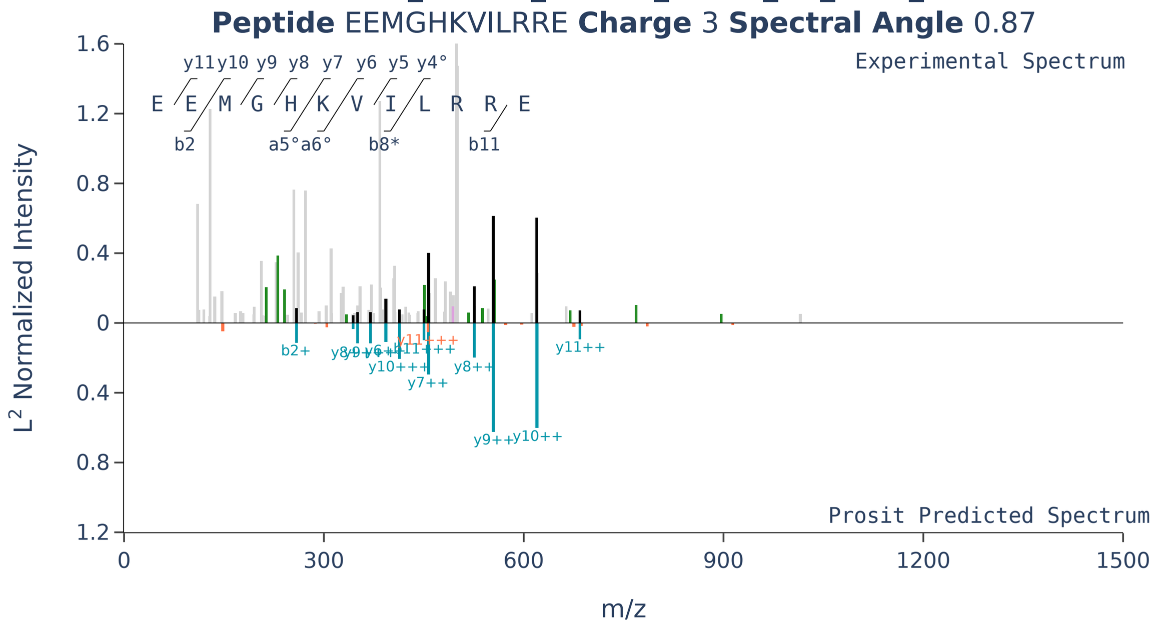


**Source** HRoetschke_211222_160123_HFGoe_IF2_b1R1_4h **Scan** 13796
**Peptide** LDTPGHAAFTSMRAR **Charge** 4 **Spectral Angle** 0.89

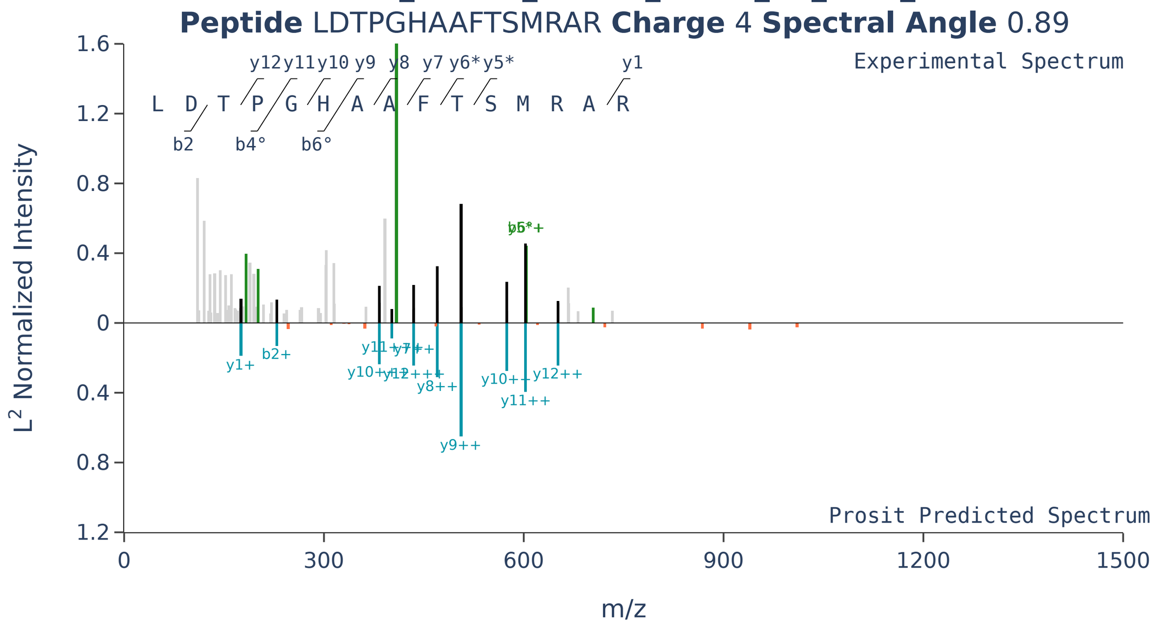


**Source** HRoetschke_211222_160123_HFGoe_IF2_b1R1_24h **Scan** 6720
**Peptide** NSGPDKLTLQRKTRST **Charge** 3 **Spectral Angle** 0.77

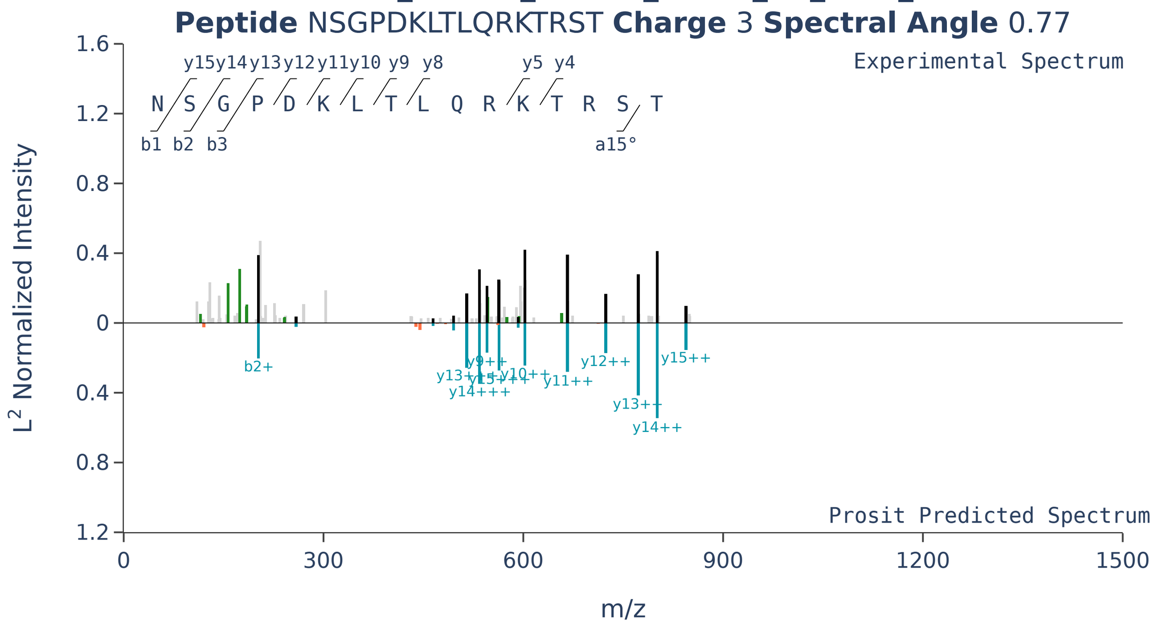


**Source** HRoetschke_211222_160123_HFGoe_IF2_b1R1_4h **Scan** 16569
**Peptide** AEVLELKAVR **Charge** 3 **Spectral Angle** 0.81

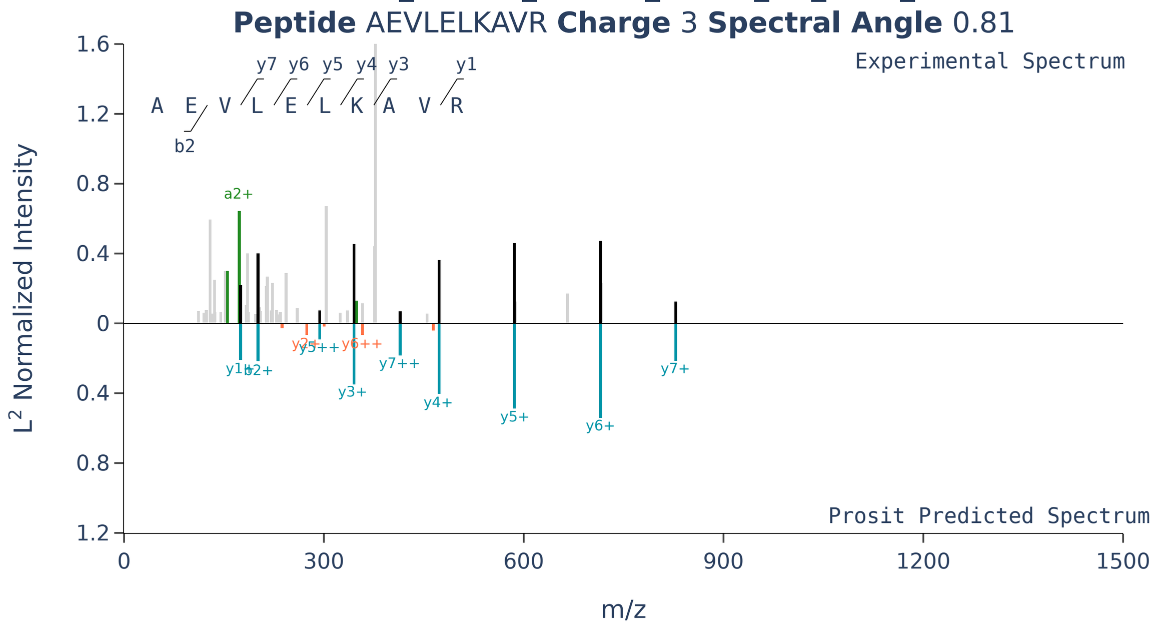


**Source** HRoetschke_211222_160123_HFGoe_IF2_b1R2_24h **Scan** 12611
**Peptide** FLDTPGH **Charge** 2 **Spectral Angle** 0.82

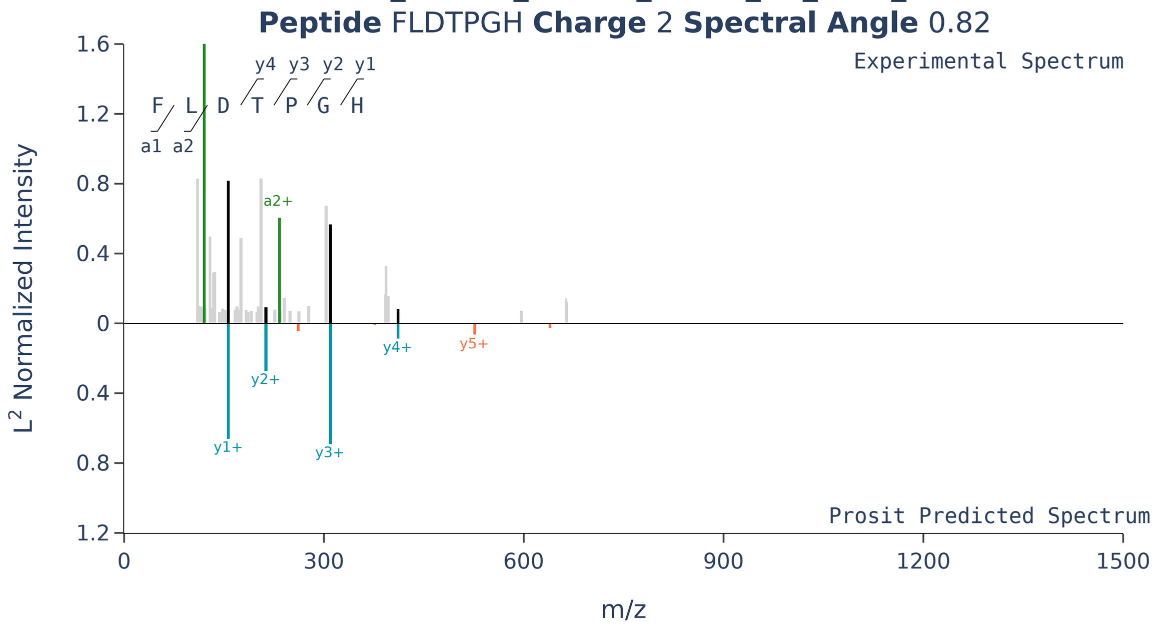


**Source** HRoetschke_211222_160123_HFGoe_IF2_b1R2_1h **Scan** 15283
**Peptide** KQTLIDHL **Charge** 2 **Spectral Angle** 0.81

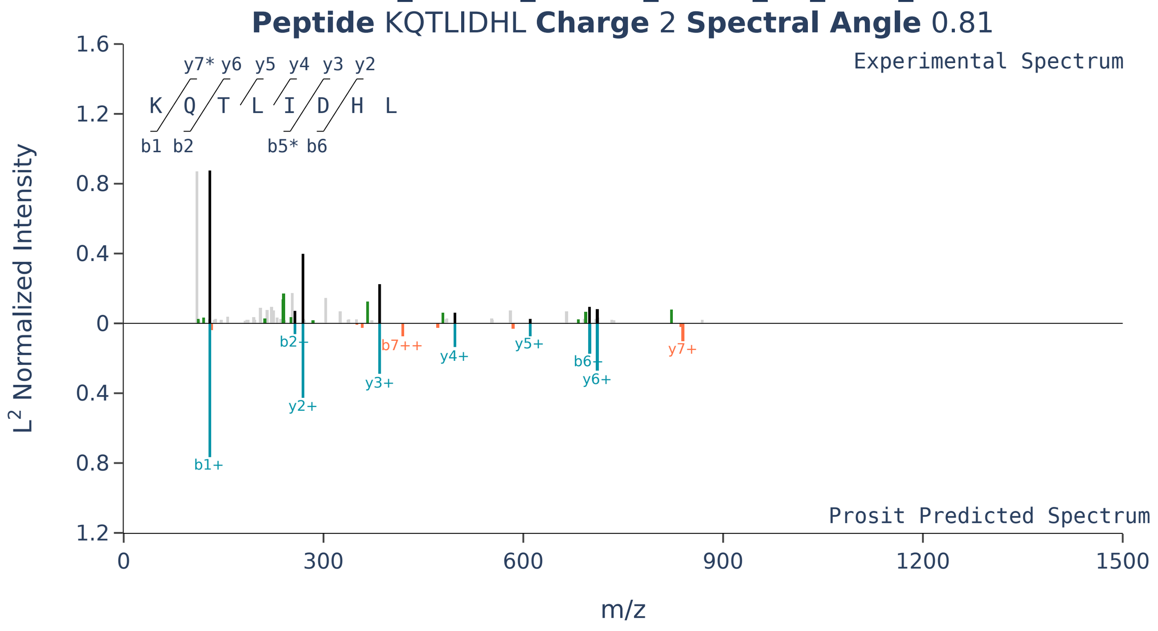


**Source** HRoetschke_211222_160123_HFGoe_IF2_b1R1_4h **Scan** 11512
**Peptide** FLDKGRGPVAT **Charge** 2 **Spectral Angle** 0.77

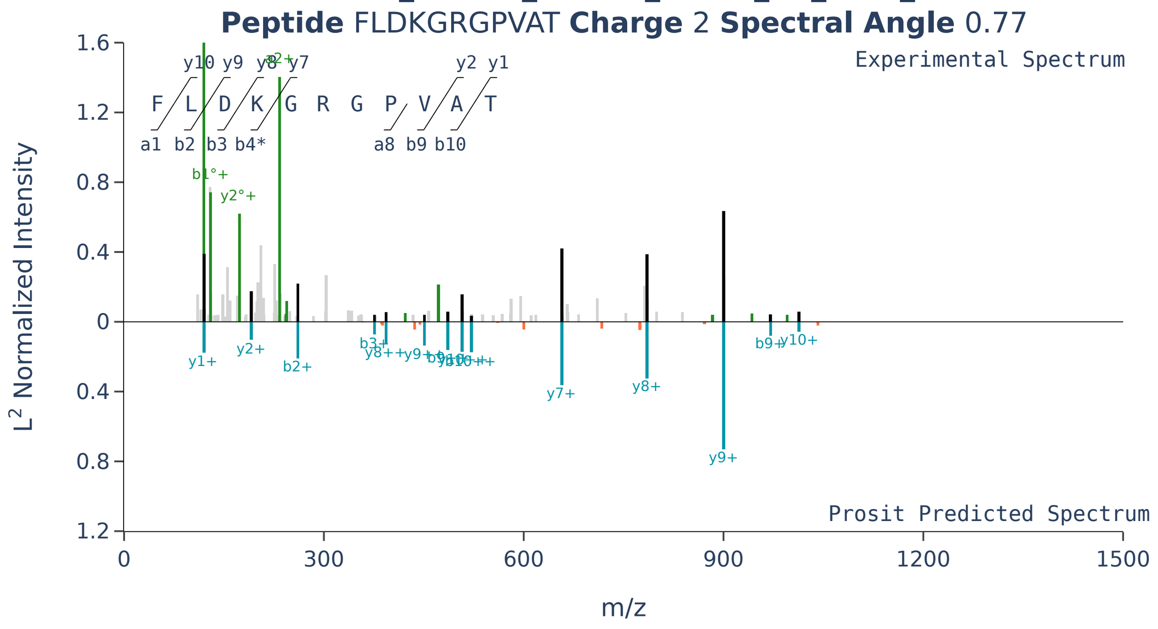


**Source** HRoetschke_211222_160123_HFGoe_IF2_b2R2_24h **Scan** 8154
**Peptide** SGPDKLTLQRKT **Charge** 2 **Spectral Angle** 0.84

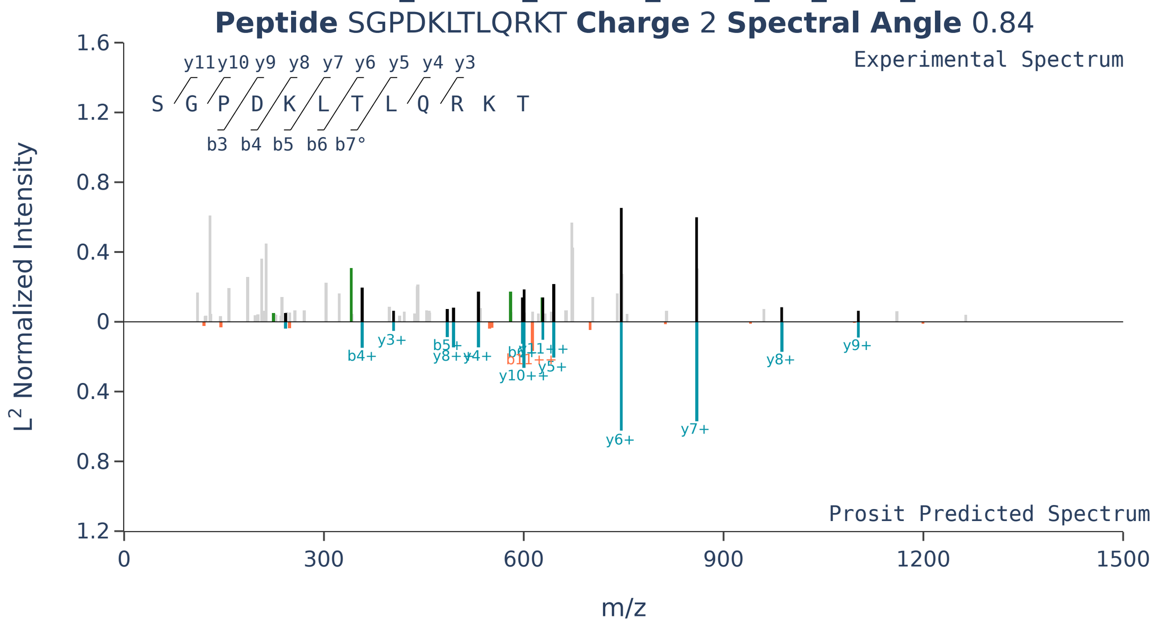


**Source** HRoetschke_211222_160123_HFGoe_IF2_b1R2_24h **Scan** 11453
**Peptide** TIKTLAA **Charge** 2 **Spectral Angle** 0.72

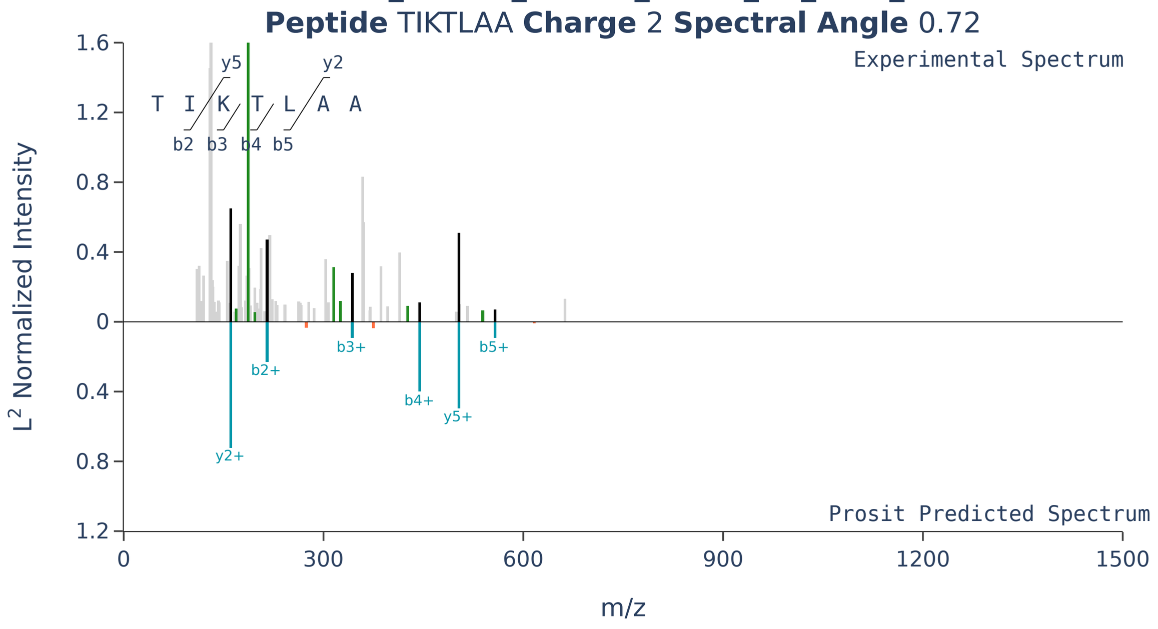


**Source** HRoetschke_211222_160123_HFGoe_IF2_b1R2_2h **Scan** 12146
**Peptide** GSGVGGITE **Charge** 1 **Spectral Angle** 0.91

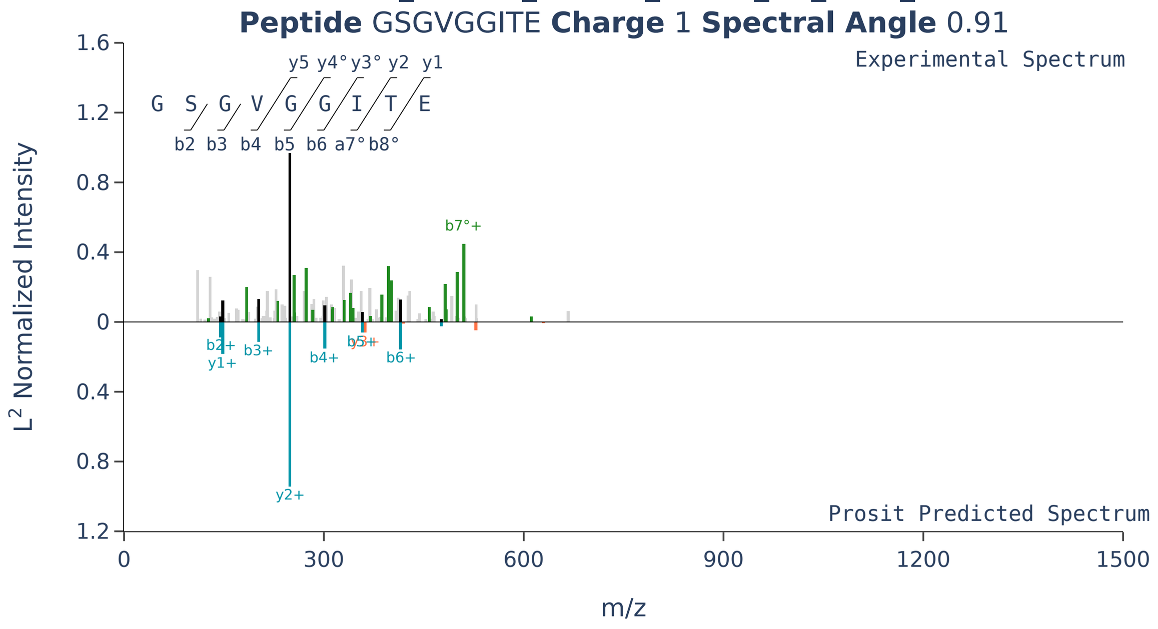


**Source** HRoetschke_211222_160123_HFGoe_IF2_b1R1_24h **Scan** 8687
**Peptide** DEVTVVRDEKKAREV **Charge** 4 **Spectral Angle** 0.88

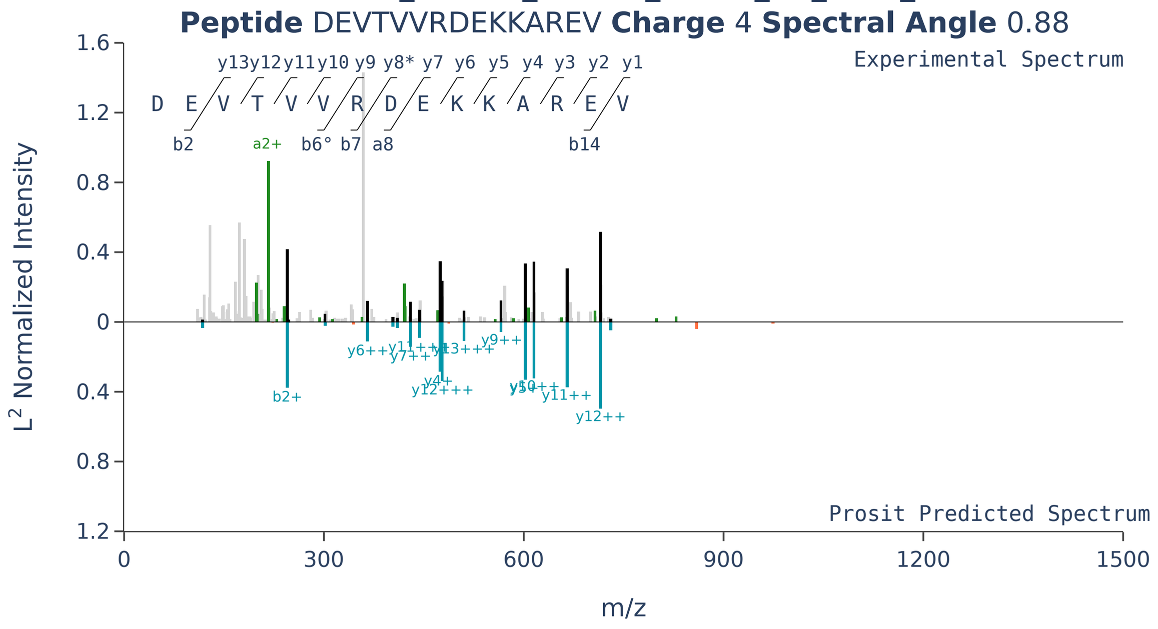


**Source** HRoetschke_211222_160123_HFGoe_IF2_b1R1_4h **Scan** 9358
**Peptide** TPGHAAFTSMRARGA **Charge** 3 **Spectral Angle** 0.75

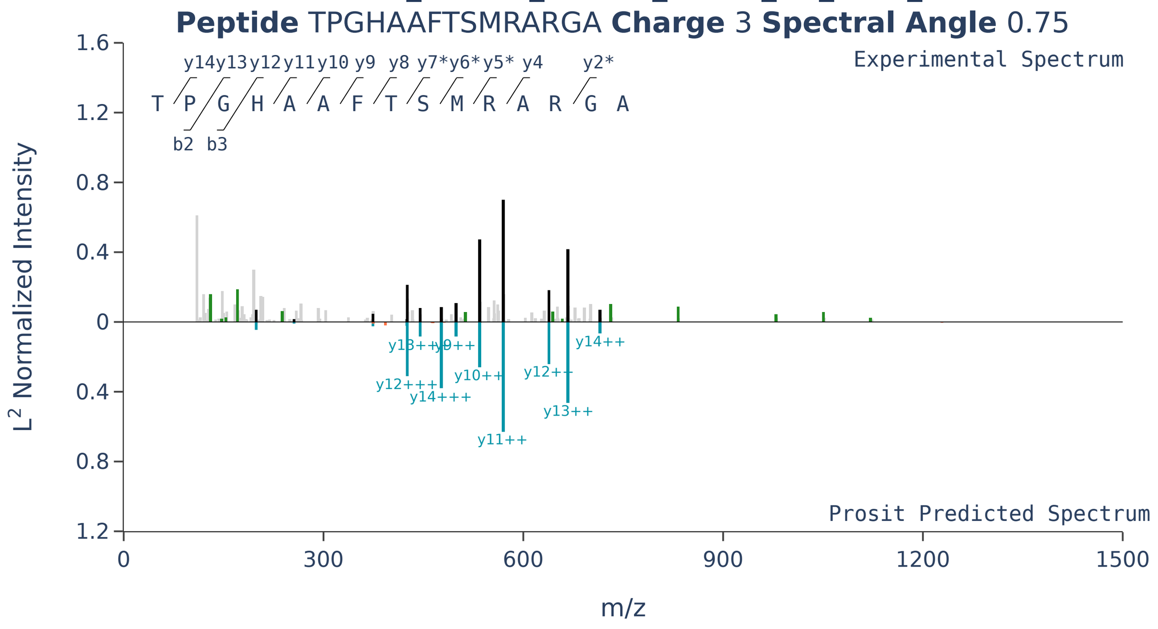

**Source** HRoetschke_211222_160123_HFGoe_IF2_b2R1_24h **Scan** 7823
**Peptide** VLDQETA **Charge** 1 **Spectral Angle** 0.91

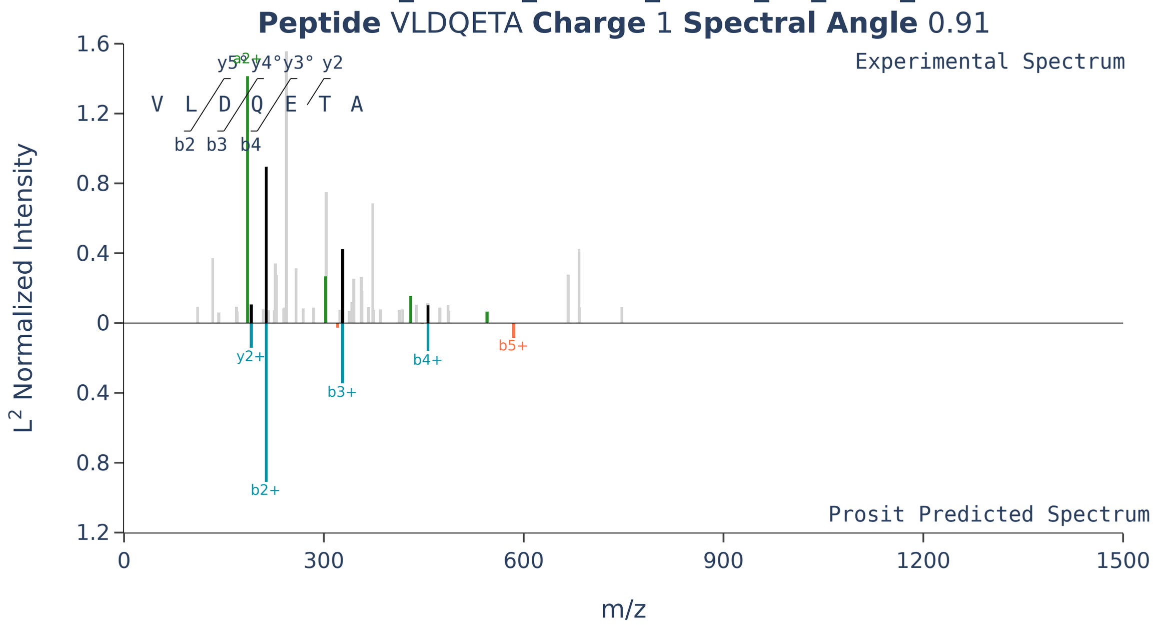


**Source** HRoetschke_211222_160123_HFGoe_IF2_b1R2_2h **Scan** 9174
**Peptide** QFADAGIRKSAD **Charge** 3 **Spectral Angle** 0.83

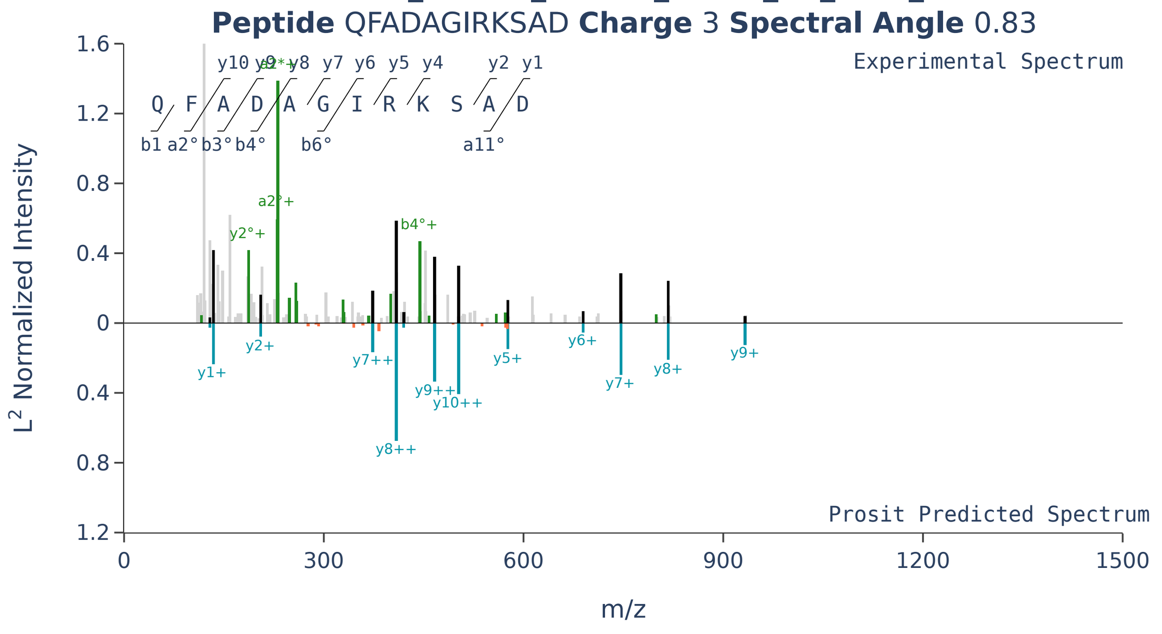


**Source** HRoetschke_211222_160123_HFGoe_IF2_b1R2_2h **Scan** 7540
**Peptide** AVKGSQVIKAMM **Charge** 3 **Spectral Angle** 0.81

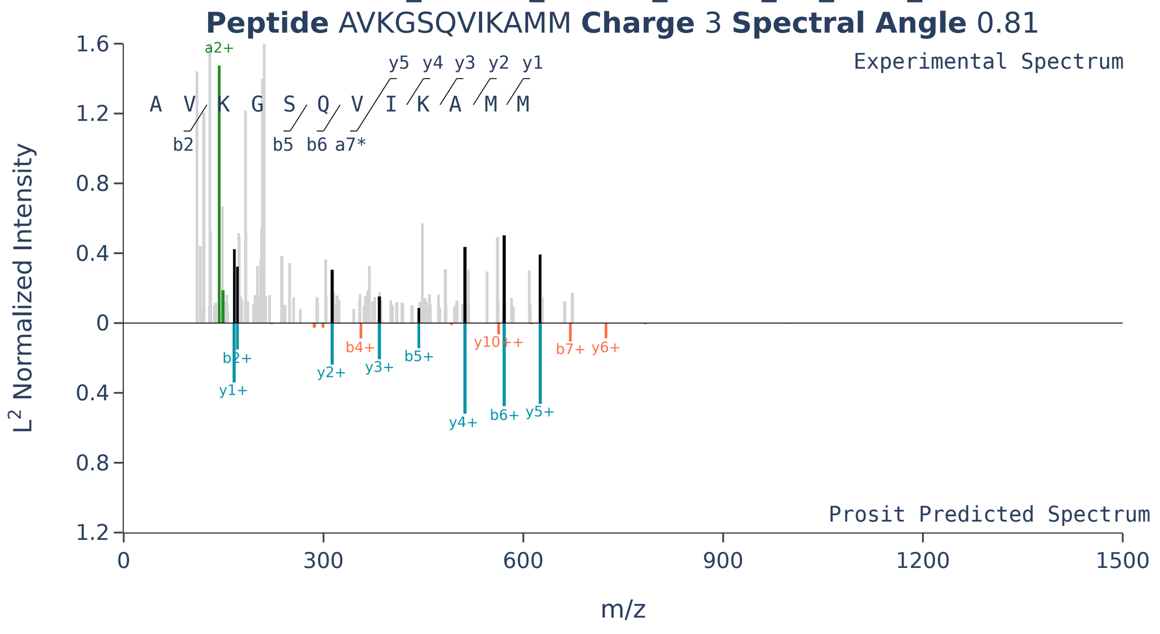


**Source** HRoetschke_211222_160123_HFGoe_IF2_b1R1_24h **Scan** 8424
**Peptide** LLRRENELE **Charge** 3 **Spectral Angle** 0.92

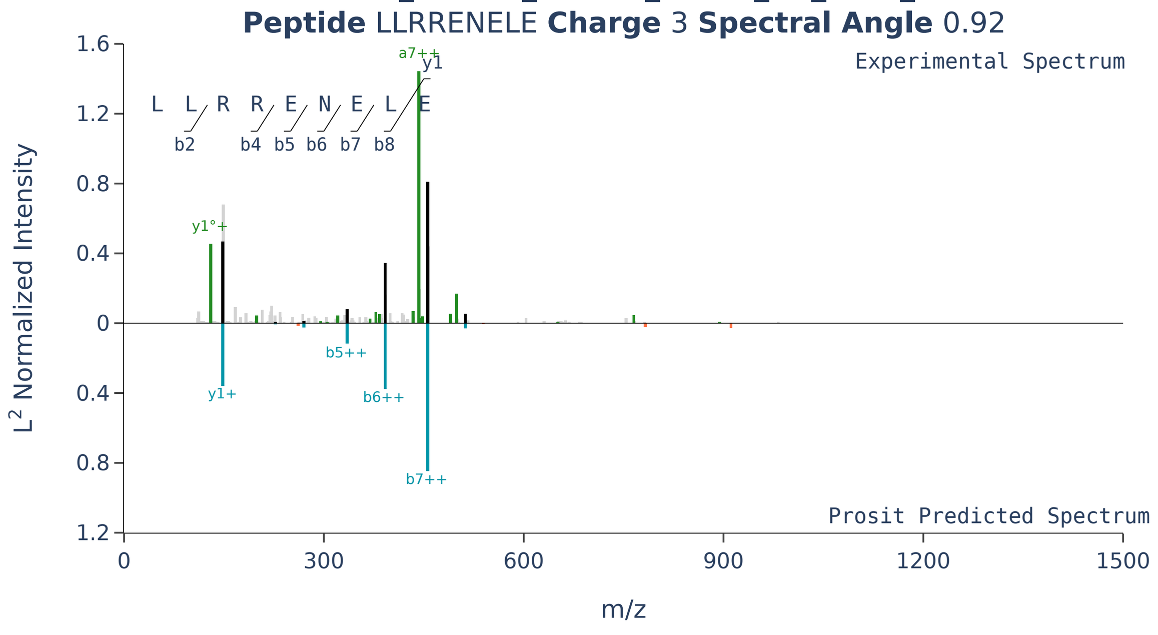


**Source** HRoetschke_211222_160123_HFGoe_IF2_b1R2_2h **Scan** 13036
**Peptide** VREGTLHKGDIVLC **Charge** 3 **Spectral Angle** 0.84

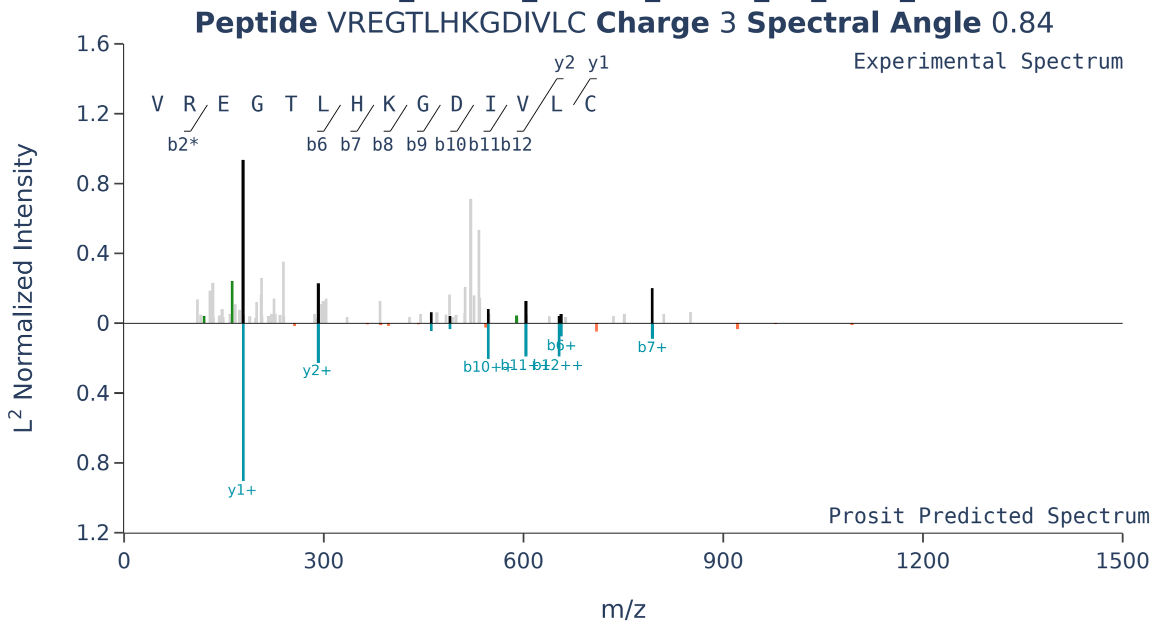


**Source** HRoetschke_211222_160123_HFGoe_IF2_b2R1_24h **Scan** 14341
**Peptide** KAGTGIDEL **Charge** 2 **Spectral Angle** 0.74

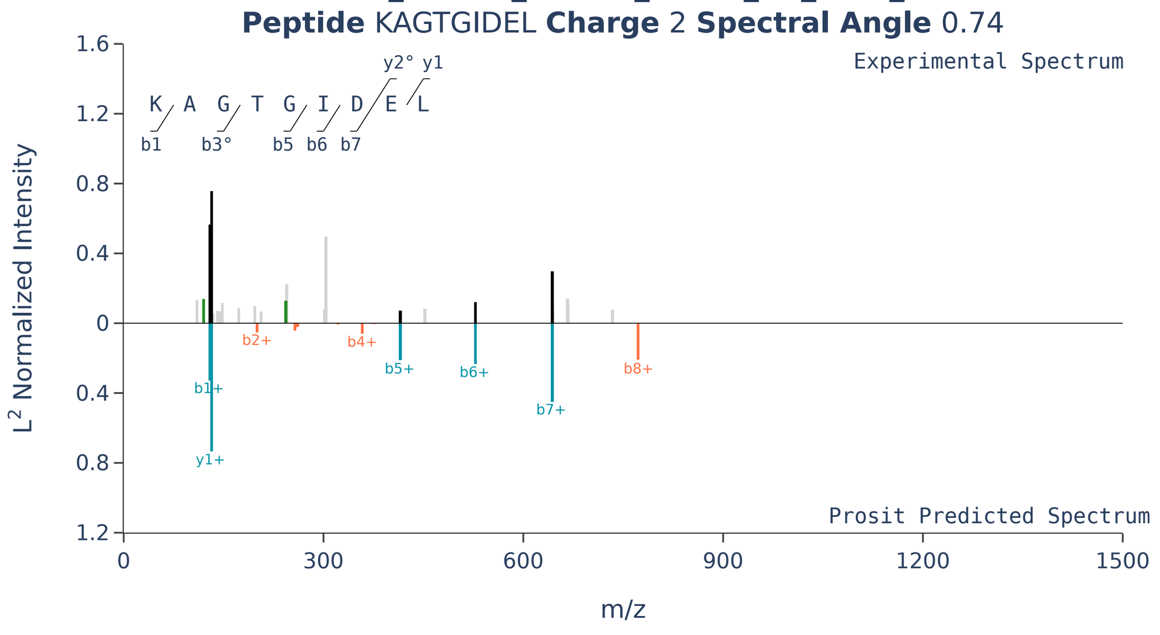


**Source** HRoetschke_211222_160123_HFGoe_IF2_b2R2_24h **Scan** 7411
**Peptide** SDYHVTTSQHA **Charge** 2 **Spectral Angle** 0.73

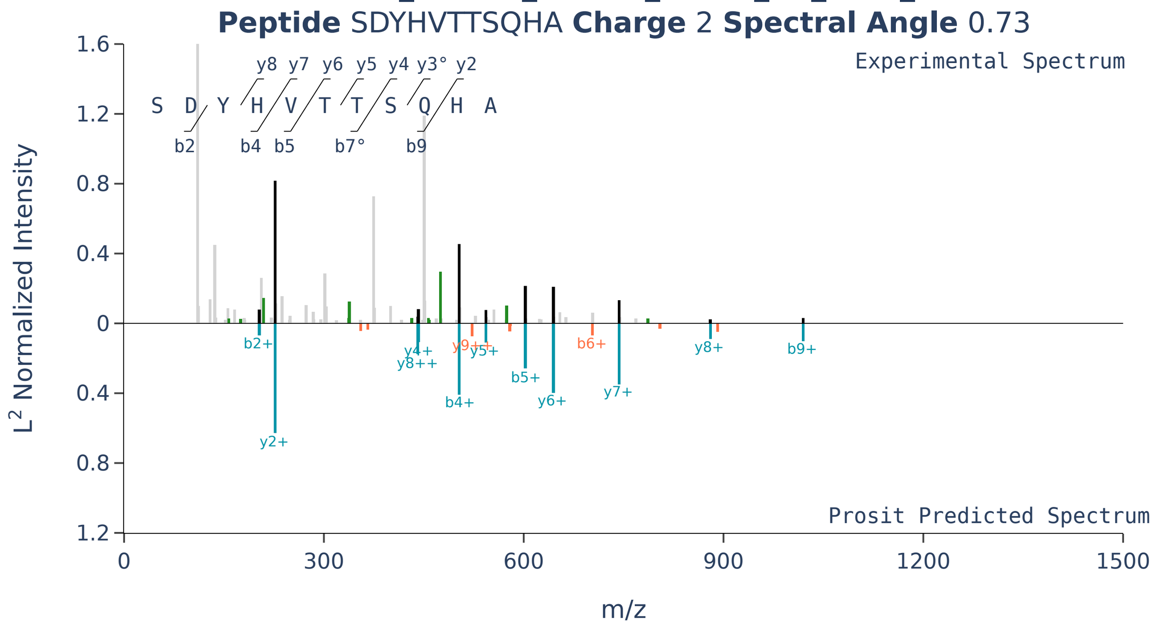


**Source** HRoetschke_211222_160123_HFGoe_IF2_b1R1_4h **Scan** 13449
**Peptide** SVSAQEKQTLID **Charge** 2 **Spectral Angle** 0.79

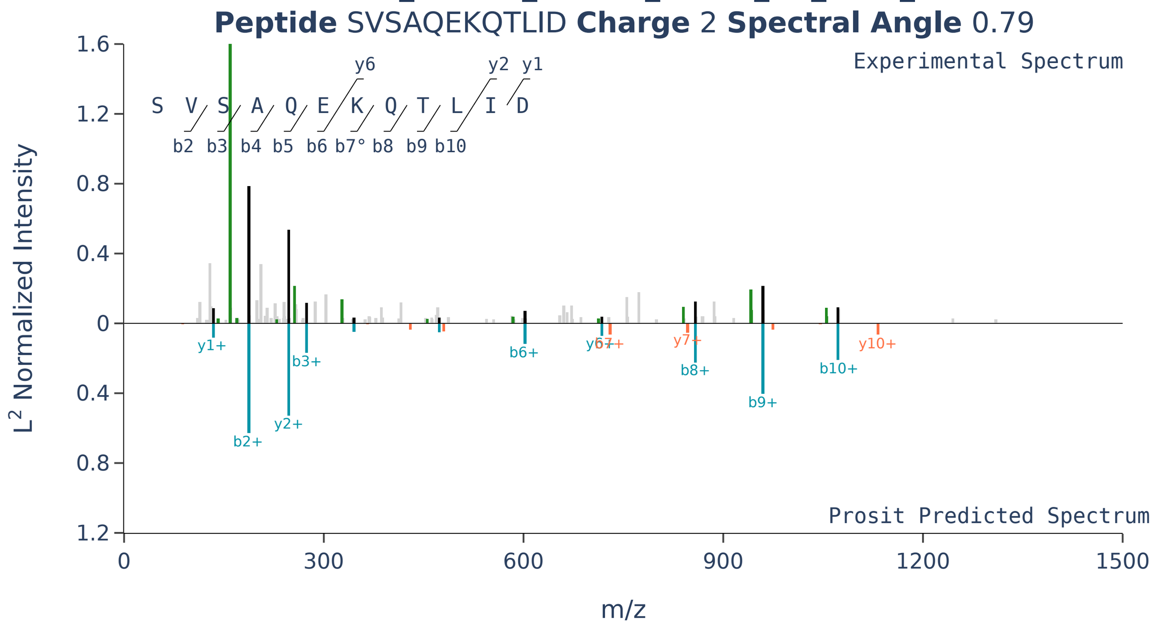


**Source** HRoetschke_211222_160123_HFGoe_IF2_b2R1_24h **Scan** 11338
**Peptide** EENKWTDNAEPTE **Charge** 2 **Spectral Angle** 0.94

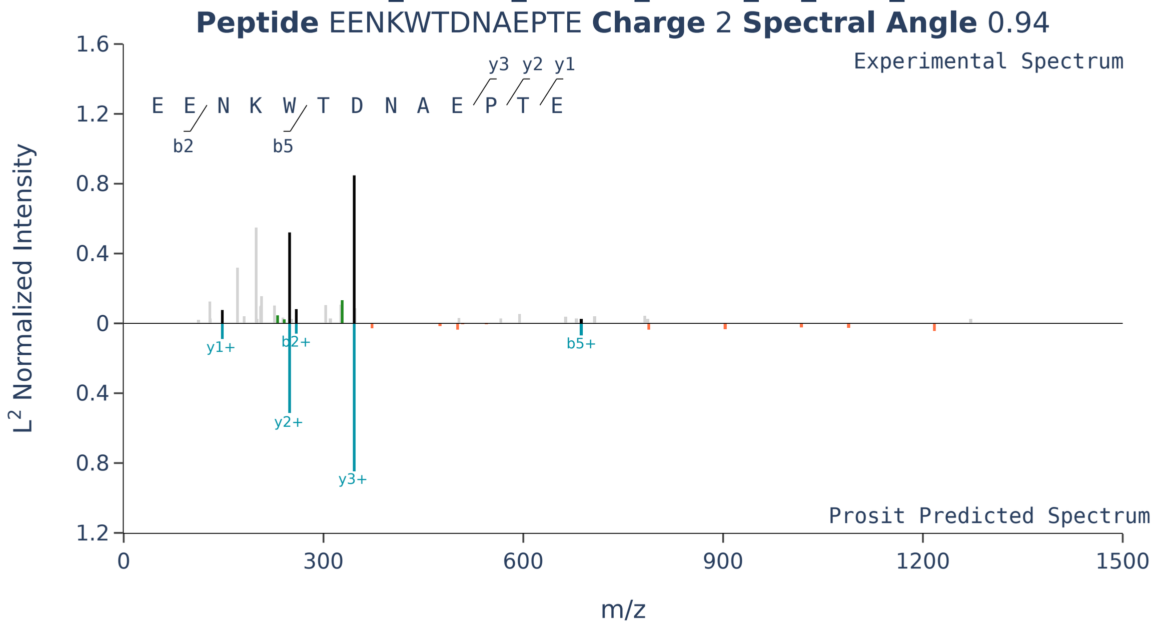


**Source** HRoetschke_211222_160123_HFGoe_IF2_b1R1_4h **Scan** 15316
**Peptide** PAQAVNRDVVIGE **Charge** 2 **Spectral Angle** 0.78

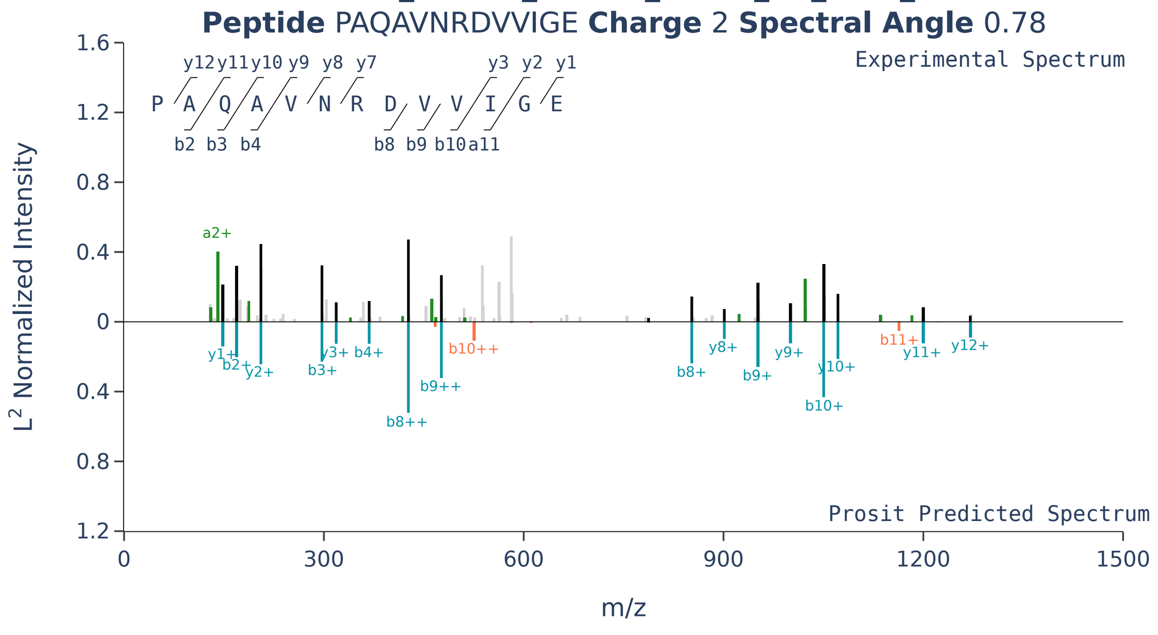


**Source** HRoetschke_211222_160123_HFGoe_IF2_b1R1_24h **Scan** 10681
**Peptide** DHGKTSLLD **Charge** 2 **Spectral Angle** 0.82

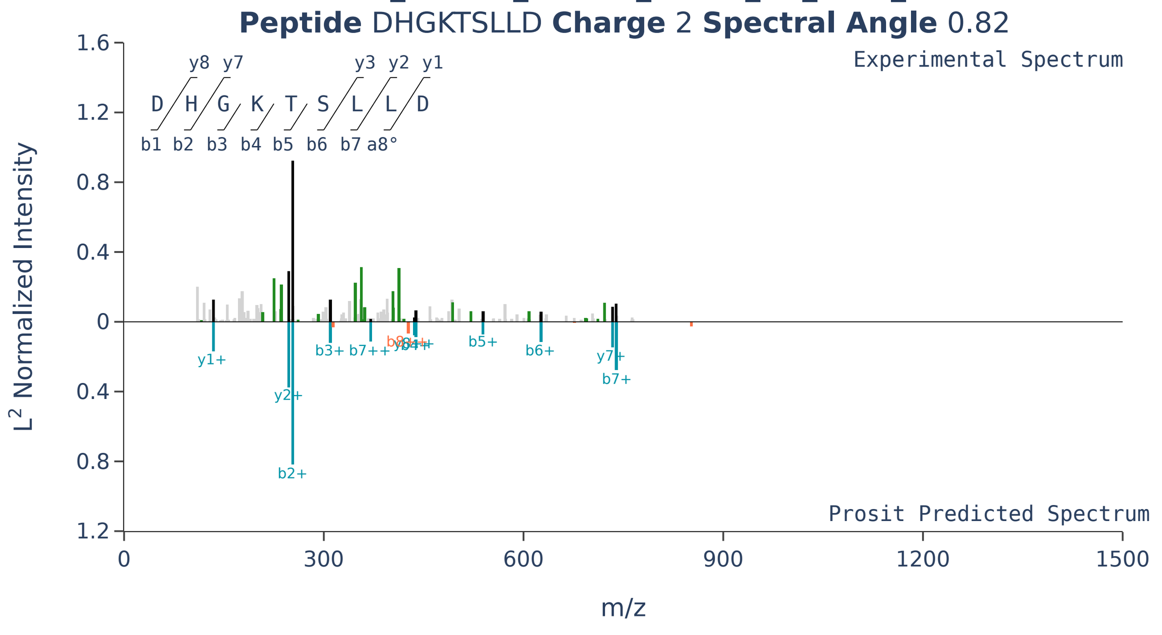


**Source** HRoetschke_211222_160123_HFGoe_IF2_b1R1_2h **Scan** 8594
**Peptide** KNSGPDKLTLQR **Charge** 3 **Spectral Angle** 0.75

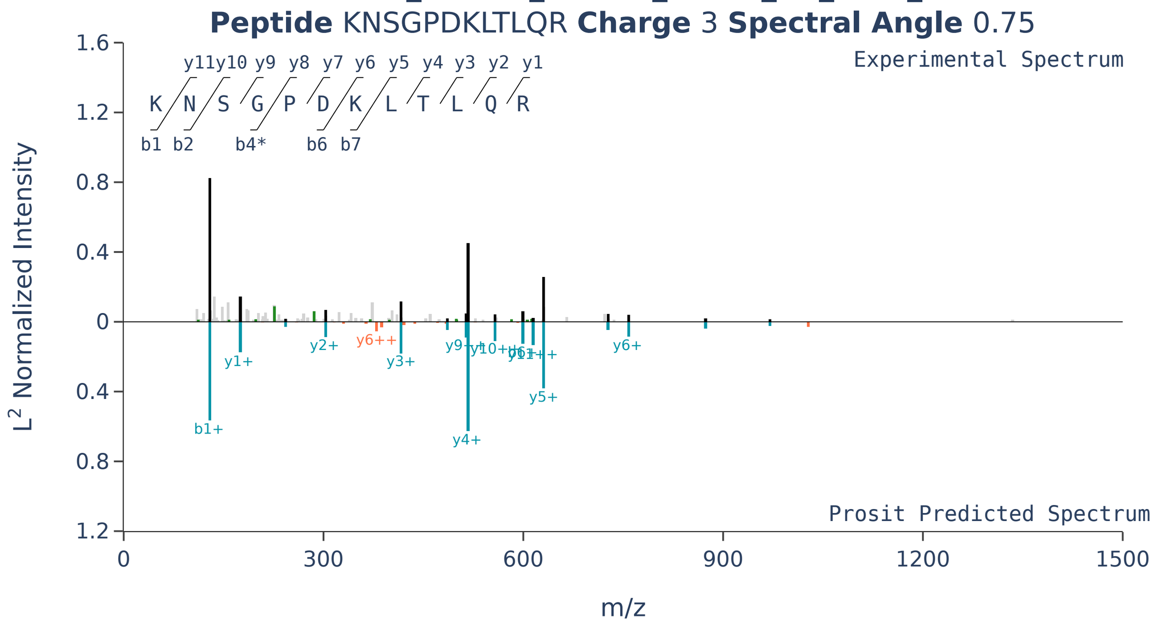


**Source** HRoetschke_211222_160123_HFGoe_IF2_b1R1_4h **Scan** 16230
**Peptide** EGTLHKGDIVL **Charge** 2 **Spectral Angle** 0.79

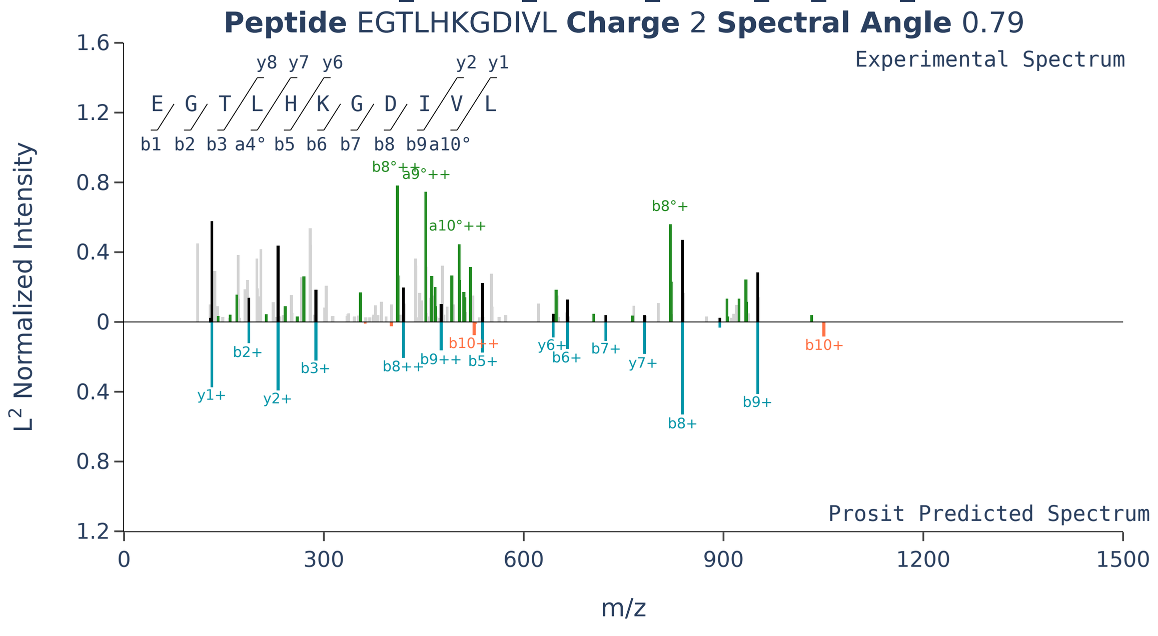


**Source** HRoetschke_211222_160123_HFGoe_IF2_b1R2_4h **Scan** 15140
**Peptide** GRGPVATVLVREG **Charge** 3 **Spectral Angle** 0.9

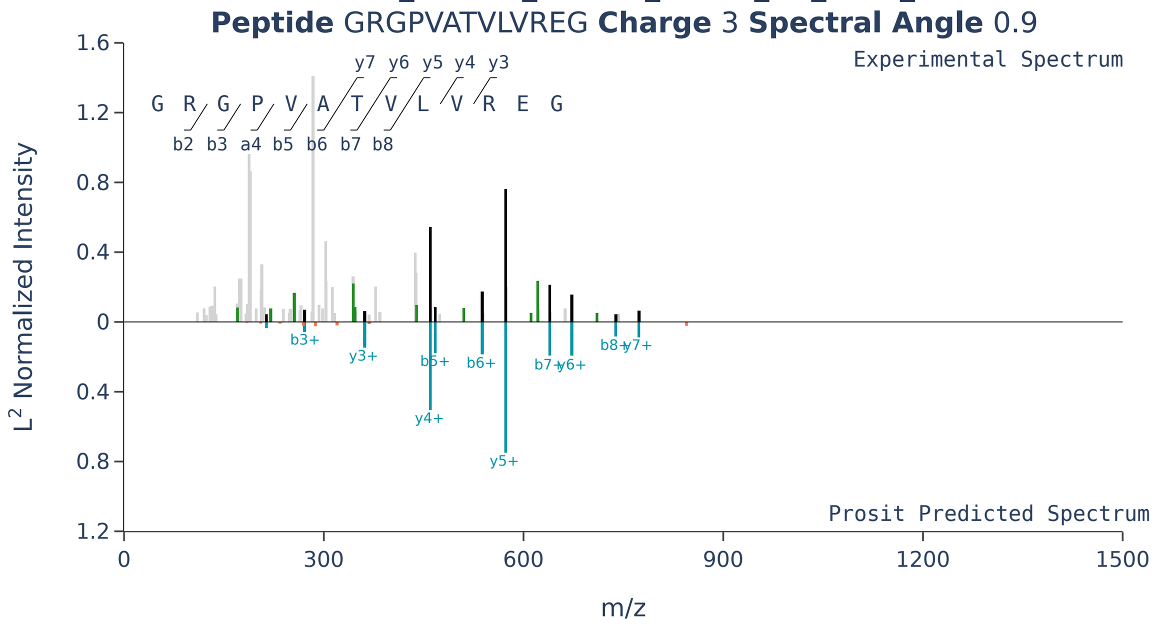


**Source** HRoetschke_211222_160123_HFGoe_IF2_b1R2_24h **Scan** 7063
**Peptide** RFKDDVNEVRN **Charge** 3 **Spectral Angle** 0.86

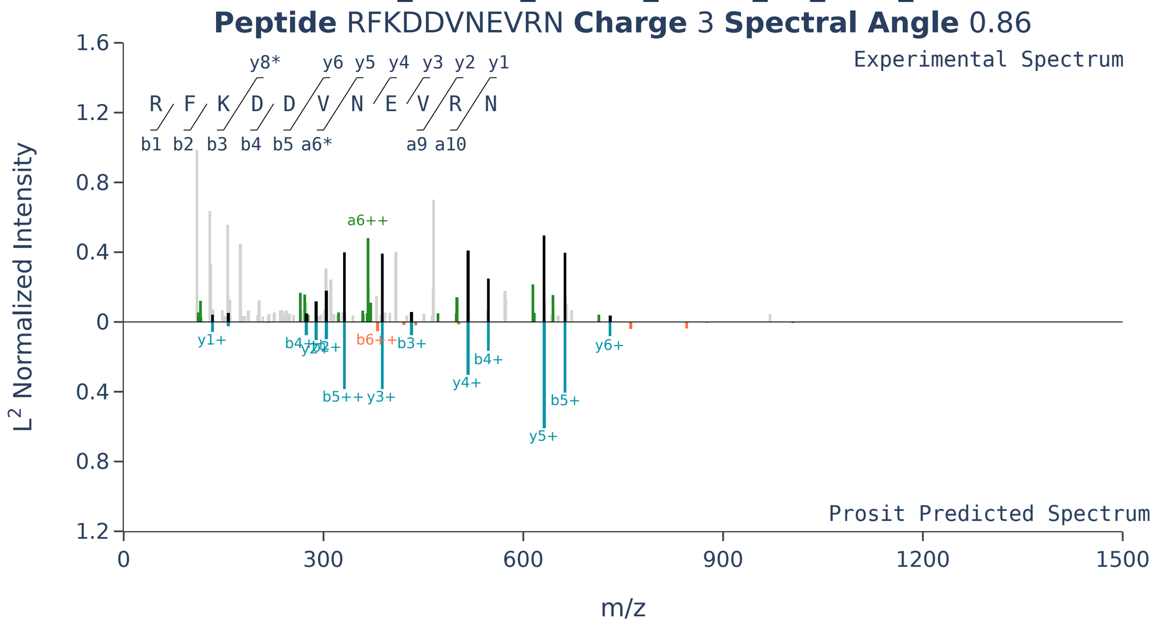

**Source** HRoetschke_211222_160123_HFGoe_IF2_b1R1_2h **Scan** 12130
**Peptide** REGTLHKGDIVLC **Charge** 3 **Spectral Angle** 0.89

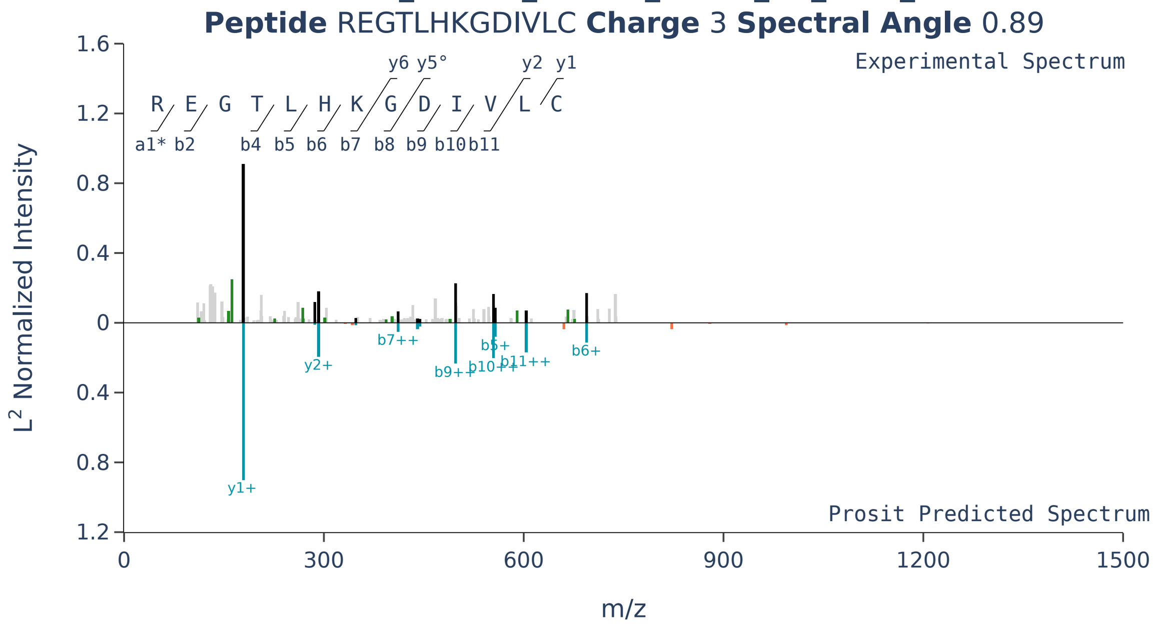


**Source** HRoetschke_211222_160123_HFGoe_IF2_b1R2_24h **Scan** 15689
**Peptide** GVMPQTIEAIQH **Charge** 2 **Spectral Angle** 0.71

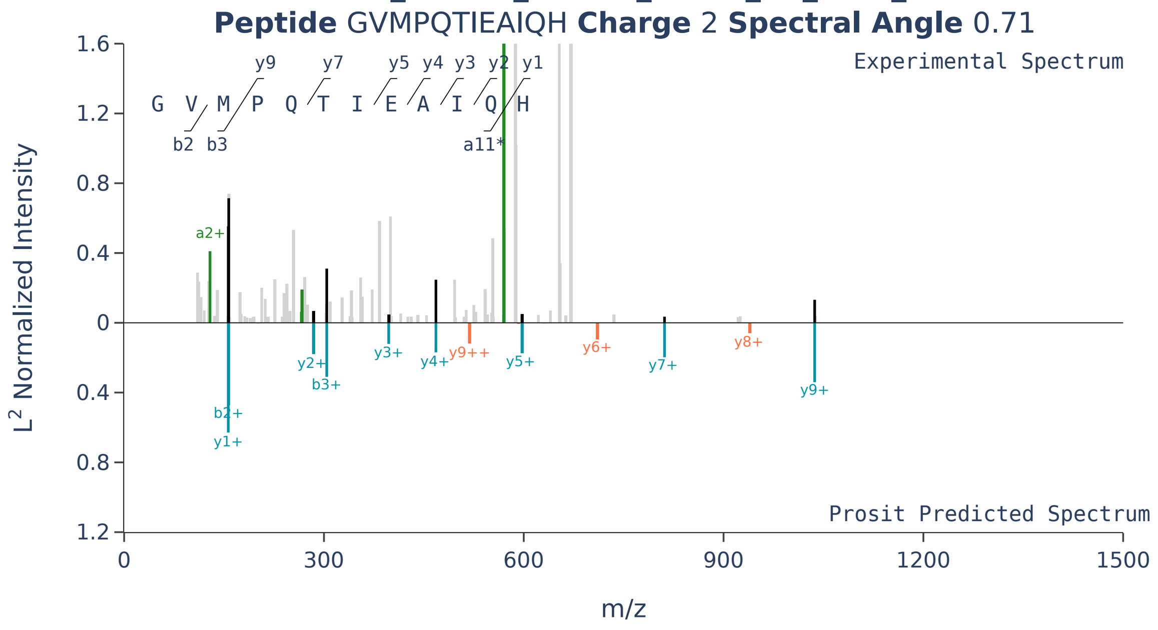


**Source** HRoetschke_211222_160123_HFGoe_IF2_b2R1_1h **Scan** 7739
**Peptide** LAAERQTSV **Charge** 2 **Spectral Angle** 0.79

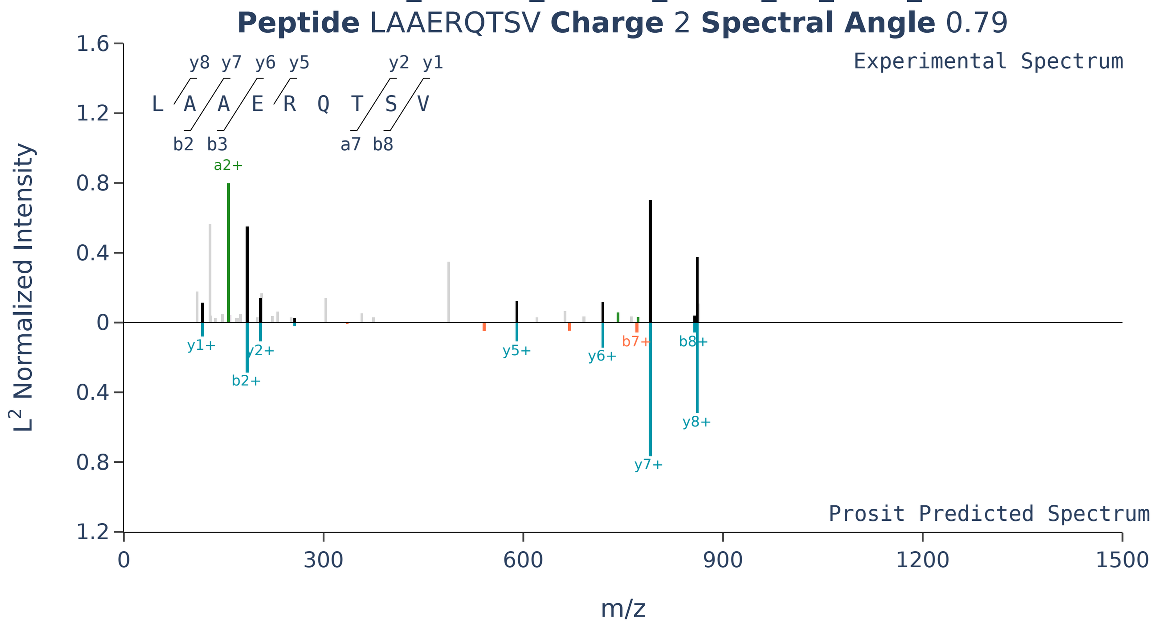


**Source** HRoetschke_211222_160123_HFGoe_IF2_b1R1_4h **Scan** 13260
**Peptide** DGVMPQT **Charge** 1 **Spectral Angle** 0.87

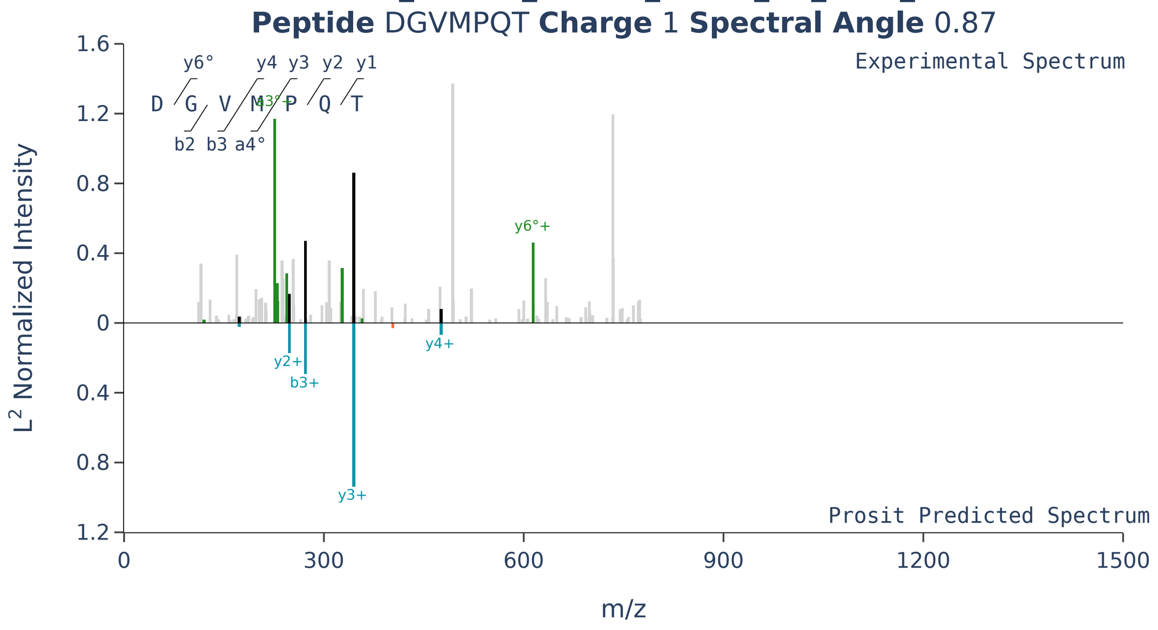


**Source** HRoetschke_211222_160123_HFGoe_IF2_b2R2_4h **Scan** 17223
**Peptide** AMATINQVID **Charge** 2 **Spectral Angle** 0.79

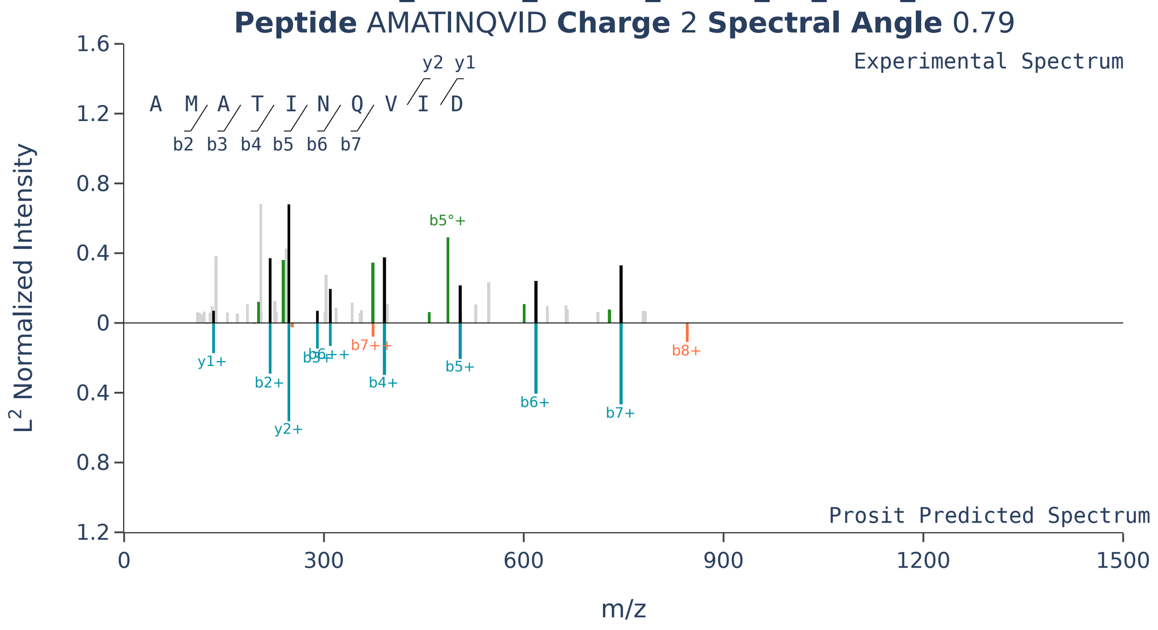


**Source** HRoetschke_211222_160123_HFGoe_IF2_b1R1_4h **Scan** 16853
**Peptide** ENELEEAVM **Charge** 2 **Spectral Angle** 0.92

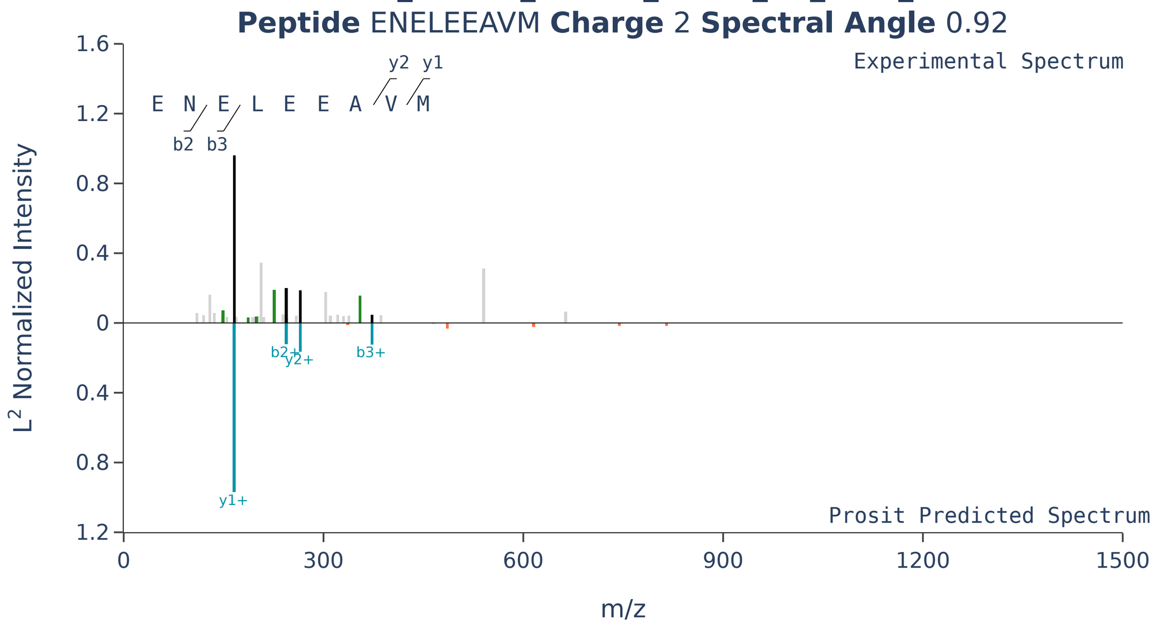


**Source** HRoetschke_211222_160123_HFGoe_IF2_b2R1_24h **Scan** 14696
**Peptide** SAKAGTGIDEL **Charge** 2 **Spectral Angle** 0.73

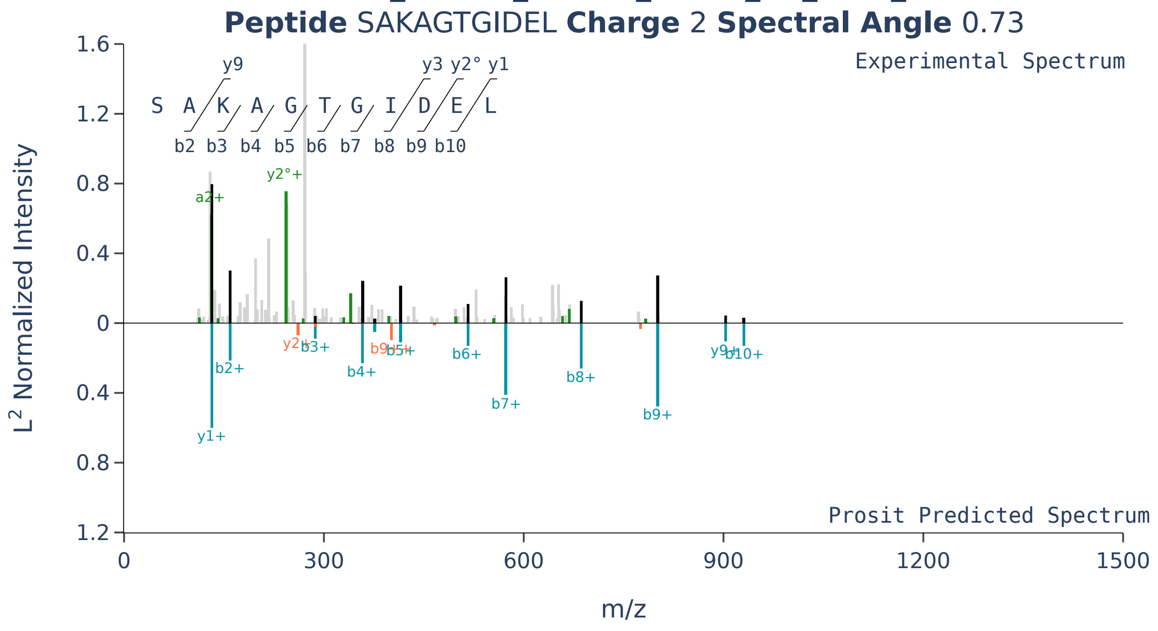


**Source** HRoetschke_211222_160123_HFGoe_IF2_b2R2_4h **Scan** 14438
**Peptide** GGESQFVHVSA **Charge** 1 **Spectral Angle** 0.84

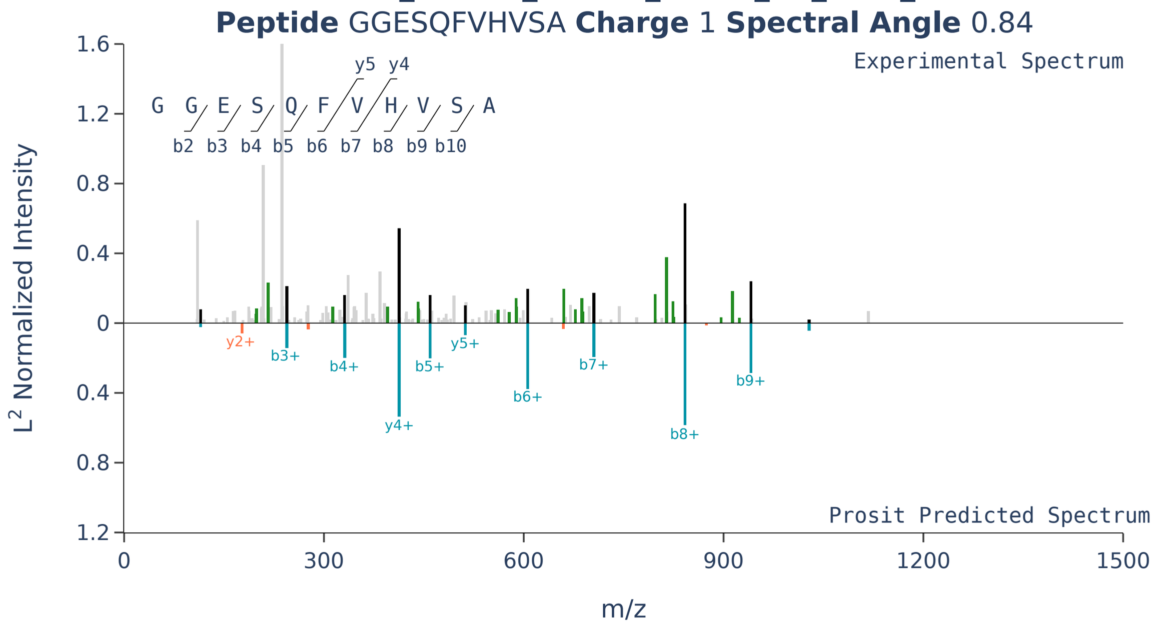


**Source** HRoetschke_211222_160123_HFGoe_IF2_b1R2_1h **Scan** 7210
**Peptide** GAYHVETEN **Charge** 2 **Spectral Angle** 0.84

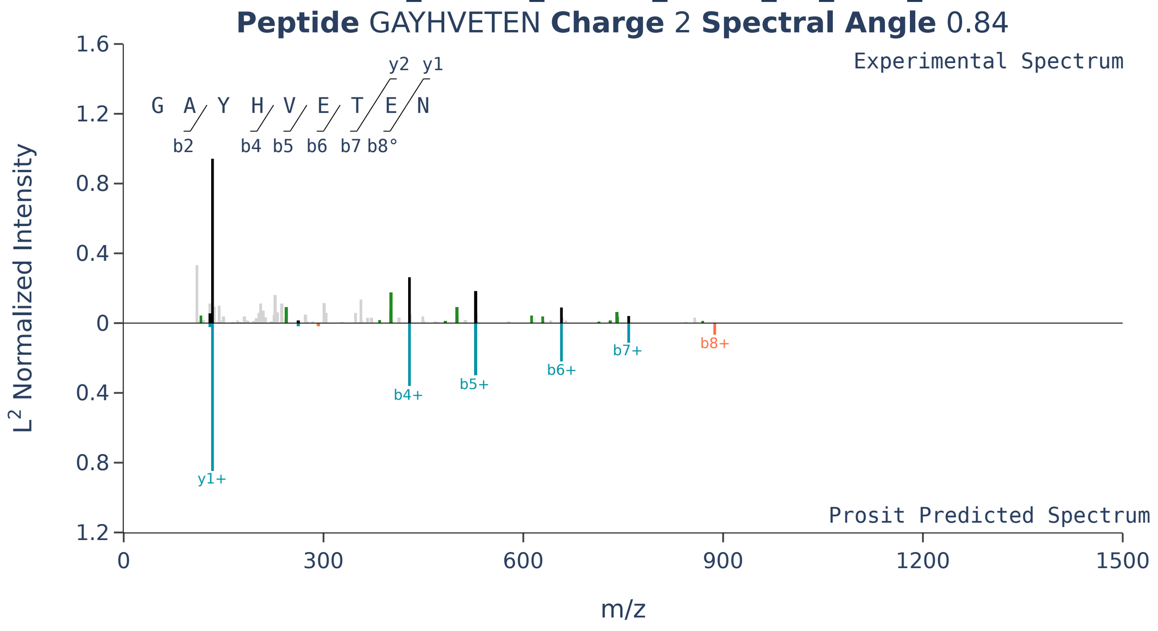


**Source** HRoetschke_211222_160123_HFGoe_IF2_b2R2_24h **Scan** 14186
**Peptide** STLNIPGTGGKSKSV **Charge** 3 **Spectral Angle** 0.8

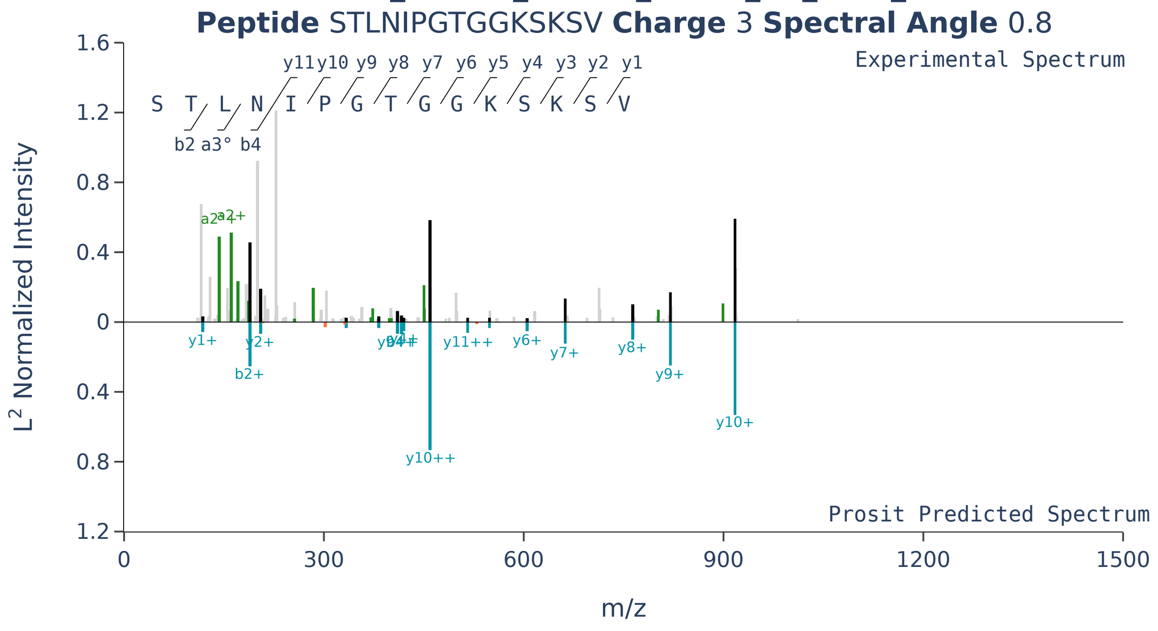


**Source** HRoetschke_211222_160123_HFGoe_IF2_b1R2_1h **Scan** 7471
**Peptide** KIDKPEADPDRV **Charge** 3 **Spectral Angle** 0.73

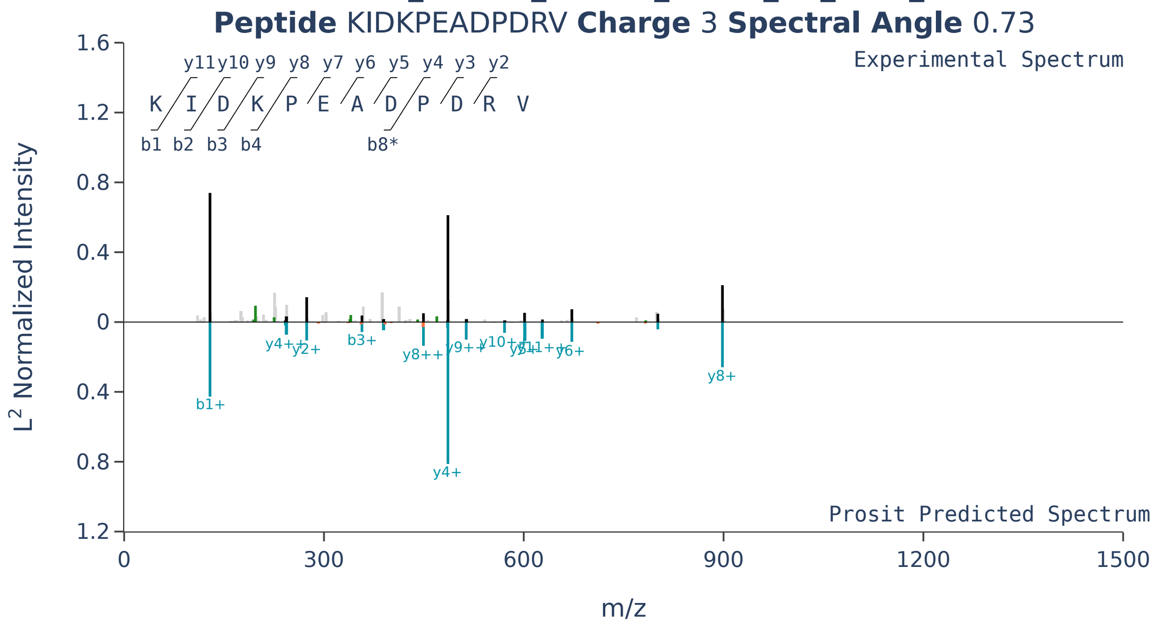


**Source** HRoetschke_211222_160123_HFGoe_IF2_b1R2_1h **Scan** 12273
**Peptide** SLQQGFQKPAQA **Charge** 2 **Spectral Angle** 0.83

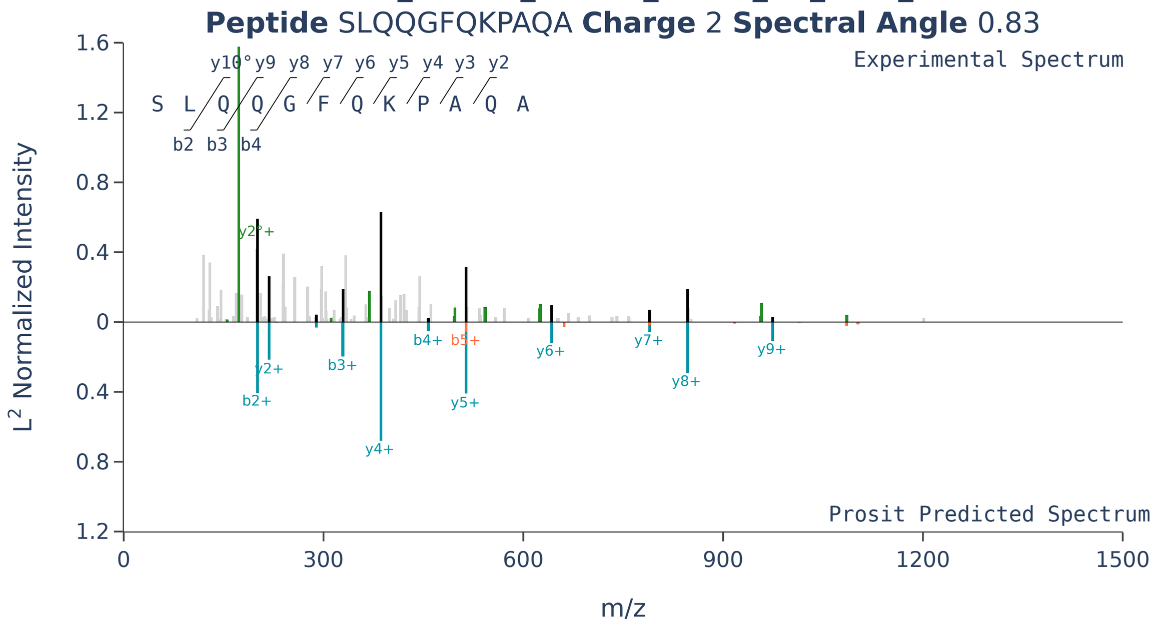


**Source** HRoetschke_211222_160123_HFGoe_IF2_b1R2_24h **Scan** 11960
**Peptide** SLQQGFQKPAQAVNRD **Charge** 3 **Spectral Angle** 0.87

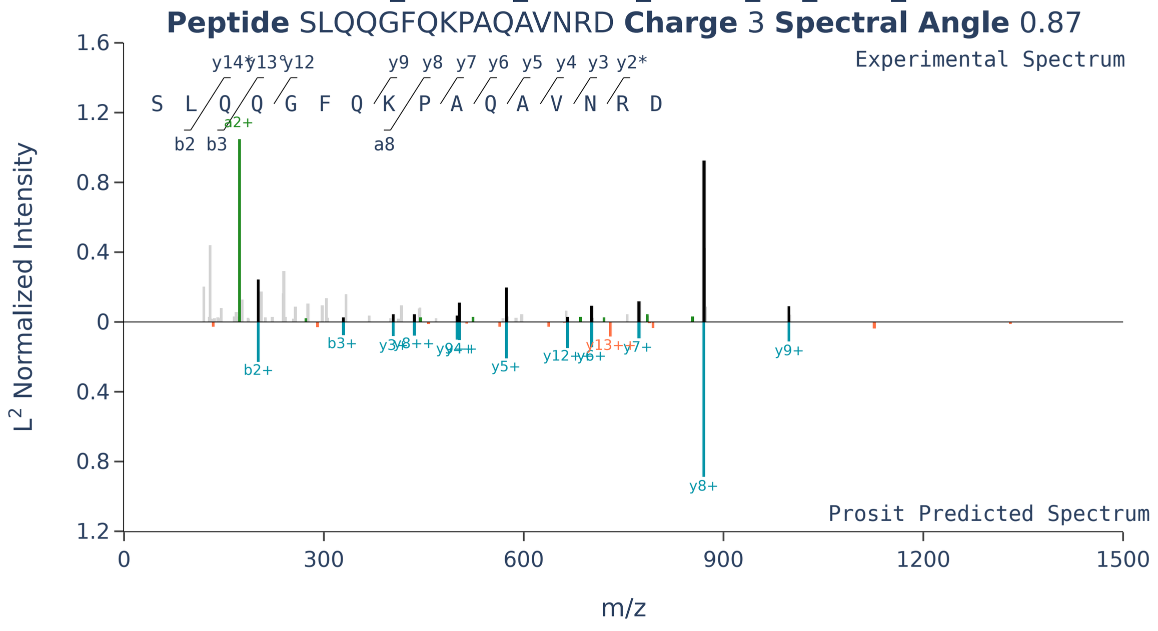


**Source** HRoetschke_211222_160123_HFGoe_IF2_b1R1_2h **Scan** 10447
**Peptide** PQEAERL **Charge** 2 **Spectral Angle** 0.87

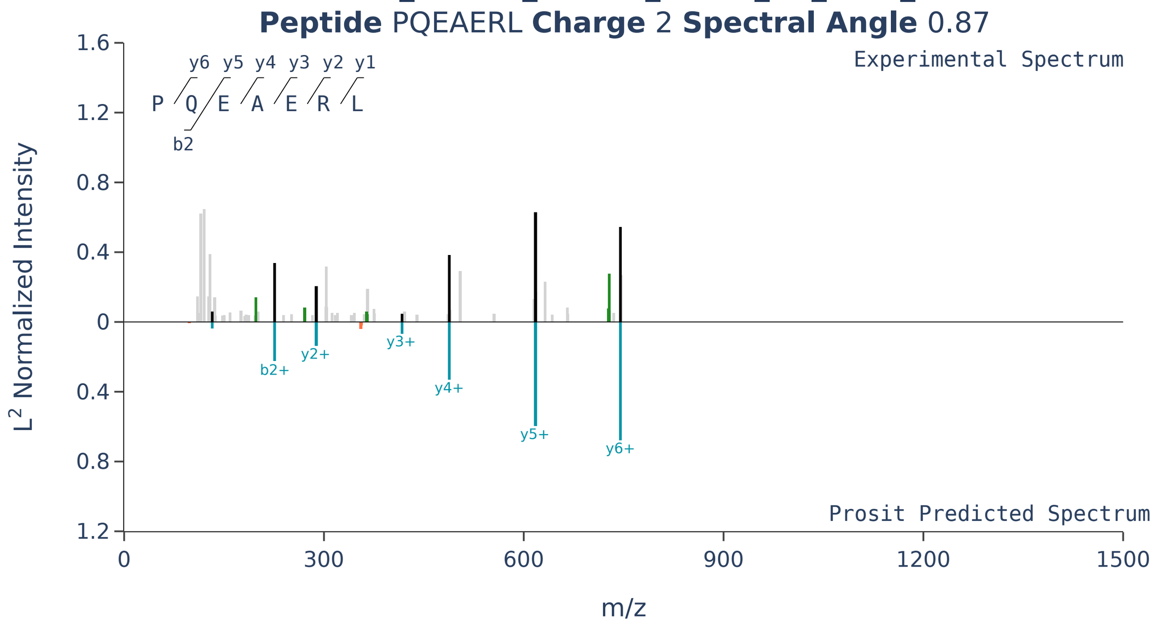


**Source** HRoetschke_211222_160123_HFGoe_IF2_b1R1_2h **Scan** 9498
**Peptide** QFADAGIRKSADD **Charge** 2 **Spectral Angle** 0.8

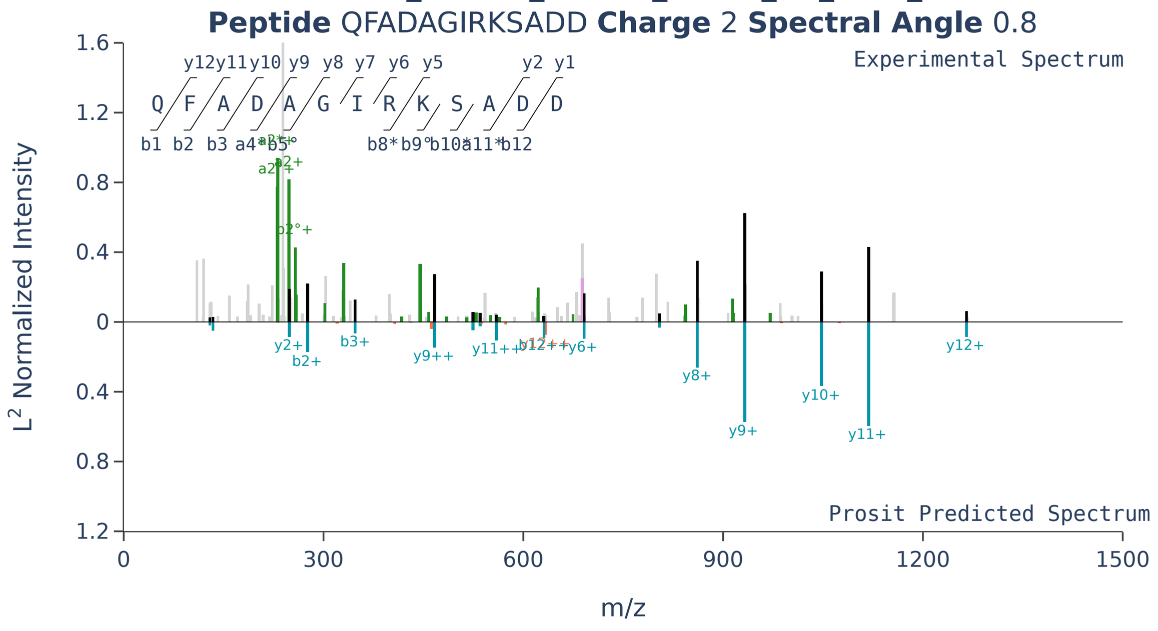

**Source** HRoetschke_211222_160123_HFGoe_IF2_b1R1_4h **Scan** 24231
**Peptide** TGIDELLD **Charge** 1 **Spectral Angle** 0.93

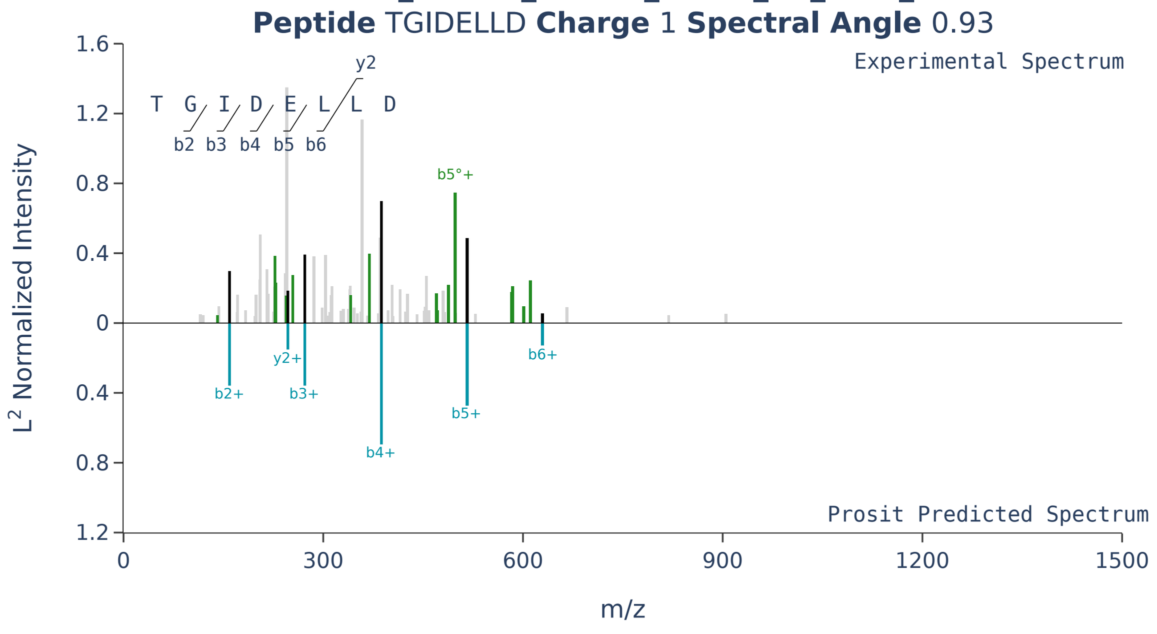


**Source** HRoetschke_211222_160123_HFGoe_IF2_b1R2_24h **Scan** 7174
**Peptide** STKVASGEAGGITQH **Charge** 3 **Spectral Angle** 0.77

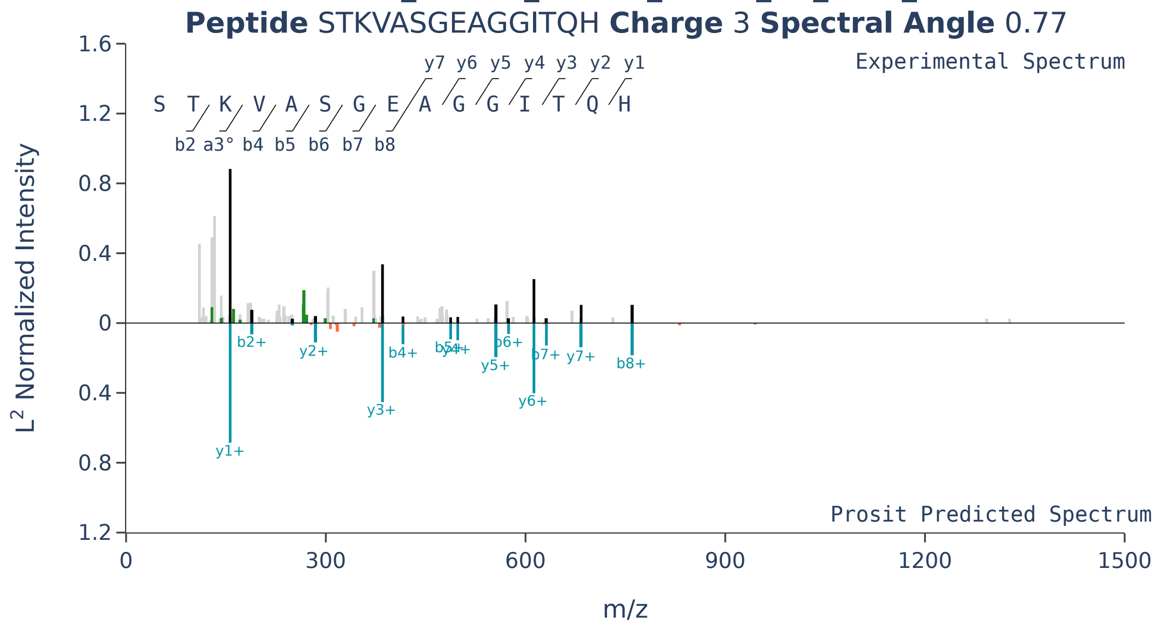


**Source** HRoetschke_211222_160123_HFGoe_IF2_b2R1_4h **Scan** 6862
**Peptide** LRRENEL **Charge** 2 **Spectral Angle** 0.9

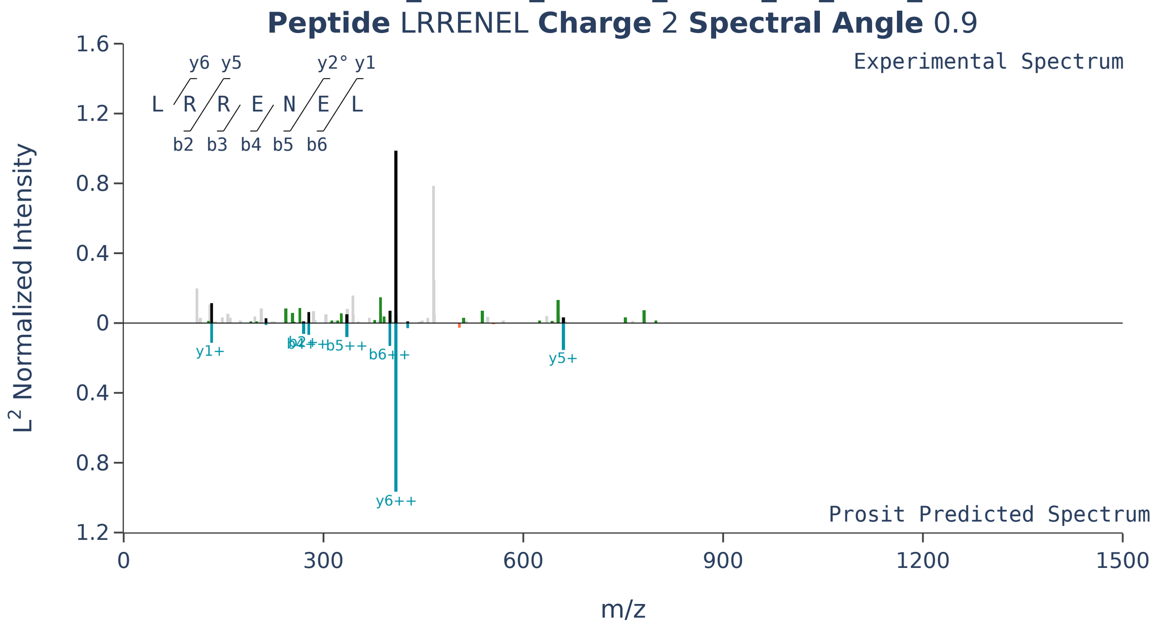


**Source** HRoetschke_211222_160123_HFGoe_IF2_b1R2_24h **Scan** 24005
**Peptide** ATDIVVL **Charge** 1 **Spectral Angle** 0.8

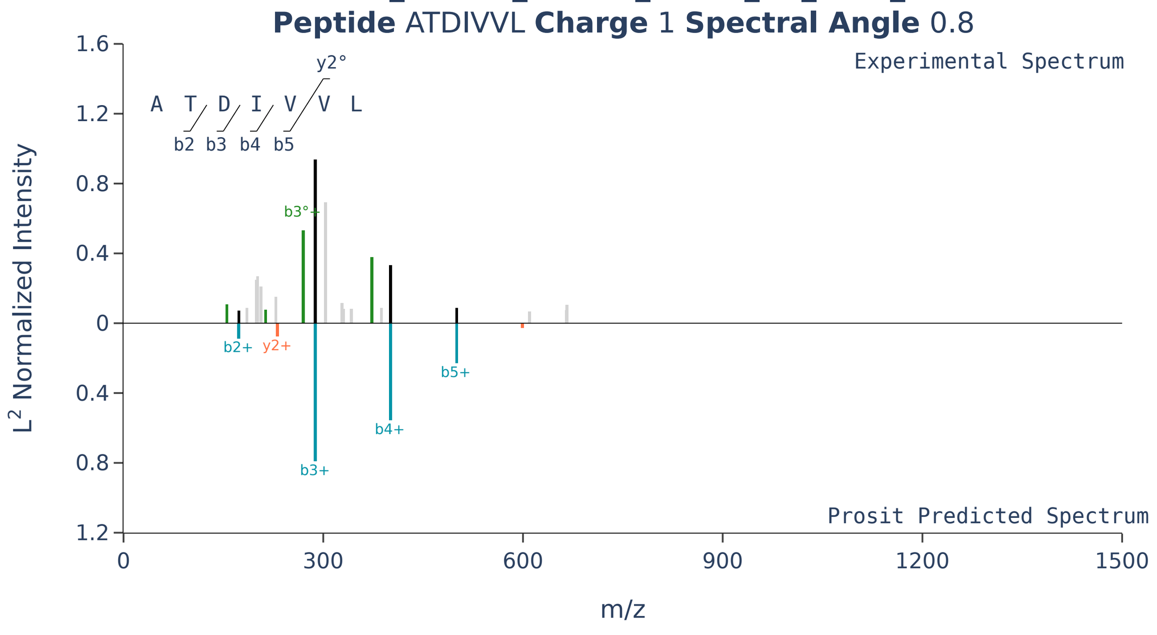


**Source** HRoetschke_211222_160123_HFGoe_IF2_b2R2_2h **Scan** 6748
**Peptide** AVNKIDKPEADPDRVKN **Charge** 3 **Spectral Angle** 0.76

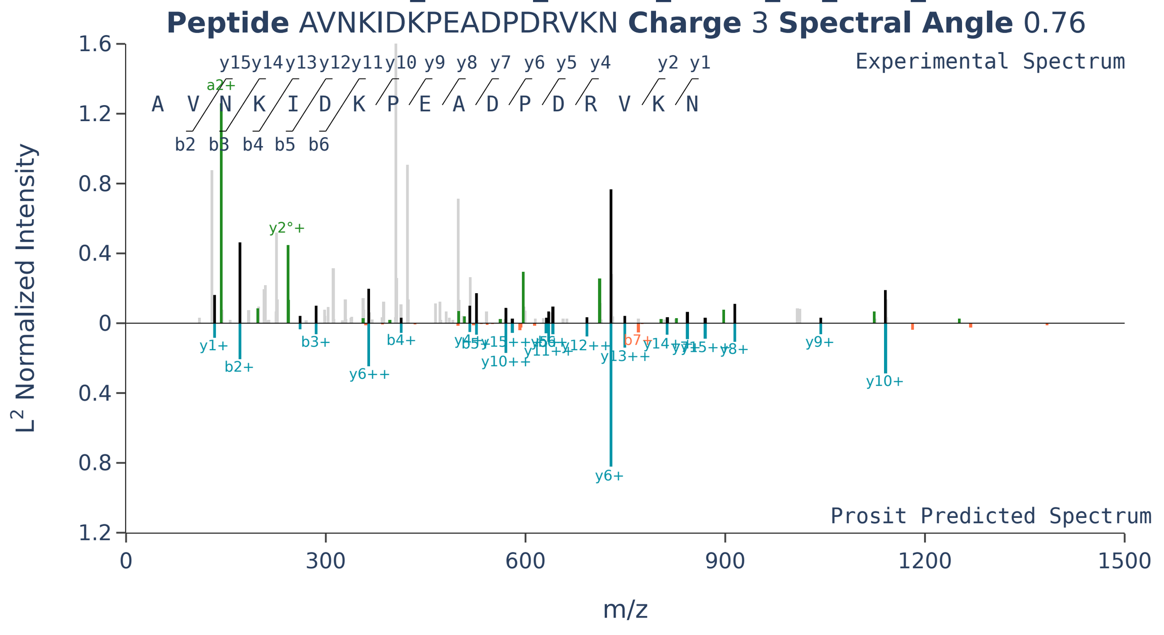


**Source** HRoetschke_211222_160123_HFGoe_IF2_b2R2_2h **Scan** 7237
**Peptide** DASARKVIE **Charge** 2 **Spectral Angle** 0.8

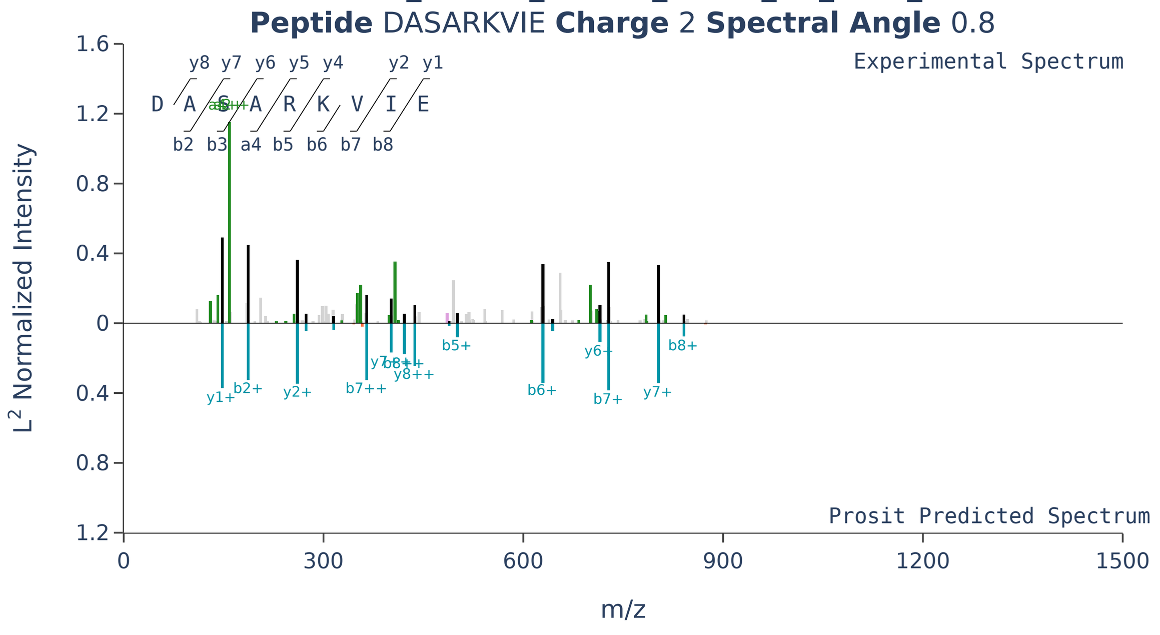


**Source** HRoetschke_211222_160123_HFGoe_IF2_b1R1_24h **Scan** 8563
**Peptide** LYRQGKFREV **Charge** 3 **Spectral Angle** 0.93

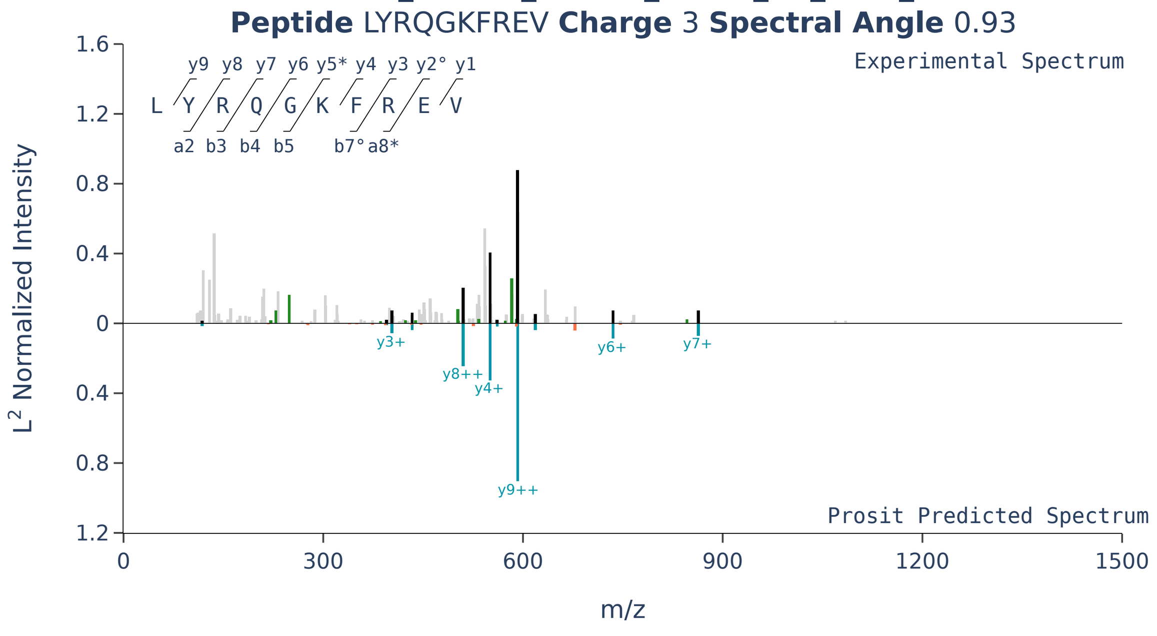


**Source** HRoetschke_211222_160123_HFGoe_IF2_b1R1_24h **Scan** 6356
**Peptide** VNKIDKPEADPDRVKN **Charge** 2 **Spectral Angle** 0.78

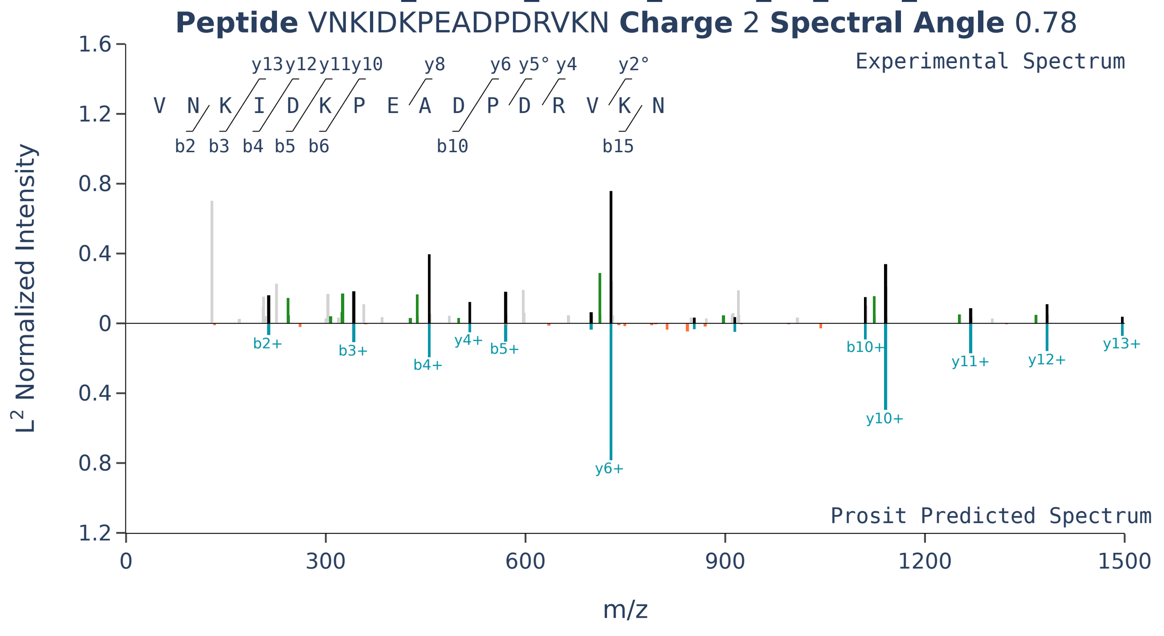


**Source** HRoetschke_211222_160123_HFGoe_IF2_b1R1_24h **Scan** 15636
**Peptide** SGVPAAGDEVTVVR **Charge** 2 **Spectral Angle** 0.84

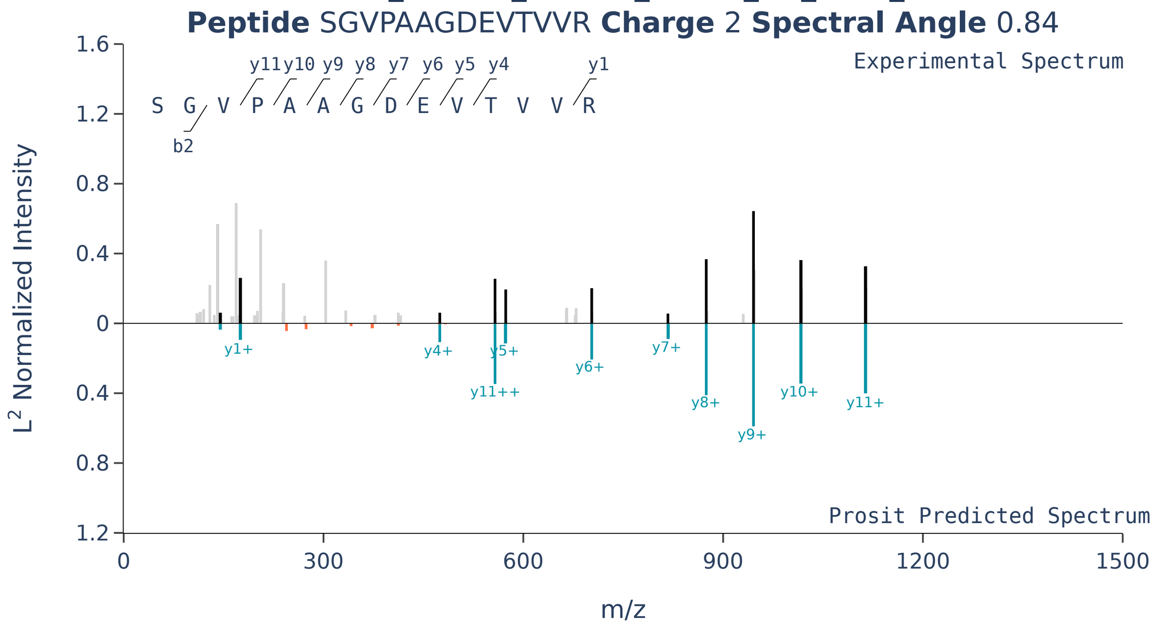


**Source** HRoetschke_211222_160123_HFGoe_IF2_b1R1_4h **Scan** 7649
**Peptide** KLDKPEADPDRV **Charge** 3 **Spectral Angle** 0.76

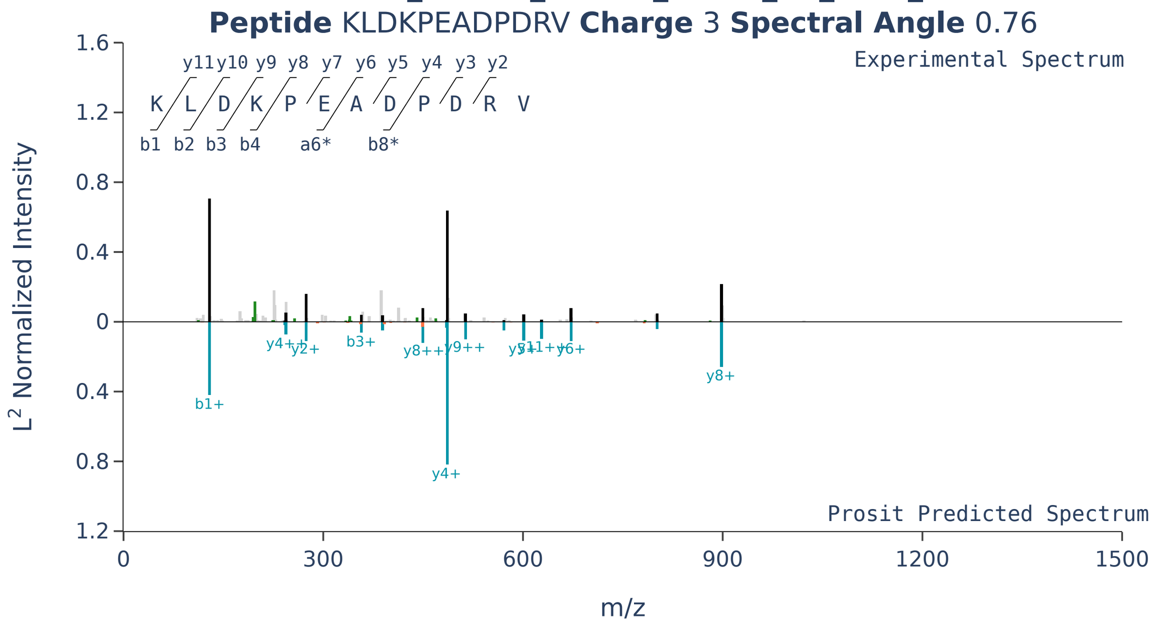


**Source** HRoetschke_211222_160123_HFGoe_IF2_b1R2_24h **Scan** 23993
**Peptide** QATDIVVL **Charge** 2 **Spectral Angle** 0.84

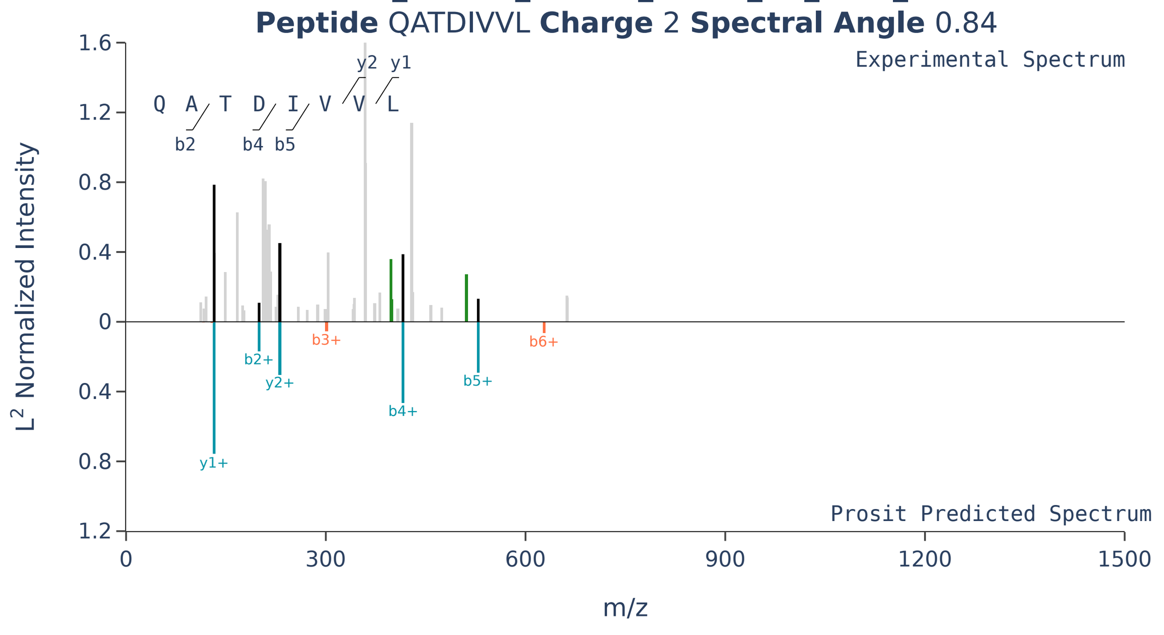


**Source** HRoetschke_211222_160123_HFGoe_IF2_b2R2_4h **Scan** 14167
**Peptide** RDNVVIYE **Charge** 2 **Spectral Angle** 0.83

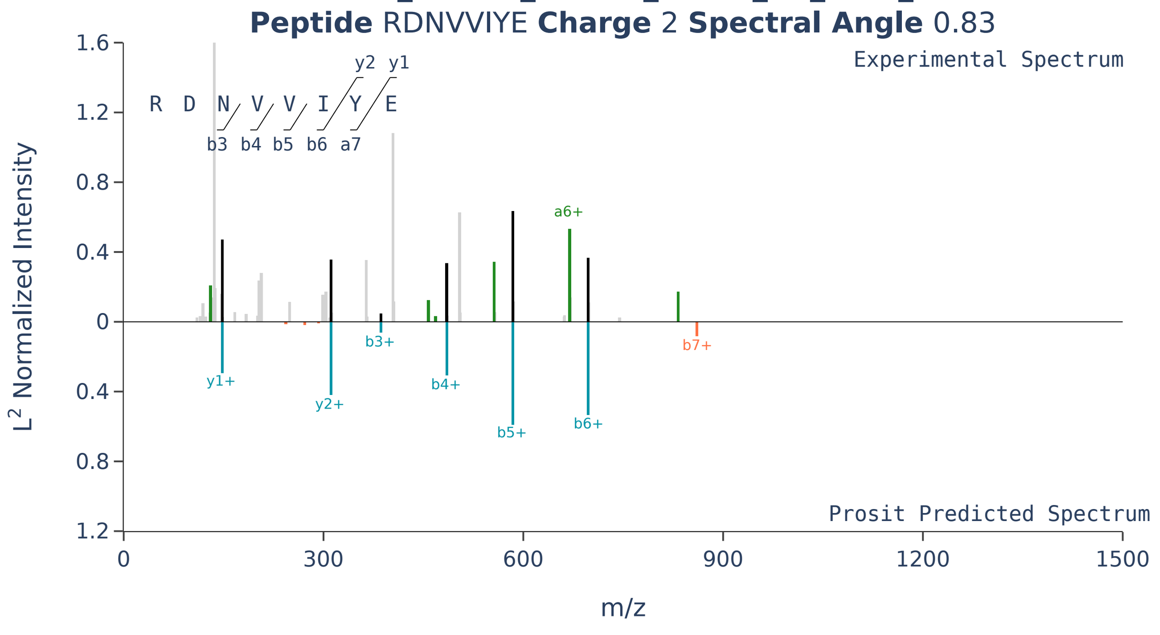


**Source** HRoetschke_211222_160123_HFGoe_IF2_b2R1_1h **Scan** 7523
**Peptide** AQEKQTLIDH **Charge** 3 **Spectral Angle** 0.91

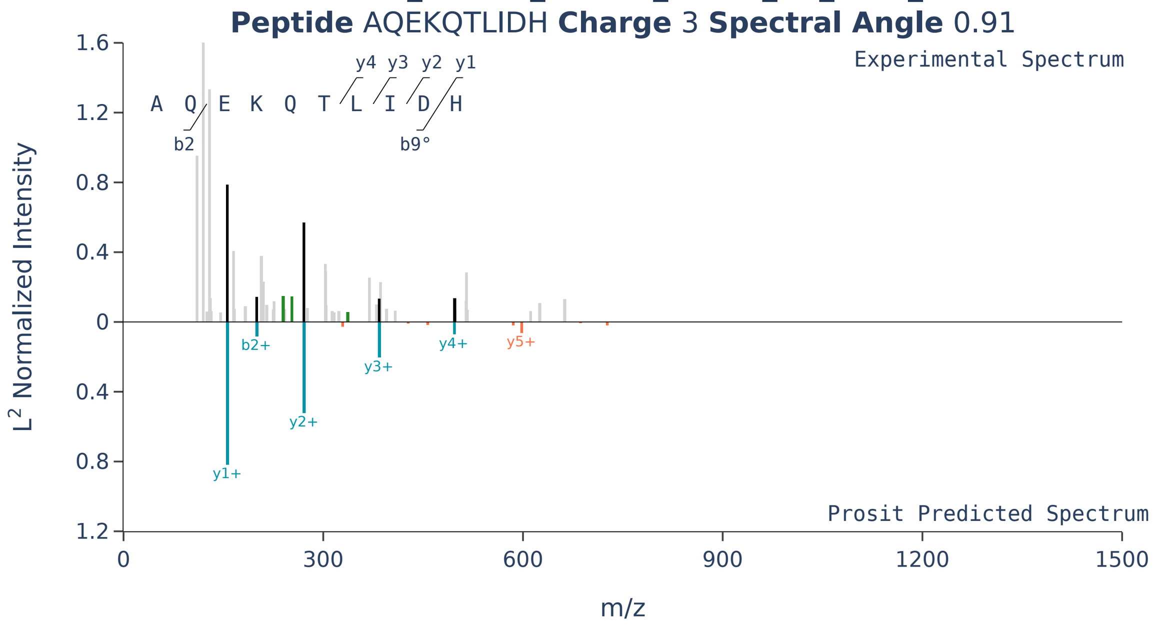


**Source** HRoetschke_211222_160123_HFGoe_IF2_b1R2_2h **Scan** 6323
**Peptide** DSSDYHVTTSQHARQAED **Charge** 2 **Spectral Angle** 0.82

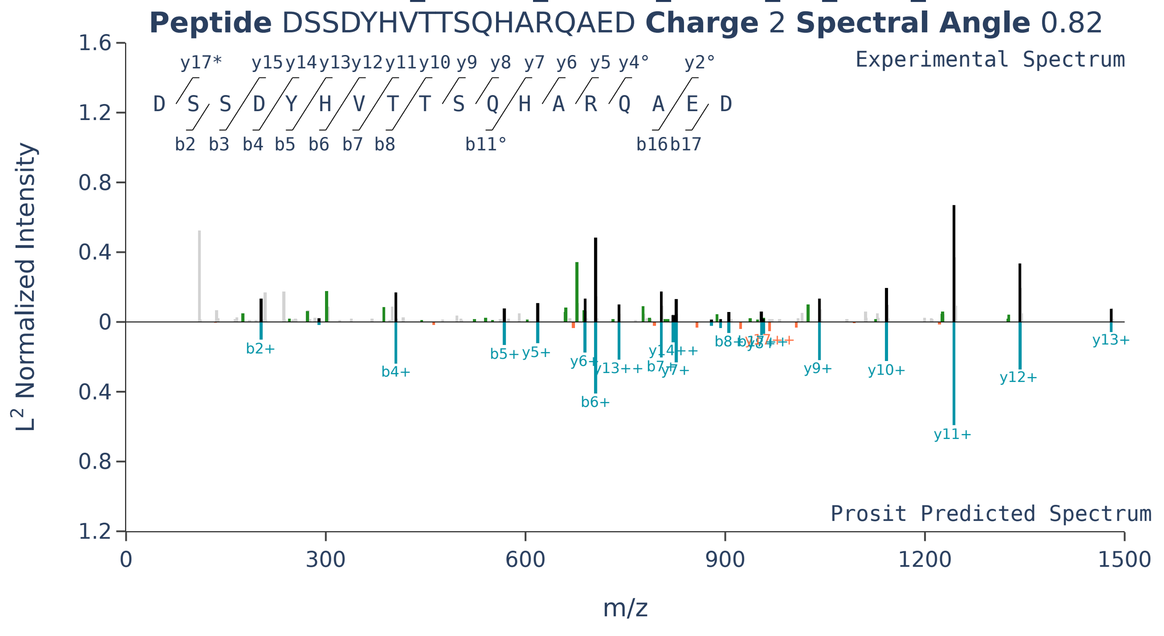


**Source** HRoetschke_211222_160123_HFGoe_IF2_b1R2_1h **Scan** 8285
**Peptide** ELANKMAVKG **Charge** 3 **Spectral Angle** 0.91

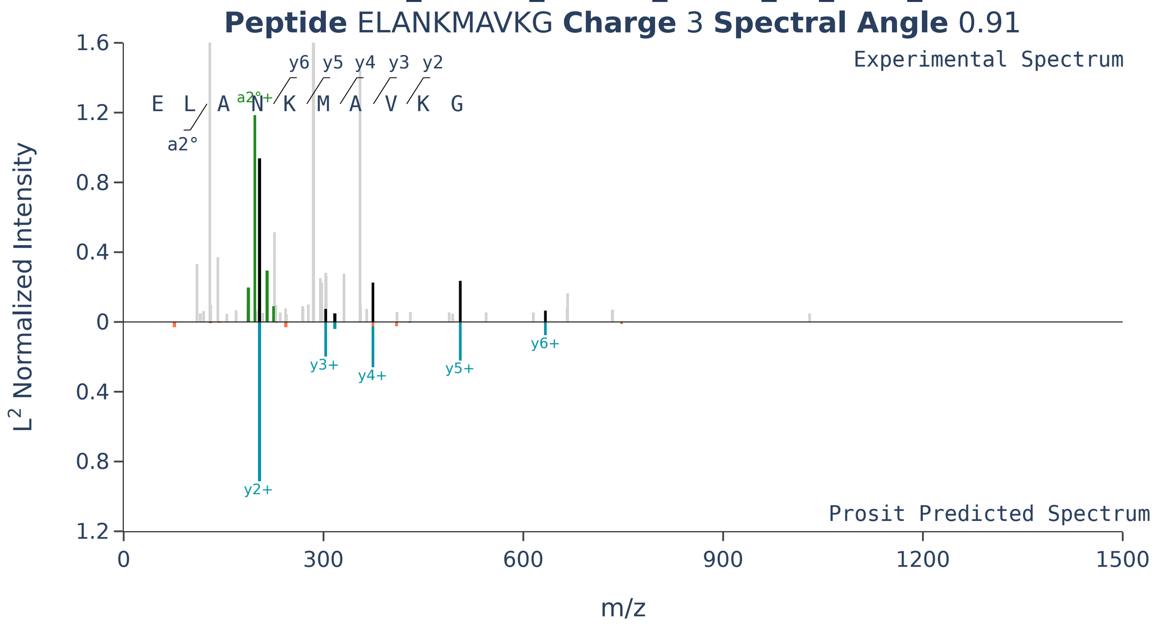

**Source** HRoetschke_211222_160123_HFGoe_IF2_b1R1_24h **Scan** 7290
**Peptide** SARKVIE **Charge** 2 **Spectral Angle** 0.89

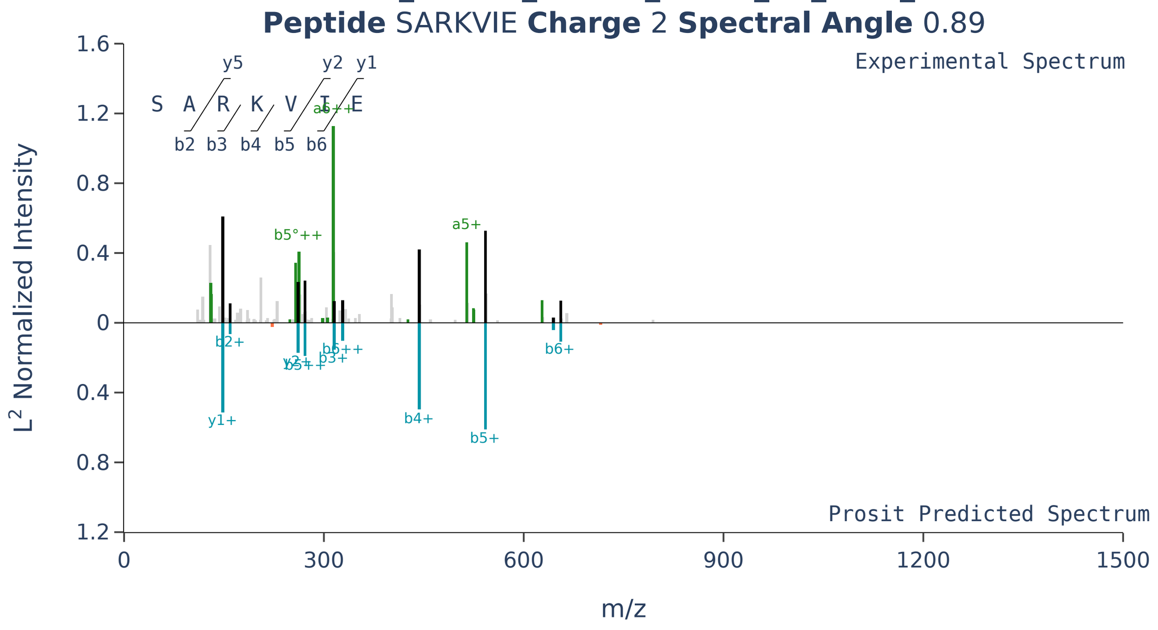


**Source** HRoetschke_211222_160123_HFGoe_IF2_b2R2_4h **Scan** 13090
**Peptide** DGVMPQTIE **Charge** 2 **Spectral Angle** 0.86

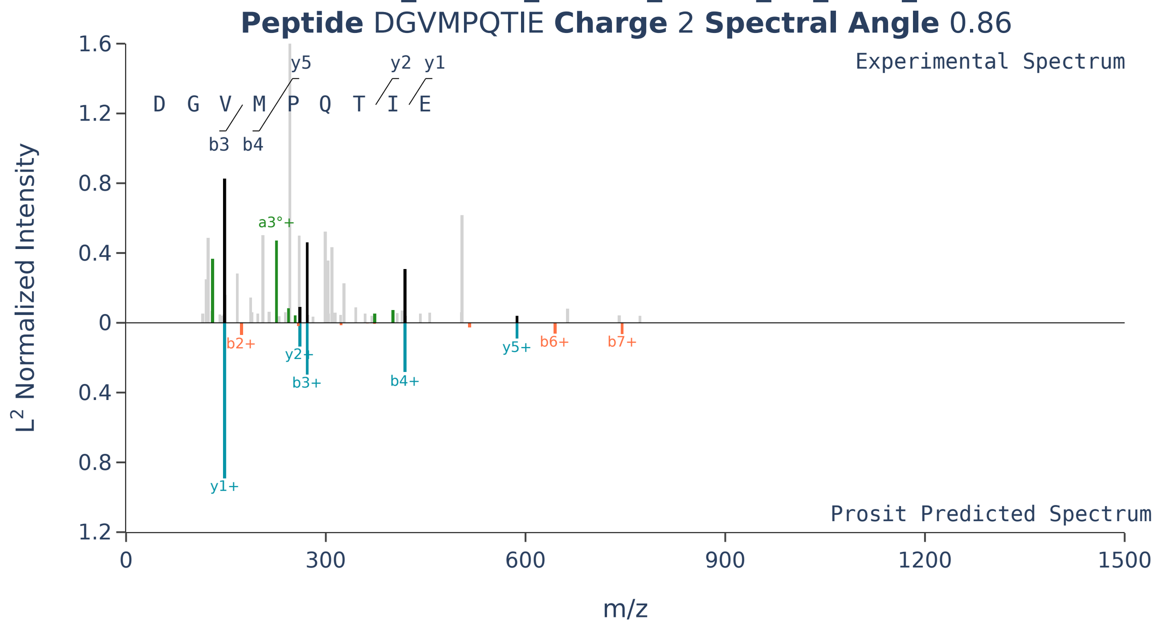


**Source** HRoetschke_211222_160123_HFGoe_IF2_b1R1_2h **Scan** 6572
**Peptide** YHVETEN **Charge** 2 **Spectral Angle** 0.87

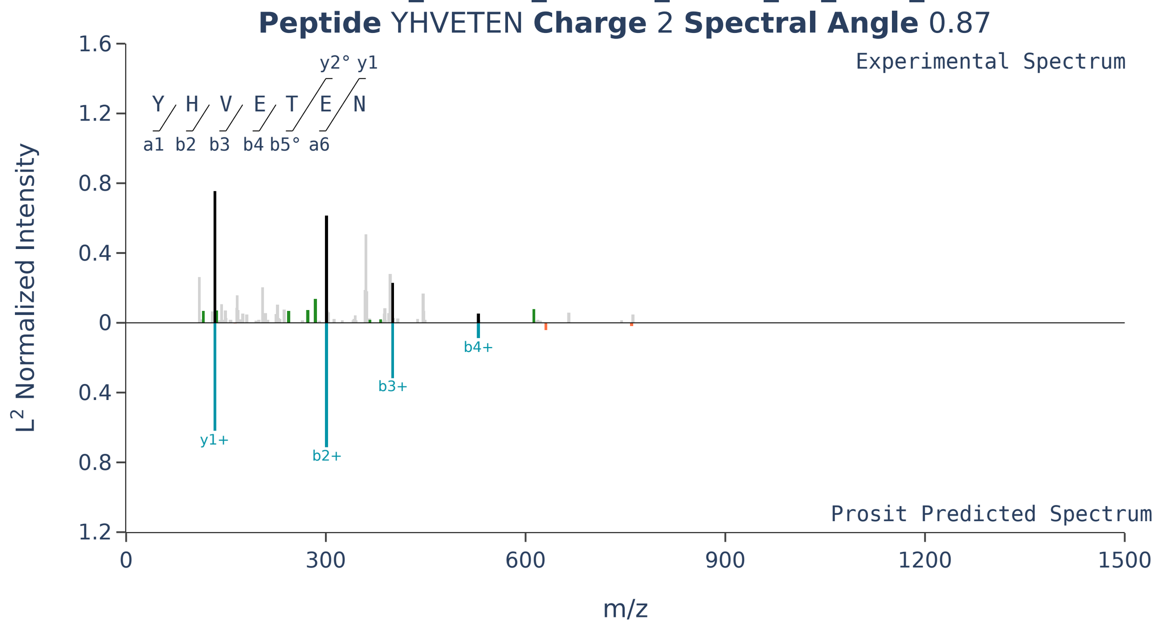


**Source** HRoetschke_211222_160123_HFGoe_IF2_b1R1_4h **Scan** 21260
**Peptide** NGMITFLDTPGH **Charge** 2 **Spectral Angle** 0.89

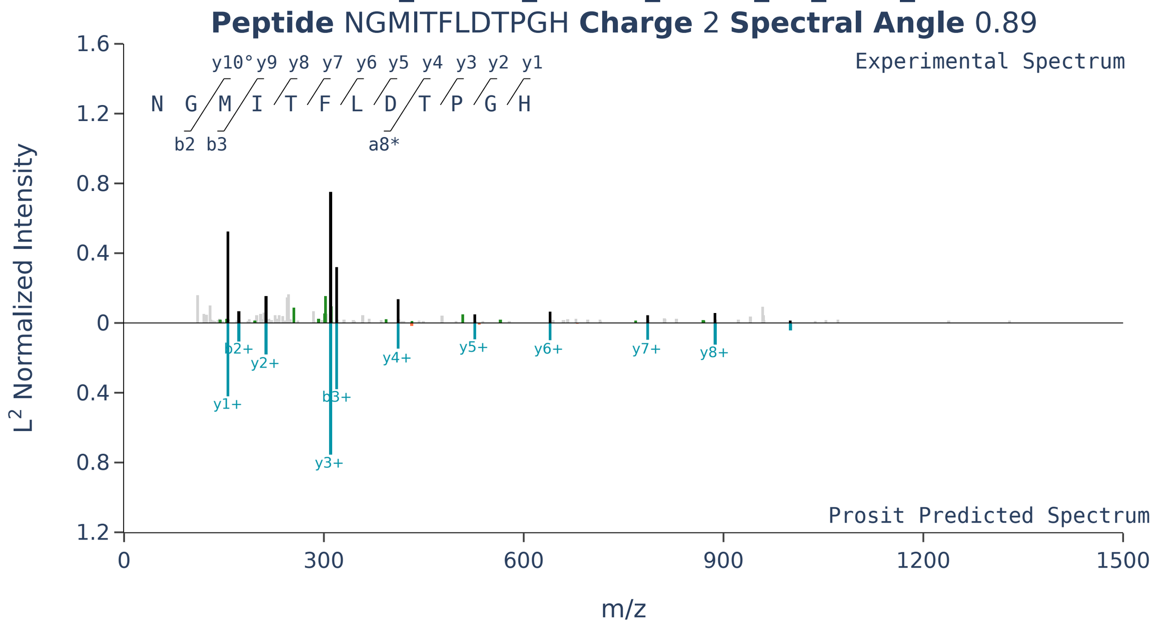


**Source** HRoetschke_211222_160123_HFGoe_IF2_b2R1_24h **Scan** 7821
**Peptide** RGAQATDL **Charge** 2 **Spectral Angle** 0.89

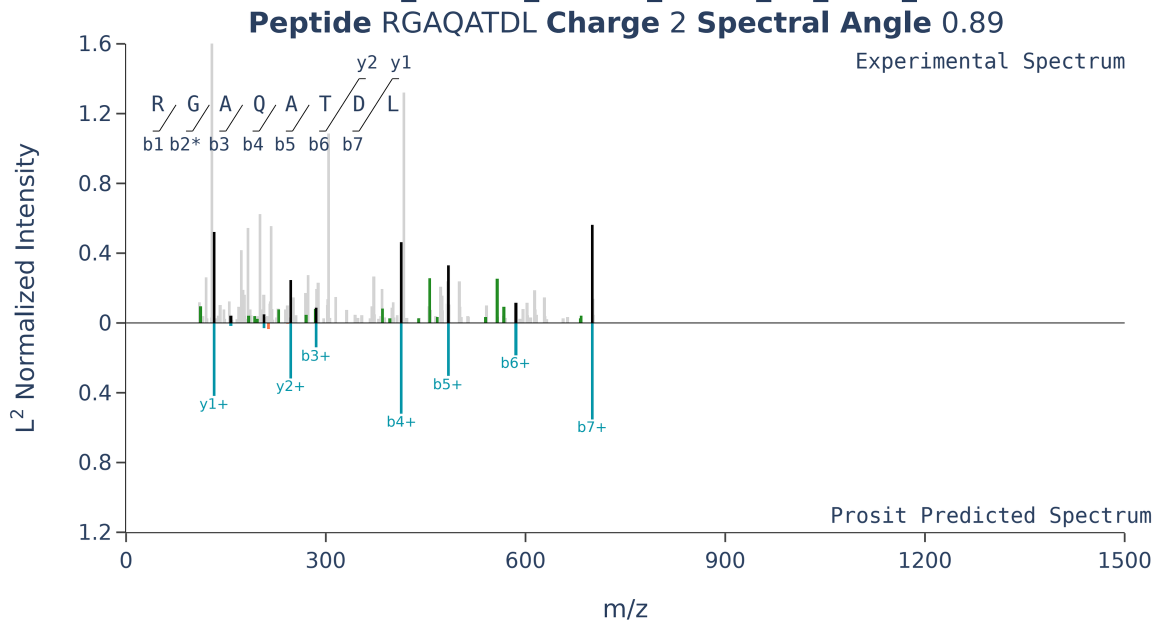


**Source** HRoetschke_211222_160123_HFGoe_IF2_b1R1_24h **Scan** 13115
**Peptide** LHKGDIVLC **Charge** 2 **Spectral Angle** 0.87

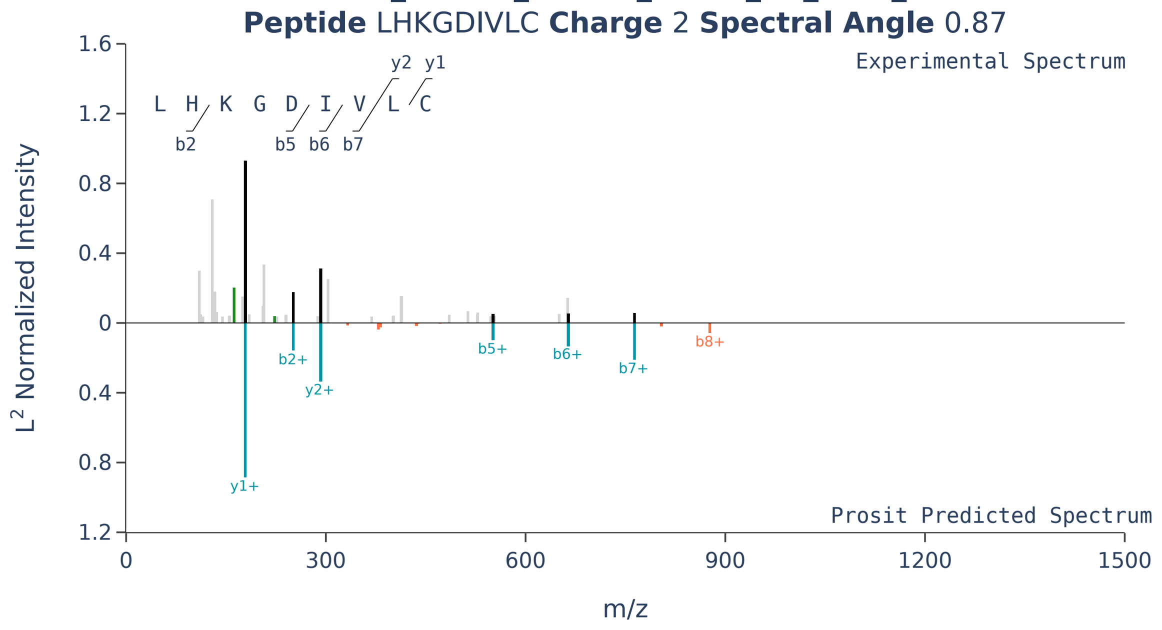


**Source** HRoetschke_211222_160123_HFGoe_IF2_b1R1_4h **Scan** 16296
**Peptide** TINQVIDQE **Charge** 2 **Spectral Angle** 0.8

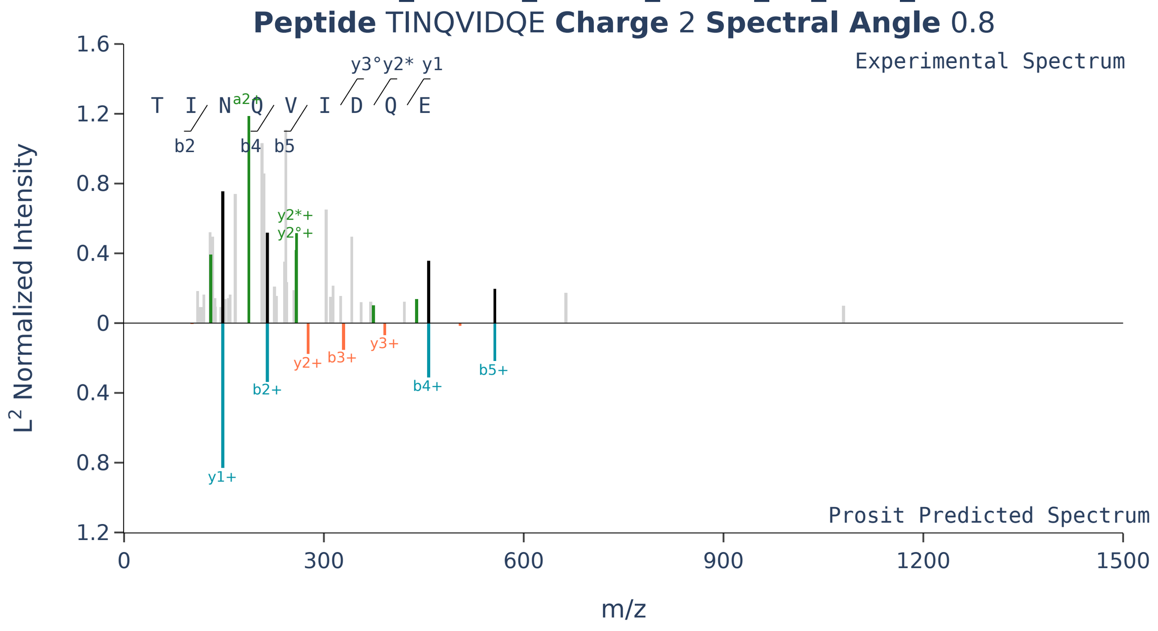


**Source** HRoetschke_211222_160123_HFGoe_IF2_b1R1_2h **Scan** 11024
**Peptide** GIGVKNYNDVRTGD **Charge** 2 **Spectral Angle** 0.76

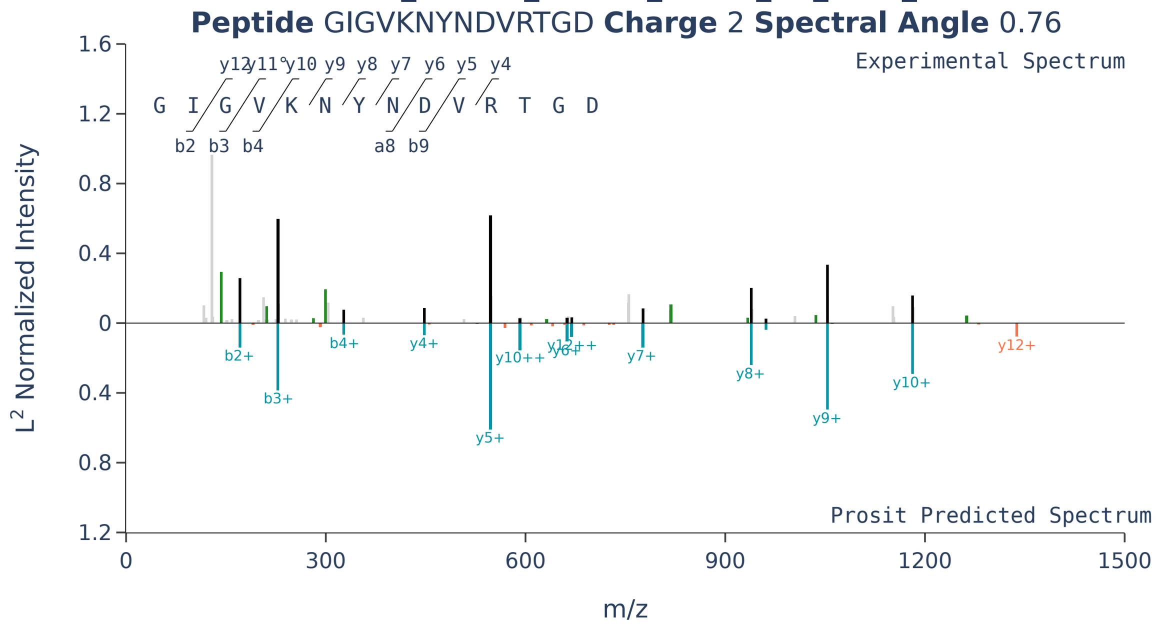


**Source** HRoetschke_211222_160123_HFGoe_IF2_b1R1_4h **Scan** 12135
**Peptide** ARKVIEAESL **Charge** 2 **Spectral Angle** 0.72

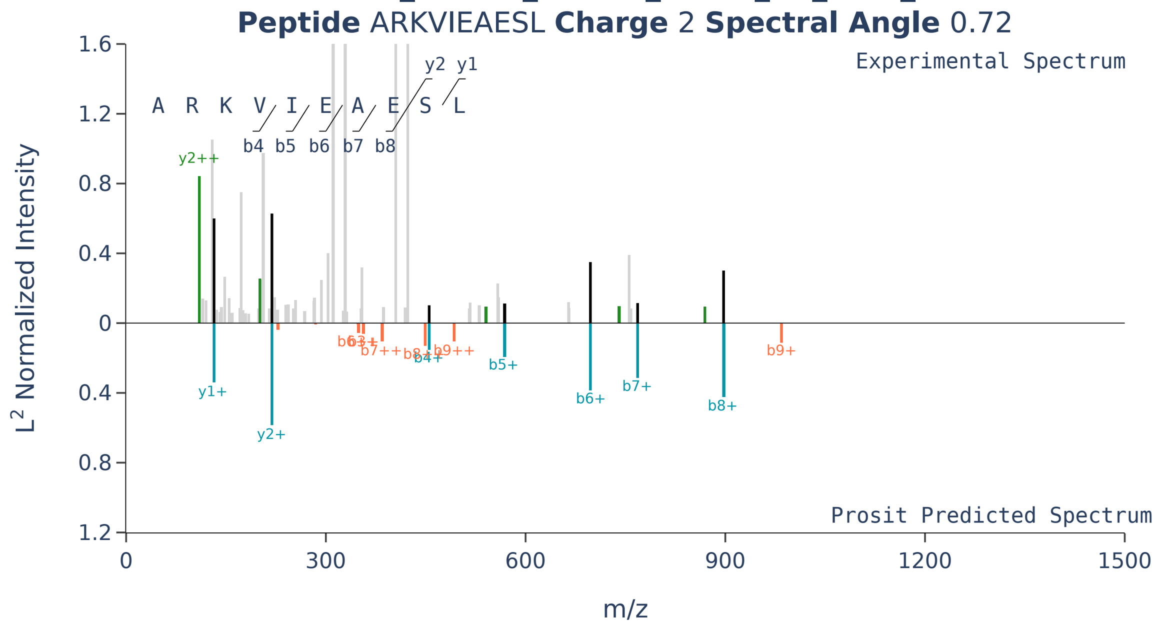


**Source** HRoetschke_211222_160123_HFGoe_IF2_b1R1_2h **Scan** 8723
**Peptide** DPDRVKNELSQ **Charge** 2 **Spectral Angle** 0.76

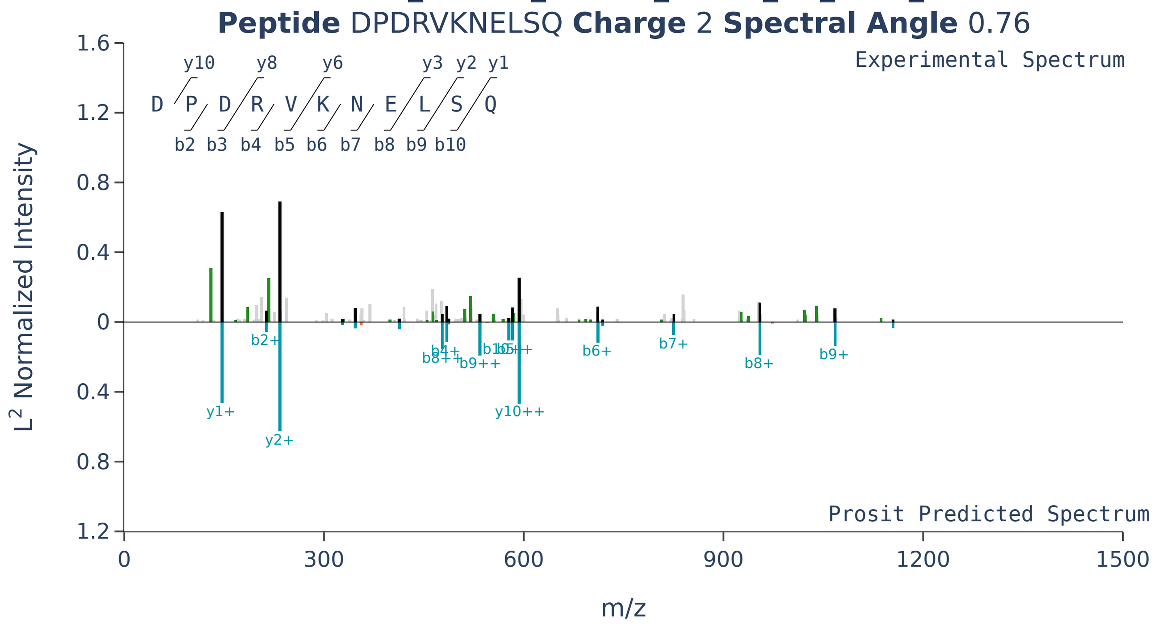


**Source** HRoetschke_211222_160123_HFGoe_IF2_b1R1_4h **Scan** 17655
**Peptide** DVFKSPKF **Charge** 2 **Spectral Angle** 0.78

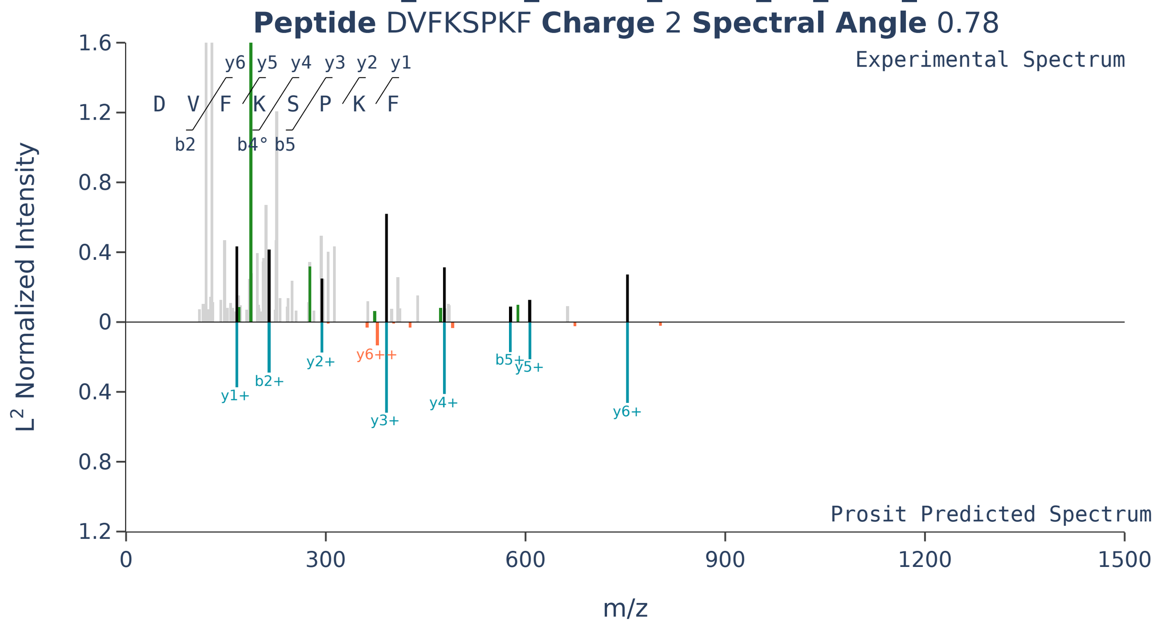


**Source** HRoetschke_211222_160123_HFGoe_IF2_b1R2_4h **Scan** 8354
**Peptide** QQGFQKPAQA **Charge** 2 **Spectral Angle** 0.74

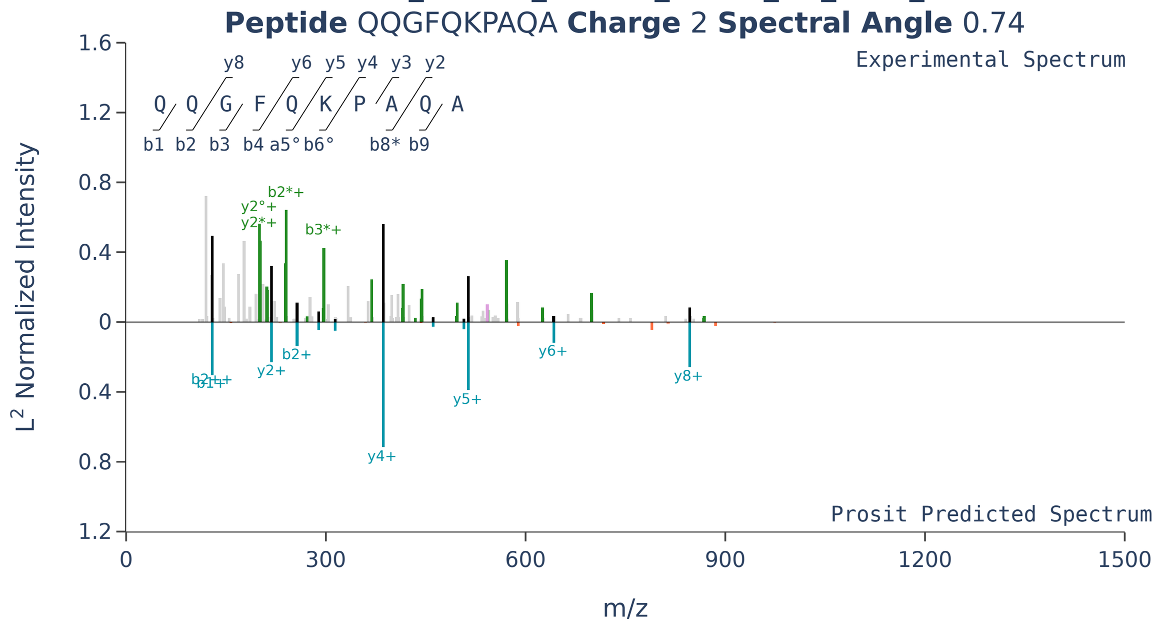


**Source** HRoetschke_211222_160123_HFGoe_IF2_b1R1_2h **Scan** 8769
**Peptide** AAERQTSVERL **Charge** 3 **Spectral Angle** 0.78

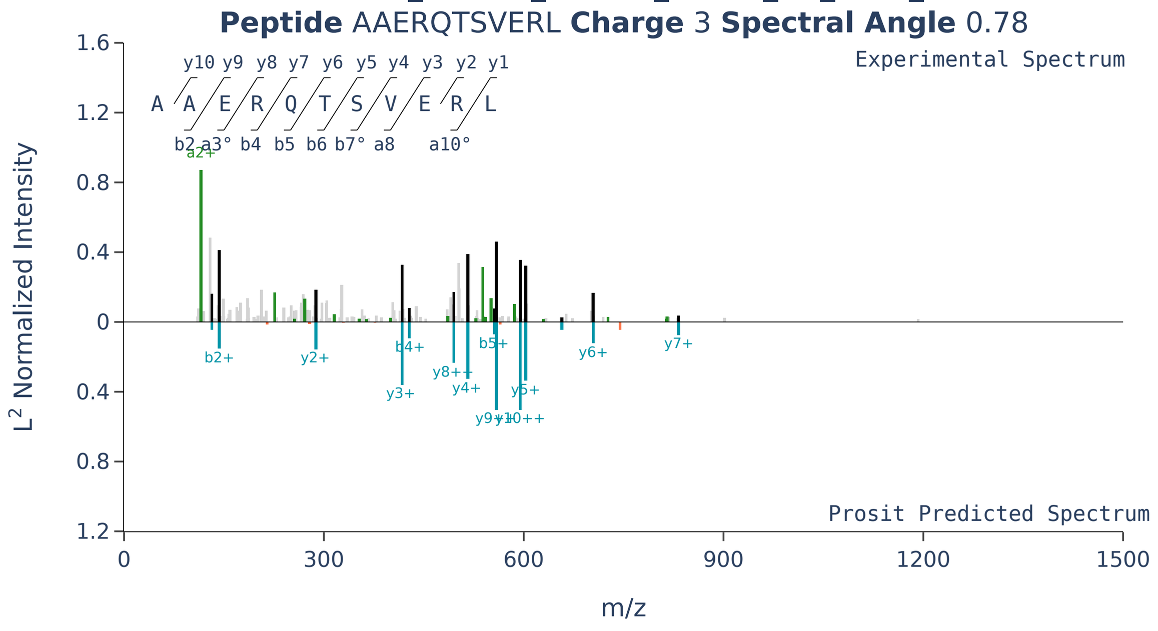


**Source** HRoetschke_211222_160123_HFGoe_IF2_b1R1_2h **Scan** 5411
**Peptide** EVTVVRDEKKAR **Charge** 4 **Spectral Angle** 0.82

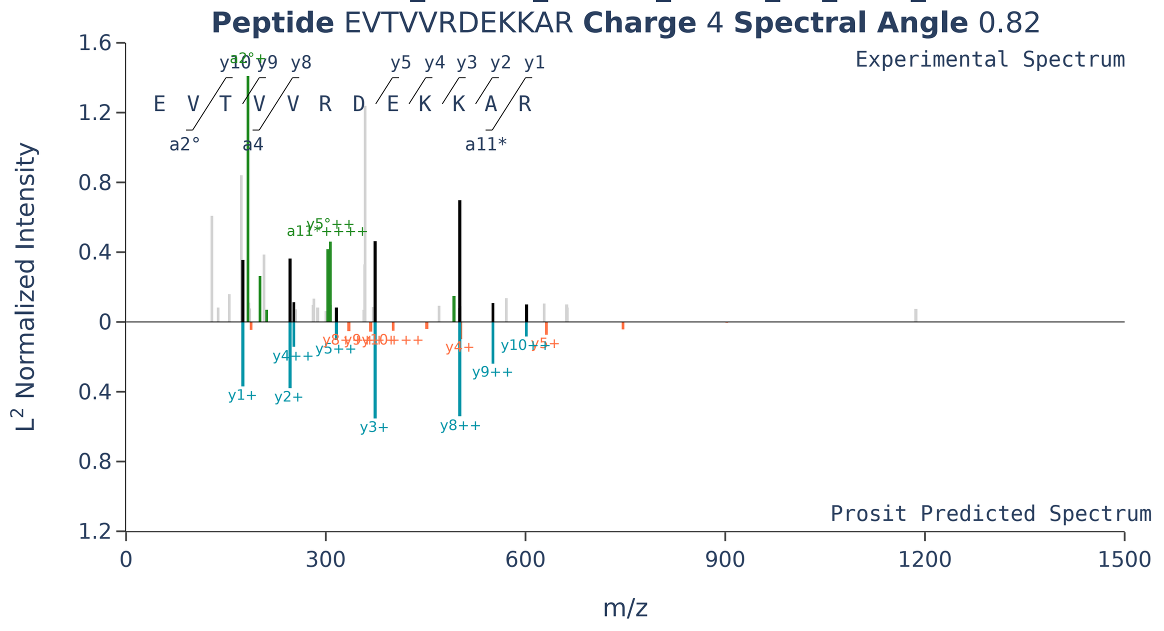


**Source** HRoetschke_211222_160123_HFGoe_IF2_b1R1_1h **Scan** 8605
**Peptide** SVSAQEKQTL **Charge** 2 **Spectral Angle** 0.8

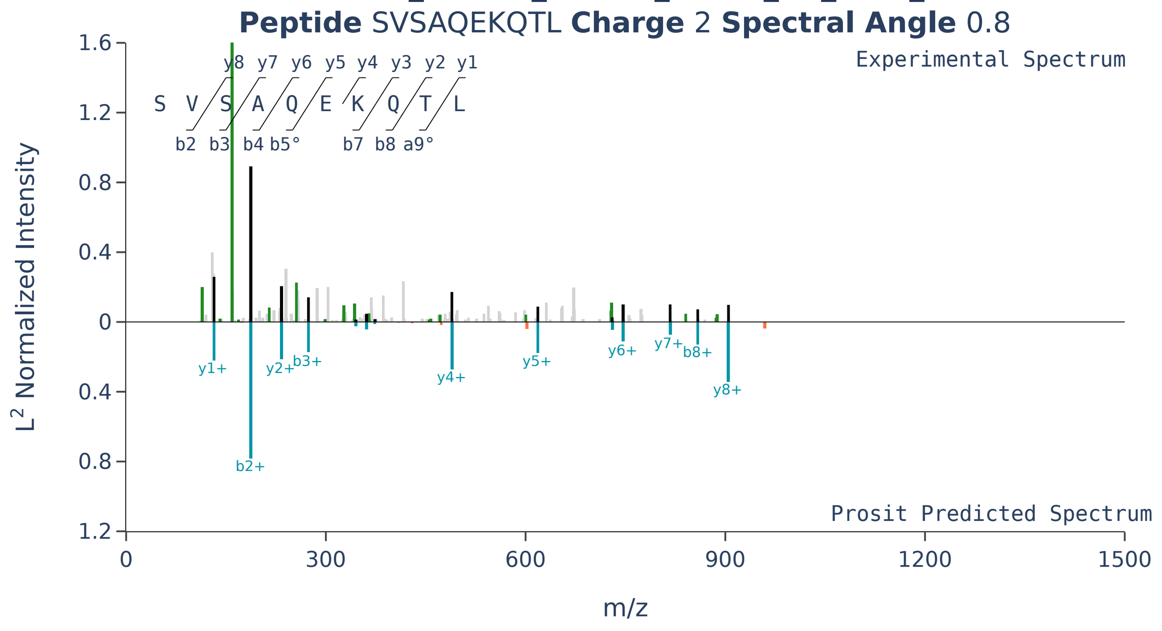

**Source** HRoetschke_211222_160123_HFGoe_IF2_b2R1_24h **Scan** 9355
**Peptide** HVDHGKTSLL **Charge** 3 **Spectral Angle** 0.83

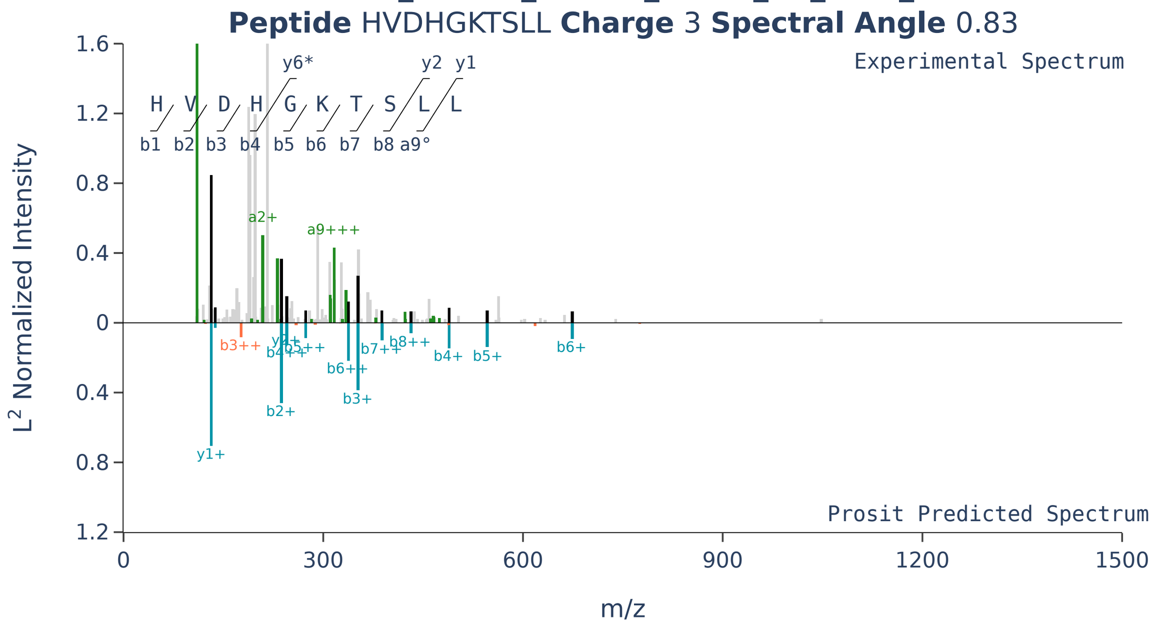


**Source** HRoetschke_211222_160123_HFGoe_IF2_b1R1_4h **Scan** 8875
**Peptide** QQFADAGIRKS **Charge** 2 **Spectral Angle** 0.81

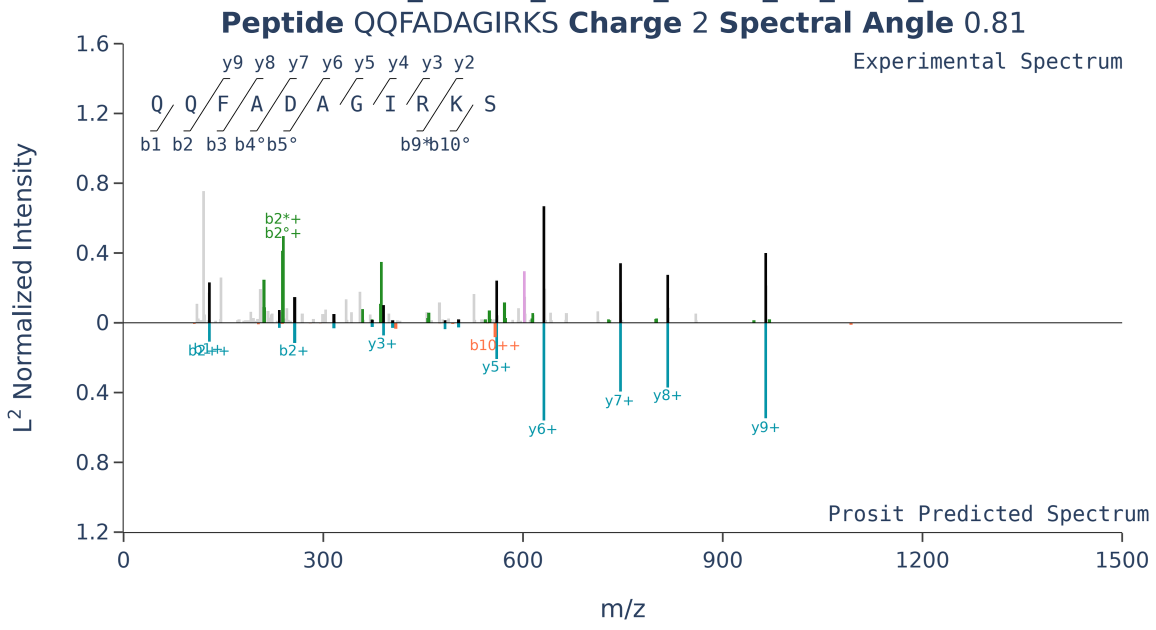


**Source** HRoetschke_211222_160123_HFGoe_IF2_b1R2_1h **Scan** 16824
**Peptide** GETITVGELANKM **Charge** 2 **Spectral Angle** 0.77

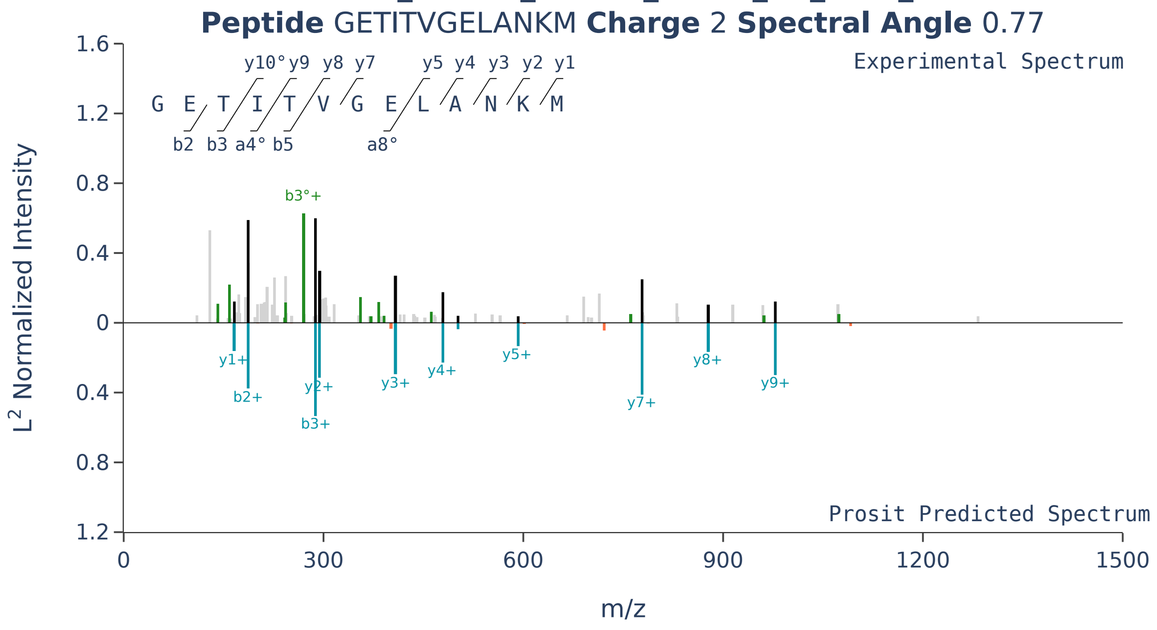


**Source** HRoetschke_211222_160123_HFGoe_IF2_b1R1_24h **Scan** 7155
**Peptide** VKGSQVIKA **Charge** 2 **Spectral Angle** 0.81

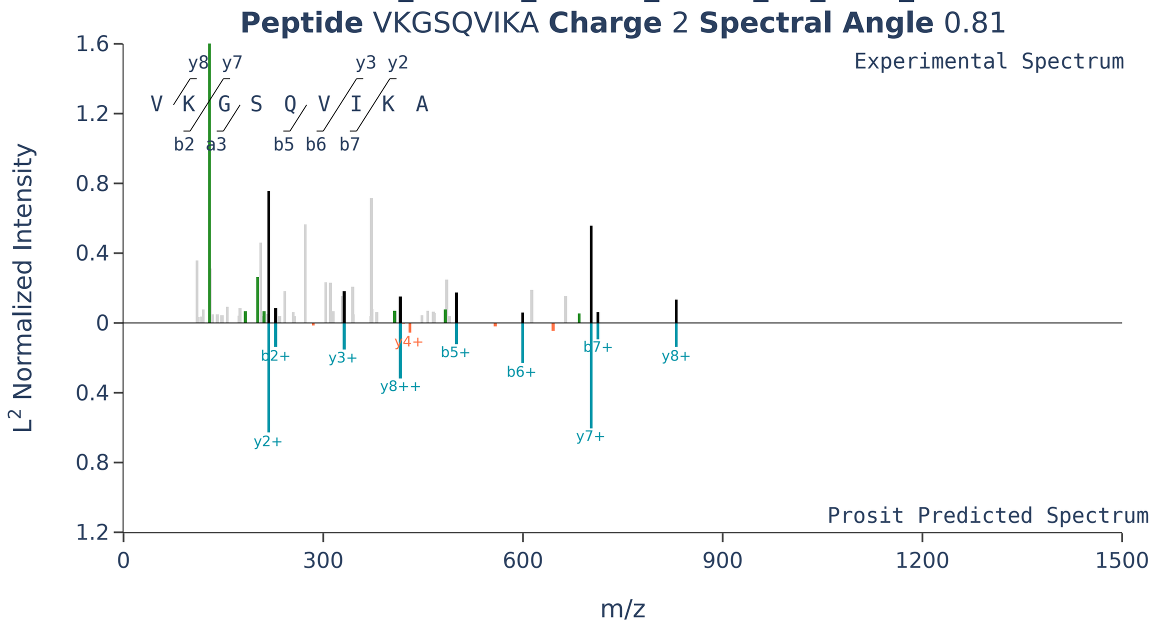


**Source** HRoetschke_211222_160123_HFGoe_IF2_b1R1_4h **Scan** 15582
**Peptide** LDTPGHAAFTSMR **Charge** 3 **Spectral Angle** 0.82

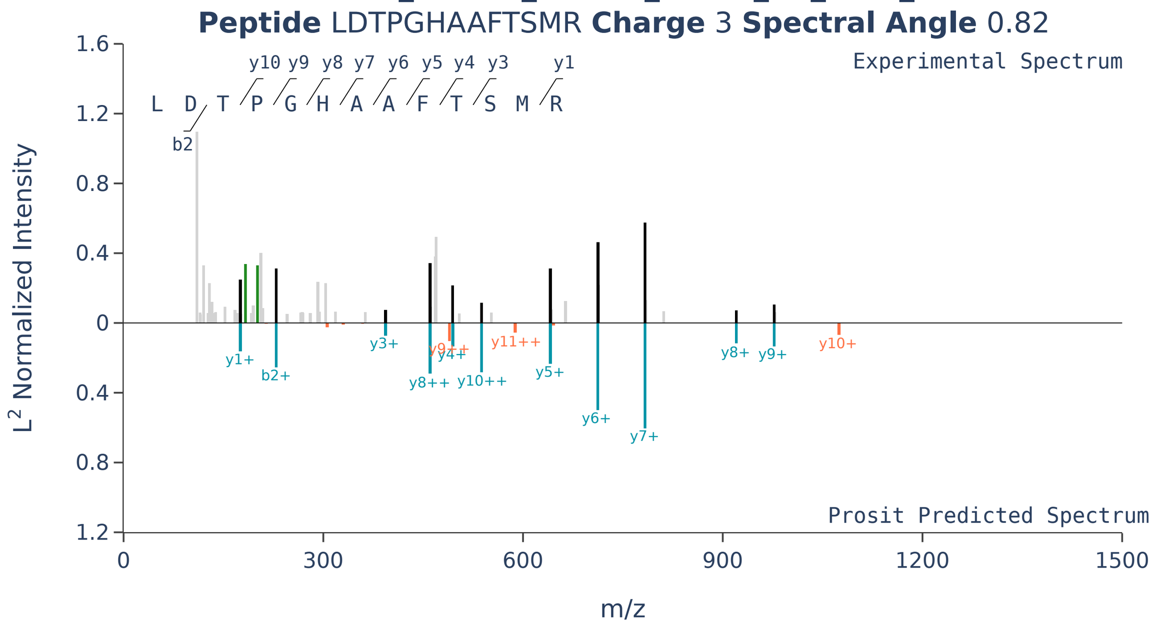


**Source** HRoetschke_211222_160123_HFGoe_IF2_b1R1_24h **Scan** 9445
**Peptide** DEVTVVRDEKKAREVA **Charge** 4 **Spectral Angle** 0.79

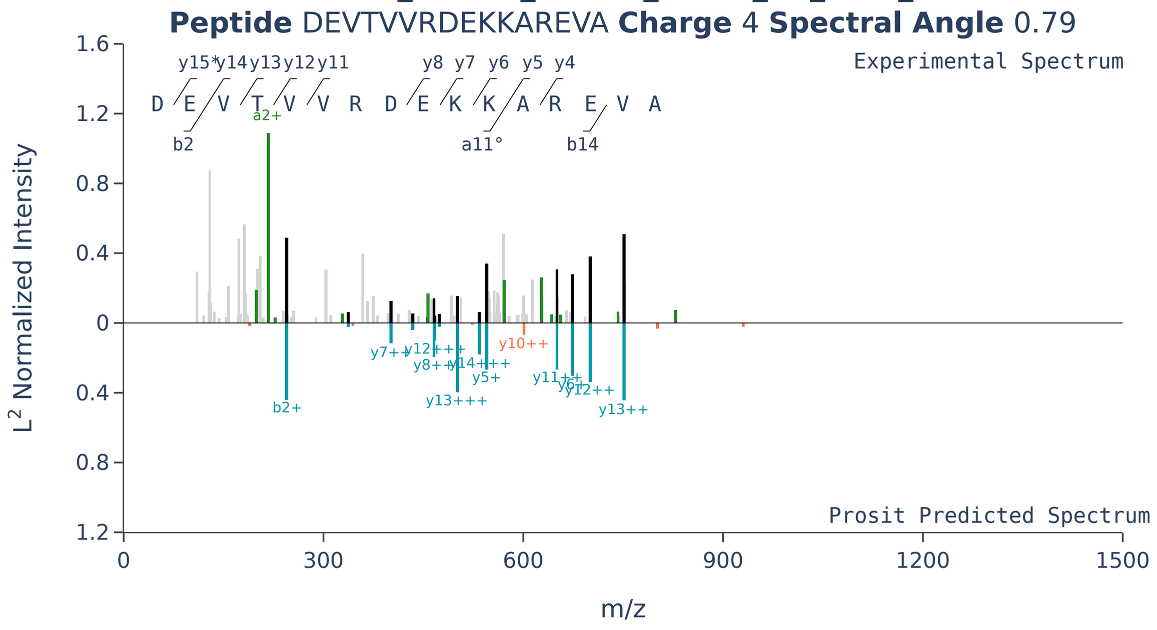


**Source** HRoetschke_211222_160123_HFGoe_IF2_b2R1_24h **Scan** 8589
**Peptide** DEVTVVRDEK **Charge** 3 **Spectral Angle** 0.78

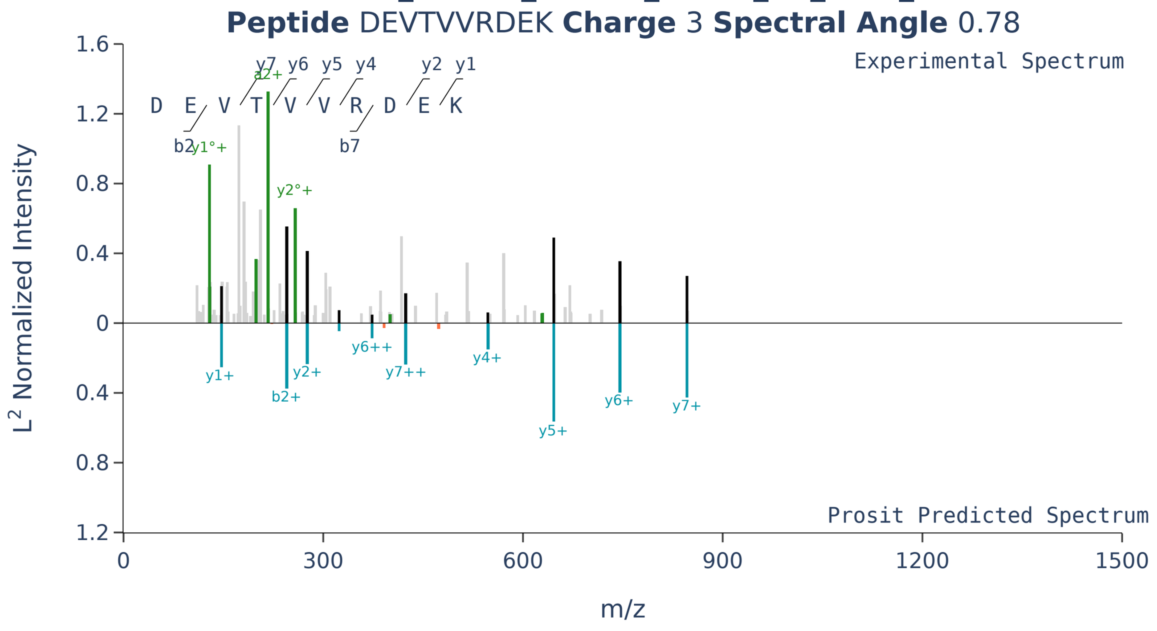


**Source** HRoetschke_211222_160123_HFGoe_IF2_b1R2_2h **Scan** 9805
**Peptide** AAFTSMRARGA **Charge** 3 **Spectral Angle** 0.85

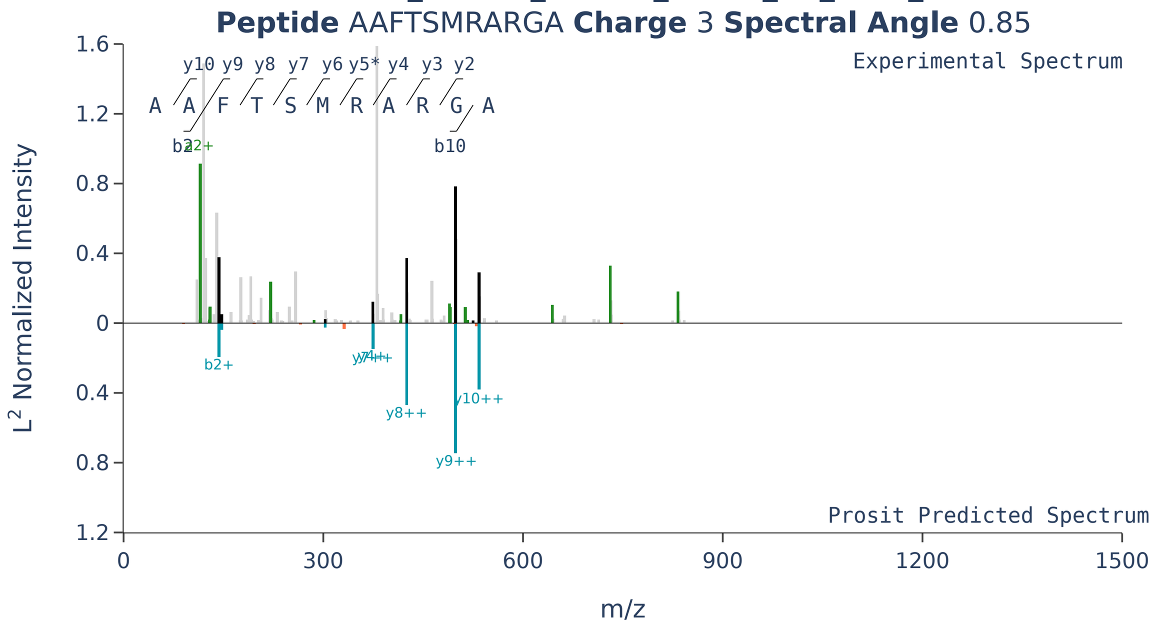


**Source** HRoetschke_211222_160123_HFGoe_IF2_b1R1_4h **Scan** 8537
**Peptide** AERLAAEEQAQREAE **Charge** 3 **Spectral Angle** 0.78

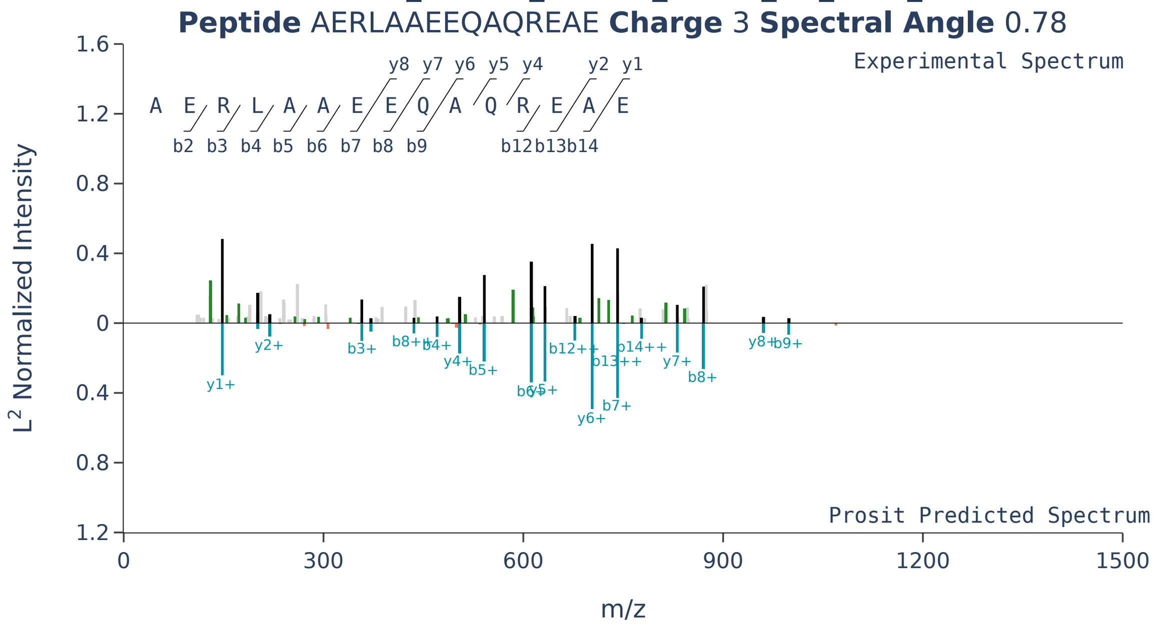


**Source** HRoetschke_211222_160123_HFGoe_IF2_b1R2_24h **Scan** 20571
**Peptide** KAGTGIDELLD **Charge** 2 **Spectral Angle** 0.81

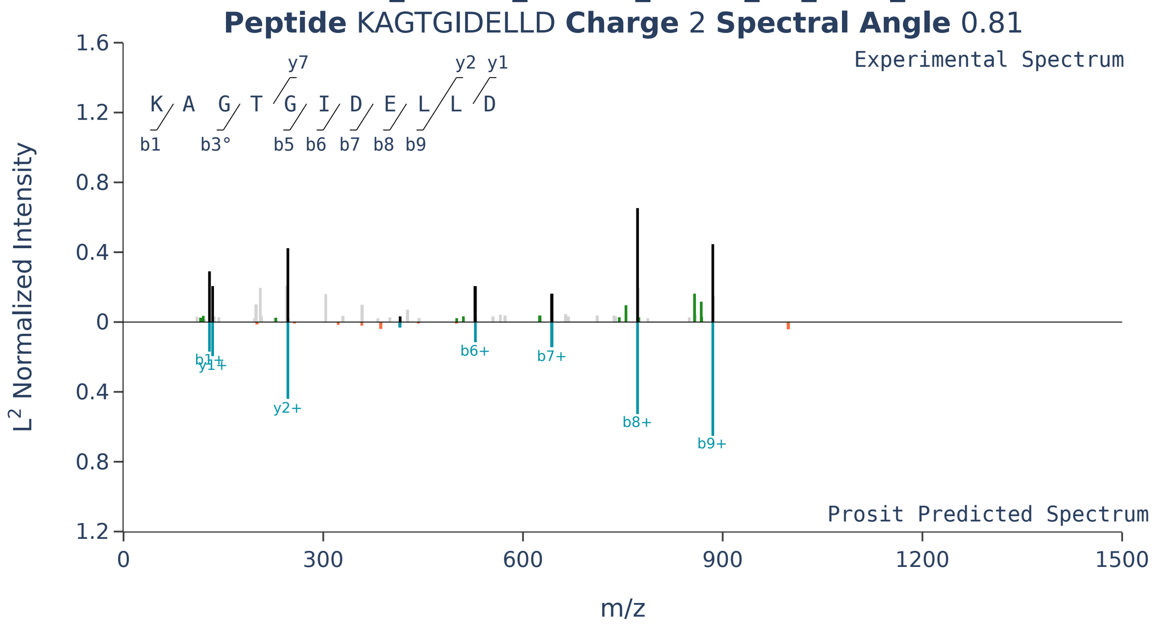


**Source** HRoetschke_211222_160123_HFGoe_IF2_b1R1_24h **Scan** 8015
**Peptide** ARQQKSKLENMF **Charge** 3 **Spectral Angle** 0.74

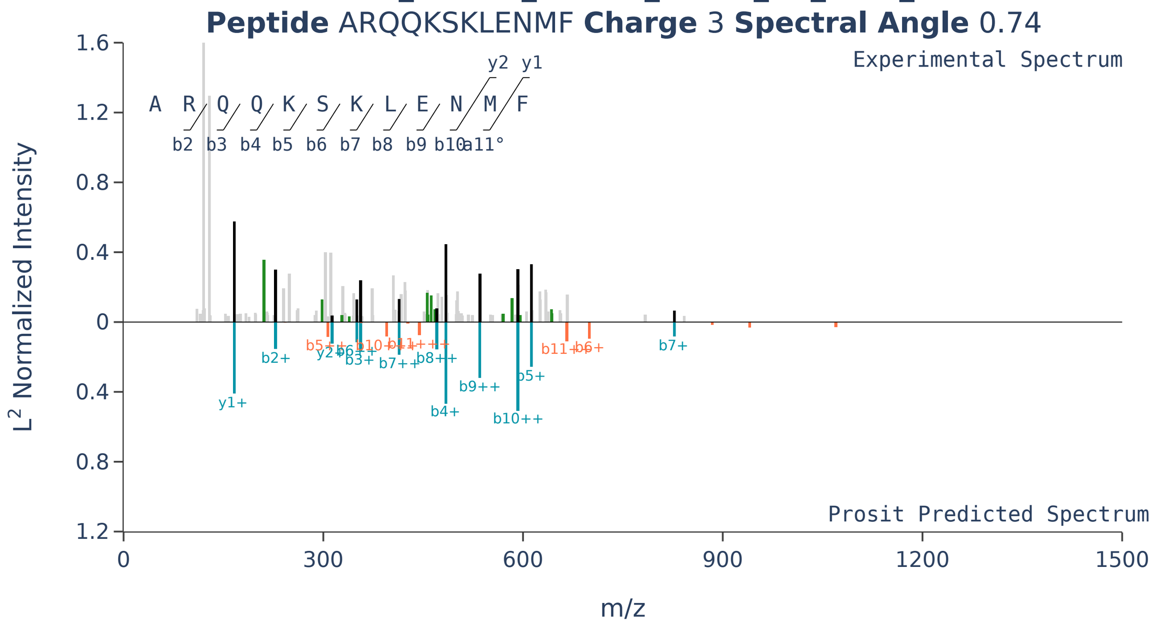


**Source** HRoetschke_211222_160123_HFGoe_IF2_b1R1_4h **Scan** 14588
**Peptide** LVQQFADAGIRKS **Charge** 3 **Spectral Angle** 0.83

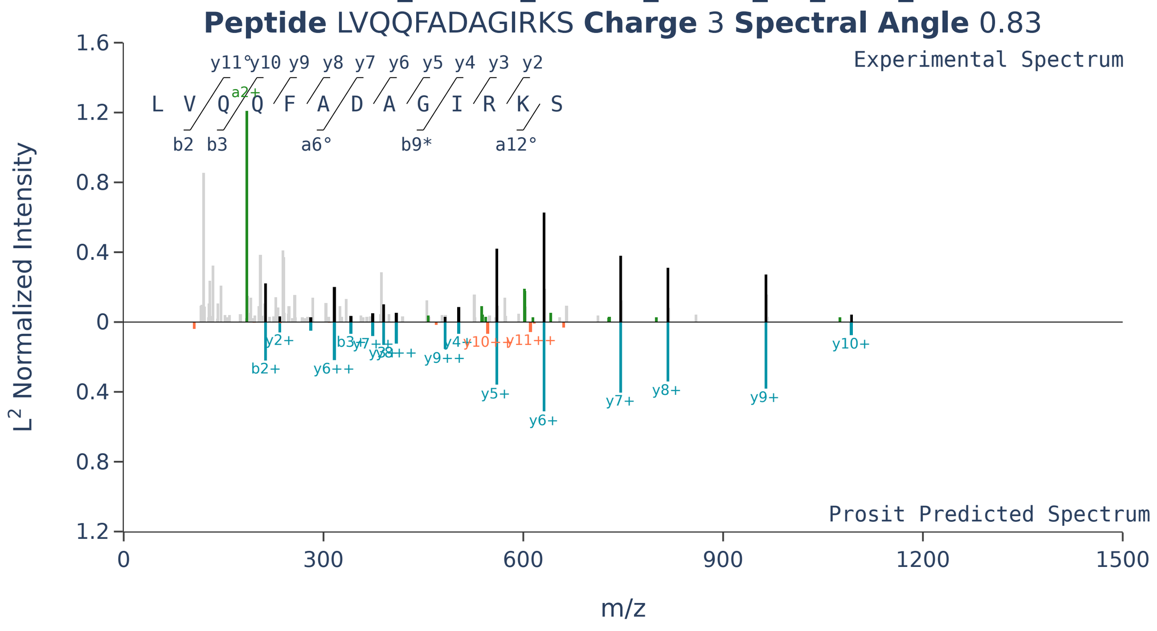


**Source** HRoetschke_211222_160123_HFGoe_IF2_b1R1_2h **Scan** 6171
**Peptide** AEEMGHKVILRRE **Charge** 4 **Spectral Angle** 0.83

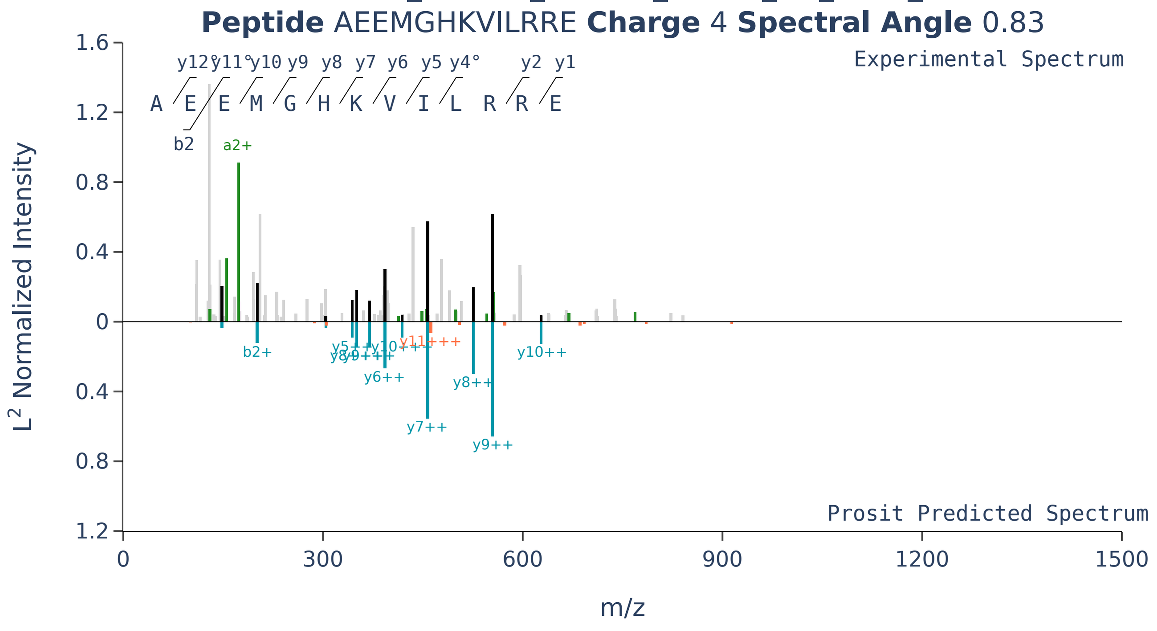


**Source** HRoetschke_211222_160123_HFGoe_IF2_b1R1_2h **Scan** 8335
**Peptide** ELANKMAVK **Charge** 2 **Spectral Angle** 0.77

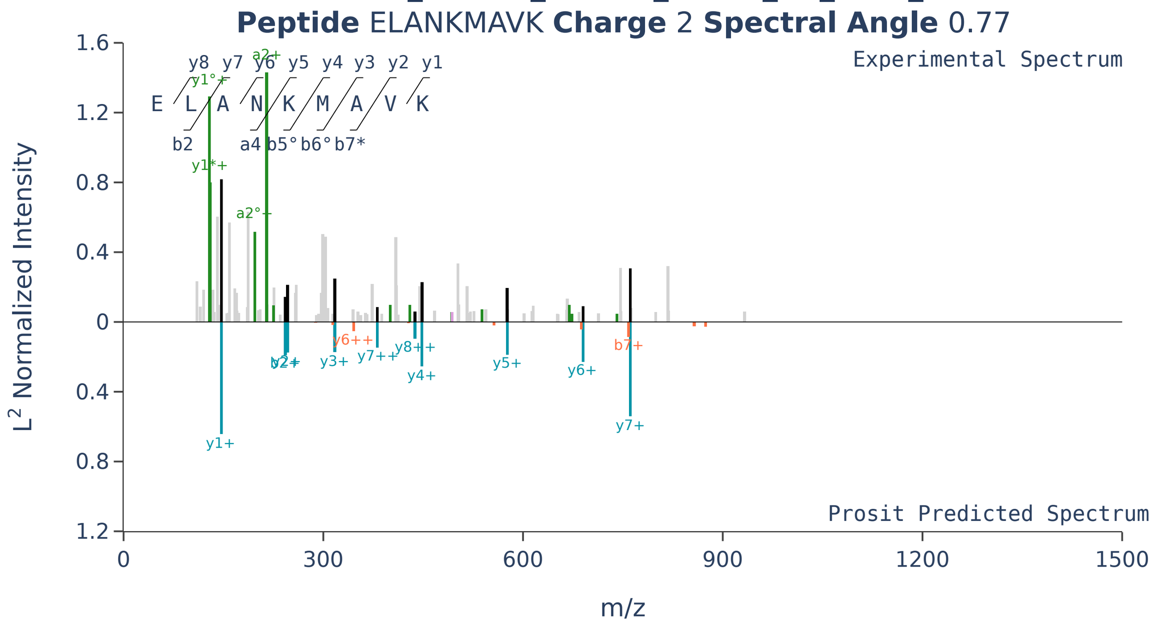


**Source** HRoetschke_211222_160123_HFGoe_IF2_b1R2_4h **Scan** 10544
**Peptide** KMAVKGSQVI **Charge** 2 **Spectral Angle** 0.82

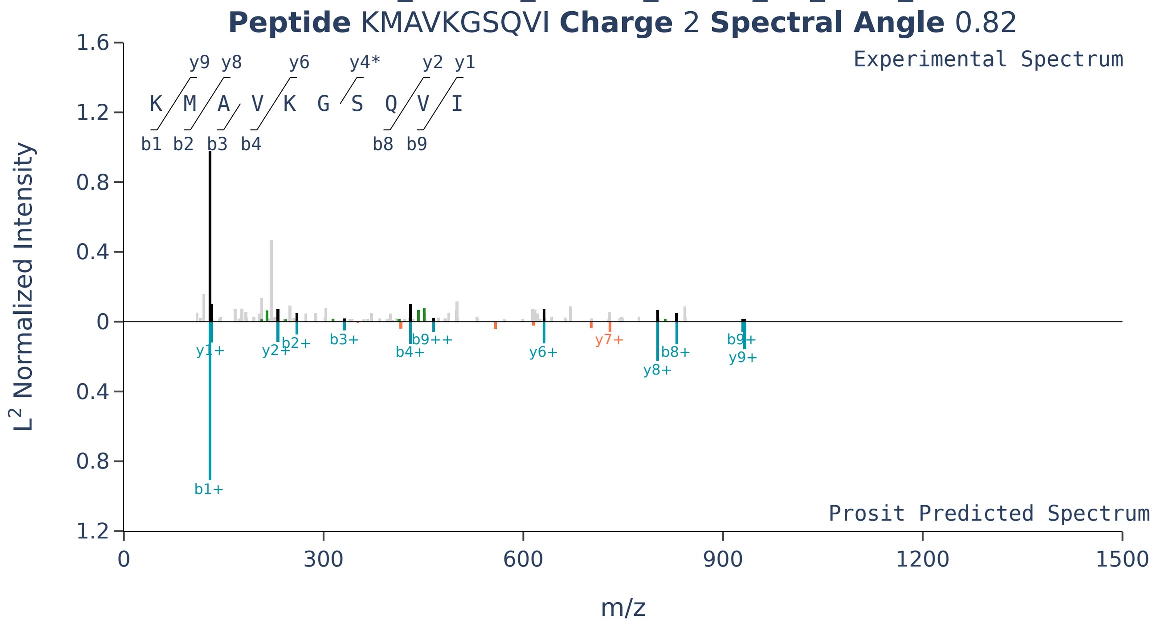

**Source** HRoetschke_211222_160123_HFGoe_IF2_b1R1_4h **Scan** 21056
**Peptide** VVIGETI **Charge** 1 **Spectral Angle** 0.91

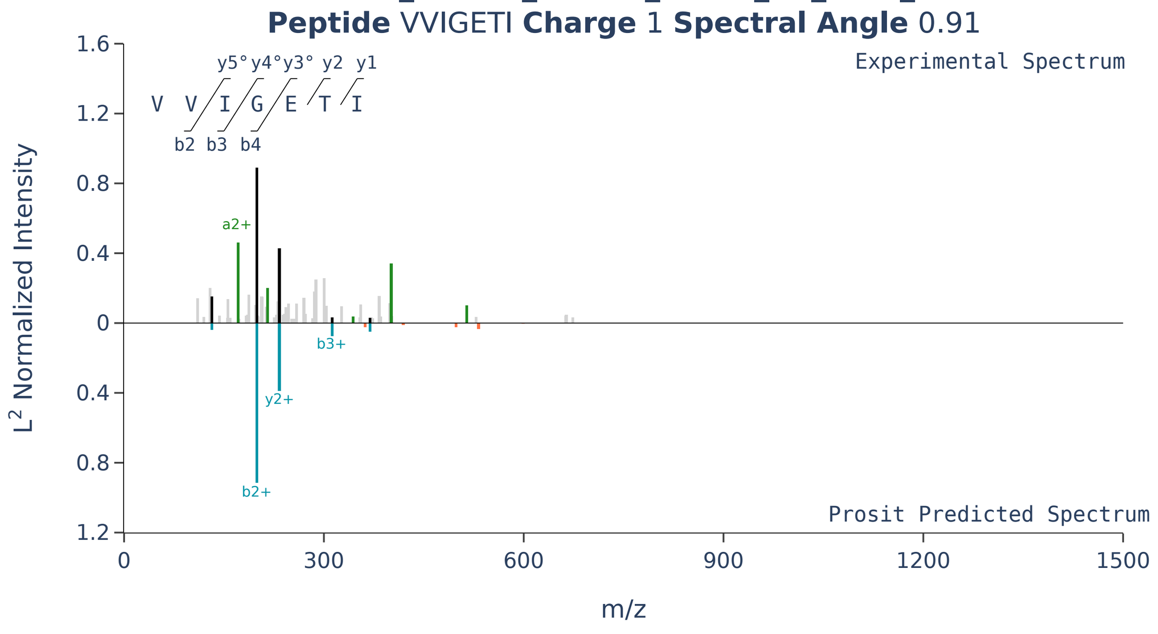


**Source** HRoetschke_211222_160123_HFGoe_IF2_b2R2_4h **Scan** 21396
**Peptide** GIDELLD **Charge** 1 **Spectral Angle** 0.81

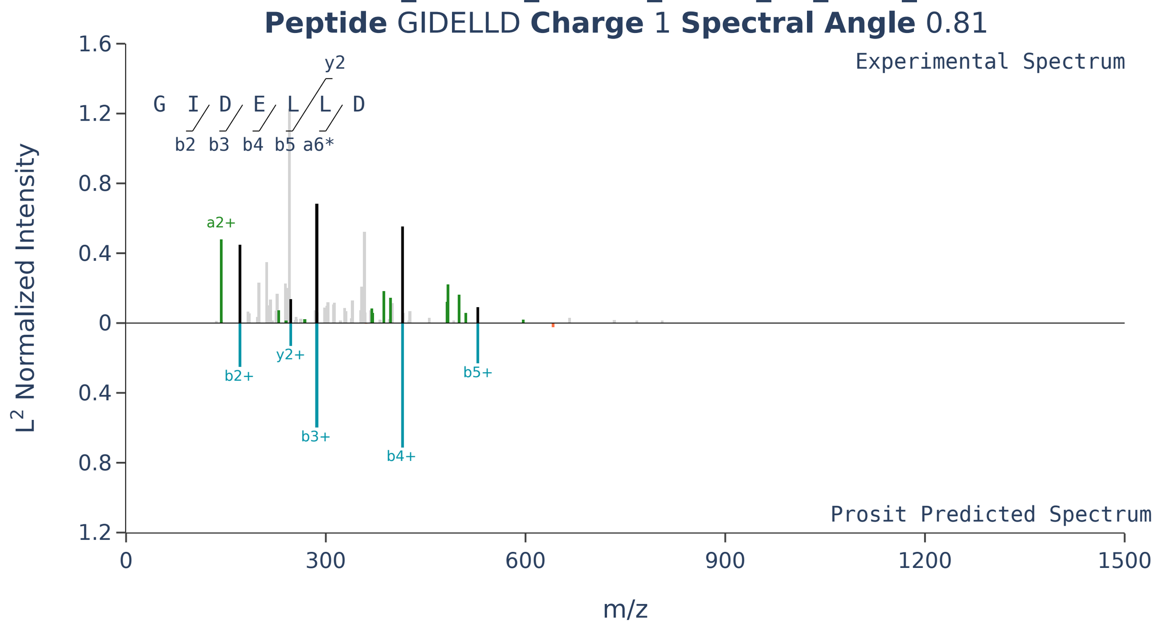


**Source** HRoetschke_211222_160123_HFGoe_IF2_b1R2_24h **Scan** 15951
**Peptide** DKGRGPVATVL **Charge** 3 **Spectral Angle** 0.85

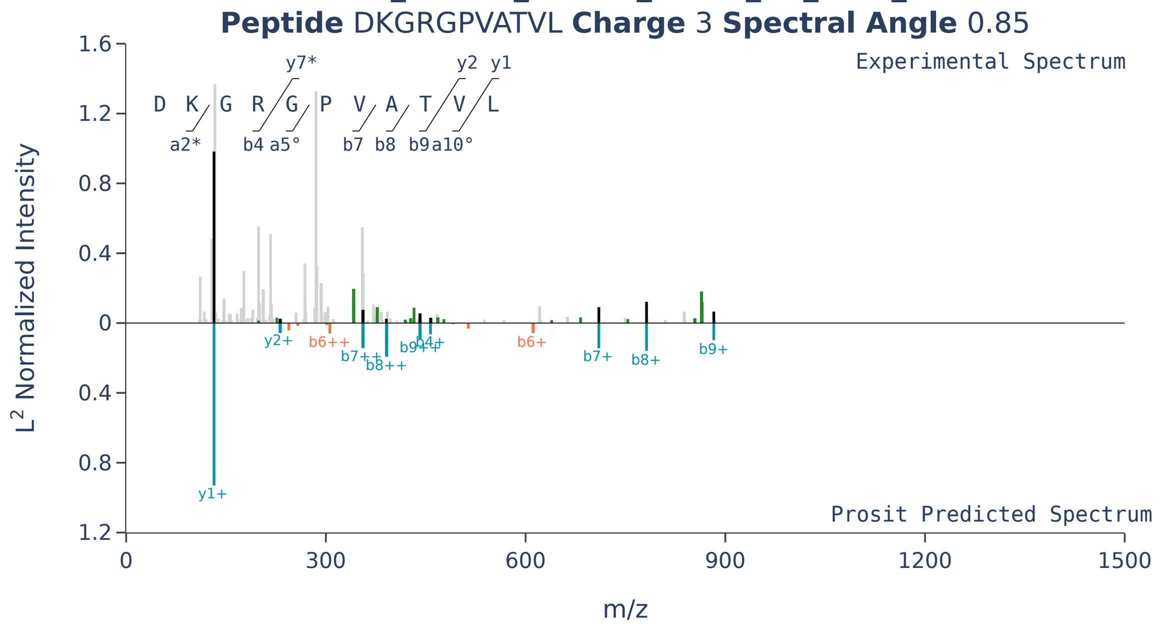


**Source** HRoetschke_211222_160123_HFGoe_IF2_b1R1_24h **Scan** 10493
**Peptide** PAQAVNRDVV **Charge** 2 **Spectral Angle** 0.75

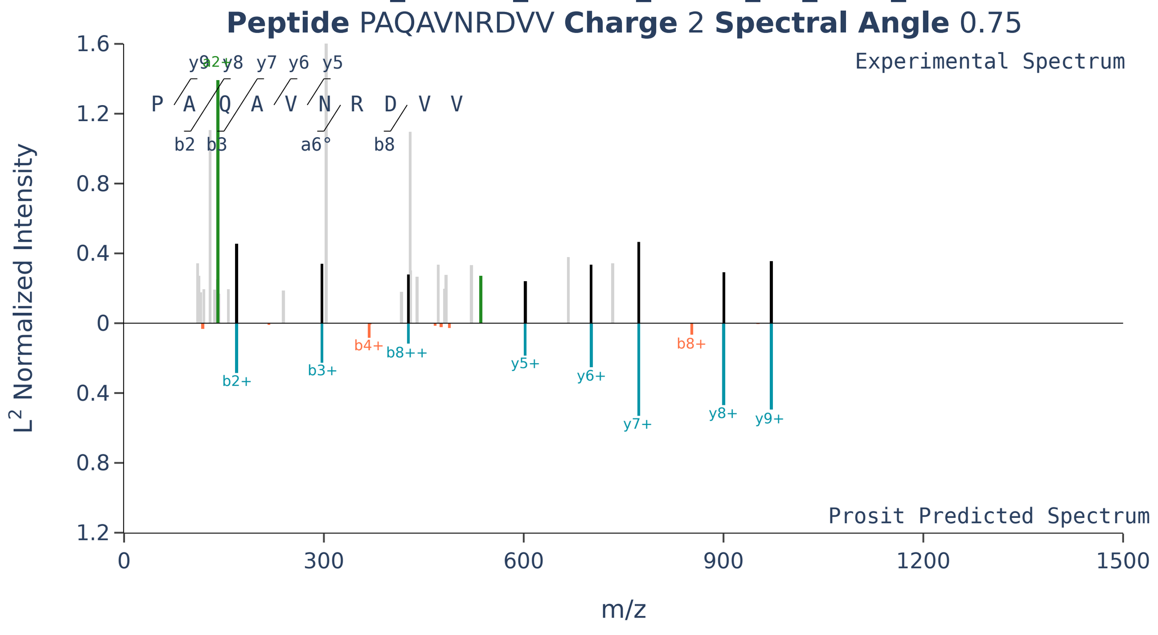


**Source** HRoetschke_211222_160123_HFGoe_IF2_b1R2_2h **Scan** 8262
**Peptide** EYGRVRAM **Charge** 2 **Spectral Angle** 0.79

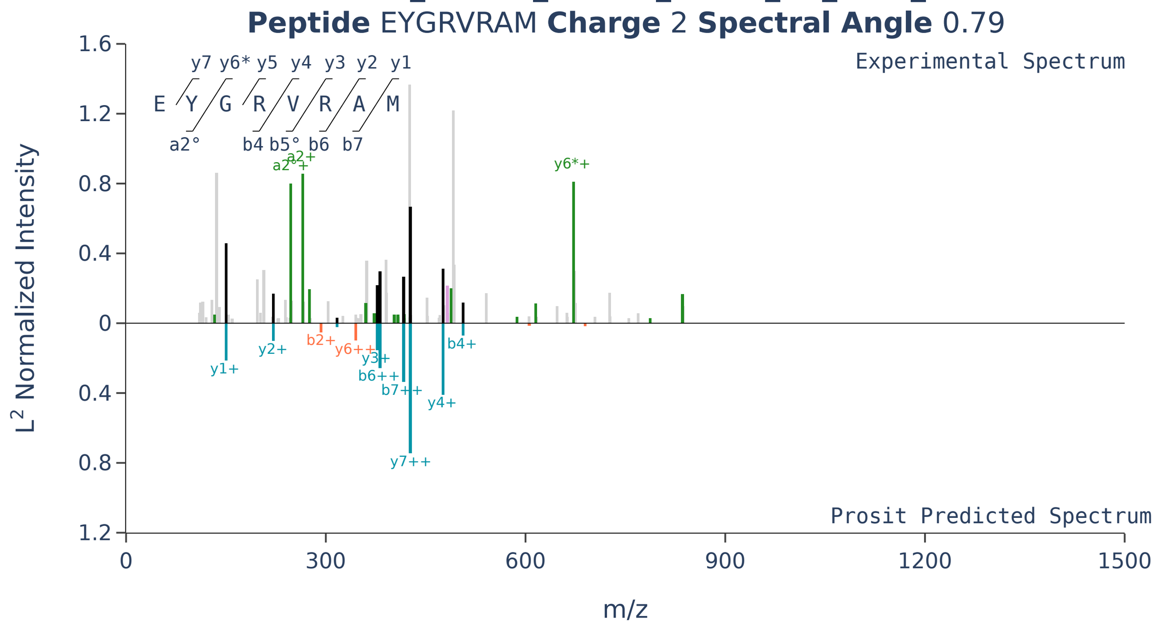


**Source** HRoetschke_211222_160123_HFGoe_IF2_b1R2_2h **Scan** 5250
**Peptide** KPEADPDRVKNE **Charge** 2 **Spectral Angle** 0.85

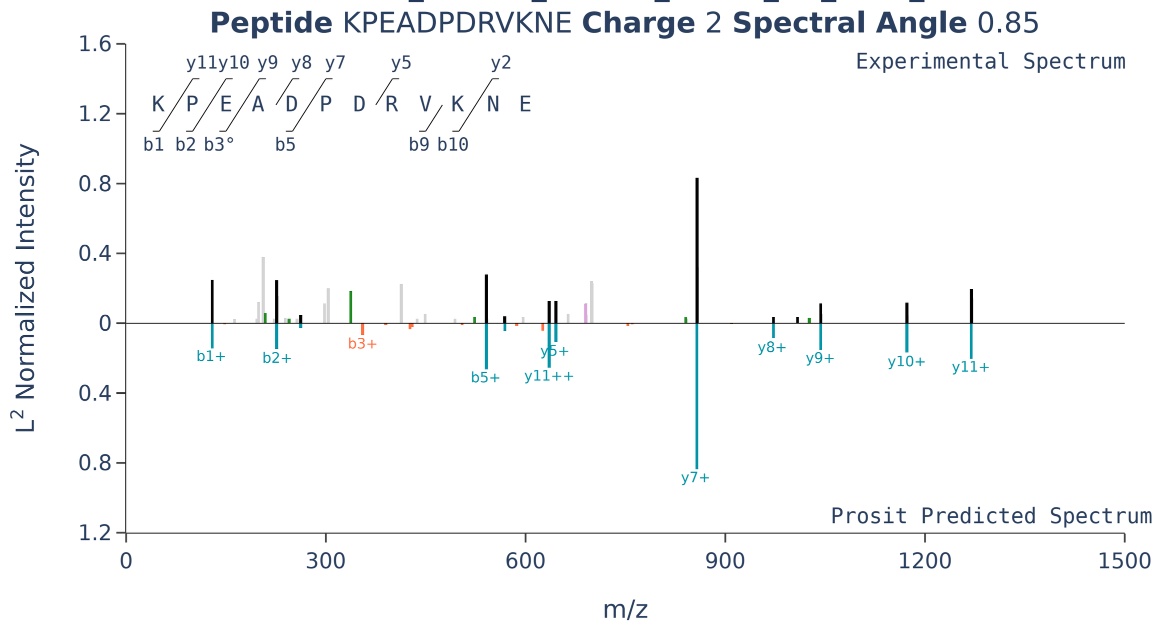


**Source** HRoetschke_211222_160123_HFGoe_IF2_b1R2_4h **Scan** 6776
**Peptide** ANKMAVKGSQVIKA **Charge** 4 **Spectral Angle** 0.92

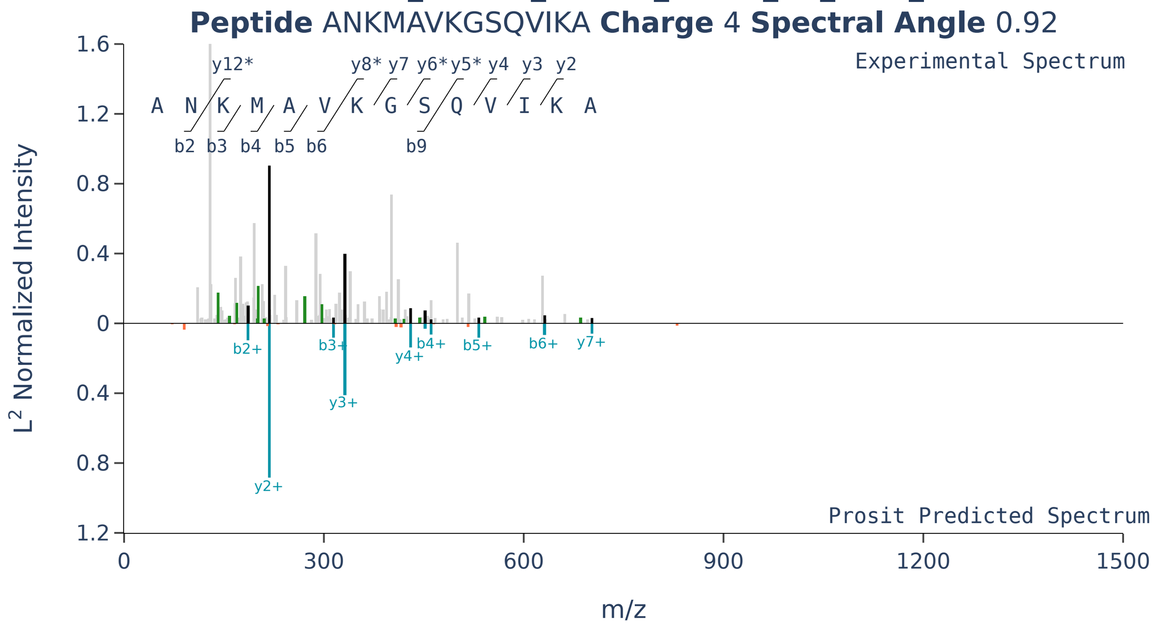


**Source** HRoetschke_211222_160123_HFGoe_IF2_b1R1_4h **Scan** 9866
**Peptide** KGSQVIKAMMK **Charge** 3 **Spectral Angle** 0.93

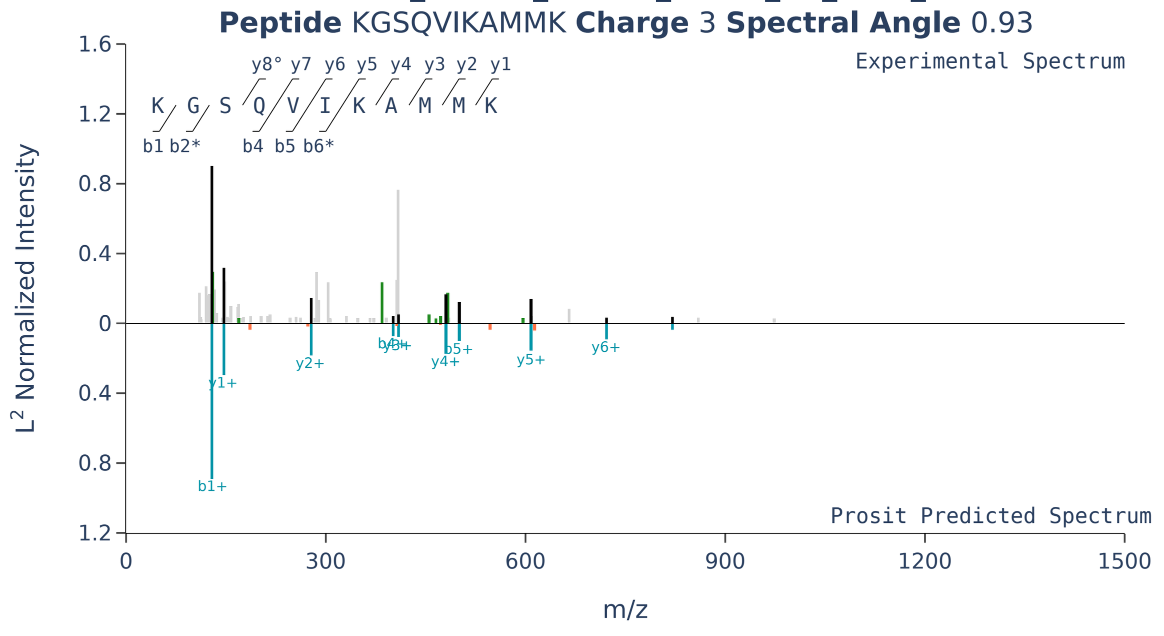


**Source** HRoetschke_211222_160123_HFGoe_IF2_b1R1_4h **Scan** 12213
**Peptide** LNIPGTGGKSKSVQ **Charge** 3 **Spectral Angle** 0.76

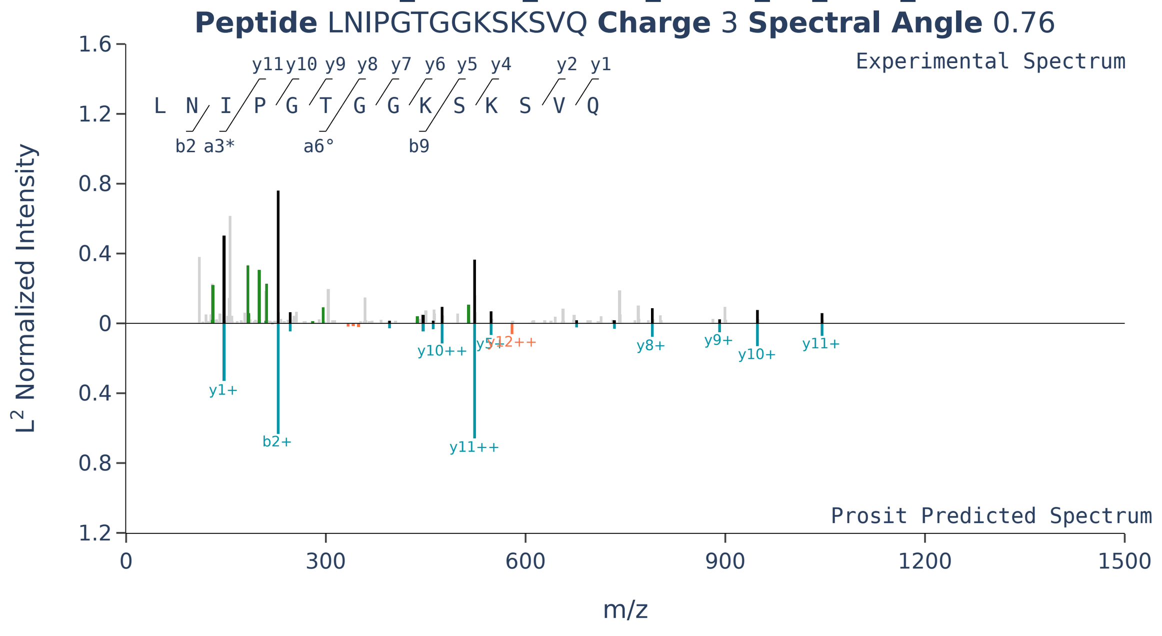


**Source** HRoetschke_211222_160123_HFGoe_IF2_b1R1_2h **Scan** 13207
**Peptide** RLVQQFADAGIRKS **Charge** 3 **Spectral Angle** 0.82

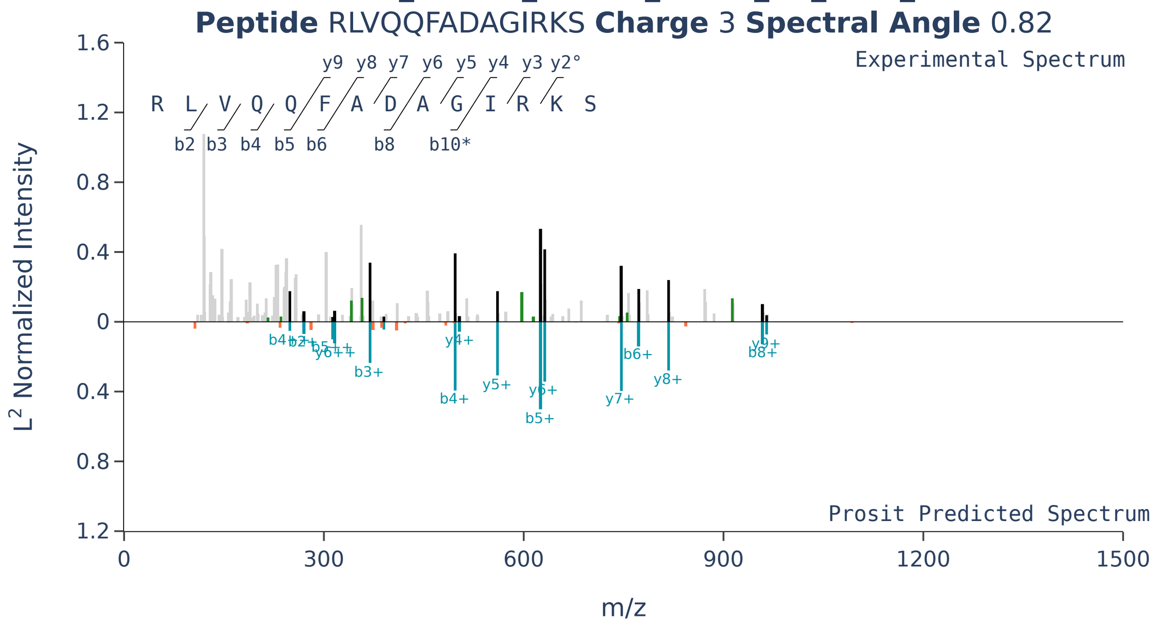


**Source** HRoetschke_211222_160123_HFGoe_IF2_b2R1_2h **Scan** 10857
**Peptide** RRENELEEAV **Charge** 3 **Spectral Angle** 0.8

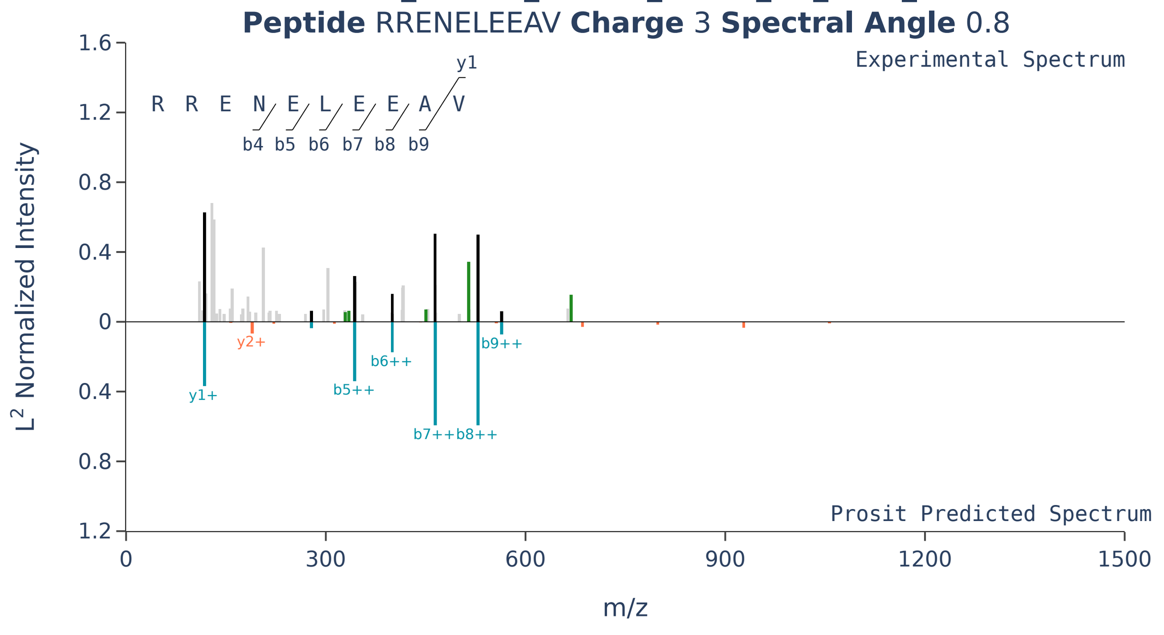


**Source** HRoetschke_211222_160123_HFGoe_IF2_b1R1_1h **Scan** 12078
**Peptide** VNRDVVLG **Charge** 2 **Spectral Angle** 0.95

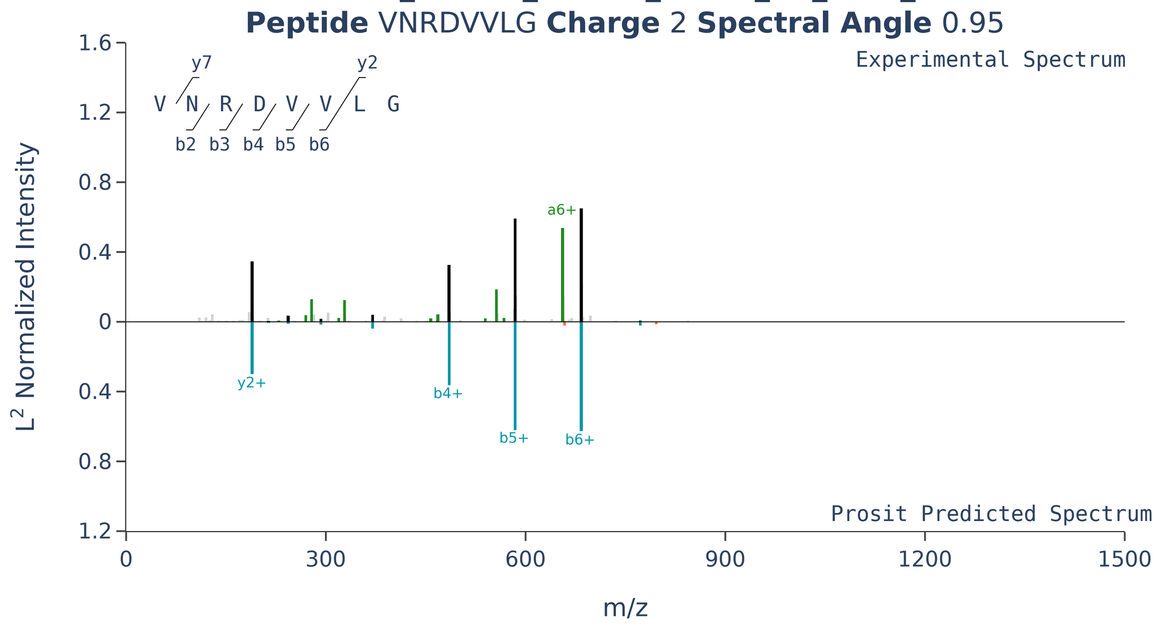


**Source** HRoetschke_211222_160123_HFGoe_IF2_b2R2_1h **Scan** 6987
**Peptide** QQGFQKPAQ **Charge** 2 **Spectral Angle** 0.79

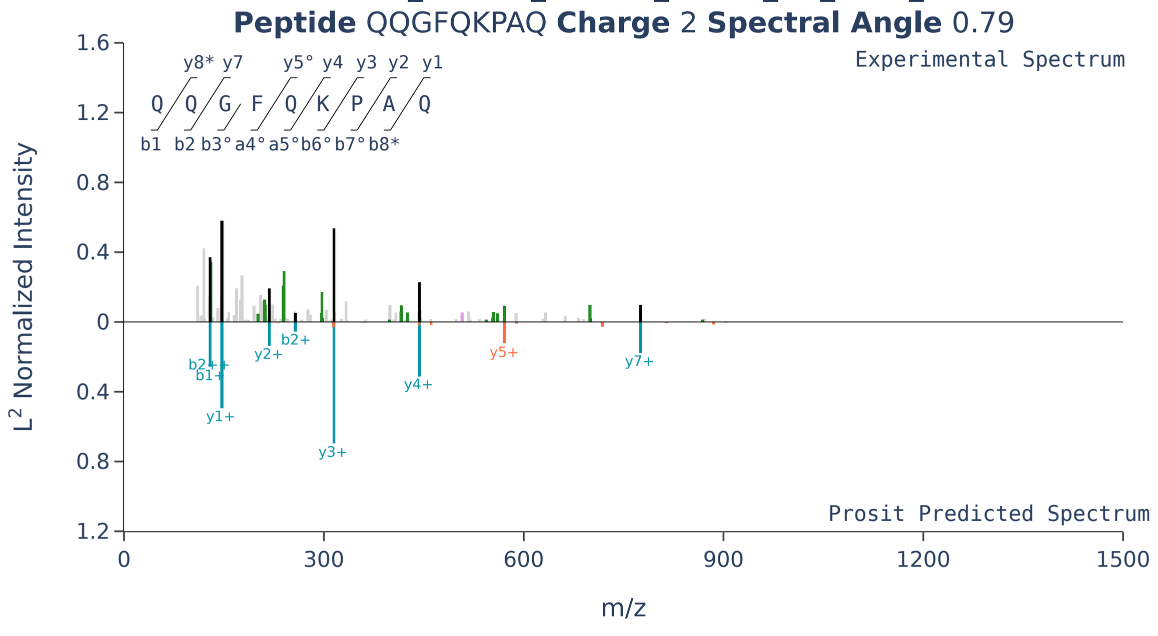


**Source** HRoetschke_211222_160123_HFGoe_IF2_b1R2_24h **Scan** 7742
**Peptide** KGSQVLKA **Charge** 2 **Spectral Angle** 0.97

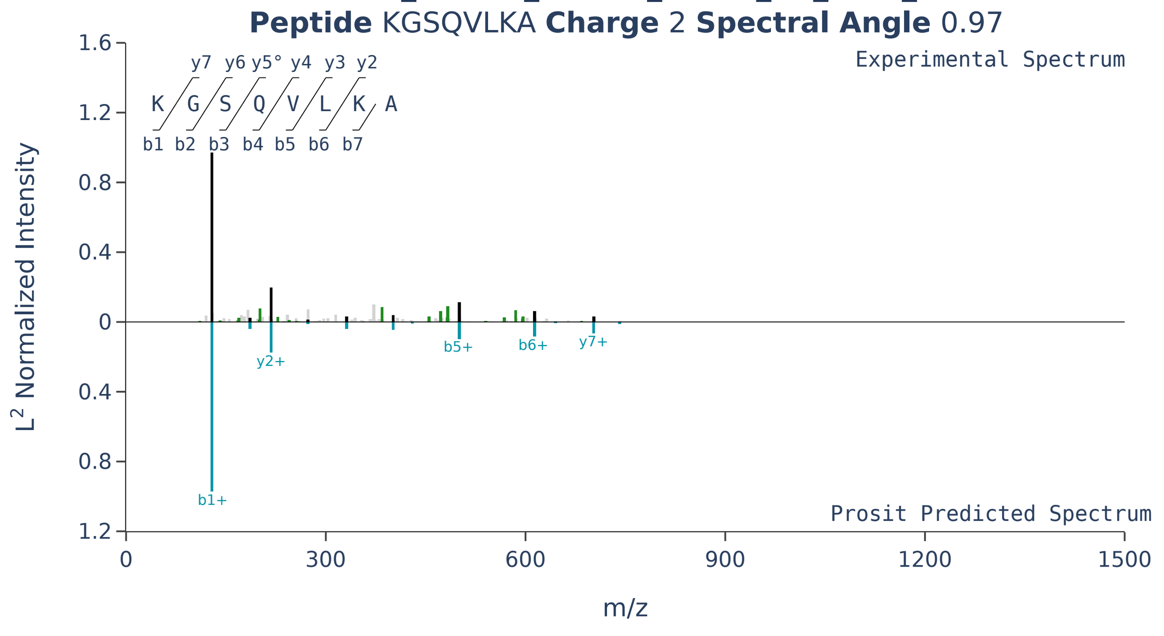


**Source** HRoetschke_211222_160123_HFGoe_IF2_b2R2_4h **Scan** 20348
**Peptide** YGILPEE **Charge** 1 **Spectral Angle** 0.91

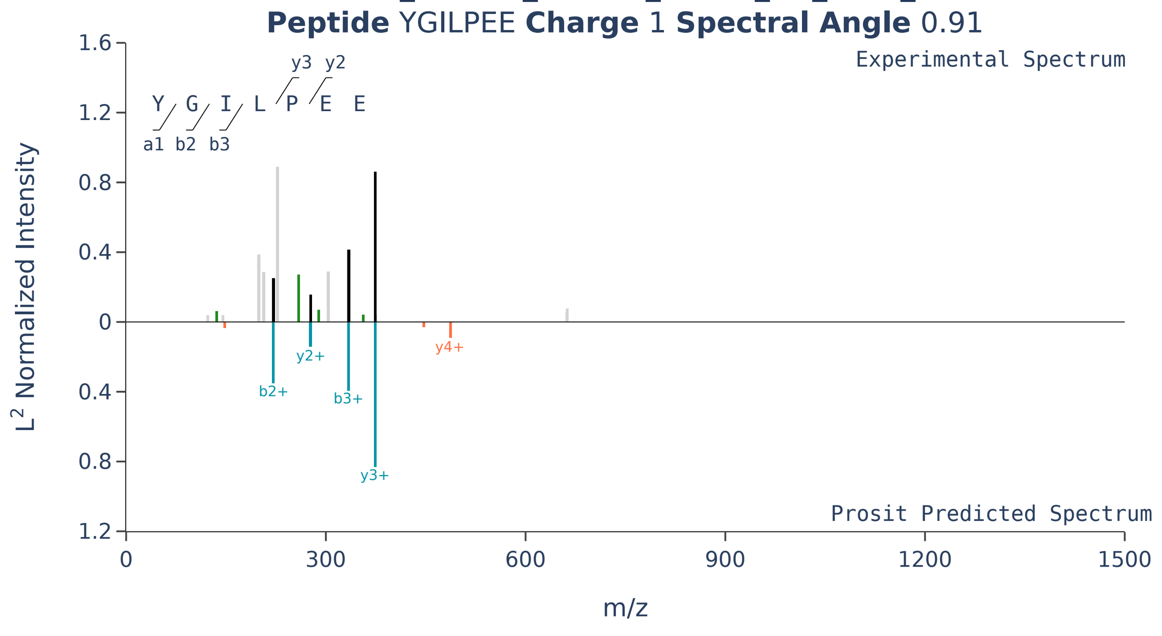

**Source** HRoetschke_211222_160123_HFGoe_IF2_b1R1_4h **Scan** 7691
**Peptide** AEEMGHKVILRRENE **Charge** 4 **Spectral Angle** 0.85

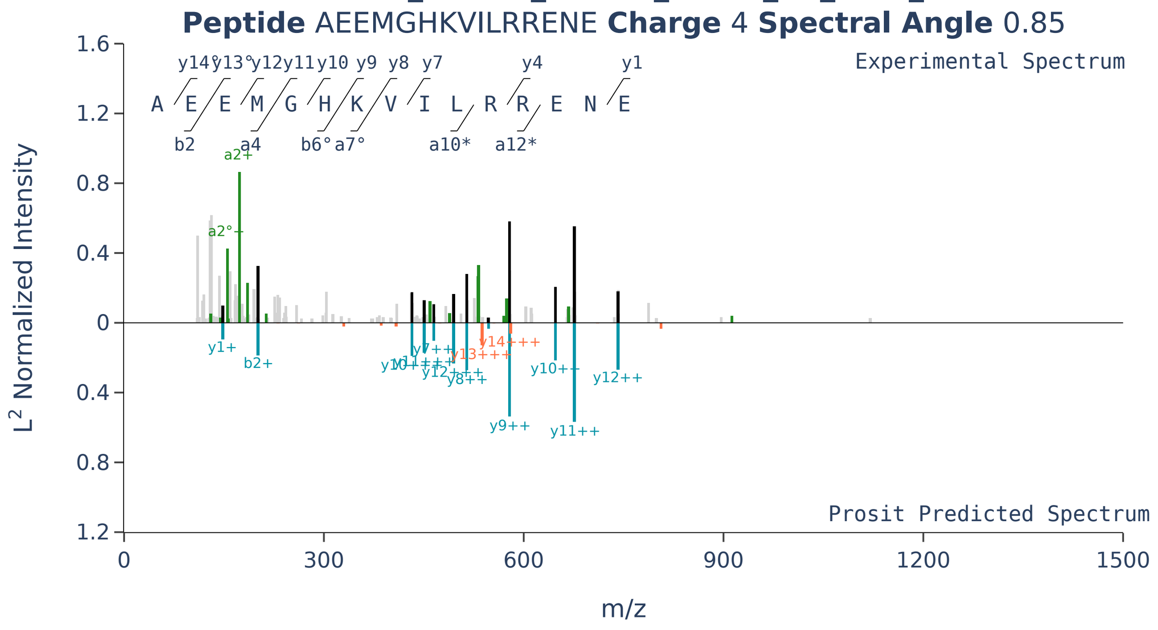


**Source** HRoetschke_211222_160123_HFGoe_IF2_b1R1_4h **Scan** 16242
**Peptide** CGFEYGRV **Charge** 2 **Spectral Angle** 0.83

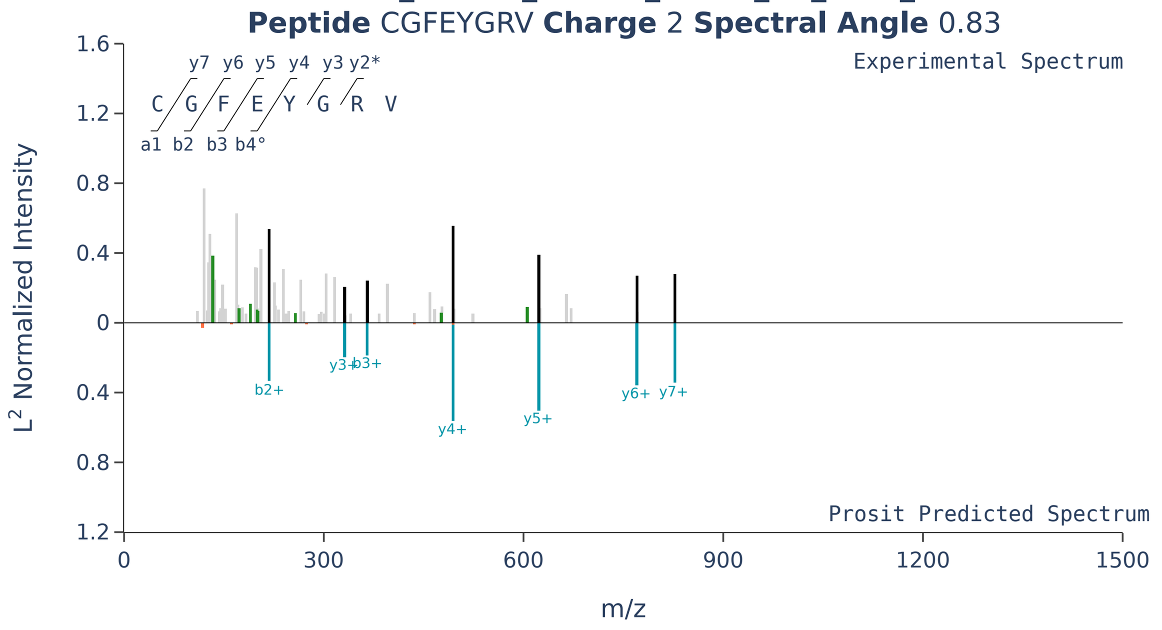


**Source** HRoetschke_211222_160123_HFGoe_IF2_b2R2_24h **Scan** 11553
**Peptide** SFLDKGRGP **Charge** 2 **Spectral Angle** 0.81

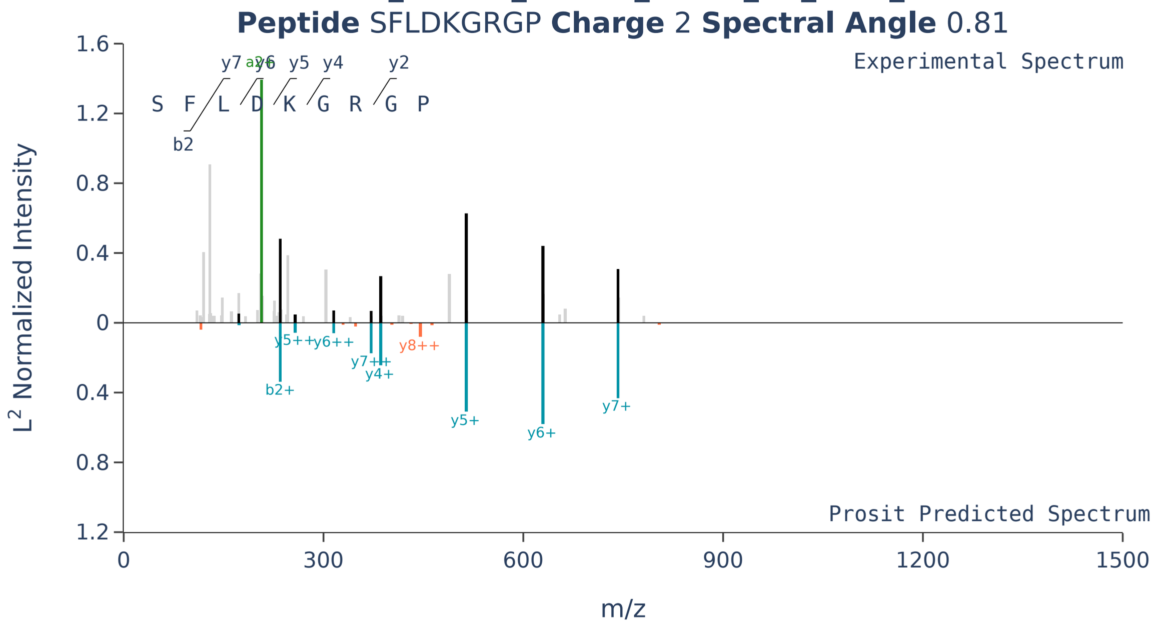


**Source** HRoetschke_211222_160123_HFGoe_IF2_b2R1_24h **Scan** 7783
**Peptide** GPDKLTLQRKT **Charge** 3 **Spectral Angle** 0.8

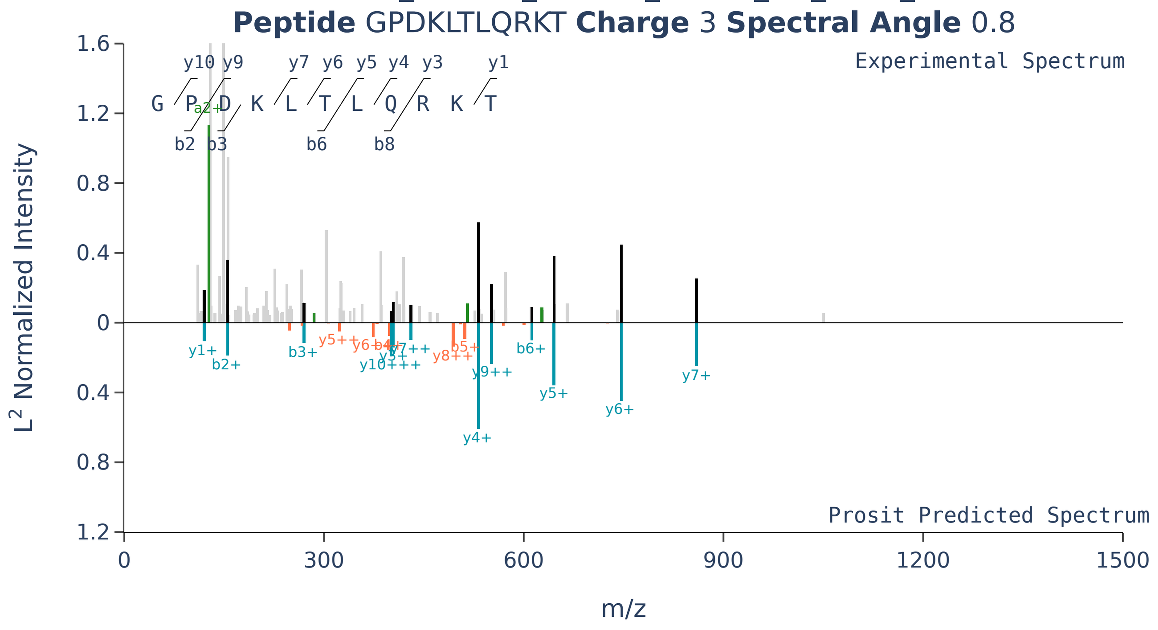


**Source** HRoetschke_211222_160123_HFGoe_IF2_b1R2_2h **Scan** 8987
**Peptide** KVILRRENELE **Charge** 3 **Spectral Angle** 0.79

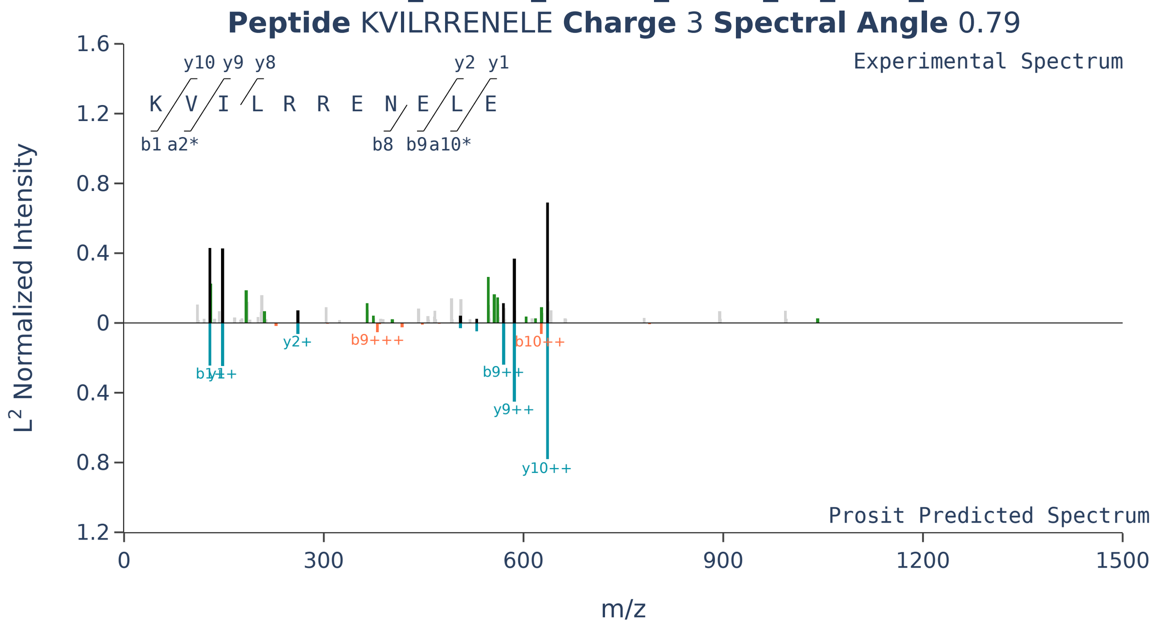


**Source** HRoetschke_211222_160123_HFGoe_IF2_b1R1_4h **Scan** 12476
**Peptide** GGESQFVHVSAKA **Charge** 2 **Spectral Angle** 0.73

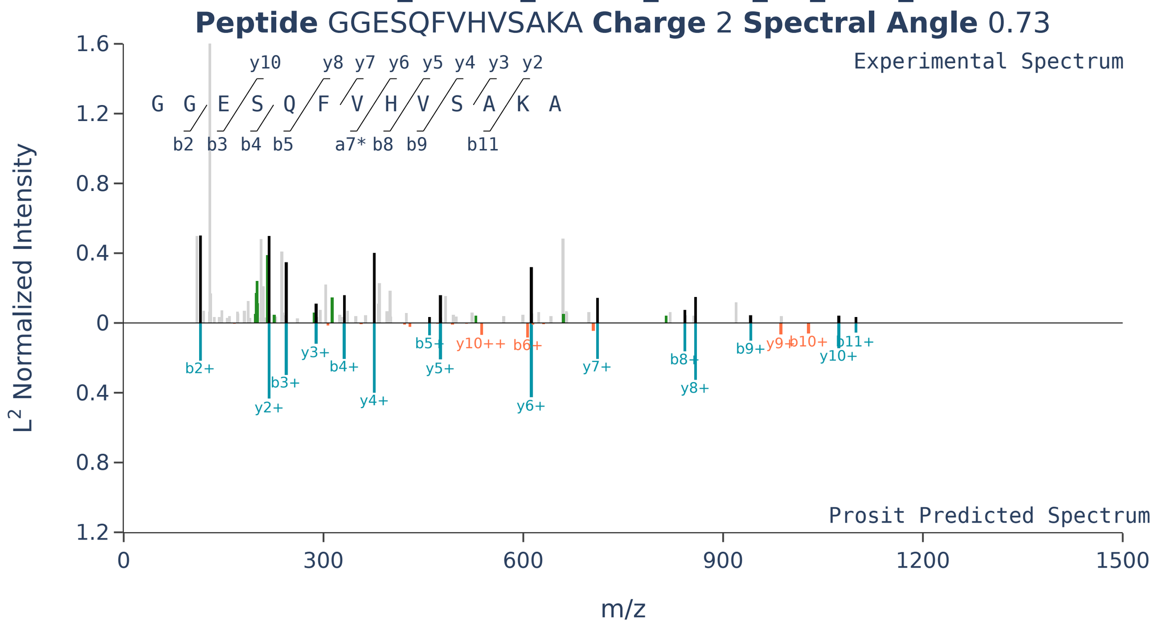


**Source** HRoetschke_211222_160123_HFGoe_IF2_b1R2_4h **Scan** 8559
**Peptide** RTFVKRDPQEAERL **Charge** 4 **Spectral Angle** 0.74

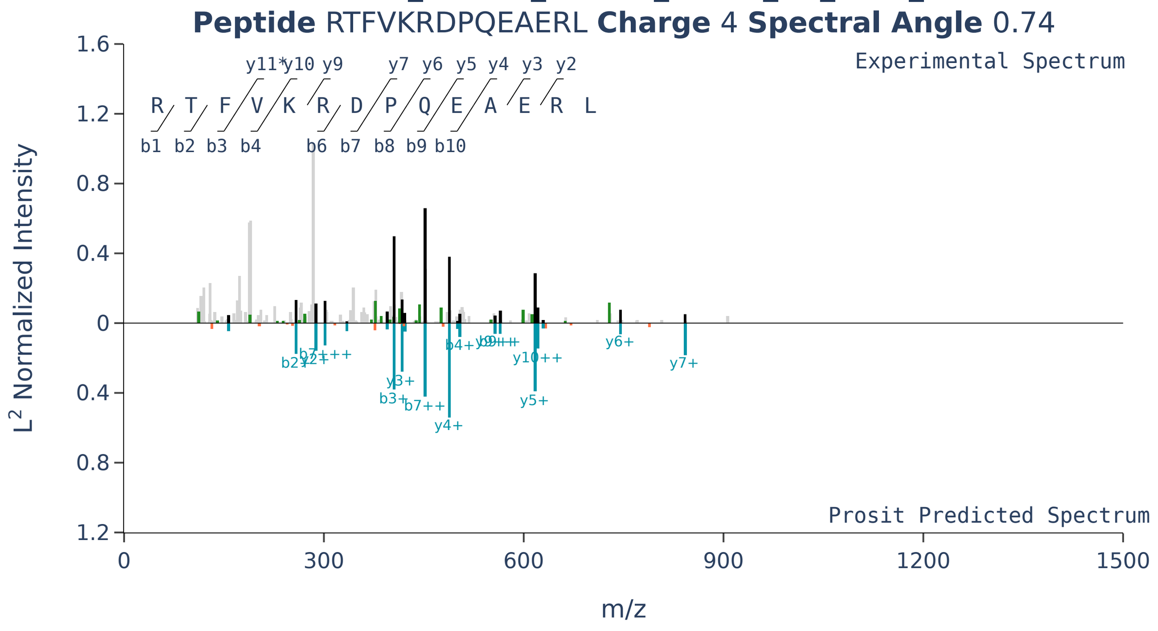


**Source** HRoetschke_211222_160123_HFGoe_IF2_b1R2_24h **Scan** 7804
**Peptide** RRFKDDVNEV **Charge** 3 **Spectral Angle** 0.82

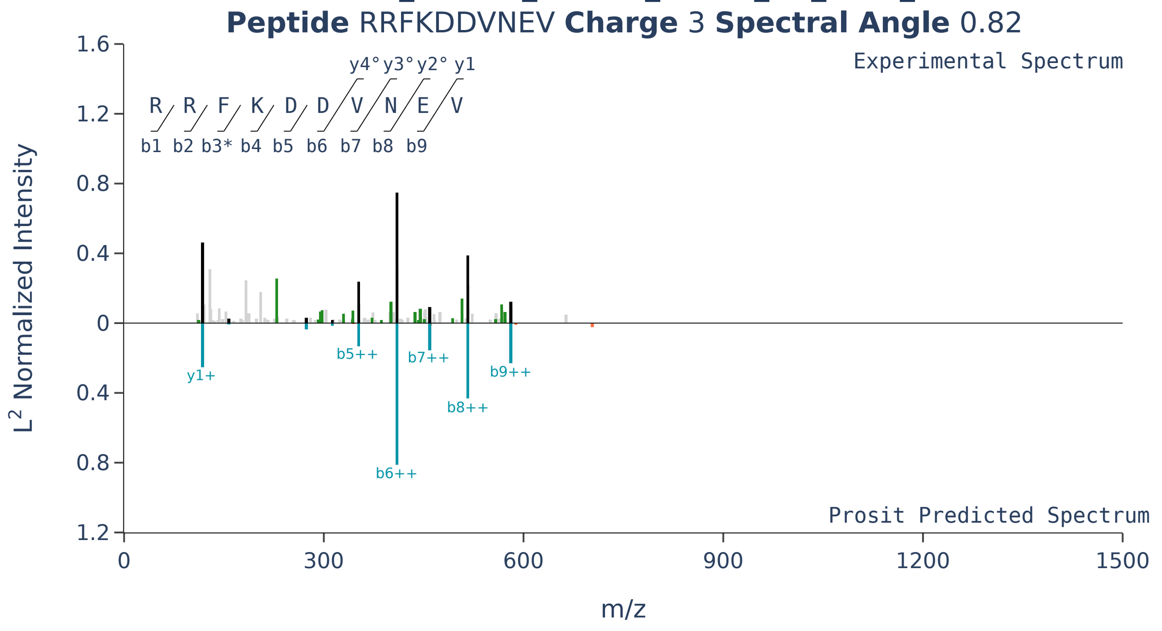


**Source** HRoetschke_211222_160123_HFGoe_IF2_b1R2_2h **Scan** 7546
**Peptide** DRVKNEL **Charge** 2 **Spectral Angle** 0.87

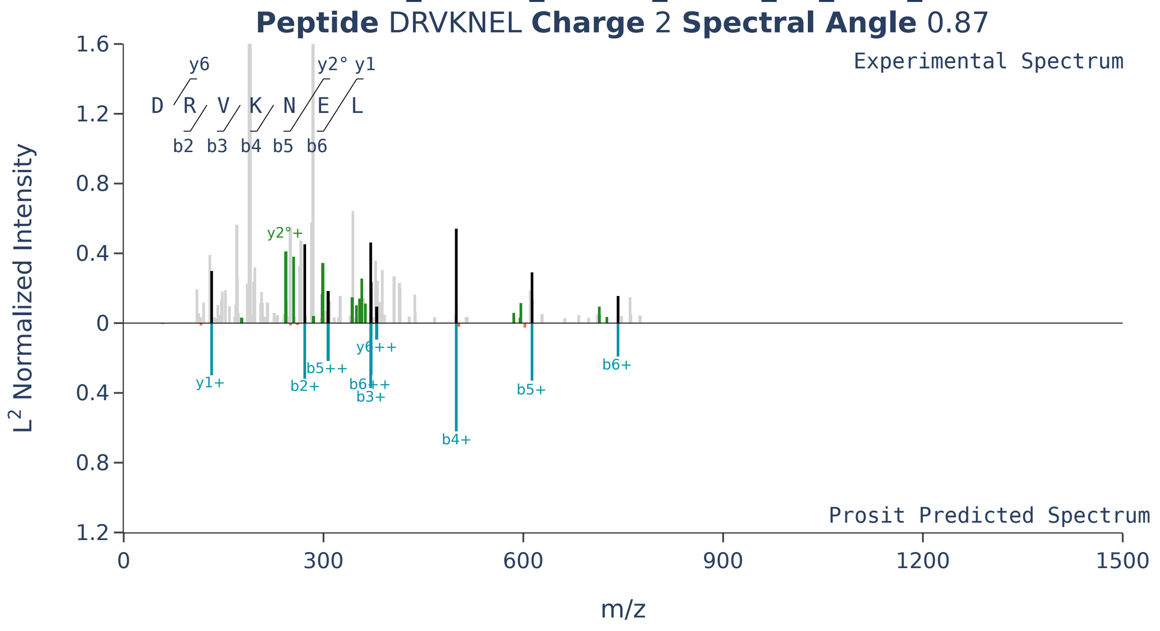


**Source** HRoetschke_211222_160123_HFGoe_IF2_b1R2_24h **Scan** 19205
**Peptide** IIEIQRTI **Charge** 2 **Spectral Angle** 0.87

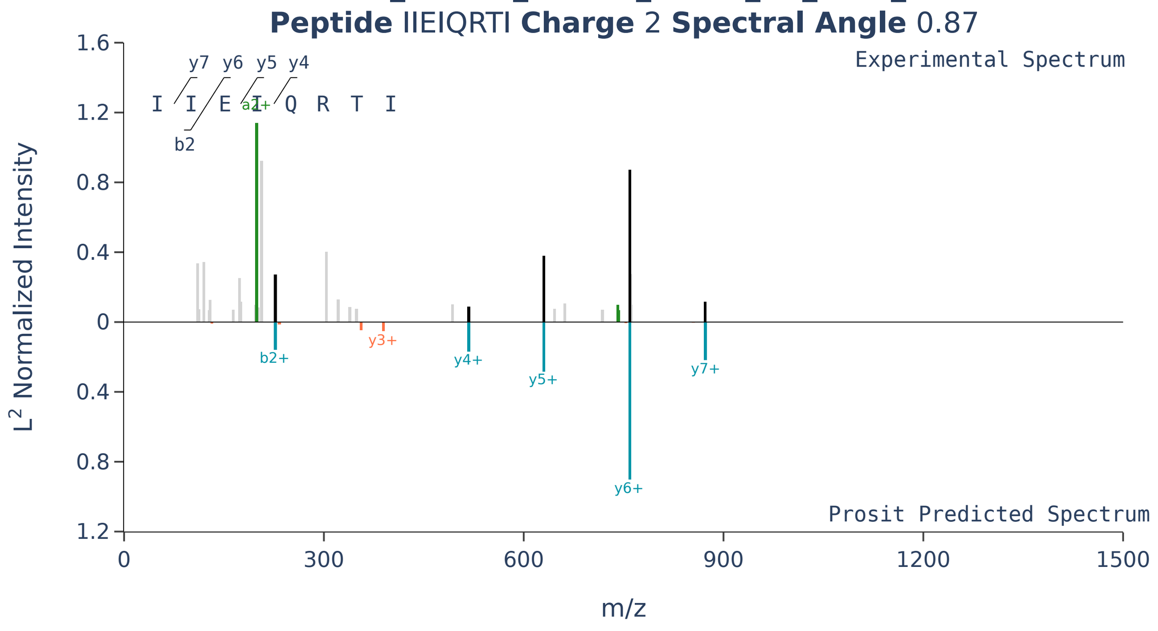


**Source** HRoetschke_211222_160123_HFGoe_IF2_b1R1_4h **Scan** 13222
**Peptide** CGFEYGRVRA **Charge** 3 **Spectral Angle** 0.87

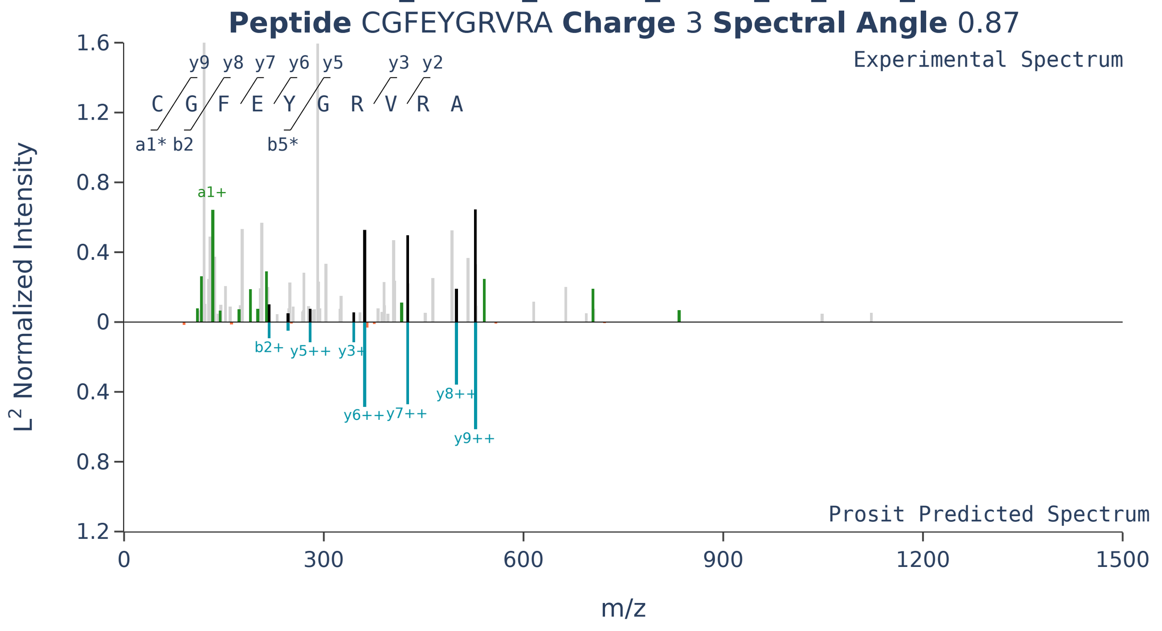


**Source** HRoetschke_211222_160123_HFGoe_IF2_b2R2_4h **Scan** 13848
**Peptide** RDNVVIYEG **Charge** 2 **Spectral Angle** 0.88

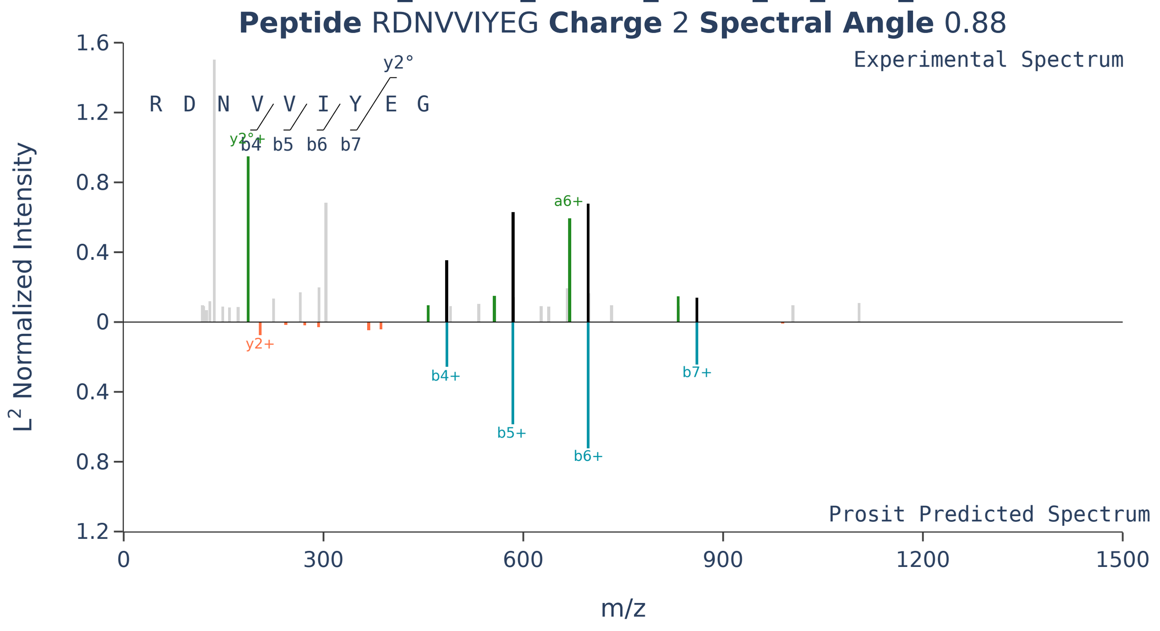


**Source** HRoetschke_211222_160123_HFGoe_IF2_b1R1_1h **Scan** 11586
**Peptide** GVGGITETD **Charge** 1 **Spectral Angle** 0.78

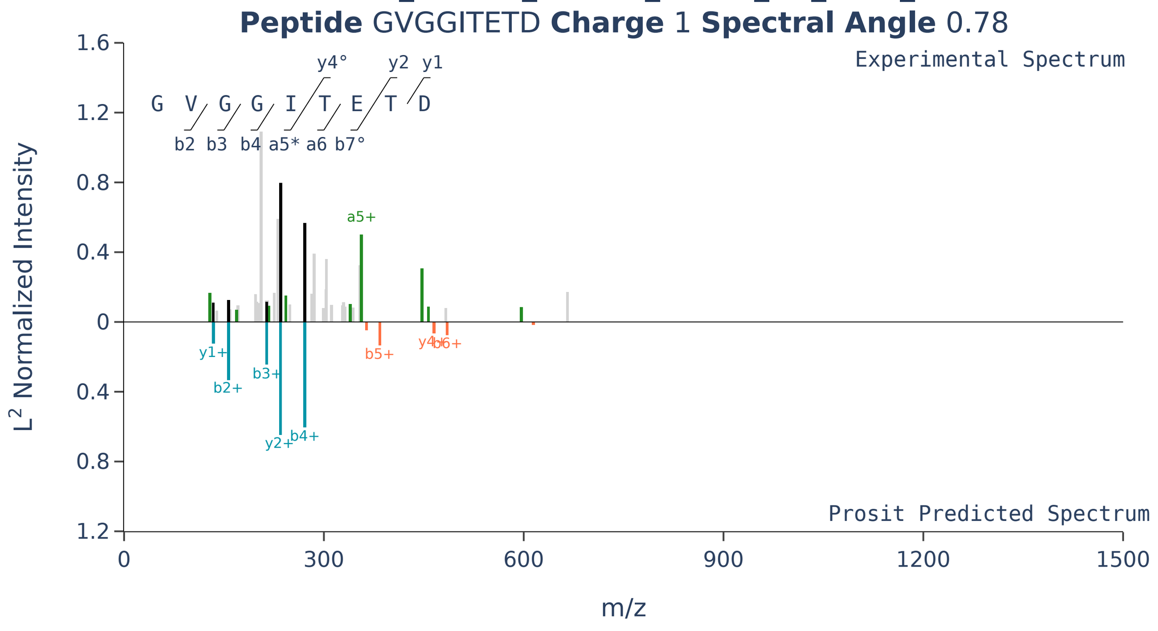


**Source** HRoetschke_211222_160123_HFGoe_IF2_b1R2_2h **Scan** 10422
**Peptide** TQHIGAYHVETE **Charge** 3 **Spectral Angle** 0.83

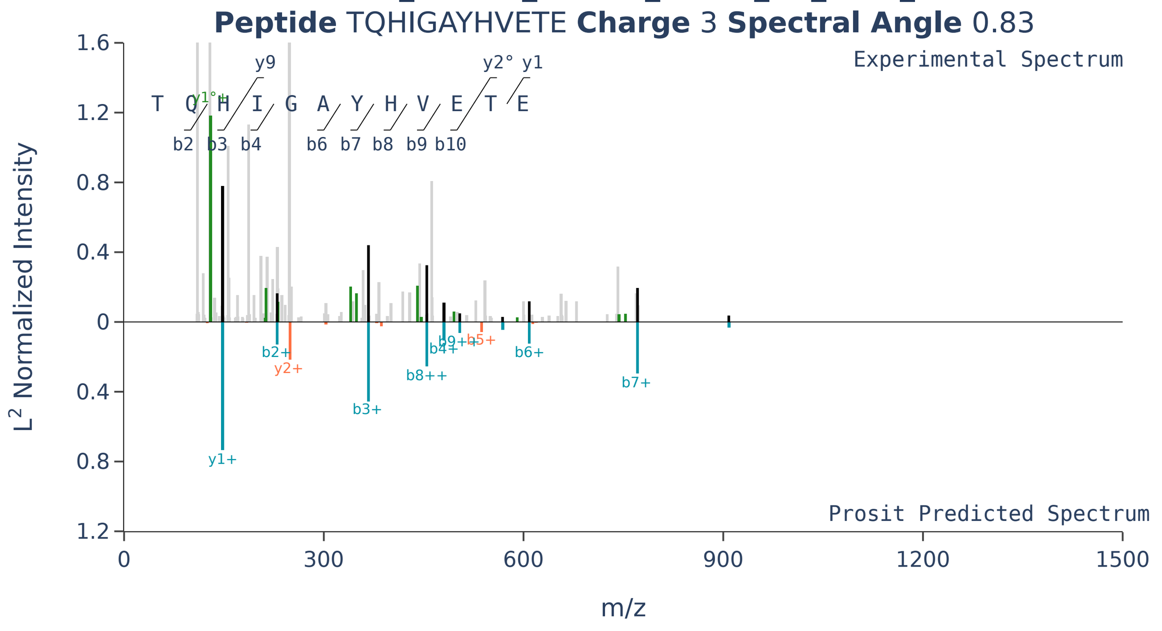


**Source** HRoetschke_211222_160123_HFGoe_IF2_b1R1_4h **Scan** 6283
**Peptide** SDYHVTTSQHARQAE **Charge** 3 **Spectral Angle** 0.7

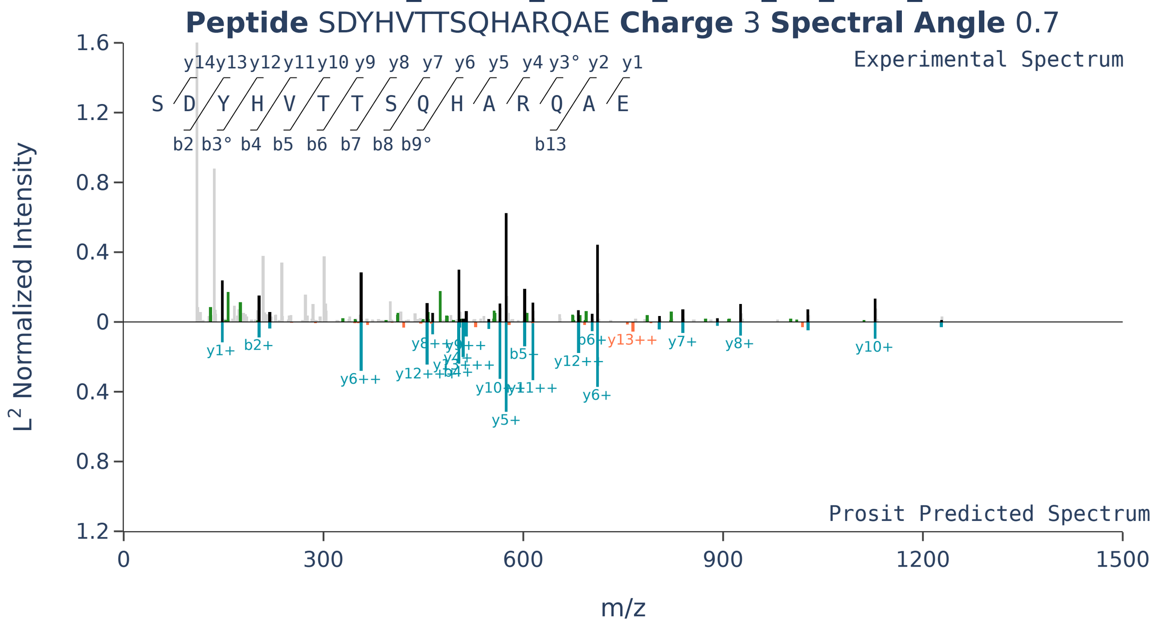

**Source** HRoetschke_211222_160123_HFGoe_IF2_b1R1_4h **Scan** 8285
**Peptide** HLNQKNSGPDKLTLQR **Charge** 4 **Spectral Angle** 0.87

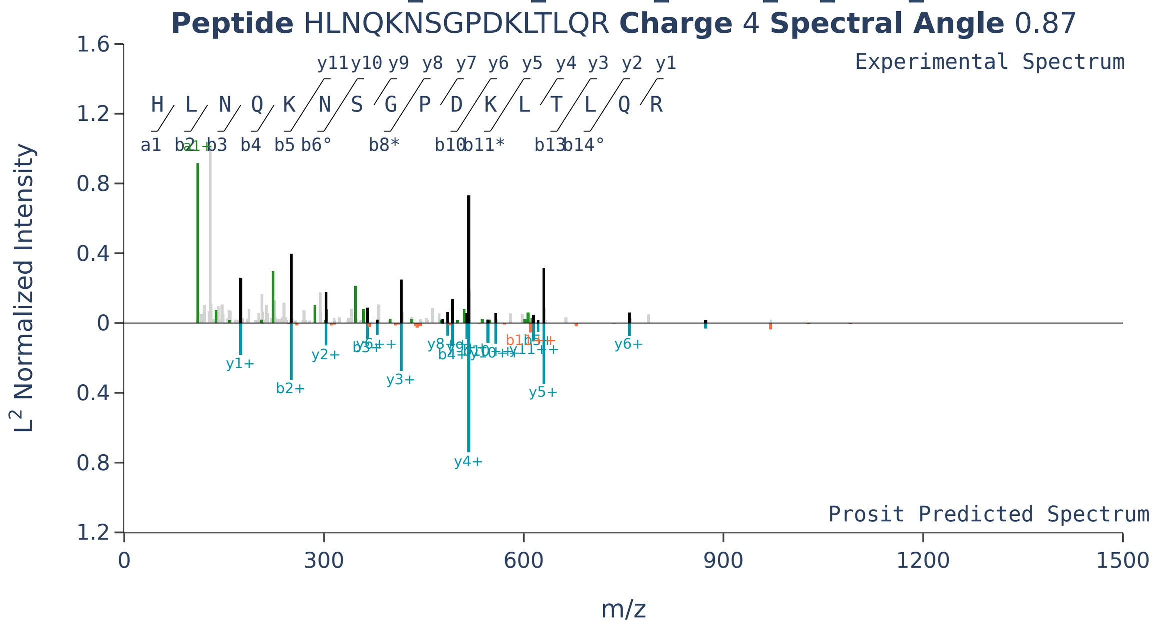


**Source** HRoetschke_211222_160123_HFGoe_IF2_b1R1_4h **Scan** 8006
**Peptide** VSAKAGTGIDE **Charge** 2 **Spectral Angle** 0.85

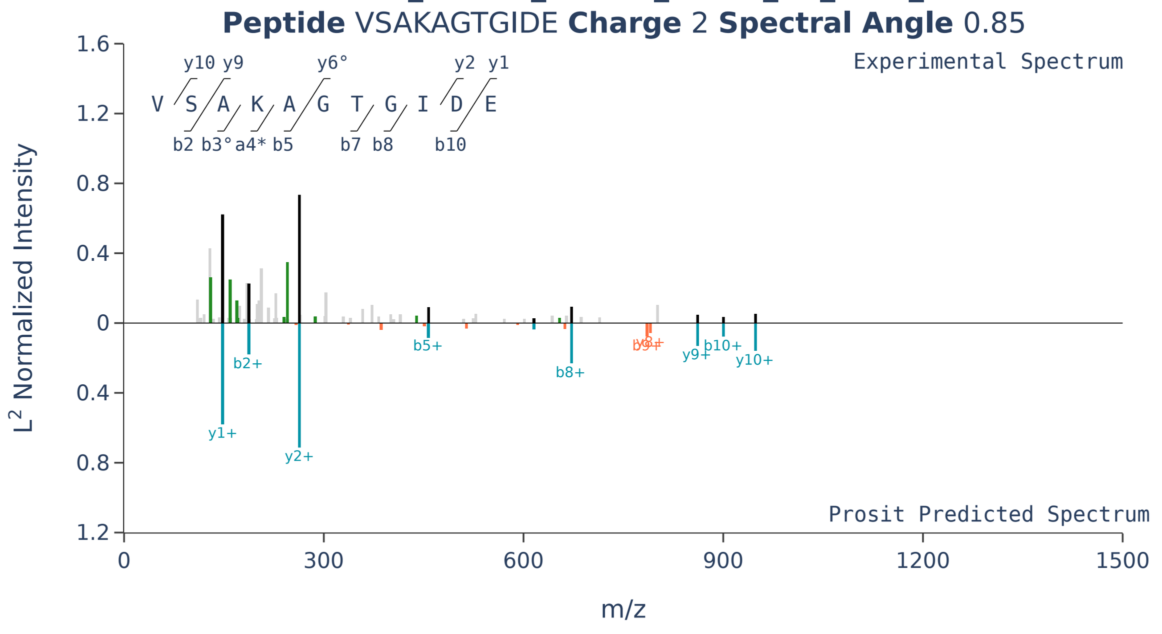


**Source** HRoetschke_211222_160123_HFGoe_IF2_b1R2_2h **Scan** 6157
**Peptide** KPEADPDRV **Charge** 2 **Spectral Angle** 0.75

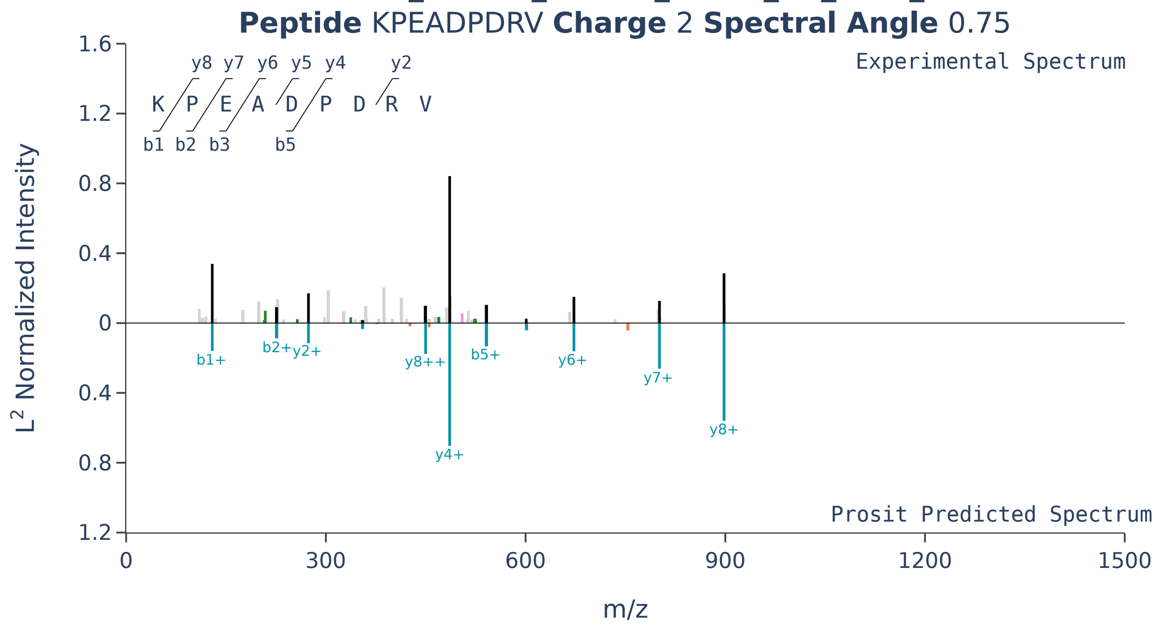


**Source** HRoetschke_211222_160123_HFGoe_IF2_b1R2_1h **Scan** 13477
**Peptide** QTSVERLVQ **Charge** 2 **Spectral Angle** 0.7

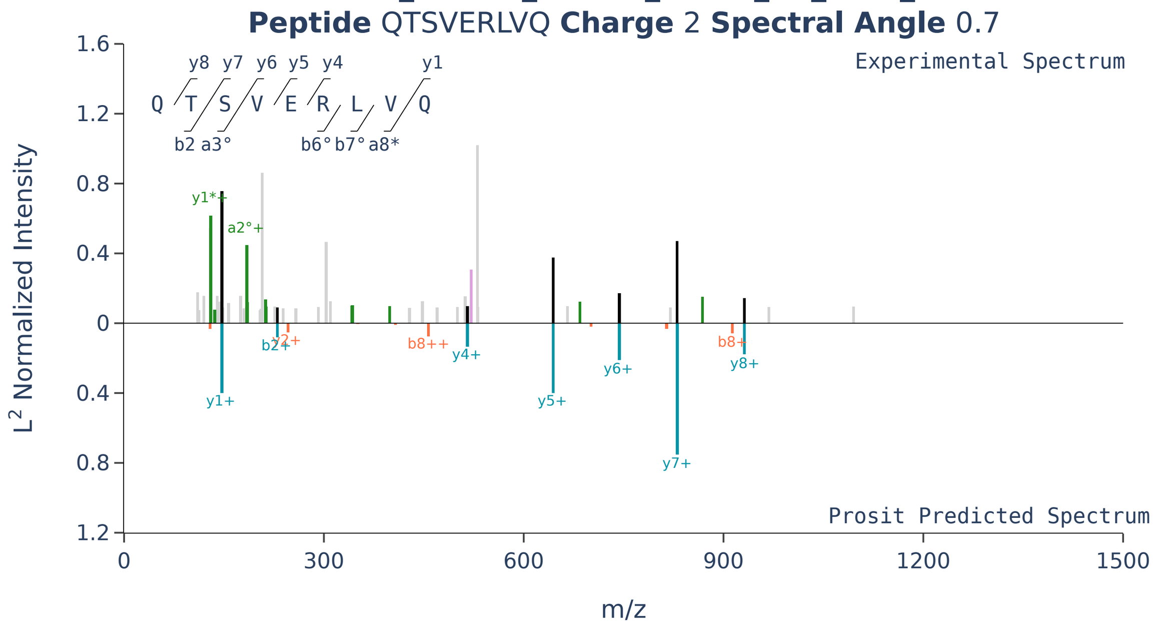


**Source** HRoetschke_211222_160123_HFGoe_IF2_b1R1_4h **Scan** 6636
**Peptide** SGPDKLTLQRKTRST **Charge** 3 **Spectral Angle** 0.74

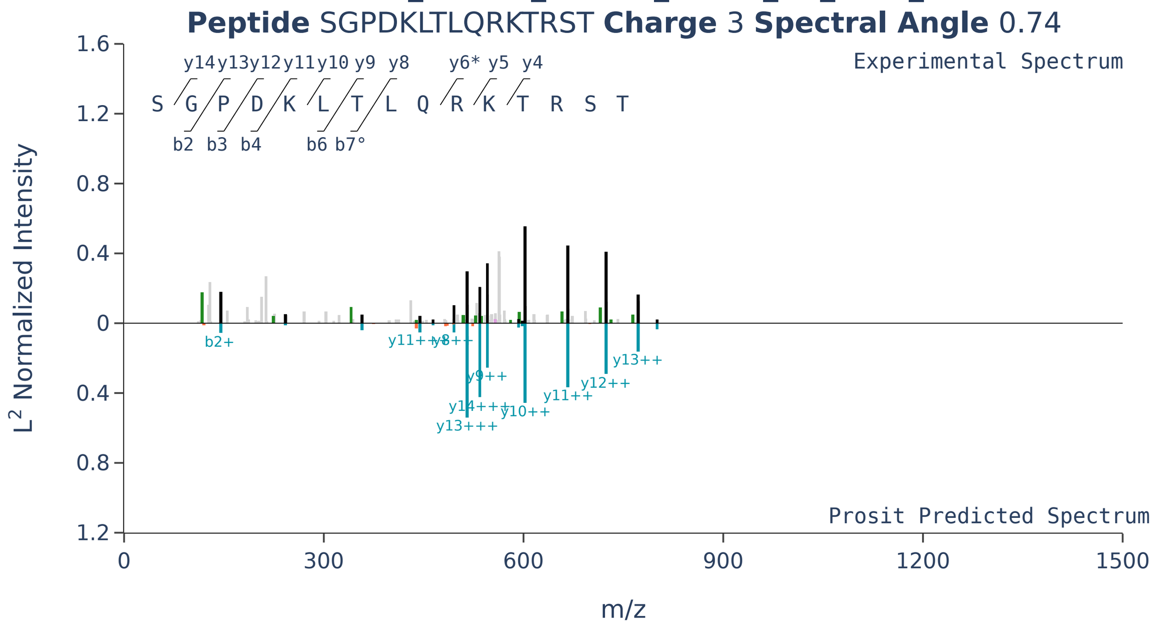


**Source** HRoetschke_211222_160123_HFGoe_IF2_b1R1_2h **Scan** 8431
**Peptide** KMAVKGSQVIKA **Charge** 2 **Spectral Angle** 0.87

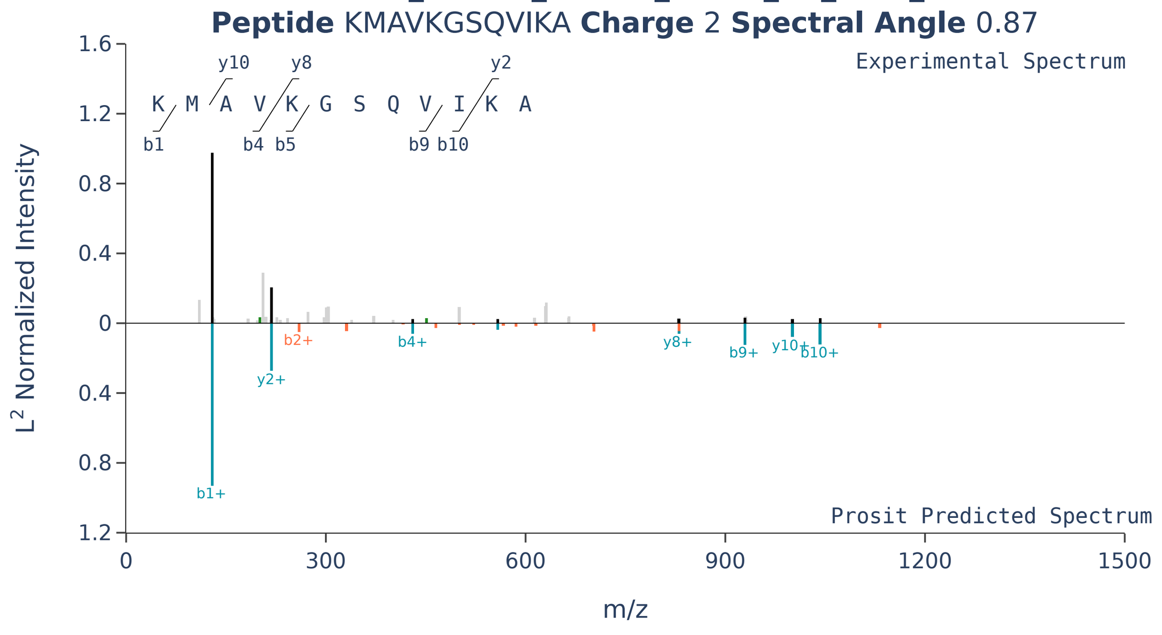


**Source** HRoetschke_211222_160123_HFGoe_IF2_b1R1_2h **Scan** 9264
**Peptide** KLSTDEV **Charge** 2 **Spectral Angle** 0.87

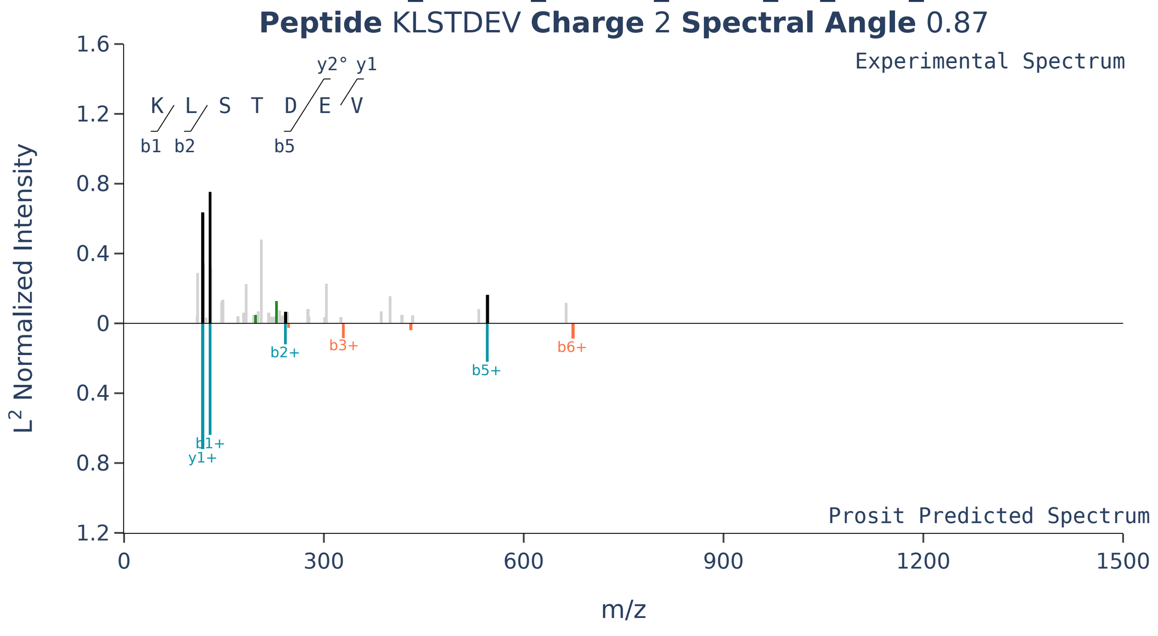


**Source** HRoetschke_211222_160123_HFGoe_IF2_b1R1_2h **Scan** 9427
**Peptide** SFLDKGR **Charge** 2 **Spectral Angle** 0.92

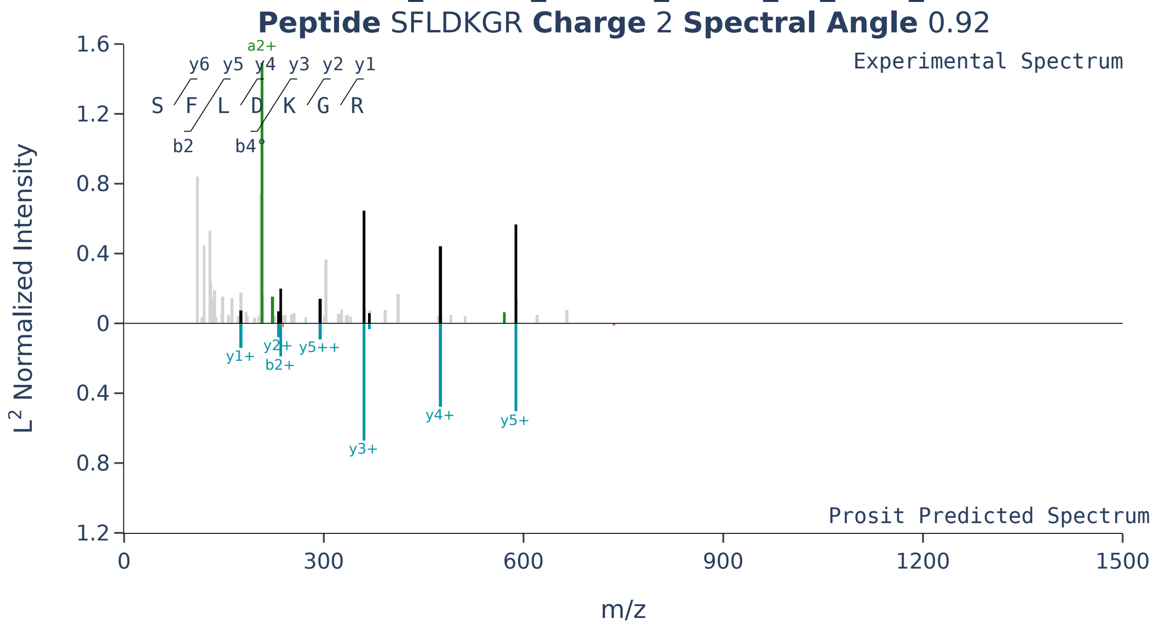


**Source** HRoetschke_211222_160123_HFGoe_IF2_b1R2_2h **Scan** 7639
**Peptide** DAGIRKSADDSVSAQE **Charge** 3 **Spectral Angle** 0.85

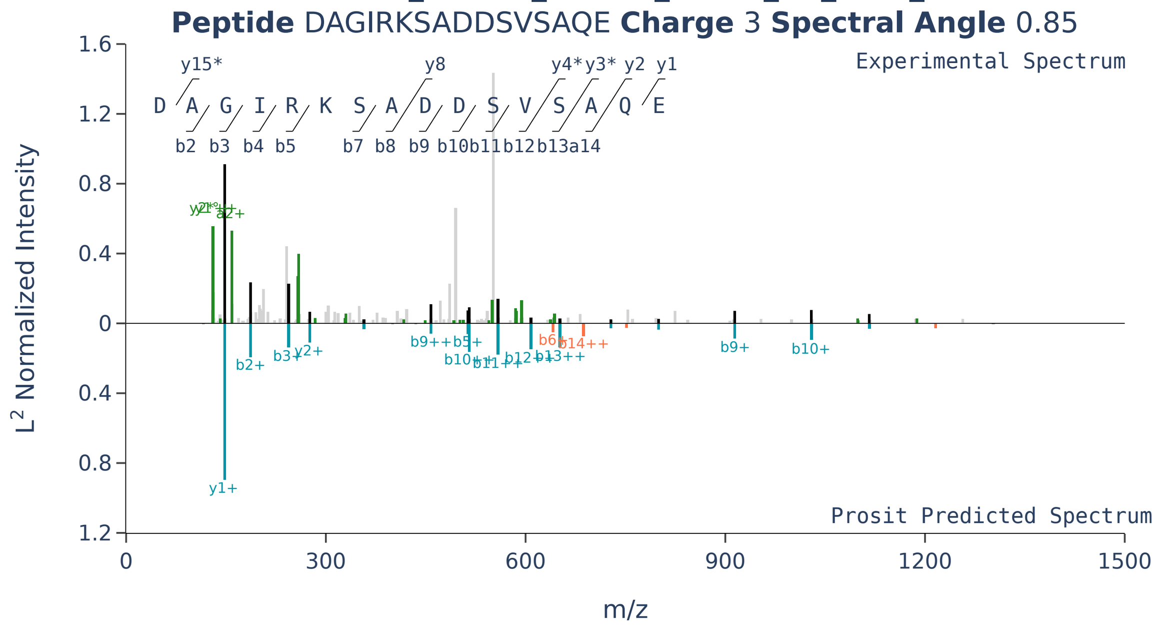


**Source** HRoetschke_211222_160123_HFGoe_IF2_b2R2_1h **Scan** 16153
**Peptide** MATINQVID **Charge** 2 **Spectral Angle** 0.82

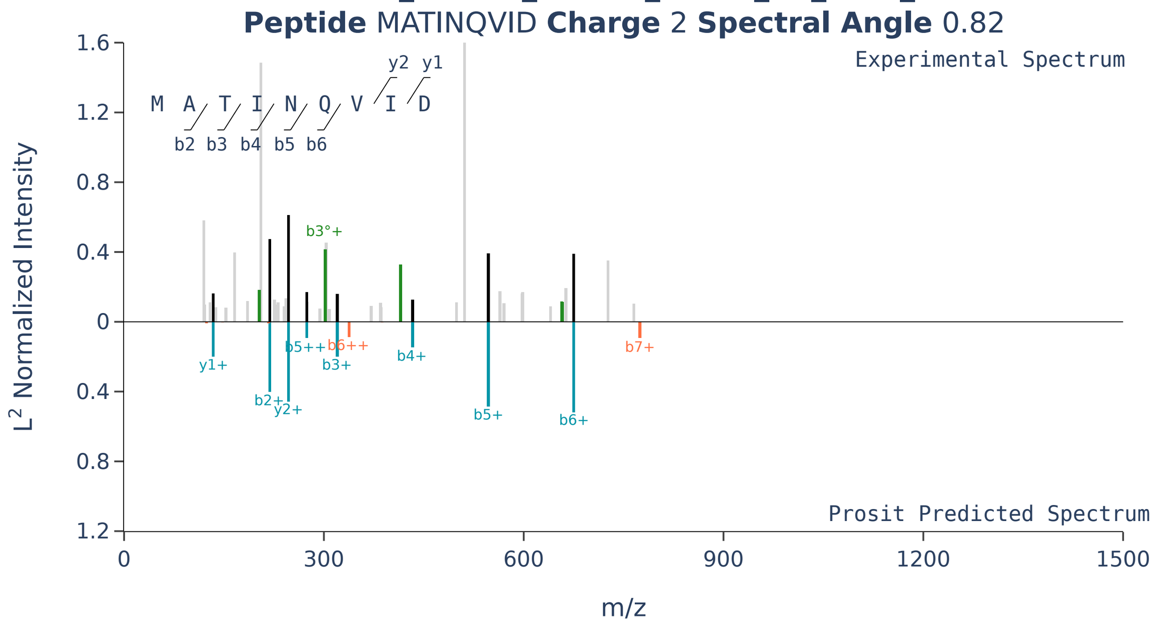


**Source** HRoetschke_211222_160123_HFGoe_IF2_b1R2_24h **Scan** 10945
**Peptide** GLGVKNYNDVRTGD **Charge** 3 **Spectral Angle** 0.78

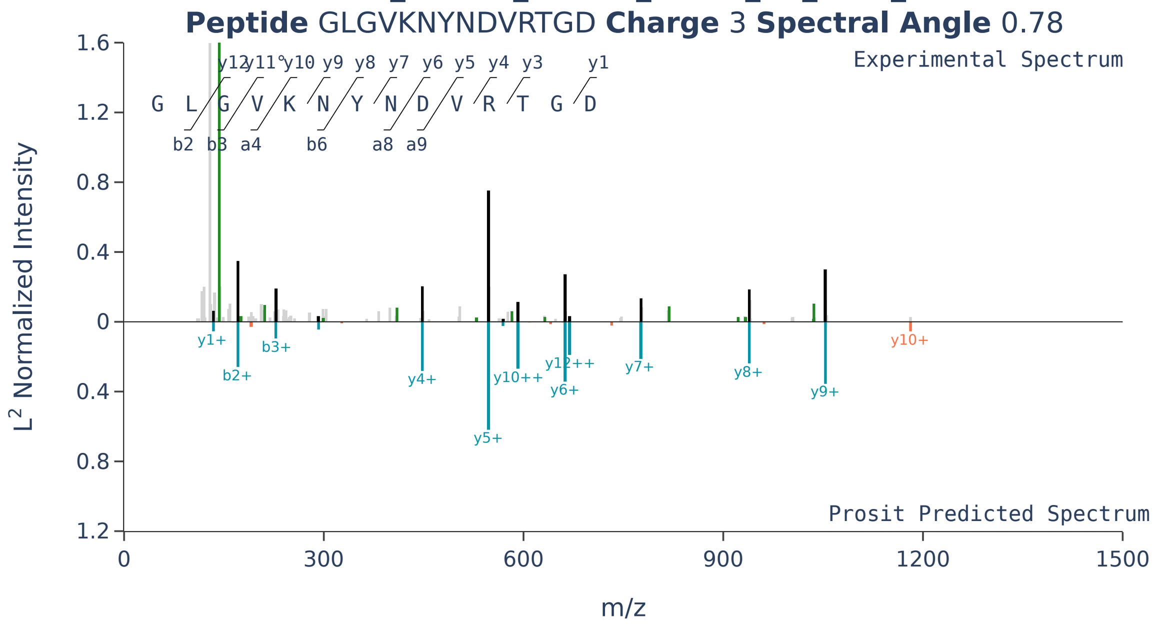


**Source** HRoetschke_211222_160123_HFGoe_IF2_b1R1_24h **Scan** 6990
**Peptide** PGTGGKSKSV **Charge** 2 **Spectral Angle** 0.75

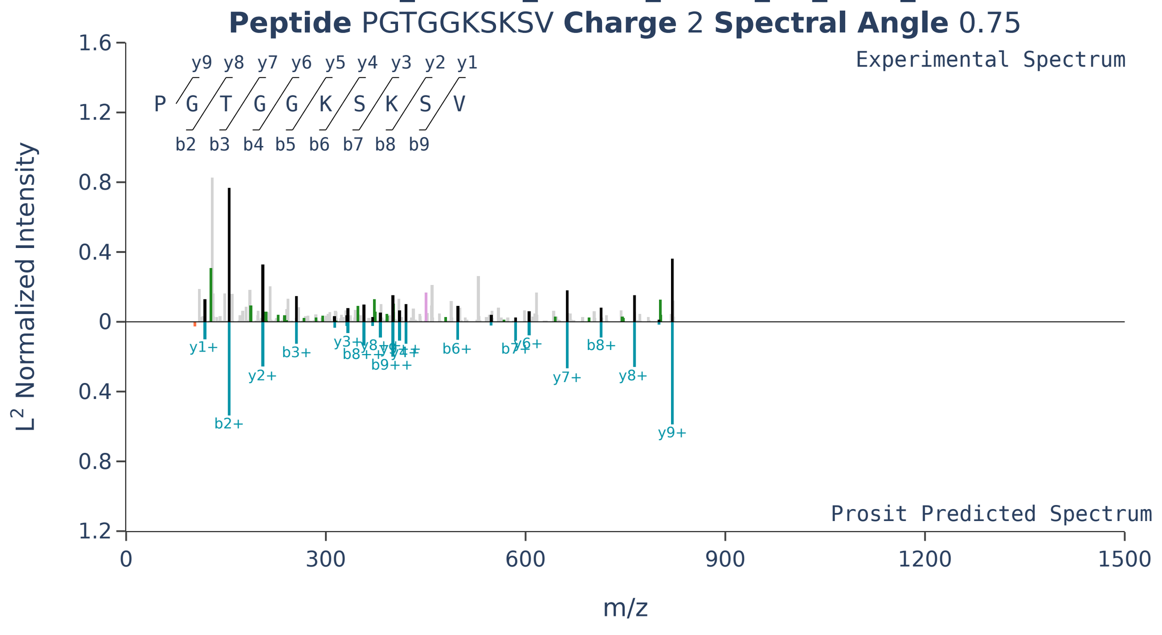


**Source** HRoetschke_211222_160123_HFGoe_IF2_b1R2_1h **Scan** 17063
**Peptide** AGPSIPVE **Charge** 1 **Spectral Angle** 0.88

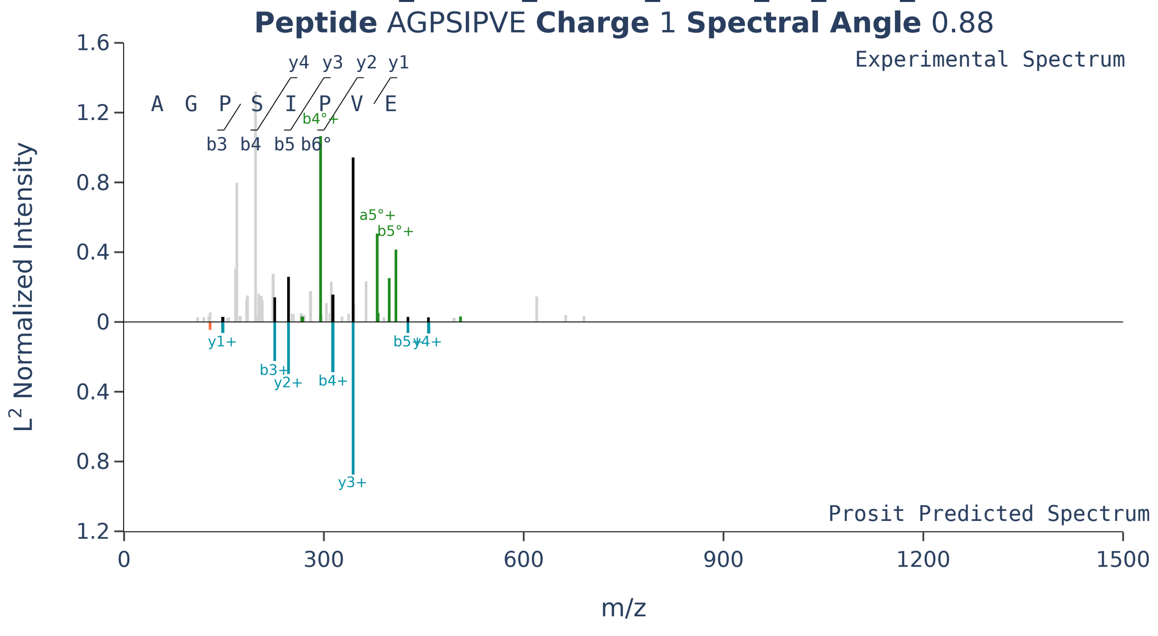


**Source** HRoetschke_211222_160123_HFGoe_IF2_b2R1_24h **Scan** 13841
**Peptide** QTSVERLVQQ **Charge** 2 **Spectral Angle** 0.81

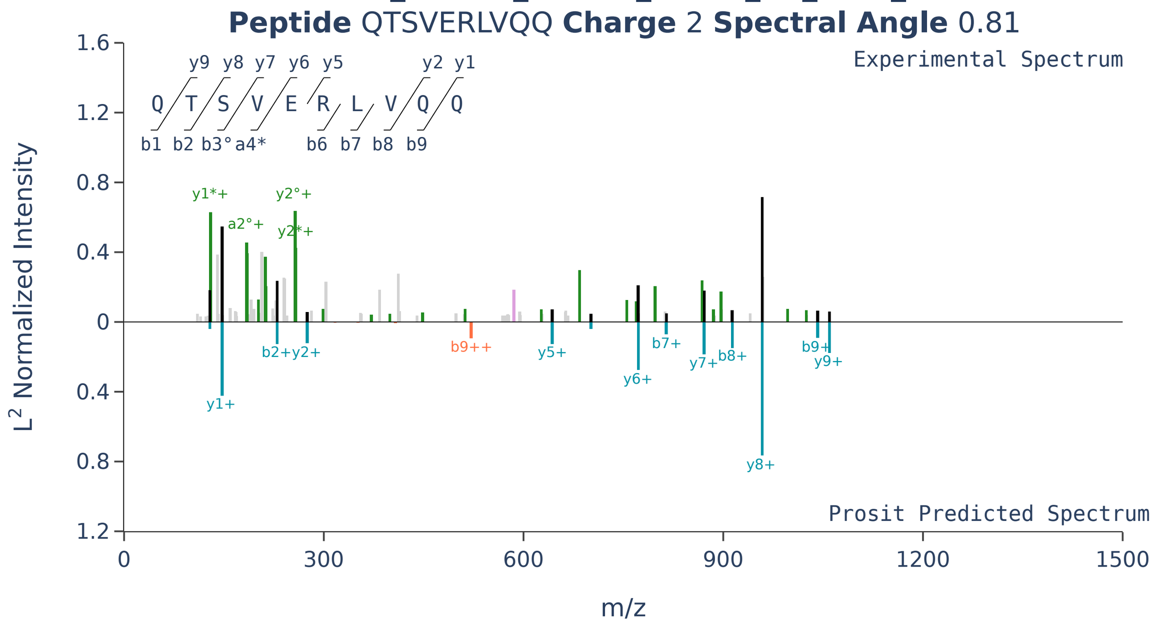


**Source** HRoetschke_211222_160123_HFGoe_IF2_b1R2_24h **Scan** 12639
**Peptide** RDTGAAAEPRAPVV **Charge** 3 **Spectral Angle** 0.79

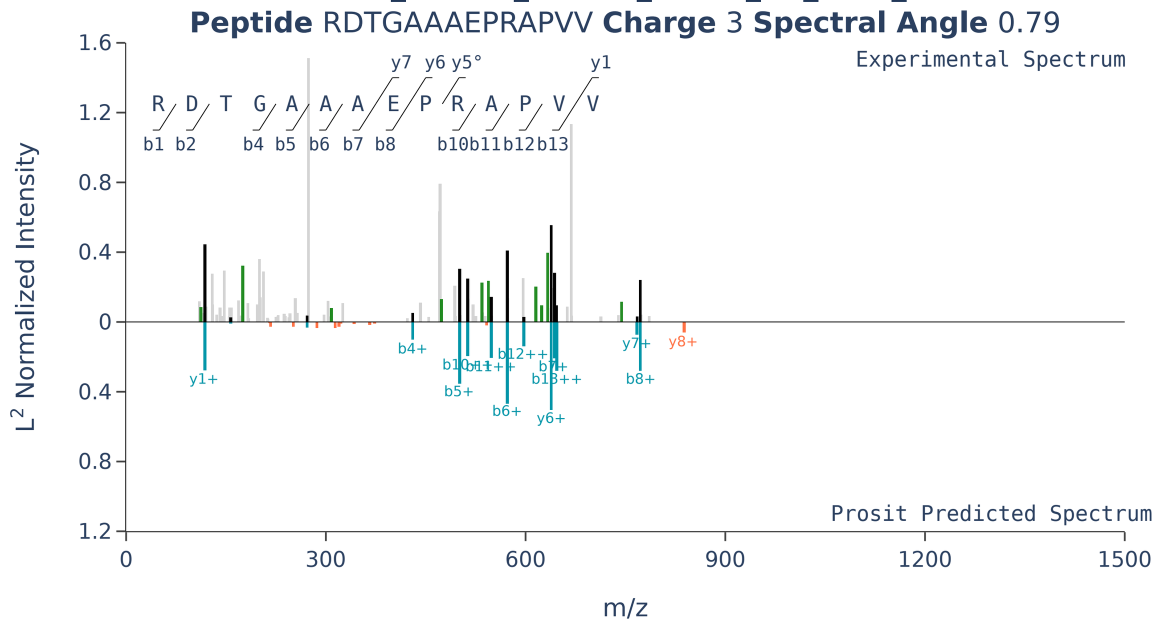

**Source** HRoetschke_211222_160123_HFGoe_IF2_b1R1_24h **Scan** 12524
**Peptide** RDVFKSPKFG **Charge** 3 **Spectral Angle** 0.81

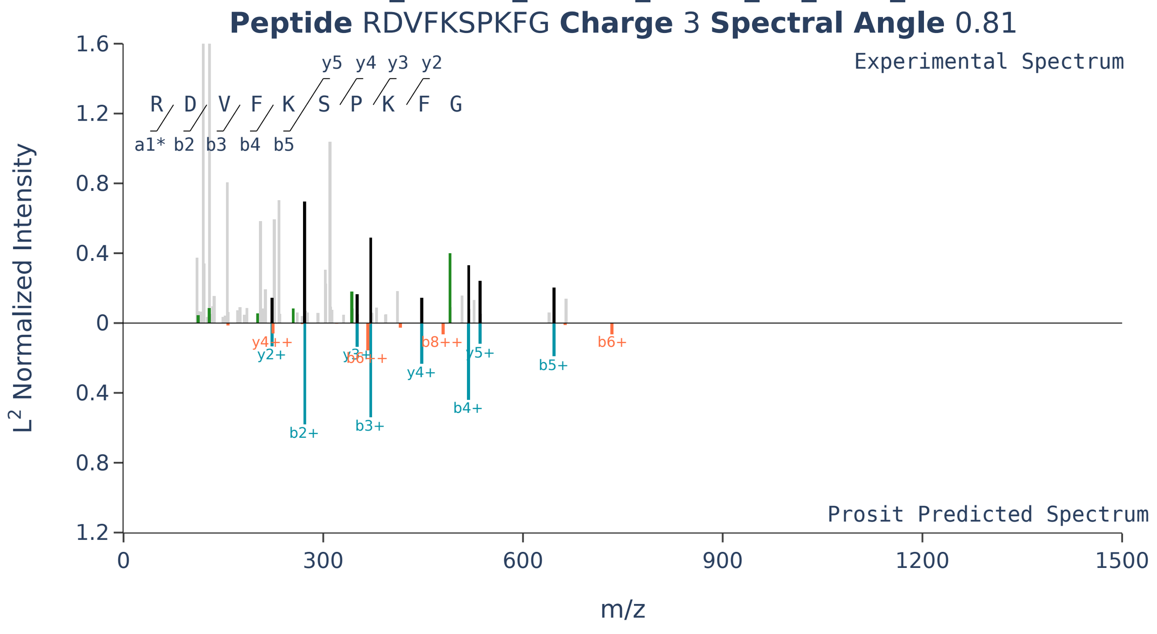


**Source** HRoetschke_211222_160123_HFGoe_IF2_b1R1_4h **Scan** 7970
**Peptide** VLVREGTLHK **Charge** 2 **Spectral Angle** 0.84

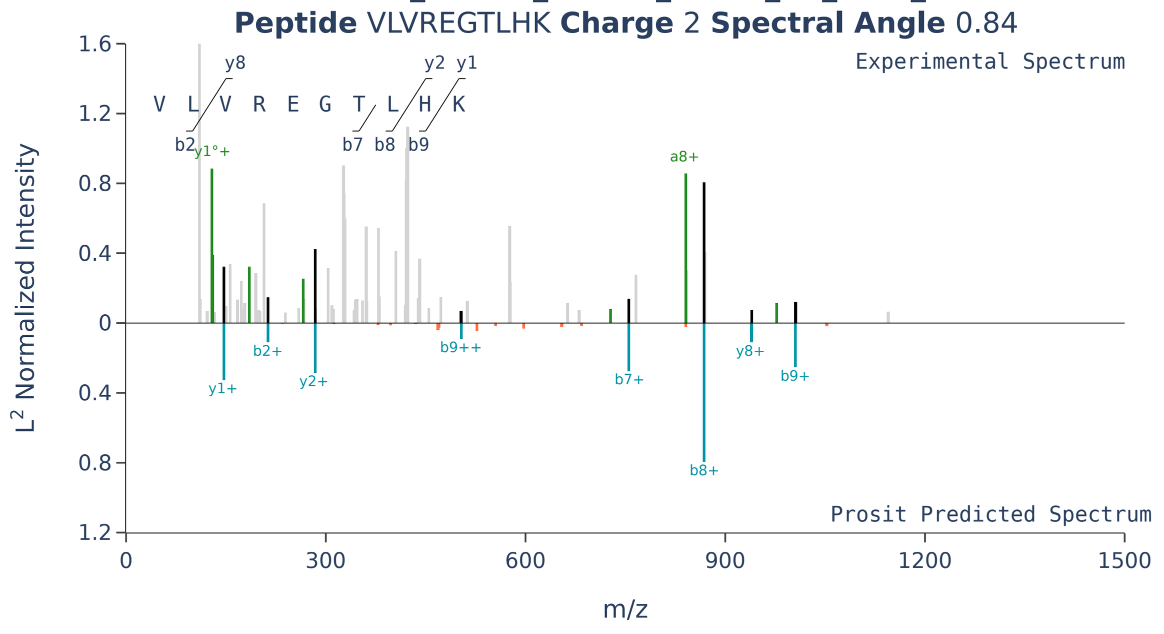


**Source** HRoetschke_211222_160123_HFGoe_IF2_b1R2_24h **Scan** 15001
**Peptide** TDVTIKTLAAERQ **Charge** 3 **Spectral Angle** 0.85

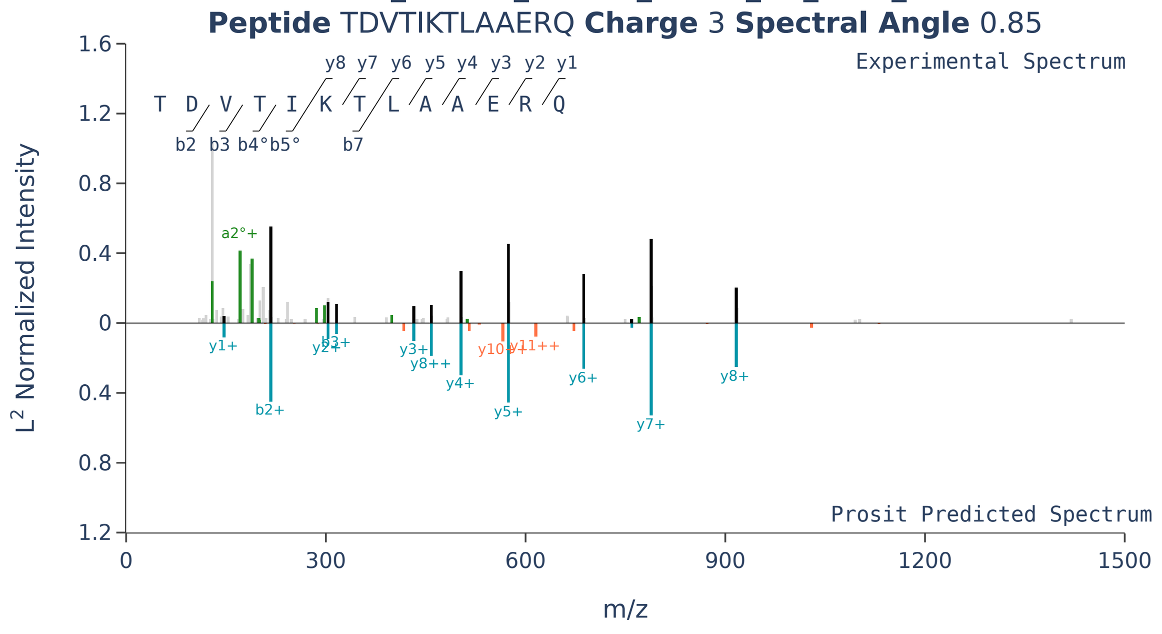


**Source** HRoetschke_211222_160123_HFGoe_IF2_b2R2_1h **Scan** 11809
**Peptide** KADVQGSVEAISD **Charge** 2 **Spectral Angle** 0.78

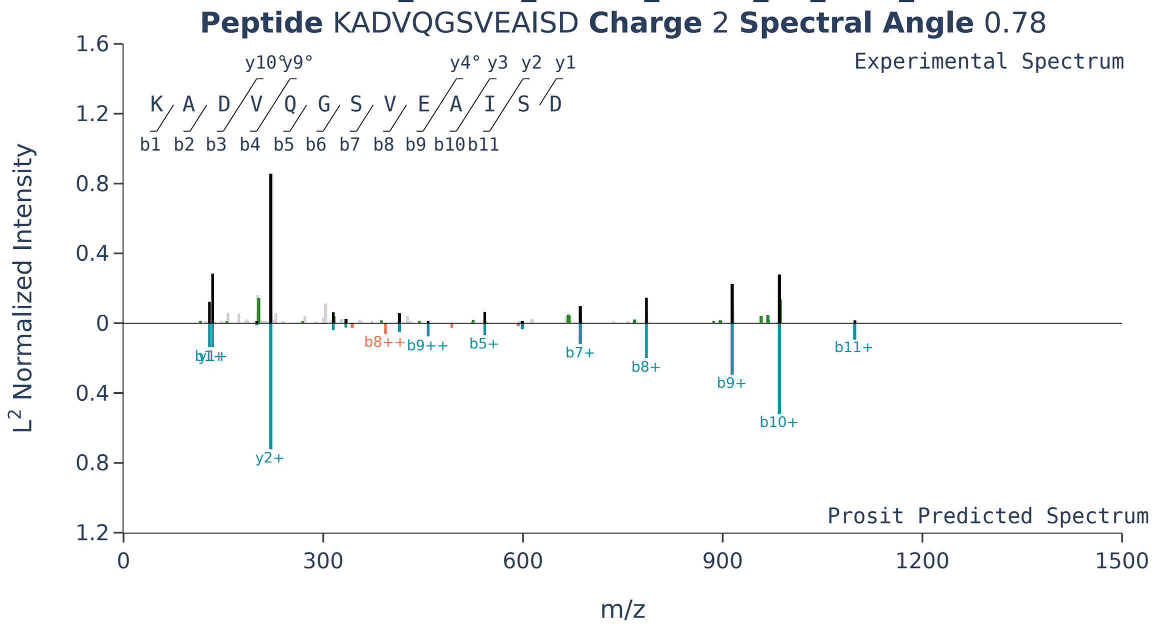


**Source** HRoetschke_211222_160123_HFGoe_IF2_b2R2_4h **Scan** 15998
**Peptide** KAAQVPVVVA **Charge** 2 **Spectral Angle** 0.86

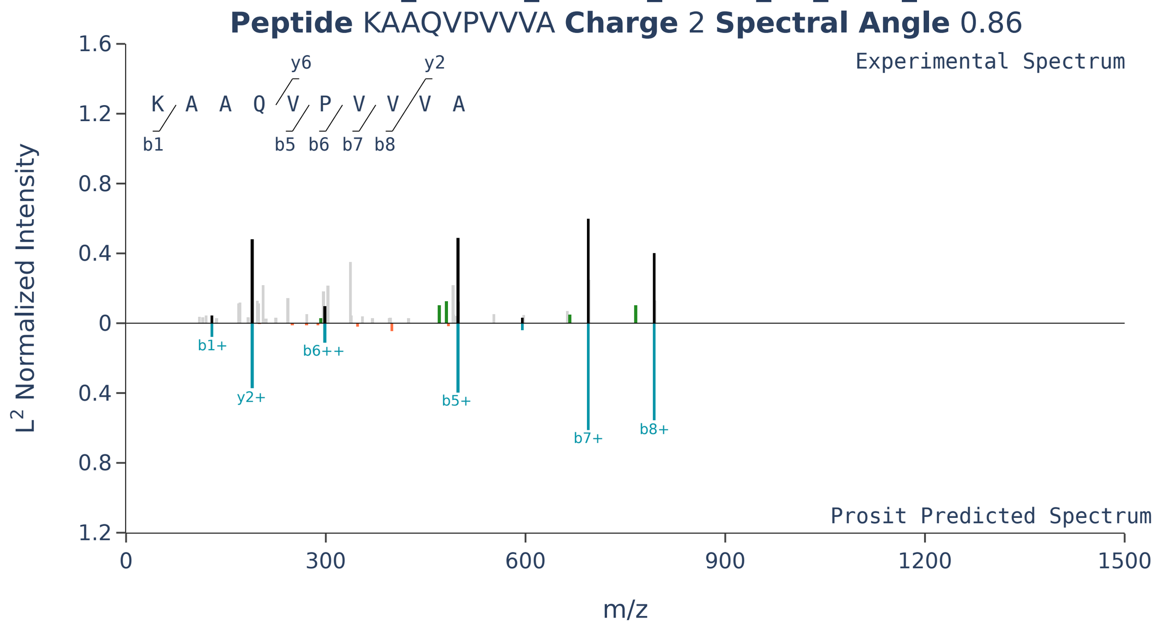


**Source** HRoetschke_211222_160123_HFGoe_IF2_b2R2_24h **Scan** 7709
**Peptide** NKIDKPEADPDRV **Charge** 4 **Spectral Angle** 0.83

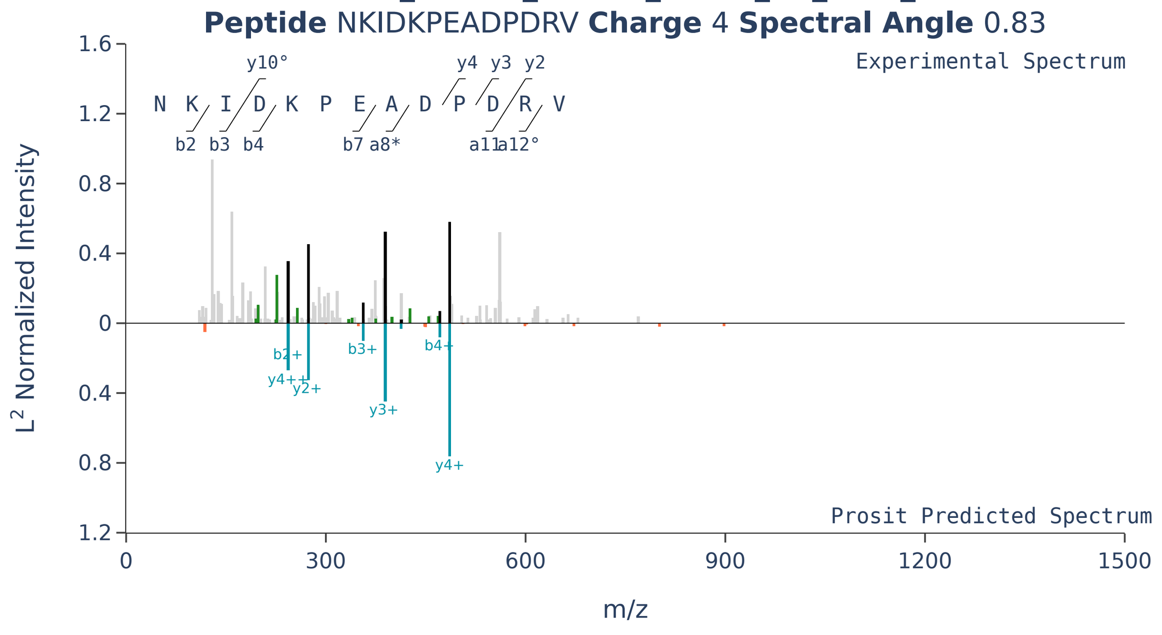


**Source** HRoetschke_211222_160123_HFGoe_IF2_b1R1_4h **Scan** 18434
**Peptide** AEVLELKA **Charge** 2 **Spectral Angle** 0.9

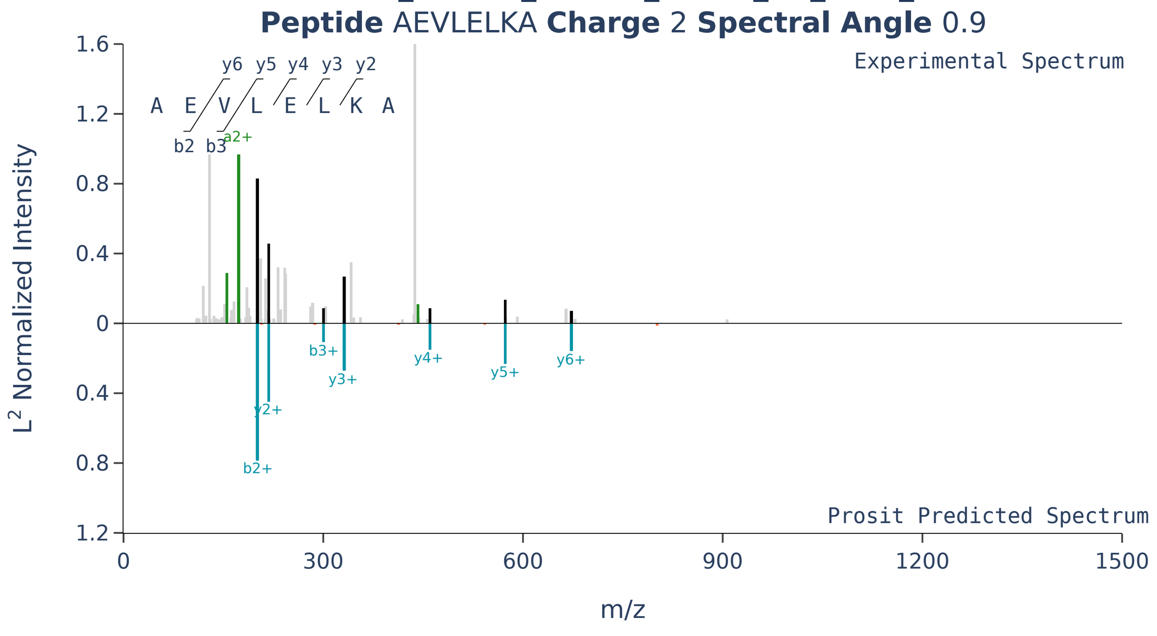


**Source** HRoetschke_211222_160123_HFGoe_IF2_b2R2_4h **Scan** 9143
**Peptide** HLGAYHV **Charge** 2 **Spectral Angle** 0.79

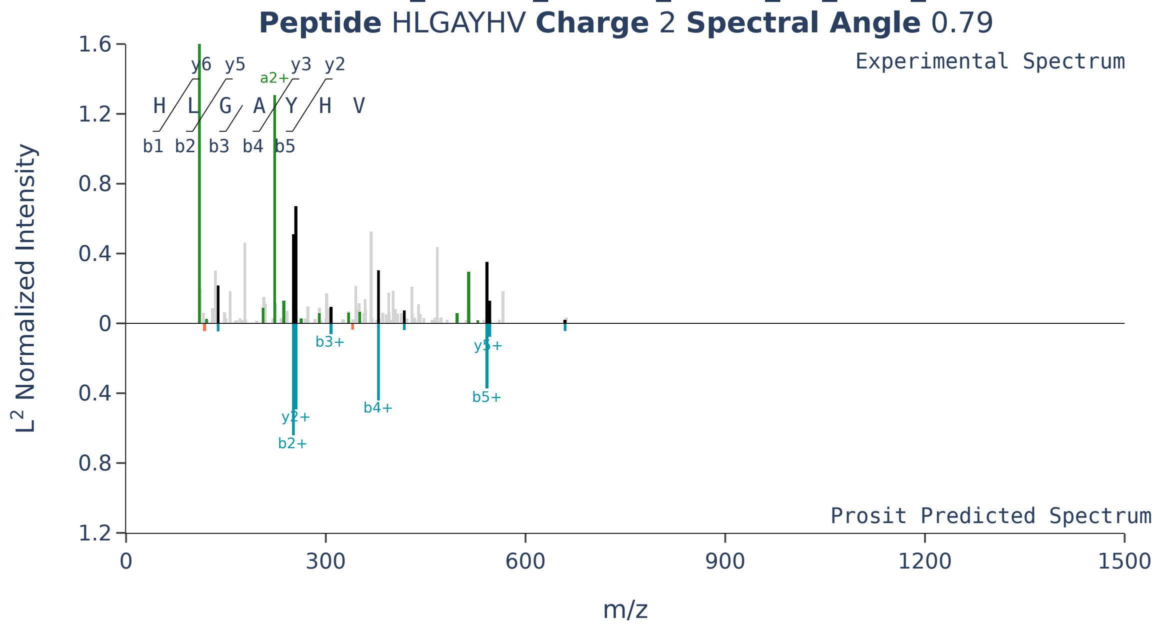


**Source** HRoetschke_211222_160123_HFGoe_IF2_b1R1_2h **Scan** 5964
**Peptide** NQKNSGPDKLTLQRKTR **Charge** 4 **Spectral Angle** 0.79

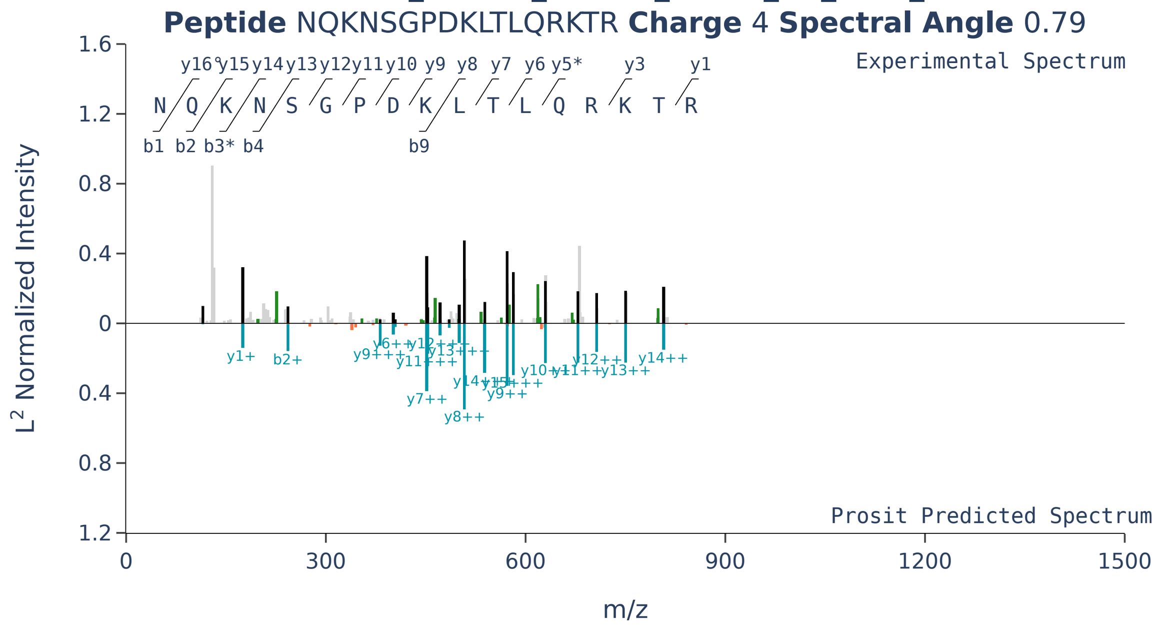


**Source** HRoetschke_211222_160123_HFGoe_IF2_b1R1_4h **Scan** 20488
**Peptide** YEGELESL **Charge** 1 **Spectral Angle** 0.92

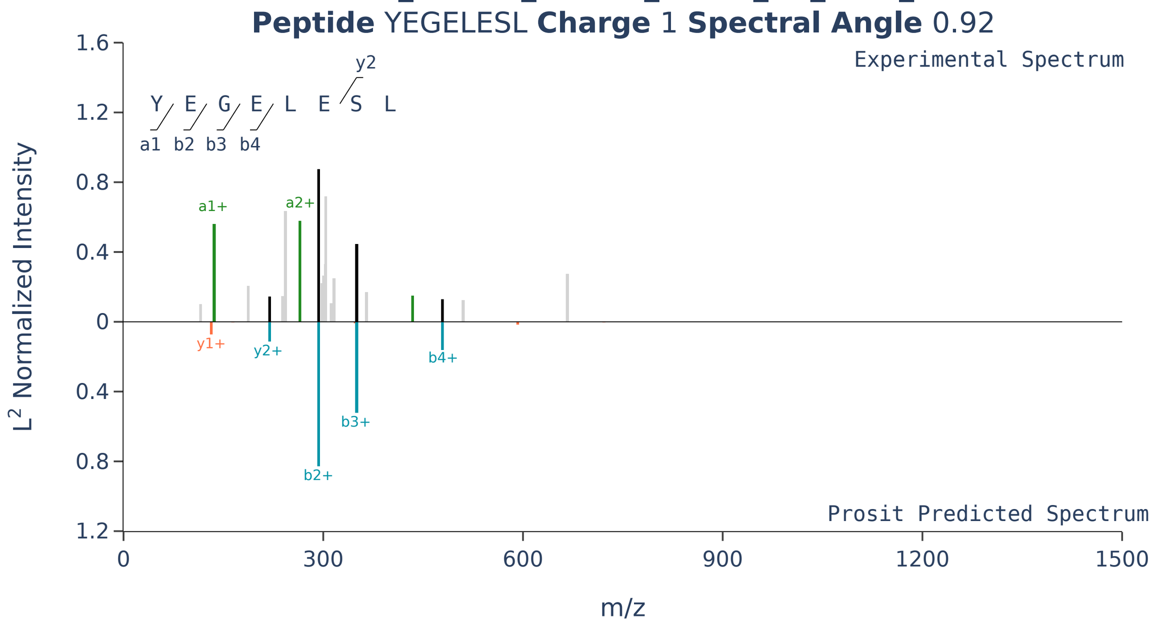


**Source** HRoetschke_211222_160123_HFGoe_IF2_b1R1_24h **Scan** 12140
**Peptide** KVASGEAGGITQHI **Charge** 3 **Spectral Angle** 0.8

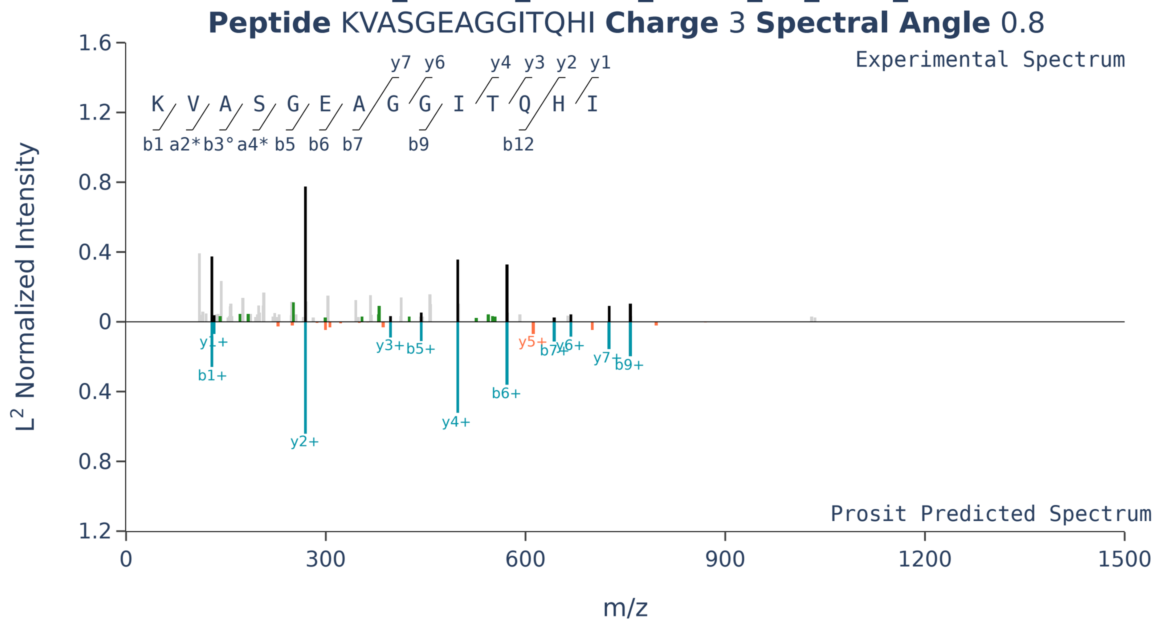


**Source** HRoetschke_211222_160123_HFGoe_IF2_b1R1_24h **Scan** 7530
**Peptide** ADAGIRKS **Charge** 2 **Spectral Angle** 0.86

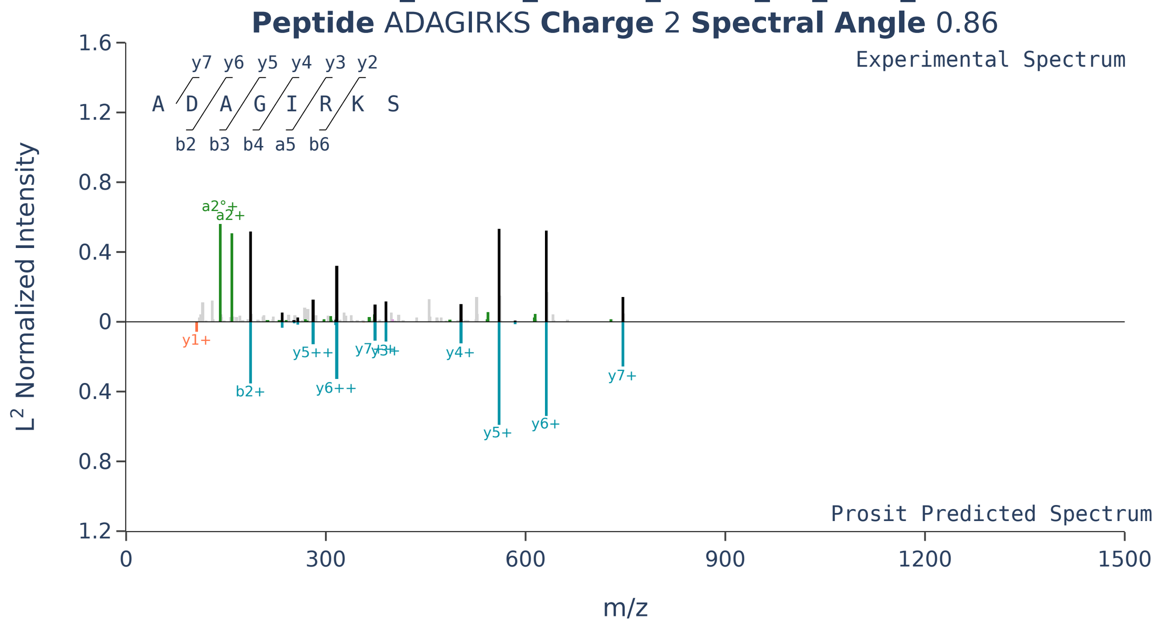


**Source** HRoetschke_211222_160123_HFGoe_IF2_b1R1_1h **Scan** 9320
**Peptide** QQFADAGIRKSAD **Charge** 2 **Spectral Angle** 0.83

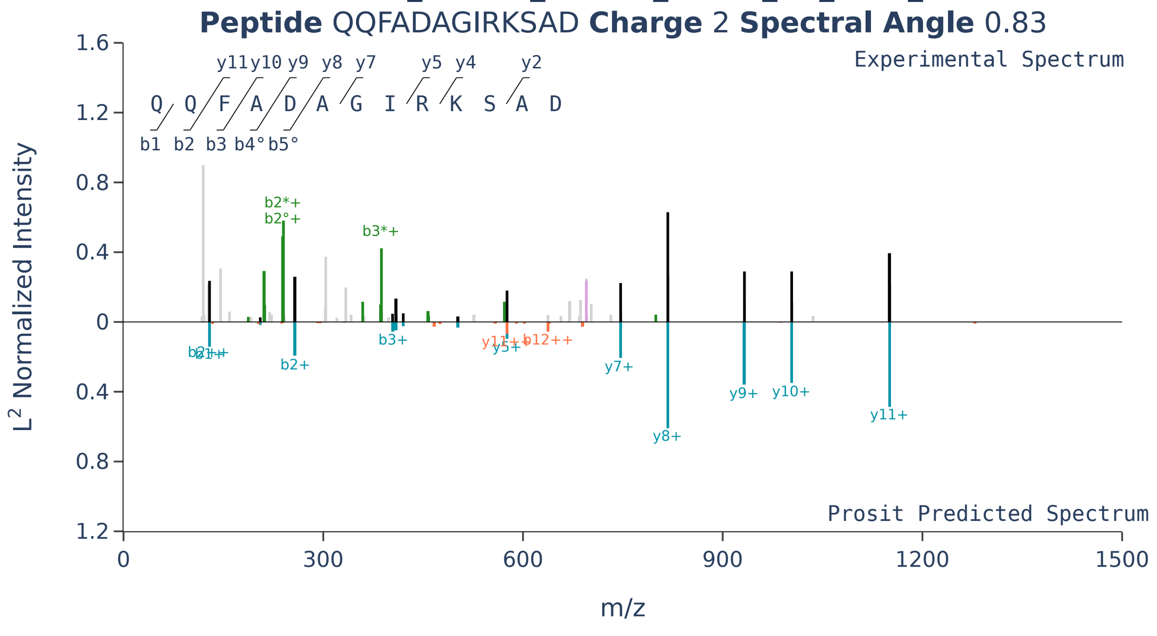


**Source** HRoetschke_211222_160123_HFGoe_IF2_b2R1_24h **Scan** 7073
**Peptide** LDKPEADPDRVKNE **Charge** 2 **Spectral Angle** 0.91

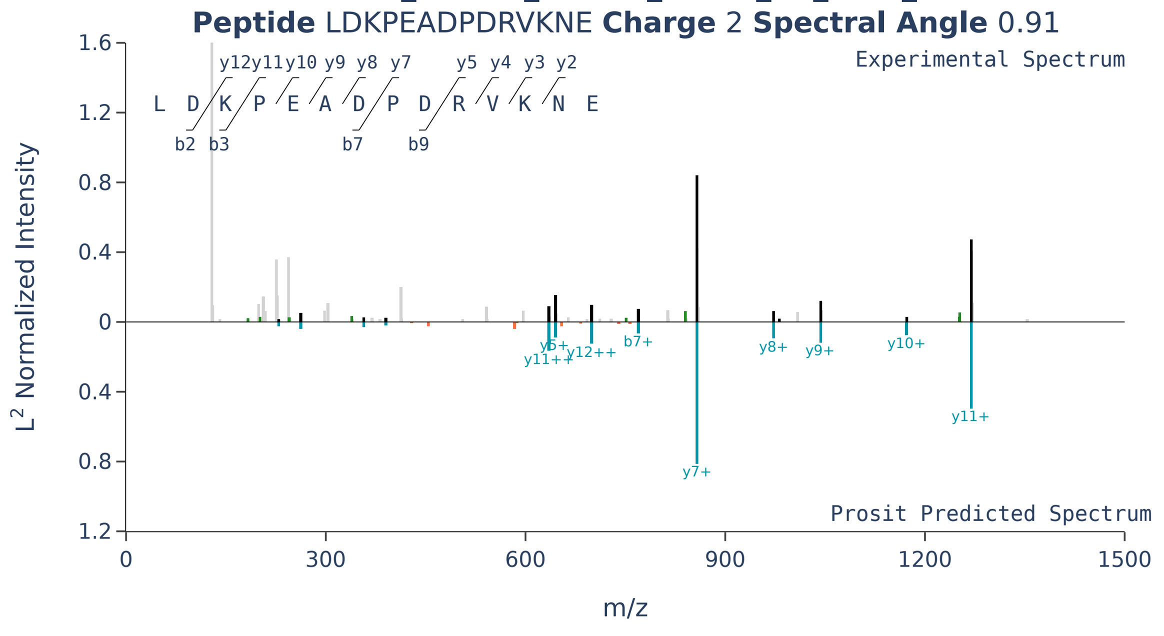


**Source** HRoetschke_211222_160123_HFGoe_IF2_b1R2_24h **Scan** 8873
**Peptide** HVDHGKTSLLD **Charge** 3 **Spectral Angle** 0.85

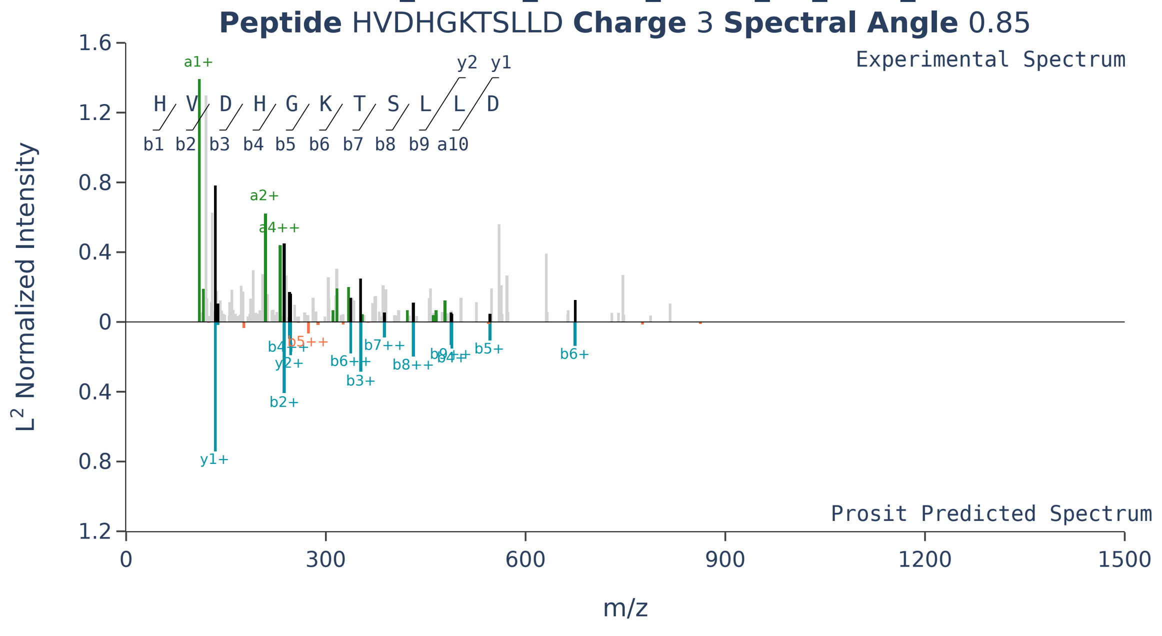

**Source** HRoetschke_211222_160123_HFGoe_IF2_b1R1_4h **Scan** 6340
**Peptide** ADDSVSAQE **Charge** 2 **Spectral Angle** 0.91

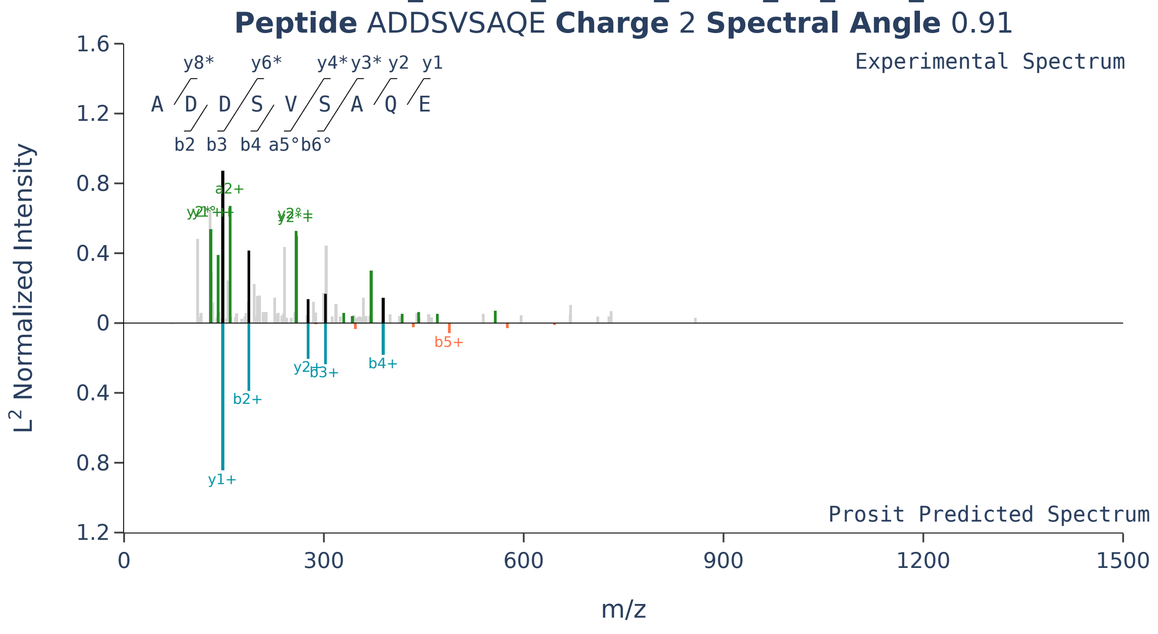


**Source** HRoetschke_211222_160123_HFGoe_IF2_b1R2_4h **Scan** 7242
**Peptide** SSDYHVTTSQ **Charge** 2 **Spectral Angle** 0.84

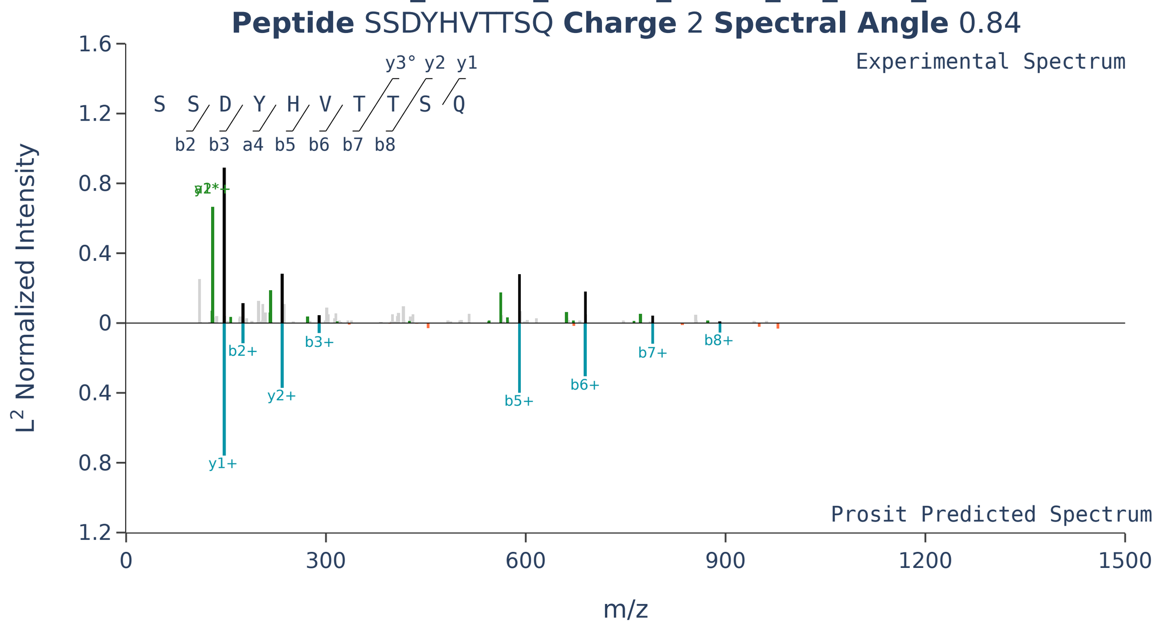


**Source** HRoetschke_211222_160123_HFGoe_IF2_b1R1_4h **Scan** 11580
**Peptide** QQGFQKPAQAV **Charge** 2 **Spectral Angle** 0.76

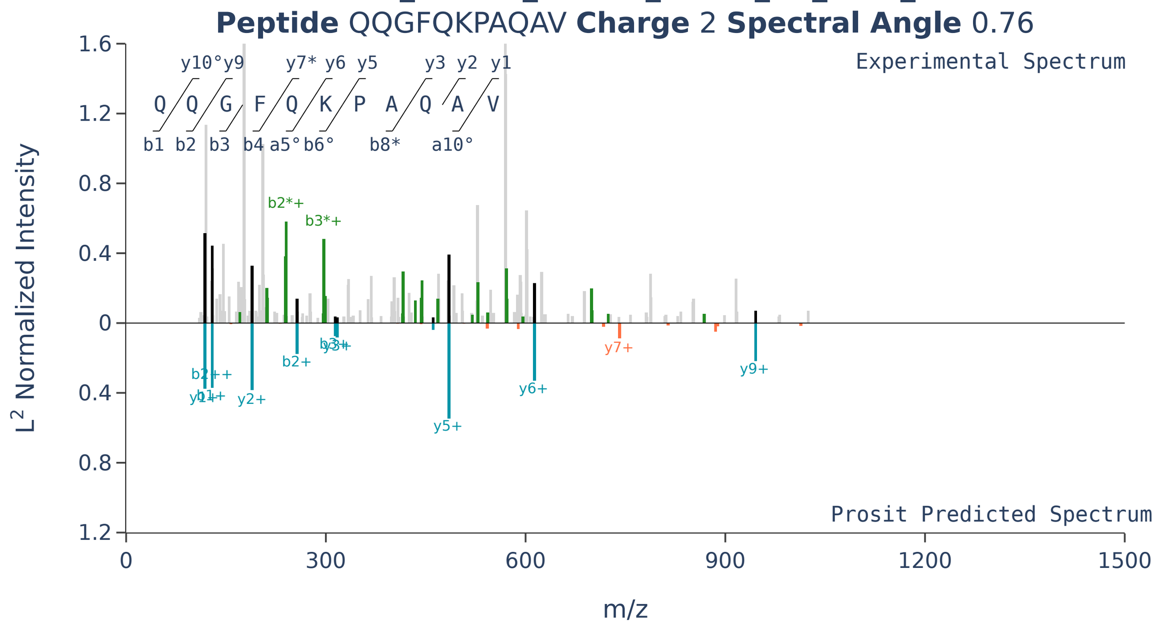


**Source** HRoetschke_211222_160123_HFGoe_IF2_b2R2_24h **Scan** 14328
**Peptide** LNIPGTGGKS **Charge** 2 **Spectral Angle** 0.85

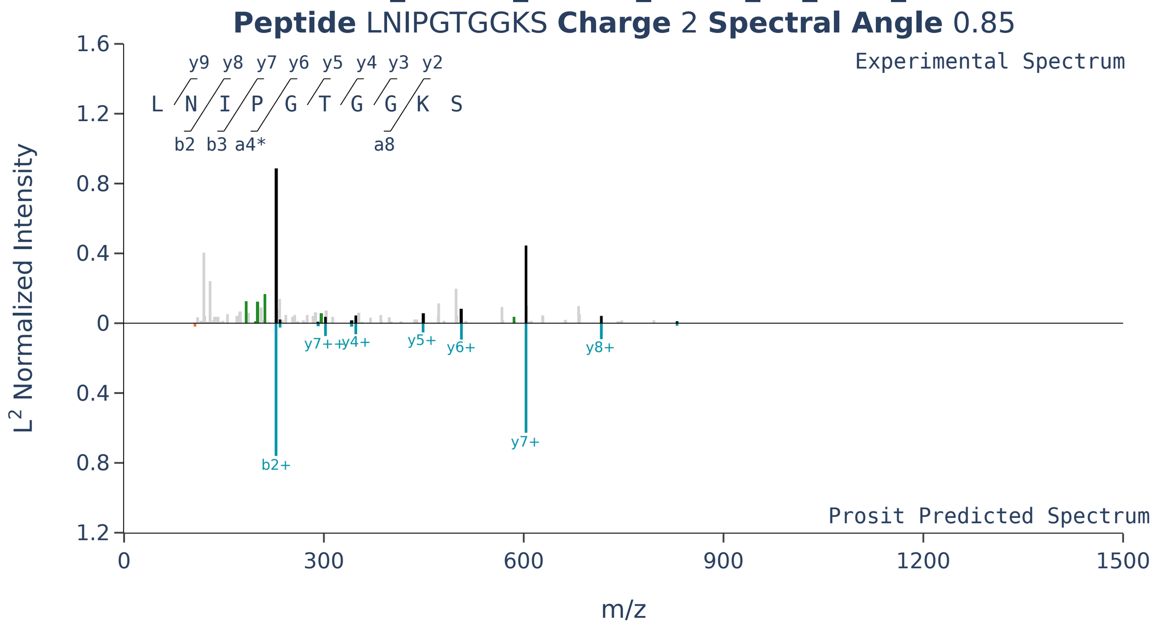


**Source** HRoetschke_211222_160123_HFGoe_IF2_b1R2_24h **Scan** 6406
**Peptide** ELKAVRKGM **Charge** 2 **Spectral Angle** 0.85

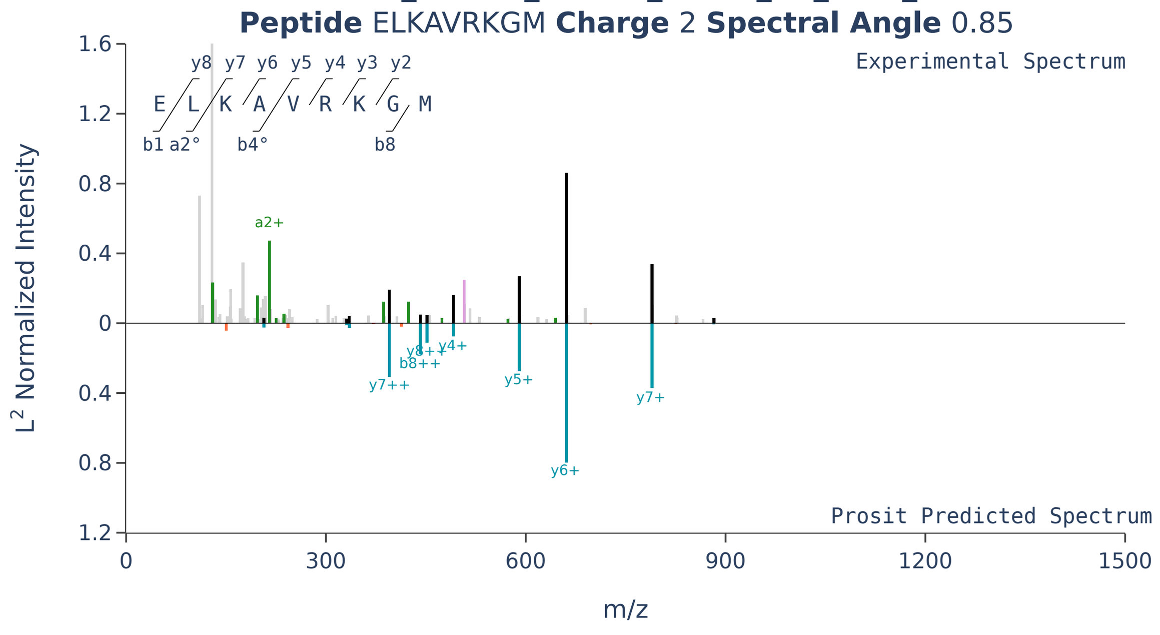


**Source** HRoetschke_211222_160123_HFGoe_IF2_b1R1_4h **Scan** 17934
**Peptide** KAAQVPVVV **Charge** 2 **Spectral Angle** 0.81

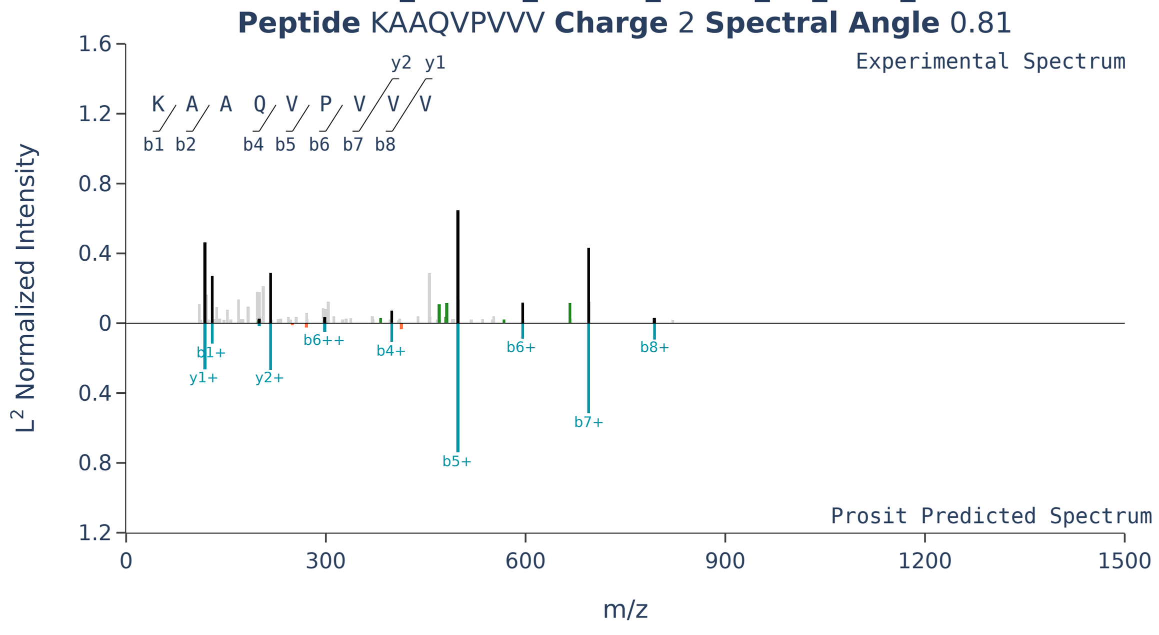


**Source** HRoetschke_211222_160123_HFGoe_IF2_b1R1_4h **Scan** 8949
**Peptide** RNGMECGIGVKN **Charge** 3 **Spectral Angle** 0.91

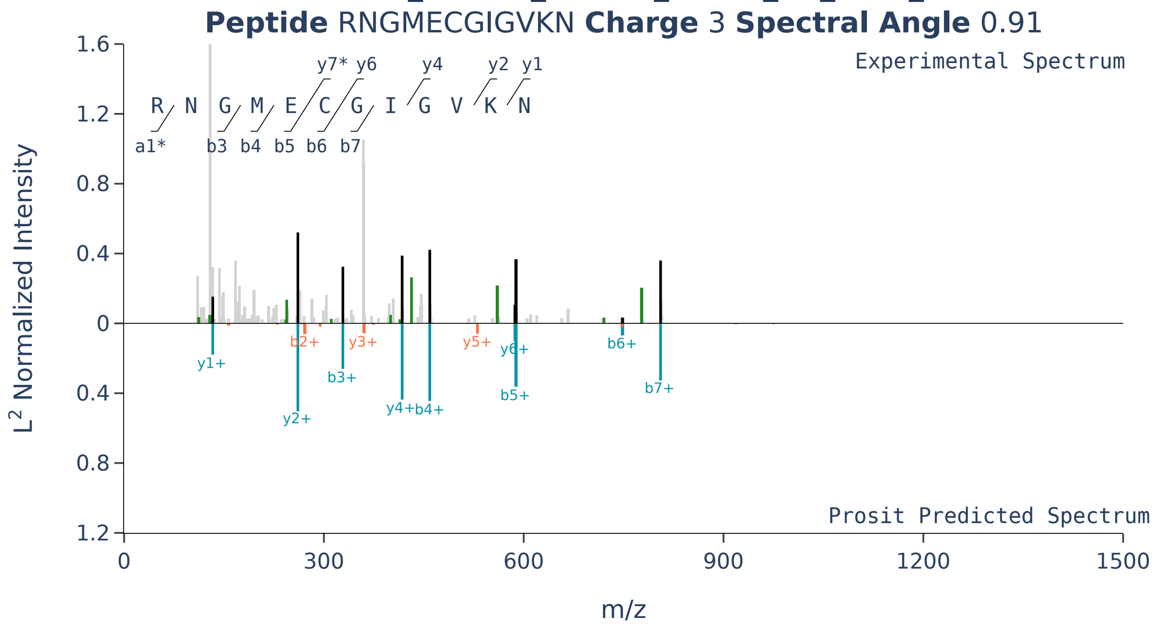


**Source** HRoetschke_211222_160123_HFGoe_IF2_b1R2_2h **Scan** 8547
**Peptide** KVASGEAGGL **Charge** 2 **Spectral Angle** 0.78

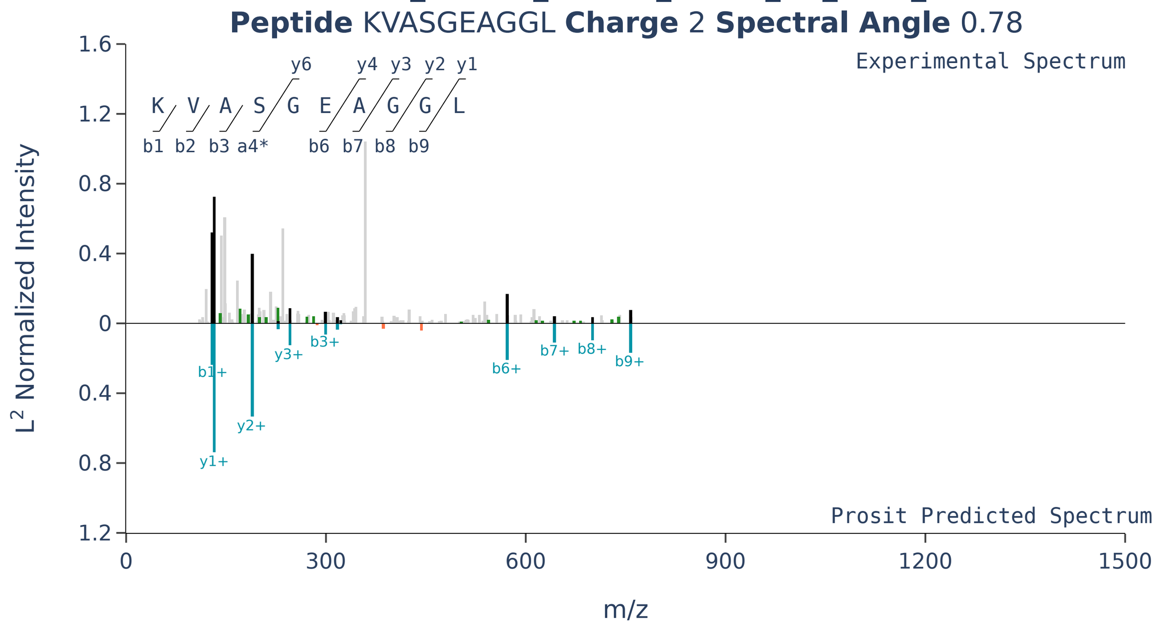


**Source** HRoetschke_211222_160123_HFGoe_IF2_b1R2_24h **Scan** 7042
**Peptide** MTEGEVHE **Charge** 2 **Spectral Angle** 0.89

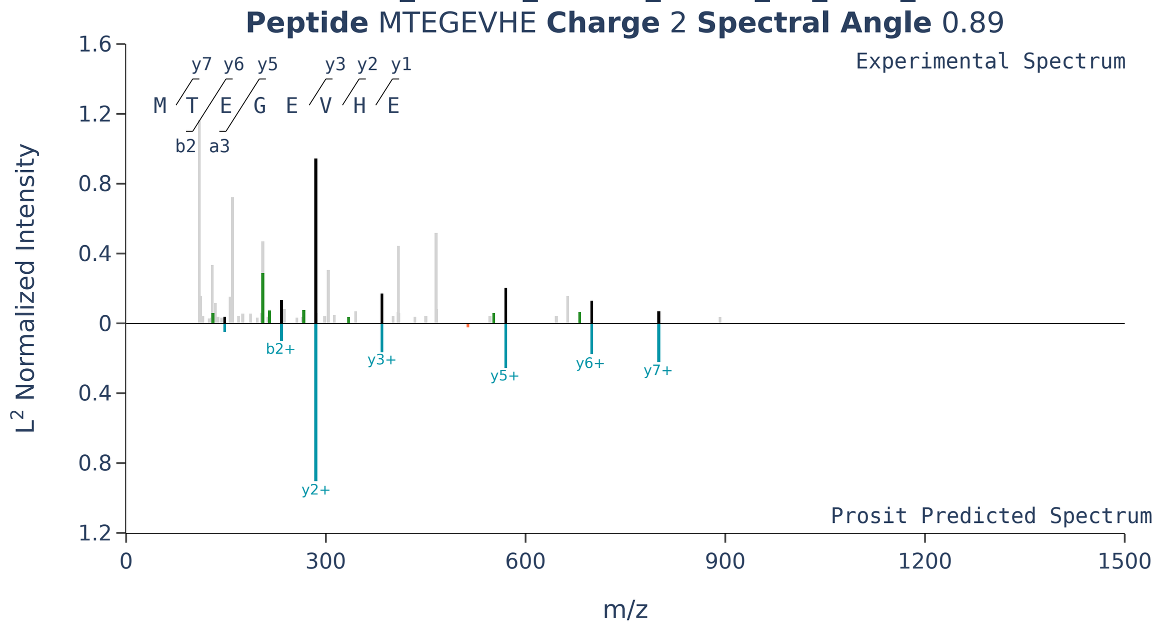


**Source** HRoetschke_211222_160123_HFGoe_IF2_b1R2_24h **Scan** 17665
**Peptide** TDVTIKTLAA **Charge** 1 **Spectral Angle** 0.83

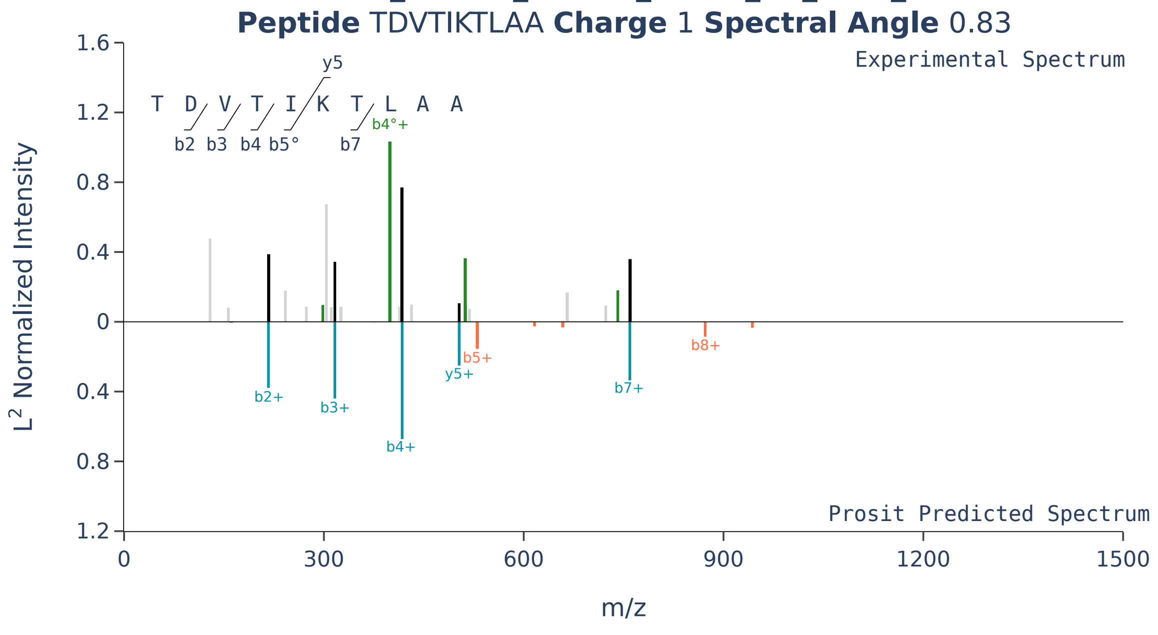


**Source** HRoetschke_211222_160123_HFGoe_IF2_b1R1_4h **Scan** 6120
**Peptide** MGHKVILRRENE **Charge** 4 **Spectral Angle** 0.83

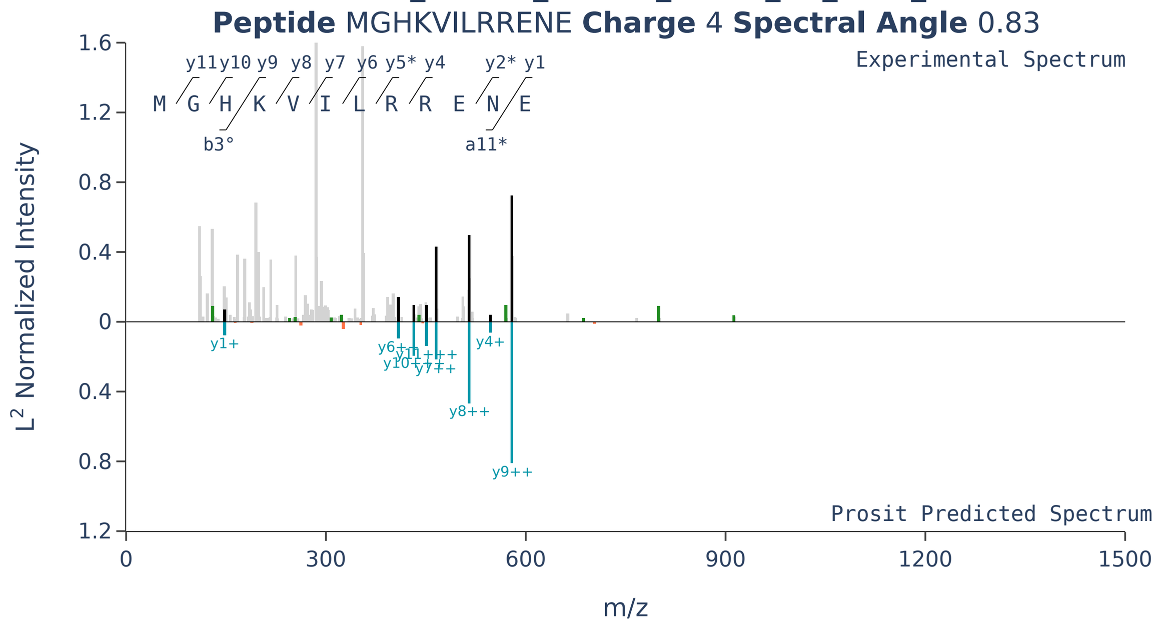


**Source** HRoetschke_211222_160123_HFGoe_IF2_b1R1_4h **Scan** 13466
**Peptide** QAEVLELKAVRK **Charge** 3 **Spectral Angle** 0.79

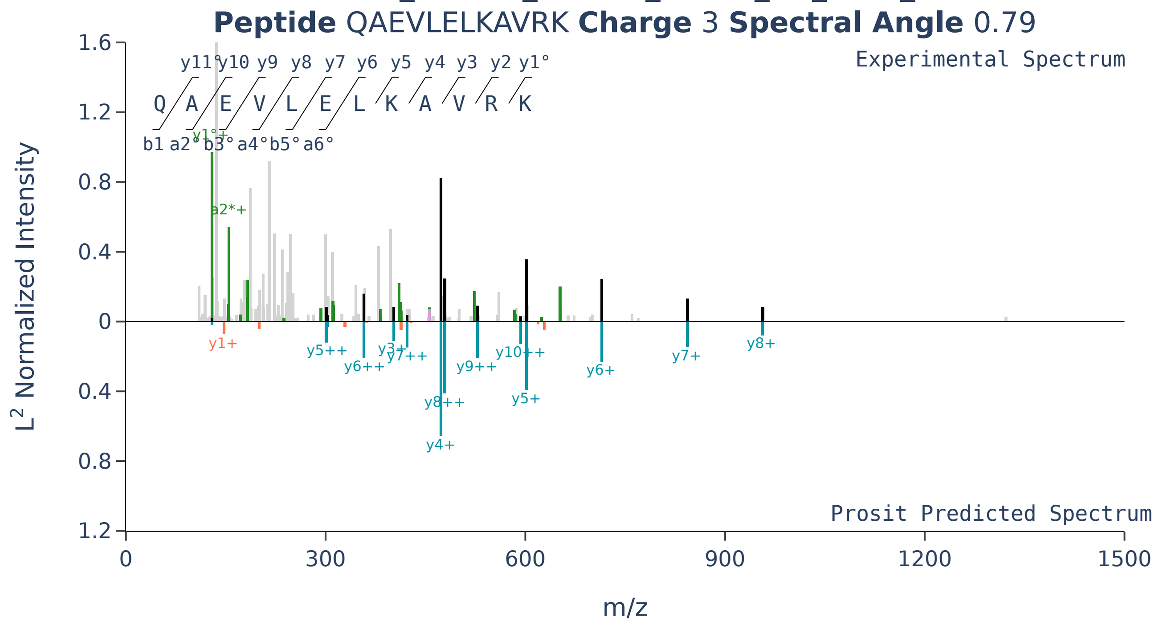


**Source** HRoetschke_211222_160123_HFGoe_IF2_b1R1_4h **Scan** 7982
**Peptide** GHKVILRRENELEE **Charge** 4 **Spectral Angle** 0.84

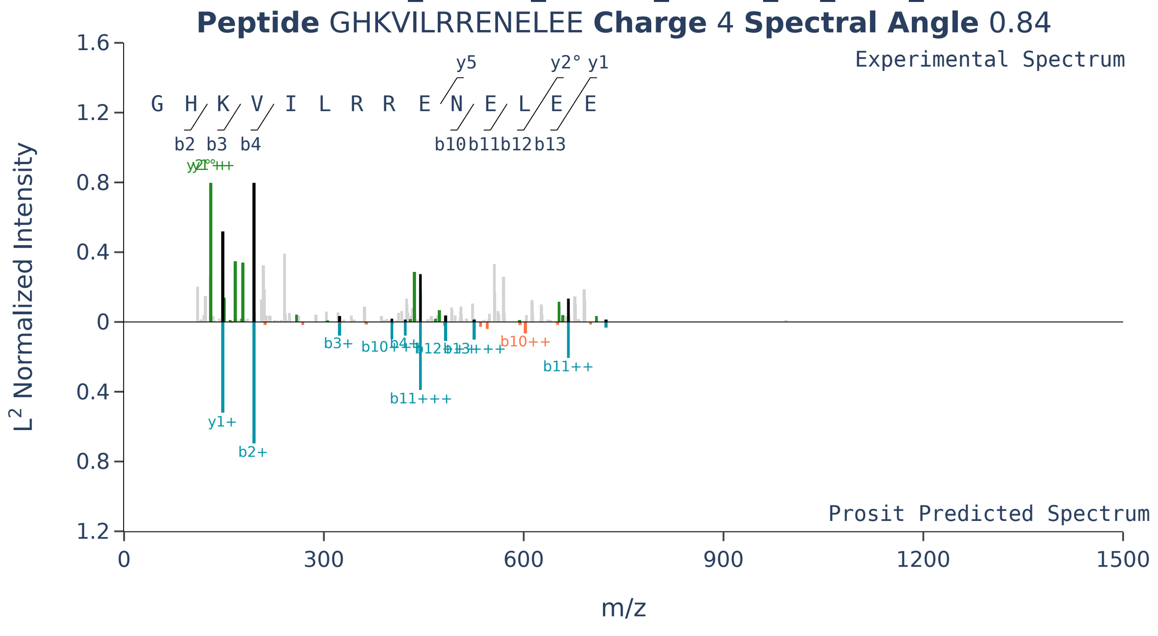


**Source** HRoetschke_211222_160123_HFGoe_IF2_b1R1_4h **Scan** 13818
**Peptide** KGSQVIKAMM **Charge** 2 **Spectral Angle** 0.76

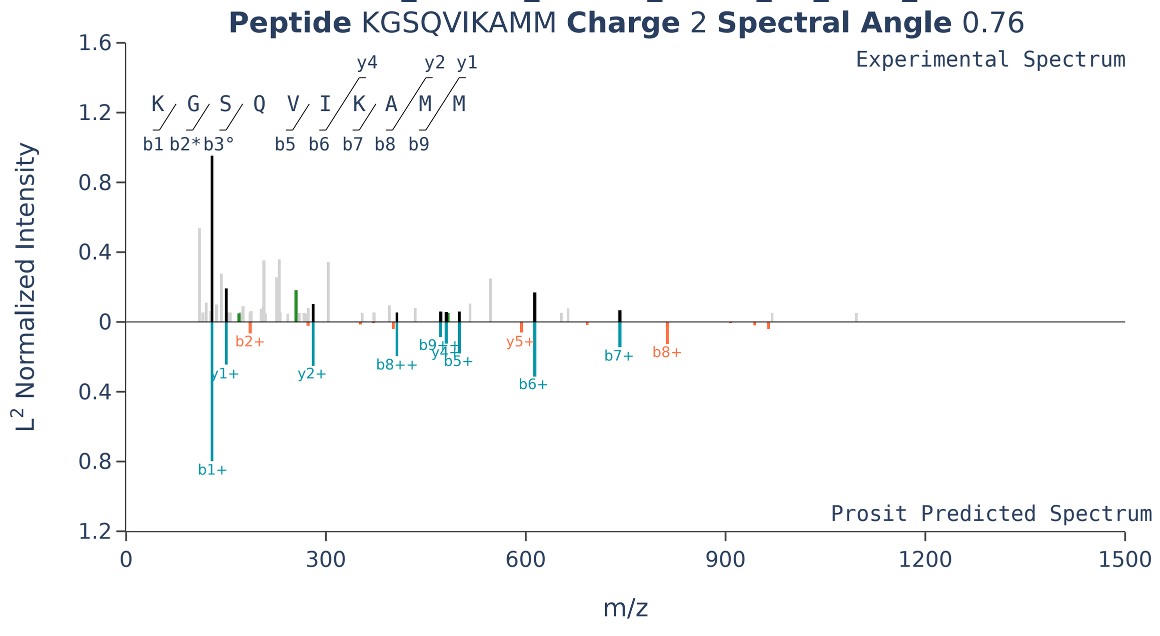


**Source** HRoetschke_211222_160123_HFGoe_IF2_b1R2_1h **Scan** 7918
**Peptide** SDYHVTTS **Charge** 1 **Spectral Angle** 0.94

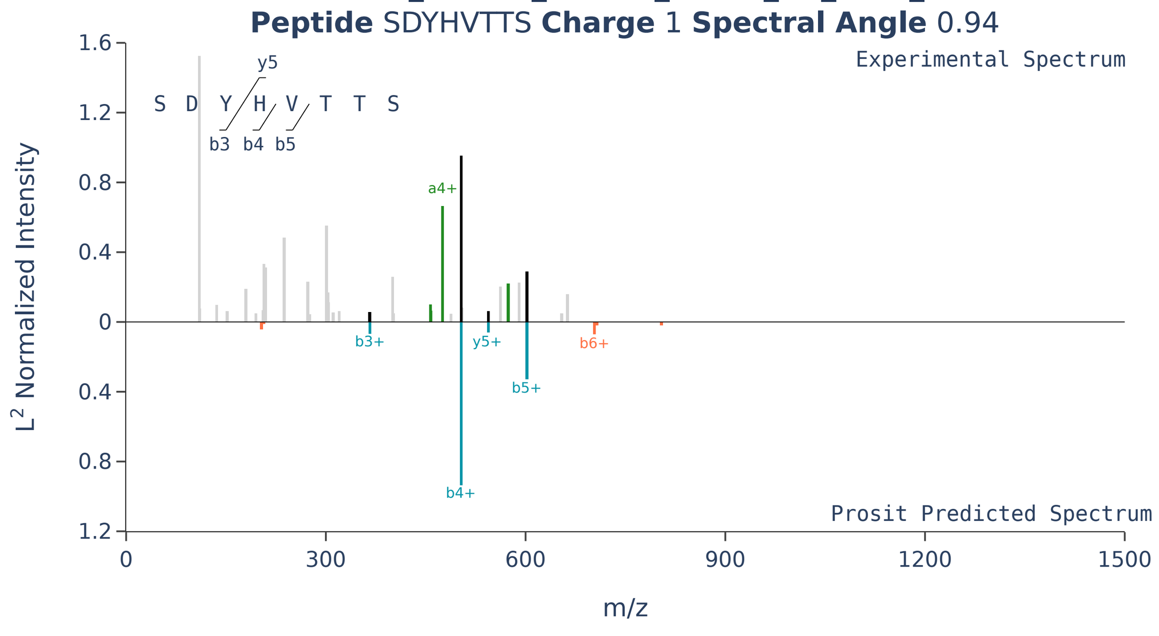

**Source** HRoetschke_211222_160123_HFGoe_IF2_b2R2_24h **Scan** 6420
**Peptide** VNKIDKPEA **Charge** 2 **Spectral Angle** 0.68

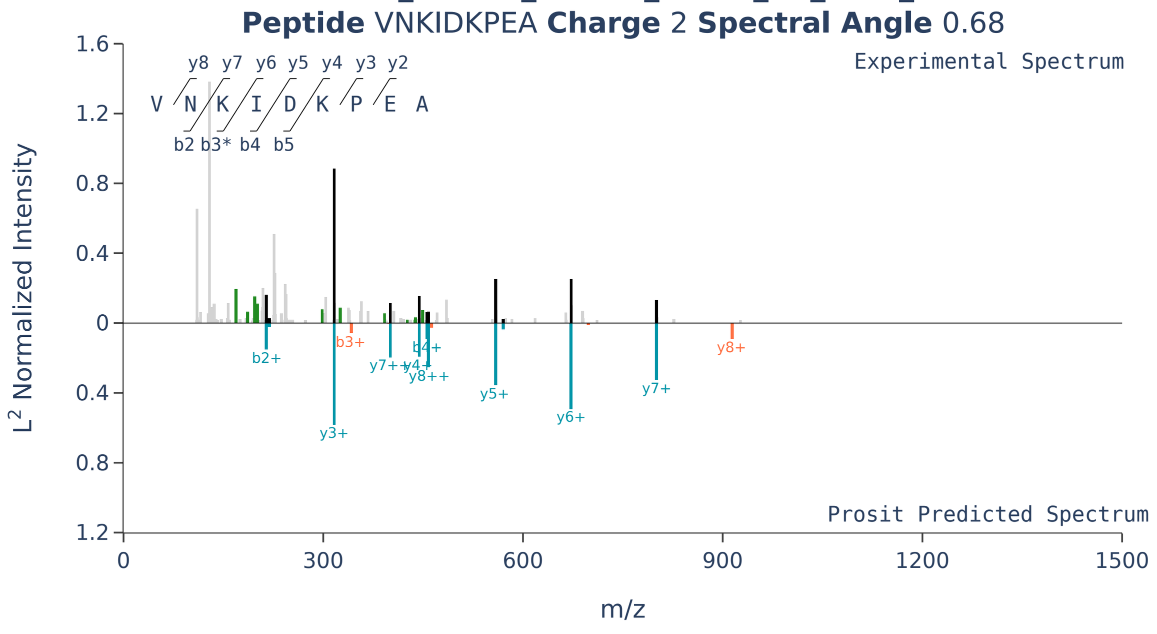


**Source** HRoetschke_211222_160123_HFGoe_IF2_b1R1_4h **Scan** 9351
**Peptide** AAFTSMRAR **Charge** 2 **Spectral Angle** 0.68

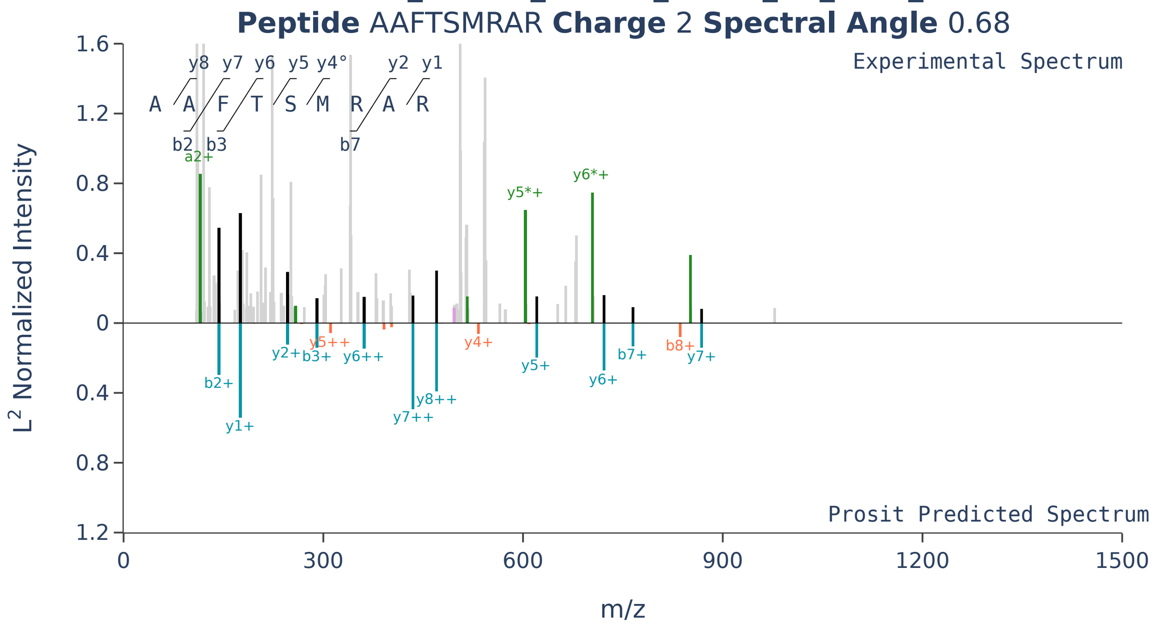


**Source** HRoetschke_211222_160123_HFGoe_IF2_b1R1_4h **Scan** 8587
**Peptide** NKMAVKGSQVIKA **Charge** 3 **Spectral Angle** 0.85

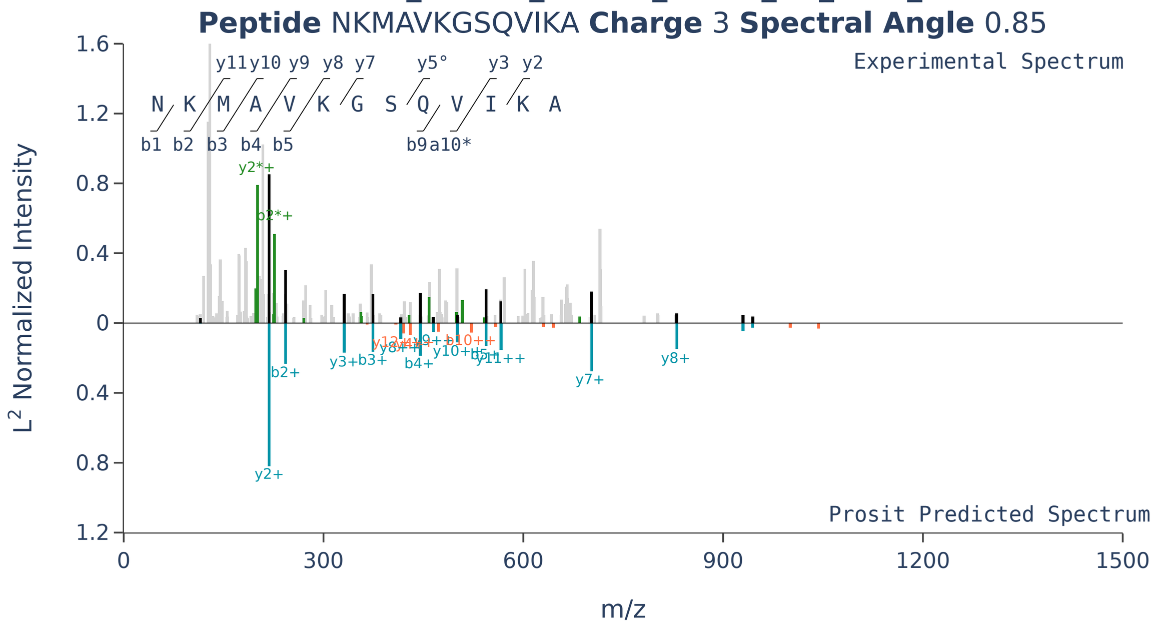


**Source** HRoetschke_211222_160123_HFGoe_IF2_b1R1_24h **Scan** 8145
**Peptide** RENELEEA **Charge** 2 **Spectral Angle** 0.86

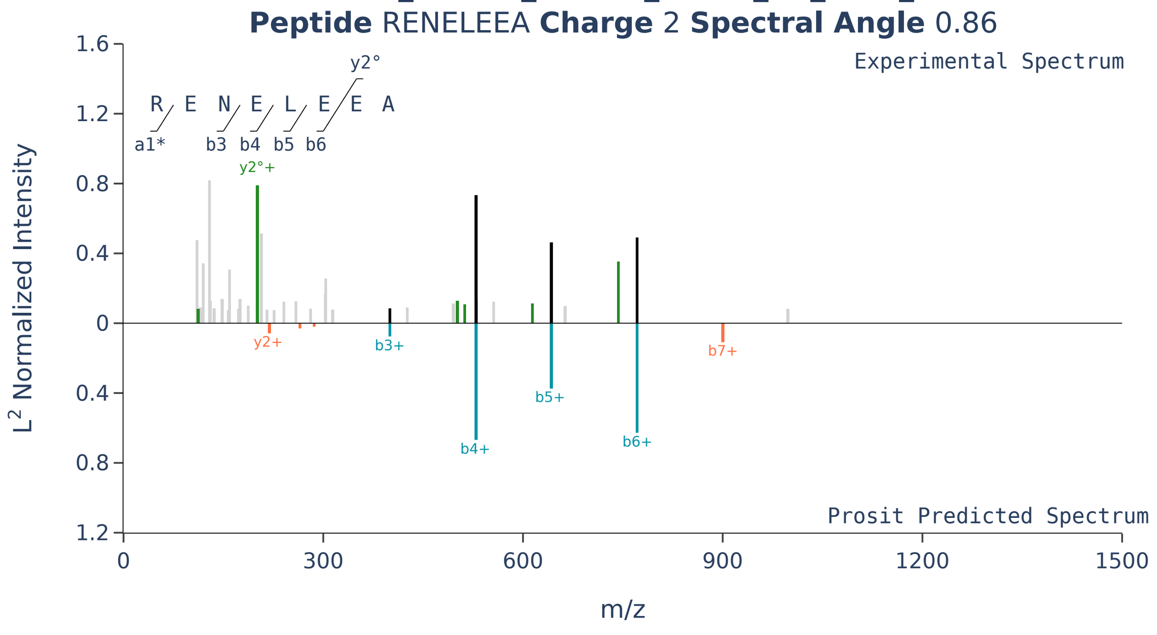


**Source** HRoetschke_211222_160123_HFGoe_IF2_b2R2_4h **Scan** 20068
**Peptide** VVLVVAAD **Charge** 1 **Spectral Angle** 0.95

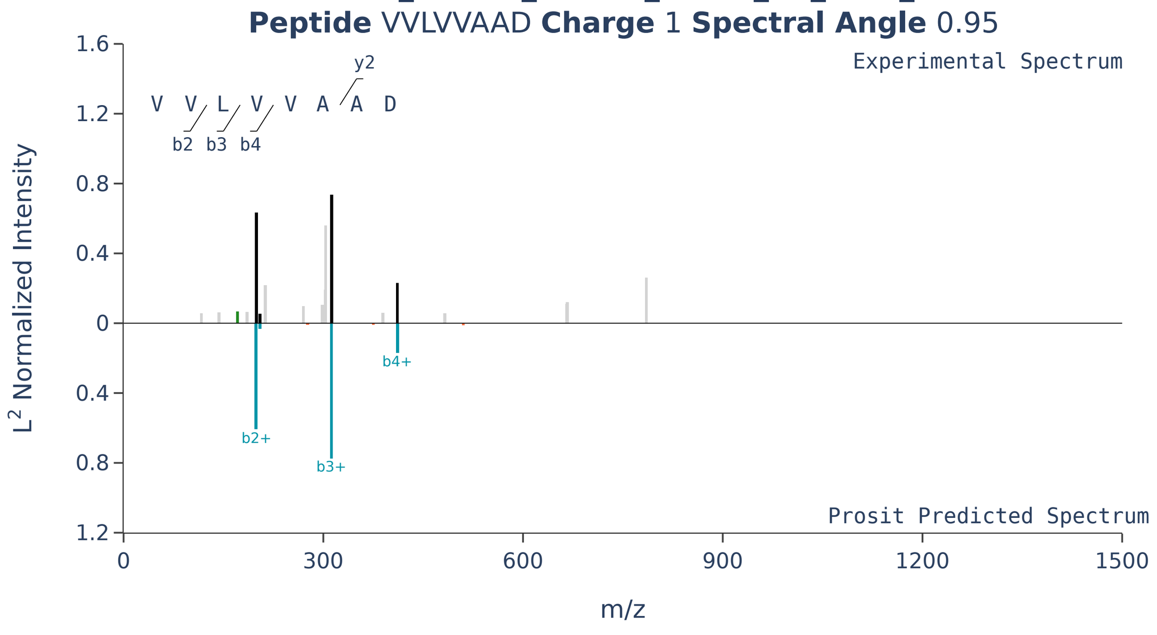


**Source** HRoetschke_211222_160123_HFGoe_IF2_b2R2_24h **Scan** 8972
**Peptide** GELANKMA **Charge** 2 **Spectral Angle** 0.87

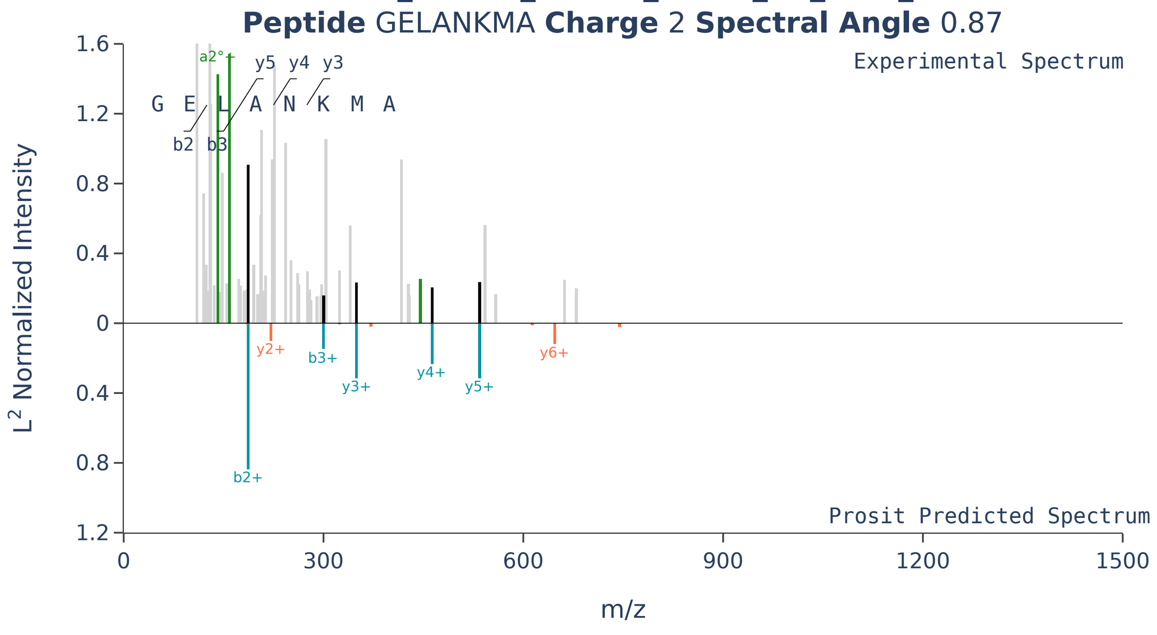


**Source** HRoetschke_211222_160123_HFGoe_IF2_b1R1_4h **Scan** 21166
**Peptide** AKAGTGIDELLD **Charge** 2 **Spectral Angle** 0.79

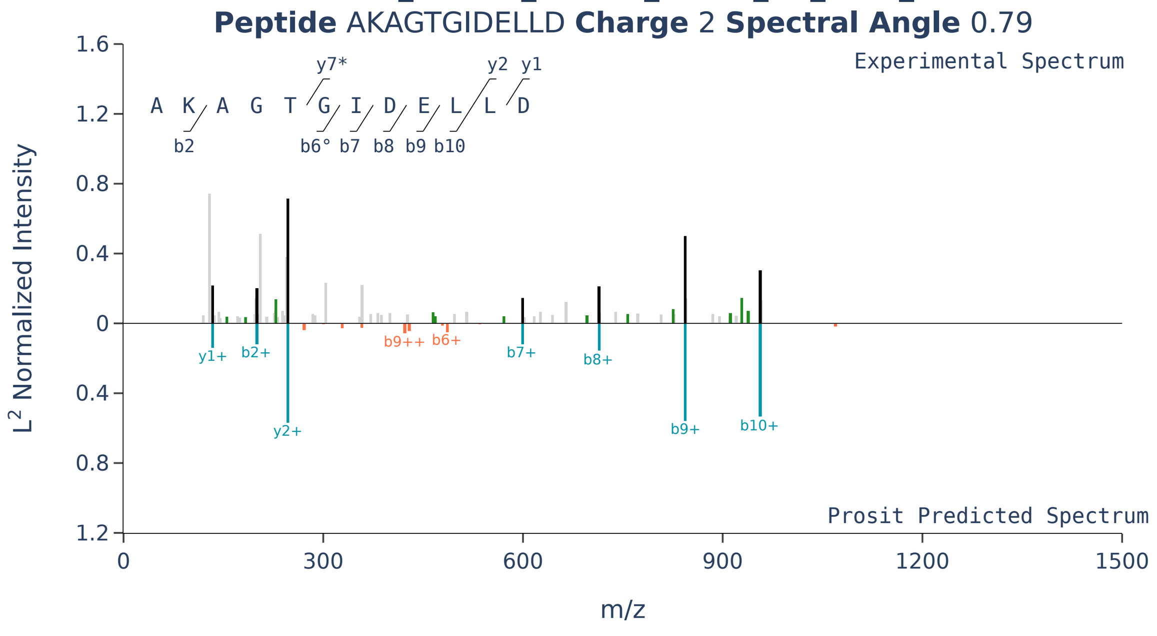


**Source** HRoetschke_211222_160123_HFGoe_IF2_b1R1_4h **Scan** 13973
**Peptide** QKPAQAVNRDVVI **Charge** 3 **Spectral Angle** 0.84

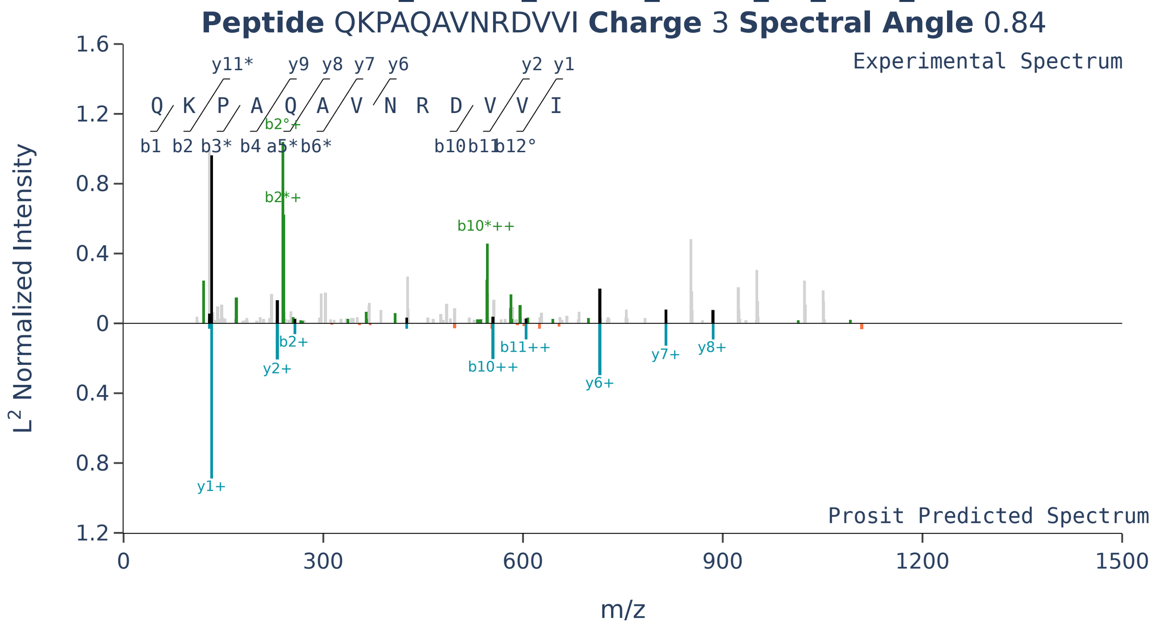


**Source** HRoetschke_211222_160123_HFGoe_IF2_b1R2_24h **Scan** 10800
**Peptide** RQTSVERLVQQ **Charge** 3 **Spectral Angle** 0.74

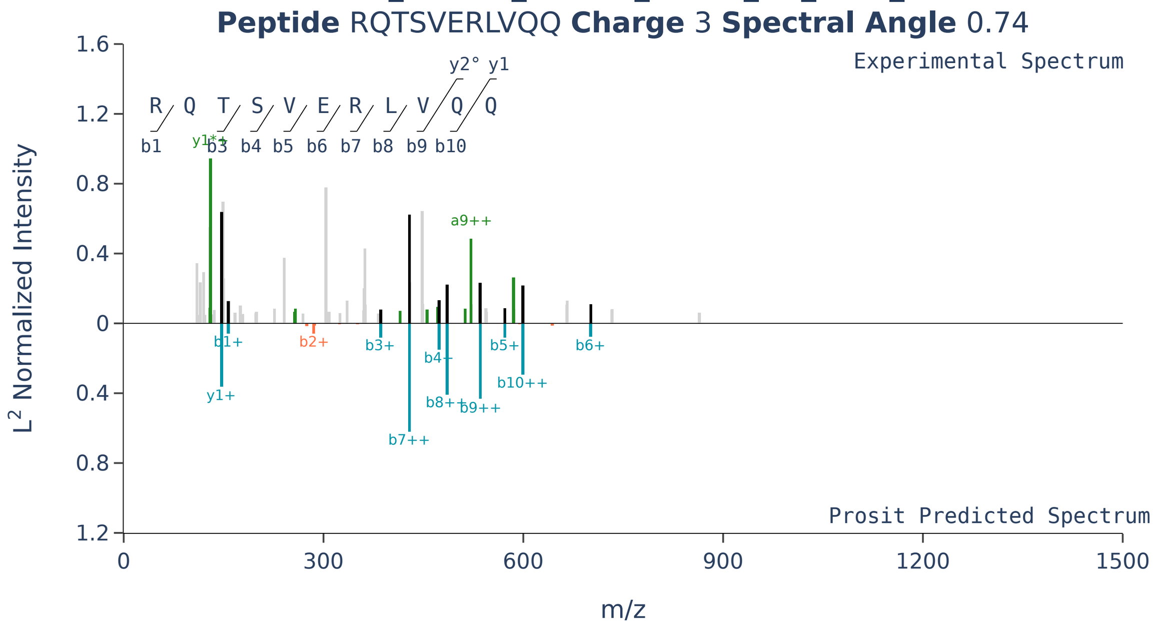


**Source** HRoetschke_211222_160123_HFGoe_IF2_b1R2_1h **Scan** 15406
**Peptide** DVTIKTLAAERQT **Charge** 3 **Spectral Angle** 0.85

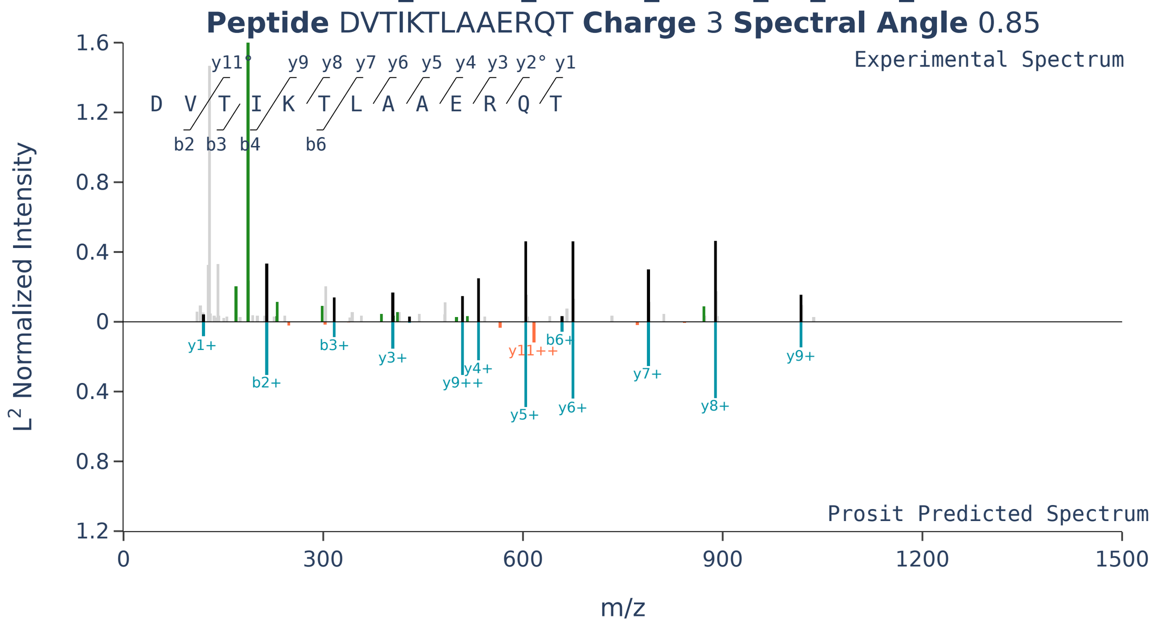


**Source** HRoetschke_211222_160123_HFGoe_IF2_b1R1_4h **Scan** 8083
**Peptide** ARGAQATDL **Charge** 2 **Spectral Angle** 0.84

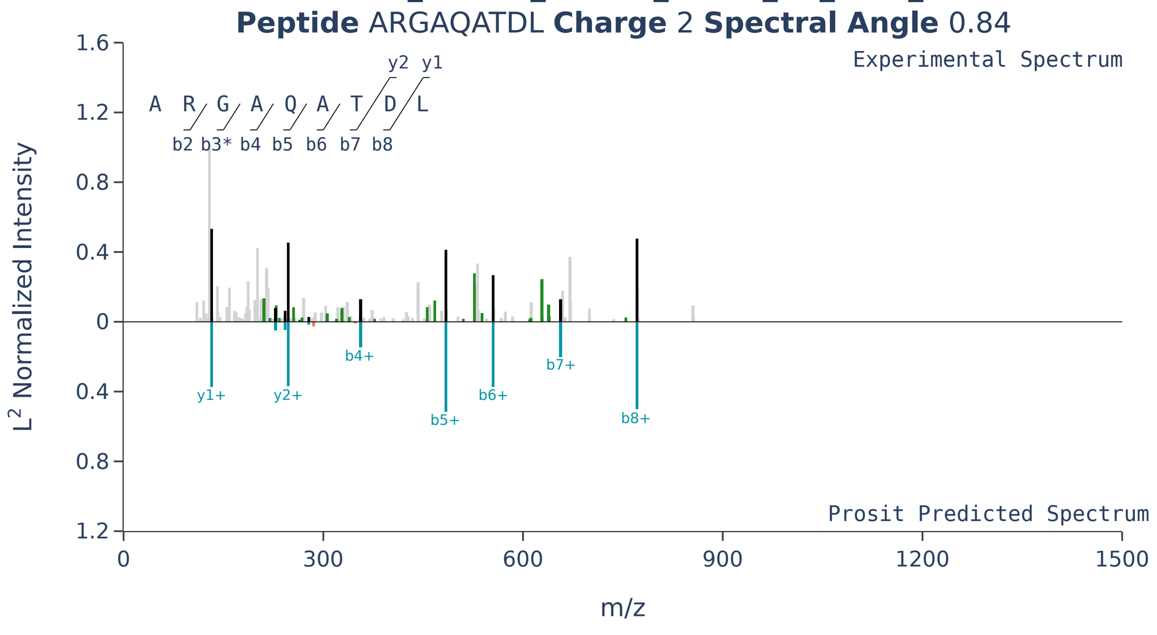


**Source** HRoetschke_211222_160123_HFGoe_IF2_b1R2_24h **Scan** 16758
**Peptide** TDEVKVKII **Charge** 2 **Spectral Angle** 0.78

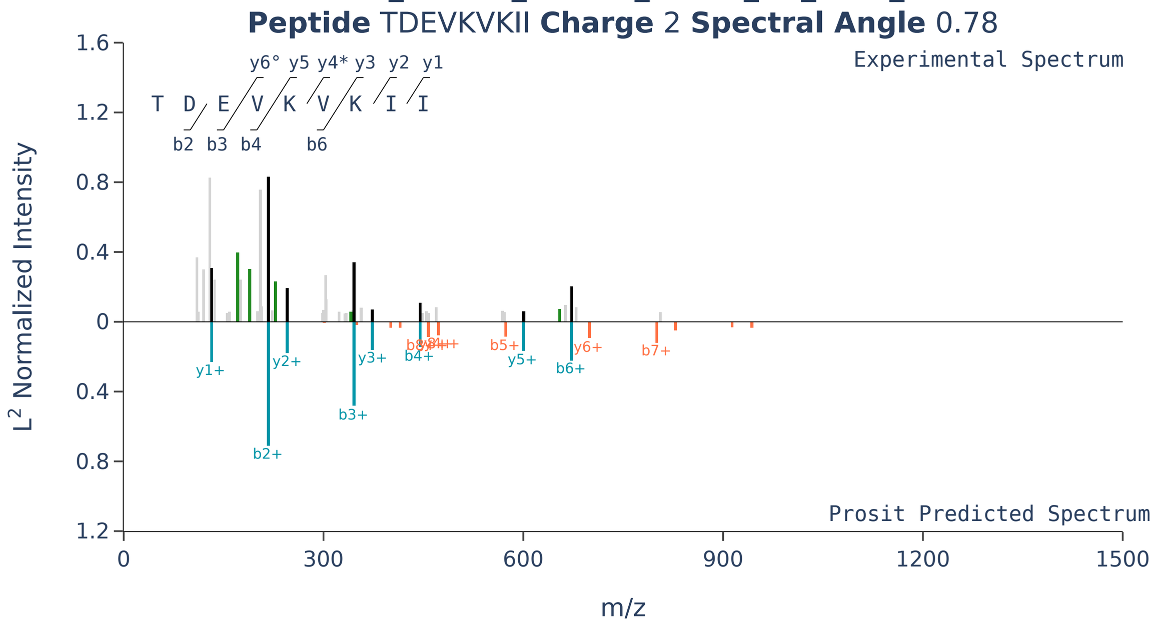


**Source** HRoetschke_211222_160123_HFGoe_IF2_b1R1_4h **Scan** 12970
**Peptide** LDYIRSTKVASGE **Charge** 2 **Spectral Angle** 0.72

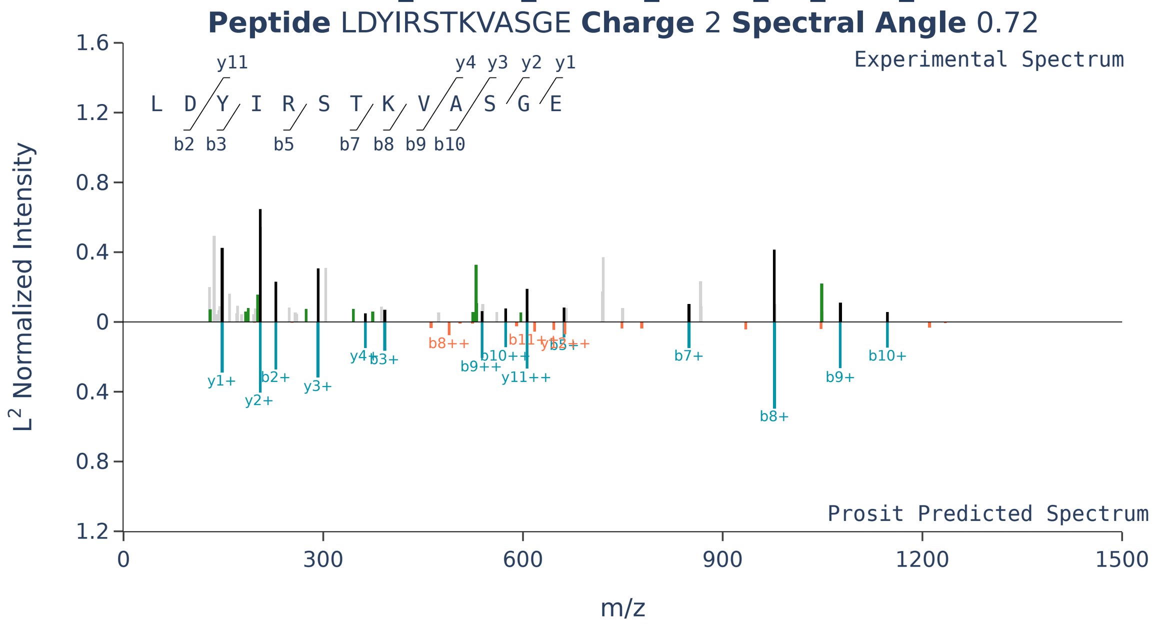


**Source** HRoetschke_211222_160123_HFGoe_IF2_b1R2_2h **Scan** 7520
**Peptide** KPAQAVNR **Charge** 2 **Spectral Angle** 0.84

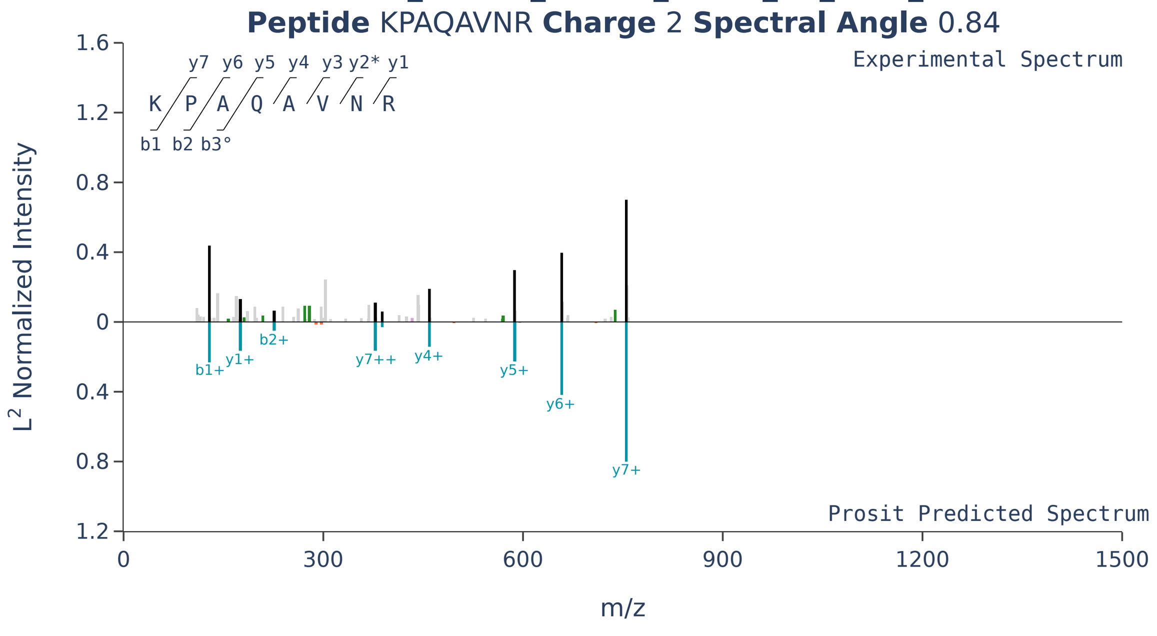


**Source** HRoetschke_211222_160123_HFGoe_IF2_b2R2_4h **Scan** 22237
**Peptide** NDVRTGDVIEVFE **Charge** 2 **Spectral Angle** 0.86

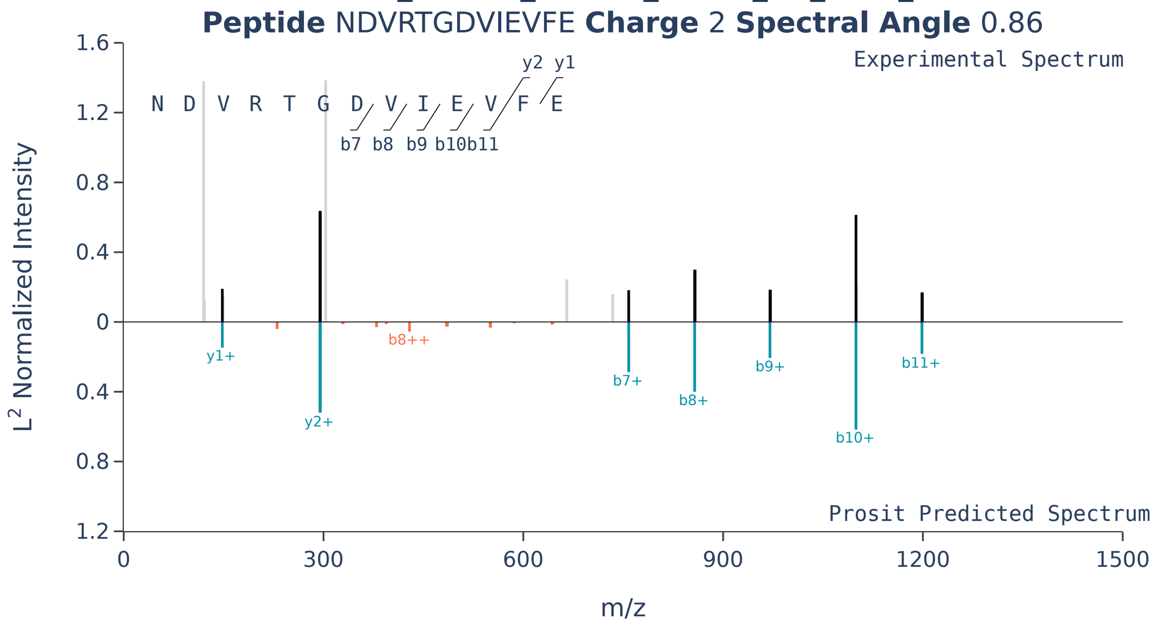

**Source** HRoetschke_211222_160123_HFGoe_IF2_b1R1_4h **Scan** 14903
**Peptide** PAQAVNRDVVIG **Charge** 2 **Spectral Angle** 0.8

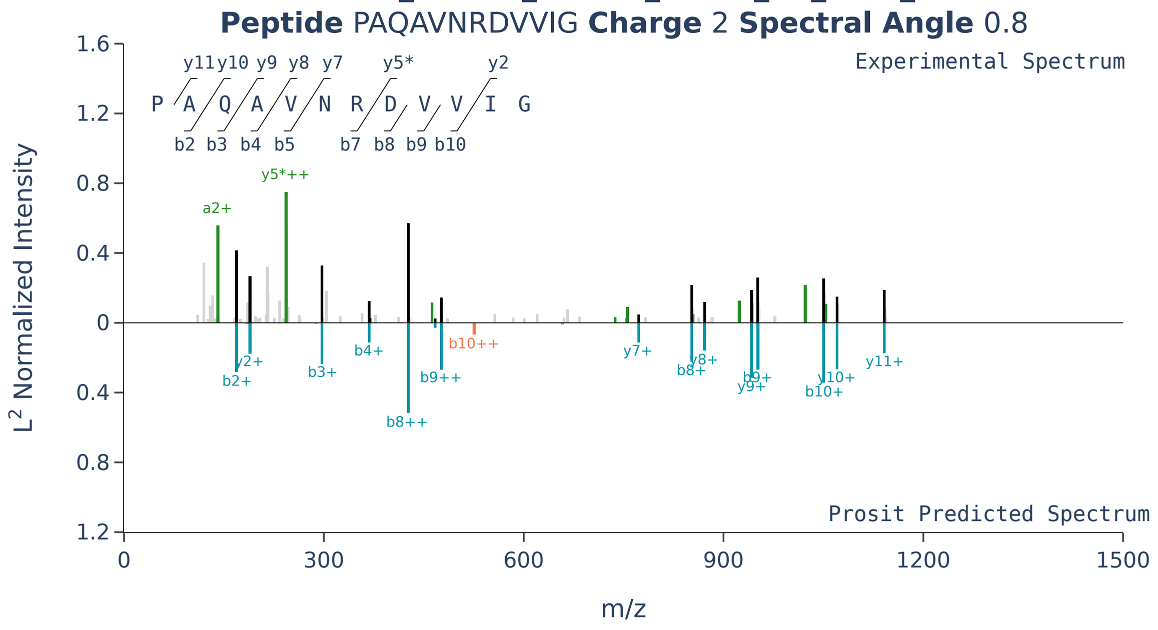


**Source** HRoetschke_211222_160123_HFGoe_IF2_b2R2_2h **Scan** 16932
**Peptide** EAGPSIPVE **Charge** 2 **Spectral Angle** 0.92

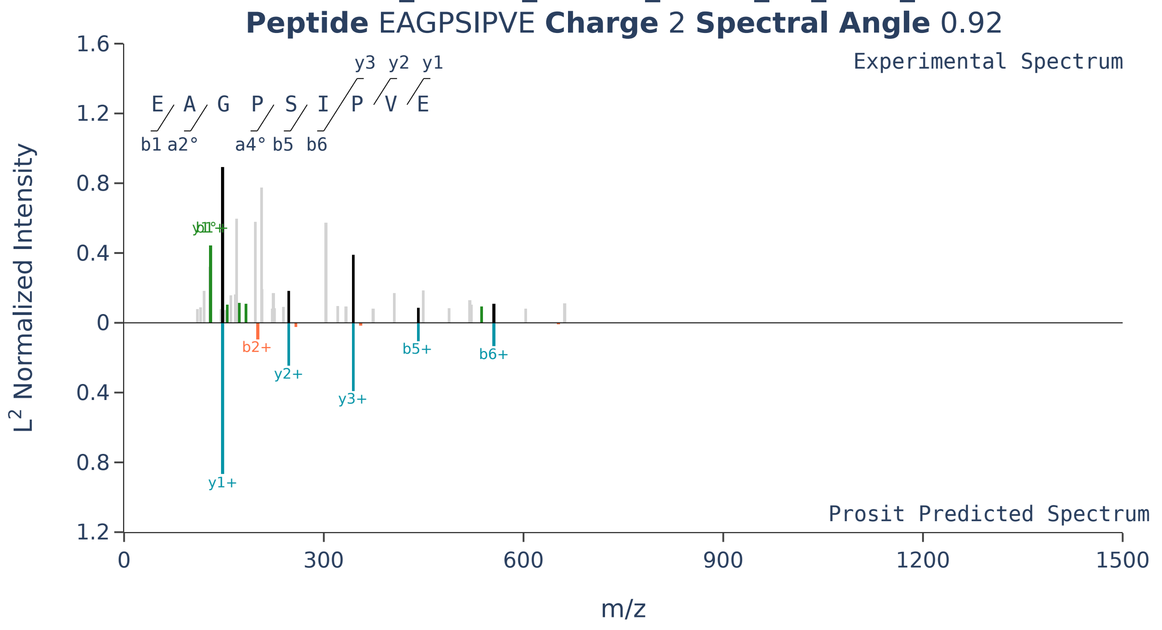


**Source** HRoetschke_211222_160123_HFGoe_IF2_b1R2_24h **Scan** 13491
**Peptide** GGESQFVHVS **Charge** 2 **Spectral Angle** 0.66

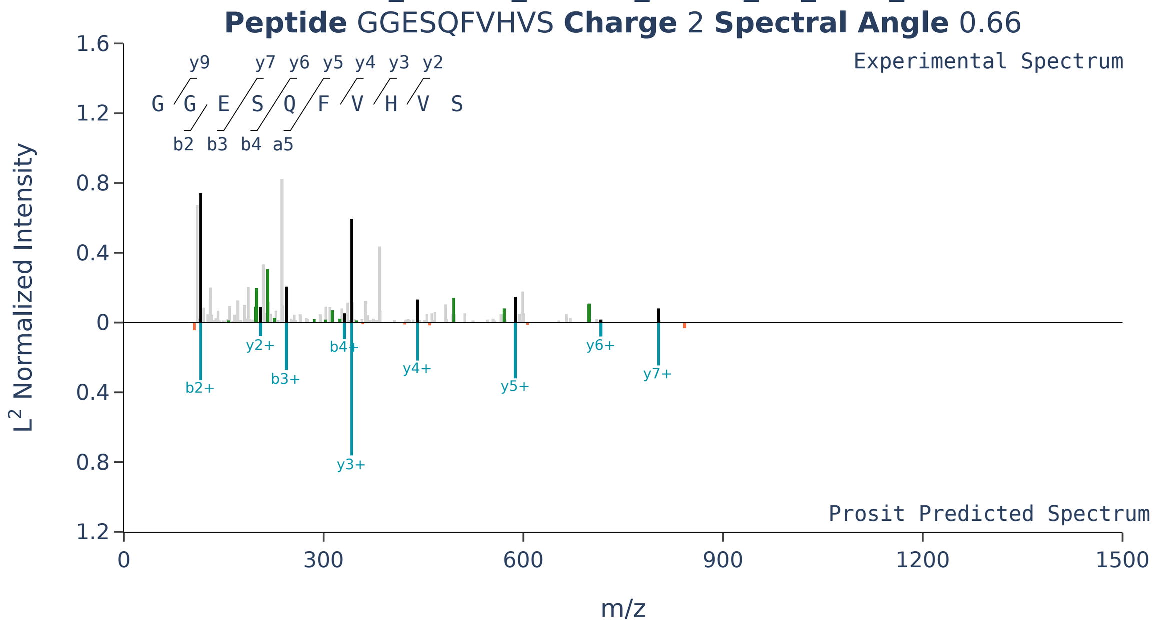


**Source** HRoetschke_211222_160123_HFGoe_IF2_b1R1_2h **Scan** 9983
**Peptide** DYIRSTKV **Charge** 2 **Spectral Angle** 0.77

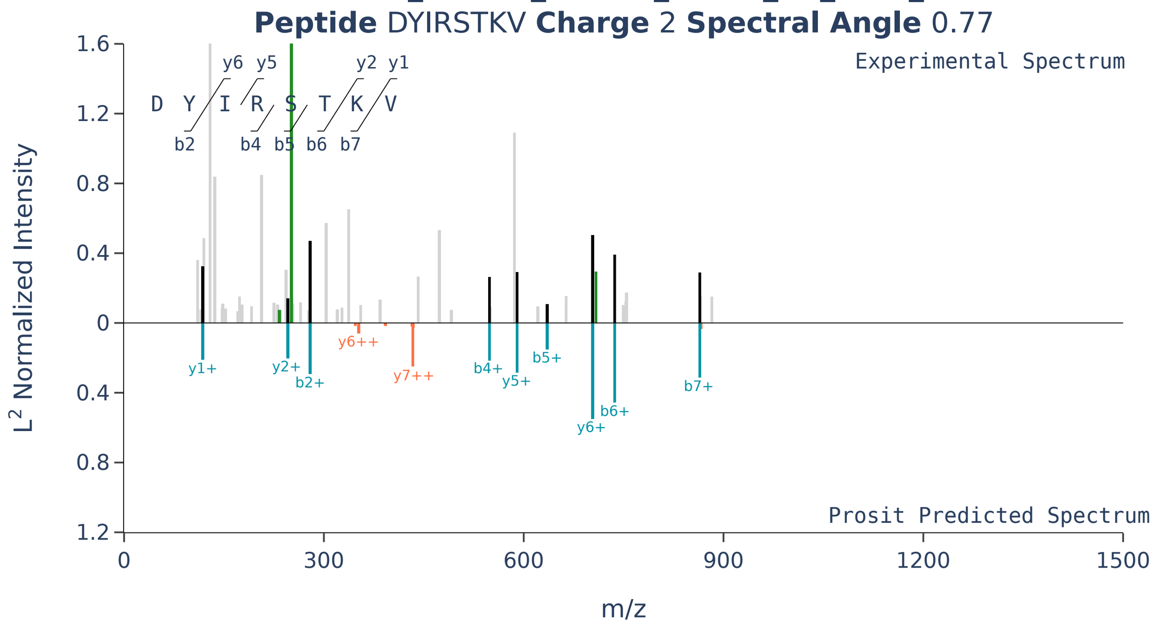


**Source** HRoetschke_211222_160123_HFGoe_IF2_b2R1_1h **Scan** 6342
**Peptide** AGIRKSADDSVSAQE **Charge** 3 **Spectral Angle** 0.77

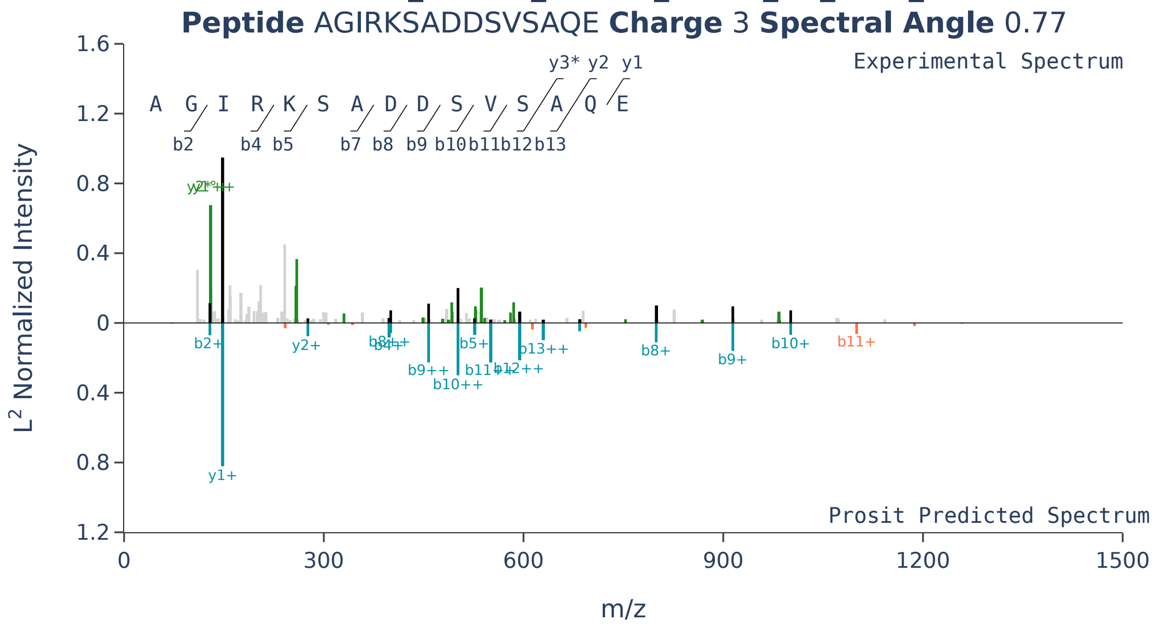


**Source** HRoetschke_211222_160123_HFGoe_IF2_b1R1_24h **Scan** 13800
**Peptide** GAAAEPRAPVV **Charge** 2 **Spectral Angle** 0.85

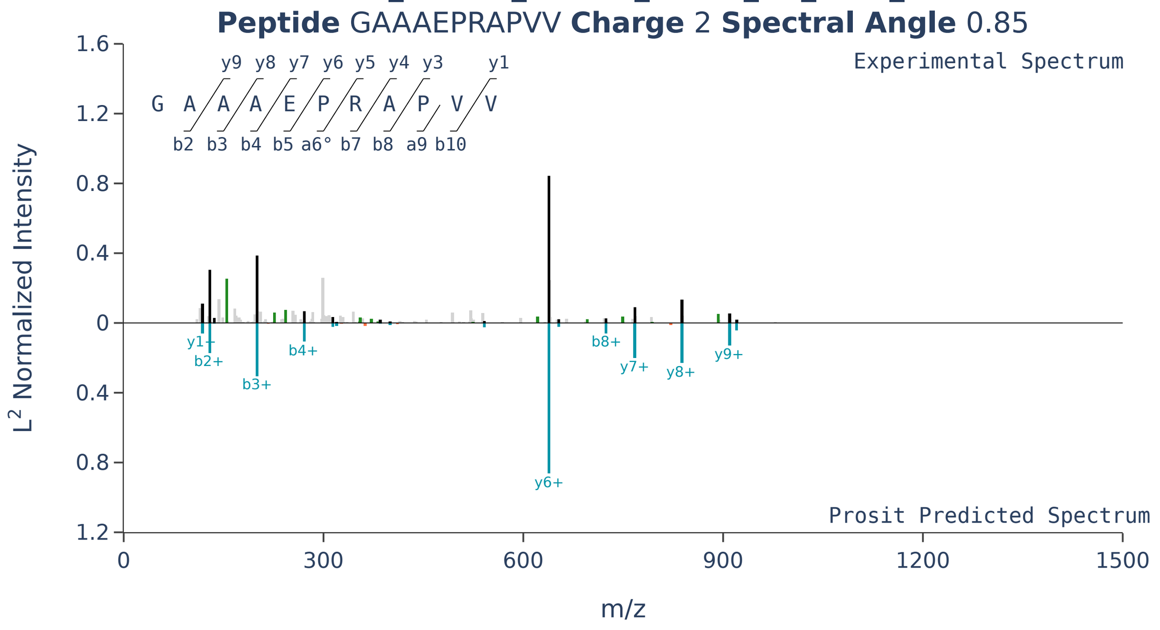


**Source** HRoetschke_211222_160123_HFGoe_IF2_b1R1_4h **Scan** 14877
**Peptide** GLSGVPAAGDE **Charge** 1 **Spectral Angle** 0.82

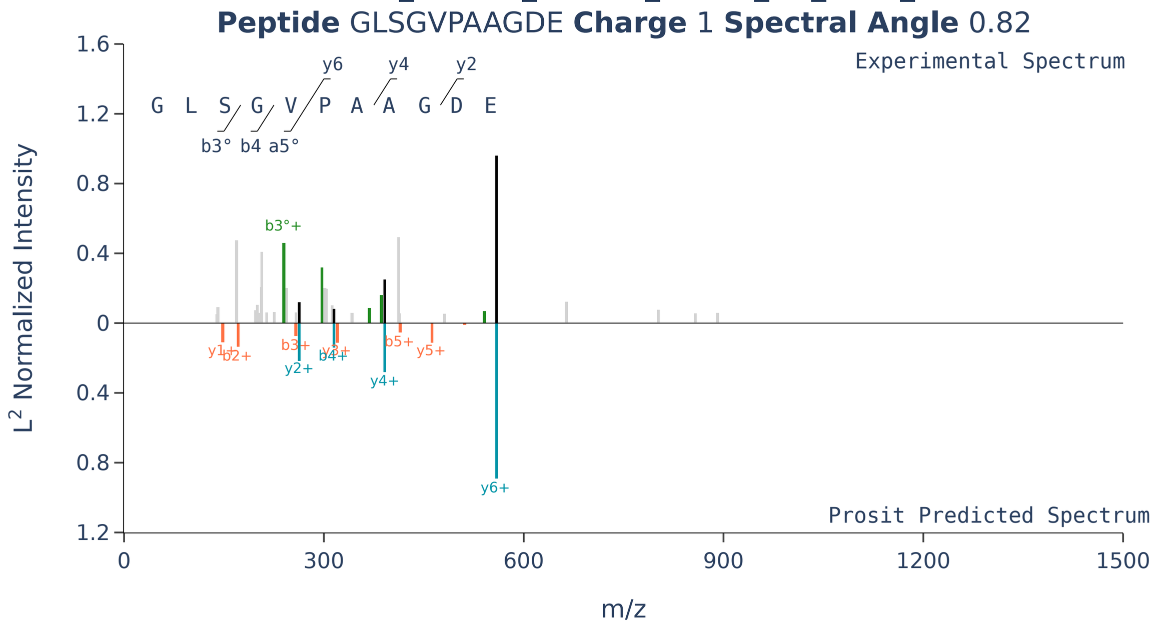


**Source** HRoetschke_211222_160123_HFGoe_IF2_b1R1_24h **Scan** 18019
**Peptide** NRDVVIGETI **Charge** 2 **Spectral Angle** 0.83

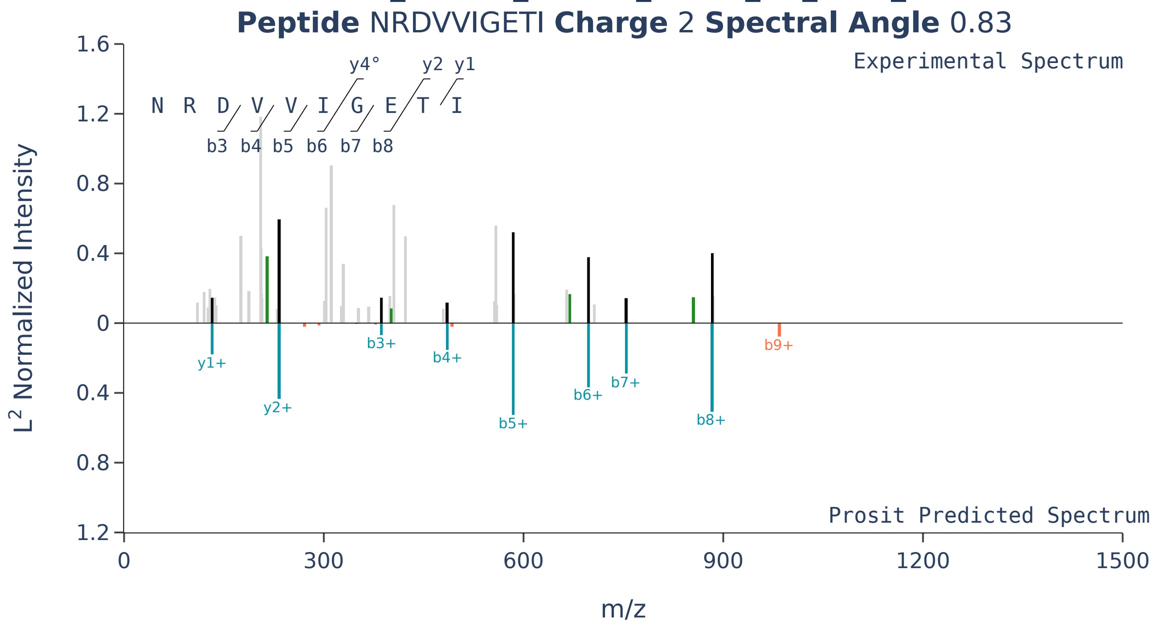


**Source** HRoetschke_211222_160123_HFGoe_IF2_b1R2_24h **Scan** 18326
**Peptide** SDRDTGAAAEPRAPVVTI **Charge** 3 **Spectral Angle** 0.78

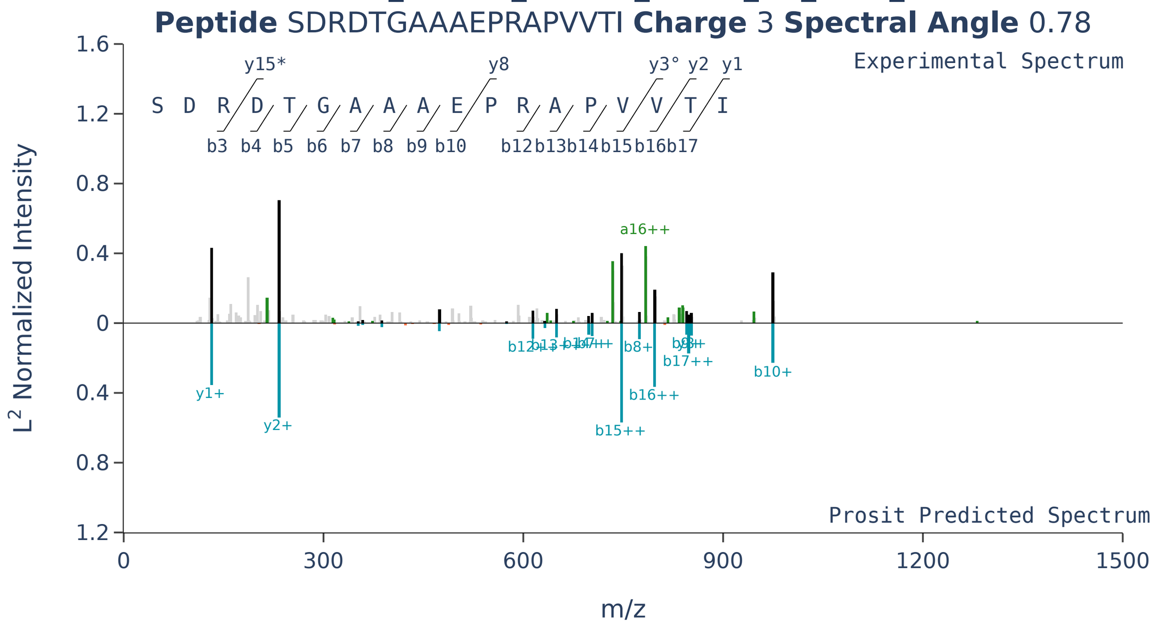


**Source** HRoetschke_211222_160123_HFGoe_IF2_b1R1_4h **Scan** 10156
**Peptide** KTLAAERQTSVERL **Charge** 4 **Spectral Angle** 0.84

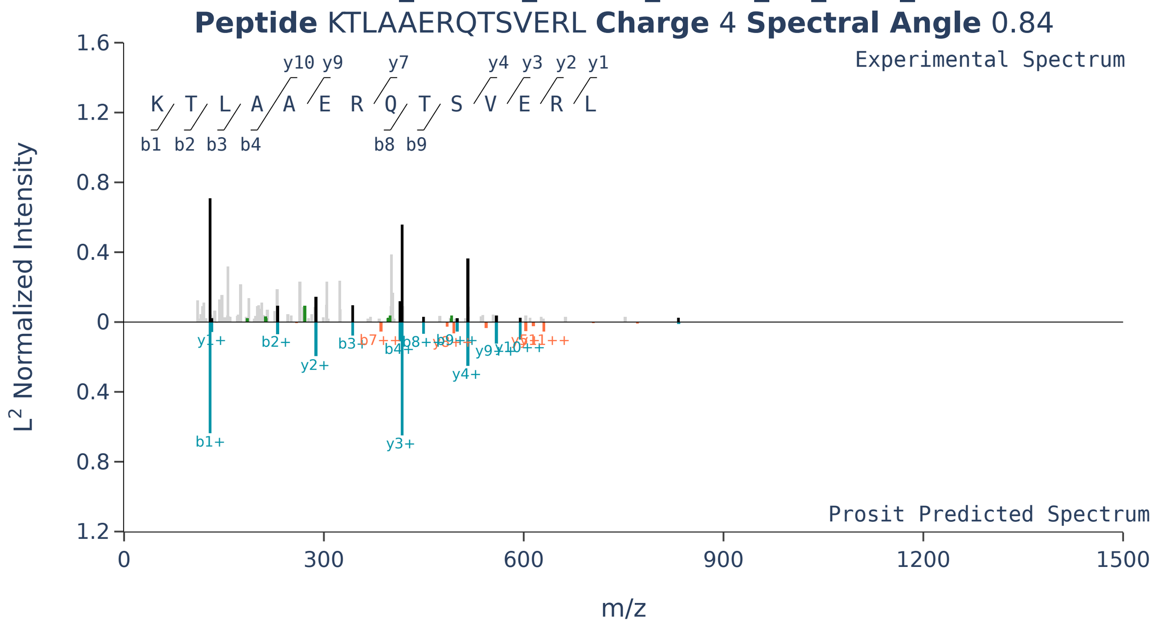


**Source** HRoetschke_211222_160123_HFGoe_IF2_b1R2_2h **Scan** 10625
**Peptide** SQFVHVSAK **Charge** 2 **Spectral Angle** 0.85

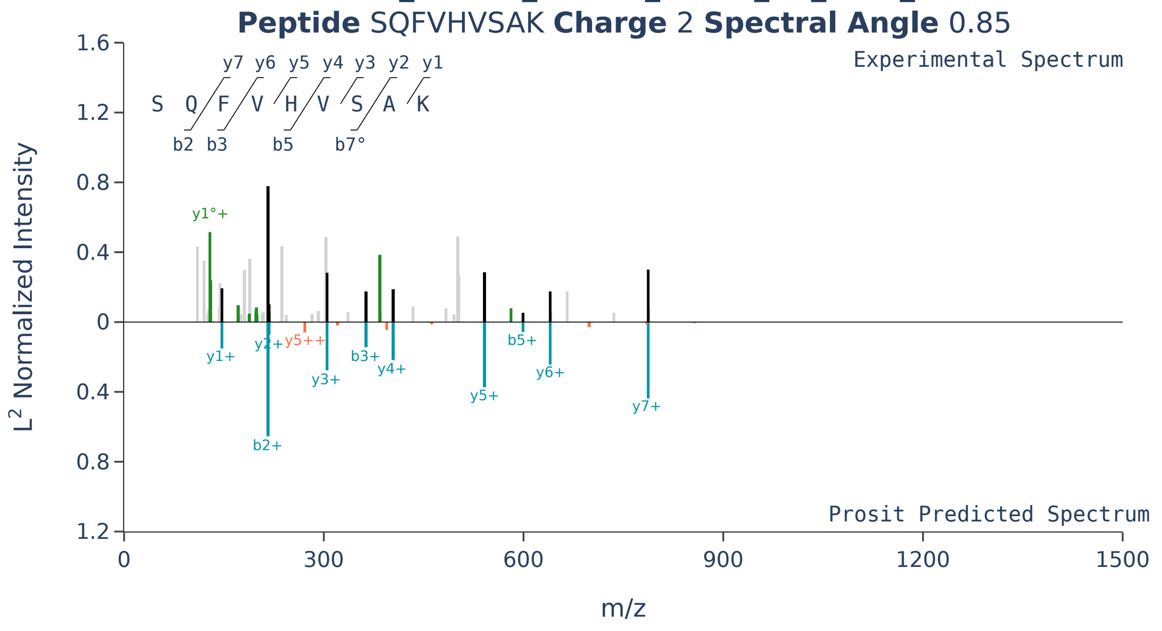


**Source** HRoetschke_211222_160123_HFGoe_IF2_b1R2_24h **Scan** 18945
**Peptide** VNRDVVIGETI **Charge** 2 **Spectral Angle** 0.8

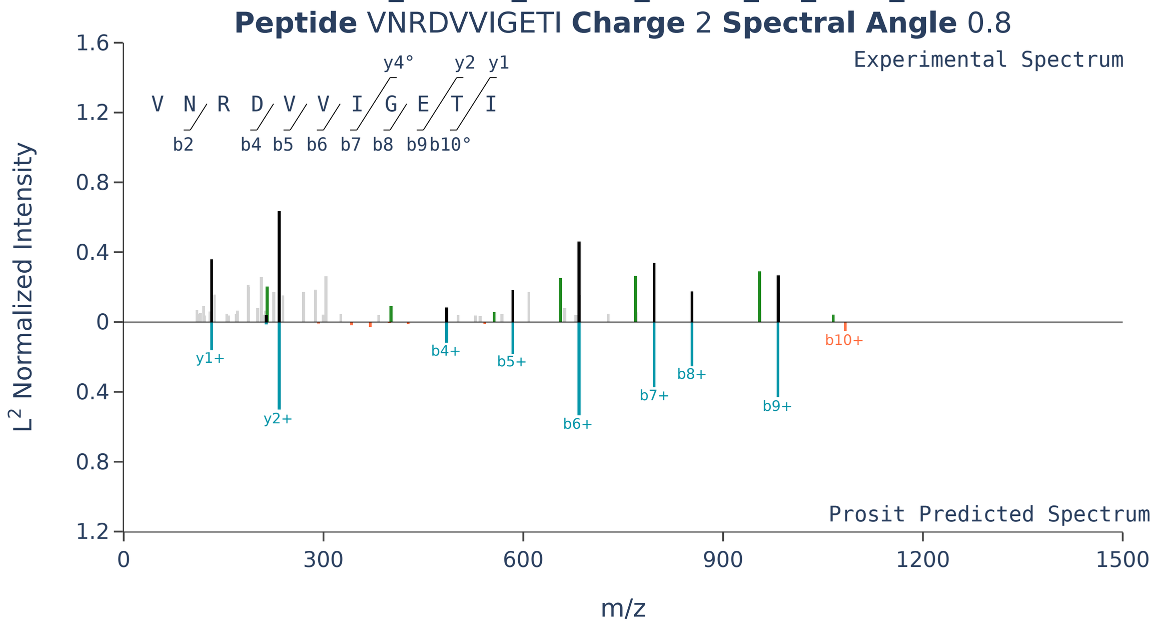


**Source** HRoetschke_211222_160123_HFGoe_IF2_b1R2_24h **Scan** 17202
**Peptide** AKAAQVPVVVA **Charge** 2 **Spectral Angle** 0.87

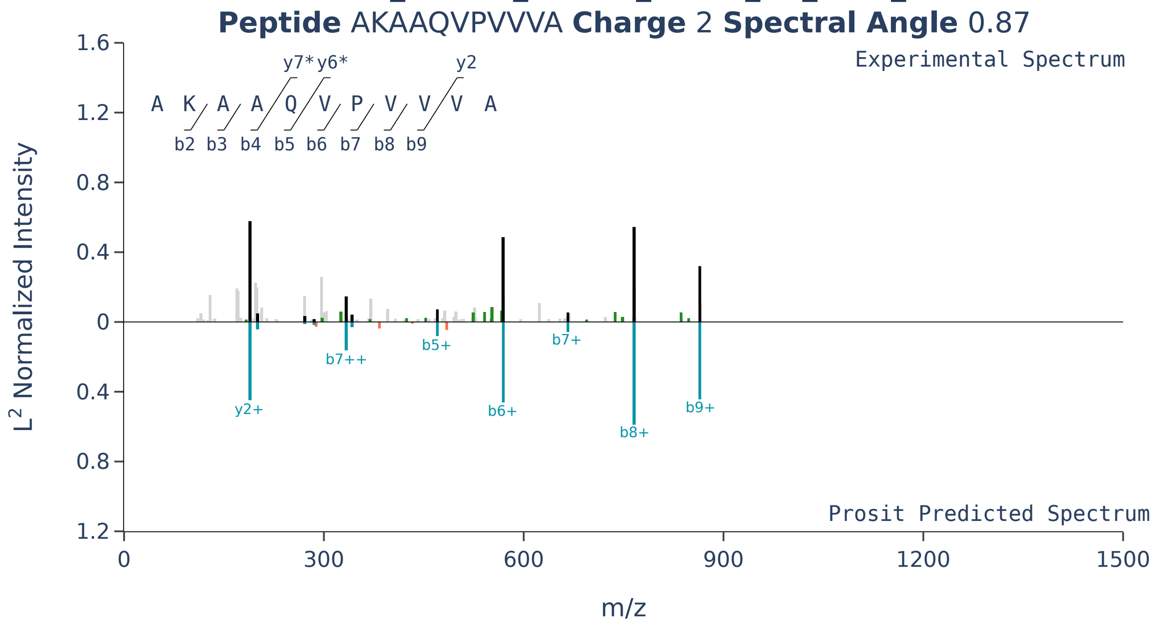


**Source** HRoetschke_211222_160123_HFGoe_IF2_b1R1_1h **Scan** 11452
**Peptide** CGIGVKNYNDVRTGD **Charge** 3 **Spectral Angle** 0.81

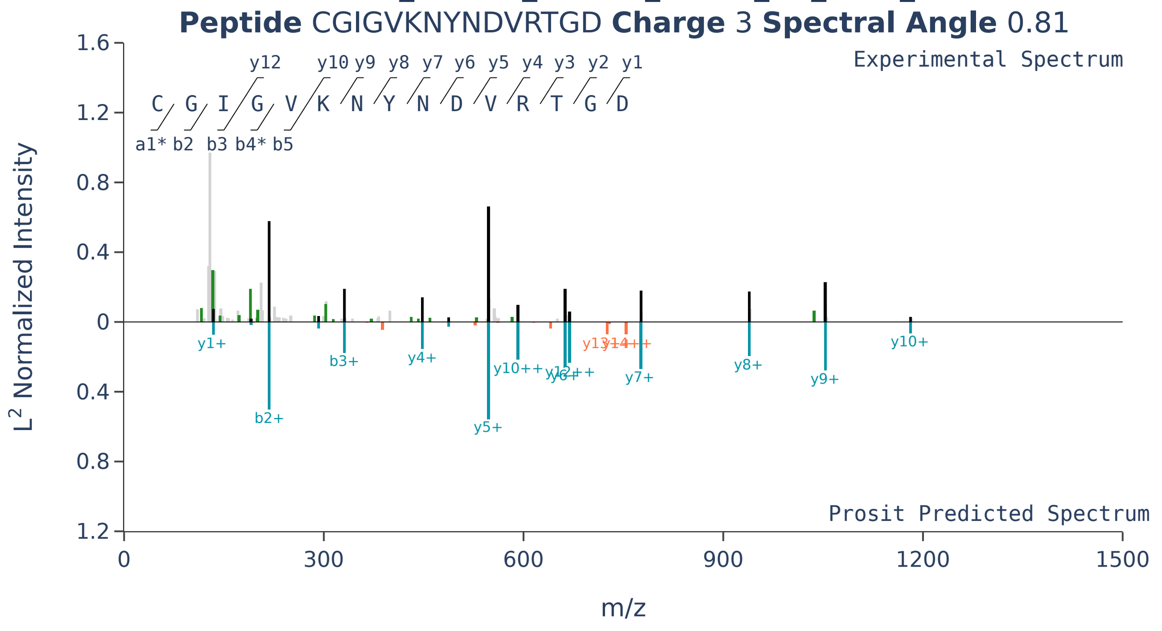


**Source** HRoetschke_211222_160123_HFGoe_IF2_b1R1_24h **Scan** 7142
**Peptide** IRSTKVASGEAGGITQH **Charge** 3 **Spectral Angle** 0.81

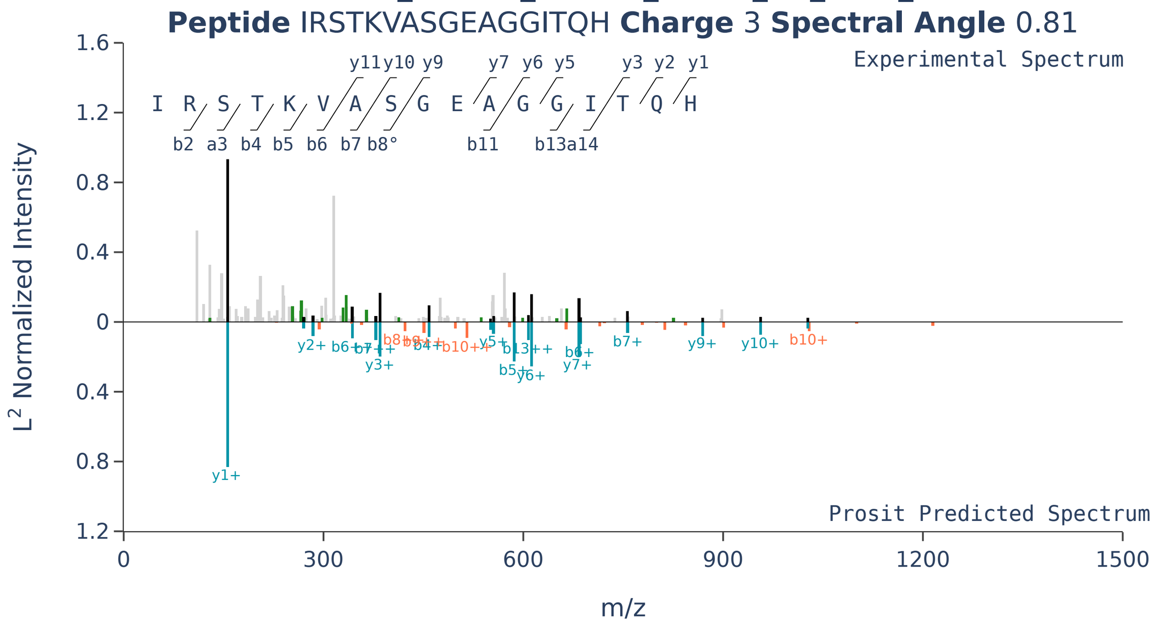

**Source** HRoetschke_211222_160123_HFGoe_IF2_b1R2_24h **Scan** 16727
**Peptide** LEAGPSI **Charge** 1 **Spectral Angle** 0.87

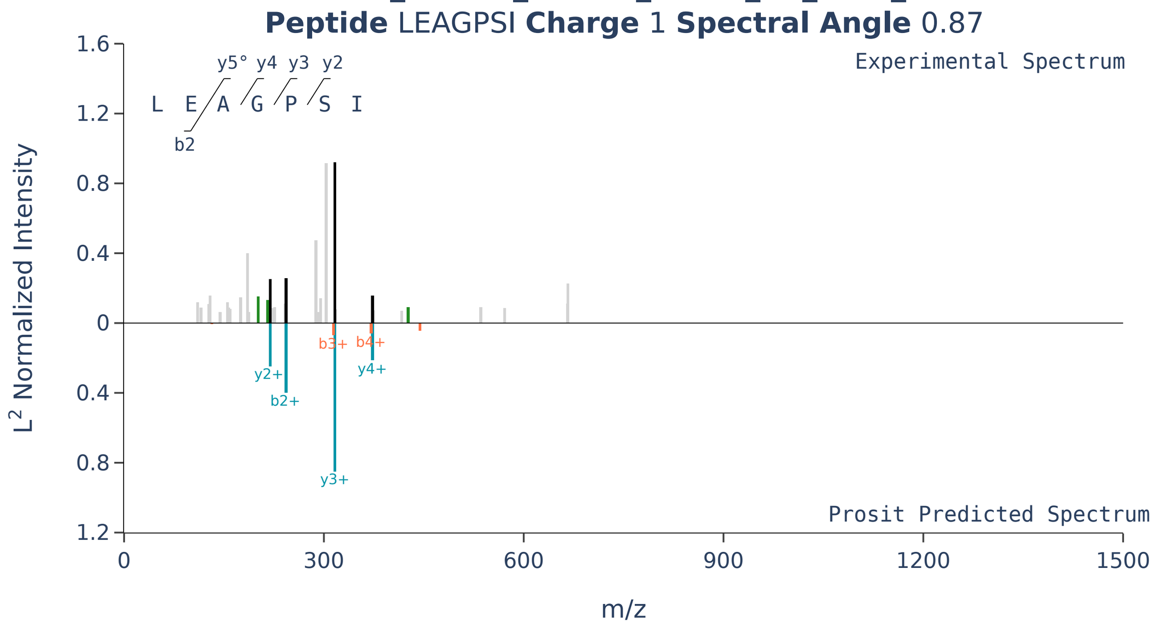


**Source** HRoetschke_211222_160123_HFGoe_IF2_b2R1_24h **Scan** 5911
**Peptide** NQKNSGPDKLTLQRKTRST **Charge** 4 **Spectral Angle** 0.76

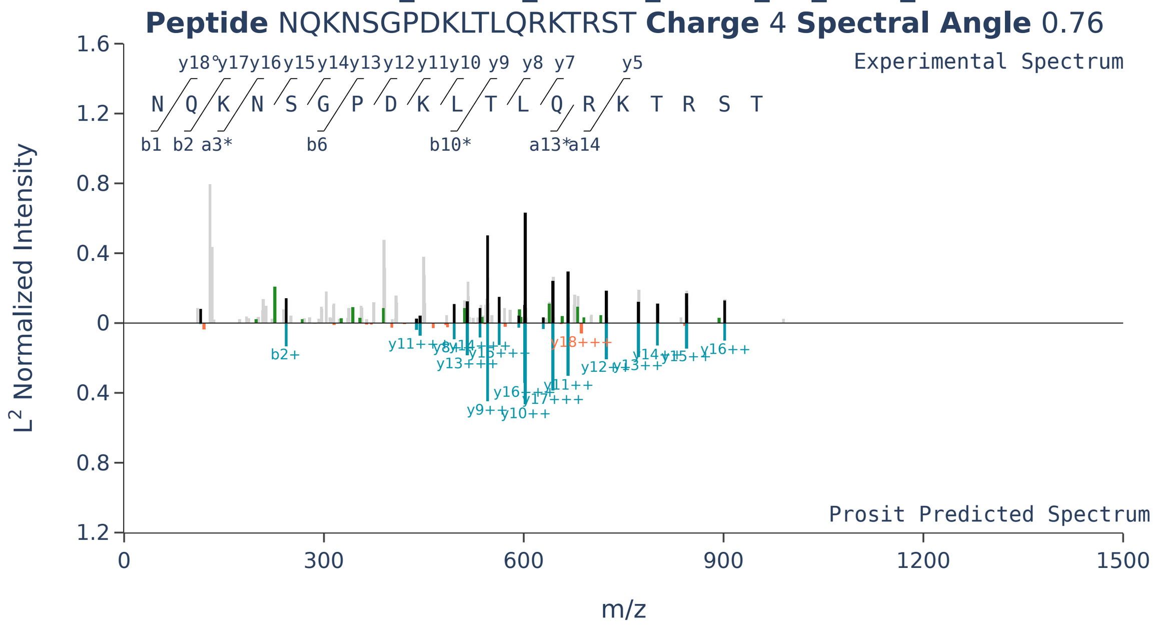


**Source** HRoetschke_211222_160123_HFGoe_IF2_b1R2_24h **Scan** 16776
**Peptide** DEVKVKII **Charge** 2 **Spectral Angle** 0.76

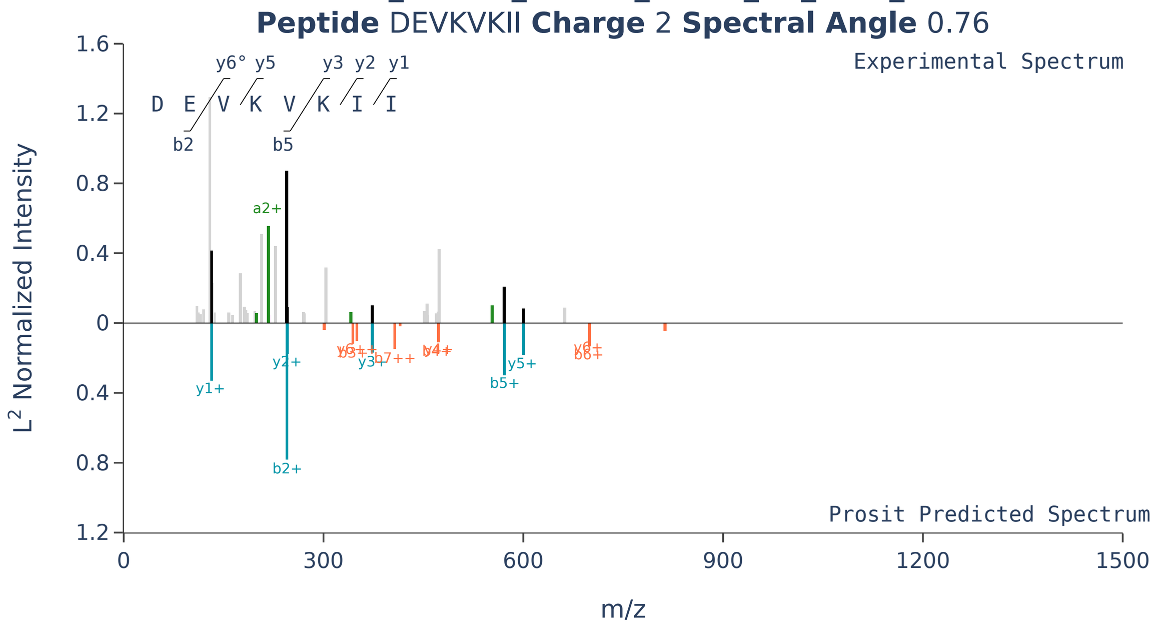


**Source** HRoetschke_211222_160123_HFGoe_IF2_b1R2_24h **Scan** 6390
**Peptide** NKIDKPEA **Charge** 2 **Spectral Angle** 0.83

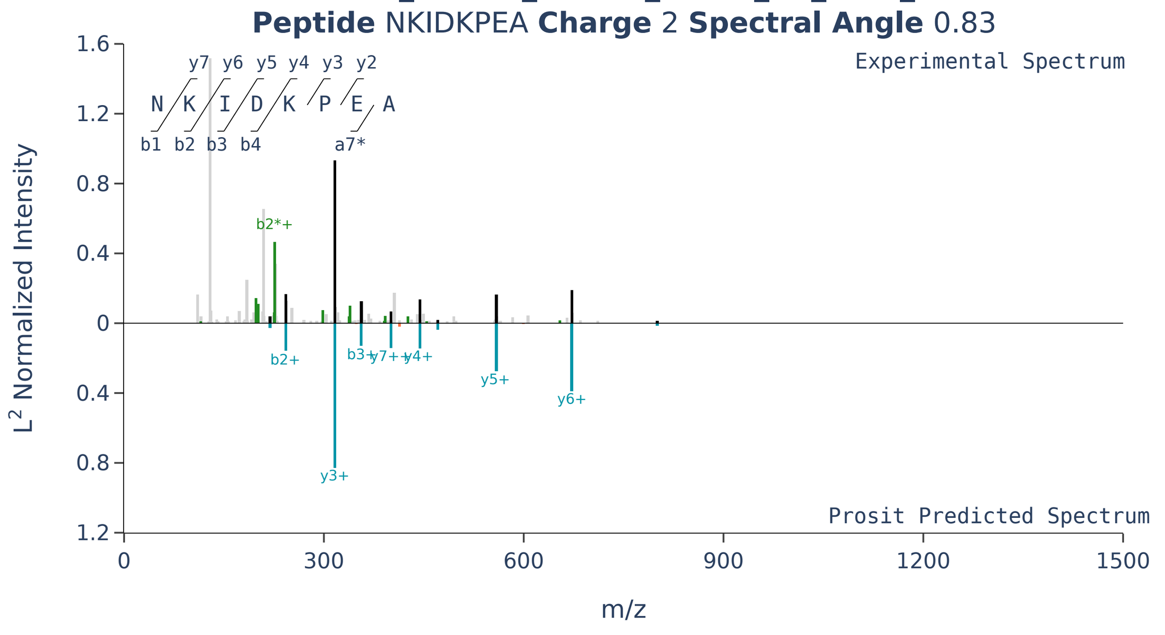


**Source** HRoetschke_211222_160123_HFGoe_IF2_b1R1_4h **Scan** 5768
**Peptide** SSDYHVTTSQHARQAED **Charge** 2 **Spectral Angle** 0.81

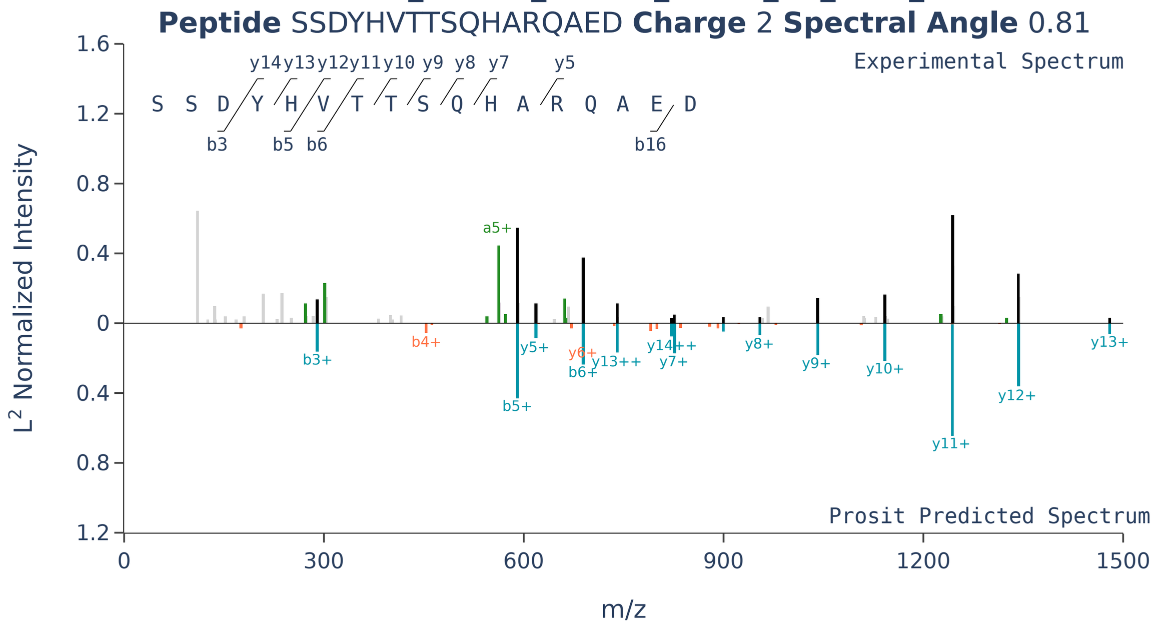


**Source** HRoetschke_211222_160123_HFGoe_IF2_b1R2_24h **Scan** 20722
**Peptide** WGGESQFV **Charge** 1 **Spectral Angle** 0.84

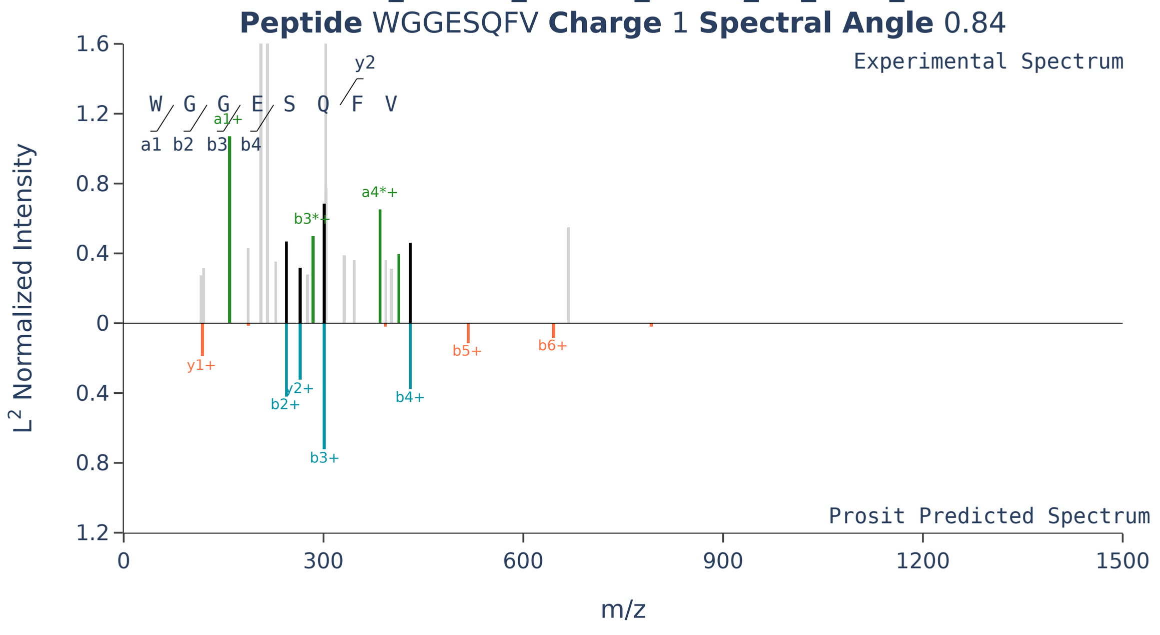


**Source** HRoetschke_211222_160123_HFGoe_IF2_b1R1_4h **Scan** 11814
**Peptide** MAEENKWTDNAEPTE **Charge** 2 **Spectral Angle** 0.91

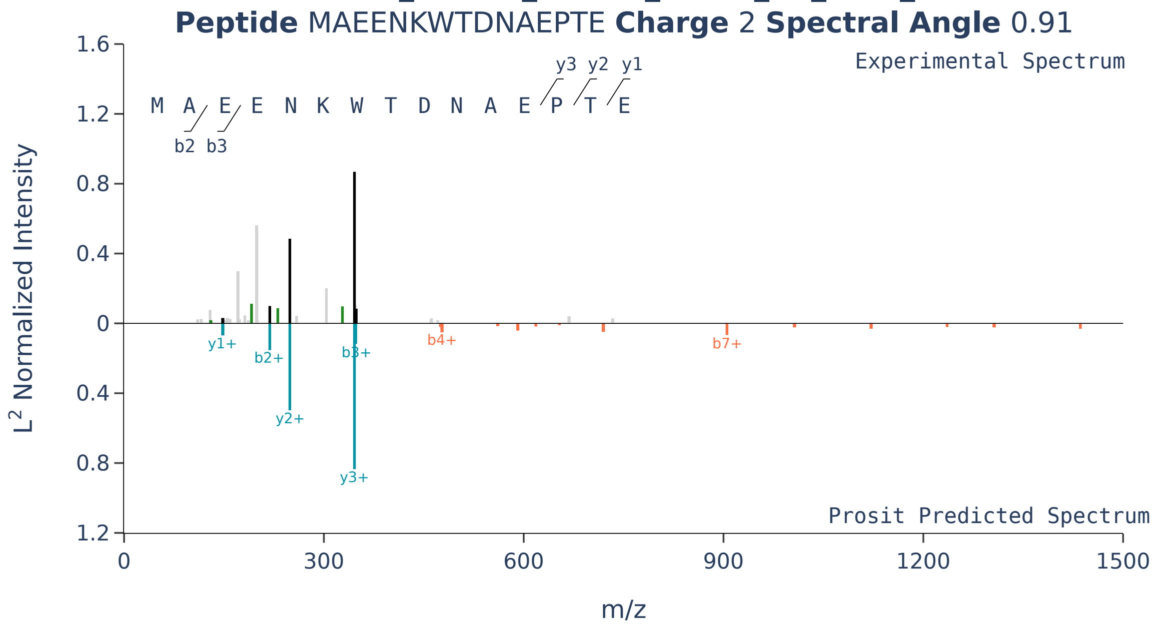


**Source** HRoetschke_211222_160123_HFGoe_IF2_b1R2_1h **Scan** 13528
**Peptide** RENELEEAV **Charge** 2 **Spectral Angle** 0.73

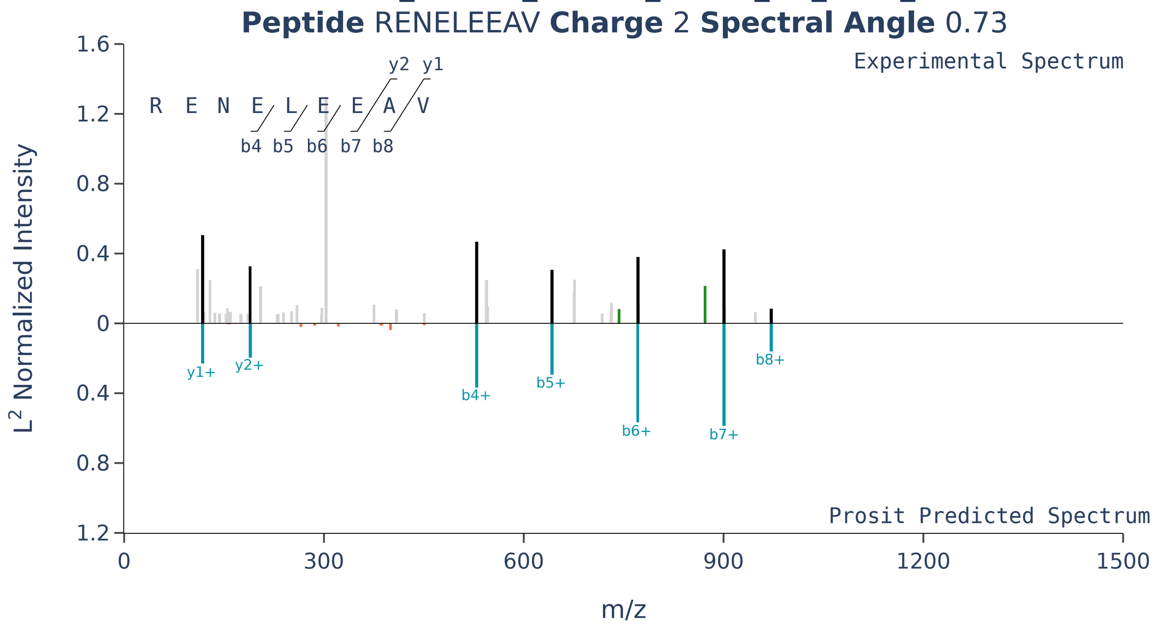


**Source** HRoetschke_211222_160123_HFGoe_IF2_b1R1_4h **Scan** 12116
**Peptide** ADDSVSAQEKQTLIDH **Charge** 3 **Spectral Angle** 0.86

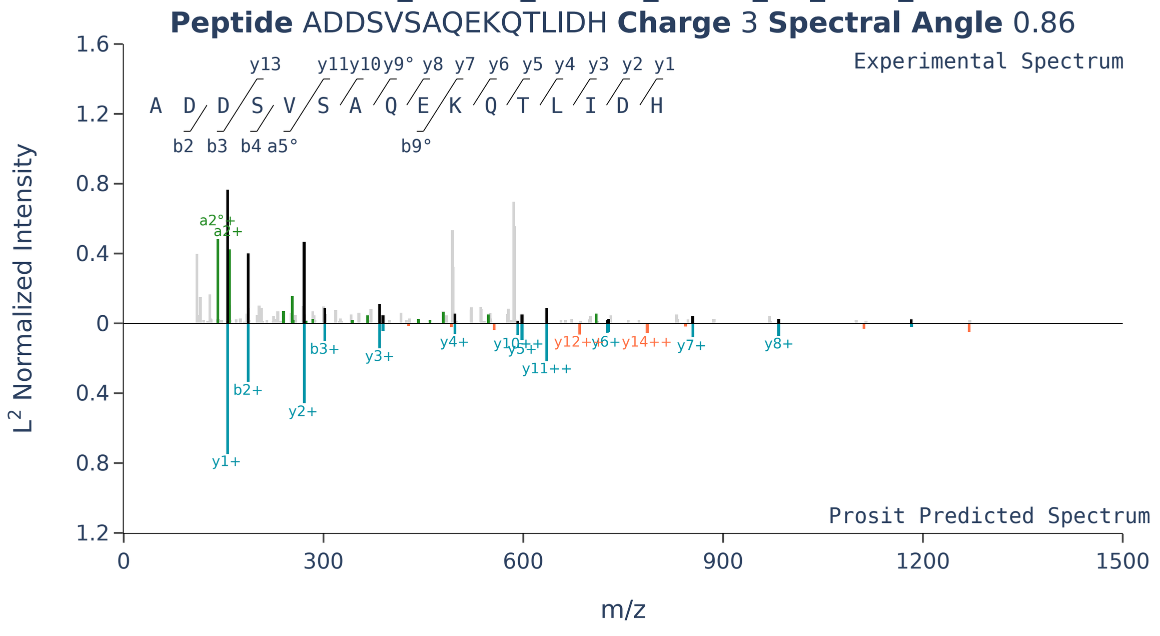


**Source** HRoetschke_211222_160123_HFGoe_IF2_b1R1_4h **Scan** 7926
**Peptide** ATLAAASN **Charge** 1 **Spectral Angle** 0.83

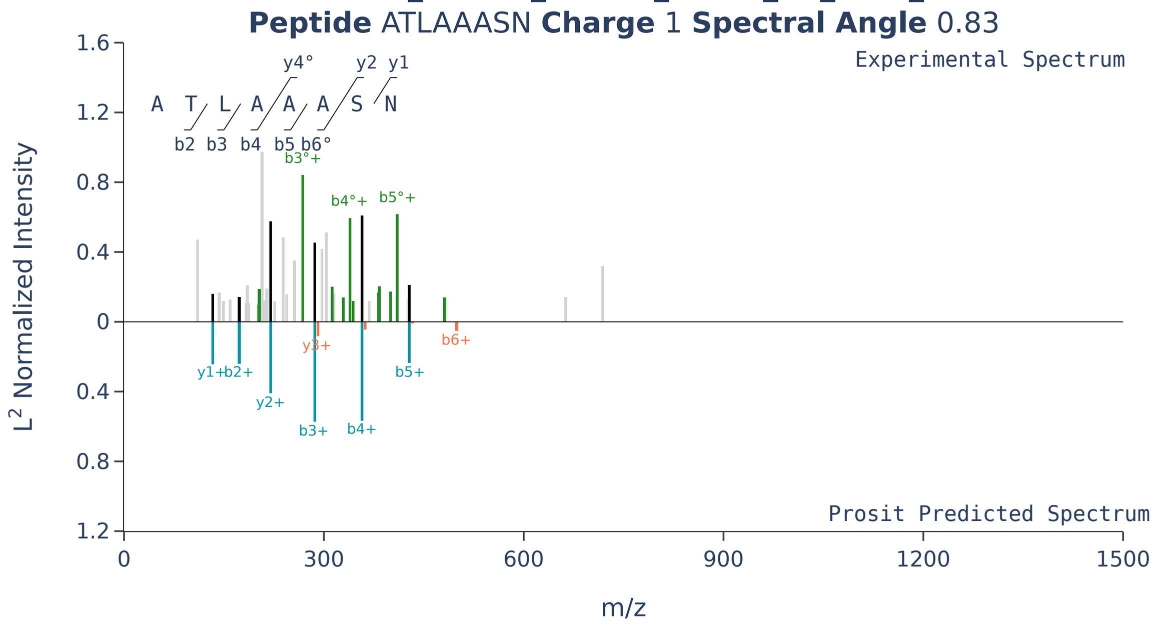


**Source** HRoetschke_211222_160123_HFGoe_IF2_b1R1_2h **Scan** 11647
**Peptide** NDVRTGDVLE **Charge** 2 **Spectral Angle** 0.88

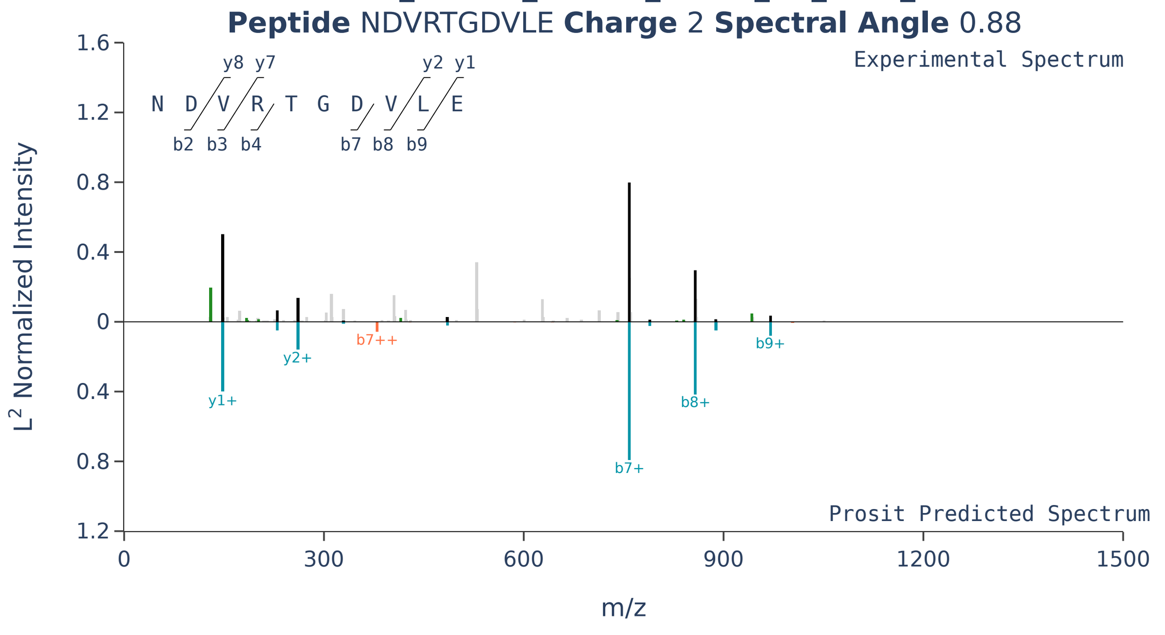


**Source** HRoetschke_211222_160123_HFGoe_IF2_b1R1_4h **Scan** 5951
**Peptide** EVRKKRTFV **Charge** 3 **Spectral Angle** 0.75

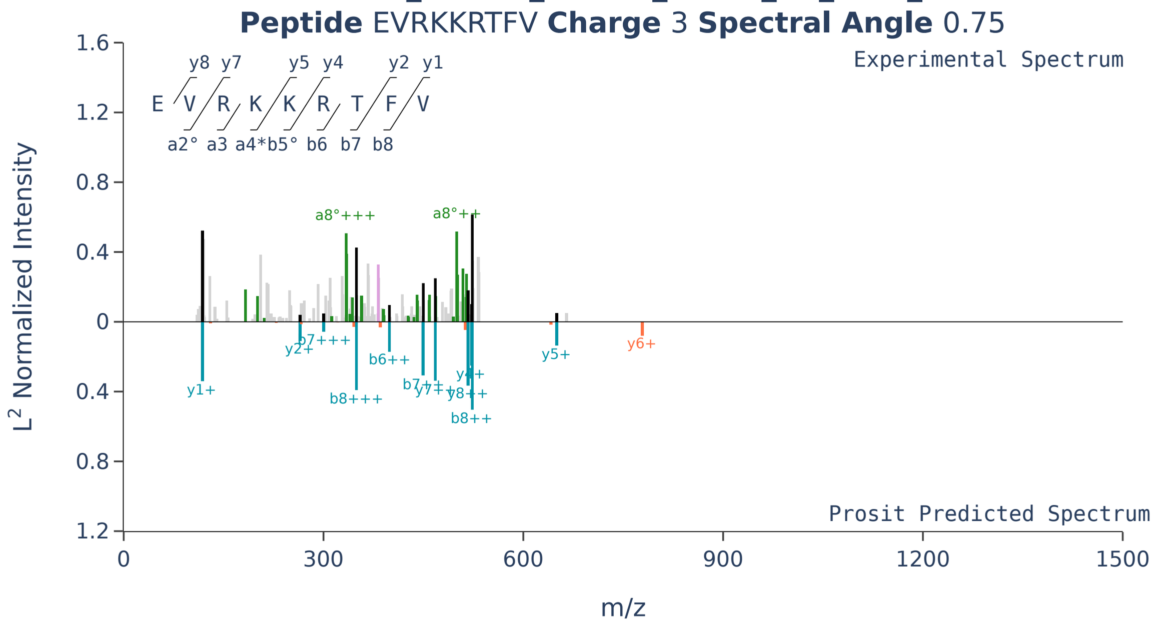


**Source** HRoetschke_211222_160123_HFGoe_IF2_b1R1_1h **Scan** 9337
**Peptide** KGSSLQQGFQKPAQ **Charge** 3 **Spectral Angle** 0.81

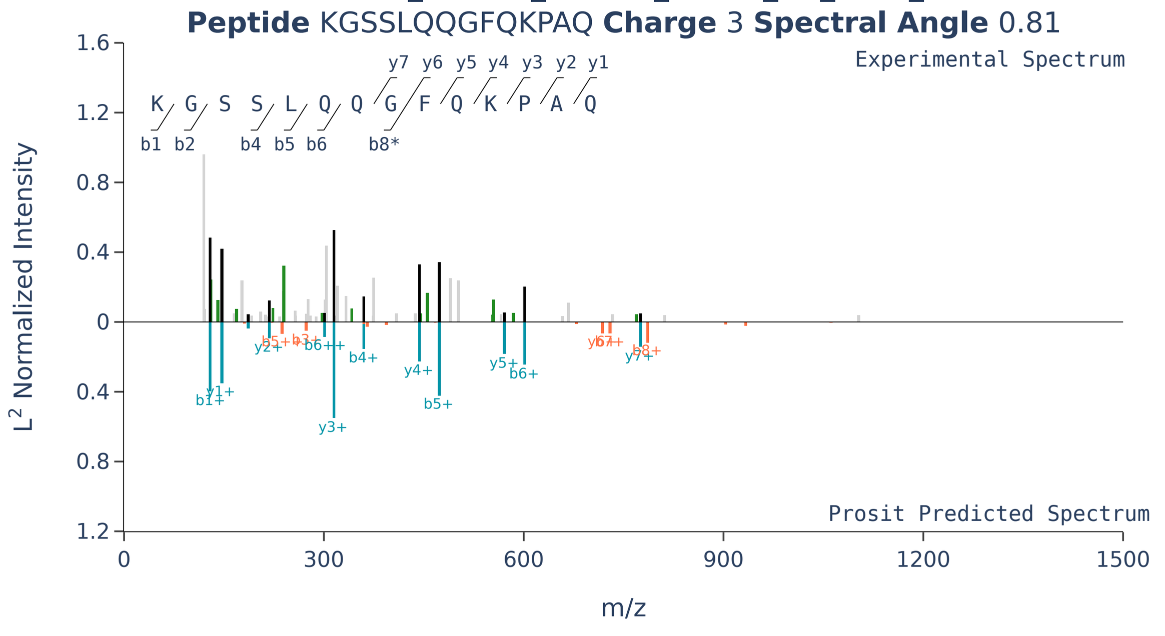


**Source** HRoetschke_211222_160123_HFGoe_IF2_b1R1_4h **Scan** 8675
**Peptide** CMVTEGVVKRH **Charge** 3 **Spectral Angle** 0.74

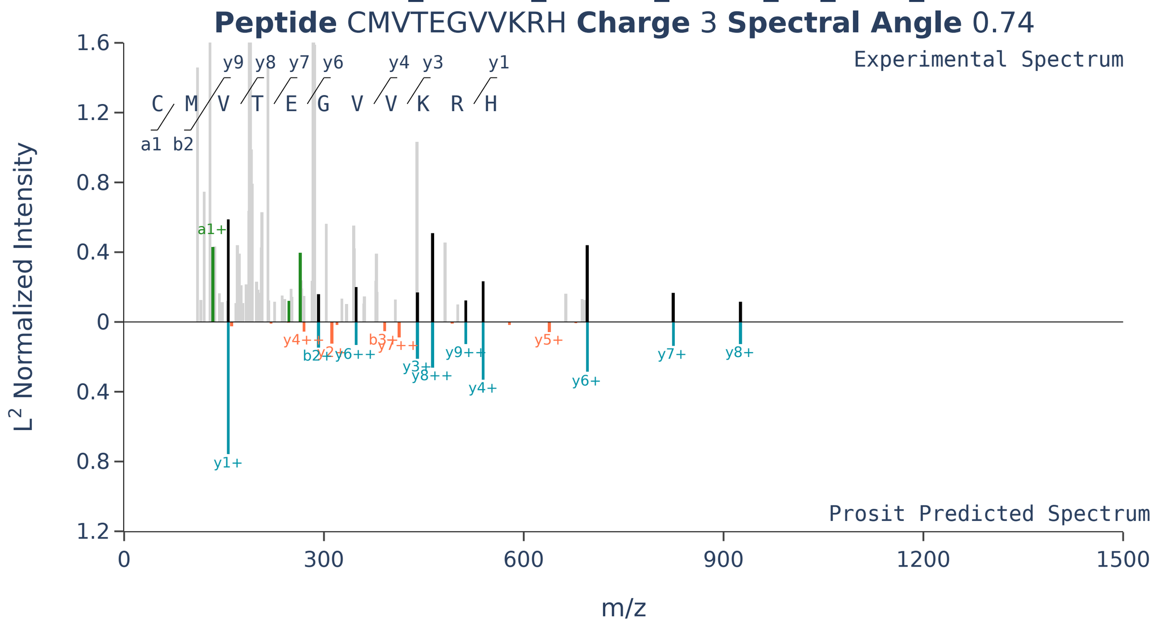


**Source** HRoetschke_211222_160123_HFGoe_IF2_b1R2_1h **Scan** 11598
**Peptide** TKVASGEAGGITQHIG **Charge** 3 **Spectral Angle** 0.79

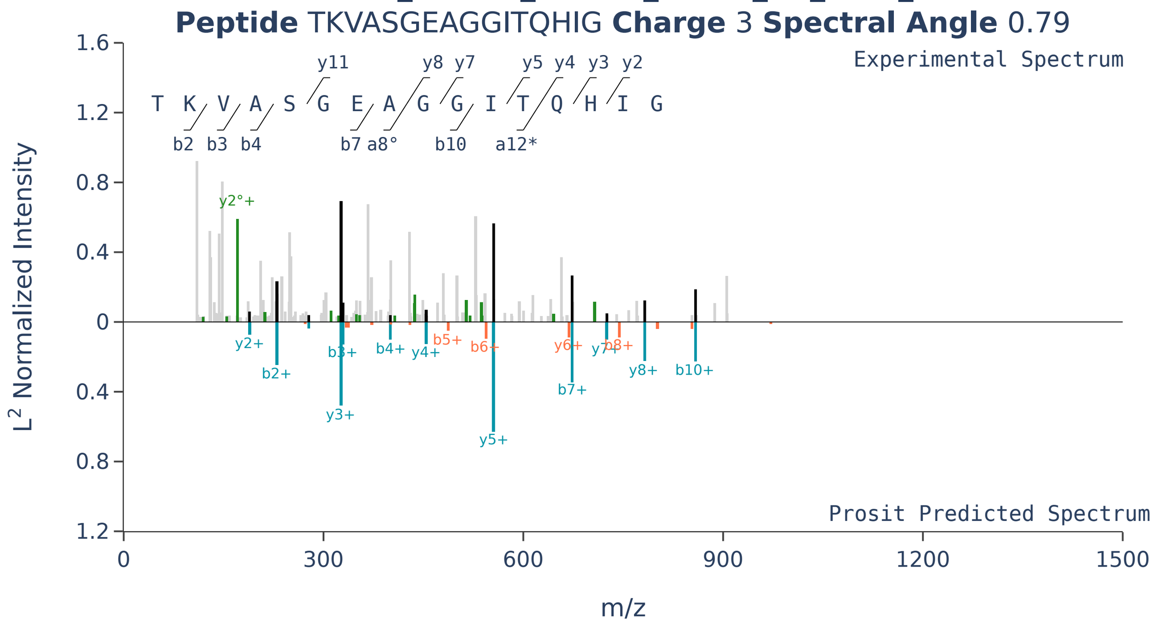

**Source** HRoetschke_211222_160123_HFGoe_IF2_b1R1_2h **Scan** 6599
**Peptide** HVSAKAGTGIDE **Charge** 2 **Spectral Angle** 0.81

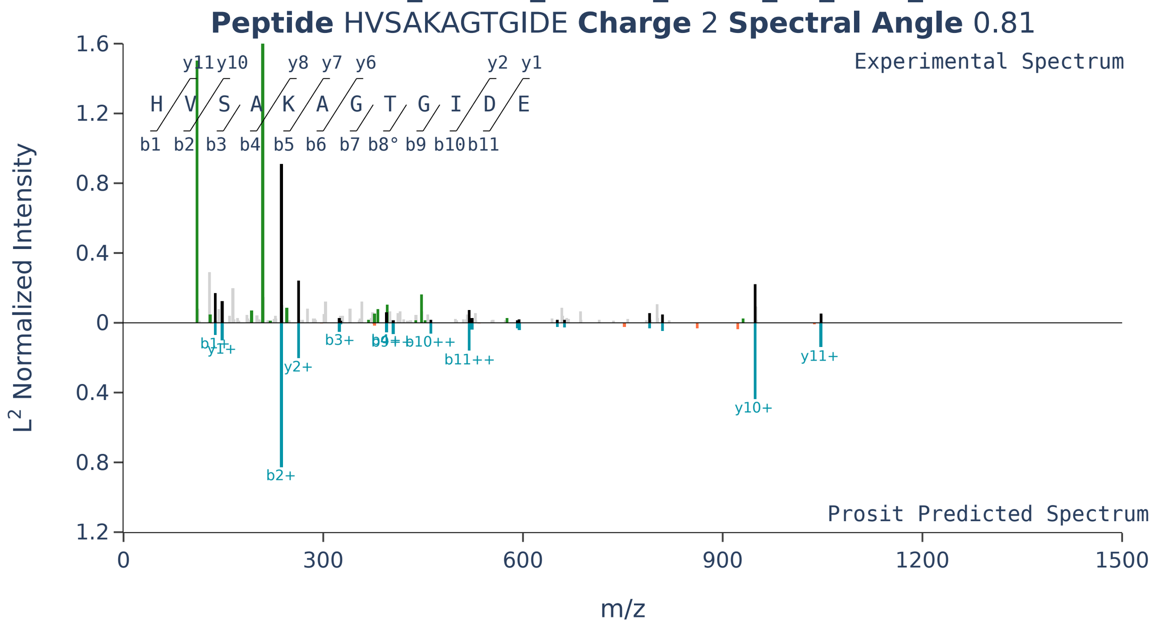


**Source** HRoetschke_211222_160123_HFGoe_IF2_b1R1_2h **Scan** 8603
**Peptide** HLGAYHVE **Charge** 2 **Spectral Angle** 0.86

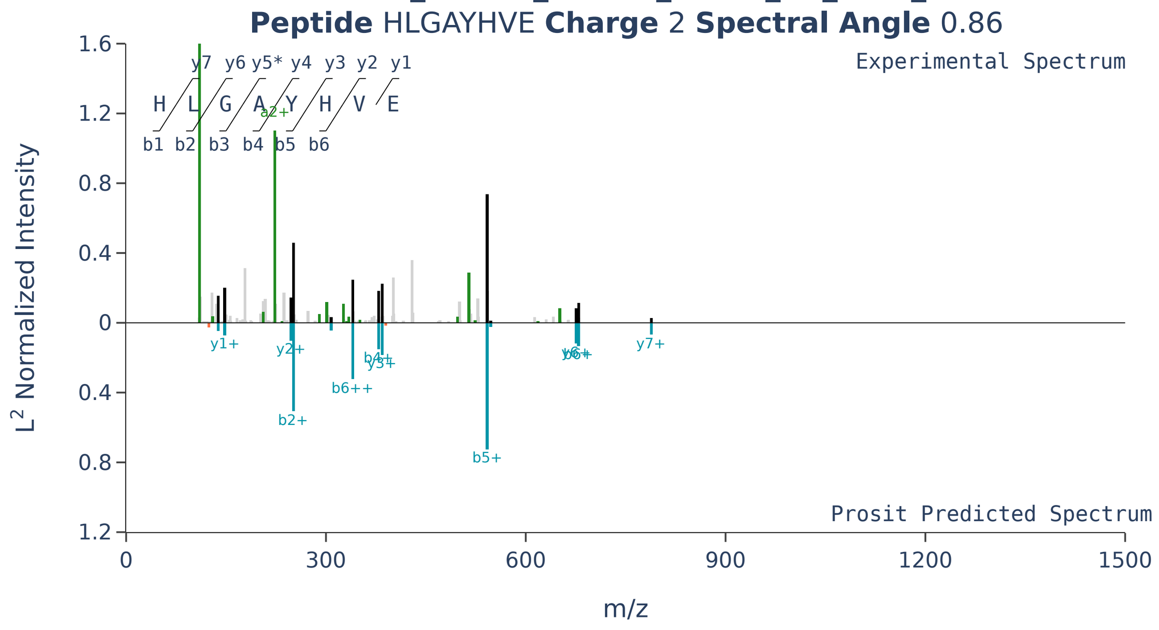


**Source** HRoetschke_211222_160123_HFGoe_IF2_b1R2_4h **Scan** 11714
**Peptide** AEVRDVFKS **Charge** 2 **Spectral Angle** 0.82

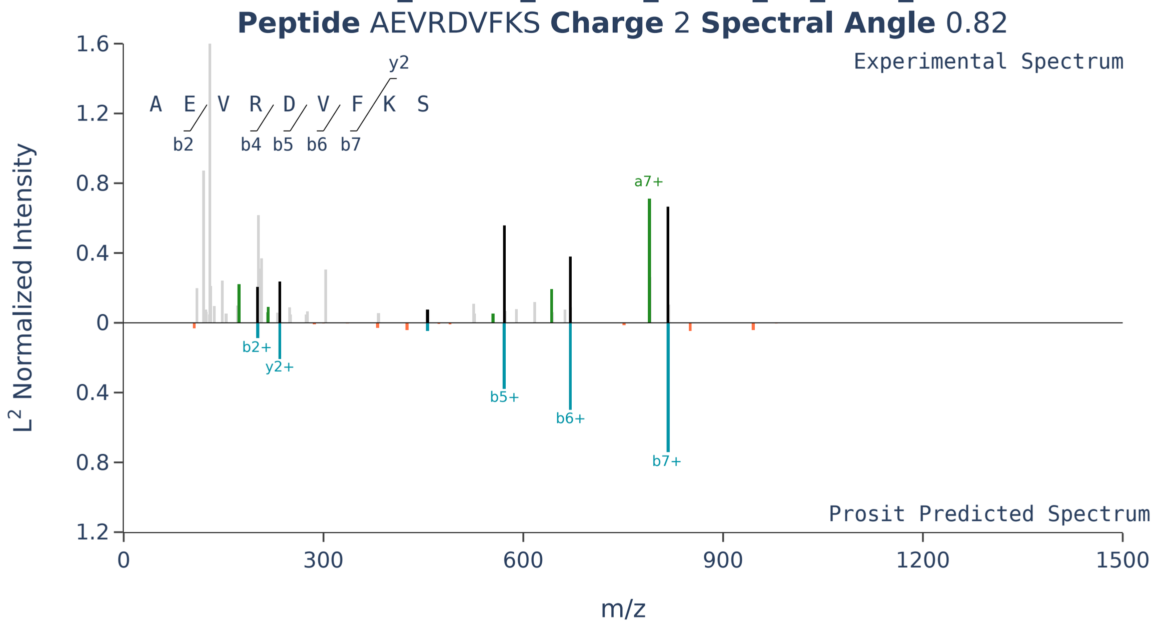


**Source** HRoetschke_211222_160123_HFGoe_IF2_b1R1_24h **Scan** 14723
**Peptide** LDTPGHAAFT **Charge** 2 **Spectral Angle** 0.81

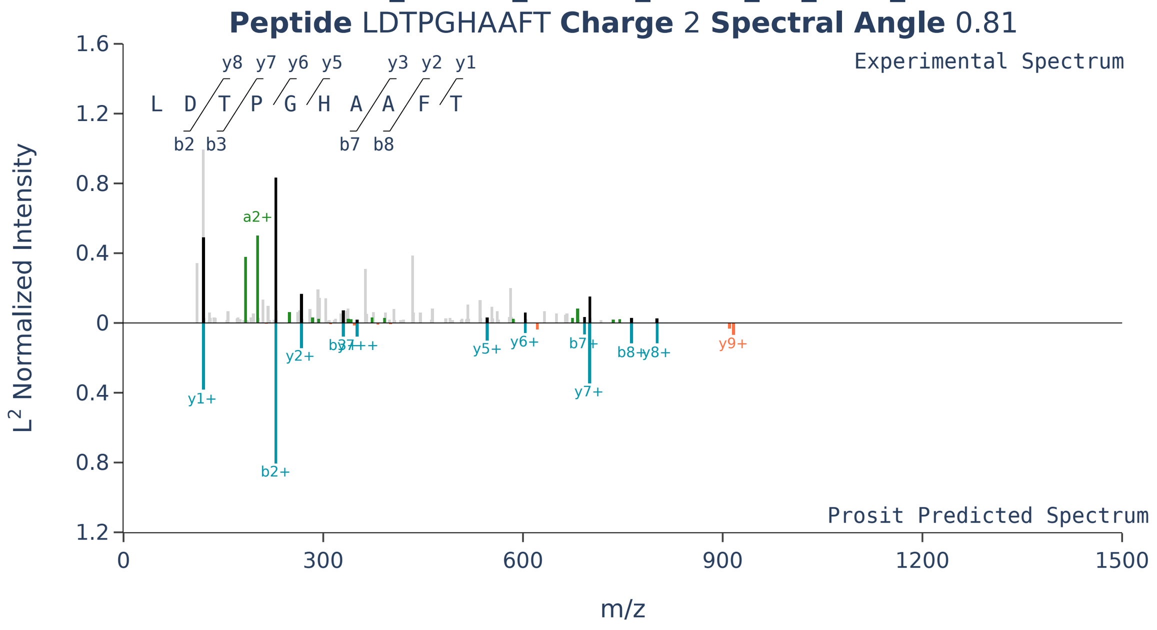


**Source** HRoetschke_211222_160123_HFGoe_IF2_b2R1_24h **Scan** 6525
**Peptide** MGHVDHGKTSL **Charge** 1 **Spectral Angle** 0.8

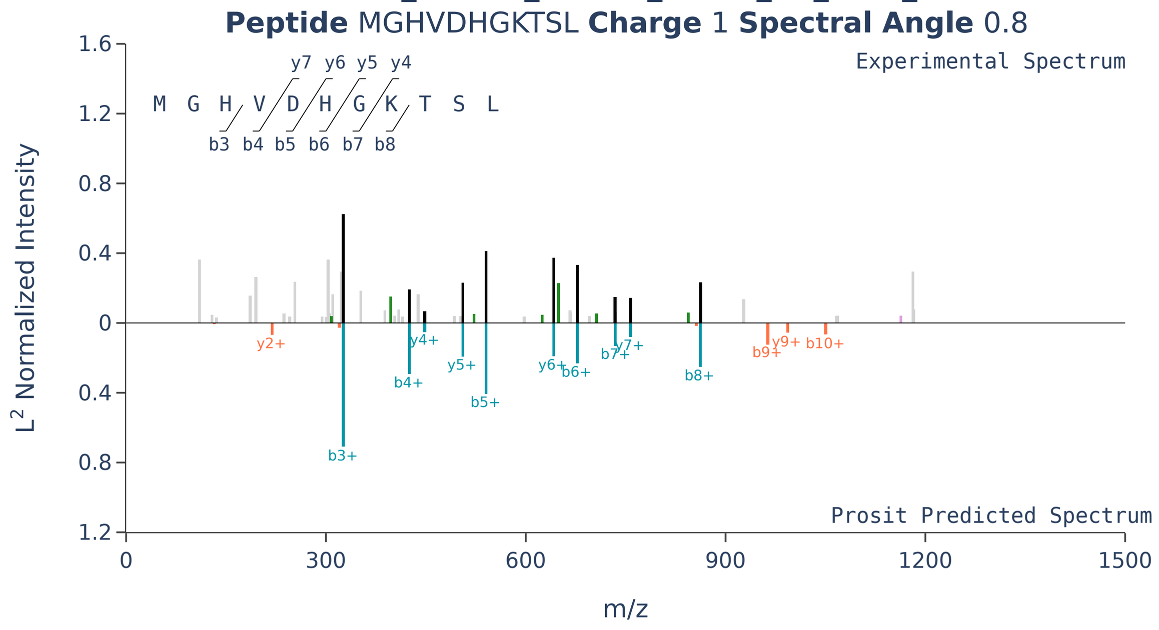


**Source** HRoetschke_211222_160123_HFGoe_IF2_b1R2_4h **Scan** 10310
**Peptide** CMVTEGVVKR **Charge** 3 **Spectral Angle** 0.83

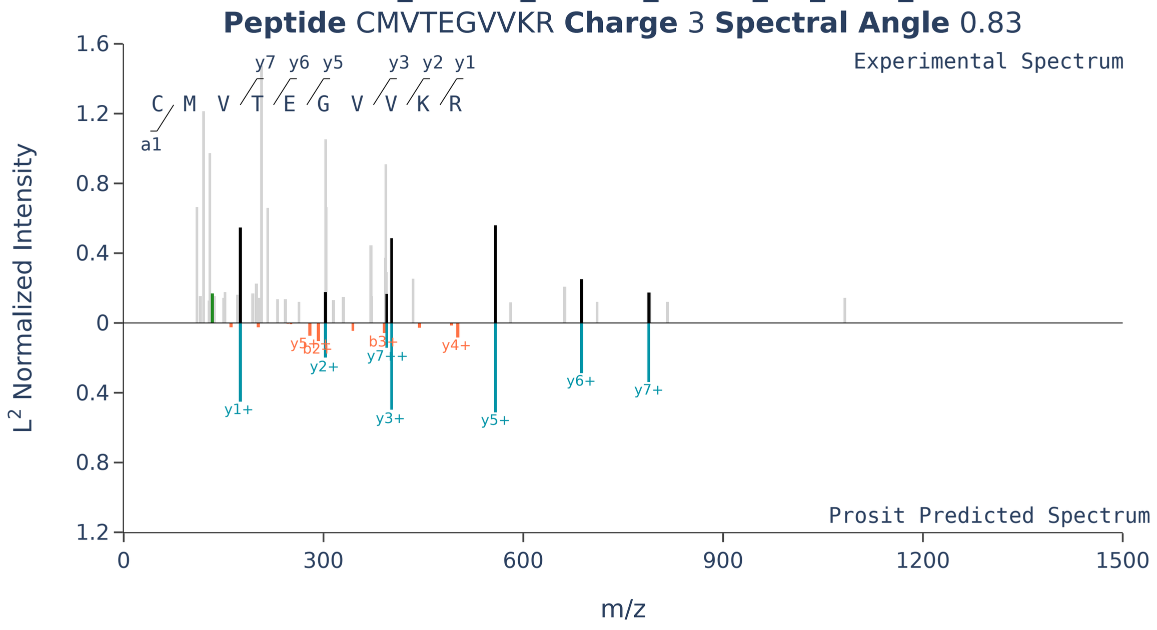


**Source** HRoetschke_211222_160123_HFGoe_IF2_b1R1_1h **Scan** 6969
**Peptide** TVVRDEKKAREV **Charge** 3 **Spectral Angle** 0.83

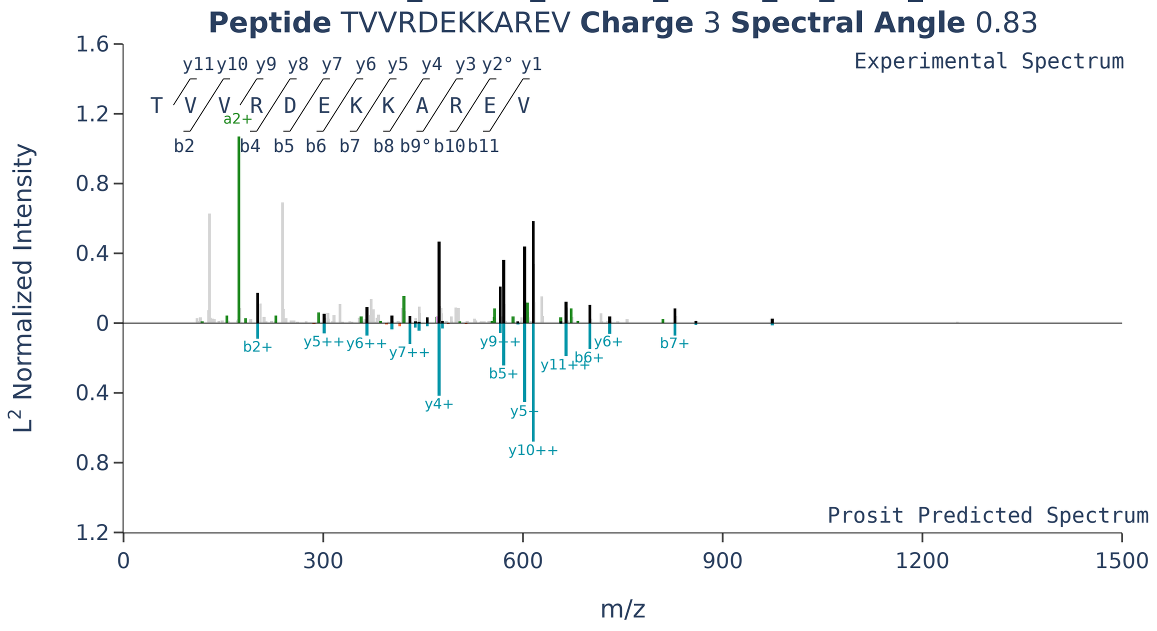


**Source** HRoetschke_211222_160123_HFGoe_IF2_b1R1_4h **Scan** 5250
**Peptide** VRDEKKAREV **Charge** 3 **Spectral Angle** 0.86

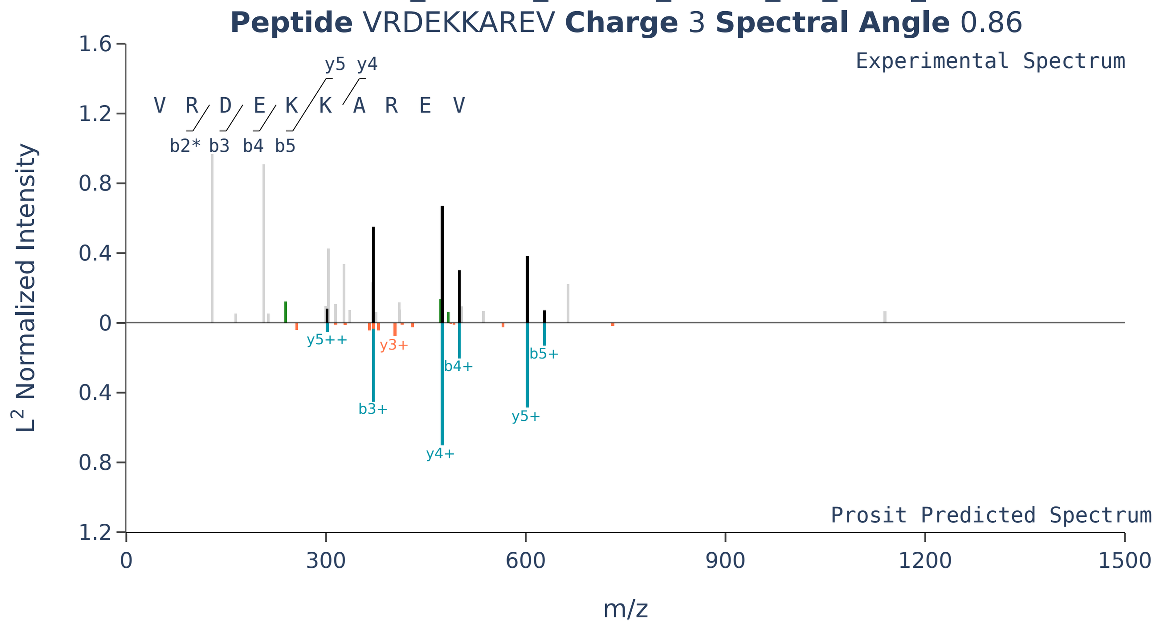


**Source** HRoetschke_211222_160123_HFGoe_IF2_b1R1_4h **Scan** 8775
**Peptide** HIGAYHVETEN **Charge** 3 **Spectral Angle** 0.78

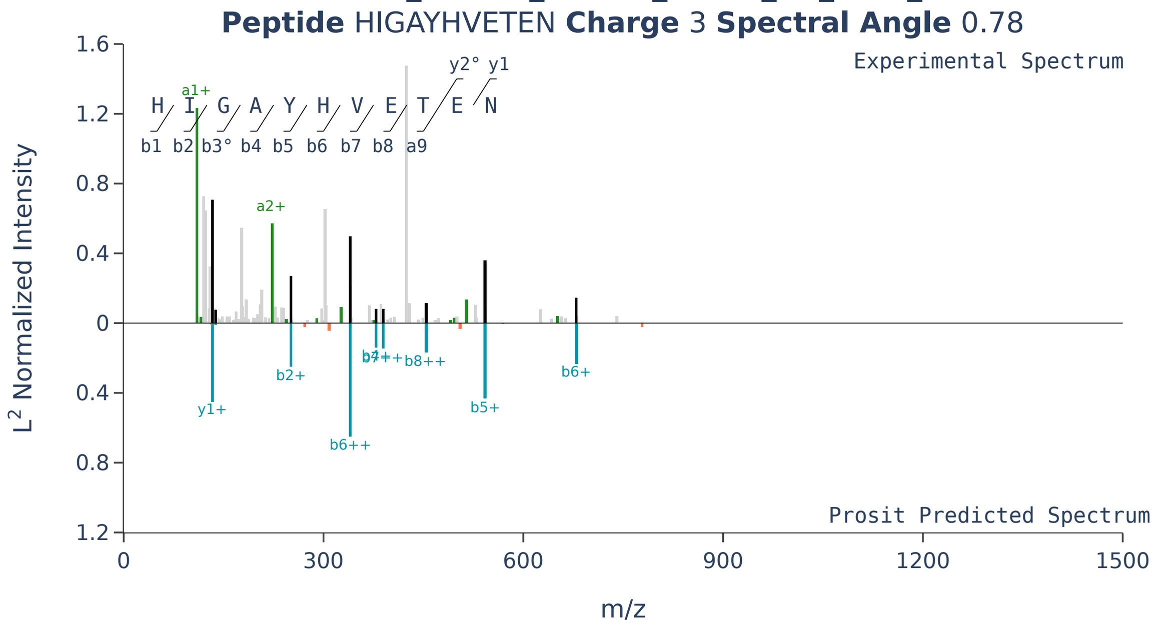


**Source** HRoetschke_211222_160123_HFGoe_IF2_b1R2_1h **Scan** 16934
**Peptide** SPELKQQII **Charge** 2 **Spectral Angle** 0.79

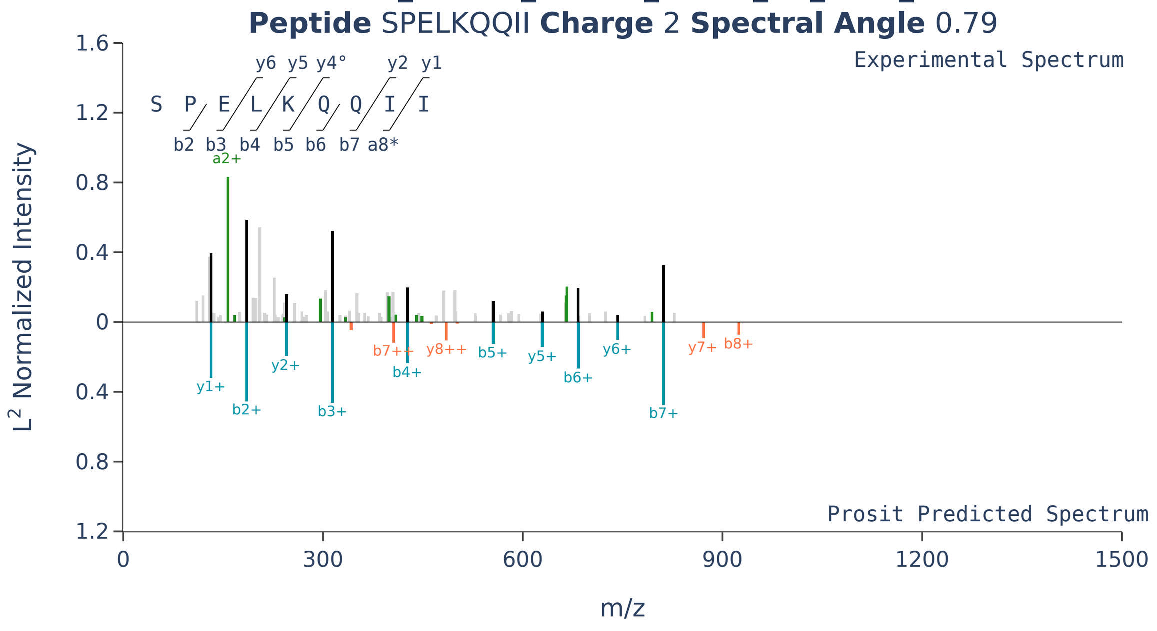


**Source** HRoetschke_211222_160123_HFGoe_IF2_b1R1_2h **Scan** 6156
**Peptide** PDKLTLQRKTR **Charge** 3 **Spectral Angle** 0.84

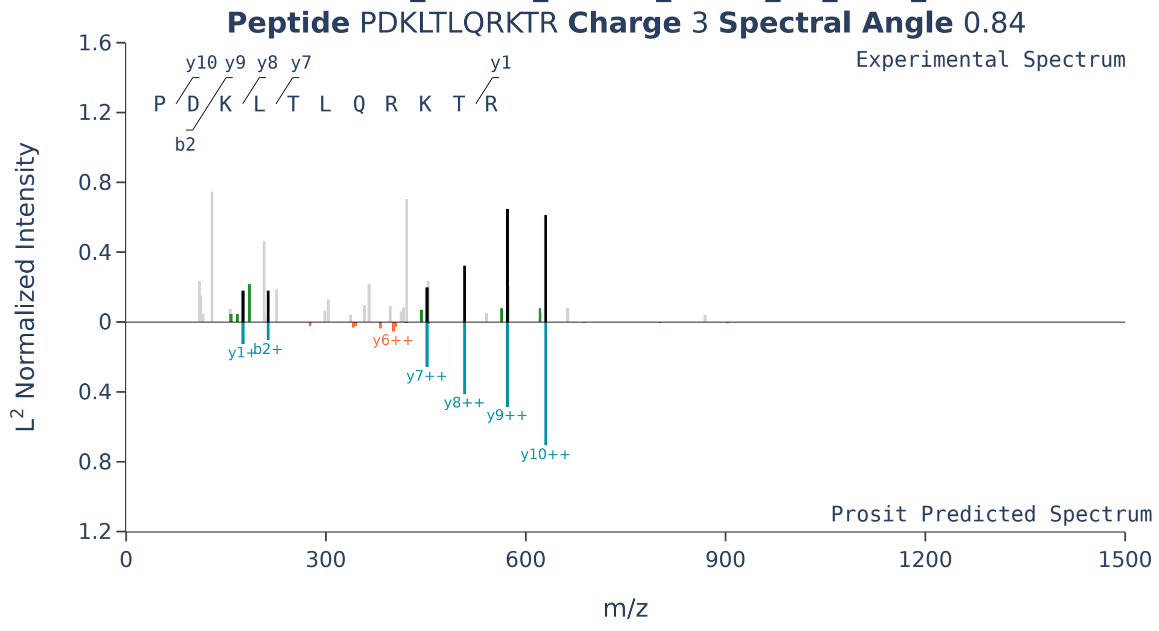


**Source** HRoetschke_211222_160123_HFGoe_IF2_b1R1_4h **Scan** 14043
**Peptide** VLELKAVR **Charge** 2 **Spectral Angle** 0.77

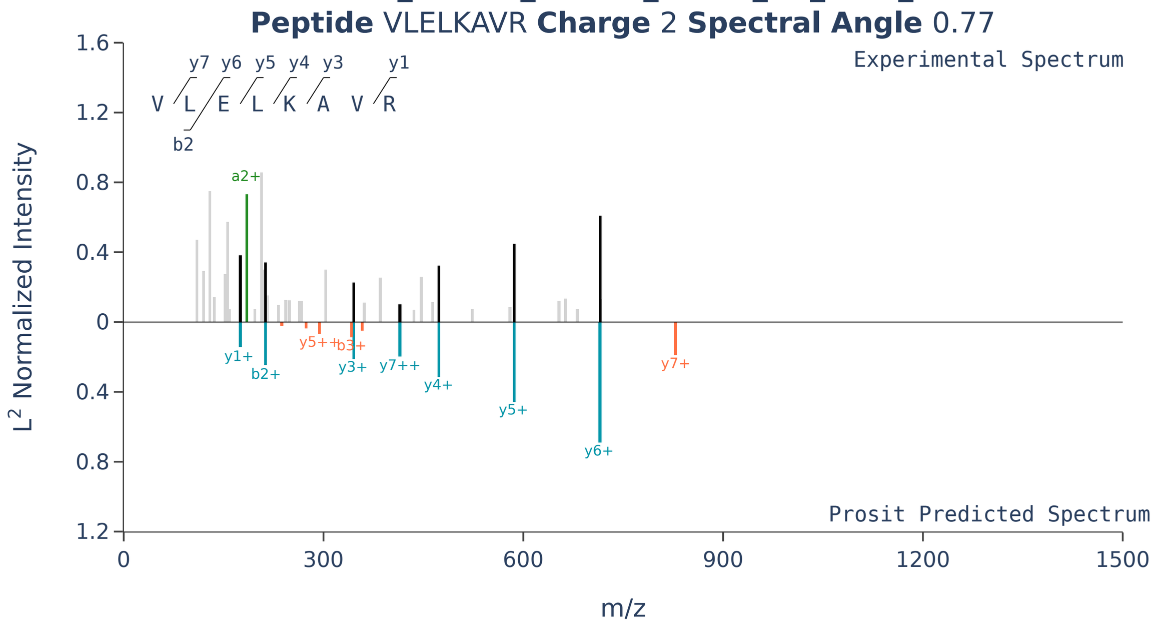


**Source** HRoetschke_211222_160123_HFGoe_IF2_b1R2_24h **Scan** 20738
**Peptide** SQYGILPEE **Charge** 2 **Spectral Angle** 0.85

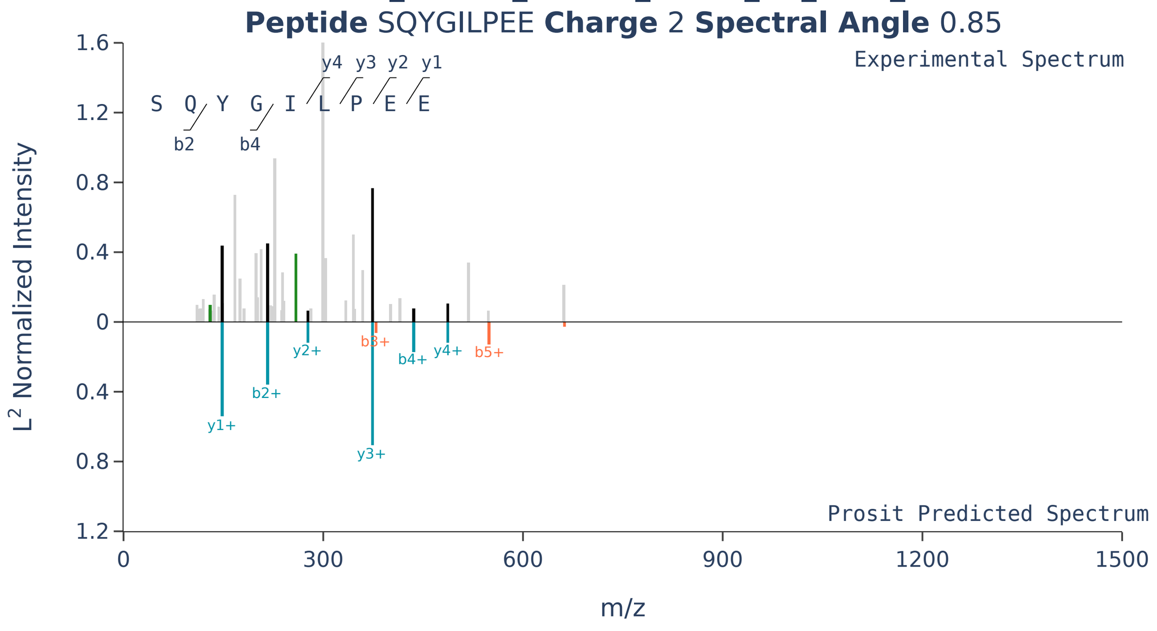


**Source** HRoetschke_211222_160123_HFGoe_IF2_b2R1_24h **Scan** 11458
**Peptide** LIDHLNQ **Charge** 2 **Spectral Angle** 0.71

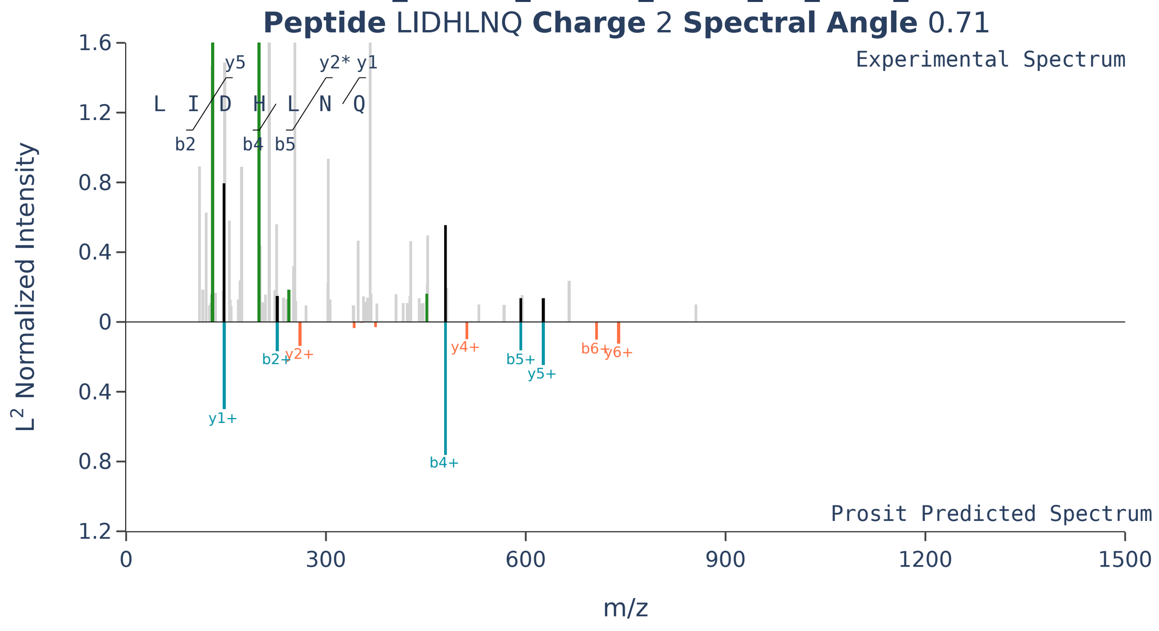


**Source** HRoetschke_211222_160123_HFGoe_IF2_b1R1_4h **Scan** 14882
**Peptide** AKAAQVPVV **Charge** 2 **Spectral Angle** 0.81

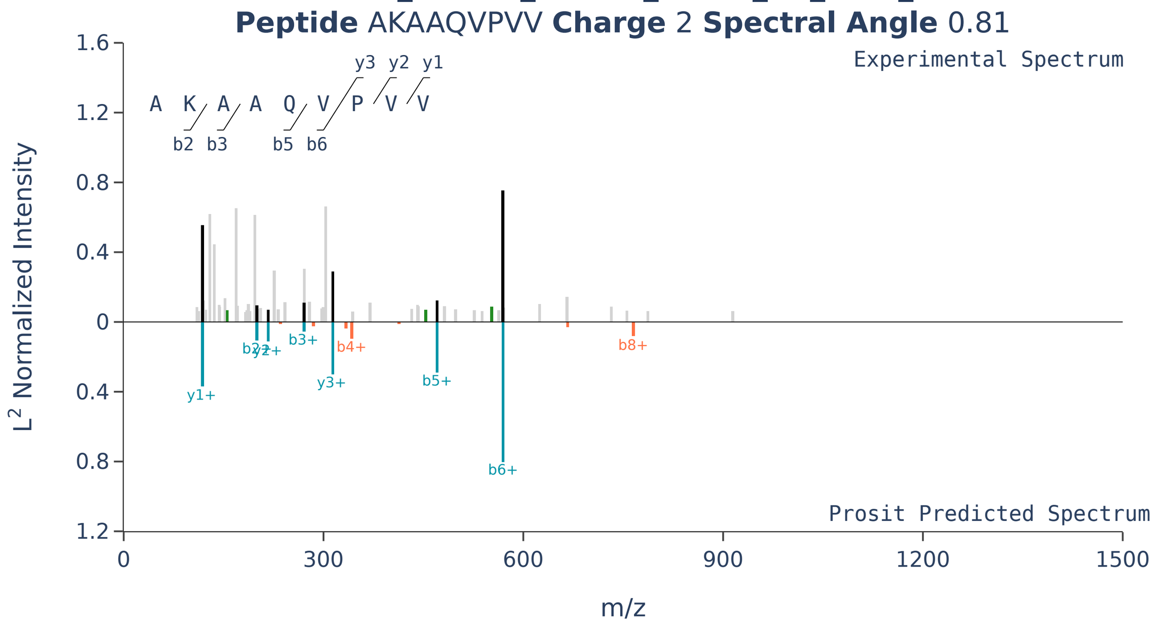

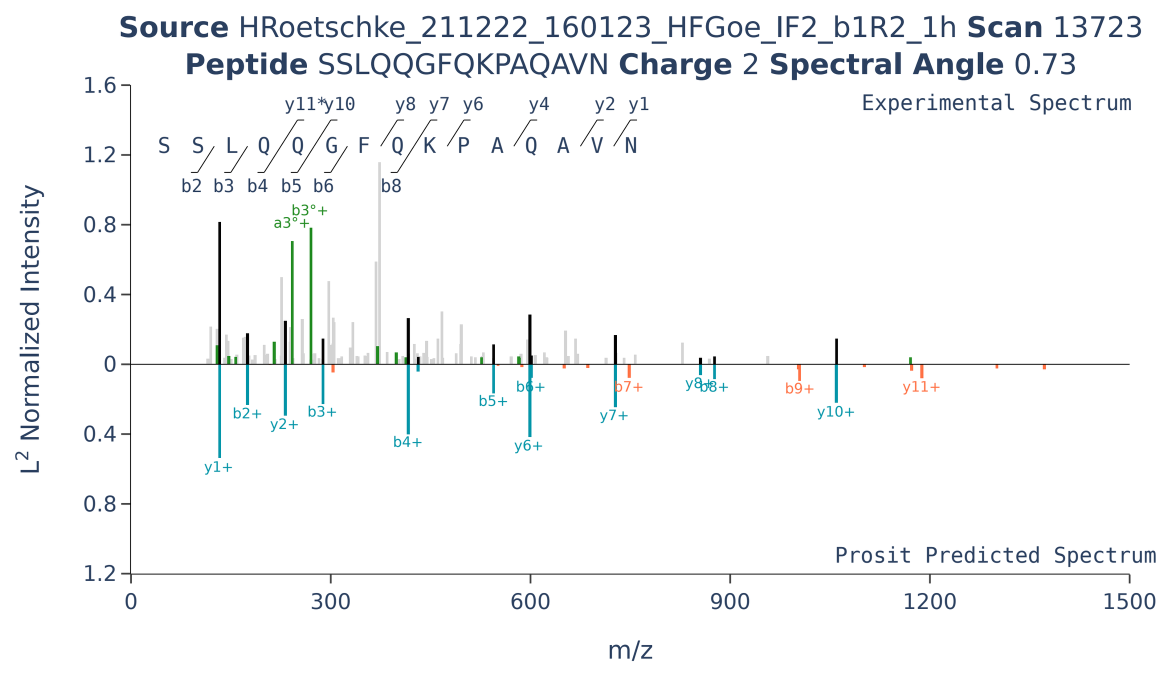
**Source** HRoetschke_211222_160123_HFGoe_IF2_b1R2_1h **Scan** 13723
**Peptide** SSLQQGFQKPAQAVN **Charge** 2 **Spectral Angle** 0.73
Experimental Spectrum
Prosit Predicted Spectrum
L² Normalized Intensity
m/z

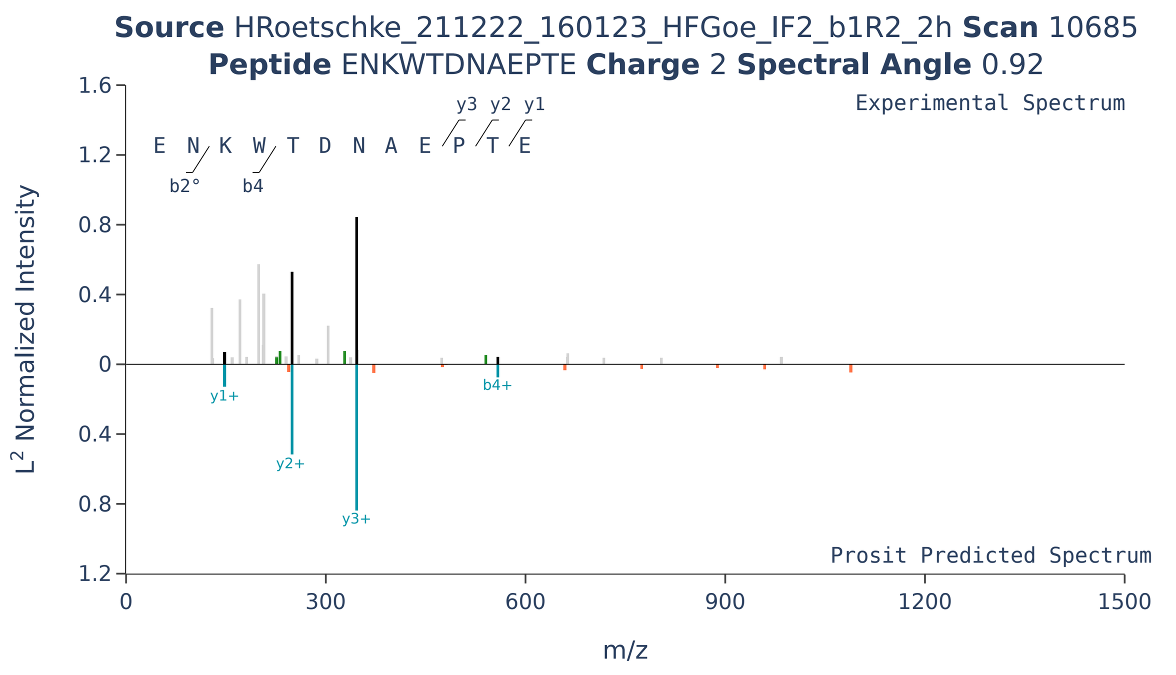
**Source** HRoetschke_211222_160123_HFGoe_IF2_b1R2_2h **Scan** 10685
**Peptide** ENKWTDNAEPTE **Charge** 2 **Spectral Angle** 0.92
Experimental Spectrum
Prosit Predicted Spectrum
L² Normalized Intensity
m/z

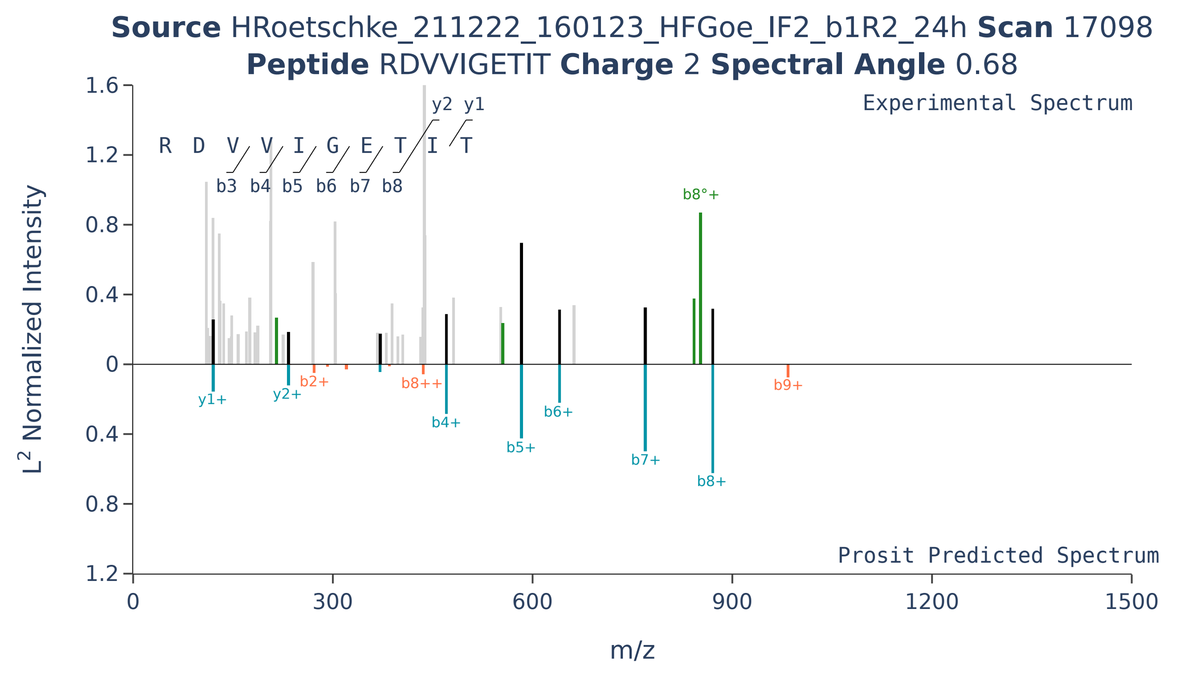
**Source** HRoetschke_211222_160123_HFGoe_IF2_b1R2_24h **Scan** 17098
**Peptide** RDVVIGETIT **Charge** 2 **Spectral Angle** 0.68
Experimental Spectrum
Prosit Predicted Spectrum
L² Normalized Intensity
m/z

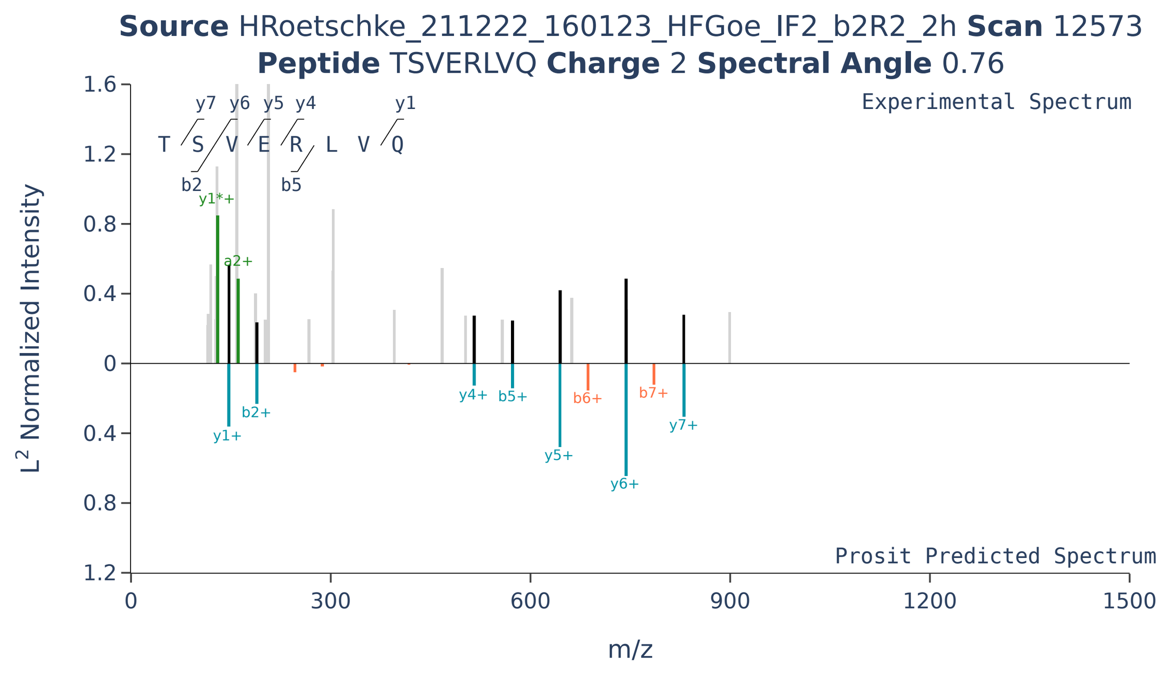
**Source** HRoetschke_211222_160123_HFGoe_IF2_b2R2_2h **Scan** 12573
**Peptide** TSVERLVQ **Charge** 2 **Spectral Angle** 0.76
Experimental Spectrum
Prosit Predicted Spectrum
L² Normalized Intensity
m/z

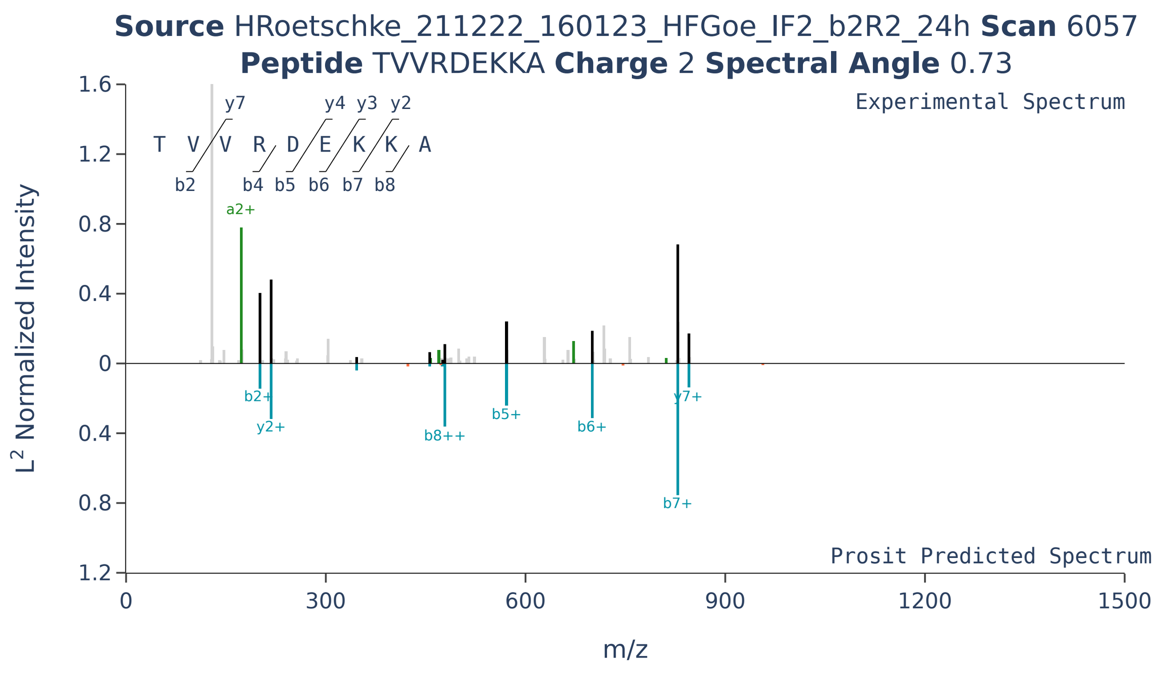
**Source** HRoetschke_211222_160123_HFGoe_IF2_b2R2_24h **Scan** 6057
**Peptide** TVVRDEKKA **Charge** 2 **Spectral Angle** 0.73
Experimental Spectrum
Prosit Predicted Spectrum
L² Normalized Intensity
m/z

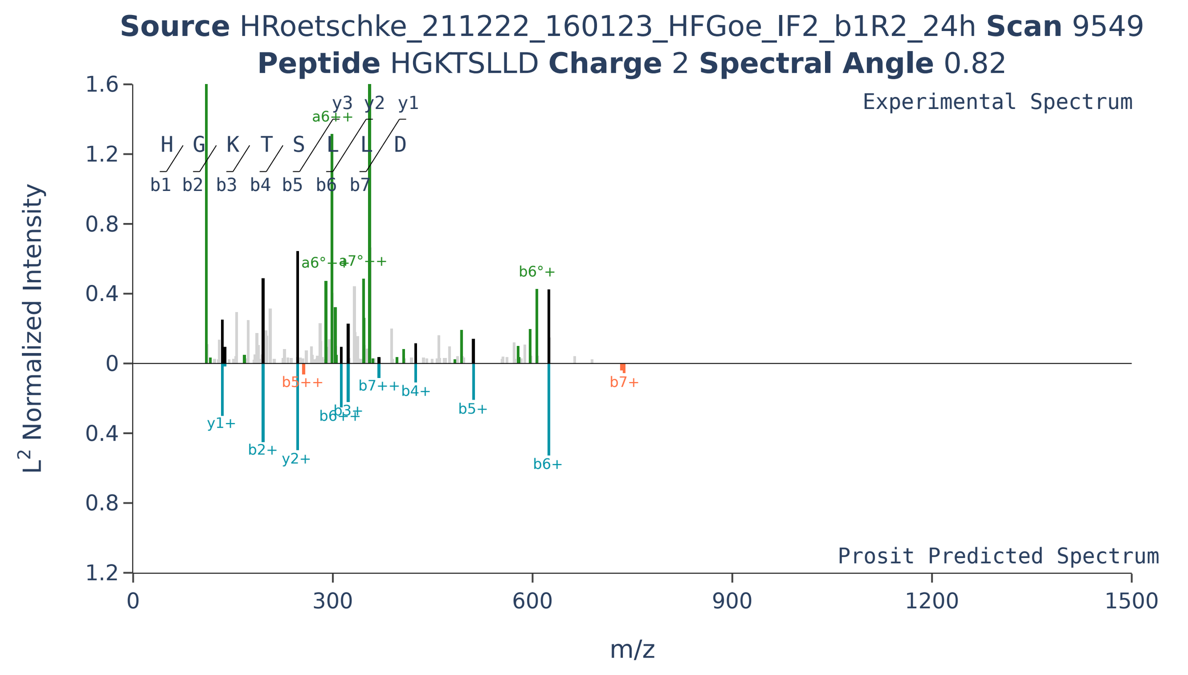
**Source** HRoetschke_211222_160123_HFGoe_IF2_b1R2_24h **Scan** 9549
**Peptide** HGKTSLLD **Charge** 2 **Spectral Angle** 0.82
Experimental Spectrum
Prosit Predicted Spectrum
L² Normalized Intensity
m/z

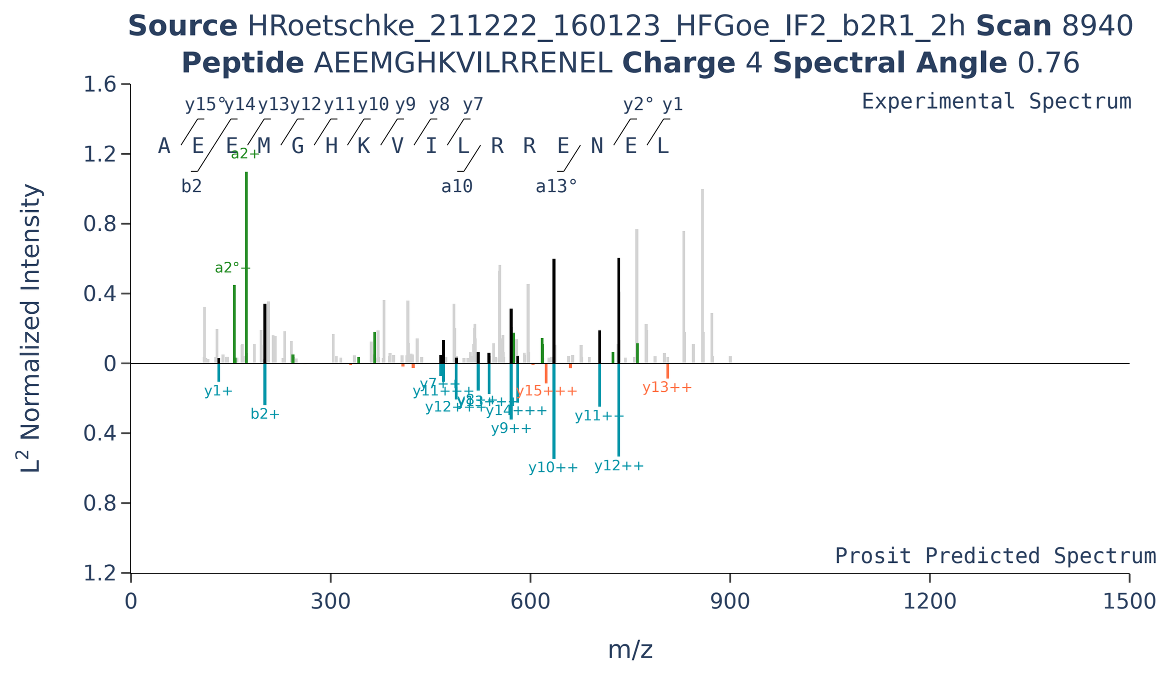
**Source** HRoetschke_211222_160123_HFGoe_IF2_b2R1_2h **Scan** 8940
**Peptide** AEEMGHKVILRRENEL **Charge** 4 **Spectral Angle** 0.76
Experimental Spectrum
Prosit Predicted Spectrum
L² Normalized Intensity
m/z

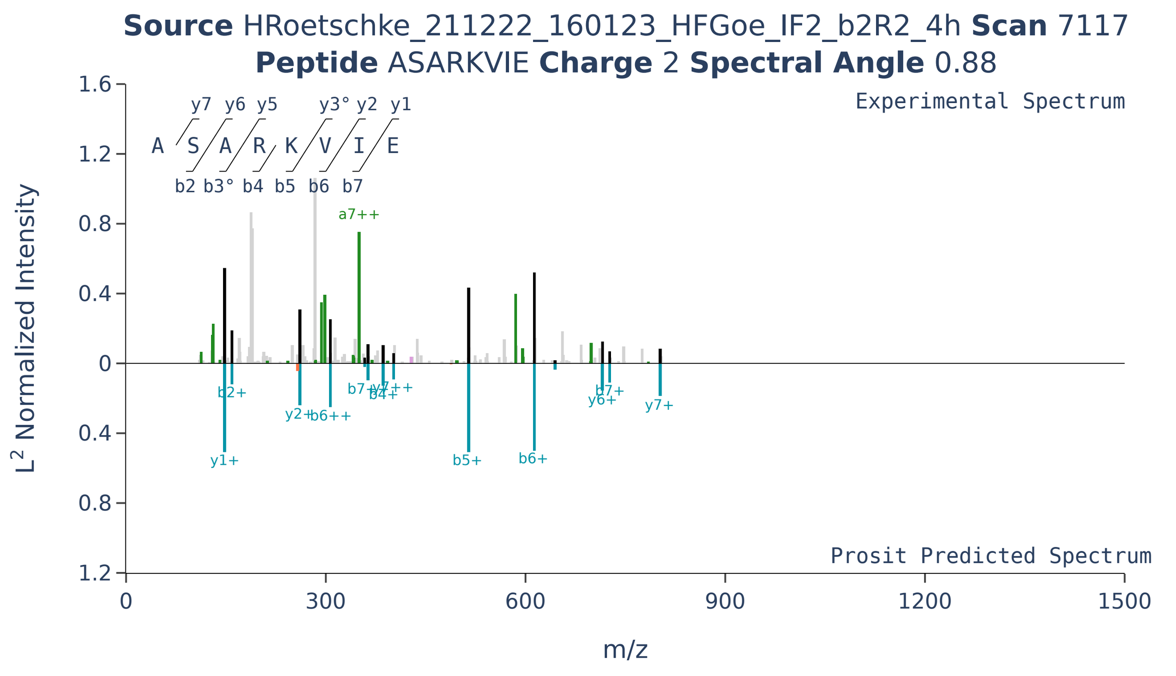
**Source** HRoetschke_211222_160123_HFGoe_IF2_b2R2_4h **Scan** 7117
**Peptide** ASARKVIE **Charge** 2 **Spectral Angle** 0.88
Experimental Spectrum
Prosit Predicted Spectrum
L² Normalized Intensity
m/z

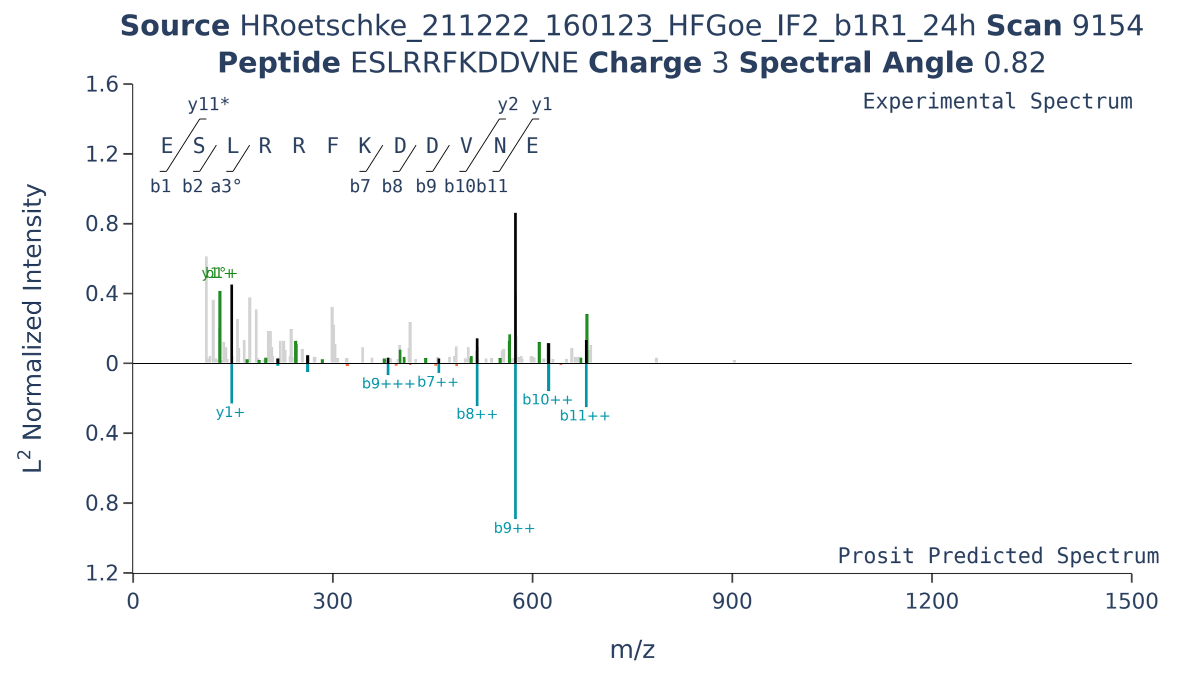
**Source** HRoetschke_211222_160123_HFGoe_IF2_b1R1_24h **Scan** 9154
**Peptide** ESLRRFKDDVNE **Charge** 3 **Spectral Angle** 0.82
Experimental Spectrum
Prosit Predicted Spectrum
L² Normalized Intensity
m/z

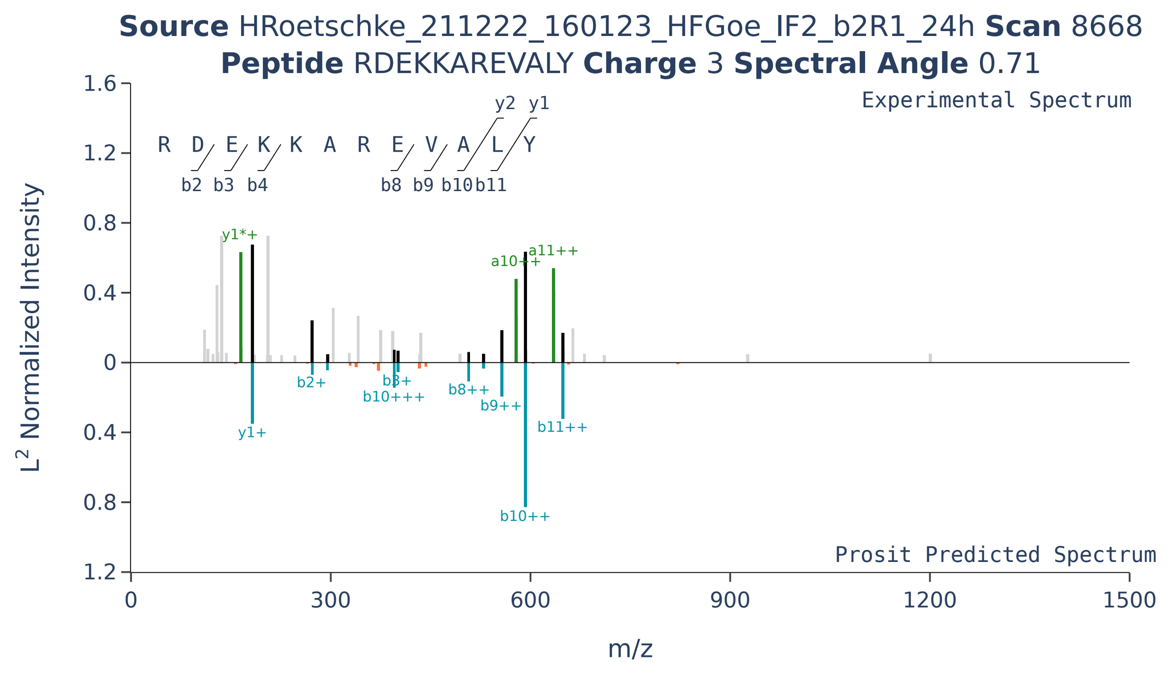
**Source** HRoetschke_211222_160123_HFGoe_IF2_b2R1_24h **Scan** 8668
**Peptide** RDEKKAREVALY **Charge** 3 **Spectral Angle** 0.71
Experimental Spectrum
Prosit Predicted Spectrum
L² Normalized Intensity
m/z

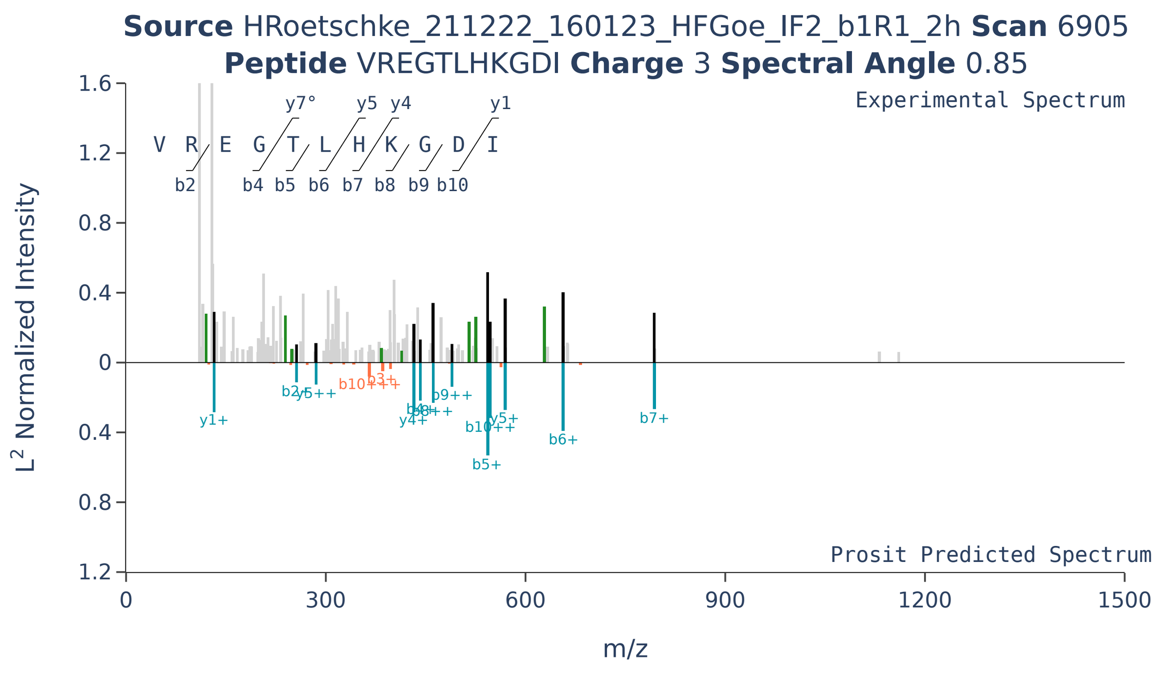
**Source** HRoetschke_211222_160123_HFGoe_IF2_b1R1_2h **Scan** 6905
**Peptide** VREGTLHKGDI **Charge** 3 **Spectral Angle** 0.85
Experimental Spectrum
Prosit Predicted Spectrum
L² Normalized Intensity
m/z

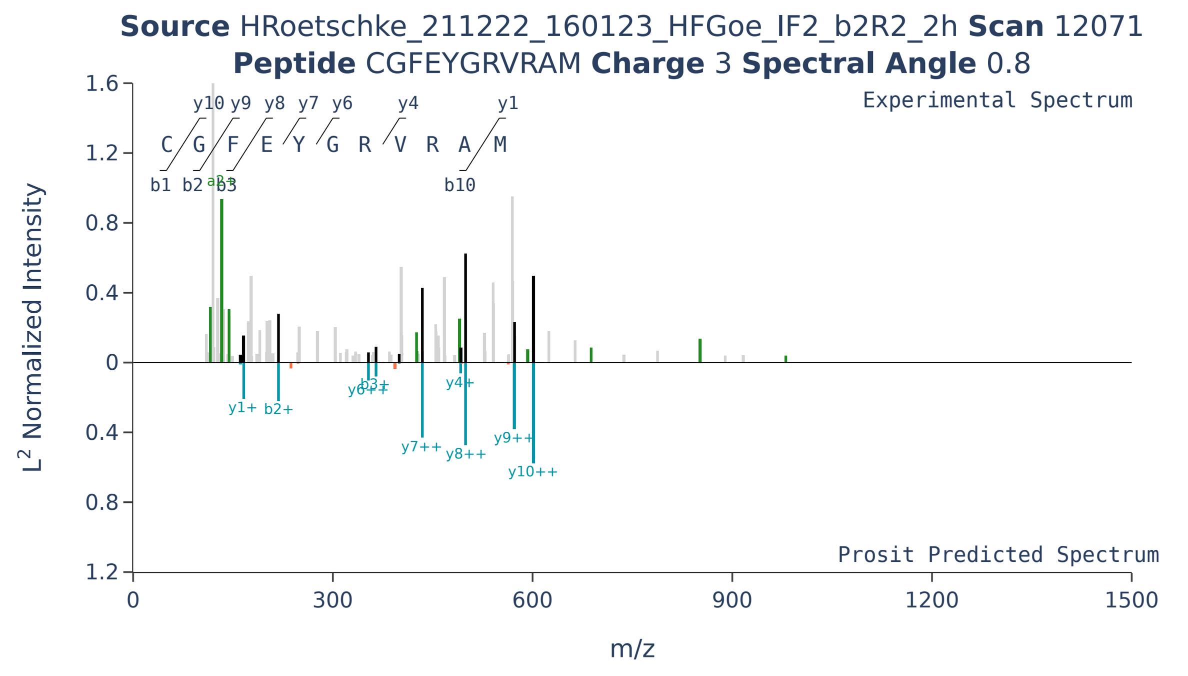
**Source** HRoetschke_211222_160123_HFGoe_IF2_b2R2_2h **Scan** 12071
**Peptide** CGFEYGRVRAM **Charge** 3 **Spectral Angle** 0.8
Experimental Spectrum
Prosit Predicted Spectrum
L² Normalized Intensity
m/z

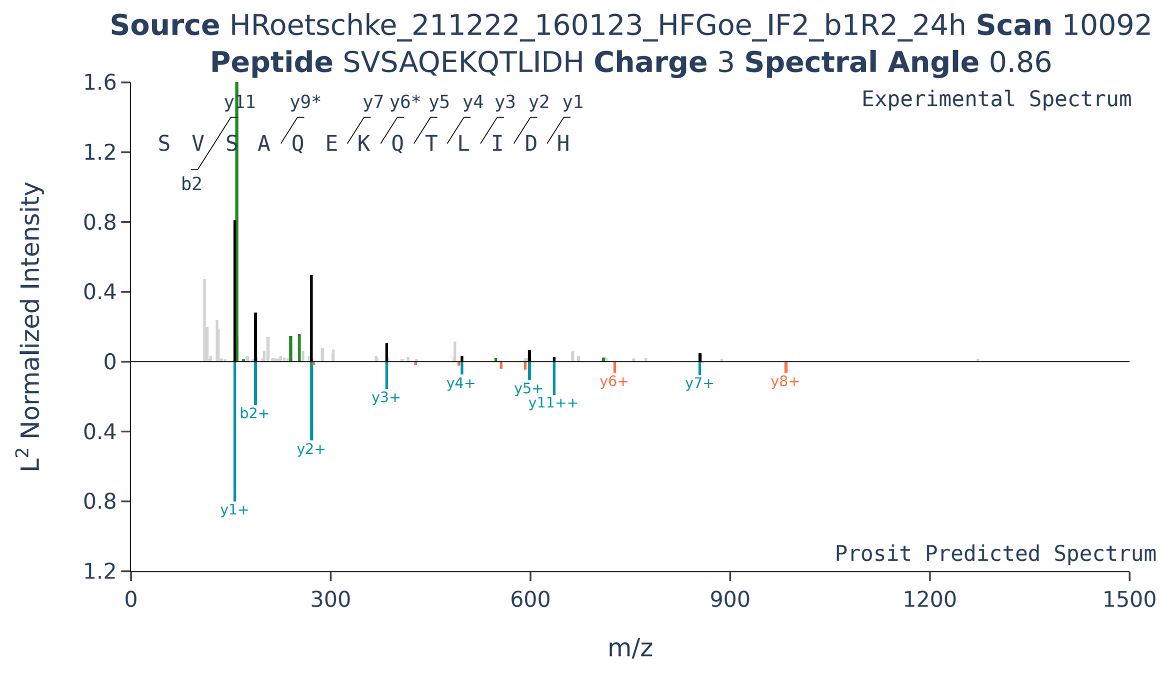
**Source** HRoetschke_211222_160123_HFGoe_IF2_b1R2_24h **Scan** 10092
**Peptide** SVSAQEKQTLIDH **Charge** 3 **Spectral Angle** 0.86
Experimental Spectrum
Prosit Predicted Spectrum
L² Normalized Intensity
m/z

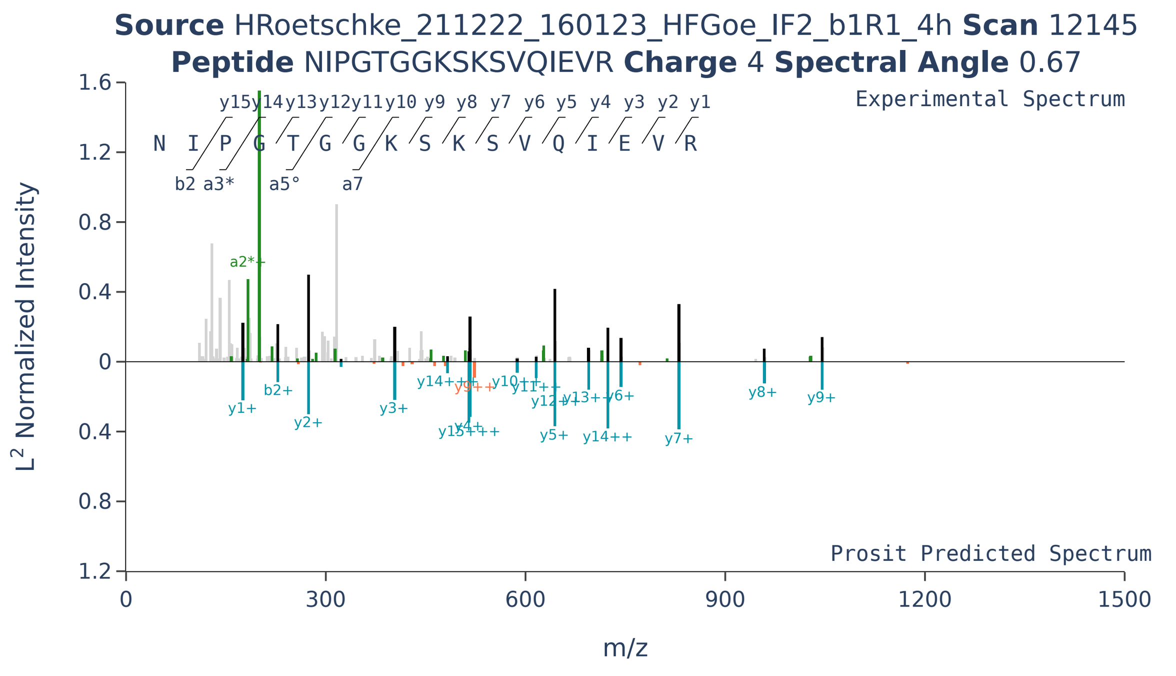
**Source** HRoetschke_211222_160123_HFGoe_IF2_b1R1_4h **Scan** 12145
**Peptide** NIPGTGGKSKSVQIEVR **Charge** 4 **Spectral Angle** 0.67
Experimental Spectrum
Prosit Predicted Spectrum
L² Normalized Intensity
m/z

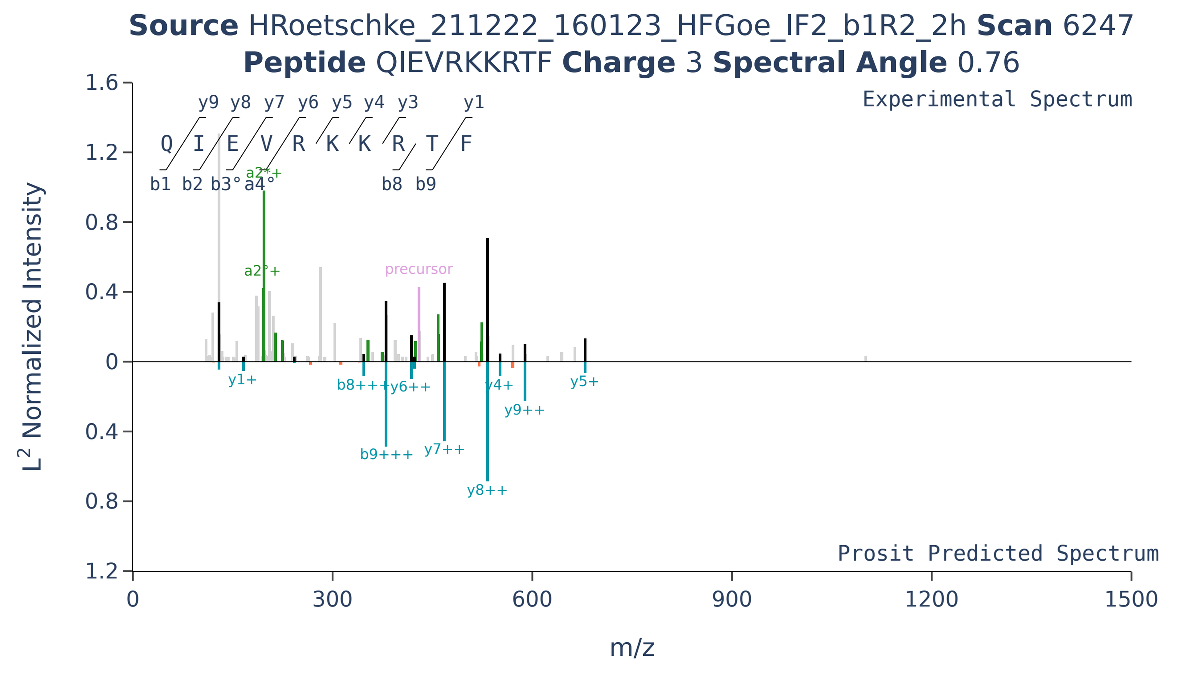
**Source** HRoetschke_211222_160123_HFGoe_IF2_b1R2_2h **Scan** 6247
**Peptide** QIEVRKKRTF **Charge** 3 **Spectral Angle** 0.76
Experimental Spectrum
Prosit Predicted Spectrum
L² Normalized Intensity
m/z

**Source** HRoetschke_211222_160123_HFGoe_IF2_b1R1_4h **Scan** 14652
**Peptide** RENELEEAVMSD **Charge** 2 **Spectral Angle** 0.78

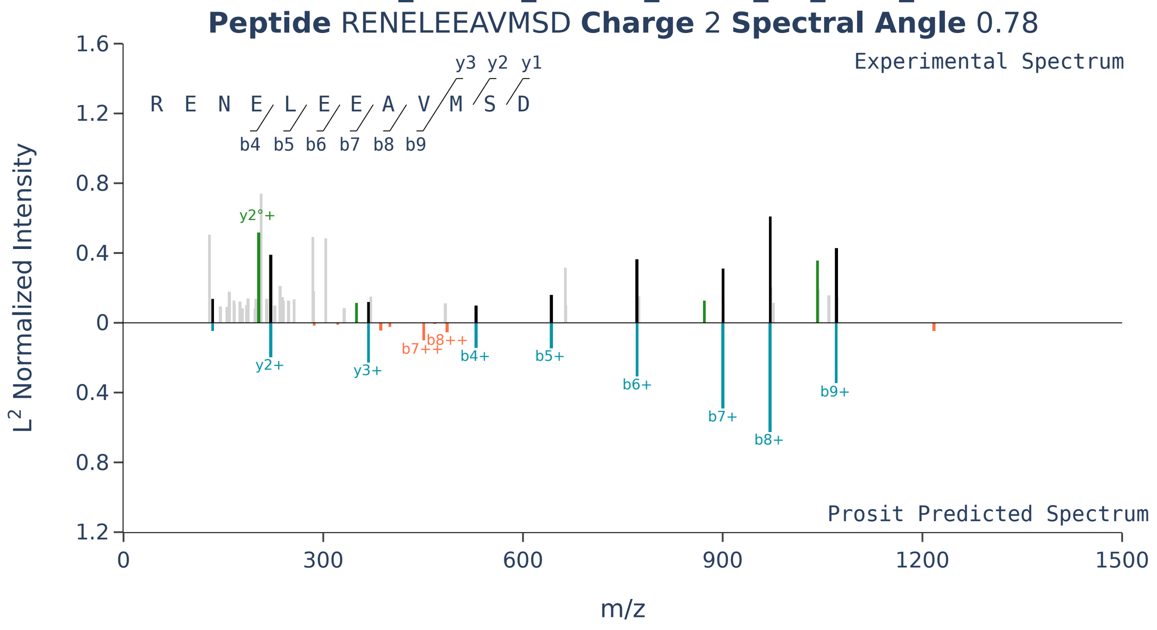


**Source** HRoetschke_211222_160123_HFGoe_IF2_b1R2_24h **Scan** 8587
**Peptide** HIGAYHVE **Charge** 2 **Spectral Angle** 0.85

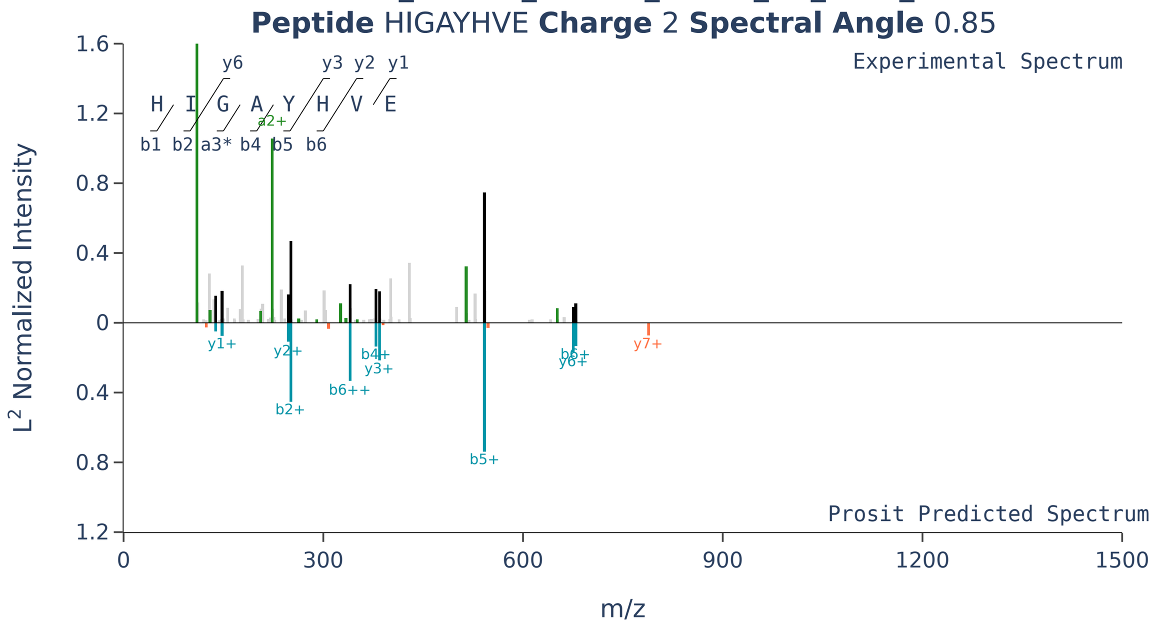


**Source** HRoetschke_211222_160123_HFGoe_IF2_b1R1_4h **Scan** 8123
**Peptide** LAAEEQAQREAE **Charge** 2 **Spectral Angle** 0.8

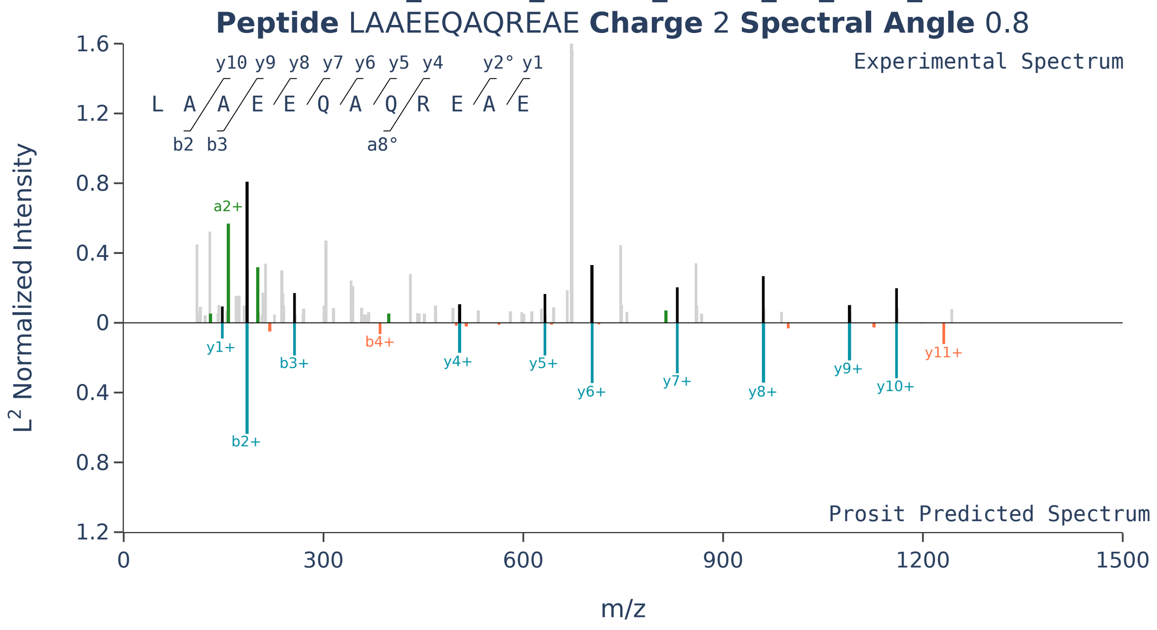


**Source** HRoetschke_211222_160123_HFGoe_IF2_b1R1_4h **Scan** 16150
**Peptide** VTIKTLAA **Charge** 2 **Spectral Angle** 0.73

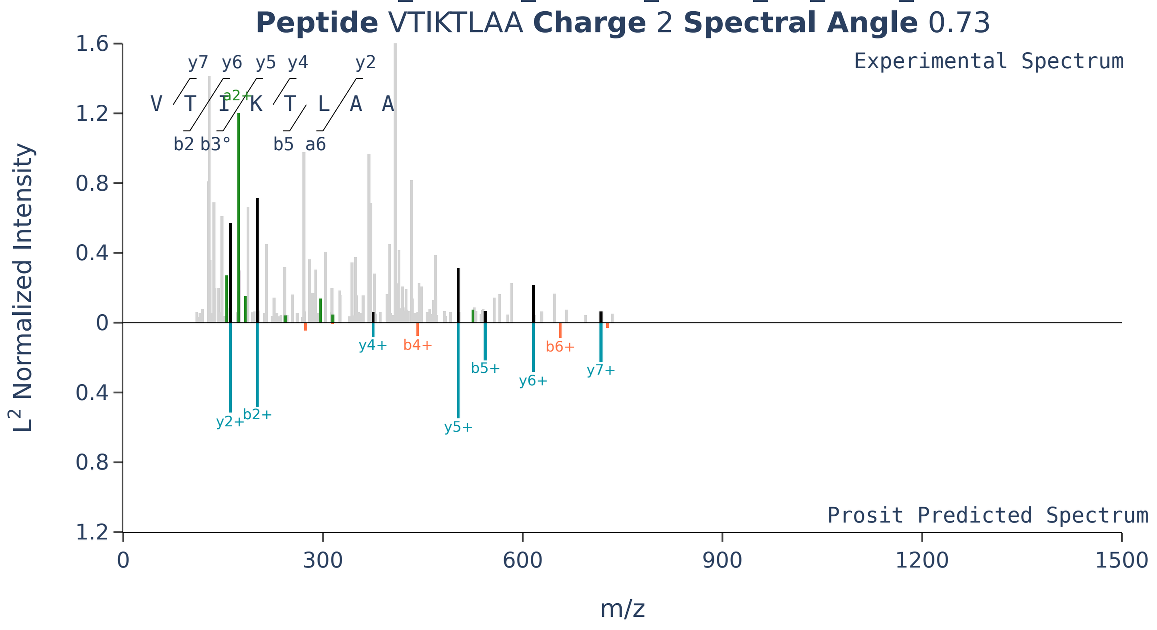


**Source** HRoetschke_211222_160123_HFGoe_IF2_b1R1_4h **Scan** 10022
**Peptide** GAQATDI **Charge** 1 **Spectral Angle** 0.88

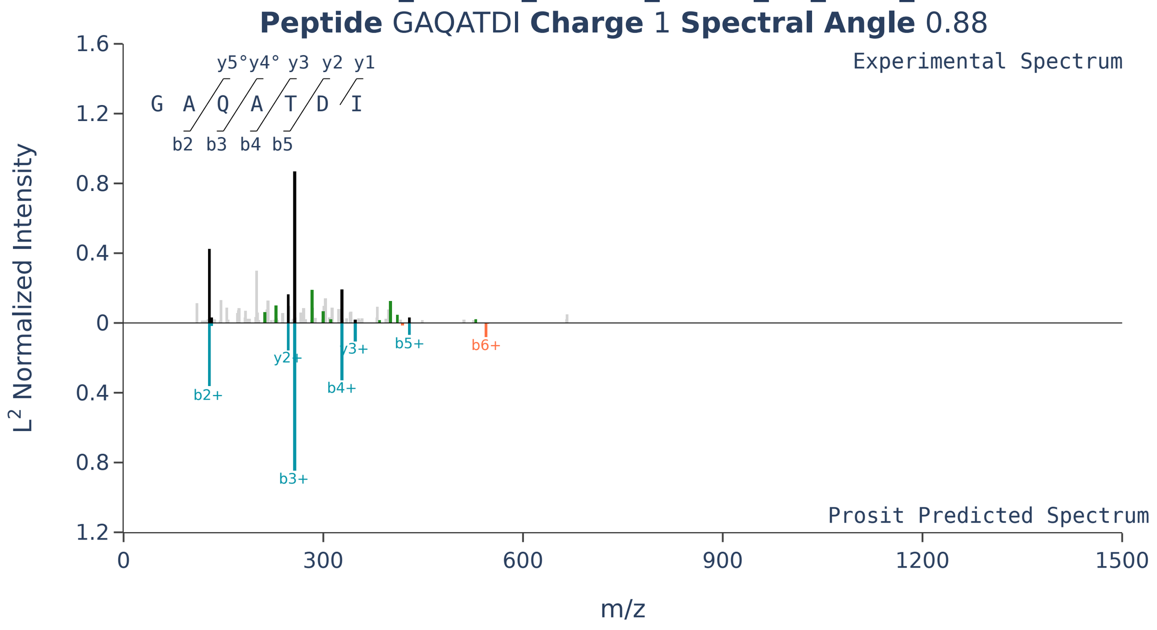


**Source** HRoetschke_211222_160123_HFGoe_IF2_b1R2_24h **Scan** 11529
**Peptide** AEEMGHKVIL **Charge** 2 **Spectral Angle** 0.71

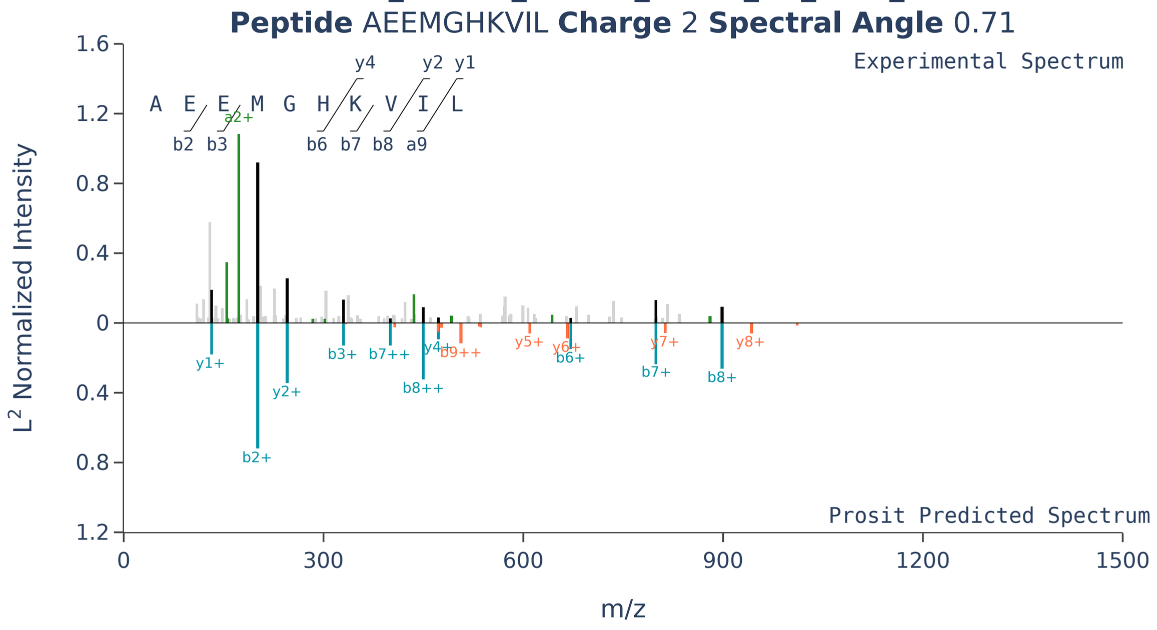


**Source** HRoetschke_211222_160123_HFGoe_IF2_b1R1_2h **Scan** 9771
**Peptide** HLGAYHVET **Charge** 2 **Spectral Angle** 0.83

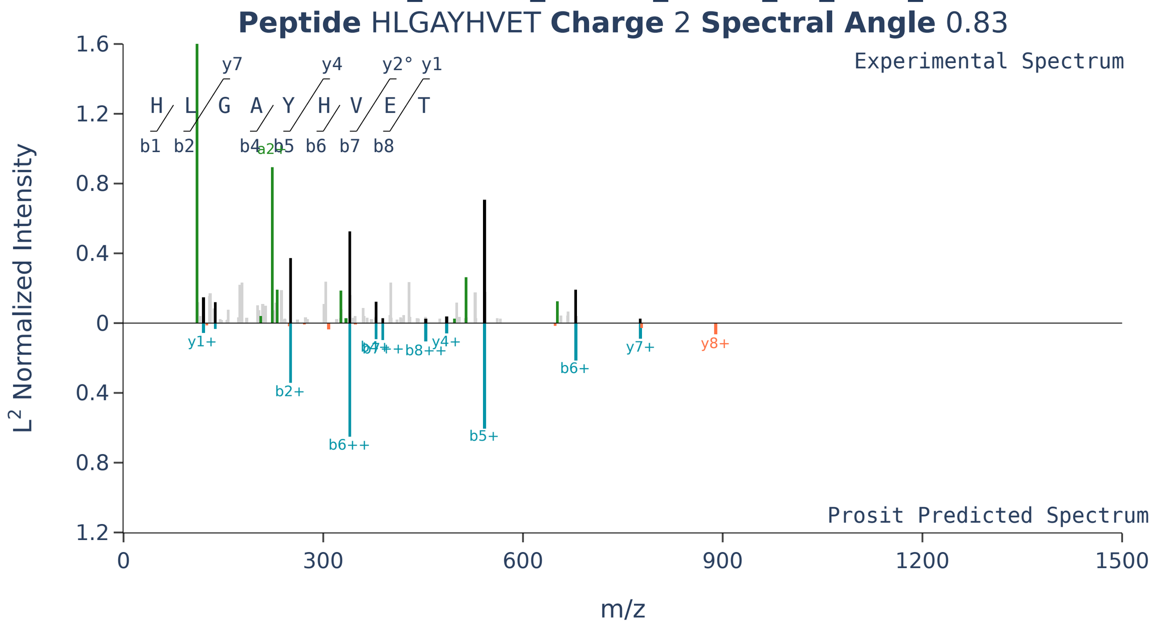


**Source** HRoetschke_211222_160123_HFGoe_IF2_b1R2_4h **Scan** 6446
**Peptide** IDKPEADPDRVKN **Charge** 4 **Spectral Angle** 0.78

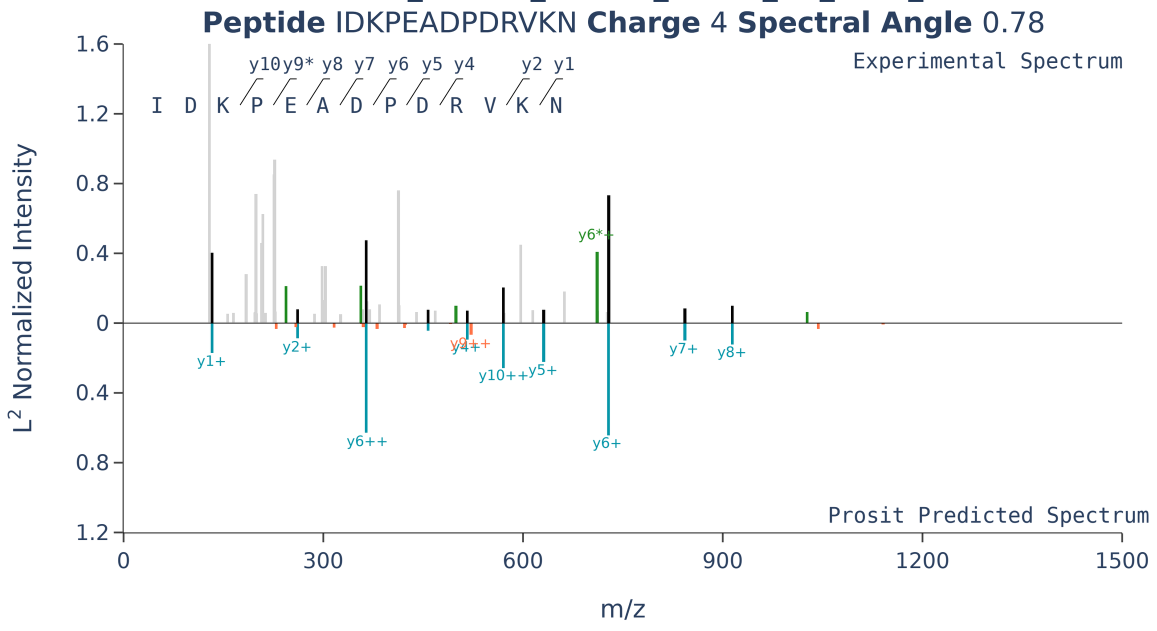


**Source** HRoetschke_211222_160123_HFGoe_IF2_b1R1_4h **Scan** 8626
**Peptide** YIRSTKVASGEAGGITQH **Charge** 3 **Spectral Angle** 0.84

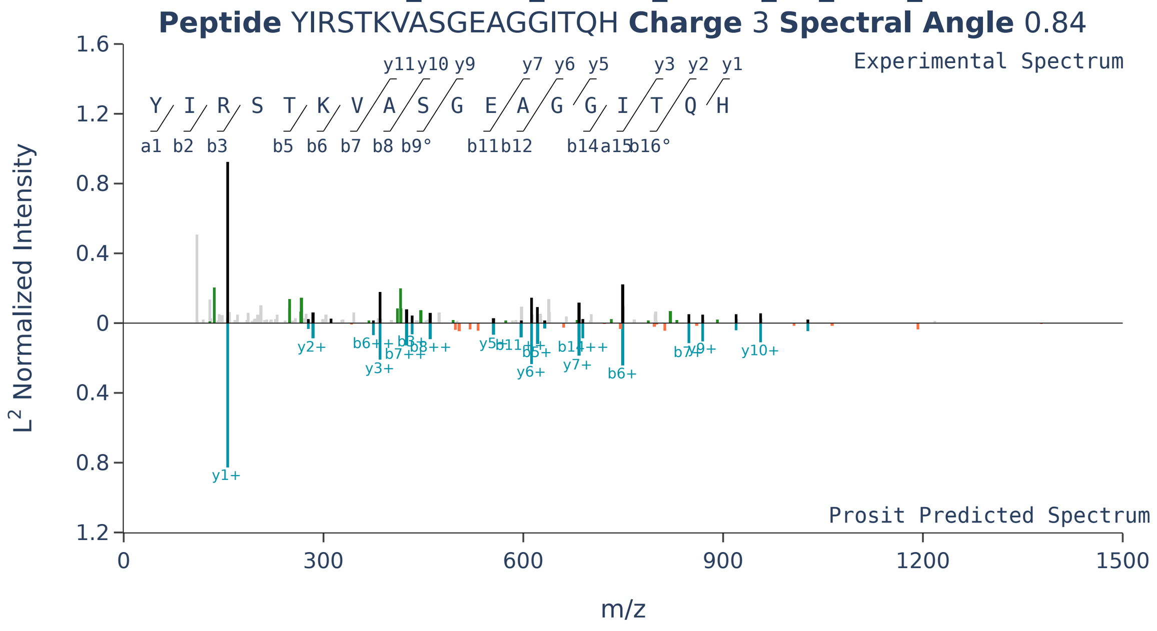


**Source** HRoetschke_211222_160123_HFGoe_IF2_b1R1_4h **Scan** 5712
**Peptide** TLQRKTRSTL **Charge** 3 **Spectral Angle** 0.86

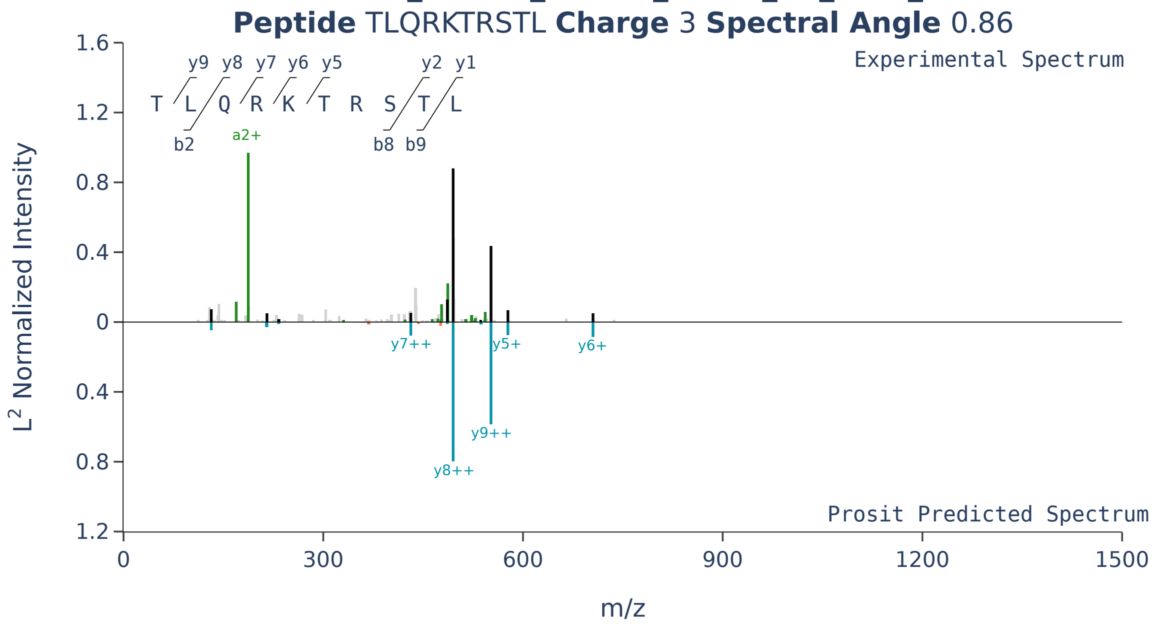


**Source** HRoetschke_211222_160123_HFGoe_IF2_b1R1_24h **Scan** 9183
**Peptide** VAVNKID **Charge** 2 **Spectral Angle** 0.79

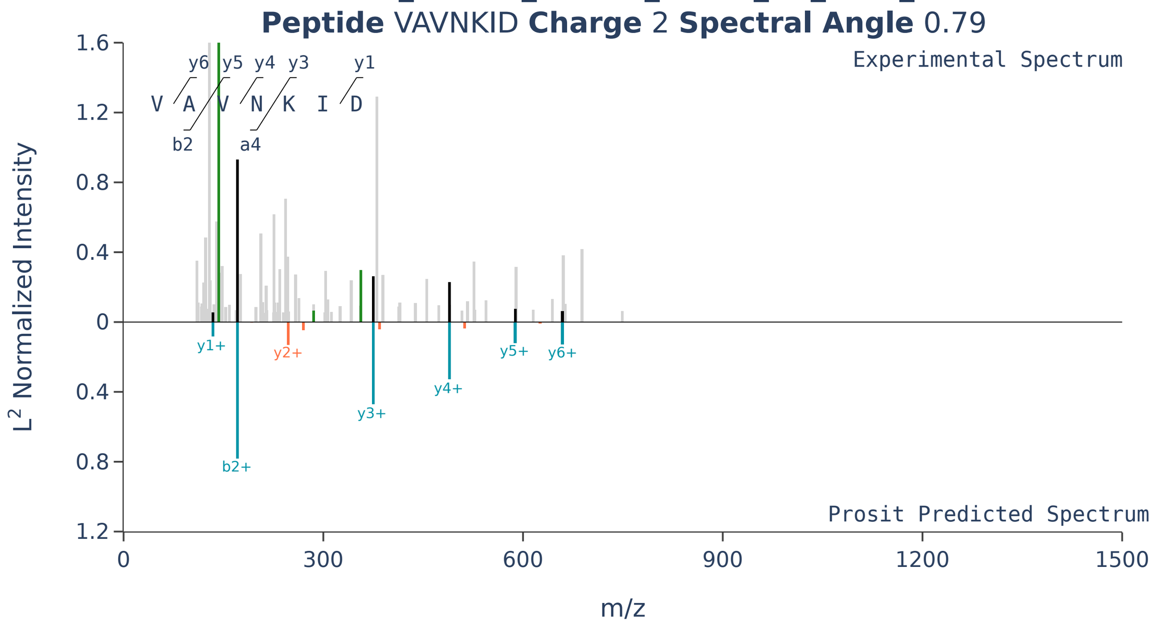


**Source** HRoetschke_211222_160123_HFGoe_IF2_b1R1_4h **Scan** 12833
**Peptide** VTIKTLAAERQTSVER **Charge** 4 **Spectral Angle** 0.82

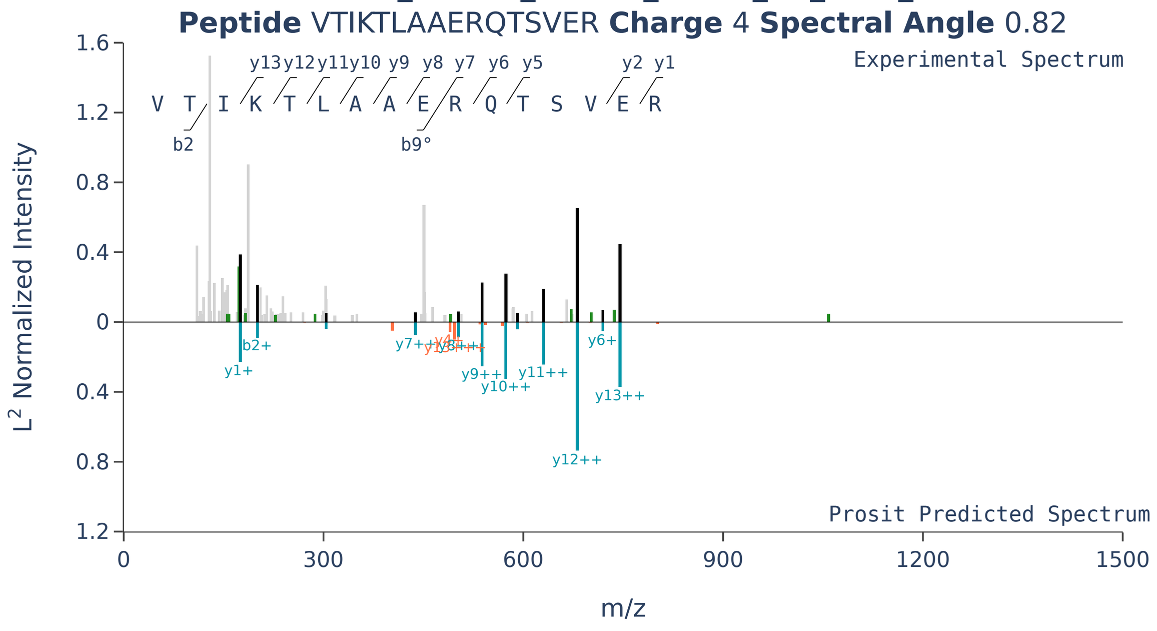


**Source** HRoetschke_211222_160123_HFGoe_IF2_b2R2_24h **Scan** 8586
**Peptide** KVASGEAGGI **Charge** 2 **Spectral Angle** 0.81

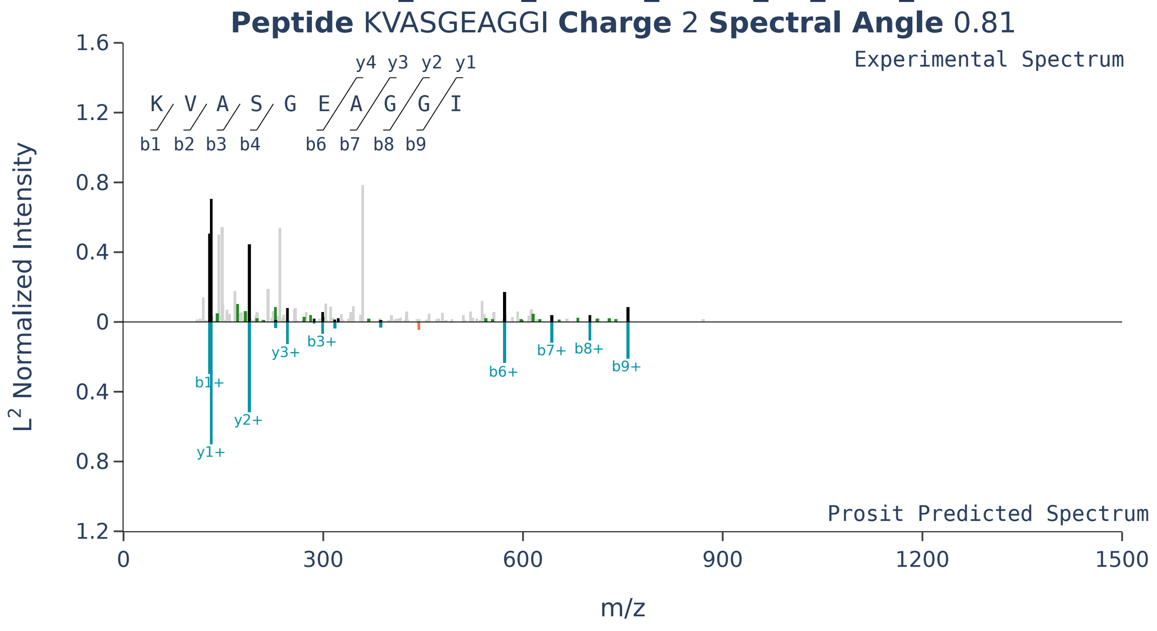


**Source** HRoetschke_211222_160123_HFGoe_IF2_b1R1_4h **Scan** 9086
**Peptide** FVKRDPQEAERL **Charge** 4 **Spectral Angle** 0.79

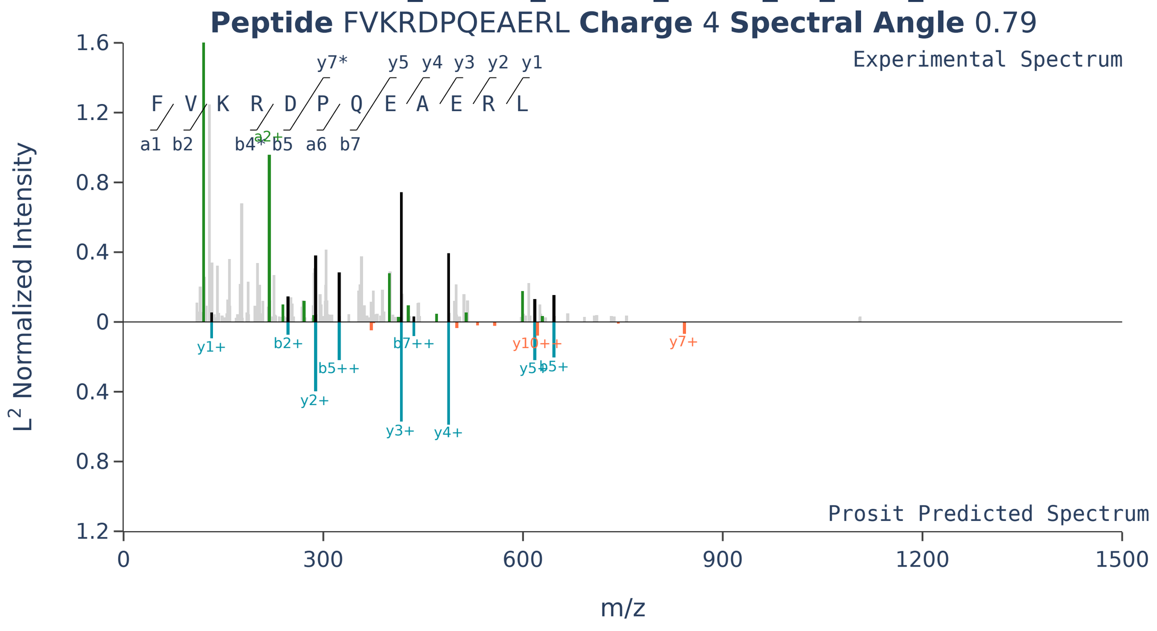


**Source** HRoetschke_211222_160123_HFGoe_IF2_b1R1_4h **Scan** 16862
**Peptide** ASGEAGGITQHIGAY **Charge** 2 **Spectral Angle** 0.81

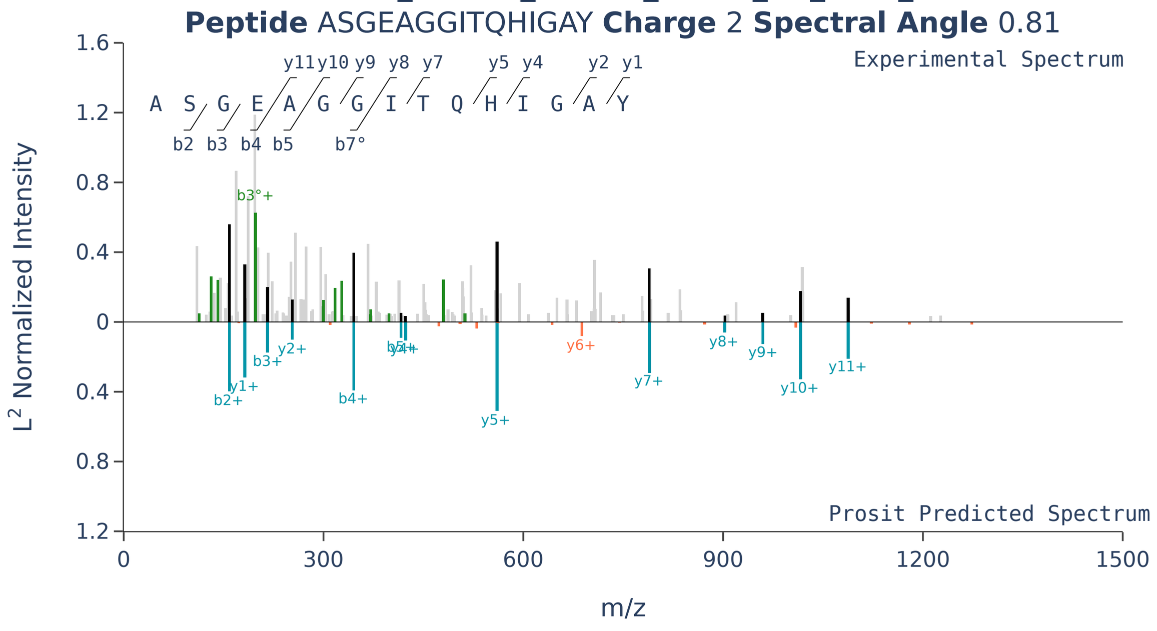

**Source** HRoetschke_211222_160123_HFGoe_IF2_b1R1_4h **Scan** 9345
**Peptide** TVLVREGTLHK **Charge** 3 **Spectral Angle** 0.79

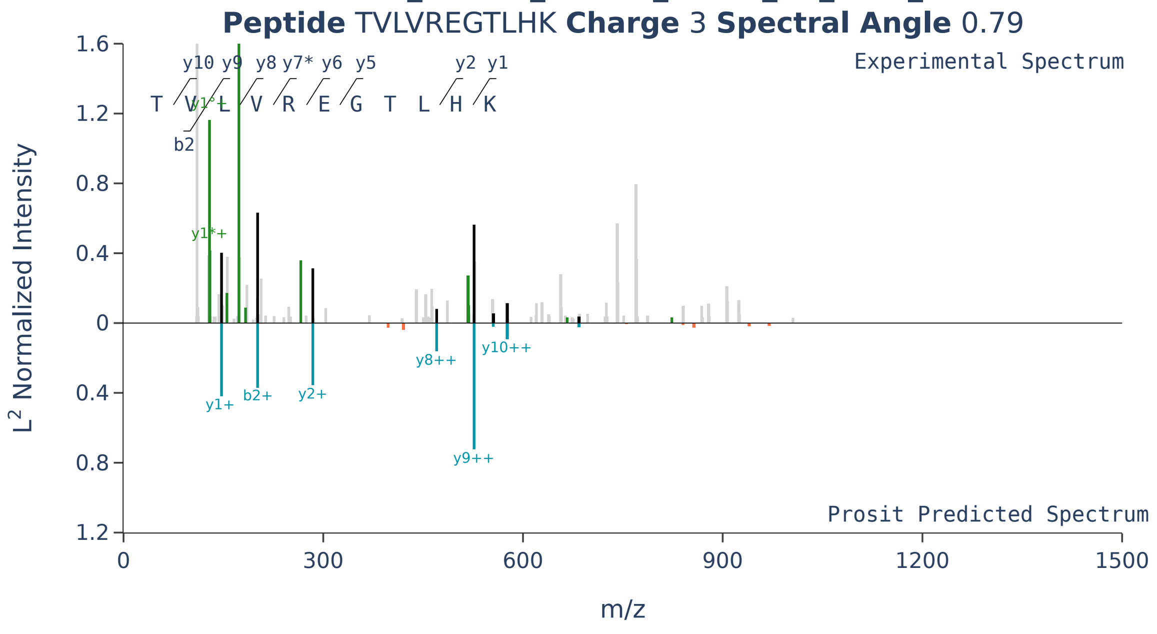


**Source** HRoetschke_211222_160123_HFGoe_IF2_b1R1_4h **Scan** 17047
**Peptide** RGPVATVL **Charge** 2 **Spectral Angle** 0.77

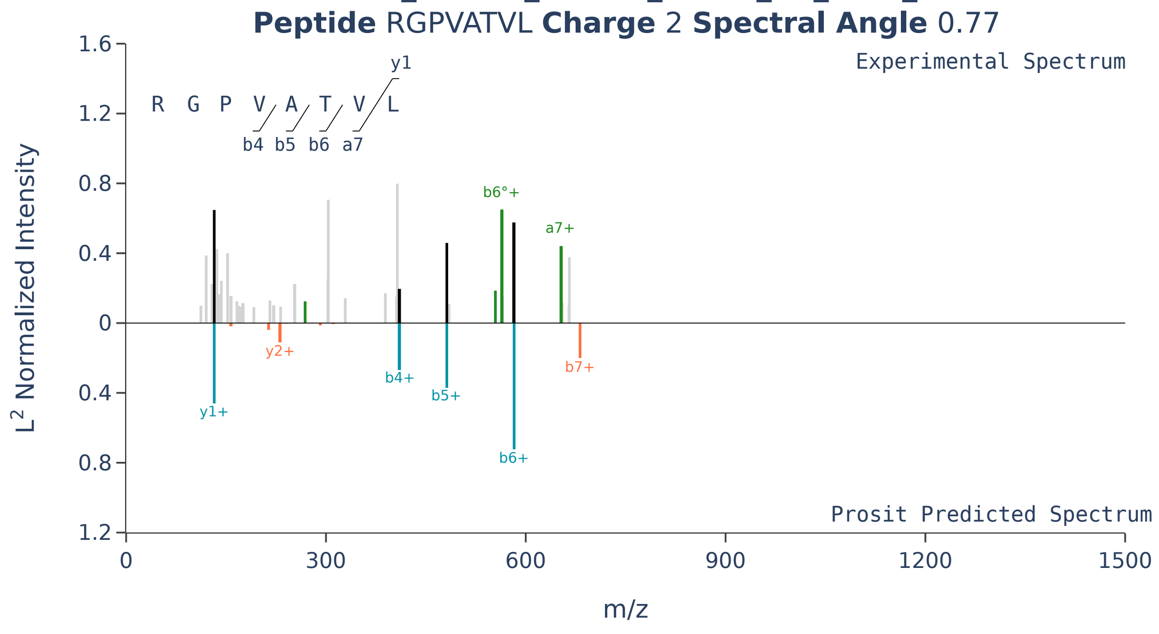


**Source** HRoetschke_211222_160123_HFGoe_IF2_b1R2_4h **Scan** 6308
**Peptide** ELKAVRKGMASG **Charge** 2 **Spectral Angle** 0.85

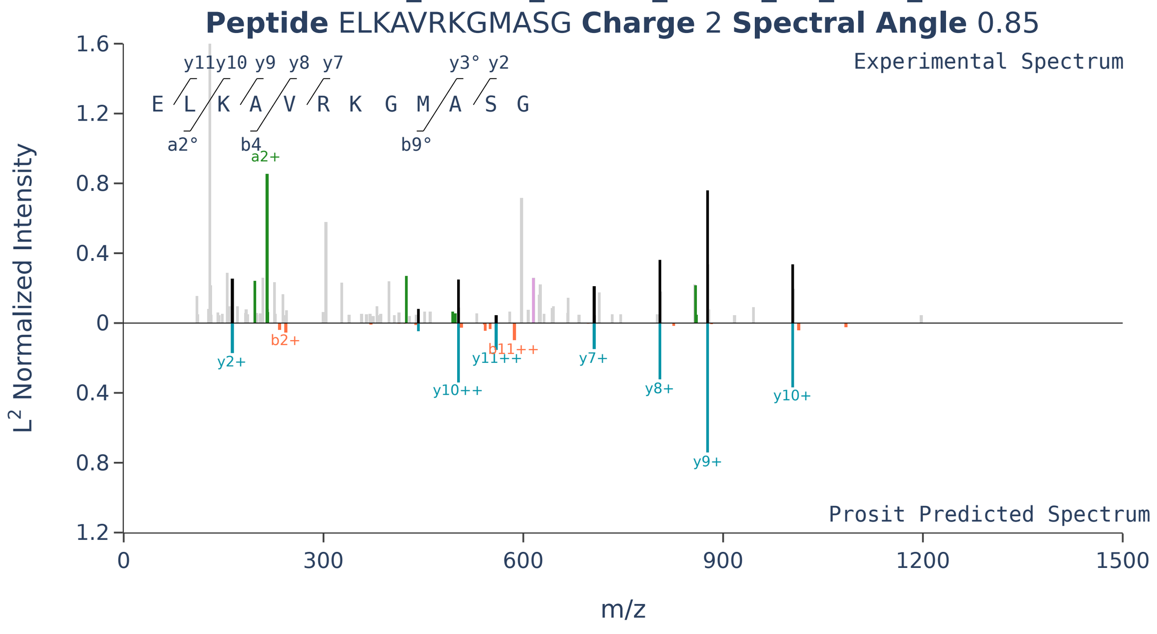


**Source** HRoetschke_211222_160123_HFGoe_IF2_b1R1_2h **Scan** 11944
**Peptide** GESQFVHVSAKA **Charge** 3 **Spectral Angle** 0.79

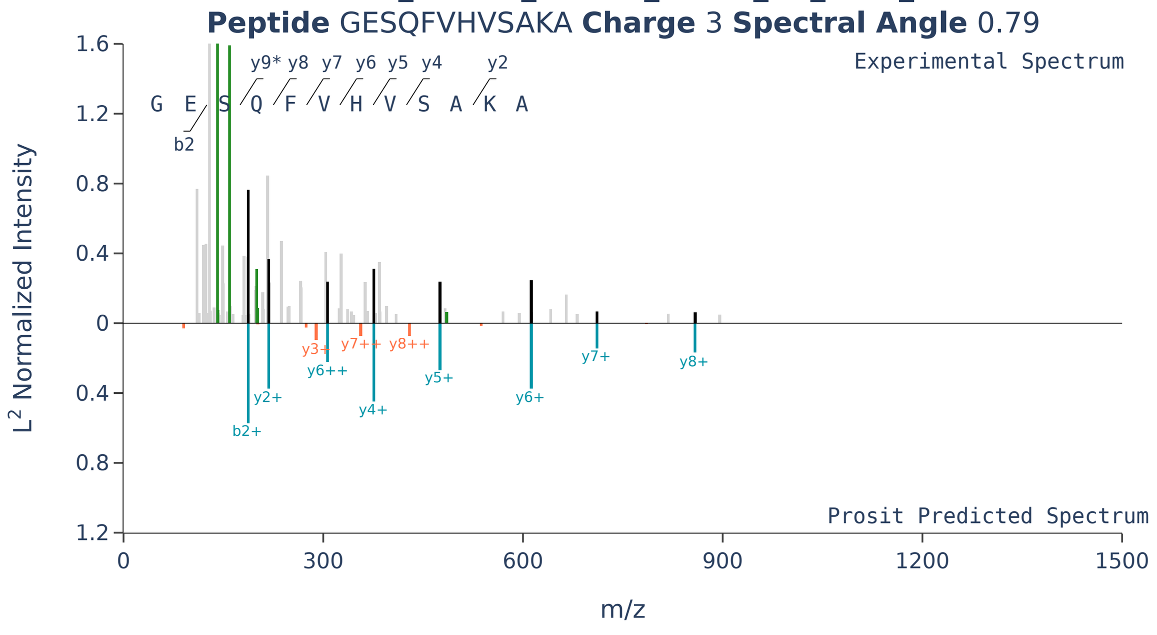


**Source** HRoetschke_211222_160123_HFGoe_IF2_b1R1_2h **Scan** 6430
**Peptide** RLAAEEQAQ **Charge** 2 **Spectral Angle** 0.79

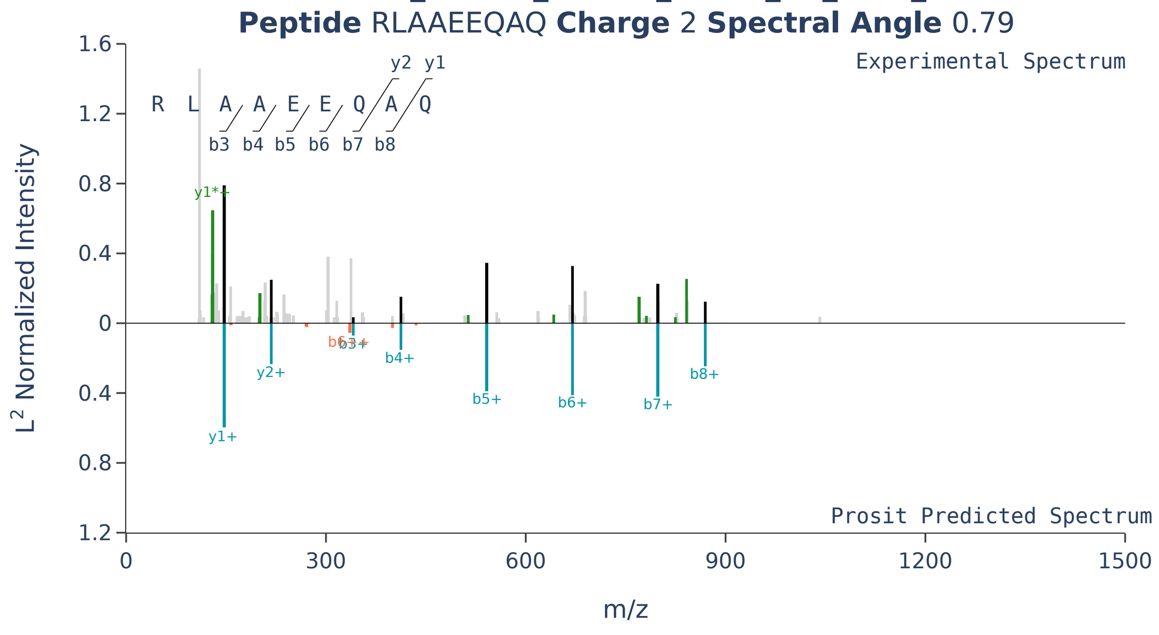


**Source** HRoetschke_211222_160123_HFGoe_IF2_b1R1_24h **Scan** 7809
**Peptide** GHKVILRRENEL **Charge** 4 **Spectral Angle** 0.8

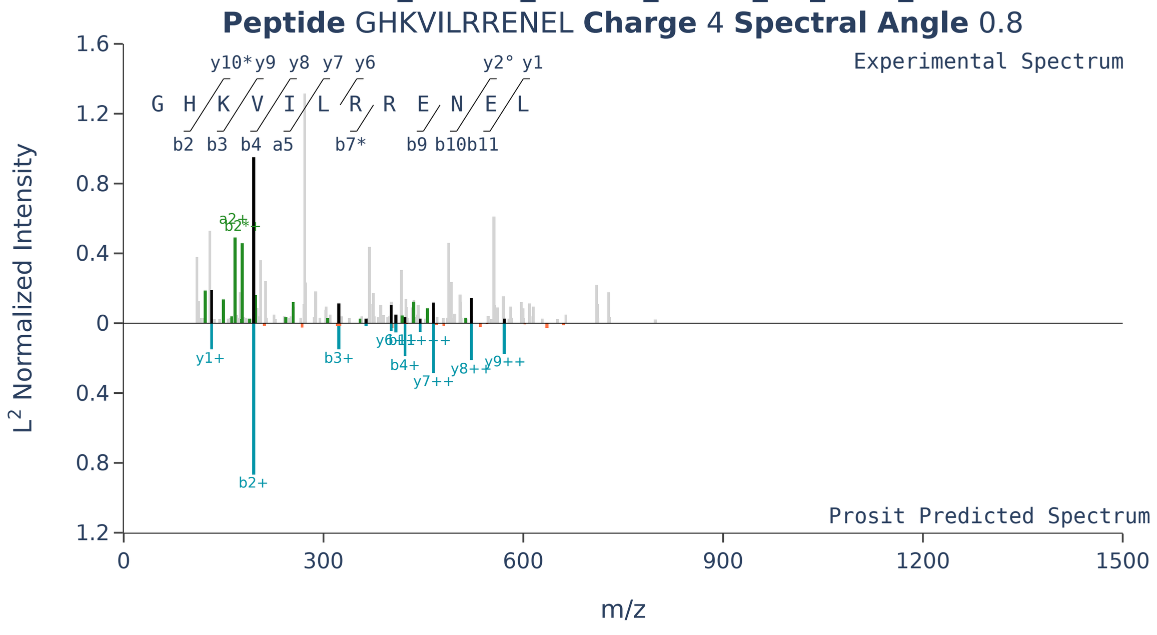


**Source** HRoetschke_211222_160123_HFGoe_IF2_b2R2_1h **Scan** 8501
**Peptide** HLGAYHVETEN **Charge** 2 **Spectral Angle** 0.8

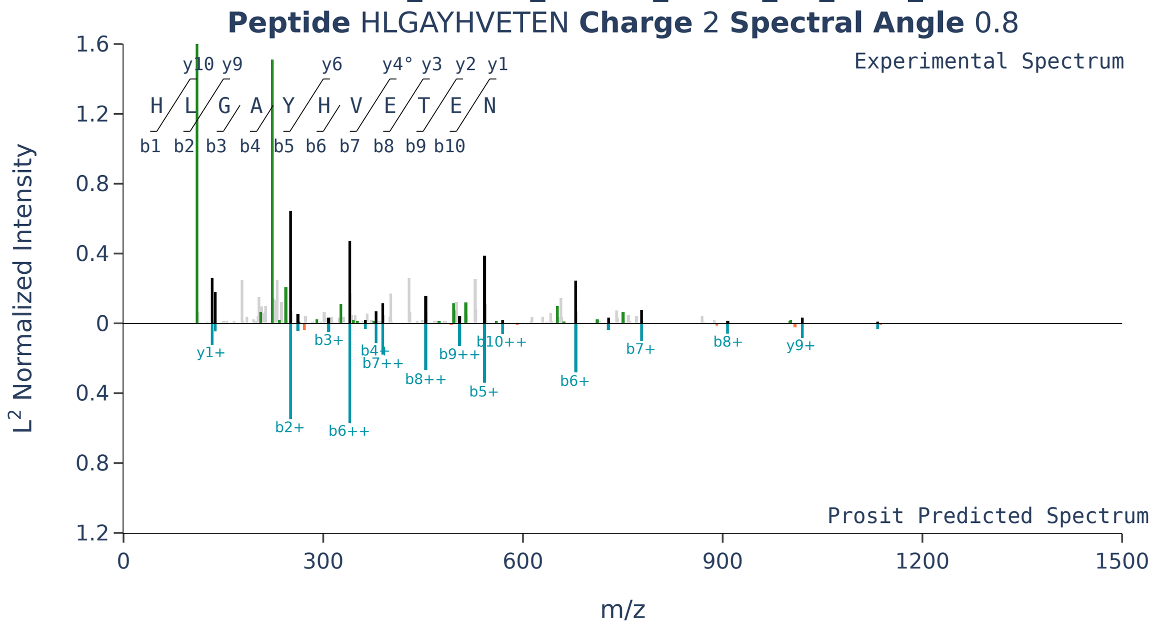


**Source** HRoetschke_211222_160123_HFGoe_IF2_b1R2_24h **Scan** 11684
**Peptide** DKPEADPDRVKNEL **Charge** 2 **Spectral Angle** 0.86

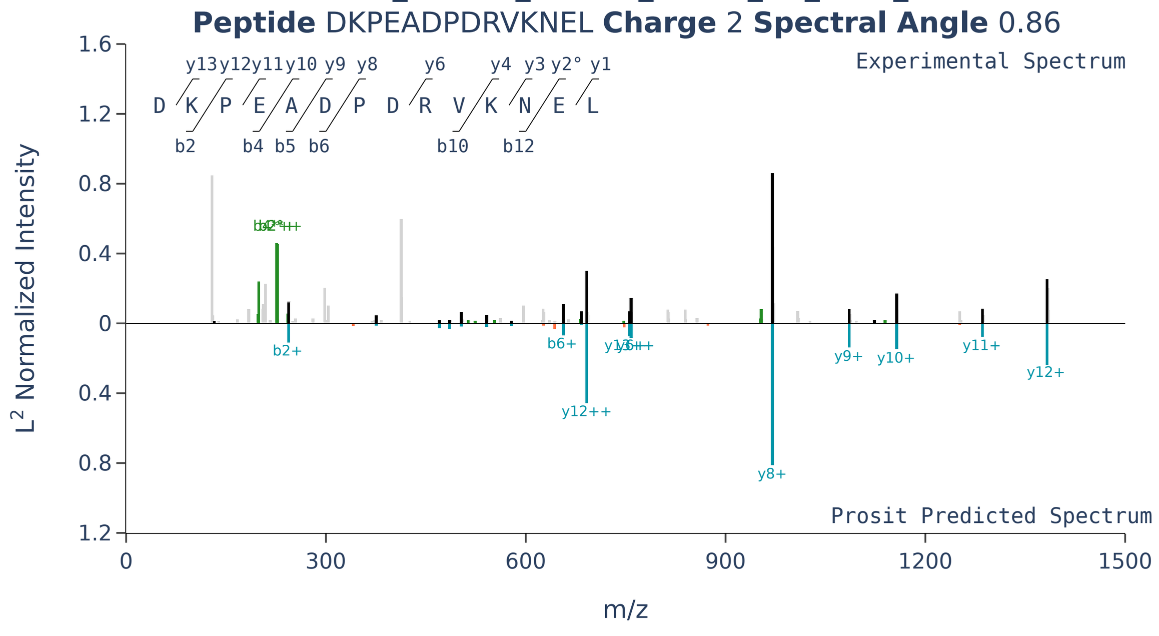


**Source** HRoetschke_211222_160123_HFGoe_IF2_b1R2_24h **Scan** 18851
**Peptide** TITVGELAN **Charge** 1 **Spectral Angle** 0.78

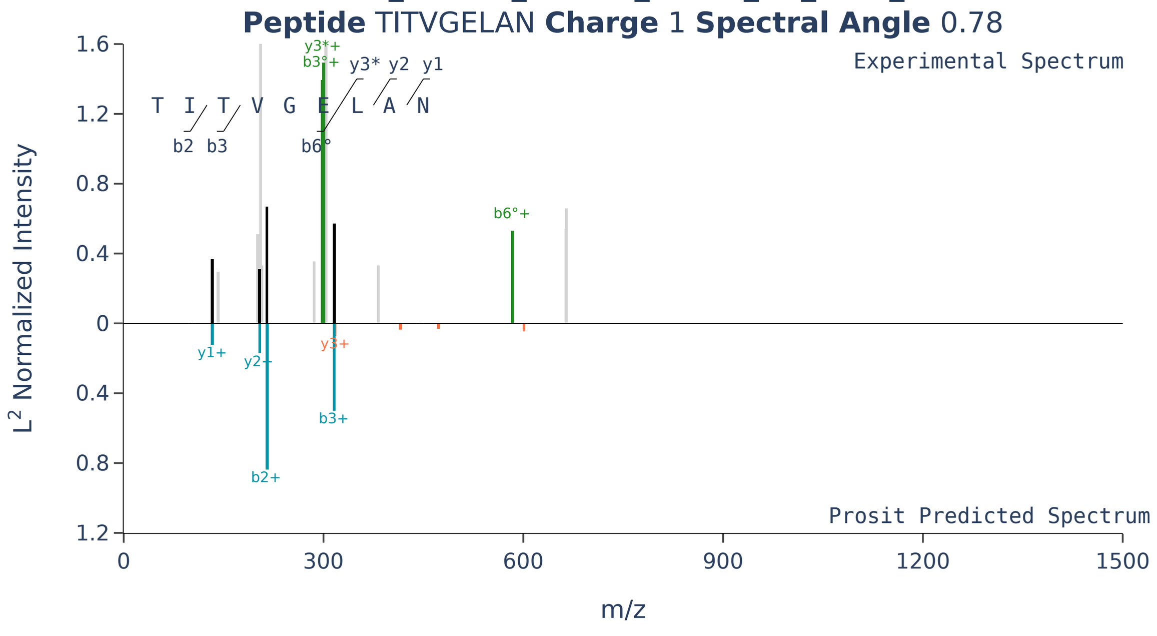


**Source** HRoetschke_211222_160123_HFGoe_IF2_b1R1_4h **Scan** 5930
**Peptide** SVQIEVRKKR **Charge** 3 **Spectral Angle** 0.82

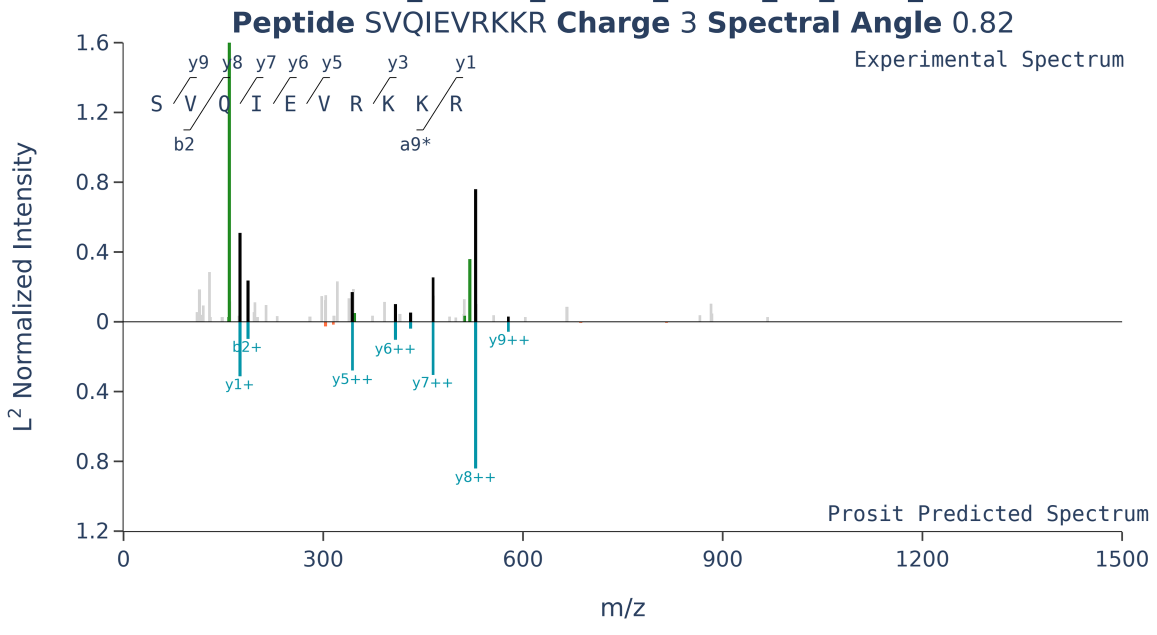


**Source** HRoetschke_211222_160123_HFGoe_IF2_b1R2_2h **Scan** 7007
**Peptide** FQKPAQAVNR **Charge** 2 **Spectral Angle** 0.87

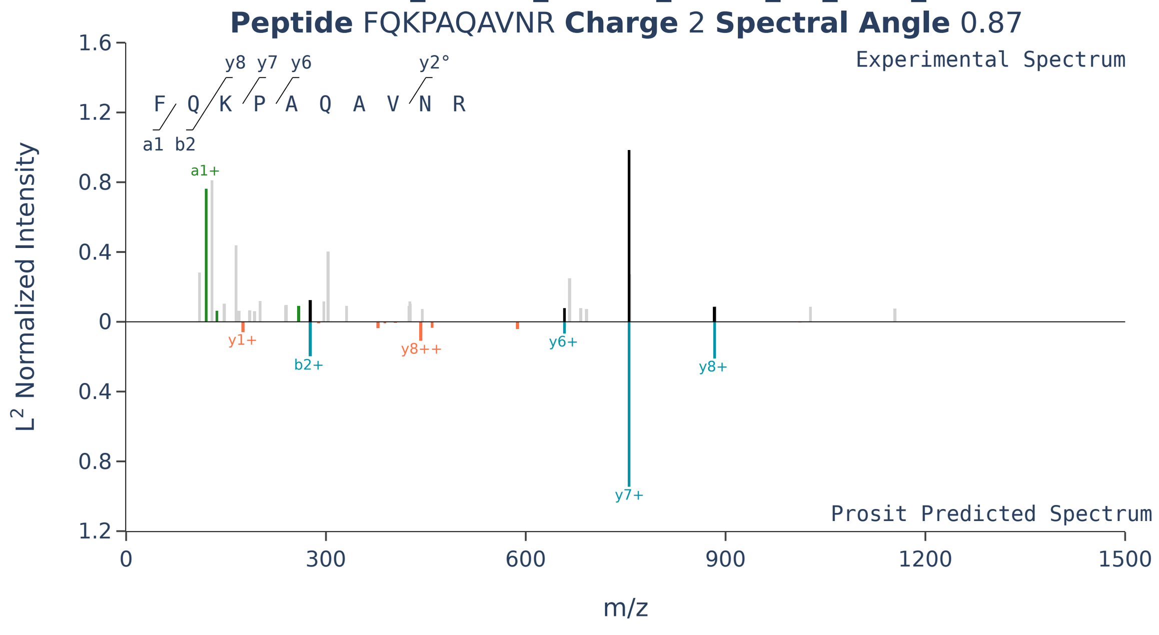


**Source** HRoetschke_211222_160123_HFGoe_IF2_b2R1_24h **Scan** 7333
**Peptide** GAYHVETENG **Charge** 2 **Spectral Angle** 0.8

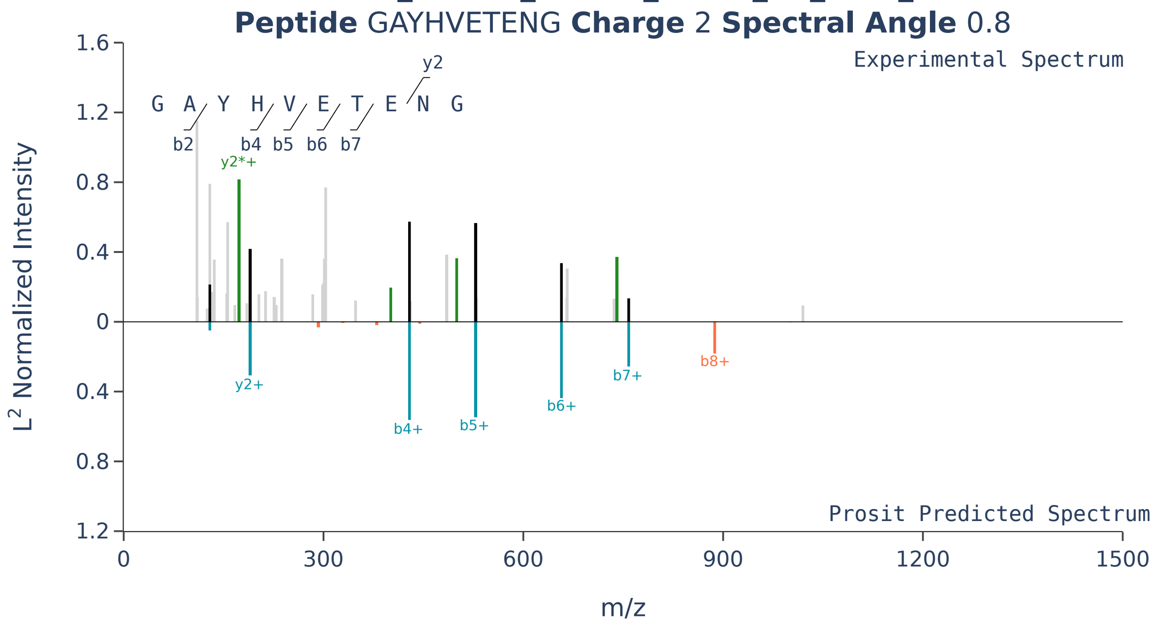


**Source** HRoetschke_211222_160123_HFGoe_IF2_b1R1_4h **Scan** 7423
**Peptide** DEVTVVRDEKKA **Charge** 3 **Spectral Angle** 0.76

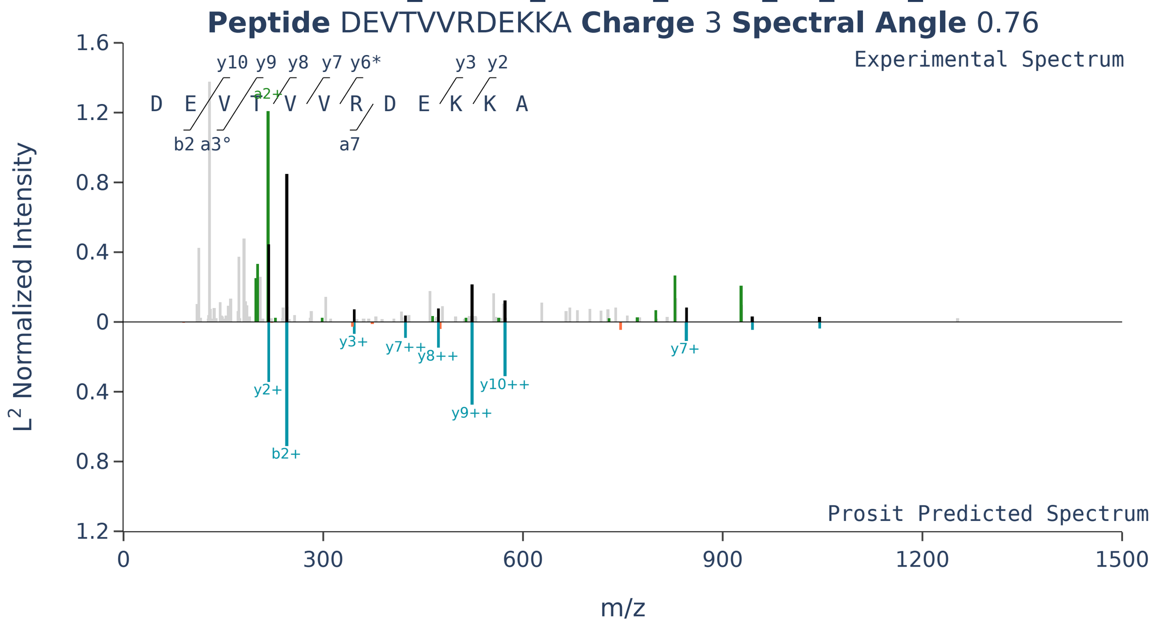


**Source** HRoetschke_211222_160123_HFGoe_IF2_b1R1_2h **Scan** 7808
**Peptide** AVMSDRDTGAAAE **Charge** 2 **Spectral Angle** 0.73

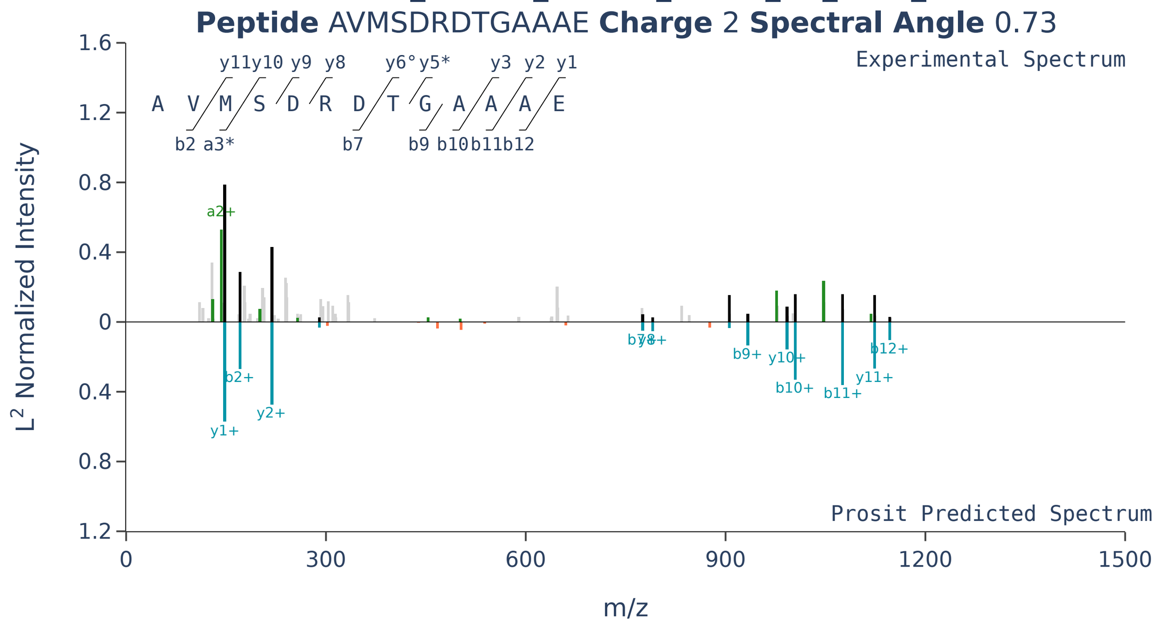


**Source** HRoetschke_211222_160123_HFGoe_IF2_b1R2_24h **Scan** 20510
**Peptide** AQVPVVV **Charge** 1 **Spectral Angle** 0.9

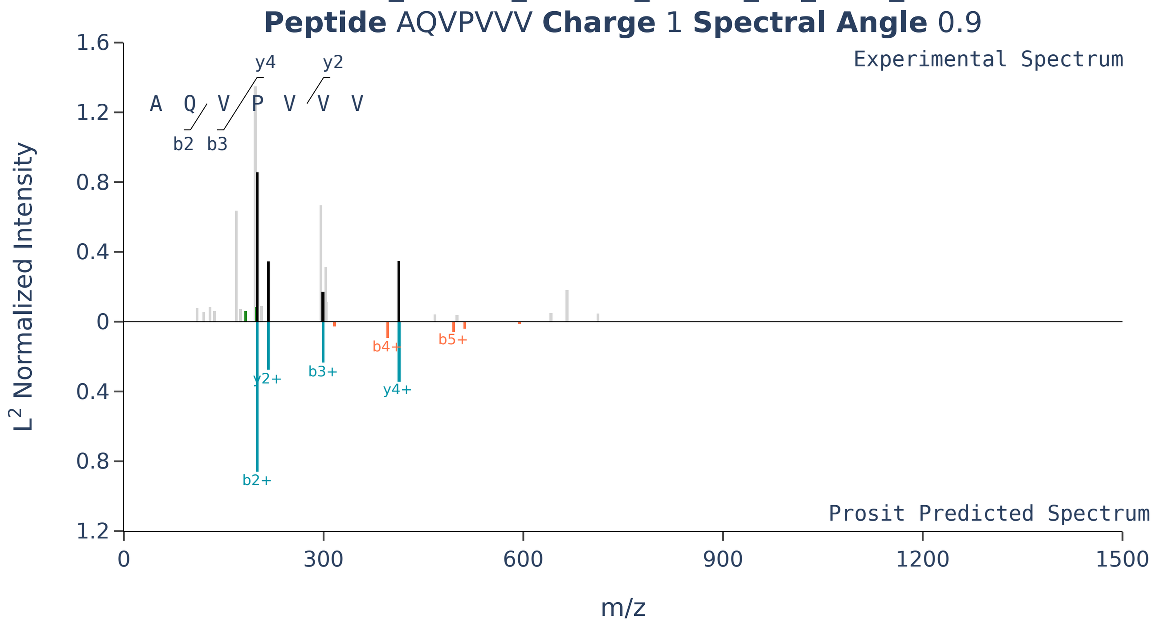

**Source** HRoetschke_211222_160123_HFGoe_IF2_b1R2_24h **Scan** 13546
**Peptide** DHLNQKNSGPDKLTL **Charge** 4 **Spectral Angle** 0.89

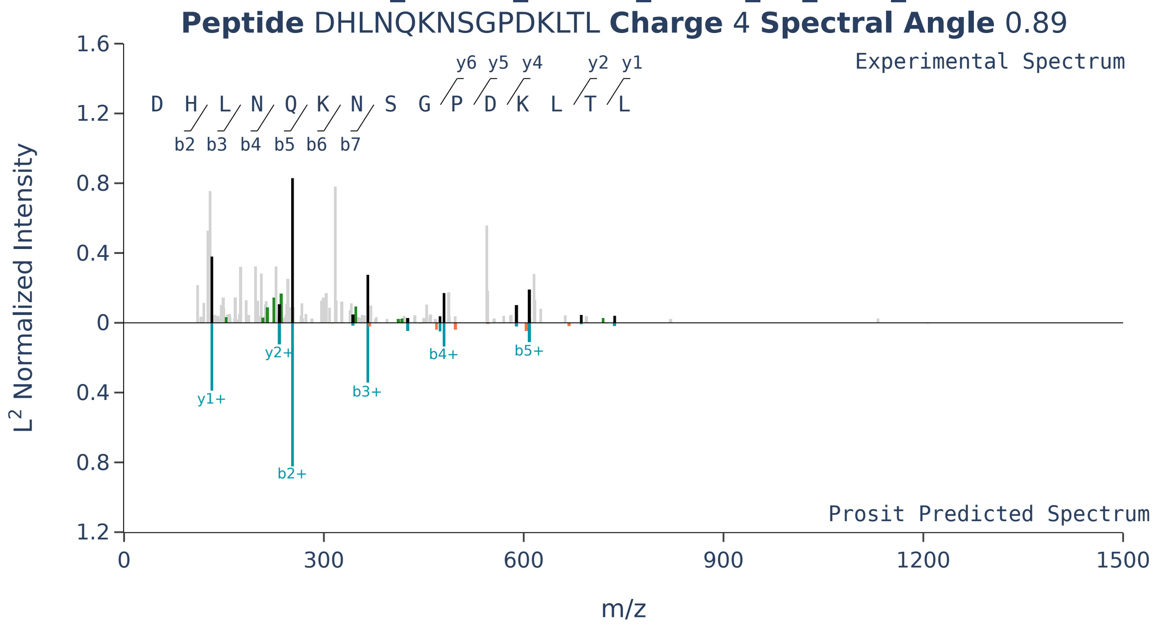


**Source** HRoetschke_211222_160123_HFGoe_IF2_b1R2_4h **Scan** 12186
**Peptide** AAEPRAPVVT **Charge** 2 **Spectral Angle** 0.76

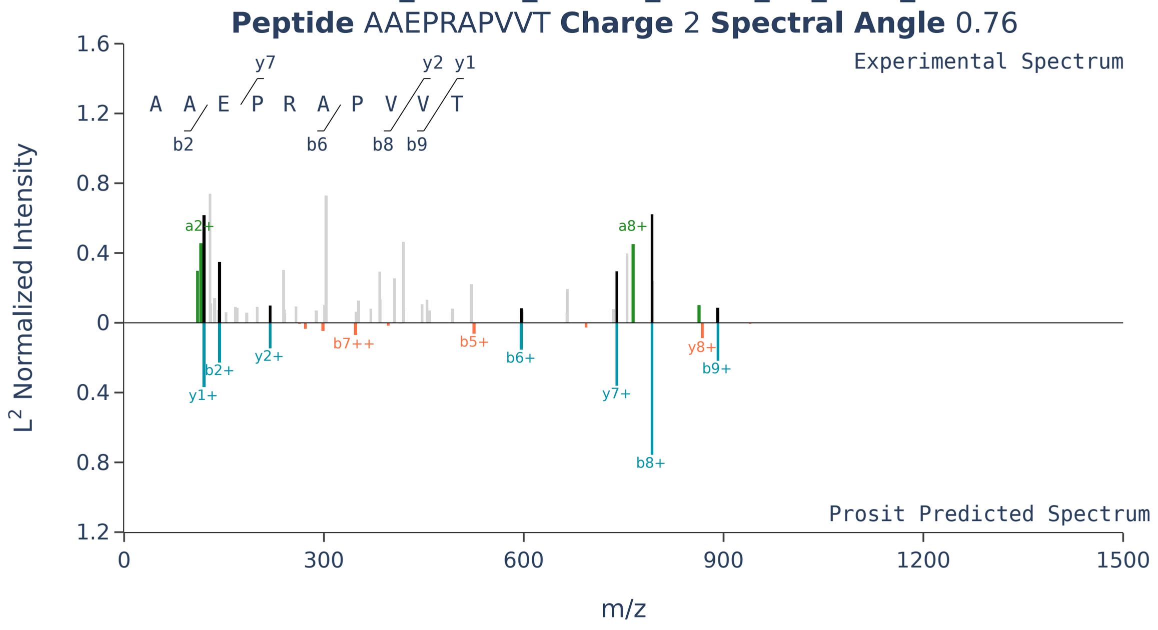


**Source** HRoetschke_211222_160123_HFGoe_IF2_b1R1_2h **Scan** 6876
**Peptide** HLNQKNSGPDKLTLQRKT **Charge** 5 **Spectral Angle** 0.85

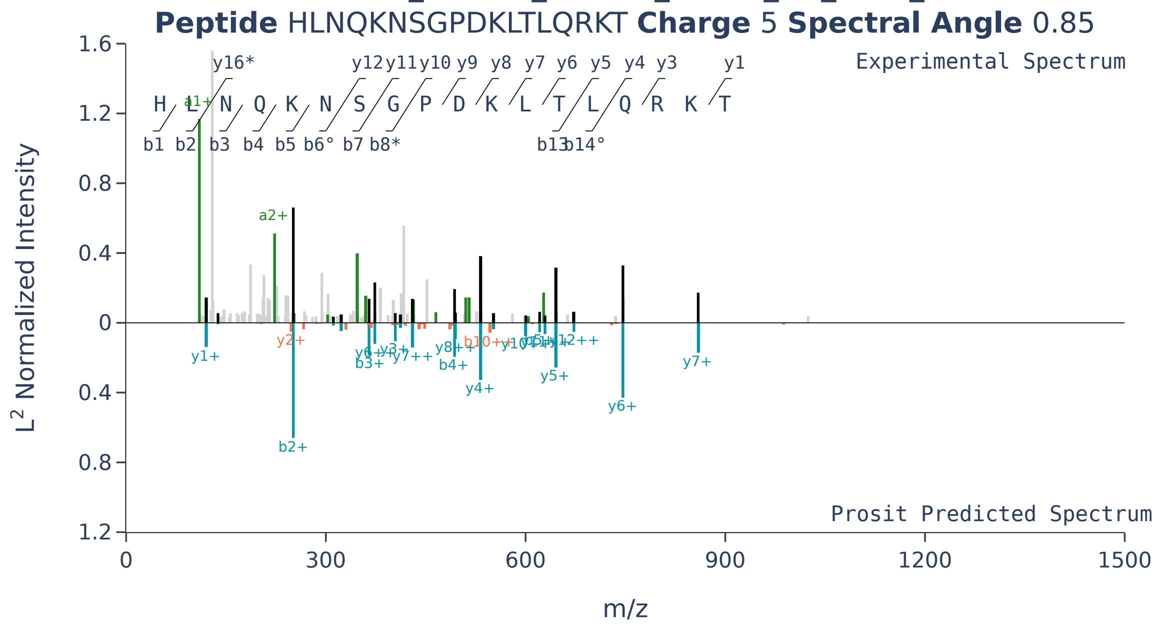


**Source** HRoetschke_211222_160123_HFGoe_IF2_b1R1_4h **Scan** 8294
**Peptide** QRKTRSTLNI **Charge** 3 **Spectral Angle** 0.78

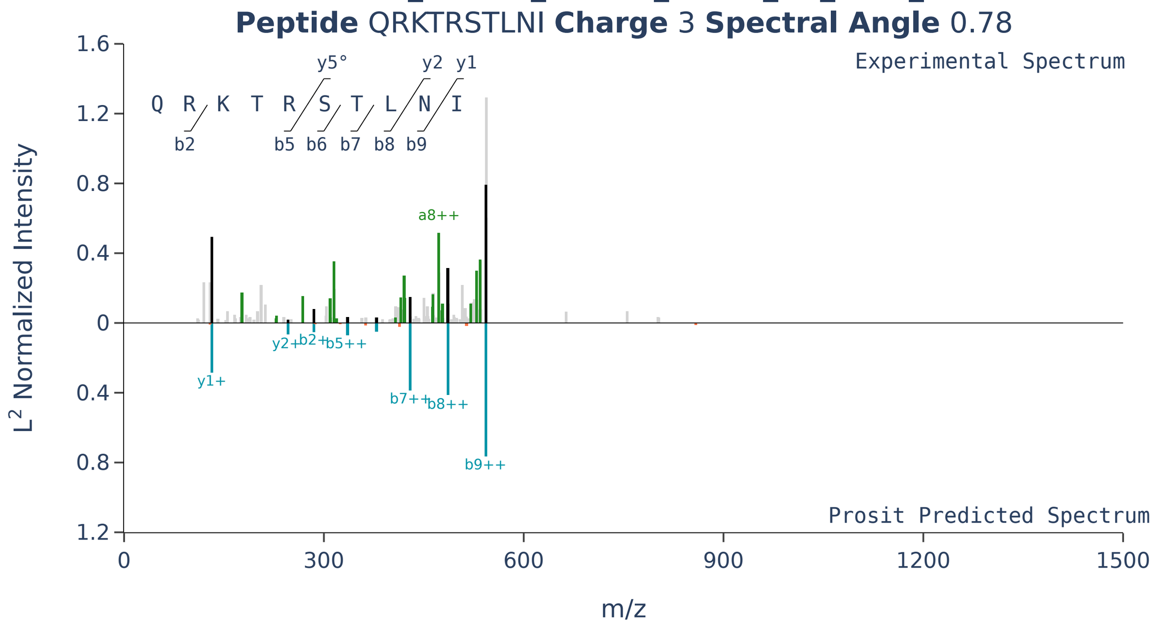


**Source** HRoetschke_211222_160123_HFGoe_IF2_b2R2_24h **Scan** 12586
**Peptide** NLPGTGGKSKSVQI **Charge** 2 **Spectral Angle** 0.72

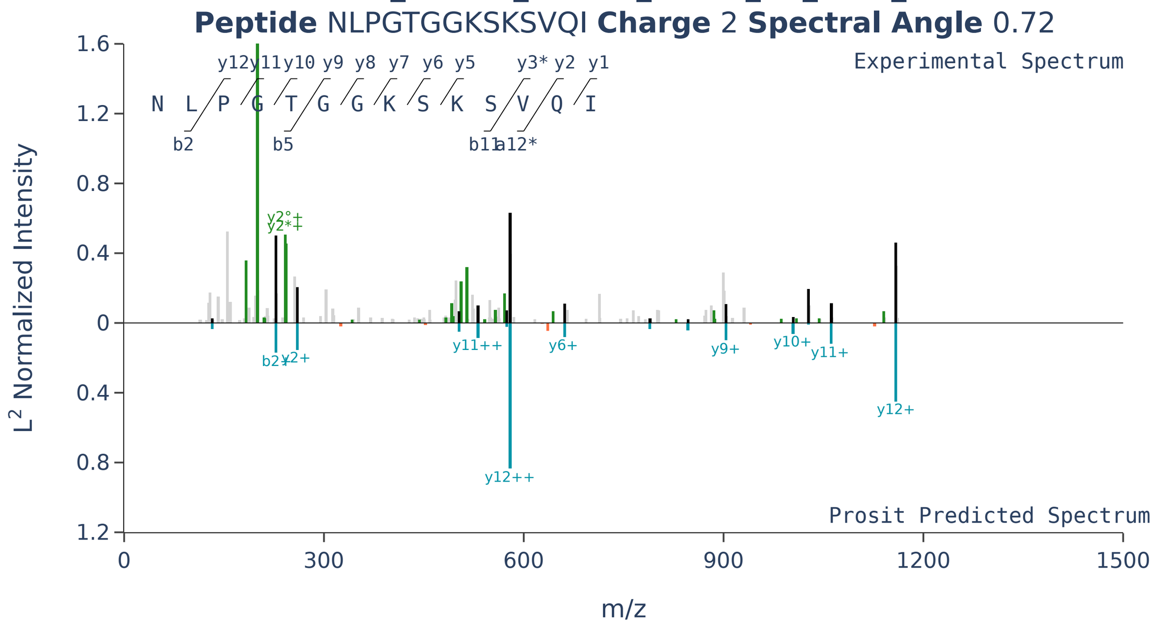


**Source** HRoetschke_211222_160123_HFGoe_IF2_b2R2_4h **Scan** 16114
**Peptide** TDVTIKTLA **Charge** 2 **Spectral Angle** 0.83

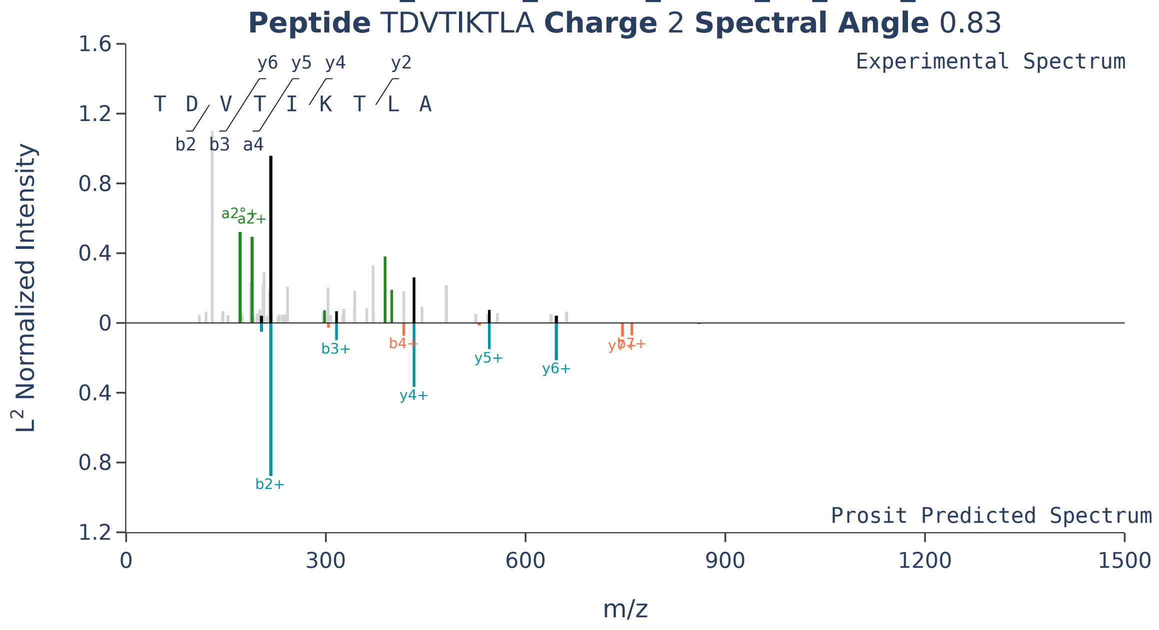


**Source** HRoetschke_211222_160123_HFGoe_IF2_b2R1_24h **Scan** 12245
**Peptide** GSSLQQGFQKPAQAVNR **Charge** 2 **Spectral Angle** 0.82

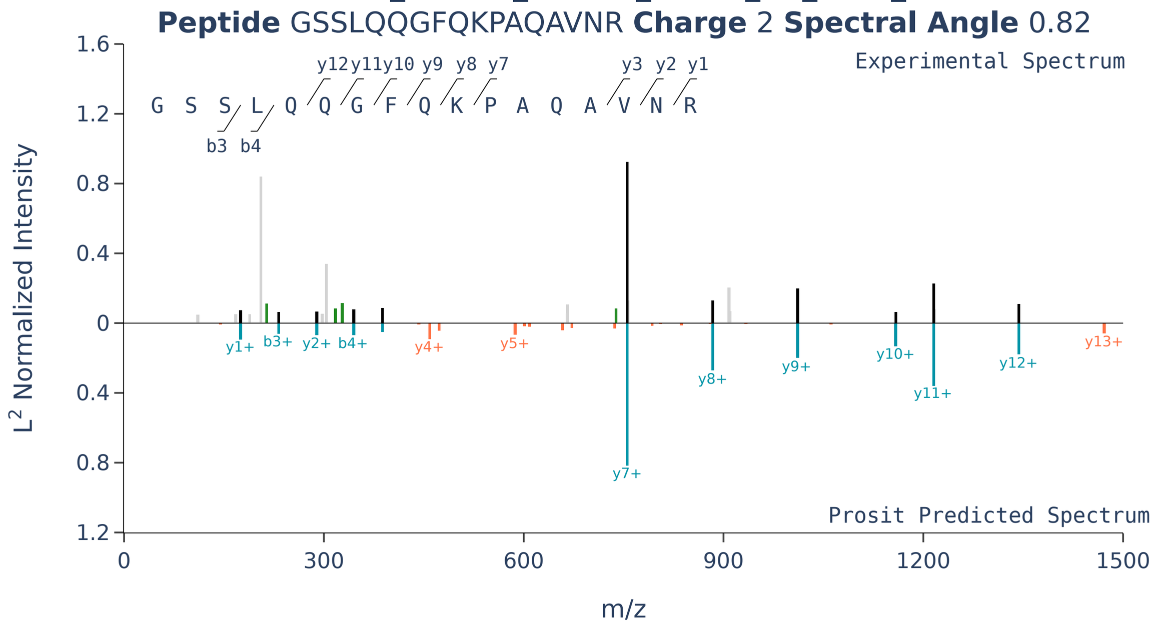


**Source** HRoetschke_211222_160123_HFGoe_IF2_b2R2_24h **Scan** 10181
**Peptide** MGHKVIL **Charge** 2 **Spectral Angle** 0.72

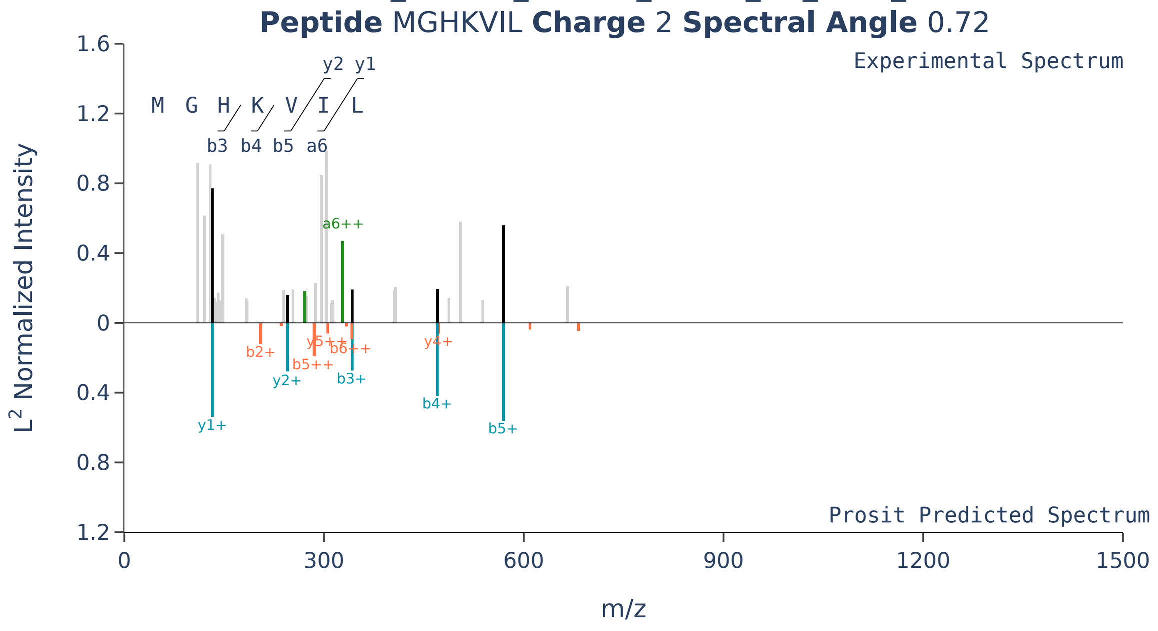


**Source** HRoetschke_211222_160123_HFGoe_IF2_b1R1_4h **Scan** 13100
**Peptide** SAQEKQTLL **Charge** 2 **Spectral Angle** 0.71

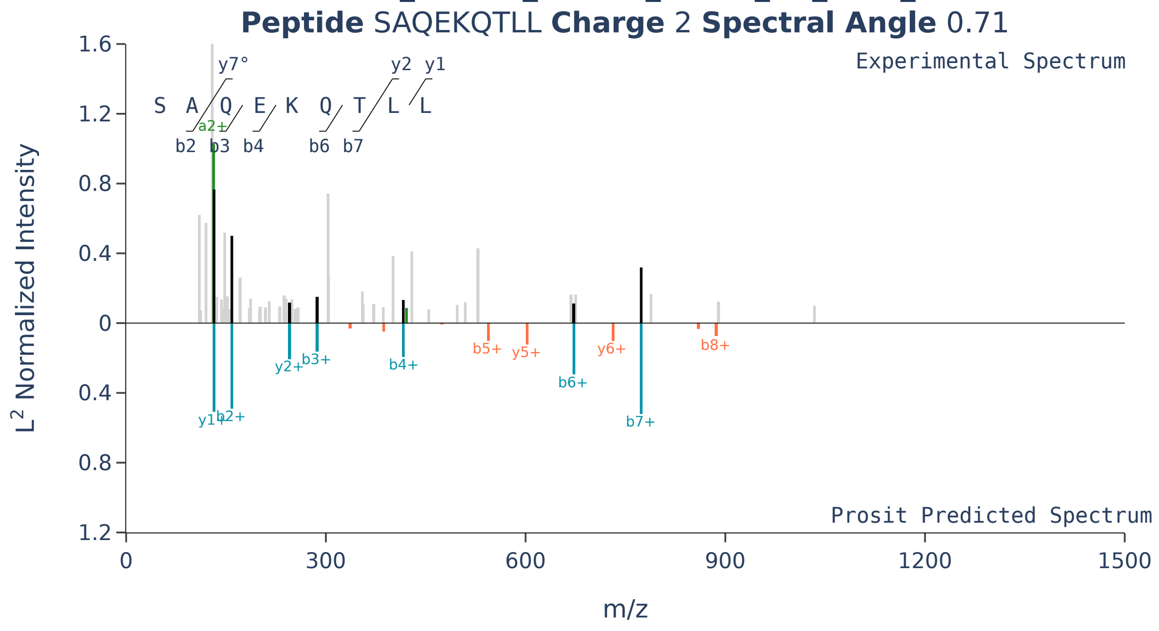


**Source** HRoetschke_211222_160123_HFGoe_IF2_b1R1_4h **Scan** 21201
**Peptide** AEVLELKAV **Charge** 2 **Spectral Angle** 0.87

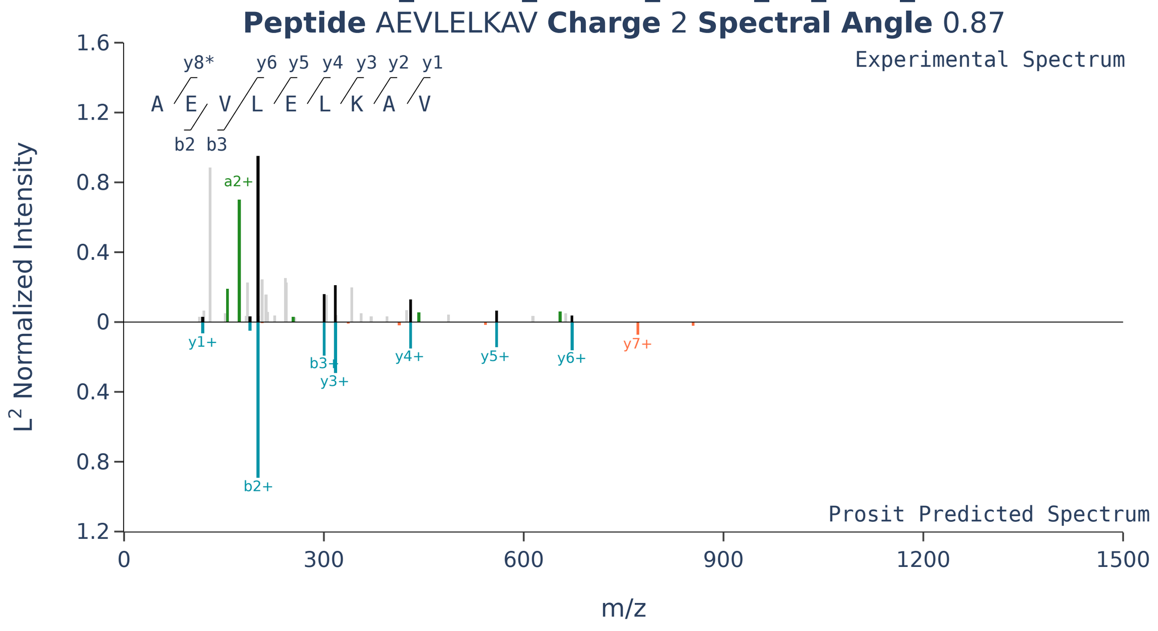


**Source** HRoetschke_211222_160123_HFGoe_IF2_b1R2_2h **Scan** 12962
**Peptide** SDRDTGAAAEPRAPVV **Charge** 3 **Spectral Angle** 0.76

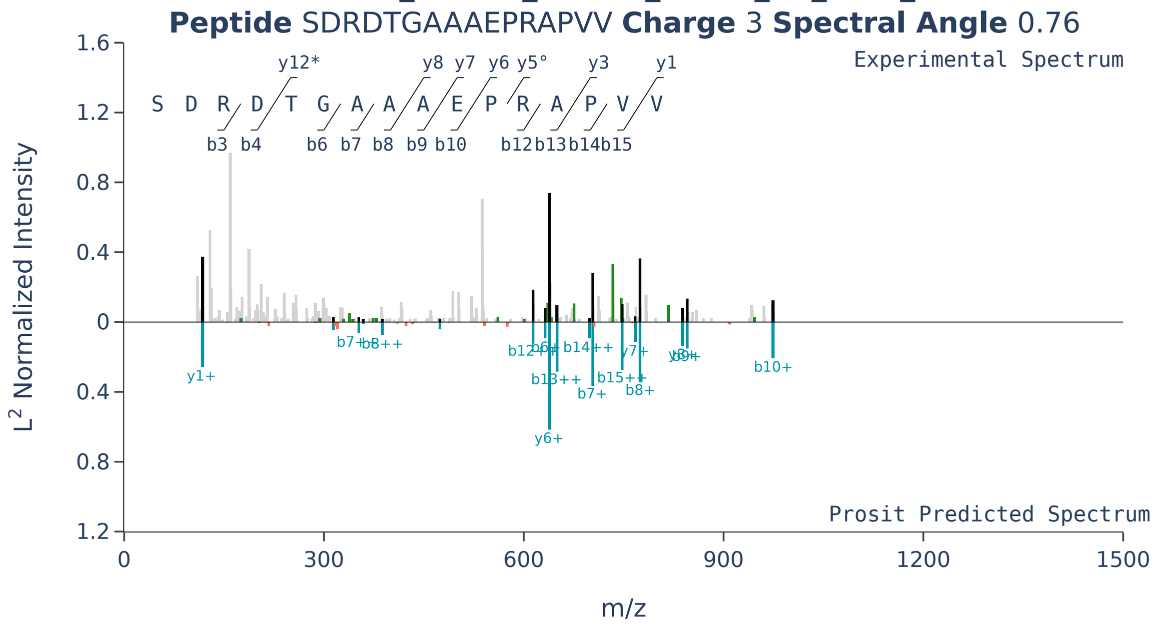


**Source** HRoetschke_211222_160123_HFGoe_IF2_b2R1_24h **Scan** 8162
**Peptide** QQGFQKPAQAVNRD **Charge** 3 **Spectral Angle** 0.76

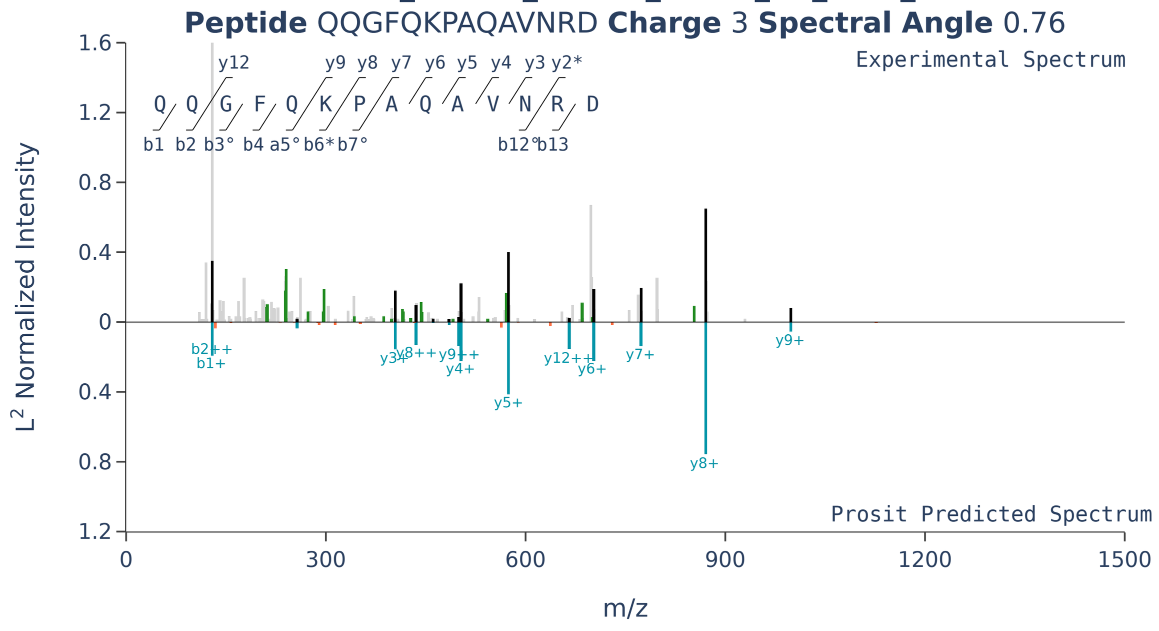


**Source** HRoetschke_211222_160123_HFGoe_IF2_b2R2_4h **Scan** 19781
**Peptide** VVLVVAADD **Charge** 1 **Spectral Angle** 0.95

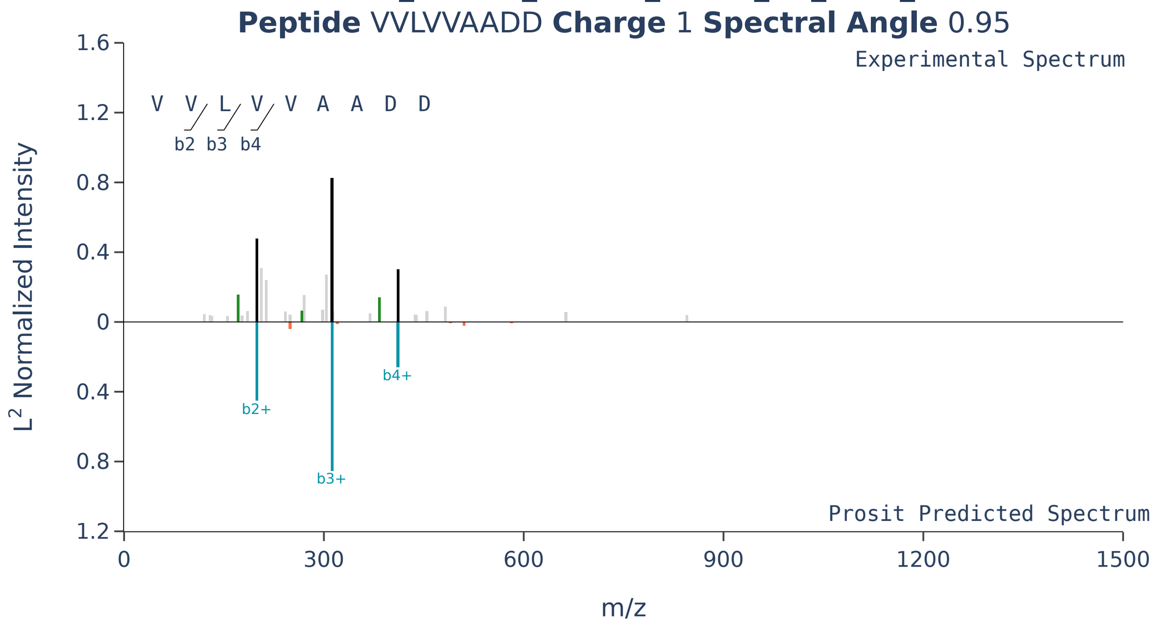


**Source** HRoetschke_211222_160123_HFGoe_IF2_b1R1_4h **Scan** 12341
**Peptide** KPAQAVNRDVVIGE **Charge** 3 **Spectral Angle** 0.77

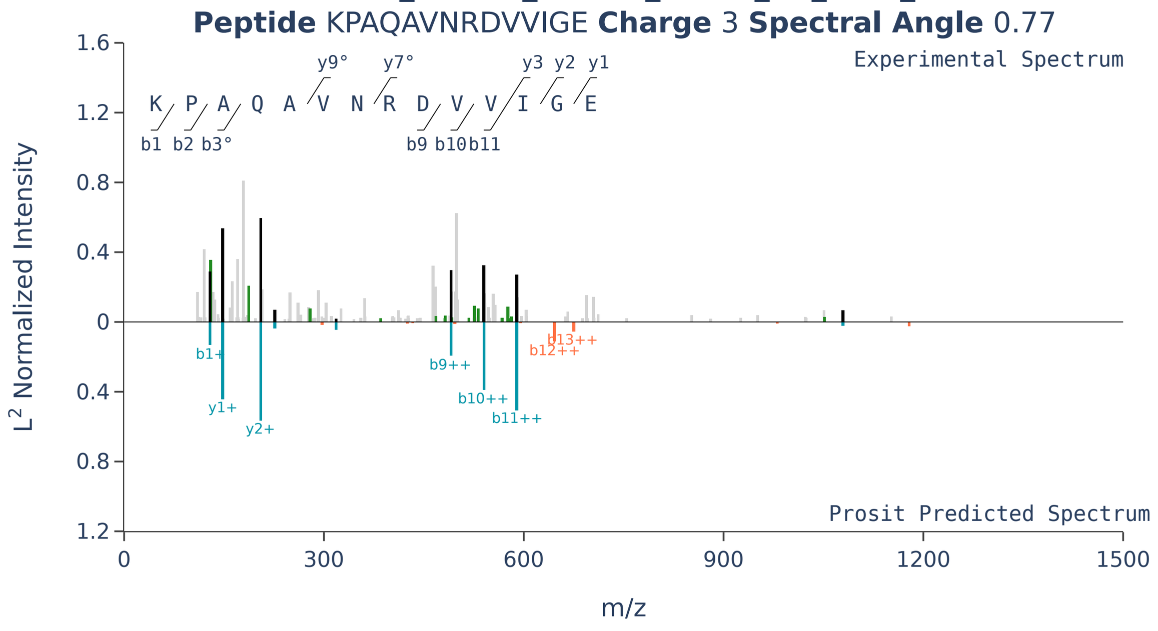


**Source** HRoetschke_211222_160123_HFGoe_IF2_b1R1_4h **Scan** 15965
**Peptide** AKAAQVPVVVAVNK **Charge** 3 **Spectral Angle** 0.83

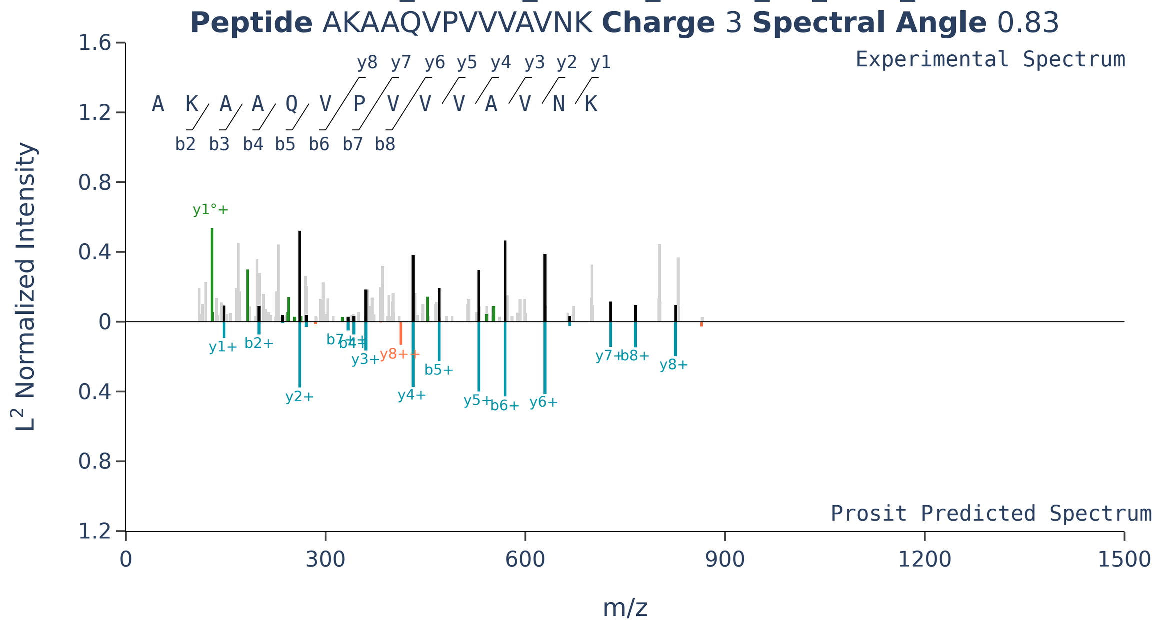

**Source** HRoetschke_211222_160123_HFGoe_IF2_b1R1_24h **Scan** 15430
**Peptide** GSSLQQGF **Charge** 1 **Spectral Angle** 0.76

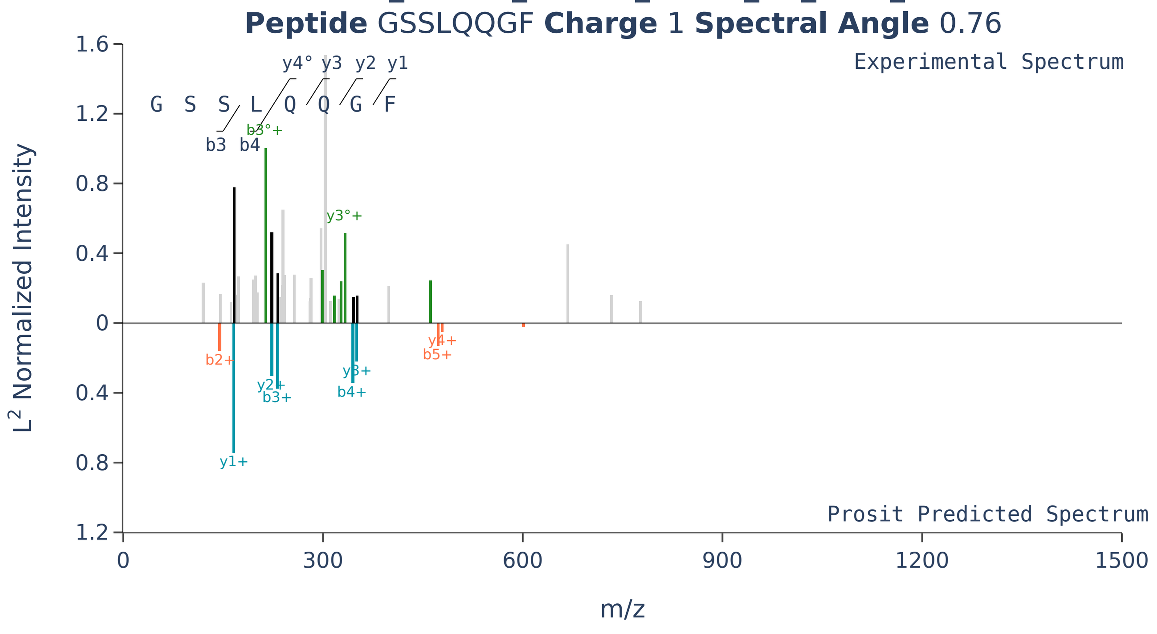


**Source** HRoetschke_211222_160123_HFGoe_IF2_b1R1_4h **Scan** 13527
**Peptide** GAYHVETENGMI **Charge** 2 **Spectral Angle** 0.75

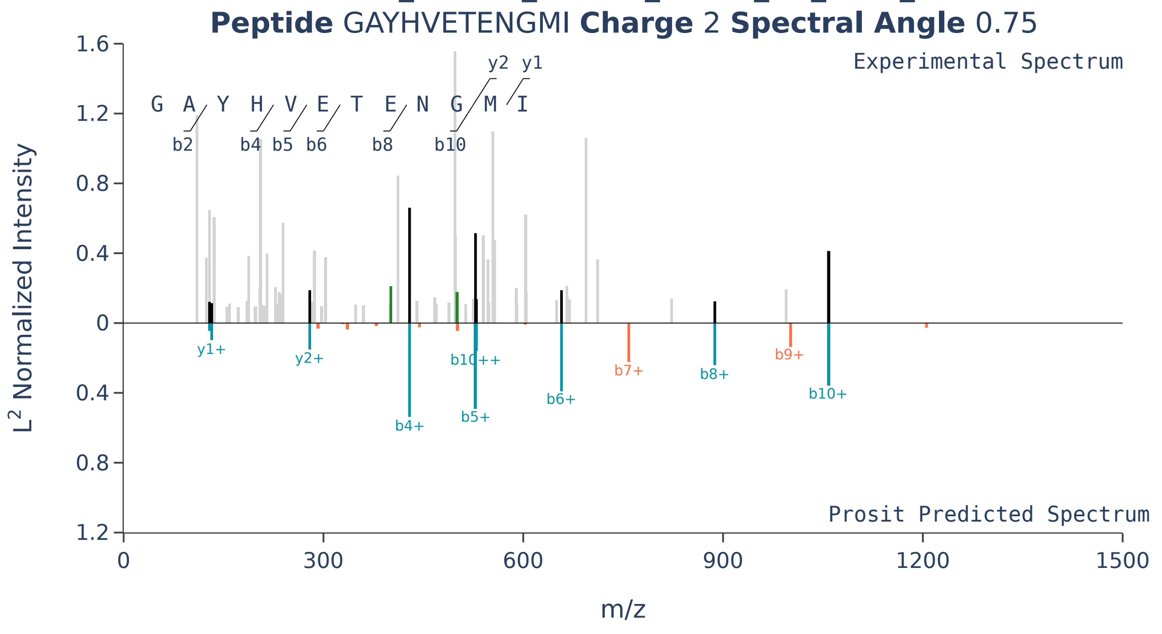


**Source** HRoetschke_211222_160123_HFGoe_IF2_b1R1_4h **Scan** 14094
**Peptide** MGHVDHGKTSLLDY **Charge** 3 **Spectral Angle** 0.85

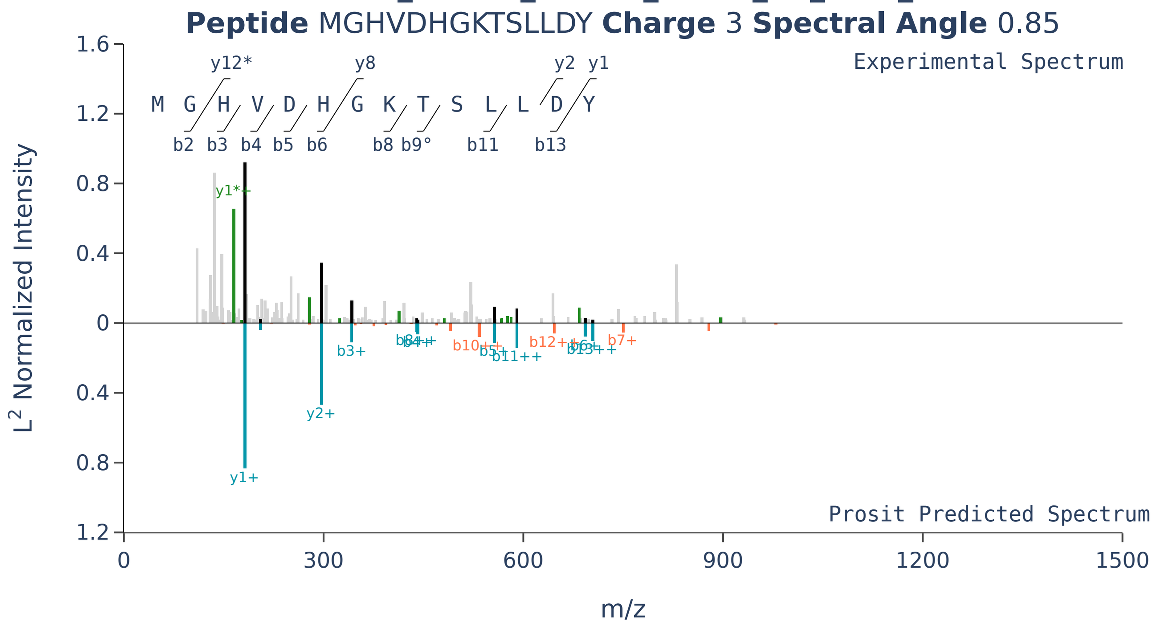


**Source** HRoetschke_211222_160123_HFGoe_IF2_b1R1_4h **Scan** 6068
**Peptide** KPAQAVNRDV **Charge** 2 **Spectral Angle** 0.67

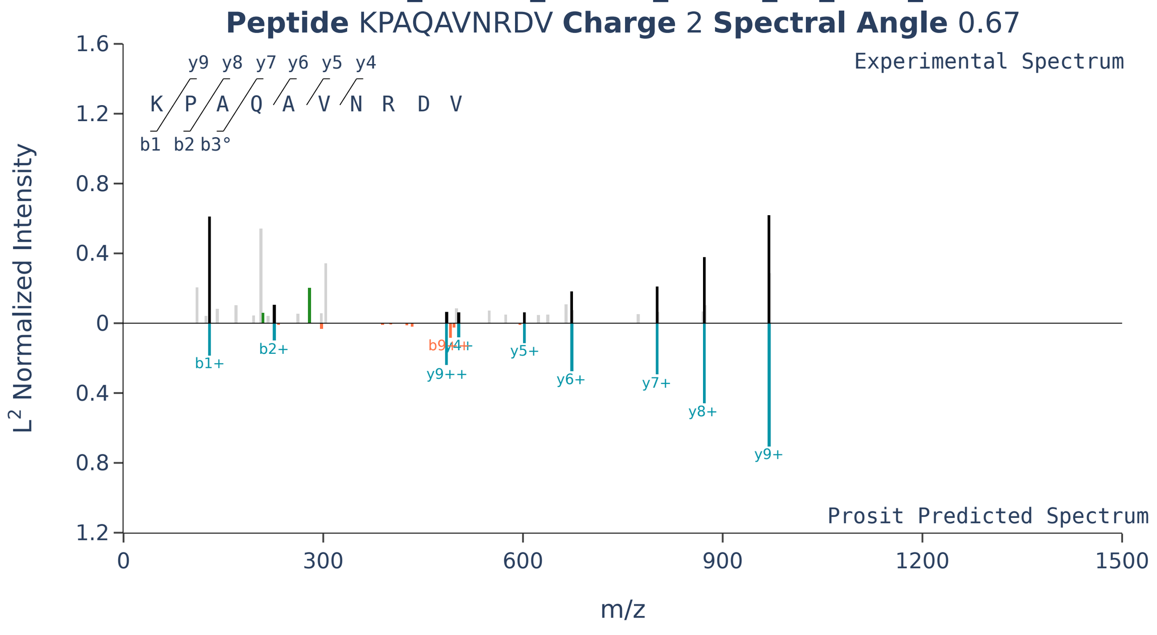


**Source** HRoetschke_211222_160123_HFGoe_IF2_b1R2_2h **Scan** 10701
**Peptide** KMAVKGSQVL **Charge** 2 **Spectral Angle** 0.83

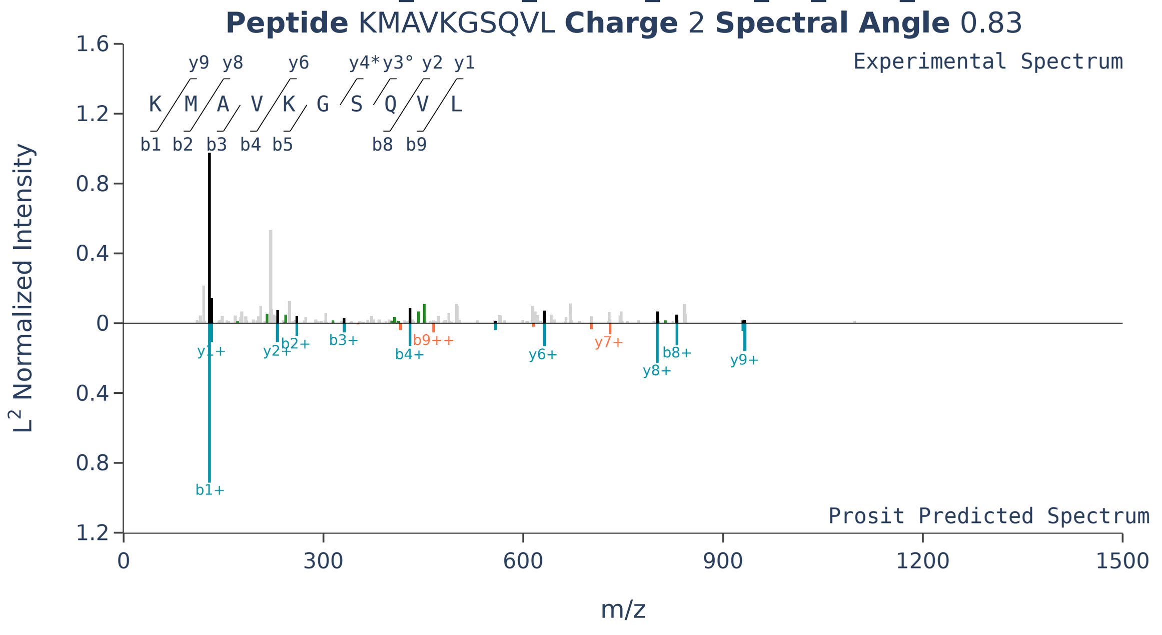


**Source** HRoetschke_211222_160123_HFGoe_IF2_b2R2_2h **Scan** 10182
**Peptide** KGSSLQQGFQKPAQA **Charge** 2 **Spectral Angle** 0.77

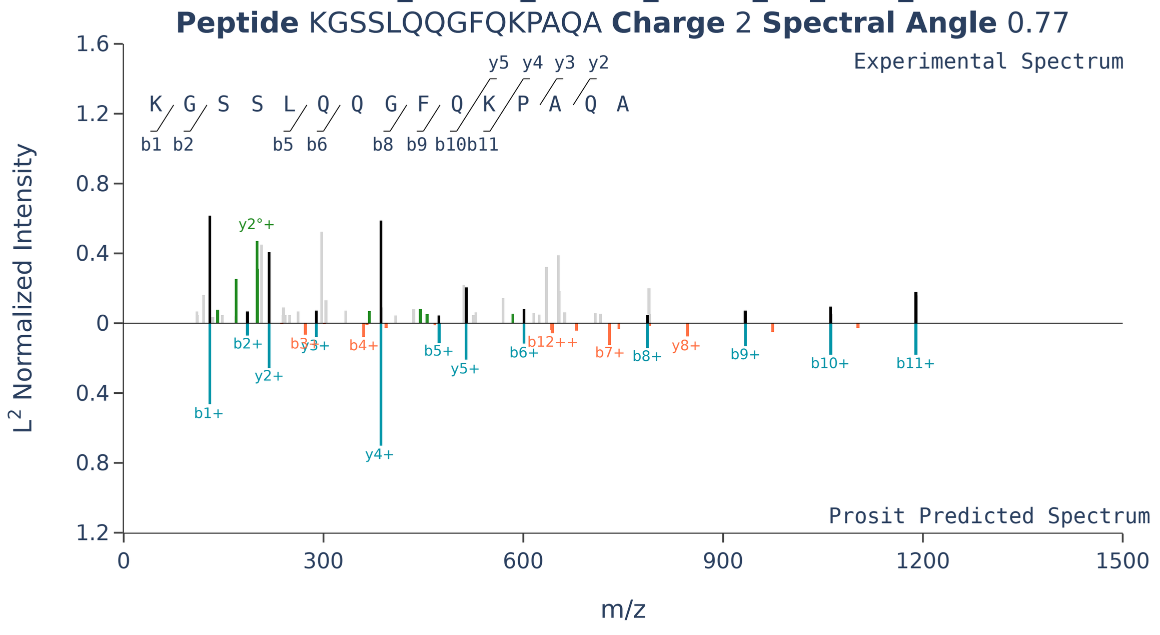


**Source** HRoetschke_211222_160123_HFGoe_IF2_b2R2_24h **Scan** 6235
**Peptide** IPGTGGKSKSV **Charge** 2 **Spectral Angle** 0.85

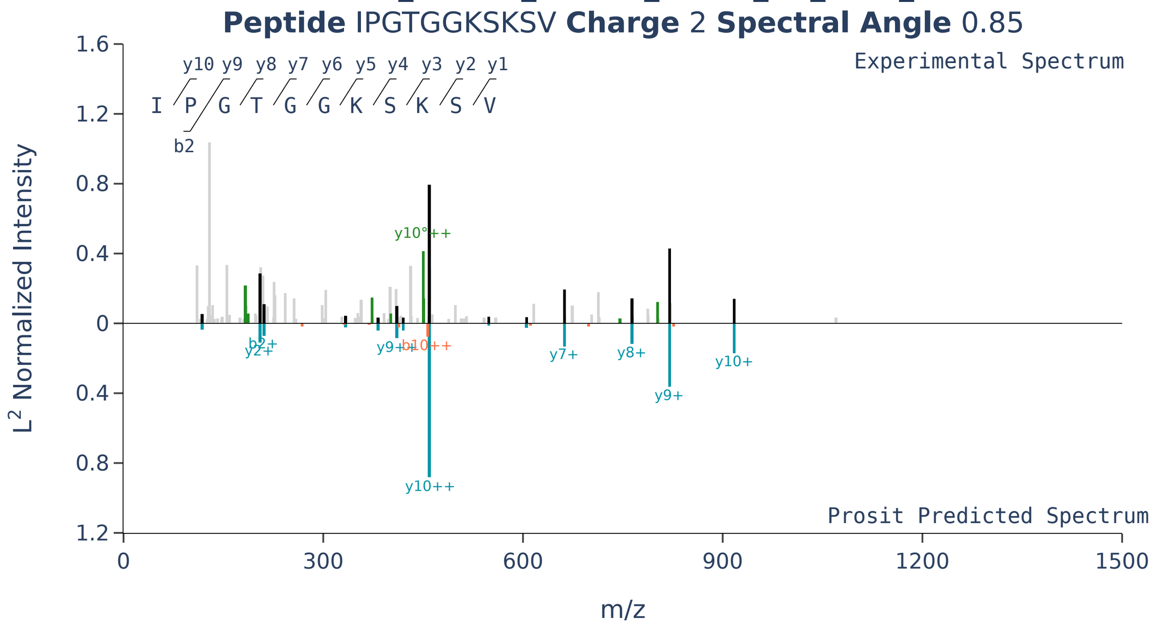


**Source** HRoetschke_211222_160123_HFGoe_IF2_b1R1_4h **Scan** 7998
**Peptide** QQGFQKPAQAVNR **Charge** 2 **Spectral Angle** 0.89

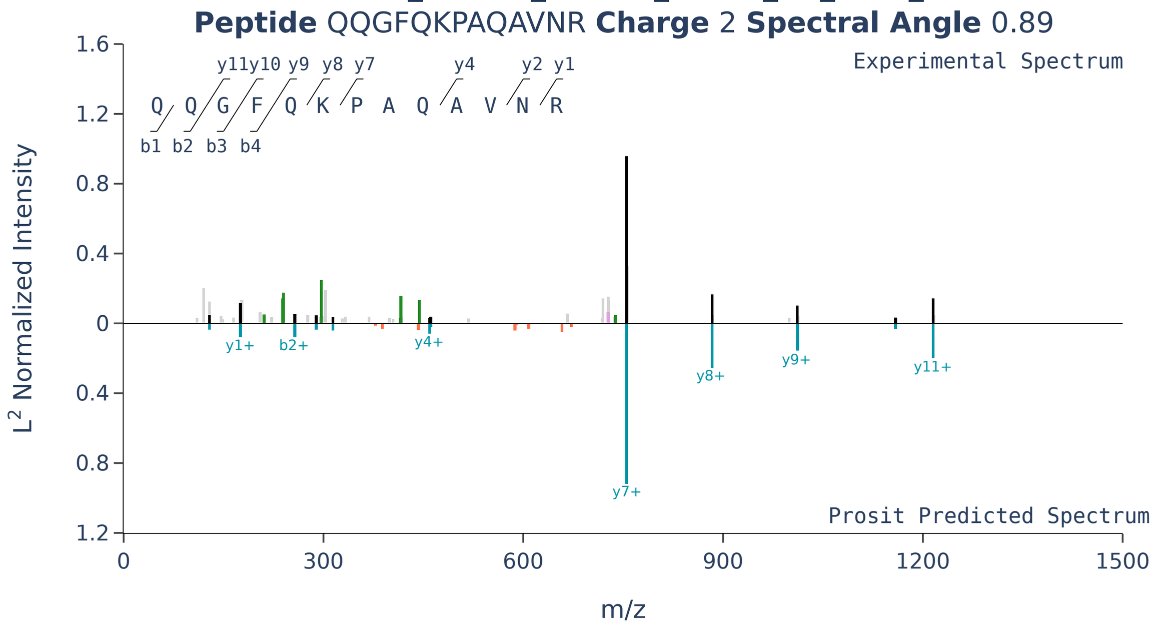


**Source** HRoetschke_211222_160123_HFGoe_IF2_b1R2_4h **Scan** 9697
**Peptide** KSKLENMFANM **Charge** 2 **Spectral Angle** 0.83

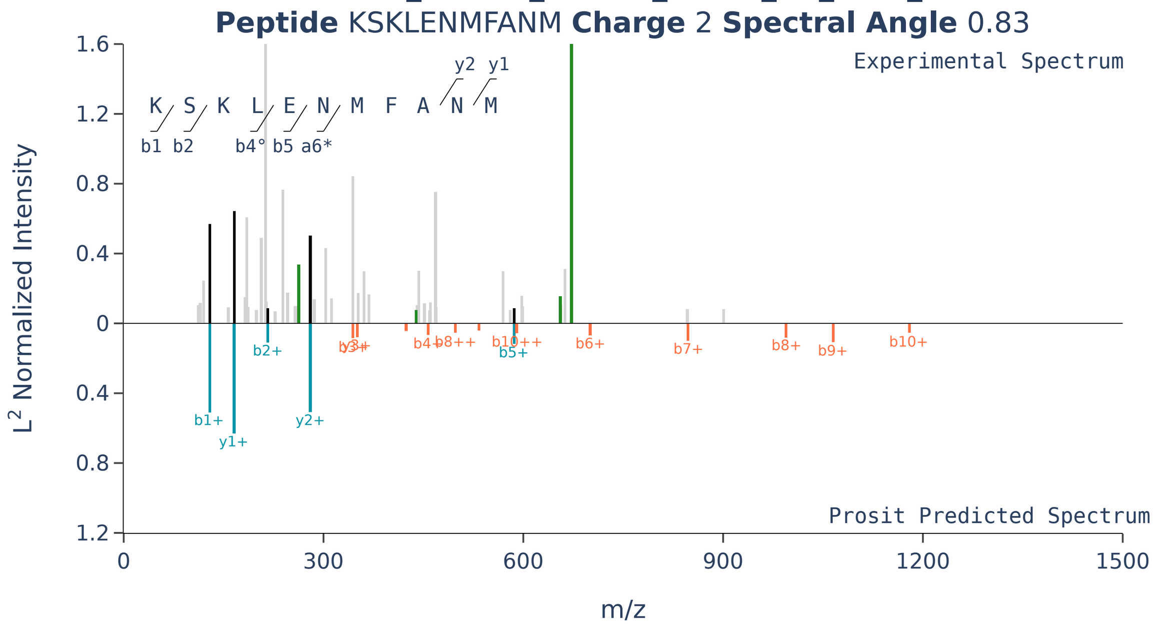


**Source** HRoetschke_211222_160123_HFGoe_IF2_b1R2_1h **Scan** 12683
**Peptide** SAQEKQTLI **Charge** 2 **Spectral Angle** 0.69

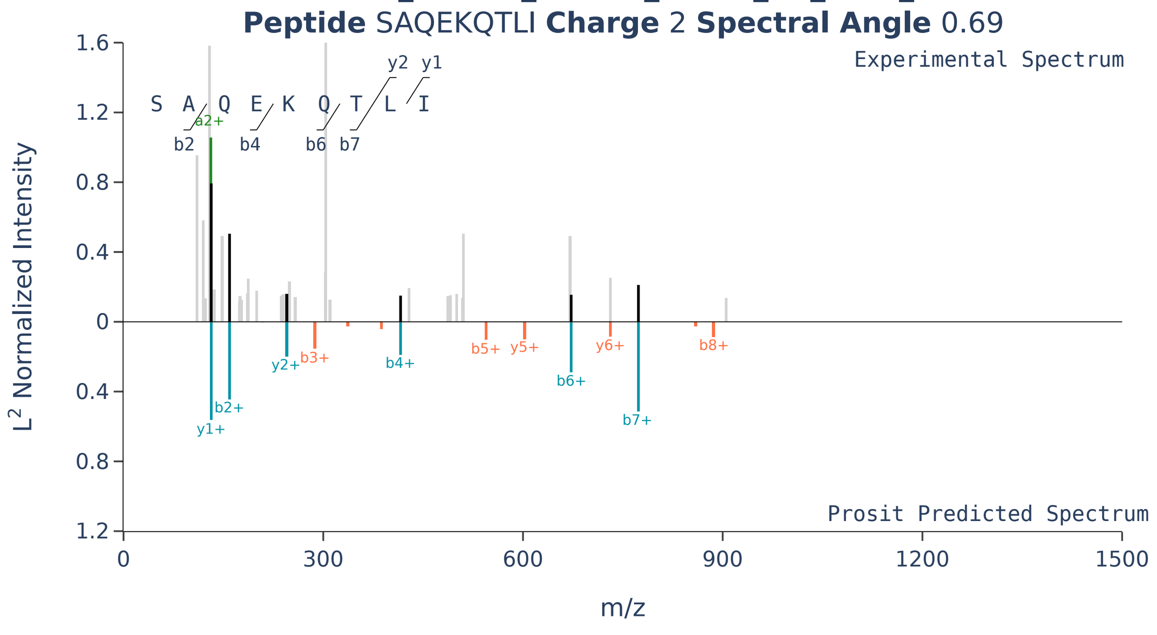


**Source** HRoetschke_211222_160123_HFGoe_IF2_b2R2_4h **Scan** 18978
**Peptide** INQVIDQETAQL **Charge** 2 **Spectral Angle** 0.87

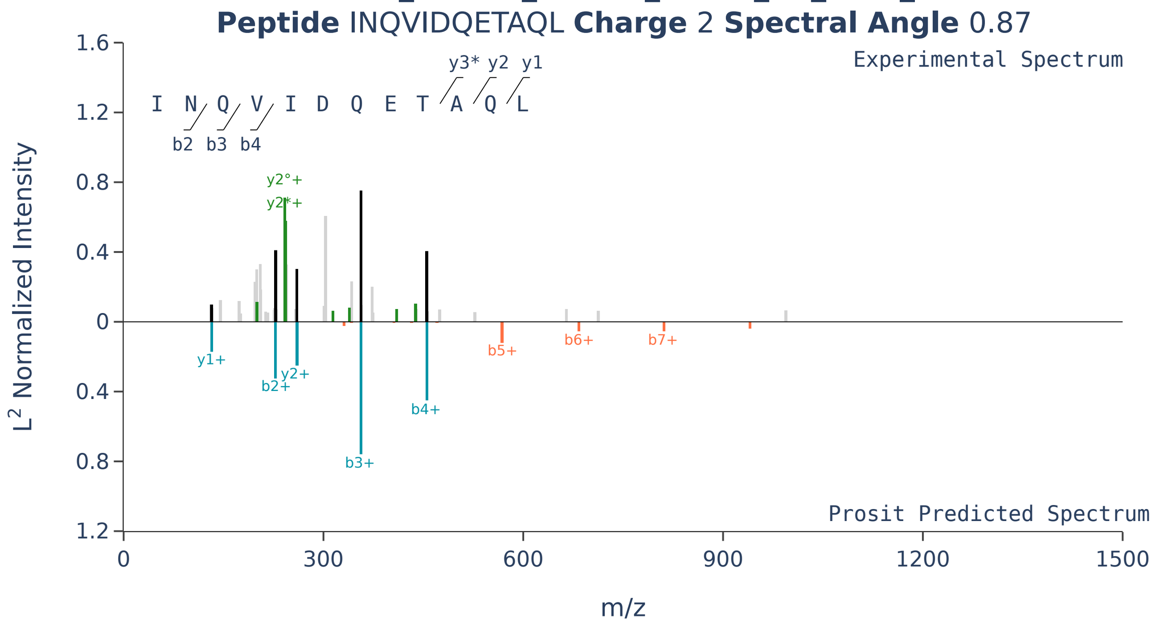


**Source** HRoetschke_211222_160123_HFGoe_IF2_b1R2_2h **Scan** 9533
**Peptide** DYIRSTKVASGE **Charge** 2 **Spectral Angle** 0.74

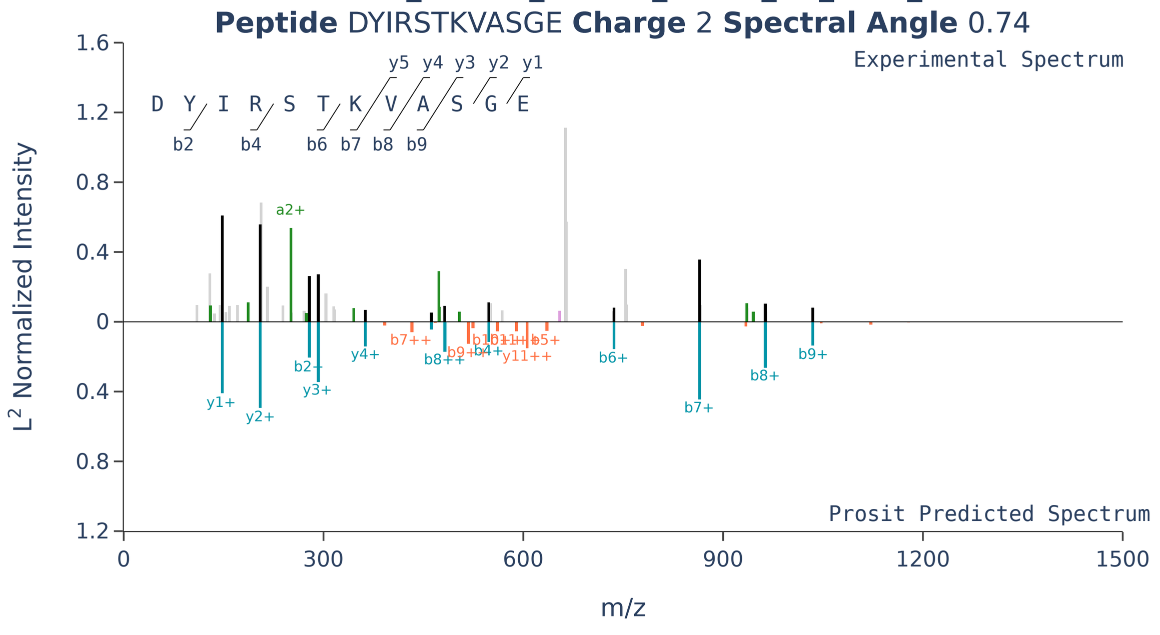


**Source** HRoetschke_211222_160123_HFGoe_IF2_b2R2_24h **Scan** 9953
**Peptide** KNELSQY **Charge** 2 **Spectral Angle** 0.68

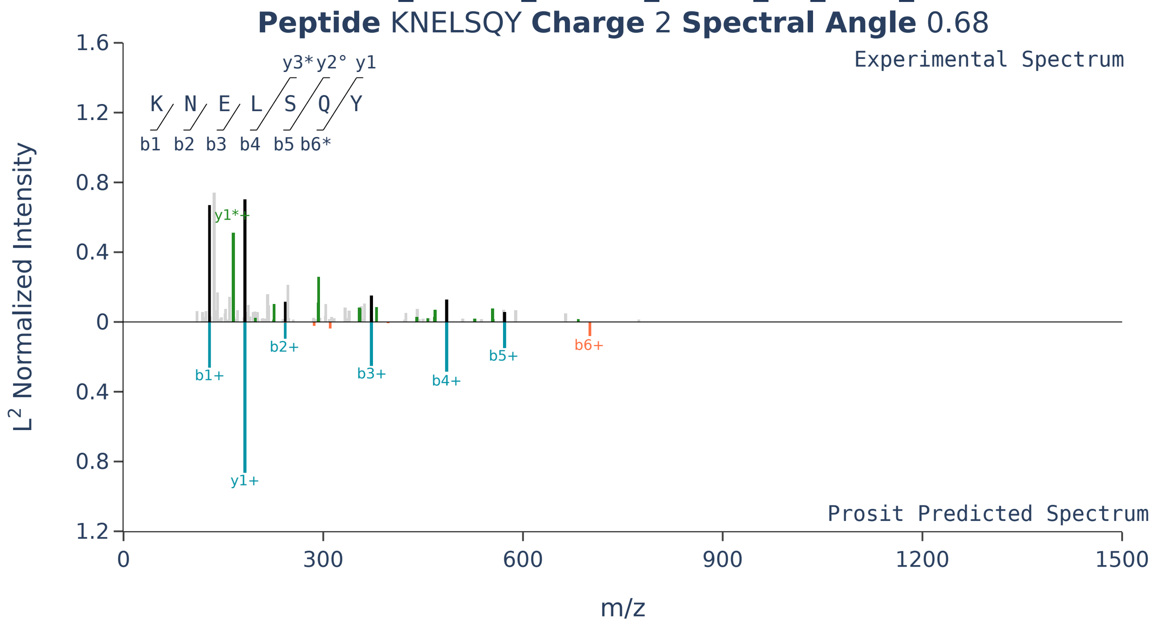


**Source** HRoetschke_211222_160123_HFGoe_IF2_b1R2_24h **Scan** 19427
**Peptide** AAEPRAPVVTI **Charge** 2 **Spectral Angle** 0.77

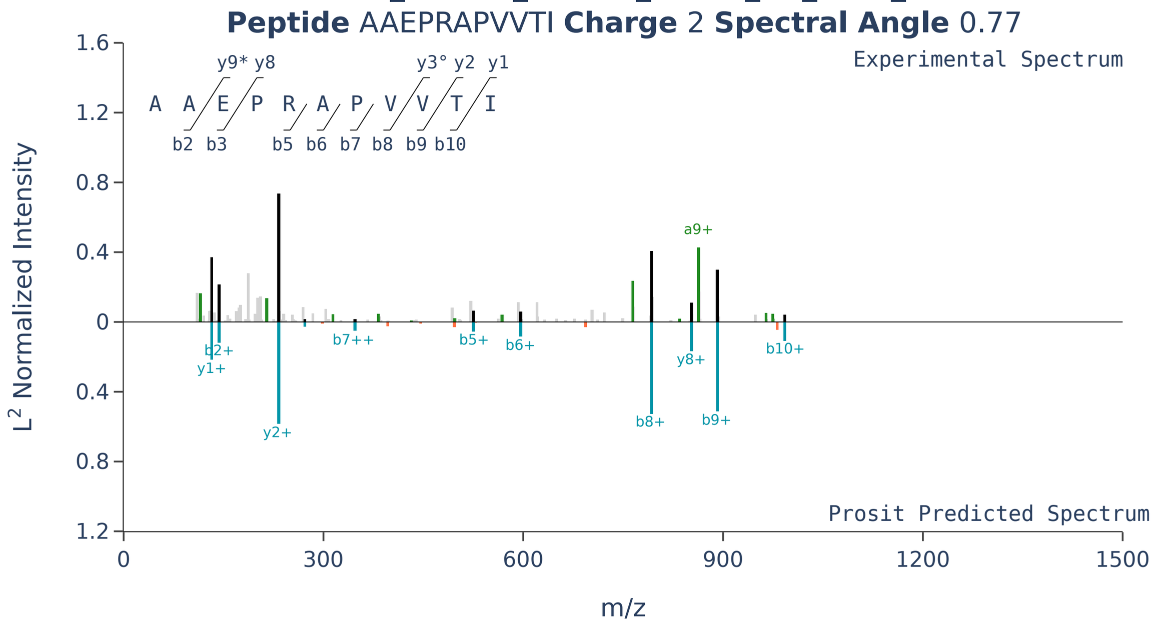


**Source** HRoetschke_211222_160123_HFGoe_IF2_b2R2_24h **Scan** 5792
**Peptide** NKIDKPEADPDRVKN **Charge** 2 **Spectral Angle** 0.77

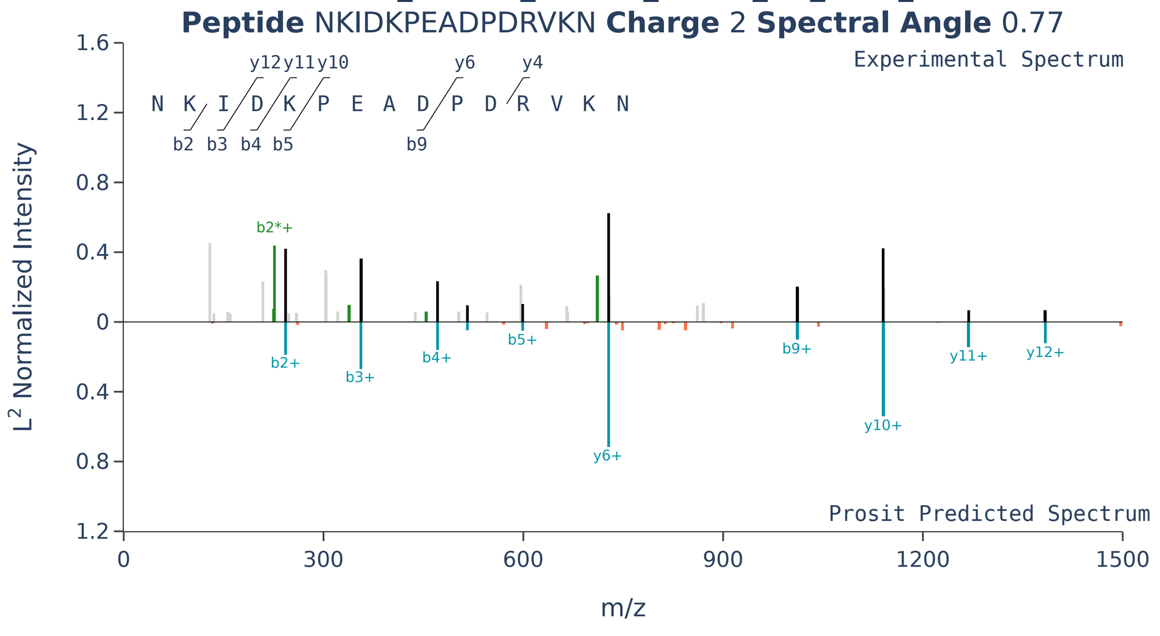

**Source** HRoetschke_211222_160123_HFGoe_IF2_b1R2_1h **Scan** 8706
**Peptide** HIGAYHVETENG **Charge** 2 **Spectral Angle** 0.74

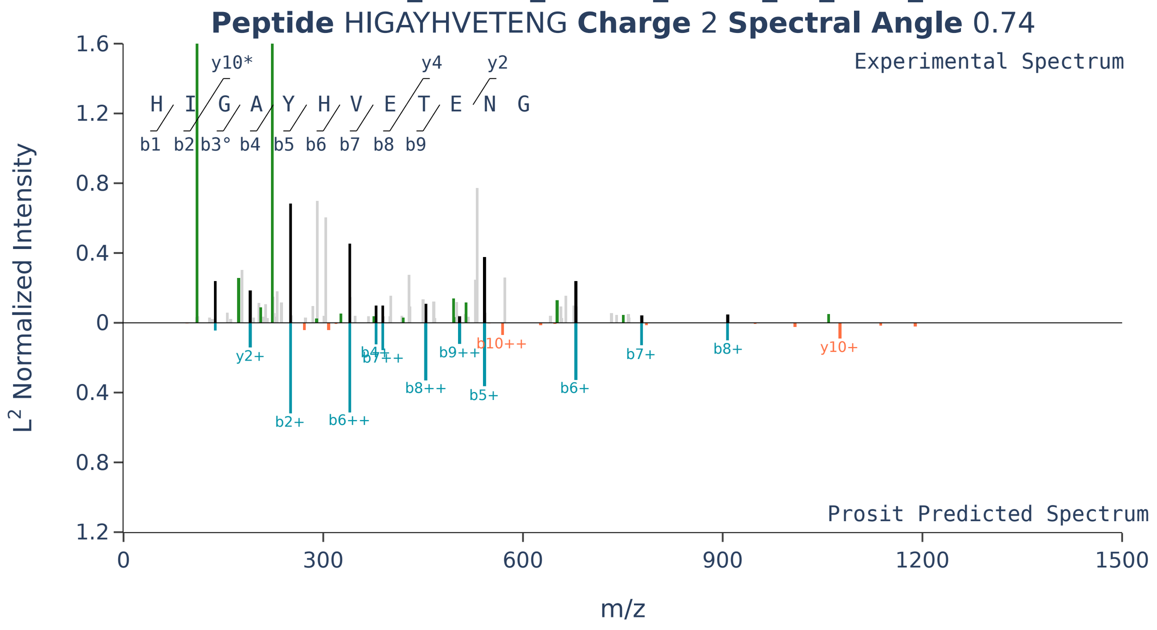


**Source** HRoetschke_211222_160123_HFGoe_IF2_b1R2_4h **Scan** 11945
**Peptide** AAERQTSVERLVQ **Charge** 3 **Spectral Angle** 0.68

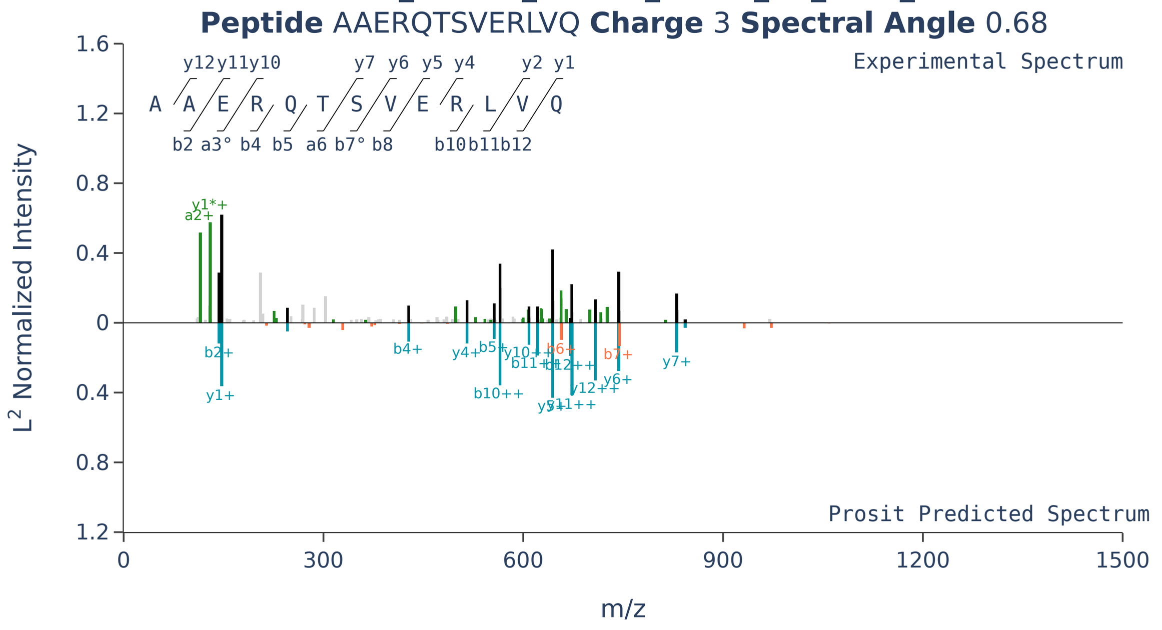


**Source** HRoetschke_211222_160123_HFGoe_IF2_b1R1_4h **Scan** 15583
**Peptide** KAAQVPVVVAVNK **Charge** 3 **Spectral Angle** 0.82

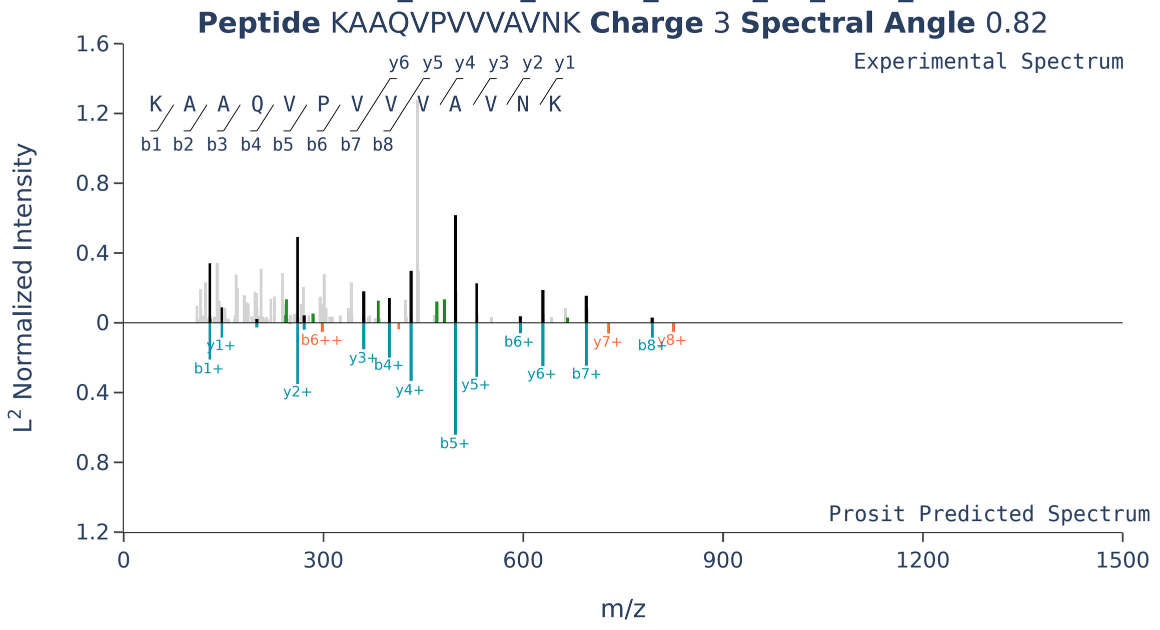


**Source** HRoetschke_211222_160123_HFGoe_IF2_b1R2_24h **Scan** 7842
**Peptide** PGTGGKSKSVQI **Charge** 3 **Spectral Angle** 0.85

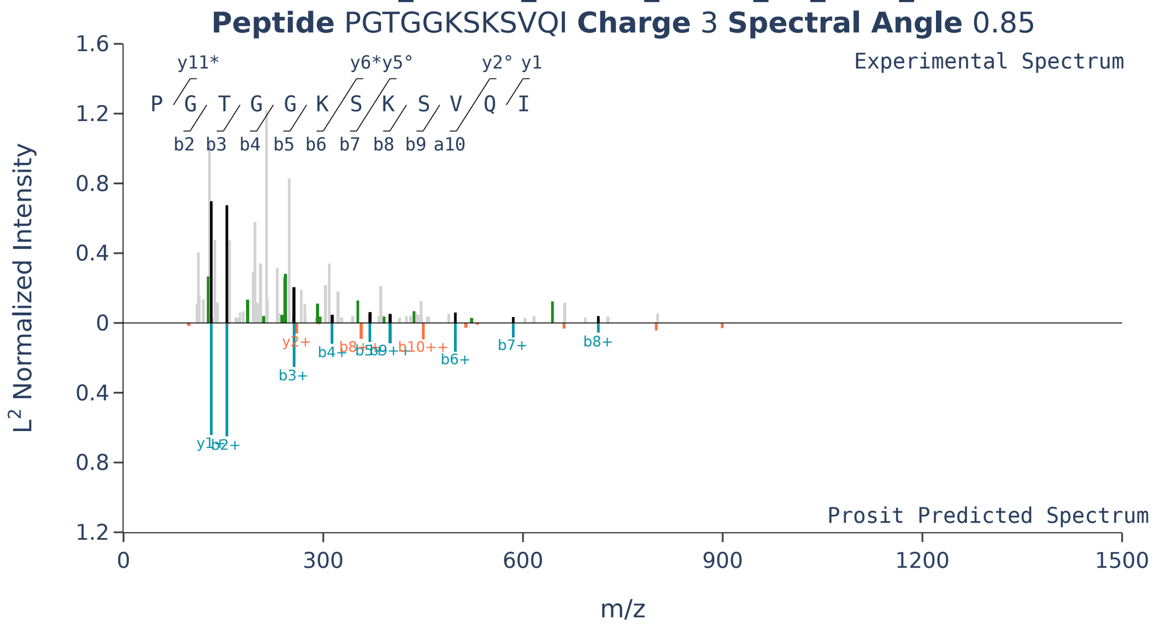


**Source** HRoetschke_211222_160123_HFGoe_IF2_b2R1_24h **Scan** 7650
**Peptide** RQTSVERL **Charge** 2 **Spectral Angle** 0.77

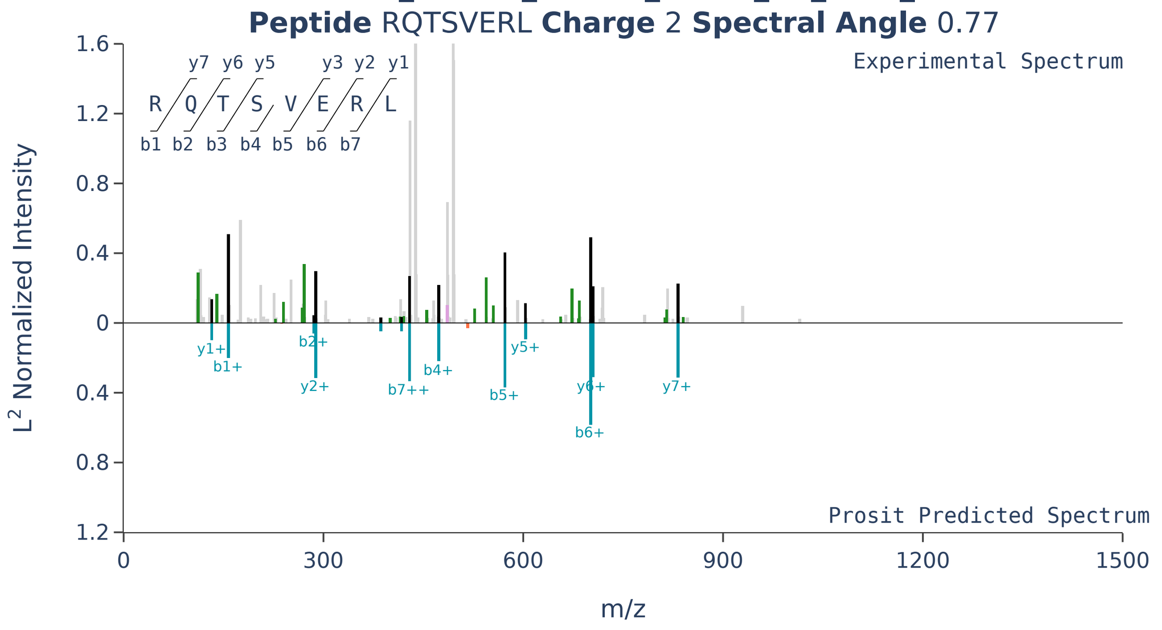


**Source** HRoetschke_211222_160123_HFGoe_IF2_b1R1_2h **Scan** 7691
**Peptide** TEGEVHEV **Charge** 2 **Spectral Angle** 0.86

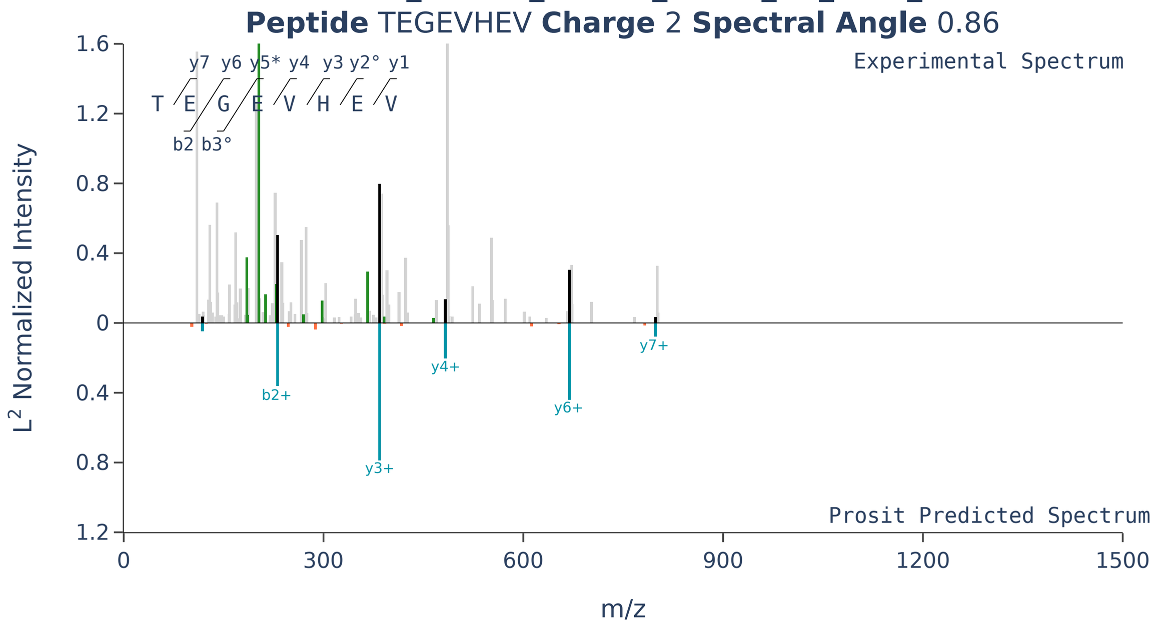


**Source** HRoetschke_211222_160123_HFGoe_IF2_b2R1_1h **Scan** 5659
**Peptide** KIDKPEADPDRVKN **Charge** 3 **Spectral Angle** 0.65

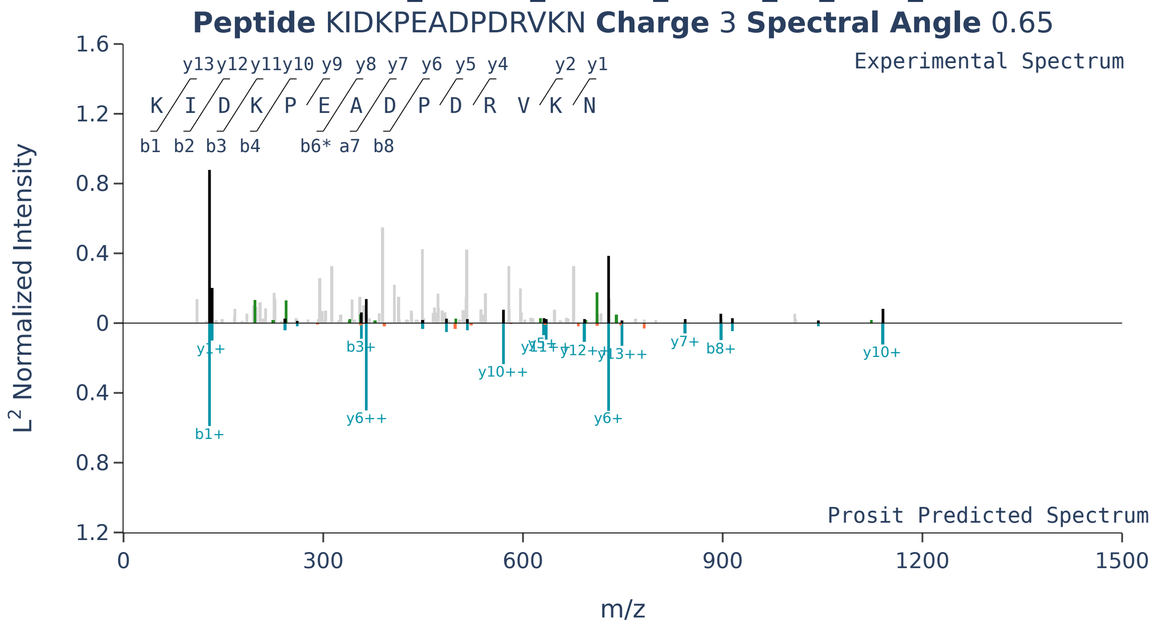


**Source** HRoetschke_211222_160123_HFGoe_IF2_b1R1_4h **Scan** 6449
**Peptide** GPDKLTLQRKTRST **Charge** 3 **Spectral Angle** 0.76

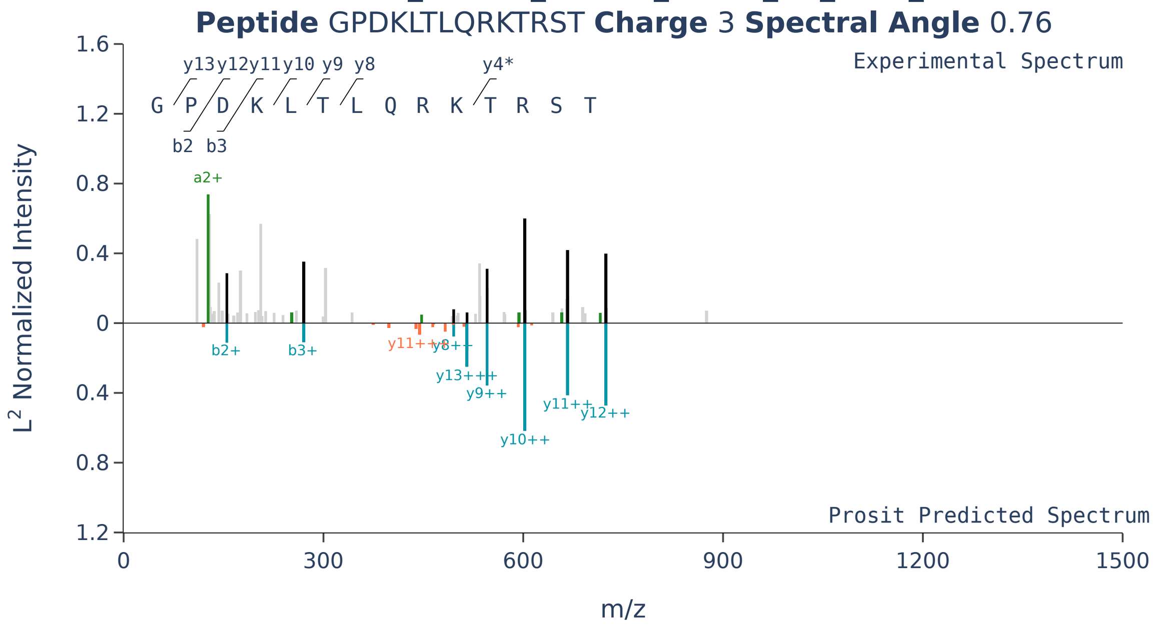


**Source** HRoetschke_211222_160123_HFGoe_IF2_b1R1_4h **Scan** 8299
**Peptide** AEENKWTD **Charge** 2 **Spectral Angle** 0.82

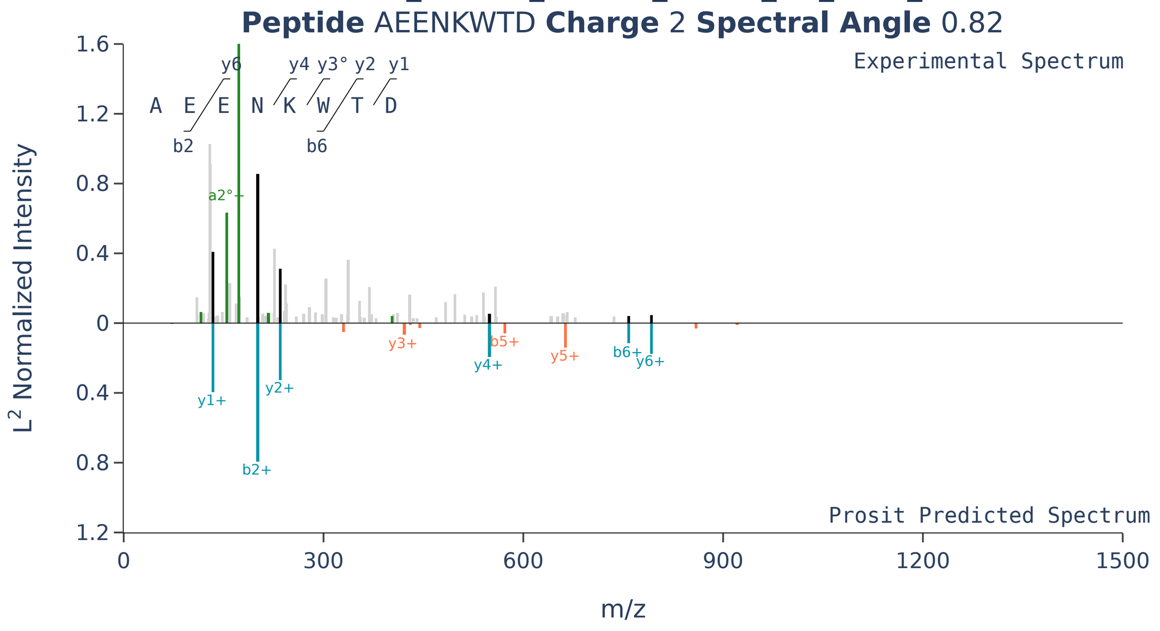


**Source** HRoetschke_211222_160123_HFGoe_IF2_b1R2_24h **Scan** 6330
**Peptide** DSSDYHVTTSQHARQAE **Charge** 2 **Spectral Angle** 0.76

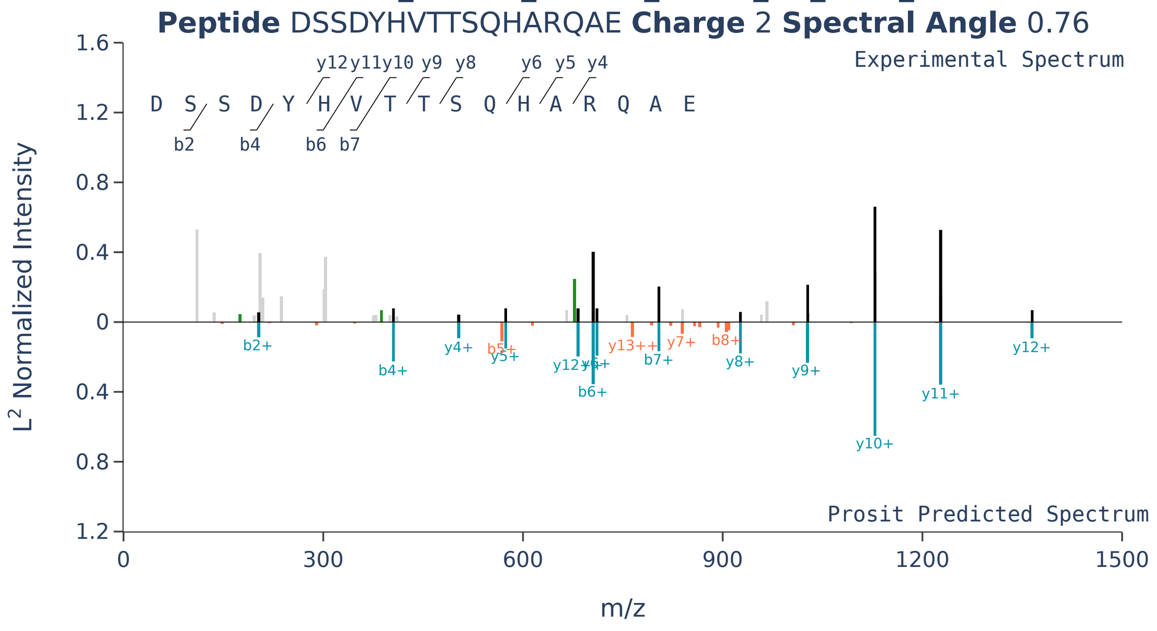


**Source** HRoetschke_211222_160123_HFGoe_IF2_b1R1_24h **Scan** 7302
**Peptide** DASARKVLE **Charge** 2 **Spectral Angle** 0.81

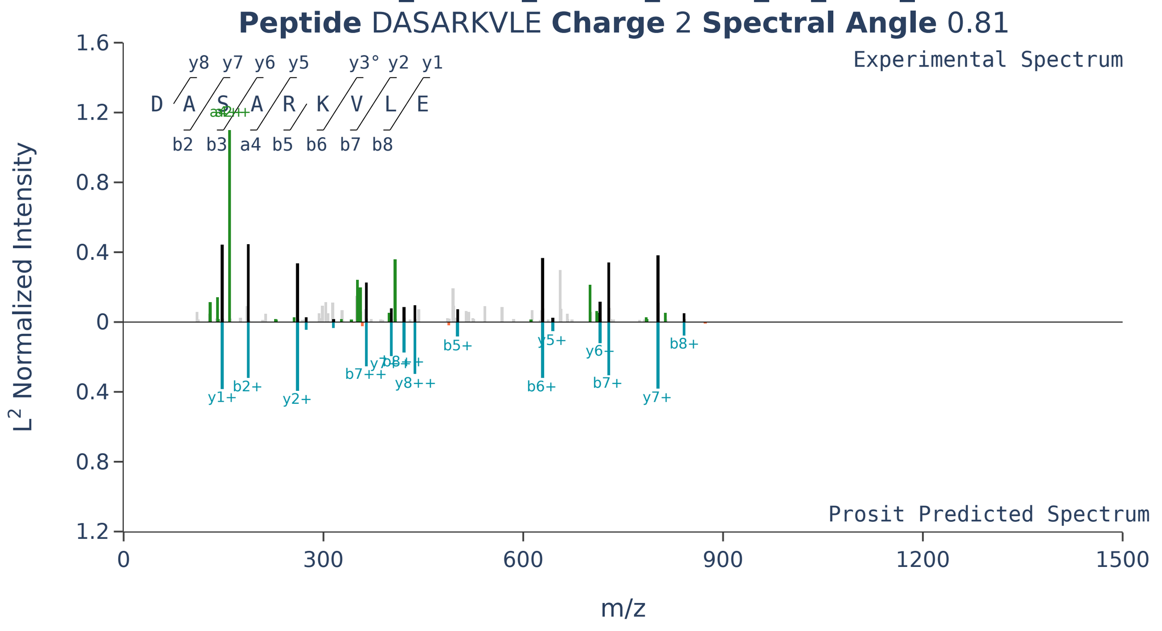


**Source** HRoetschke_211222_160123_HFGoe_IF2_b1R1_1h **Scan** 11014
**Peptide** PDRVKNELSQY **Charge** 2 **Spectral Angle** 0.74

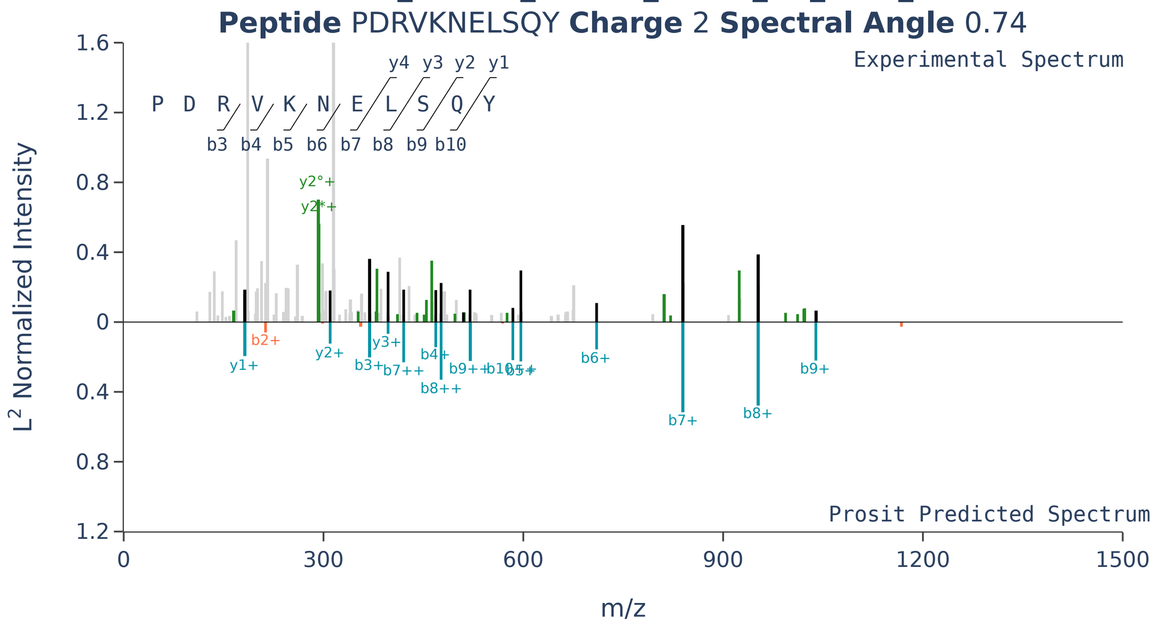


**Source** HRoetschke_211222_160123_HFGoe_IF2_b1R1_4h **Scan** 14124
**Peptide** ILRRENELEEAVM **Charge** 3 **Spectral Angle** 0.77

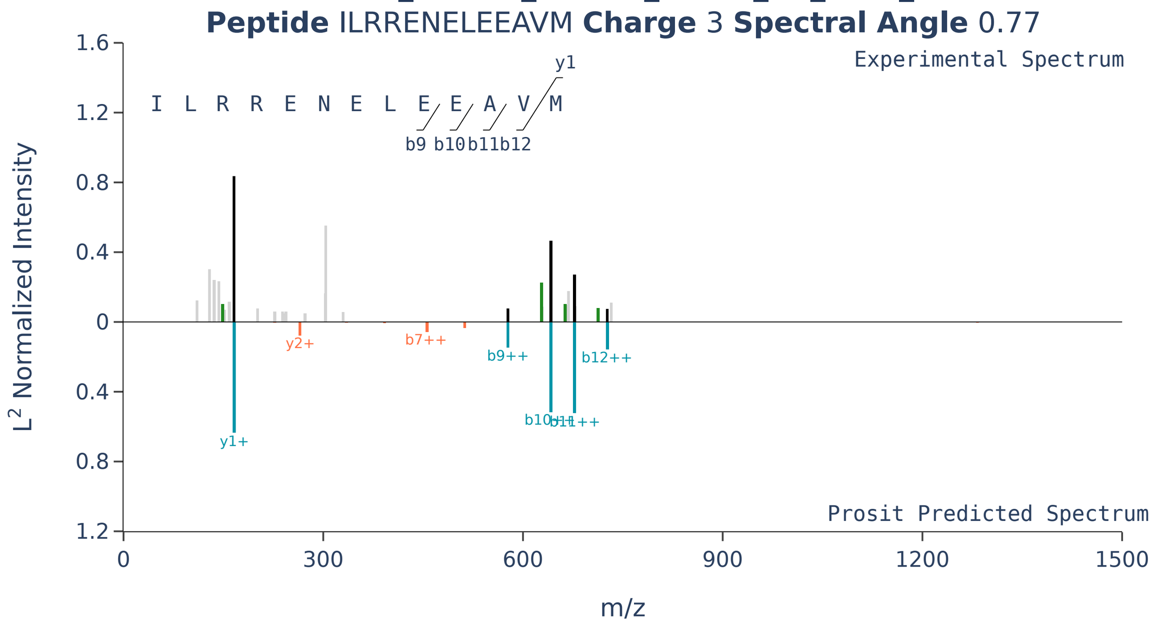


**Source** HRoetschke_211222_160123_HFGoe_IF2_b1R1_4h **Scan** 13990
**Peptide** TLNIPGTGGKSKSV **Charge** 3 **Spectral Angle** 0.83

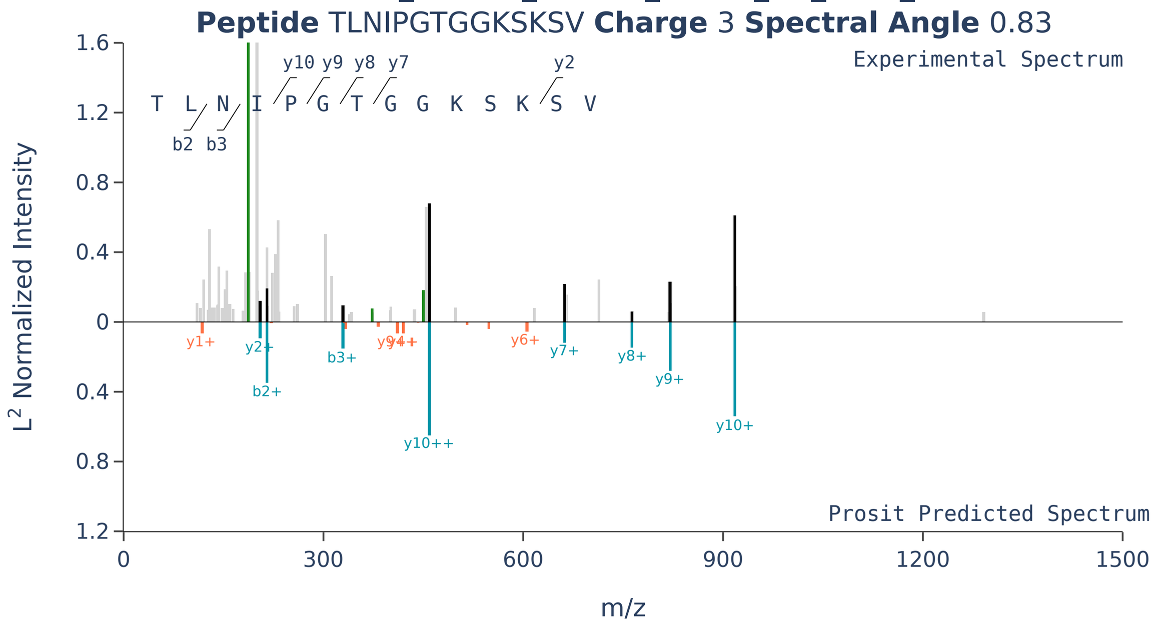


**Source** HRoetschke_211222_160123_HFGoe_IF2_b1R1_4h **Scan** 16578
**Peptide** RYYSVIYN **Charge** 2 **Spectral Angle** 0.72

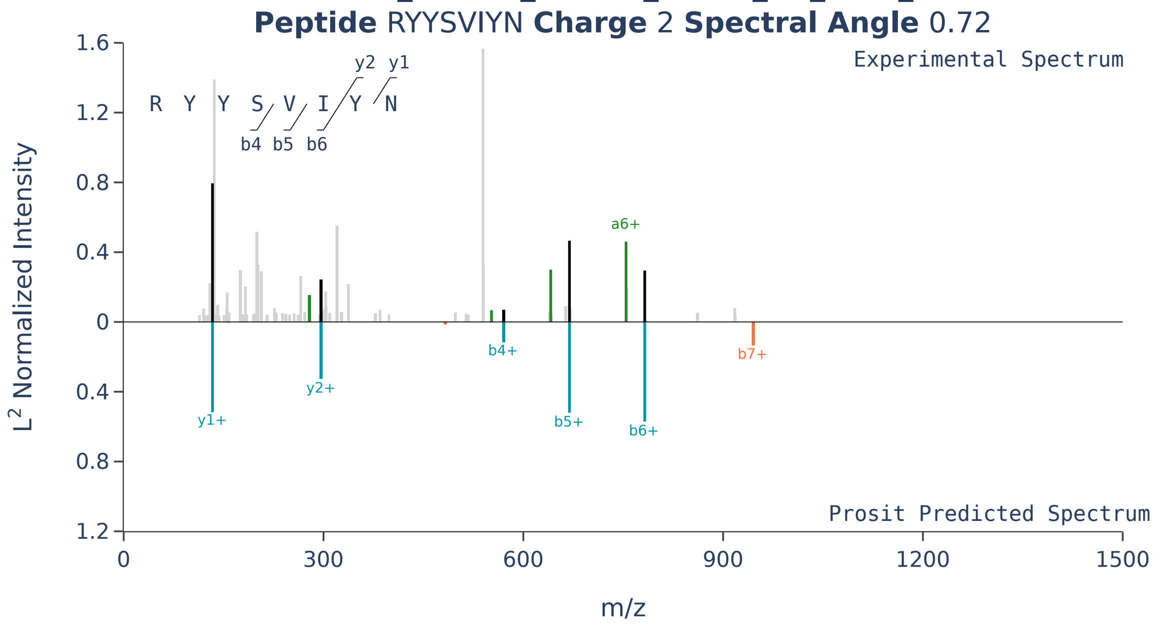

**Source** HRoetschke_211222_160123_HFGoe_IF2_b2R1_1h **Scan** 7496
**Peptide** FQKPAQA **Charge** 2 **Spectral Angle** 0.87

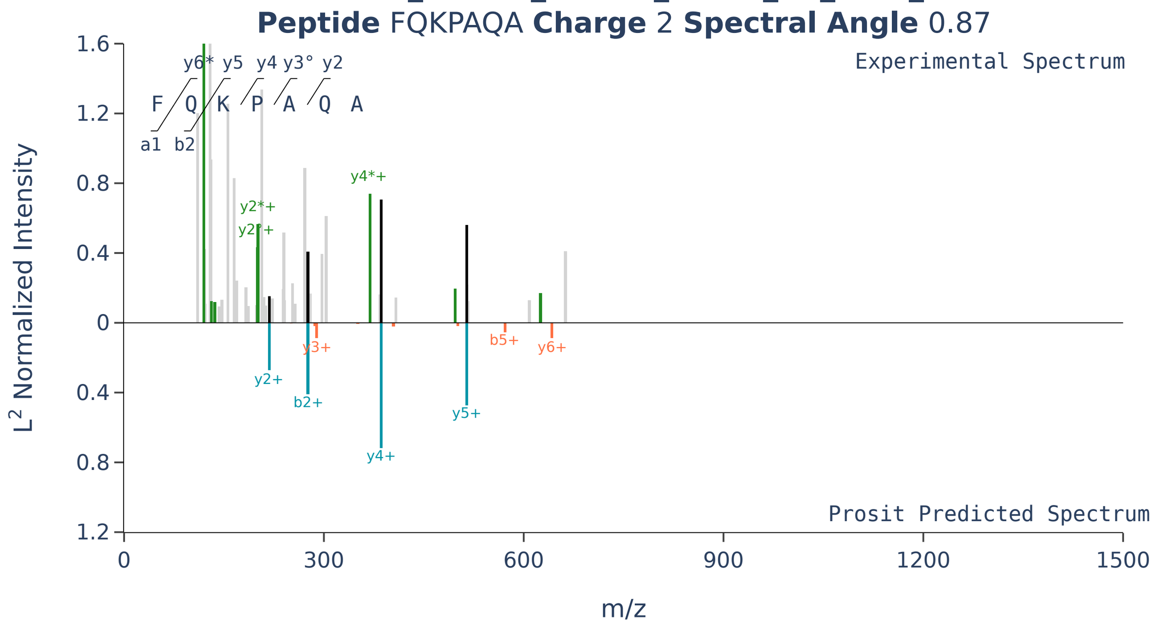


**Source** HRoetschke_211222_160123_HFGoe_IF2_b1R1_4h **Scan** 16348
**Peptide** WGGESQFVHVSAK **Charge** 3 **Spectral Angle** 0.8

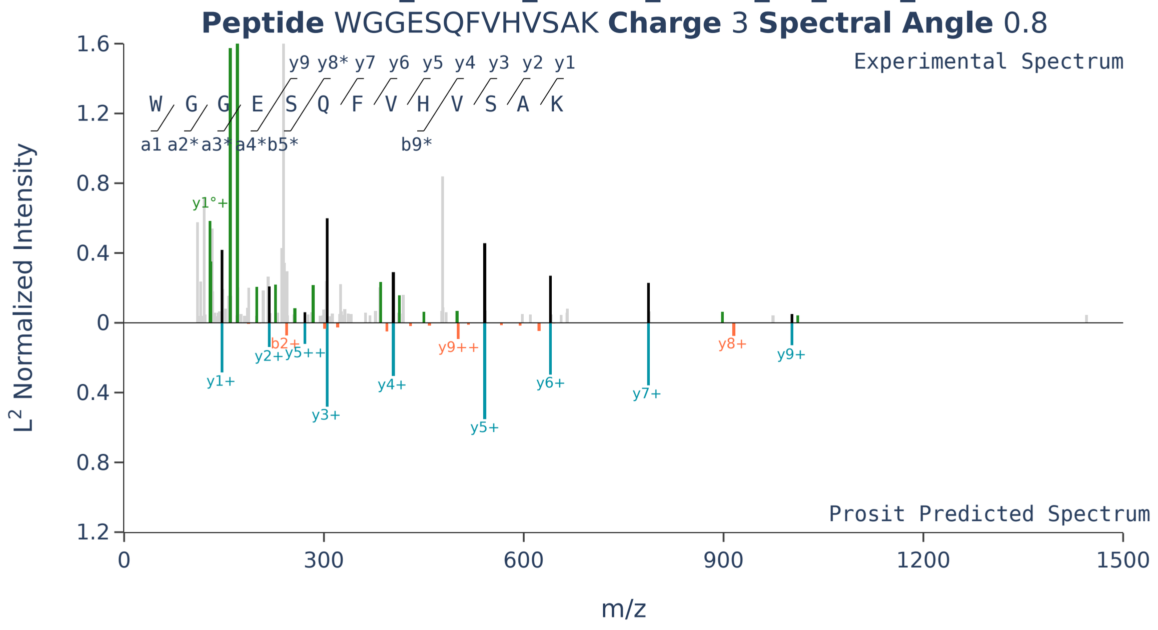


**Source** HRoetschke_211222_160123_HFGoe_IF2_b1R1_1h **Scan** 9783
**Peptide** DSVSAQEKQTL **Charge** 2 **Spectral Angle** 0.74

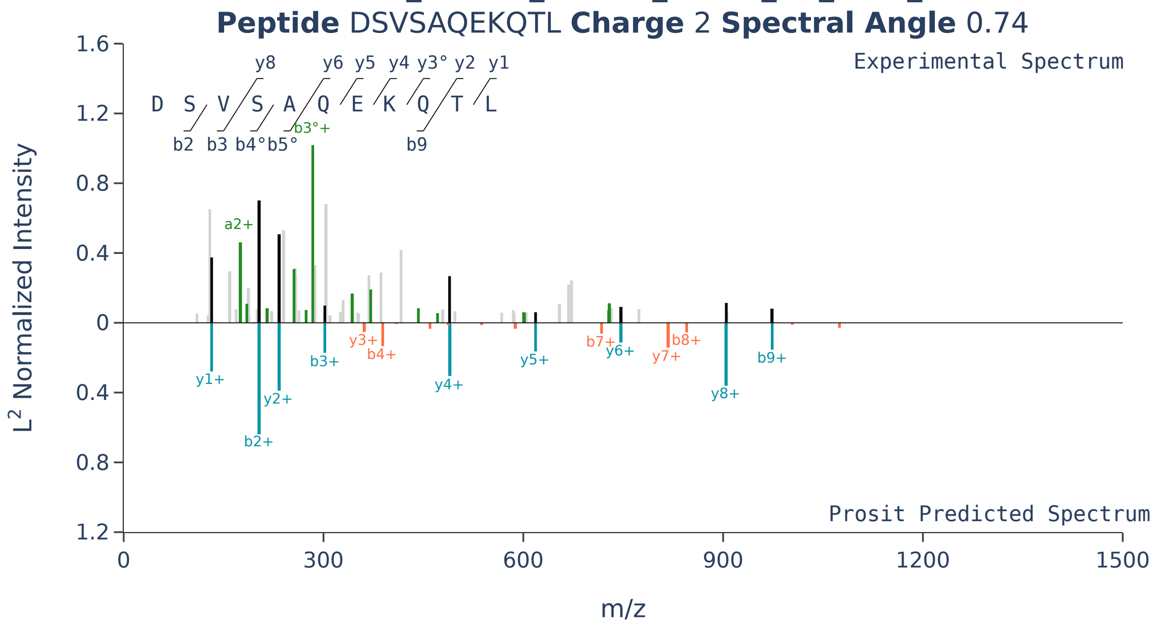


**Source** HRoetschke_211222_160123_HFGoe_IF2_b2R2_4h **Scan** 11108
**Peptide** QGFQKPAQAV **Charge** 2 **Spectral Angle** 0.8

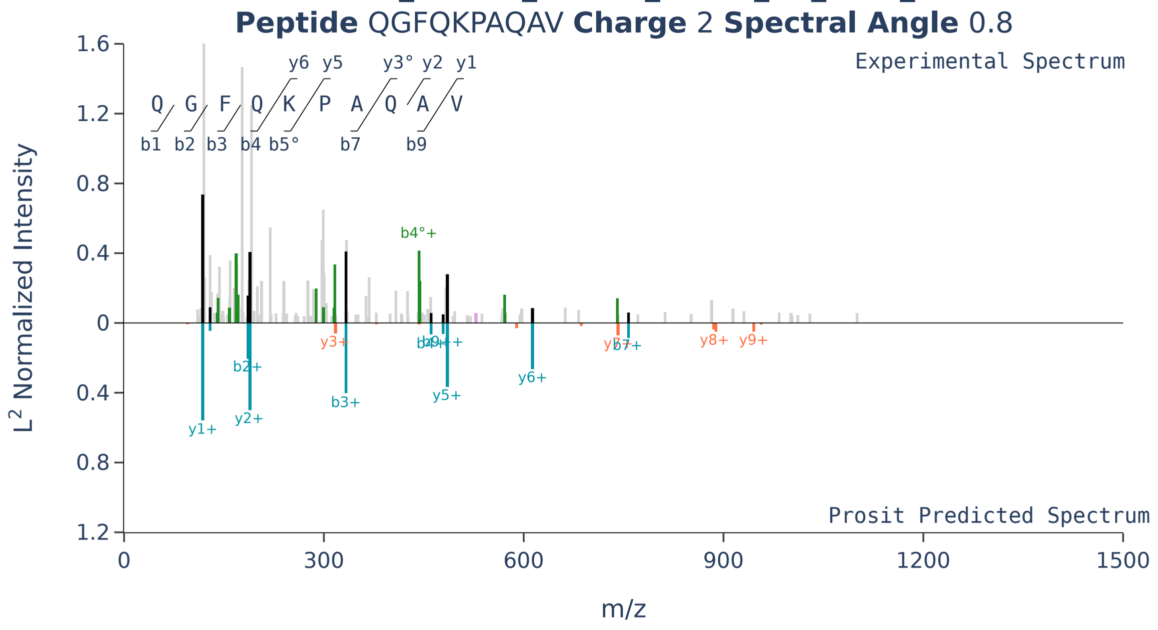


**Source** HRoetschke_211222_160123_HFGoe_IF2_b1R2_2h **Scan** 10534
**Peptide** VQQFADAGIRK **Charge** 3 **Spectral Angle** 0.78

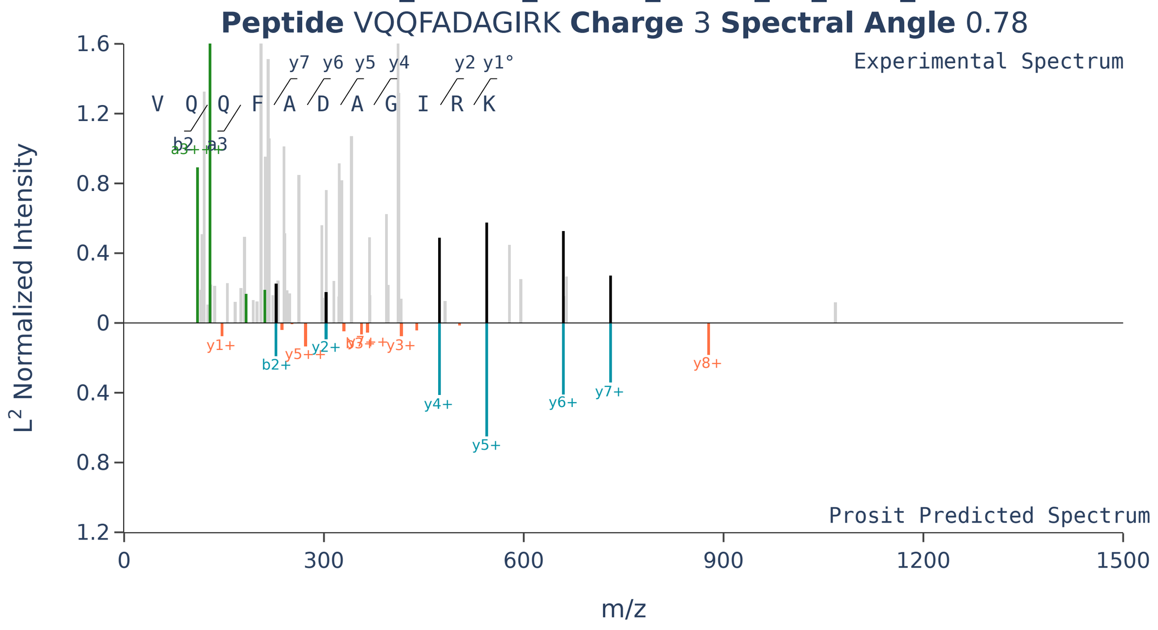


**Source** HRoetschke_211222_160123_HFGoe_IF2_b1R2_2h **Scan** 14053
**Peptide** VREGTLHKGDIVL **Charge** 3 **Spectral Angle** 0.77

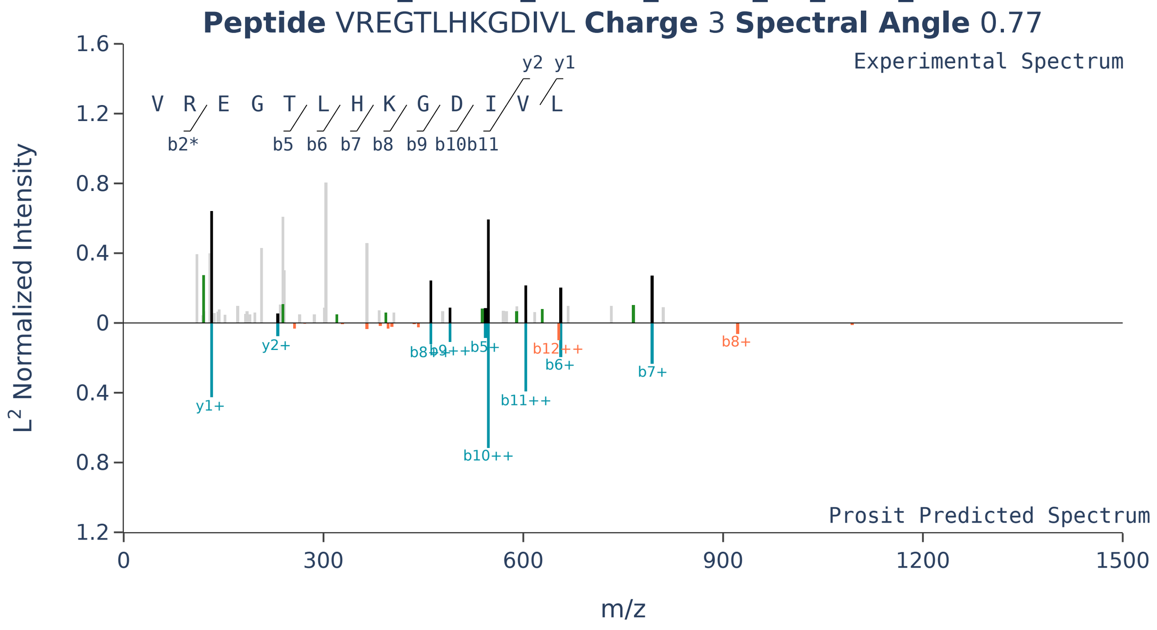


**Source** HRoetschke_211222_160123_HFGoe_IF2_b2R2_4h **Scan** 16397
**Peptide** LRDNVVIYE **Charge** 2 **Spectral Angle** 0.83

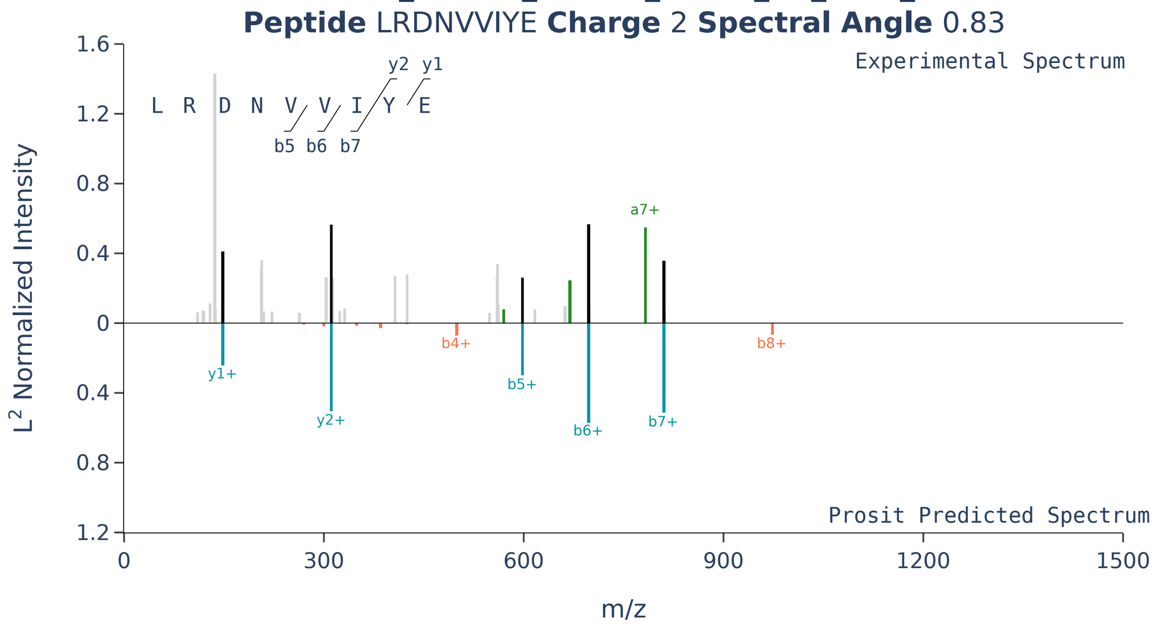


**Source** HRoetschke_211222_160123_HFGoe_IF2_b1R2_2h **Scan** 10388
**Peptide** RKGMASGAVI **Charge** 2 **Spectral Angle** 0.81

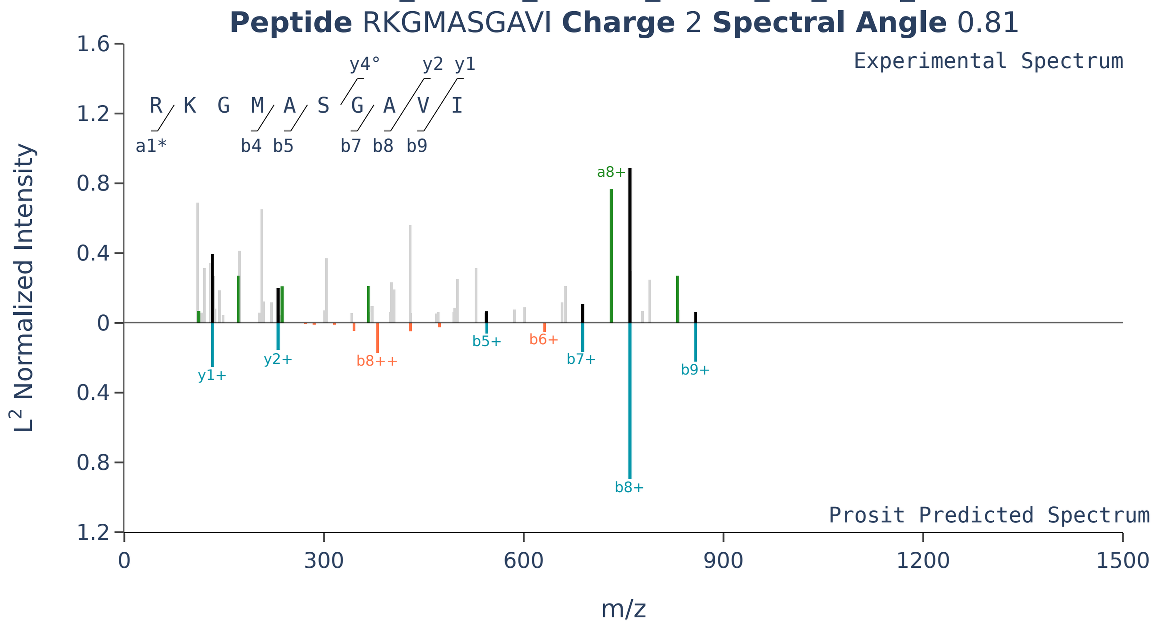


**Source** HRoetschke_211222_160123_HFGoe_IF2_b2R2_24h **Scan** 7400
**Peptide** AVNKIDKPEADPDRVKNE **Charge** 2 **Spectral Angle** 0.86

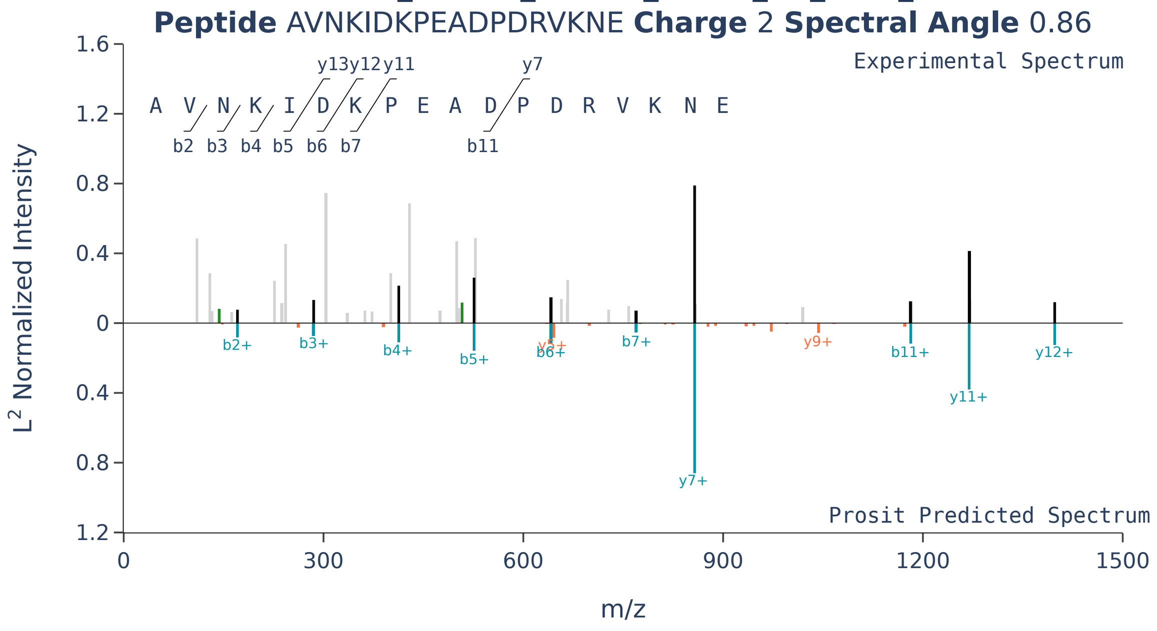


**Source** HRoetschke_211222_160123_HFGoe_IF2_b1R1_4h **Scan** 5539
**Peptide** AEEARRMAEE **Charge** 2 **Spectral Angle** 0.86

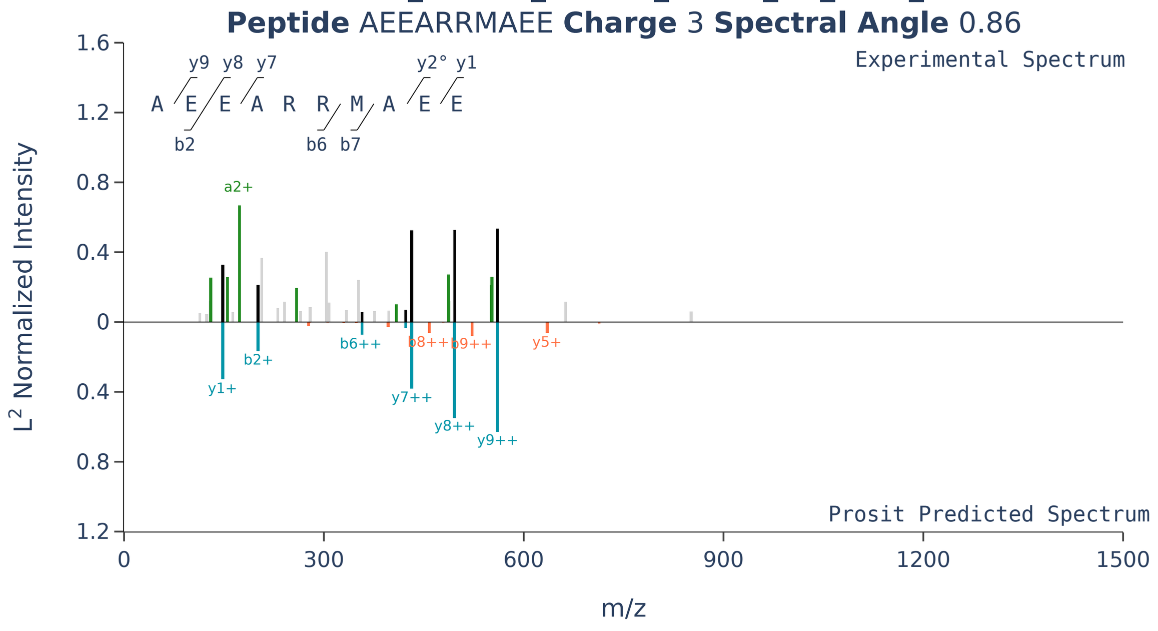


**Source** HRoetschke_211222_160123_HFGoe_IF2_b2R2_24h **Scan** 6819
**Peptide** ANKMAVKGSQV **Charge** 3 **Spectral Angle** 0.79

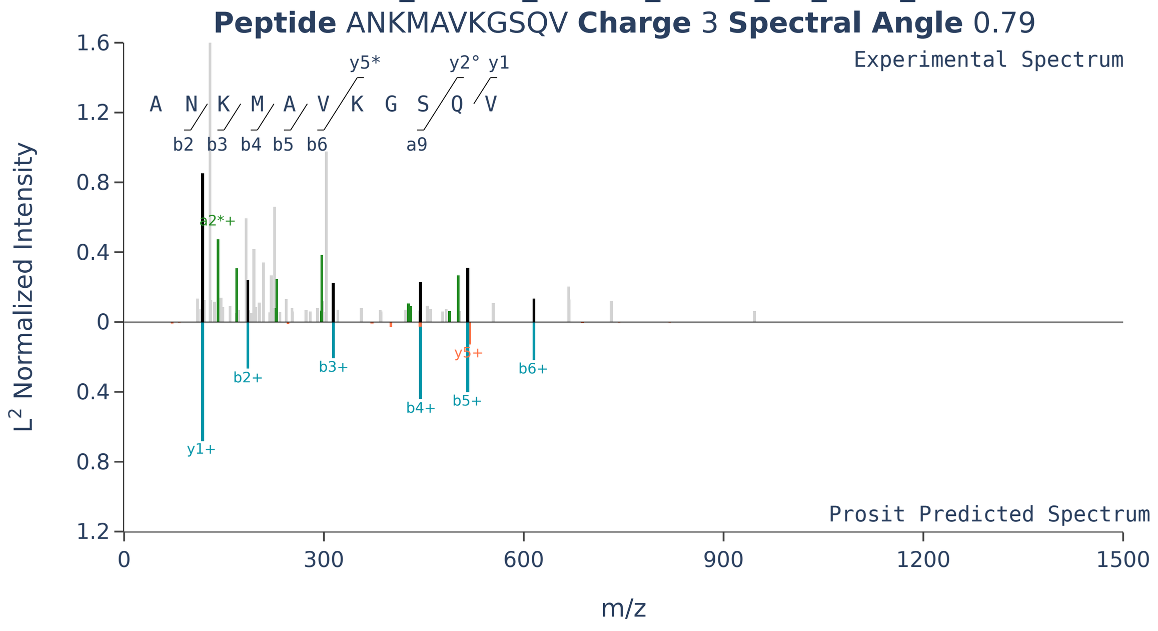


**Source** HRoetschke_211222_160123_HFGoe_IF2_b2R2_24h **Scan** 8068
**Peptide** FKDDVNEVRN **Charge** 3 **Spectral Angle** 0.79

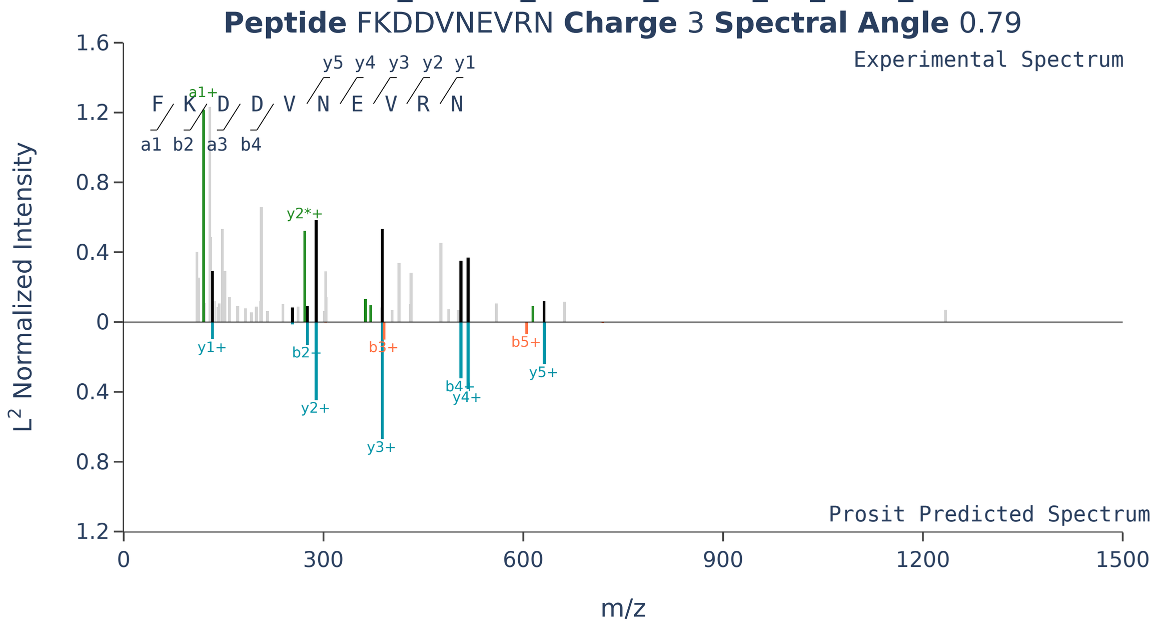


**Source** HRoetschke_211222_160123_HFGoe_IF2_b1R1_2h **Scan** 8212
**Peptide** DAGIRKSAD **Charge** 2 **Spectral Angle** 0.85

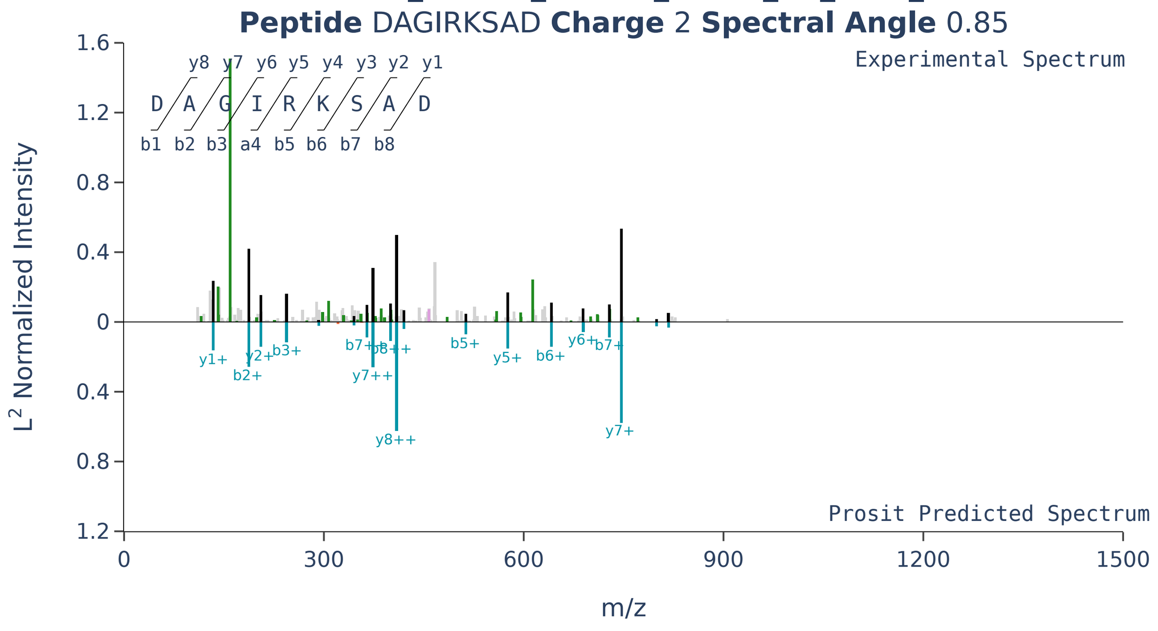


**Source** HRoetschke_211222_160123_HFGoe_IF2_b1R1_4h **Scan** 7992
**Peptide** DTPGHAAFTSMRARGA **Charge** 3 **Spectral Angle** 0.61

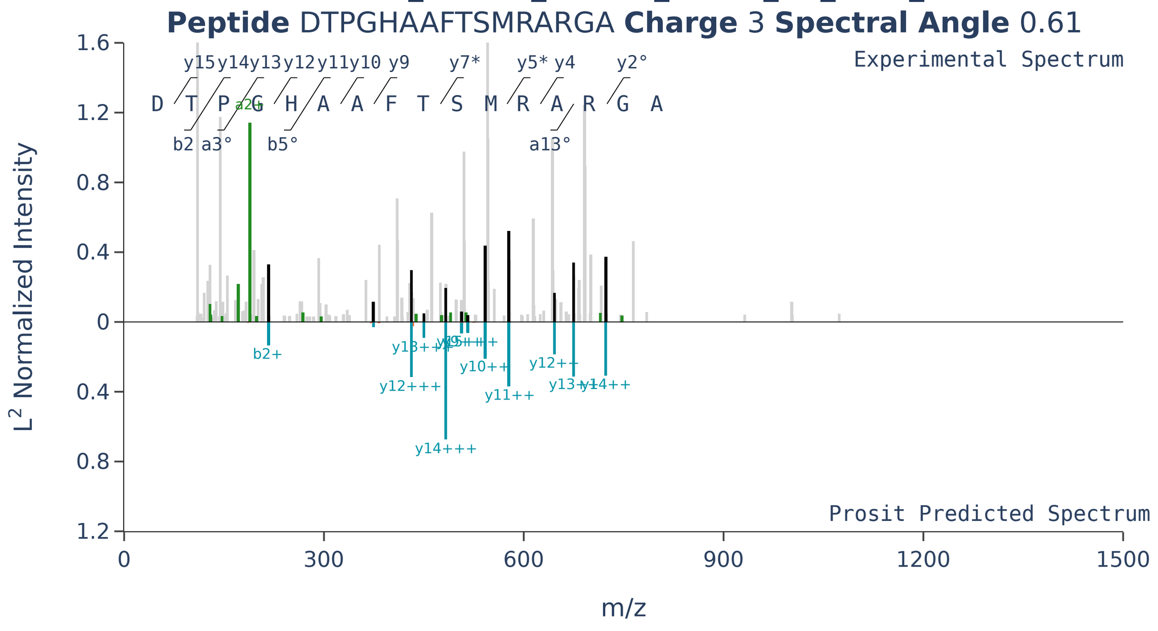


**Source** HRoetschke_211222_160123_HFGoe_IF2_b1R1_24h **Scan** 12387
**Peptide** NIPGTGGKSKSVQI **Charge** 2 **Spectral Angle** 0.75

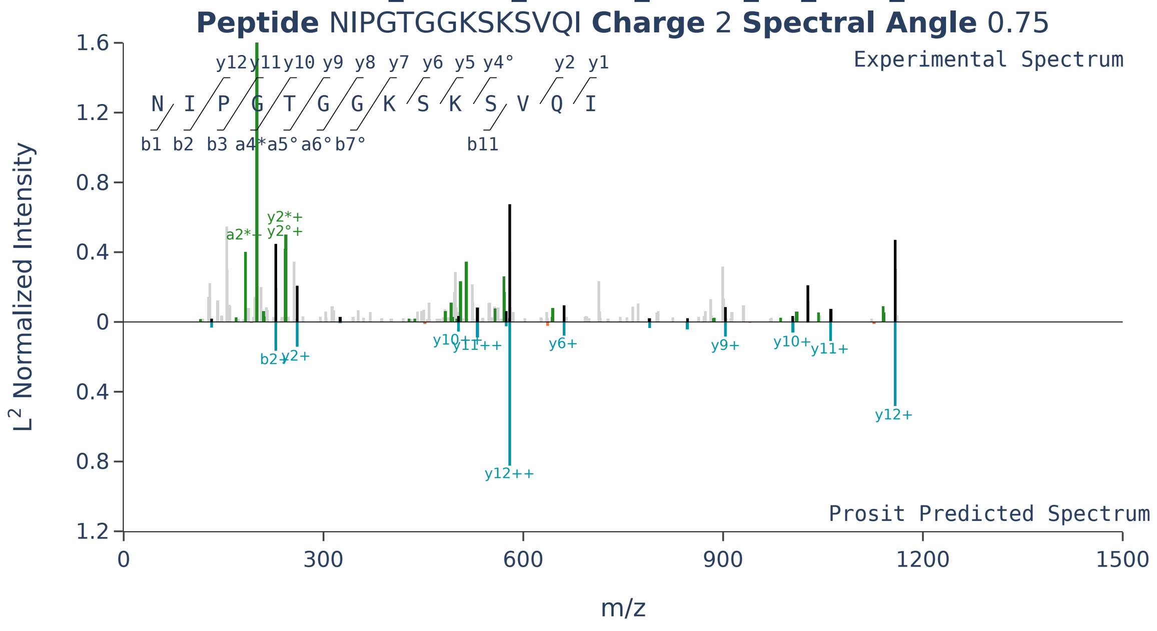

**Source** HRoetschke_211222_160123_HFGoe_IF2_b1R1_2h **Scan** 10167
**Peptide** LRRFKDDVNEV **Charge** 3 **Spectral Angle** 0.8

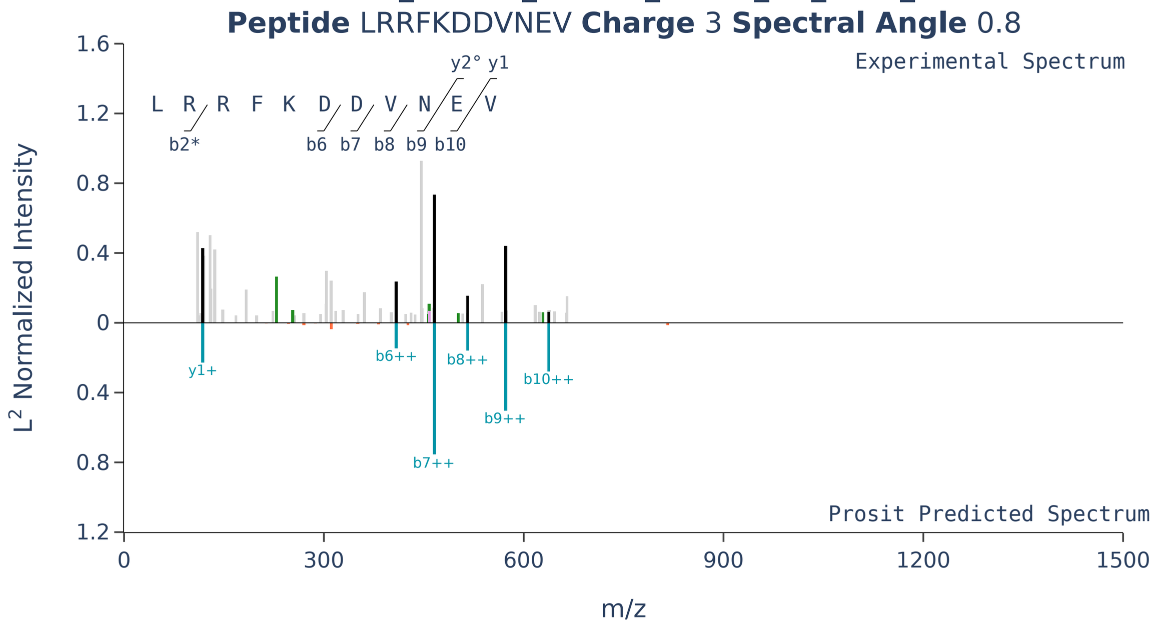


**Source** HRoetschke_211222_160123_HFGoe_IF2_b1R1_24h **Scan** 6085
**Peptide** RRFKDDVNEVRN **Charge** 3 **Spectral Angle** 0.68

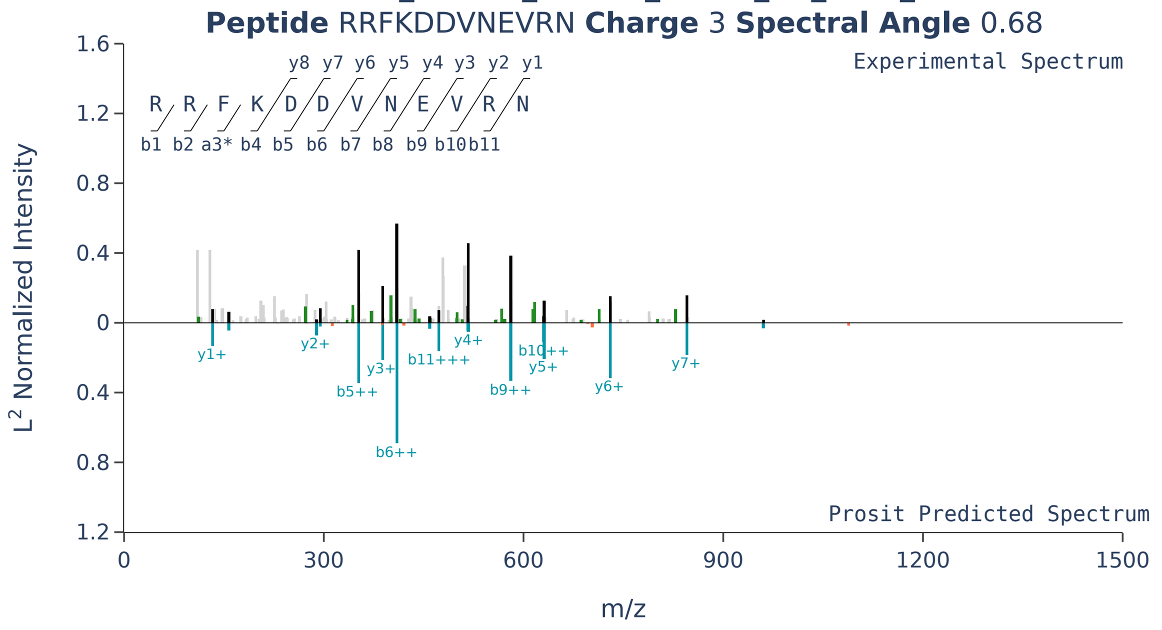


**Source** HRoetschke_211222_160123_HFGoe_IF2_b1R1_4h **Scan** 6876
**Peptide** VKRDPQEAERL **Charge** 2 **Spectral Angle** 0.84

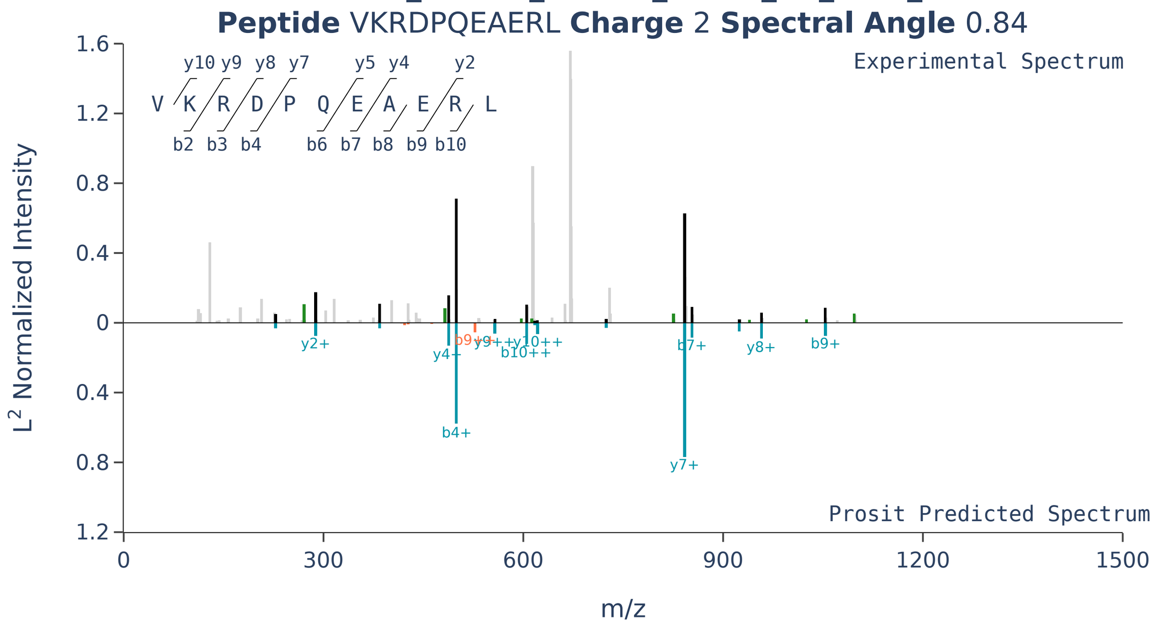


**Source** HRoetschke_211222_160123_HFGoe_IF2_b1R1_4h **Scan** 15239
**Peptide** GESQFVHVSA **Charge** 1 **Spectral Angle** 0.71

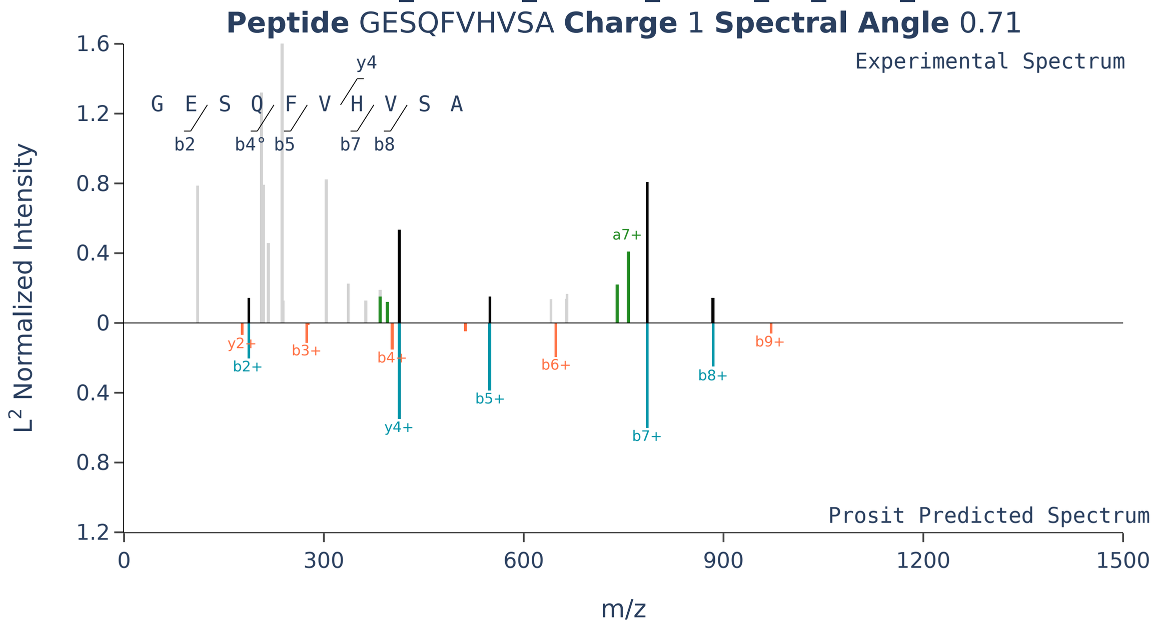


**Source** HRoetschke_211222_160123_HFGoe_IF2_b1R2_24h **Scan** 16409
**Peptide** QEKQTLIDHL **Charge** 3 **Spectral Angle** 0.81

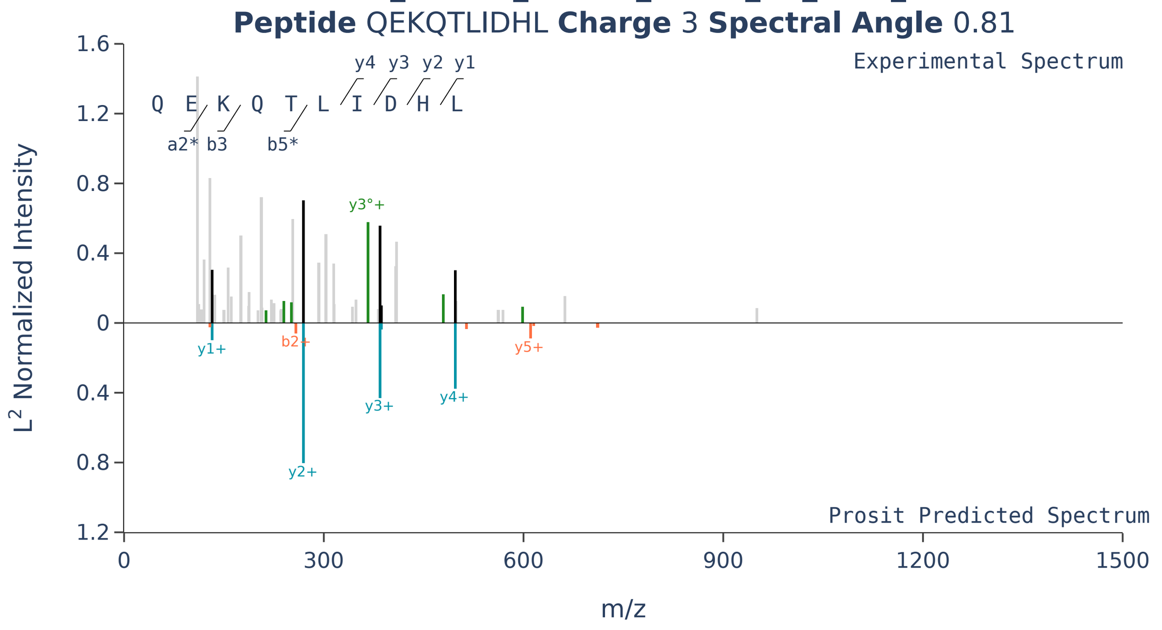


**Source** HRoetschke_211222_160123_HFGoe_IF2_b1R1_4h **Scan** 12653
**Peptide** QETAQLVAEEMGH **Charge** 2 **Spectral Angle** 0.67

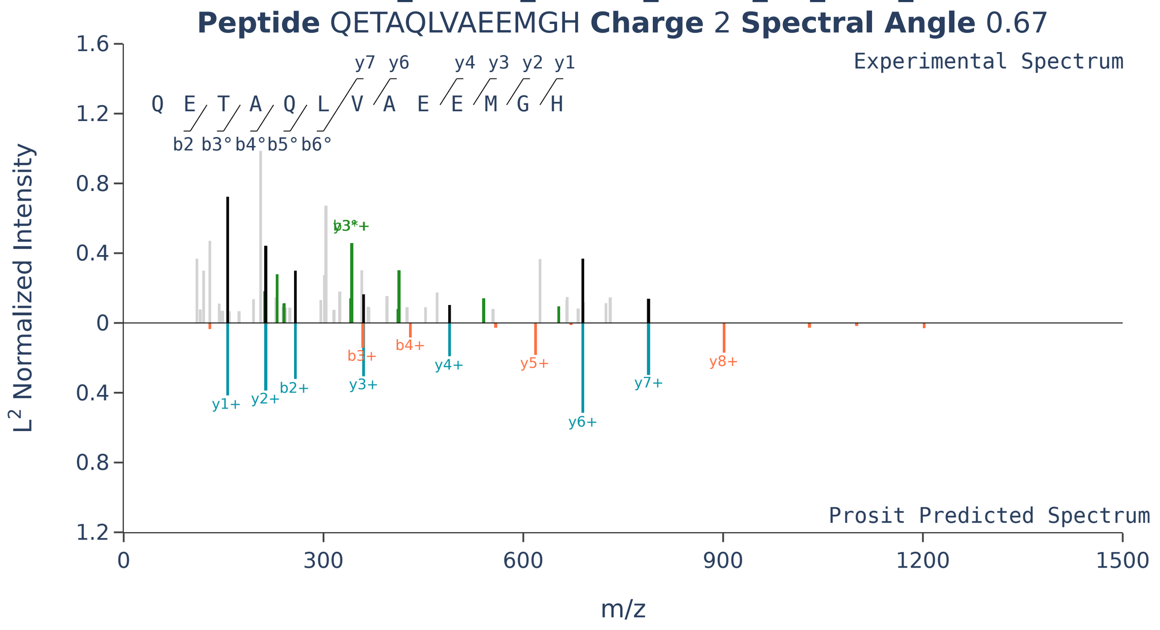


**Source** HRoetschke_211222_160123_HFGoe_IF2_b2R1_24h **Scan** 9123
**Peptide** DKPEADPDRV **Charge** 2 **Spectral Angle** 0.77

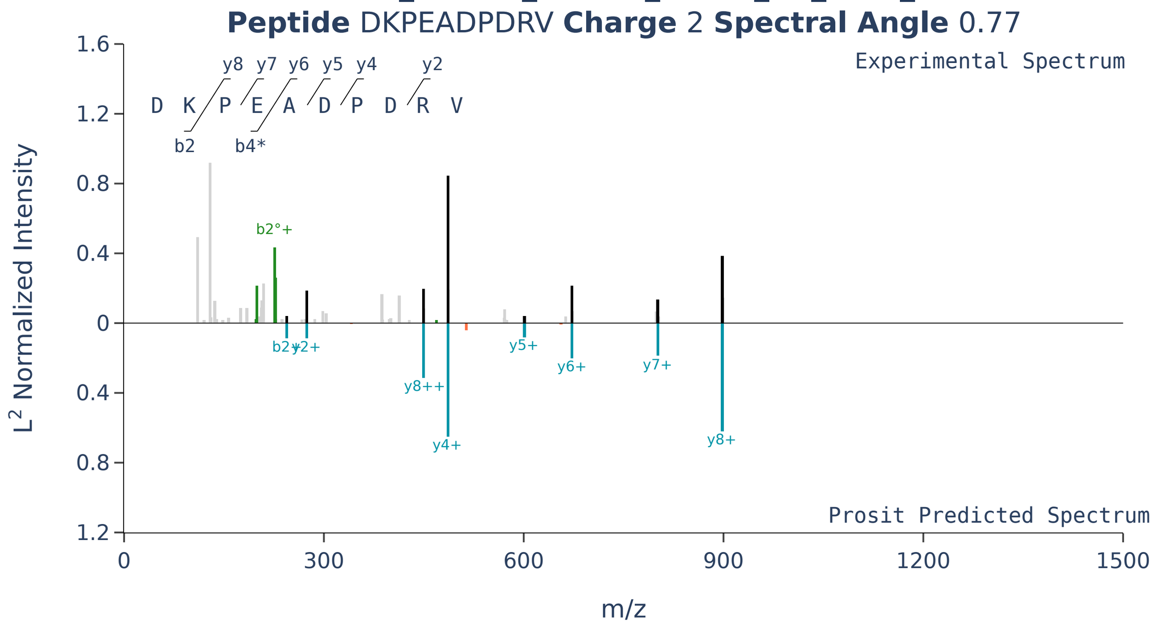


**Source** HRoetschke_211222_160123_HFGoe_IF2_b1R2_24h **Scan** 6626
**Peptide** ESLRRFKD **Charge** 3 **Spectral Angle** 0.76

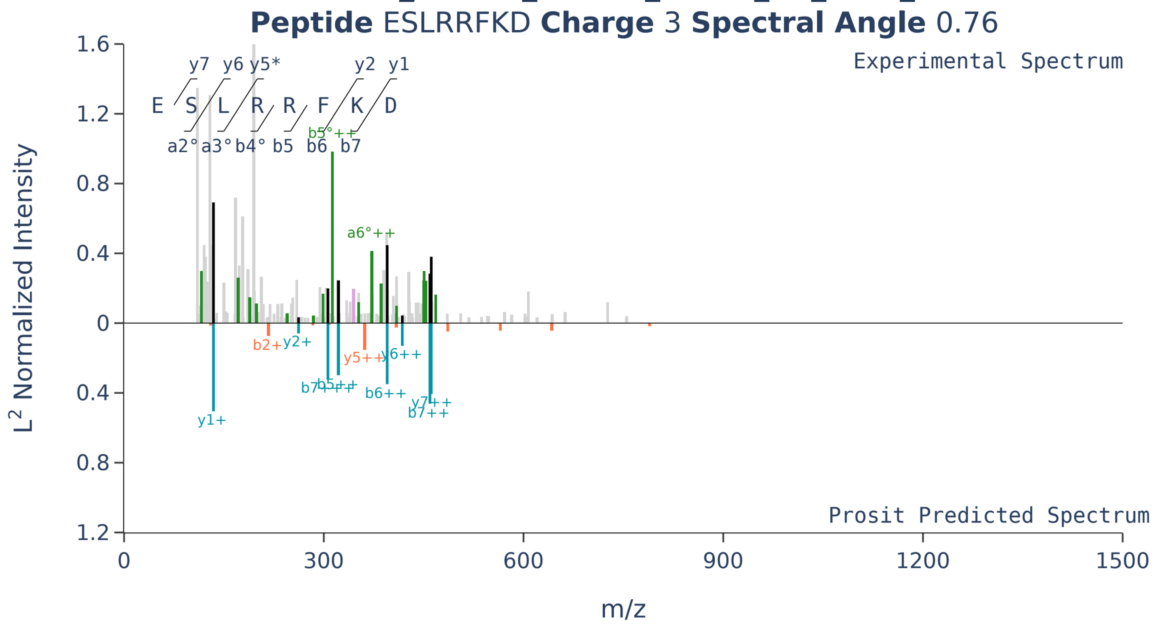


**Source** HRoetschke_211222_160123_HFGoe_IF2_b1R1_1h **Scan** 9060
**Peptide** GELANKMAVKG **Charge** 3 **Spectral Angle** 0.83

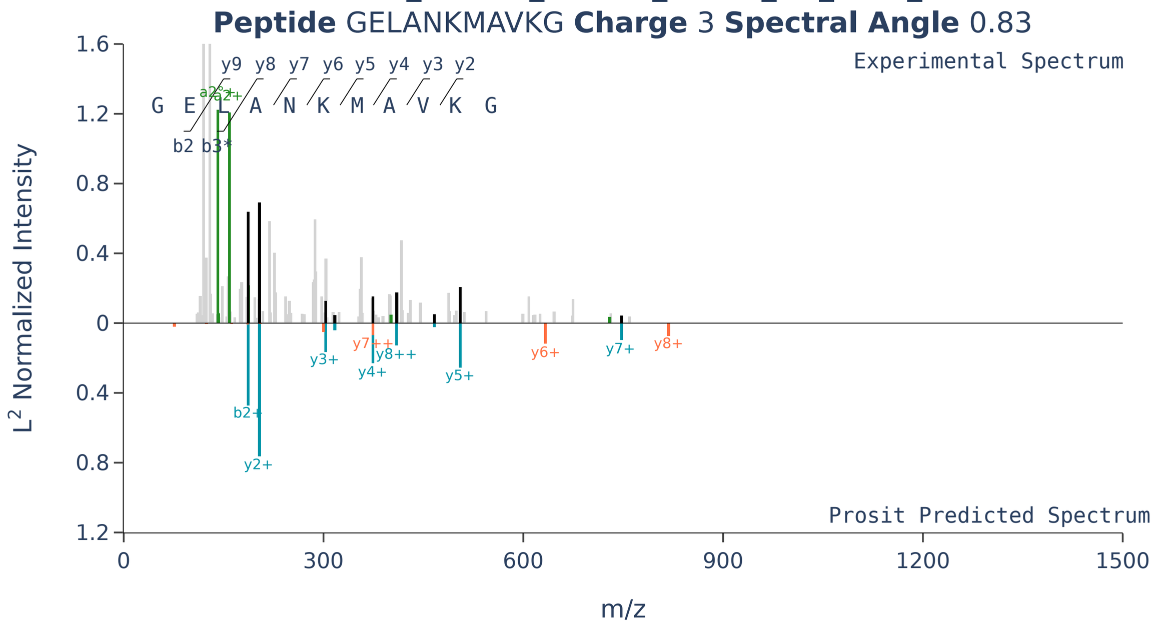


**Source** HRoetschke_211222_160123_HFGoe_IF2_b1R1_24h **Scan** 6497
**Peptide** SAKAGTGIDE **Charge** 2 **Spectral Angle** 0.9

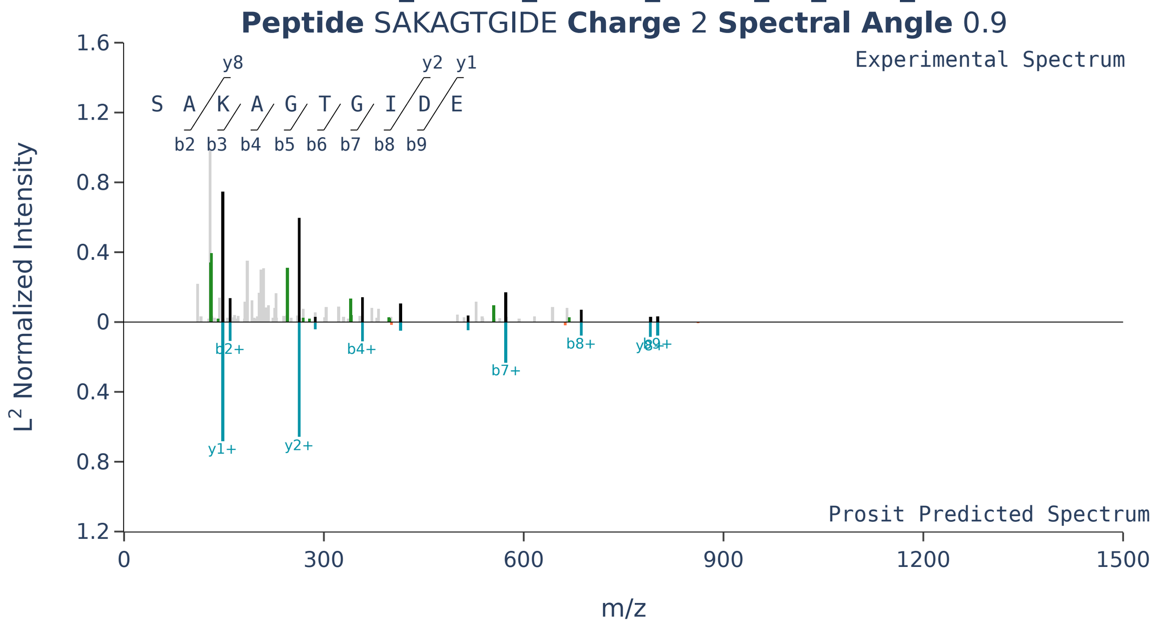


**Source** HRoetschke_211222_160123_HFGoe_IF2_b2R1_24h **Scan** 9535
**Peptide** RSTLNIPGTGGKSKS **Charge** 3 **Spectral Angle** 0.84

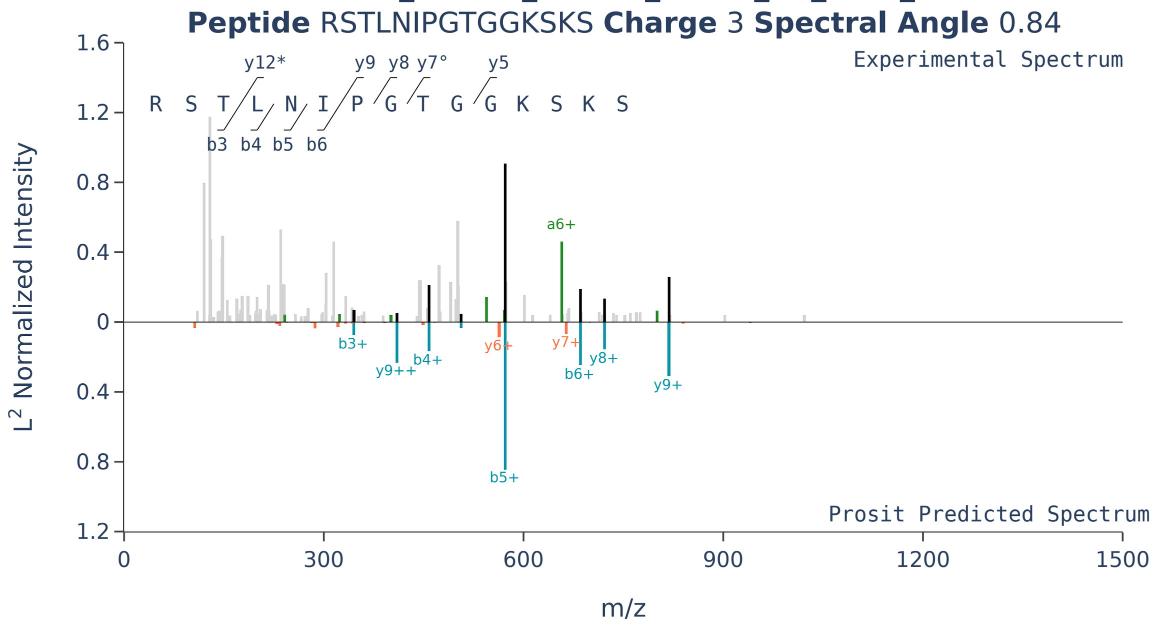


**Source** HRoetschke_211222_160123_HFGoe_IF2_b1R1_4h **Scan** 14113
**Peptide** PVVVAVNKIDK **Charge** 3 **Spectral Angle** 0.7

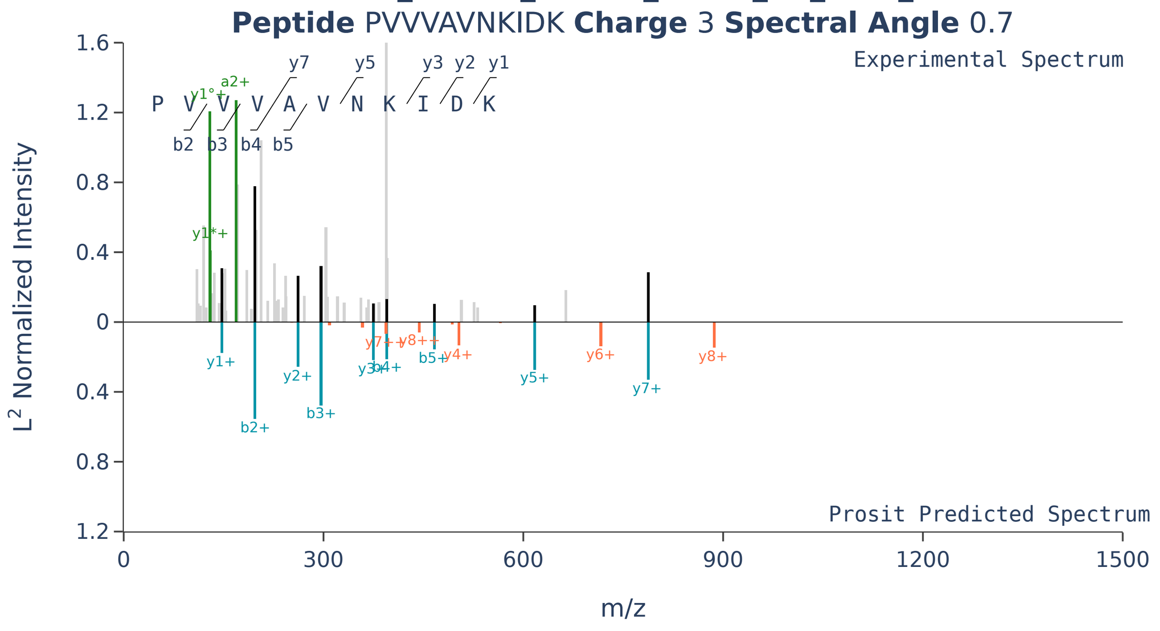


**Source** HRoetschke_211222_160123_HFGoe_IF2_b2R1_24h **Scan** 11351
**Peptide** AEENKWTDNAEPTED **Charge** 2 **Spectral Angle** 0.84

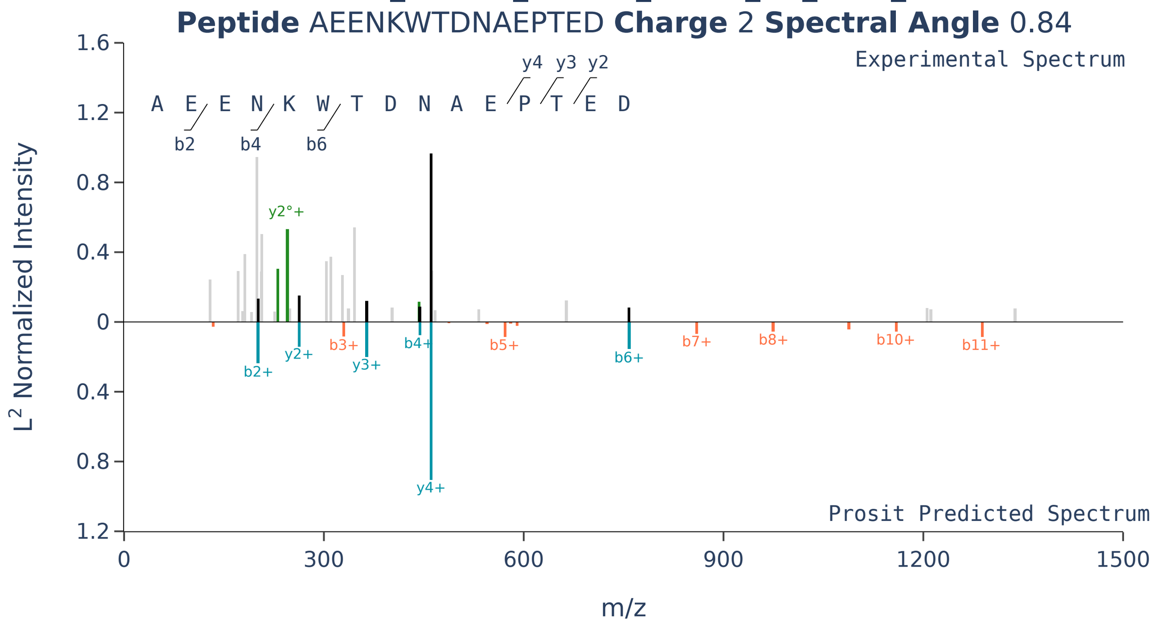


**Source** HRoetschke_211222_160123_HFGoe_IF2_b1R1_4h **Scan** 19993
**Peptide** RDNVVIYEGELE **Charge** 2 **Spectral Angle** 0.81

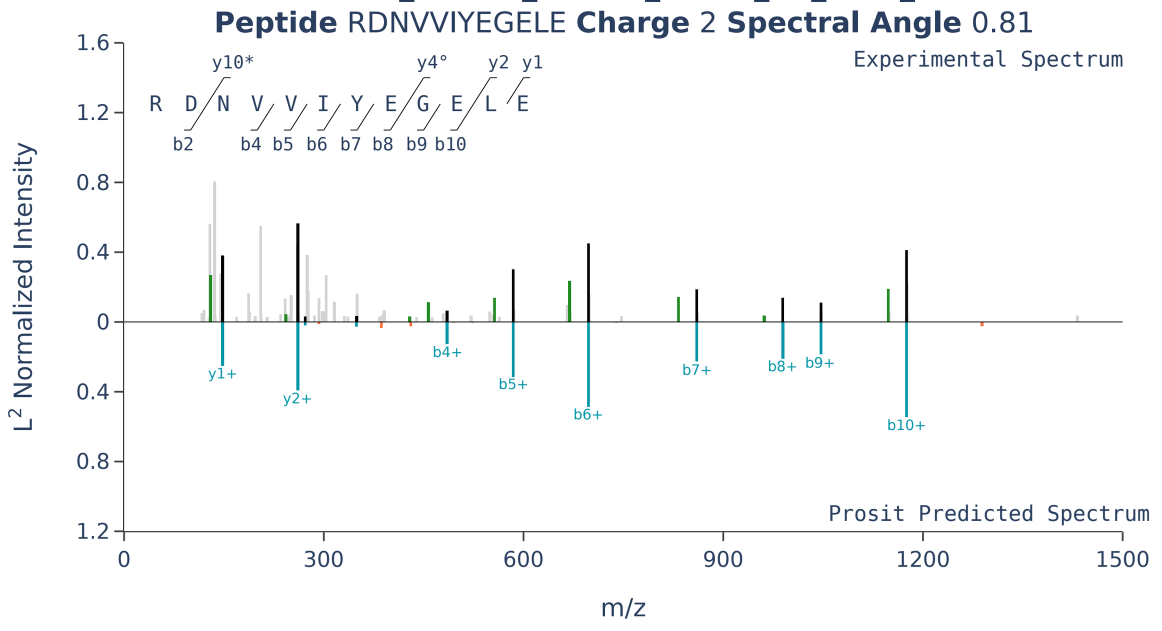


**Source** HRoetschke_211222_160123_HFGoe_IF2_b1R1_2h **Scan** 10678
**Peptide** TFVKRDPQEAERL **Charge** 3 **Spectral Angle** 0.73

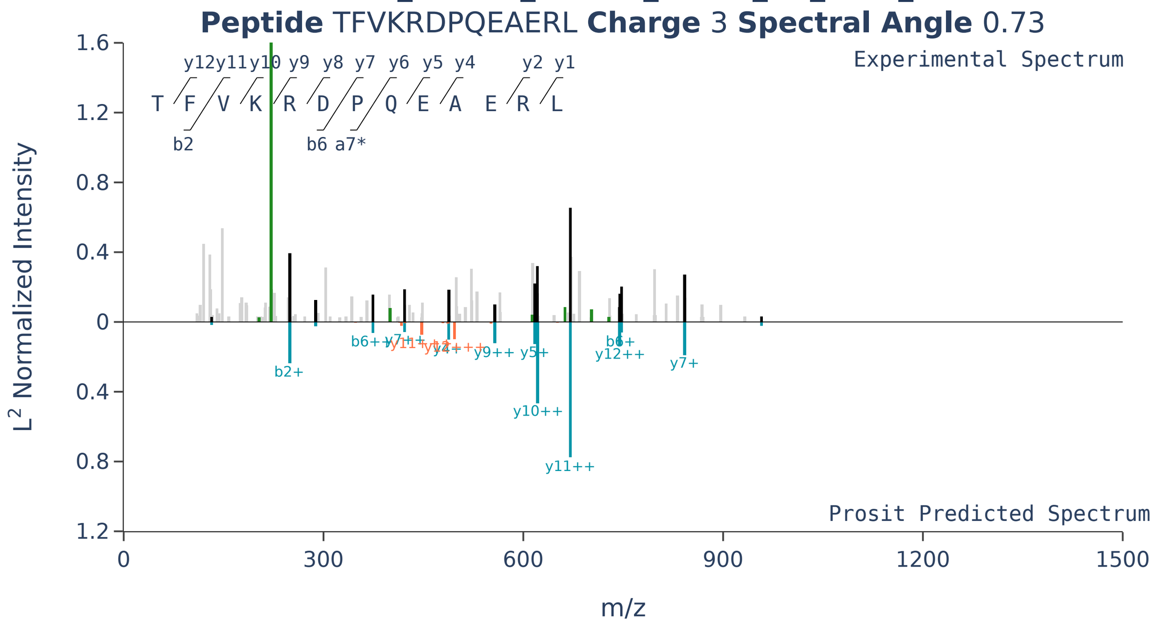

**Source** HRoetschke_211222_160123_HFGoe_IF2_b1R2_4h **Scan** 17465
**Peptide** AMATINQVLD **Charge** 2 **Spectral Angle** 0.74

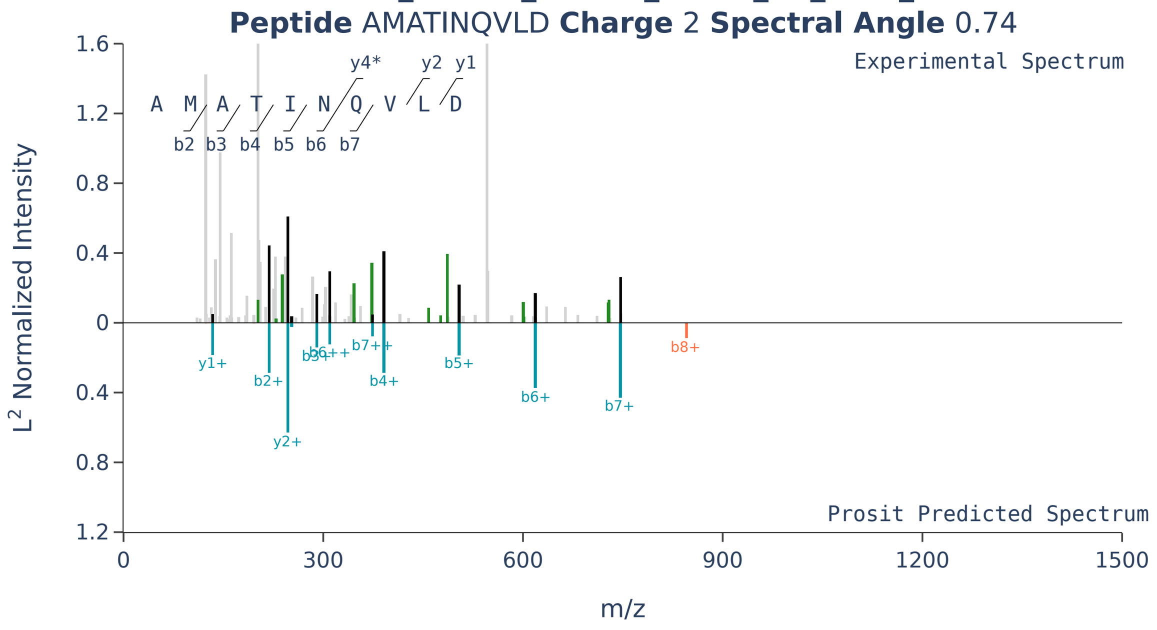

**Source** HRoetschke_211222_160123_HFGoe_IF2_b1R1_4h **Scan** 7921
**Peptide** TEGVVKRHNPIRV **Charge** 4 **Spectral Angle** 0.8

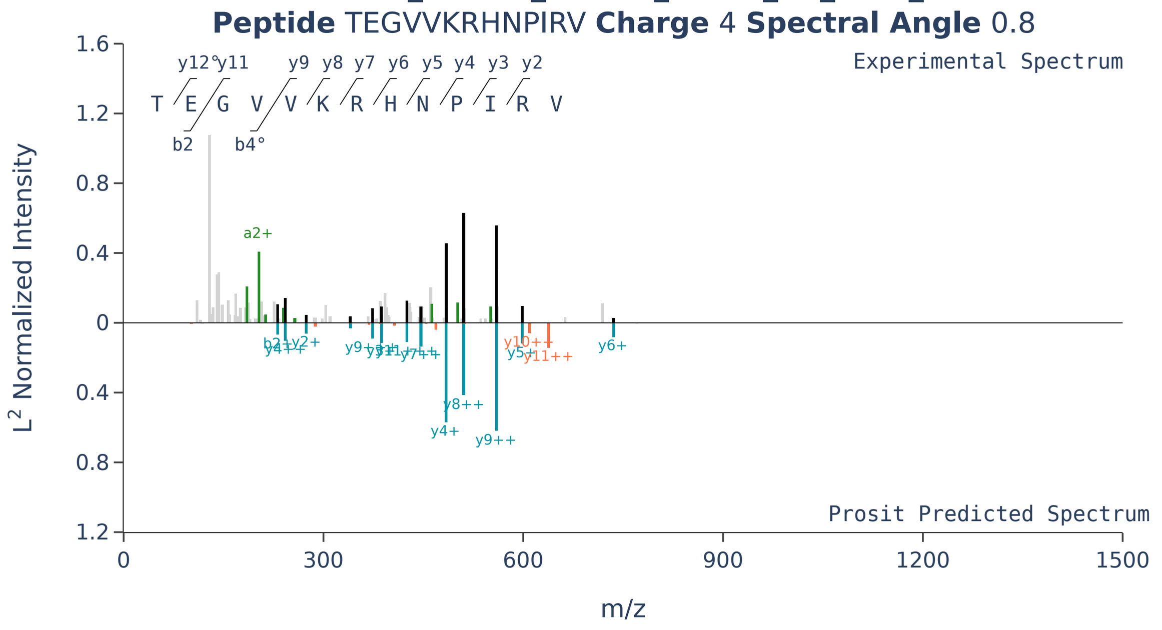

**Source** HRoetschke_211222_160123_HFGoe_IF2_b1R1_24h **Scan** 9524
**Peptide** DTPGHAAFTSMRA **Charge** 3 **Spectral Angle** 0.73

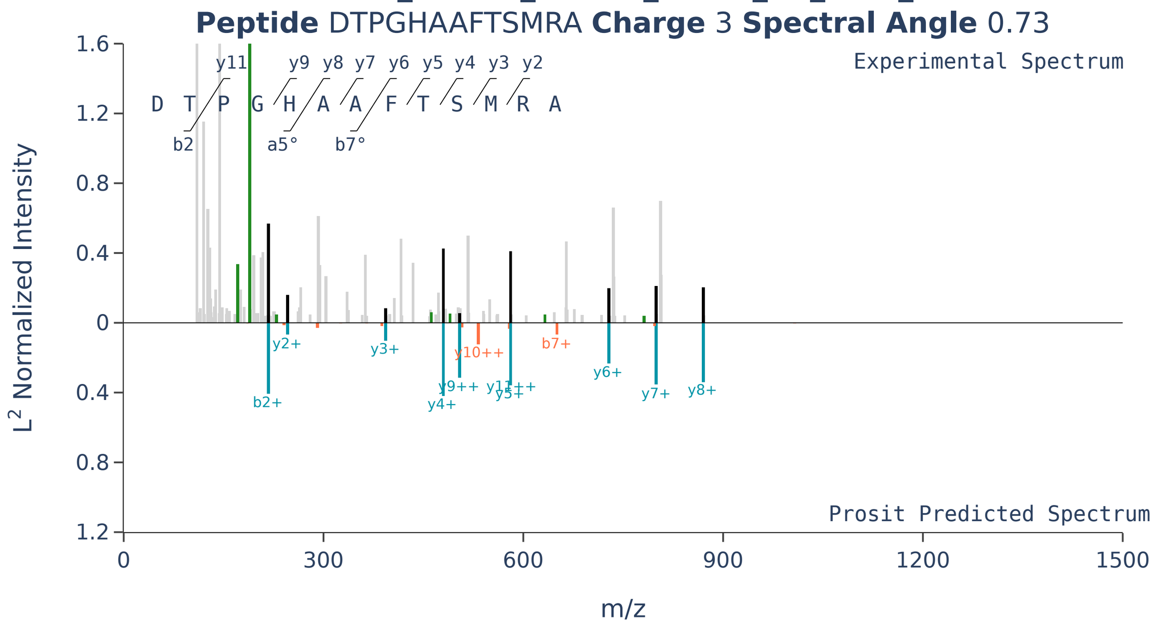

**Source** HRoetschke_211222_160123_HFGoe_IF2_b1R1_24h **Scan** 15304
**Peptide** VTIKTLA **Charge** 2 **Spectral Angle** 0.67

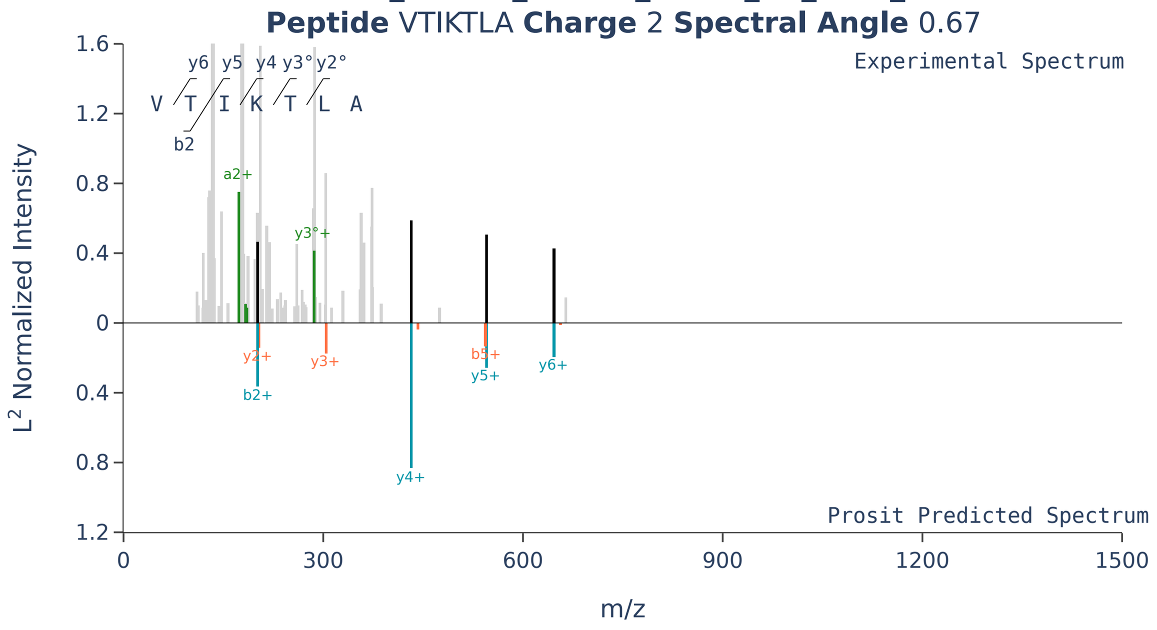

**Source** HRoetschke_211222_160123_HFGoe_IF2_b2R2_4h **Scan** 15510
**Peptide** ADDSVSAQEKQTLI **Charge** 2 **Spectral Angle** 0.85

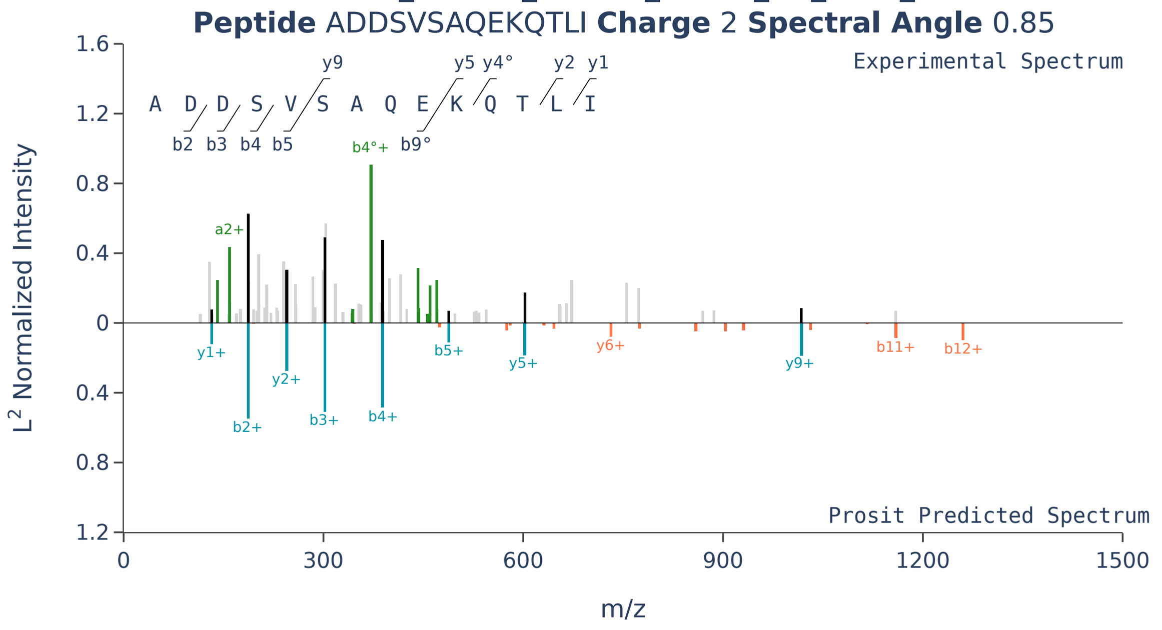

**Source** HRoetschke_211222_160123_HFGoe_IF2_b1R2_4h **Scan** 8014
**Peptide** ELANKMAVKGS **Charge** 3 **Spectral Angle** 0.82

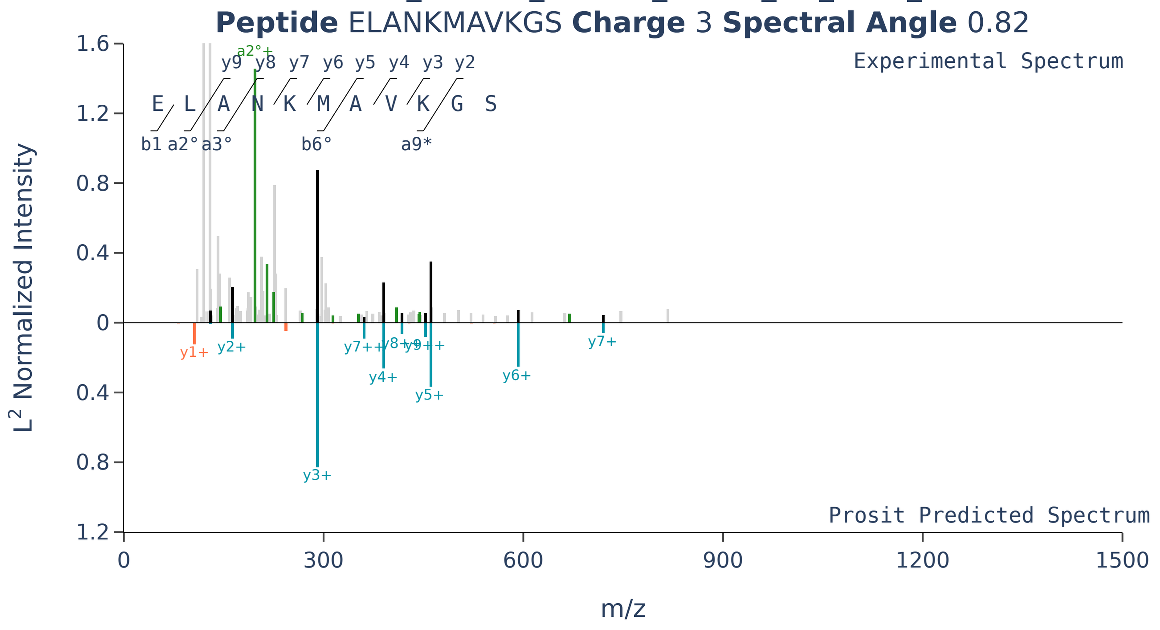

**Source** HRoetschke_211222_160123_HFGoe_IF2_b2R1_24h **Scan** 11886
**Peptide** TLNIPGTGGKSKS **Charge** 3 **Spectral Angle** 0.87

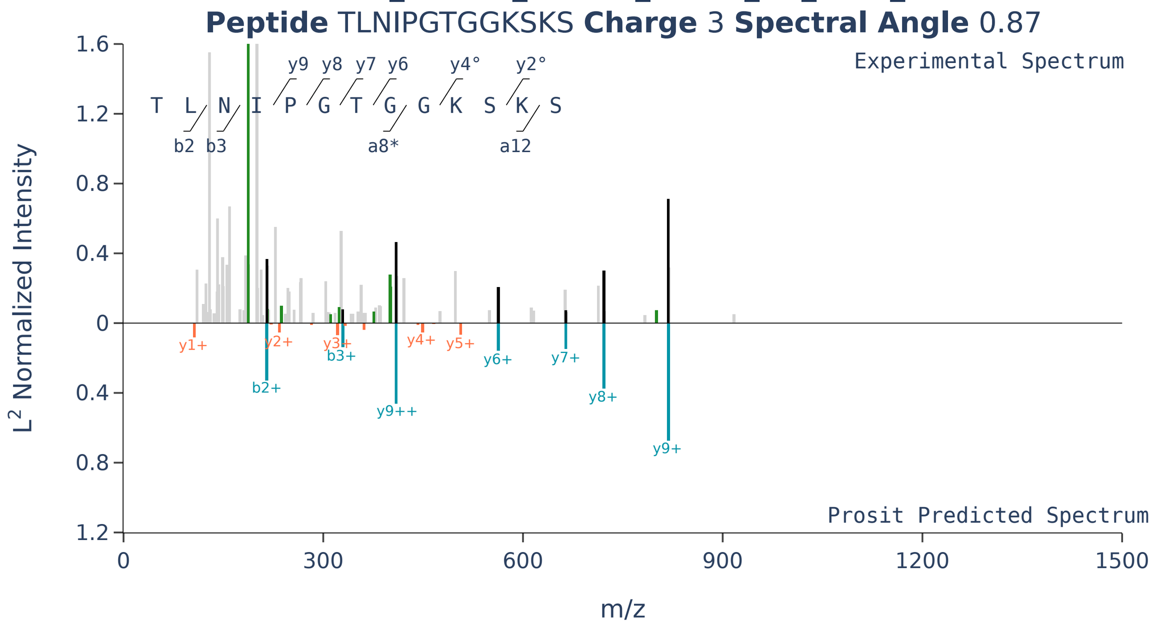

**Source** HRoetschke_211222_160123_HFGoe_IF2_b2R1_24h **Scan** 9983
**Peptide** AAFTSMRARGAQATD **Charge** 3 **Spectral Angle** 0.65

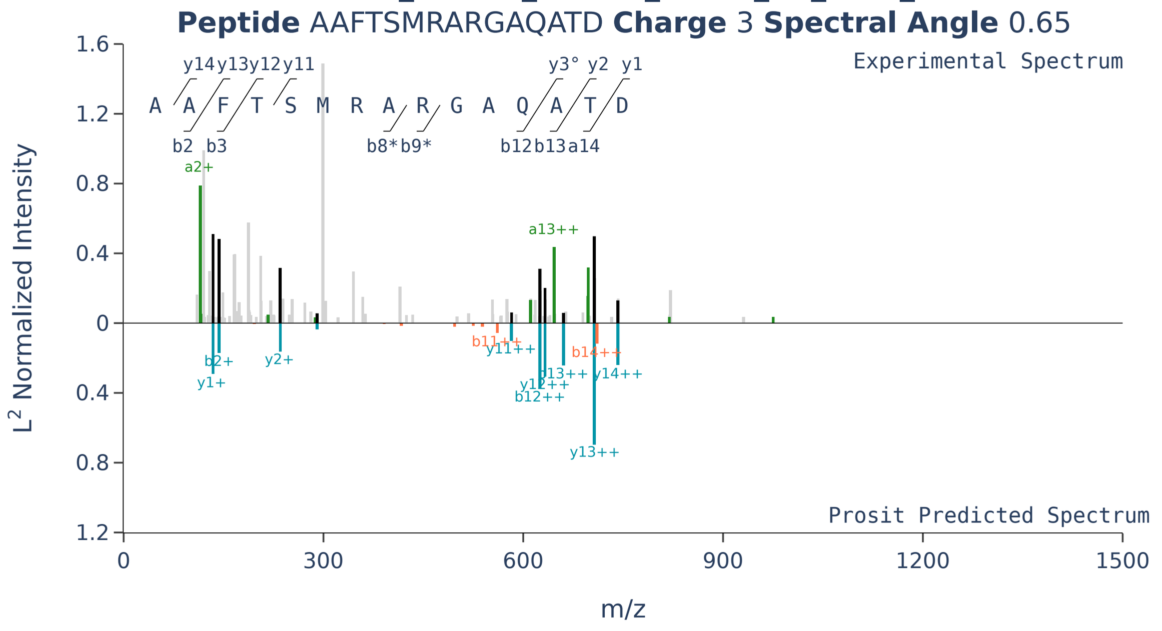

**Source** HRoetschke_211222_160123_HFGoe_IF2_b1R1_4h **Scan** 8049
**Peptide** KAVRKGMASGAVIE **Charge** 2 **Spectral Angle** 0.76

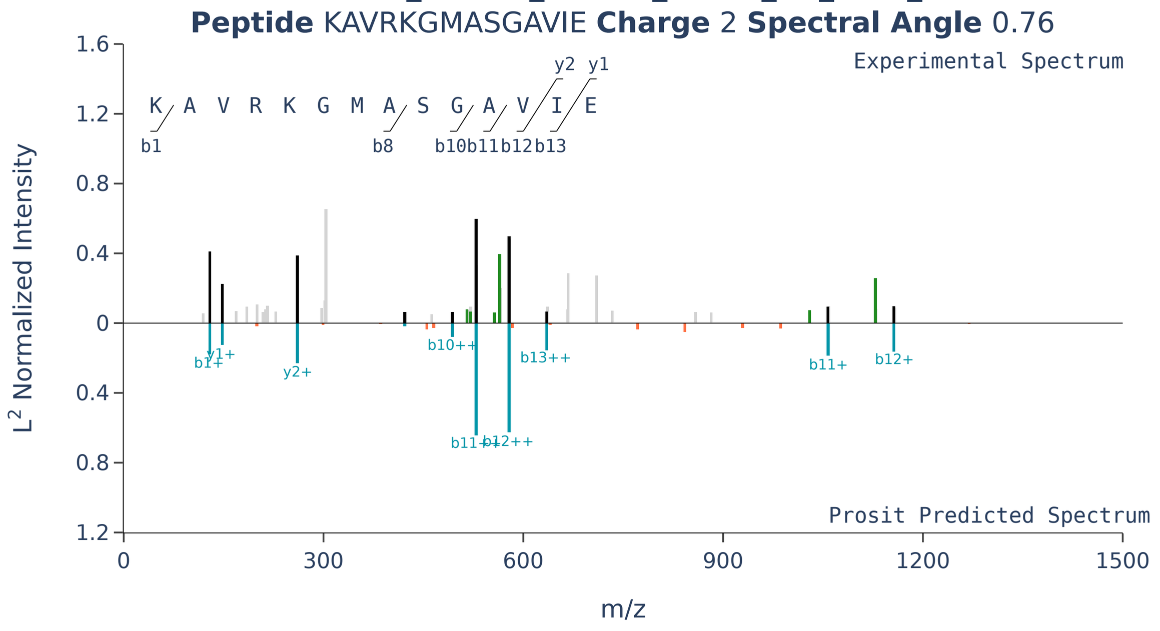

**Source** HRoetschke_211222_160123_HFGoe_IF2_b1R1_2h **Scan** 8072
**Peptide** QIEVRKKRTFV **Charge** 3 **Spectral Angle** 0.73

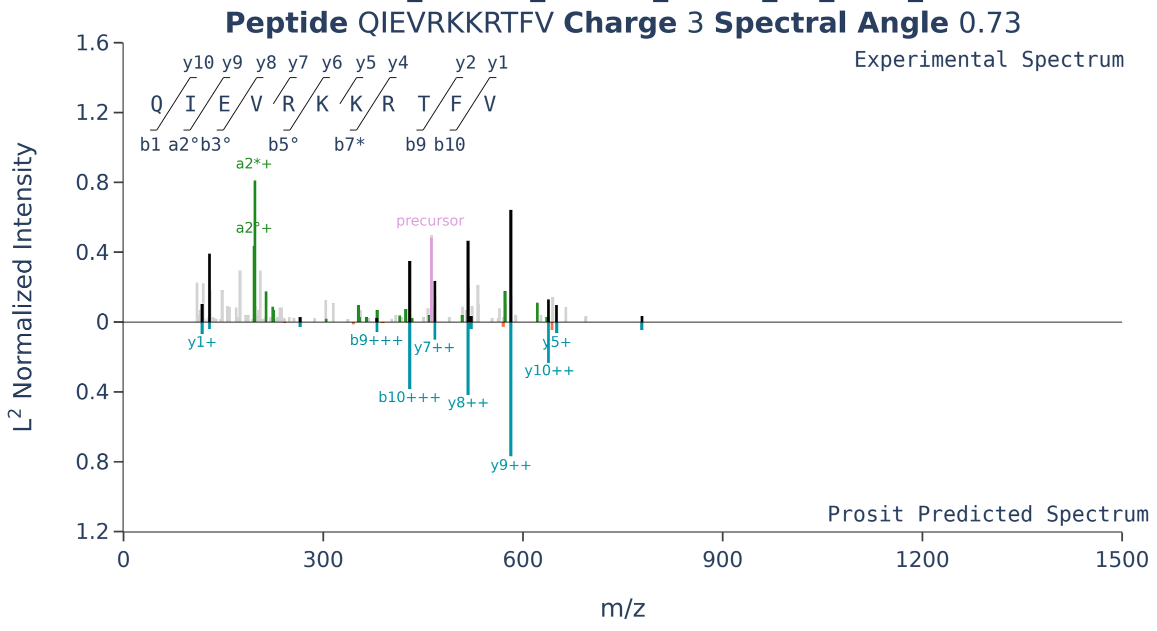

**Source** HRoetschke_211222_160123_HFGoe_IF2_b2R1_24h **Scan** 6224
**Peptide** KIDKPEADPDRVKNE **Charge** 2 **Spectral Angle** 0.71

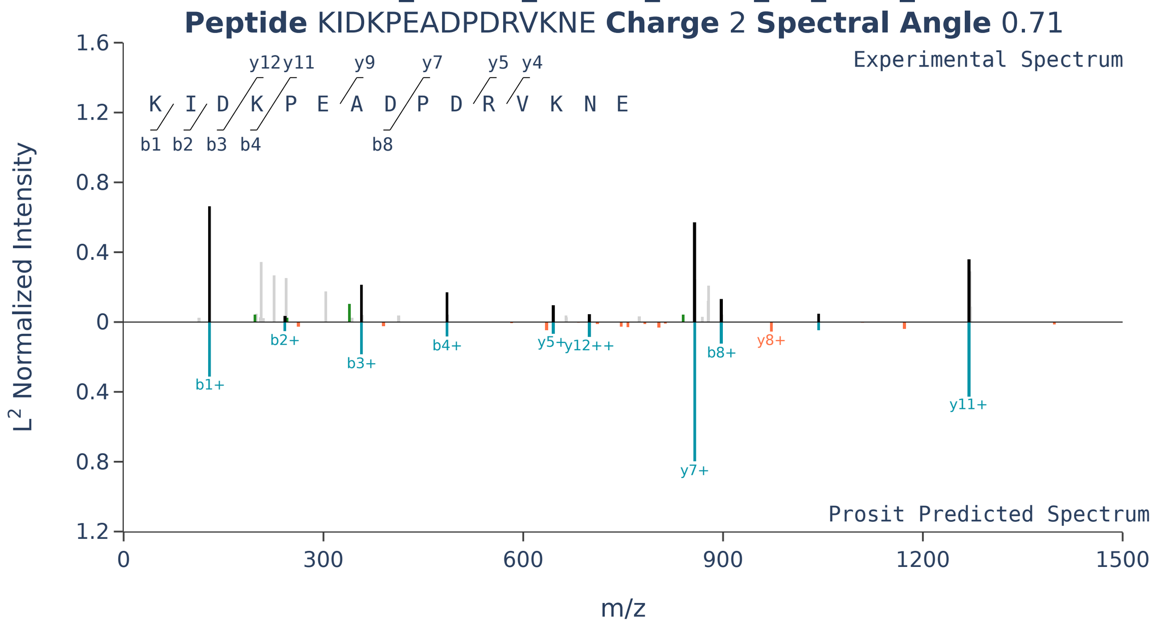

**Source** HRoetschke_211222_160123_HFGoe_IF2_b2R1_24h **Scan** 6744
**Peptide** KNSGPDKLTLQRKT **Charge** 3 **Spectral Angle** 0.79

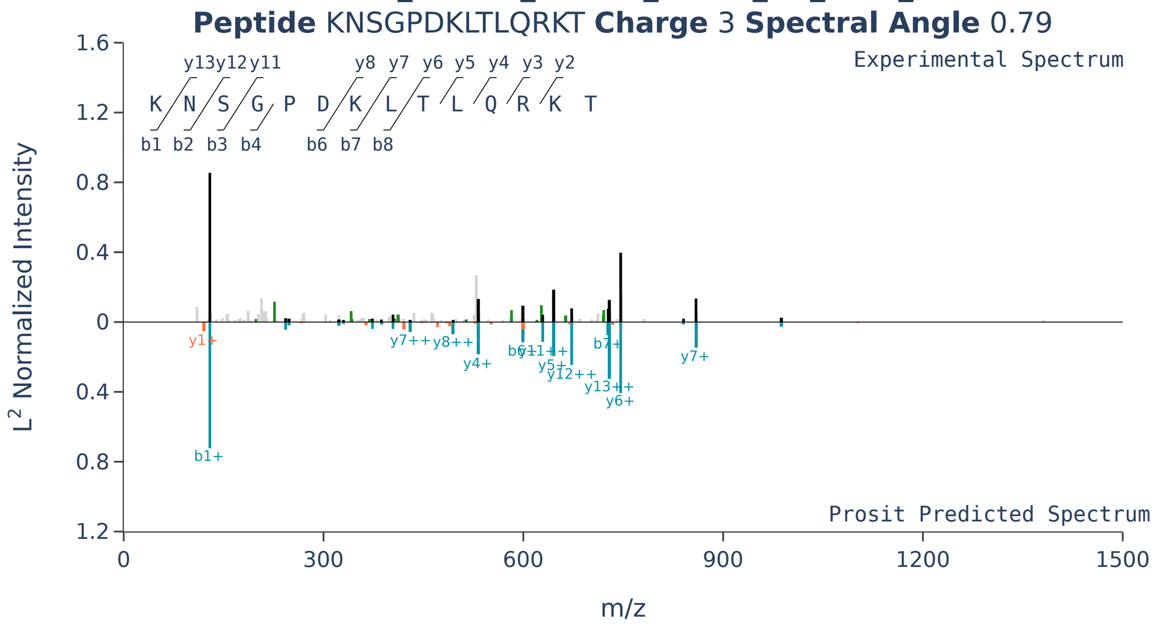

**Source** HRoetschke_211222_160123_HFGoe_IF2_b1R1_2h **Scan** 10935
**Peptide** QGSVEAISD **Charge** 2 **Spectral Angle** 0.89

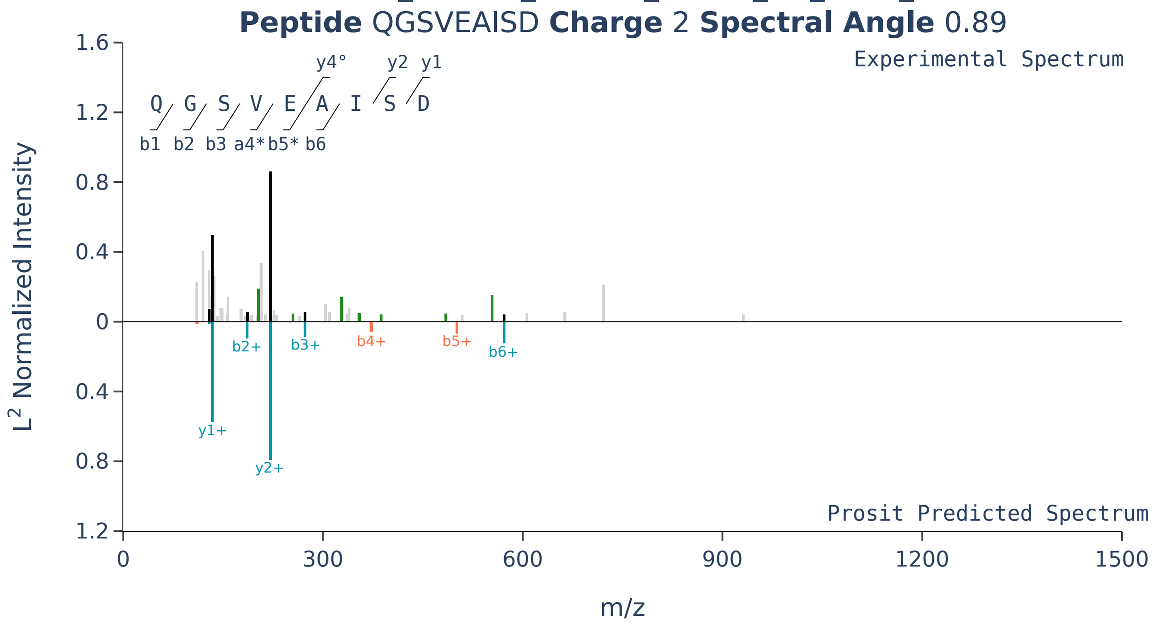

**Source** HRoetschke_211222_160123_HFGoe_IF2_b1R1_2h **Scan** 10560
**Peptide** TPGHAAFTSMR **Charge** 3 **Spectral Angle** 0.7

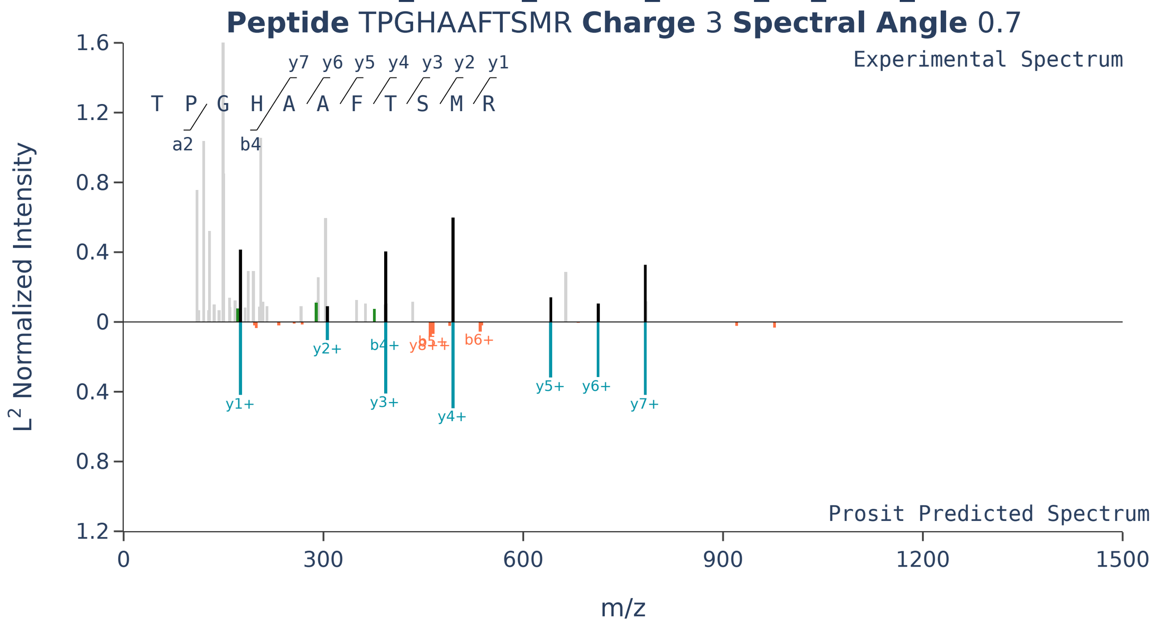

**Source** HRoetschke_211222_160123_HFGoe_IF2_b1R2_2h **Scan** 9879
**Peptide** PTEDSSDYHVTT **Charge** 2 **Spectral Angle** 0.75

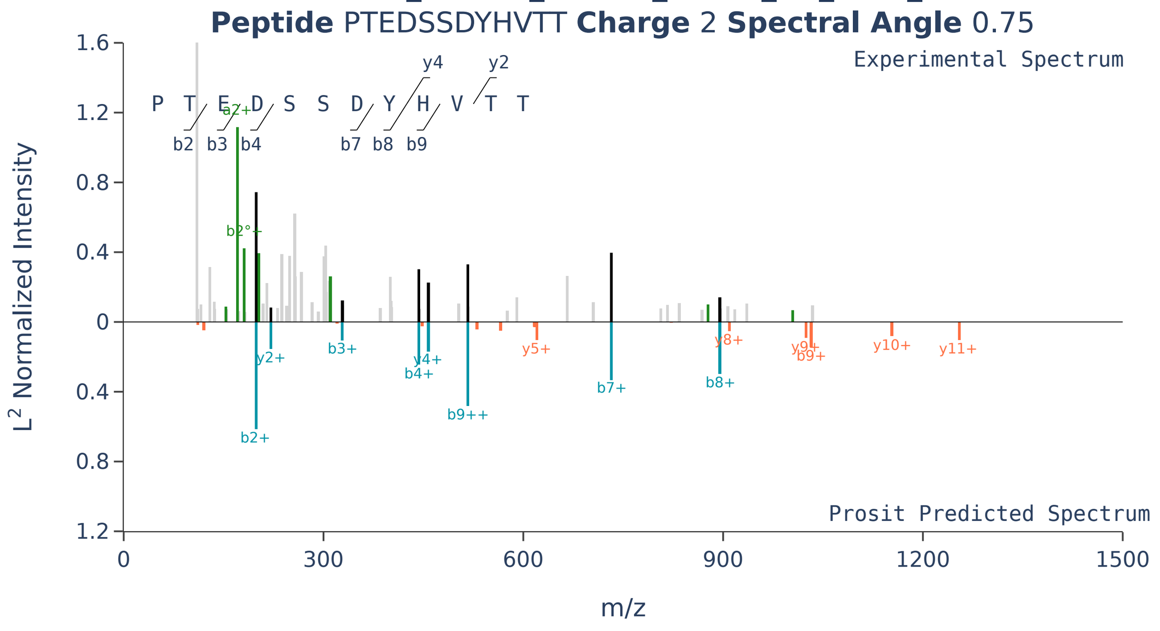

**Source** HRoetschke_211222_160123_HFGoe_IF2_b2R1_4h **Scan** 14628
**Peptide** LDTPGHAAF **Charge** 2 **Spectral Angle** 0.82

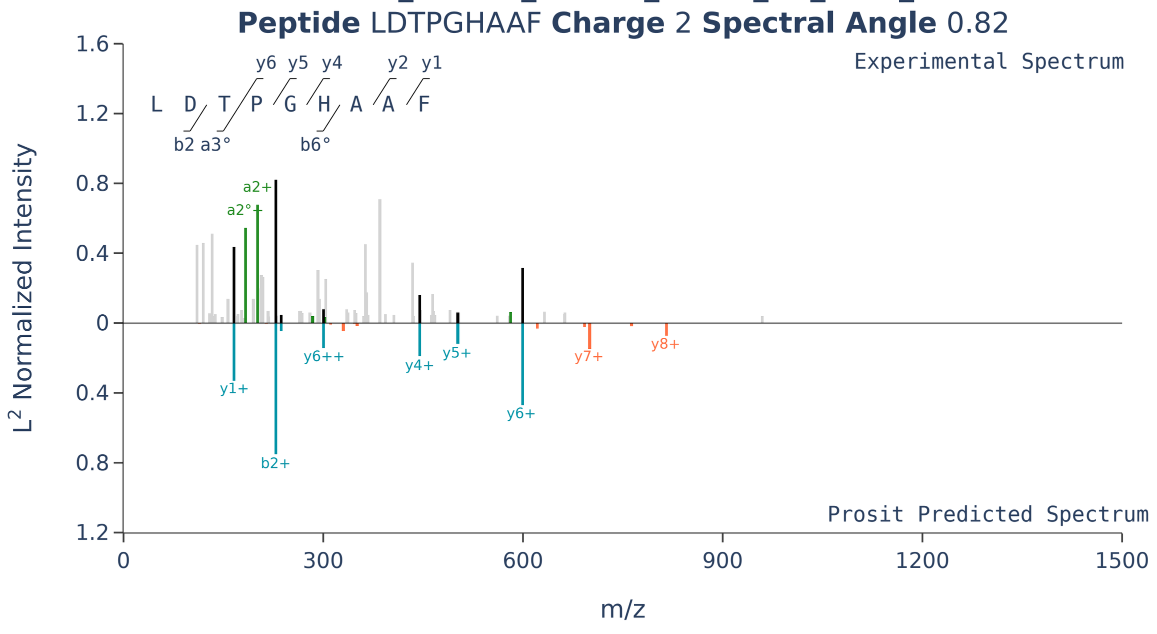


**Source** HRoetschke_211222_160123_HFGoe_IF2_b1R1_4h **Scan** 12015
**Peptide** RDTGAAAEPRAPVVT **Charge** 3 **Spectral Angle** 0.62

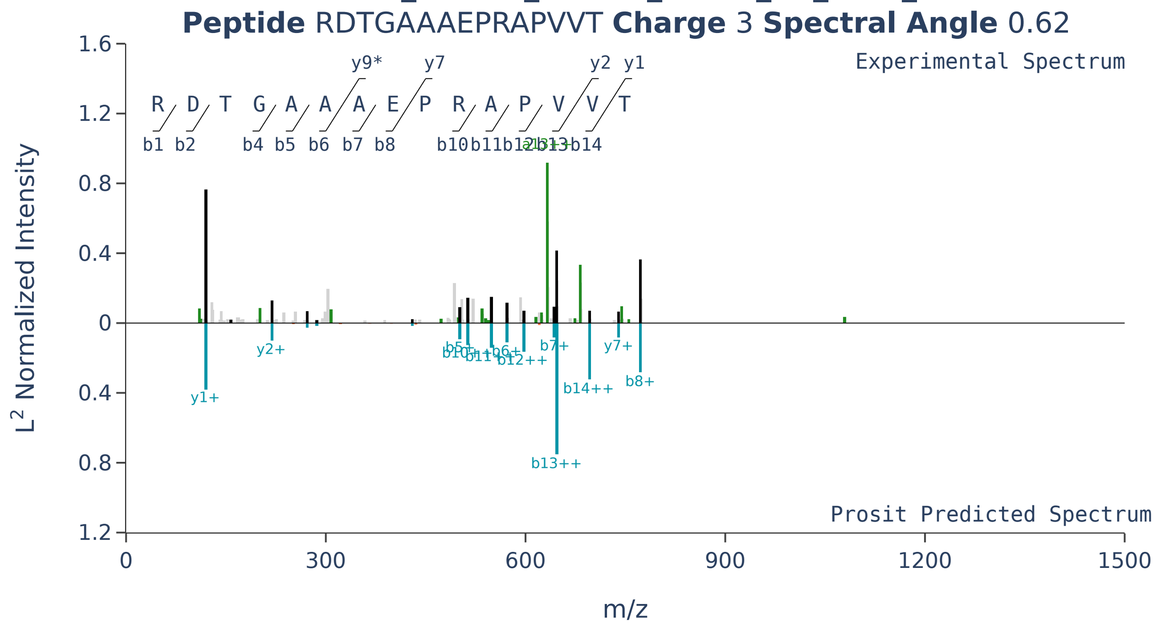


**Source** HRoetschke_211222_160123_HFGoe_IF2_b1R1_4h **Scan** 6586
**Peptide** SGPDKLTLQRKTRS **Charge** 4 **Spectral Angle** 0.73

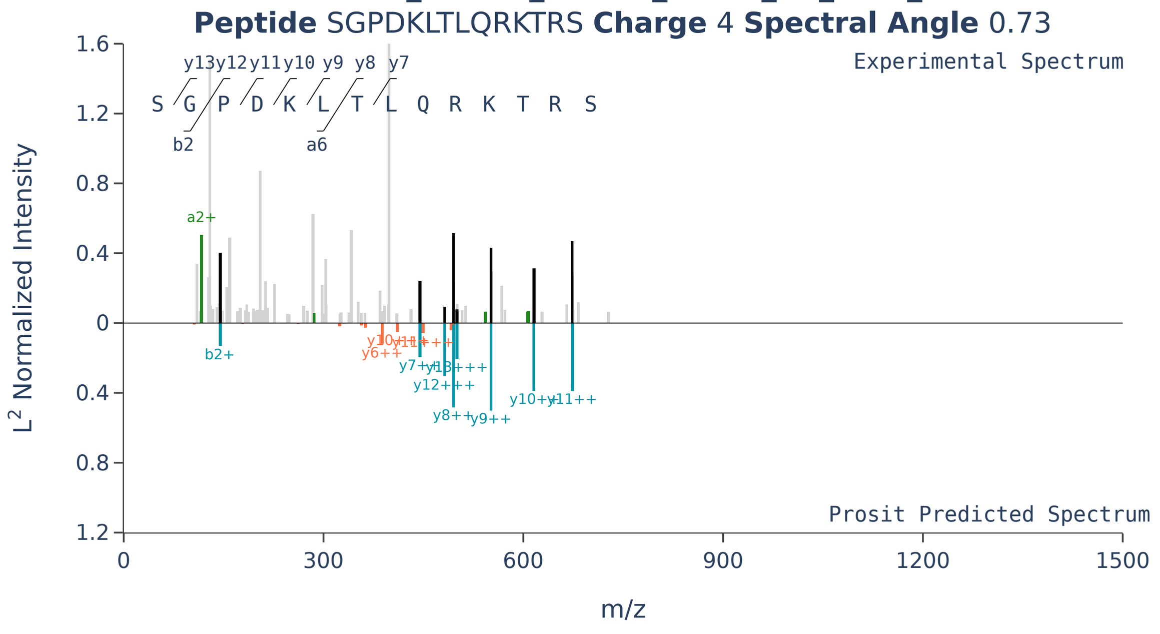


**Source** HRoetschke_211222_160123_HFGoe_IF2_b1R1_2h **Scan** 6084
**Peptide** HLNQKNSGPDKLTLQRKTR **Charge** 5 **Spectral Angle** 0.88

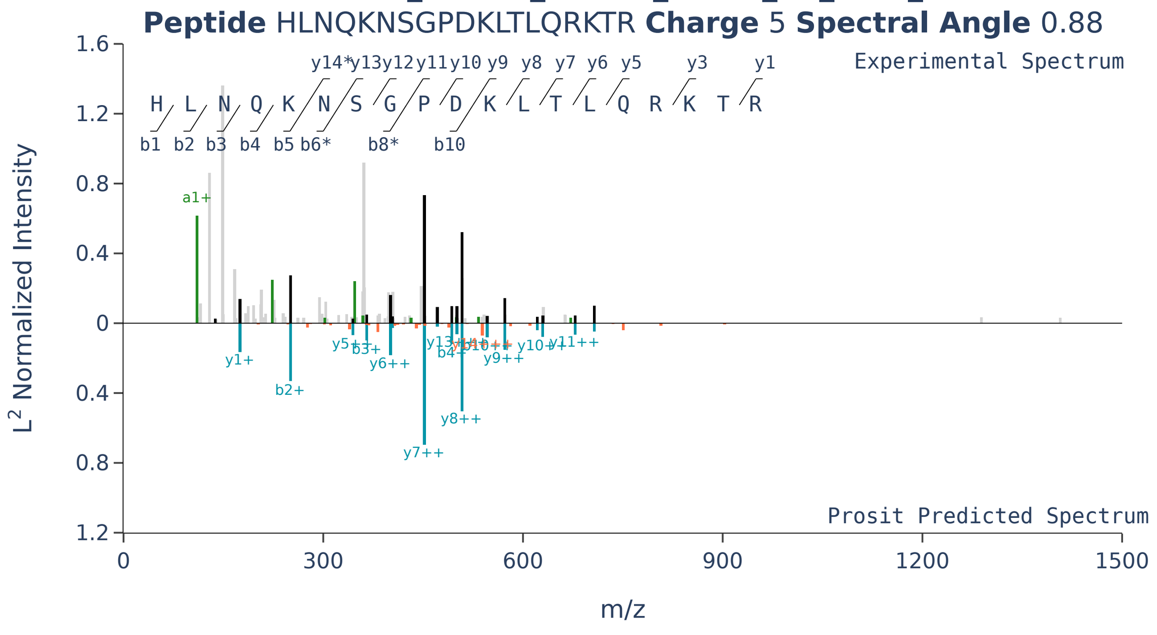


**Source** HRoetschke_211222_160123_HFGoe_IF2_b1R1_24h **Scan** 15729
**Peptide** VTIKTLAAE **Charge** 2 **Spectral Angle** 0.87

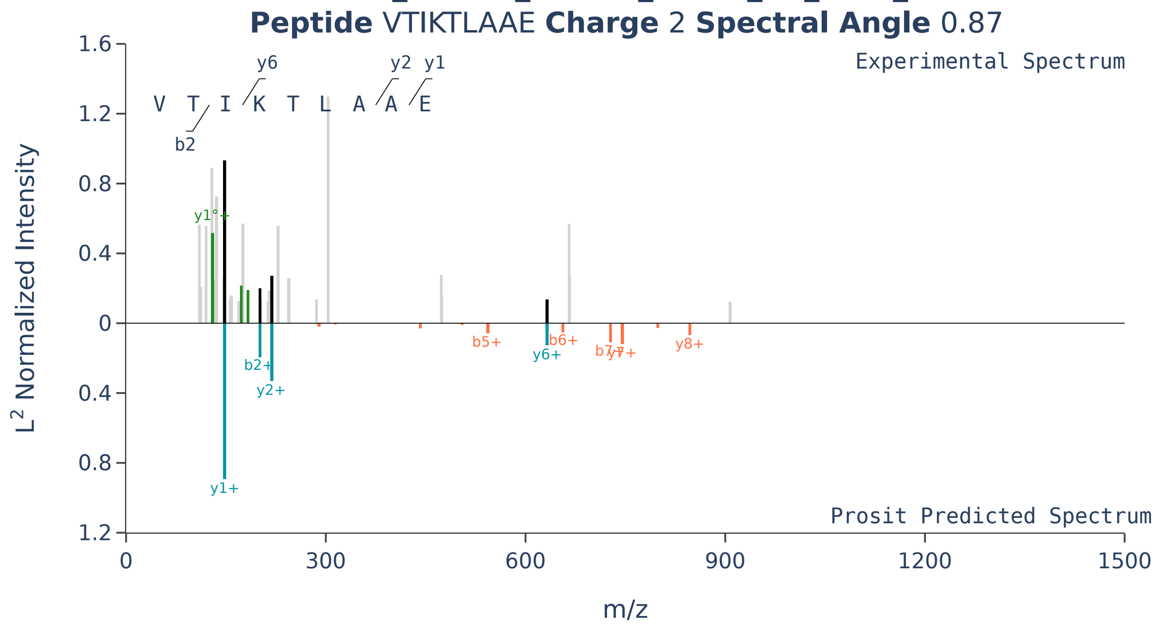


**Source** HRoetschke_211222_160123_HFGoe_IF2_b2R2_2h **Scan** 11824
**Peptide** HLNQKNSGPDKLTL **Charge** 3 **Spectral Angle** 0.81

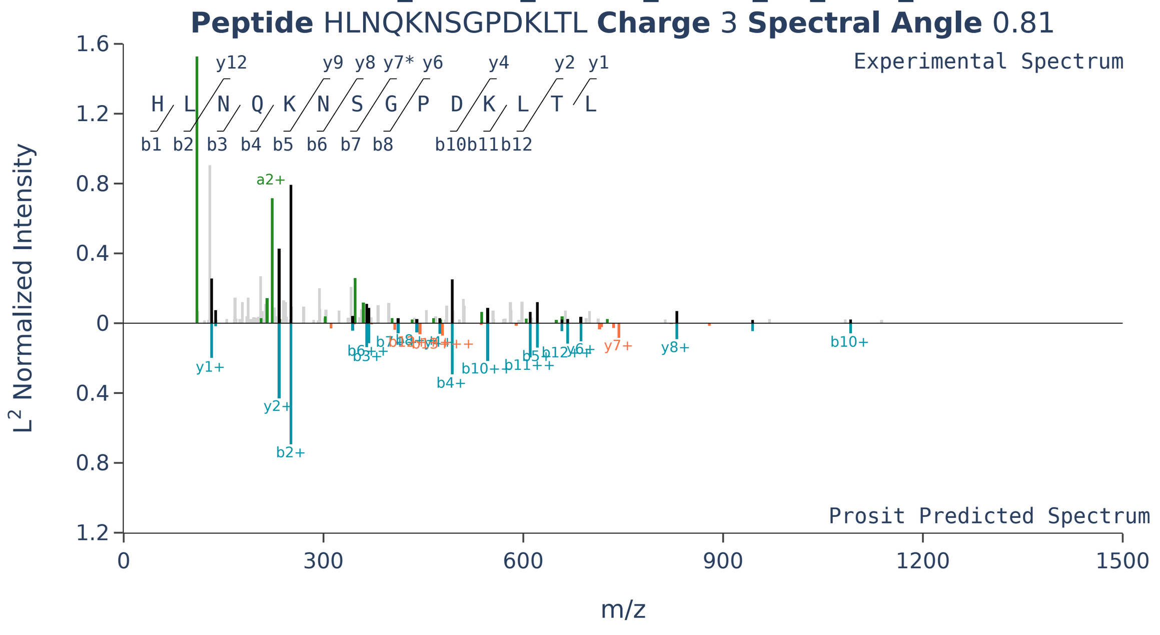


**Source** HRoetschke_211222_160123_HFGoe_IF2_b1R2_1h **Scan** 10767
**Peptide** IPGTGGKSK **Charge** 2 **Spectral Angle** 0.93

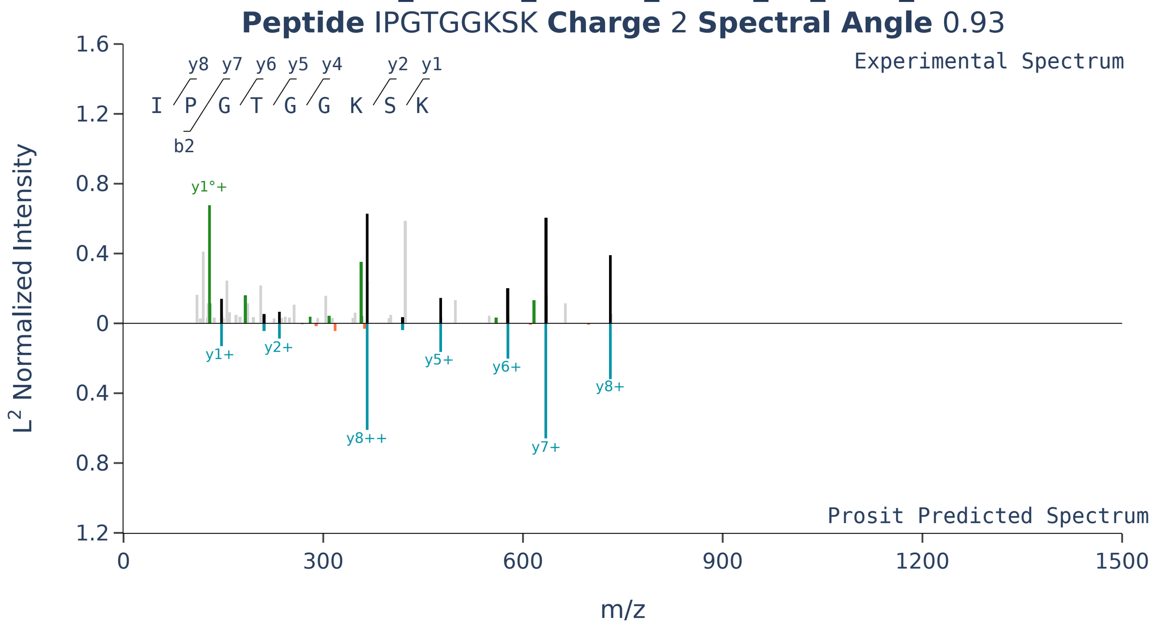


**Source** HRoetschke_211222_160123_HFGoe_IF2_b1R2_1h **Scan** 10515
**Peptide** VQQFADAGIRKSAD **Charge** 3 **Spectral Angle** 0.7

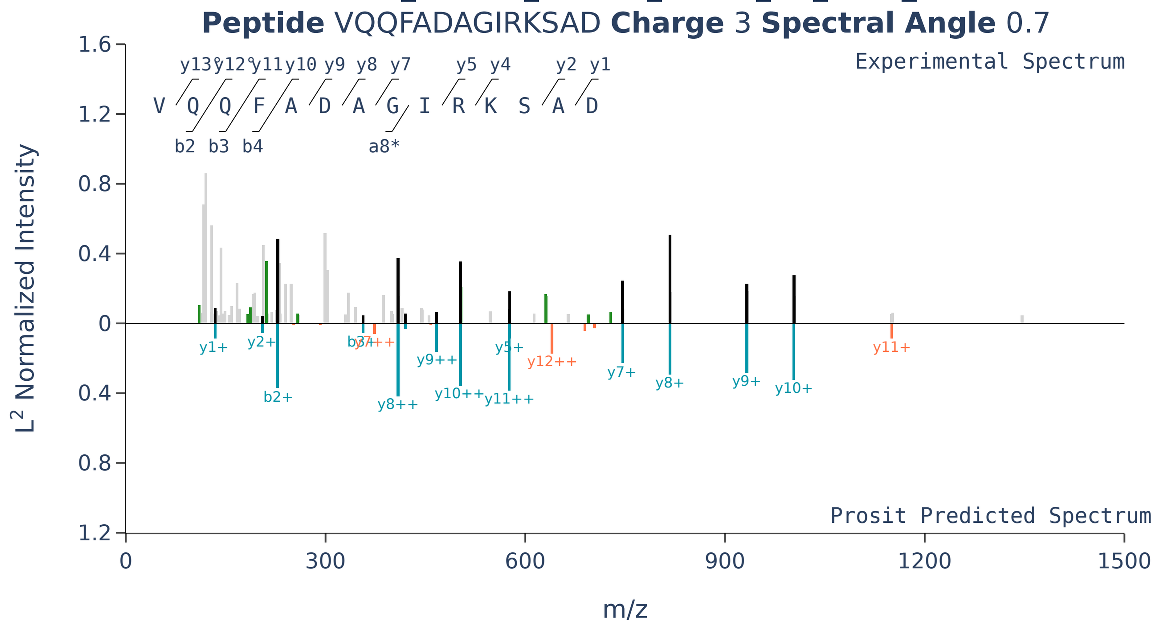


**Source** HRoetschke_211222_160123_HFGoe_IF2_b1R1_2h **Scan** 11114
**Peptide** AYHVETENGM **Charge** 2 **Spectral Angle** 0.75

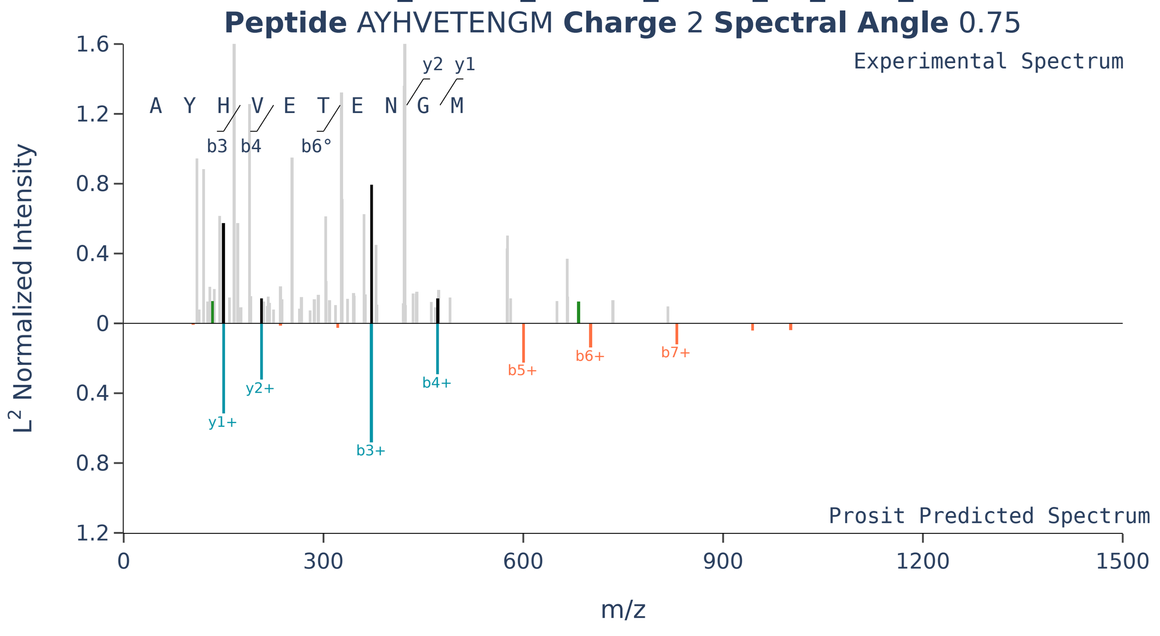


**Source** HRoetschke_211222_160123_HFGoe_IF2_b2R1_24h **Scan** 10209
**Peptide** HLNQKNSGPDKLTLQ **Charge** 4 **Spectral Angle** 0.78

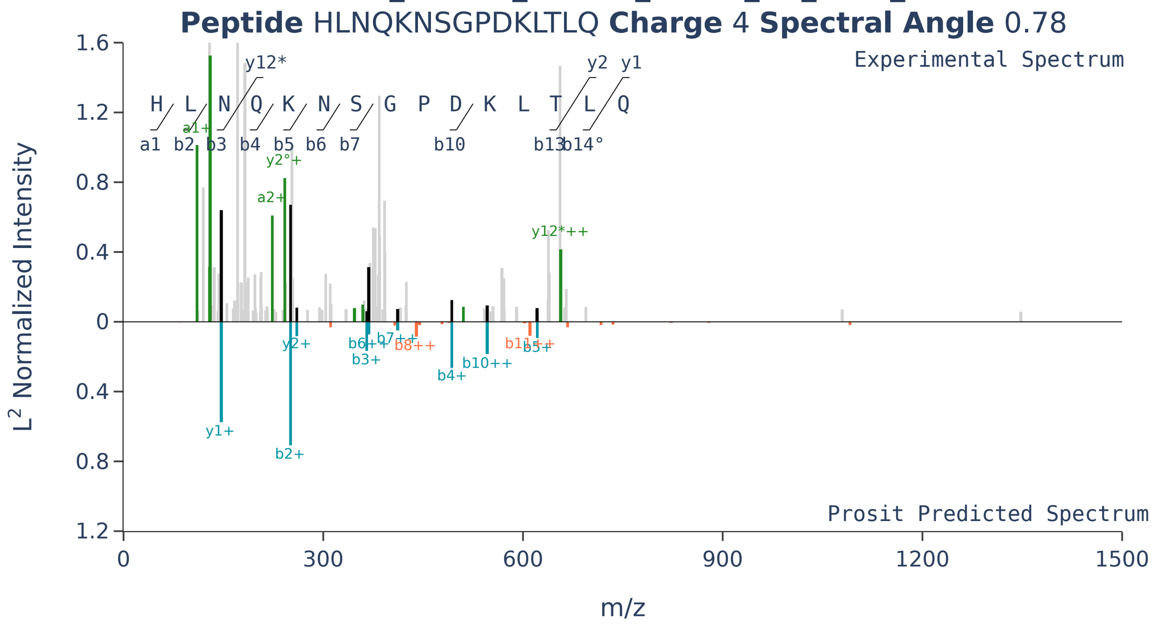


**Source** HRoetschke_211222_160123_HFGoe_IF2_b1R2_1h **Scan** 12912
**Peptide** GEAGGITQHL **Charge** 2 **Spectral Angle** 0.8

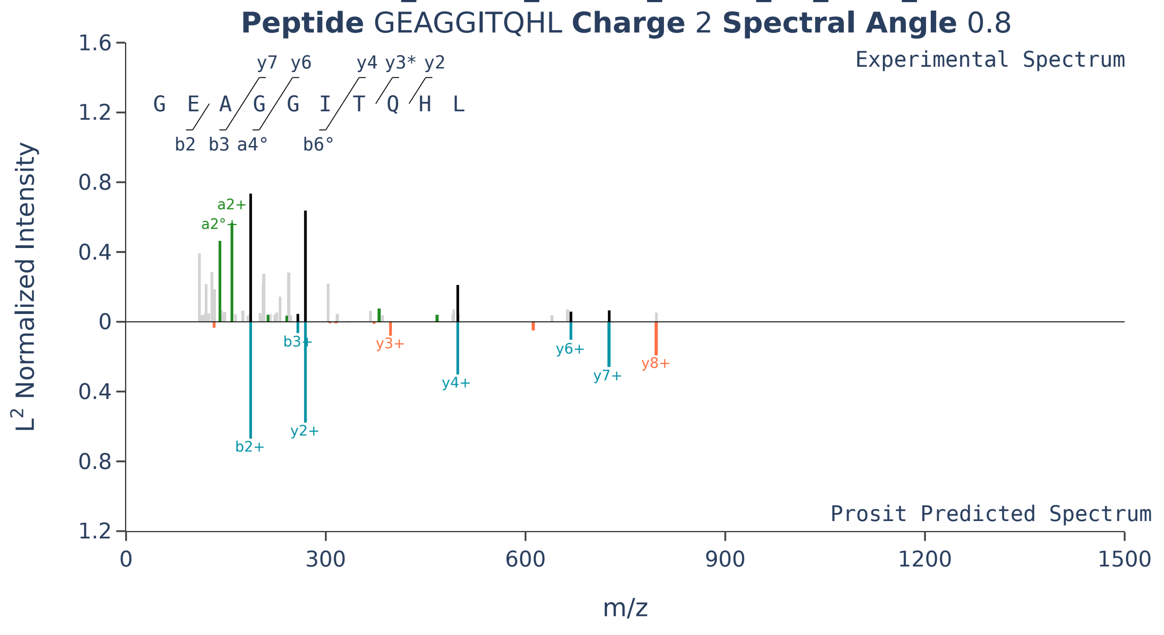


**Source** HRoetschke_211222_160123_HFGoe_IF2_b2R2_24h **Scan** 8633
**Peptide** NKMAVKGSQVLKA **Charge** 3 **Spectral Angle** 0.82

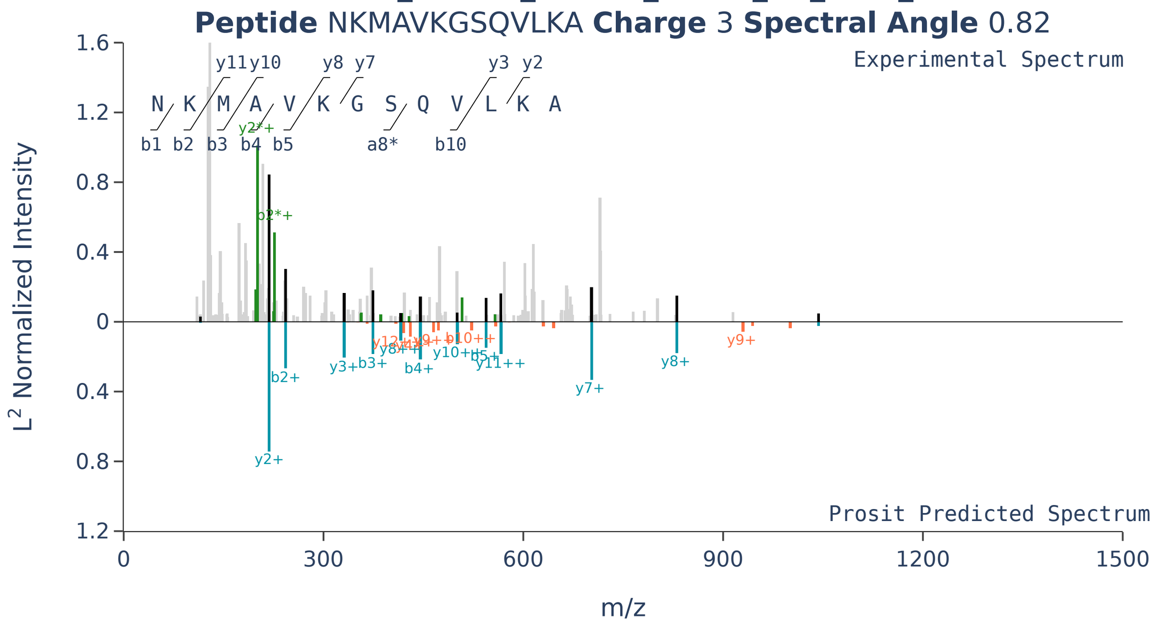


**Source** HRoetschke_211222_160123_HFGoe_IF2_b1R1_24h **Scan** 17599
**Peptide** AESLDLRY **Charge** 2 **Spectral Angle** 0.84

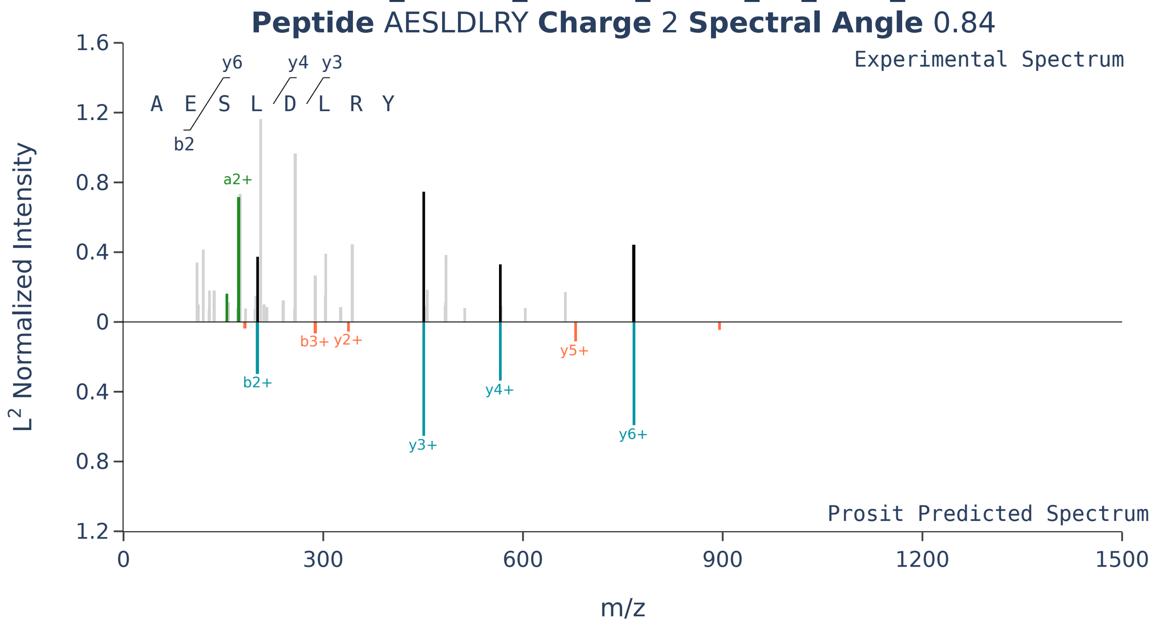


**Source** HRoetschke_211222_160123_HFGoe_IF2_b2R1_24h **Scan** 13471
**Peptide** YNDVRTGDVIE **Charge** 2 **Spectral Angle** 0.86

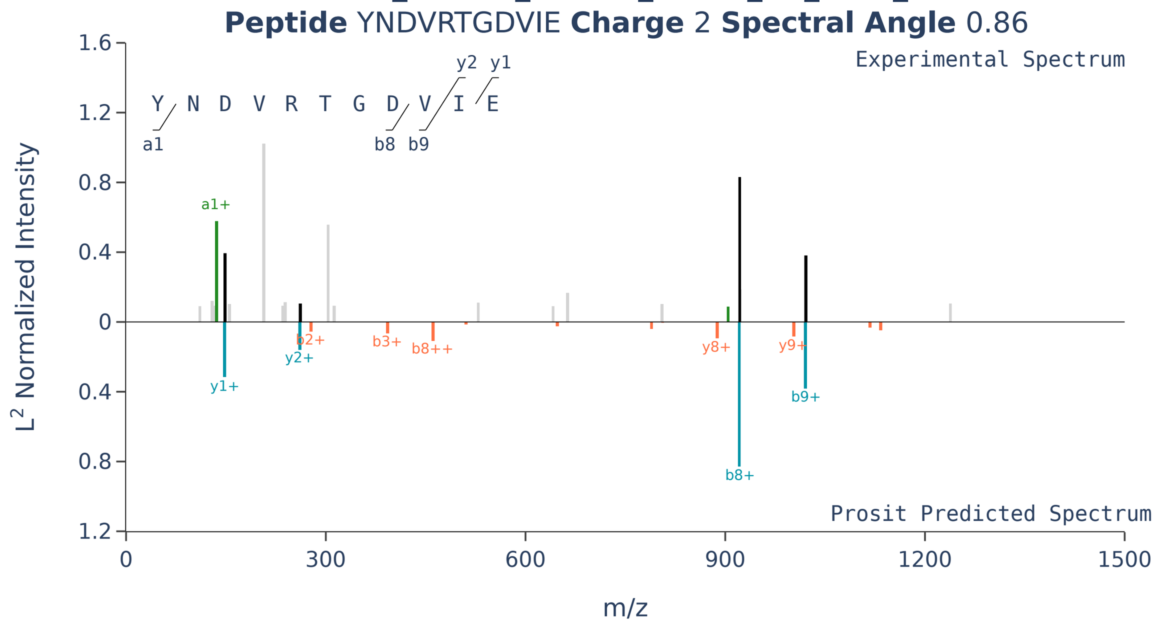


**Source** HRoetschke_211222_160123_HFGoe_IF2_b1R1_4h **Scan** 17136
**Peptide** MRNELGQEVL **Charge** 2 **Spectral Angle** 0.73

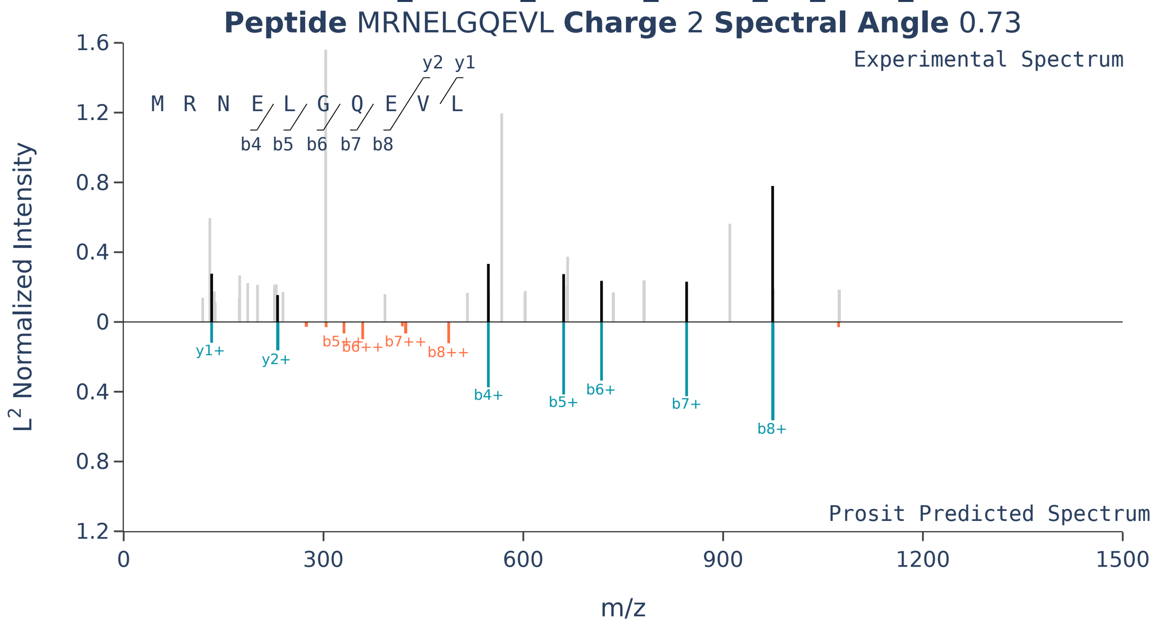

**Source** HRoetschke_211222_160123_HFGoe_IF2_b1R1_4h **Scan** 6248
**Peptide** DREEARAAV **Charge** 2 **Spectral Angle** 0.8

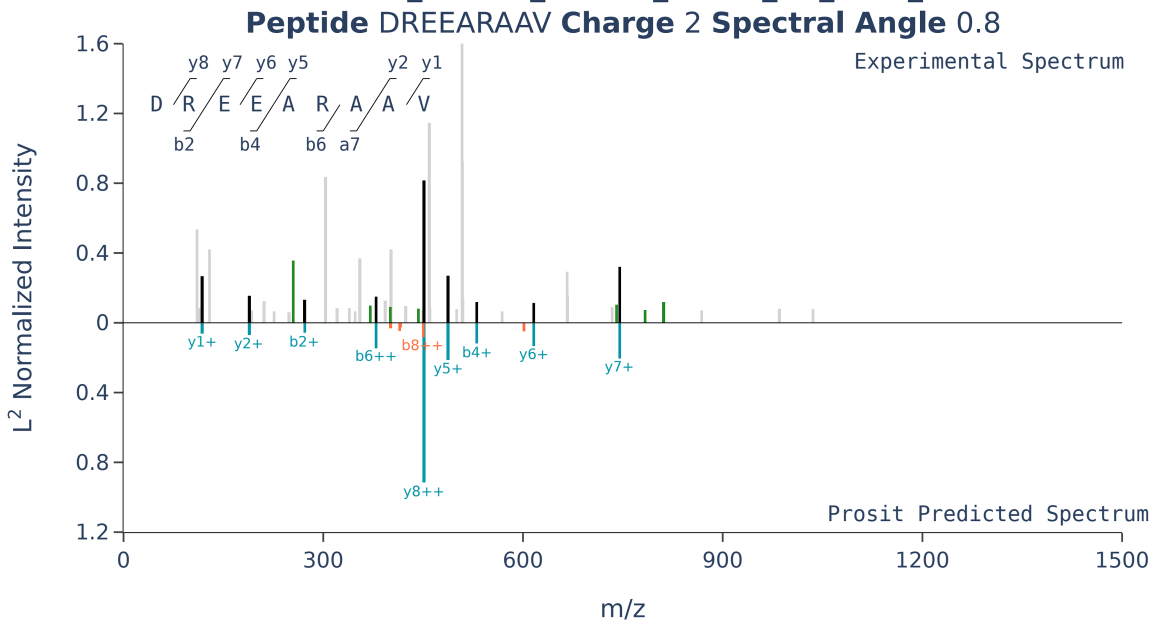


**Source** HRoetschke_211222_160123_HFGoe_IF2_b1R2_2h **Scan** 11088
**Peptide** SVEAISD **Charge** 1 **Spectral Angle** 0.82

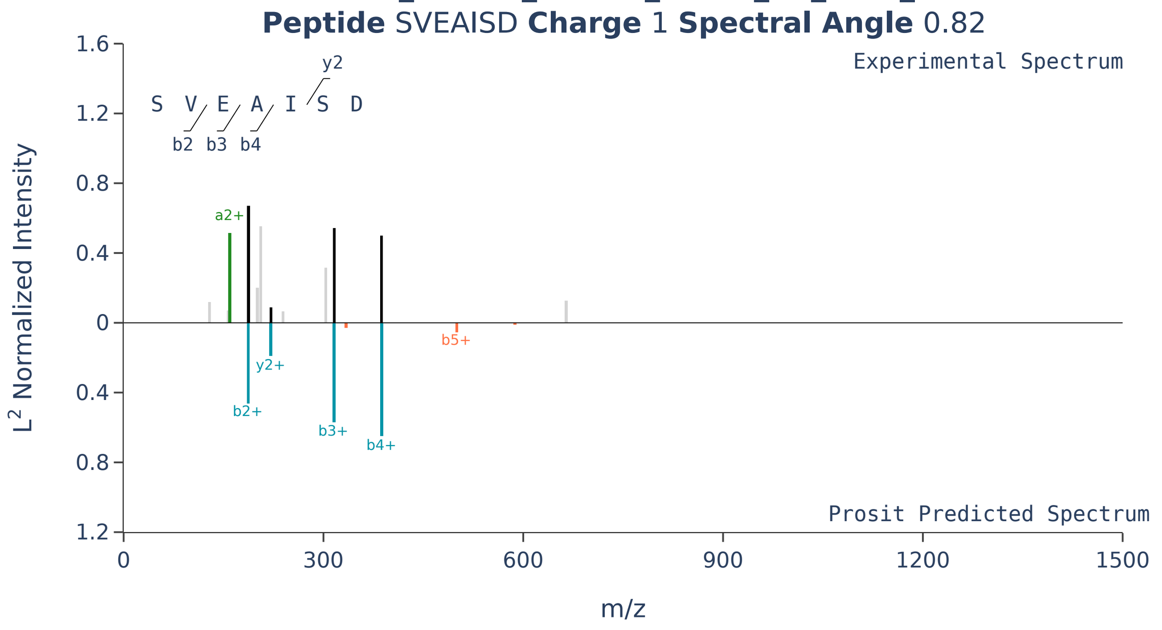


**Source** HRoetschke_211222_160123_HFGoe_IF2_b2R1_2h **Scan** 10635
**Peptide** VQQFADAGIRKSADD **Charge** 3 **Spectral Angle** 0.69

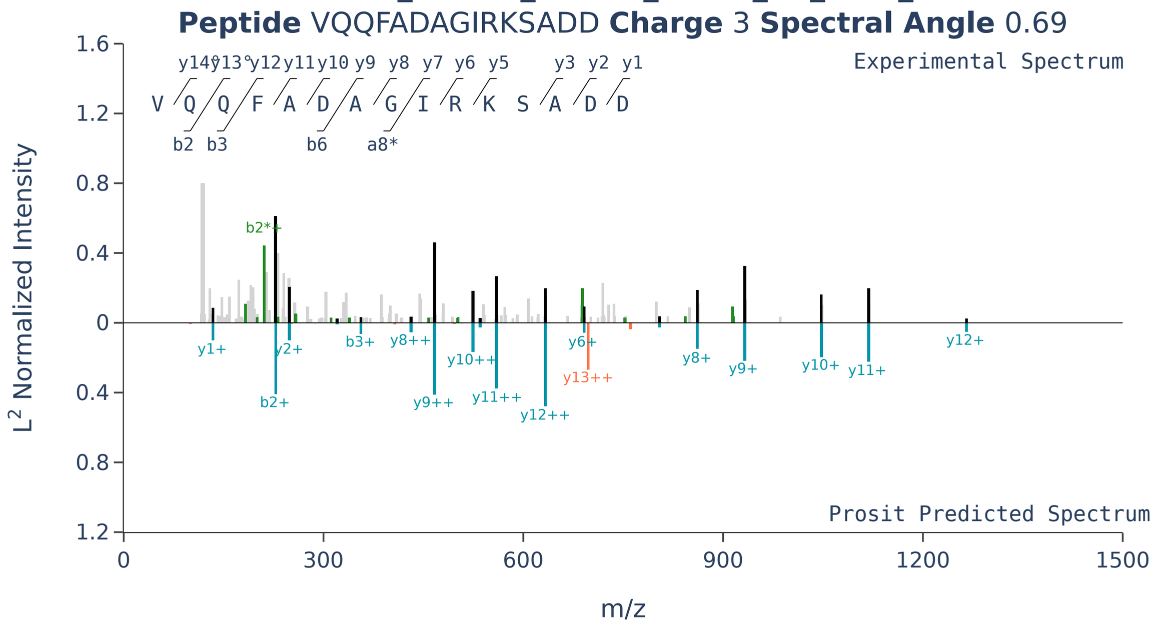


**Source** HRoetschke_211222_160123_HFGoe_IF2_b1R1_4h **Scan** 13456
**Peptide** SGEAGGITQHIGAYH **Charge** 3 **Spectral Angle** 0.81

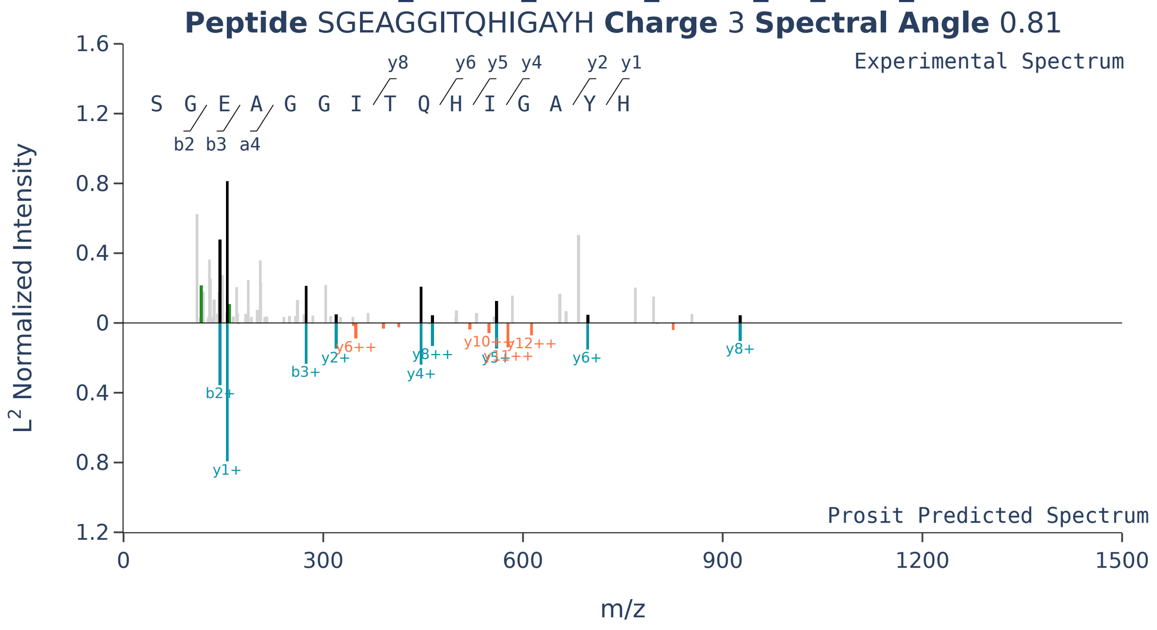


**Source** HRoetschke_211222_160123_HFGoe_IF2_b1R2_2h **Scan** 5780
**Peptide** KLDKPEADPDRVKN **Charge** 2 **Spectral Angle** 0.69

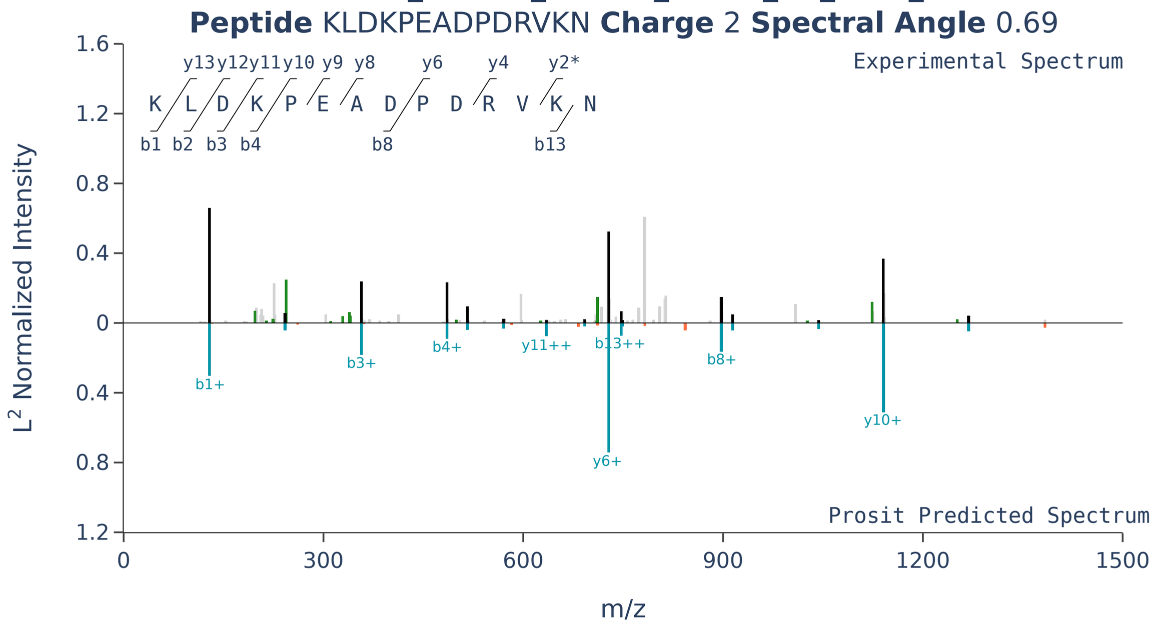


**Source** HRoetschke_211222_160123_HFGoe_IF2_b1R1_4h **Scan** 19342
**Peptide** DGVMPQTIEAIQHA **Charge** 2 **Spectral Angle** 0.79

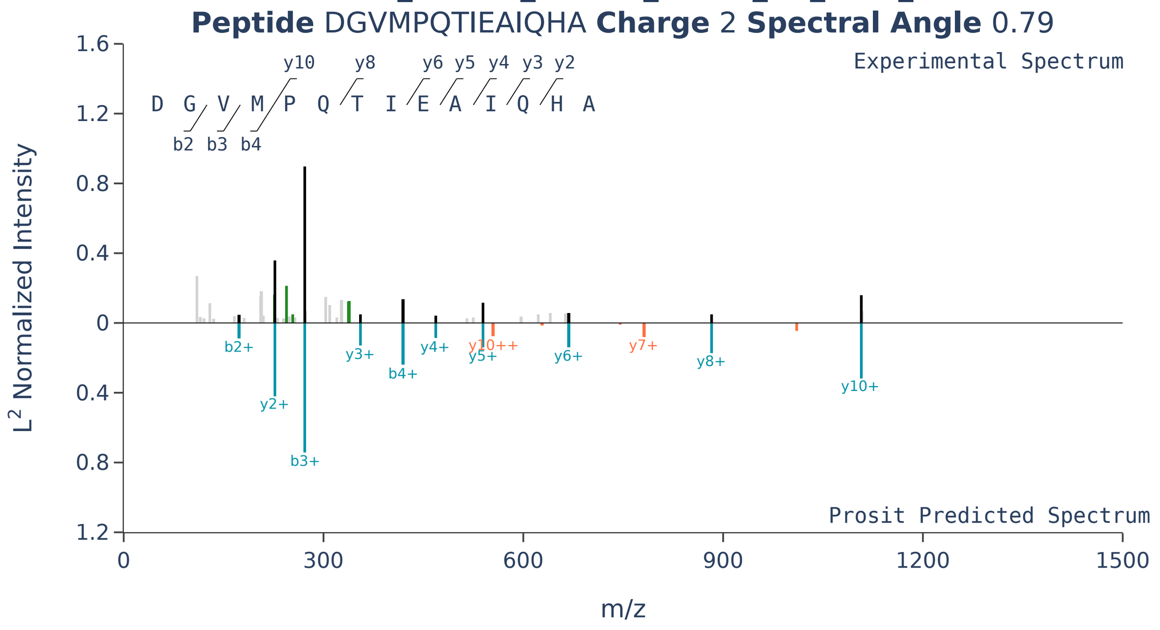


**Source** HRoetschke_211222_160123_HFGoe_IF2_b1R1_4h **Scan** 9484
**Peptide** TPGHAAFTSMRARGAQA **Charge** 4 **Spectral Angle** 0.84

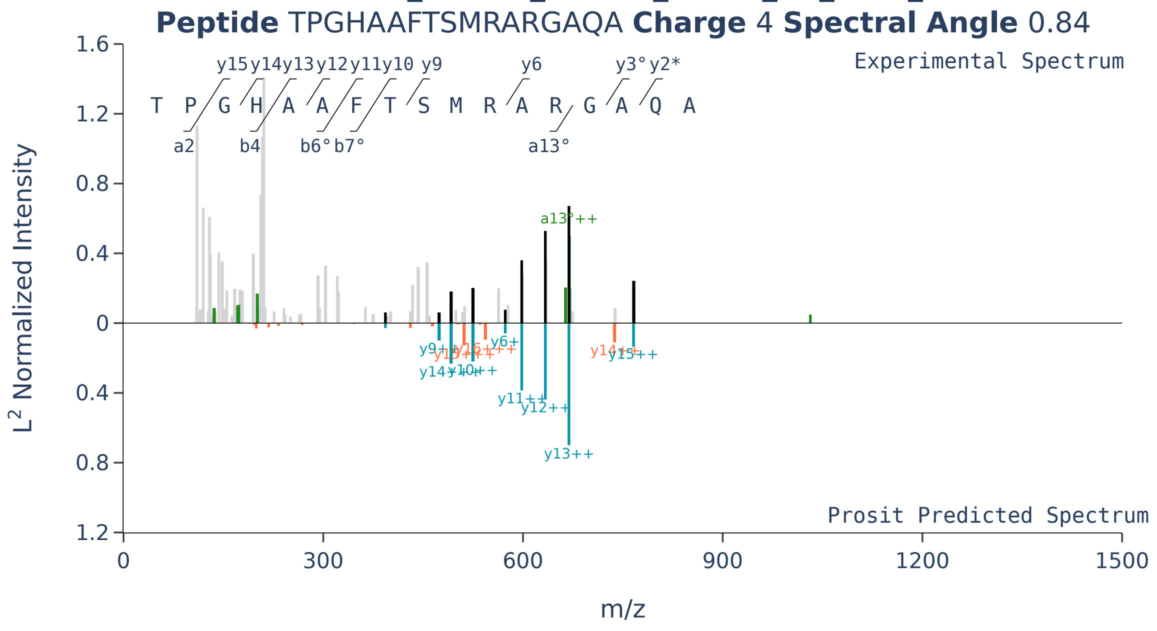


**Source** HRoetschke_211222_160123_HFGoe_IF2_b1R1_2h **Scan** 8784
**Peptide** TVLVREGTLHKG **Charge** 2 **Spectral Angle** 0.75

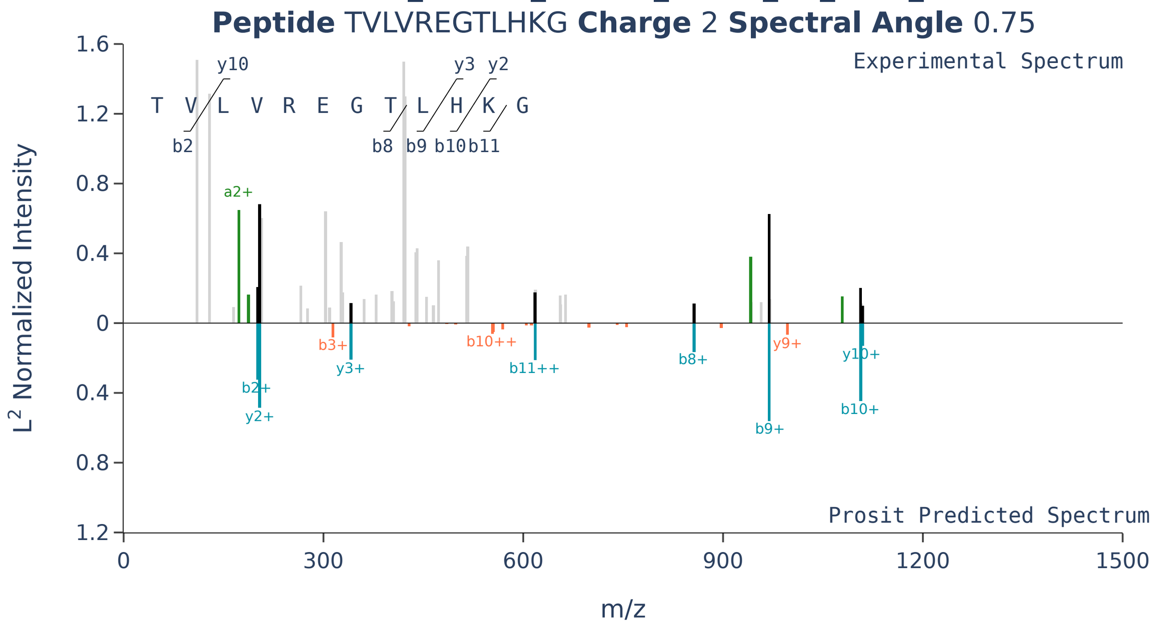


**Source** HRoetschke_211222_160123_HFGoe_IF2_b2R2_24h **Scan** 12431
**Peptide** VSAQEKQTLID **Charge** 2 **Spectral Angle** 0.73

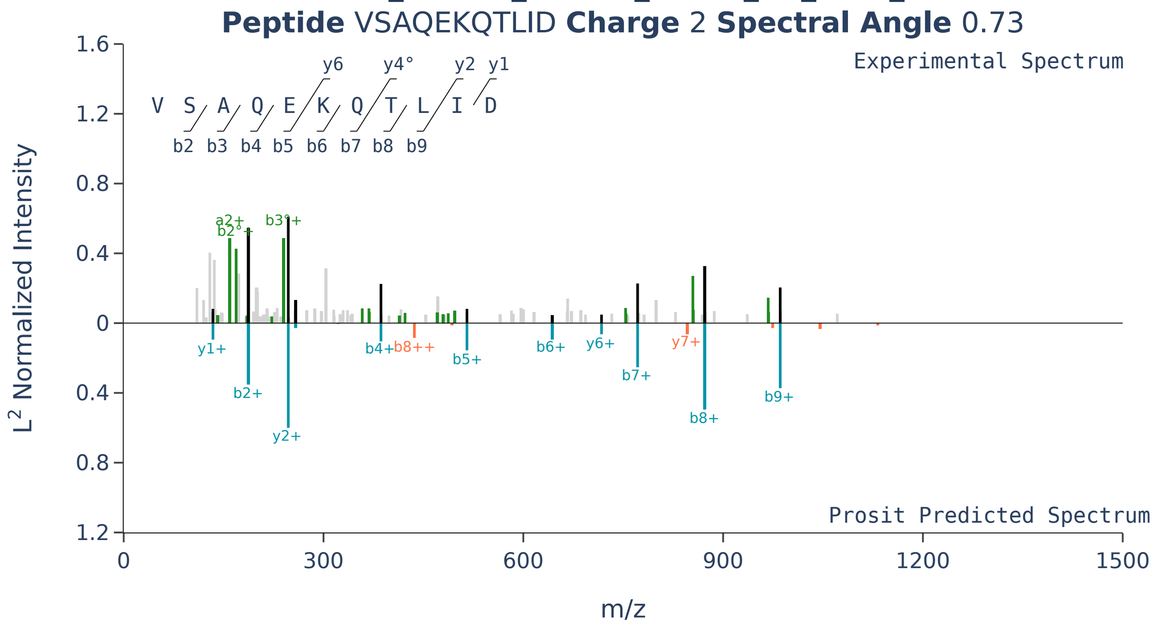


**Source** HRoetschke_211222_160123_HFGoe_IF2_b2R1_24h **Scan** 12012
**Peptide** AERQTSVERLVQQ **Charge** 3 **Spectral Angle** 0.65

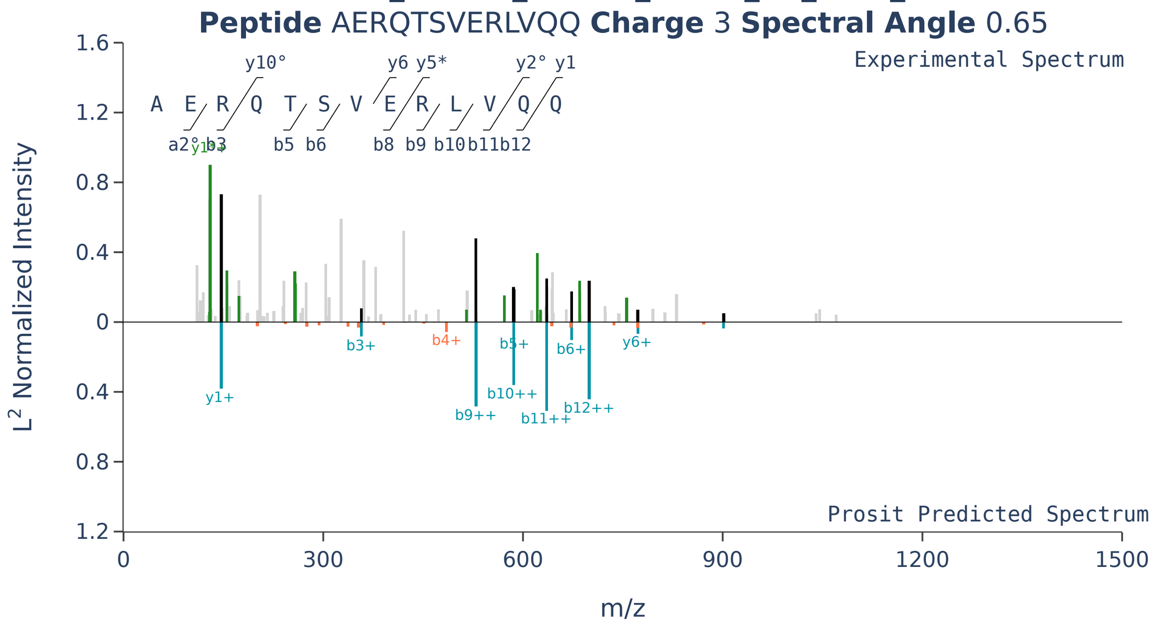


**Source** HRoetschke_211222_160123_HFGoe_IF2_b1R2_1h **Scan** 11849
**Peptide** GFEYGRVRA **Charge** 3 **Spectral Angle** 0.89

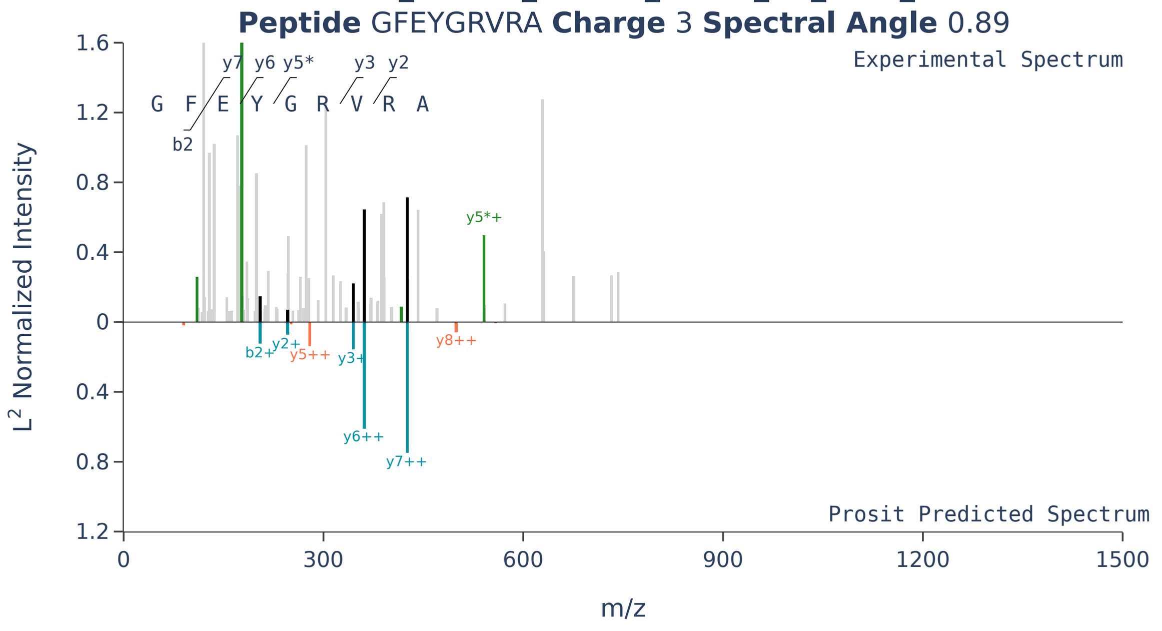


**Source** HRoetschke_211222_160123_HFGoe_IF2_b2R2_4h **Scan** 11754
**Peptide** SDRDTGAAAEPRAPVVT **Charge** 3 **Spectral Angle** 0.66

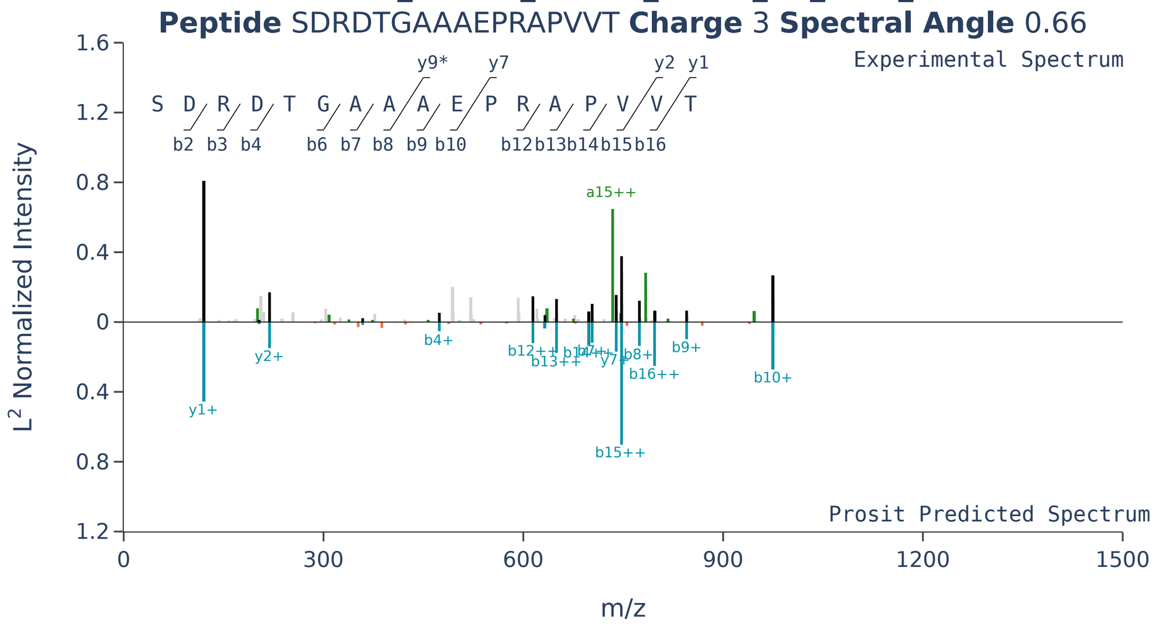


**Source** HRoetschke_211222_160123_HFGoe_IF2_b1R1_4h **Scan** 15437
**Peptide** STLNIPGTGGKS **Charge** 2 **Spectral Angle** 0.9

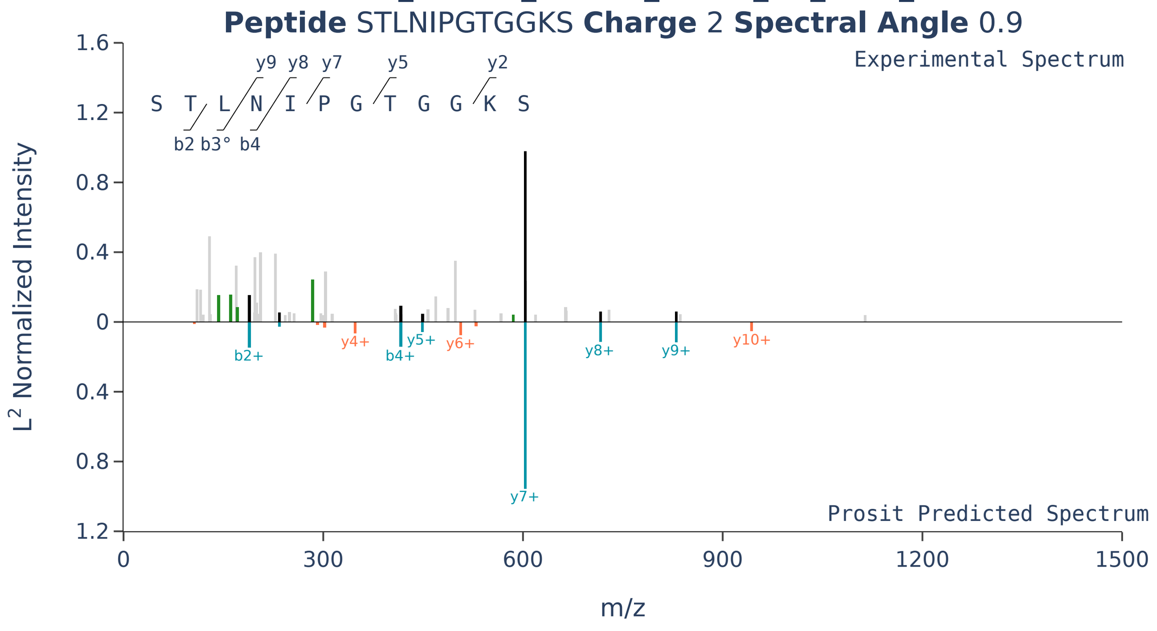


**Source** HRoetschke_211222_160123_HFGoe_IF2_b2R2_4h **Scan** 6225
**Peptide** LAAERQTSVER **Charge** 3 **Spectral Angle** 0.76

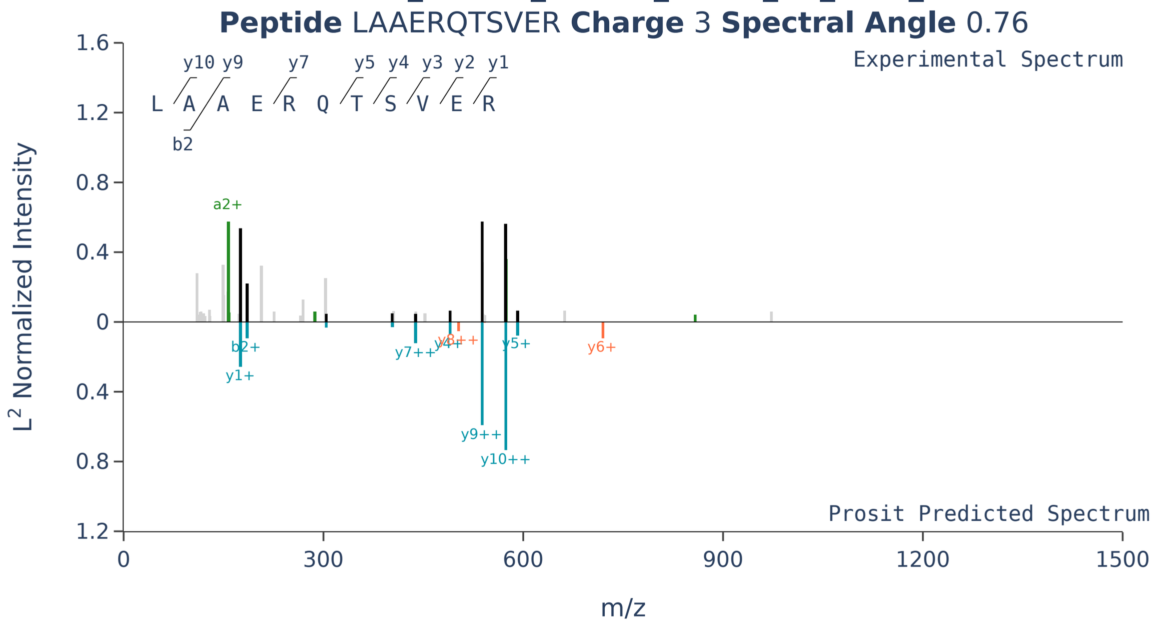


**Source** HRoetschke_211222_160123_HFGoe_IF2_b2R1_24h **Scan** 6863
**Peptide** NQKNSGPDKLTLQRKT **Charge** 5 **Spectral Angle** 0.67

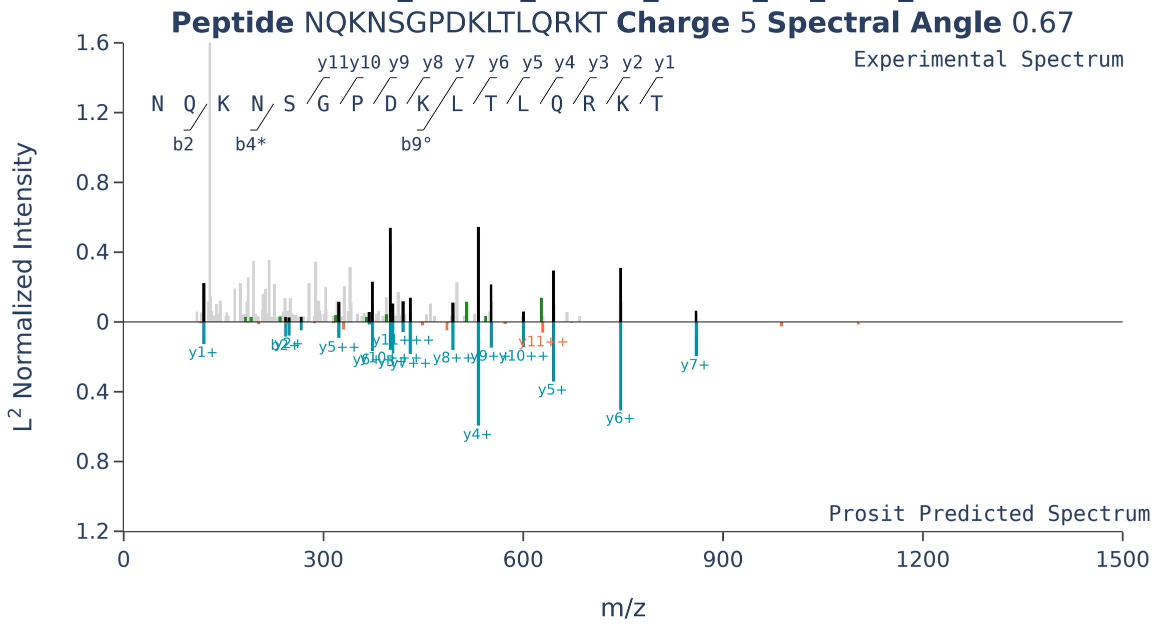

**Source** HRoetschke_211222_160123_HFGoe_IF2_b1R1_4h **Scan** 15753
**Peptide** IQHAKAAQVPVVVA **Charge** 3 **Spectral Angle** 0.8

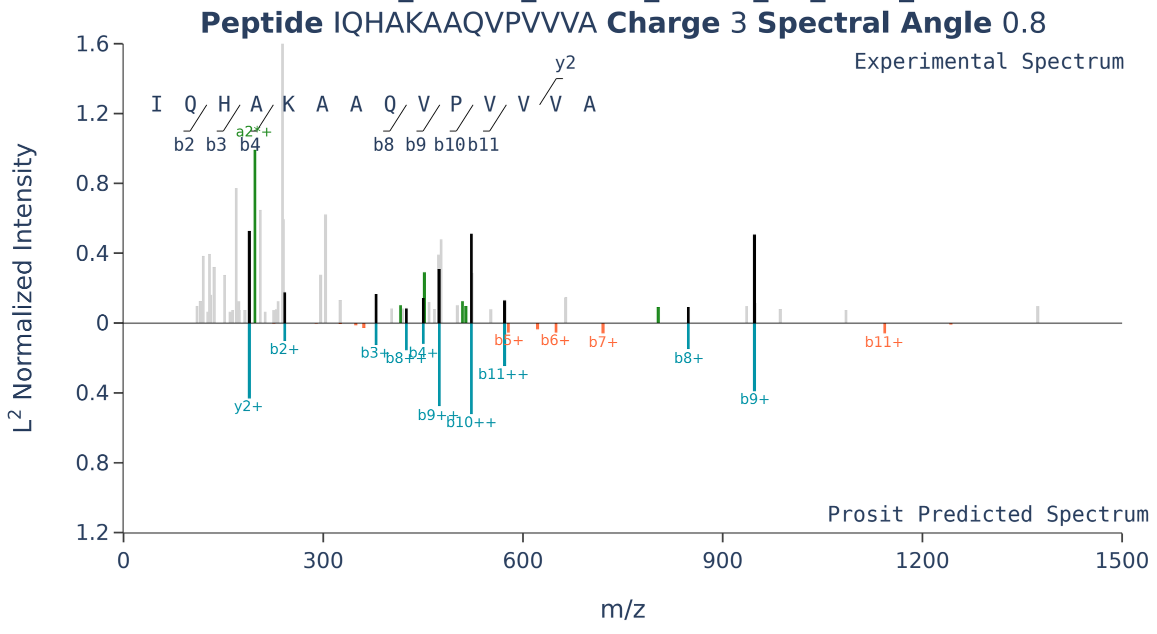


**Source** HRoetschke_211222_160123_HFGoe_IF2_b1R1_24h **Scan** 5308
**Peptide** YHVTTSQHARQAE **Charge** 2 **Spectral Angle** 0.83

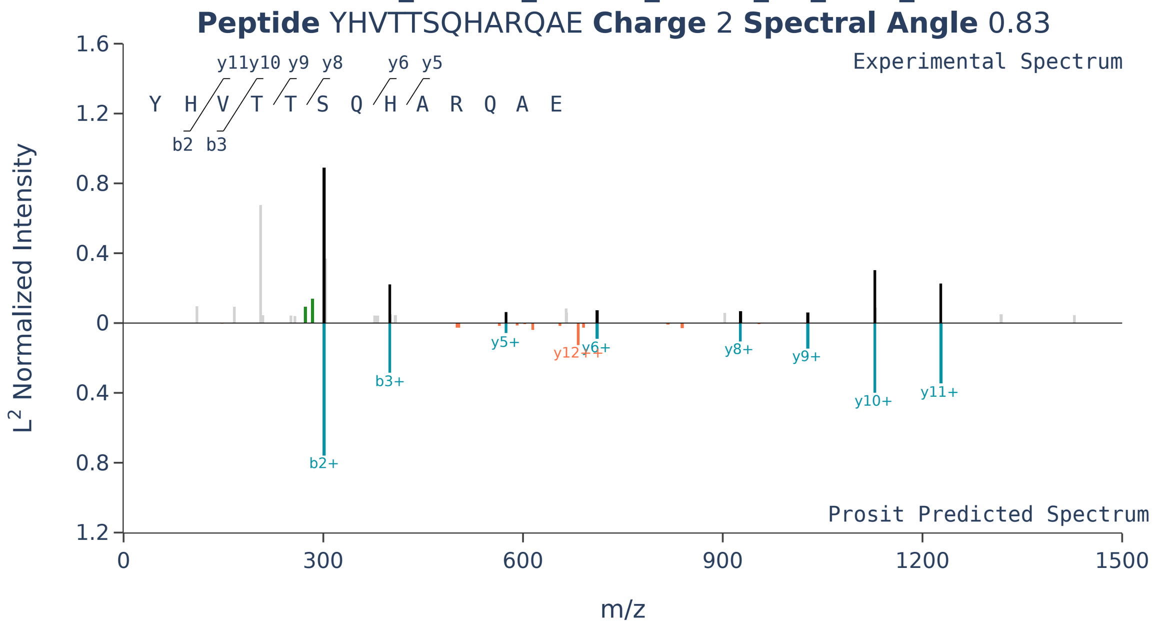


**Source** HRoetschke_211222_160123_HFGoe_IF2_b1R2_2h **Scan** 8183
**Peptide** PGHAAFTSMRAR **Charge** 3 **Spectral Angle** 0.83

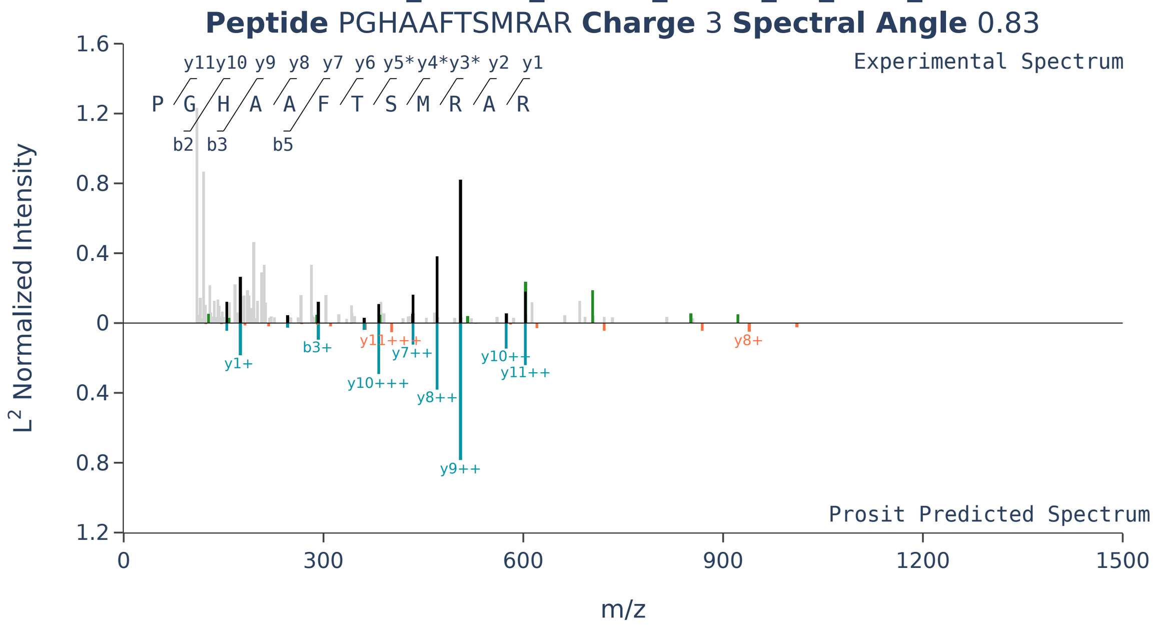


**Source** HRoetschke_211222_160123_HFGoe_IF2_b1R1_24h **Scan** 7477
**Peptide** EVTVVRDEKKAREVA **Charge** 3 **Spectral Angle** 0.86

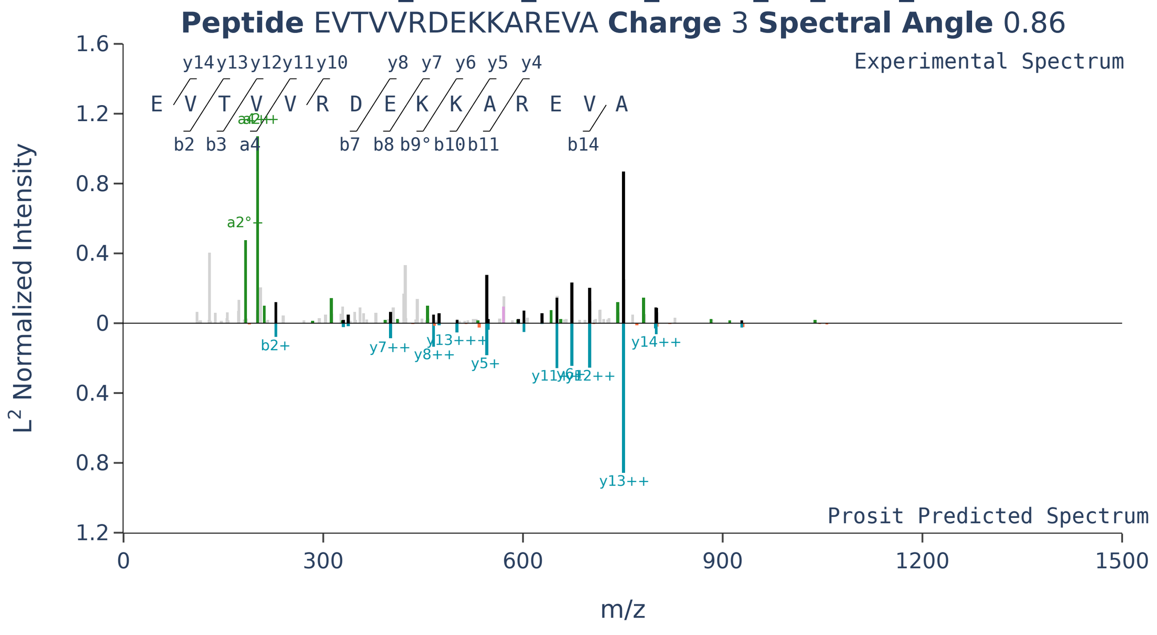


**Source** HRoetschke_211222_160123_HFGoe_IF2_b1R2_4h **Scan** 7178
**Peptide** IEAIQHAKA **Charge** 2 **Spectral Angle** 0.73

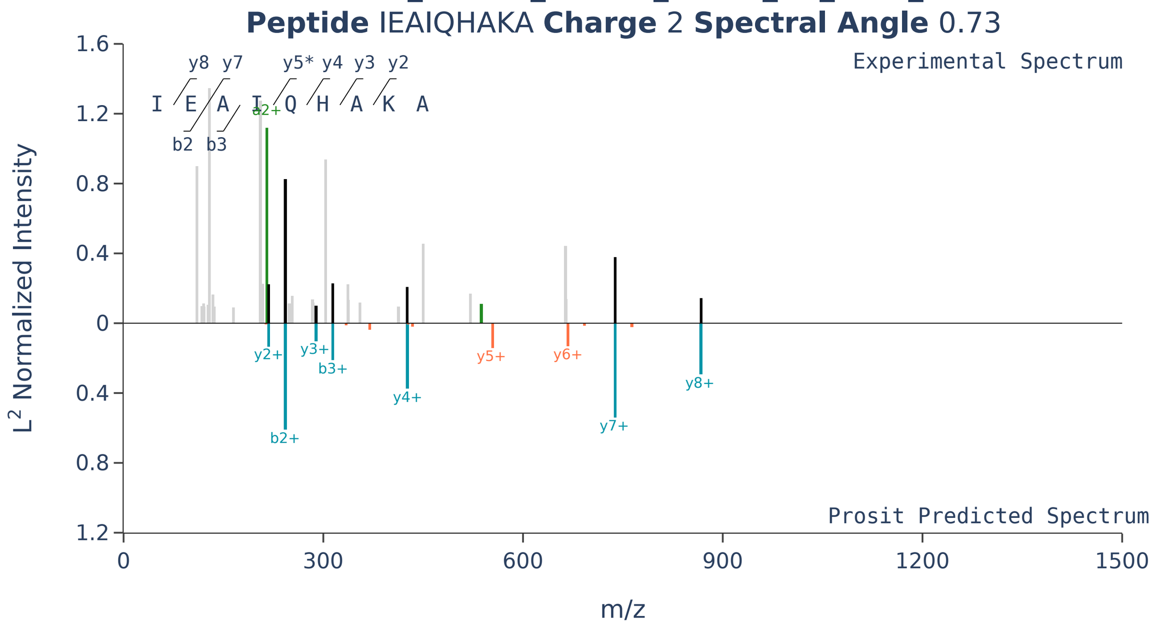


**Source** HRoetschke_211222_160123_HFGoe_IF2_b1R1_4h **Scan** 8091
**Peptide** NVRADASARKVIE **Charge** 2 **Spectral Angle** 0.79

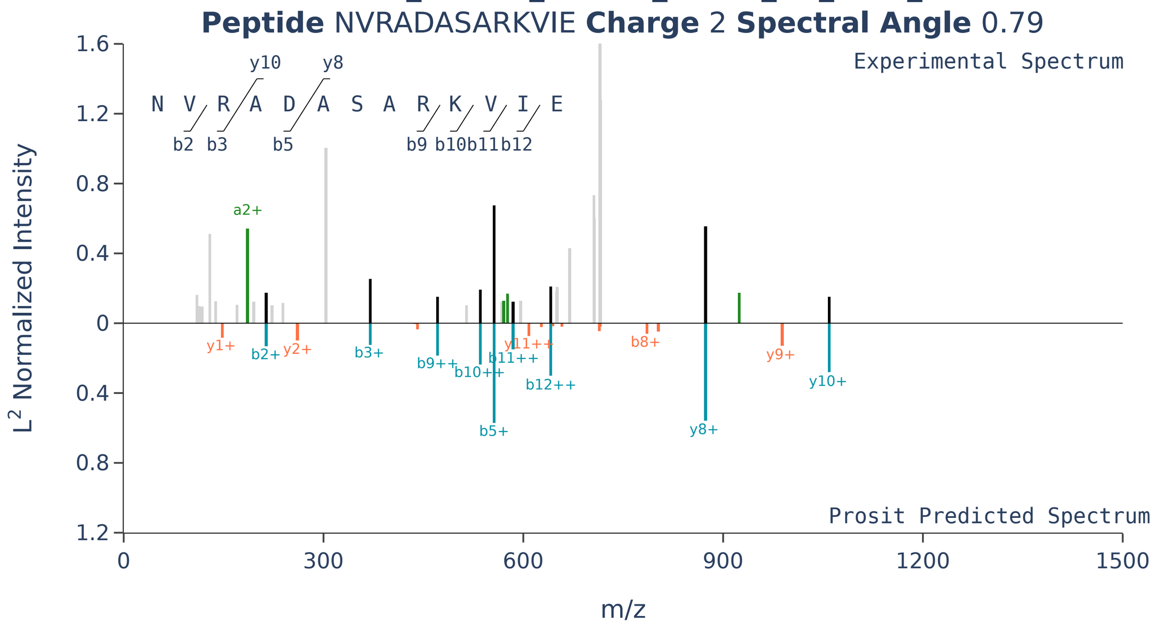


**Source** HRoetschke_211222_160123_HFGoe_IF2_b1R1_4h **Scan** 12388
**Peptide** LRRENELEEAVM **Charge** 3 **Spectral Angle** 0.81

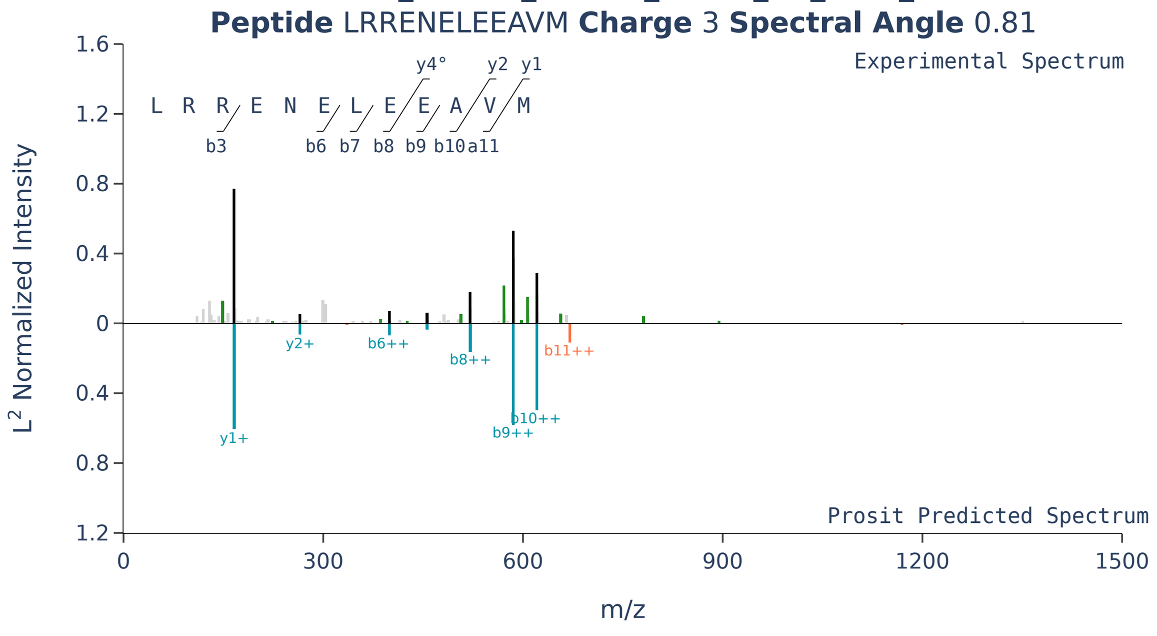


**Source** HRoetschke_211222_160123_HFGoe_IF2_b2R1_2h **Scan** 8062
**Peptide** DAGIRKSADD **Charge** 2 **Spectral Angle** 0.7

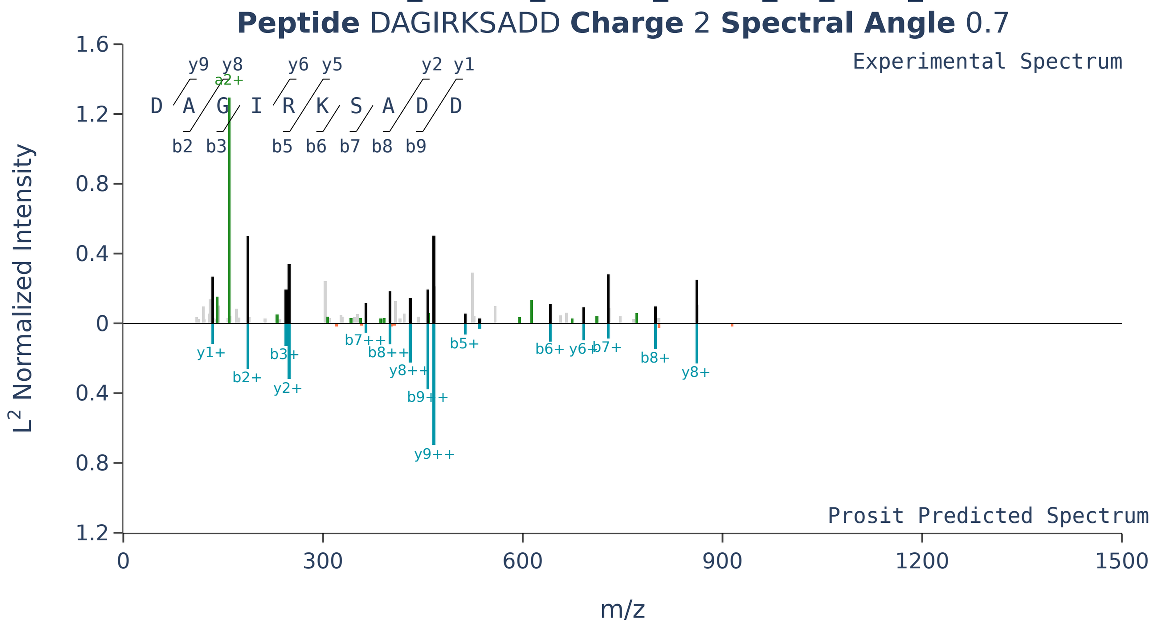


**Source** HRoetschke_211222_160123_HFGoe_IF2_b1R2_2h **Scan** 6512
**Peptide** GPDKLTLQRKTR **Charge** 3 **Spectral Angle** 0.79

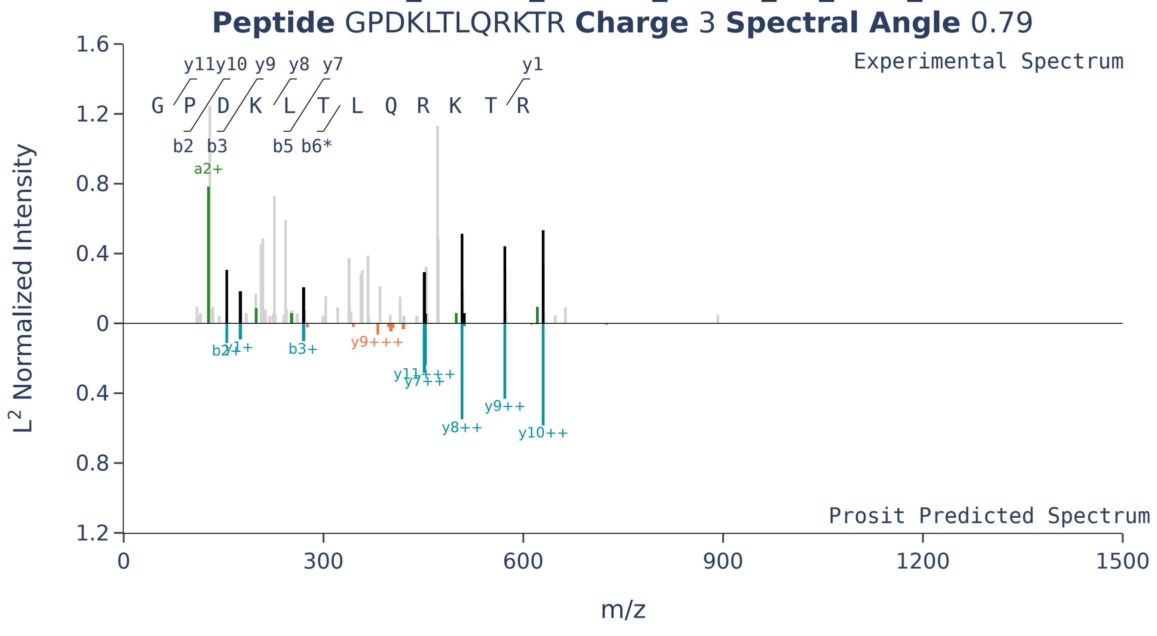


**Source** HRoetschke_211222_160123_HFGoe_IF2_b2R1_24h **Scan** 6577
**Peptide** AVMSDRDTGAAAEPRA **Charge** 3 **Spectral Angle** 0.56

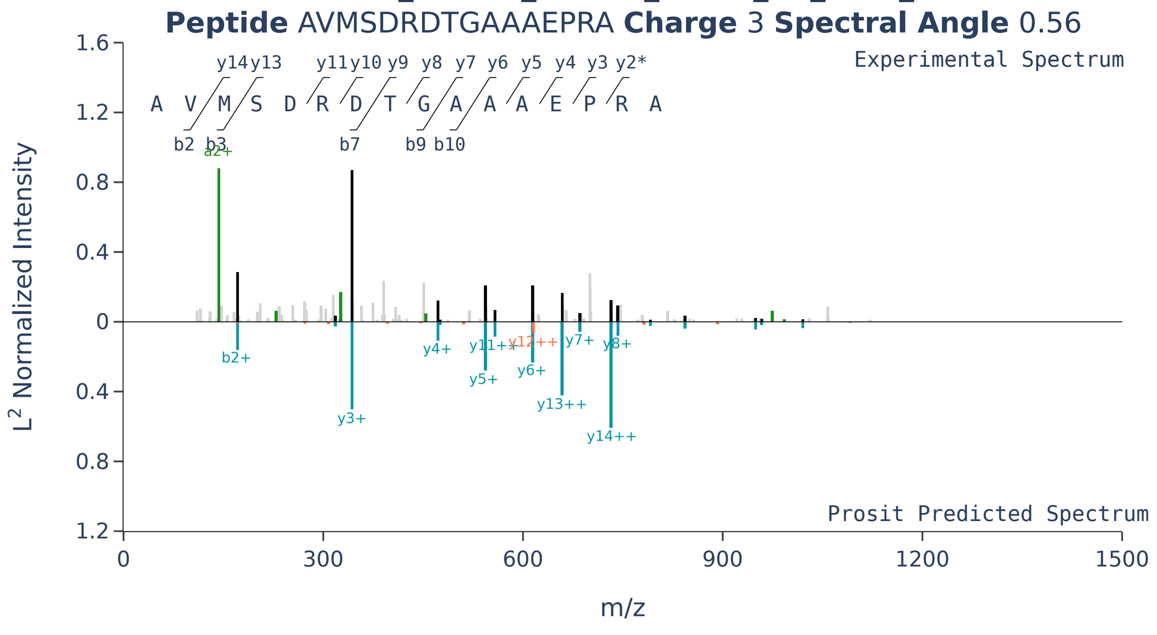


**Source** HRoetschke_211222_160123_HFGoe_IF2_b1R1_4h **Scan** 17696
**Peptide** IESFLDKGRGPVA **Charge** 3 **Spectral Angle** 0.77

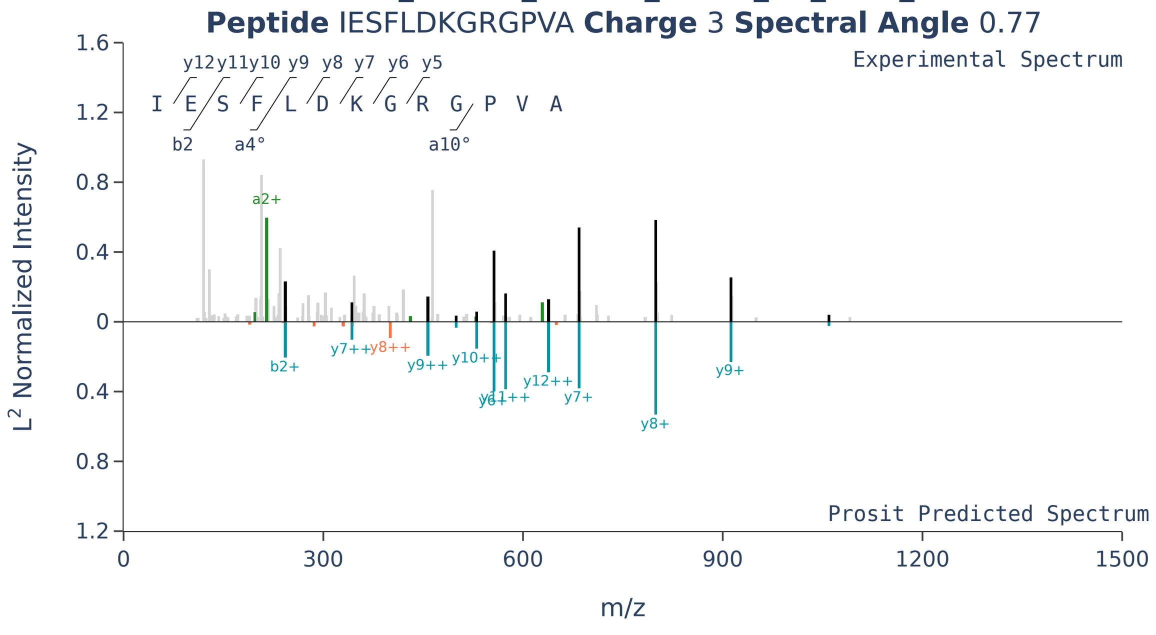


**Source** HRoetschke_211222_160123_HFGoe_IF2_b1R1_4h **Scan** 18204
**Peptide** WGGESQF **Charge** 1 **Spectral Angle** 0.8

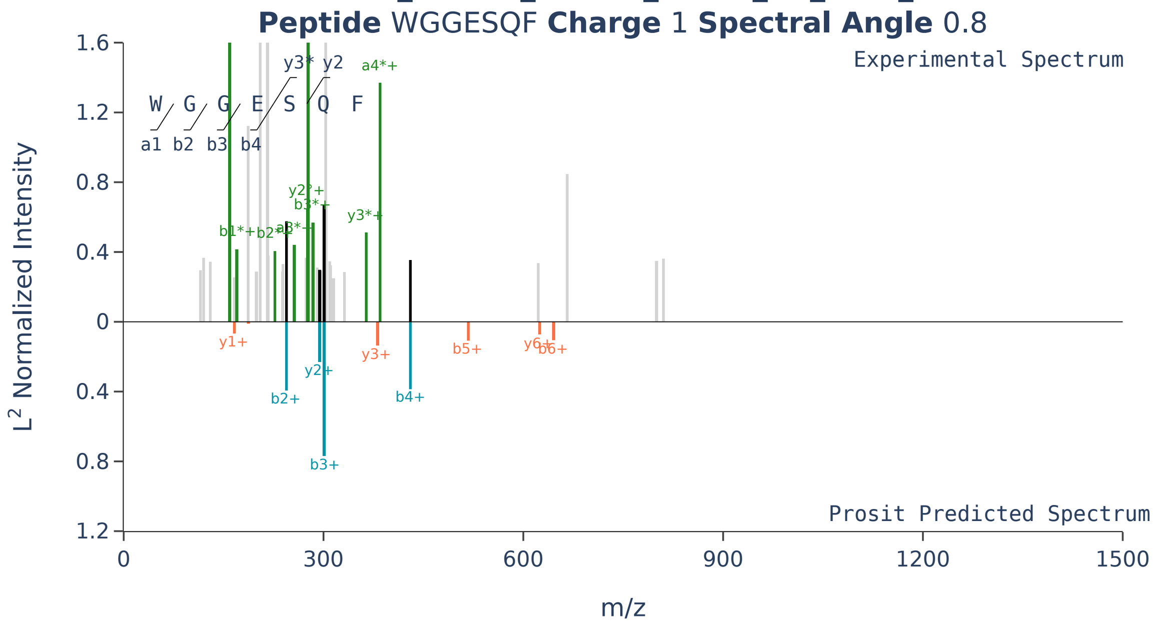


**Source** HRoetschke_211222_160123_HFGoe_IF2_b1R1_4h **Scan** 7341
**Peptide** EEMGHKVILRR **Charge** 3 **Spectral Angle** 0.8

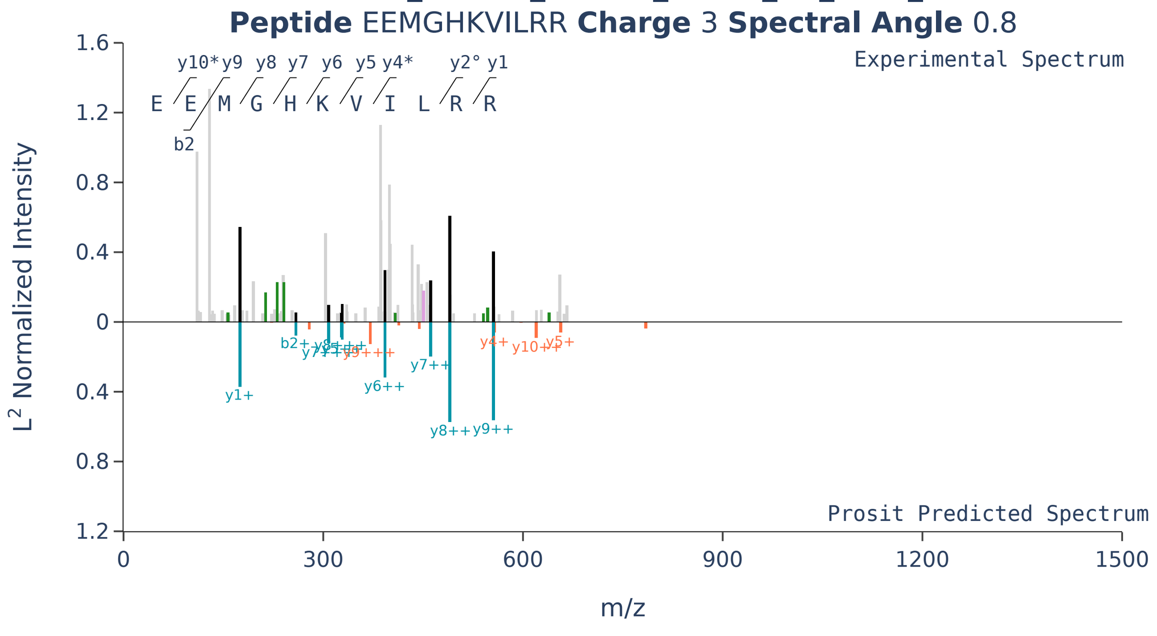


**Source** HRoetschke_211222_160123_HFGoe_IF2_b1R1_4h **Scan** 11062
**Peptide** GMECGIGVKN **Charge** 2 **Spectral Angle** 0.8

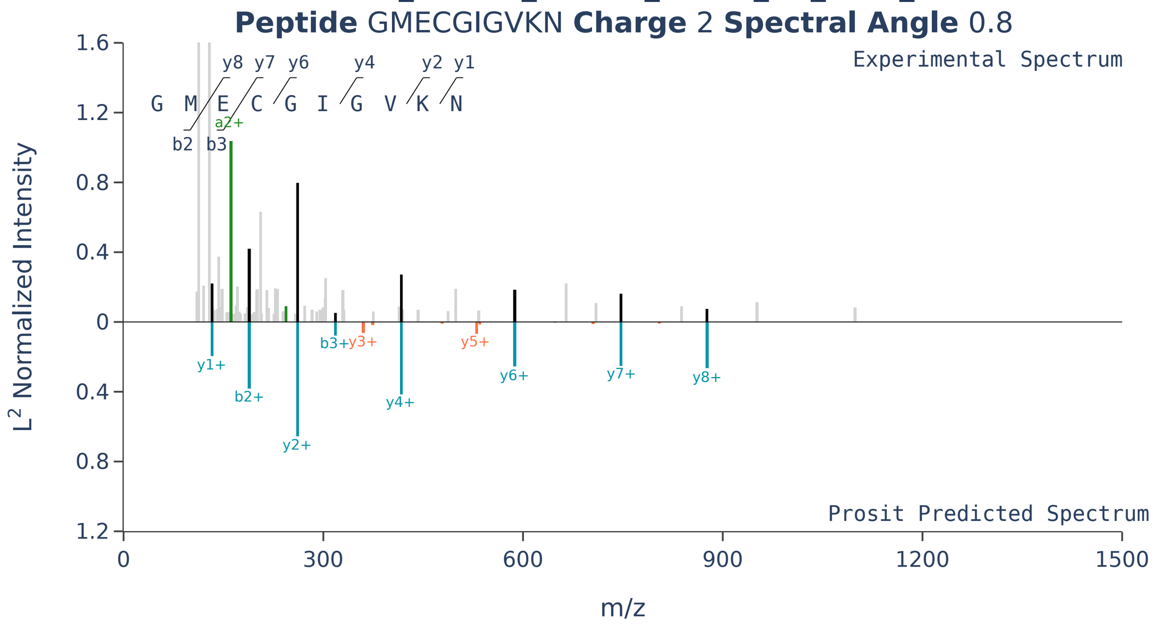


**Source** HRoetschke_211222_160123_HFGoe_IF2_b1R1_1h **Scan** 9504
**Peptide** QQFADAGIRKSADD **Charge** 2 **Spectral Angle** 0.72

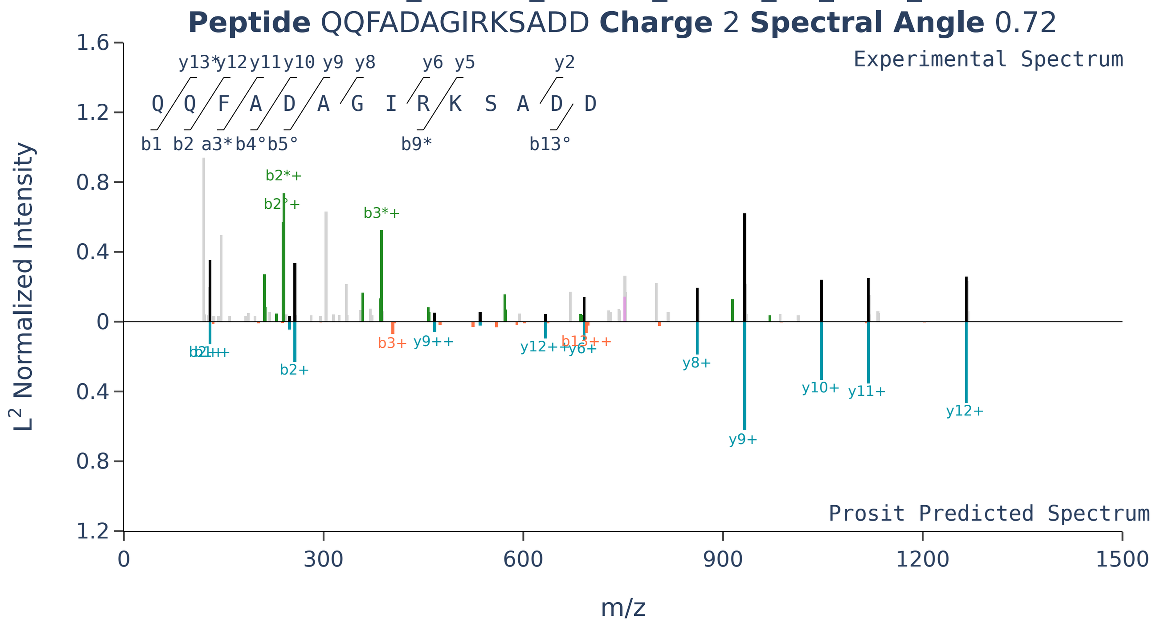

**Source** HRoetschke_211222_160123_HFGoe_IF2_b1R1_4h **Scan** 16363
**Peptide** DSVSAQEKQTLL **Charge** 2 **Spectral Angle** 0.75

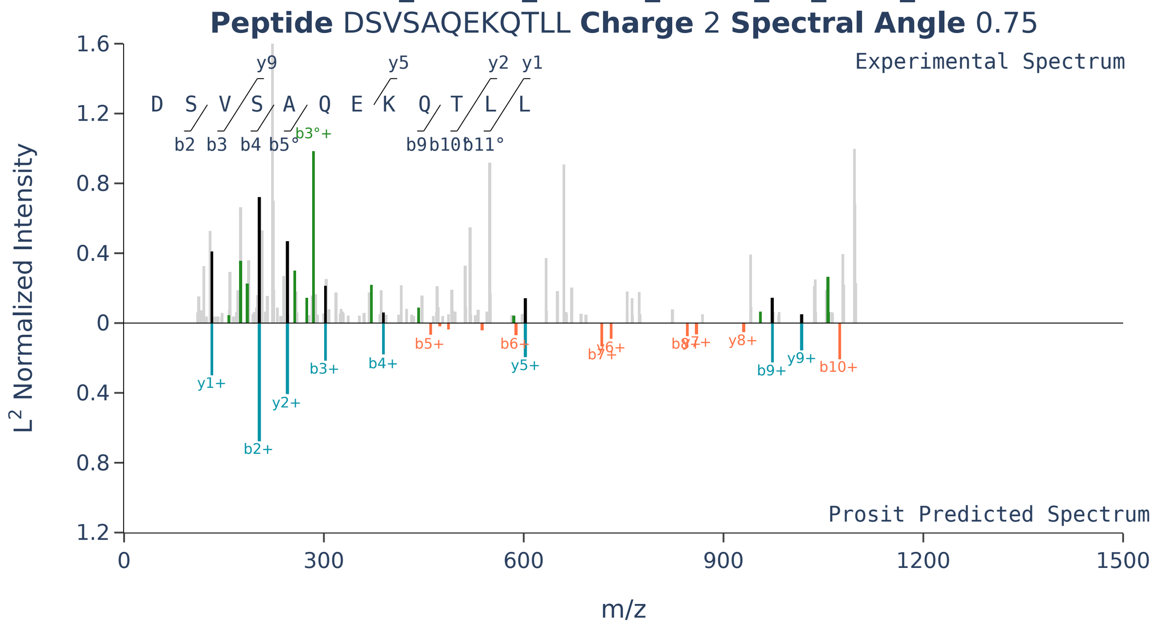


**Source** HRoetschke_211222_160123_HFGoe_IF2_b2R2_24h **Scan** 8210
**Peptide** TKVASGEAGGITQ **Charge** 2 **Spectral Angle** 0.83

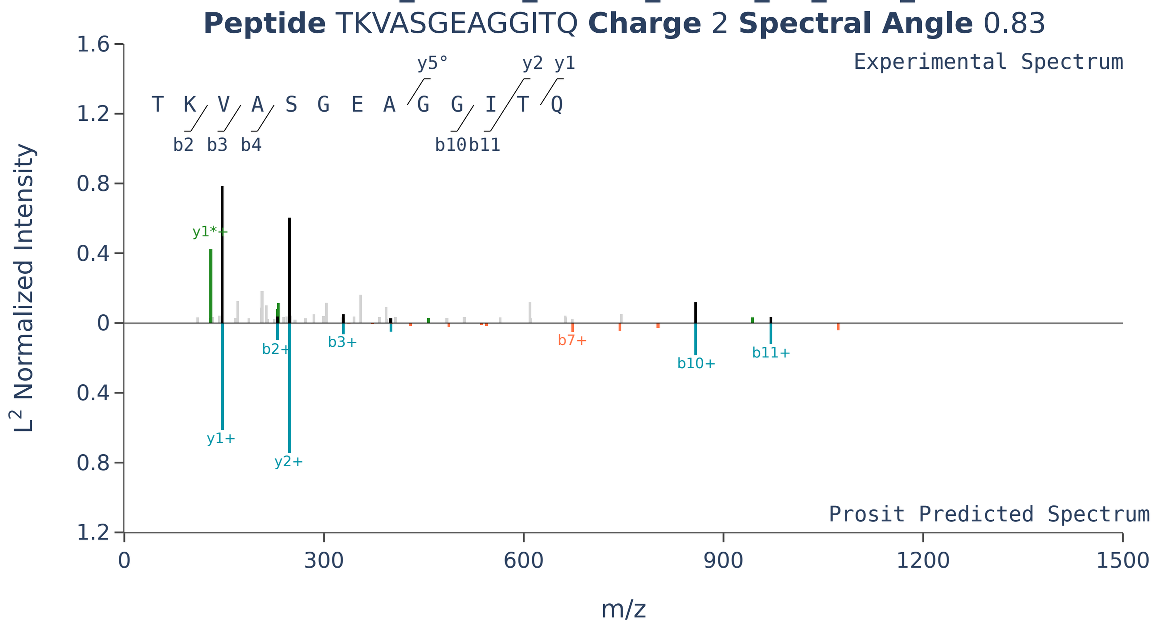


**Source** HRoetschke_211222_160123_HFGoe_IF2_b2R1_24h **Scan** 8412
**Peptide** GDEVTVVRDEKKAREV **Charge** 3 **Spectral Angle** 0.8

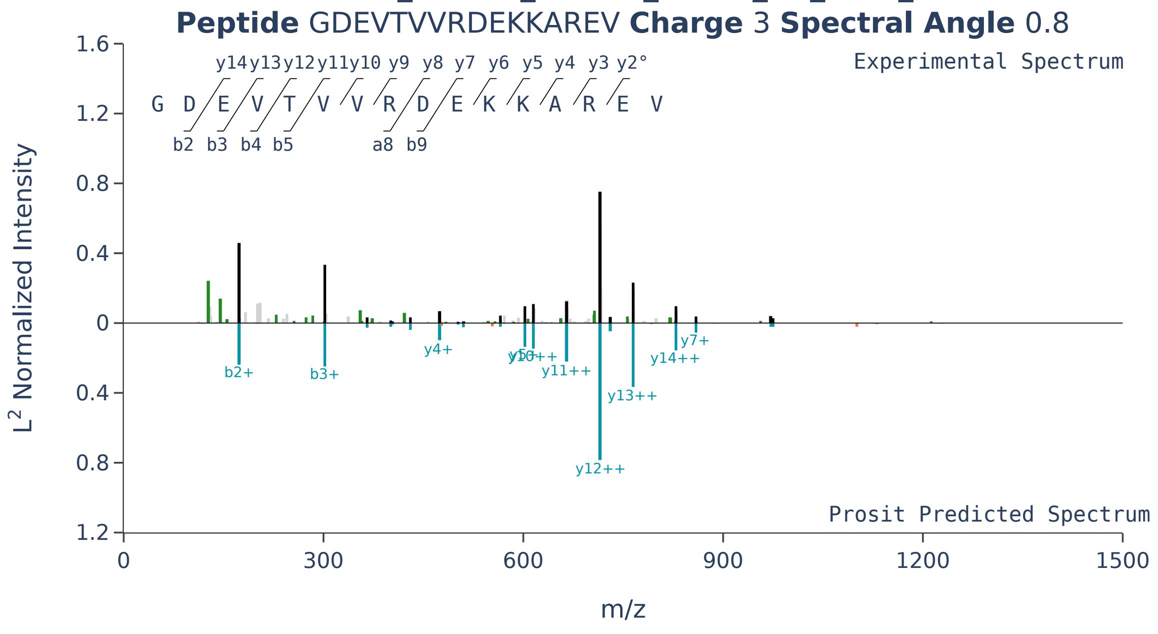


**Source** HRoetschke_211222_160123_HFGoe_IF2_b1R1_4h **Scan** 11218
**Peptide** QGFQKPAQAVNRDV **Charge** 3 **Spectral Angle** 0.85

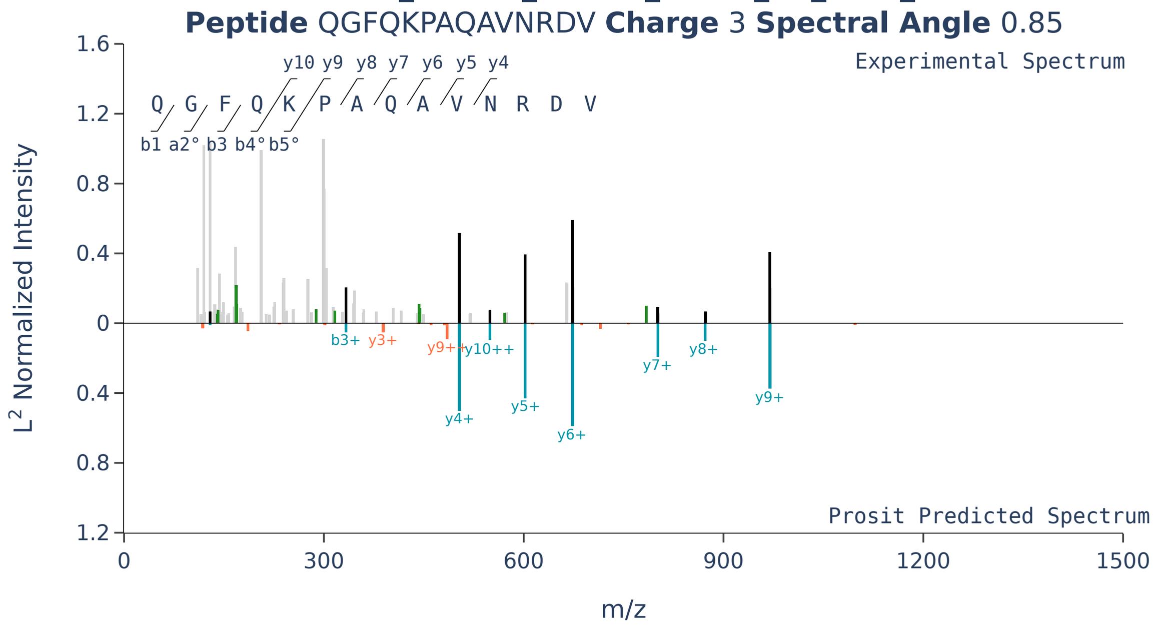


**Source** HRoetschke_211222_160123_HFGoe_IF2_b1R1_2h **Scan** 8171
**Peptide** DSSDYHVTTS **Charge** 1 **Spectral Angle** 0.82

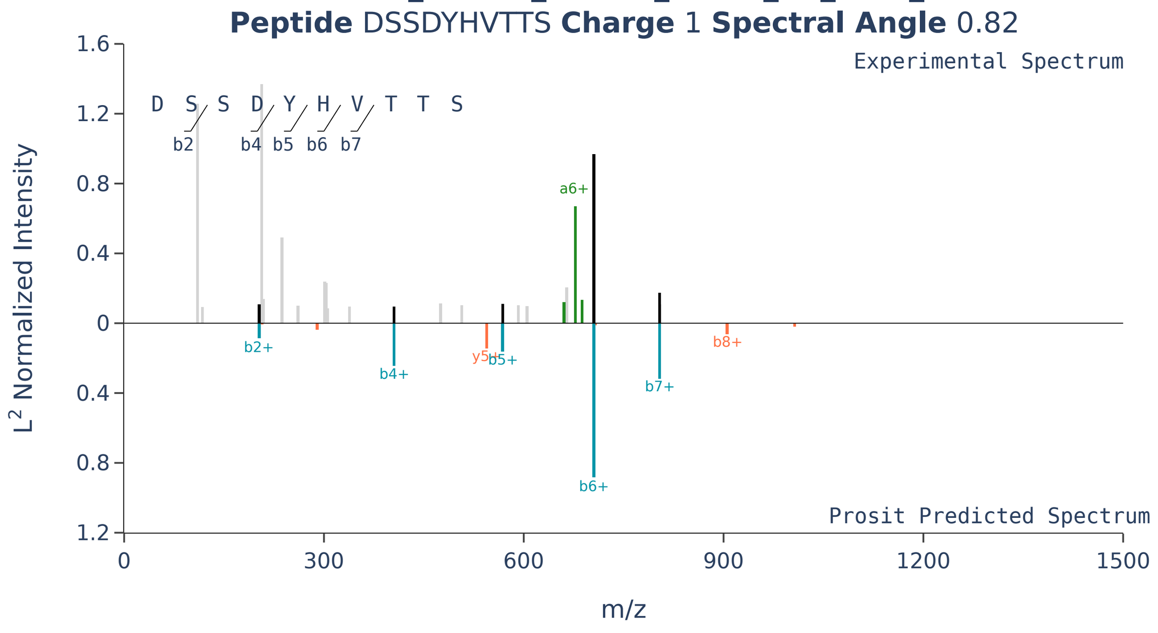


**Source** HRoetschke_211222_160123_HFGoe_IF2_b1R2_24h **Scan** 18183
**Peptide** RDTGAAAEPRAPVVTI **Charge** 3 **Spectral Angle** 0.69

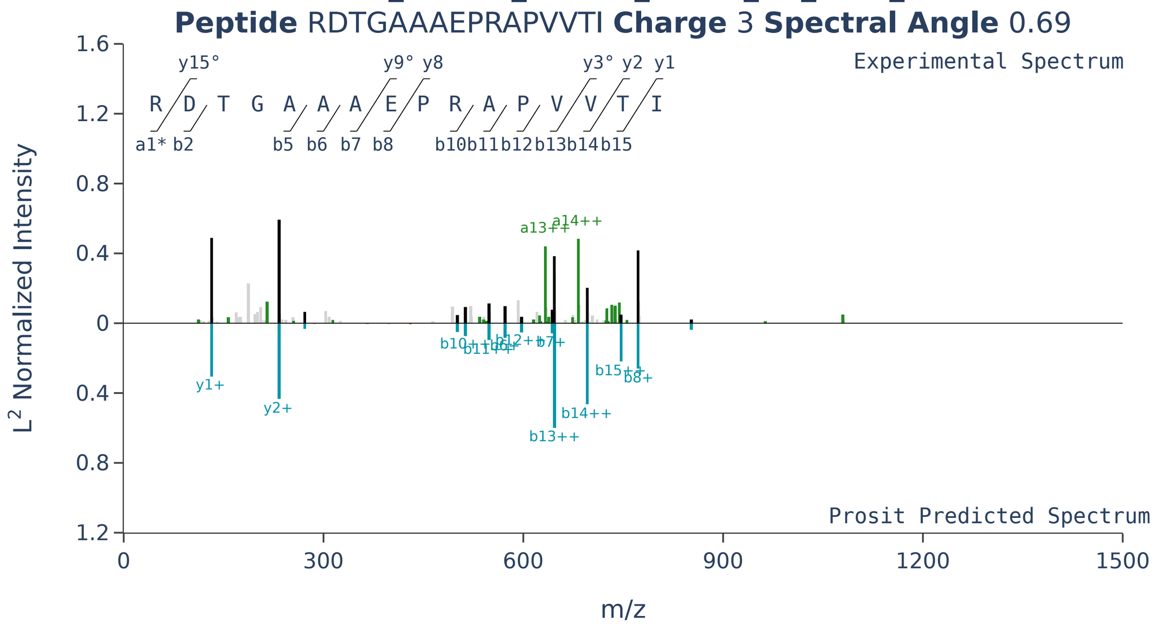


**Source** HRoetschke_211222_160123_HFGoe_IF2_b1R1_2h **Scan** 5677
**Peptide** LQHAKAAQV **Charge** 2 **Spectral Angle** 0.75

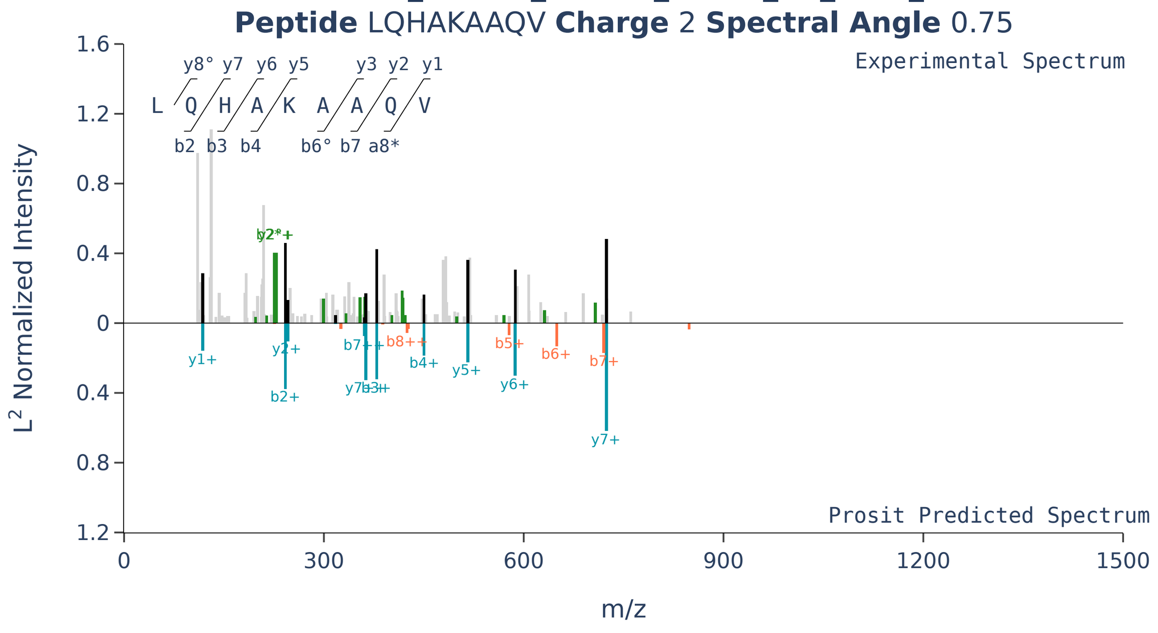


**Source** HRoetschke_211222_160123_HFGoe_IF2_b2R1_2h **Scan** 13692
**Peptide** EGTLHKGDIVLC **Charge** 3 **Spectral Angle** 0.95

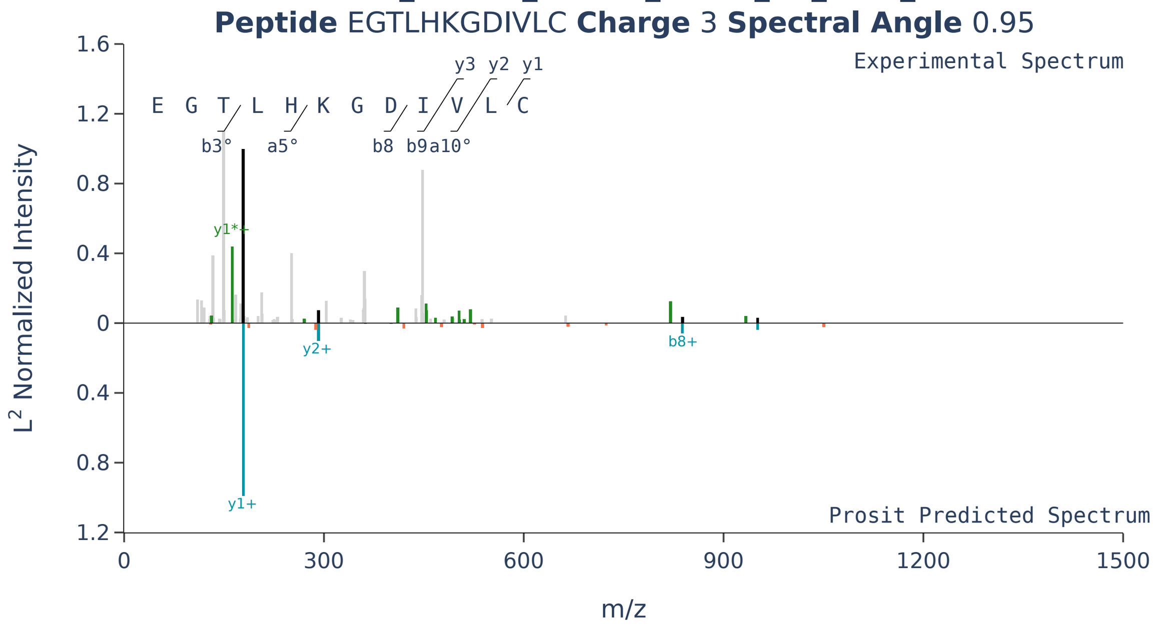


**Source** HRoetschke_211222_160123_HFGoe_IF2_b1R2_1h **Scan** 6365
**Peptide** QKPAQAVNRDV **Charge** 3 **Spectral Angle** 0.9

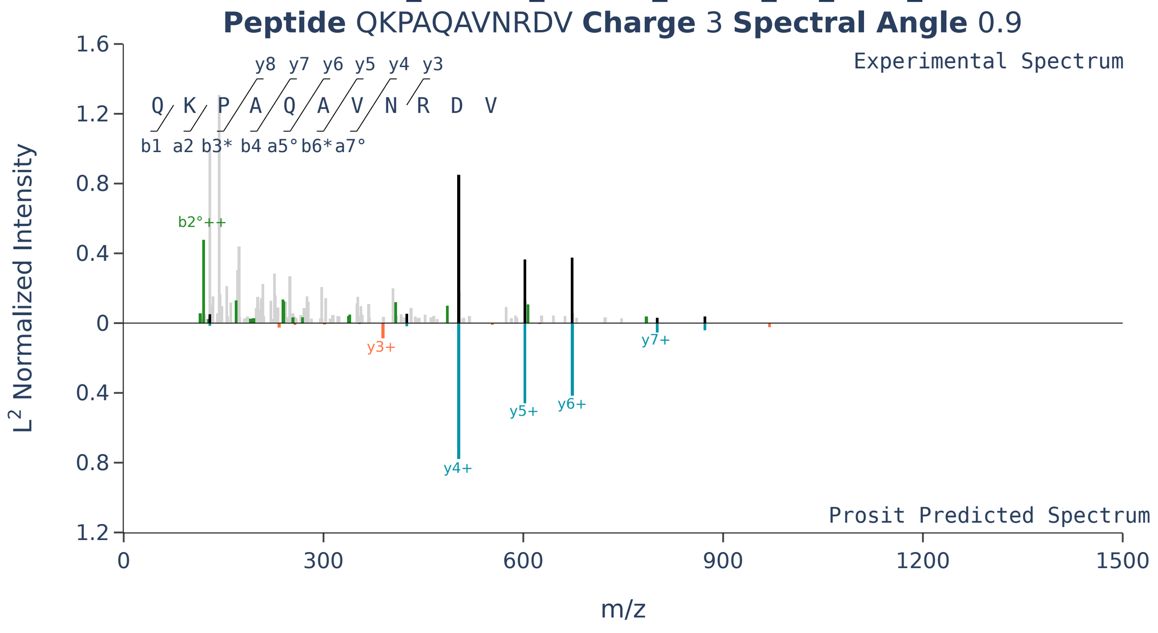


**Source** HRoetschke_211222_160123_HFGoe_IF2_b1R1_4h **Scan** 13560
**Peptide** ASGEAGGITQHIGAYH **Charge** 3 **Spectral Angle** 0.75

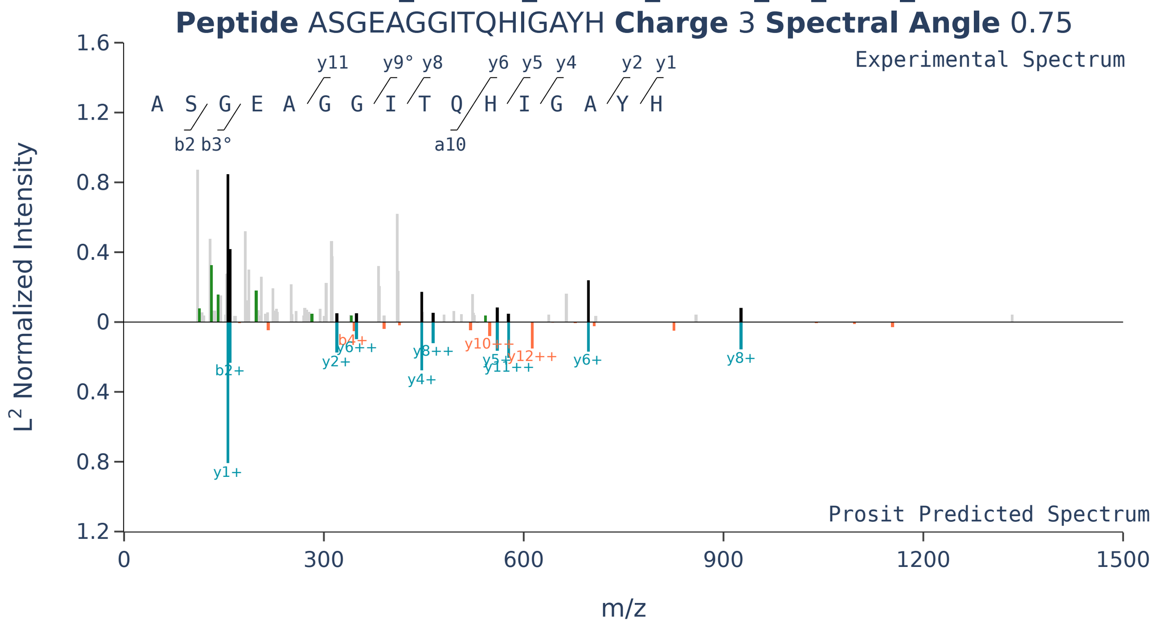


**Source** HRoetschke_211222_160123_HFGoe_IF2_b1R1_2h **Scan** 5988
**Peptide** RSTKVASGEAGGITQH **Charge** 3 **Spectral Angle** 0.8

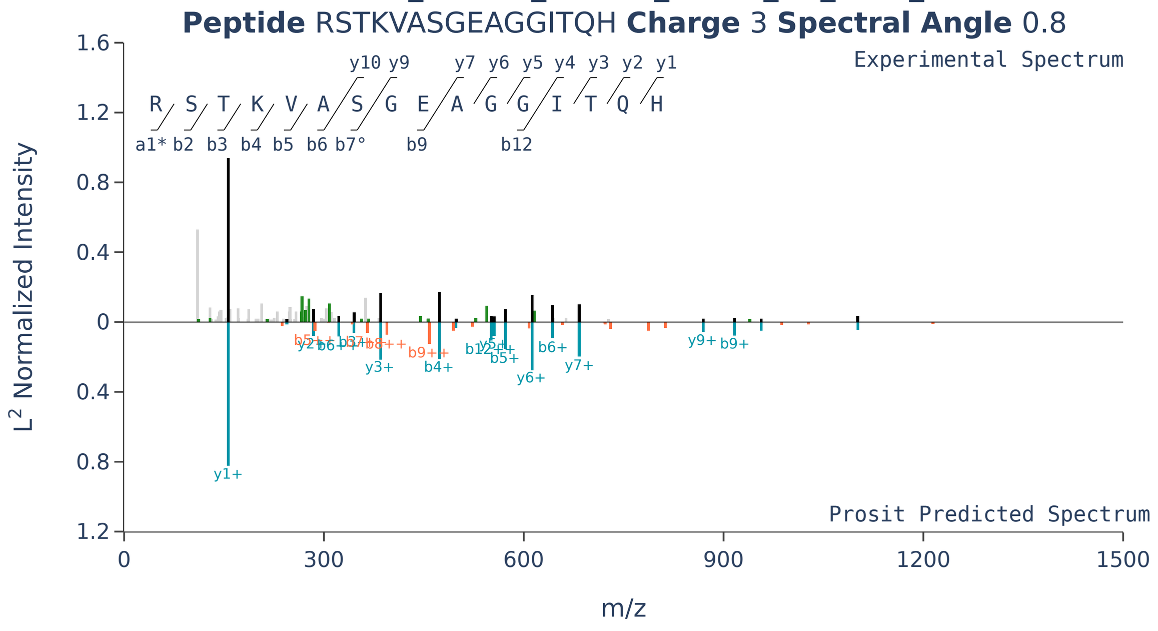


**Source** HRoetschke_211222_160123_HFGoe_IF2_b2R1_24h **Scan** 9502
**Peptide** TLAAERQTSV **Charge** 2 **Spectral Angle** 0.82

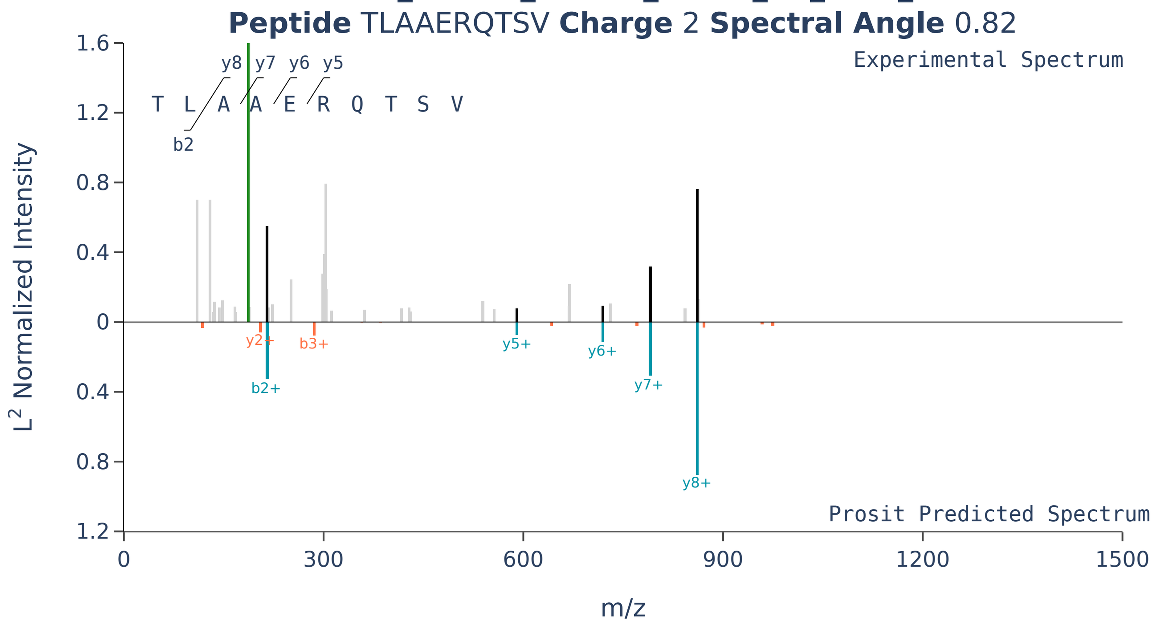


**Source** HRoetschke_211222_160123_HFGoe_IF2_b1R2_24h **Scan** 6585
**Peptide** GDEVTVVRDEKKARE **Charge** 4 **Spectral Angle** 0.78

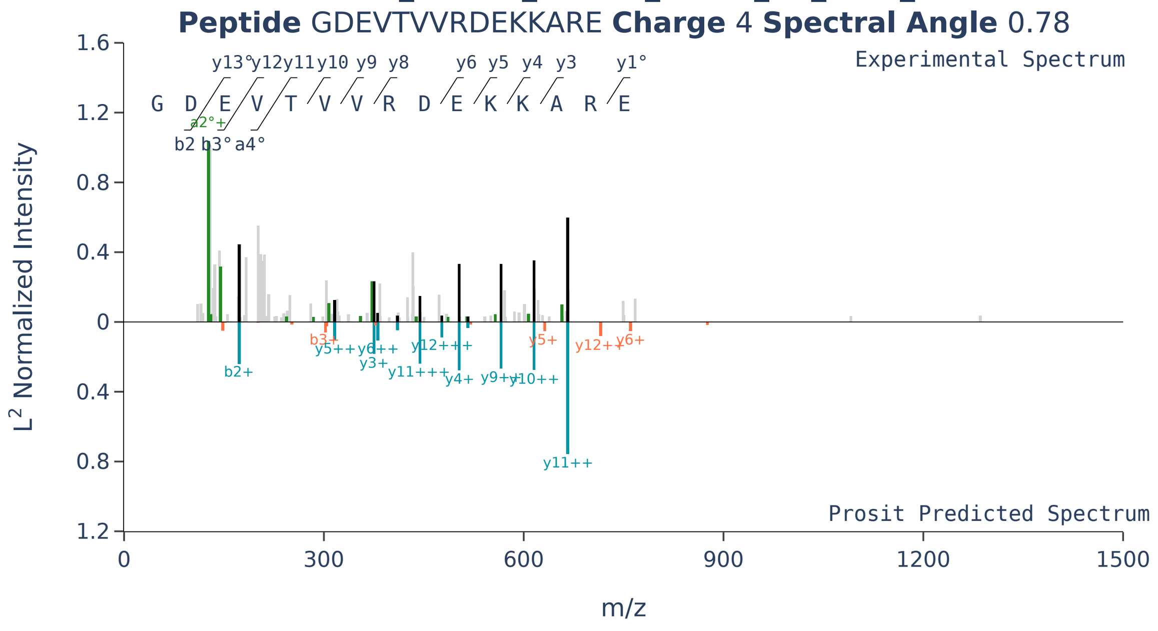


**Source** HRoetschke_211222_160123_HFGoe_IF2_b1R1_4h **Scan** 23144
**Peptide** GLDELLD **Charge** 1 **Spectral Angle** 0.79

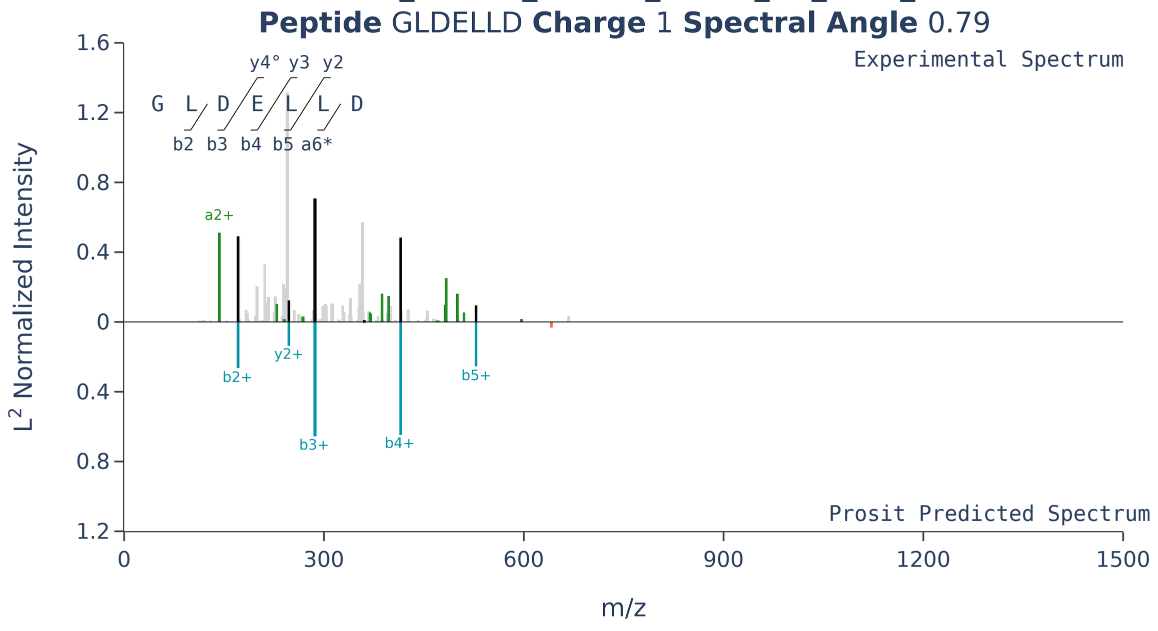


**Source** HRoetschke_211222_160123_HFGoe_IF2_b1R2_4h **Scan** 11488
**Peptide** GGESQFVHVSAKAG **Charge** 3 **Spectral Angle** 0.64

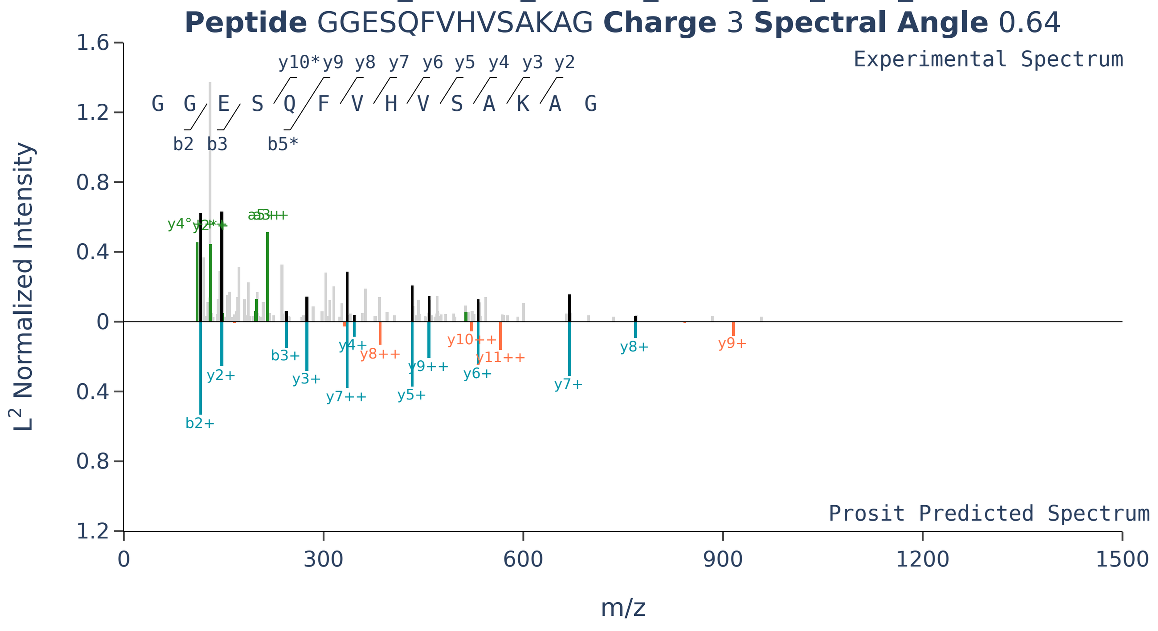

**Source** HRoetschke_211222_160123_HFGoe_IF2_b1R2_24h **Scan** 12816
**Peptide** AQEKQTLI **Charge** 2 **Spectral Angle** 0.71

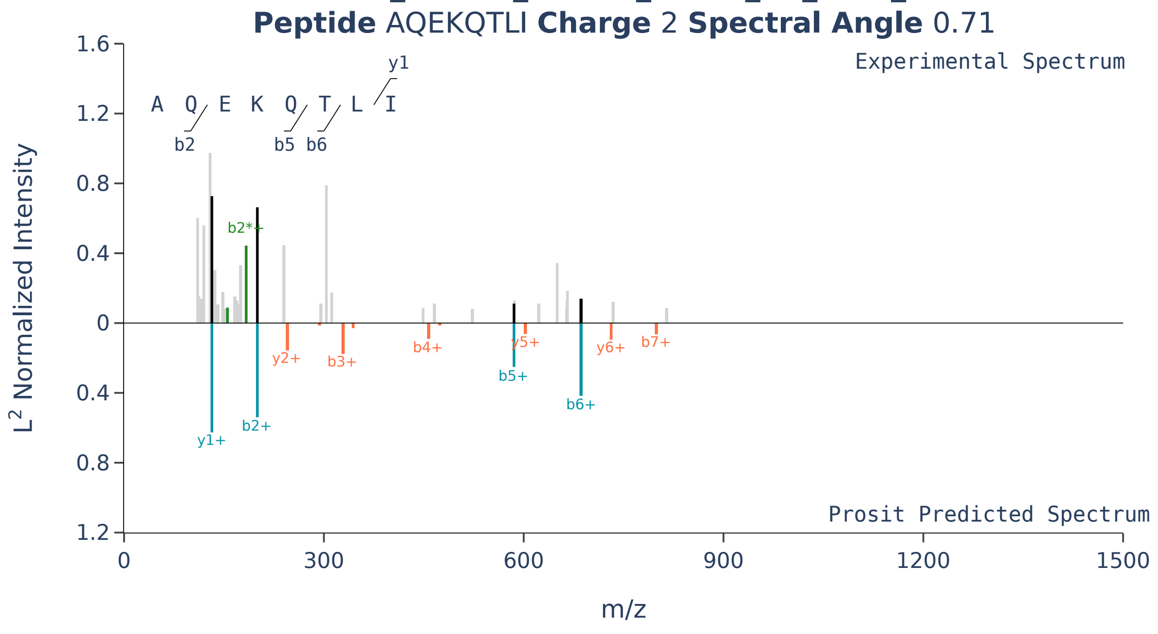


**Source** HRoetschke_211222_160123_HFGoe_IF2_b1R1_4h **Scan** 11444
**Peptide** GESQFVHVSAKAG **Charge** 3 **Spectral Angle** 0.72

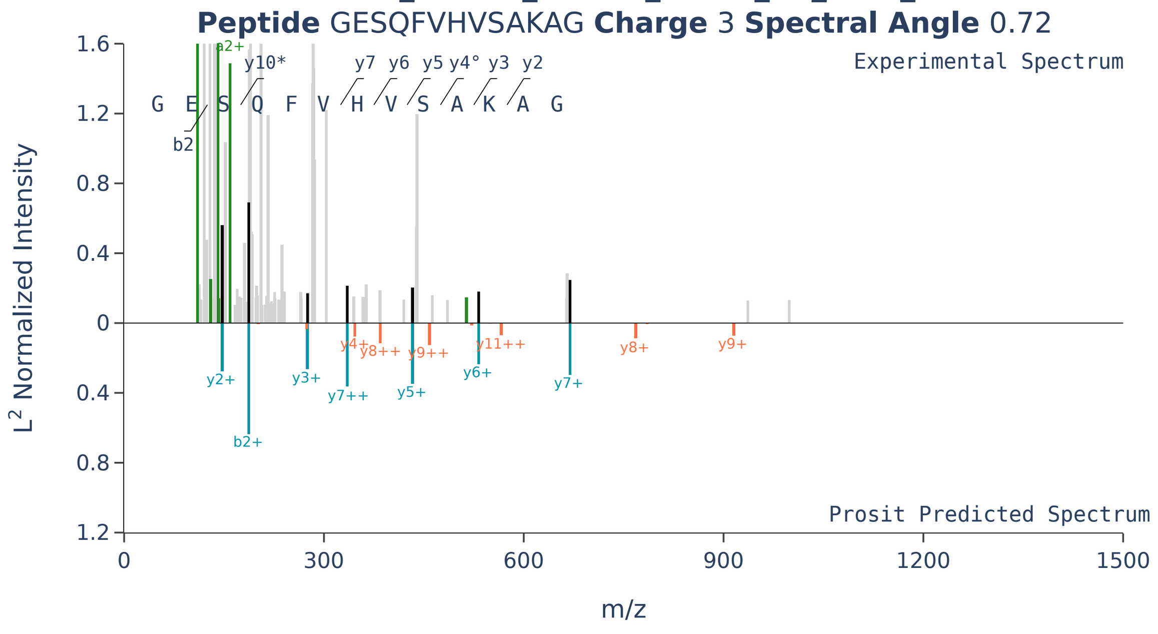


**Source** HRoetschke_211222_160123_HFGoe_IF2_b1R2_2h **Scan** 10398
**Peptide** VREGTLH **Charge** 2 **Spectral Angle** 0.92

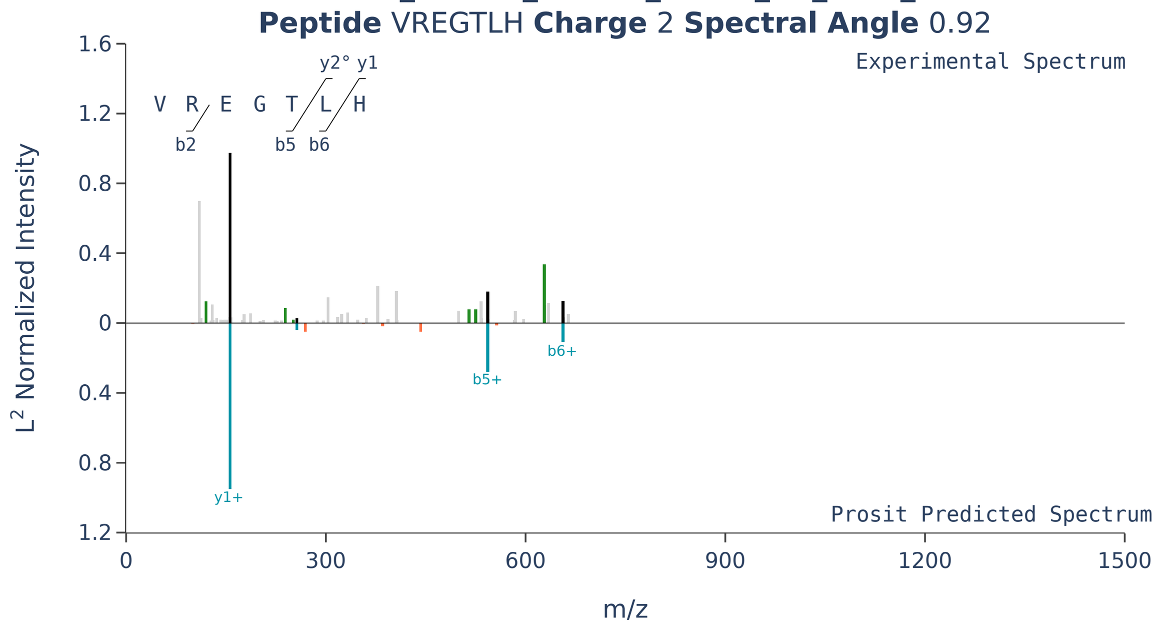


**Source** HRoetschke_211222_160123_HFGoe_IF2_b1R2_24h **Scan** 6964
**Peptide** RSTKVASGEAGGITQ **Charge** 3 **Spectral Angle** 0.79

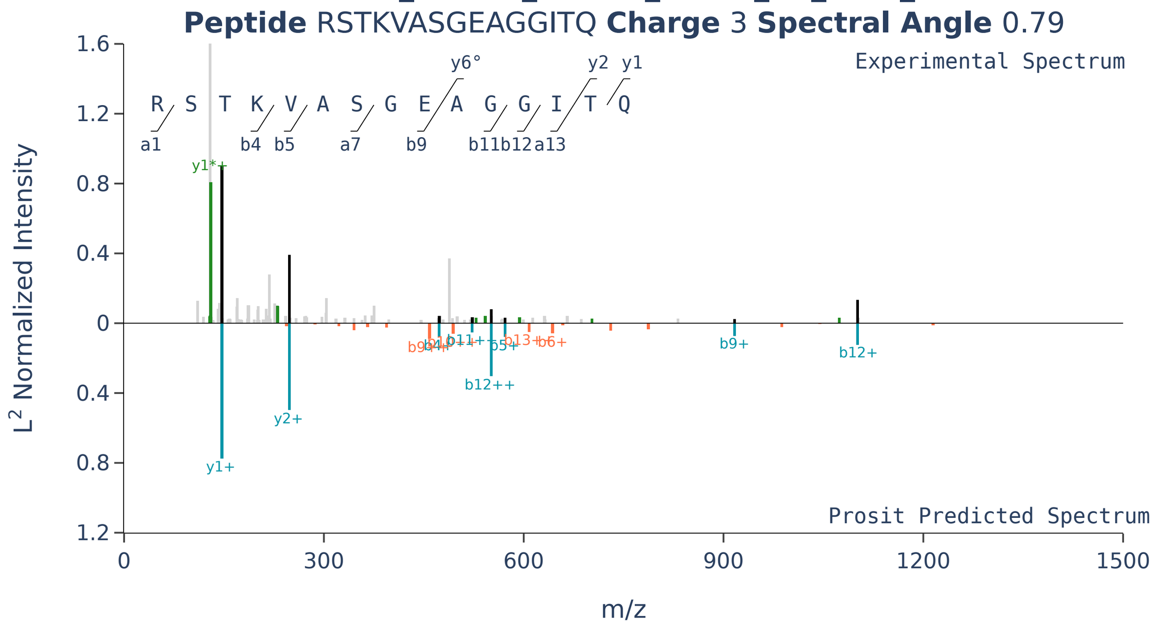


**Source** HRoetschke_211222_160123_HFGoe_IF2_b1R1_4h **Scan** 6833
**Peptide** HVSAKAGTGL **Charge** 2 **Spectral Angle** 0.73

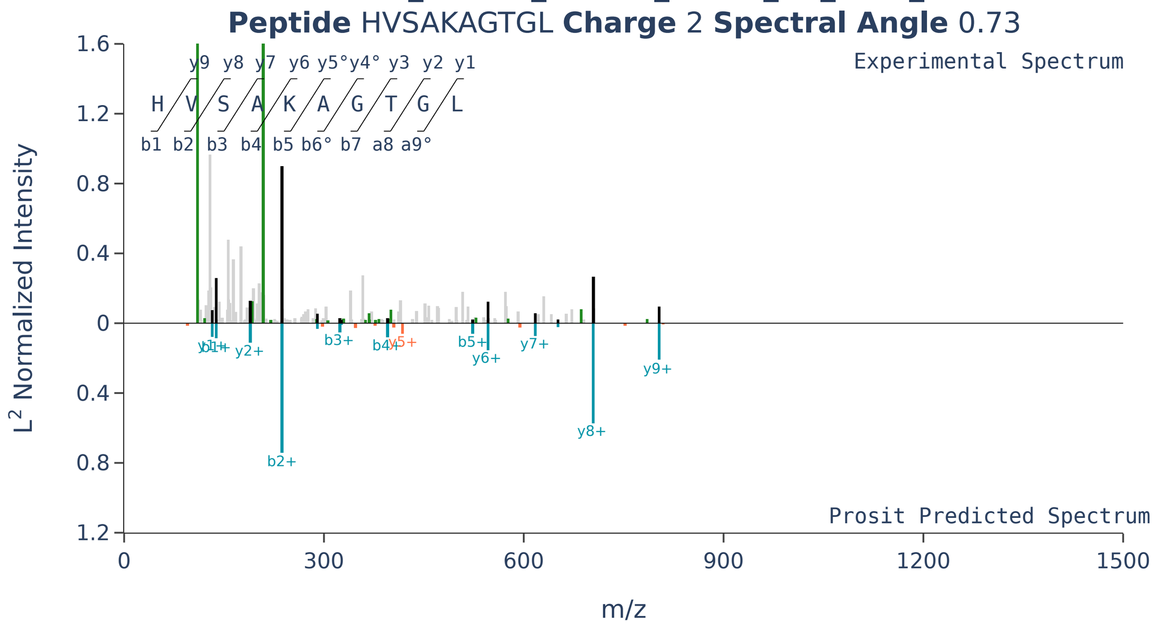


**Source** HRoetschke_211222_160123_HFGoe_IF2_b1R1_2h **Scan** 5373
**Peptide** KTLAAERQT **Charge** 2 **Spectral Angle** 0.88

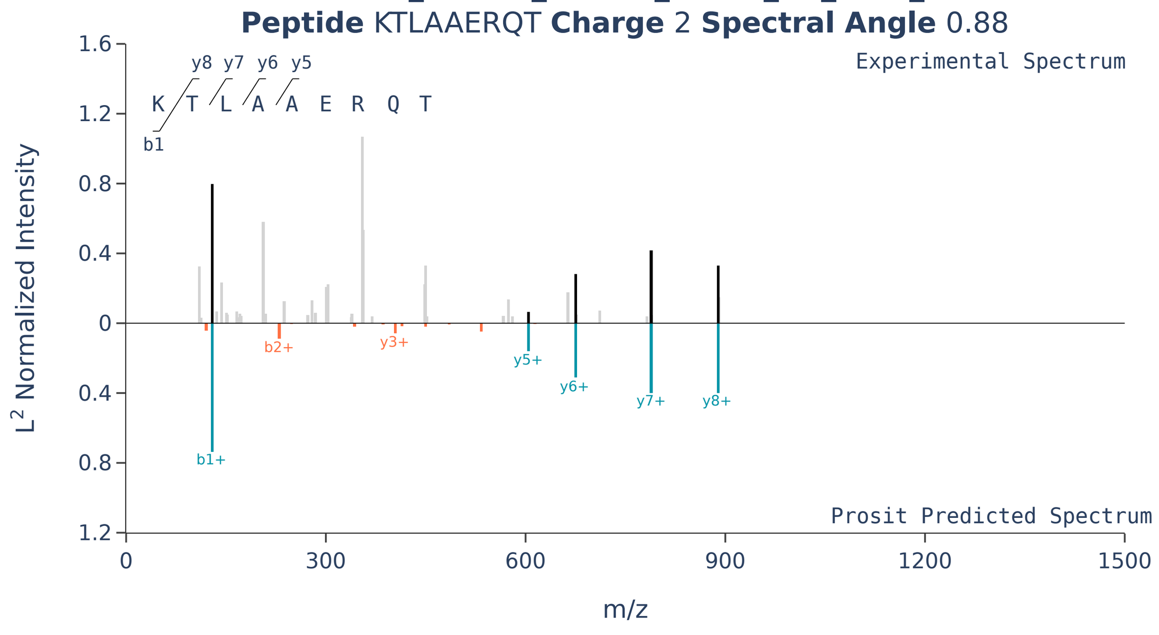


**Source** HRoetschke_211222_160123_HFGoe_IF2_b1R2_24h **Scan** 19093
**Peptide** EPRAPVVTI **Charge** 2 **Spectral Angle** 0.78

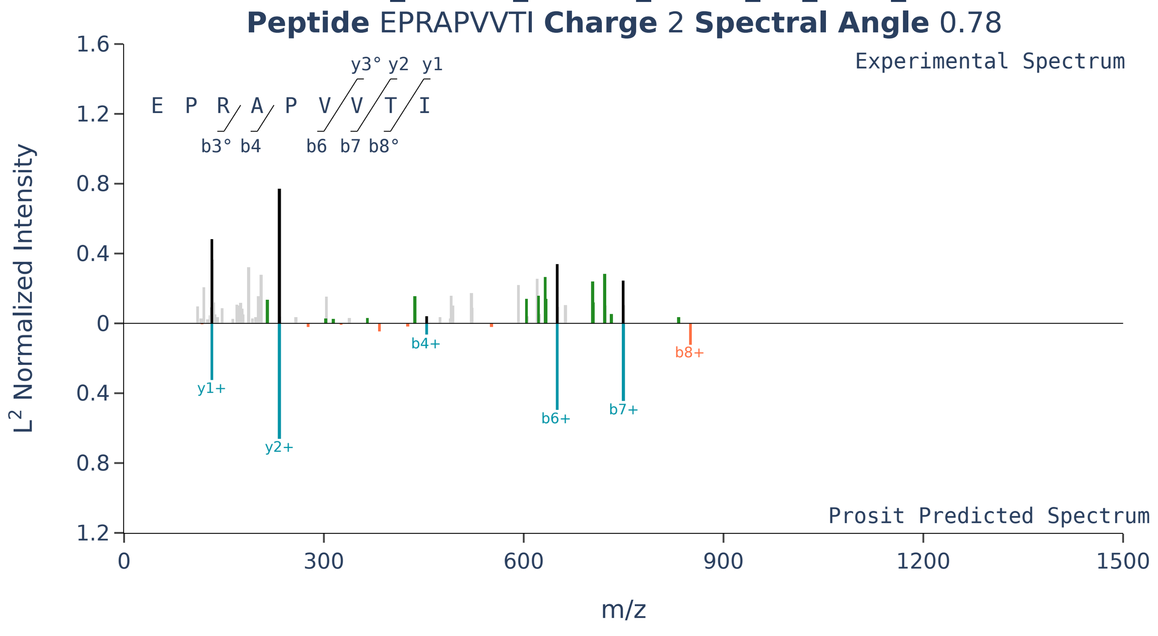


**Source** HRoetschke_211222_160123_HFGoe_IF2_b2R1_4h **Scan** 14597
**Peptide** SLQQGFQKPAQAV **Charge** 2 **Spectral Angle** 0.83

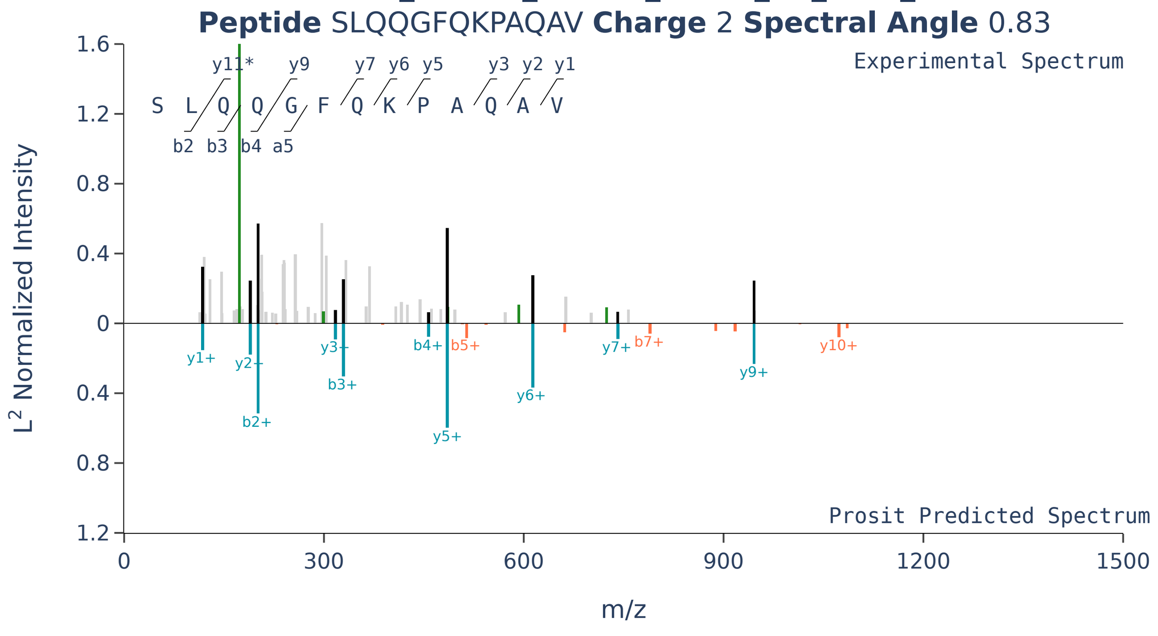


**Source** HRoetschke_211222_160123_HFGoe_IF2_b2R1_1h **Scan** 7858
**Peptide** AERLAAEEQA **Charge** 2 **Spectral Angle** 0.63

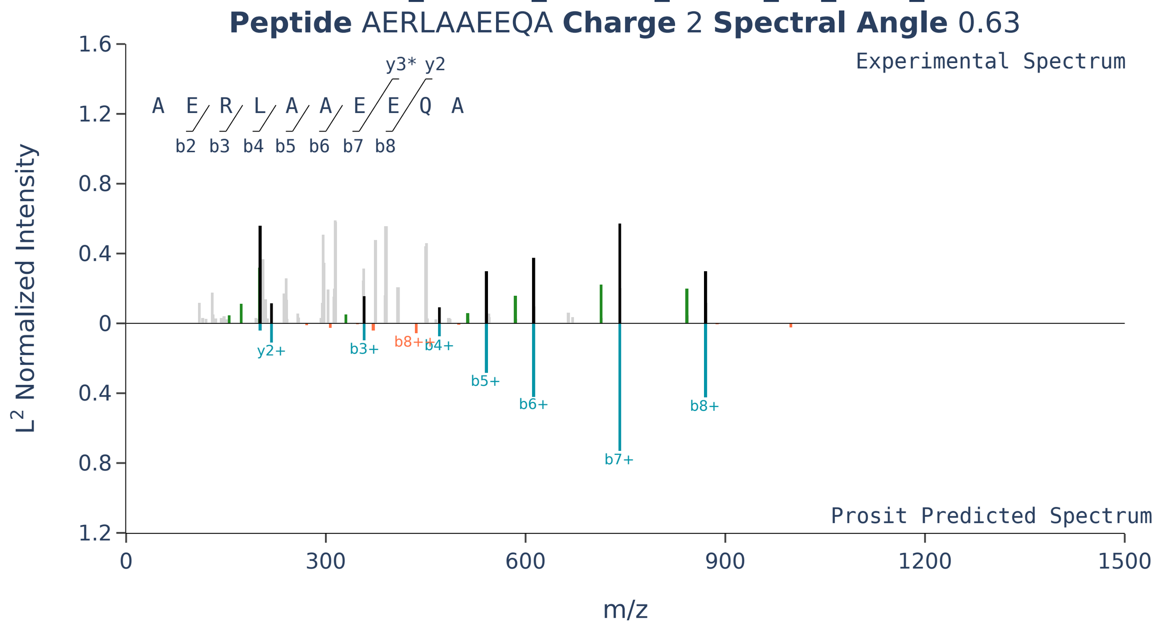


**Source** HRoetschke_211222_160123_HFGoe_IF2_b1R1_24h **Scan** 17726
**Peptide** EGELESL **Charge** 1 **Spectral Angle** 0.85

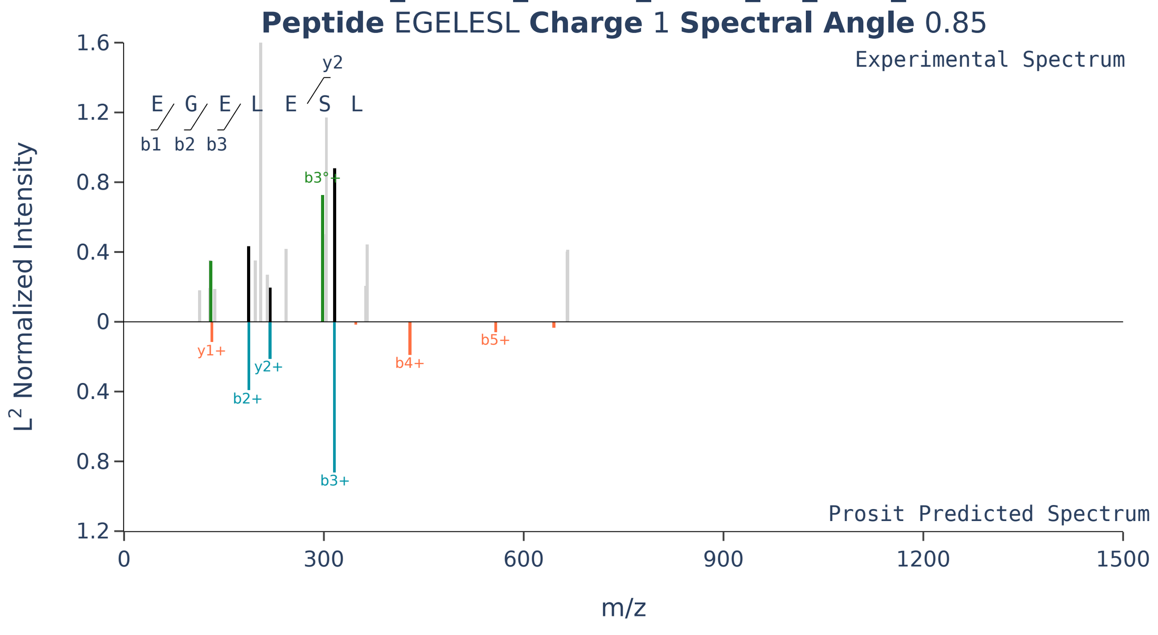


**Source** HRoetschke_211222_160123_HFGoe_IF2_b2R1_24h **Scan** 8480
**Peptide** VNKIDKPEADPDRV **Charge** 2 **Spectral Angle** 0.74

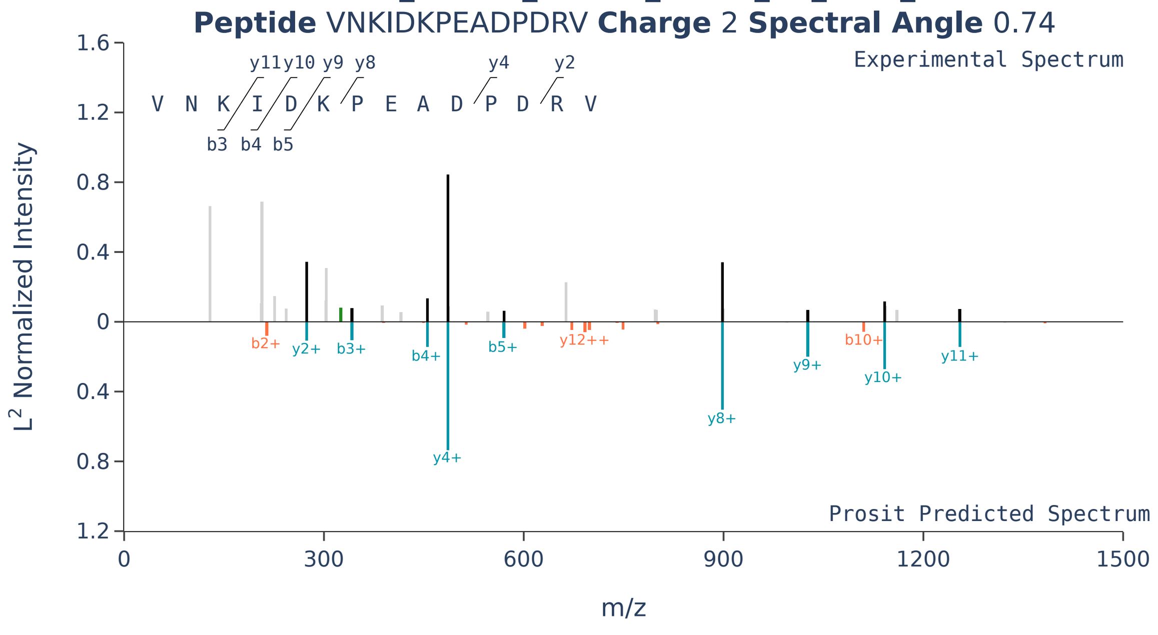


**Source** HRoetschke_211222_160123_HFGoe_IF2_b1R1_4h **Scan** 5783
**Peptide** EVTVVRDEKK **Charge** 3 **Spectral Angle** 0.81

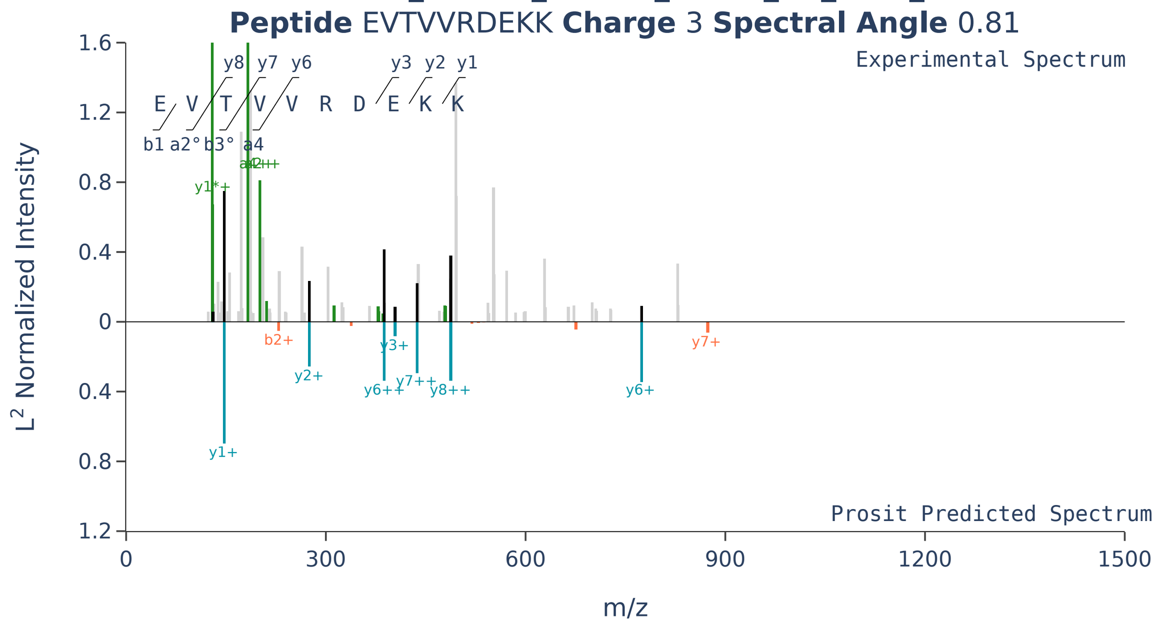


**Source** HRoetschke_211222_160123_HFGoe_IF2_b1R1_4h **Scan** 12631
**Peptide** TLIDHLNQKN **Charge** 2 **Spectral Angle** 0.72

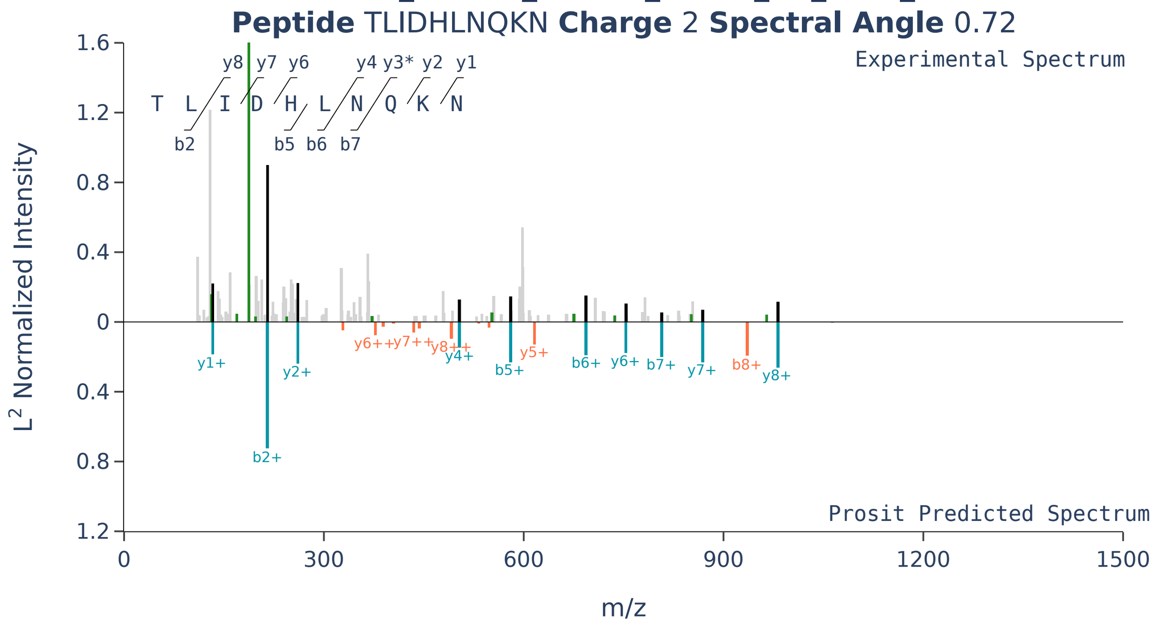


**Source** HRoetschke_211222_160123_HFGoe_IF2_b2R2_24h **Scan** 13674
**Peptide** SGPDKLTLQ **Charge** 2 **Spectral Angle** 0.82

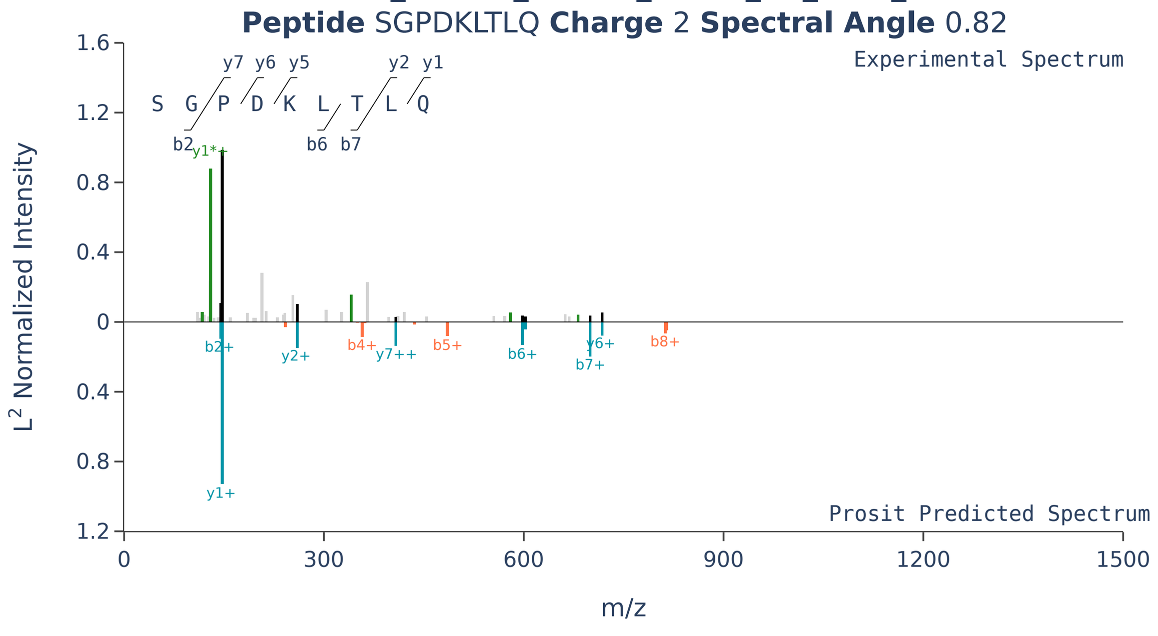


**Source** HRoetschke_211222_160123_HFGoe_IF2_b2R1_24h **Scan** 15270
**Peptide** IEIQRTIA **Charge** 2 **Spectral Angle** 0.87

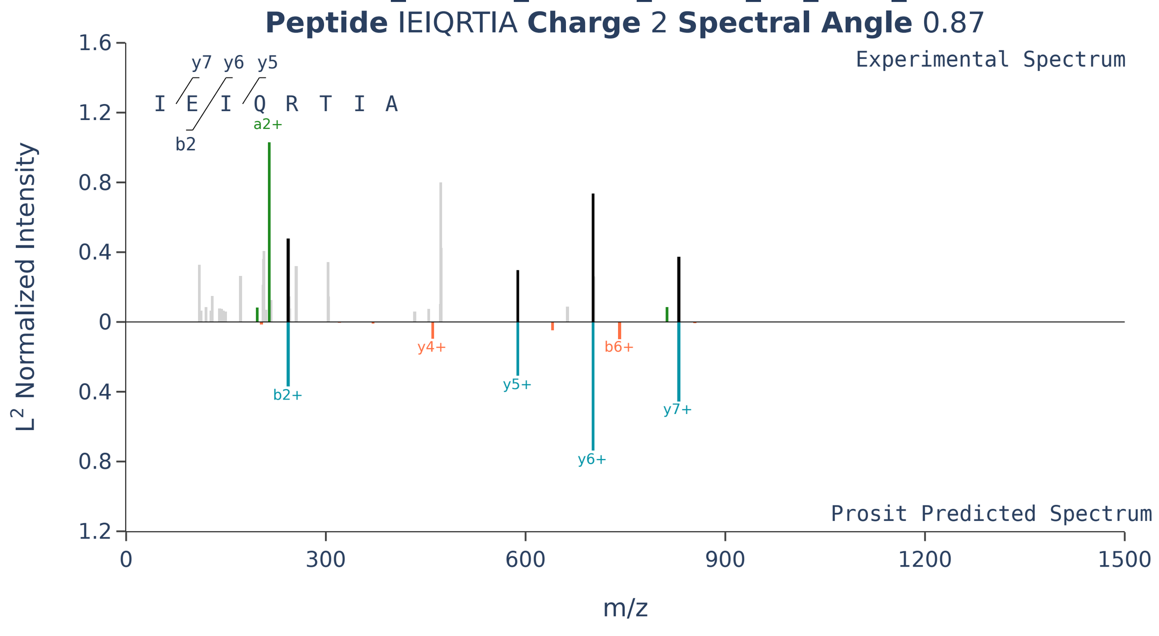

**Source** HRoetschke_211222_160123_HFGoe_IF2_b2R2_2h **Scan** 16450
**Peptide** VERLVQQFAD **Charge** 2 **Spectral Angle** 0.83

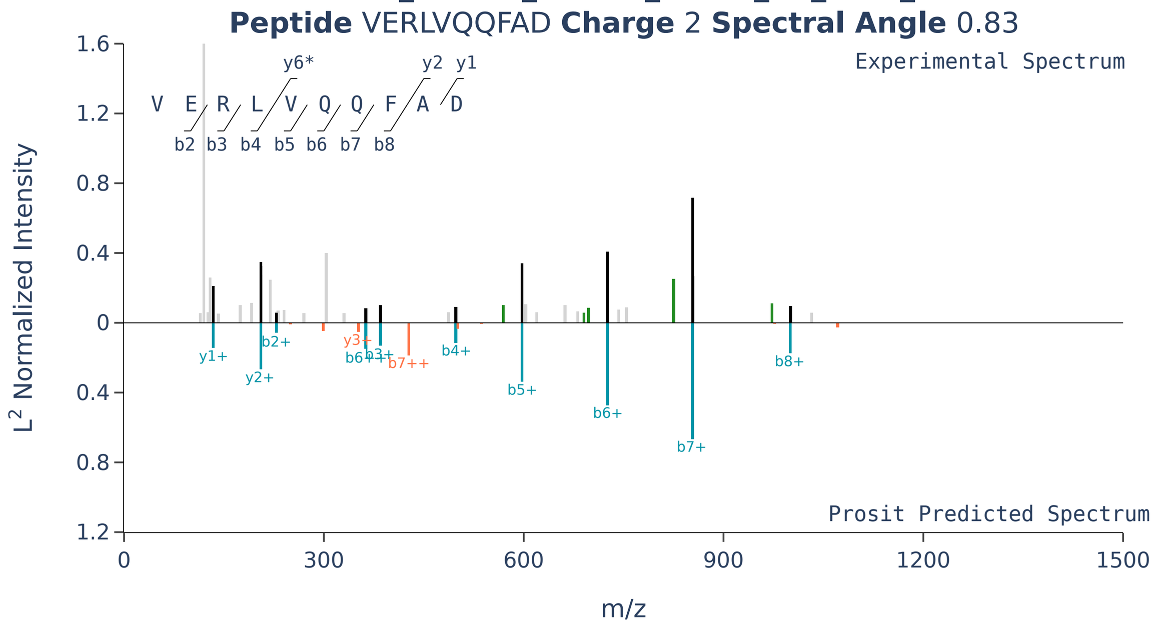


**Source** HRoetschke_211222_160123_HFGoe_IF2_b1R1_24h **Scan** 6760
**Peptide** VAEEMGHKVILRRE **Charge** 4 **Spectral Angle** 0.83

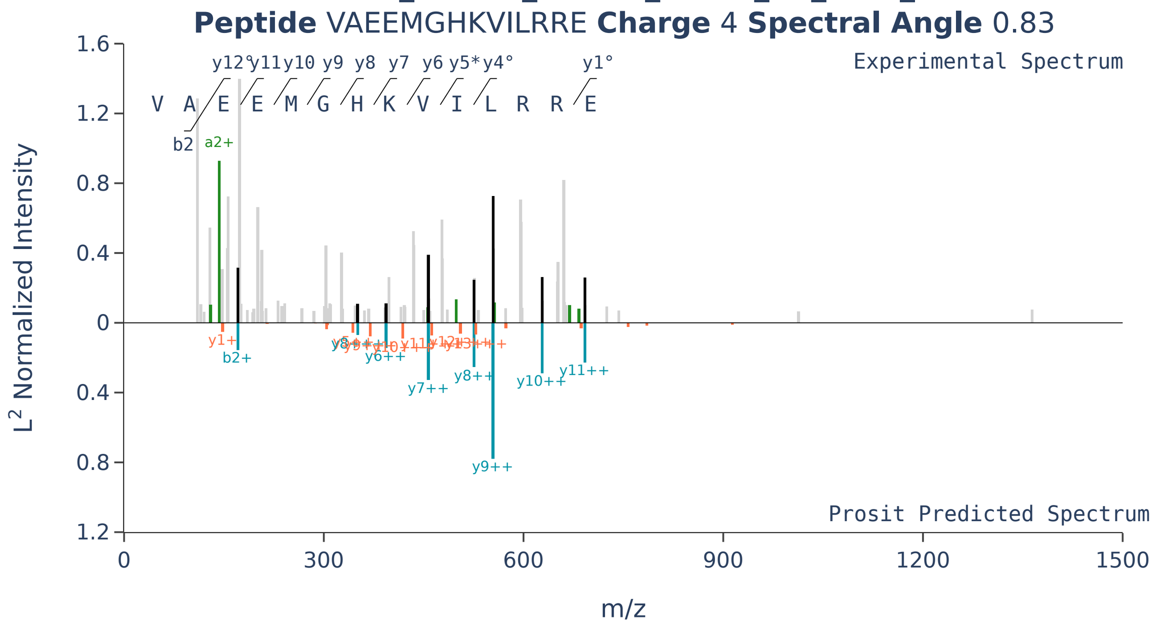


**Source** HRoetschke_211222_160123_HFGoe_IF2_b1R1_24h **Scan** 16098
**Peptide** NSGPDKLTL **Charge** 2 **Spectral Angle** 0.82

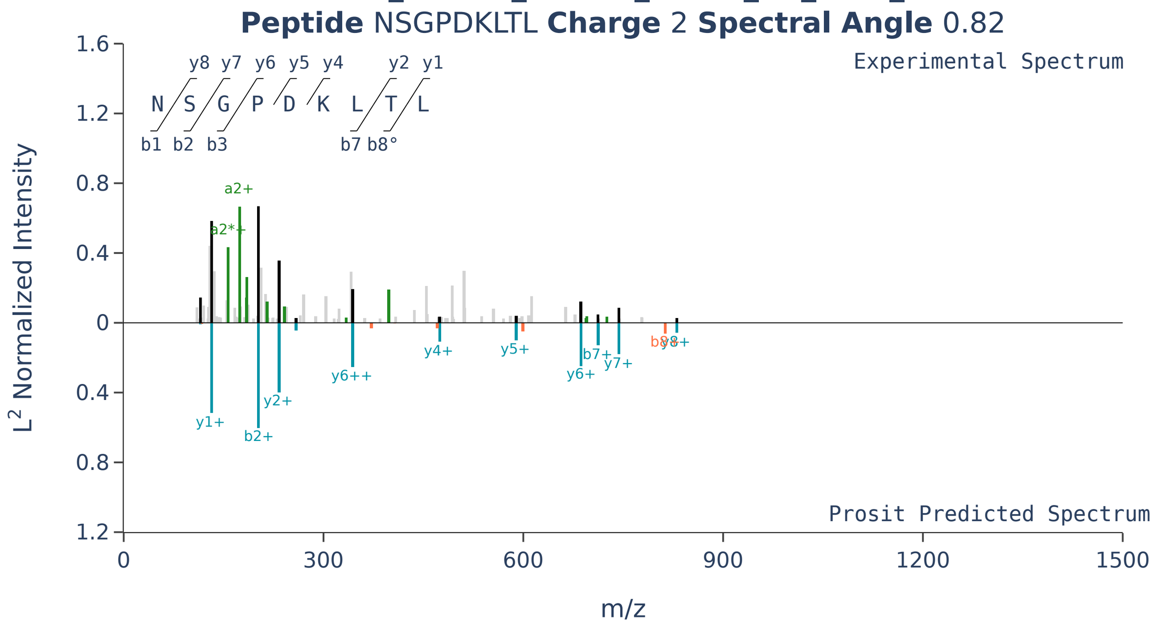


**Source** HRoetschke_211222_160123_HFGoe_IF2_b2R2_24h **Scan** 13624
**Peptide** GETITVGE **Charge** 2 **Spectral Angle** 0.67

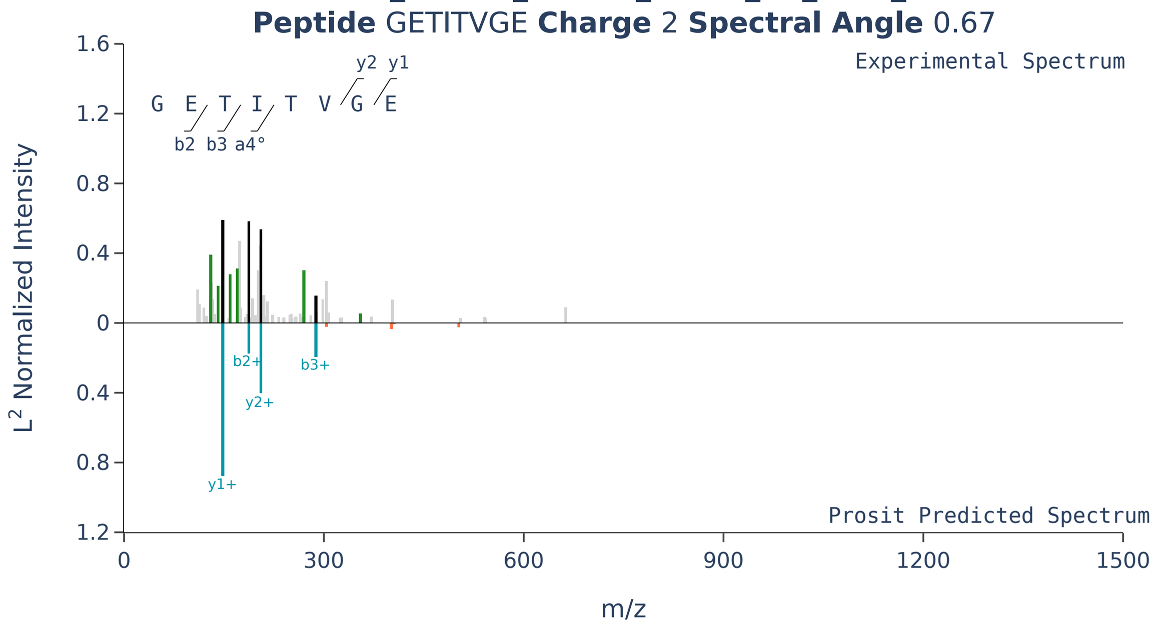


**Source** HRoetschke_211222_160123_HFGoe_IF2_b1R1_4h **Scan** 5445
**Peptide** RDTGAAAEPRA **Charge** 2 **Spectral Angle** 0.85

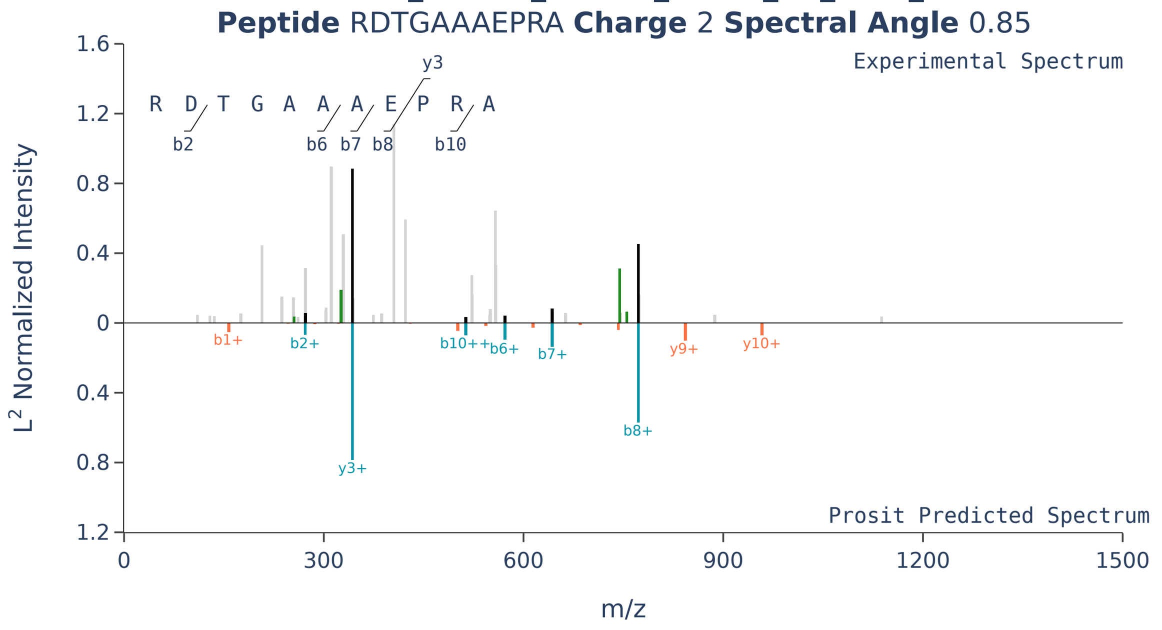


**Source** HRoetschke_211222_160123_HFGoe_IF2_b1R1_24h **Scan** 16328
**Peptide** QAEVLELK **Charge** 2 **Spectral Angle** 0.78

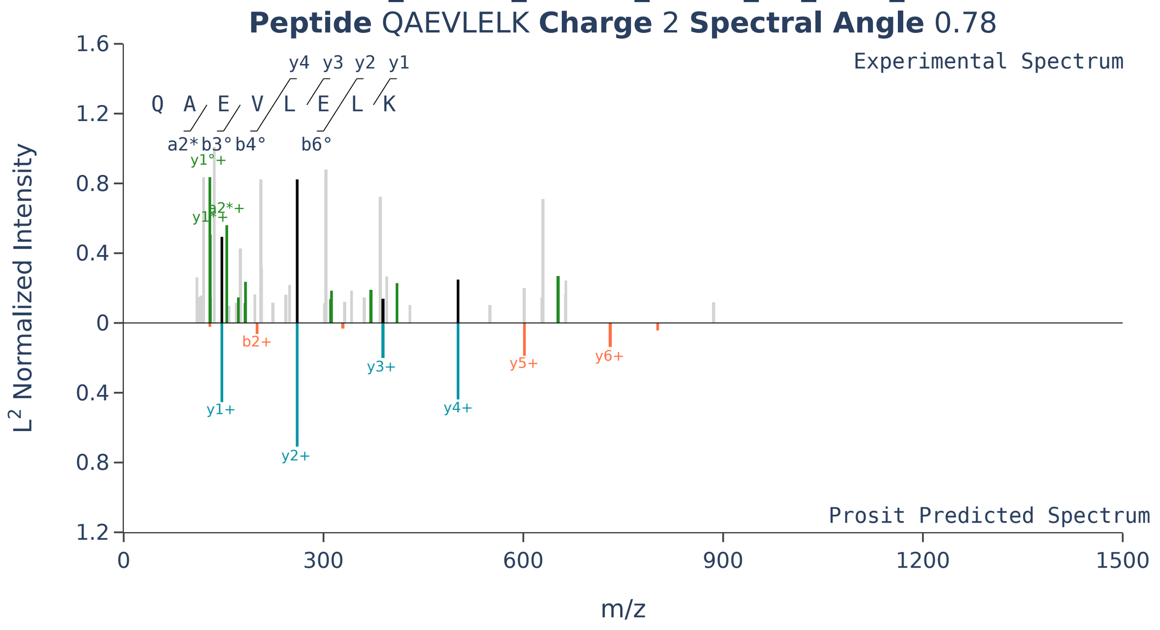


**Source** HRoetschke_211222_160123_HFGoe_IF2_b1R2_2h **Scan** 13869
**Peptide** ASGEAGGITQHL **Charge** 2 **Spectral Angle** 0.72

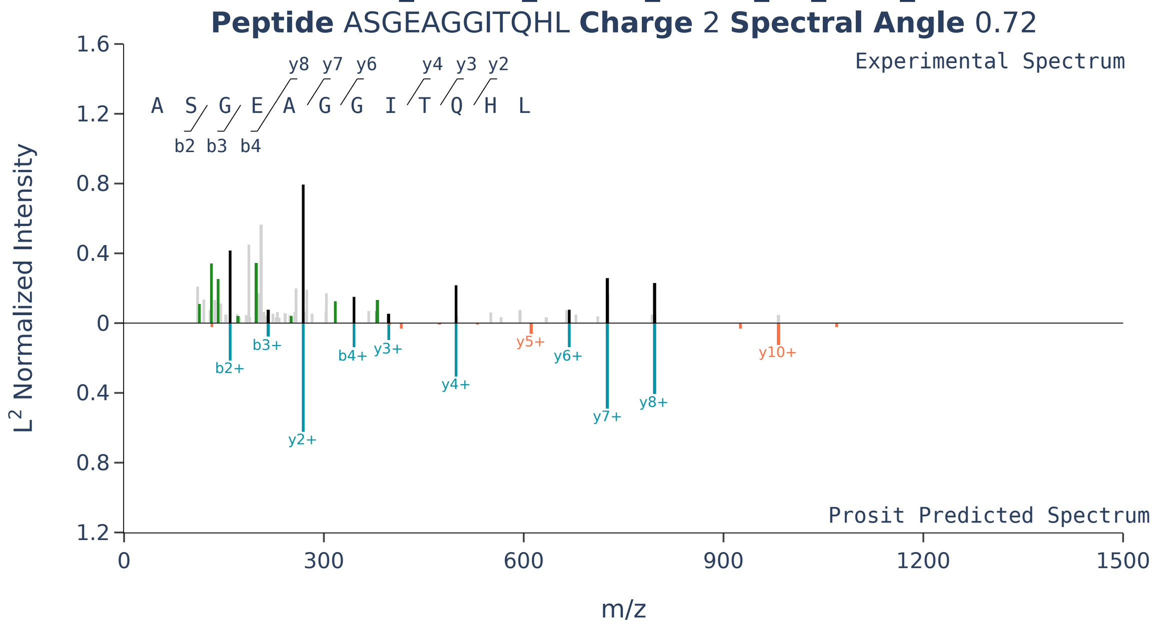


**Source** HRoetschke_211222_160123_HFGoe_IF2_b1R2_2h **Scan** 6845
**Peptide** HVSAKAGTGI **Charge** 2 **Spectral Angle** 0.73

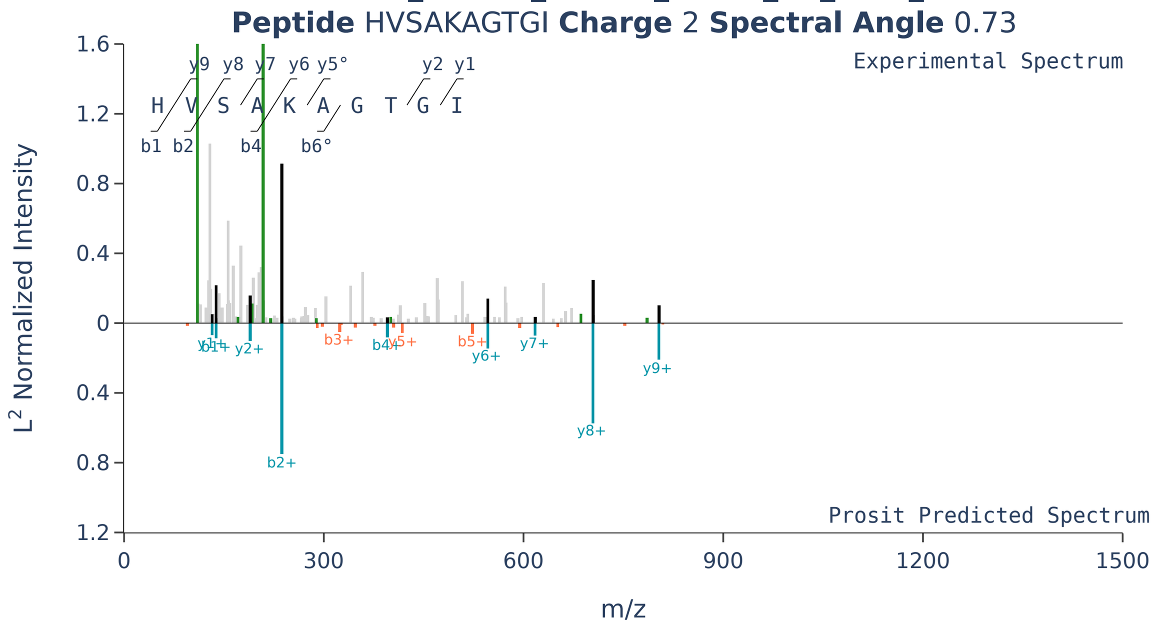


**Source** HRoetschke_211222_160123_HFGoe_IF2_b2R2_24h **Scan** 13244
**Peptide** MASGAVIES **Charge** 1 **Spectral Angle** 0.76

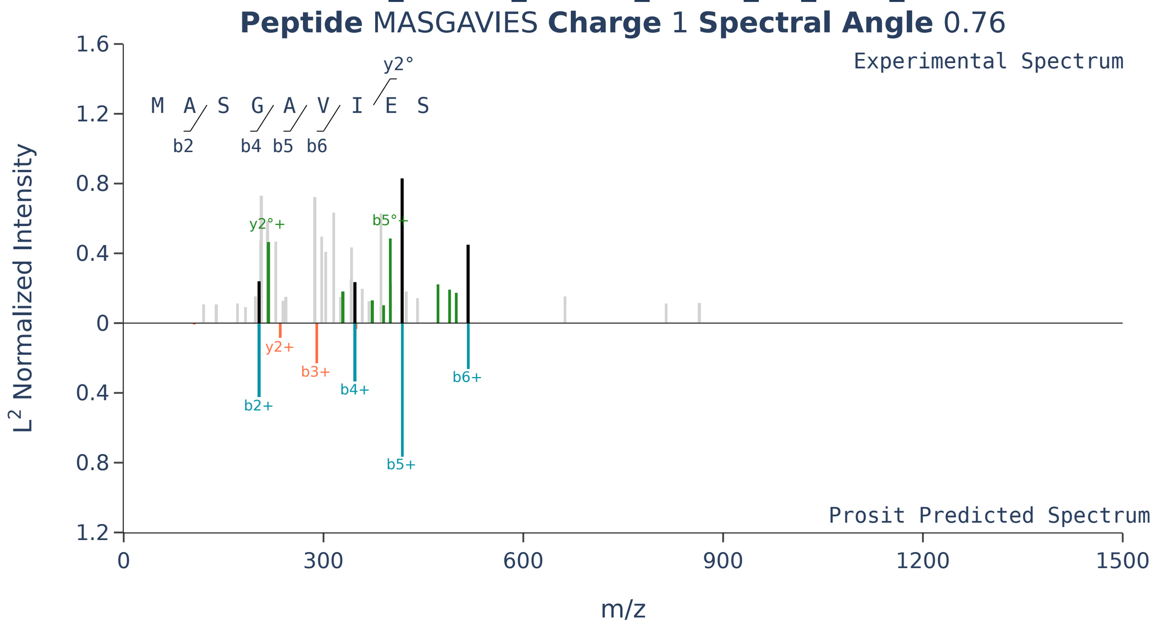


**Source** HRoetschke_211222_160123_HFGoe_IF2_b1R1_1h **Scan** 12172
**Peptide** AAERQTSVERLVQQ **Charge** 3 **Spectral Angle** 0.62

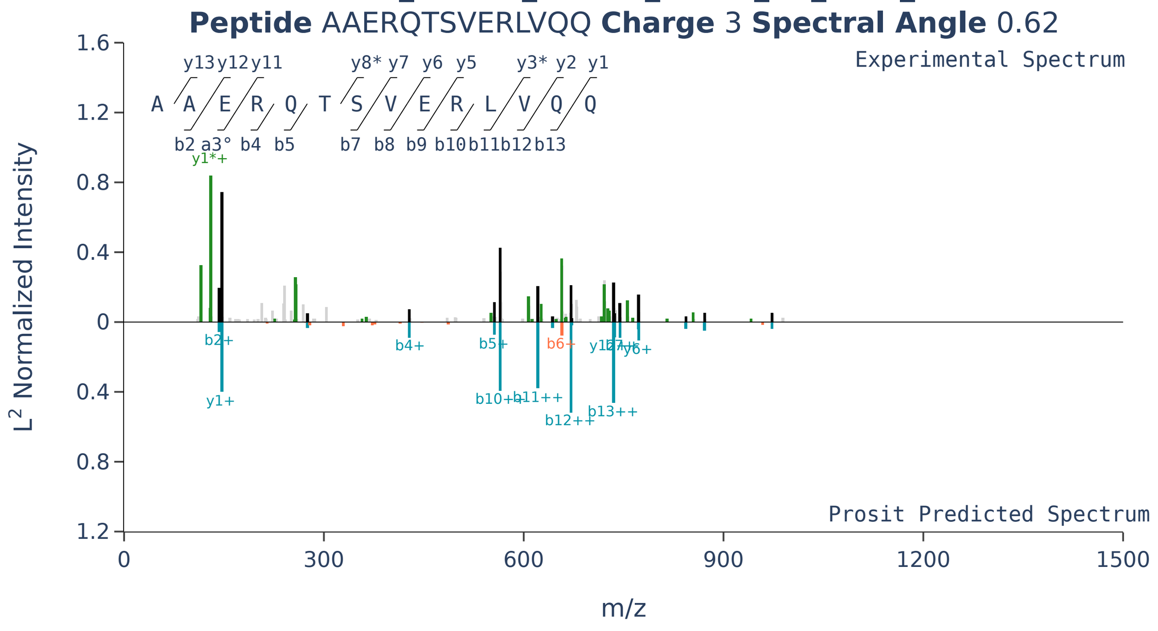


**Source** HRoetschke_211222_160123_HFGoe_IF2_b1R1_4h **Scan** 6548
**Peptide** RARGAQATDL **Charge** 3 **Spectral Angle** 0.75

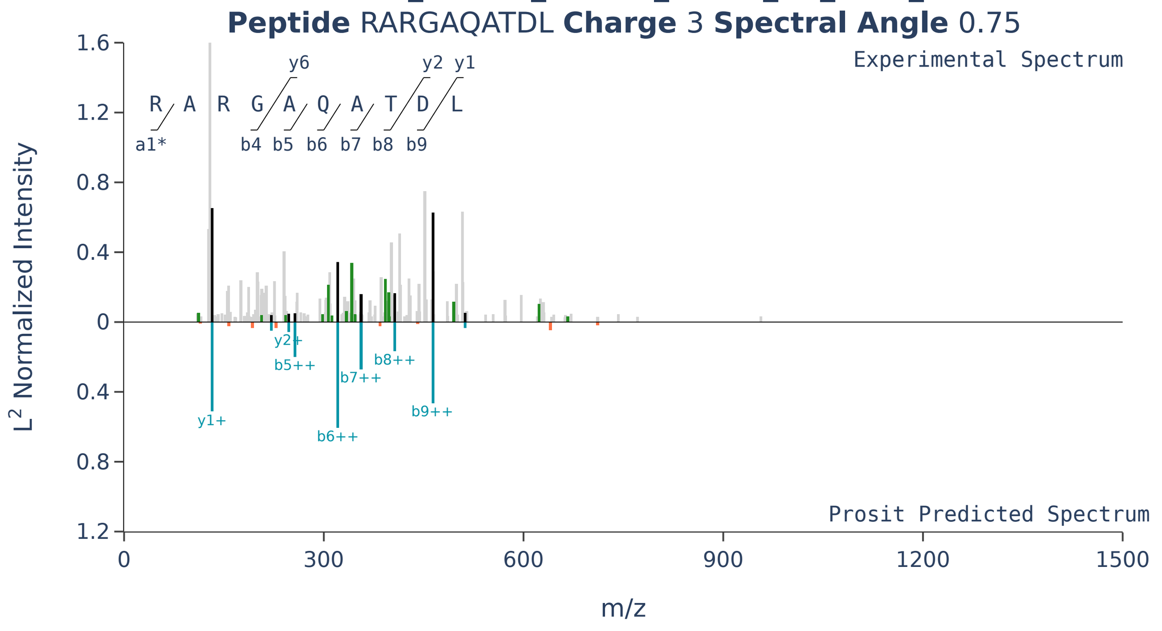


**Source** HRoetschke_211222_160123_HFGoe_IF2_b1R2_24h **Scan** 13425
**Peptide** LDYIRSTKV **Charge** 3 **Spectral Angle** 0.67

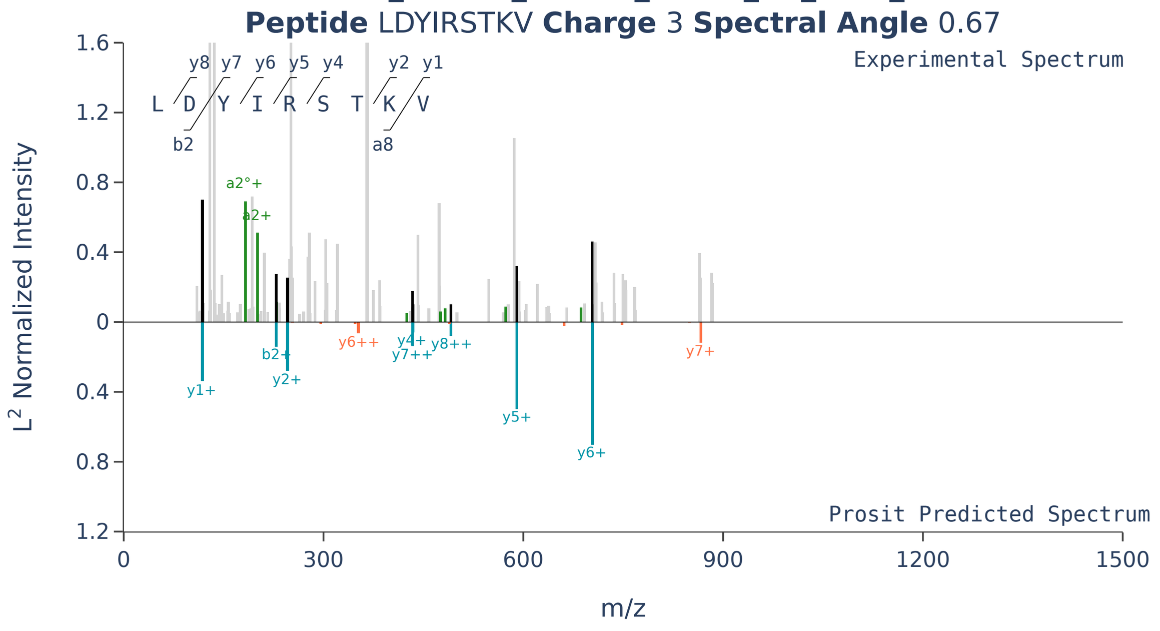


**Source** HRoetschke_211222_160123_HFGoe_IF2_b1R1_24h **Scan** 6676
**Peptide** RDEKKAREVALYRQG **Charge** 4 **Spectral Angle** 0.73

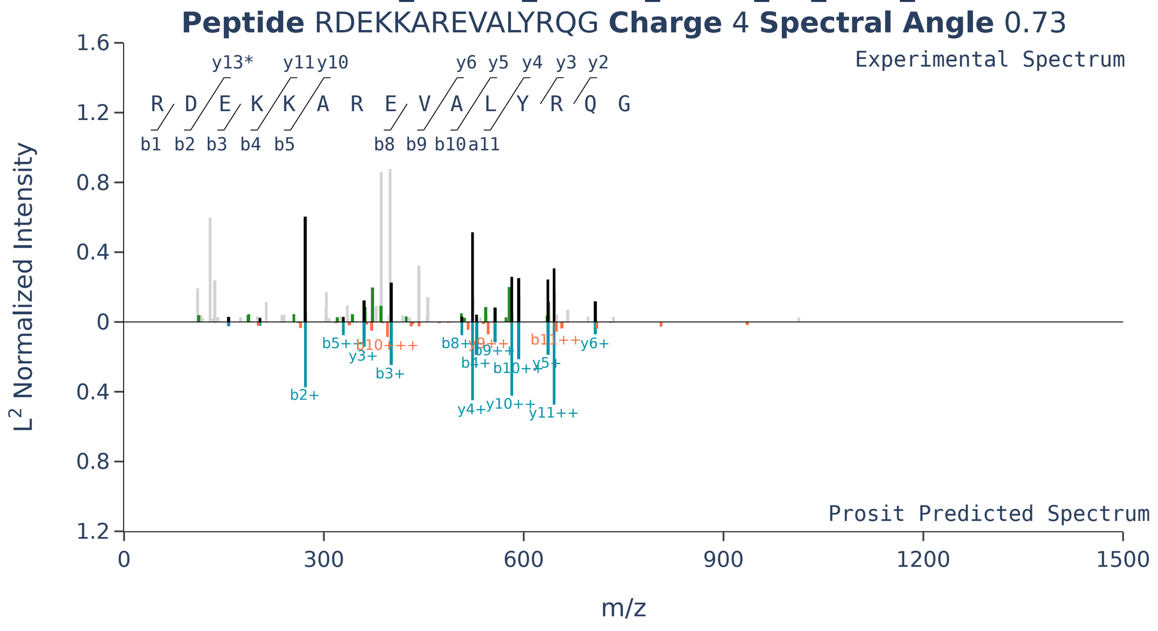


**Source** HRoetschke_211222_160123_HFGoe_IF2_b2R2_24h **Scan** 13064
**Peptide** GAAAEPRAPVVT **Charge** 2 **Spectral Angle** 0.74

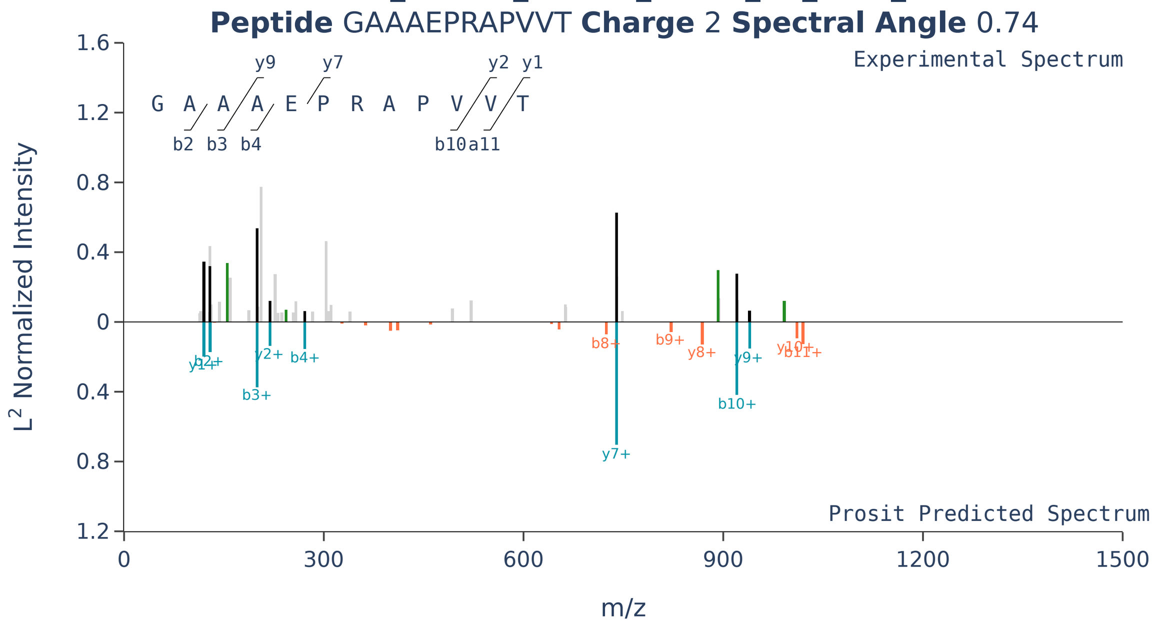


**Source** HRoetschke_211222_160123_HFGoe_IF2_b1R1_24h **Scan** 11660
**Peptide** PQTIEAIQH **Charge** 2 **Spectral Angle** 0.83

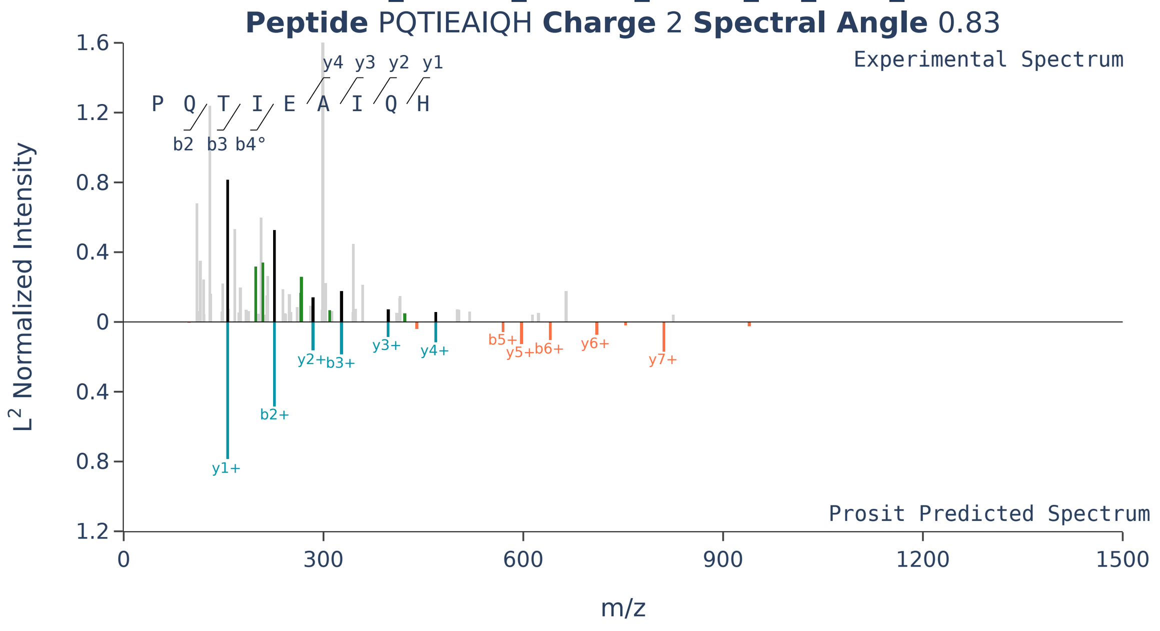

**Source** HRoetschke_211222_160123_HFGoe_IF2_b1R1_4h **Scan** 22112
**Peptide** TITVGEL **Charge** 1 **Spectral Angle** 0.94

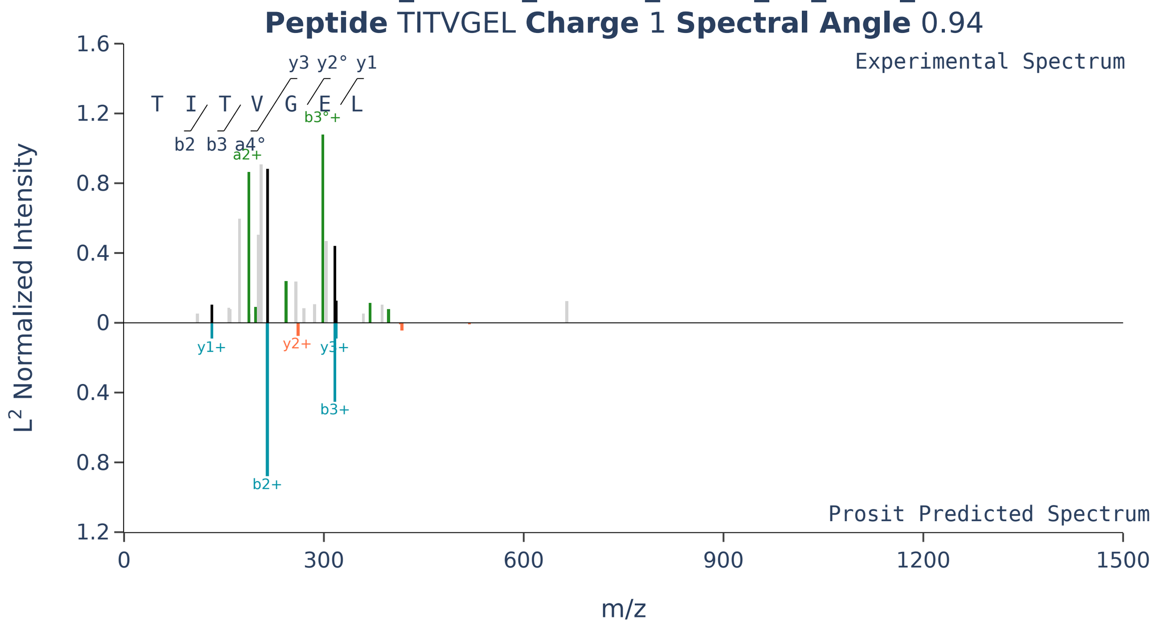


**Source** HRoetschke_211222_160123_HFGoe_IF2_b1R1_4h **Scan** 10402
**Peptide** MFANMTEGEVHE **Charge** 2 **Spectral Angle** 0.78

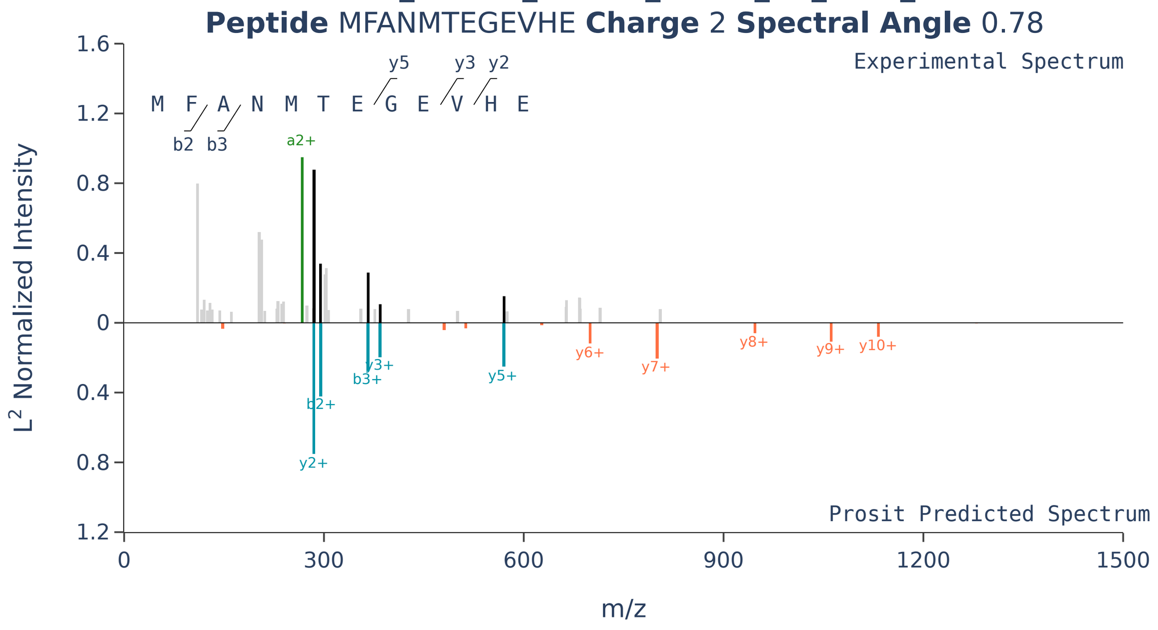


**Source** HRoetschke_211222_160123_HFGoe_IF2_b2R1_2h **Scan** 6810
**Peptide** QGKFREV **Charge** 2 **Spectral Angle** 0.83

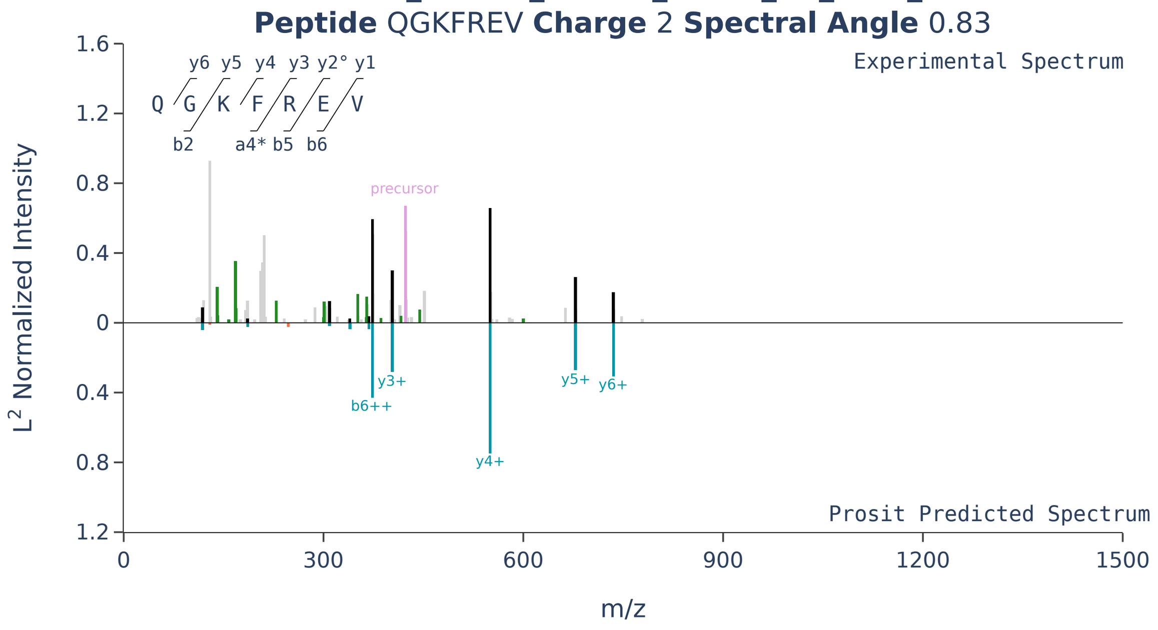


**Source** HRoetschke_211222_160123_HFGoe_IF2_b2R1_2h **Scan** 7933
**Peptide** MGHKVILRRENELEE **Charge** 3 **Spectral Angle** 0.76

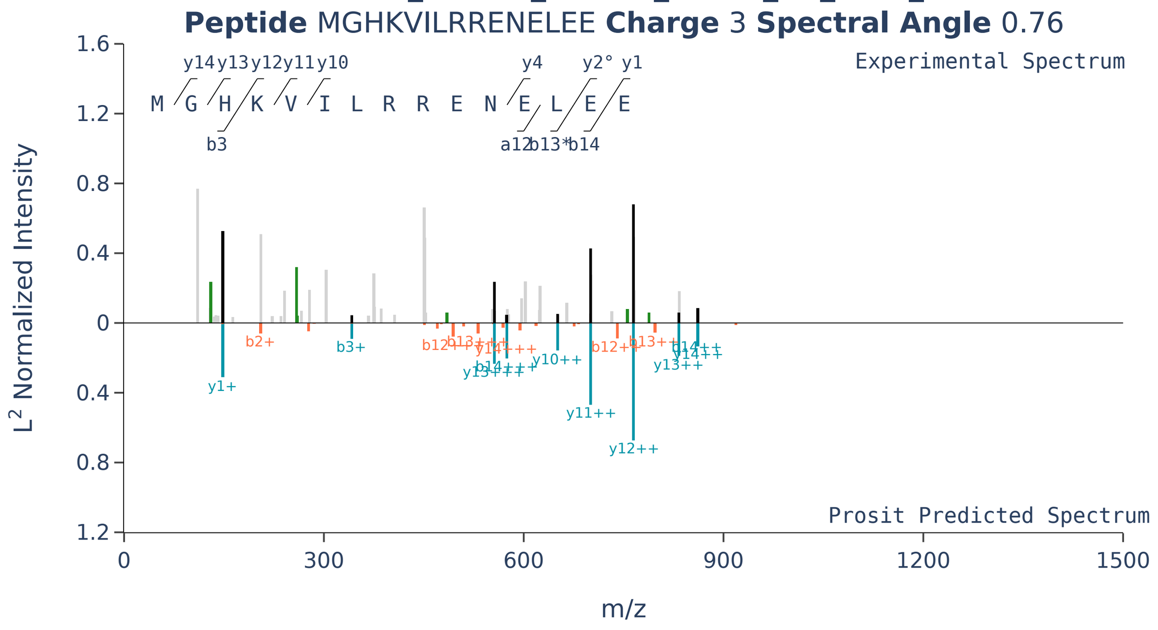


**Source** HRoetschke_211222_160123_HFGoe_IF2_b1R1_4h **Scan** 21153
**Peptide** ERQTSVERLVQQF **Charge** 3 **Spectral Angle** 0.7

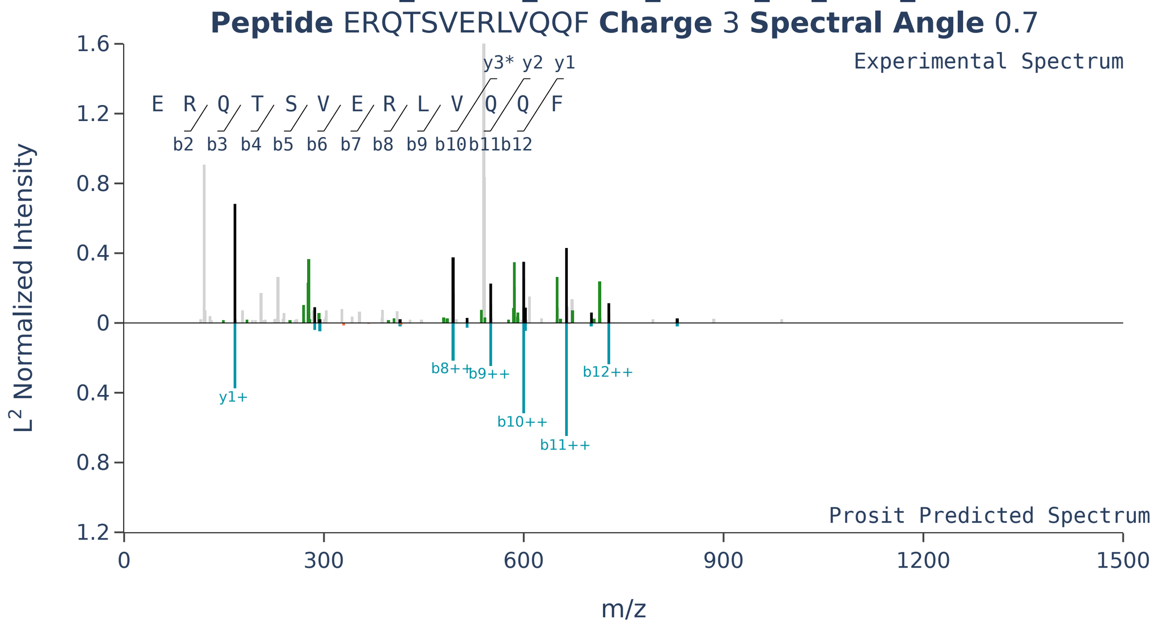


**Source** HRoetschke_211222_160123_HFGoe_IF2_b1R1_24h **Scan** 12392
**Peptide** LDTPGHAAFTSMRA **Charge** 3 **Spectral Angle** 0.77

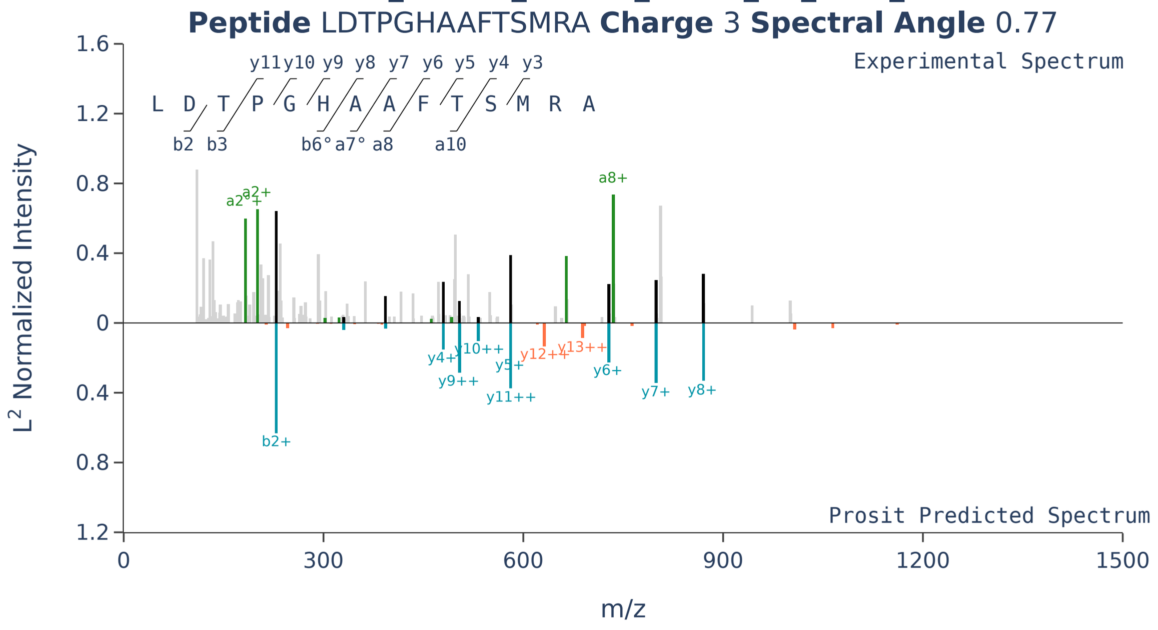


**Source** HRoetschke_211222_160123_HFGoe_IF2_b1R1_4h **Scan** 11572
**Peptide** NKIDKPEADPDRVKNEL **Charge** 2 **Spectral Angle** 0.85

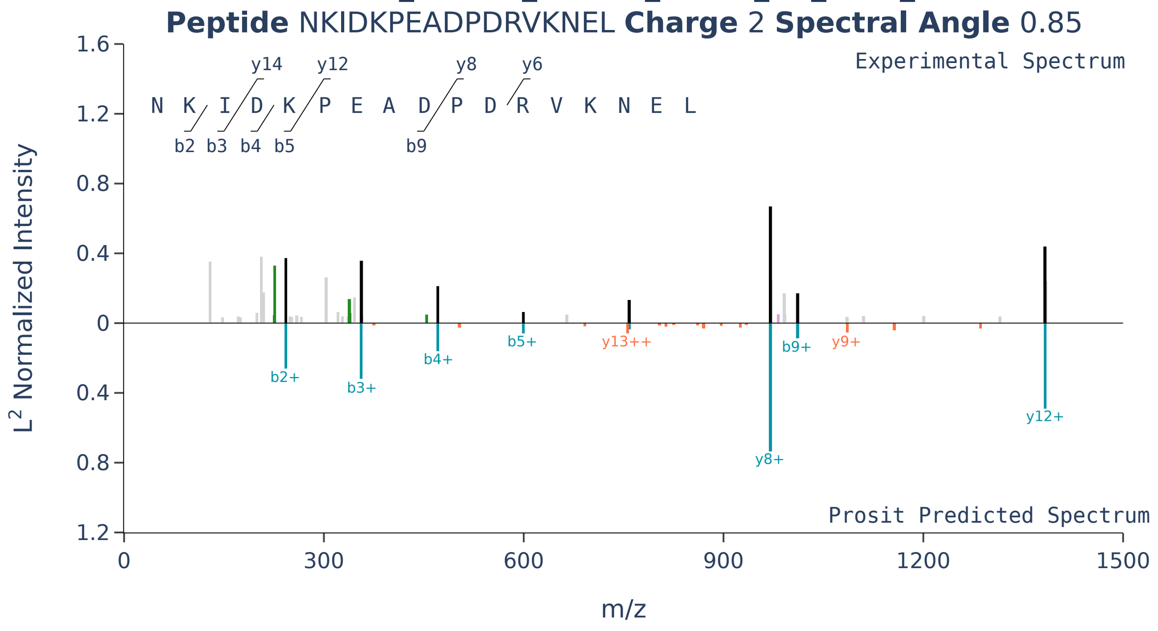


**Source** HRoetschke_211222_160123_HFGoe_IF2_b1R1_4h **Scan** 11293
**Peptide** NKIDKPEADPDRVKNELSQ **Charge** 3 **Spectral Angle** 0.79

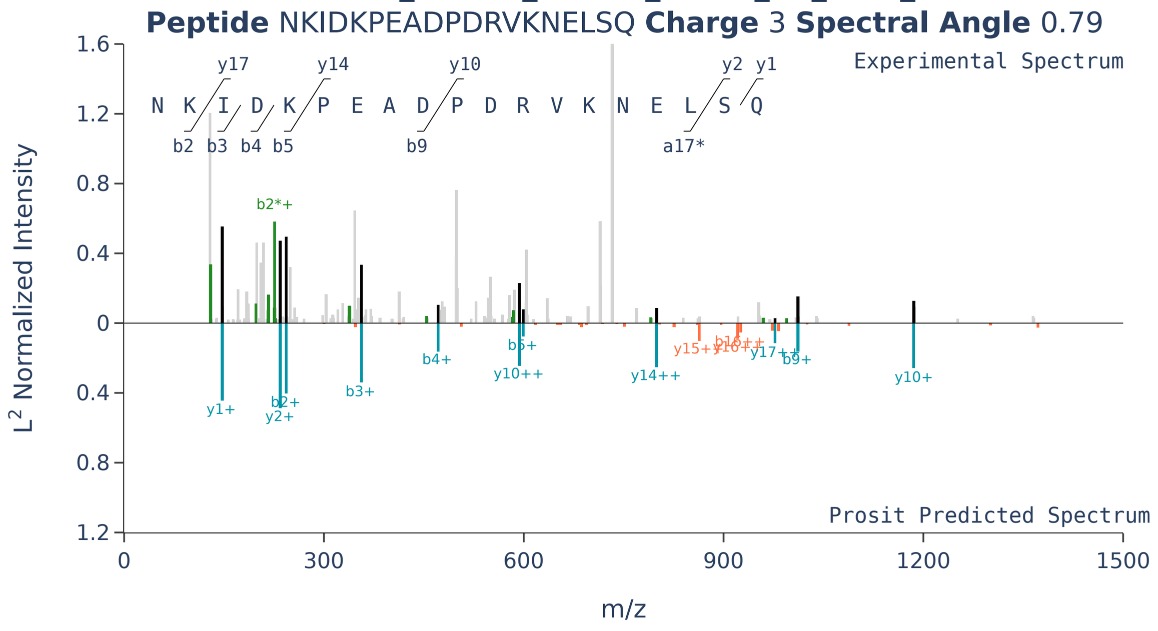


**Source** HRoetschke_211222_160123_HFGoe_IF2_b1R1_4h **Scan** 13783
**Peptide** DRDTGAAAEPRAPVV **Charge** 3 **Spectral Angle** 0.74

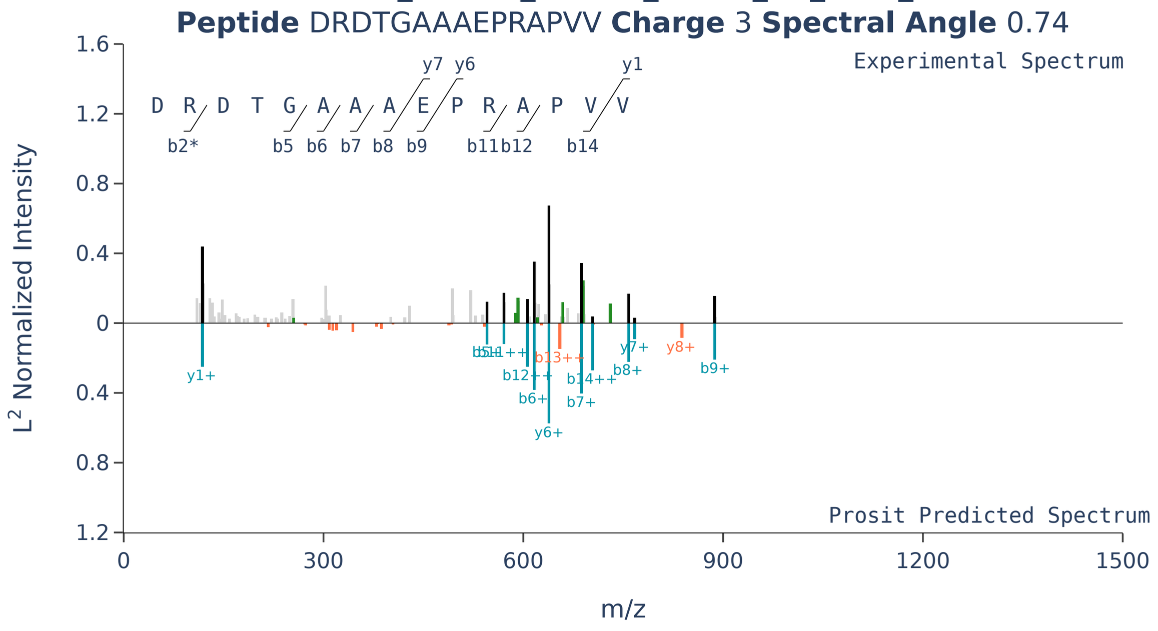


**Source** HRoetschke_211222_160123_HFGoe_IF2_b1R1_2h **Scan** 7472
**Peptide** EEMGHKVILRRENE **Charge** 3 **Spectral Angle** 0.79

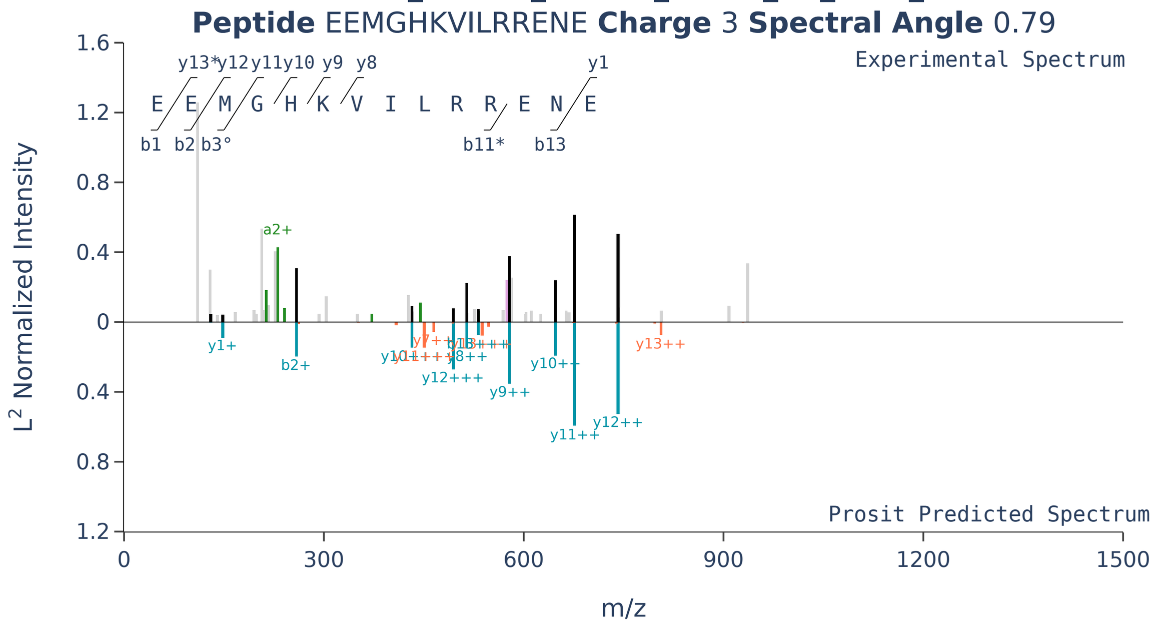


**Source** HRoetschke_211222_160123_HFGoe_IF2_b1R2_24h **Scan** 15727
**Peptide** GSSLQQGFQKPAQAV **Charge** 2 **Spectral Angle** 0.77

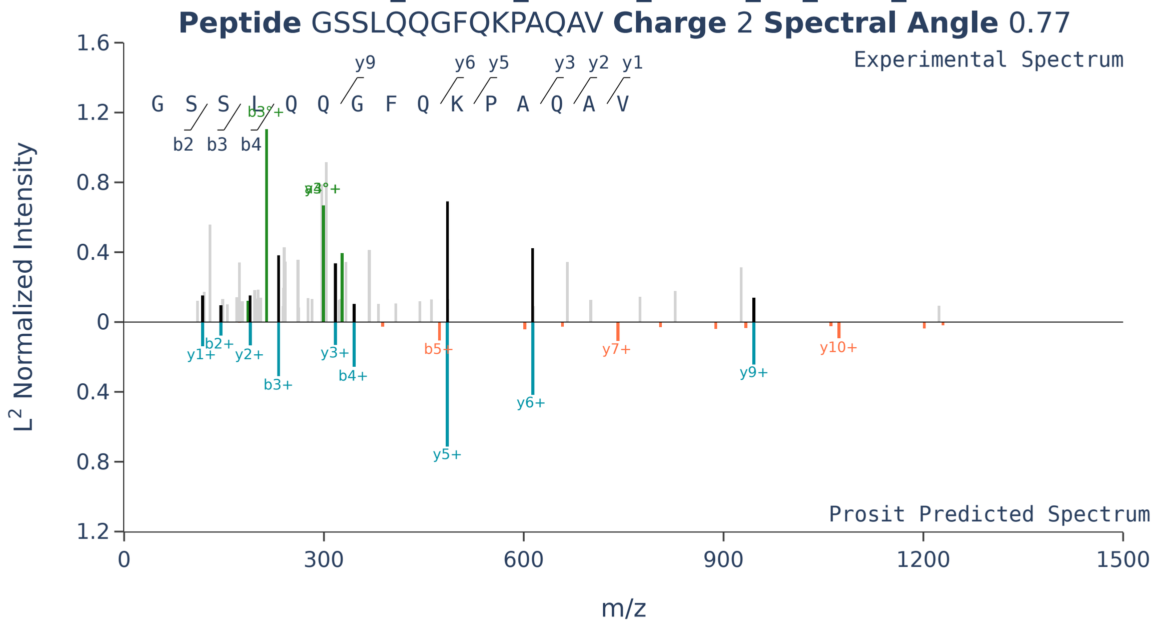


**Source** HRoetschke_211222_160123_HFGoe_IF2_b1R1_4h **Scan** 6761
**Peptide** ANKMAVKGSQVLKA **Charge** 3 **Spectral Angle** 0.76

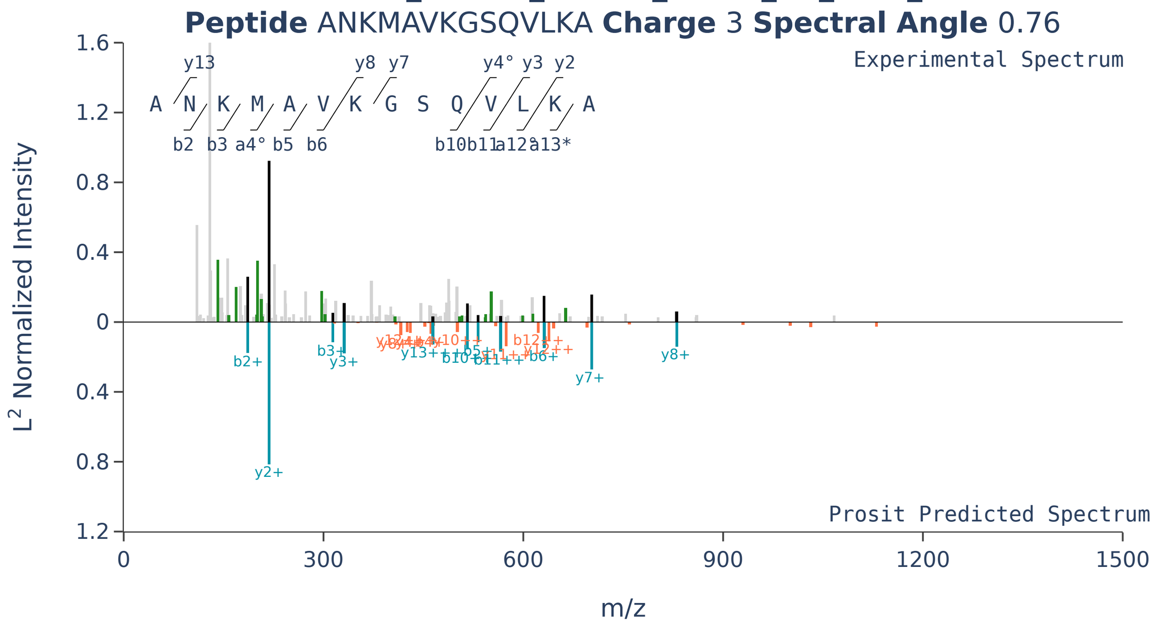


**Source** HRoetschke_211222_160123_HFGoe_IF2_b1R1_2h **Scan** 8803
**Peptide** GELANKMAVKGS **Charge** 3 **Spectral Angle** 0.67

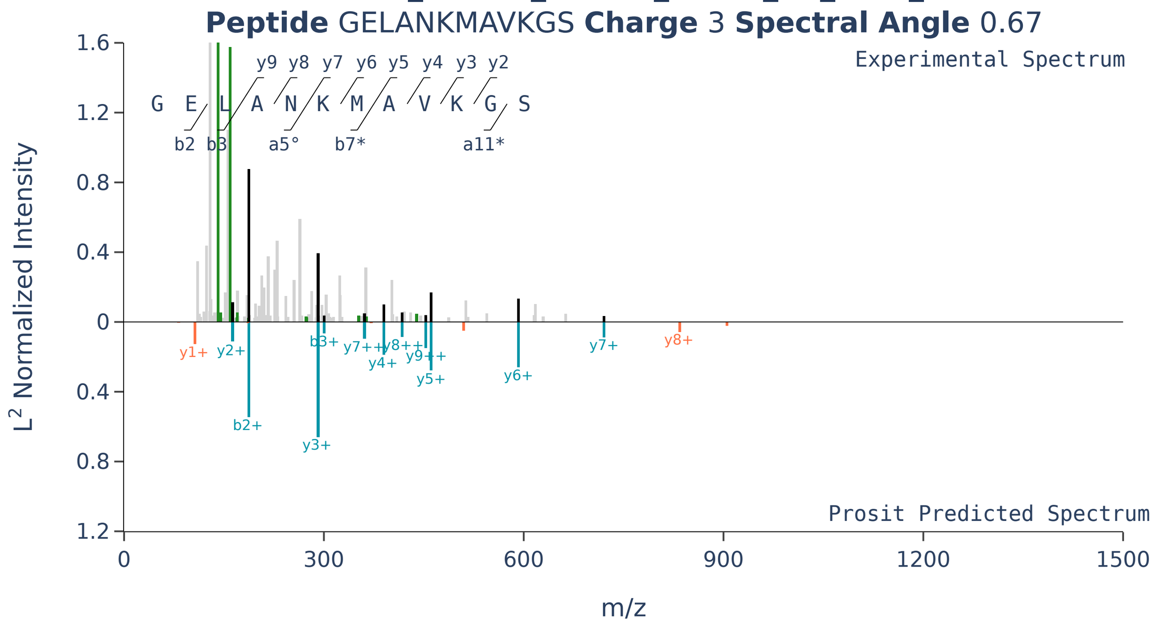


**Source** HRoetschke_211222_160123_HFGoe_IF2_b1R1_1h **Scan** 9381
**Peptide** QQGFQKPAQAVN **Charge** 2 **Spectral Angle** 0.8

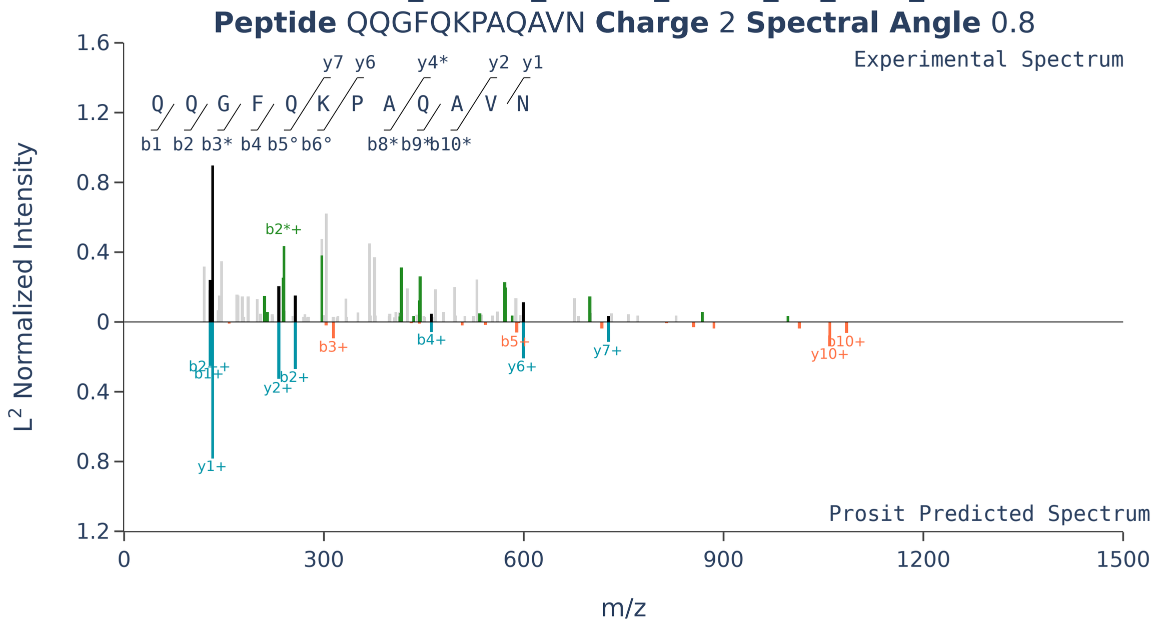


**Source** HRoetschke_211222_160123_HFGoe_IF2_b1R1_24h **Scan** 8791
**Peptide** VRDEKKAREVALY **Charge** 4 **Spectral Angle** 0.75

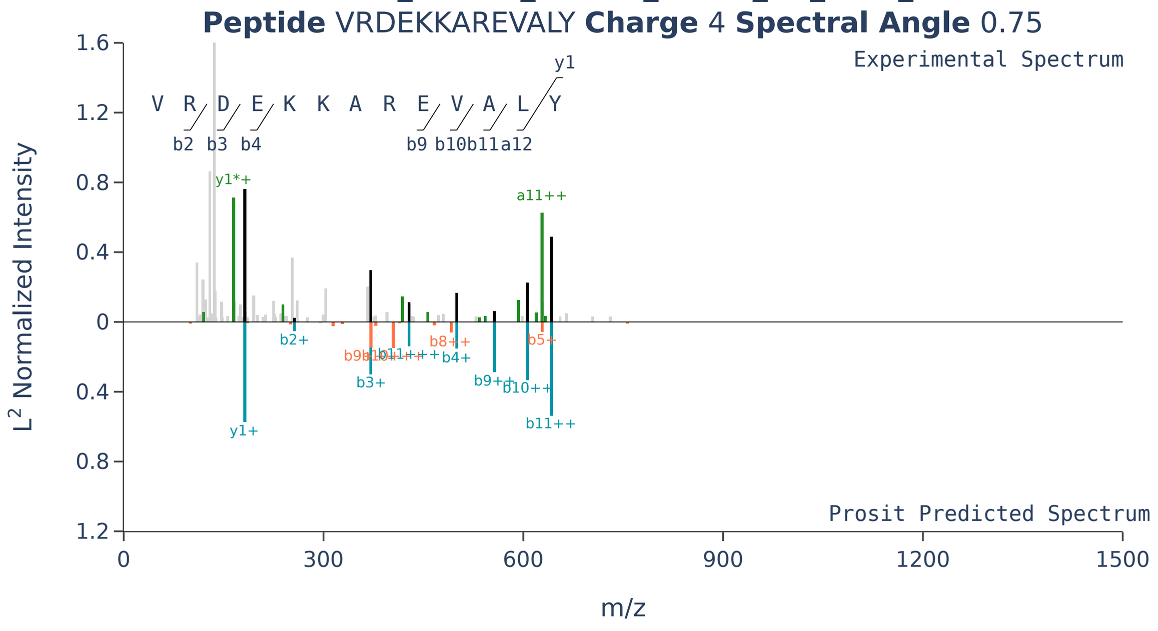

**Source** HRoetschke_211222_160123_HFGoe_IF2_b2R2_24h **Scan** 6857
**Peptide** ELKAVRKGMASGA **Charge** 2 **Spectral Angle** 0.84

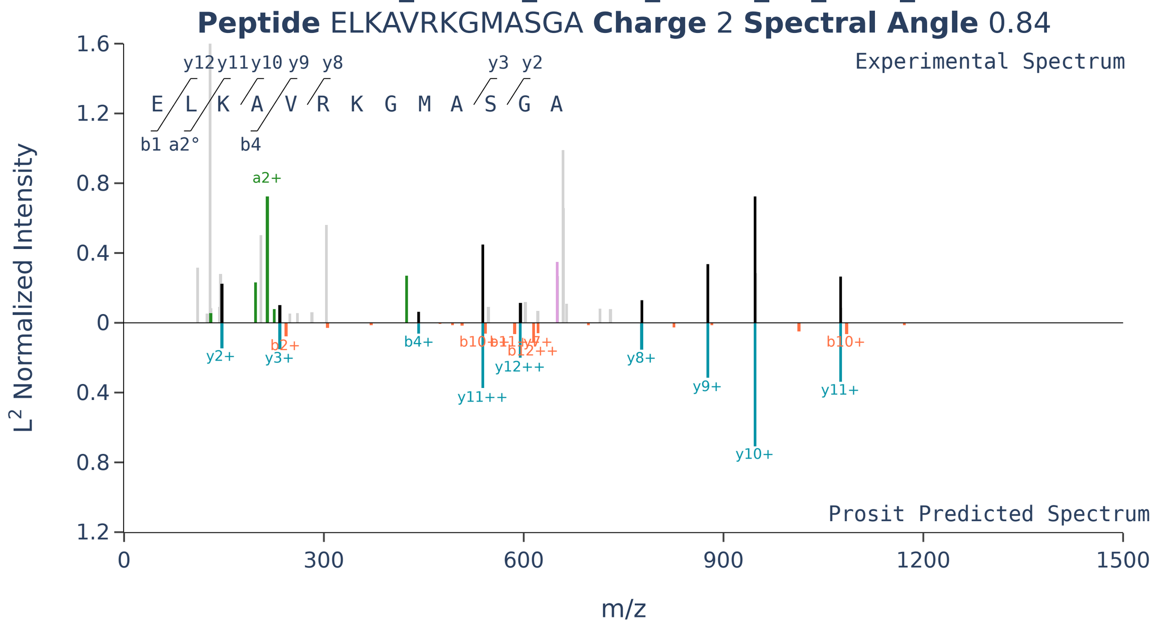


**Source** HRoetschke_211222_160123_HFGoe_IF2_b1R1_2h **Scan** 8024
**Peptide** HKVILRRENELEE **Charge** 3 **Spectral Angle** 0.8

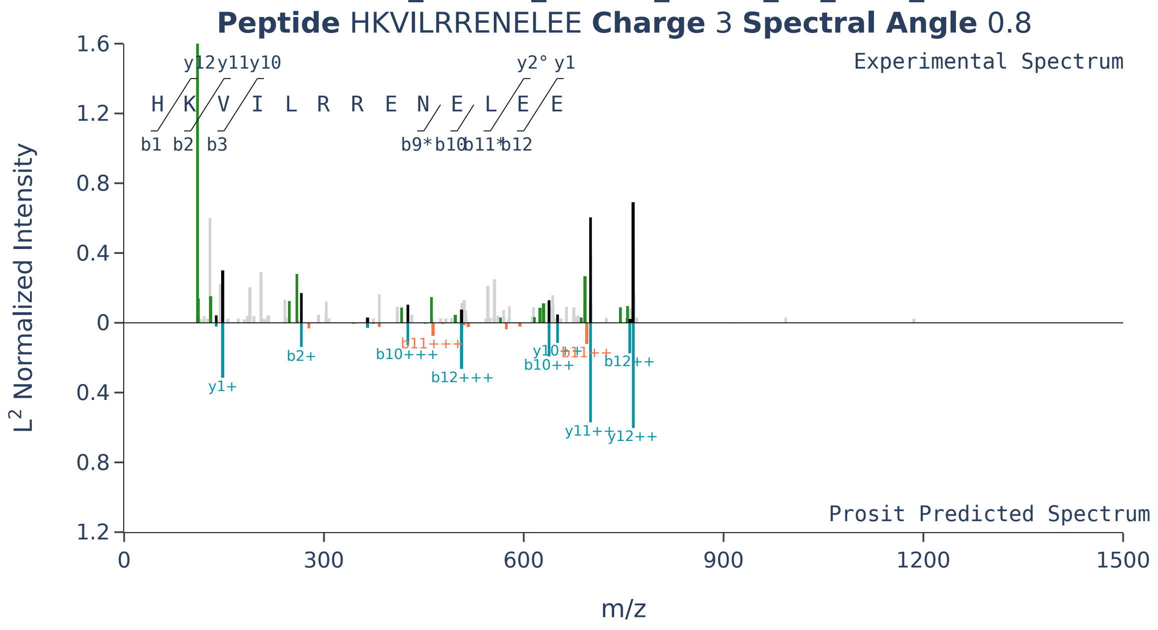


**Source** HRoetschke_211222_160123_HFGoe_IF2_b1R1_2h **Scan** 6575
**Peptide** RNELGQE **Charge** 2 **Spectral Angle** 0.86

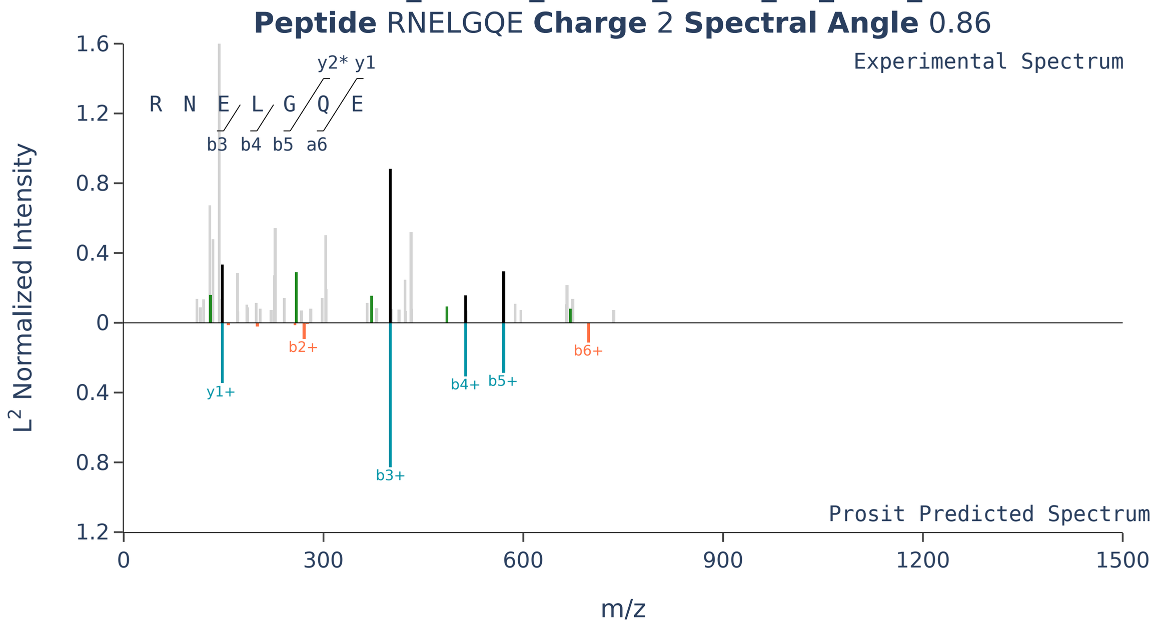


**Source** HRoetschke_211222_160123_HFGoe_IF2_b2R2_24h **Scan** 6102
**Peptide** RTFVKRDPQEAE **Charge** 3 **Spectral Angle** 0.74

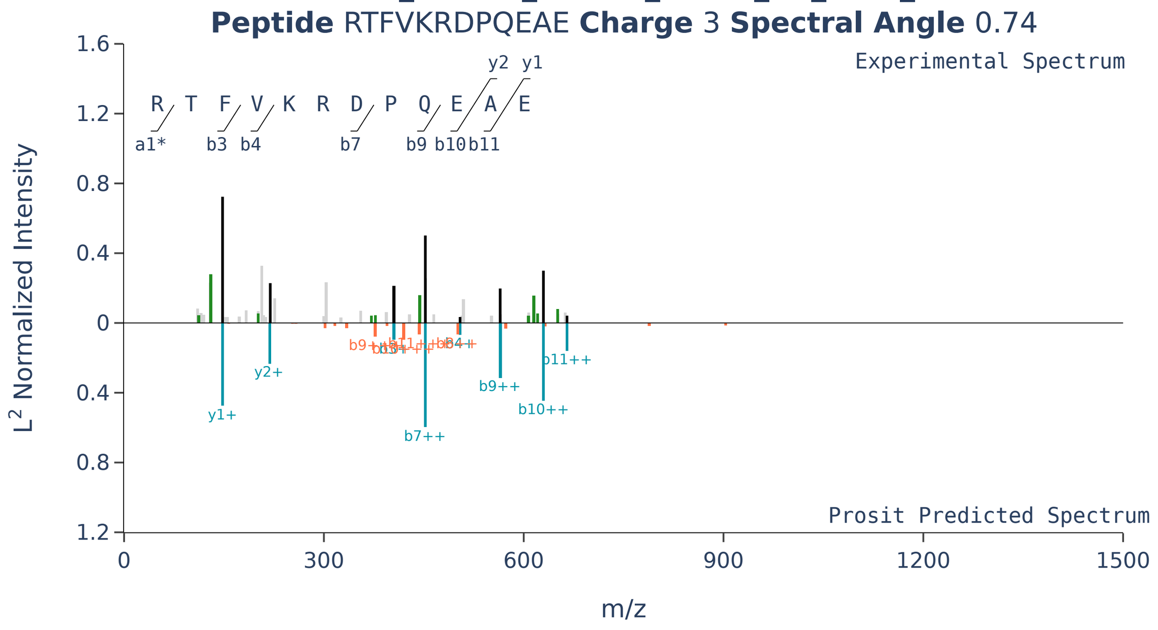


**Source** HRoetschke_211222_160123_HFGoe_IF2_b2R1_24h **Scan** 10396
**Peptide** DTPGHAAFTS **Charge** 1 **Spectral Angle** 0.66

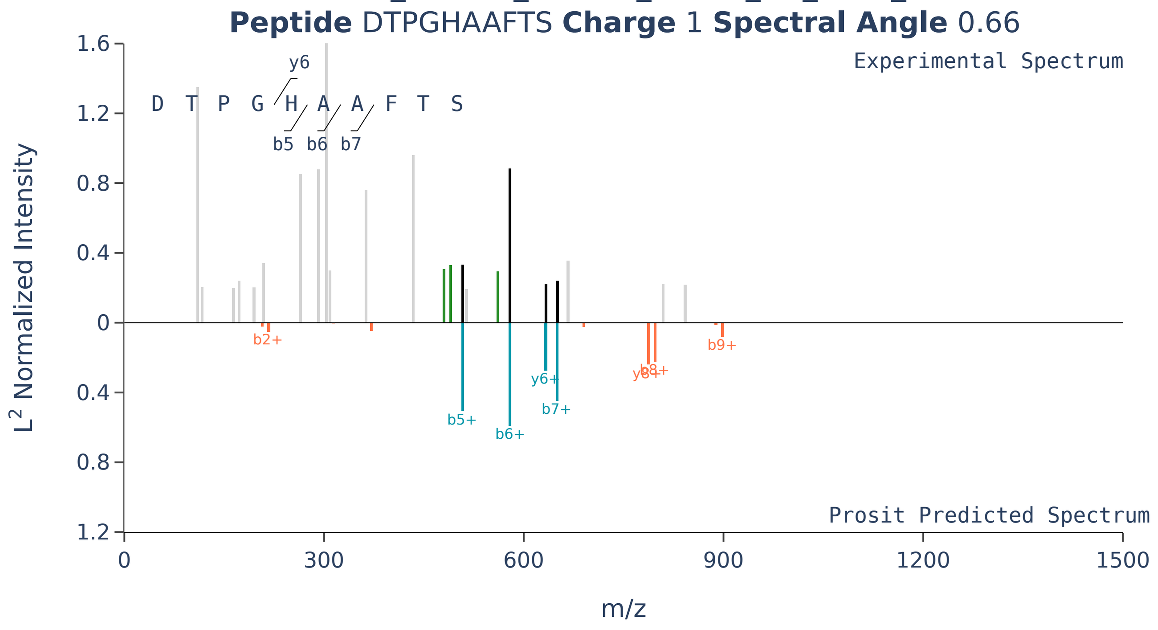


**Source** HRoetschke_211222_160123_HFGoe_IF2_b1R1_4h **Scan** 13875
**Peptide** AIQHAKAAQVPVV **Charge** 3 **Spectral Angle** 0.75

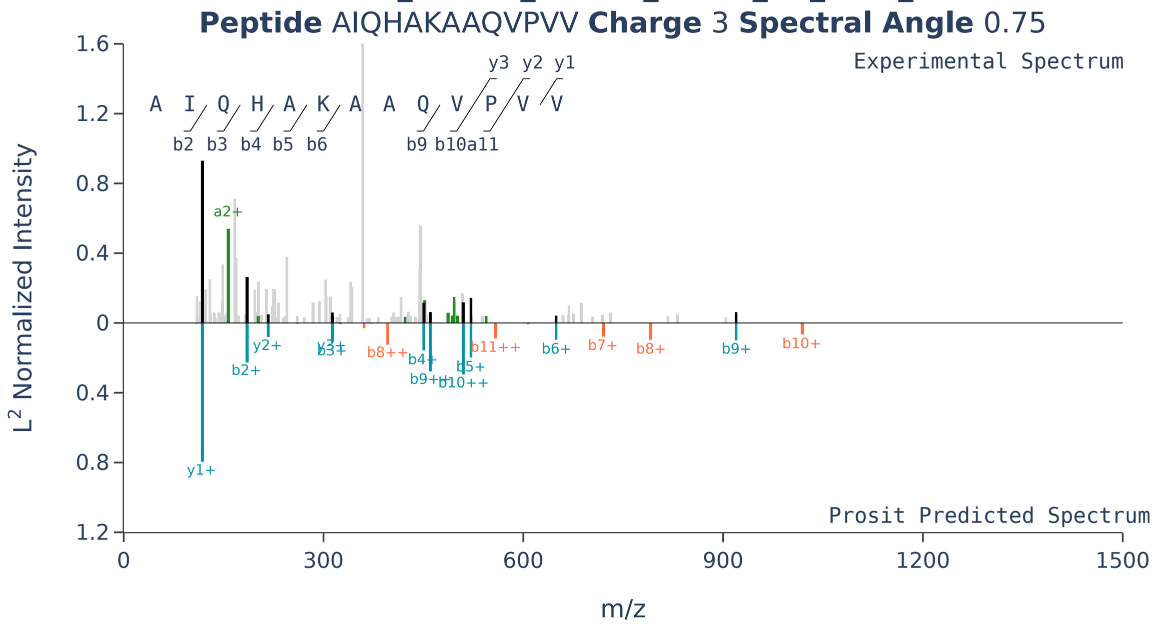


**Source** HRoetschke_211222_160123_HFGoe_IF2_b1R1_1h **Scan** 14798
**Peptide** SVSAQEKQTLI **Charge** 2 **Spectral Angle** 0.8

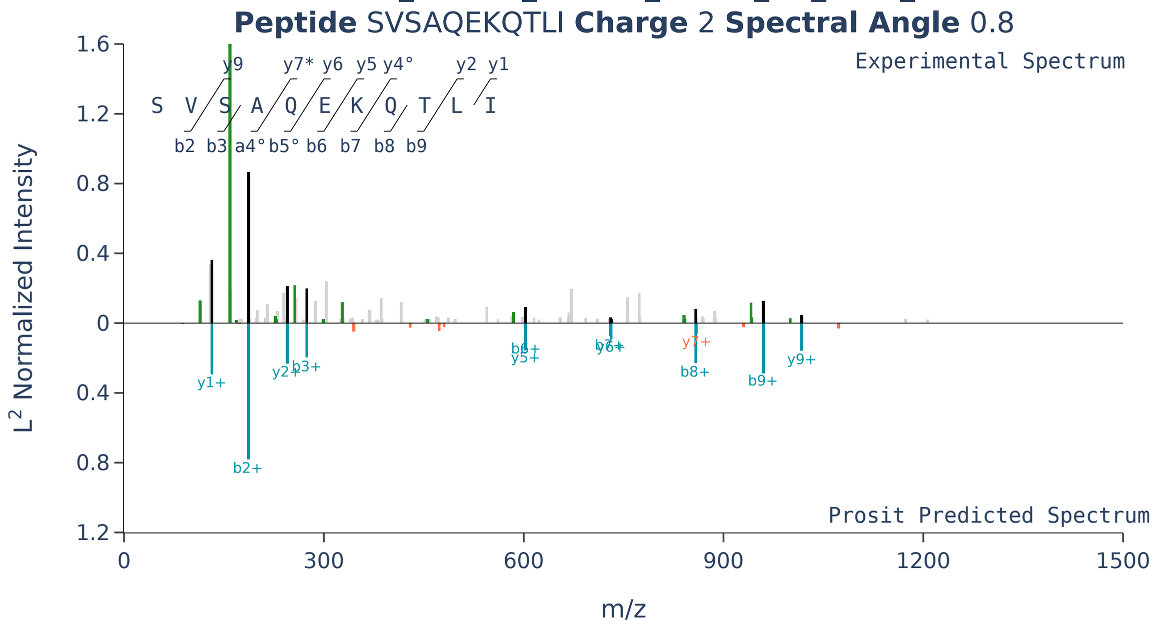


**Source** HRoetschke_211222_160123_HFGoe_IF2_b1R2_24h **Scan** 16807
**Peptide** SDRDTGAAAEPRAPVVTIM **Charge** 3 **Spectral Angle** 0.82

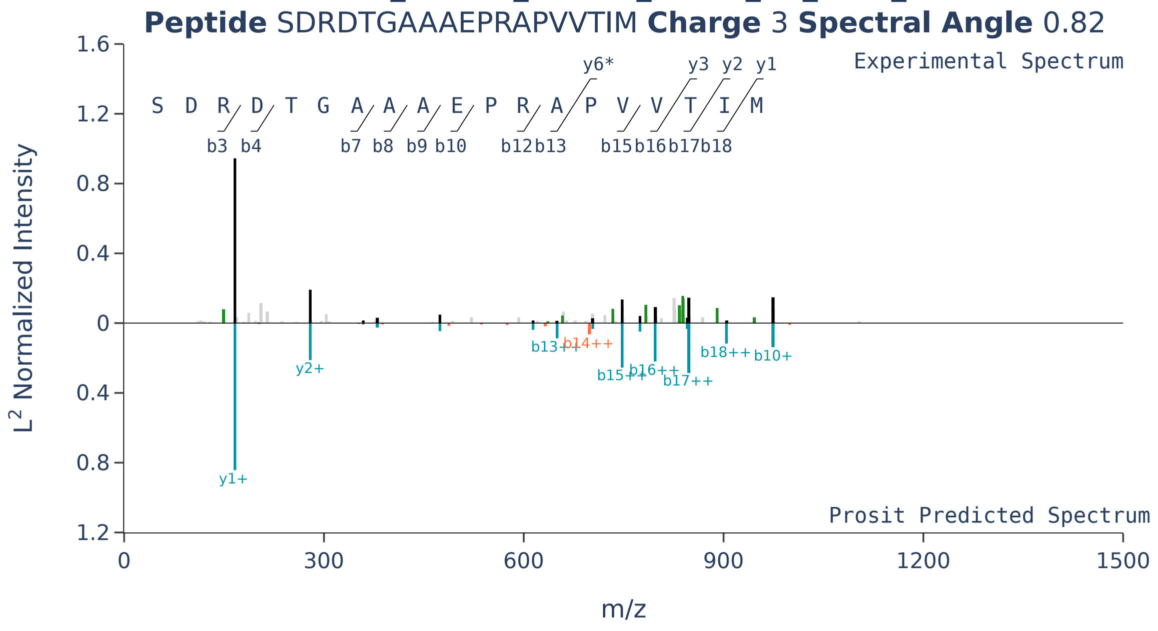


**Source** HRoetschke_211222_160123_HFGoe_IF2_b2R2_2h **Scan** 16638
**Peptide** VERLVQQF **Charge** 2 **Spectral Angle** 0.8

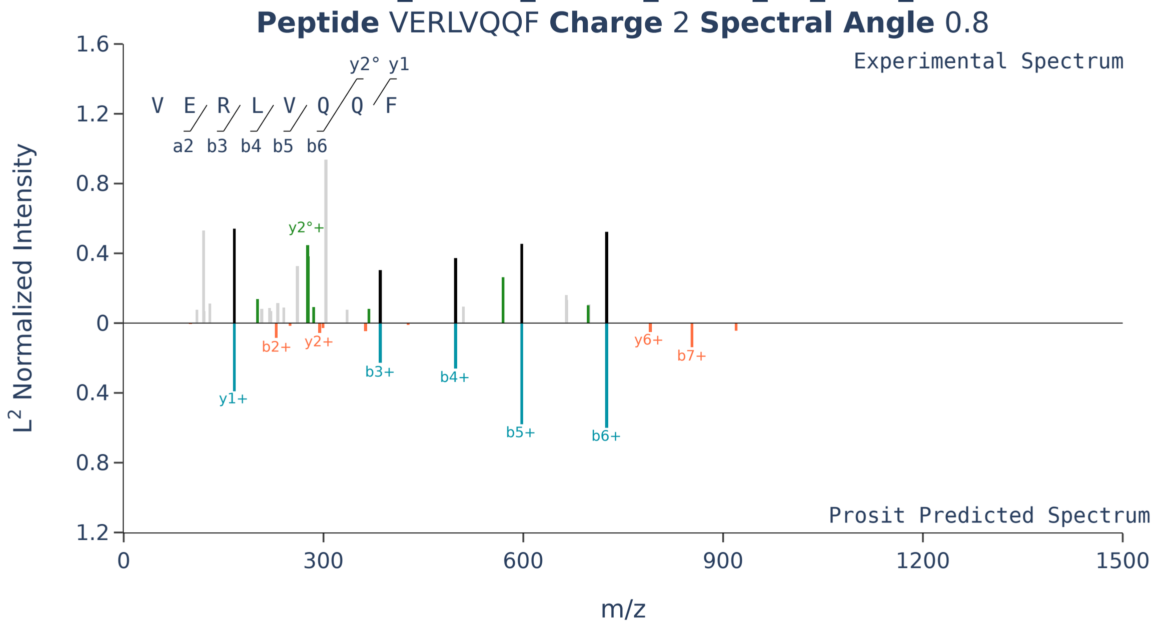


**Source** HRoetschke_211222_160123_HFGoe_IF2_b1R2_2h **Scan** 6647
**Peptide** LRRENELE **Charge** 3 **Spectral Angle** 0.85

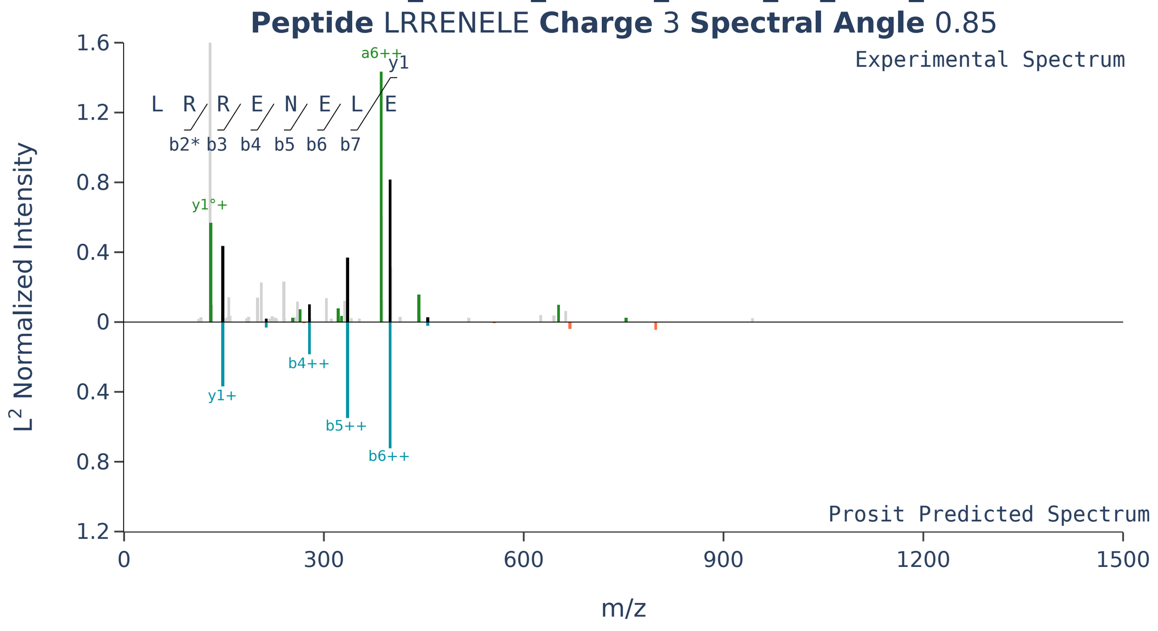


**Source** HRoetschke_211222_160123_HFGoe_IF2_b1R1_24h **Scan** 7178
**Peptide** ADAGIRKSADDSV **Charge** 2 **Spectral Angle** 0.66

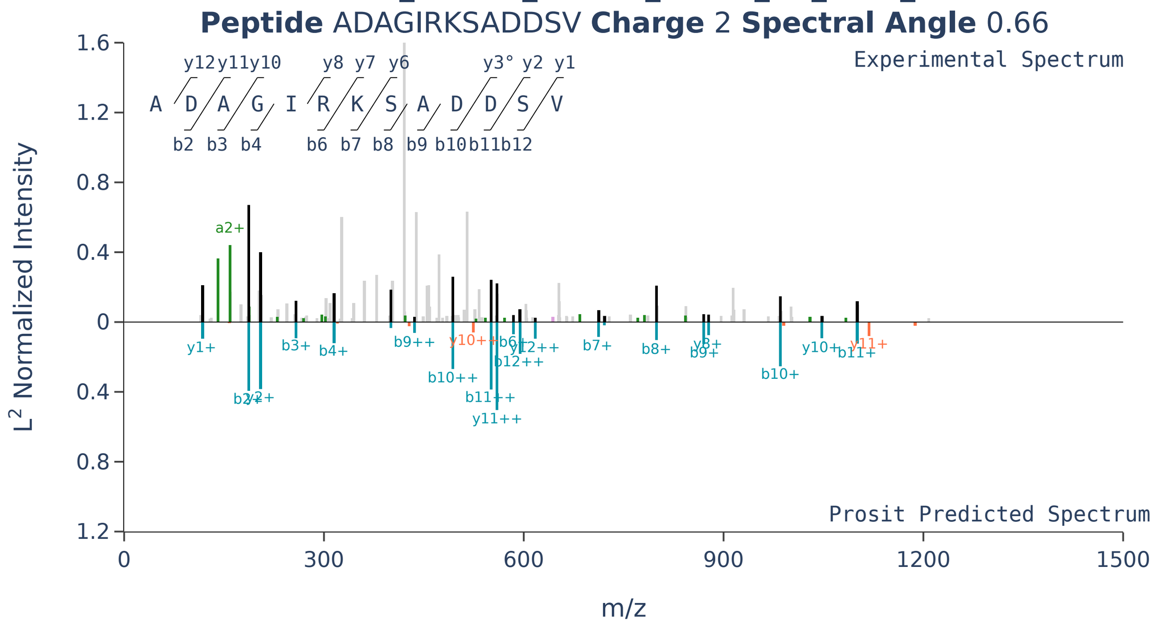


**Source** HRoetschke_211222_160123_HFGoe_IF2_b2R2_24h **Scan** 7501
**Peptide** ADAGIRKSADDSVSA **Charge** 2 **Spectral Angle** 0.68

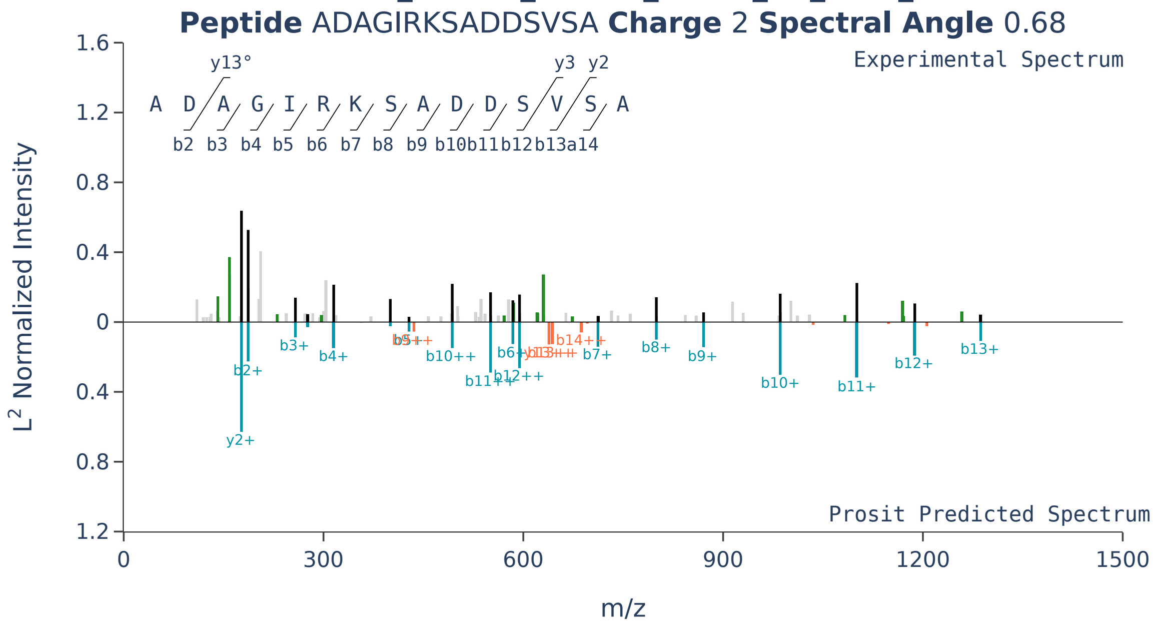


**Source** HRoetschke_211222_160123_HFGoe_IF2_b1R2_1h **Scan** 19667
**Peptide** AESLDLRYY **Charge** 2 **Spectral Angle** 0.76

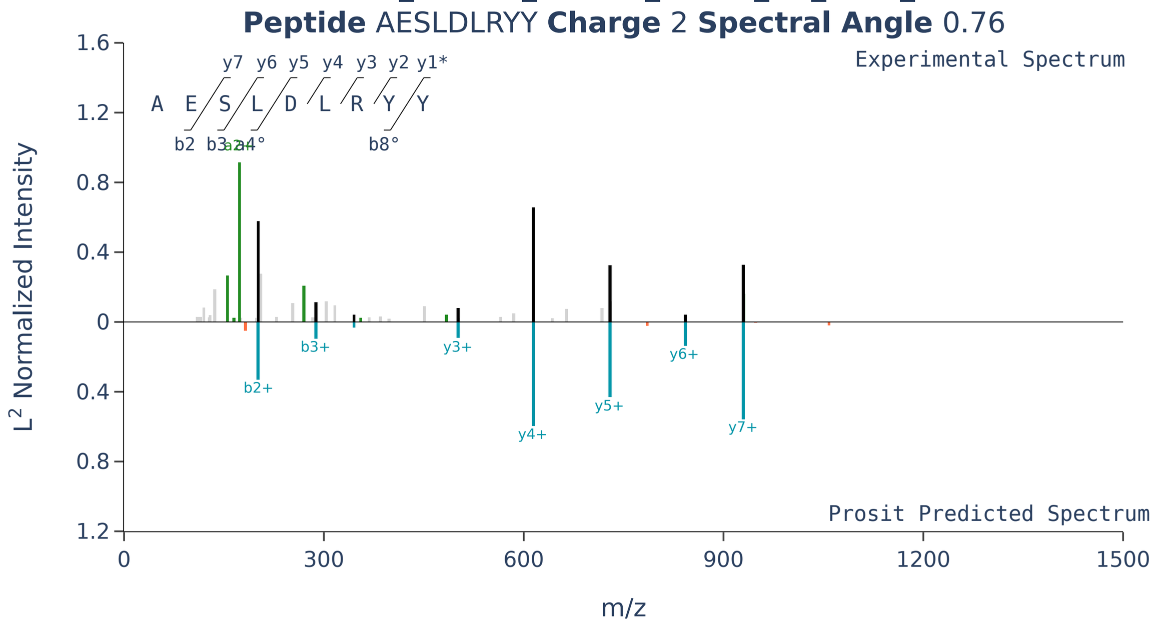


**Source** HRoetschke_211222_160123_HFGoe_IF2_b1R2_4h **Scan** 12774
**Peptide** TKVASGEAGGITQHI **Charge** 3 **Spectral Angle** 0.76

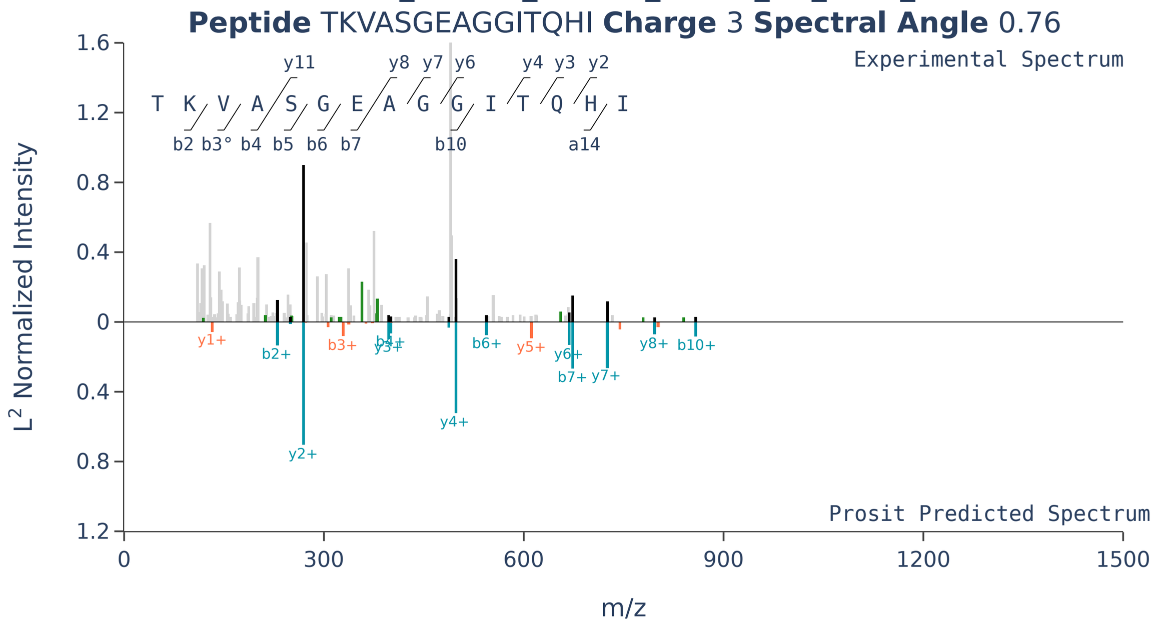


**Source** HRoetschke_211222_160123_HFGoe_IF2_b1R2_24h **Scan** 20659
**Peptide** NQVIDQETAQLV **Charge** 2 **Spectral Angle** 0.85

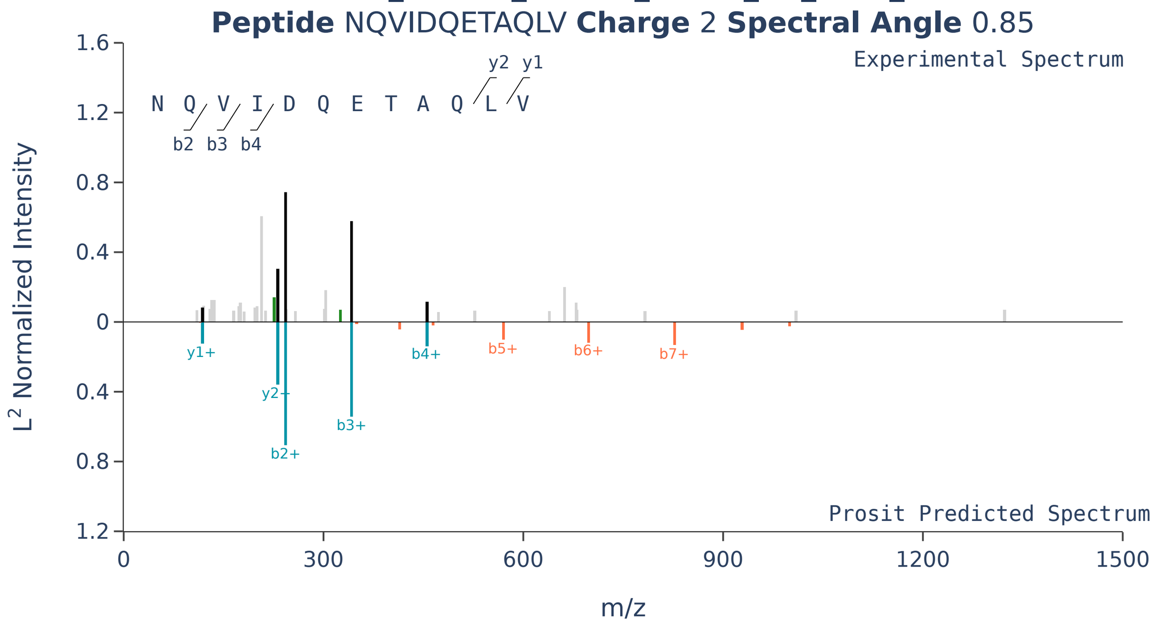

**Source** HRoetschke_211222_160123_HFGoe_IF2_b1R1_4h **Scan** 17617
**Peptide** QQGFQKPAQAVNRDVVL **Charge** 3 **Spectral Angle** 0.76

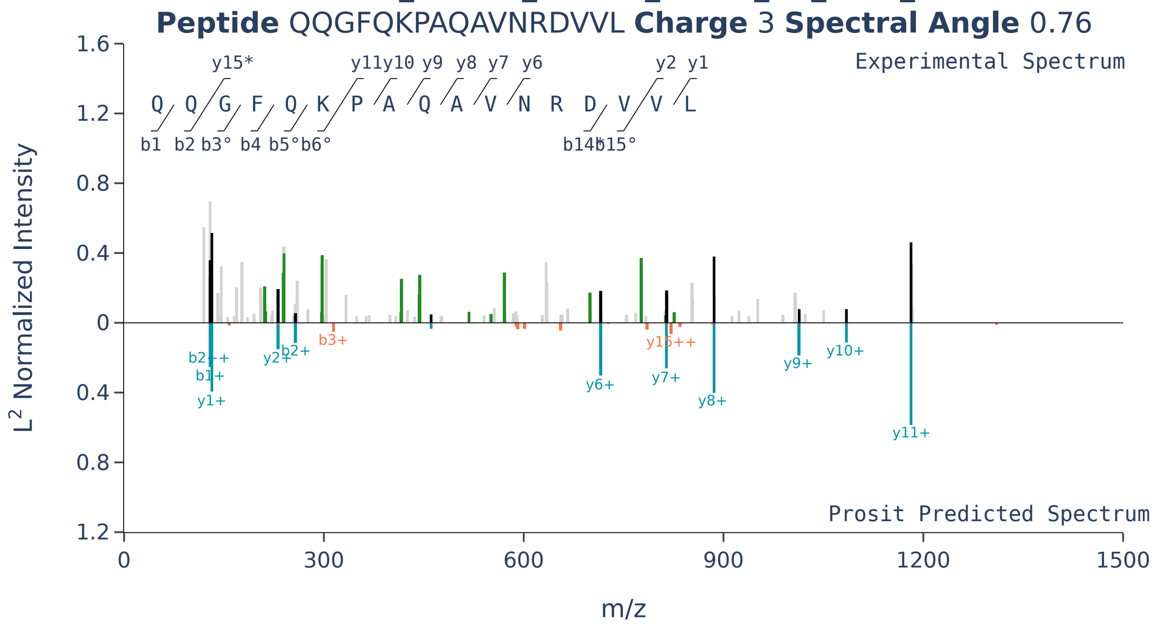


**Source** HRoetschke_211222_160123_HFGoe_IF2_b1R1_24h **Scan** 18024
**Peptide** DVTIKTLAA **Charge** 1 **Spectral Angle** 0.86

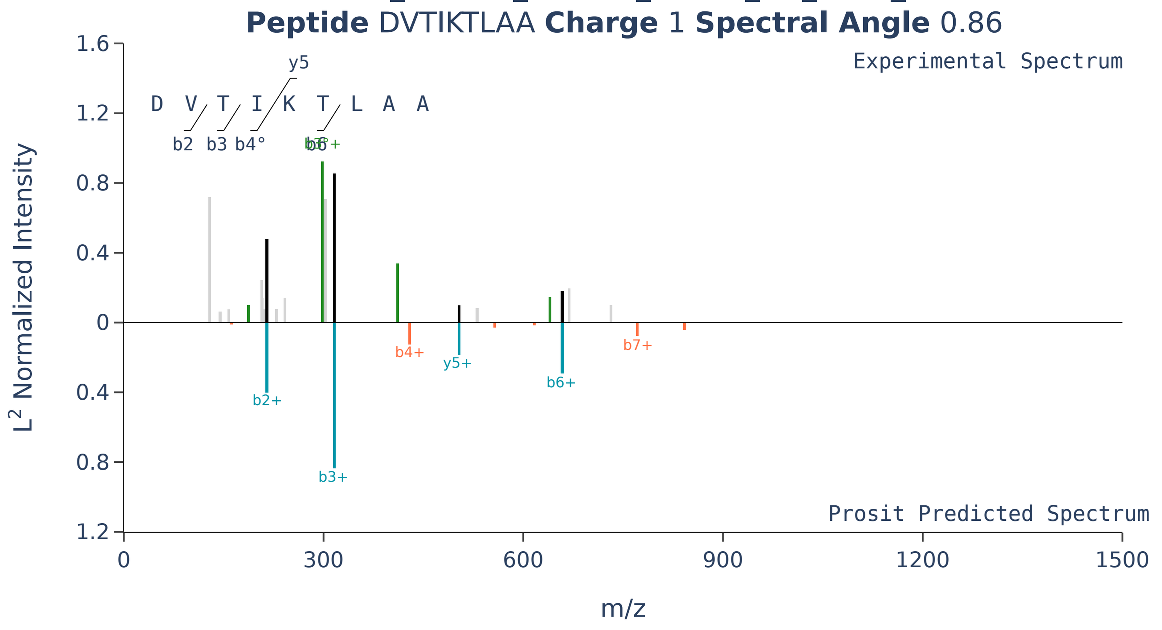


**Source** HRoetschke_211222_160123_HFGoe_IF2_b1R1_4h **Scan** 5692
**Peptide** AESKADREEARAAV **Charge** 3 **Spectral Angle** 0.83

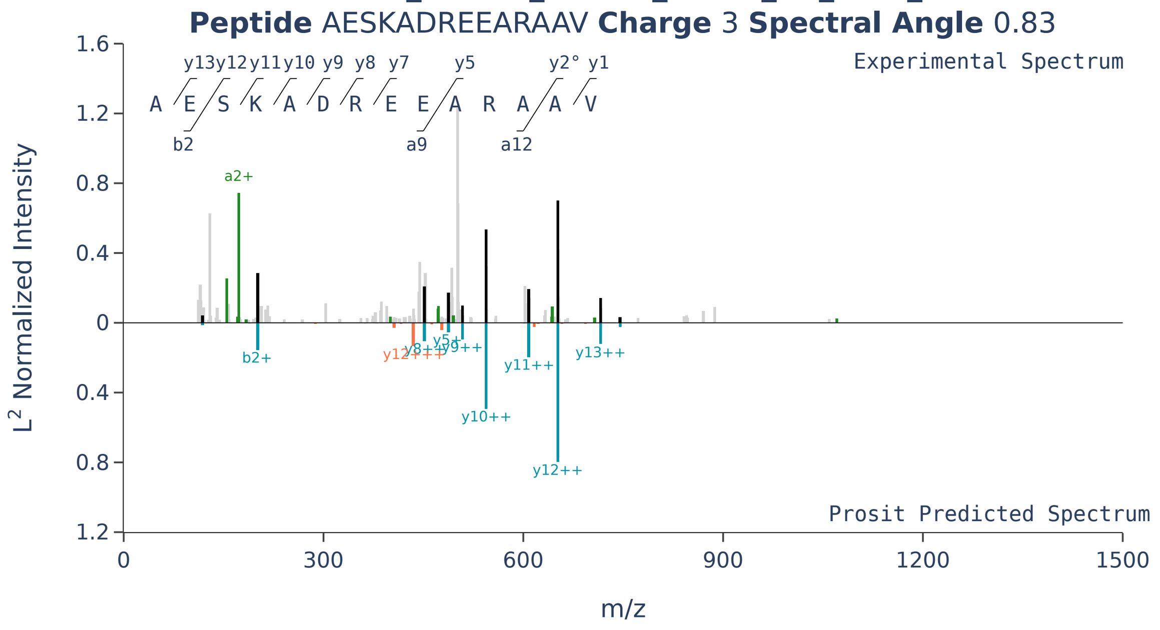


**Source** HRoetschke_211222_160123_HFGoe_IF2_b1R2_2h **Scan** 10240
**Peptide** KGSSLQQGFQKPAQAVNR **Charge** 3 **Spectral Angle** 0.84

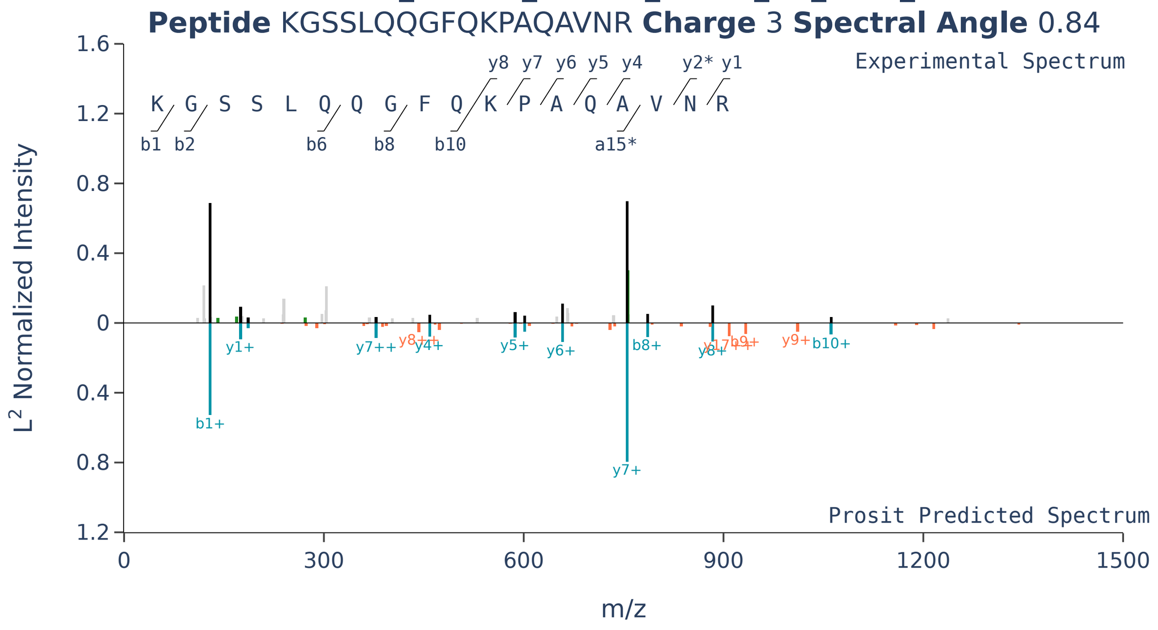


**Source** HRoetschke_211222_160123_HFGoe_IF2_b1R1_4h **Scan** 18575
**Peptide** SLLKLSTD **Charge** 2 **Spectral Angle** 0.66

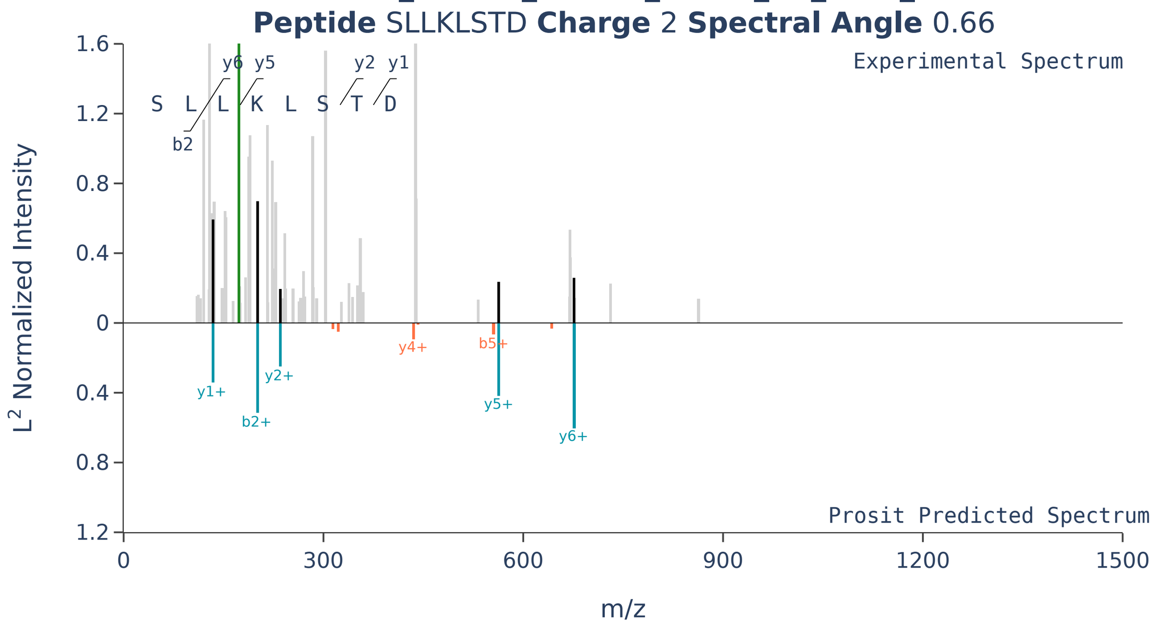


**Source** HRoetschke_211222_160123_HFGoe_IF2_b1R1_4h **Scan** 9298
**Peptide** GELESLRR **Charge** 2 **Spectral Angle** 0.73

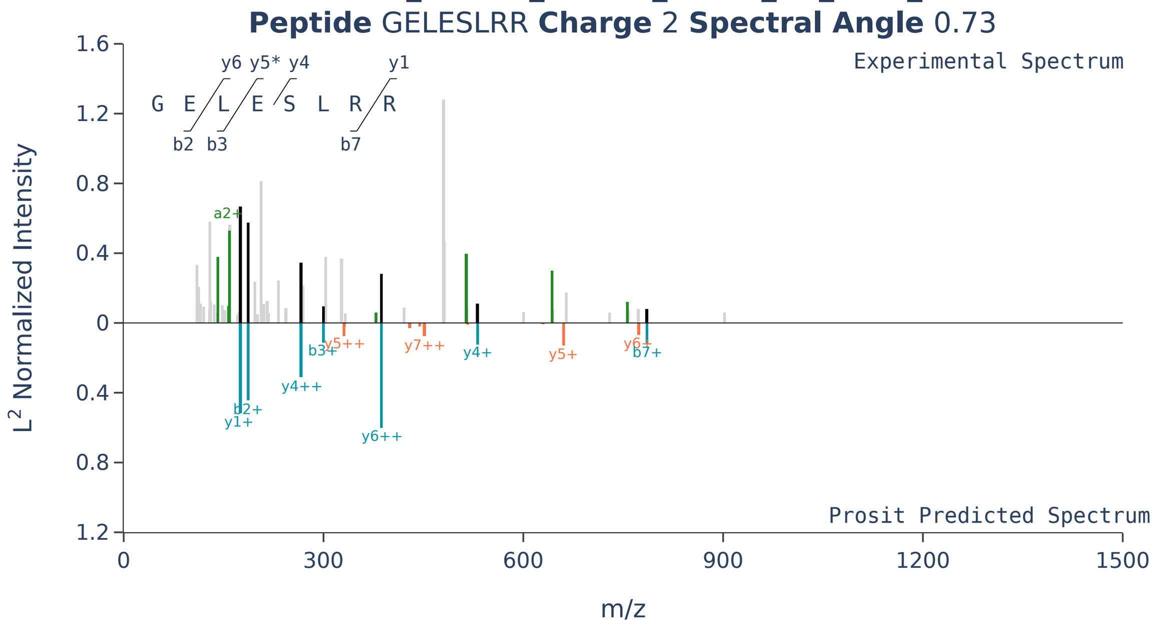


**Source** HRoetschke_211222_160123_HFGoe_IF2_b1R1_4h **Scan** 13302
**Peptide** LDYIRSTKVA **Charge** 2 **Spectral Angle** 0.72

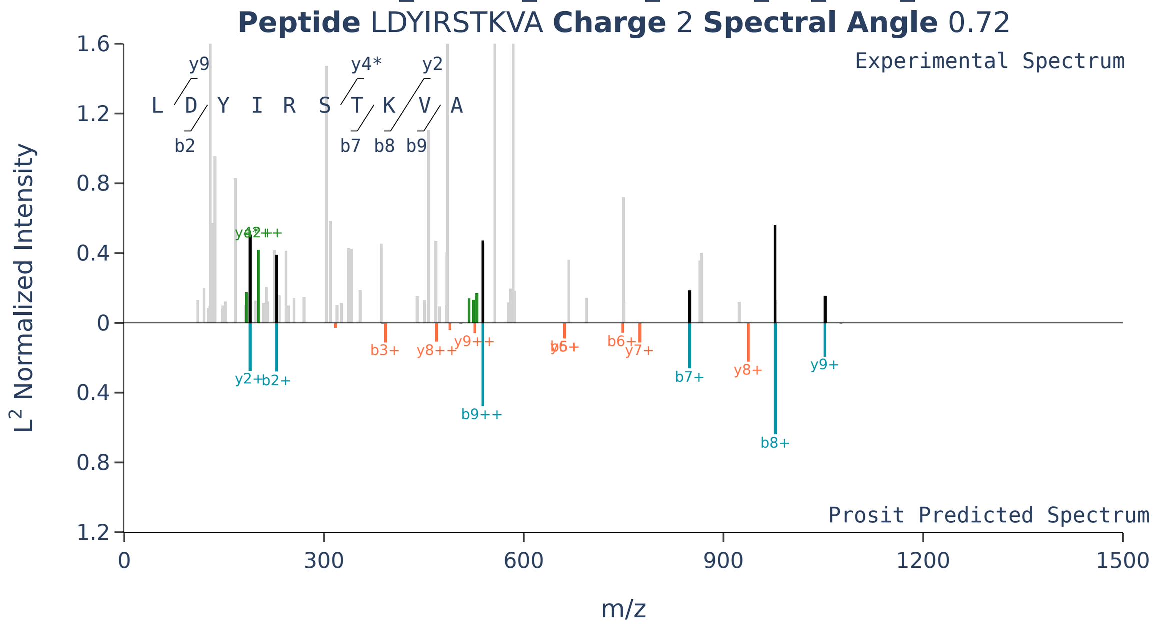


**Source** HRoetschke_211222_160123_HFGoe_IF2_b2R2_24h **Scan** 11723
**Peptide** KGSSLQQGFQKPAQAVN **Charge** 3 **Spectral Angle** 0.82

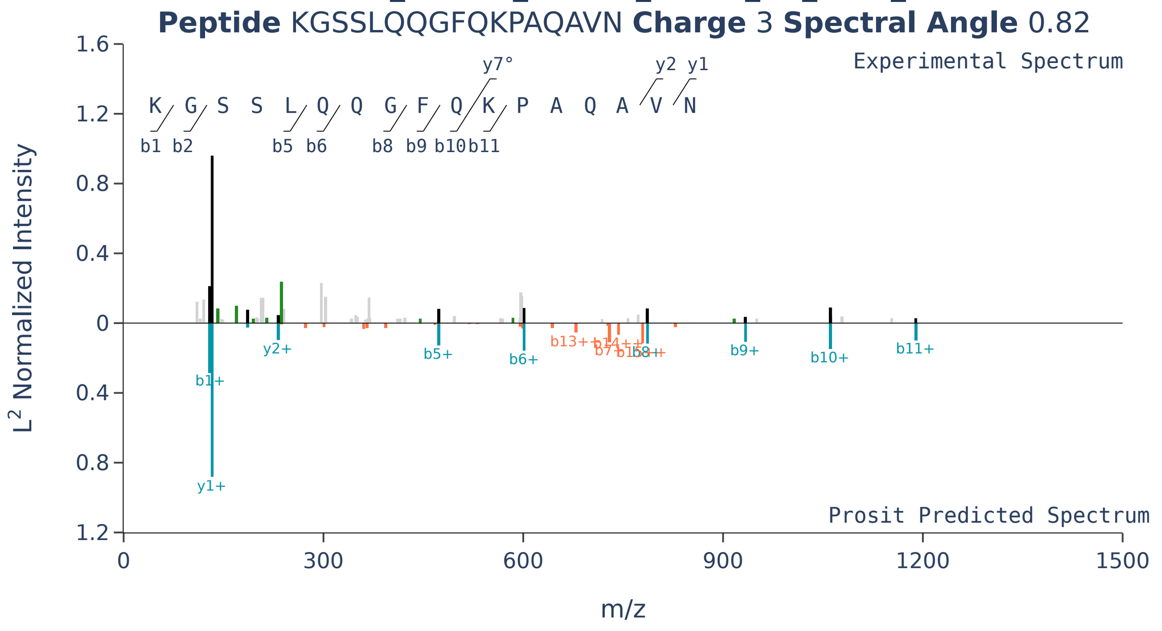


**Source** HRoetschke_211222_160123_HFGoe_IF2_b1R2_24h **Scan** 12122
**Peptide** LGAYHVET **Charge** 2 **Spectral Angle** 0.68

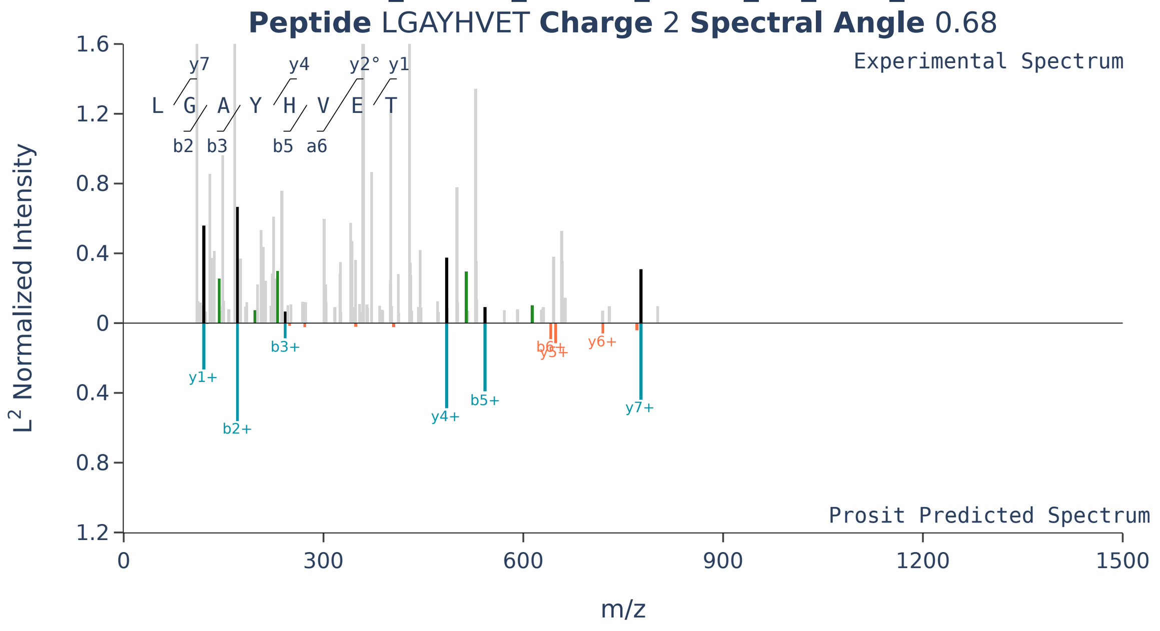


**Source** HRoetschke_211222_160123_HFGoe_IF2_b2R1_24h **Scan** 15509
**Peptide** DQETAQLVA **Charge** 2 **Spectral Angle** 0.66

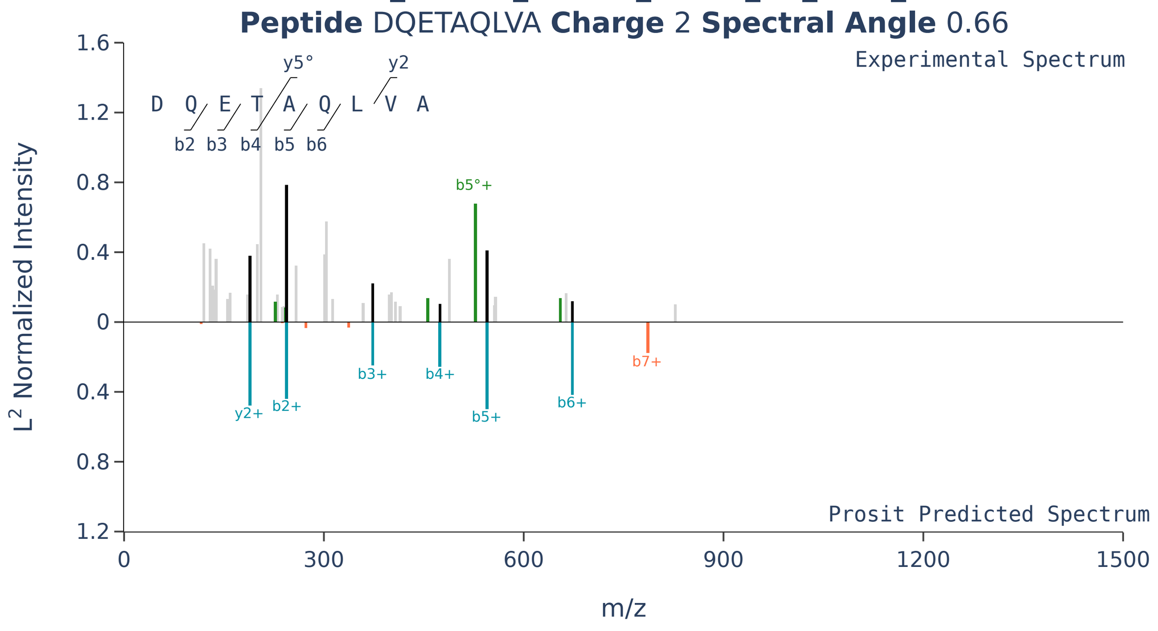


**Source** HRoetschke_211222_160123_HFGoe_IF2_b1R2_24h **Scan** 8344
**Peptide** KDDVNEVRNGM **Charge** 3 **Spectral Angle** 0.62

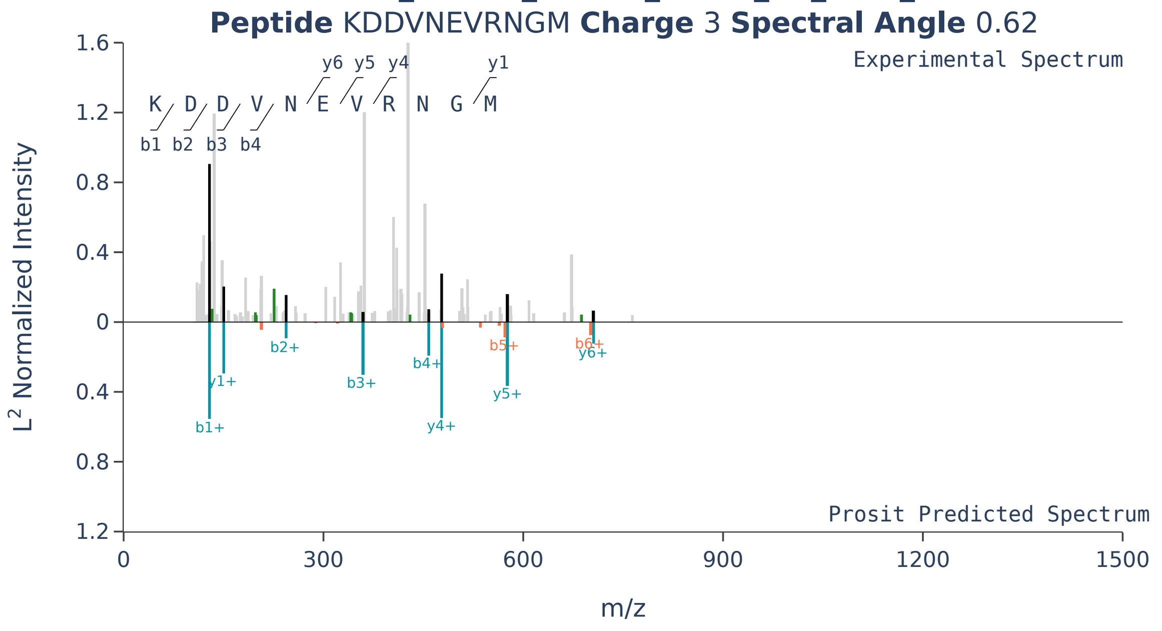


**Source** HRoetschke_211222_160123_HFGoe_IF2_b1R1_4h **Scan** 17467
**Peptide** GETITVGELANK **Charge** 2 **Spectral Angle** 0.65

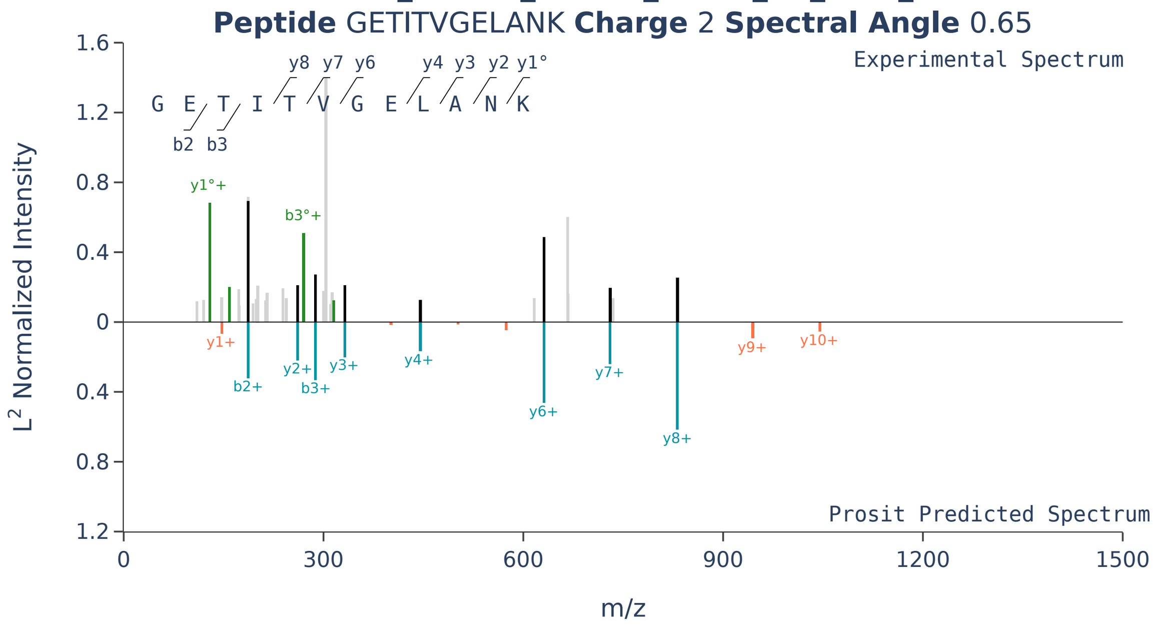


**Source** HRoetschke_211222_160123_HFGoe_IF2_b1R2_24h **Scan** 10538
**Peptide** RDPQEAERLAAEEQAQRE **Charge** 4 **Spectral Angle** 0.79

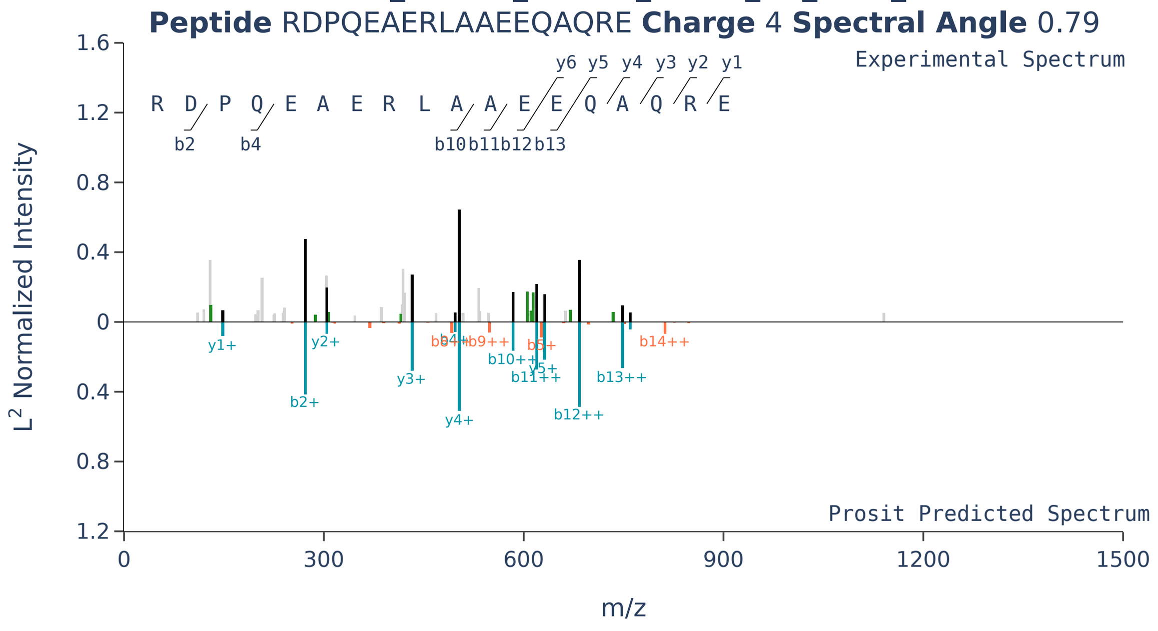


**Source** HRoetschke_211222_160123_HFGoe_IF2_b2R1_24h **Scan** 5487
**Peptide** SKADREEARAAV **Charge** 3 **Spectral Angle** 0.95

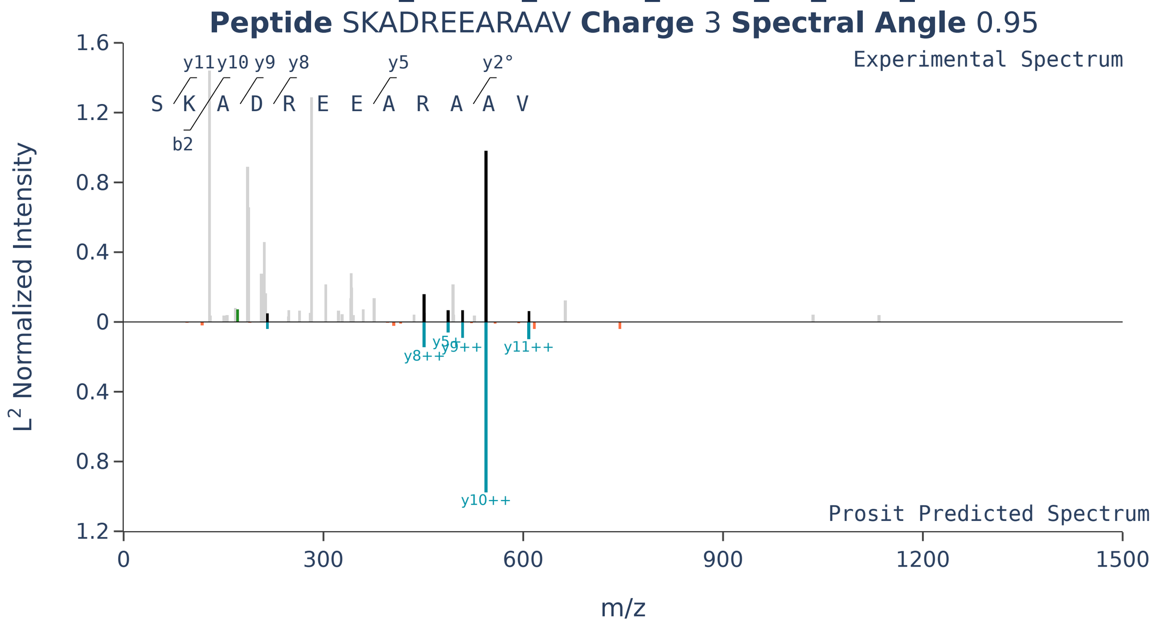


**Source** HRoetschke_211222_160123_HFGoe_IF2_b1R1_4h **Scan** 7652
**Peptide** GHKVILRRENELE **Charge** 3 **Spectral Angle** 0.73

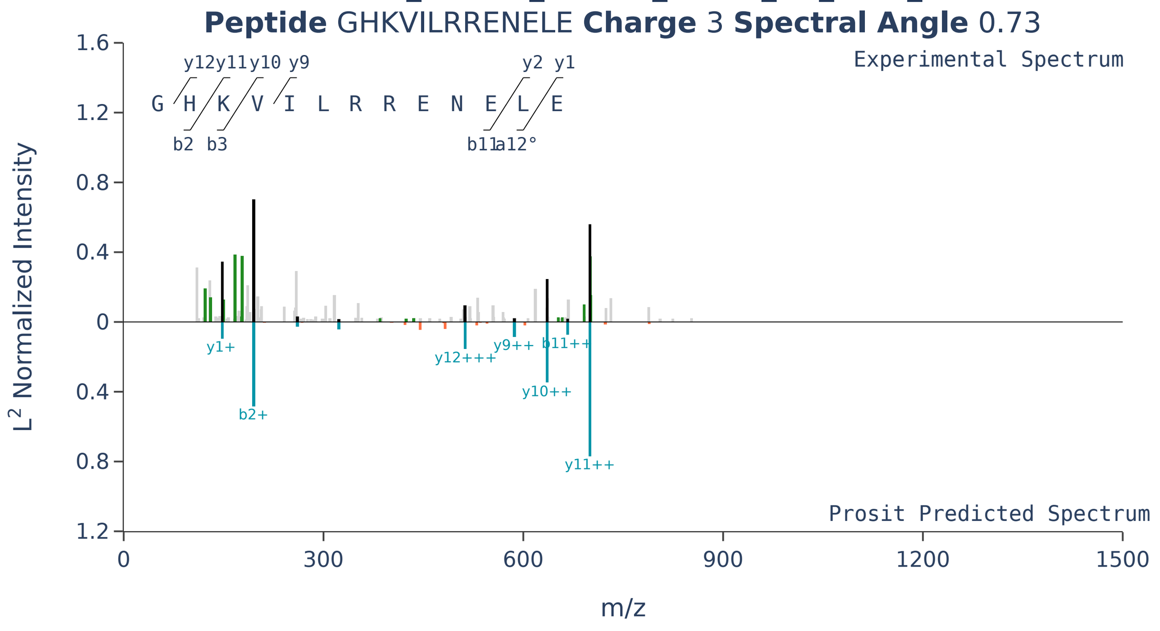

**Source** HRoetschke_211222_160123_HFGoe_IF2_b1R1_4h **Scan** 17598
**Peptide** IQHAKAAQVPVVVAVN **Charge** 3 **Spectral Angle** 0.7

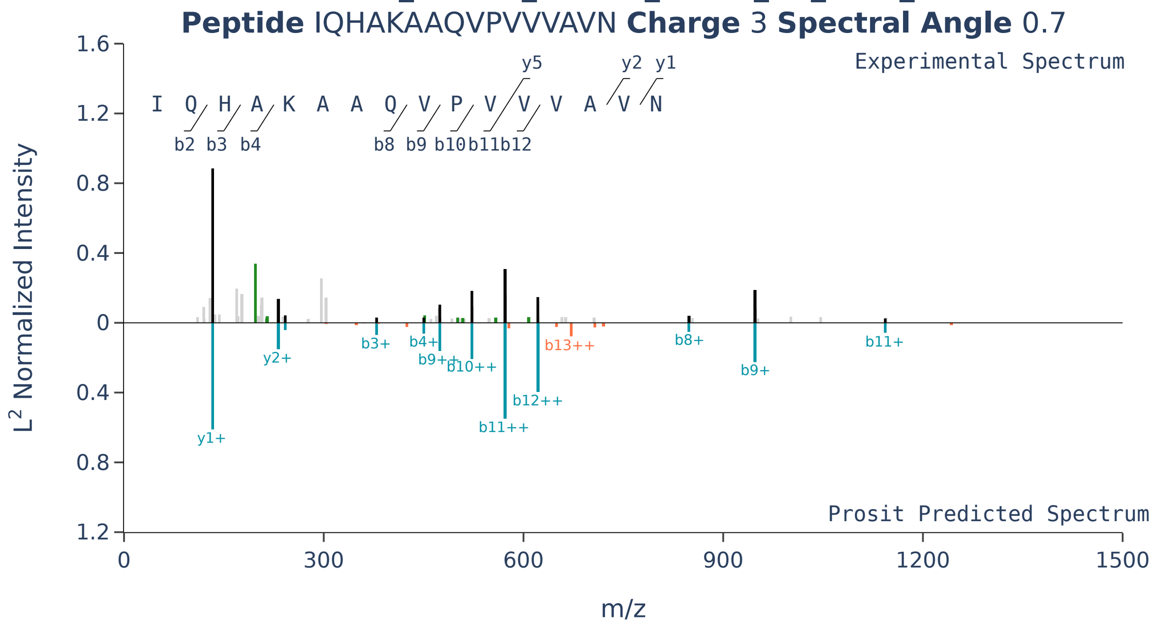


**Source** HRoetschke_211222_160123_HFGoe_IF2_b1R1_4h **Scan** 6402
**Peptide** SDYHVTTSQHARQAED **Charge** 3 **Spectral Angle** 0.69

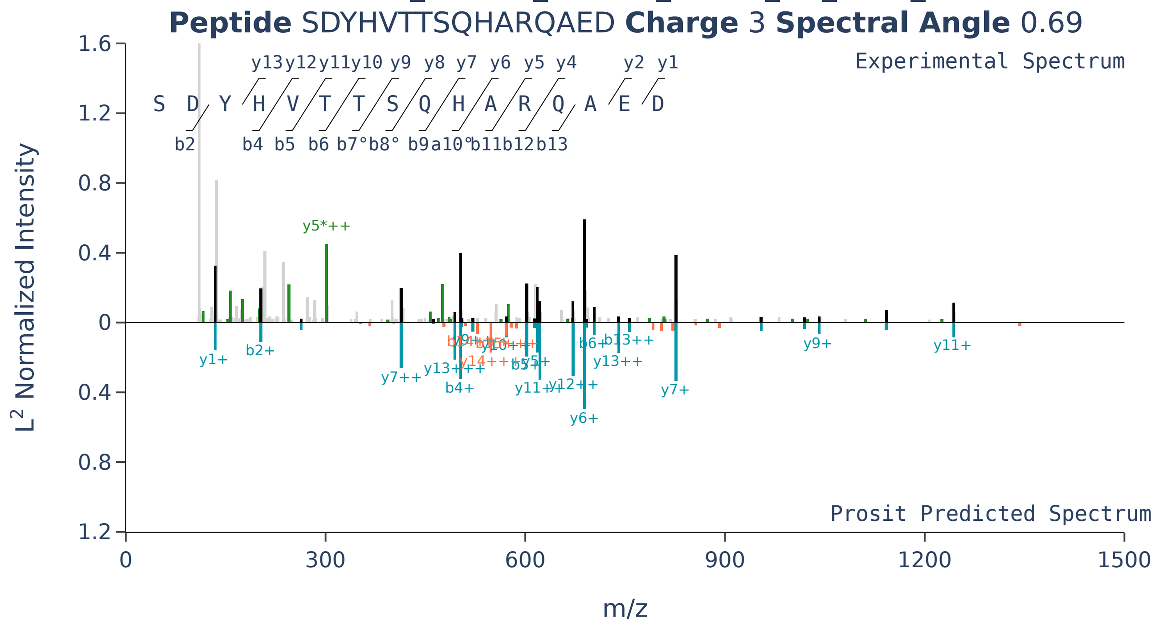


**Source** HRoetschke_211222_160123_HFGoe_IF2_b1R2_2h **Scan** 16349
**Peptide** ADDSVSAQEKQTLL **Charge** 2 **Spectral Angle** 0.83

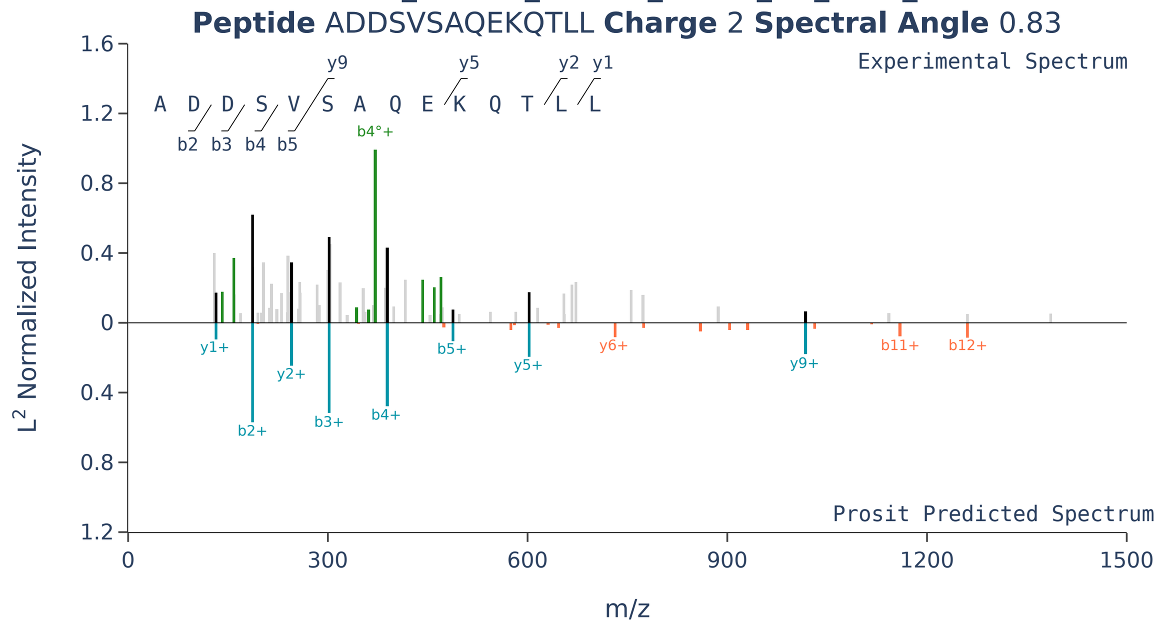


**Source** HRoetschke_211222_160123_HFGoe_IF2_b1R1_4h **Scan** 5283
**Peptide** DKPEADPDRVKN **Charge** 3 **Spectral Angle** 0.67

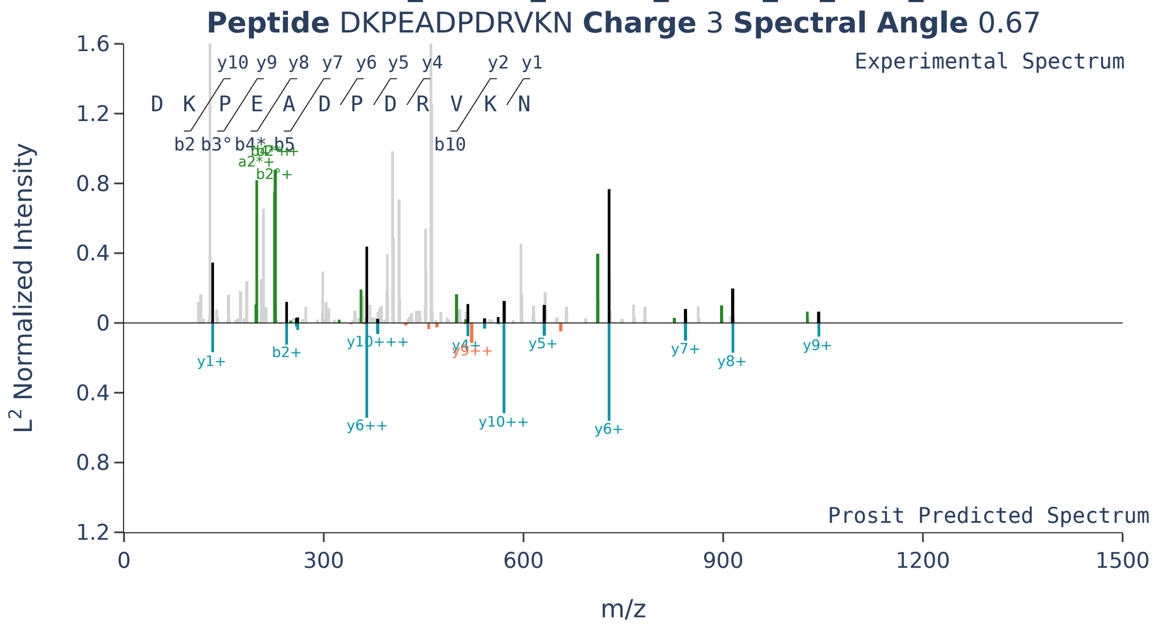


**Source** HRoetschke_211222_160123_HFGoe_IF2_b1R2_2h **Scan** 7196
**Peptide** KFREVKLARQQ **Charge** 3 **Spectral Angle** 0.8

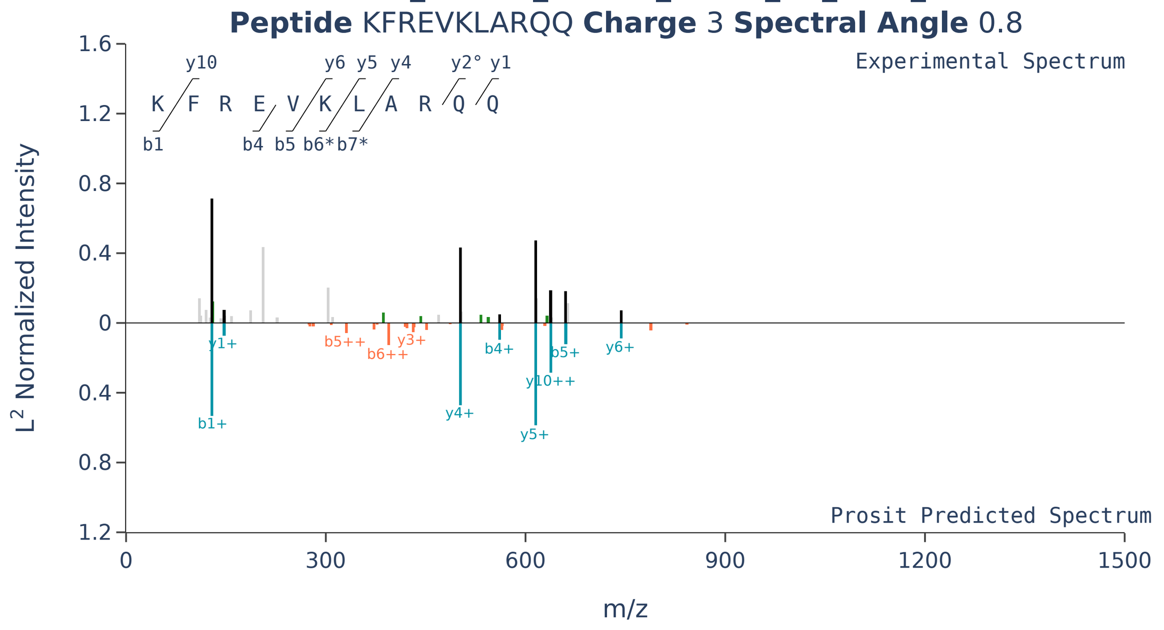


**Source** HRoetschke_211222_160123_HFGoe_IF2_b1R1_24h **Scan** 12839
**Peptide** SGVPAAGDEVTVVRDEKKAREV **Charge** 3 **Spectral Angle** 0.79

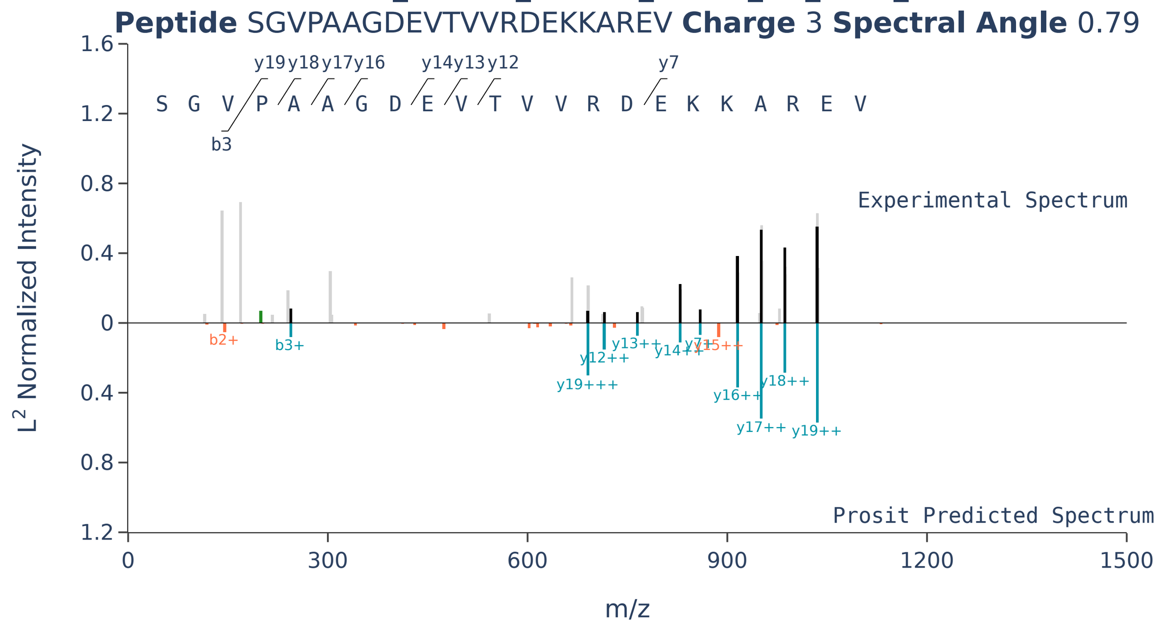


**Source** HRoetschke_211222_160123_HFGoe_IF2_b1R1_4h **Scan** 14690
**Peptide** DTGAAAEPRAPVVT **Charge** 2 **Spectral Angle** 0.77

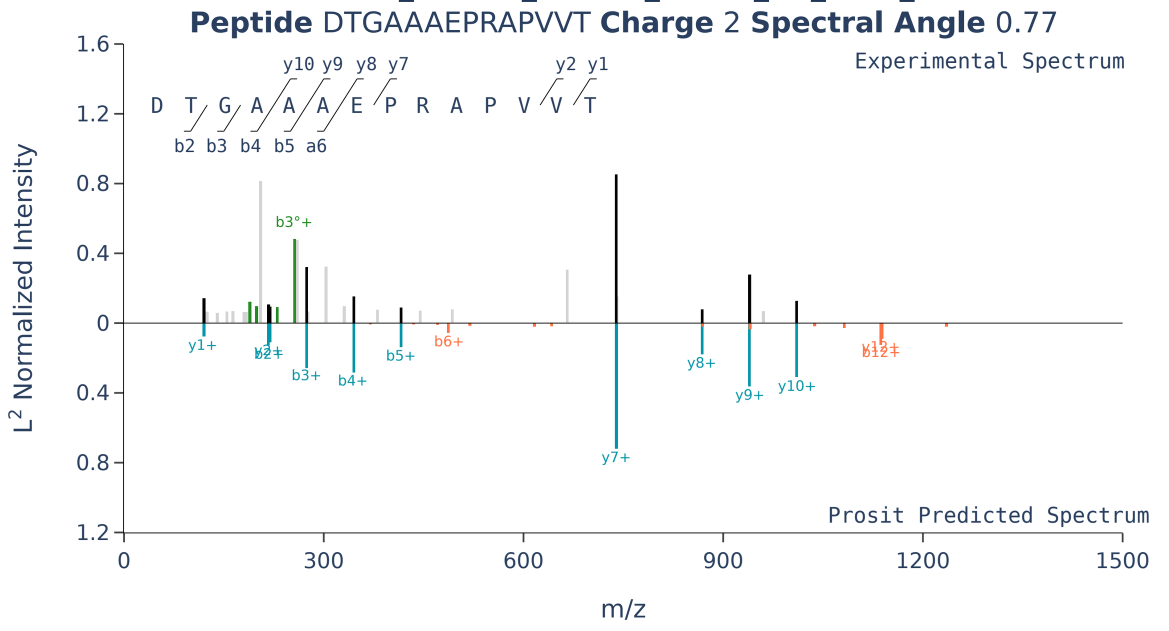


**Source** HRoetschke_211222_160123_HFGoe_IF2_b2R1_4h **Scan** 6919
**Peptide** VRDEKKAREVAL **Charge** 3 **Spectral Angle** 0.67

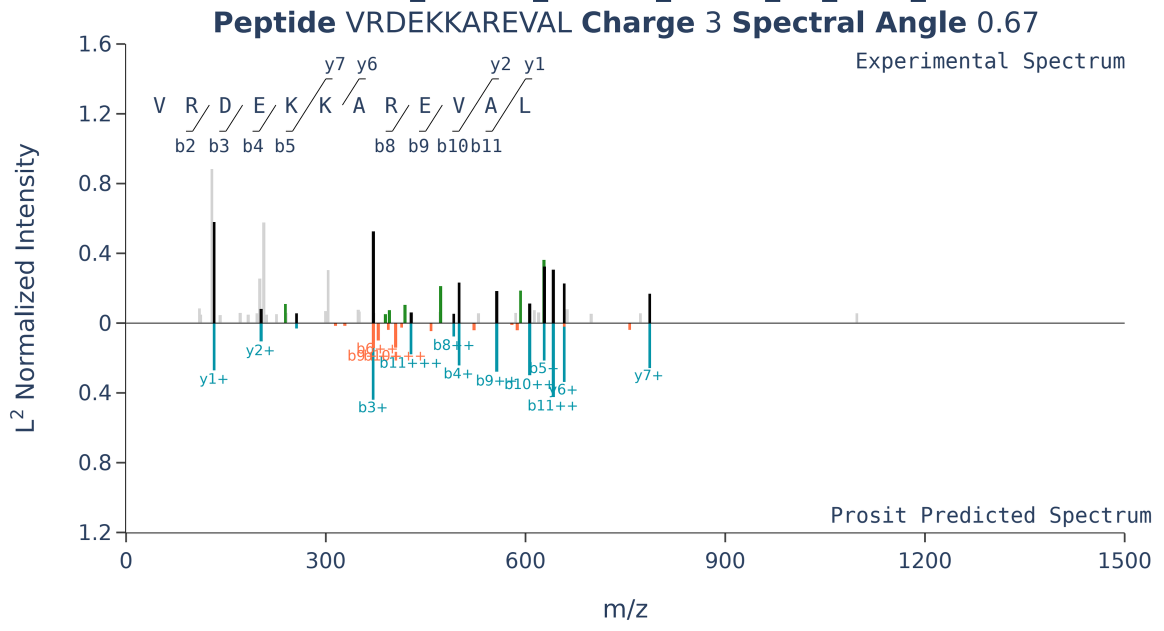


**Source** HRoetschke_211222_160123_HFGoe_IF2_b1R2_4h **Scan** 14441
**Peptide** DKGRGPVATVLVREG **Charge** 3 **Spectral Angle** 0.87

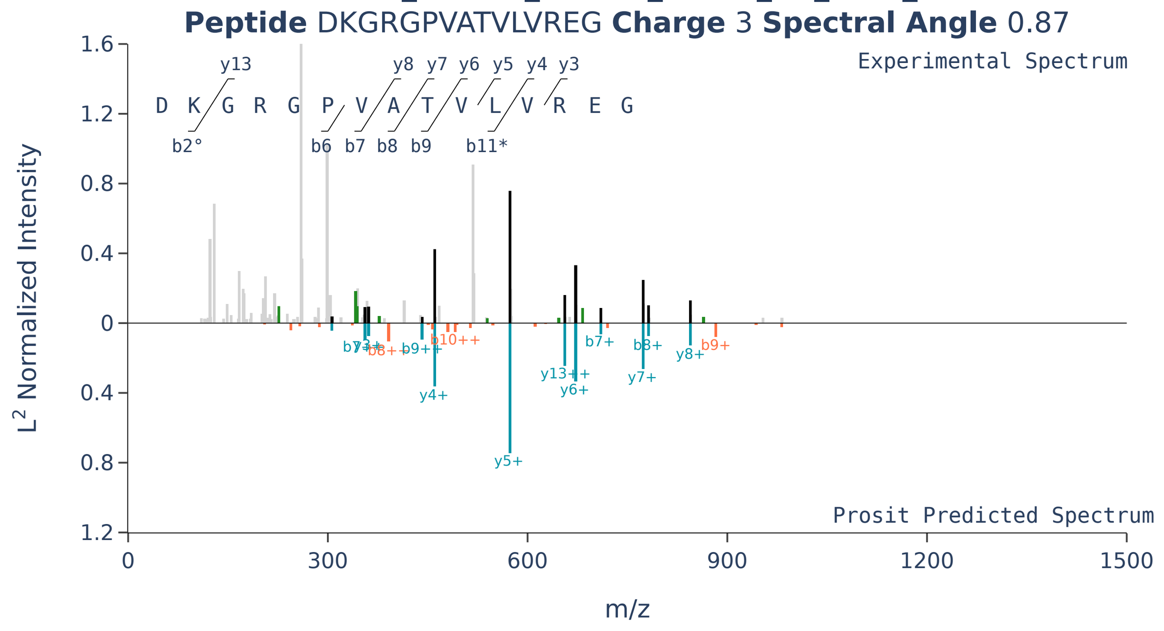


**Source** HRoetschke_211222_160123_HFGoe_IF2_b1R2_1h **Scan** 18261
**Peptide** KAAQVPVVVAVN **Charge** 2 **Spectral Angle** 0.74

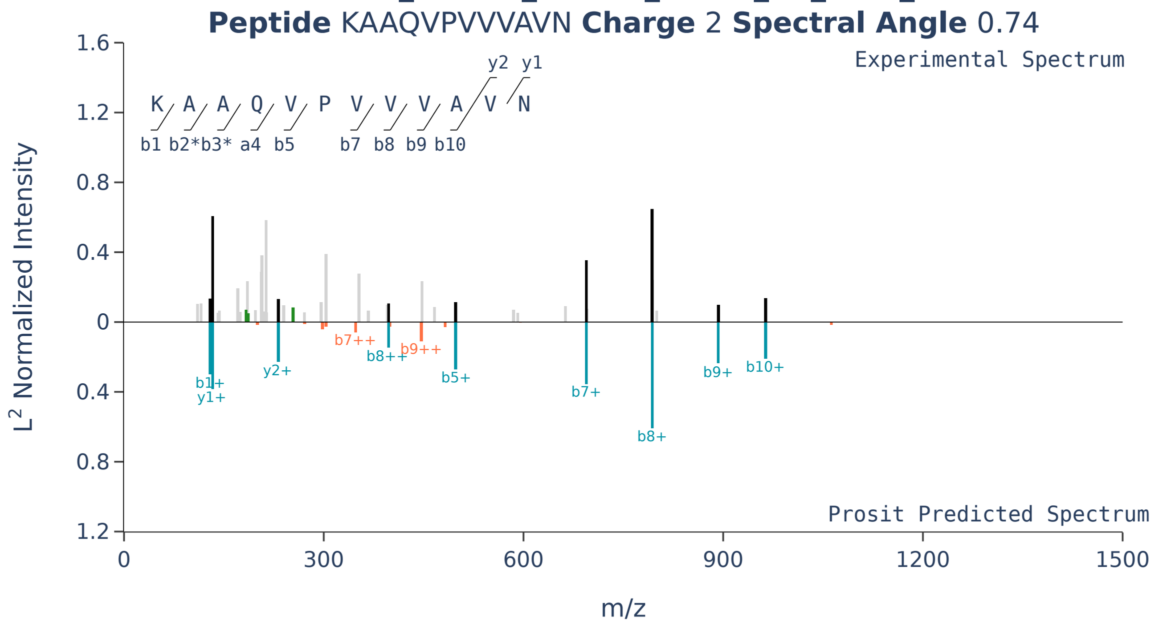


**Source** HRoetschke_211222_160123_HFGoe_IF2_b2R1_24h **Scan** 9937
**Peptide** LSPELKQQ **Charge** 2 **Spectral Angle** 0.67

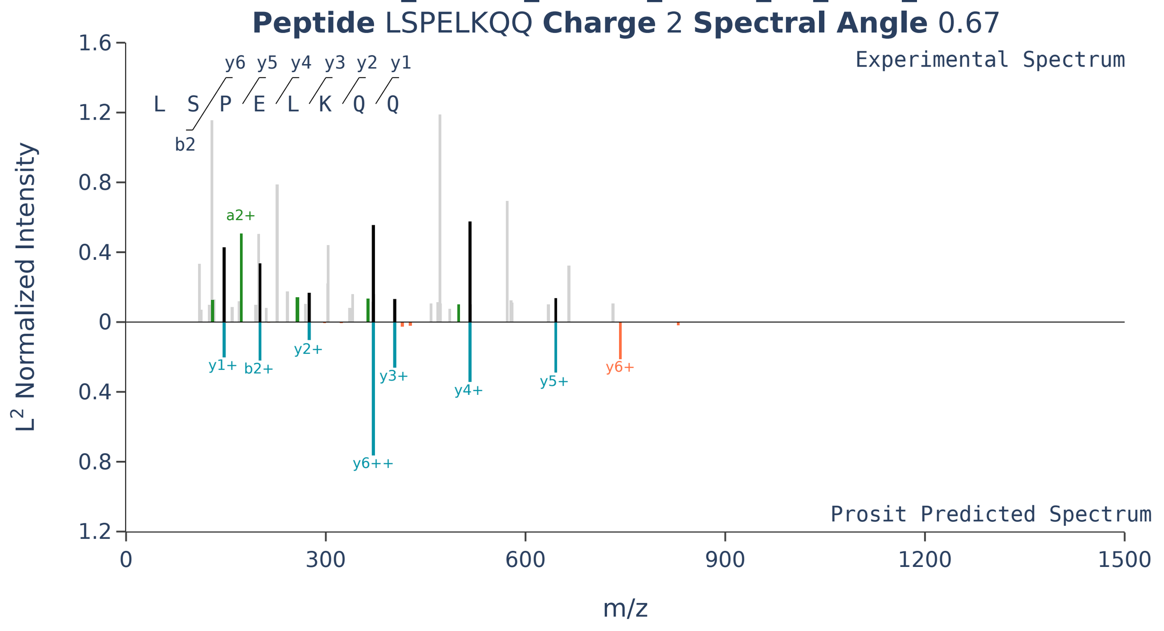


**Source** HRoetschke_211222_160123_HFGoe_IF2_b1R1_4h **Scan** 16342
**Peptide** IQHAKAAQVPVVV **Charge** 3 **Spectral Angle** 0.74

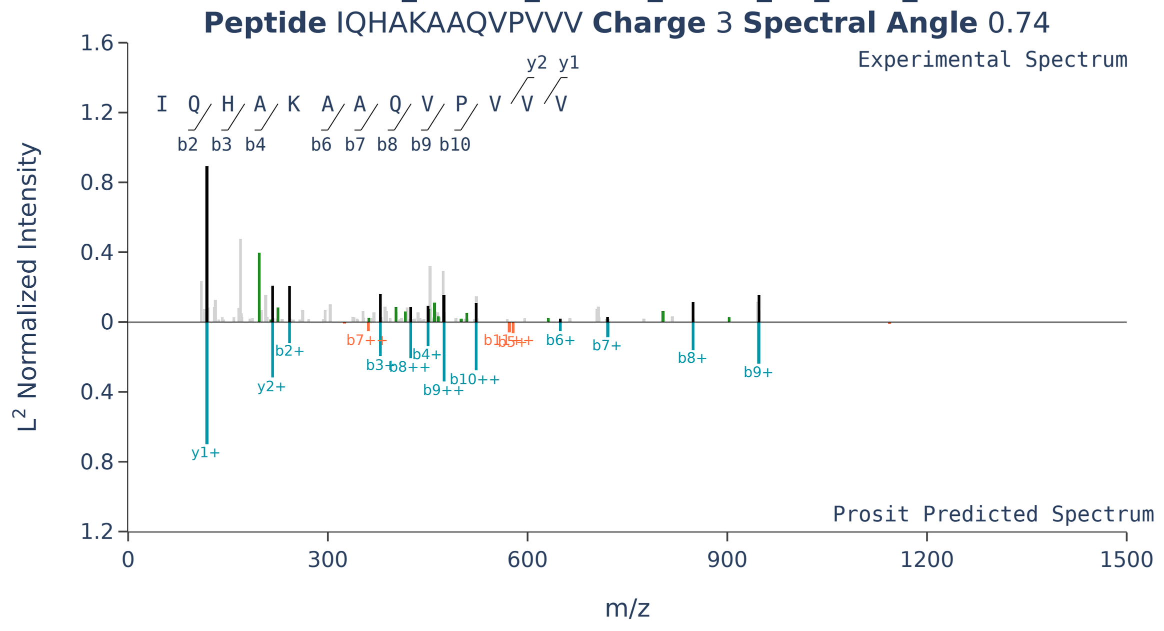


**Source** HRoetschke_211222_160123_HFGoe_IF2_b1R1_24h **Scan** 12109
**Peptide** KSPKFGAIAGC **Charge** 3 **Spectral Angle** 0.81

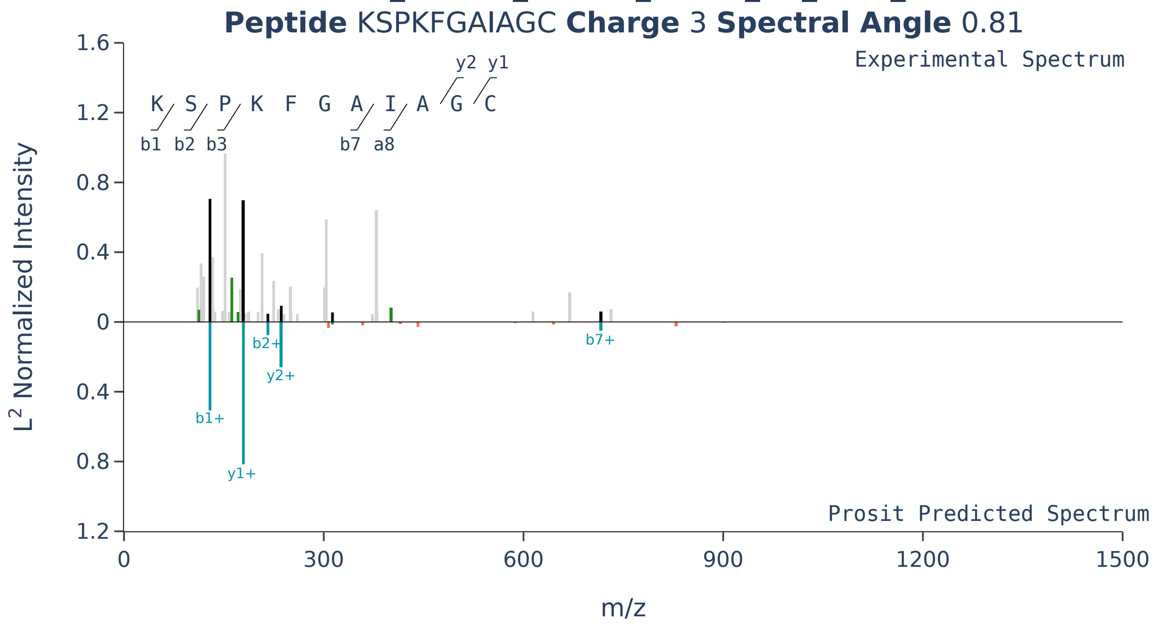


**Source** HRoetschke_211222_160123_HFGoe_IF2_b1R1_24h **Scan** 11006
**Peptide** GFNVRADAS **Charge** 2 **Spectral Angle** 0.82

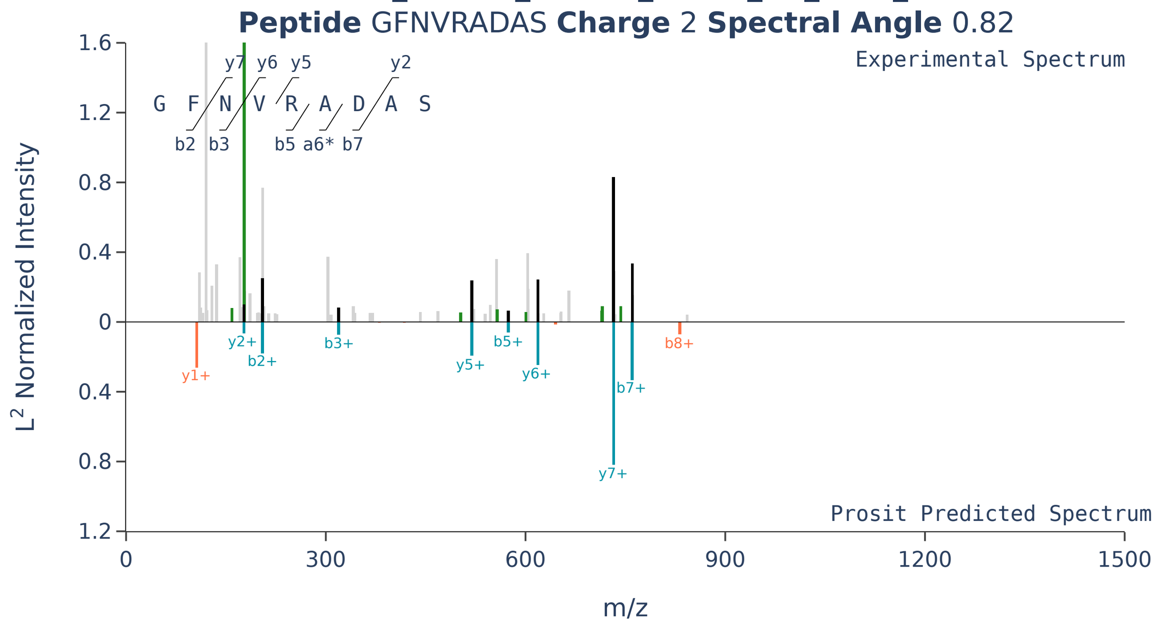


**Source** HRoetschke_211222_160123_HFGoe_IF2_b1R1_4h **Scan** 20672
**Peptide** SVERLVQQF **Charge** 2 **Spectral Angle** 0.73

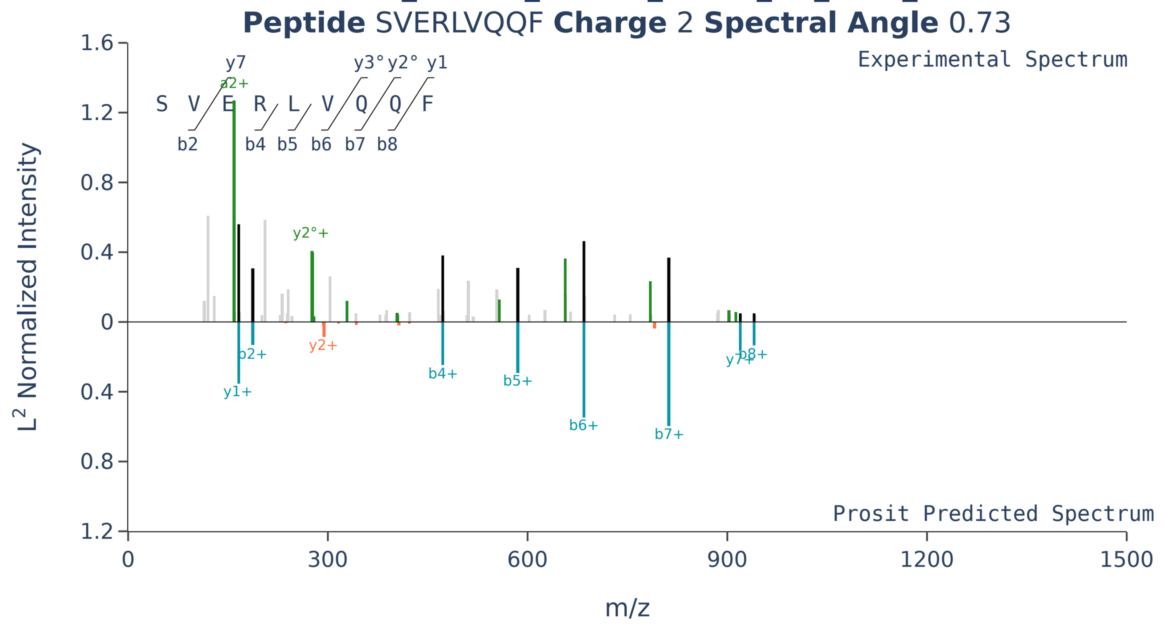

**Source** HRoetschke_211222_160123_HFGoe_IF2_b2R1_24h **Scan** 11528
**Peptide** KIDKPEADPDRVKNEL **Charge** 2 **Spectral Angle** 0.79

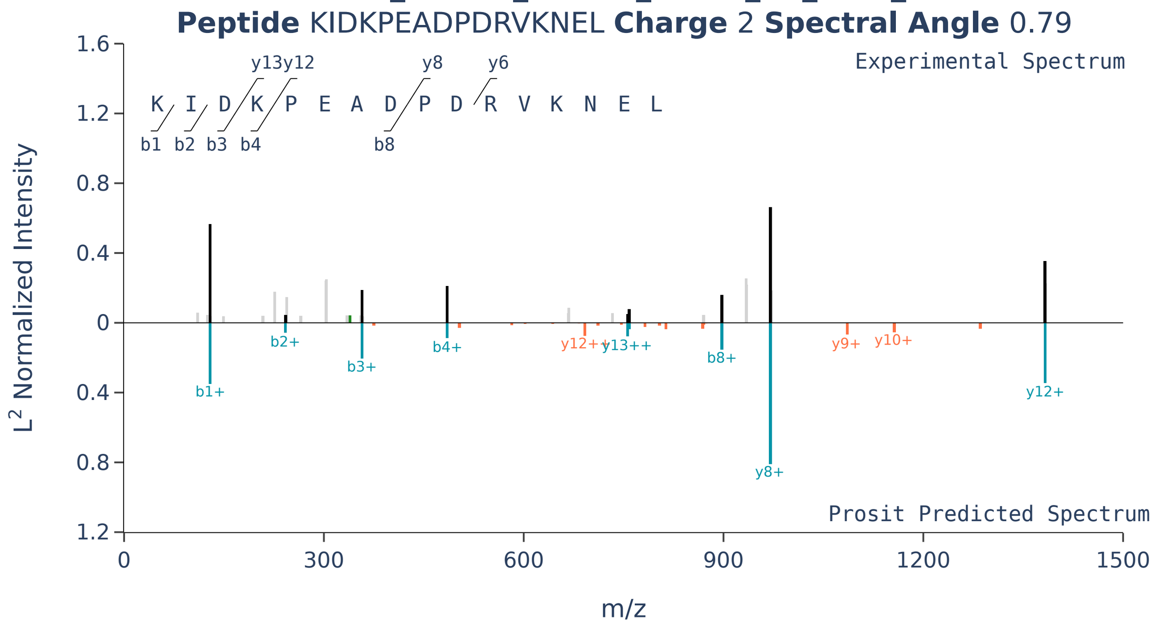


**Source** HRoetschke_211222_160123_HFGoe_IF2_b2R2_24h **Scan** 11588
**Peptide** ERQTSVERLVQ **Charge** 2 **Spectral Angle** 0.7

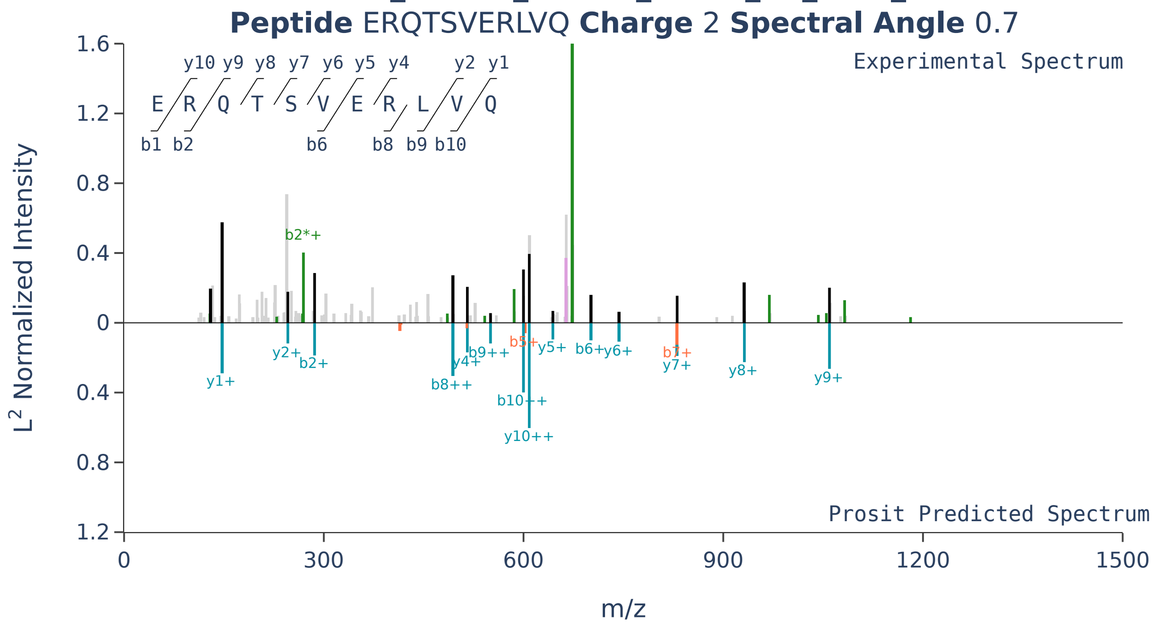


**Source** HRoetschke_211222_160123_HFGoe_IF2_b2R2_24h **Scan** 9755
**Peptide** HIGAYHVET **Charge** 2 **Spectral Angle** 0.82

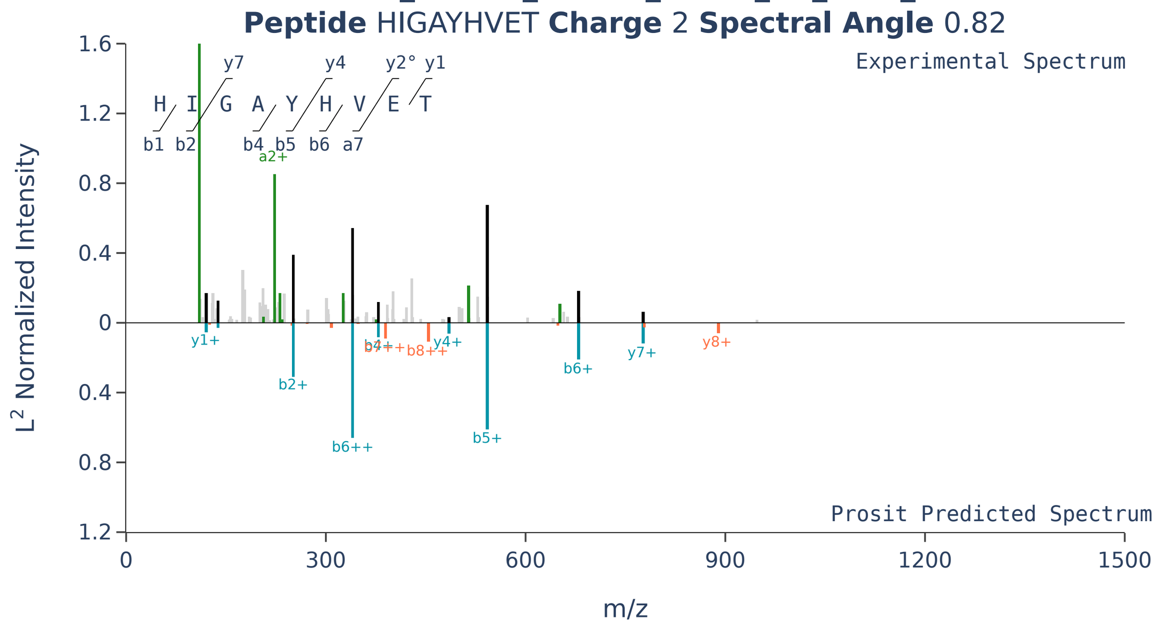


**Source** HRoetschke_211222_160123_HFGoe_IF2_b1R1_4h **Scan** 11513
**Peptide** TEGVVKRHNPIRVL **Charge** 3 **Spectral Angle** 0.81

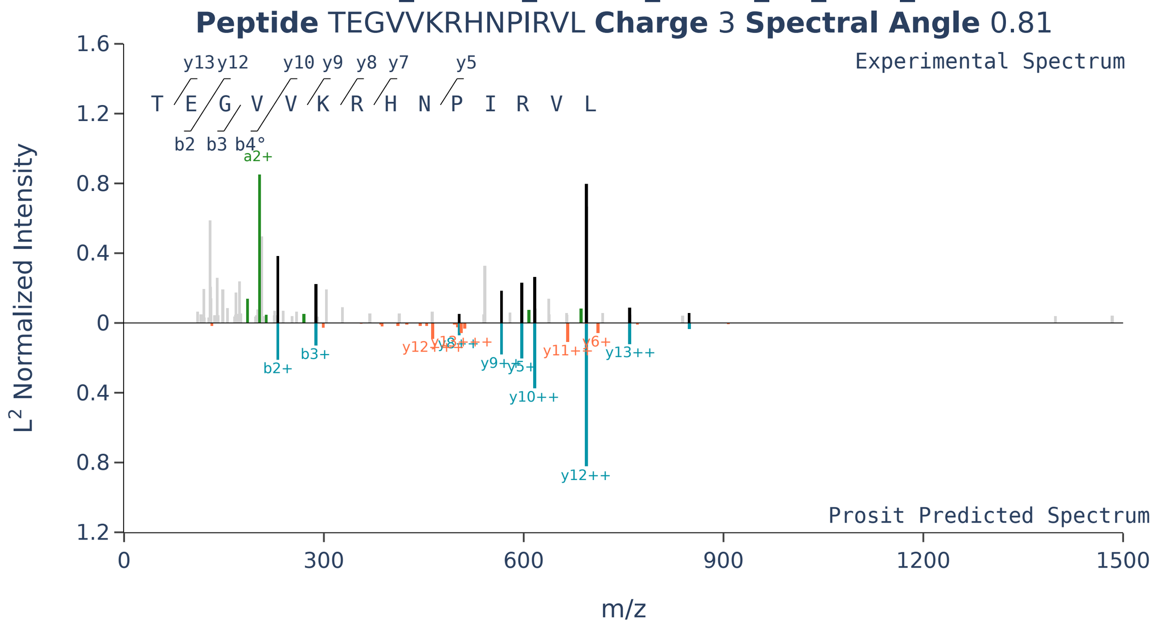


**Source** HRoetschke_211222_160123_HFGoe_IF2_b2R1_24h **Scan** 9251
**Peptide** EIQRTIA **Charge** 2 **Spectral Angle** 0.87

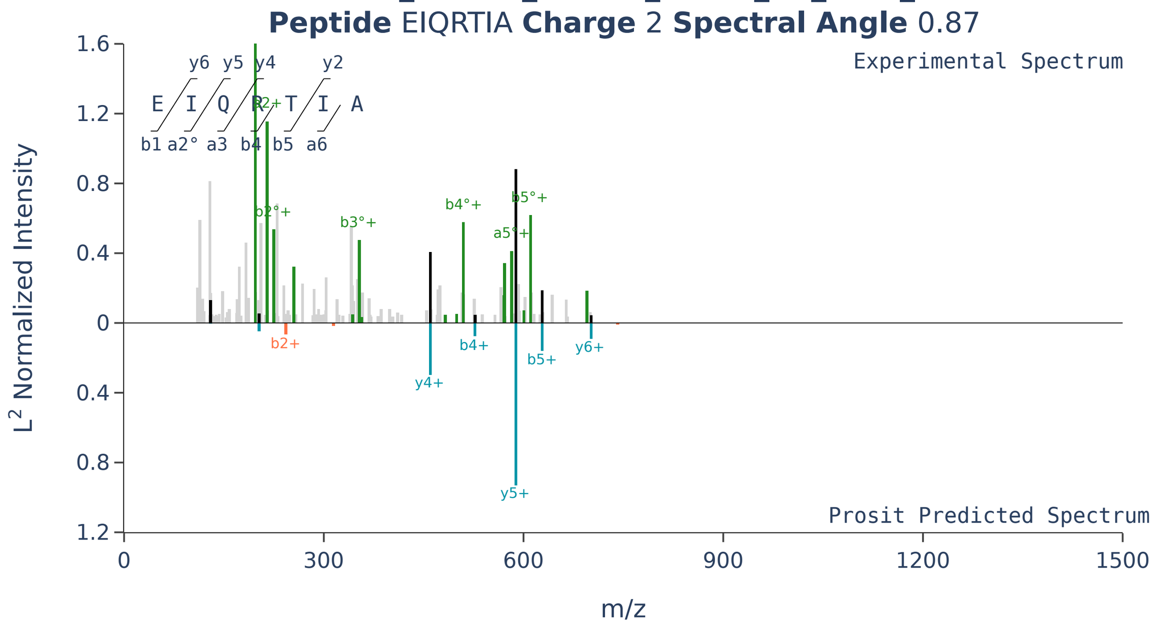


**Source** HRoetschke_211222_160123_HFGoe_IF2_b1R1_2h **Scan** 9600
**Peptide** EKKAREVALY **Charge** 2 **Spectral Angle** 0.67

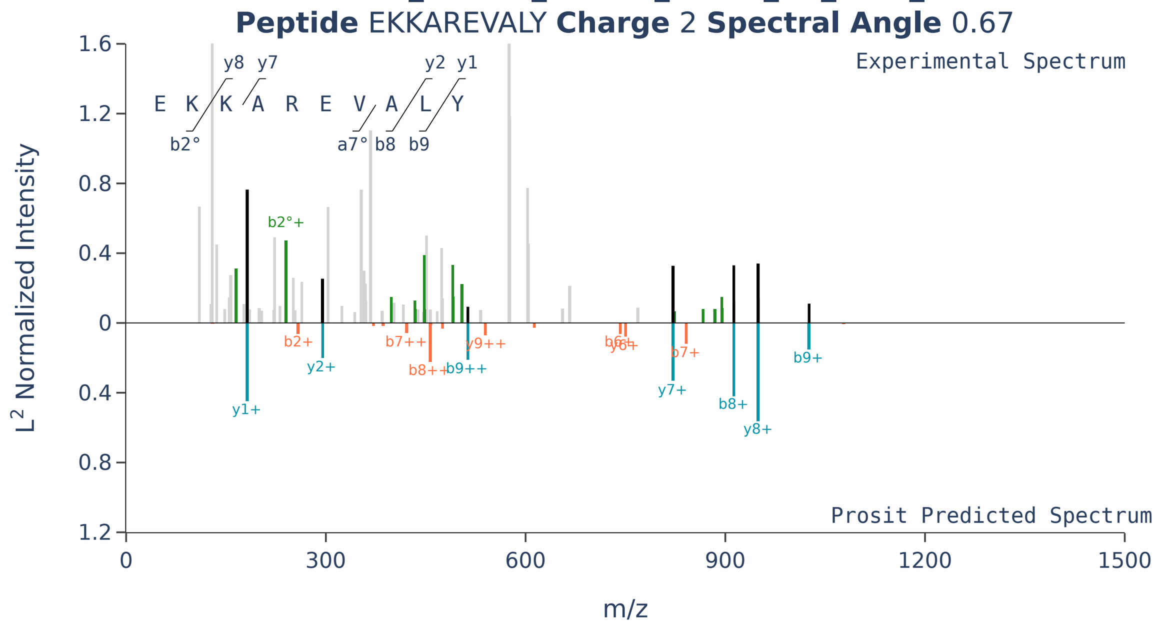


**Source** HRoetschke_211222_160123_HFGoe_IF2_b2R1_24h **Scan** 16853
**Peptide** DEVKVKLL **Charge** 2 **Spectral Angle** 0.74

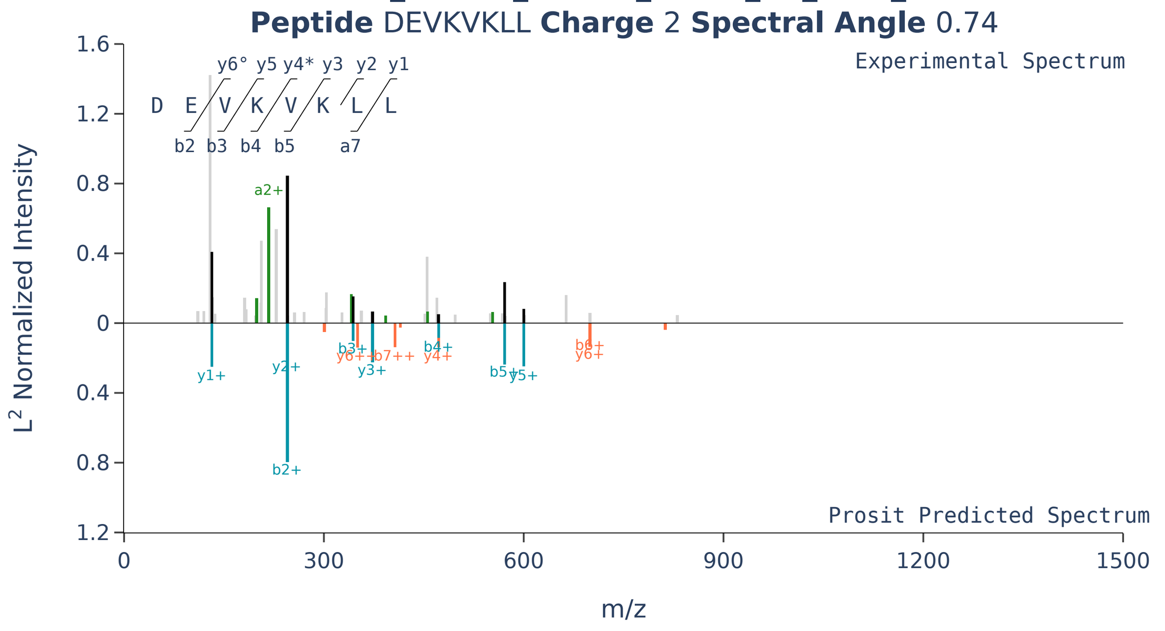


**Source** HRoetschke_211222_160123_HFGoe_IF2_b2R2_1h **Scan** 6214
**Peptide** KLDKPEADPDRVKNE **Charge** 2 **Spectral Angle** 0.81

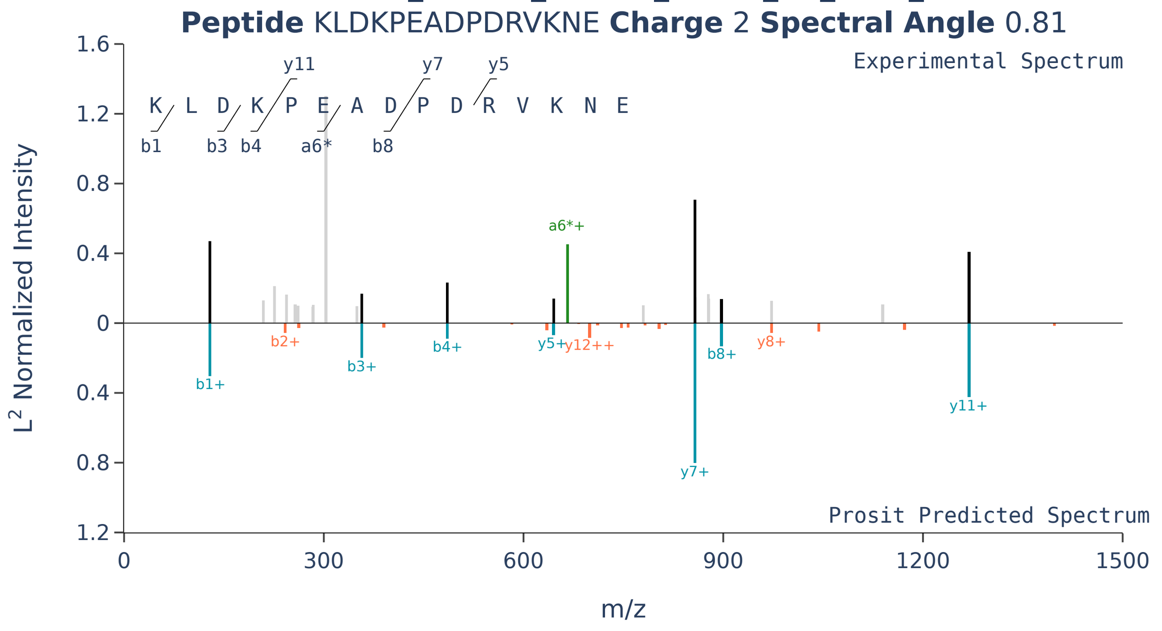


**Source** HRoetschke_211222_160123_HFGoe_IF2_b1R1_24h **Scan** 18086
**Peptide** QAEVLELKA **Charge** 2 **Spectral Angle** 0.83

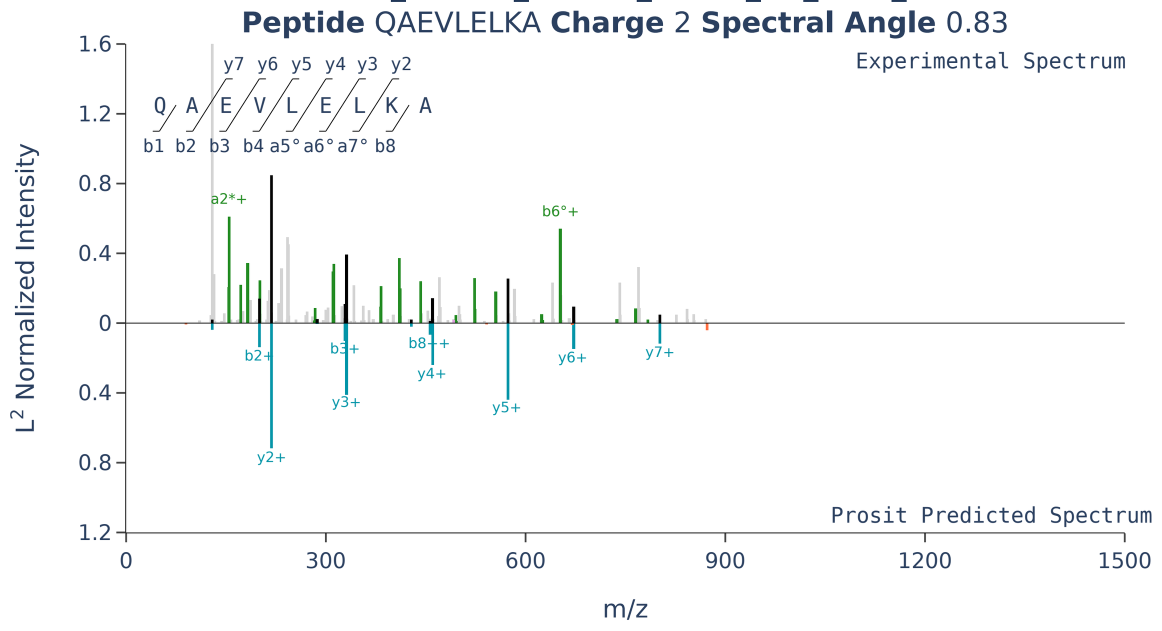


**Source** HRoetschke_211222_160123_HFGoe_IF2_b2R1_24h **Scan** 11479
**Peptide** KSPKFGAIAG **Charge** 2 **Spectral Angle** 0.82

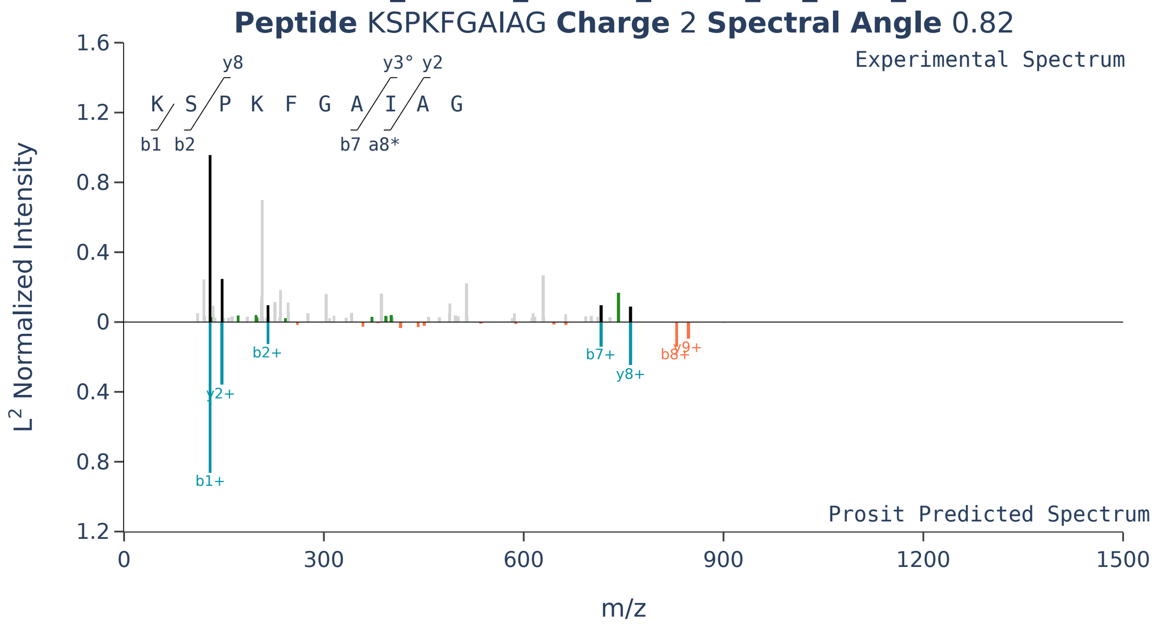


**Source** HRoetschke_211222_160123_HFGoe_IF2_b1R1_2h **Scan** 10162
**Peptide** YIRSTKVASGEAGGITQ **Charge** 3 **Spectral Angle** 0.8

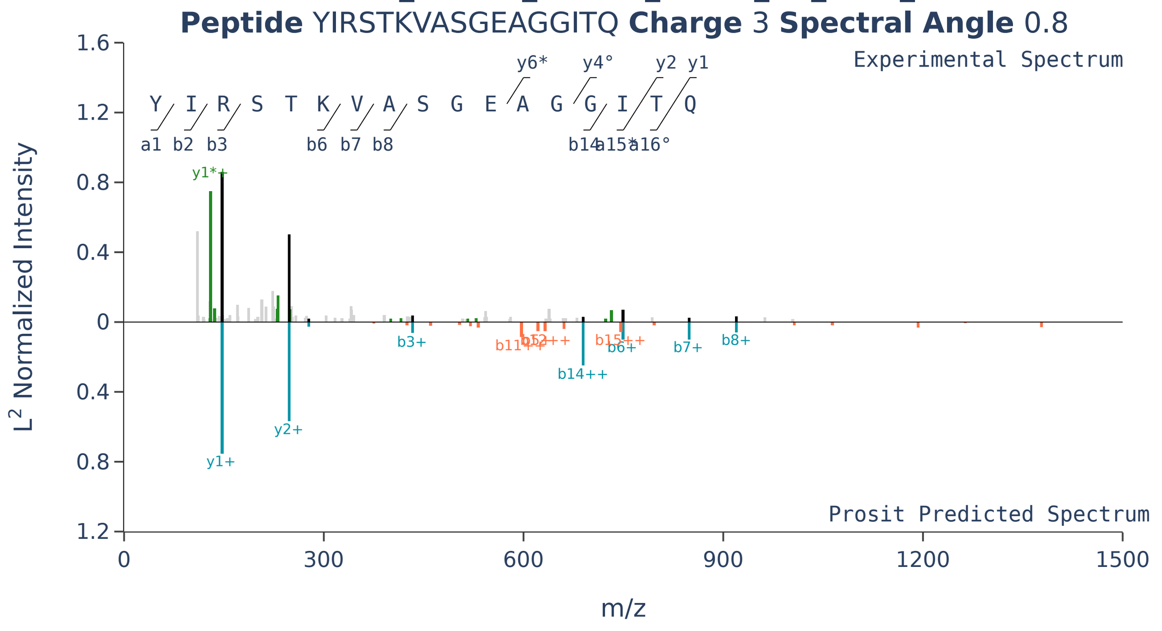


**Source** HRoetschke_211222_160123_HFGoe_IF2_b1R1_4h **Scan** 14434
**Peptide** TGAAAEPRAPVV **Charge** 2 **Spectral Angle** 0.74

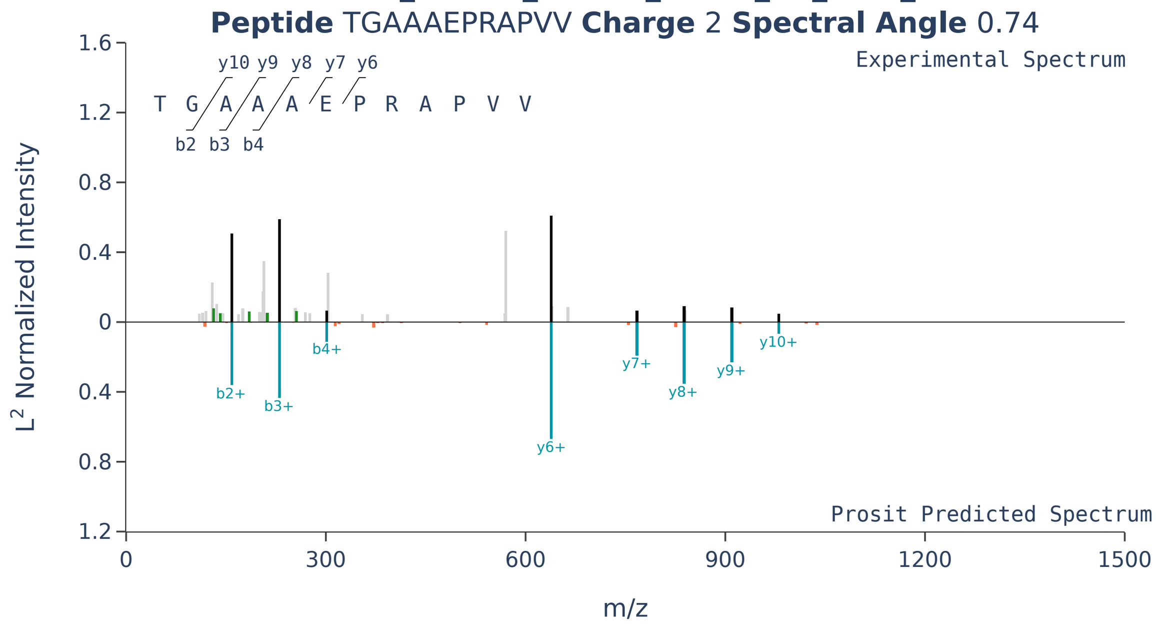


**Source** HRoetschke_211222_160123_HFGoe_IF2_b1R2_4h **Scan** 12081
**Peptide** KGSSLQQGF **Charge** 2 **Spectral Angle** 0.61

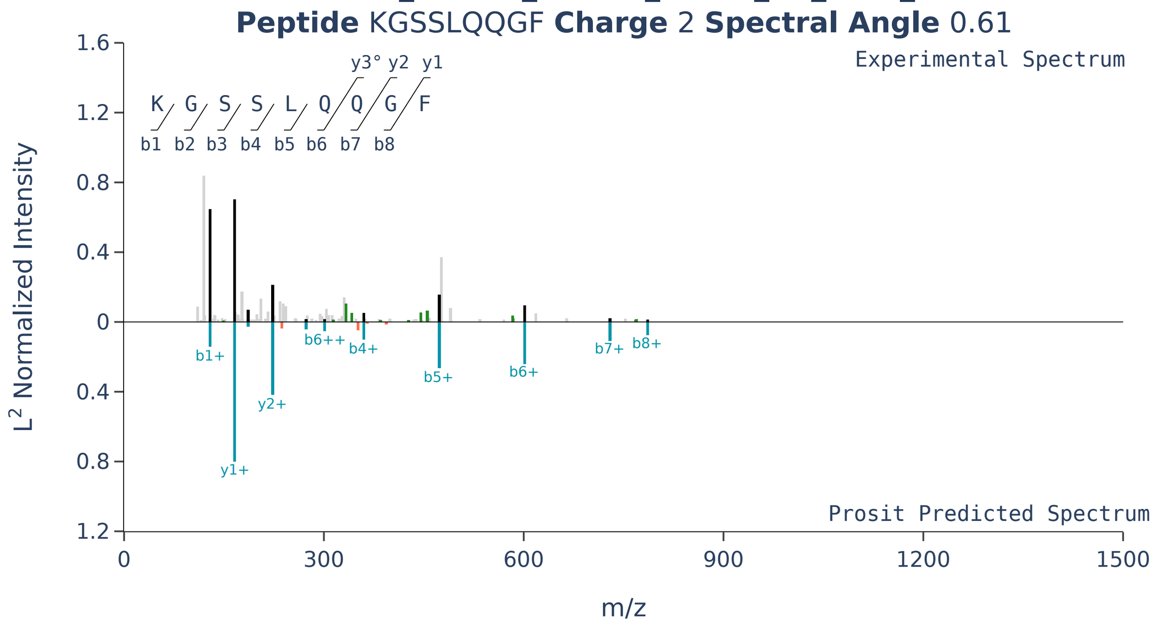


**Source** HRoetschke_211222_160123_HFGoe_IF2_b1R2_1h **Scan** 12450
**Peptide** QEKQTLI **Charge** 2 **Spectral Angle** 0.84

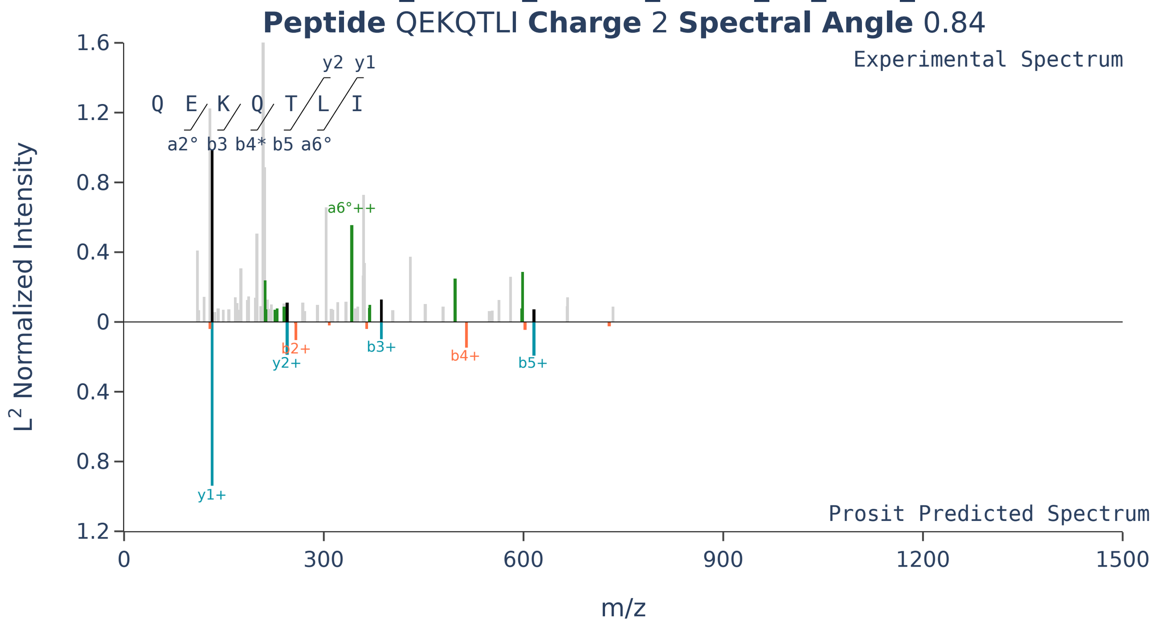


**Source** HRoetschke_211222_160123_HFGoe_IF2_b1R1_4h **Scan** 14206
**Peptide** GELANKMAV **Charge** 2 **Spectral Angle** 0.73

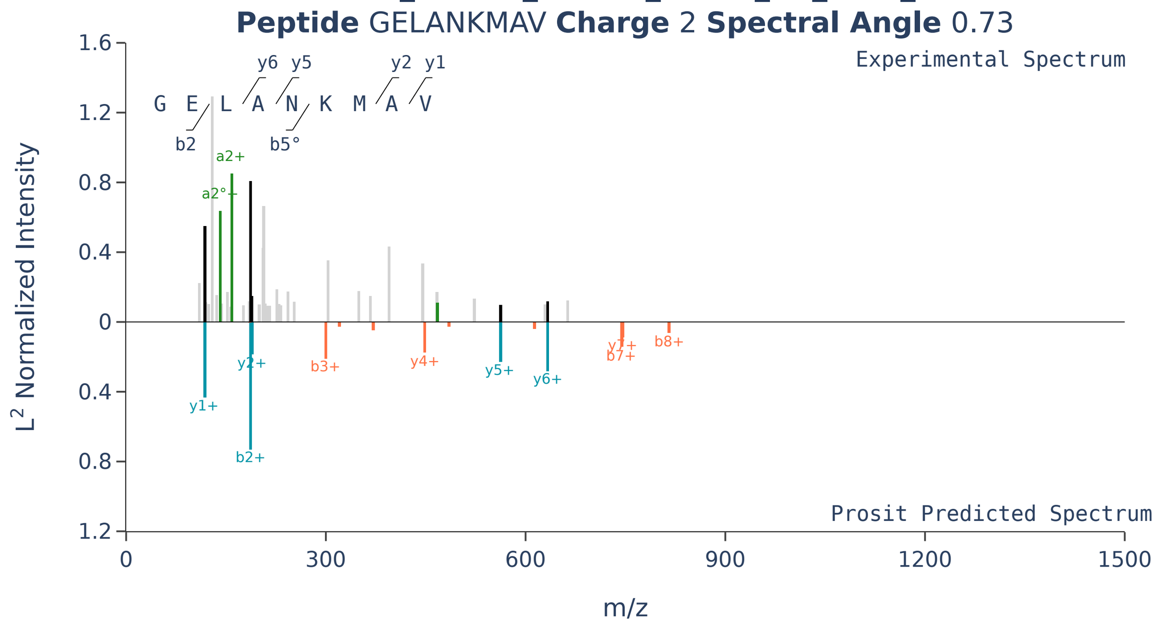

**Source** HRoetschke_211222_160123_HFGoe_IF2_b2R2_4h **Scan** 10659
**Peptide** IGAYHVETENGM **Charge** 2 **Spectral Angle** 0.77

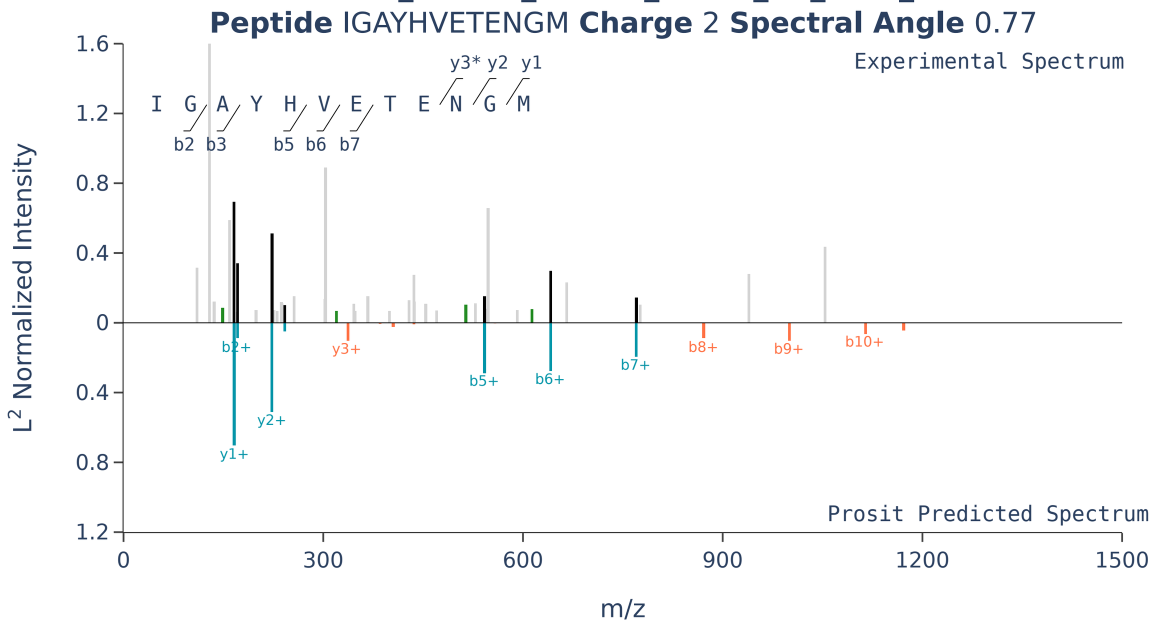


**Source** HRoetschke_211222_160123_HFGoe_IF2_b1R2_24h **Scan** 9573
**Peptide** ELKAVRKGMASGAVIE **Charge** 3 **Spectral Angle** 0.71

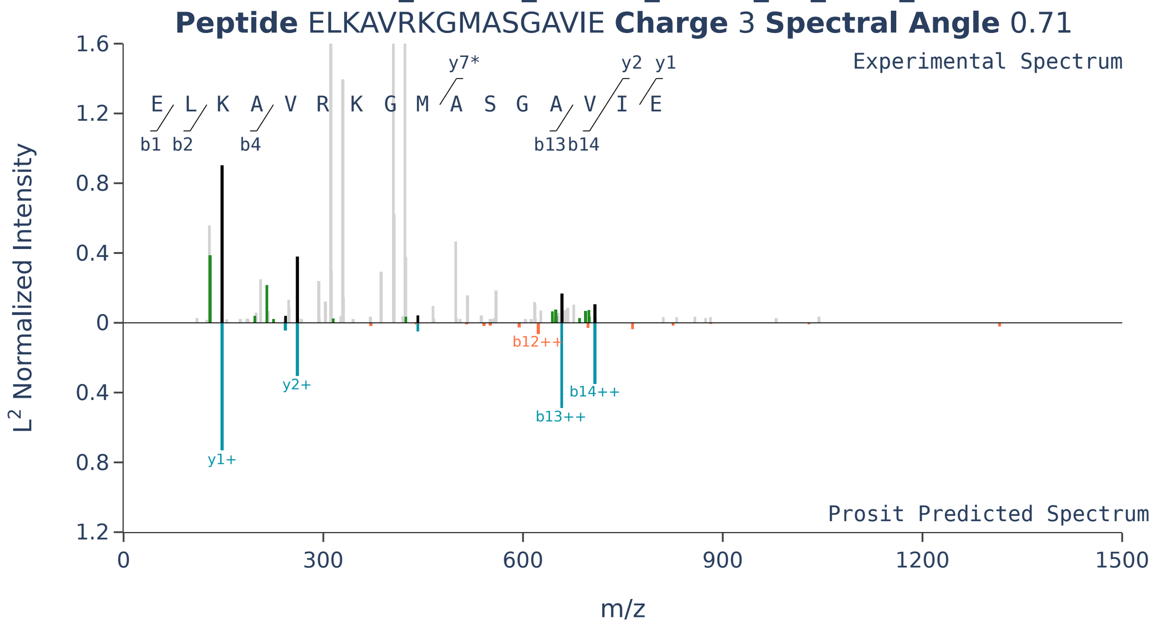


**Source** HRoetschke_211222_160123_HFGoe_IF2_b1R1_4h **Scan** 12879
**Peptide** VTIKTLAAERQTS **Charge** 2 **Spectral Angle** 0.78

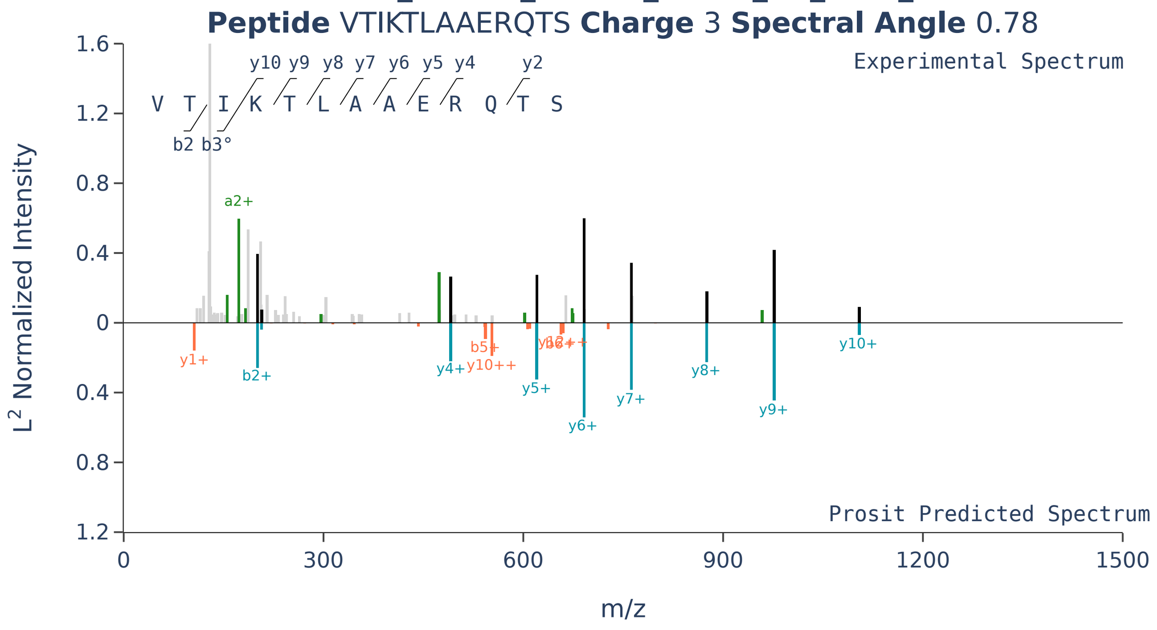


**Source** HRoetschke_211222_160123_HFGoe_IF2_b1R2_2h **Scan** 6245
**Peptide** HVSAKAGTGID **Charge** 3 **Spectral Angle** 0.89

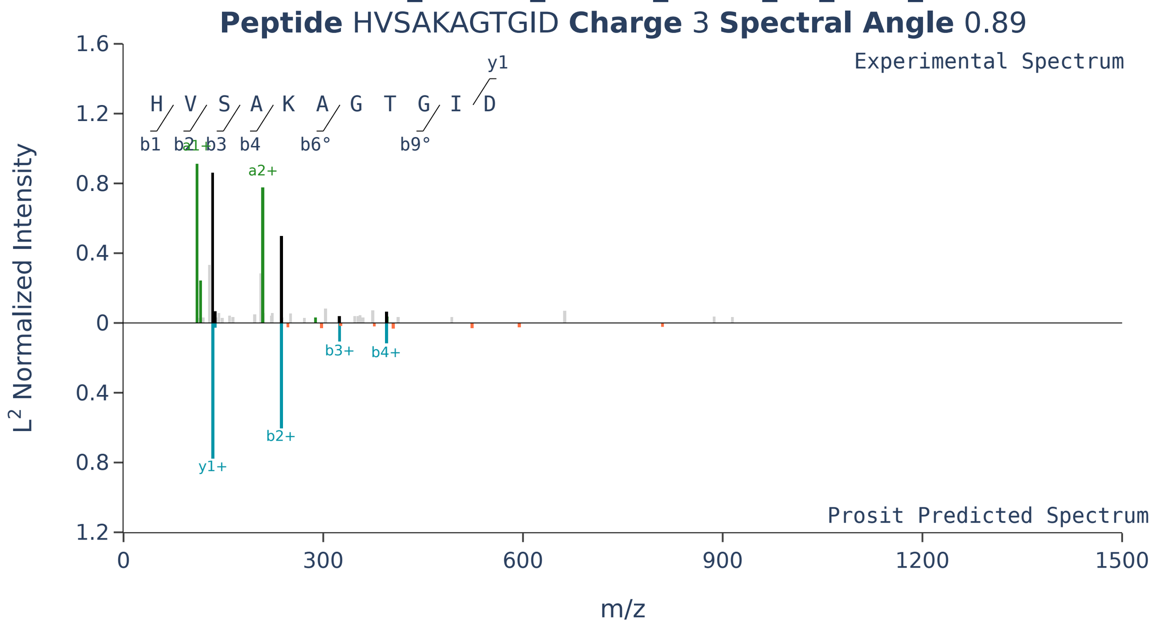


**Source** HRoetschke_211222_160123_HFGoe_IF2_b1R1_4h **Scan** 7714
**Peptide** AVMSDRDTGAAA **Charge** 2 **Spectral Angle** 0.68

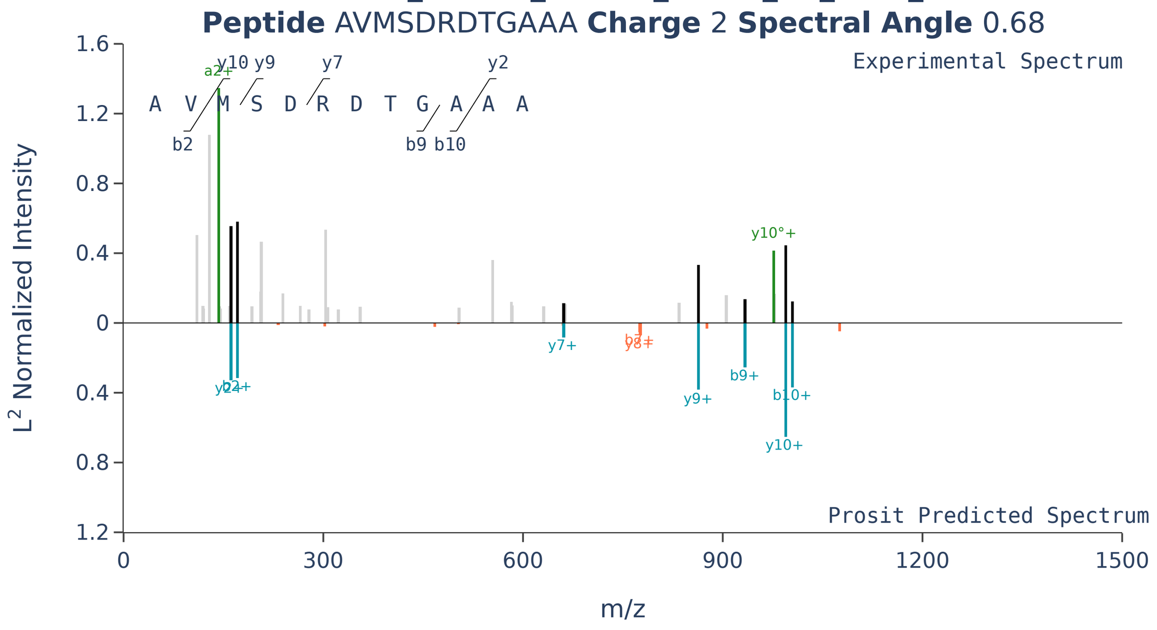


**Source** HRoetschke_211222_160123_HFGoe_IF2_b2R2_4h **Scan** 17174
**Peptide** GAMATINQVID **Charge** 2 **Spectral Angle** 0.69

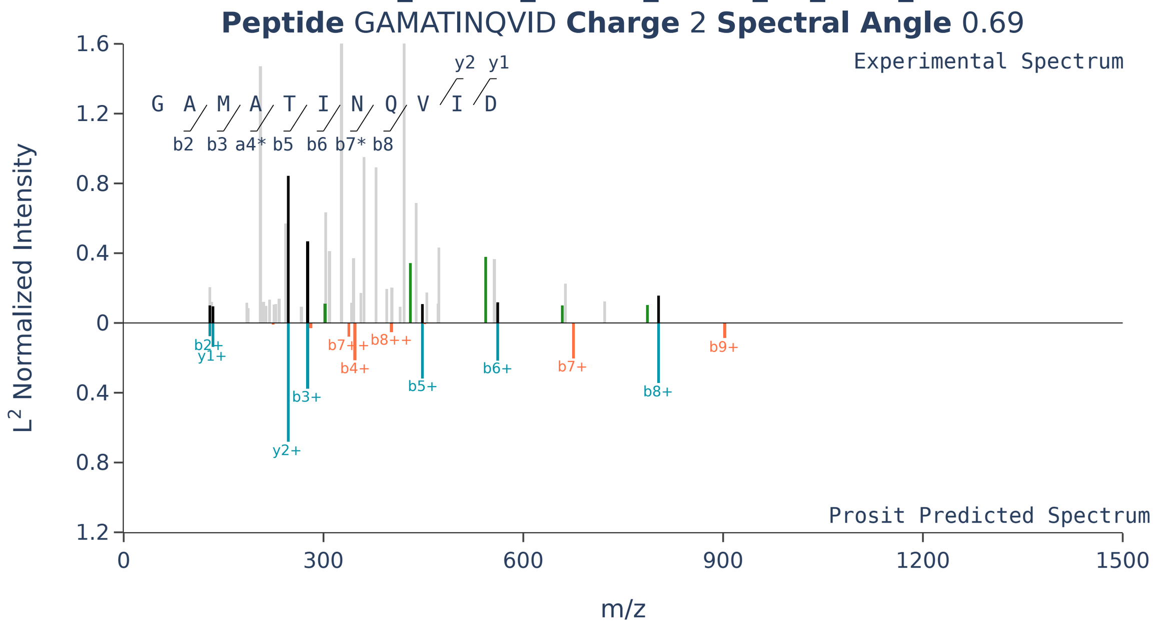


**Source** HRoetschke_211222_160123_HFGoe_IF2_b1R1_2h **Scan** 11437
**Peptide** FADAGIRKSADDSVSAQE **Charge** 3 **Spectral Angle** 0.78

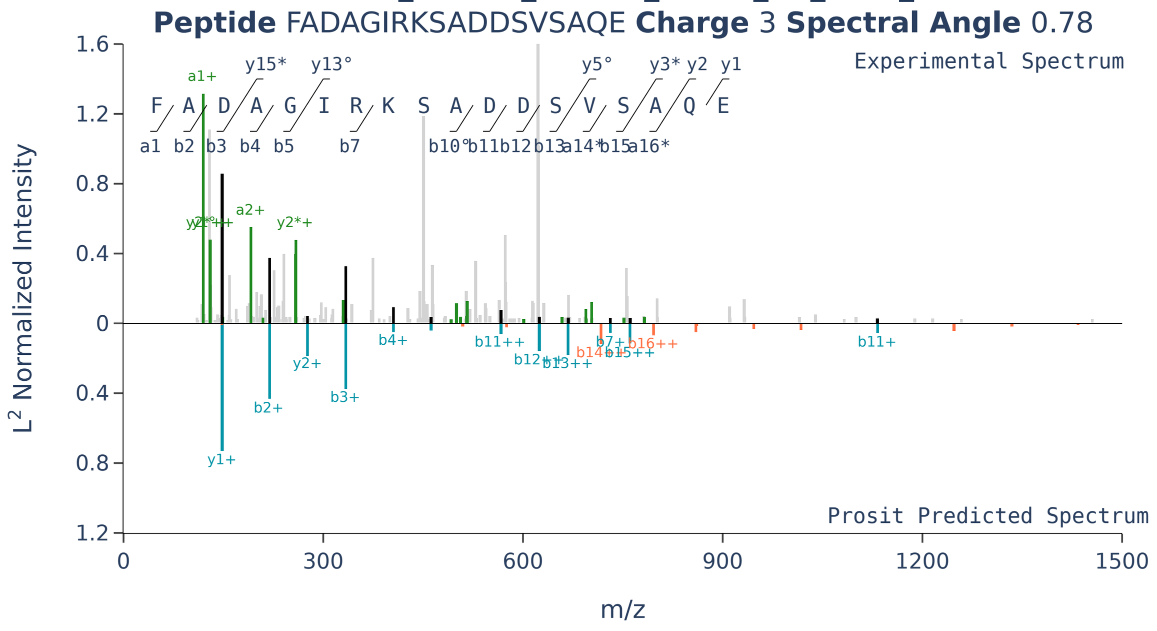


**Source** HRoetschke_211222_160123_HFGoe_IF2_b1R1_4h **Scan** 8830
**Peptide** PGHAAFTSMRARGAQATD **Charge** 4 **Spectral Angle** 0.73

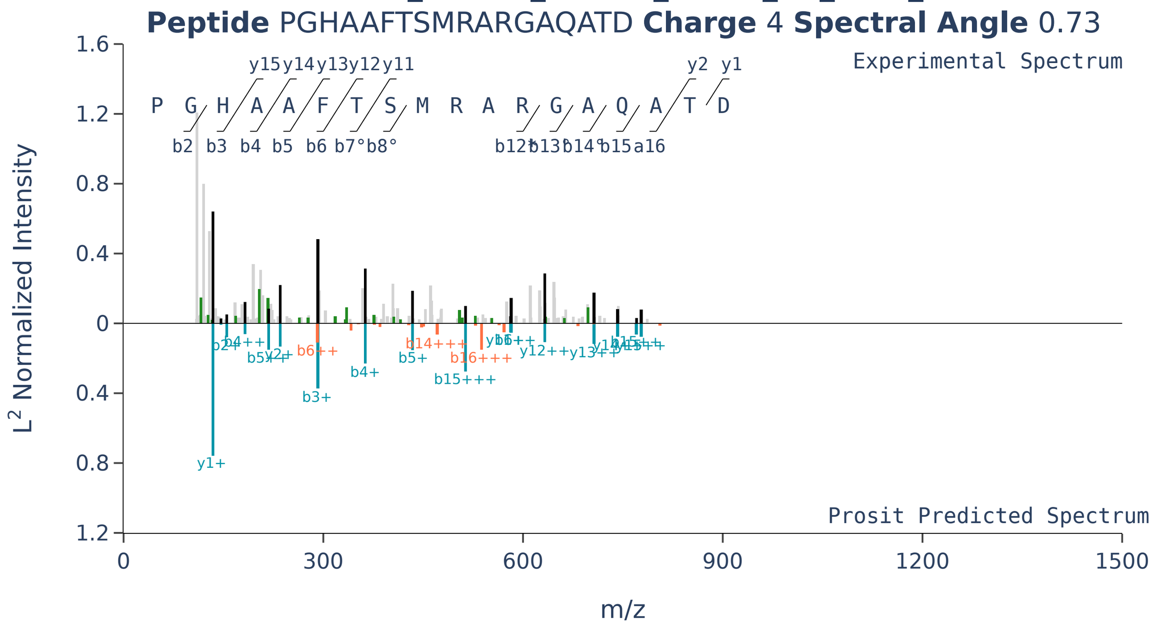


**Source** HRoetschke_211222_160123_HFGoe_IF2_b1R1_4h **Scan** 20299
**Peptide** SLDLRYY **Charge** 2 **Spectral Angle** 0.83

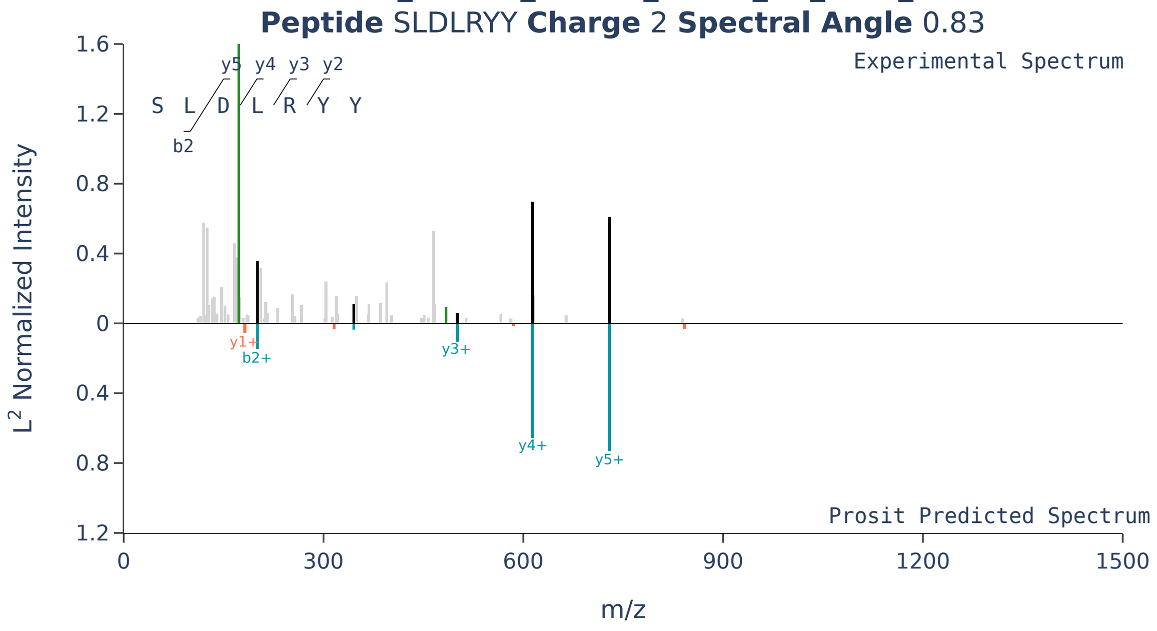


**Source** HRoetschke_211222_160123_HFGoe_IF2_b2R2_2h **Scan** 8595
**Peptide** VLKADVQG **Charge** 2 **Spectral Angle** 0.72

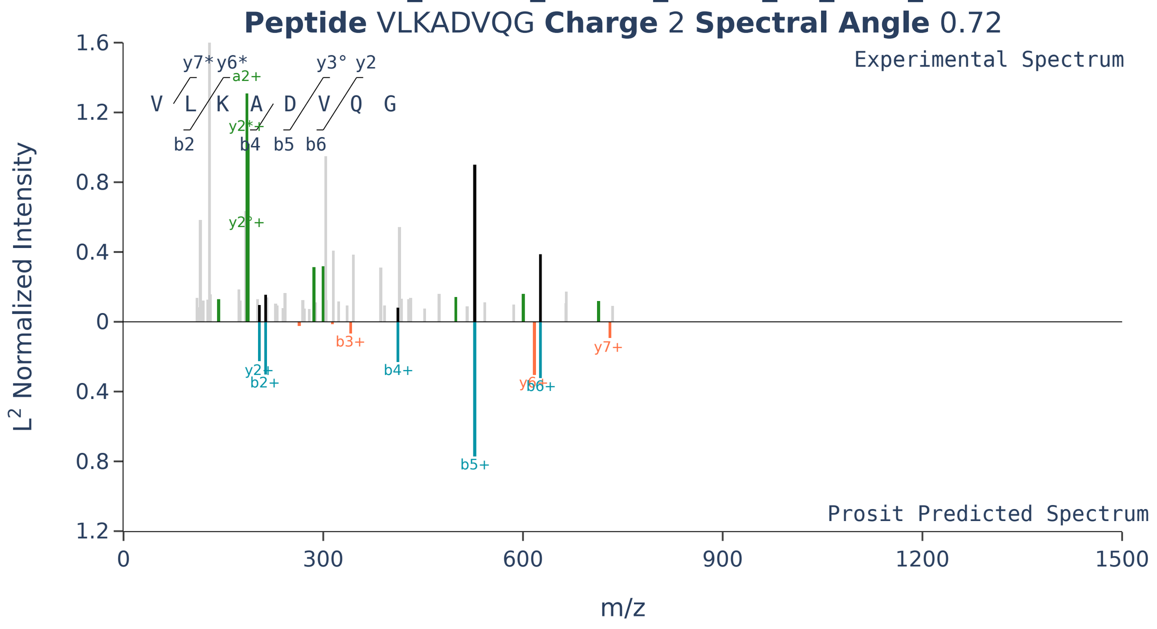


**Source** HRoetschke_211222_160123_HFGoe_IF2_b1R1_2h **Scan** 7536
**Peptide** PAQAVNR **Charge** 1 **Spectral Angle** 0.71

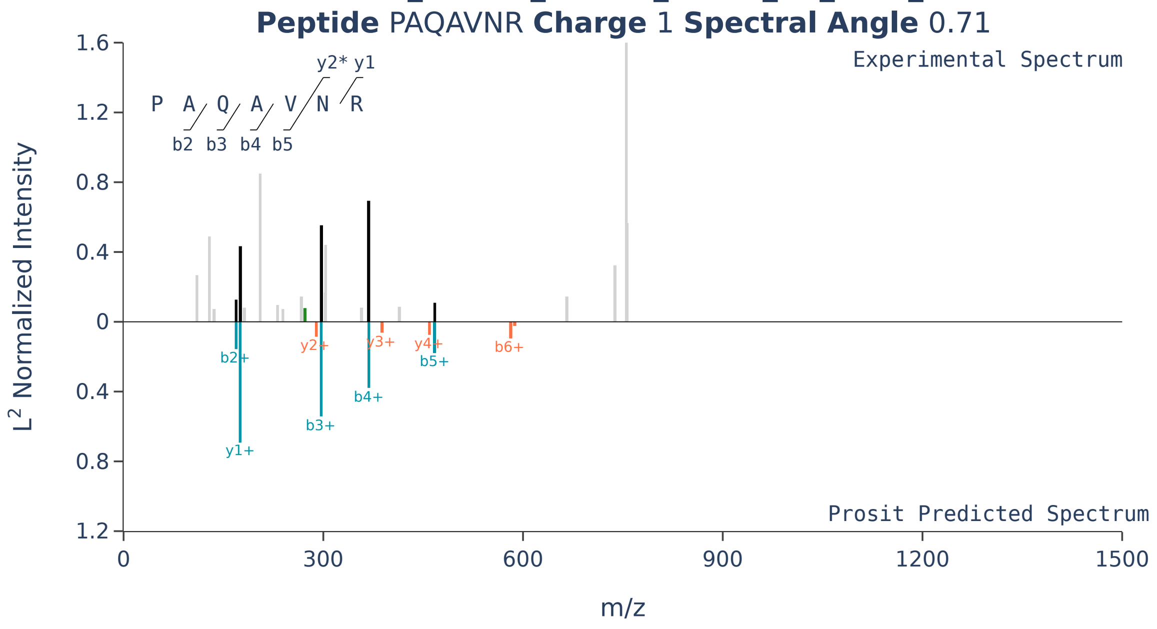


**Source** HRoetschke_211222_160123_HFGoe_IF2_b1R1_24h **Scan** 16598
**Peptide** SDRDTGAAAEPRAPVVTIMG **Charge** 3 **Spectral Angle** 0.78

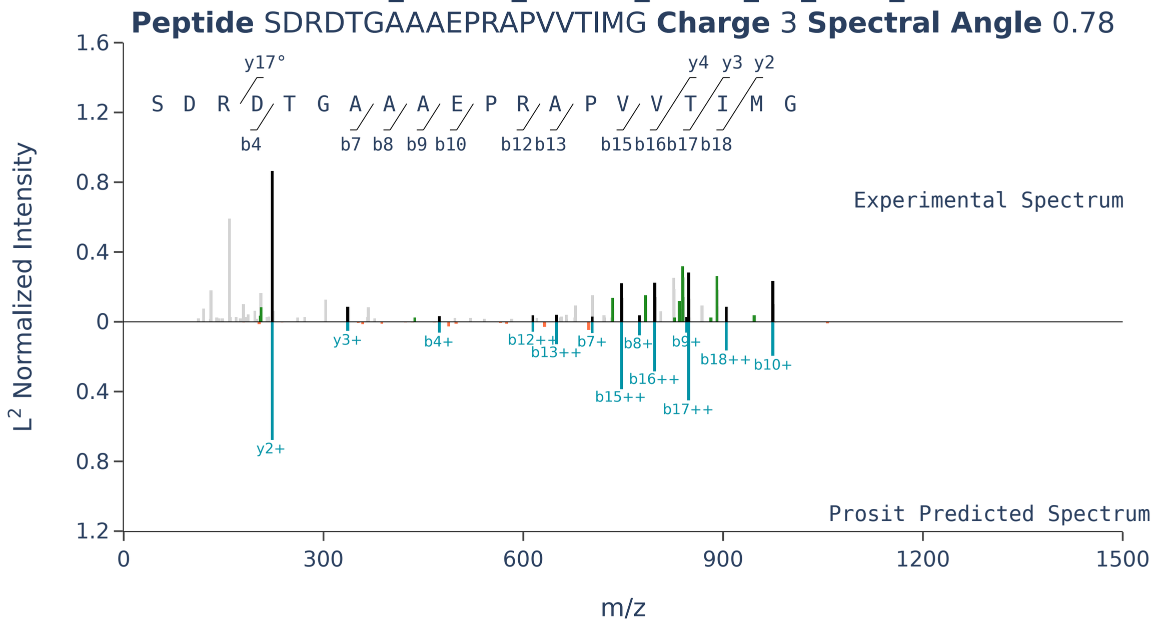


**Source** HRoetschke_211222_160123_HFGoe_IF2_b1R1_4h **Scan** 11294
**Peptide** QQGFQKPAQAVNRDV **Charge** 3 **Spectral Angle** 0.74

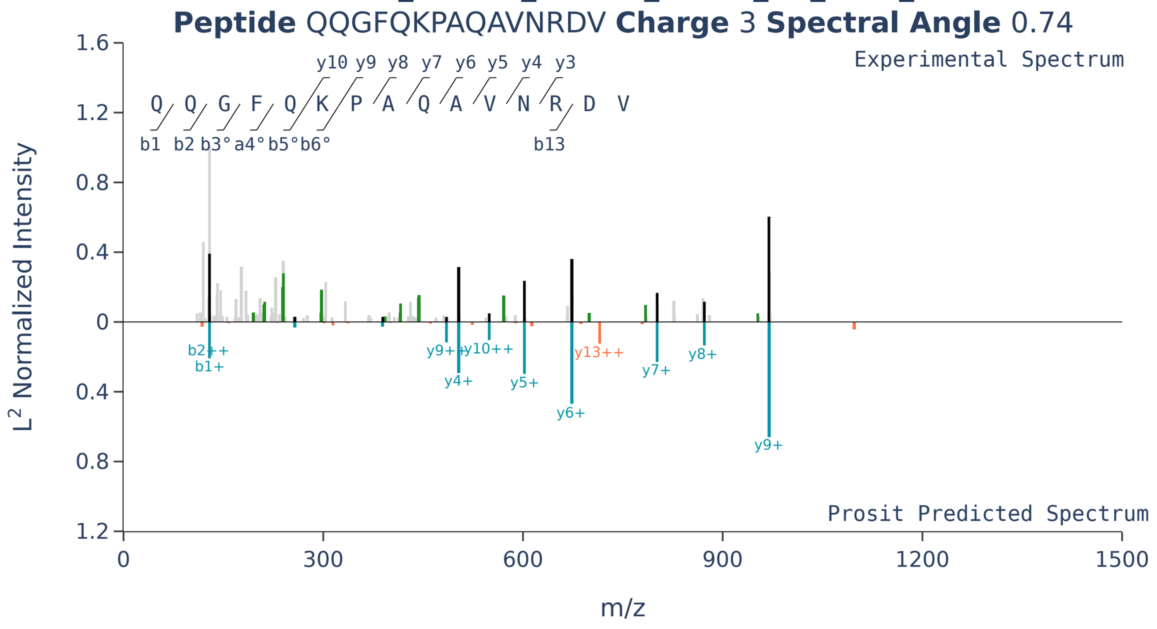


**Source** HRoetschke_211222_160123_HFGoe_IF2_b1R2_2h **Scan** 18027
**Peptide** PRAPVVTL **Charge** 2 **Spectral Angle** 0.82

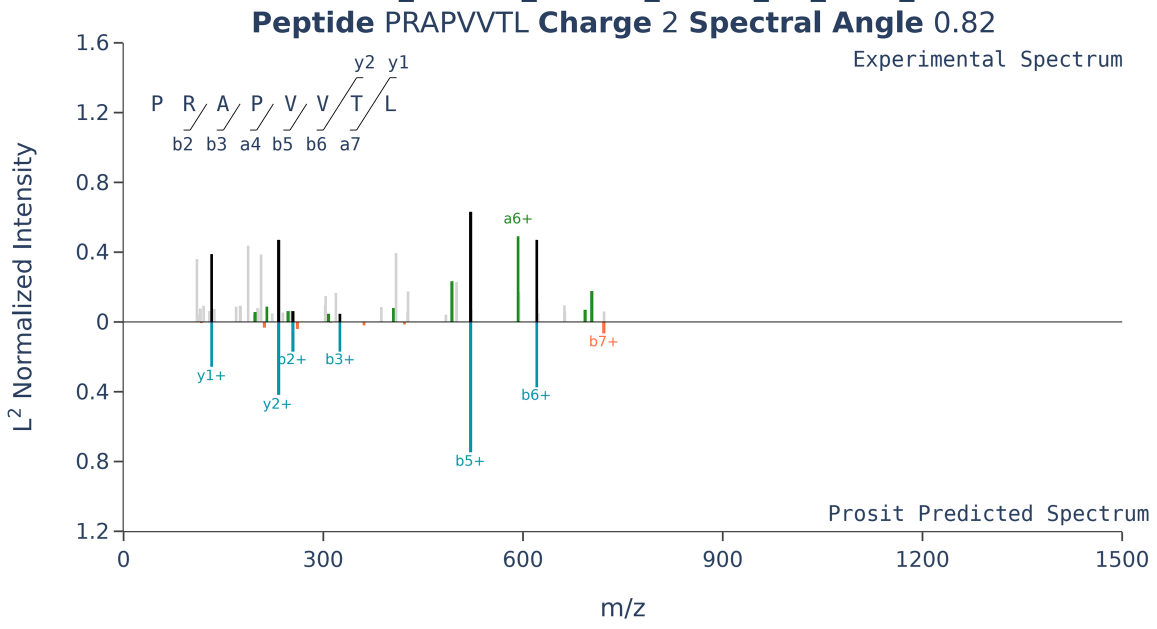


**Source** HRoetschke_211222_160123_HFGoe_IF2_b2R1_24h **Scan** 7557
**Peptide** EGVVKRHNPIRV **Charge** 3 **Spectral Angle** 0.76

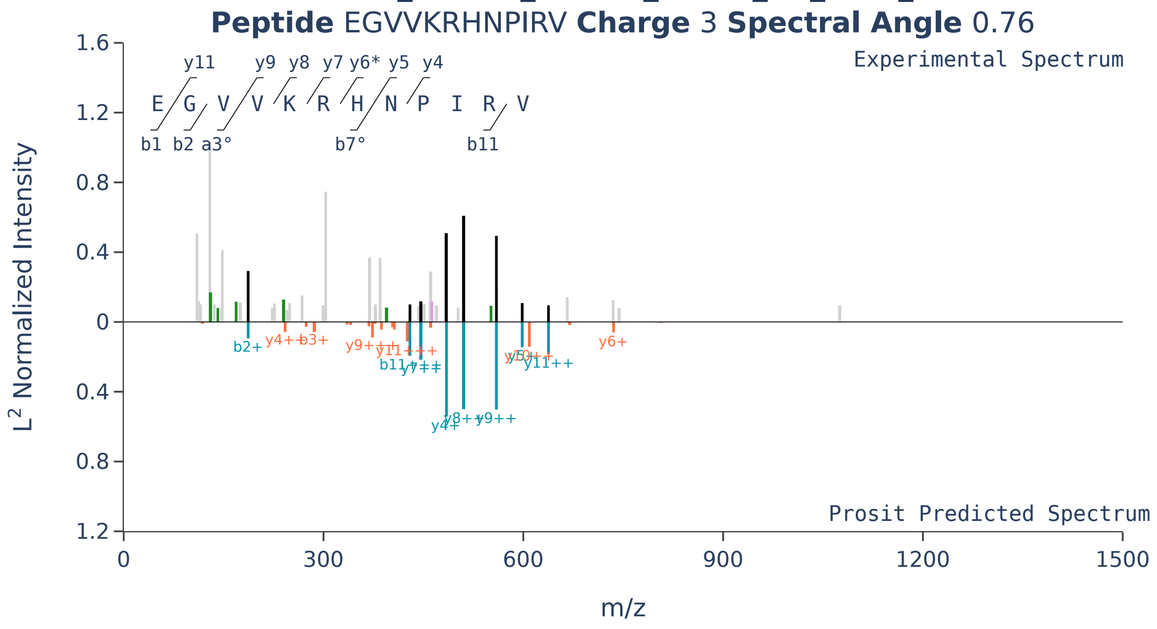

**Source** HRoetschke_211222_160123_HFGoe_IF2_b1R1_4h **Scan** 10362
**Peptide** ALYRQGKFRE **Charge** 3 **Spectral Angle** 0.93

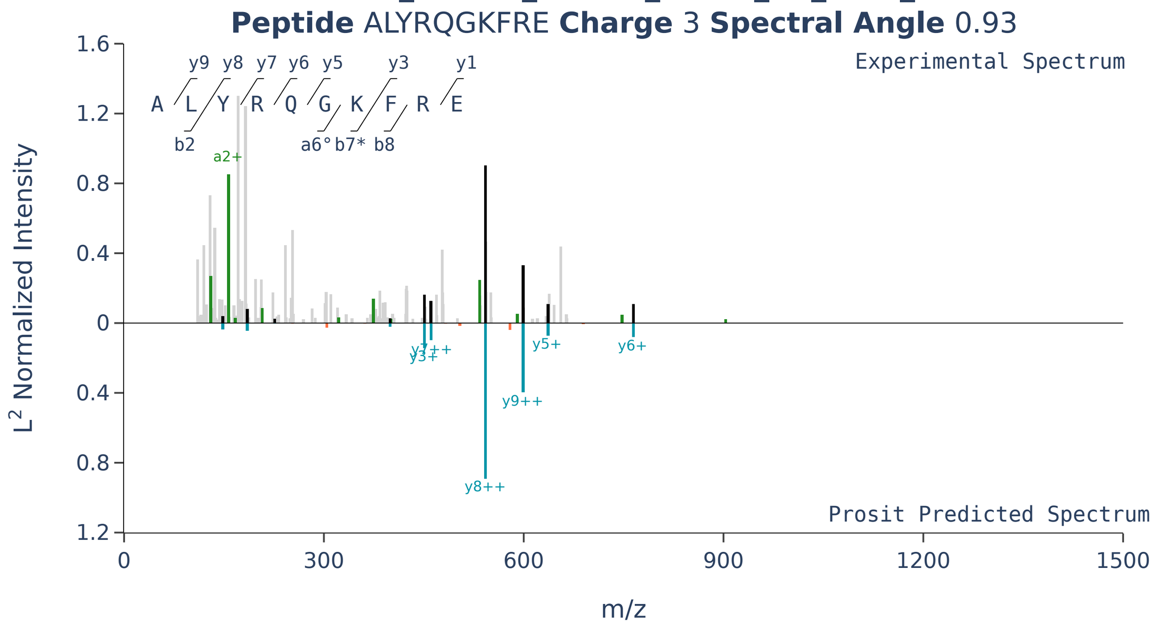


**Source** HRoetschke_211222_160123_HFGoe_IF2_b1R1_4h **Scan** 8926
**Peptide** AEEMGHKVILRRENELE **Charge** 4 **Spectral Angle** 0.7

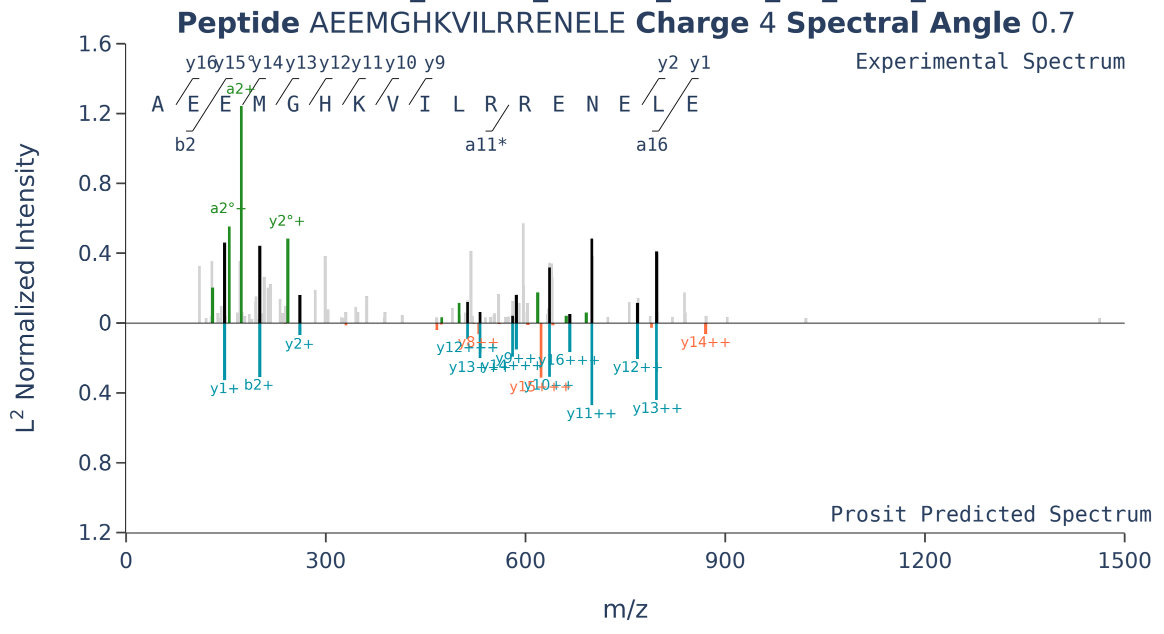


**Source** HRoetschke_211222_160123_HFGoe_IF2_b1R1_4h **Scan** 11382
**Peptide** GAYHVETENGM **Charge** 2 **Spectral Angle** 0.75

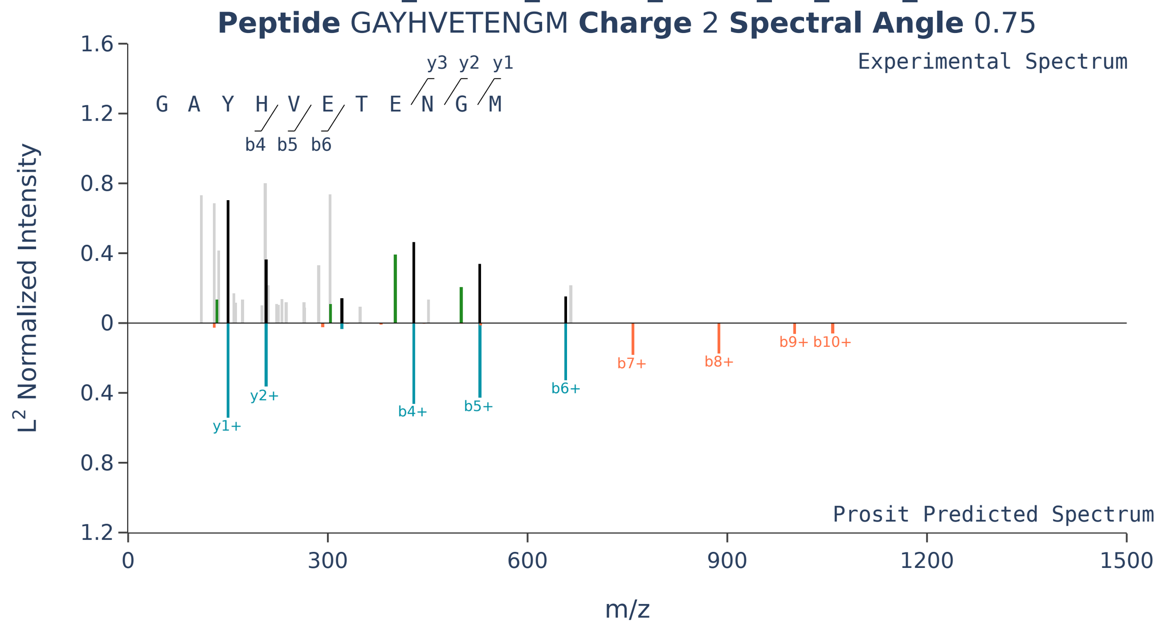


**Source** HRoetschke_211222_160123_HFGoe_IF2_b2R1_1h **Scan** 23349
**Peptide** AGPSIPVEI **Charge** 1 **Spectral Angle** 0.83

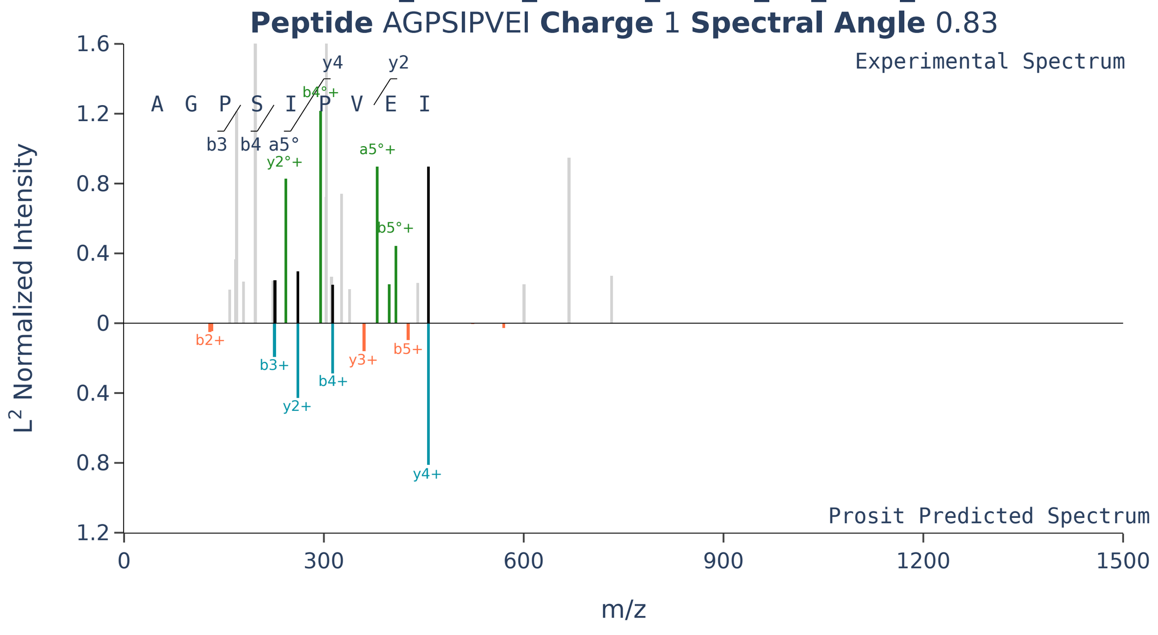


**Source** HRoetschke_211222_160123_HFGoe_IF2_b1R1_4h **Scan** 10122
**Peptide** GVVKRHNPIRVL **Charge** 3 **Spectral Angle** 0.84

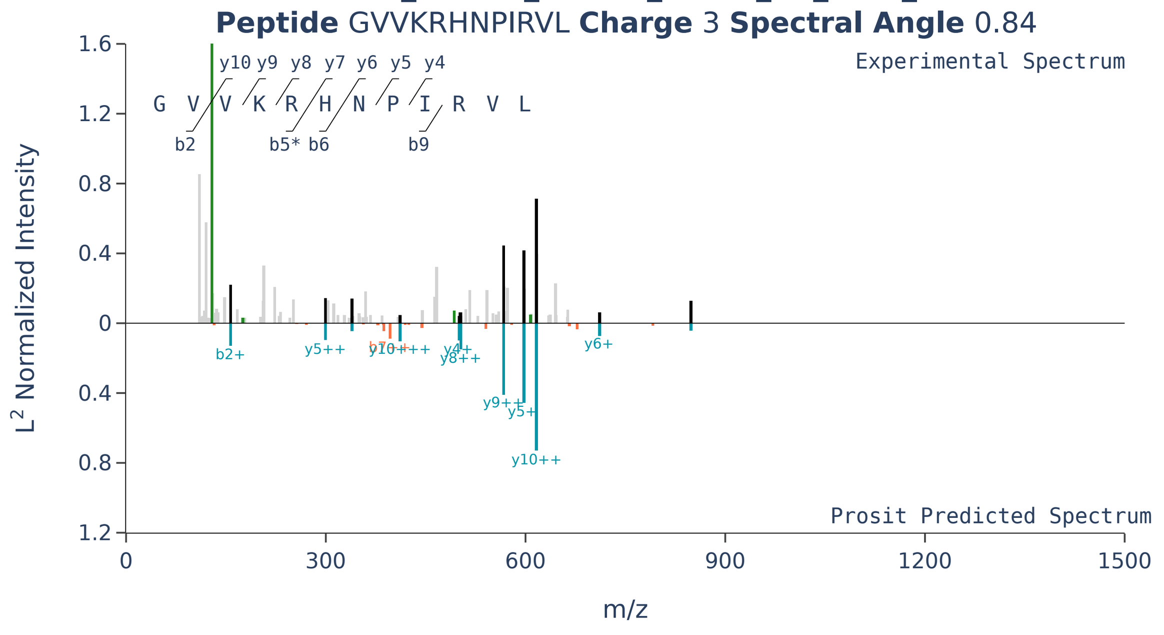


**Source** HRoetschke_211222_160123_HFGoe_IF2_b2R2_4h **Scan** 7040
**Peptide** ASARKVIEAE **Charge** 2 **Spectral Angle** 0.74

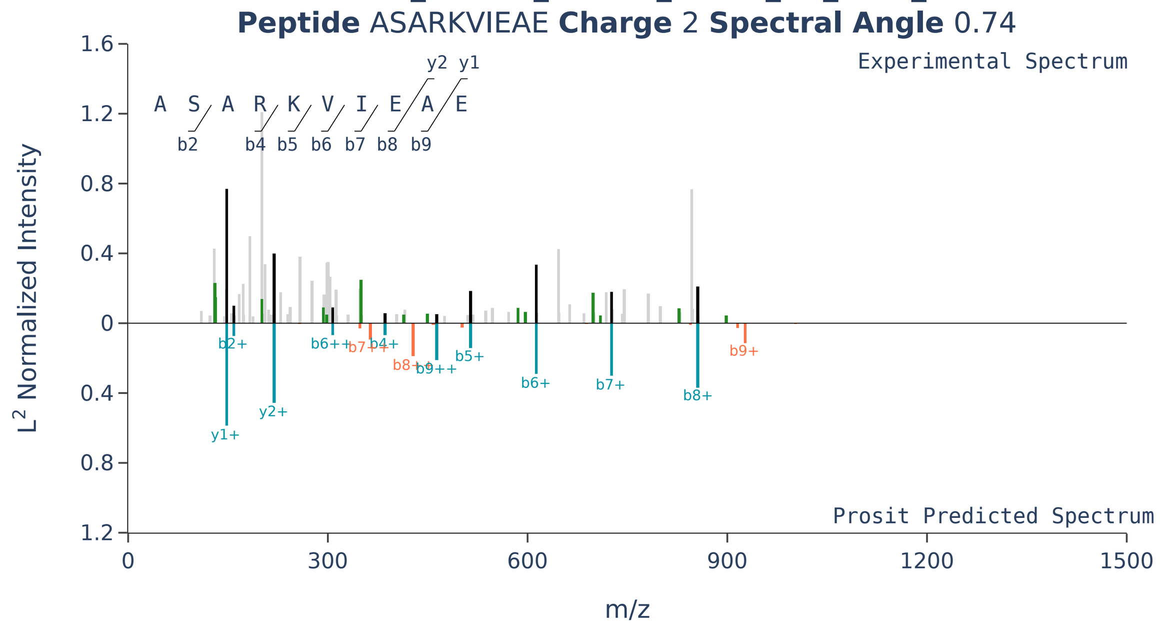


**Source** HRoetschke_211222_160123_HFGoe_IF2_b1R1_24h **Scan** 17538
**Peptide** ESLDLRY **Charge** 2 **Spectral Angle** 0.8

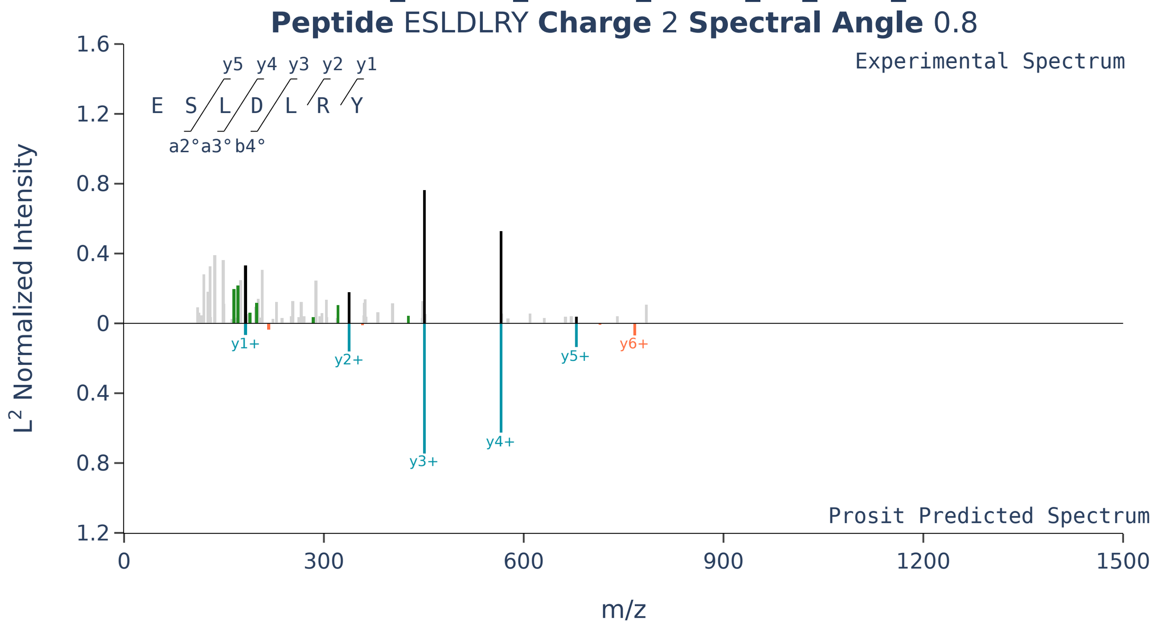


**Source** HRoetschke_211222_160123_HFGoe_IF2_b2R2_24h **Scan** 7166
**Peptide** ADASARKVLE **Charge** 1 **Spectral Angle** 0.86

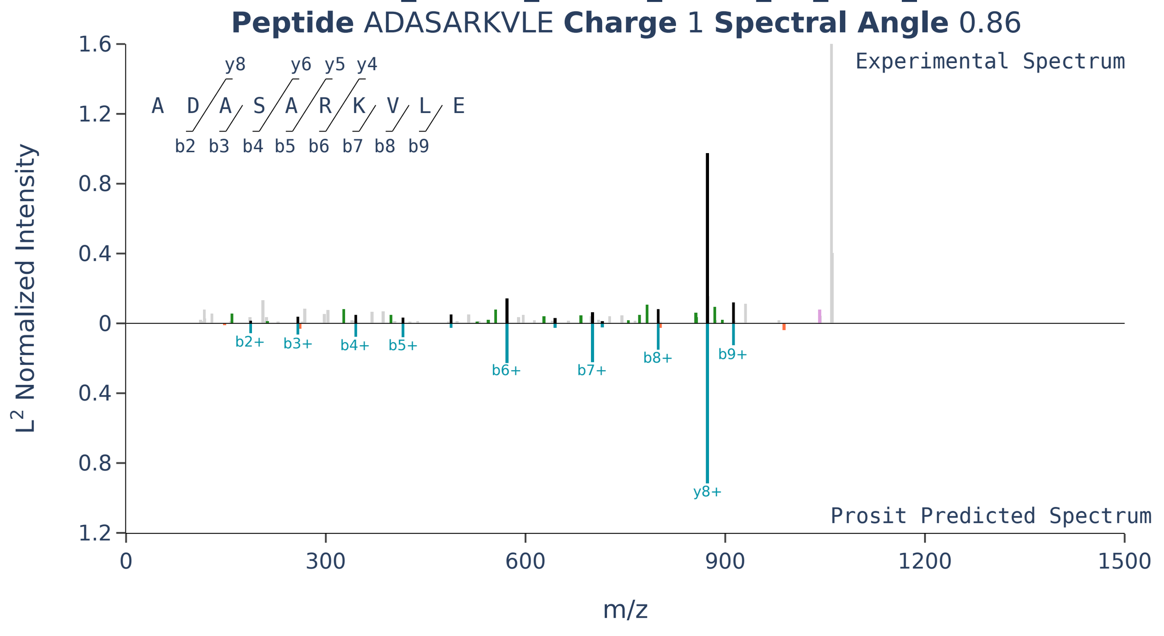


**Source** HRoetschke_211222_160123_HFGoe_IF2_b1R2_24h **Scan** 19671
**Peptide** AQVPVVVA **Charge** 1 **Spectral Angle** 0.88

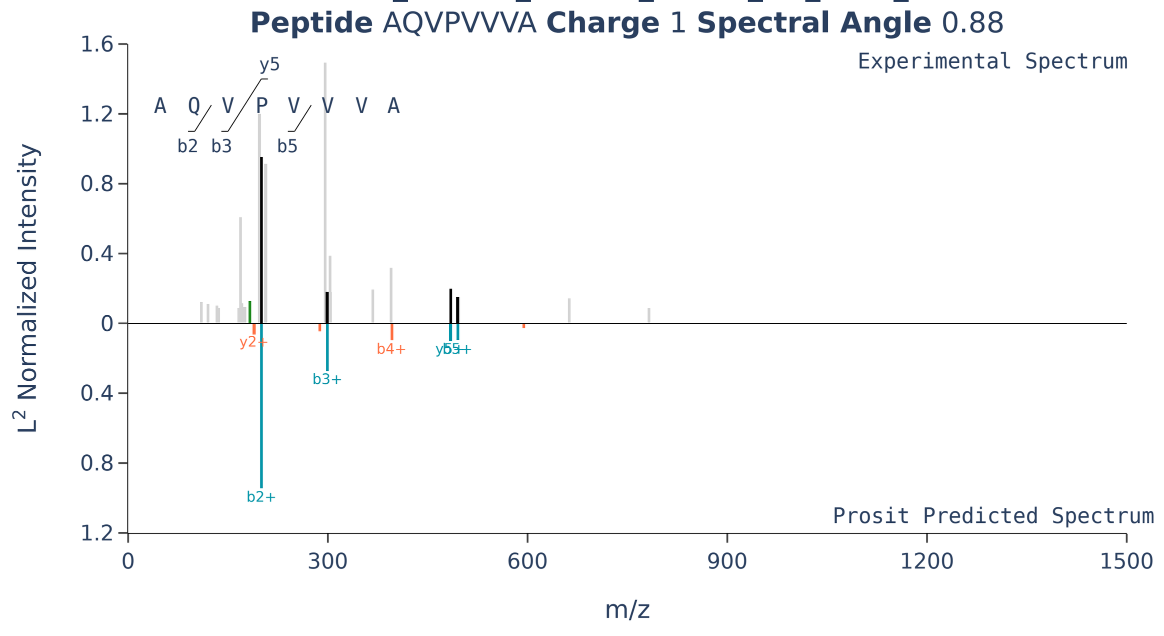


**Source** HRoetschke_211222_160123_HFGoe_IF2_b1R1_4h **Scan** 12247
**Peptide** AGIRKSADDSVSAQEKQTLLD **Charge** 3 **Spectral Angle** 0.75

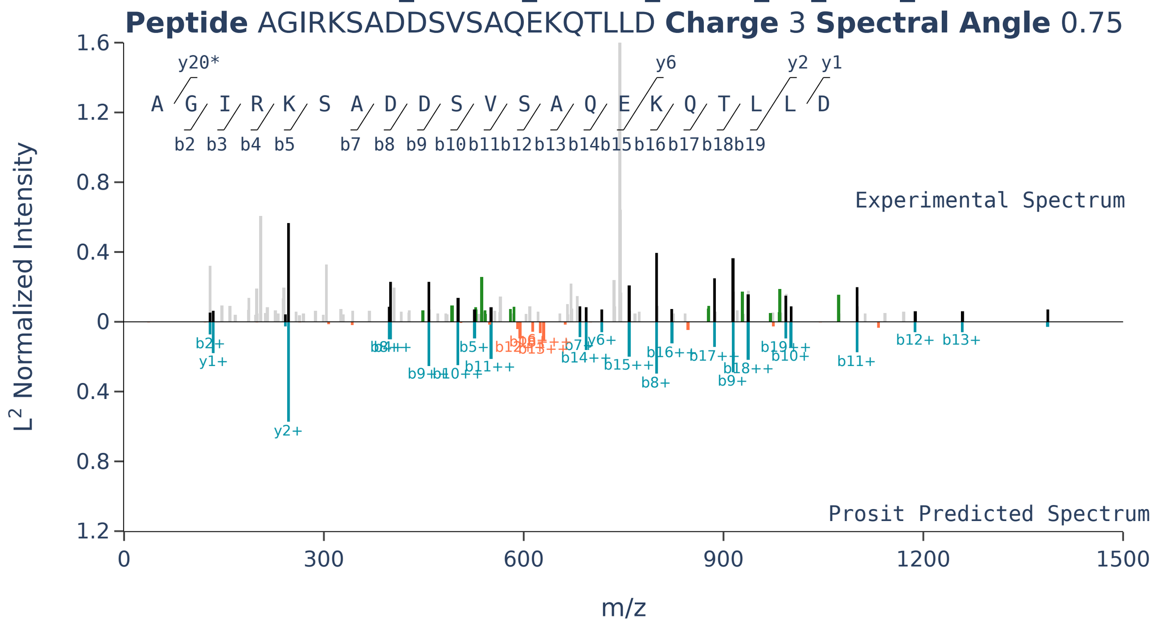


**Source** HRoetschke_211222_160123_HFGoe_IF2_b1R2_2h **Scan** 12407
**Peptide** VSAQEKQTLLD **Charge** 2 **Spectral Angle** 0.79

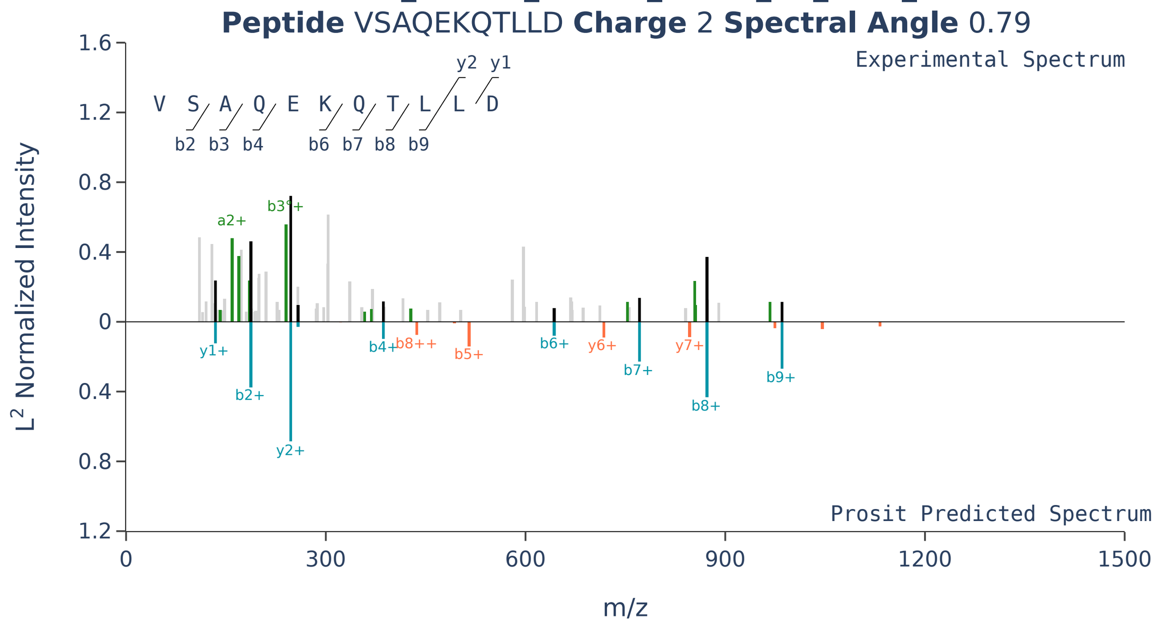


**Source** HRoetschke_211222_160123_HFGoe_IF2_b1R1_24h **Scan** 7600
**Peptide** QEKQTLIDH **Charge** 2 **Spectral Angle** 0.84

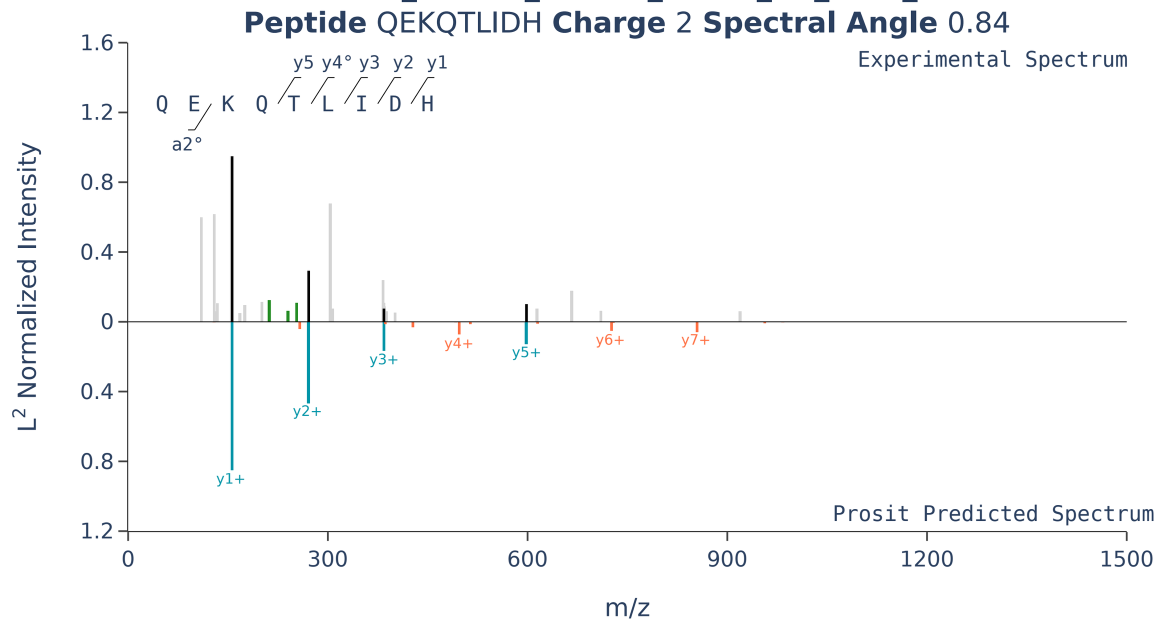


**Source** HRoetschke_211222_160123_HFGoe_IF2_b1R1_2h **Scan** 7338
**Peptide** EKKAREVAL **Charge** 2 **Spectral Angle** 0.83

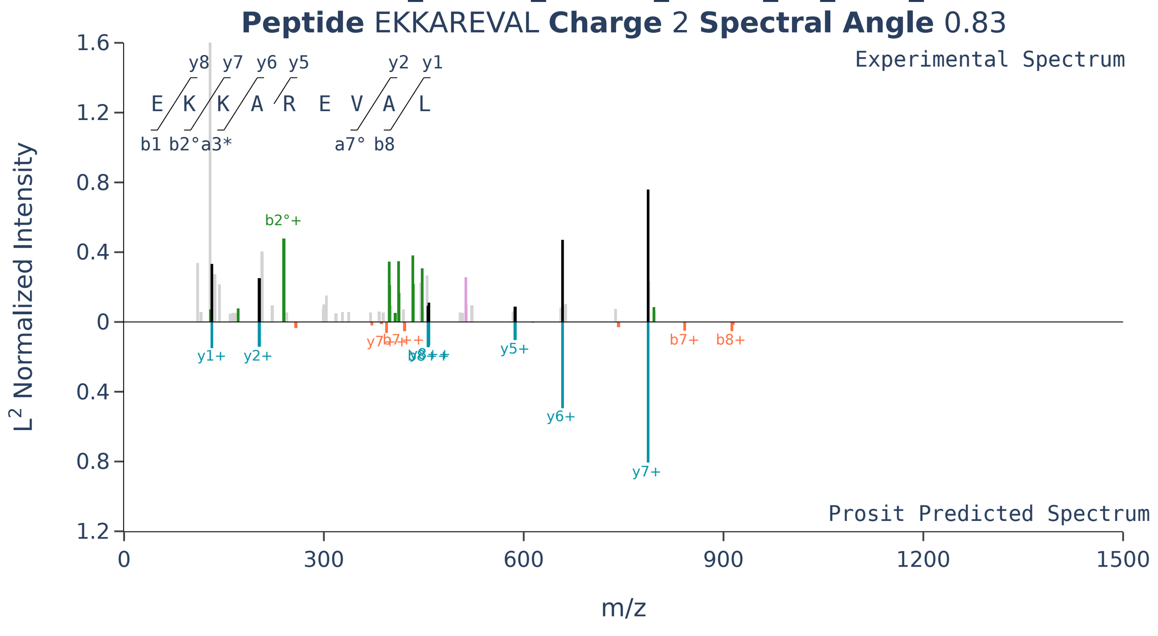


**Source** HRoetschke_211222_160123_HFGoe_IF2_b1R1_4h **Scan** 15019
**Peptide** DKGRGPVATVLVRE **Charge** 4 **Spectral Angle** 0.87

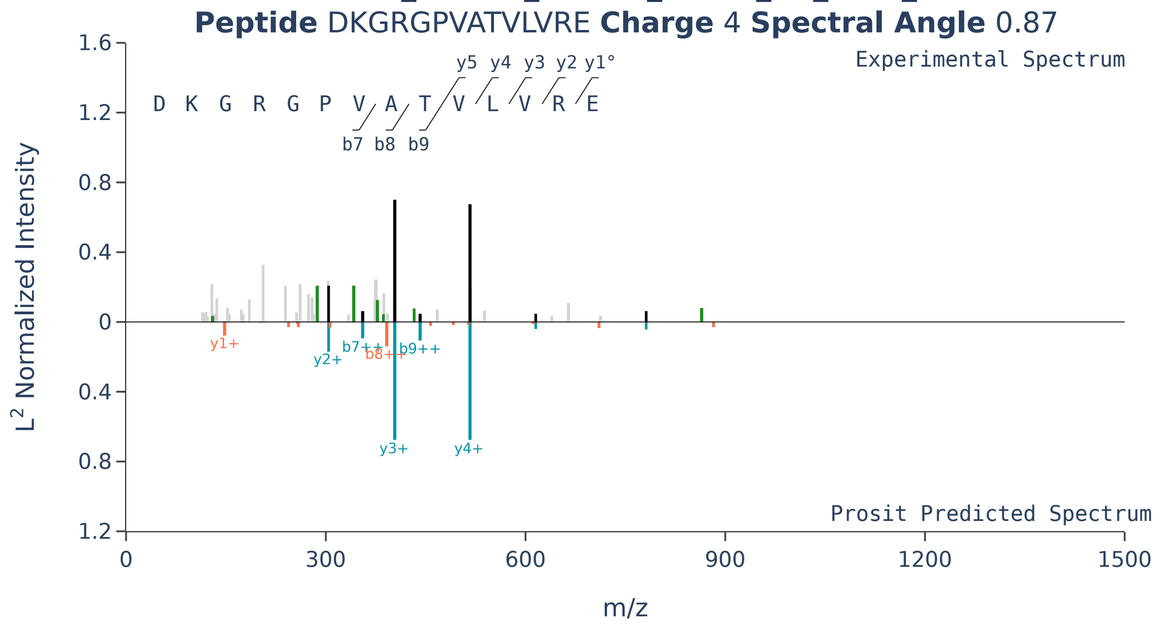


**Source** HRoetschke_211222_160123_HFGoe_IF2_b2R1_24h **Scan** 6348
**Peptide** ADAGIRKSADDSVSAQEKQ **Charge** 3 **Spectral Angle** 0.75

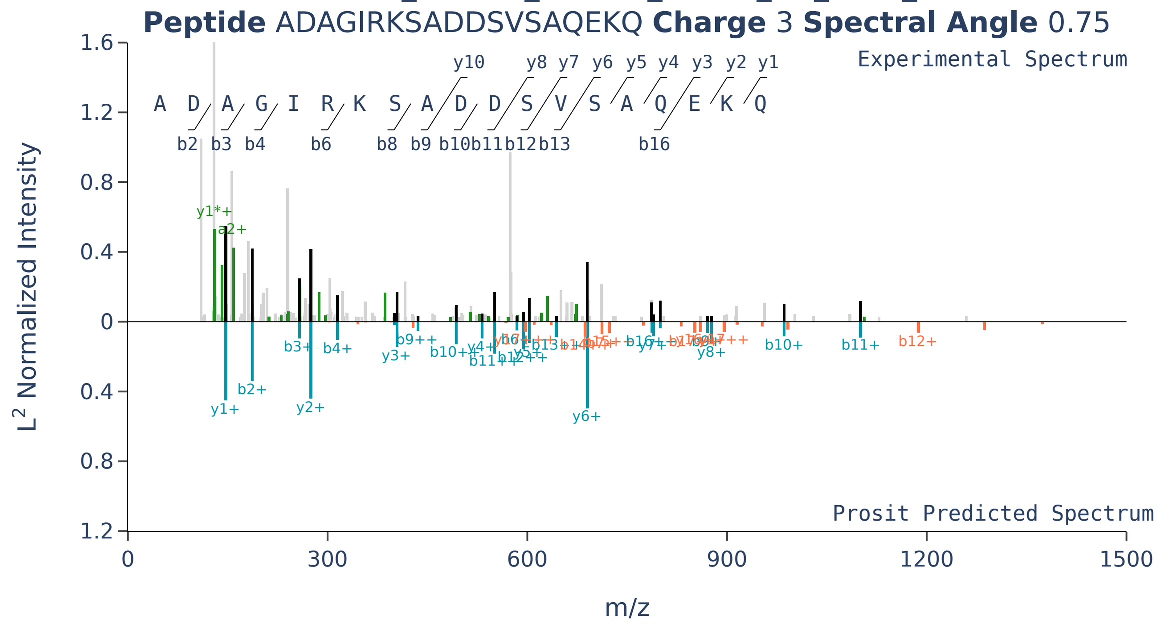

**Source** HRoetschke_211222_160123_HFGoe_IF2_b2R2_24h **Scan** 10901
**Peptide** KSKLENMFANMTE **Charge** 3 **Spectral Angle** 0.67

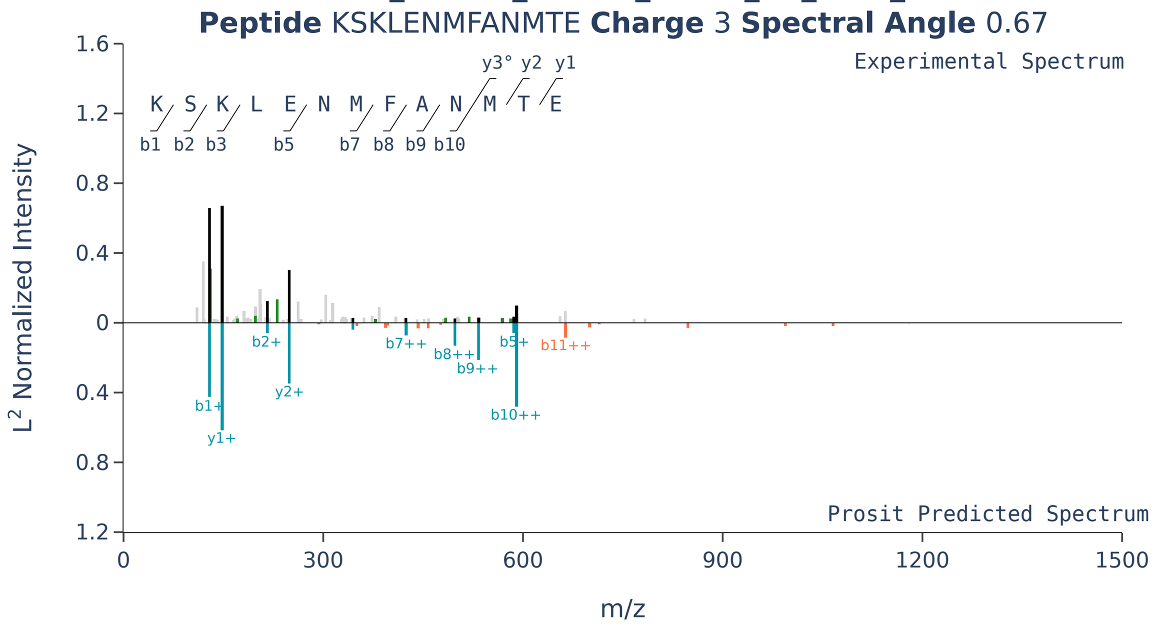


**Source** HRoetschke_211222_160123_HFGoe_IF2_b1R2_24h **Scan** 16254
**Peptide** DSVSAQEKQTLI **Charge** 2 **Spectral Angle** 0.71

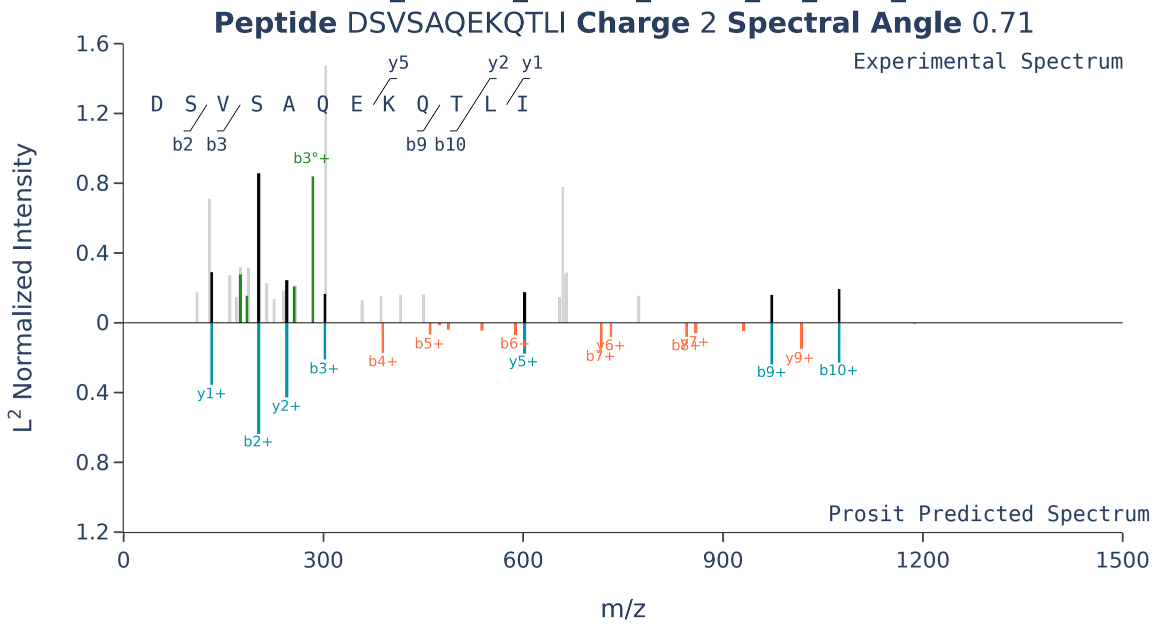


**Source** HRoetschke_211222_160123_HFGoe_IF2_b1R1_2h **Scan** 10862
**Peptide** KGSSLQQGFQ **Charge** 2 **Spectral Angle** 0.72

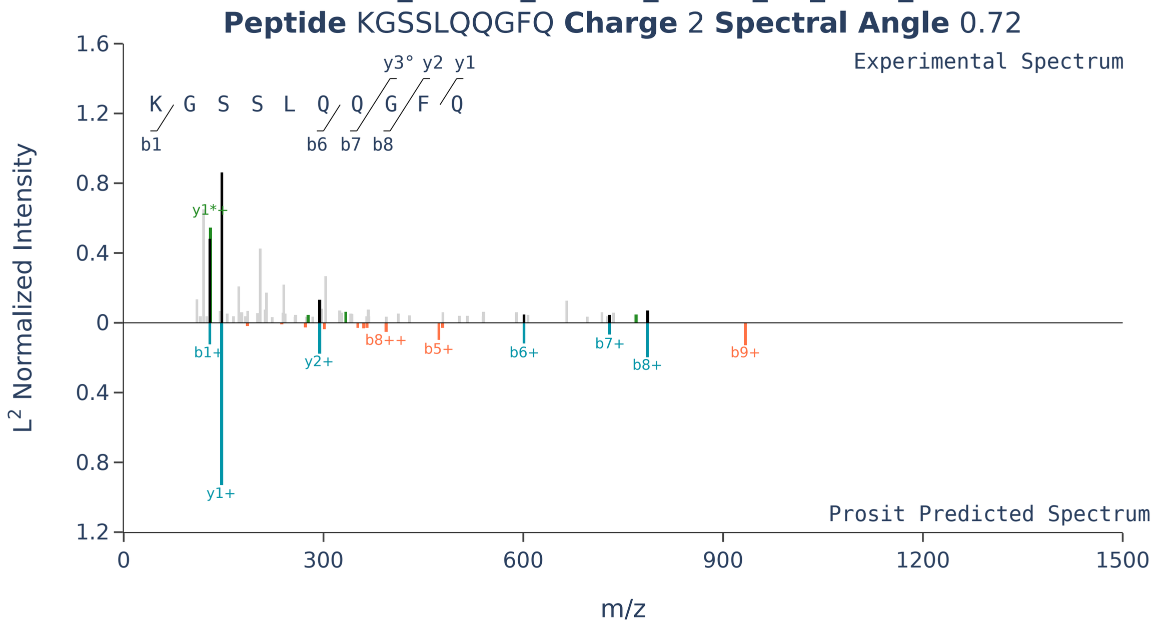


**Source** HRoetschke_211222_160123_HFGoe_IF2_b2R2_4h **Scan** 8213
**Peptide** FQKPAQAVN **Charge** 2 **Spectral Angle** 0.74

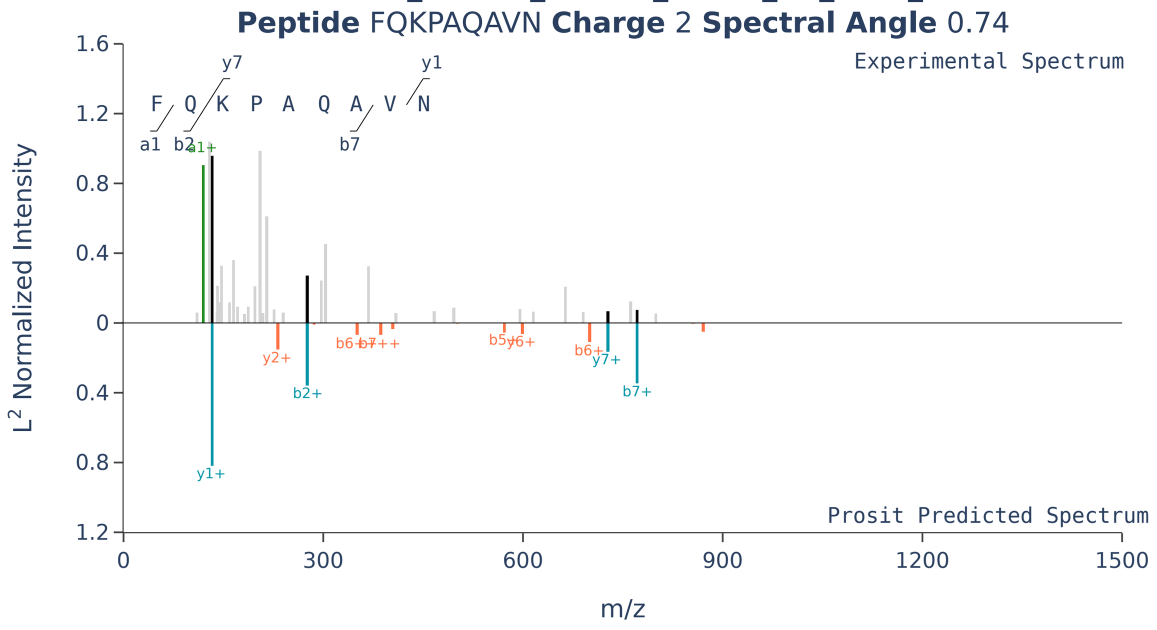


**Source** HRoetschke_211222_160123_HFGoe_IF2_b1R1_4h **Scan** 7939
**Peptide** NAEPTEDSSDYHVTTSQHARQAE **Charge** 4 **Spectral Angle** 0.75

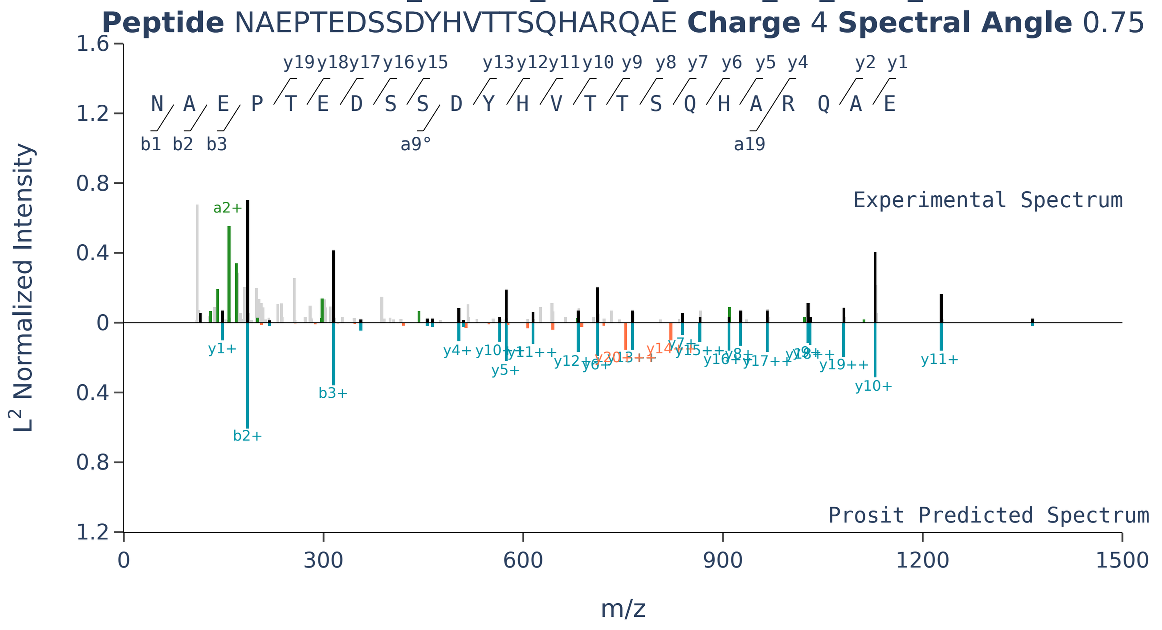


**Source** HRoetschke_211222_160123_HFGoe_IF2_b2R1_24h **Scan** 10479
**Peptide** LDTPGHAAFTSMRARGA **Charge** 4 **Spectral Angle** 0.68

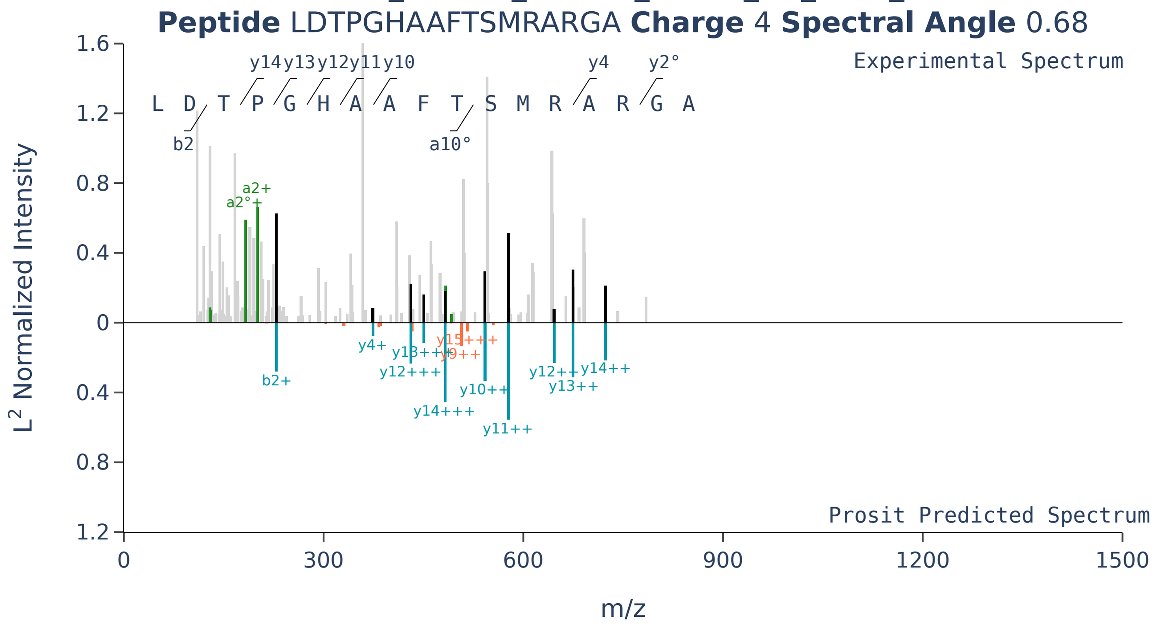


**Source** HRoetschke_211222_160123_HFGoe_IF2_b1R1_4h **Scan** 8956
**Peptide** VHVSAKAGTGIDE **Charge** 2 **Spectral Angle** 0.86

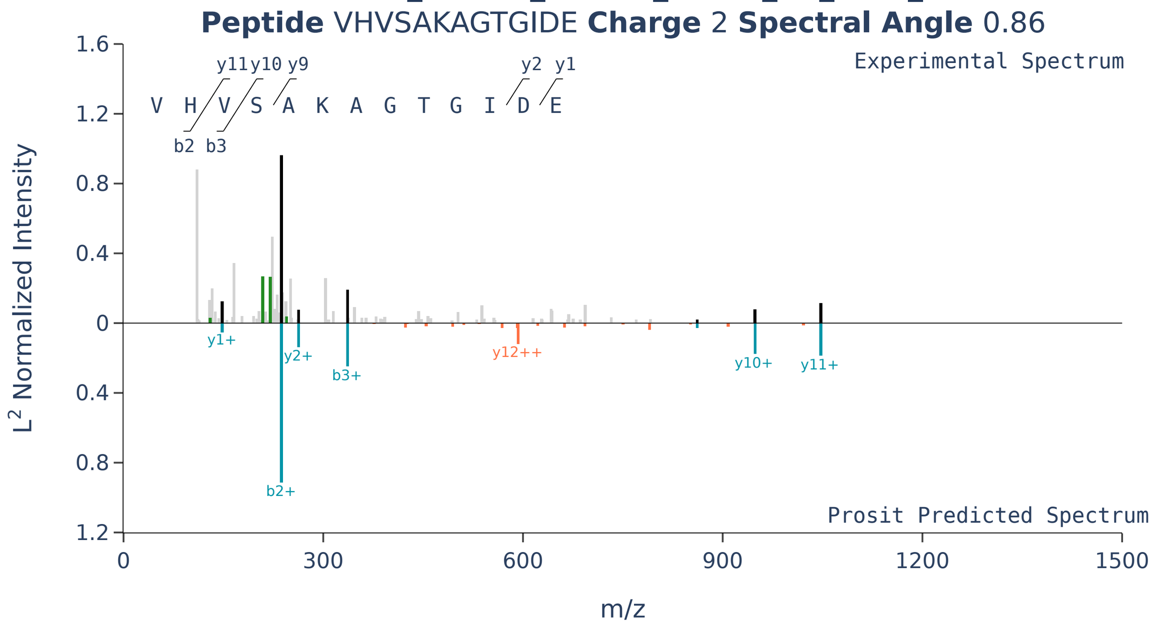


**Source** HRoetschke_211222_160123_HFGoe_IF2_b1R1_4h **Scan** 20236
**Peptide** LGLSGVPAAGD **Charge** 1 **Spectral Angle** 0.89

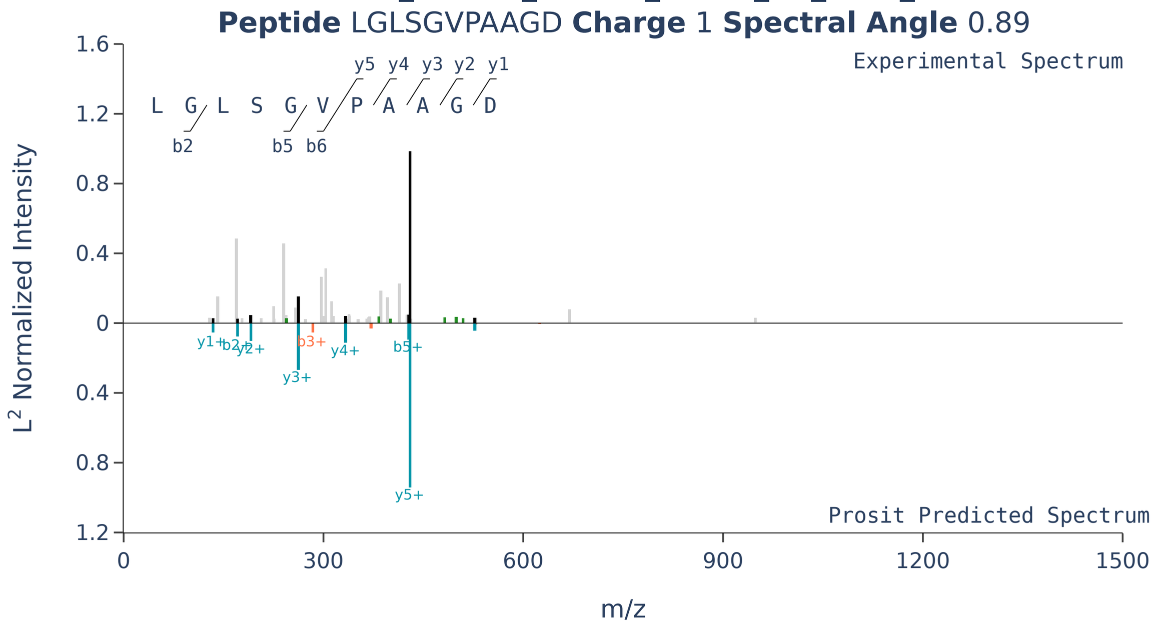


**Source** HRoetschke_211222_160123_HFGoe_IF2_b1R1_4h **Scan** 11957
**Peptide** TDNAEPTEDSSDYHVTT **Charge** 2 **Spectral Angle** 0.79

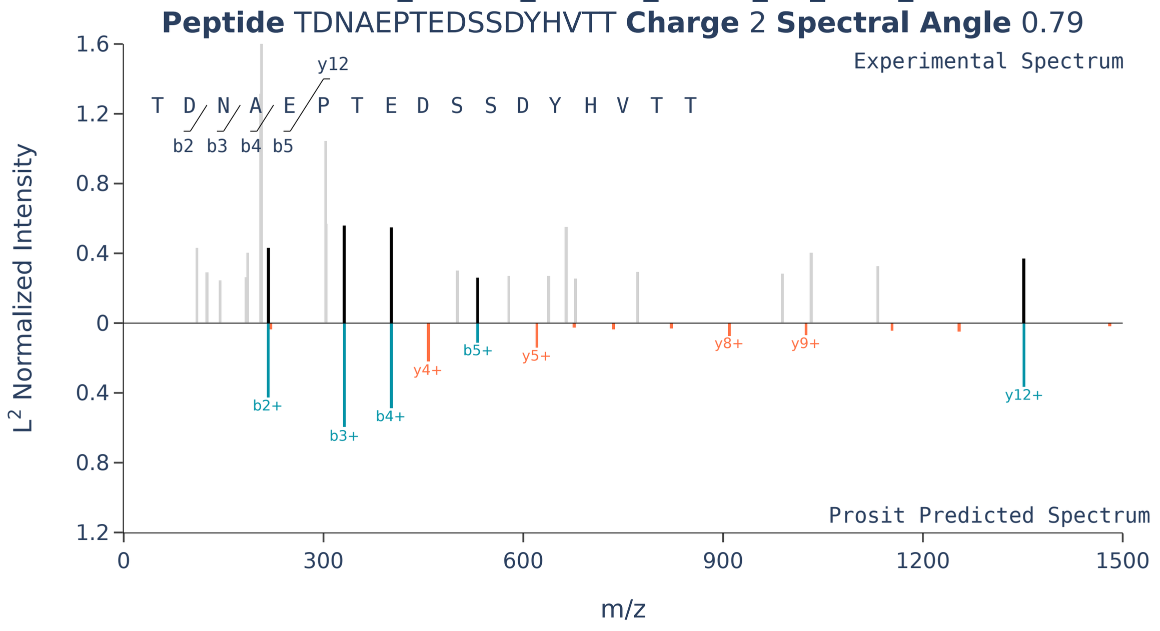


**Source** HRoetschke_211222_160123_HFGoe_IF2_b1R1_4h **Scan** 5152
**Peptide** DMTKNAQAEKARREQE **Charge** 3 **Spectral Angle** 0.81

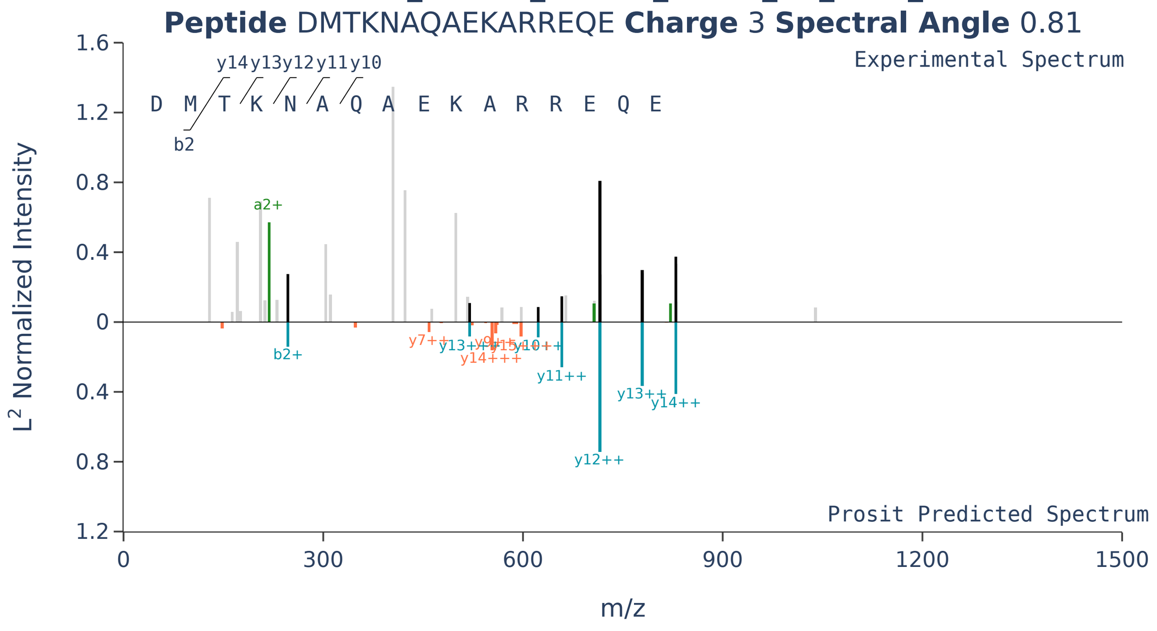


**Source** HRoetschke_211222_160123_HFGoe_IF2_b1R1_4h **Scan** 18785
**Peptide** ELGQEVLE **Charge** 1 **Spectral Angle** 0.75

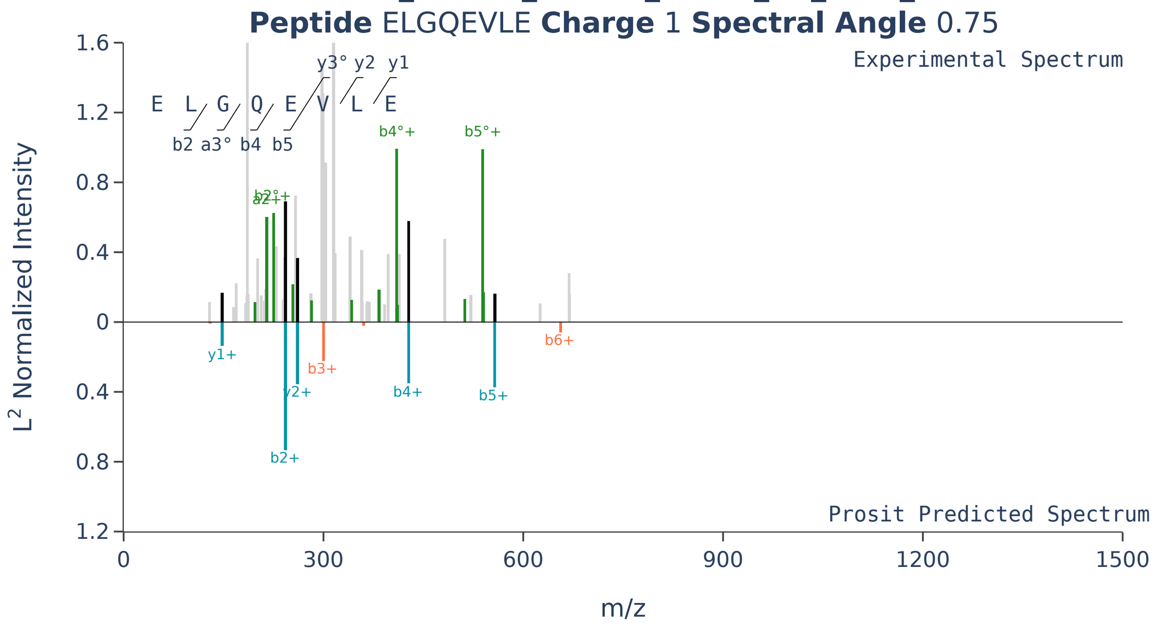


**Source** HRoetschke_211222_160123_HFGoe_IF2_b1R1_4h **Scan** 20566
**Peptide** MATINQVIDQETA **Charge** 2 **Spectral Angle** 0.81

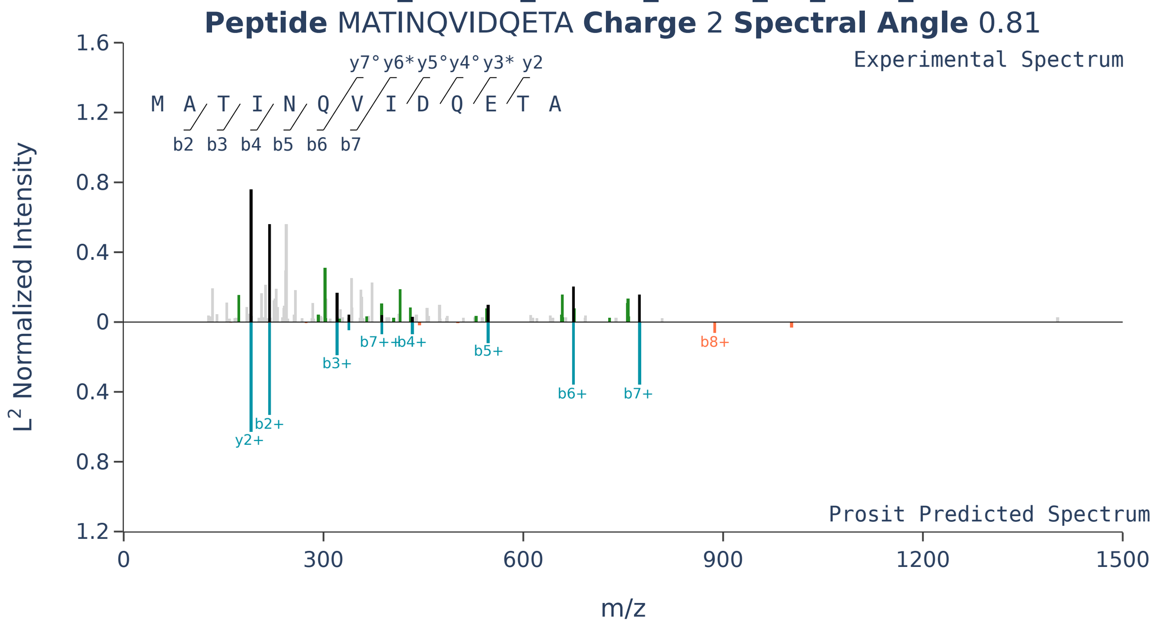


**Source** HRoetschke_211222_160123_HFGoe_IF2_b1R1_4h **Scan** 11393
**Peptide** KIDKPEADPDRVKNELSQ **Charge** 3 **Spectral Angle** 0.87

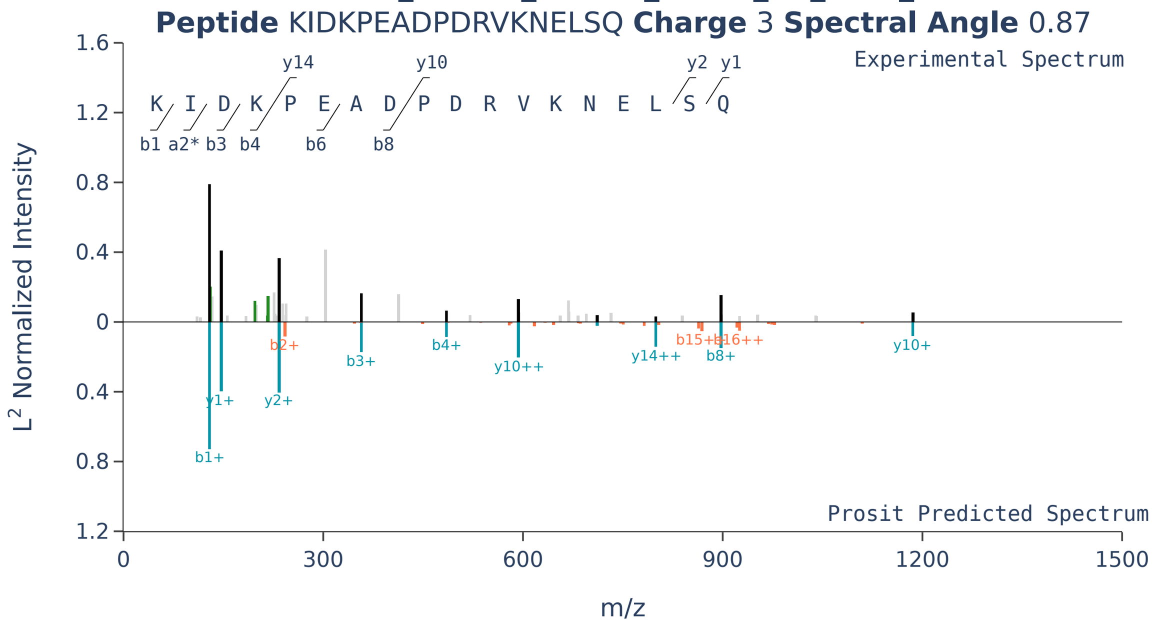


**Source** HRoetschke_211222_160123_HFGoe_IF2_b1R1_2h **Scan** 16523
**Peptide** PAQAVNRDVVL **Charge** 2 **Spectral Angle** 0.81

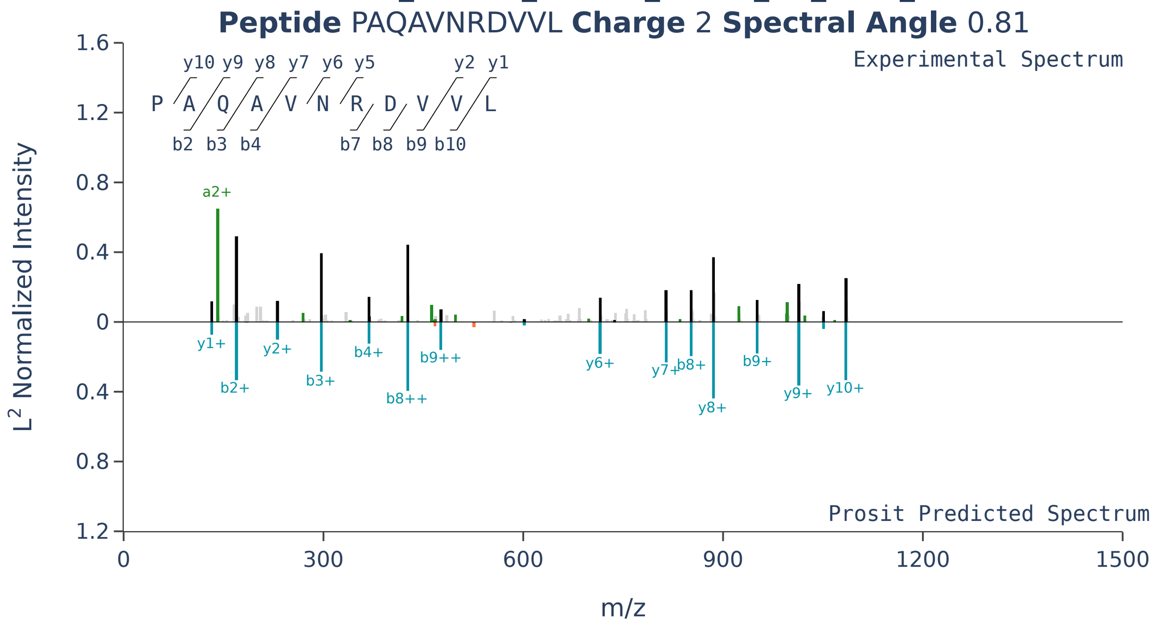


**Source** HRoetschke_211222_160123_HFGoe_IF2_b1R1_24h **Scan** 6527
**Peptide** PDRVKNELS **Charge** 2 **Spectral Angle** 0.78

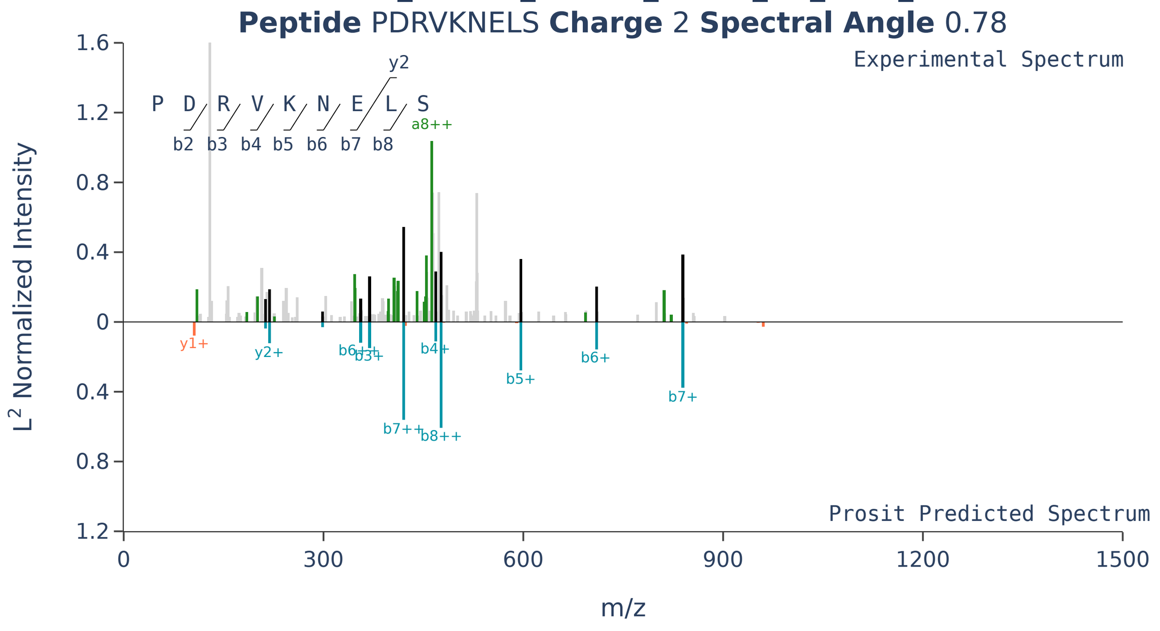

**Source** HRoetschke_211222_160123_HFGoe_IF2_b2R2_2h **Scan** 7464
**Peptide** DASARKV **Charge** 2 **Spectral Angle** 0.82

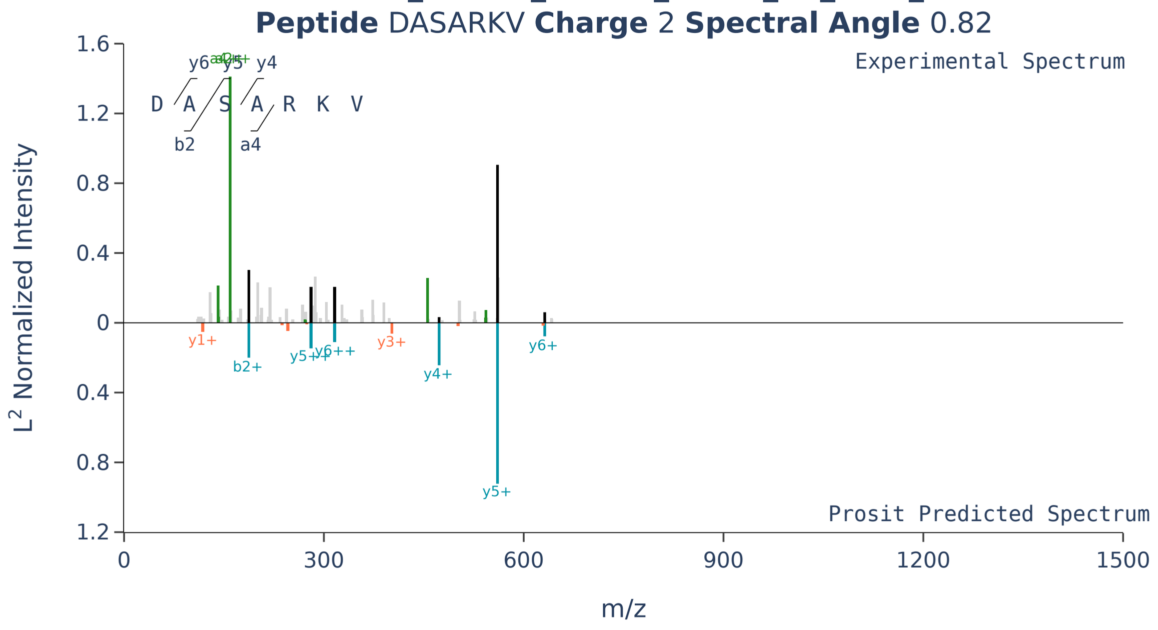


**Source** HRoetschke_211222_160123_HFGoe_IF2_b1R1_2h **Scan** 5157
**Peptide** VTVVRDEKKARE **Charge** 3 **Spectral Angle** 0.88

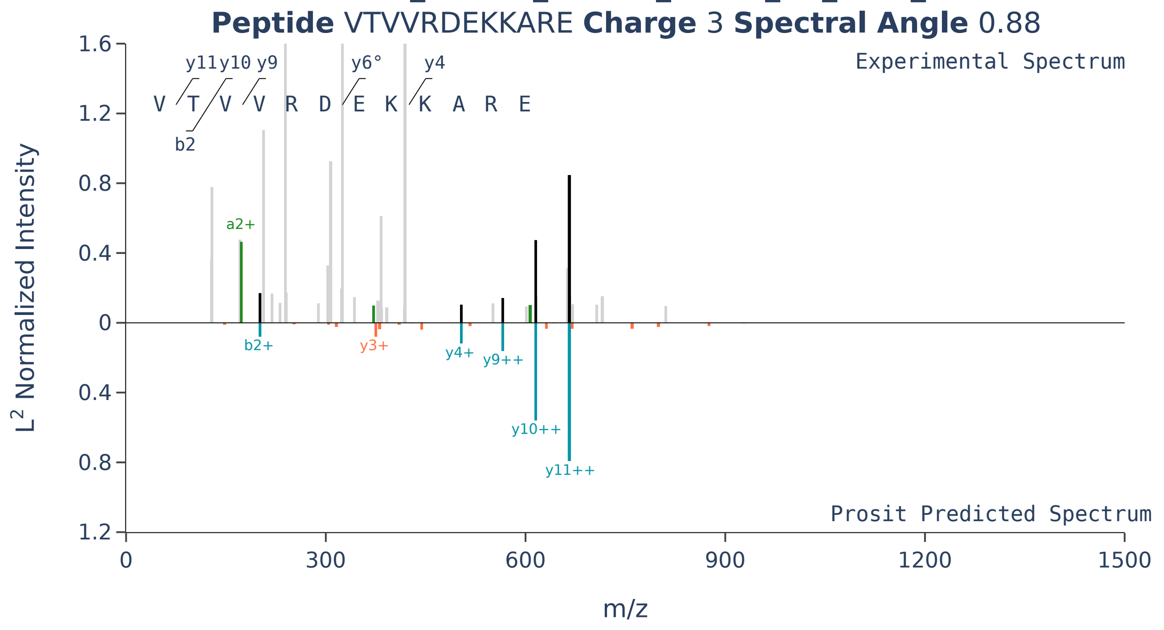


**Source** HRoetschke_211222_160123_HFGoe_IF2_b1R1_4h **Scan** 9877
**Peptide** TQHIGAYHVETEN **Charge** 3 **Spectral Angle** 0.8

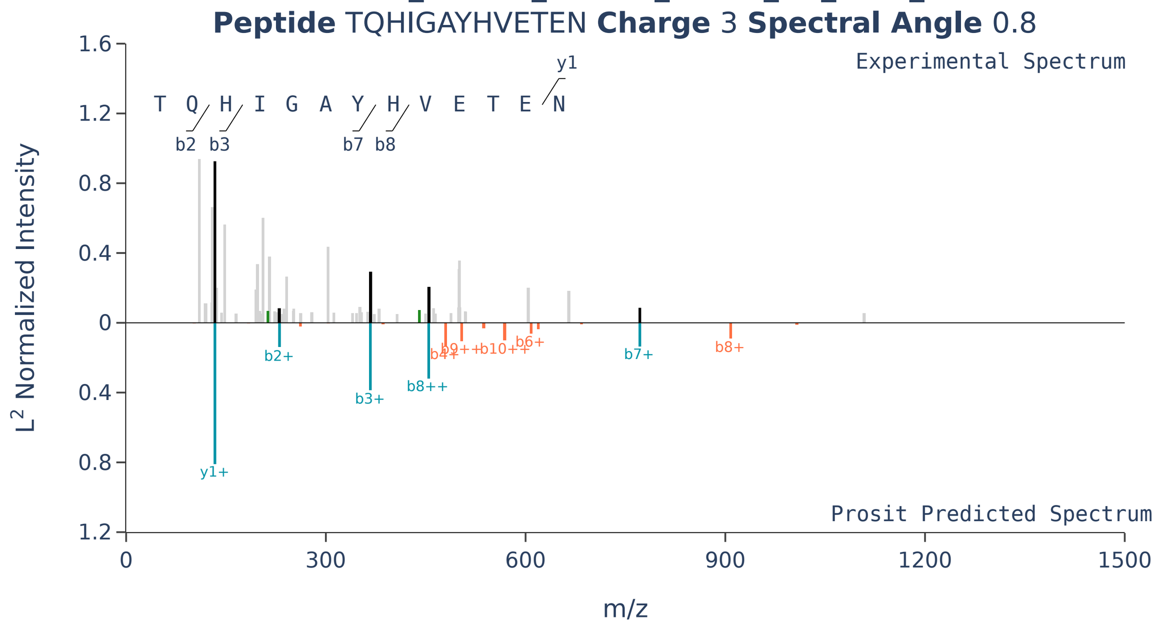


**Source** HRoetschke_211222_160123_HFGoe_IF2_b2R1_1h **Scan** 8668
**Peptide** SDYHVTT **Charge** 1 **Spectral Angle** 0.92

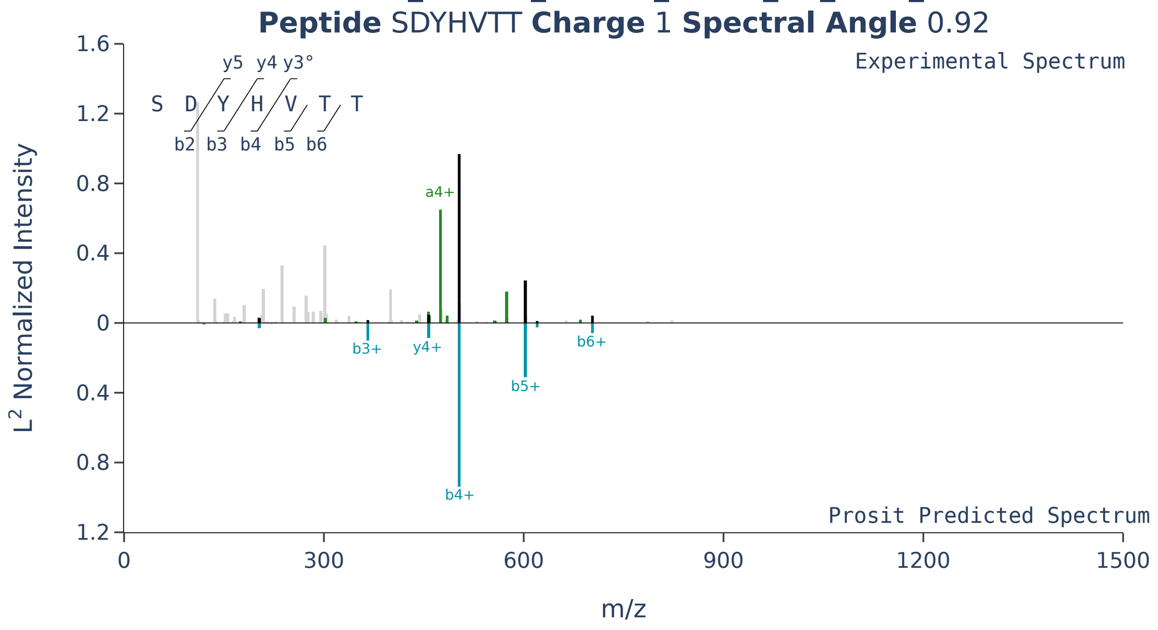


**Source** HRoetschke_211222_160123_HFGoe_IF2_b1R1_4h **Scan** 9815
**Peptide** VKGSQVI **Charge** 2 **Spectral Angle** 0.92

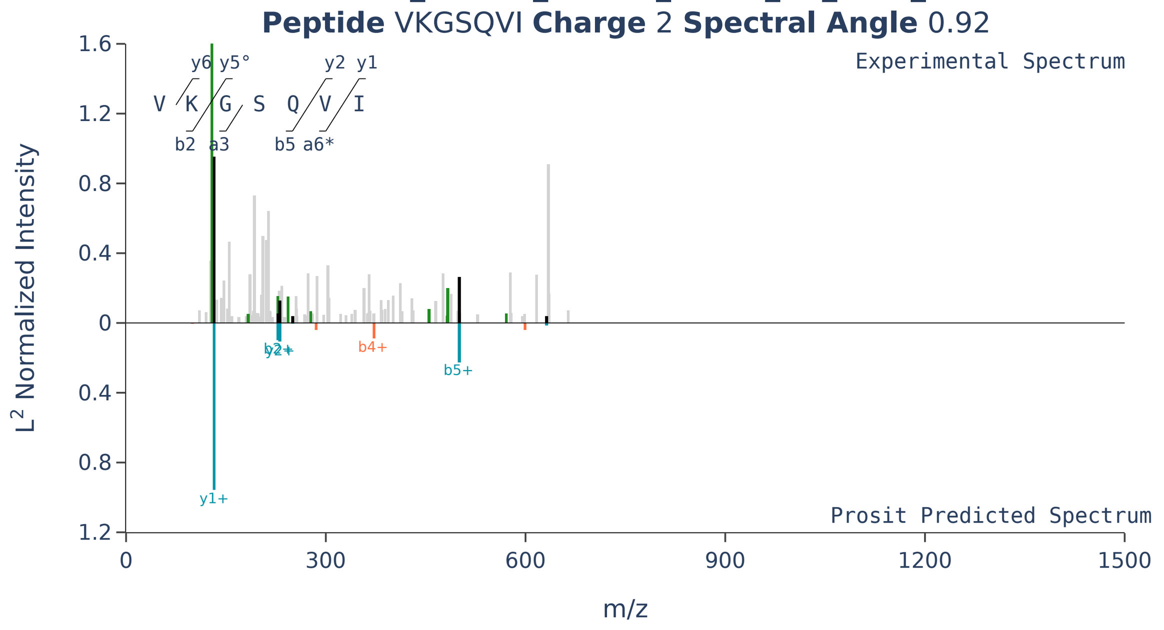


**Source** HRoetschke_211222_160123_HFGoe_IF2_b1R1_2h **Scan** 9533
**Peptide** DHLNQKNSGPDKLTLQR **Charge** 4 **Spectral Angle** 0.82

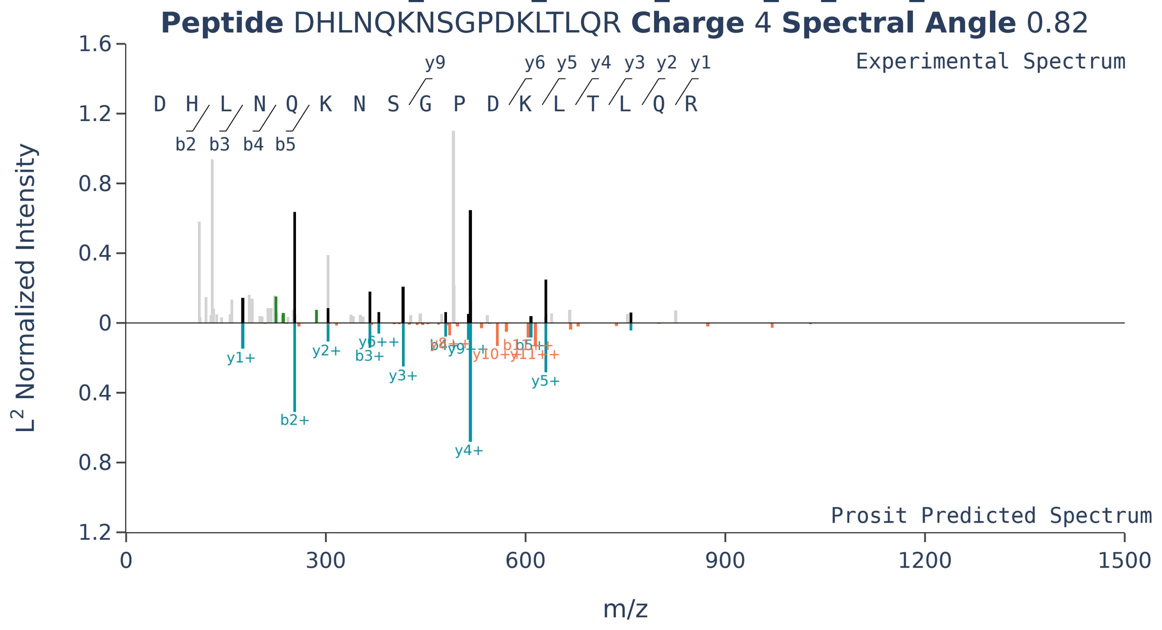


**Source** HRoetschke_211222_160123_HFGoe_IF2_b1R1_4h **Scan** 11298
**Peptide** QEKQTLIDHLNQK **Charge** 4 **Spectral Angle** 0.68

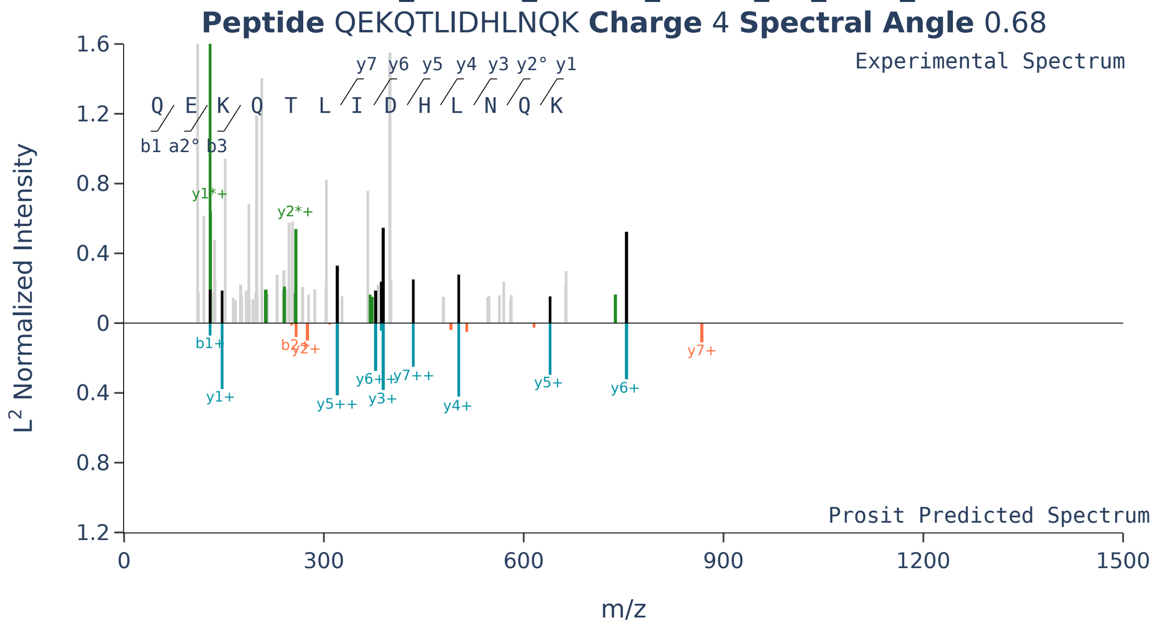


**Source** HRoetschke_211222_160123_HFGoe_IF2_b1R1_24h **Scan** 5304
**Peptide** KAEEEARRKLEE **Charge** 3 **Spectral Angle** 0.86

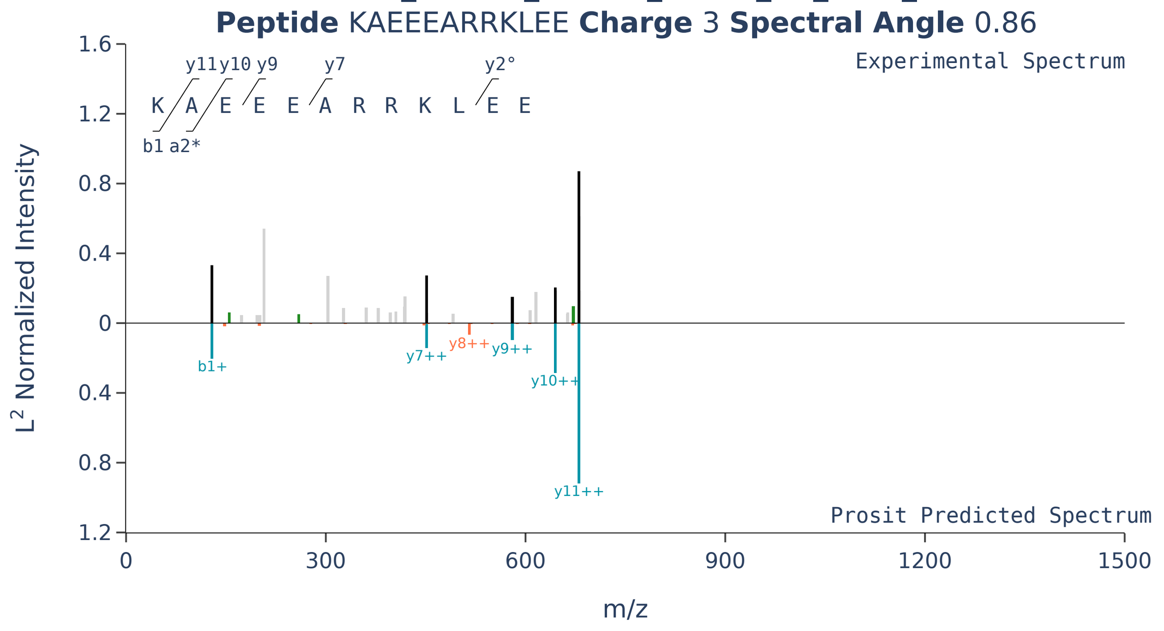


**Source** HRoetschke_211222_160123_HFGoe_IF2_b2R1_1h **Scan** 13692
**Peptide** ESFLDKGRGPVA **Charge** 2 **Spectral Angle** 0.8

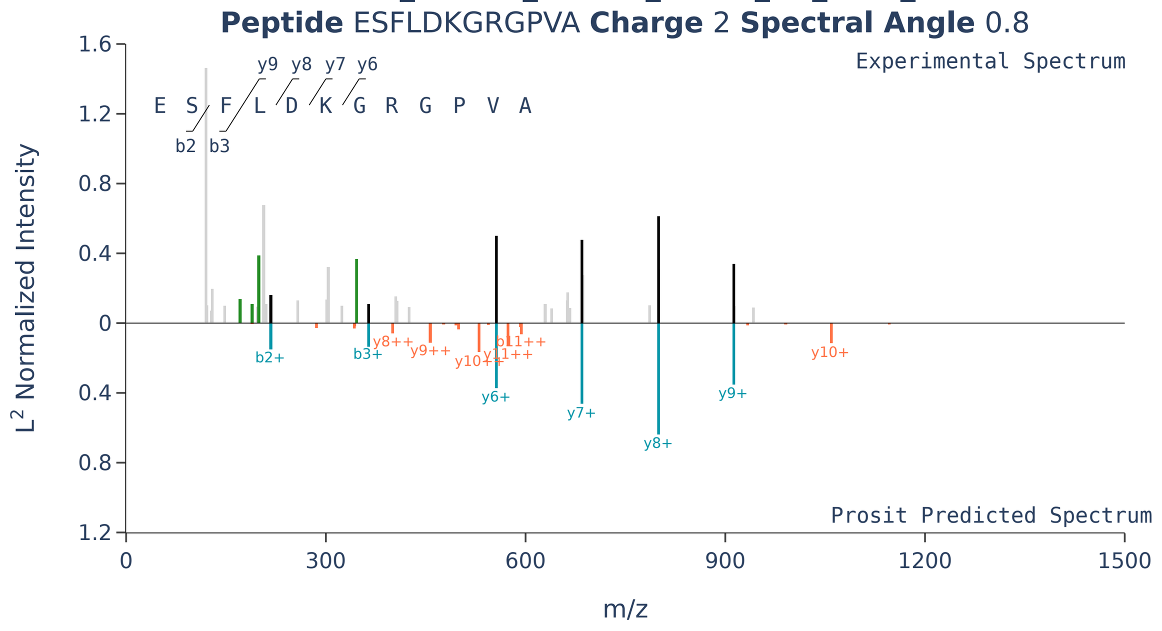


**Source** HRoetschke_211222_160123_HFGoe_IF2_b1R1_4h **Scan** 13390
**Peptide** KTLAAERQTSVERLVQQ **Charge** 3 **Spectral Angle** 0.75

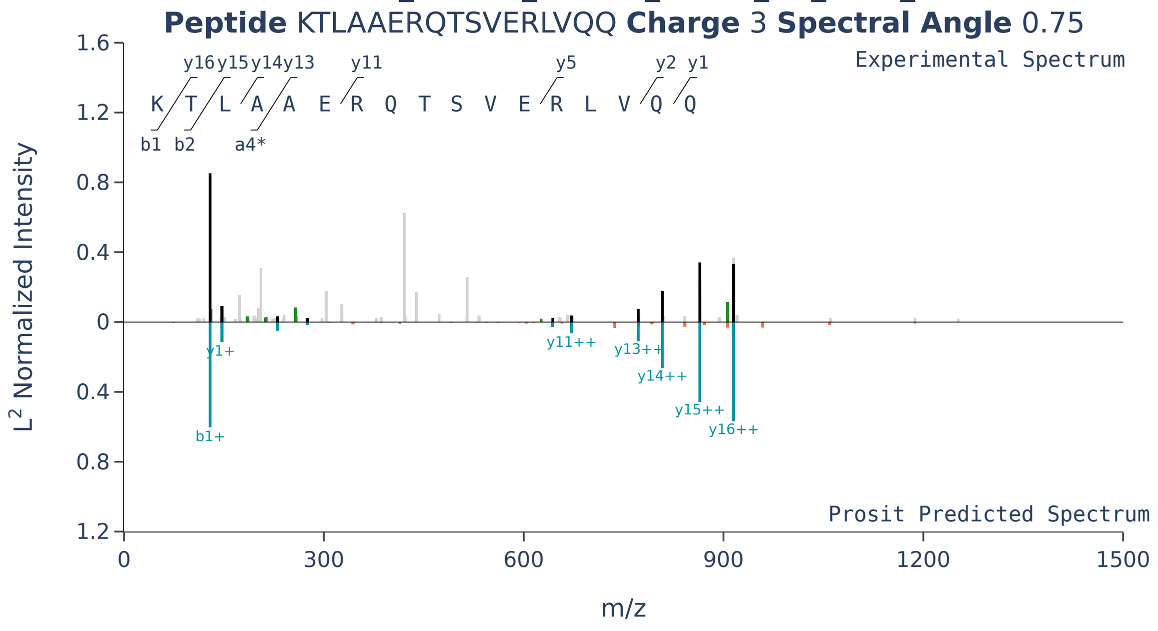


**Source** HRoetschke_211222_160123_HFGoe_IF2_b2R1_24h **Scan** 10941
**Peptide** LNIPGTGGKSKS **Charge** 2 **Spectral Angle** 0.82

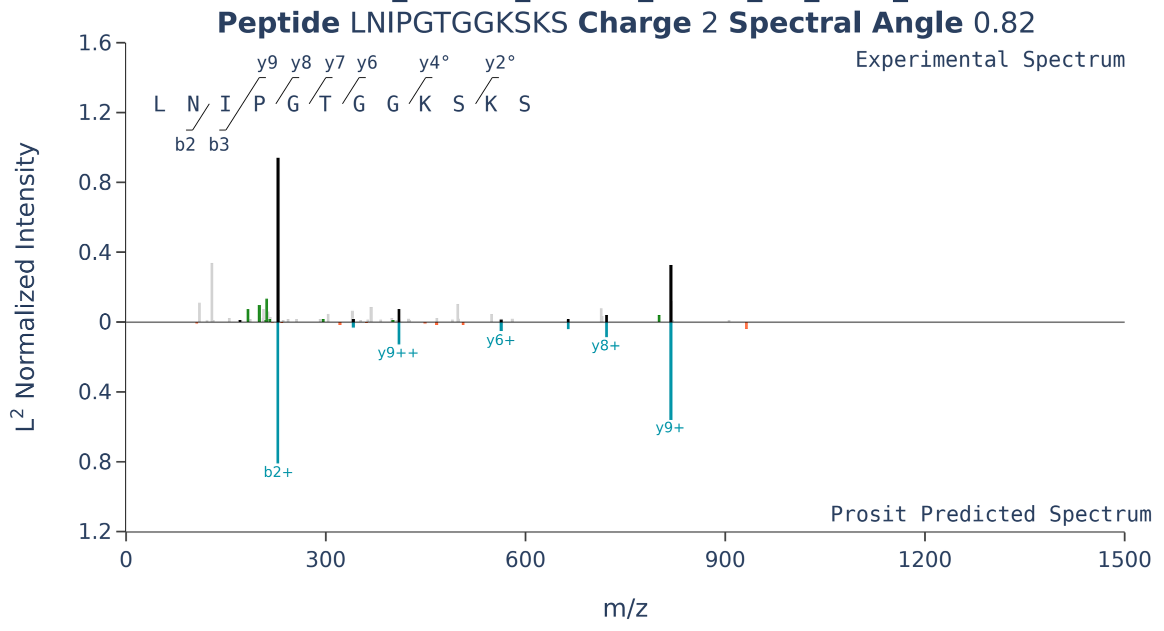


**Source** HRoetschke_211222_160123_HFGoe_IF2_b1R2_24h **Scan** 6519
**Peptide** DAGIRKSADDSVS **Charge** 2 **Spectral Angle** 0.59

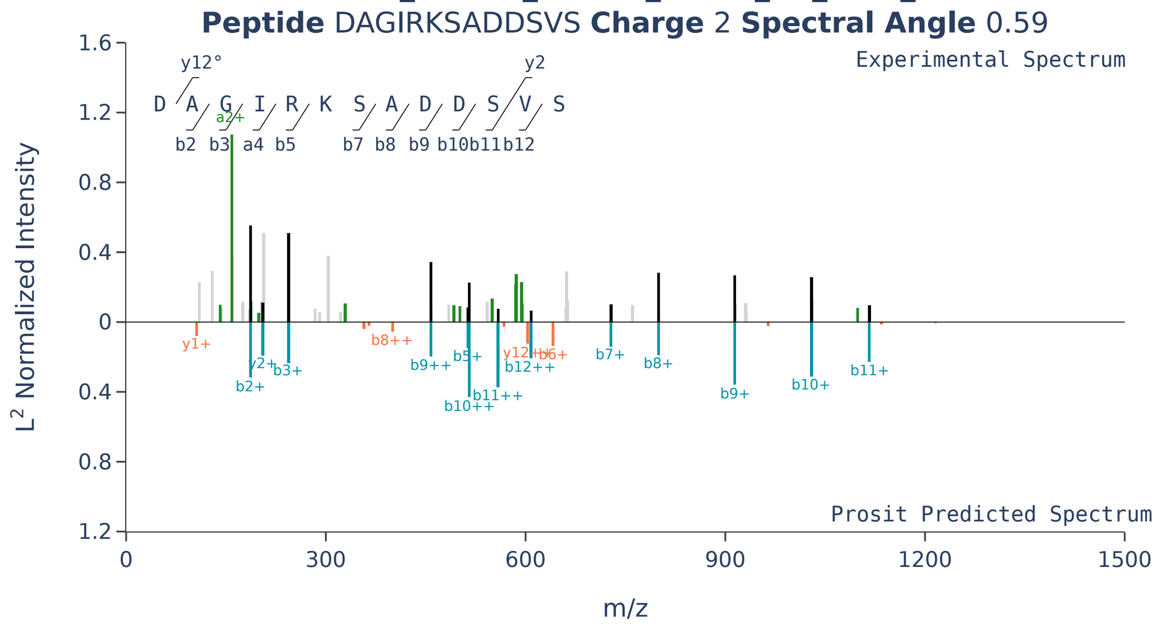


**Source** HRoetschke_211222_160123_HFGoe_IF2_b2R2_24h **Scan** 12863
**Peptide** DRDTGAAAEPRAPVVT **Charge** 3 **Spectral Angle** 0.58

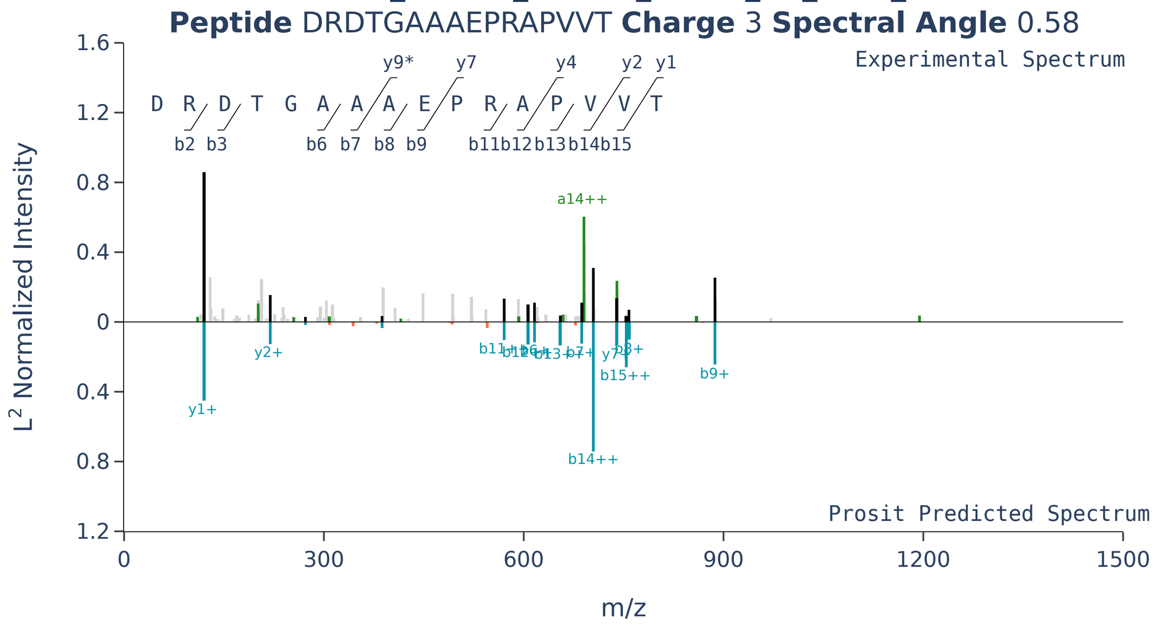


**Source** HRoetschke_211222_160123_HFGoe_IF2_b2R1_24h **Scan** 7886
**Peptide** RKGMASGAVL **Charge** 2 **Spectral Angle** 0.76

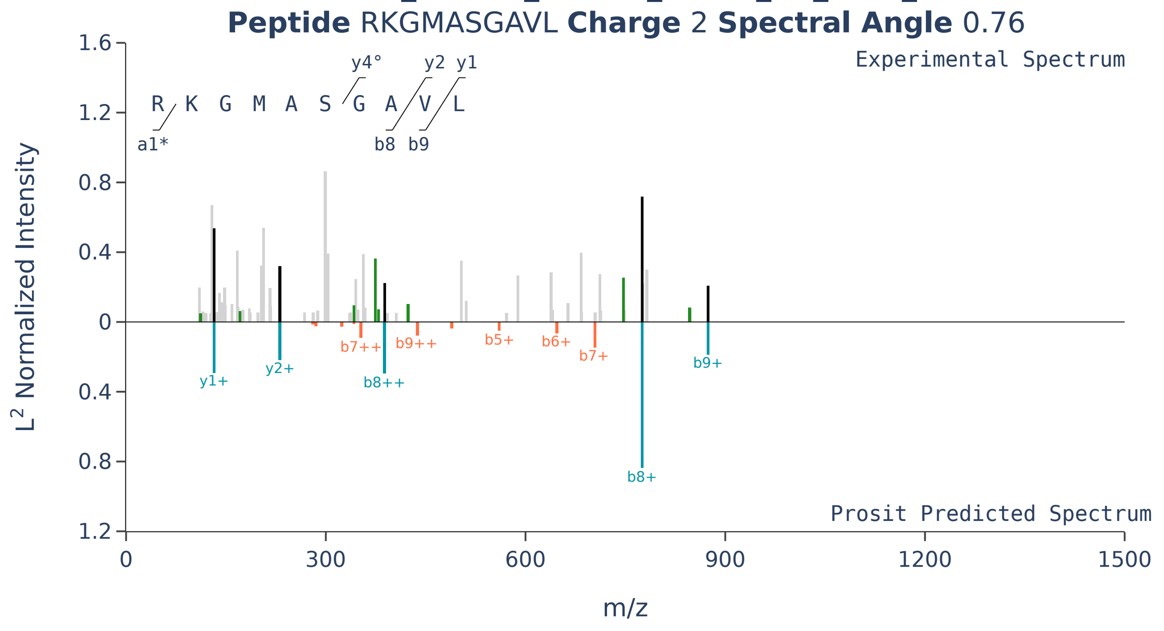


**Source** HRoetschke_211222_160123_HFGoe_IF2_b1R1_4h **Scan** 6164
**Peptide** DSSDYHVTTSQHARQA **Charge** 2 **Spectral Angle** 0.79

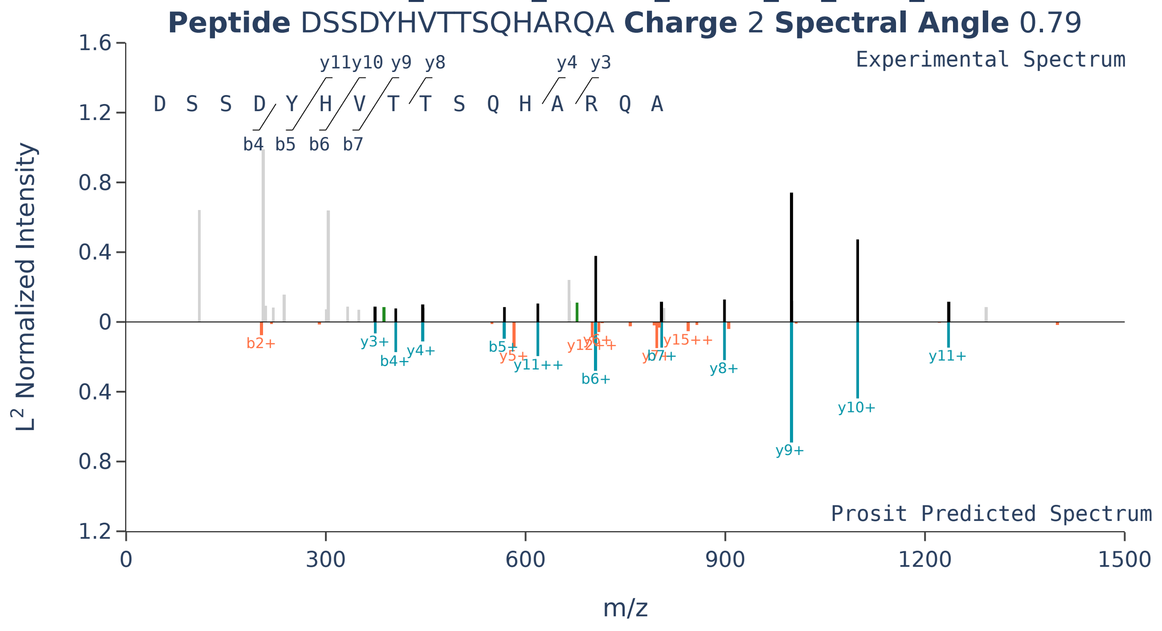

**Source** HRoetschke_211222_160123_HFGoe_IF2_b1R1_4h **Scan** 8222
**Peptide** DTPGHAAFTSMRARGAQA **Charge** 3 **Spectral Angle** 0.6

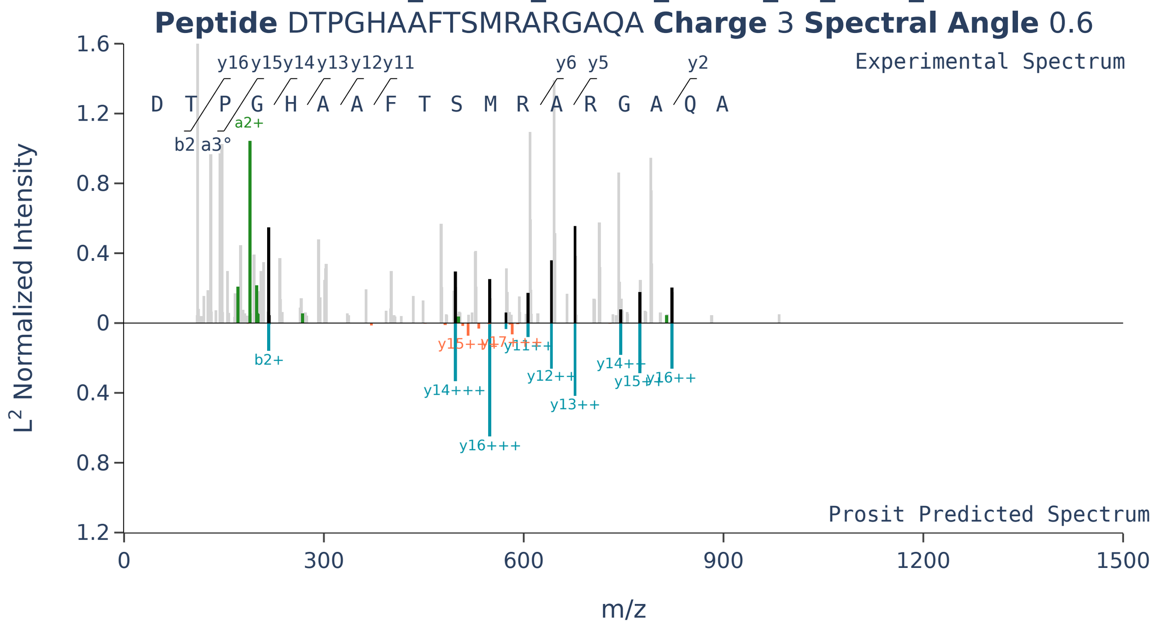


**Source** HRoetschke_211222_160123_HFGoe_IF2_b2R1_24h **Scan** 6899
**Peptide** PGTGGKSKSVQIE **Charge** 3 **Spectral Angle** 0.84

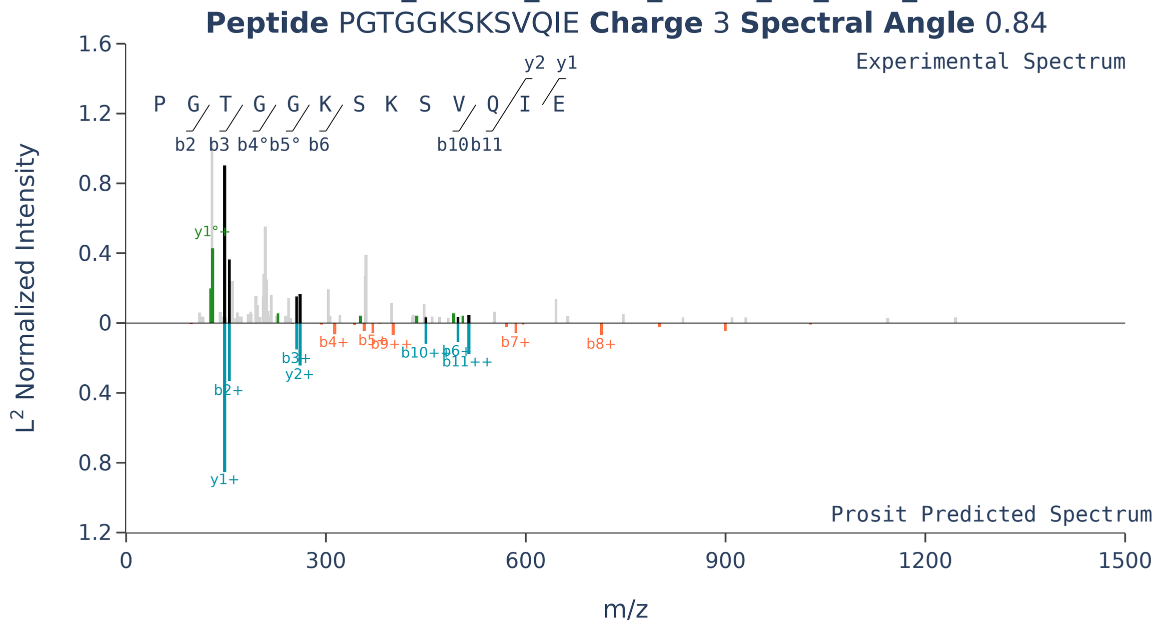


**Source** HRoetschke_211222_160123_HFGoe_IF2_b2R1_24h **Scan** 10500
**Peptide** LQQGFQKPAQA **Charge** 2 **Spectral Angle** 0.81

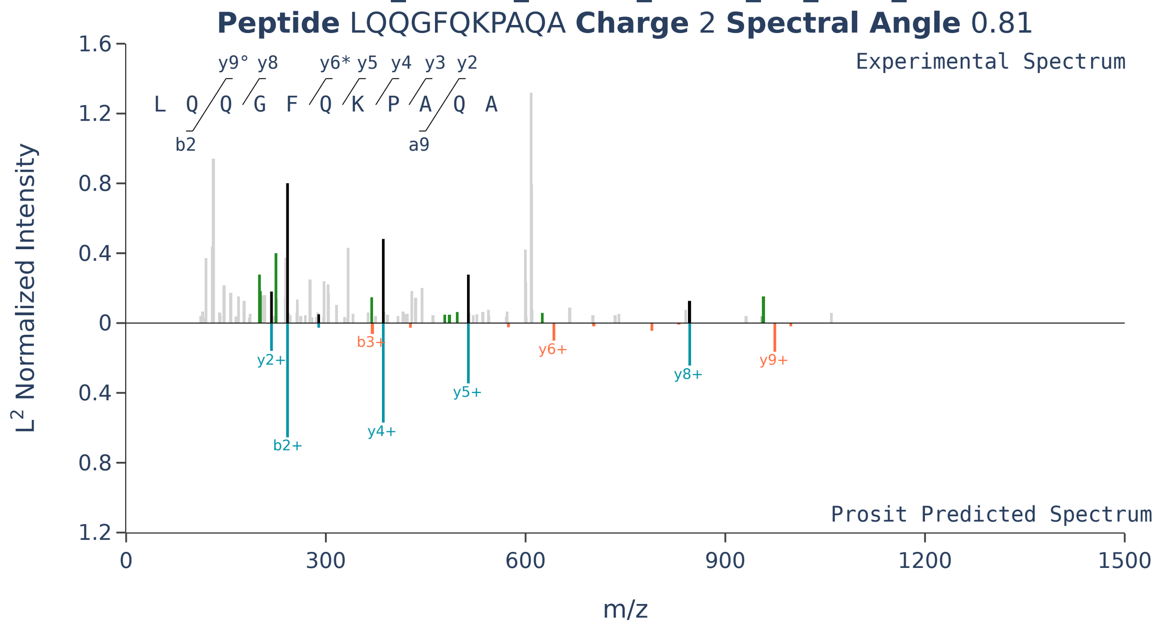


**Source** HRoetschke_211222_160123_HFGoe_IF2_b1R1_4h **Scan** 21674
**Peptide** AAQVPVVV **Charge** 1 **Spectral Angle** 0.88

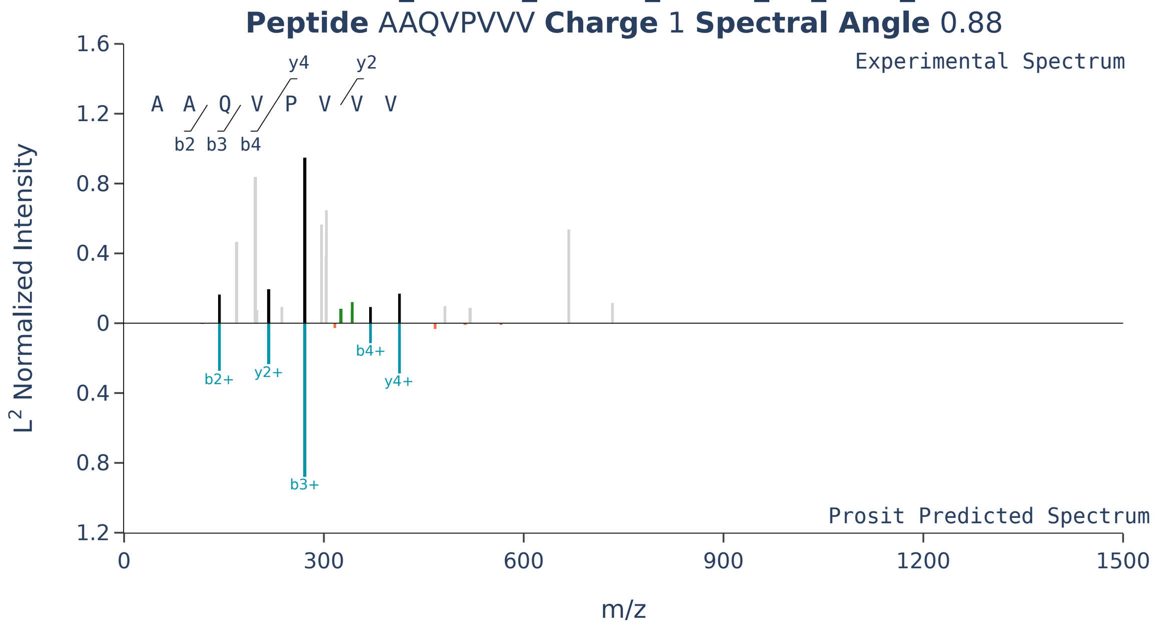


**Source** HRoetschke_211222_160123_HFGoe_IF2_b1R2_4h **Scan** 6258
**Peptide** AVKGSQVIK **Charge** 2 **Spectral Angle** 0.85

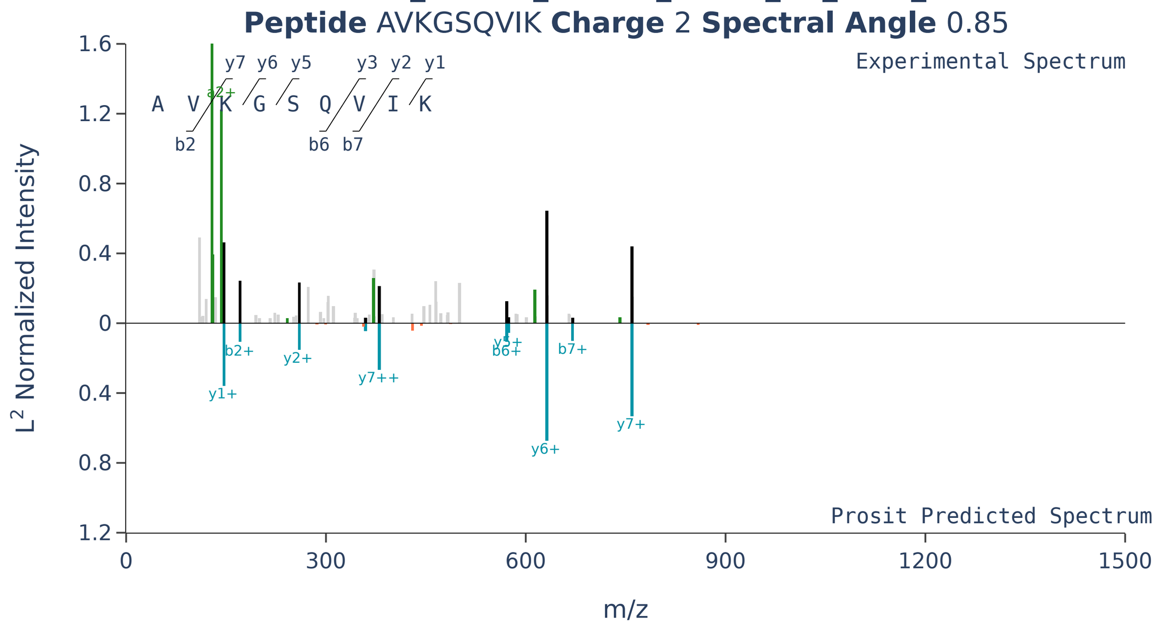


**Source** HRoetschke_211222_160123_HFGoe_IF2_b1R1_4h **Scan** 14070
**Peptide** RDTGAAAEPRAPVVTIMGH **Charge** 4 **Spectral Angle** 0.76

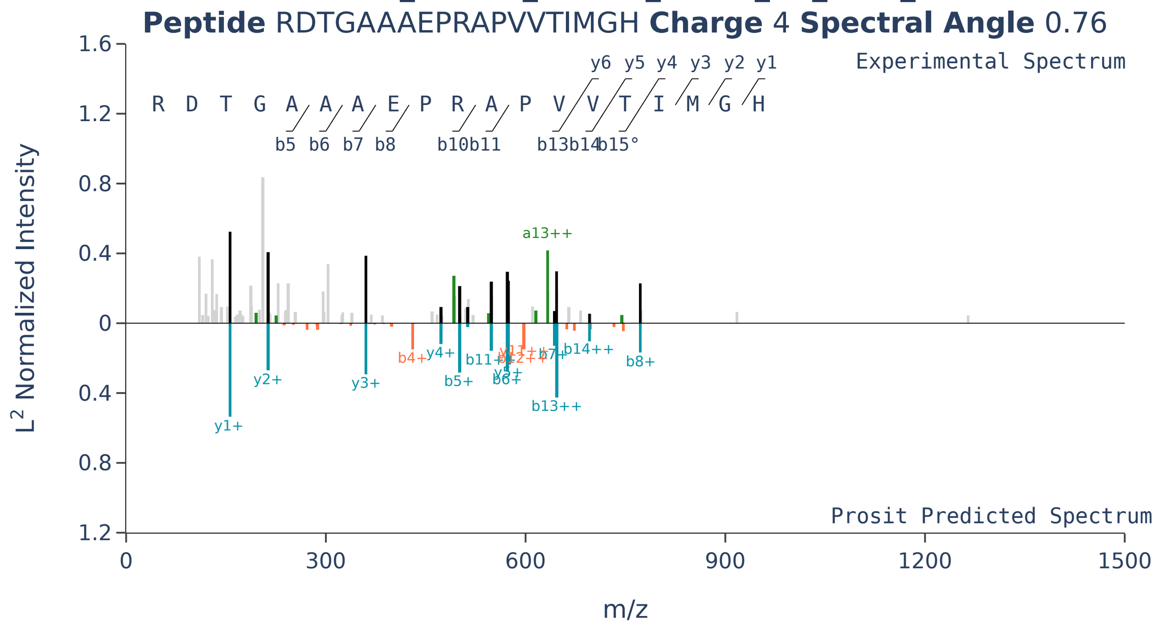


**Source** HRoetschke_211222_160123_HFGoe_IF2_b2R1_24h **Scan** 12151
**Peptide** GMECGIGVKNYNDVRTGD **Charge** 3 **Spectral Angle** 0.72

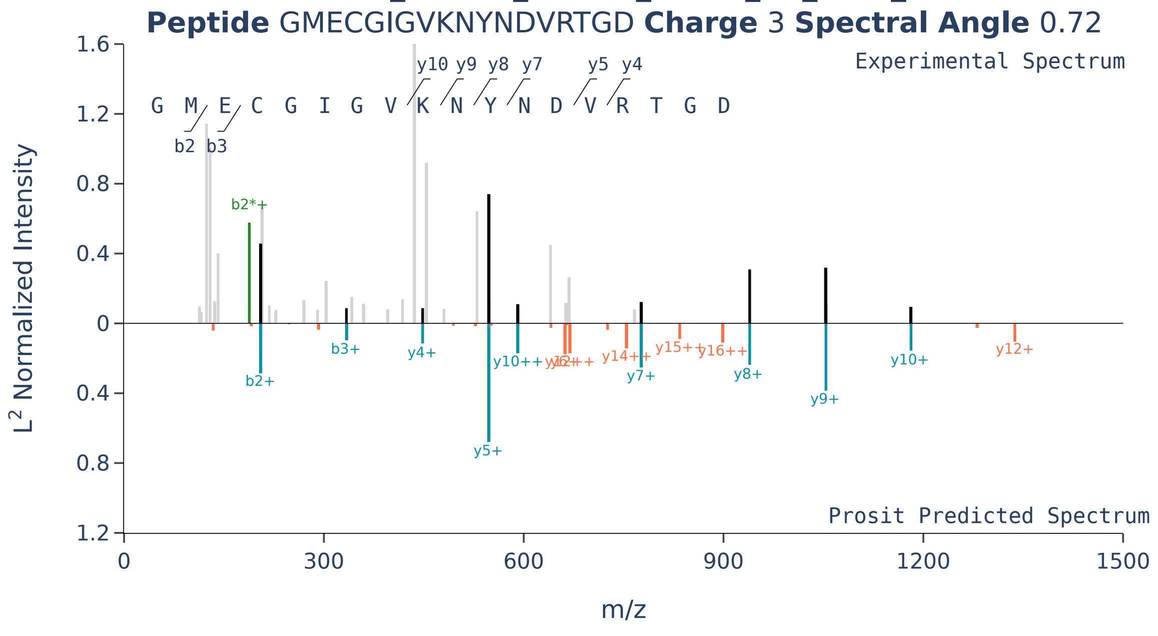


**Source** HRoetschke_211222_160123_HFGoe_IF2_b1R1_4h **Scan** 13949
**Peptide** FANMTEGEVHE **Charge** 2 **Spectral Angle** 0.74

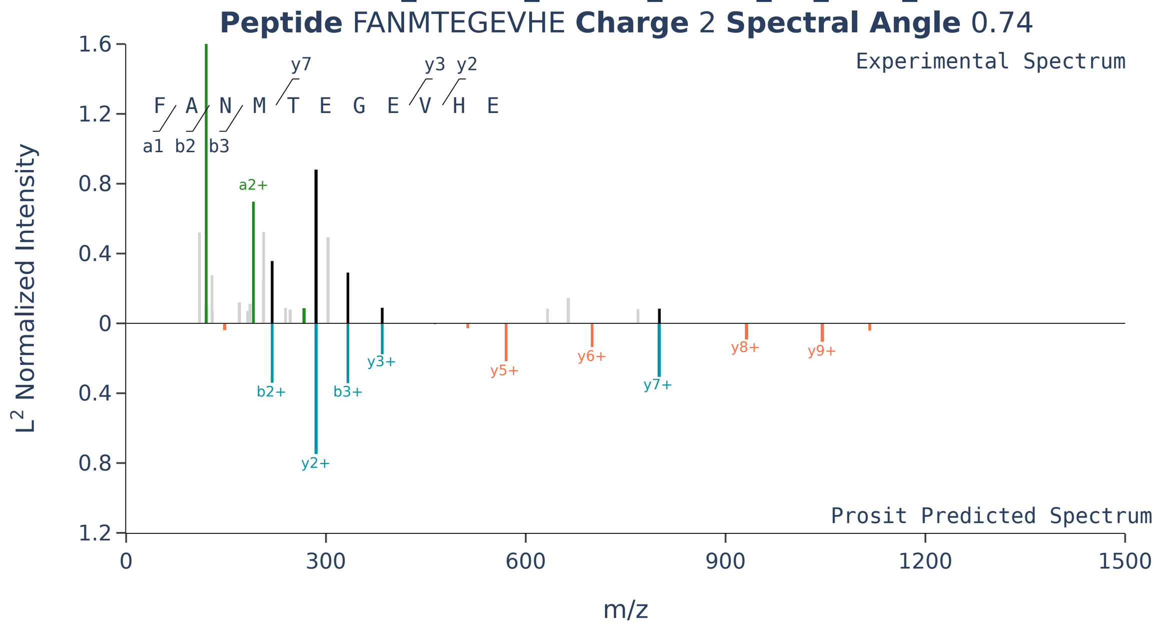


**Source** HRoetschke_211222_160123_HFGoe_IF2_b2R1_24h **Scan** 12615
**Peptide** ESLRRFKDDVNEV **Charge** 3 **Spectral Angle** 0.68

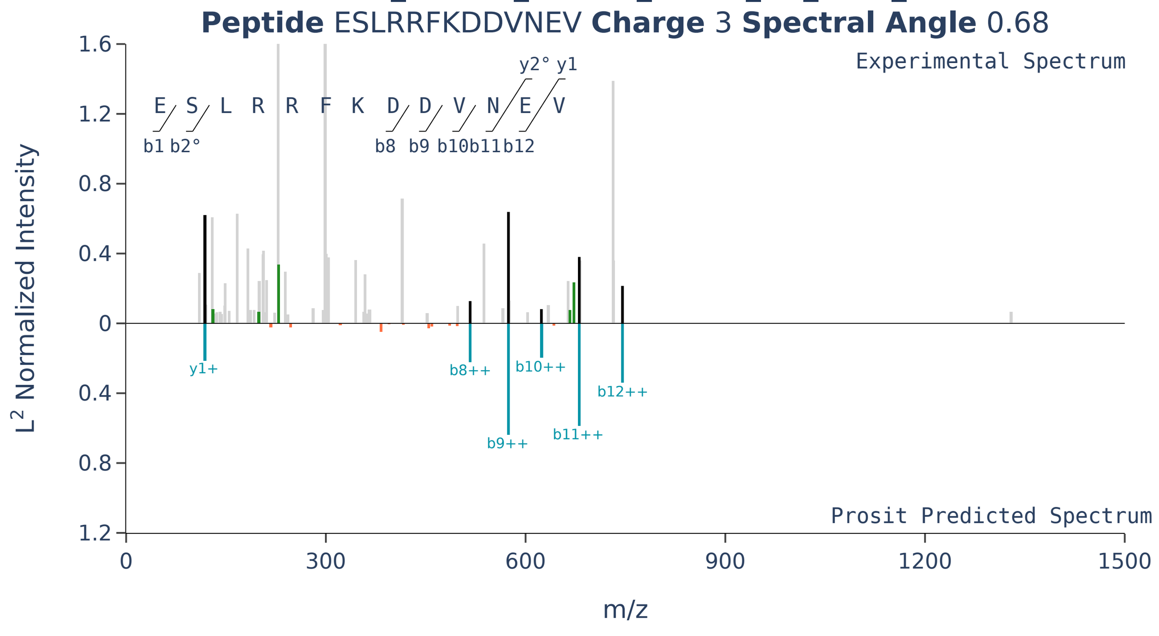


**Source** HRoetschke_211222_160123_HFGoe_IF2_b1R1_2h **Scan** 13313
**Peptide** GESQFVHVS **Charge** 2 **Spectral Angle** 0.8

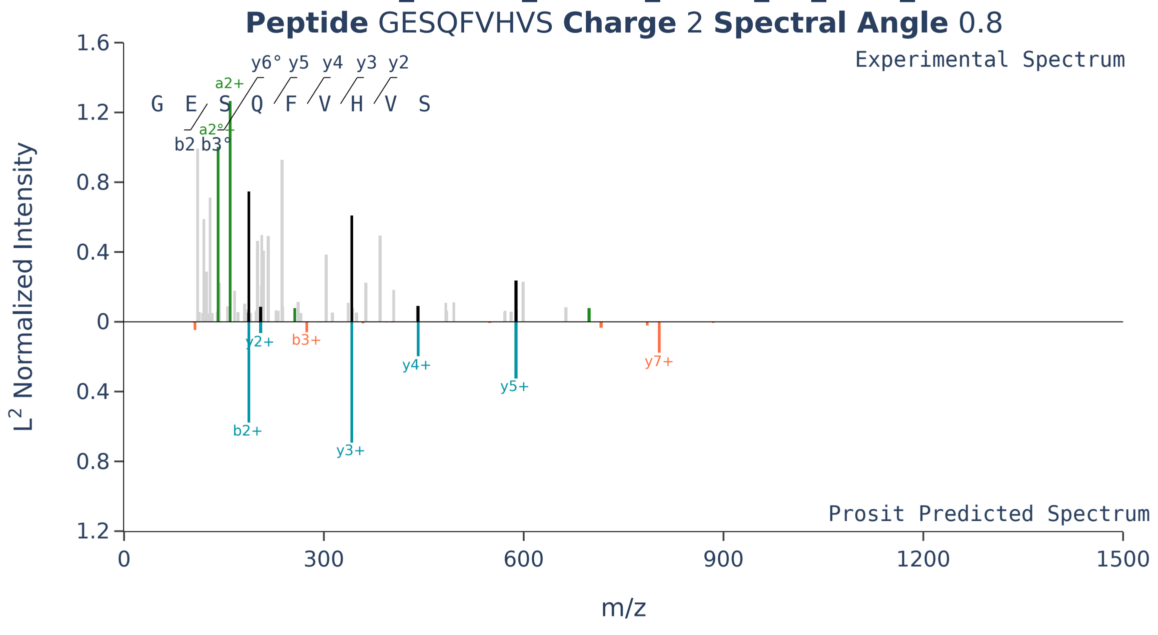


**Source** HRoetschke_211222_160123_HFGoe_IF2_b1R1_2h **Scan** 5869
**Peptide** DSSDYHVTTSQHARQ **Charge** 4 **Spectral Angle** 0.74

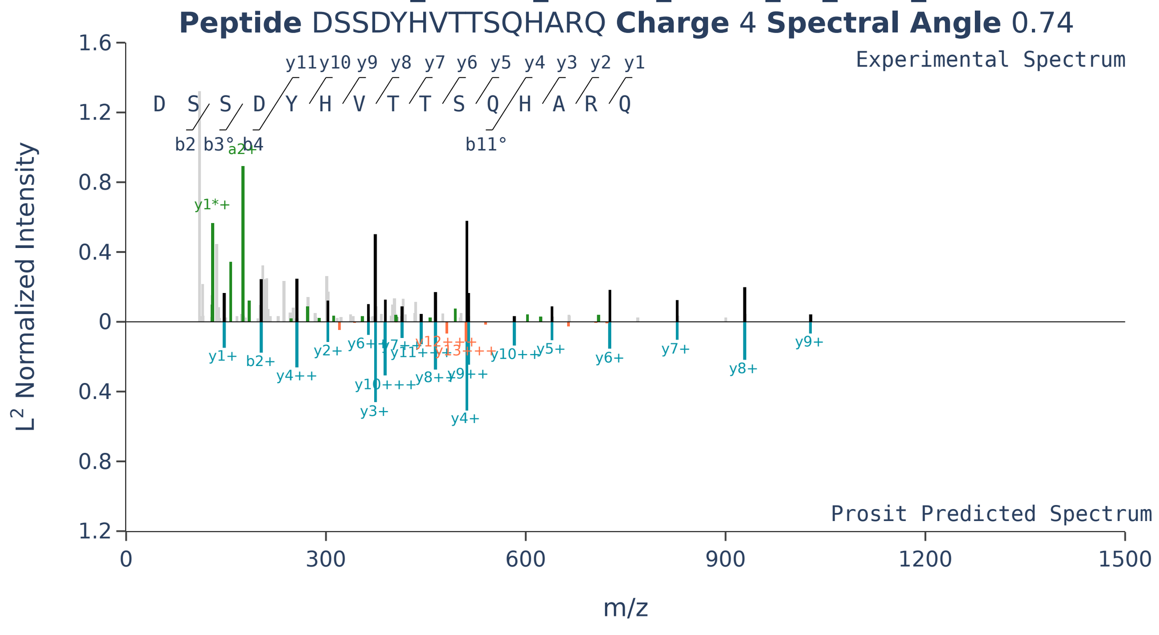


**Source** HRoetschke_211222_160123_HFGoe_IF2_b1R1_4h **Scan** 5741
**Peptide** AGIRKSADDSVSAQEKQT **Charge** 3 **Spectral Angle** 0.68

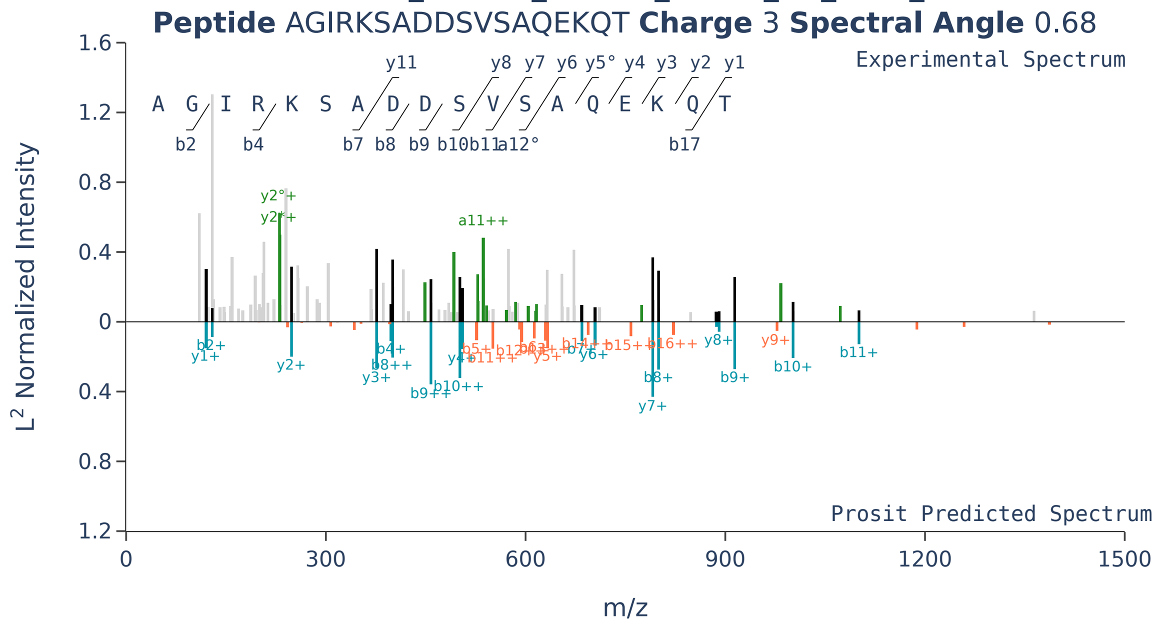


**Source** HRoetschke_211222_160123_HFGoe_IF2_b1R1_4h **Scan** 9704
**Peptide** TPGHAAFTSMRARGAQATD **Charge** 4 **Spectral Angle** 0.79

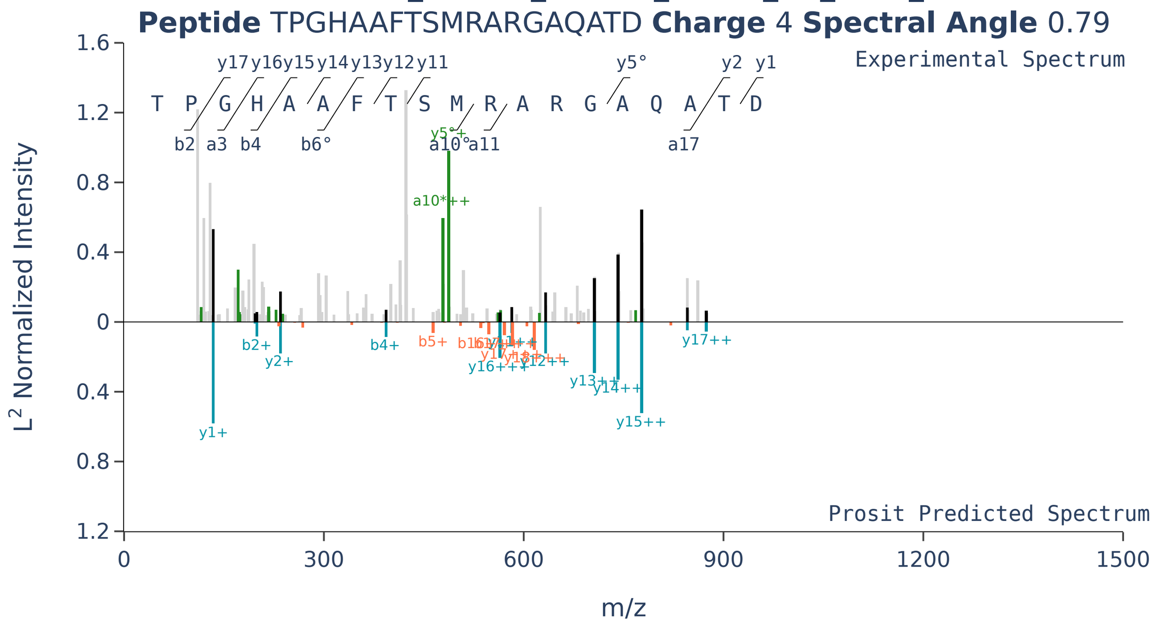


**Source** HRoetschke_211222_160123_HFGoe_IF2_b1R1_4h **Scan** 19427
**Peptide** VLEAGPSI **Charge** 1 **Spectral Angle** 0.77

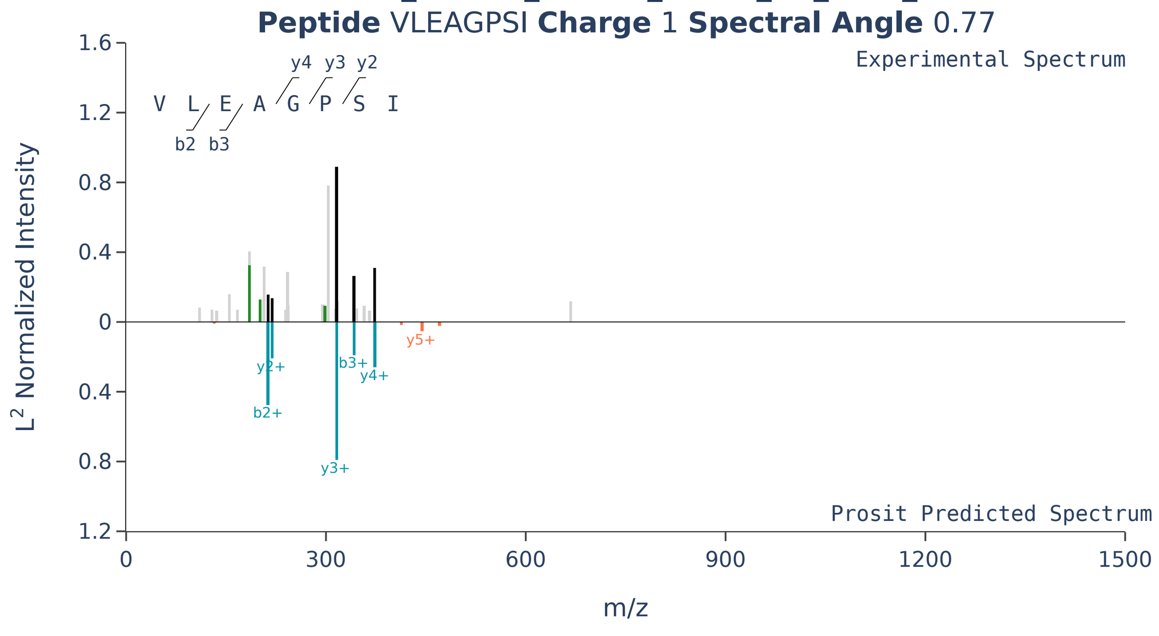


**Source** HRoetschke_211222_160123_HFGoe_IF2_b2R1_24h **Scan** 8143
**Peptide** PGTGGKSKSVQIEVR **Charge** 3 **Spectral Angle** 0.65

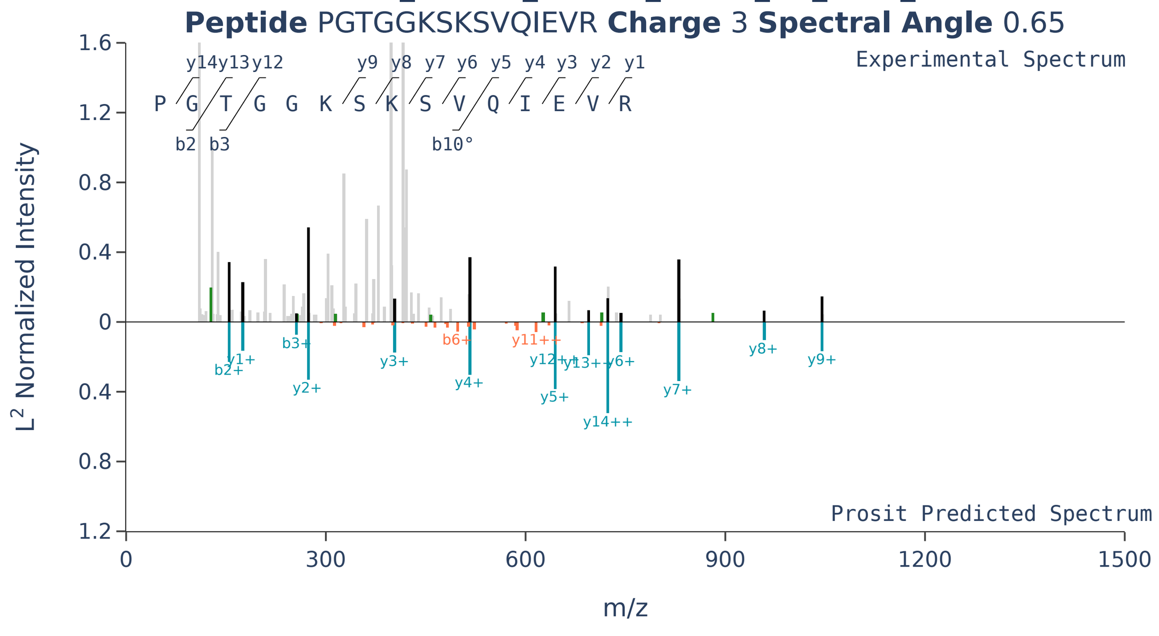

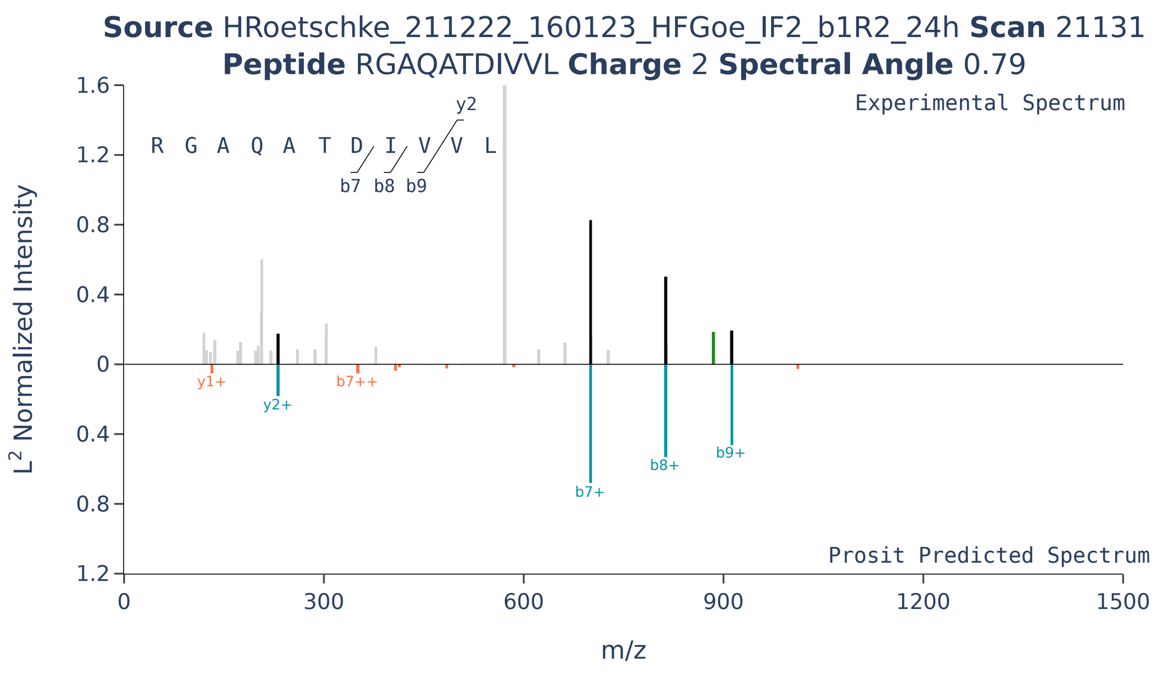
Source HRoetschke_211222_160123_HFGoe_IF2_b1R2_24h Scan 21131
Peptide RGAQATDIVVL Charge 2 Spectral Angle 0.79
Experimental Spectrum
Prosit Predicted Spectrum
L² Normalized Intensity
m/z

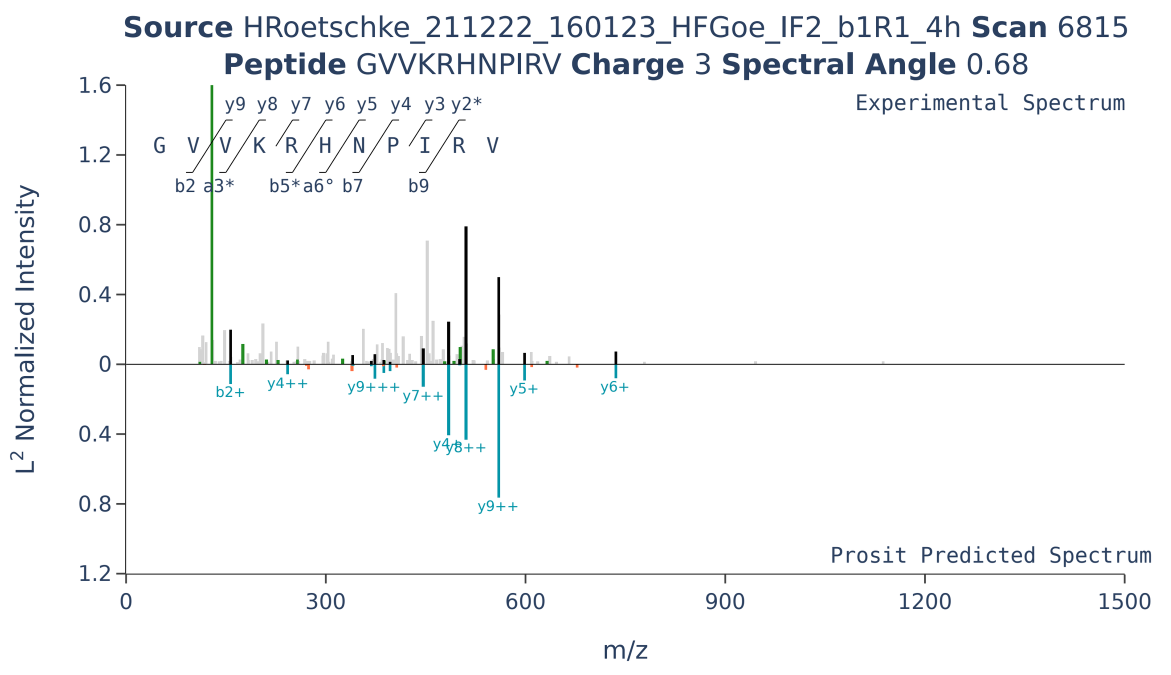
Source HRoetschke_211222_160123_HFGoe_IF2_b1R1_4h Scan 6815
Peptide GVVKRHNPIRV Charge 3 Spectral Angle 0.68
Experimental Spectrum
Prosit Predicted Spectrum
L² Normalized Intensity
m/z

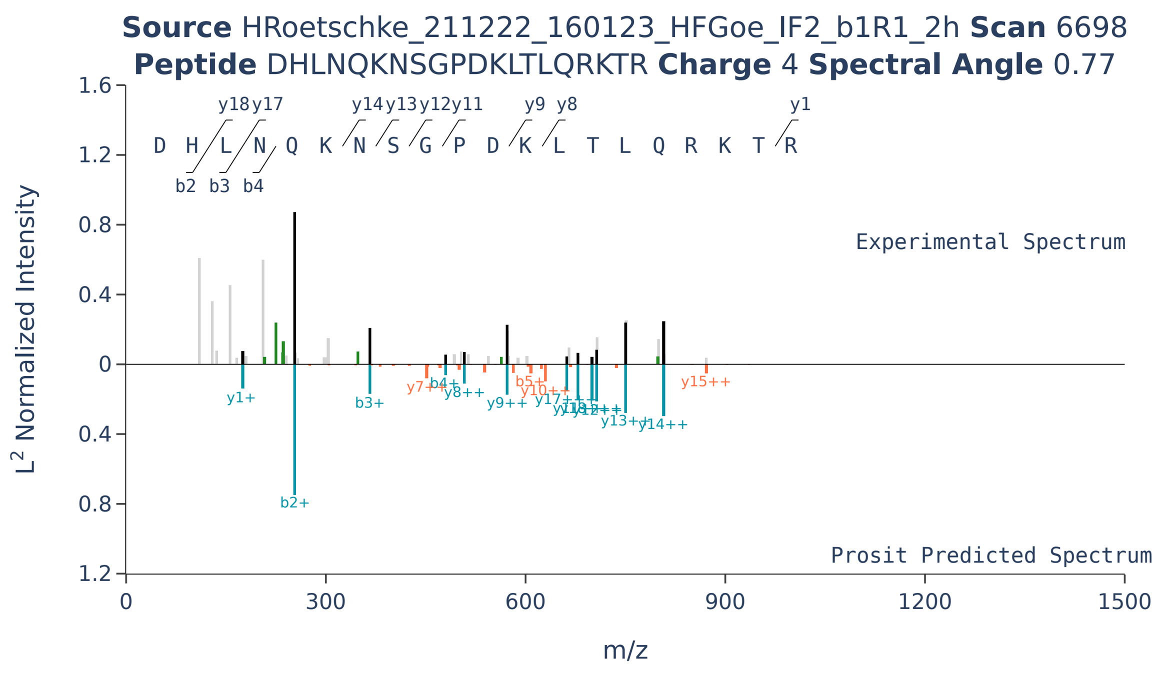
Source HRoetschke_211222_160123_HFGoe_IF2_b1R1_2h Scan 6698
Peptide DHLNQKNSGPDKLTLQRKTR Charge 4 Spectral Angle 0.77
Experimental Spectrum
Prosit Predicted Spectrum
L² Normalized Intensity
m/z

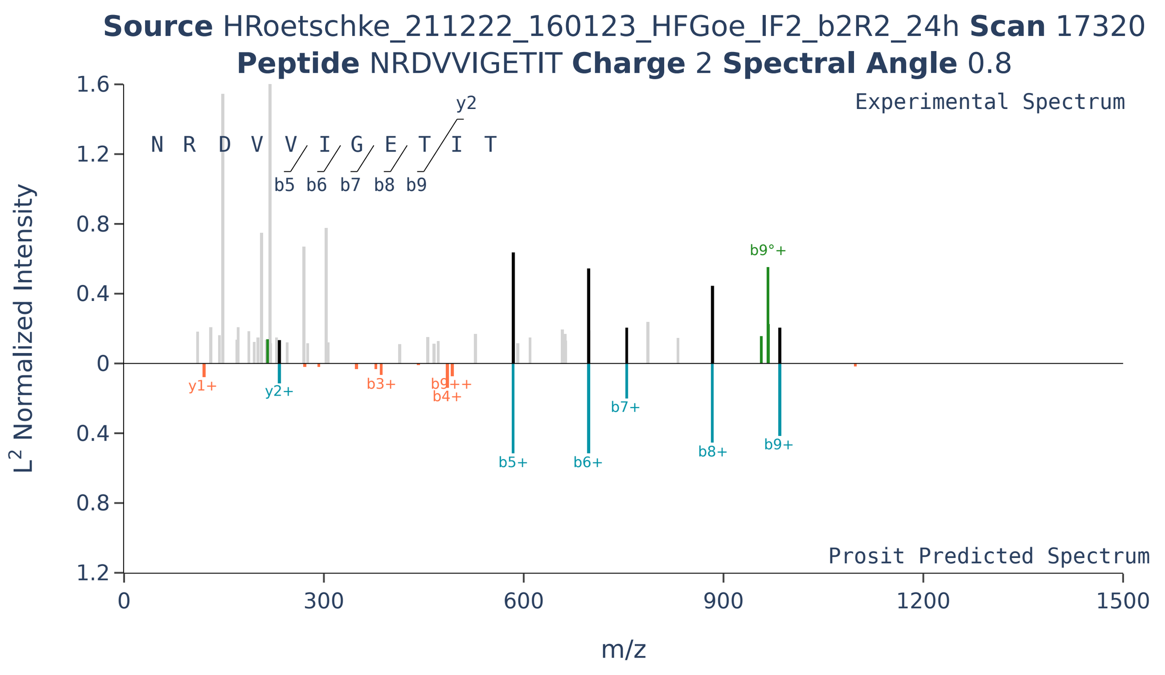
Source HRoetschke_211222_160123_HFGoe_IF2_b2R2_24h Scan 17320
Peptide NRDVVIGETIT Charge 2 Spectral Angle 0.8
Experimental Spectrum
Prosit Predicted Spectrum
L² Normalized Intensity
m/z

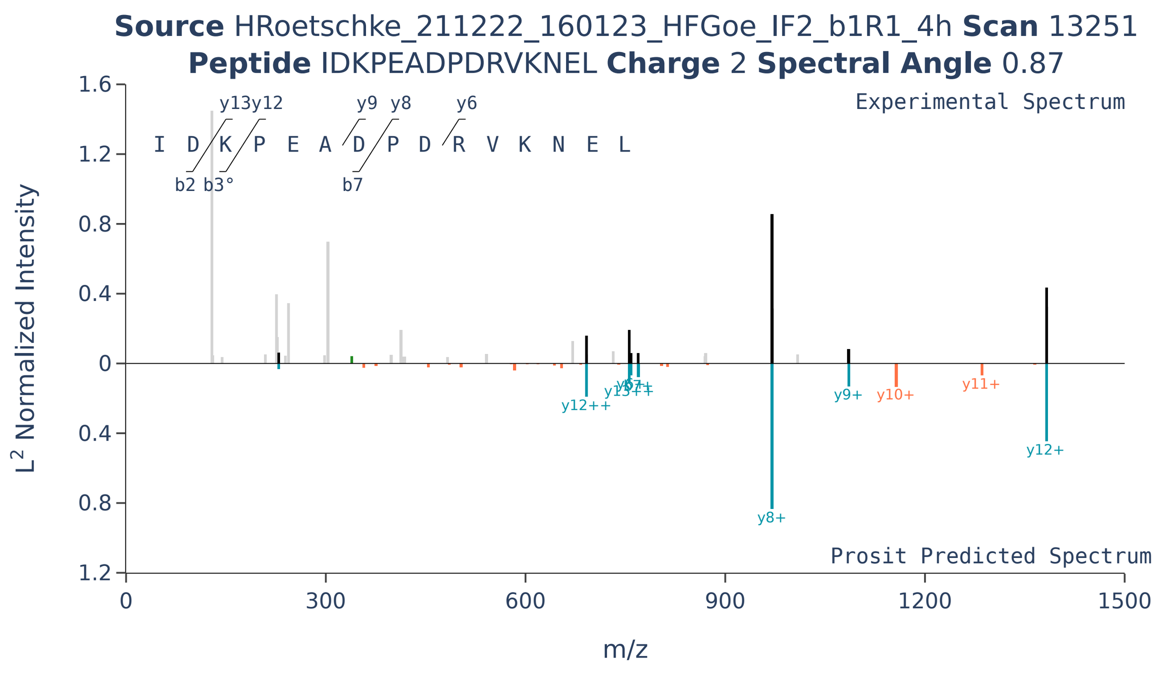
Source HRoetschke_211222_160123_HFGoe_IF2_b1R1_4h Scan 13251
Peptide IDKPEADPDRVKNEL Charge 2 Spectral Angle 0.87
Experimental Spectrum
Prosit Predicted Spectrum
L² Normalized Intensity
m/z

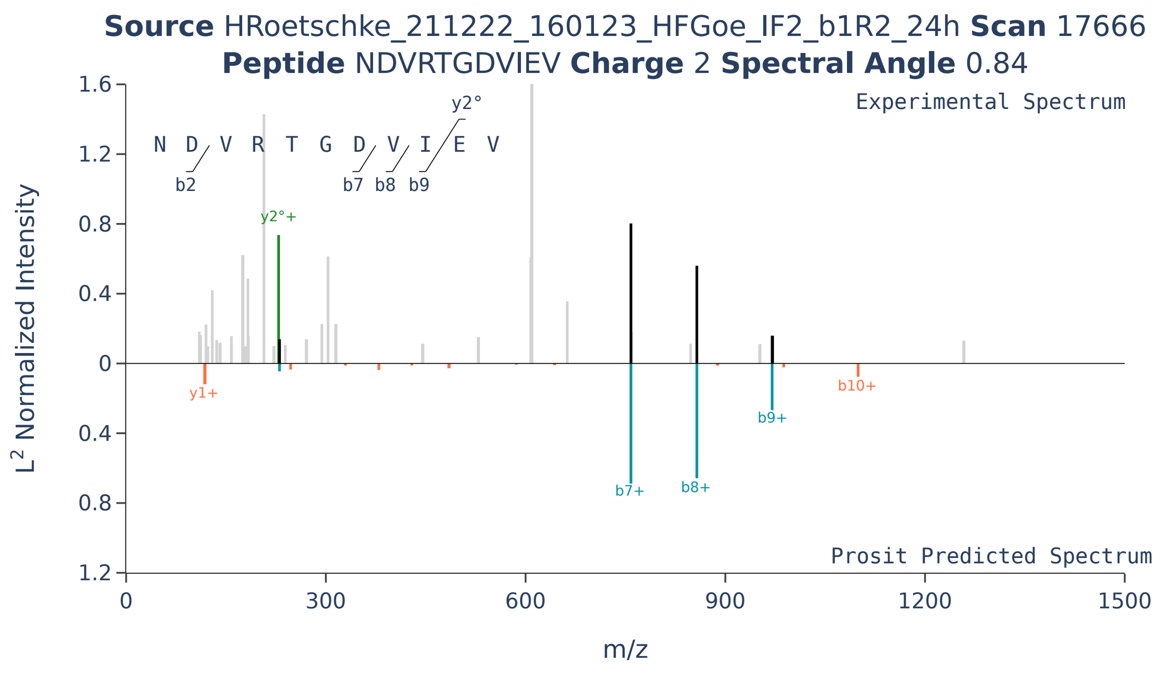
Source HRoetschke_211222_160123_HFGoe_IF2_b1R2_24h Scan 17666
Peptide NDVRTGDVIEV Charge 2 Spectral Angle 0.84
Experimental Spectrum
Prosit Predicted Spectrum
L² Normalized Intensity
m/z

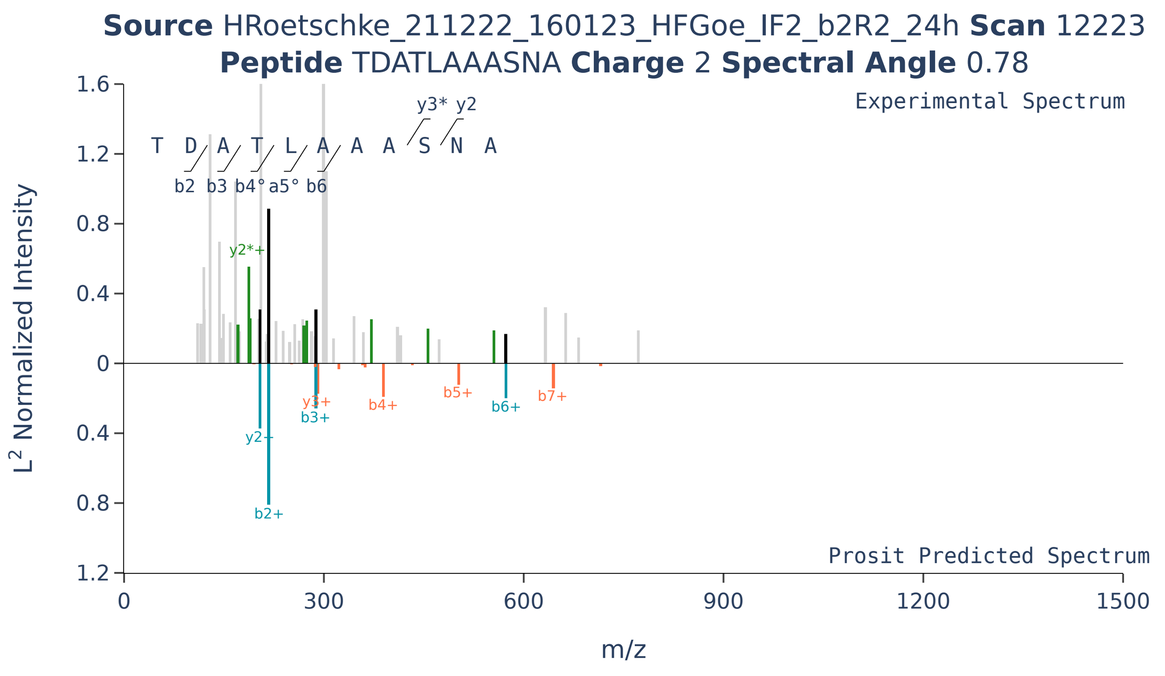
Source HRoetschke_211222_160123_HFGoe_IF2_b2R2_24h Scan 12223
Peptide TDATLAAASNA Charge 2 Spectral Angle 0.78
Experimental Spectrum
Prosit Predicted Spectrum
L² Normalized Intensity
m/z

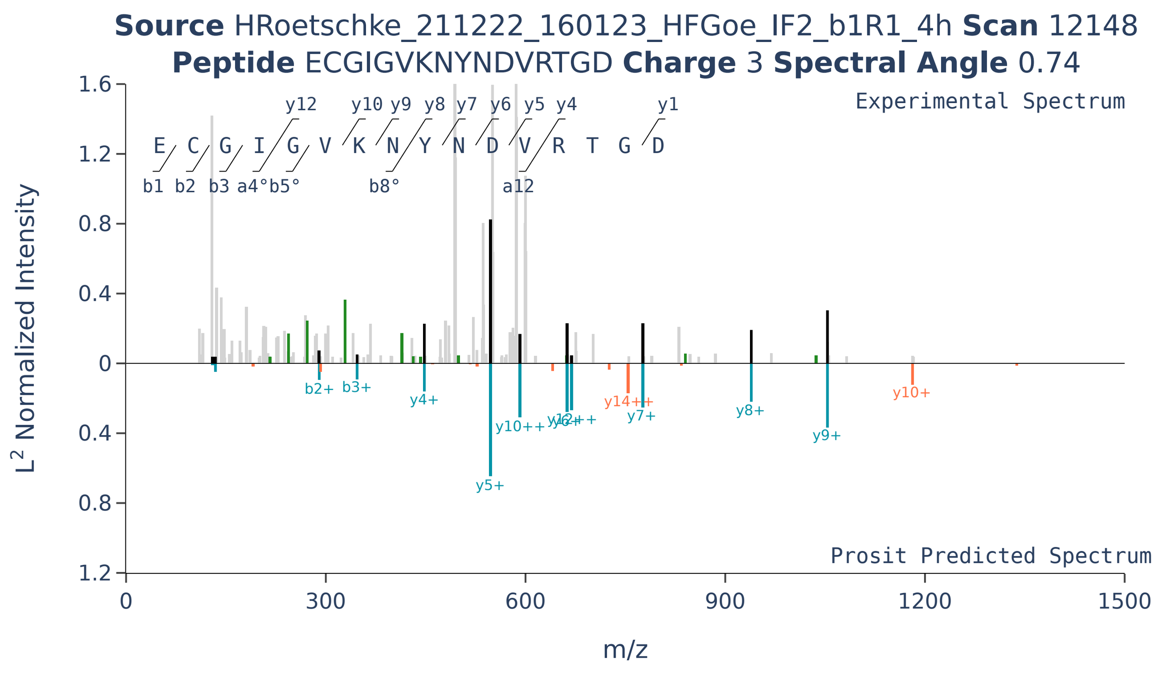
Source HRoetschke_211222_160123_HFGoe_IF2_b1R1_4h Scan 12148
Peptide ECGIGVKNYNDVRTGD Charge 3 Spectral Angle 0.74
Experimental Spectrum
Prosit Predicted Spectrum
L² Normalized Intensity
m/z

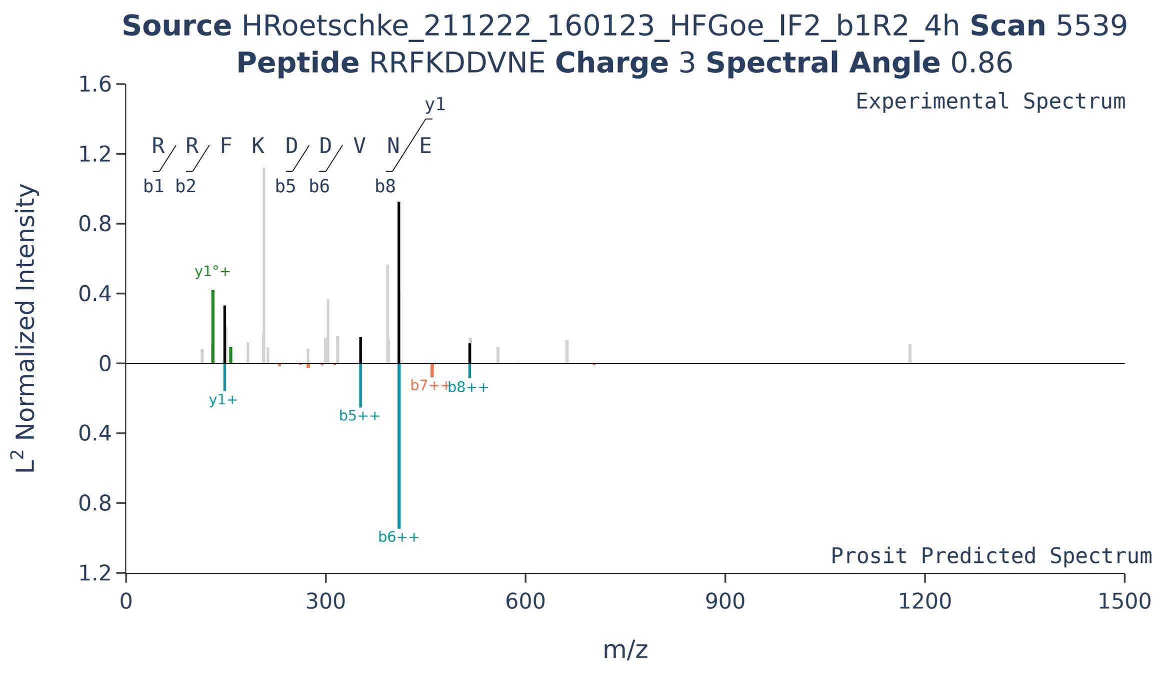
Source HRoetschke_211222_160123_HFGoe_IF2_b1R2_4h Scan 5539
Peptide RRFKDDVNE Charge 3 Spectral Angle 0.86
Experimental Spectrum
Prosit Predicted Spectrum
L² Normalized Intensity
m/z

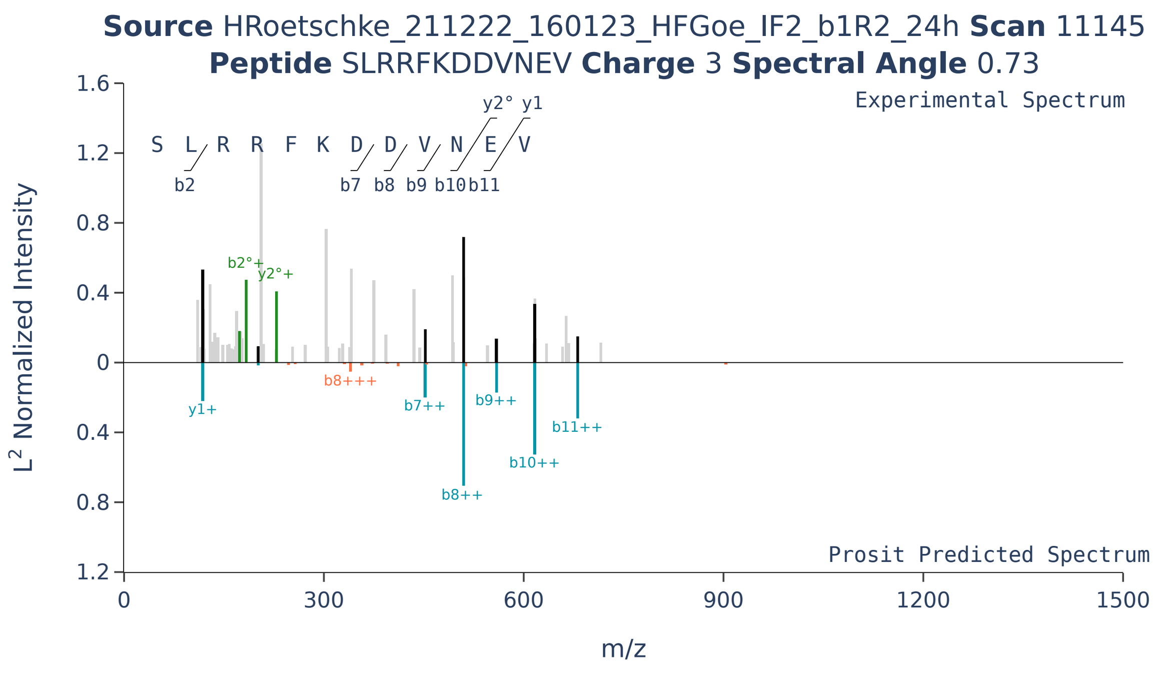
Source HRoetschke_211222_160123_HFGoe_IF2_b1R2_24h Scan 11145
Peptide SLRRFKDDVNEV Charge 3 Spectral Angle 0.73
Experimental Spectrum
Prosit Predicted Spectrum
L² Normalized Intensity
m/z

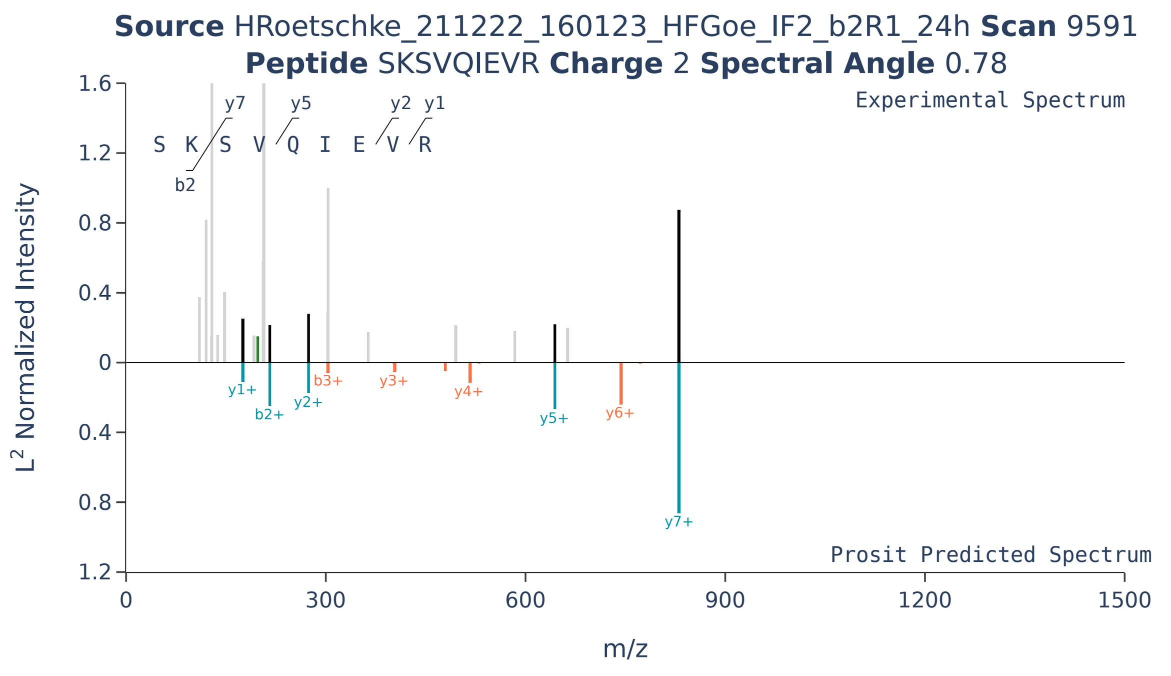
Source HRoetschke_211222_160123_HFGoe_IF2_b2R1_24h Scan 9591
Peptide SKSVQIEVR Charge 2 Spectral Angle 0.78
Experimental Spectrum
Prosit Predicted Spectrum
L² Normalized Intensity
m/z

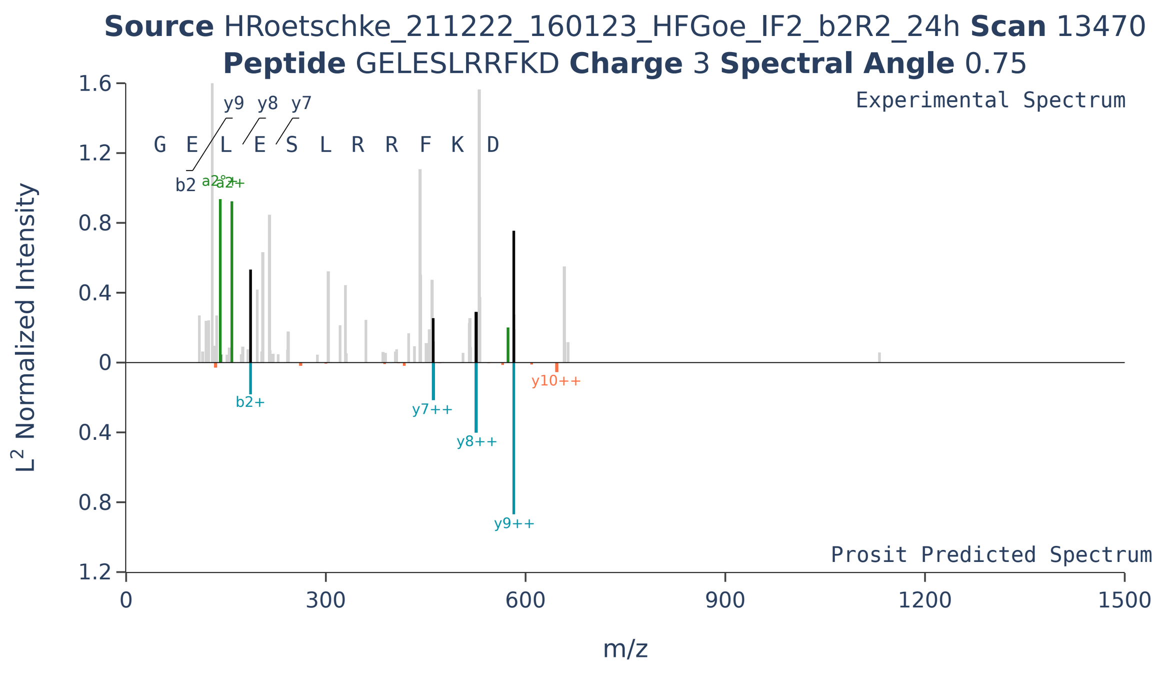
Source HRoetschke_211222_160123_HFGoe_IF2_b2R2_24h Scan 13470
Peptide GELESLRRFKD Charge 3 Spectral Angle 0.75
Experimental Spectrum
Prosit Predicted Spectrum
L² Normalized Intensity
m/z

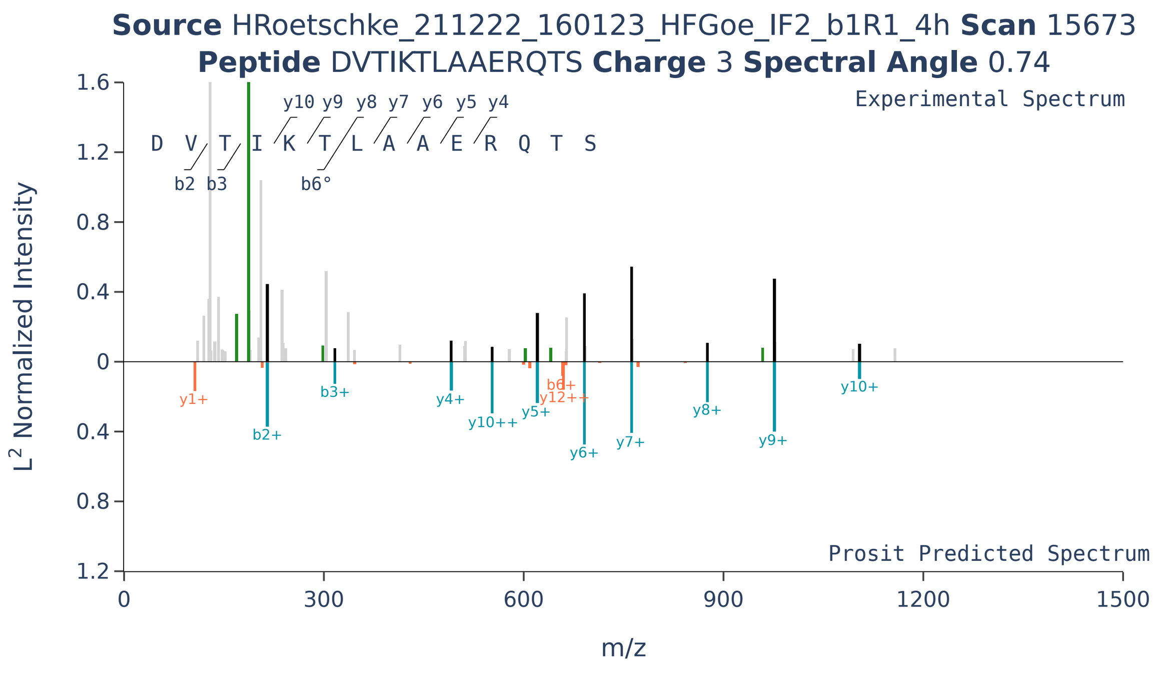
Source HRoetschke_211222_160123_HFGoe_IF2_b1R1_4h Scan 15673
Peptide DVTIKTLAAERQTS Charge 3 Spectral Angle 0.74
Experimental Spectrum
Prosit Predicted Spectrum
L² Normalized Intensity
m/z

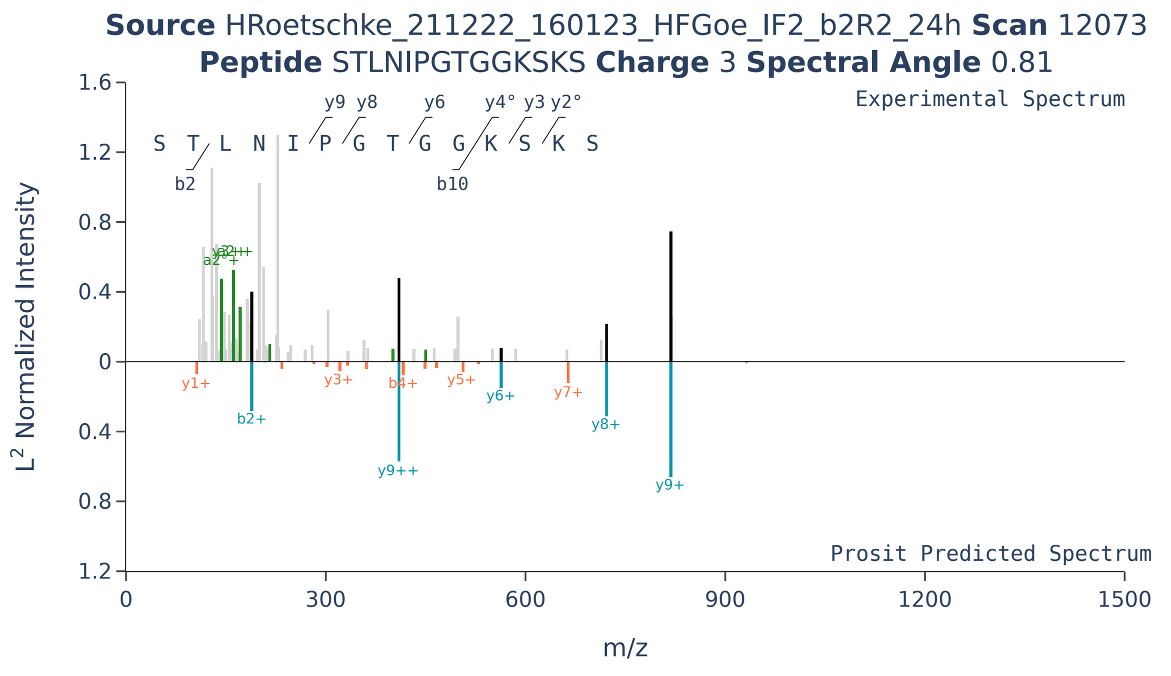
Source HRoetschke_211222_160123_HFGoe_IF2_b2R2_24h Scan 12073
Peptide STLNIPGTGGKSKS Charge 3 Spectral Angle 0.81
Experimental Spectrum
Prosit Predicted Spectrum
L² Normalized Intensity
m/z

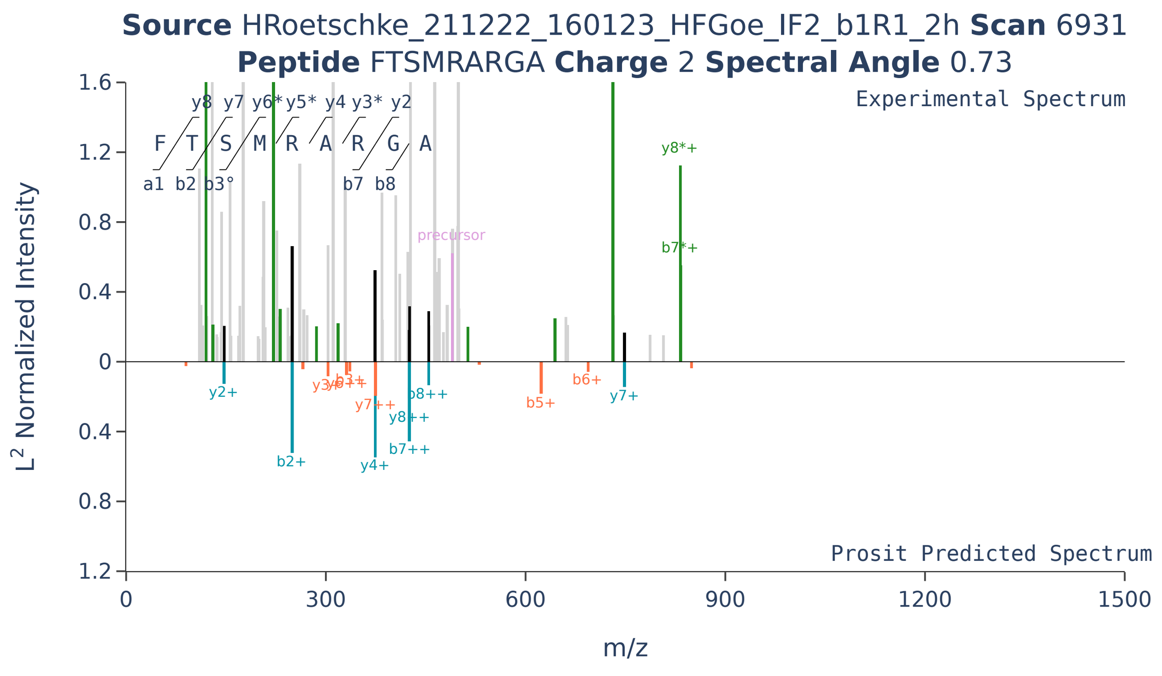
Source HRoetschke_211222_160123_HFGoe_IF2_b1R1_2h Scan 6931
Peptide FTSMRARGA Charge 2 Spectral Angle 0.73
Experimental Spectrum
Prosit Predicted Spectrum
L² Normalized Intensity
m/z

**Source** HRoetschke_211222_160123_HFGoe_IF2_b1R1_4h **Scan** 12181
**Peptide** TPGHAAFTSMRA **Charge** 2 **Spectral Angle** 0.72

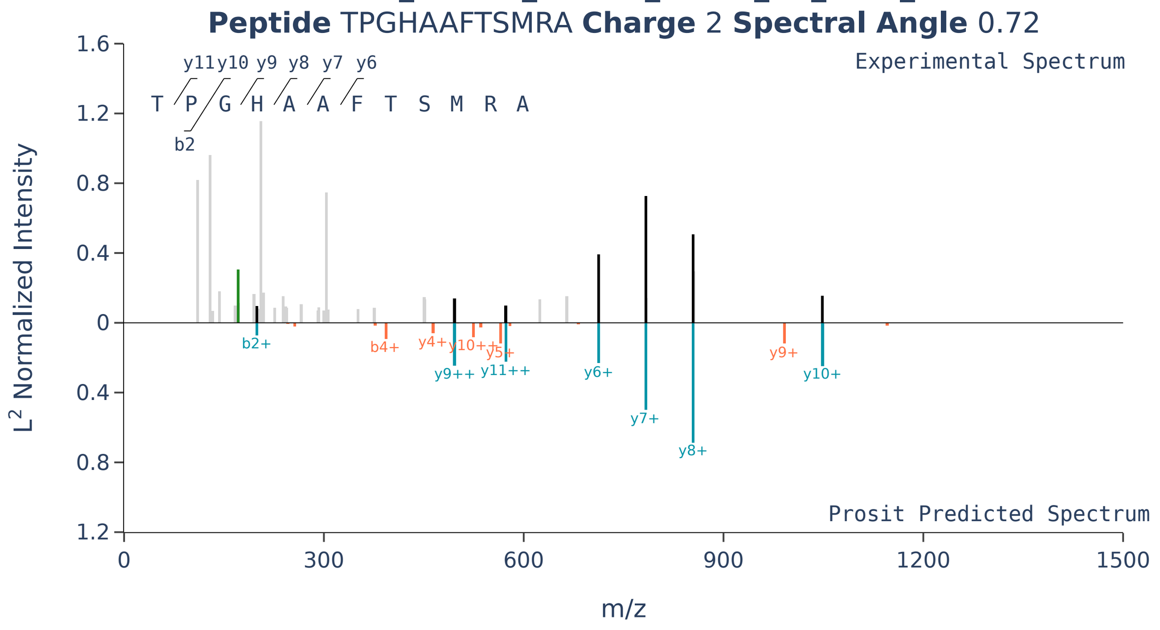

**Source** HRoetschke_211222_160123_HFGoe_IF2_b2R1_4h **Scan** 6096
**Peptide** DYHVTTSQH **Charge** 2 **Spectral Angle** 0.76

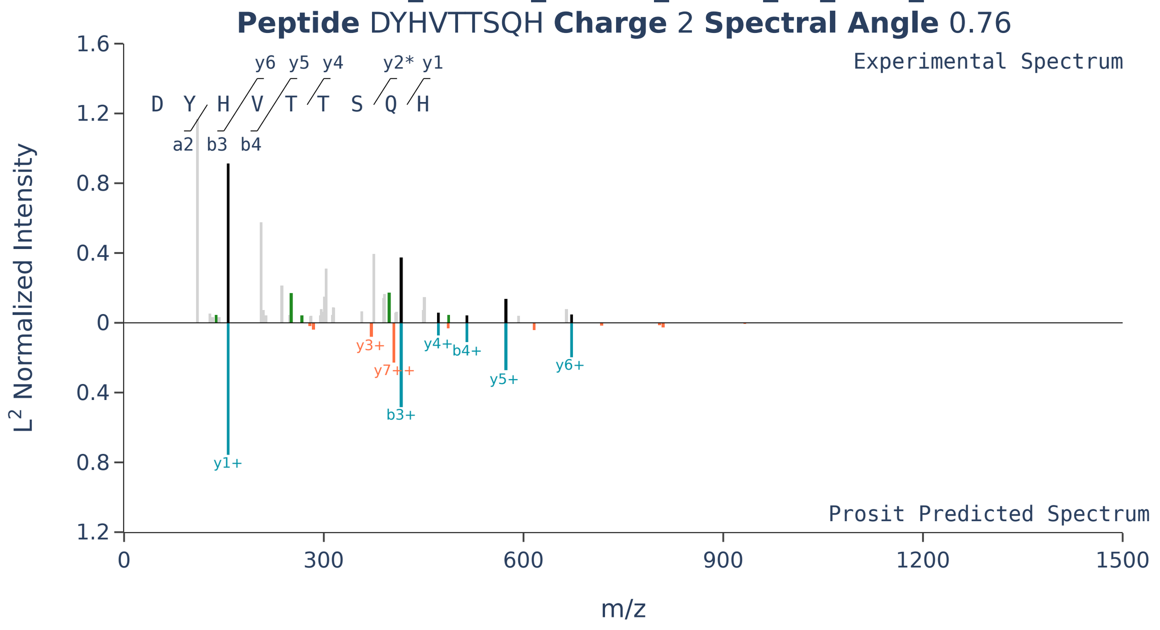

**Source** HRoetschke_211222_160123_HFGoe_IF2_b1R1_4h **Scan** 20879
**Peptide** DGVMPQTL **Charge** 2 **Spectral Angle** 0.76

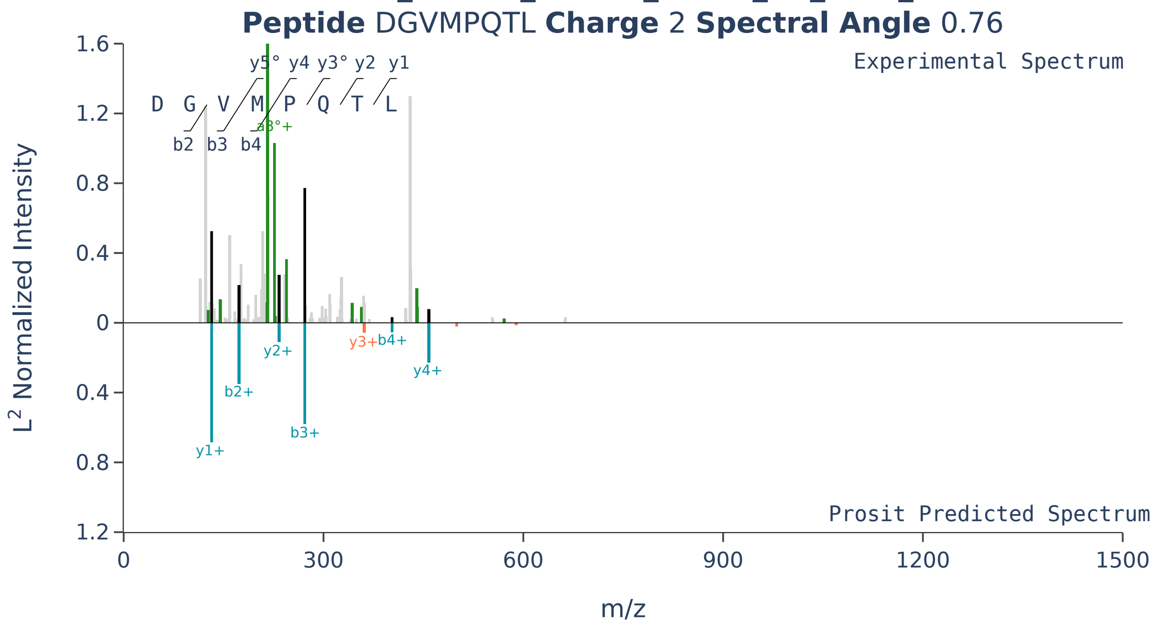

**Source** HRoetschke_211222_160123_HFGoe_IF2_b1R2_2h **Scan** 16881
**Peptide** AADDGVMPQTIEAIQH **Charge** 2 **Spectral Angle** 0.73

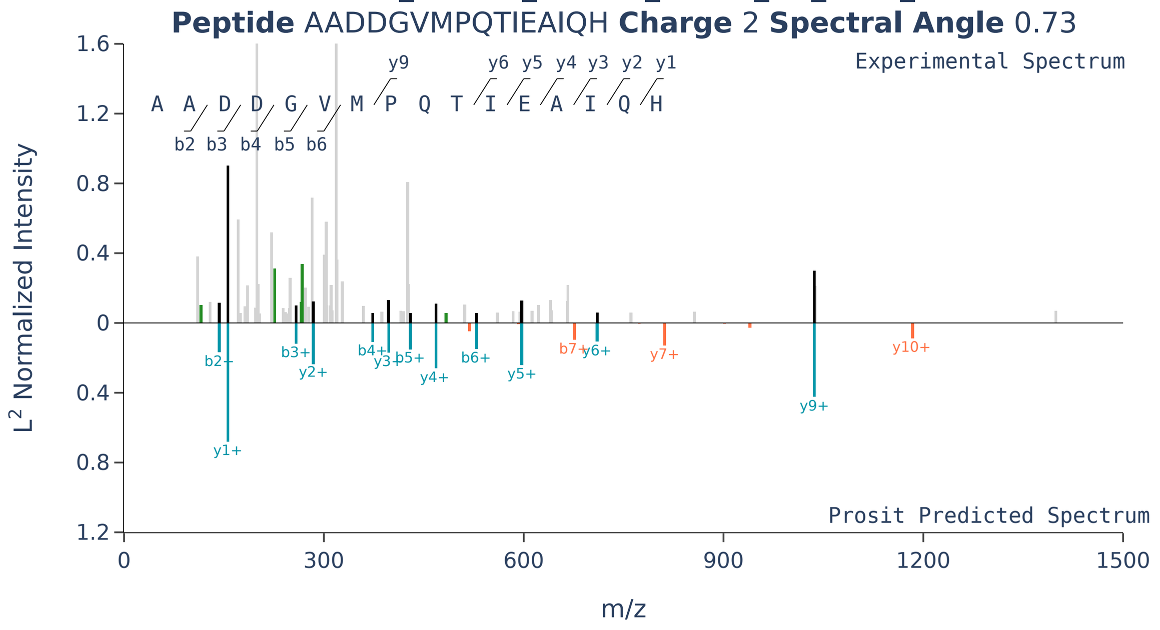

**Source** HRoetschke_211222_160123_HFGoe_IF2_b1R2_4h **Scan** 16318
**Peptide** SAQEKQTLIDHL **Charge** 3 **Spectral Angle** 0.76

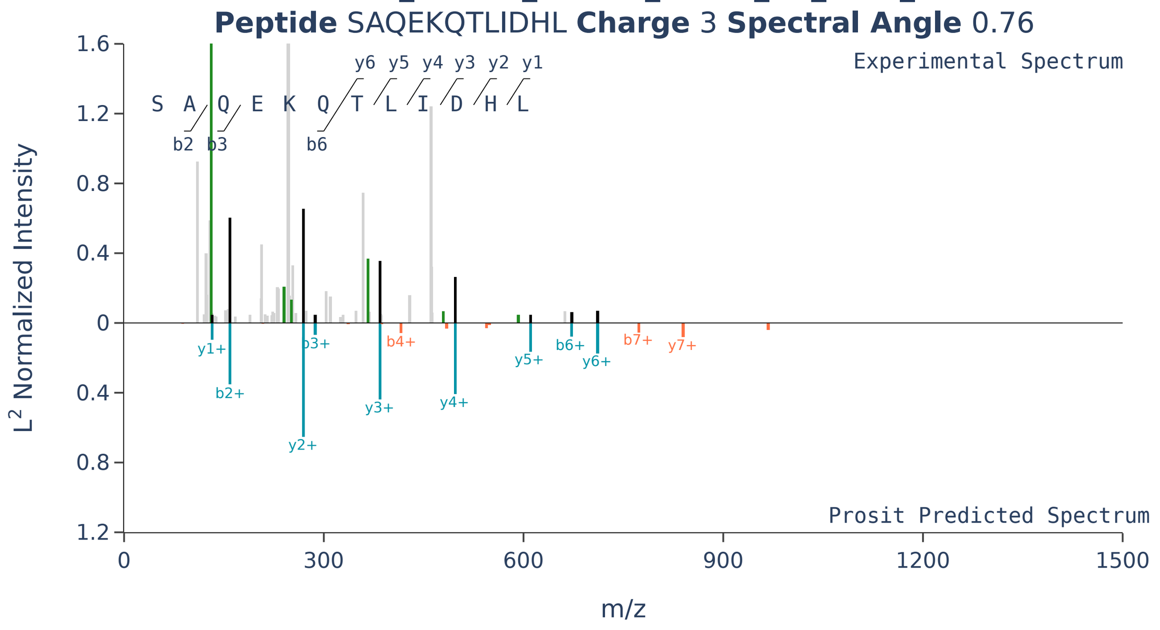

**Source** HRoetschke_211222_160123_HFGoe_IF2_b1R2_24h **Scan** 19518
**Peptide** AAAEPRAPVVTI **Charge** 2 **Spectral Angle** 0.75

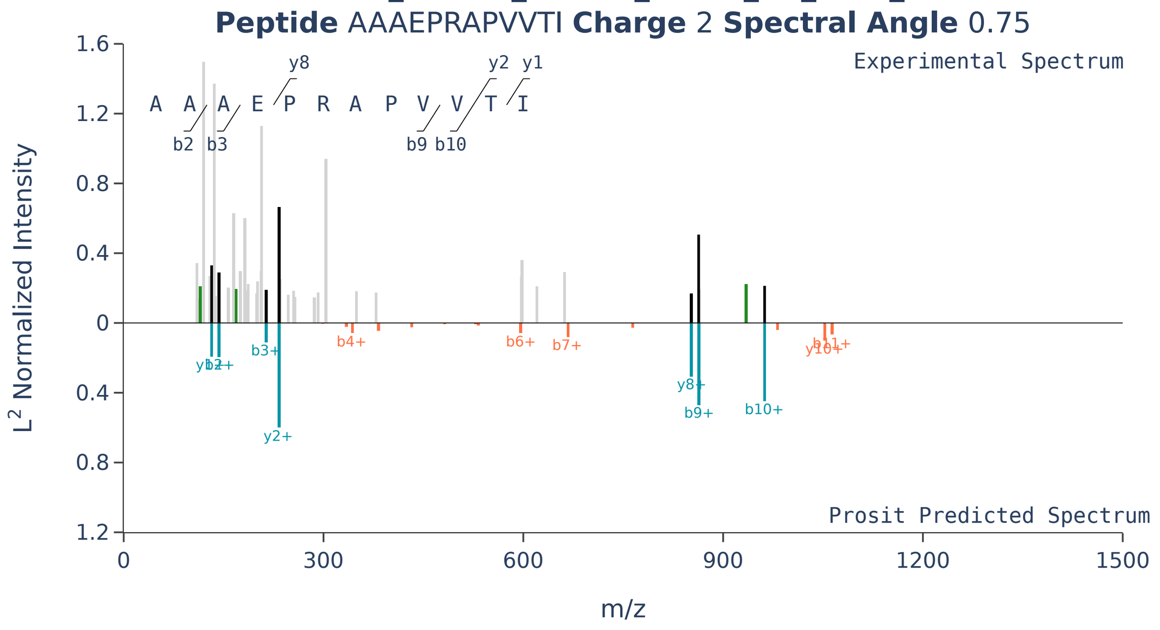

**Source** HRoetschke_211222_160123_HFGoe_IF2_b1R1_24h **Scan** 12075
**Peptide** ELESLRRFKD **Charge** 3 **Spectral Angle** 0.83

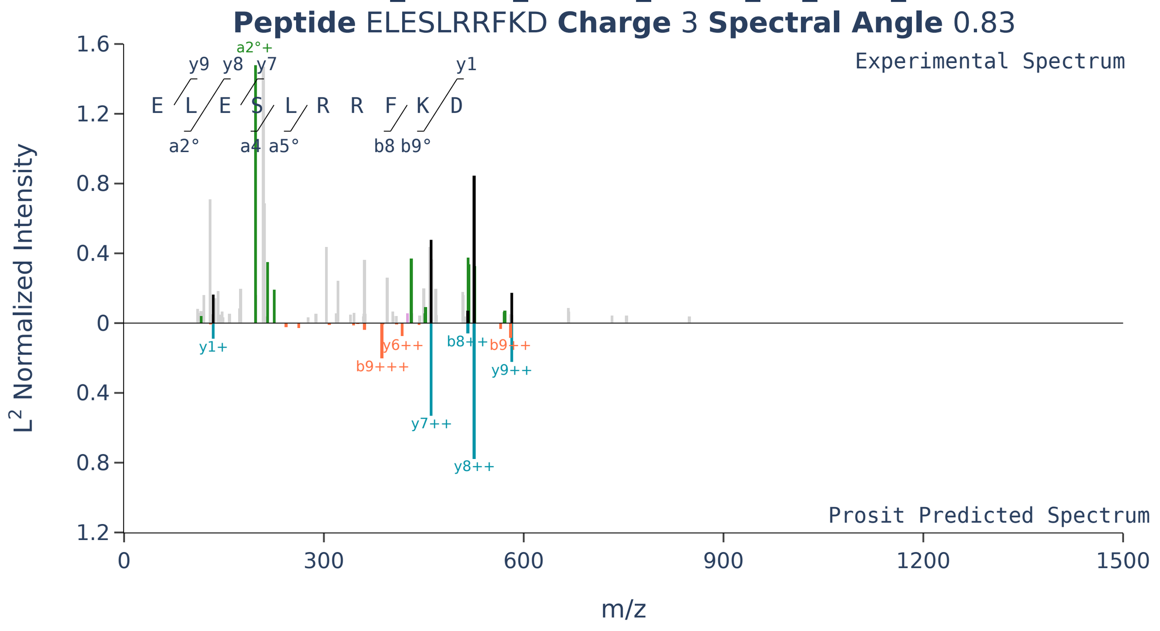

**Source** HRoetschke_211222_160123_HFGoe_IF2_b2R1_24h **Scan** 9192
**Peptide** GTLHKGDIV **Charge** 2 **Spectral Angle** 0.65

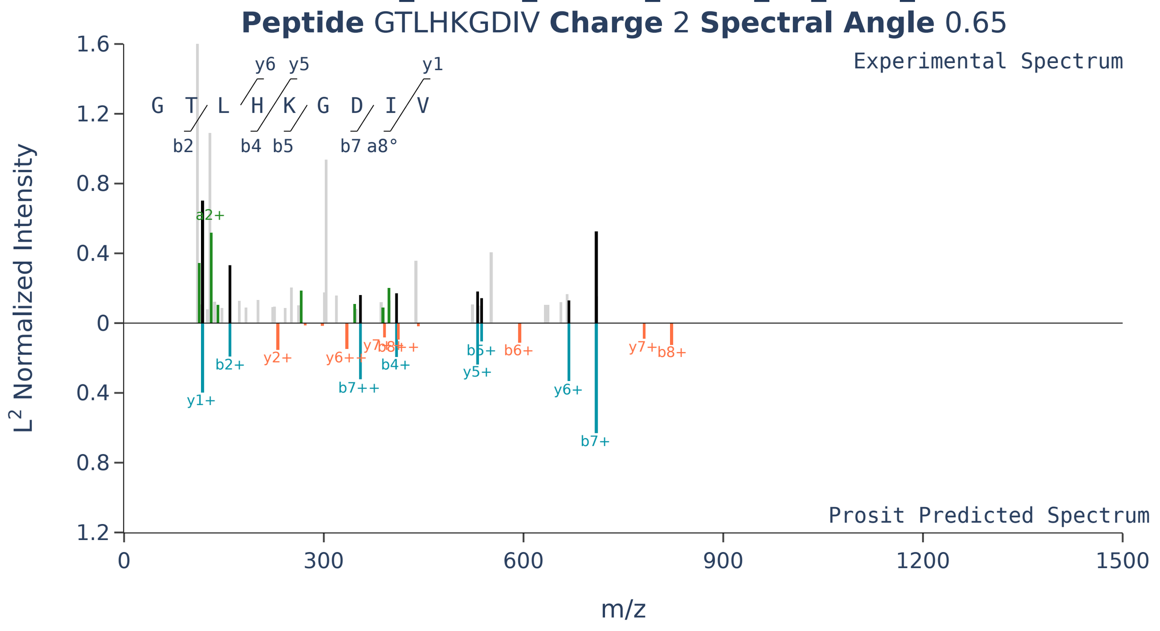

**Source** HRoetschke_211222_160123_HFGoe_IF2_b1R1_4h **Scan** 10112
**Peptide** LDTPGHAAFTSMRARG **Charge** 4 **Spectral Angle** 0.66

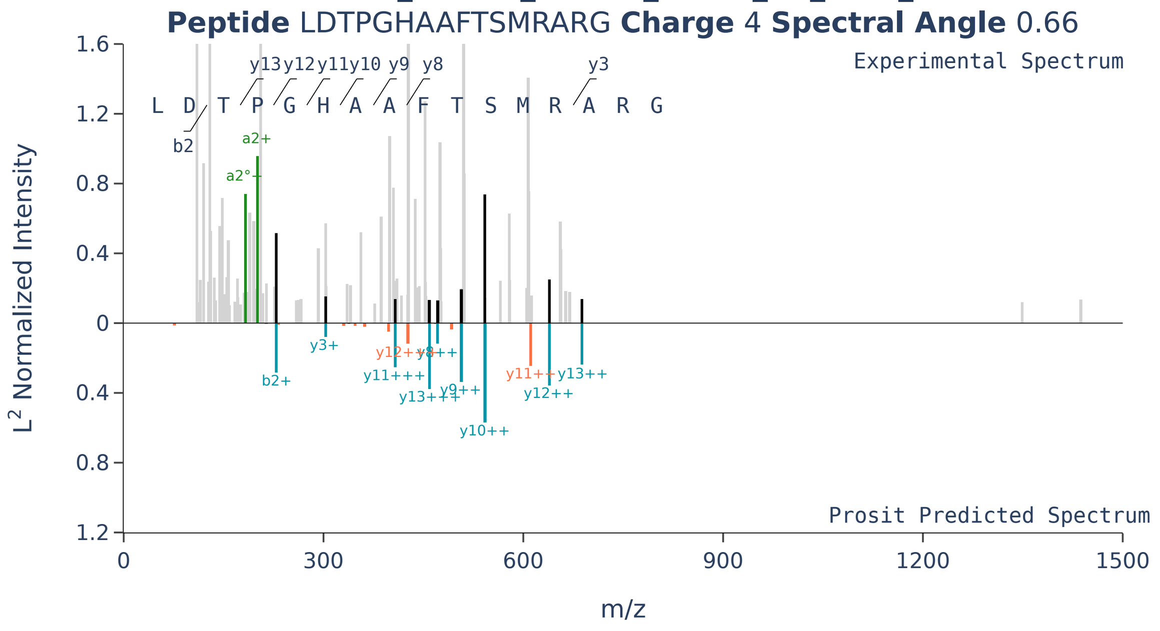

**Source** HRoetschke_211222_160123_HFGoe_IF2_b2R2_24h **Scan** 7115
**Peptide** VVRDEKKAREV **Charge** 3 **Spectral Angle** 0.84

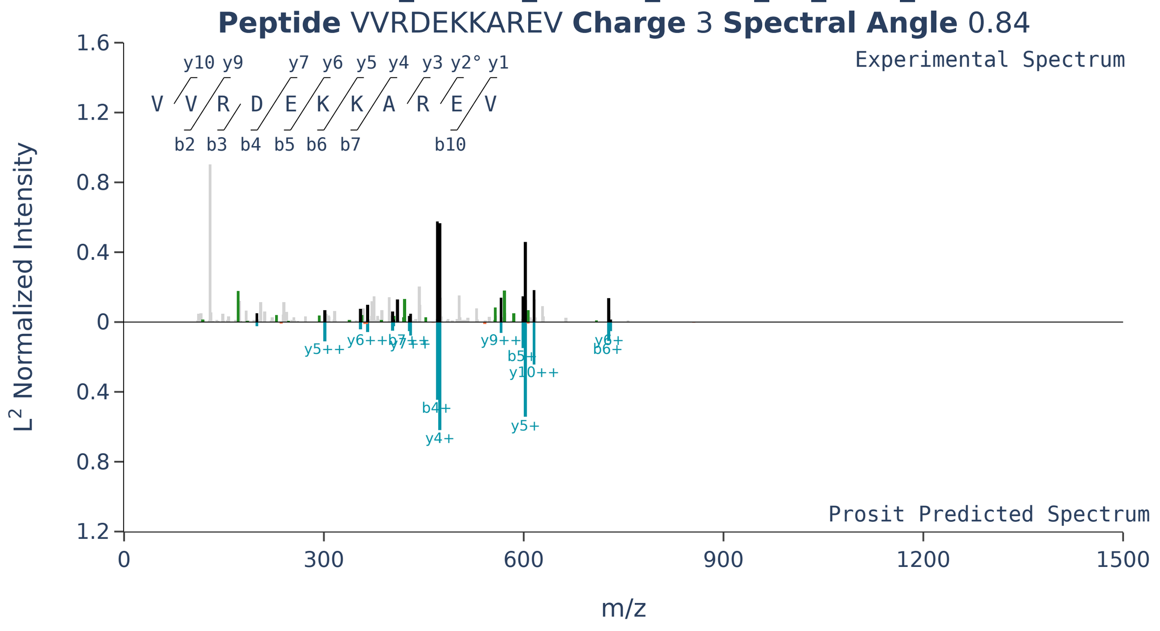

**Source** HRoetschke_211222_160123_HFGoe_IF2_b1R1_4h **Scan** 8964
**Peptide** KAVRKGMASGAVI **Charge** 2 **Spectral Angle** 0.62

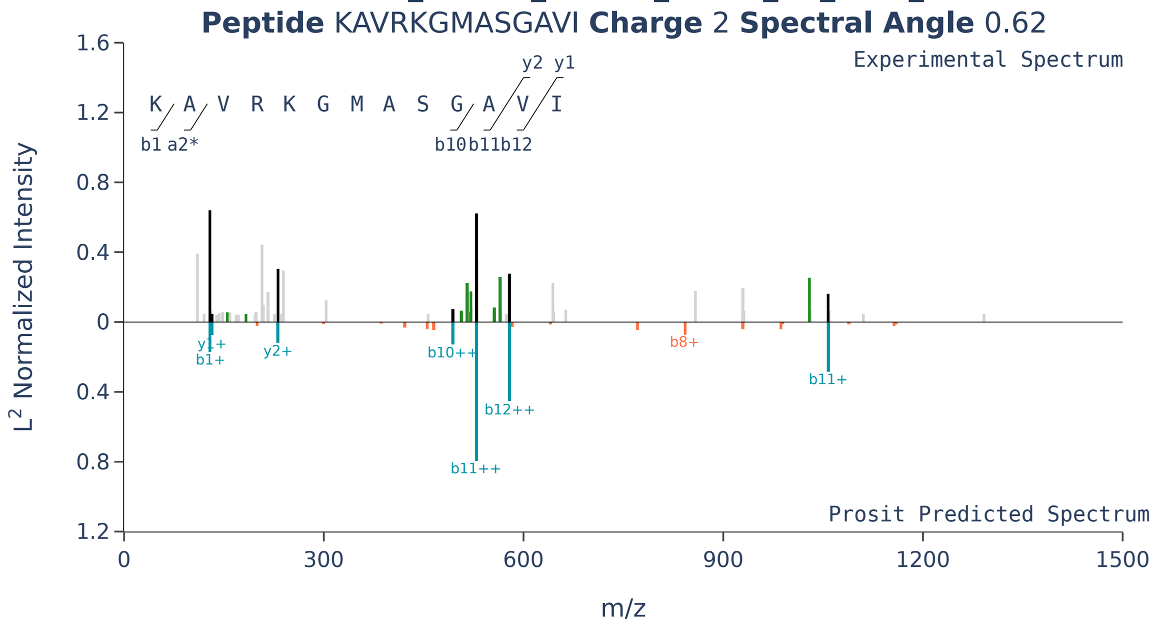

**Source** HRoetschke_211222_160123_HFGoe_IF2_b2R2_2h **Scan** 9674
**Peptide** KPEADPDRVKNELSQ **Charge** 3 **Spectral Angle** 0.78

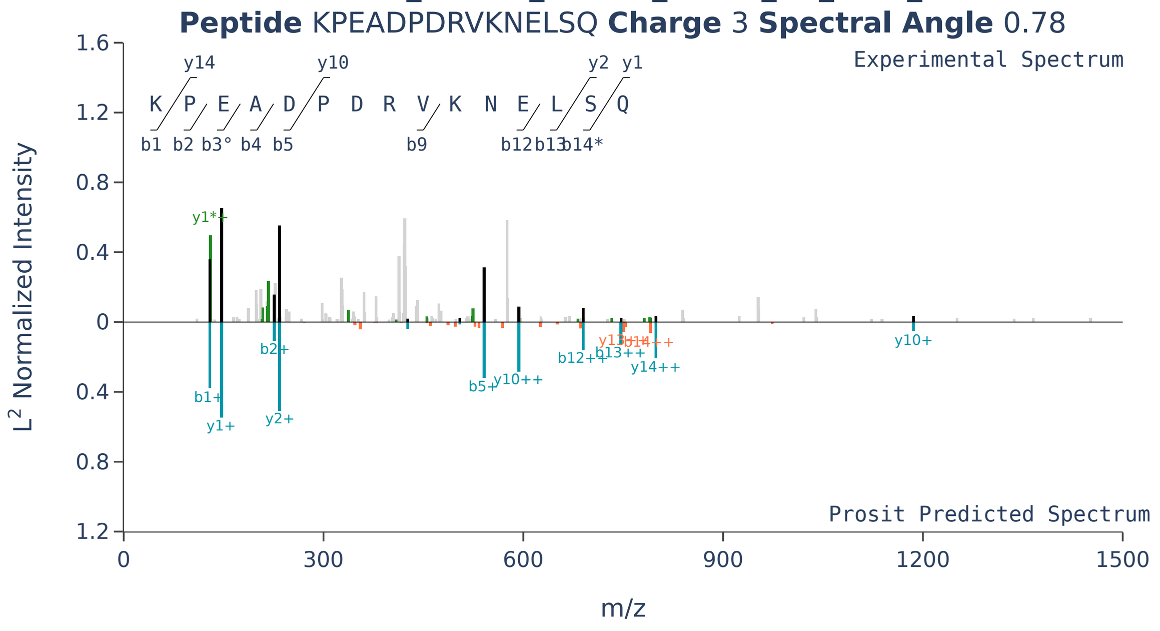

**Source** HRoetschke_211222_160123_HFGoe_IF2_b1R1_4h **Scan** 14475
**Peptide** LNQKNSGPDKLTL **Charge** 3 **Spectral Angle** 0.77

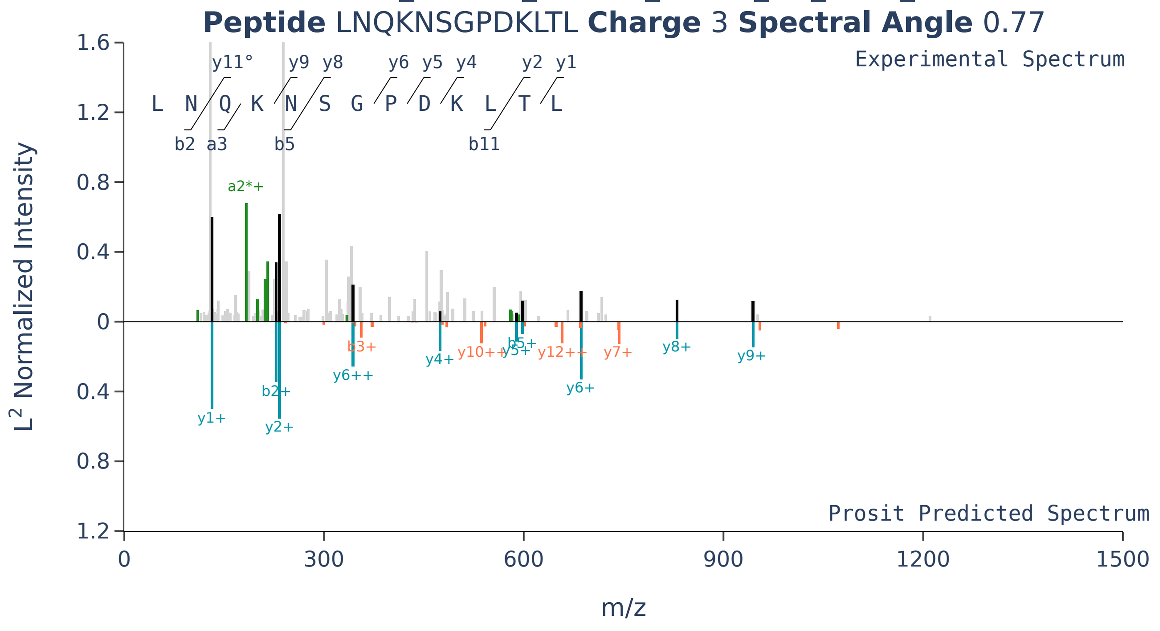

**Source** HRoetschke_211222_160123_HFGoe_IF2_b1R2_24h **Scan** 17351
**Peptide** AAEPRAPVVTIM **Charge** 2 **Spectral Angle** 0.77

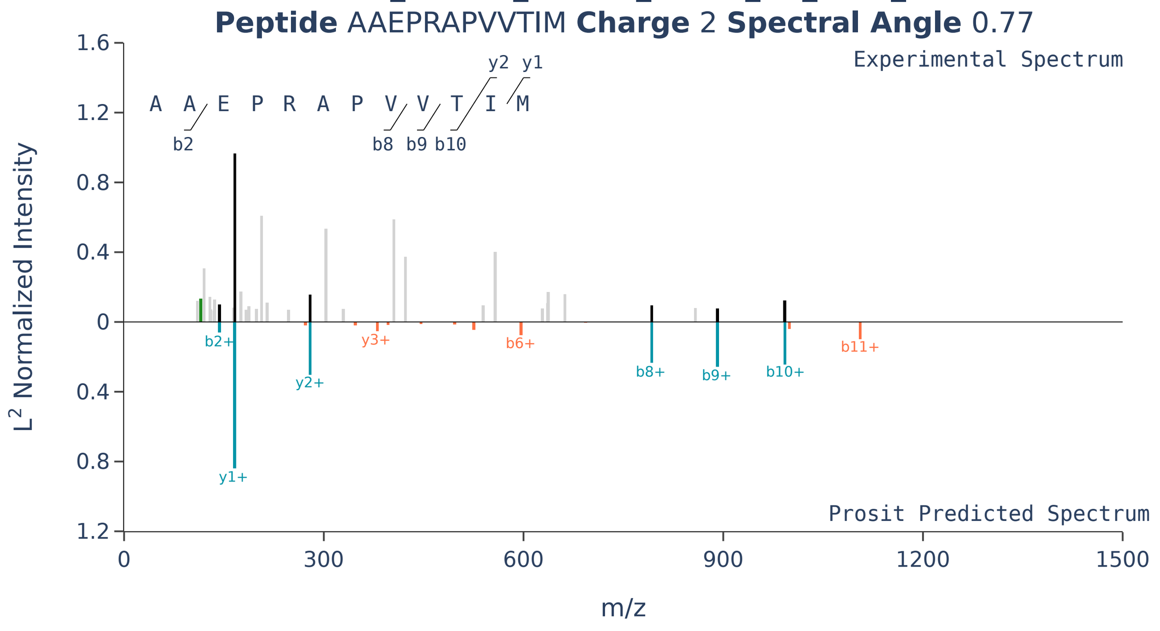

**Source** HRoetschke_211222_160123_HFGoe_IF2_b1R1_4h **Scan** 13237
**Peptide** AEVLELKAVRKGMA **Charge** 3 **Spectral Angle** 0.56

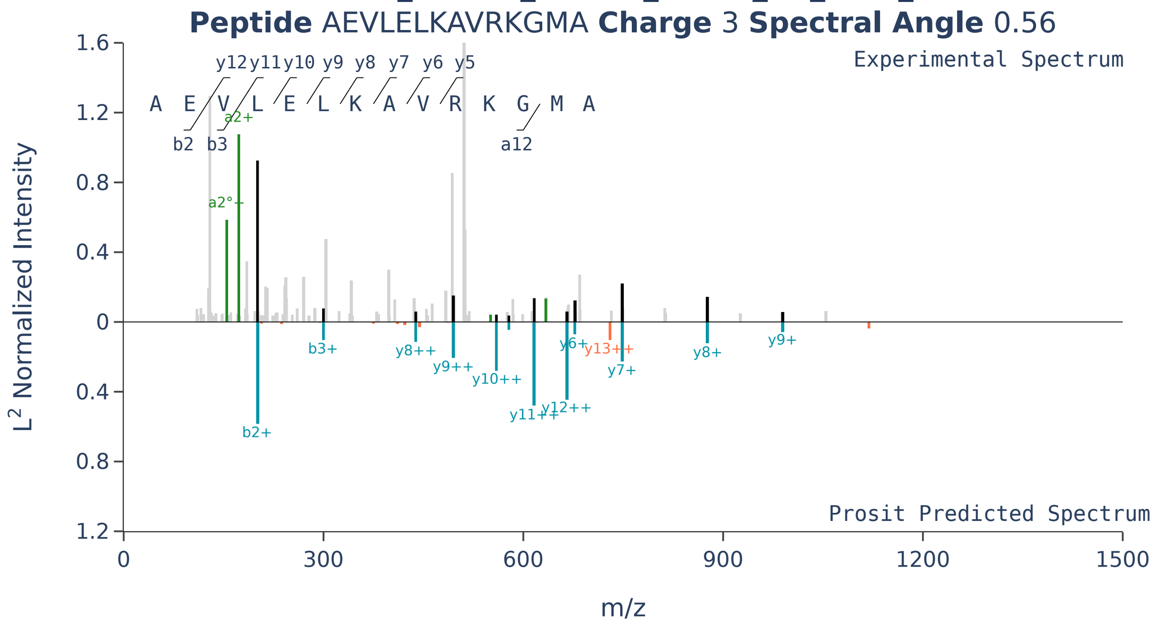

**Source** HRoetschke_211222_160123_HFGoe_IF2_b1R1_4h **Scan** 5913
**Peptide** KNSGPDKLTLQRKTR **Charge** 3 **Spectral Angle** 0.74

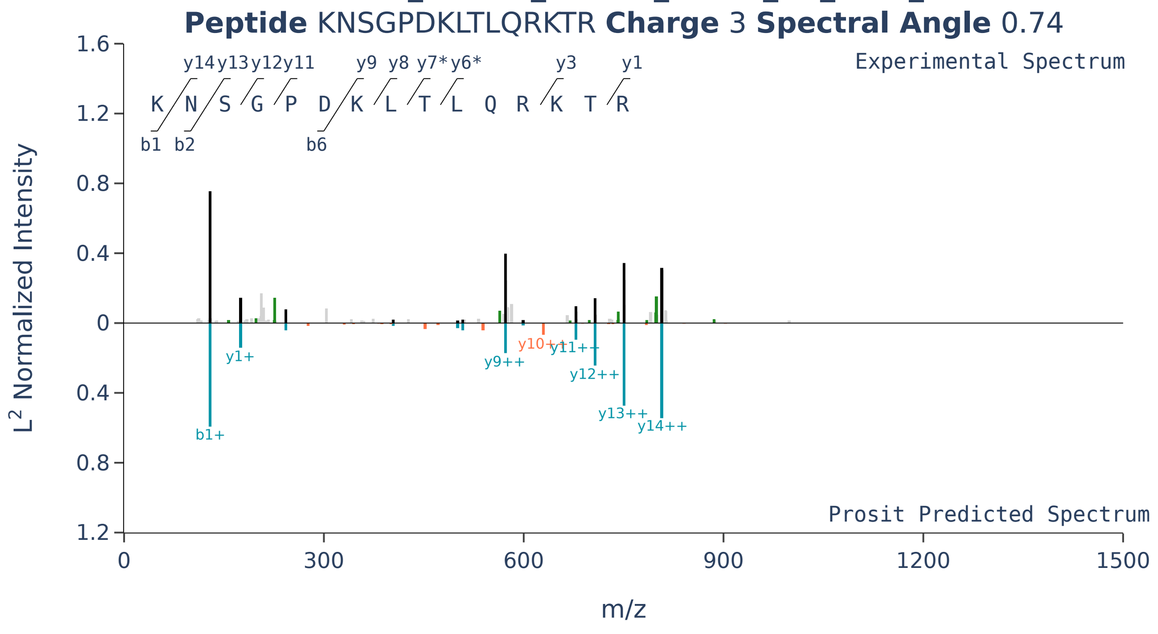

**Source** HRoetschke_211222_160123_HFGoe_IF2_b1R2_24h **Scan** 13432
**Peptide** DYIRSTKVASGEAGGITQ **Charge** 3 **Spectral Angle** 0.84

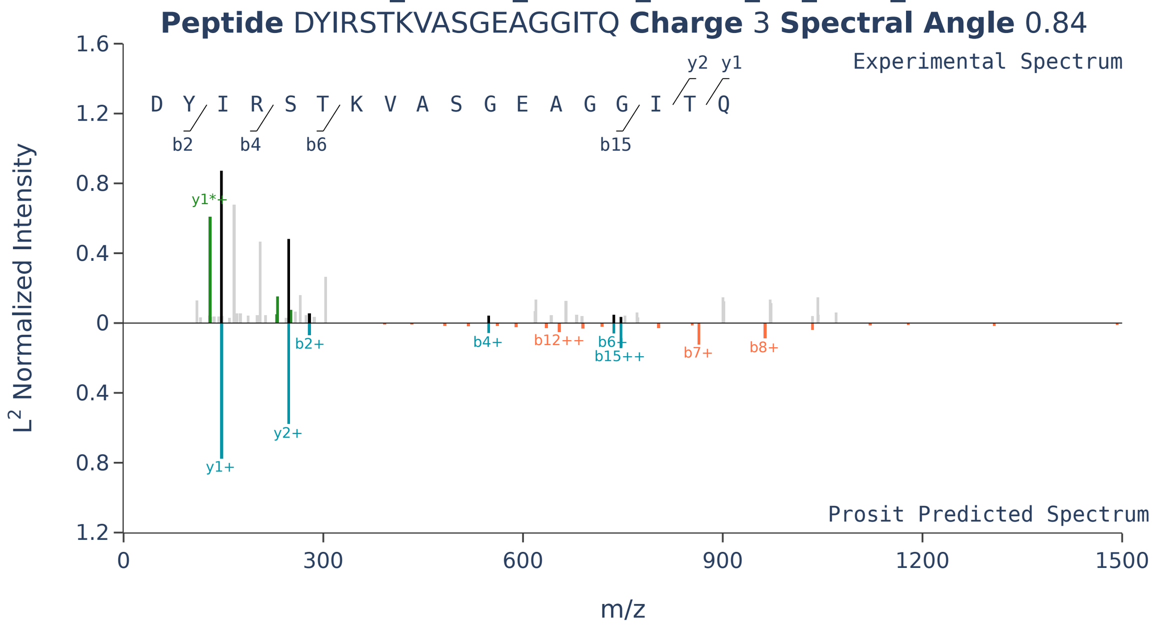

**Source** HRoetschke_211222_160123_HFGoe_IF2_b2R2_4h **Scan** 10998
**Peptide** DSVSAQEKQTLIDH **Charge** 3 **Spectral Angle** 0.79

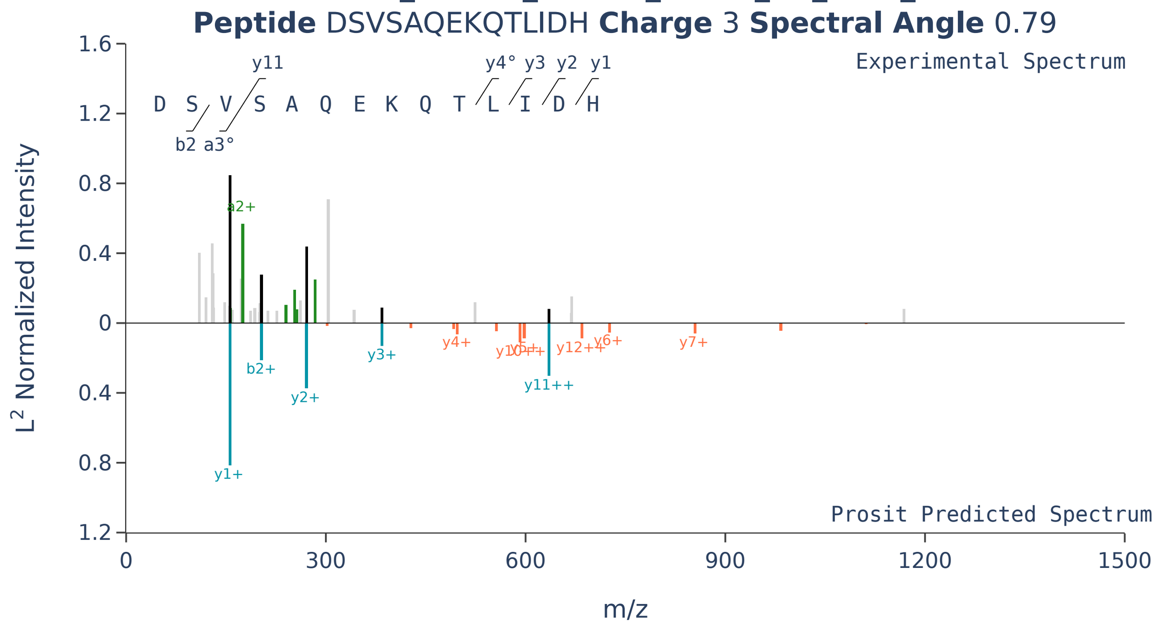

**Source** HRoetschke_211222_160123_HFGoe_IF2_b1R1_2h **Scan** 7348
**Peptide** TFVKRDPQEAE **Charge** 2 **Spectral Angle** 0.68

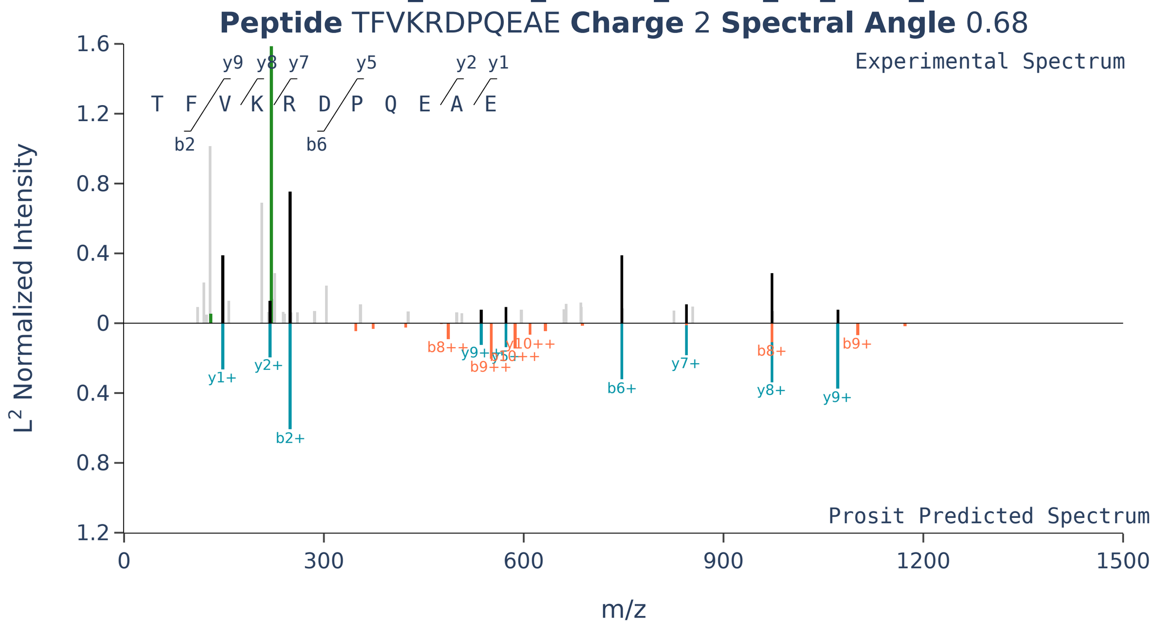

**Source** HRoetschke_211222_160123_HFGoe_IF2_b1R1_4h **Scan** 13759
**Peptide** AQEKQTLIDHLNQ **Charge** 3 **Spectral Angle** 0.72

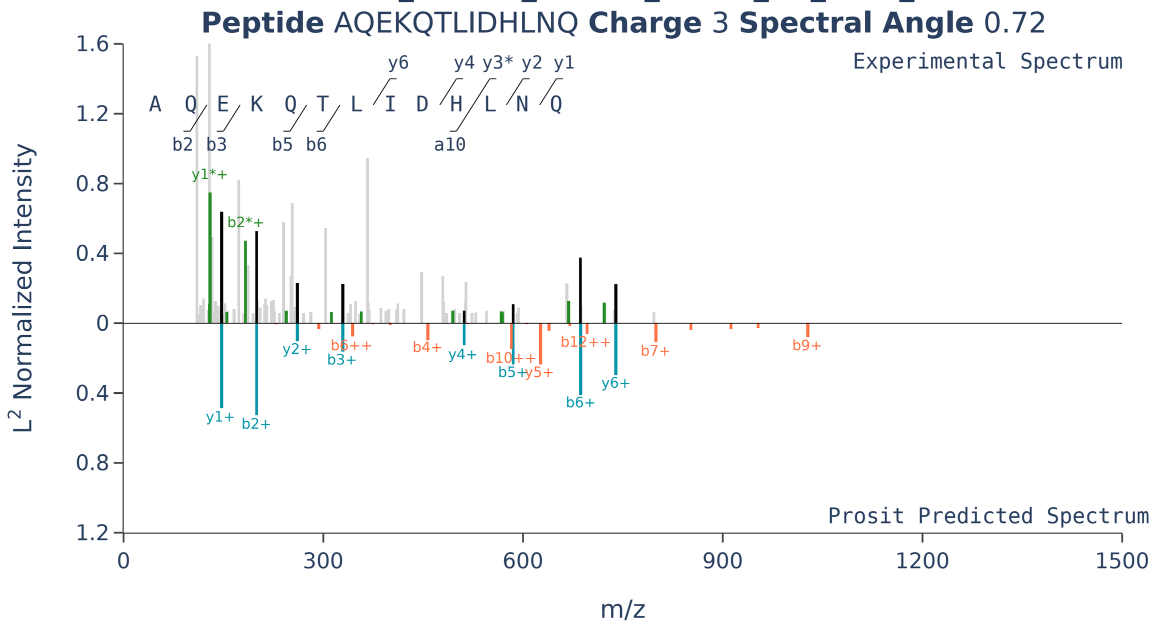

**Source** HRoetschke_211222_160123_HFGoe_IF2_b2R1_24h **Scan** 6391
**Peptide** TIMGHVDHGKTS **Charge** 3 **Spectral Angle** 0.62

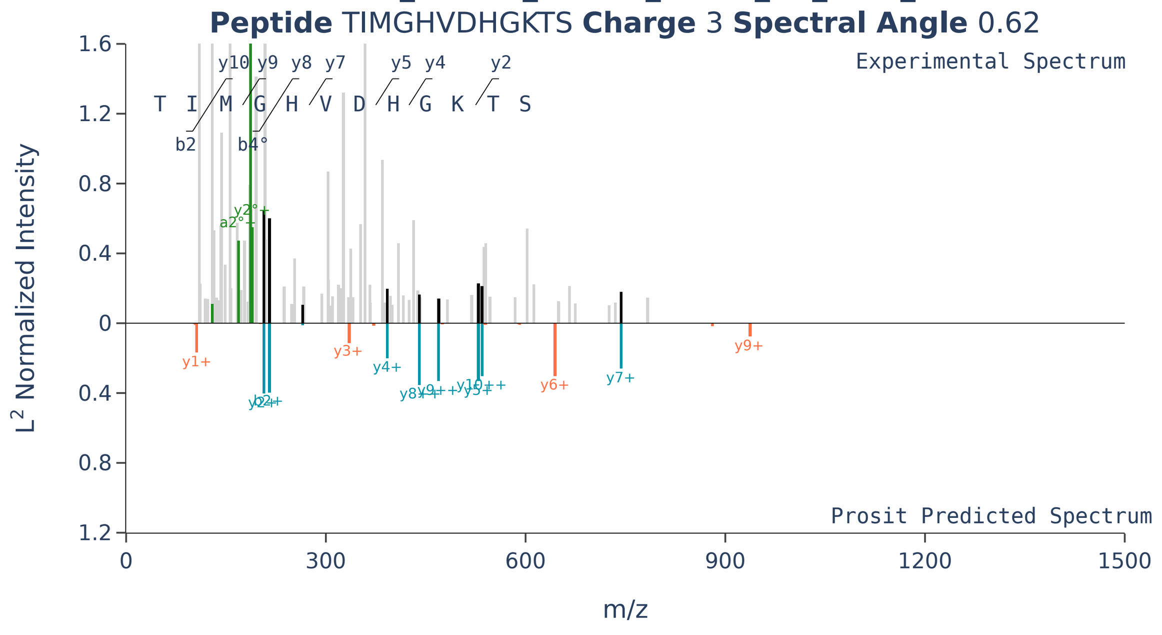

**Source** HRoetschke_211222_160123_HFGoe_IF2_b1R1_4h **Scan** 9050
**Peptide** DYIRSTKVASG **Charge** 2 **Spectral Angle** 0.71

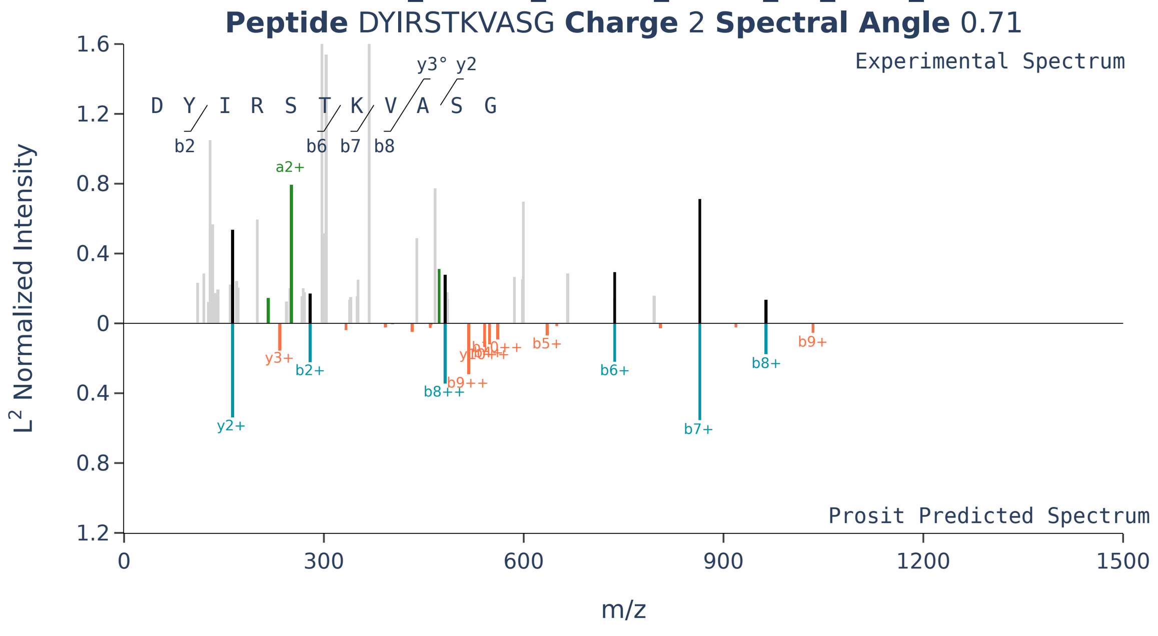

**Source** HRoetschke_211222_160123_HFGoe_IF2_b1R1_24h **Scan** 8378
**Peptide** TVVRDEKKAREVAL **Charge** 3 **Spectral Angle** 0.66

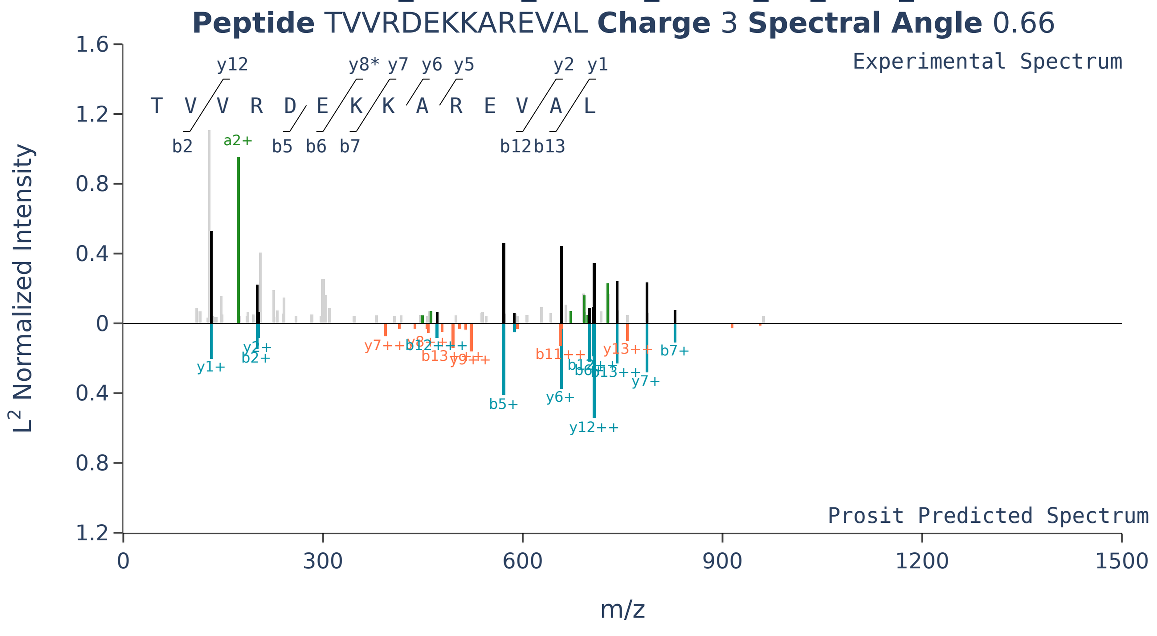

**Source** HRoetschke_211222_160123_HFGoe_IF2_b1R1_24h **Scan** 16242
**Peptide** RDTGAAAEPRAPVVTIMG **Charge** 3 **Spectral Angle** 0.74

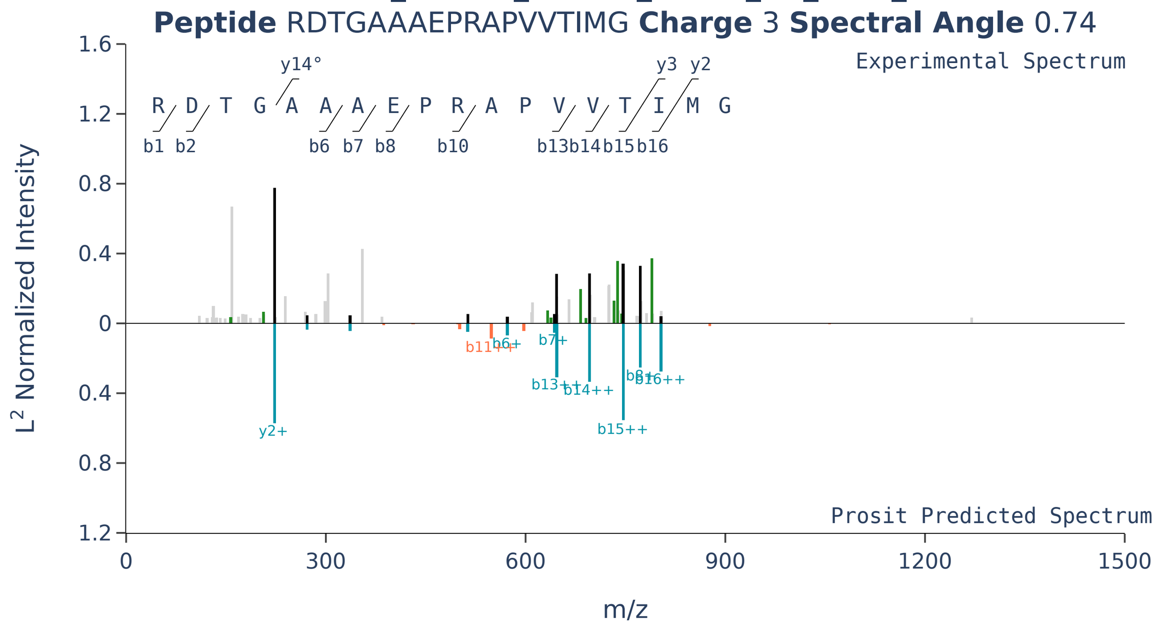

**Source** HRoetschke_211222_160123_HFGoe_IF2_b2R2_24h **Scan** 9729
**Peptide** RFKDDVNEV **Charge** 3 **Spectral Angle** 0.62

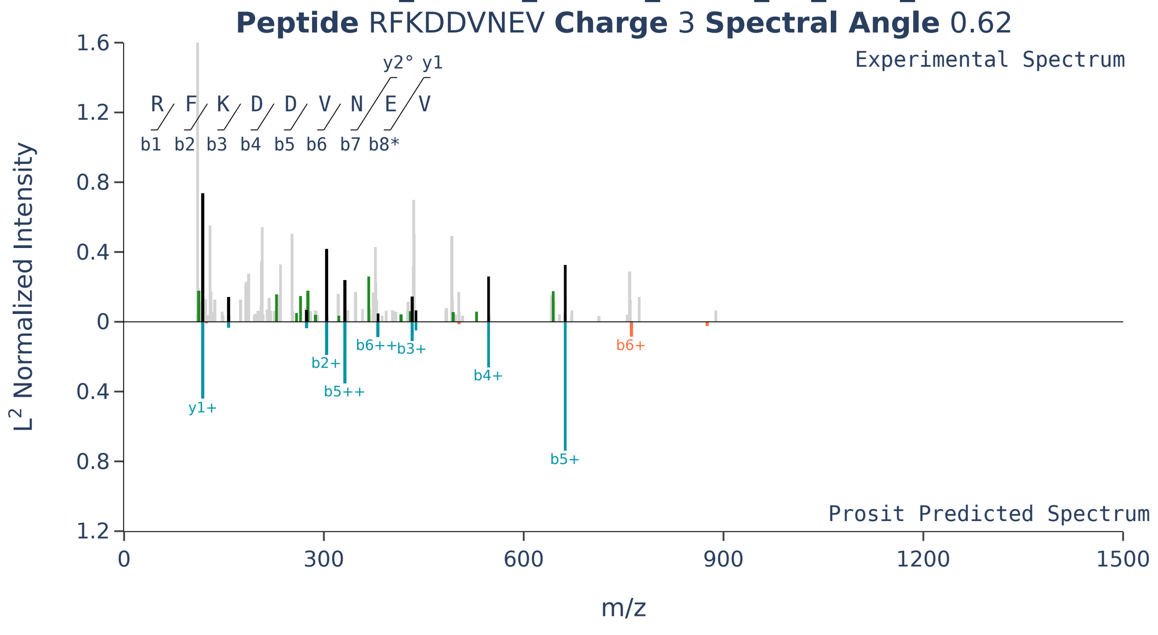

**Source** HRoetschke_211222_160123_HFGoe_IF2_b2R1_24h **Scan** 7003
**Peptide** VNKIDKPEADPD **Charge** 3 **Spectral Angle** 0.79

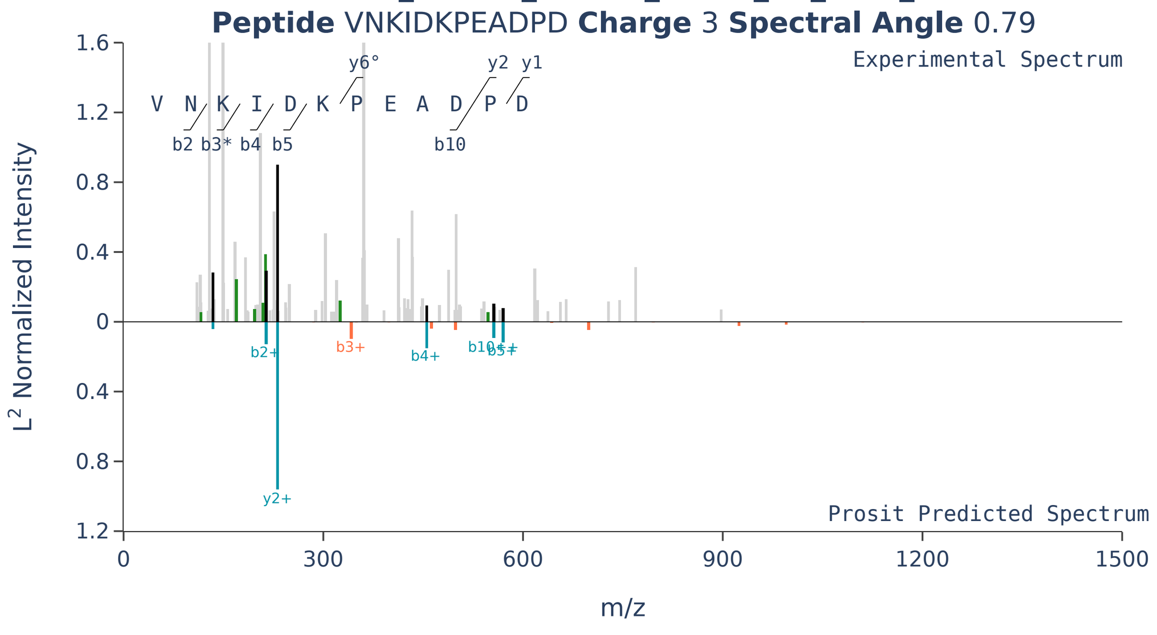

**Source** HRoetschke_211222_160123_HFGoe_IF2_b1R1_2h **Scan** 6544
**Peptide** RARGAQATDI **Charge** 3 **Spectral Angle** 0.75

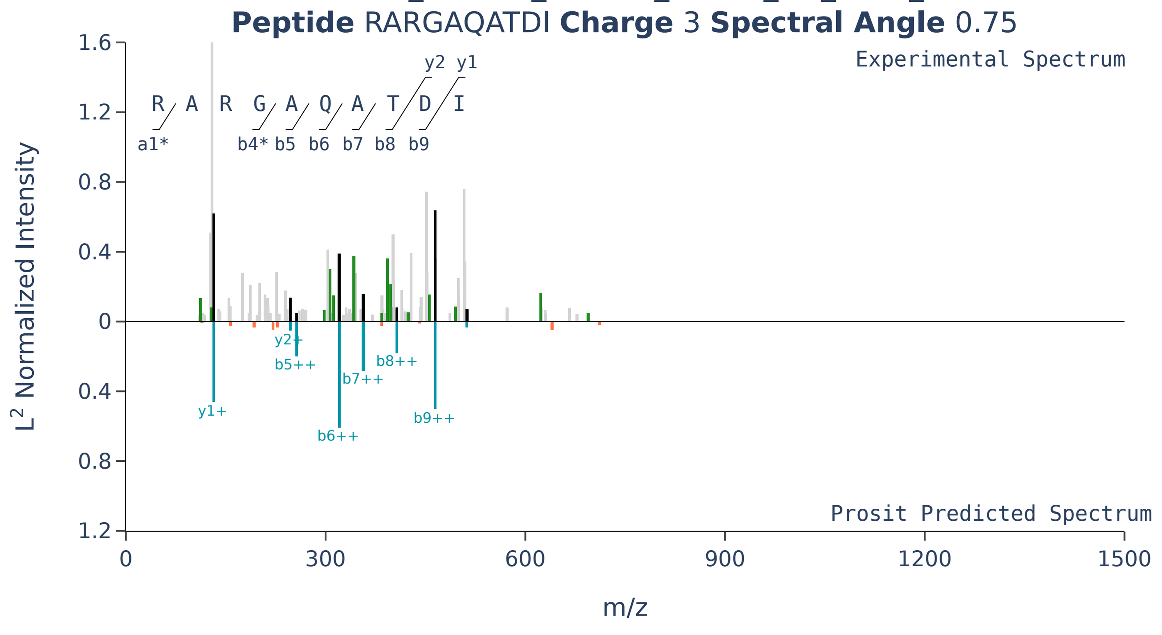

**Source** HRoetschke_211222_160123_HFGoe_IF2_b2R2_4h **Scan** 12934
**Peptide** SLQQGFQKPAQAVN **Charge** 3 **Spectral Angle** 0.86

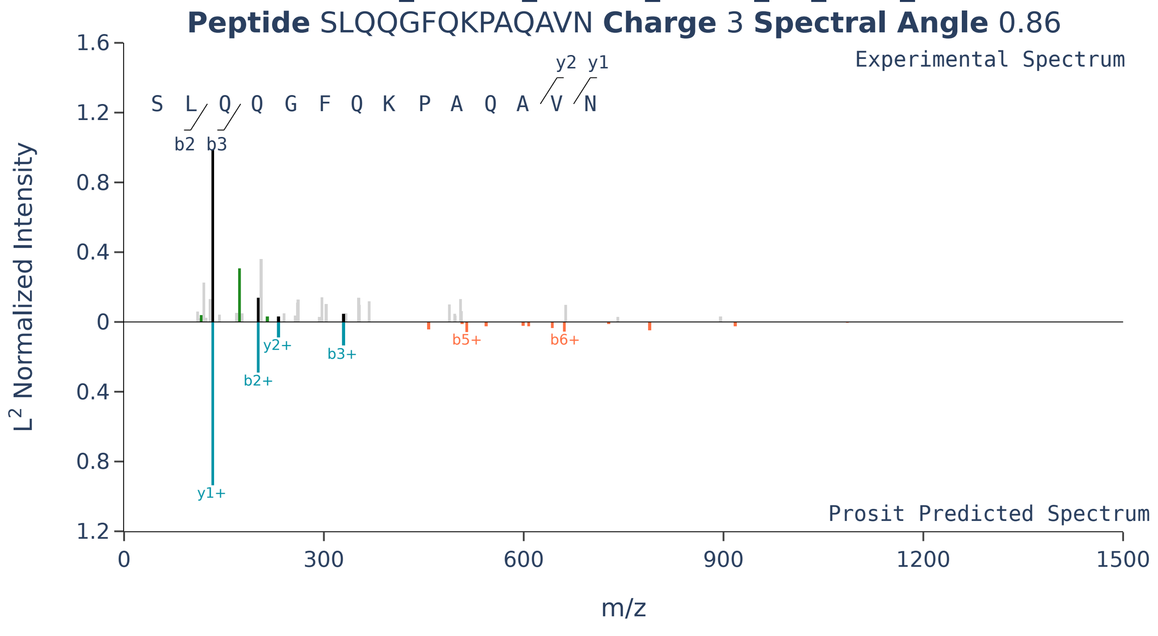

**Source** HRoetschke_211222_160123_HFGoe_IF2_b2R1_24h **Scan** 9408
**Peptide** MGHVDHGKTSLL **Charge** 3 **Spectral Angle** 0.8

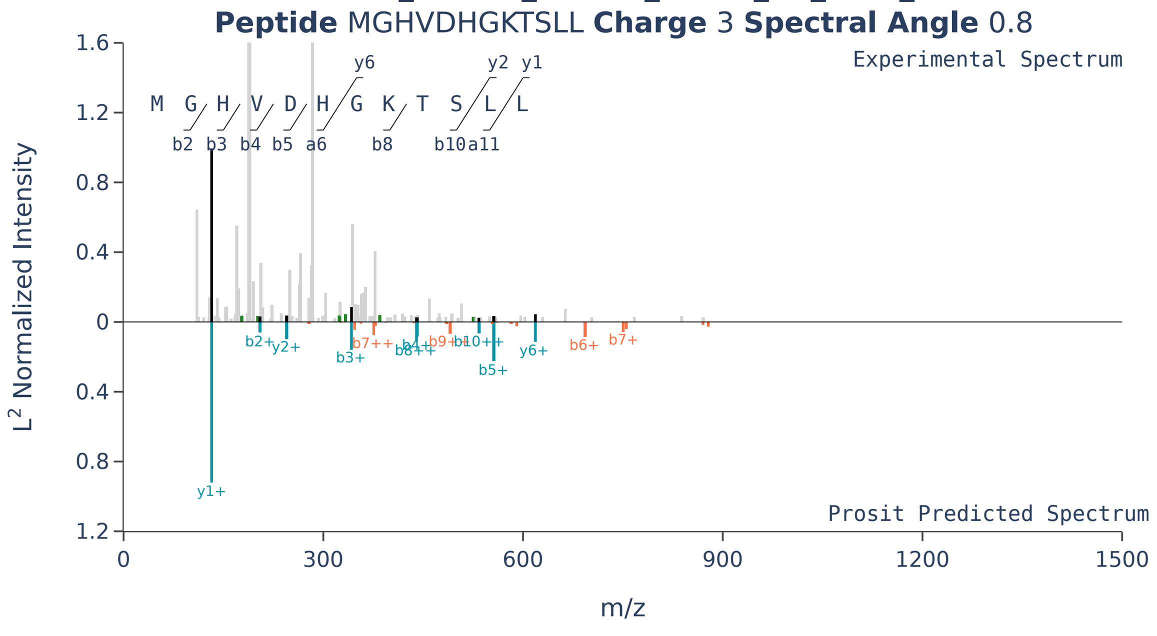

**Source** HRoetschke_211222_160123_HFGoe_IF2_b1R1_4h **Scan** 11478
**Peptide** IPGTGGKSKSVQI **Charge** 2 **Spectral Angle** 0.83

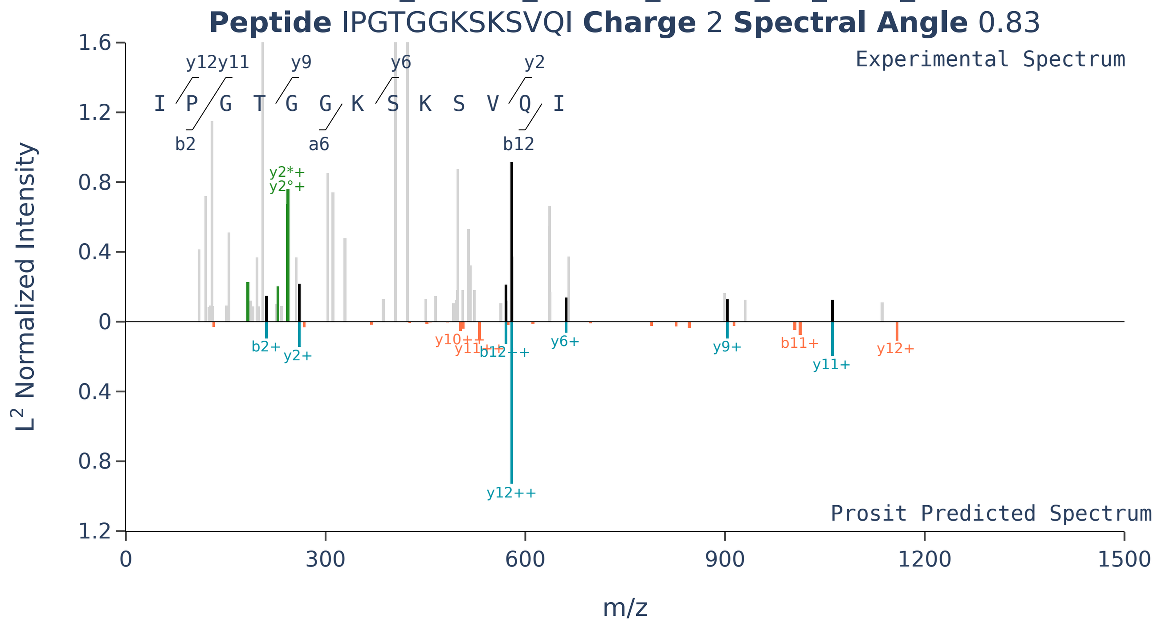

**Source** HRoetschke_211222_160123_HFGoe_IF2_b1R1_4h **Scan** 7295
**Peptide** DSSDYHVTTSQHA **Charge** 2 **Spectral Angle** 0.71

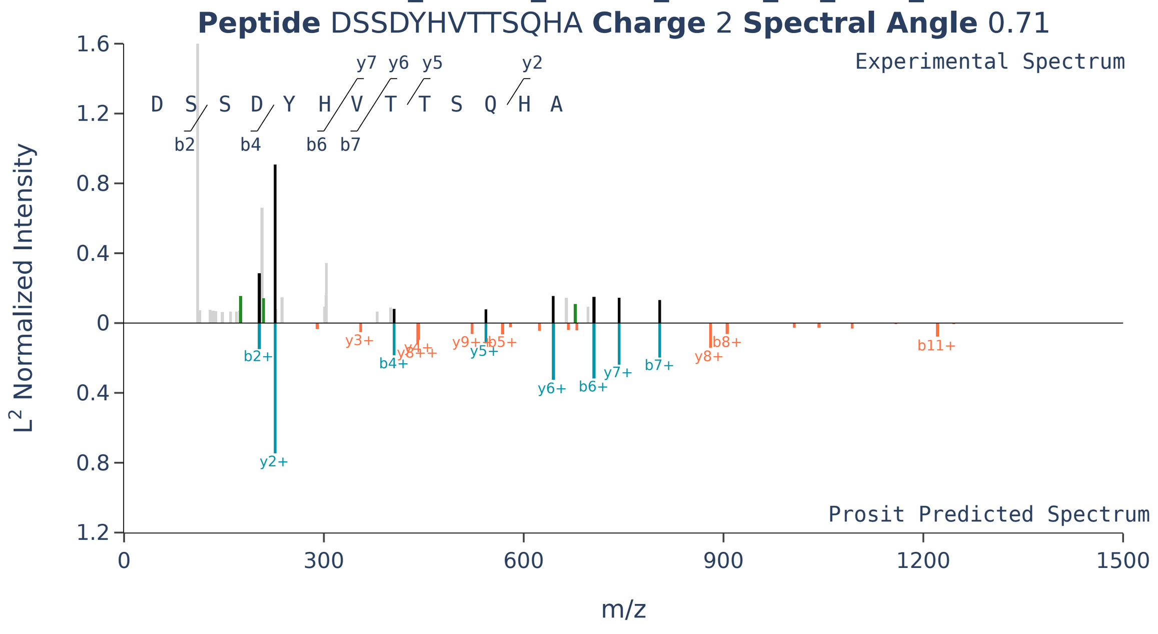


**Source** HRoetschke_211222_160123_HFGoe_IF2_b2R1_24h **Scan** 6444
**Peptide** LDKPEADPDRVKN **Charge** 4 **Spectral Angle** 0.73

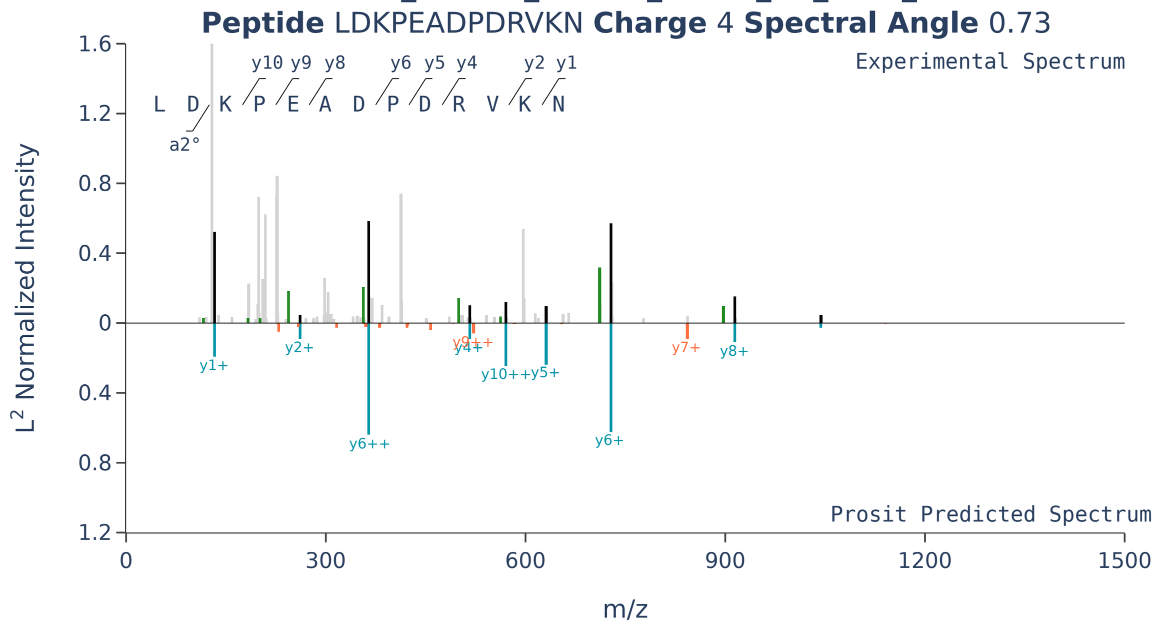


**Source** HRoetschke_211222_160123_HFGoe_IF2_b2R1_24h **Scan** 6395
**Peptide** DAGIRKSADDSVSAQEKQ **Charge** 3 **Spectral Angle** 0.71

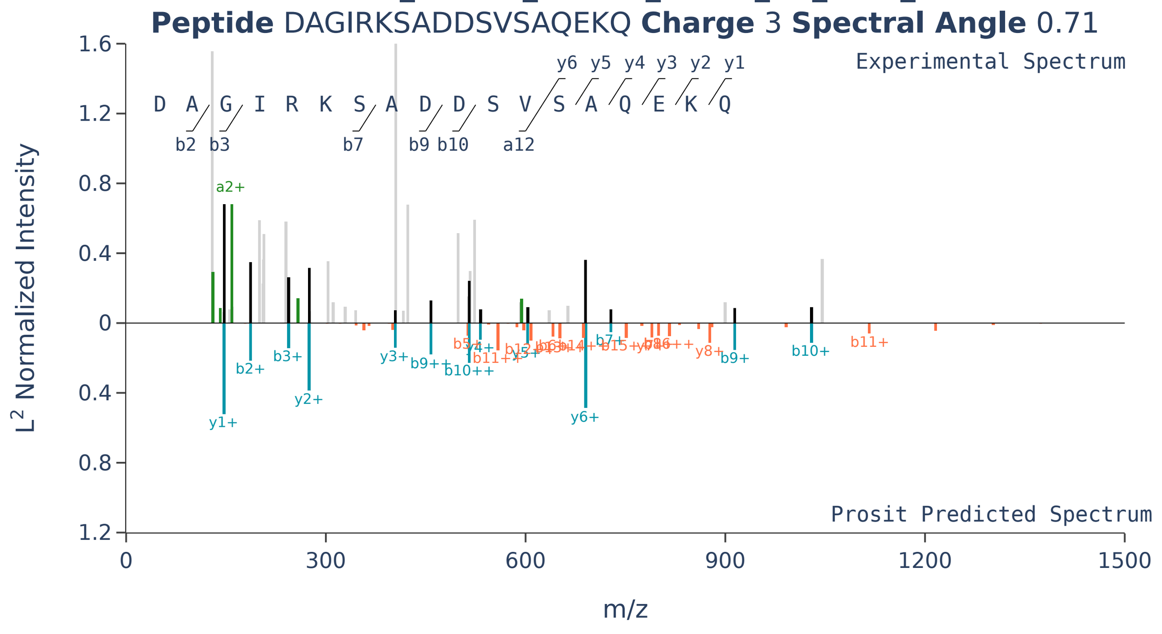


**Source** HRoetschke_211222_160123_HFGoe_IF2_b1R1_4h **Scan** 12219
**Peptide** VNKIDKPEADPDRVKNEL **Charge** 3 **Spectral Angle** 0.69

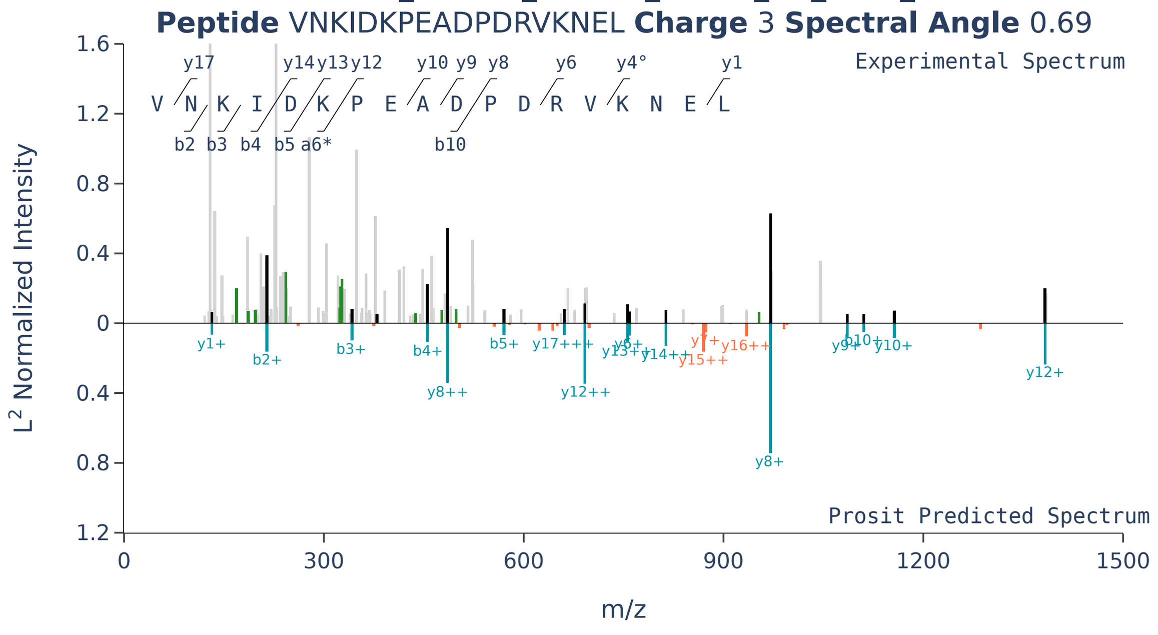


**Source** HRoetschke_211222_160123_HFGoe_IF2_b1R2_1h **Scan** 17301
**Peptide** IDQETAQLVAEE **Charge** 2 **Spectral Angle** 0.73

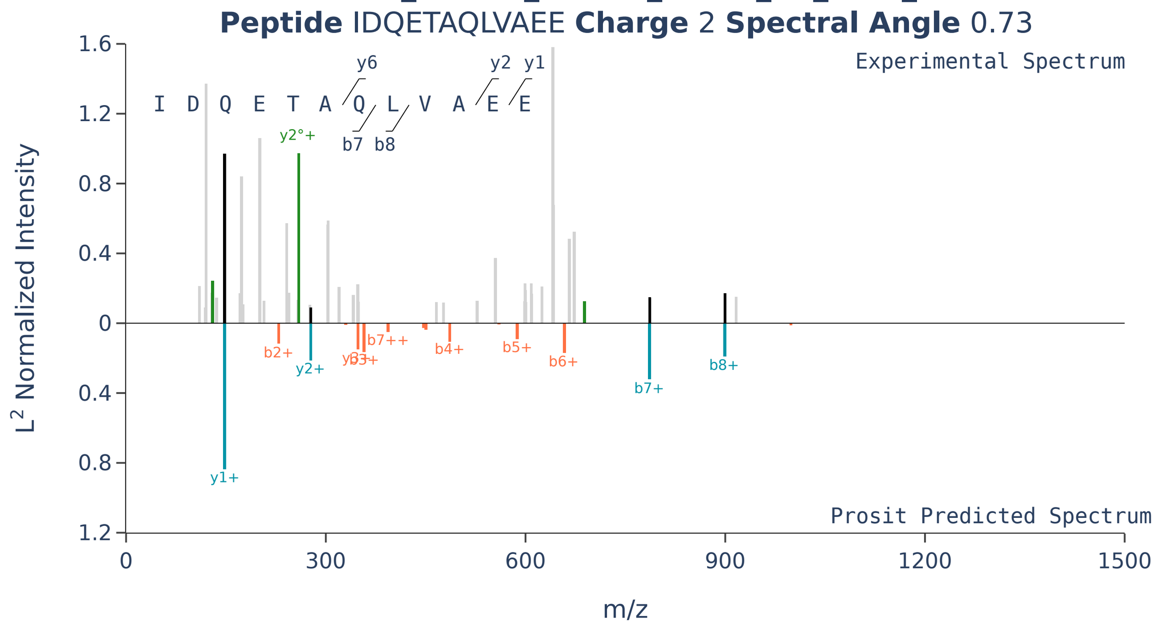


**Source** HRoetschke_211222_160123_HFGoe_IF2_b1R2_24h **Scan** 20611
**Peptide** RDNVVIYEGEL **Charge** 2 **Spectral Angle** 0.77

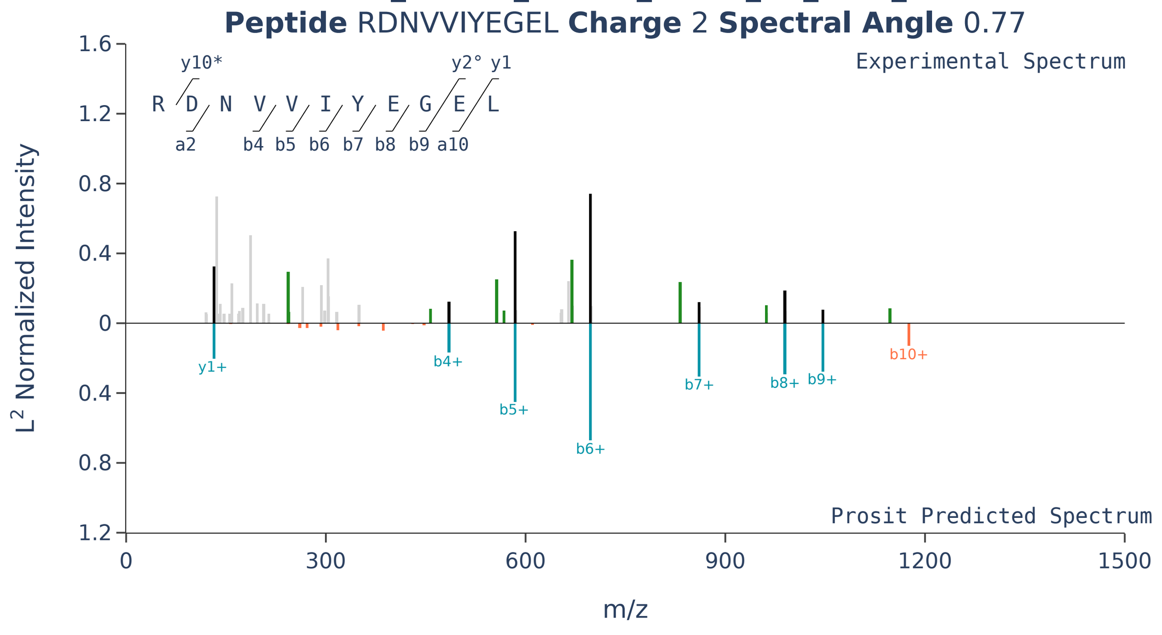


**Source** HRoetschke_211222_160123_HFGoe_IF2_b1R1_4h **Scan** 15132
**Peptide** TDVTIKTLAAERQTSVER **Charge** 3 **Spectral Angle** 0.82

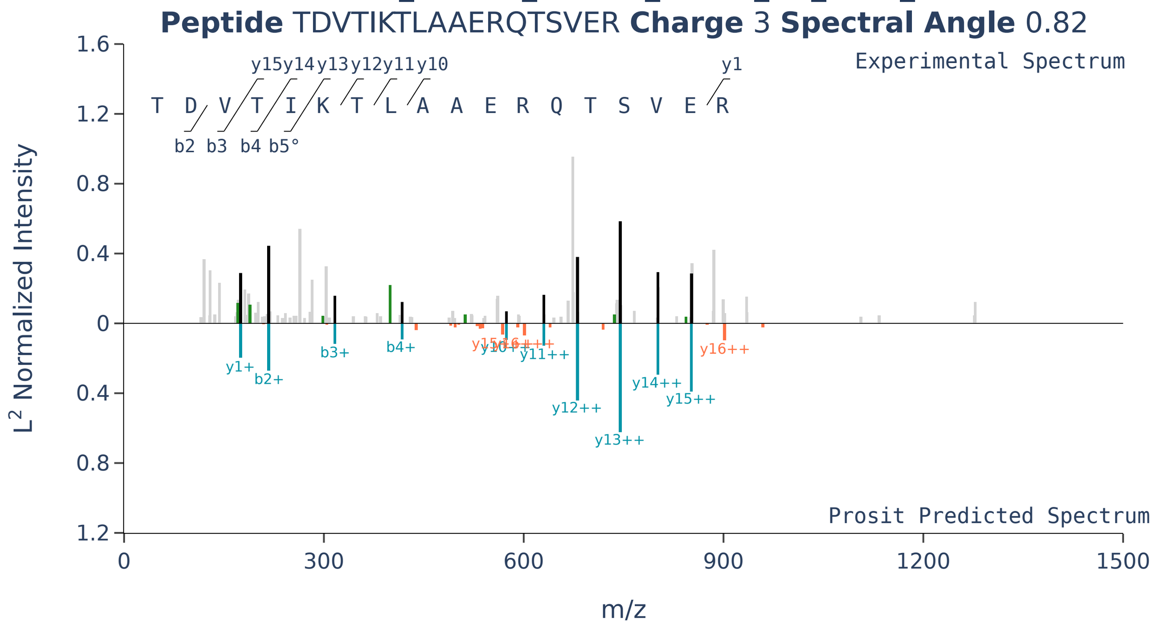


**Source** HRoetschke_211222_160123_HFGoe_IF2_b1R1_2h **Scan** 9388
**Peptide** PQEAERLAA **Charge** 2 **Spectral Angle** 0.67

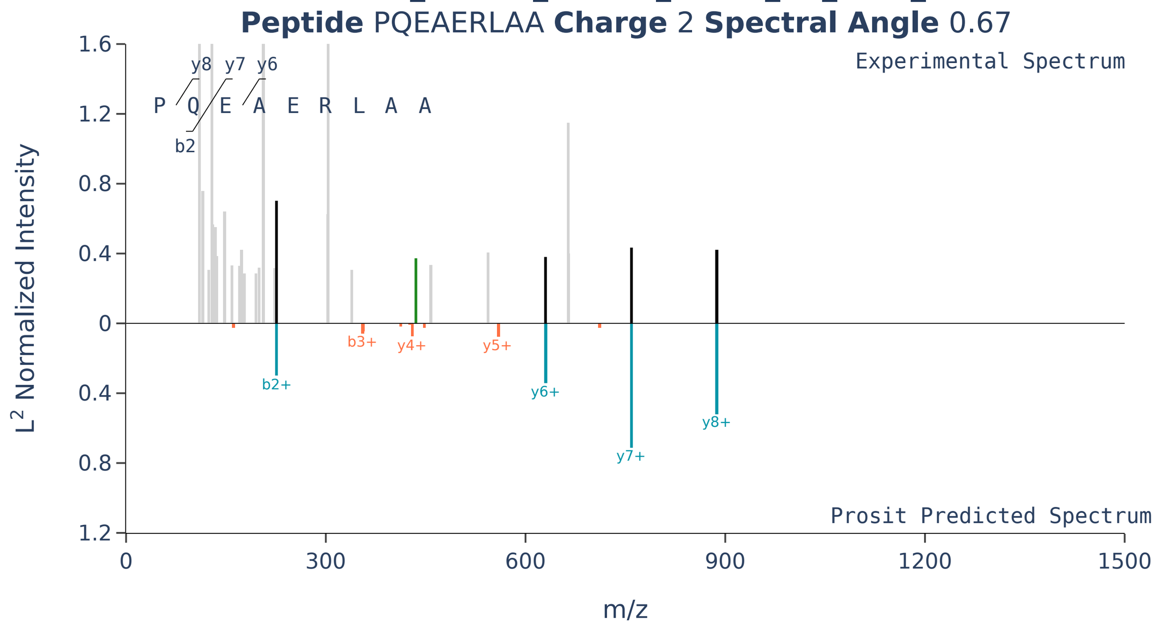


**Source** HRoetschke_211222_160123_HFGoe_IF2_b2R2_4h **Scan** 18366
**Peptide** VIDQETAQLV **Charge** 2 **Spectral Angle** 0.76

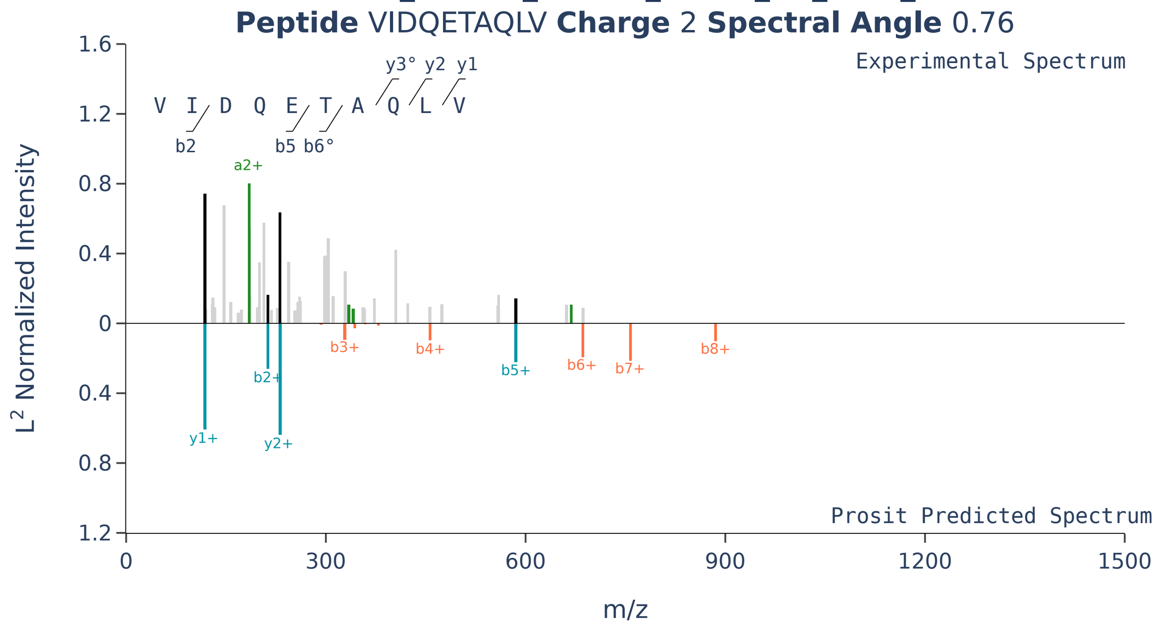


**Source** HRoetschke_211222_160123_HFGoe_IF2_b1R2_24h **Scan** 14990
**Peptide** TDVTIKTLAAERQTS **Charge** 2 **Spectral Angle** 0.77

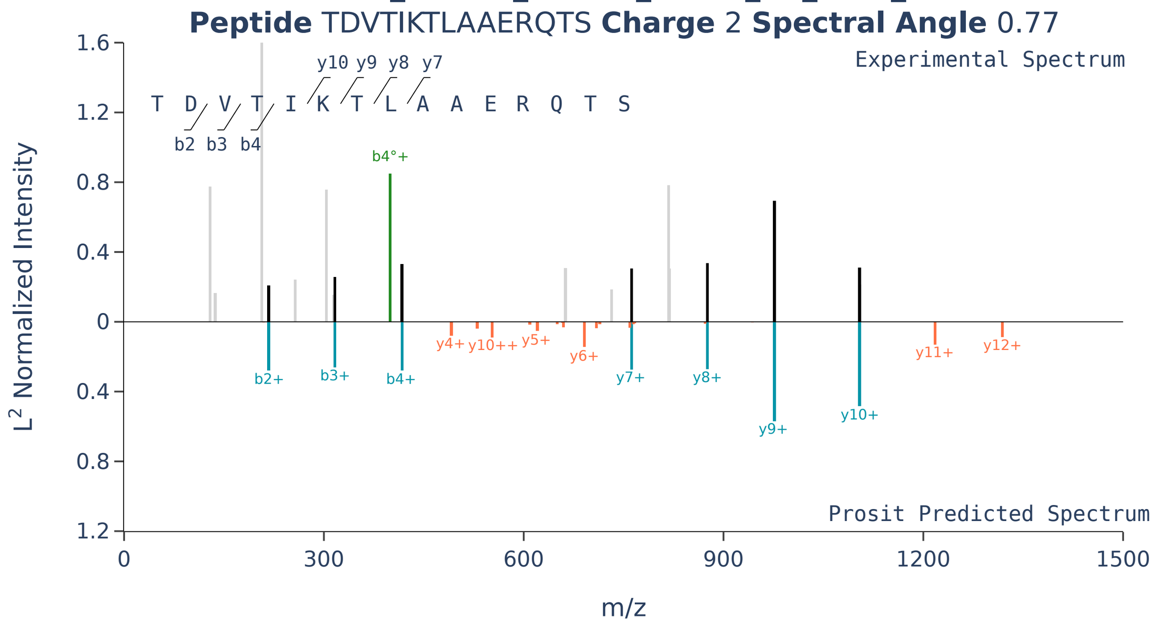


**Source** HRoetschke_211222_160123_HFGoe_IF2_b1R2_24h **Scan** 13099
**Peptide** NQKNSGPDKLTL **Charge** 2 **Spectral Angle** 0.74

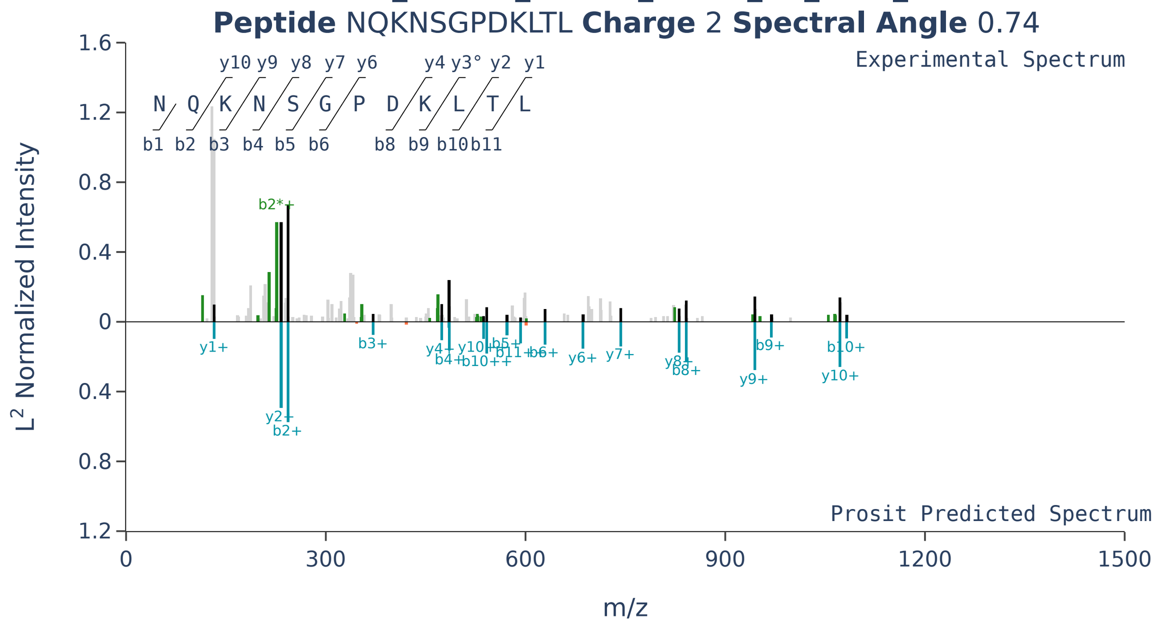


**Source** HRoetschke_211222_160123_HFGoe_IF2_b1R2_2h **Scan** 11410
**Peptide** EAGGITQHIG **Charge** 2 **Spectral Angle** 0.76

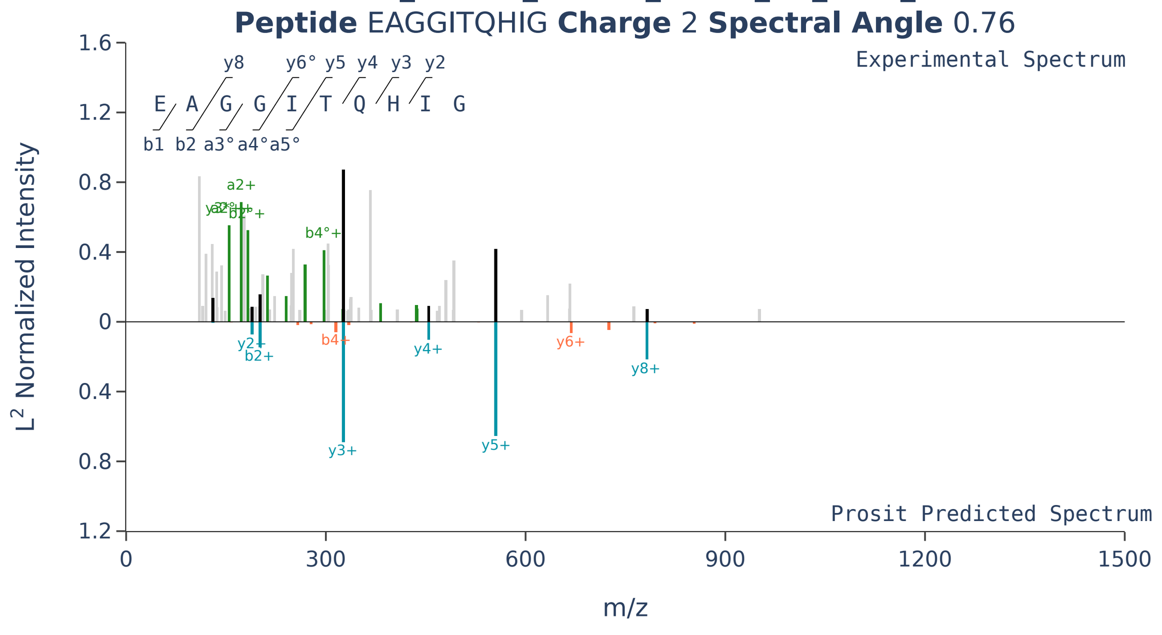


**Source** HRoetschke_211222_160123_HFGoe_IF2_b1R2_1h **Scan** 14979
**Peptide** NELEEAVMSD **Charge** 2 **Spectral Angle** 0.68

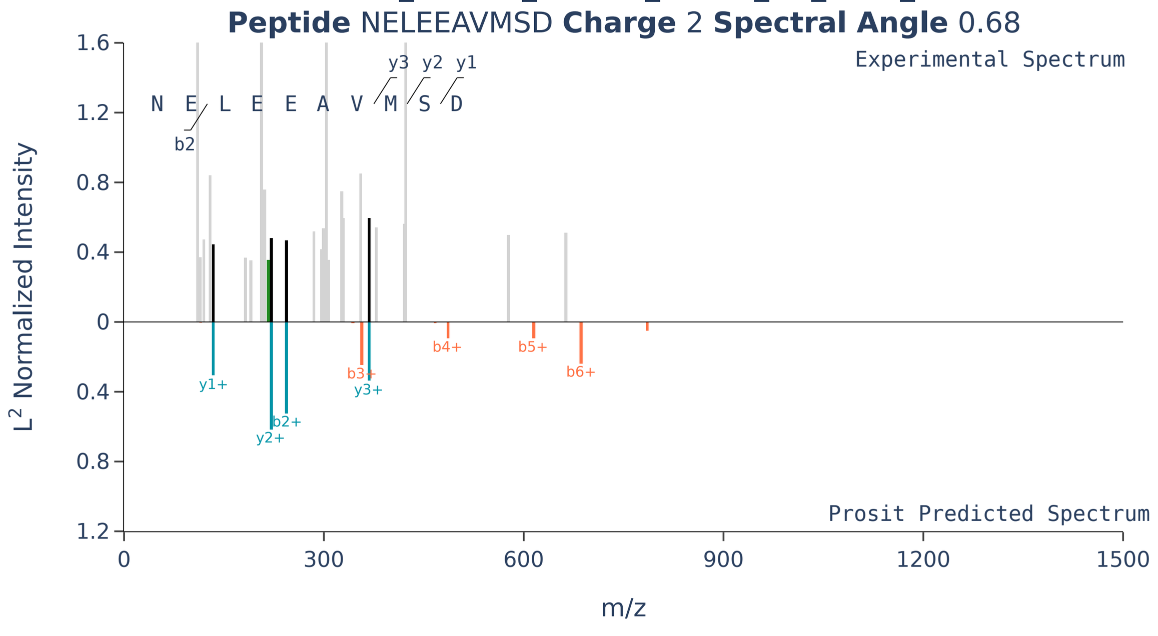


**Source** HRoetschke_211222_160123_HFGoe_IF2_b1R1_4h **Scan** 14762
**Peptide** SDRDTGAAAEPRAPVVTIMGH **Charge** 3 **Spectral Angle** 0.79

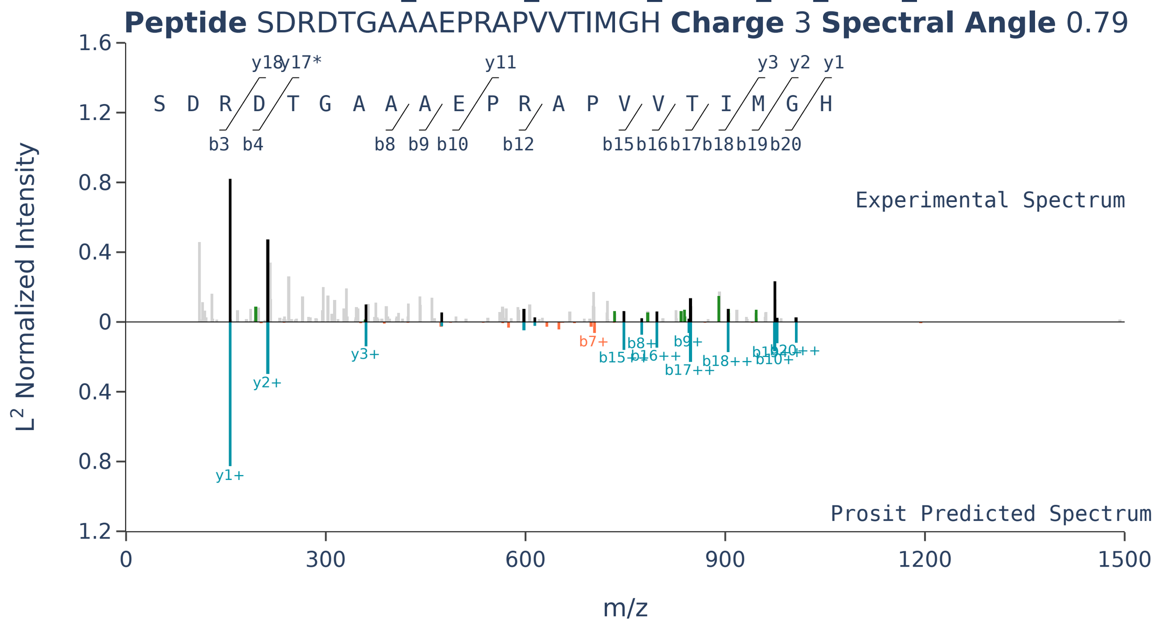


**Source** HRoetschke_211222_160123_HFGoe_IF2_b1R1_4h **Scan** 5892
**Peptide** AEEMGHKV **Charge** 2 **Spectral Angle** 0.72

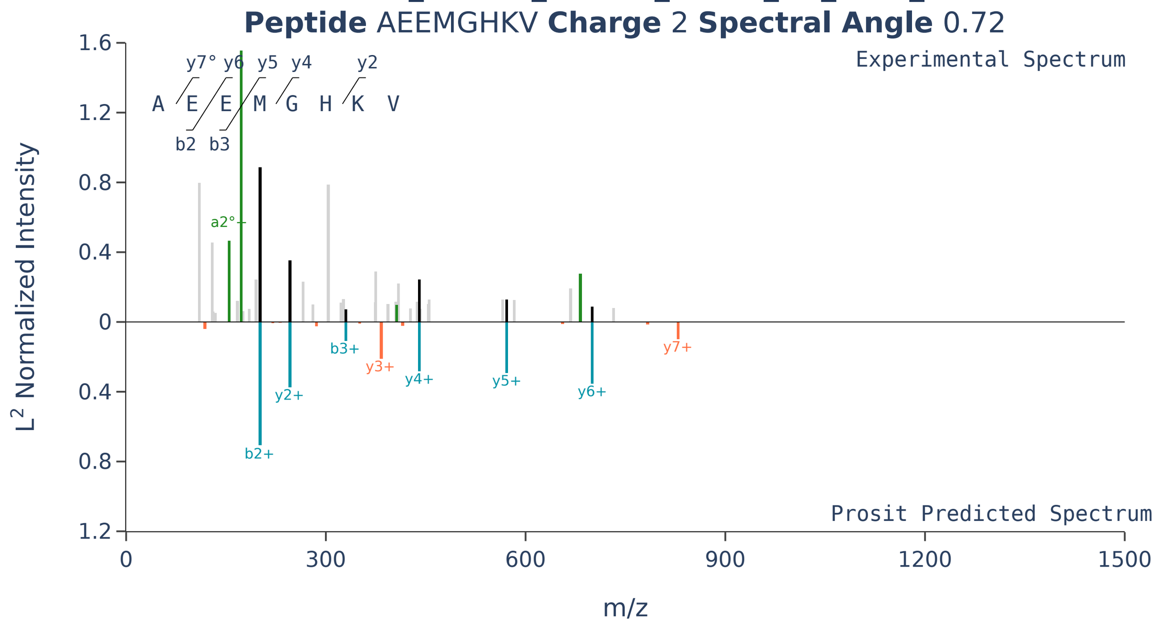

**Source** HRoetschke_211222_160123_HFGoe_IF2_b2R2_1h **Scan** 6556
**Peptide** FVKRDPQE **Charge** 2 **Spectral Angle** 0.75

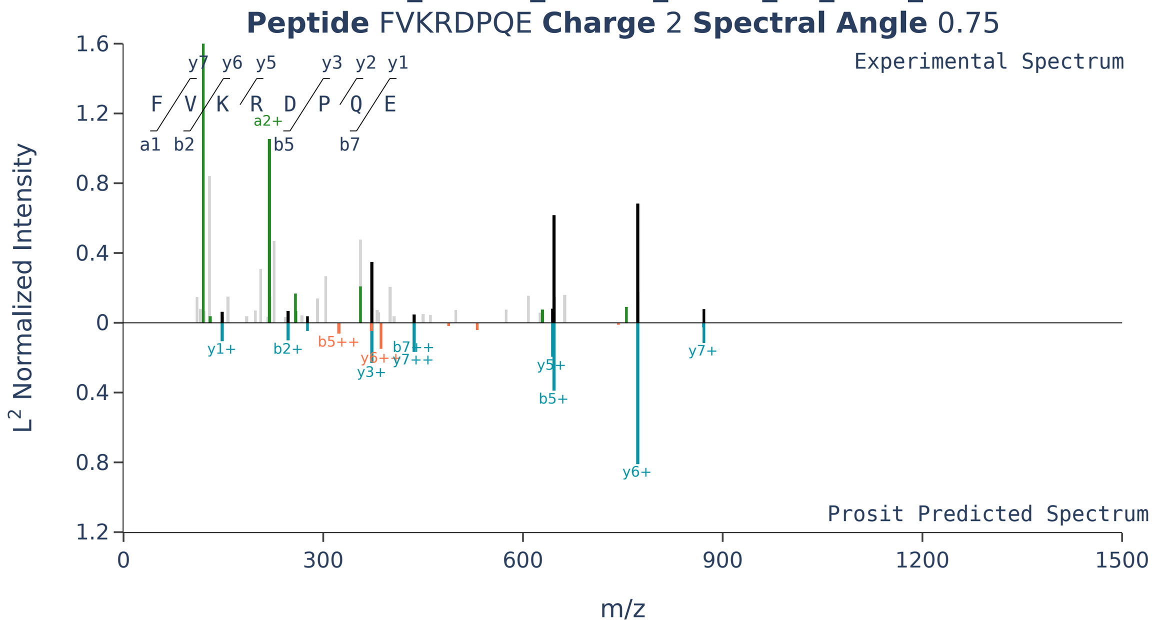


**Source** HRoetschke_211222_160123_HFGoe_IF2_b2R2_24h **Scan** 12628
**Peptide** KVASGEAGGITQHL **Charge** 3 **Spectral Angle** 0.72

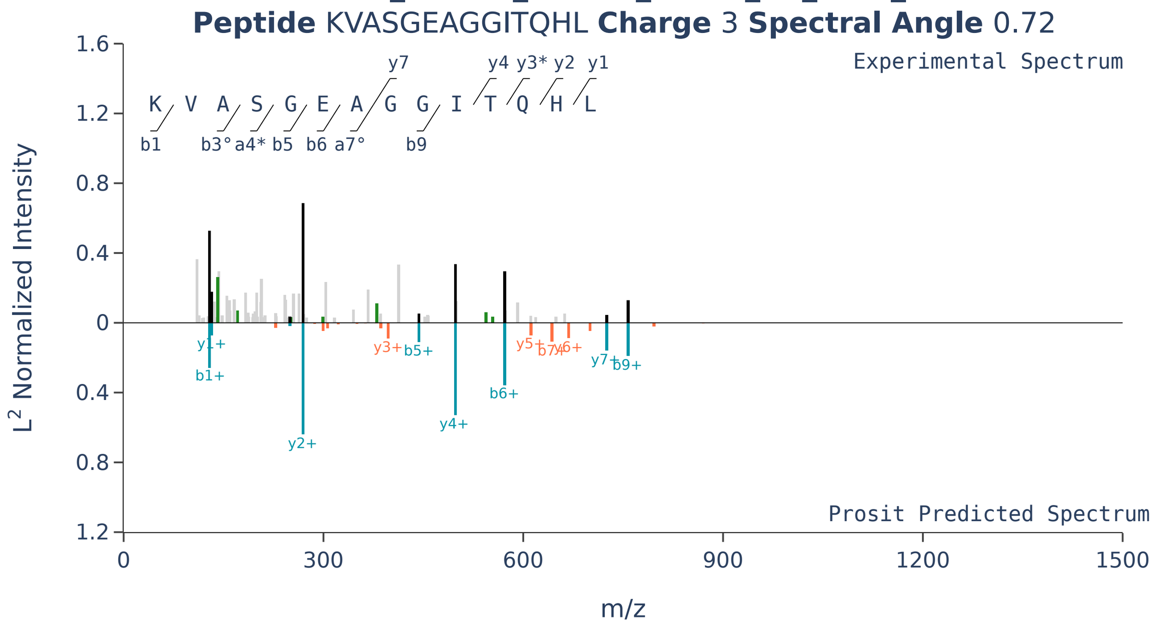


**Source** HRoetschke_211222_160123_HFGoe_IF2_b2R1_24h **Scan** 9552
**Peptide** TIEAIQH **Charge** 2 **Spectral Angle** 0.83

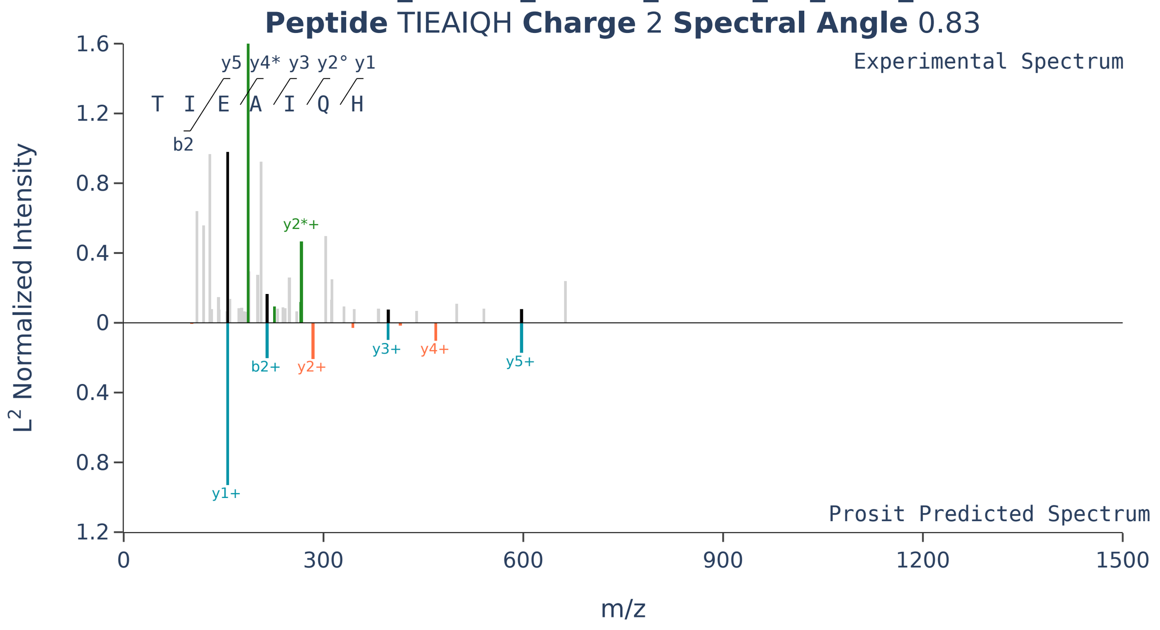


**Source** HRoetschke_211222_160123_HFGoe_IF2_b2R2_4h **Scan** 12811
**Peptide** ASGEAGGITQHIGA **Charge** 2 **Spectral Angle** 0.72

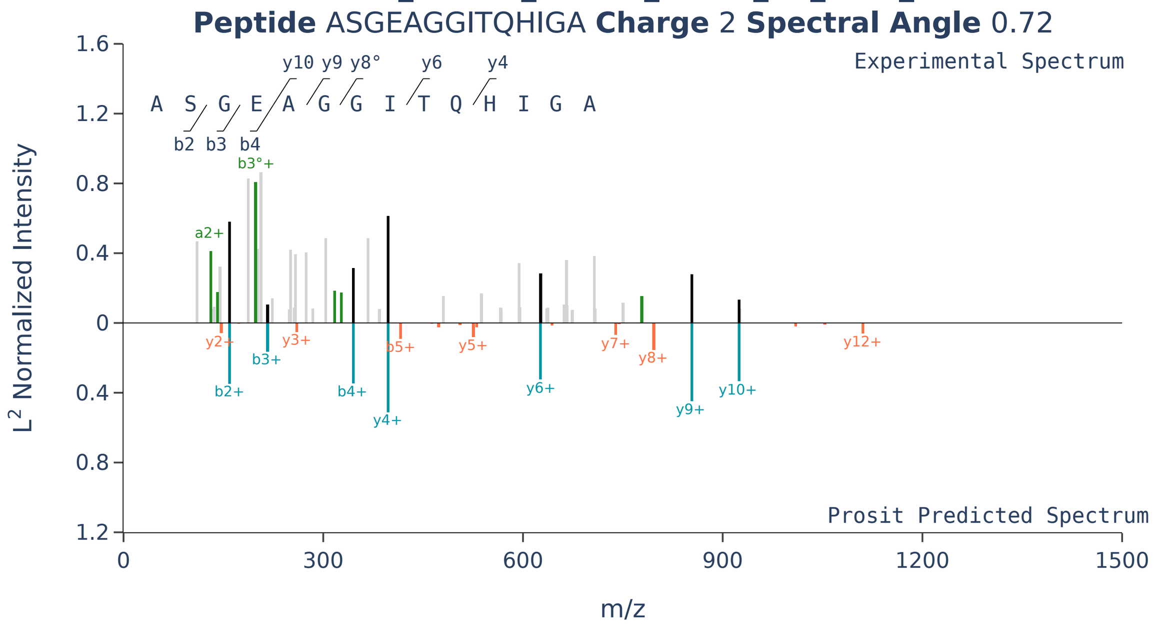


**Source** HRoetschke_211222_160123_HFGoe_IF2_b2R1_4h **Scan** 9182
**Peptide** QGFQKPAQAVN **Charge** 2 **Spectral Angle** 0.8

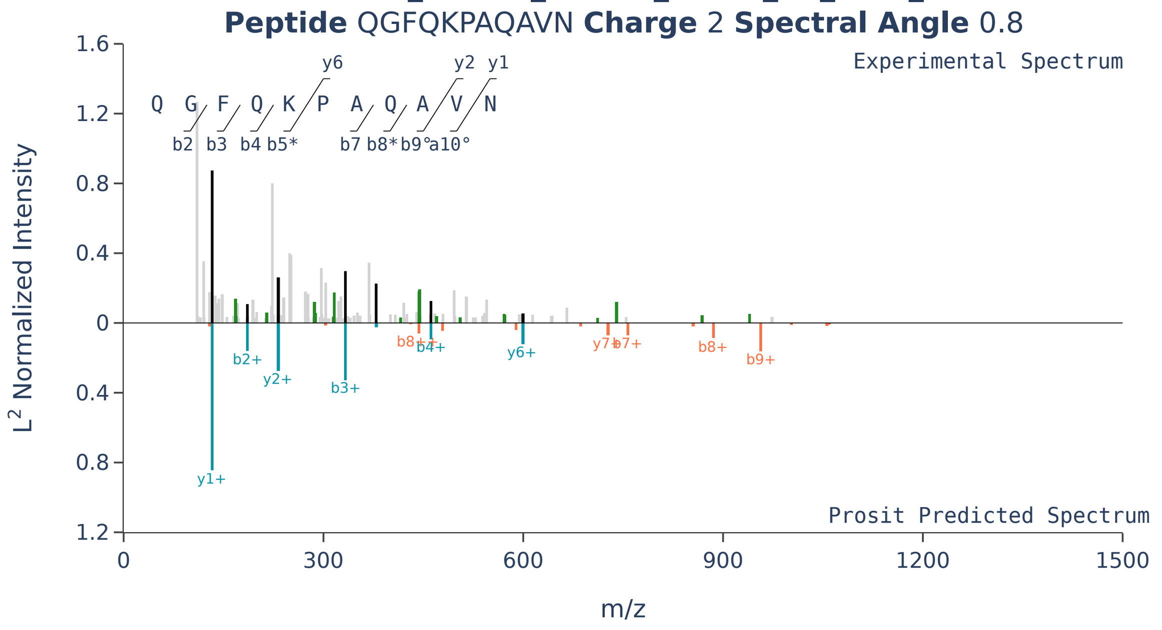


**Source** HRoetschke_211222_160123_HFGoe_IF2_b1R1_4h **Scan** 19327
**Peptide** AKAAQVPVVVAVN **Charge** 2 **Spectral Angle** 0.73

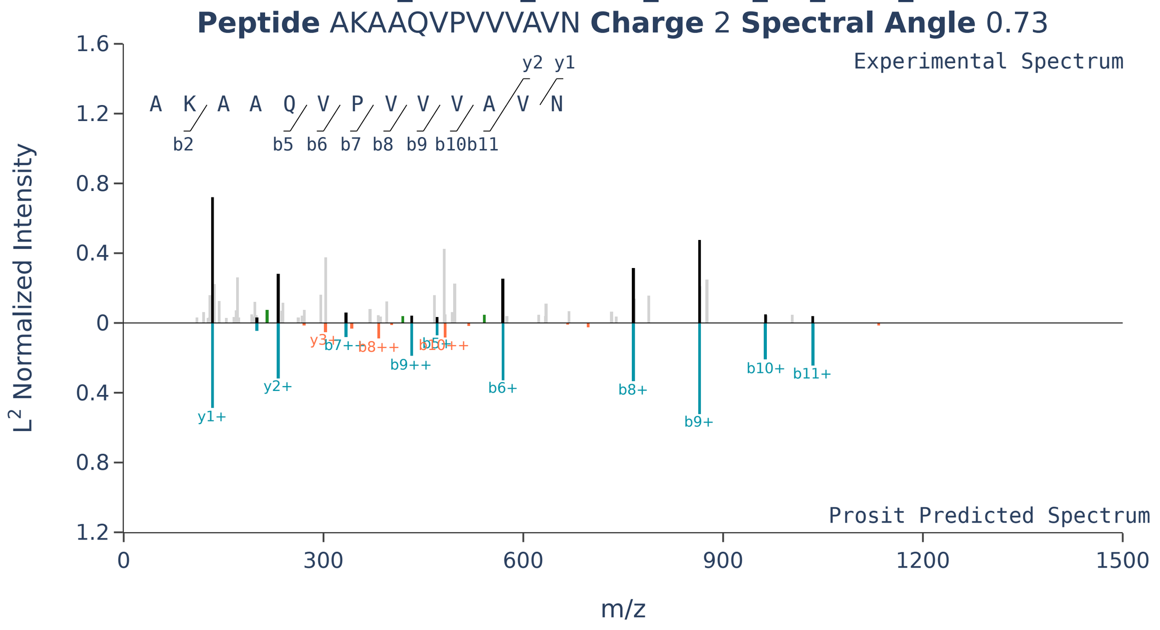


**Source** HRoetschke_211222_160123_HFGoe_IF2_b1R2_4h **Scan** 6570
**Peptide** GTLHKGDI **Charge** 2 **Spectral Angle** 0.8

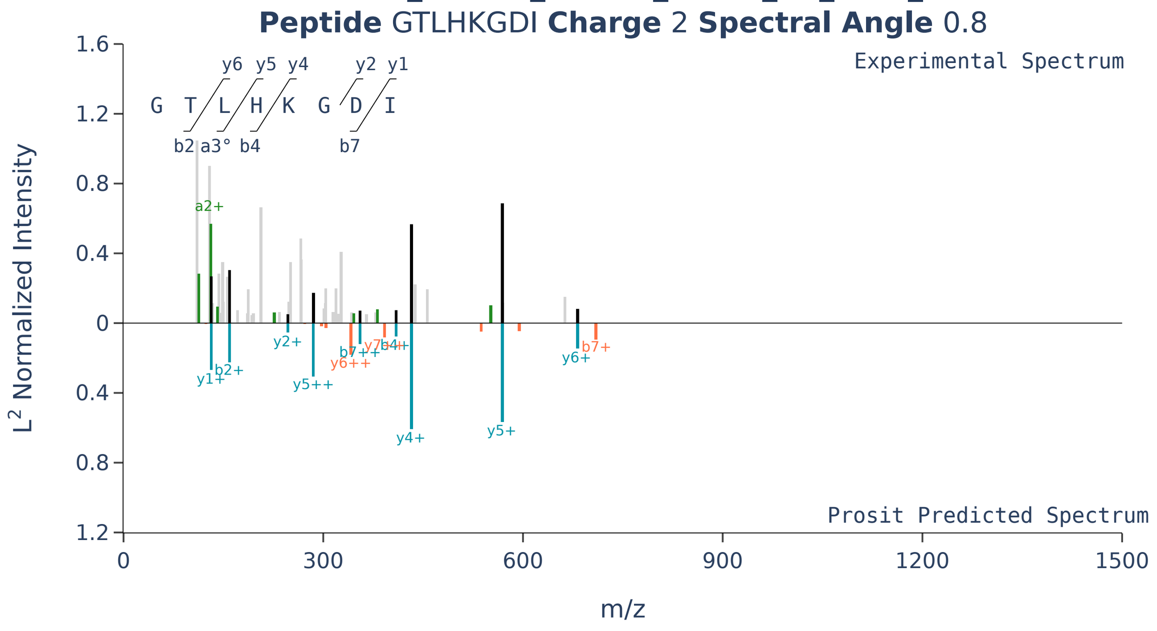


**Source** HRoetschke_211222_160123_HFGoe_IF2_b1R1_4h **Scan** 9255
**Peptide** AEEMGHKVILRRENELEE **Charge** 4 **Spectral Angle** 0.68

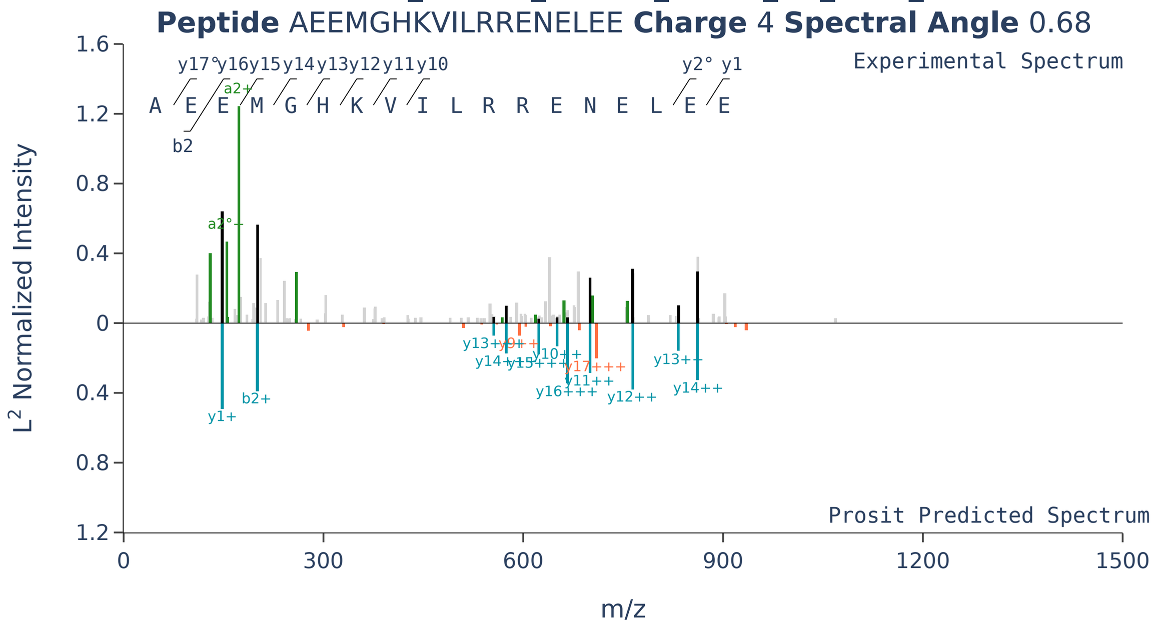


**Source** HRoetschke_211222_160123_HFGoe_IF2_b1R1_4h **Scan** 8631
**Peptide** PGHAAFTSMRARGAQA **Charge** 3 **Spectral Angle** 0.73

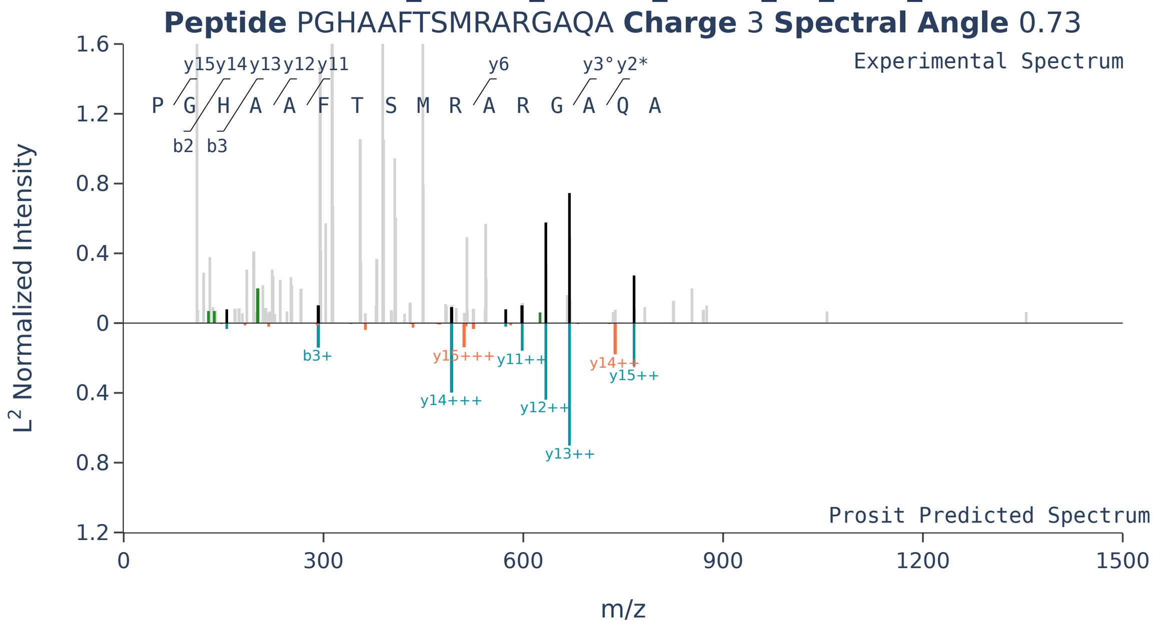


**Source** HRoetschke_211222_160123_HFGoe_IF2_b1R1_4h **Scan** 21203
**Peptide** NGMITFLDTPGHAA **Charge** 2 **Spectral Angle** 0.78

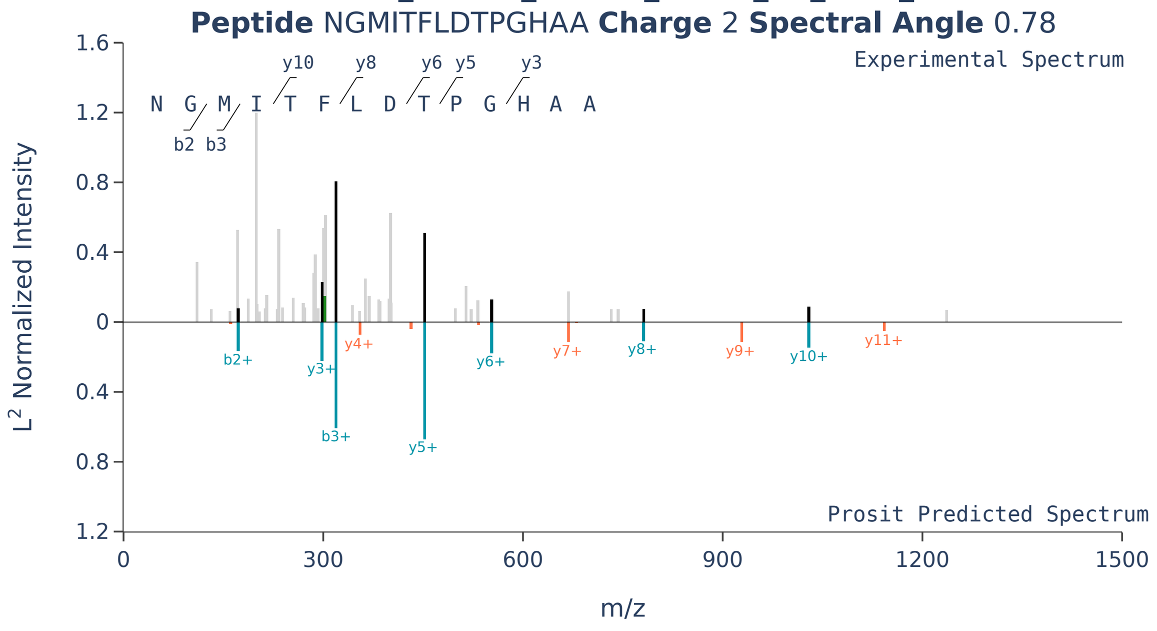


**Source** HRoetschke_211222_160123_HFGoe_IF2_b1R1_4h **Scan** 12569
**Peptide** GMECGIGVKNY **Charge** 2 **Spectral Angle** 0.71

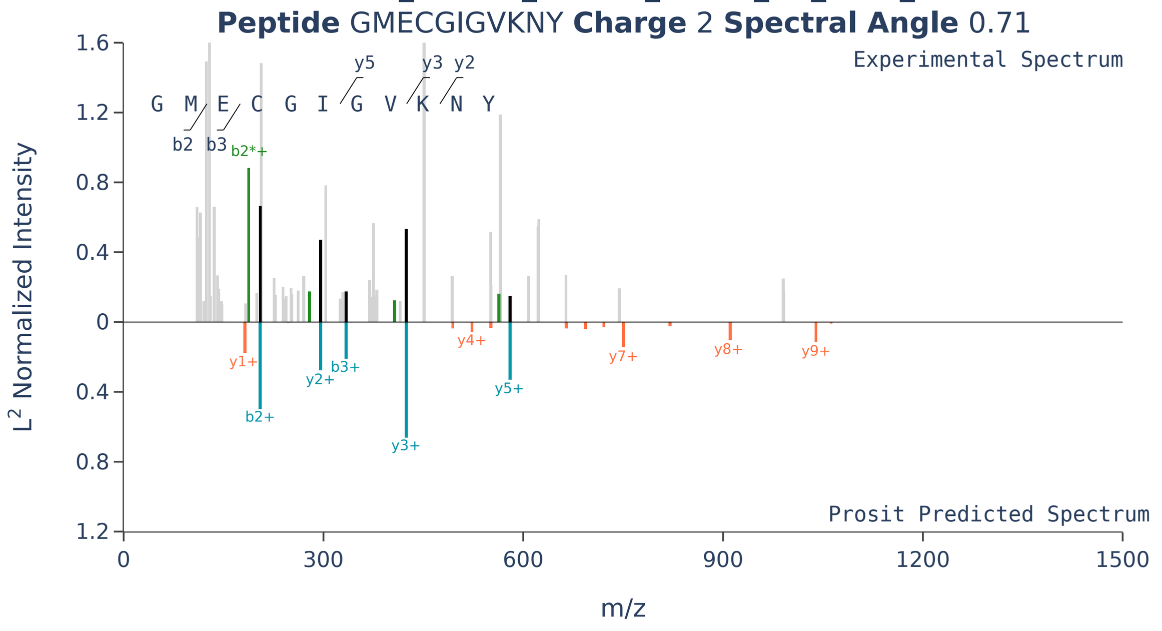


**Source** HRoetschke_211222_160123_HFGoe_IF2_b1R1_4h **Scan** 7563
**Peptide** DTPGHAAFTSMRARG **Charge** 3 **Spectral Angle** 0.6

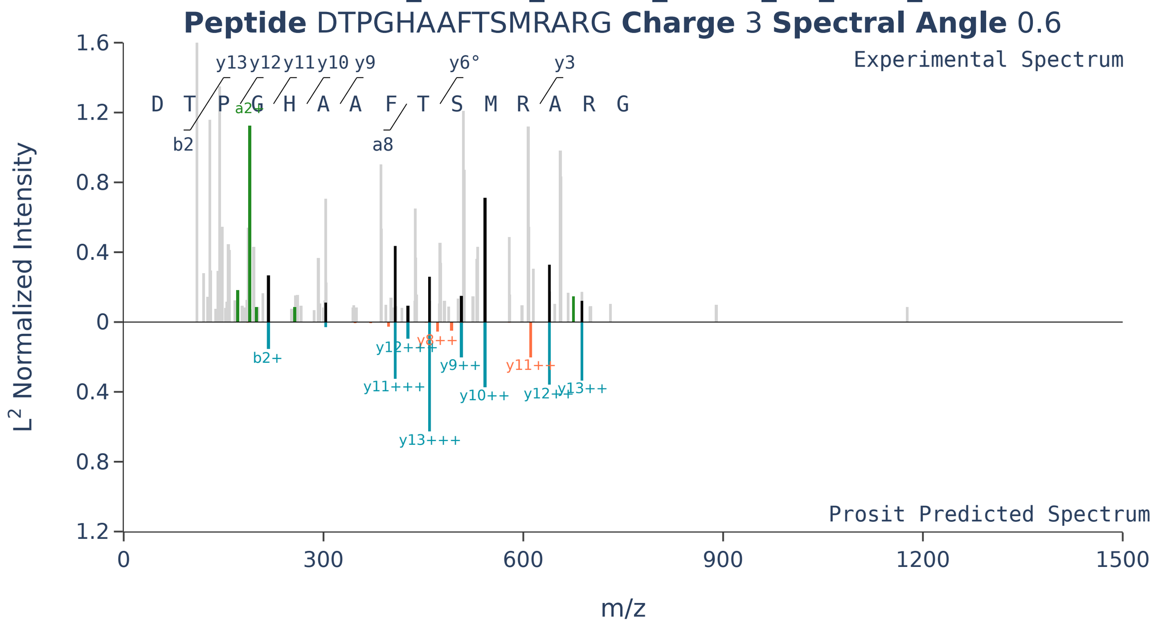


**Source** HRoetschke_211222_160123_HFGoe_IF2_b1R1_4h **Scan** 7942
**Peptide** LVREGTLHK **Charge** 2 **Spectral Angle** 0.79

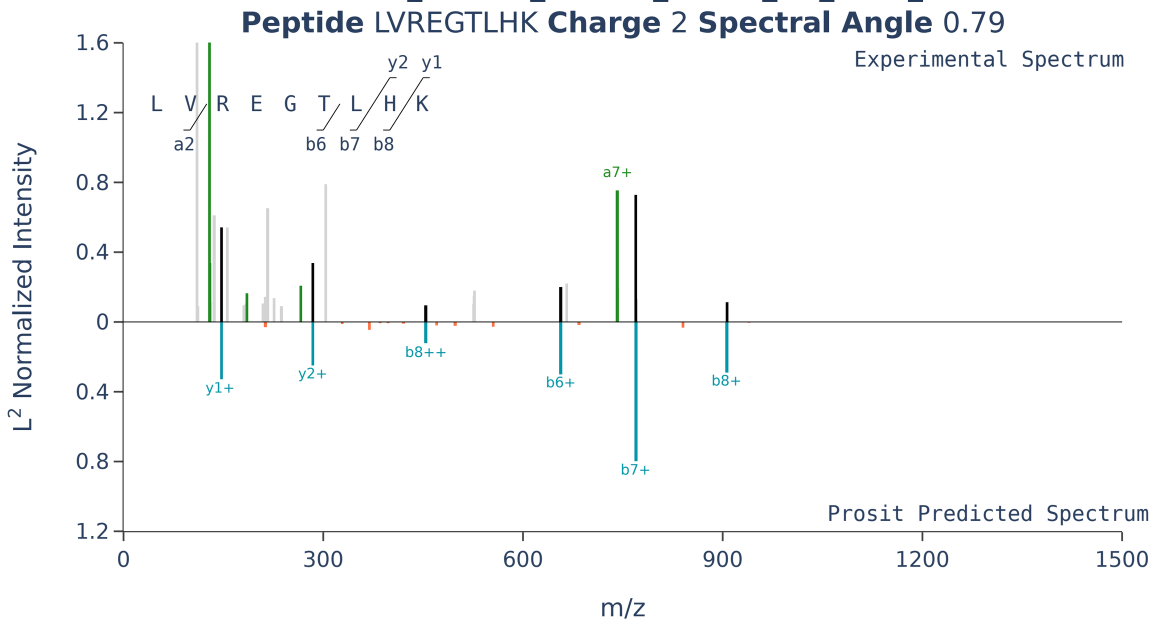


**Source** HRoetschke_211222_160123_HFGoe_IF2_b2R1_2h **Scan** 12365
**Peptide** TKVASGEAGGITQHL **Charge** 3 **Spectral Angle** 0.77

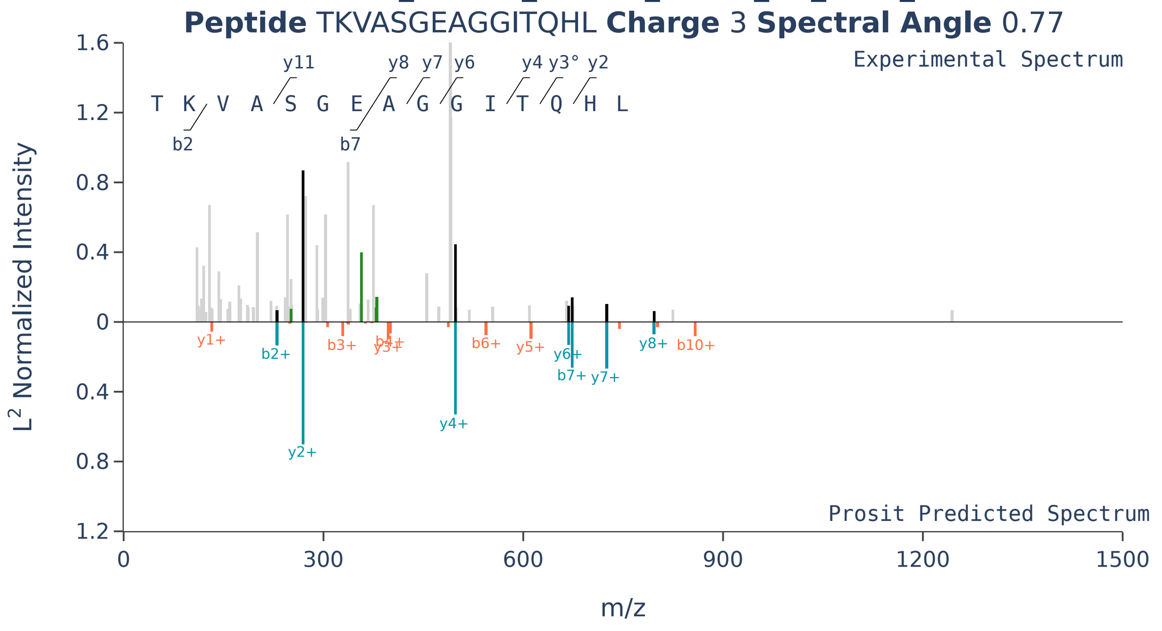


**Source** HRoetschke_211222_160123_HFGoe_IF2_b1R1_4h **Scan** 25185
**Peptide** GPSIPVEI **Charge** 1 **Spectral Angle** 0.83

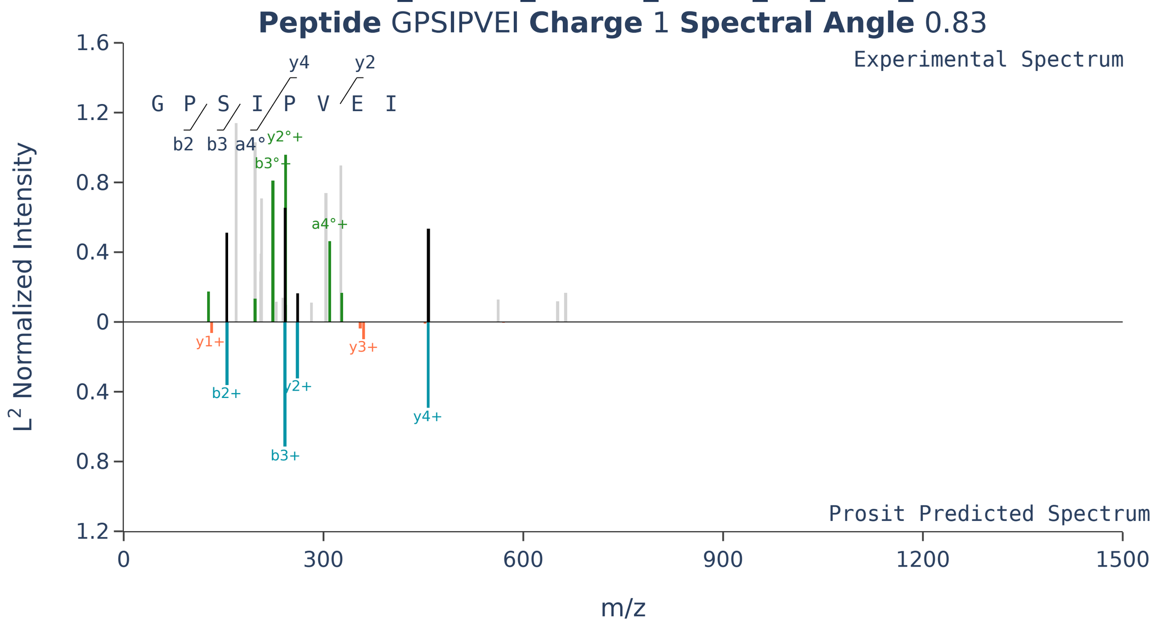

**Source** HRoetschke_211222_160123_HFGoe_IF2_b1R1_1h **Scan** 11042
**Peptide** NAEPTEDSSDYHVTT **Charge** 2 **Spectral Angle** 0.81

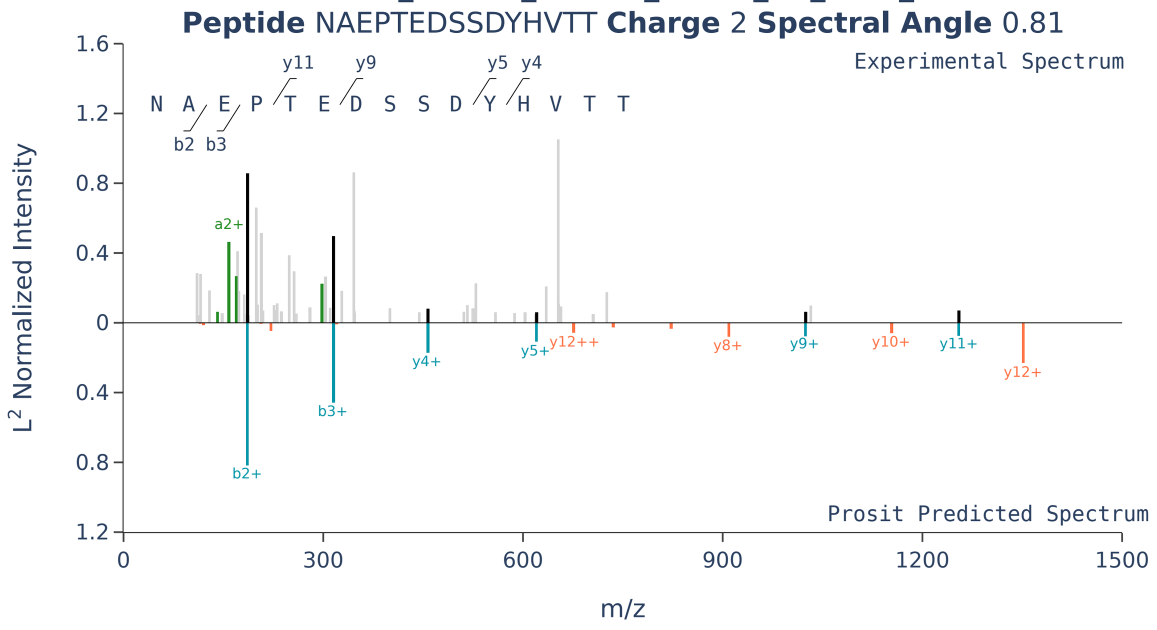


**Source** HRoetschke_211222_160123_HFGoe_IF2_b2R1_24h **Scan** 7069
**Peptide** ASARKVLE **Charge** 2 **Spectral Angle** 0.83

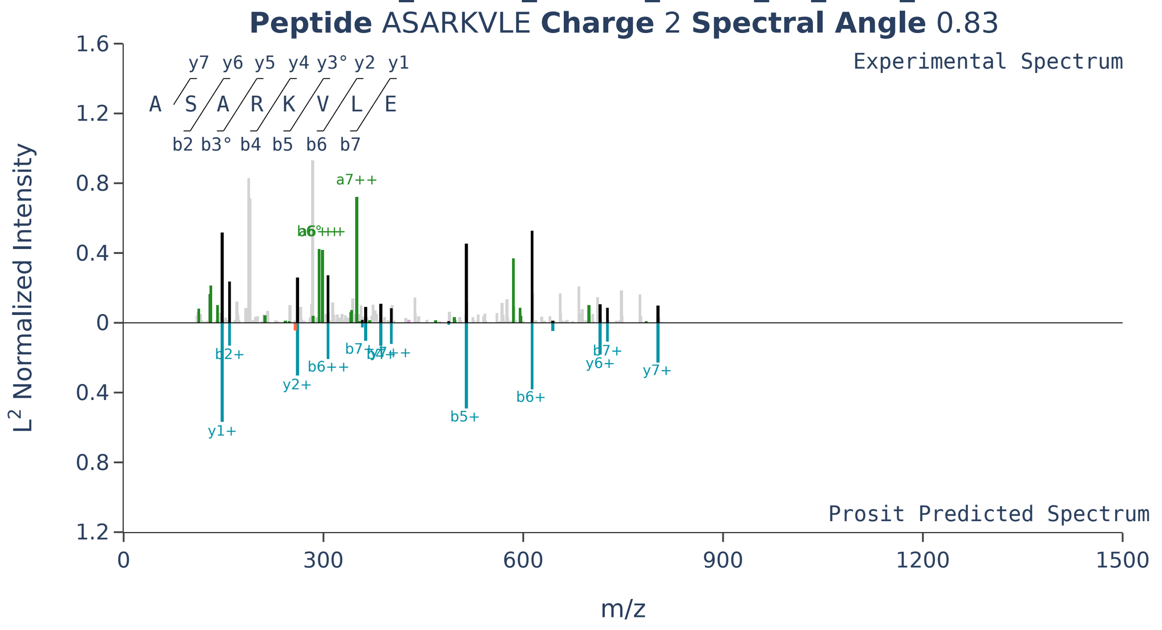


**Source** HRoetschke_211222_160123_HFGoe_IF2_b1R2_2h **Scan** 14719
**Peptide** QETAQLVAEE **Charge** 1 **Spectral Angle** 0.64

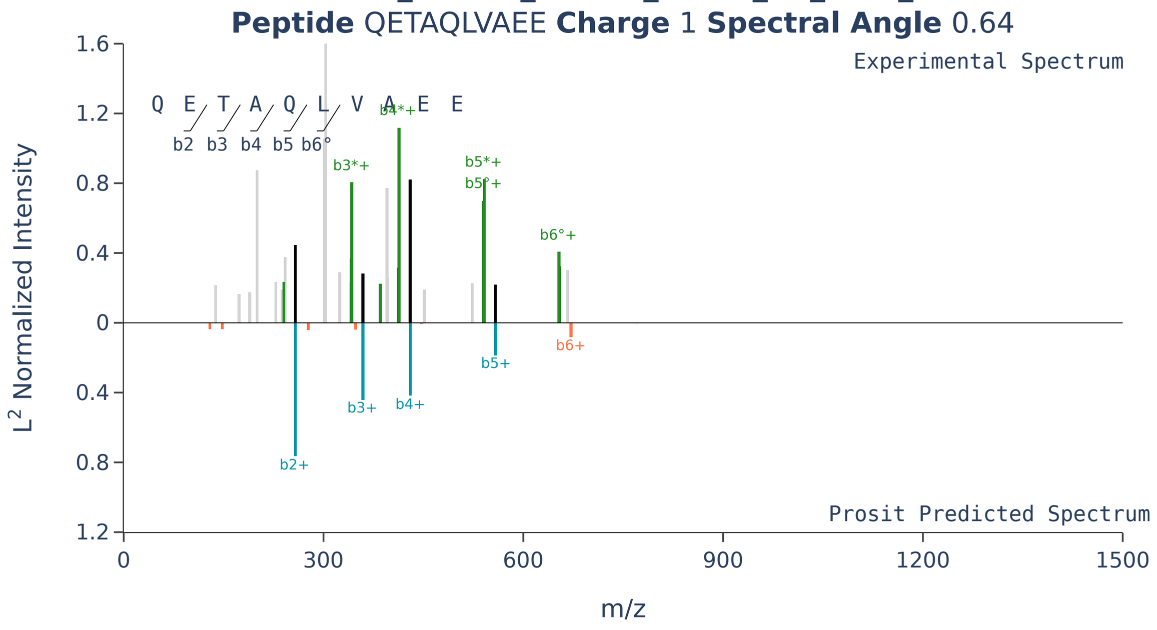


**Source** HRoetschke_211222_160123_HFGoe_IF2_b1R2_2h **Scan** 12110
**Peptide** IGAYHVET **Charge** 2 **Spectral Angle** 0.66

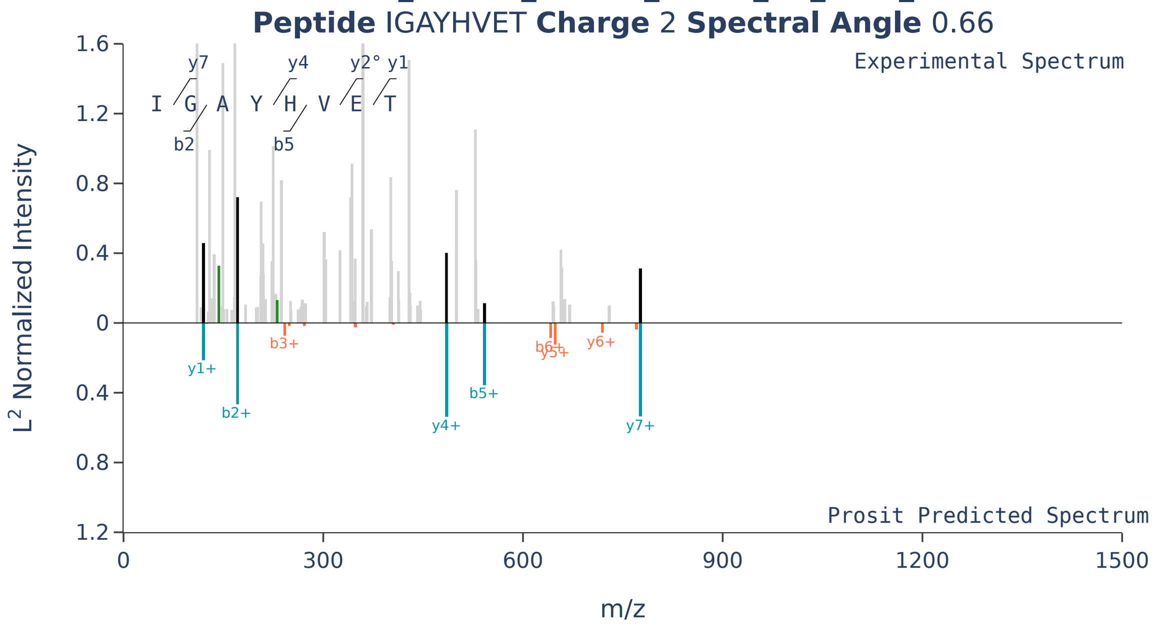


**Source** HRoetschke_211222_160123_HFGoe_IF2_b2R1_24h **Scan** 12365
**Peptide** SGVPAAGDEVTVVRDEKKA **Charge** 4 **Spectral Angle** 0.63

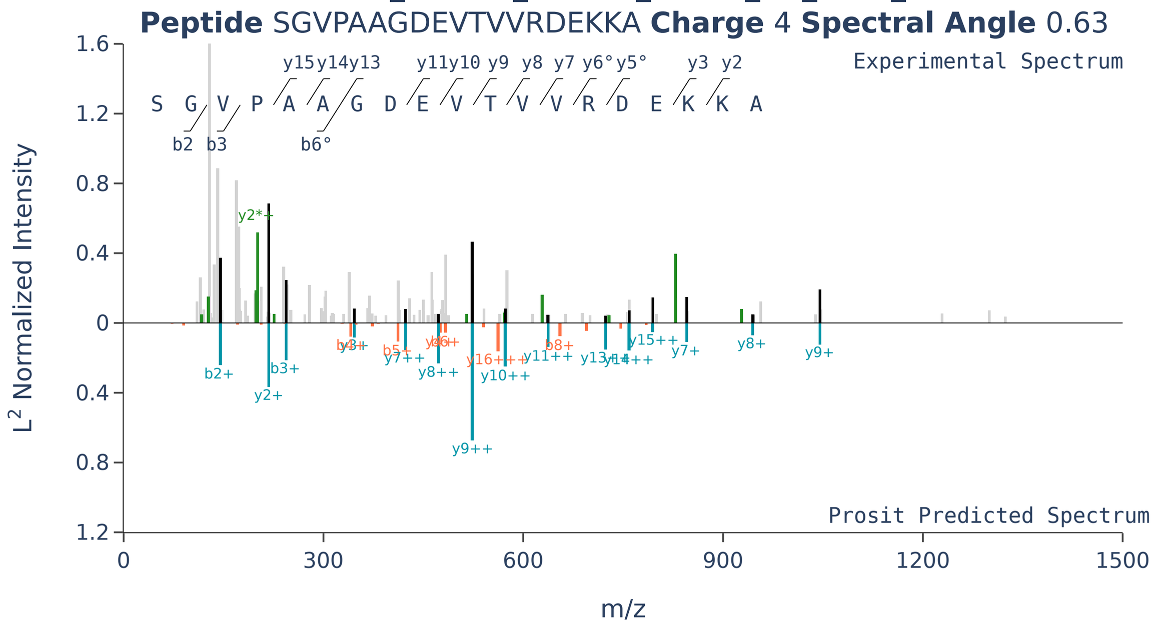


**Source** HRoetschke_211222_160123_HFGoe_IF2_b1R1_4h **Scan** 6256
**Peptide** QLEVRKKRTF **Charge** 3 **Spectral Angle** 0.73

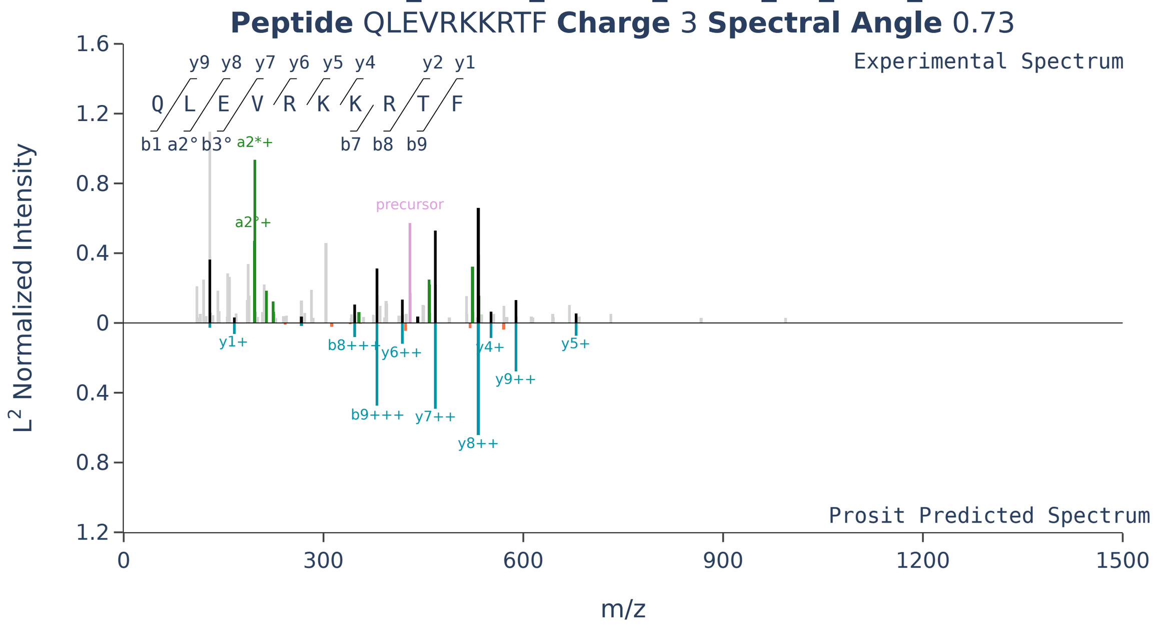


**Source** HRoetschke_211222_160123_HFGoe_IF2_b1R1_4h **Scan** 21949
**Peptide** ARGAQATDIVVL **Charge** 2 **Spectral Angle** 0.9

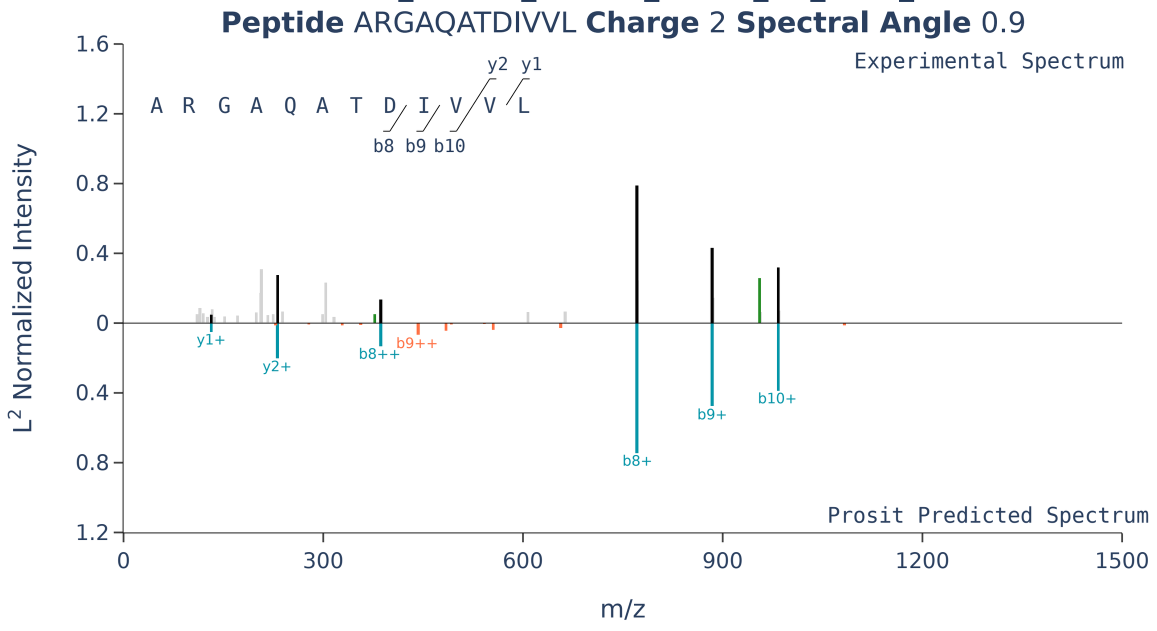


**Source** HRoetschke_211222_160123_HFGoe_IF2_b2R1_1h **Scan** 7173
**Peptide** VRKGMASGAVLE **Charge** 3 **Spectral Angle** 0.85

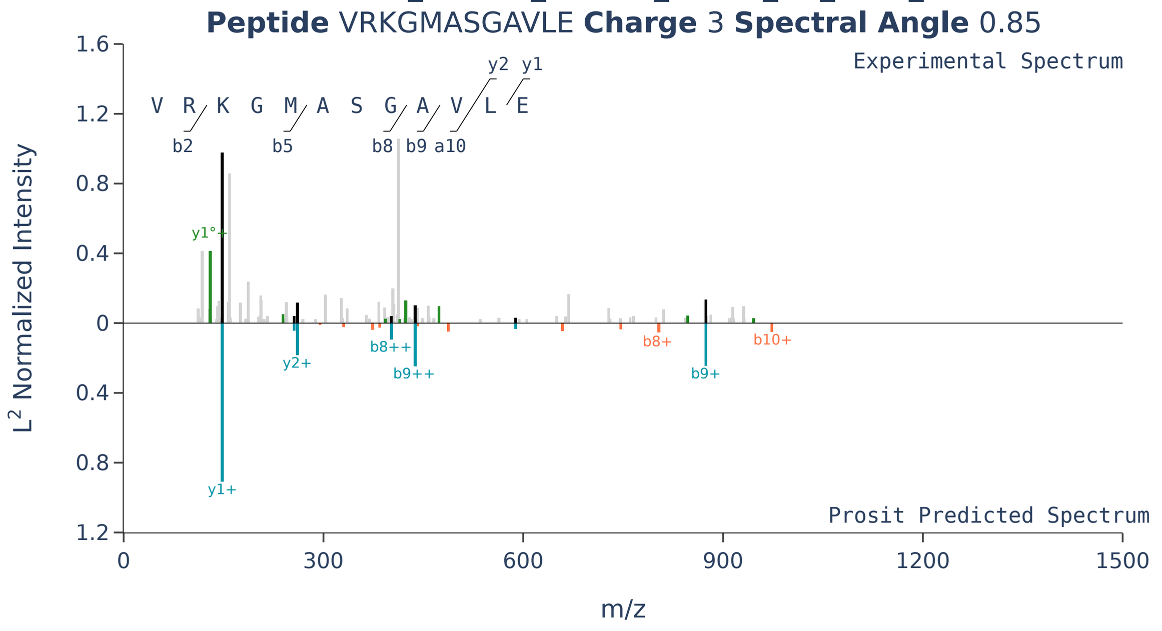


**Source** HRoetschke_211222_160123_HFGoe_IF2_b1R1_4h **Scan** 9441
**Peptide** AERLAAEEQAQREAEE **Charge** 3 **Spectral Angle** 0.7

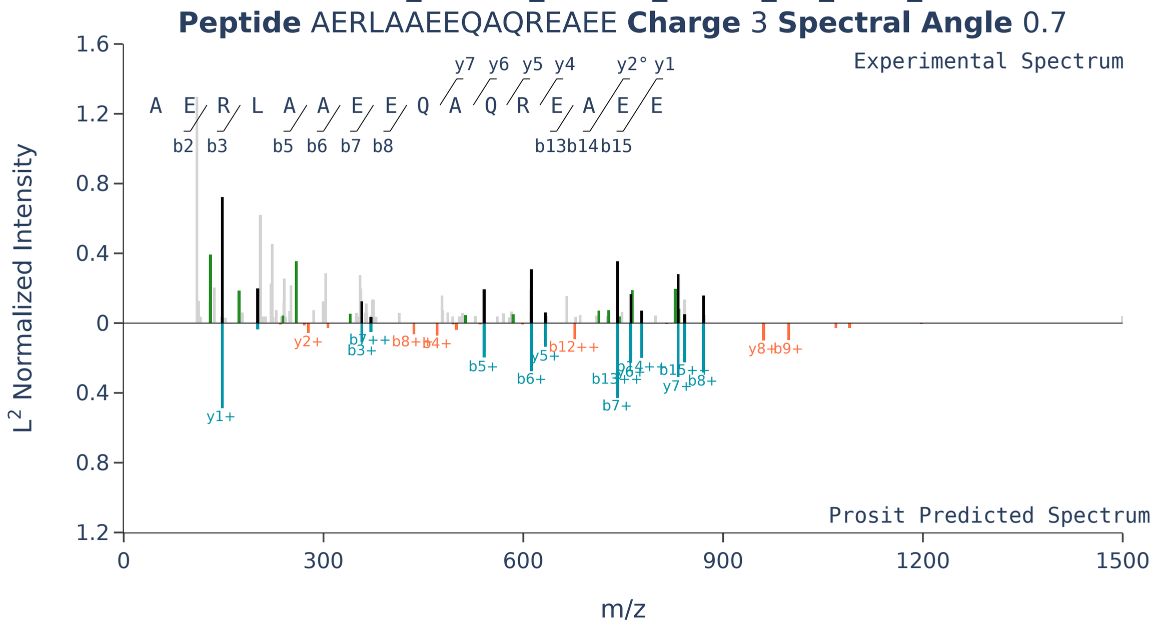


**Source** HRoetschke_211222_160123_HFGoe_IF2_b2R2_24h **Scan** 14928
**Peptide** VASGEAGGITQHI **Charge** 2 **Spectral Angle** 0.69

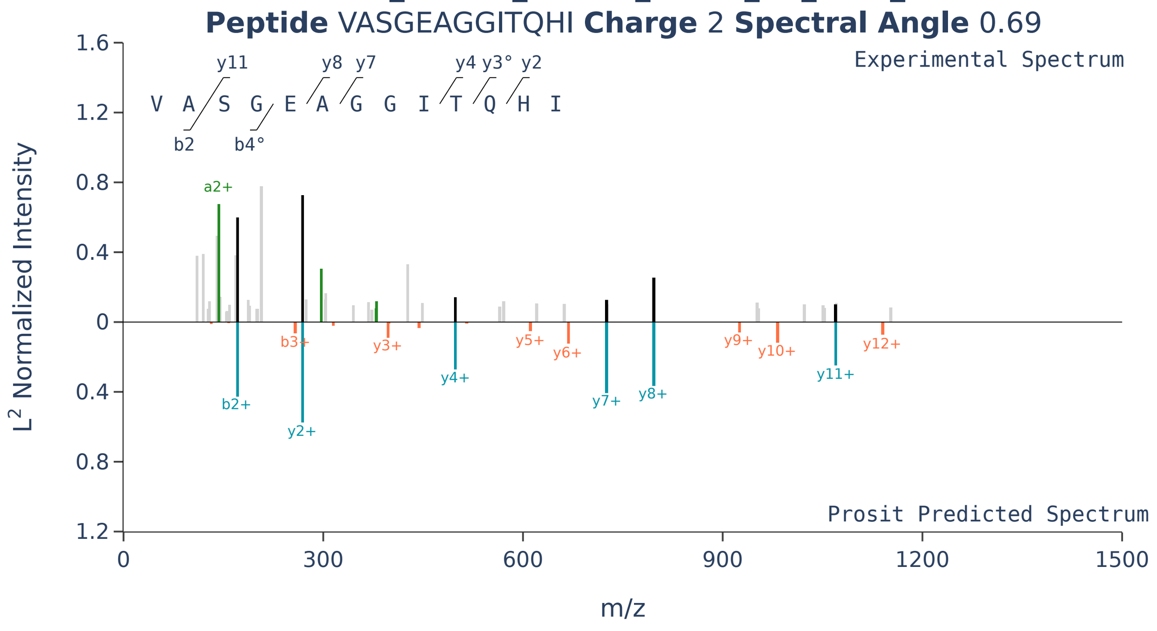


**Source** HRoetschke_211222_160123_HFGoe_IF2_b1R1_4h **Scan** 11188
**Peptide** NMFANMTEGEVHE **Charge** 2 **Spectral Angle** 0.78

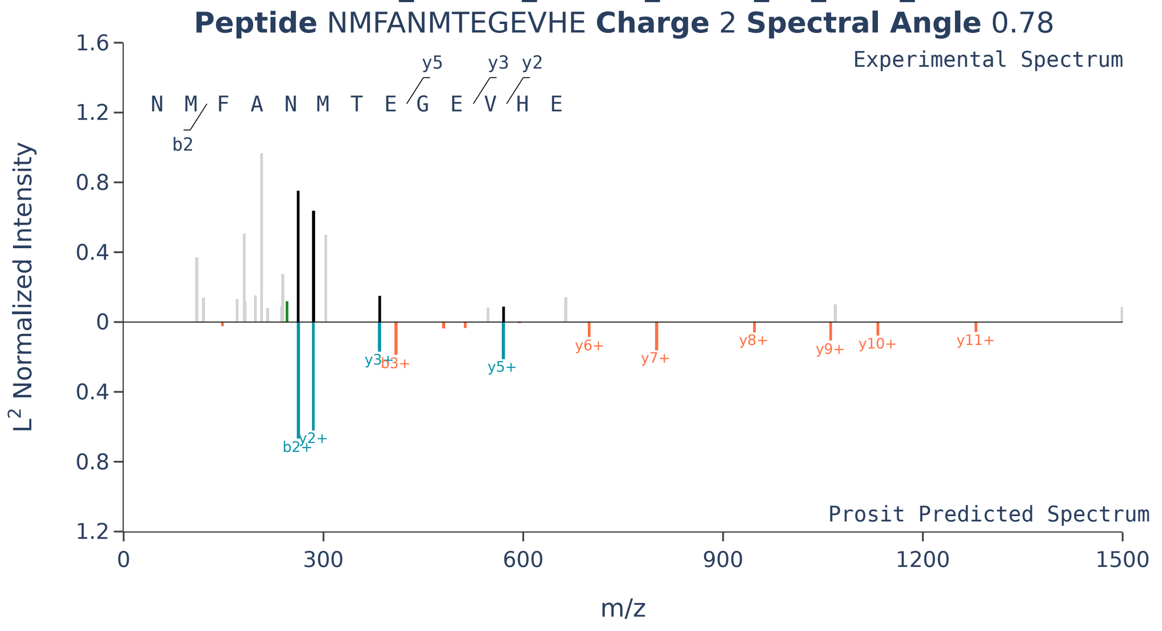


**Source** HRoetschke_211222_160123_HFGoe_IF2_b2R2_24h **Scan** 16326
**Peptide** KIIGSGVGGITE **Charge** 2 **Spectral Angle** 0.82

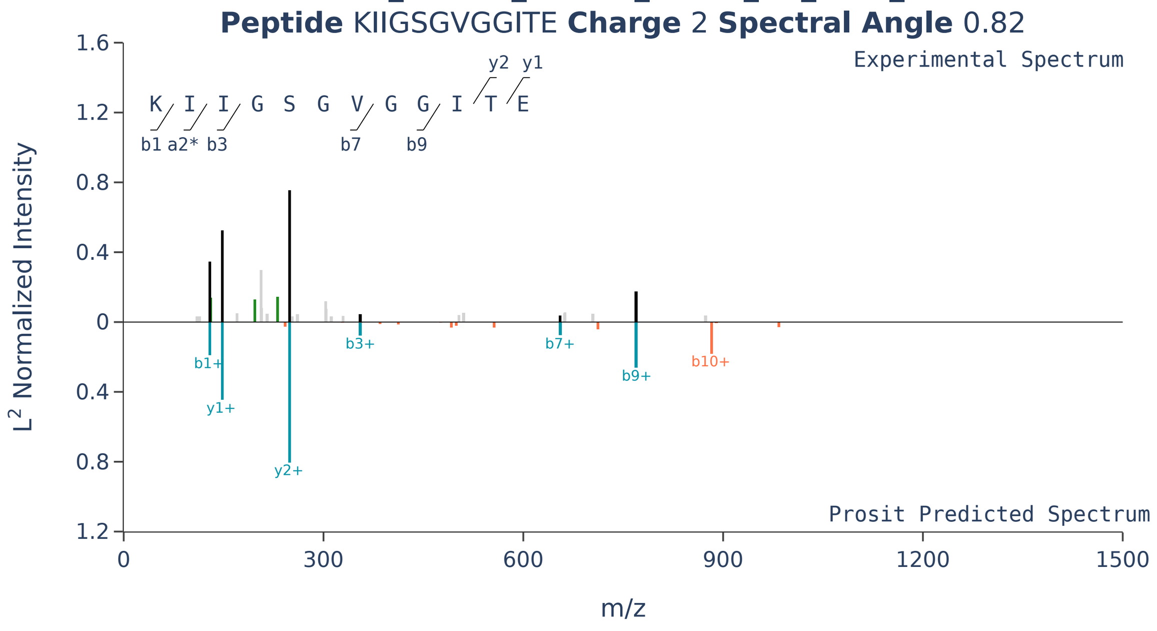


**Source** HRoetschke_211222_160123_HFGoe_IF2_b1R2_1h **Scan** 16935
**Peptide** DGVMPQTIEAIQHAKAA **Charge** 3 **Spectral Angle** 0.73

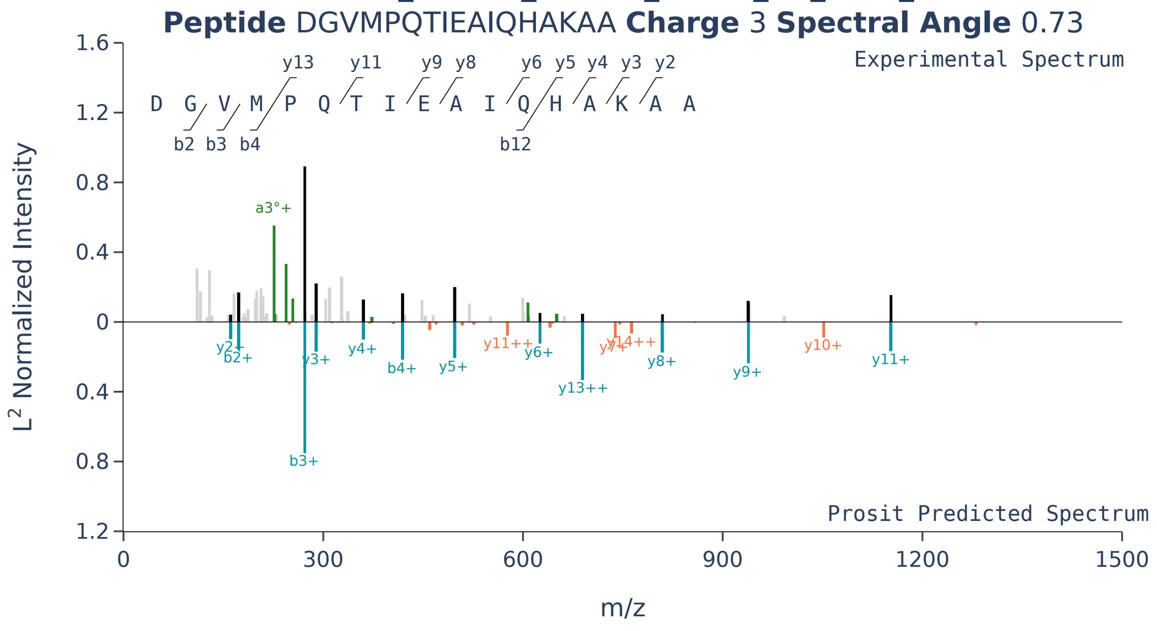


**Source** HRoetschke_211222_160123_HFGoe_IF2_b2R1_24h **Scan** 13483
**Peptide** AAAEPRAPVV **Charge** 2 **Spectral Angle** 0.77

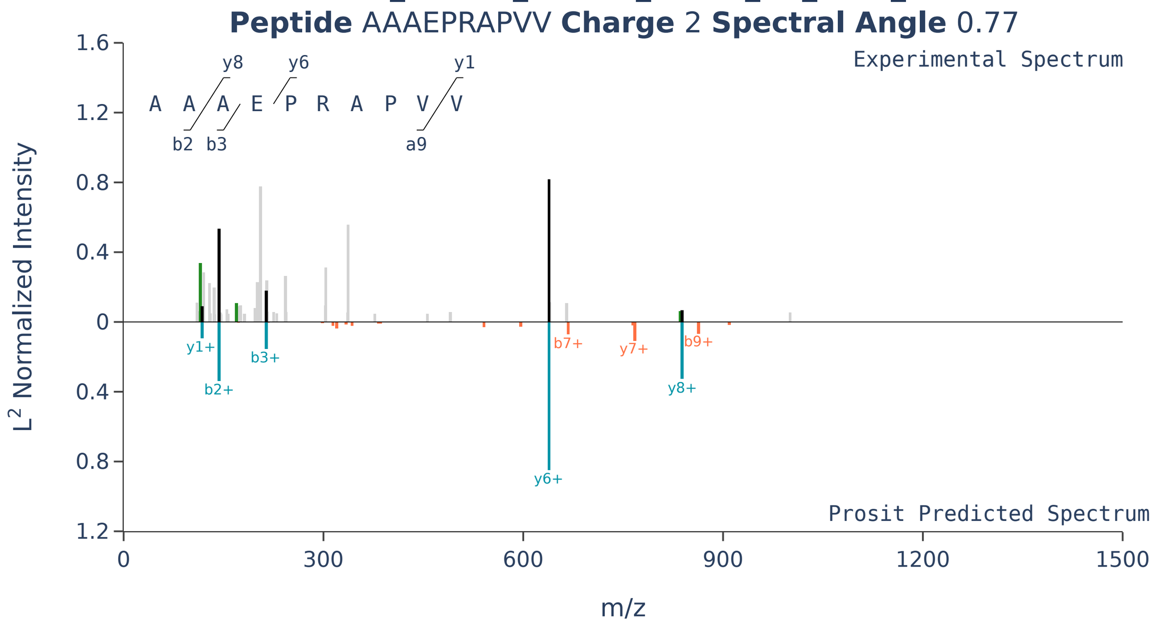


**Source** HRoetschke_211222_160123_HFGoe_IF2_b1R1_4h **Scan** 21054
**Peptide** VVAADDGVMPQTIE **Charge** 2 **Spectral Angle** 0.7

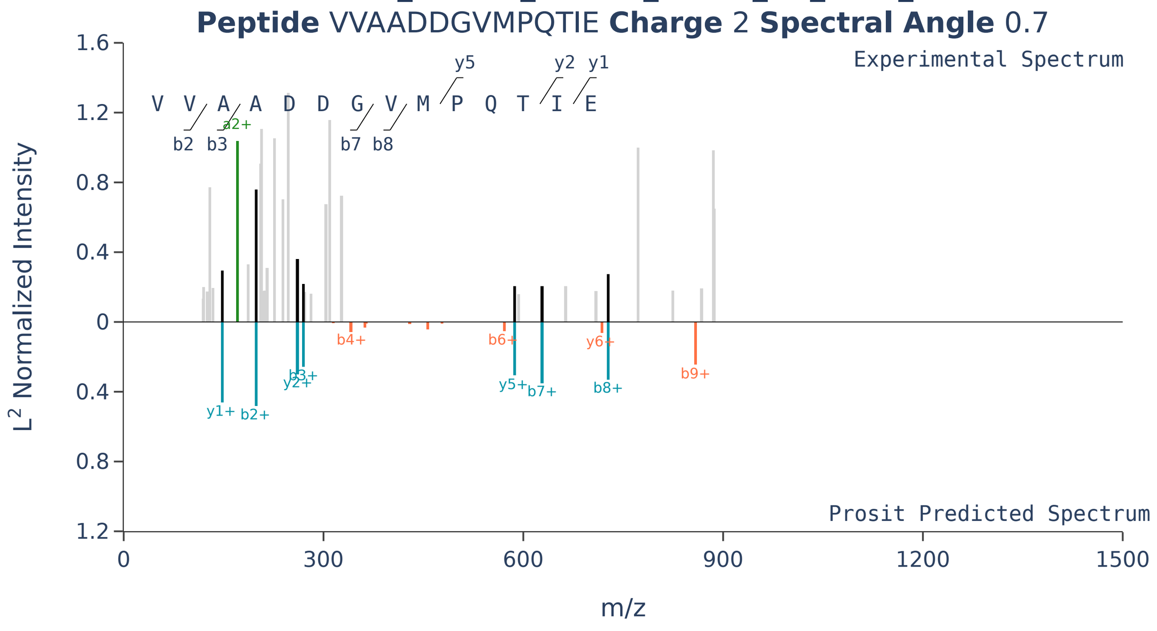

**Source** HRoetschke_211222_160123_HFGoe_IF2_b1R1_24h **Scan** 21329
**Peptide** GEVHEVNIVL **Charge** 2 **Spectral Angle** 0.78

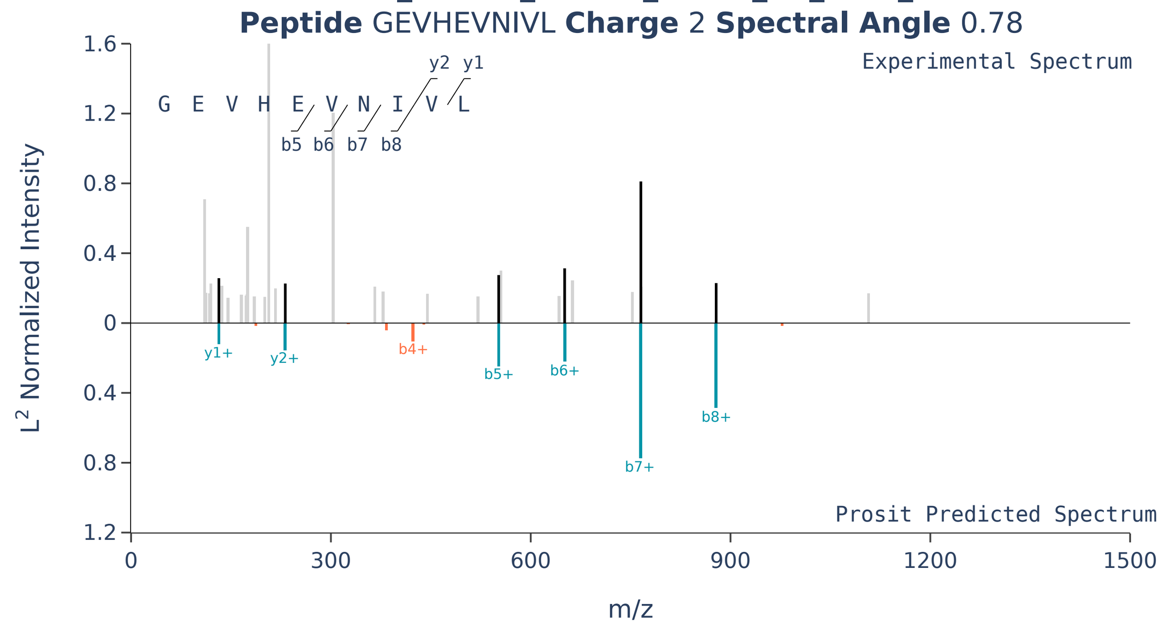


**Source** HRoetschke_211222_160123_HFGoe_IF2_b1R1_1h **Scan** 8185
**Peptide** LIDHLNQKN **Charge** 2 **Spectral Angle** 0.59

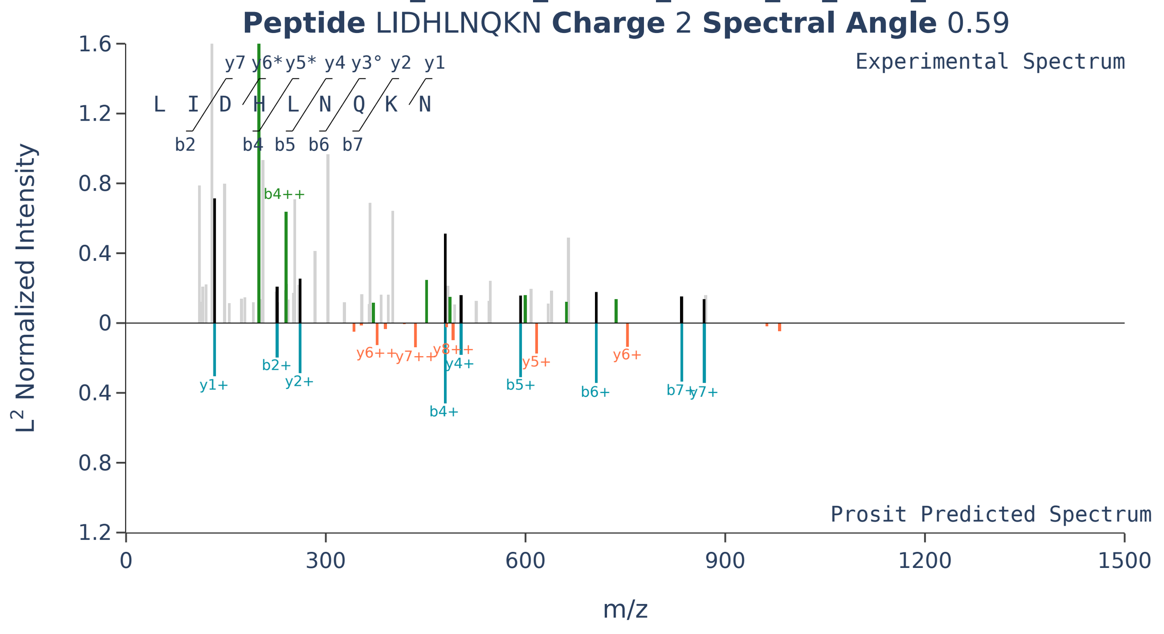


**Source** HRoetschke_211222_160123_HFGoe_IF2_b2R1_24h **Scan** 12509
**Peptide** RSTLNIPGTGGK **Charge** 2 **Spectral Angle** 0.73

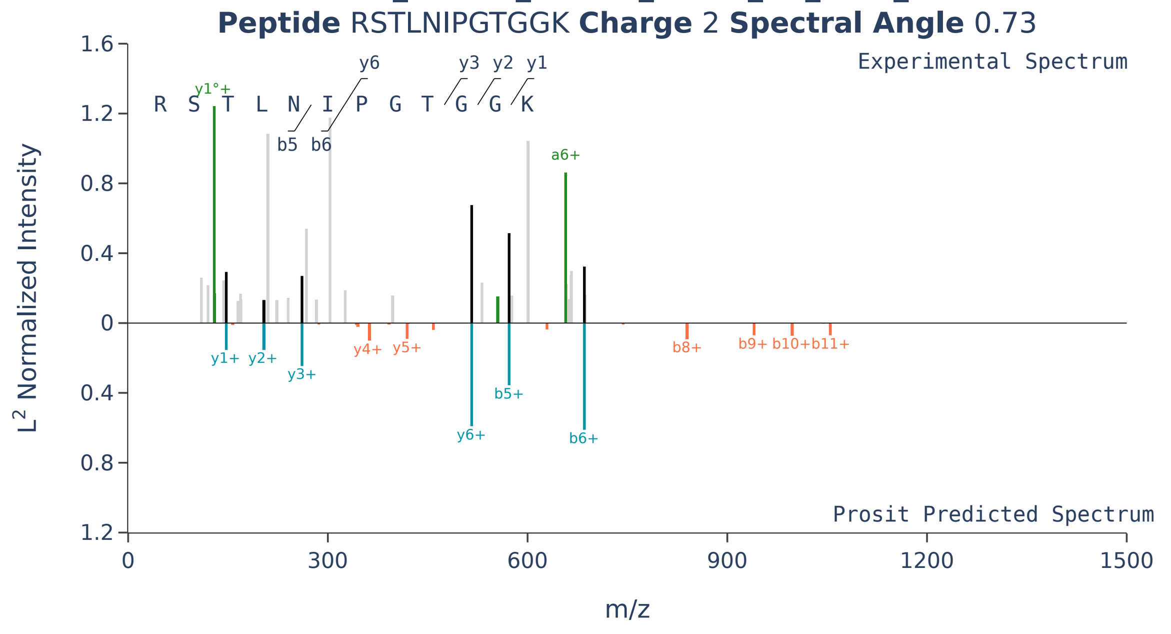


**Source** HRoetschke_211222_160123_HFGoe_IF2_b2R2_24h **Scan** 16734
**Peptide** DHGKTSLLDY **Charge** 2 **Spectral Angle** 0.78

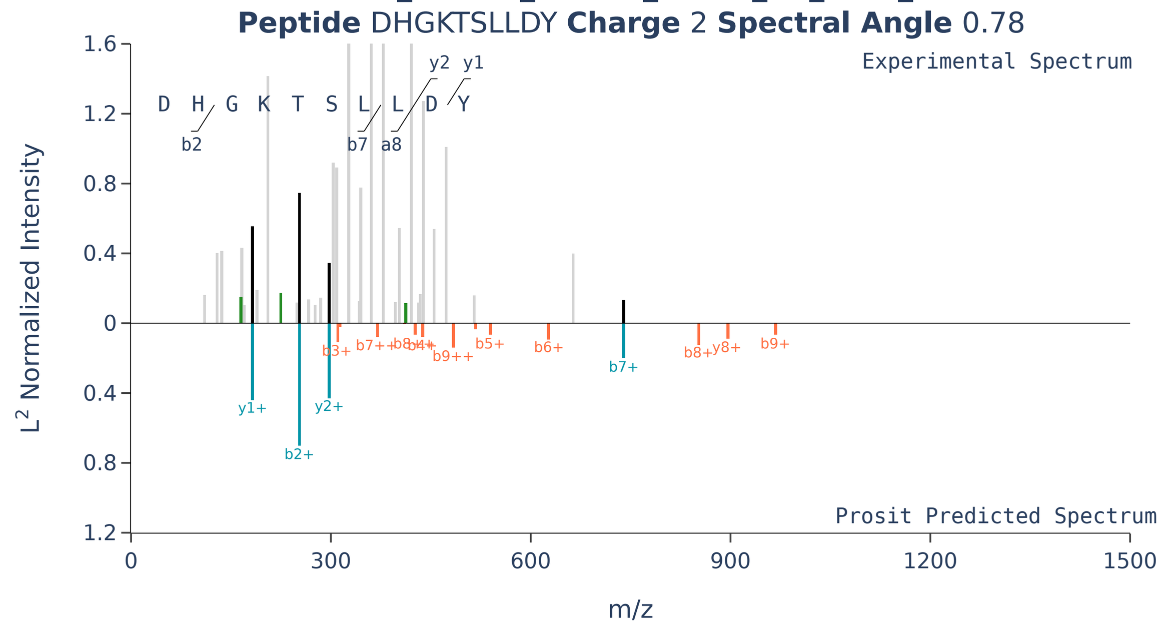


**Source** HRoetschke_211222_160123_HFGoe_IF2_b2R2_24h **Scan** 16439
**Peptide** EKQTLIDHL **Charge** 3 **Spectral Angle** 0.85

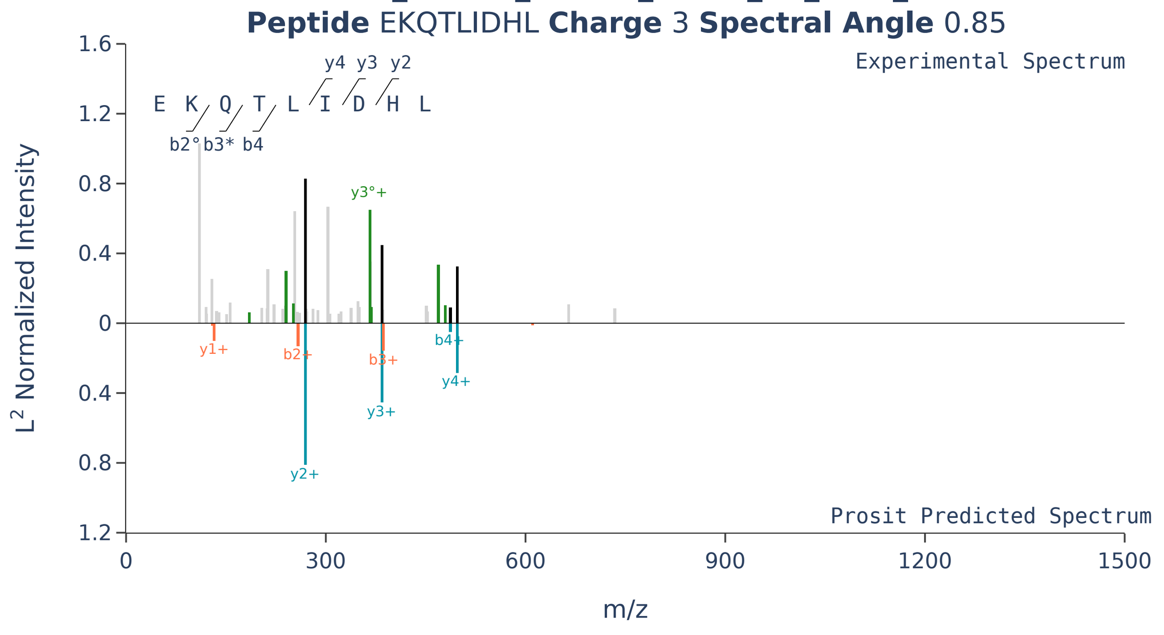


**Source** HRoetschke_211222_160123_HFGoe_IF2_b2R2_24h **Scan** 7846
**Peptide** KVASGEAGGLT **Charge** 2 **Spectral Angle** 0.73

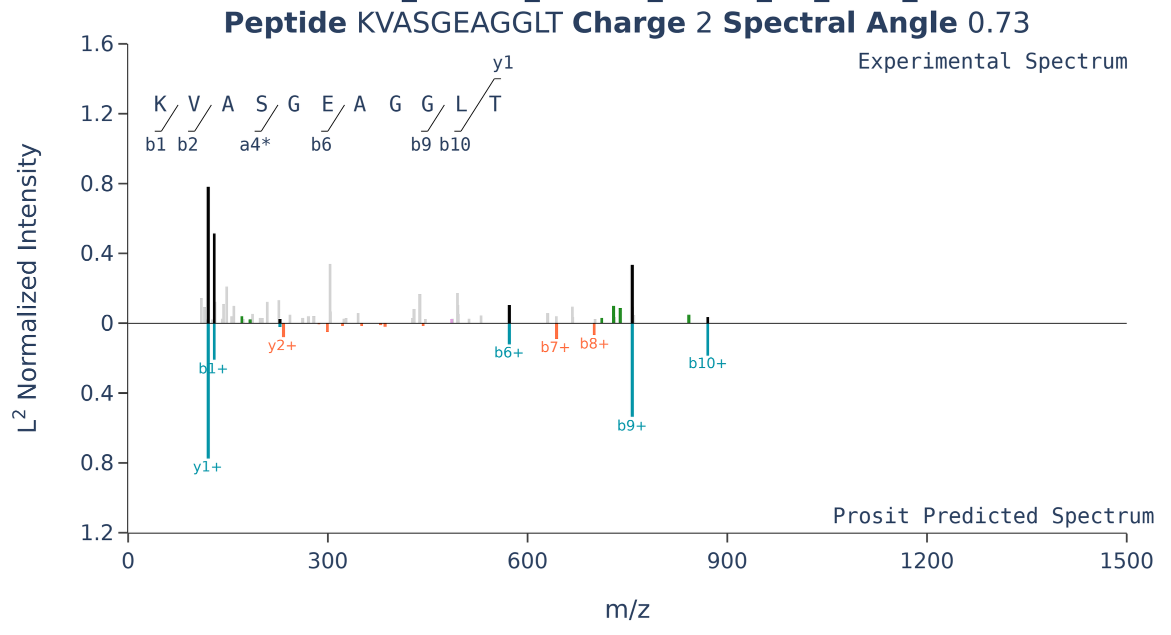


**Source** HRoetschke_211222_160123_HFGoe_IF2_b1R2_24h **Scan** 8105
**Peptide** QLEVRKKRTFV **Charge** 3 **Spectral Angle** 0.69

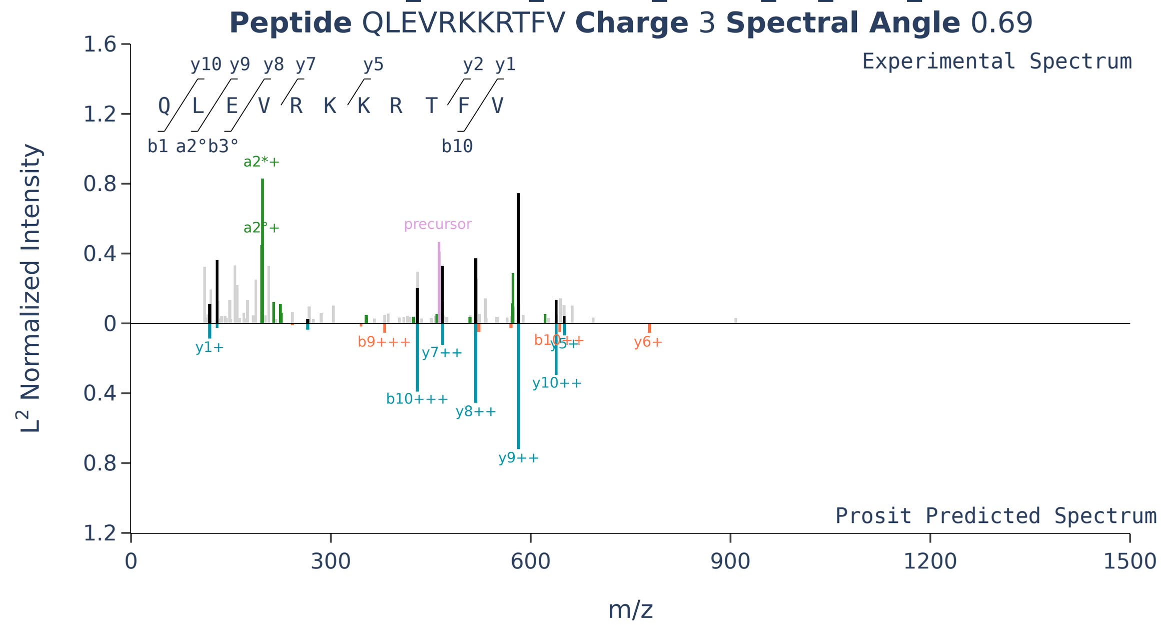


**Source** HRoetschke_211222_160123_HFGoe_IF2_b1R2_1h **Scan** 11387
**Peptide** AERQTSVERLVQ **Charge** 3 **Spectral Angle** 0.63

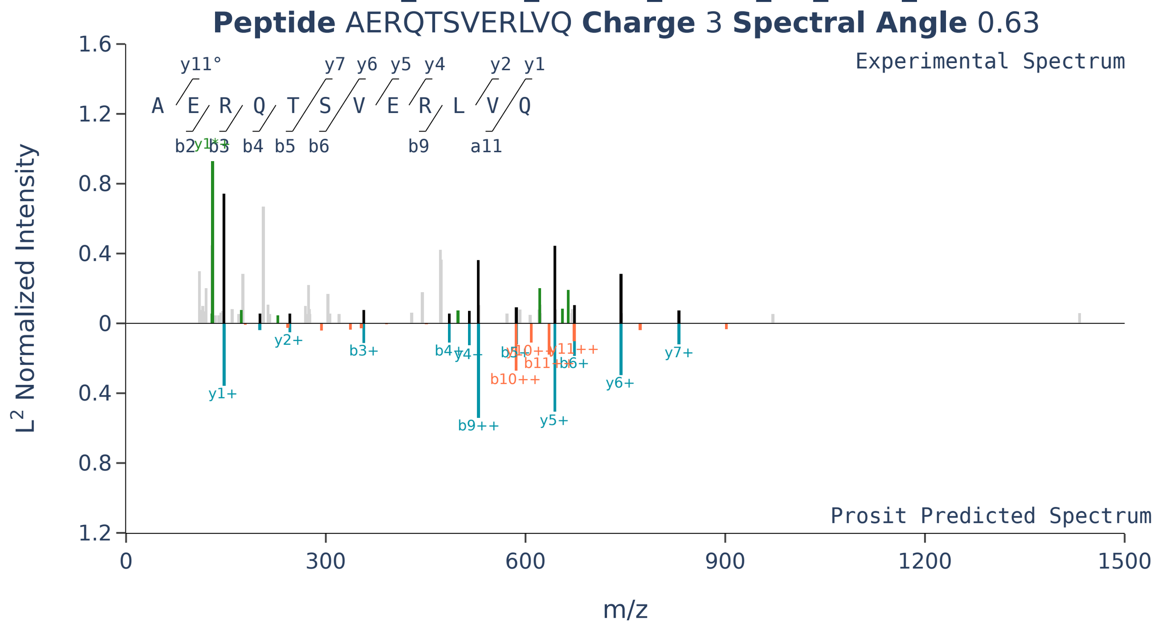


**Source** HRoetschke_211222_160123_HFGoe_IF2_b1R2_4h **Scan** 11240
**Peptide** DKPEADPDRVKNELSQ **Charge** 3 **Spectral Angle** 0.75

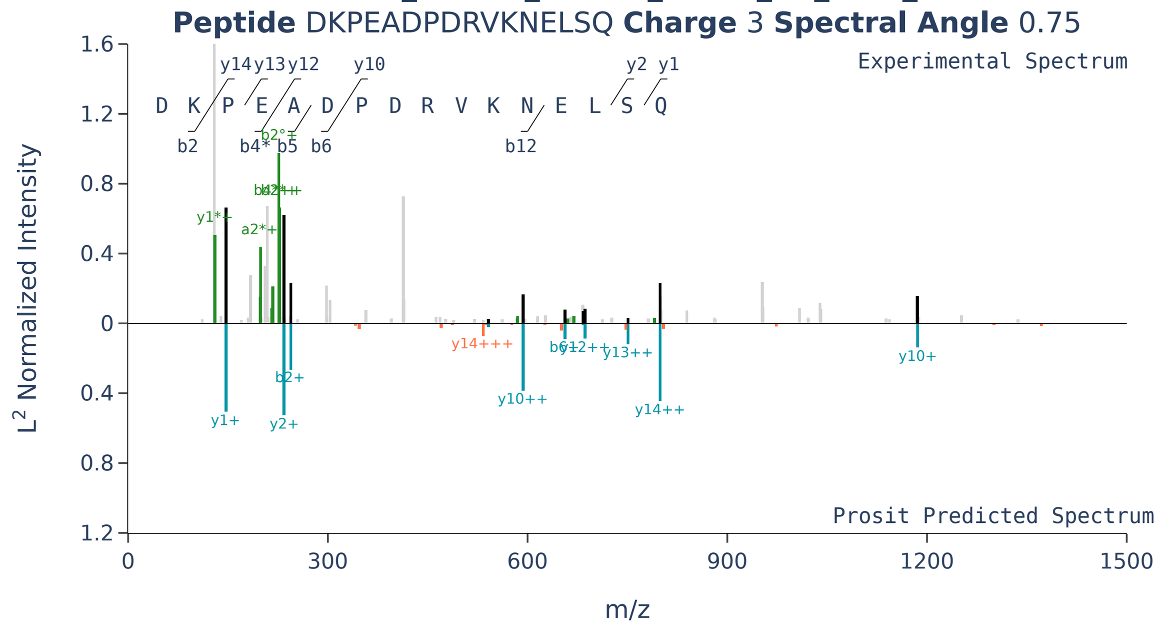


**Source** HRoetschke_211222_160123_HFGoe_IF2_b1R1_4h **Scan** 11253
**Peptide** VRKGMASGAVI **Charge** 2 **Spectral Angle** 0.63

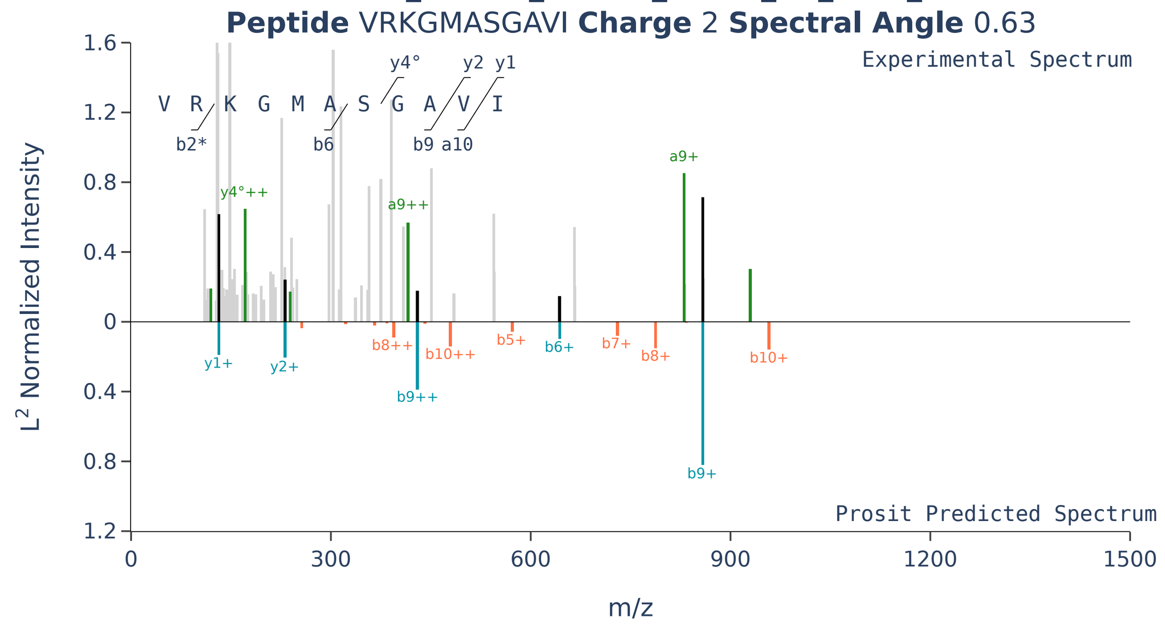


**Source** HRoetschke_211222_160123_HFGoe_IF2_b2R2_24h **Scan** 11878
**Peptide** KSKSVQIEV **Charge** 2 **Spectral Angle** 0.65

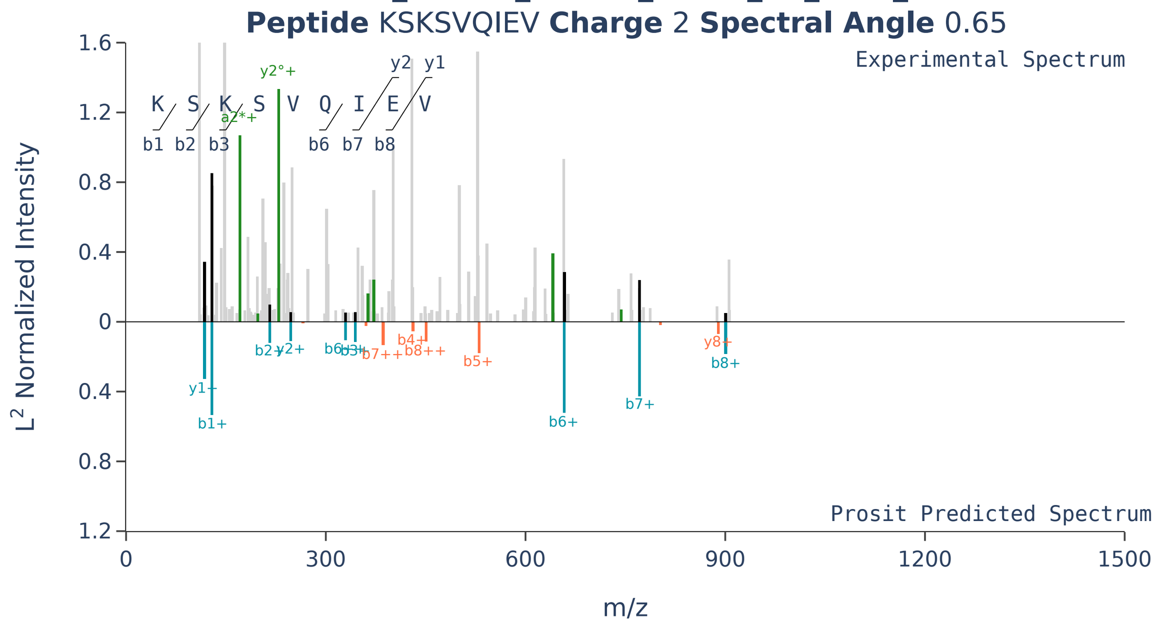


**Source** HRoetschke_211222_160123_HFGoe_IF2_b1R1_4h **Scan** 17438
**Peptide** KQQIIGLAE **Charge** 2 **Spectral Angle** 0.8

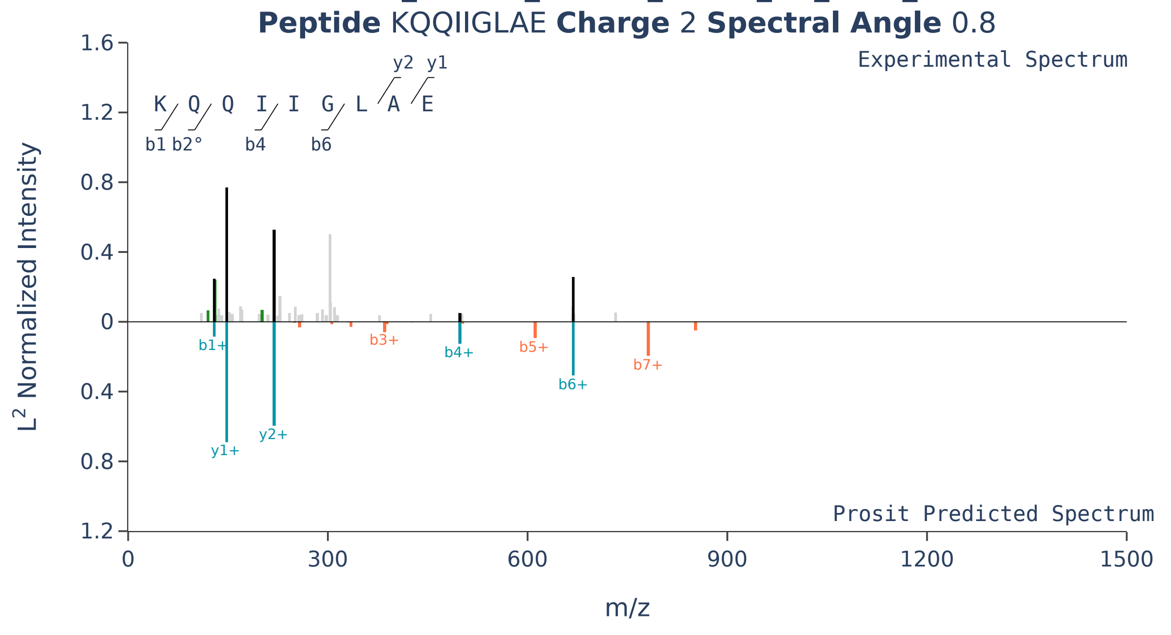


**Source** HRoetschke_211222_160123_HFGoe_IF2_b2R2_4h **Scan** 18390
**Peptide** AVNRDVVIGETI **Charge** 2 **Spectral Angle** 0.68

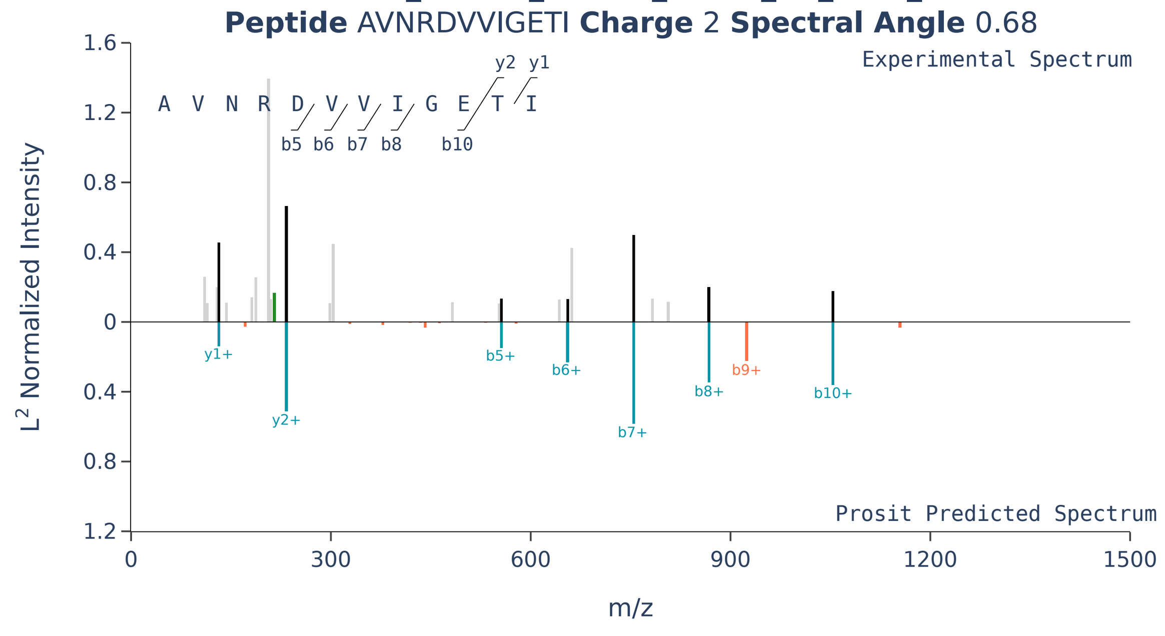


**Source** HRoetschke_211222_160123_HFGoe_IF2_b1R1_4h **Scan** 18125
**Peptide** TINQVIDQETA **Charge** 2 **Spectral Angle** 0.77

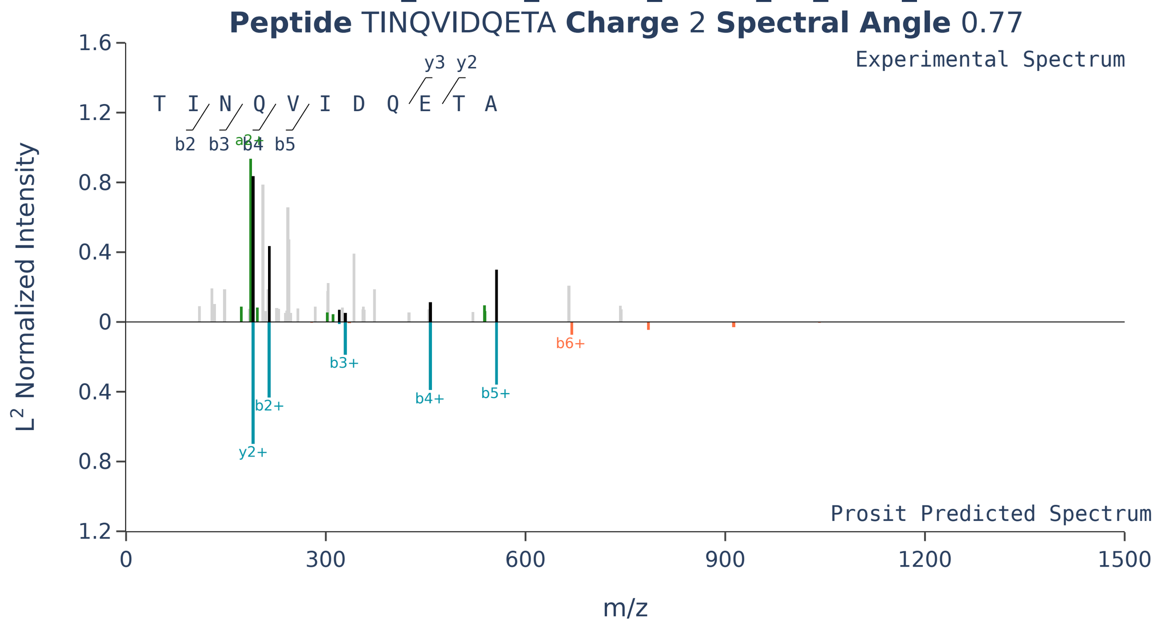


**Source** HRoetschke_211222_160123_HFGoe_IF2_b1R2_1h **Scan** 12396
**Peptide** EAGGITQHIGAYH **Charge** 3 **Spectral Angle** 0.82

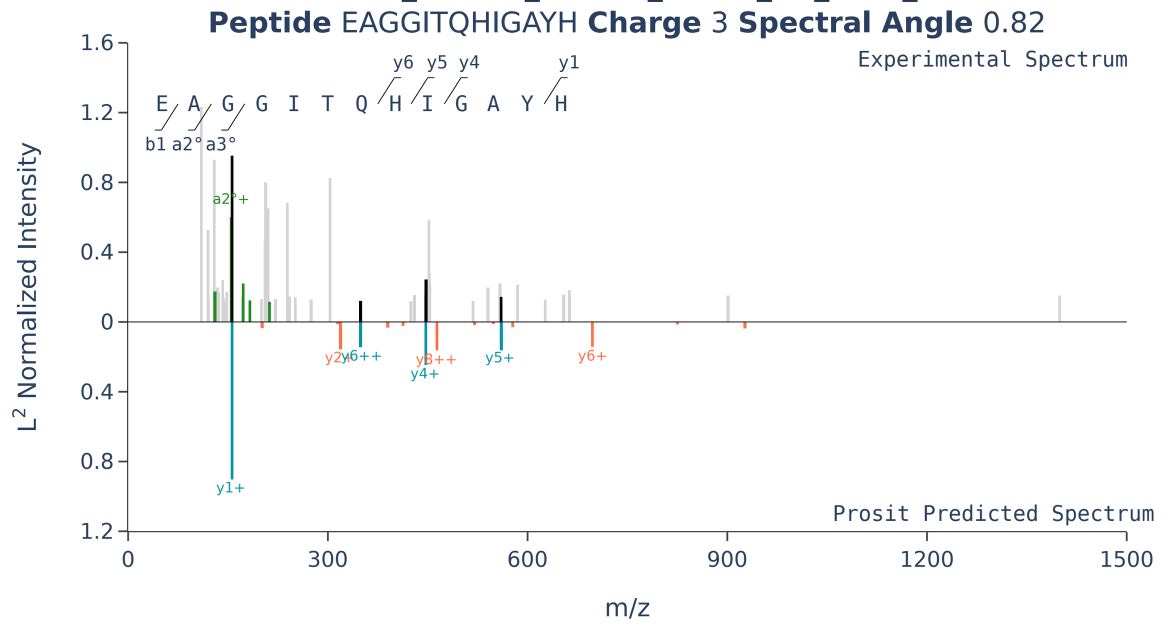

**Source** HRoetschke_211222_160123_HFGoe_IF2_b1R1_4h **Scan** 8058
**Peptide** NAEPTEDSSDYHVTTSQHARQAED **Charge** 4 **Spectral Angle** 0.75

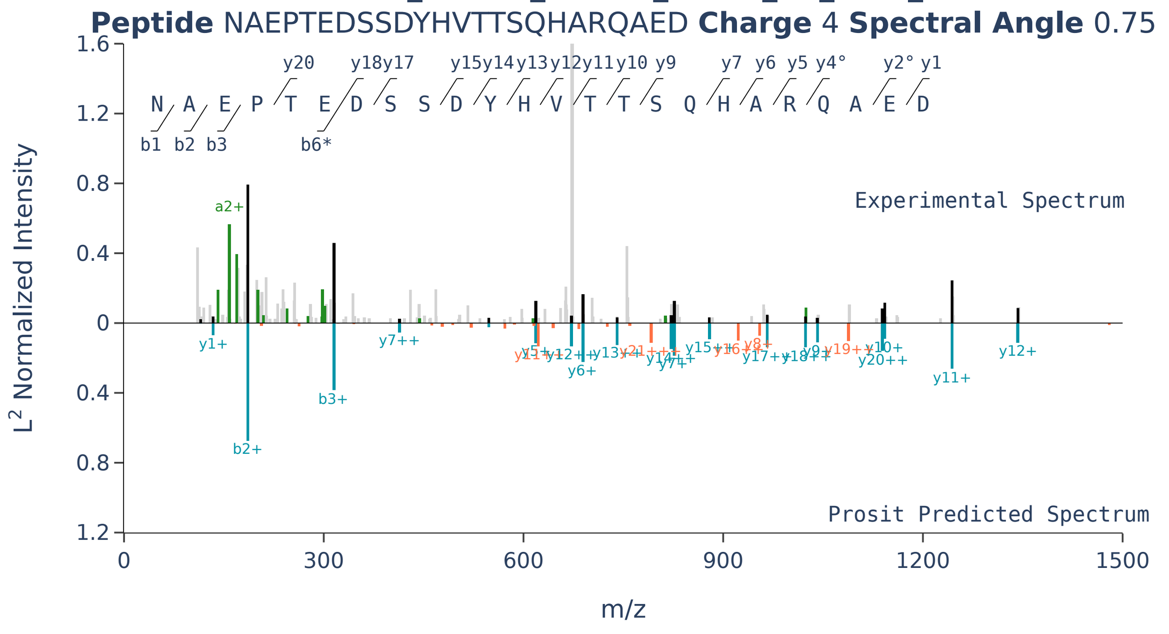


**Source** HRoetschke_211222_160123_HFGoe_IF2_b1R1_4h **Scan** 25175
**Peptide** AQATDIVVL **Charge** 2 **Spectral Angle** 0.82

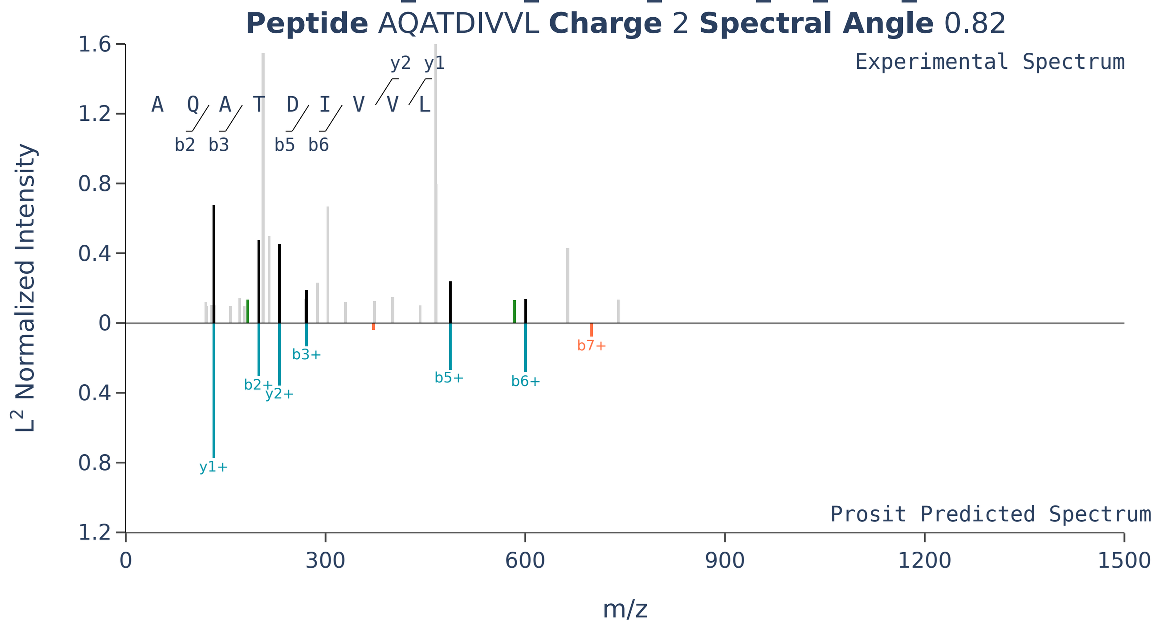


**Source** HRoetschke_211222_160123_HFGoe_IF2_b2R2_4h **Scan** 19478
**Peptide** GQEVLEAGPSI **Charge** 2 **Spectral Angle** 0.75

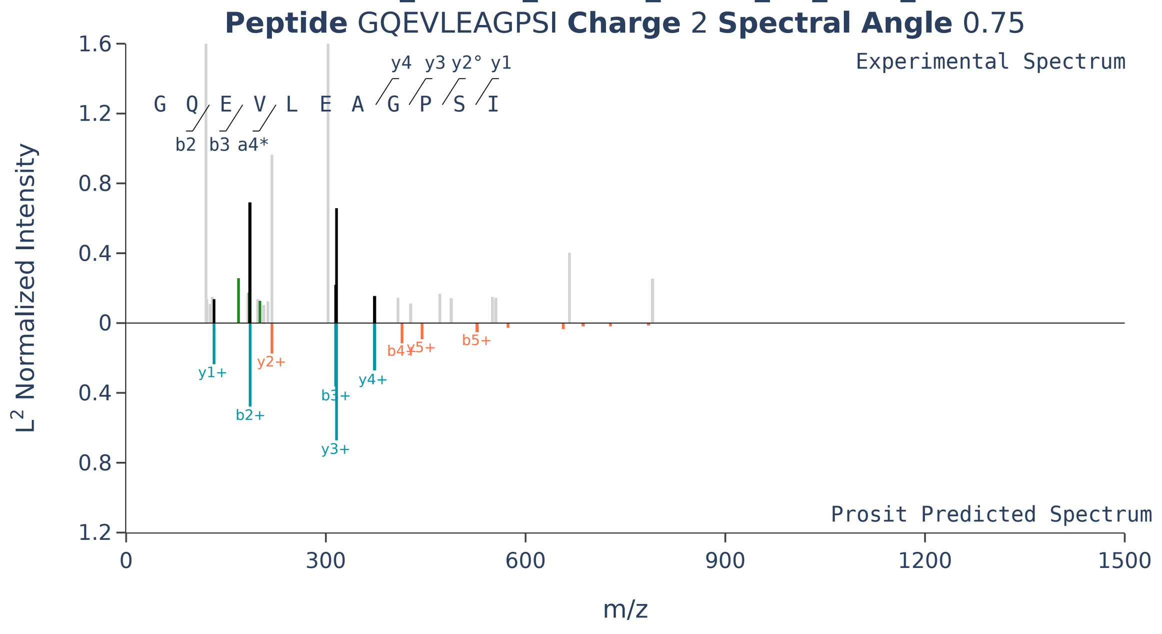


**Source** HRoetschke_211222_160123_HFGoe_IF2_b1R1_1h **Scan** 9351
**Peptide** DPDRVKNEL **Charge** 2 **Spectral Angle** 0.73

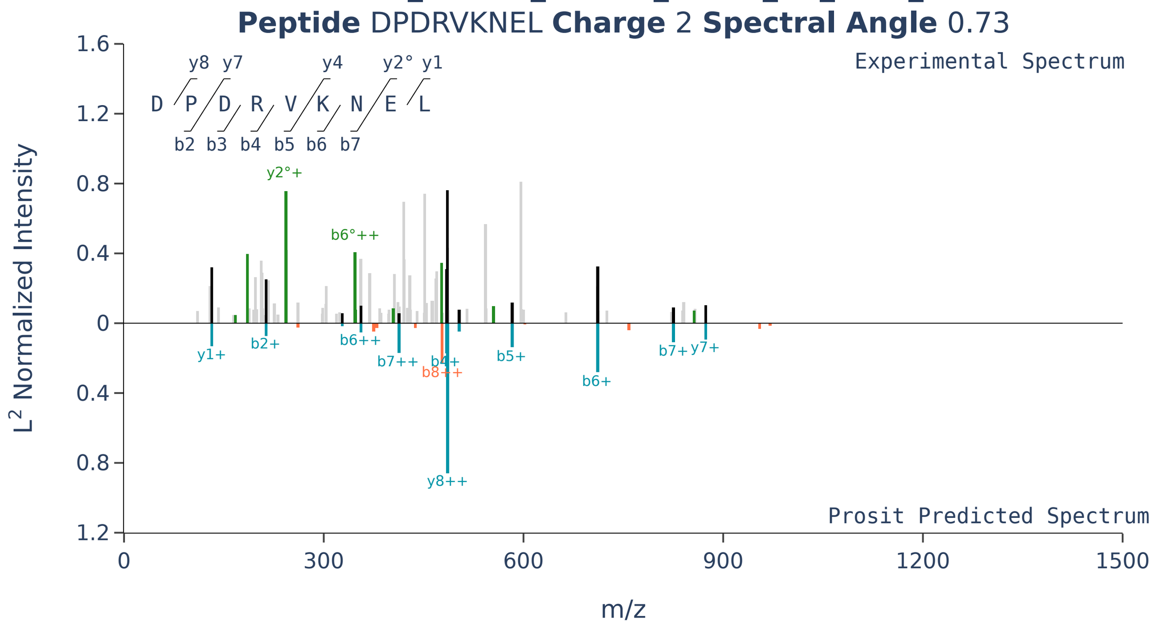


**Source** HRoetschke_211222_160123_HFGoe_IF2_b1R1_4h **Scan** 8219
**Peptide** VKRDPQEAERLAAE **Charge** 3 **Spectral Angle** 0.64

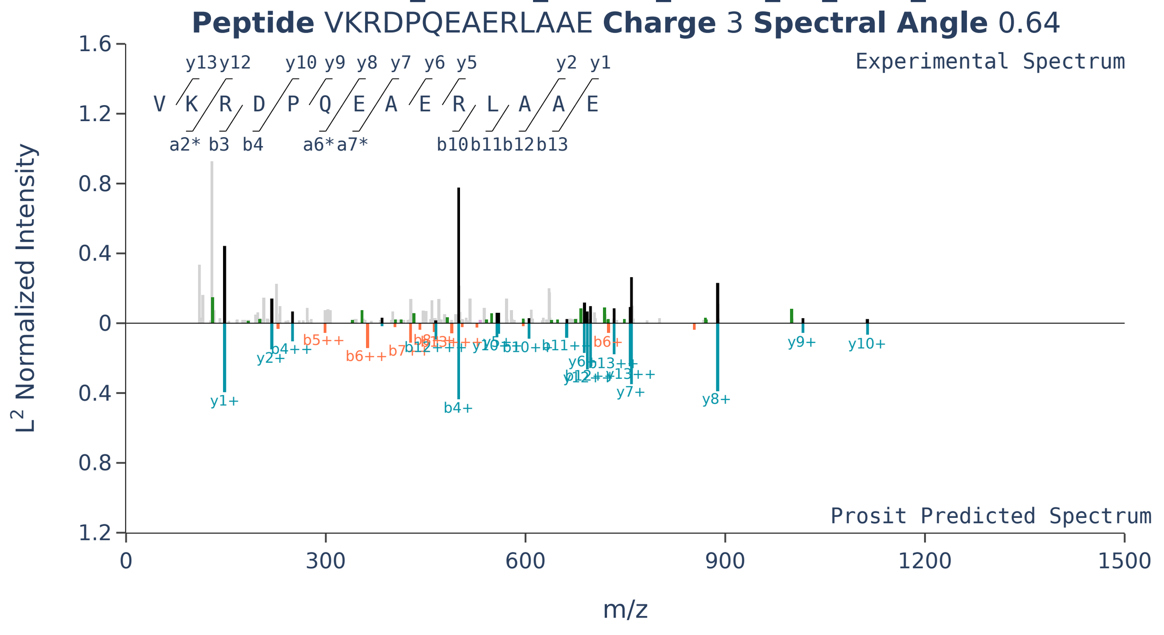


**Source** HRoetschke_211222_160123_HFGoe_IF2_b2R2_2h **Scan** 6470
**Peptide** KAVRKGMASGAVLE **Charge** 3 **Spectral Angle** 0.63

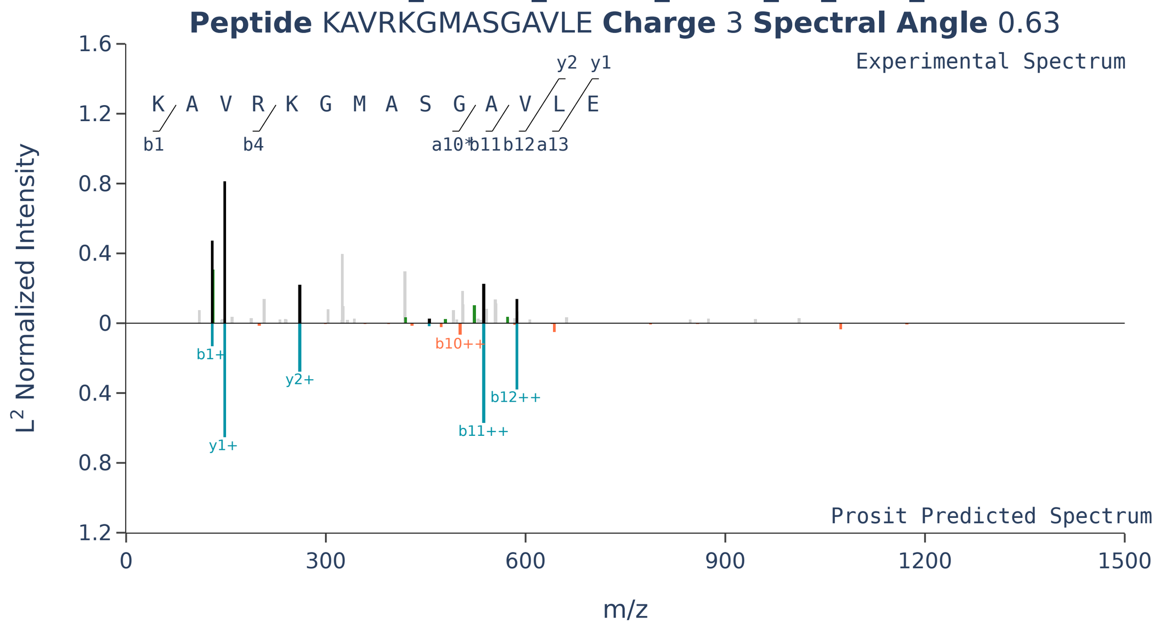


**Source** HRoetschke_211222_160123_HFGoe_IF2_b1R2_4h **Scan** 7193
**Peptide** AVMSDRDTGAA **Charge** 2 **Spectral Angle** 0.76

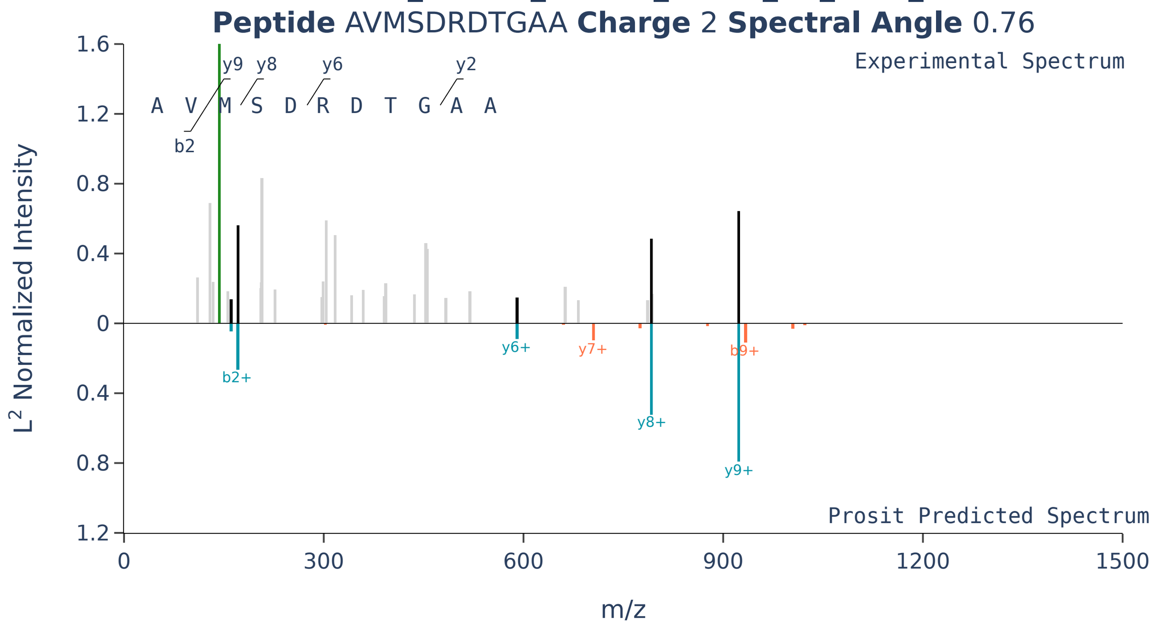


**Source** HRoetschke_211222_160123_HFGoe_IF2_b1R1_4h **Scan** 18081
**Peptide** SGVPAAGDEVTV **Charge** 2 **Spectral Angle** 0.84

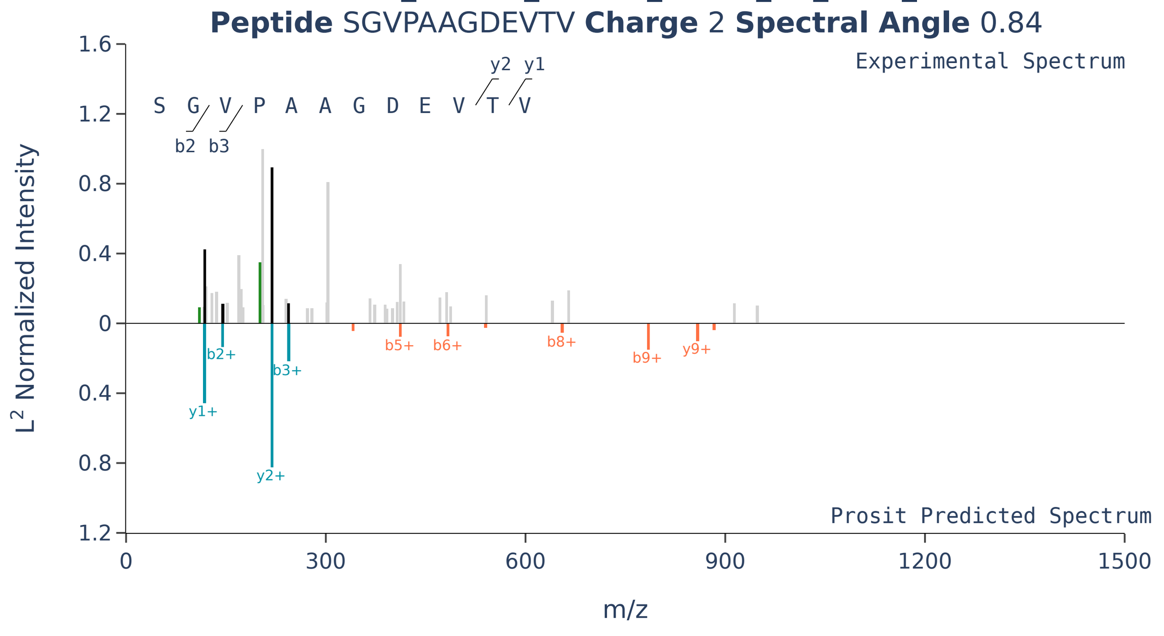


**Source** HRoetschke_211222_160123_HFGoe_IF2_b2R2_24h **Scan** 12269
**Peptide** EPRAPVV **Charge** 2 **Spectral Angle** 0.82

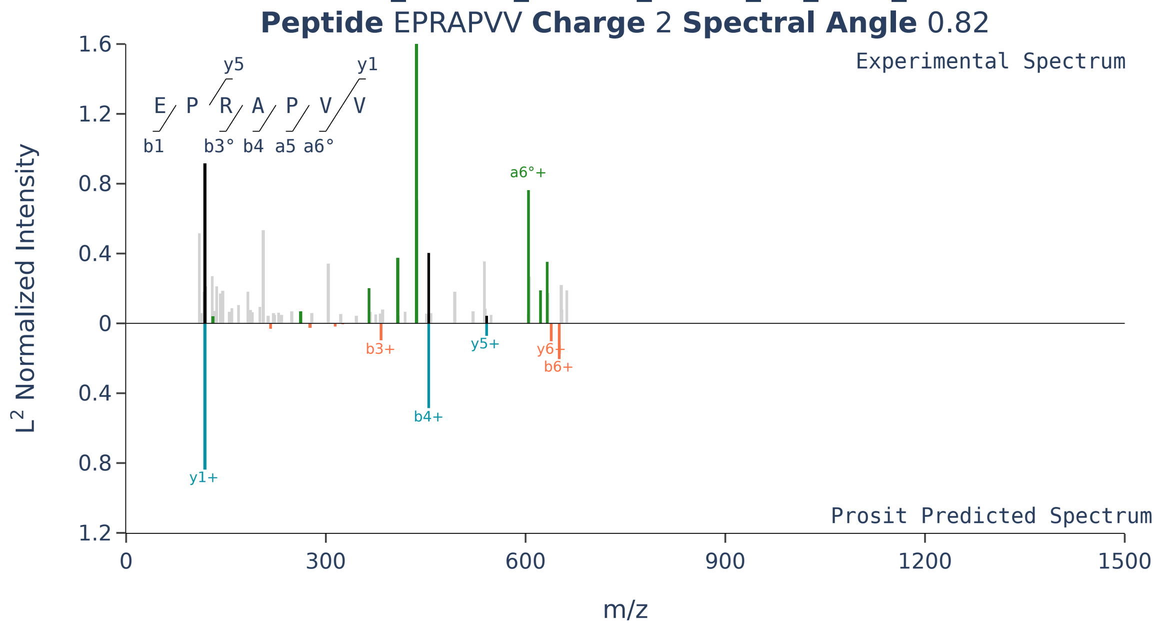


**Source** HRoetschke_211222_160123_HFGoe_IF2_b1R2_24h **Scan** 6979
**Peptide** VRDEKKAREVALYRQG **Charge** 4 **Spectral Angle** 0.68

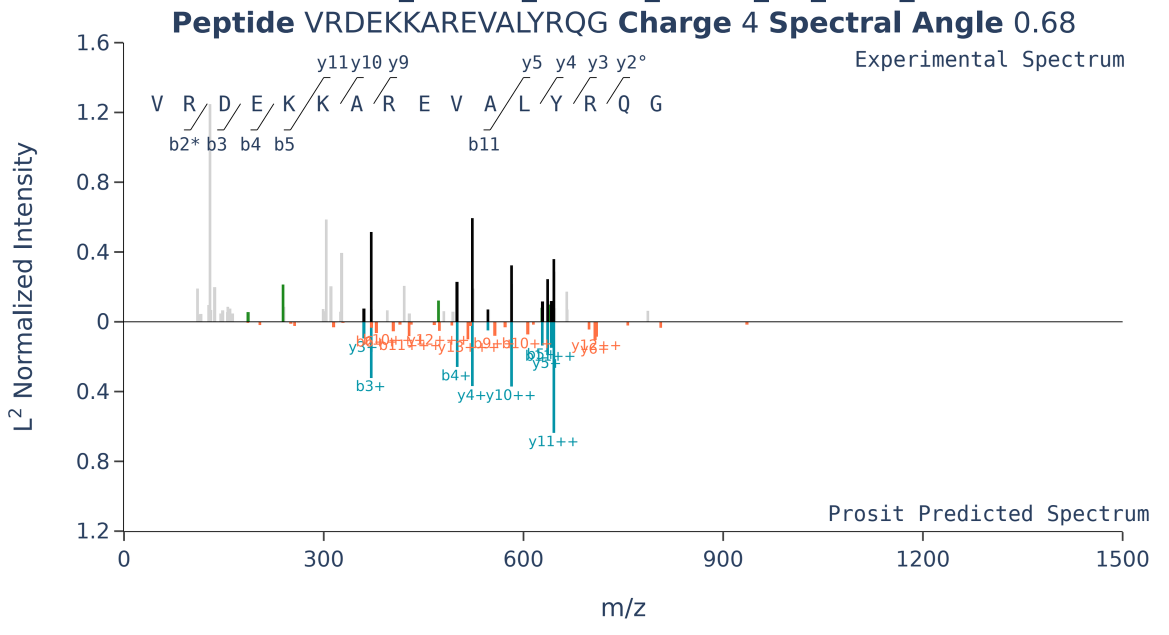


**Source** HRoetschke_211222_160123_HFGoe_IF2_b1R2_24h **Scan** 19881
**Peptide** EVLELKAV **Charge** 2 **Spectral Angle** 0.71

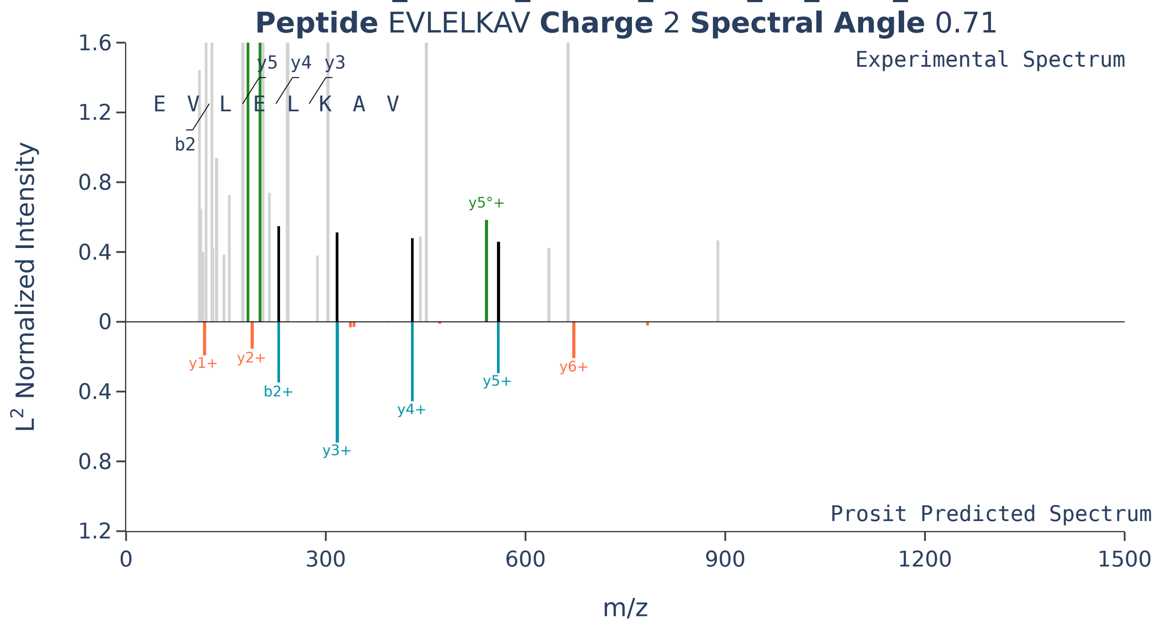


**Source** HRoetschke_211222_160123_HFGoe_IF2_b2R2_24h **Scan** 14773
**Peptide** EAIQHAKAAQVPVV **Charge** 2 **Spectral Angle** 0.82

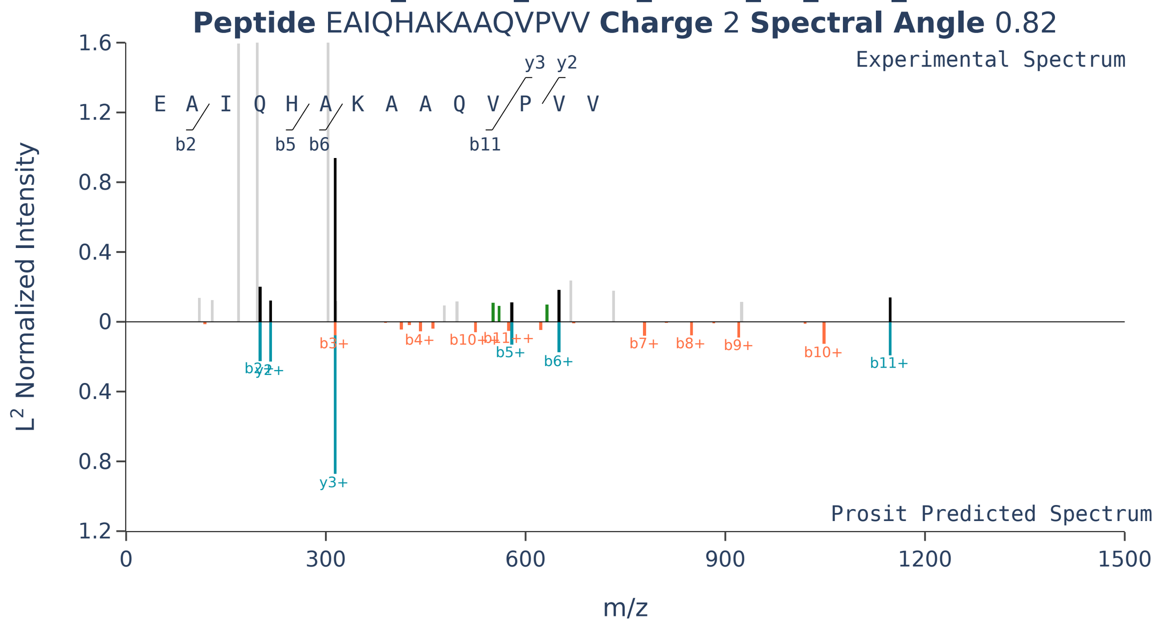


**Source** HRoetschke_211222_160123_HFGoe_IF2_b2R2_24h **Scan** 8677
**Peptide** TIKTLAAERQTS **Charge** 3 **Spectral Angle** 0.71

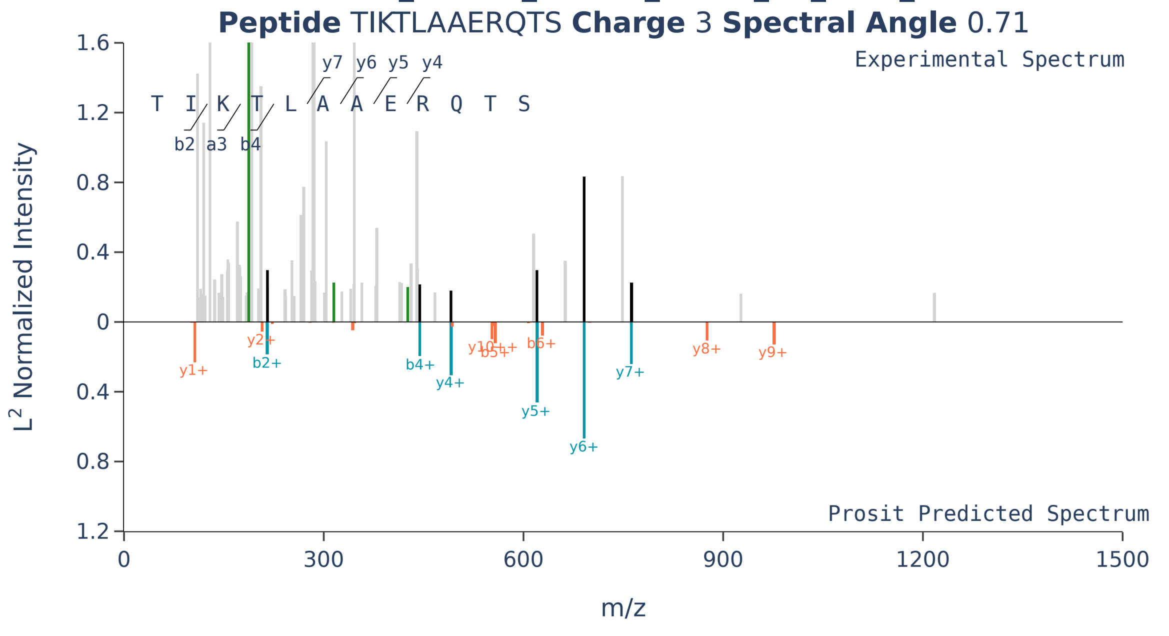


**Source** HRoetschke_211222_160123_HFGoe_IF2_b1R1_4h **Scan** 19160
**Peptide** WGGESQFVHVS **Charge** 2 **Spectral Angle** 0.76

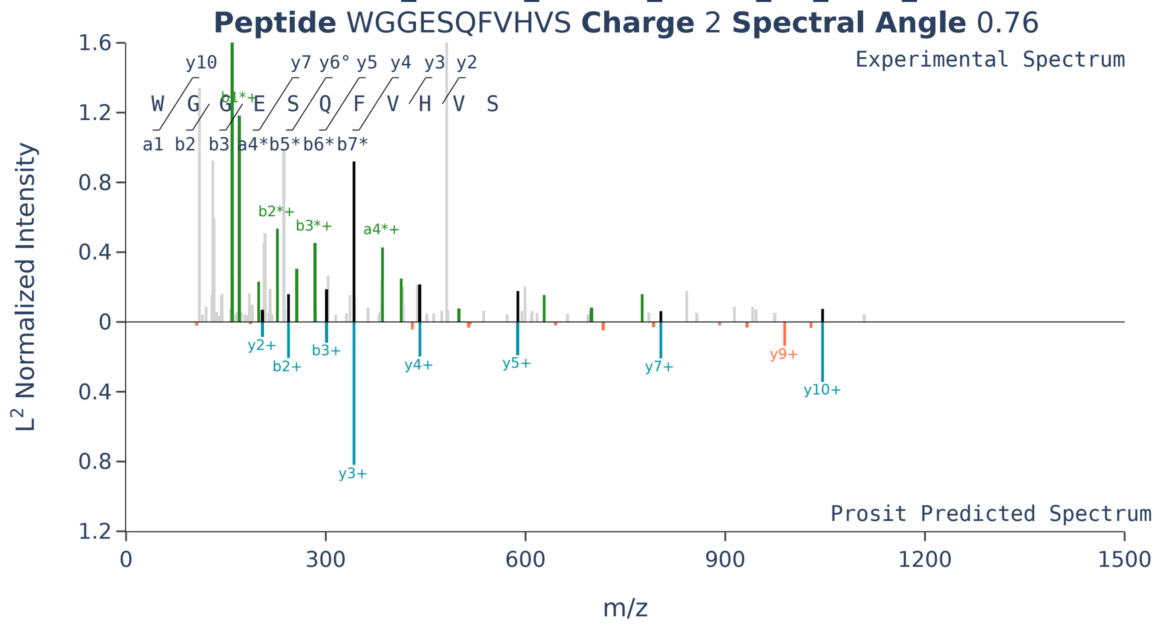


**Source** HRoetschke_211222_160123_HFGoe_IF2_b1R2_4h **Scan** 6132
**Peptide** SSDYHVTTSQHARQAEDE **Charge** 3 **Spectral Angle** 0.69

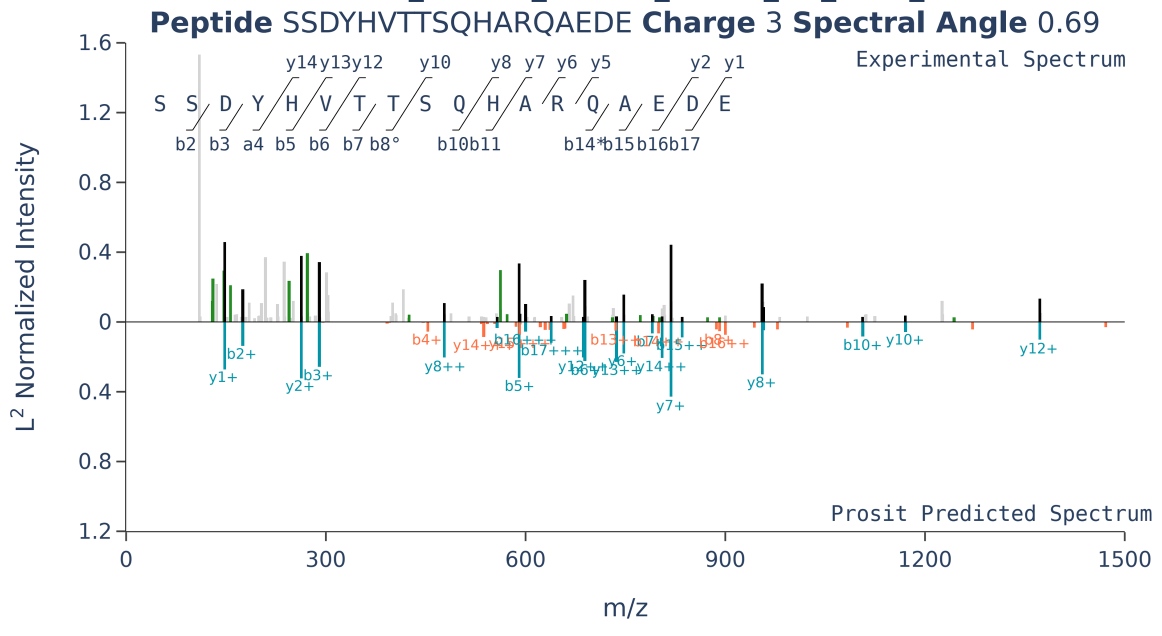

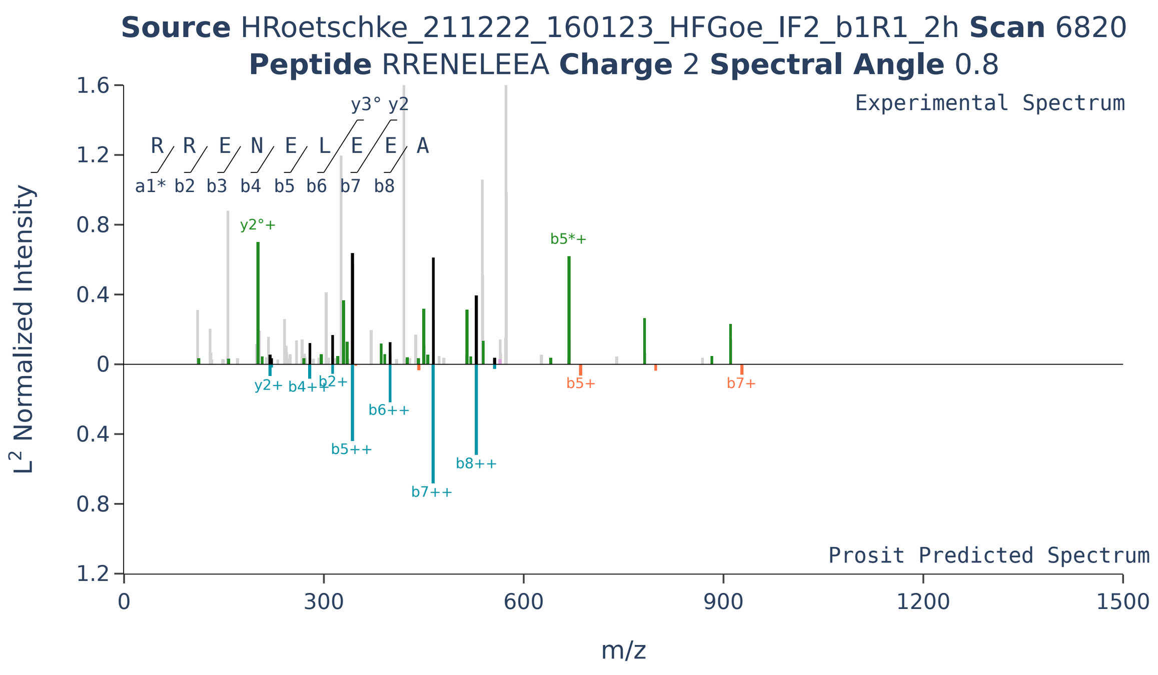
**Source** HRoetschke_211222_160123_HFGoe_IF2_b1R1_2h **Scan** 6820
**Peptide** RRENELEEA **Charge** 2 **Spectral Angle** 0.8
Experimental Spectrum
Prosit Predicted Spectrum
L² Normalized Intensity
m/z

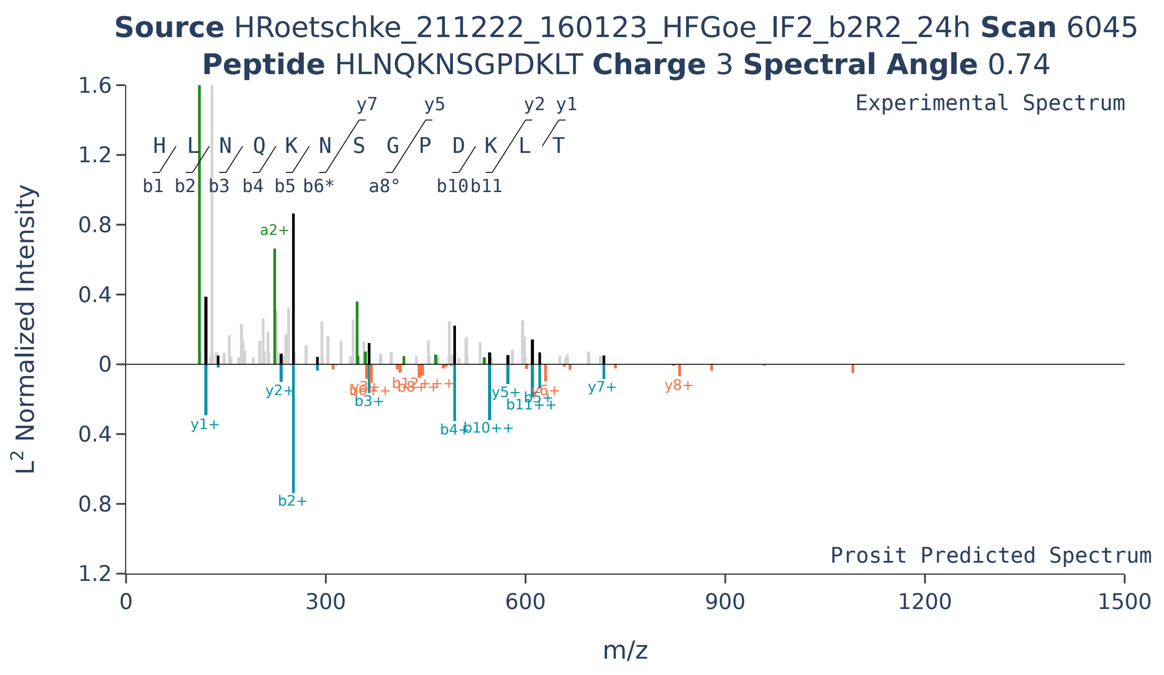
**Source** HRoetschke_211222_160123_HFGoe_IF2_b2R2_24h **Scan** 6045
**Peptide** HLNQKNSGPDKLT **Charge** 3 **Spectral Angle** 0.74
Experimental Spectrum
Prosit Predicted Spectrum
L² Normalized Intensity
m/z

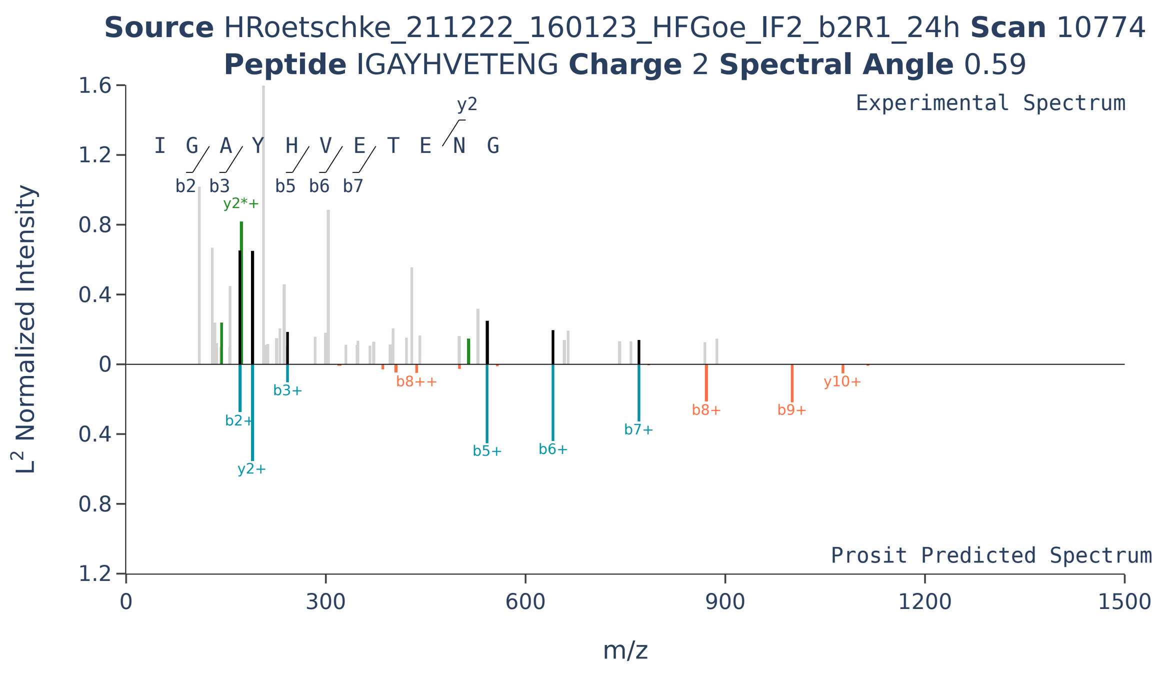
**Source** HRoetschke_211222_160123_HFGoe_IF2_b2R1_24h **Scan** 10774
**Peptide** IGAYHVETENG **Charge** 2 **Spectral Angle** 0.59
Experimental Spectrum
Prosit Predicted Spectrum
L² Normalized Intensity
m/z

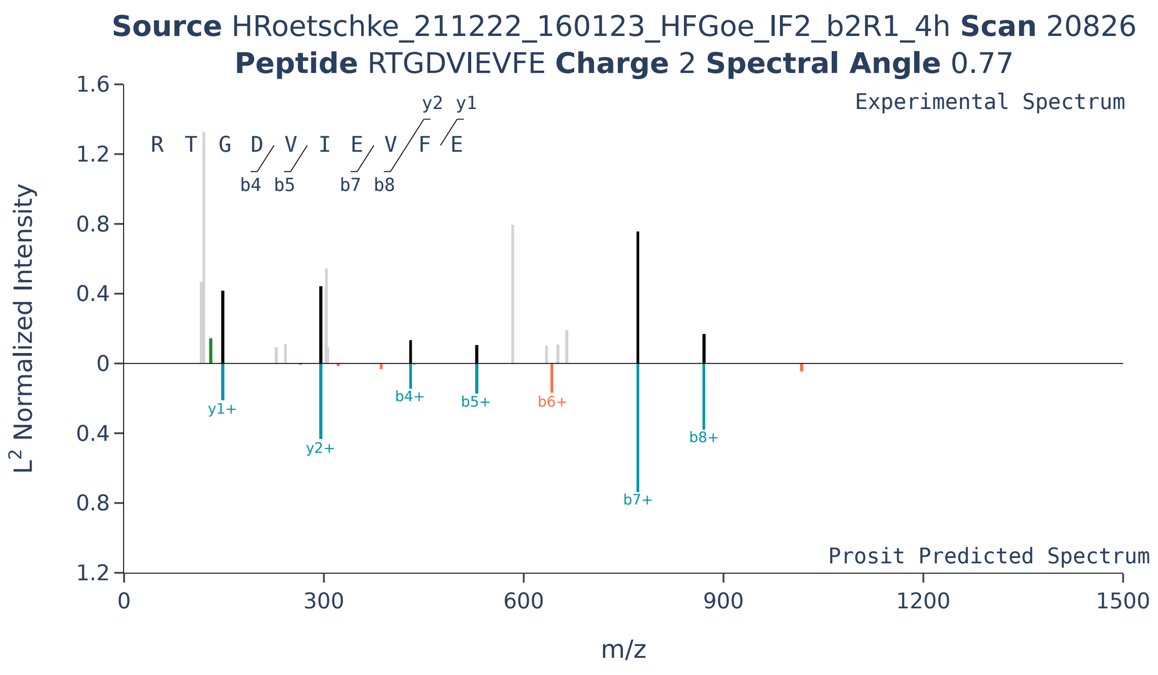
**Source** HRoetschke_211222_160123_HFGoe_IF2_b2R1_4h **Scan** 20826
**Peptide** RTGDVIEVFE **Charge** 2 **Spectral Angle** 0.77
Experimental Spectrum
Prosit Predicted Spectrum
L² Normalized Intensity
m/z

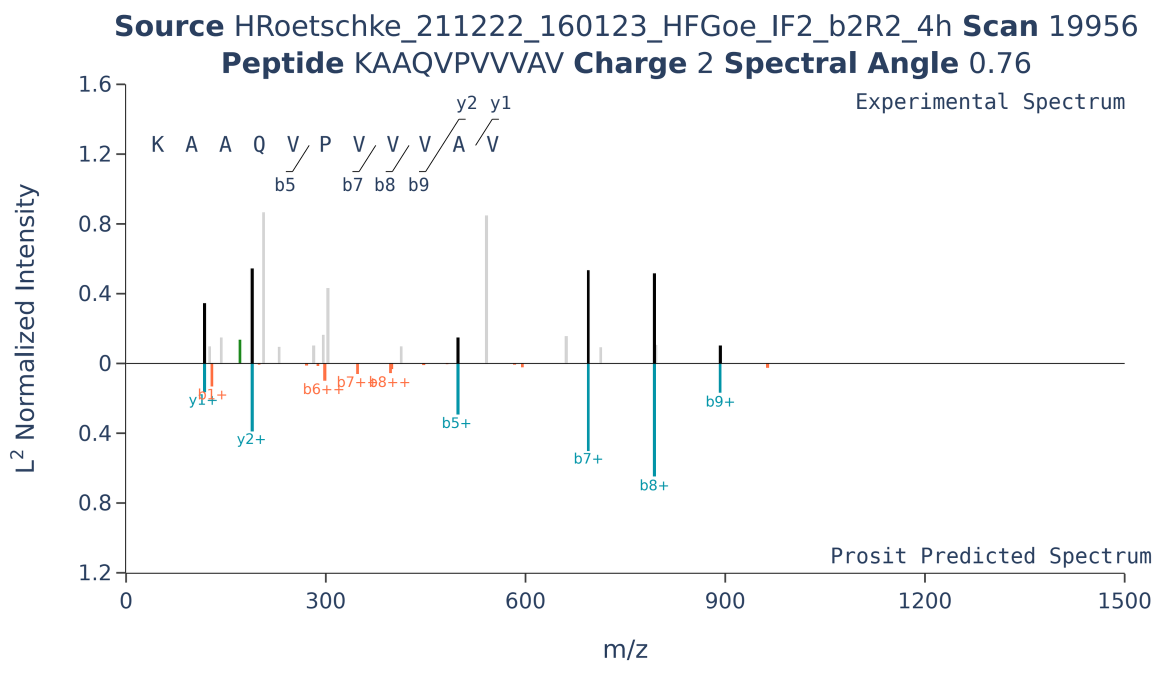
**Source** HRoetschke_211222_160123_HFGoe_IF2_b2R2_4h **Scan** 19956
**Peptide** KAAQVPVVVAV **Charge** 2 **Spectral Angle** 0.76
Experimental Spectrum
Prosit Predicted Spectrum
L² Normalized Intensity
m/z

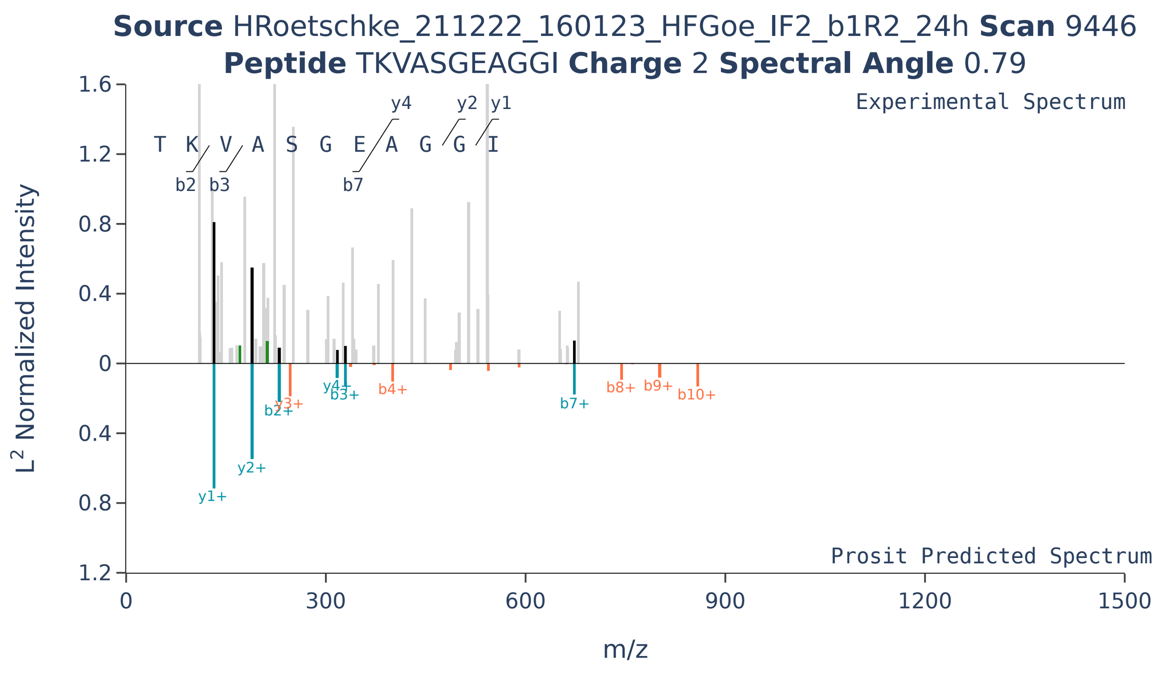
**Source** HRoetschke_211222_160123_HFGoe_IF2_b1R2_24h **Scan** 9446
**Peptide** TKVASGEAGGI **Charge** 2 **Spectral Angle** 0.79
Experimental Spectrum
Prosit Predicted Spectrum
L² Normalized Intensity
m/z

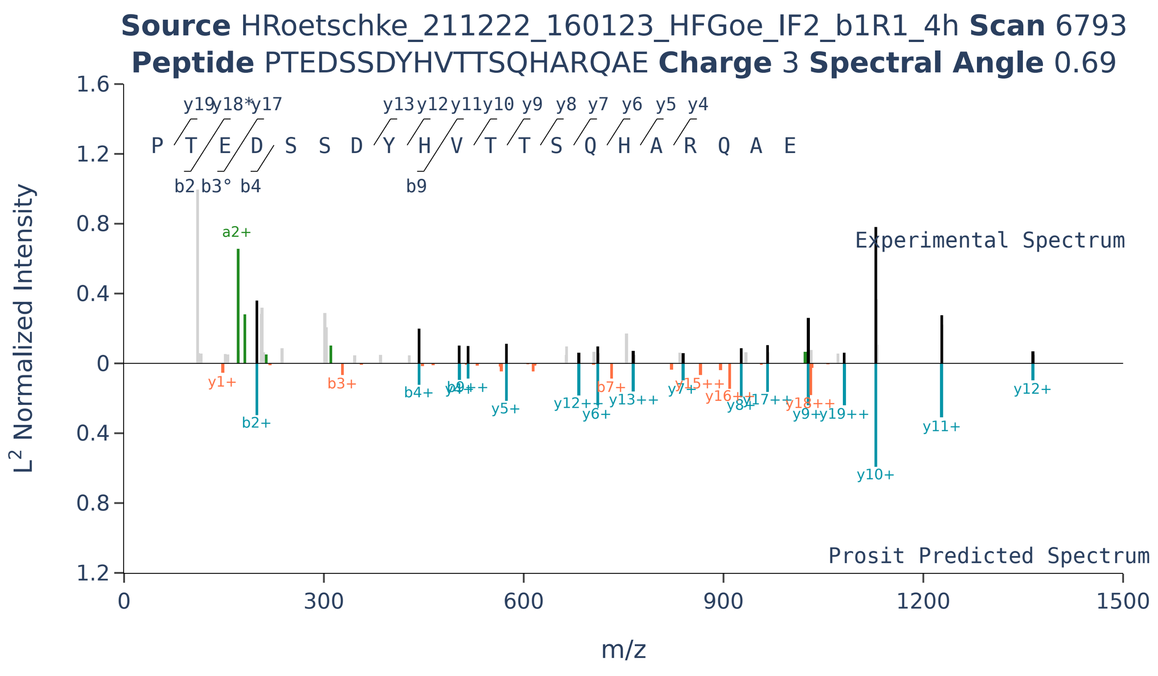
**Source** HRoetschke_211222_160123_HFGoe_IF2_b1R1_4h **Scan** 6793
**Peptide** PTEDSSDYHVTTSQHARQAE **Charge** 3 **Spectral Angle** 0.69
Experimental Spectrum
Prosit Predicted Spectrum
L² Normalized Intensity
m/z

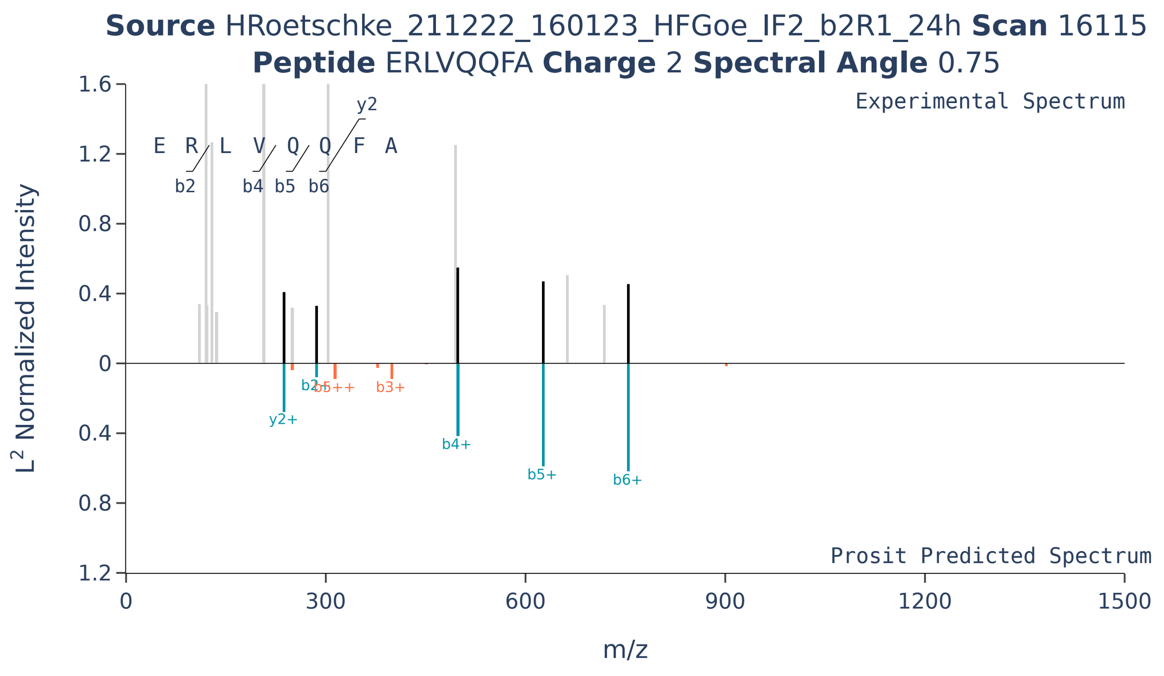
**Source** HRoetschke_211222_160123_HFGoe_IF2_b2R1_24h **Scan** 16115
**Peptide** ERLVQQFA **Charge** 2 **Spectral Angle** 0.75
Experimental Spectrum
Prosit Predicted Spectrum
L² Normalized Intensity
m/z

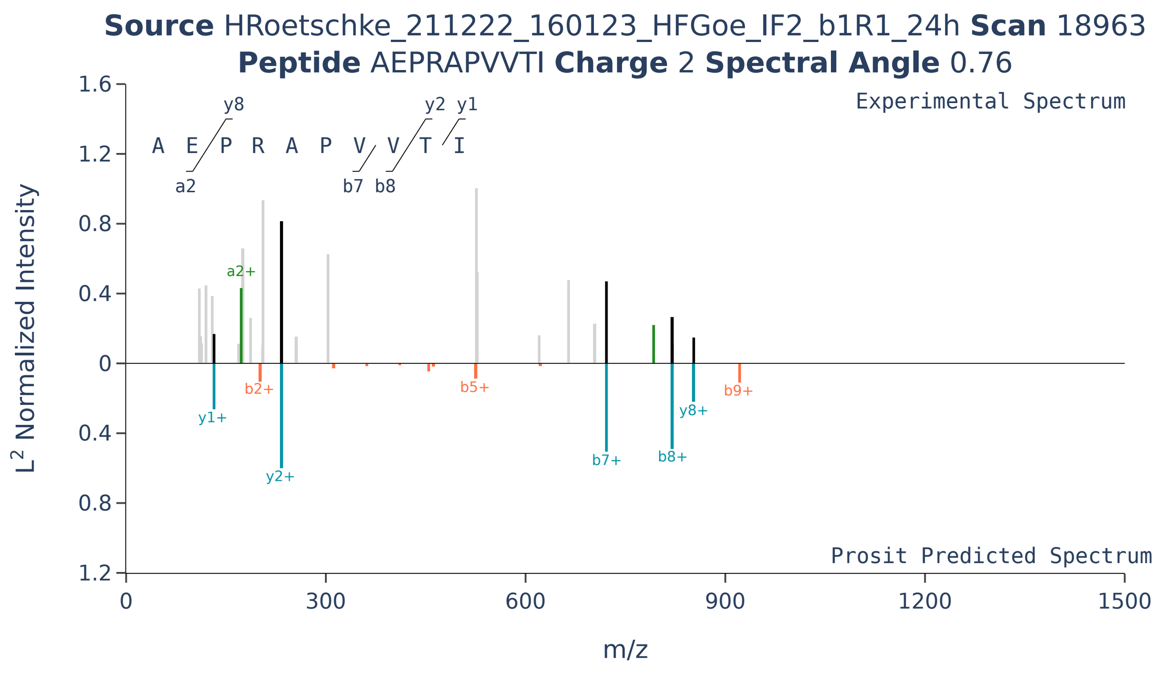
**Source** HRoetschke_211222_160123_HFGoe_IF2_b1R1_24h **Scan** 18963
**Peptide** AEPRAPVVTI **Charge** 2 **Spectral Angle** 0.76
Experimental Spectrum
Prosit Predicted Spectrum
L² Normalized Intensity
m/z

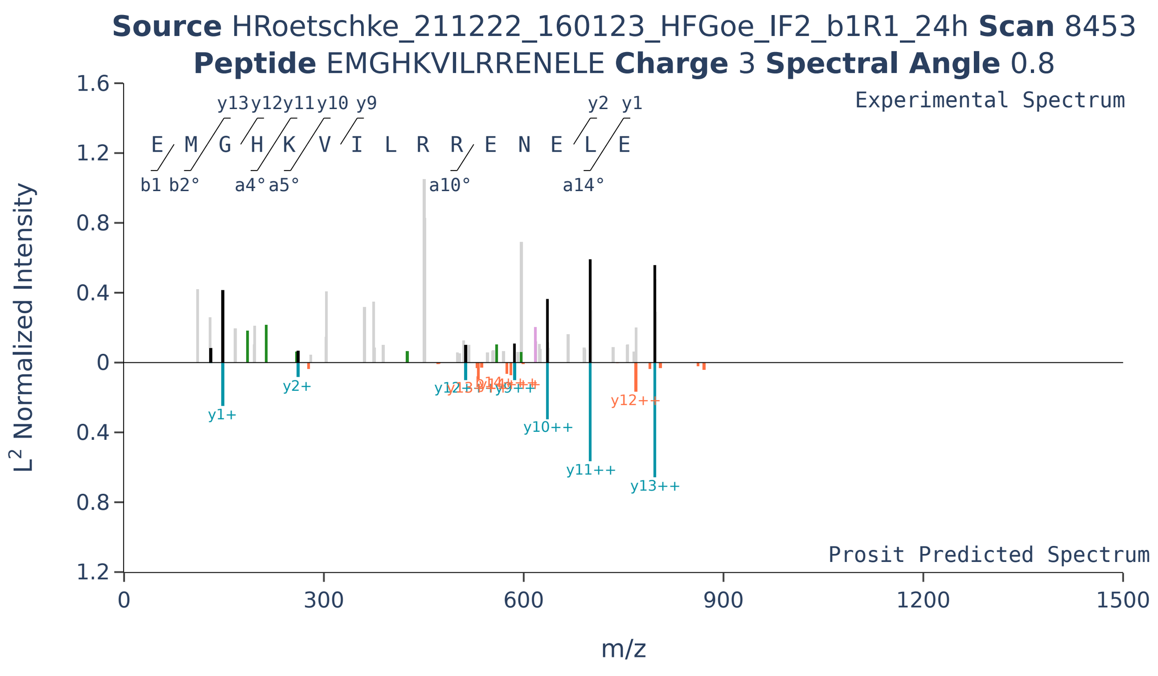
**Source** HRoetschke_211222_160123_HFGoe_IF2_b1R1_24h **Scan** 8453
**Peptide** EMGHKVILRRENELE **Charge** 3 **Spectral Angle** 0.8
Experimental Spectrum
Prosit Predicted Spectrum
L² Normalized Intensity
m/z

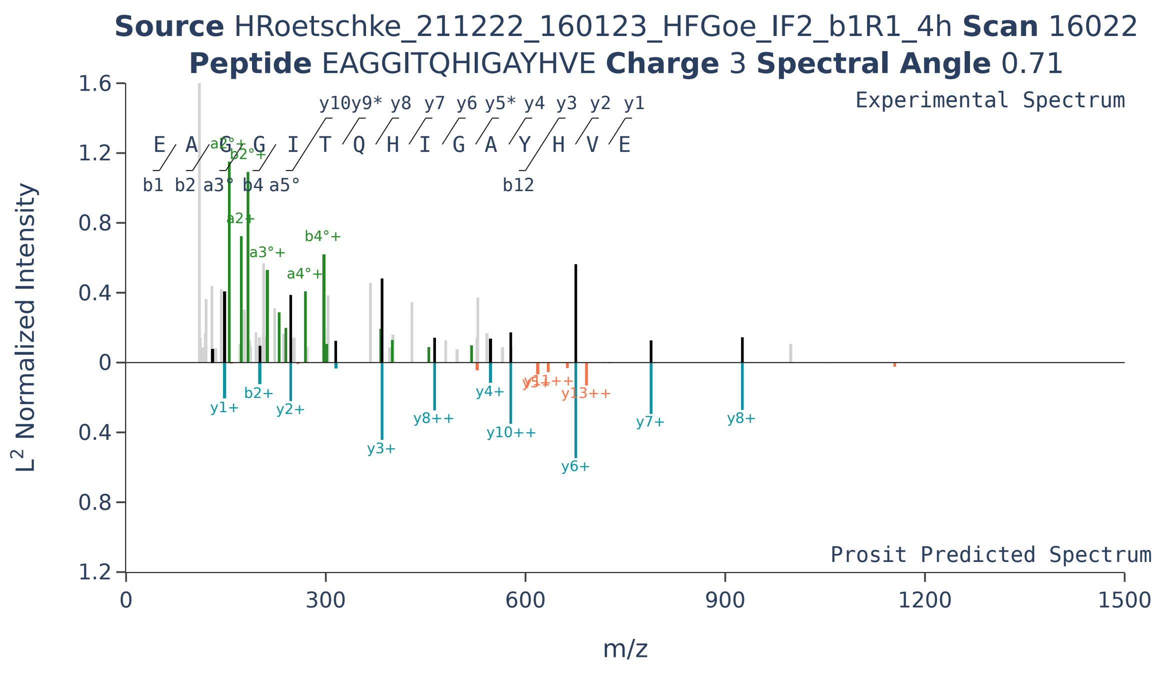
**Source** HRoetschke_211222_160123_HFGoe_IF2_b1R1_4h **Scan** 16022
**Peptide** EAGGITQHIGAYHVE **Charge** 3 **Spectral Angle** 0.71
Experimental Spectrum
Prosit Predicted Spectrum
L² Normalized Intensity
m/z

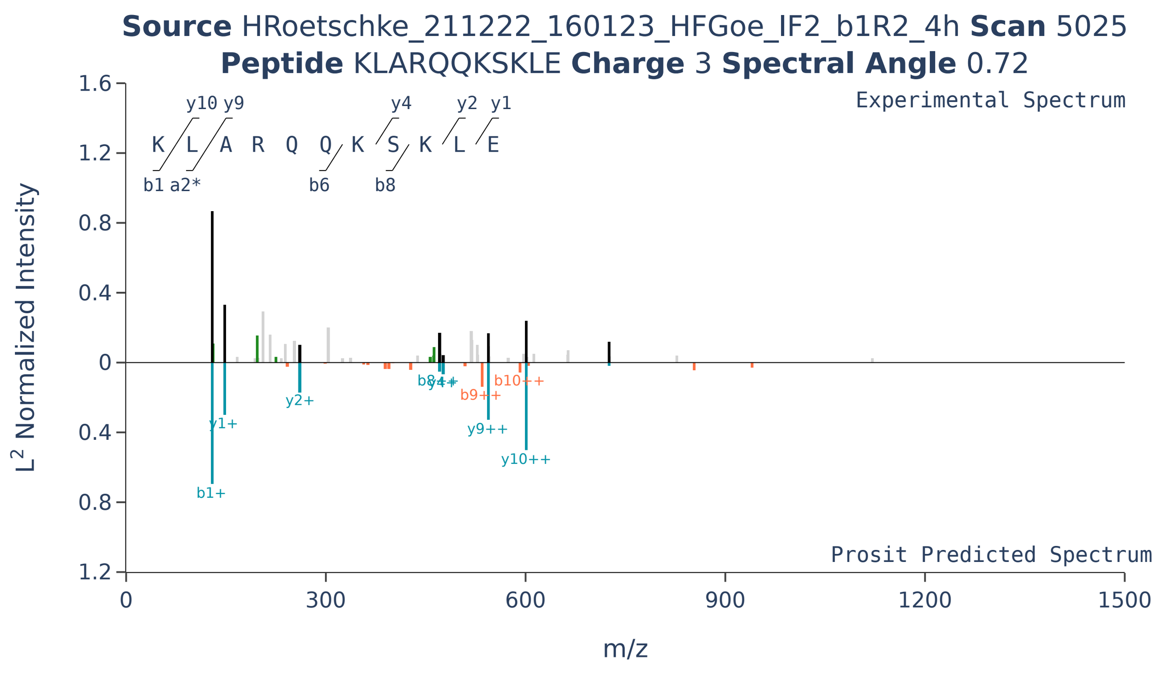
**Source** HRoetschke_211222_160123_HFGoe_IF2_b1R2_4h **Scan** 5025
**Peptide** KLARQQKSKLE **Charge** 3 **Spectral Angle** 0.72
Experimental Spectrum
Prosit Predicted Spectrum
L² Normalized Intensity
m/z

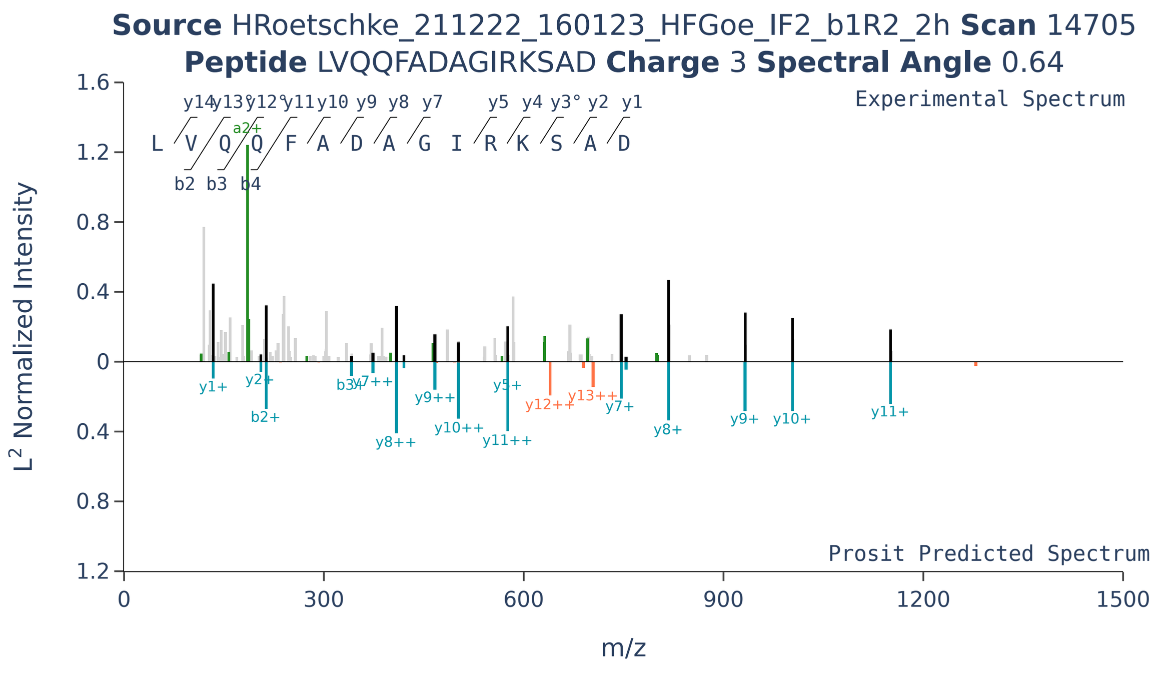
**Source** HRoetschke_211222_160123_HFGoe_IF2_b1R2_2h **Scan** 14705
**Peptide** LVQQFADAGIRKSAD **Charge** 3 **Spectral Angle** 0.64
Experimental Spectrum
Prosit Predicted Spectrum
L² Normalized Intensity
m/z

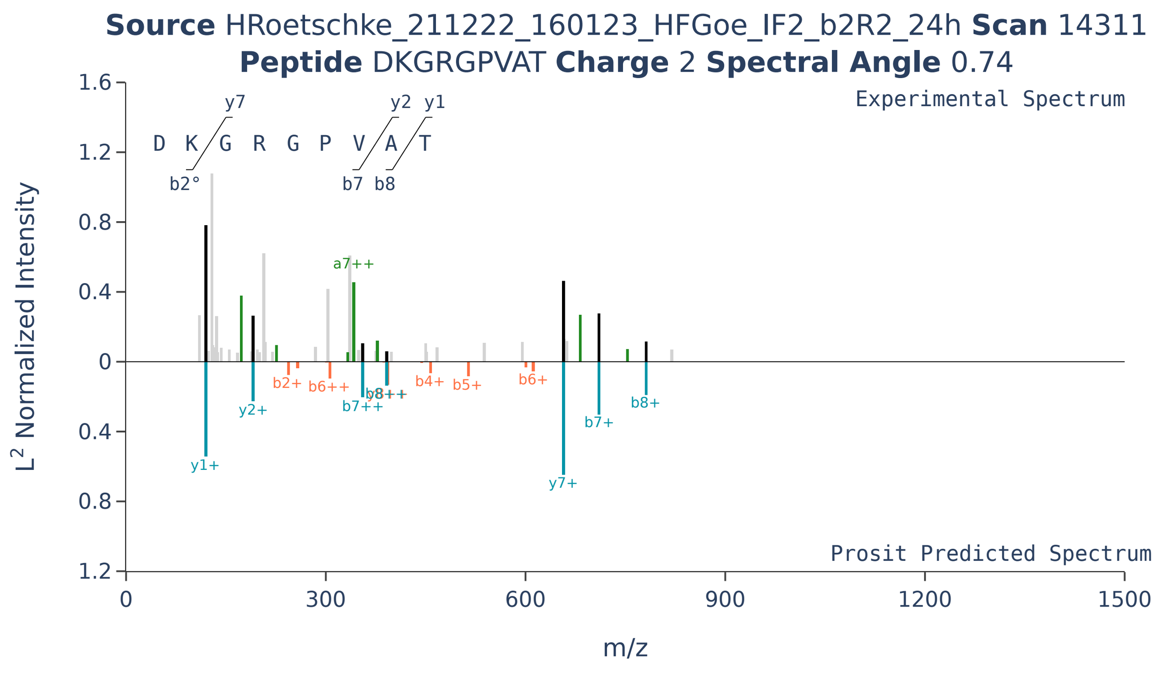
**Source** HRoetschke_211222_160123_HFGoe_IF2_b2R2_24h **Scan** 14311
**Peptide** DKGRGPVAT **Charge** 2 **Spectral Angle** 0.74
Experimental Spectrum
Prosit Predicted Spectrum
L² Normalized Intensity
m/z

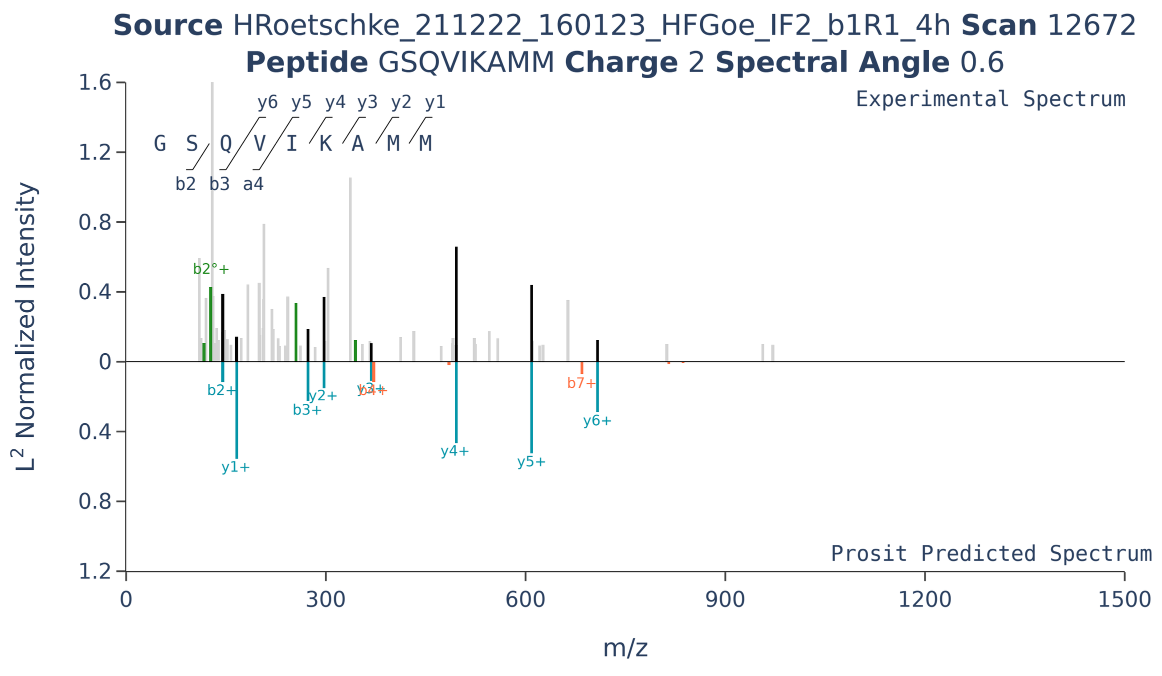
**Source** HRoetschke_211222_160123_HFGoe_IF2_b1R1_4h **Scan** 12672
**Peptide** GSQVIKAMM **Charge** 2 **Spectral Angle** 0.6
Experimental Spectrum
Prosit Predicted Spectrum
L² Normalized Intensity
m/z

**Source** HRoetschke_211222_160123_HFGoe_IF2_b2R1_2h **Scan** 7212
**Peptide** ADAGIRKSADDSVSAQE **Charge** 3 **Spectral Angle** 0.72

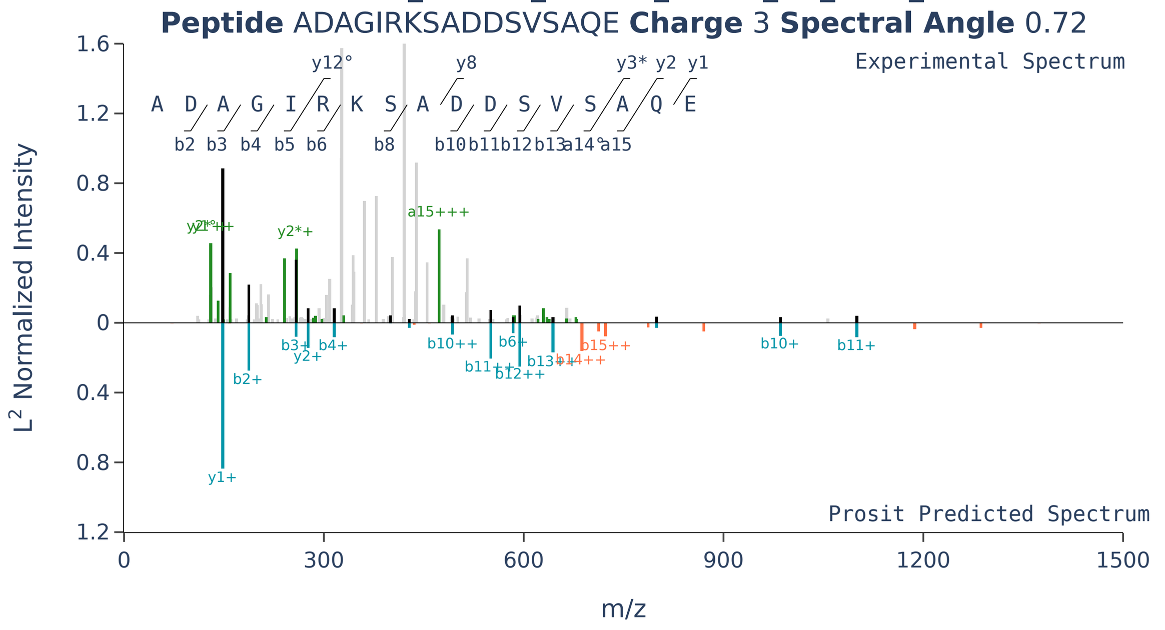


**Source** HRoetschke_211222_160123_HFGoe_IF2_b1R2_1h **Scan** 15467
**Peptide** PVVVAVNKIDKPEAD **Charge** 2 **Spectral Angle** 0.88

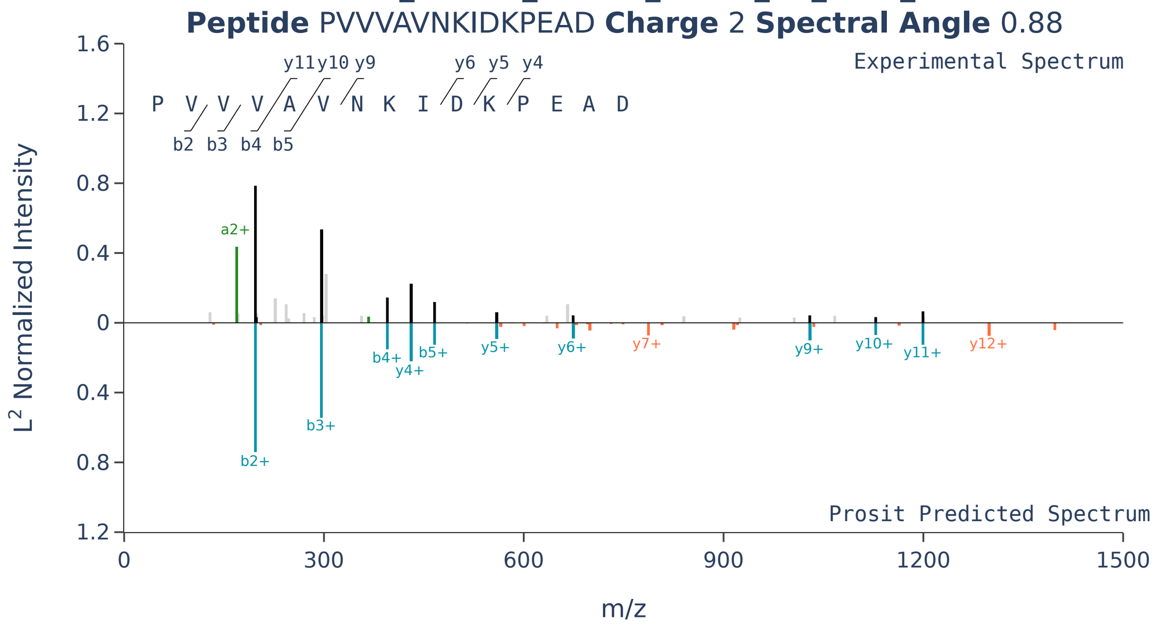


**Source** HRoetschke_211222_160123_HFGoe_IF2_b1R2_24h **Scan** 6597
**Peptide** NKIDKPEAD **Charge** 2 **Spectral Angle** 0.72

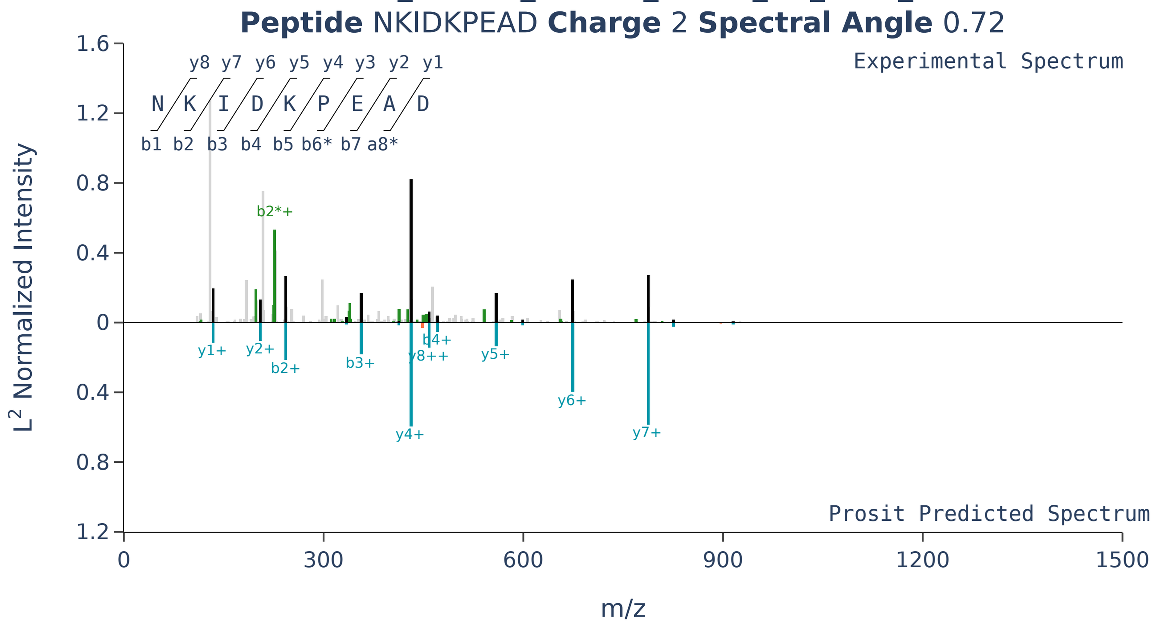


**Source** HRoetschke_211222_160123_HFGoe_IF2_b1R2_1h **Scan** 16151
**Peptide** DVFKSPKFG **Charge** 2 **Spectral Angle** 0.62

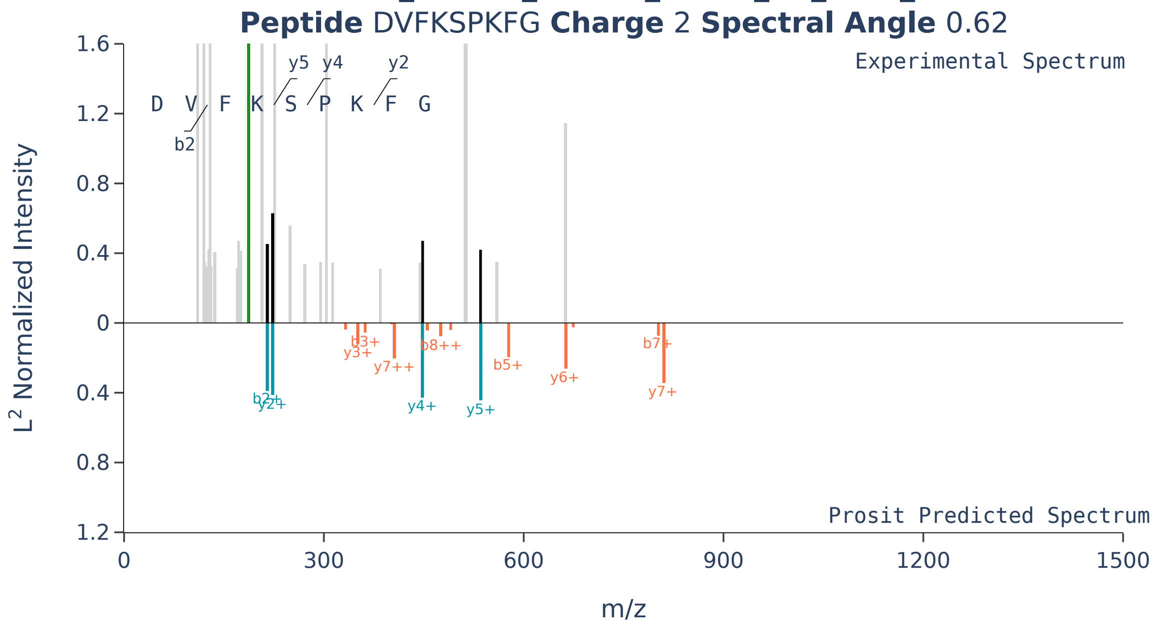


**Source** HRoetschke_211222_160123_HFGoe_IF2_b2R2_24h **Scan** 8533
**Peptide** AGCMVTEGVVKR **Charge** 3 **Spectral Angle** 0.66

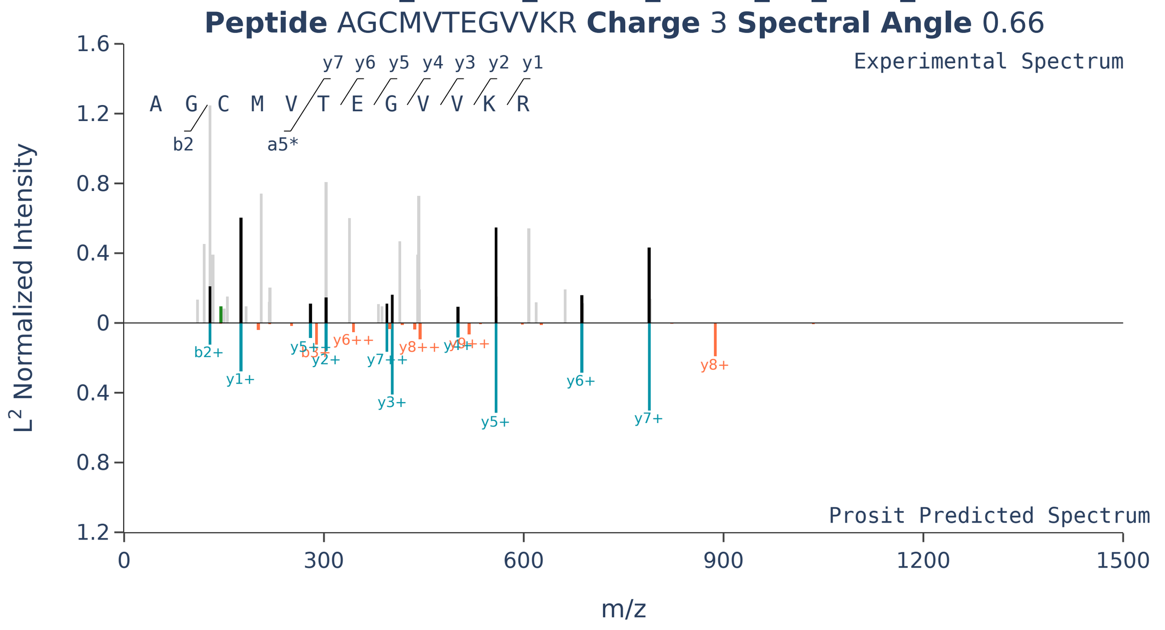


**Source** HRoetschke_211222_160123_HFGoe_IF2_b1R1_4h **Scan** 10446
**Peptide** IEAIQHAKAAQV **Charge** 2 **Spectral Angle** 0.79

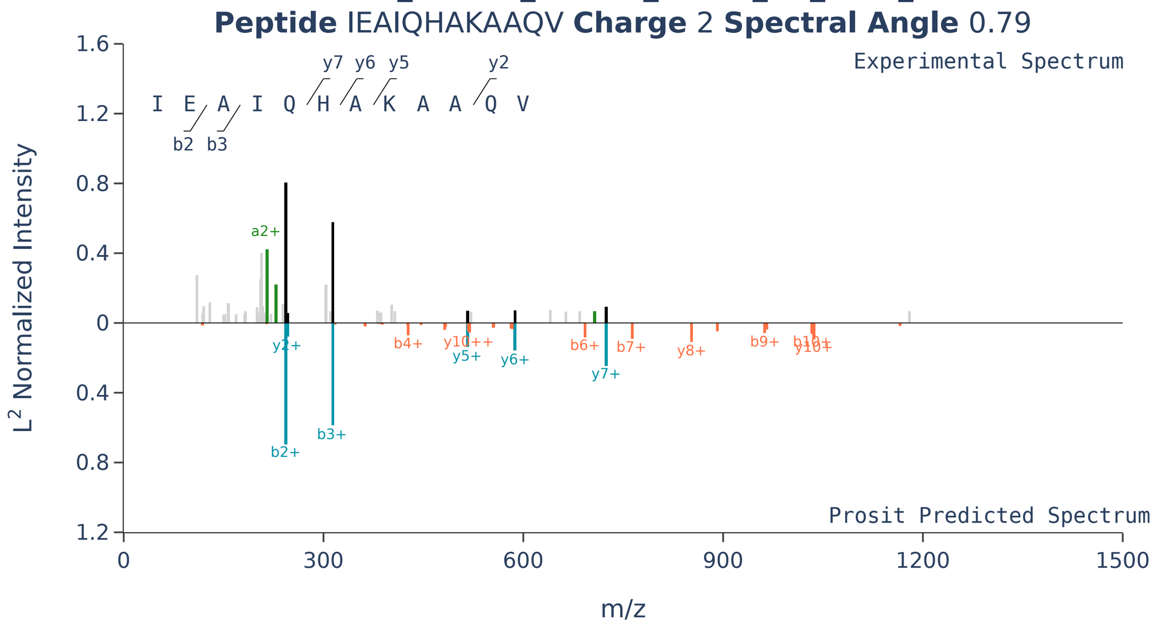


**Source** HRoetschke_211222_160123_HFGoe_IF2_b1R1_4h **Scan** 7200
**Peptide** GDEVTVVRDEKKA **Charge** 3 **Spectral Angle** 0.63

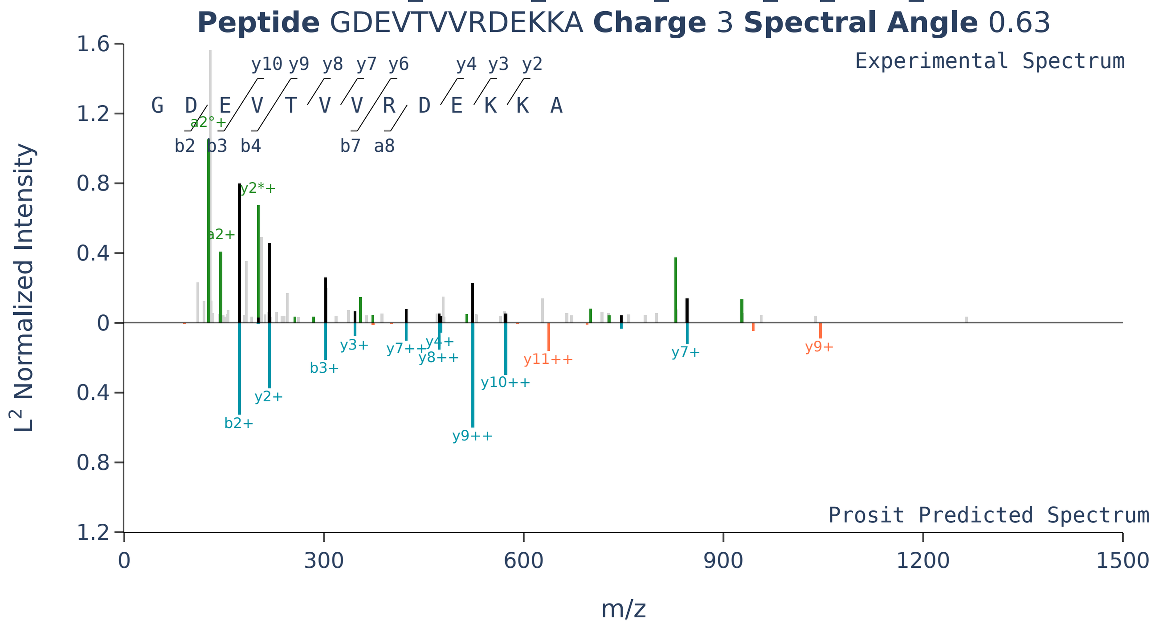


**Source** HRoetschke_211222_160123_HFGoe_IF2_b2R1_24h **Scan** 11401
**Peptide** TDNAEPTE **Charge** 1 **Spectral Angle** 0.88

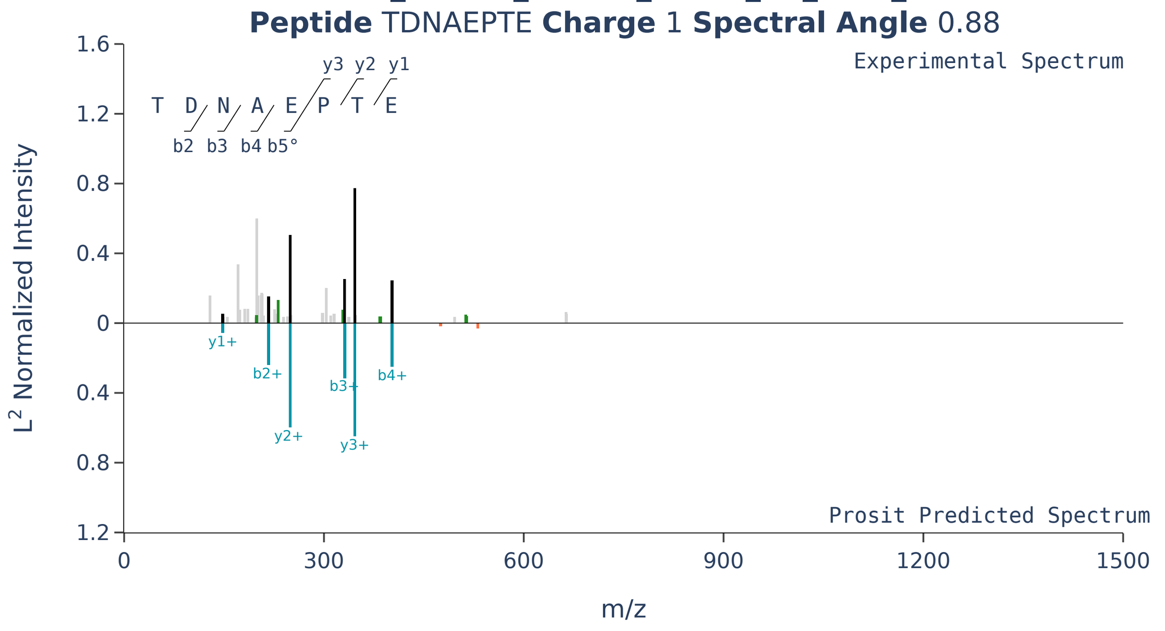


**Source** HRoetschke_211222_160123_HFGoe_IF2_b1R2_1h **Scan** 16795
**Peptide** TINQVIDQET **Charge** 2 **Spectral Angle** 0.74

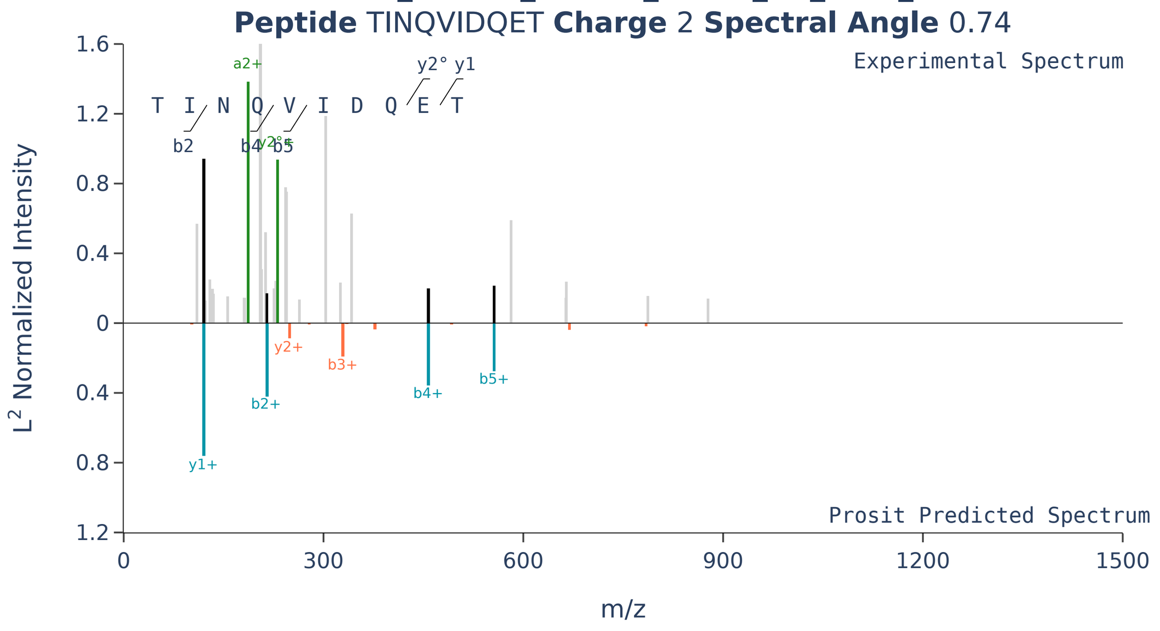


**Source** HRoetschke_211222_160123_HFGoe_IF2_b1R1_4h **Scan** 6718
**Peptide** ADAGIRKSADDSVSAQEKQT **Charge** 3 **Spectral Angle** 0.68

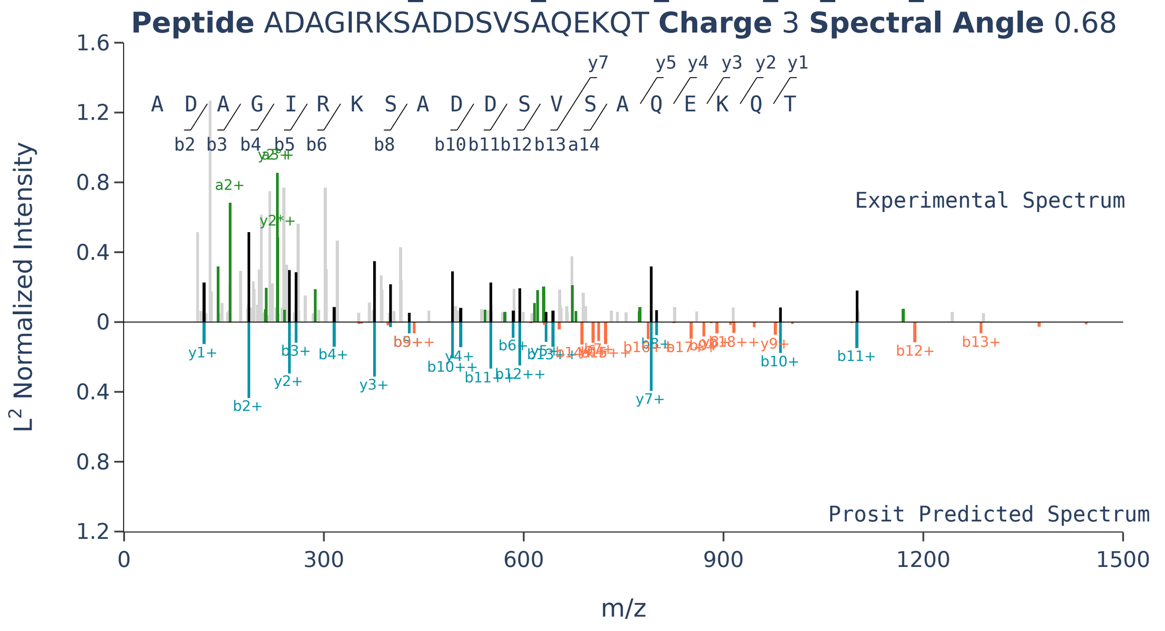


**Source** HRoetschke_211222_160123_HFGoe_IF2_b1R1_4h **Scan** 13699
**Peptide** QAEVLELKAVRKGMA **Charge** 4 **Spectral Angle** 0.67

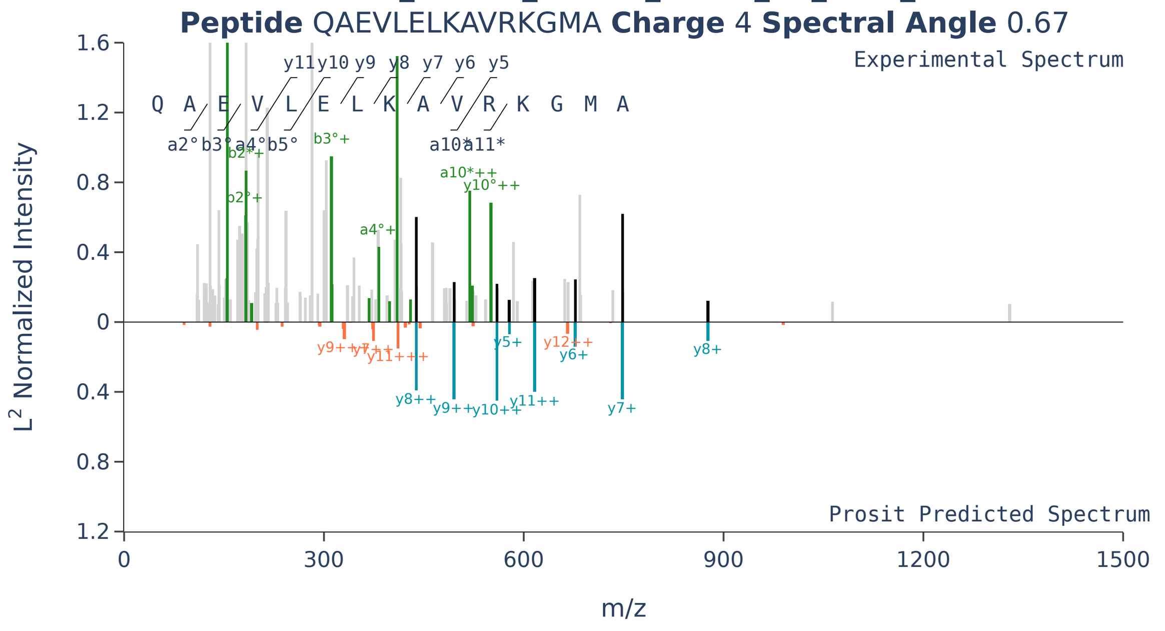


**Source** HRoetschke_211222_160123_HFGoe_IF2_b1R2_24h **Scan** 23304
**Peptide** GTGIDELLD **Charge** 1 **Spectral Angle** 0.81

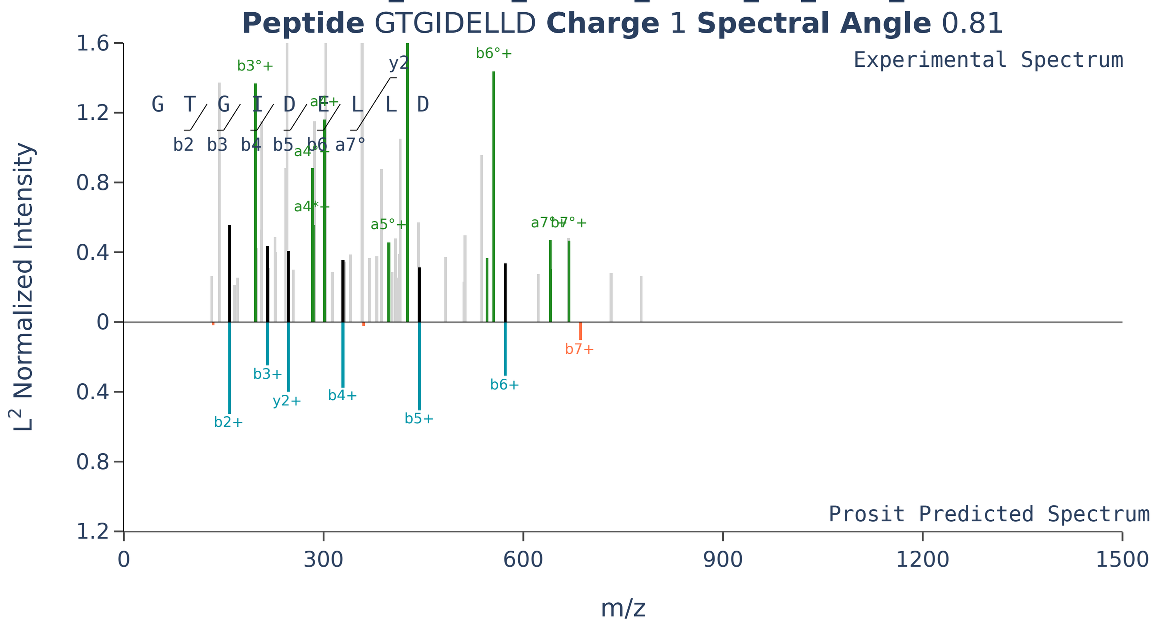


**Source** HRoetschke_211222_160123_HFGoe_IF2_b1R1_4h **Scan** 8563
**Peptide** ARQQKSKLENMFA **Charge** 3 **Spectral Angle** 0.55

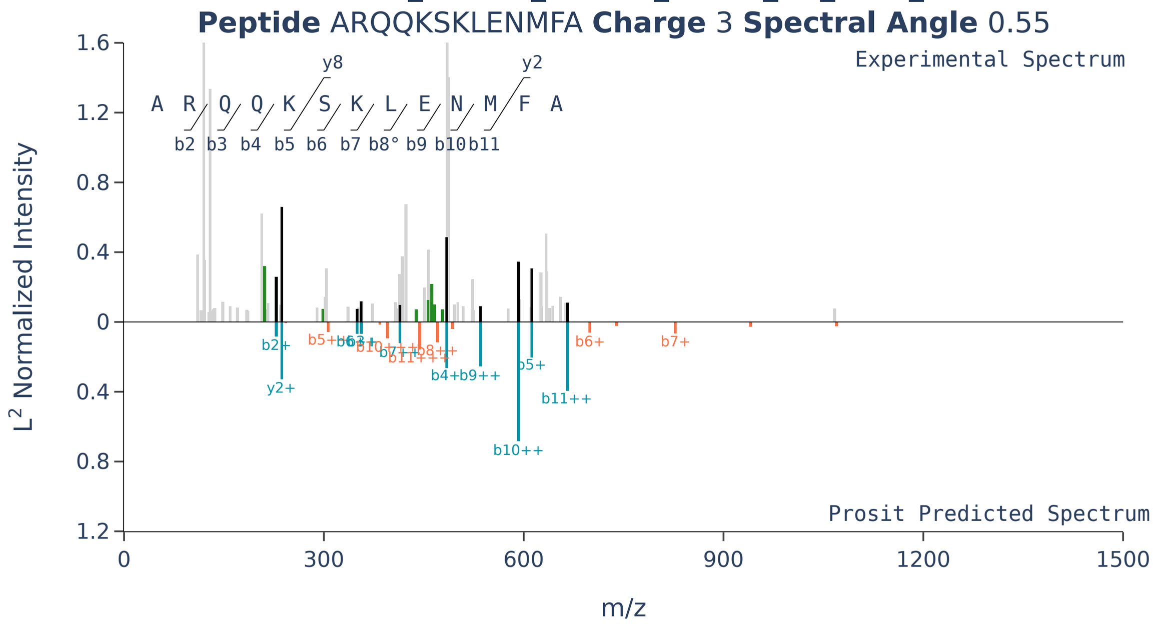


**Source** HRoetschke_211222_160123_HFGoe_IF2_b1R1_4h **Scan** 6109
**Peptide** AVKGSQVIKAMMK **Charge** 3 **Spectral Angle** 0.57

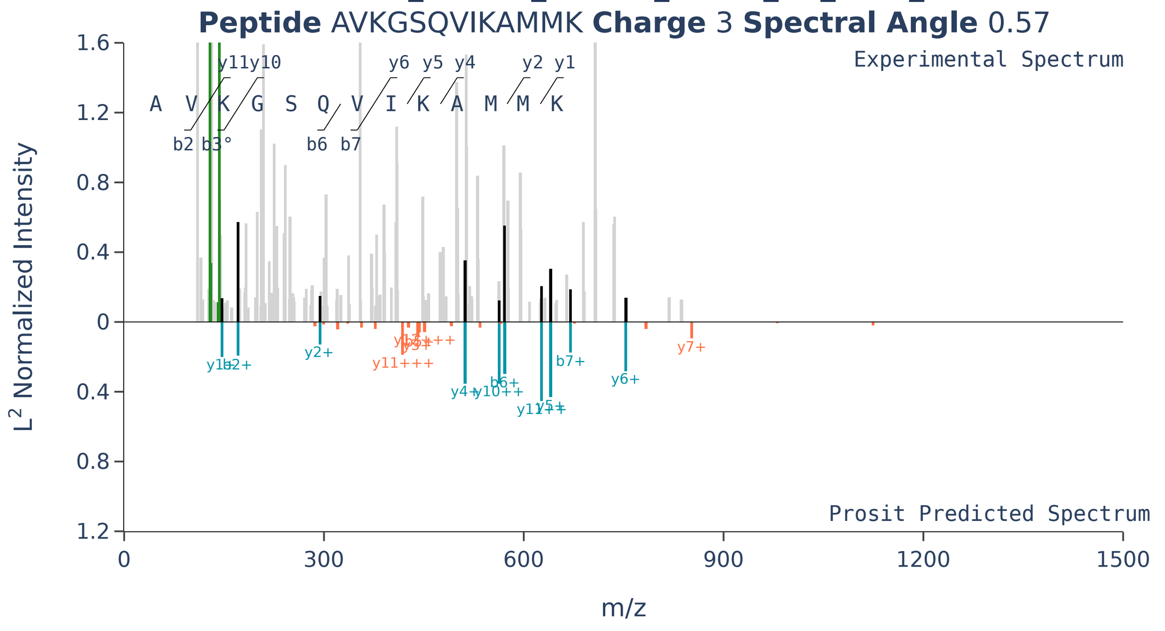


**Source** HRoetschke_211222_160123_HFGoe_IF2_b1R1_24h **Scan** 17705
**Peptide** VNRDVVIGETIT **Charge** 2 **Spectral Angle** 0.73

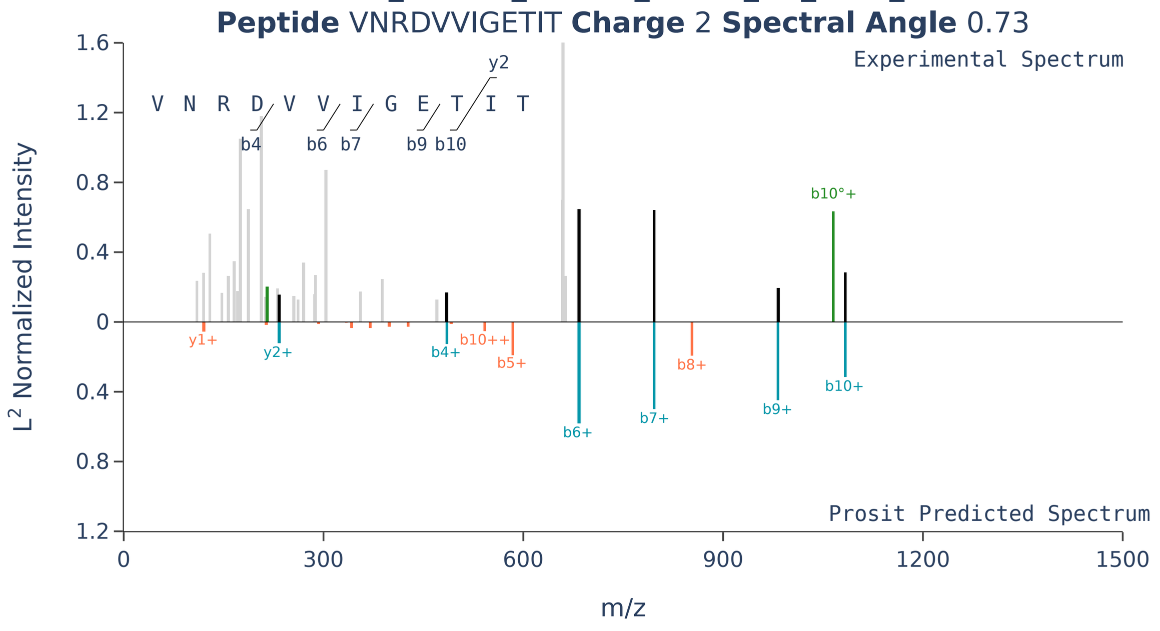

**Source** HRoetschke_211222_160123_HFGoe_IF2_b2R2_24h **Scan** 10117
**Peptide** LDKGRGPVATV **Charge** 2 **Spectral Angle** 0.63

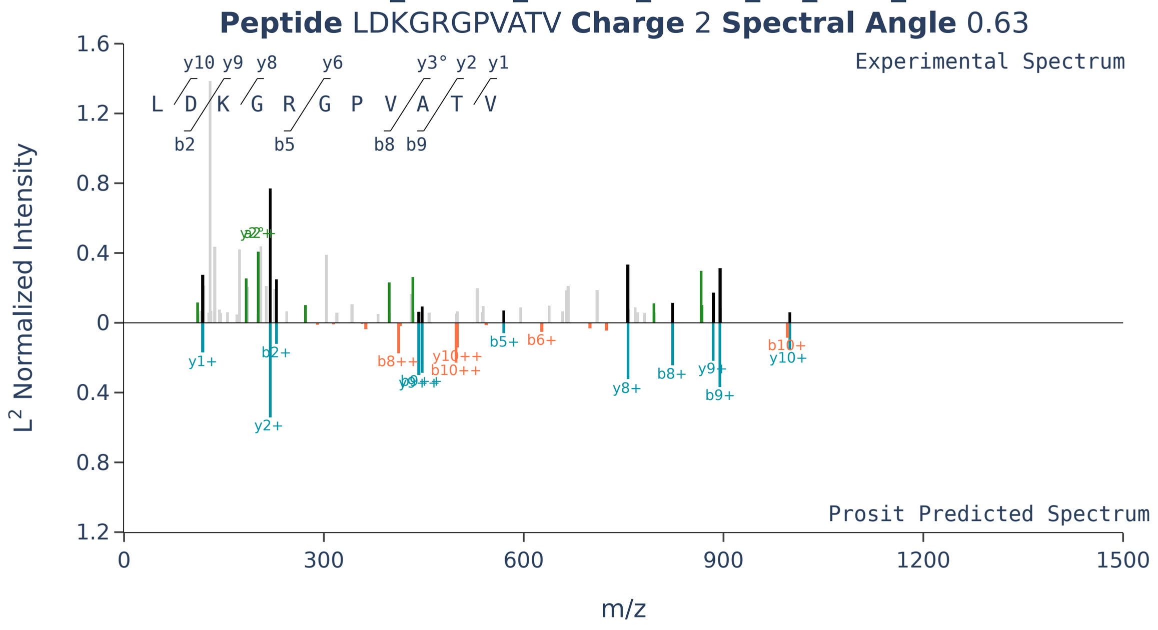


**Source** HRoetschke_211222_160123_HFGoe_IF2_b1R2_24h **Scan** 16487
**Peptide** AEVRDVFKSPKFG **Charge** 4 **Spectral Angle** 0.75

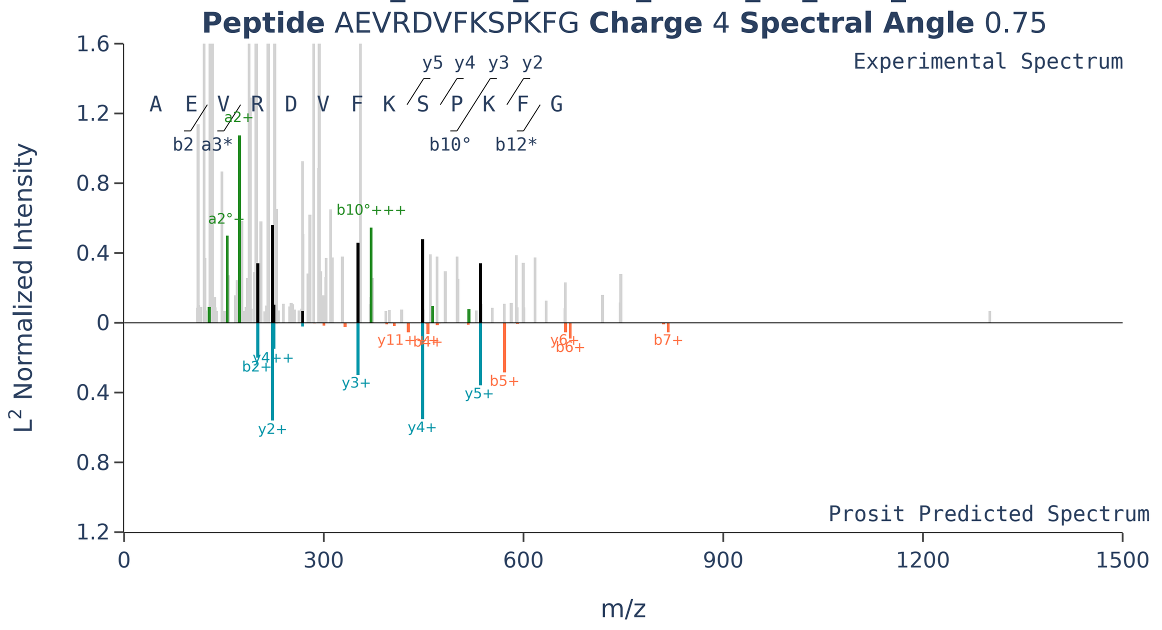


**Source** HRoetschke_211222_160123_HFGoe_IF2_b1R1_2h **Scan** 5968
**Peptide** YHVTTSQHAR **Charge** 3 **Spectral Angle** 0.63

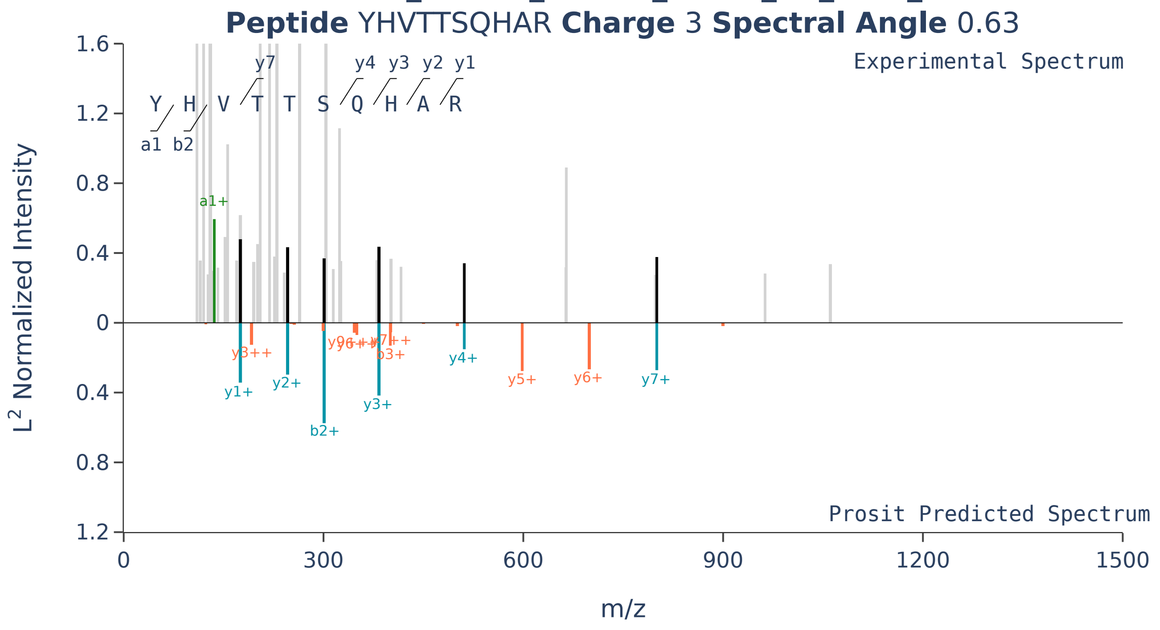


**Source** HRoetschke_211222_160123_HFGoe_IF2_b1R1_4h **Scan** 16955
**Peptide** RDTGAAAEPRAPVVTIM **Charge** 3 **Spectral Angle** 0.73

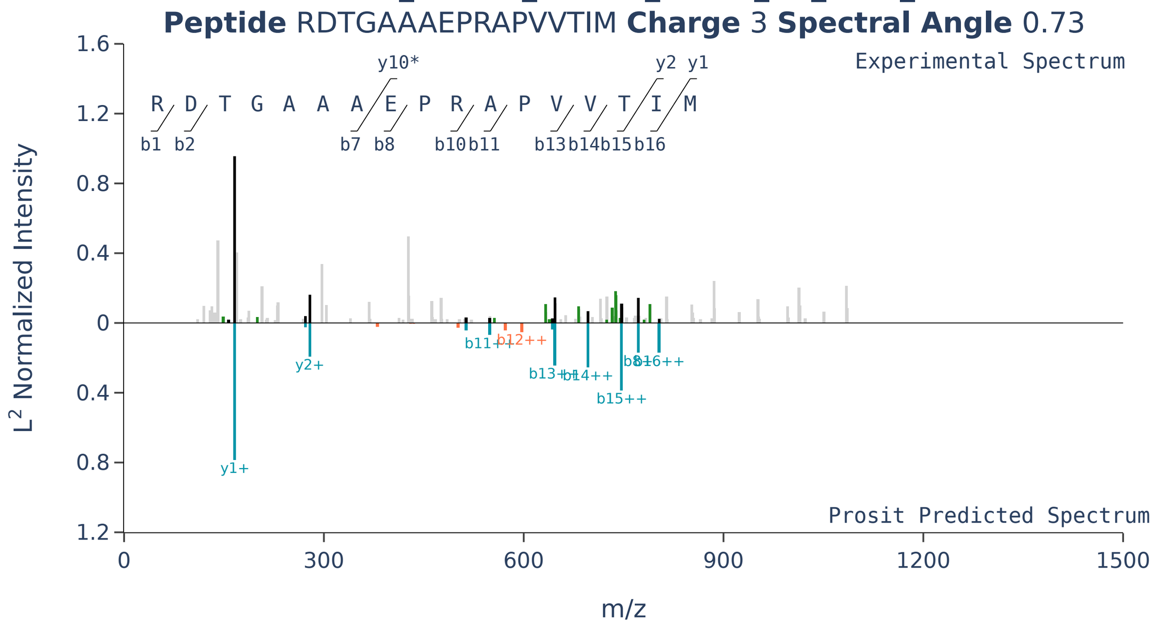


**Source** HRoetschke_211222_160123_HFGoe_IF2_b2R2_24h **Scan** 16019
**Peptide** AIQHAKAAQVPVVVA **Charge** 3 **Spectral Angle** 0.64

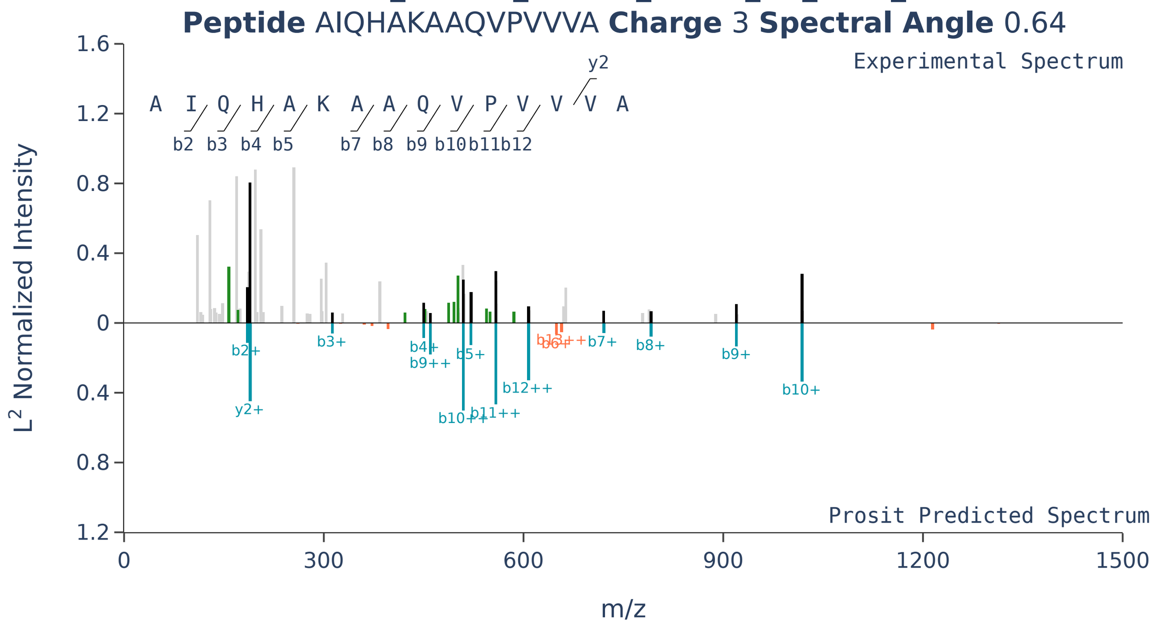


**Source** HRoetschke_211222_160123_HFGoe_IF2_b1R2_4h **Scan** 14425
**Peptide** PAQAVNRDVVLG **Charge** 2 **Spectral Angle** 0.66

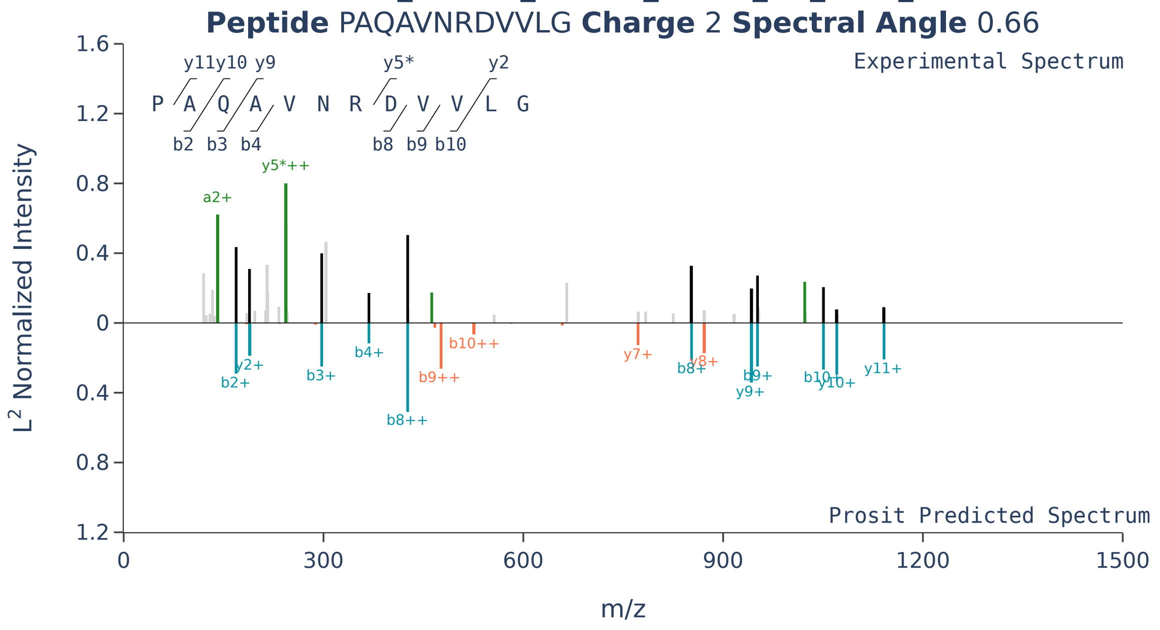


**Source** HRoetschke_211222_160123_HFGoe_IF2_b2R1_24h **Scan** 11494
**Peptide** FADAGIRKSADDSVSA **Charge** 3 **Spectral Angle** 0.9

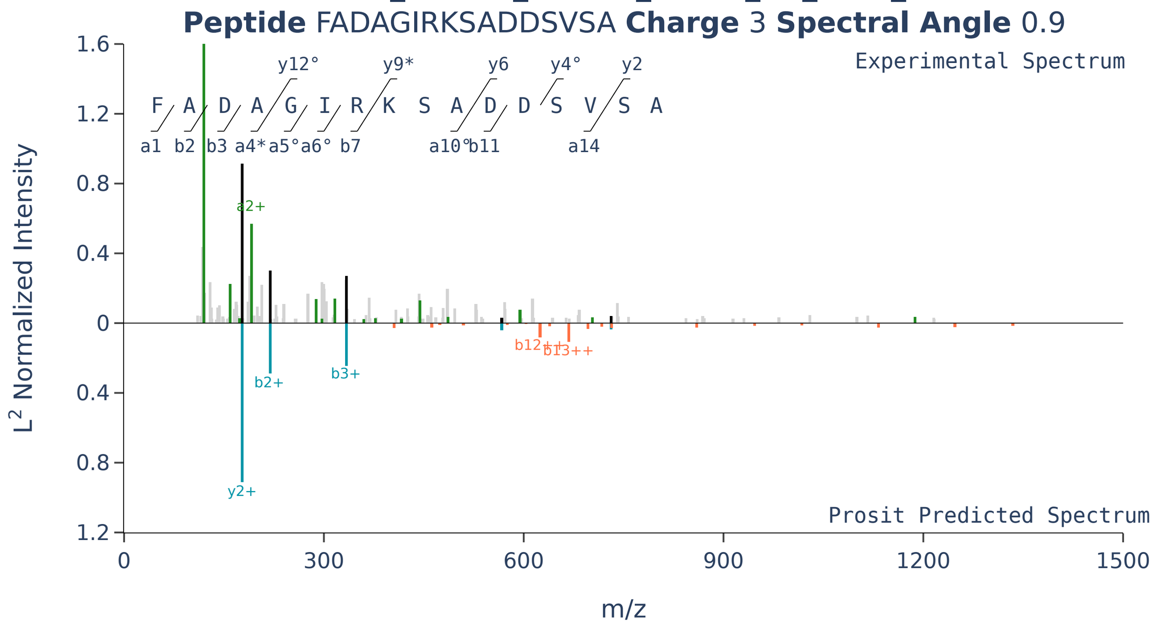


**Source** HRoetschke_211222_160123_HFGoe_IF2_b1R2_24h **Scan** 9511
**Peptide** AEEMGHKVI **Charge** 2 **Spectral Angle** 0.77

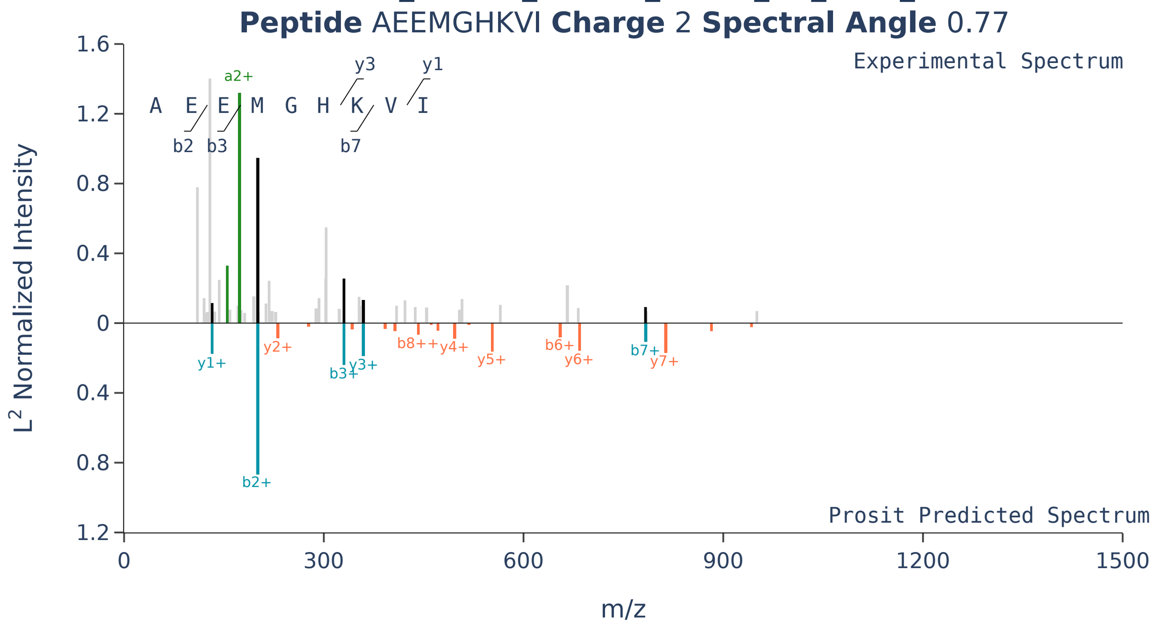


**Source** HRoetschke_211222_160123_HFGoe_IF2_b1R1_4h **Scan** 17660
**Peptide** ADDSVSAQEKQTLIDHLNQ **Charge** 3 **Spectral Angle** 0.75

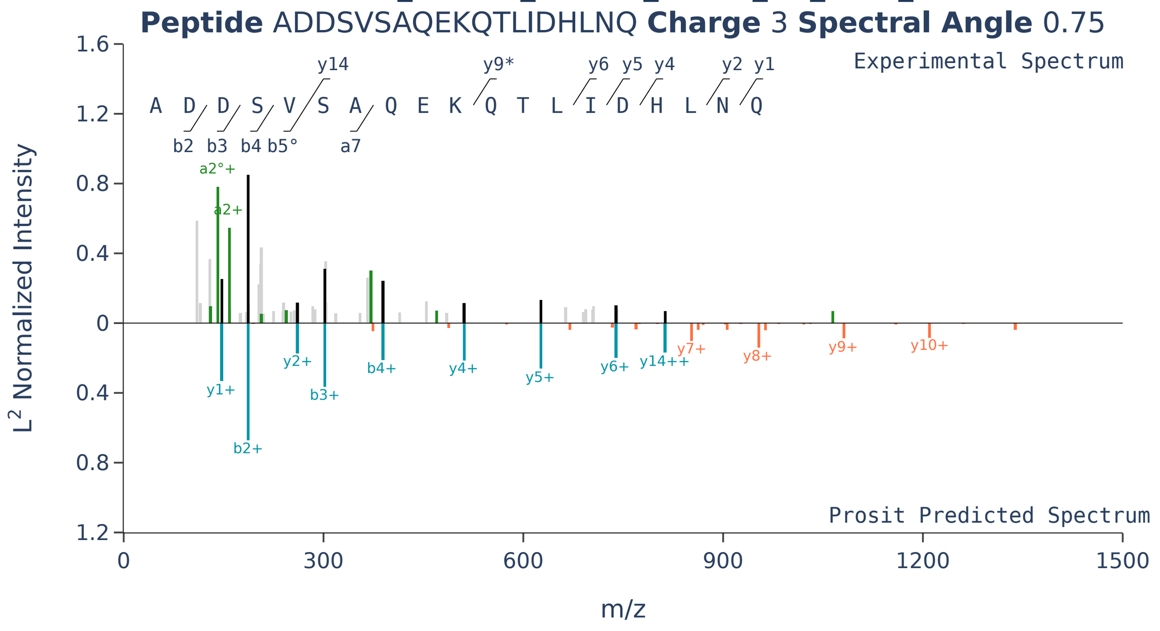


**Source** HRoetschke_211222_160123_HFGoe_IF2_b1R1_4h **Scan** 6537
**Peptide** GHVDHGKTSL **Charge** 3 **Spectral Angle** 0.55

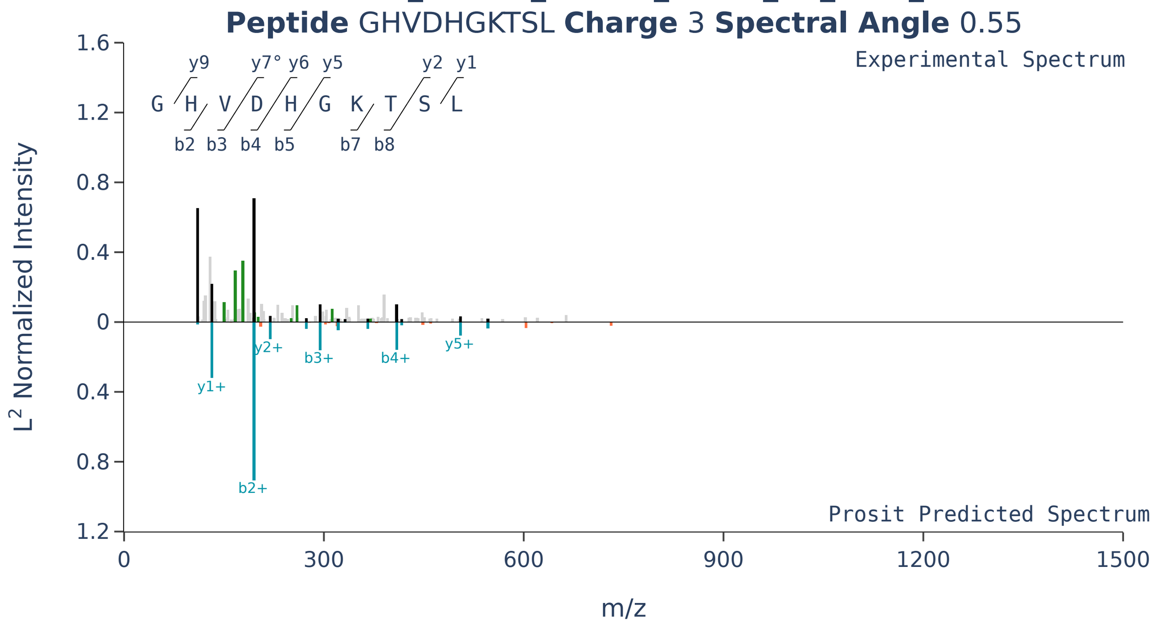


**Source** HRoetschke_211222_160123_HFGoe_IF2_b1R1_4h **Scan** 7978
**Peptide** RQQKSKLENMF **Charge** 3 **Spectral Angle** 0.65

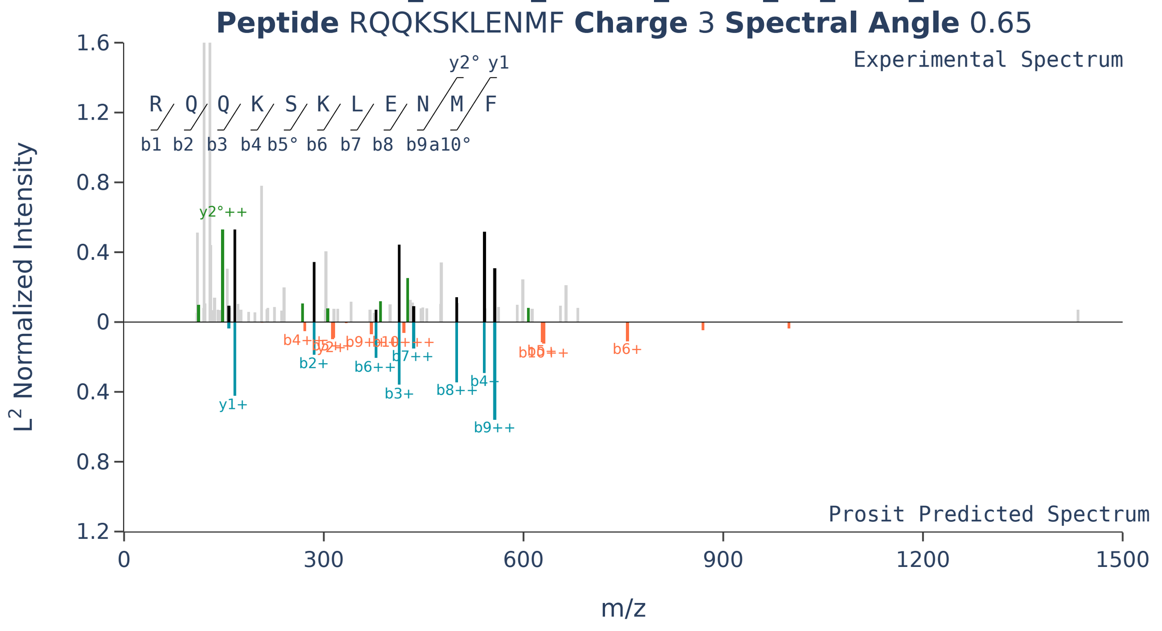


**Source** HRoetschke_211222_160123_HFGoe_IF2_b1R2_1h **Scan** 17968
**Peptide** DVTIKTLAAE **Charge** 2 **Spectral Angle** 0.73

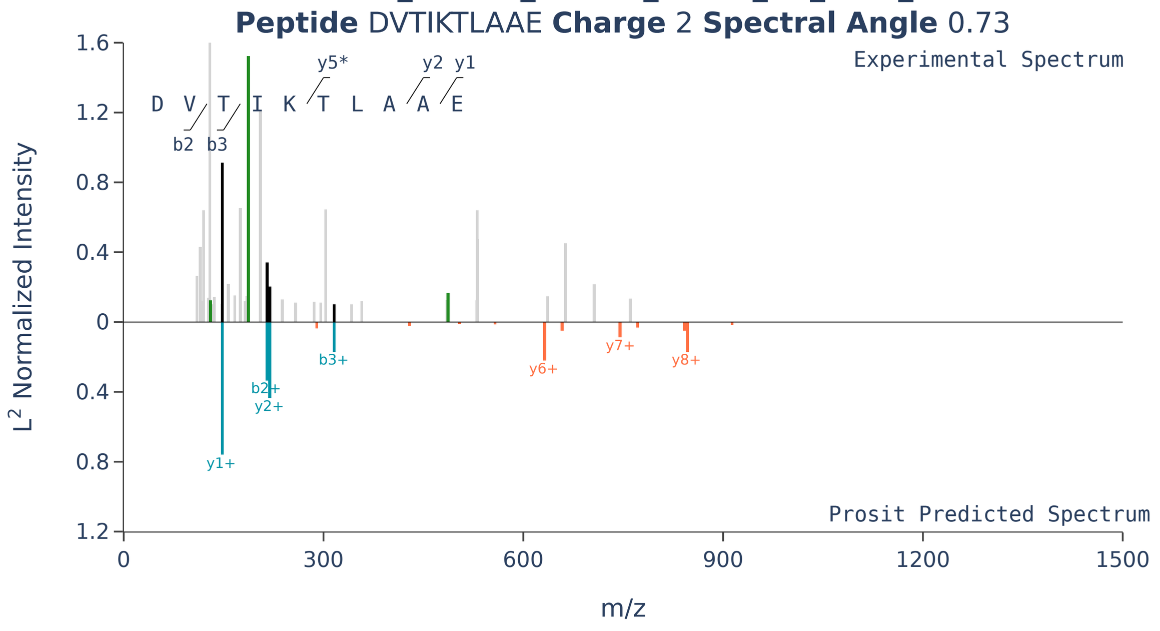


**Source** HRoetschke_211222_160123_HFGoe_IF2_b1R1_4h **Scan** 21317
**Peptide** SGAVIESF **Charge** 1 **Spectral Angle** 0.85

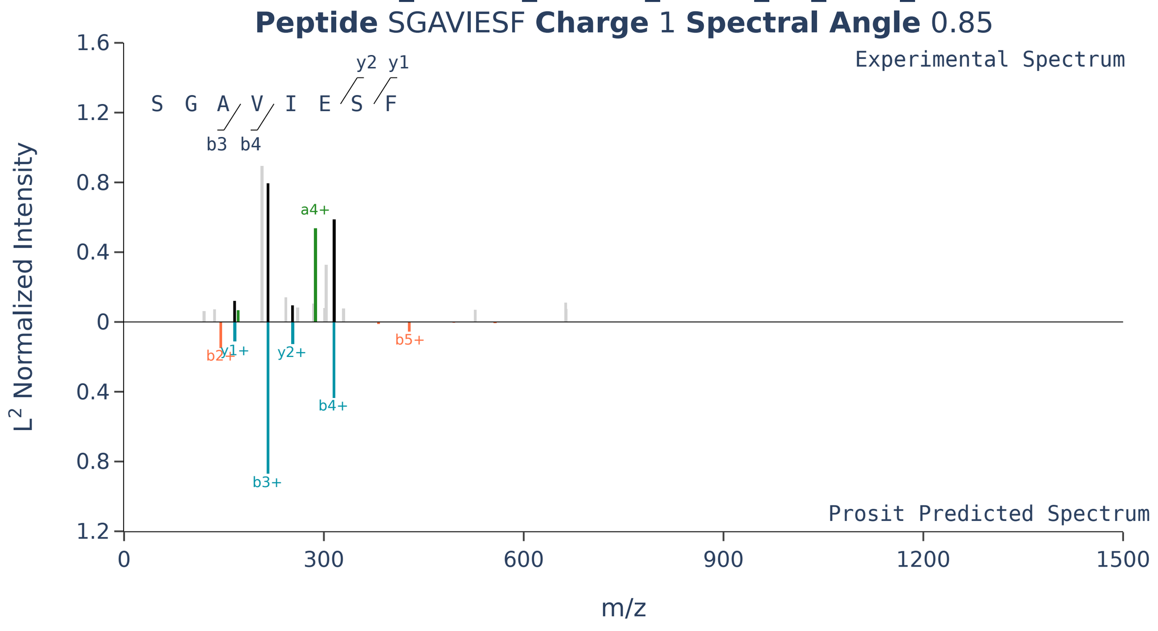


**Source** HRoetschke_211222_160123_HFGoe_IF2_b1R2_24h **Scan** 12130
**Peptide** AGIRKSADDSVSAQEKQTLID **Charge** 3 **Spectral Angle** 0.67

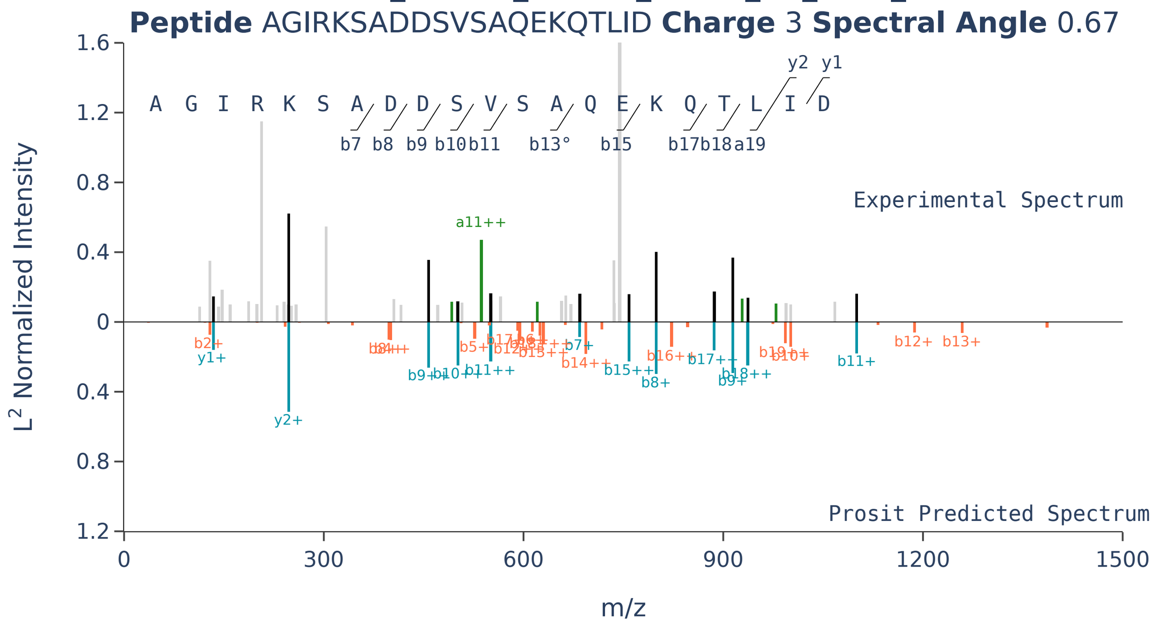


**Source** HRoetschke_211222_160123_HFGoe_IF2_b1R1_2h **Scan** 7322
**Peptide** VKRDPQEAERLA **Charge** 3 **Spectral Angle** 0.57

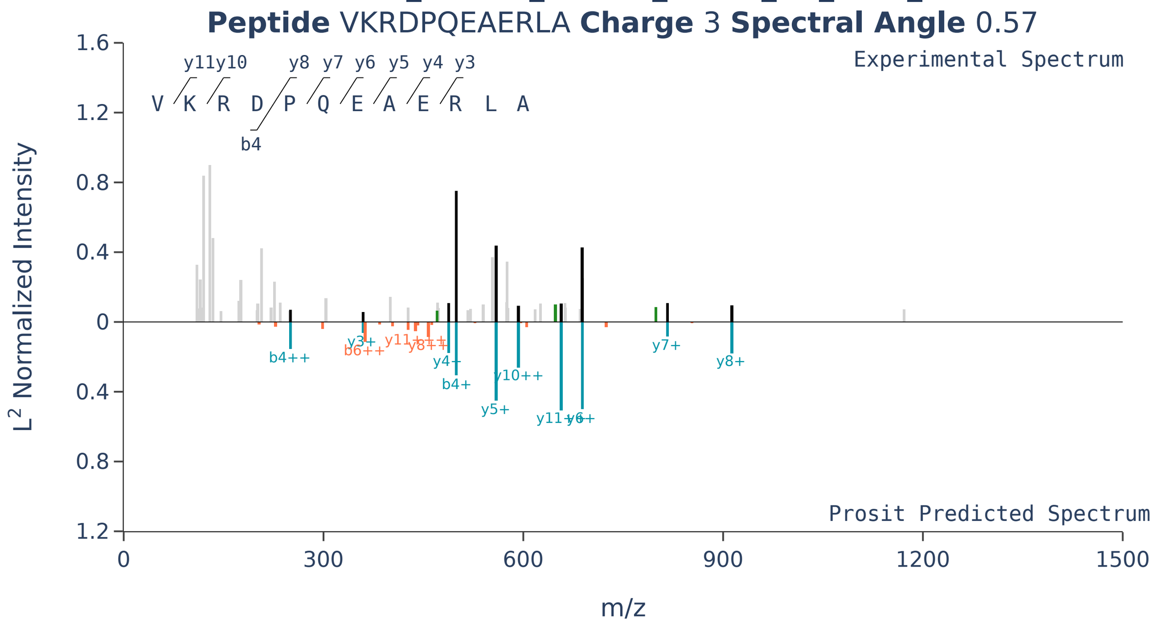

**Source** HRoetschke_211222_160123_HFGoe_IF2_b1R1_2h **Scan** 13191
**Peptide** QKNSGPDKLTL **Charge** 2 **Spectral Angle** 0.72

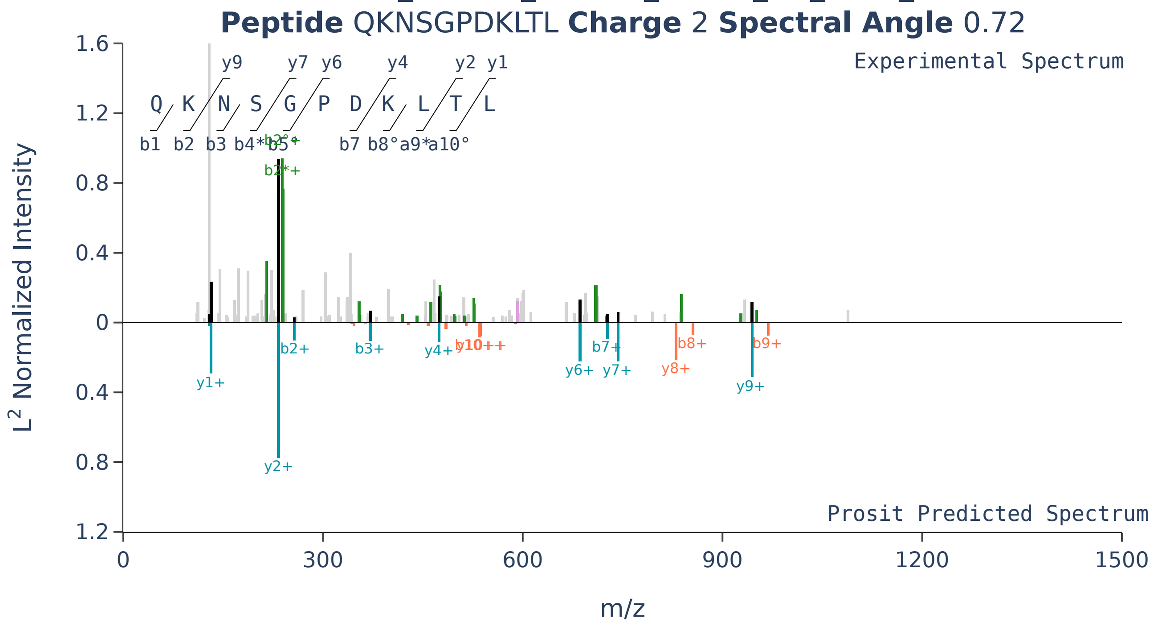


**Source** HRoetschke_211222_160123_HFGoe_IF2_b2R1_24h **Scan** 13203
**Peptide** SGEAGGITQHIGA **Charge** 2 **Spectral Angle** 0.78

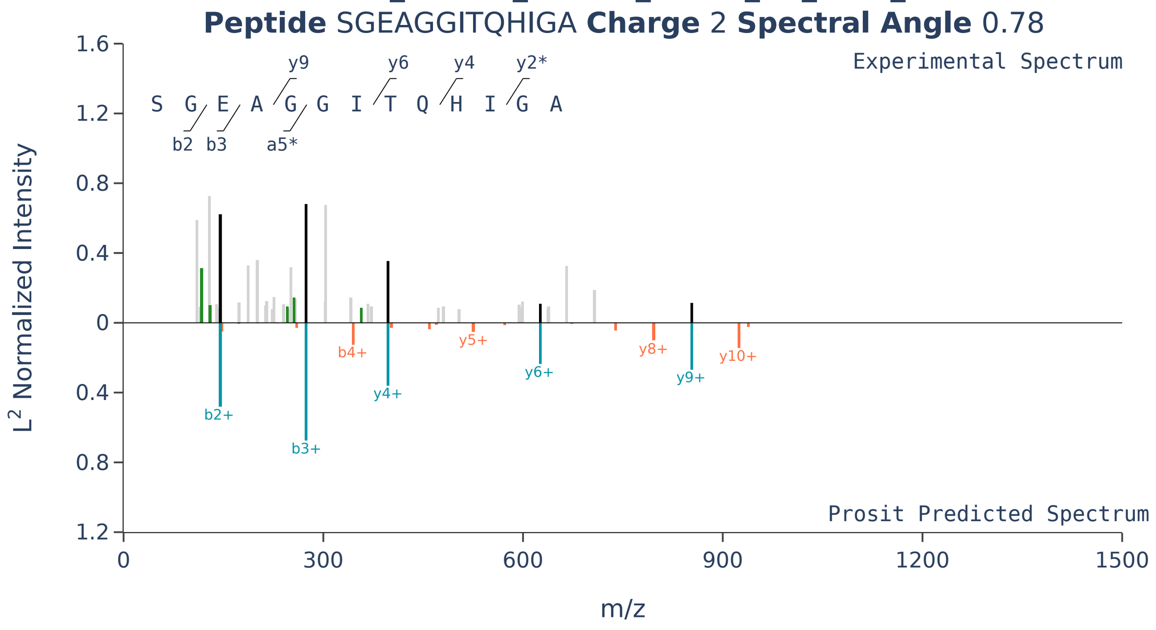


**Source** HRoetschke_211222_160123_HFGoe_IF2_b1R1_4h **Scan** 9490
**Peptide** RKGSSLQQGF **Charge** 2 **Spectral Angle** 0.79

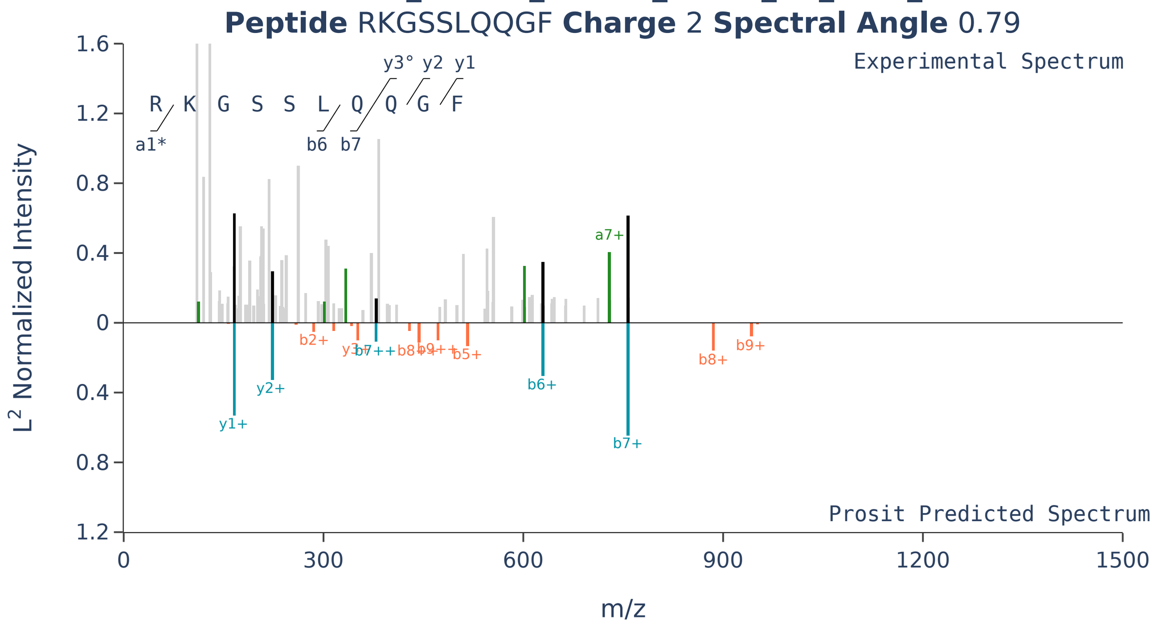


**Source** HRoetschke_211222_160123_HFGoe_IF2_b1R2_24h **Scan** 11171
**Peptide** GTGGKSK **Charge** 1 **Spectral Angle** 0.74

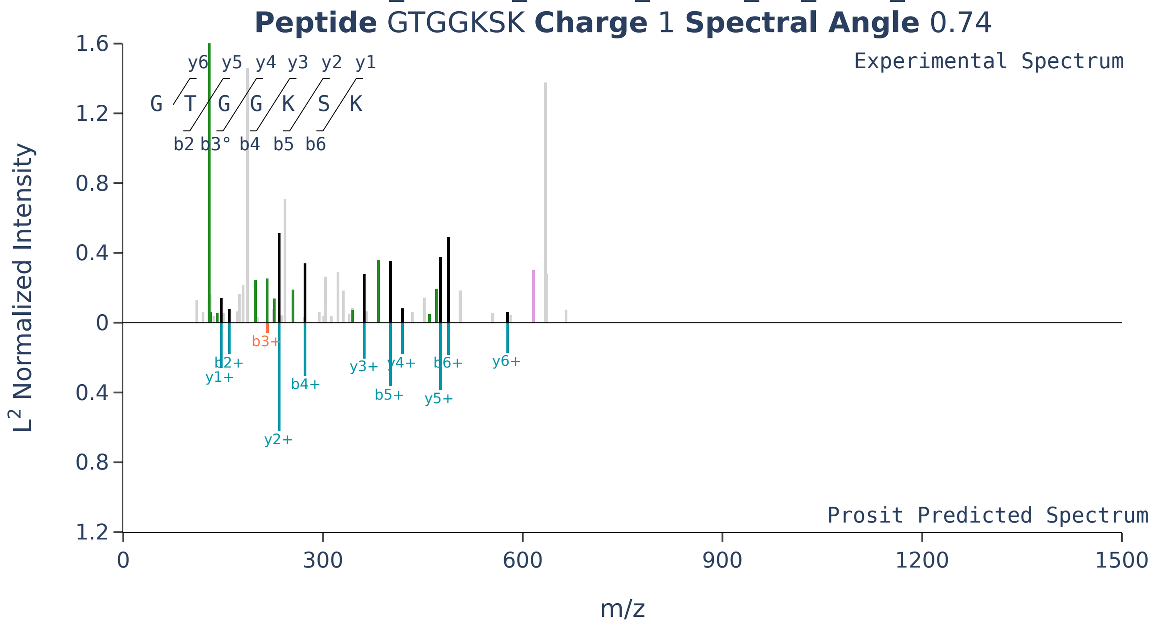


**Source** HRoetschke_211222_160123_HFGoe_IF2_b1R1_2h **Scan** 6617
**Peptide** LANKMAVKGS **Charge** 2 **Spectral Angle** 0.65

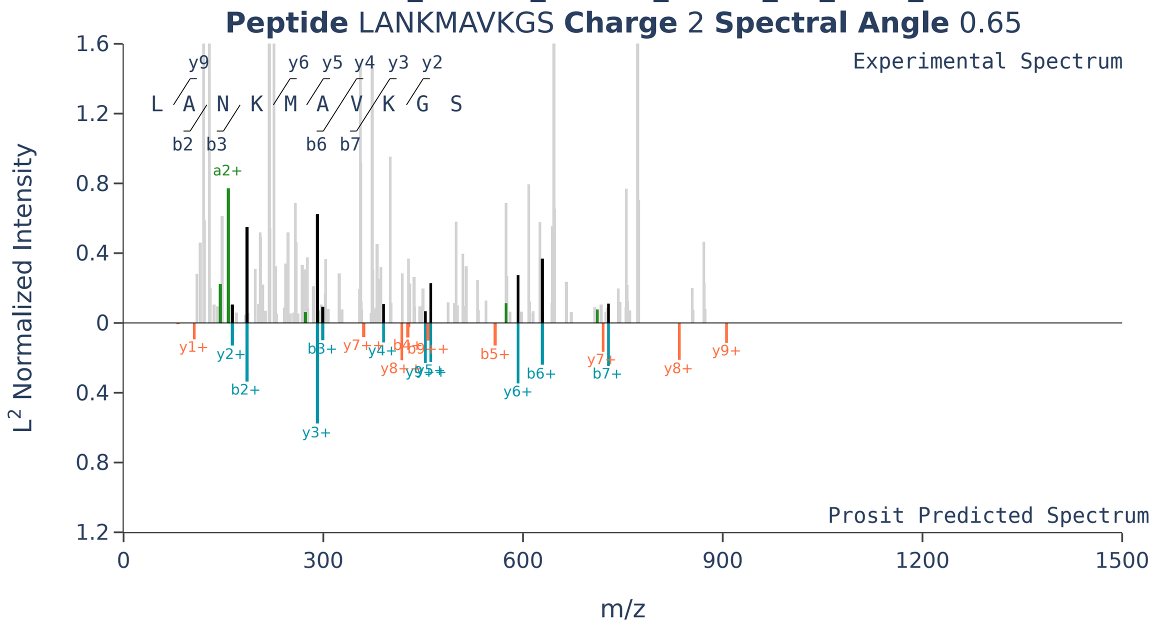


**Source** HRoetschke_211222_160123_HFGoe_IF2_b1R1_4h **Scan** 22856
**Peptide** KGDIVLCGF **Charge** 2 **Spectral Angle** 0.75

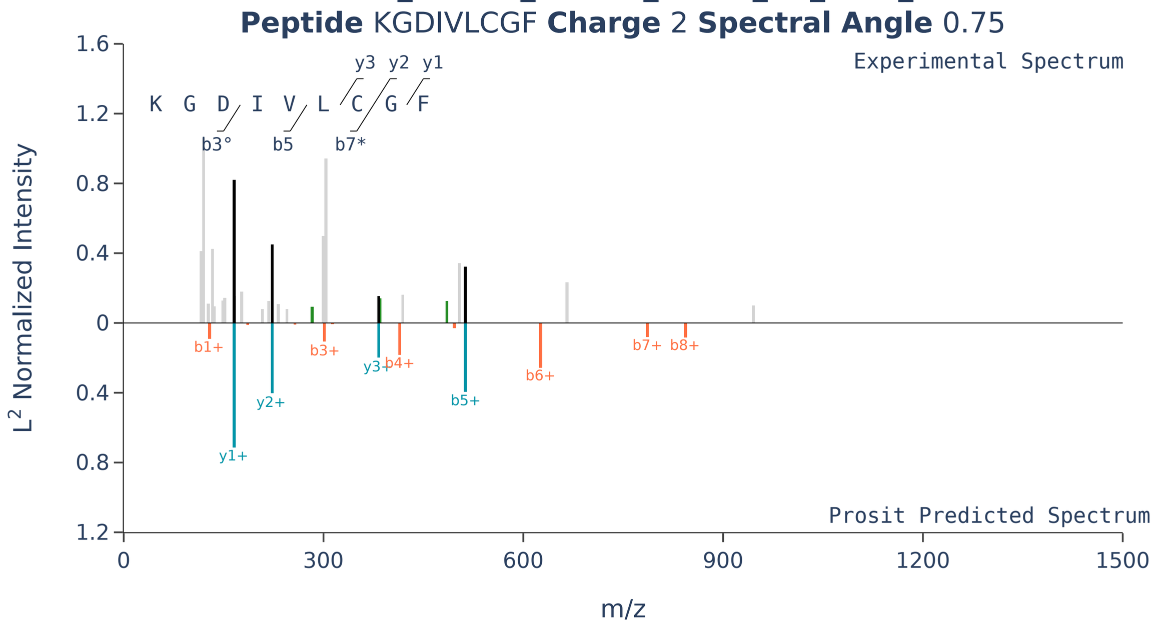


**Source** HRoetschke_211222_160123_HFGoe_IF2_b1R1_24h **Scan** 5748
**Peptide** KVSNQQDDMTKNAQAEKA **Charge** 3 **Spectral Angle** 0.9

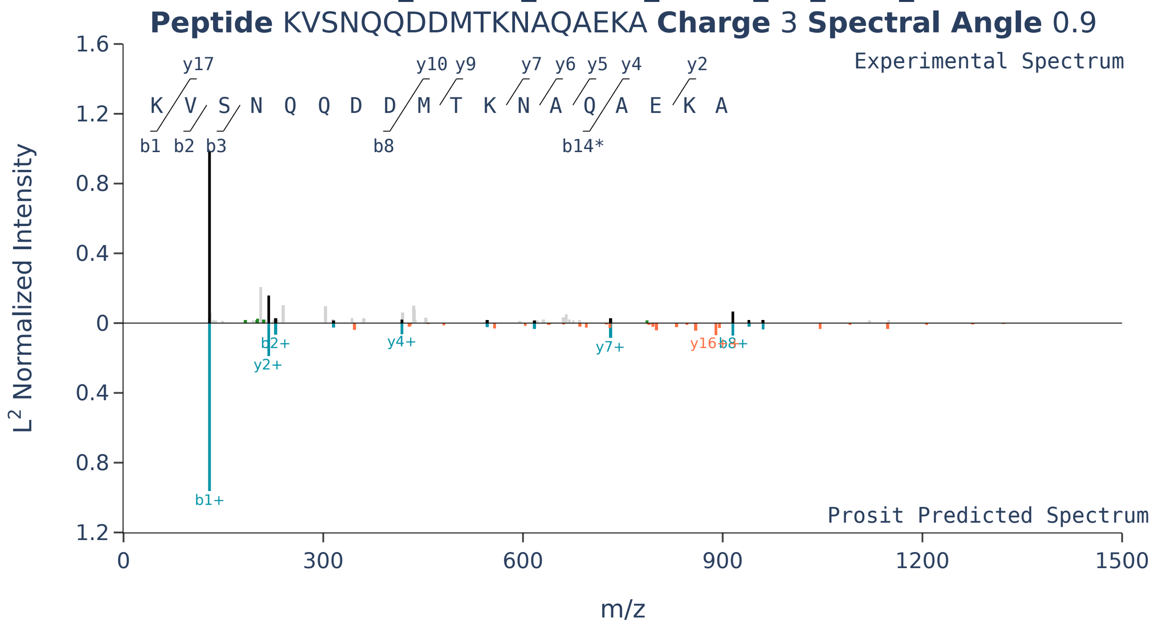


**Source** HRoetschke_211222_160123_HFGoe_IF2_b1R1_4h **Scan** 17576
**Peptide** DVTIKTLAAERQTSVE **Charge** 3 **Spectral Angle** 0.88

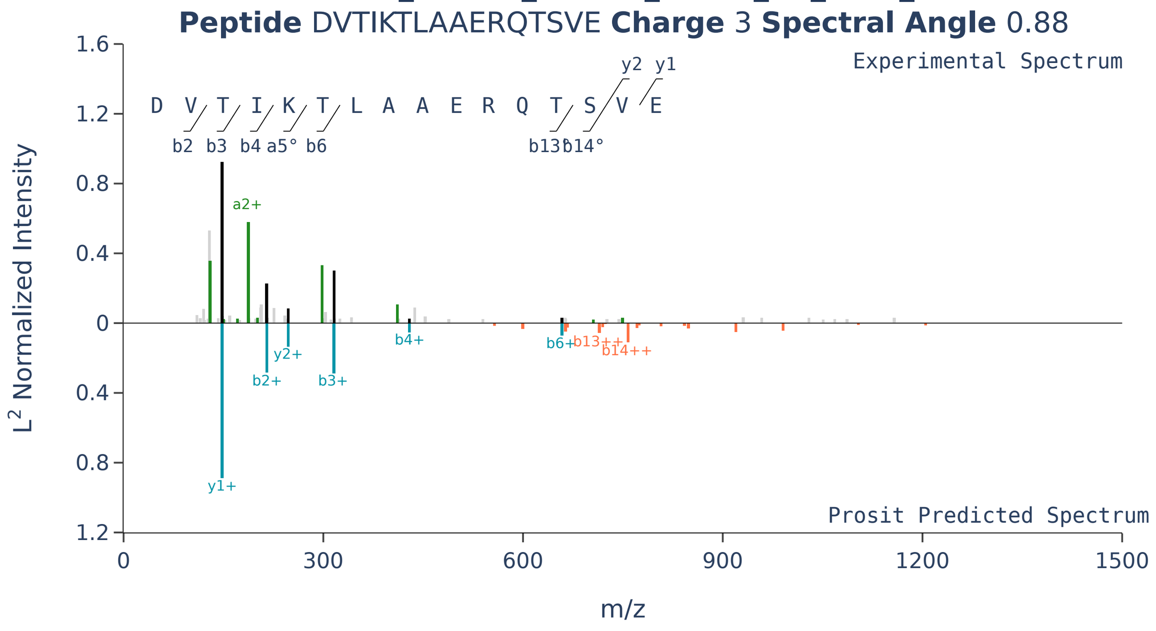


**Source** HRoetschke_211222_160123_HFGoe_IF2_b1R1_2h **Scan** 5340
**Peptide** YHVTTSQHARQAED **Charge** 2 **Spectral Angle** 0.79

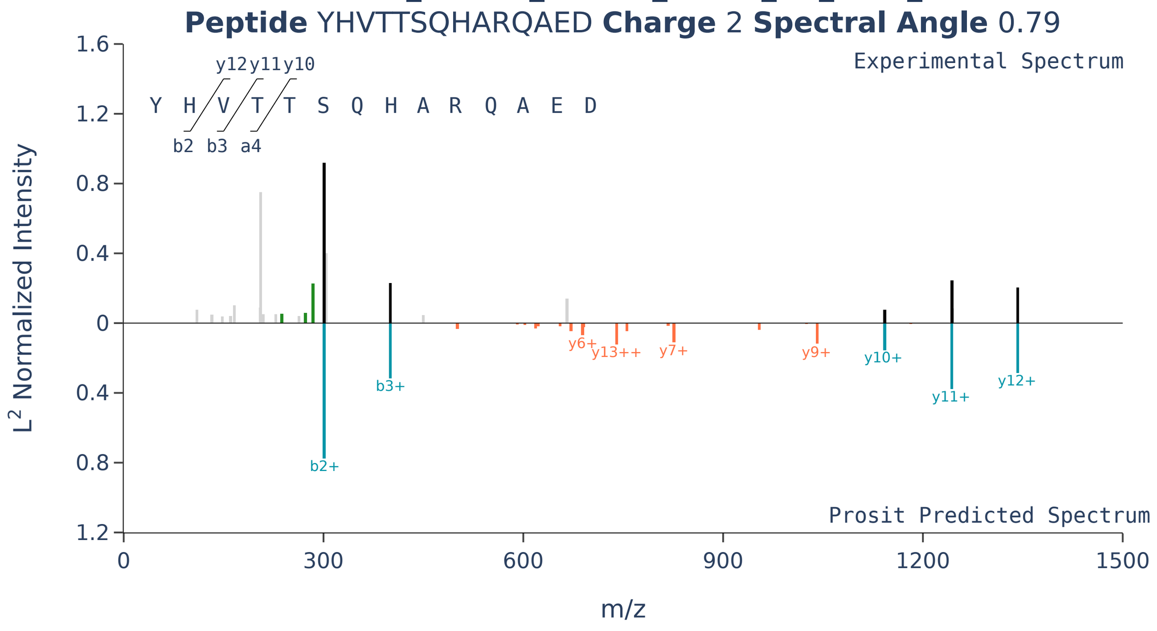


**Source** HRoetschke_211222_160123_HFGoe_IF2_b2R2_4h **Scan** 13096
**Peptide** NSGPDKLTLQ **Charge** 2 **Spectral Angle** 0.82

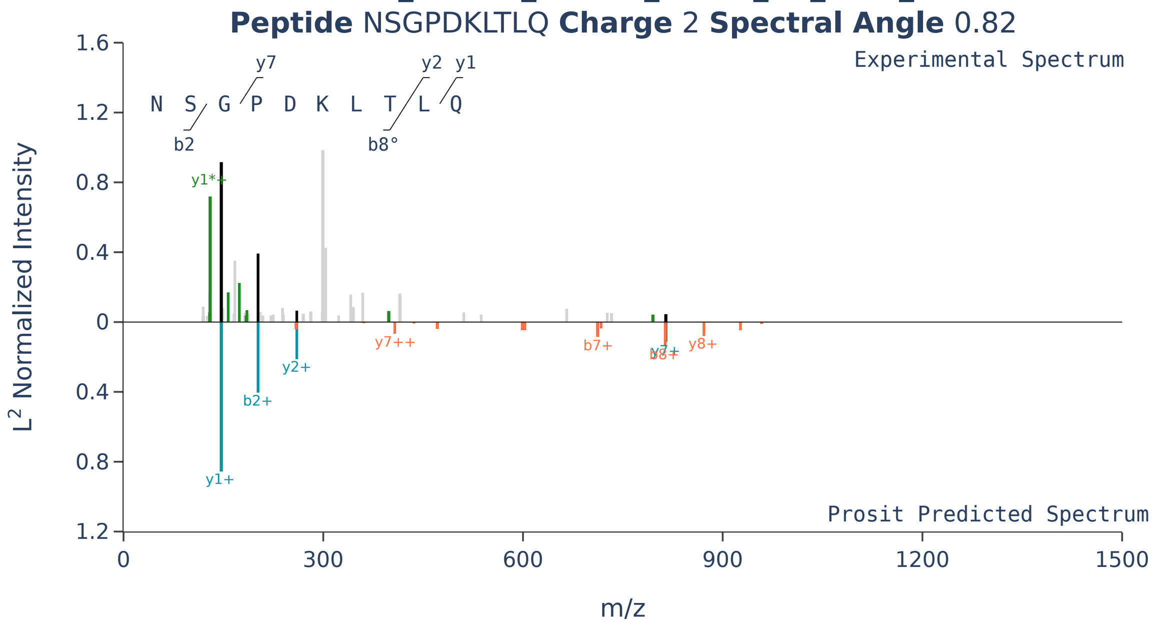


**Source** HRoetschke_211222_160123_HFGoe_IF2_b1R2_2h **Scan** 8401
**Peptide** IRSTKVASGEAGGITQ **Charge** 3 **Spectral Angle** 0.77

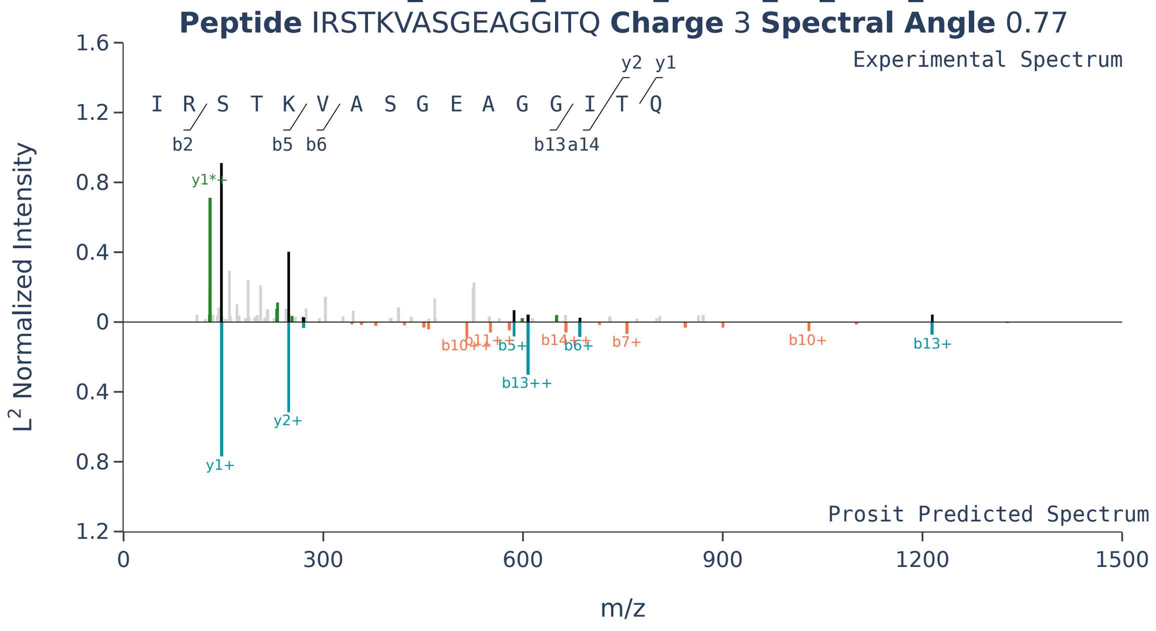


**Source** HRoetschke_211222_160123_HFGoe_IF2_b1R1_1h **Scan** 7262
**Peptide** DAGIRKSADDSV **Charge** 3 **Spectral Angle** 0.8

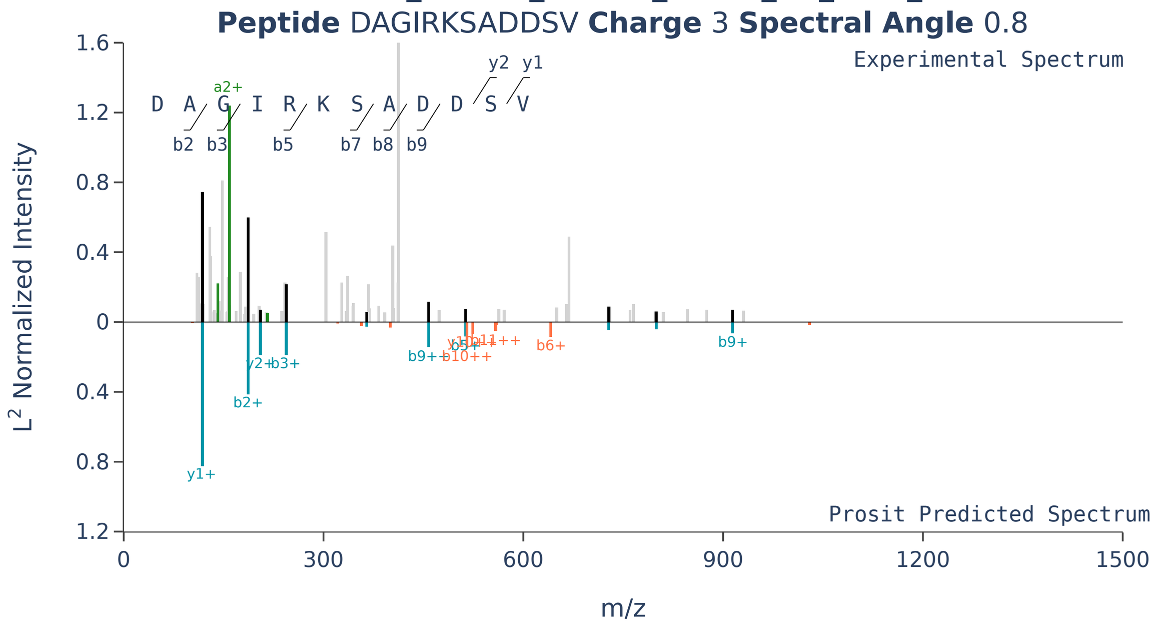


**Source** HRoetschke_211222_160123_HFGoe_IF2_b1R1_4h **Scan** 17354
**Peptide** KQQIIGLAEVR **Charge** 2 **Spectral Angle** 0.59

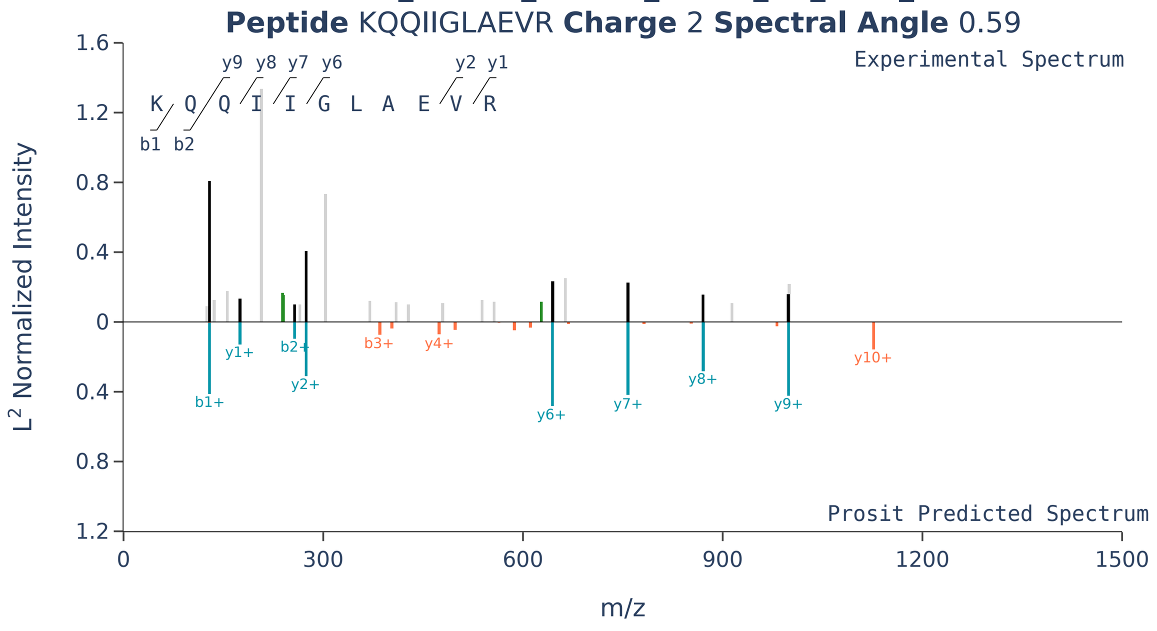


**Source** HRoetschke_211222_160123_HFGoe_IF2_b1R2_2h **Scan** 8970
**Peptide** EARRMAEENKWTDNAEPTE **Charge** 3 **Spectral Angle** 0.84

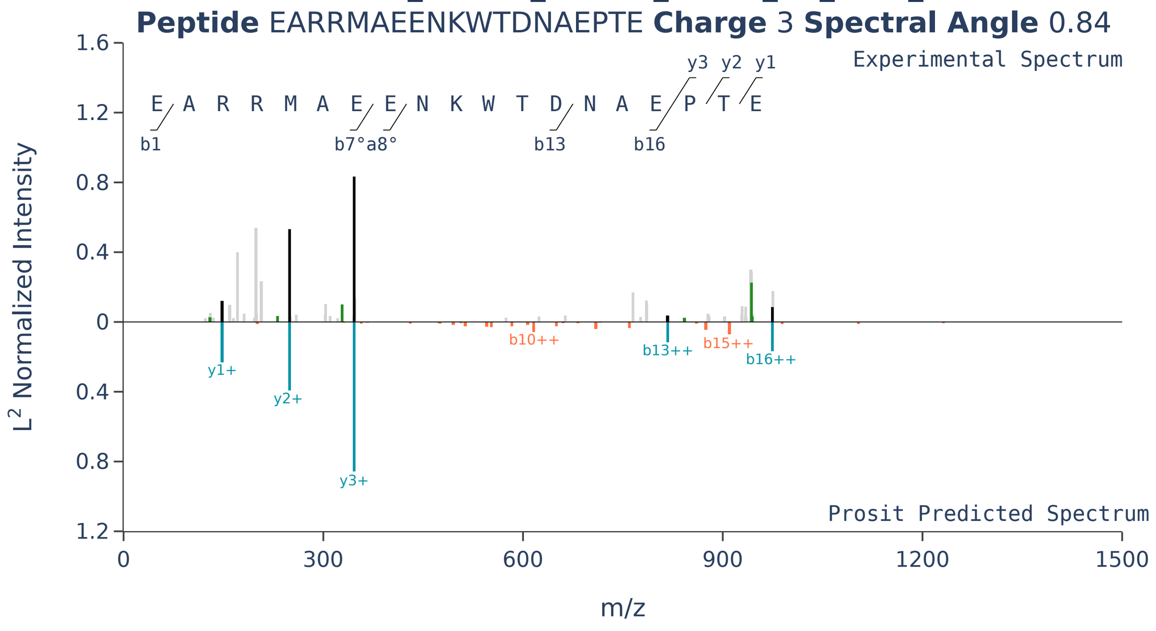


**Source** HRoetschke_211222_160123_HFGoe_IF2_b1R1_4h **Scan** 14261
**Peptide** KLGAMATINQ **Charge** 2 **Spectral Angle** 0.5

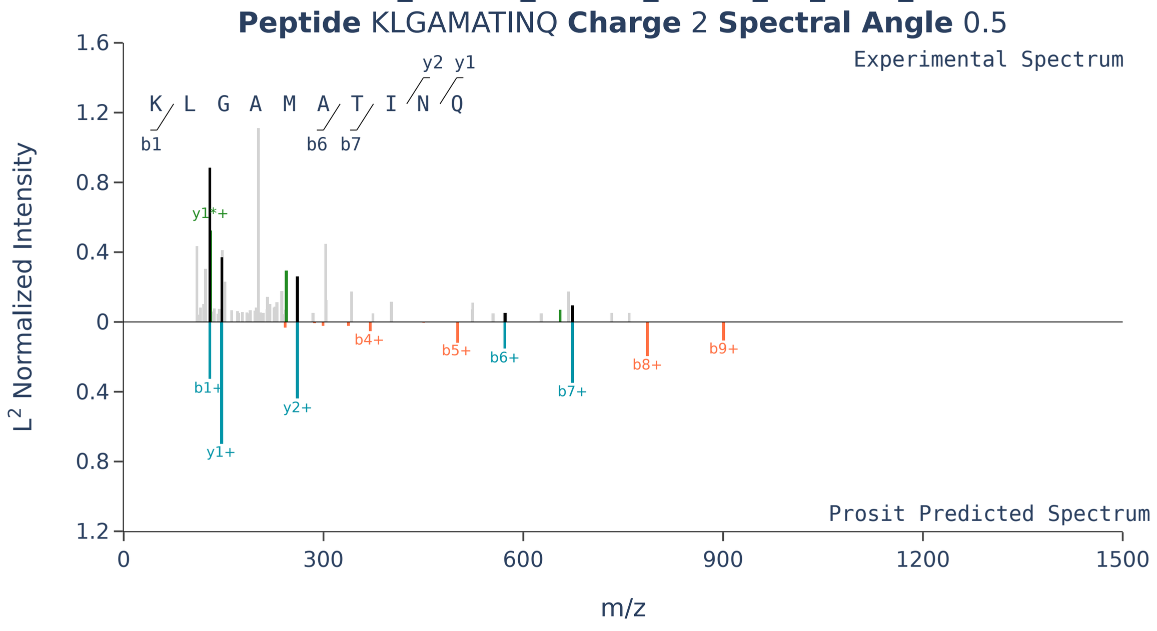

**Source** HRoetschke_211222_160123_HFGoe_IF2_b1R1_4h **Scan** 22723
**Peptide** AESLDLRYYSV **Charge** 2 **Spectral Angle** 0.74

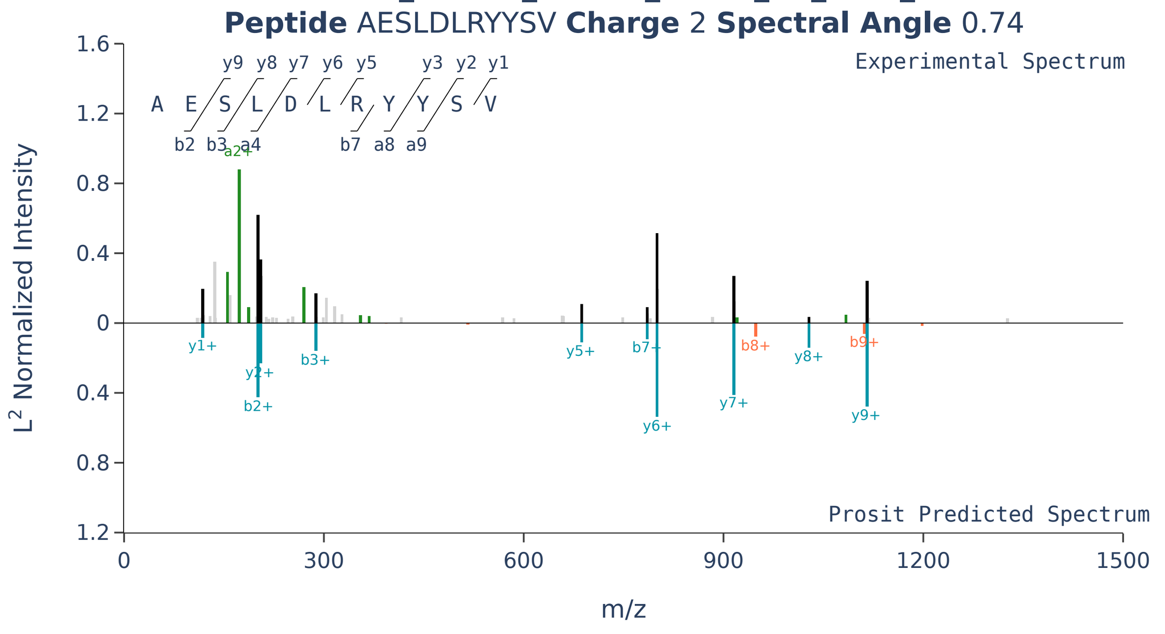


**Source** HRoetschke_211222_160123_HFGoe_IF2_b1R1_4h **Scan** 7897
**Peptide** KVASGEAGGIT **Charge** 2 **Spectral Angle** 0.64

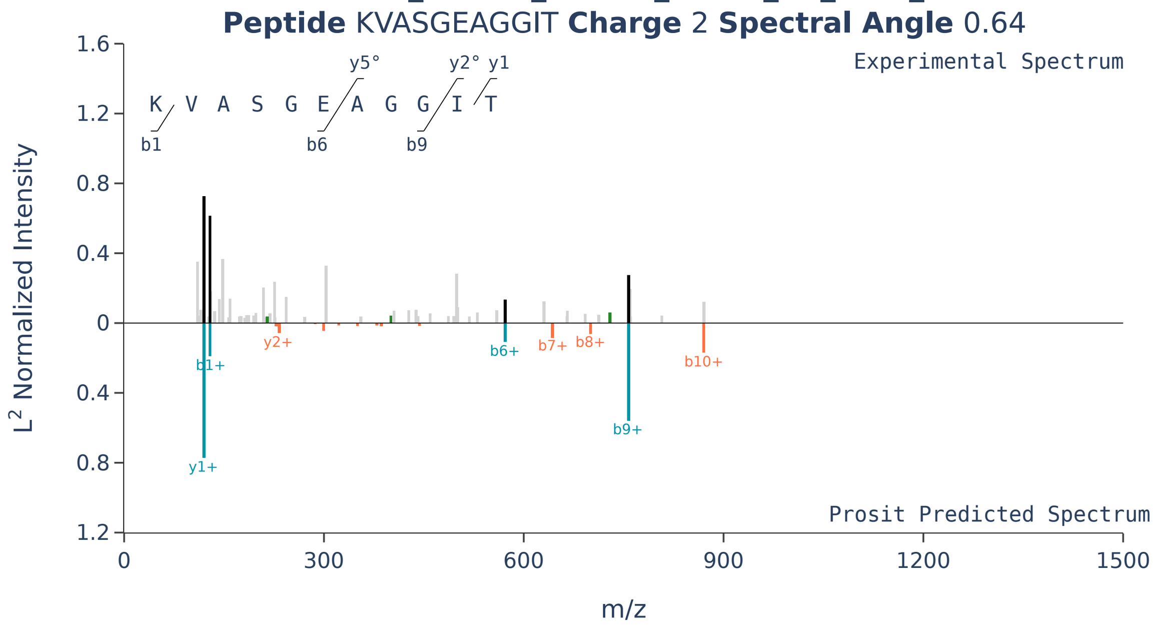


**Source** HRoetschke_211222_160123_HFGoe_IF2_b1R2_24h **Scan** 19016
**Peptide** DDGVMPQTIEAIQHA **Charge** 2 **Spectral Angle** 0.73

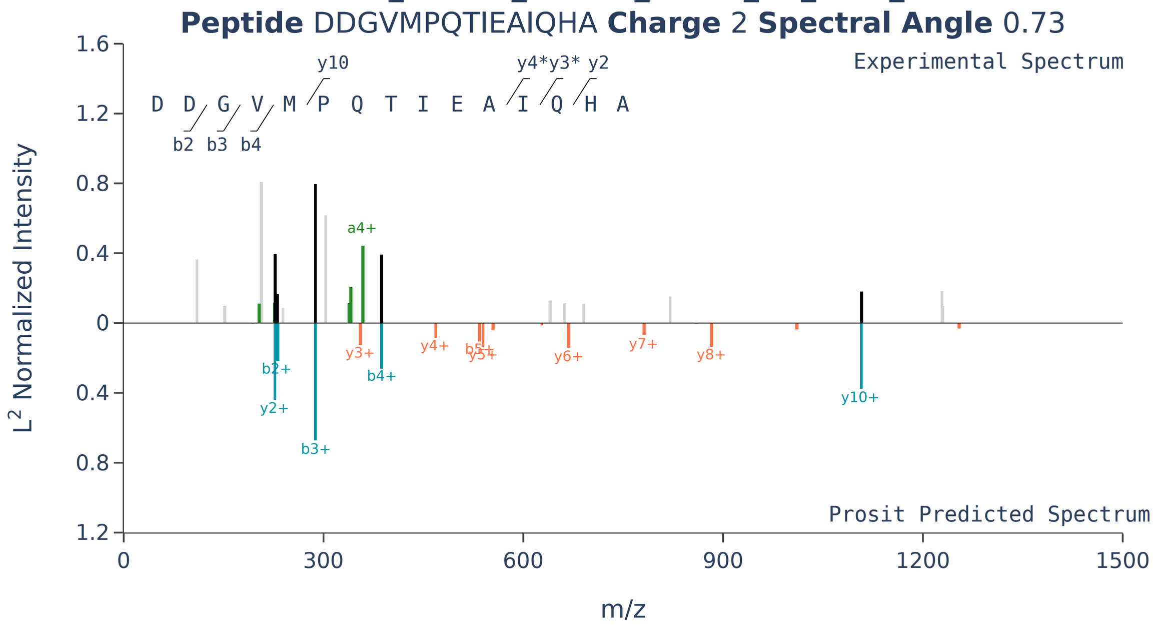


**Source** HRoetschke_211222_160123_HFGoe_IF2_b1R2_4h **Scan** 8298
**Peptide** KMAVKGSQVLKA **Charge** 2 **Spectral Angle** 0.87

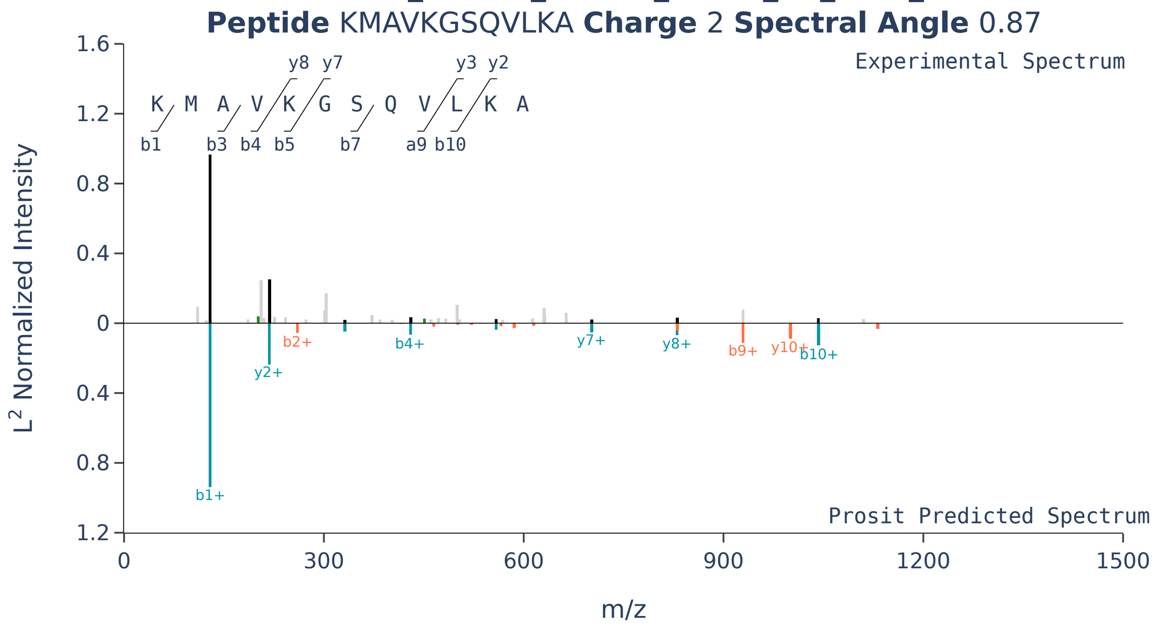


**Source** HRoetschke_211222_160123_HFGoe_IF2_b2R1_24h **Scan** 12244
**Peptide** AVNKIDKPEADPDRVKNELSQ **Charge** 3 **Spectral Angle** 0.76

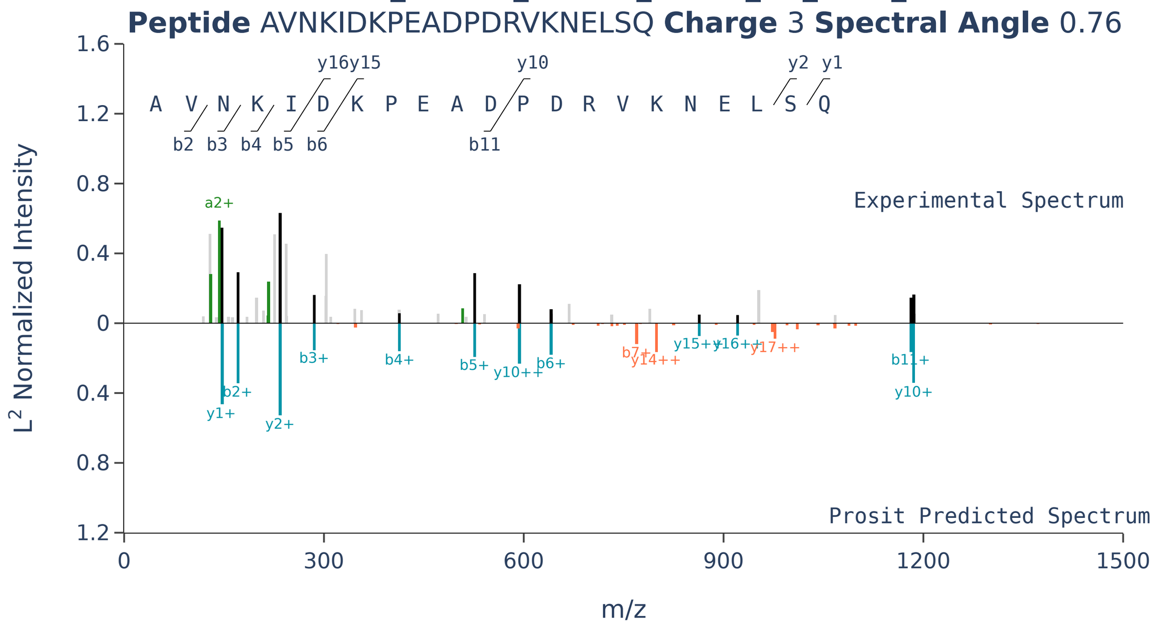


**Source** HRoetschke_211222_160123_HFGoe_IF2_b1R1_2h **Scan** 5901
**Peptide** NQKNSGPDKLTLQRKTRS **Charge** 3 **Spectral Angle** 0.73

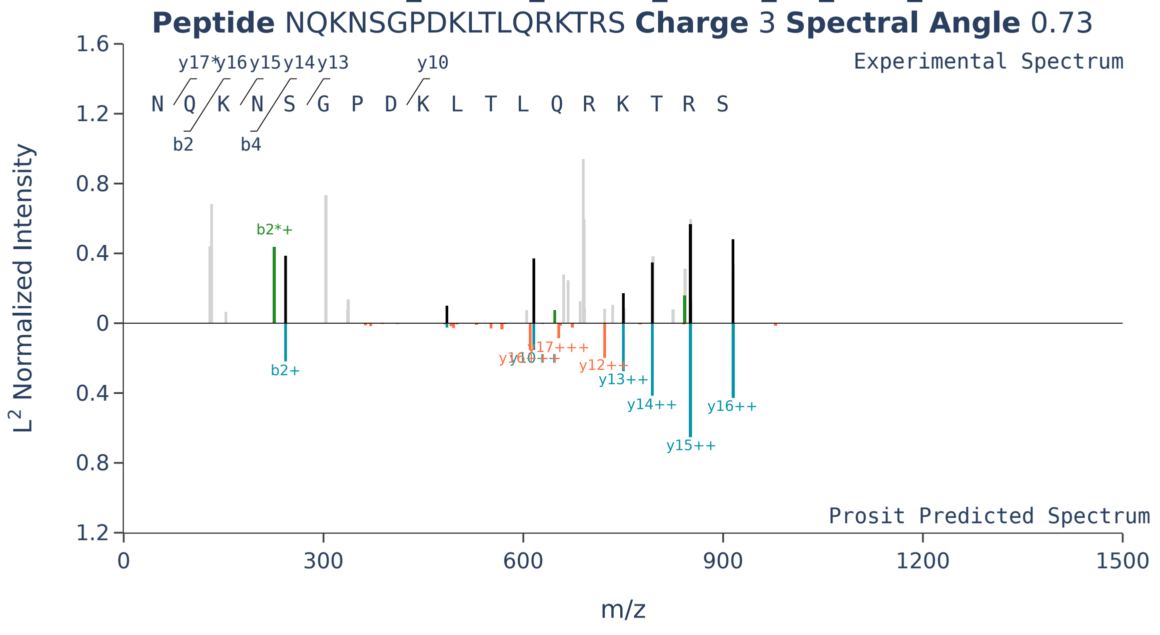


**Source** HRoetschke_211222_160123_HFGoe_IF2_b1R2_1h **Scan** 17982
**Peptide** RDTGAAAEPRAPVVTL **Charge** 3 **Spectral Angle** 0.66

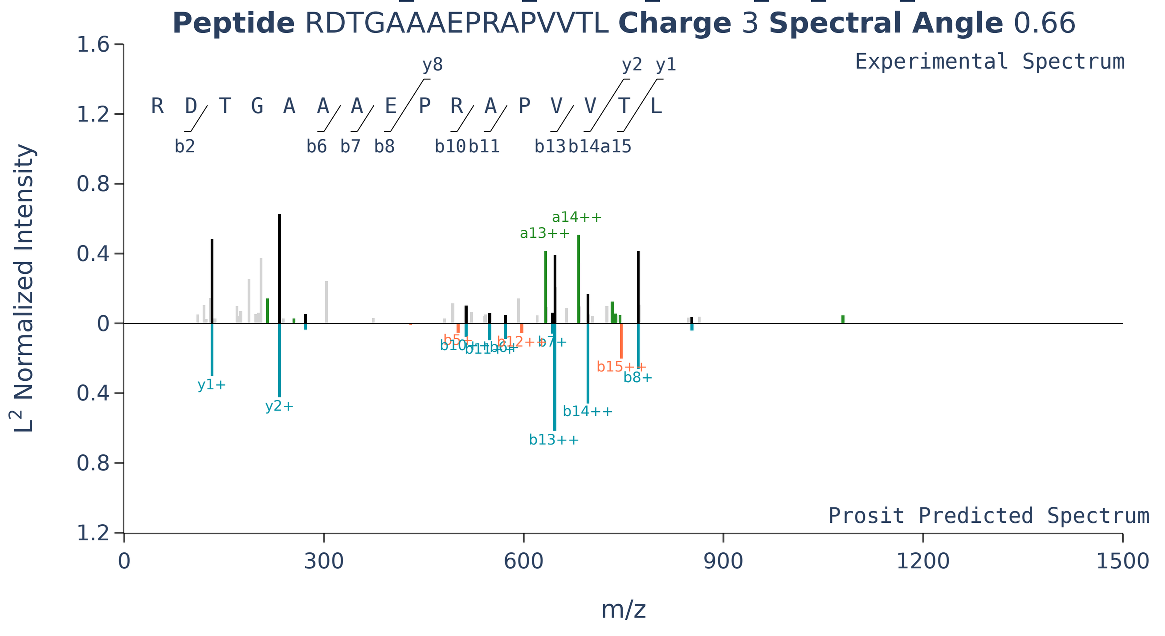


**Source** HRoetschke_211222_160123_HFGoe_IF2_b1R1_24h **Scan** 13837
**Peptide** DEVTVVRDEKKAREVAL **Charge** 3 **Spectral Angle** 0.81

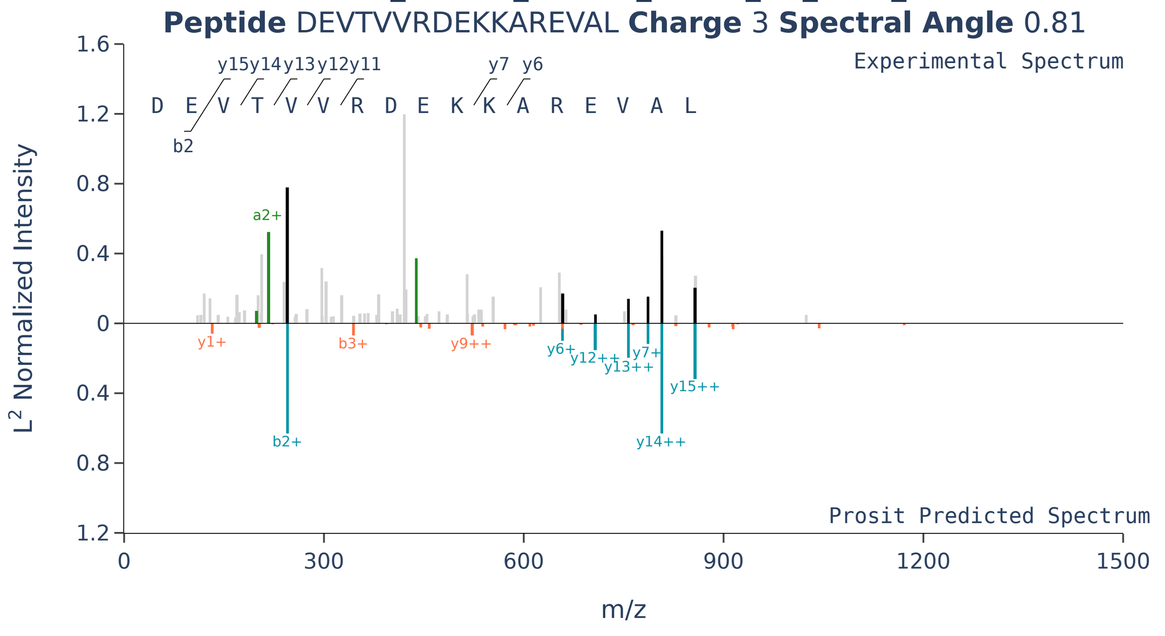


**Source** HRoetschke_211222_160123_HFGoe_IF2_b1R1_4h **Scan** 16764
**Peptide** SGEAGGITQHIGAY **Charge** 2 **Spectral Angle** 0.82

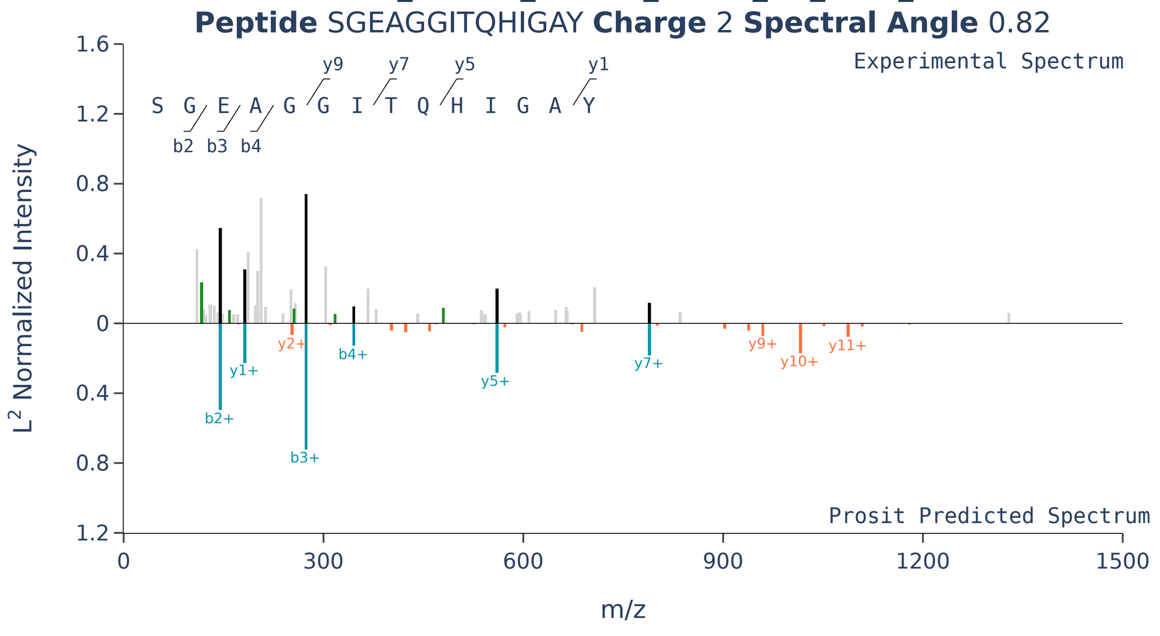


**Source** HRoetschke_211222_160123_HFGoe_IF2_b1R1_4h **Scan** 18059
**Peptide** AQVPVVVAVNK **Charge** 2 **Spectral Angle** 0.79

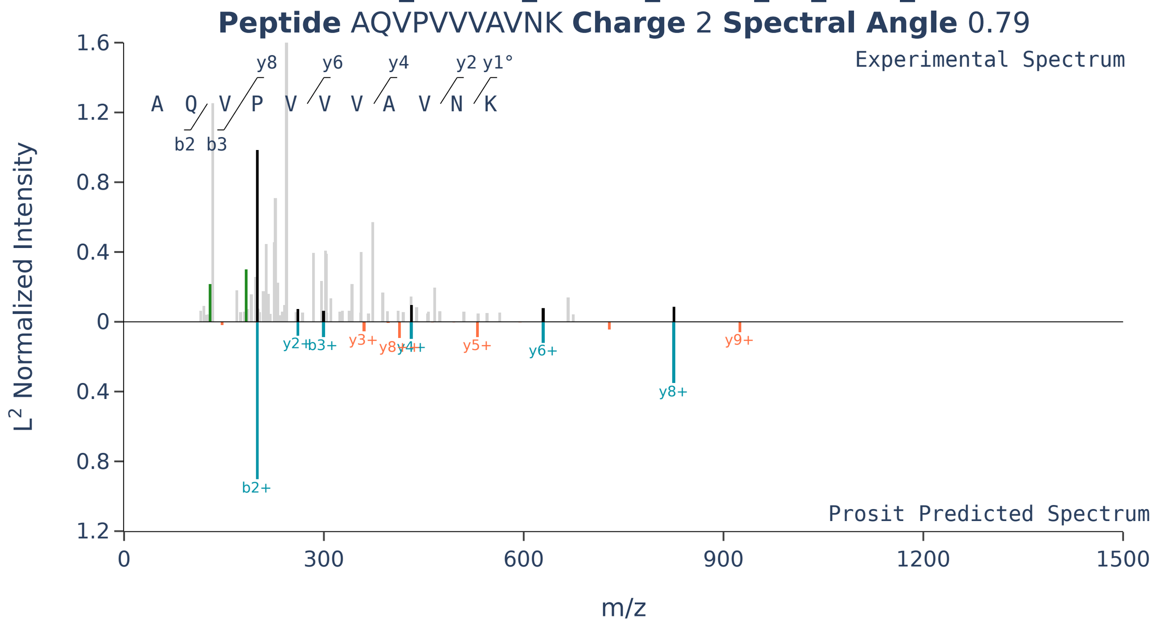


**Source** HRoetschke_211222_160123_HFGoe_IF2_b2R2_4h **Scan** 19044
**Peptide** AMATINQVIDQETA **Charge** 2 **Spectral Angle** 0.74

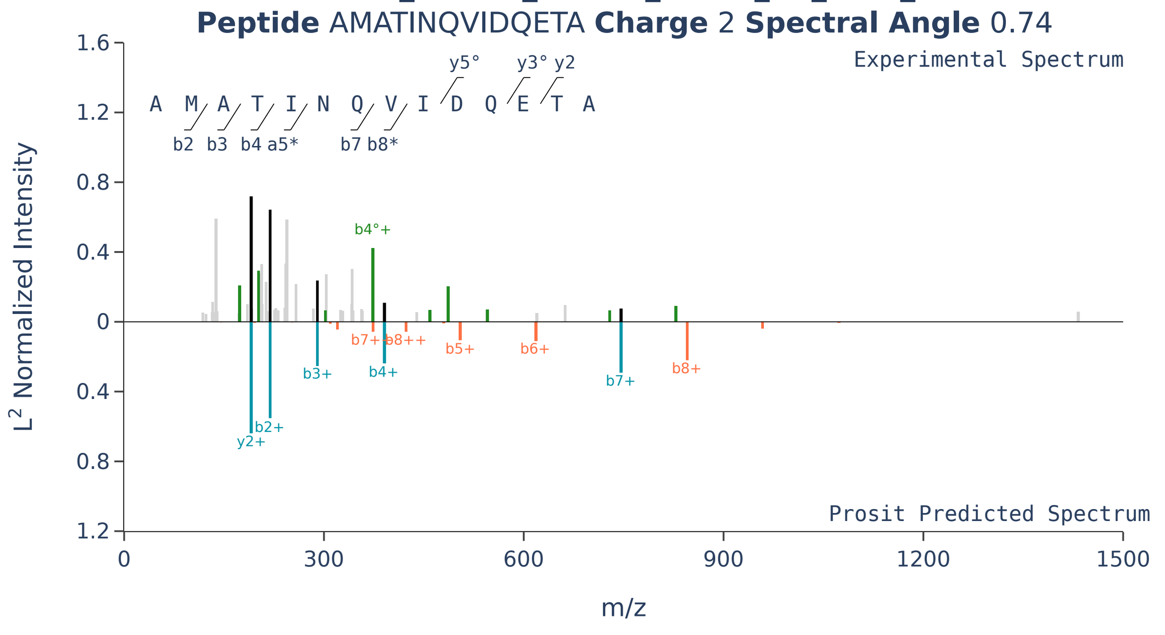


**Source** HRoetschke_211222_160123_HFGoe_IF2_b1R1_24h **Scan** 13137
**Peptide** ADAGIRKSADDSVSAQEKQTLID **Charge** 4 **Spectral Angle** 0.74

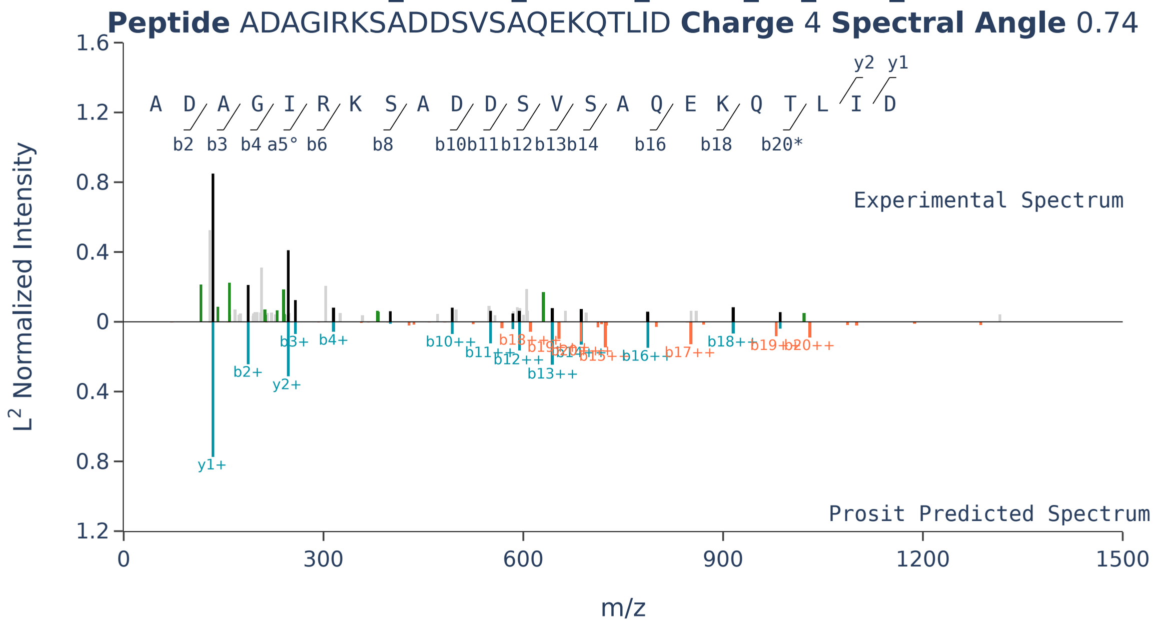


**Source** HRoetschke_211222_160123_HFGoe_IF2_b2R1_2h **Scan** 10718
**Peptide** TIKTLAAERQTSVE **Charge** 3 **Spectral Angle** 0.88

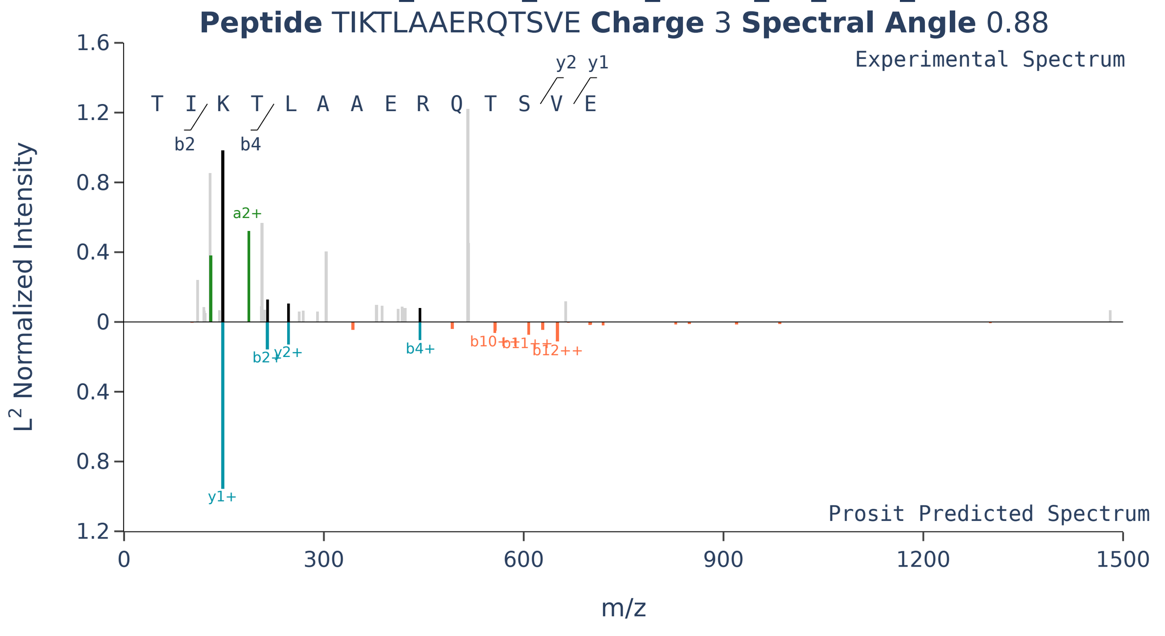


**Source** HRoetschke_211222_160123_HFGoe_IF2_b1R2_24h **Scan** 15085
**Peptide** QHAKAAQVPVVV **Charge** 2 **Spectral Angle** 0.68

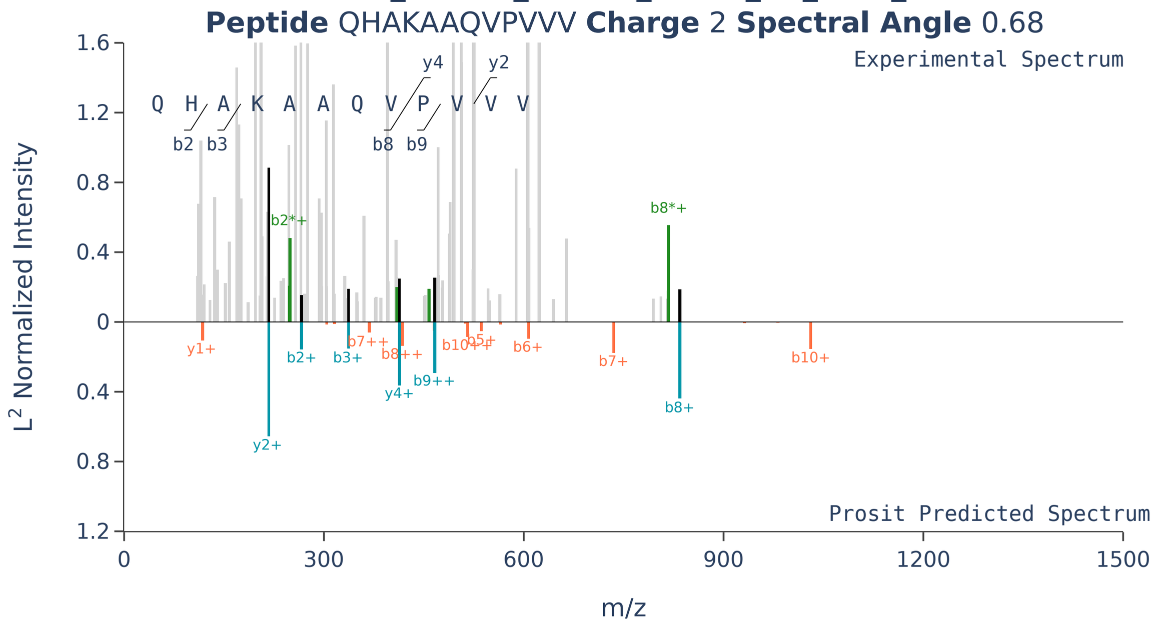


**Source** HRoetschke_211222_160123_HFGoe_IF2_b1R1_4h **Scan** 24794
**Peptide** AQVPVVVAV **Charge** 1 **Spectral Angle** 0.91

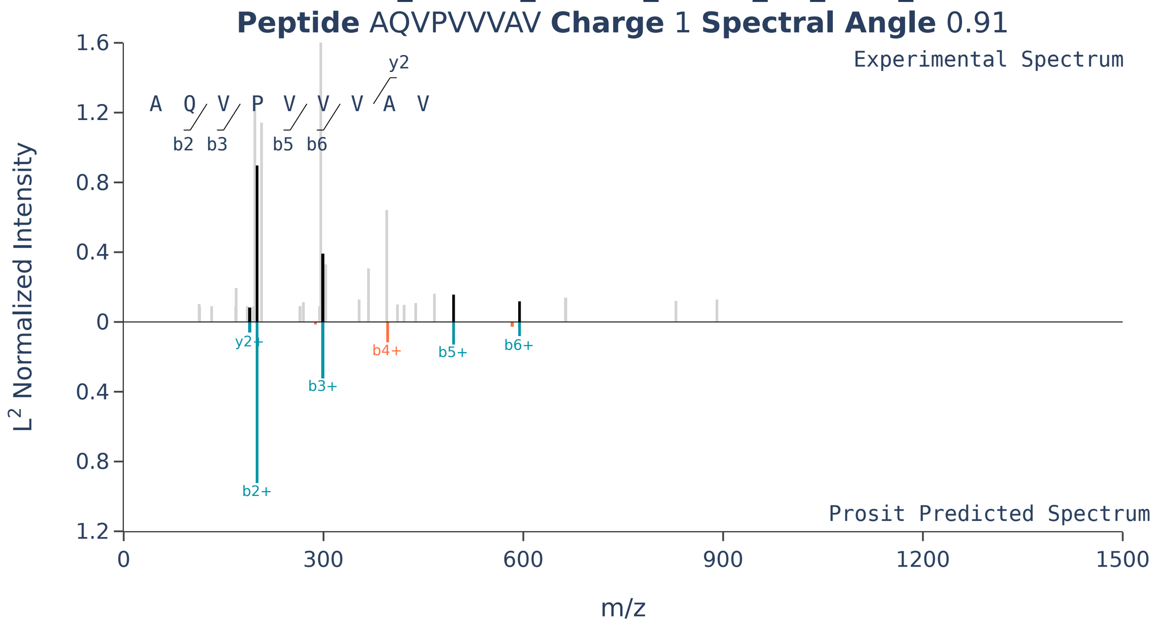

**Source** HRoetschke_211222_160123_HFGoe_IF2_b1R1_24h **Scan** 15640
**Peptide** WGGESQFVH **Charge** 2 **Spectral Angle** 0.77

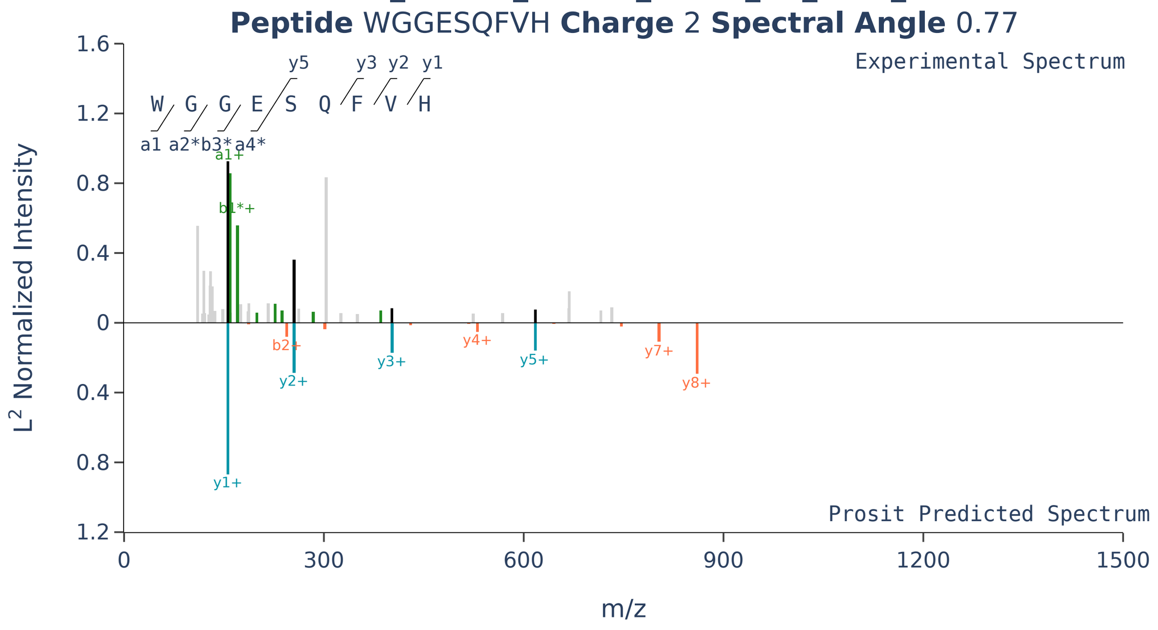

**Source** HRoetschke_211222_160123_HFGoe_IF2_b1R1_2h **Scan** 5485
**Peptide** RTFVKRDPQEAER **Charge** 4 **Spectral Angle** 0.67

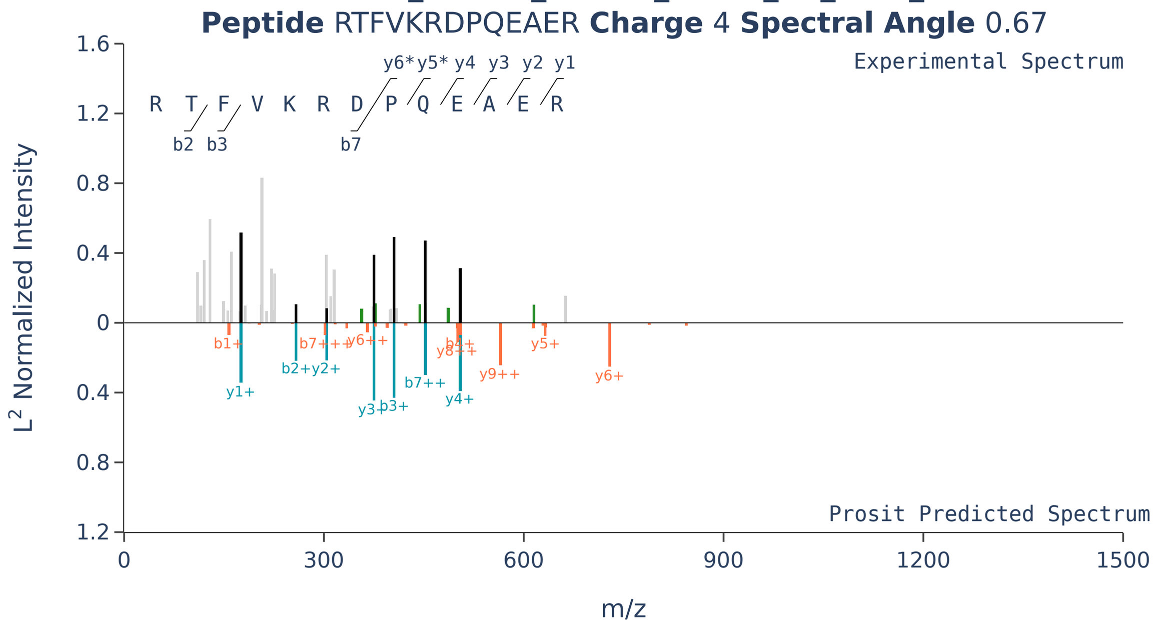

**Source** HRoetschke_211222_160123_HFGoe_IF2_b2R2_24h **Scan** 6311
**Peptide** ELKAVRKGMAS **Charge** 2 **Spectral Angle** 0.73

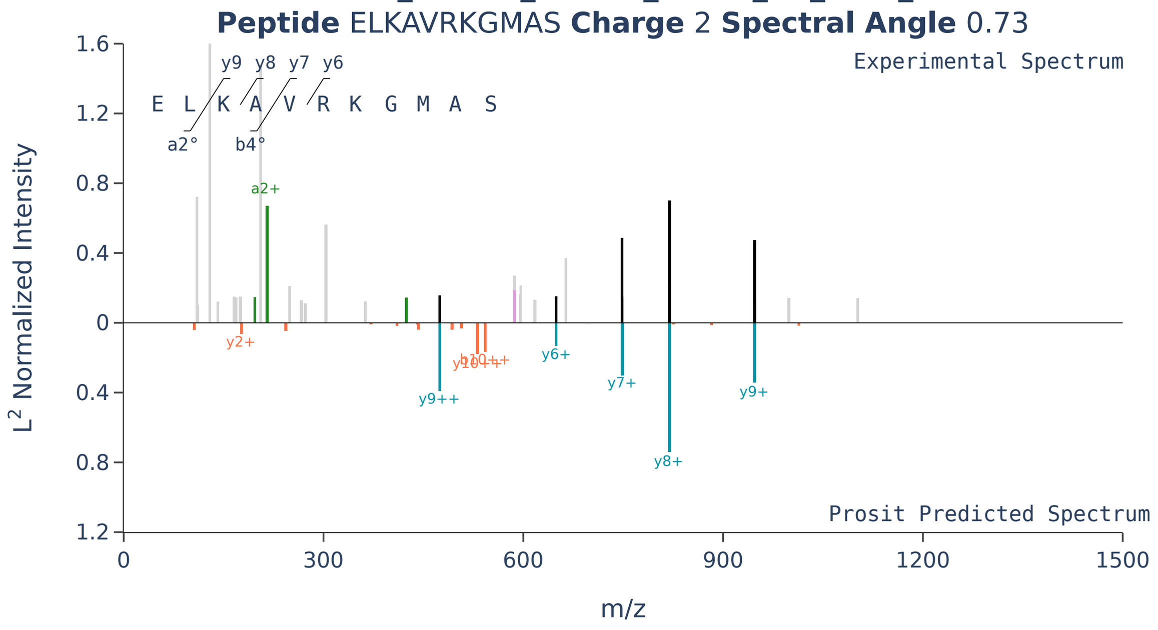

**Source** HRoetschke_211222_160123_HFGoe_IF2_b2R1_24h **Scan** 11824
**Peptide** ERQTSVERLVQQ **Charge** 3 **Spectral Angle** 0.48

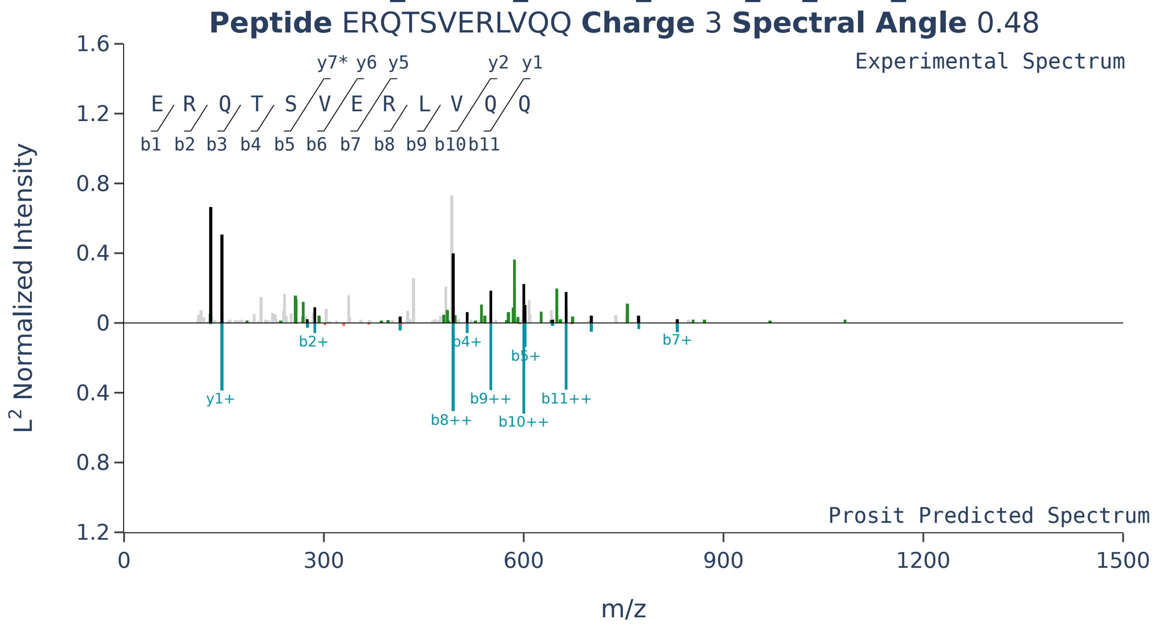

**Source** HRoetschke_211222_160123_HFGoe_IF2_b2R2_24h **Scan** 19326
**Peptide** EPRAPVVTL **Charge** 2 **Spectral Angle** 0.77

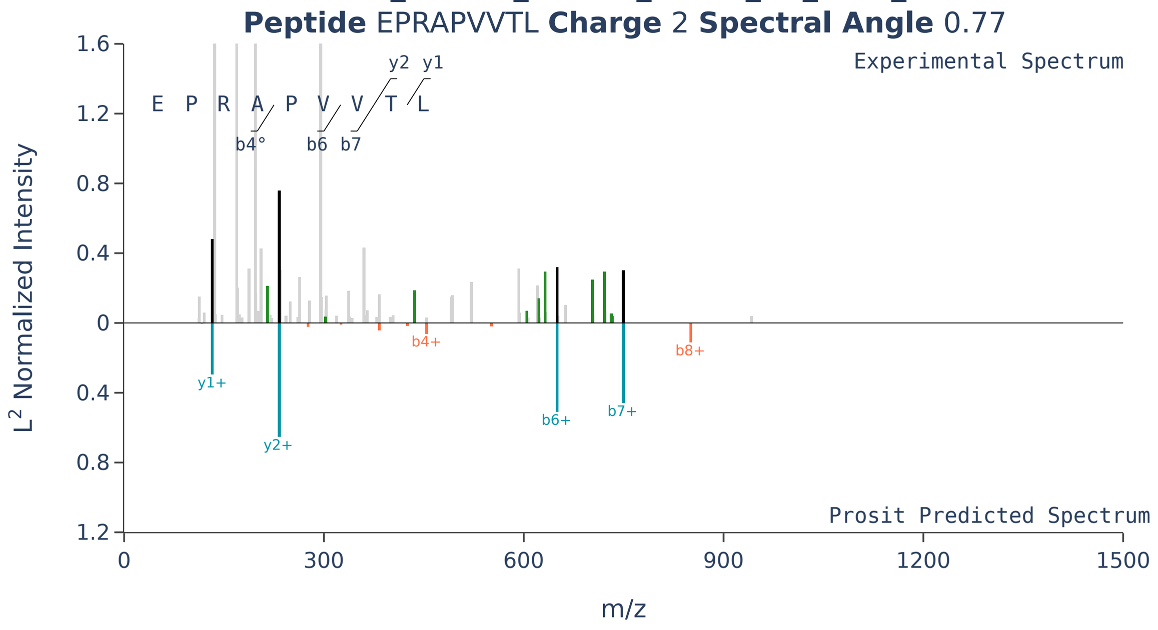

**Source** HRoetschke_211222_160123_HFGoe_IF2_b1R1_4h **Scan** 17547
**Peptide** ASGEAGGITQHIGAYHV **Charge** 3 **Spectral Angle** 0.66

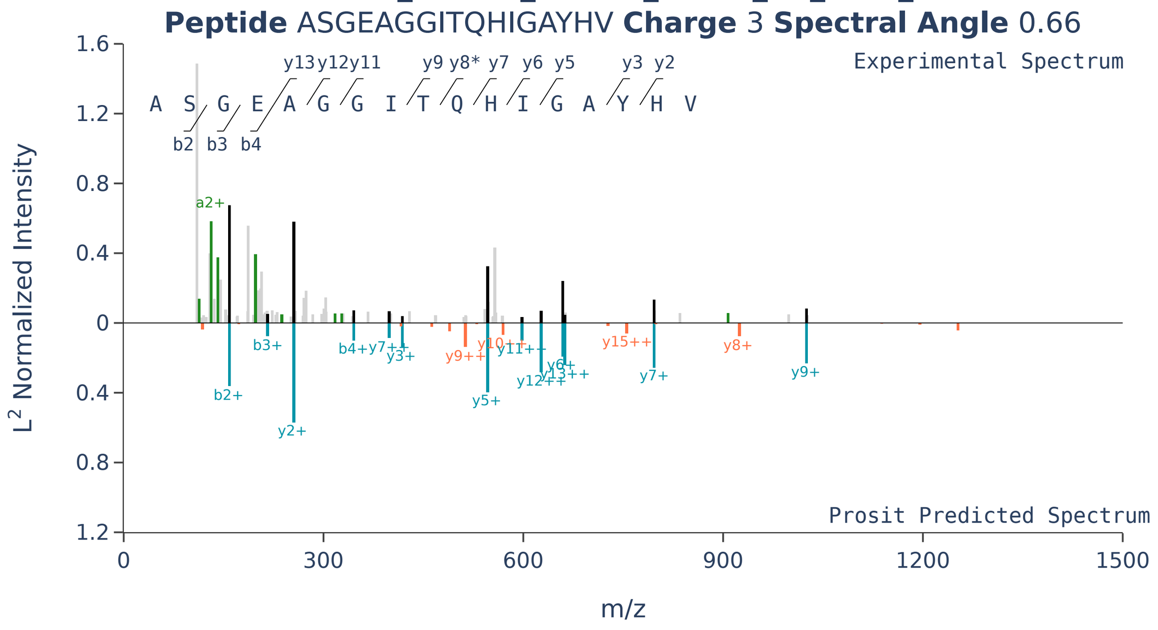

**Source** HRoetschke_211222_160123_HFGoe_IF2_b1R1_4h **Scan** 20290
**Peptide** GRGPVATVLV **Charge** 2 **Spectral Angle** 0.79

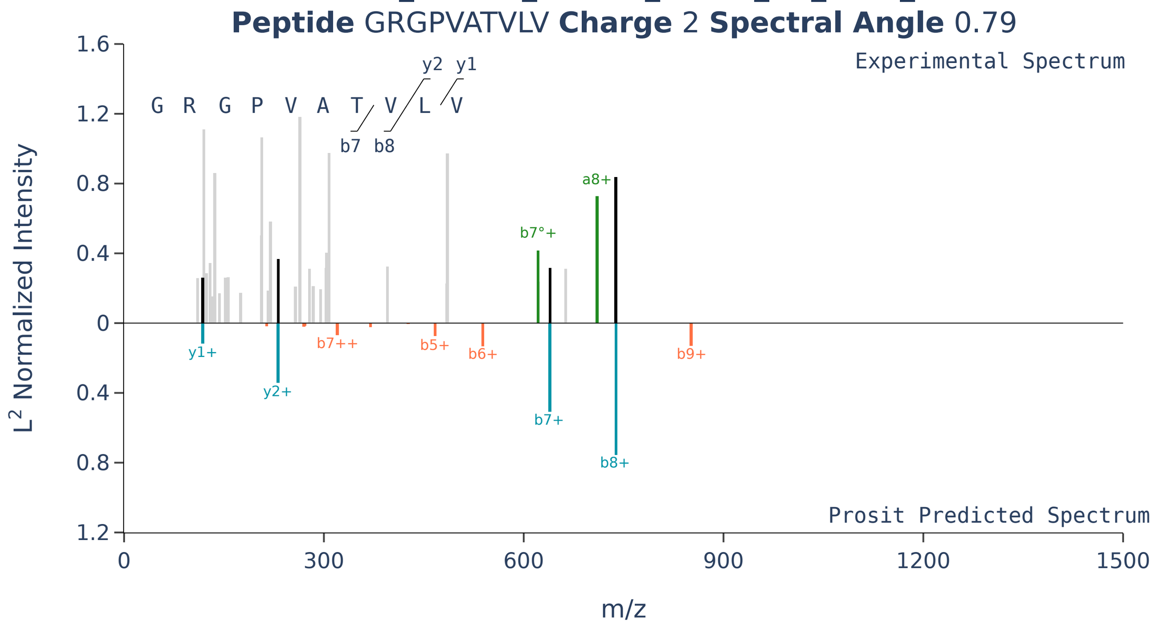

**Source** HRoetschke_211222_160123_HFGoe_IF2_b1R1_4h **Scan** 9381
**Peptide** AEEARRMAEENKWTDNAEPTE **Charge** 4 **Spectral Angle** 0.77

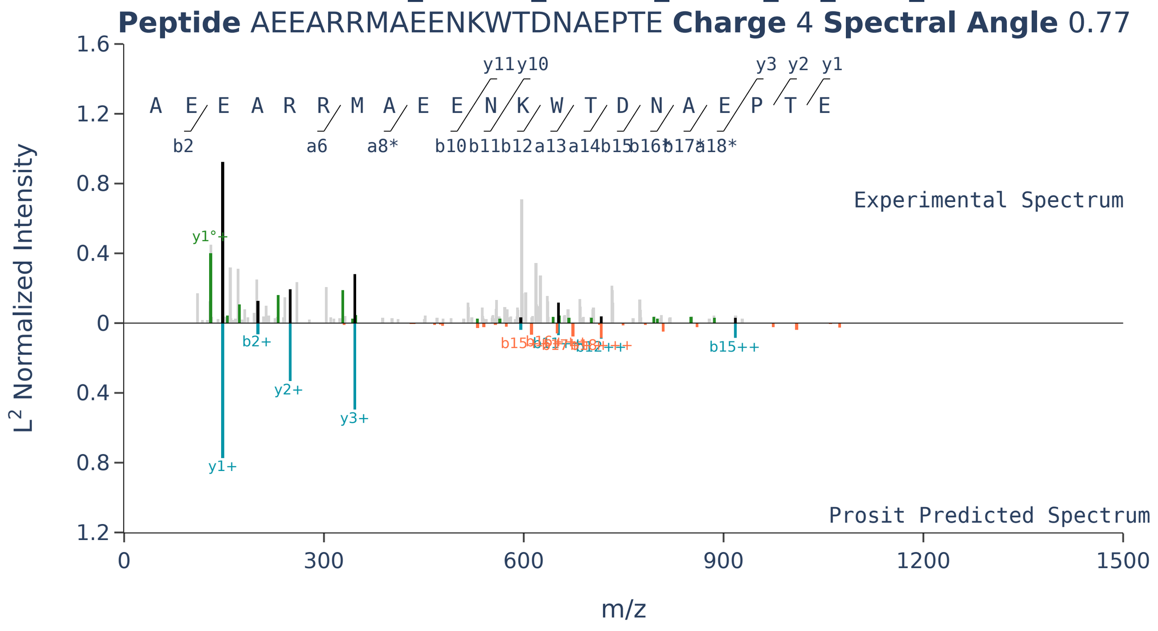

**Source** HRoetschke_211222_160123_HFGoe_IF2_b1R1_4h **Scan** 12420
**Peptide** KVASGEAGGITQHIGA **Charge** 3 **Spectral Angle** 0.61

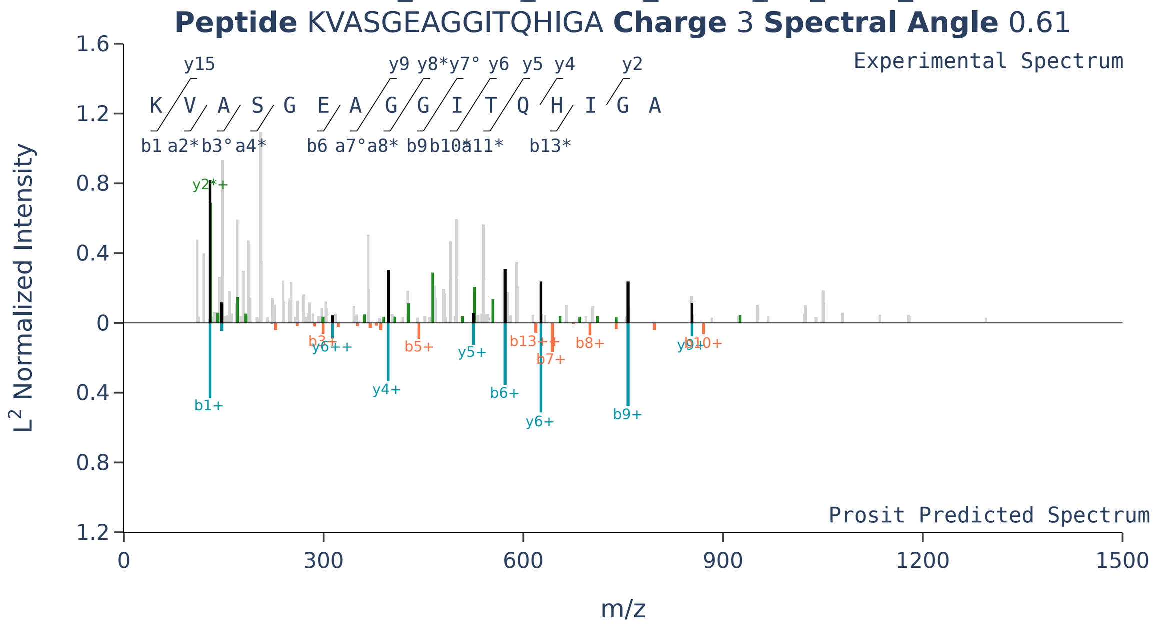

**Source** HRoetschke_211222_160123_HFGoe_IF2_b1R1_4h **Scan** 13318
**Peptide** KAVRKGMASGAVIESF **Charge** 3 **Spectral Angle** 0.69

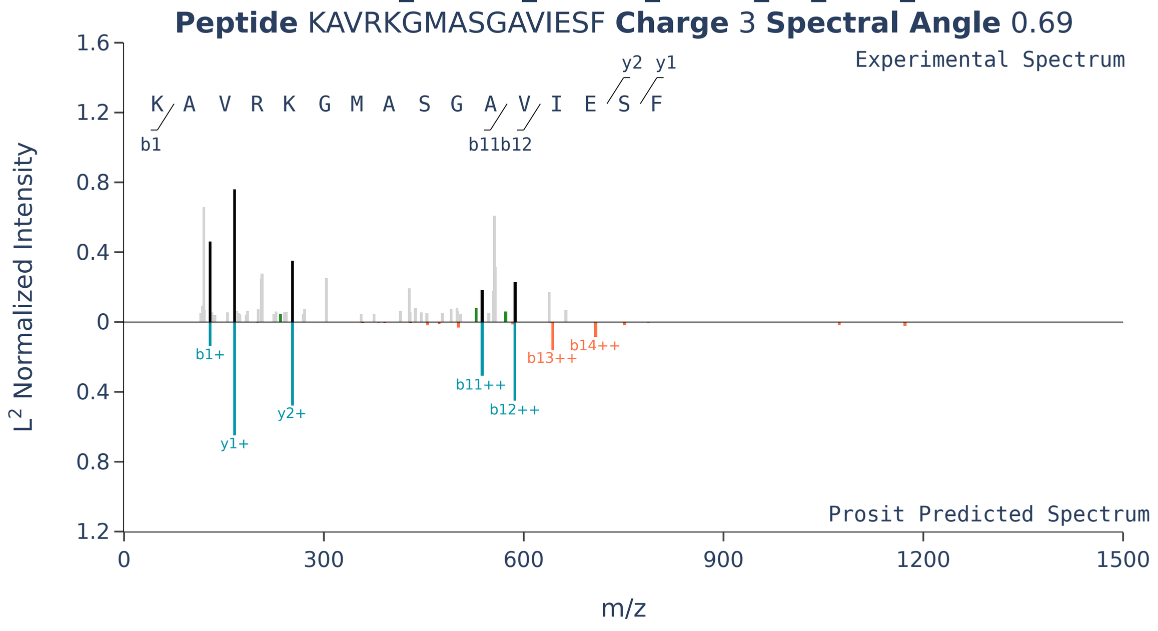

**Source** HRoetschke_211222_160123_HFGoe_IF2_b1R1_4h **Scan** 10514
**Peptide** KTRSTLNL **Charge** 2 **Spectral Angle** 0.51

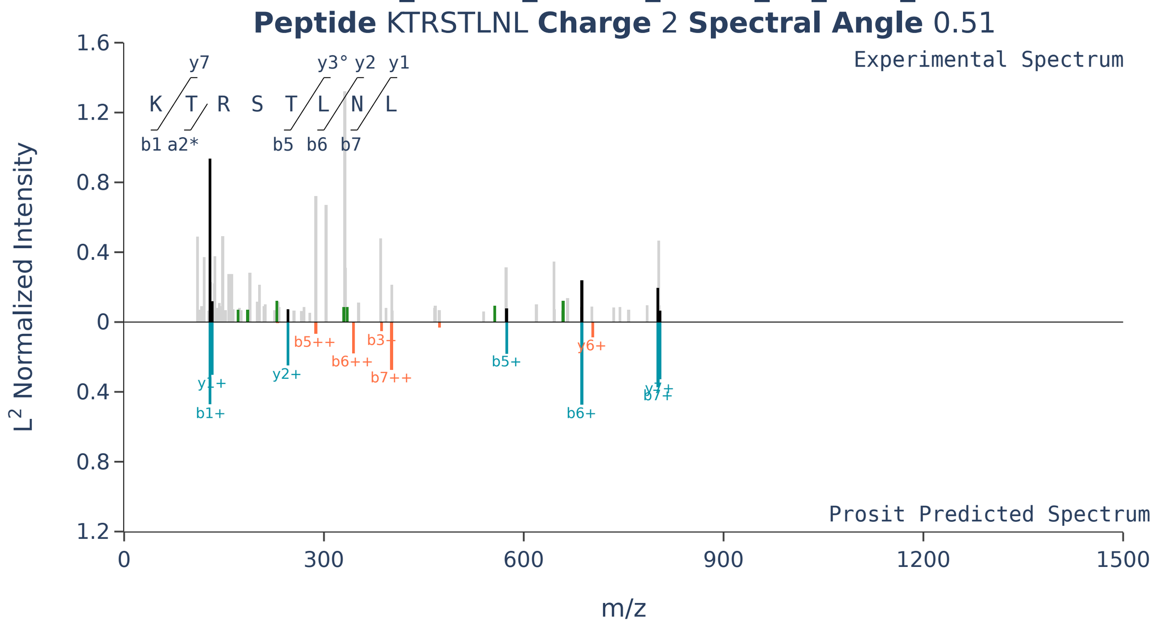

**Source** HRoetschke_211222_160123_HFGoe_IF2_b2R2_2h **Scan** 15677
**Peptide** AIQHAKAAQVPVVV **Charge** 3 **Spectral Angle** 0.65

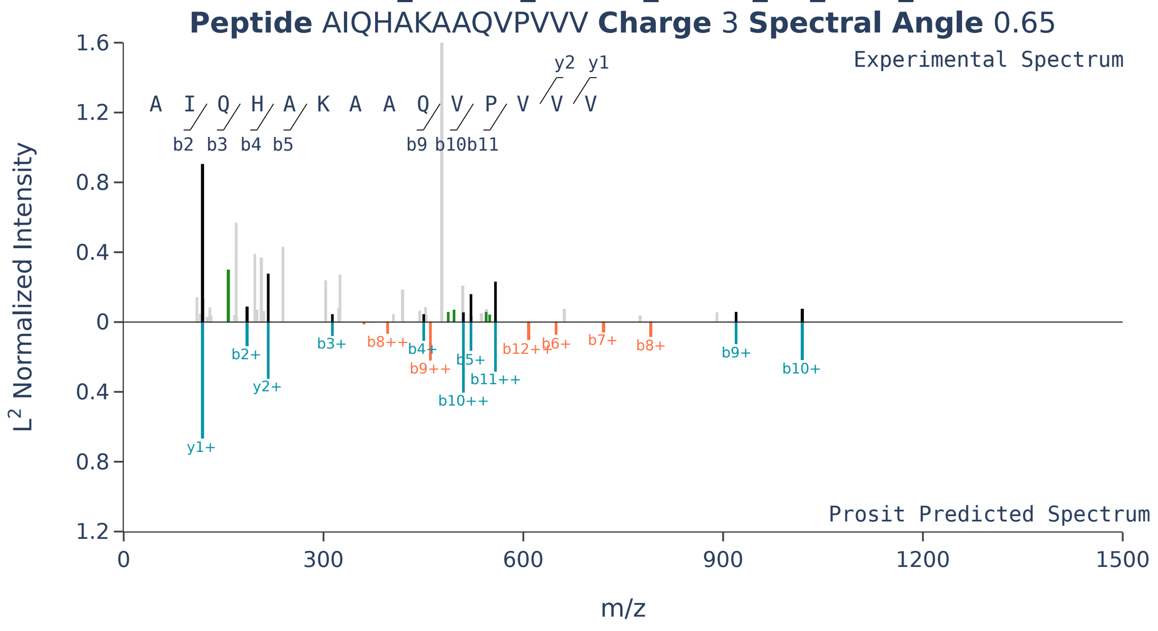

**Source** HRoetschke_211222_160123_HFGoe_IF2_b1R1_2h **Scan** 5830
**Peptide** GIRKSADDSV **Charge** 2 **Spectral Angle** 0.66

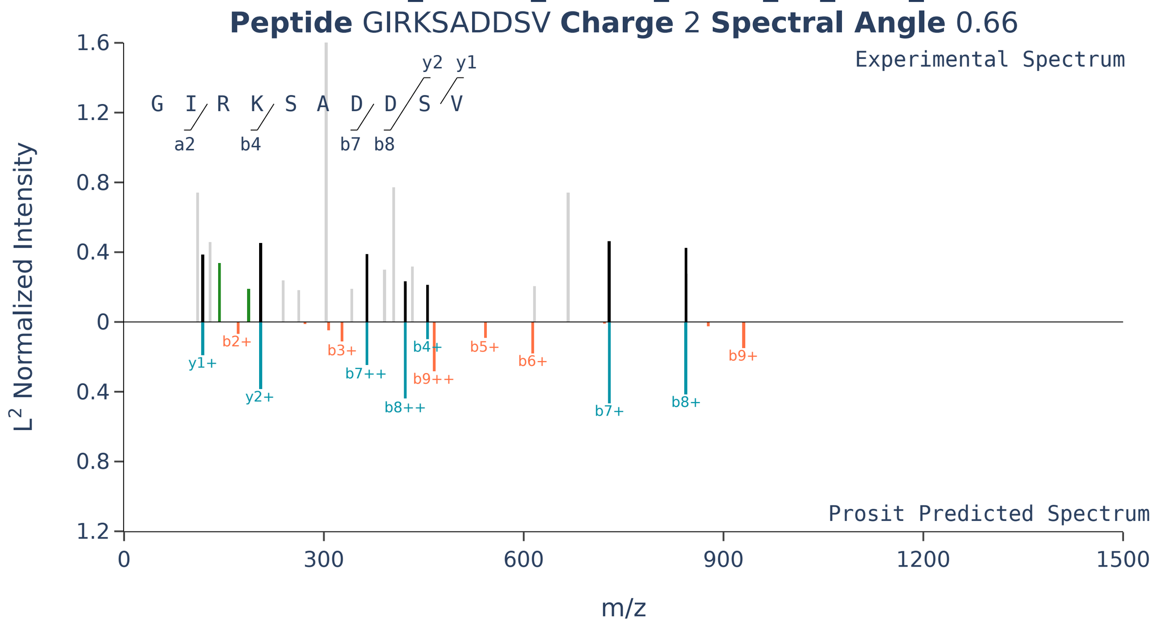

**Source** HRoetschke_211222_160123_HFGoe_IF2_b2R2_4h **Scan** 18572
**Peptide** GETITVGELAN **Charge** 2 **Spectral Angle** 0.62

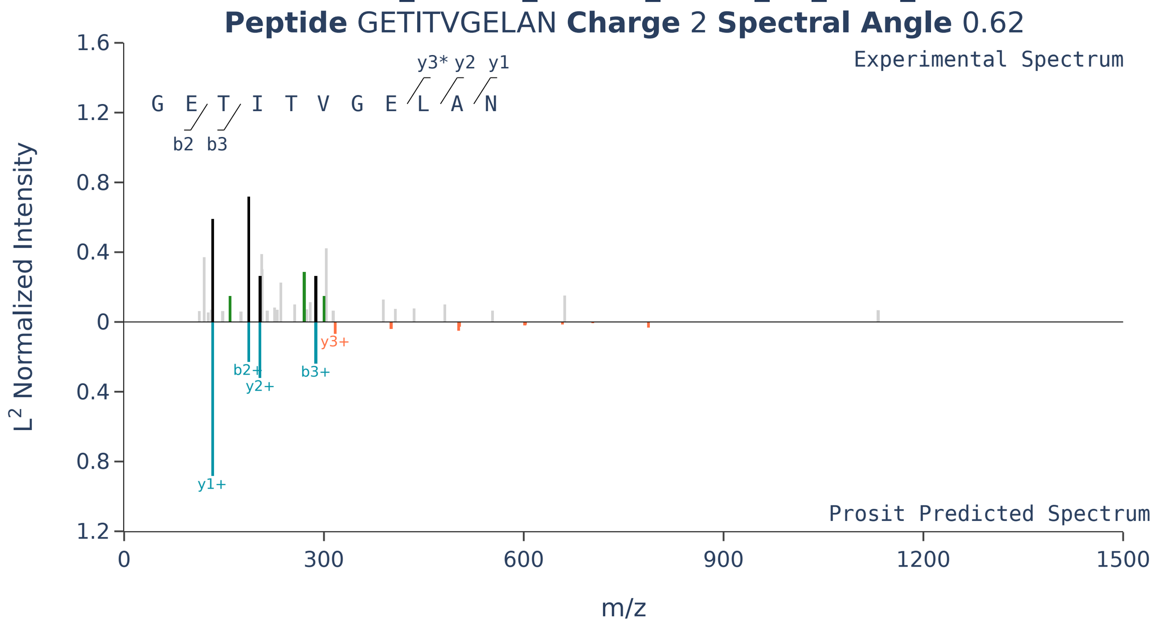

**Source** HRoetschke_211222_160123_HFGoe_IF2_b2R2_4h **Scan** 16320
**Peptide** DRDTGAAAEPRAPVVTIM **Charge** 3 **Spectral Angle** 0.73

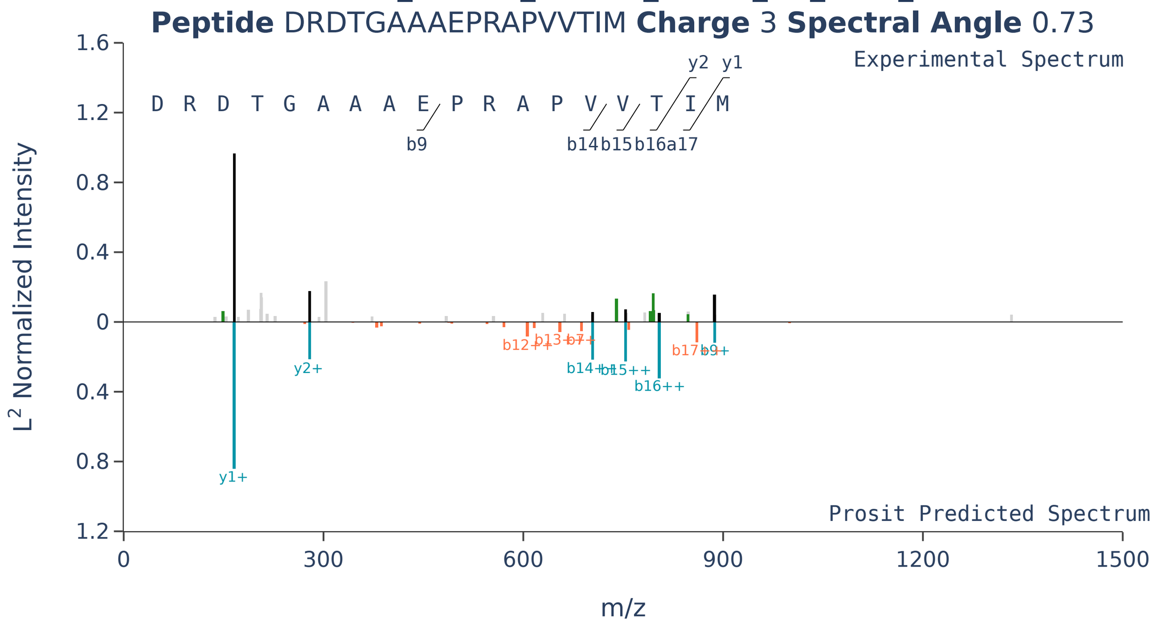

**Source** HRoetschke_211222_160123_HFGoe_IF2_b1R1_24h **Scan** 19251
**Peptide** DGVMPQTIEAIQ **Charge** 2 **Spectral Angle** 0.87

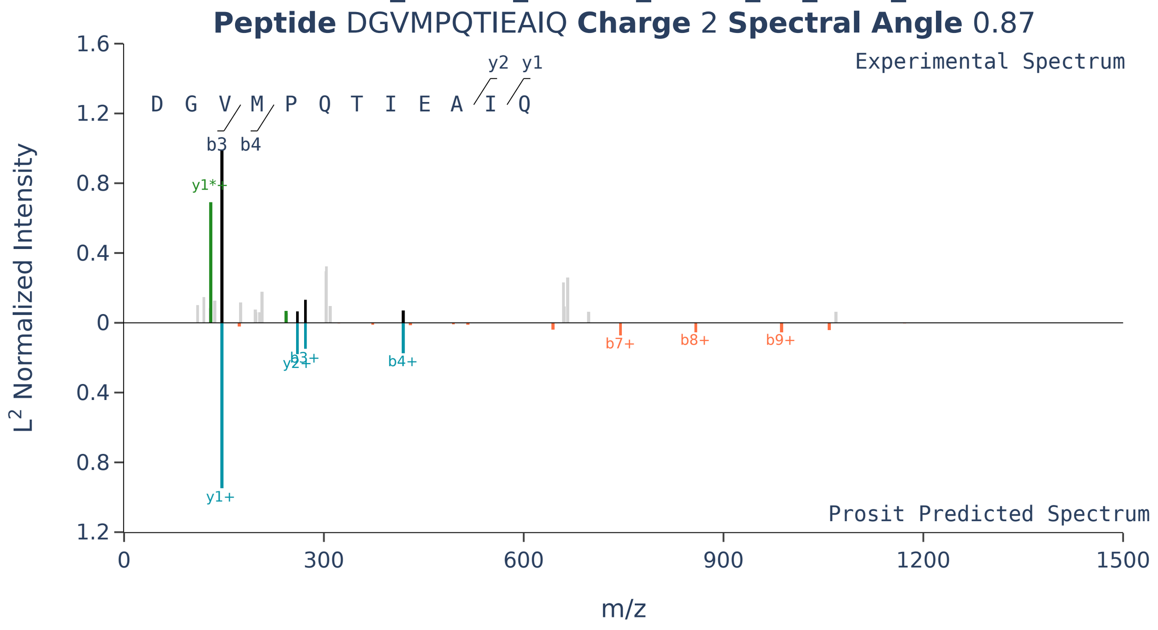


**Source** HRoetschke_211222_160123_HFGoe_IF2_b2R2_4h **Scan** 20185
**Peptide** AKAAQVPVVVAV **Charge** 2 **Spectral Angle** 0.78

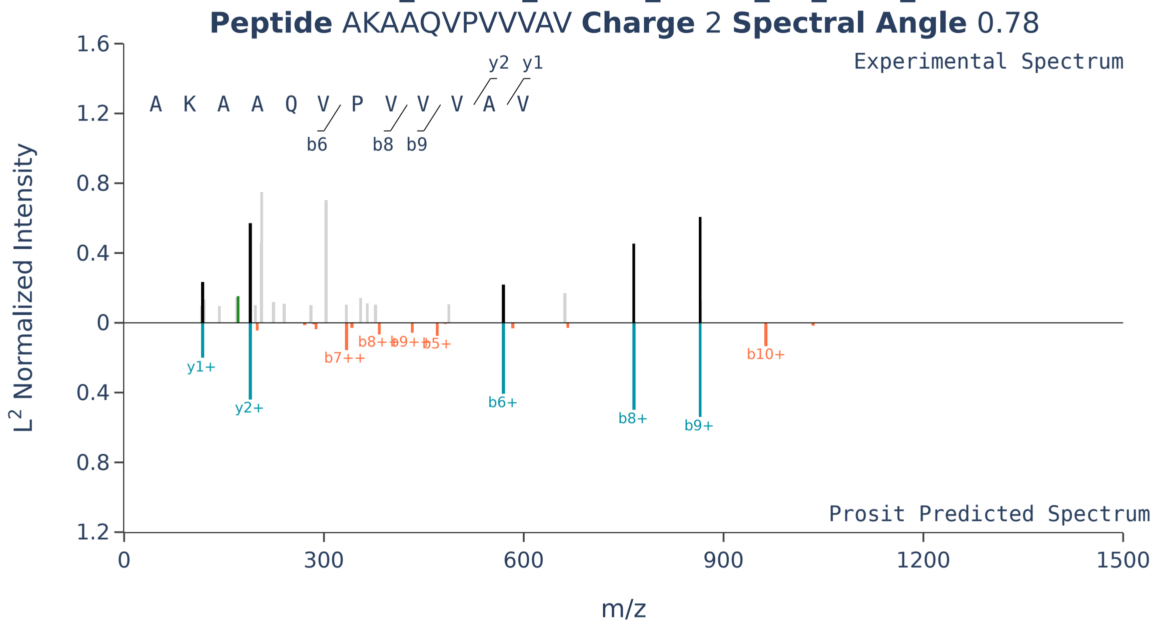


**Source** HRoetschke_211222_160123_HFGoe_IF2_b1R1_2h **Scan** 15156
**Peptide** HGKTSLLDY **Charge** 2 **Spectral Angle** 0.6

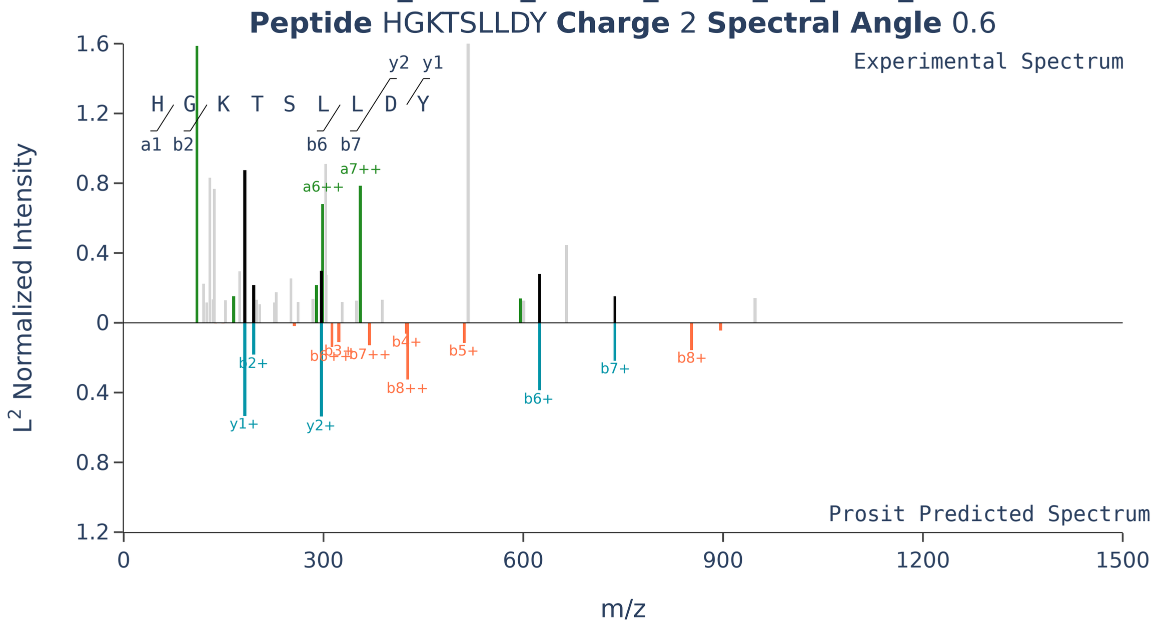


**Source** HRoetschke_211222_160123_HFGoe_IF2_b1R2_24h **Scan** 8249
**Peptide** NSGPDKLTLQRKT **Charge** 4 **Spectral Angle** 0.65

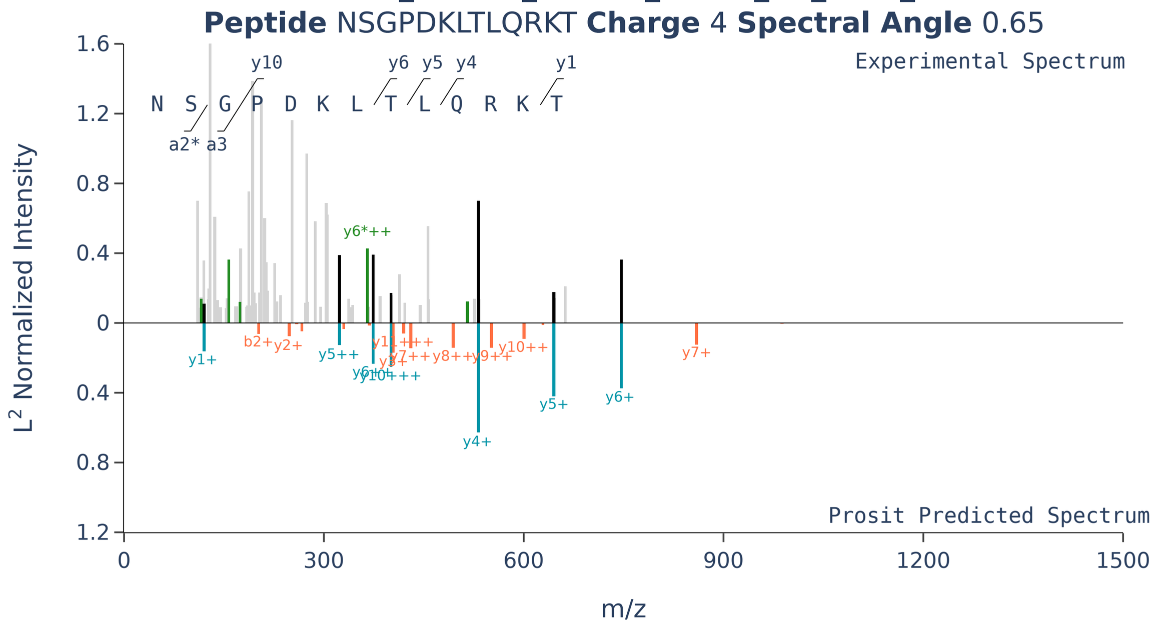


**Source** HRoetschke_211222_160123_HFGoe_IF2_b1R1_4h **Scan** 9659
**Peptide** ELANKMAVKGSQVIKA **Charge** 4 **Spectral Angle** 0.84

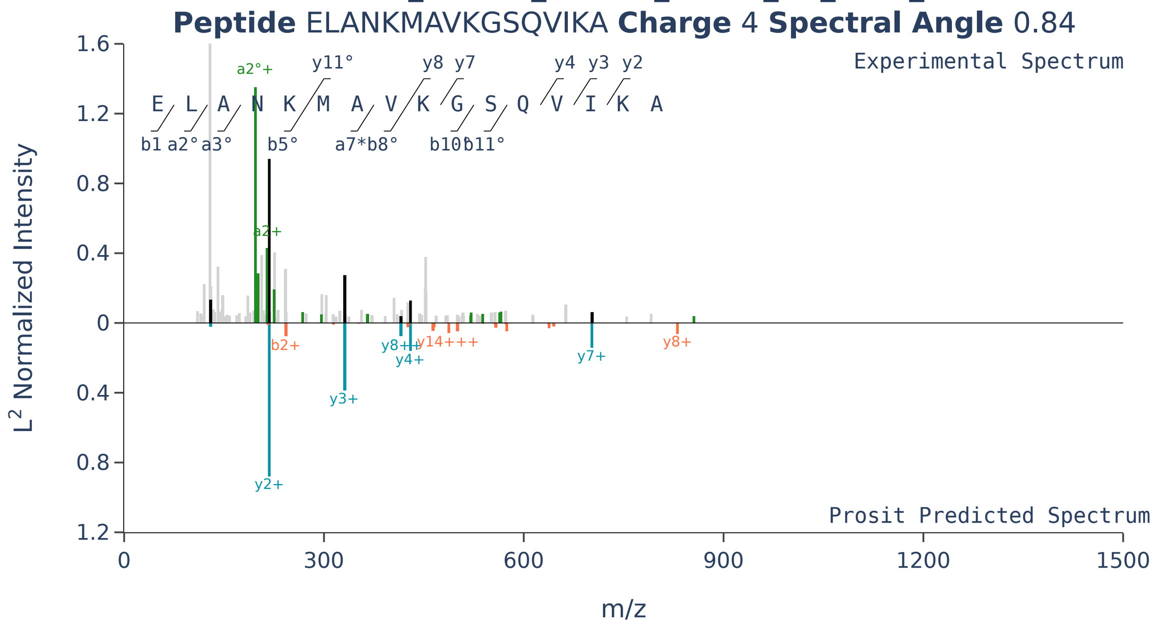


**Source** HRoetschke_211222_160123_HFGoe_IF2_b1R1_4h **Scan** 12927
**Peptide** GSQVIKAMMK **Charge** 2 **Spectral Angle** 0.57

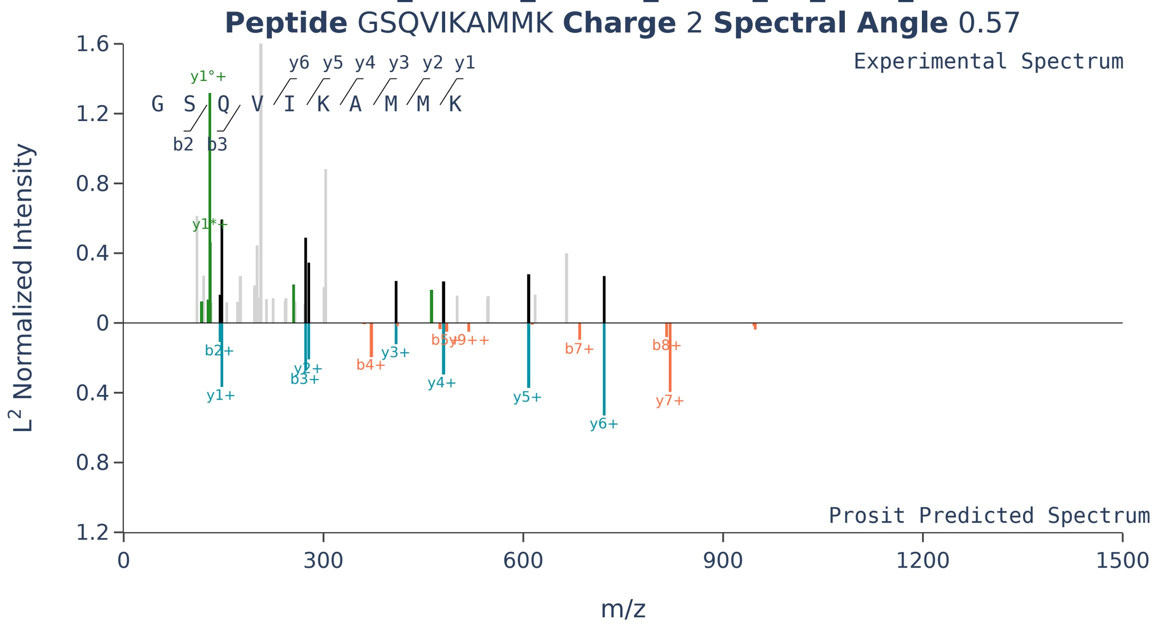


**Source** HRoetschke_211222_160123_HFGoe_IF2_b2R1_24h **Scan** 13450
**Peptide** GETITVG **Charge** 1 **Spectral Angle** 0.82

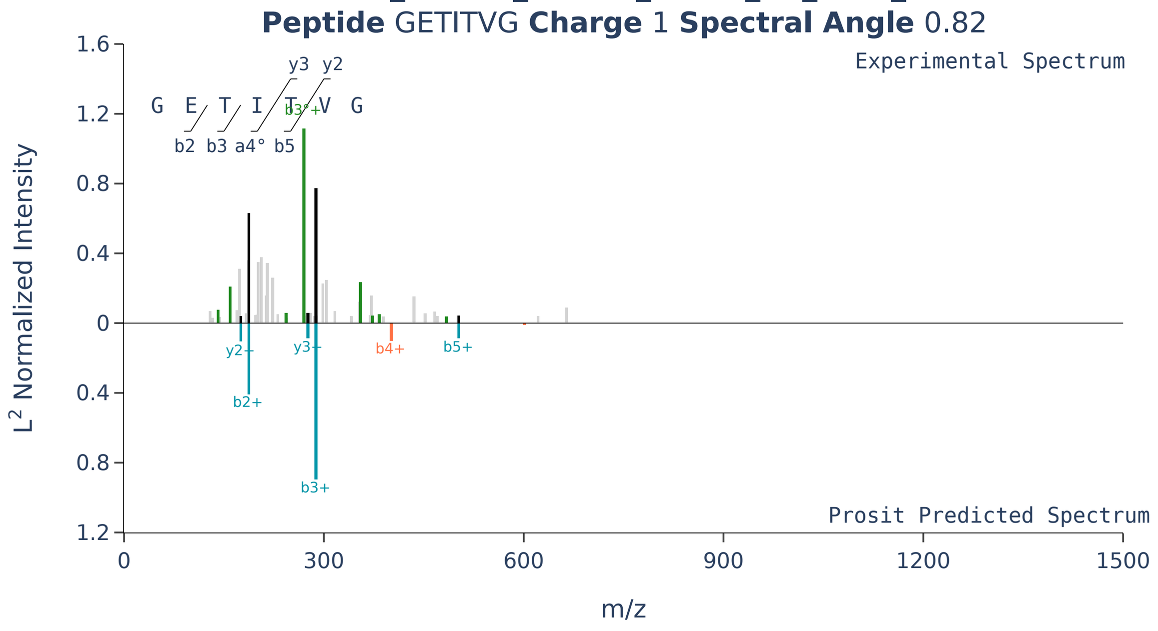


**Source** HRoetschke_211222_160123_HFGoe_IF2_b1R1_4h **Scan** 10559
**Peptide** ELKAVRKGMASGAVI **Charge** 3 **Spectral Angle** 0.67

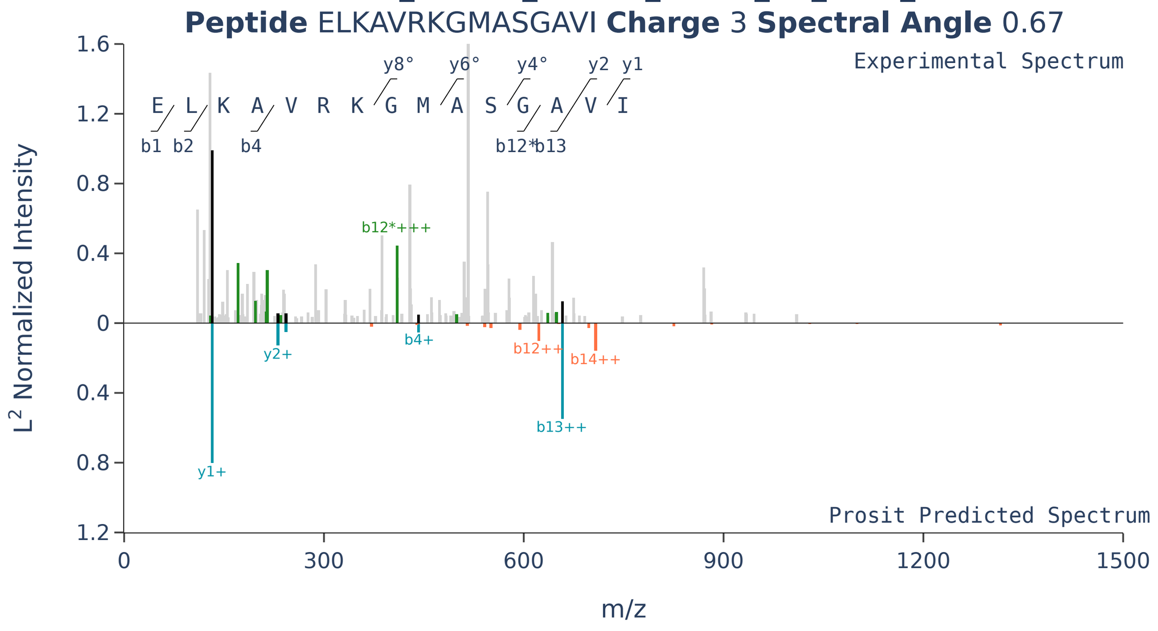


**Source** HRoetschke_211222_160123_HFGoe_IF2_b1R1_4h **Scan** 12986
**Peptide** VALYRQGKFREV **Charge** 4 **Spectral Angle** 0.85

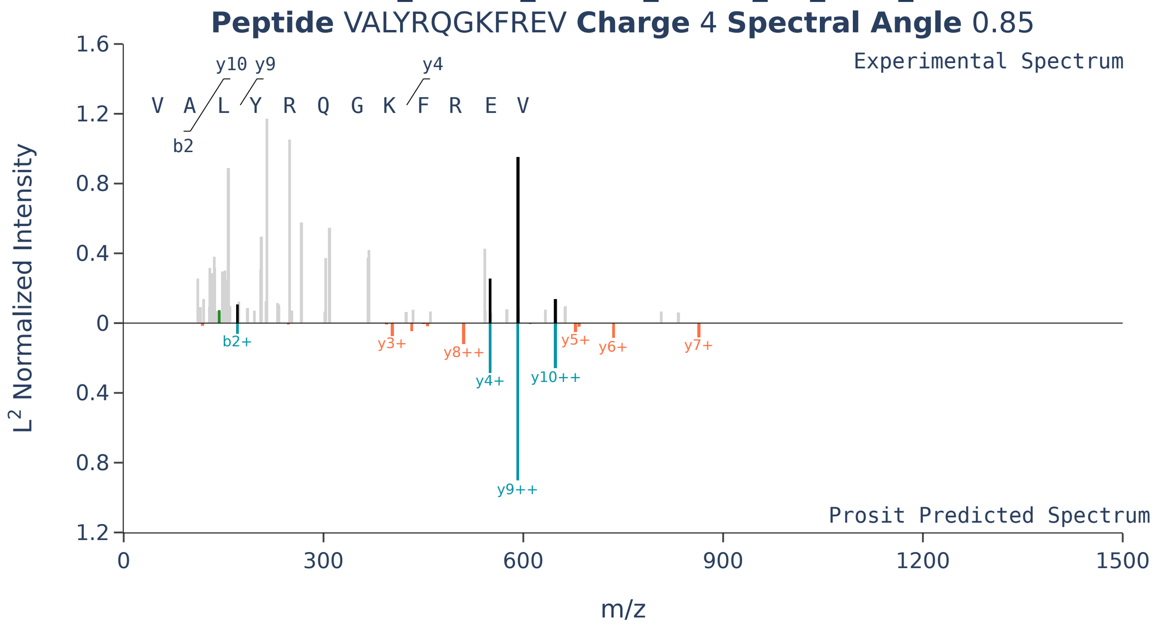


**Source** HRoetschke_211222_160123_HFGoe_IF2_b1R1_4h **Scan** 17900
**Peptide** SFLDKGRGPVATV **Charge** 3 **Spectral Angle** 0.83

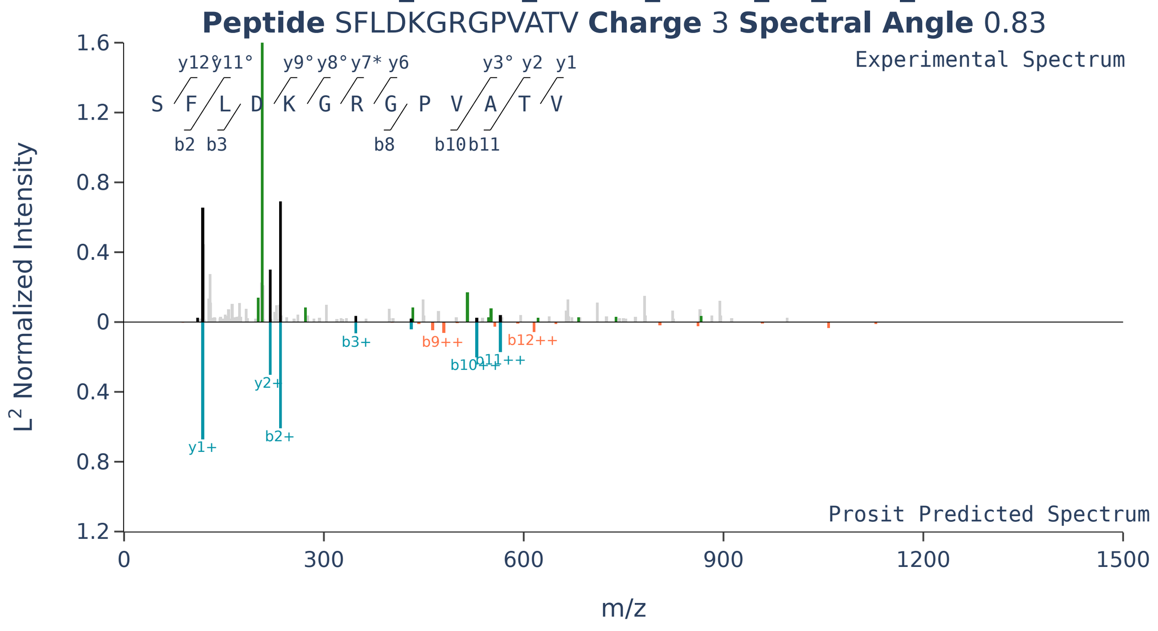


**Source** HRoetschke_211222_160123_HFGoe_IF2_b1R2_4h **Scan** 15034
**Peptide** LDKGRGPVATVLVRE **Charge** 3 **Spectral Angle** 0.77

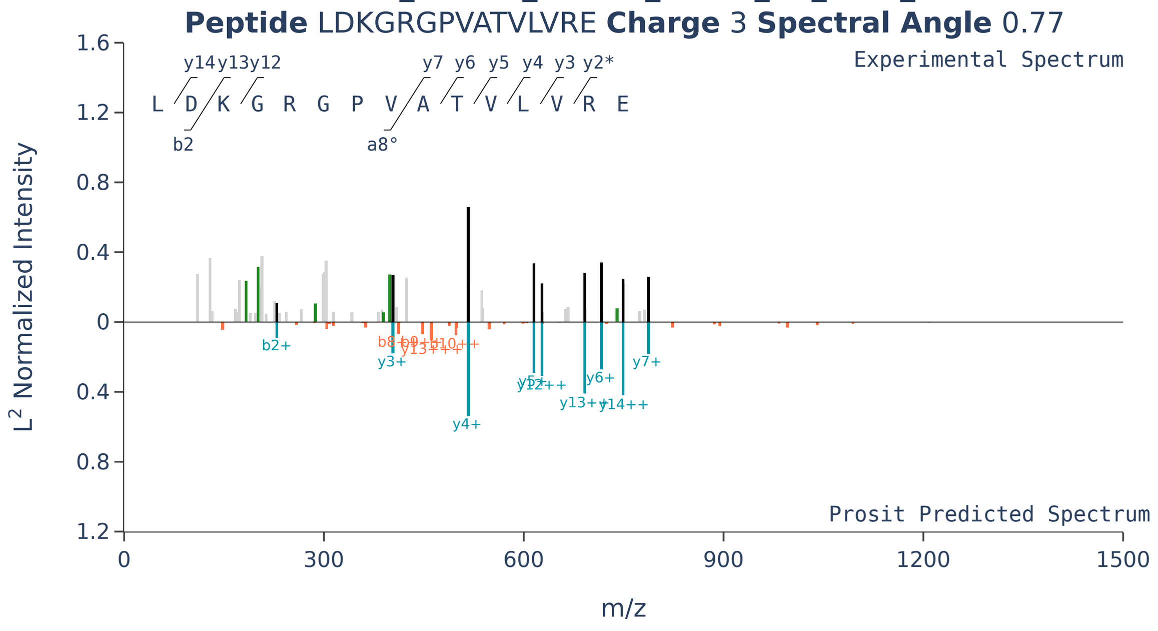


**Source** HRoetschke_211222_160123_HFGoe_IF2_b1R1_4h **Scan** 19474
**Peptide** PVVVAVNKI **Charge** 2 **Spectral Angle** 0.76

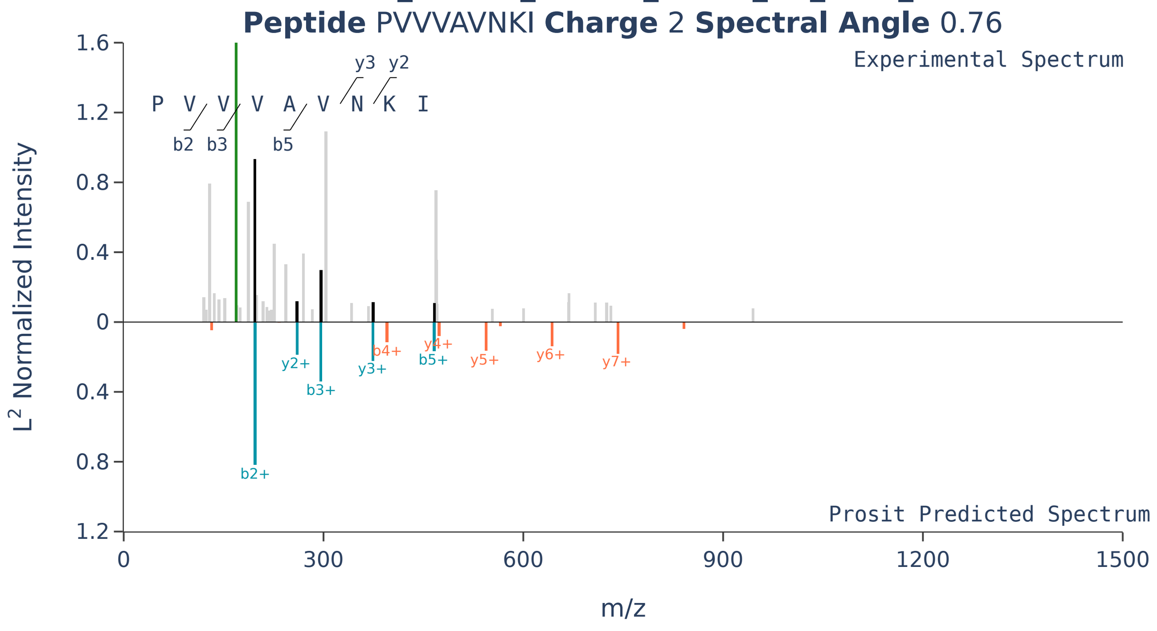


**Source** HRoetschke_211222_160123_HFGoe_IF2_b1R1_4h **Scan** 11880
**Peptide** NPIRVLRD **Charge** 2 **Spectral Angle** 0.47

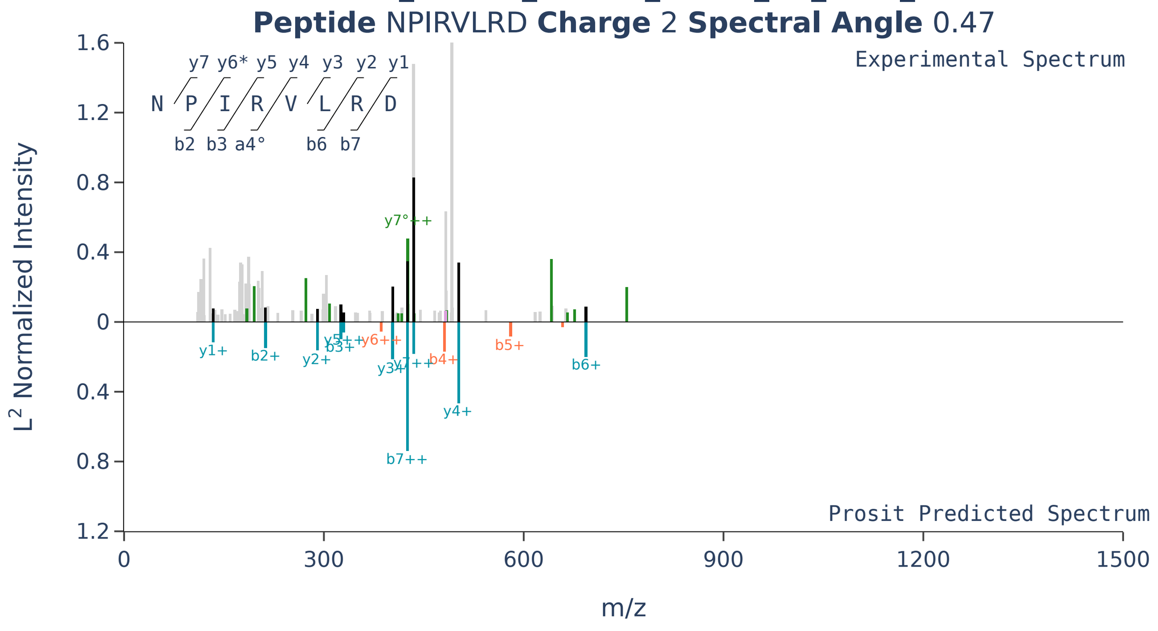


**Source** HRoetschke_211222_160123_HFGoe_IF2_b1R2_2h **Scan** 12659
**Peptide** QKPAQAVNRDVVIGE **Charge** 2 **Spectral Angle** 0.81

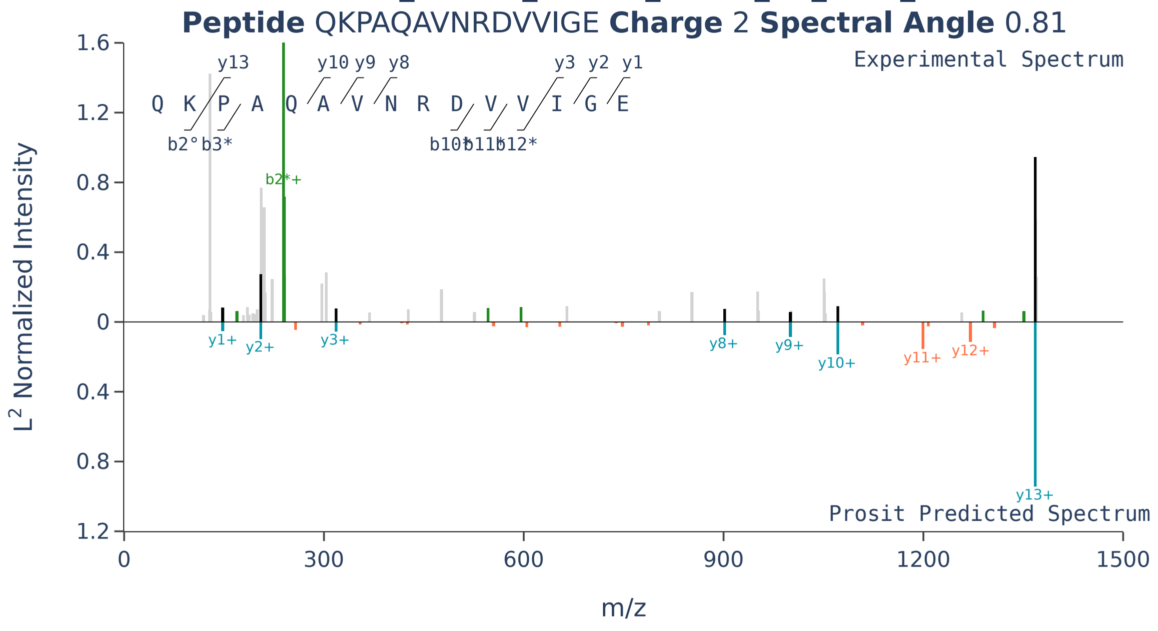


**Source** HRoetschke_211222_160123_HFGoe_IF2_b2R1_24h **Scan** 14066
**Peptide** HVDHGKTSLLDY **Charge** 3 **Spectral Angle** 0.76

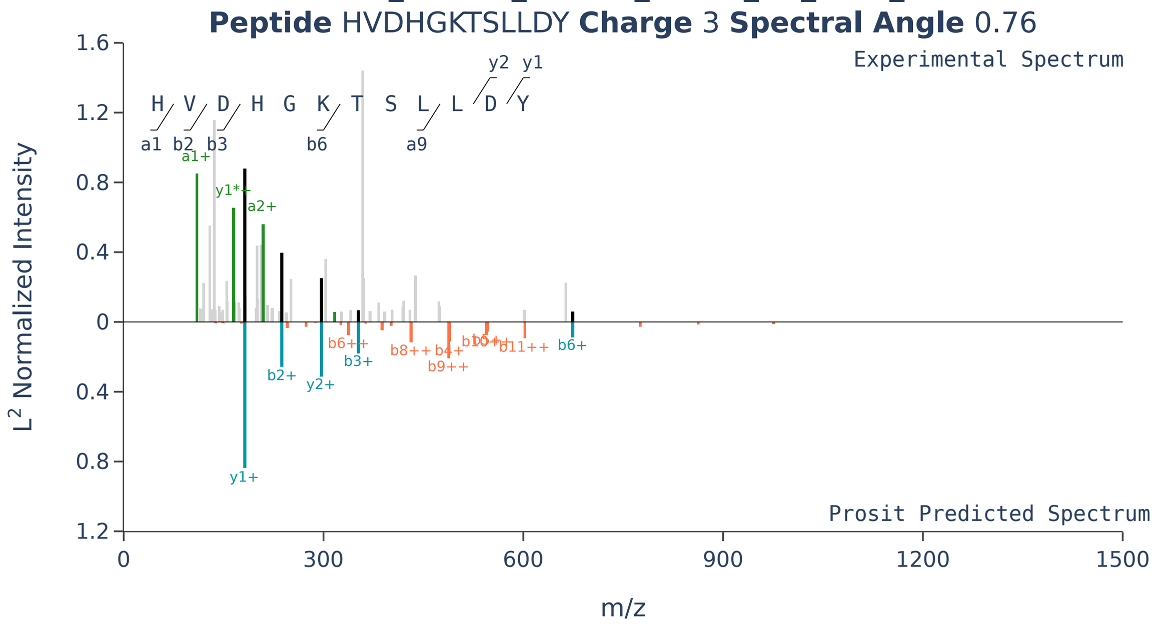

**Source** HRoetschke_211222_160123_HFGoe_IF2_b1R2_24h **Scan** 6120
**Peptide** AGIRKSADDSVSAQ **Charge** 2 **Spectral Angle** 0.68

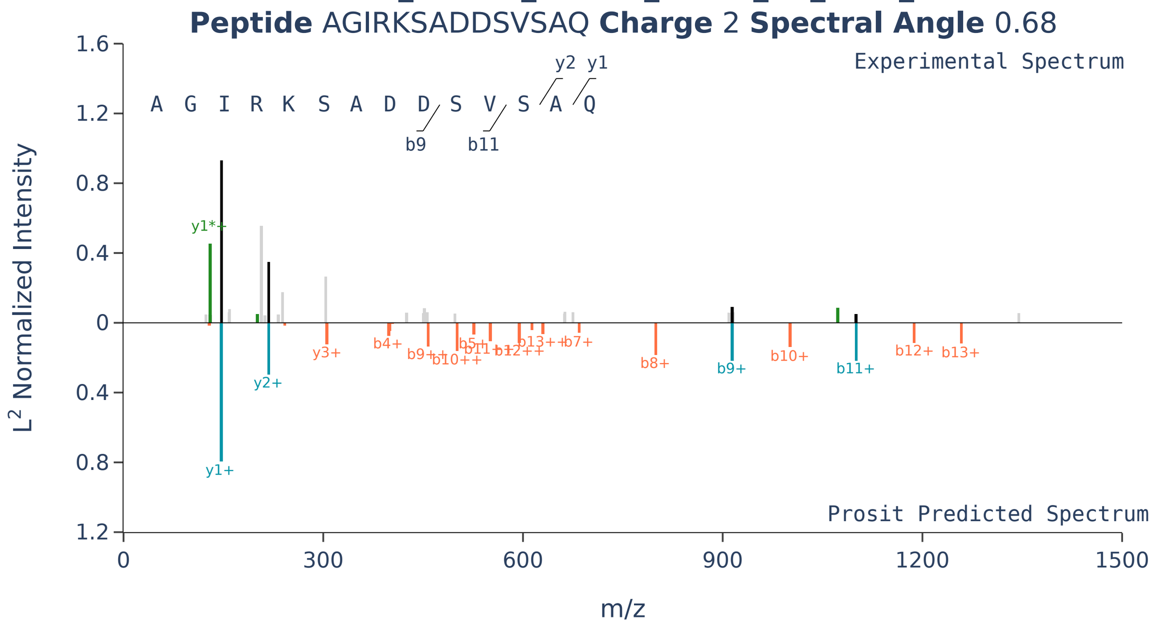


**Source** HRoetschke_211222_160123_HFGoe_IF2_b1R1_4h **Scan** 11232
**Peptide** LELKAVRKGMA **Charge** 3 **Spectral Angle** 0.73

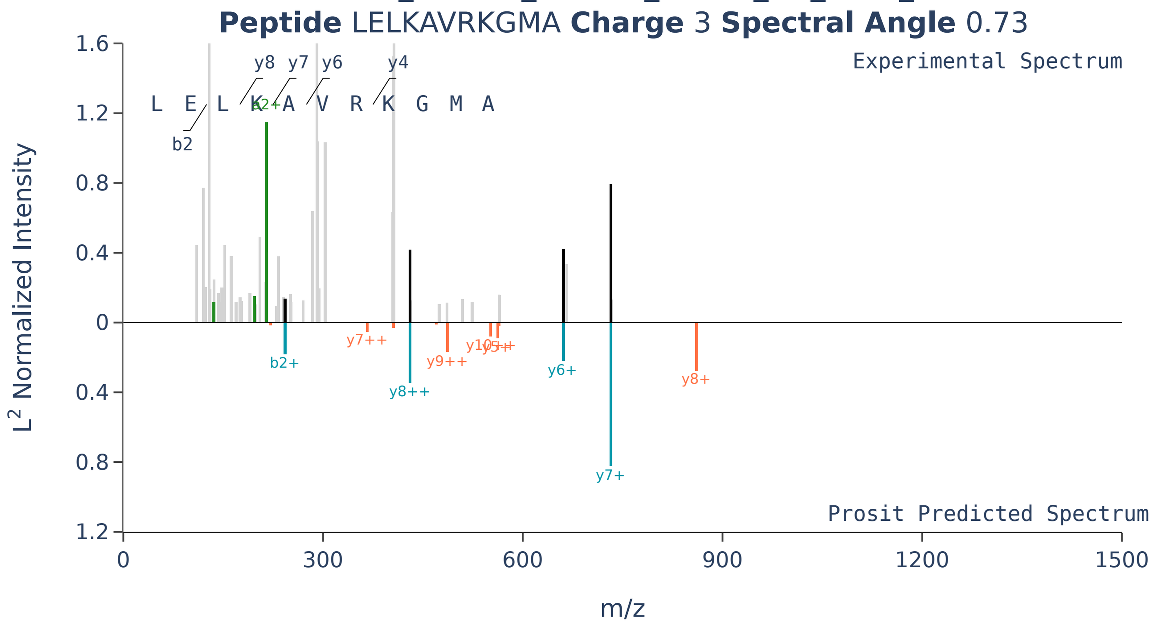


**Source** HRoetschke_211222_160123_HFGoe_IF2_b1R1_4h **Scan** 15798
**Peptide** AVMSDRDTGAAAEPRAPVV **Charge** 3 **Spectral Angle** 0.61

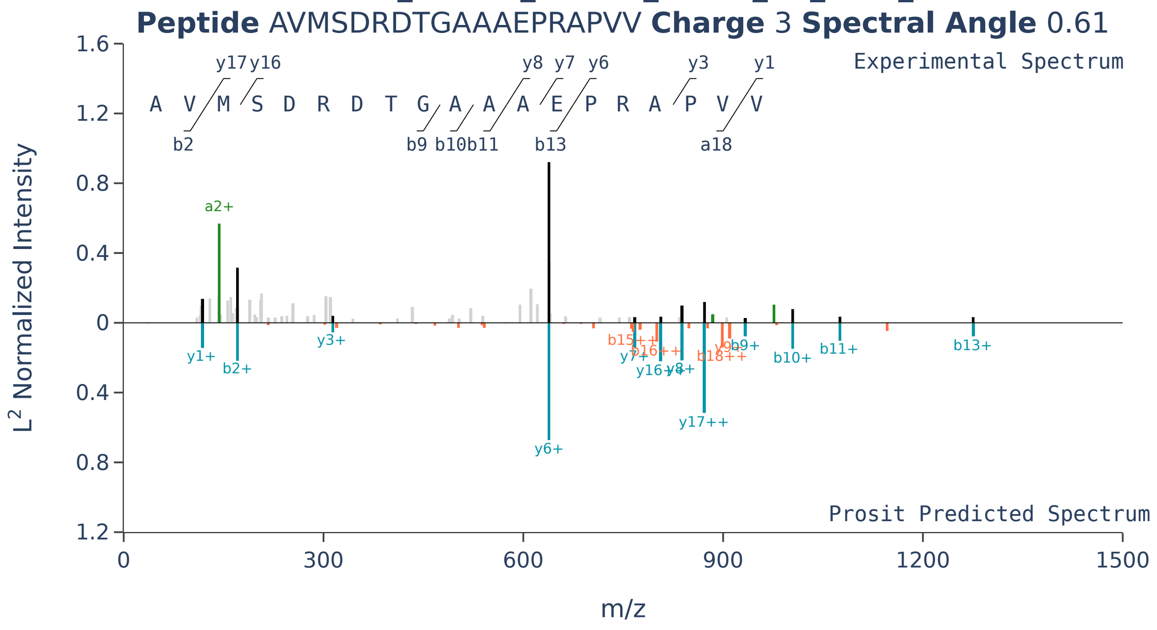


**Source** HRoetschke_211222_160123_HFGoe_IF2_b1R1_2h **Scan** 9166
**Peptide** HIGAYHVETENGM **Charge** 3 **Spectral Angle** 0.67

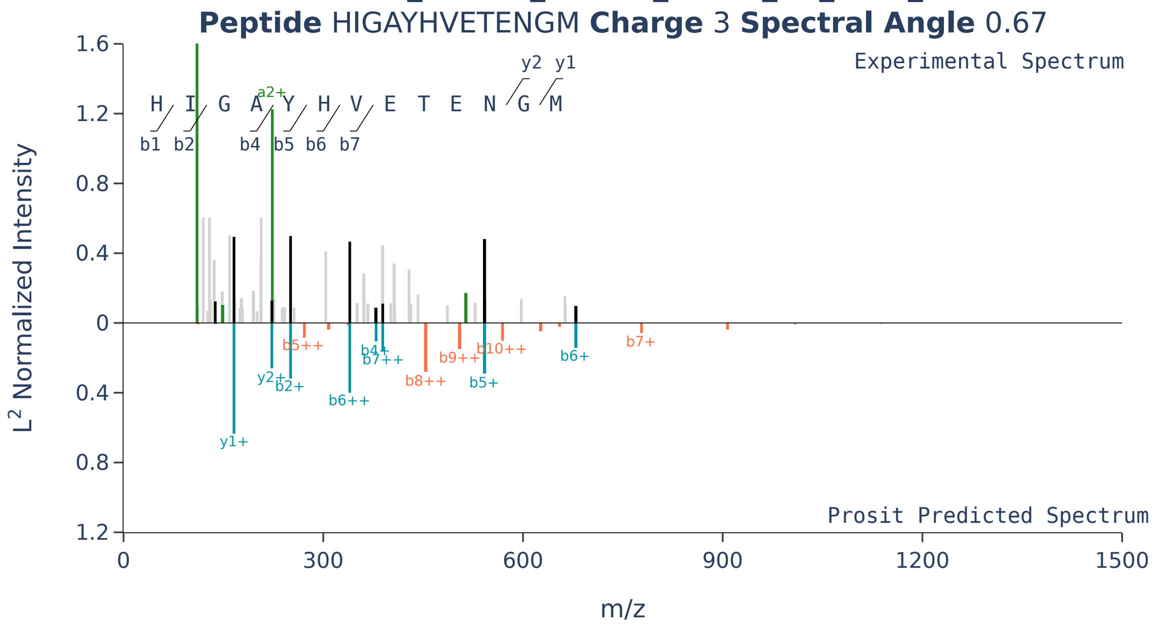


**Source** HRoetschke_211222_160123_HFGoe_IF2_b2R1_24h **Scan** 15034
**Peptide** TLNIPGTGGKS **Charge** 2 **Spectral Angle** 0.83

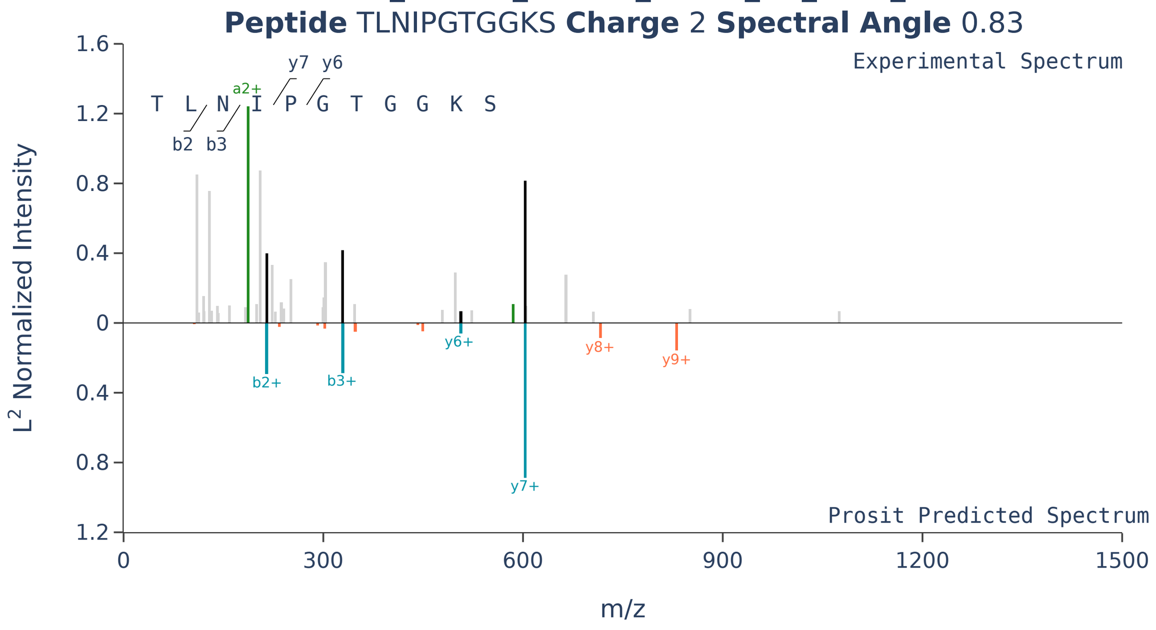


**Source** HRoetschke_211222_160123_HFGoe_IF2_b1R2_24h **Scan** 22421
**Peptide** INQVIDQETAQLV **Charge** 2 **Spectral Angle** 0.77

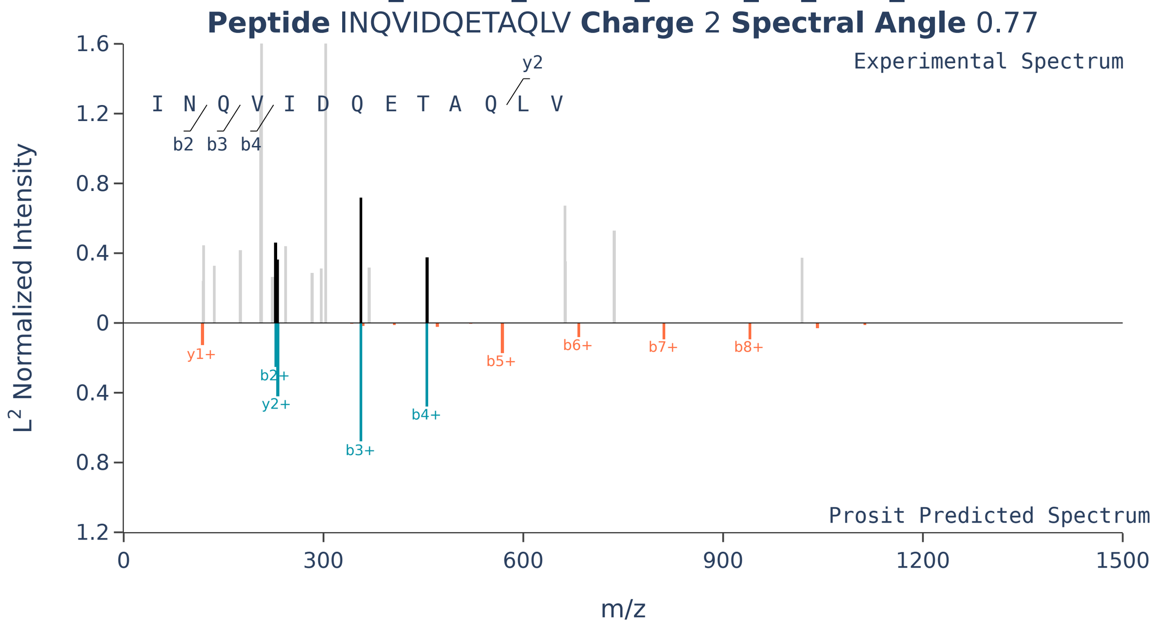


**Source** HRoetschke_211222_160123_HFGoe_IF2_b1R1_4h **Scan** 16543
**Peptide** VDHGKTSLLDY **Charge** 3 **Spectral Angle** 0.83

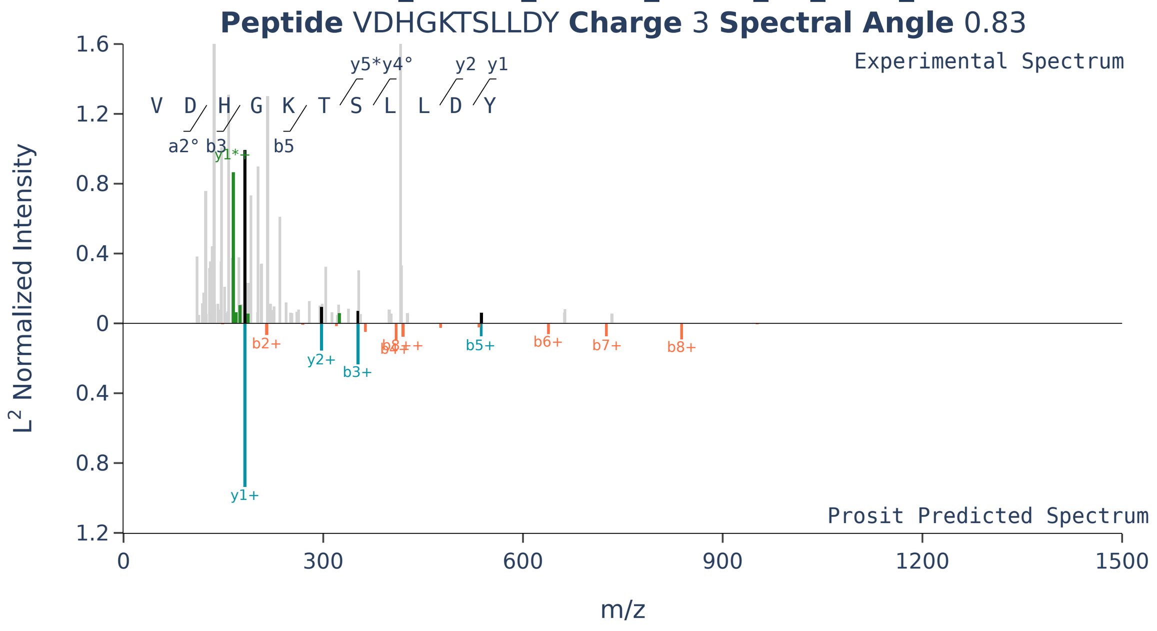


**Source** HRoetschke_211222_160123_HFGoe_IF2_b1R1_2h **Scan** 5440
**Peptide** SSDYHVTTSQHARQ **Charge** 2 **Spectral Angle** 0.76

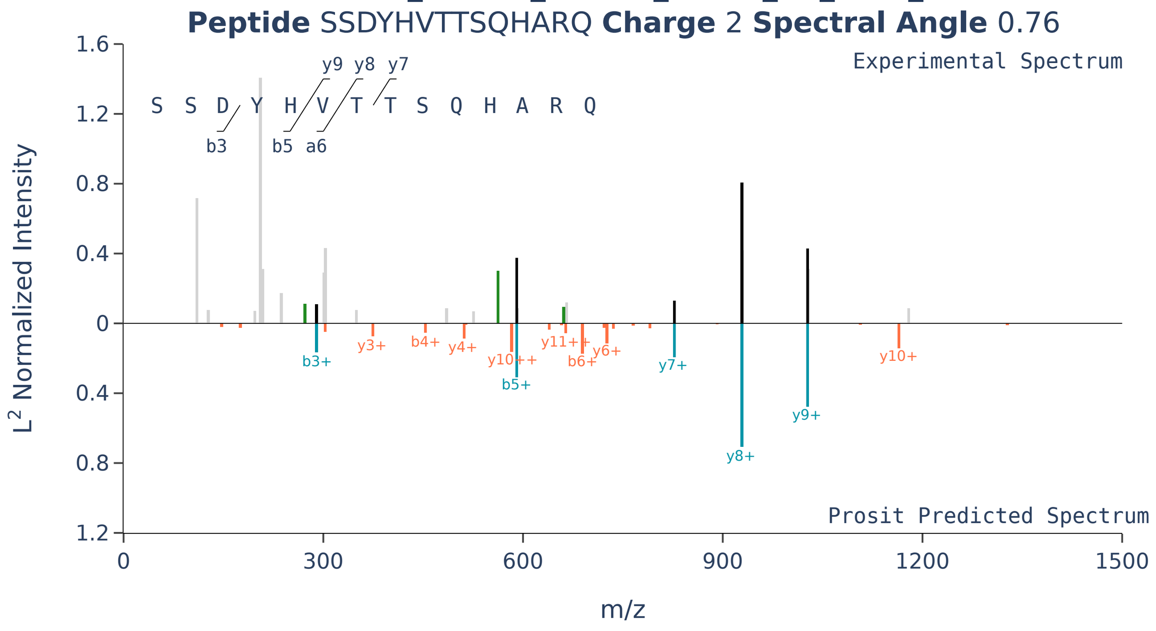


**Source** HRoetschke_211222_160123_HFGoe_IF2_b1R1_2h **Scan** 12478
**Peptide** DTPGHAAFTSMRARGAQATD **Charge** 3 **Spectral Angle** 0.78

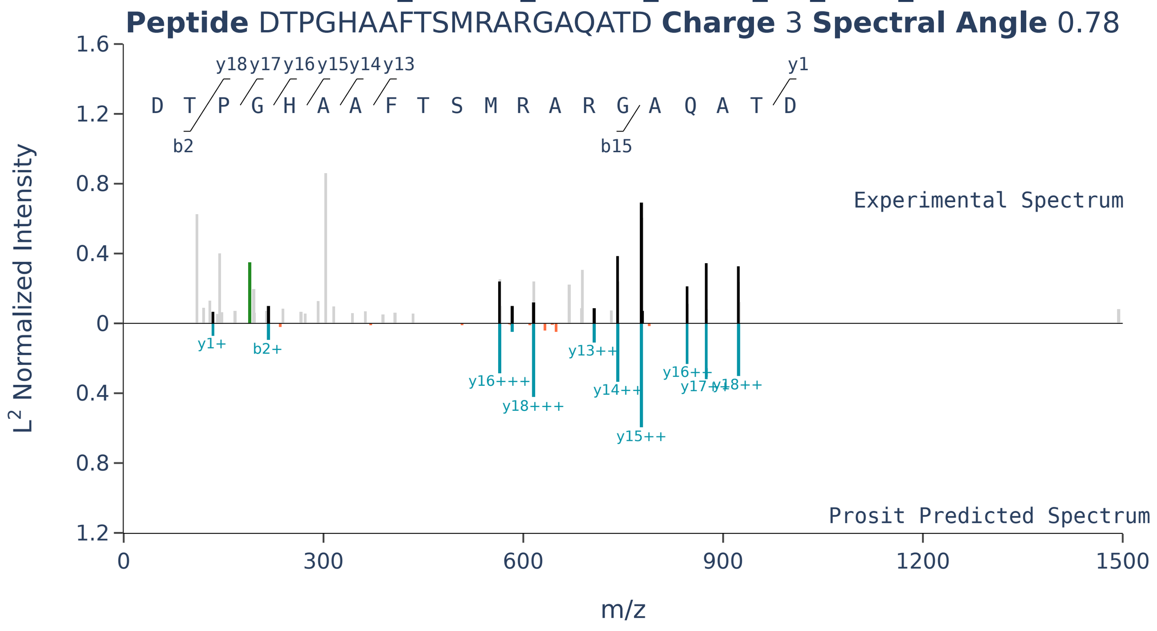


**Source** HRoetschke_211222_160123_HFGoe_IF2_b1R1_4h **Scan** 24320
**Peptide** AGTGIDELLD **Charge** 1 **Spectral Angle** 0.71

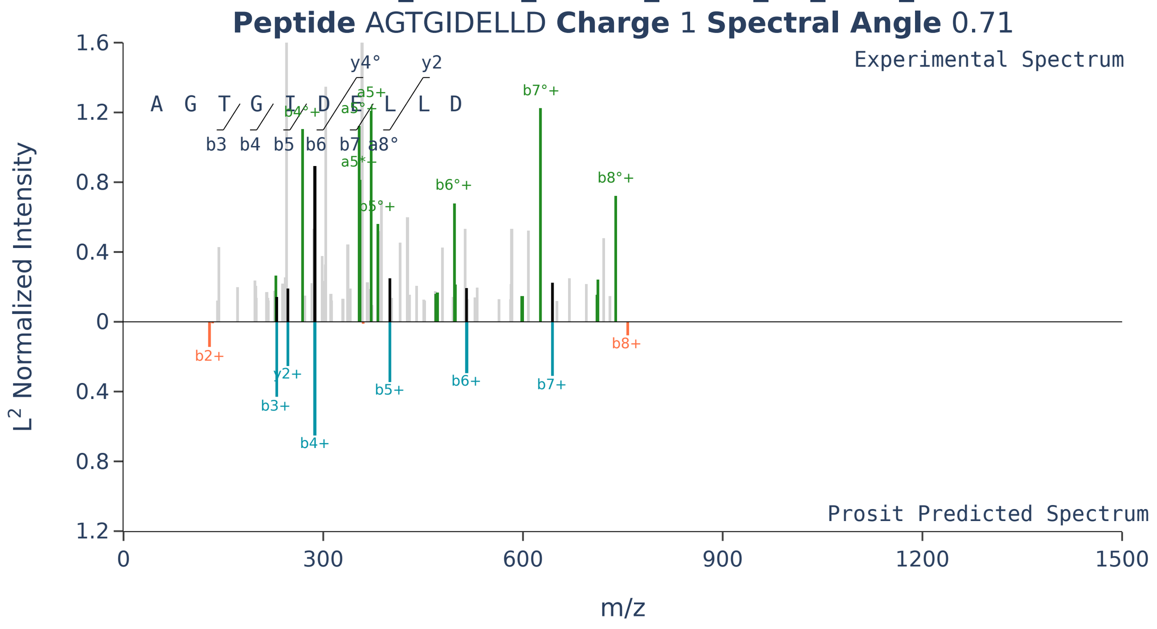


**Source** HRoetschke_211222_160123_HFGoe_IF2_b2R2_4h **Scan** 19306
**Peptide** NQVIDQETAQLVAE **Charge** 2 **Spectral Angle** 0.73

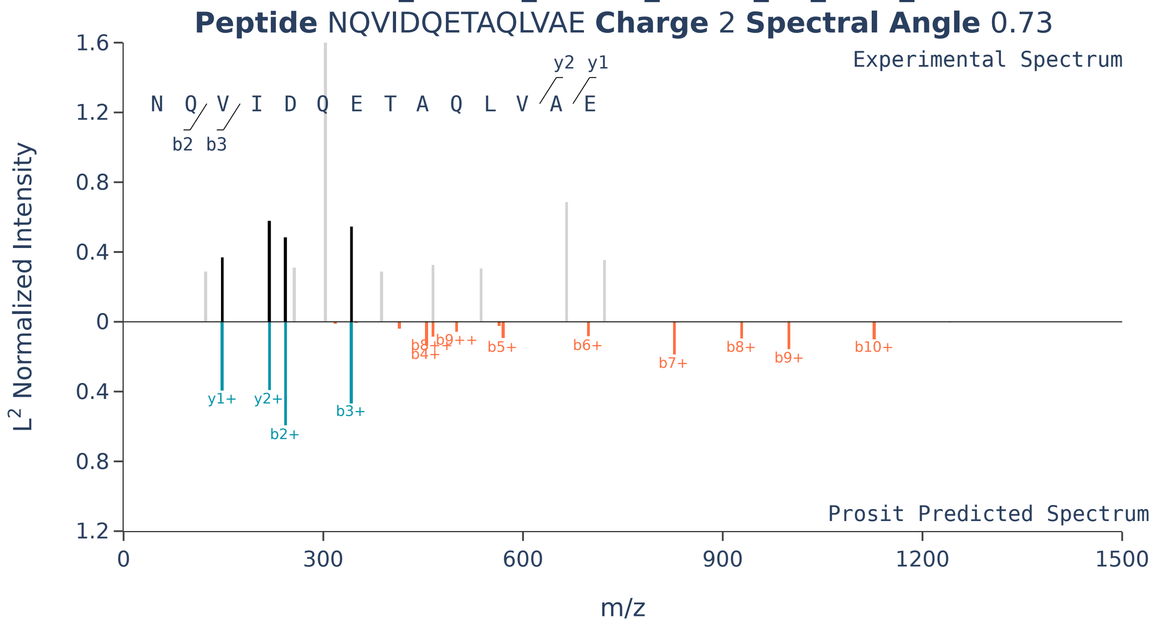


**Source** HRoetschke_211222_160123_HFGoe_IF2_b2R2_24h **Scan** 10922
**Peptide** NKMAVKGSQVI **Charge** 3 **Spectral Angle** 0.81

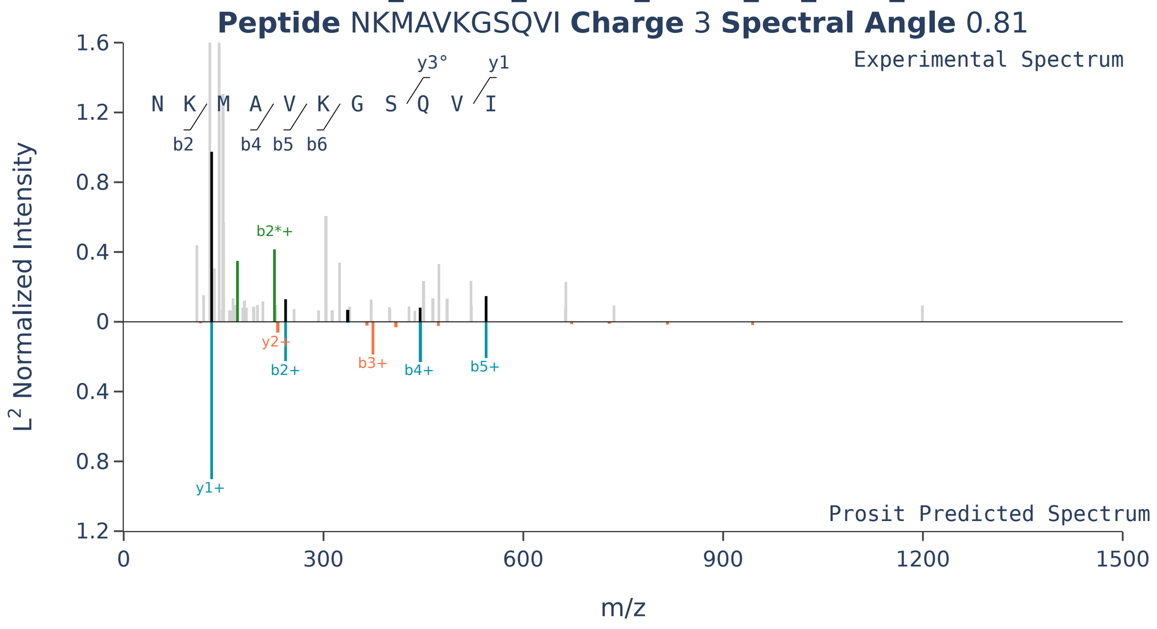


**Source** HRoetschke_211222_160123_HFGoe_IF2_b2R1_4h **Scan** 8908
**Peptide** KSKLENMFAN **Charge** 2 **Spectral Angle** 0.73

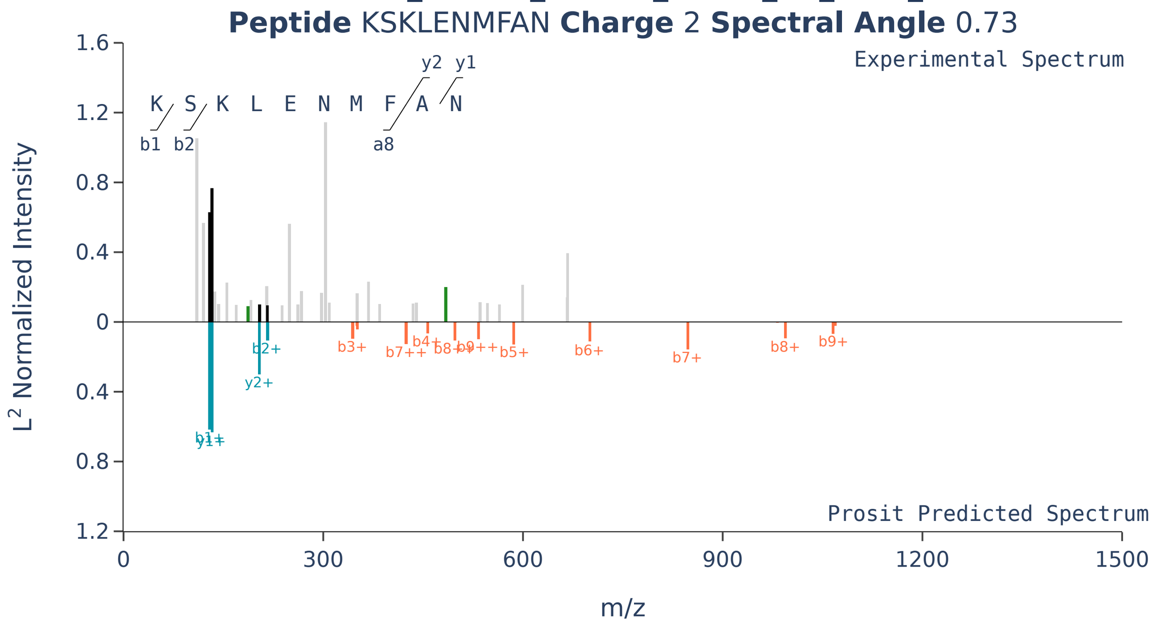


**Source** HRoetschke_211222_160123_HFGoe_IF2_b1R2_2h **Scan** 6318
**Peptide** HVTTSQHARQAE **Charge** 3 **Spectral Angle** 0.8

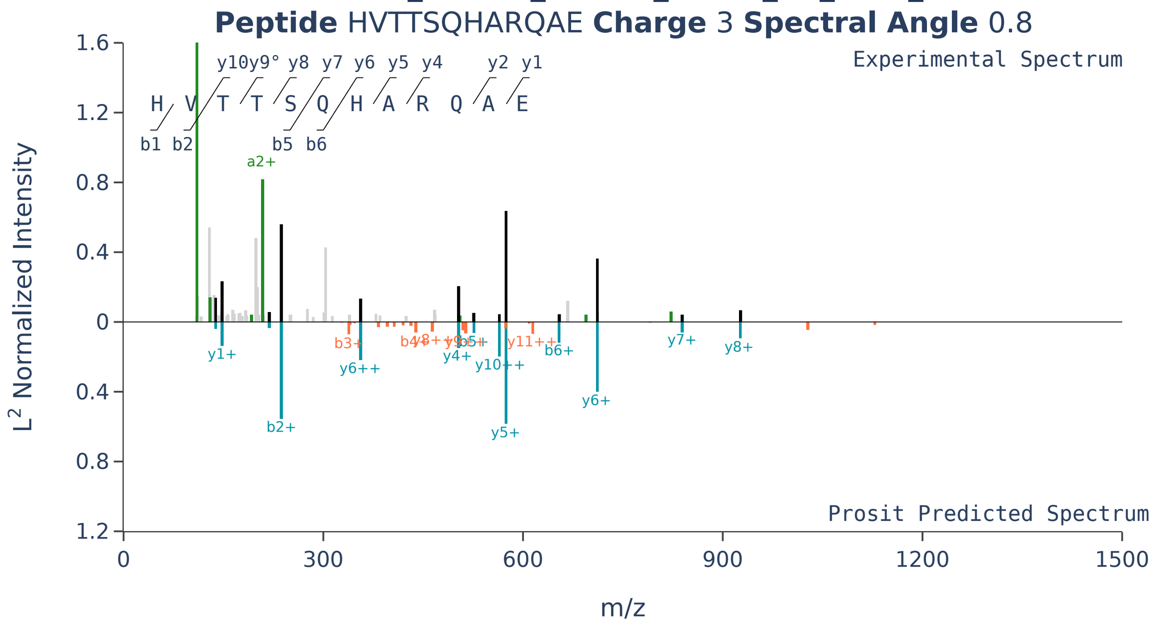


**Source** HRoetschke_211222_160123_HFGoe_IF2_b1R1_4h **Scan** 10910
**Peptide** RTFVKRDPQEAERLAAEE **Charge** 4 **Spectral Angle** 0.67

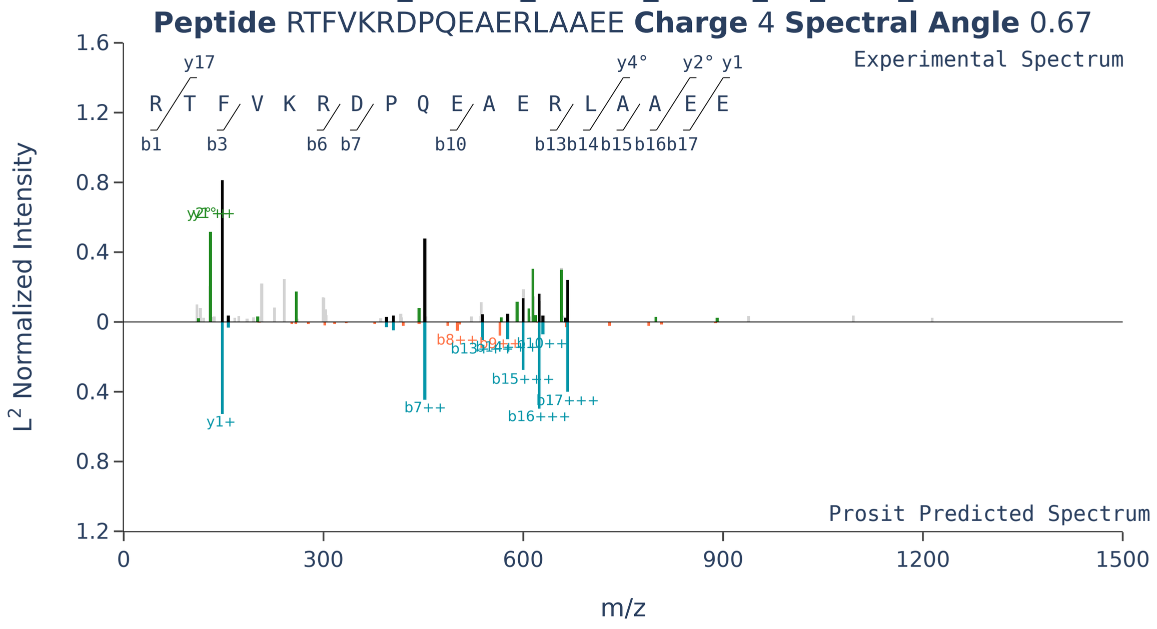

**Source** HRoetschke_211222_160123_HFGoe_IF2_b2R1_24h **Scan** 7464
**Peptide** ANKMAVKGSQVIK **Charge** 3 **Spectral Angle** 0.71

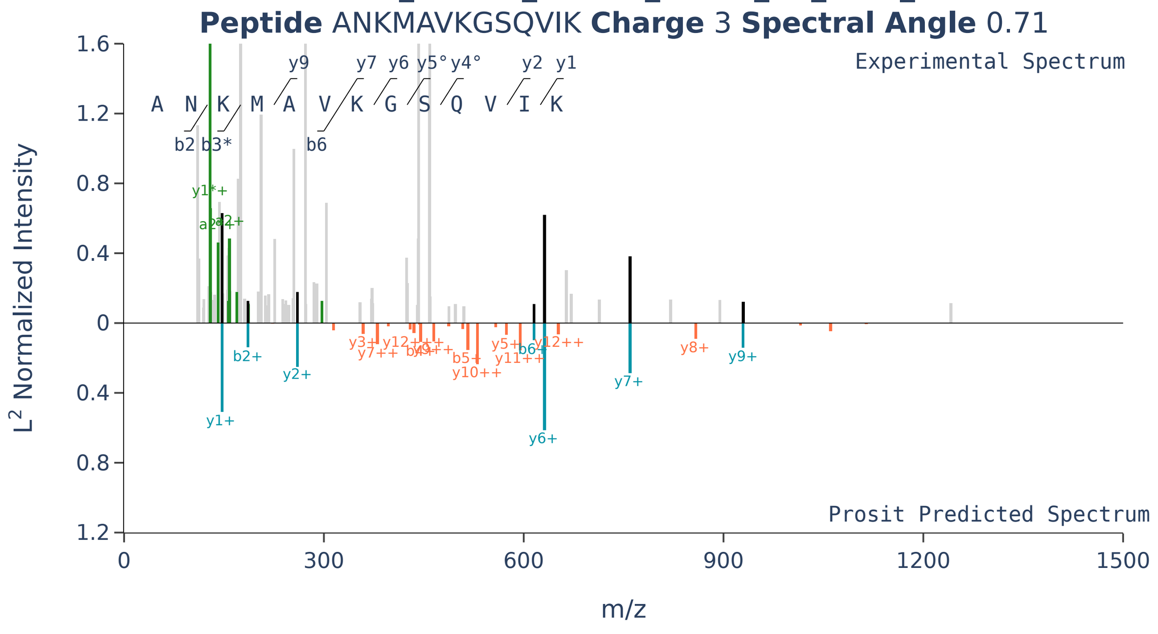


**Source** HRoetschke_211222_160123_HFGoe_IF2_b1R1_4h **Scan** 10202
**Peptide** IDHLNQKN **Charge** 2 **Spectral Angle** 0.7

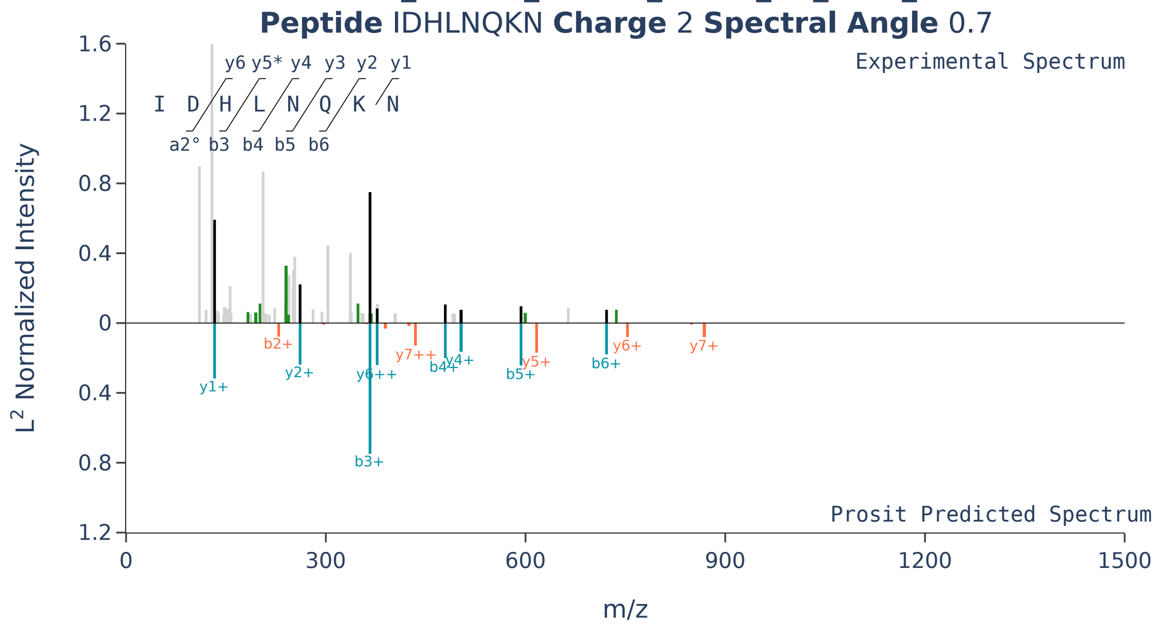


**Source** HRoetschke_211222_160123_HFGoe_IF2_b2R1_4h **Scan** 7075
**Peptide** LRSTKVASGEAGGITQH **Charge** 3 **Spectral Angle** 0.76

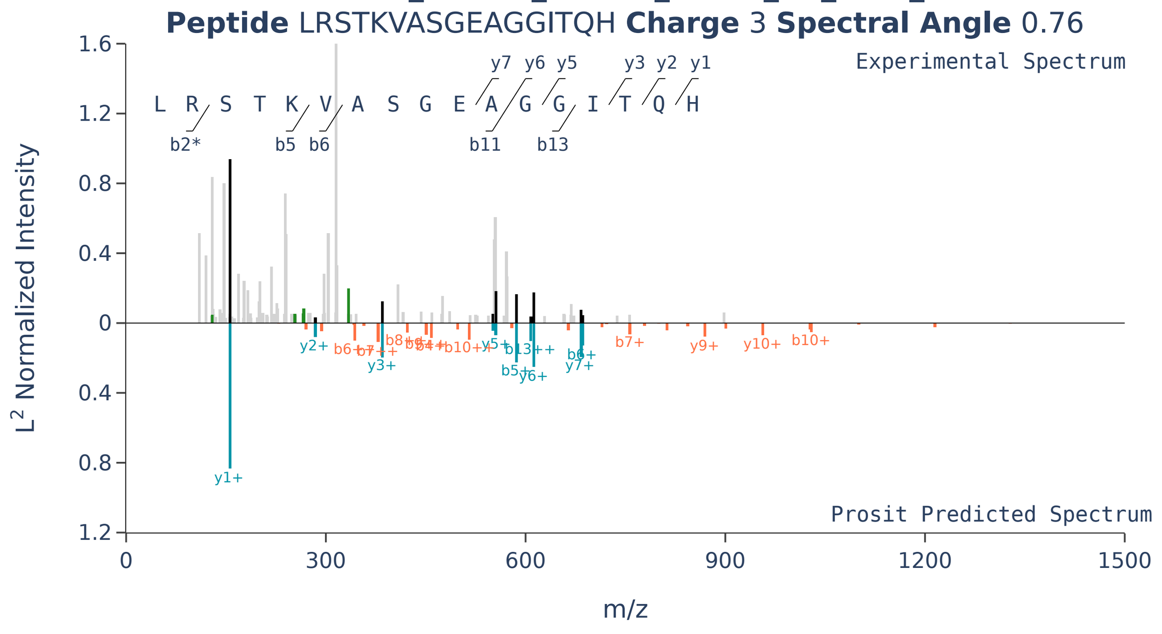


**Source** HRoetschke_211222_160123_HFGoe_IF2_b1R1_4h **Scan** 5976
**Peptide** DSSDYHVTTSQHAR **Charge** 4 **Spectral Angle** 0.64

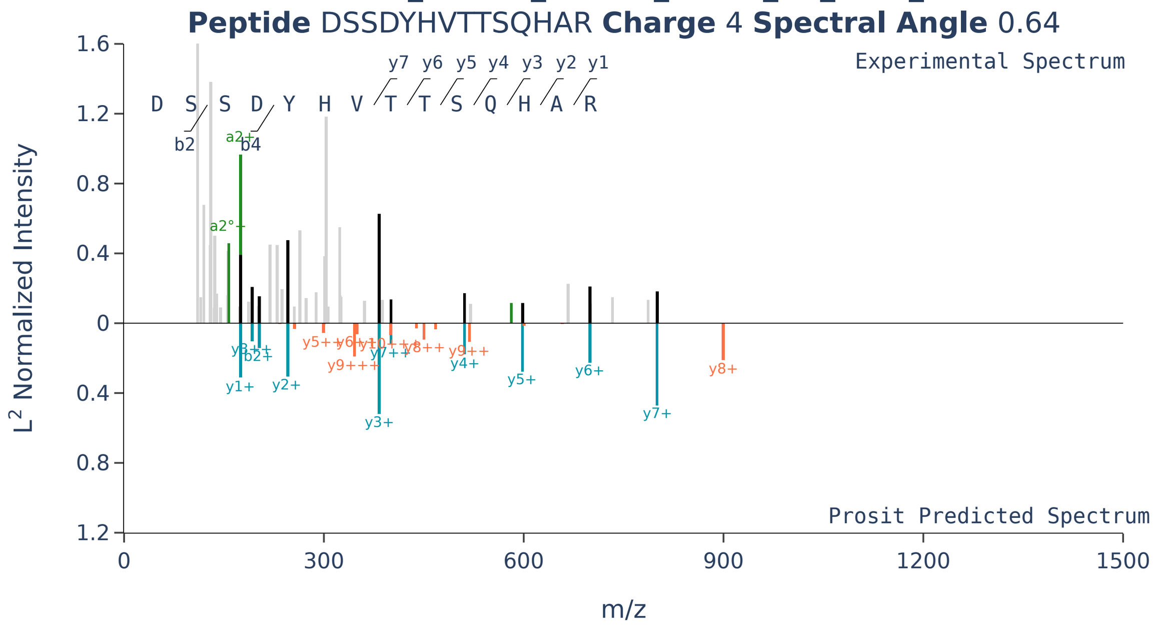


**Source** HRoetschke_211222_160123_HFGoe_IF2_b1R1_4h **Scan** 12597
**Peptide** EVLELKAVRKGMA **Charge** 3 **Spectral Angle** 0.58

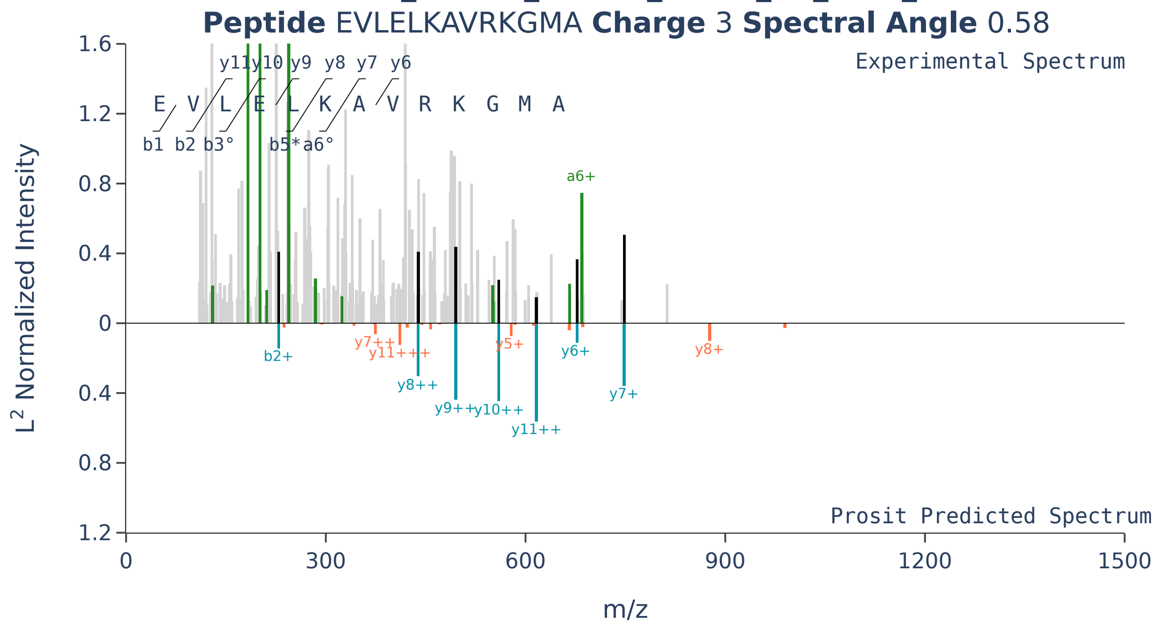


**Source** HRoetschke_211222_160123_HFGoe_IF2_b1R1_2h **Scan** 8604
**Peptide** LIDHLNQK **Charge** 2 **Spectral Angle** 0.69

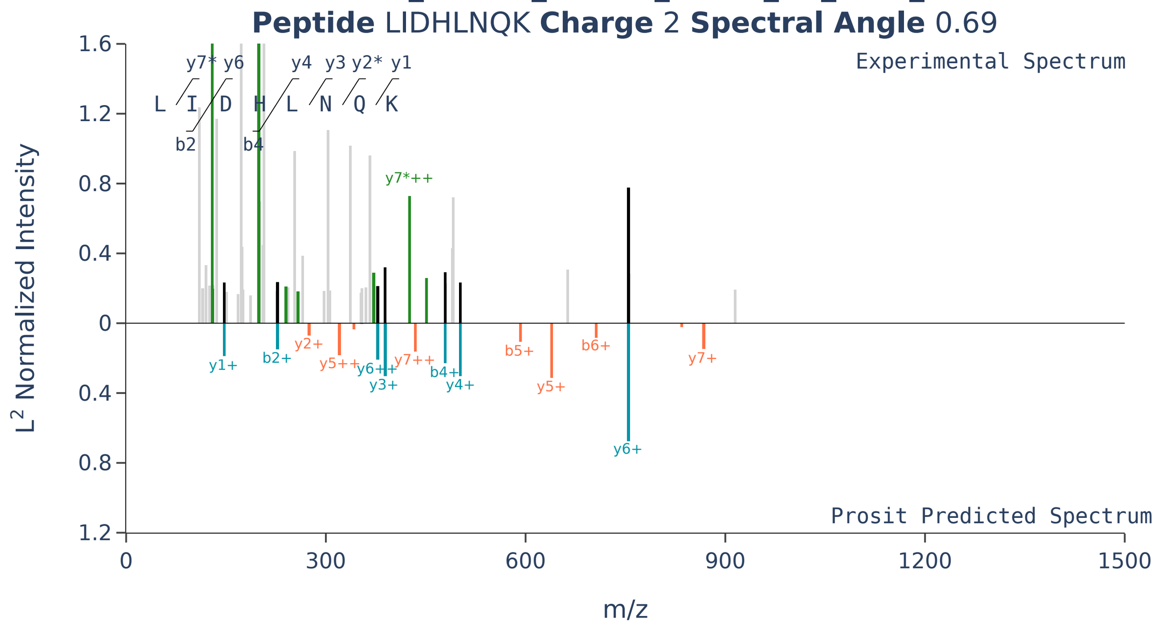


**Source** HRoetschke_211222_160123_HFGoe_IF2_b1R2_24h **Scan** 12512
**Peptide** AVNKIDKPEADPDRVKNEL **Charge** 2 **Spectral Angle** 0.74

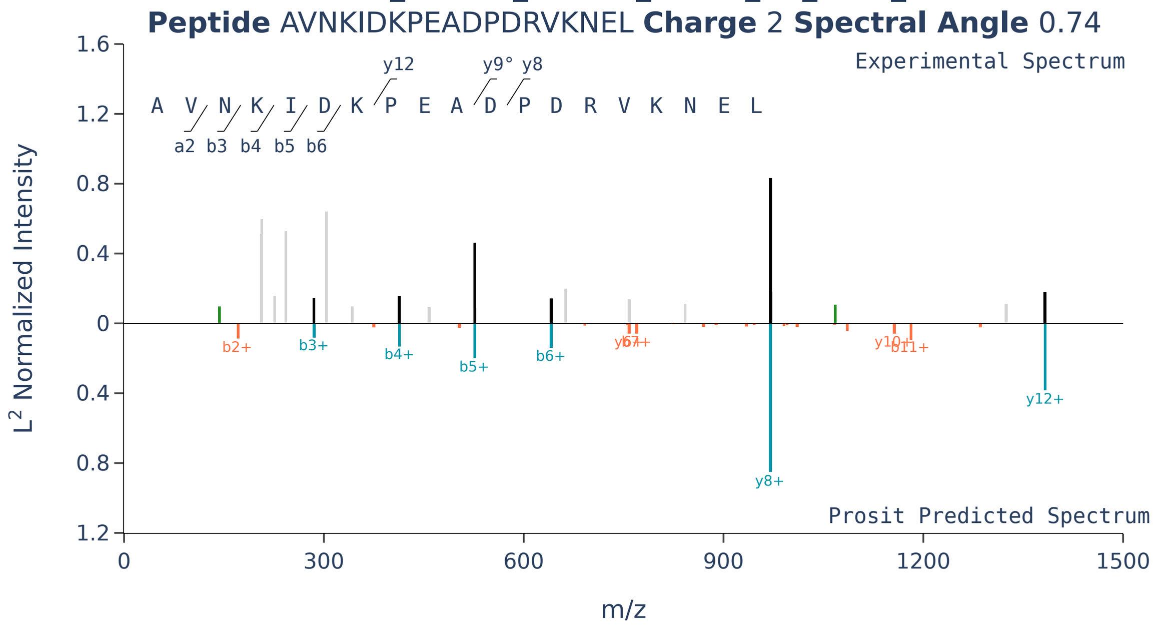


**Source** HRoetschke_211222_160123_HFGoe_IF2_b1R1_4h **Scan** 7516
**Peptide** QGKFREVKLARQQ **Charge** 3 **Spectral Angle** 0.72

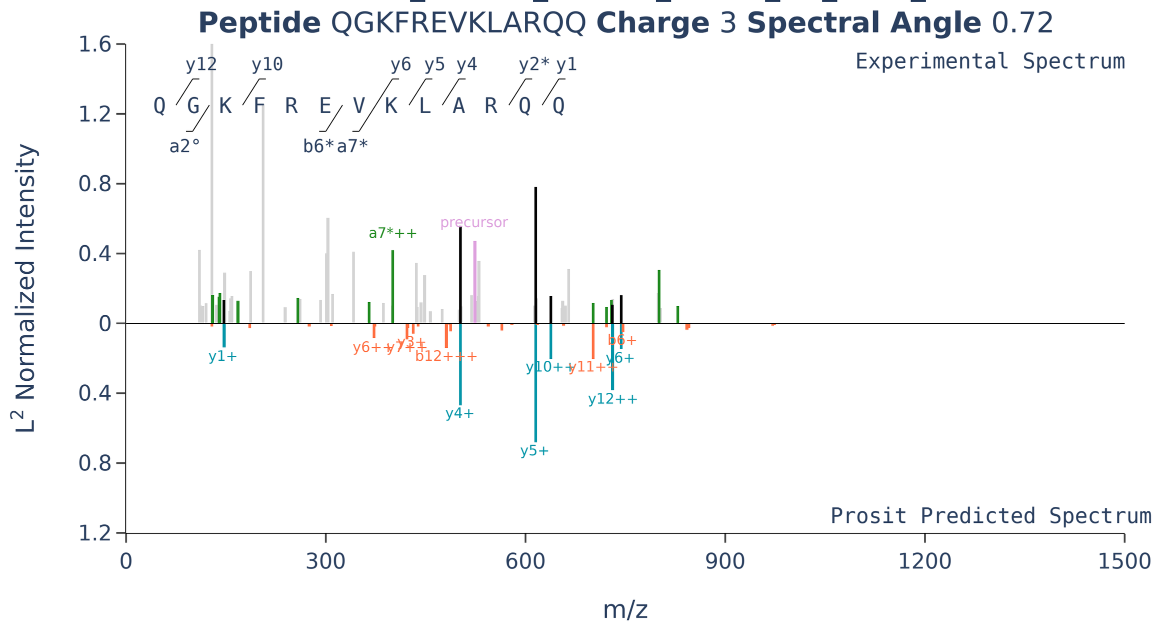


**Source** HRoetschke_211222_160123_HFGoe_IF2_b1R1_4h **Scan** 15180
**Peptide** AGGITQHIGAYHVE **Charge** 3 **Spectral Angle** 0.67

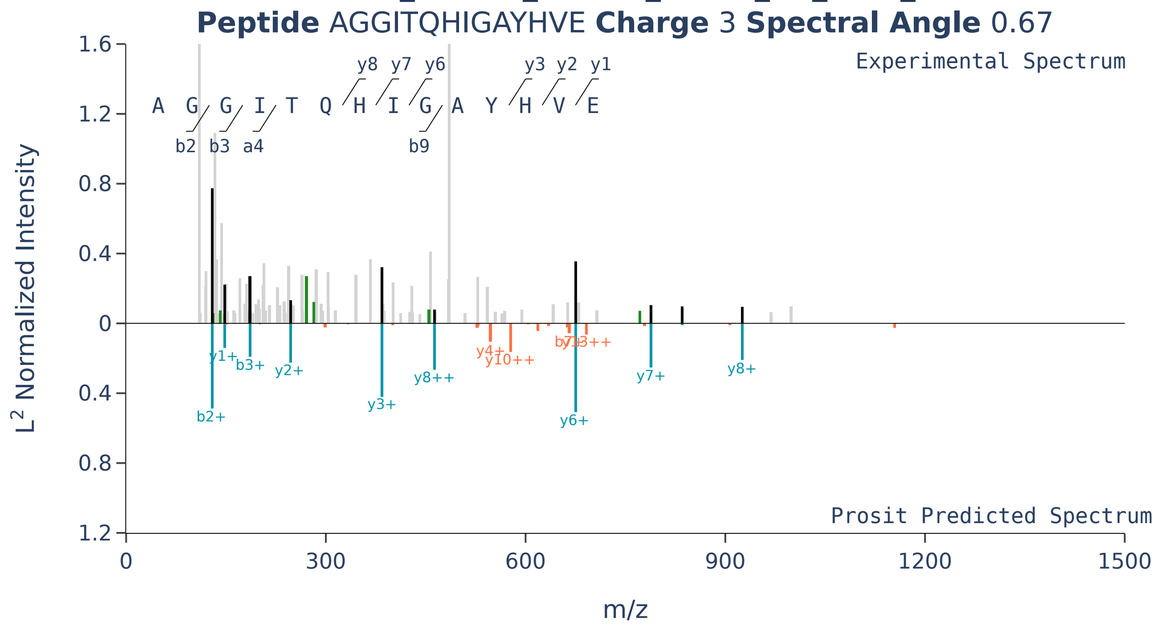


**Source** HRoetschke_211222_160123_HFGoe_IF2_b2R1_24h **Scan** 13265
**Peptide** KGSSLQQGFQKPAQAV **Charge** 3 **Spectral Angle** 0.66

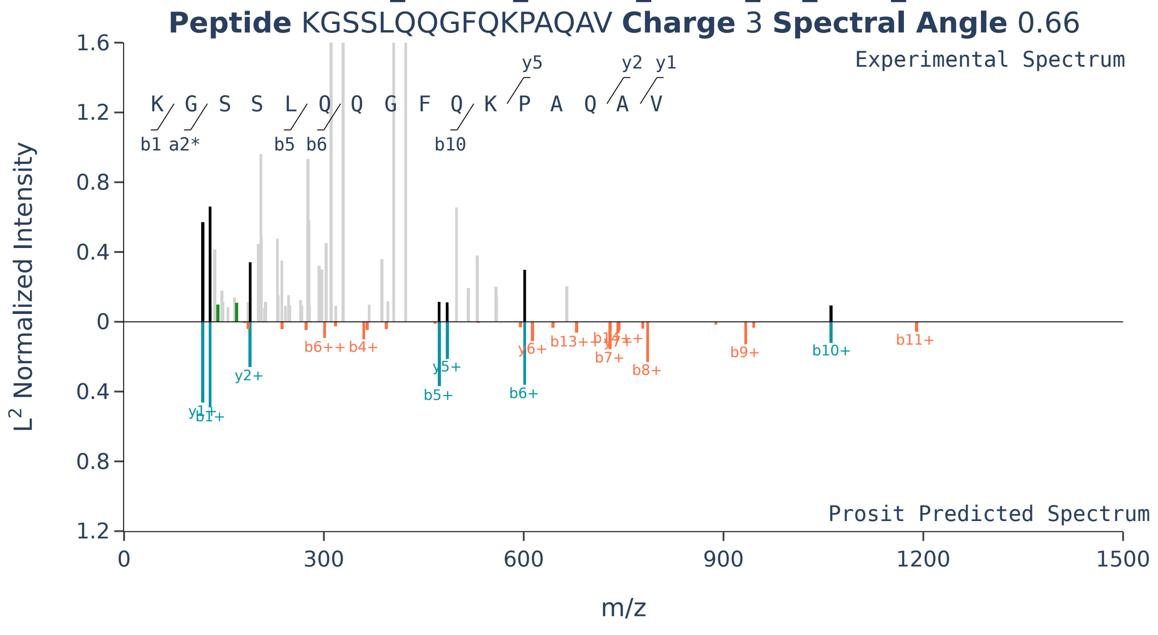


**Source** HRoetschke_211222_160123_HFGoe_IF2_b1R1_4h **Scan** 14848
**Peptide** IDHLNQKNSGPDKLTL **Charge** 4 **Spectral Angle** 0.78

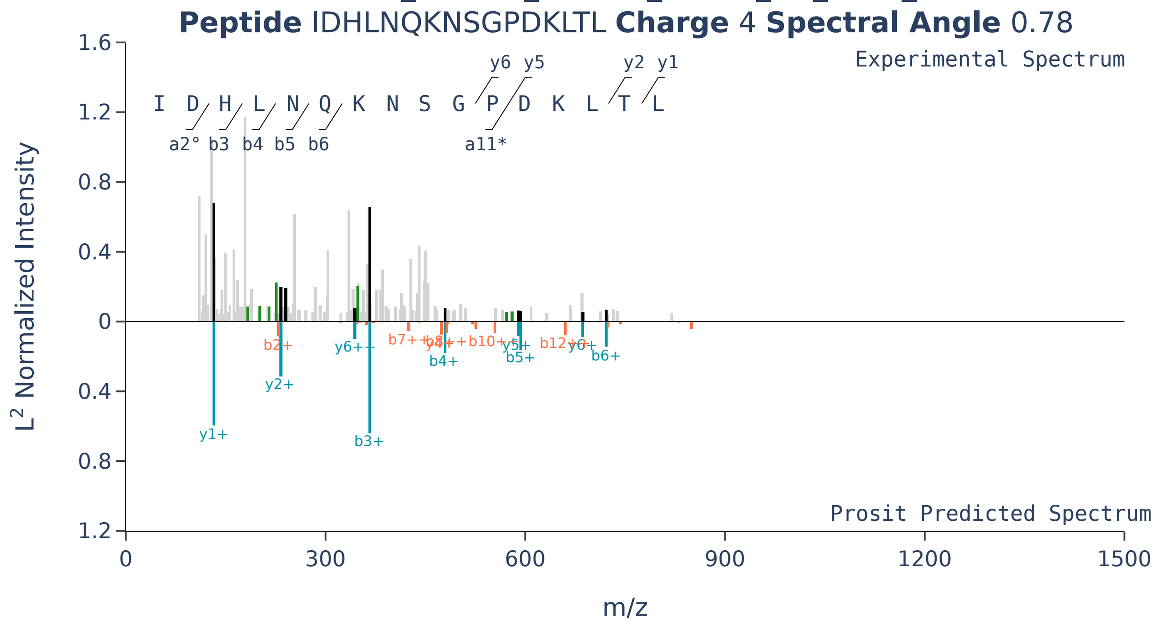


**Source** HRoetschke_211222_160123_HFGoe_IF2_b2R2_2h **Scan** 14968
**Peptide** DGVMPQTLE **Charge** 2 **Spectral Angle** 0.83

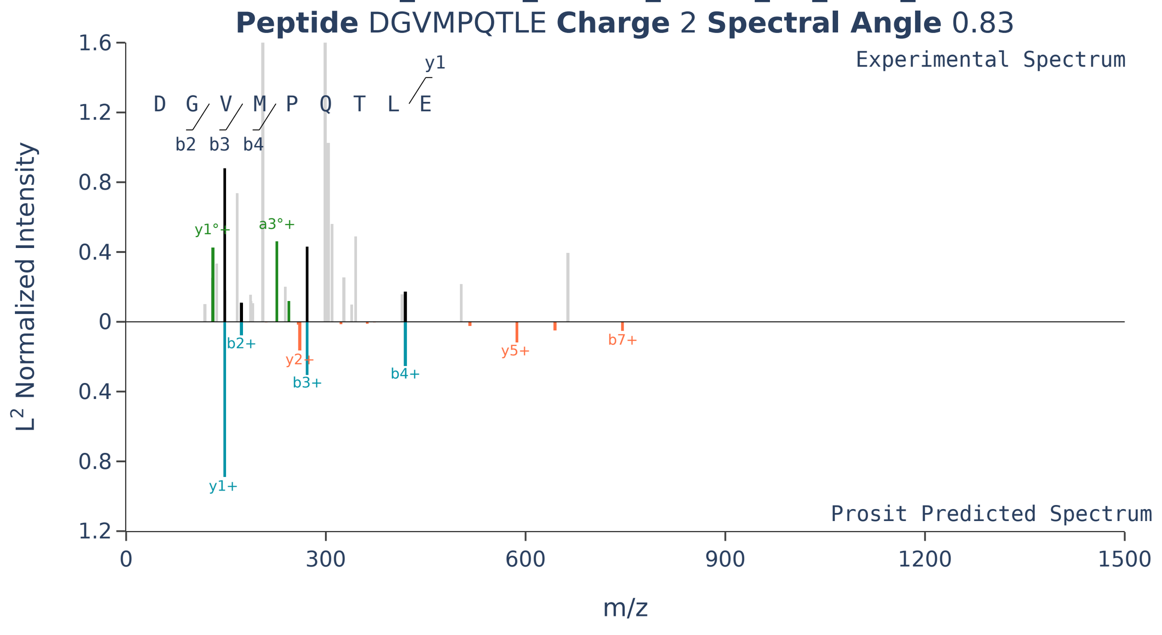


**Source** HRoetschke_211222_160123_HFGoe_IF2_b2R1_4h **Scan** 18008
**Peptide** QVPVVVA **Charge** 1 **Spectral Angle** 0.75

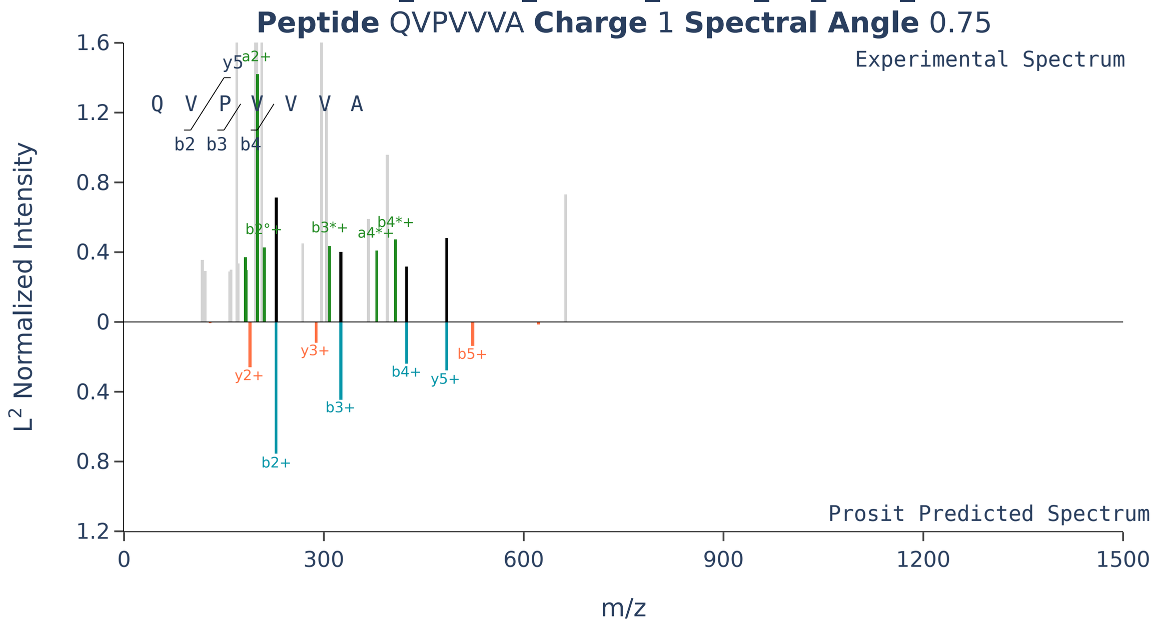


**Source** HRoetschke_211222_160123_HFGoe_IF2_b1R2_2h **Scan** 17501
**Peptide** LDQETAQLVAEE **Charge** 2 **Spectral Angle** 0.78

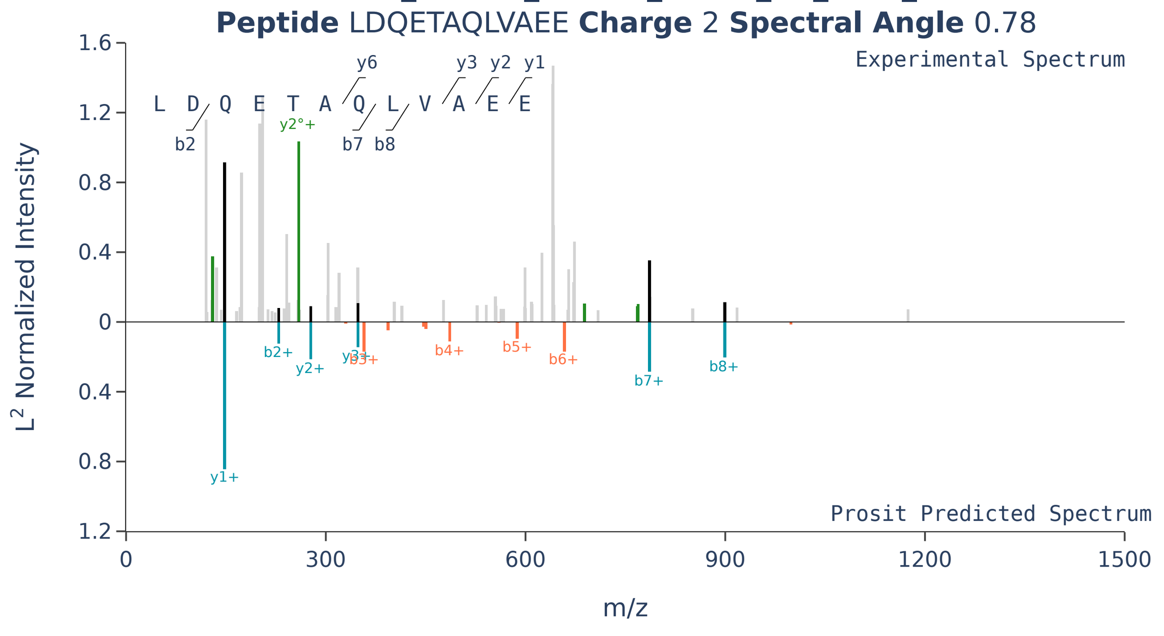


**Source** HRoetschke_211222_160123_HFGoe_IF2_b1R1_4h **Scan** 16651
**Peptide** GEAGGITQHIGAY **Charge** 2 **Spectral Angle** 0.81

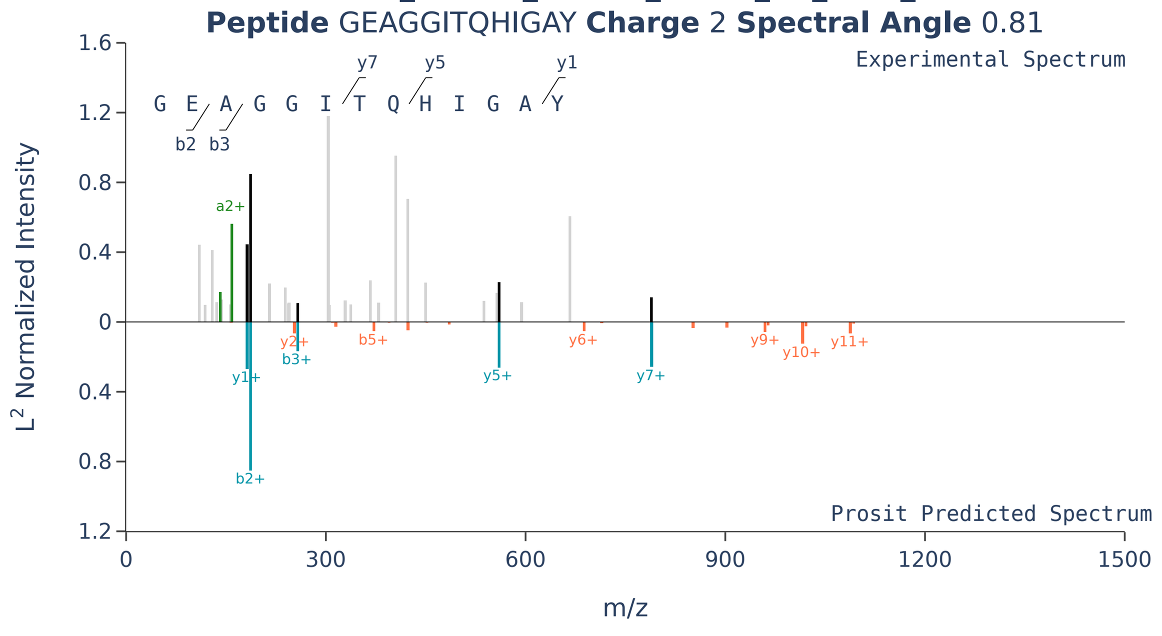

**Source** HRoetschke_211222_160123_HFGoe_IF2_b1R1_24h **Scan** 5550
**Peptide** AGIRKSADDSVS **Charge** 2 **Spectral Angle** 0.62

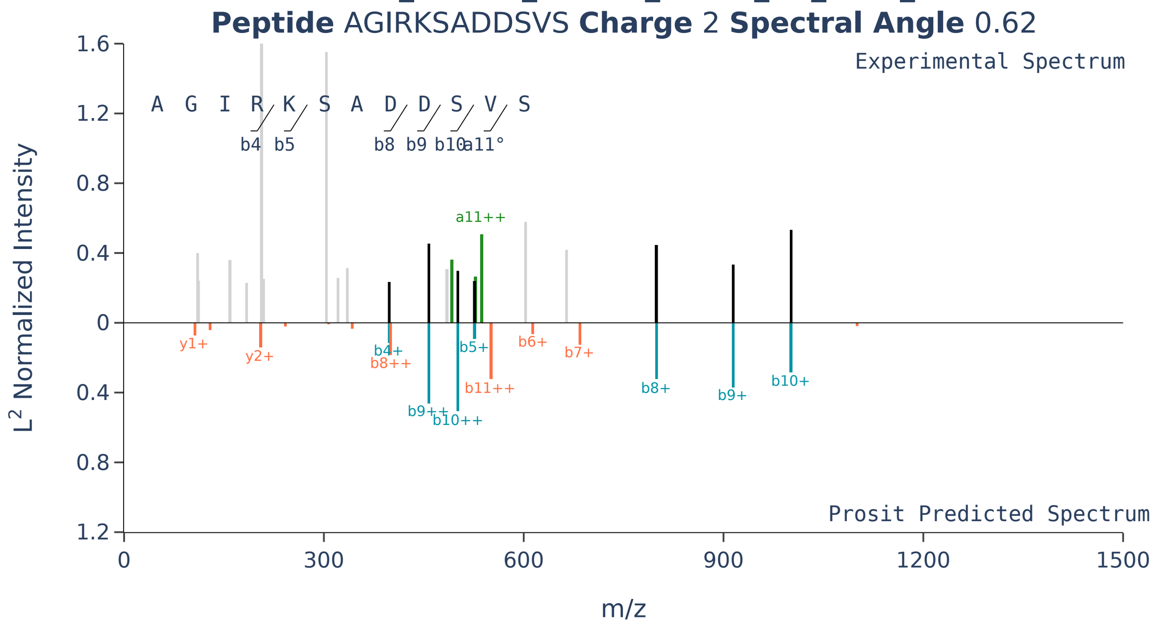


**Source** HRoetschke_211222_160123_HFGoe_IF2_b1R1_4h **Scan** 10091
**Peptide** NVRADASARKVIEAE **Charge** 3 **Spectral Angle** 0.64

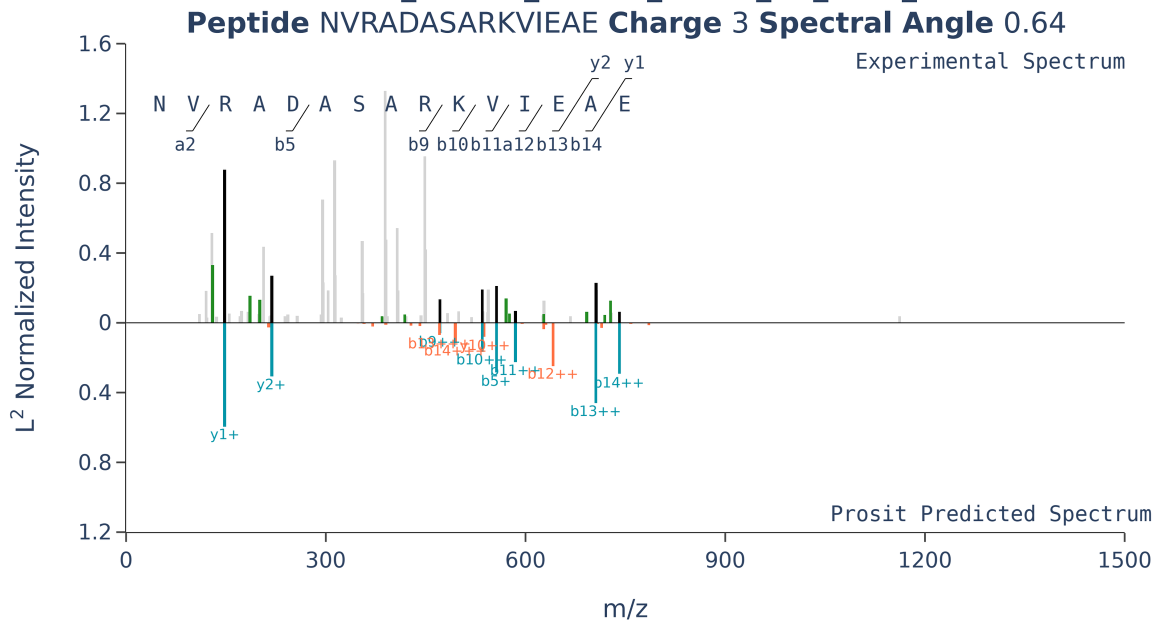


**Source** HRoetschke_211222_160123_HFGoe_IF2_b1R1_4h **Scan** 13776
**Peptide** GIGVKNYNDV **Charge** 2 **Spectral Angle** 0.58

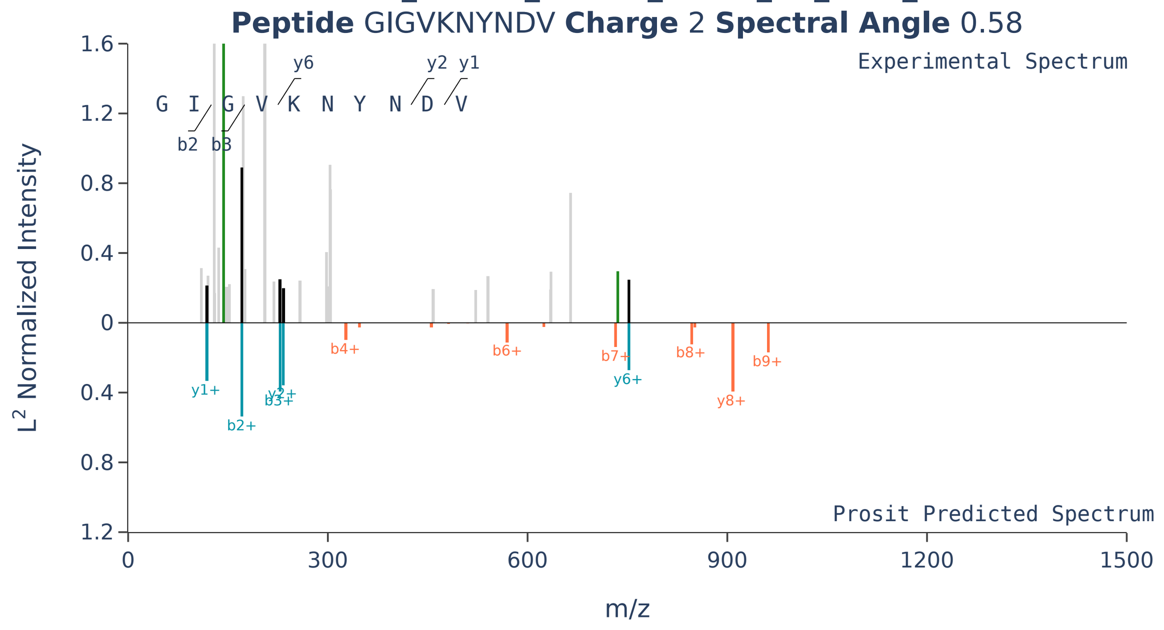


**Source** HRoetschke_211222_160123_HFGoe_IF2_b1R2_24h **Scan** 6422
**Peptide** ADAGIRKSADDSVS **Charge** 2 **Spectral Angle** 0.5

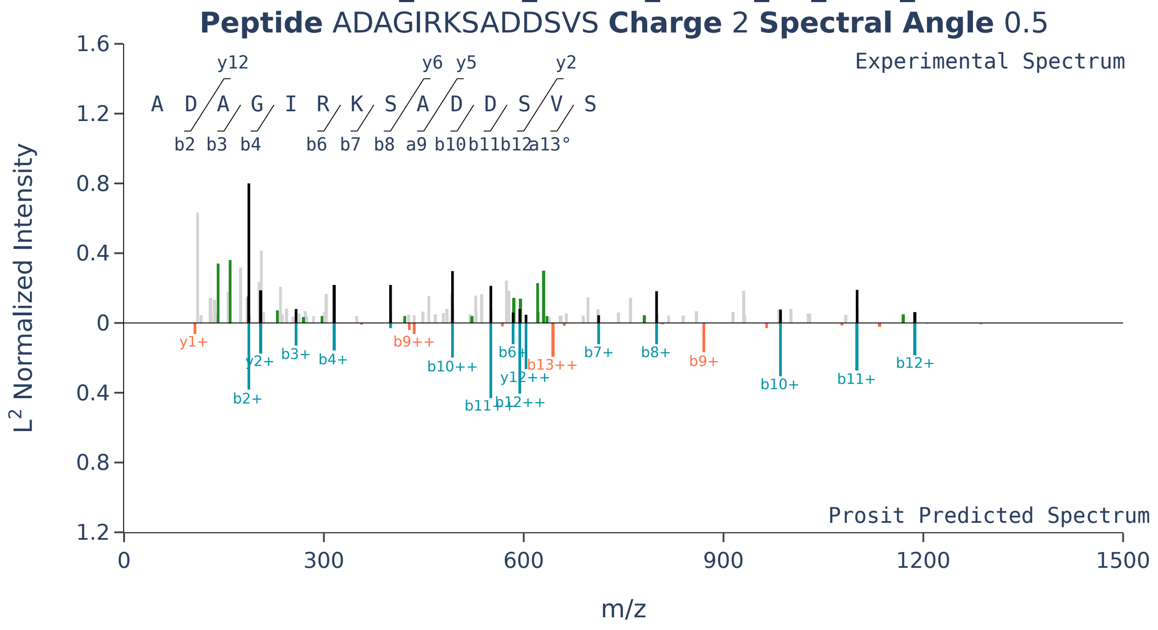


**Source** HRoetschke_211222_160123_HFGoe_IF2_b1R2_2h **Scan** 10505
**Peptide** KGSSLQQGFQKPAQAVNRD **Charge** 4 **Spectral Angle** 0.72

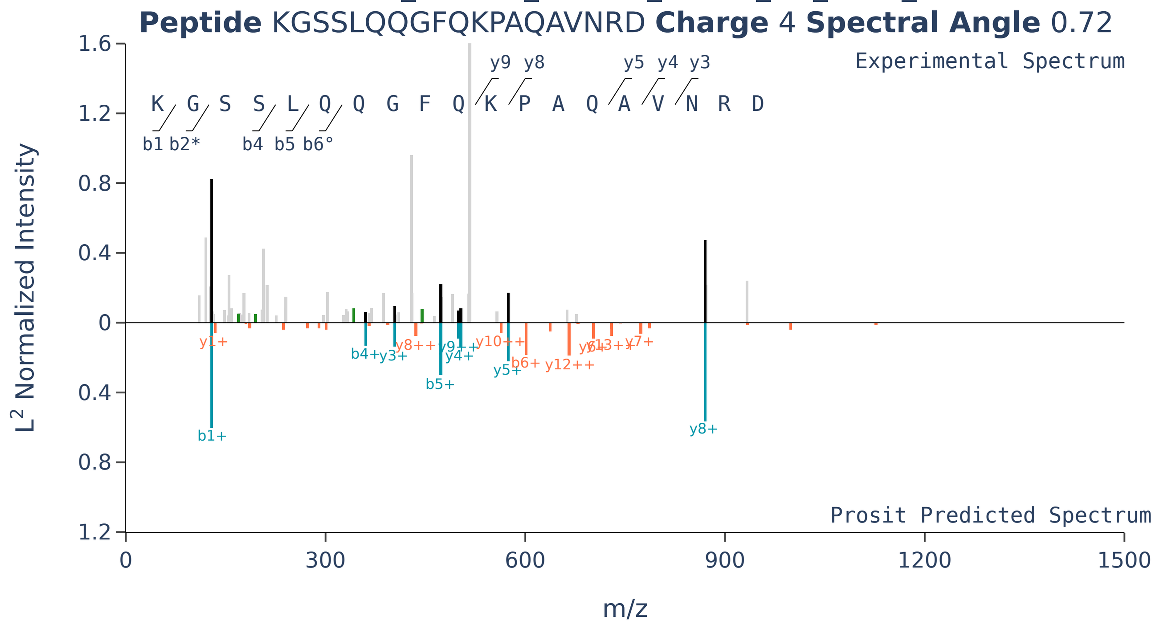


**Source** HRoetschke_211222_160123_HFGoe_IF2_b1R2_1h **Scan** 15711
**Peptide** ALQHAKAAQVPVVVA **Charge** 3 **Spectral Angle** 0.6

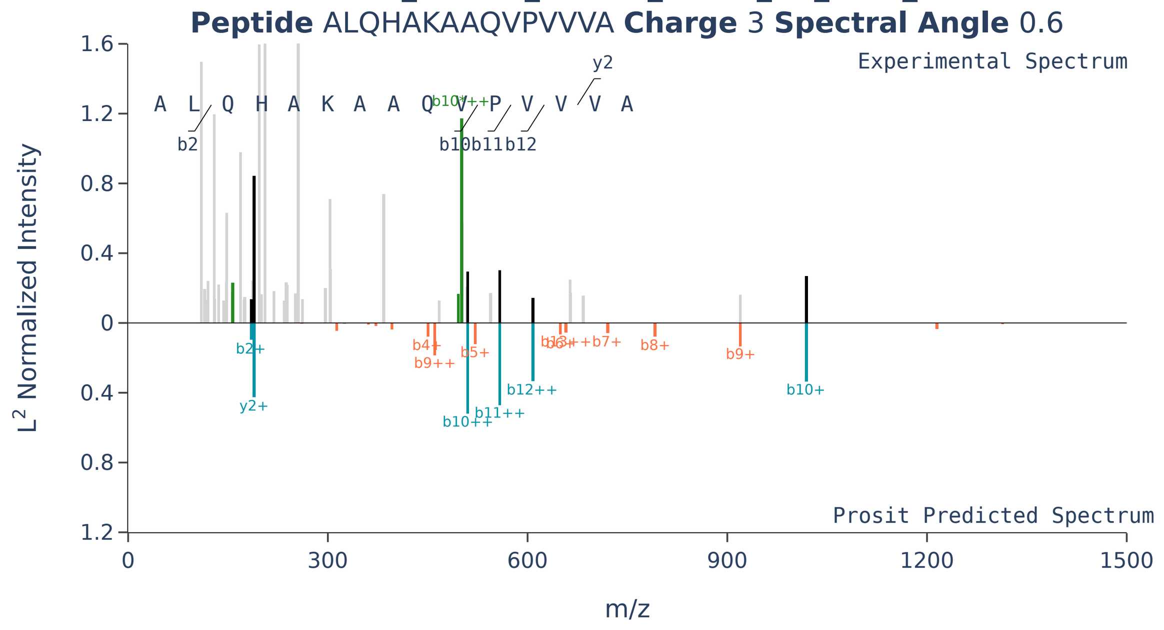


**Source** HRoetschke_211222_160123_HFGoe_IF2_b1R1_24h **Scan** 12613
**Peptide** IDKPEADPDRVKNELSQ **Charge** 3 **Spectral Angle** 0.75

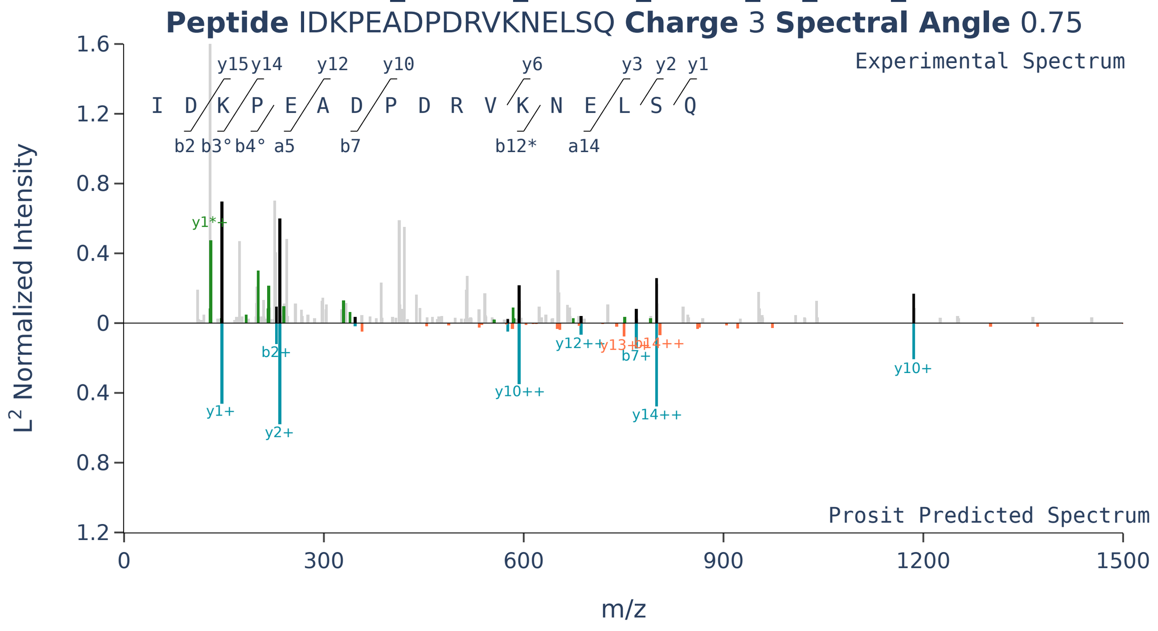


**Source** HRoetschke_211222_160123_HFGoe_IF2_b1R1_4h **Scan** 14180
**Peptide** DKGRGPVATVLVR **Charge** 3 **Spectral Angle** 0.67

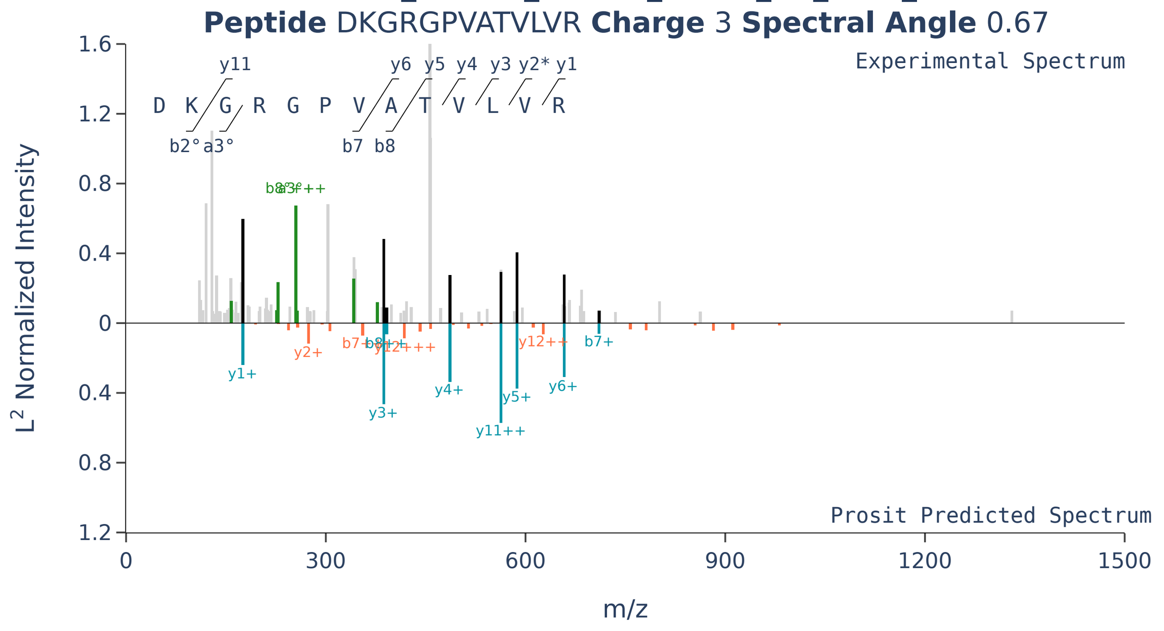


**Source** HRoetschke_211222_160123_HFGoe_IF2_b1R1_4h **Scan** 17092
**Peptide** TDVTIKTLAAERQTSVE **Charge** 2 **Spectral Angle** 0.78

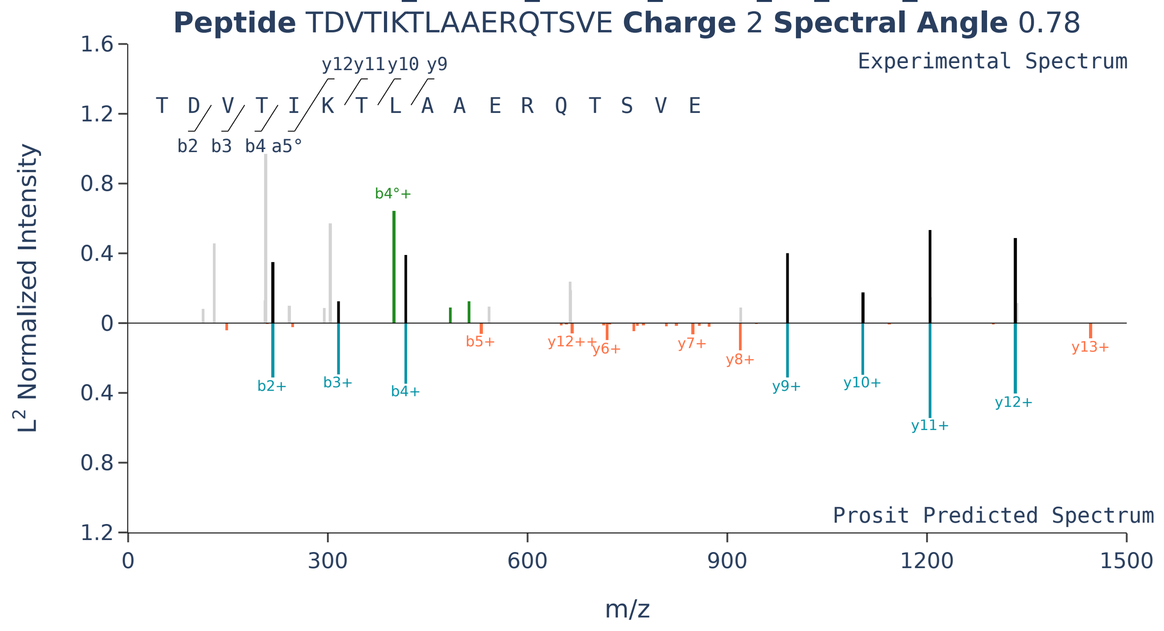


**Source** HRoetschke_211222_160123_HFGoe_IF2_b2R2_2h **Scan** 5885
**Peptide** KVSNQQDDMTKNAQAE **Charge** 3 **Spectral Angle** 0.84

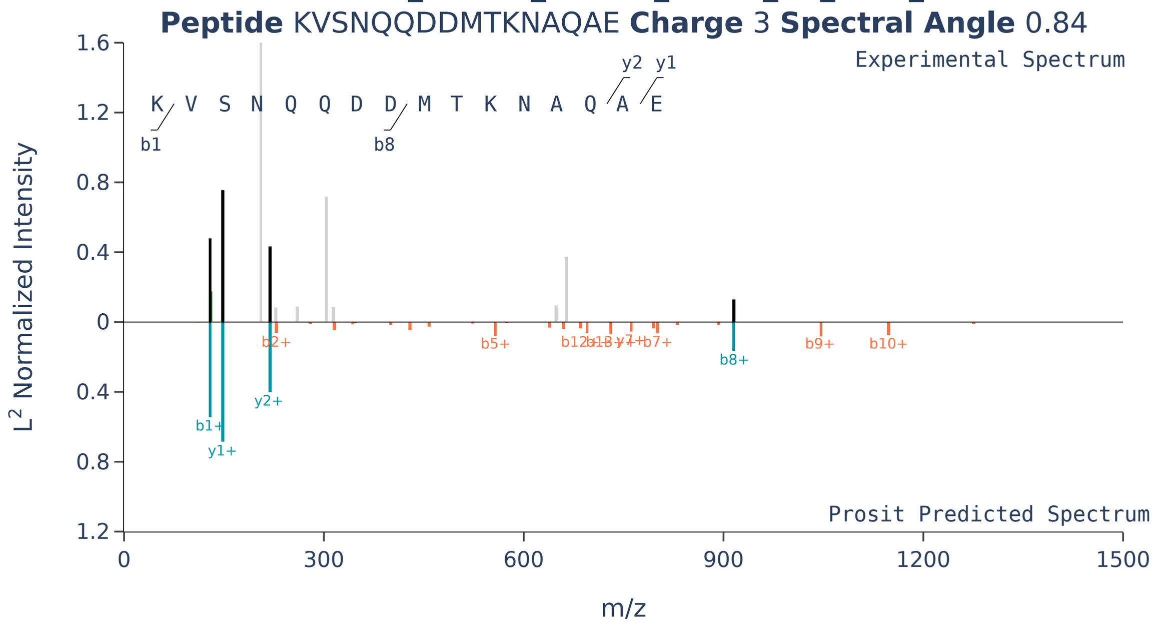


**Source** HRoetschke_211222_160123_HFGoe_IF2_b1R1_4h **Scan** 8415
**Peptide** ENKWTDNAE **Charge** 2 **Spectral Angle** 0.79

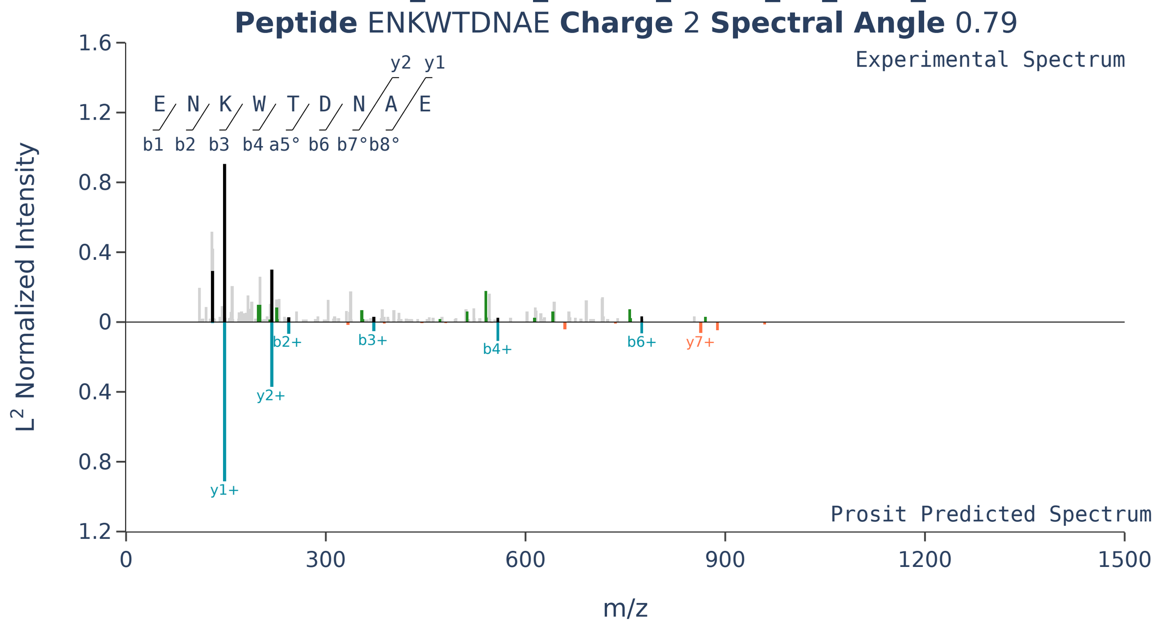


**Source** HRoetschke_211222_160123_HFGoe_IF2_b1R2_24h **Scan** 11138
**Peptide** SGVPAAGDEVTVVRDEKKARE **Charge** 3 **Spectral Angle** 0.78

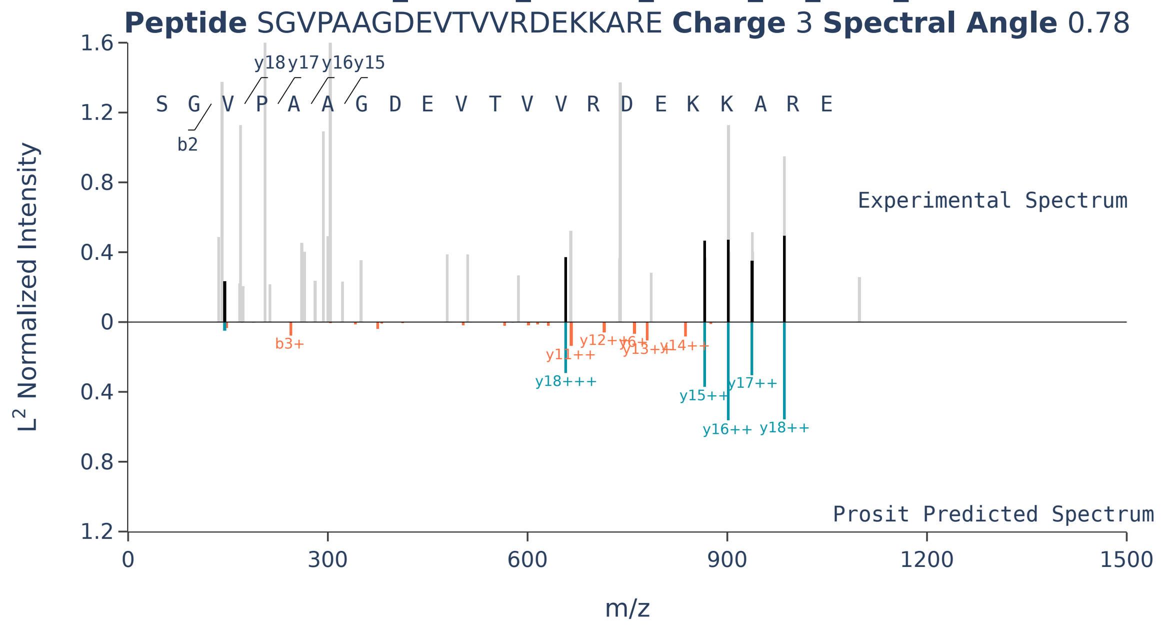


**Source** HRoetschke_211222_160123_HFGoe_IF2_b1R1_1h **Scan** 15547
**Peptide** SSLQQGFQKPAQAV **Charge** 2 **Spectral Angle** 0.78

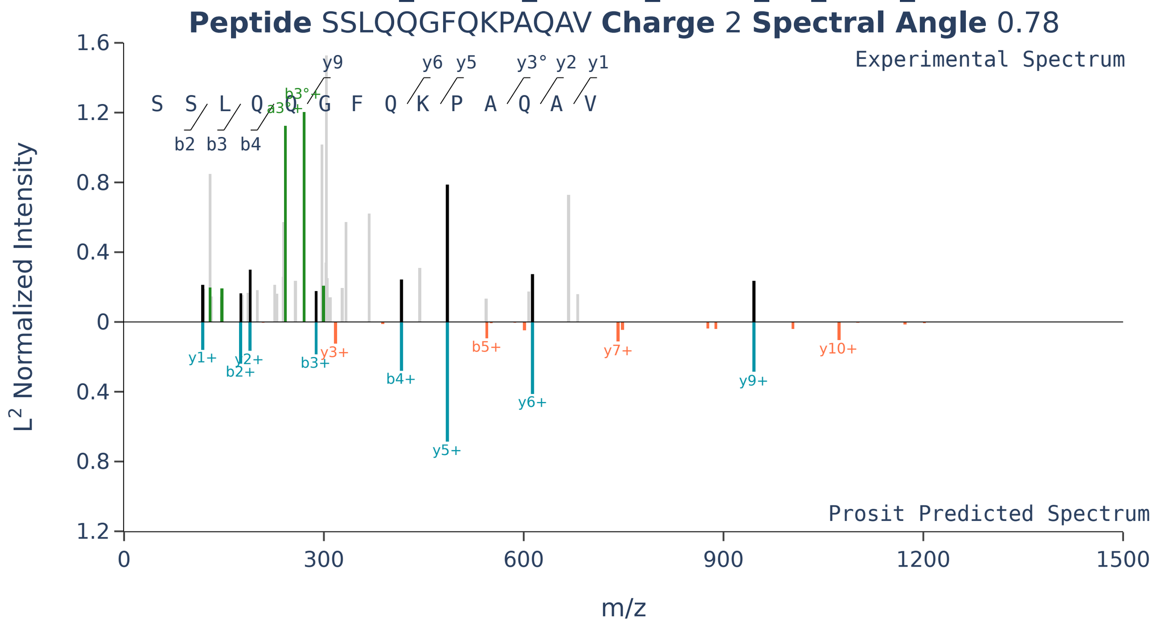


**Source** HRoetschke_211222_160123_HFGoe_IF2_b1R1_2h **Scan** 7845
**Peptide** HKVILRRENEL **Charge** 3 **Spectral Angle** 0.91

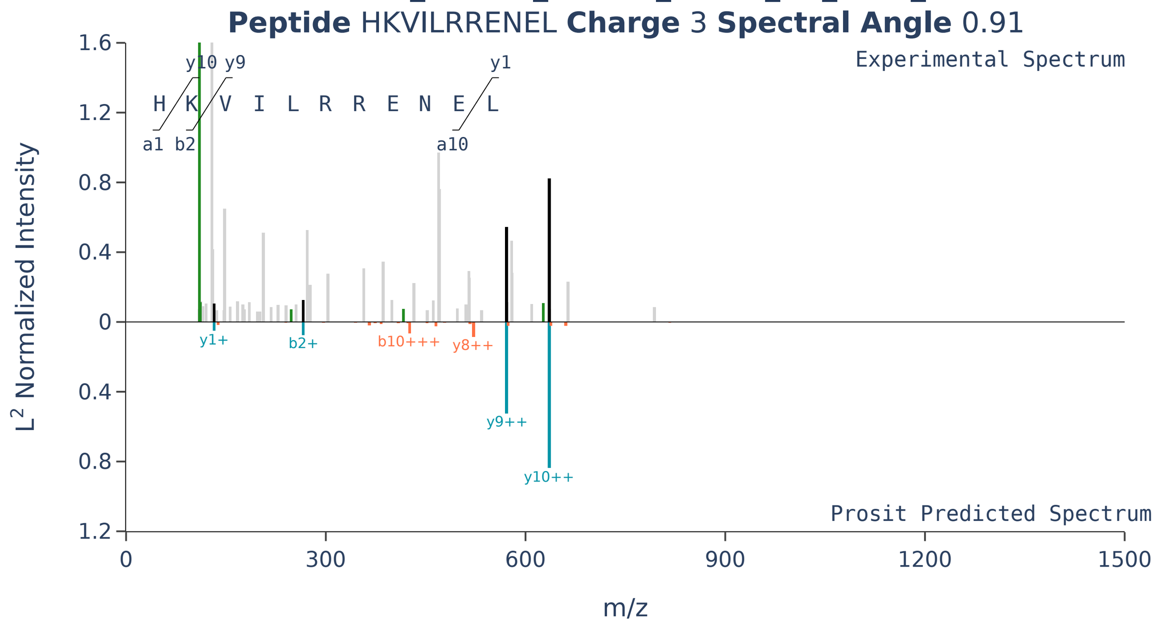


**Source** HRoetschke_211222_160123_HFGoe_IF2_b1R1_4h **Scan** 17483
**Peptide** DGVMPQTIEAIQHAKA **Charge** 2 **Spectral Angle** 0.71

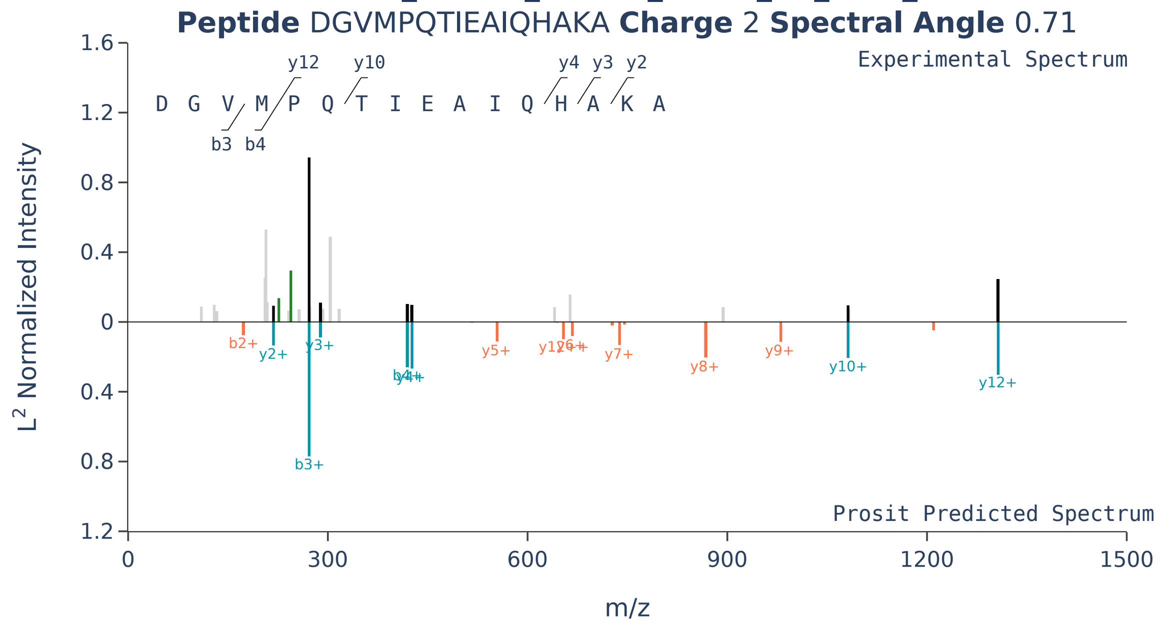

**Source** HRoetschke_211222_160123_HFGoe_IF2_b1R1_24h **Scan** 10928
**Peptide** QEKQTLIDHLNQKN **Charge** 4 **Spectral Angle** 0.66

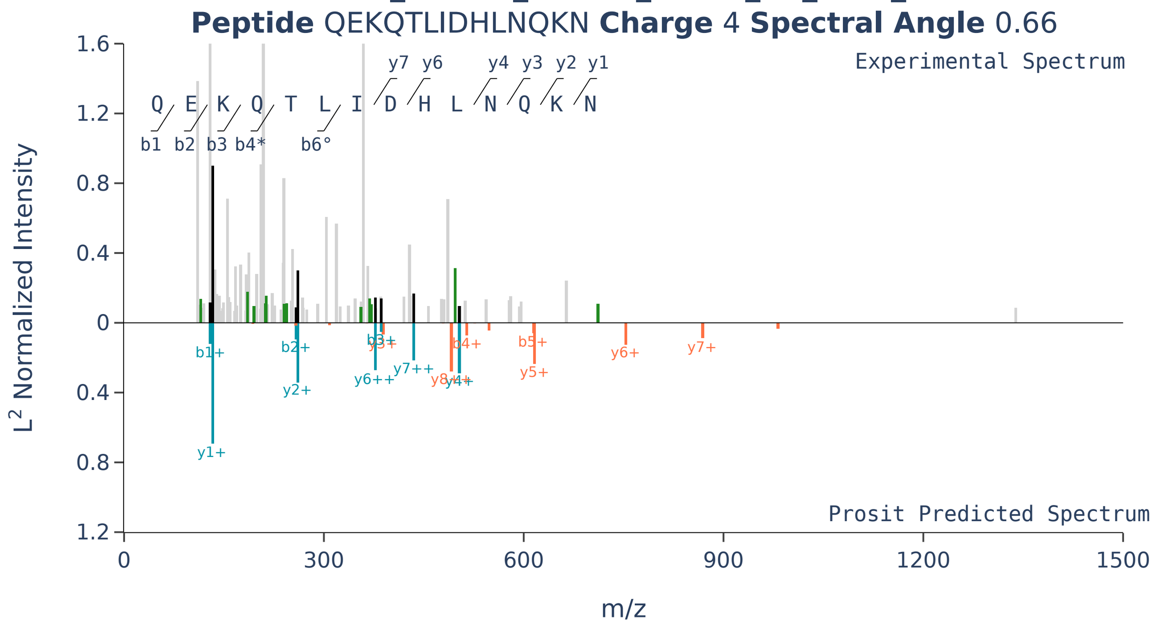


**Source** HRoetschke_211222_160123_HFGoe_IF2_b1R1_4h **Scan** 14192
**Peptide** GSSLQQGFQKPAQAVN **Charge** 2 **Spectral Angle** 0.62

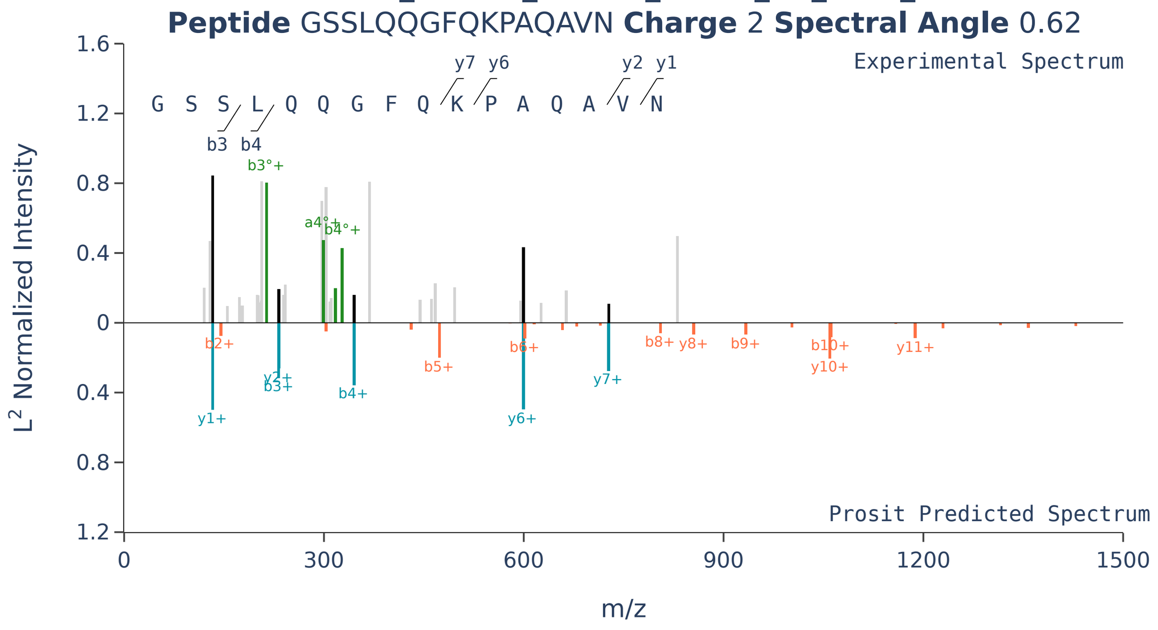


**Source** HRoetschke_211222_160123_HFGoe_IF2_b2R2_24h **Scan** 20795
**Peptide** TENGMITFLDTPGH **Charge** 2 **Spectral Angle** 0.83

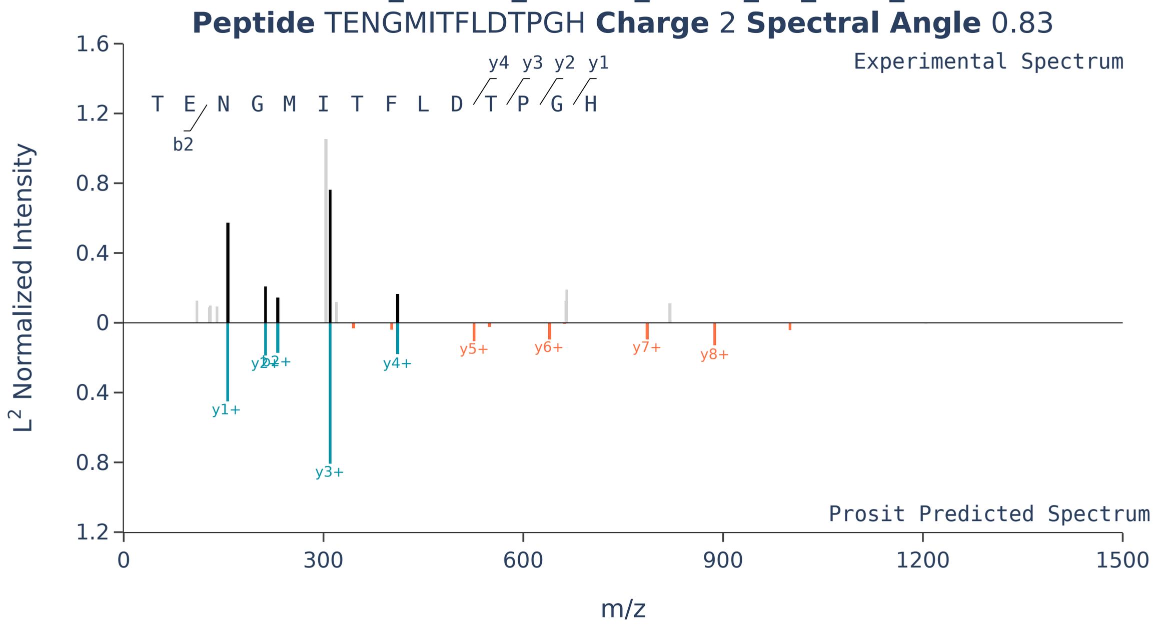


**Source** HRoetschke_211222_160123_HFGoe_IF2_b1R1_2h **Scan** 7005
**Peptide** EAIQHAKAAQV **Charge** 2 **Spectral Angle** 0.69

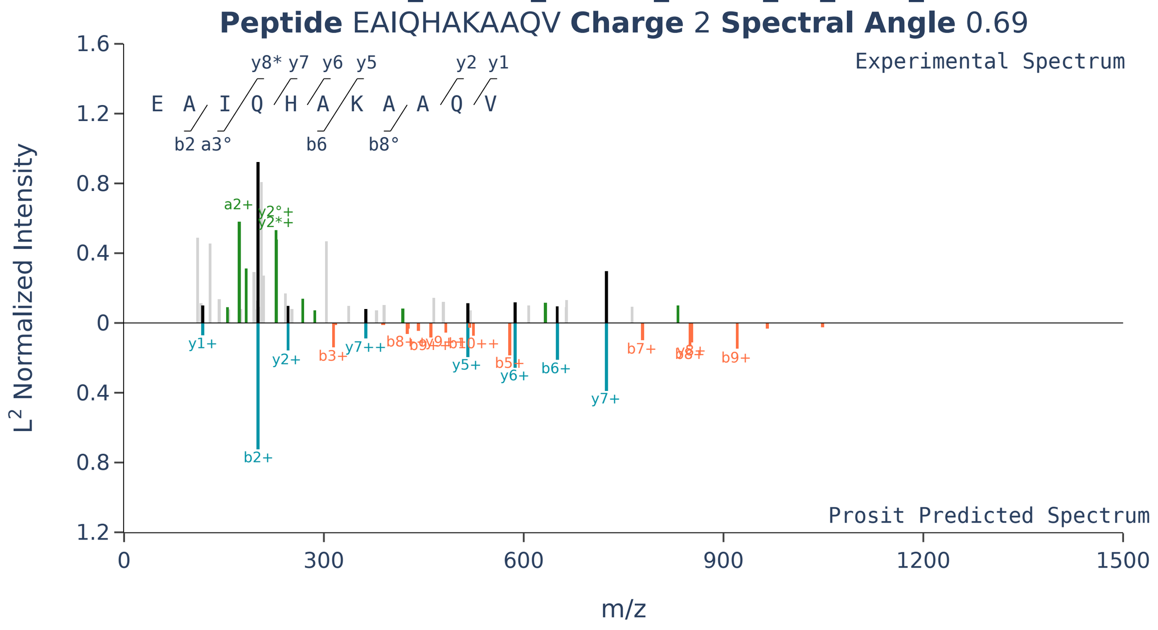


**Source** HRoetschke_211222_160123_HFGoe_IF2_b1R1_24h **Scan** 16156
**Peptide** KIIGSGVGGITETD **Charge** 2 **Spectral Angle** 0.72

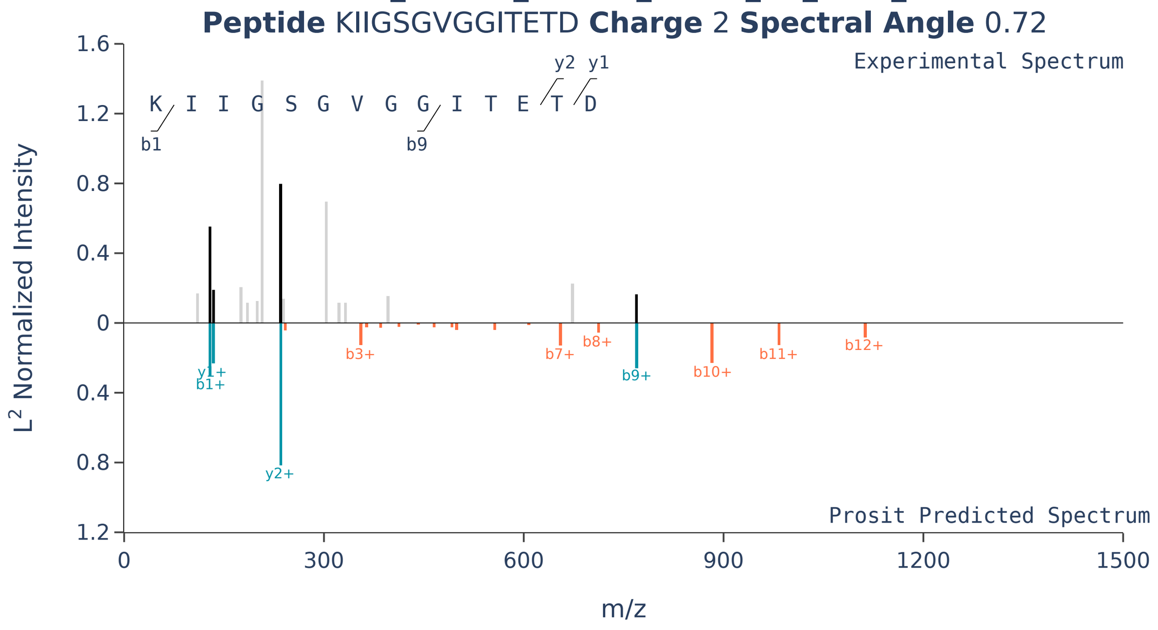


**Source** HRoetschke_211222_160123_HFGoe_IF2_b2R2_2h **Scan** 12307
**Peptide** LDKPEADPDRVKNELSQ **Charge** 3 **Spectral Angle** 0.72

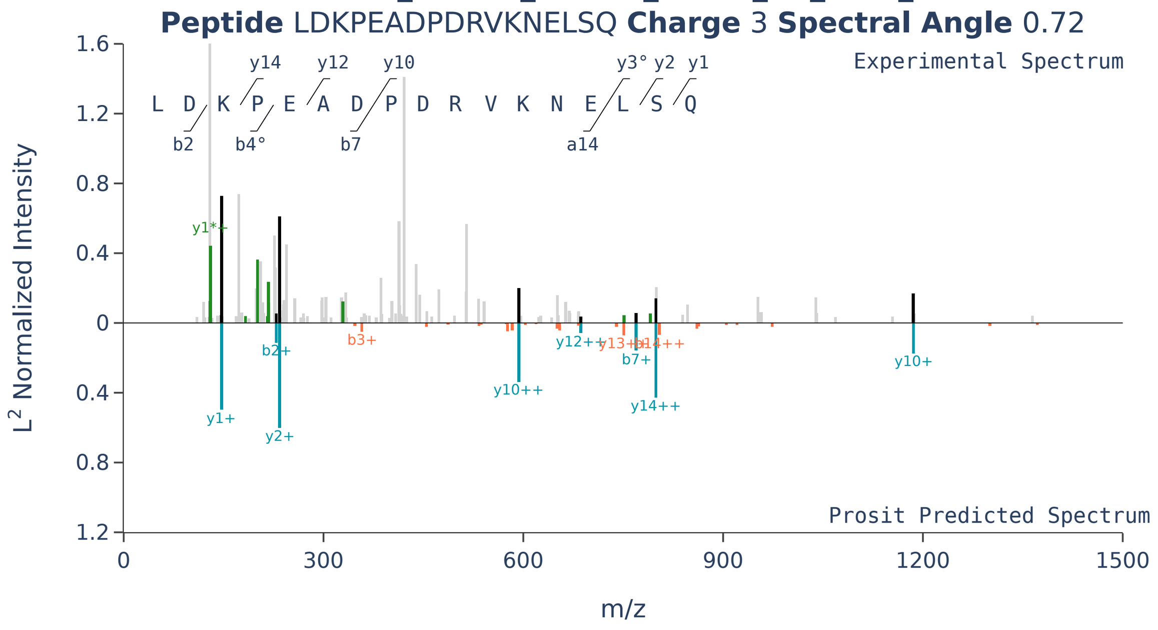


**Source** HRoetschke_211222_160123_HFGoe_IF2_b1R1_4h **Scan** 15984
**Peptide** GVMPQTIEAIQHAKA **Charge** 3 **Spectral Angle** 0.62

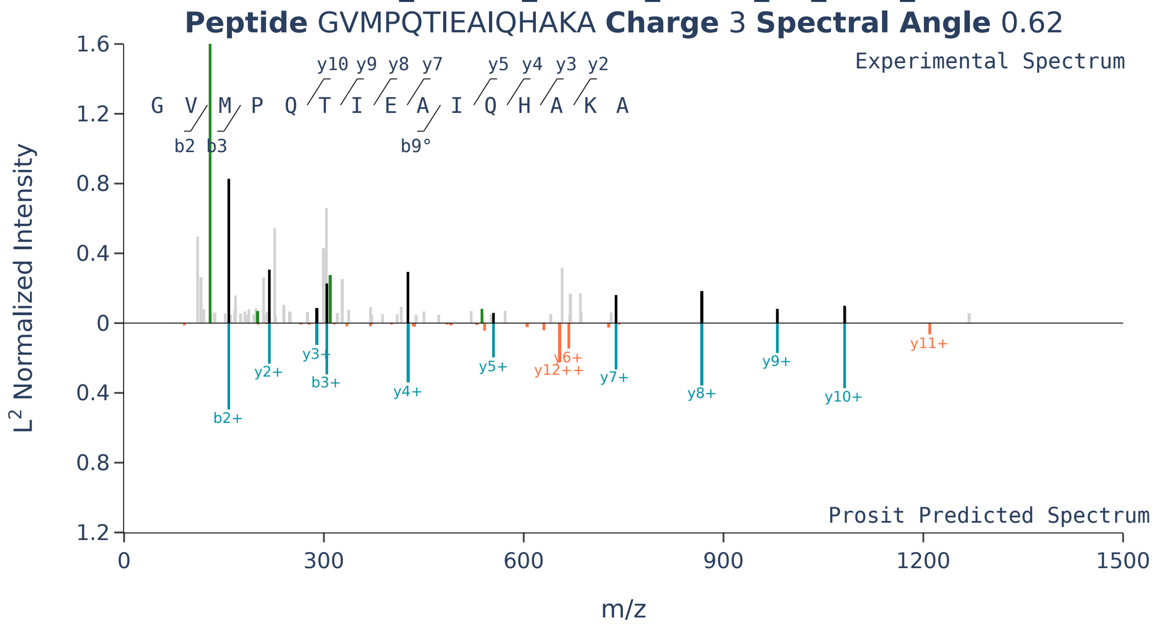


**Source** HRoetschke_211222_160123_HFGoe_IF2_b1R1_4h **Scan** 14987
**Peptide** ESFLDKGRGPVAT **Charge** 2 **Spectral Angle** 0.72

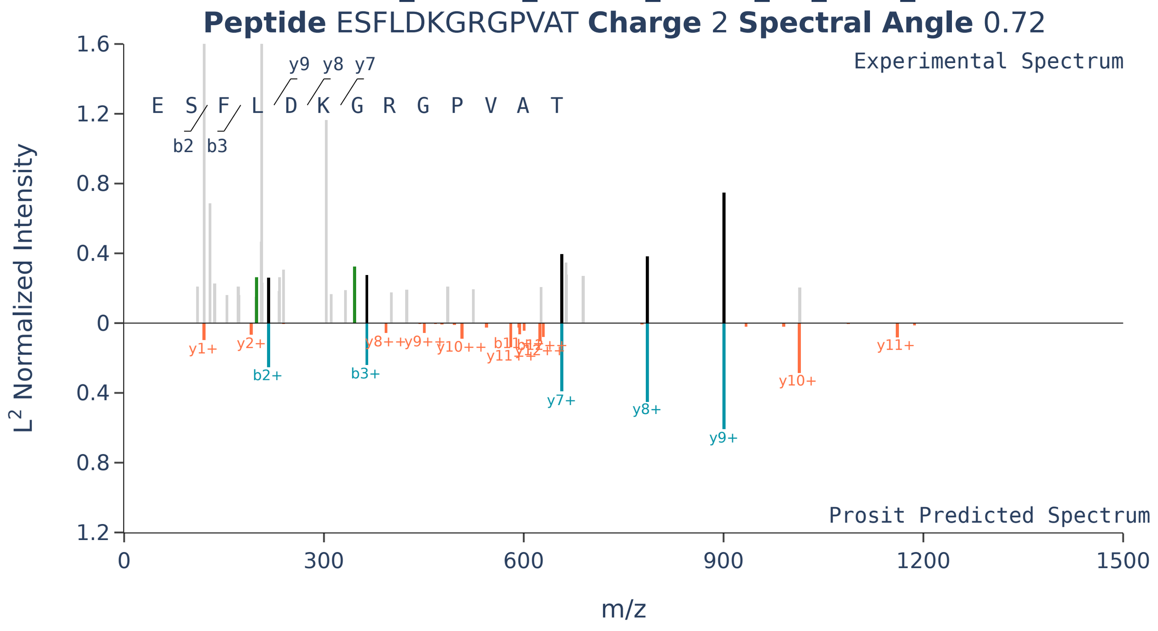


**Source** HRoetschke_211222_160123_HFGoe_IF2_b2R2_2h **Scan** 16969
**Peptide** AKAAQVPVVV **Charge** 2 **Spectral Angle** 0.78

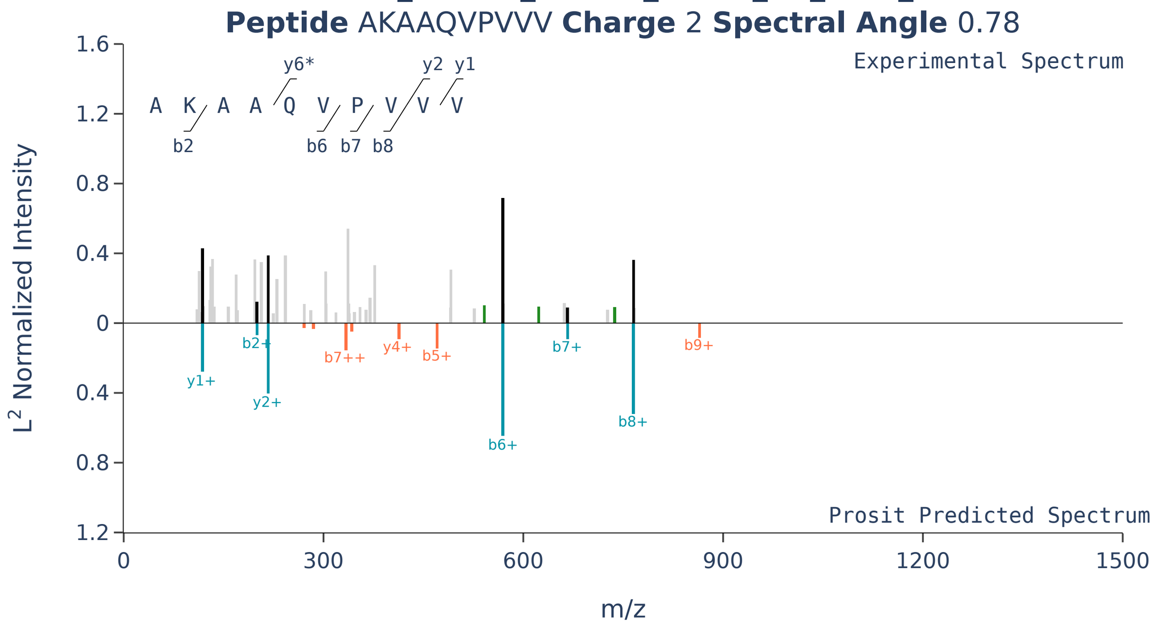


**Source** HRoetschke_211222_160123_HFGoe_IF2_b2R2_2h **Scan** 10564
**Peptide** NQKNSGPDKLTLQ **Charge** 3 **Spectral Angle** 0.87

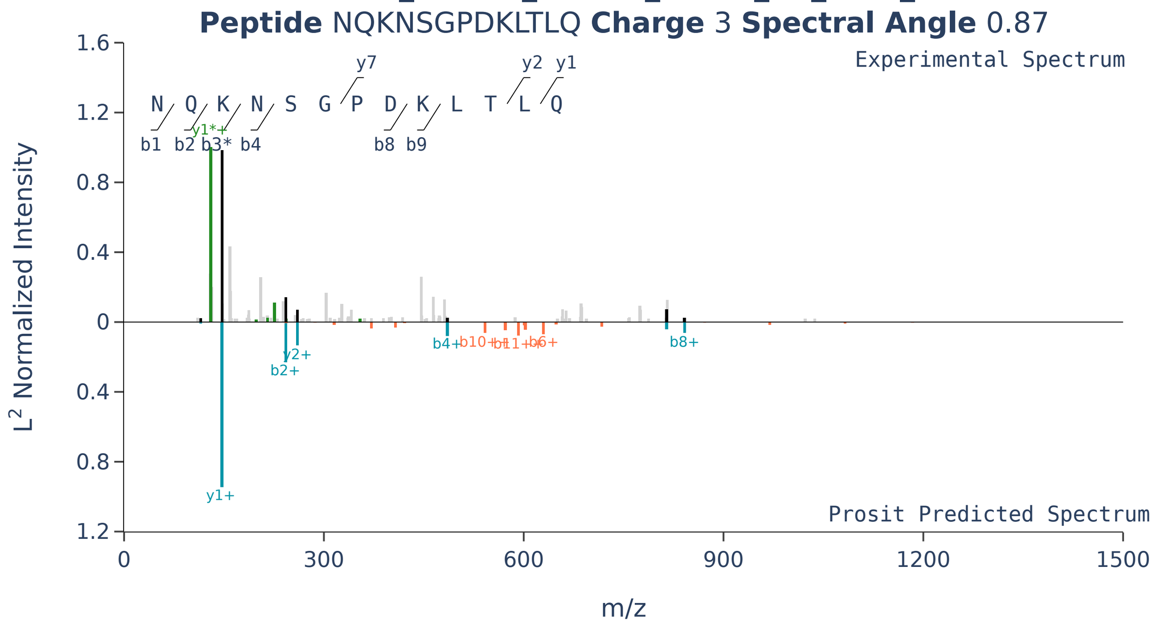


**Source** HRoetschke_211222_160123_HFGoe_IF2_b1R1_4h **Scan** 7797
**Peptide** RADASARKVIEAE **Charge** 3 **Spectral Angle** 0.64

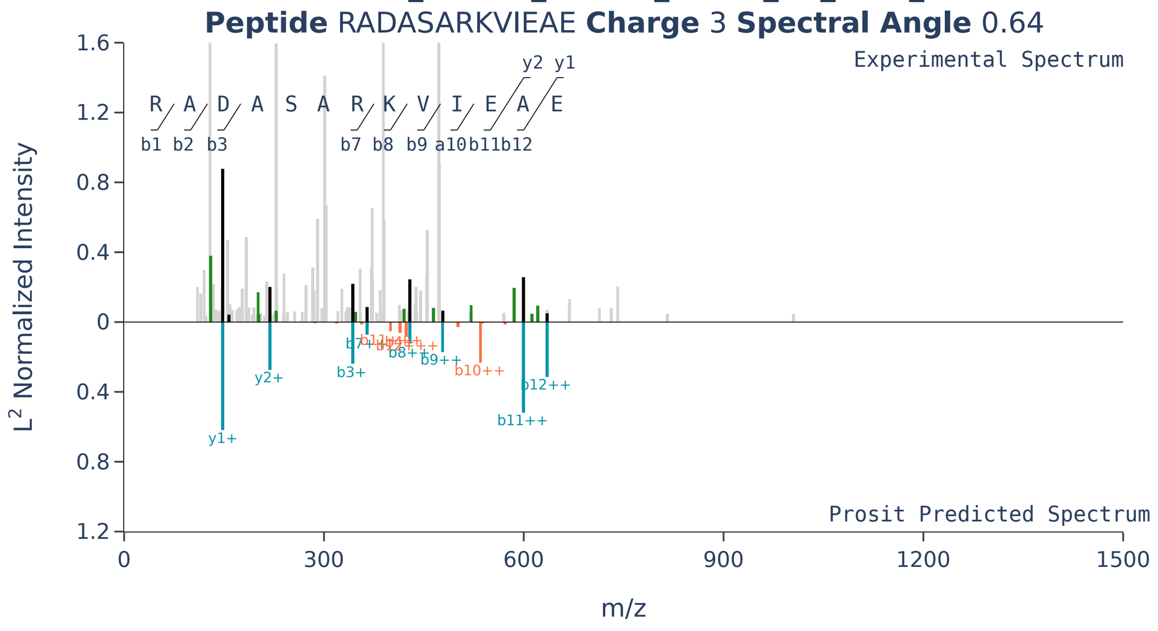


**Source** HRoetschke_211222_160123_HFGoe_IF2_b2R2_4h **Scan** 16440
**Peptide** HVETENGMITF **Charge** 2 **Spectral Angle** 0.59

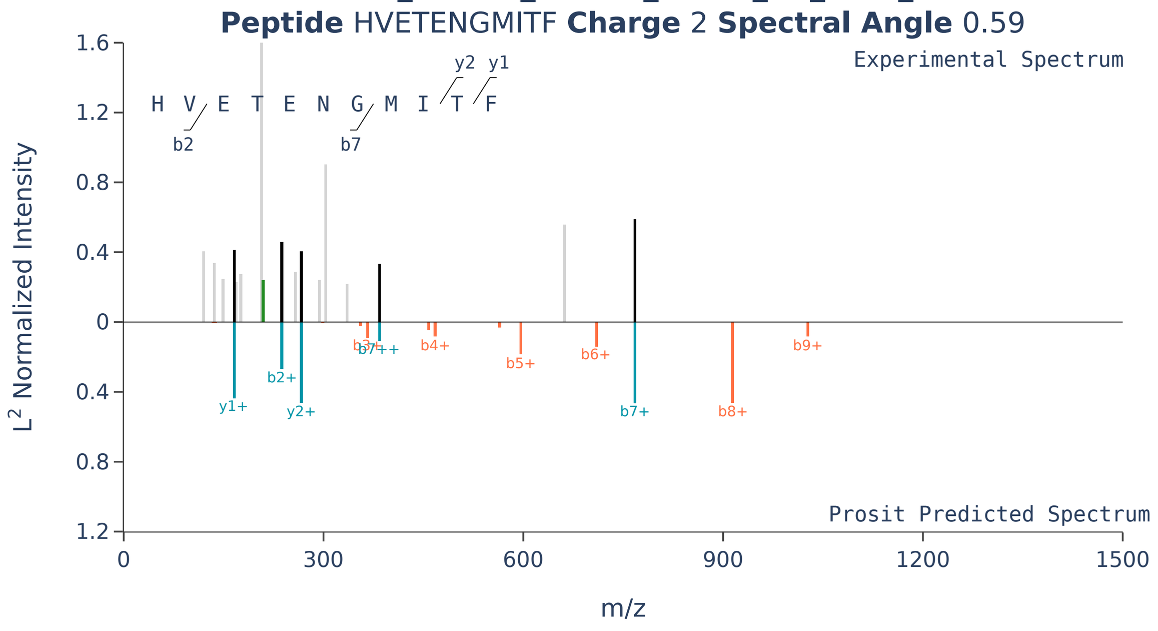


**Source** HRoetschke_211222_160123_HFGoe_IF2_b1R1_4h **Scan** 17247
**Peptide** ETITVGELANKM **Charge** 2 **Spectral Angle** 0.64

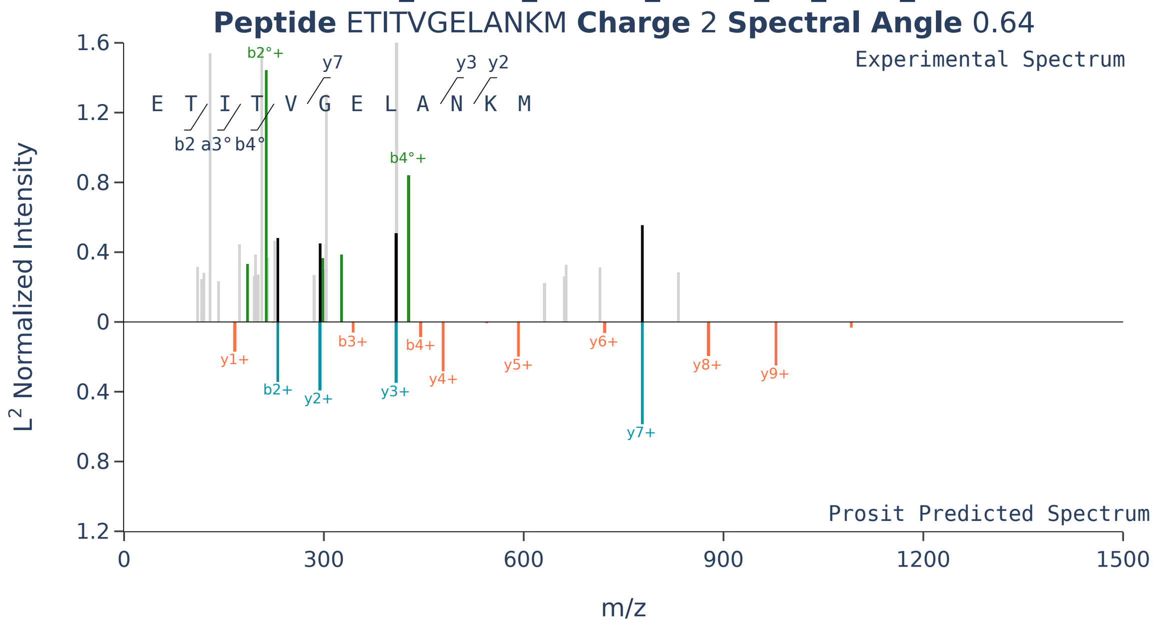


**Source** HRoetschke_211222_160123_HFGoe_IF2_b1R1_4h **Scan** 11910
**Peptide** PIRVLRD **Charge** 2 **Spectral Angle** 0.62

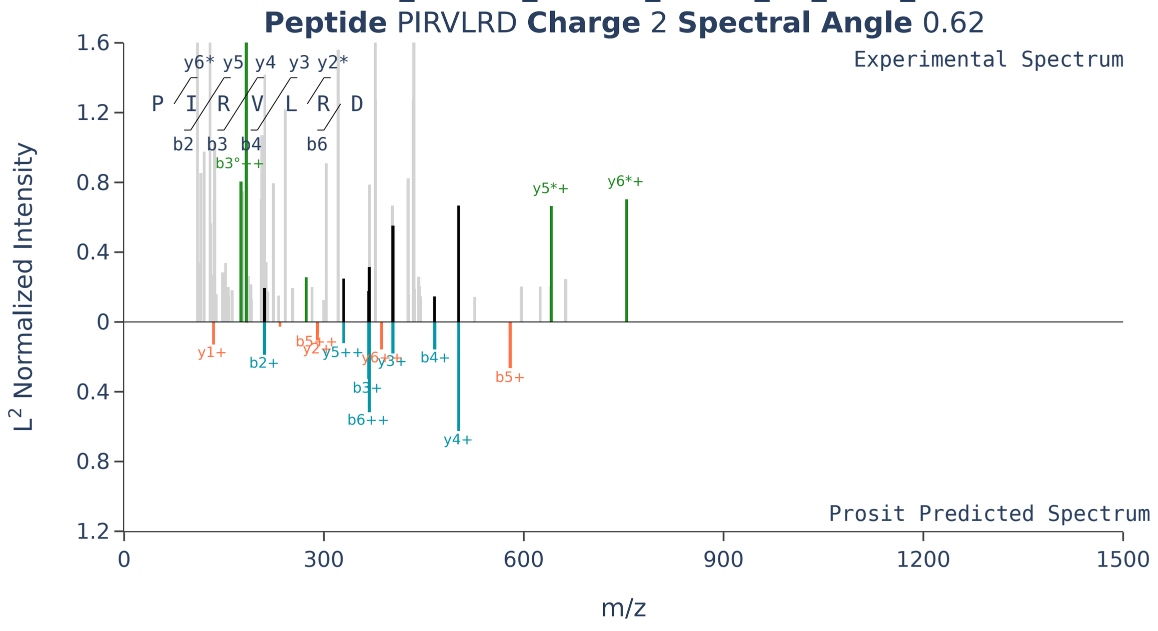


**Source** HRoetschke_211222_160123_HFGoe_IF2_b1R1_4h **Scan** 13836
**Peptide** RLVQQFADAGIRKSADD **Charge** 3 **Spectral Angle** 0.68

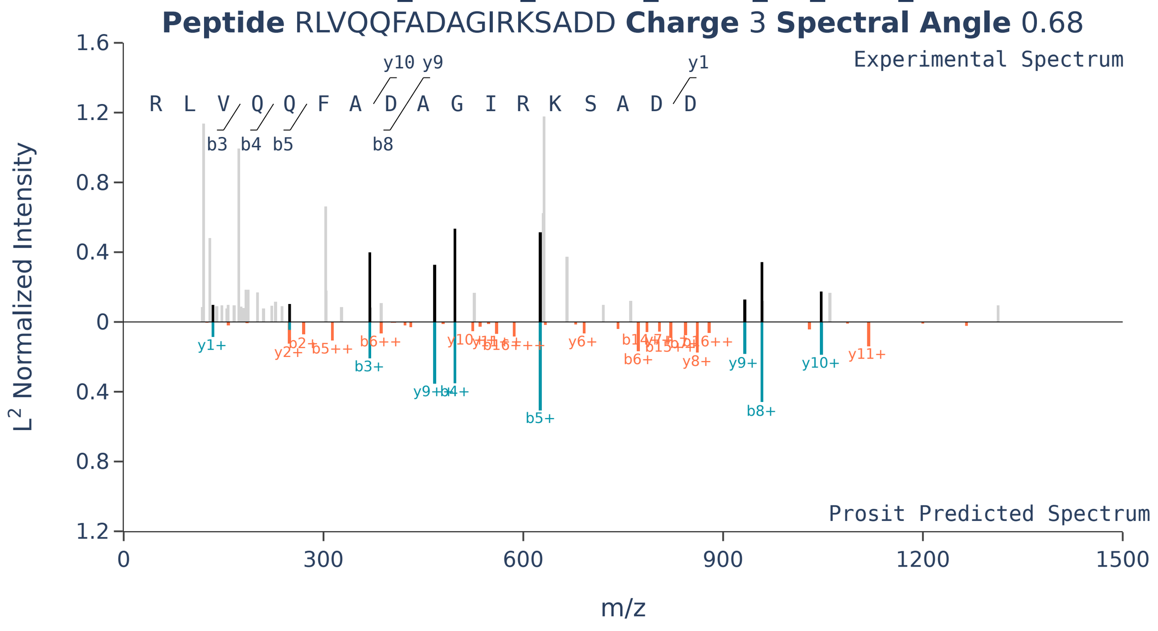

**Source** HRoetschke_211222_160123_HFGoe_IF2_b2R1_24h **Scan** 7959
**Peptide** DSSDYHVTTSQ **Charge** 2 **Spectral Angle** 0.85

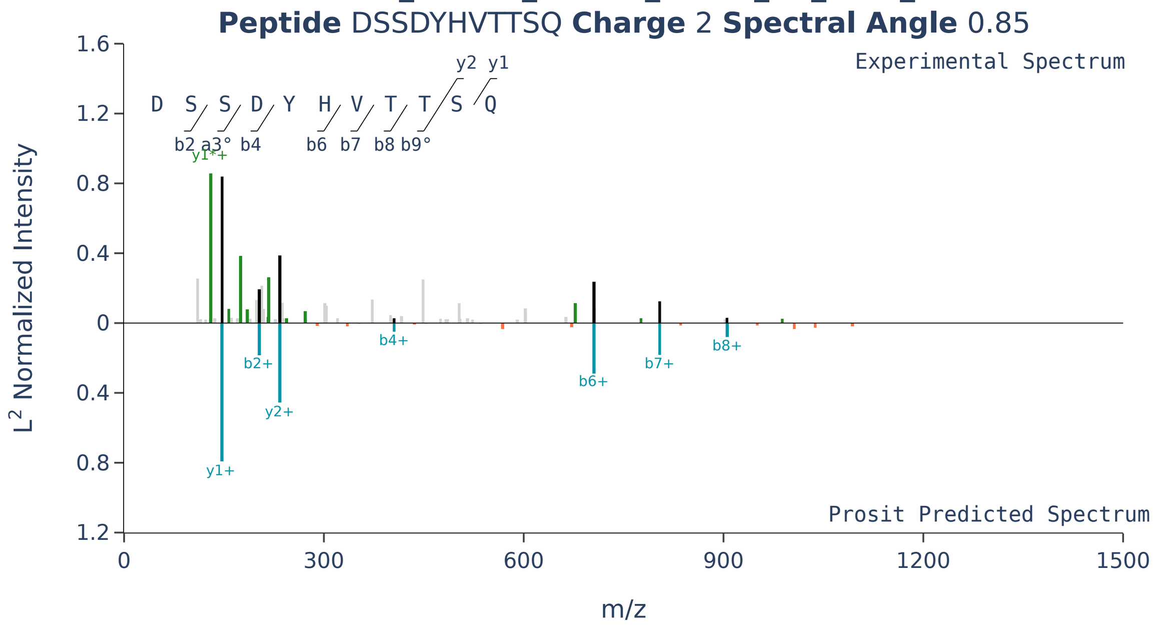


**Source** HRoetschke_211222_160123_HFGoe_IF2_b1R1_4h **Scan** 5346
**Peptide** EEEARRKLEE **Charge** 3 **Spectral Angle** 0.52

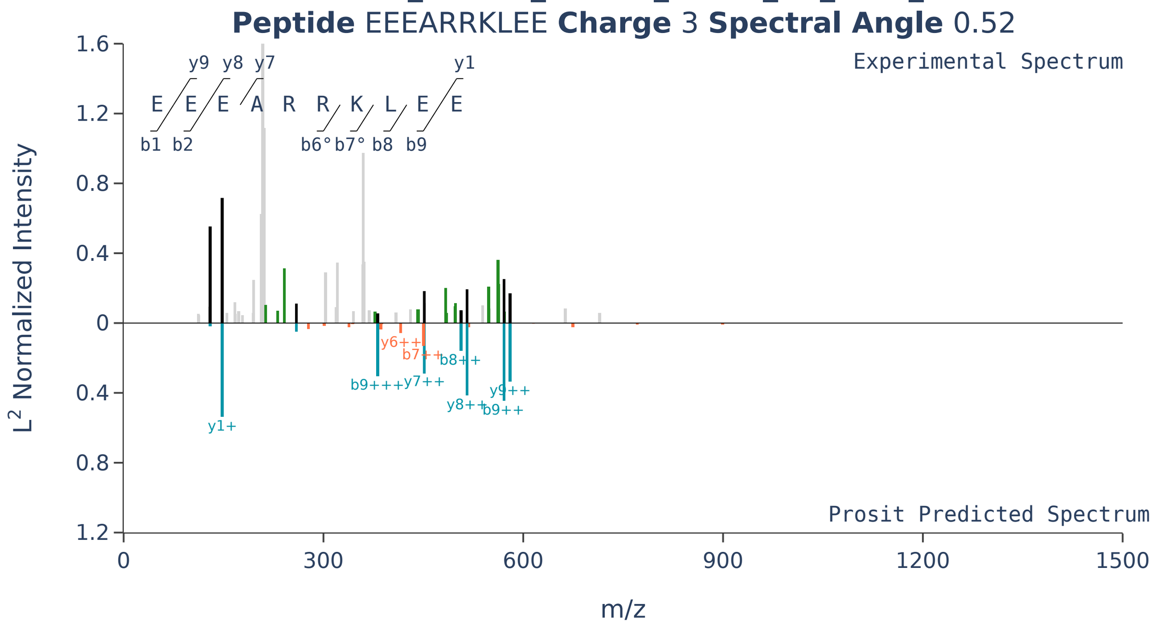


**Source** HRoetschke_211222_160123_HFGoe_IF2_b2R2_24h **Scan** 5926
**Peptide** SDYHVTTSQHARQ **Charge** 3 **Spectral Angle** 0.53

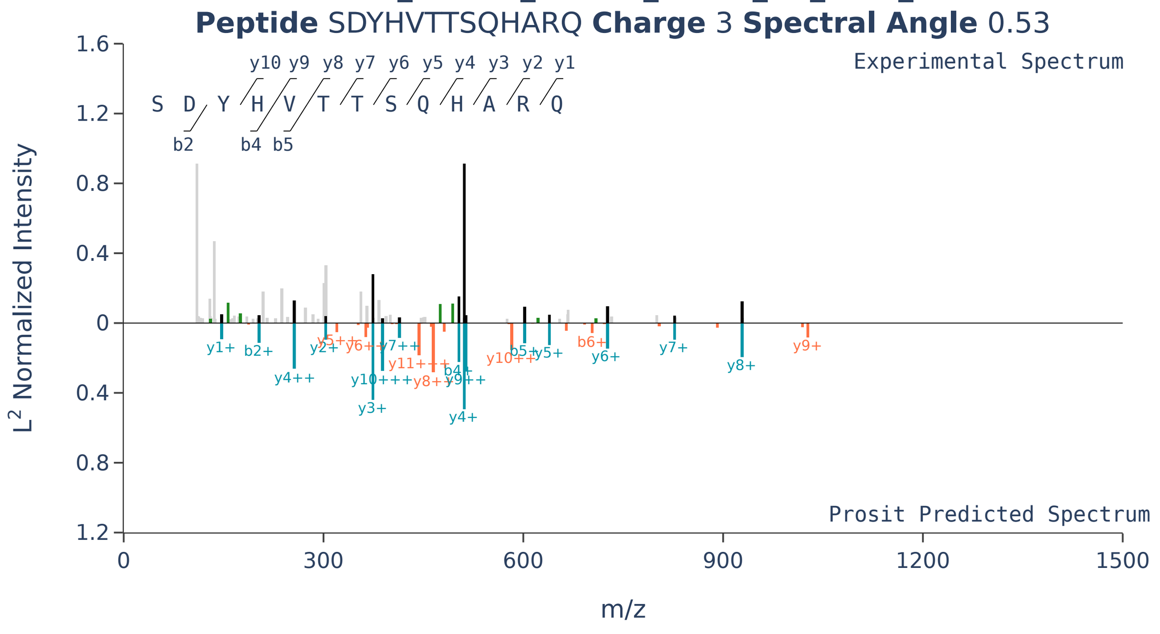


**Source** HRoetschke_211222_160123_HFGoe_IF2_b2R2_24h **Scan** 9883
**Peptide** GELANKMAVKGSQVIKA **Charge** 3 **Spectral Angle** 0.76

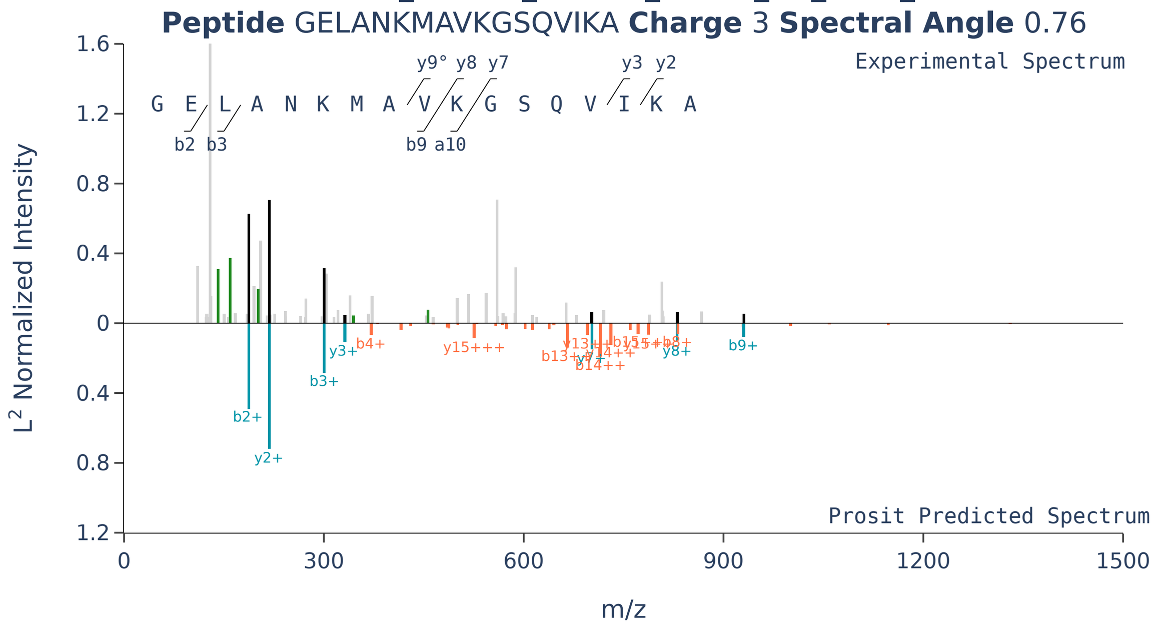


**Source** HRoetschke_211222_160123_HFGoe_IF2_b1R1_4h **Scan** 17235
**Peptide** QVPVVVAVNK **Charge** 2 **Spectral Angle** 0.59

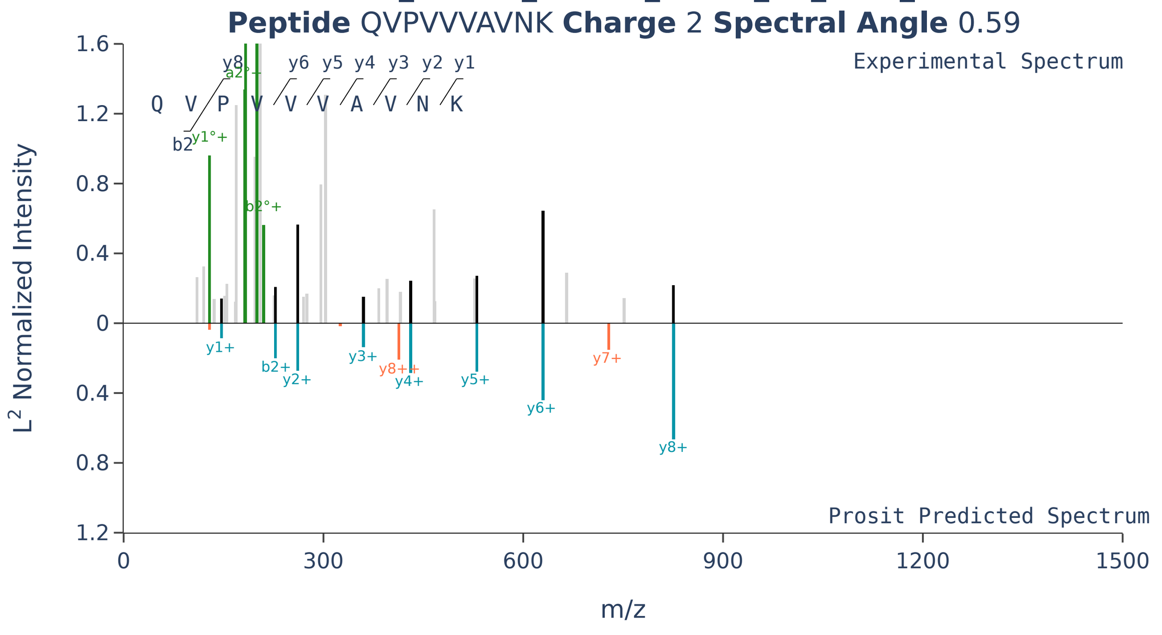


**Source** HRoetschke_211222_160123_HFGoe_IF2_b2R1_24h **Scan** 6957
**Peptide** DAGIRKSADDSVSAQ **Charge** 3 **Spectral Angle** 0.86

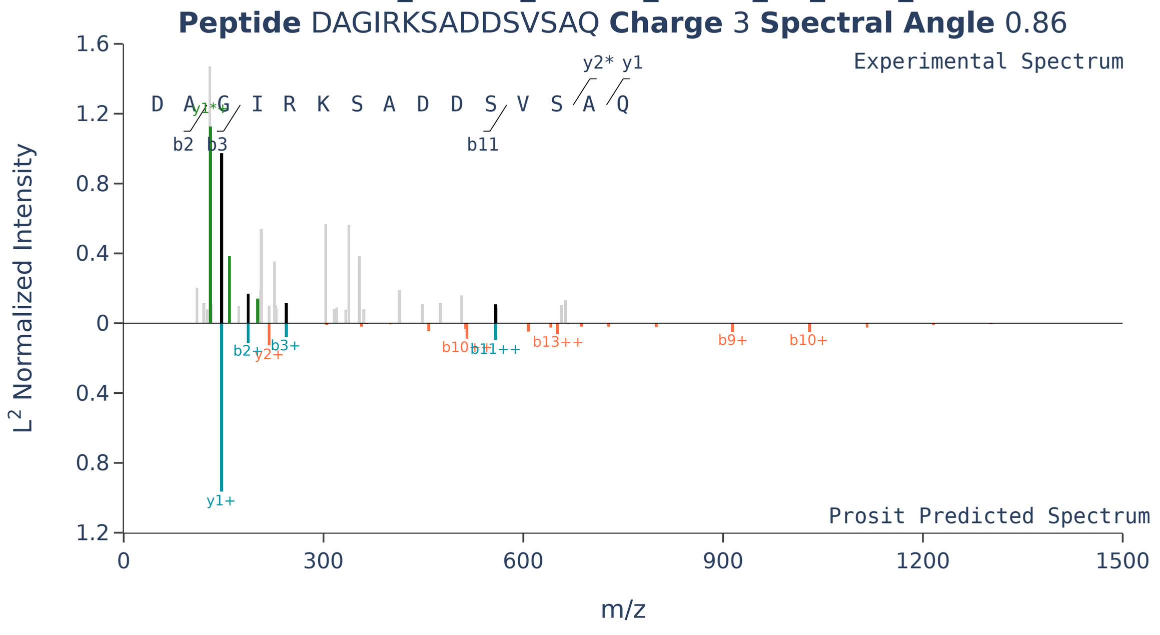


**Source** HRoetschke_211222_160123_HFGoe_IF2_b1R2_24h **Scan** 11862
**Peptide** VNKIDKPEADPDRVKNELSQ **Charge** 4 **Spectral Angle** 0.75

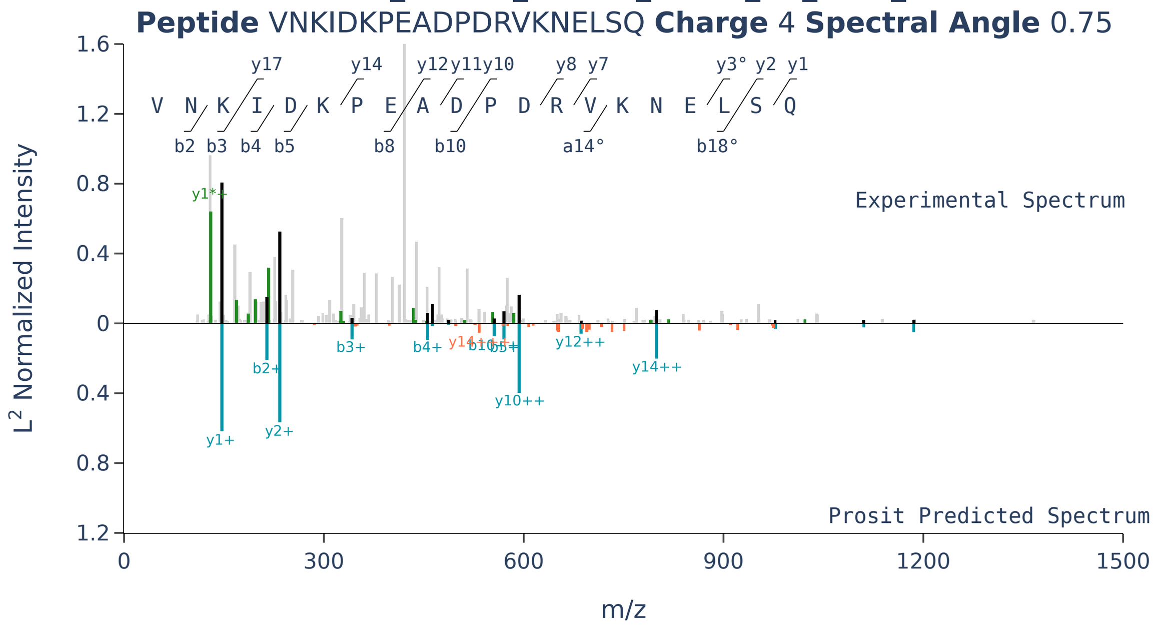


**Source** HRoetschke_211222_160123_HFGoe_IF2_b1R1_4h **Scan** 7984
**Peptide** NIPGTGGKSKSVQIEVRKK **Charge** 5 **Spectral Angle** 0.6

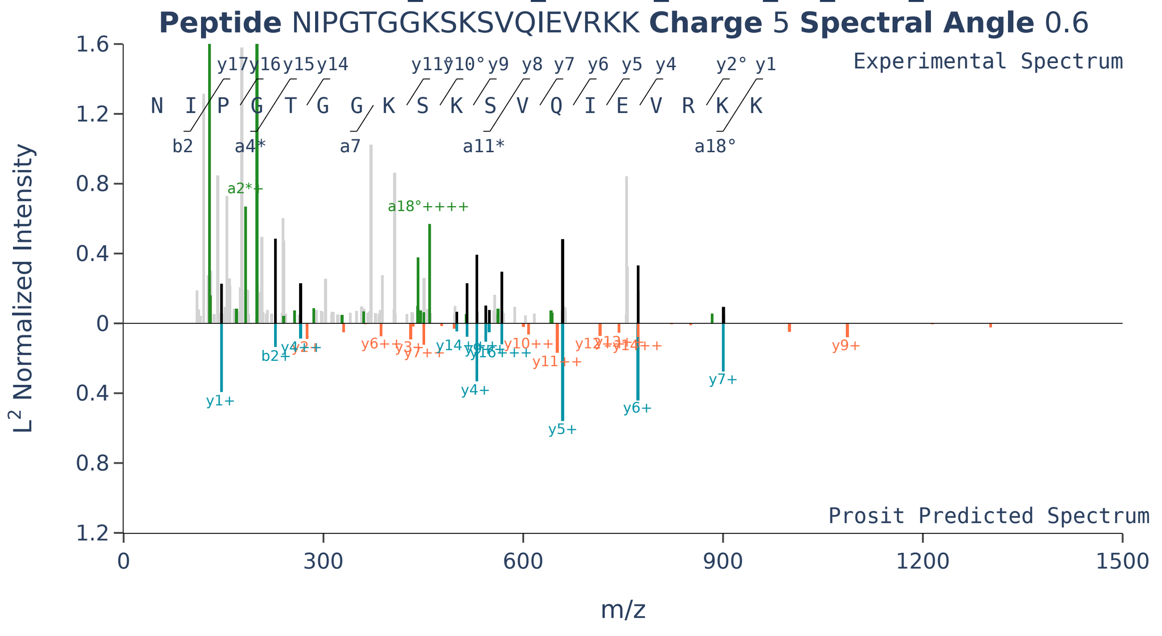


**Source** HRoetschke_211222_160123_HFGoe_IF2_b1R1_4h **Scan** 7178
**Peptide** FTSMRARGAQATD **Charge** 3 **Spectral Angle** 0.62

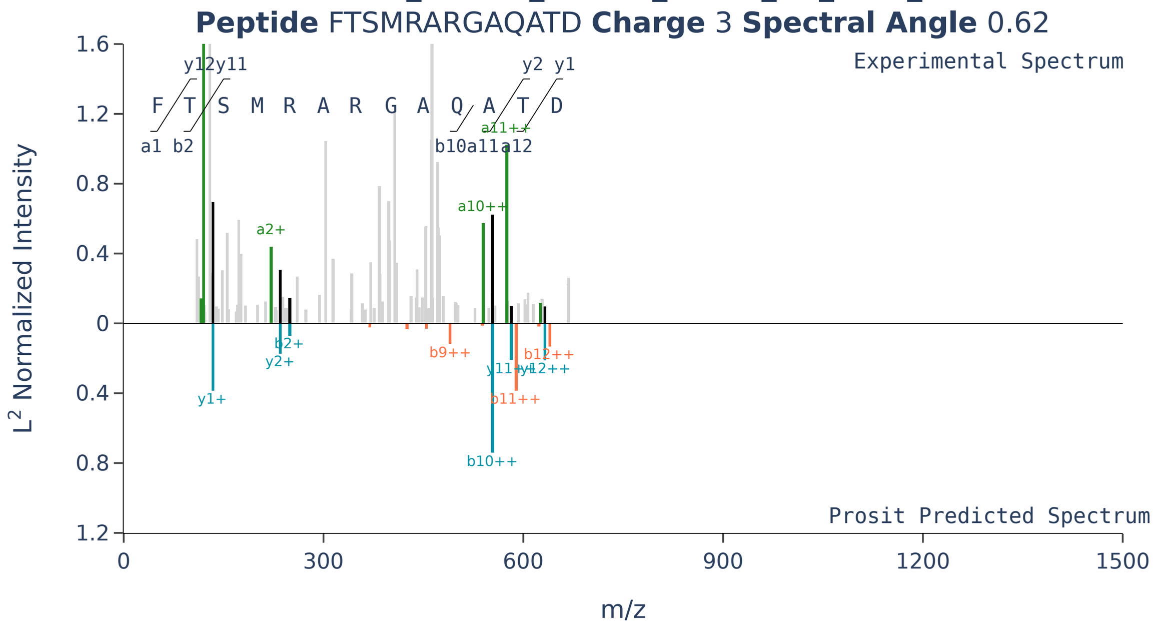


**Source** HRoetschke_211222_160123_HFGoe_IF2_b1R1_4h **Scan** 4920
**Peptide** DMTKNAQAEKA **Charge** 2 **Spectral Angle** 0.71

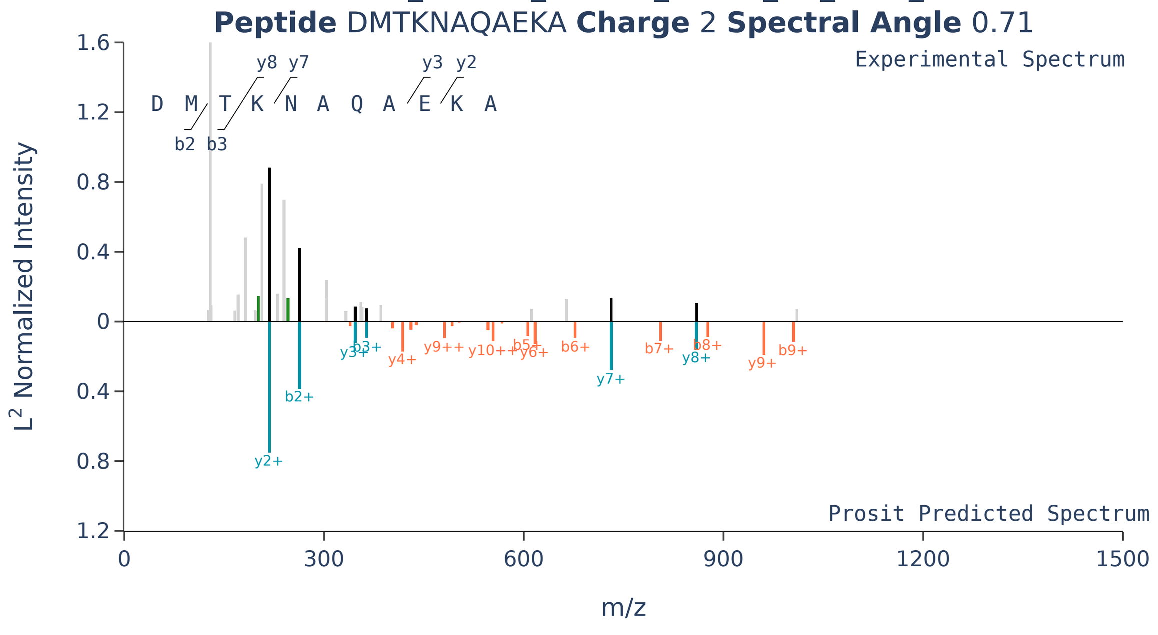


**Source** HRoetschke_211222_160123_HFGoe_IF2_b1R1_24h **Scan** 6343
**Peptide** GGKGGKRKGSSLQQGF **Charge** 3 **Spectral Angle** 0.63

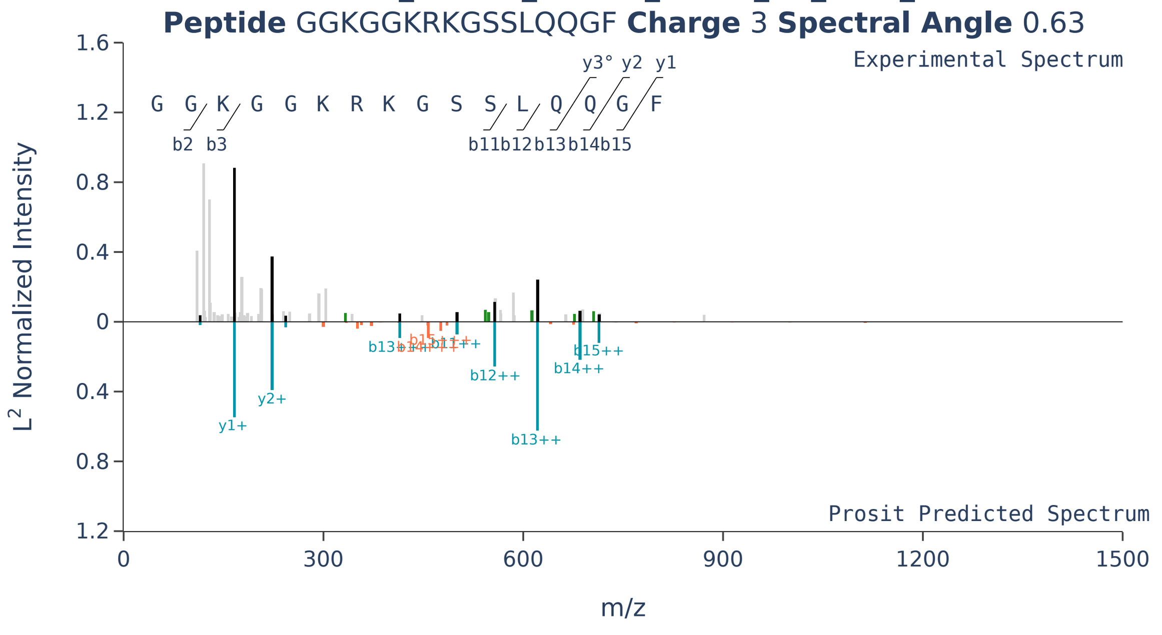


**Source** HRoetschke_211222_160123_HFGoe_IF2_b1R1_4h **Scan** 19223
**Peptide** AVMSDRDTGAAAEPRAPVVTI **Charge** 3 **Spectral Angle** 0.76

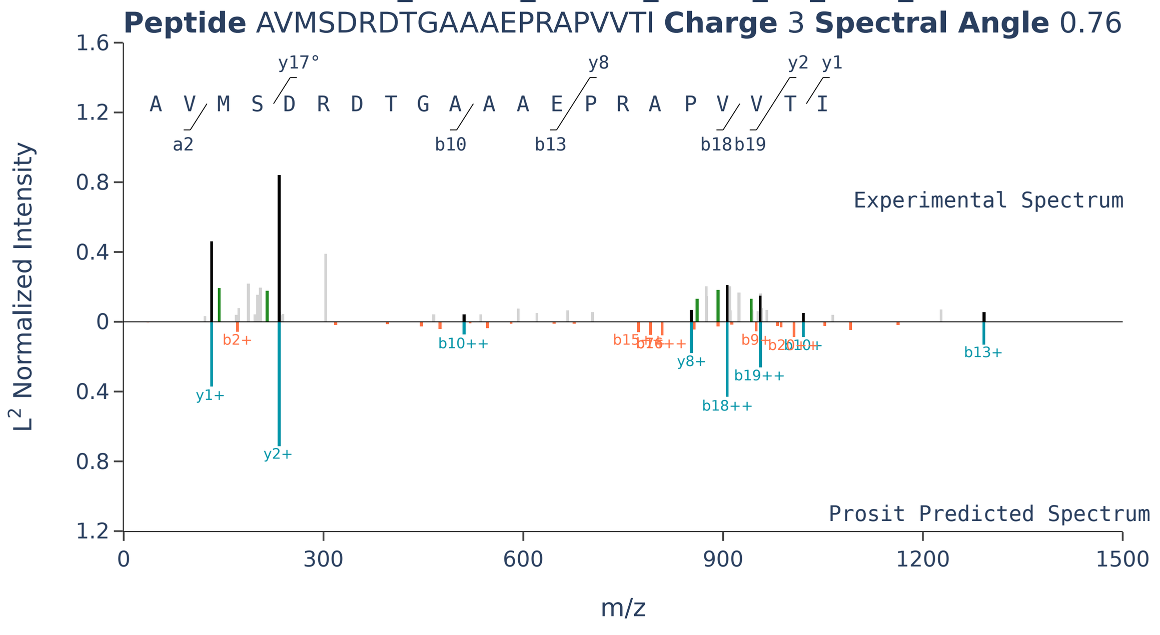


**Source** HRoetschke_211222_160123_HFGoe_IF2_b1R1_24h **Scan** 9114
**Peptide** VKRDPQEAERLAAEE **Charge** 3 **Spectral Angle** 0.66

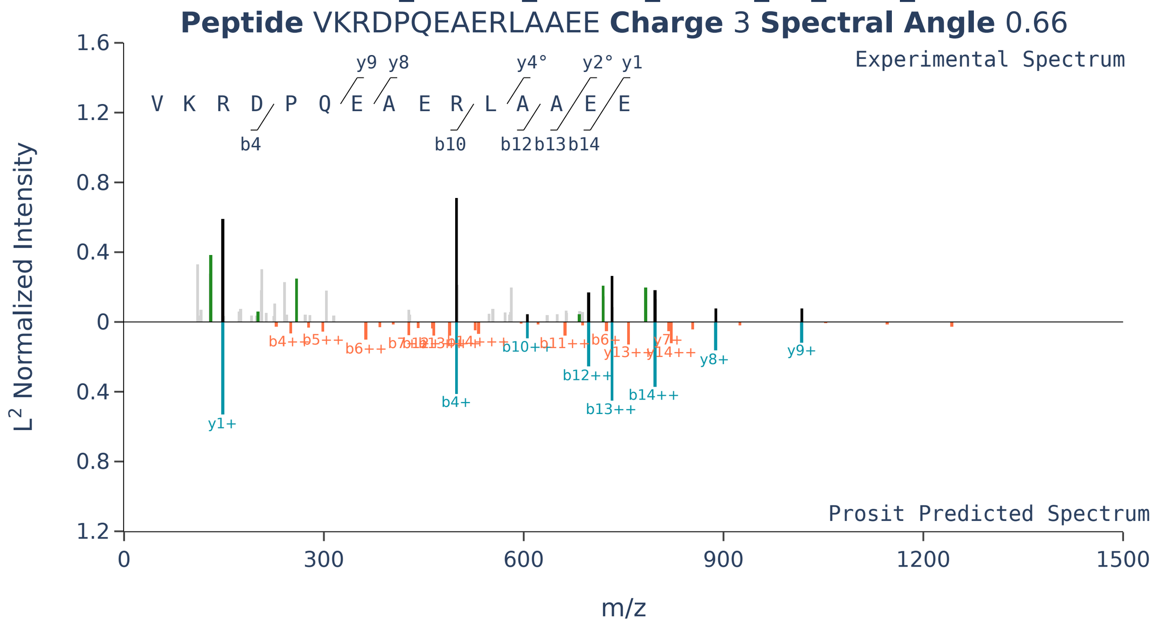


**Source** HRoetschke_211222_160123_HFGoe_IF2_b2R2_24h **Scan** 11555
**Peptide** KLDKPEADPDRVKNEL **Charge** 3 **Spectral Angle** 0.69

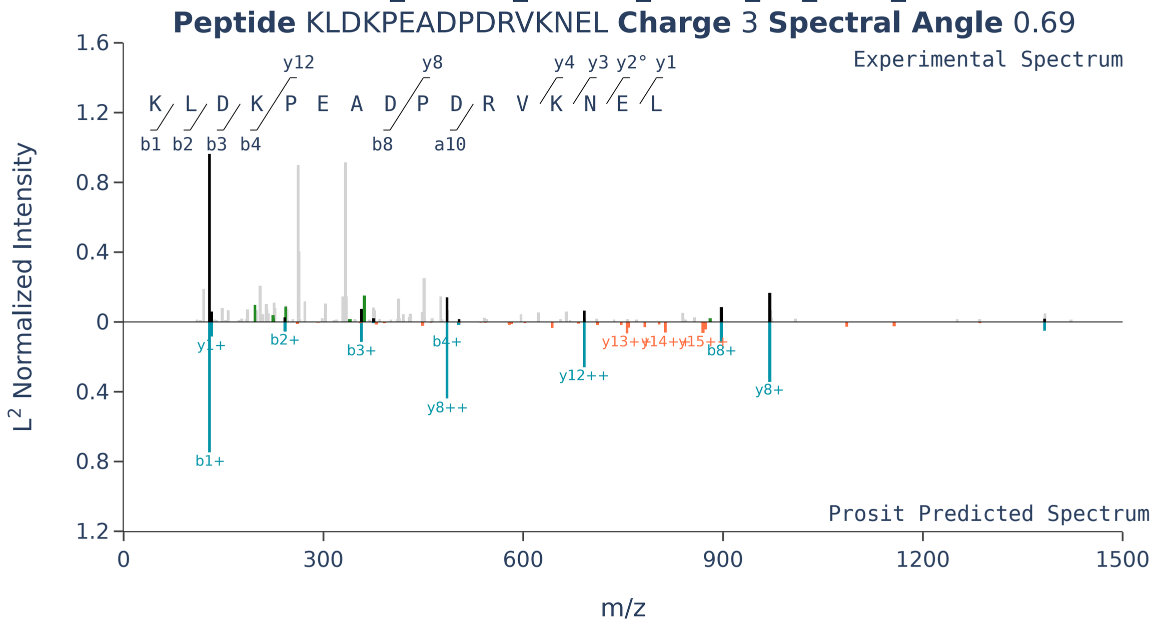


**Source** HRoetschke_211222_160123_HFGoe_IF2_b1R1_4h **Scan** 19676
**Peptide** DRDTGAAAEPRAPVVTI **Charge** 2 **Spectral Angle** 0.74

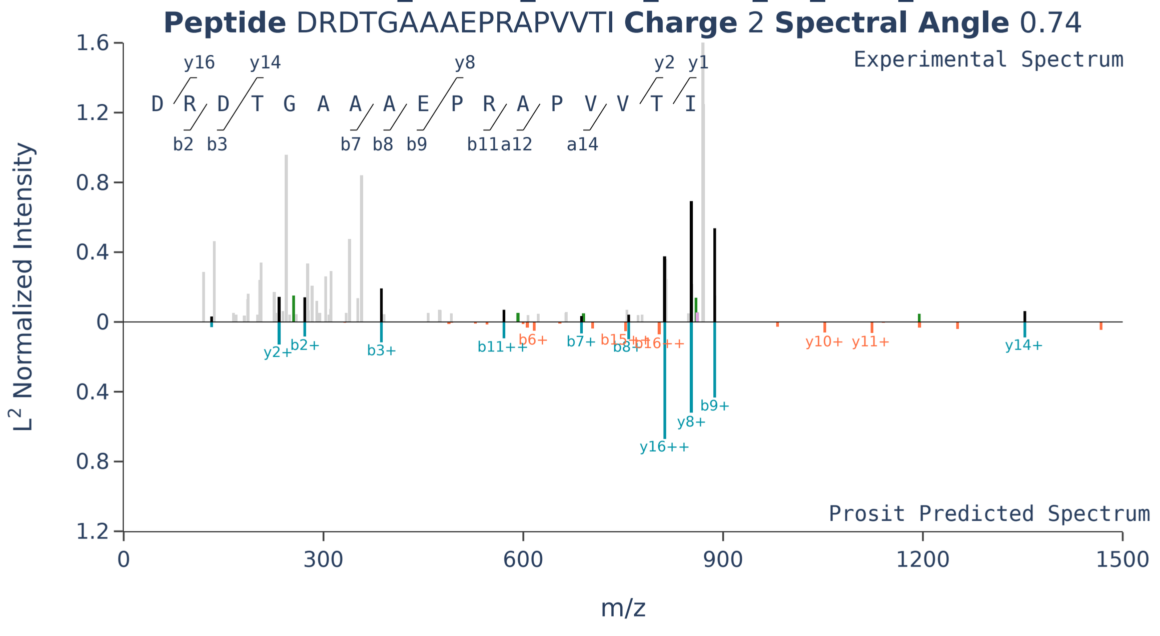

**Source** HRoetschke_211222_160123_HFGoe_IF2_b1R1_4h **Scan** 5102
**Peptide** AESKADREEARAAVR **Charge** 4 **Spectral Angle** 0.62

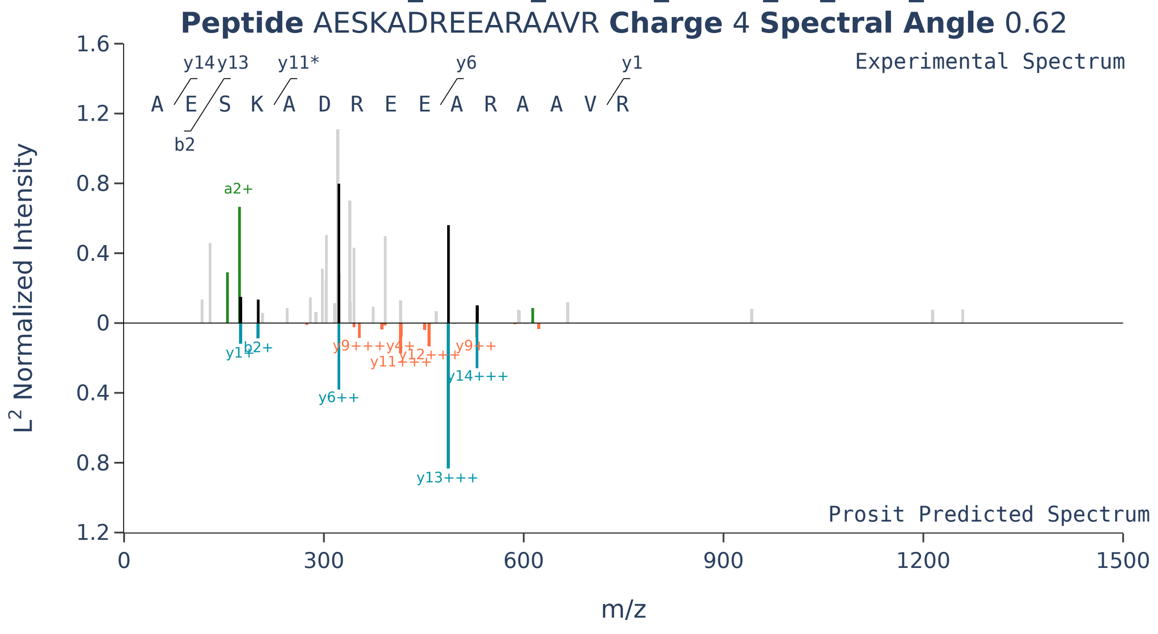


**Source** HRoetschke_211222_160123_HFGoe_IF2_b2R2_24h **Scan** 9045
**Peptide** RVKNELSQY **Charge** 2 **Spectral Angle** 0.6

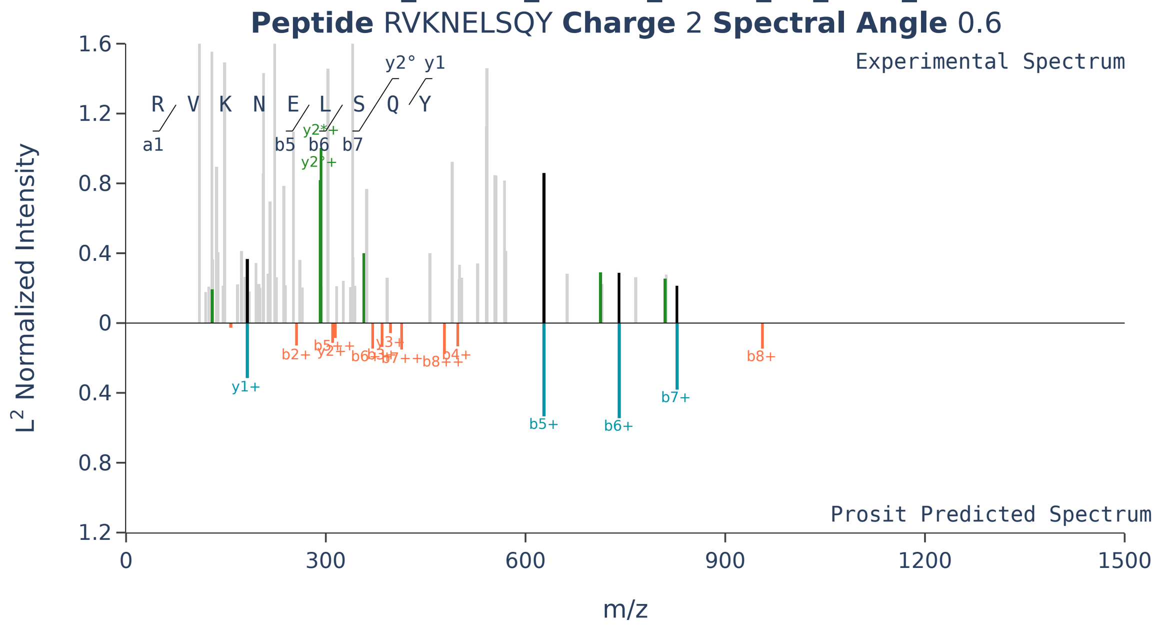


**Source** HRoetschke_211222_160123_HFGoe_IF2_b1R1_1h **Scan** 8878
**Peptide** KAVRKGMASGAVL **Charge** 3 **Spectral Angle** 0.54

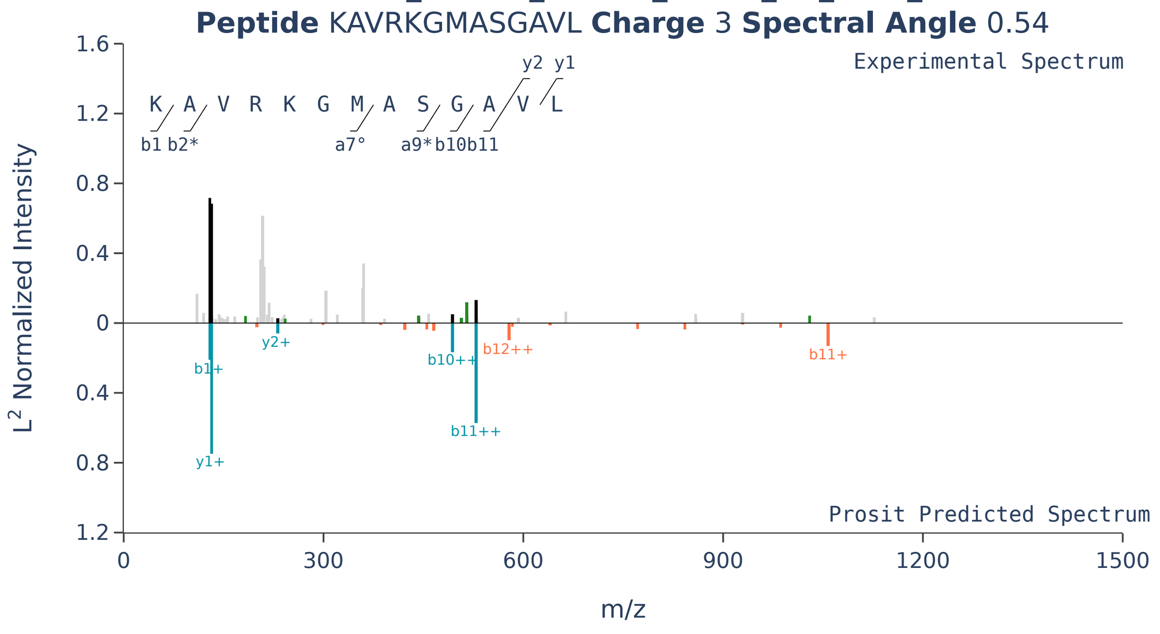


**Source** HRoetschke_211222_160123_HFGoe_IF2_b1R1_4h **Scan** 5522
**Peptide** SSDYHVTTSQHAR **Charge** 3 **Spectral Angle** 0.66

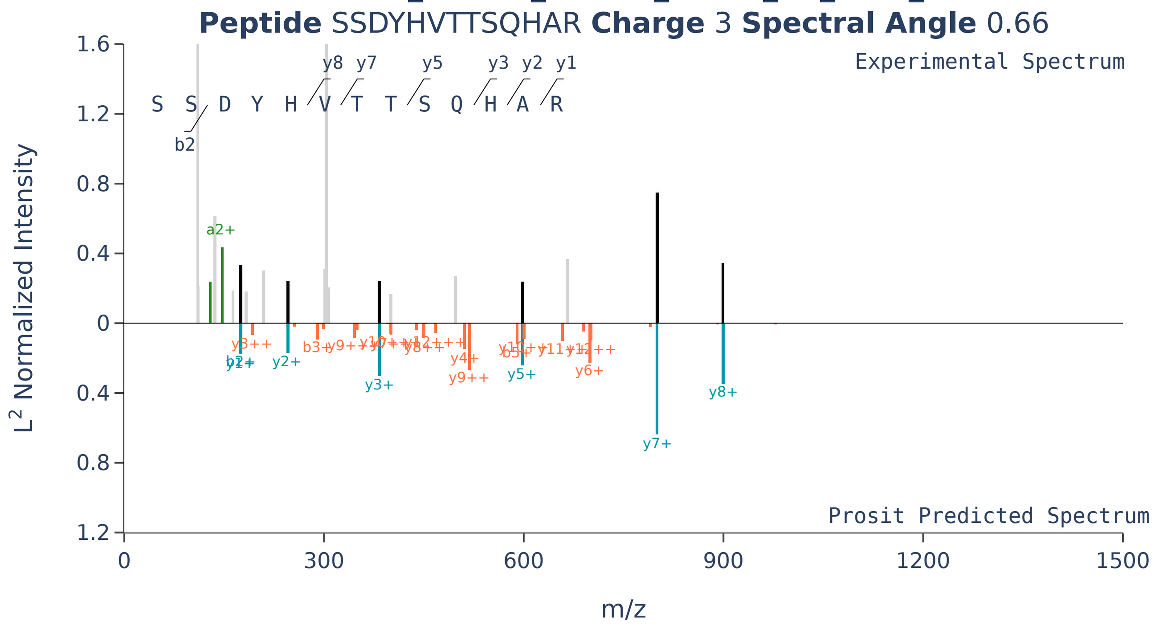


**Source** HRoetschke_211222_160123_HFGoe_IF2_b1R1_4h **Scan** 19002
**Peptide** DKGRGPVATVLV **Charge** 2 **Spectral Angle** 0.68

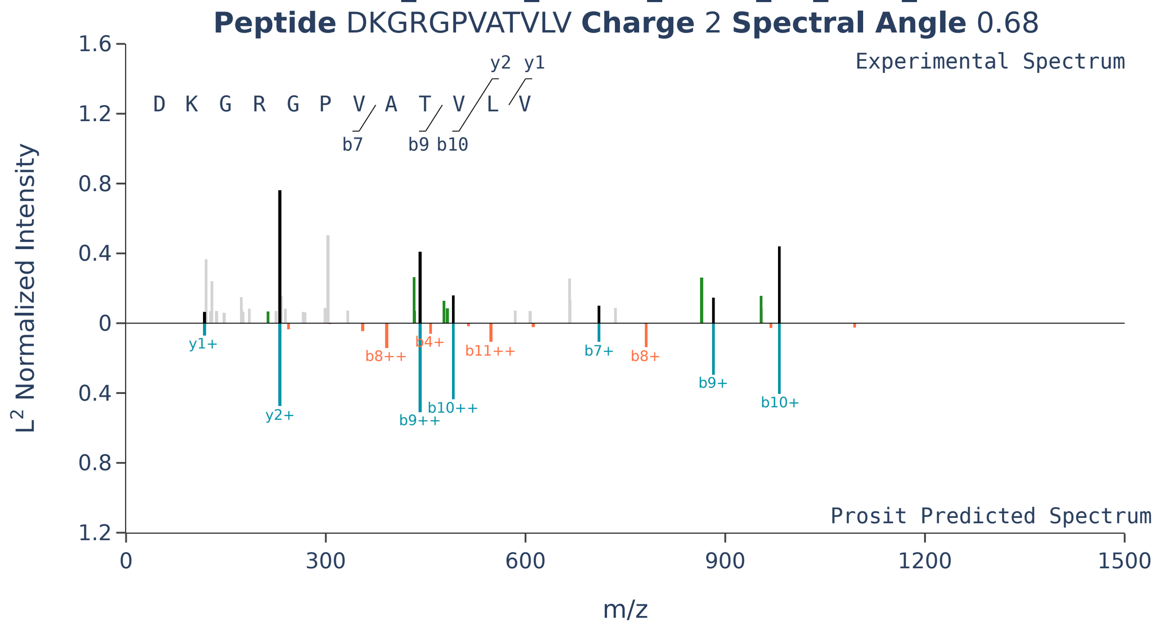


**Source** HRoetschke_211222_160123_HFGoe_IF2_b1R2_24h **Scan** 6985
**Peptide** SVQIEVRKK **Charge** 3 **Spectral Angle** 0.82

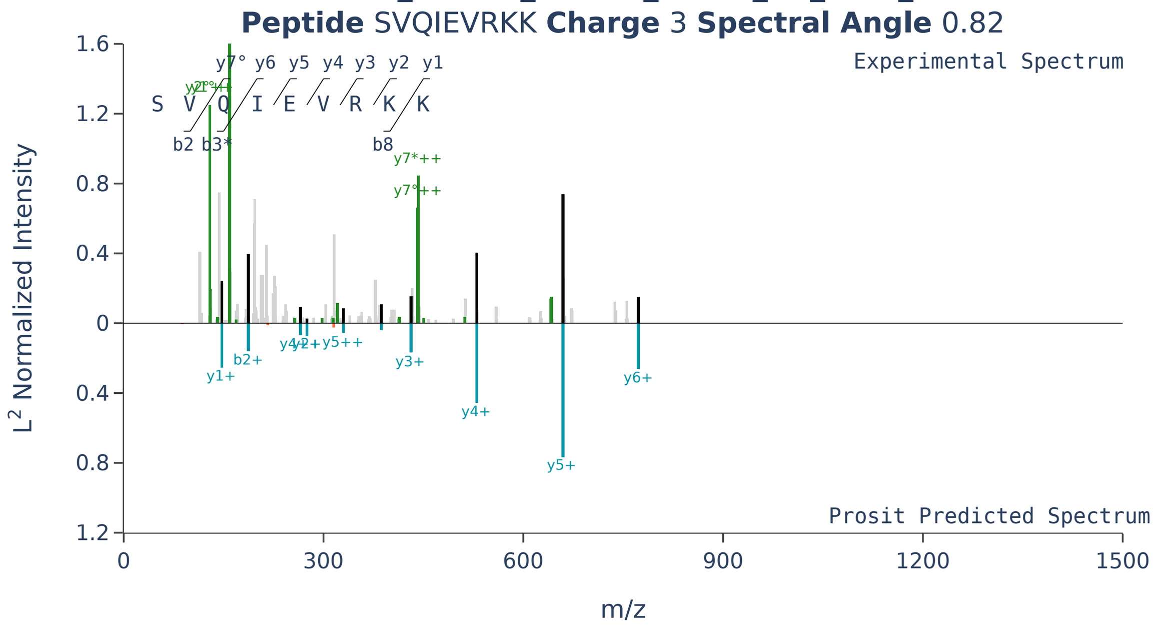


**Source** HRoetschke_211222_160123_HFGoe_IF2_b1R2_1h **Scan** 17359
**Peptide** GVMPQTIEAIQHA **Charge** 2 **Spectral Angle** 0.63

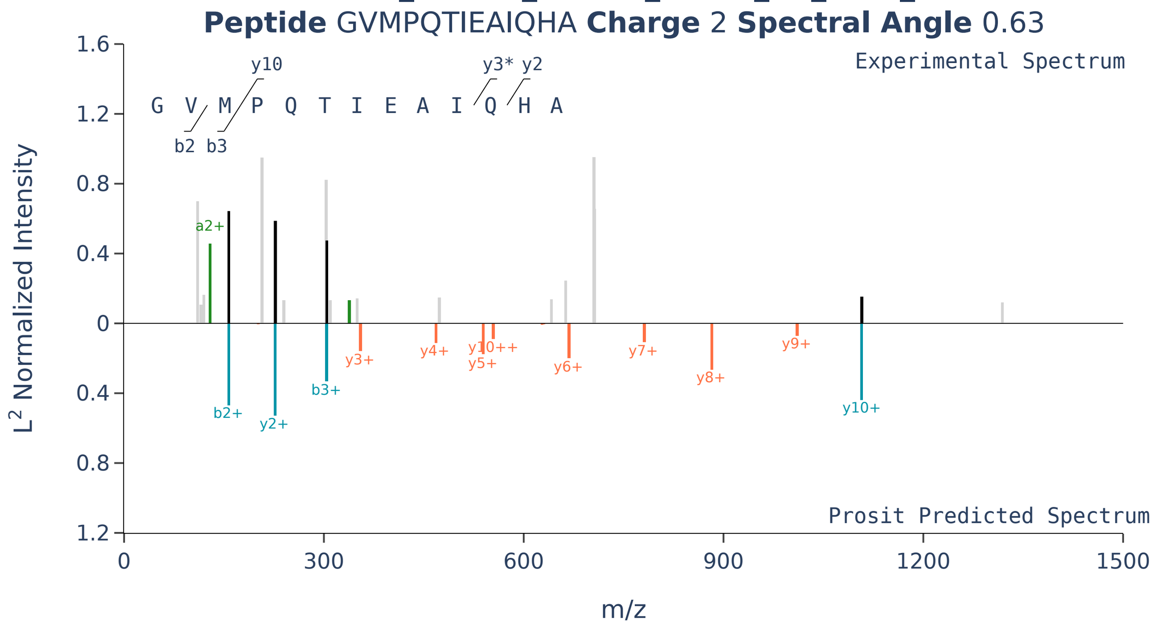


**Source** HRoetschke_211222_160123_HFGoe_IF2_b1R1_24h **Scan** 6610
**Peptide** RADASARKVI **Charge** 3 **Spectral Angle** 0.62

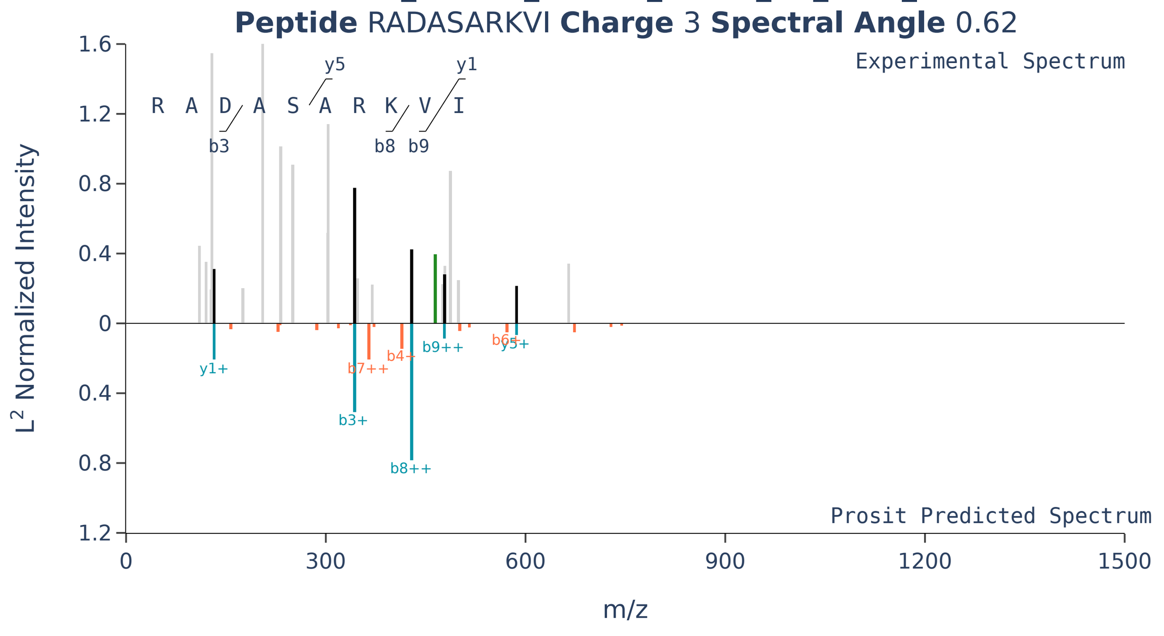


**Source** HRoetschke_211222_160123_HFGoe_IF2_b1R1_4h **Scan** 13610
**Peptide** RLVQQFADAGIRKSAD **Charge** 3 **Spectral Angle** 0.66

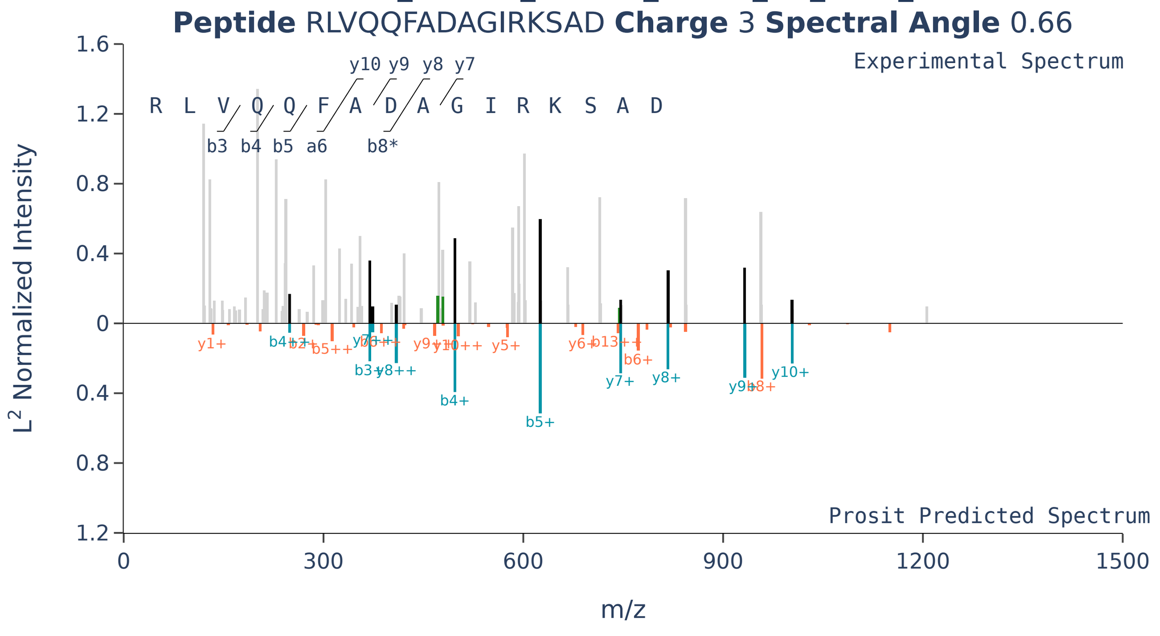


**Source** HRoetschke_211222_160123_HFGoe_IF2_b1R1_2h **Scan** 6062
**Peptide** HLNQKNSGPDKLTLQRKTRST **Charge** 4 **Spectral Angle** 0.65

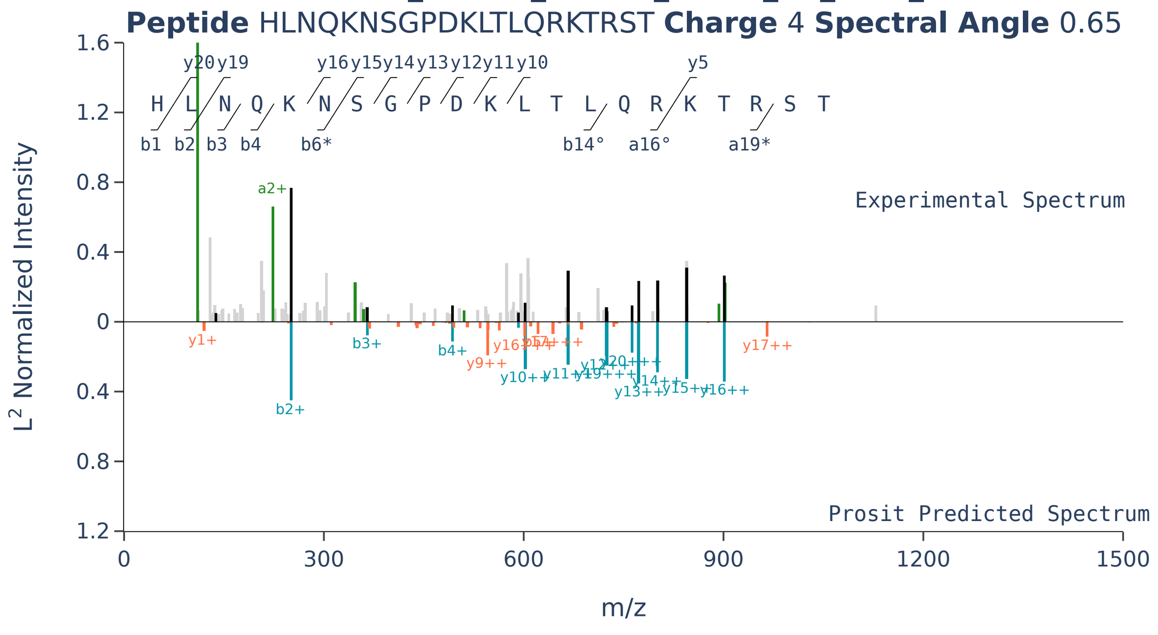


**Source** HRoetschke_211222_160123_HFGoe_IF2_b1R1_4h **Scan** 17806
**Peptide** DDGVMPQTIEAIQHAKA **Charge** 3 **Spectral Angle** 0.68

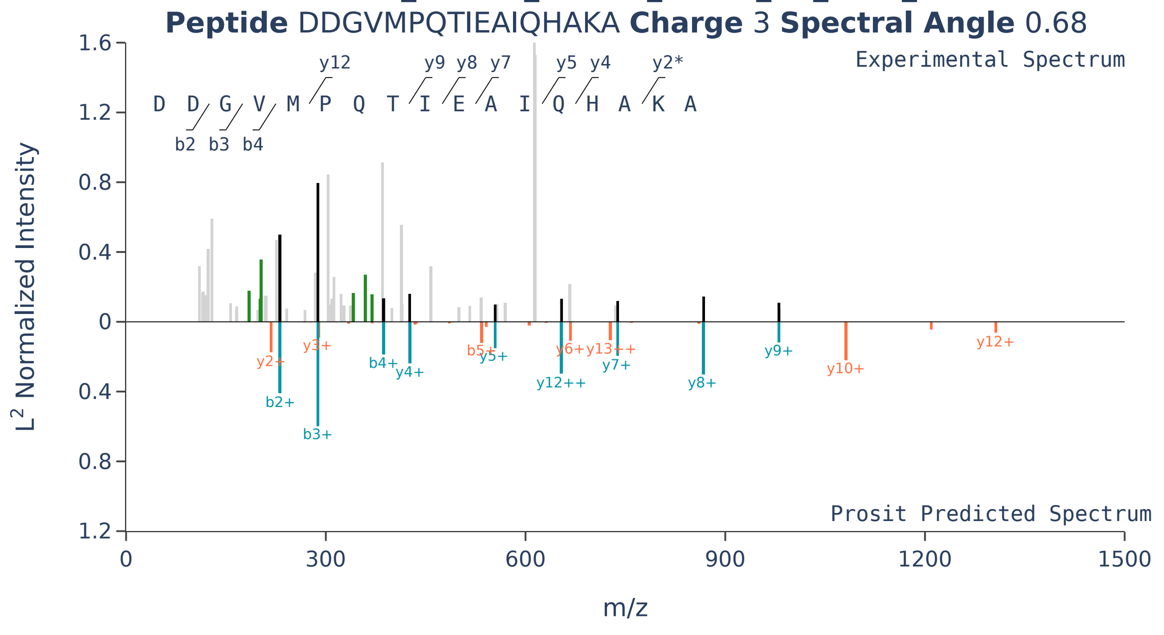


**Source** HRoetschke_211222_160123_HFGoe_IF2_b1R2_2h **Scan** 15943
**Peptide** GFQKPAQAVNRDVVIGE **Charge** 3 **Spectral Angle** 0.77

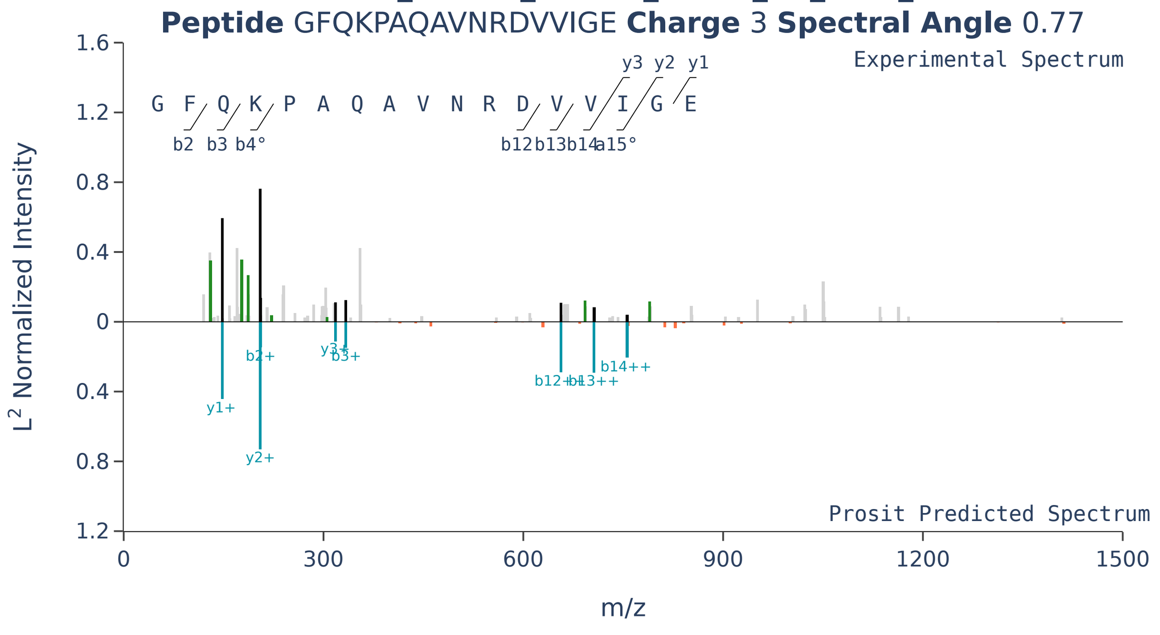


**Source** HRoetschke_211222_160123_HFGoe_IF2_b2R1_24h **Scan** 11562
**Peptide** DHLNQKNSGPDKLTLQ **Charge** 3 **Spectral Angle** 0.8

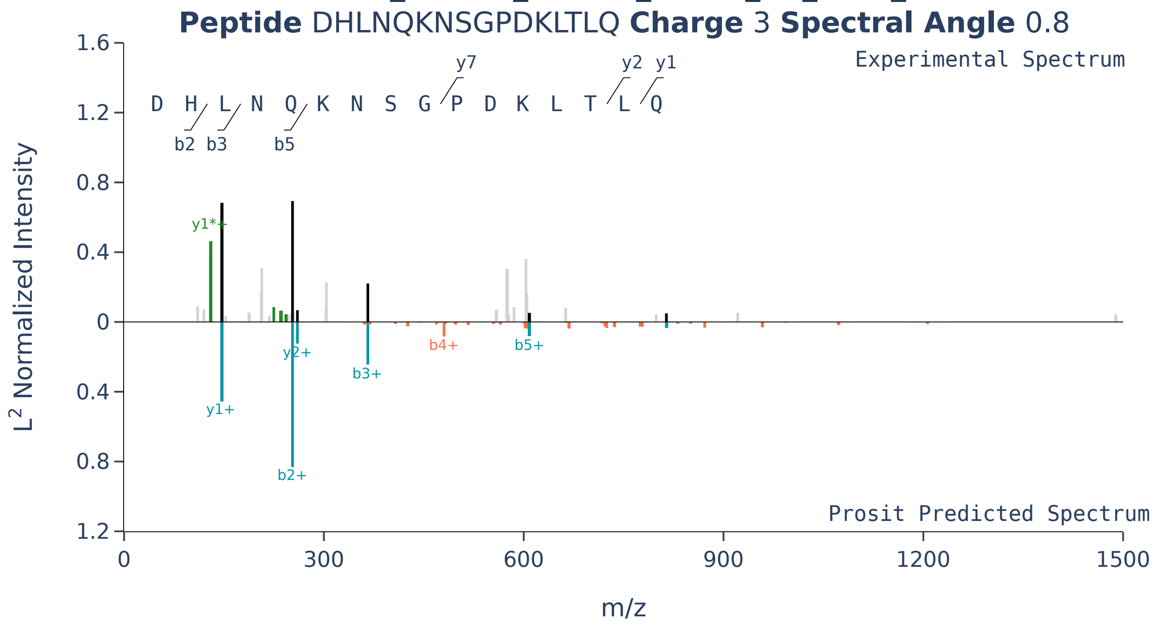


**Source** HRoetschke_211222_160123_HFGoe_IF2_b2R2_2h **Scan** 13604
**Peptide** LDTPGHAAFTS **Charge** 2 **Spectral Angle** 0.73

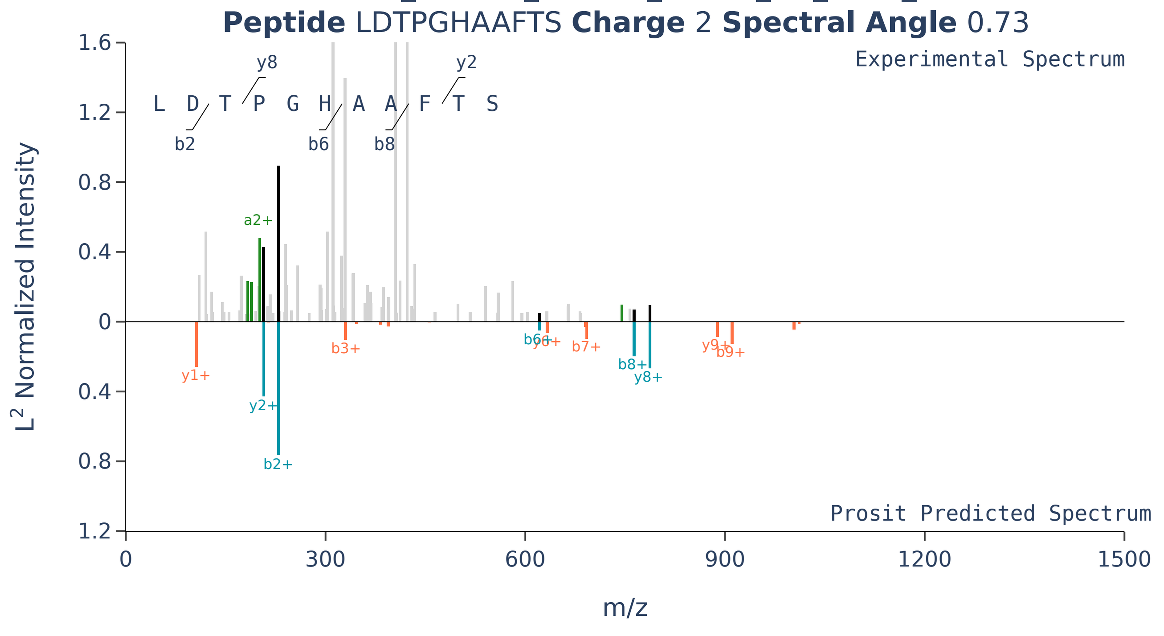


**Source** HRoetschke_211222_160123_HFGoe_IF2_b2R1_24h **Scan** 8502
**Peptide** VAVNKIDKPEADPDRVKN **Charge** 4 **Spectral Angle** 0.65

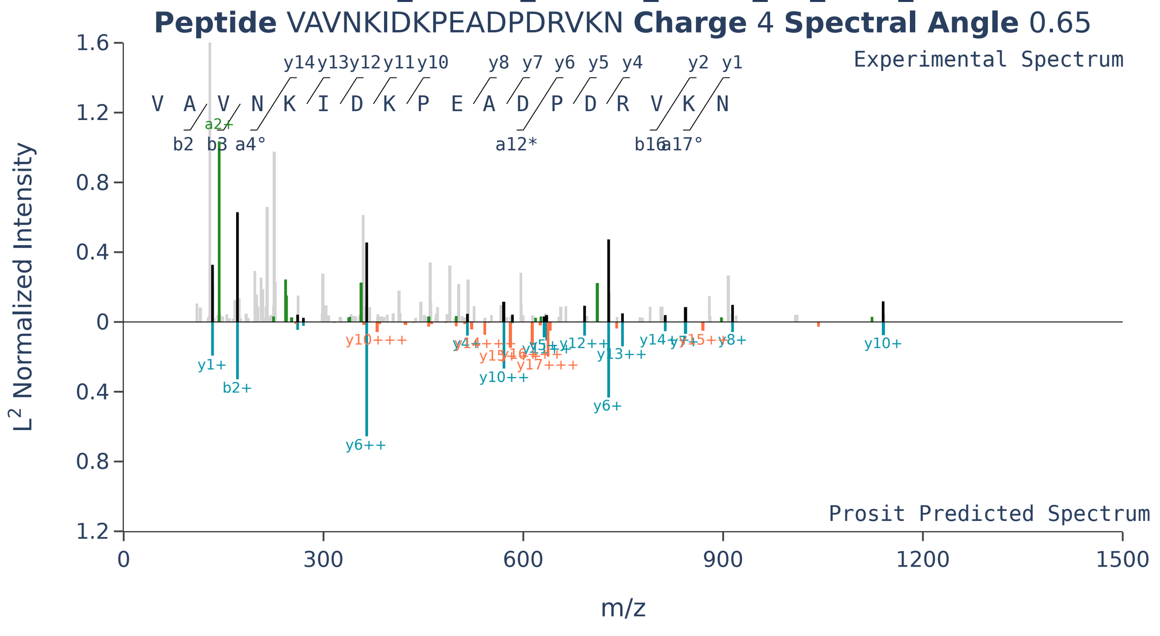

**Source** HRoetschke_211222_160123_HFGoe_IF2_b1R1_4h **Scan** 10718
**Peptide** LDTPGHAAFTSMRARGAQATD **Charge** 3 **Spectral Angle** 0.61

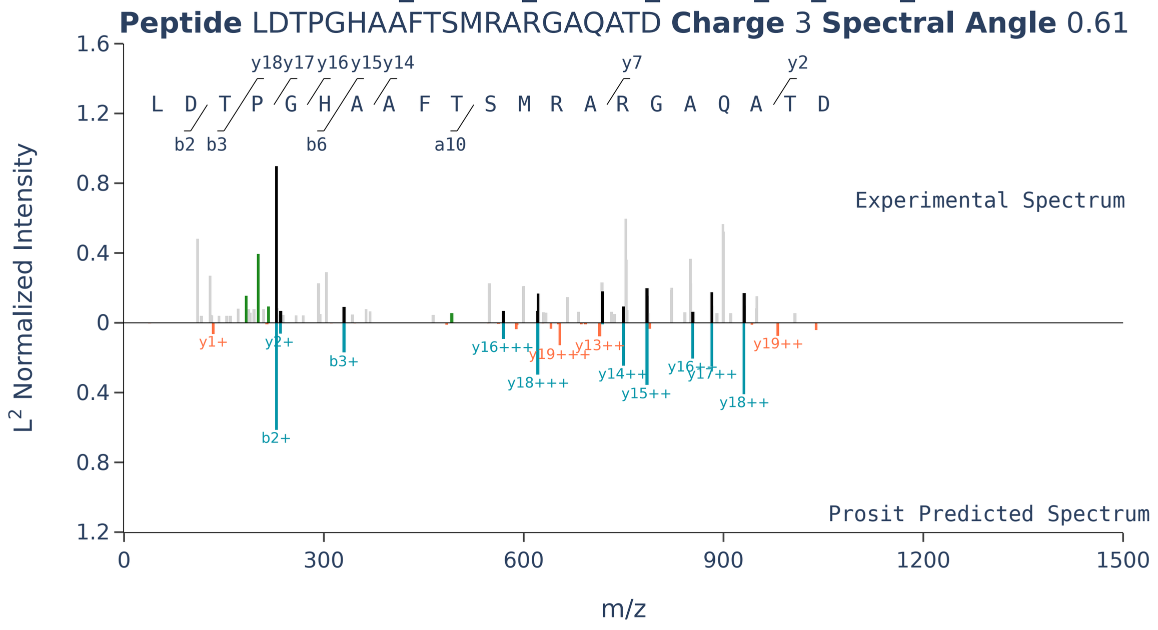


**Source** HRoetschke_211222_160123_HFGoe_IF2_b1R1_24h **Scan** 5915
**Peptide** RTFVKRDPQEA **Charge** 3 **Spectral Angle** 0.69

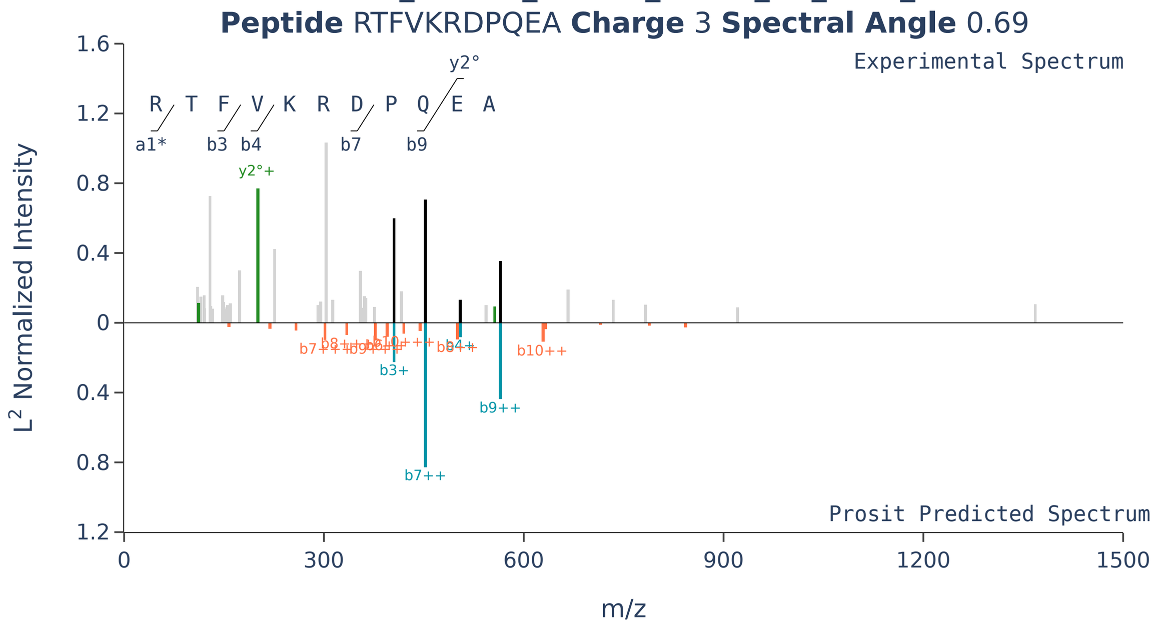


**Source** HRoetschke_211222_160123_HFGoe_IF2_b1R2_24h **Scan** 28776
**Peptide** YGILPEEW **Charge** 1 **Spectral Angle** 0.78

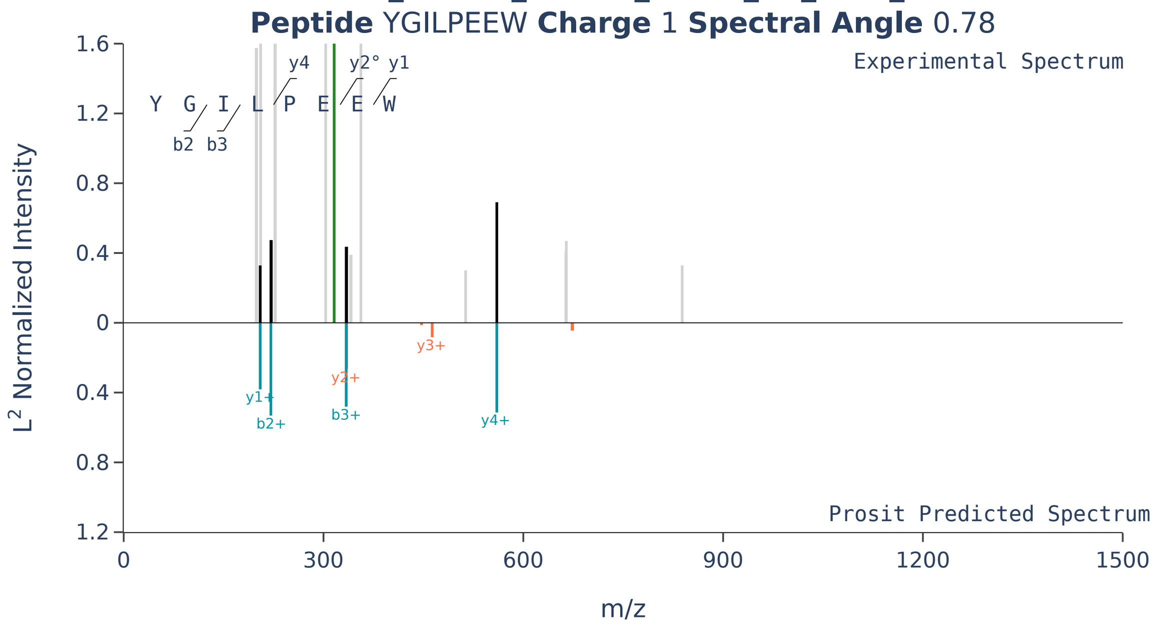


**Source** HRoetschke_211222_160123_HFGoe_IF2_b2R1_2h **Scan** 8562
**Peptide** EMGHKVILRRENELEE **Charge** 3 **Spectral Angle** 0.66

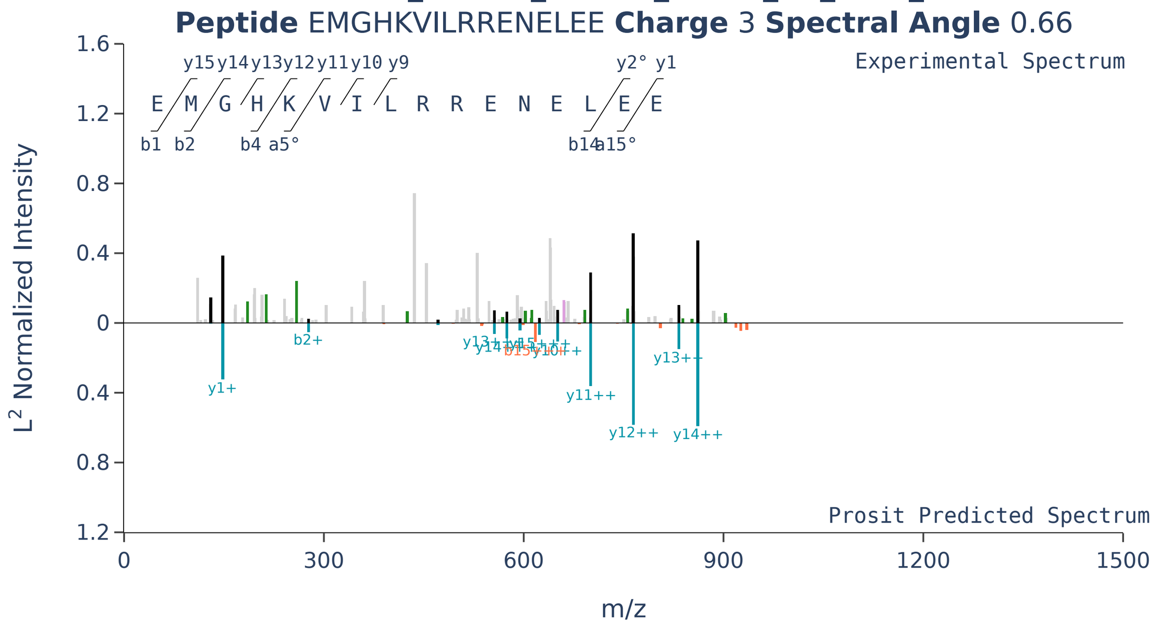


**Source** HRoetschke_211222_160123_HFGoe_IF2_b2R2_24h **Scan** 6869
**Peptide** ERLAAEEQAQRE **Charge** 2 **Spectral Angle** 0.56

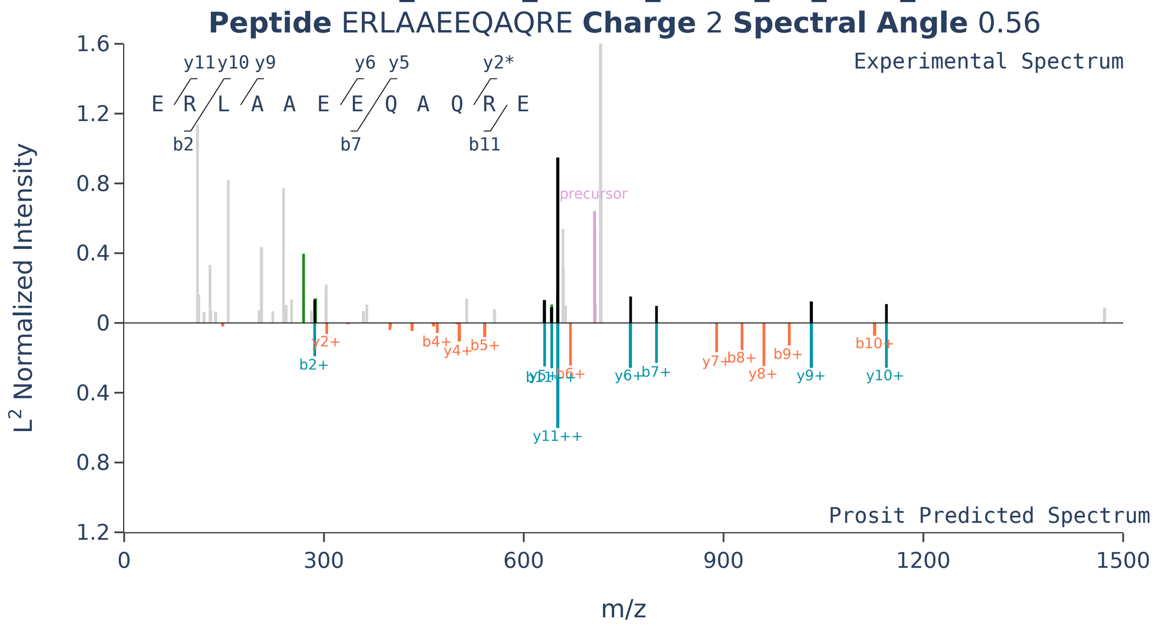


**Source** HRoetschke_211222_160123_HFGoe_IF2_b1R1_4h **Scan** 19127
**Peptide** AADDGVMPQTIE **Charge** 2 **Spectral Angle** 0.66

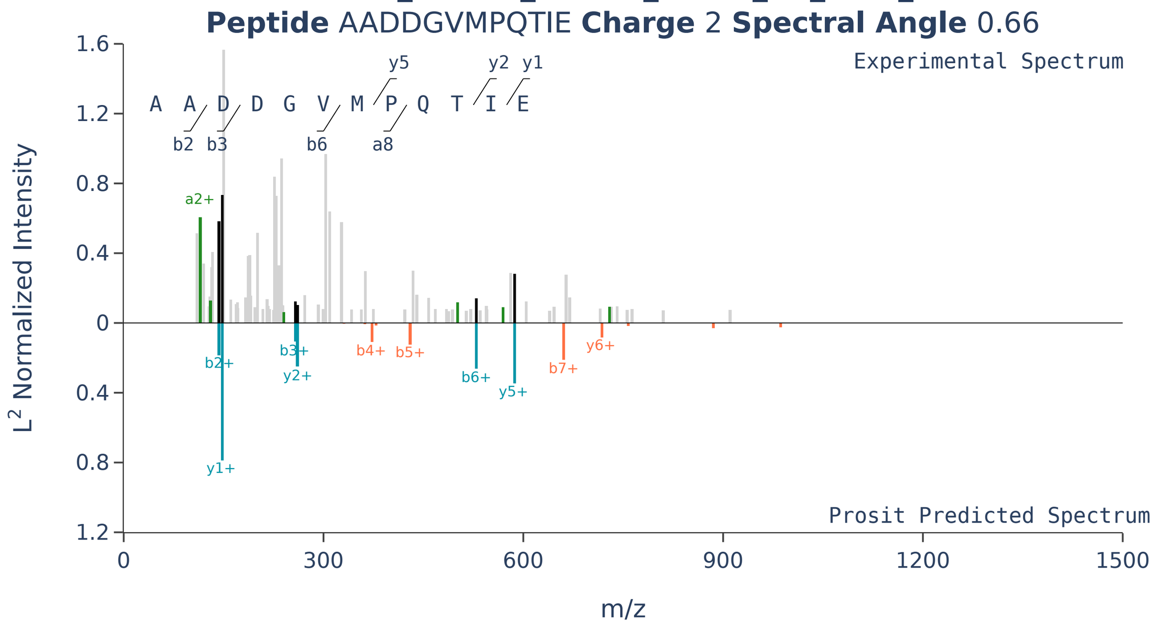


**Source** HRoetschke_211222_160123_HFGoe_IF2_b1R1_4h **Scan** 12269
**Peptide** LIDHLNQKNSGPDKL **Charge** 4 **Spectral Angle** 0.67

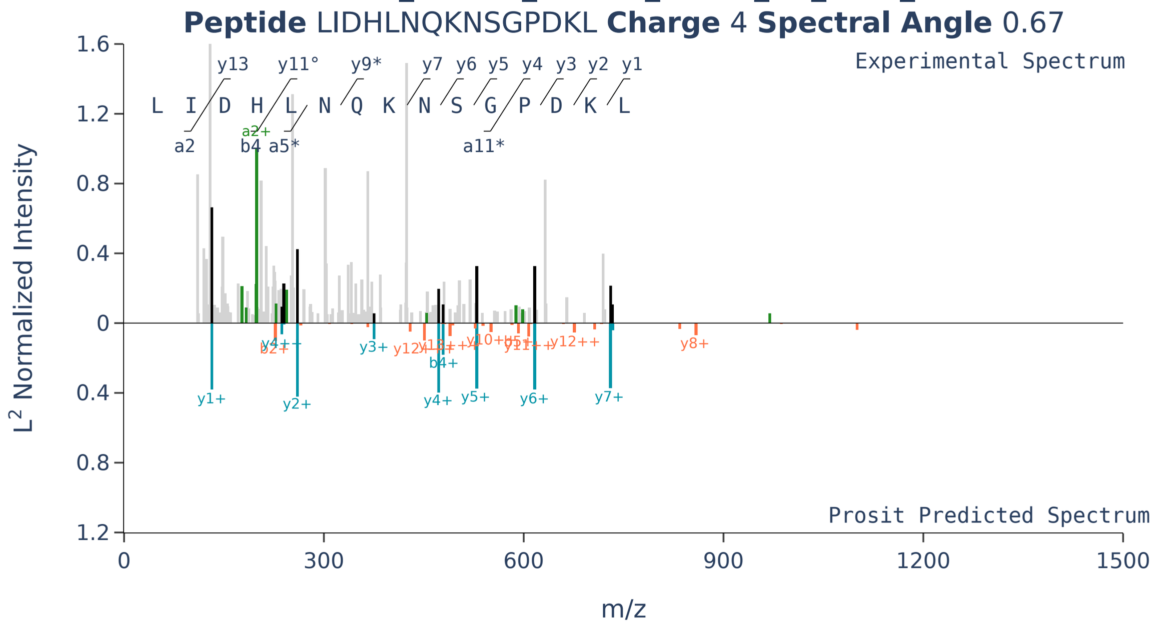


**Source** HRoetschke_211222_160123_HFGoe_IF2_b2R2_24h **Scan** 9337
**Peptide** EEMGHKVILRRENEL **Charge** 4 **Spectral Angle** 0.59

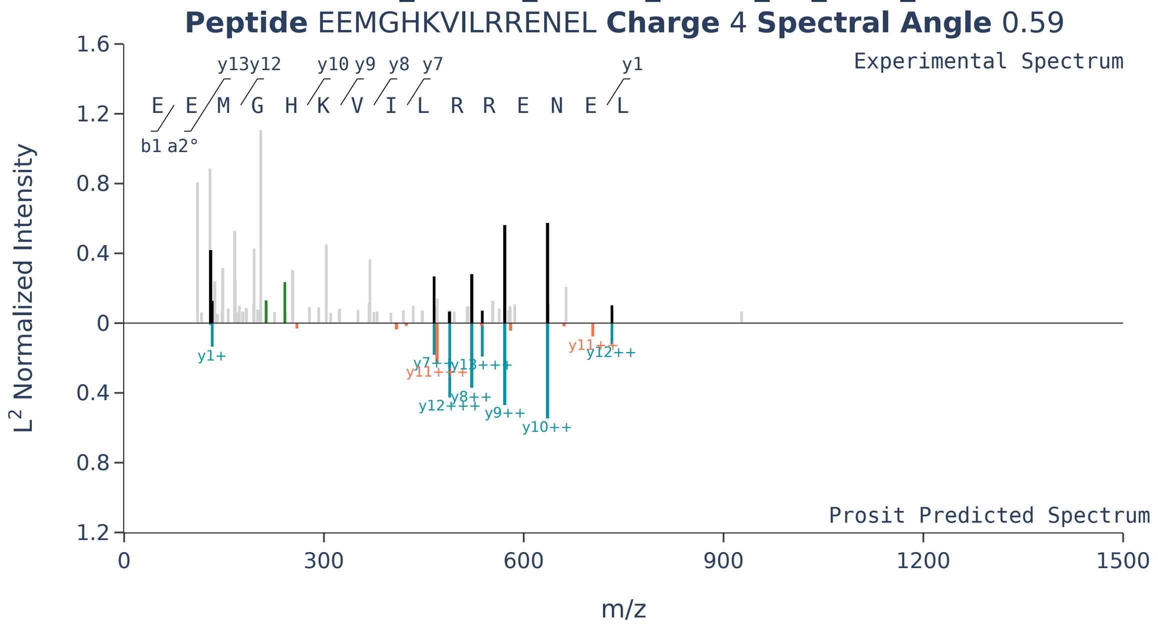


**Source** HRoetschke_211222_160123_HFGoe_IF2_b1R1_2h **Scan** 5939
**Peptide** QQDDMTKNAQAE **Charge** 2 **Spectral Angle** 0.75

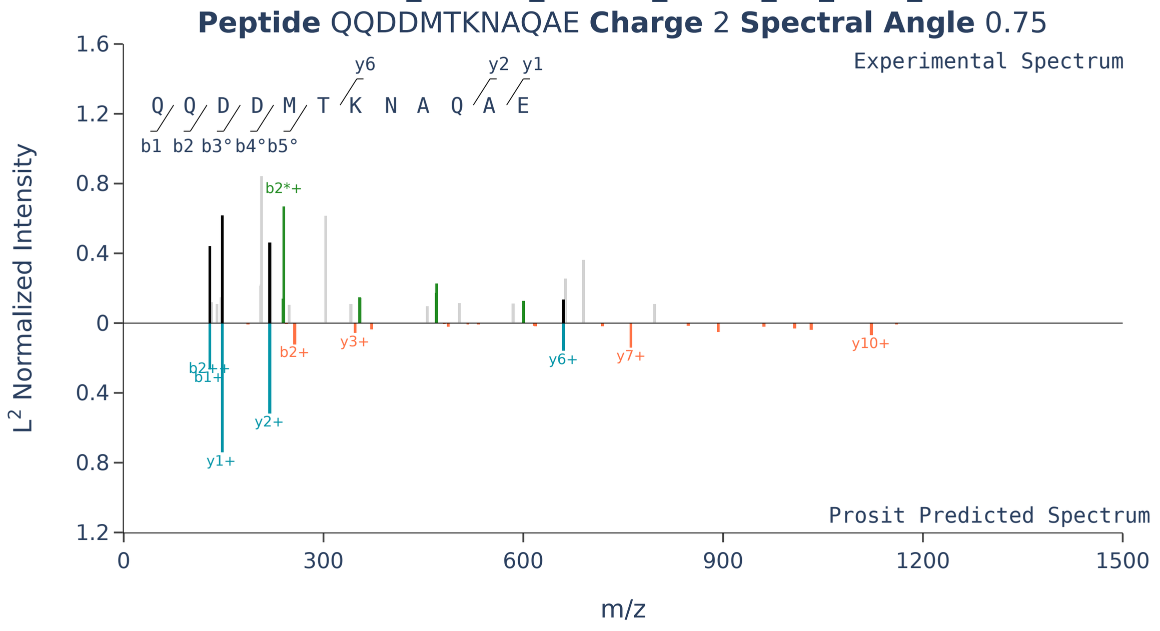


**Source** HRoetschke_211222_160123_HFGoe_IF2_b1R2_4h **Scan** 12782
**Peptide** KTLAAERQTSVERLVQ **Charge** 3 **Spectral Angle** 0.68

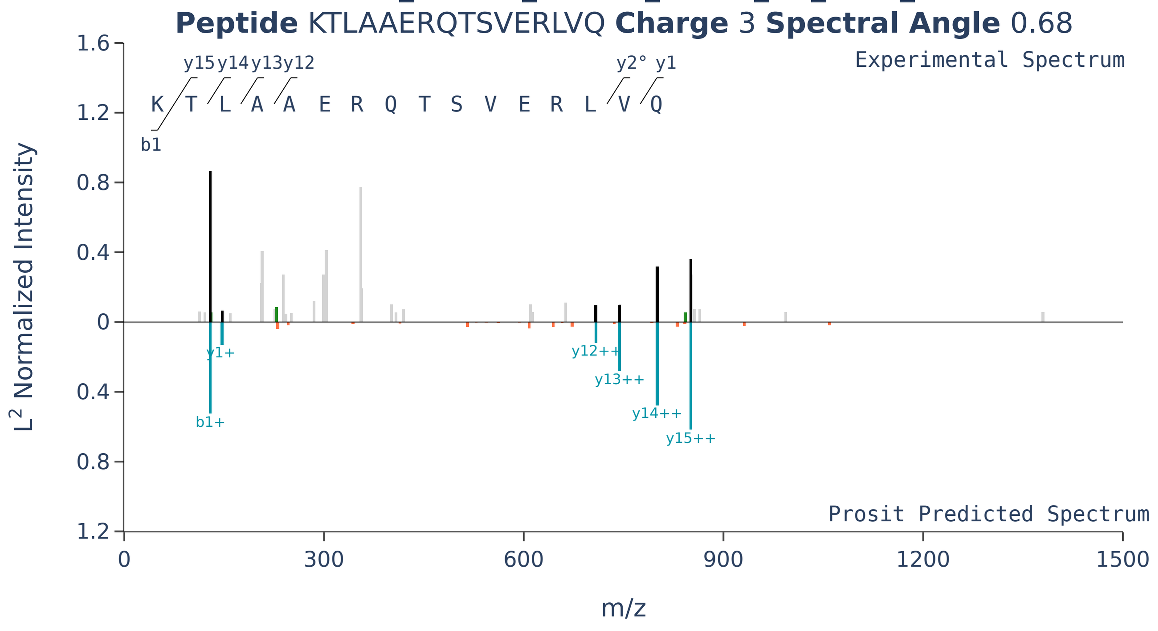


**Source** HRoetschke_211222_160123_HFGoe_IF2_b1R1_2h **Scan** 10089
**Peptide** RTFVKRDPQEAERLAAE **Charge** 3 **Spectral Angle** 0.84

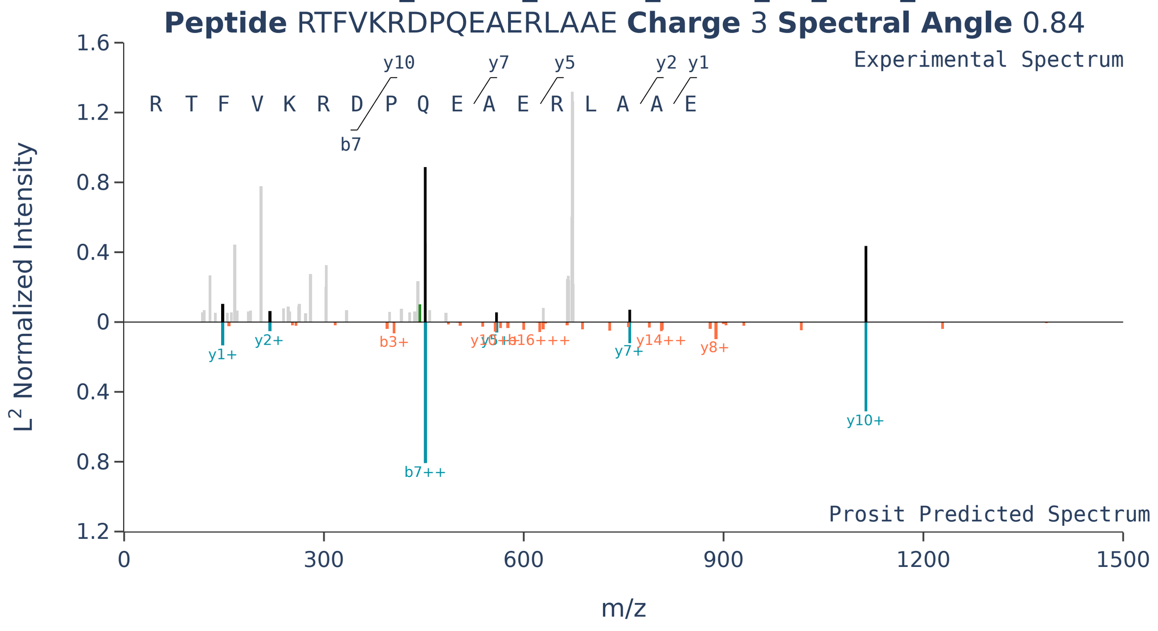


**Source** HRoetschke_211222_160123_HFGoe_IF2_b1R1_4h **Scan** 21837
**Peptide** SVERLVQQFAD **Charge** 2 **Spectral Angle** 0.8

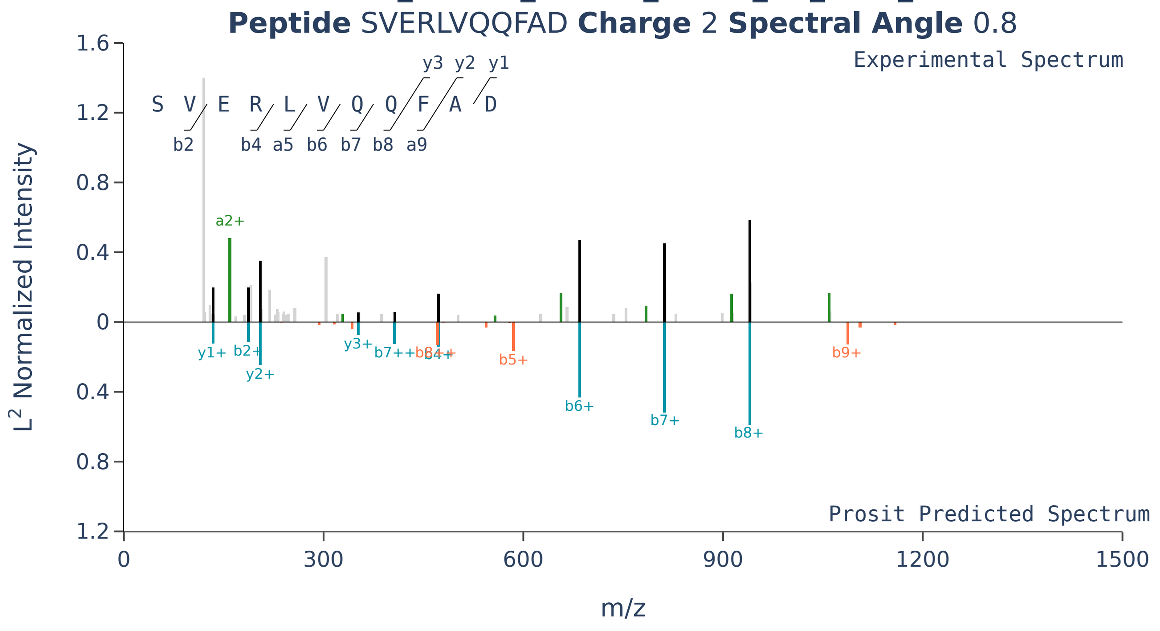


**Source** HRoetschke_211222_160123_HFGoe_IF2_b1R1_2h **Scan** 13153
**Peptide** KAMMKLGAMATI **Charge** 3 **Spectral Angle** 0.78

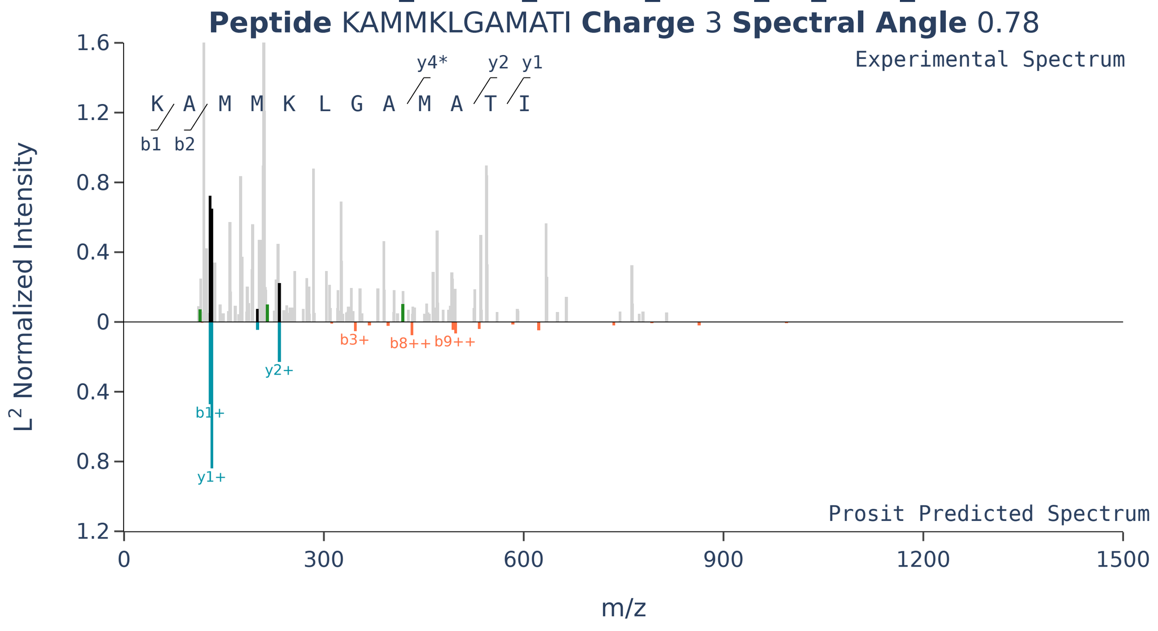


**Source** HRoetschke_211222_160123_HFGoe_IF2_b1R2_24h **Scan** 12621
**Peptide** VAEEMGHKVIL **Charge** 3 **Spectral Angle** 0.75

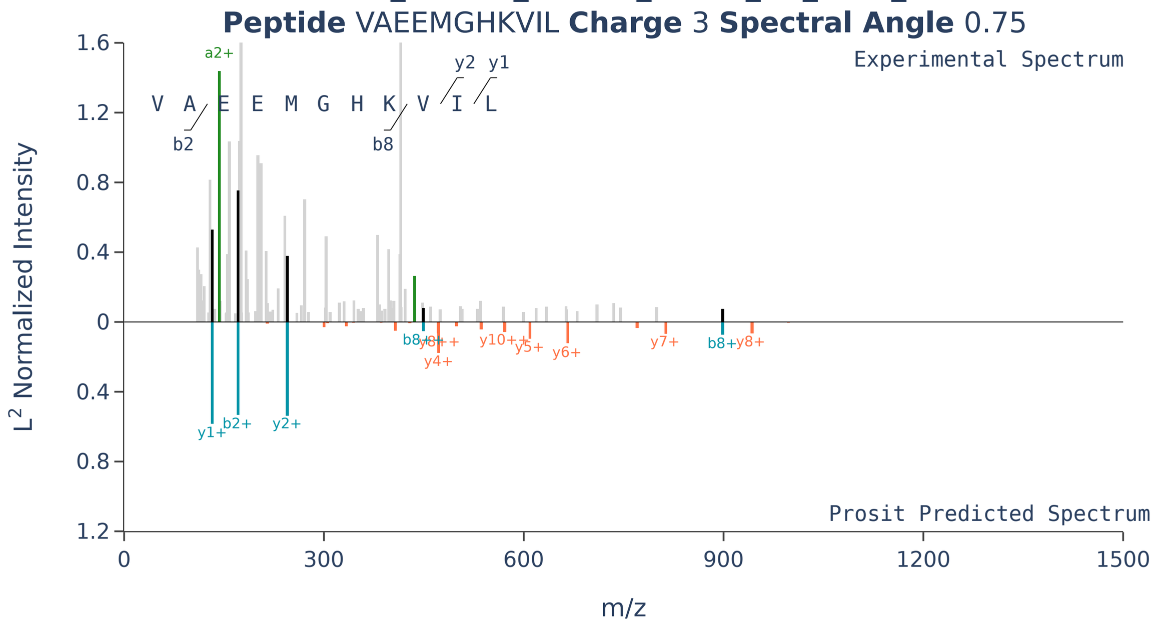


**Source** HRoetschke_211222_160123_HFGoe_IF2_b1R1_24h **Scan** 14965
**Peptide** DVTIKTLAAERQTSVER **Charge** 3 **Spectral Angle** 0.74

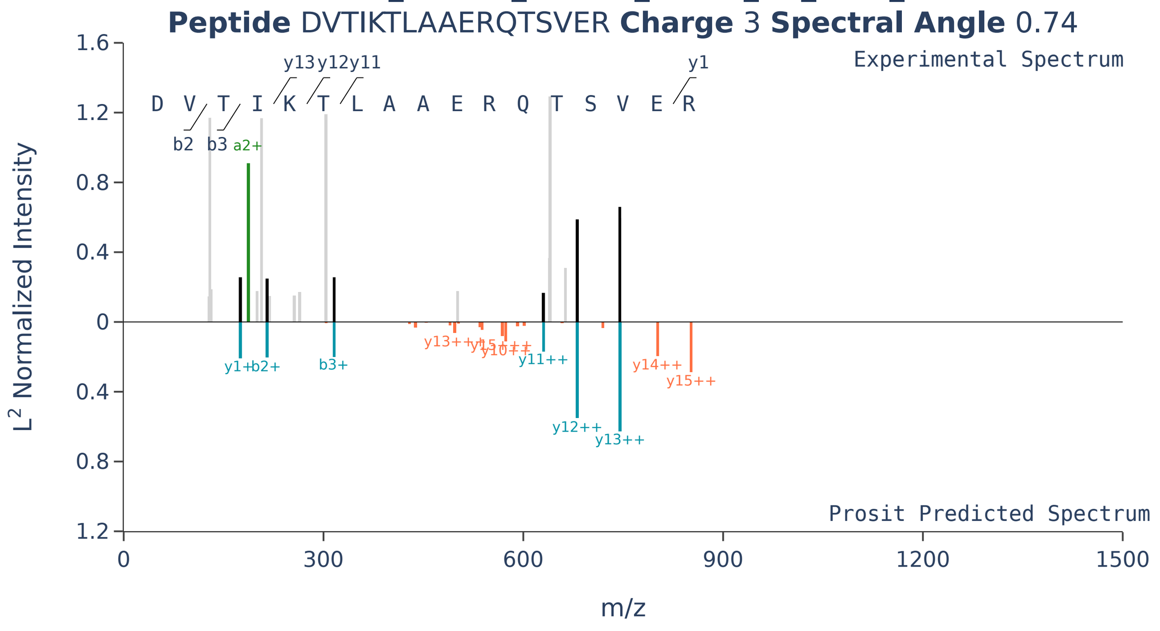

**Source** HRoetschke_211222_160123_HFGoe_IF2_b1R1_4h **Scan** 7415
**Peptide** KRDPQEAERLAAE **Charge** 3 **Spectral Angle** 0.63

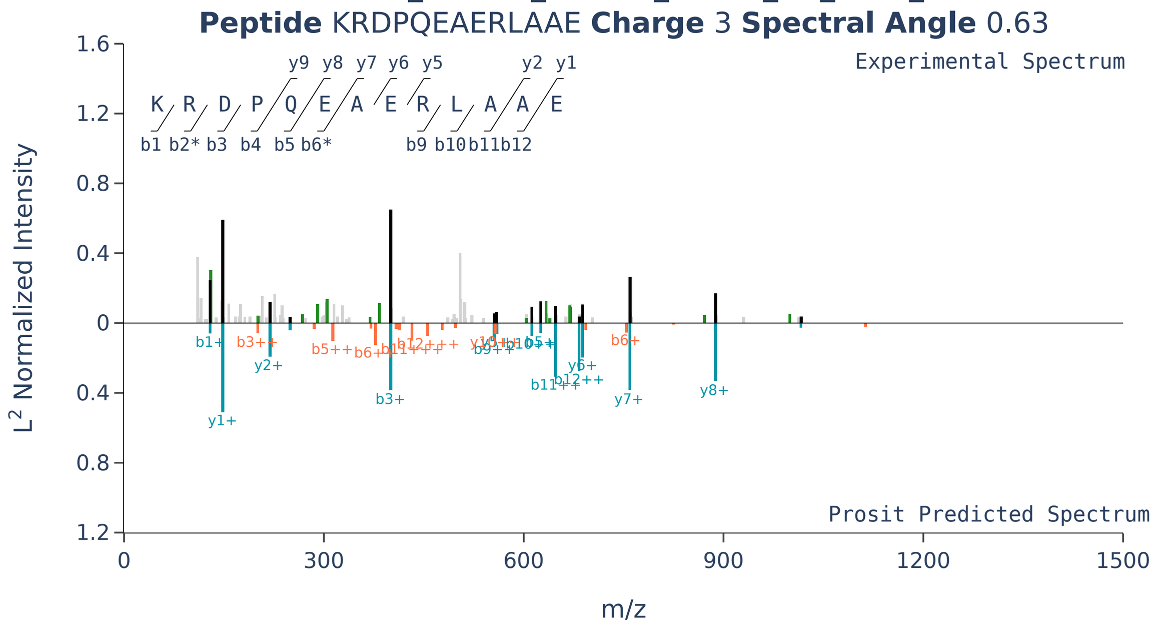


**Source** HRoetschke_211222_160123_HFGoe_IF2_b1R1_4h **Scan** 9231
**Peptide** AGCMVTEGVVKRH **Charge** 3 **Spectral Angle** 0.52

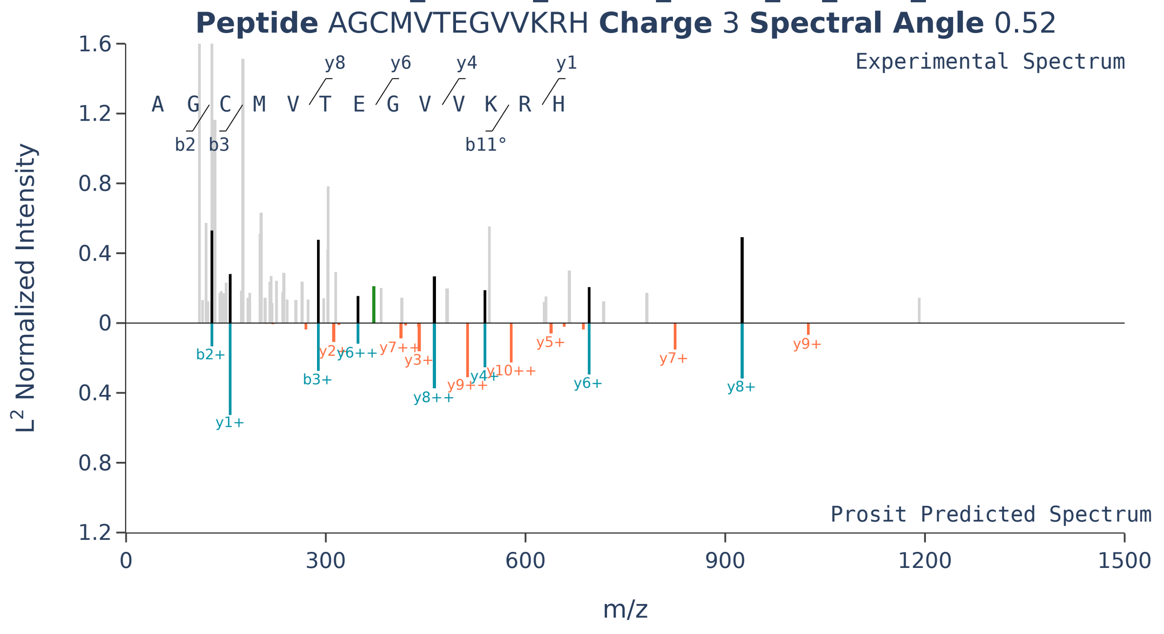


**Source** HRoetschke_211222_160123_HFGoe_IF2_b1R1_4h **Scan** 13079
**Peptide** QETAQLVAEEMGHKV **Charge** 3 **Spectral Angle** 0.63

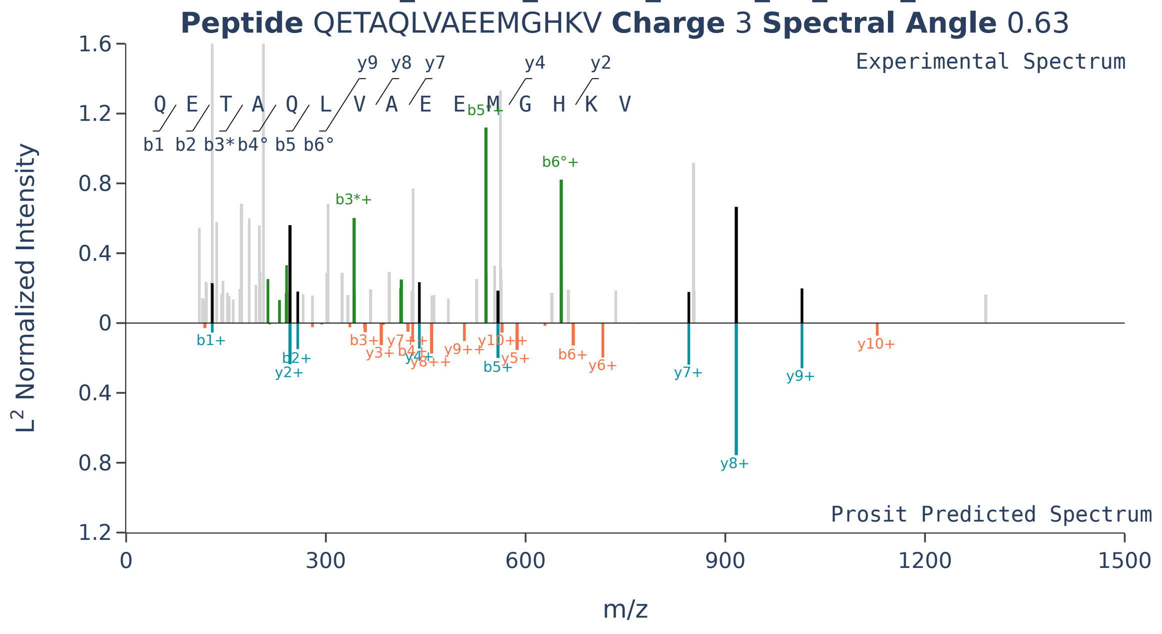


**Source** HRoetschke_211222_160123_HFGoe_IF2_b1R2_2h **Scan** 14677
**Peptide** DSVSAQEKQTLLD **Charge** 2 **Spectral Angle** 0.71

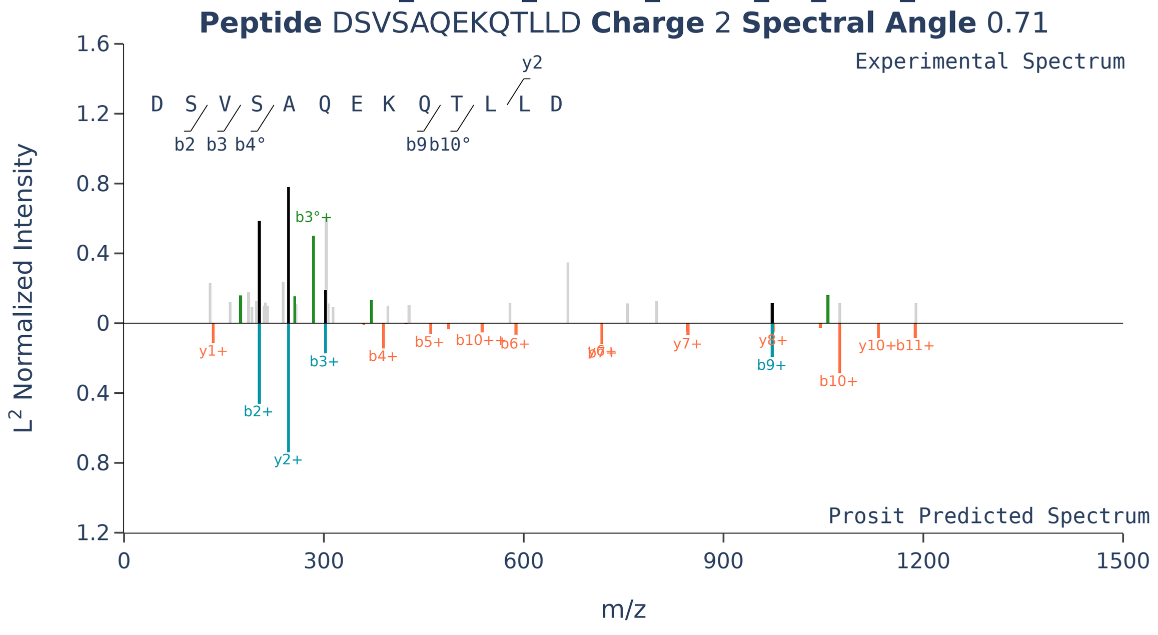


**Source** HRoetschke_211222_160123_HFGoe_IF2_b1R1_24h **Scan** 14068
**Peptide** EGEVHEVNI **Charge** 2 **Spectral Angle** 0.69

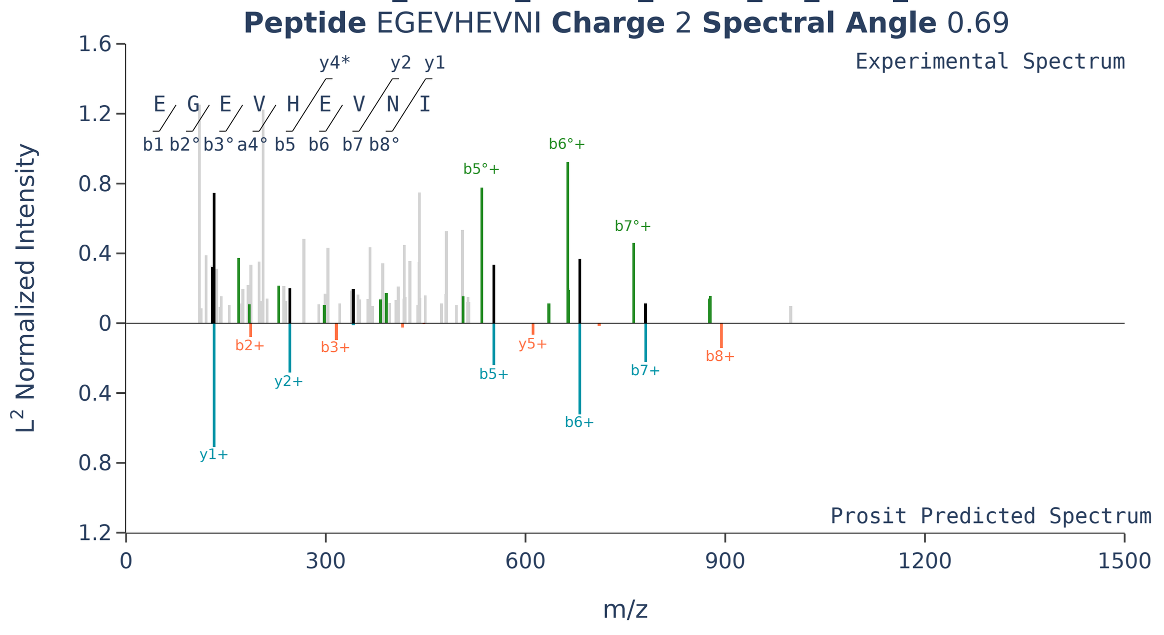


**Source** HRoetschke_211222_160123_HFGoe_IF2_b1R1_2h **Scan** 10500
**Peptide** EEAVMSDRDTGAAAEPRA **Charge** 3 **Spectral Angle** 0.58

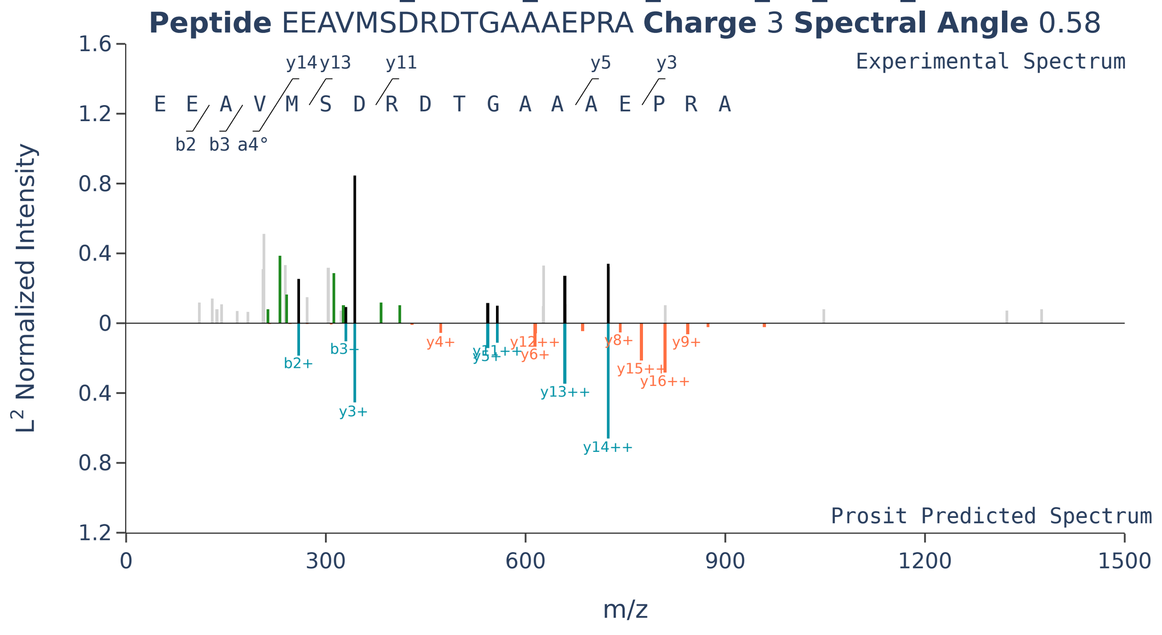


**Source** HRoetschke_211222_160123_HFGoe_IF2_b1R1_4h **Scan** 16051
**Peptide** GVMPQTIEAIQHAKAA **Charge** 3 **Spectral Angle** 0.62

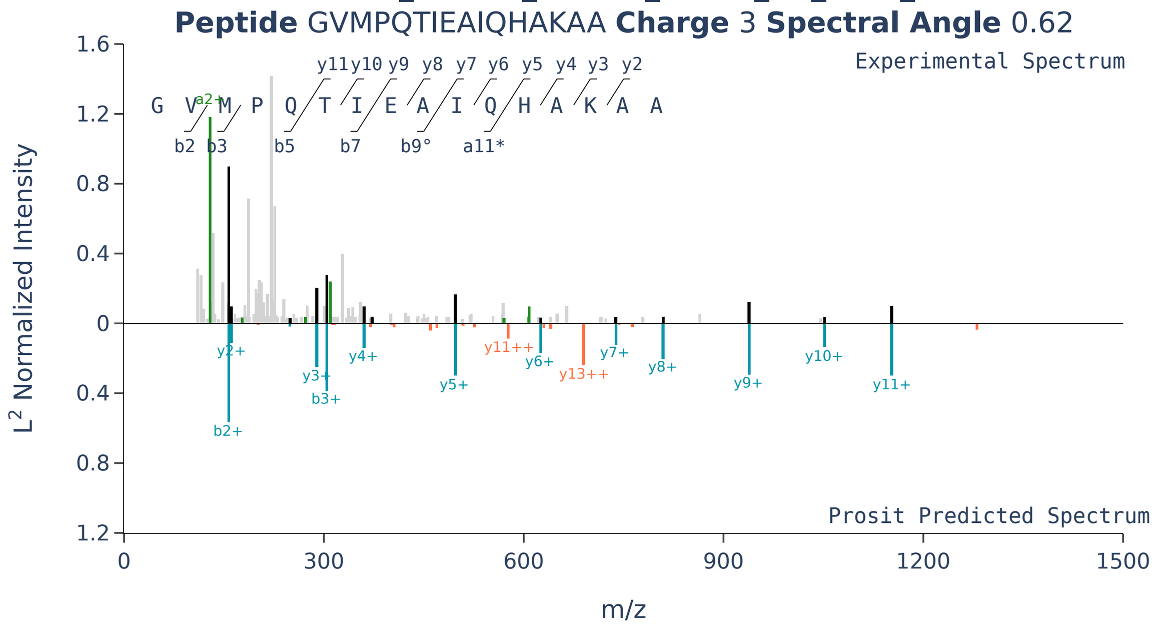


**Source** HRoetschke_211222_160123_HFGoe_IF2_b1R2_24h **Scan** 19020
**Peptide** DRDTGAAAEPRAPVVTL **Charge** 2 **Spectral Angle** 0.73

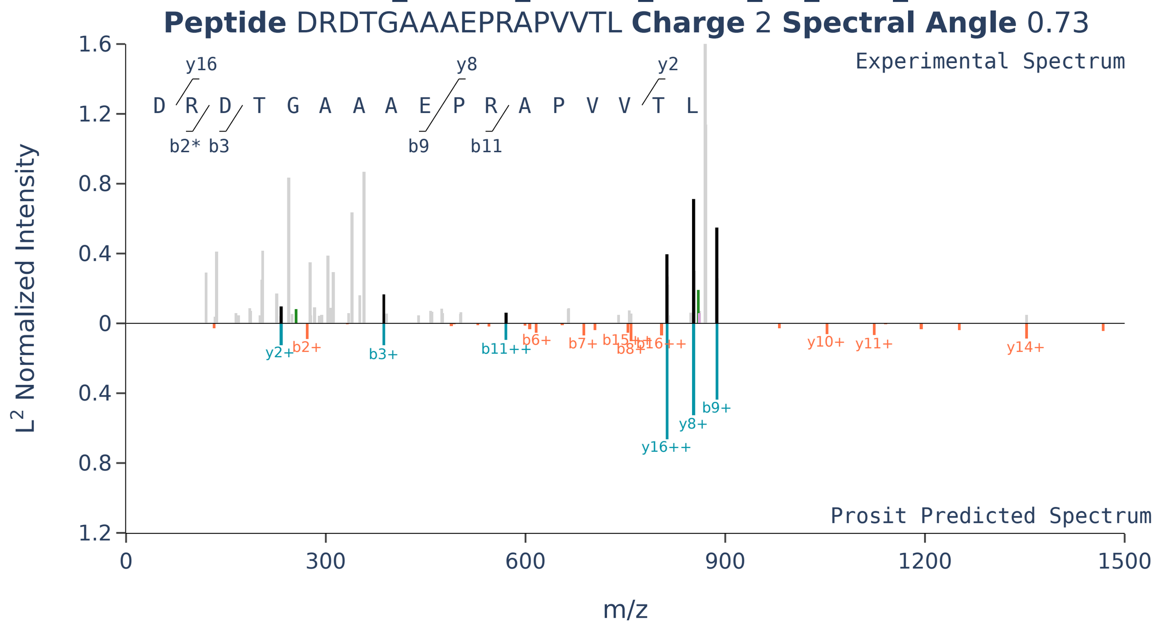


**Source** HRoetschke_211222_160123_HFGoe_IF2_b1R1_4h **Scan** 20300
**Peptide** HKGDIVLCGF **Charge** 2 **Spectral Angle** 0.66

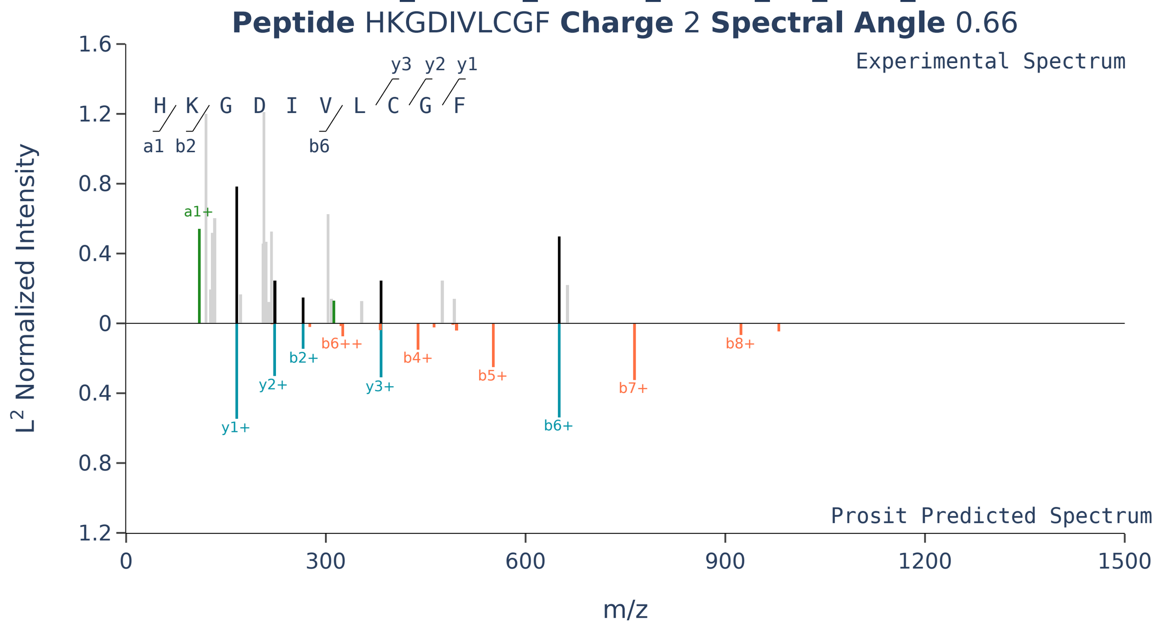


**Source** HRoetschke_211222_160123_HFGoe_IF2_b1R2_24h **Scan** 14030
**Peptide** DPDRVKNELSQY **Charge** 3 **Spectral Angle** 0.76

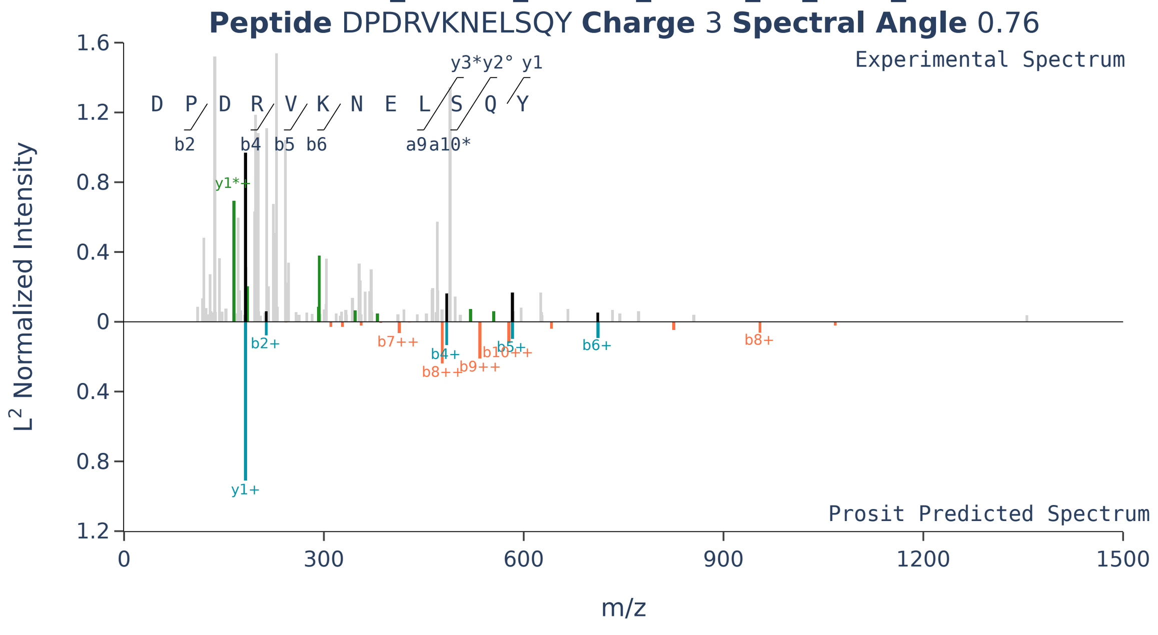


**Source** HRoetschke_211222_160123_HFGoe_IF2_b1R1_4h **Scan** 14324
**Peptide** EEMGHKVIL **Charge** 2 **Spectral Angle** 0.6

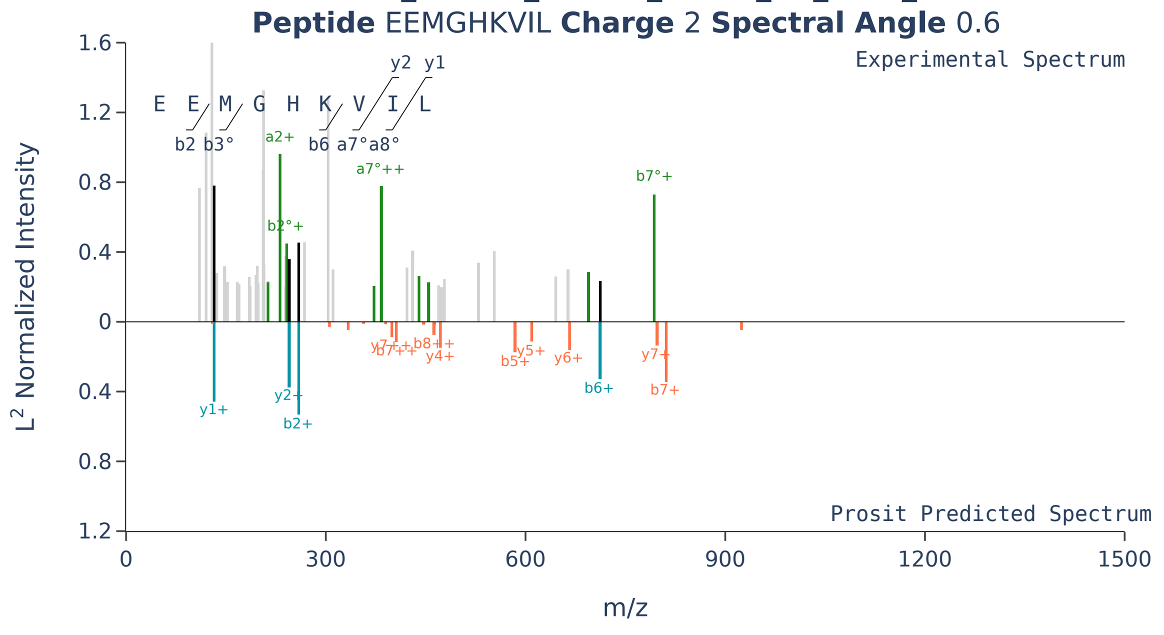


**Source** HRoetschke_211222_160123_HFGoe_IF2_b1R1_4h **Scan** 15761
**Peptide** SVSAQEKQTLIDHLNQ **Charge** 3 **Spectral Angle** 0.68

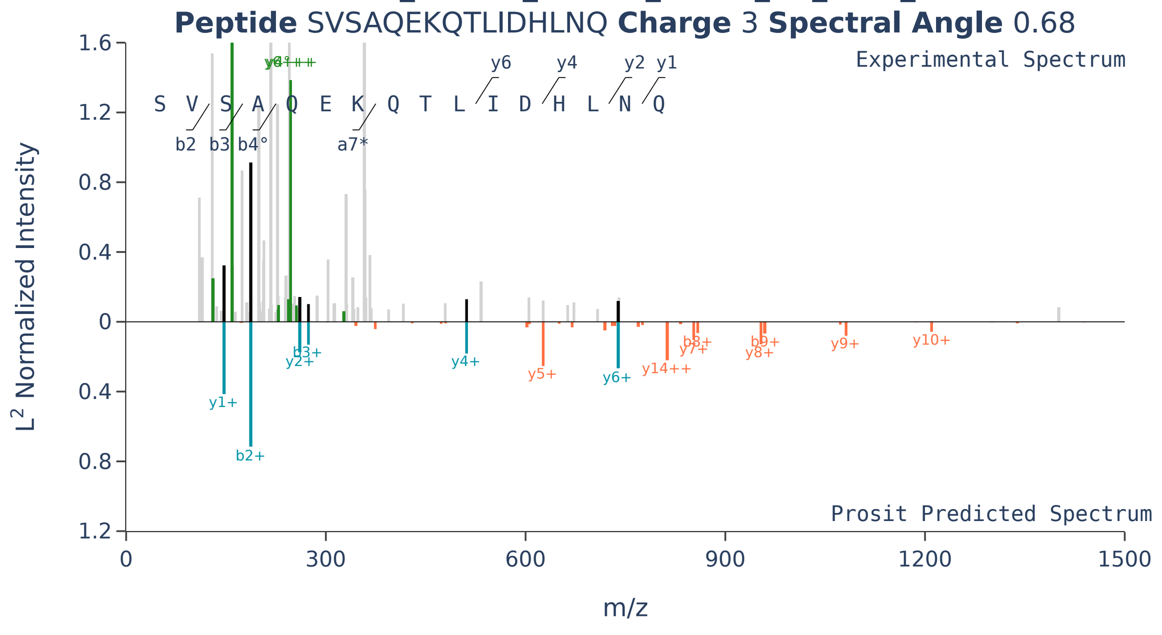


**Source** HRoetschke_211222_160123_HFGoe_IF2_b2R1_24h **Scan** 10697
**Peptide** MAEENKWTD **Charge** 2 **Spectral Angle** 0.6

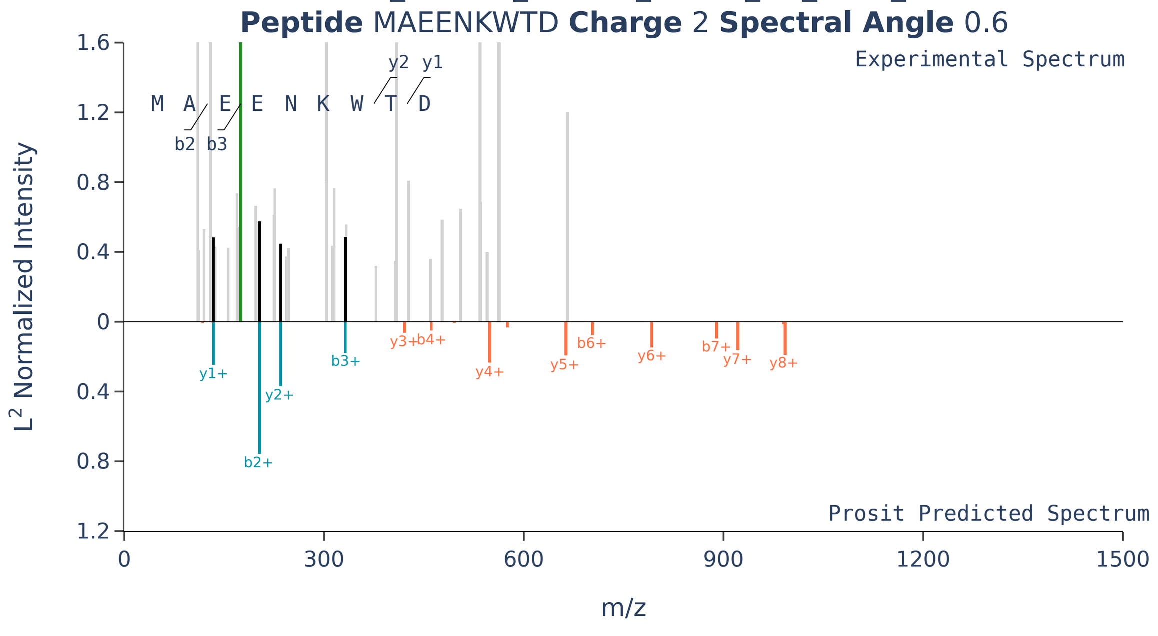


**Source** HRoetschke_211222_160123_HFGoe_IF2_b1R1_2h **Scan** 10783
**Peptide** QGKFREVKL **Charge** 3 **Spectral Angle** 0.73

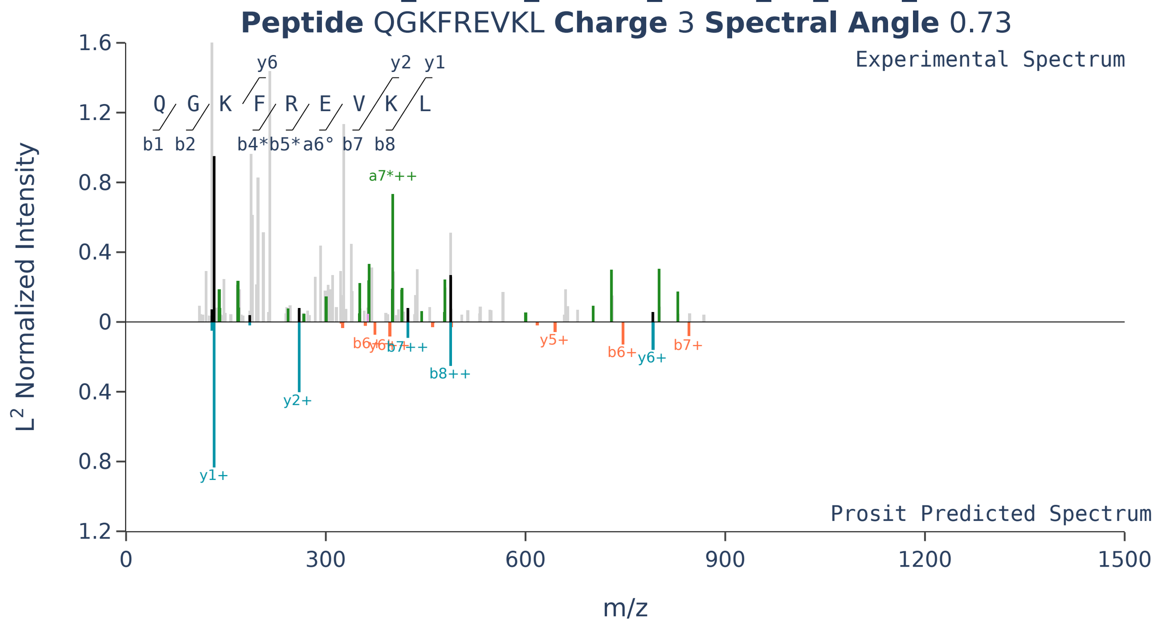


**Source** HRoetschke_211222_160123_HFGoe_IF2_b1R2_2h **Scan** 12007
**Peptide** LVREGTLH **Charge** 2 **Spectral Angle** 0.88

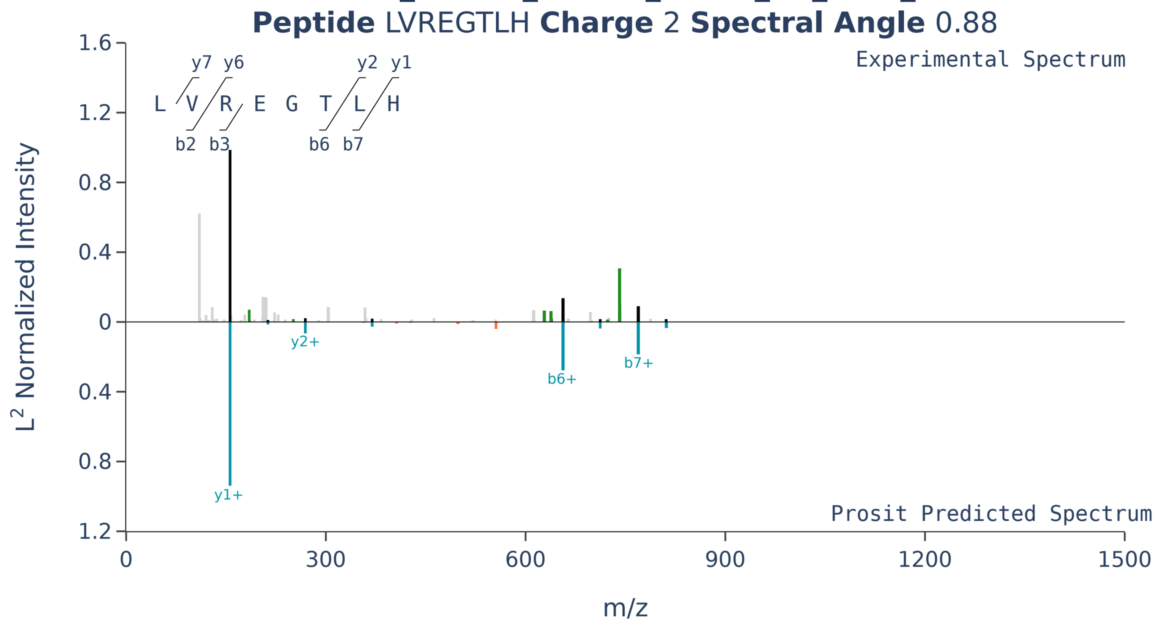

**Source** HRoetschke_211222_160123_HFGoe_IF2_b1R1_4h **Scan** 9132
**Peptide** VAVNKIDKPEADPDRVKNE **Charge** 4 **Spectral Angle** 0.7

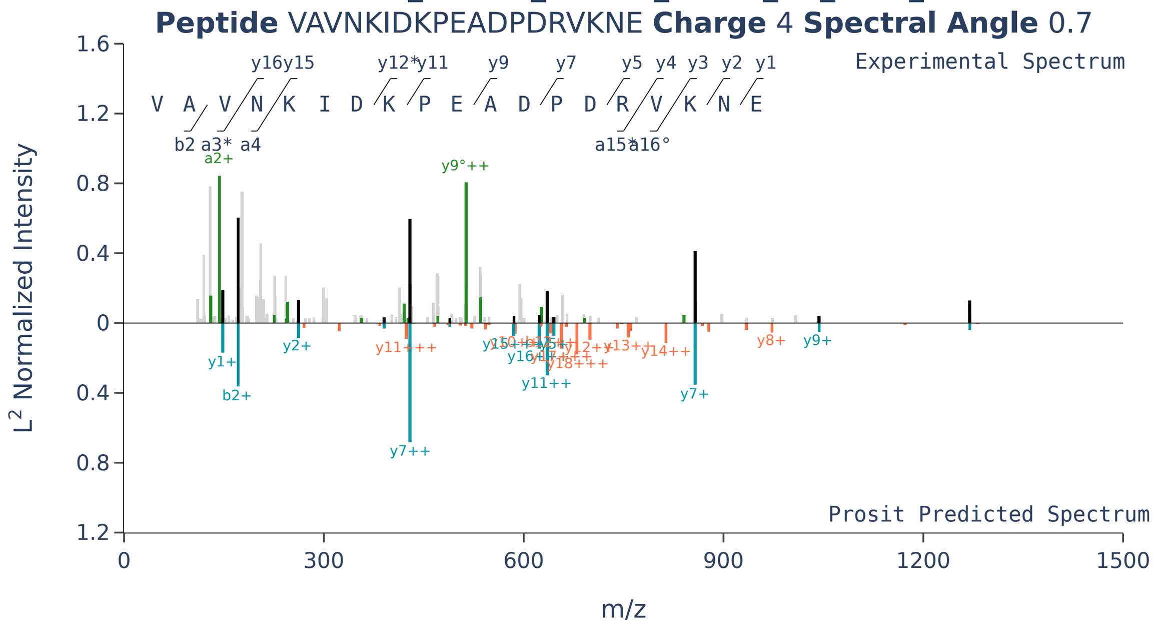


**Source** HRoetschke_211222_160123_HFGoe_IF2_b1R1_2h **Scan** 5711
**Peptide** PGHAAFTSMRARG **Charge** 3 **Spectral Angle** 0.55

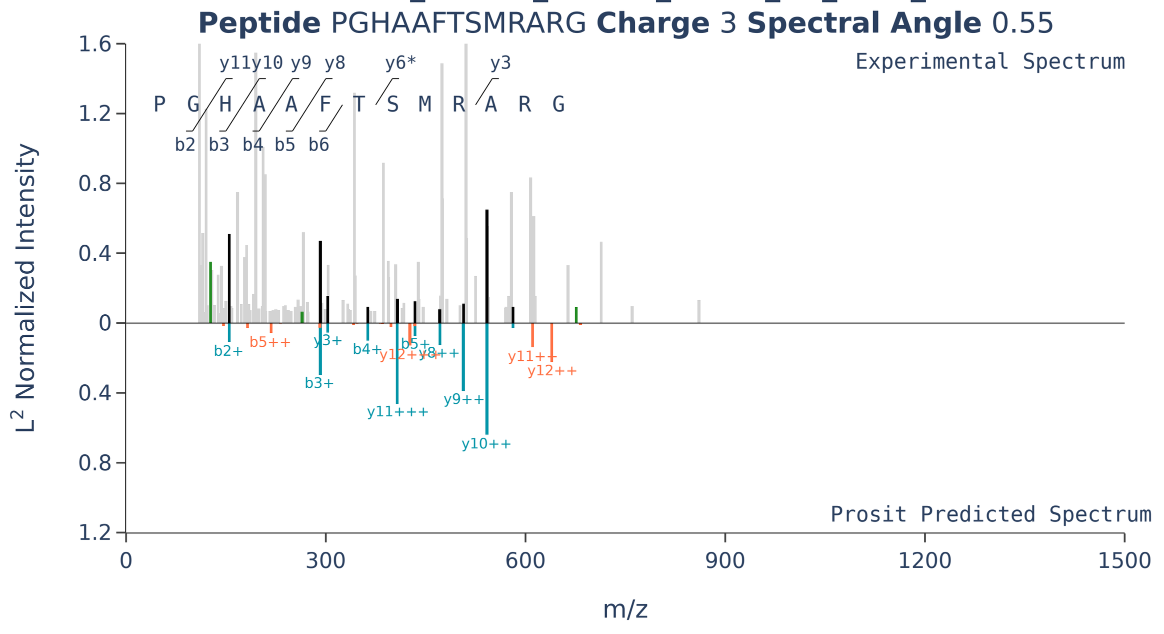


**Source** HRoetschke_211222_160123_HFGoe_IF2_b1R1_4h **Scan** 17485
**Peptide** AEVRDVFKSPKF **Charge** 4 **Spectral Angle** 0.78

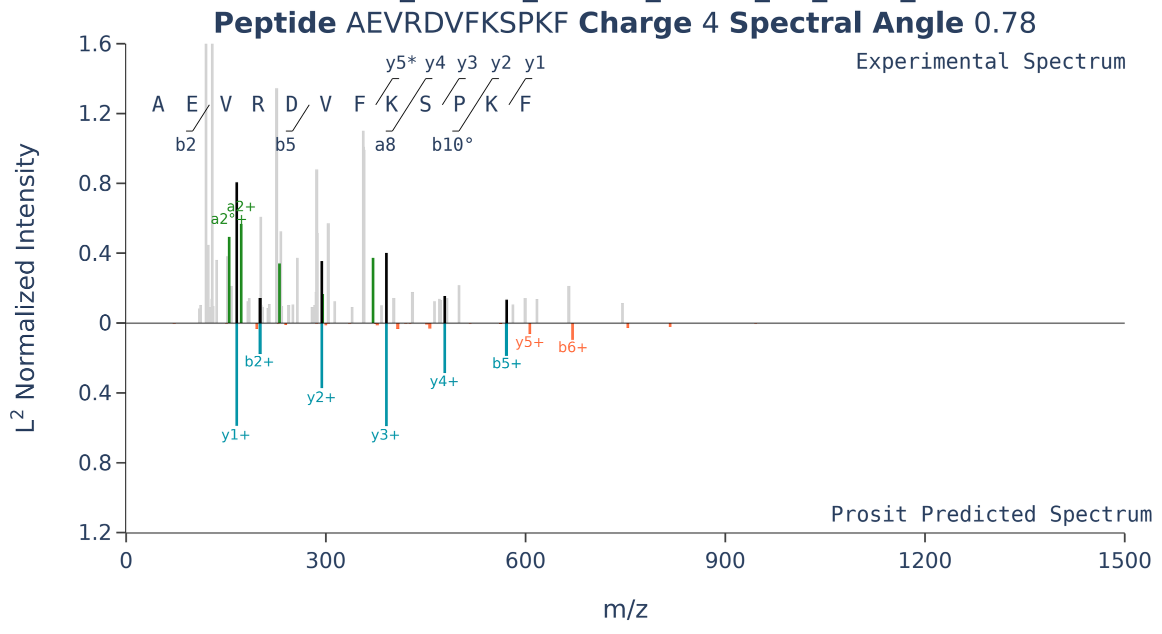


**Source** HRoetschke_211222_160123_HFGoe_IF2_b1R2_24h **Scan** 21385
**Peptide** KAGTGIDELL **Charge** 2 **Spectral Angle** 0.61

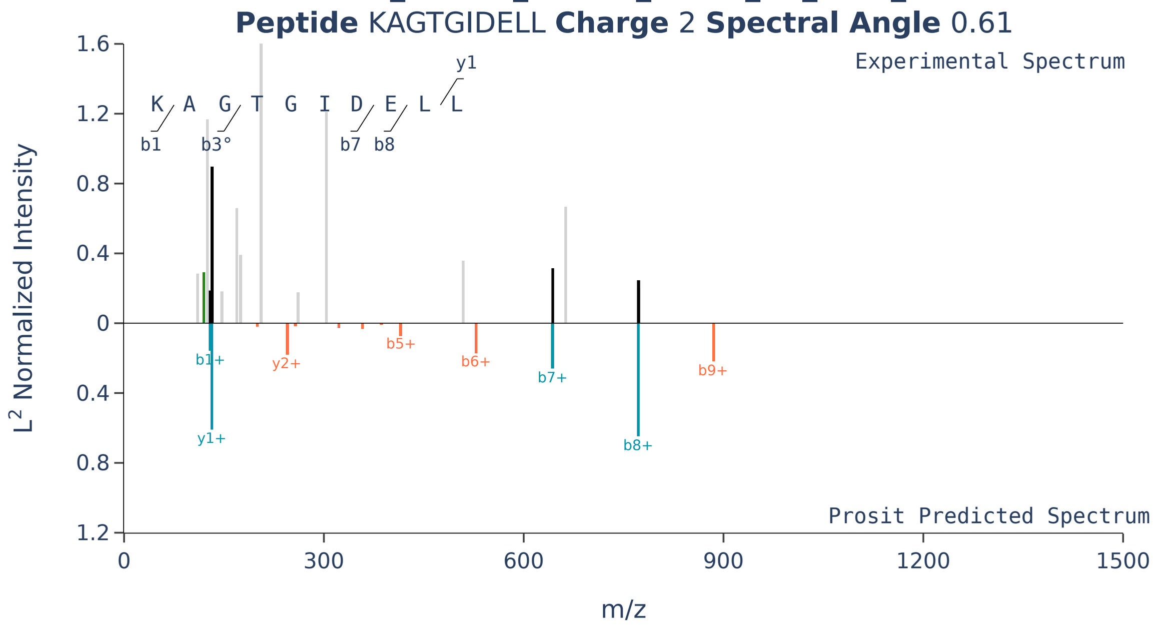


**Source** HRoetschke_211222_160123_HFGoe_IF2_b1R1_4h **Scan** 30503
**Peptide** TDIVVLVV **Charge** 1 **Spectral Angle** 0.91

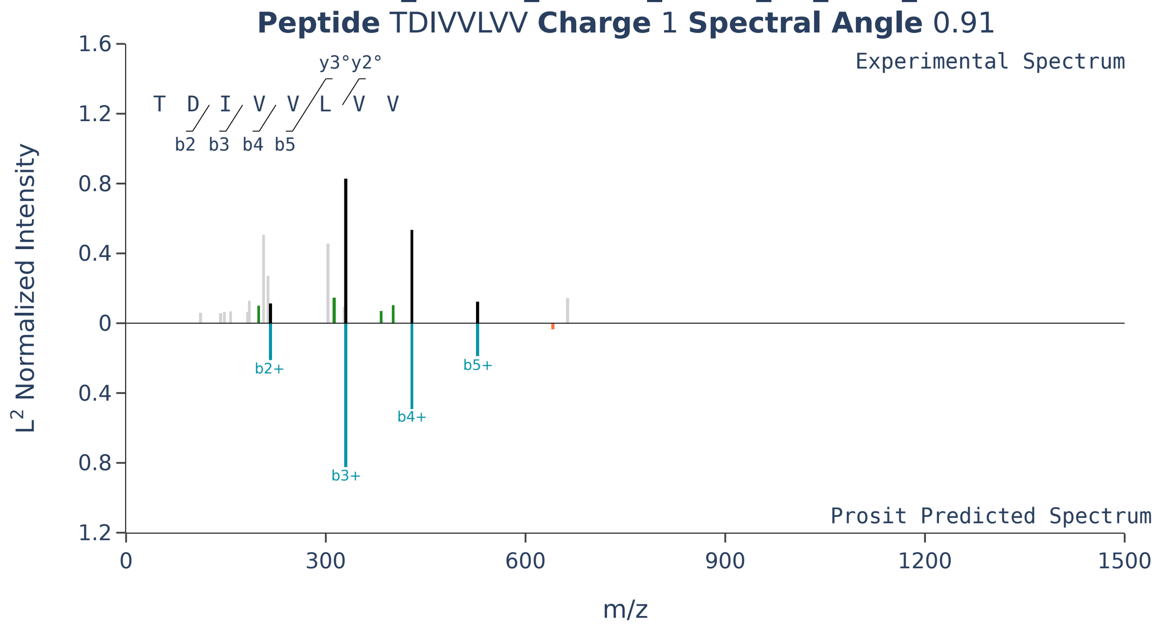


**Source** HRoetschke_211222_160123_HFGoe_IF2_b1R1_2h **Scan** 13431
**Peptide** KPAQAVNRDVVI **Charge** 2 **Spectral Angle** 0.58

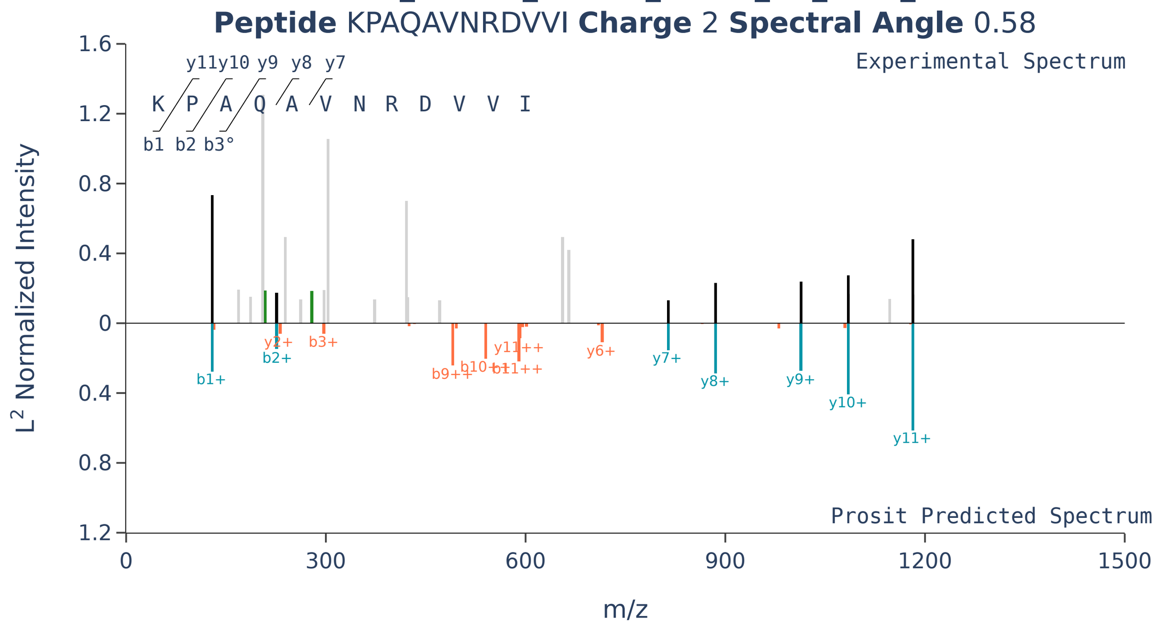


**Source** HRoetschke_211222_160123_HFGoe_IF2_b1R2_24h **Scan** 5597
**Peptide** VVRDEKKARE **Charge** 3 **Spectral Angle** 0.83

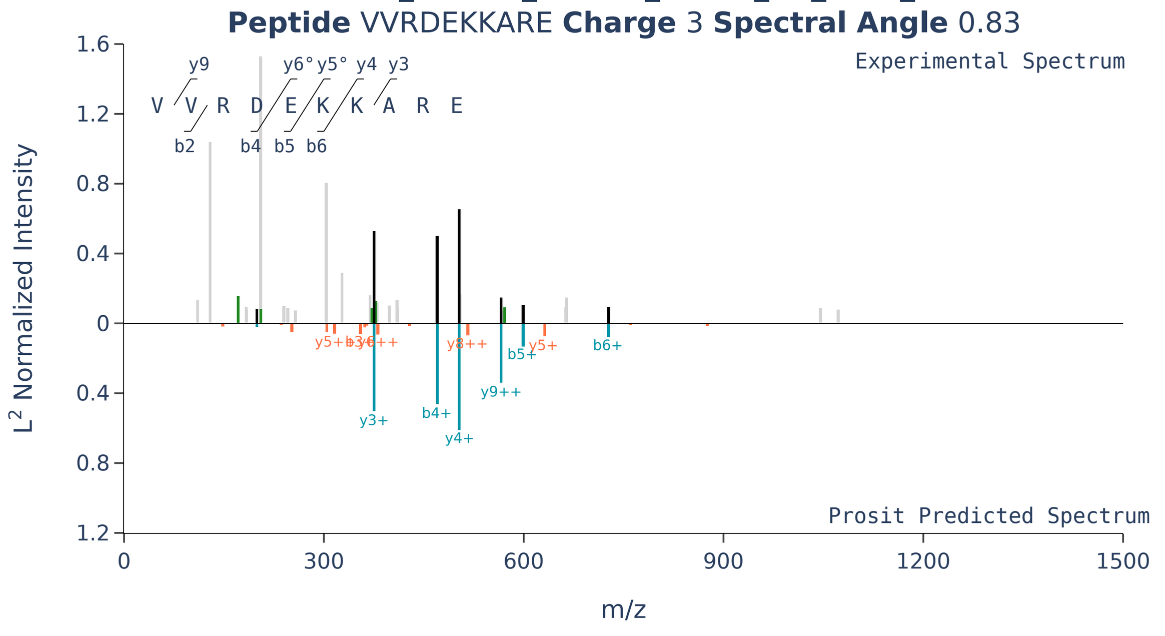


**Source** HRoetschke_211222_160123_HFGoe_IF2_b1R1_4h **Scan** 5321
**Peptide** TVVRDEKKAREVA **Charge** 3 **Spectral Angle** 0.73

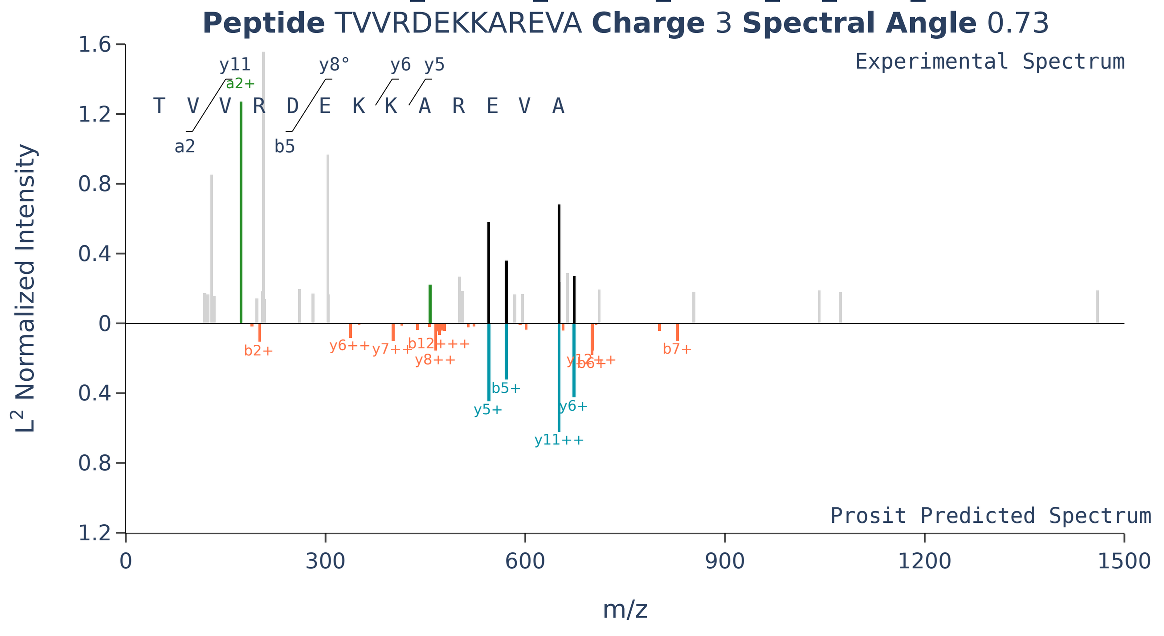


**Source** HRoetschke_211222_160123_HFGoe_IF2_b1R1_24h **Scan** 11866
**Peptide** EVTVVRDEKKAREVAL **Charge** 4 **Spectral Angle** 0.68

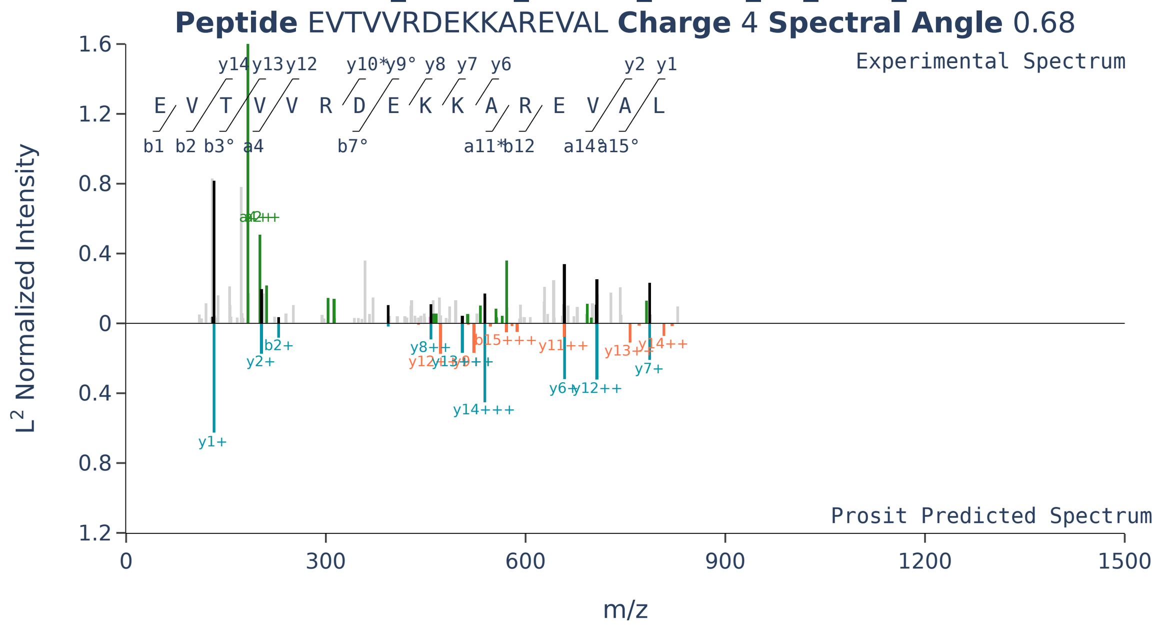


**Source** HRoetschke_211222_160123_HFGoe_IF2_b1R1_4h **Scan** 20968
**Peptide** YNLIDEVKA **Charge** 2 **Spectral Angle** 0.74

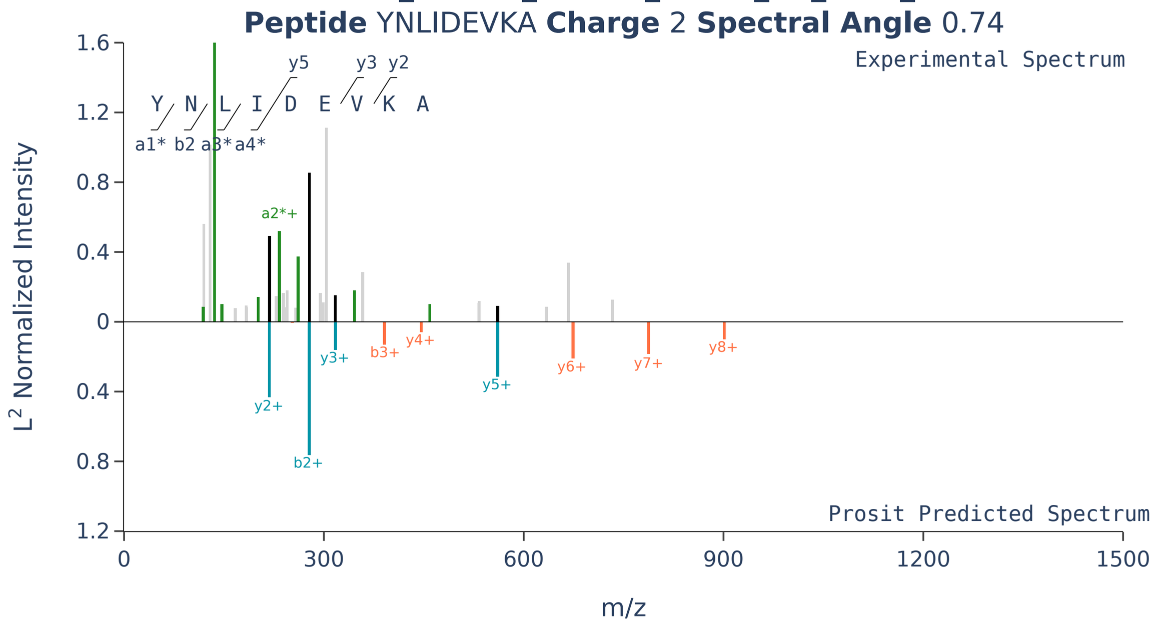


**Source** HRoetschke_211222_160123_HFGoe_IF2_b1R1_2h **Scan** 8322
**Peptide** YGRVRAM **Charge** 2 **Spectral Angle** 0.61

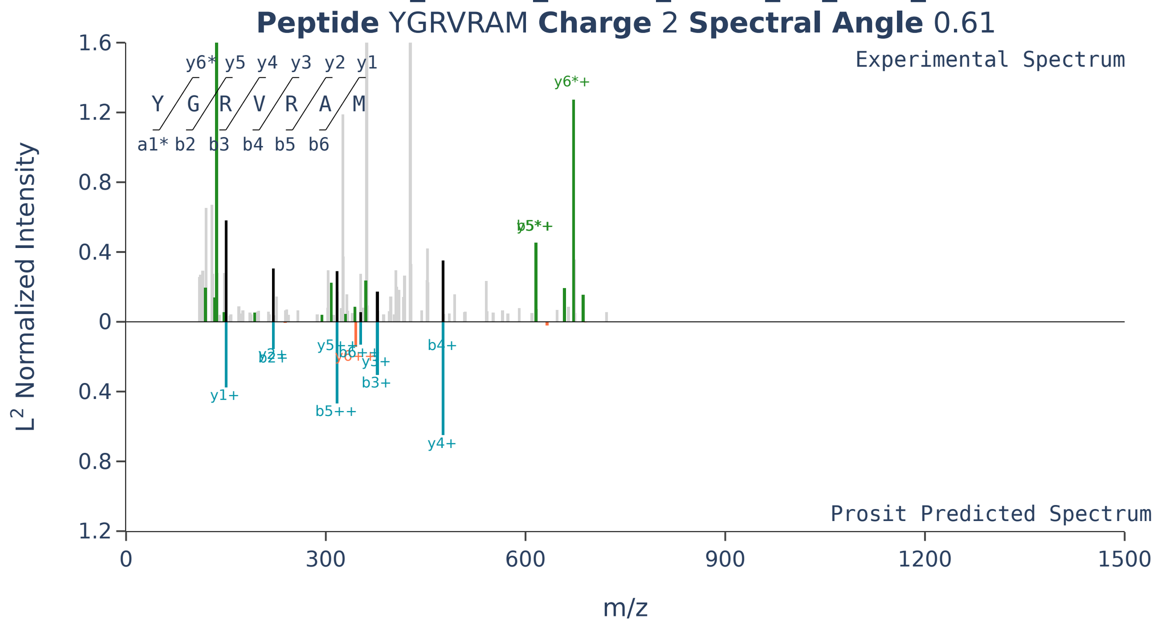


**Source** HRoetschke_211222_160123_HFGoe_IF2_b1R1_2h **Scan** 9233
**Peptide** RTFVKRDPQEAERLA **Charge** 4 **Spectral Angle** 0.68

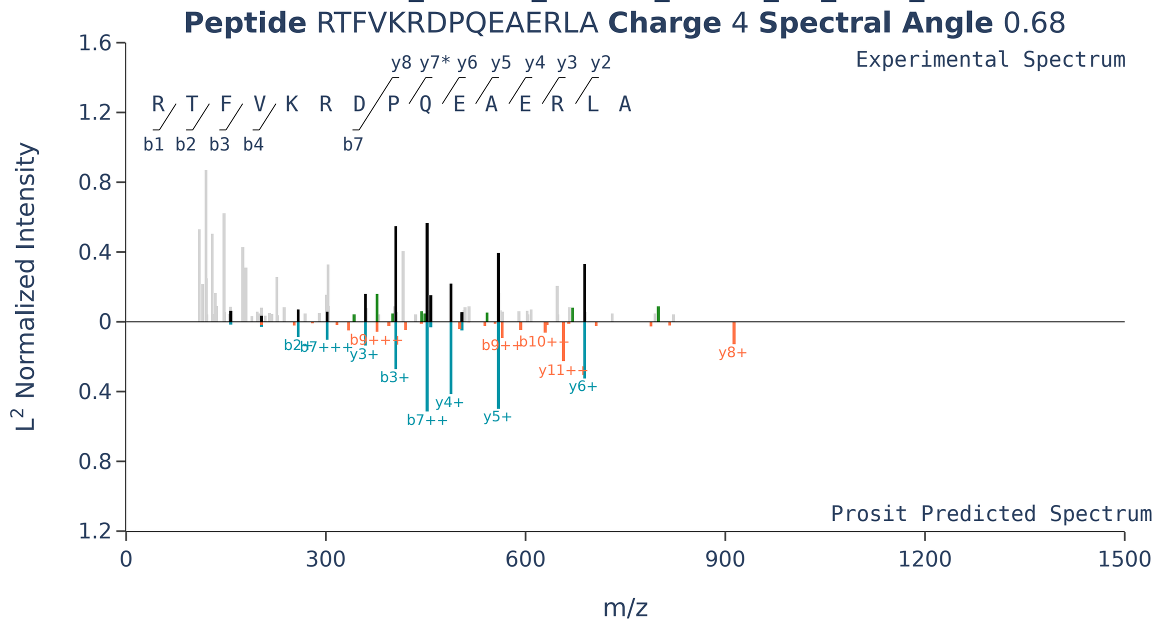


**Source** HRoetschke_211222_160123_HFGoe_IF2_b1R2_24h **Scan** 7338
**Peptide** SSDYHVTTS **Charge** 2 **Spectral Angle** 0.56

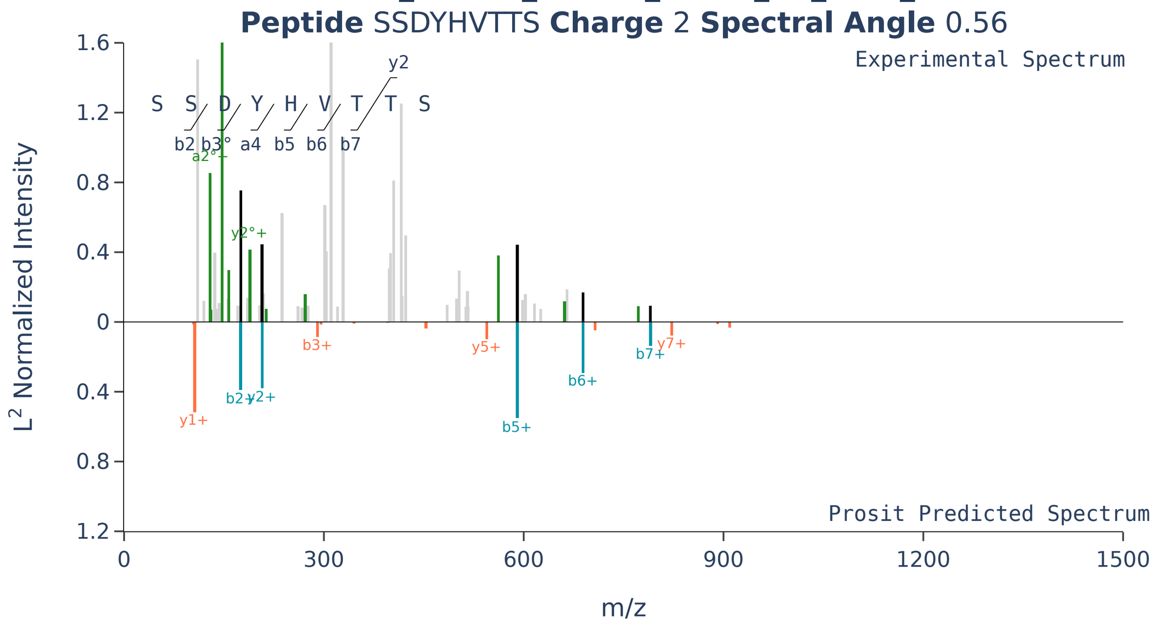


**Source** HRoetschke_211222_160123_HFGoe_IF2_b1R1_4h **Scan** 20620
**Peptide** PAQAVNRDVVIGETI **Charge** 2 **Spectral Angle** 0.62

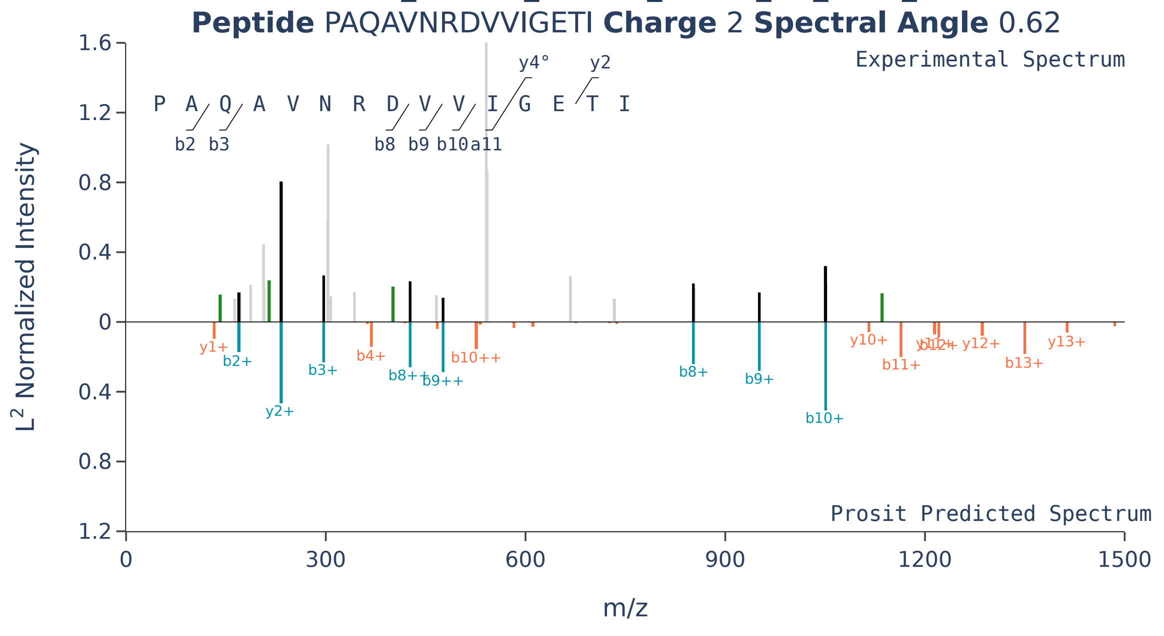


**Source** HRoetschke_211222_160123_HFGoe_IF2_b1R1_4h **Scan** 23396
**Peptide** LSQYGILPEE **Charge** 2 **Spectral Angle** 0.83

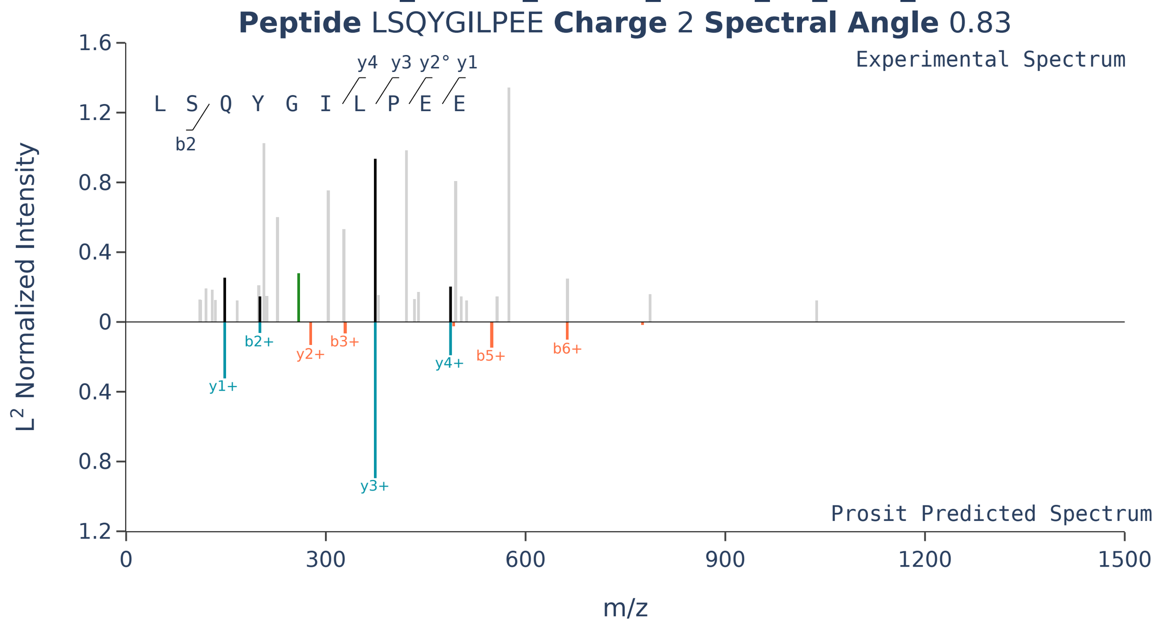

**Source** HRoetschke_211222_160123_HFGoe_IF2_b1R1_4h **Scan** 7969
**Peptide** KAVRKGMASGAVIES **Charge** 3 **Spectral Angle** 0.51

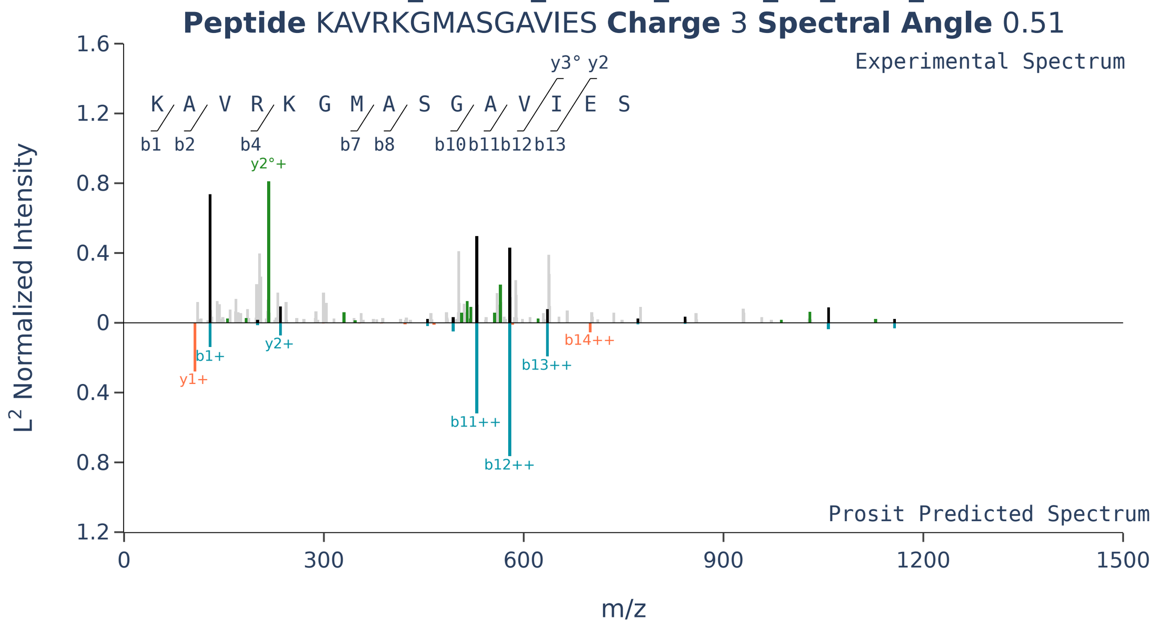


**Source** HRoetschke_211222_160123_HFGoe_IF2_b1R1_4h **Scan** 5324
**Peptide** PEADPDRVKN **Charge** 2 **Spectral Angle** 0.7

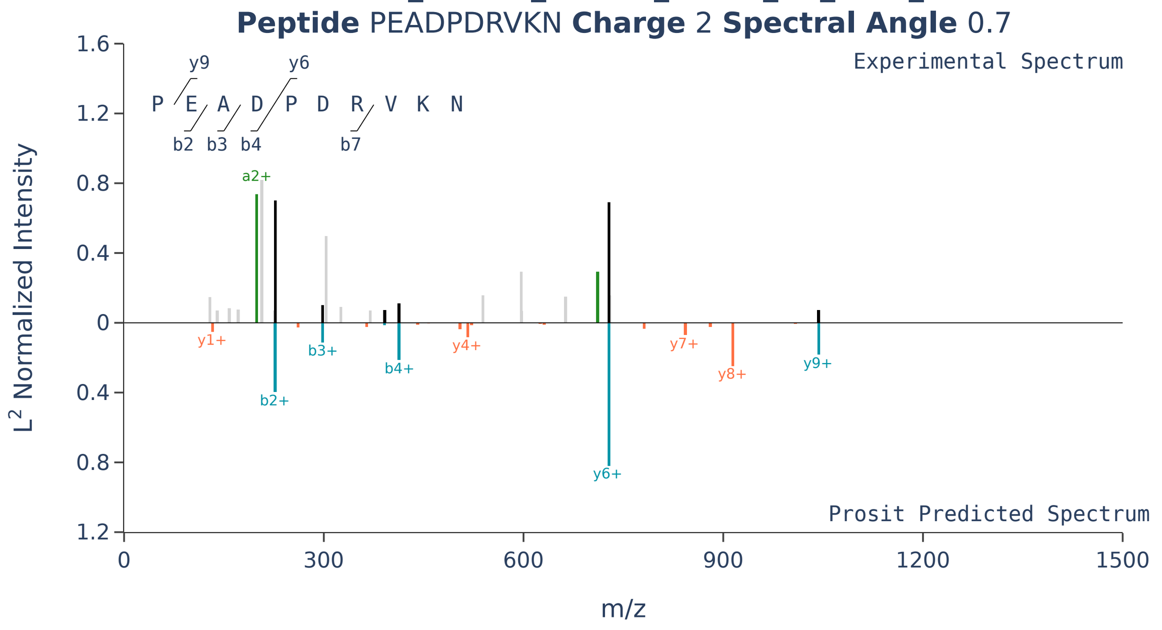


**Source** HRoetschke_211222_160123_HFGoe_IF2_b1R1_4h **Scan** 13548
**Peptide** EMGHKVIL **Charge** 2 **Spectral Angle** 0.73

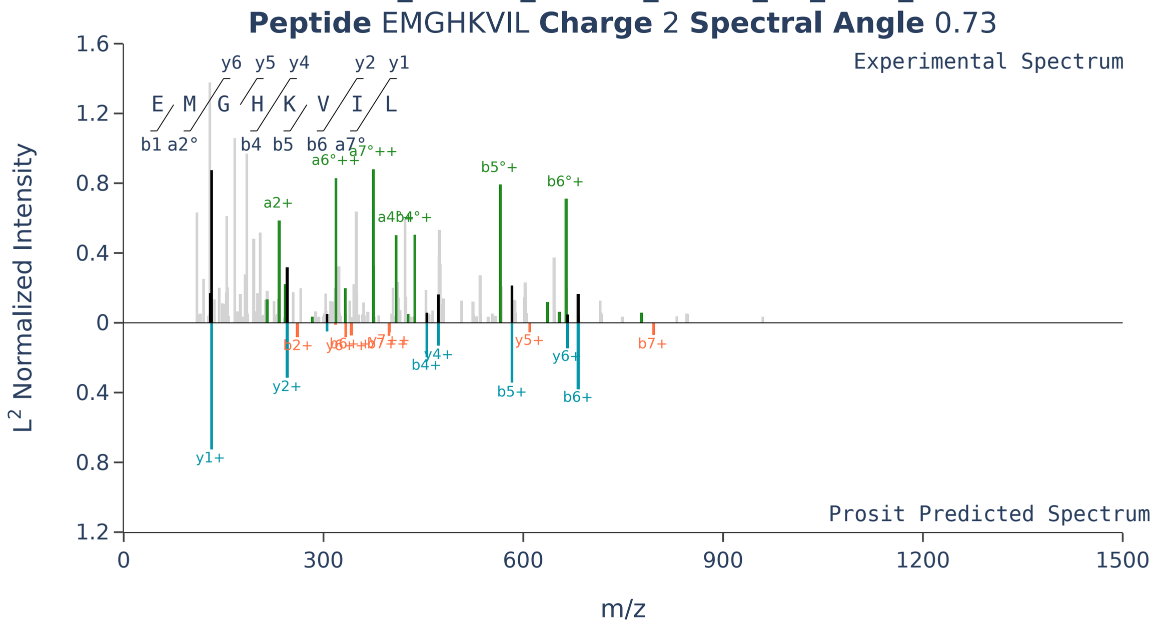


**Source** HRoetschke_211222_160123_HFGoe_IF2_b1R1_4h **Scan** 8338
**Peptide** TDNAEPTEDSSDYHVTTSQHARQAE **Charge** 4 **Spectral Angle** 0.63

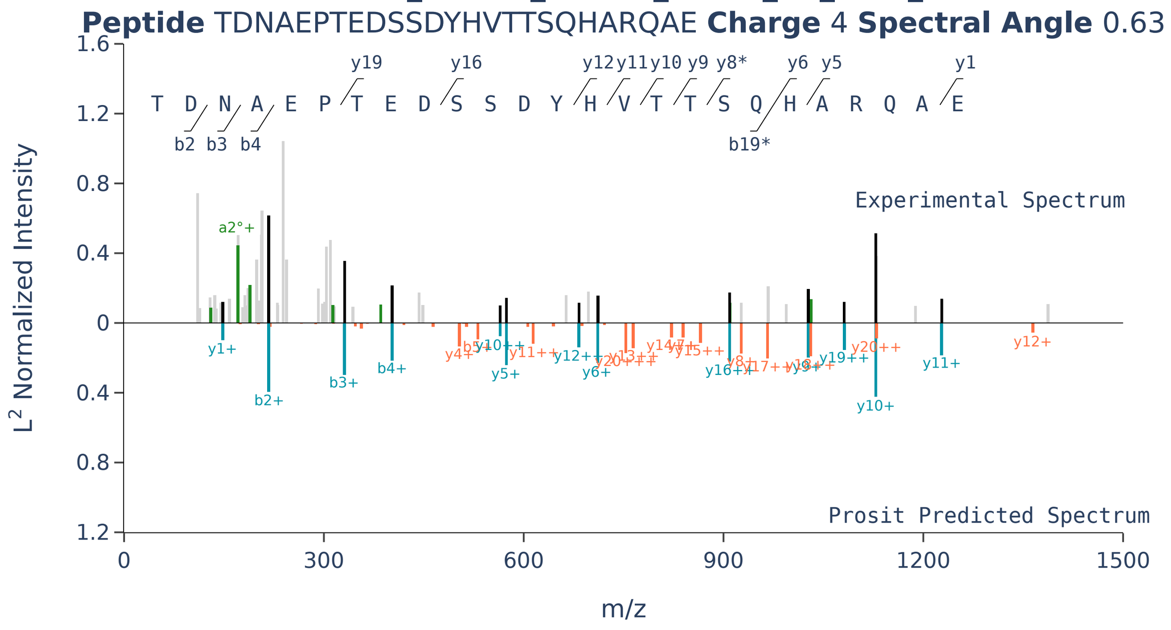


**Source** HRoetschke_211222_160123_HFGoe_IF2_b1R2_24h **Scan** 16744
**Peptide** ADDGVMPQTIEAIQH **Charge** 3 **Spectral Angle** 0.73

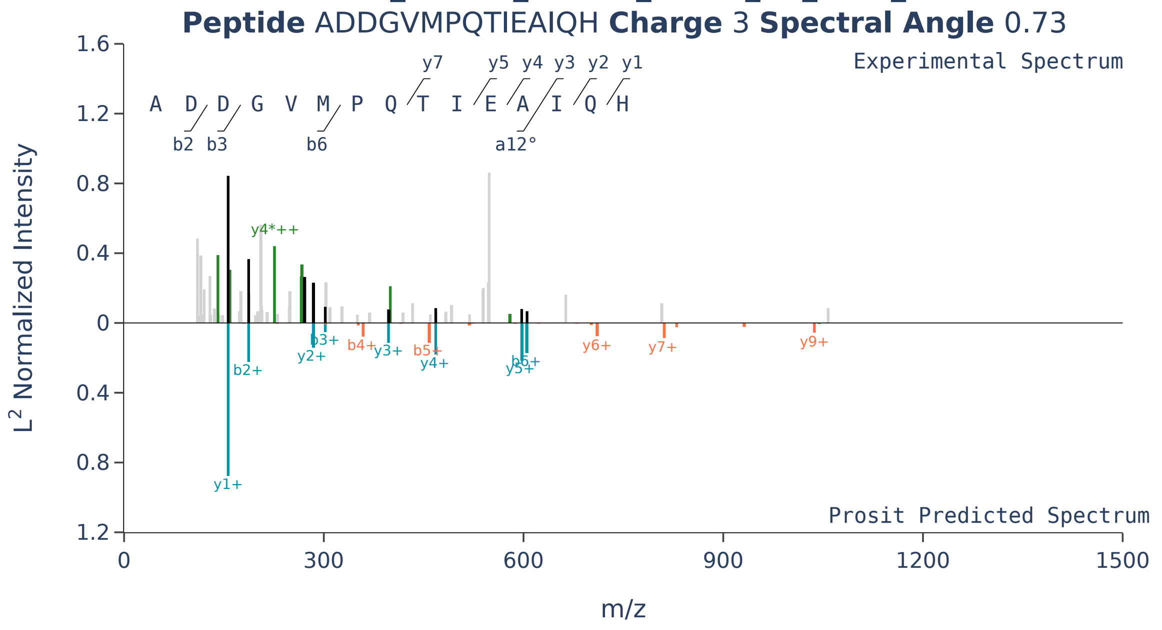


**Source** HRoetschke_211222_160123_HFGoe_IF2_b1R1_4h **Scan** 11096
**Peptide** ESQFVHVSAK **Charge** 3 **Spectral Angle** 0.65

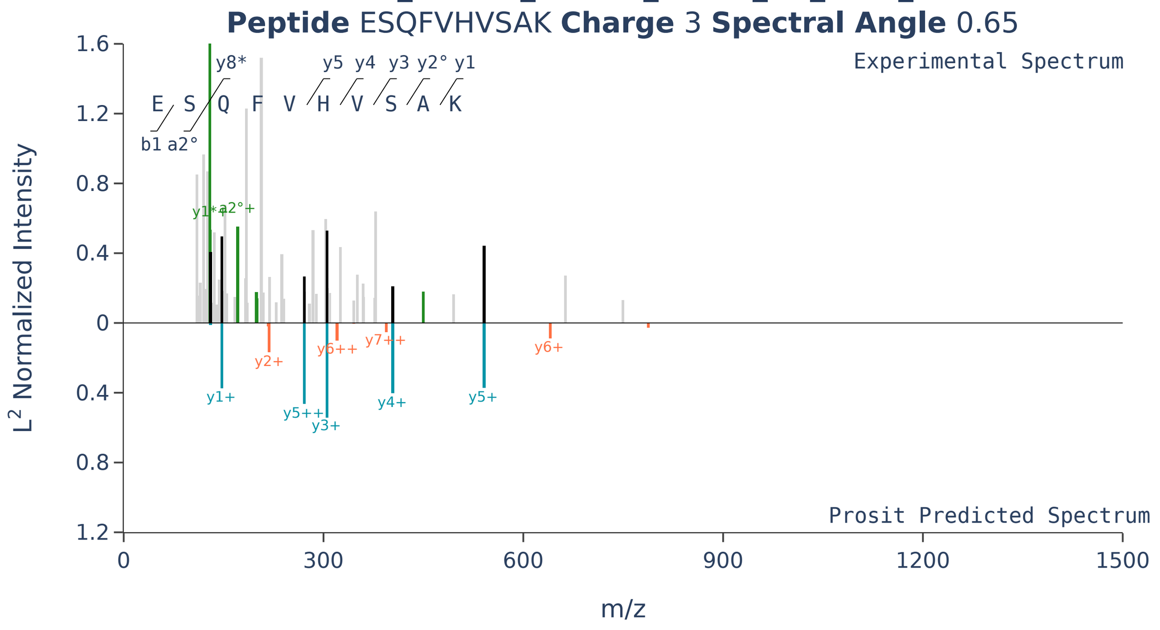


**Source** HRoetschke_211222_160123_HFGoe_IF2_b1R2_24h **Scan** 26409
**Peptide** GILPEEW **Charge** 1 **Spectral Angle** 0.71

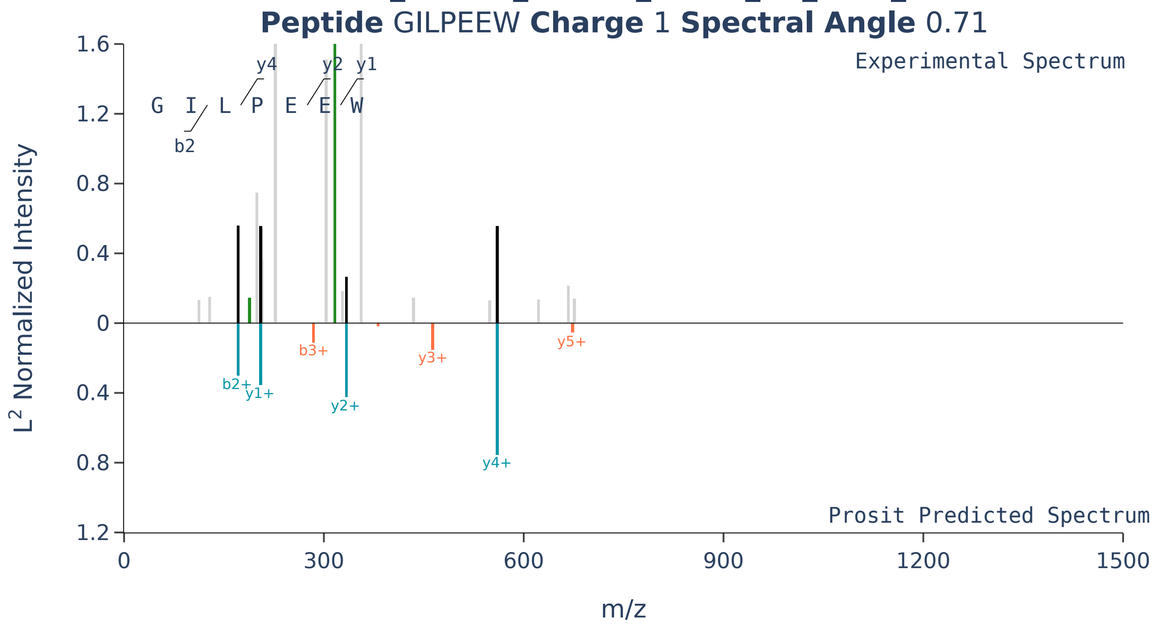


**Source** HRoetschke_211222_160123_HFGoe_IF2_b1R1_2h **Scan** 12854
**Peptide** KGRGPVATVLVRE **Charge** 3 **Spectral Angle** 0.63

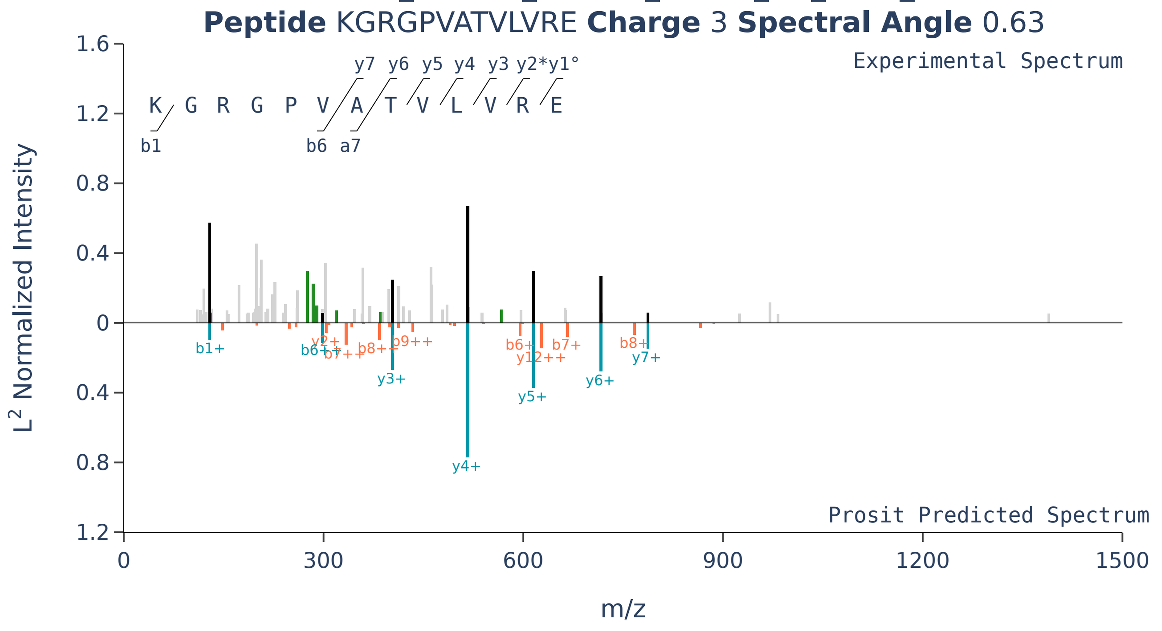


**Source** HRoetschke_211222_160123_HFGoe_IF2_b1R1_24h **Scan** 12433
**Peptide** KSADDSVSAQEKQTLID **Charge** 3 **Spectral Angle** 0.62

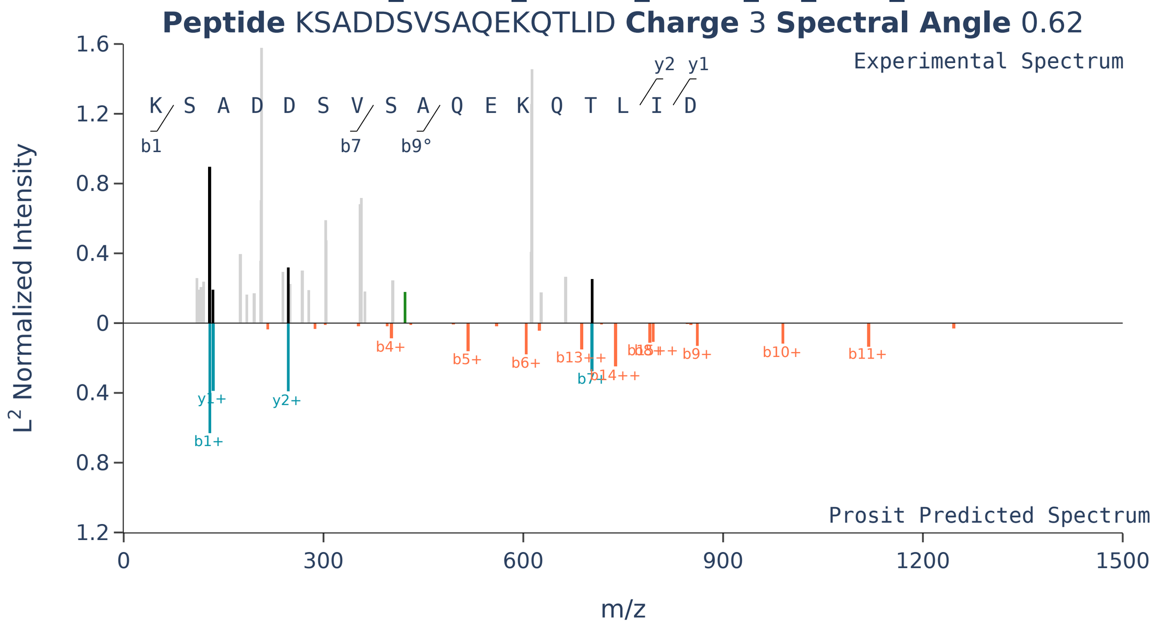


**Source** HRoetschke_211222_160123_HFGoe_IF2_b1R1_1h **Scan** 12858
**Peptide** STLNIPGTGGKSKSVQ **Charge** 3 **Spectral Angle** 0.7

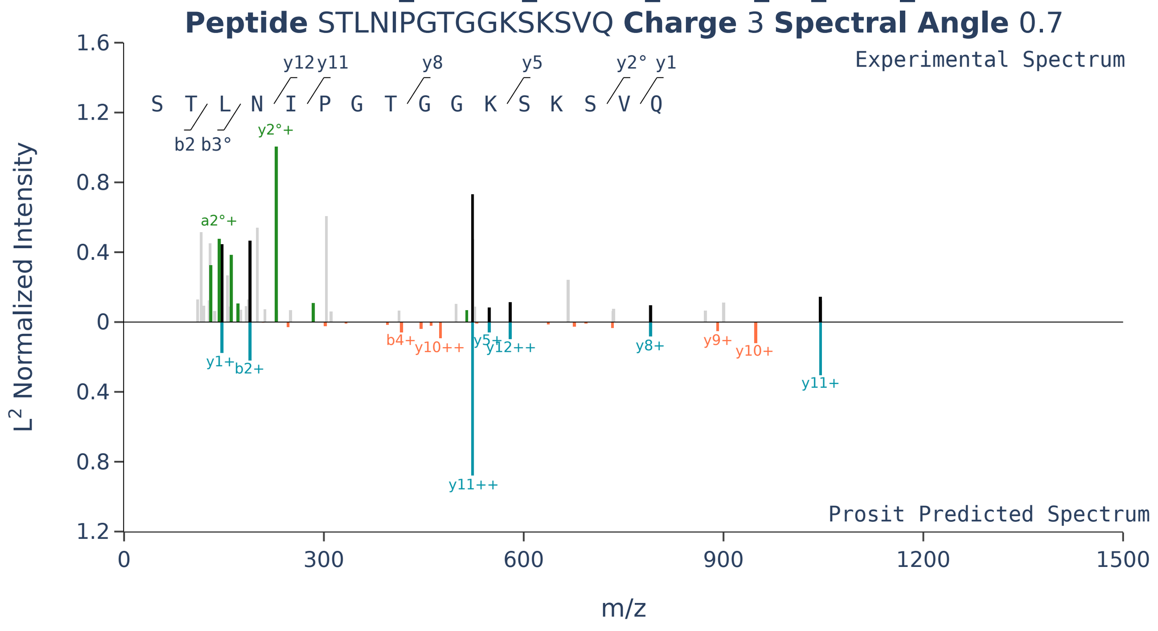


**Source** HRoetschke_211222_160123_HFGoe_IF2_b2R2_4h **Scan** 18802
**Peptide** TGAAAEPRAPVVTI **Charge** 2 **Spectral Angle** 0.68

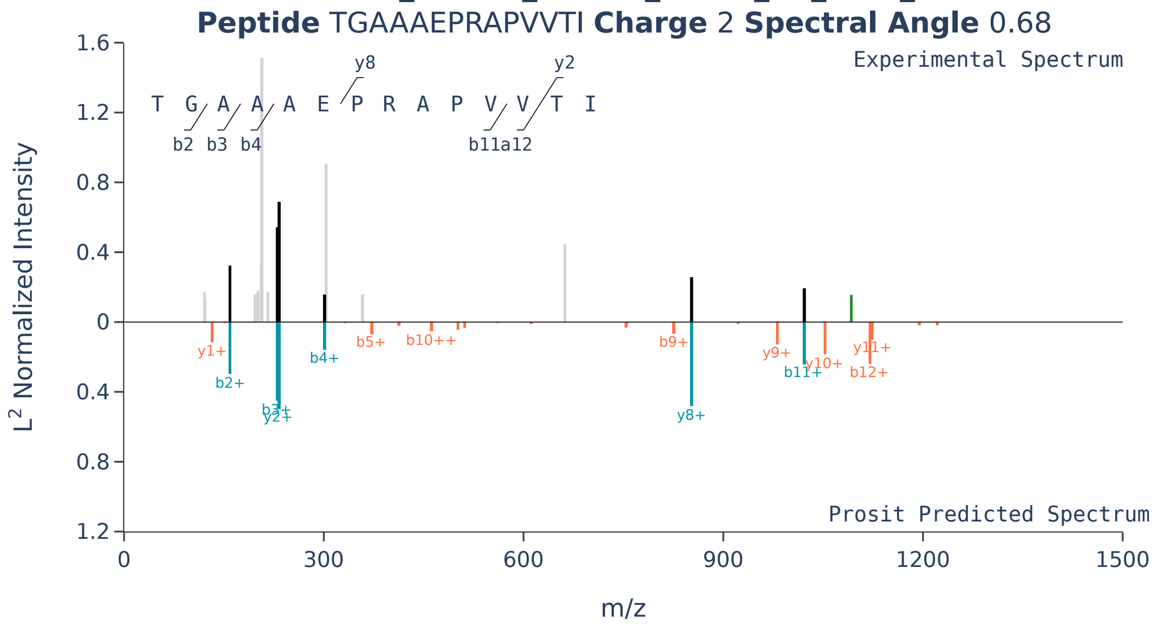


**Source** HRoetschke_211222_160123_HFGoe_IF2_b1R1_4h **Scan** 10244
**Peptide** PQEAERLAAEEQAQRE **Charge** 3 **Spectral Angle** 0.62

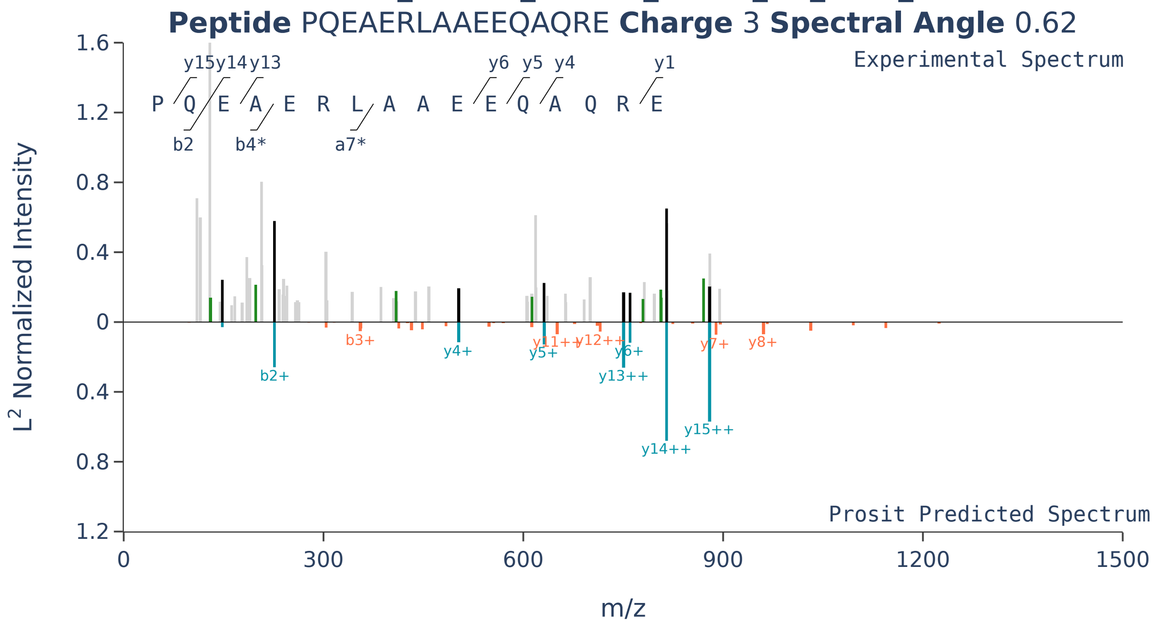


**Source** HRoetschke_211222_160123_HFGoe_IF2_b1R1_4h **Scan** 11429
**Peptide** AQEKQTLIDHLNQKN **Charge** 3 **Spectral Angle** 0.7

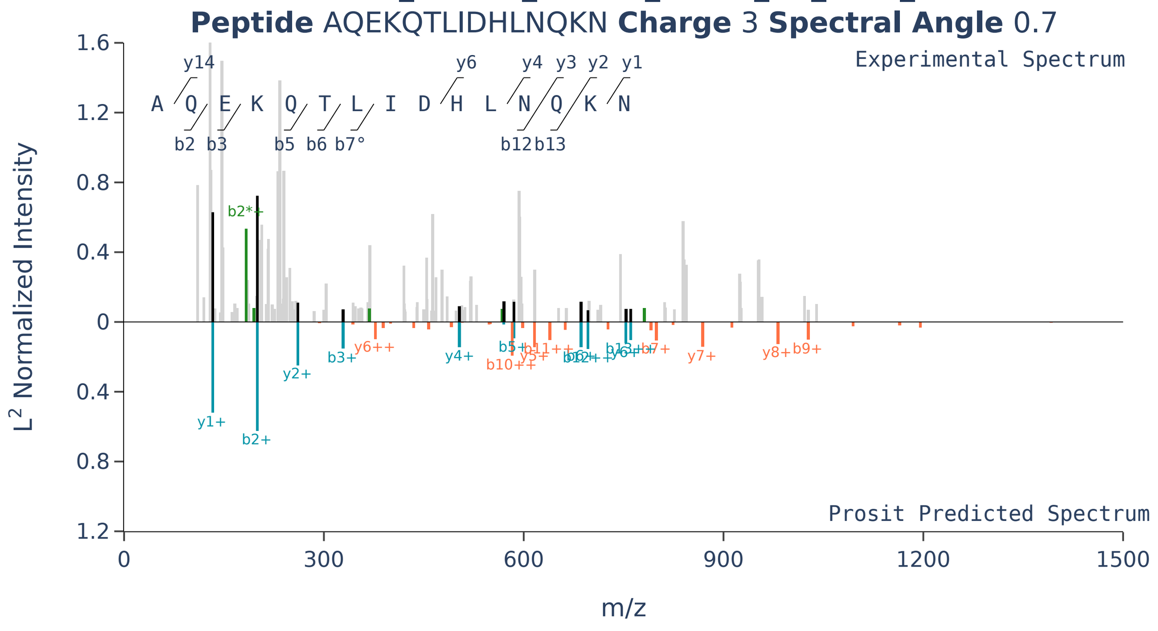


**Source** HRoetschke_211222_160123_HFGoe_IF2_b2R2_24h **Scan** 12184
**Peptide** LLDHLNQKNSGPDKL **Charge** 4 **Spectral Angle** 0.62

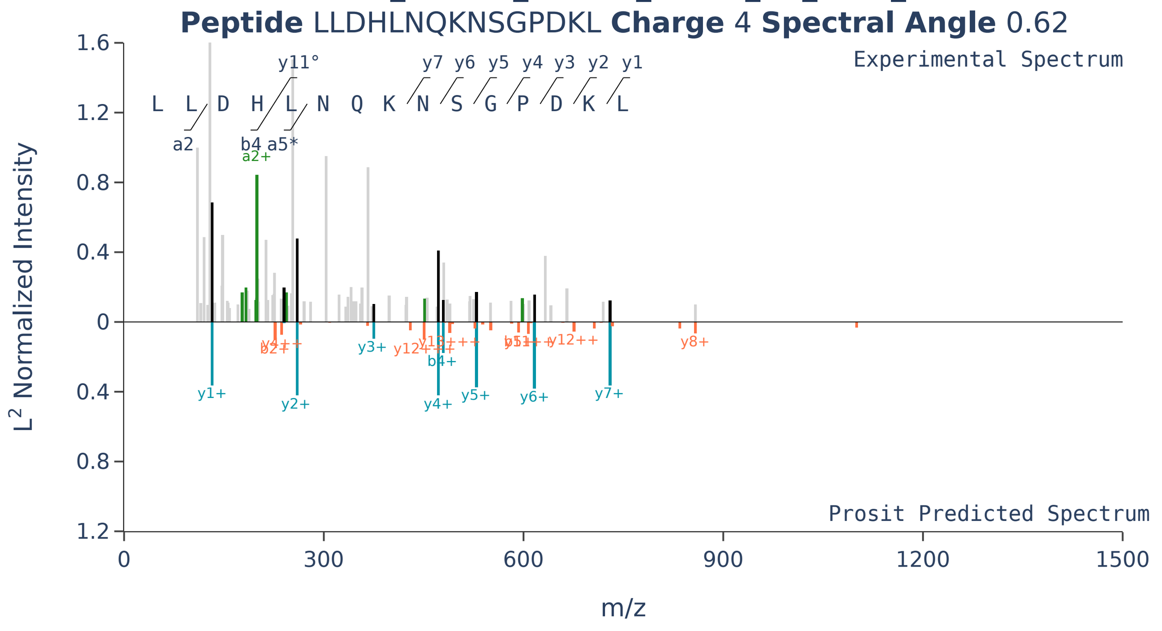


**Source** HRoetschke_211222_160123_HFGoe_IF2_b1R1_24h **Scan** 10827
**Peptide** EADPDRVKNEL **Charge** 3 **Spectral Angle** 0.83

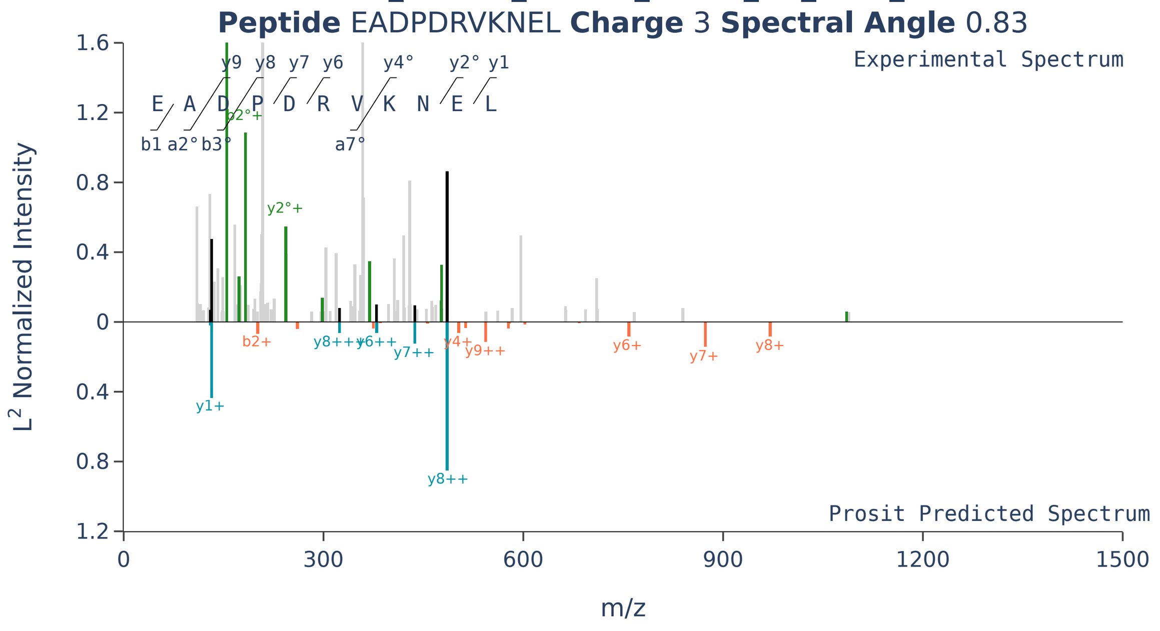

**Source** HRoetschke_211222_160123_HFGoe_IF2_b1R1_4h **Scan** 8456
**Peptide** ARRMAEENKWTDNAEPTE **Charge** 3 **Spectral Angle** 0.79

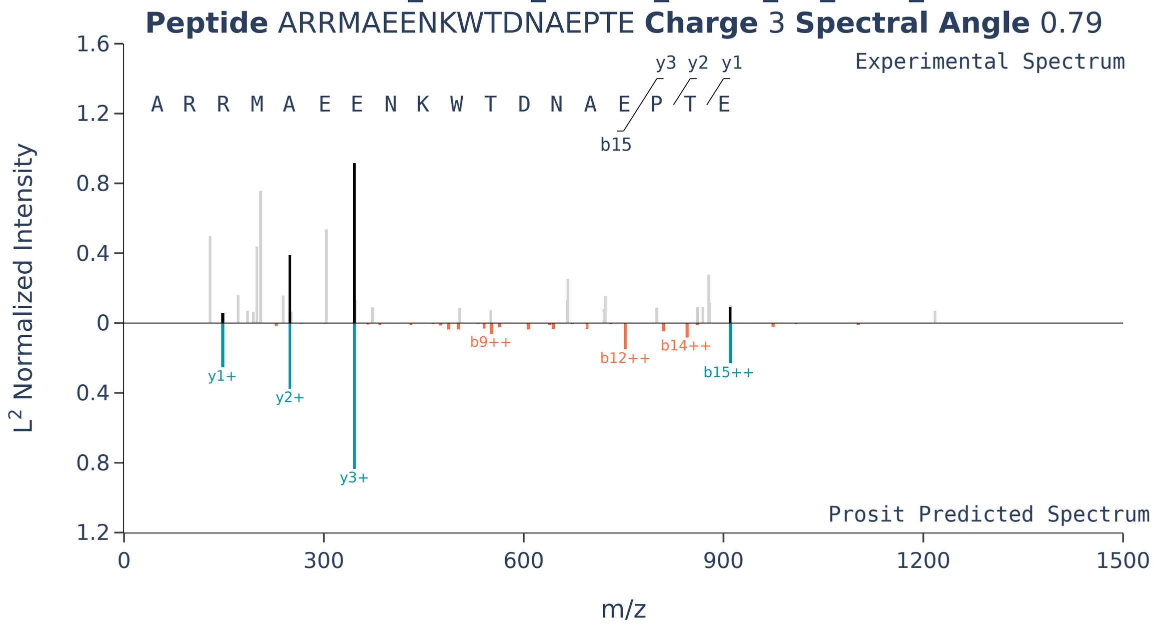


**Source** HRoetschke_211222_160123_HFGoe_IF2_b1R1_4h **Scan** 9787
**Peptide** VAEEMGHKVILRRENELE **Charge** 4 **Spectral Angle** 0.58

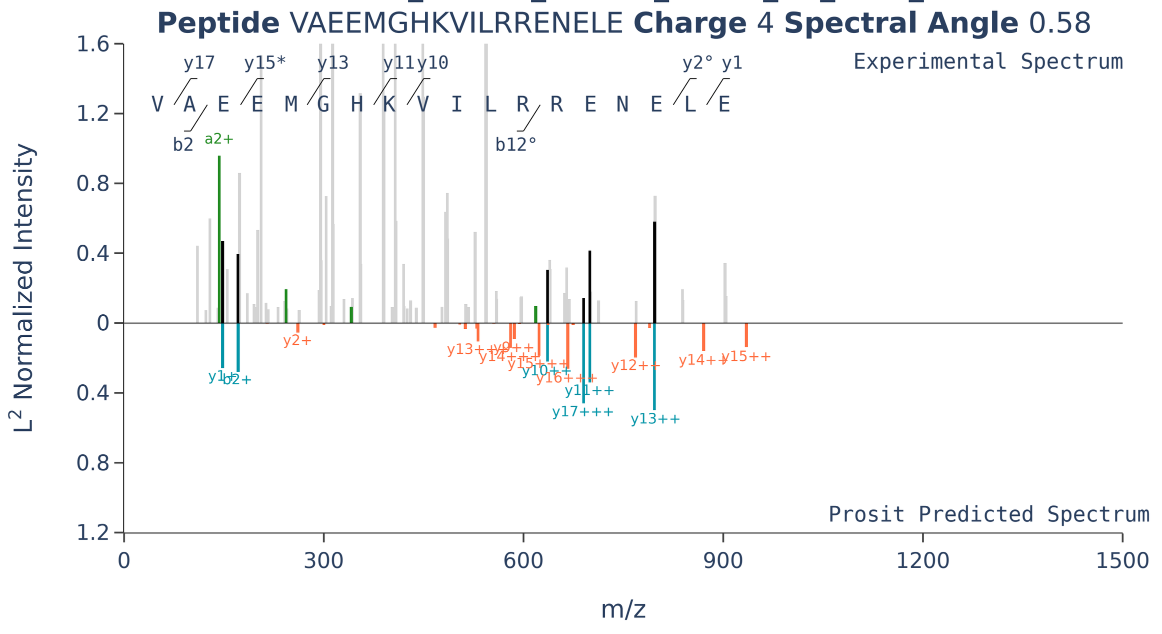


**Source** HRoetschke_211222_160123_HFGoe_IF2_b1R1_4h **Scan** 19255
**Peptide** ADDGVMPQTIEAIQHA **Charge** 2 **Spectral Angle** 0.7

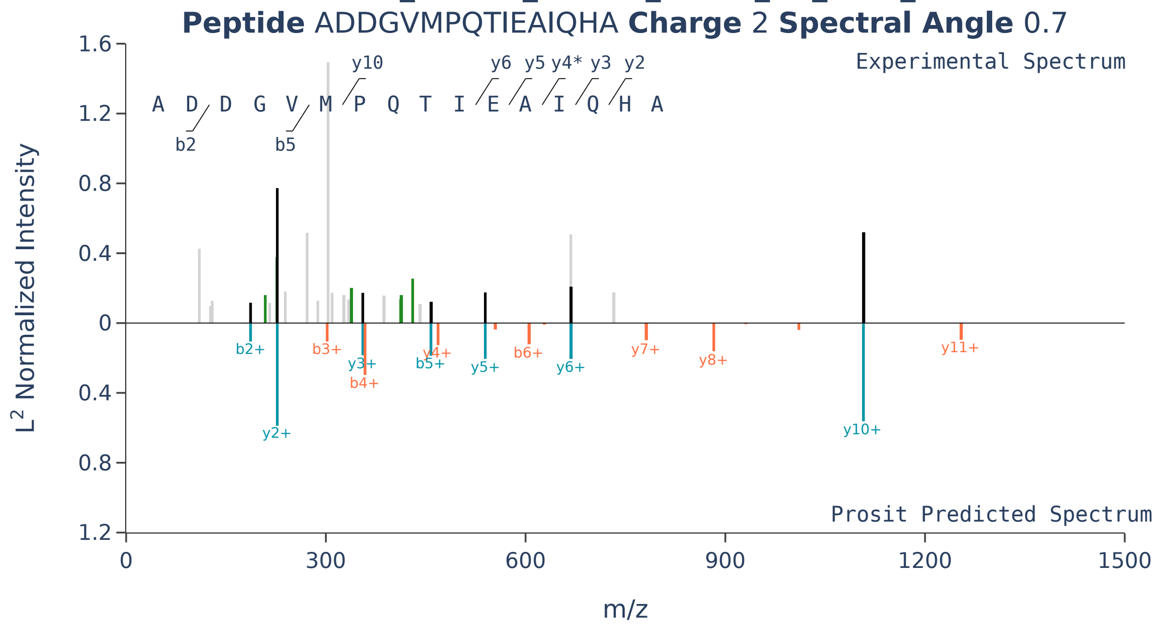


**Source** HRoetschke_211222_160123_HFGoe_IF2_b1R1_4h **Scan** 17603
**Peptide** EAIQHAKAAQVPVVV **Charge** 2 **Spectral Angle** 0.62

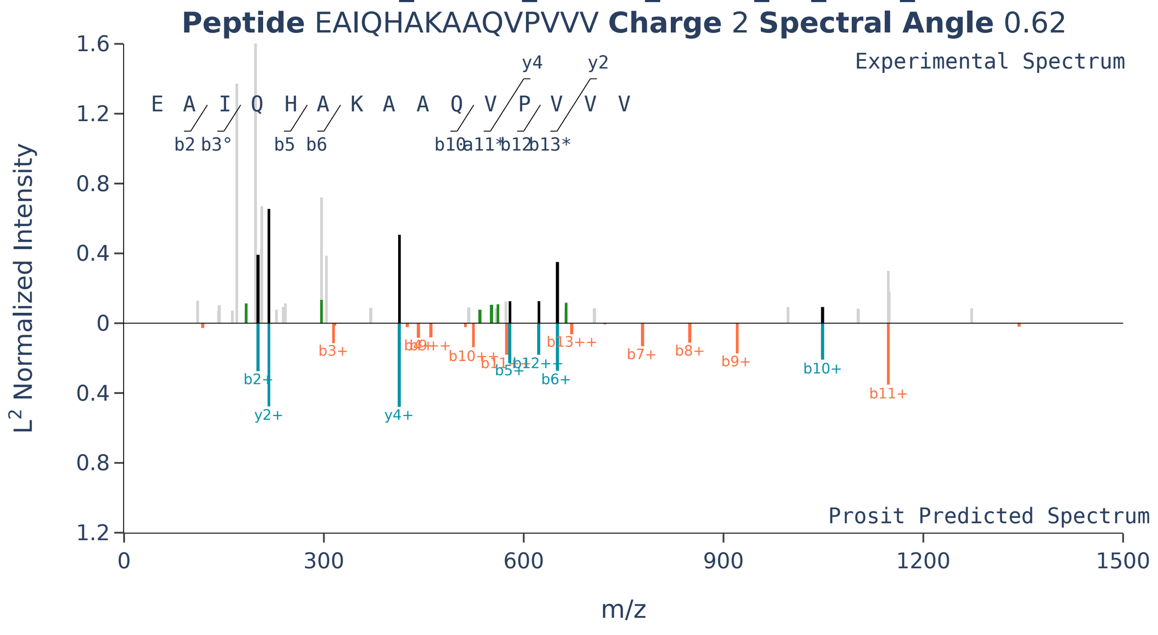


**Source** HRoetschke_211222_160123_HFGoe_IF2_b1R1_4h **Scan** 8241
**Peptide** SQVIKAMMK **Charge** 2 **Spectral Angle** 0.77

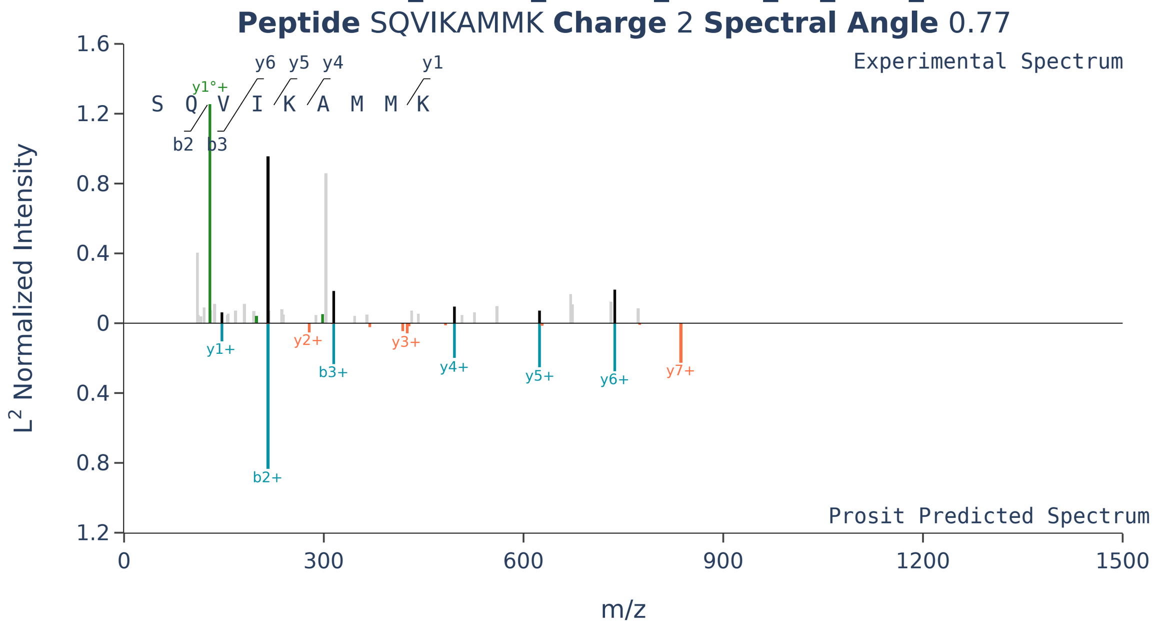


**Source** HRoetschke_211222_160123_HFGoe_IF2_b1R2_24h **Scan** 19643
**Peptide** GAAAEPRAPVVTI **Charge** 2 **Spectral Angle** 0.71

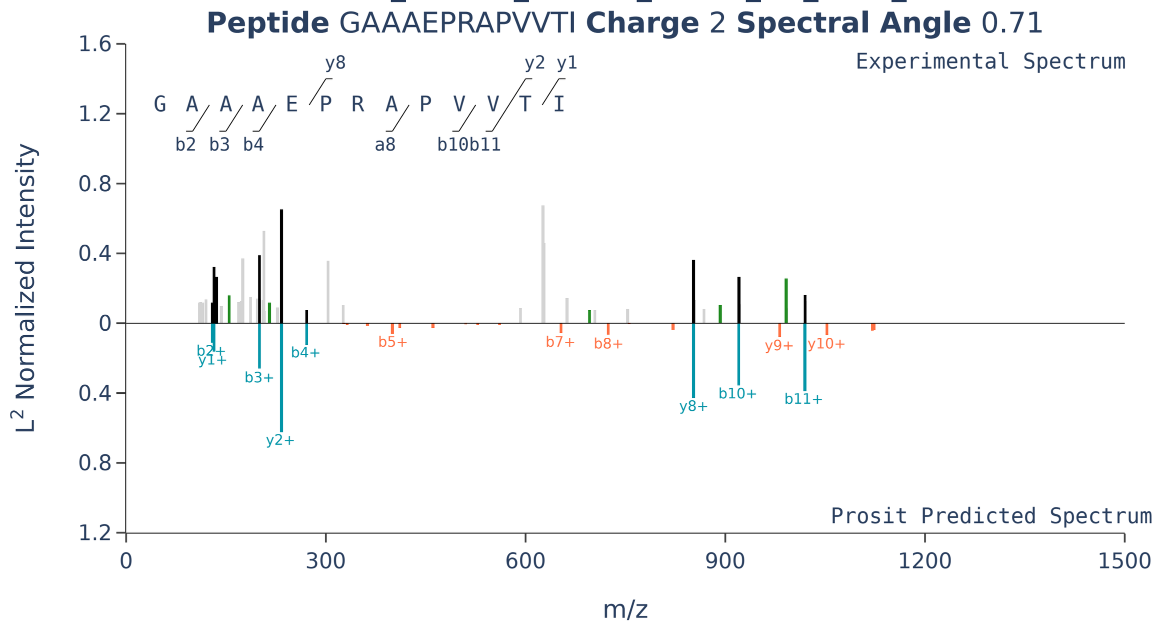


**Source** HRoetschke_211222_160123_HFGoe_IF2_b1R2_24h **Scan** 11887
**Peptide** FKDDVNEV **Charge** 2 **Spectral Angle** 0.59

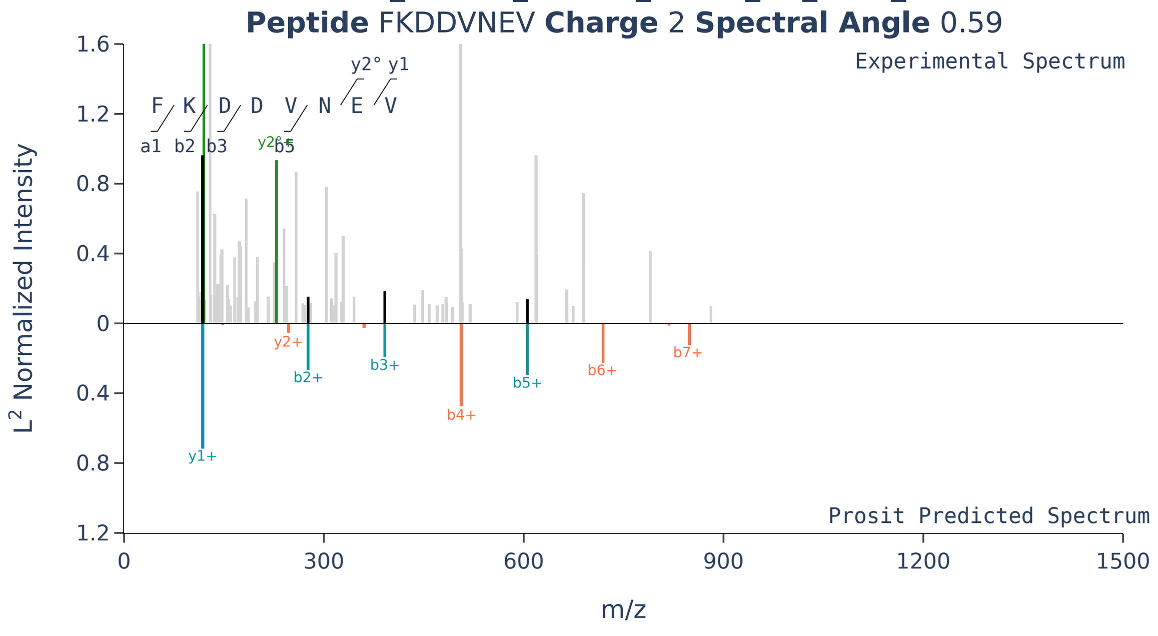


**Source** HRoetschke_211222_160123_HFGoe_IF2_b2R2_4h **Scan** 15916
**Peptide** STDEVKVKII **Charge** 2 **Spectral Angle** 0.6

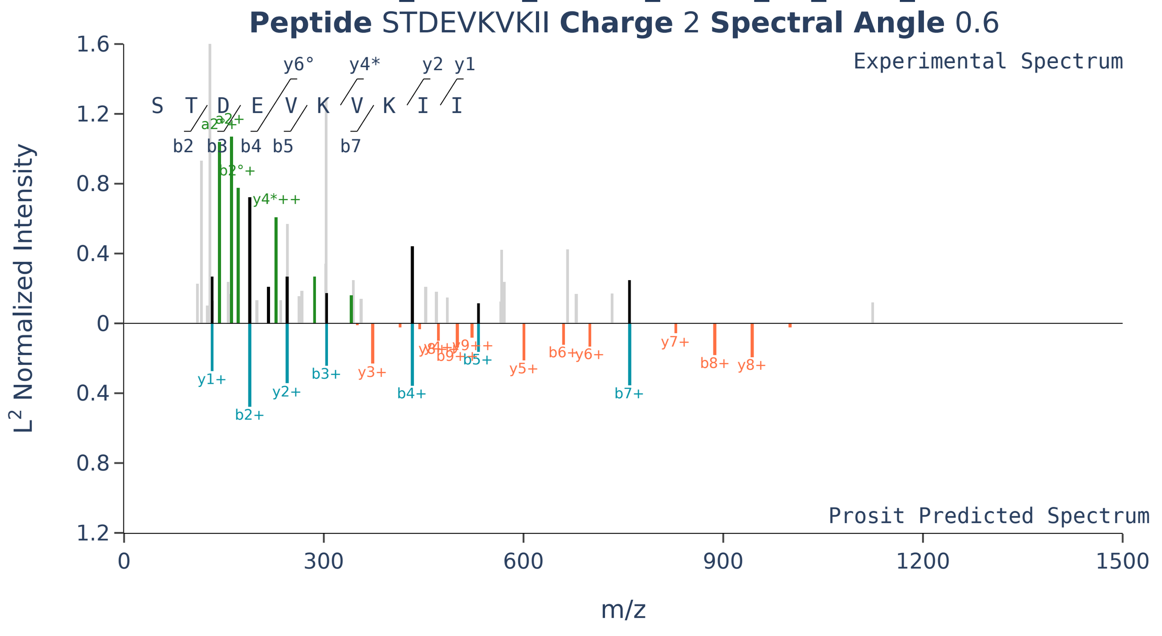


**Source** HRoetschke_211222_160123_HFGoe_IF2_b1R2_4h **Scan** 11857
**Peptide** GGESQFVHVSAKAGT **Charge** 3 **Spectral Angle** 0.59

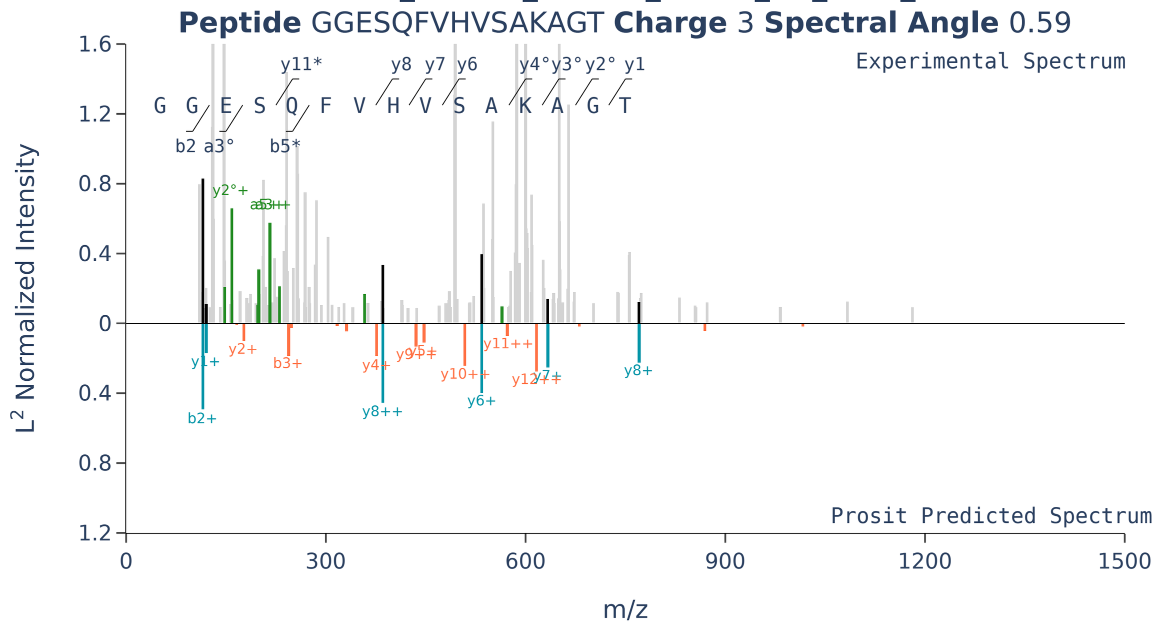


**Source** HRoetschke_211222_160123_HFGoe_IF2_b2R2_24h **Scan** 10997
**Peptide** DDSVSAQEKQTL **Charge** 2 **Spectral Angle** 0.72

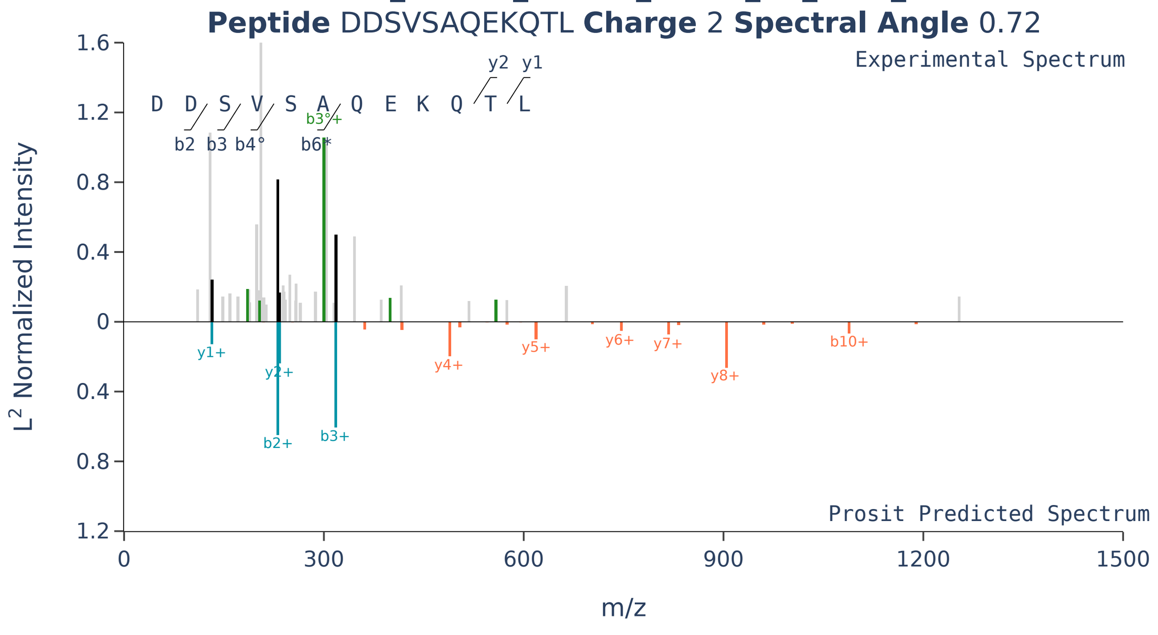


**Source** HRoetschke_211222_160123_HFGoe_IF2_b1R1_24h **Scan** 10002
**Peptide** VAEEMGHKVILRRENELEE **Charge** 3 **Spectral Angle** 0.66

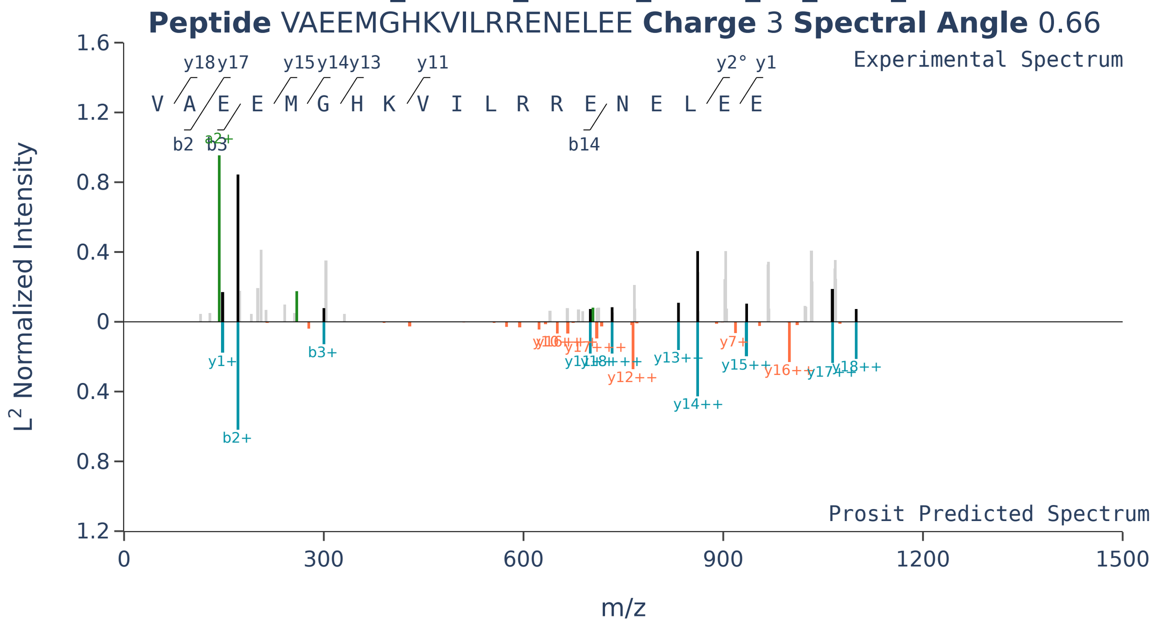


**Source** HRoetschke_211222_160123_HFGoe_IF2_b1R1_24h **Scan** 11095
**Peptide** NIPGTGGKSKSVQIE **Charge** 3 **Spectral Angle** 0.57

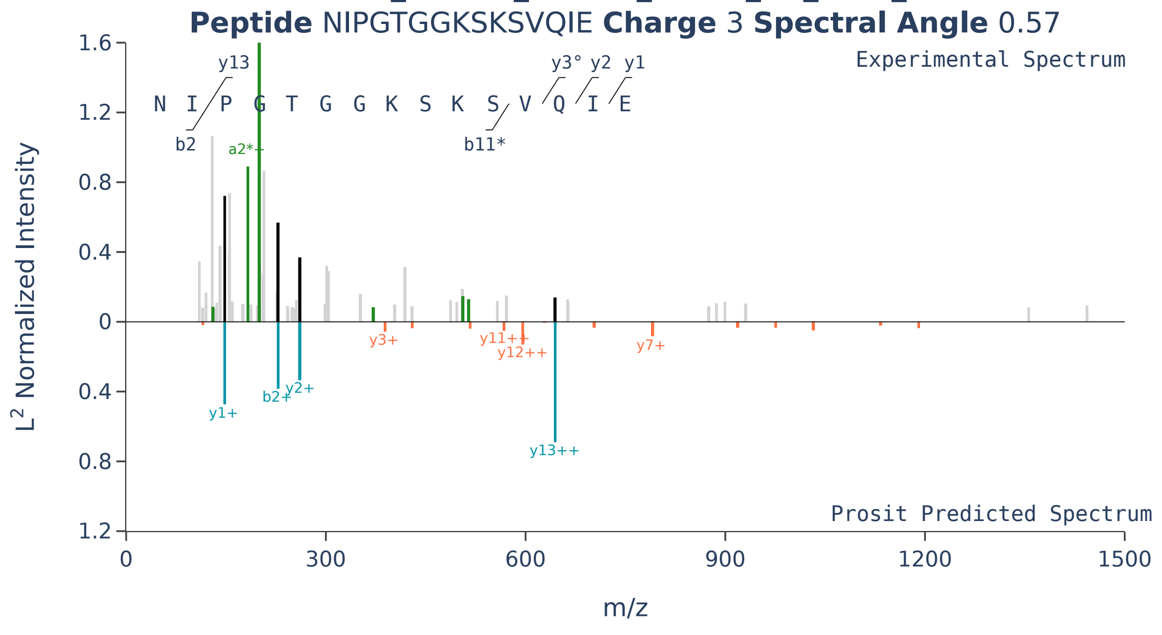


**Source** HRoetschke_211222_160123_HFGoe_IF2_b2R1_24h **Scan** 8056
**Peptide** EMGHKVI **Charge** 2 **Spectral Angle** 0.76

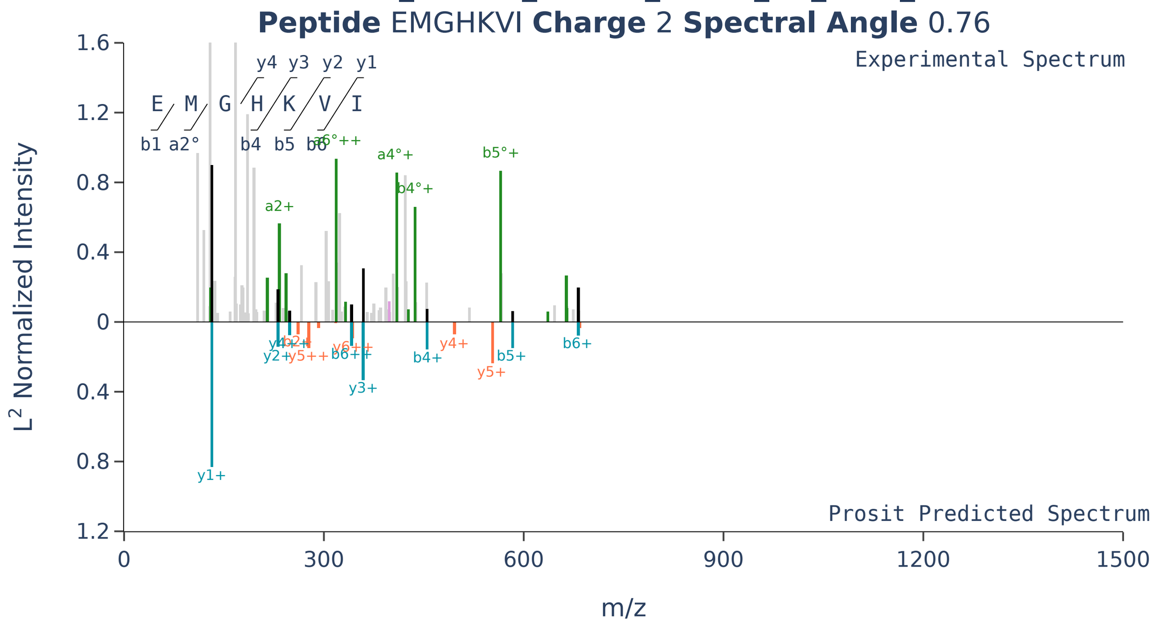


**Source** HRoetschke_211222_160123_HFGoe_IF2_b1R2_24h **Scan** 6887
**Peptide** DHLNQKNSGPDKLT **Charge** 3 **Spectral Angle** 0.79

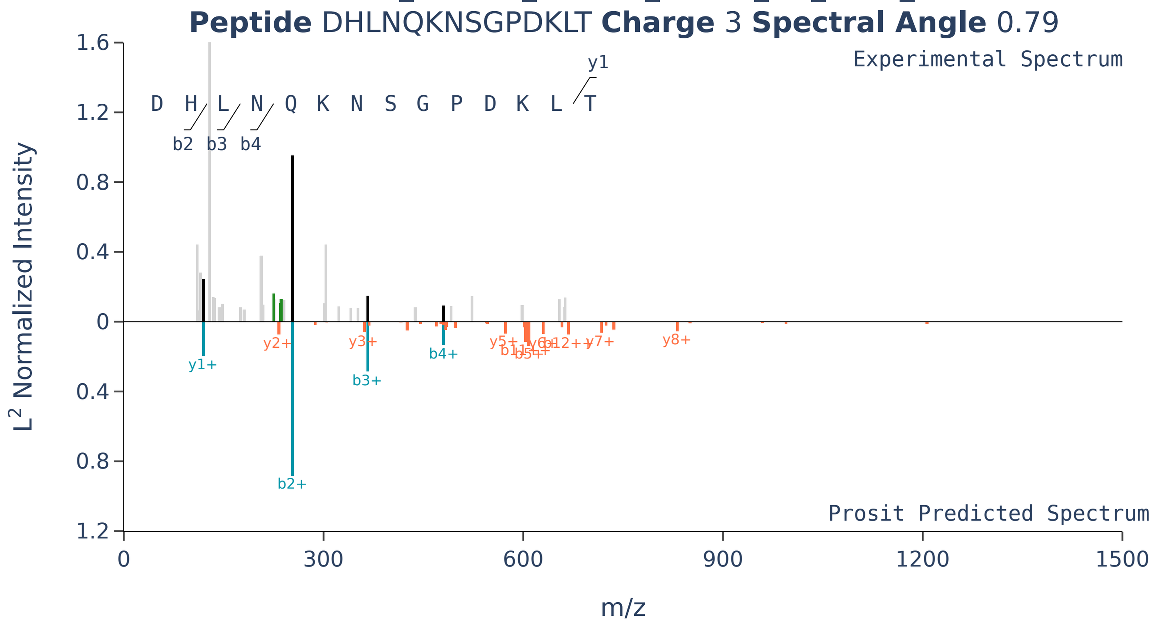


**Source** HRoetschke_211222_160123_HFGoe_IF2_b2R2_24h **Scan** 7766
**Peptide** LNQKNSGPDKLTLQRKT **Charge** 3 **Spectral Angle** 0.66

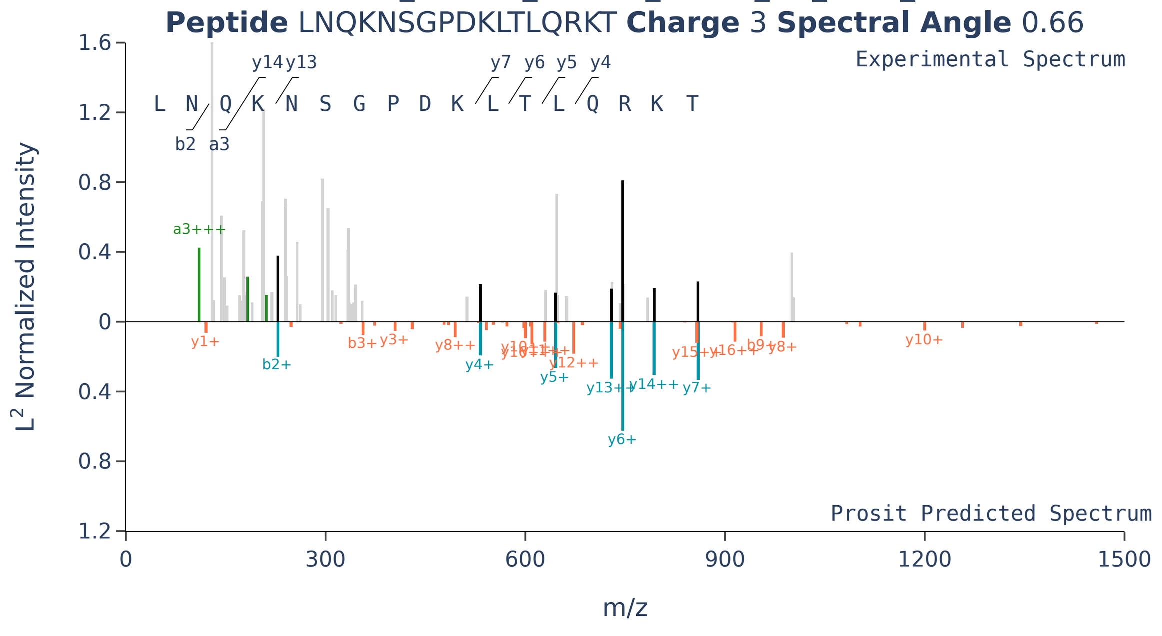

**Source** HRoetschke_211222_160123_HFGoe_IF2_b2R1_24h **Scan** 8009
**Peptide** NVRADASARKVLE **Charge** 3 **Spectral Angle** 0.71

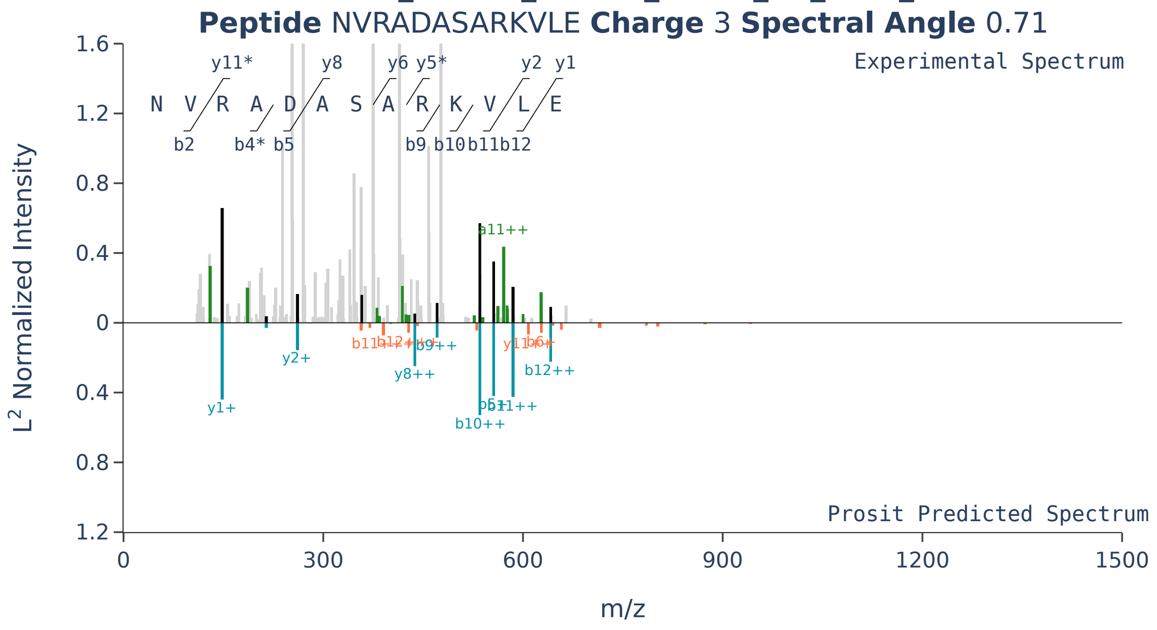


**Source** HRoetschke_211222_160123_HFGoe_IF2_b1R1_4h **Scan** 20681
**Peptide** AYHVETENGMITF **Charge** 2 **Spectral Angle** 0.68

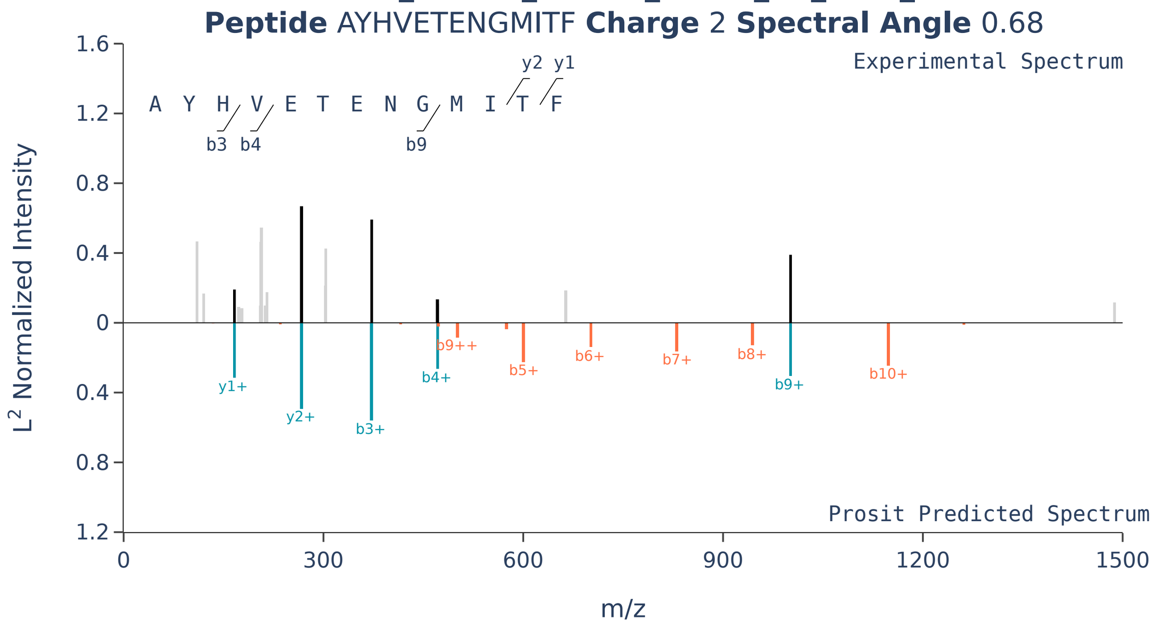


**Source** HRoetschke_211222_160123_HFGoe_IF2_b1R1_2h **Scan** 6926
**Peptide** PTEDSSDYHVTTSQHARQAED **Charge** 3 **Spectral Angle** 0.66

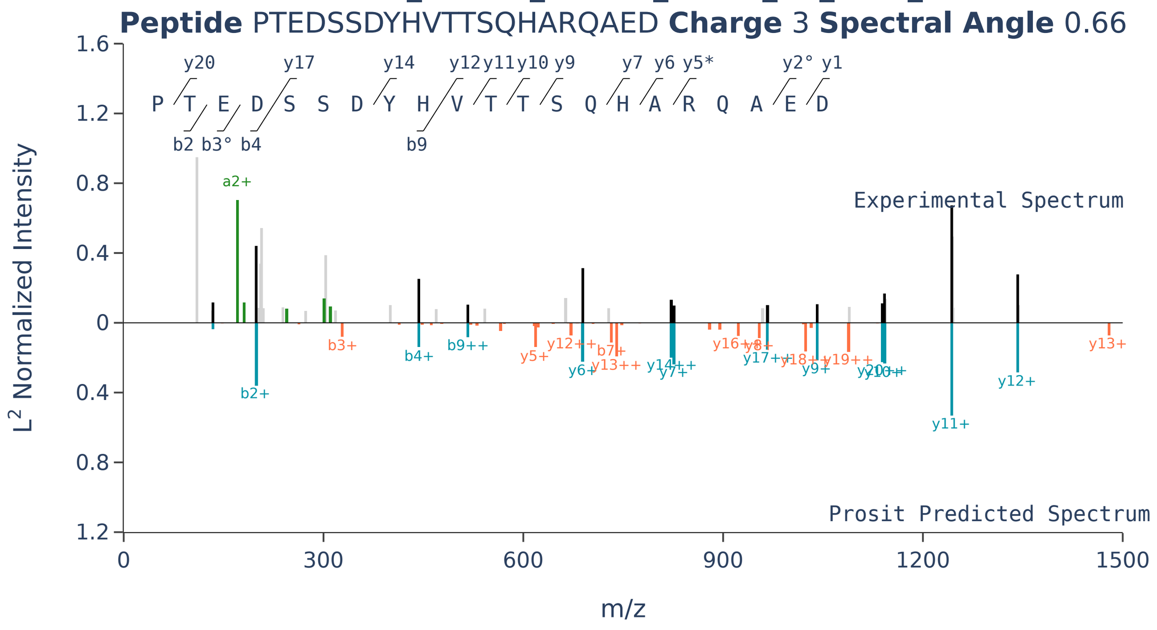


**Source** HRoetschke_211222_160123_HFGoe_IF2_b1R2_24h **Scan** 18123
**Peptide** KAAQVPVVVAVNKIDKPEAD **Charge** 3 **Spectral Angle** 0.78

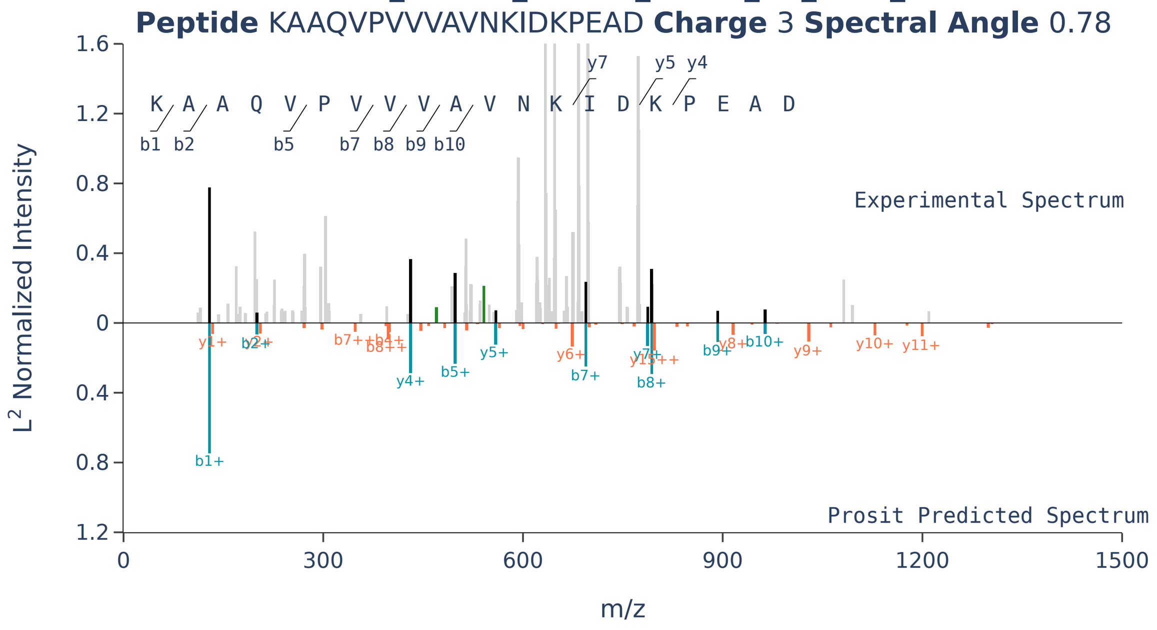


**Source** HRoetschke_211222_160123_HFGoe_IF2_b1R1_4h **Scan** 15022
**Peptide** SADDSVSAQEKQTLID **Charge** 3 **Spectral Angle** 0.61

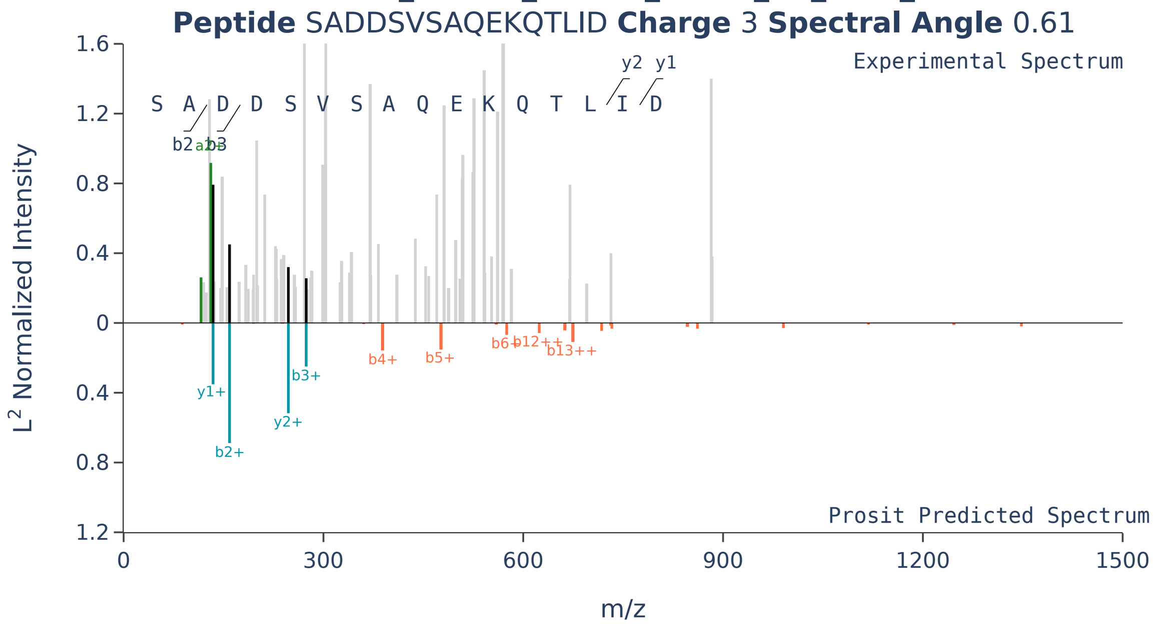


**Source** HRoetschke_211222_160123_HFGoe_IF2_b1R1_4h **Scan** 10602
**Peptide** LDTPGHAAFTSMRARGAQA **Charge** 3 **Spectral Angle** 0.56

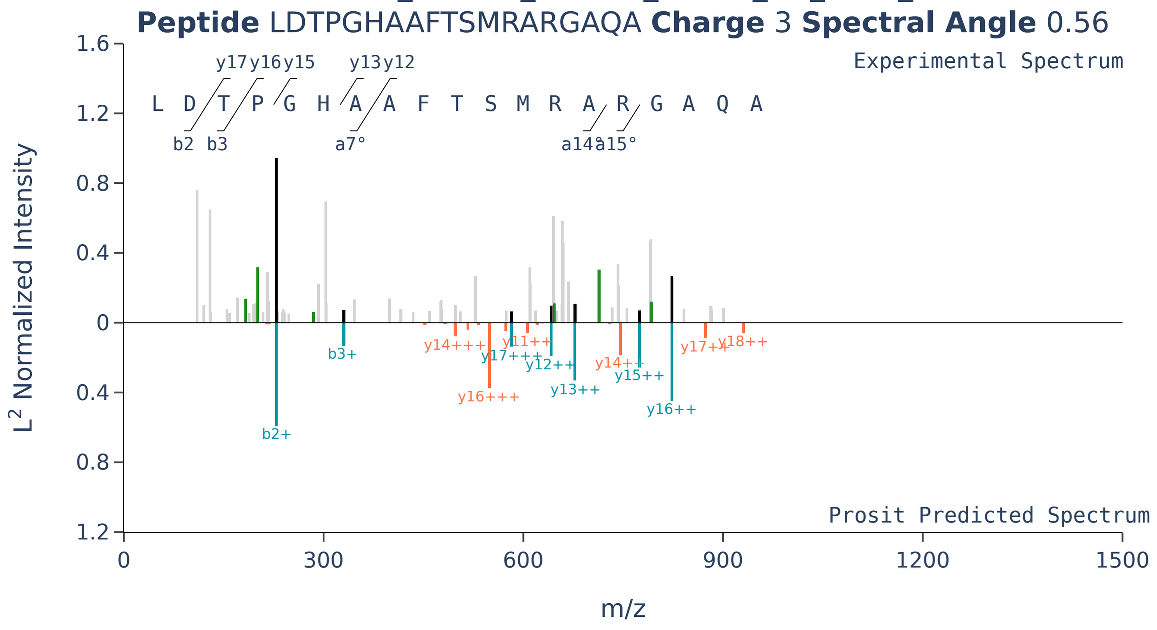


**Source** HRoetschke_211222_160123_HFGoe_IF2_b2R2_24h **Scan** 8793
**Peptide** IDHLNQKNSGPDKL **Charge** 4 **Spectral Angle** 0.73

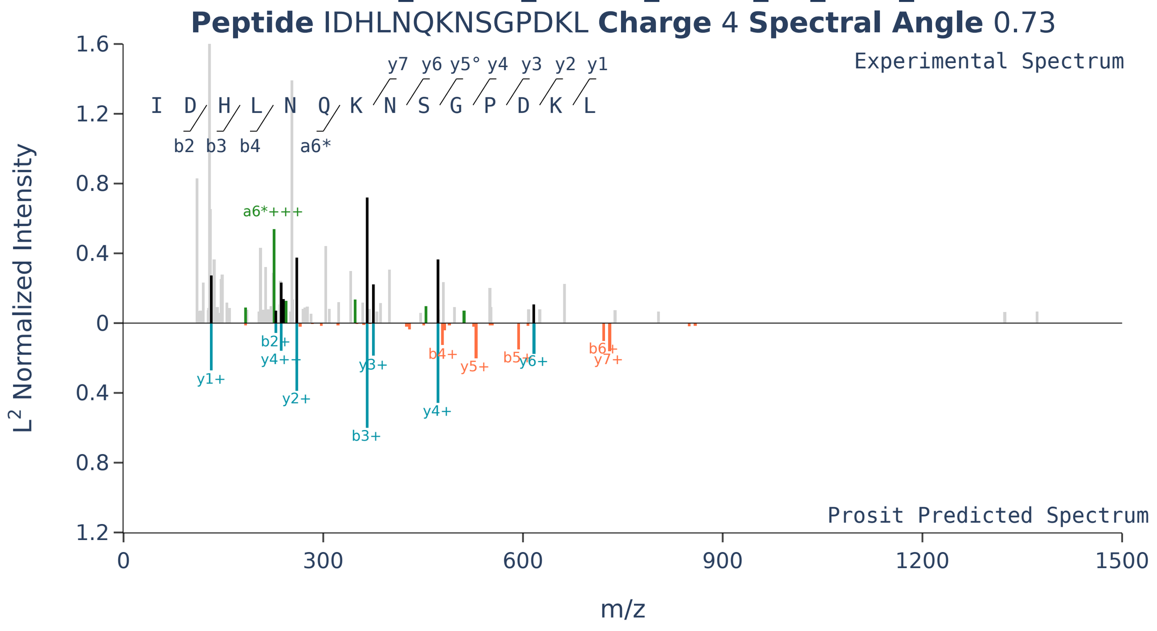


**Source** HRoetschke_211222_160123_HFGoe_IF2_b2R2_24h **Scan** 6750
**Peptide** NQKNSGPDKLTLQRK **Charge** 4 **Spectral Angle** 0.68

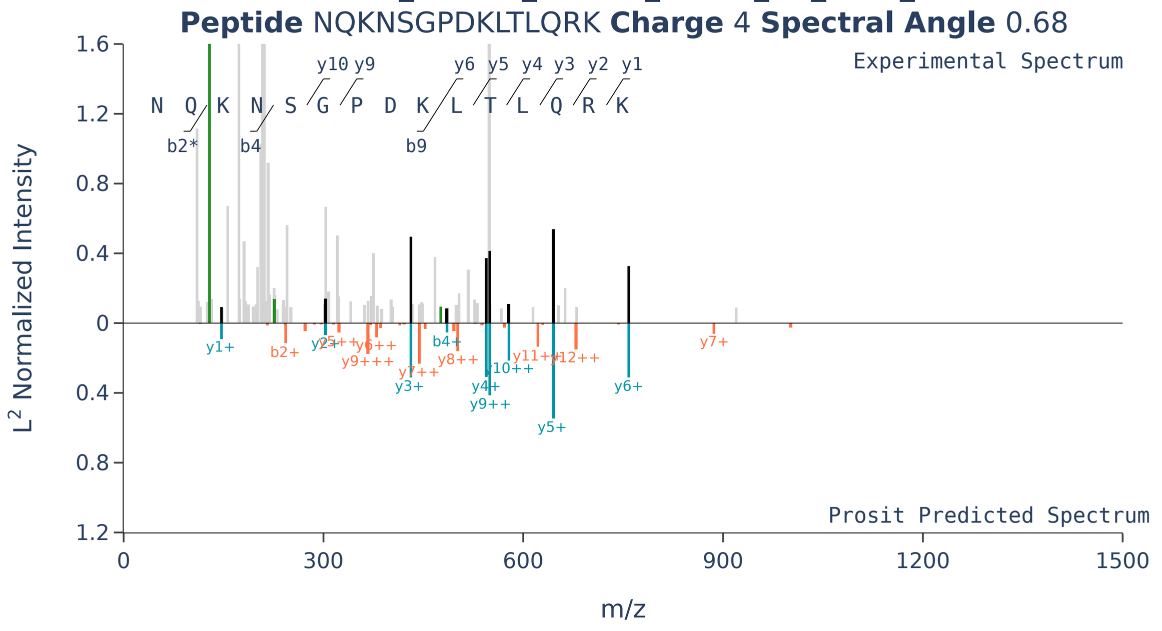


**Source** HRoetschke_211222_160123_HFGoe_IF2_b1R2_2h **Scan** 6793
**Peptide** DSSDYHVTTSQHARQAEDESD **Charge** 3 **Spectral Angle** 0.64

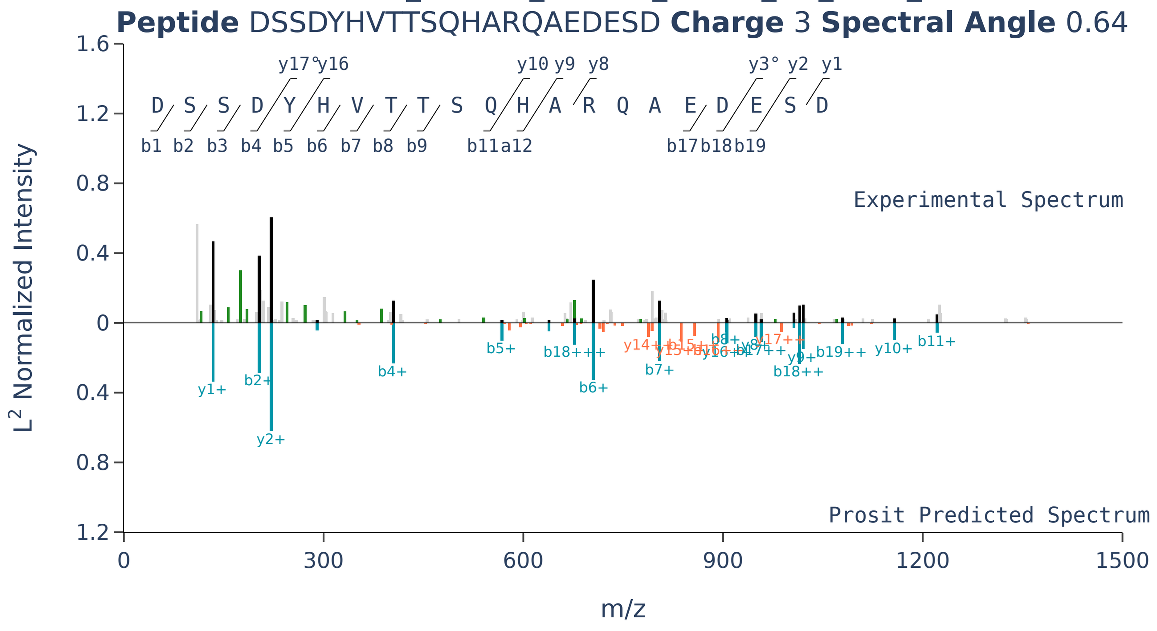


**Source** HRoetschke_211222_160123_HFGoe_IF2_b1R1_4h **Scan** 9075
**Peptide** GFEYGRVRAMRNE **Charge** 4 **Spectral Angle** 0.51

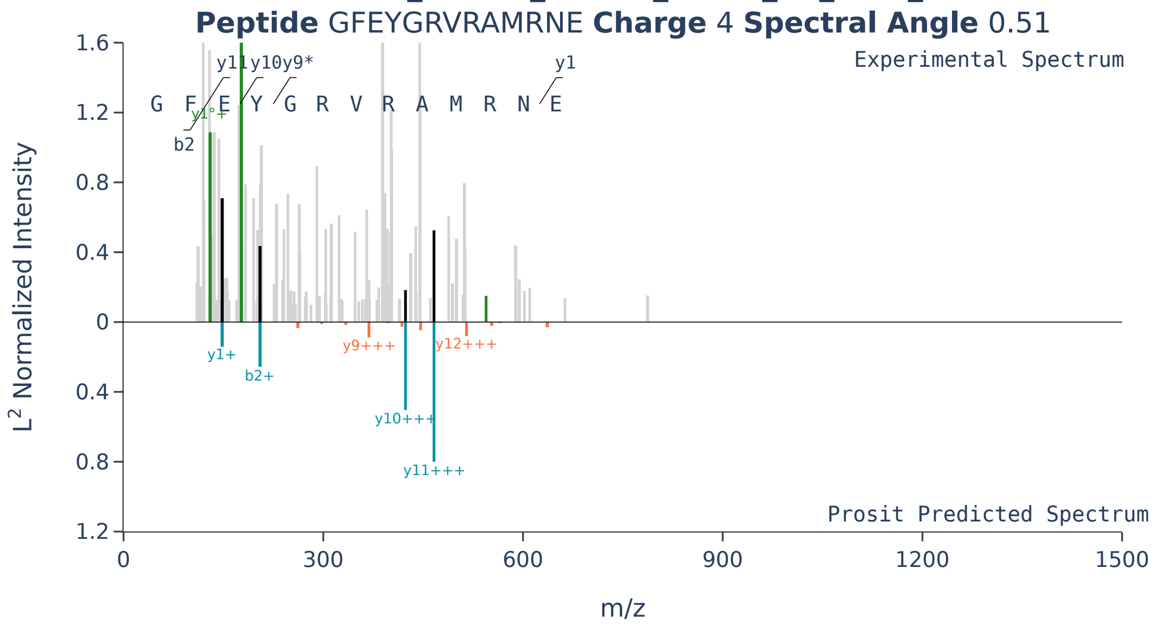


**Source** HRoetschke_211222_160123_HFGoe_IF2_b1R1_4h **Scan** 16129
**Peptide** LDYIRSTKVASGEAGGITQ **Charge** 3 **Spectral Angle** 0.84

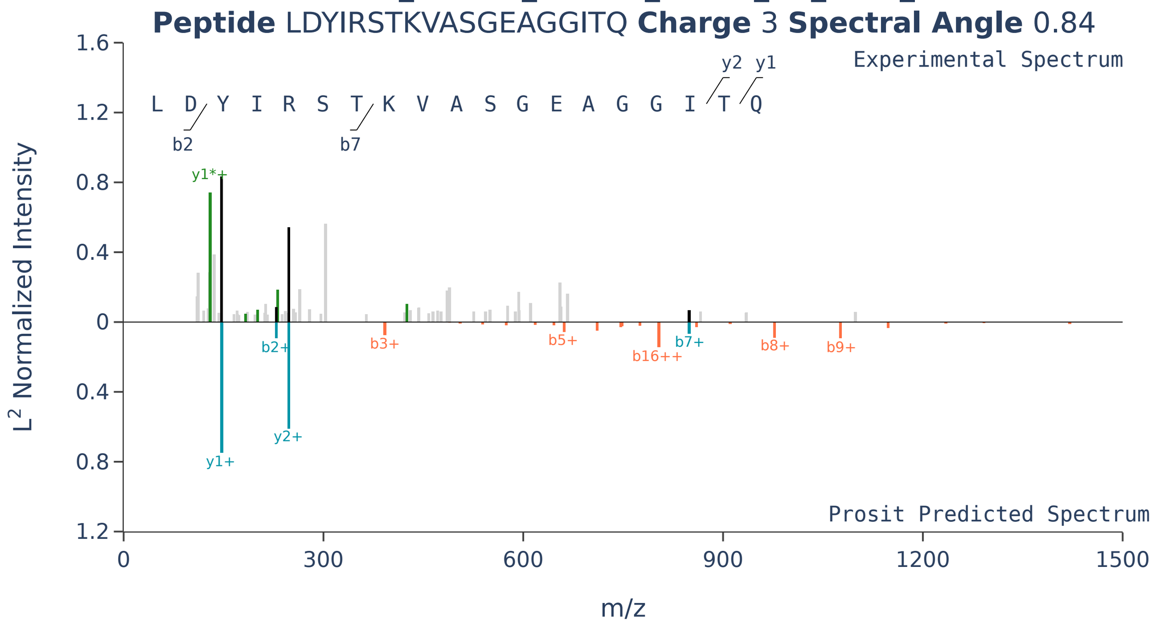


**Source** HRoetschke_211222_160123_HFGoe_IF2_b1R1_2h **Scan** 5192
**Peptide** KRTFVKRDPQE **Charge** 3 **Spectral Angle** 0.61

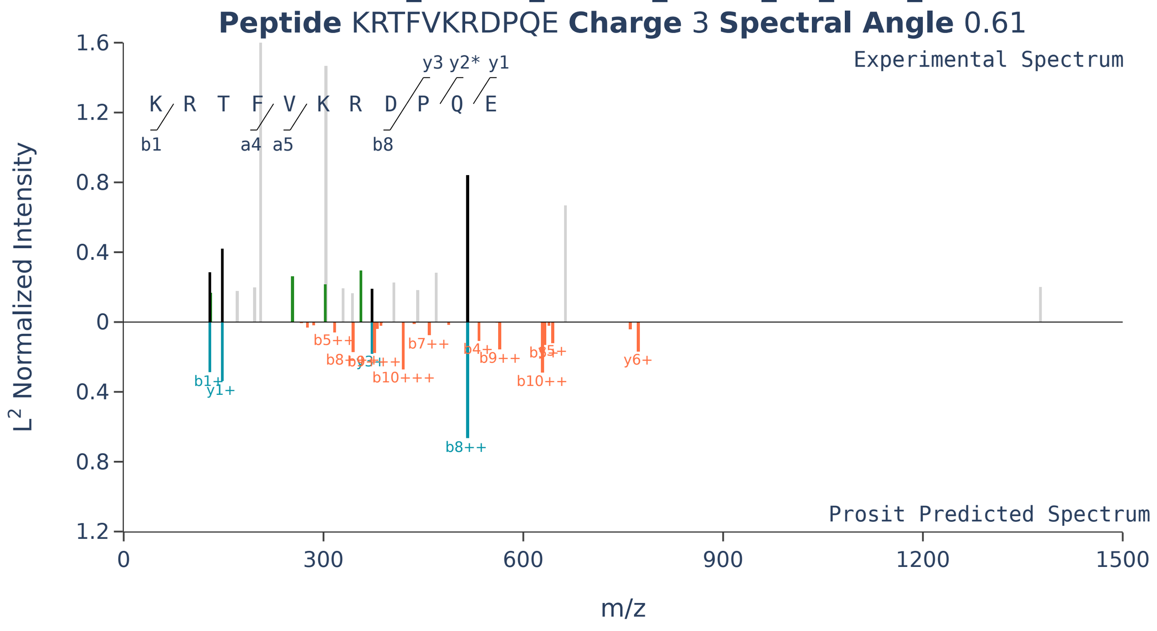


**Source** HRoetschke_211222_160123_HFGoe_IF2_b2R1_24h **Scan** 6213
**Peptide** KAGTGIDE **Charge** 2 **Spectral Angle** 0.78

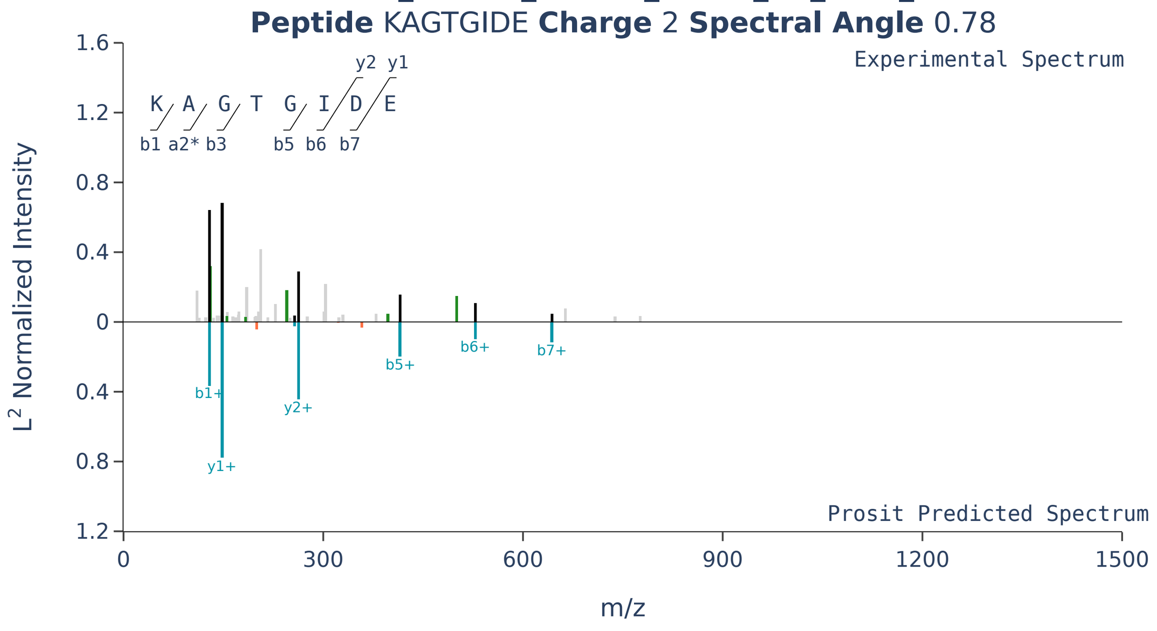


**Source** HRoetschke_211222_160123_HFGoe_IF2_b1R1_4h **Scan** 18296
**Peptide** SVSAQEKQTLIDHL **Charge** 3 **Spectral Angle** 0.61

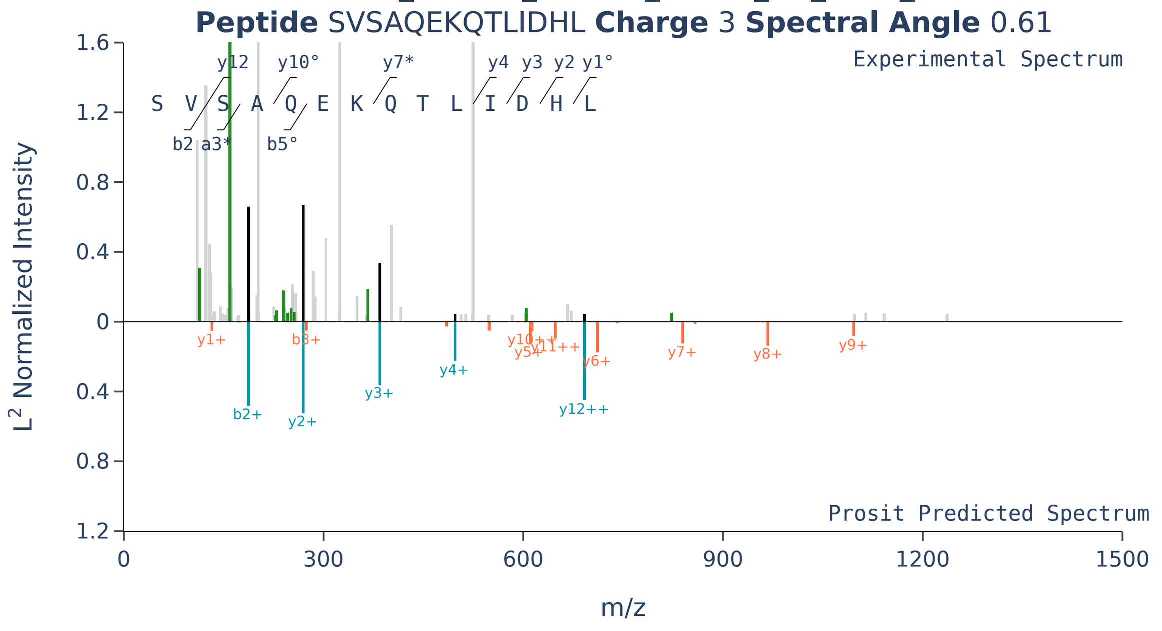


**Source** HRoetschke_211222_160123_HFGoe_IF2_b1R1_2h **Scan** 13500
**Peptide** ELKAVRK **Charge** 2 **Spectral Angle** 0.88

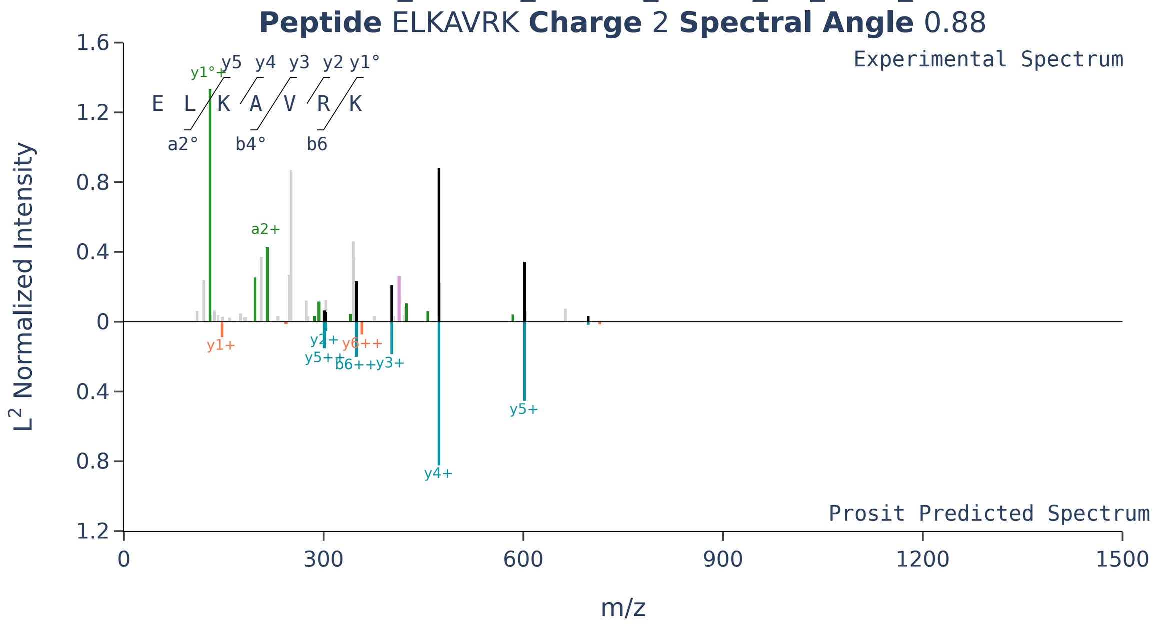

**Source** HRoetschke_211222_160123_HFGoe_IF2_b1R1_4h **Scan** 15061
**Peptide** VTIKTLAAERQTSVE **Charge** 2 **Spectral Angle** 0.73

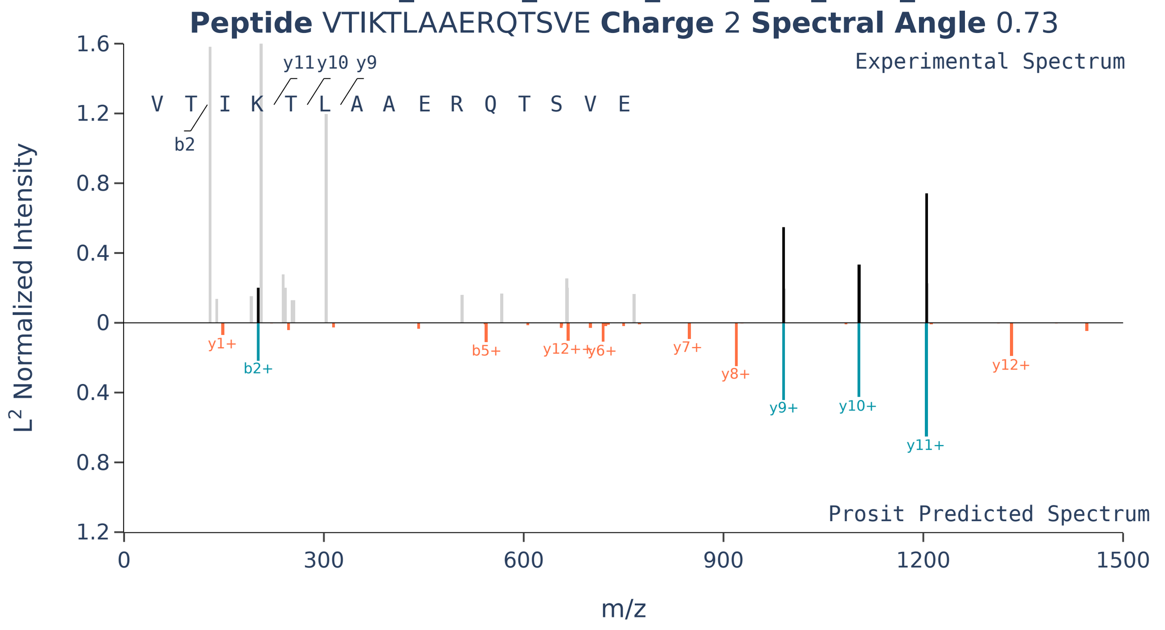


**Source** HRoetschke_211222_160123_HFGoe_IF2_b2R2_24h **Scan** 5582
**Peptide** QEAAELKRKAEE **Charge** 2 **Spectral Angle** 0.62

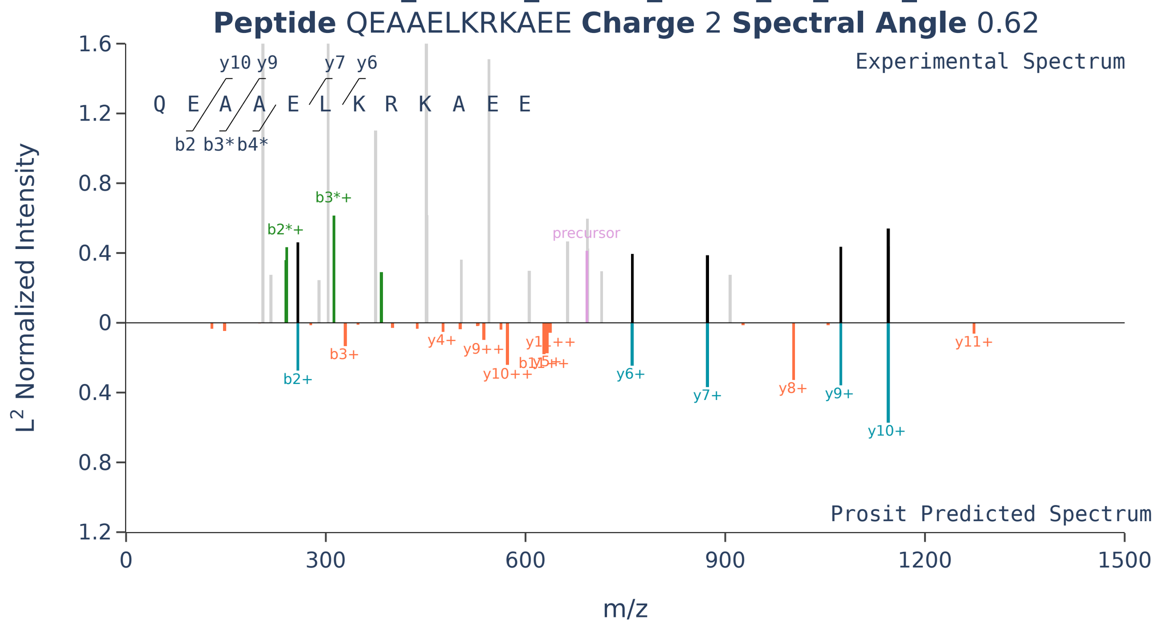


**Source** HRoetschke_211222_160123_HFGoe_IF2_b1R1_4h **Scan** 17575
**Peptide** ADDGVMPQTIEAIQHAKAA **Charge** 3 **Spectral Angle** 0.57

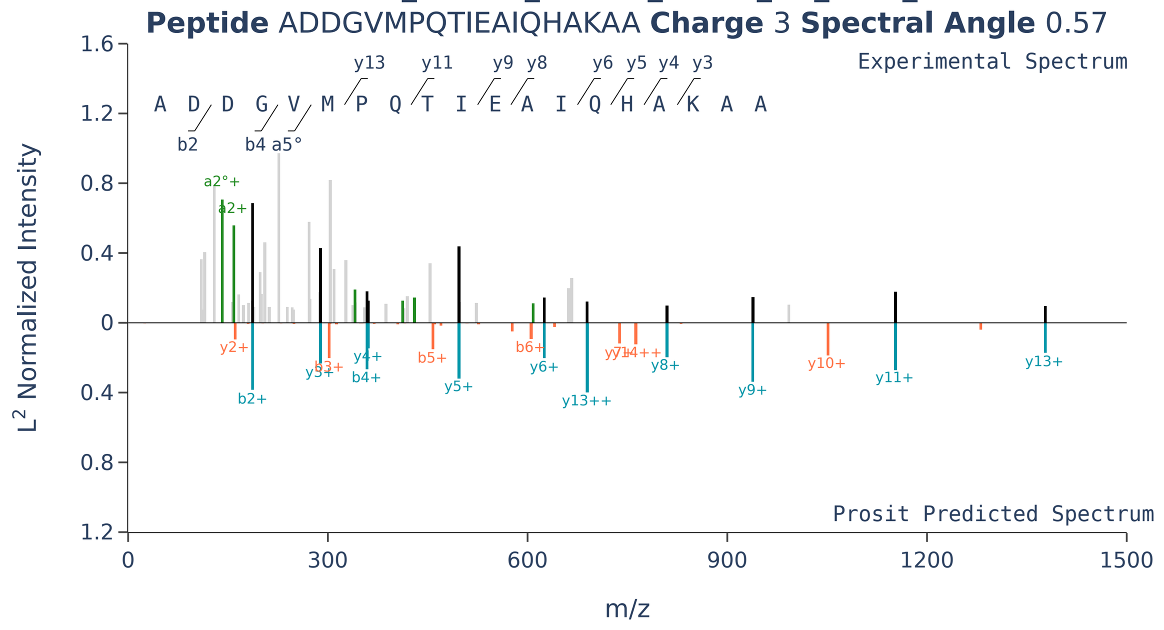


**Source** HRoetschke_211222_160123_HFGoe_IF2_b2R2_24h **Scan** 17341
**Peptide** QQGFQKPAQAVNRDVVI **Charge** 3 **Spectral Angle** 0.66

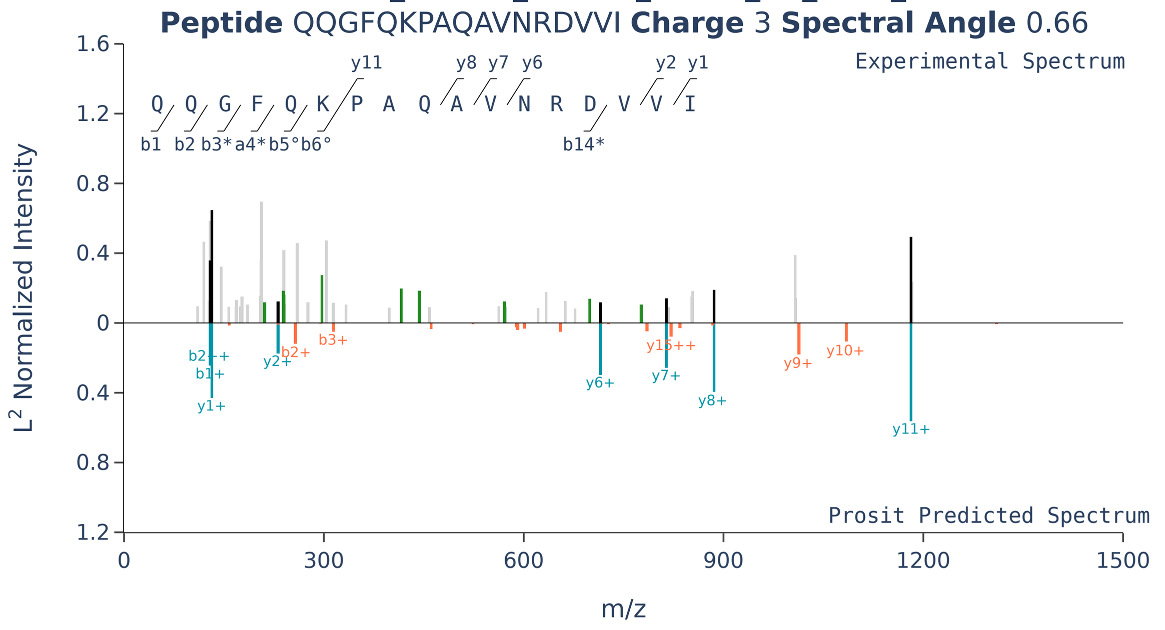


**Source** HRoetschke_211222_160123_HFGoe_IF2_b2R1_2h **Scan** 7937
**Peptide** AERLAAEEQAQREA **Charge** 3 **Spectral Angle** 0.61

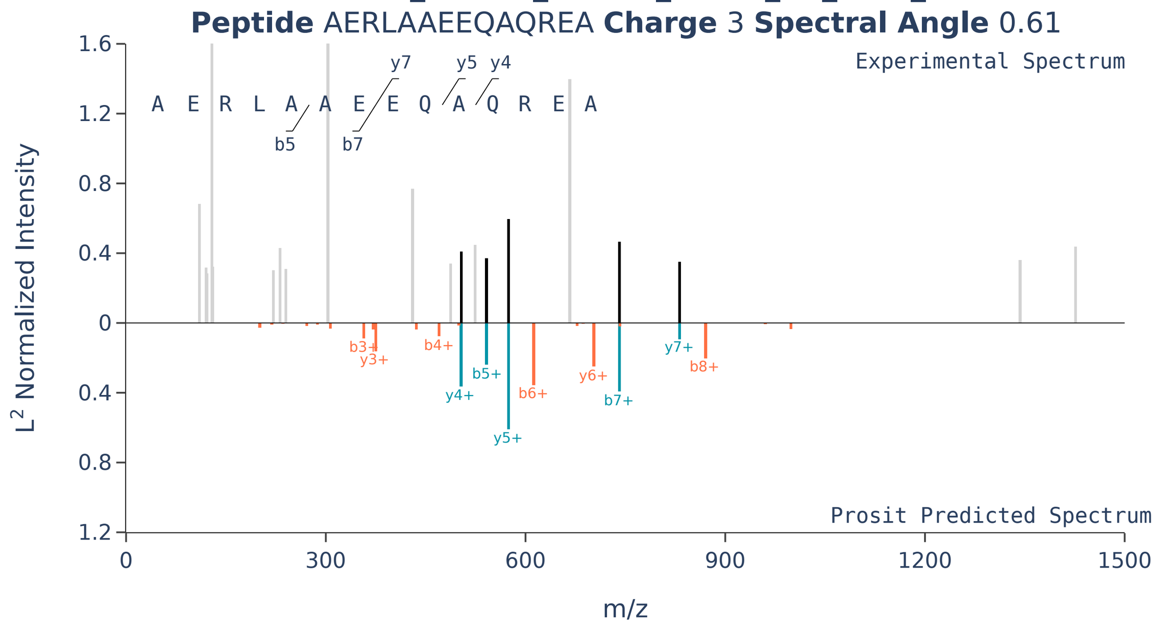


**Source** HRoetschke_211222_160123_HFGoe_IF2_b1R2_24h **Scan** 17066
**Peptide** GFQKPAQAVNRDVVI **Charge** 3 **Spectral Angle** 0.57

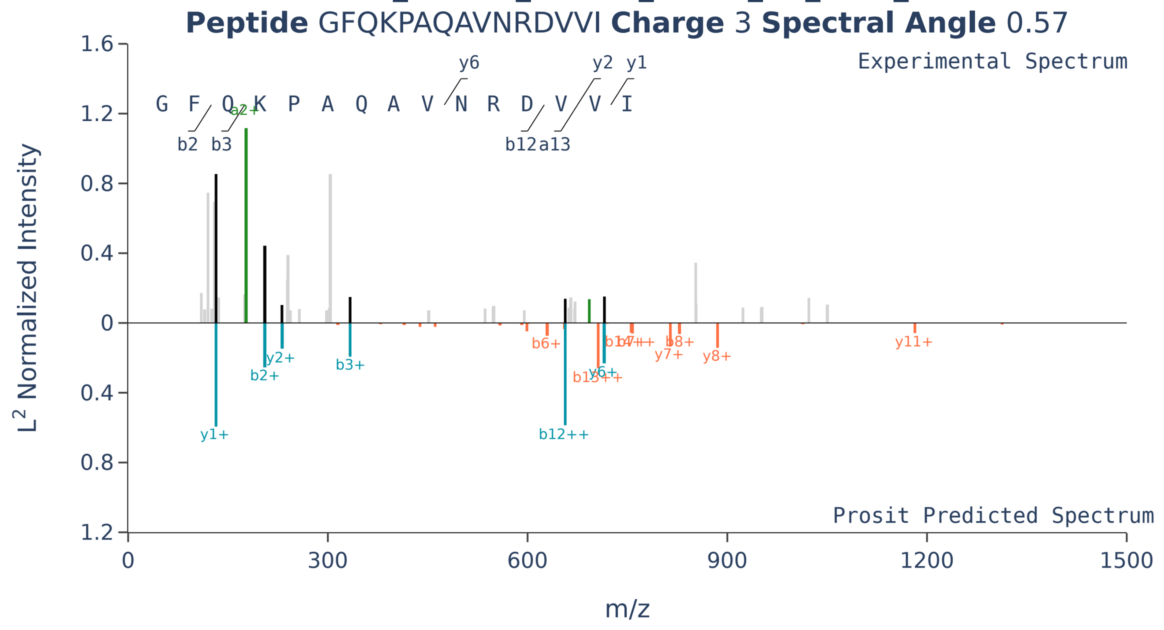


**Source** HRoetschke_211222_160123_HFGoe_IF2_b1R1_2h **Scan** 5377
**Peptide** GHKVILRRENE **Charge** 3 **Spectral Angle** 0.7

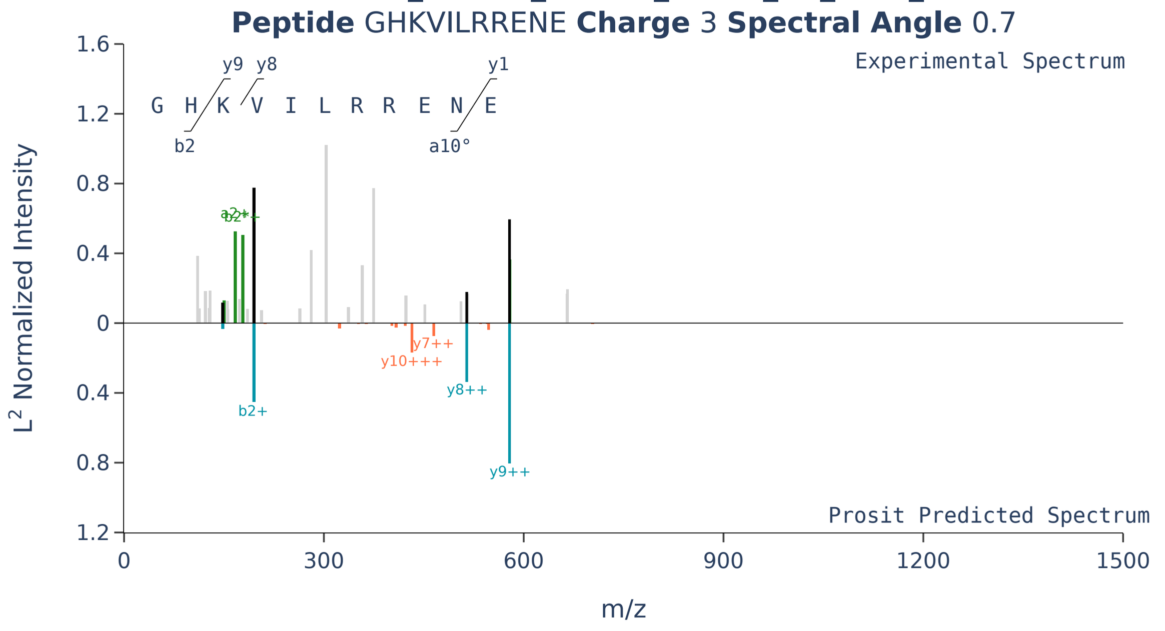


**Source** HRoetschke_211222_160123_HFGoe_IF2_b1R1_4h **Scan** 9277
**Peptide** EEMGHKVILRRENELEE **Charge** 3 **Spectral Angle** 0.6

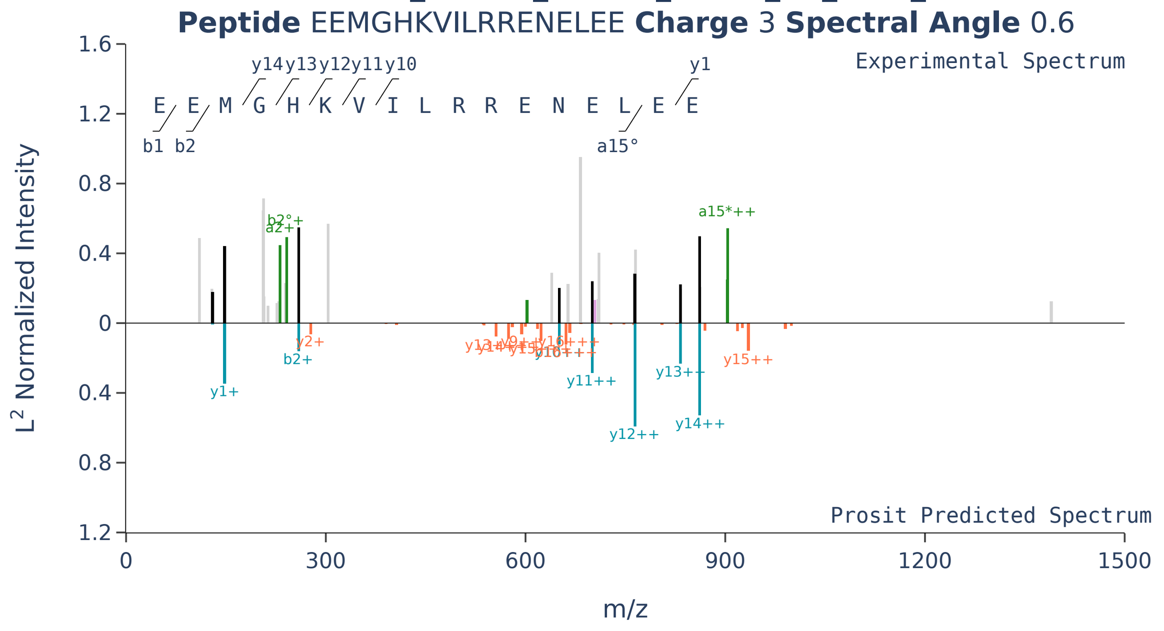


**Source** HRoetschke_211222_160123_HFGoe_IF2_b1R1_24h **Scan** 8433
**Peptide** KAREVAL **Charge** 2 **Spectral Angle** 0.37

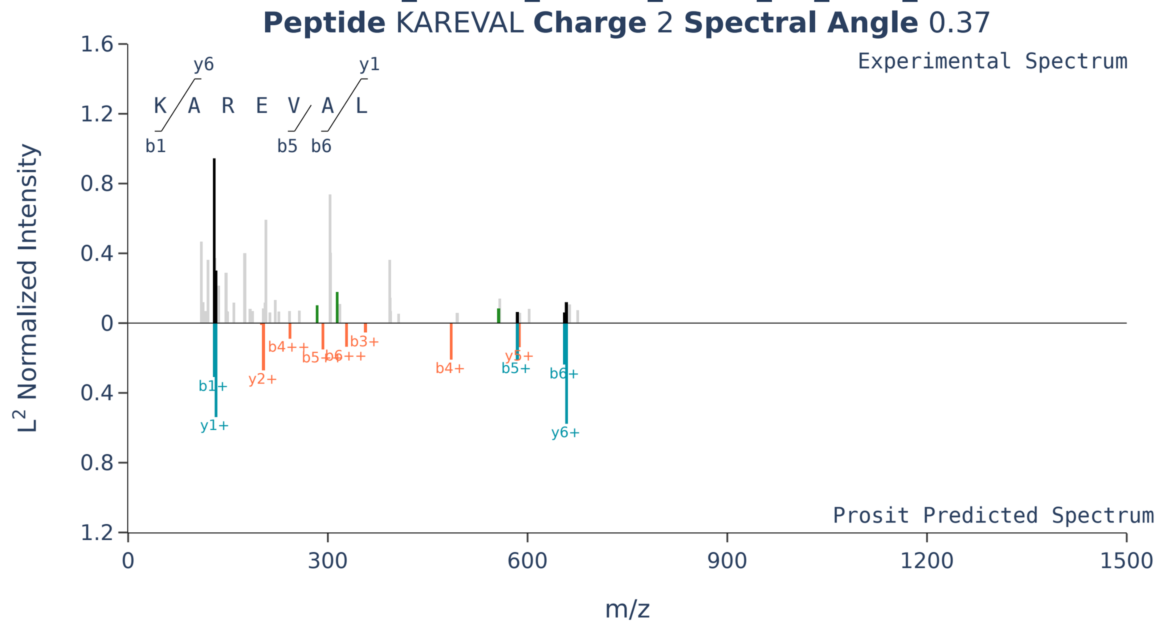


**Source** HRoetschke_211222_160123_HFGoe_IF2_b1R1_4h **Scan** 13858
**Peptide** SVSAQEKQTLIDHLNQKN **Charge** 3 **Spectral Angle** 0.67

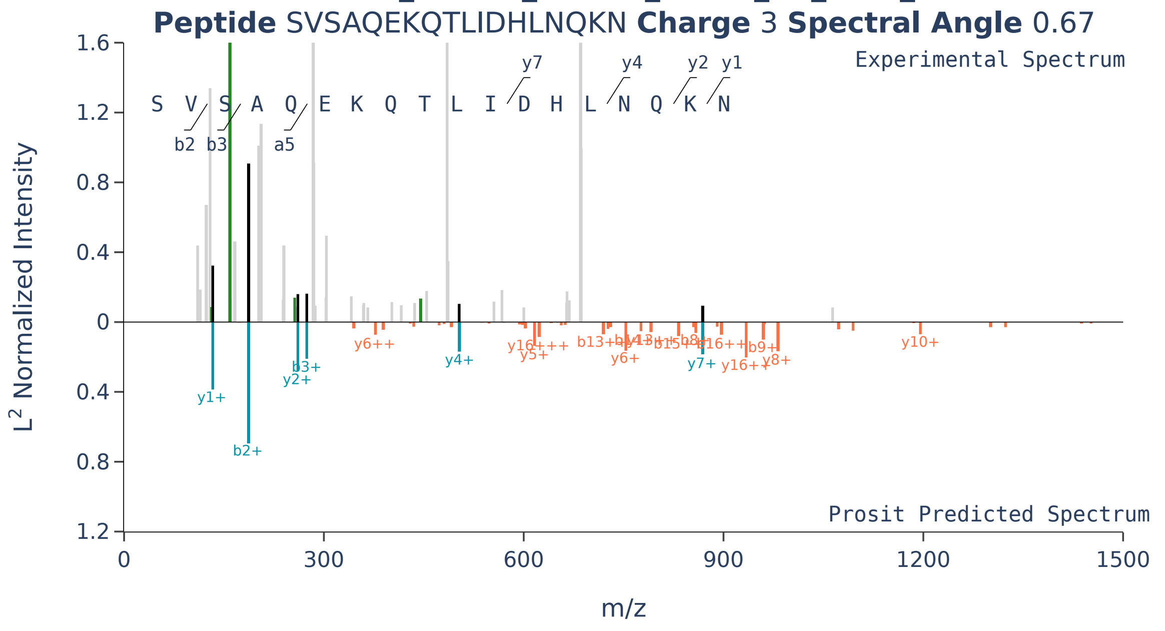


**Source** HRoetschke_211222_160123_HFGoe_IF2_b1R1_4h **Scan** 4952
**Peptide** AEESAKREAQQKAERE **Charge** 3 **Spectral Angle** 0.66

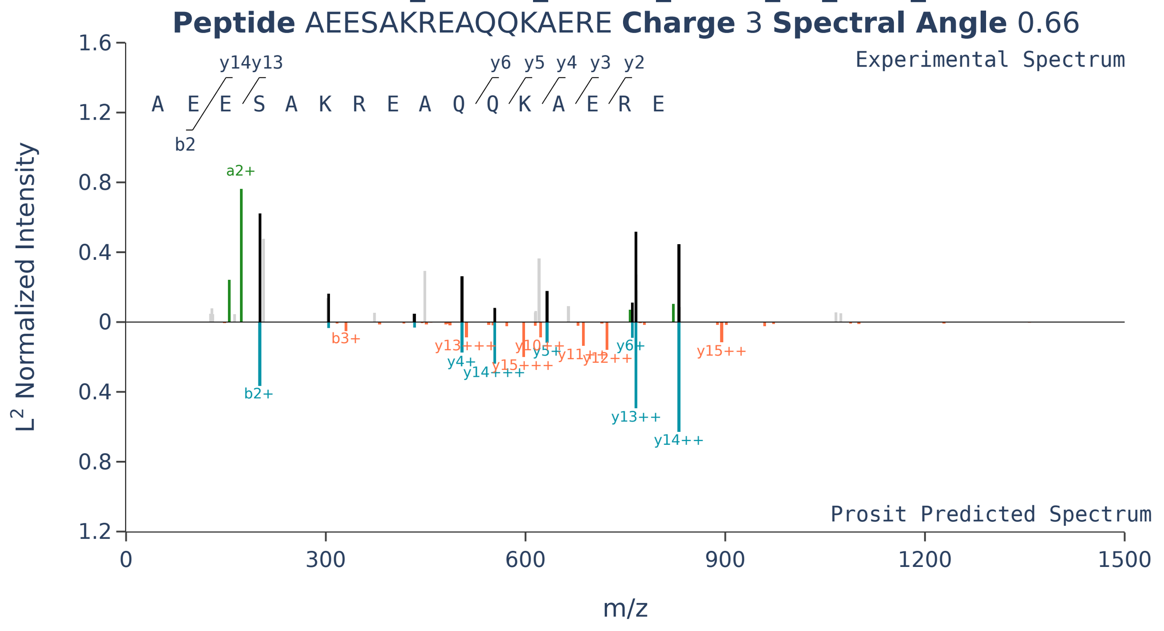


**Source** HRoetschke_211222_160123_HFGoe_IF2_b1R2_4h **Scan** 8680
**Peptide** HLGAYHVETENG **Charge** 2 **Spectral Angle** 0.68

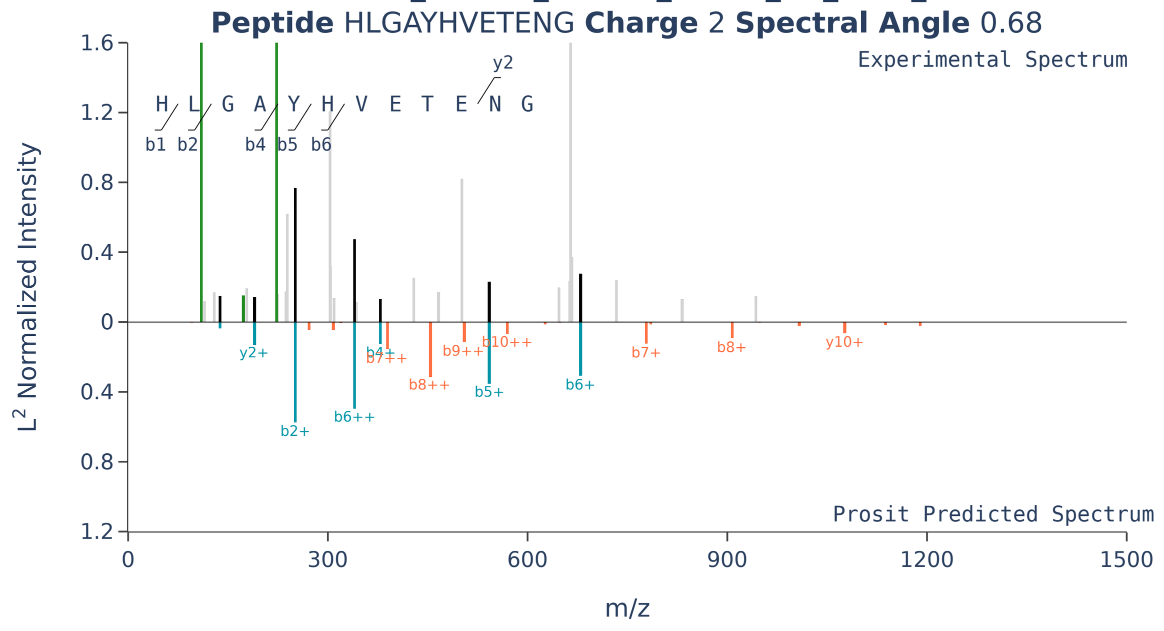


**Source** HRoetschke_211222_160123_HFGoe_IF2_b2R1_24h **Scan** 7302
**Peptide** KADVQGSVEA **Charge** 2 **Spectral Angle** 0.27

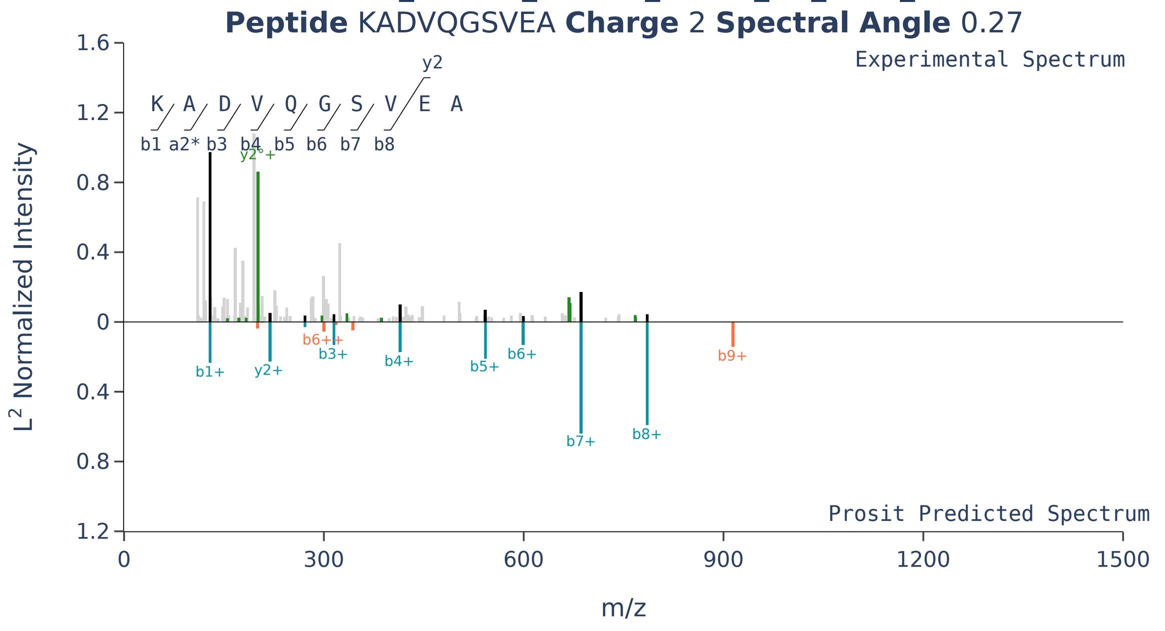


**Source** HRoetschke_211222_160123_HFGoe_IF2_b2R2_2h **Scan** 15515
**Peptide** SGEAGGITQHIGAYHVETE **Charge** 3 **Spectral Angle** 0.79

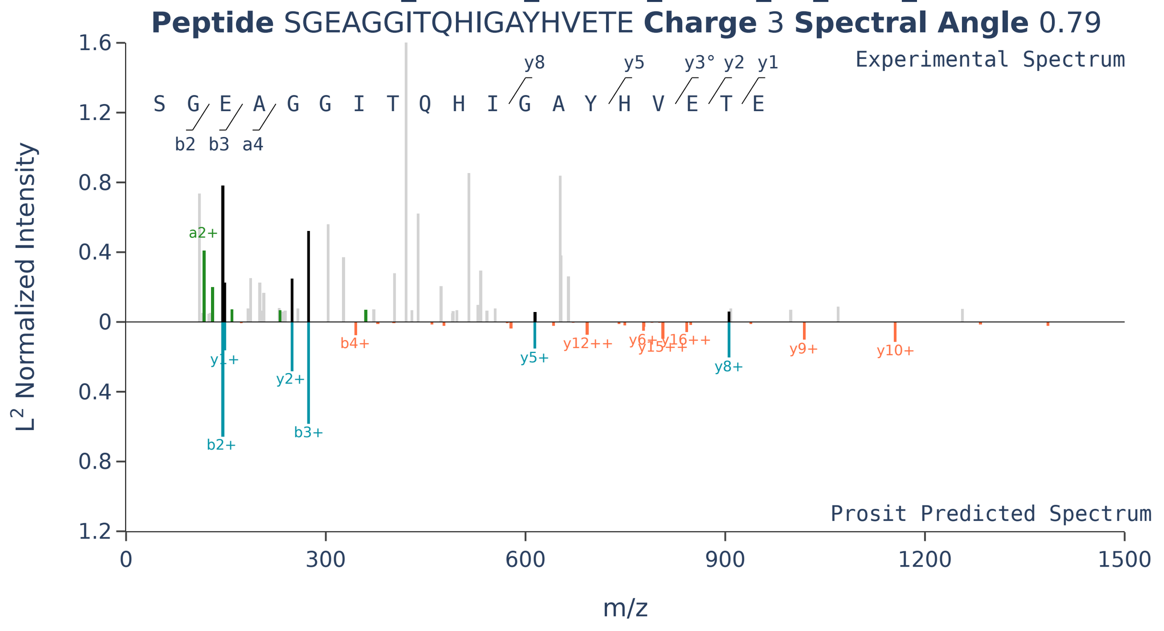


**Source** HRoetschke_211222_160123_HFGoe_IF2_b1R1_4h **Scan** 24212
**Peptide** NDVRTGDVIEVF **Charge** 2 **Spectral Angle** 0.67

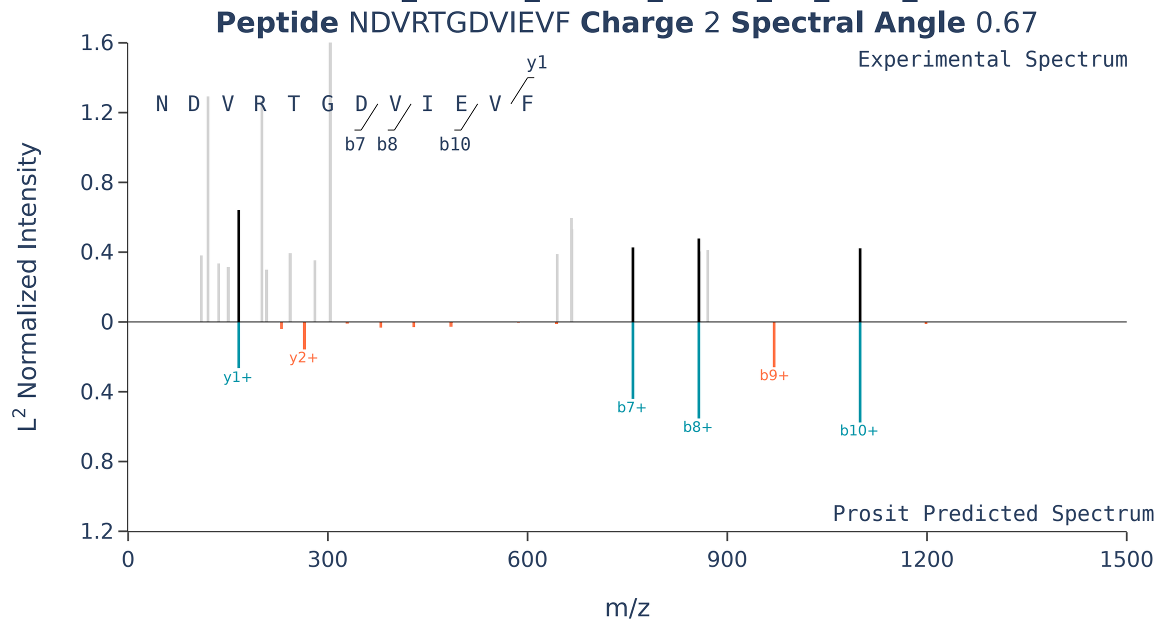

**Source** HRoetschke_211222_160123_HFGoe_IF2_b1R1_4h **Scan** 5547
**Peptide** AGIRKSADDSVSAQEKQ **Charge** 4 **Spectral Angle** 0.53

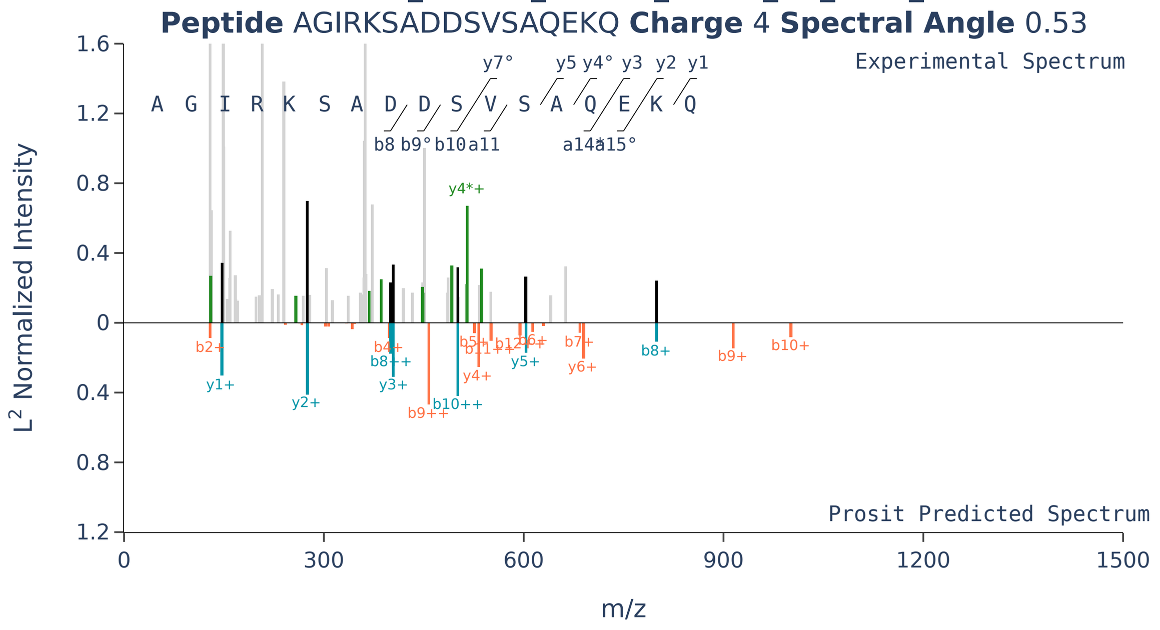


**Source** HRoetschke_211222_160123_HFGoe_IF2_b2R2_24h **Scan** 15755
**Peptide** AADDGVMPQTIEA **Charge** 2 **Spectral Angle** 0.65

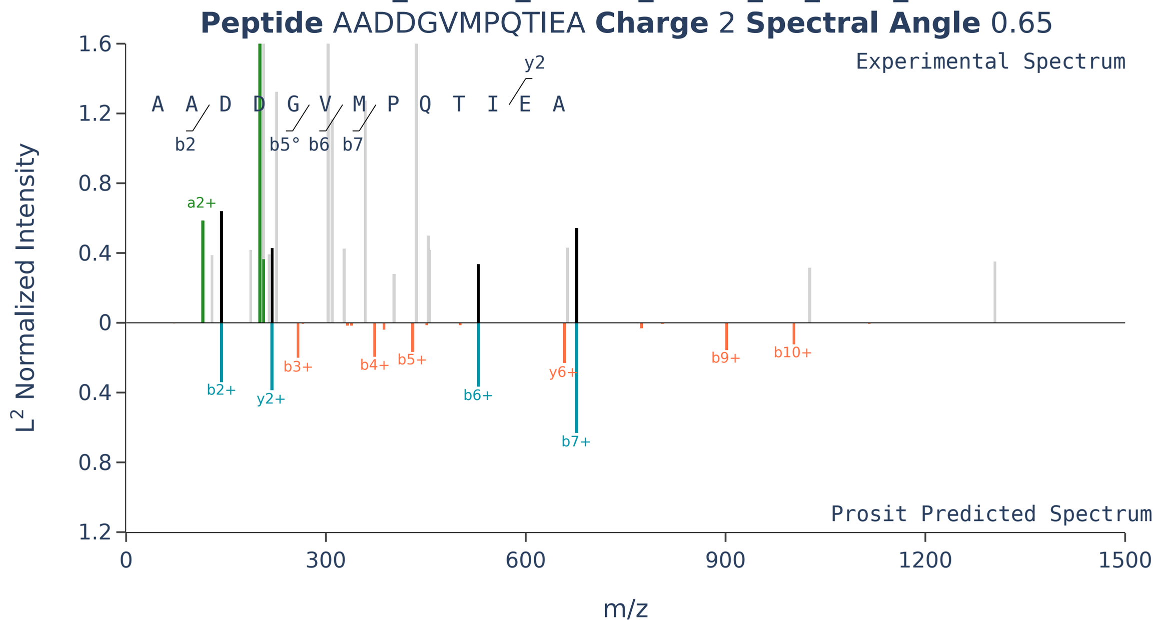


**Source** HRoetschke_211222_160123_HFGoe_IF2_b1R1_4h **Scan** 11674
**Peptide** FADAGIRKSADDSV **Charge** 2 **Spectral Angle** 0.58

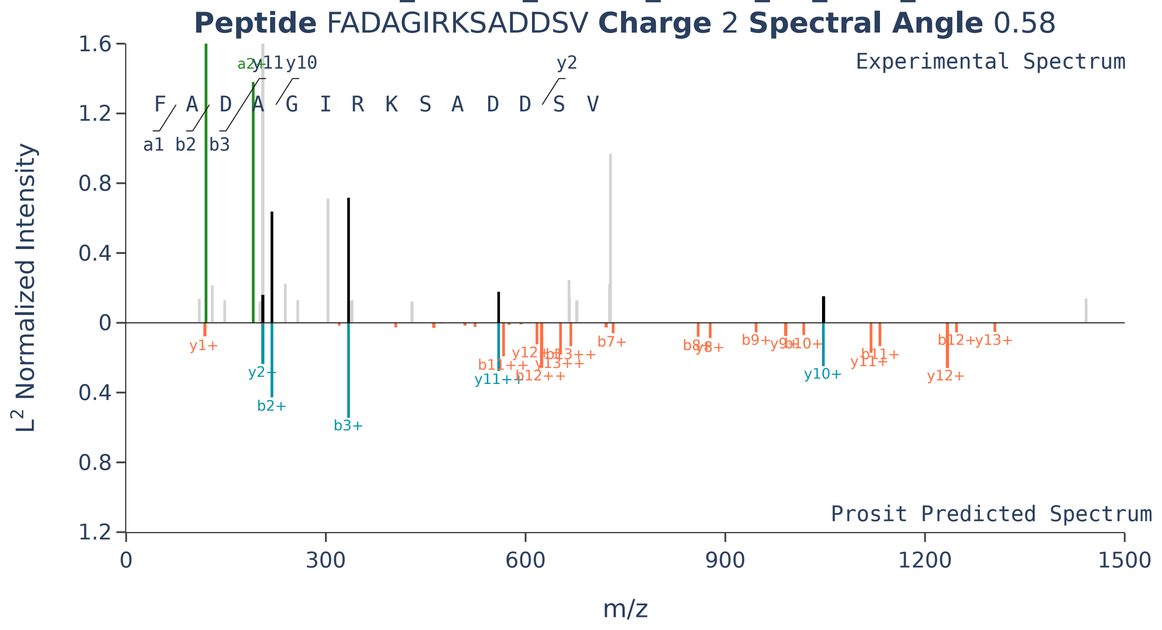


**Source** HRoetschke_211222_160123_HFGoe_IF2_b1R1_4h **Scan** 6412
**Peptide** YIRSTKVA **Charge** 2 **Spectral Angle** 0.9

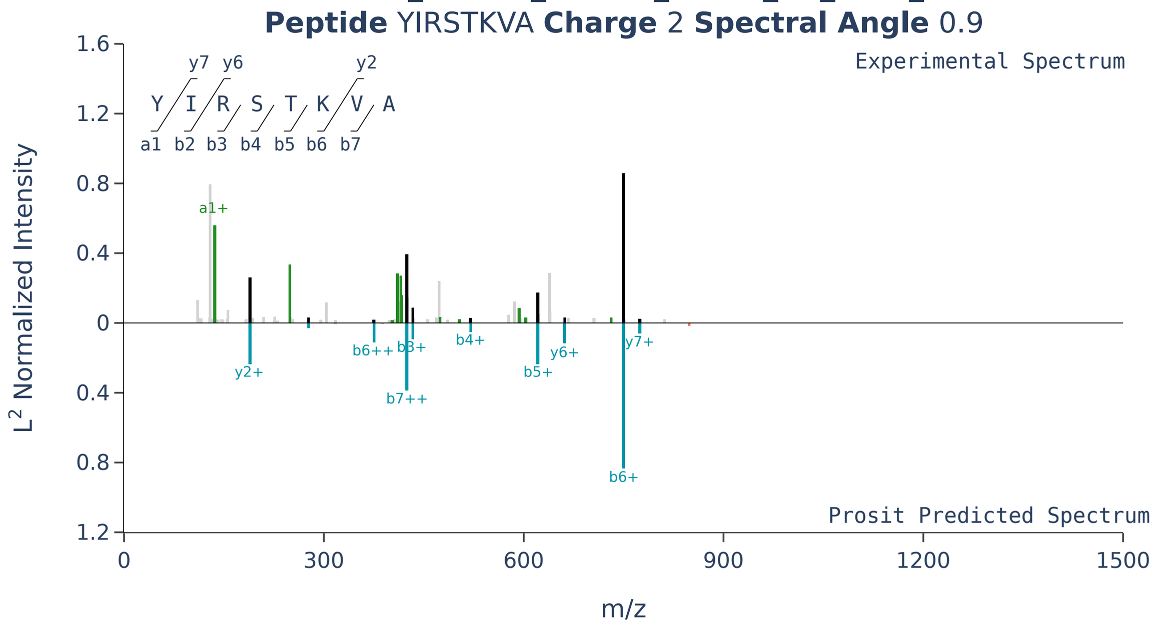


**Source** HRoetschke_211222_160123_HFGoe_IF2_b1R1_4h **Scan** 8520
**Peptide** ADASARKVIEA **Charge** 2 **Spectral Angle** 0.56

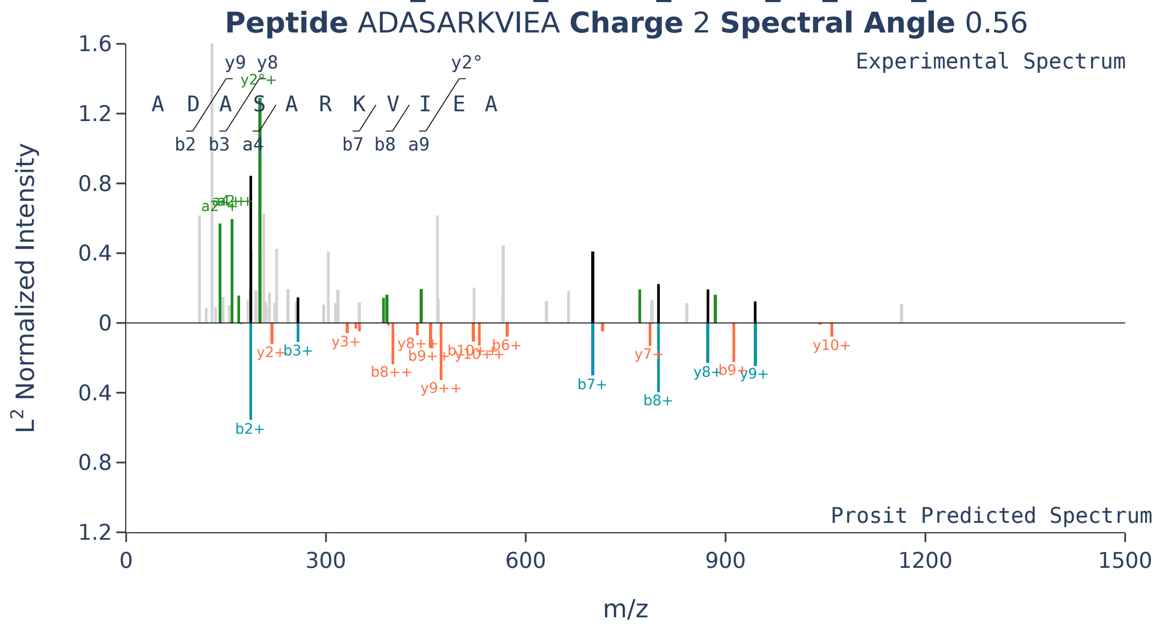


**Source** HRoetschke_211222_160123_HFGoe_IF2_b2R2_24h **Scan** 7441
**Peptide** VLVREGTLHKG **Charge** 3 **Spectral Angle** 0.69

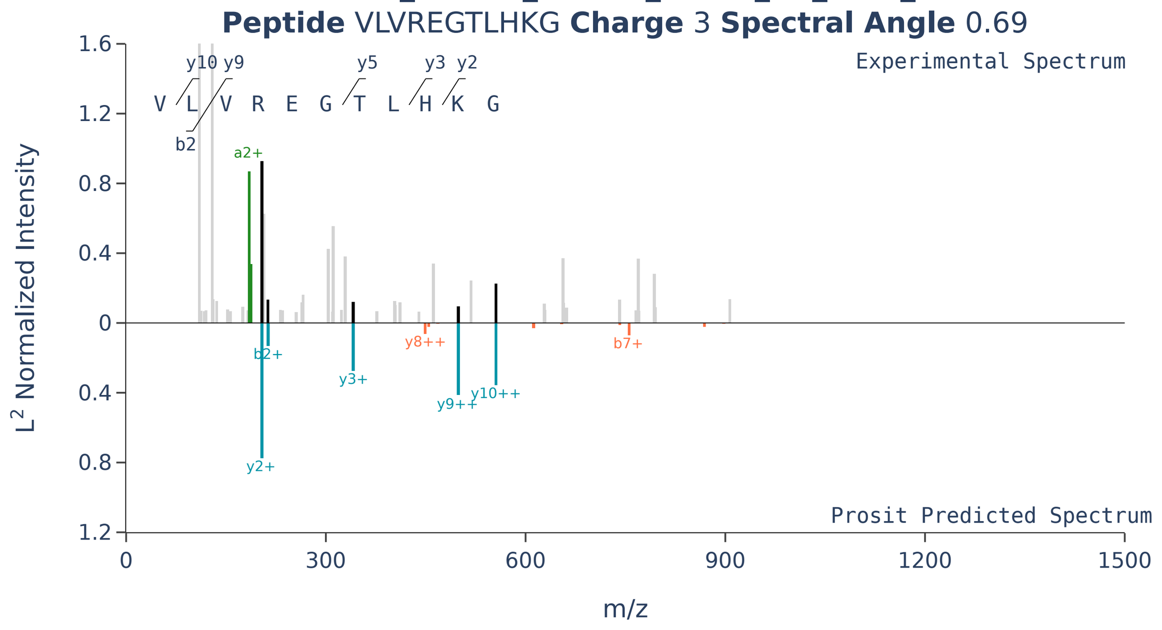


**Source** HRoetschke_211222_160123_HFGoe_IF2_b1R2_2h **Scan** 10480
**Peptide** KTRSTLNI **Charge** 2 **Spectral Angle** 0.37

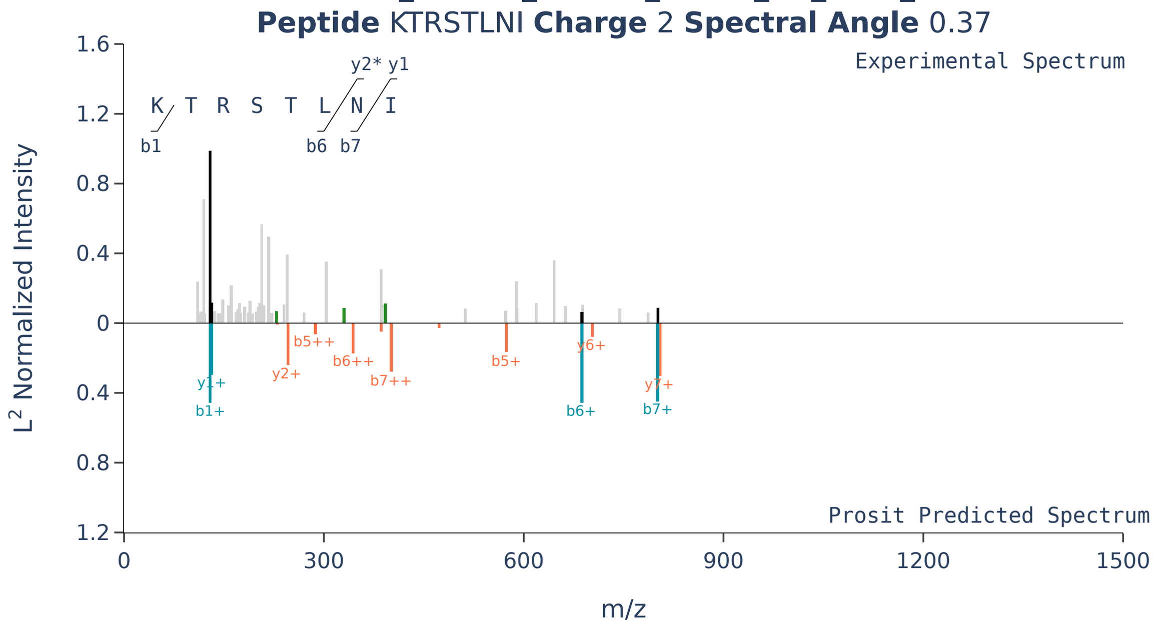


**Source** HRoetschke_211222_160123_HFGoe_IF2_b1R1_4h **Scan** 15404
**Peptide** LDKGRGPVATVLVREG **Charge** 3 **Spectral Angle** 0.62

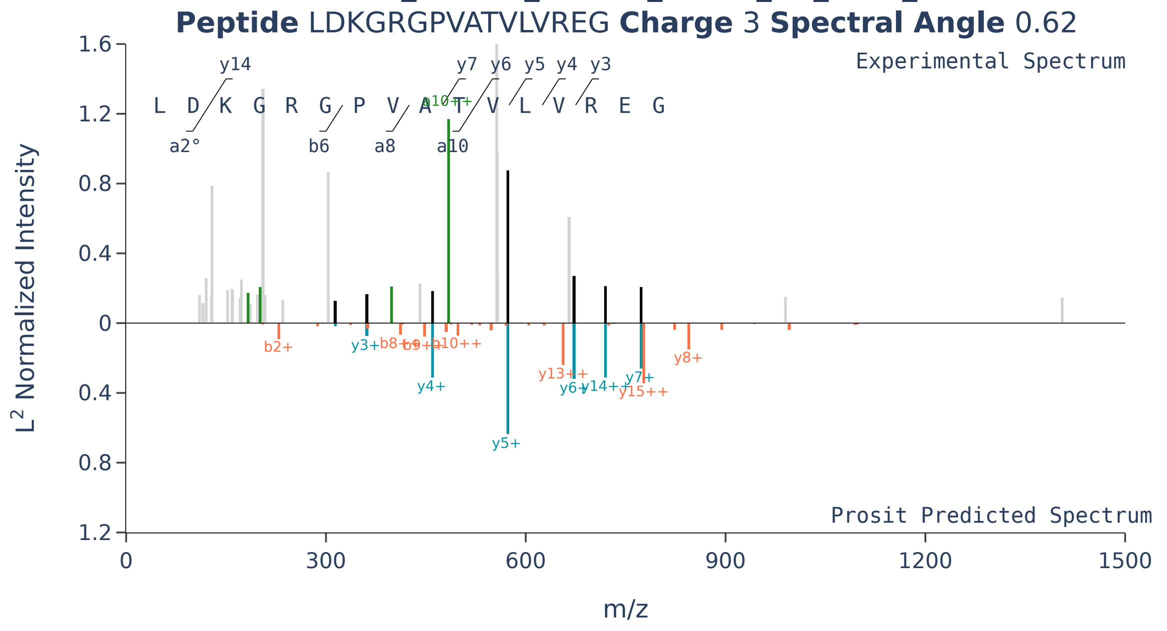


**Source** HRoetschke_211222_160123_HFGoe_IF2_b2R1_24h **Scan** 13045
**Peptide** LDKPEADPDRVKNEL **Charge** 3 **Spectral Angle** 0.72

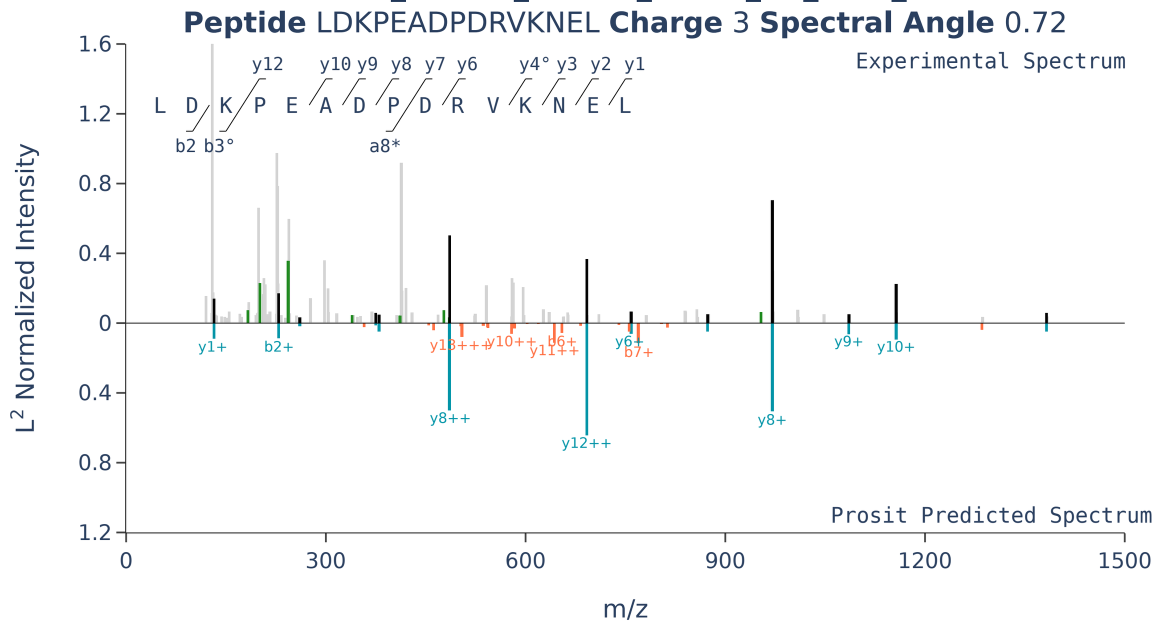


**Source** HRoetschke_211222_160123_HFGoe_IF2_b1R2_24h **Scan** 14340
**Peptide** KPEADPDRVKNELSQY **Charge** 4 **Spectral Angle** 0.67

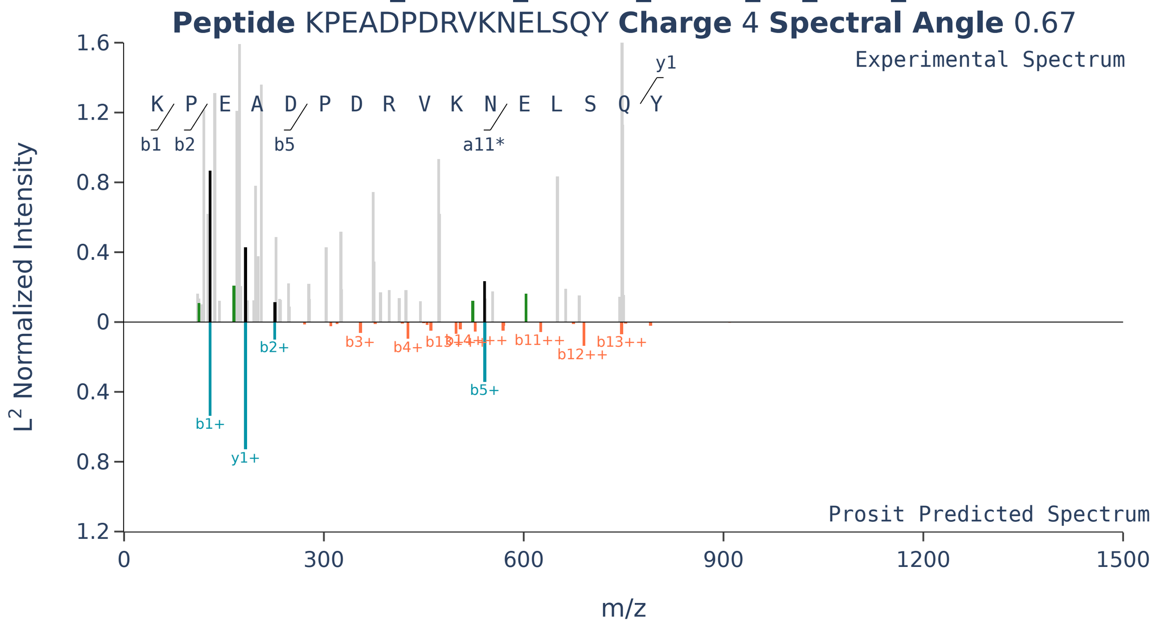


**Source** HRoetschke_211222_160123_HFGoe_IF2_b1R1_4h **Scan** 17501
**Peptide** ADDGVMPQTIEAIQHAKA **Charge** 3 **Spectral Angle** 0.55

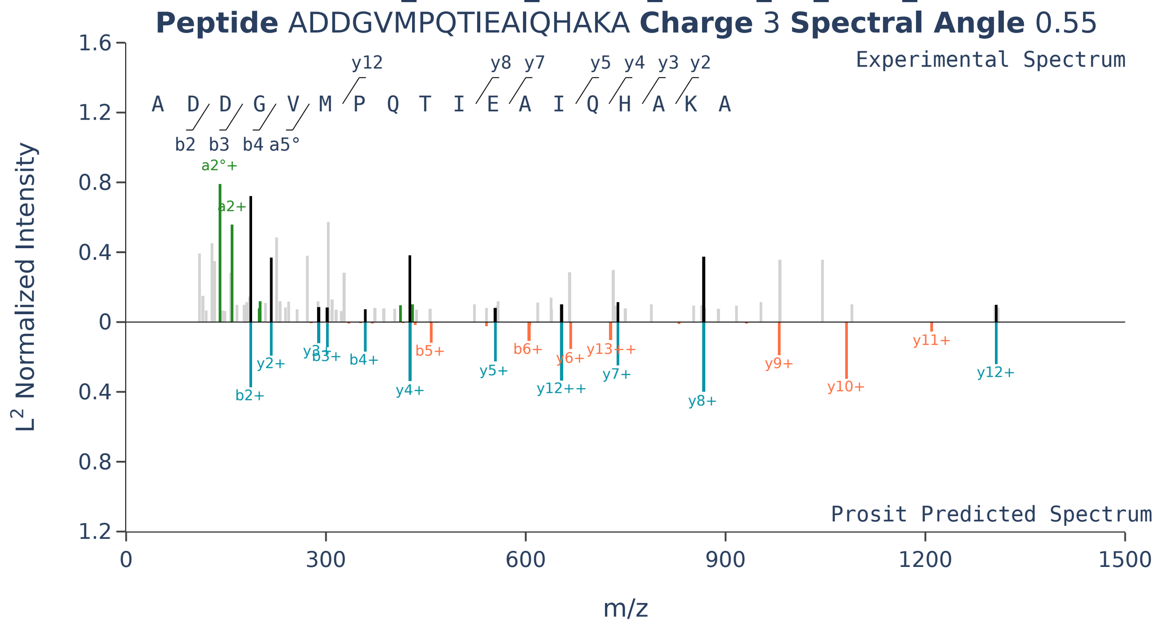


**Source** HRoetschke_211222_160123_HFGoe_IF2_b1R1_2h **Scan** 11917
**Peptide** ELANKMAVKGSQVI **Charge** 3 **Spectral Angle** 0.87

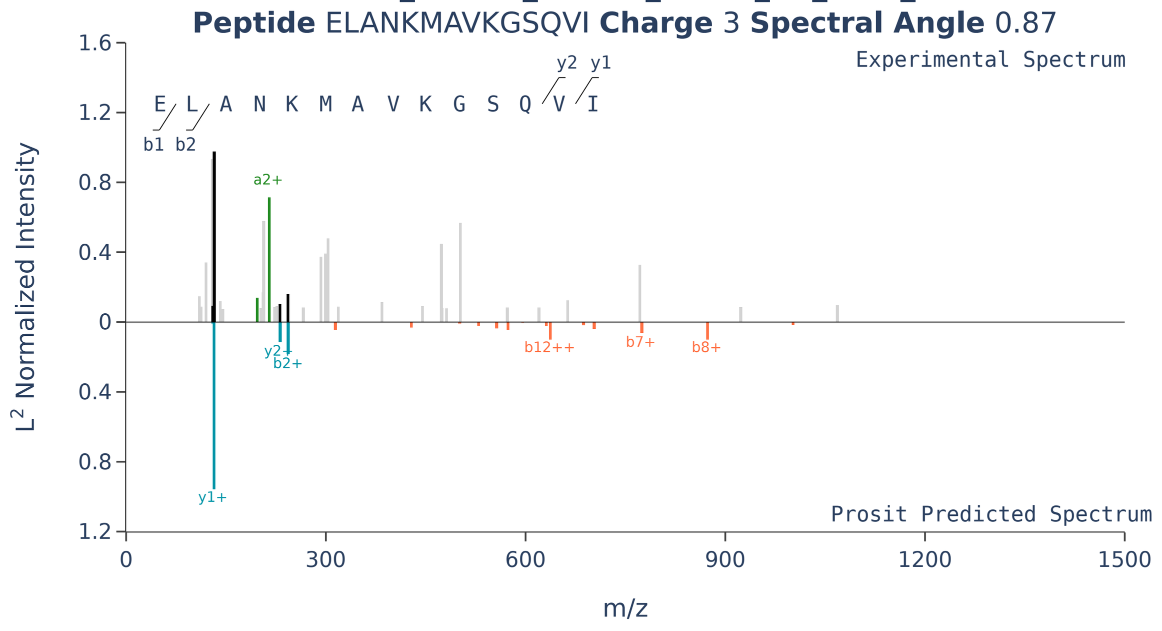


**Source** HRoetschke_211222_160123_HFGoe_IF2_b1R1_4h **Scan** 6103
**Peptide** SSDYHVTTSQHARQAEDESD **Charge** 3 **Spectral Angle** 0.67

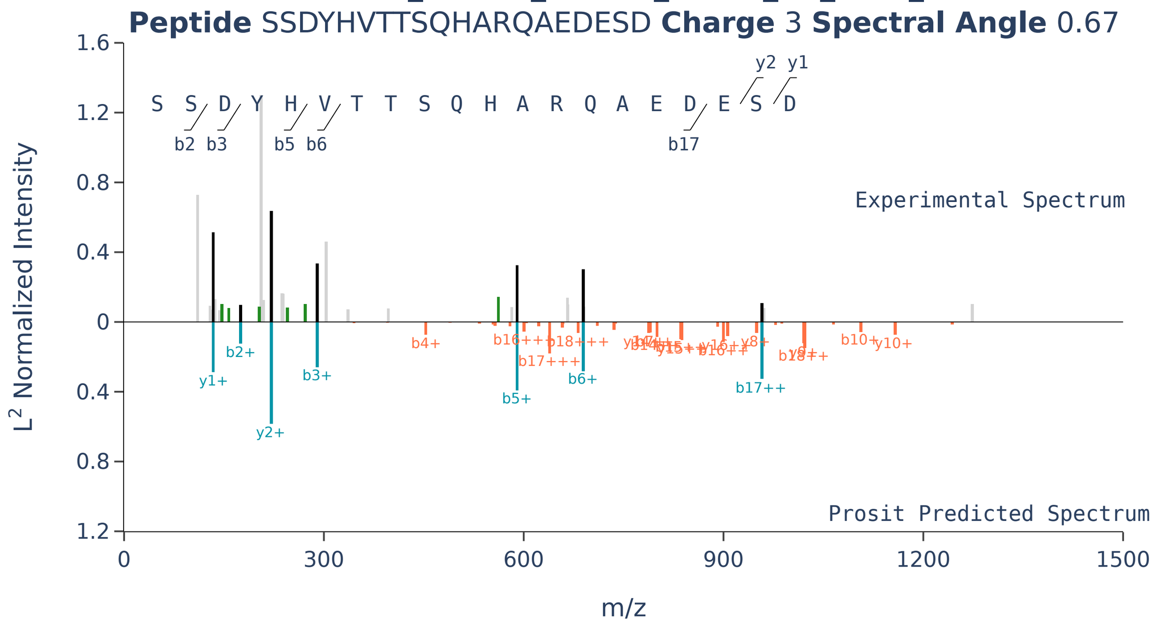


**Source** HRoetschke_211222_160123_HFGoe_IF2_b1R1_4h **Scan** 22656
**Peptide** TEGEVHEVNIVL **Charge** 2 **Spectral Angle** 0.75

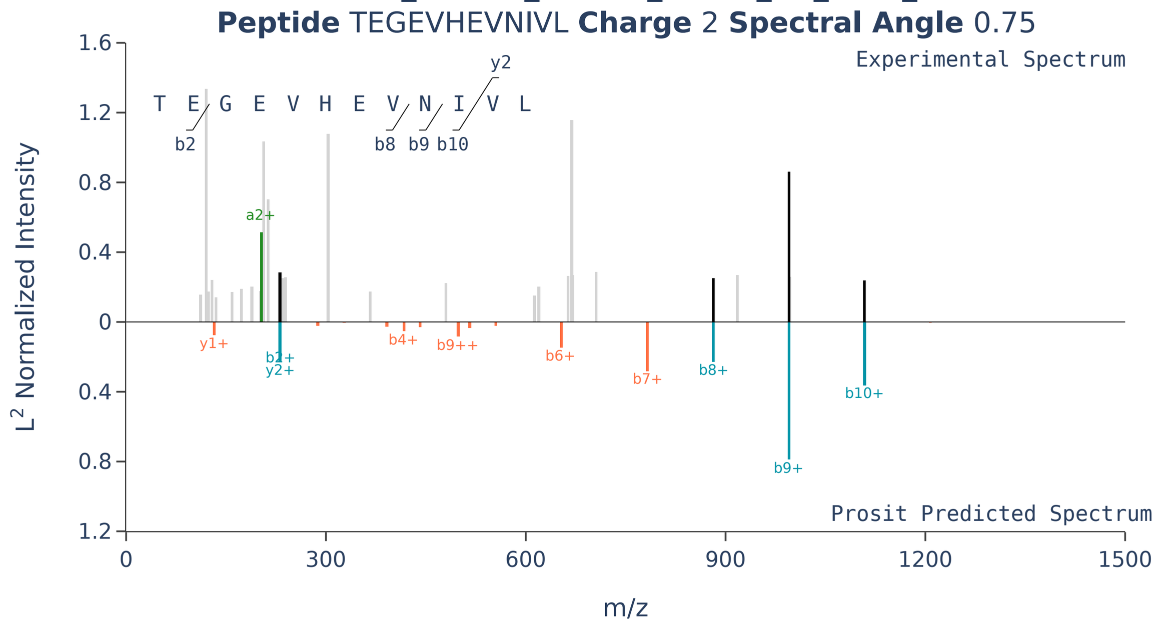


**Source** HRoetschke_211222_160123_HFGoe_IF2_b1R1_4h **Scan** 5731
**Peptide** AAEQAKREAAEQAKREAAE **Charge** 3 **Spectral Angle** 0.55

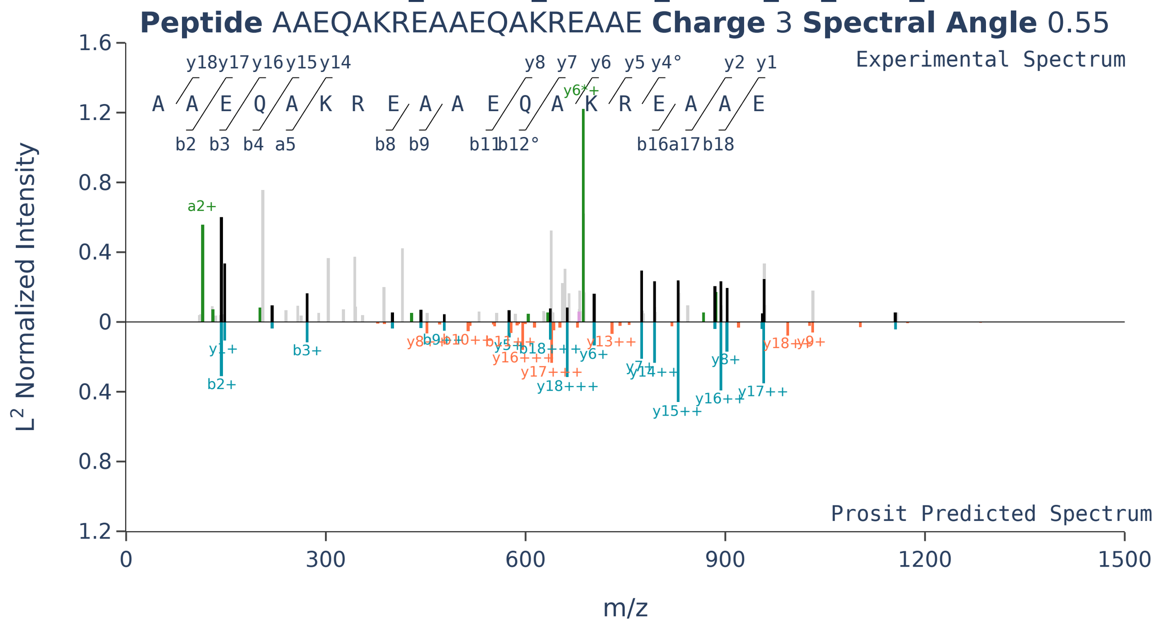

**Source** HRoetschke_211222_160123_HFGoe_IF2_b2R2_24h **Scan** 16396
**Peptide** KVKIIGSGVGGITE **Charge** 2 **Spectral Angle** 0.69

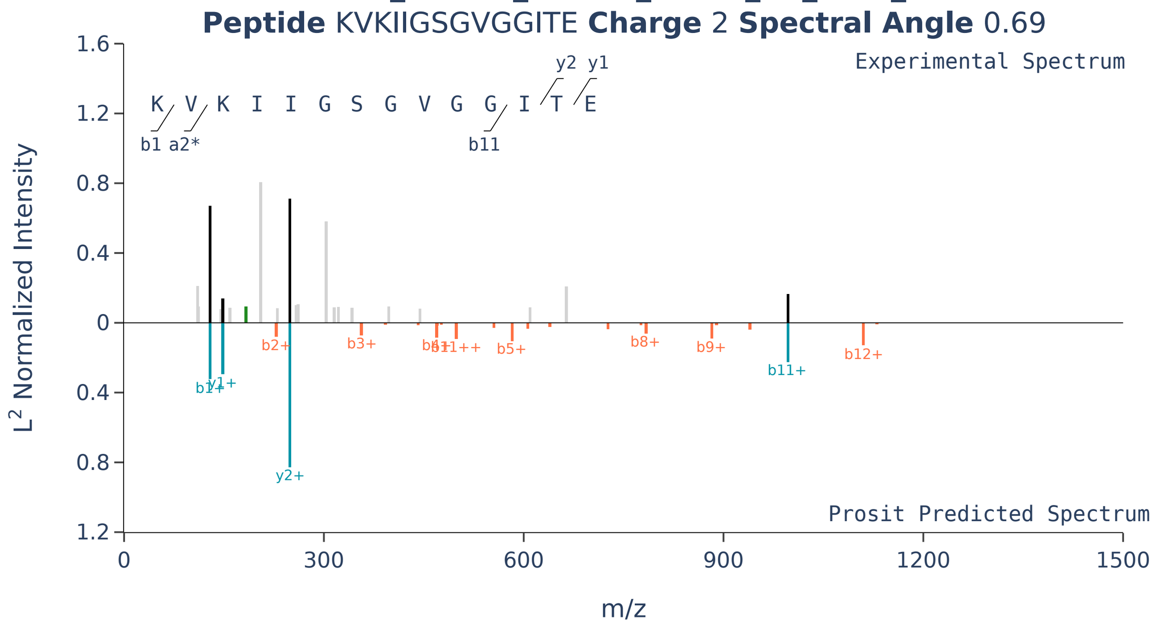


**Source** HRoetschke_211222_160123_HFGoe_IF2_b1R2_4h **Scan** 9366
**Peptide** REQEAAEL **Charge** 2 **Spectral Angle** 0.55

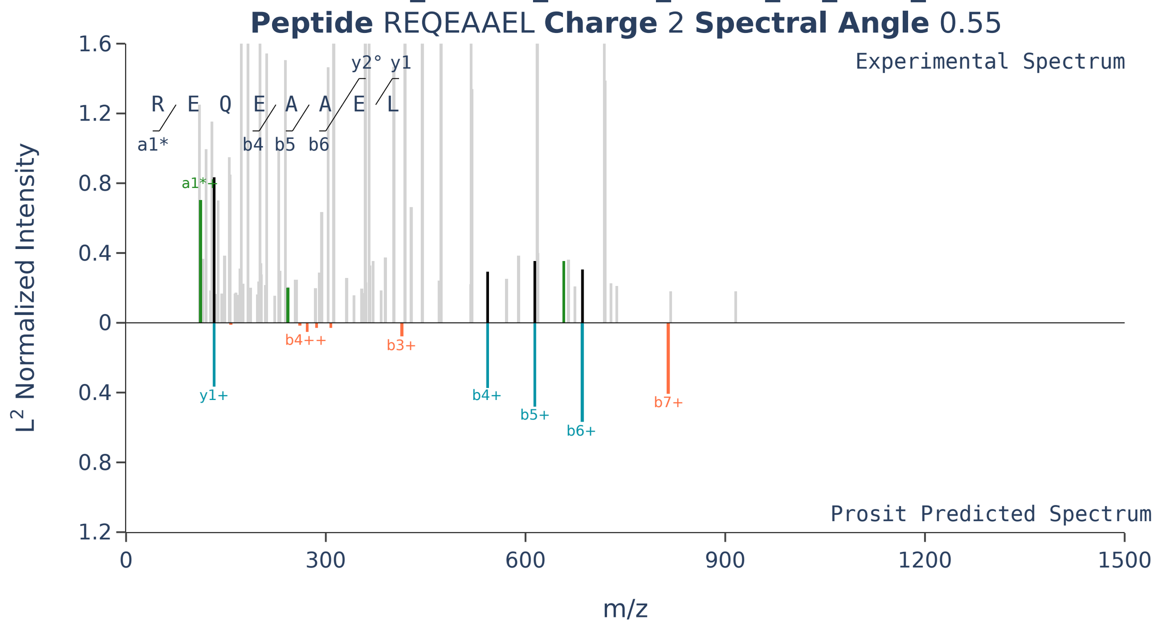


**Source** HRoetschke_211222_160123_HFGoe_IF2_b2R2_24h **Scan** 12561
**Peptide** PGTGGKSKSVQL **Charge** 2 **Spectral Angle** 0.62

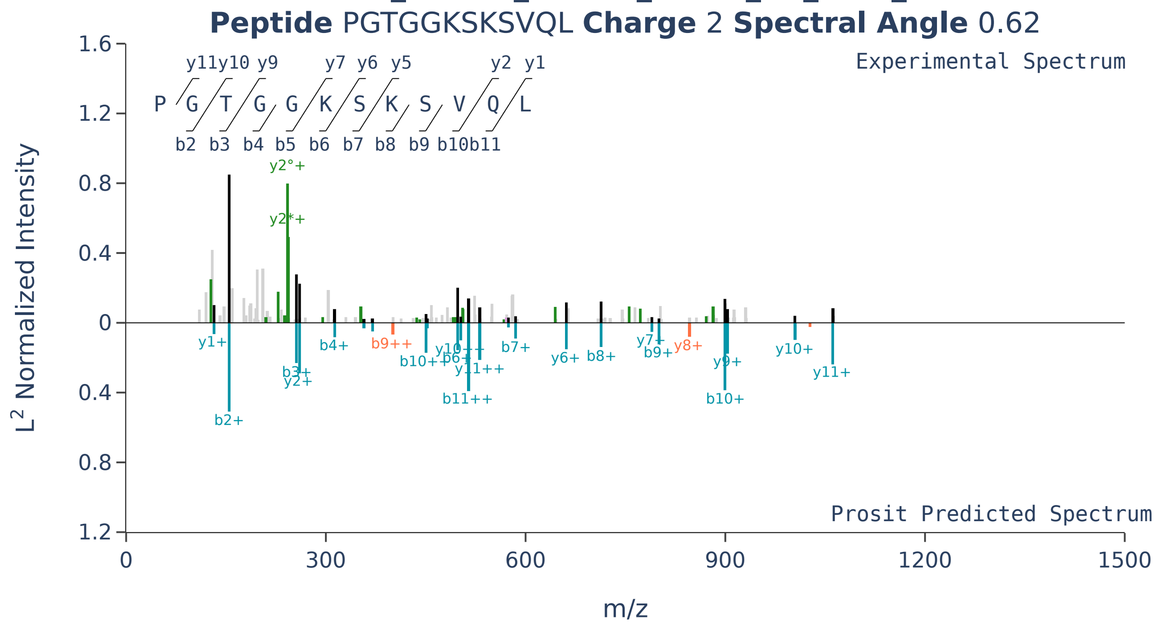


**Source** HRoetschke_211222_160123_HFGoe_IF2_b2R1_24h **Scan** 10428
**Peptide** RNGMECGIGVKNYNDVRTGD **Charge** 4 **Spectral Angle** 0.57

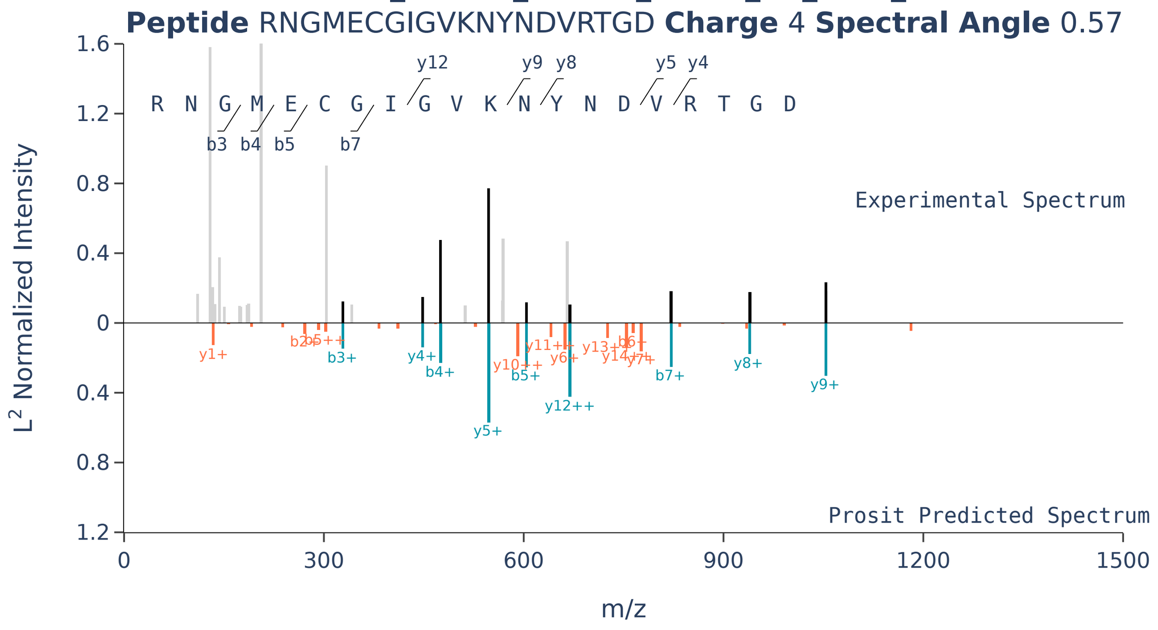


**Source** HRoetschke_211222_160123_HFGoe_IF2_b1R1_4h **Scan** 8041
**Peptide** GSQVIKA **Charge** 1 **Spectral Angle** 0.86

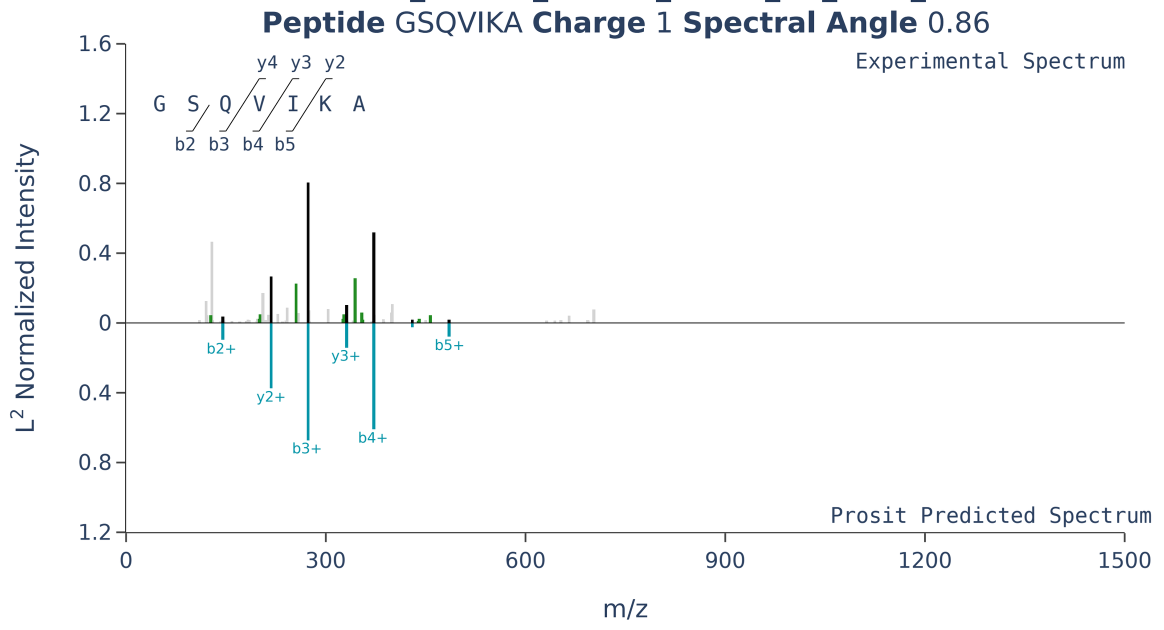


**Source** HRoetschke_211222_160123_HFGoe_IF2_b1R1_4h **Scan** 10824
**Peptide** IDHLNQKNSGPDKLTLQR **Charge** 4 **Spectral Angle** 0.76

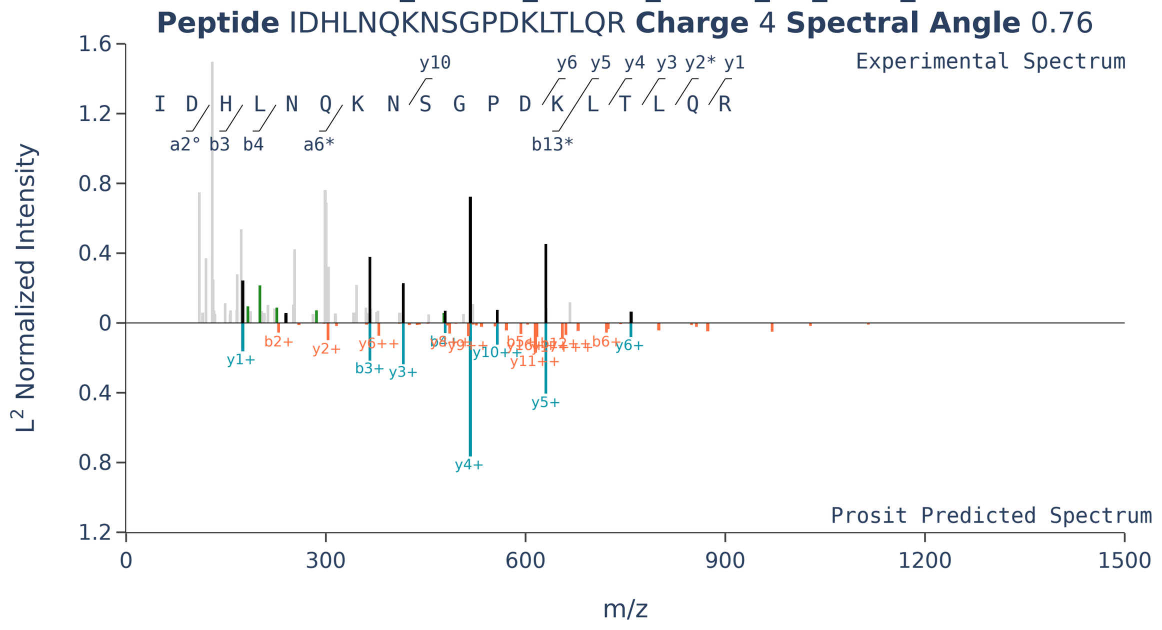


**Source** HRoetschke_211222_160123_HFGoe_IF2_b1R2_2h **Scan** 15101
**Peptide** KVASGEAGGITQHIGAY **Charge** 3 **Spectral Angle** 0.74

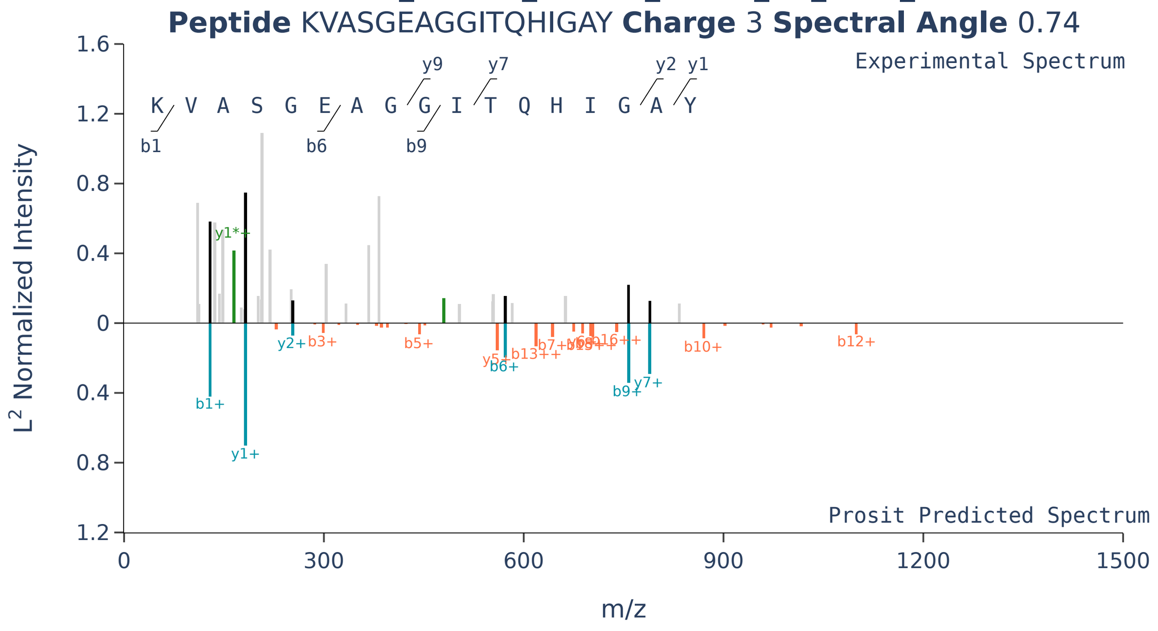


**Source** HRoetschke_211222_160123_HFGoe_IF2_b2R2_2h **Scan** 19958
**Peptide** DTGAAAEPRAPVVTI **Charge** 2 **Spectral Angle** 0.66

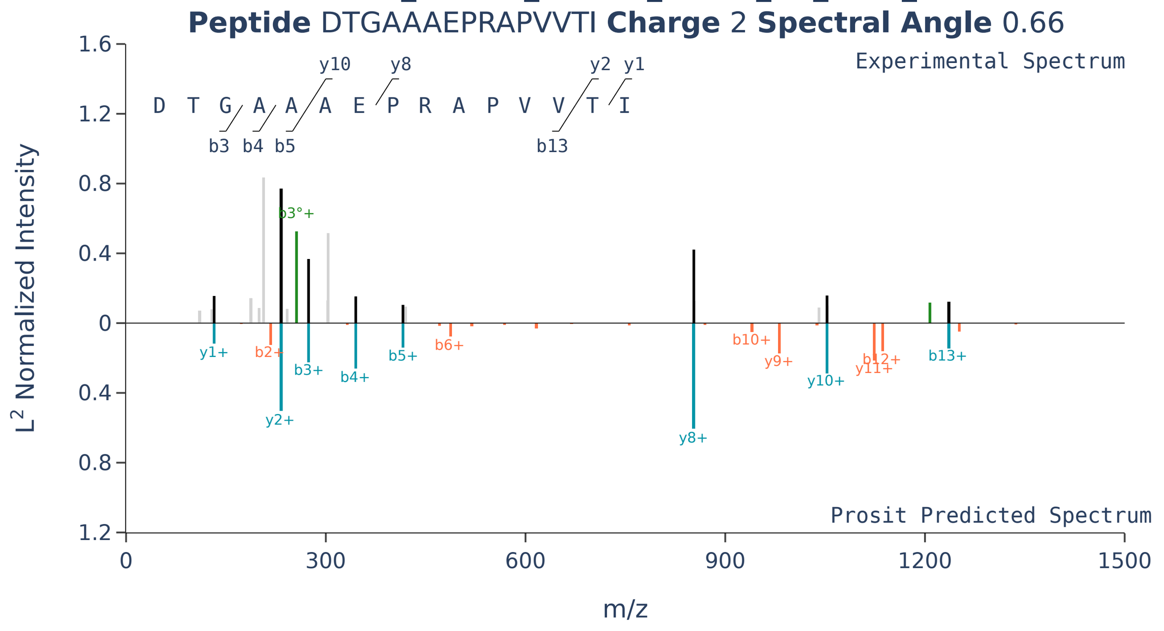


**Source** HRoetschke_211222_160123_HFGoe_IF2_b1R1_24h **Scan** 5740
**Peptide** DDMTKNAQAEKA **Charge** 2 **Spectral Angle** 0.59

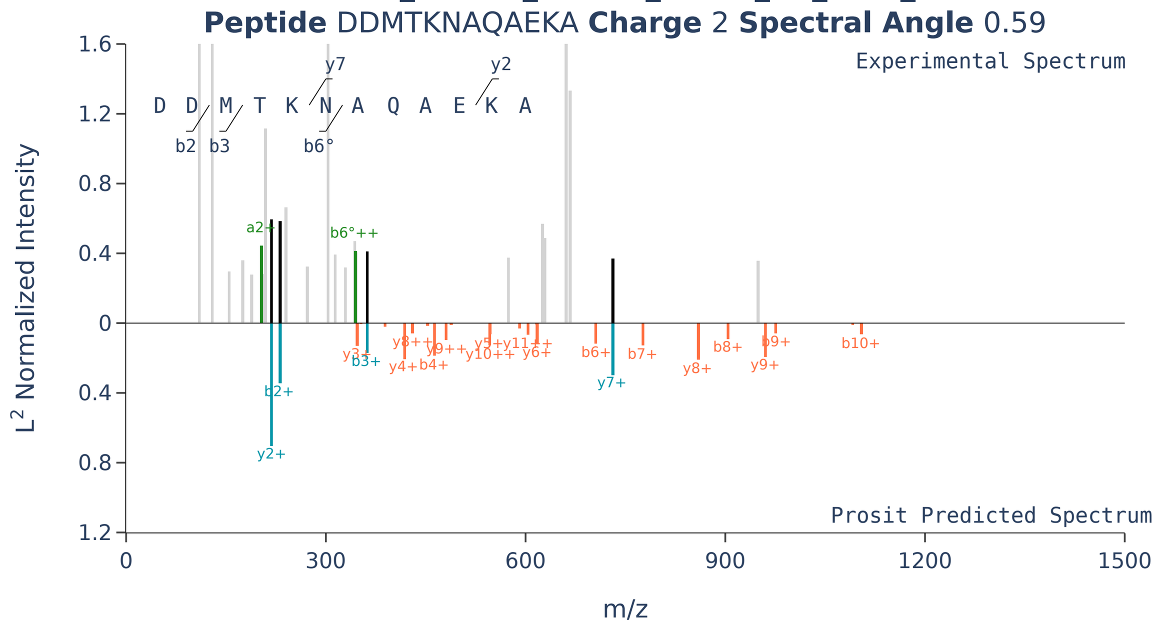


**Source** HRoetschke_211222_160123_HFGoe_IF2_b2R1_24h **Scan** 7687
**Peptide** DHLNQKNSGPDKLTLQRKT **Charge** 4 **Spectral Angle** 0.67

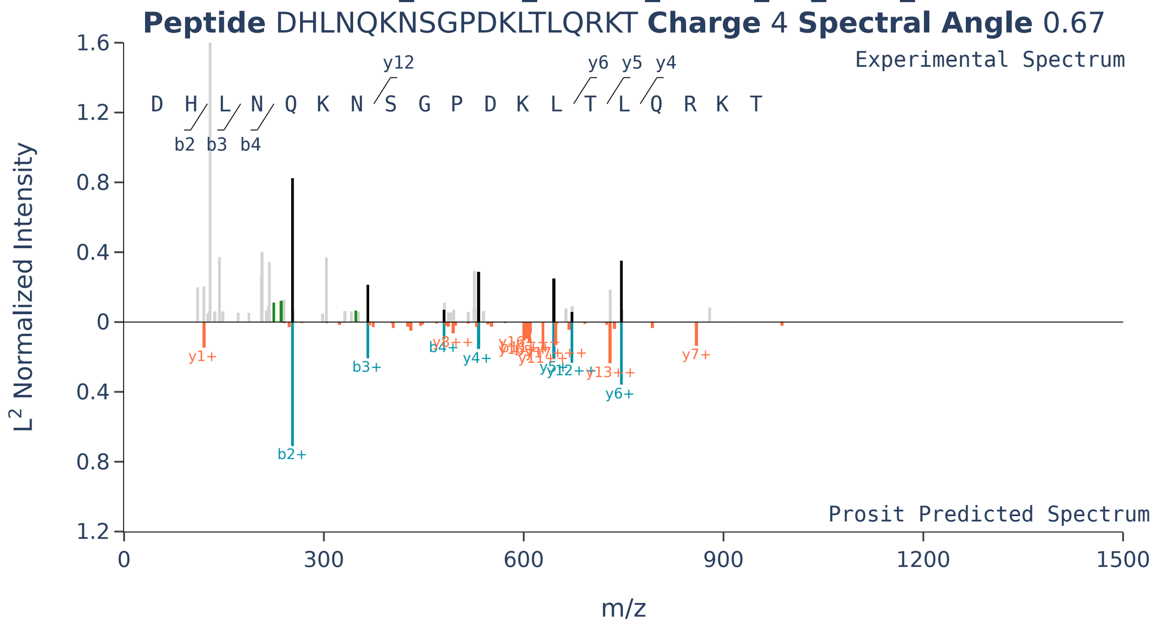


**Source** HRoetschke_211222_160123_HFGoe_IF2_b2R1_24h **Scan** 8919
**Peptide** QLVAEEMGHKV **Charge** 3 **Spectral Angle** 0.47

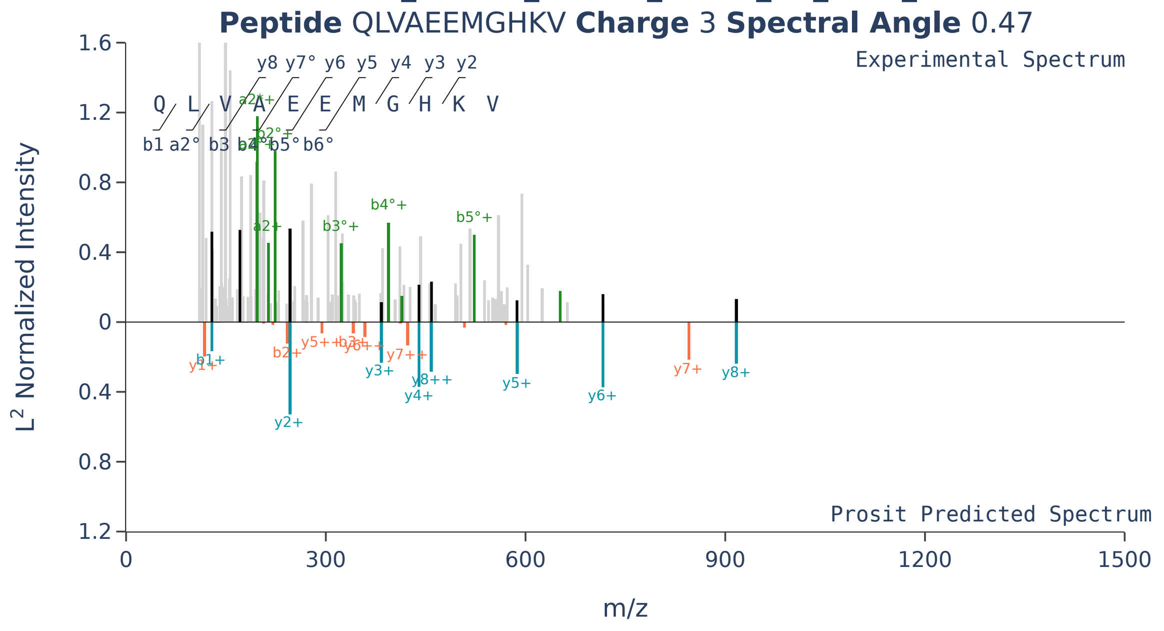


**Source** HRoetschke_211222_160123_HFGoe_IF2_b1R1_4h **Scan** 17685
**Peptide** QGFQKPAQAVNRDVVI **Charge** 3 **Spectral Angle** 0.7

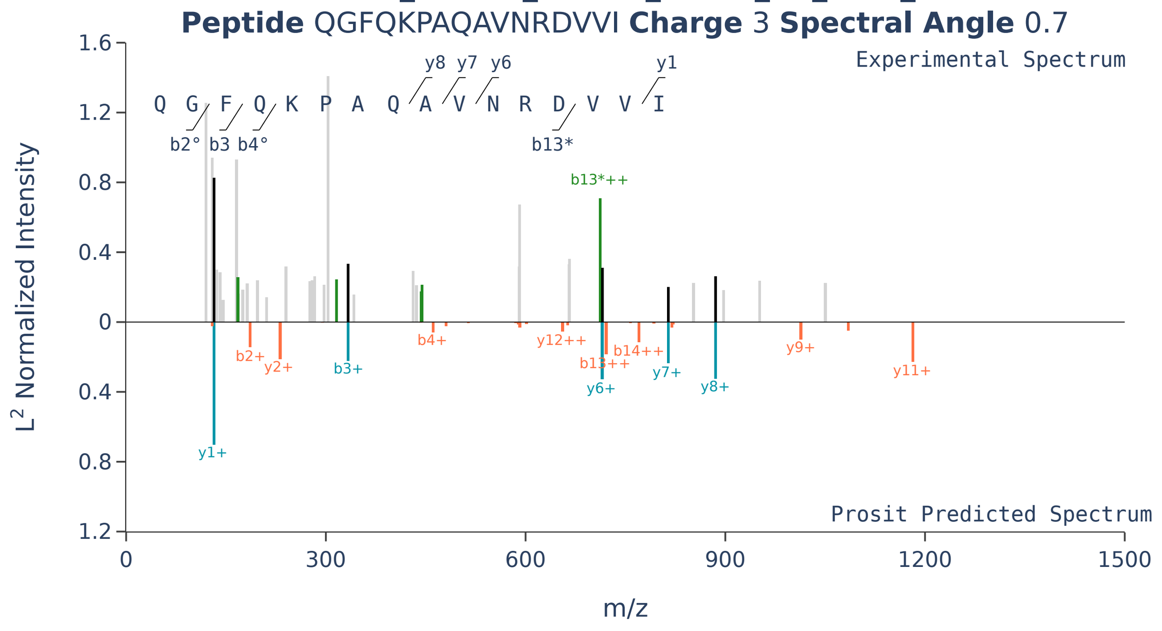


**Source** HRoetschke_211222_160123_HFGoe_IF2_b1R2_24h **Scan** 15455
**Peptide** STDEVKVKIIGS **Charge** 2 **Spectral Angle** 0.6

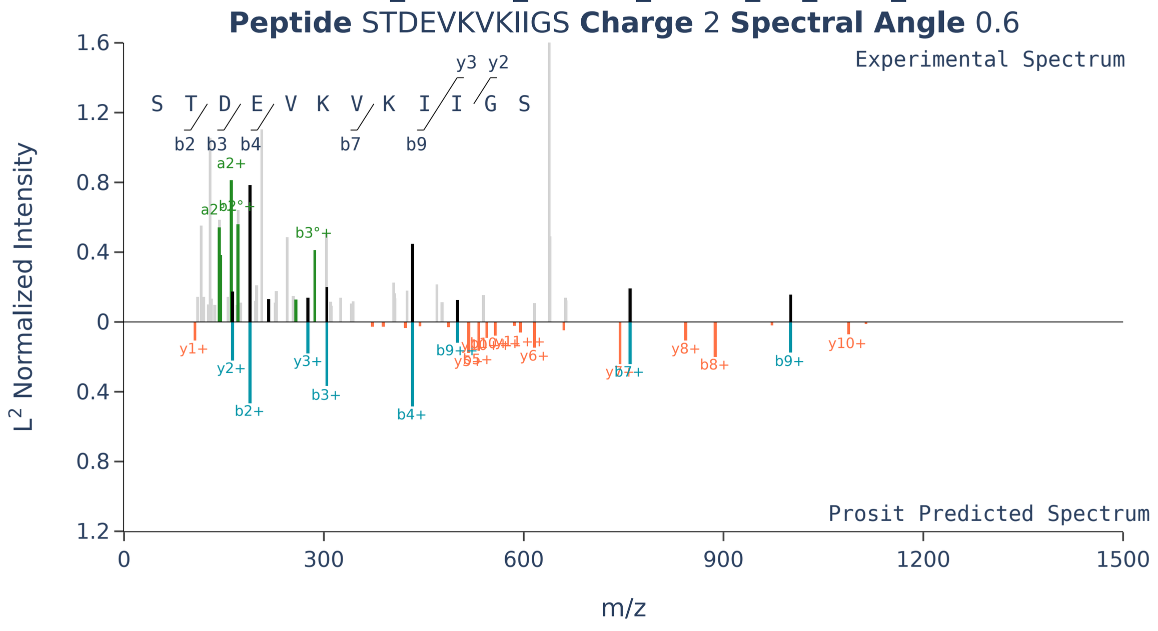


**Source** HRoetschke_211222_160123_HFGoe_IF2_b1R1_4h **Scan** 17090
**Peptide** DGVMPQTIEAIQHAKAAQ **Charge** 3 **Spectral Angle** 0.72

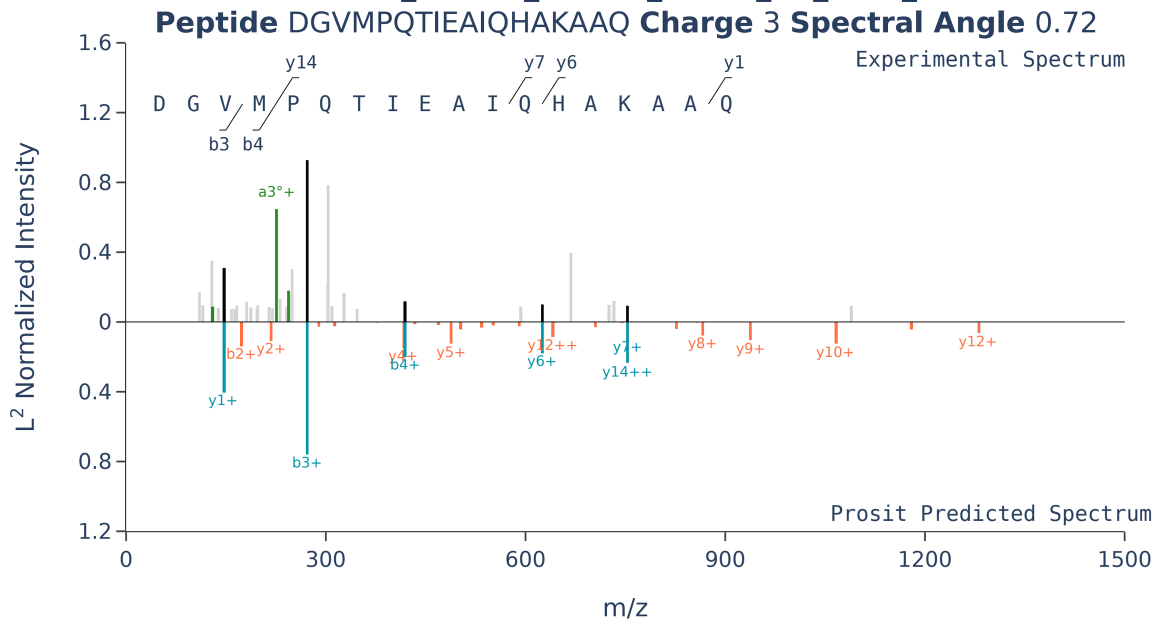


**Source** HRoetschke_211222_160123_HFGoe_IF2_b1R1_4h **Scan** 12609
**Peptide** KADVQGSVEAIS **Charge** 2 **Spectral Angle** 0.28

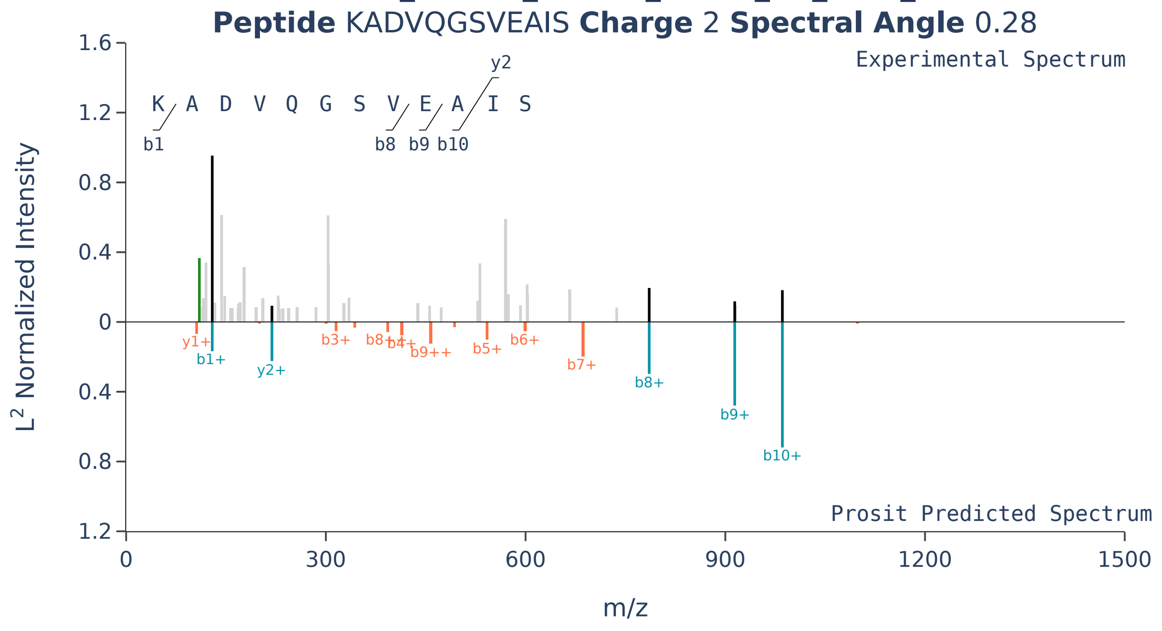

**Source** HRoetschke_211222_160123_HFGoe_IF2_b1R1_2h **Scan** 8919
**Peptide** GFEYGRVRAMRN **Charge** 3 **Spectral Angle** 0.59

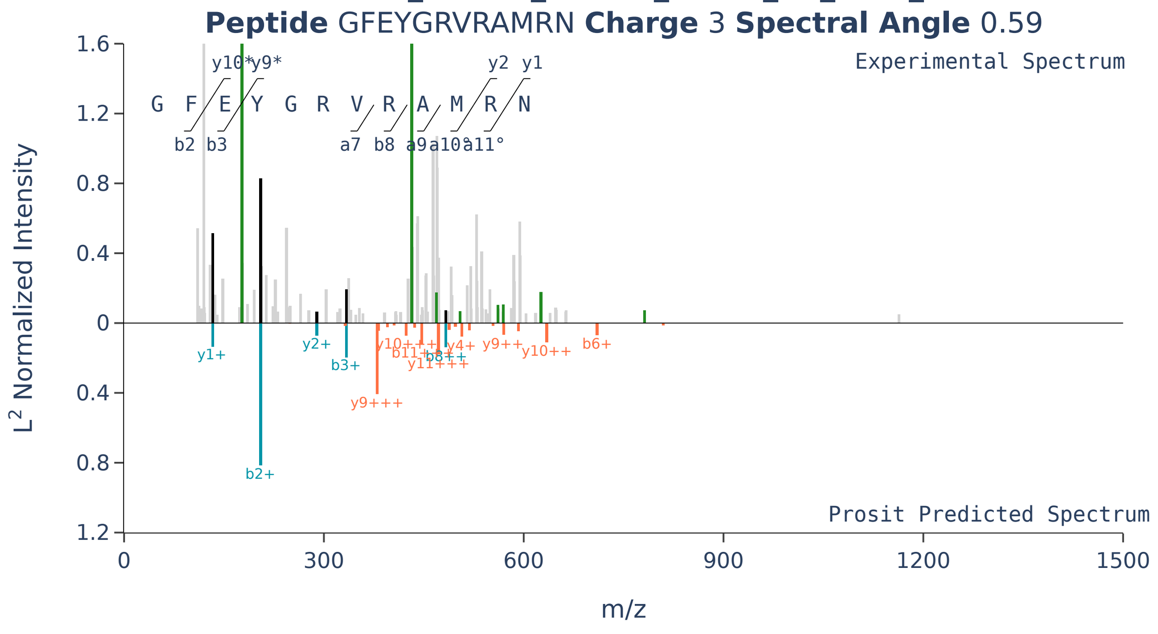


**Source** HRoetschke_211222_160123_HFGoe_IF2_b1R1_4h **Scan** 20175
**Peptide** AAAEPRAPVVTL **Charge** 2 **Spectral Angle** 0.54

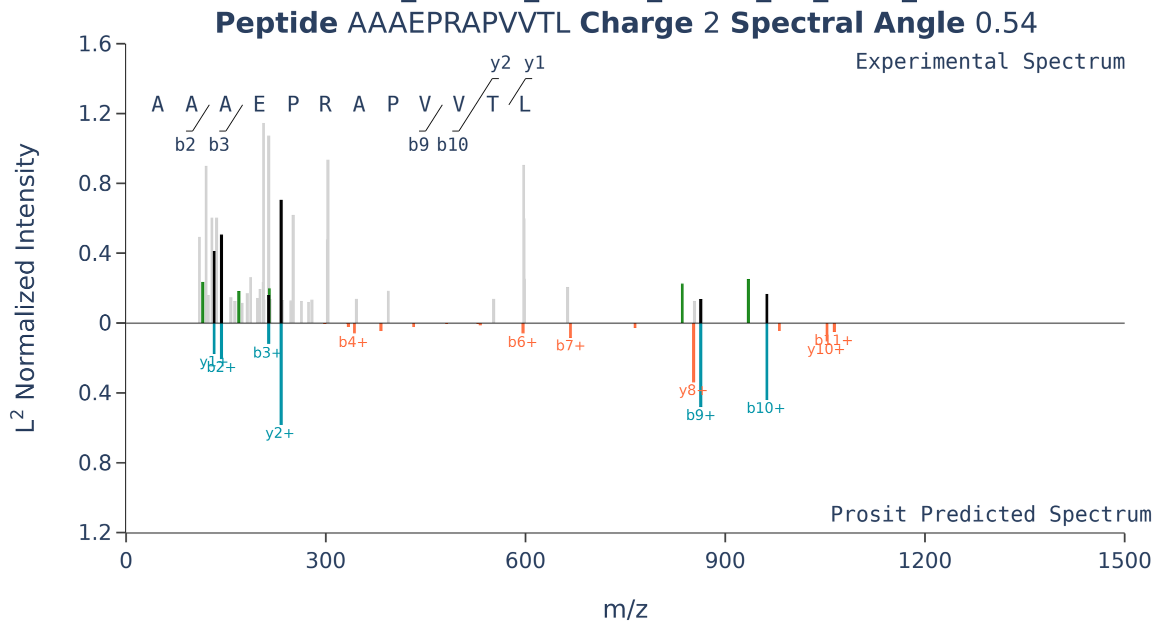


**Source** HRoetschke_211222_160123_HFGoe_IF2_b1R2_4h **Scan** 14096
**Peptide** LAAERQTSVERLVQ **Charge** 3 **Spectral Angle** 0.68

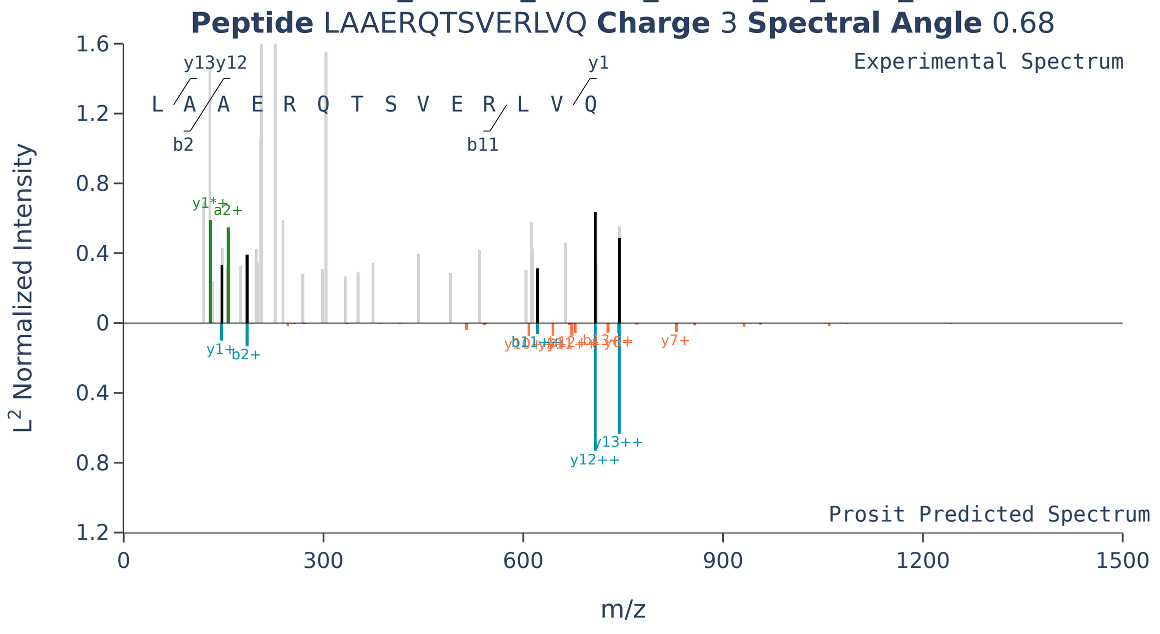


**Source** HRoetschke_211222_160123_HFGoe_IF2_b1R2_24h **Scan** 14358
**Peptide** LDYIRSTKVASGEAGGITQH **Charge** 3 **Spectral Angle** 0.78

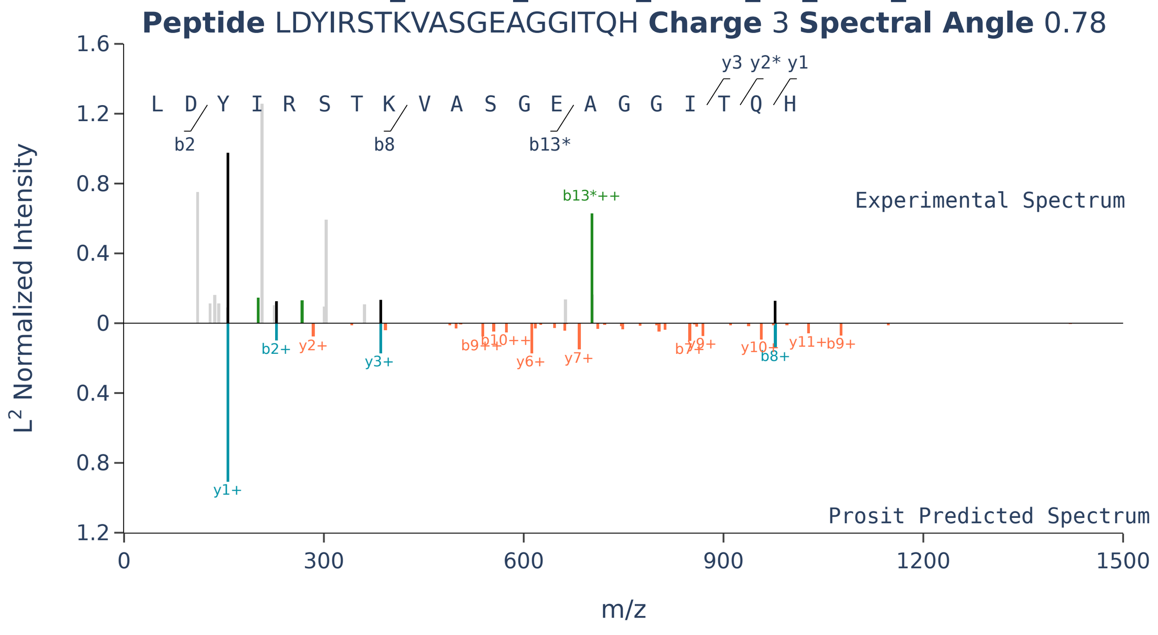


**Source** HRoetschke_211222_160123_HFGoe_IF2_b1R2_2h **Scan** 7866
**Peptide** VKRDPQEAERLAA **Charge** 3 **Spectral Angle** 0.6

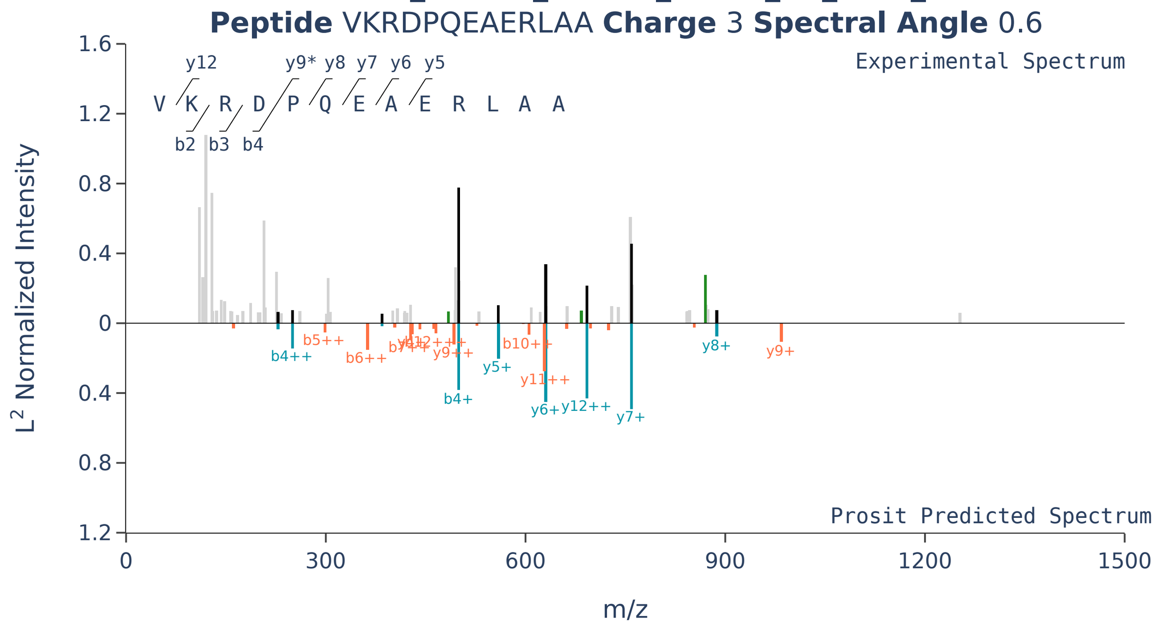


**Source** HRoetschke_211222_160123_HFGoe_IF2_b2R2_4h **Scan** 22422
**Peptide** ELSQYGILPEE **Charge** 2 **Spectral Angle** 0.85

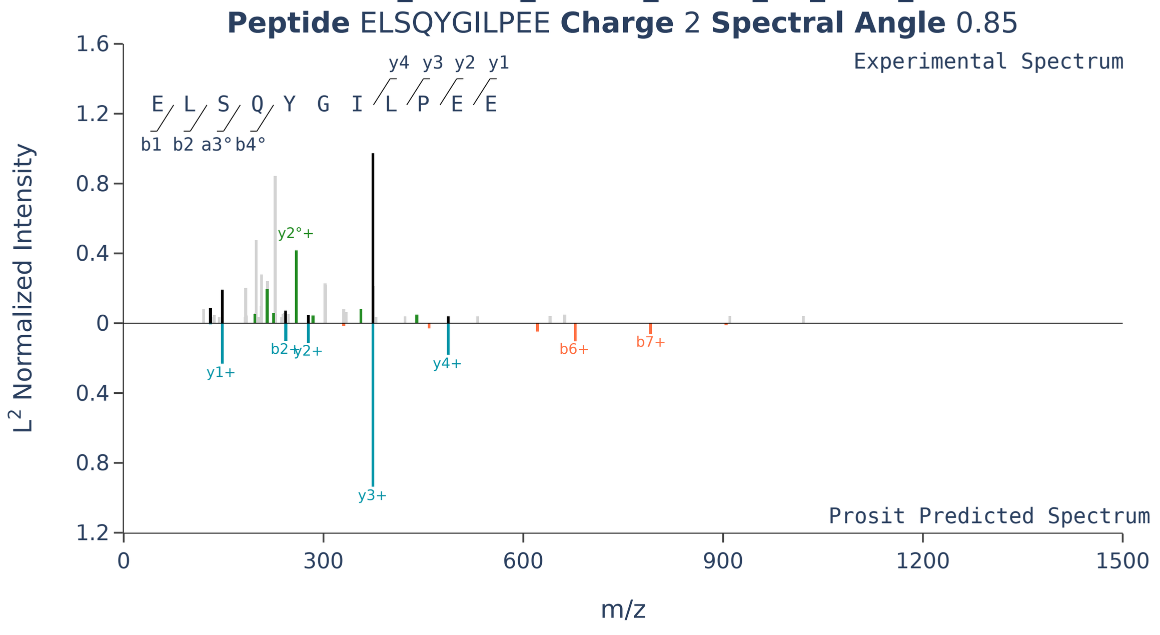


**Source** HRoetschke_211222_160123_HFGoe_IF2_b1R2_2h **Scan** 6383
**Peptide** DYHVTTSQHARQAE **Charge** 3 **Spectral Angle** 0.52

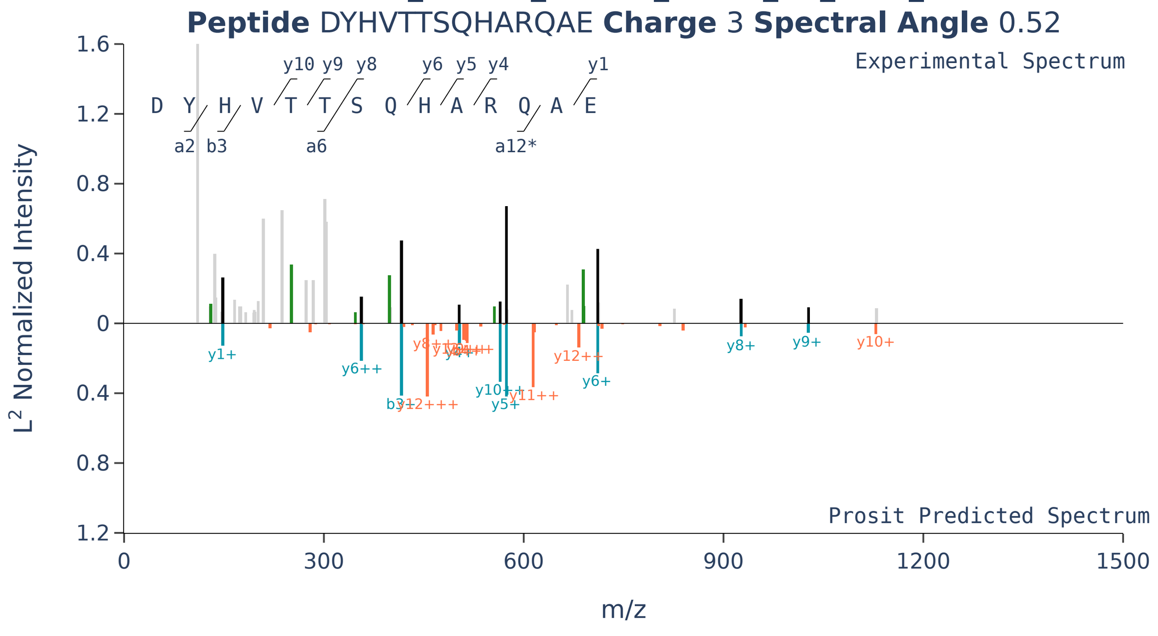


**Source** HRoetschke_211222_160123_HFGoe_IF2_b1R1_4h **Scan** 18920
**Peptide** MSDRDTGAAAEPRAPVVTI **Charge** 3 **Spectral Angle** 0.64

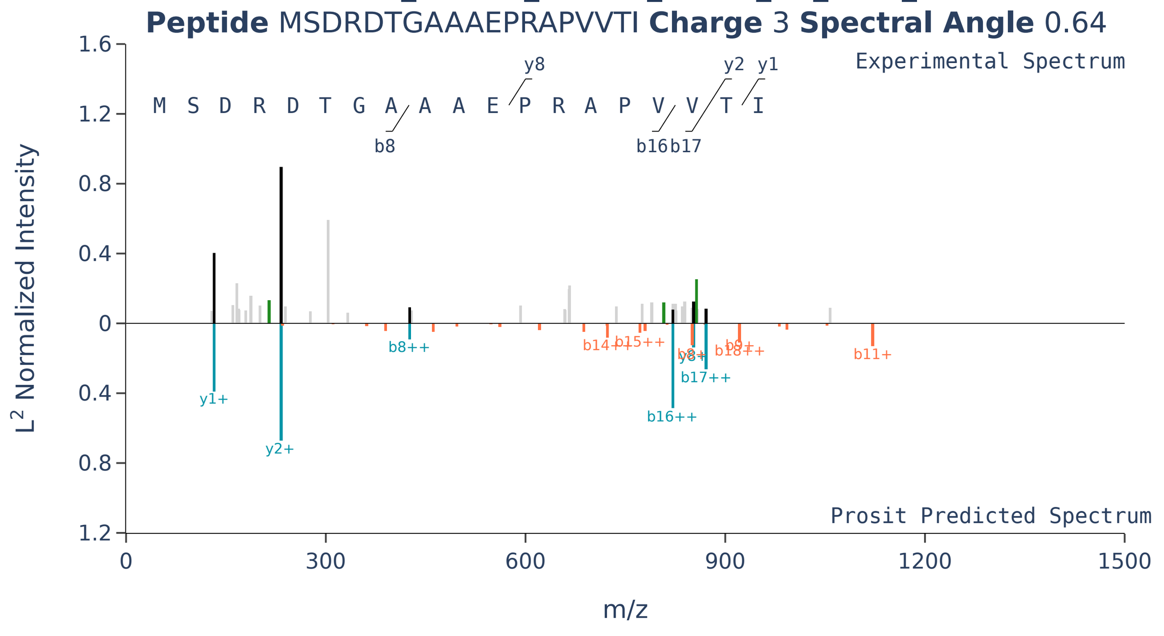


**Source** HRoetschke_211222_160123_HFGoe_IF2_b1R1_4h **Scan** 17260
**Peptide** RDVFKSPKFGAIAG **Charge** 3 **Spectral Angle** 0.52

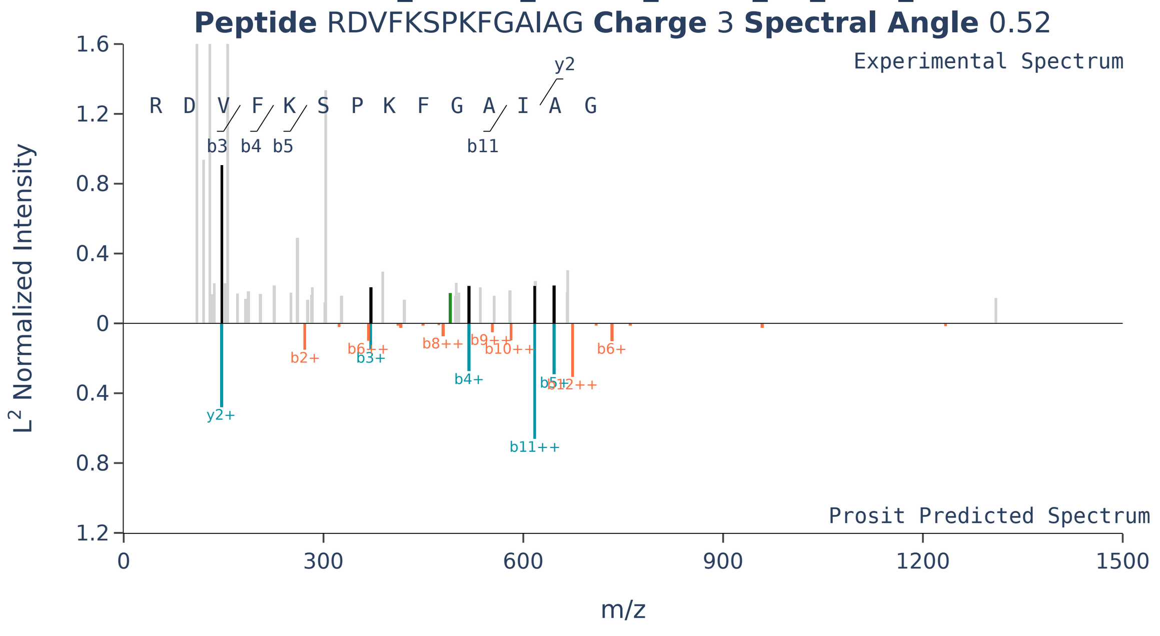


**Source** HRoetschke_211222_160123_HFGoe_IF2_b1R1_2h **Scan** 11396
**Peptide** KLDKPEADPDRVKNELSQ **Charge** 4 **Spectral Angle** 0.74

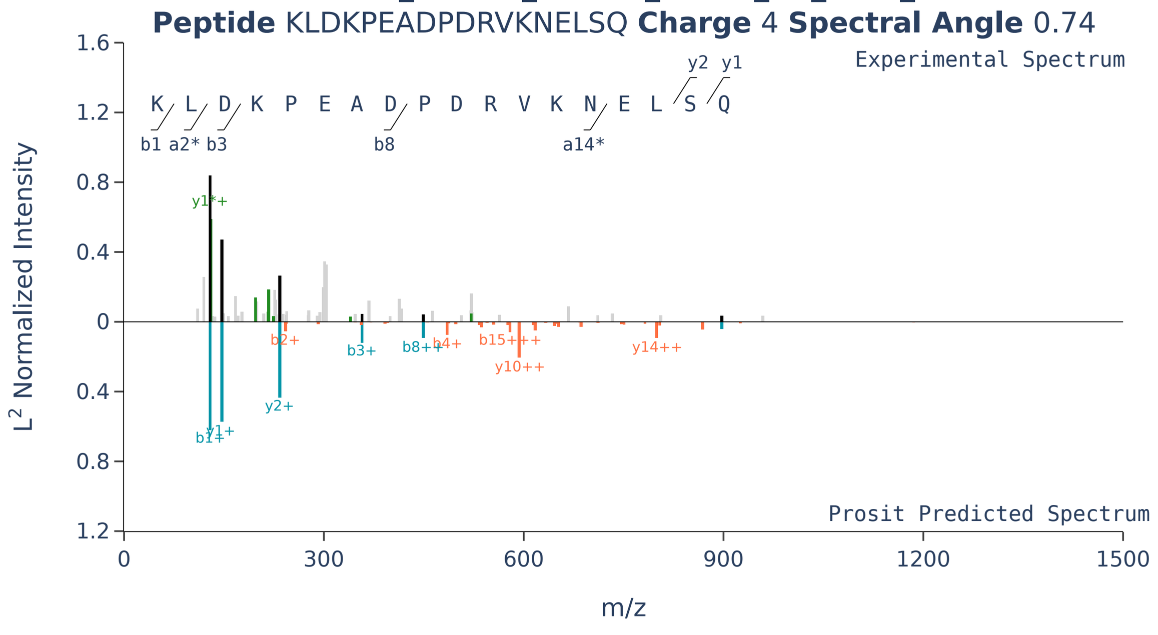


**Source** HRoetschke_211222_160123_HFGoe_IF2_b1R2_2h **Scan** 7141
**Peptide** KRDPQEAERLAA **Charge** 3 **Spectral Angle** 0.53

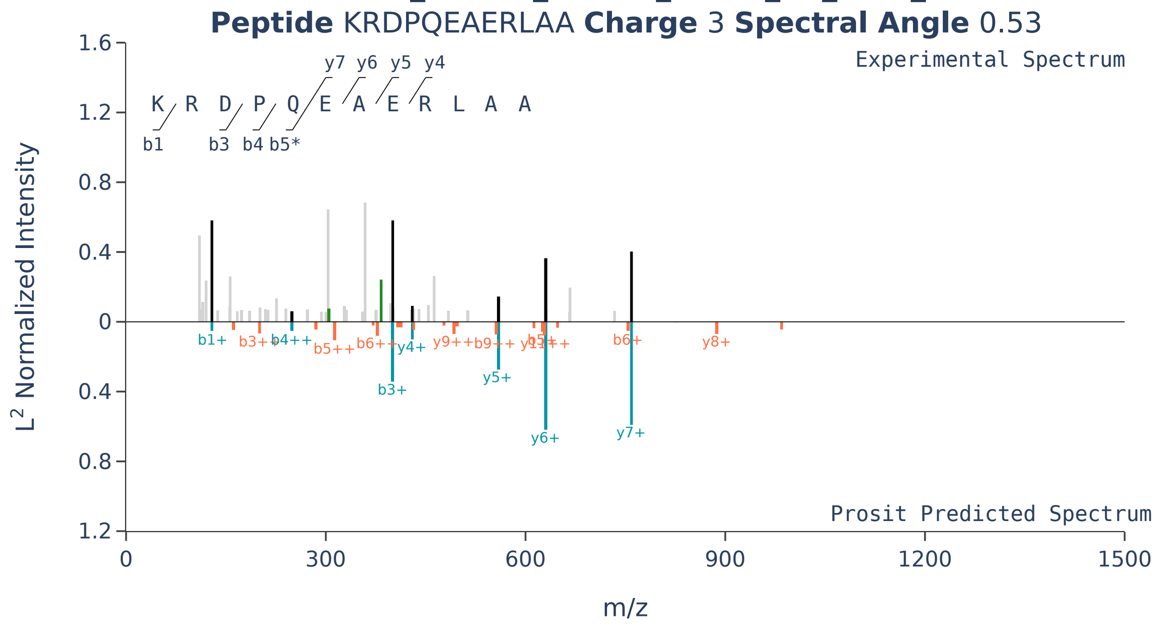


**Source** HRoetschke_211222_160123_HFGoe_IF2_b1R2_4h **Scan** 6376
**Peptide** HVTTSQHARQAED **Charge** 3 **Spectral Angle** 0.68

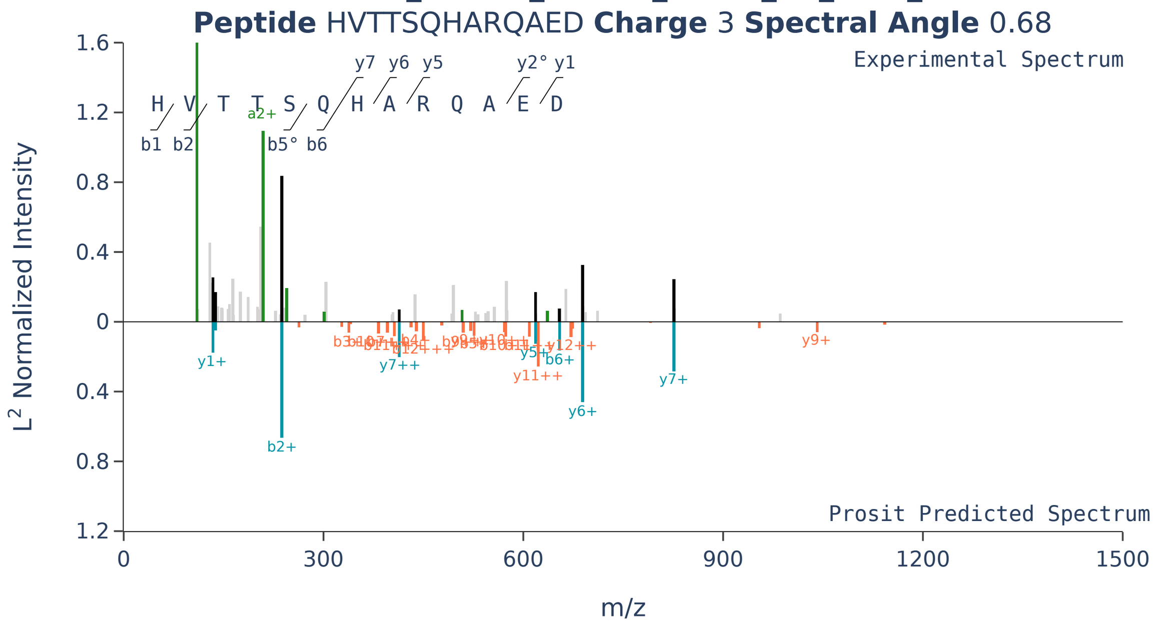


**Source** HRoetschke_211222_160123_HFGoe_IF2_b1R1_4h **Scan** 10632
**Peptide** ADAGIRKSADDSVSAQEKQTL **Charge** 3 **Spectral Angle** 0.52

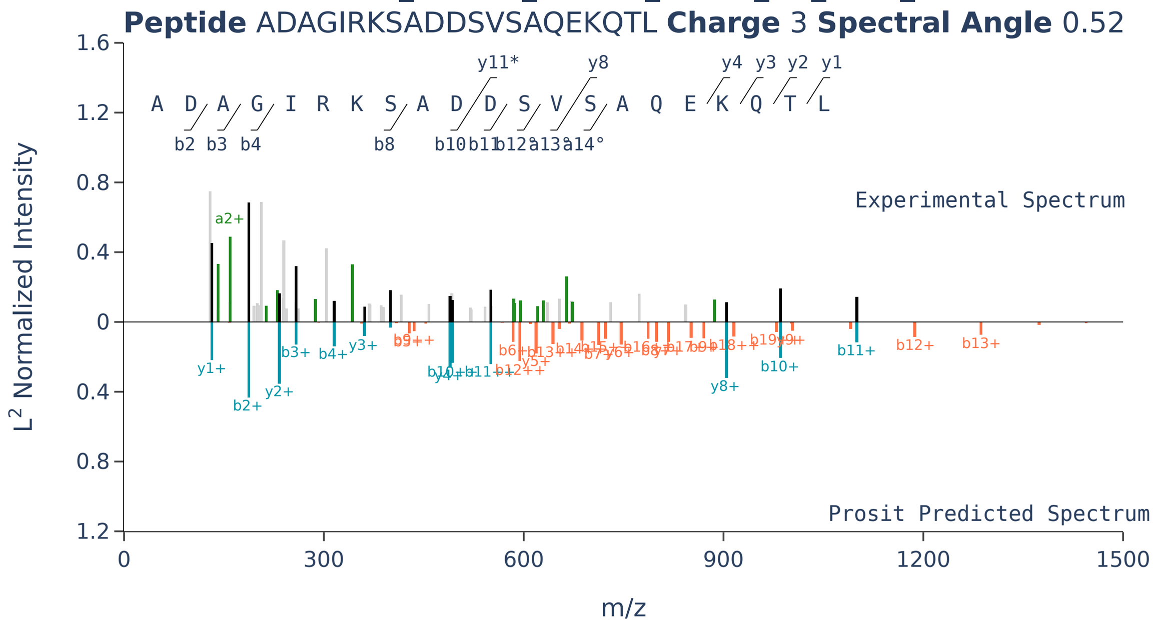


**Source** HRoetschke_211222_160123_HFGoe_IF2_b1R1_4h **Scan** 14904
**Peptide** AIQHAKAAQVPVVVAVNK **Charge** 3 **Spectral Angle** 0.65

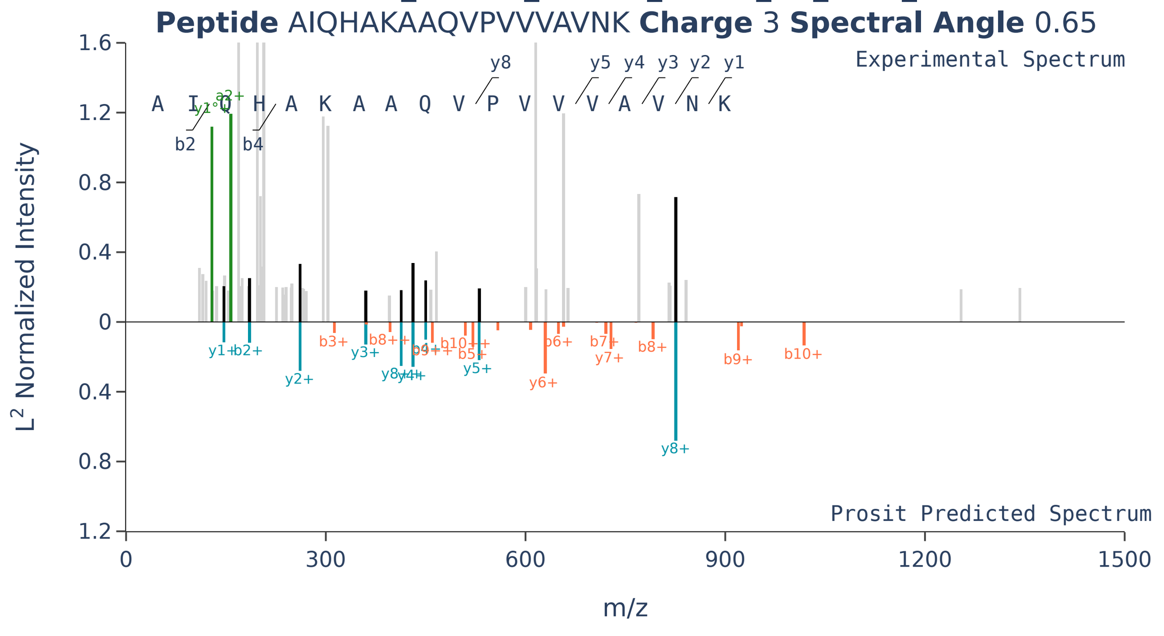


**Source** HRoetschke_211222_160123_HFGoe_IF2_b1R1_4h **Scan** 18451
**Peptide** TLNIPGTGGKSKSVQI **Charge** 3 **Spectral Angle** 0.73

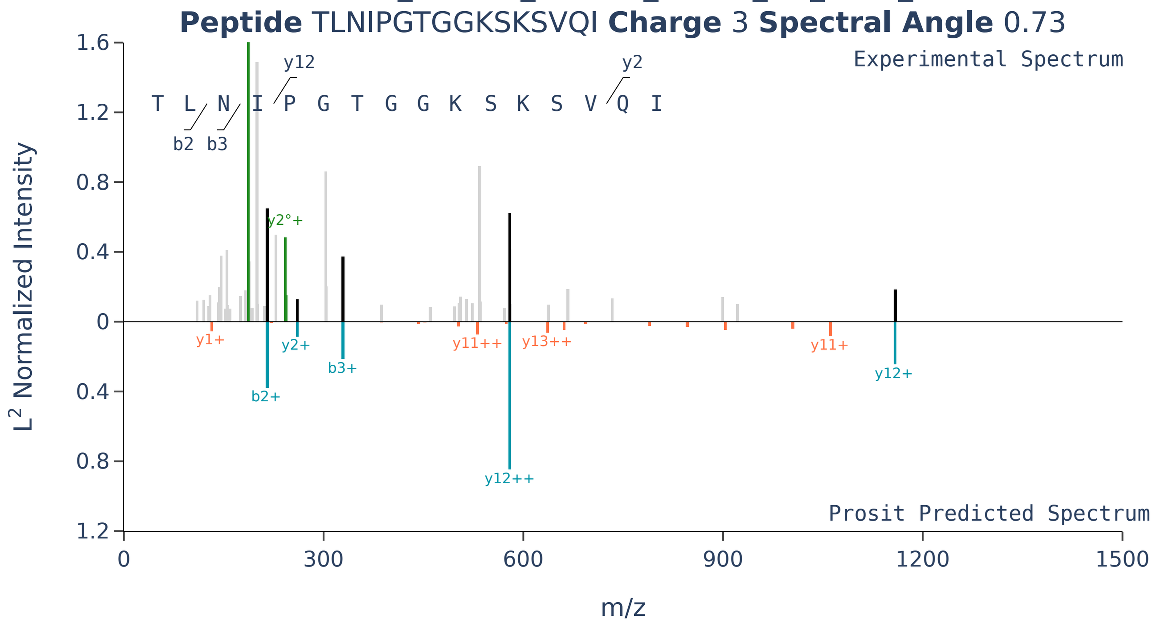

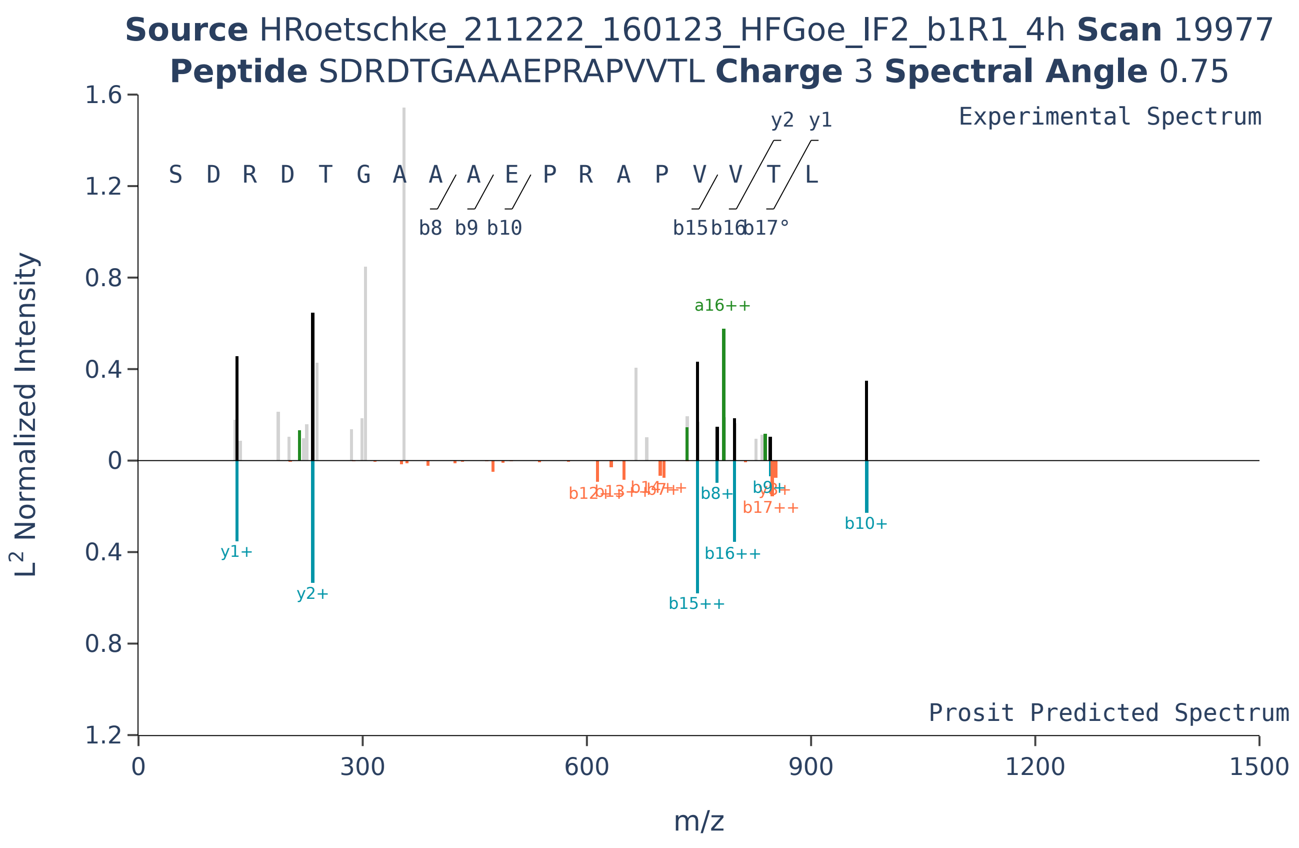

Source HRoetschke_211222_160123_HFGoe_IF2_b1R1_4h Scan 19977
Peptide SDRDTGAAAEPRAPVVTL Charge 3 Spectral Angle 0.75
Experimental Spectrum
Prosit Predicted Spectrum
L² Normalized Intensity
m/z
y2 y1
S D R D T G A A A E P R A P V V T L
b8 b9 b10
b15 b16 b17°
a16++
y1+
y2+
b8+
b16++
b15++
b17++
b10+

# inSPIRE Spectral Plotting for IL37b

Experimental Spectrum Colour Code:

- Experimental peak matched to a Prosit predicted peak.
- Possible ion unknown to Prosit.
- Precursor matched peak.
- Experimental peak not matched to any potential ion.

Prosit Spectrum Colour Code:

- Prosit predicted peak matched to experimental spectrum.
- Prosit predicted peak not matched to experimental spectrum.

Loading [MathJax]/extensions/MathMenu.js

**Source** WSoh_101121_151121_HFGoe_G1_IL37b_24h_R2 **Scan** 8946
**Peptide** AVPDKNYIR **Charge** 2 **Spectral Angle** 0.92

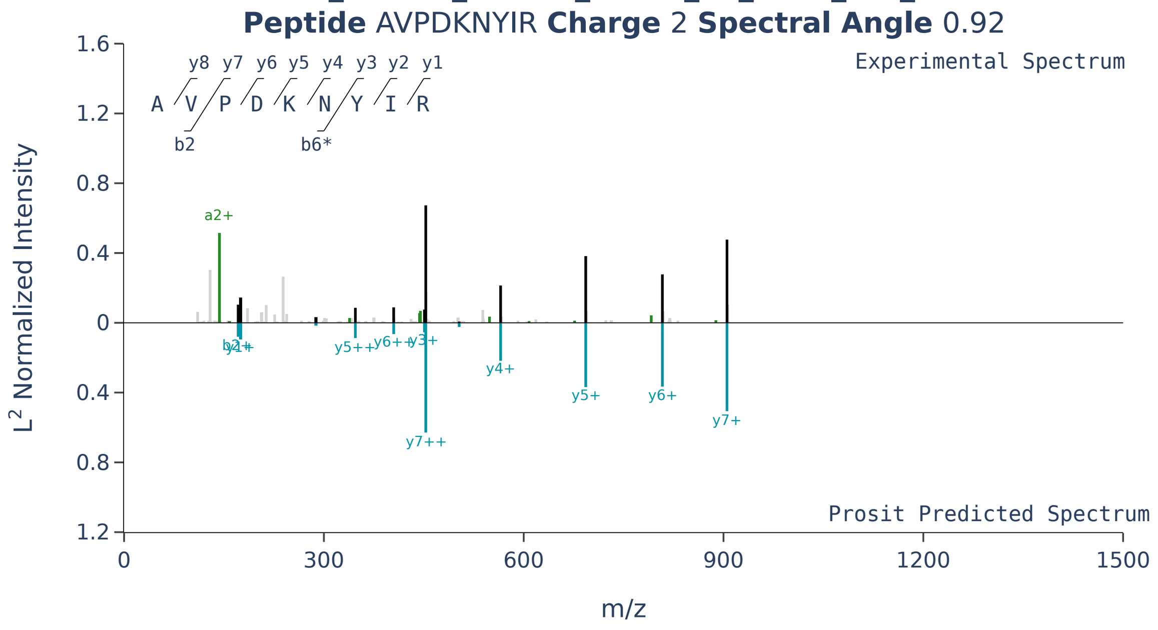


**Source** WSoh_101121_151121_HFGoe_G2_IL37b_24h_R1 **Scan** 6447
**Peptide** VTDKFENR **Charge** 2 **Spectral Angle** 0.94

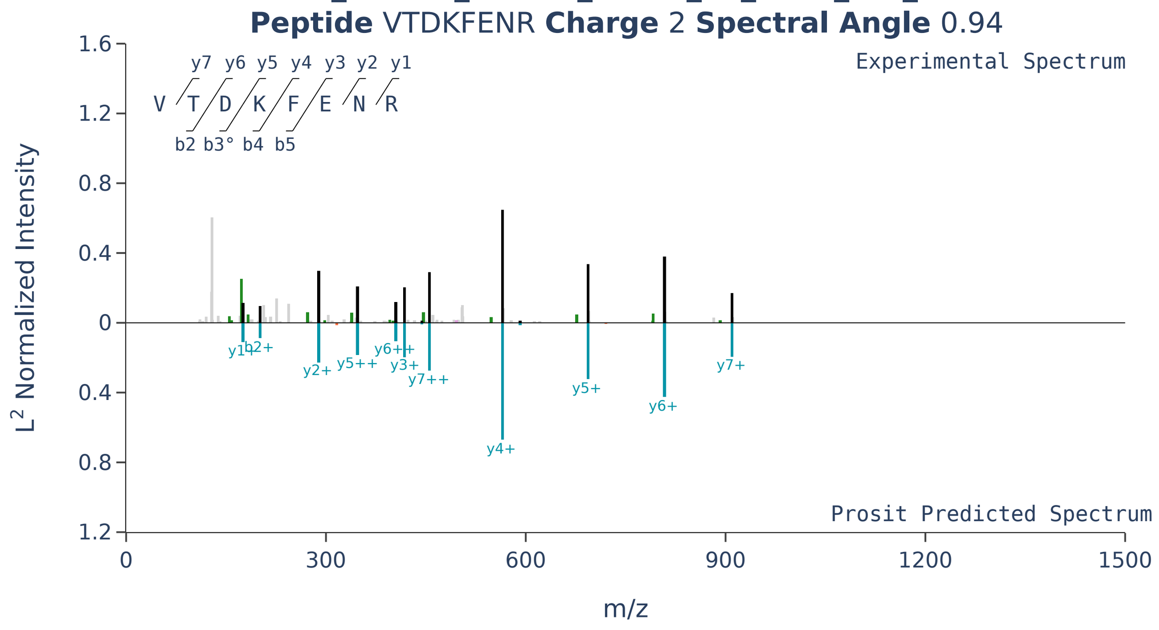


**Source** WSoh_101121_151121_HFGoe_G1_IL37b_24h_R2 **Scan** 8002
**Peptide** PDKNYIRPE **Charge** 2 **Spectral Angle** 0.94

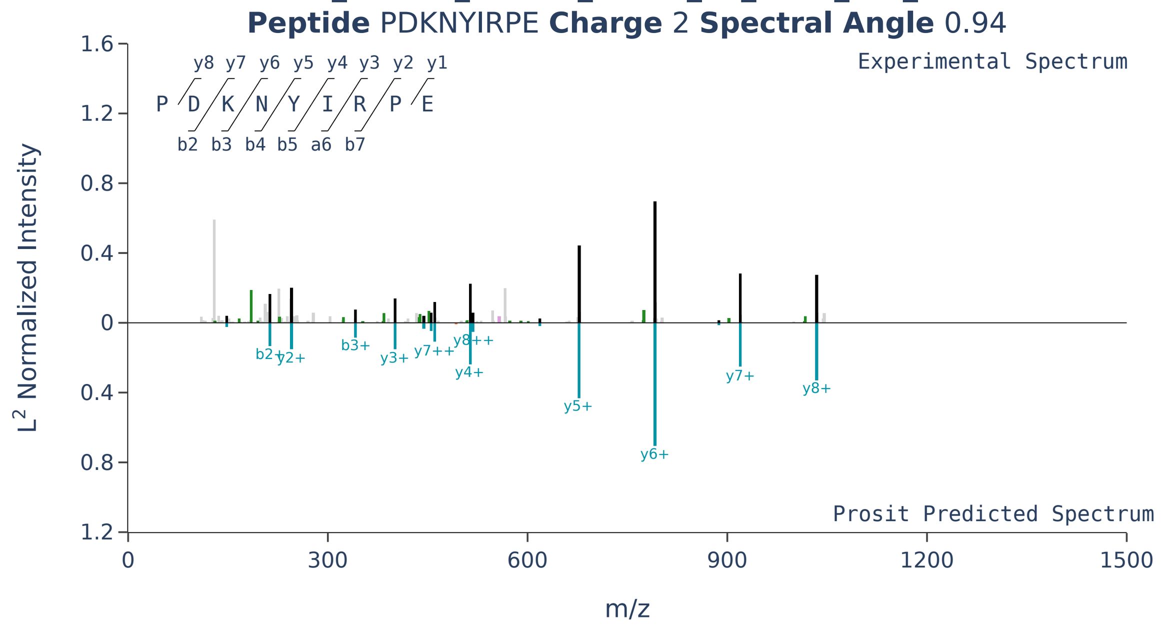


**Source** WSoh_101121_151121_HFGoe_G1_IL37b_24h_R2 **Scan** 10662
**Peptide** NMLESAAH **Charge** 2 **Spectral Angle** 0.96

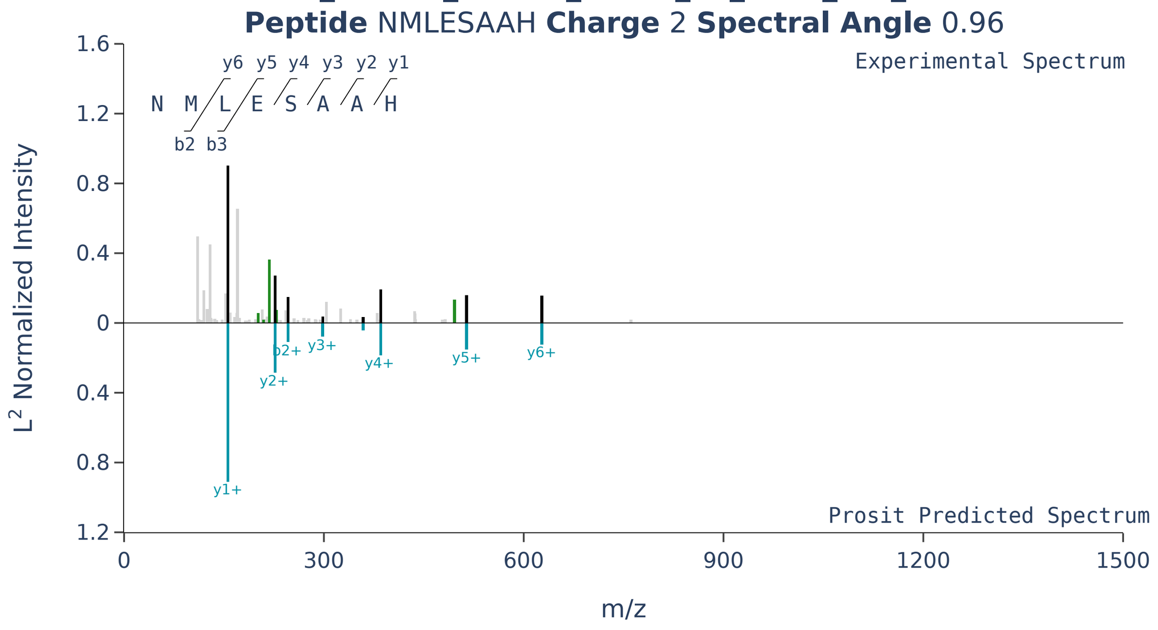


**Source** WSoh_101121_151121_HFGoe_G2_IL37b_24h_R2 **Scan** 6461
**Peptide** DKDKGQSHPSL **Charge** 2 **Spectral Angle** 0.95

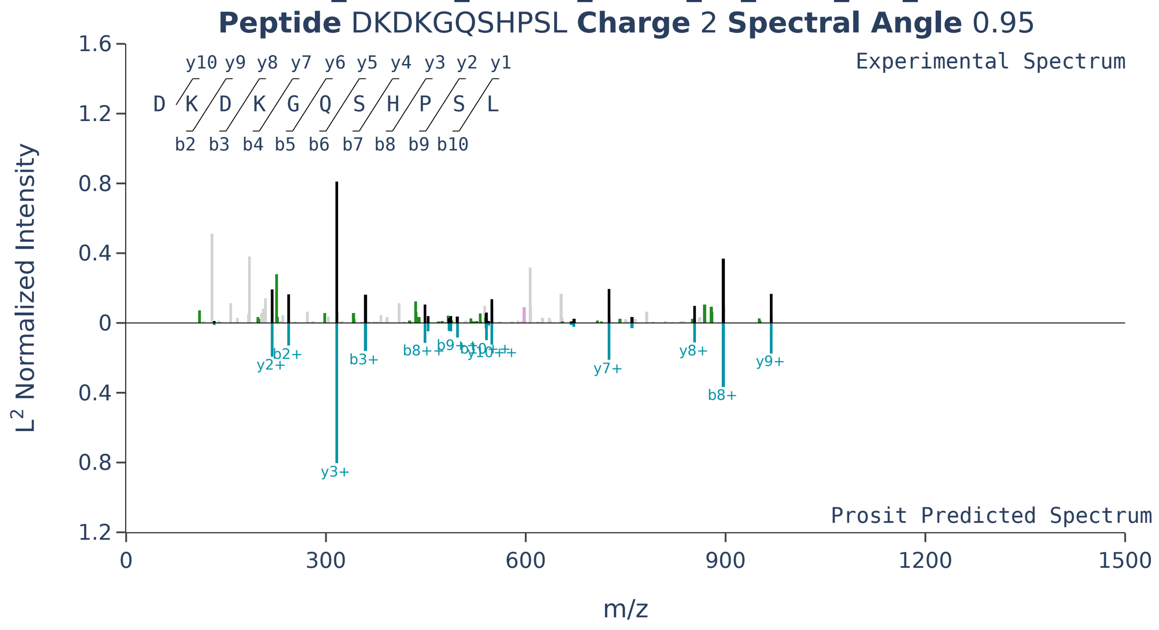


**Source** WSoh_101121_151121_HFGoe_G2_IL37b_2h_R2 **Scan** 20419
**Peptide** PDKNYIRPEIF **Charge** 2 **Spectral Angle** 0.92

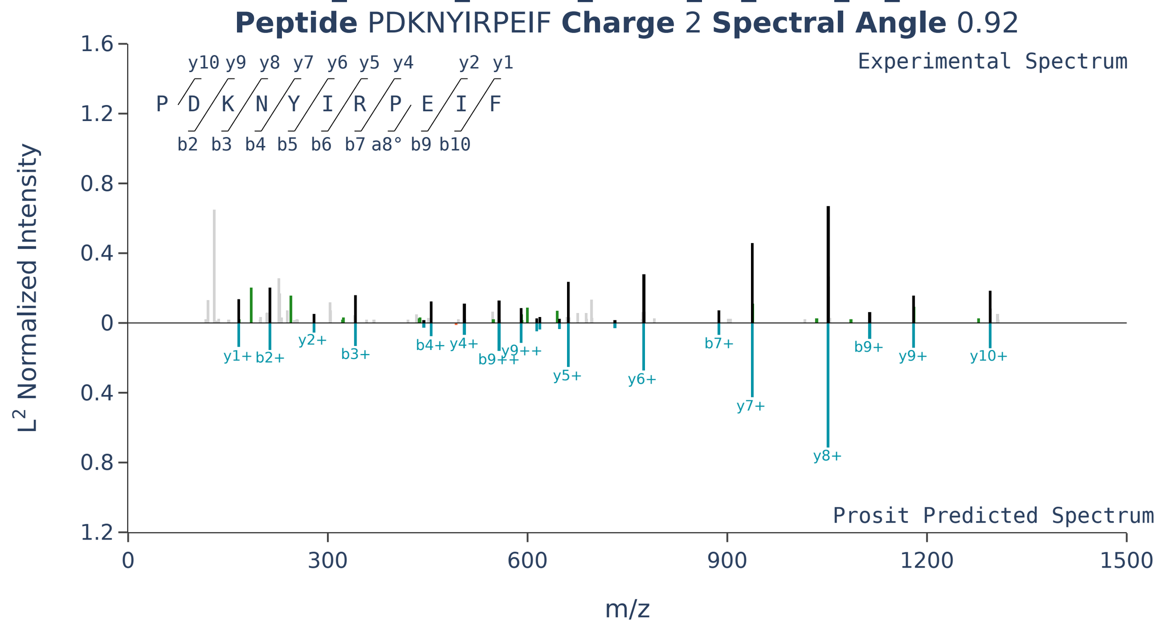


**Source** WSoh_101121_151121_HFGoe_G1_IL37b_24h_R2 **Scan** 8835
**Peptide** IAVPDKN **Charge** 1 **Spectral Angle** 0.96

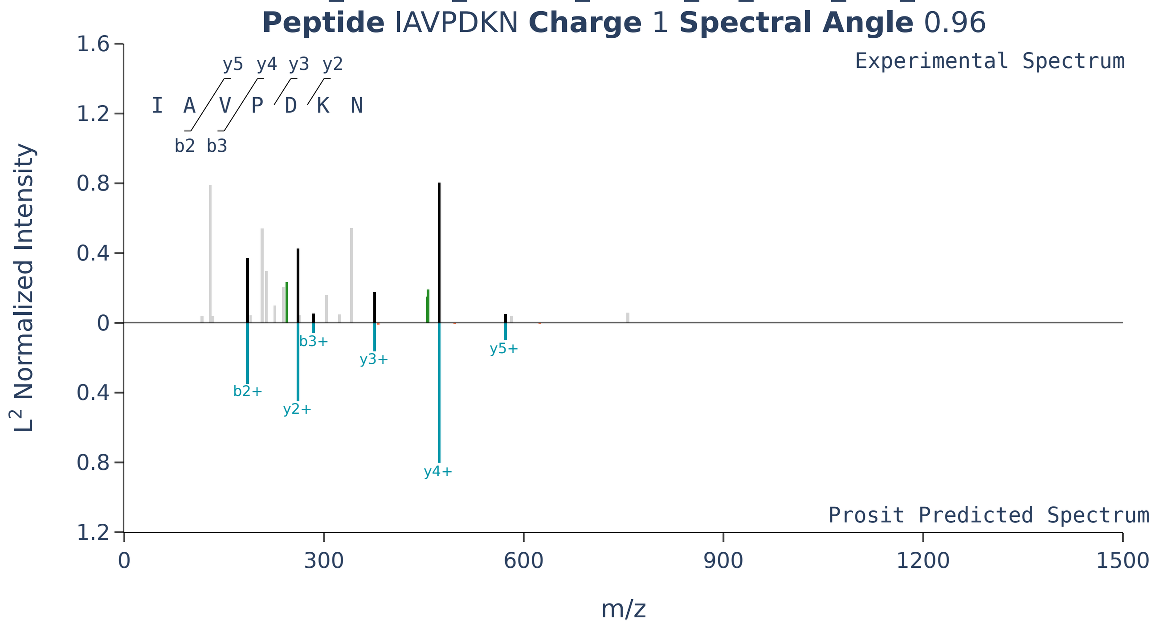


**Source** WSoh_101121_151121_HFGoe_G1_IL37b_2h_R1 **Scan** 13059
**Peptide** SCNCNEPVGV **Charge** 2 **Spectral Angle** 0.93

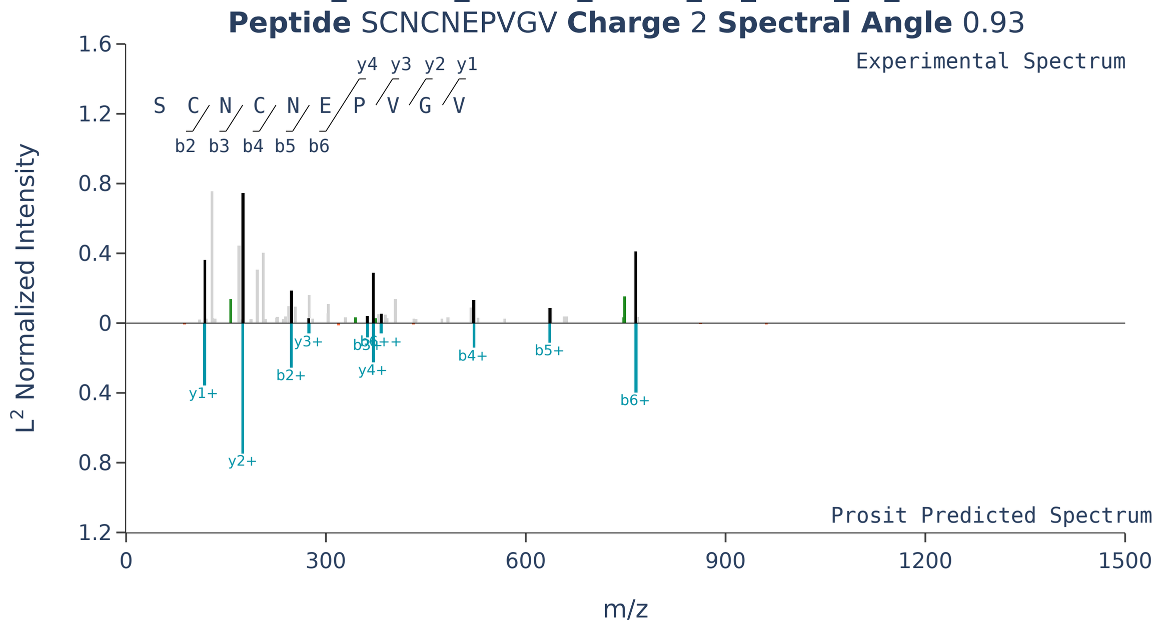


**Source** WSoh_101121_151121_HFGoe_G2_IL37b_24h_R2 **Scan** 7975
**Peptide** VPDKNYIR **Charge** 2 **Spectral Angle** 0.9

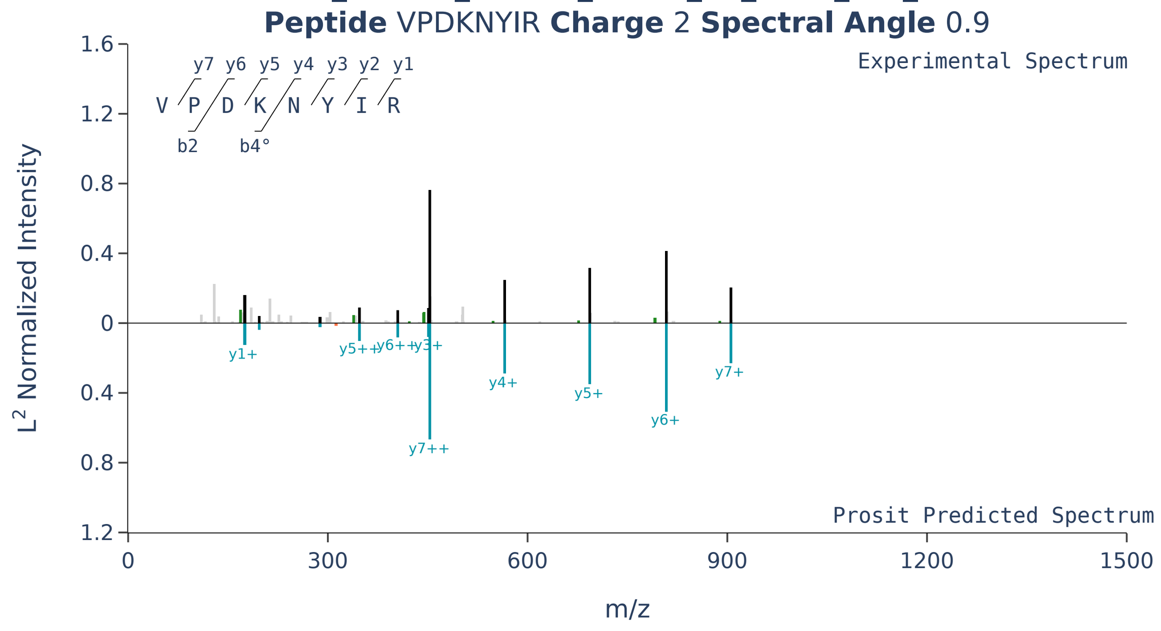


**Source** WSoh_101121_151121_HFGoe_G2_IL37b_24h_R1 **Scan** 16628
**Peptide** SHPSLQL **Charge** 2 **Spectral Angle** 0.93

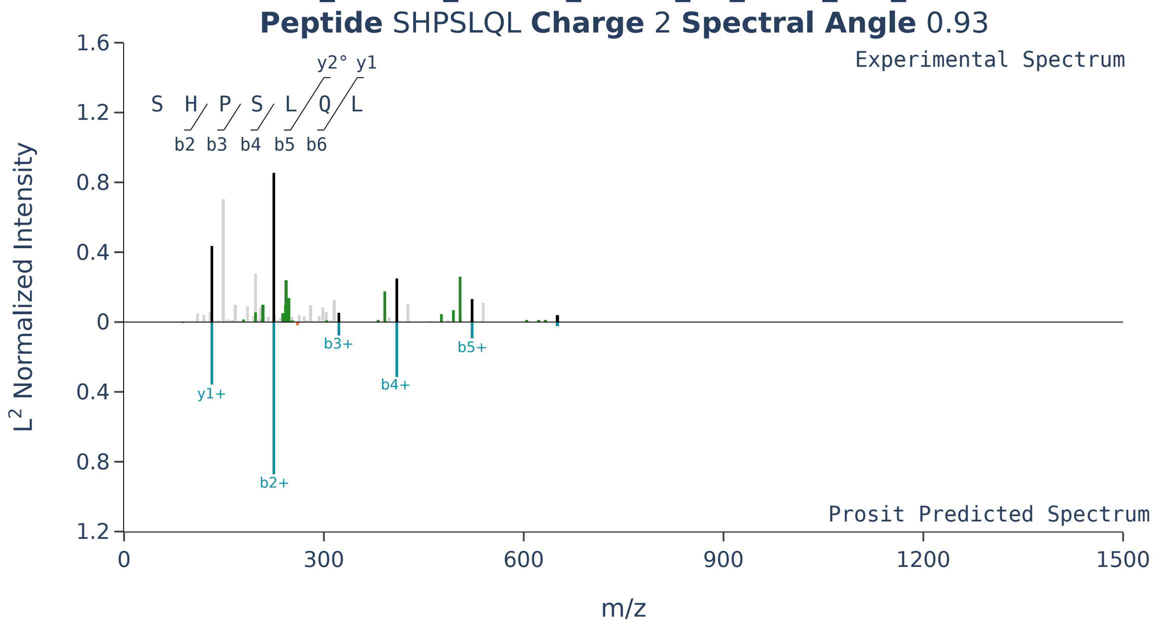


**Source** WSoh_101121_151121_HFGoe_G1_IL37b_24h_R2 **Scan** 12300
**Peptide** ALASSLSSASAEKG **Charge** 2 **Spectral Angle** 0.91

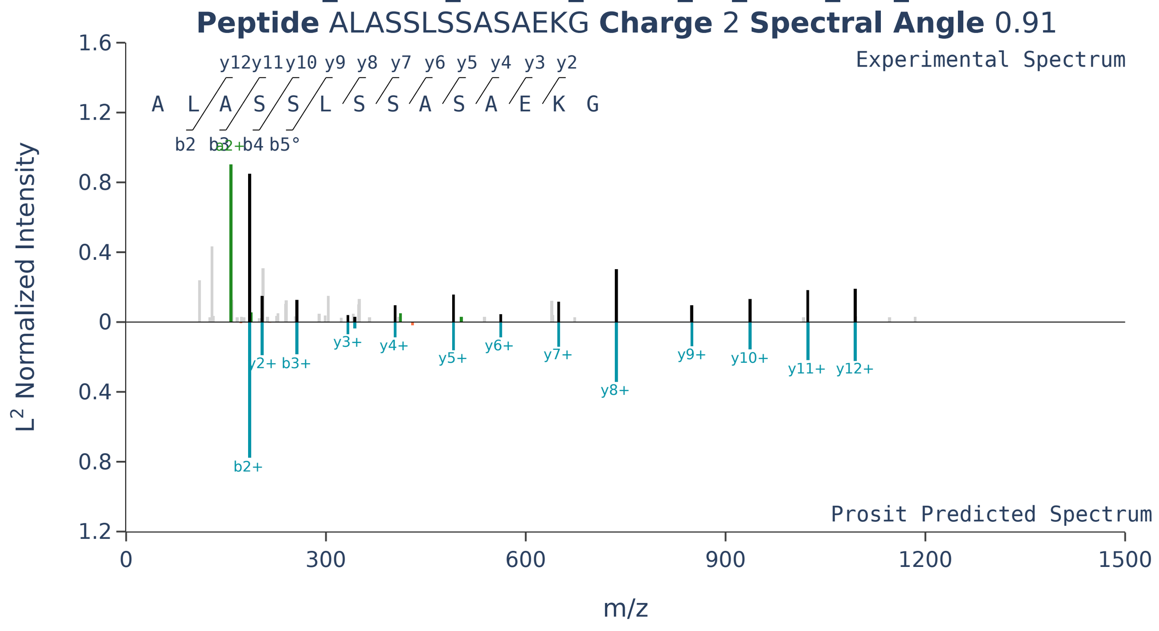


**Source** WSoh_101121_151121_HFGoe_G2_IL37b_24h_R1 **Scan** 10962
**Peptide** AVPDKNYIRPE **Charge** 2 **Spectral Angle** 0.91

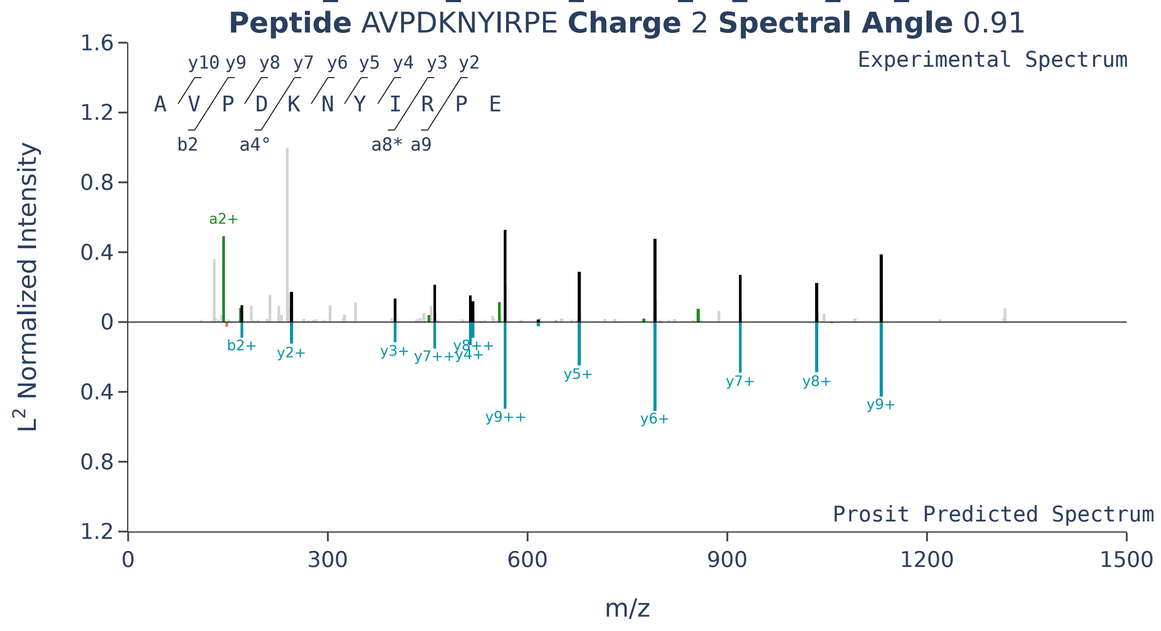


**Source** WSoh_101121_151121_HFGoe_G1_IL37b_24h_R2 **Scan** 15053
**Peptide** PDKNYIRPEI **Charge** 2 **Spectral Angle** 0.92

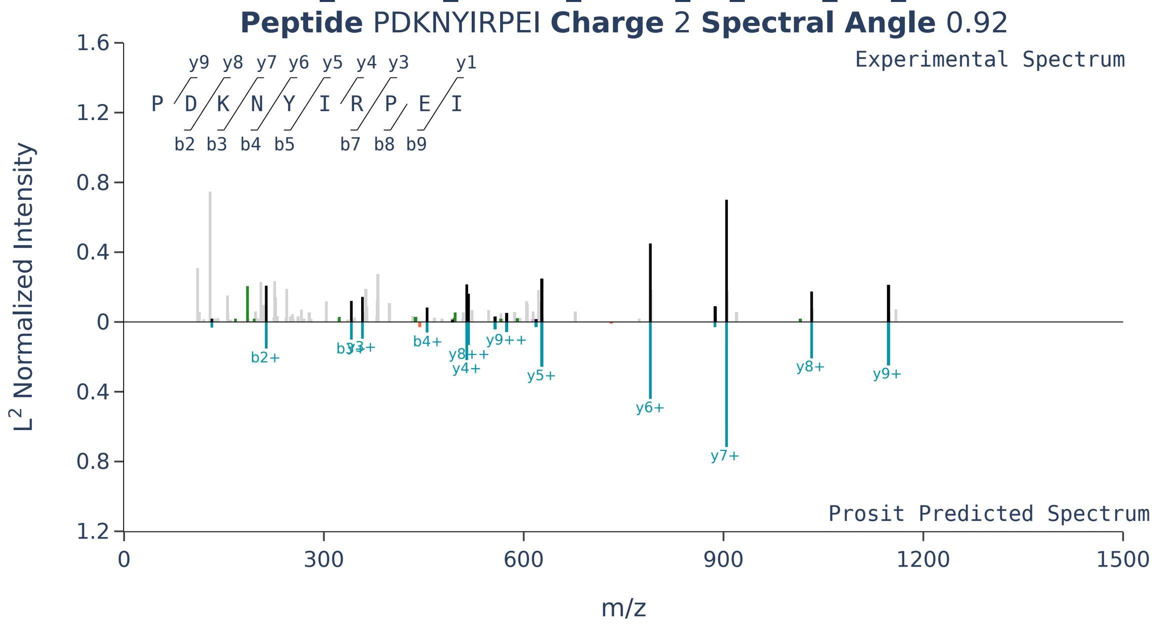


**Source** WSoh_101121_151121_HFGoe_G2_IL37b_24h_R1 **Scan** 18819
**Peptide** FSFQPVCK **Charge** 2 **Spectral Angle** 0.94

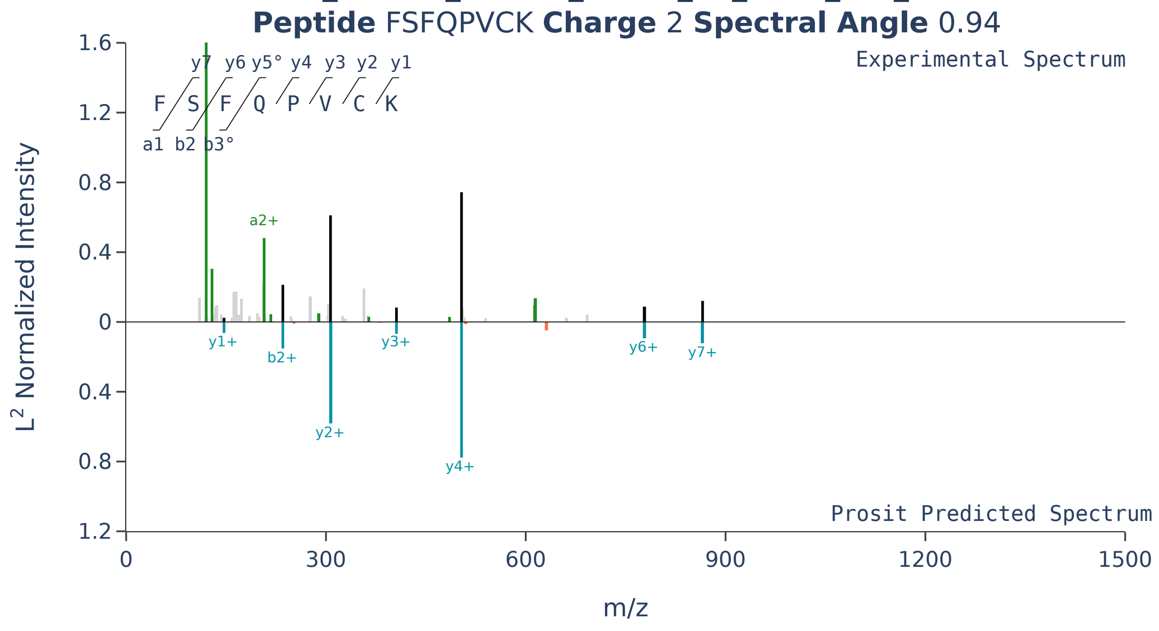


**Source** WSoh_101121_151121_HFGoe_G2_IL37b_24h_R2 **Scan** 10997
**Peptide** SFQPVCKA **Charge** 2 **Spectral Angle** 0.94

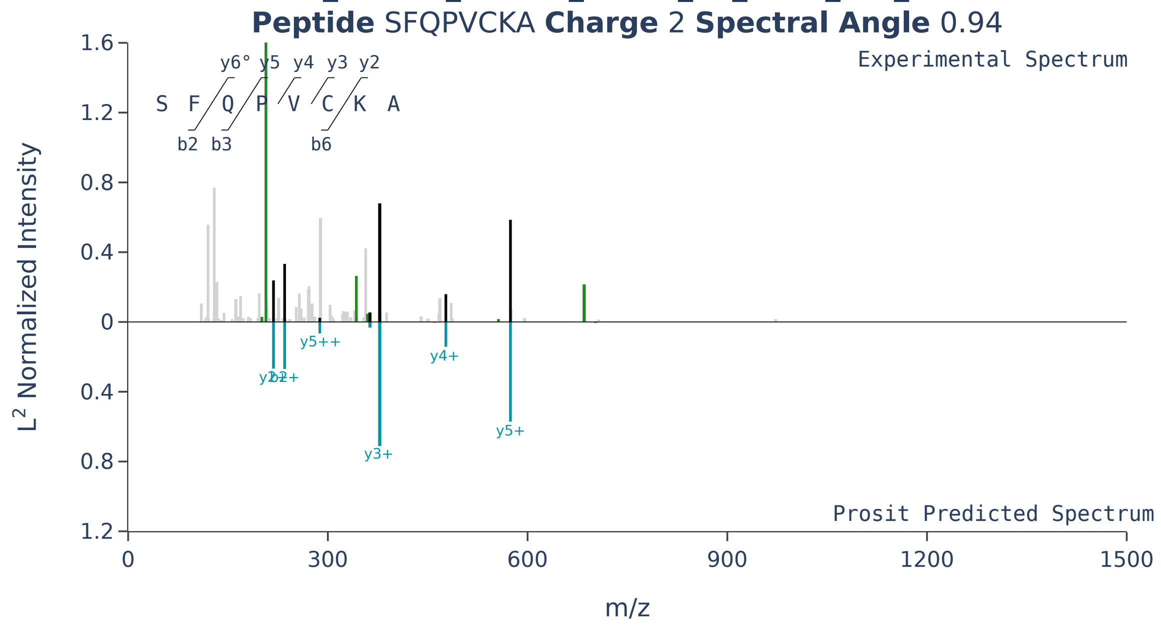

**Source** WSoh_101121_151121_HFGoe_G1_IL37b_4h_R1 **Scan** 13469
**Peptide** LIAVPDKN **Charge** 2 **Spectral Angle** 0.91

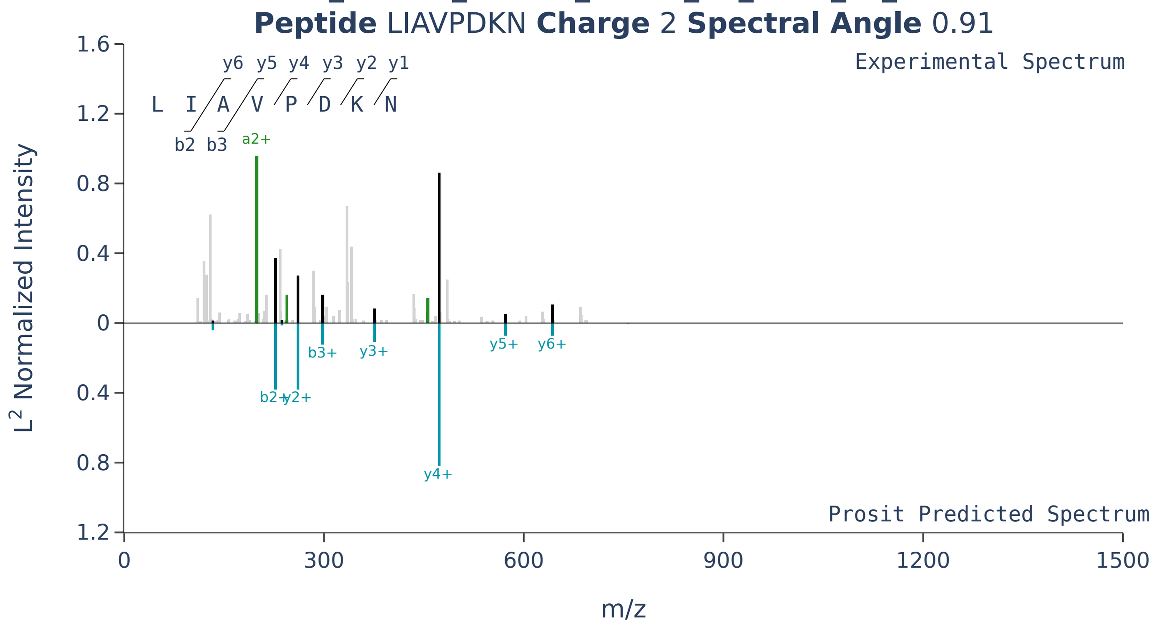


**Source** WSoh_101121_151121_HFGoe_G2_IL37b_24h_R2 **Scan** 7902
**Peptide** LMKLAAQKESAR **Charge** 3 **Spectral Angle** 0.91

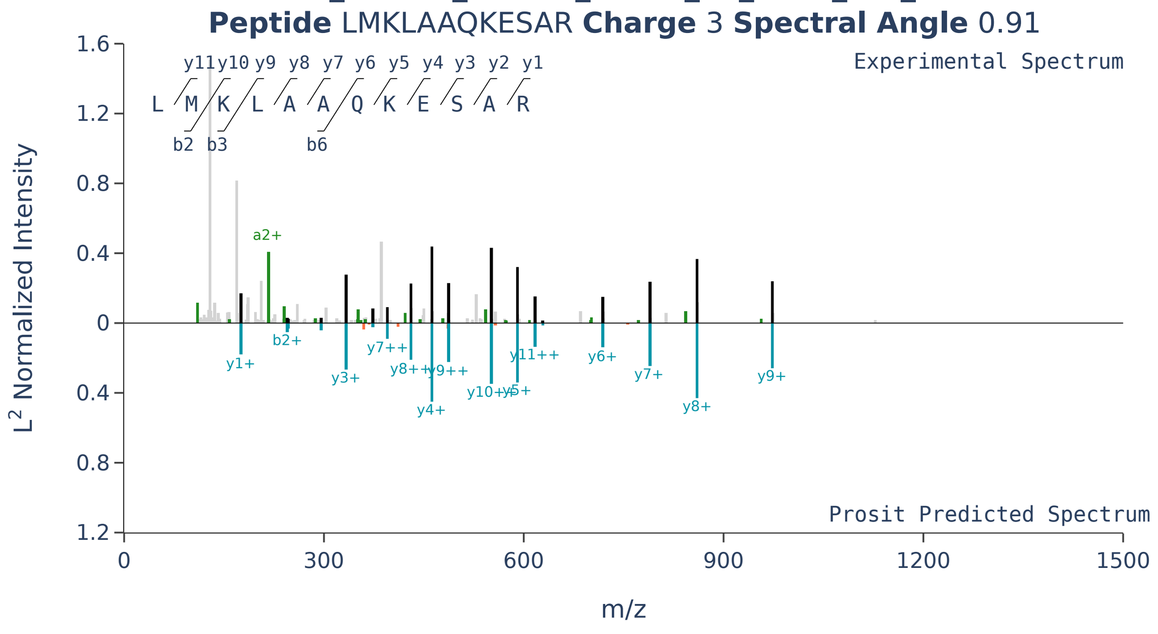


**Source** WSoh_101121_151121_HFGoe_G2_IL37b_2h_R1 **Scan** 16056
**Peptide** NLIAVPDKN **Charge** 2 **Spectral Angle** 0.93

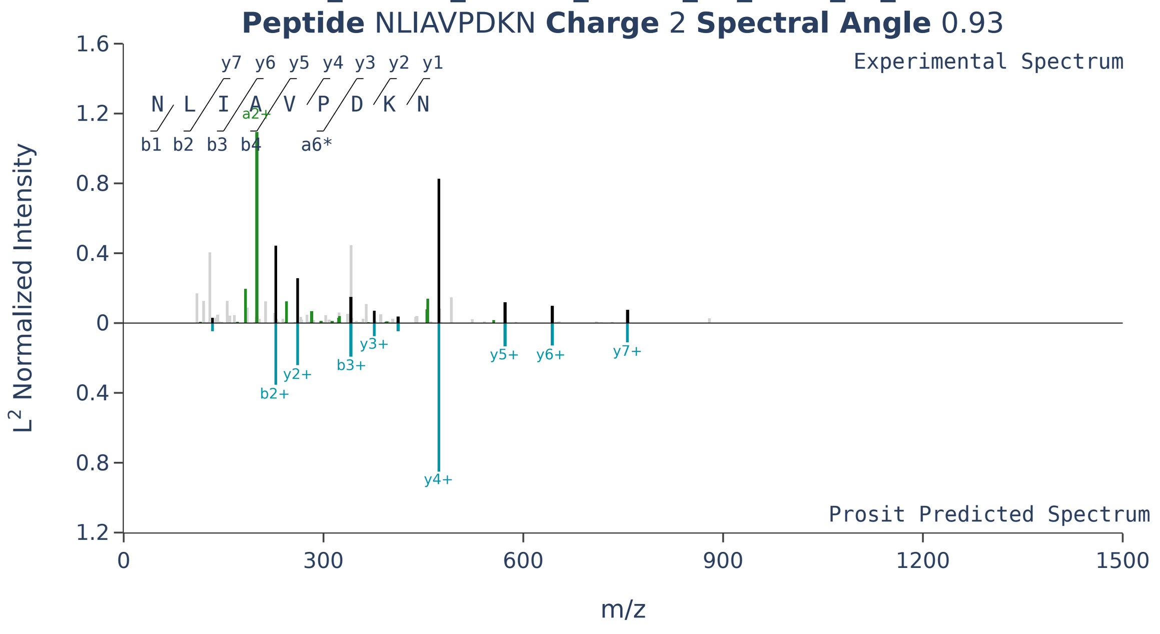


**Source** WSoh_101121_151121_HFGoe_G2_IL37b_4h_R1 **Scan** 6273
**Peptide** SSLSSASAEKG **Charge** 2 **Spectral Angle** 0.89

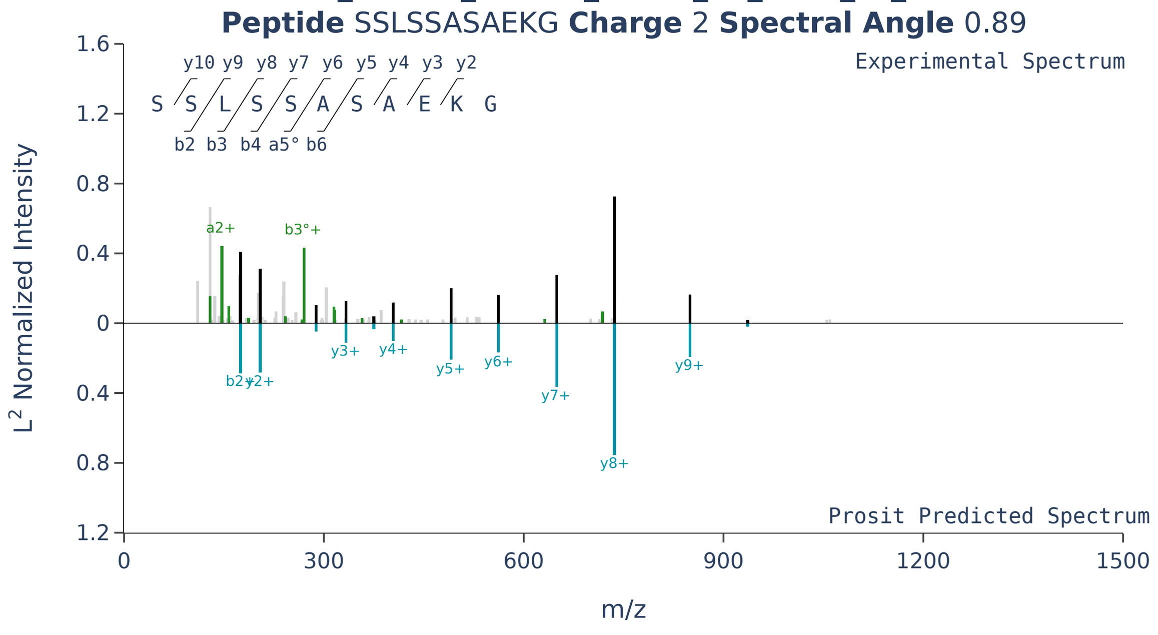


**Source** WSoh_101121_151121_HFGoe_G1_IL37b_24h_R2 **Scan** 11324
**Peptide** PVGVTDKFENR **Charge** 3 **Spectral Angle** 0.89

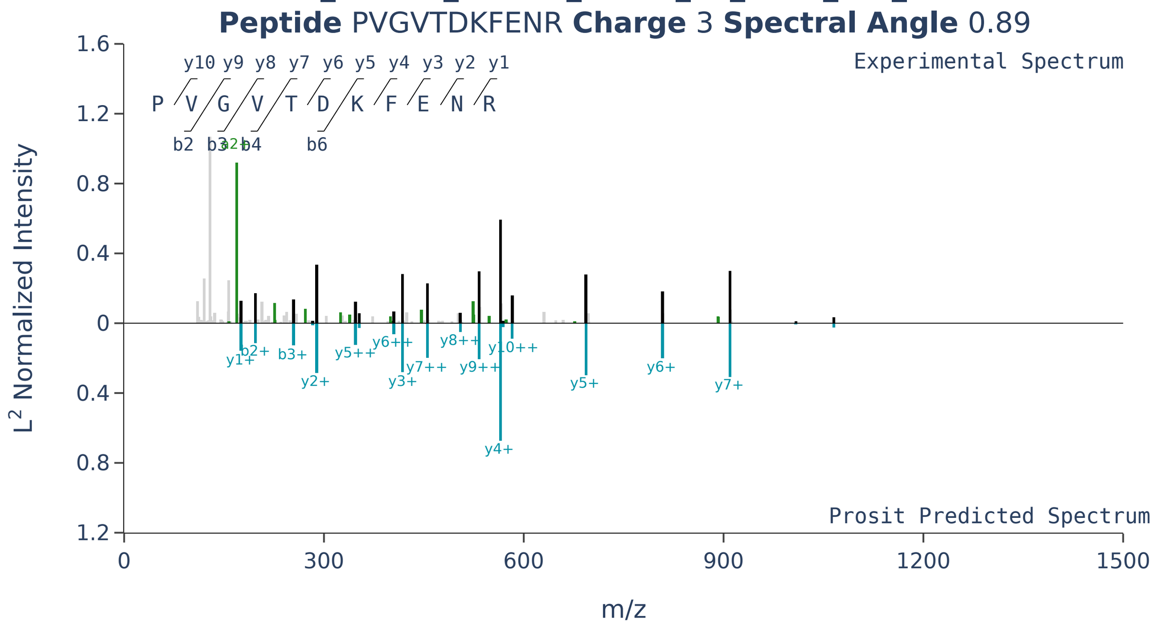


**Source** WSoh_101121_151121_HFGoe_G2_IL37b_24h_R1 **Scan** 11636
**Peptide** NCNEPVGVTD **Charge** 2 **Spectral Angle** 0.92

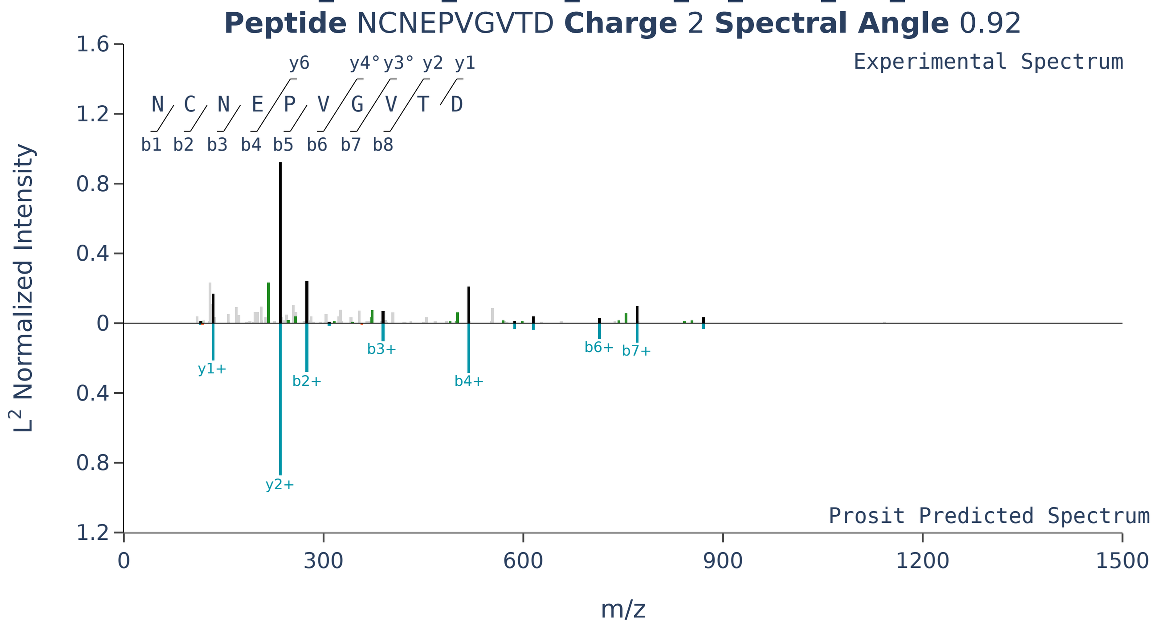


**Source** WSoh_101121_151121_HFGoe_G1_IL37b_24h_R2 **Scan** 16001
**Peptide** SGNLIAVPDKN **Charge** 2 **Spectral Angle** 0.94

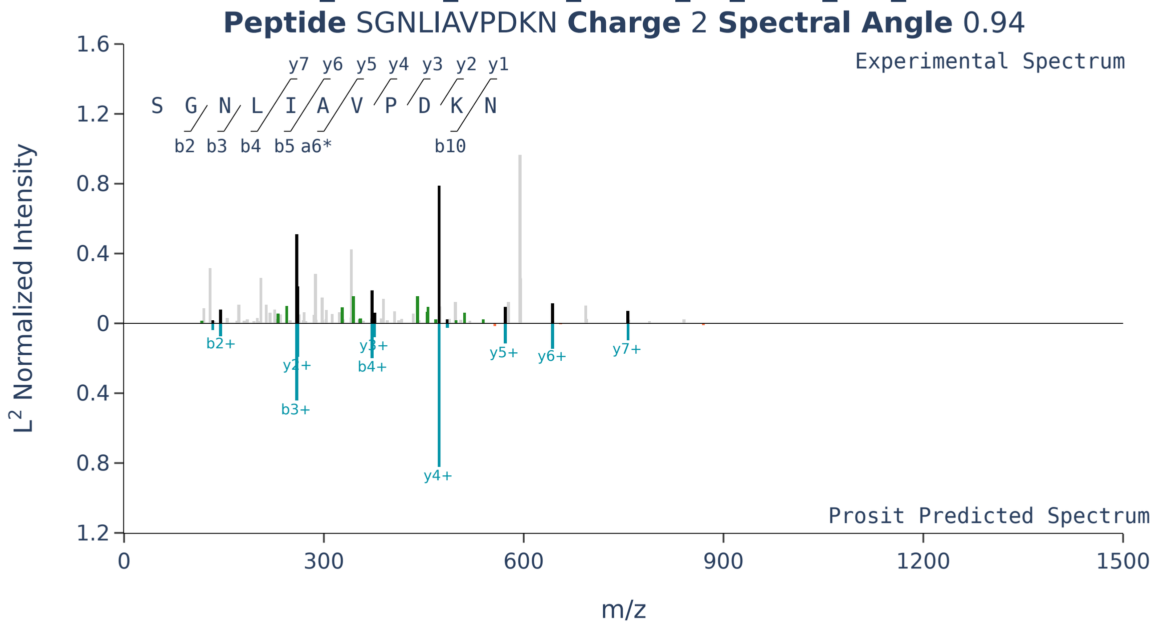


**Source** WSoh_101121_151121_HFGoe_G2_IL37b_24h_R2 **Scan** 11271
**Peptide** PSLQLKKEKL **Charge** 2 **Spectral Angle** 0.91

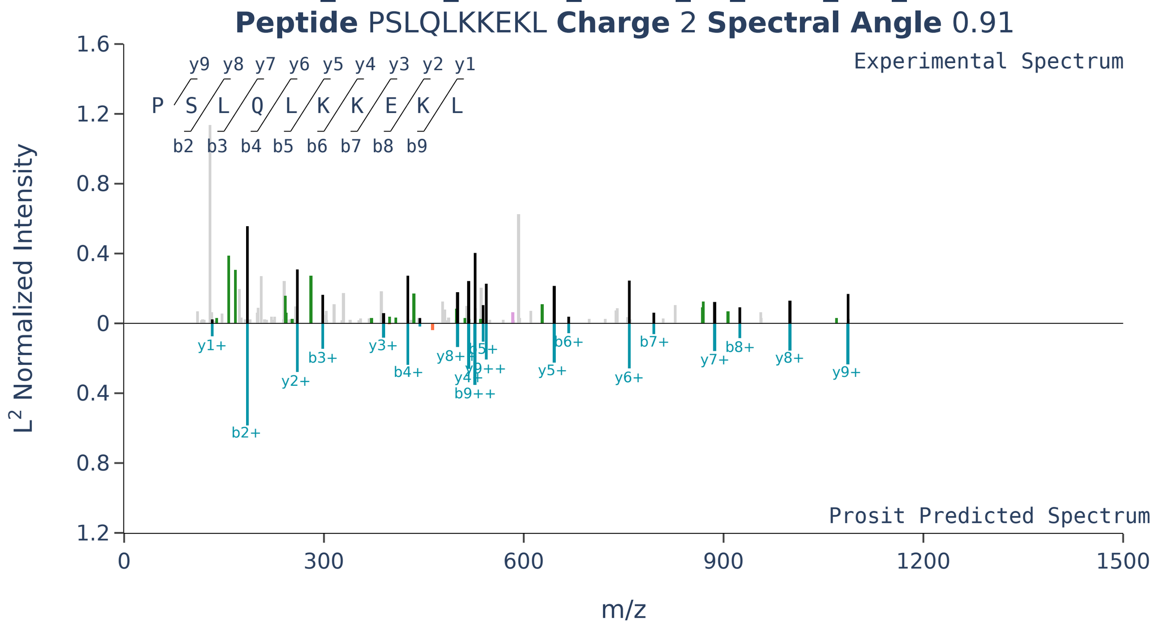


**Source** WSoh_101121_151121_HFGoe_G1_IL37b_1h_R1 **Scan** 15472
**Peptide** PVGVTDKF **Charge** 2 **Spectral Angle** 0.89

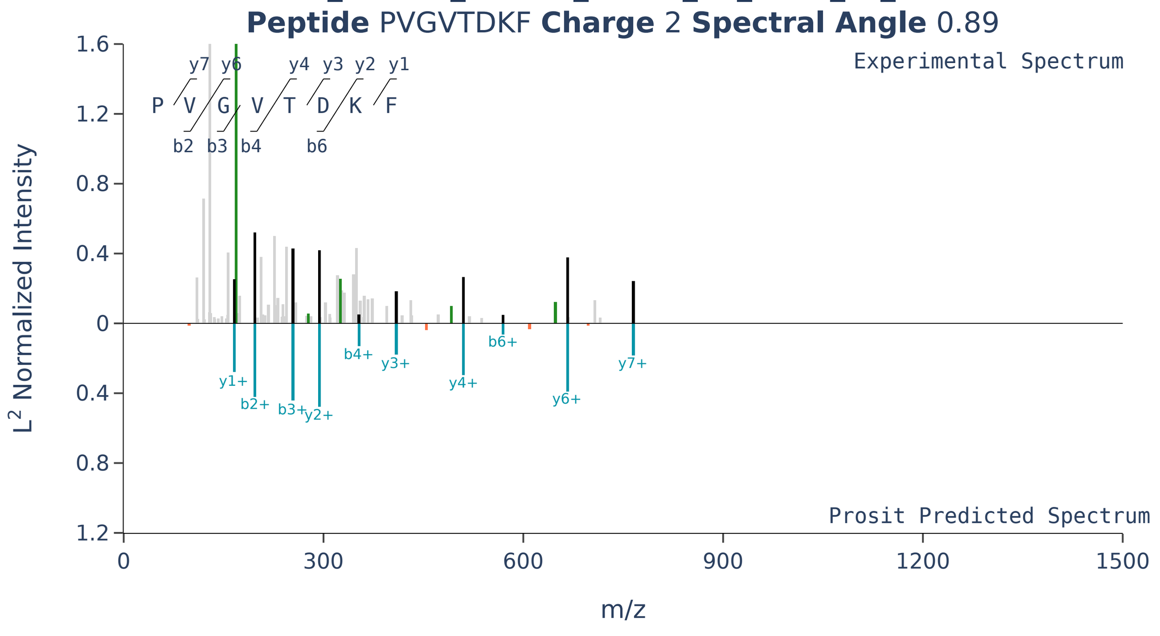


**Source** WSoh_101121_151121_HFGoe_G2_IL37b_1h_R2 **Scan** 13673
**Peptide** SAEKGSPIL **Charge** 2 **Spectral Angle** 0.92

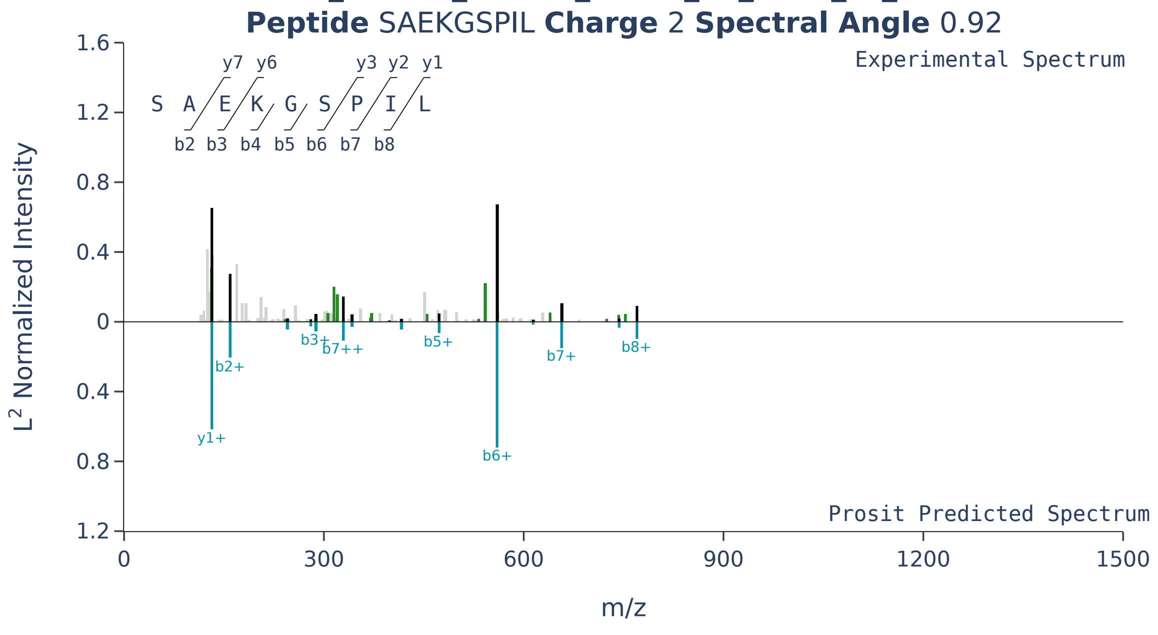


**Source** WSoh_101121_151121_HFGoe_G2_IL37b_24h_R1 **Scan** 9741
**Peptide** SFQPVCK **Charge** 1 **Spectral Angle** 0.94

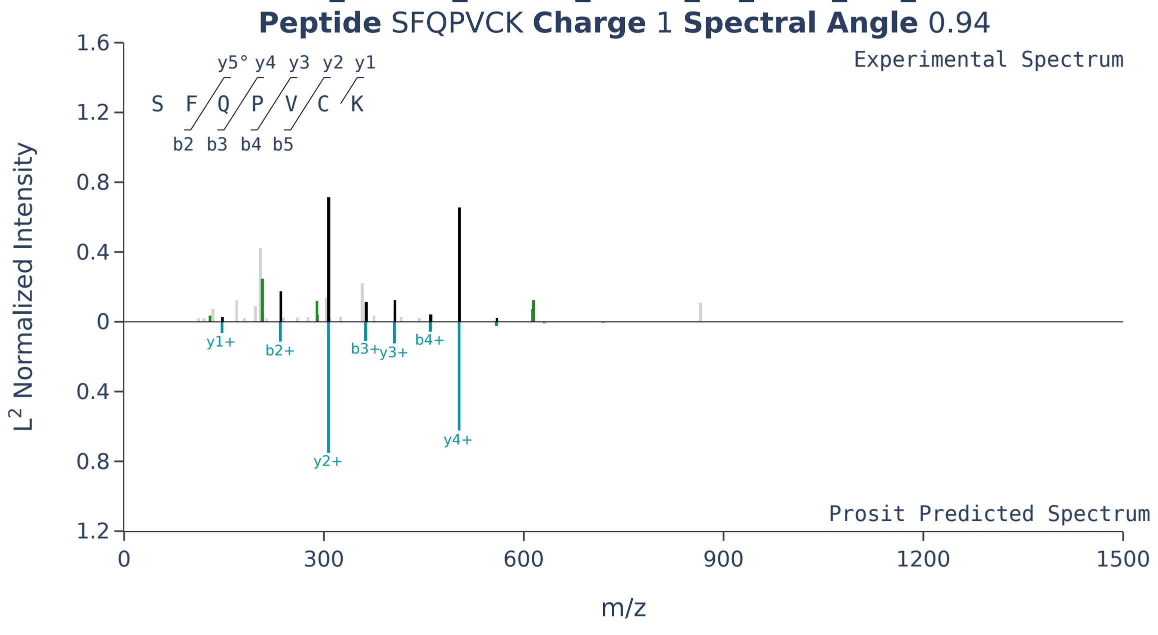


**Source** WSoh_101121_151121_HFGoe_G1_IL37b_1h_R1 **Scan** 17281
**Peptide** DSGNLIAVPDKN **Charge** 2 **Spectral Angle** 0.96

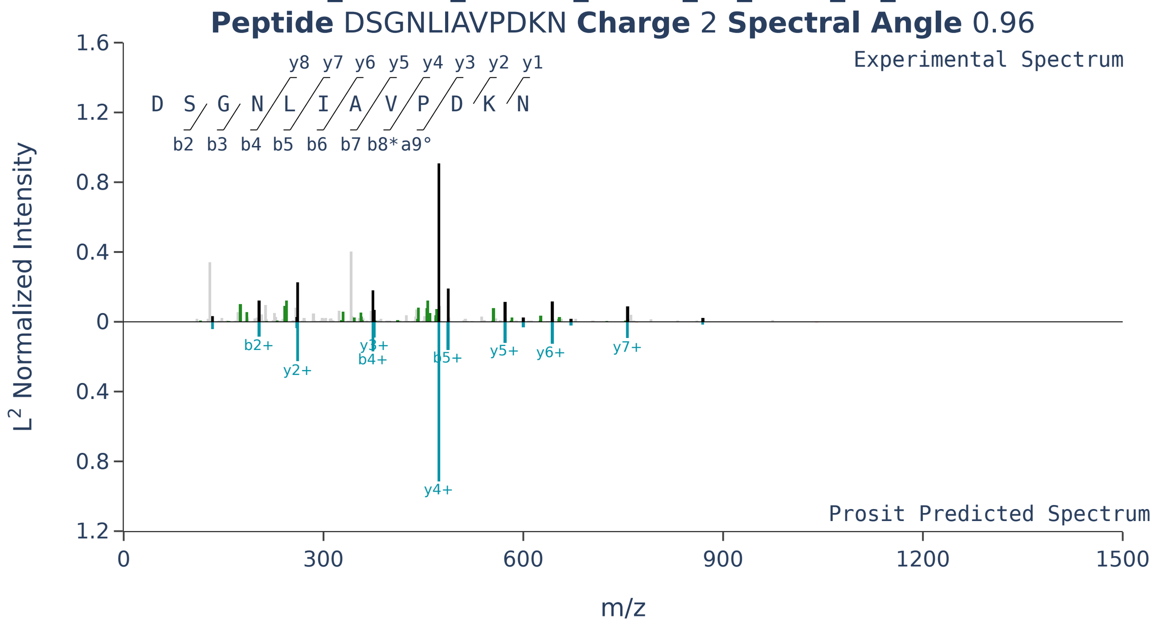


**Source** WSoh_101121_151121_HFGoe_G1_IL37b_1h_R2 **Scan** 7742
**Peptide** VTDKFEN **Charge** 2 **Spectral Angle** 0.89

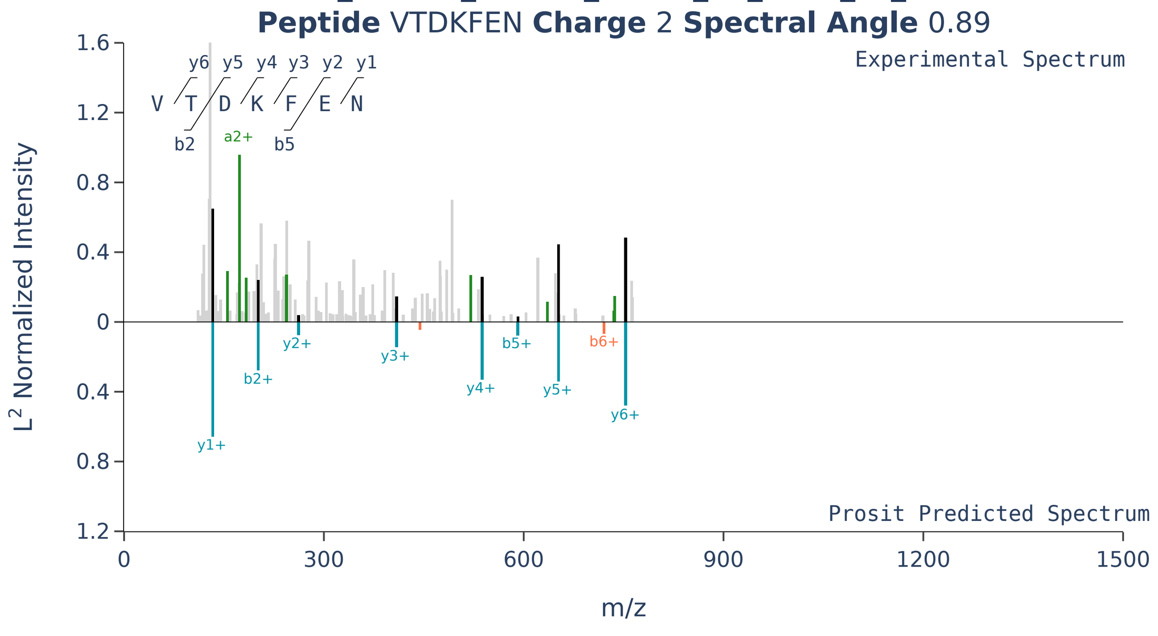


**Source** WSoh_101121_151121_HFGoe_G2_IL37b_4h_R1 **Scan** 10924
**Peptide** KLMKLAA **Charge** 2 **Spectral Angle** 0.92

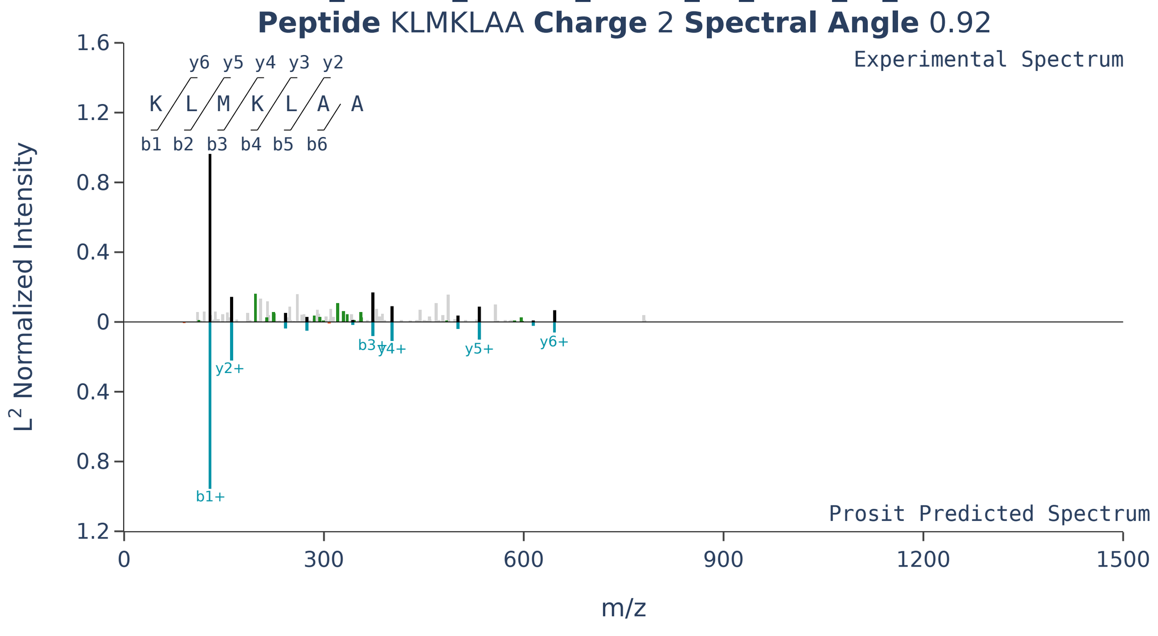


**Source** WSoh_101121_151121_HFGoe_G1_IL37b_24h_R1 **Scan** 18998
**Peptide** SKGEFCLYC **Charge** 2 **Spectral Angle** 0.96

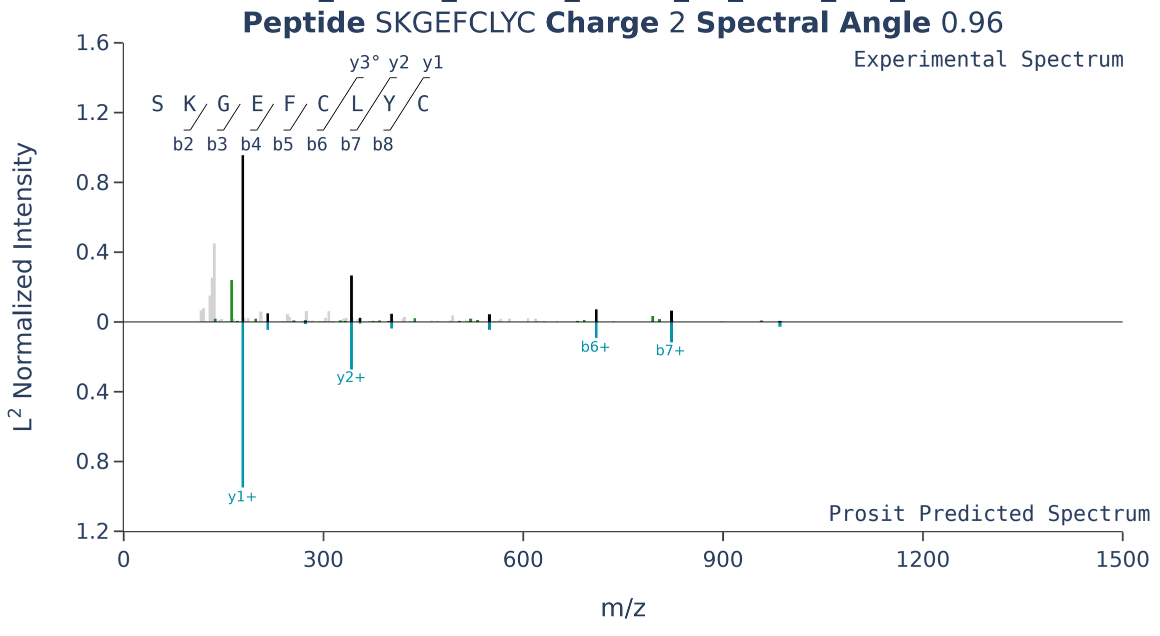

**Source** WSoh_101121_151121_HFGoe_G1_IL37b_1h_R1 **Scan** 20599
**Peptide** EKGSPILL **Charge** 2 **Spectral Angle** 0.92

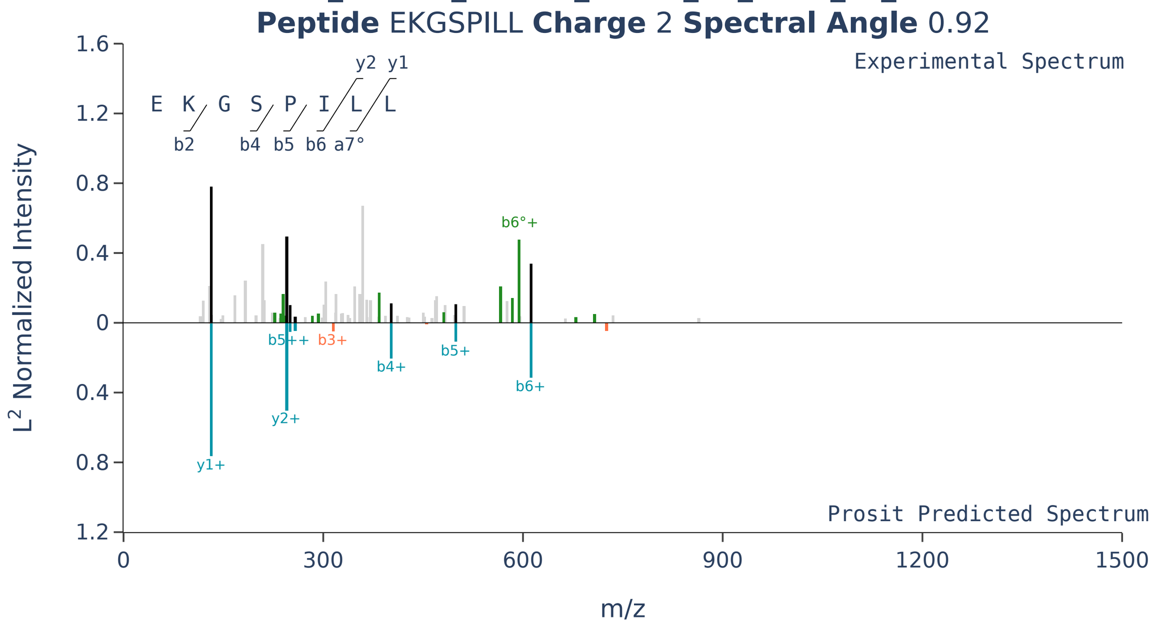


**Source** WSoh_101121_151121_HFGoe_G2_IL37b_24h_R1 **Scan** 8987
**Peptide** KAEMSPSEVSD **Charge** 1 **Spectral Angle** 0.92

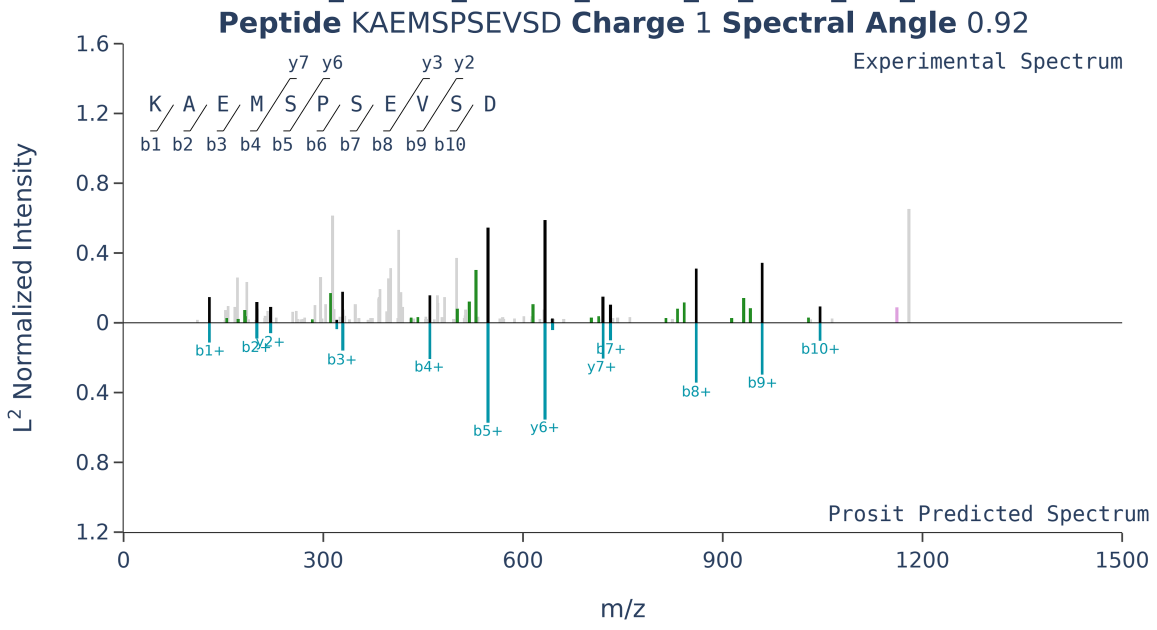


**Source** WSoh_101121_151121_HFGoe_G2_IL37b_24h_R1 **Scan** 9753
**Peptide** LASSLSSASAEKG **Charge** 2 **Spectral Angle** 0.89

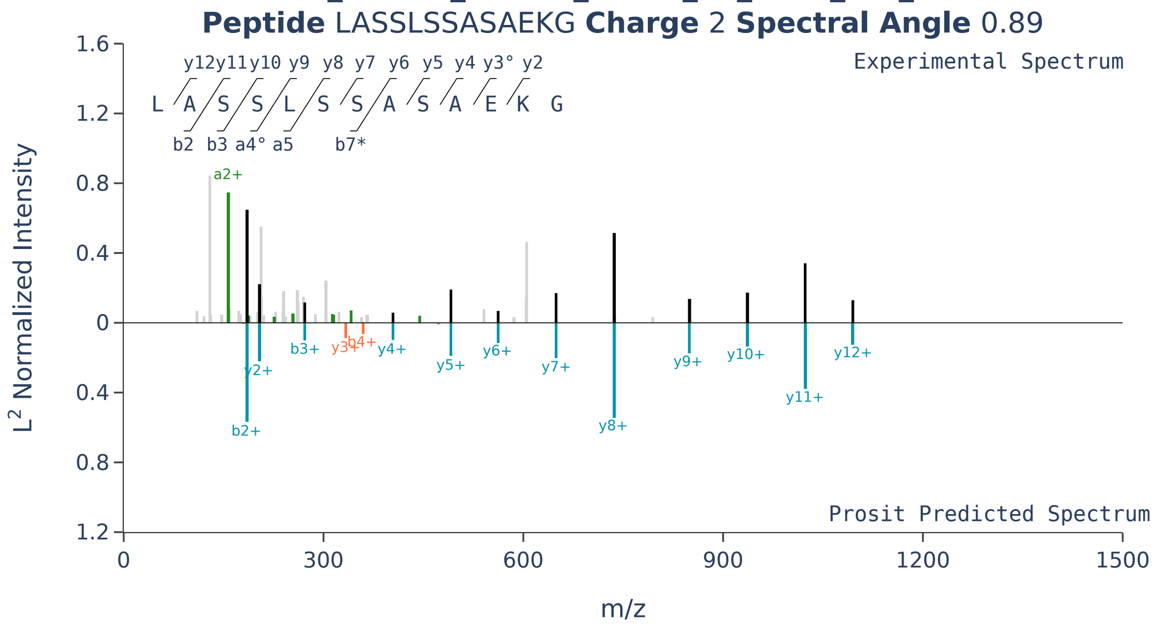


**Source** WSoh_101121_151121_HFGoe_G1_IL37b_1h_R1 **Scan** 22612
**Peptide** SAAHPGWFICTS **Charge** 2 **Spectral Angle** 0.93

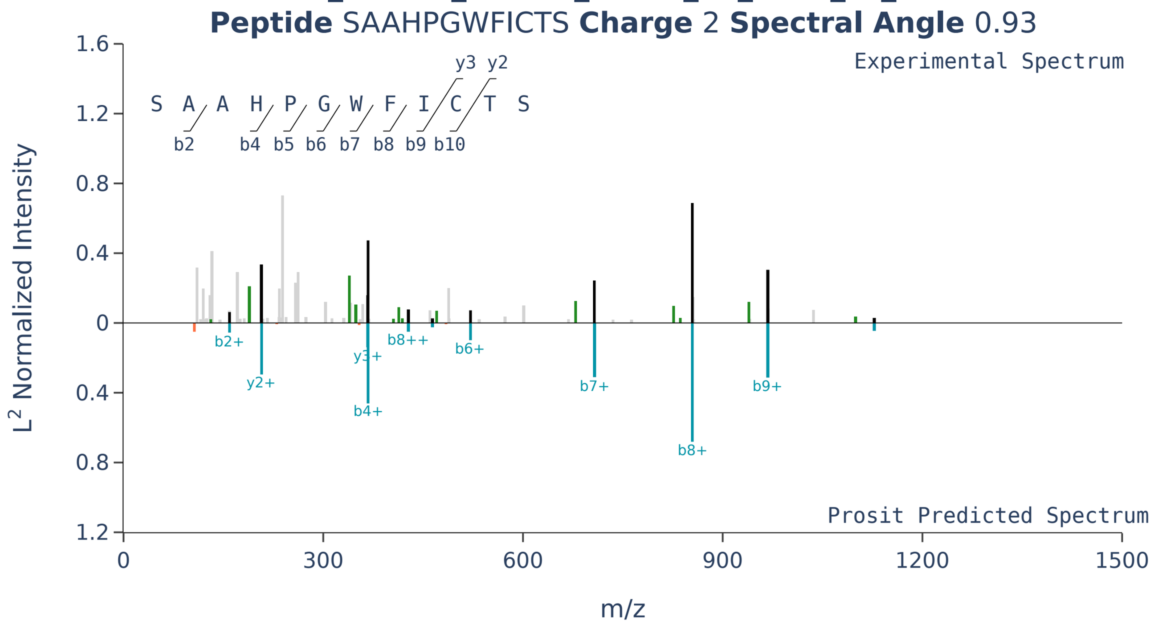


**Source** WSoh_101121_151121_HFGoe_G1_IL37b_24h_R1 **Scan** 13067
**Peptide** NCNEPVGV **Charge** 1 **Spectral Angle** 0.87

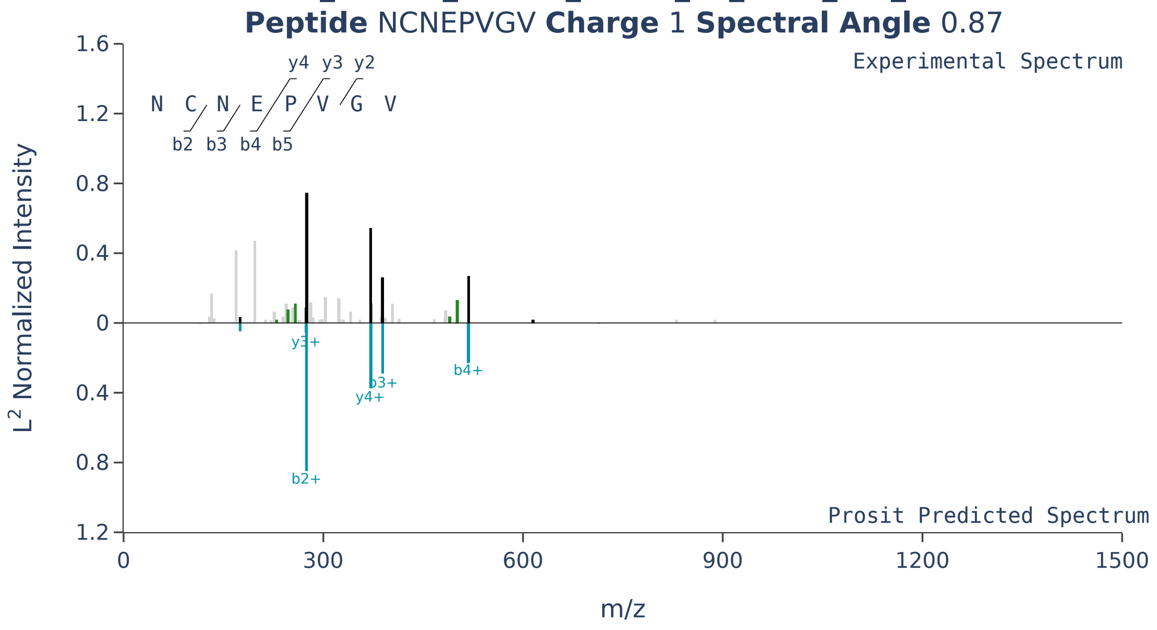


**Source** WSoh_101121_151121_HFGoe_G1_IL37b_24h_R1 **Scan** 16732
**Peptide** NEPVGVTDKF **Charge** 2 **Spectral Angle** 0.92

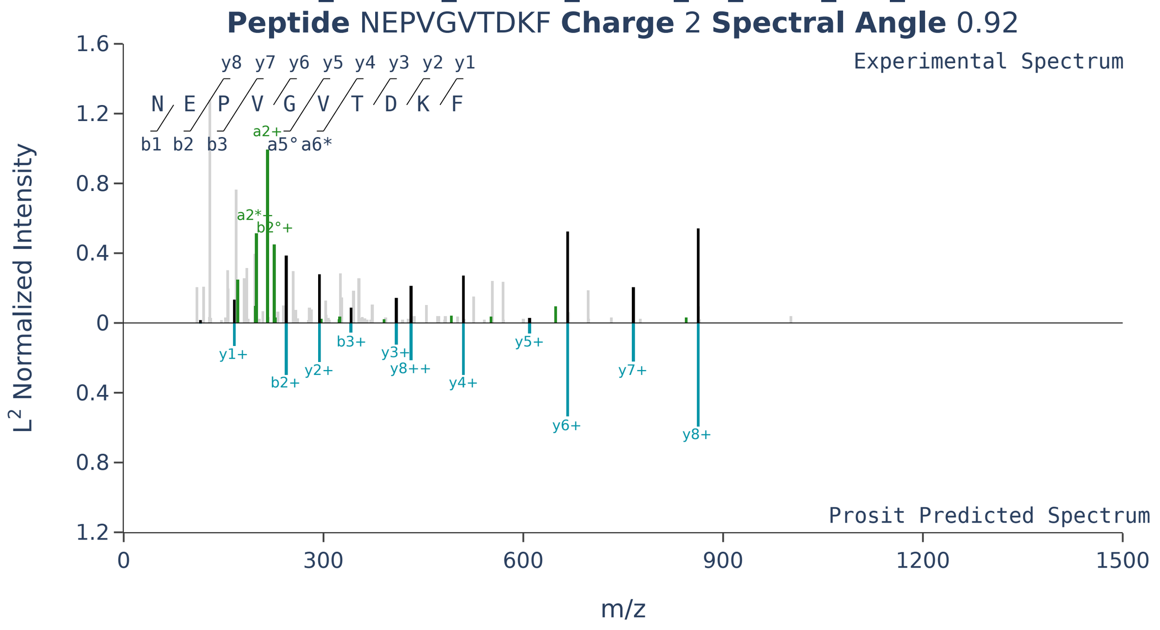


**Source** WSoh_101121_151121_HFGoe_G1_IL37b_4h_R1 **Scan** 9318
**Peptide** NLNPKKF **Charge** 2 **Spectral Angle** 0.87

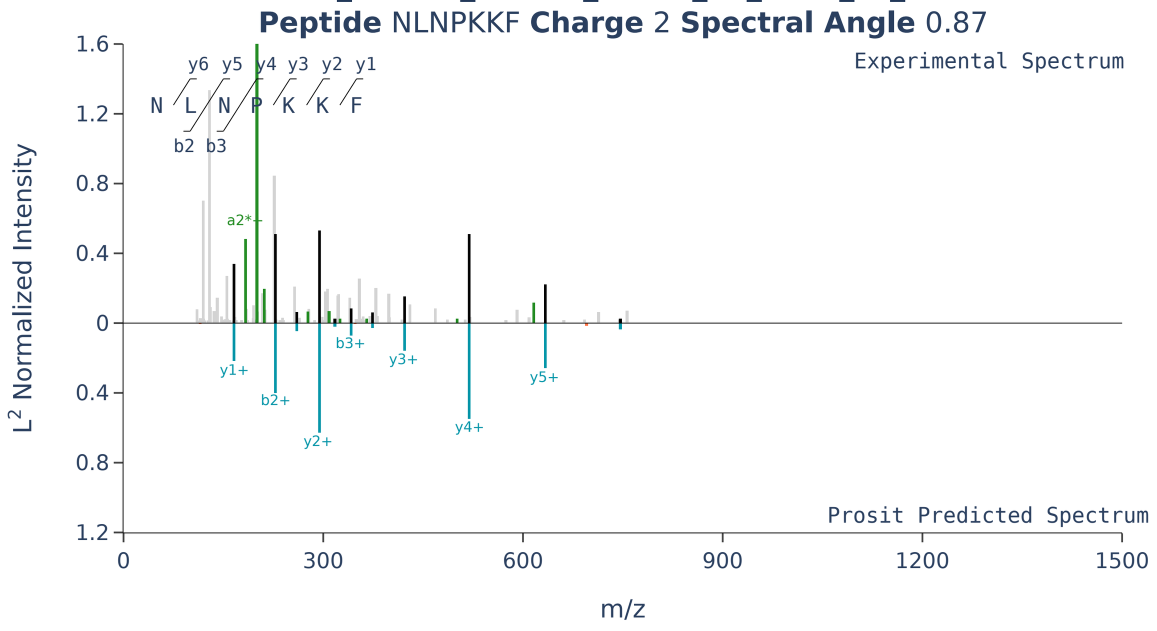


**Source** WSoh_101121_151121_HFGoe_G2_IL37b_24h_R1 **Scan** 13647
**Peptide** SCNCNEPVGVTDKFENR **Charge** 3 **Spectral Angle** 0.89

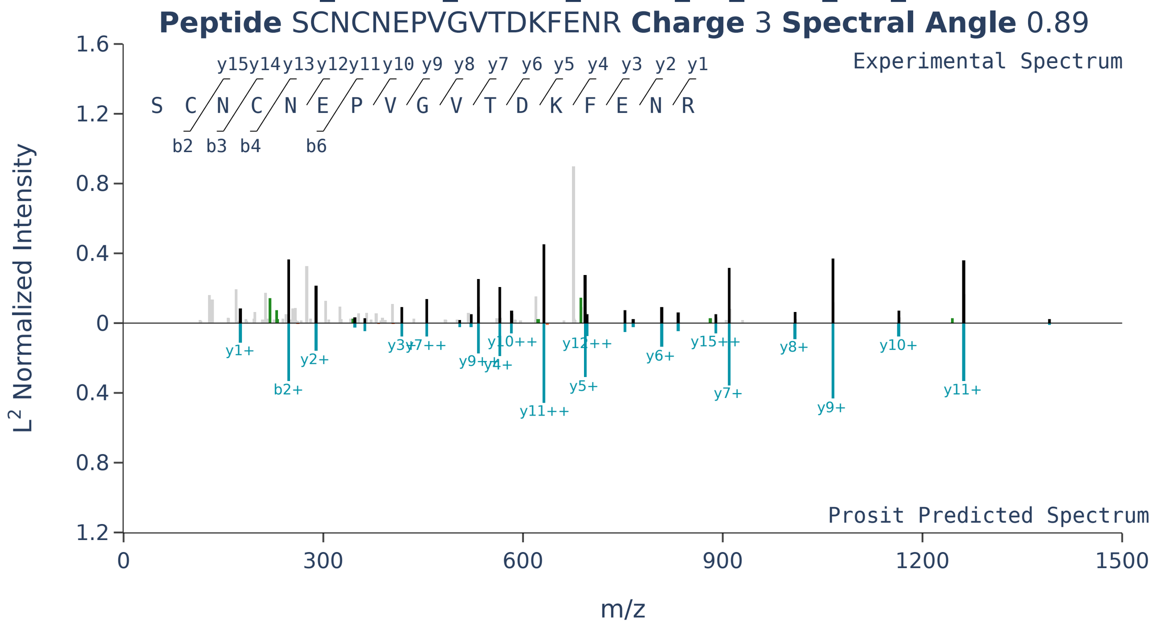


**Source** WSoh_101121_151121_HFGoe_G1_IL37b_1h_R2 **Scan** 16966
**Peptide** SCNCNEPVGVTDKF **Charge** 2 **Spectral Angle** 0.93

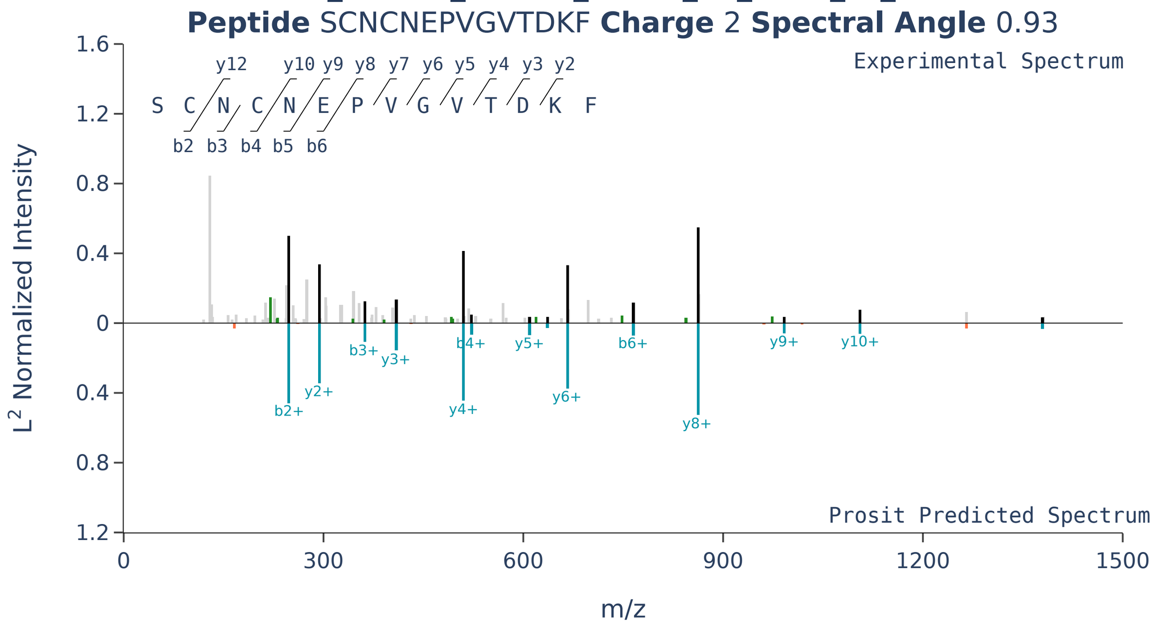


**Source** WSoh_101121_151121_HFGoe_G1_IL37b_2h_R1 **Scan** 16112
**Peptide** KGSPILLGVSK **Charge** 3 **Spectral Angle** 0.86

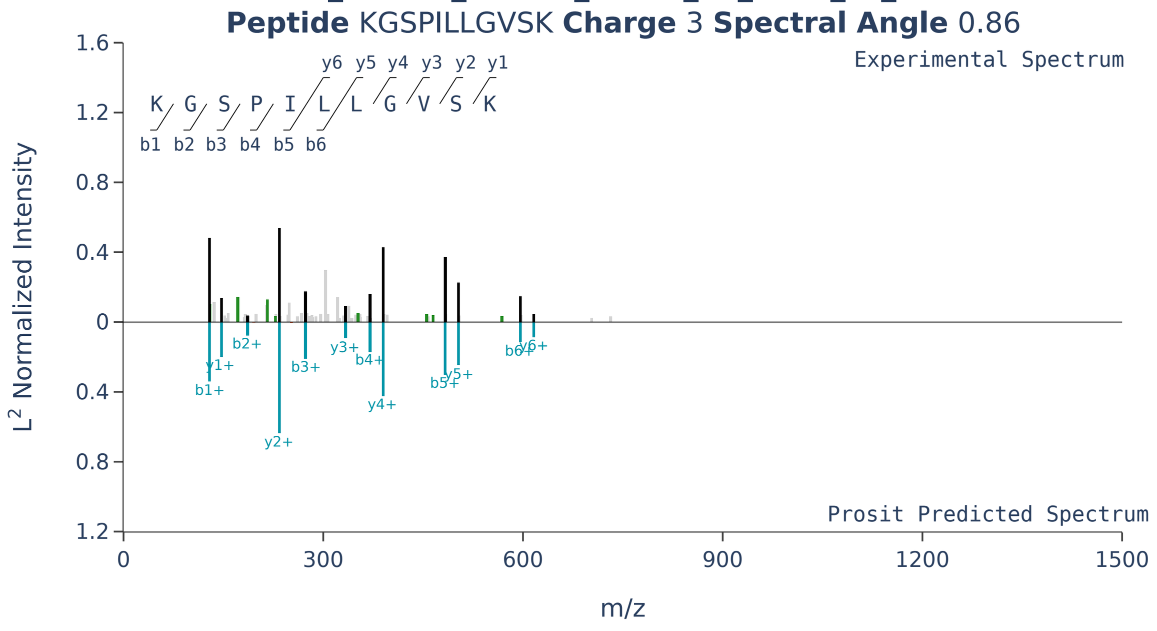


**Source** WSoh_101121_151121_HFGoe_G1_IL37b_24h_R2 **Scan** 6858
**Peptide** YCDKDKGQSHPSL **Charge** 2 **Spectral Angle** 0.94

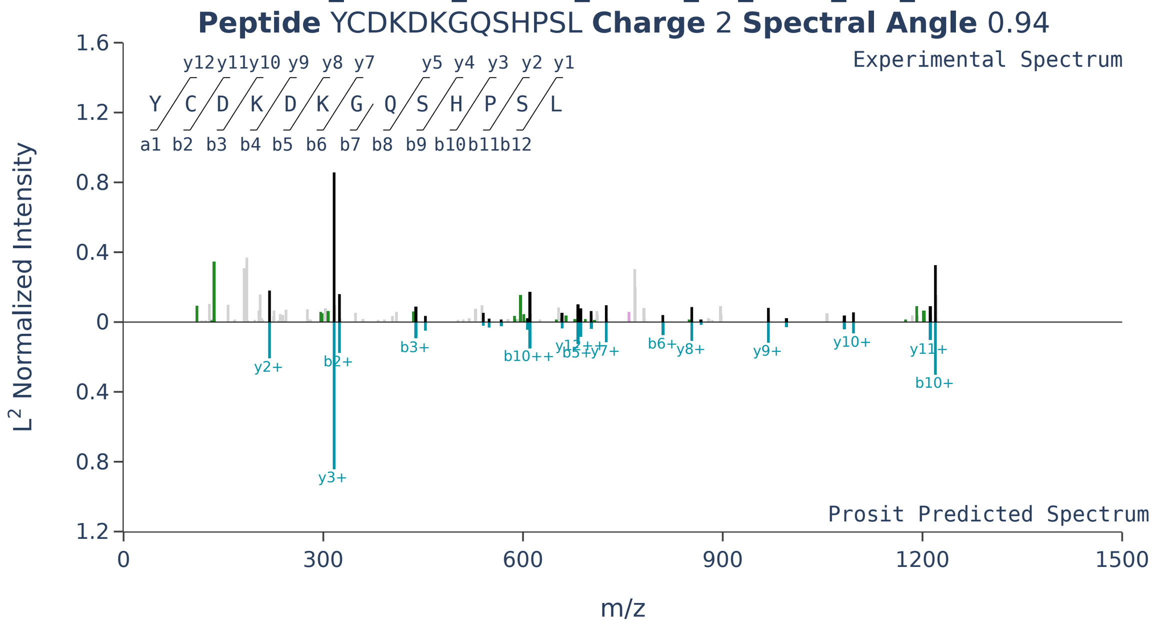


**Source** WSoh_101121_151121_HFGoe_G2_IL37b_24h_R2 **Scan** 10257
**Peptide** RAQVGSWN **Charge** 2 **Spectral Angle** 0.89

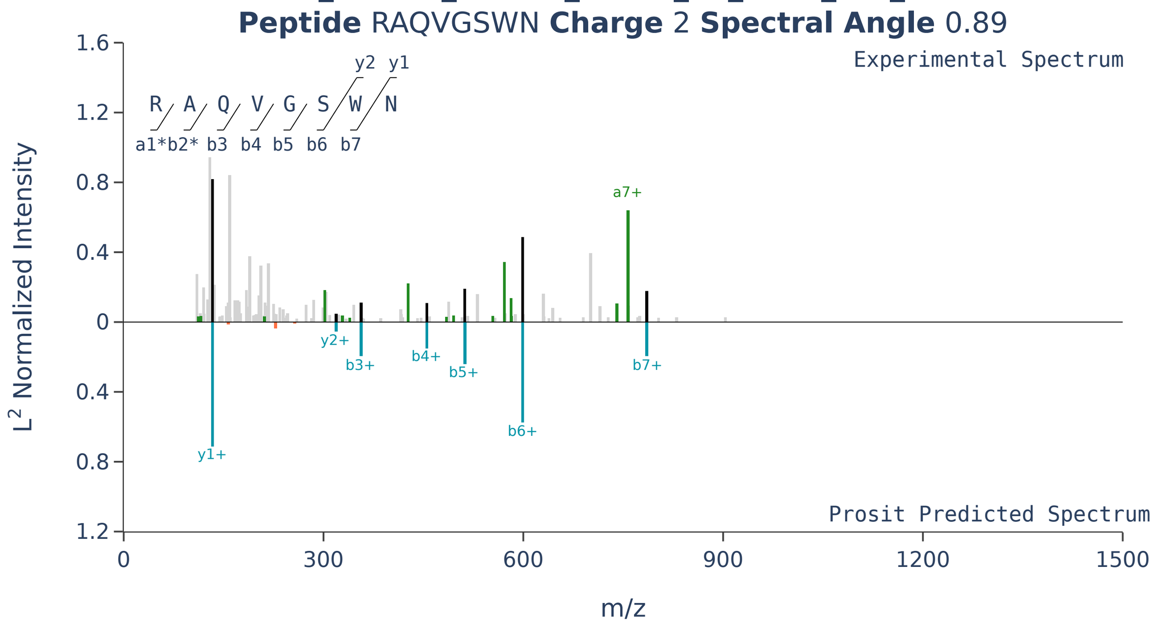


**Source** WSoh_101121_151121_HFGoe_G1_IL37b_4h_R1 **Scan** 7859
**Peptide** SSLSSASAE **Charge** 2 **Spectral Angle** 0.95

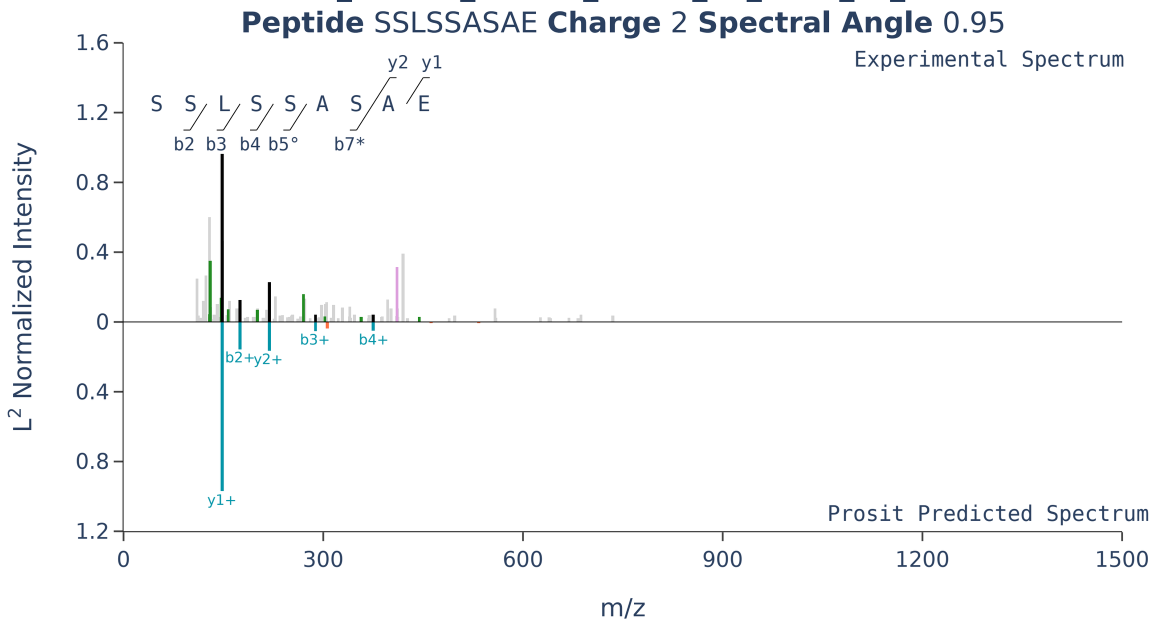


**Source** WSoh_101121_151121_HFGoe_G1_IL37b_2h_R1 **Scan** 7510
**Peptide** PVGVTDKFENRKH **Charge** 2 **Spectral Angle** 0.88

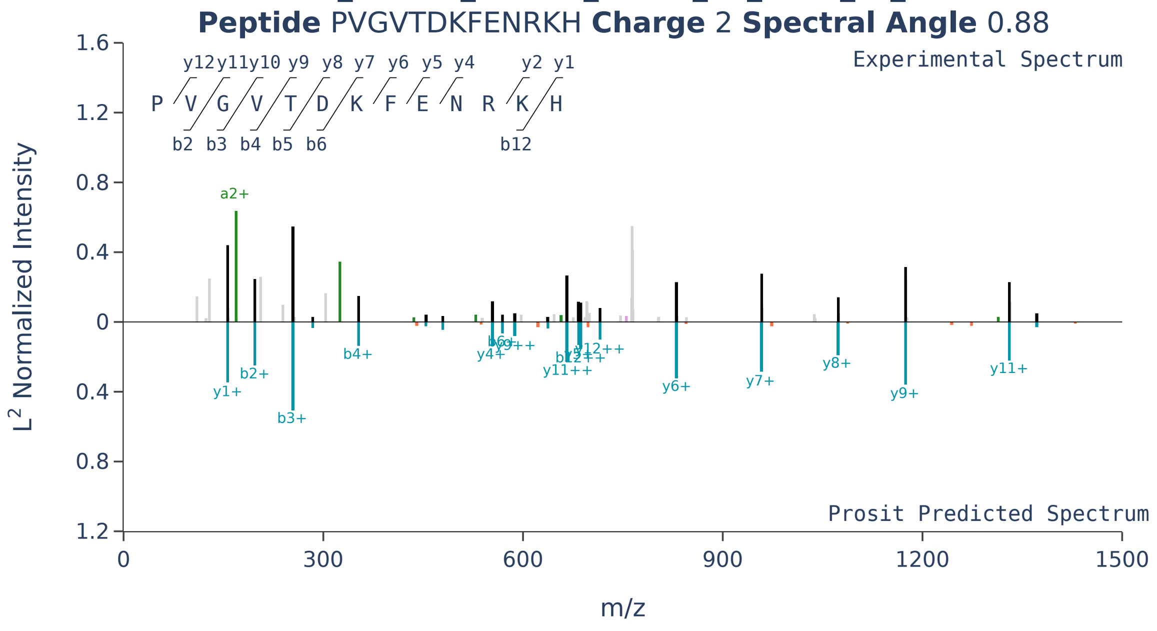


**Source** WSoh_101121_151121_HFGoe_G1_IL37b_1h_R1 **Scan** 13687
**Peptide** HKVLVLD **Charge** 2 **Spectral Angle** 0.92

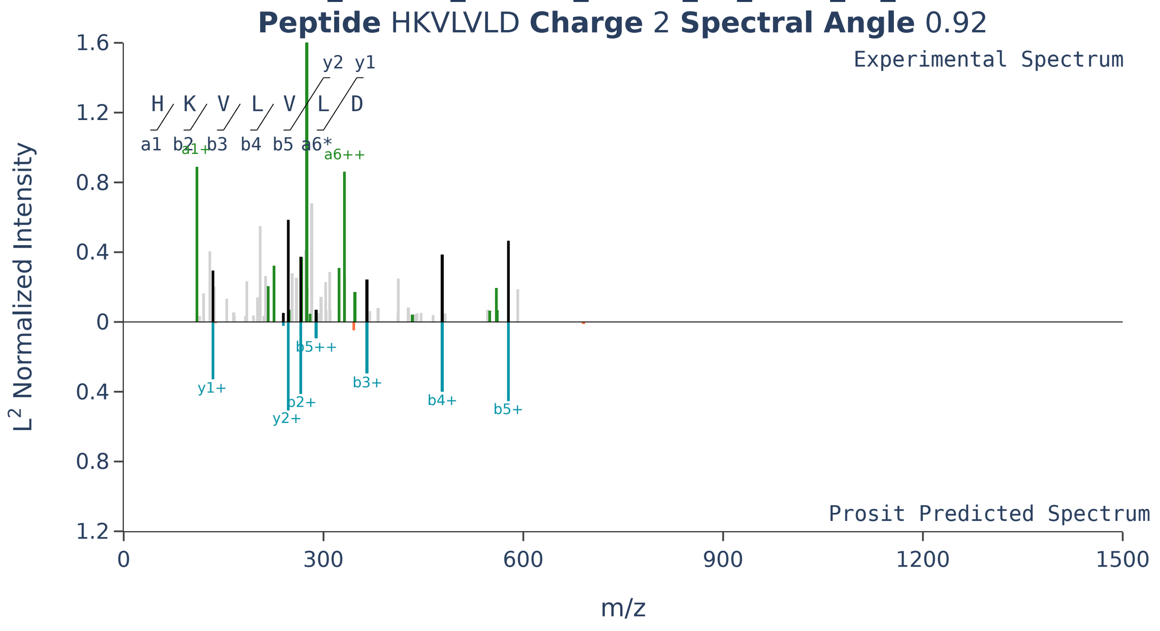

**Source** WSoh_101121_151121_HFGoe_G2_IL37b_24h_R2 **Scan** 9083
**Peptide** PSLQLKKE **Charge** 1 **Spectral Angle** 0.88

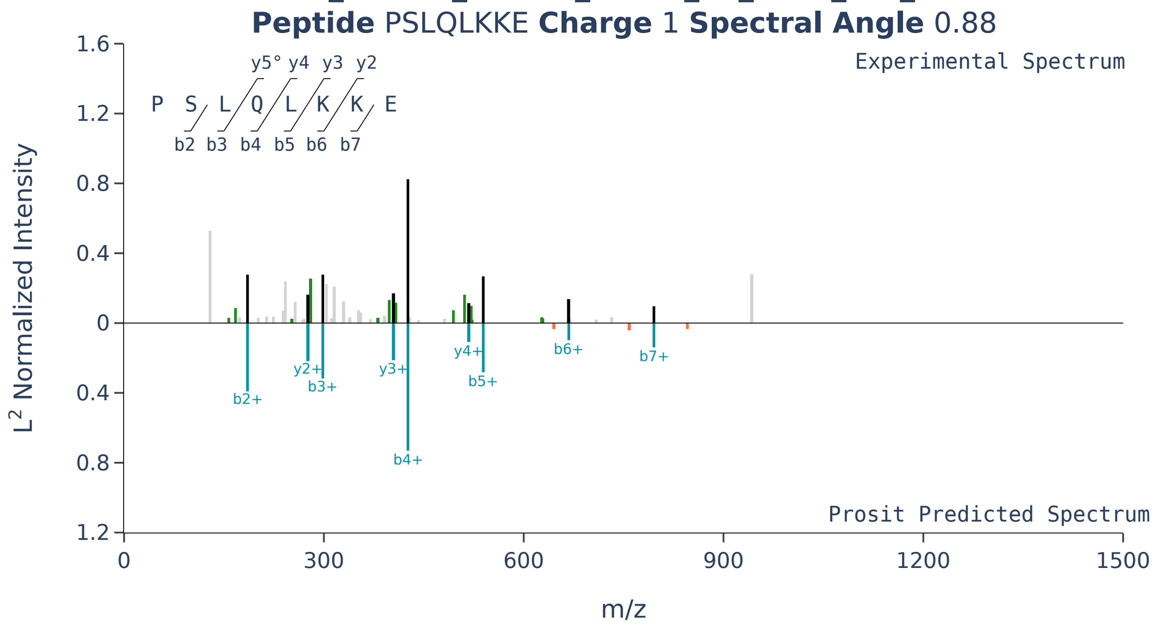


**Source** WSoh_101121_151121_HFGoe_G2_IL37b_24h_R1 **Scan** 20162
**Peptide** KGSPILL **Charge** 2 **Spectral Angle** 0.89

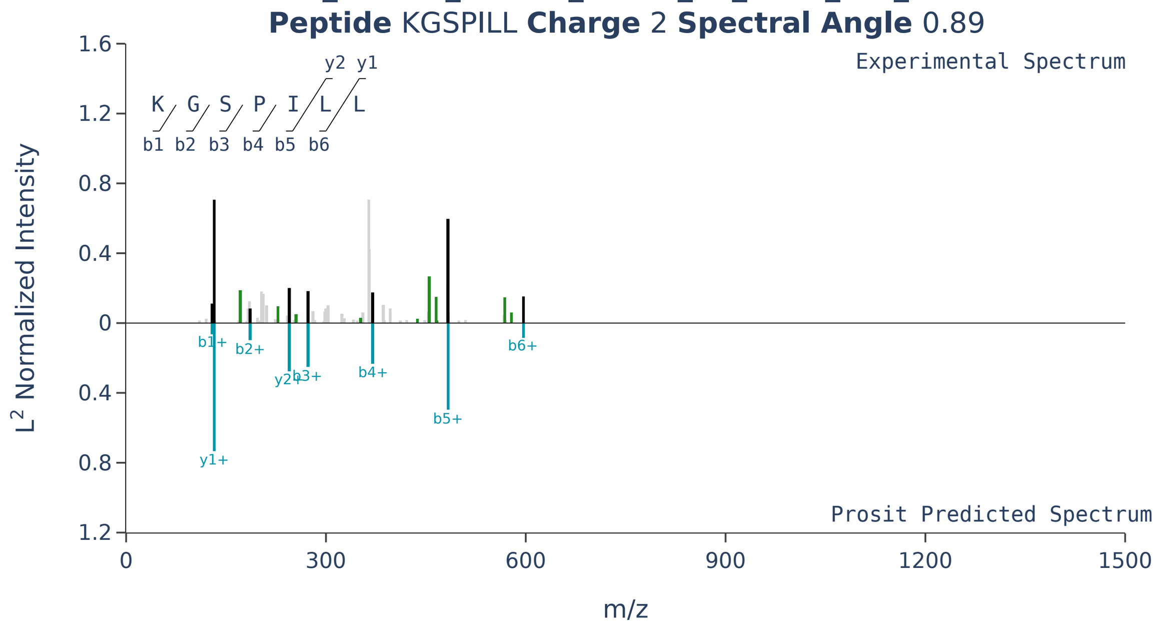


**Source** WSoh_101121_151121_HFGoe_G2_IL37b_24h_R2 **Scan** 6321
**Peptide** NPKKFSIHDQDH **Charge** 3 **Spectral Angle** 0.9

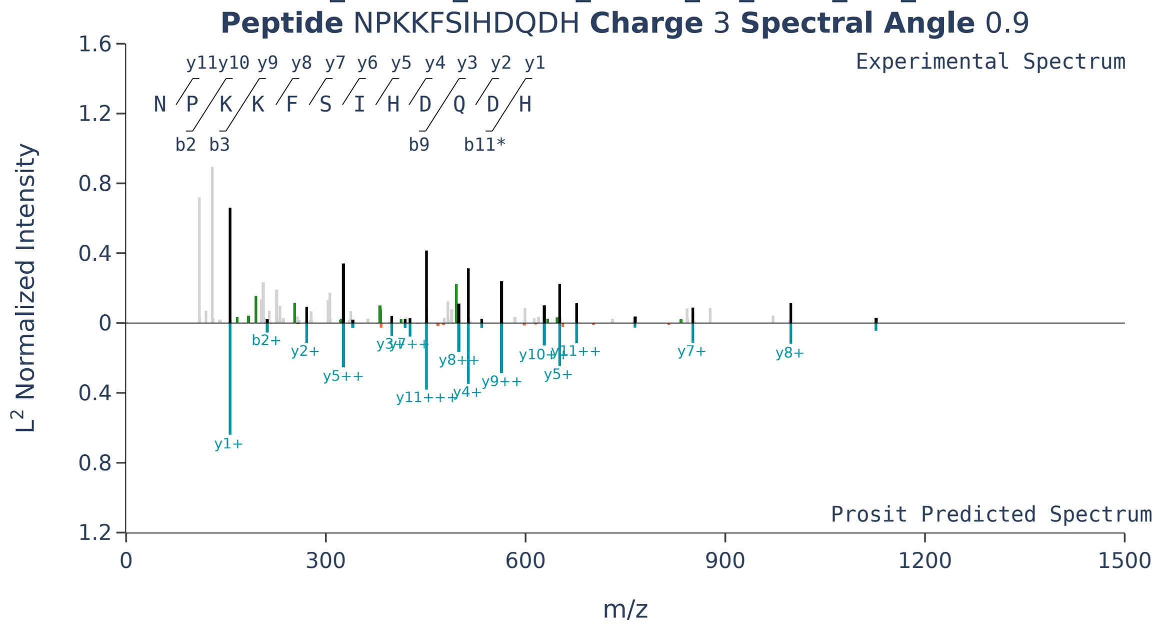


**Source** WSoh_101121_151121_HFGoe_G1_IL37b_2h_R1 **Scan** 22737
**Peptide** RPEIFFA **Charge** 2 **Spectral Angle** 0.93

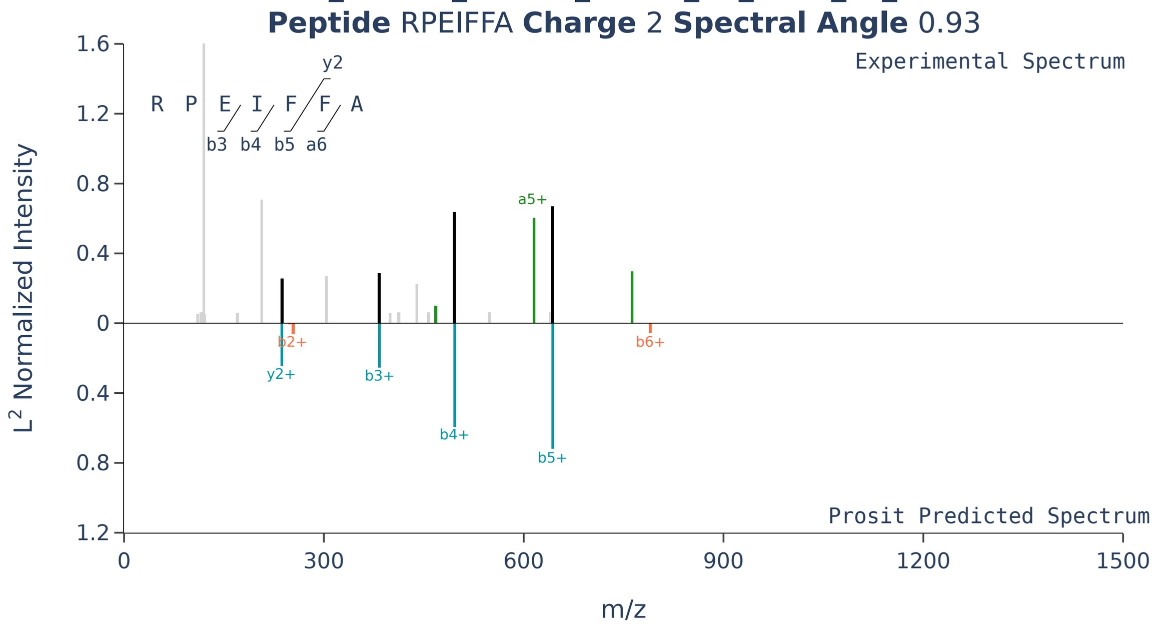


**Source** WSoh_101121_151121_HFGoe_G1_IL37b_1h_R1 **Scan** 7562
**Peptide** KKEKLMKLAA **Charge** 2 **Spectral Angle** 0.9

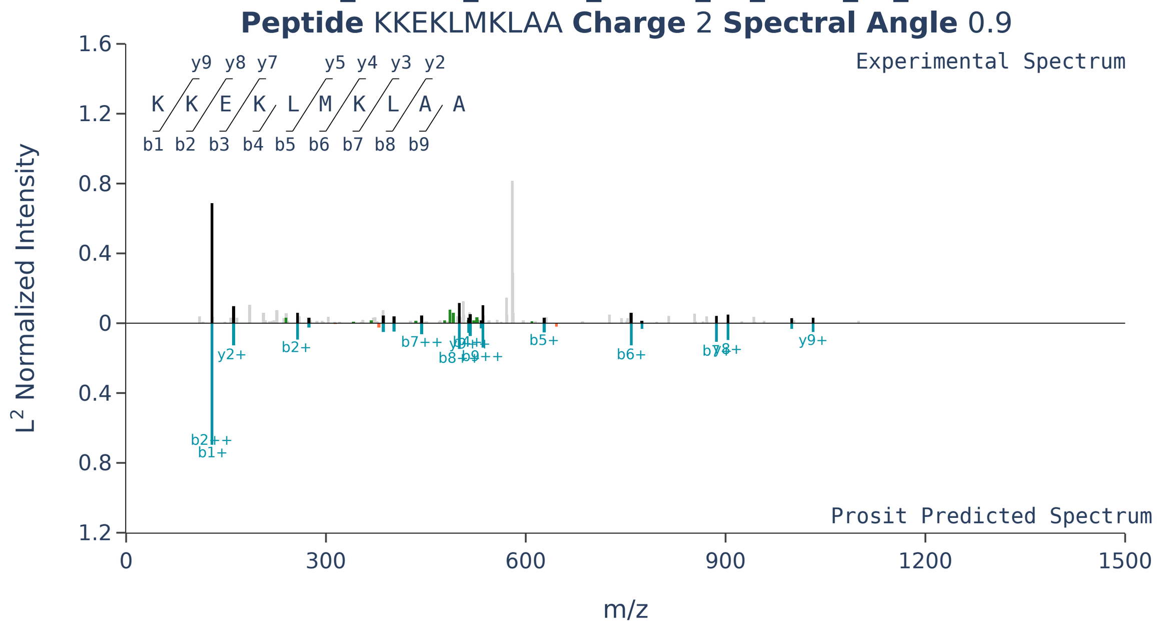


**Source** WSoh_101121_151121_HFGoe_G2_IL37b_24h_R1 **Scan** 15465
**Peptide** LNPKKFSI **Charge** 2 **Spectral Angle** 0.87

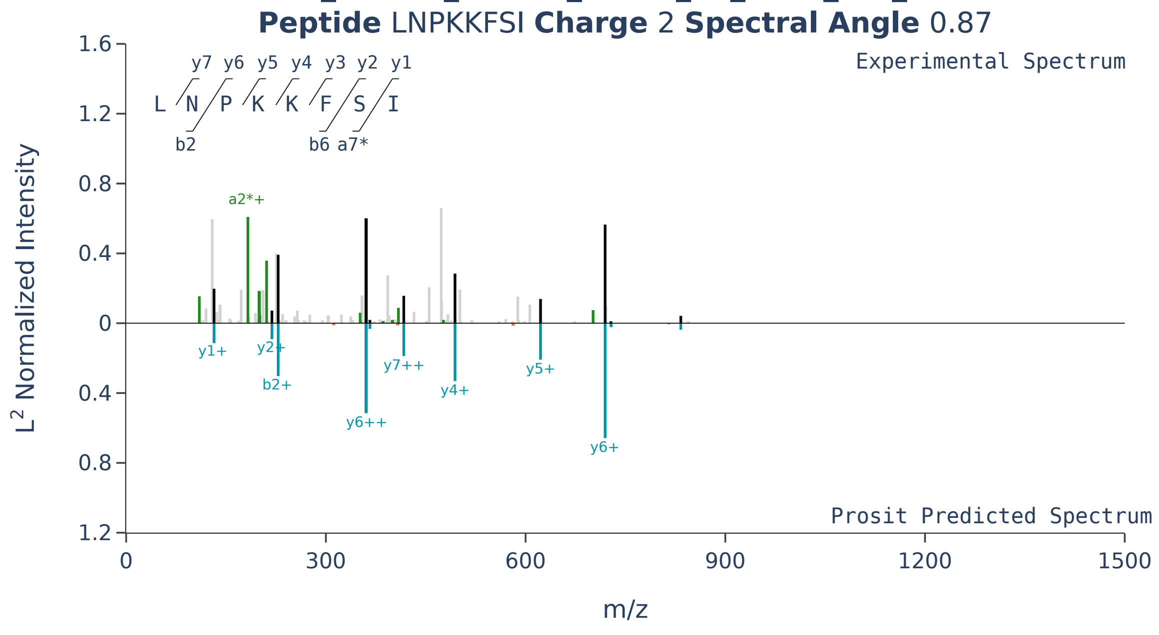


**Source** WSoh_101121_151121_HFGoe_G2_IL37b_24h_R1 **Scan** 15890
**Peptide** IAVPDKNYIRPE **Charge** 3 **Spectral Angle** 0.93

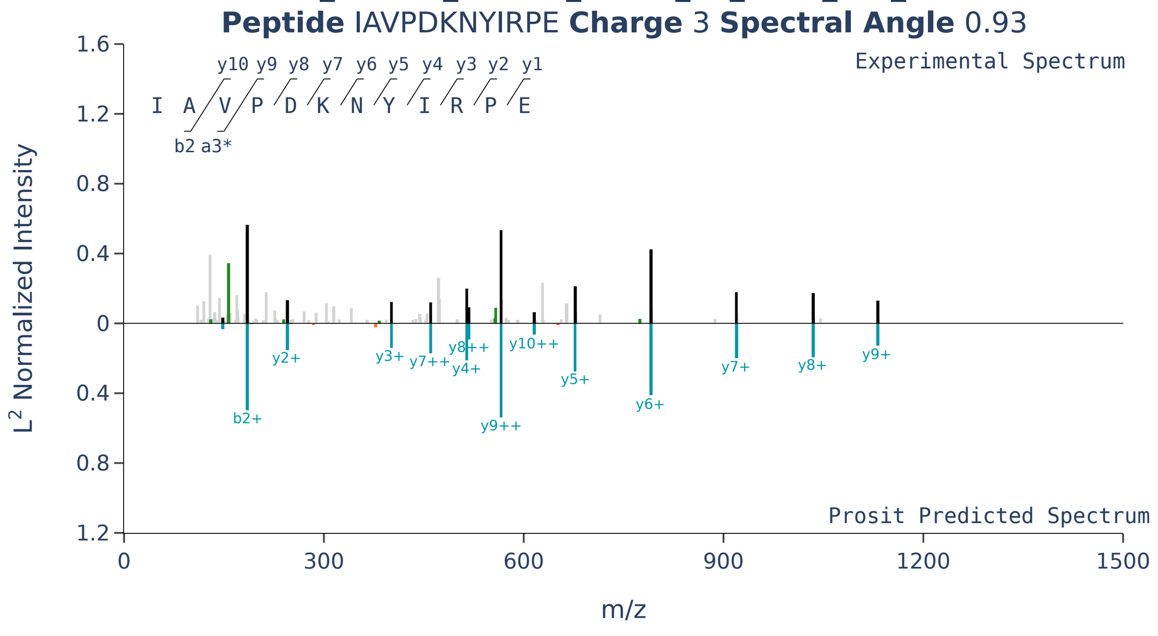


**Source** WSoh_101121_151121_HFGoe_G1_IL37b_4h_R2 **Scan** 17396
**Peptide** SKGEFCL **Charge** 2 **Spectral Angle** 0.92

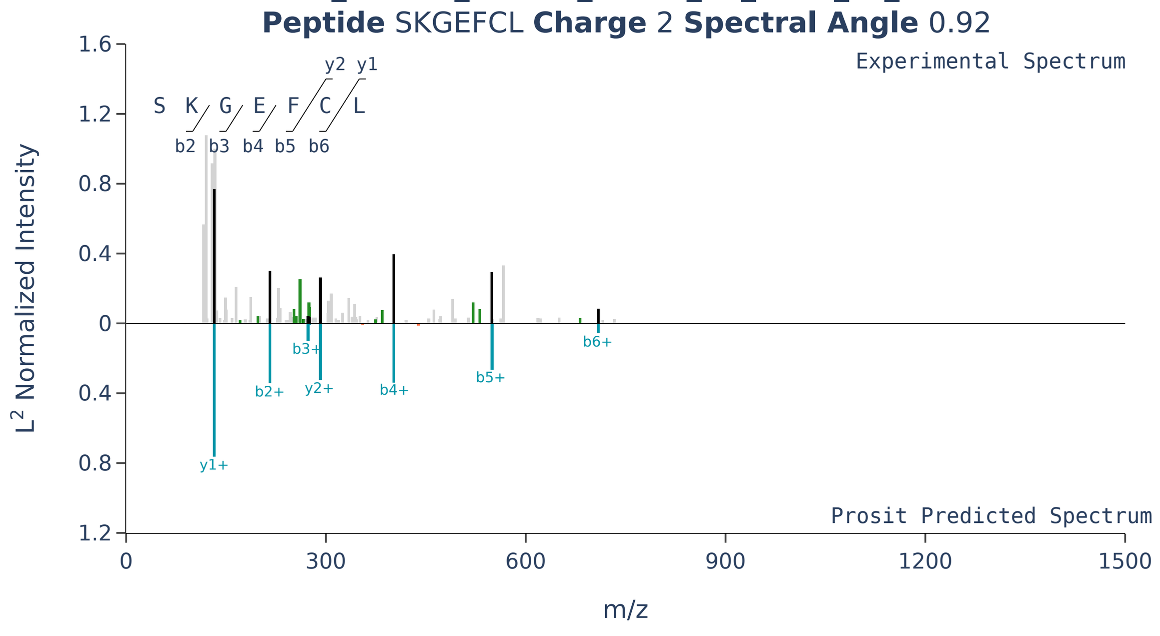


**Source** WSoh_101121_151121_HFGoe_G1_IL37b_24h_R2 **Scan** 16070
**Peptide** DHKVLVLD **Charge** 2 **Spectral Angle** 0.91

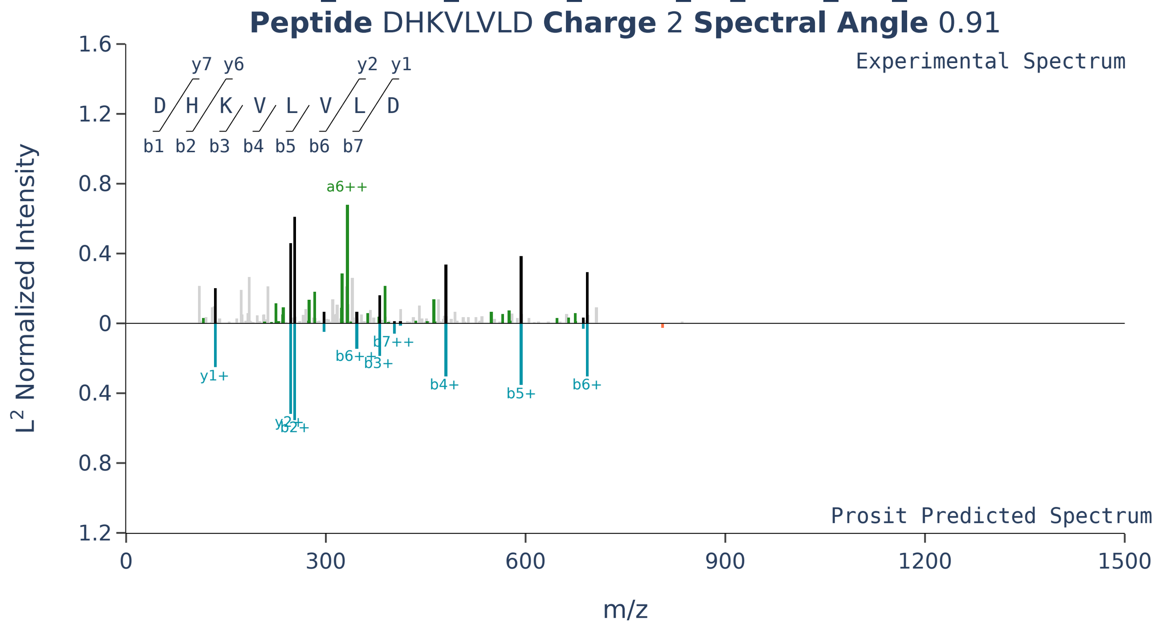


**Source** WSoh_101121_151121_HFGoe_G2_IL37b_2h_R2 **Scan** 9252
**Peptide** QKESARRPFI **Charge** 3 **Spectral Angle** 0.93

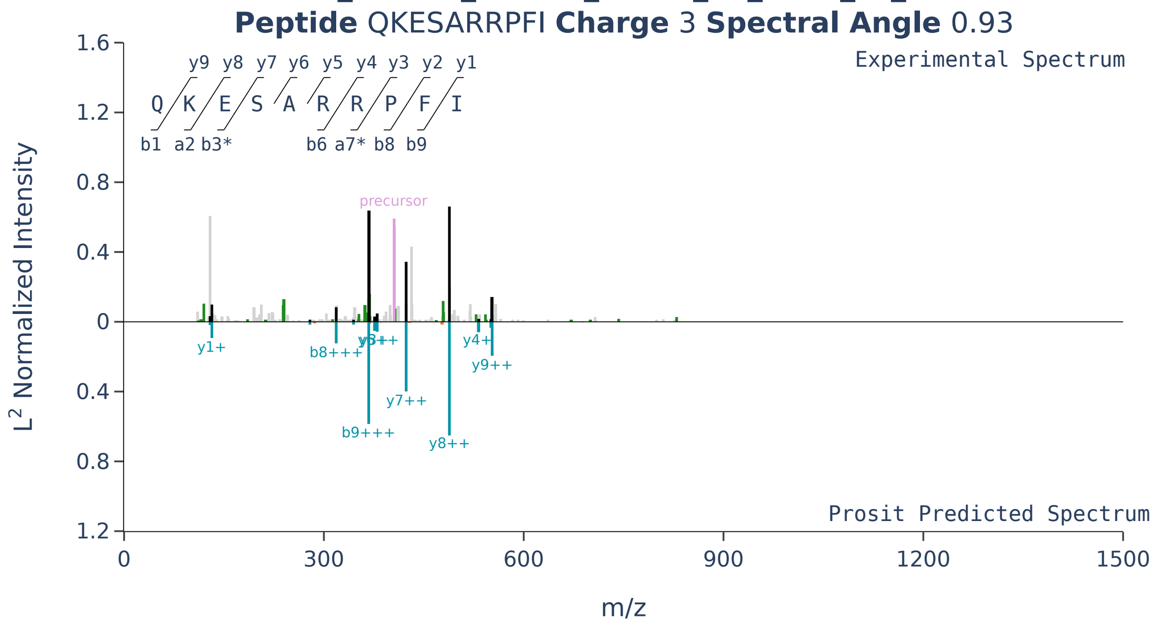


**Source** WSoh_101121_151121_HFGoe_G1_IL37b_4h_R2 **Scan** 6233
**Peptide** VTDKFENRKHIE **Charge** 2 **Spectral Angle** 0.89

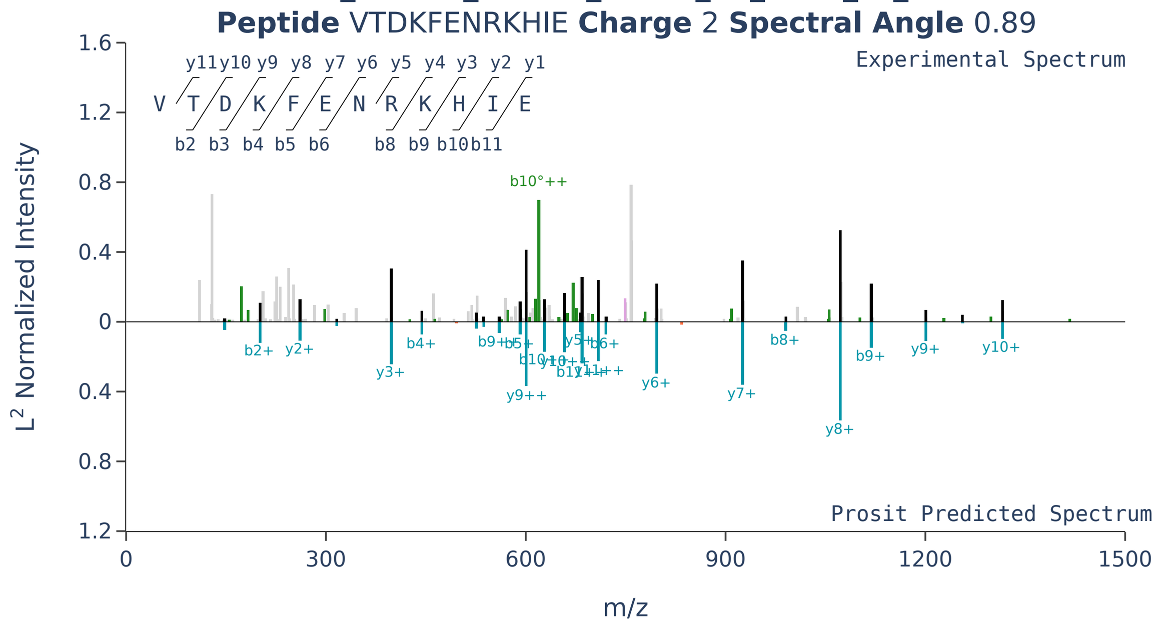


**Source** WSoh_101121_151121_HFGoe_G1_IL37b_24h_R1 **Scan** 9919
**Peptide** VPDKNYIRPE **Charge** 3 **Spectral Angle** 0.88

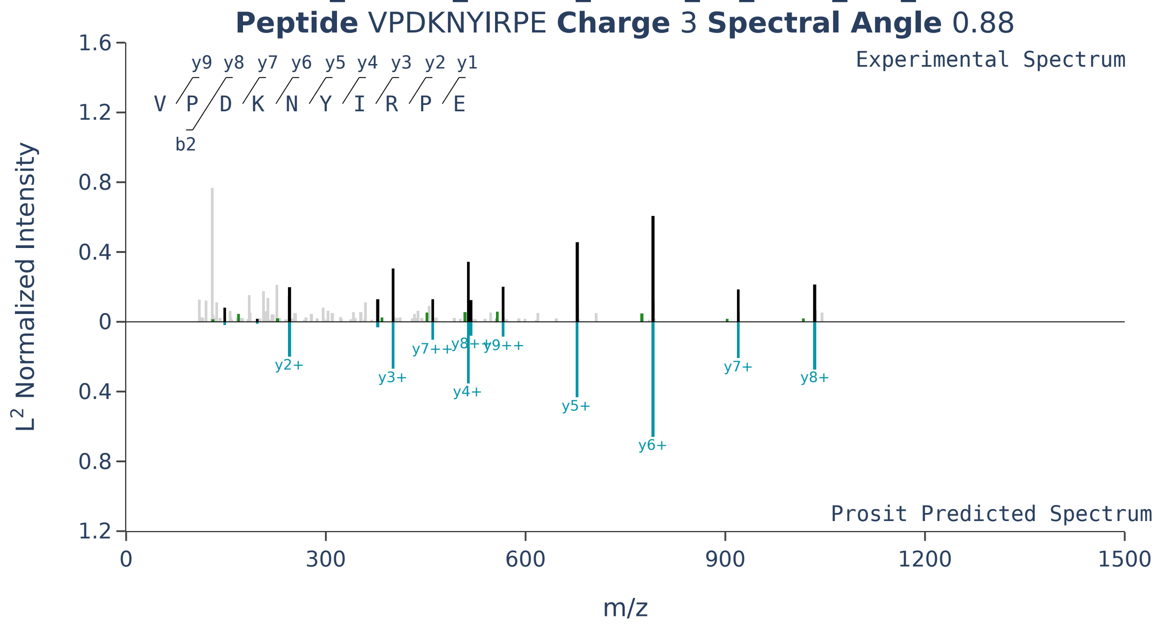


**Source** WSoh_101121_151121_HFGoe_G1_IL37b_1h_R1 **Scan** 18587
**Peptide** RAQVGSWNML **Charge** 2 **Spectral Angle** 0.91

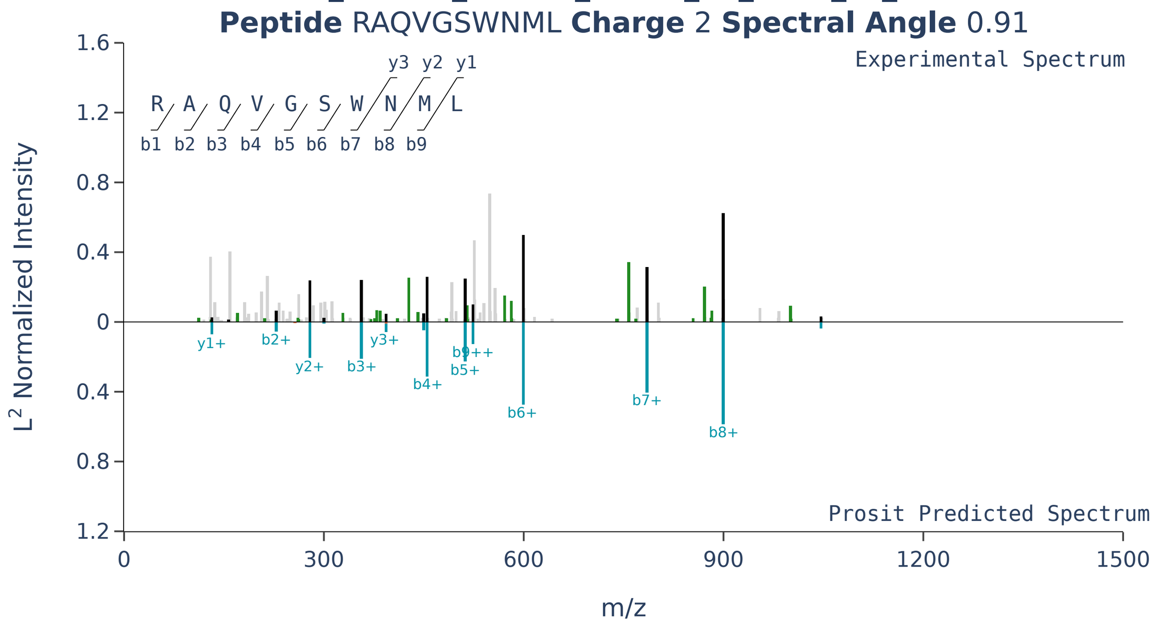


**Source** WSoh_101121_151121_HFGoe_G1_IL37b_24h_R2 **Scan** 15948
**Peptide** DSGNLIA **Charge** 1 **Spectral Angle** 0.9

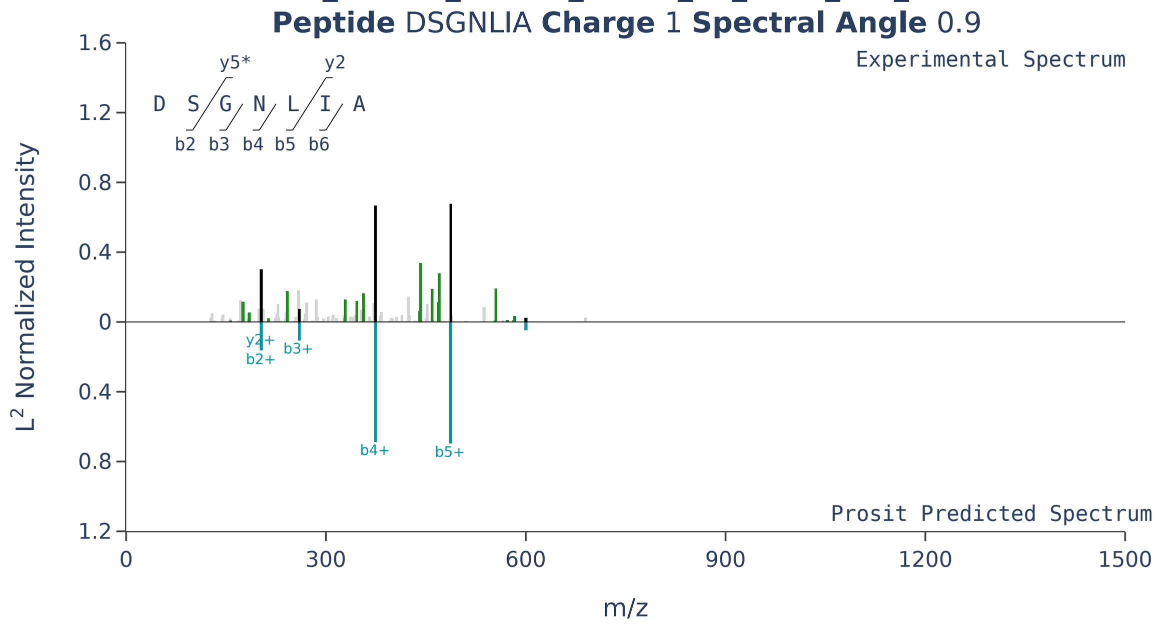


**Source** WSoh_101121_151121_HFGoe_G1_IL37b_4h_R1 **Scan** 12506
**Peptide** GEFCLYCDKDKGQSH **Charge** 3 **Spectral Angle** 0.89

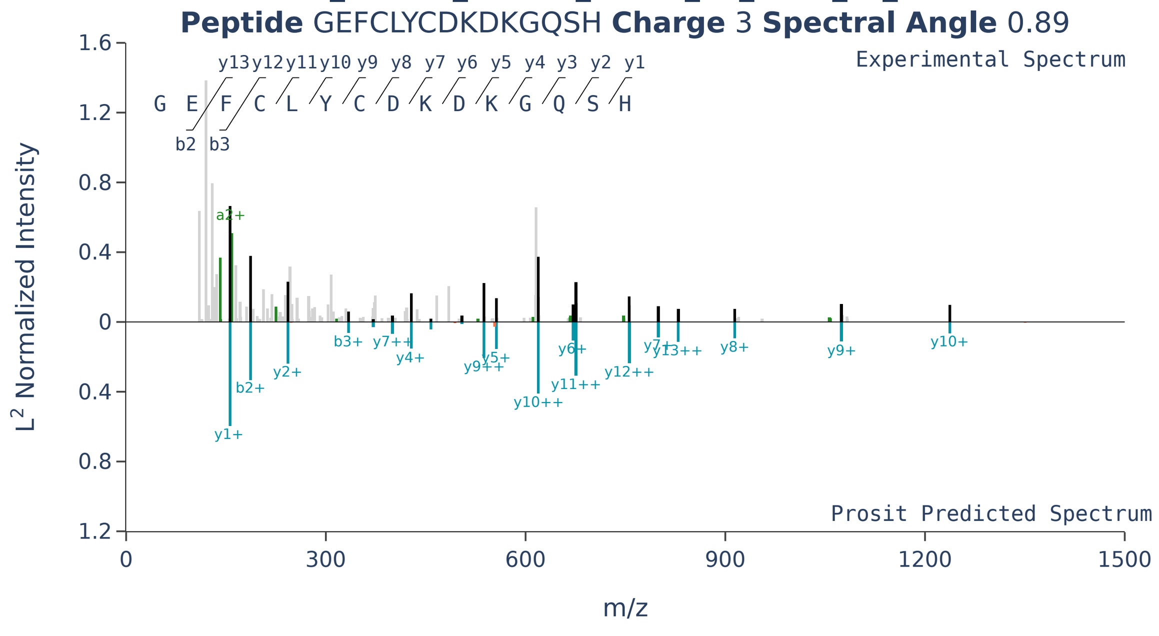

**Source** WSoh_101121_151121_HFGoe_G1_IL37b_4h_R2 **Scan** 6845
**Peptide** LMKLAAQKESARR **Charge** 4 **Spectral Angle** 0.92

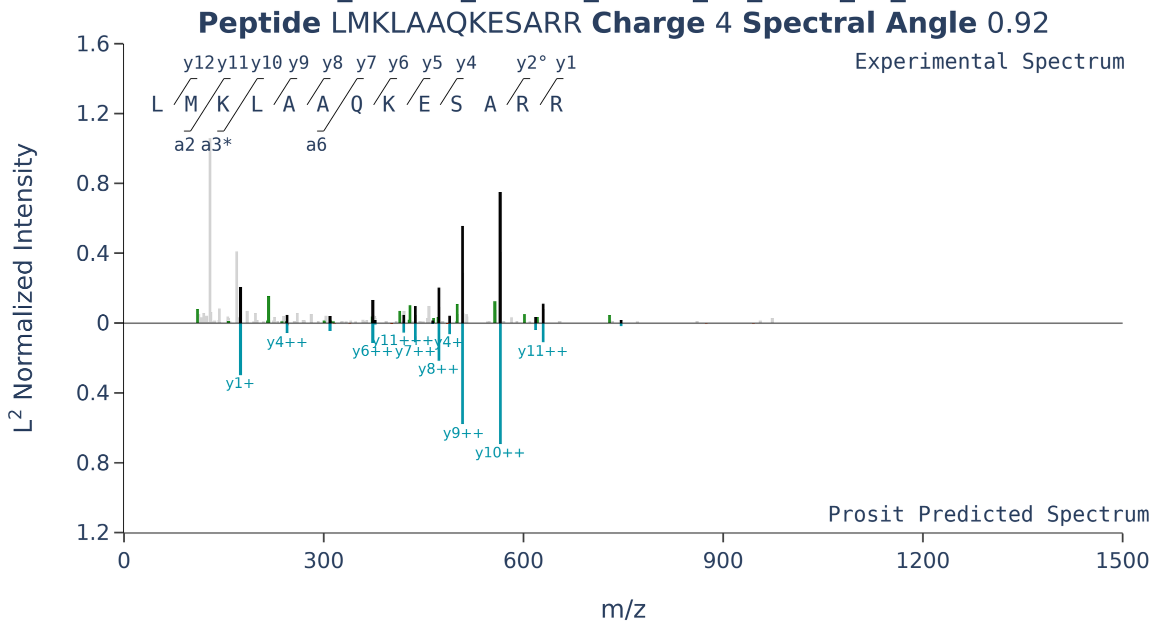


**Source** WSoh_101121_151121_HFGoe_G1_IL37b_24h_R1 **Scan** 5744
**Peptide** TDKFENRKHI **Charge** 2 **Spectral Angle** 0.89

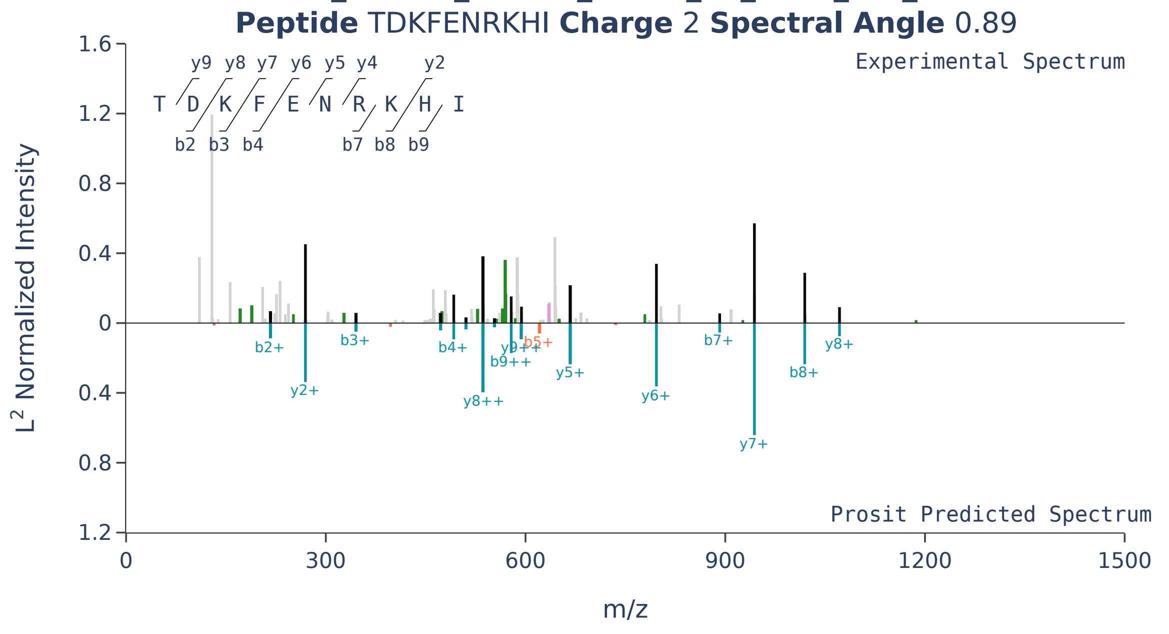


**Source** WSoh_101121_151121_HFGoe_G1_IL37b_2h_R1 **Scan** 15923
**Peptide** QDHKVLVL **Charge** 2 **Spectral Angle** 0.85

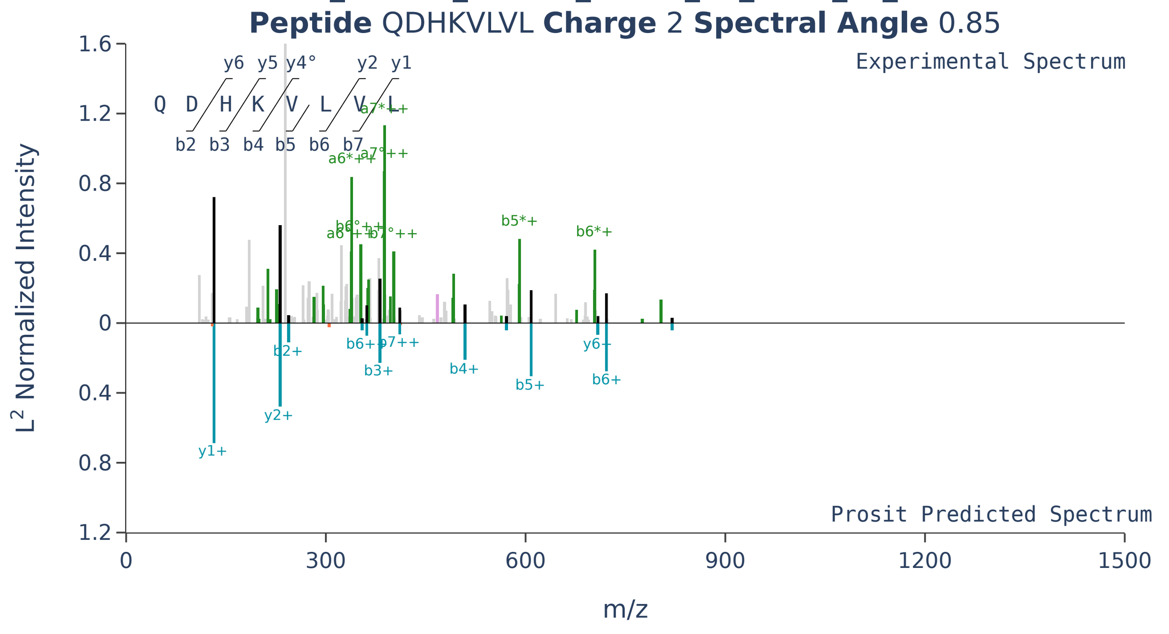


**Source** WSoh_101121_151121_HFGoe_G1_IL37b_24h_R1 **Scan** 11021
**Peptide** EMSPSEVSD **Charge** 2 **Spectral Angle** 0.95

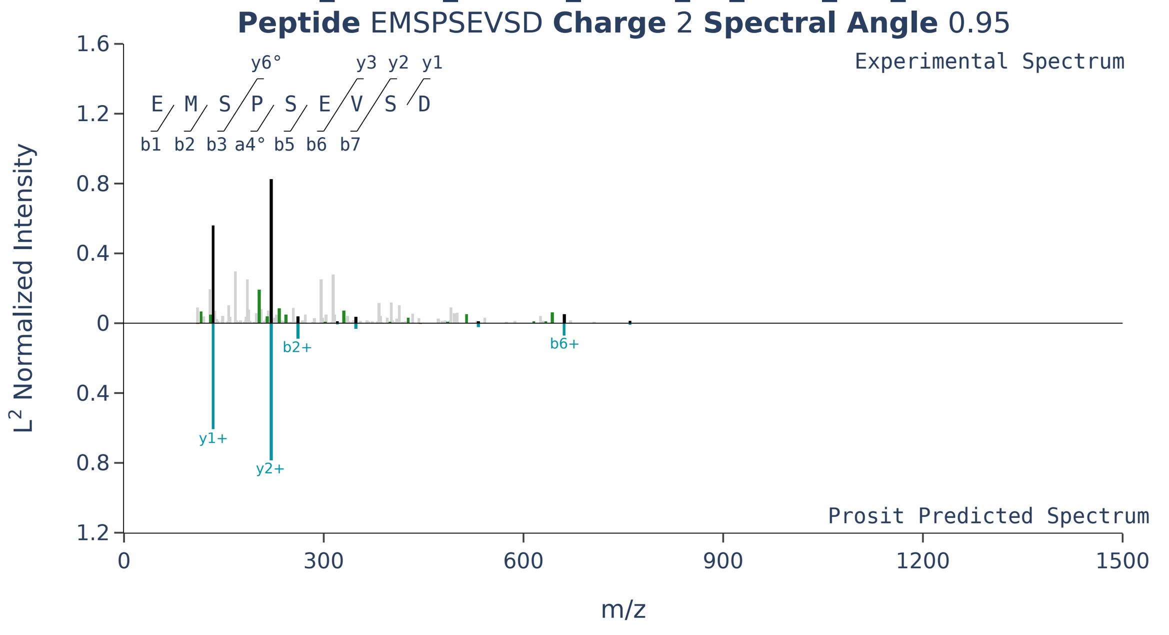


**Source** WSoh_101121_151121_HFGoe_G1_IL37b_4h_R1 **Scan** 6570
**Peptide** ASSLSSASAEKG **Charge** 2 **Spectral Angle** 0.87

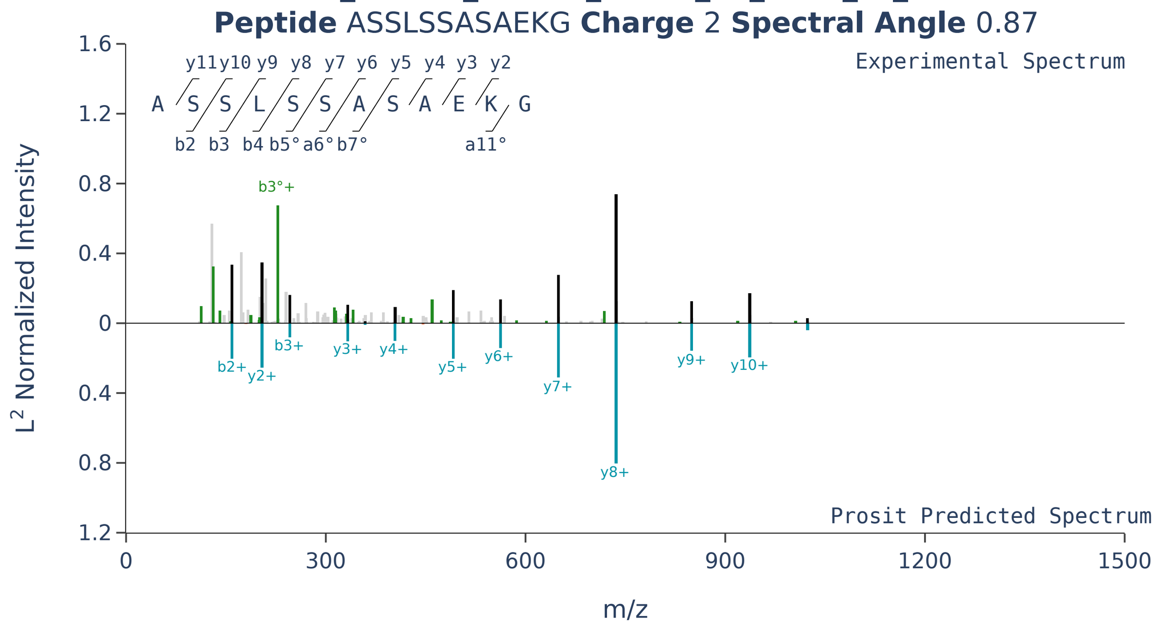


**Source** WSoh_101121_151121_HFGoe_G1_IL37b_4h_R1 **Scan** 6835
**Peptide** LMKLAAQKE **Charge** 2 **Spectral Angle** 0.87

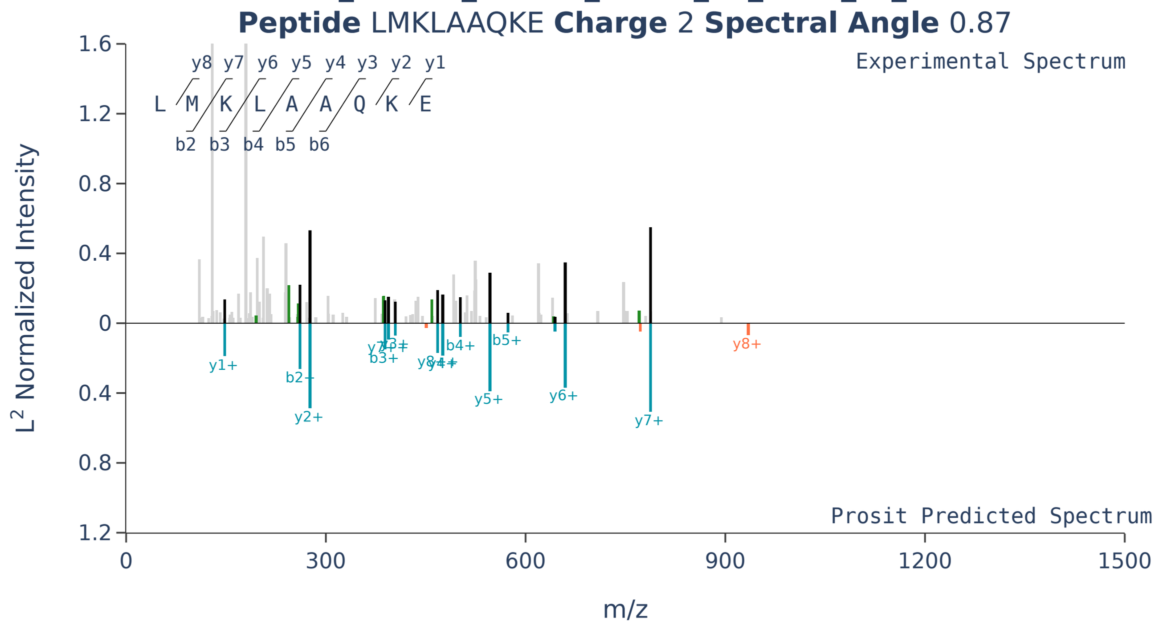


**Source** WSoh_101121_151121_HFGoe_G1_IL37b_24h_R2 **Scan** 11249
**Peptide** ESAAHPGW **Charge** 2 **Spectral Angle** 0.9

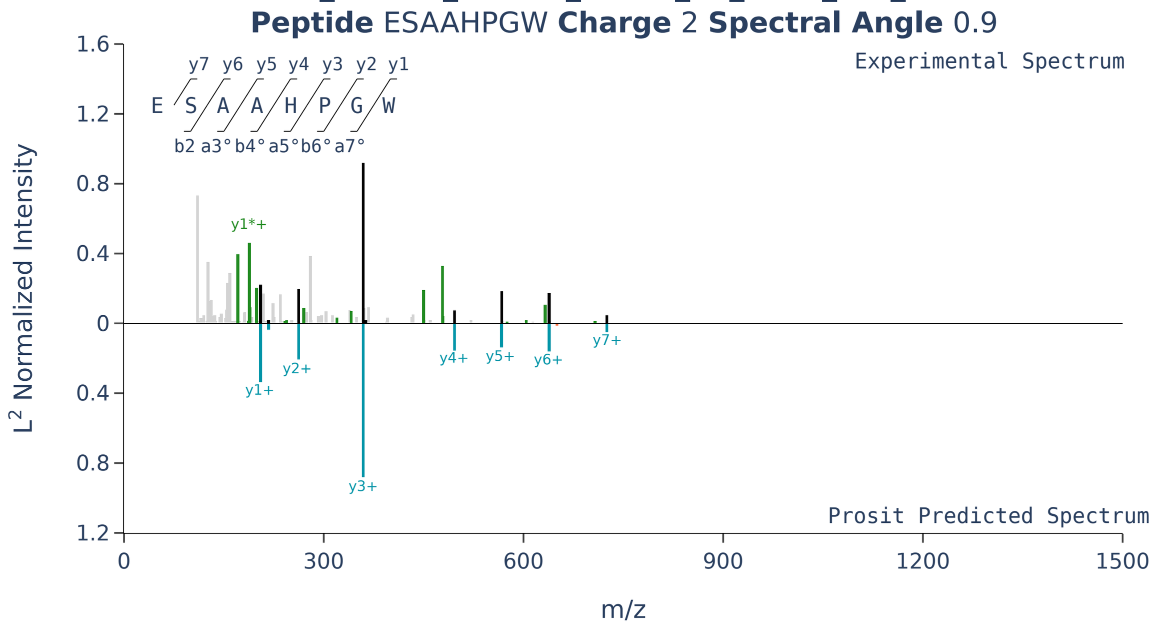


**Source** WSoh_101121_151121_HFGoe_G1_IL37b_4h_R2 **Scan** 5434
**Peptide** VTDKFENRKH **Charge** 3 **Spectral Angle** 0.88

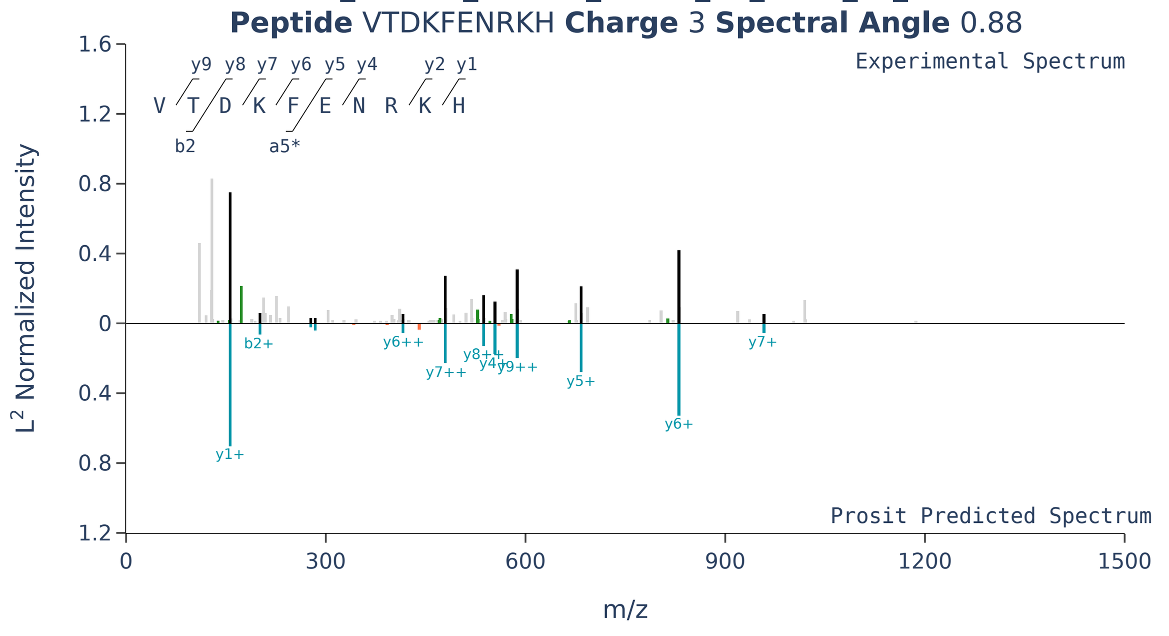


**Source** WSoh_101121_151121_HFGoe_G1_IL37b_24h_R1 **Scan** 6571
**Peptide** VHTSPKVKN **Charge** 2 **Spectral Angle** 0.86

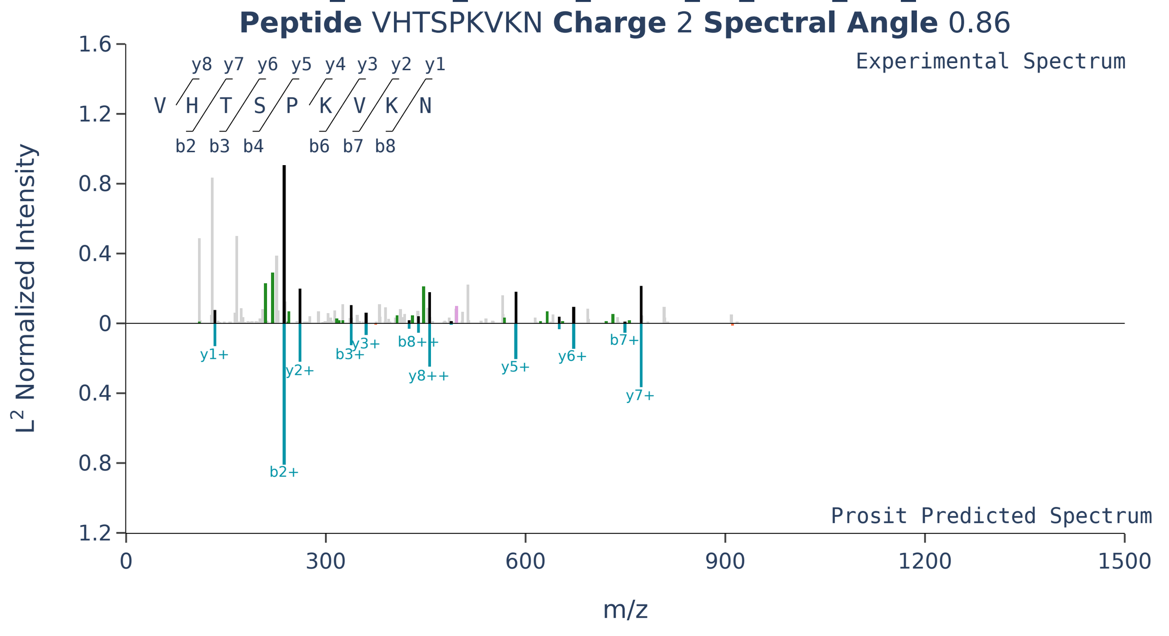


**Source** WSoh_101121_151121_HFGoe_G1_IL37b_24h_R1 **Scan** 7389
**Peptide** KLMKLAAQKES **Charge** 2 **Spectral Angle** 0.91

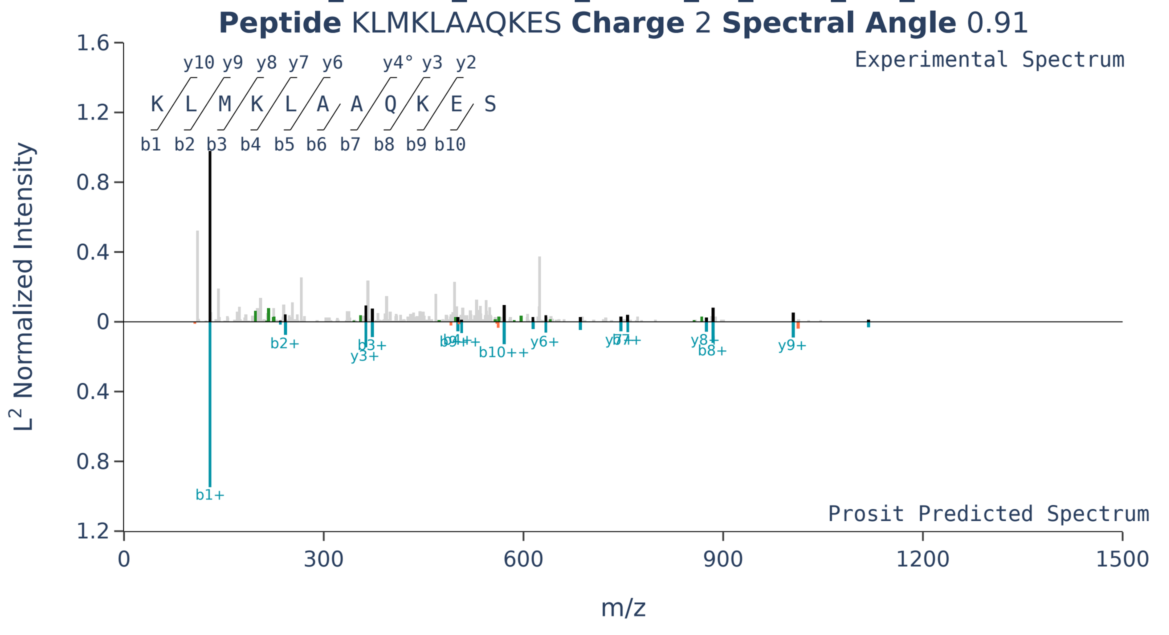


**Source** WSoh_101121_151121_HFGoe_G2_IL37b_24h_R1 **Scan** 6949
**Peptide** KKEKLMK **Charge** 2 **Spectral Angle** 0.89

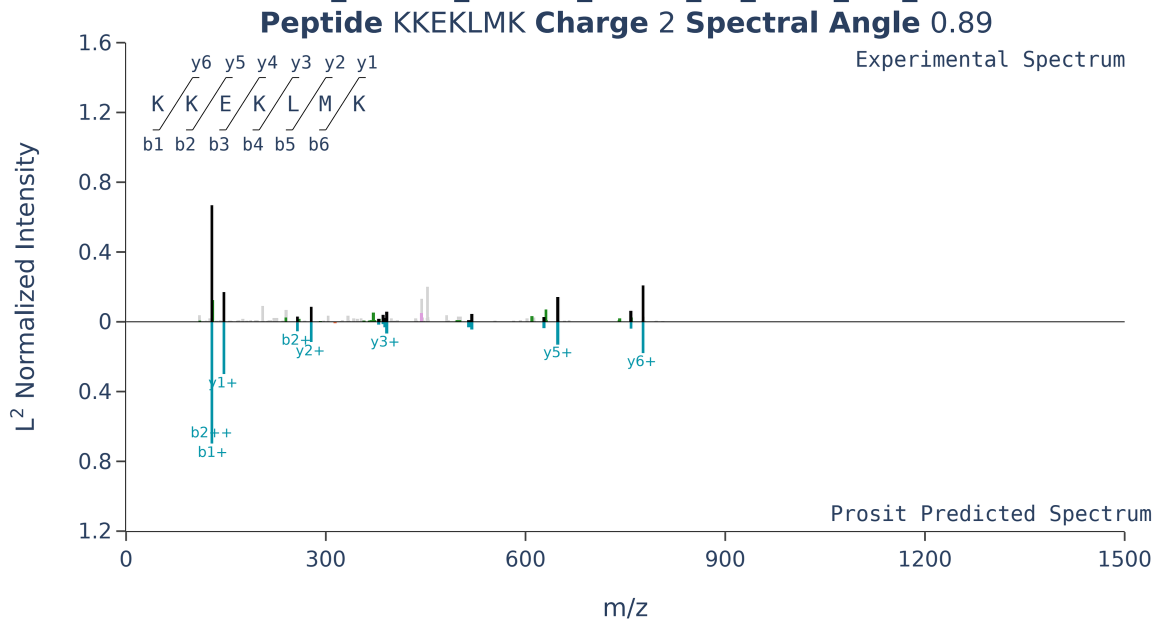


**Source** WSoh_101121_151121_HFGoe_G2_IL37b_24h_R1 **Scan** 8420
**Peptide** PDKNYIRP **Charge** 2 **Spectral Angle** 0.9

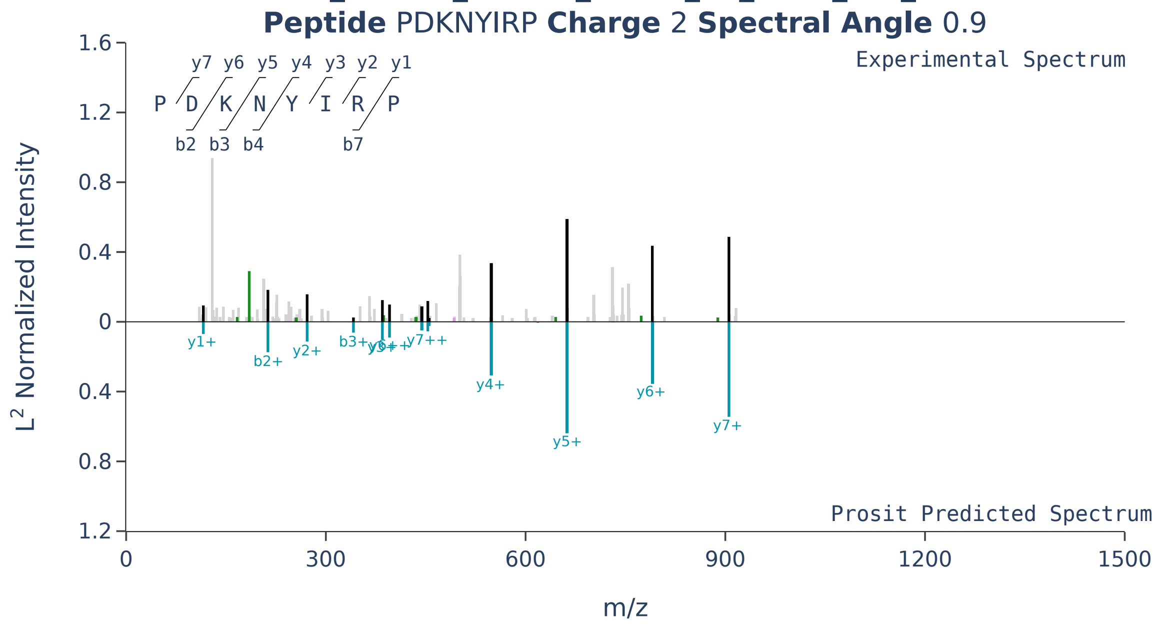


**Source** WSoh_101121_151121_HFGoe_G1_IL37b_4h_R2 **Scan** 6077
**Peptide** MKLAAQKESAR **Charge** 3 **Spectral Angle** 0.9

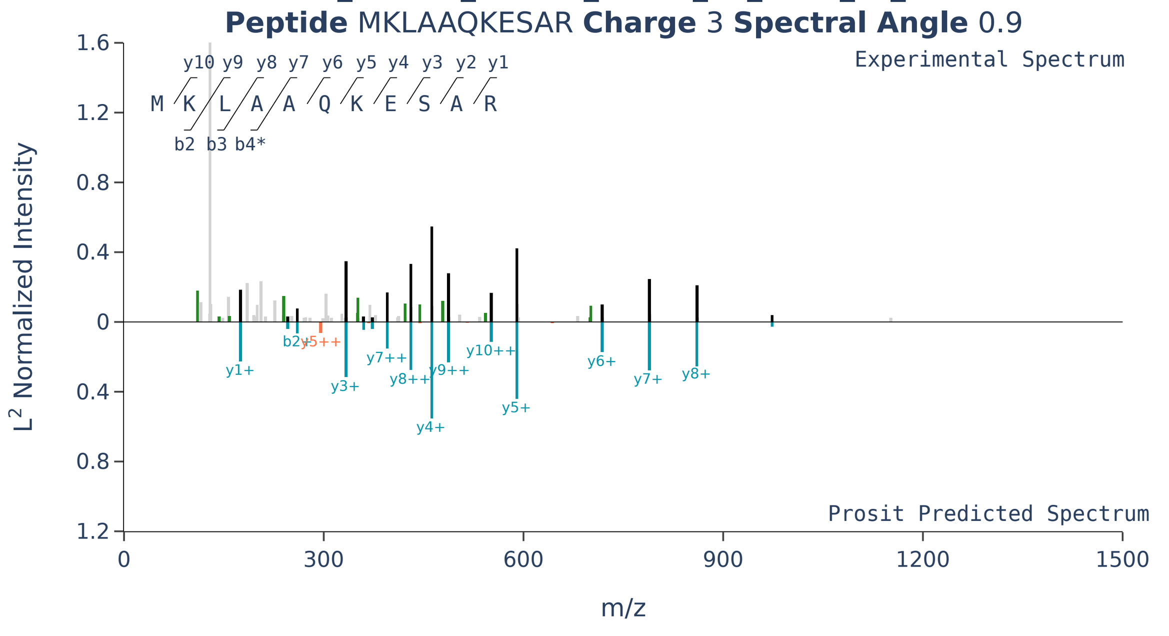


**Source** WSoh_101121_151121_HFGoe_G1_IL37b_4h_R2 **Scan** 5436
**Peptide** KFENRKHIE **Charge** 2 **Spectral Angle** 0.9

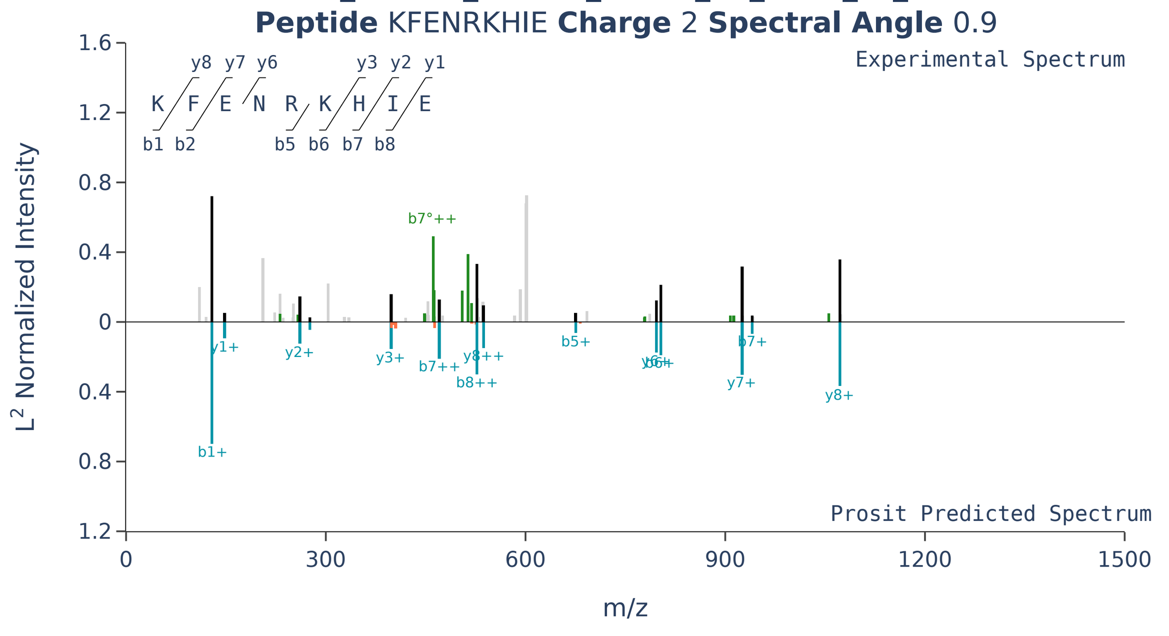


**Source** WSoh_101121_151121_HFGoe_G2_IL37b_24h_R1 **Scan** 20685
**Peptide** AEKGSPILL **Charge** 2 **Spectral Angle** 0.87

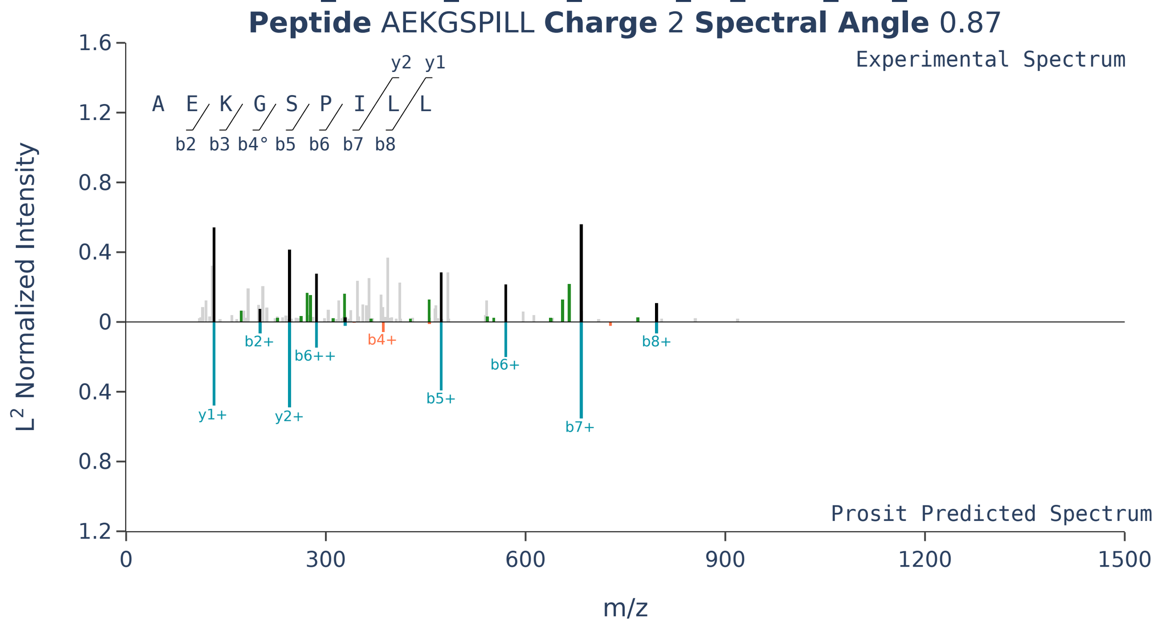

**Source** WSoh_101121_151121_HFGoe_G2_IL37b_24h_R1 **Scan** 16995
**Peptide** SAEKGSPILLGVSK **Charge** 3 **Spectral Angle** 0.91

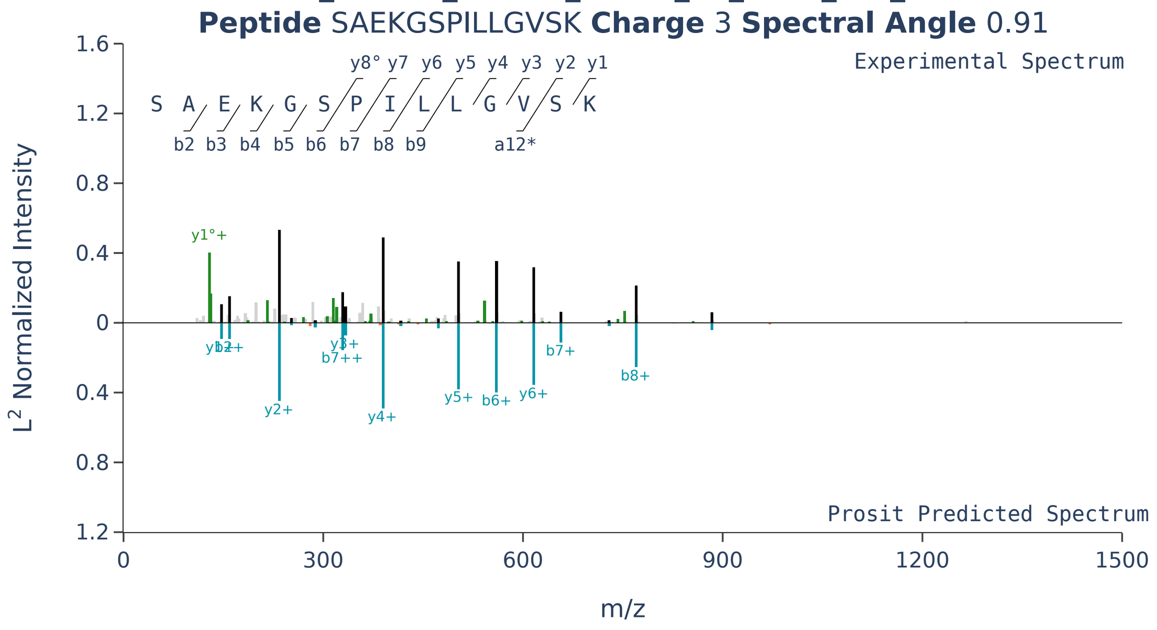

**Source** WSoh_101121_151121_HFGoe_G2_IL37b_4h_R2 **Scan** 7546
**Peptide** PKKFSIHD **Charge** 2 **Spectral Angle** 0.87

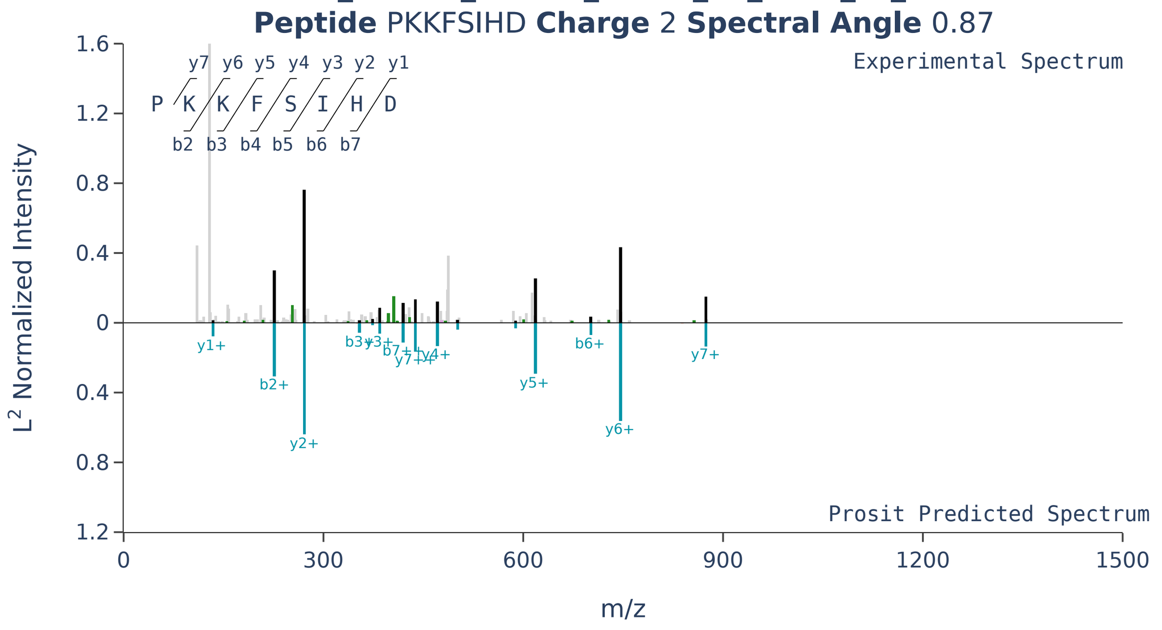

**Source** WSoh_101121_151121_HFGoe_G1_IL37b_24h_R2 **Scan** 16176
**Peptide** GNLIAVPDKN **Charge** 2 **Spectral Angle** 0.87

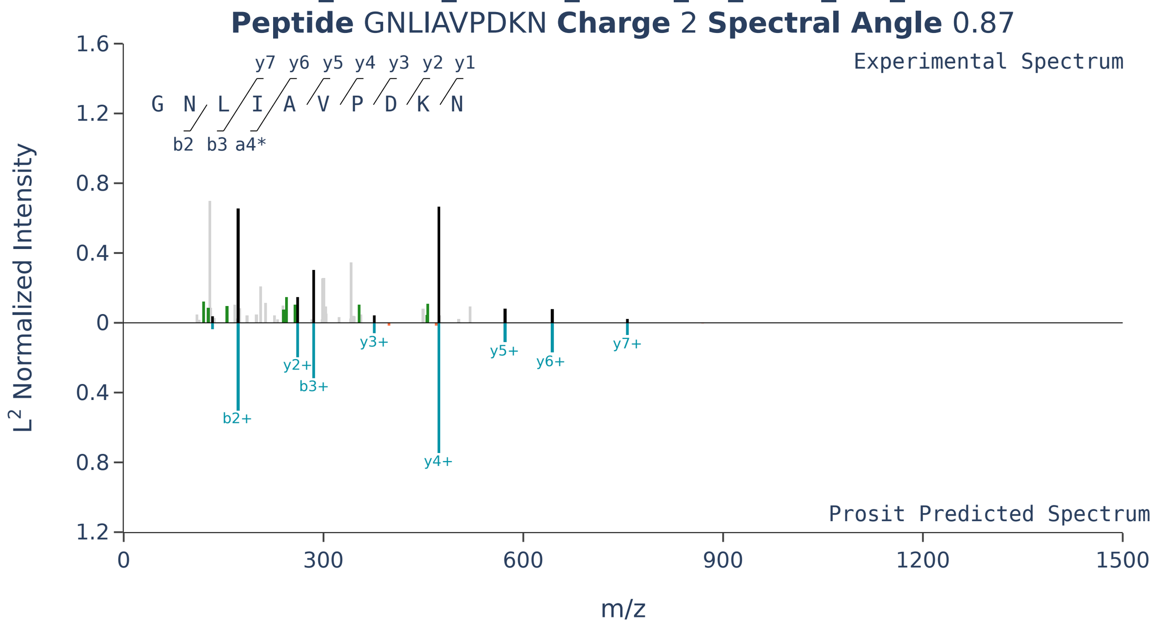

**Source** WSoh_101121_151121_HFGoe_G2_IL37b_2h_R1 **Scan** 9747
**Peptide** SKGEFCLYCDKDKGQSH **Charge** 3 **Spectral Angle** 0.9

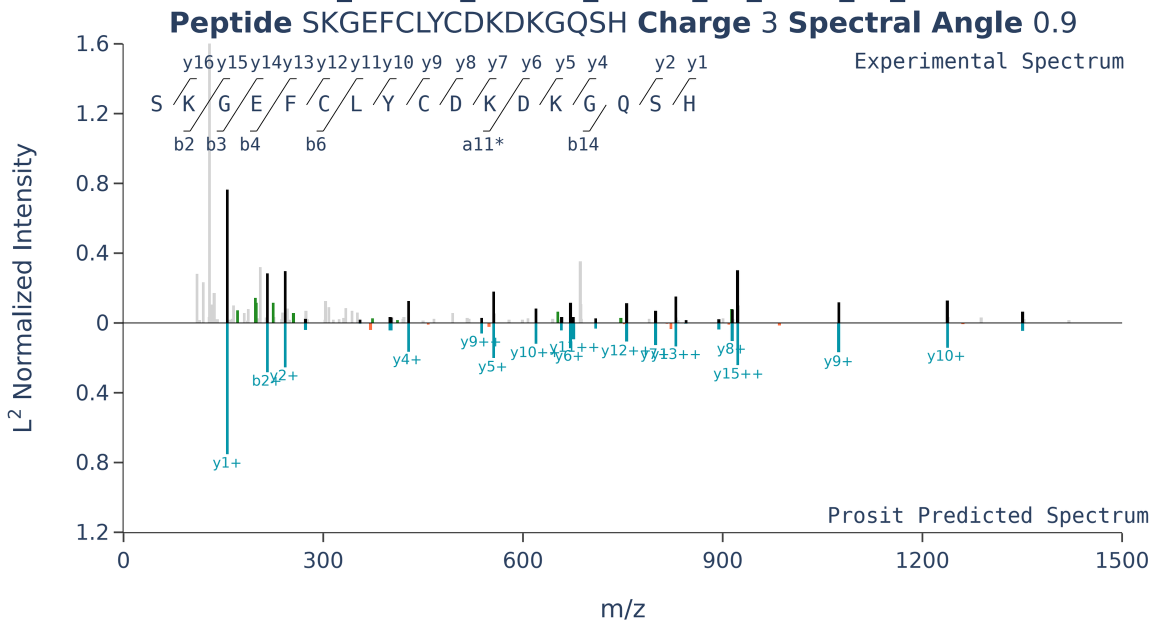

**Source** WSoh_101121_151121_HFGoe_G2_IL37b_24h_R2 **Scan** 7570
**Peptide** AEMSPSEVSD **Charge** 2 **Spectral Angle** 0.95

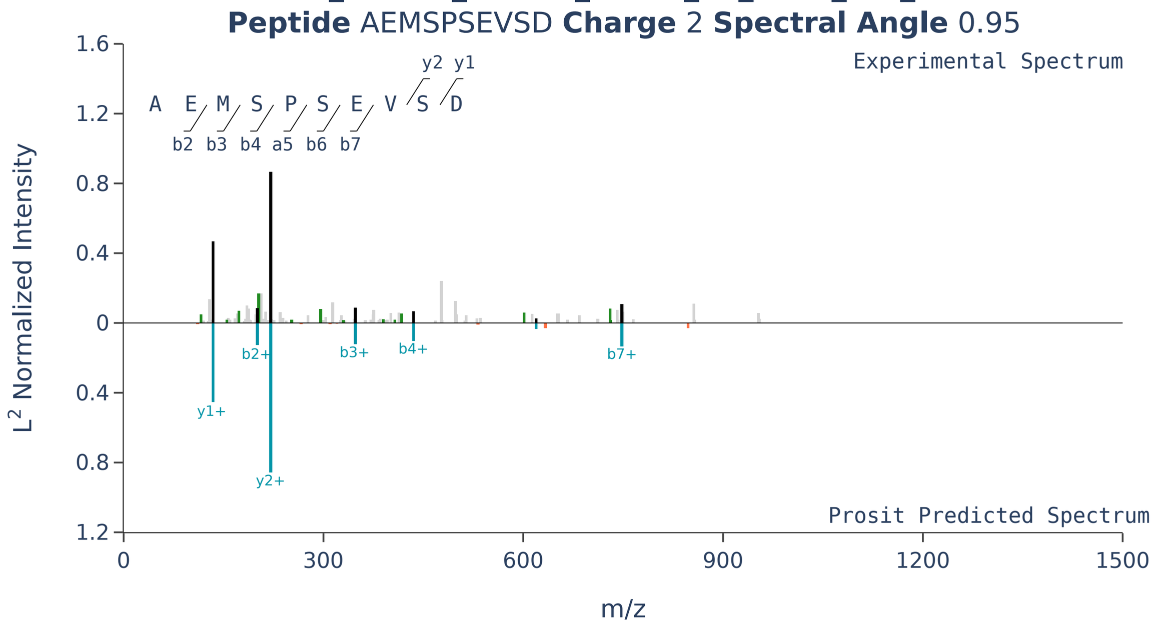

**Source** WSoh_101121_151121_HFGoe_G2_IL37b_24h_R2 **Scan** 14384
**Peptide** HDQDHKVLVL **Charge** 2 **Spectral Angle** 0.88

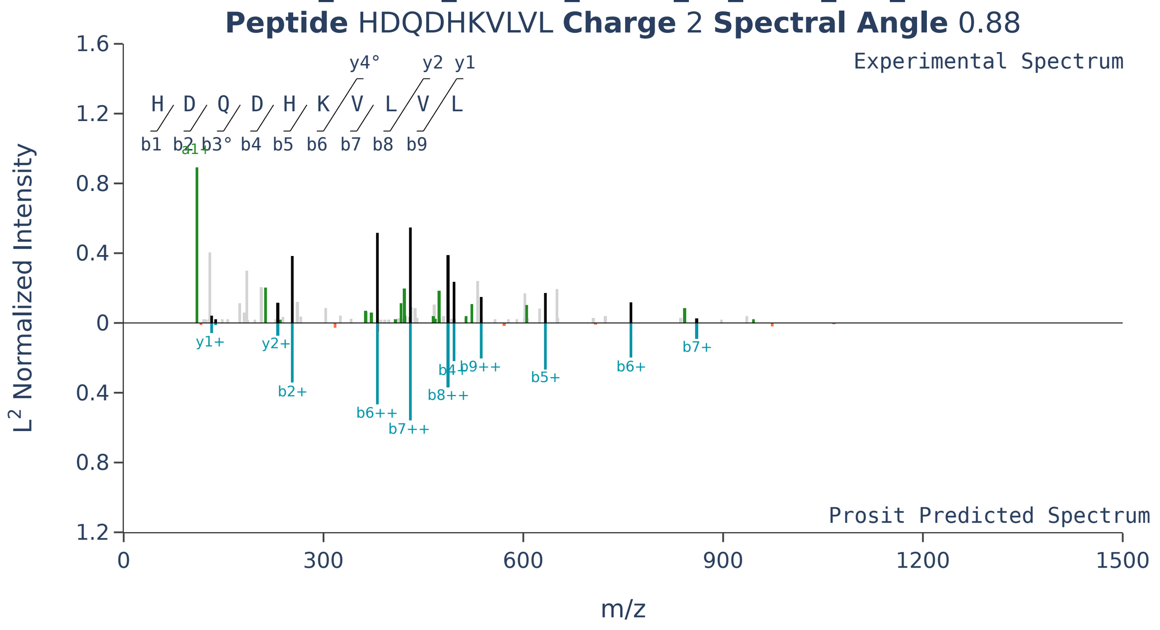

**Source** WSoh_101121_151121_HFGoe_G1_IL37b_24h_R2 **Scan** 21057
**Peptide** SAEKGSPILL **Charge** 2 **Spectral Angle** 0.92

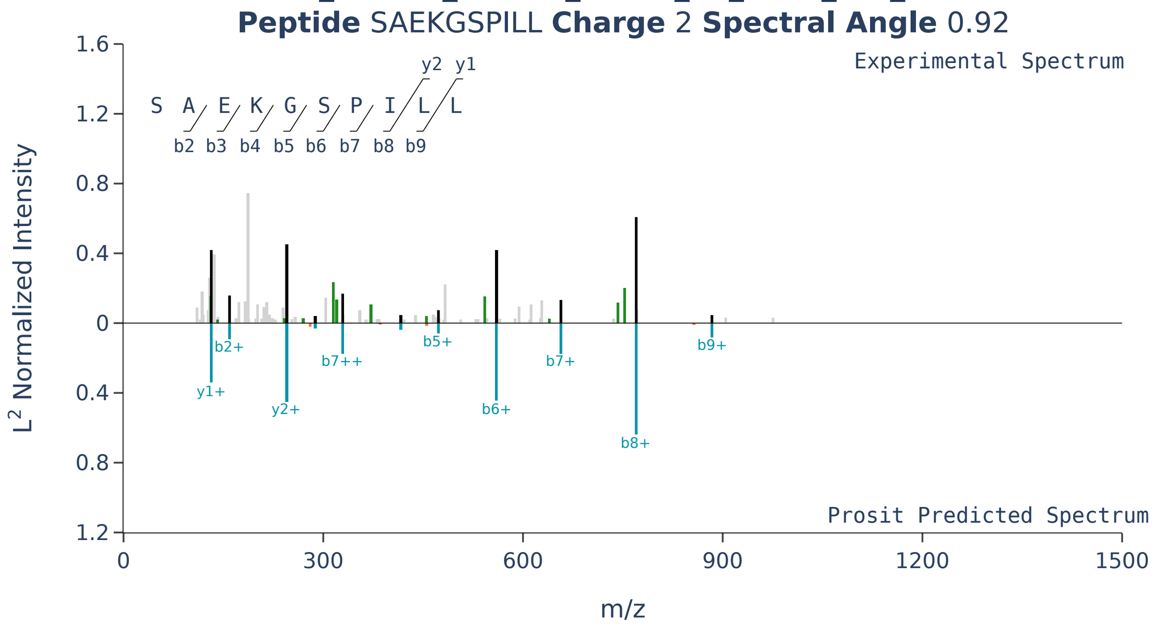

**Source** WSoh_101121_151121_HFGoe_G1_IL37b_1h_R1 **Scan** 14577
**Peptide** SIHDQDHKVLVLD **Charge** 3 **Spectral Angle** 0.89

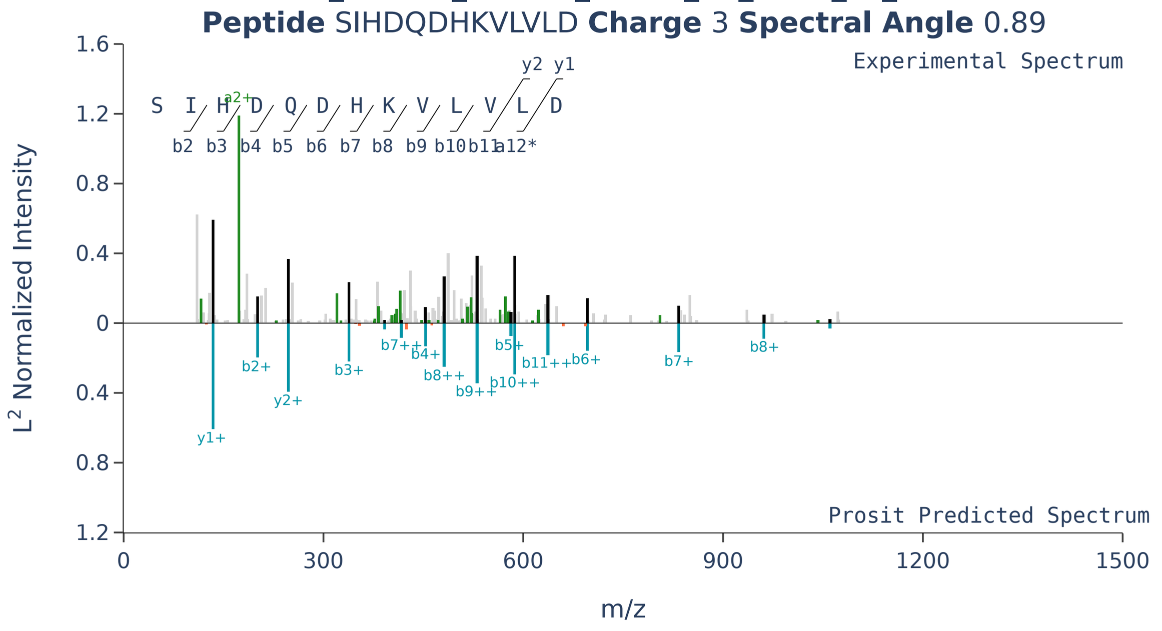

**Source** WSoh_101121_151121_HFGoe_G1_IL37b_1h_R2 **Scan** 22653
**Peptide** SAAHPGWFICT **Charge** 2 **Spectral Angle** 0.94

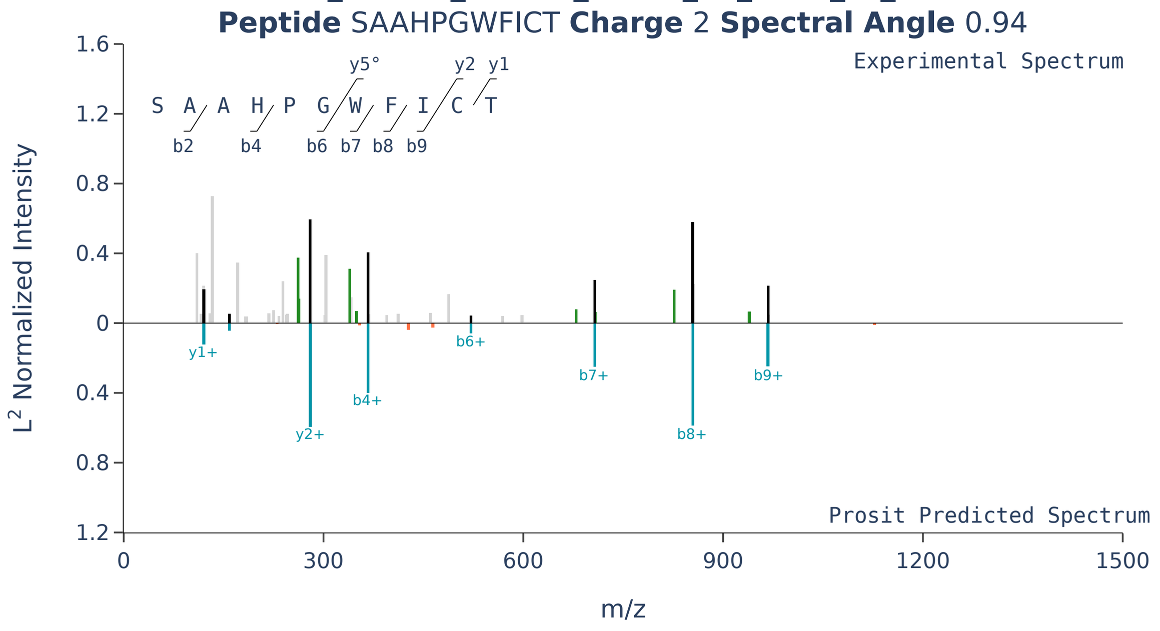

**Source** WSoh_101121_151121_HFGoe_G2_IL37b_4h_R2 **Scan** 8464
**Peptide** ENRKHIEF **Charge** 2 **Spectral Angle** 0.88

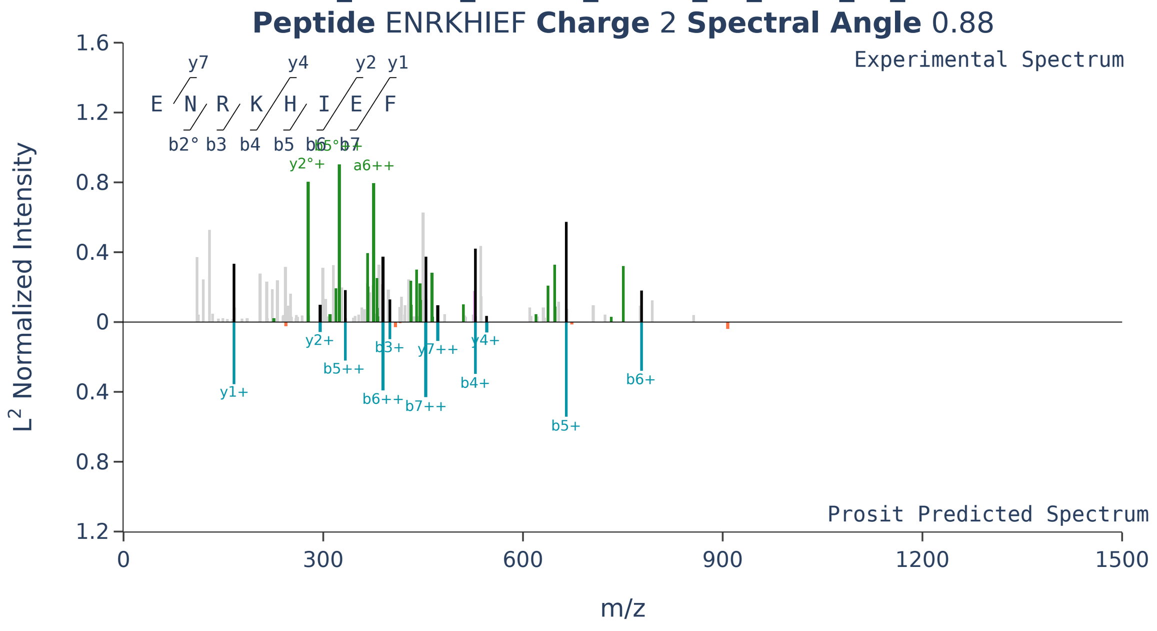

**Source** WSoh_101121_151121_HFGoe_G1_IL37b_1h_R2 **Scan** 22836
**Peptide** PFIFYRA **Charge** 2 **Spectral Angle** 0.95

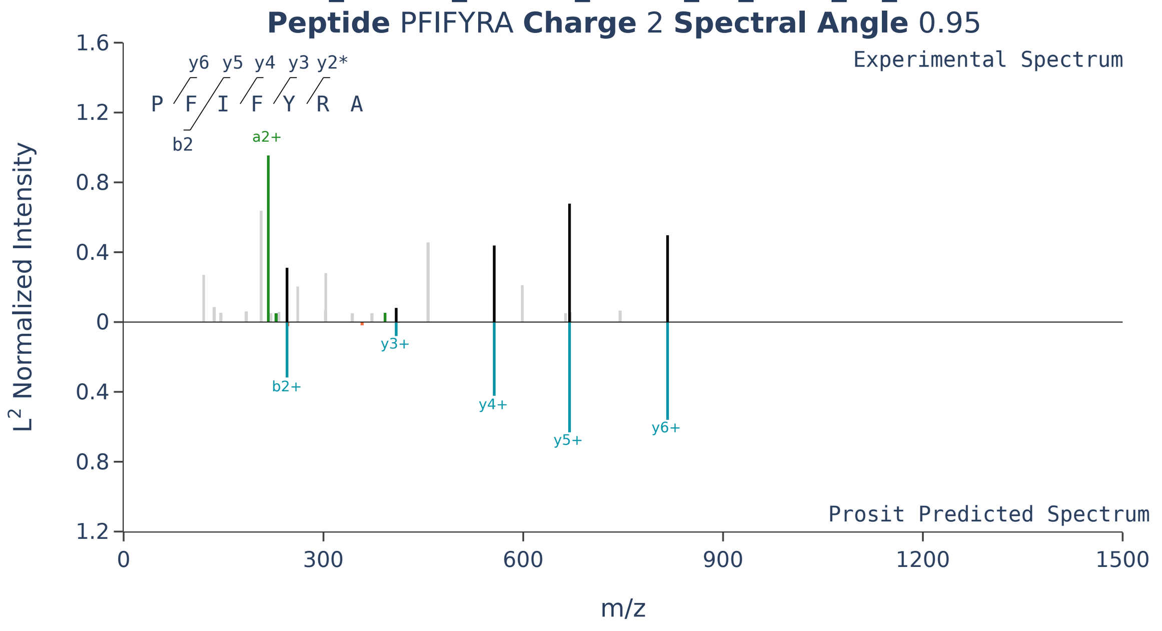

**Source** WSoh_101121_151121_HFGoe_G2_IL37b_24h_R2 **Scan** 7268
**Peptide** NCNEPVG **Charge** 1 **Spectral Angle** 0.94

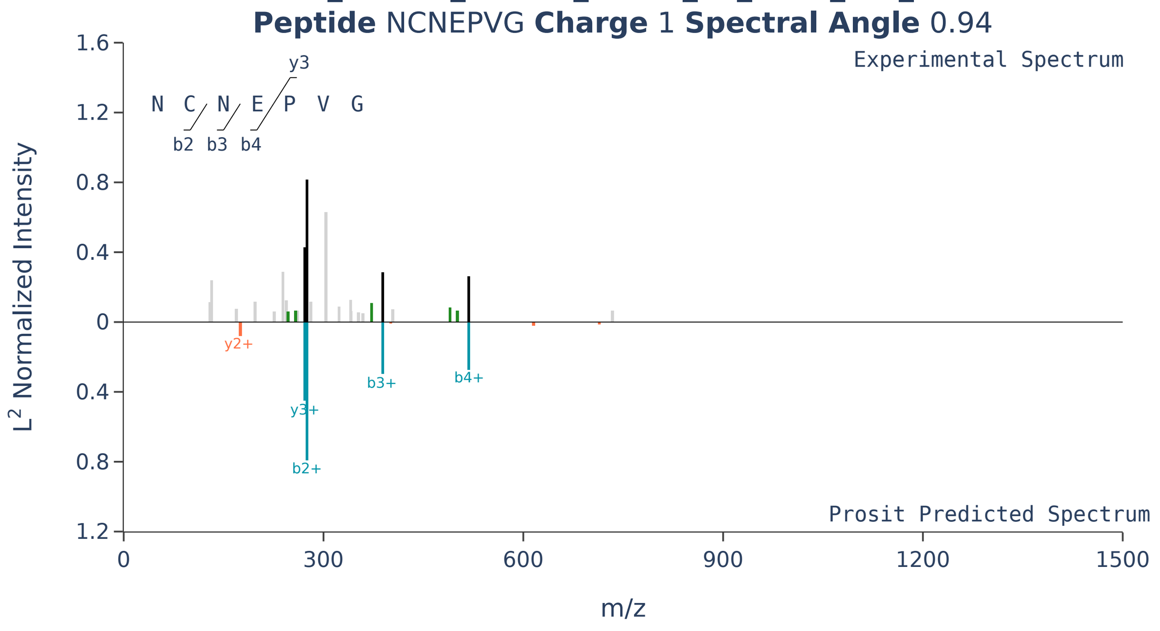

**Source** WSoh_101121_151121_HFGoe_G2_IL37b_24h_R2 **Scan** 6532
**Peptide** VHTSPKVKNL **Charge** 1 **Spectral Angle** 0.84

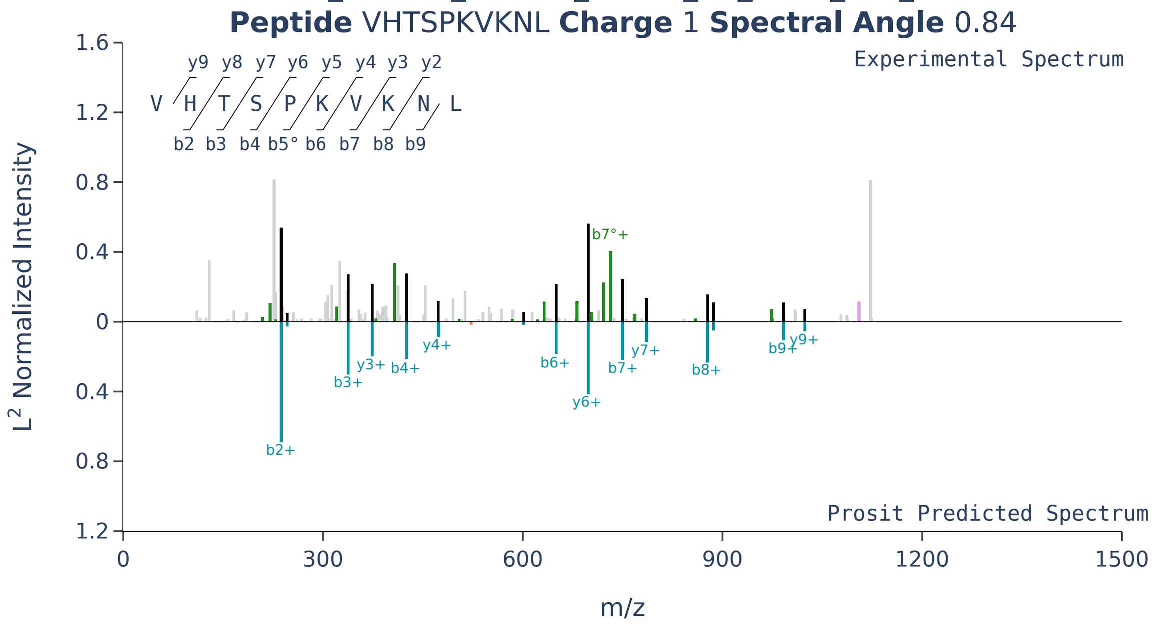

**Source** WSoh_101121_151121_HFGoe_G1_IL37b_24h_R2 **Scan** 8190
**Peptide** GQSHPSL **Charge** 2 **Spectral Angle** 0.94

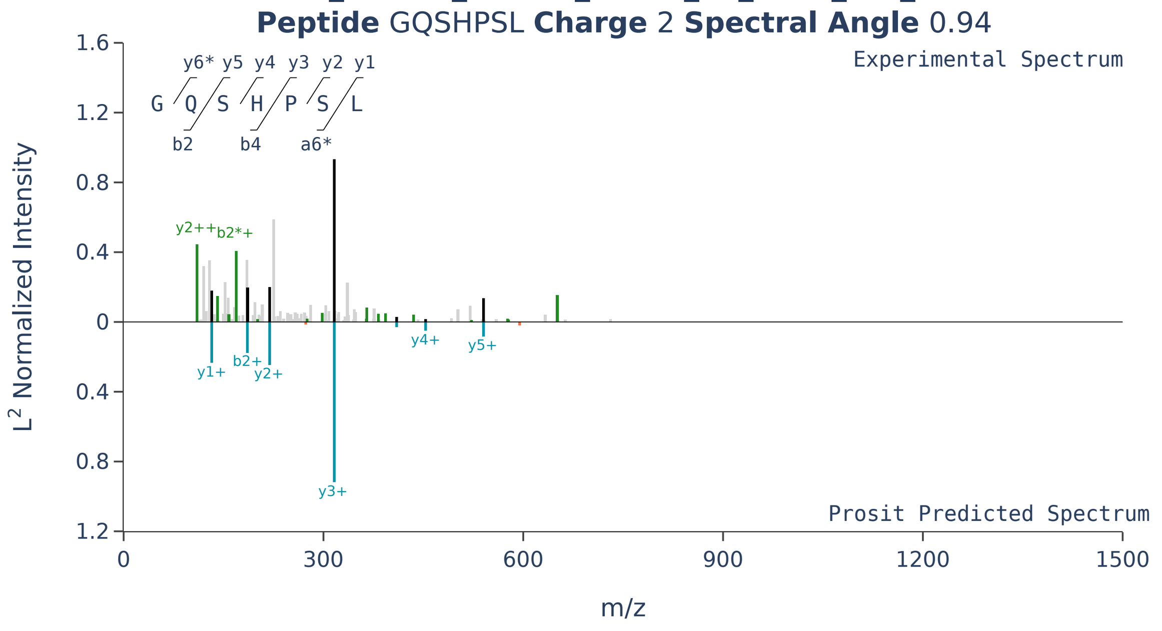

**Source** WSoh_101121_151121_HFGoe_G1_IL37b_24h_R1 **Scan** 11424
**Peptide** LNPKKFSIHD **Charge** 2 **Spectral Angle** 0.84

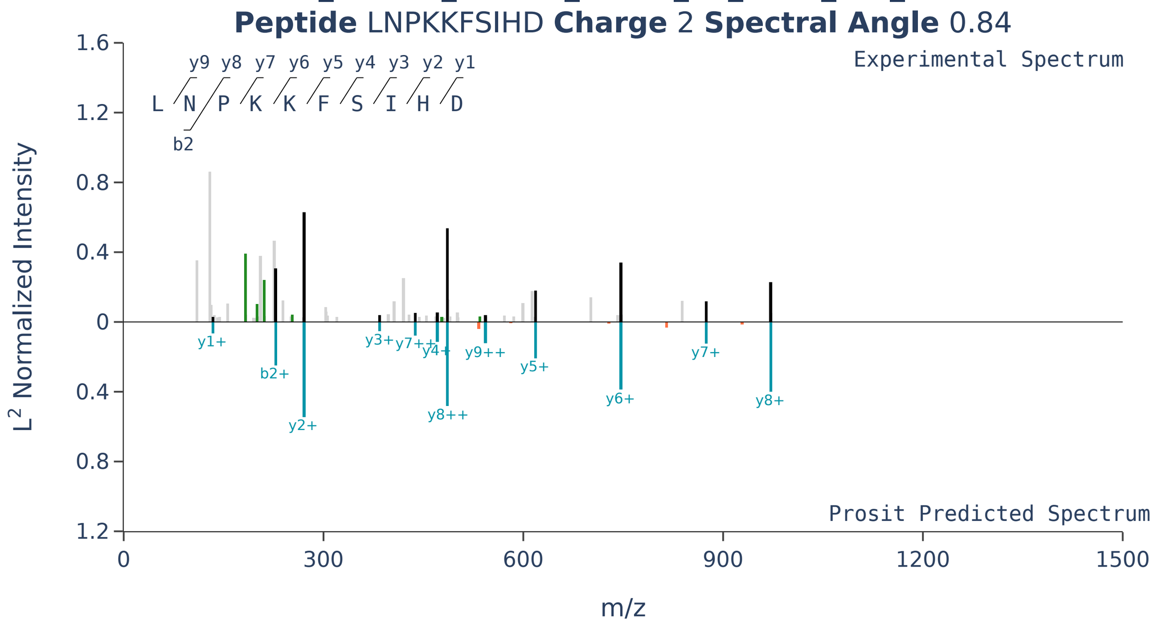

**Source** WSoh_101121_151121_HFGoe_G1_IL37b_24h_R1 **Scan** 13487
**Peptide** HDQDHKVLVLD **Charge** 2 **Spectral Angle** 0.88

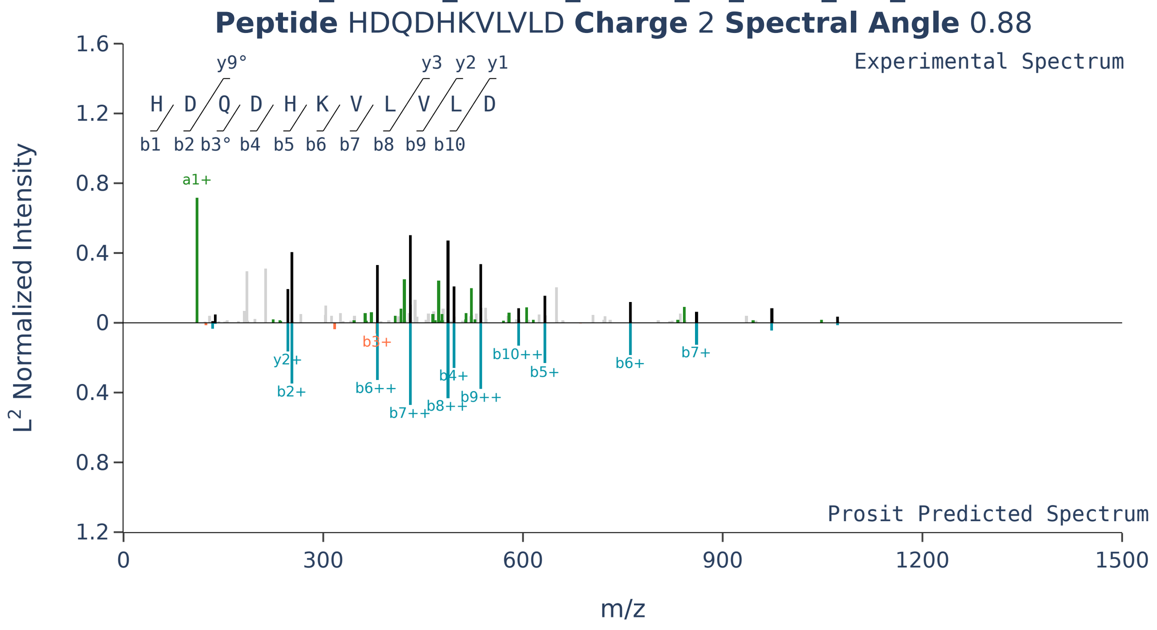


**Source** WSoh_101121_151121_HFGoe_G1_IL37b_24h_R1 **Scan** 9126
**Peptide** KNLNPKKFSIHD **Charge** 2 **Spectral Angle** 0.88

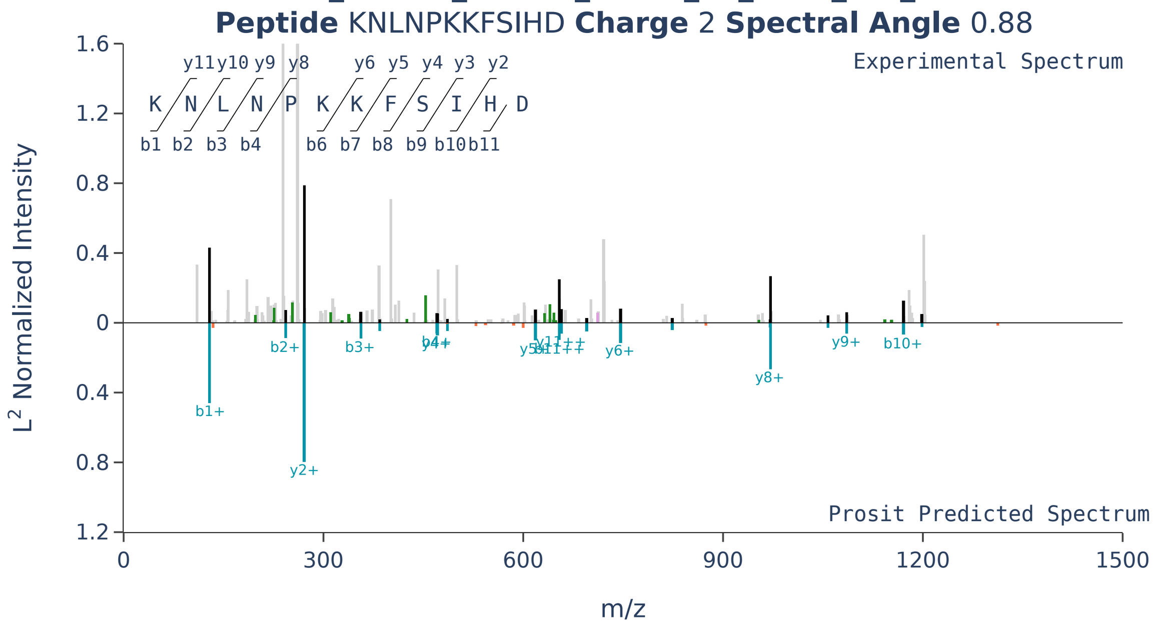


**Source** WSoh_101121_151121_HFGoe_G2_IL37b_24h_R1 **Scan** 6351
**Peptide** VTDKFENRKHI **Charge** 2 **Spectral Angle** 0.84

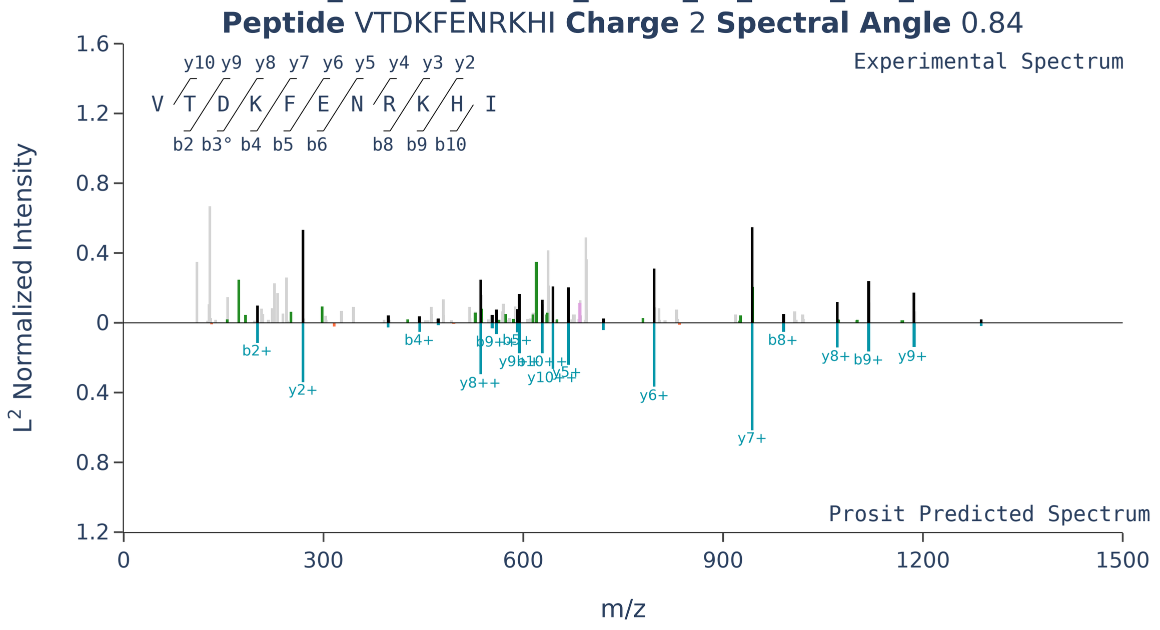


**Source** WSoh_101121_151121_HFGoe_G2_IL37b_24h_R1 **Scan** 9926
**Peptide** KLMKLAAQ **Charge** 2 **Spectral Angle** 0.88

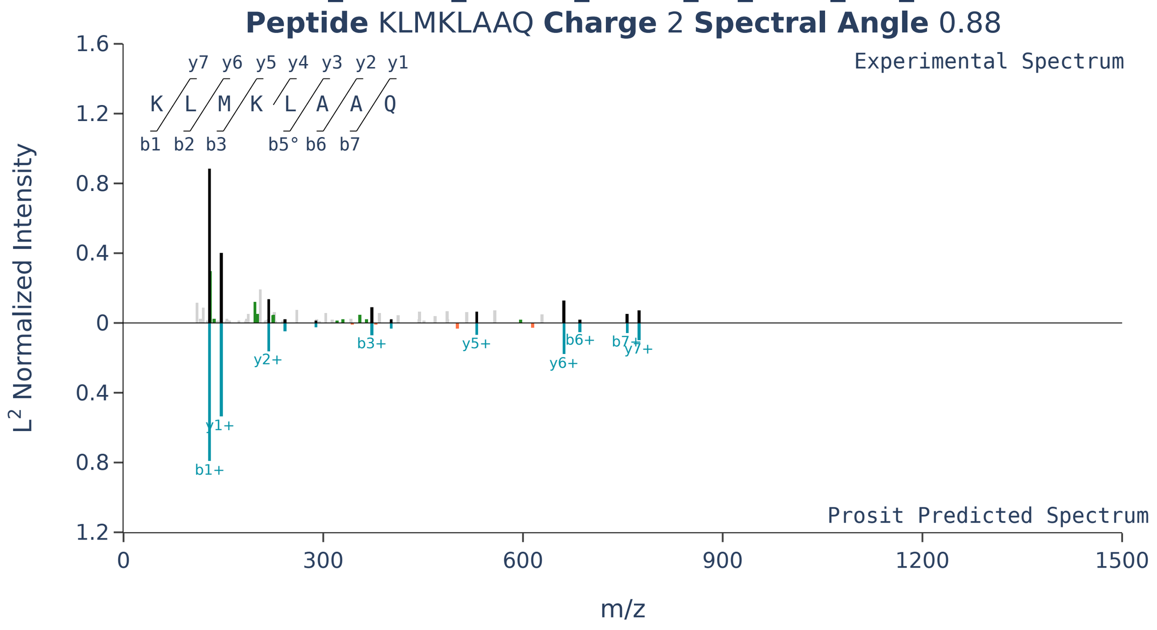


**Source** WSoh_101121_151121_HFGoe_G1_IL37b_1h_R1 **Scan** 19054
**Peptide** RPFIFYRA **Charge** 3 **Spectral Angle** 0.9

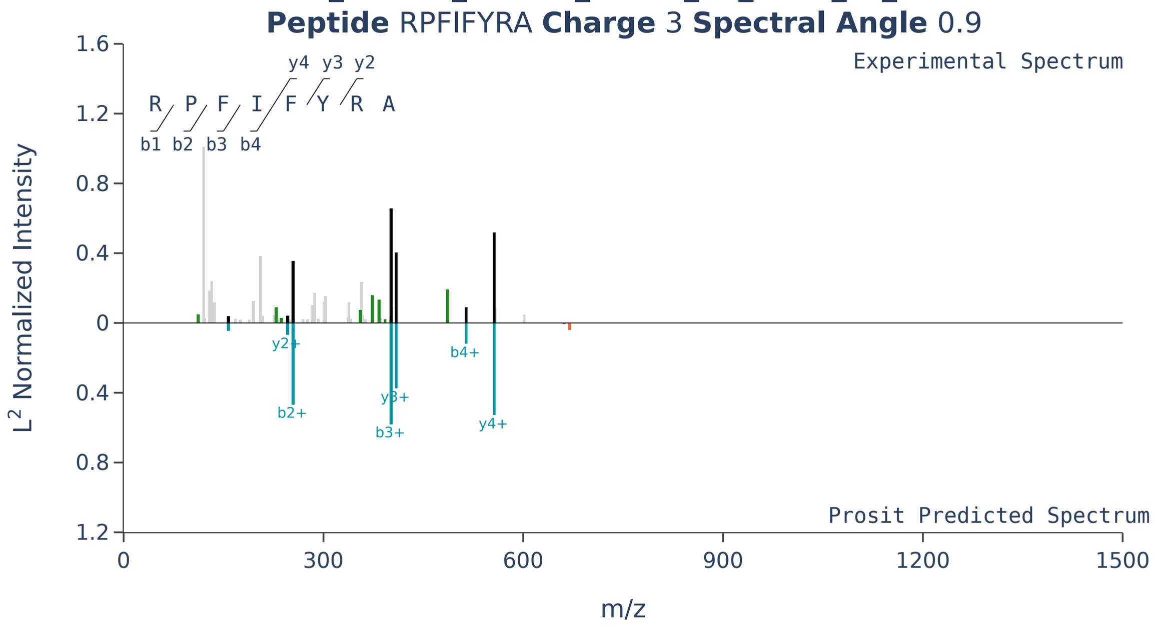


**Source** WSoh_101121_151121_HFGoe_G2_IL37b_24h_R1 **Scan** 26833
**Peptide** IRPEIFF **Charge** 2 **Spectral Angle** 0.91

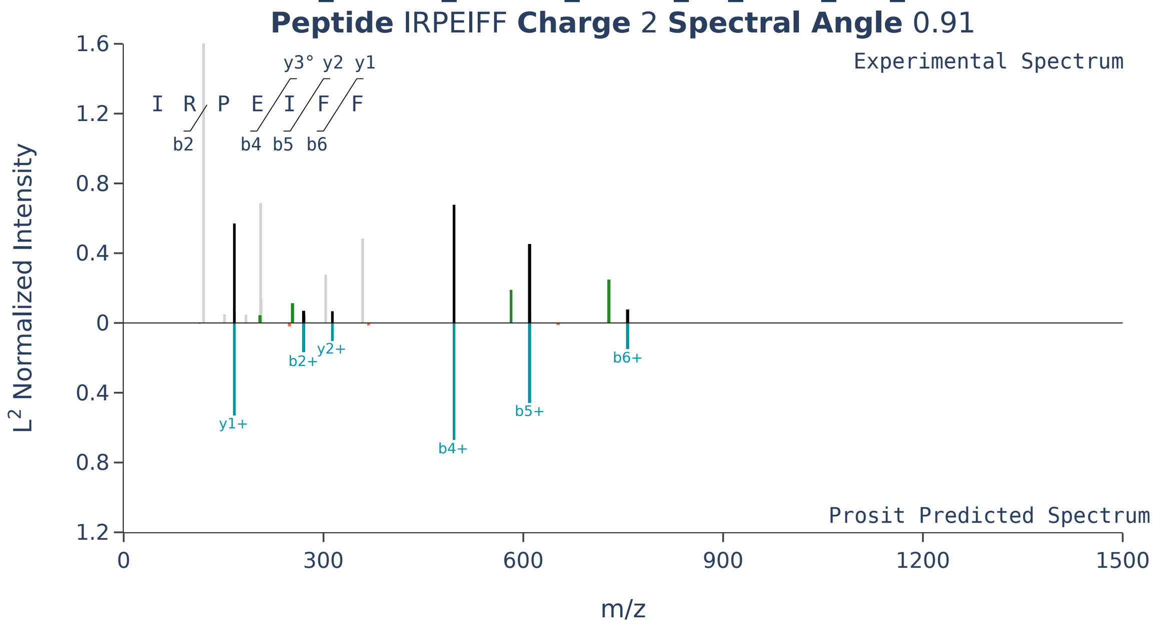


**Source** WSoh_101121_151121_HFGoe_G1_IL37b_4h_R2 **Scan** 13002
**Peptide** DKDKGQSHPSLQL **Charge** 2 **Spectral Angle** 0.9

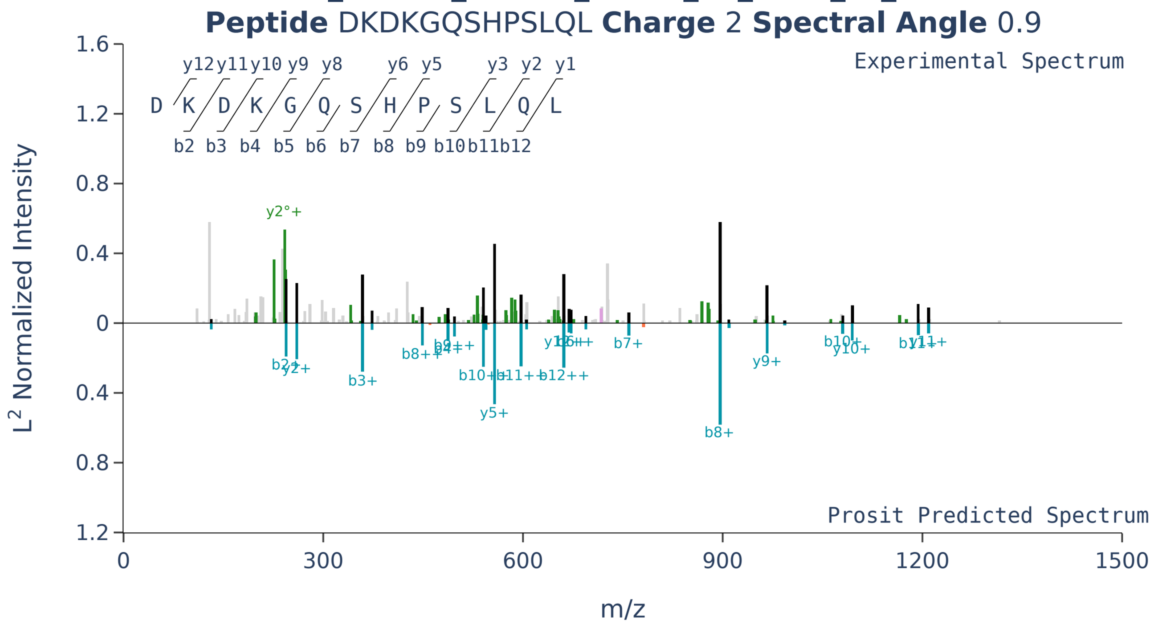


**Source** WSoh_101121_151121_HFGoe_G1_IL37b_24h_R2 **Scan** 12788
**Peptide** NEPVGVTDKFENR **Charge** 2 **Spectral Angle** 0.81

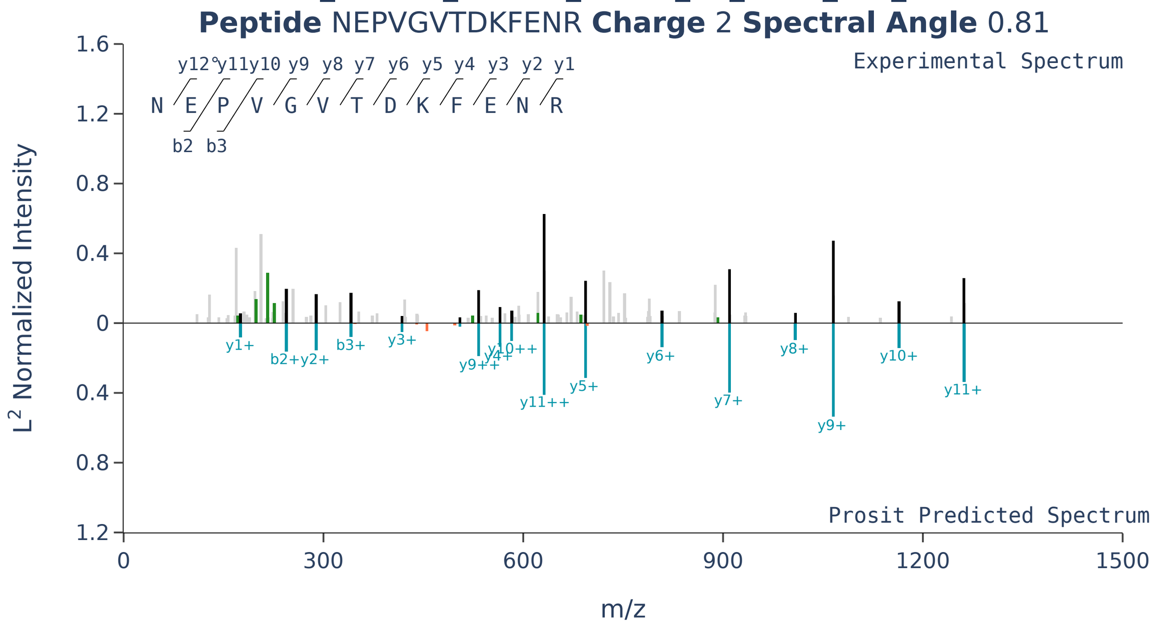


**Source** WSoh_101121_151121_HFGoe_G1_IL37b_1h_R2 **Scan** 7045
**Peptide** TSPKVKNL **Charge** 2 **Spectral Angle** 0.85

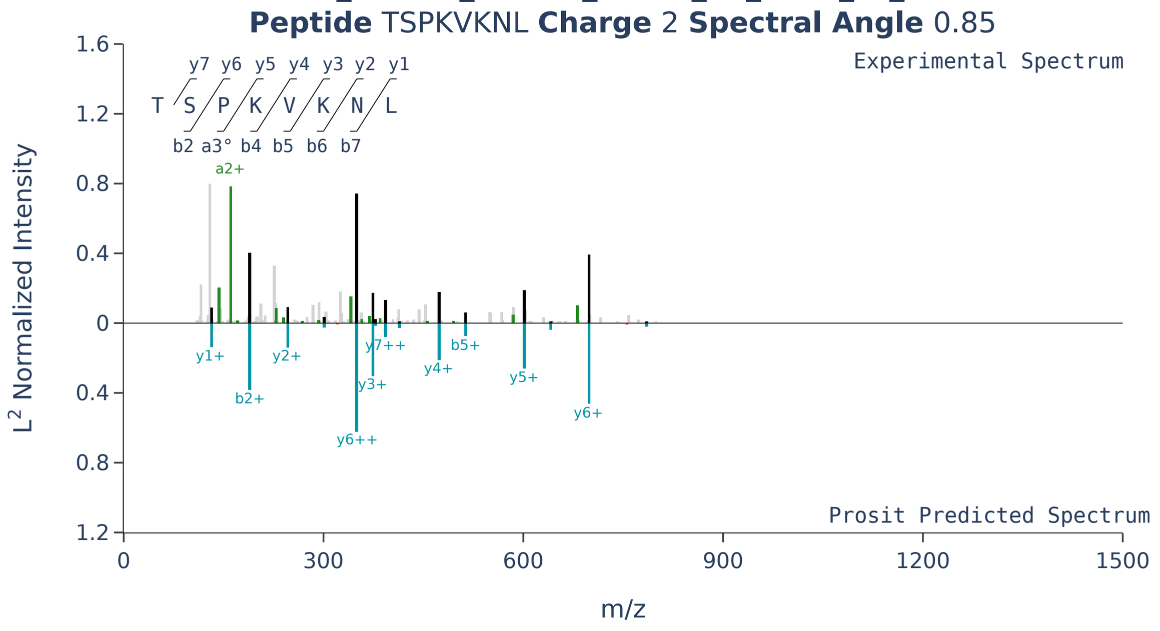


**Source** WSoh_101121_151121_HFGoe_G1_IL37b_24h_R2 **Scan** 11578
**Peptide** AVPDKNYIRP **Charge** 2 **Spectral Angle** 0.88

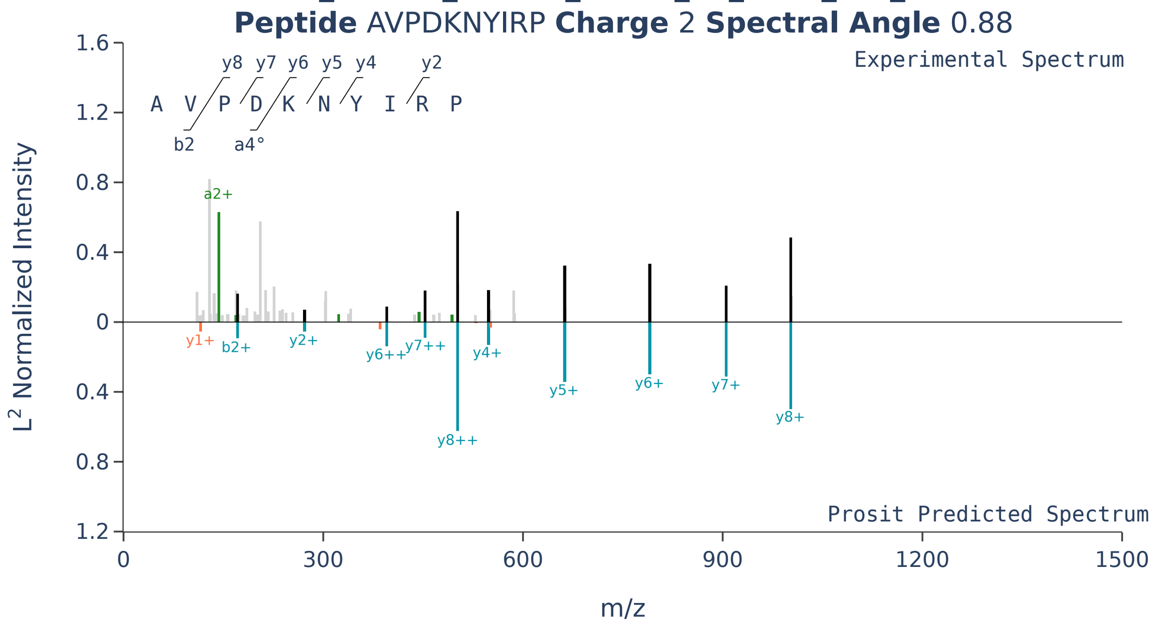


**Source** WSoh_101121_151121_HFGoe_G1_IL37b_24h_R2 **Scan** 7510
**Peptide** LNPKKFS **Charge** 2 **Spectral Angle** 0.89

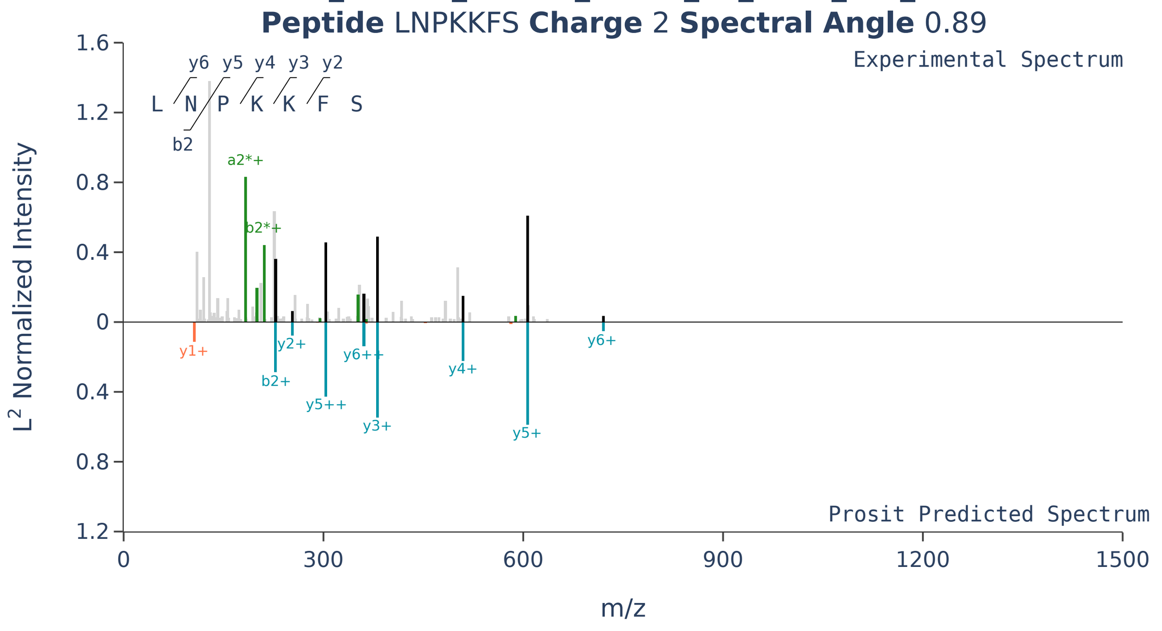


**Source** WSoh_101121_151121_HFGoe_G1_IL37b_1h_R1 **Scan** 6845
**Peptide** PSLQLKKEK **Charge** 2 **Spectral Angle** 0.85

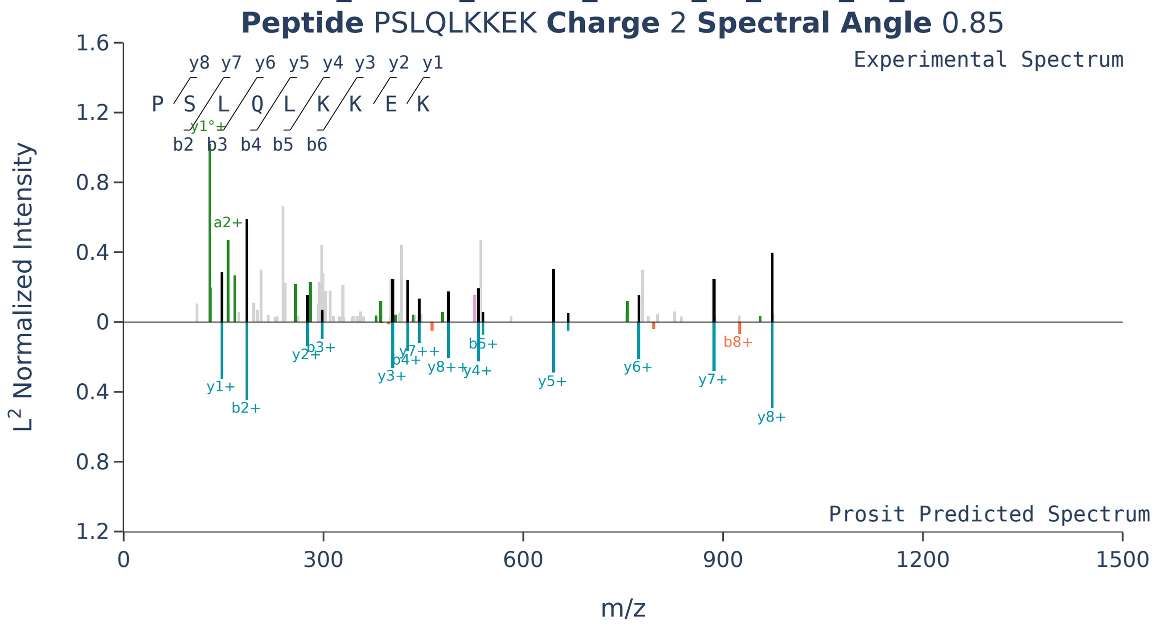


**Source** WSoh_101121_151121_HFGoe_G2_IL37b_4h_R2 **Scan** 7140
**Peptide** AVPDKNY **Charge** 2 **Spectral Angle** 0.8

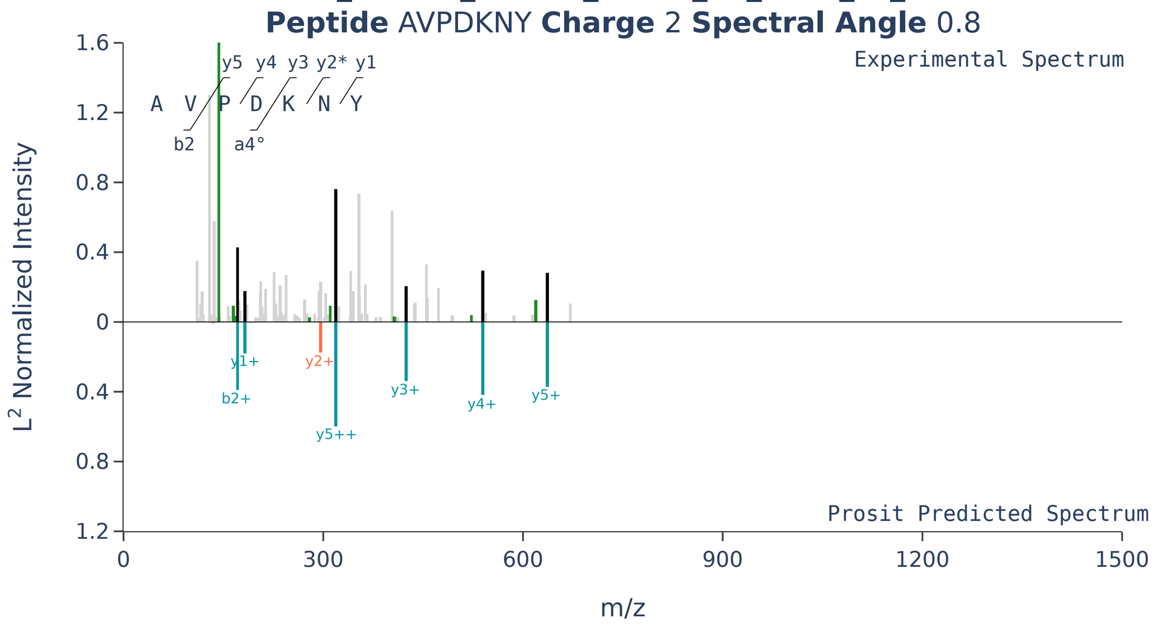


**Source** WSoh_101121_151121_HFGoe_G2_IL37b_24h_R1 **Scan** 13394
**Peptide** NCNEPVGVTDKFENR **Charge** 3 **Spectral Angle** 0.87

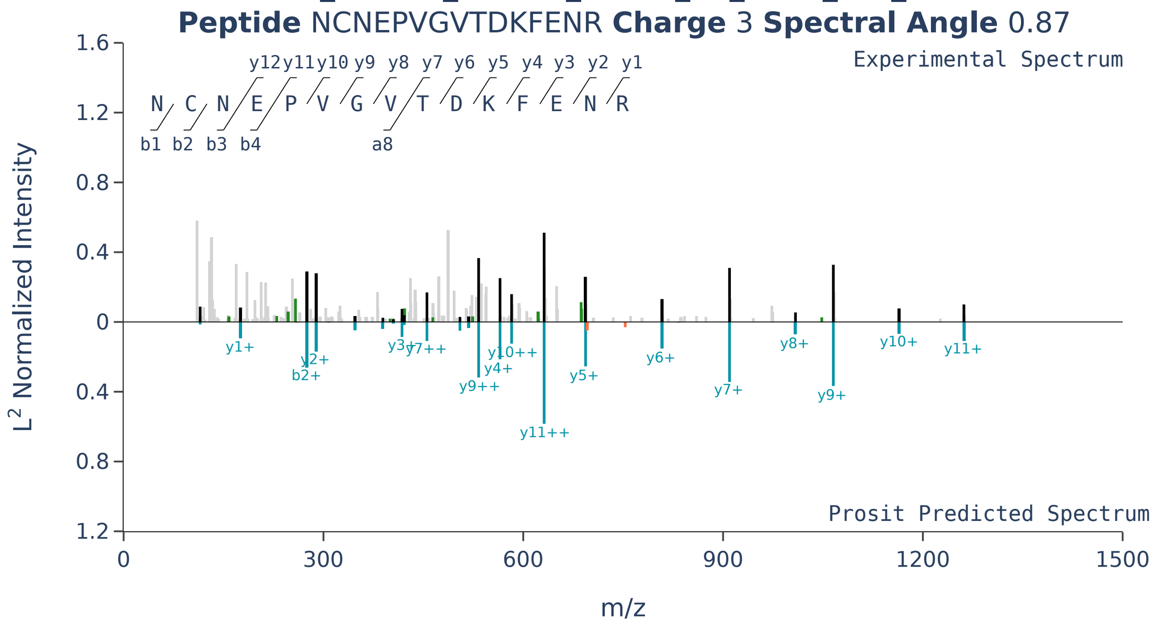


**Source** WSoh_101121_151121_HFGoe_G1_IL37b_24h_R1 **Scan** 10792
**Peptide** NEPVGVTD **Charge** 1 **Spectral Angle** 0.82

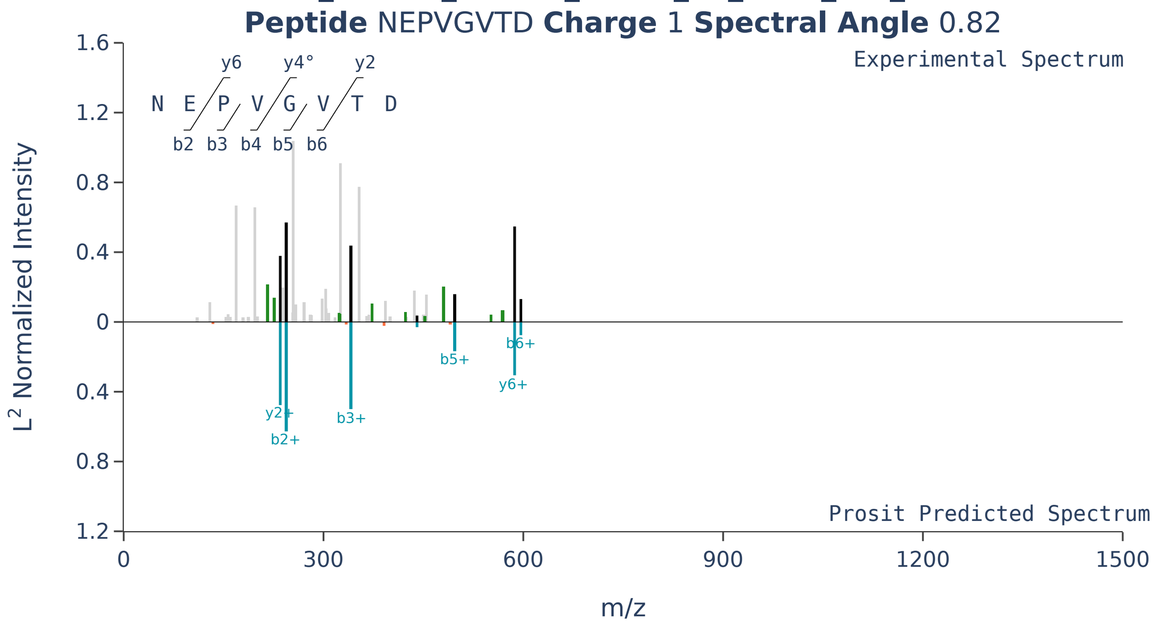

**Source** WSoh_101121_151121_HFGoe_G1_IL37b_1h_R2 **Scan** 7642
**Peptide** AEMSPSE **Charge** 1 **Spectral Angle** 0.91

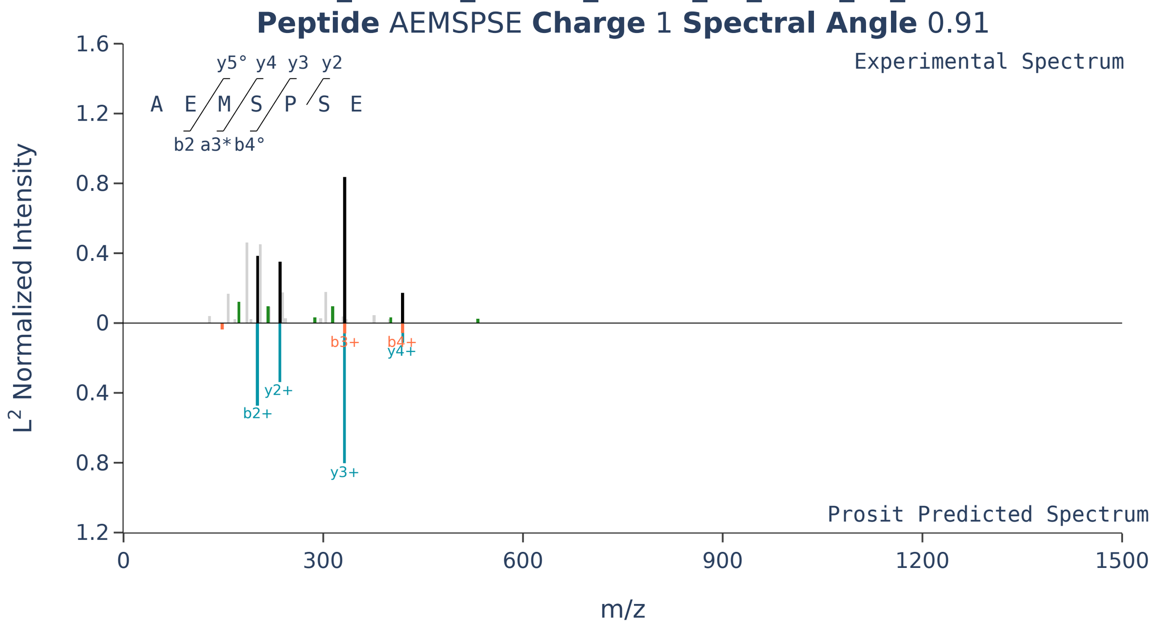


**Source** WSoh_101121_151121_HFGoe_G1_IL37b_24h_R1 **Scan** 15528
**Peptide** SIHDQDHKVLVL **Charge** 2 **Spectral Angle** 0.85

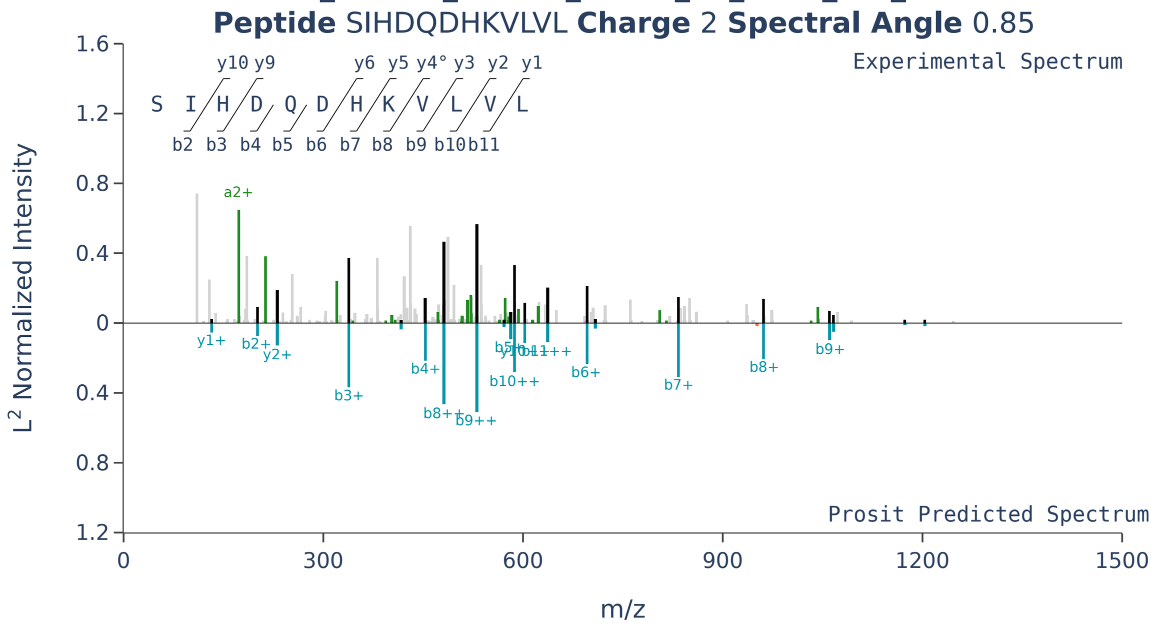


**Source** WSoh_101121_151121_HFGoe_G1_IL37b_4h_R1 **Scan** 17118
**Peptide** ASAEKGSPILLGVSK **Charge** 2 **Spectral Angle** 0.9

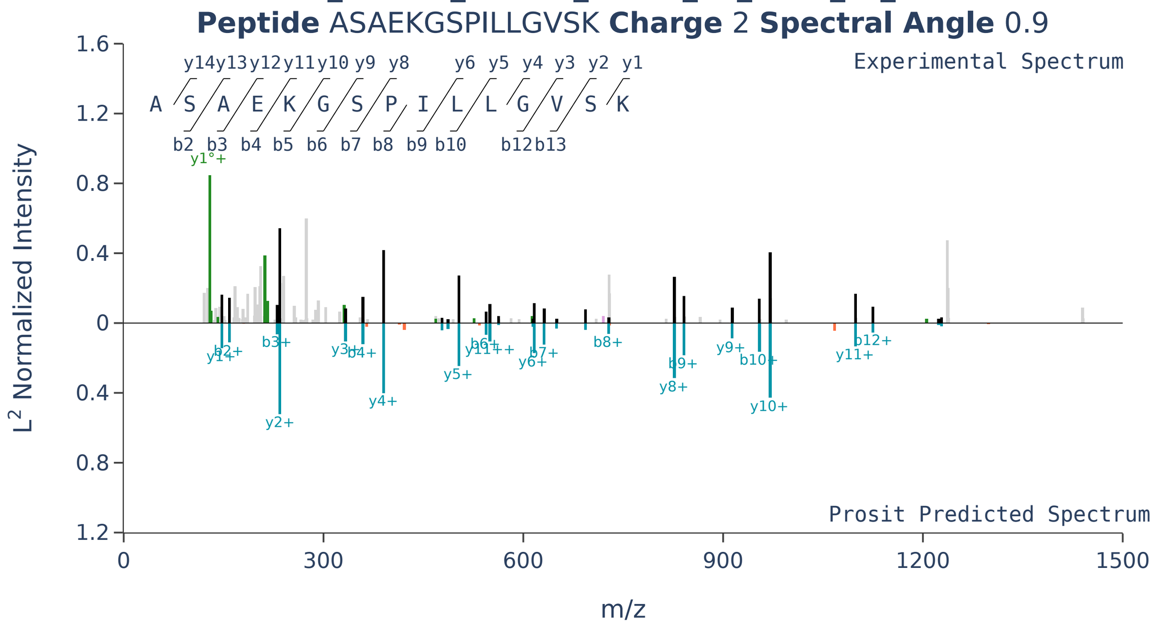


**Source** WSoh_101121_151121_HFGoe_G1_IL37b_2h_R1 **Scan** 17094
**Peptide** NCNEPVGVTDKF **Charge** 2 **Spectral Angle** 0.92

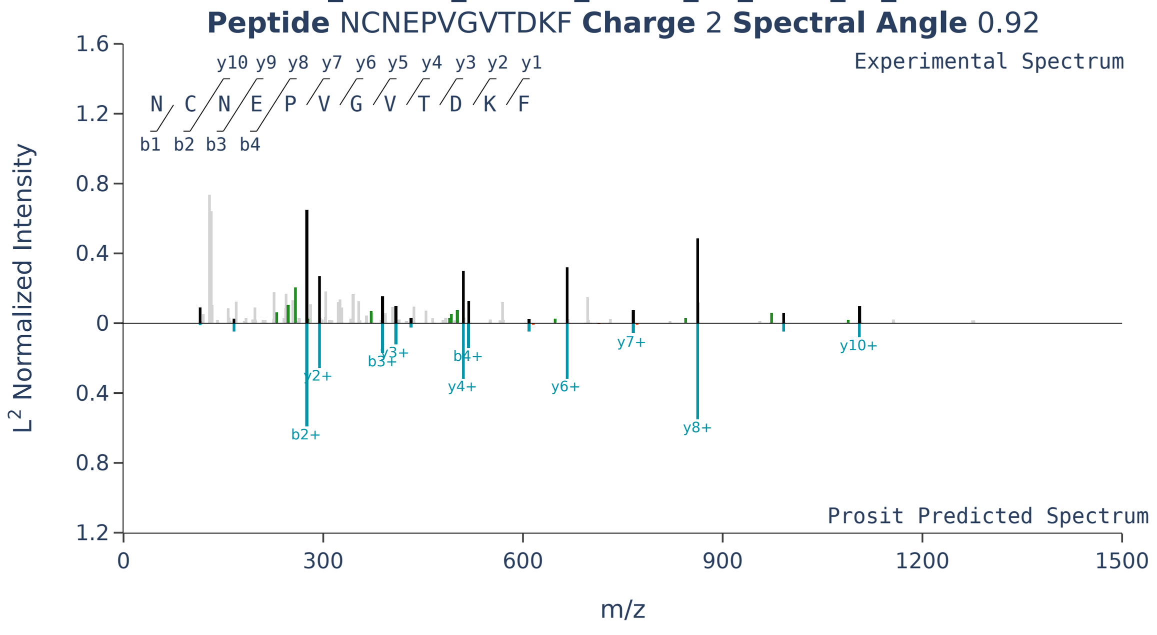


**Source** WSoh_101121_151121_HFGoe_G1_IL37b_4h_R1 **Scan** 11638
**Peptide** KDKGQSHPSLQL **Charge** 3 **Spectral Angle** 0.85

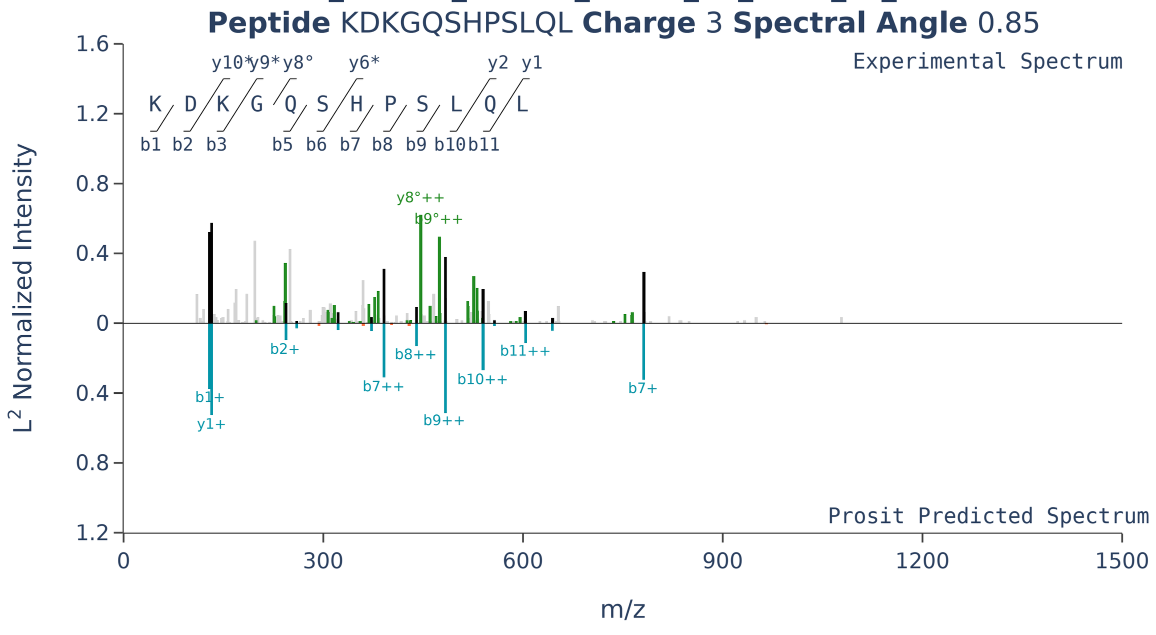


**Source** WSoh_101121_151121_HFGoe_G2_IL37b_24h_R1 **Scan** 9596
**Peptide** NCNEPVGVTDKFENRKH **Charge** 3 **Spectral Angle** 0.81

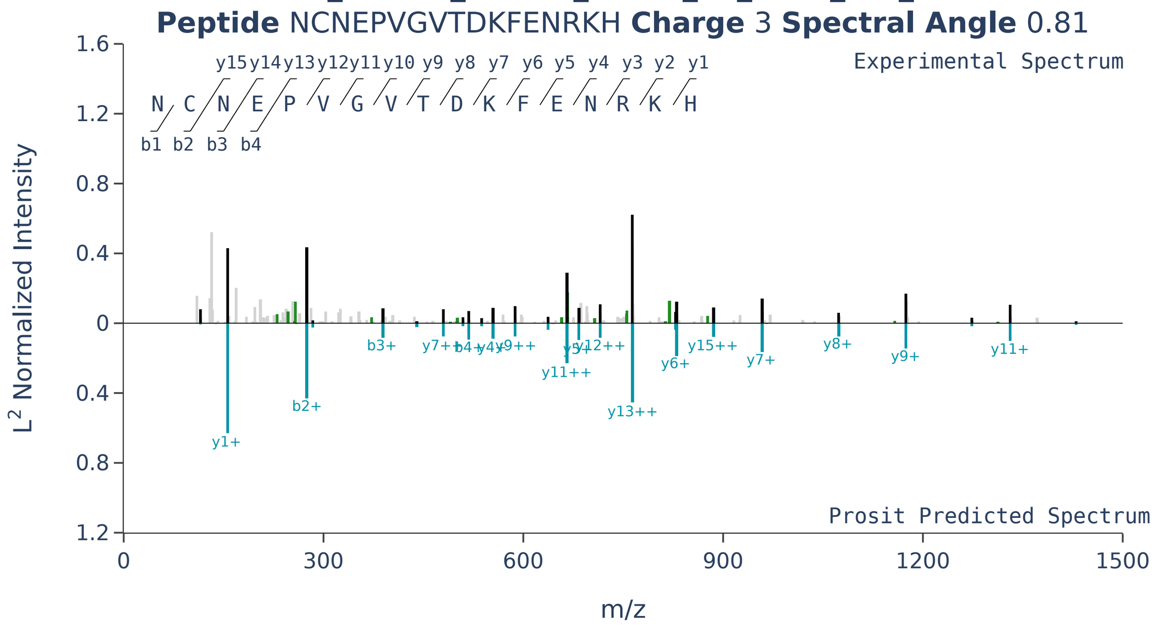


**Source** WSoh_101121_151121_HFGoe_G1_IL37b_24h_R2 **Scan** 19994
**Peptide** SKGEFCLY **Charge** 2 **Spectral Angle** 0.93

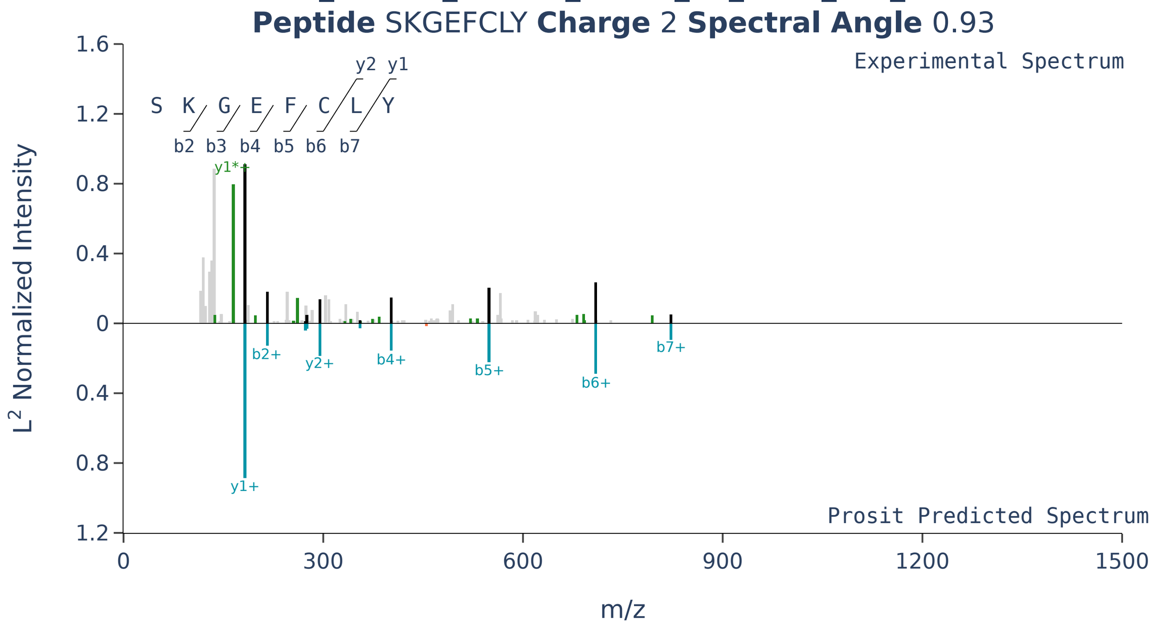


**Source** WSoh_101121_151121_HFGoe_G1_IL37b_1h_R2 **Scan** 24587
**Peptide** PDKNYIRPEIFF **Charge** 3 **Spectral Angle** 0.9

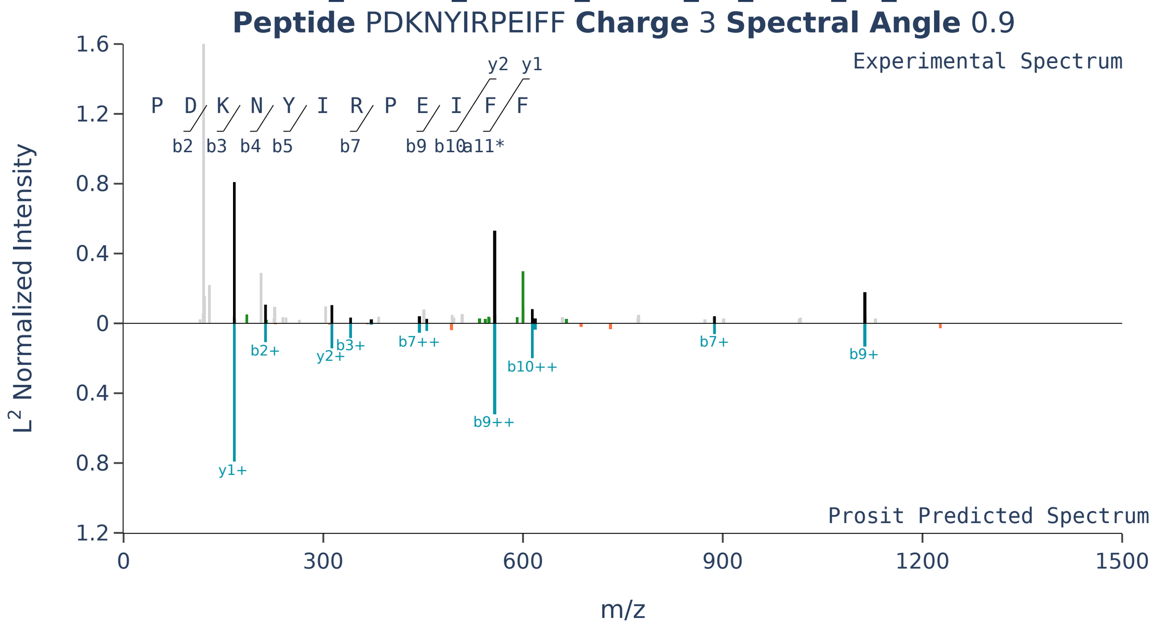


**Source** WSoh_101121_151121_HFGoe_G1_IL37b_4h_R1 **Scan** 5672
**Peptide** TDKFENRKHIE **Charge** 2 **Spectral Angle** 0.85

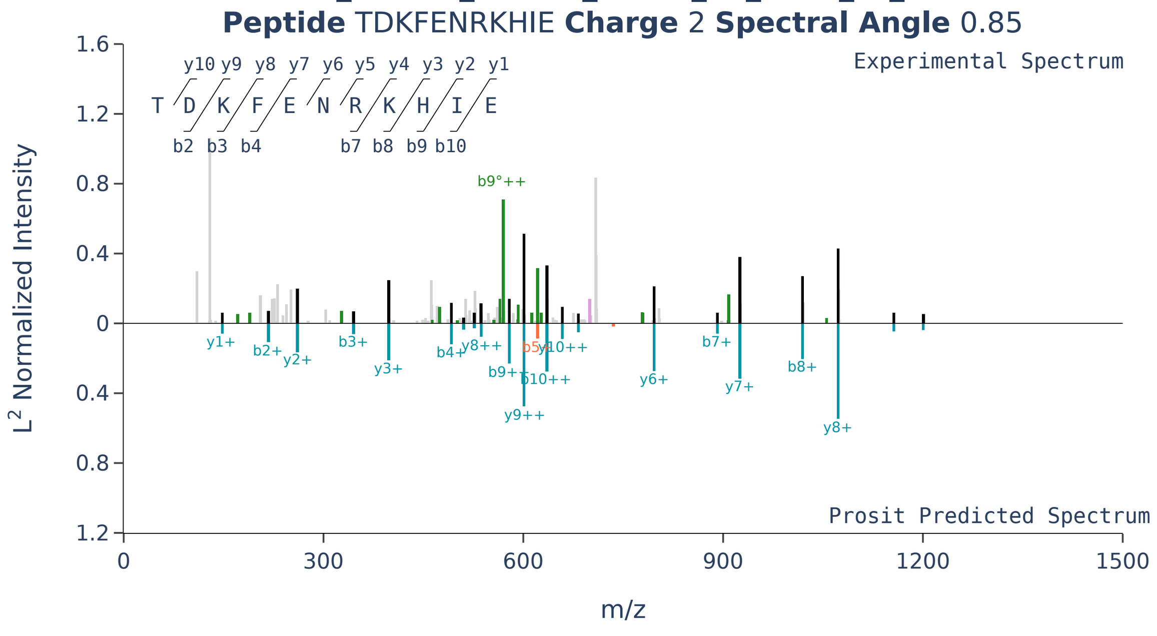


**Source** WSoh_101121_151121_HFGoe_G2_IL37b_1h_R1 **Scan** 11969
**Peptide** RAQVGSW **Charge** 2 **Spectral Angle** 0.91

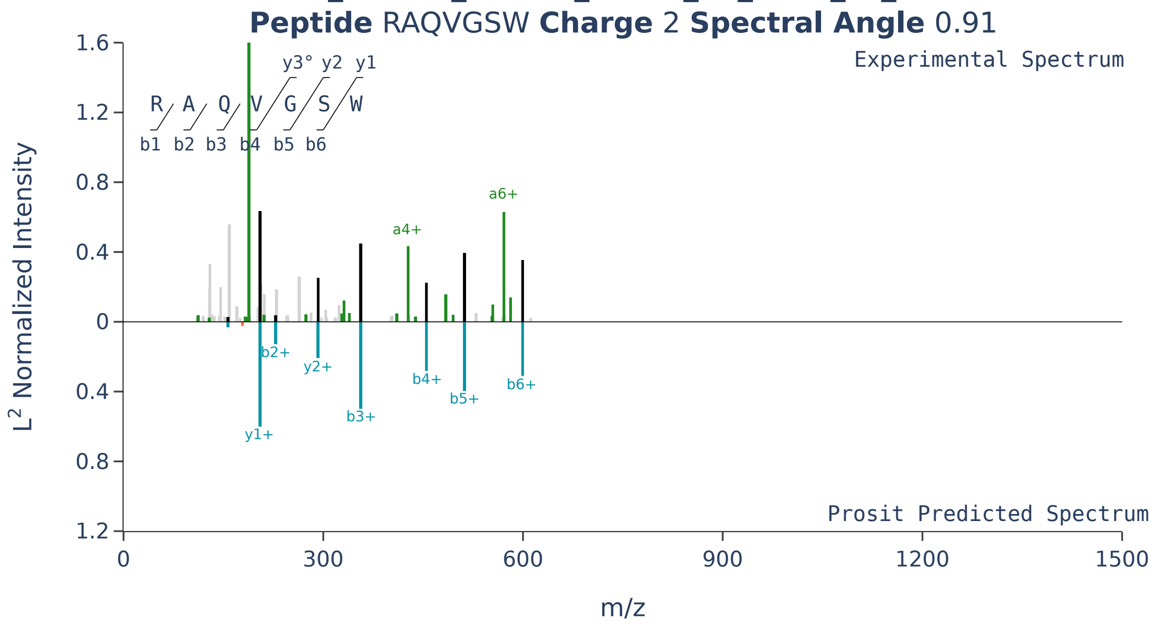


**Source** WSoh_101121_151121_HFGoe_G1_IL37b_24h_R2 **Scan** 12937
**Peptide** KGQSHPSLQL **Charge** 3 **Spectral Angle** 0.89

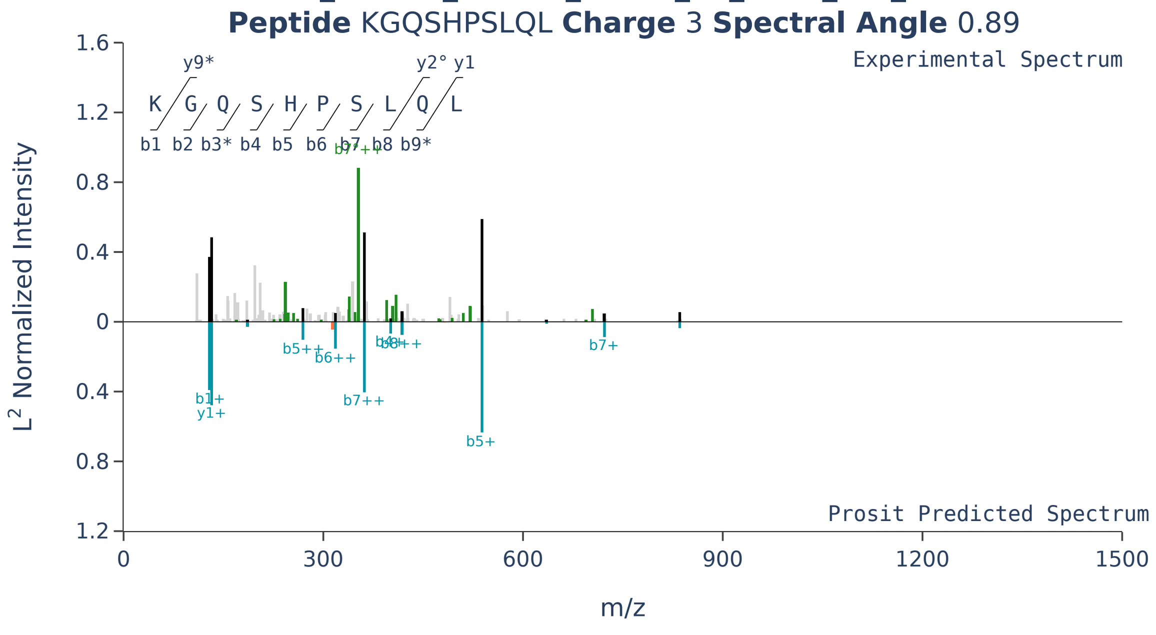


**Source** WSoh_101121_151121_HFGoe_G1_IL37b_24h_R2 **Scan** 8892
**Peptide** NEPVGVTDKFENRKH **Charge** 2 **Spectral Angle** 0.84

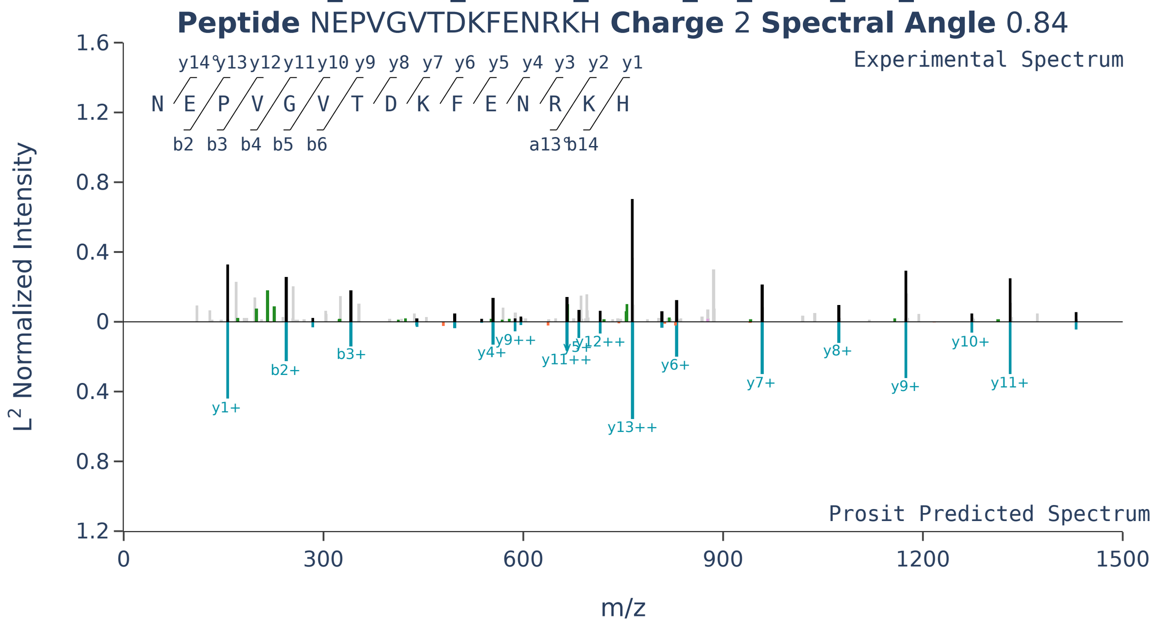


**Source** WSoh_101121_151121_HFGoe_G1_IL37b_24h_R2 **Scan** 8050
**Peptide** DKNYIRPE **Charge** 2 **Spectral Angle** 0.91

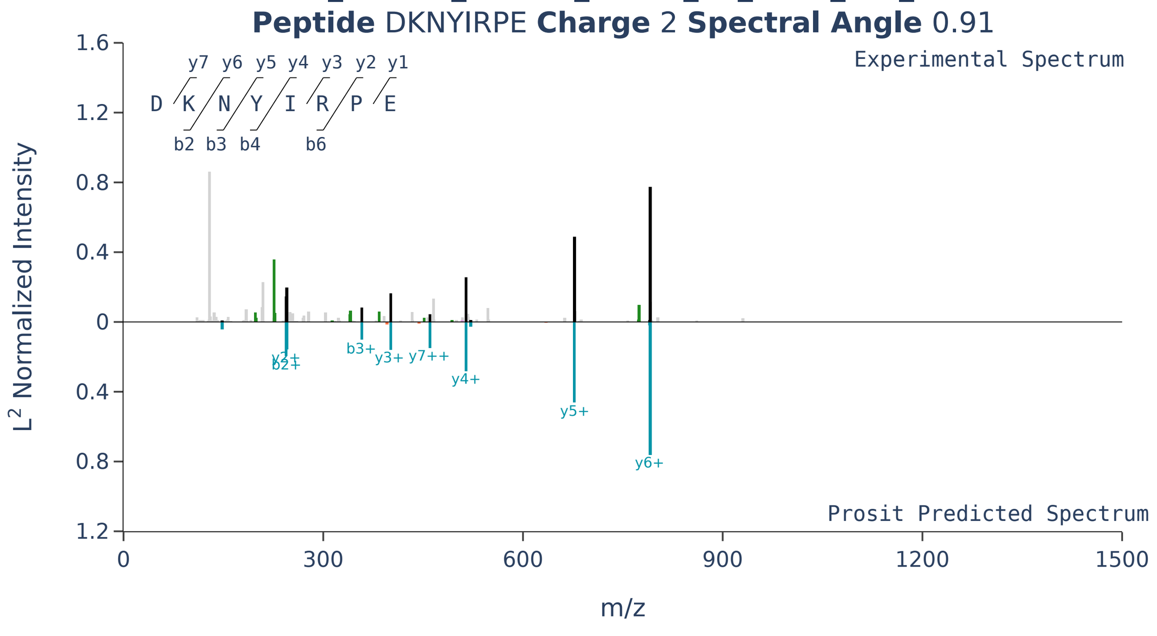


**Source** WSoh_101121_151121_HFGoe_G1_IL37b_24h_R1 **Scan** 14949
**Peptide** IHDQDHKVLVL **Charge** 2 **Spectral Angle** 0.82

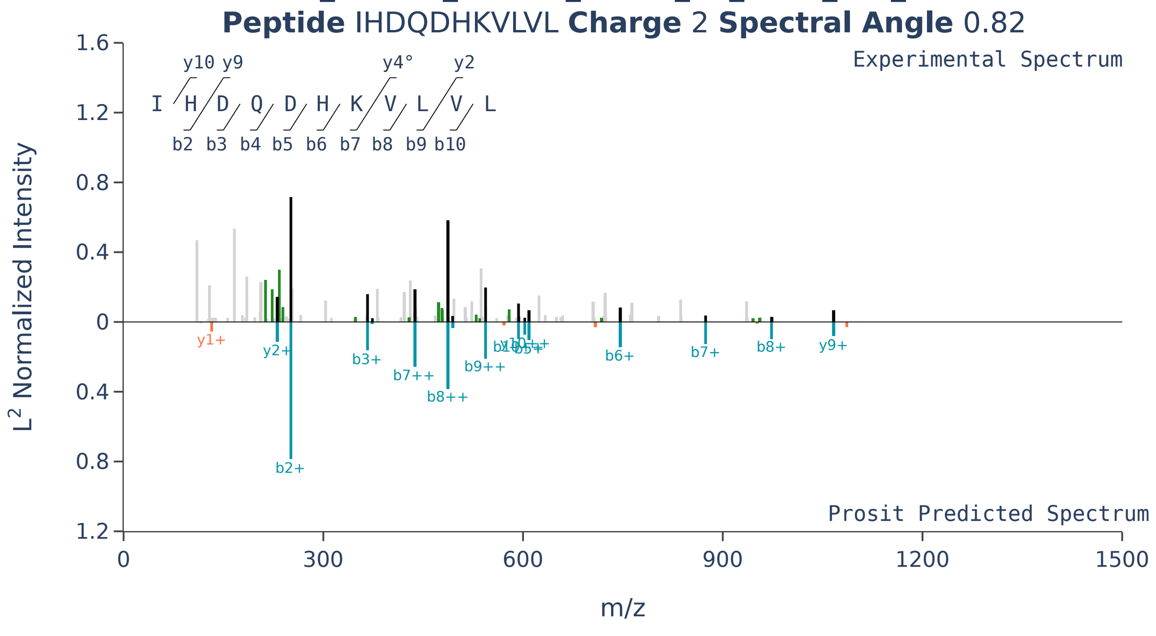


**Source** WSoh_101121_151121_HFGoe_G1_IL37b_1h_R2 **Scan** 21974
**Peptide** DSGNLIAV **Charge** 2 **Spectral Angle** 0.93

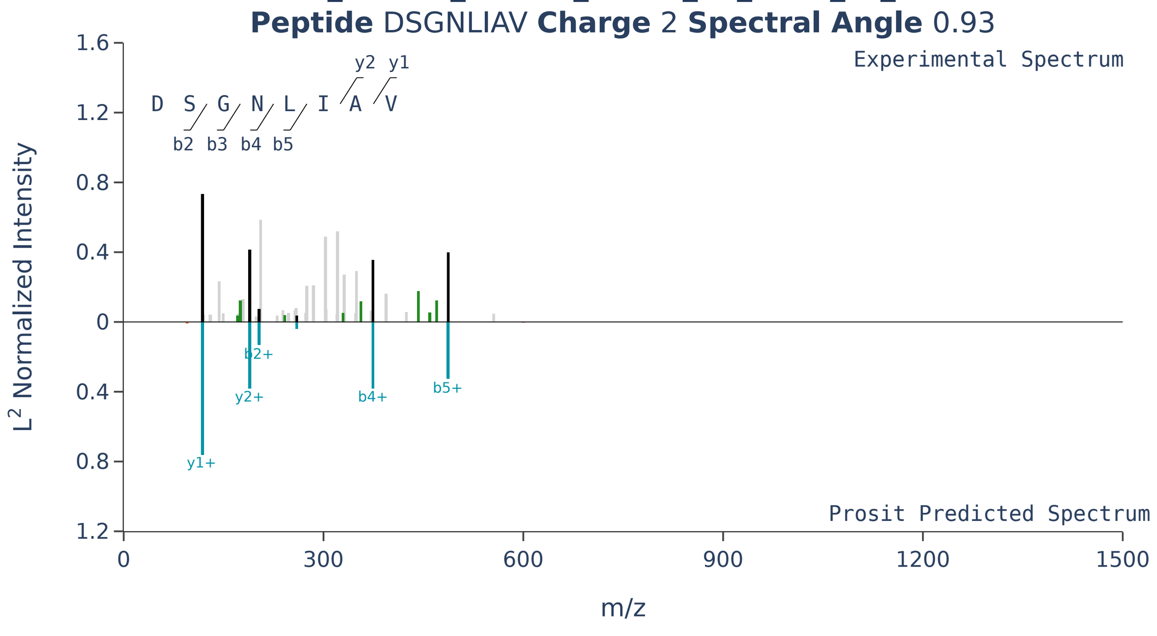

**Source** WSoh_101121_151121_HFGoe_G1_IL37b_1h_R1 **Scan** 14919
**Peptide** QDHKVLVLD **Charge** 1 **Spectral Angle** 0.81

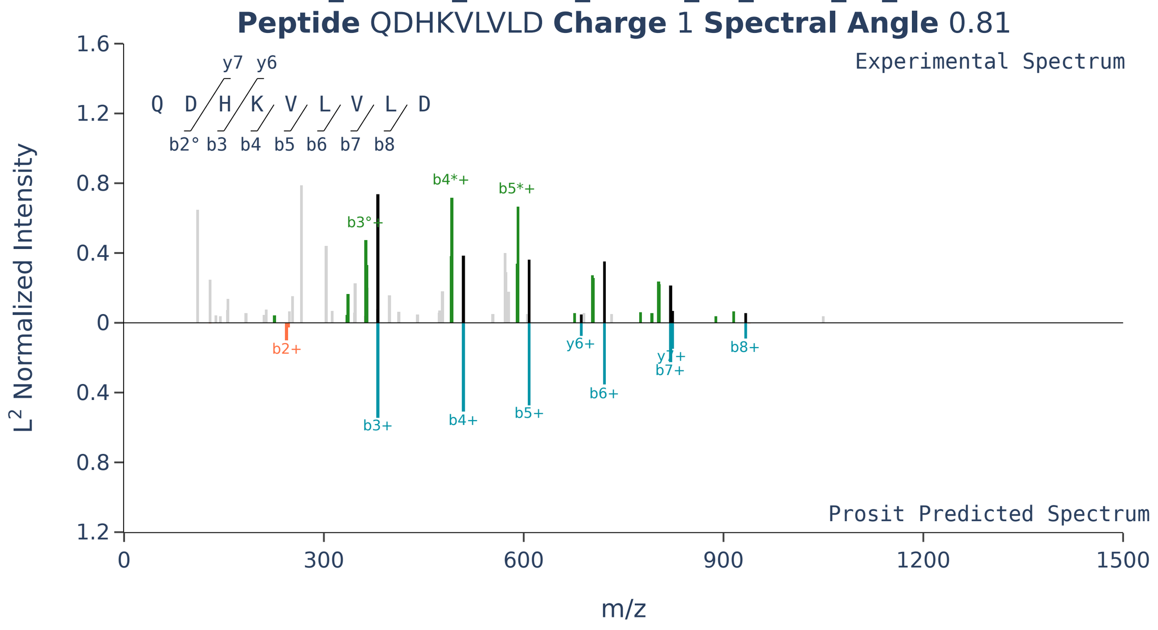


**Source** WSoh_101121_151121_HFGoe_G2_IL37b_24h_R1 **Scan** 13008
**Peptide** QVGSWNM **Charge** 2 **Spectral Angle** 0.9

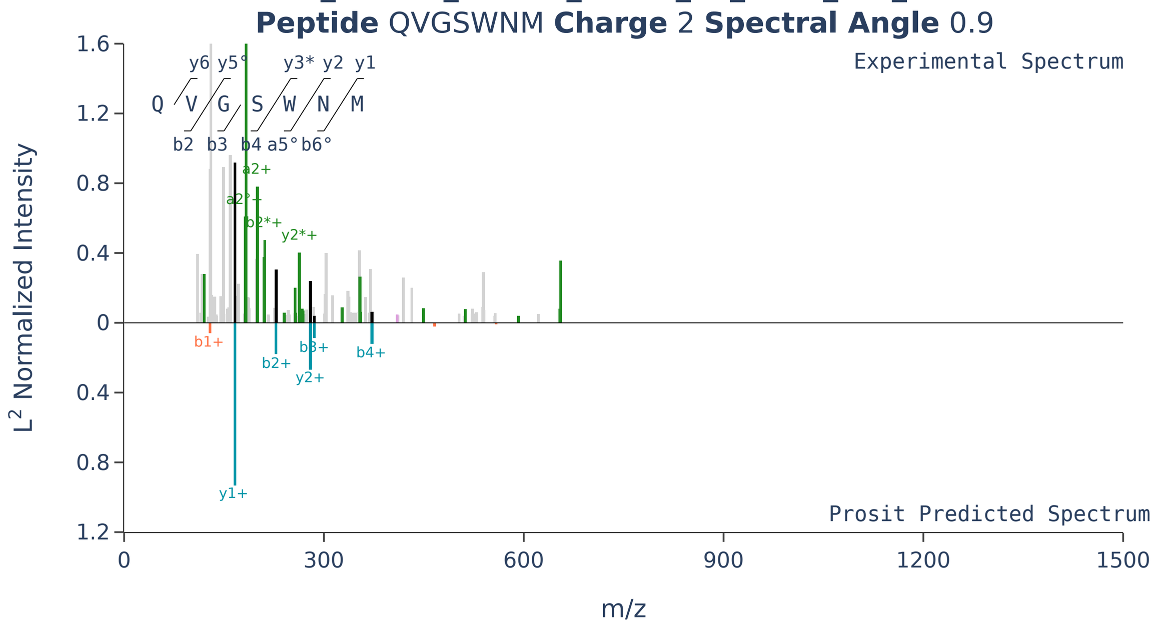


**Source** WSoh_101121_151121_HFGoe_G1_IL37b_4h_R2 **Scan** 9364
**Peptide** AQKESARRPFI **Charge** 3 **Spectral Angle** 0.94

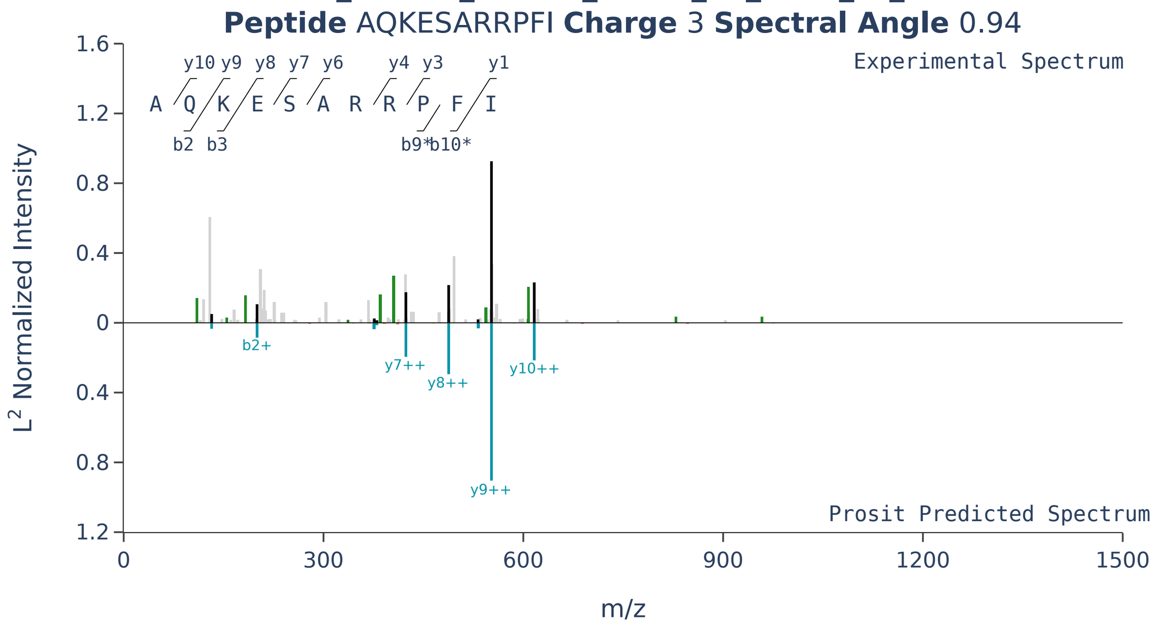


**Source** WSoh_101121_151121_HFGoe_G1_IL37b_24h_R1 **Scan** 13491
**Peptide** TSPKVKNLNPKKFSI **Charge** 4 **Spectral Angle** 0.9

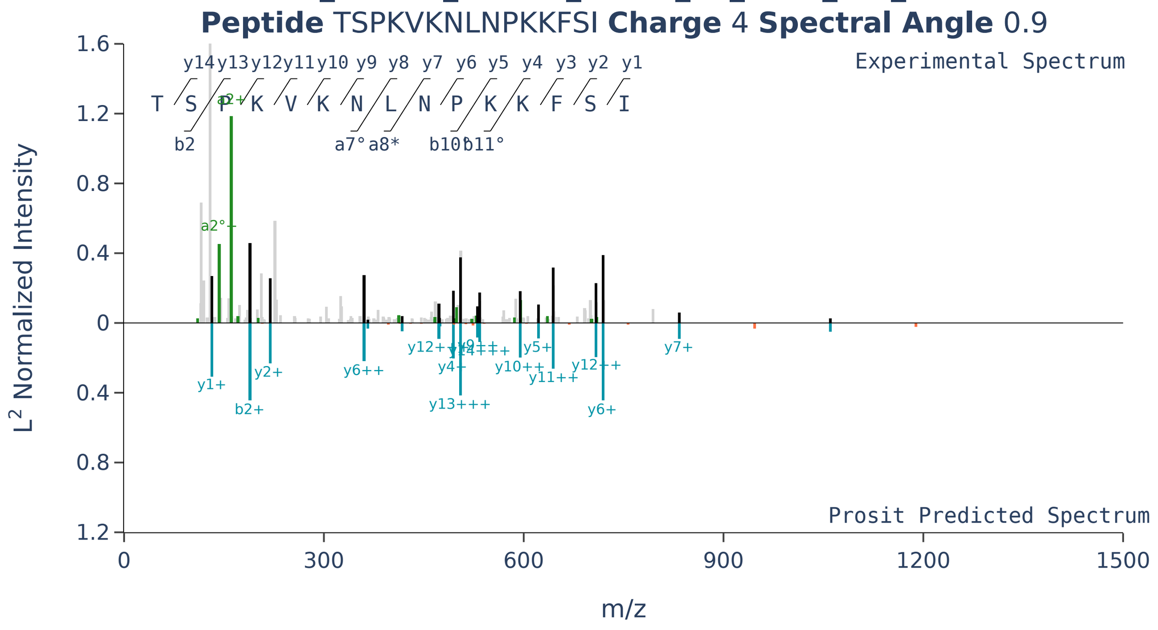


**Source** WSoh_101121_151121_HFGoe_G2_IL37b_24h_R1 **Scan** 7369
**Peptide** SIHDQDHKV **Charge** 2 **Spectral Angle** 0.88

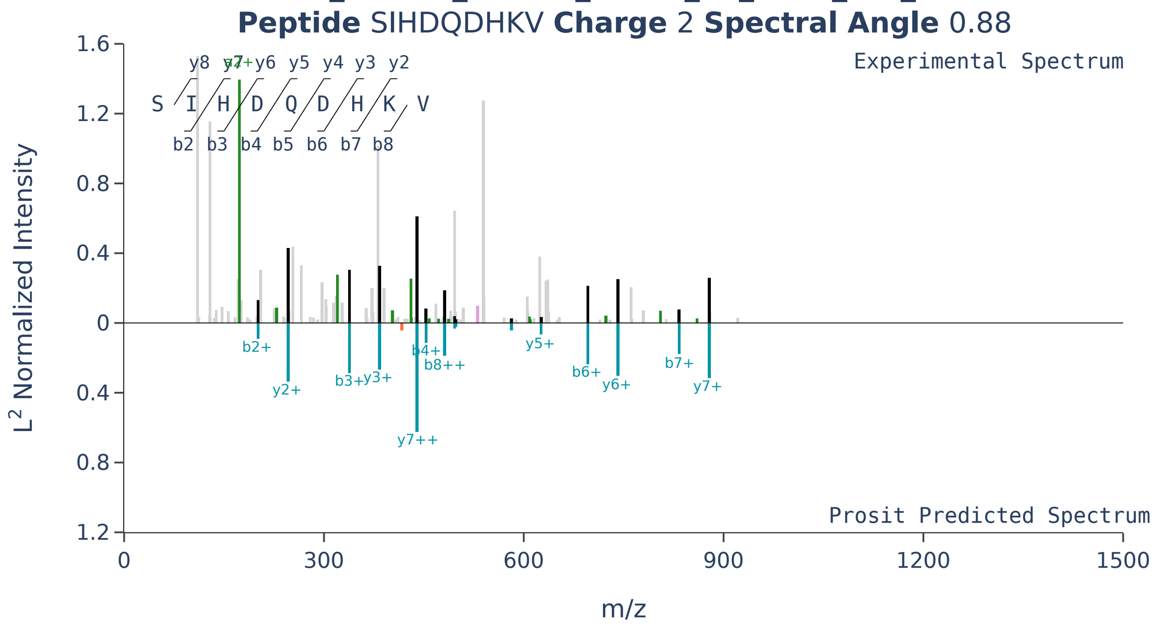


**Source** WSoh_101121_151121_HFGoe_G1_IL37b_24h_R2 **Scan** 7152
**Peptide** NPKKFSIHDQD **Charge** 2 **Spectral Angle** 0.85

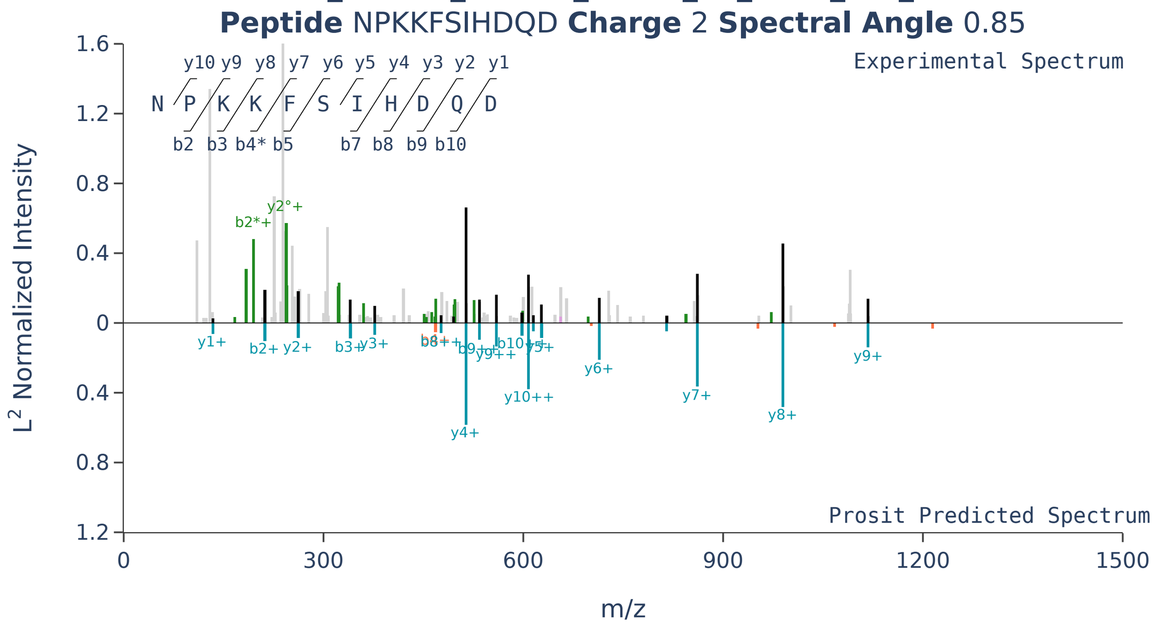


**Source** WSoh_101121_151121_HFGoe_G1_IL37b_1h_R2 **Scan** 7128
**Peptide** KKEKLMKLA **Charge** 2 **Spectral Angle** 0.91

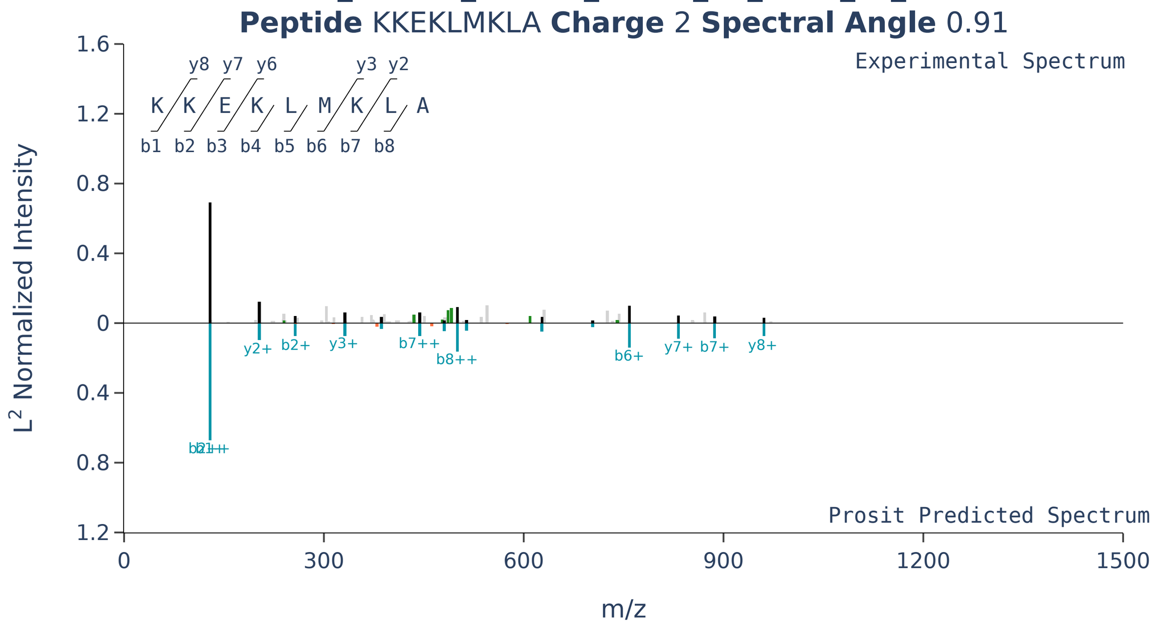


**Source** WSoh_101121_151121_HFGoe_G2_IL37b_24h_R2 **Scan** 12717
**Peptide** NLNPKKFSIHD **Charge** 3 **Spectral Angle** 0.87

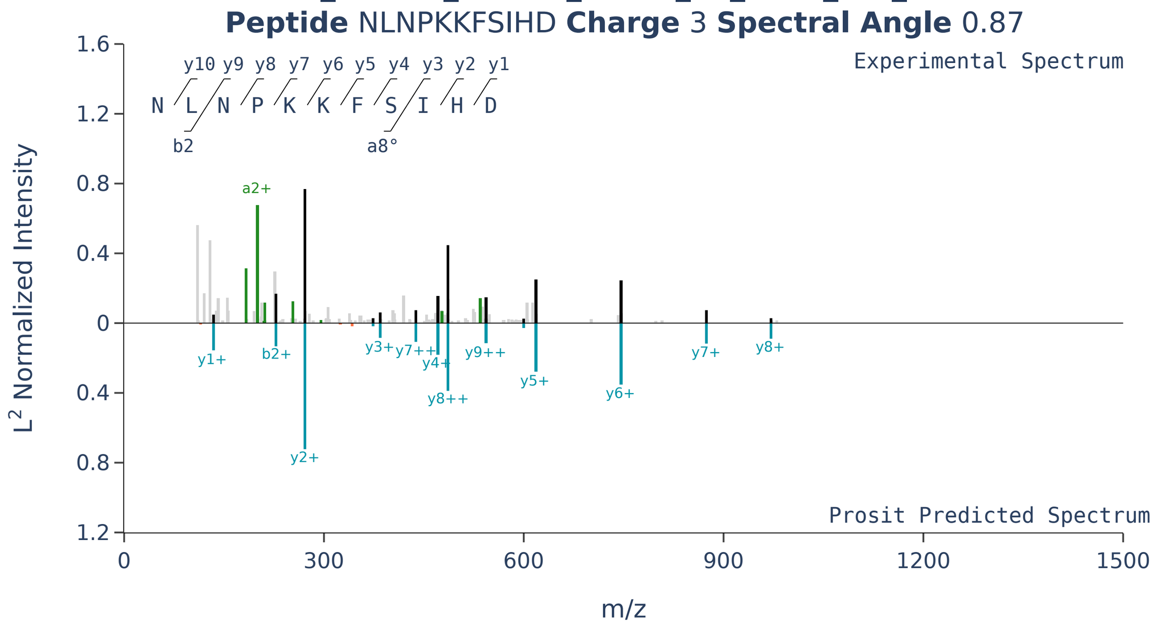


**Source** WSoh_101121_151121_HFGoe_G1_IL37b_4h_R2 **Scan** 9518
**Peptide** AAQKESARRPFI **Charge** 3 **Spectral Angle** 0.93

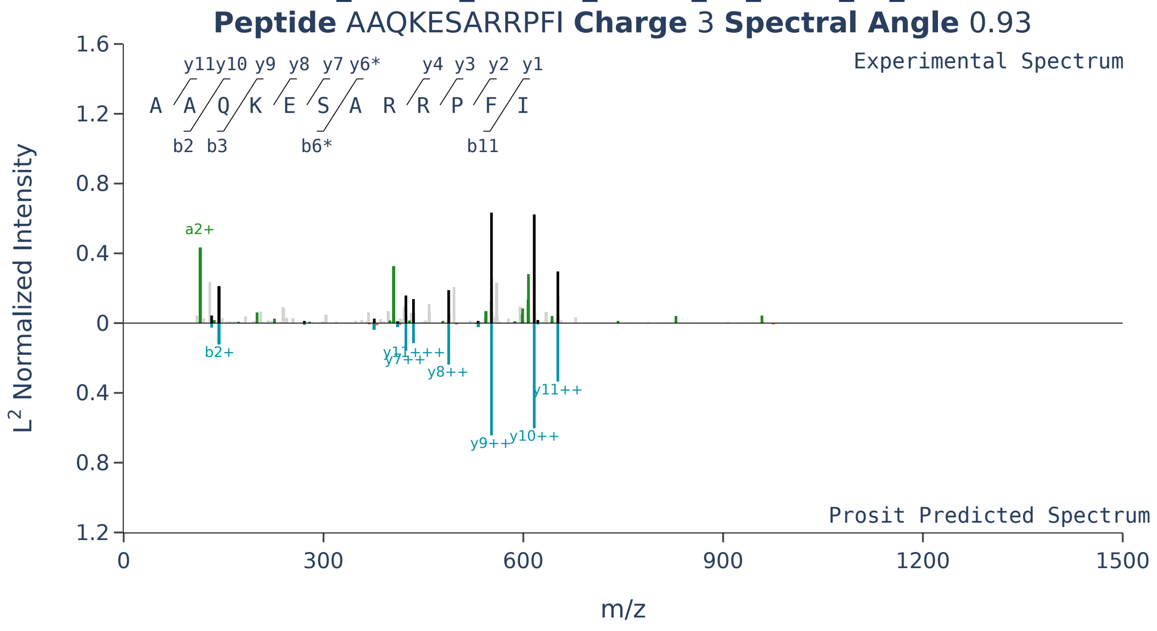


**Source** WSoh_101121_151121_HFGoe_G1_IL37b_1h_R2 **Scan** 16476
**Peptide** RAQVGSWNM **Charge** 2 **Spectral Angle** 0.83

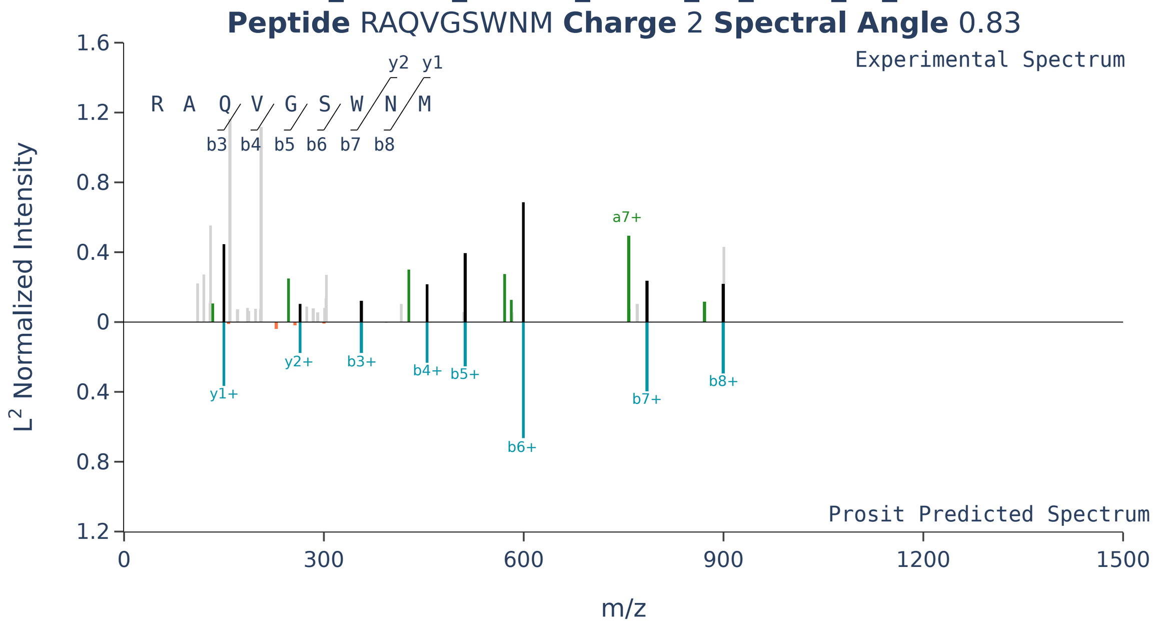


**Source** WSoh_101121_151121_HFGoe_G1_IL37b_24h_R1 **Scan** 7306
**Peptide** SLSSASAEKGSP **Charge** 2 **Spectral Angle** 0.84

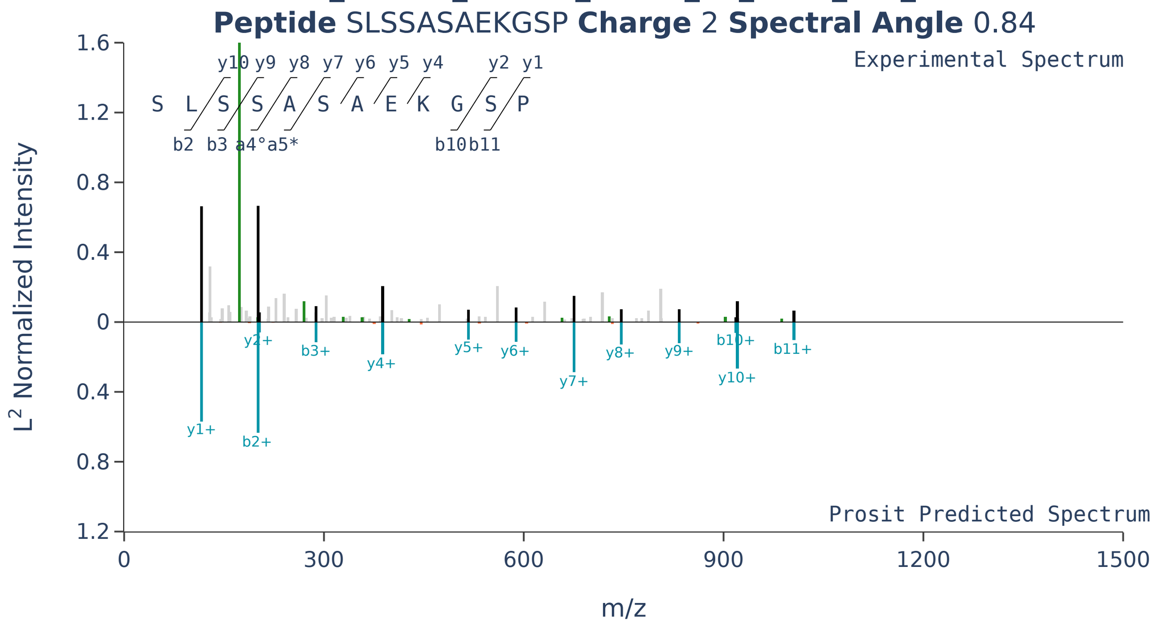


**Source** WSoh_101121_151121_HFGoe_G2_IL37b_24h_R2 **Scan** 14245
**Peptide** ASAEKGSPIL **Charge** 2 **Spectral Angle** 0.85

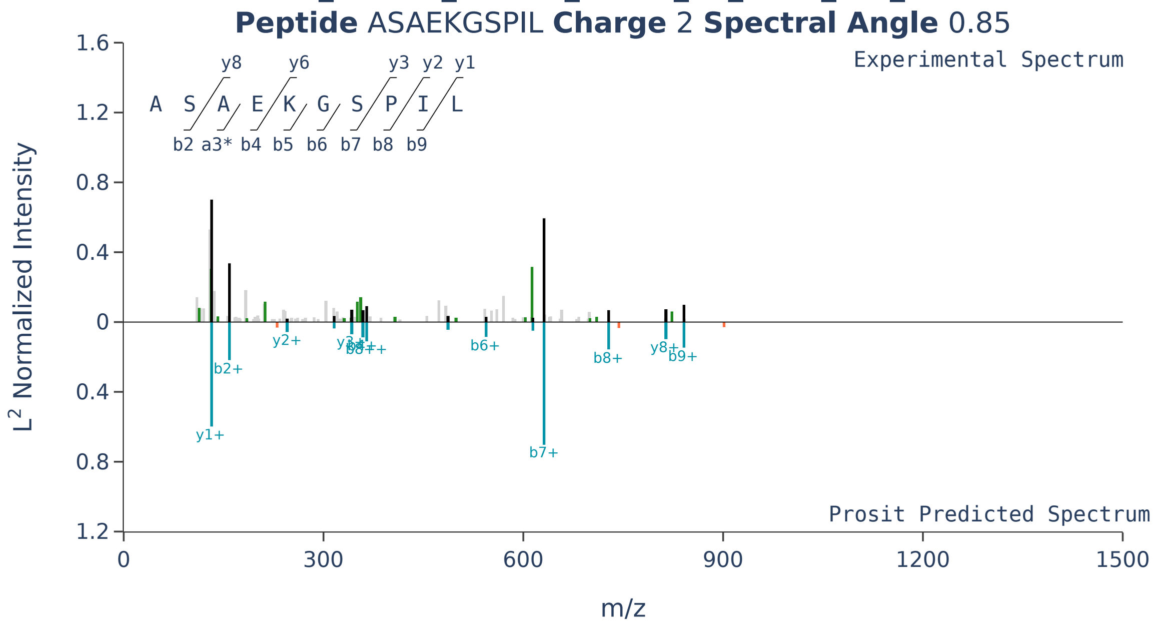


**Source** WSoh_101121_151121_HFGoe_G1_IL37b_1h_R1 **Scan** 18351
**Peptide** RPFIFYRAQ **Charge** 3 **Spectral Angle** 0.94

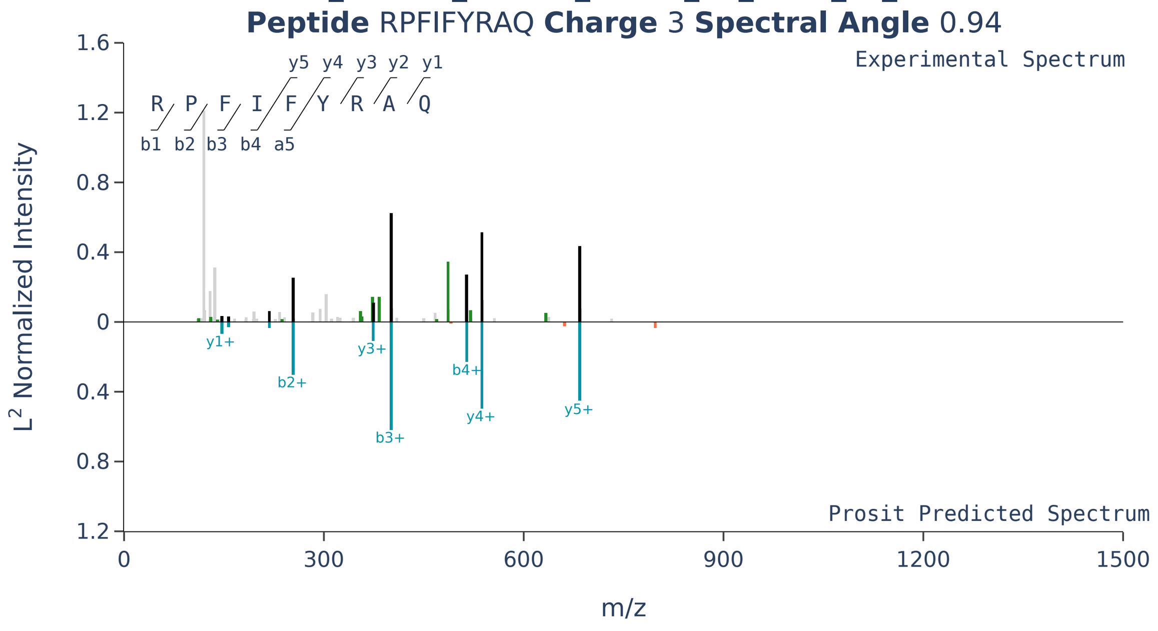


**Source** WSoh_101121_151121_HFGoe_G1_IL37b_24h_R2 **Scan** 17746
**Peptide** LIAVPDKNYIR **Charge** 3 **Spectral Angle** 0.88

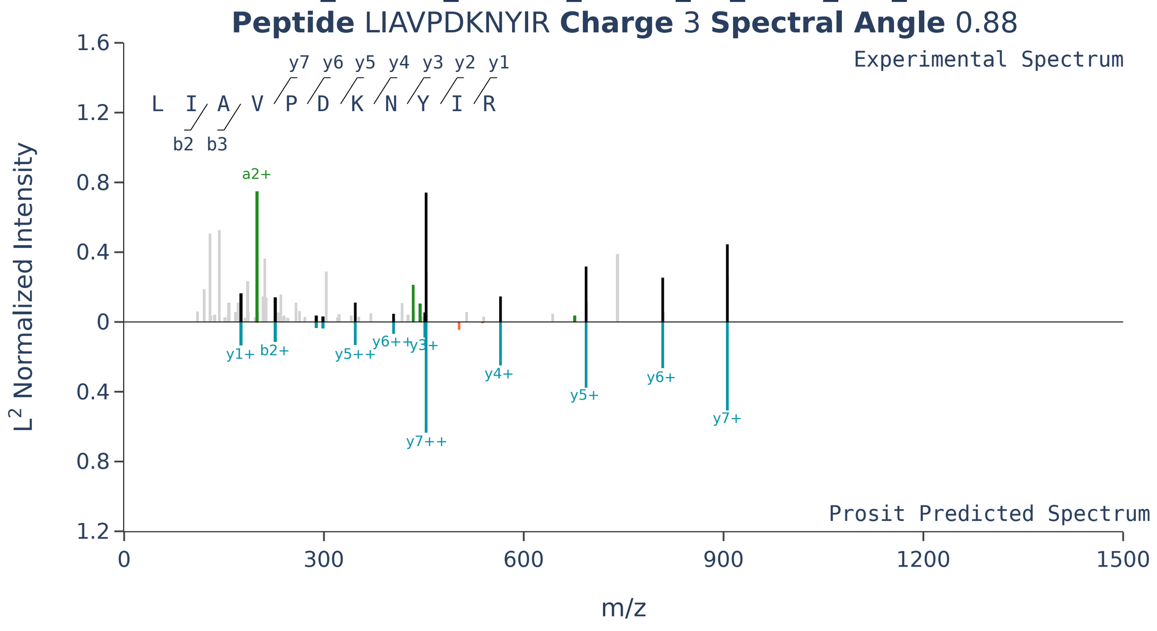


**Source** WSoh_101121_151121_HFGoe_G2_IL37b_24h_R1 **Scan** 14997
**Peptide** LGVSKGEF **Charge** 1 **Spectral Angle** 0.88

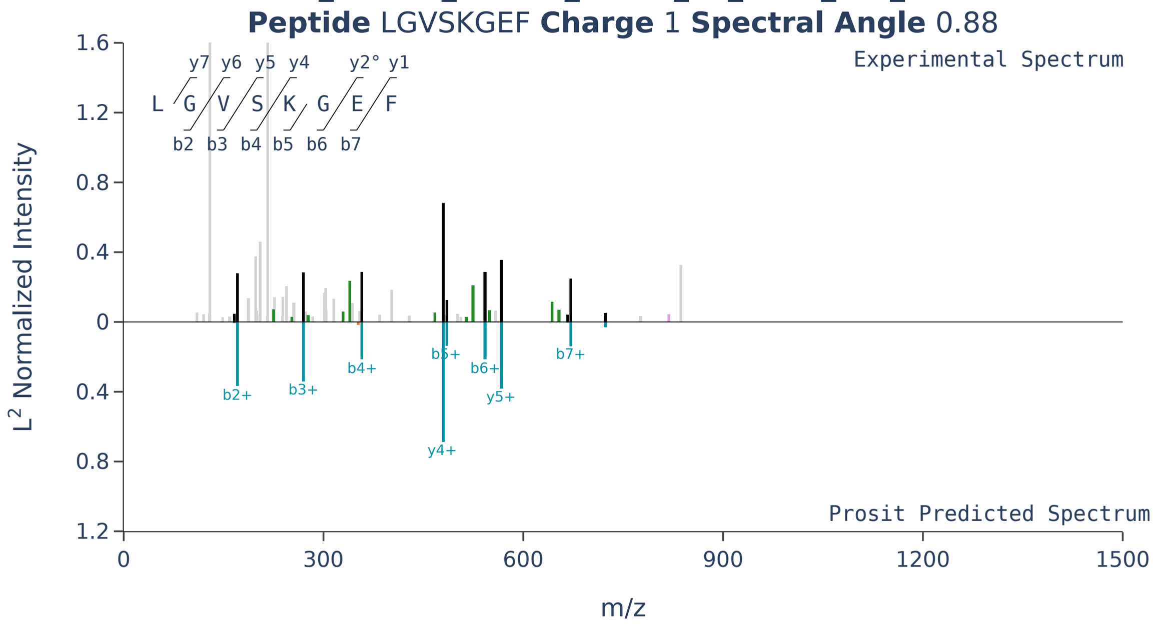

**Source** WSoh_101121_151121_HFGoe_G1_IL37b_2h_R1 **Scan** 8901
**Peptide** KFENRKHIEF **Charge** 2 **Spectral Angle** 0.83

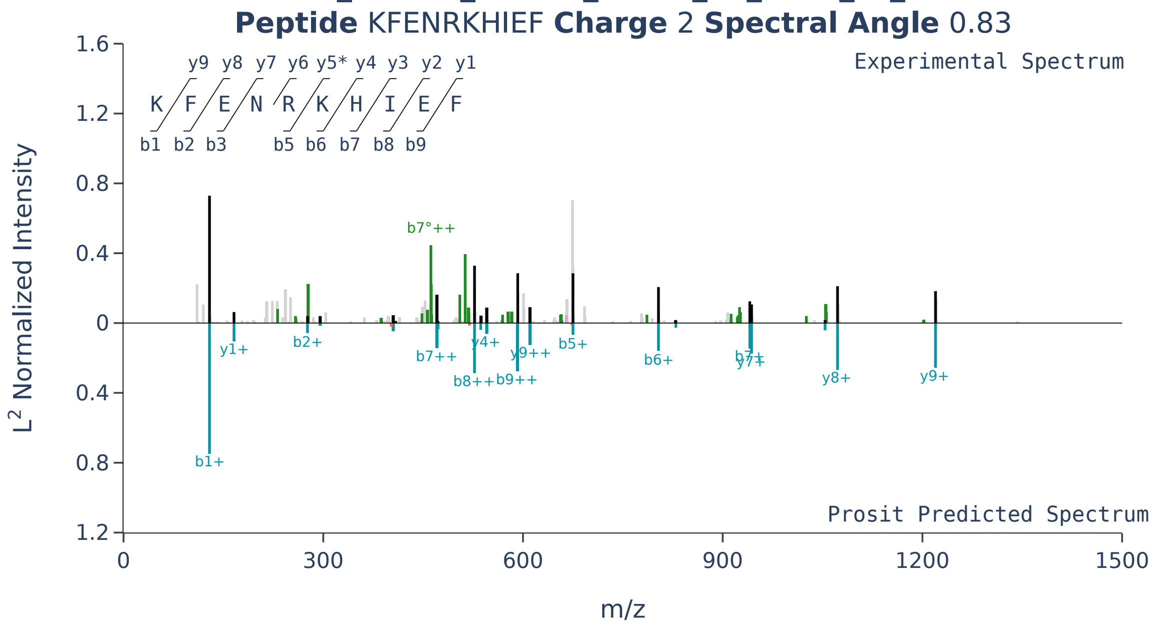


**Source** WSoh_101121_151121_HFGoe_G1_IL37b_24h_R2 **Scan** 18378
**Peptide** SSLSSASAEKGSPIL **Charge** 2 **Spectral Angle** 0.9

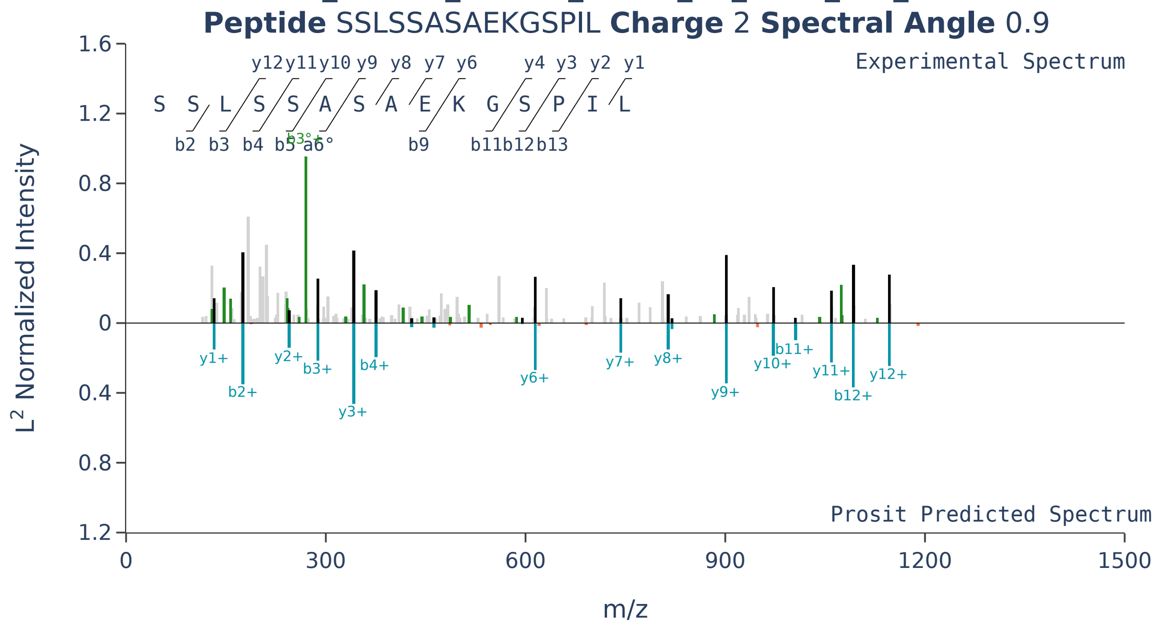


**Source** WSoh_101121_151121_HFGoe_G1_IL37b_2h_R2 **Scan** 16992
**Peptide** AVPDKNYIRPEI **Charge** 3 **Spectral Angle** 0.84

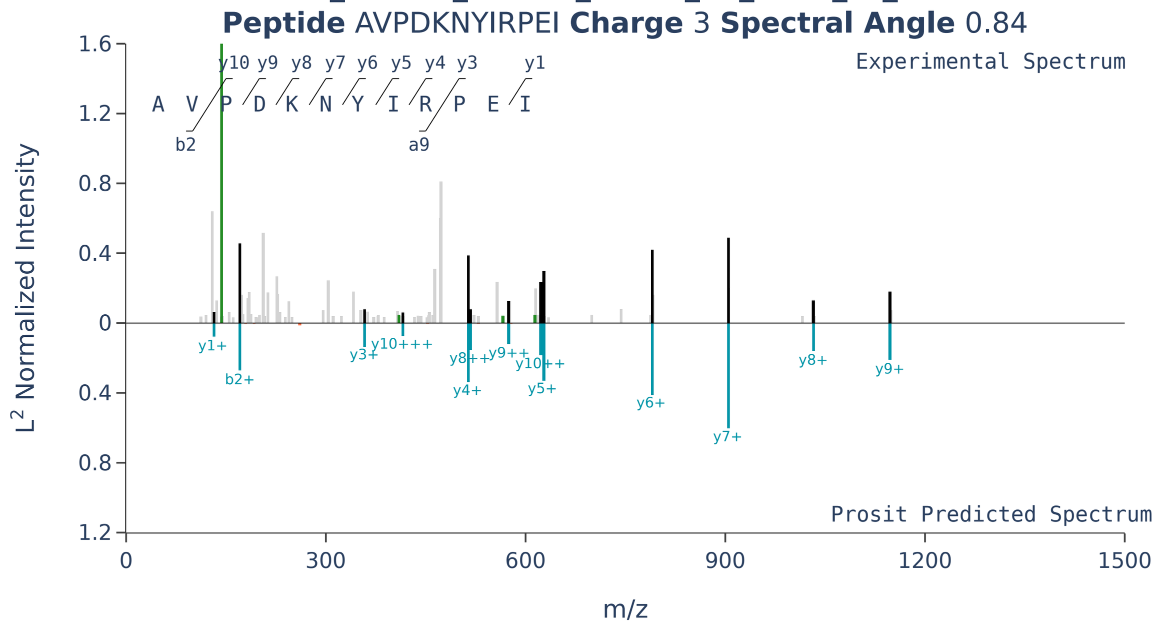


**Source** WSoh_101121_151121_HFGoe_G1_IL37b_2h_R1 **Scan** 20094
**Peptide** ASAEKGSPILL **Charge** 2 **Spectral Angle** 0.86

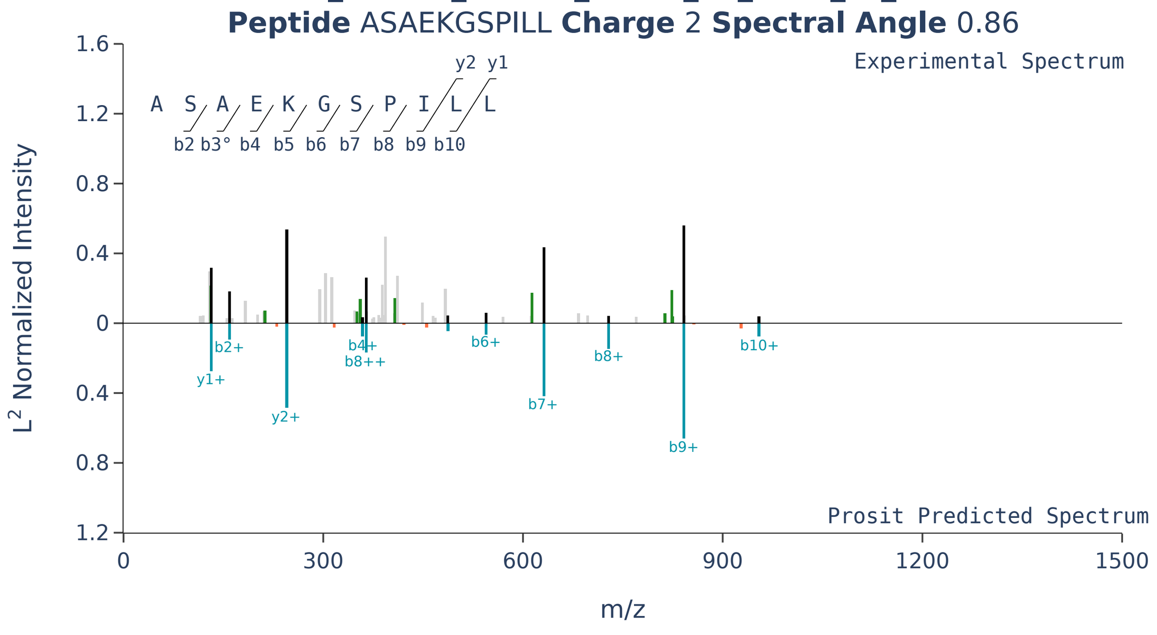


**Source** WSoh_101121_151121_HFGoe_G1_IL37b_24h_R2 **Scan** 24004
**Peptide** AAHPGWFIC **Charge** 2 **Spectral Angle** 0.85

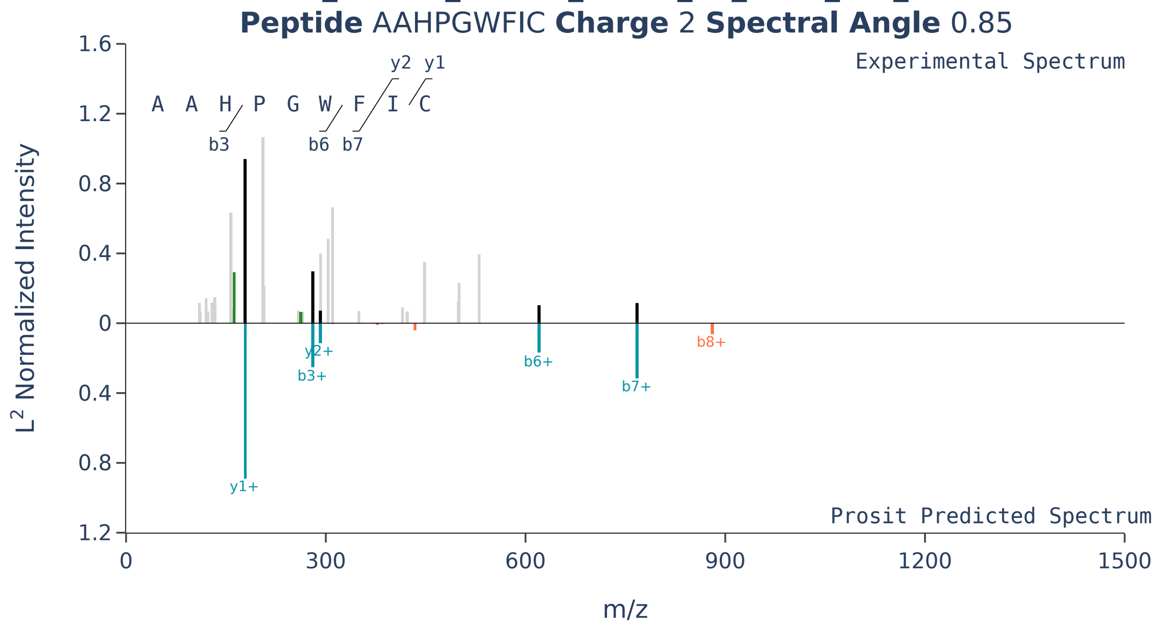


**Source** WSoh_101121_151121_HFGoe_G2_IL37b_24h_R1 **Scan** 17394
**Peptide** DHKVLVL **Charge** 2 **Spectral Angle** 0.82

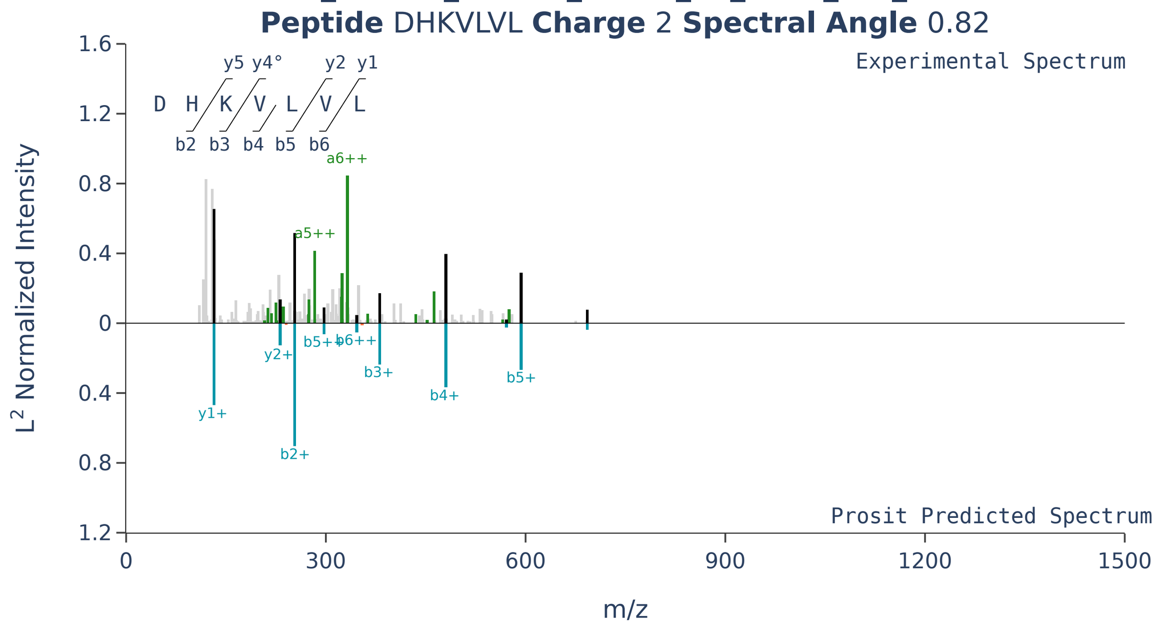


**Source** WSoh_101121_151121_HFGoe_G1_IL37b_4h_R1 **Scan** 12115
**Peptide** KNLNPKKFSI **Charge** 3 **Spectral Angle** 0.89

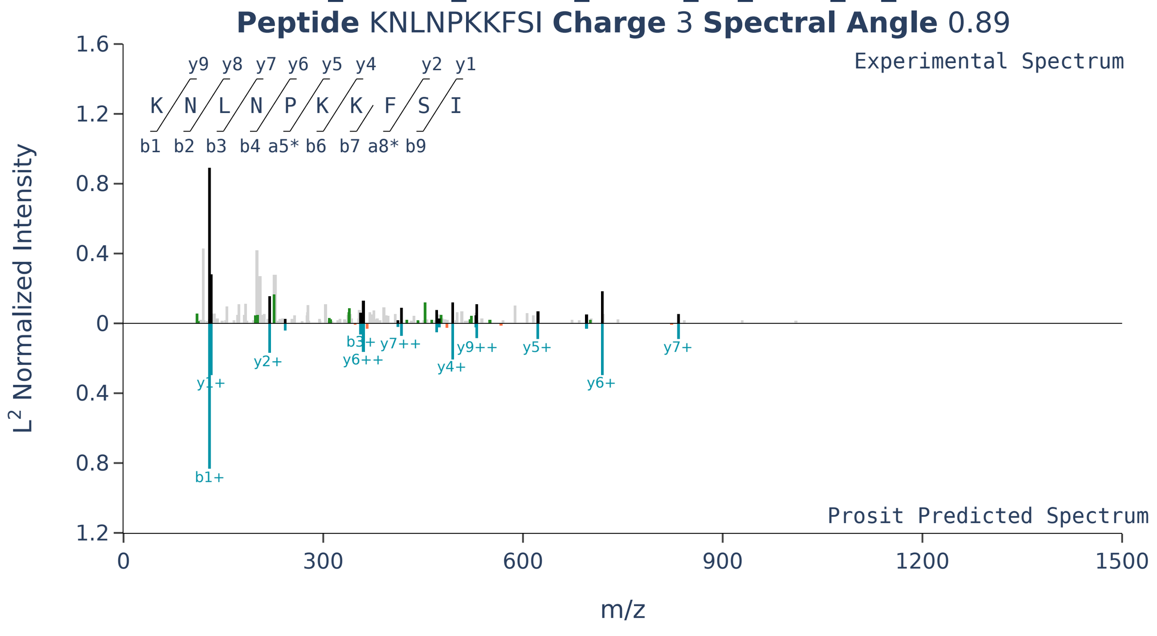


**Source** WSoh_101121_151121_HFGoe_G2_IL37b_24h_R1 **Scan** 14143
**Peptide** IHDQDHKVLVLD **Charge** 2 **Spectral Angle** 0.82

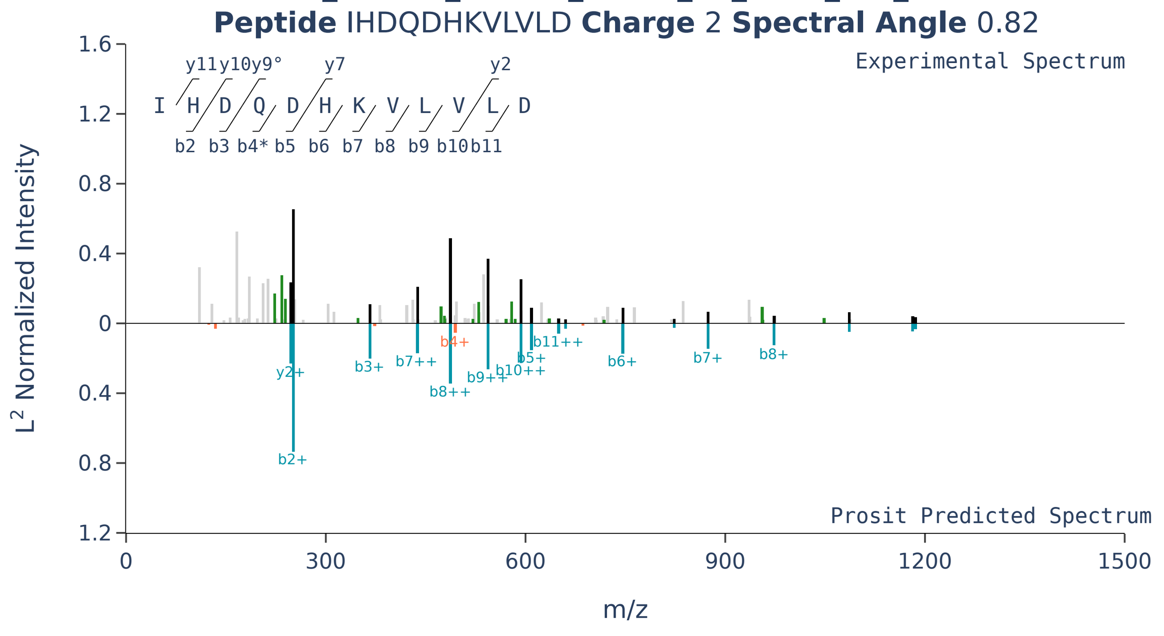


**Source** WSoh_101121_151121_HFGoe_G2_IL37b_24h_R1 **Scan** 12794
**Peptide** YCDKDKGQSHPSLQL **Charge** 3 **Spectral Angle** 0.88

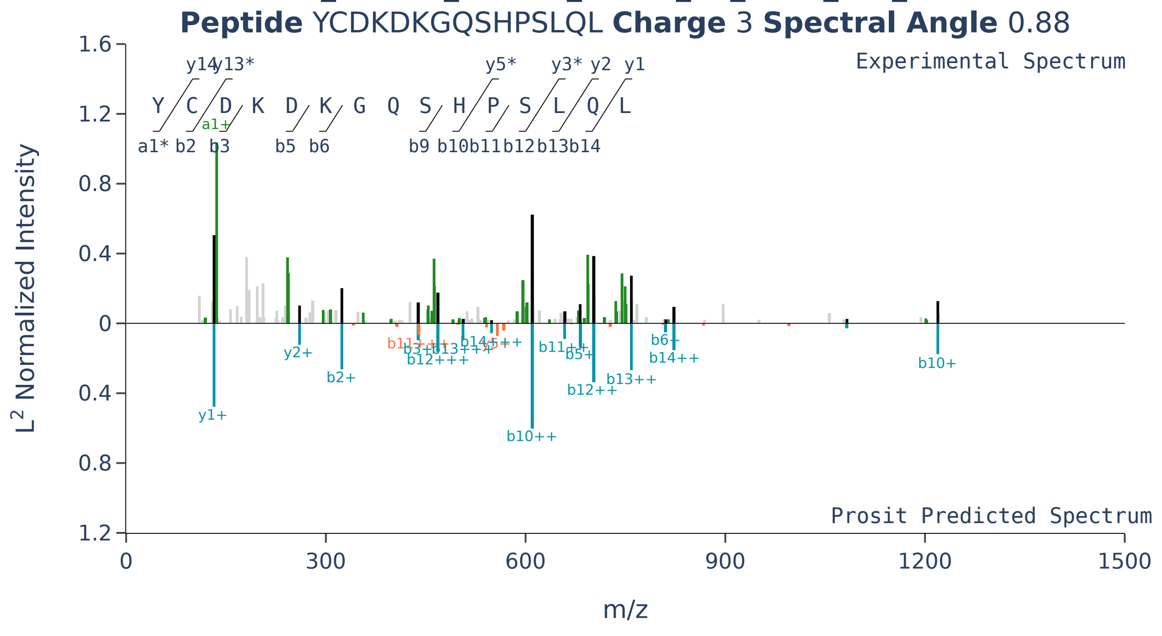


**Source** WSoh_101121_151121_HFGoe_G2_IL37b_24h_R1 **Scan** 23929
**Peptide** AAHPGWFICT **Charge** 2 **Spectral Angle** 0.88

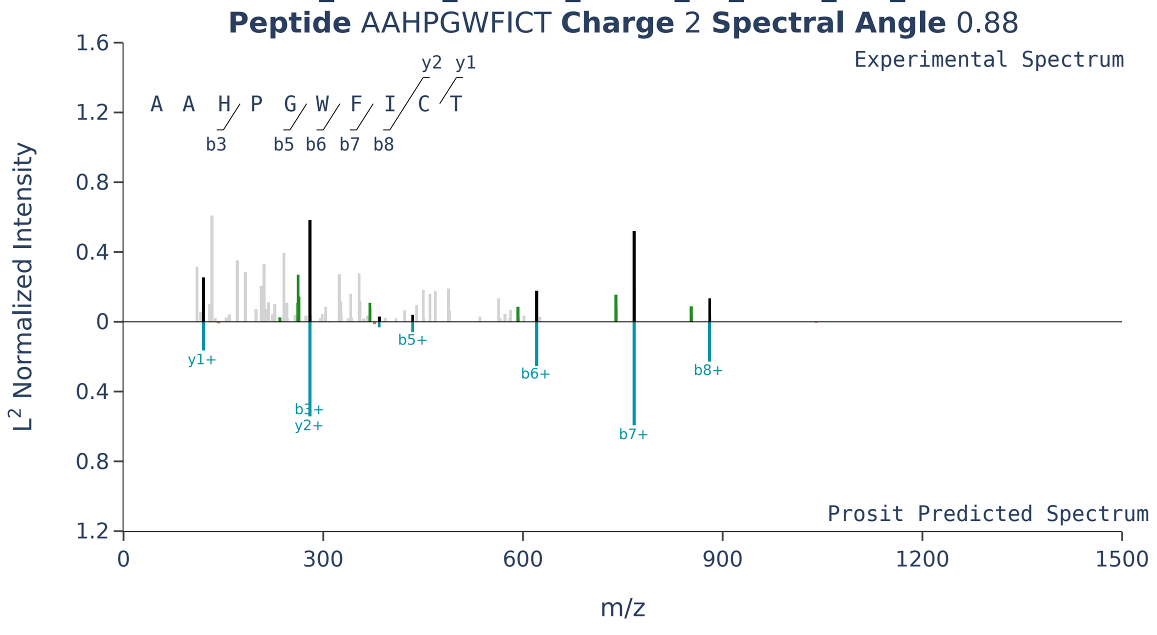


**Source** WSoh_101121_151121_HFGoe_G2_IL37b_24h_R2 **Scan** 13611
**Peptide** AEKGSPIL **Charge** 2 **Spectral Angle** 0.81

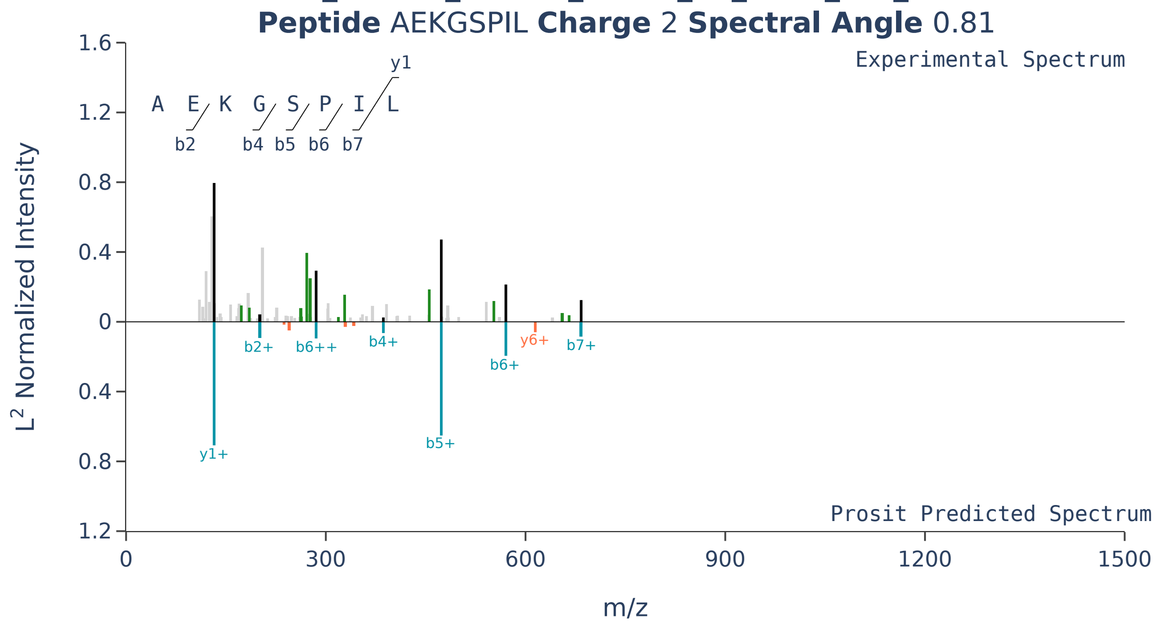


**Source** WSoh_101121_151121_HFGoe_G1_IL37b_4h_R1 **Scan** 11852
**Peptide** SCNCNEPVGVTD **Charge** 2 **Spectral Angle** 0.9

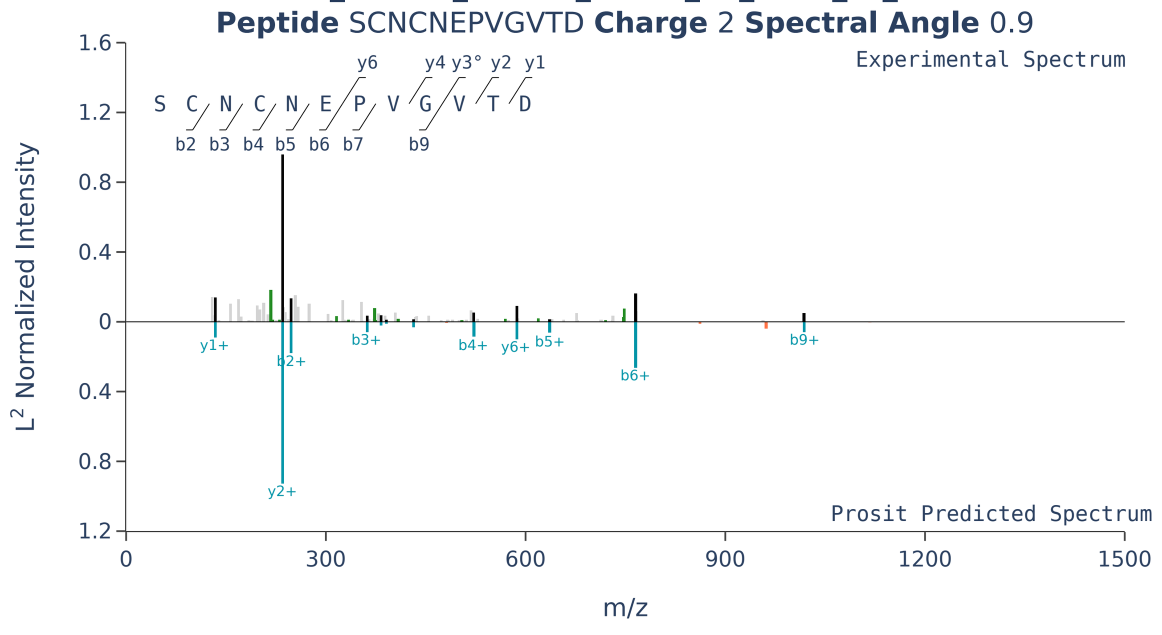


**Source** WSoh_101121_151121_HFGoe_G2_IL37b_24h_R1 **Scan** 7407
**Peptide** SCNCNEPVG **Charge** 2 **Spectral Angle** 0.82

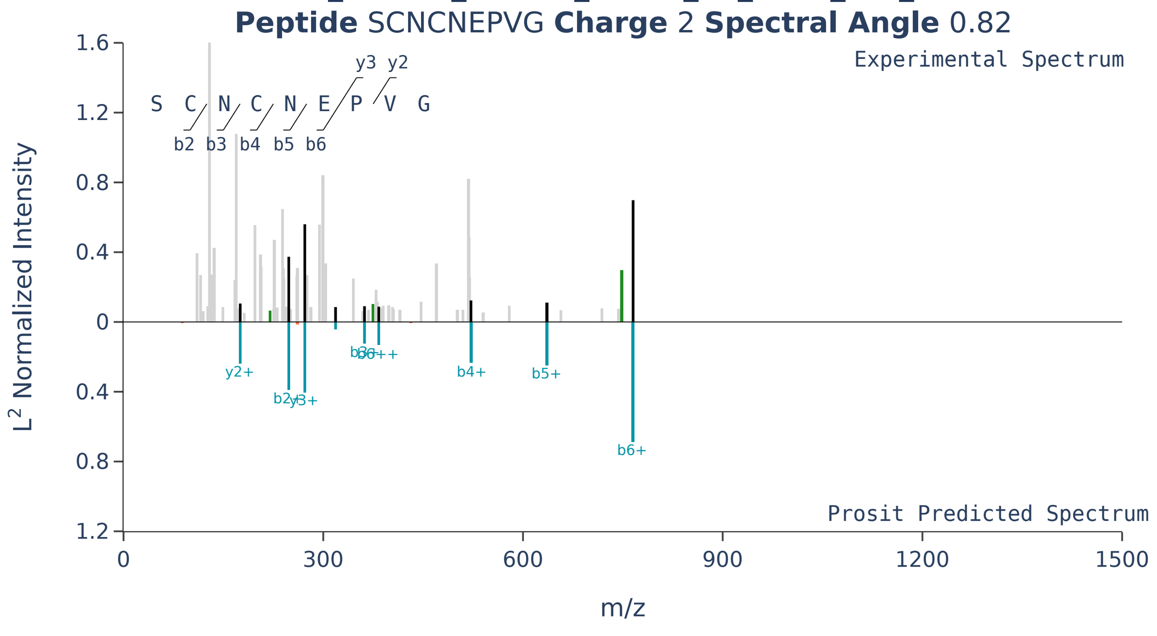


**Source** WSoh_101121_151121_HFGoe_G1_IL37b_24h_R2 **Scan** 18611
**Peptide** ASSLSSASAEKGSPIL **Charge** 2 **Spectral Angle** 0.87

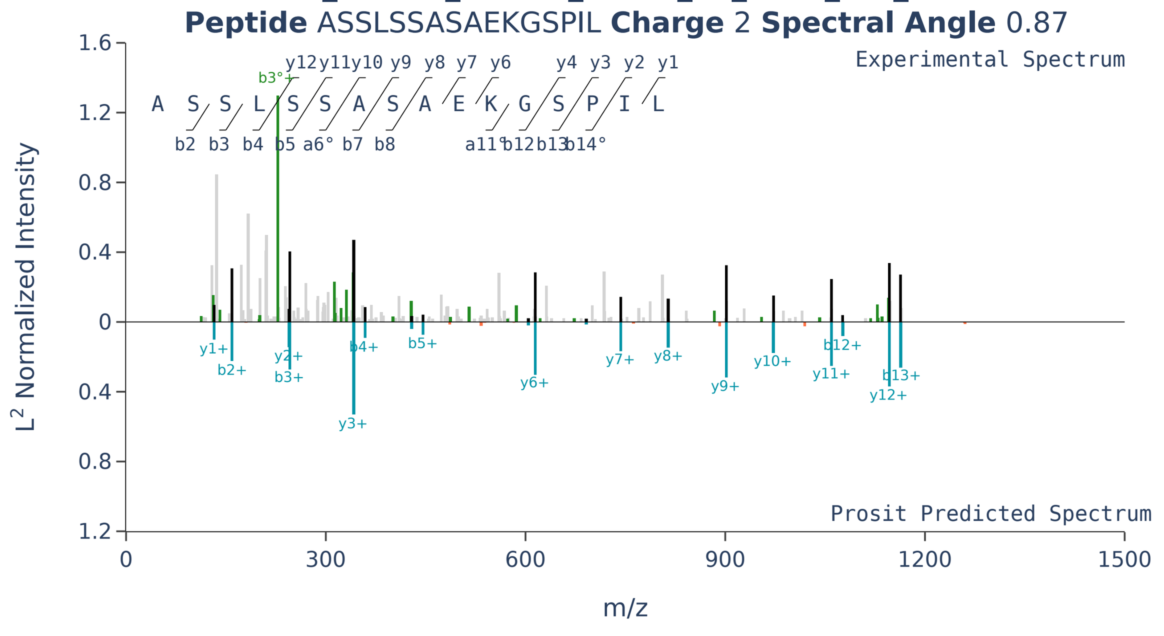


**Source** WSoh_101121_151121_HFGoe_G1_IL37b_24h_R2 **Scan** 13857
**Peptide** AVPDKNYI **Charge** 2 **Spectral Angle** 0.81

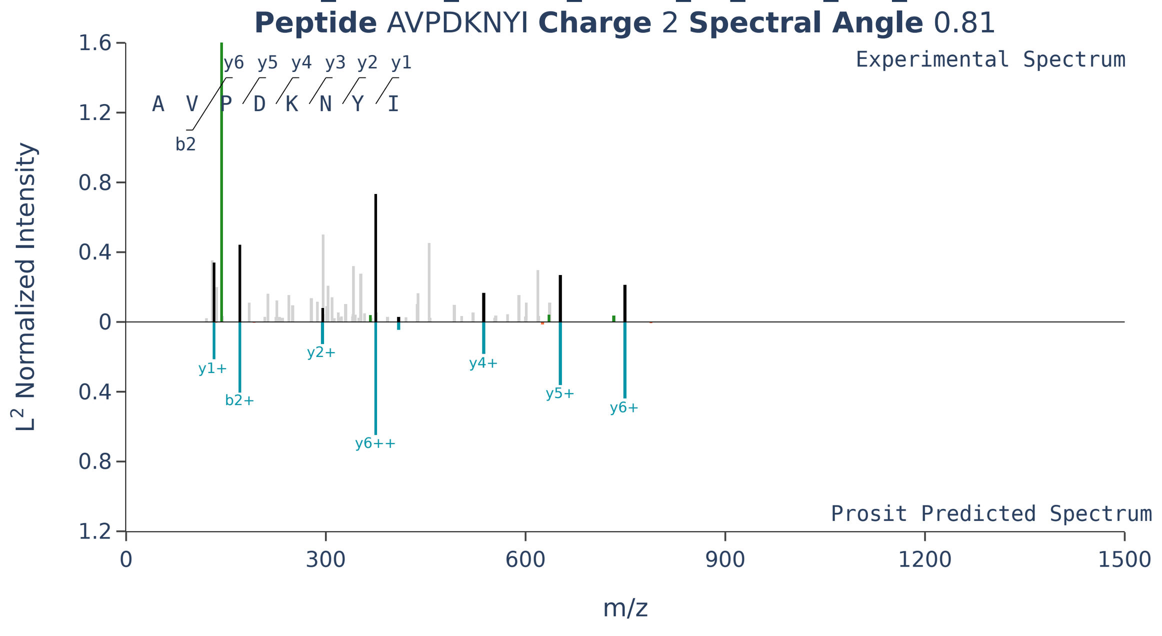

**Source** WSoh_101121_151121_HFGoe_G1_IL37b_24h_R1 **Scan** 16424
**Peptide** VPDKNYIRPEI **Charge** 2 **Spectral Angle** 0.85

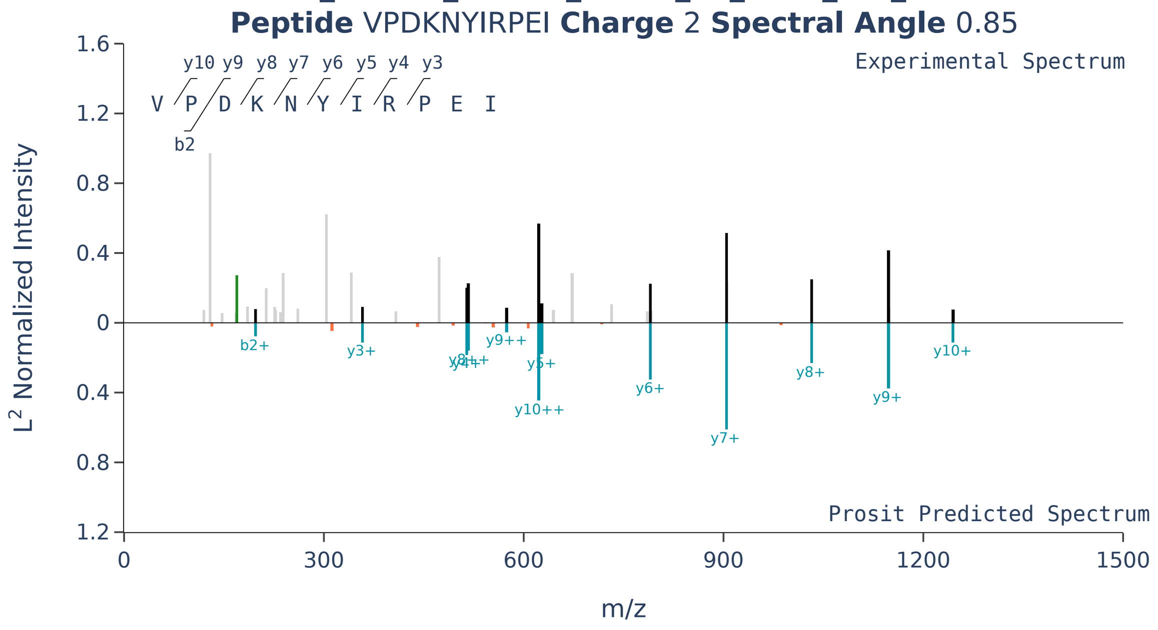


**Source** WSoh_101121_151121_HFGoe_G2_IL37b_1h_R2 **Scan** 16554
**Peptide** NLNPKKFSI **Charge** 2 **Spectral Angle** 0.84

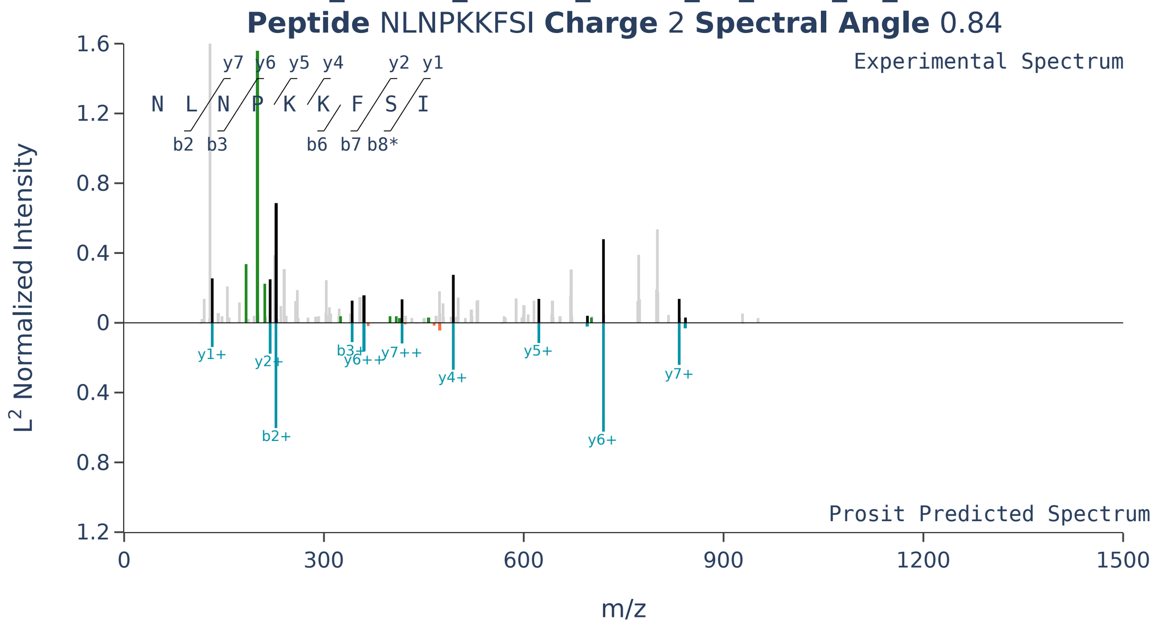


**Source** WSoh_101121_151121_HFGoe_G2_IL37b_24h_R2 **Scan** 8825
**Peptide** LYCDKDKGQSHPSL **Charge** 2 **Spectral Angle** 0.91

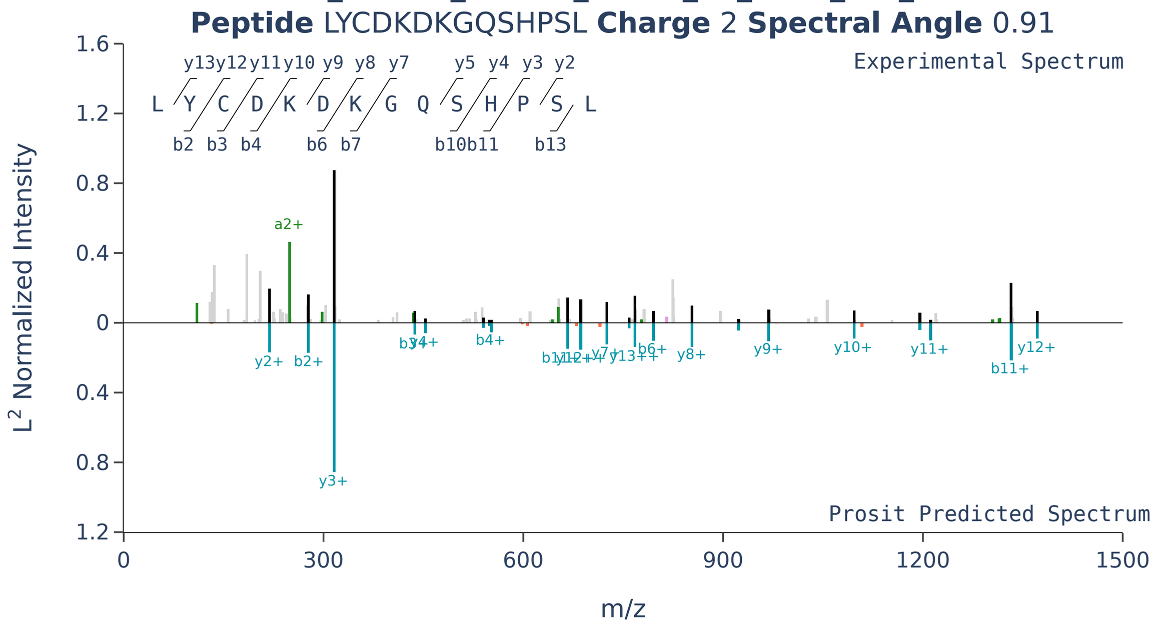


**Source** WSoh_101121_151121_HFGoe_G2_IL37b_24h_R1 **Scan** 8120
**Peptide** TSPKVKNLNPKKF **Charge** 4 **Spectral Angle** 0.83

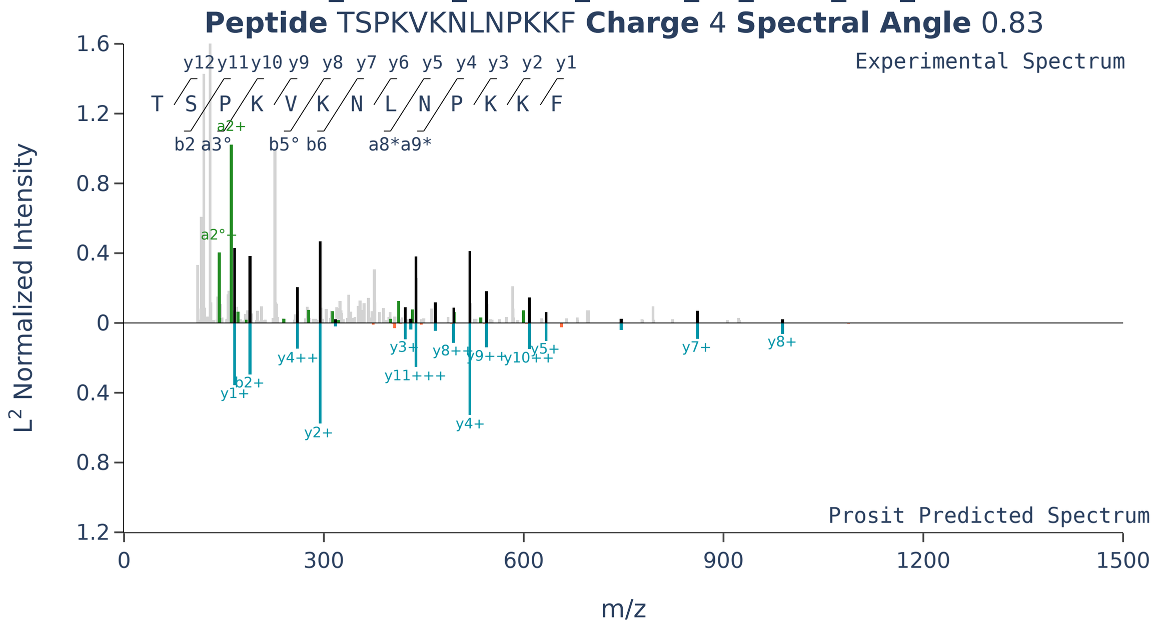


**Source** WSoh_101121_151121_HFGoe_G2_IL37b_2h_R1 **Scan** 18636
**Peptide** LIAVPDKNYIRPE **Charge** 3 **Spectral Angle** 0.88

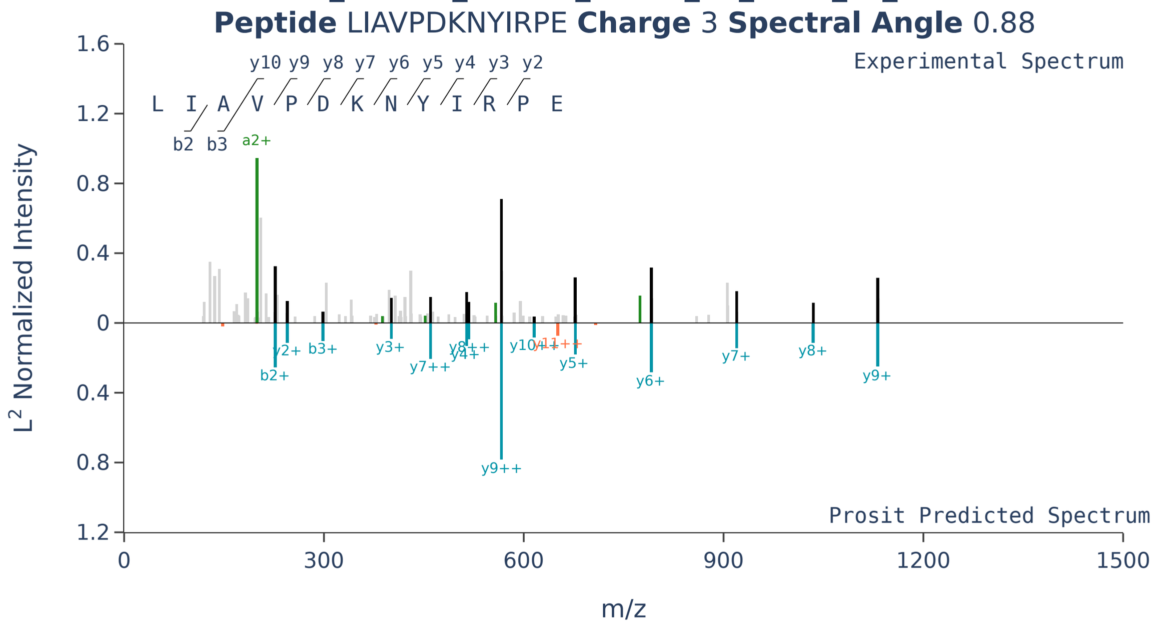


**Source** WSoh_101121_151121_HFGoe_G1_IL37b_1h_R1 **Scan** 6111
**Peptide** VTDKFENRKHLE **Charge** 3 **Spectral Angle** 0.84

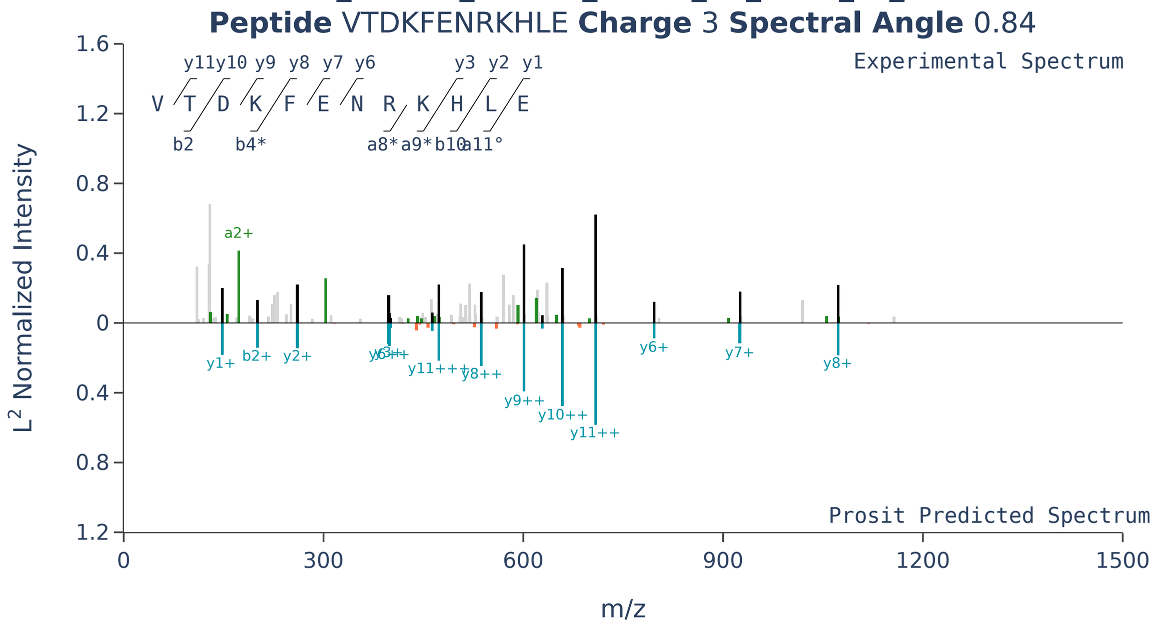


**Source** WSoh_101121_151121_HFGoe_G1_IL37b_4h_R1 **Scan** 6076
**Peptide** KLMKLAAQKESARR **Charge** 4 **Spectral Angle** 0.92

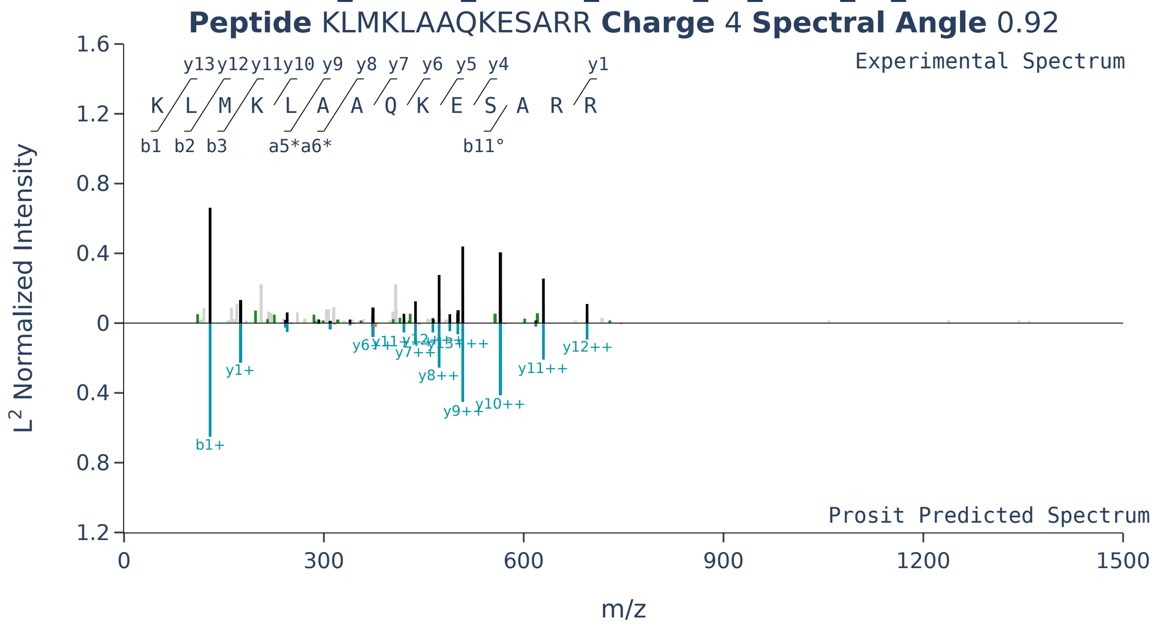


**Source** WSoh_101121_151121_HFGoe_G1_IL37b_4h_R1 **Scan** 19367
**Peptide** NLIAVPDKNYIR **Charge** 2 **Spectral Angle** 0.88

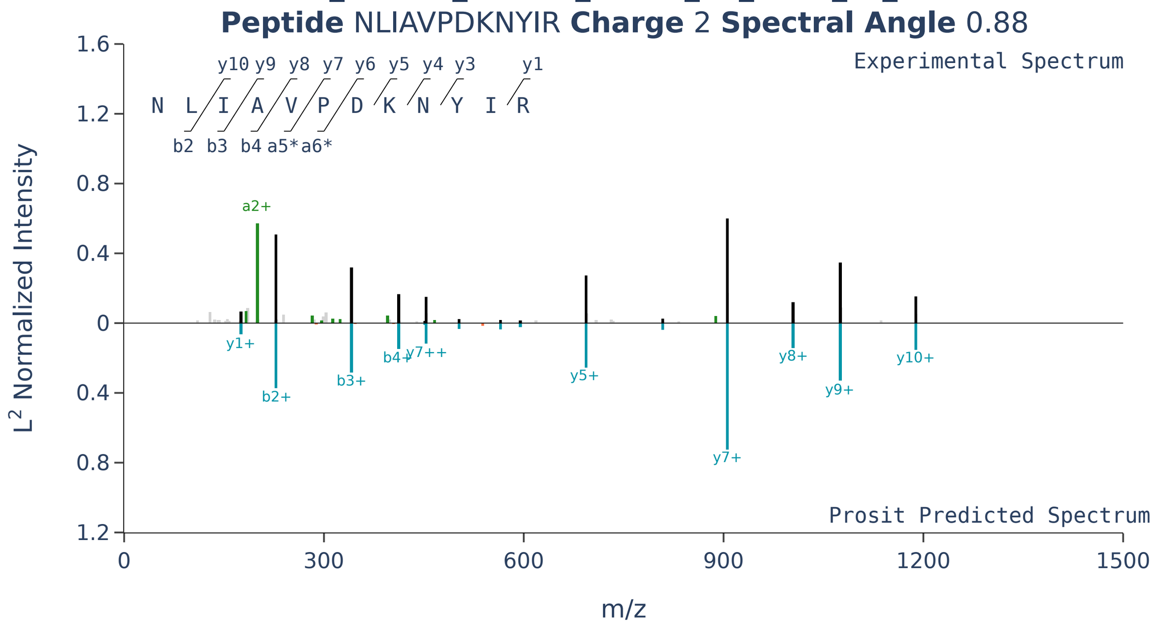


**Source** WSoh_101121_151121_HFGoe_G2_IL37b_24h_R1 **Scan** 24055
**Peptide** ASAEKGSPILLGV **Charge** 2 **Spectral Angle** 0.91

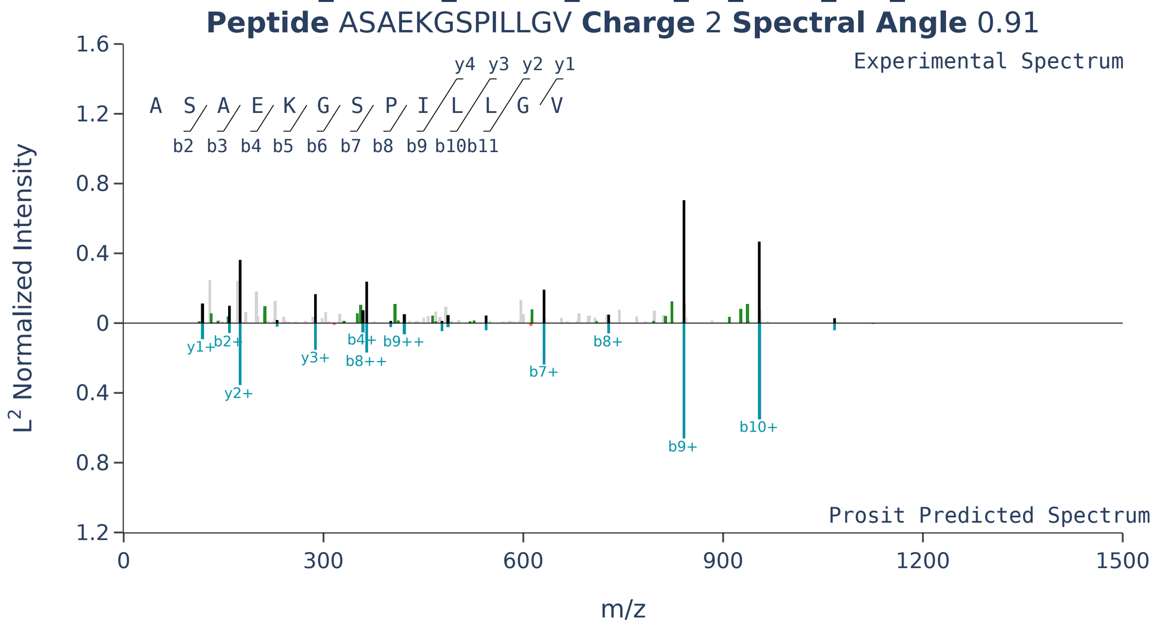


**Source** WSoh_101121_151121_HFGoe_G1_IL37b_4h_R1 **Scan** 6637
**Peptide** MKLAAQKES **Charge** 2 **Spectral Angle** 0.84

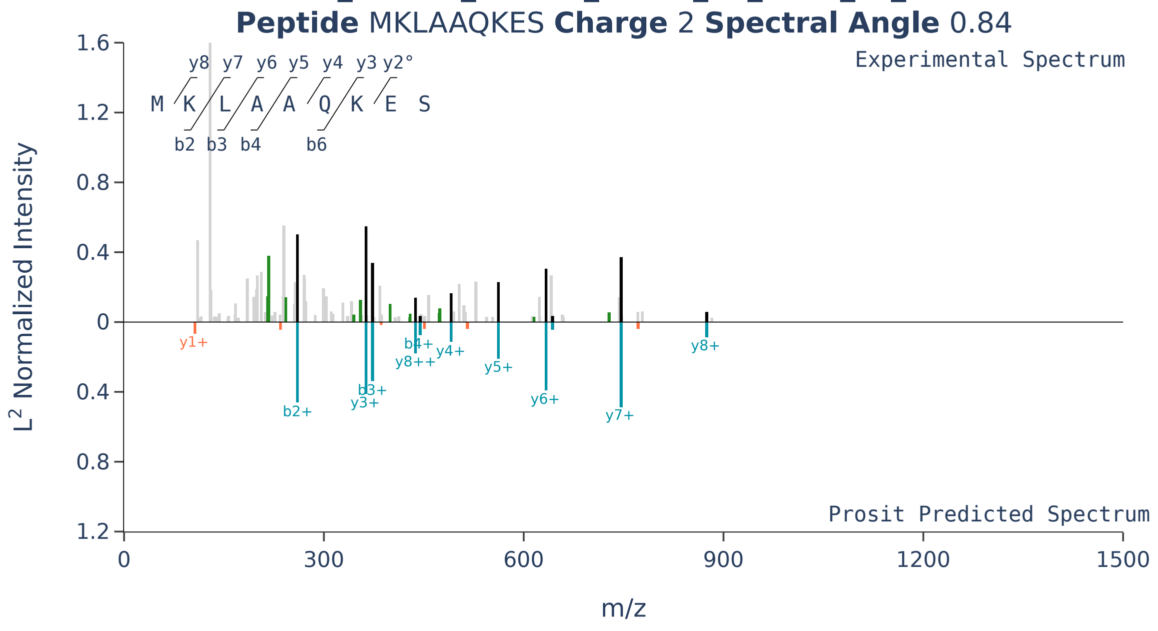


**Source** WSoh_101121_151121_HFGoe_G1_IL37b_24h_R2 **Scan** 10721
**Peptide** PSLQLKKEKLM **Charge** 3 **Spectral Angle** 0.88

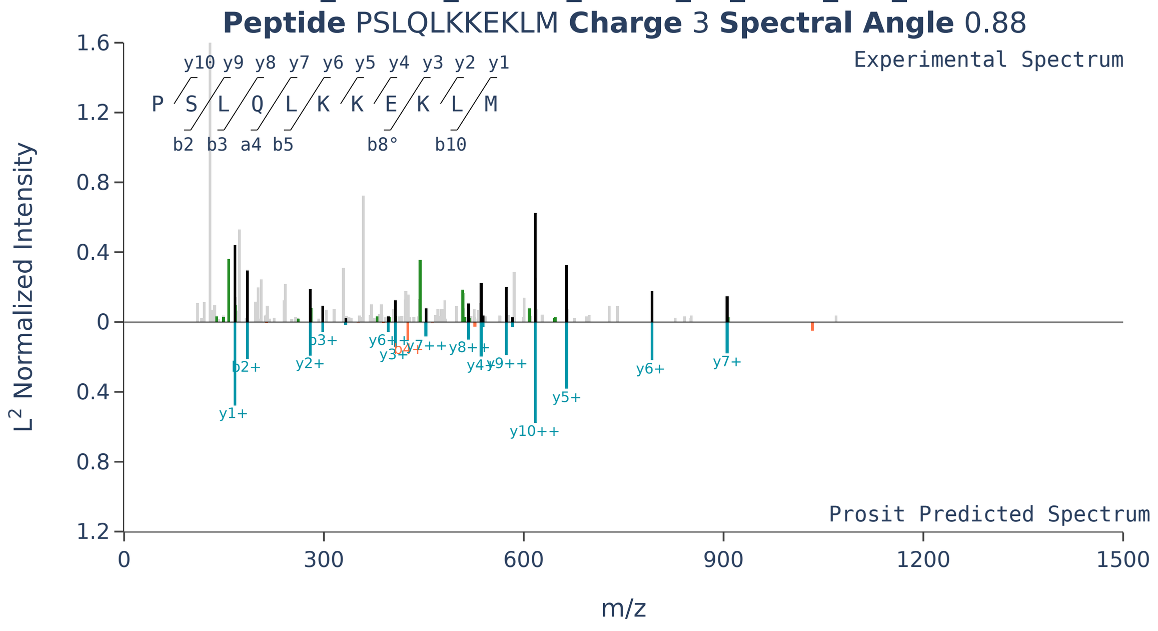


**Source** WSoh_101121_151121_HFGoe_G2_IL37b_24h_R2 **Scan** 8857
**Peptide** QDHKVLV **Charge** 1 **Spectral Angle** 0.87

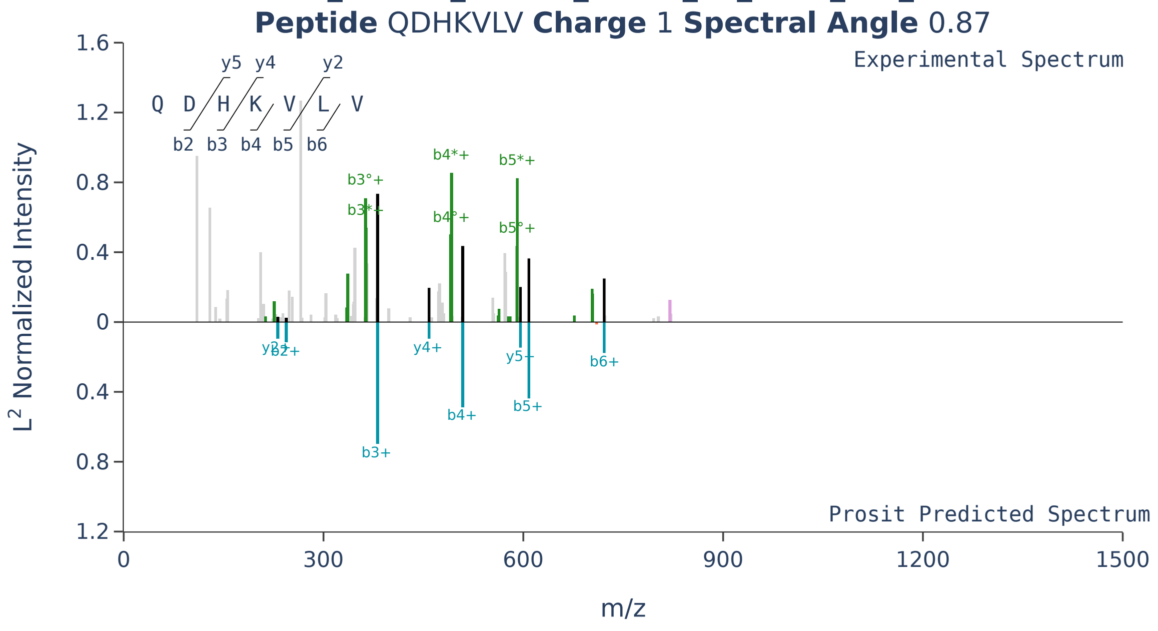


**Source** WSoh_101121_151121_HFGoe_G1_IL37b_1h_R2 **Scan** 16836
**Peptide** SSASAEKGSPILLGVSK **Charge** 3 **Spectral Angle** 0.81

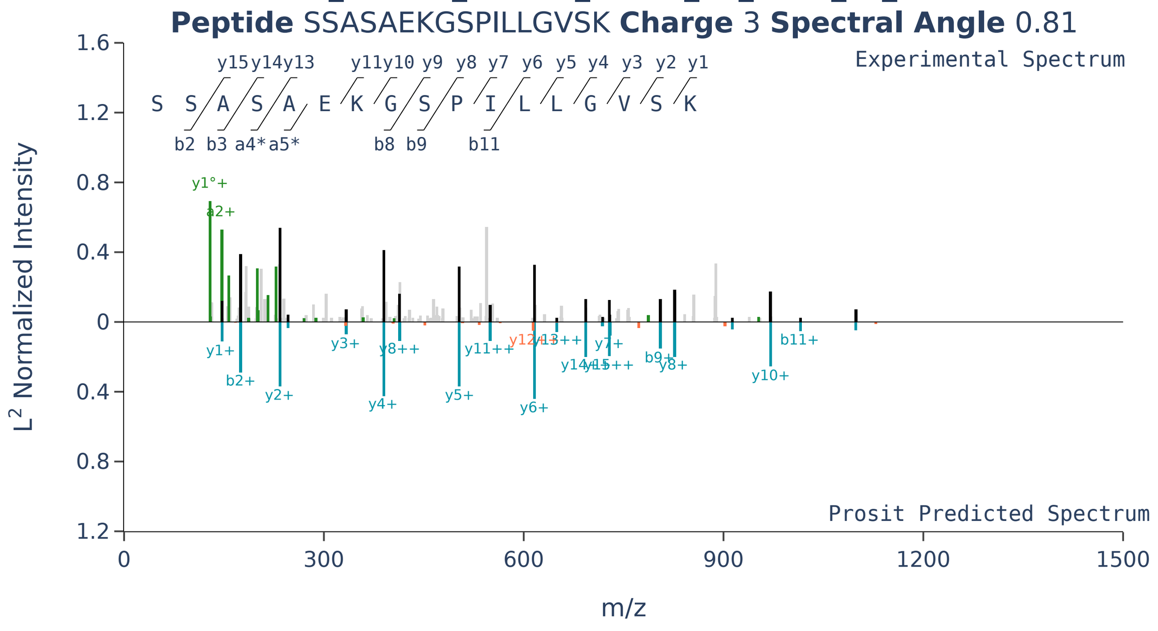


**Source** WSoh_101121_151121_HFGoe_G2_IL37b_24h_R1 **Scan** 11167
**Peptide** FCLYCDKDKGQSH **Charge** 3 **Spectral Angle** 0.78

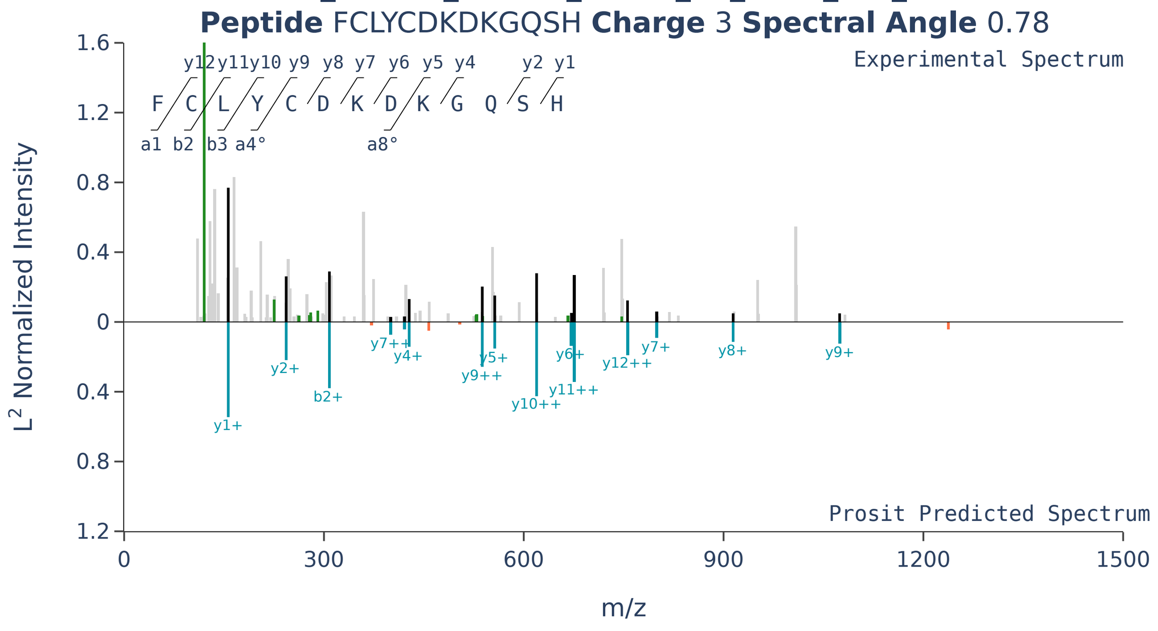


**Source** WSoh_101121_151121_HFGoe_G2_IL37b_2h_R2 **Scan** 11612
**Peptide** CNEPVGVTD **Charge** 2 **Spectral Angle** 0.89

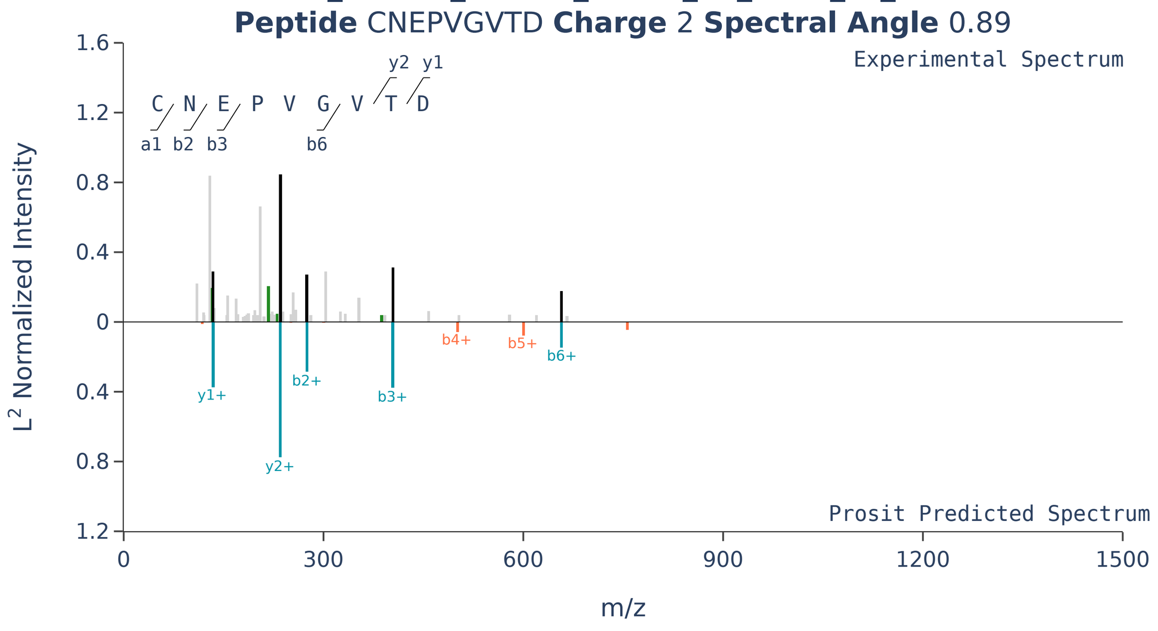

**Source** WSoh_101121_151121_HFGoe_G1_IL37b_1h_R2 **Scan** 22784
**Peptide** SSASAEKGSPILLGV **Charge** 2 **Spectral Angle** 0.91

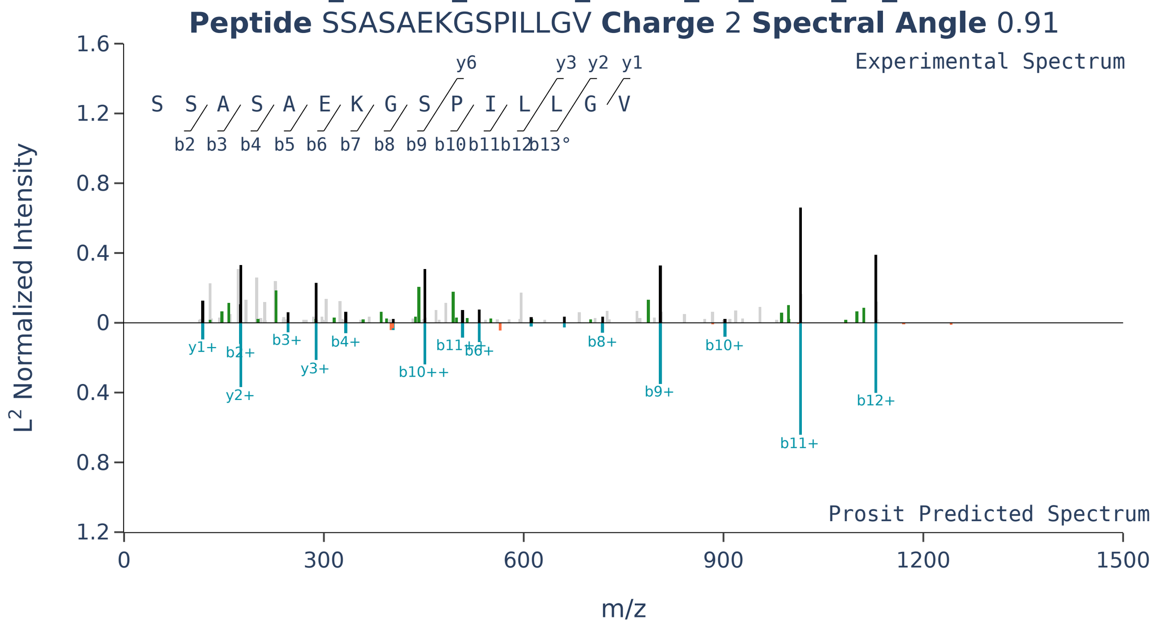


**Source** WSoh_101121_151121_HFGoe_G2_IL37b_24h_R2 **Scan** 6742
**Peptide** DKDKGQSHPSLQLKKE **Charge** 3 **Spectral Angle** 0.83

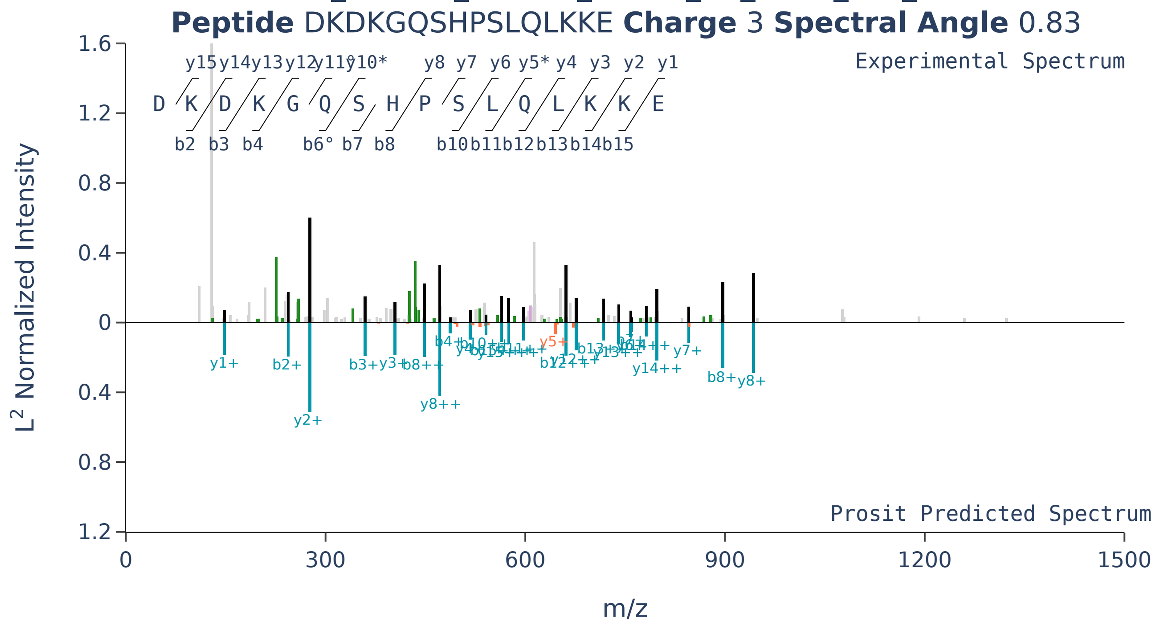


**Source** WSoh_101121_151121_HFGoe_G2_IL37b_2h_R2 **Scan** 5228
**Peptide** VHTSPKVK **Charge** 2 **Spectral Angle** 0.91

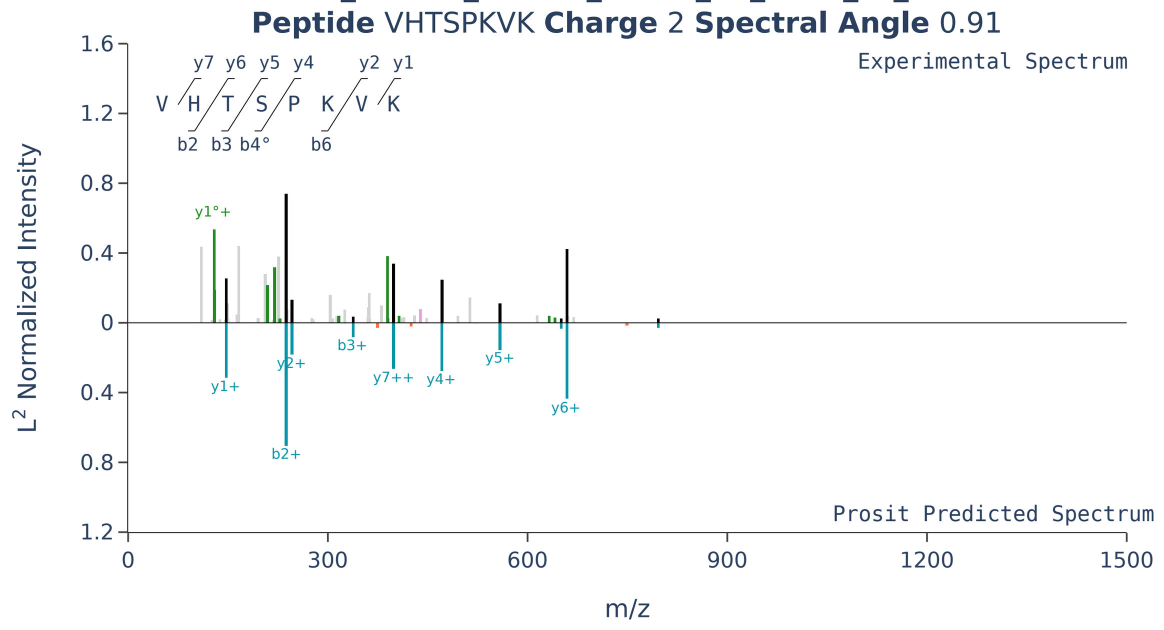


**Source** WSoh_101121_151121_HFGoe_G1_IL37b_24h_R1 **Scan** 13530
**Peptide** AQVGSWN **Charge** 1 **Spectral Angle** 0.93

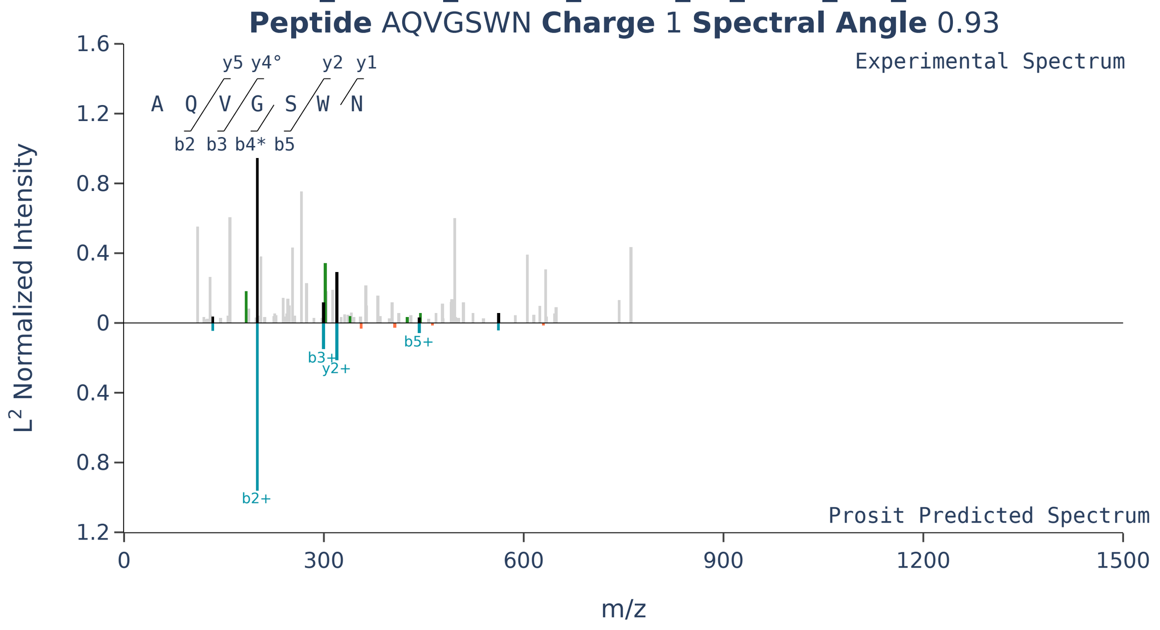


**Source** WSoh_101121_151121_HFGoe_G1_IL37b_24h_R2 **Scan** 20748
**Peptide** DSGNLIAVPDKNY **Charge** 2 **Spectral Angle** 0.9

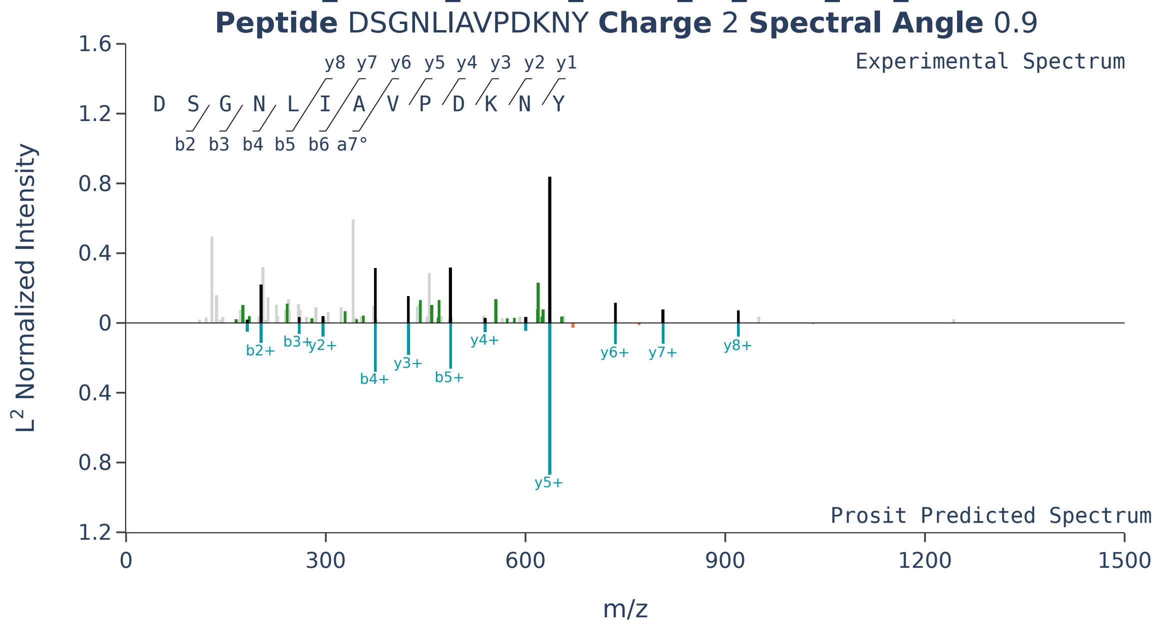


**Source** WSoh_101121_151121_HFGoe_G2_IL37b_24h_R1 **Scan** 19230
**Peptide** IAVPDKNYI **Charge** 2 **Spectral Angle** 0.91

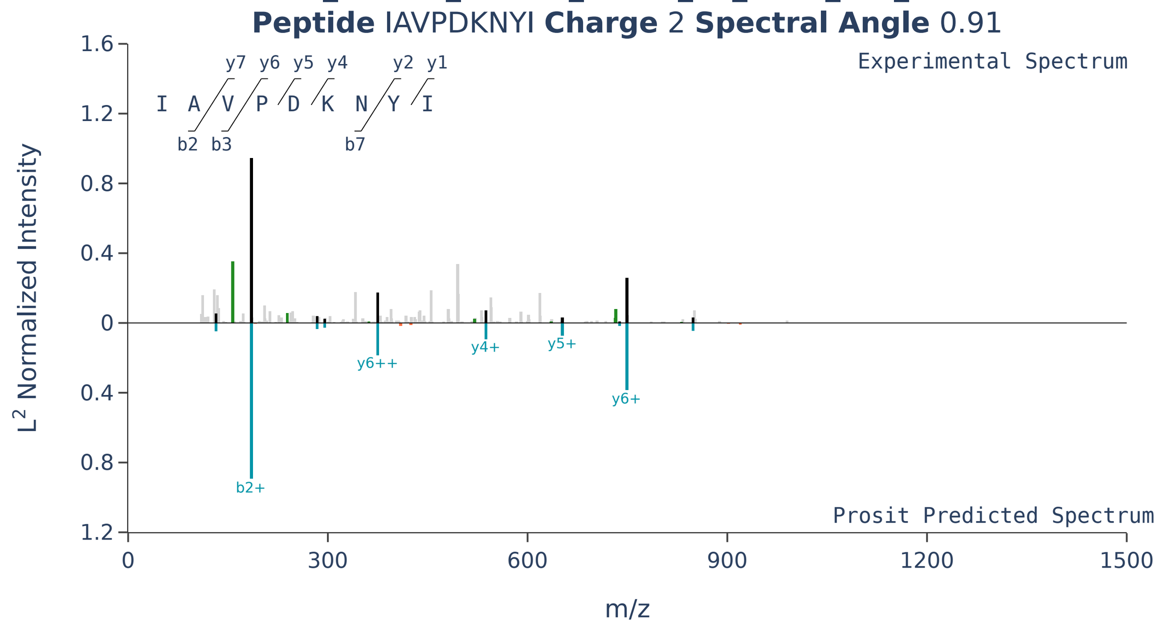


**Source** WSoh_101121_151121_HFGoe_G1_IL37b_1h_R1 **Scan** 12960
**Peptide** HDQDHKVLVLDSG **Charge** 2 **Spectral Angle** 0.83

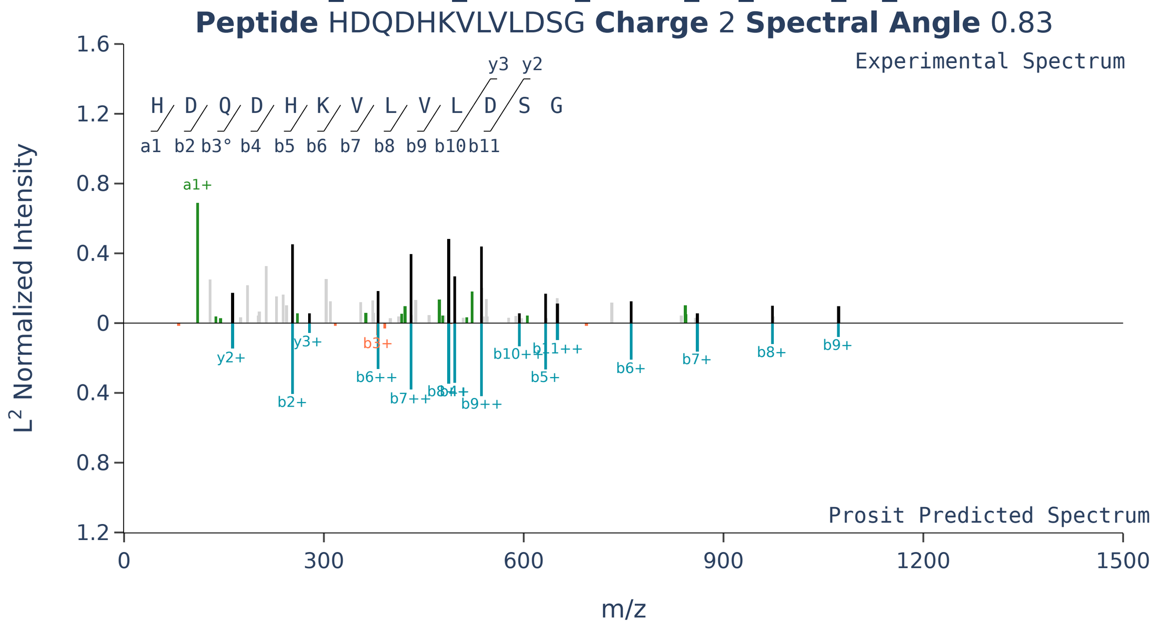


**Source** WSoh_101121_151121_HFGoe_G1_IL37b_24h_R2 **Scan** 9596
**Peptide** SIHDQDHKVLV **Charge** 2 **Spectral Angle** 0.85

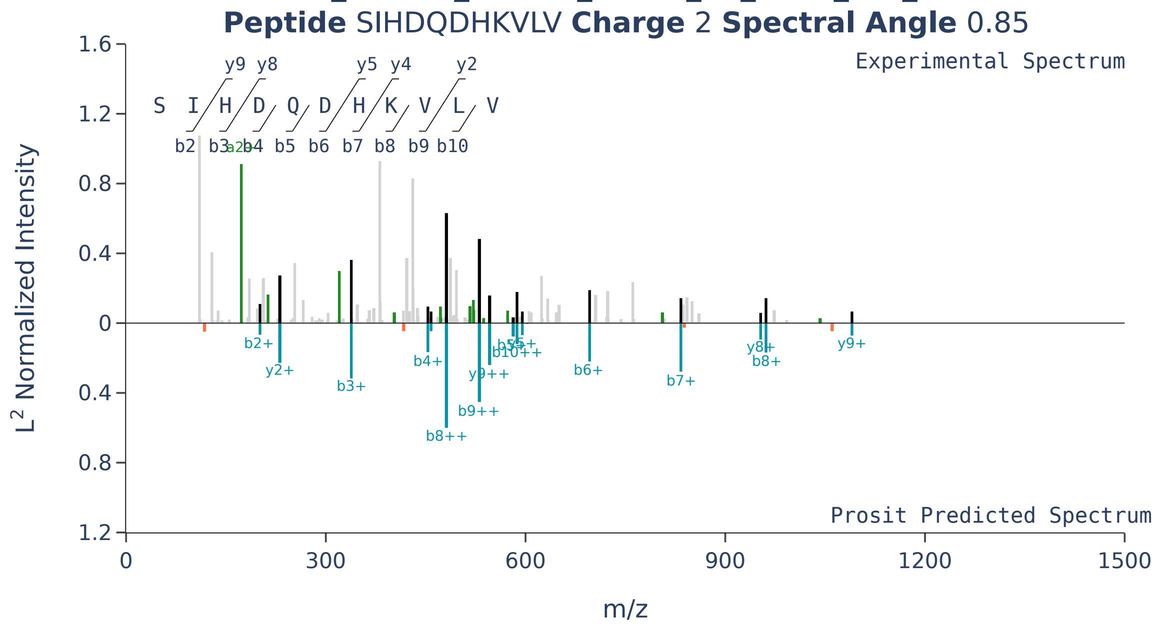


**Source** WSoh_101121_151121_HFGoe_G1_IL37b_24h_R1 **Scan** 16684
**Peptide** GQSHPSLQL **Charge** 2 **Spectral Angle** 0.82

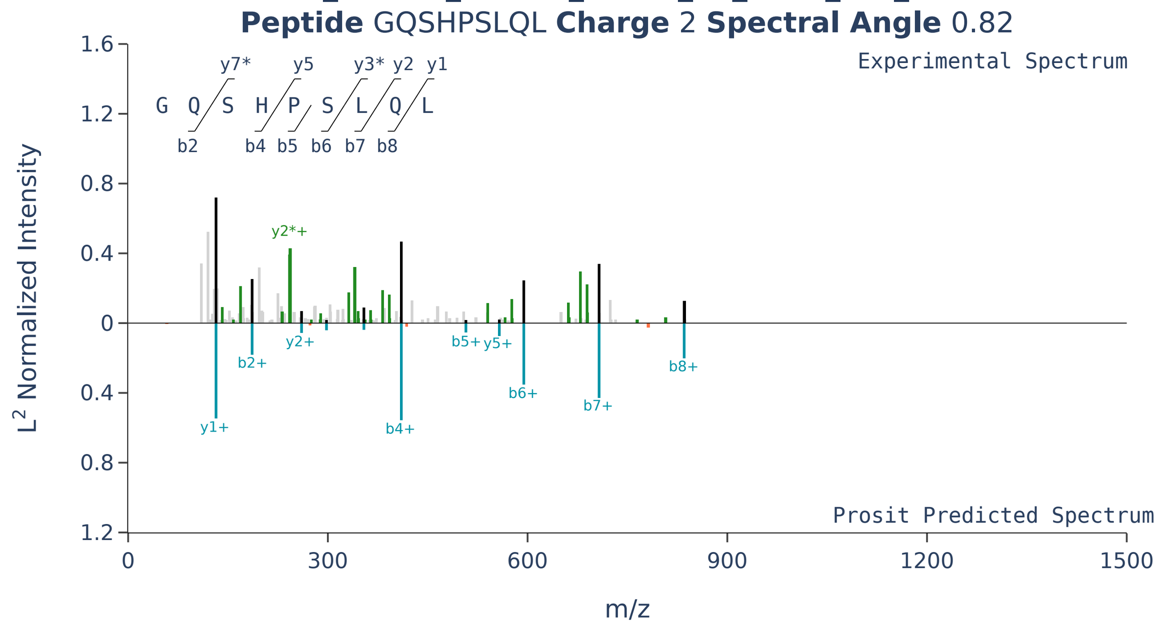


**Source** WSoh_101121_151121_HFGoe_G1_IL37b_4h_R1 **Scan** 9565
**Peptide** LMKLAAQKES **Charge** 2 **Spectral Angle** 0.81

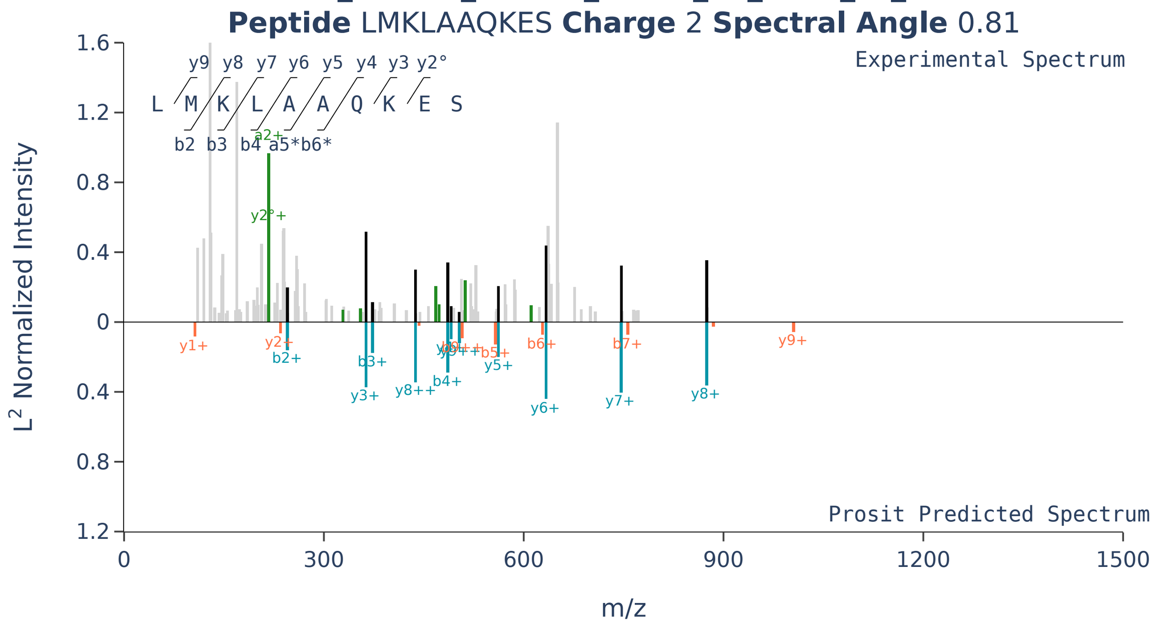


**Source** WSoh_101121_151121_HFGoe_G1_IL37b_1h_R2 **Scan** 22695
**Peptide** IEFSFQPVCK **Charge** 2 **Spectral Angle** 0.92

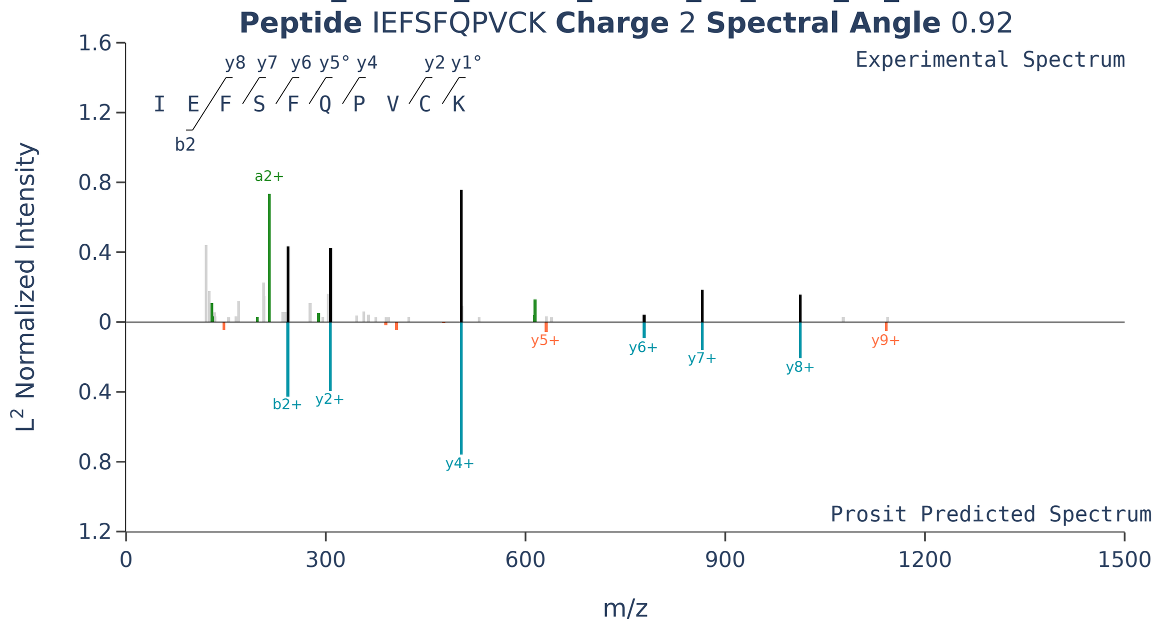


**Source** WSoh_101121_151121_HFGoe_G2_IL37b_24h_R1 **Scan** 17933
**Peptide** VGSWNMLESAAH **Charge** 2 **Spectral Angle** 0.85

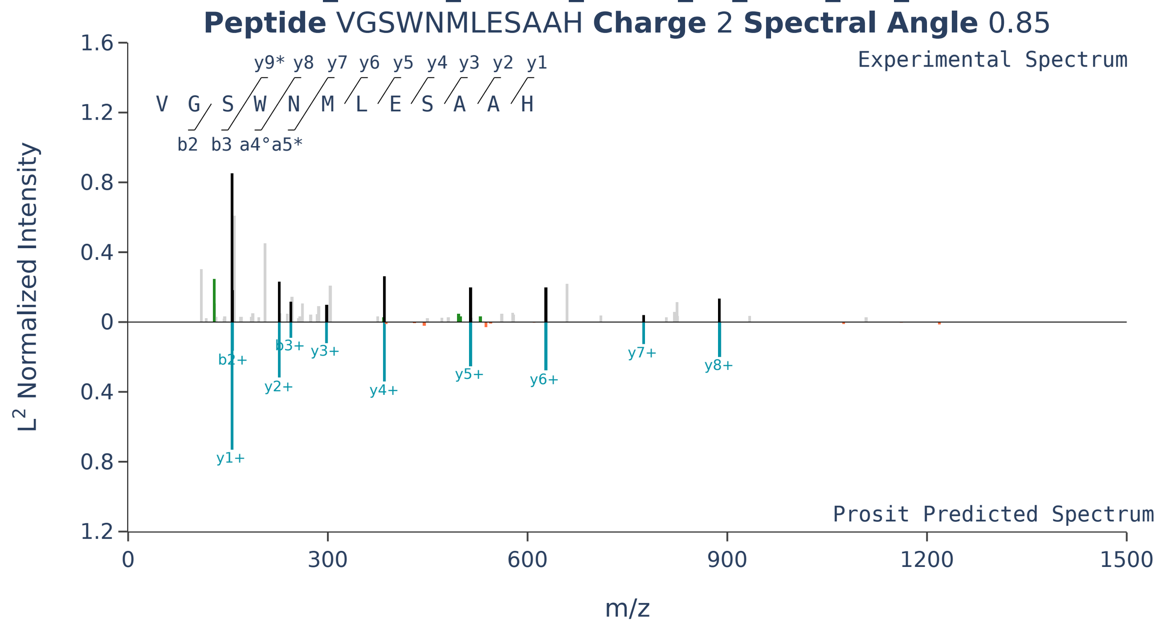


**Source** WSoh_101121_151121_HFGoe_G2_IL37b_24h_R1 **Scan** 7528
**Peptide** SSLSSASAEKGSP **Charge** 2 **Spectral Angle** 0.81

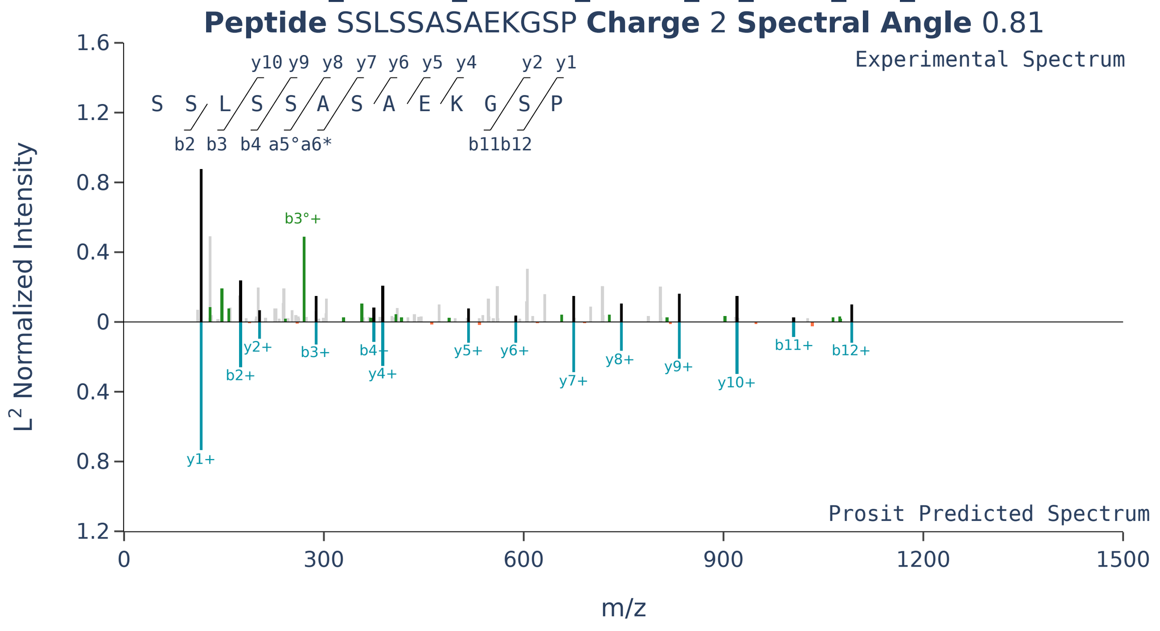


**Source** WSoh_101121_151121_HFGoe_G1_IL37b_24h_R2 **Scan** 11449
**Peptide** VTDKFENRKHIEF **Charge** 4 **Spectral Angle** 0.93

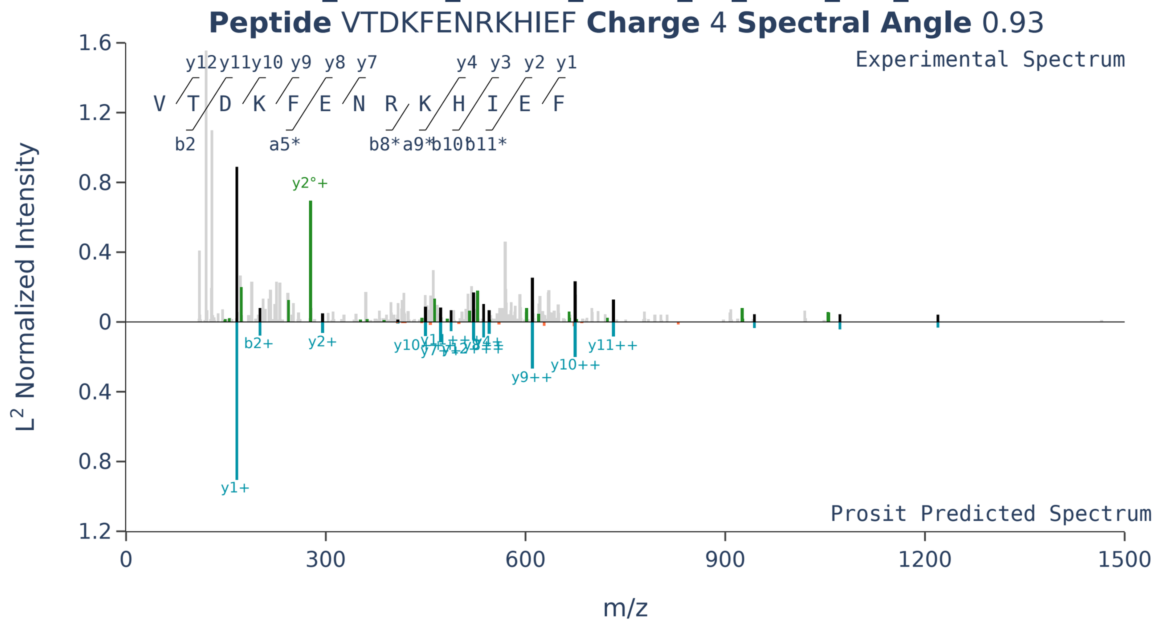


**Source** WSoh_101121_151121_HFGoe_G2_IL37b_24h_R2 **Scan** 7764
**Peptide** NEPVGVTDK **Charge** 2 **Spectral Angle** 0.79

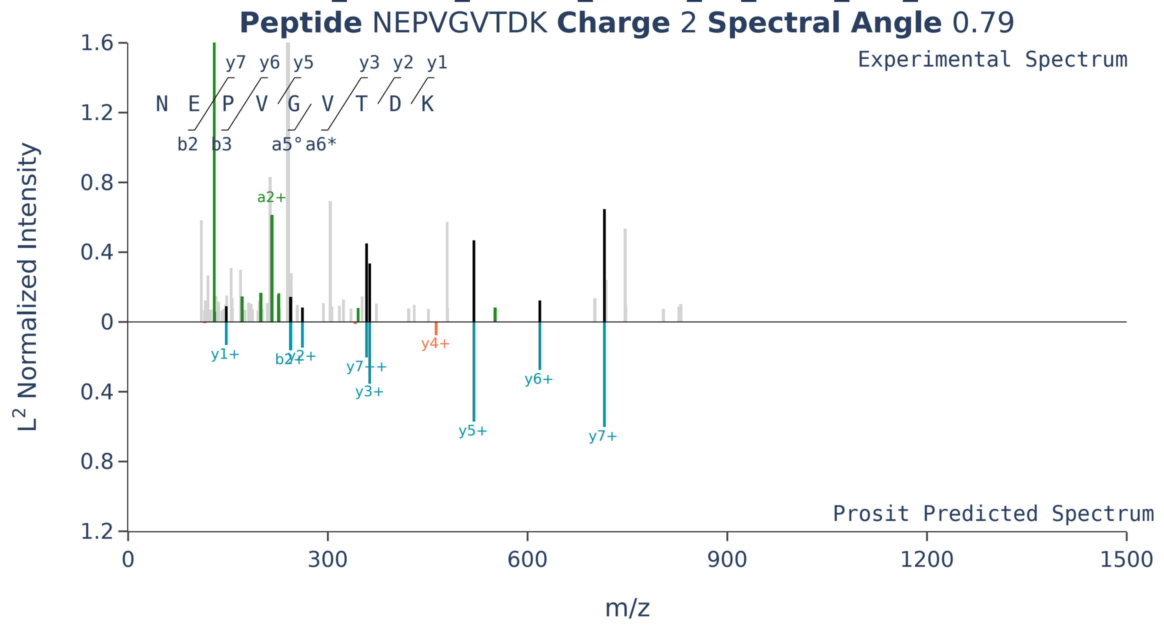

**Source** WSoh_101121_151121_HFGoe_G1_IL37b_24h_R1 **Scan** 8404
**Peptide** DKDKGQSHPSLQLKKEKL **Charge** 3 **Spectral Angle** 0.84

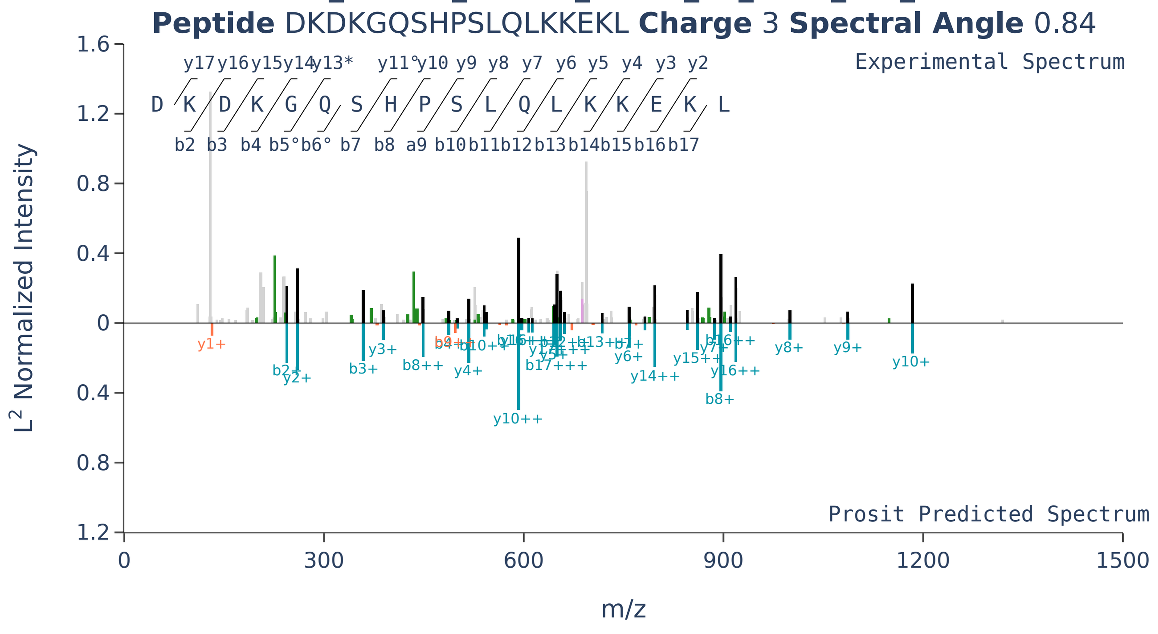


**Source** WSoh_101121_151121_HFGoe_G1_IL37b_1h_R1 **Scan** 17377
**Peptide** RAQVGSWNMLE **Charge** 2 **Spectral Angle** 0.79

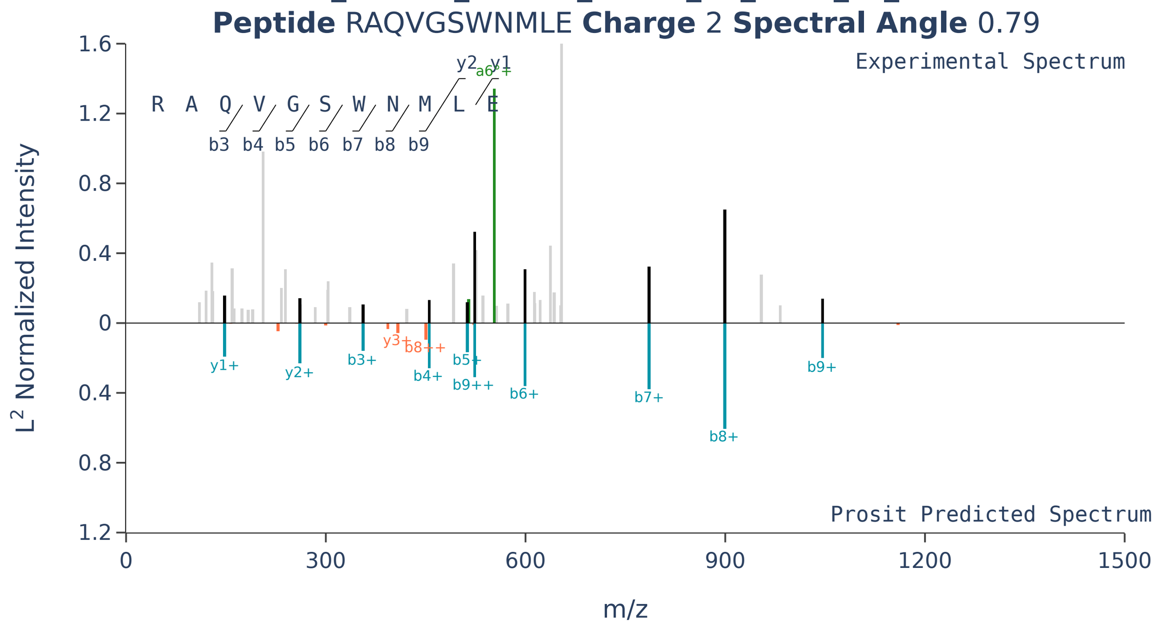


**Source** WSoh_101121_151121_HFGoe_G1_IL37b_4h_R2 **Scan** 5950
**Peptide** DKDKGQSHPSLQLKKEK **Charge** 3 **Spectral Angle** 0.86

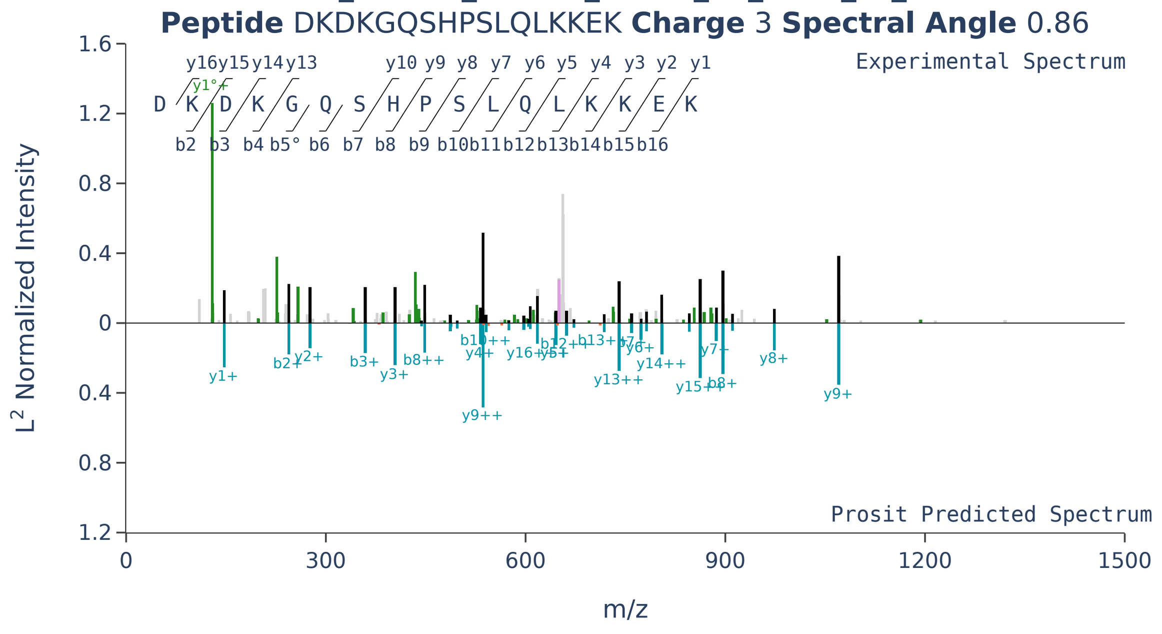


**Source** WSoh_101121_151121_HFGoe_G2_IL37b_24h_R1 **Scan** 8472
**Peptide** NCNEPVGVTDK **Charge** 2 **Spectral Angle** 0.88

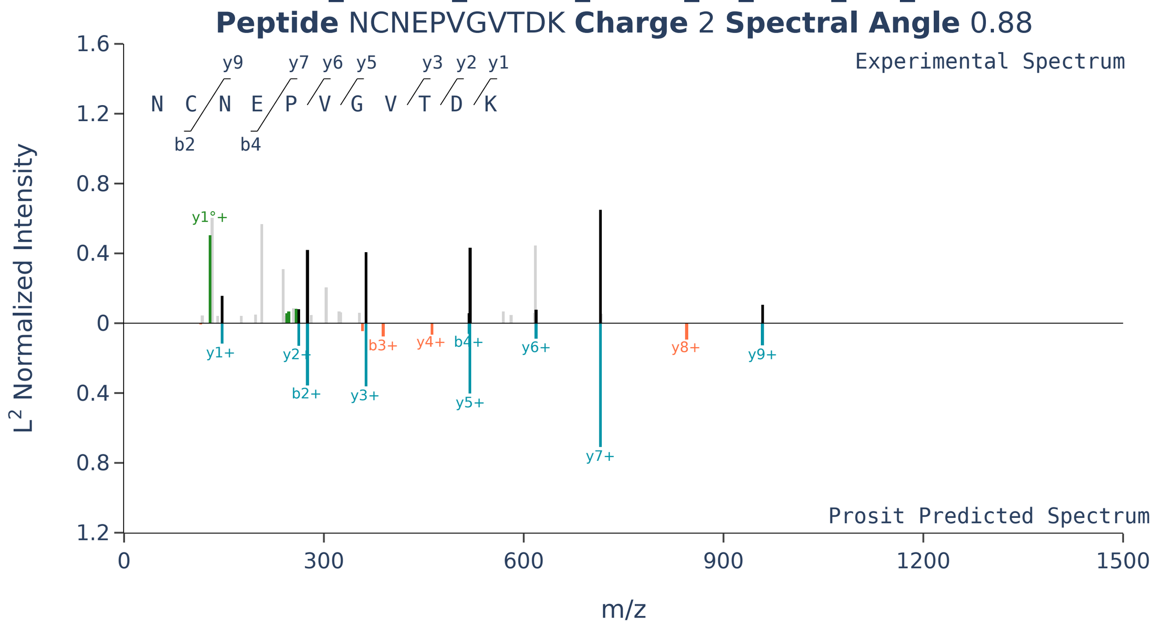


**Source** WSoh_101121_151121_HFGoe_G1_IL37b_1h_R1 **Scan** 18328
**Peptide** RPFIFYR **Charge** 2 **Spectral Angle** 0.84

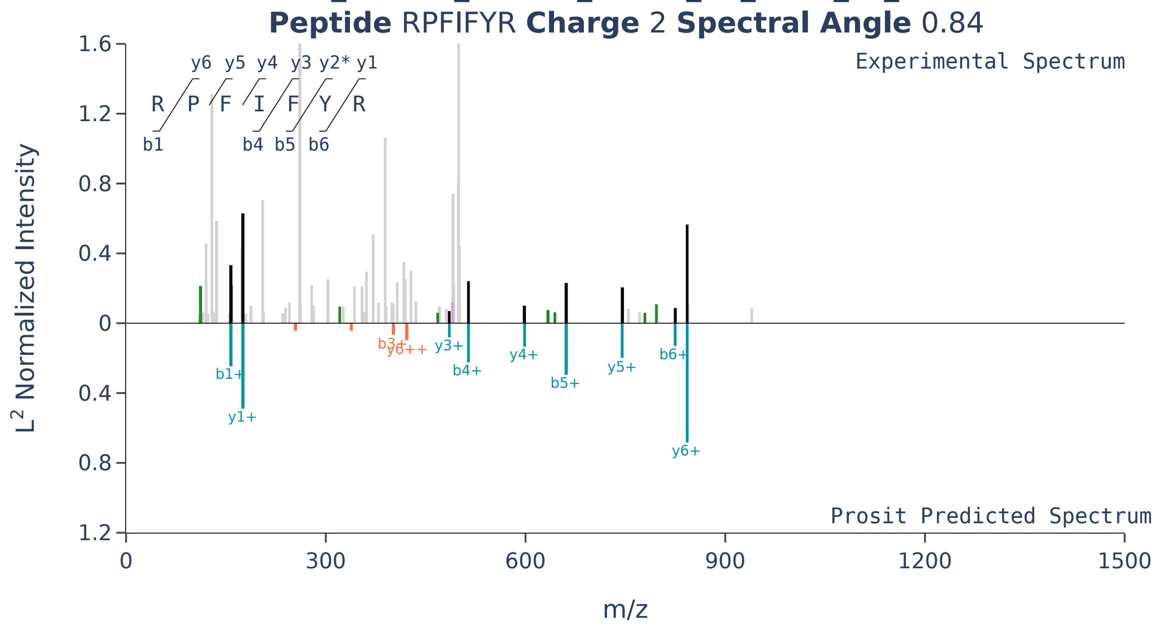


**Source** WSoh_101121_151121_HFGoe_G1_IL37b_2h_R1 **Scan** 16794
**Peptide** SAEKGSPILLGVSKG **Charge** 3 **Spectral Angle** 0.85

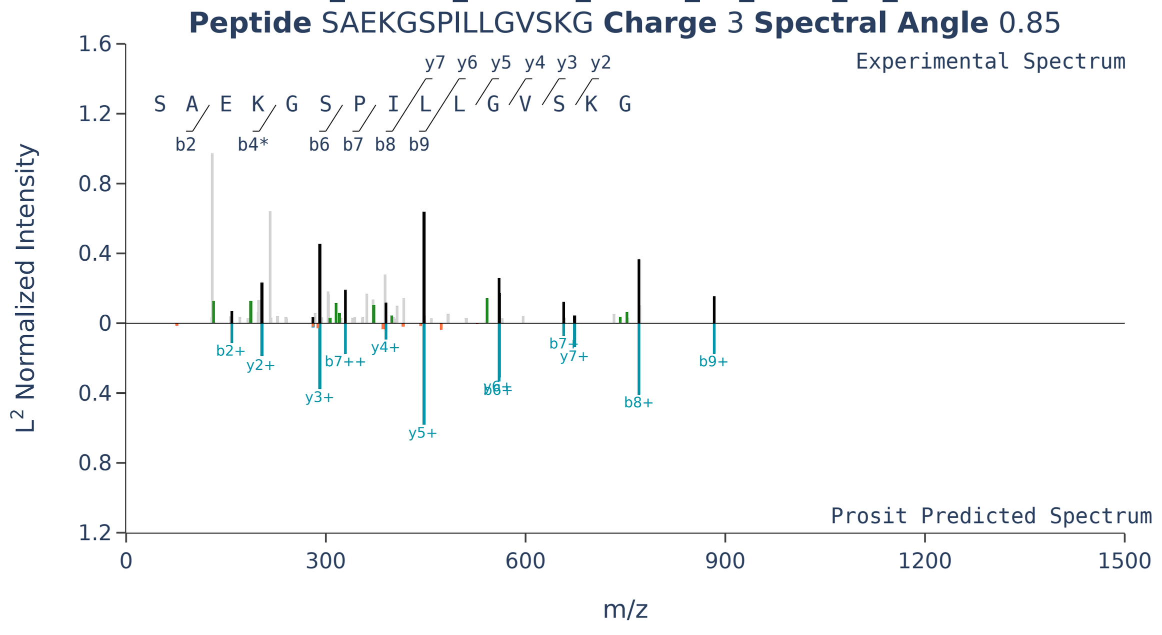


**Source** WSoh_101121_151121_HFGoe_G1_IL37b_4h_R2 **Scan** 6121
**Peptide** SLSSASAEKG **Charge** 2 **Spectral Angle** 0.89

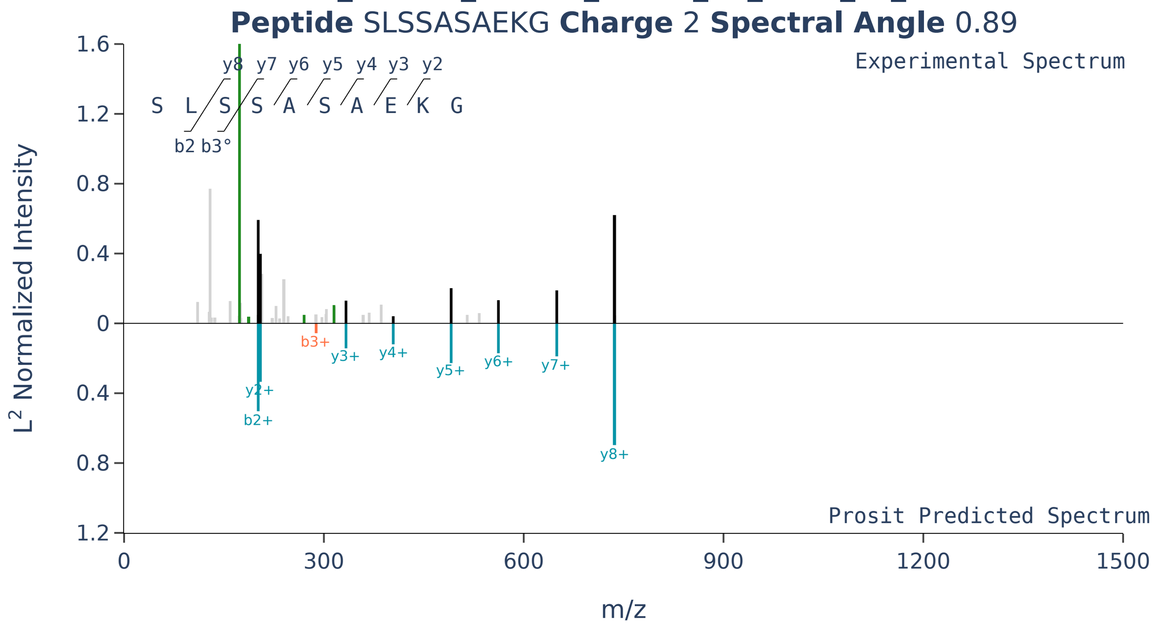


**Source** WSoh_101121_151121_HFGoe_G1_IL37b_24h_R1 **Scan** 5653
**Peptide** TDKFENRKHLE **Charge** 3 **Spectral Angle** 0.8

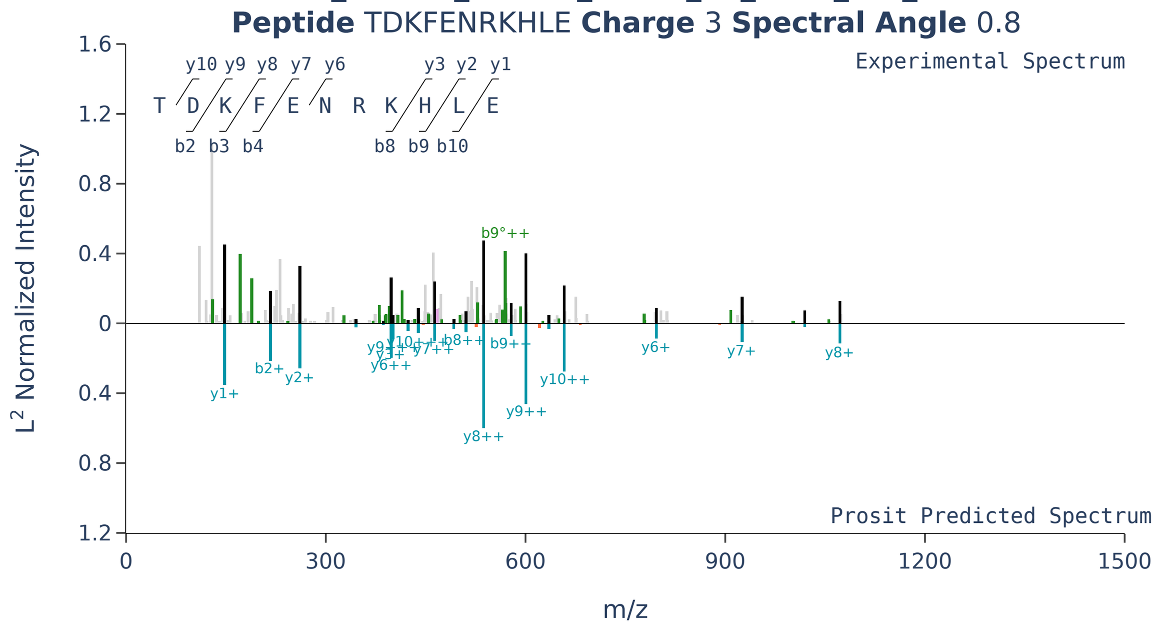


**Source** WSoh_101121_151121_HFGoe_G1_IL37b_4h_R1 **Scan** 12388
**Peptide** VPDKNYI **Charge** 2 **Spectral Angle** 0.8

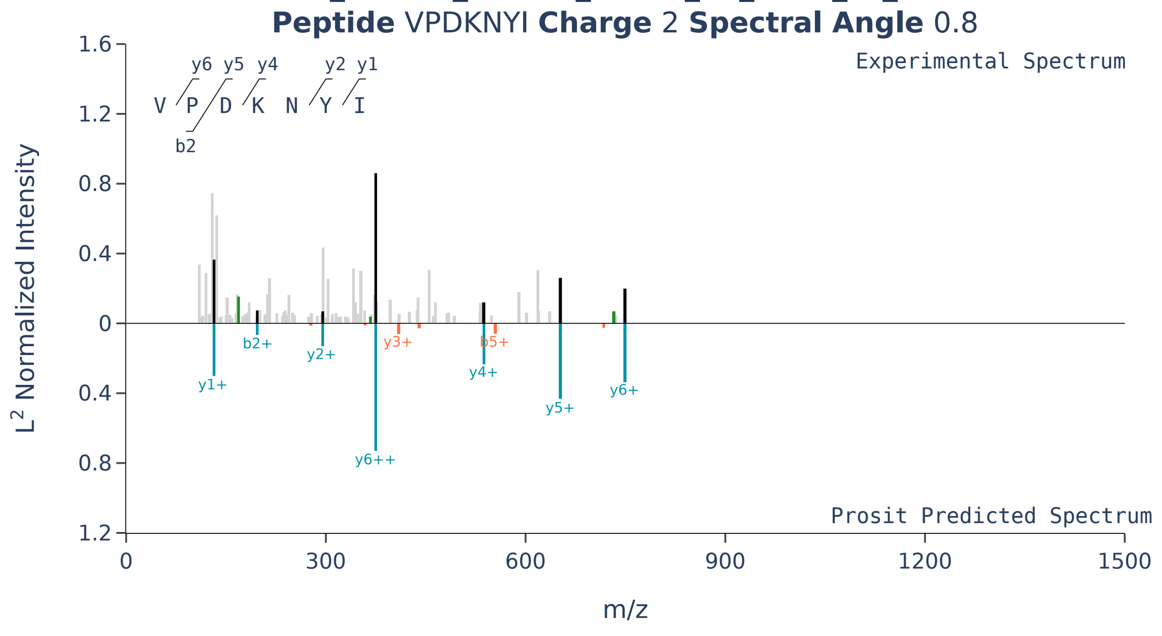


**Source** WSoh_101121_151121_HFGoe_G1_IL37b_24h_R1 **Scan** 14410
**Peptide** IAVPDKNYIR **Charge** 3 **Spectral Angle** 0.77

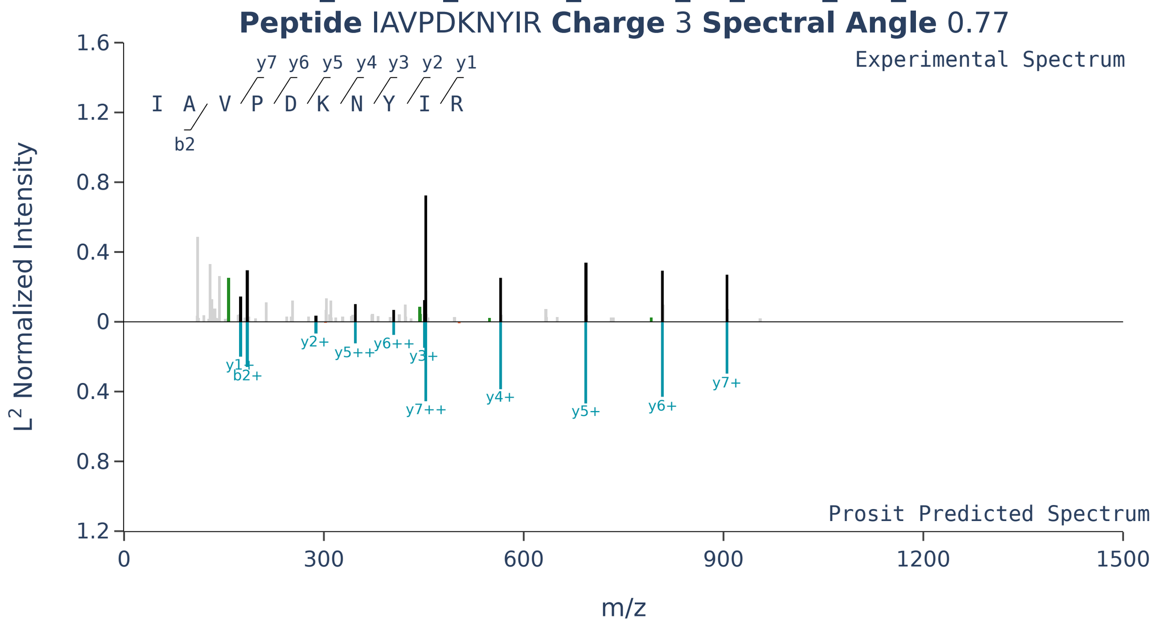


**Source** WSoh_101121_151121_HFGoe_G1_IL37b_24h_R2 **Scan** 8522
**Peptide** EKLMKLAA **Charge** 2 **Spectral Angle** 0.82

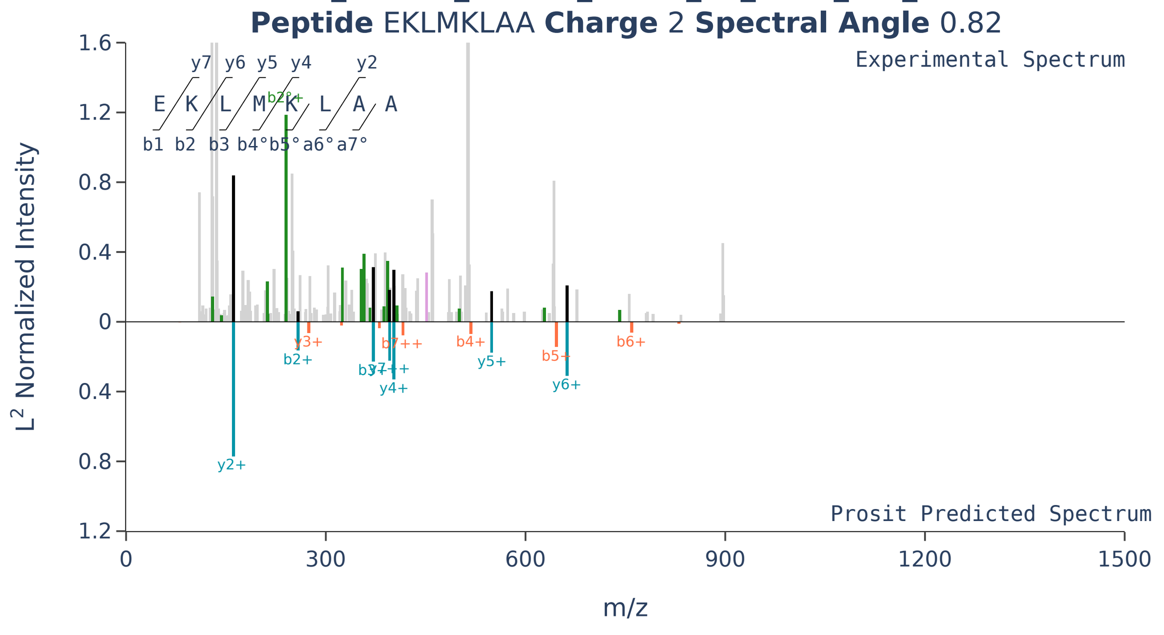


**Source** WSoh_101121_151121_HFGoe_G2_IL37b_24h_R2 **Scan** 10265
**Peptide** KAEMSPSEV **Charge** 1 **Spectral Angle** 0.82

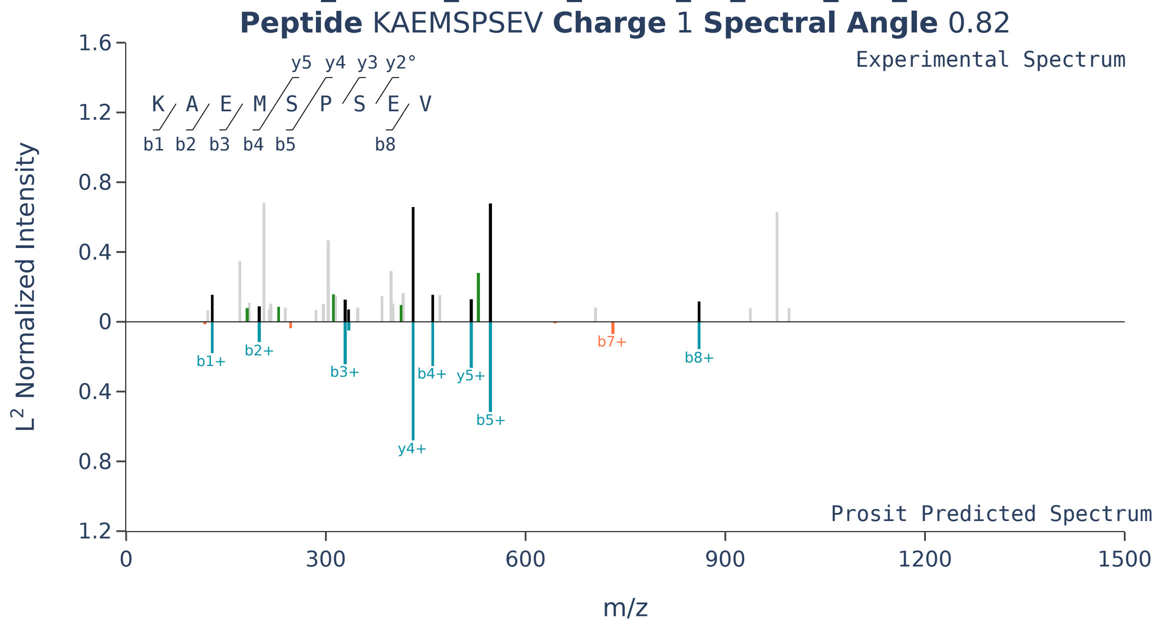


**Source** WSoh_101121_151121_HFGoe_G1_IL37b_1h_R1 **Scan** 11818
**Peptide** CNCNEPVGVTD **Charge** 2 **Spectral Angle** 0.91

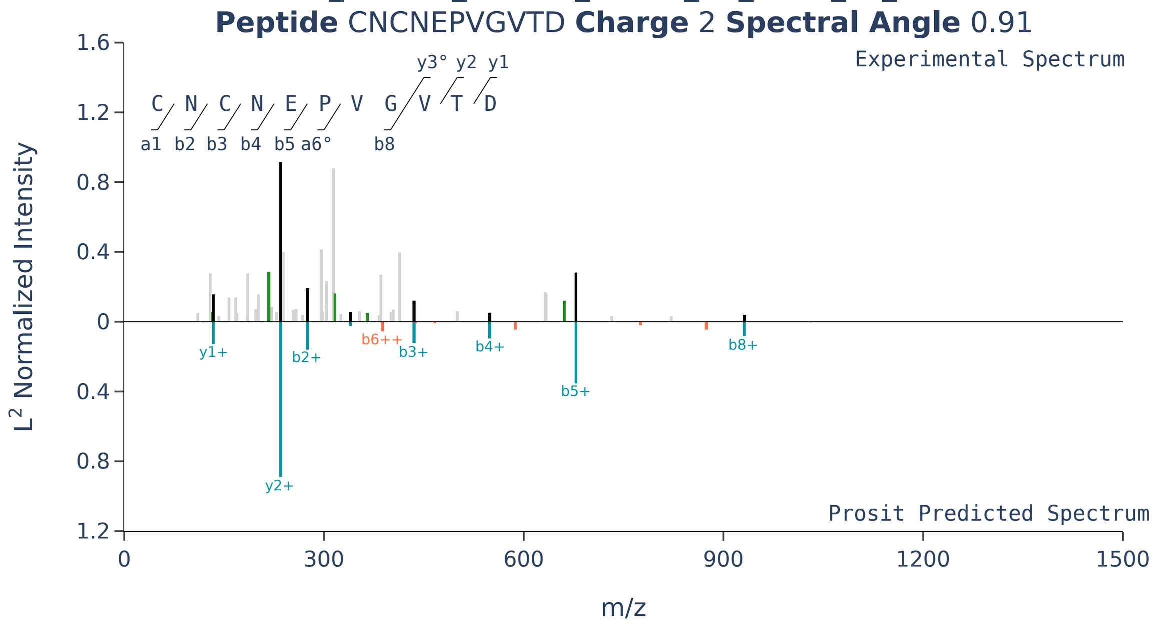


**Source** WSoh_101121_151121_HFGoe_G2_IL37b_24h_R1 **Scan** 7095
**Peptide** ENRKHIEFS **Charge** 2 **Spectral Angle** 0.84

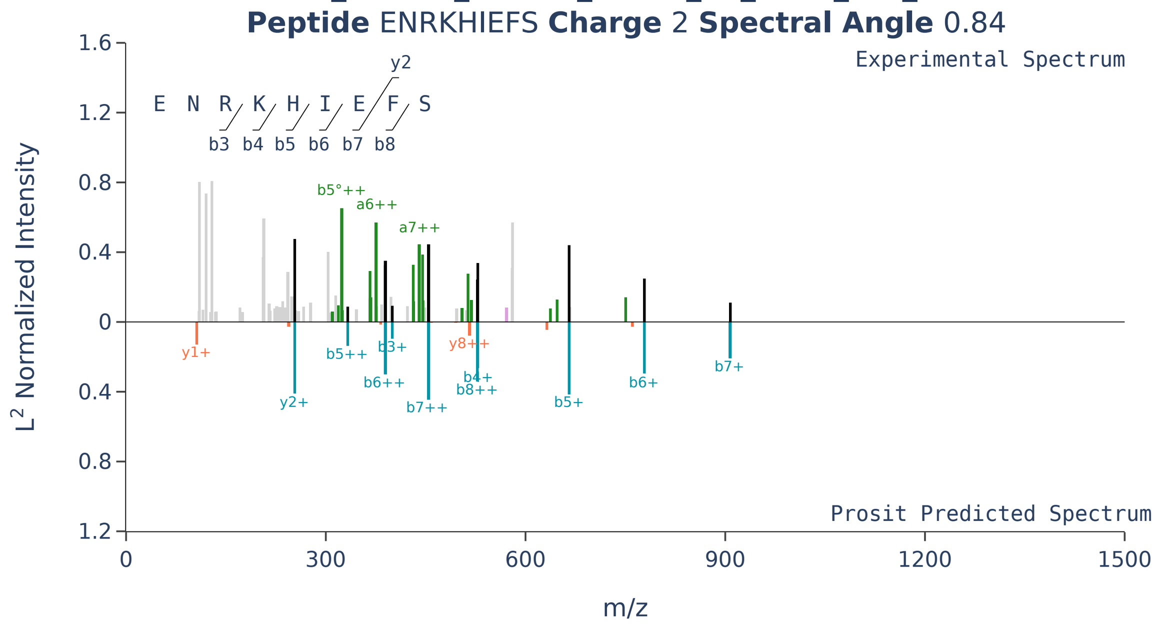


**Source** WSoh_101121_151121_HFGoe_G1_IL37b_24h_R2 **Scan** 16650
**Peptide** EPVGVTDKF **Charge** 2 **Spectral Angle** 0.88

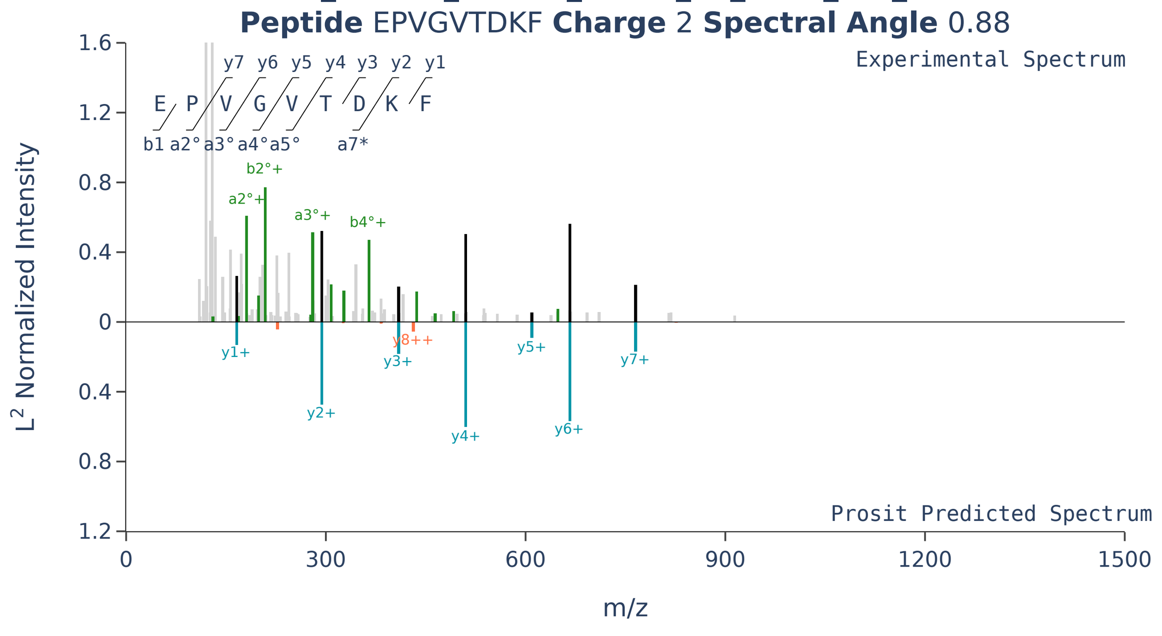

**Source** WSoh_101121_151121_HFGoe_G2_IL37b_24h_R1 **Scan** 6775
**Peptide** IHDQDHKVL **Charge** 2 **Spectral Angle** 0.8

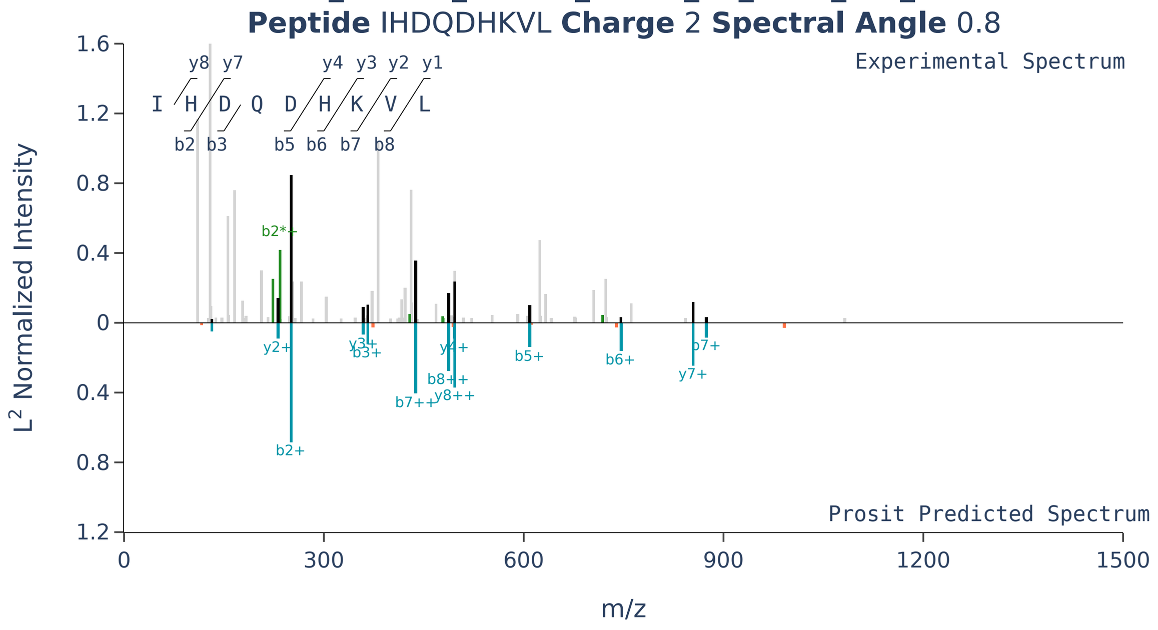


**Source** WSoh_101121_151121_HFGoe_G1_IL37b_24h_R2 **Scan** 11598
**Peptide** SKGEFCLYCDKDKG **Charge** 3 **Spectral Angle** 0.89

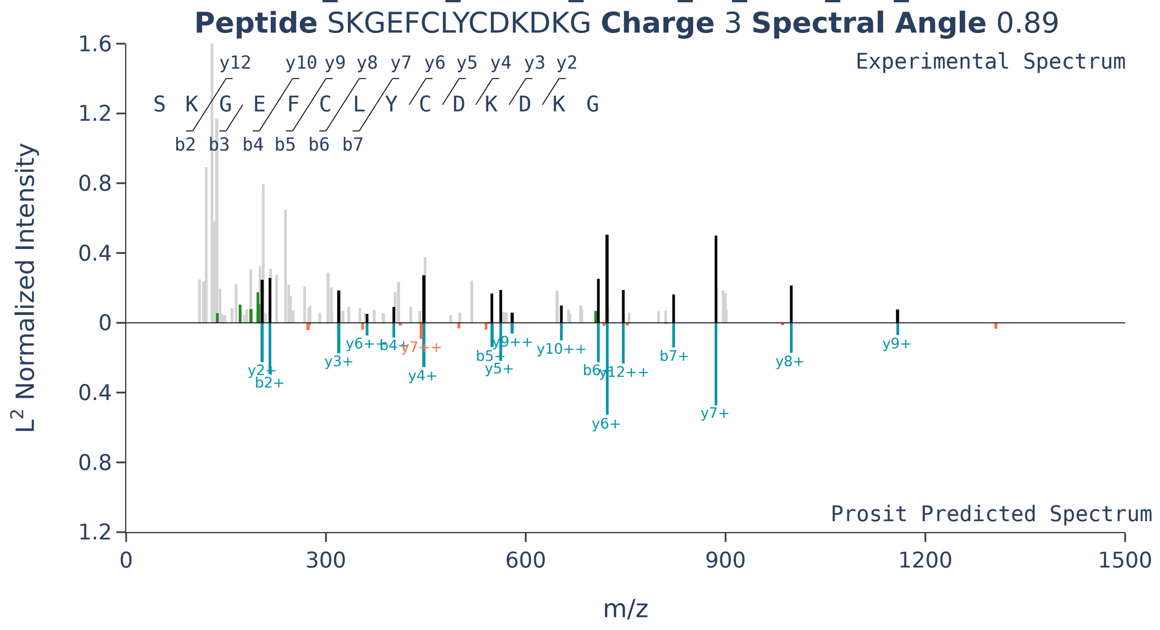


**Source** WSoh_101121_151121_HFGoe_G2_IL37b_24h_R2 **Scan** 12029
**Peptide** EKLMKLA **Charge** 2 **Spectral Angle** 0.8

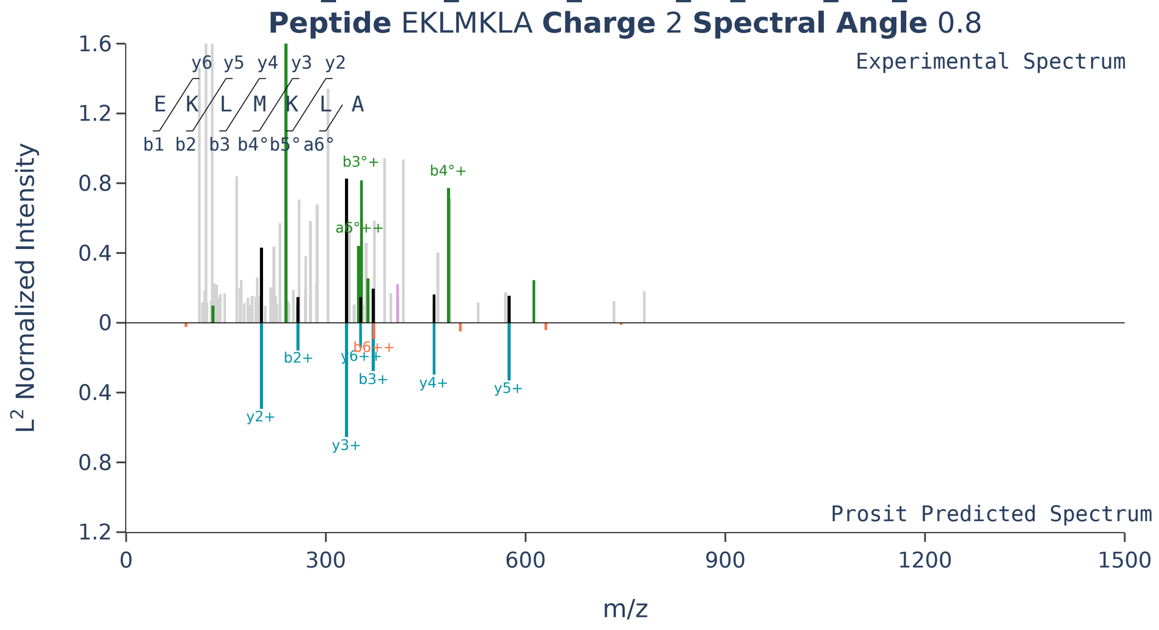


**Source** WSoh_101121_151121_HFGoe_G1_IL37b_4h_R1 **Scan** 6670
**Peptide** KLMKLAAQKESAR **Charge** 3 **Spectral Angle** 0.83

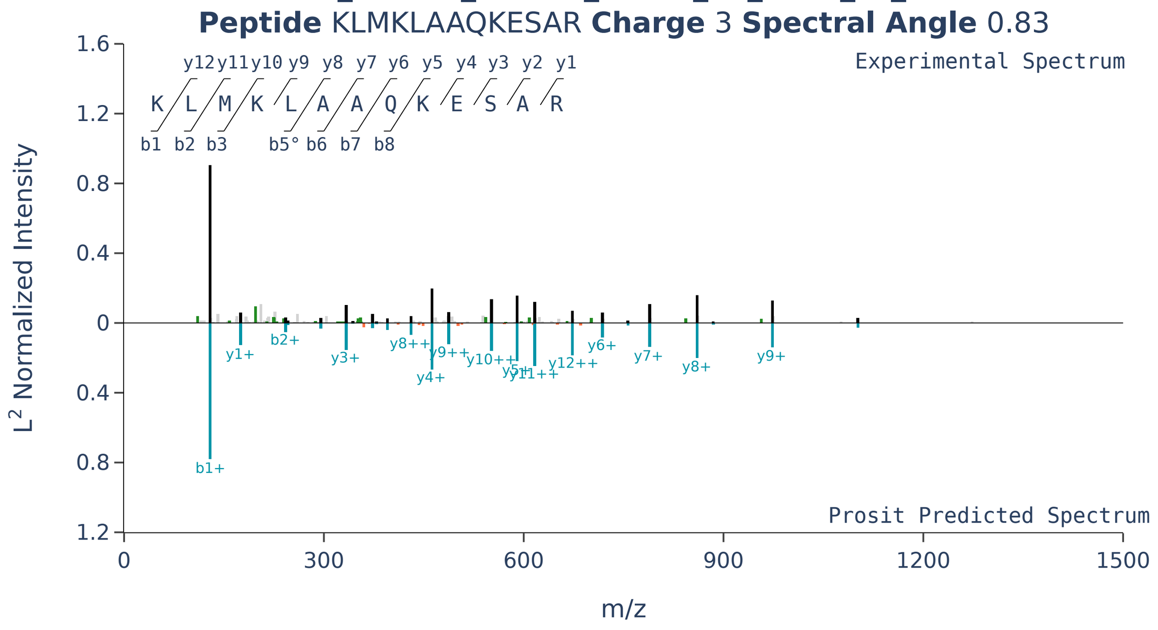


**Source** WSoh_101121_151121_HFGoe_G2_IL37b_4h_R1 **Scan** 10222
**Peptide** SAAHPGW **Charge** 2 **Spectral Angle** 0.9

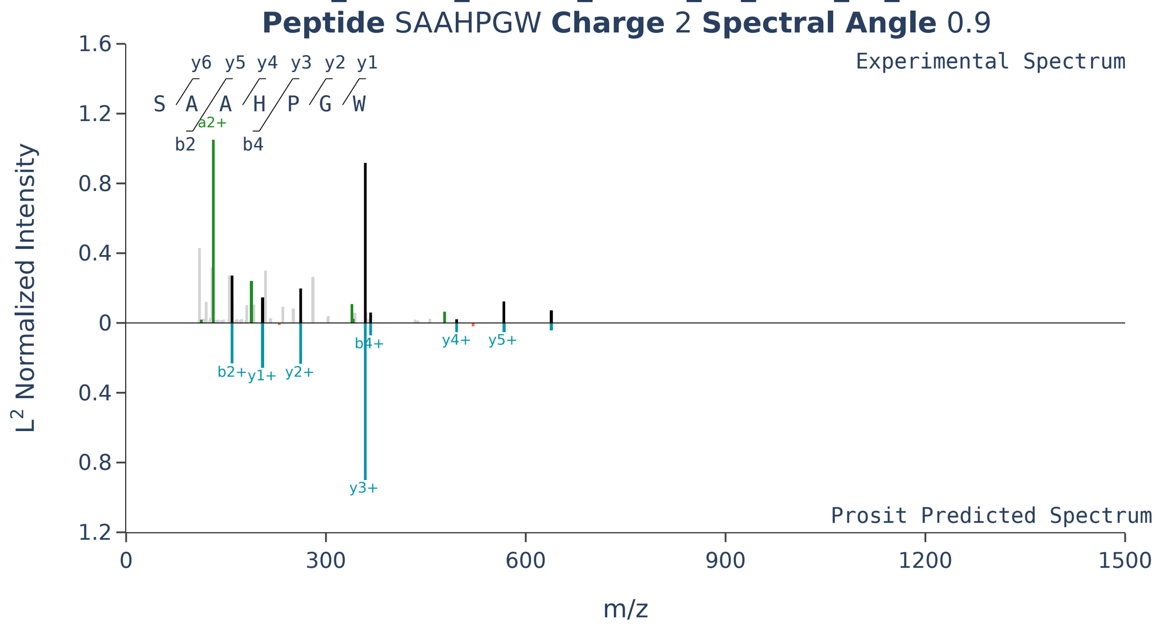


**Source** WSoh_101121_151121_HFGoe_G1_IL37b_24h_R2 **Scan** 6638
**Peptide** NPKKFSIHDQDHKV **Charge** 2 **Spectral Angle** 0.83

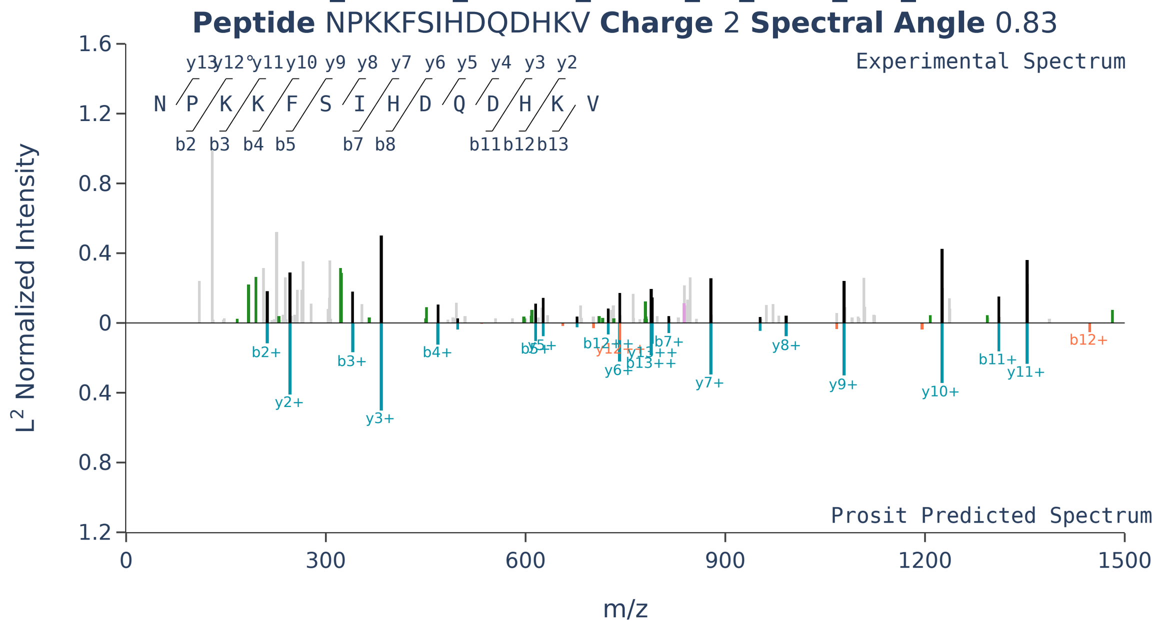


**Source** WSoh_101121_151121_HFGoe_G2_IL37b_24h_R1 **Scan** 23881
**Peptide** AEKGSPILLGV **Charge** 2 **Spectral Angle** 0.84

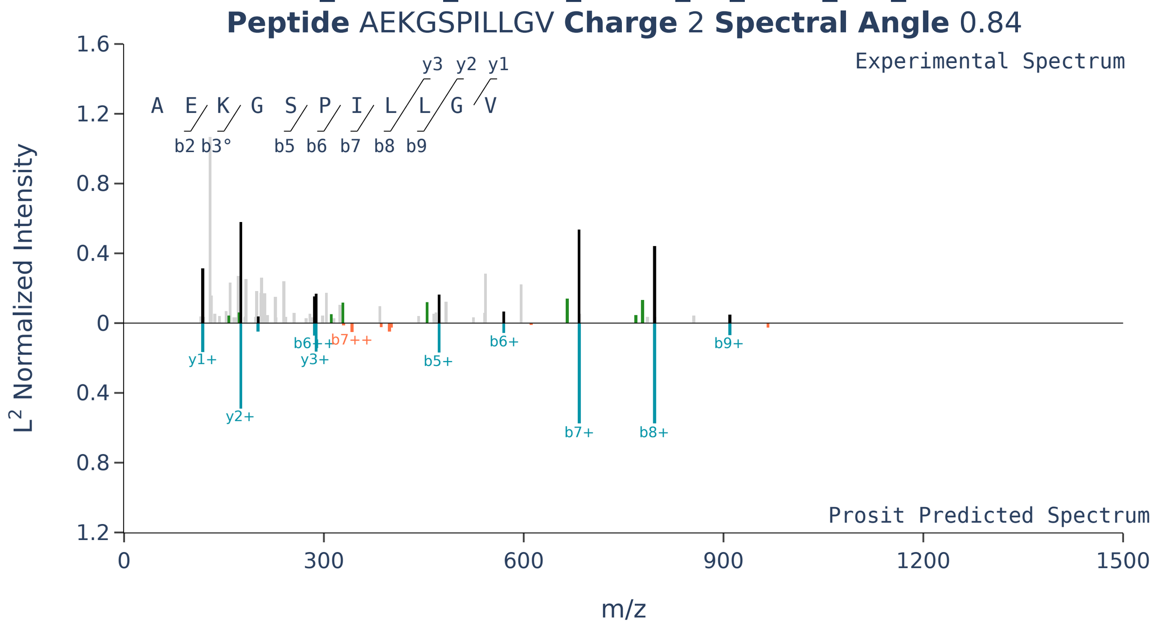


**Source** WSoh_101121_151121_HFGoe_G1_IL37b_24h_R2 **Scan** 8097
**Peptide** DKDKGQSHPSLQLKKEKLM **Charge** 4 **Spectral Angle** 0.81

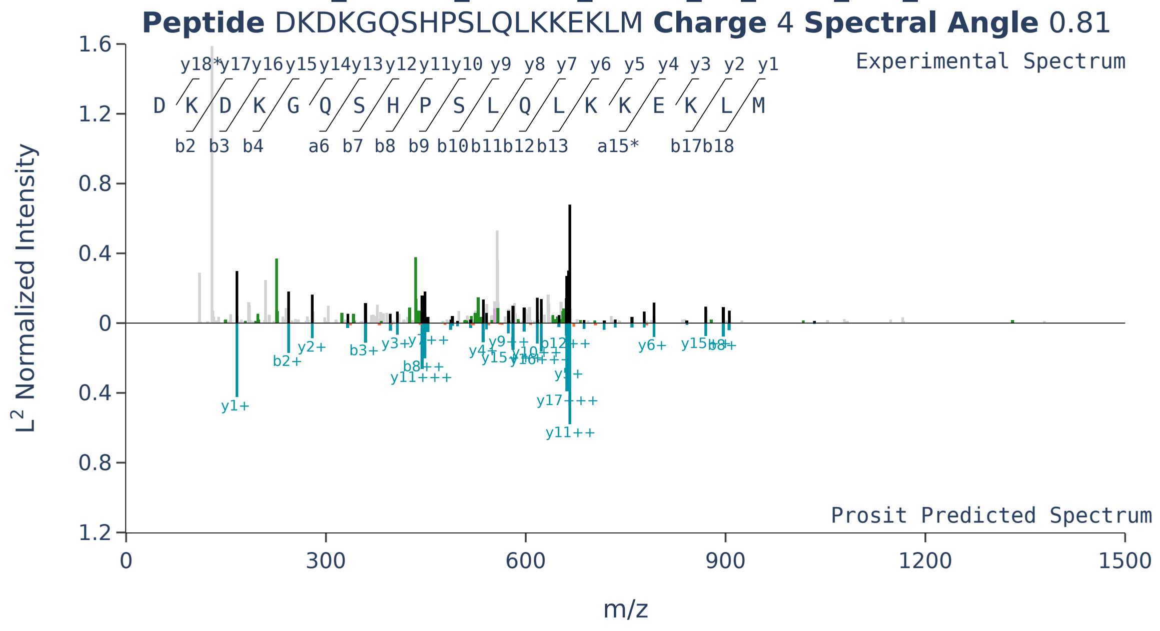


**Source** WSoh_101121_151121_HFGoe_G1_IL37b_24h_R2 **Scan** 6012
**Peptide** VHTSPKVKNLN **Charge** 2 **Spectral Angle** 0.85

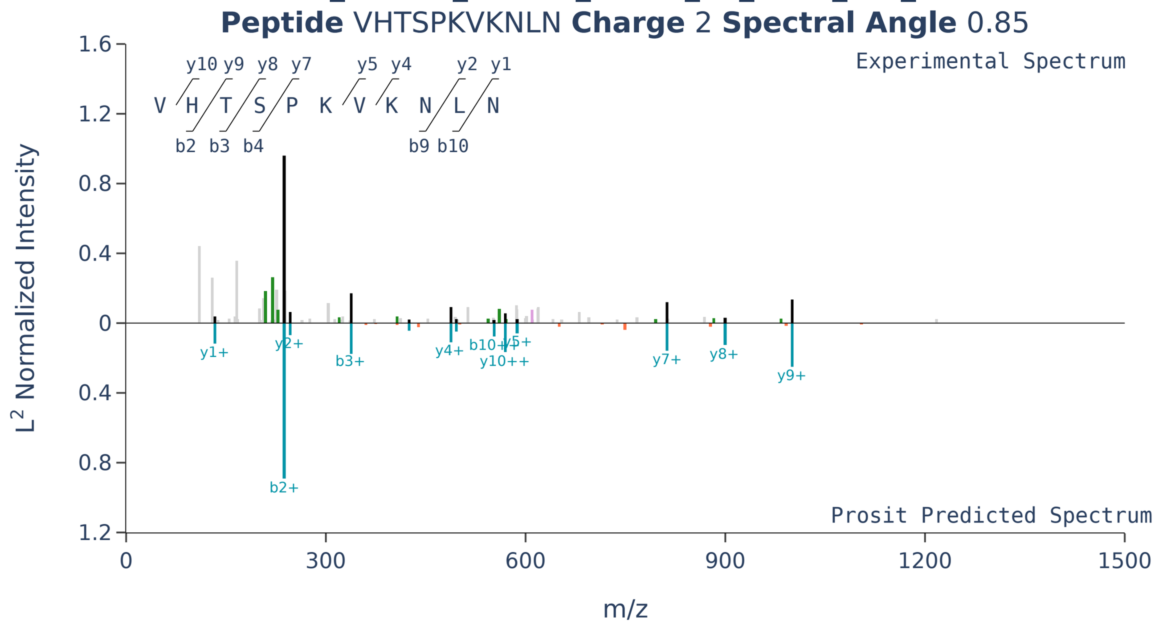


**Source** WSoh_101121_151121_HFGoe_G2_IL37b_1h_R2 **Scan** 7790
**Peptide** HDQDHKVLV **Charge** 2 **Spectral Angle** 0.87

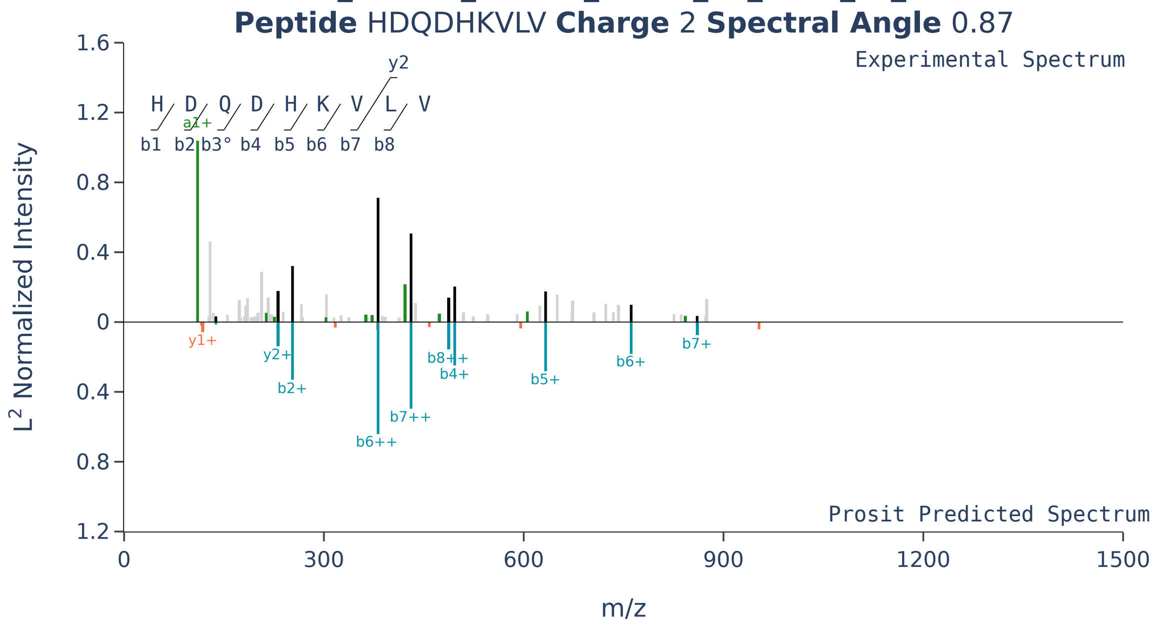


**Source** WSoh_101121_151121_HFGoe_G1_IL37b_24h_R1 **Scan** 15712
**Peptide** ENRKHIEFSFQPVCK **Charge** 4 **Spectral Angle** 0.9

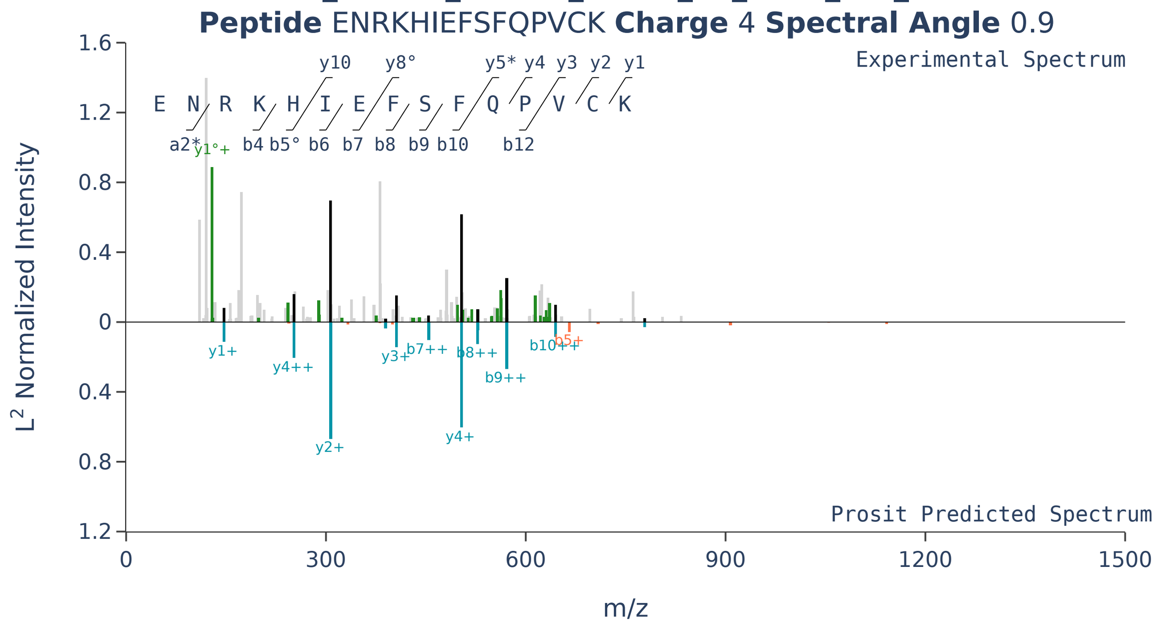


**Source** WSoh_101121_151121_HFGoe_G1_IL37b_4h_R1 **Scan** 13803
**Peptide** AQKESARRPFIFYR **Charge** 4 **Spectral Angle** 0.94

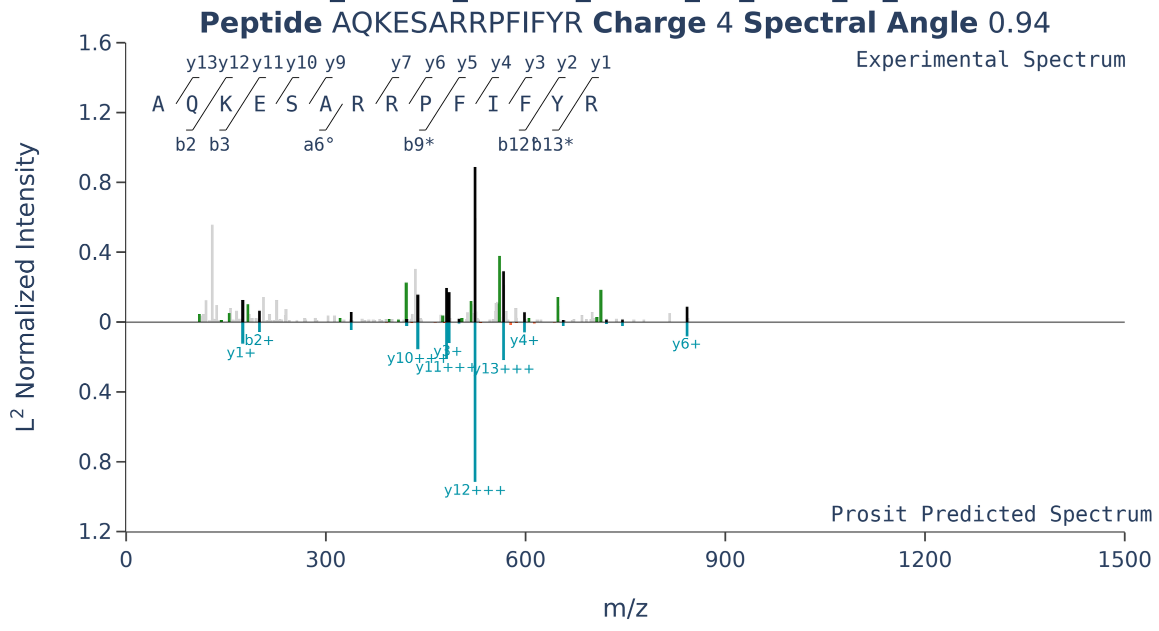


**Source** WSoh_101121_151121_HFGoe_G2_IL37b_24h_R2 **Scan** 7257
**Peptide** KKEKLMKLAAQ **Charge** 3 **Spectral Angle** 0.84

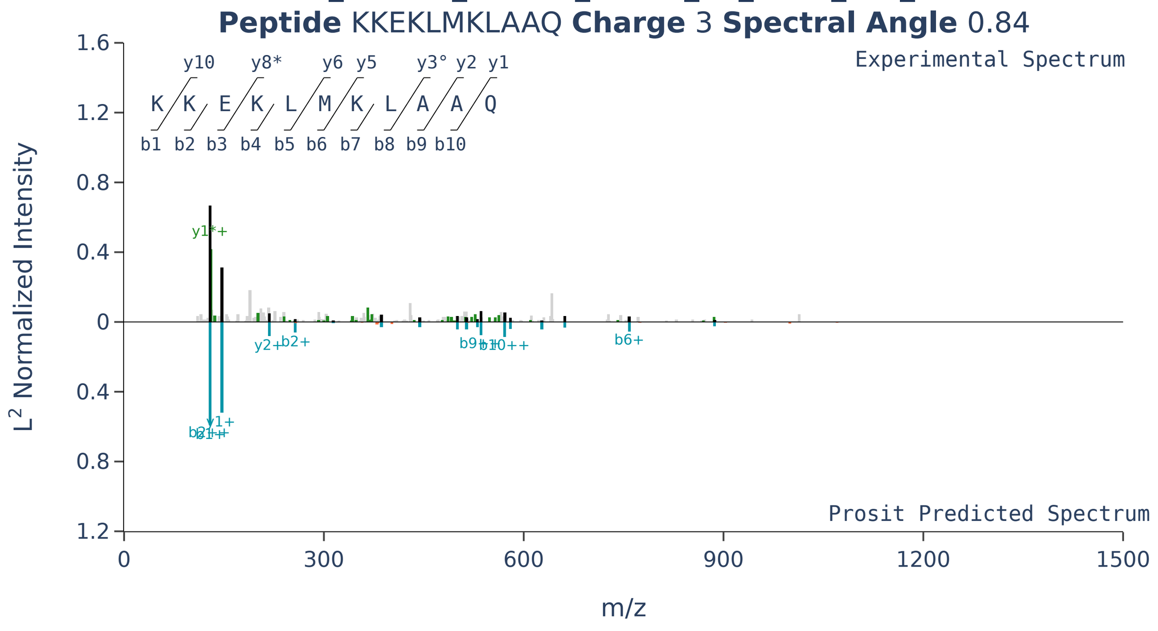


**Source** WSoh_101121_151121_HFGoe_G2_IL37b_24h_R1 **Scan** 14232
**Peptide** SIHDQDHKVLVLDSG **Charge** 3 **Spectral Angle** 0.84

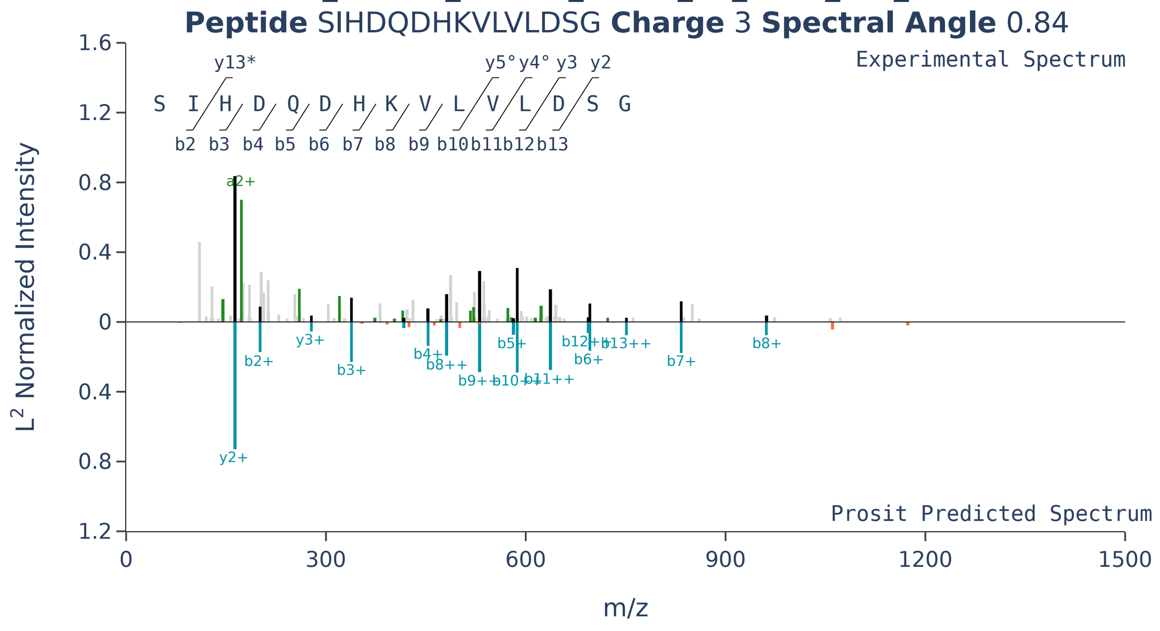


**Source** WSoh_101121_151121_HFGoe_G1_IL37b_1h_R1 **Scan** 14470
**Peptide** AAQKESARRPFIFYRA **Charge** 4 **Spectral Angle** 0.9

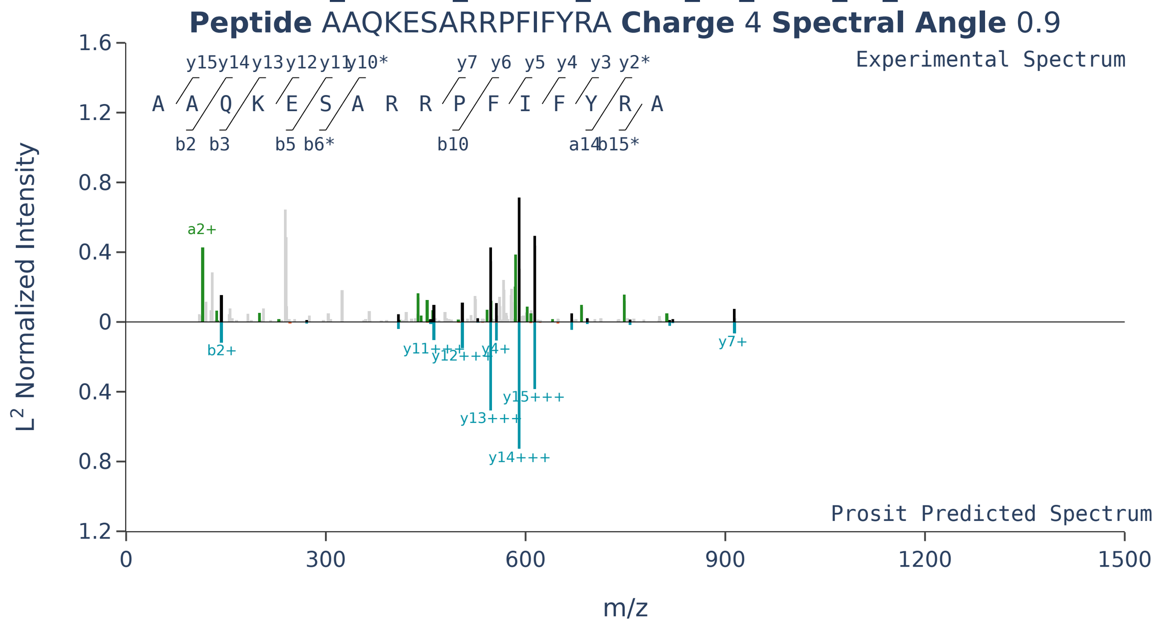

**Source** WSoh_101121_151121_HFGoe_G2_IL37b_24h_R2 **Scan** 11880
**Peptide** NCNEPVGVTDKFENRKHI **Charge** 3 **Spectral Angle** 0.88

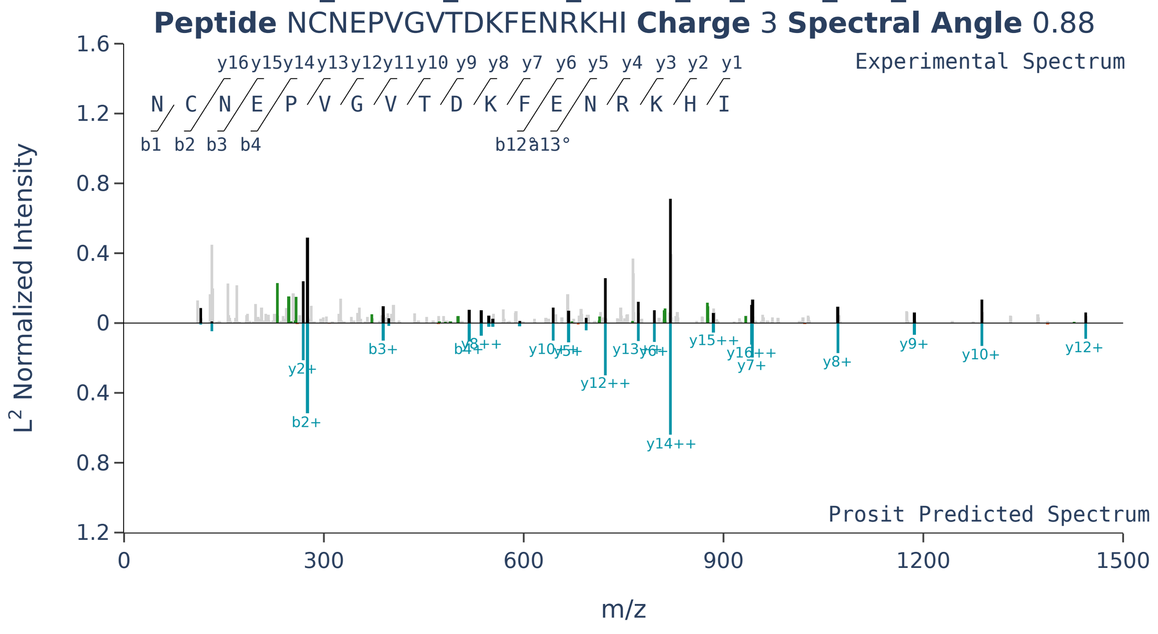

**Source** WSoh_101121_151121_HFGoe_G1_IL37b_4h_R1 **Scan** 13933
**Peptide** KESARRPFIFYR **Charge** 4 **Spectral Angle** 0.95

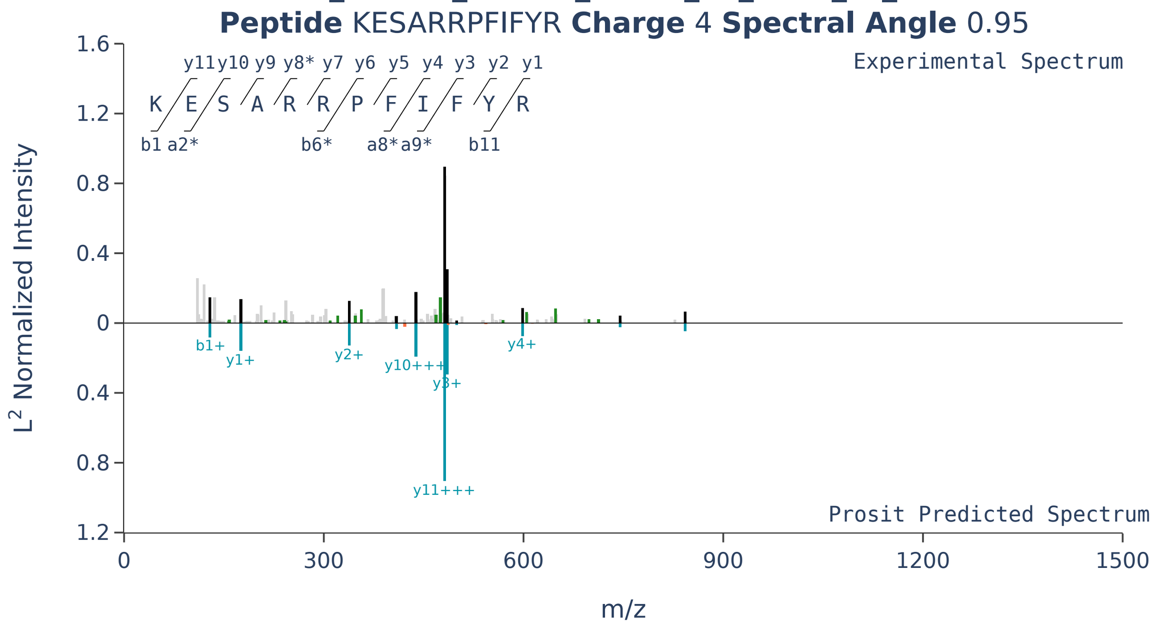

**Source** WSoh_101121_151121_HFGoe_G2_IL37b_24h_R2 **Scan** 11368
**Peptide** VGVTDKFENR **Charge** 2 **Spectral Angle** 0.88

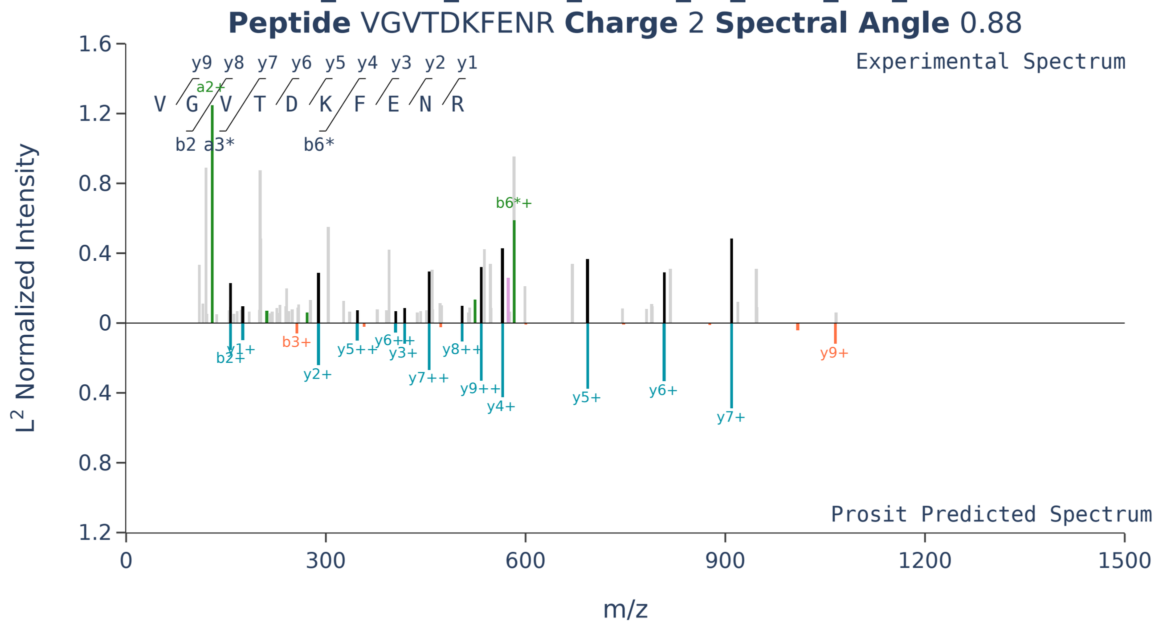

**Source** WSoh_101121_151121_HFGoe_G1_IL37b_2h_R2 **Scan** 23169
**Peptide** ESAAHPGWFICT **Charge** 2 **Spectral Angle** 0.92

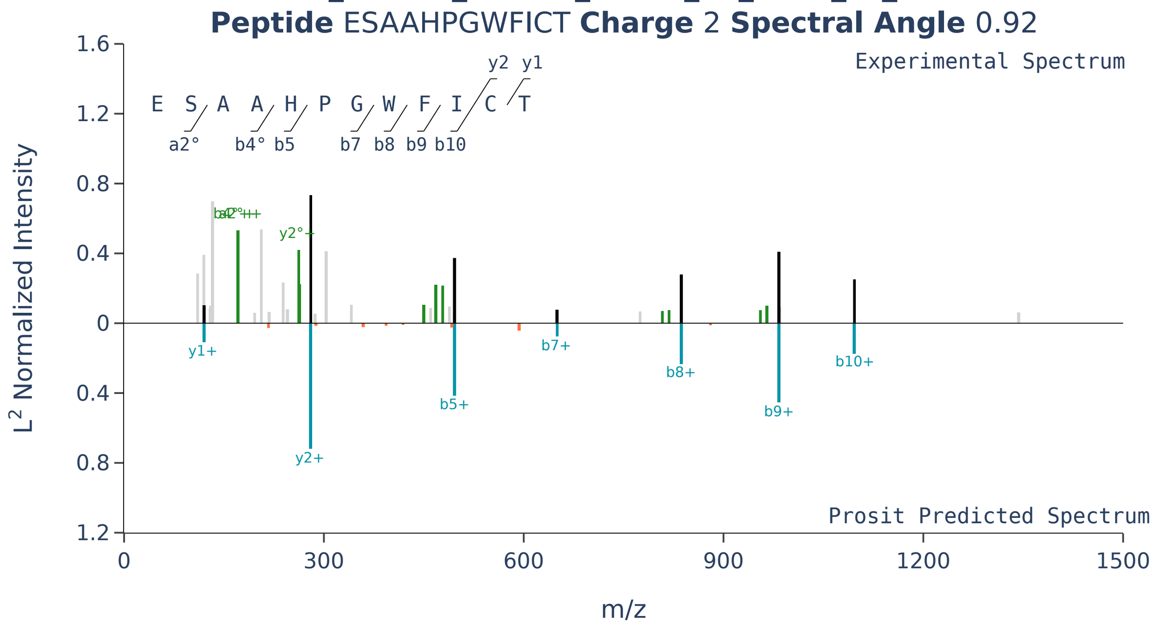

**Source** WSoh_101121_151121_HFGoe_G1_IL37b_2h_R1 **Scan** 7259
**Peptide** SIHDQDHKVL **Charge** 2 **Spectral Angle** 0.83

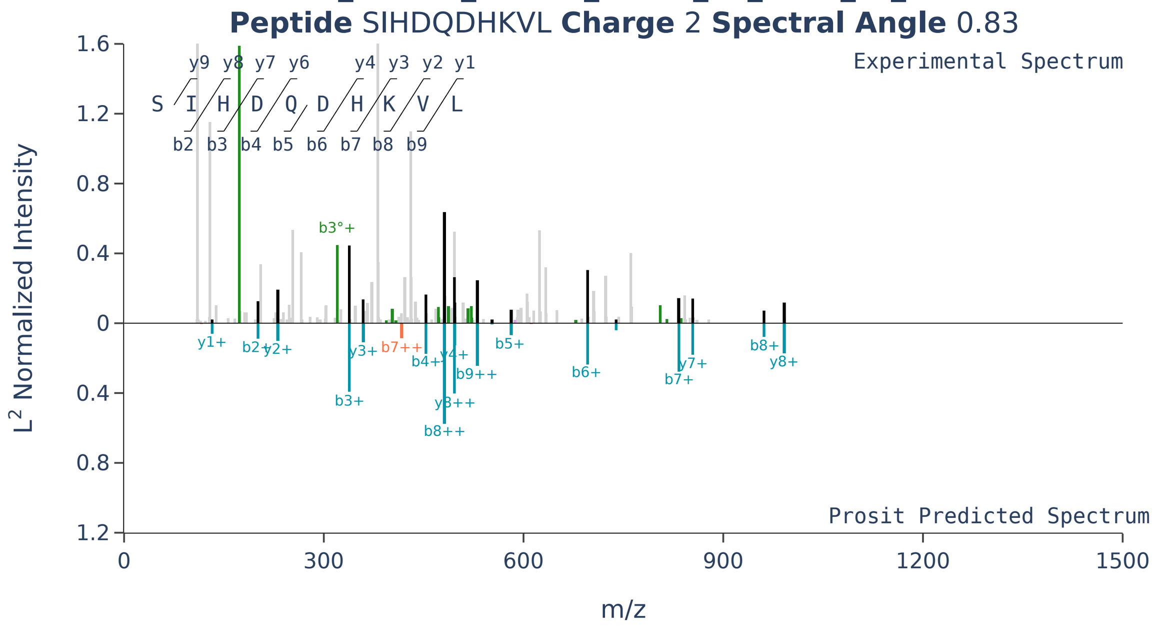

**Source** WSoh_101121_151121_HFGoe_G2_IL37b_2h_R1 **Scan** 22231
**Peptide** PFIFYRAQ **Charge** 2 **Spectral Angle** 0.96

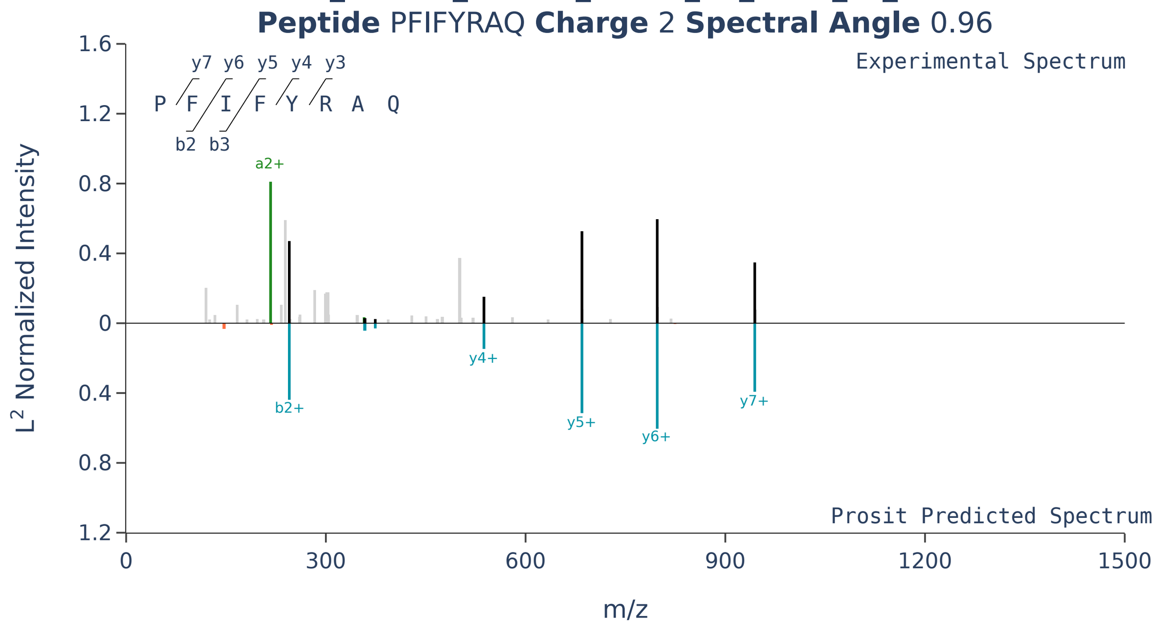

**Source** WSoh_101121_151121_HFGoe_G2_IL37b_24h_R1 **Scan** 13864
**Peptide** PVGVTDKFEN **Charge** 2 **Spectral Angle** 0.85

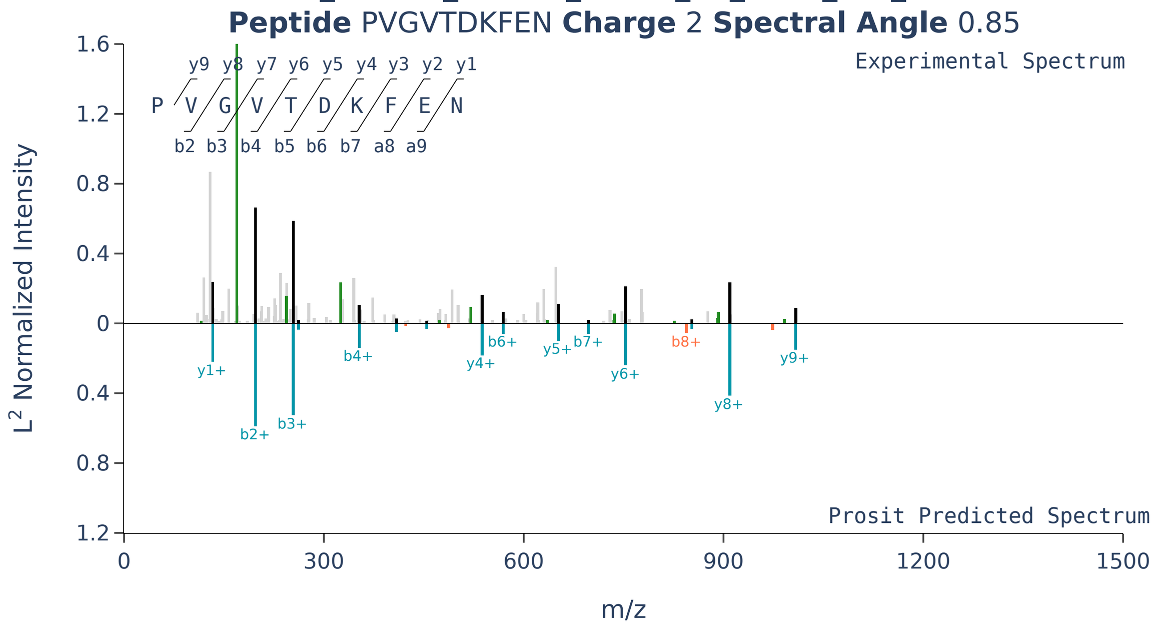

**Source** WSoh_101121_151121_HFGoe_G1_IL37b_24h_R1 **Scan** 11454
**Peptide** NCNEPVGVTDKFENRKHIE **Charge** 3 **Spectral Angle** 0.86

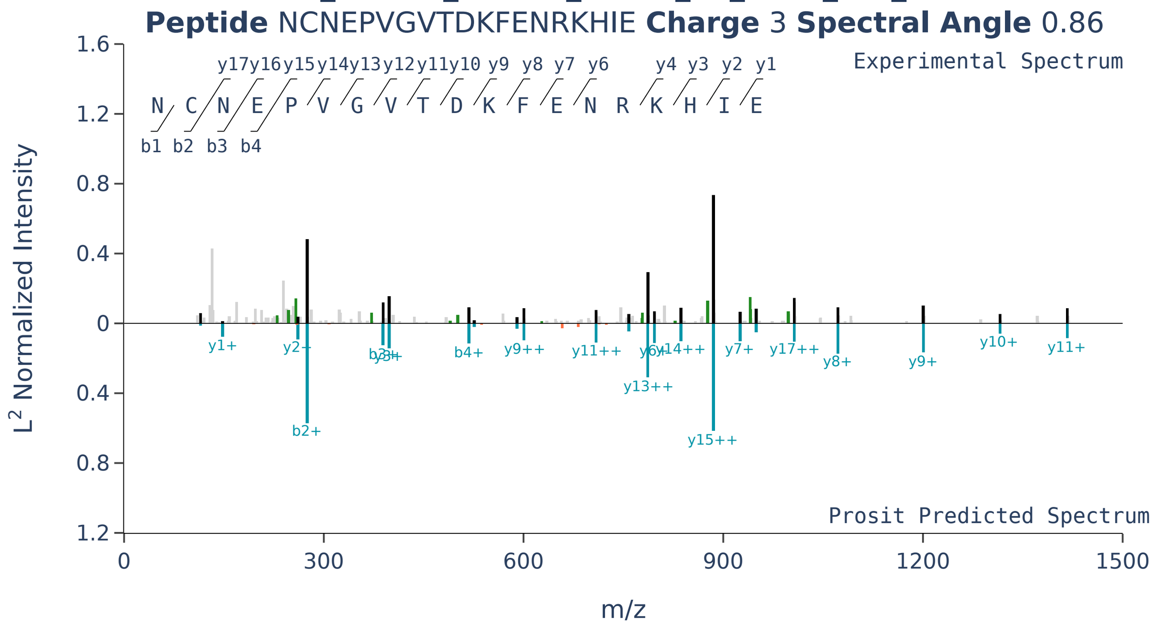

**Source** WSoh_101121_151121_HFGoe_G2_IL37b_24h_R2 **Scan** 6608
**Peptide** DKDKGQSHPSLQLKK **Charge** 3 **Spectral Angle** 0.87

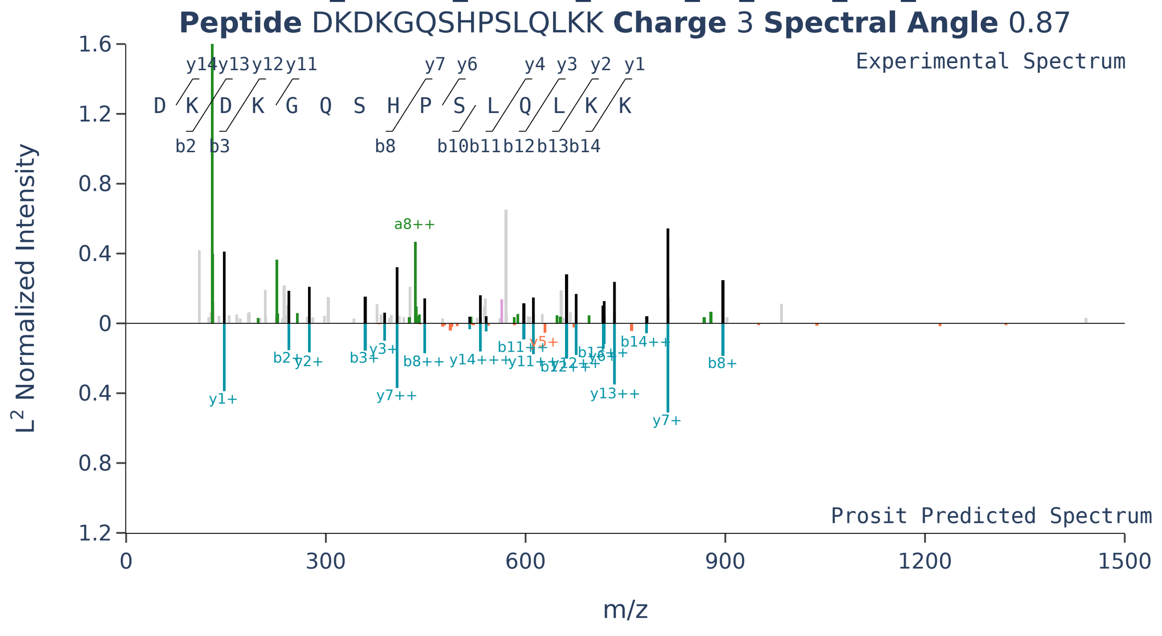

**Source** WSoh_101121_151121_HFGoe_G1_IL37b_2h_R1 **Scan** 20078
**Peptide** SSASAEKGSPILL **Charge** 2 **Spectral Angle** 0.9

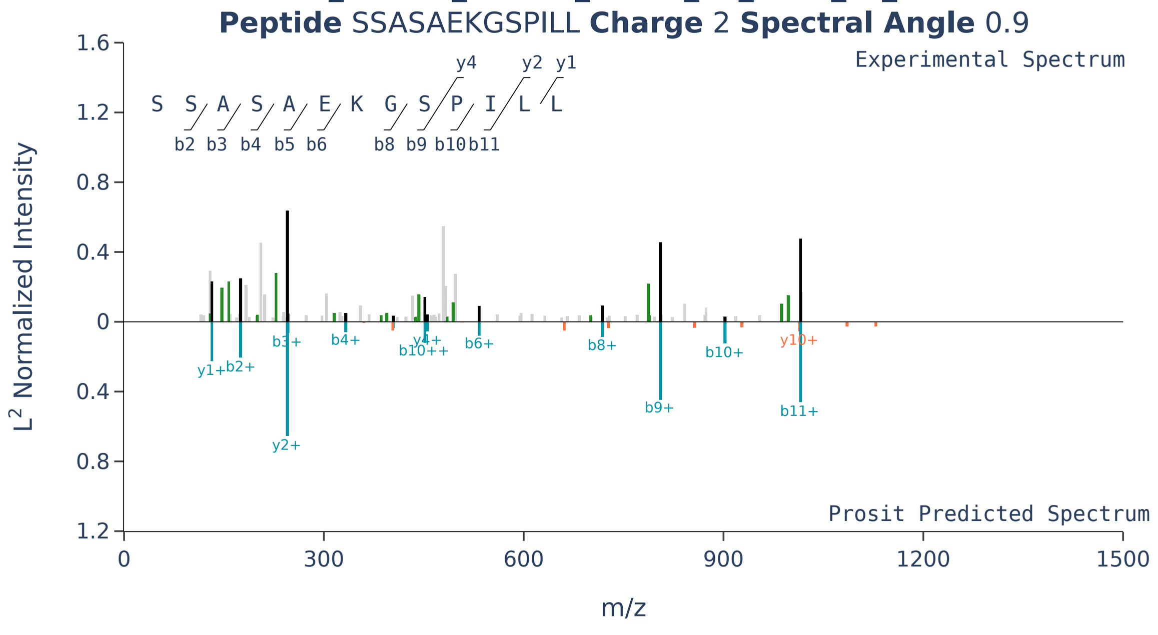

**Source** WSoh_101121_151121_HFGoe_G2_IL37b_24h_R1 **Scan** 13049
**Peptide** EMSPSEV **Charge** 1 **Spectral Angle** 0.85

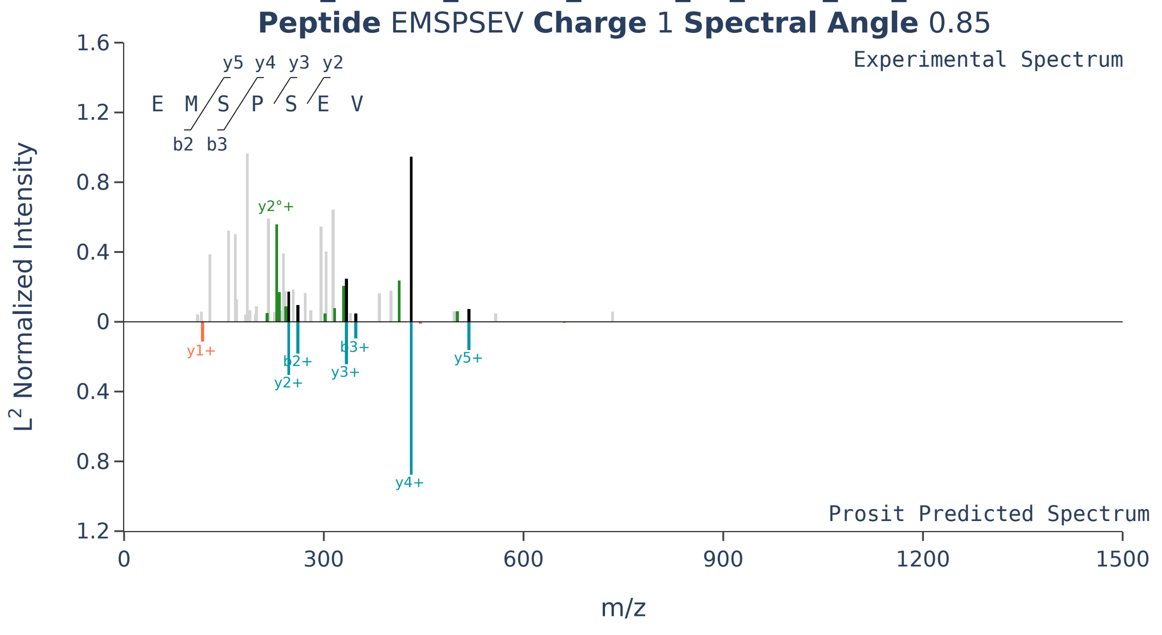

**Source** WSoh_101121_151121_HFGoe_G1_IL37b_4h_R2 **Scan** 19165
**Peptide** SGNLIAVPDKNYIR **Charge** 3 **Spectral Angle** 0.88

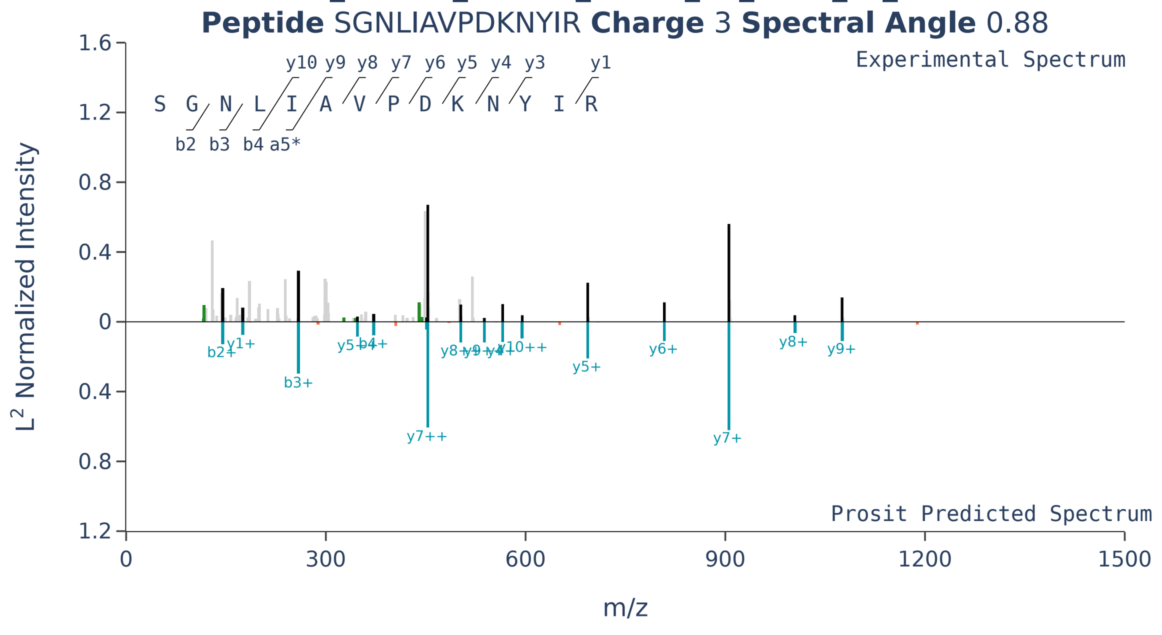

**Source** WSoh_101121_151121_HFGoe_G2_IL37b_4h_R1 **Scan** 8688
**Peptide** GVSKGEFC **Charge** 2 **Spectral Angle** 0.9

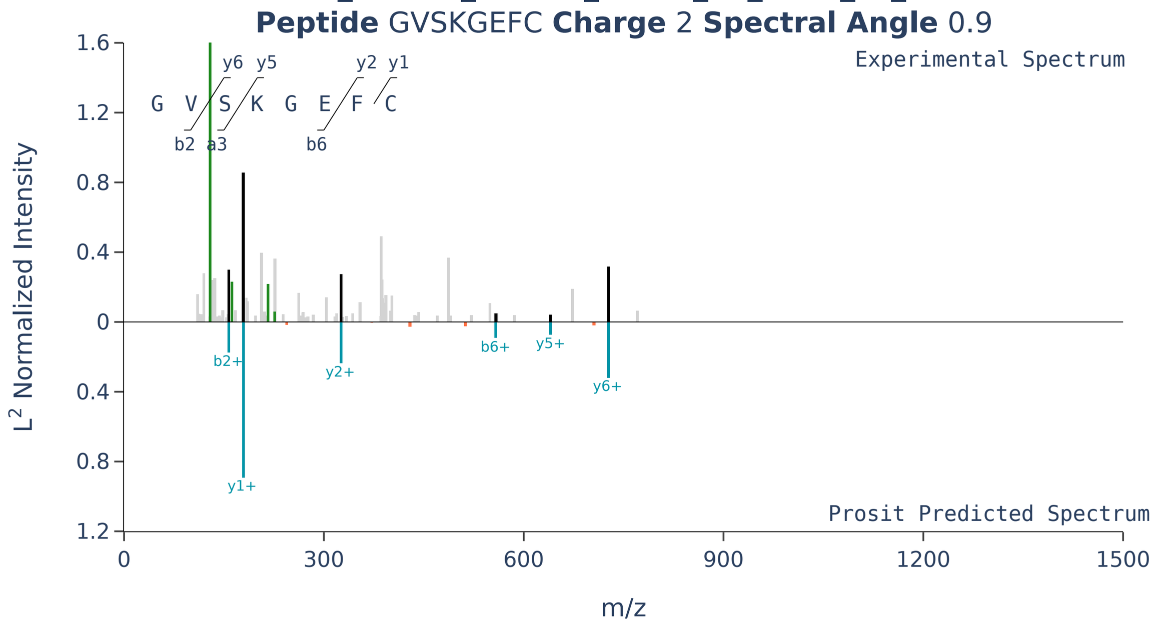

**Source** WSoh_101121_151121_HFGoe_G2_IL37b_2h_R2 **Scan** 9826
**Peptide** CNCNEPVGVTDKFENRKH **Charge** 4 **Spectral Angle** 0.83

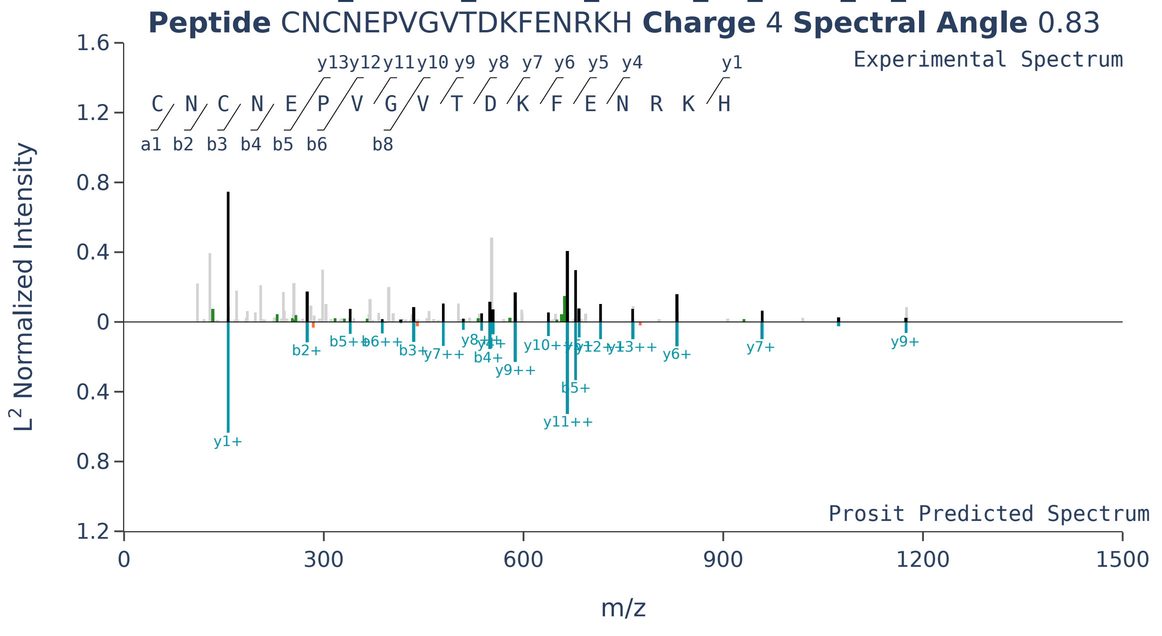

**Source** WSoh_101121_151121_HFGoe_G1_IL37b_4h_R1 **Scan** 14194
**Peptide** SSASAEKGSPIL **Charge** 2 **Spectral Angle** 0.84

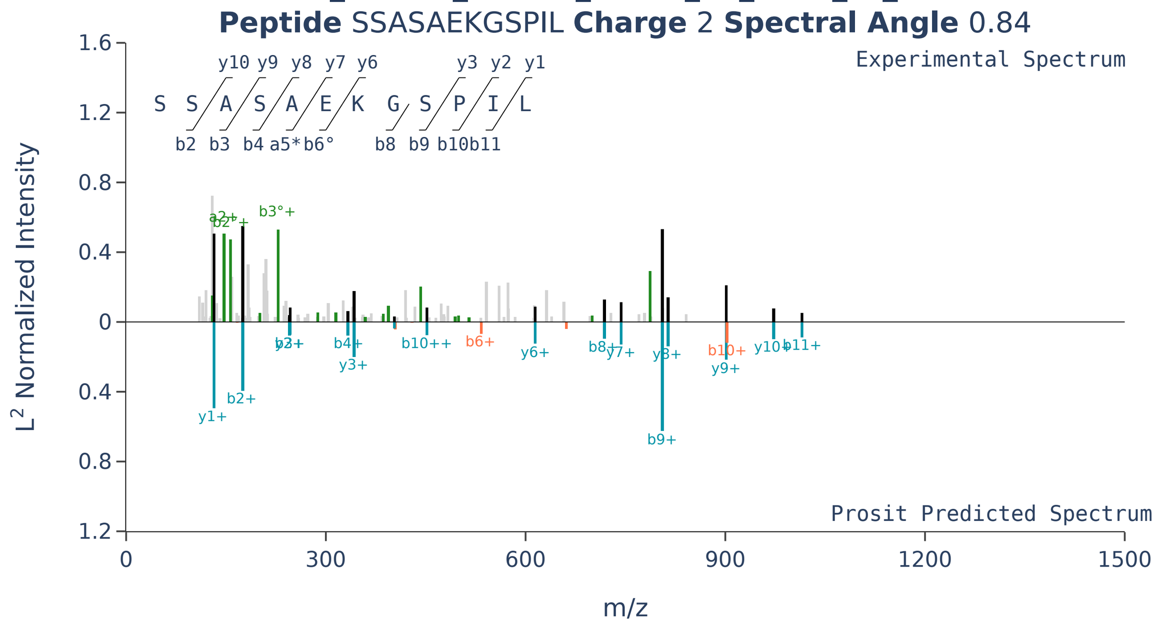

**Source** WSoh_101121_151121_HFGoe_G1_IL37b_1h_R1 **Scan** 15471
**Peptide** SFQPVCKAEM **Charge** 2 **Spectral Angle** 0.78

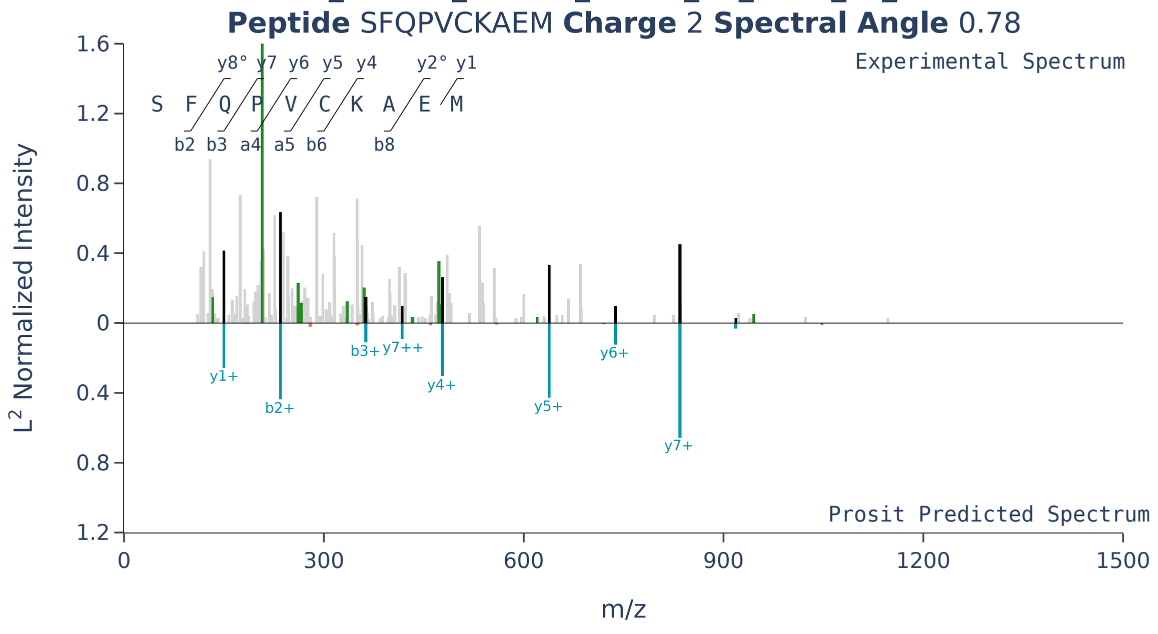


**Source** WSoh_101121_151121_HFGoe_G1_IL37b_1h_R2 **Scan** 17370
**Peptide** SFQPVCKAEMSPSEVSD **Charge** 2 **Spectral Angle** 0.82

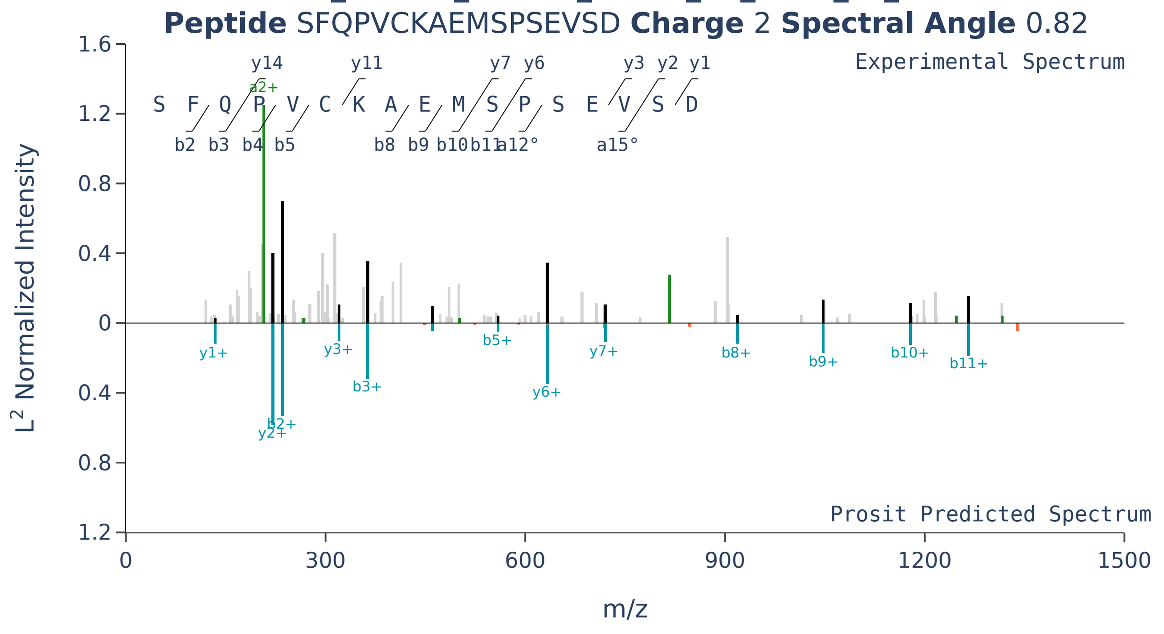


**Source** WSoh_101121_151121_HFGoe_G1_IL37b_24h_R1 **Scan** 7869
**Peptide** SPKVKNLNPKKF **Charge** 2 **Spectral Angle** 0.8

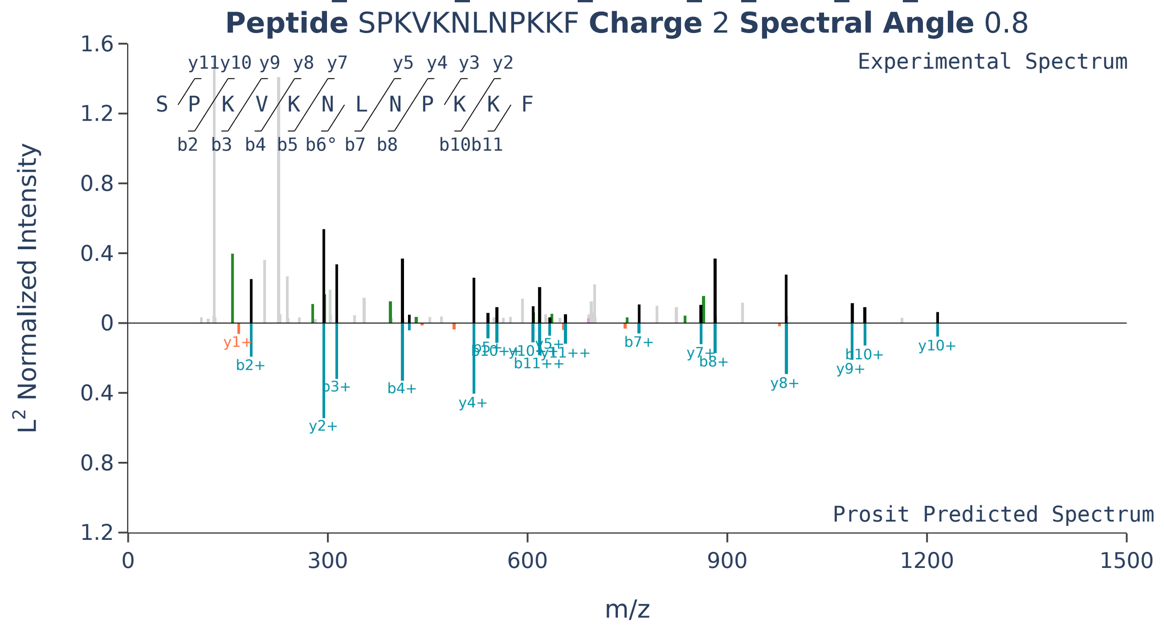


**Source** WSoh_101121_151121_HFGoe_G2_IL37b_24h_R1 **Scan** 12328
**Peptide** CDKDKGQSHPSLQL **Charge** 3 **Spectral Angle** 0.86

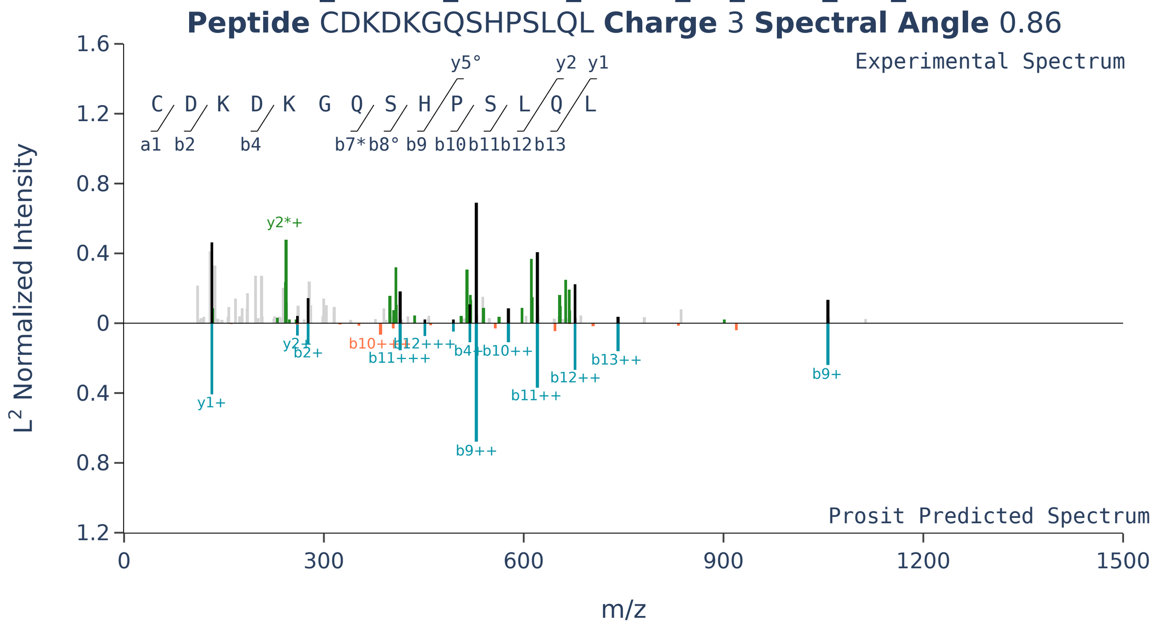


**Source** WSoh_101121_151121_HFGoe_G1_IL37b_4h_R1 **Scan** 12362
**Peptide** VHTSPKVKNLNPKKFSI **Charge** 3 **Spectral Angle** 0.91

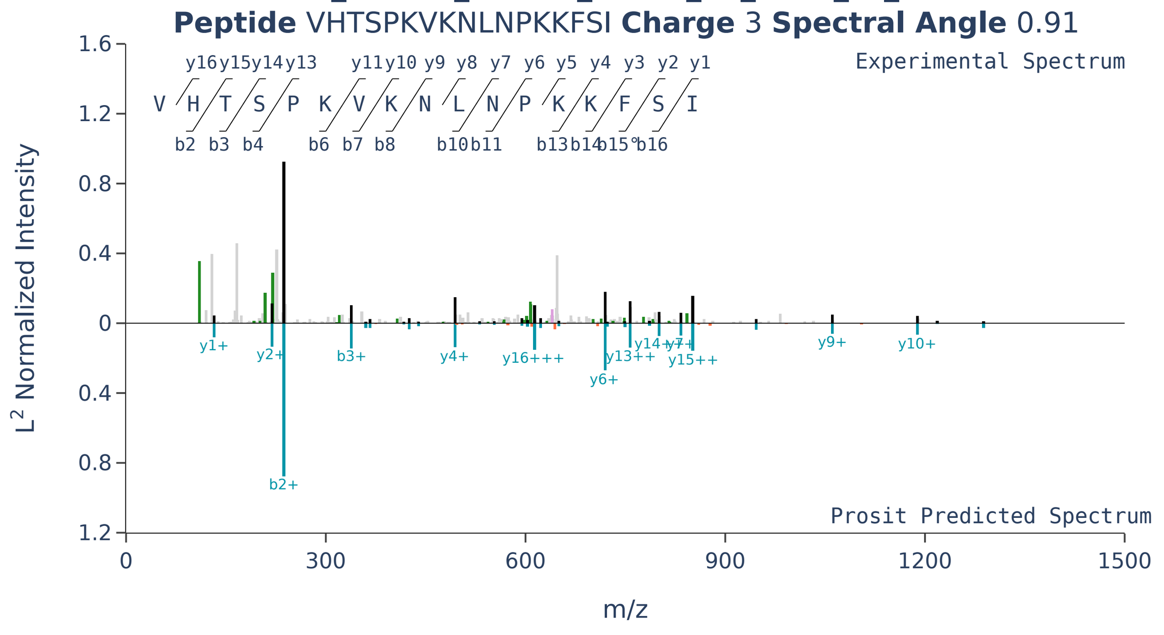


**Source** WSoh_101121_151121_HFGoe_G2_IL37b_24h_R1 **Scan** 5306
**Peptide** KAEMSPSE **Charge** 2 **Spectral Angle** 0.92

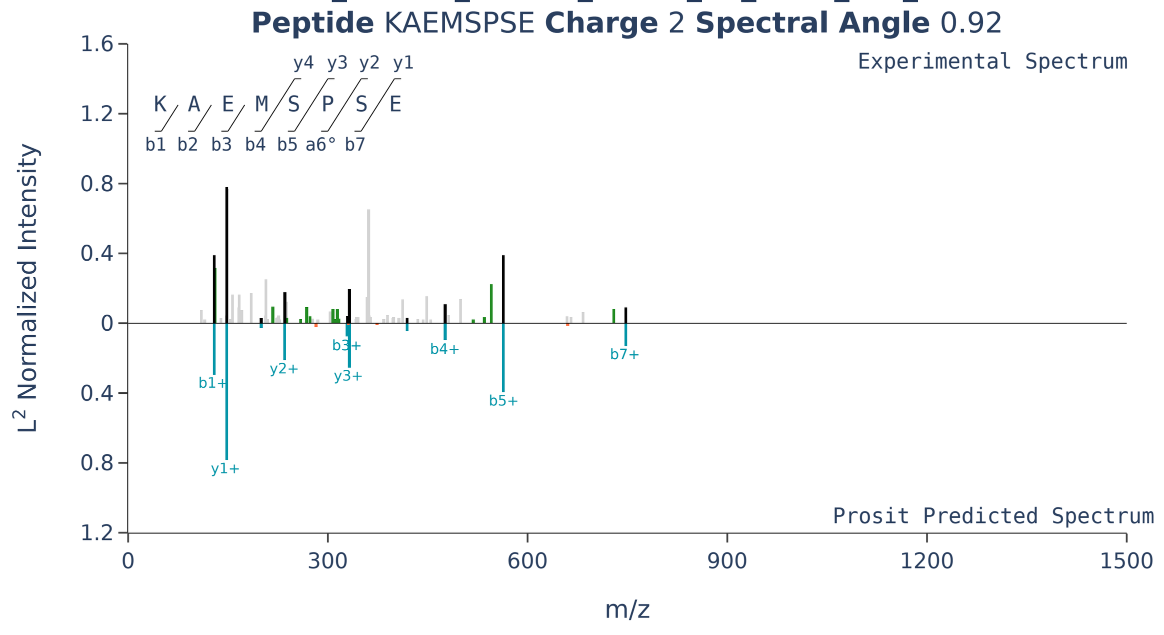


**Source** WSoh_101121_151121_HFGoe_G1_IL37b_4h_R1 **Scan** 6391
**Peptide** KDKGQSHPSLQLKKE **Charge** 3 **Spectral Angle** 0.83

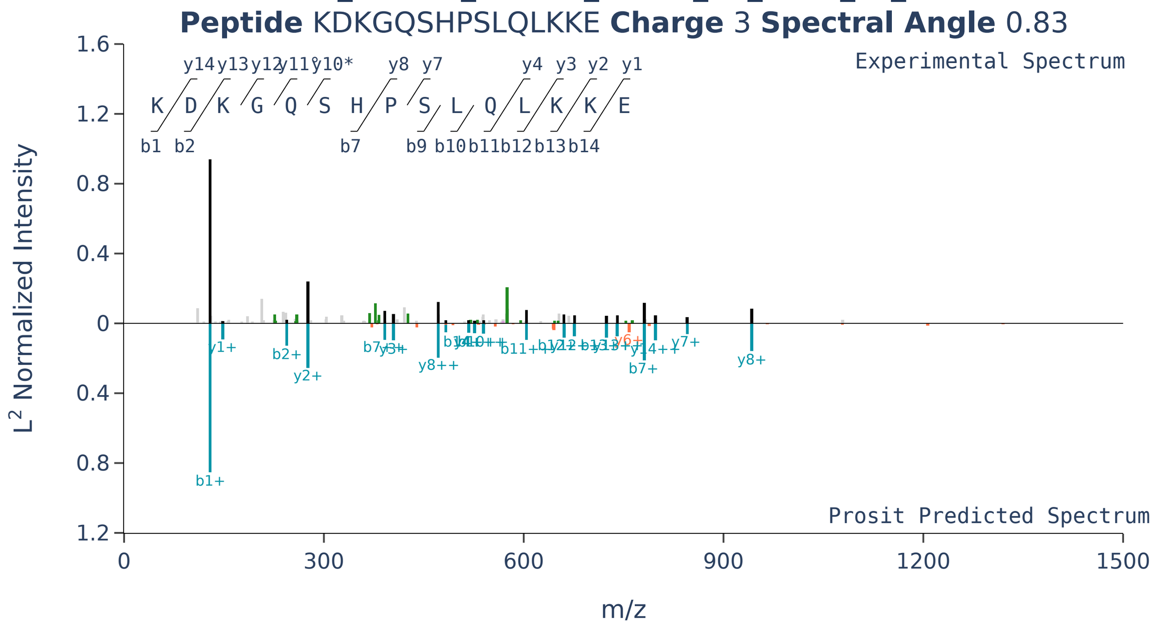


**Source** WSoh_101121_151121_HFGoe_G2_IL37b_2h_R2 **Scan** 11428
**Peptide** NCNEPVGVTDKFENRKHLE **Charge** 5 **Spectral Angle** 0.83

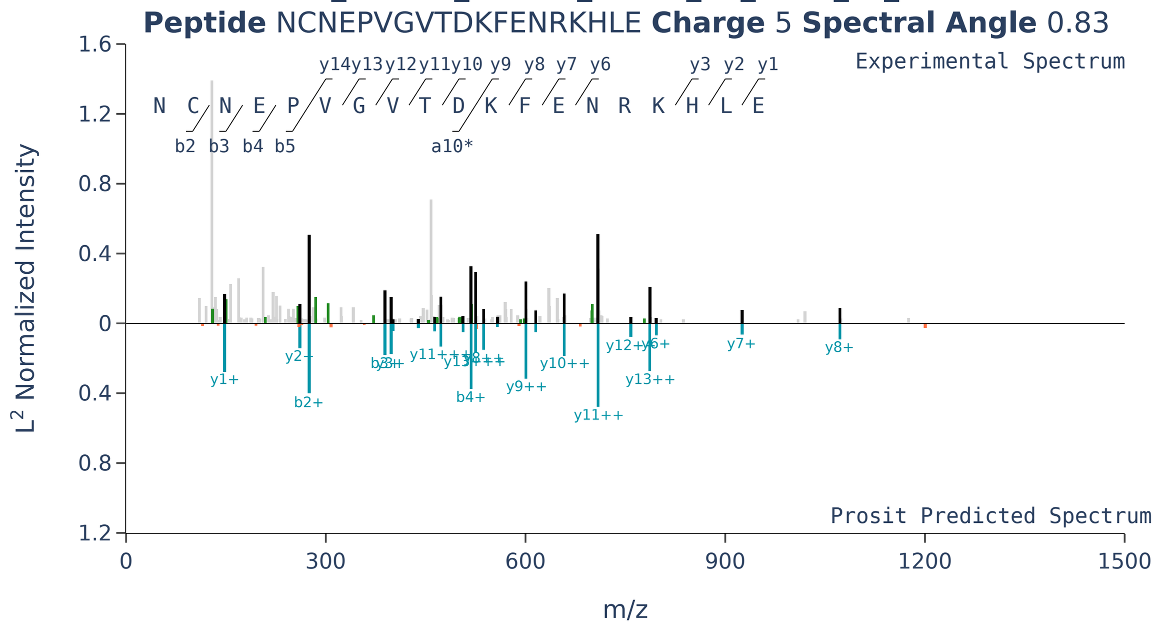


**Source** WSoh_101121_151121_HFGoe_G1_IL37b_1h_R1 **Scan** 18461
**Peptide** QVGSWNMLESAAH **Charge** 2 **Spectral Angle** 0.83

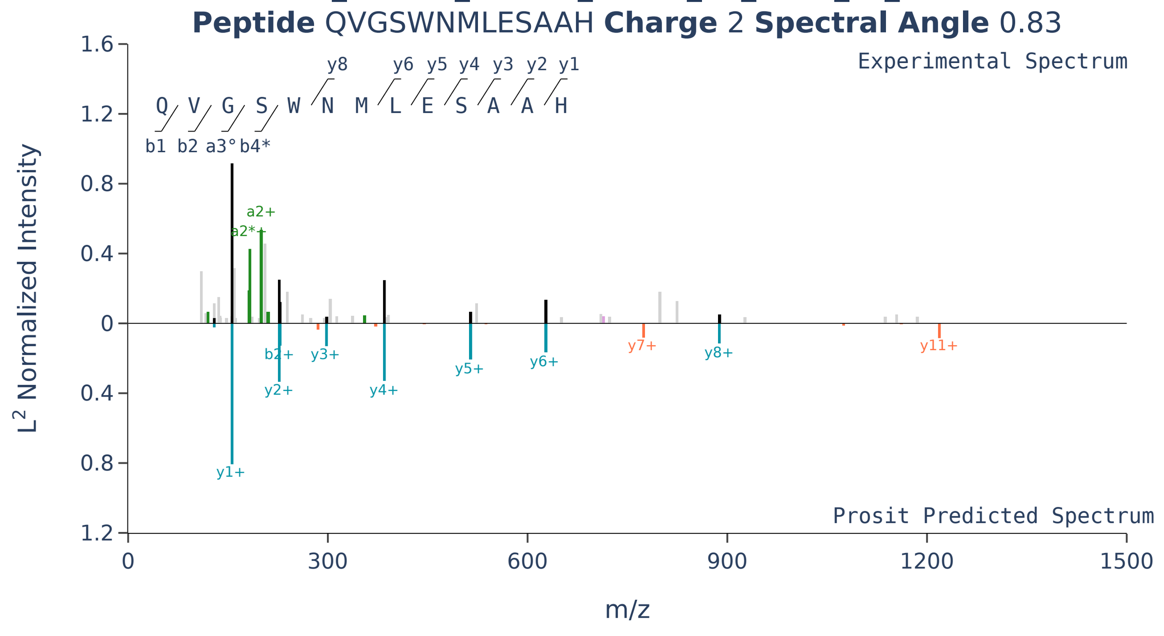


**Source** WSoh_101121_151121_HFGoe_G2_IL37b_1h_R1 **Scan** 7550
**Peptide** VHTSPKVKNLNPKKF **Charge** 4 **Spectral Angle** 0.86

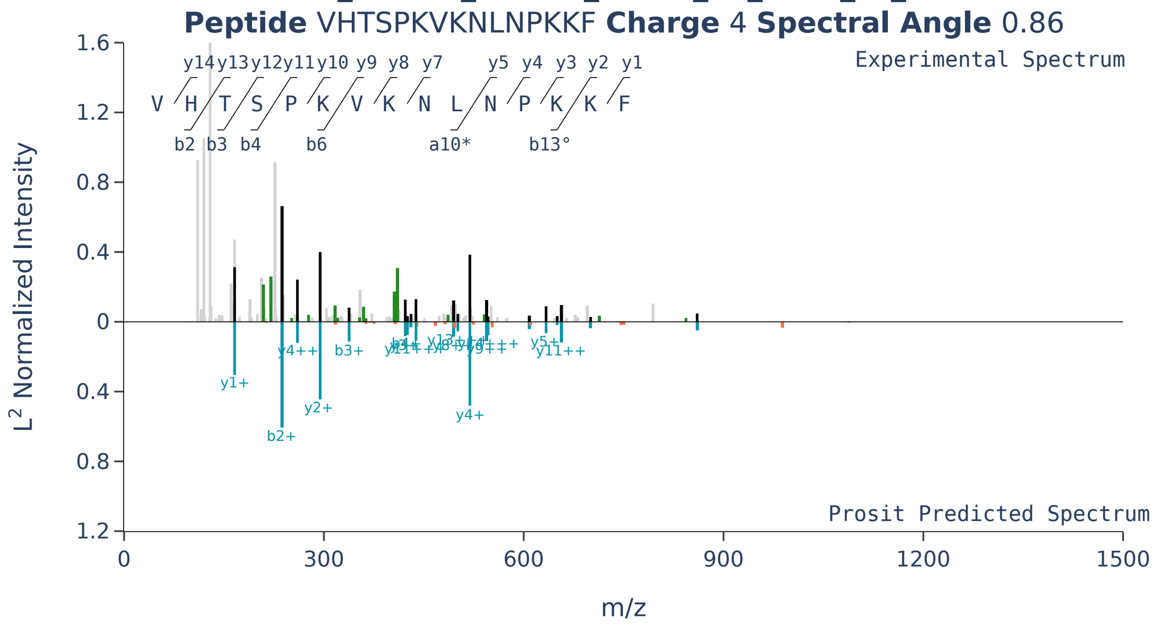


**Source** WSoh_101121_151121_HFGoe_G2_IL37b_24h_R1 **Scan** 13837
**Peptide** AEMSPSEV **Charge** 1 **Spectral Angle** 0.78

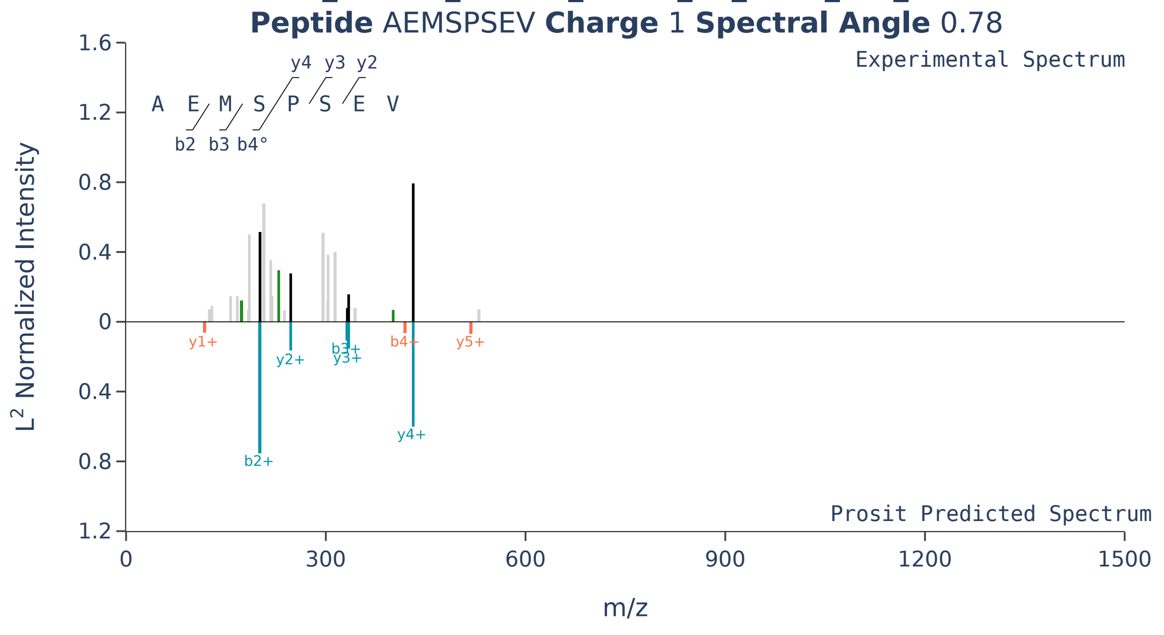


**Source** WSoh_101121_151121_HFGoe_G2_IL37b_24h_R1 **Scan** 8729
**Peptide** KEKLMKL **Charge** 2 **Spectral Angle** 0.94

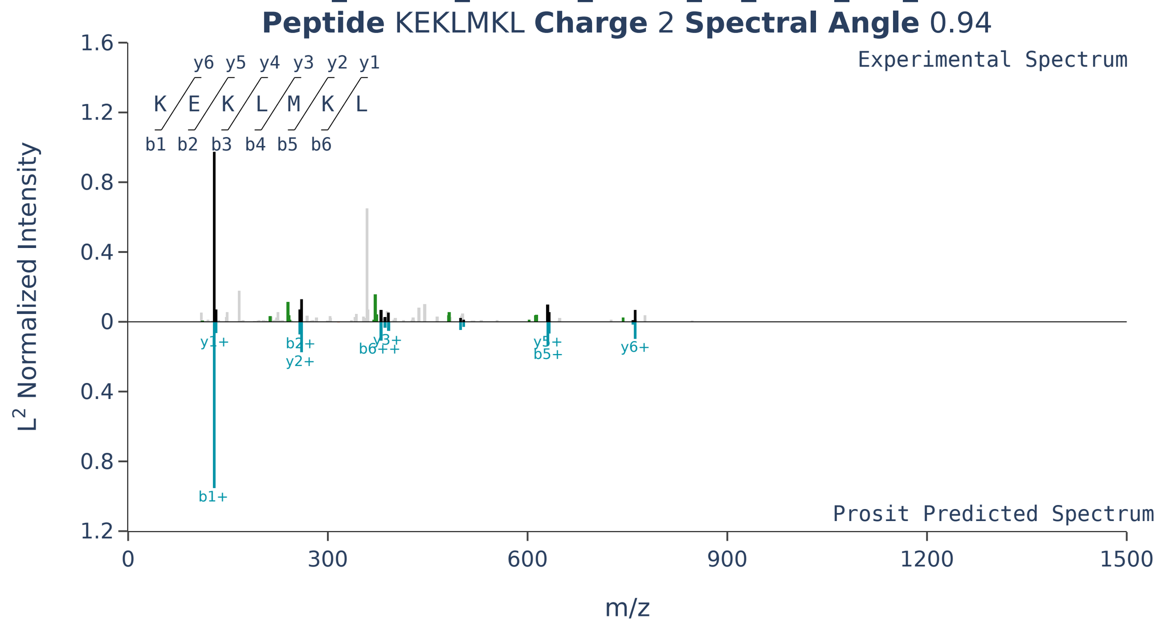


**Source** WSoh_101121_151121_HFGoe_G2_IL37b_24h_R2 **Scan** 10882
**Peptide** LNPKKFSIHDQD **Charge** 3 **Spectral Angle** 0.81

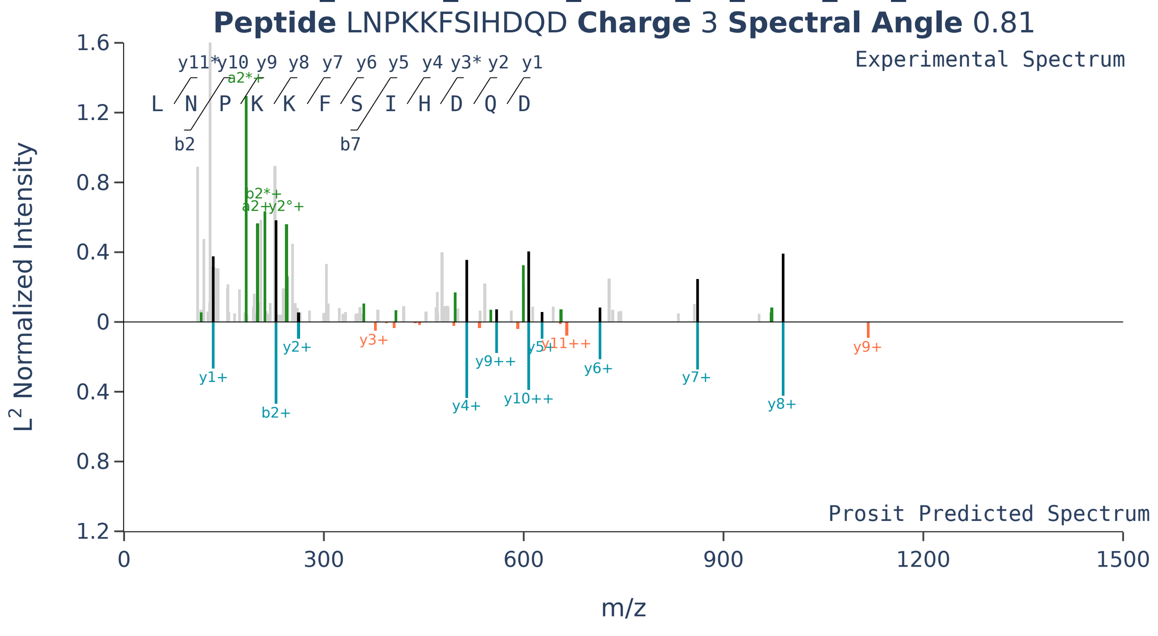


**Source** WSoh_101121_151121_HFGoe_G1_IL37b_4h_R1 **Scan** 13811
**Peptide** QKESARRPFIFYR **Charge** 4 **Spectral Angle** 0.94

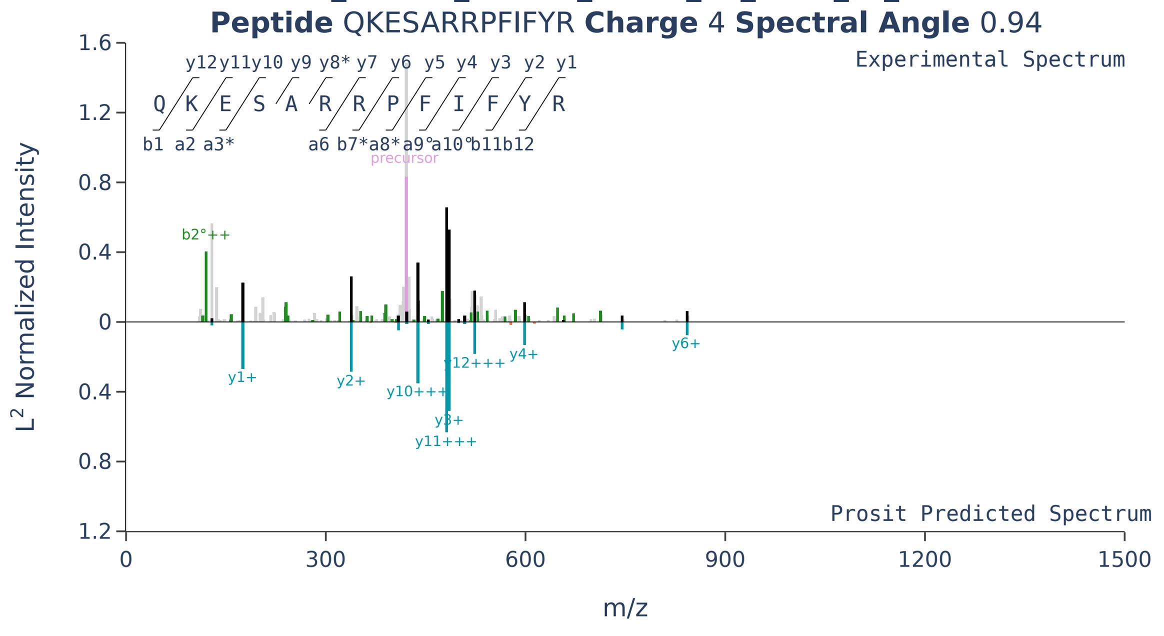


**Source** WSoh_101121_151121_HFGoe_G1_IL37b_2h_R2 **Scan** 15843
**Peptide** KVLVLDSG **Charge** 2 **Spectral Angle** 0.79

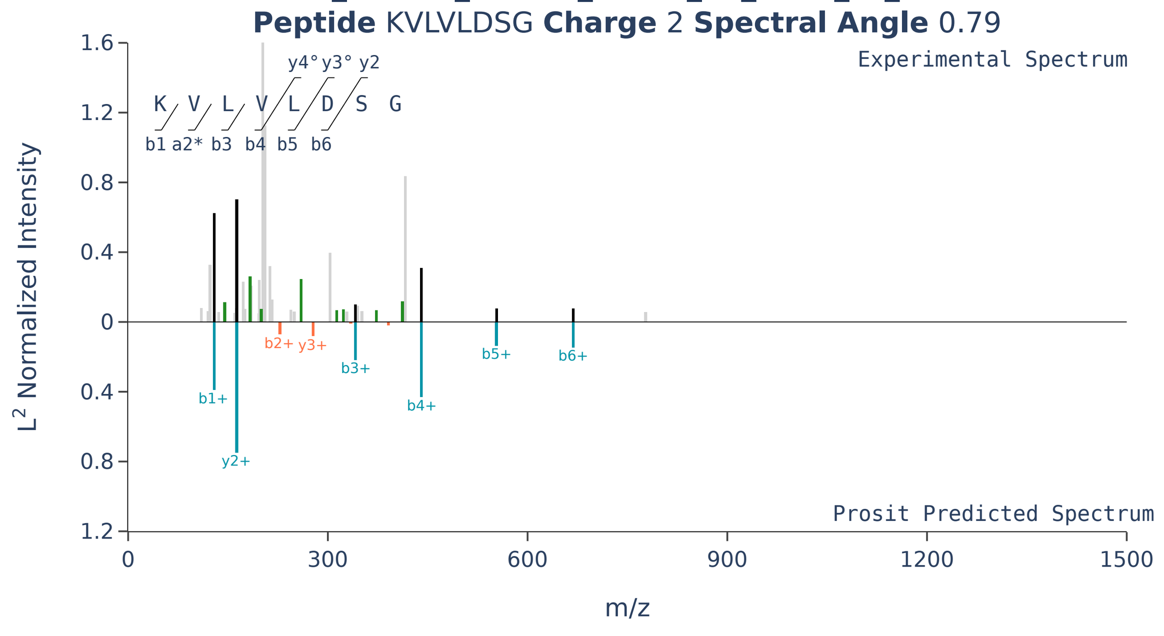

**Source** WSoh_101121_151121_HFGoe_G2_IL37b_24h_R1 **Scan** 23812
**Peptide** SAEKGSPILLGV **Charge** 2 **Spectral Angle** 0.86

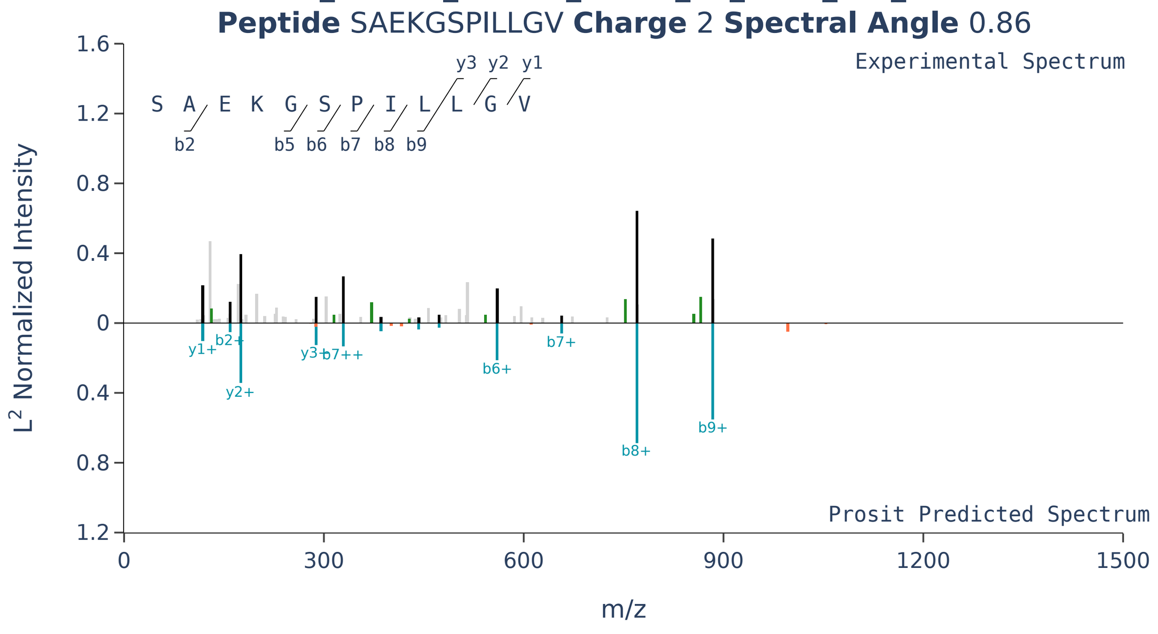


**Source** WSoh_101121_151121_HFGoe_G1_IL37b_24h_R2 **Scan** 11700
**Peptide** LQLKKEKLM **Charge** 3 **Spectral Angle** 0.86

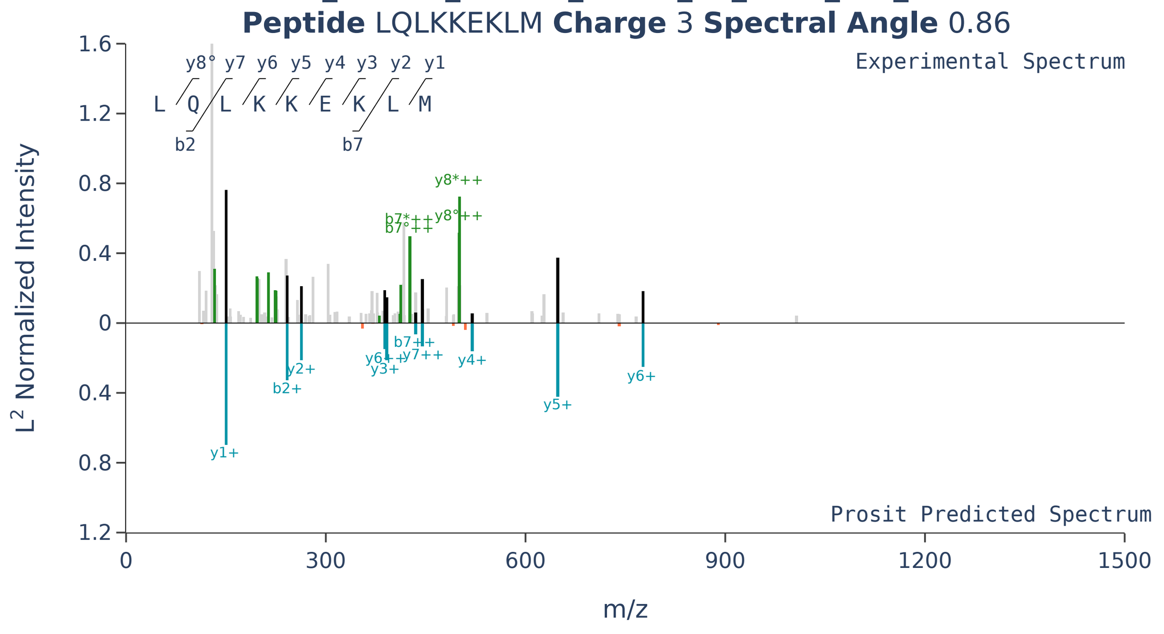


**Source** WSoh_101121_151121_HFGoe_G2_IL37b_2h_R1 **Scan** 14434
**Peptide** QKESARRPFIFYRA **Charge** 4 **Spectral Angle** 0.93

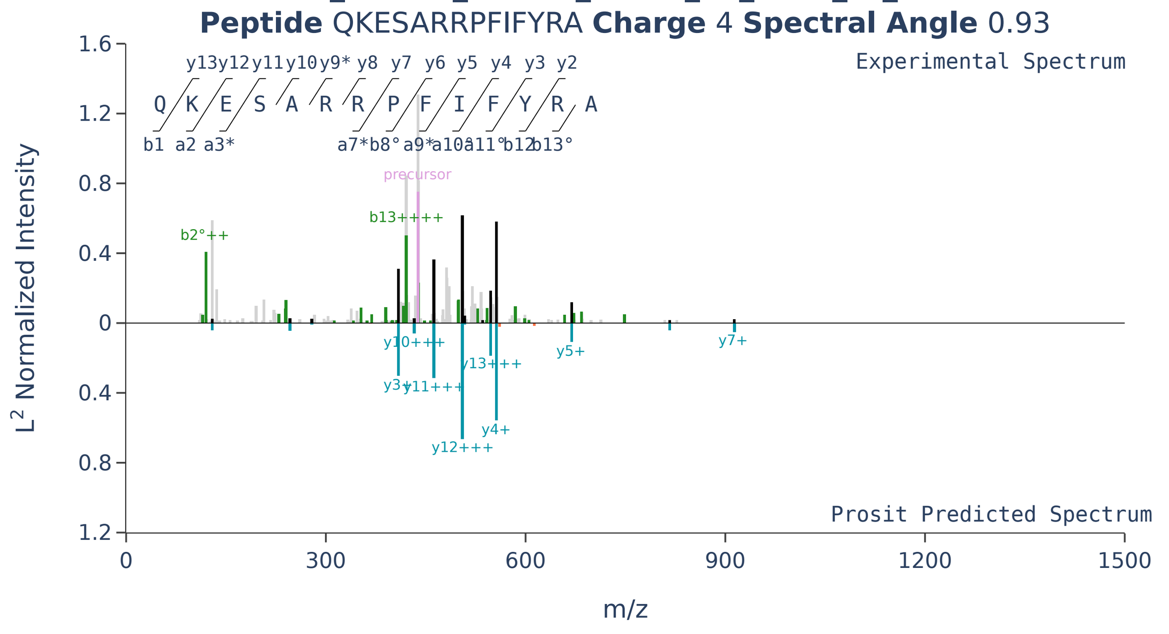


**Source** WSoh_101121_151121_HFGoe_G1_IL37b_1h_R1 **Scan** 14743
**Peptide** LYCDKDKGQSHPSLQL **Charge** 4 **Spectral Angle** 0.89

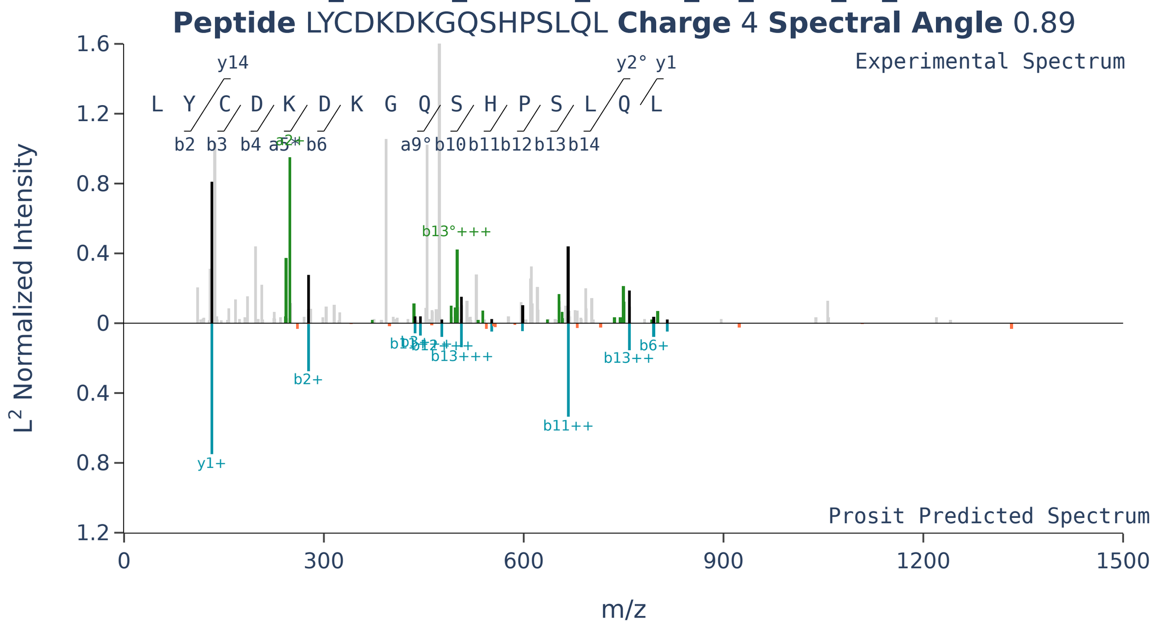


**Source** WSoh_101121_151121_HFGoe_G2_IL37b_24h_R1 **Scan** 12444
**Peptide** PSLQLKKEKLMKL **Charge** 3 **Spectral Angle** 0.8

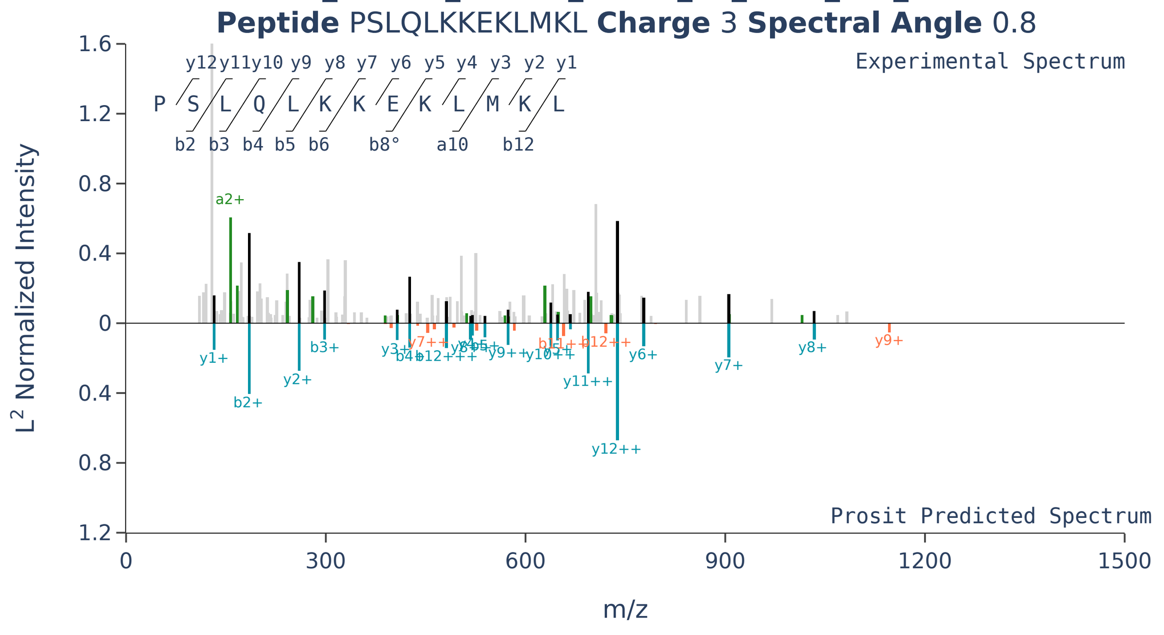


**Source** WSoh_101121_151121_HFGoe_G1_IL37b_4h_R1 **Scan** 10245
**Peptide** NLNPKKFSIHDQDHKV **Charge** 3 **Spectral Angle** 0.83

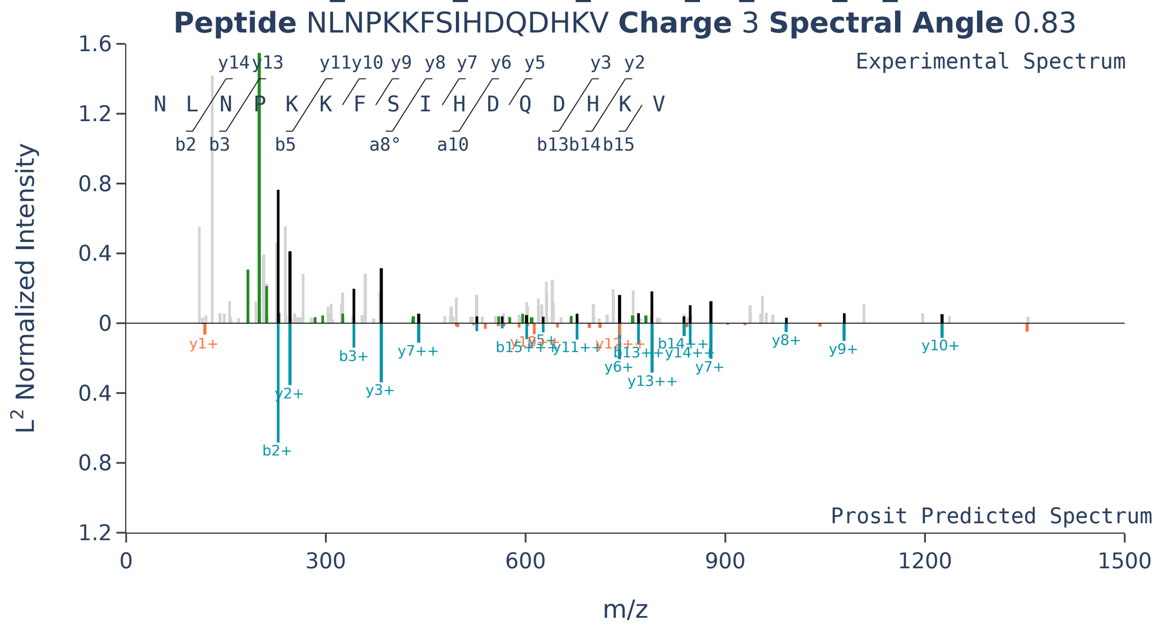


**Source** WSoh_101121_151121_HFGoe_G2_IL37b_24h_R1 **Scan** 17303
**Peptide** CNEPVGVTDKF **Charge** 2 **Spectral Angle** 0.88

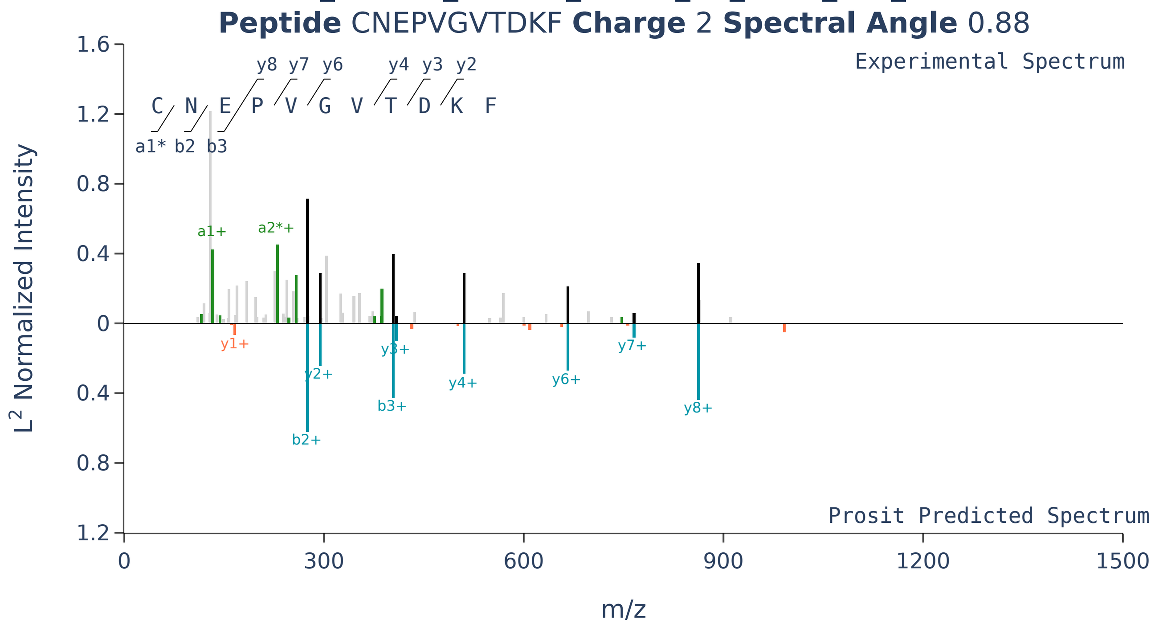


**Source** WSoh_101121_151121_HFGoe_G1_IL37b_24h_R2 **Scan** 16362
**Peptide** QKESARRPFIF **Charge** 3 **Spectral Angle** 0.92

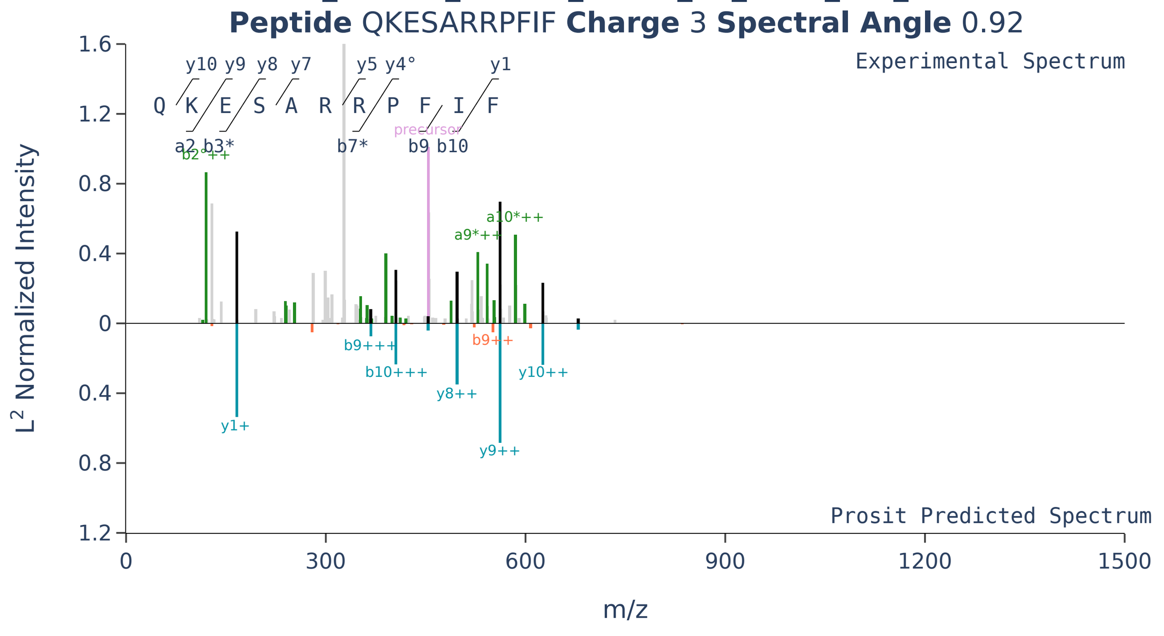


**Source** WSoh_101121_151121_HFGoe_G1_IL37b_24h_R1 **Scan** 14490
**Peptide** SKGEFCLYCDKDKGQSHPSL **Charge** 3 **Spectral Angle** 0.92

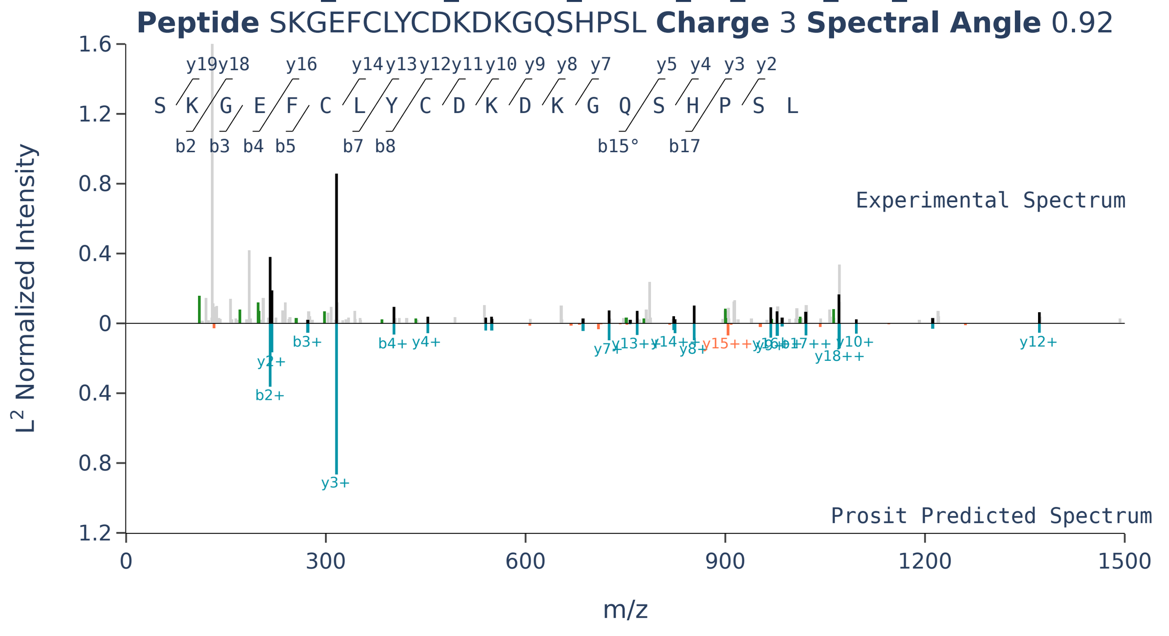


**Source** WSoh_101121_151121_HFGoe_G2_IL37b_24h_R2 **Scan** 9688
**Peptide** PVGVTDKFENRKHIE **Charge** 3 **Spectral Angle** 0.89

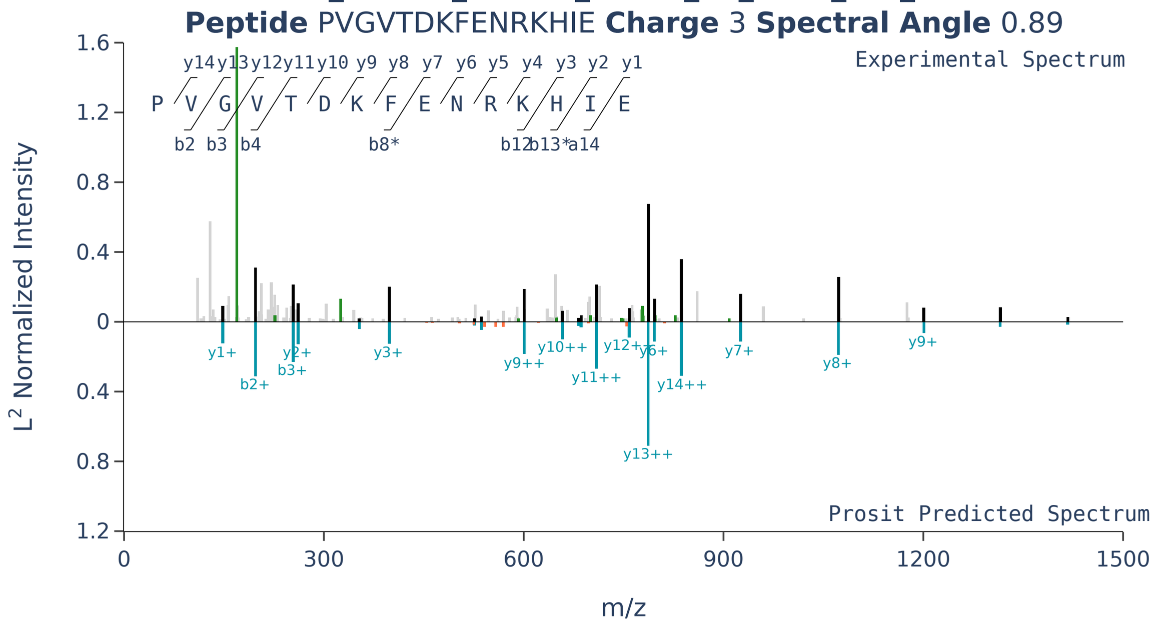


**Source** WSoh_101121_151121_HFGoe_G2_IL37b_4h_R2 **Scan** 15673
**Peptide** SFQPVCKAEMSPSE **Charge** 2 **Spectral Angle** 0.87

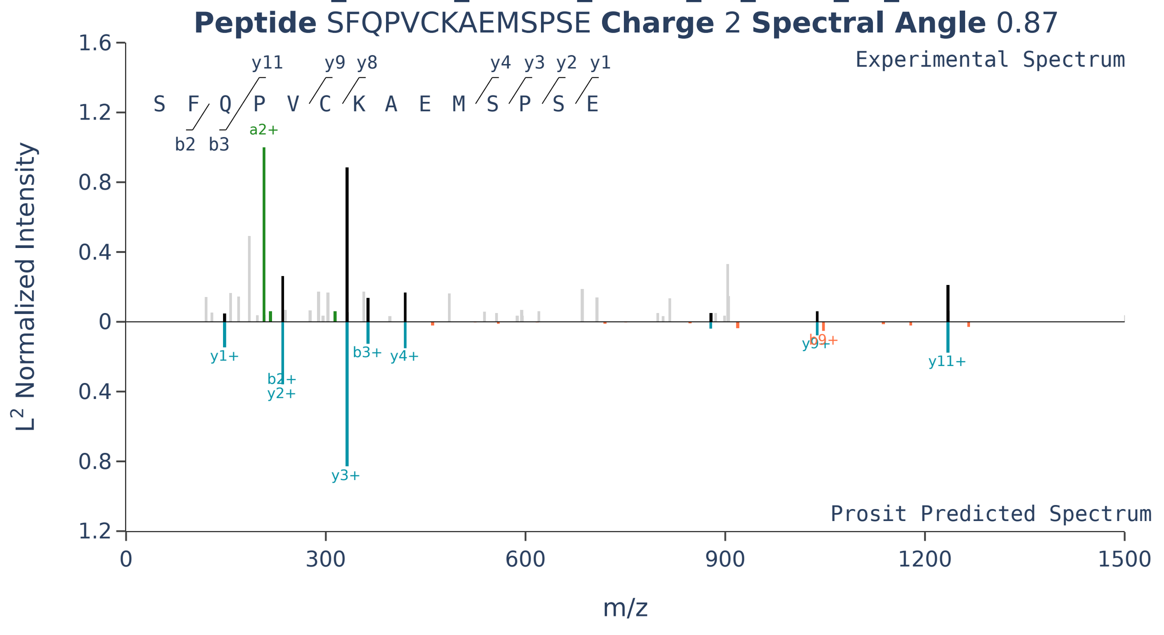


**Source** WSoh_101121_151121_HFGoe_G1_IL37b_4h_R1 **Scan** 17117
**Peptide** ASAEKGSPILLGVSKG **Charge** 3 **Spectral Angle** 0.9

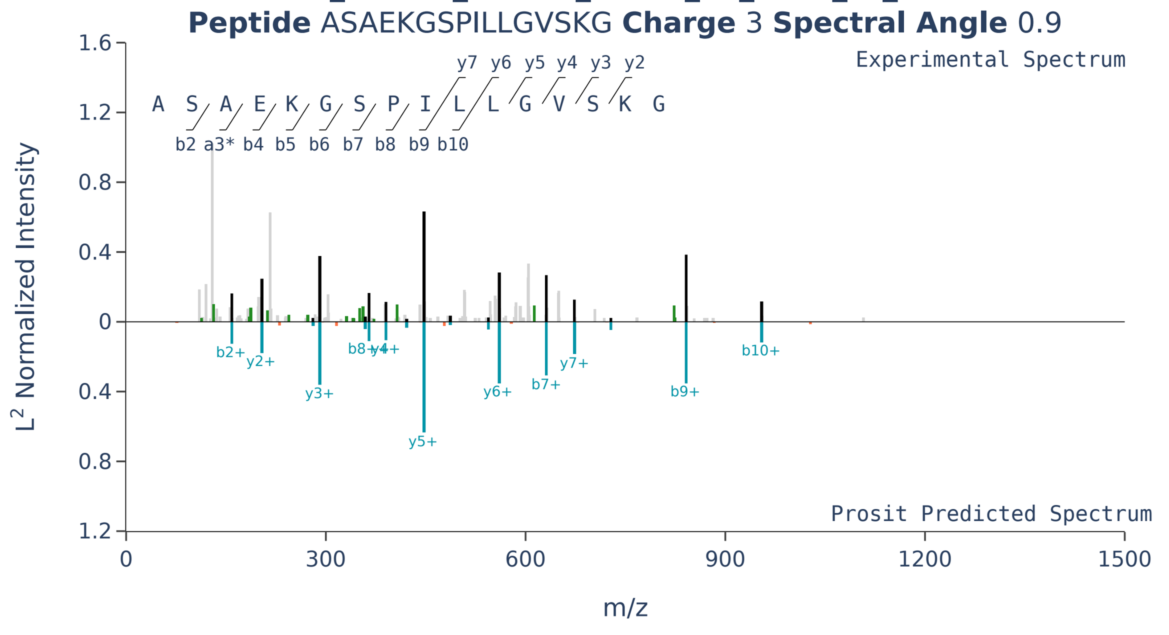


**Source** WSoh_101121_151121_HFGoe_G2_IL37b_4h_R2 **Scan** 8955
**Peptide** PVGVTDKFENRK **Charge** 3 **Spectral Angle** 0.77

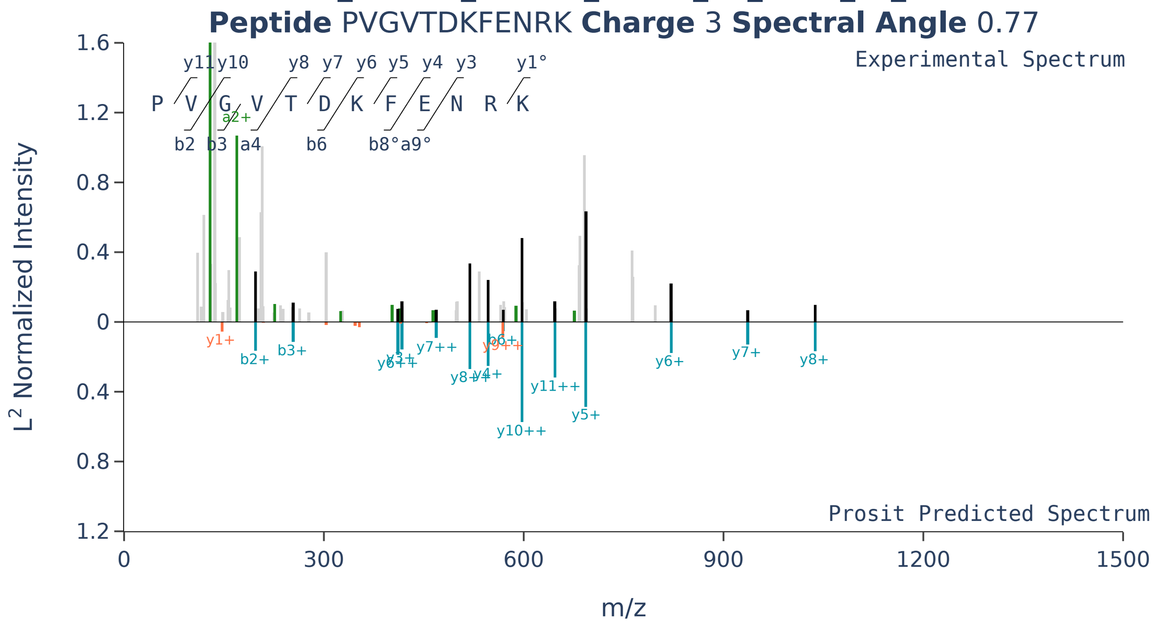


**Source** WSoh_101121_151121_HFGoe_G1_IL37b_4h_R2 **Scan** 9974
**Peptide** SCNCNEPVGVTDKFENRKH **Charge** 4 **Spectral Angle** 0.85

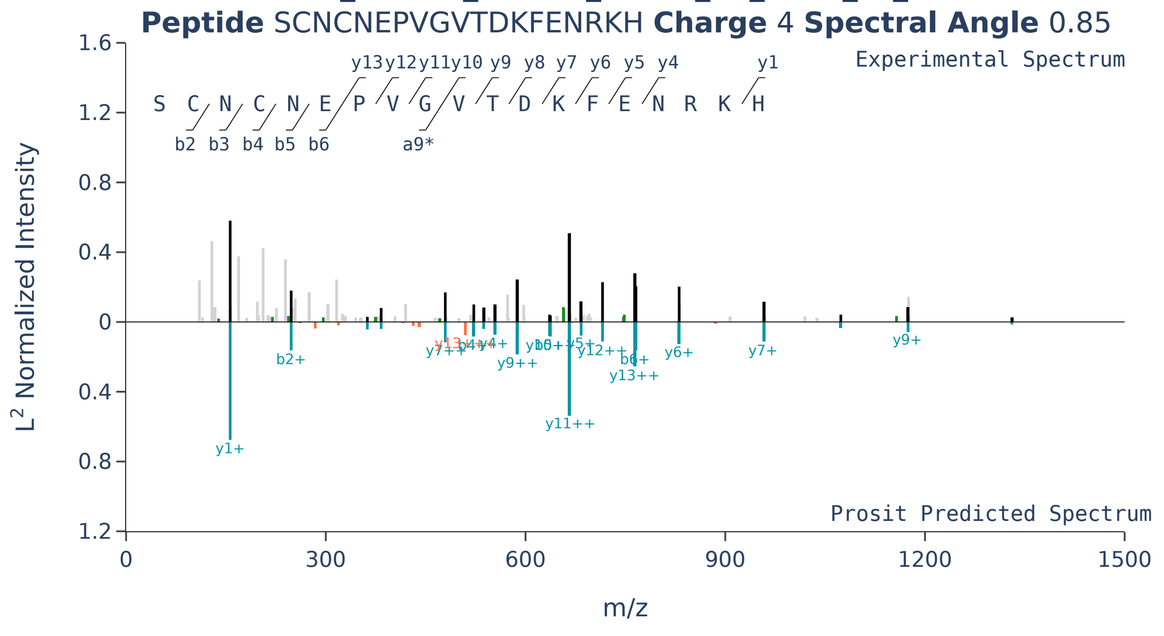


**Source** WSoh_101121_151121_HFGoe_G1_IL37b_24h_R2 **Scan** 9092
**Peptide** SLQLKKE **Charge** 2 **Spectral Angle** 0.89

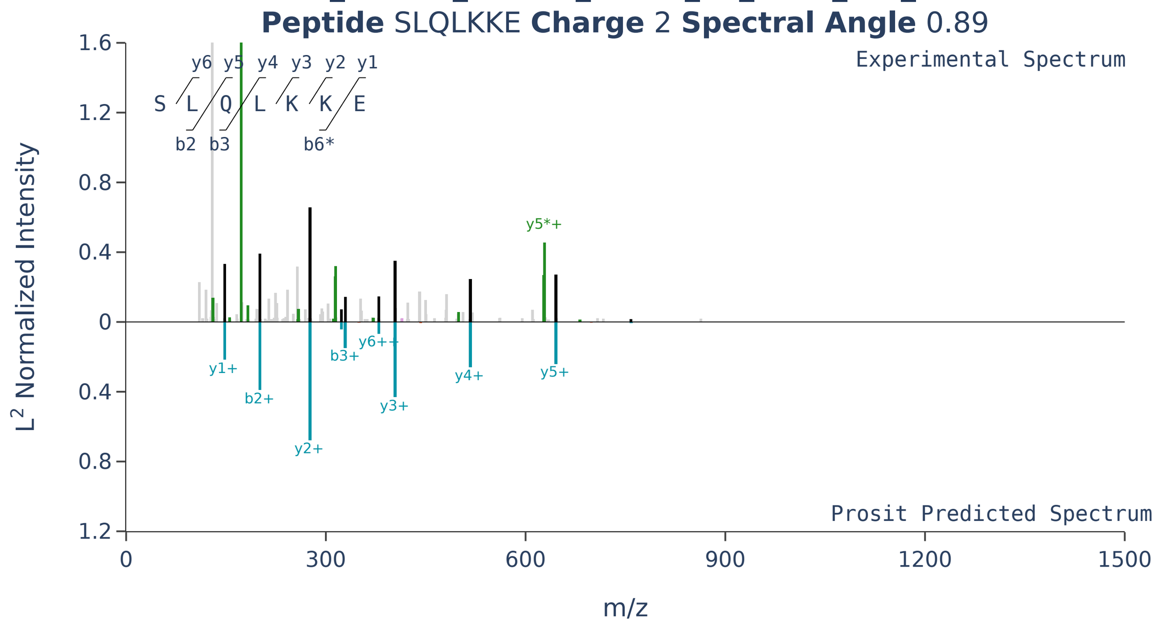

**Source** WSoh_101121_151121_HFGoe_G1_IL37b_24h_R2 **Scan** 6323
**Peptide** KNLNPKKFS **Charge** 2 **Spectral Angle** 0.83

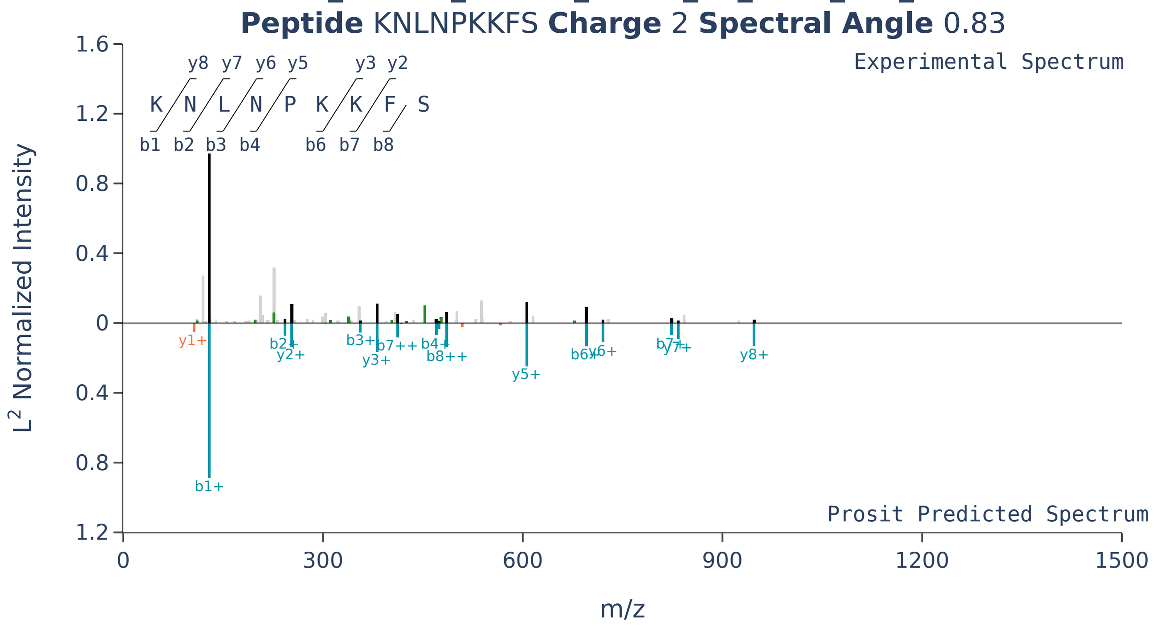


**Source** WSoh_101121_151121_HFGoe_G1_IL37b_24h_R2 **Scan** 18386
**Peptide** DQDHKVLVL **Charge** 3 **Spectral Angle** 0.85

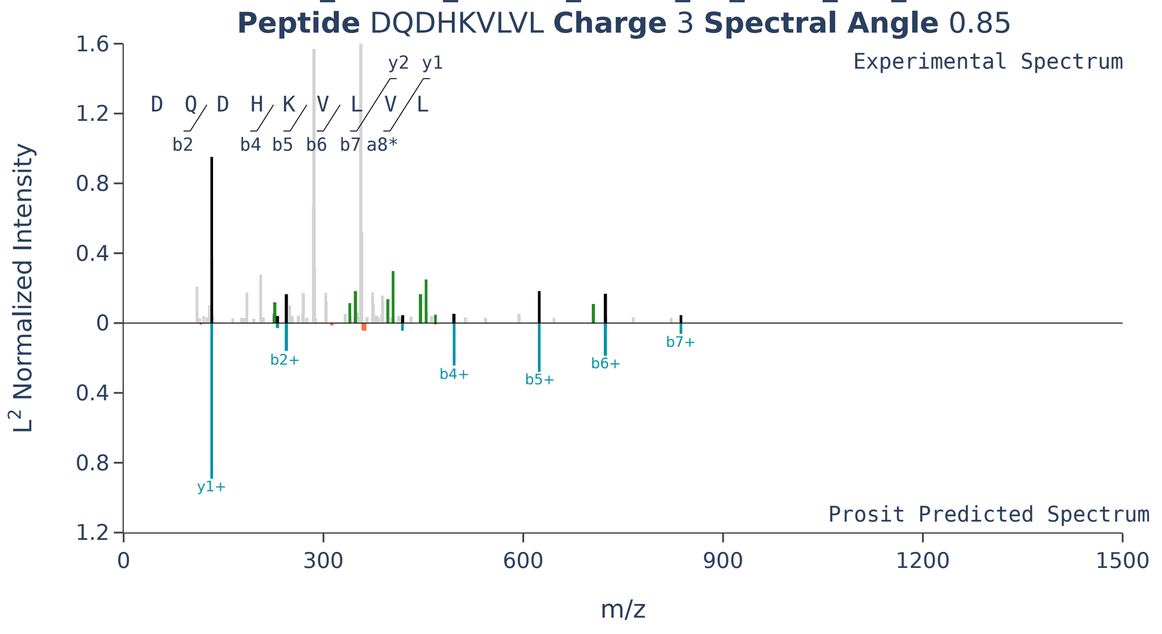


**Source** WSoh_101121_151121_HFGoe_G2_IL37b_24h_R1 **Scan** 20158
**Peptide** SGNLIAVPDKNYIRPE **Charge** 3 **Spectral Angle** 0.88

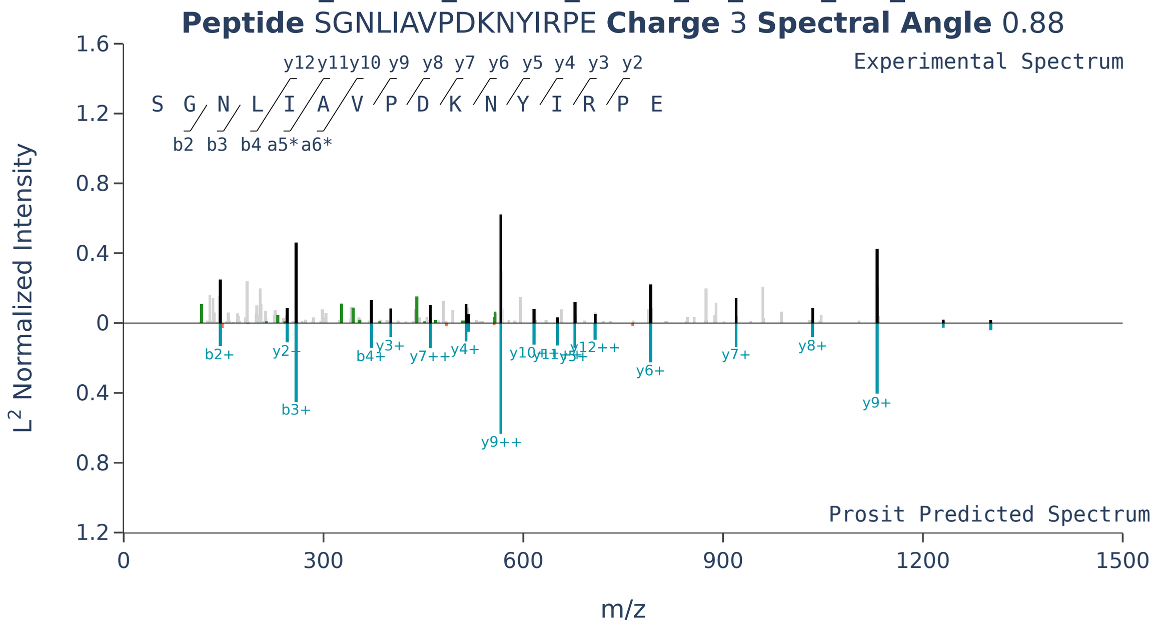


**Source** WSoh_101121_151121_HFGoe_G2_IL37b_24h_R2 **Scan** 14596
**Peptide** LGVSKGEFC **Charge** 2 **Spectral Angle** 0.79

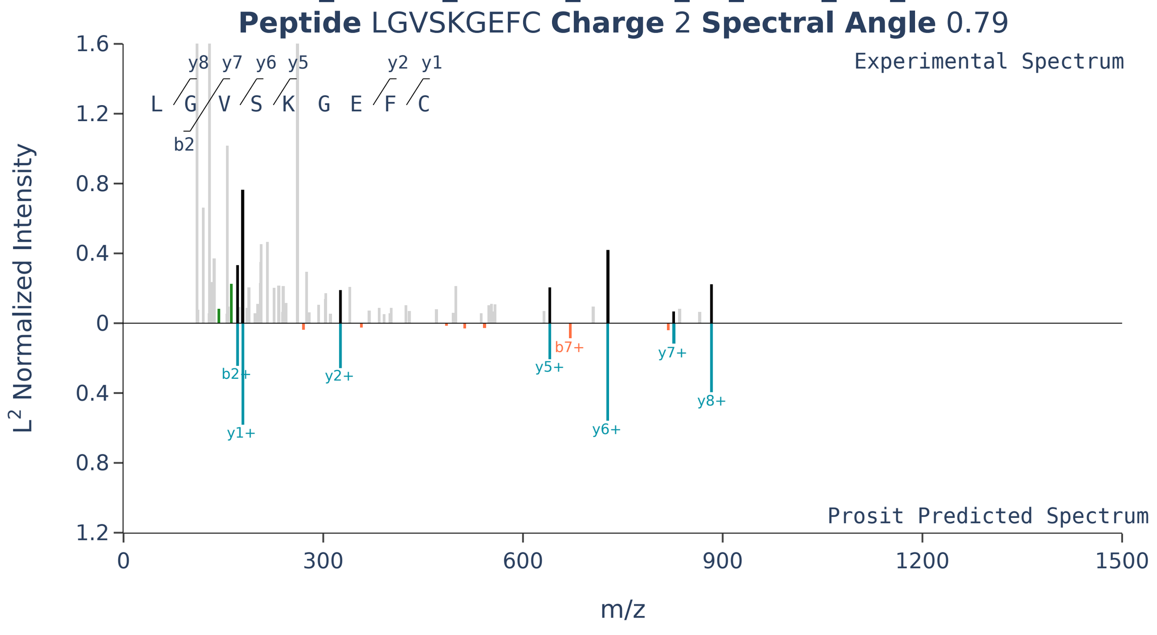


**Source** WSoh_101121_151121_HFGoe_G1_IL37b_24h_R1 **Scan** 19228
**Peptide** KNYIRPEIF **Charge** 2 **Spectral Angle** 0.83

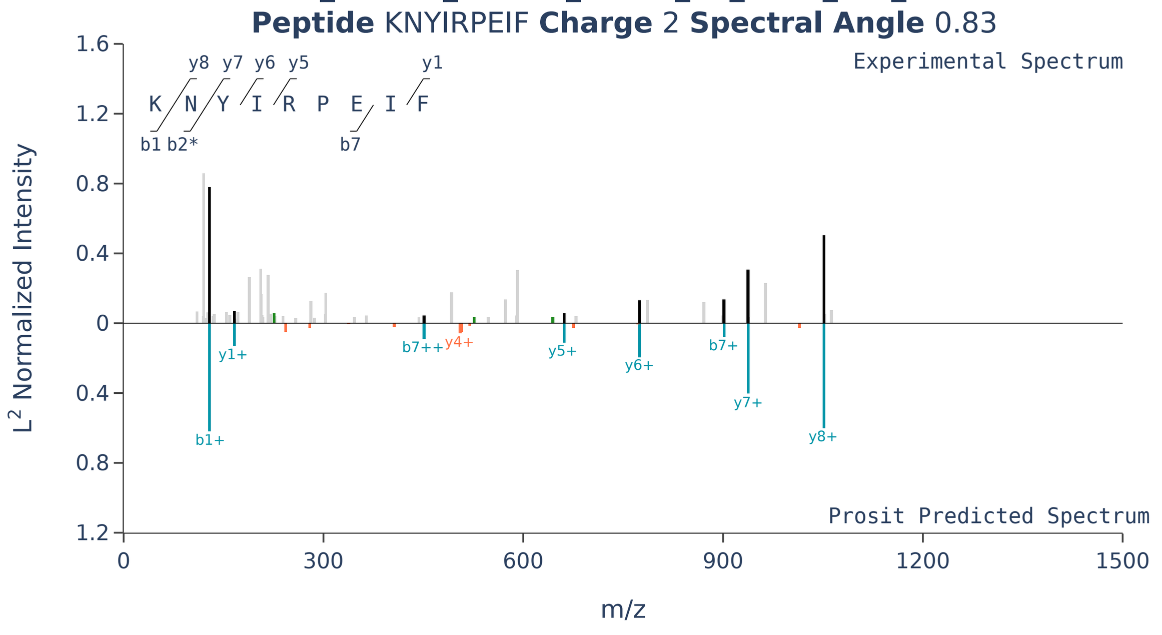


**Source** WSoh_101121_151121_HFGoe_G1_IL37b_24h_R2 **Scan** 10303
**Peptide** VHTSPKVKNLNPKKFSIHD **Charge** 5 **Spectral Angle** 0.86

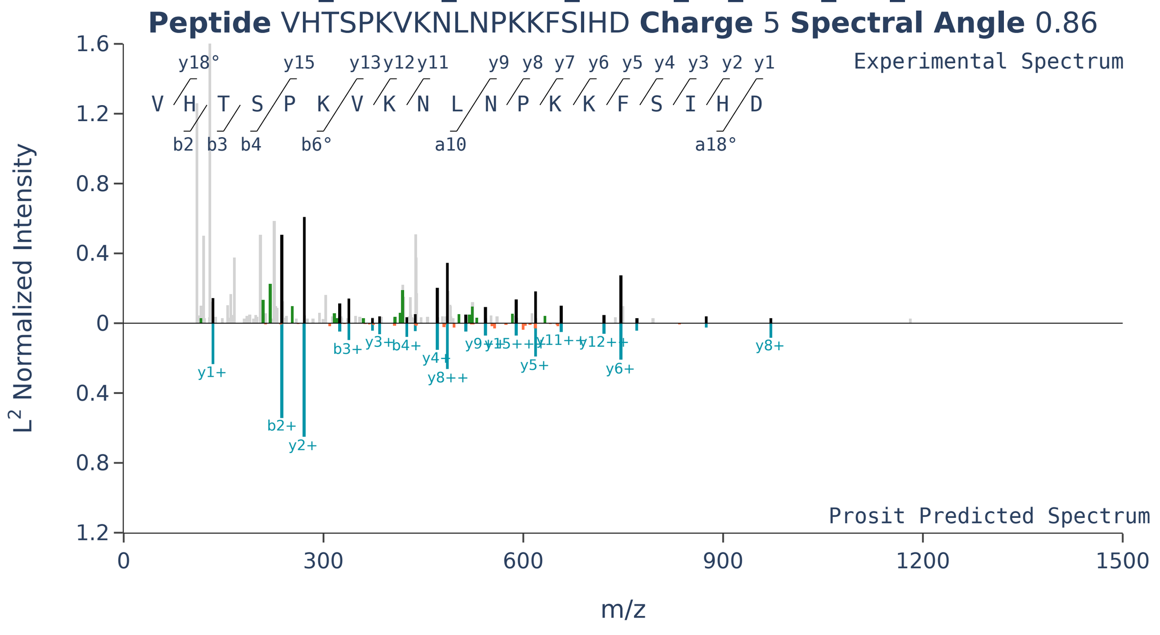


**Source** WSoh_101121_151121_HFGoe_G1_IL37b_2h_R1 **Scan** 5943
**Peptide** HTSPKVKNL **Charge** 2 **Spectral Angle** 0.86

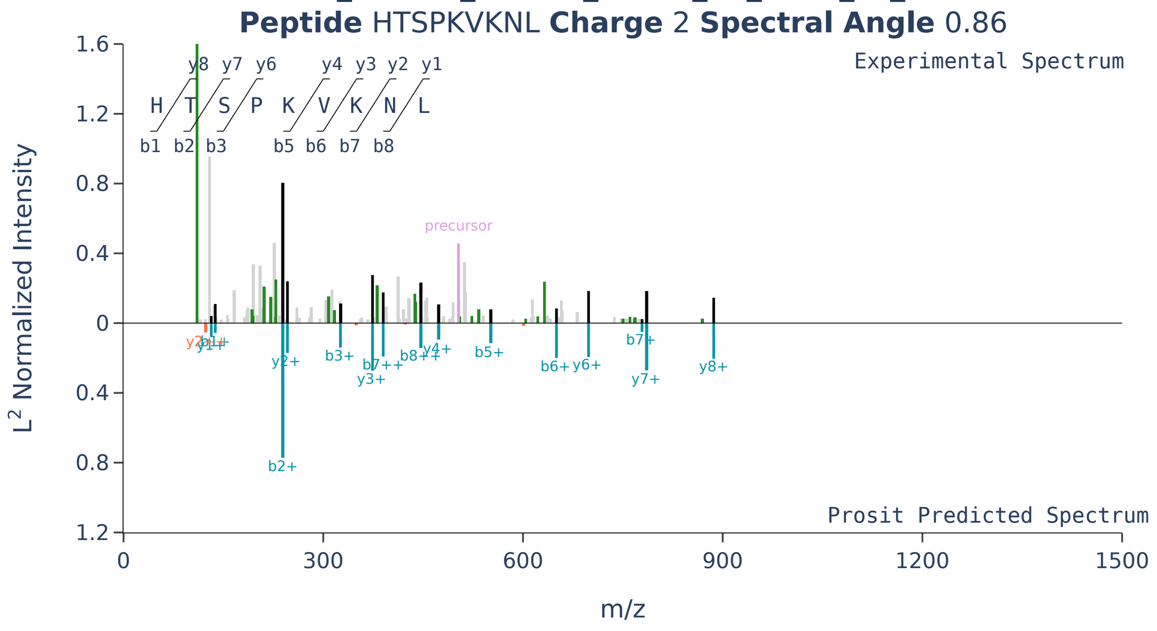


**Source** WSoh_101121_151121_HFGoe_G2_IL37b_24h_R2 **Scan** 6631
**Peptide** AAQKESARRPF **Charge** 2 **Spectral Angle** 0.83

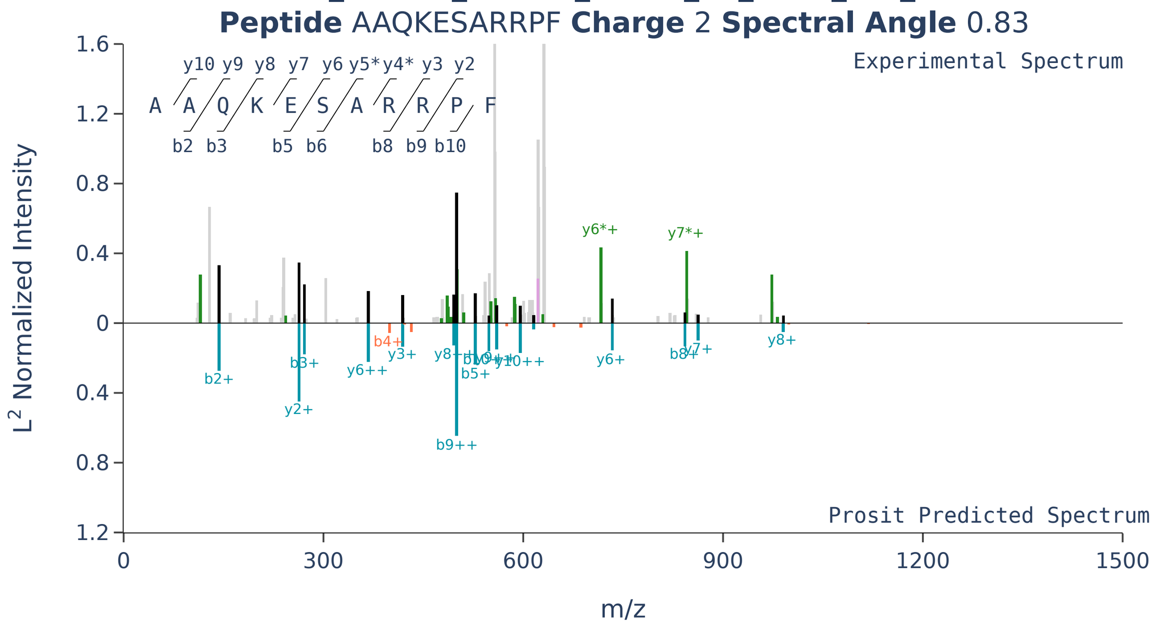


**Source** WSoh_101121_151121_HFGoe_G2_IL37b_24h_R1 **Scan** 19022
**Peptide** GVSKGEFCL **Charge** 2 **Spectral Angle** 0.8

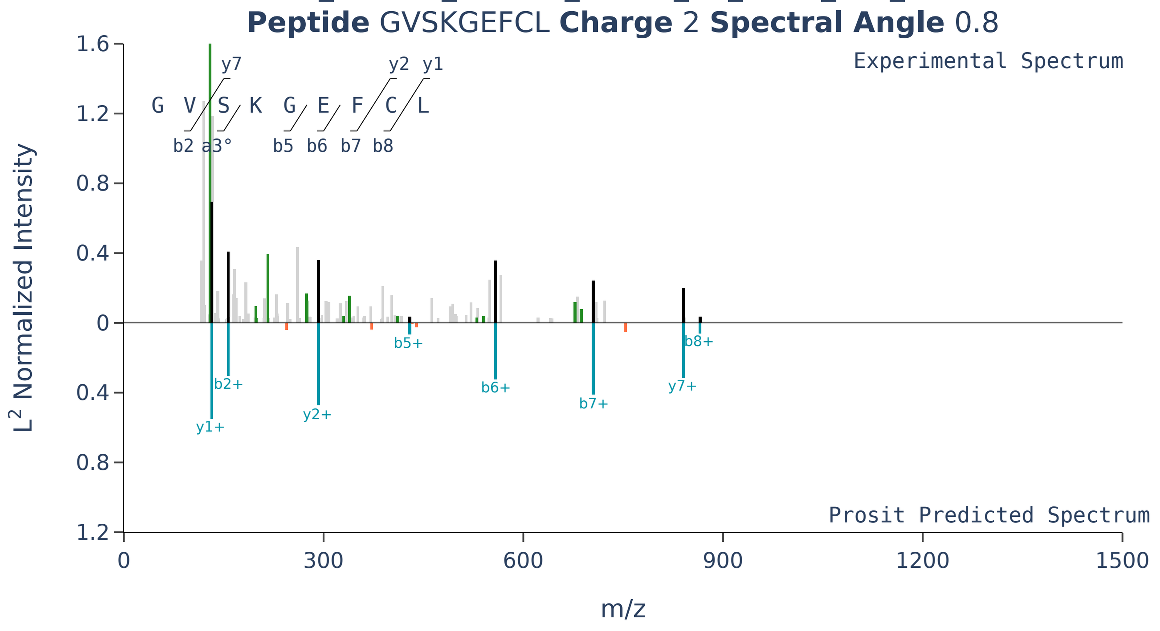


**Source** WSoh_101121_151121_HFGoe_G2_IL37b_2h_R1 **Scan** 8988
**Peptide** GVSKGEF **Charge** 2 **Spectral Angle** 0.77

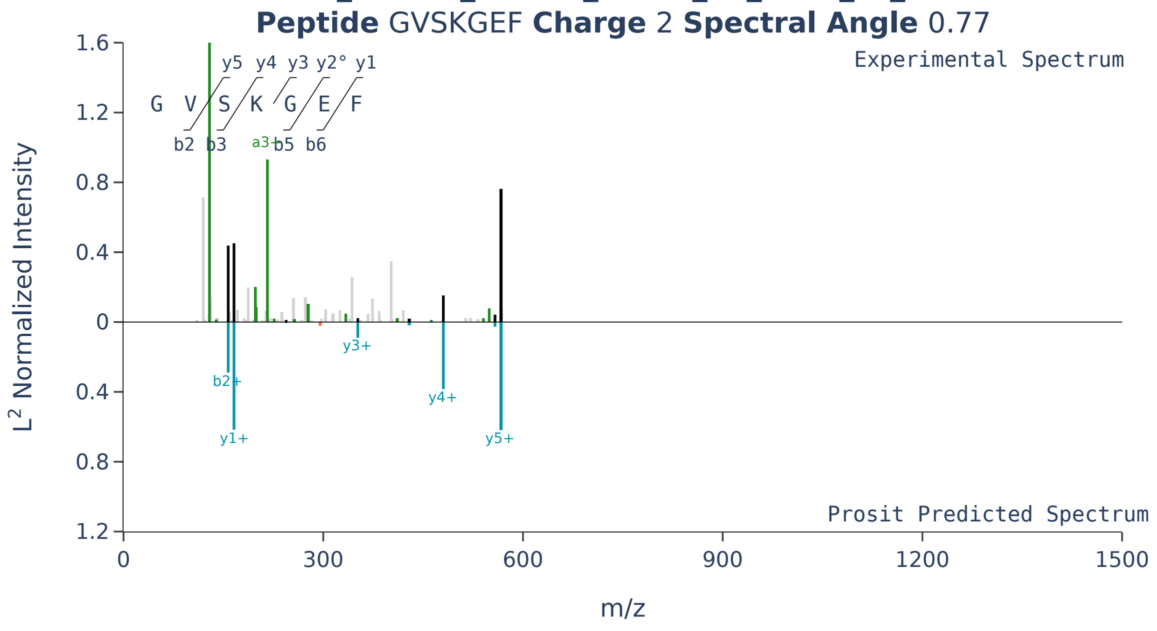


**Source** WSoh_101121_151121_HFGoe_G2_IL37b_24h_R1 **Scan** 19088
**Peptide** ASAEKGSPILLG **Charge** 2 **Spectral Angle** 0.89

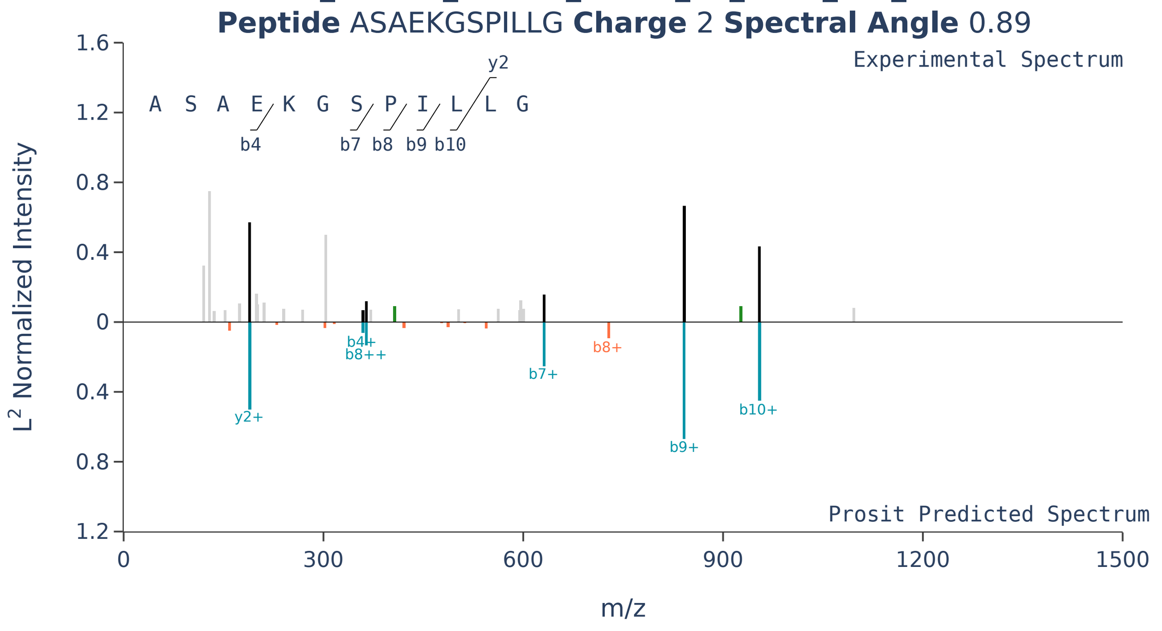


**Source** WSoh_101121_151121_HFGoe_G1_IL37b_24h_R2 **Scan** 17584
**Peptide** ASAEKGSPILLGVSKGE **Charge** 3 **Spectral Angle** 0.88

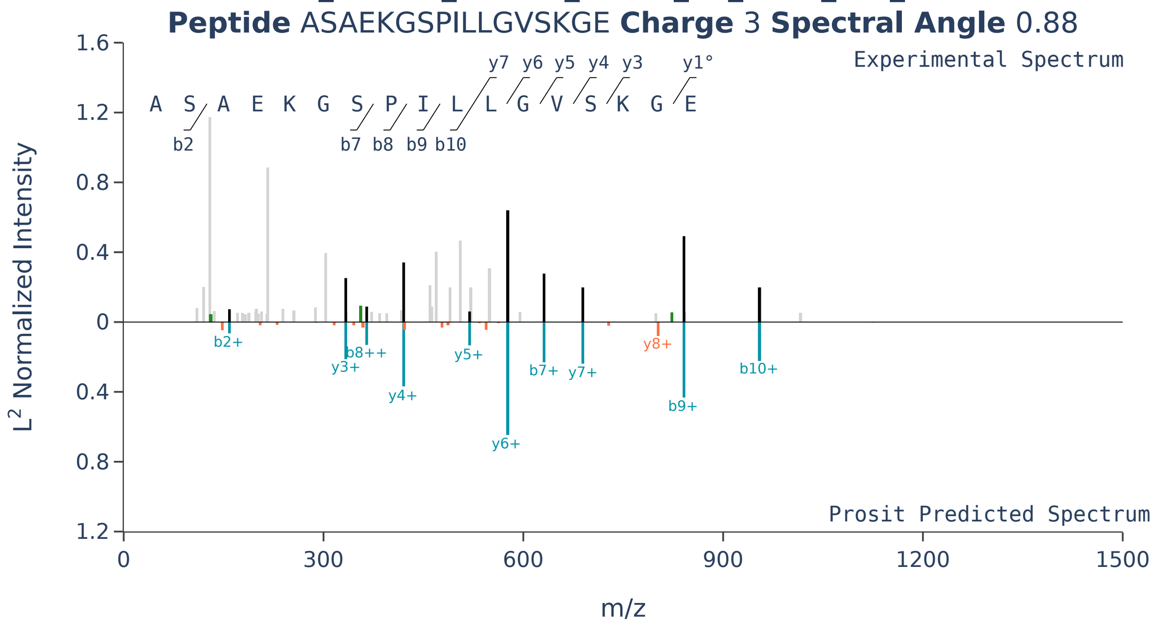


**Source** WSoh_101121_151121_HFGoe_G1_IL37b_1h_R1 **Scan** 9291
**Peptide** QLKKEKLMKL **Charge** 3 **Spectral Angle** 0.76

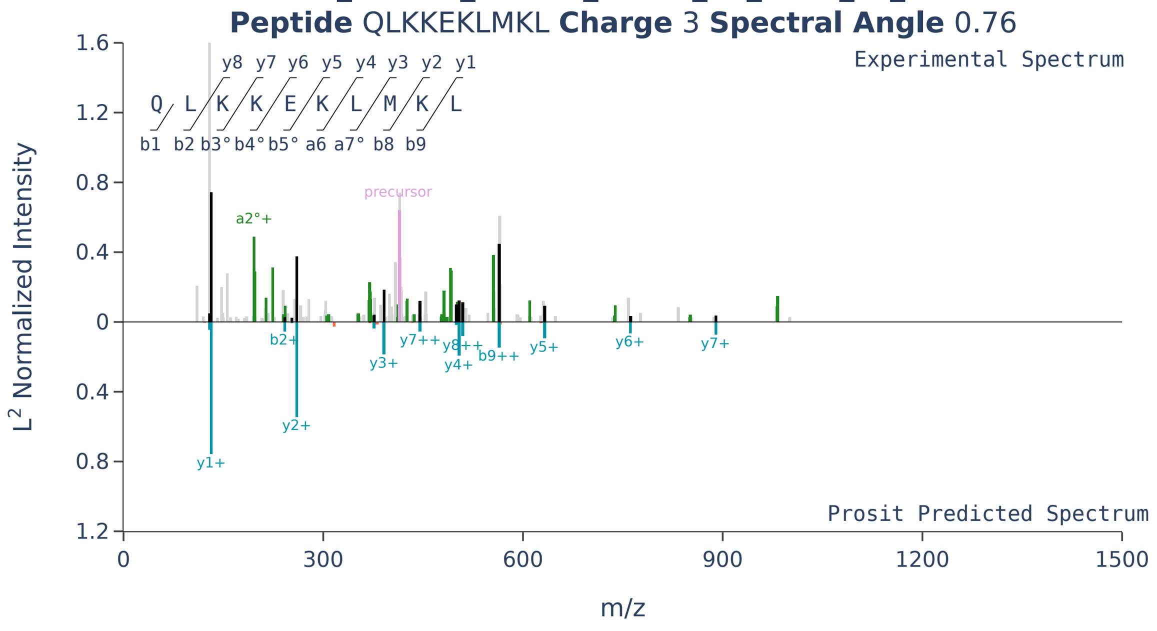


**Source** WSoh_101121_151121_HFGoe_G2_IL37b_24h_R1 **Scan** 7518
**Peptide** TSCNCNEPVG **Charge** 2 **Spectral Angle** 0.79

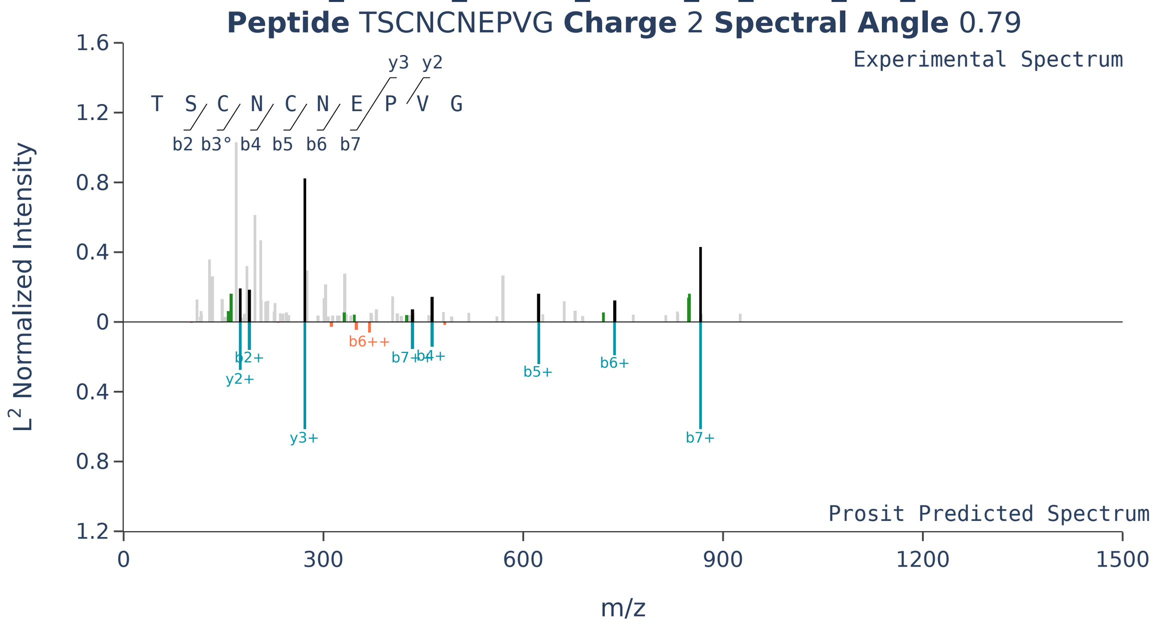


**Source** WSoh_101121_151121_HFGoe_G2_IL37b_24h_R1 **Scan** 17278
**Peptide** SSASAEKGSPILLGVSKG **Charge** 3 **Spectral Angle** 0.85

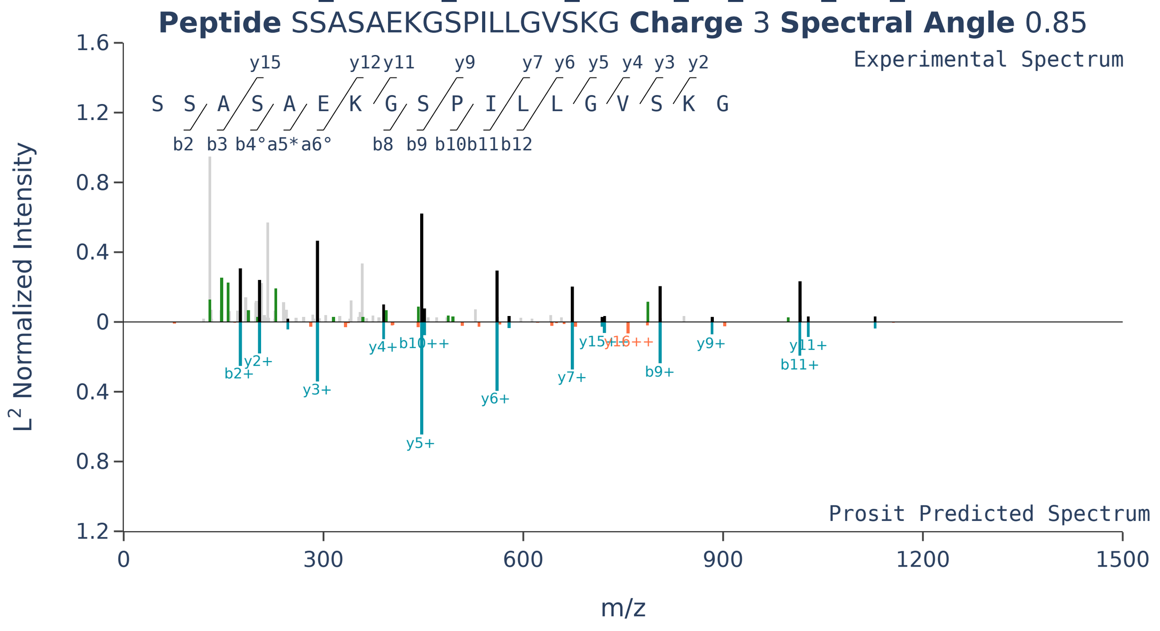

**Source** WSoh_101121_151121_HFGoe_G1_IL37b_24h_R1 **Scan** 17428
**Peptide** SAEKGSPILLGVSKGE **Charge** 3 **Spectral Angle** 0.88

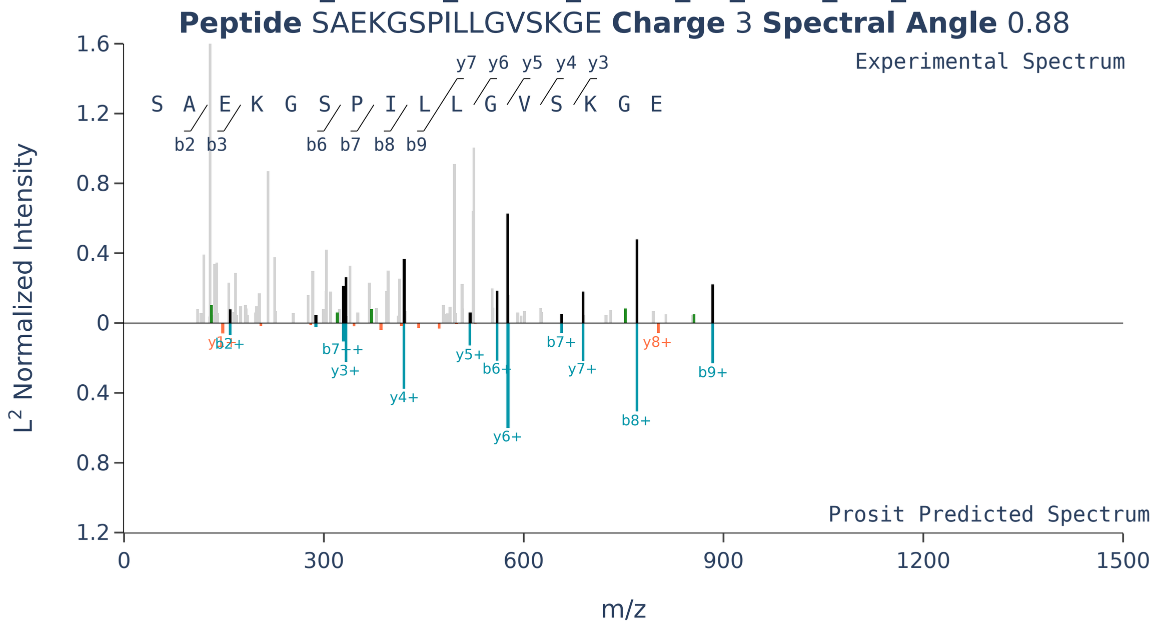


**Source** WSoh_101121_151121_HFGoe_G2_IL37b_24h_R1 **Scan** 9871
**Peptide** KGEFCLYCDKDKGQSH **Charge** 4 **Spectral Angle** 0.71

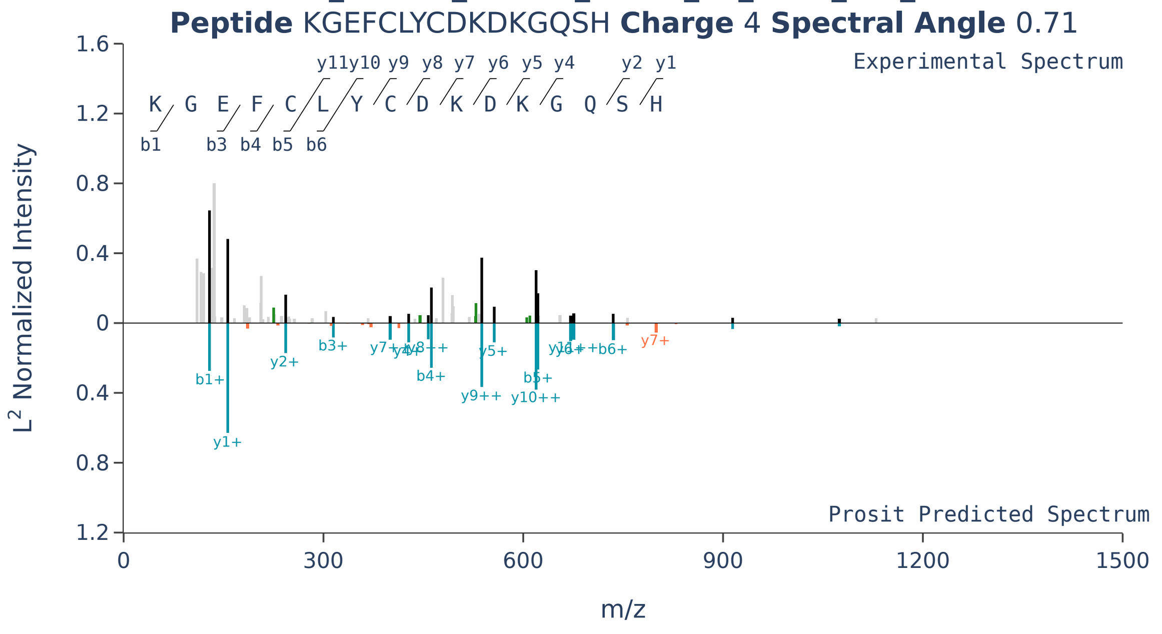


**Source** WSoh_101121_151121_HFGoe_G1_IL37b_24h_R2 **Scan** 19811
**Peptide** SAAHPGWF **Charge** 2 **Spectral Angle** 0.87

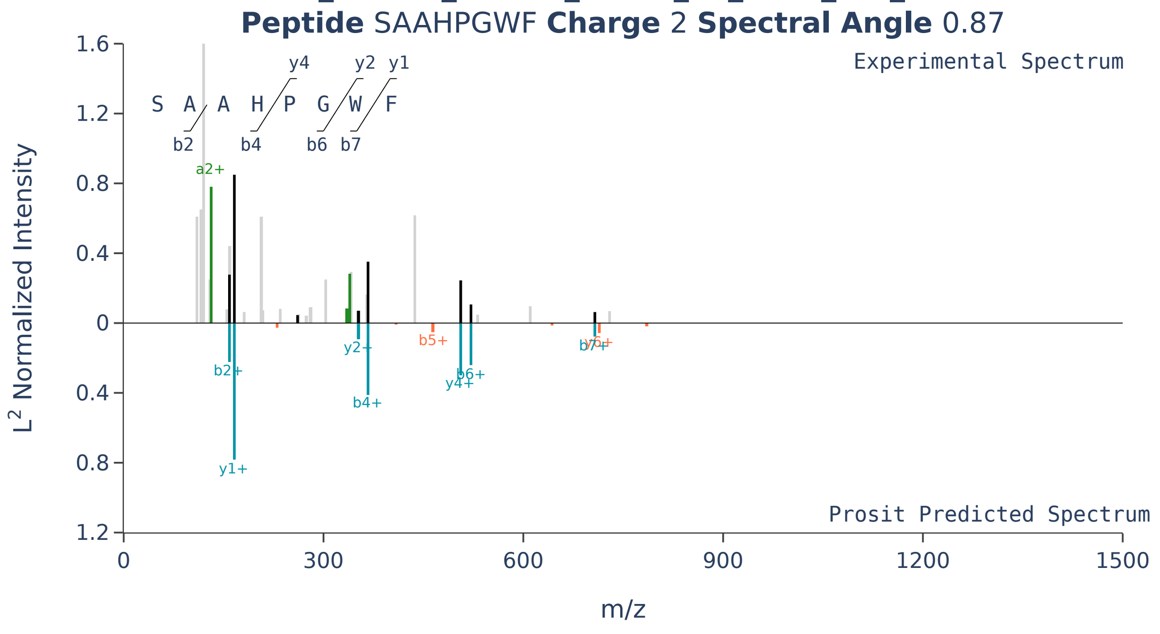


**Source** WSoh_101121_151121_HFGoe_G1_IL37b_2h_R1 **Scan** 16738
**Peptide** RAQVGSWNMLES **Charge** 2 **Spectral Angle** 0.76

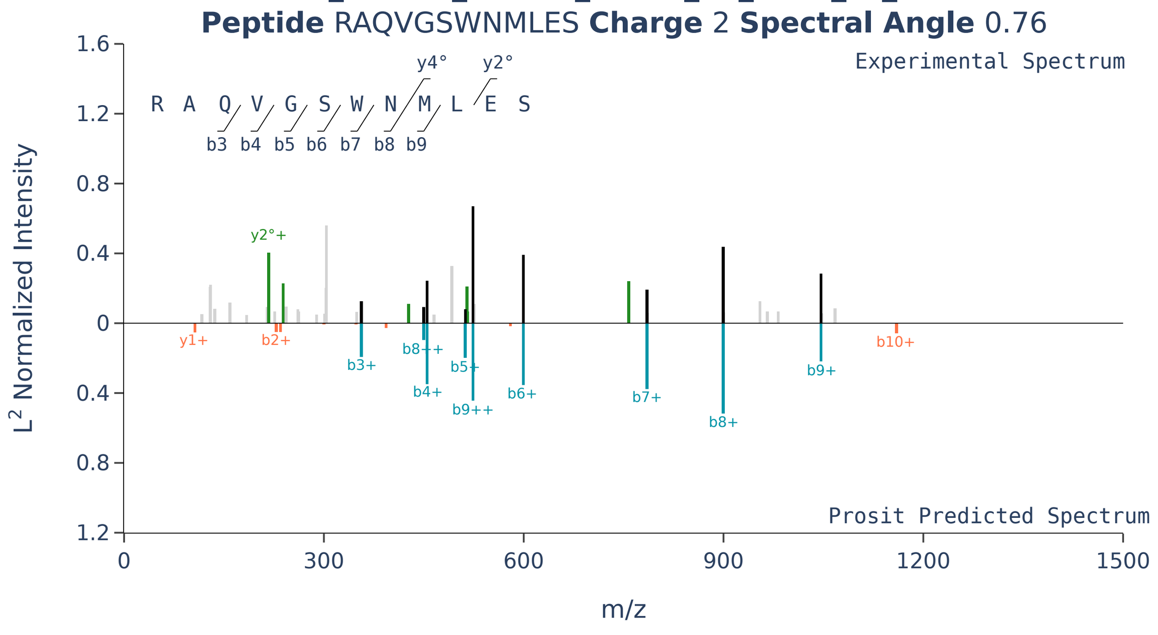


**Source** WSoh_101121_151121_HFGoe_G1_IL37b_4h_R2 **Scan** 6441
**Peptide** KKEKLMKLAAQKES **Charge** 2 **Spectral Angle** 0.86

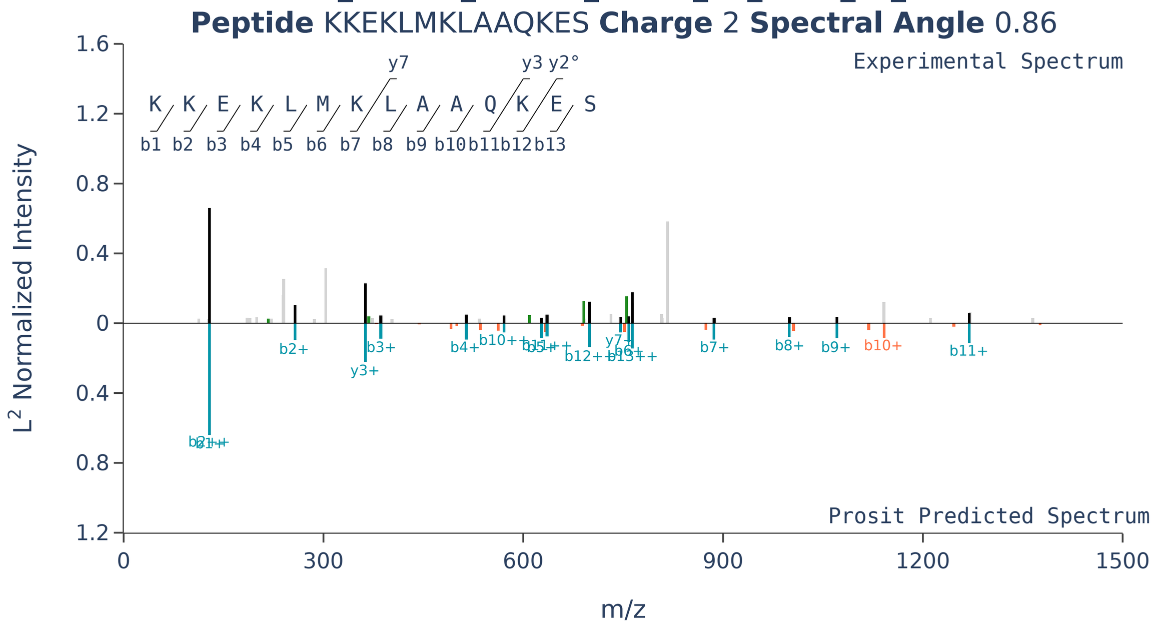


**Source** WSoh_101121_151121_HFGoe_G1_IL37b_24h_R1 **Scan** 19122
**Peptide** SAEKGSPILLG **Charge** 2 **Spectral Angle** 0.86

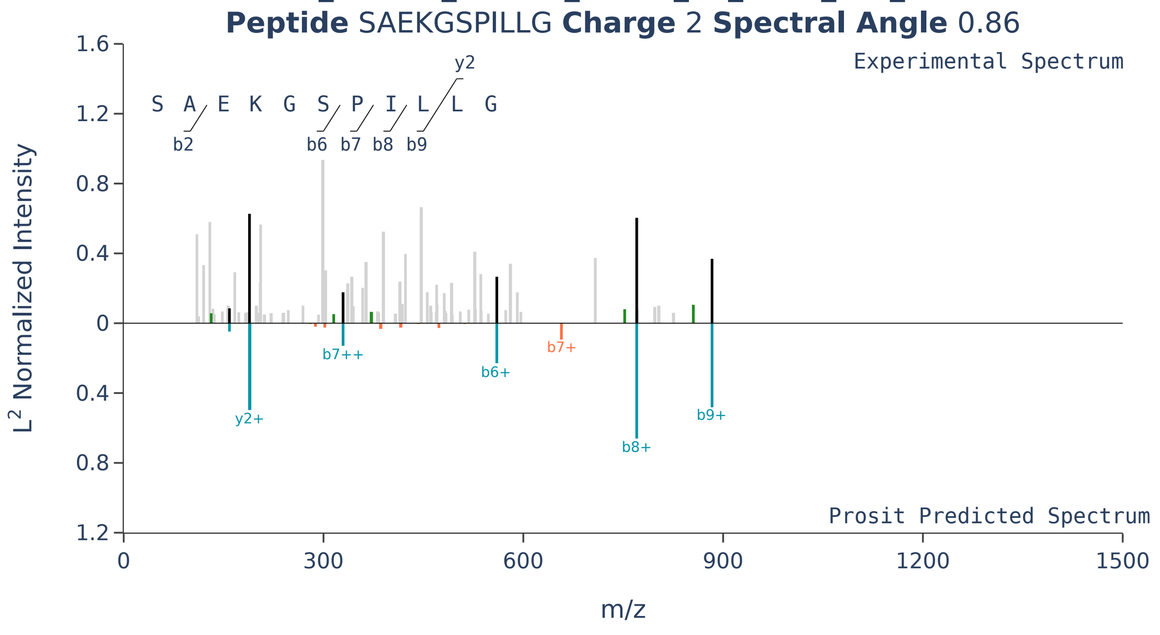


**Source** WSoh_101121_151121_HFGoe_G1_IL37b_4h_R1 **Scan** 6455
**Peptide** ASSLSSASAEKGS **Charge** 2 **Spectral Angle** 0.78

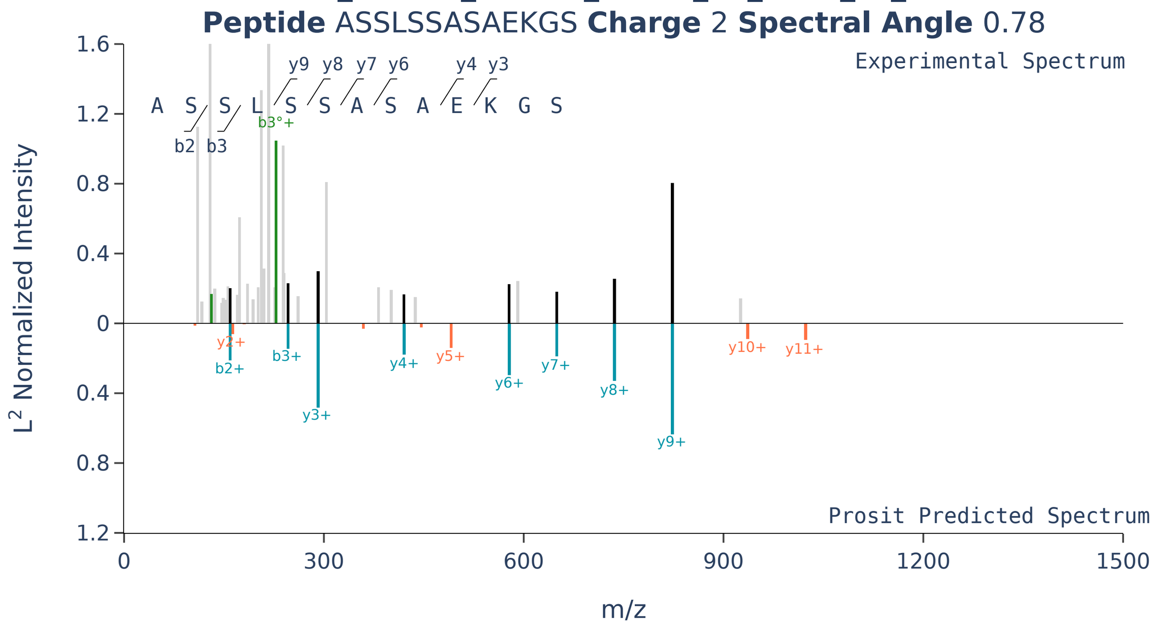


**Source** WSoh_101121_151121_HFGoe_G2_IL37b_24h_R1 **Scan** 20032
**Peptide** ASSLSSASAEKGSPILLGVSKG **Charge** 3 **Spectral Angle** 0.84

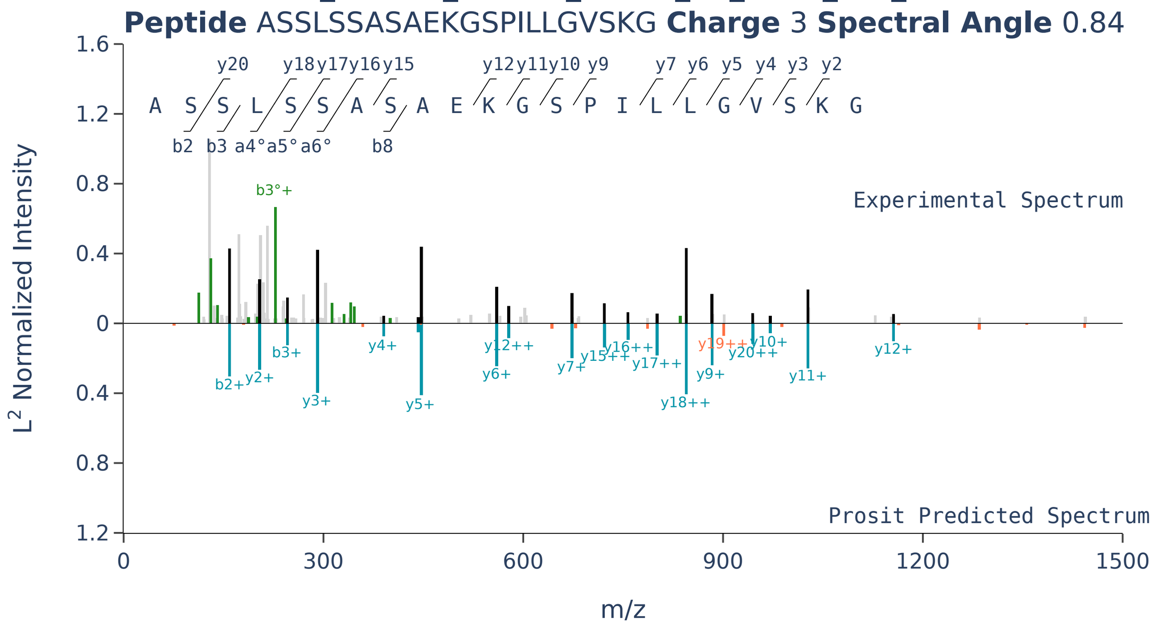


**Source** WSoh_101121_151121_HFGoe_G1_IL37b_1h_R1 **Scan** 18503
**Peptide** AAQKESARRPFIFY **Charge** 3 **Spectral Angle** 0.87

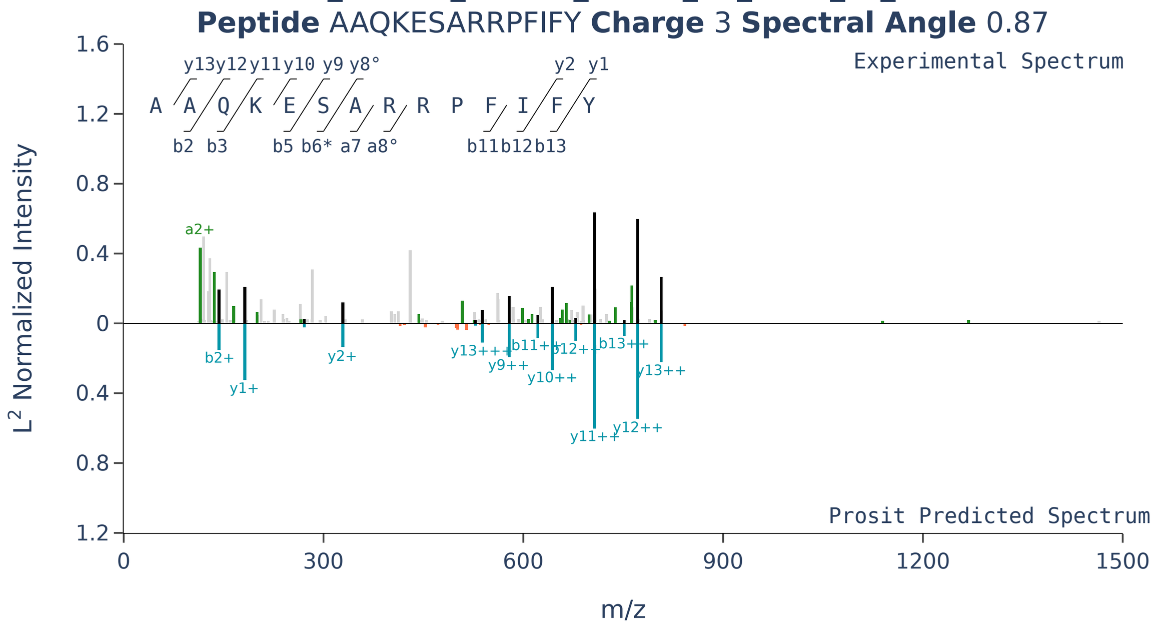


**Source** WSoh_101121_151121_HFGoe_G1_IL37b_24h_R2 **Scan** 6569
**Peptide** KGQSHPSL **Charge** 2 **Spectral Angle** 0.86

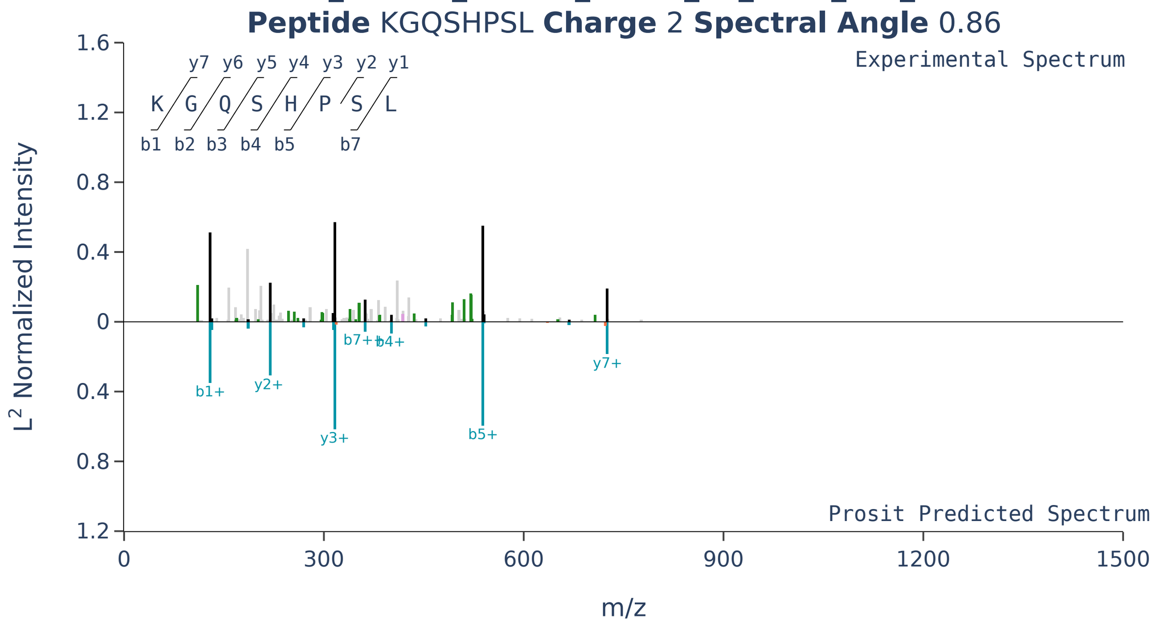


**Source** WSoh_101121_151121_HFGoe_G1_IL37b_4h_R1 **Scan** 14388
**Peptide** QDHKVLVLDSG **Charge** 2 **Spectral Angle** 0.87

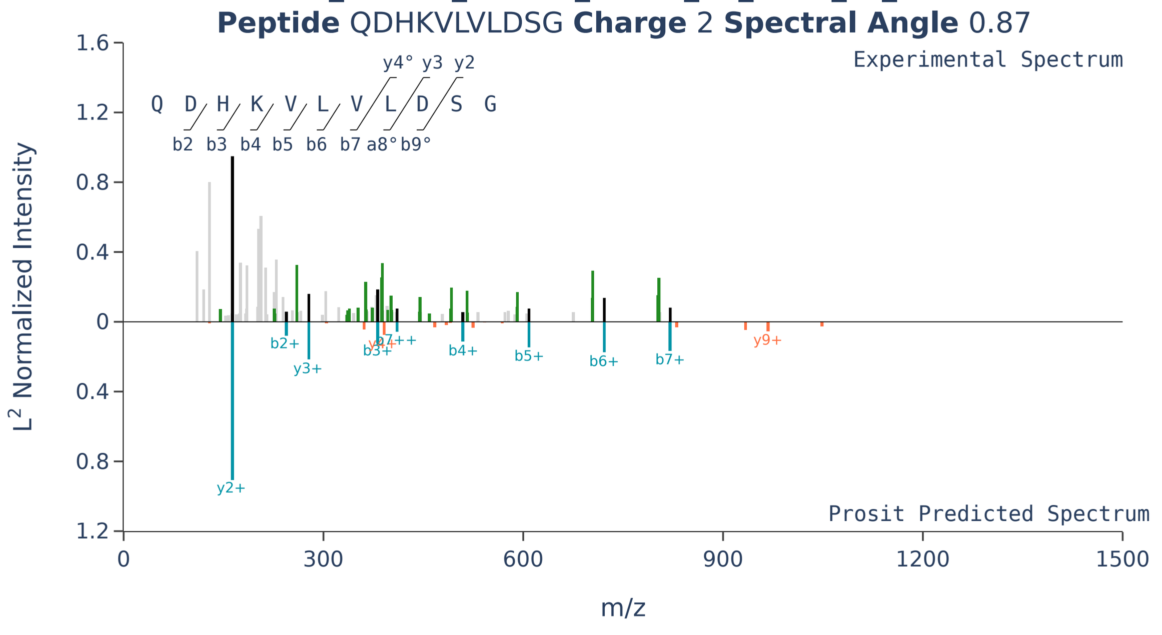


**Source** WSoh_101121_151121_HFGoe_G1_IL37b_1h_R1 **Scan** 10906
**Peptide** SFQPVCKAE **Charge** 2 **Spectral Angle** 0.87

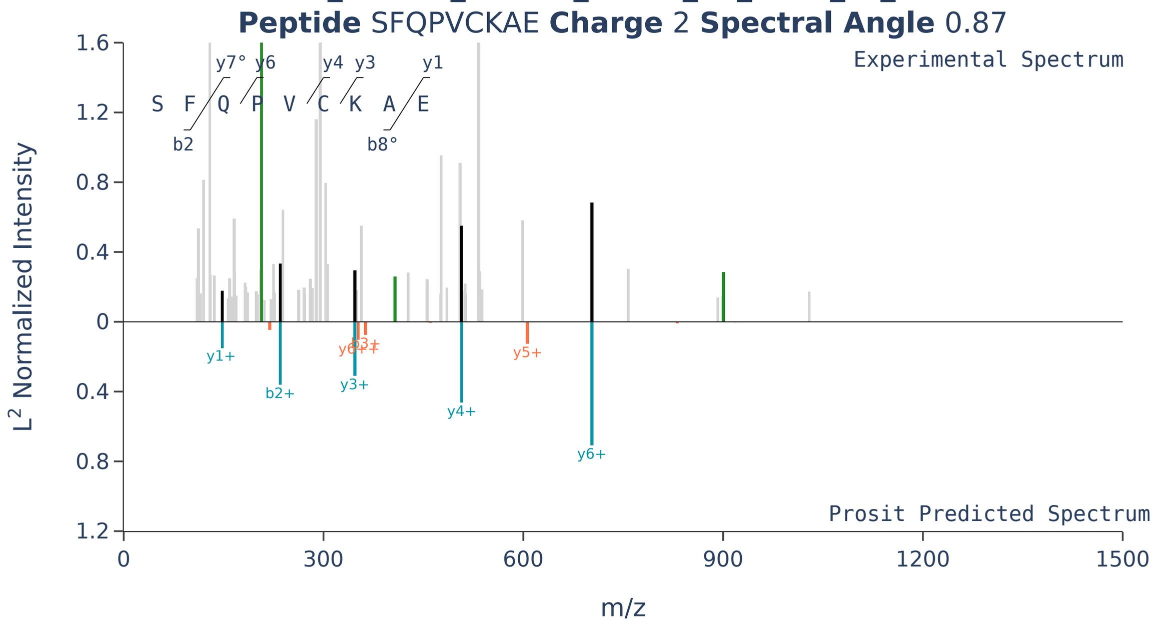


**Source** WSoh_101121_151121_HFGoe_G2_IL37b_24h_R2 **Scan** 10251
**Peptide** EPVGVTD **Charge** 1 **Spectral Angle** 0.75

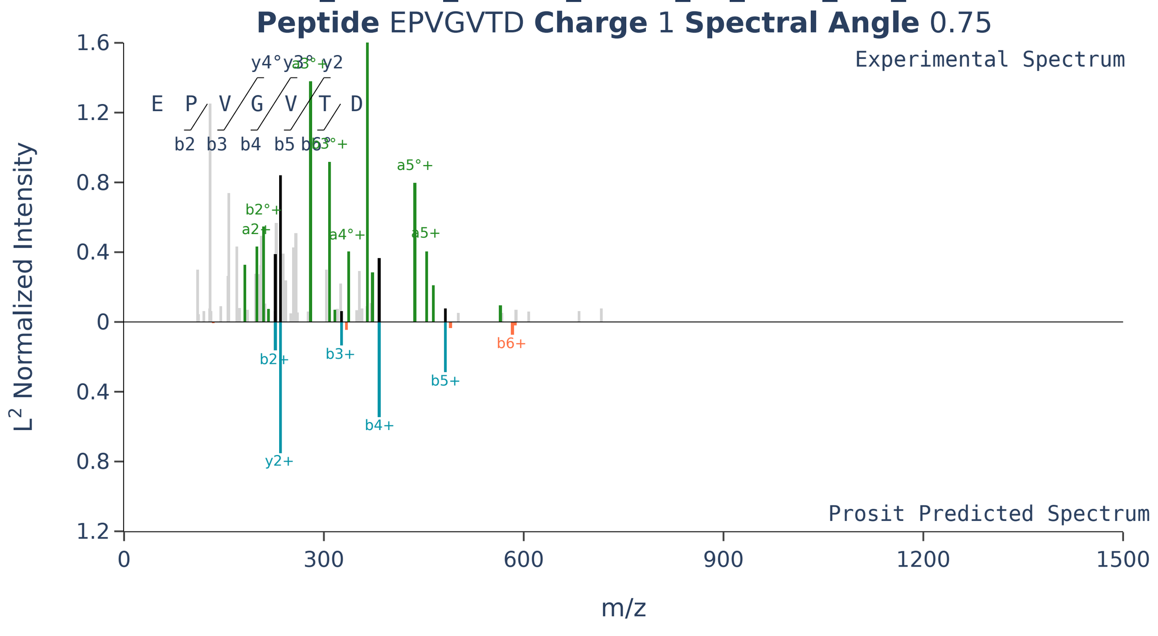


**Source** WSoh_101121_151121_HFGoe_G1_IL37b_24h_R2 **Scan** 7901
**Peptide** ASSLSSASAEKGSP **Charge** 2 **Spectral Angle** 0.78

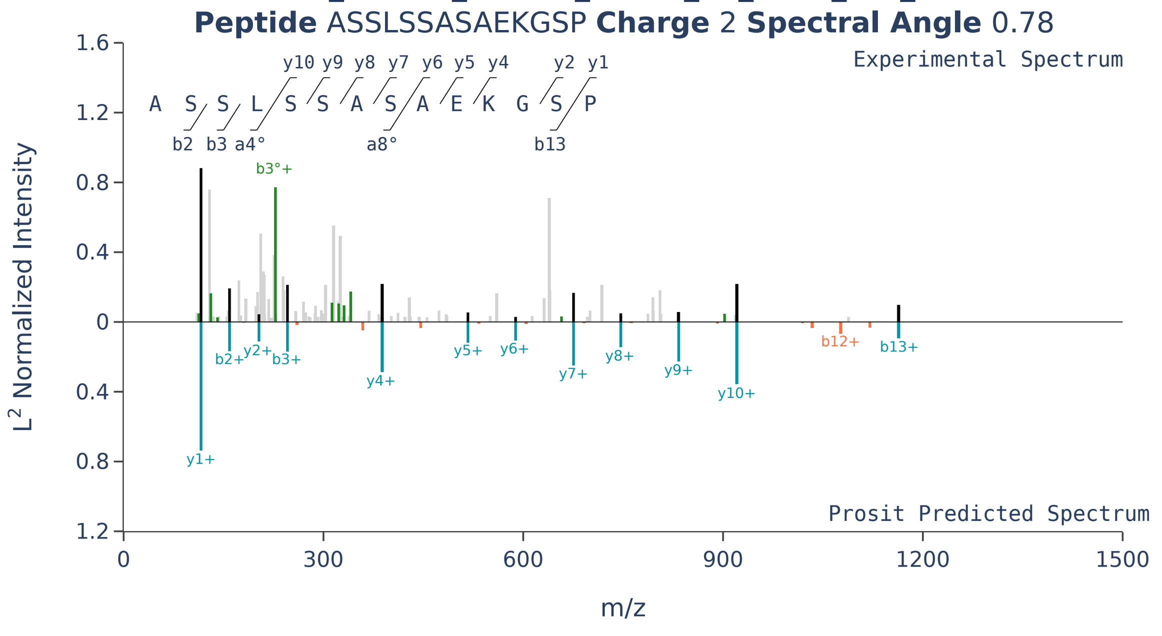


**Source** WSoh_101121_151121_HFGoe_G2_IL37b_24h_R2 **Scan** 11874
**Peptide** TSCNCNEPVGVTD **Charge** 2 **Spectral Angle** 0.89

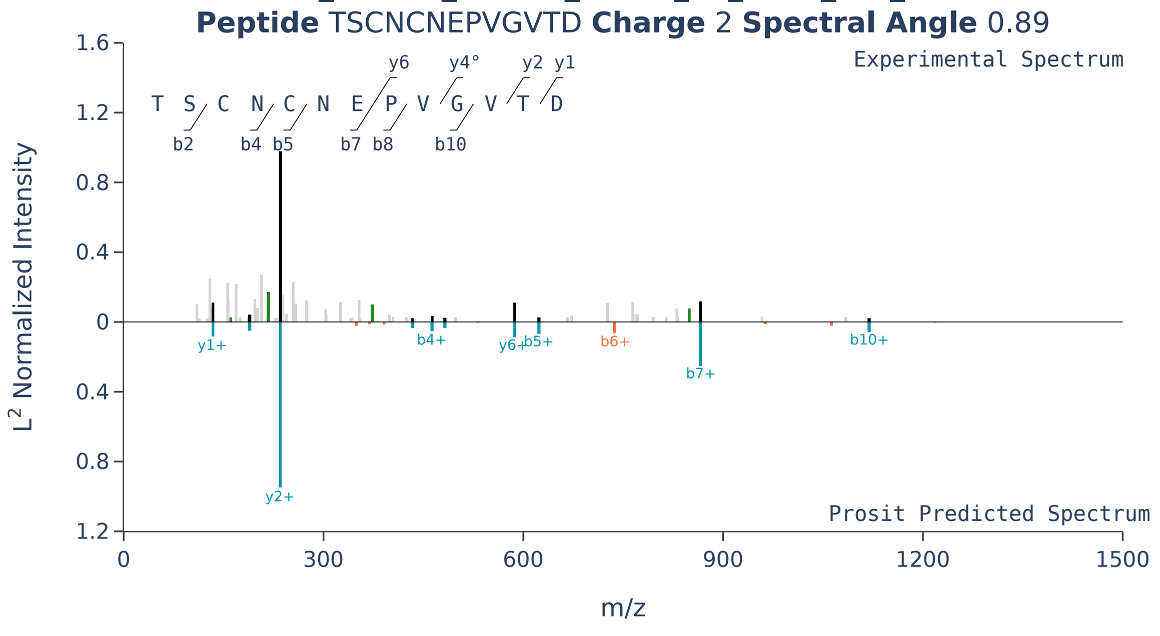

**Source** WSoh_101121_151121_HFGoe_G1_IL37b_24h_R1 **Scan** 11086
**Peptide** NCNEPVGVTDKFENRK **Charge** 3 **Spectral Angle** 0.85

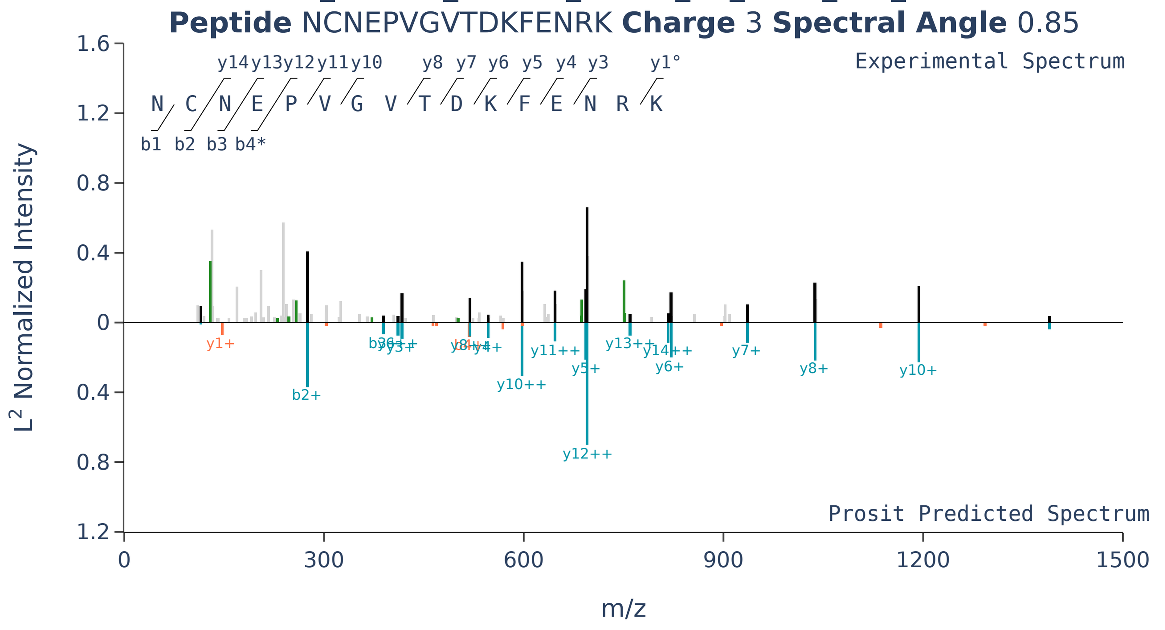


**Source** WSoh_101121_151121_HFGoe_G1_IL37b_1h_R1 **Scan** 16148
**Peptide** AQKESARRPFIF **Charge** 3 **Spectral Angle** 0.9

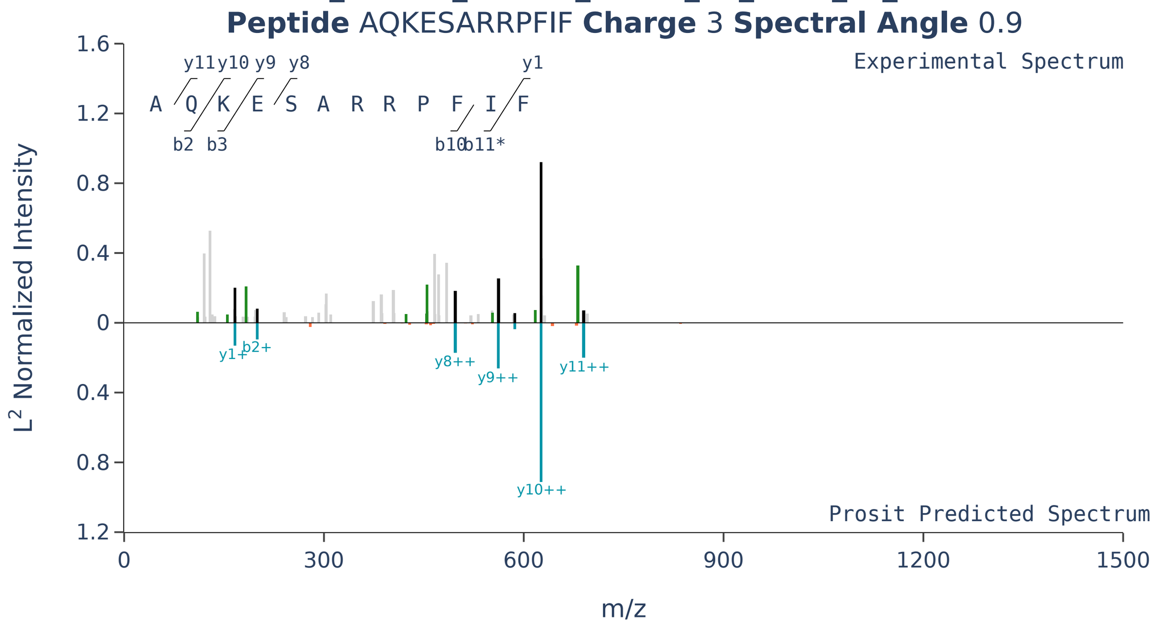


**Source** WSoh_101121_151121_HFGoe_G2_IL37b_24h_R2 **Scan** 11257
**Peptide** NEPVGVTDKFENRKHI **Charge** 3 **Spectral Angle** 0.88

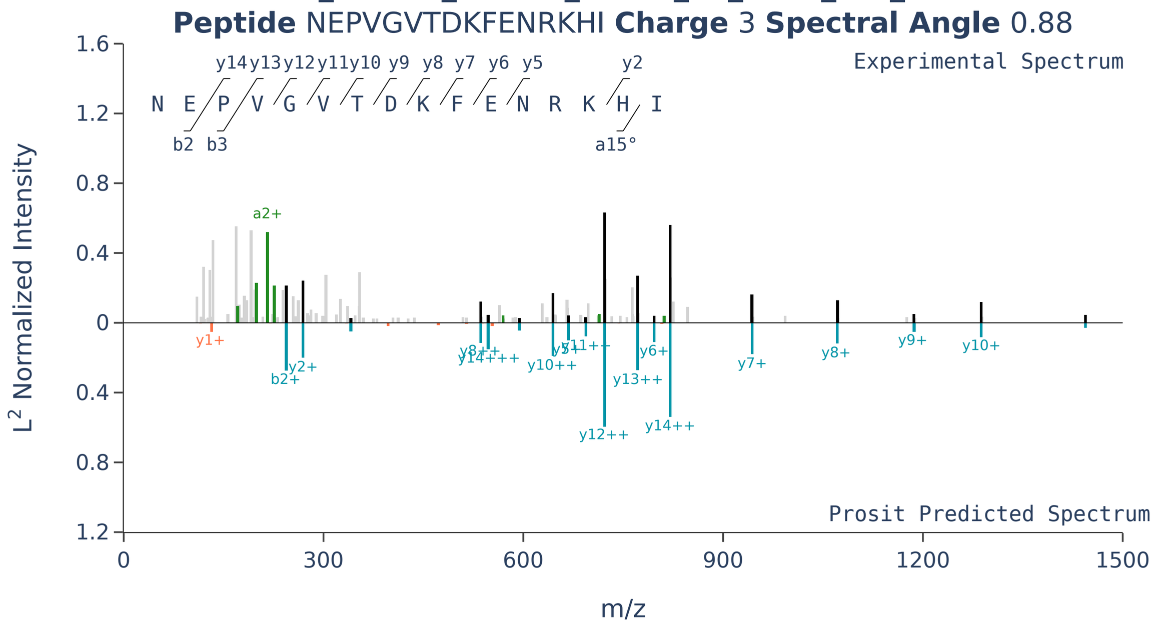


**Source** WSoh_101121_151121_HFGoe_G2_IL37b_4h_R2 **Scan** 8166
**Peptide** ASSLSSASAE **Charge** 2 **Spectral Angle** 0.89

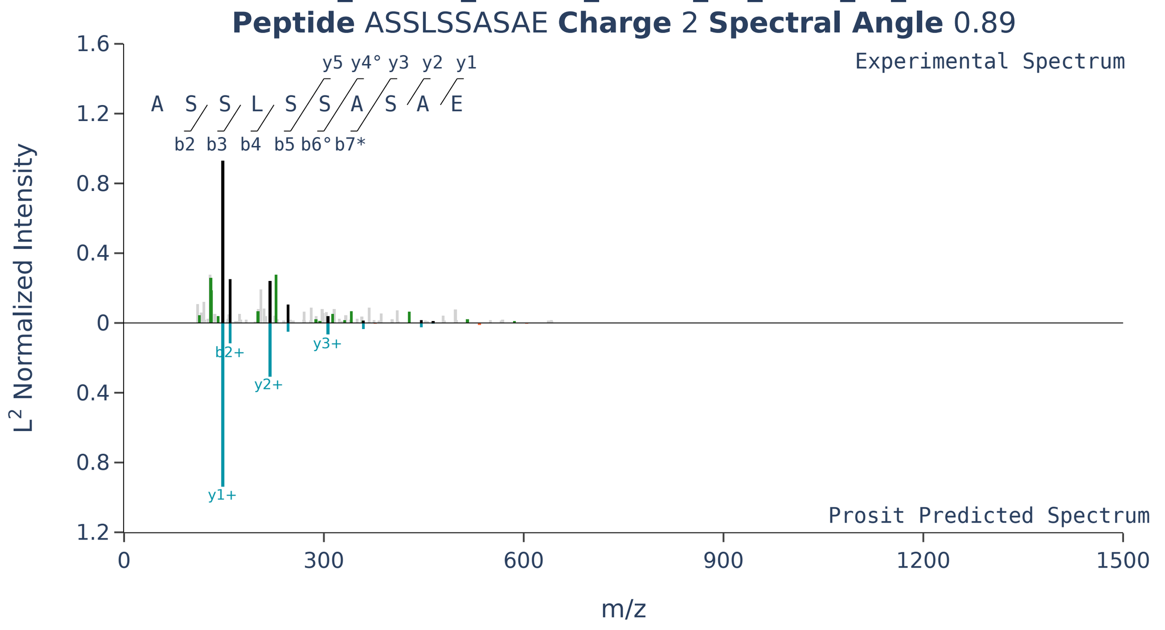


**Source** WSoh_101121_151121_HFGoe_G1_IL37b_24h_R2 **Scan** 19779
**Peptide** LDSGNLIAVPDKN **Charge** 2 **Spectral Angle** 0.92

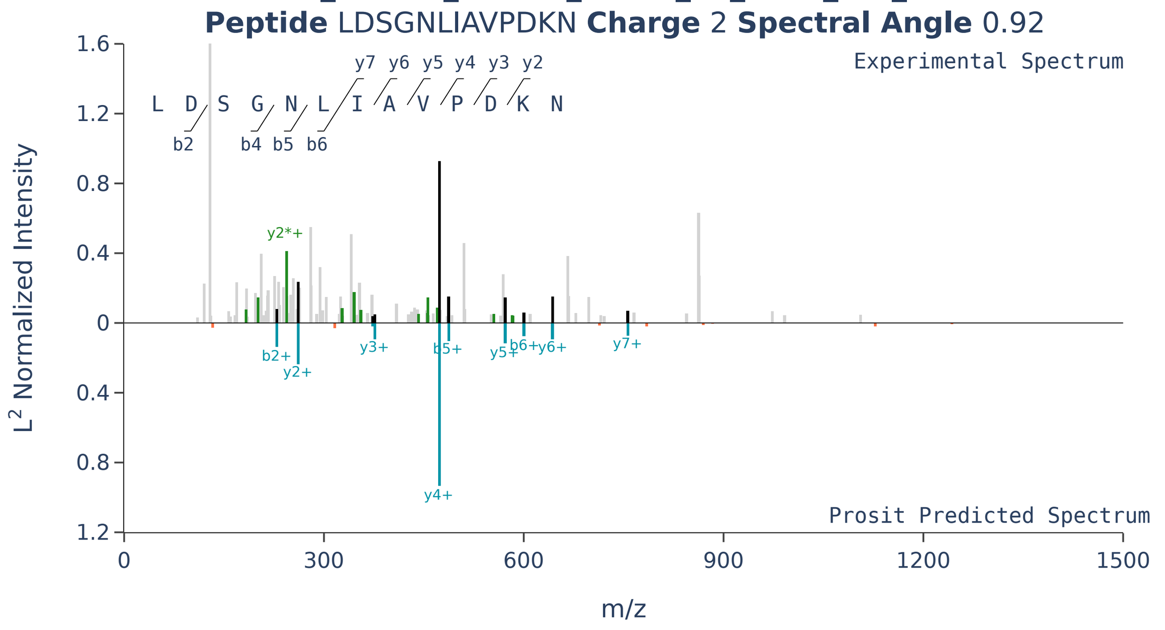


**Source** WSoh_101121_151121_HFGoe_G2_IL37b_24h_R1 **Scan** 19286
**Peptide** GNLIAVPDKNYIR **Charge** 2 **Spectral Angle** 0.91

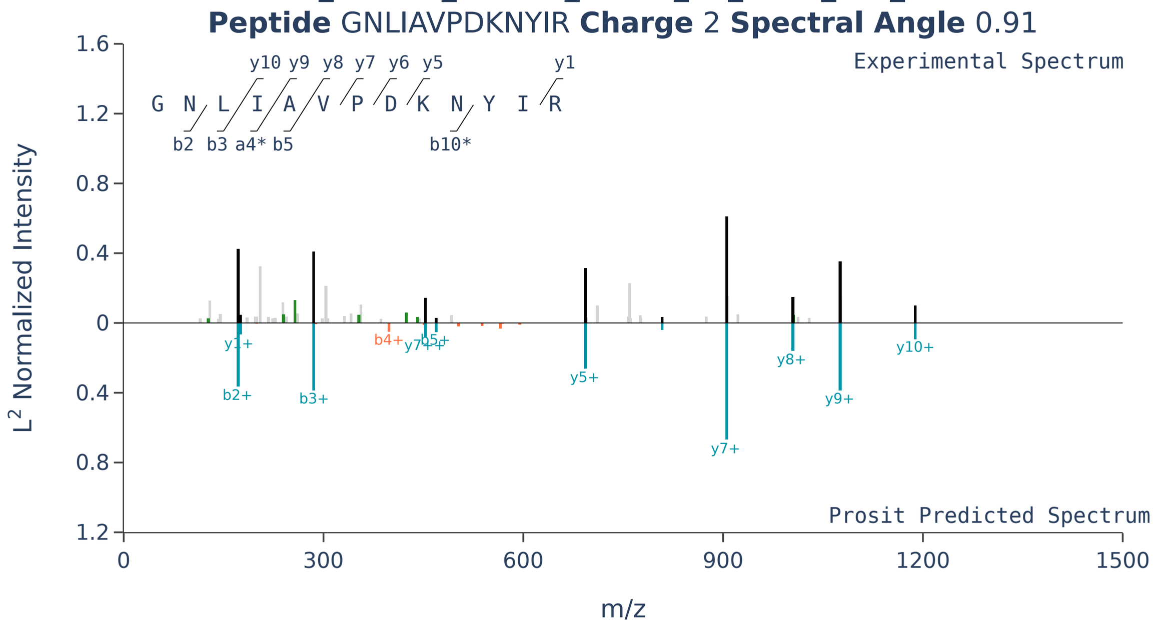


**Source** WSoh_101121_151121_HFGoe_G2_IL37b_24h_R2 **Scan** 20741
**Peptide** SASAEKGSPILL **Charge** 2 **Spectral Angle** 0.89

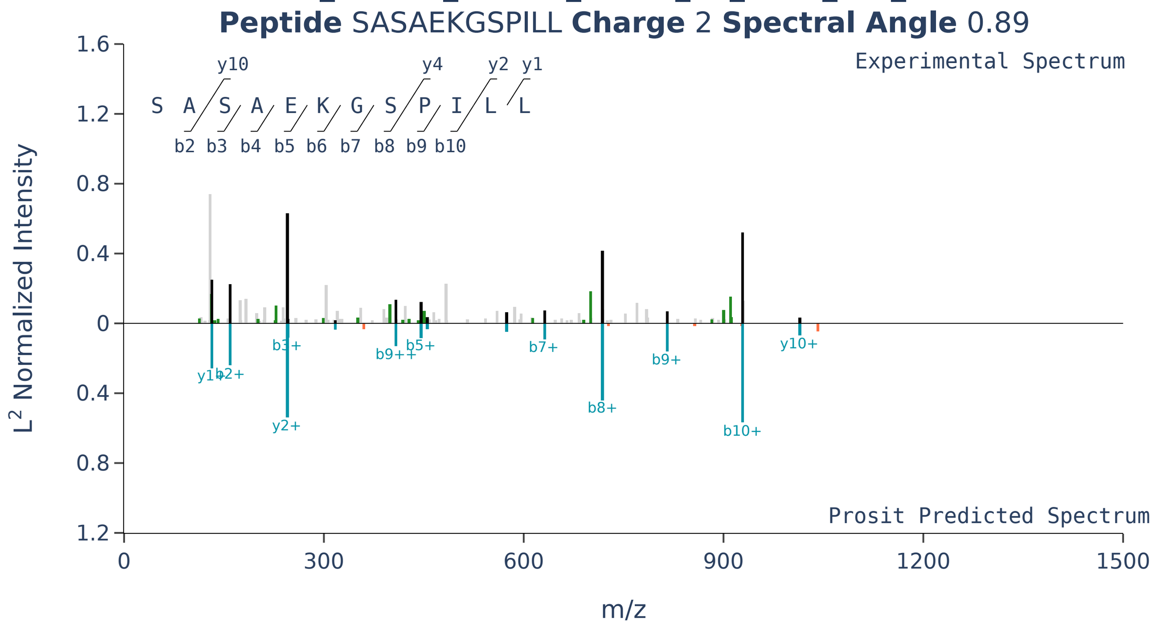


**Source** WSoh_101121_151121_HFGoe_G1_IL37b_24h_R2 **Scan** 20476
**Peptide** NLIAVPDKNYIRPE **Charge** 3 **Spectral Angle** 0.86

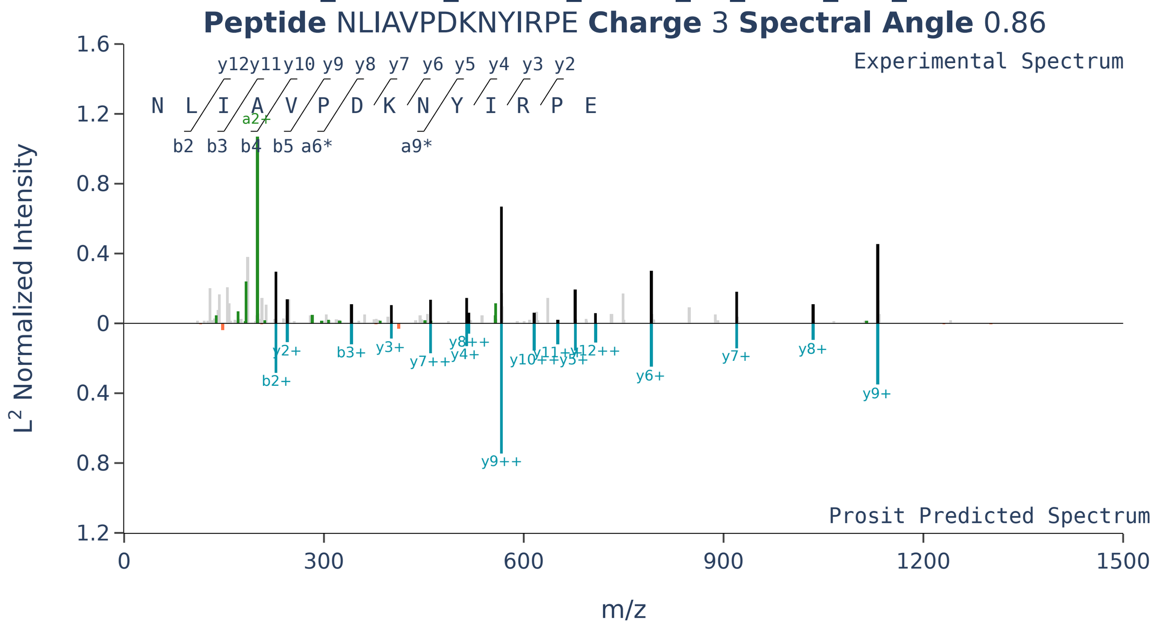


**Source** WSoh_101121_151121_HFGoe_G1_IL37b_4h_R1 **Scan** 15597
**Peptide** NCNEPVGVTDKFEN **Charge** 2 **Spectral Angle** 0.94

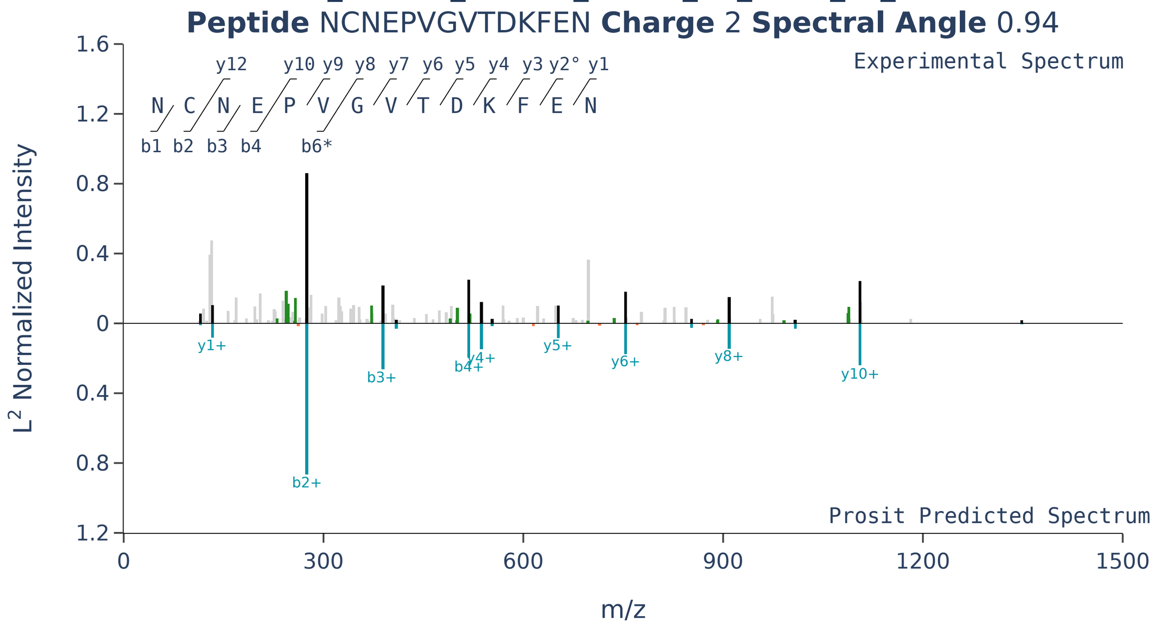


**Source** WSoh_101121_151121_HFGoe_G1_IL37b_4h_R2 **Scan** 8680
**Peptide** EPVGVTDKFENRKH **Charge** 3 **Spectral Angle** 0.87

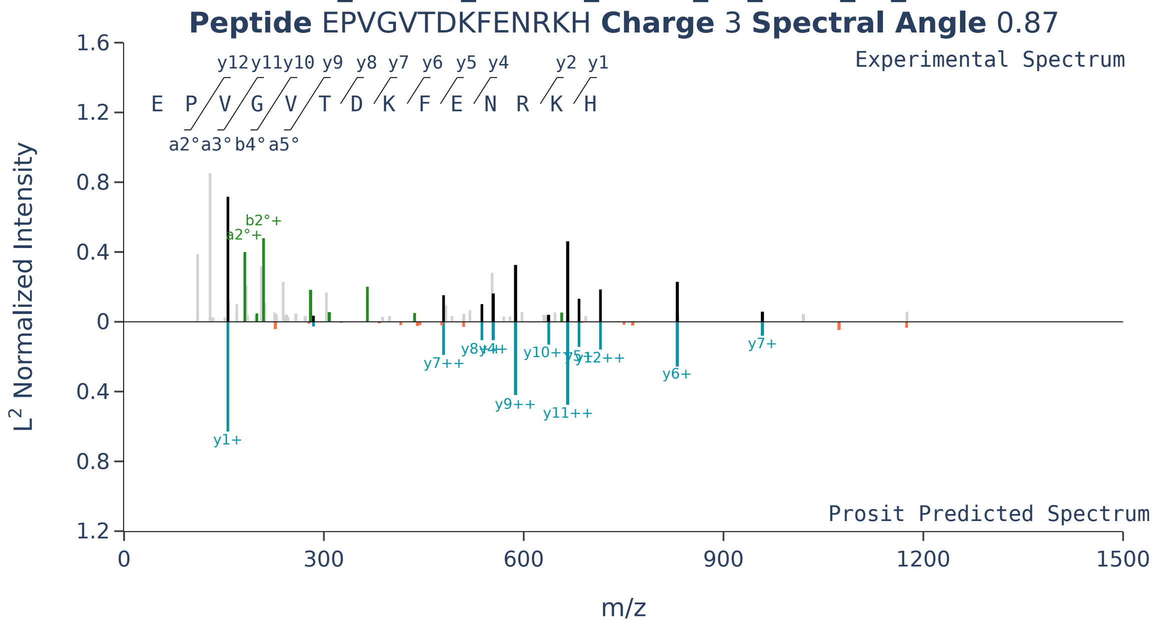


**Source** WSoh_101121_151121_HFGoe_G2_IL37b_24h_R2 **Scan** 16633
**Peptide** ENRKHIEFSF **Charge** 3 **Spectral Angle** 0.88

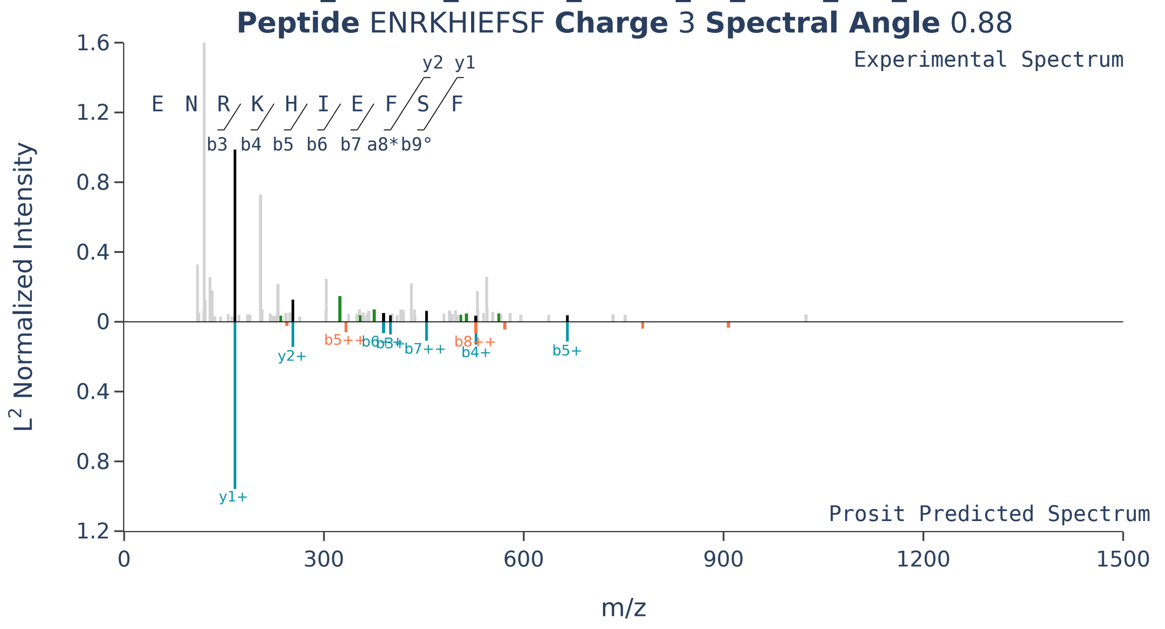


**Source** WSoh_101121_151121_HFGoe_G1_IL37b_24h_R1 **Scan** 5951
**Peptide** VHTSPKVKNLNPK **Charge** 3 **Spectral Angle** 0.85

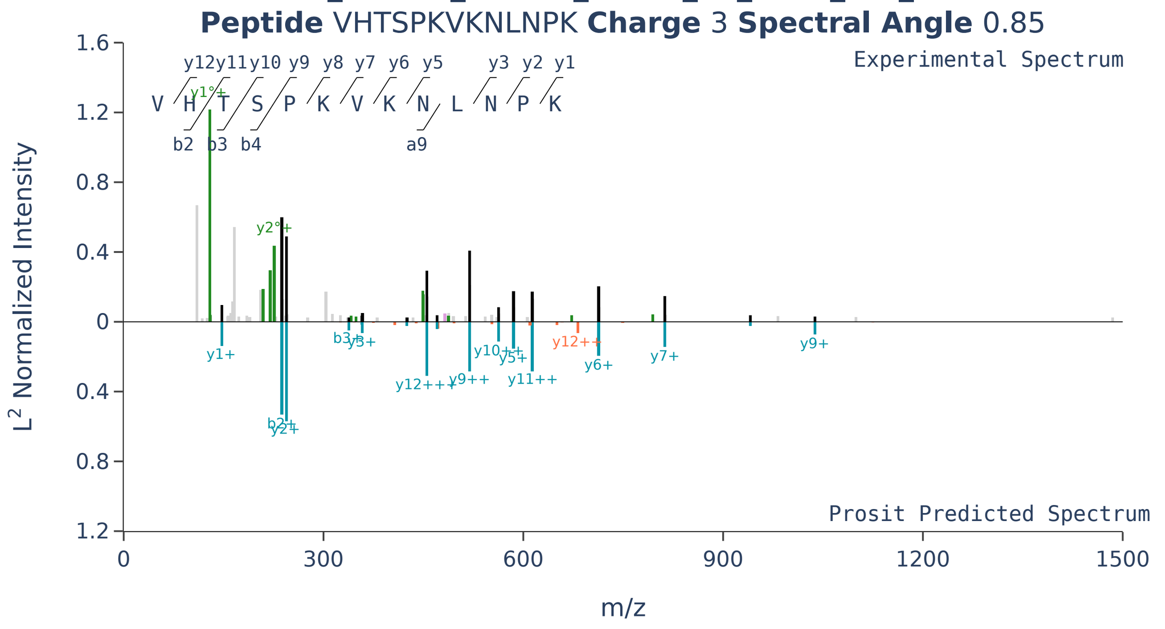


**Source** WSoh_101121_151121_HFGoe_G1_IL37b_24h_R1 **Scan** 18683
**Peptide** AQKESARRPFIFY **Charge** 3 **Spectral Angle** 0.91

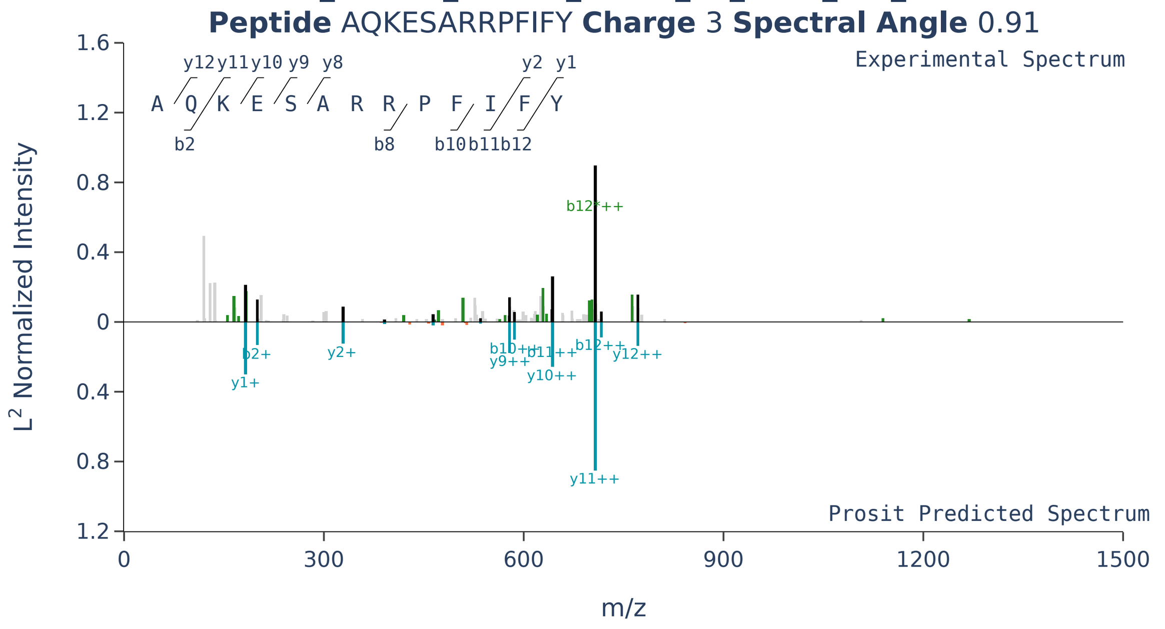


**Source** WSoh_101121_151121_HFGoe_G2_IL37b_24h_R1 **Scan** 16656
**Peptide** QSHPSLQL **Charge** 2 **Spectral Angle** 0.84

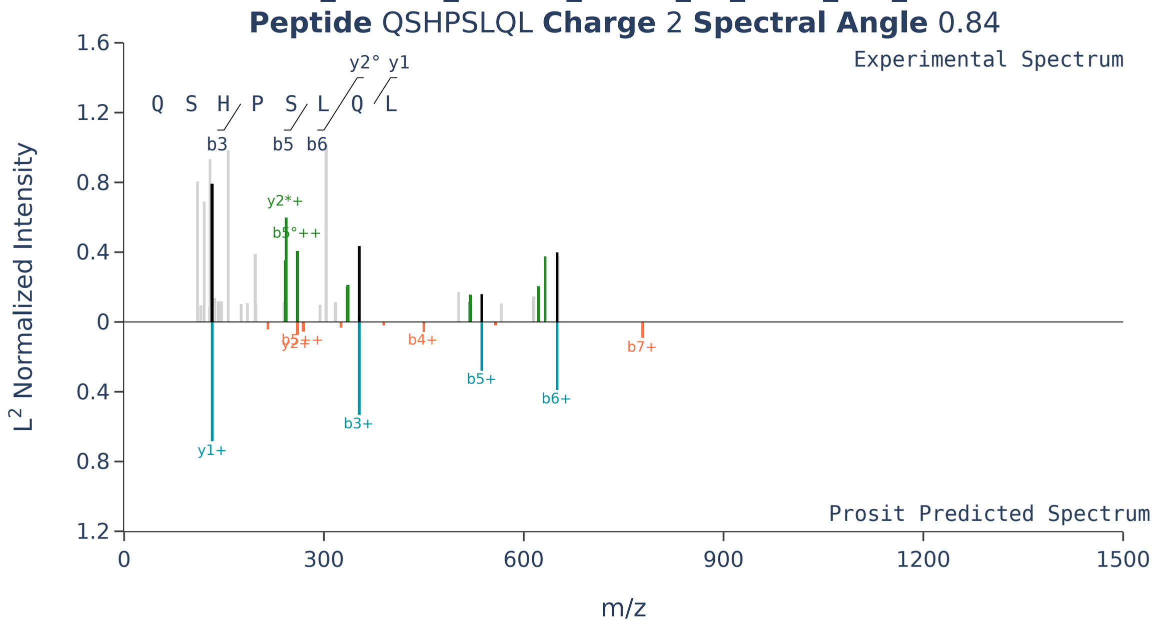


**Source** WSoh_101121_151121_HFGoe_G1_IL37b_24h_R1 **Scan** 8235
**Peptide** KGQSHPSLQLKKEKL **Charge** 4 **Spectral Angle** 0.9

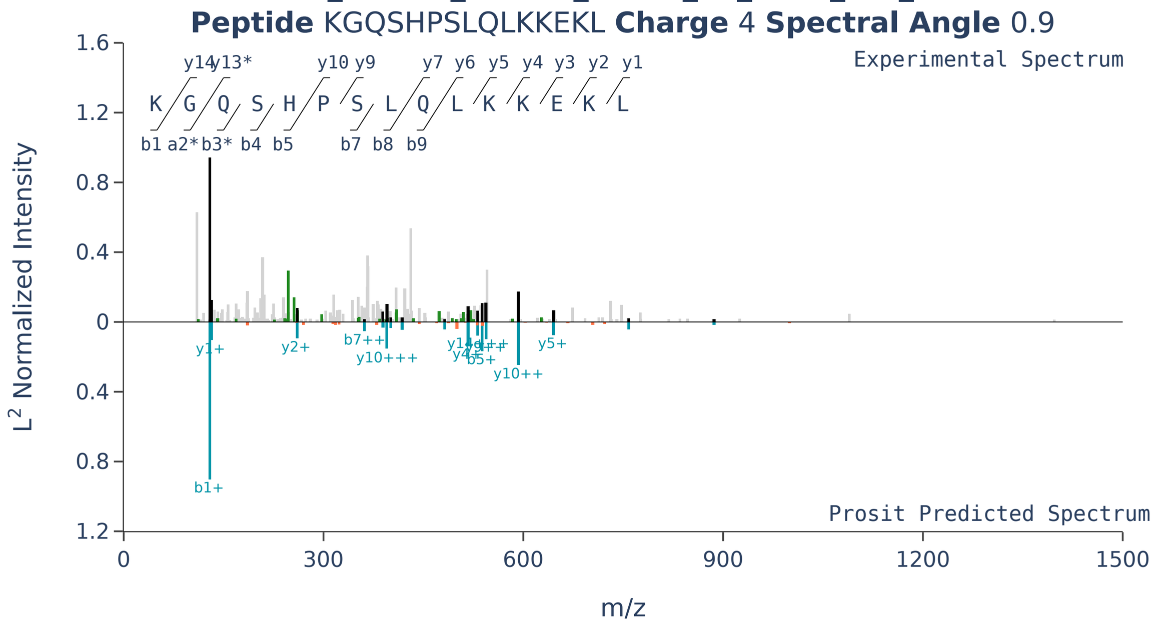

**Source** WSoh_101121_151121_HFGoe_G1_IL37b_24h_R2 **Scan** 9557
**Peptide** KEKLMKLAA **Charge** 2 **Spectral Angle** 0.81

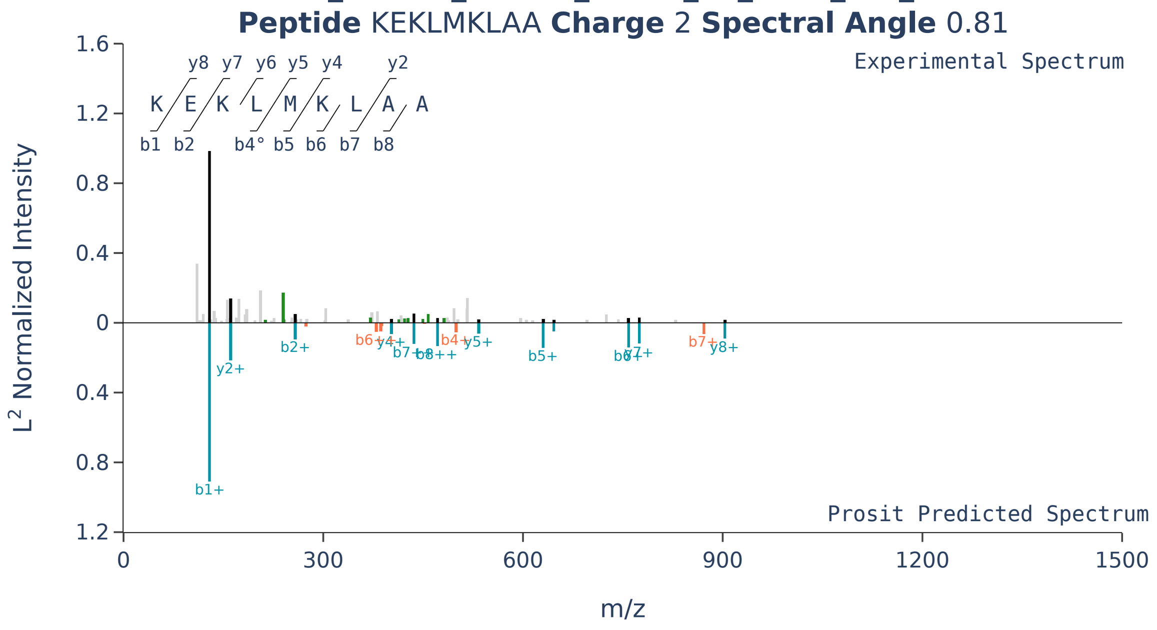


**Source** WSoh_101121_151121_HFGoe_G1_IL37b_4h_R1 **Scan** 6433
**Peptide** ASSLSSASAEK **Charge** 2 **Spectral Angle** 0.81

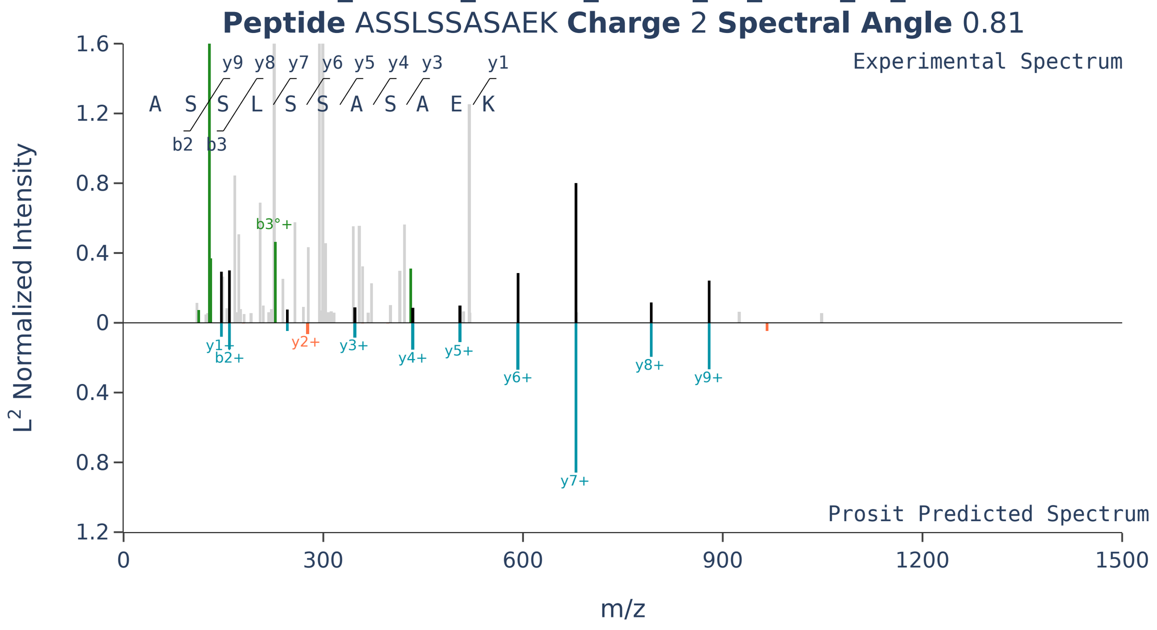


**Source** WSoh_101121_151121_HFGoe_G1_IL37b_4h_R1 **Scan** 14576
**Peptide** KESARRPFIFYRA **Charge** 4 **Spectral Angle** 0.87

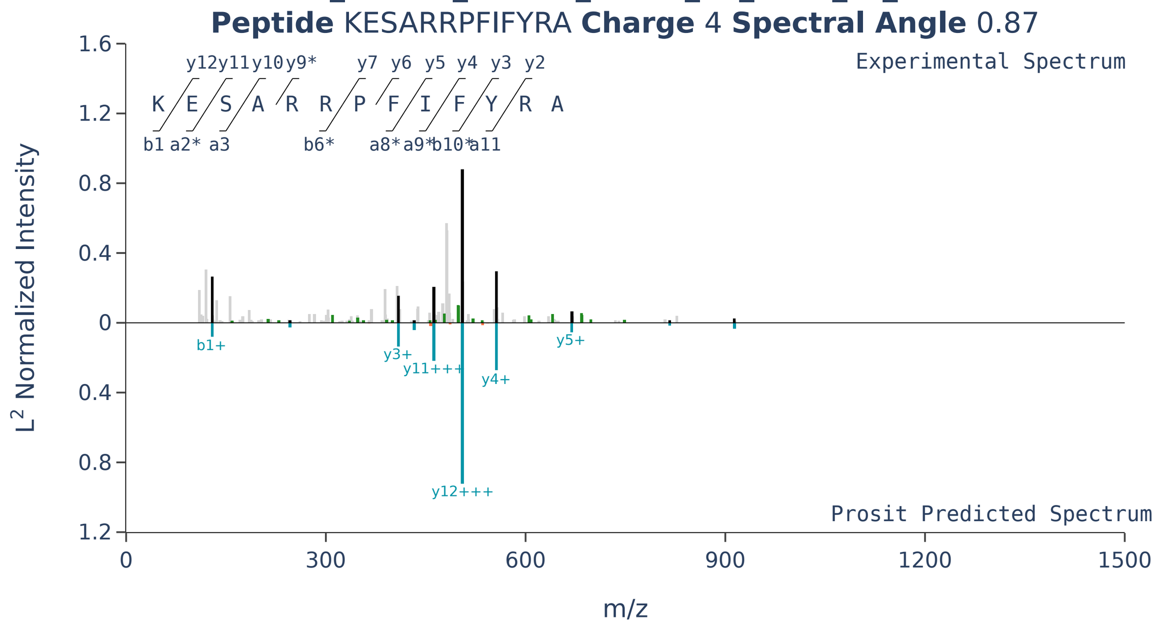


**Source** WSoh_101121_151121_HFGoe_G2_IL37b_24h_R1 **Scan** 24027
**Peptide** NLIAVPDKNYI **Charge** 2 **Spectral Angle** 0.85

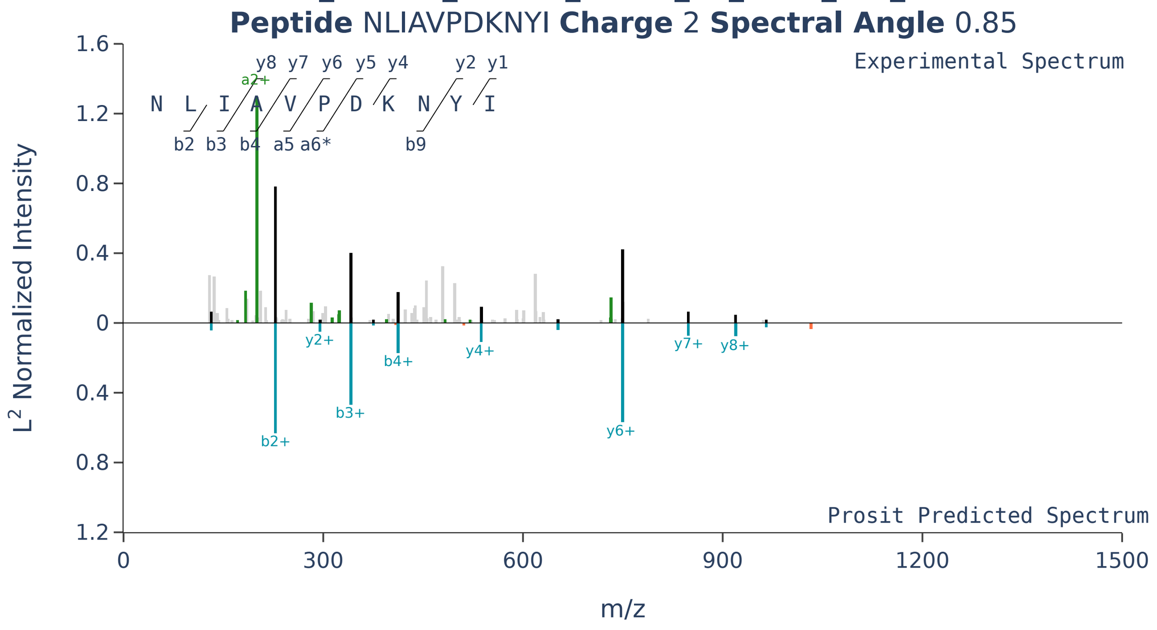


**Source** WSoh_101121_151121_HFGoe_G2_IL37b_24h_R1 **Scan** 19107
**Peptide** AEKGSPILLG **Charge** 2 **Spectral Angle** 0.89

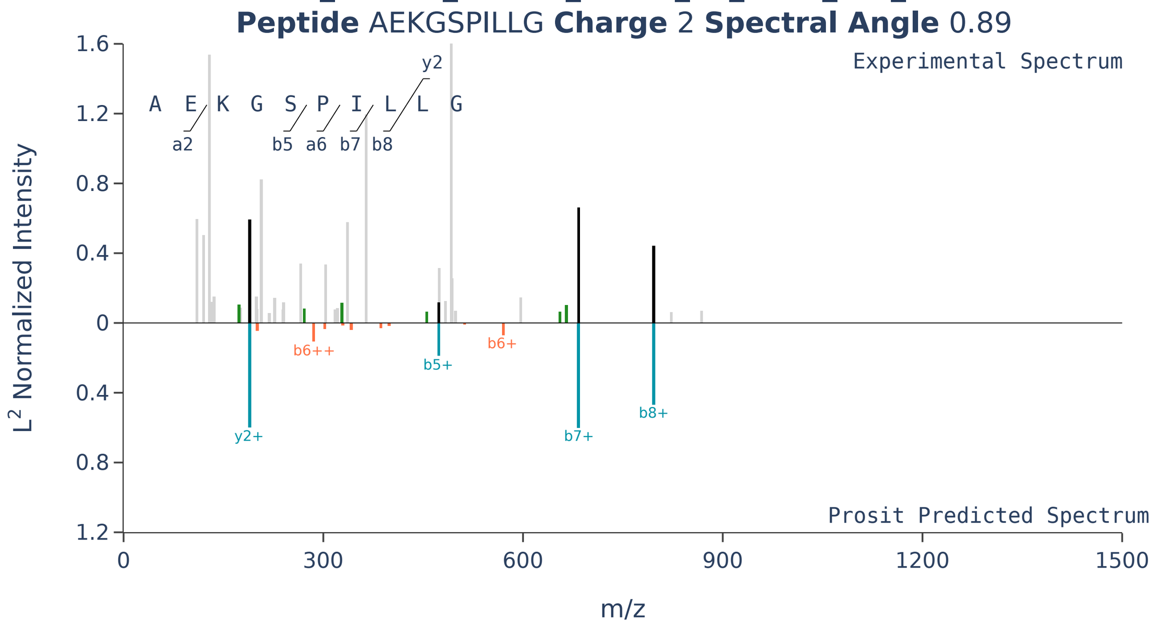


**Source** WSoh_101121_151121_HFGoe_G2_IL37b_24h_R1 **Scan** 24257
**Peptide** ESAAHPGWFIC **Charge** 2 **Spectral Angle** 0.91

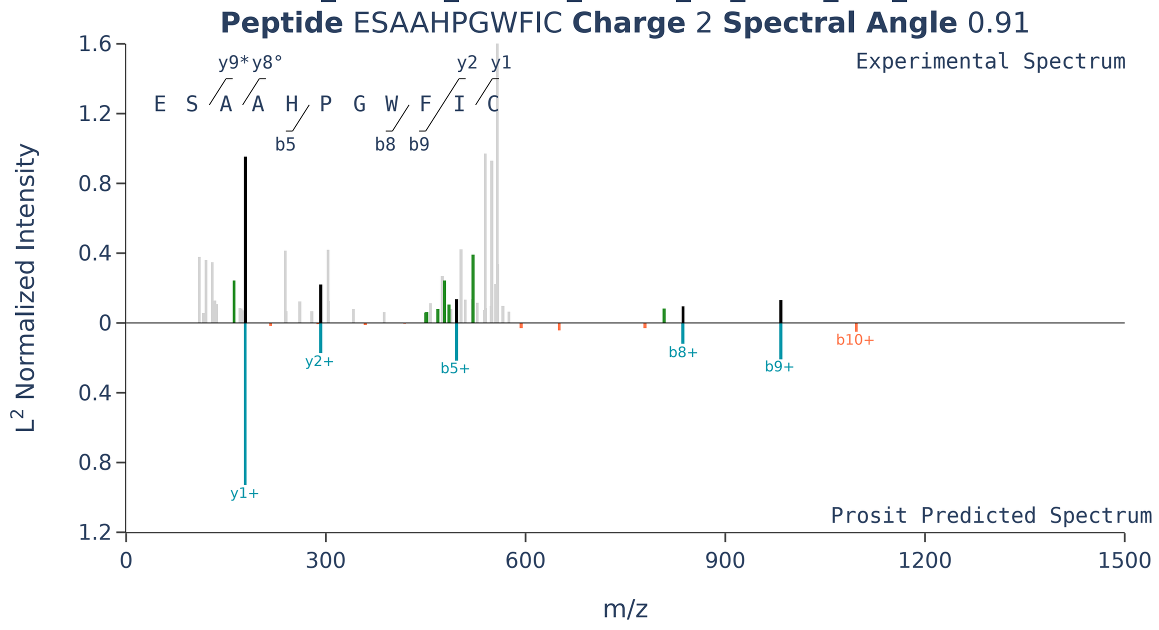


**Source** WSoh_101121_151121_HFGoe_G1_IL37b_4h_R2 **Scan** 7682
**Peptide** HTSPKVKNLNPKKF **Charge** 4 **Spectral Angle** 0.88

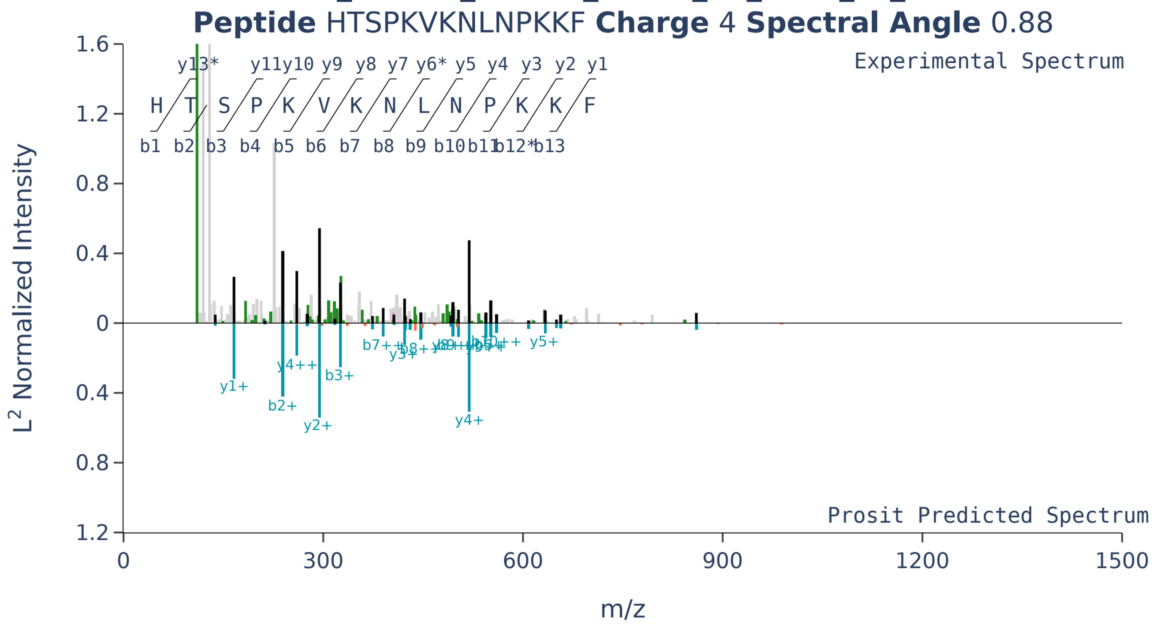


**Source** WSoh_101121_151121_HFGoe_G2_IL37b_24h_R1 **Scan** 12307
**Peptide** VHTSPKVKNLNPKKFSL **Charge** 3 **Spectral Angle** 0.9

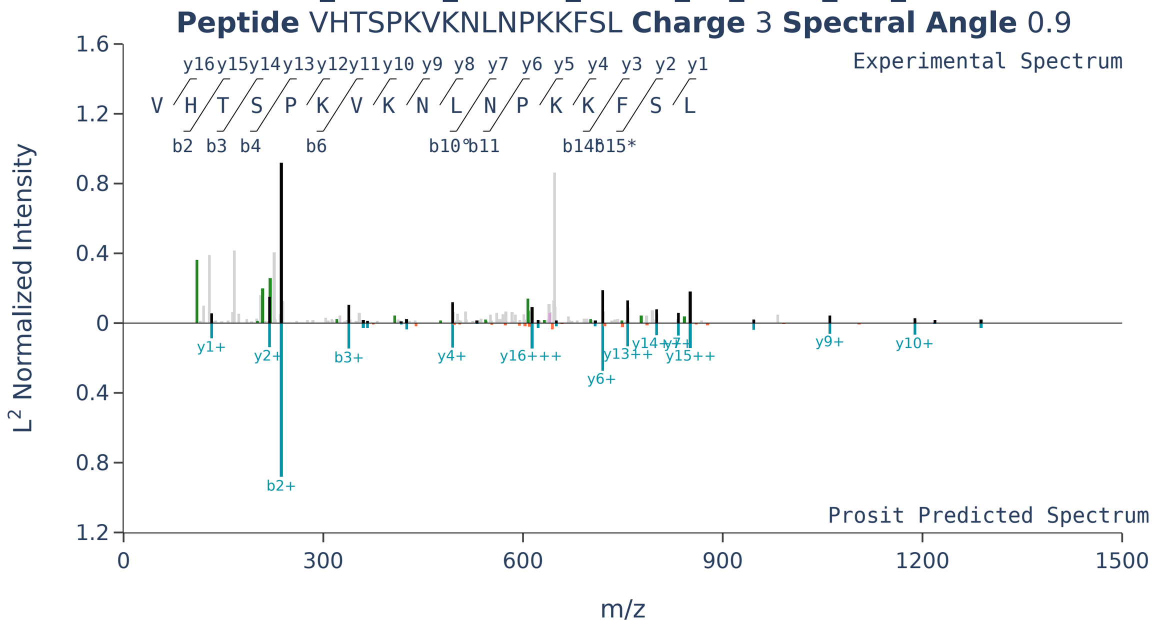


**Source** WSoh_101121_151121_HFGoe_G2_IL37b_24h_R2 **Scan** 12543
**Peptide** EFCLYCDKDKGQSH **Charge** 4 **Spectral Angle** 0.77

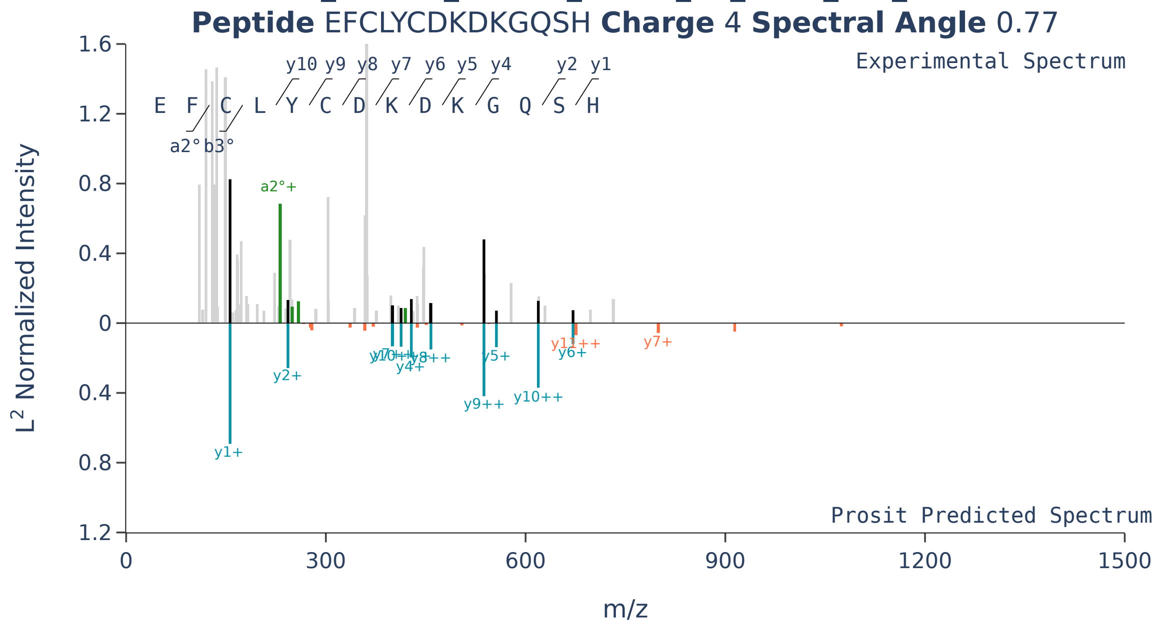


**Source** WSoh_101121_151121_HFGoe_G2_IL37b_24h_R1 **Scan** 6723
**Peptide** KGQSHPSLQLKKE **Charge** 2 **Spectral Angle** 0.87

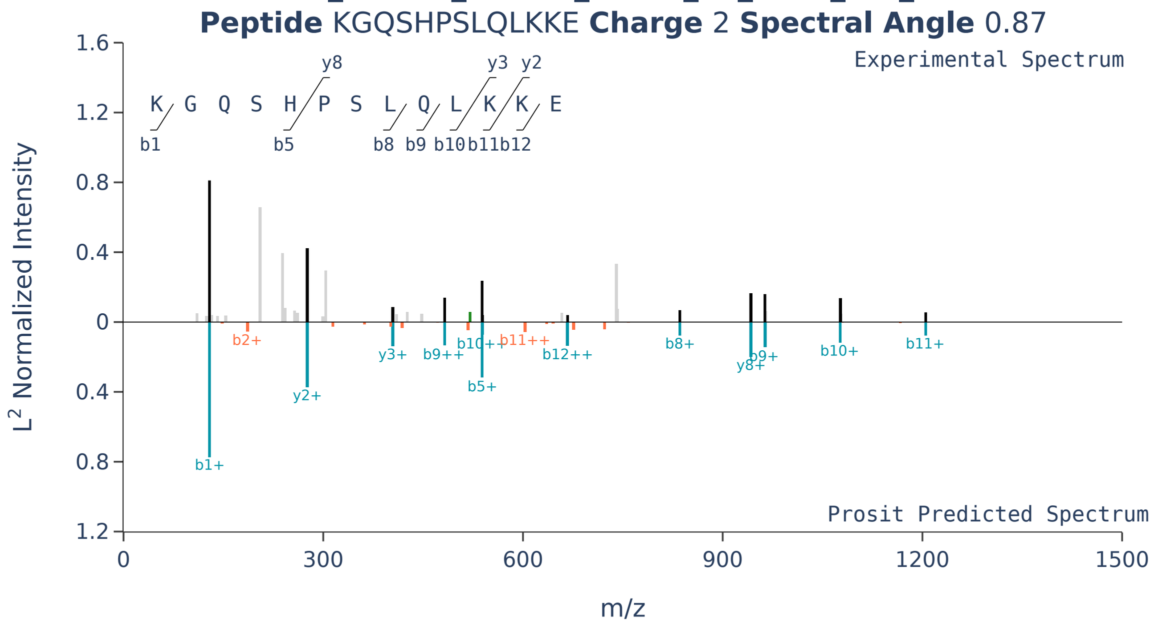


**Source** WSoh_101121_151121_HFGoe_G1_IL37b_4h_R2 **Scan** 19723
**Peptide** AAHPGWF **Charge** 2 **Spectral Angle** 0.85

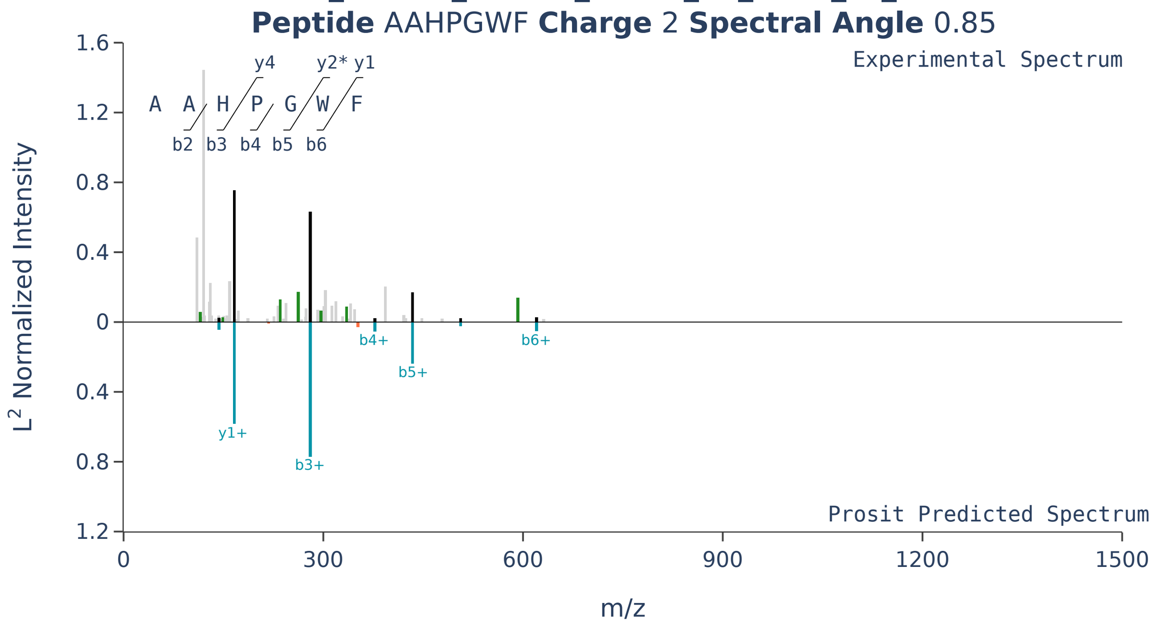


**Source** WSoh_101121_151121_HFGoe_G2_IL37b_24h_R1 **Scan** 25228
**Peptide** AAHPGWFI **Charge** 2 **Spectral Angle** 0.86

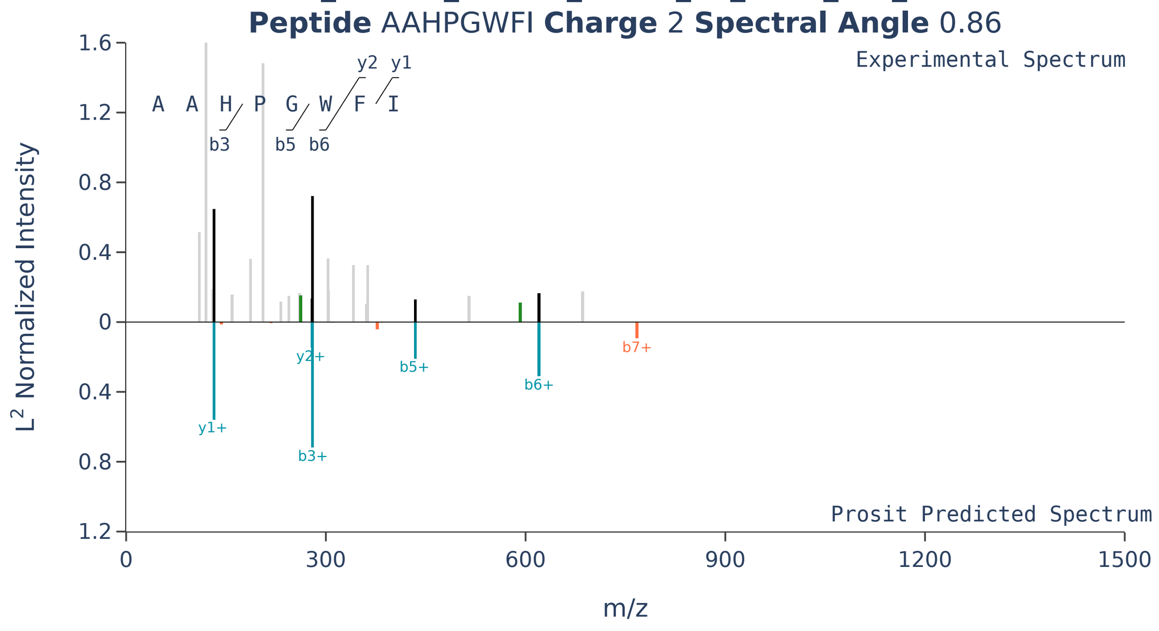


**Source** WSoh_101121_151121_HFGoe_G1_IL37b_24h_R2 **Scan** 21681
**Peptide** VPDKNYIRPEIF **Charge** 2 **Spectral Angle** 0.82

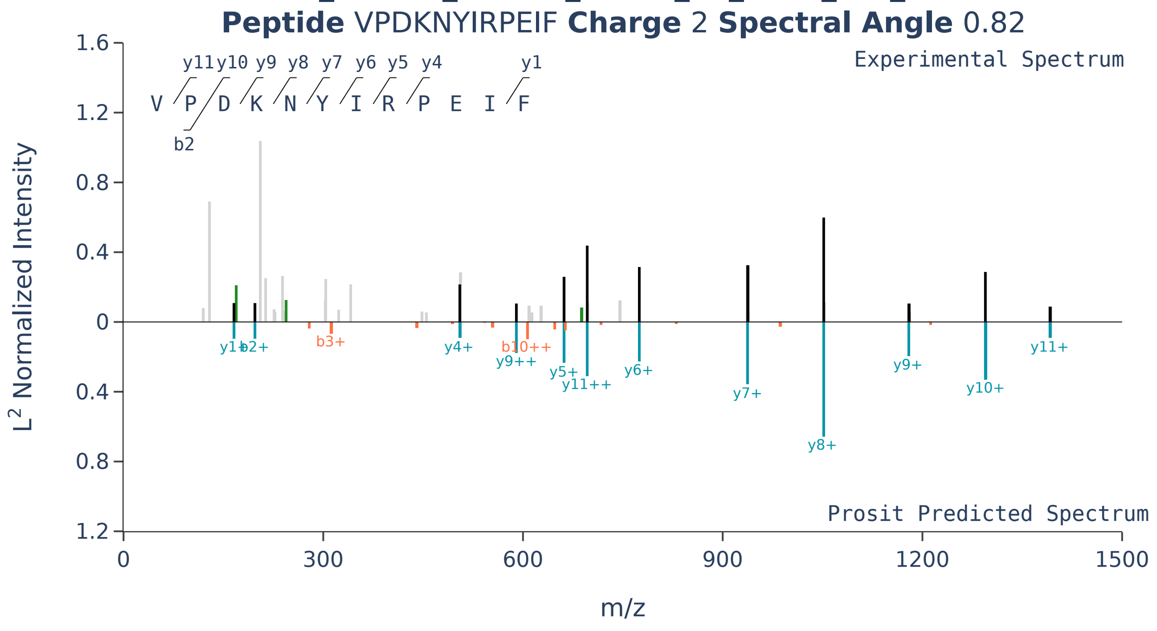


**Source** WSoh_101121_151121_HFGoe_G1_IL37b_4h_R1 **Scan** 6470
**Peptide** KKEKLMKLAAQKE **Charge** 3 **Spectral Angle** 0.88

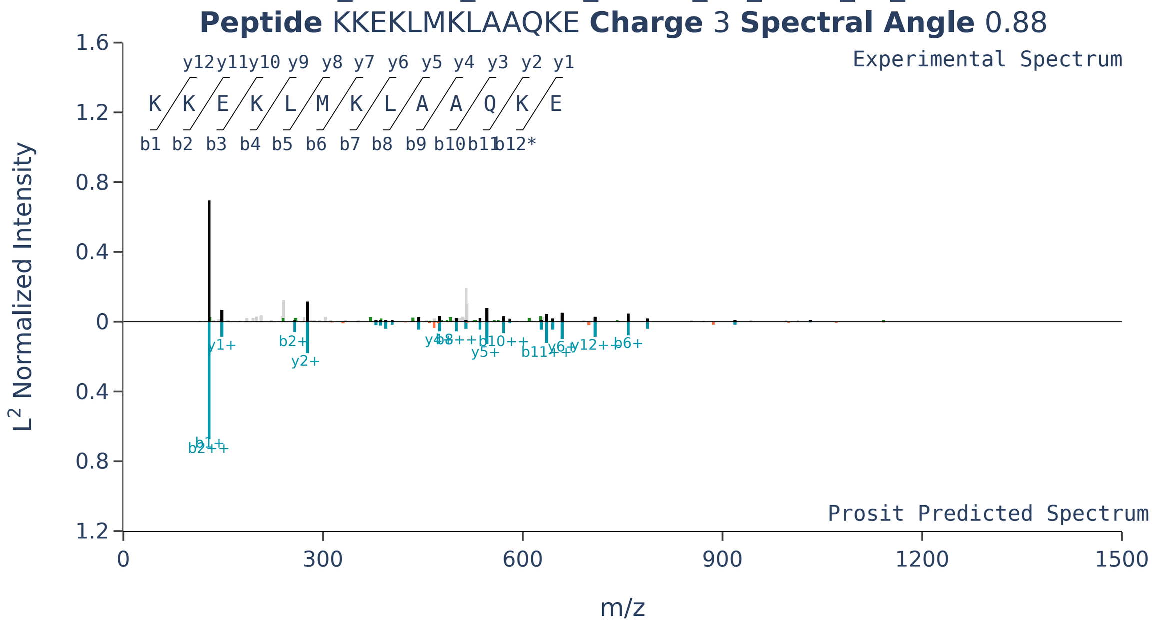


**Source** WSoh_101121_151121_HFGoe_G2_IL37b_4h_R1 **Scan** 13179
**Peptide** ALASSLSS **Charge** 1 **Spectral Angle** 0.95

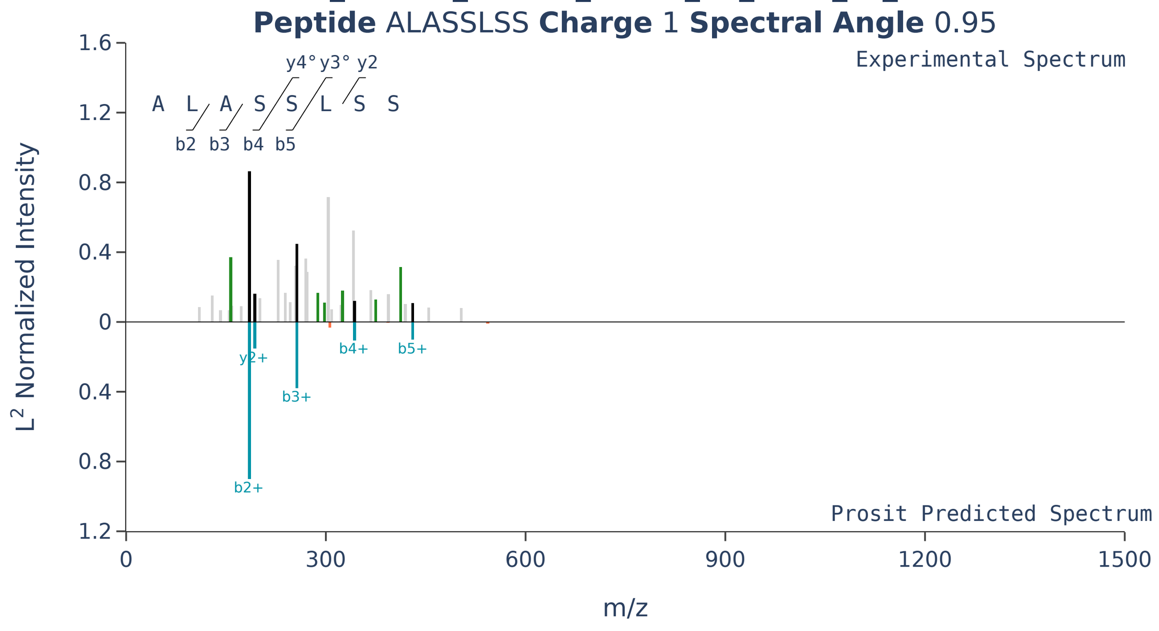

**Source** WSoh_101121_151121_HFGoe_G1_IL37b_24h_R1 **Scan** 17666
**Peptide** SSASAEKGSPILLGVSKGE **Charge** 3 **Spectral Angle** 0.88

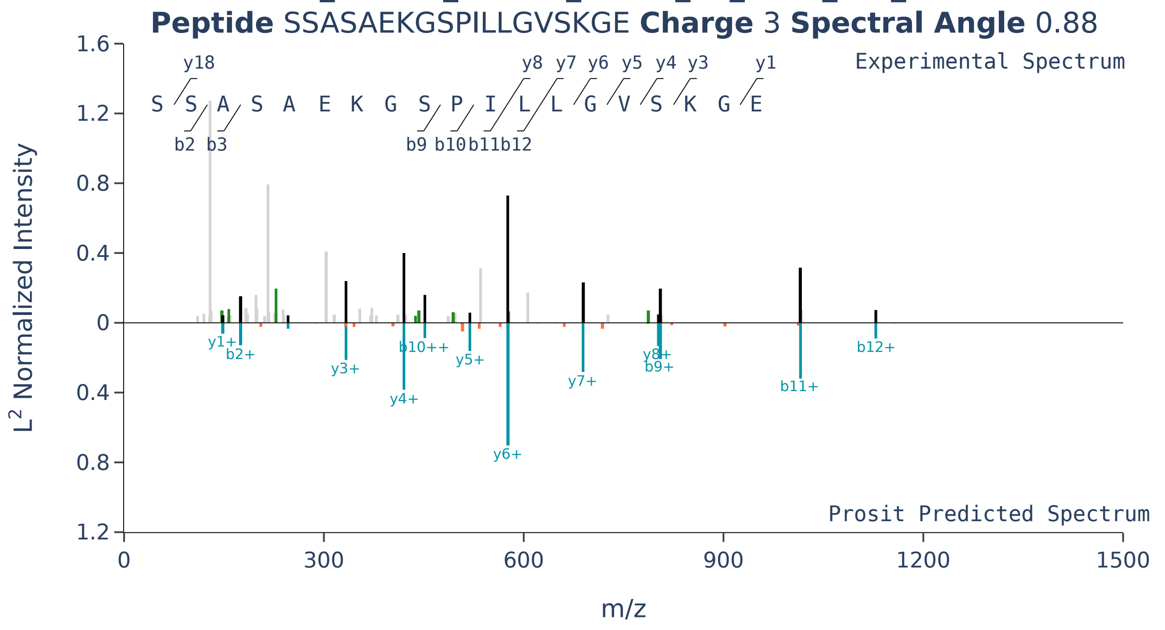

**Source** WSoh_101121_151121_HFGoe_G2_IL37b_24h_R2 **Scan** 5615
**Peptide** QLKKEKLM **Charge** 2 **Spectral Angle** 0.85

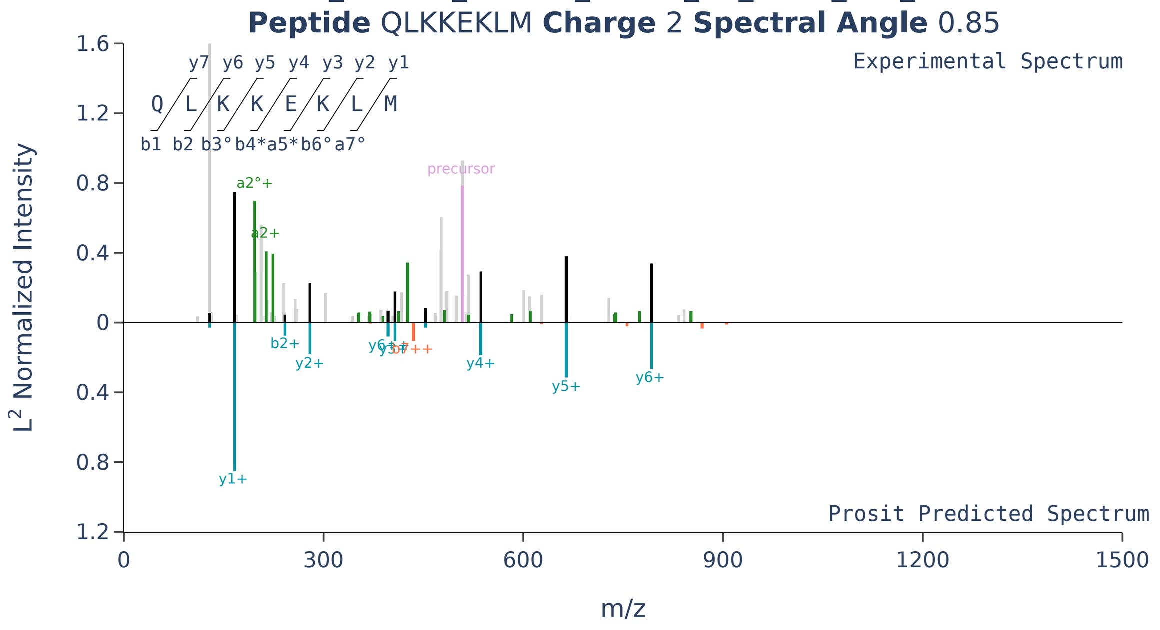

**Source** WSoh_101121_151121_HFGoe_G2_IL37b_2h_R2 **Scan** 19274
**Peptide** FSFQPVCKA **Charge** 2 **Spectral Angle** 0.88

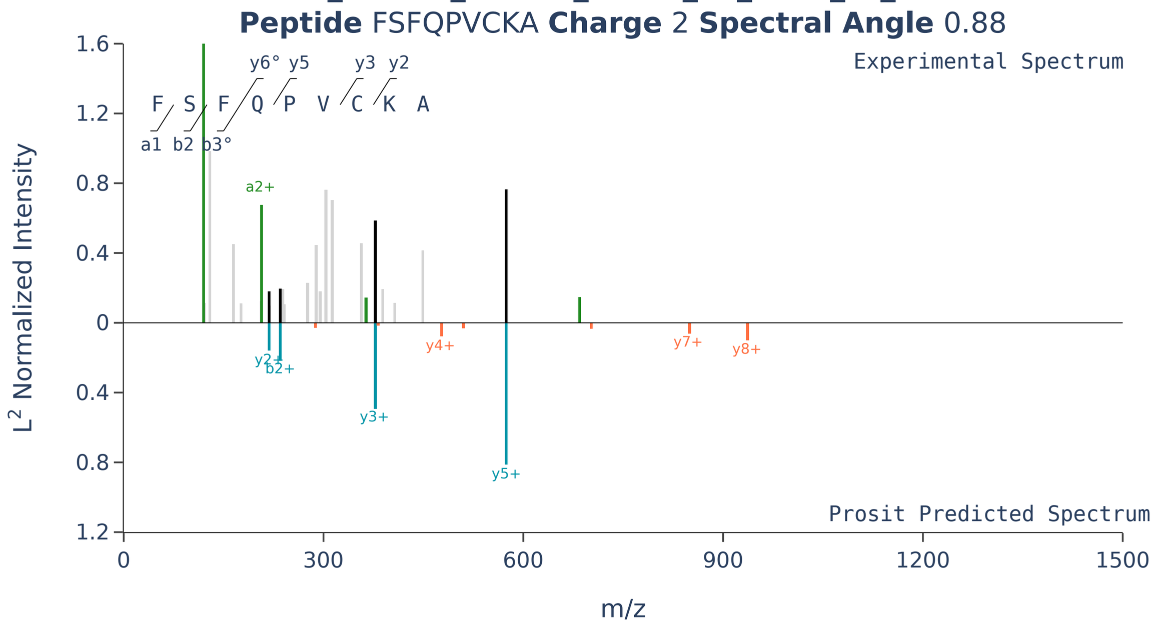

**Source** WSoh_101121_151121_HFGoe_G1_IL37b_24h_R2 **Scan** 11853
**Peptide** HTSPKVKNLNPKKFSI **Charge** 4 **Spectral Angle** 0.83

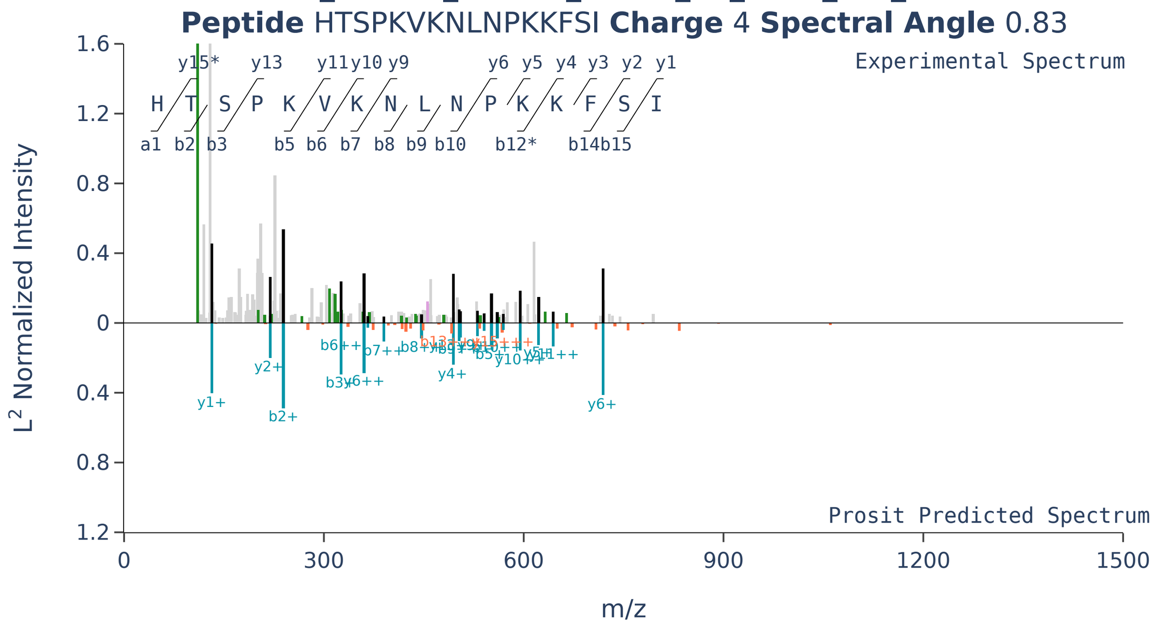

**Source** WSoh_101121_151121_HFGoe_G2_IL37b_2h_R2 **Scan** 7320
**Peptide** PKVKNLNPKKF **Charge** 2 **Spectral Angle** 0.63

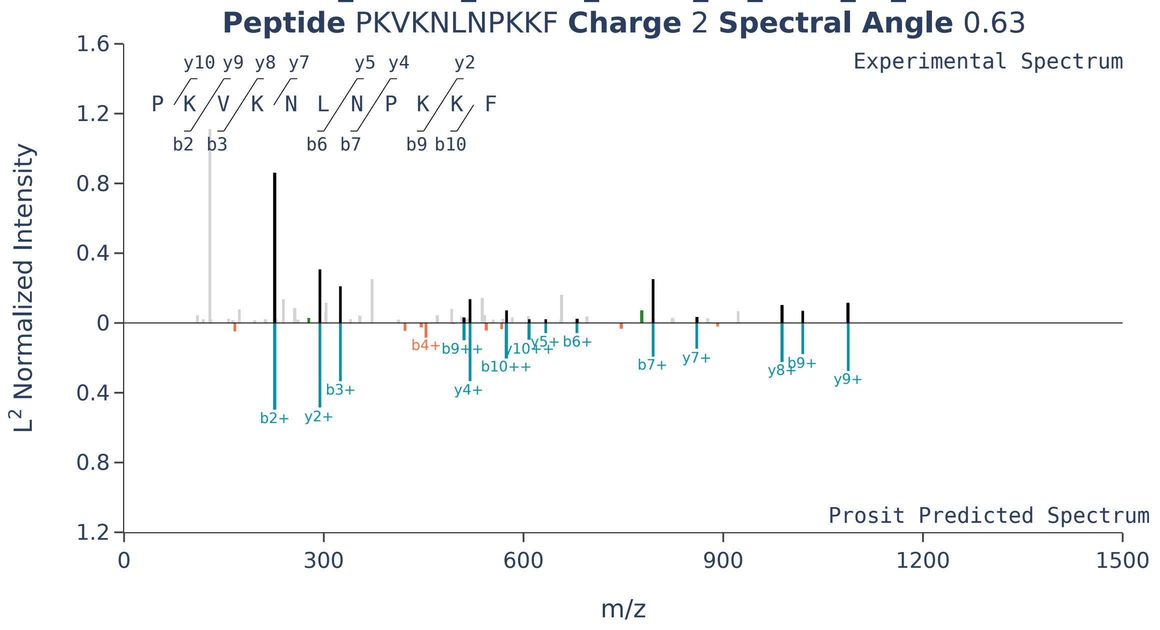

**Source** WSoh_101121_151121_HFGoe_G1_IL37b_24h_R1 **Scan** 24068
**Peptide** SASAEKGSPILLGV **Charge** 2 **Spectral Angle** 0.9

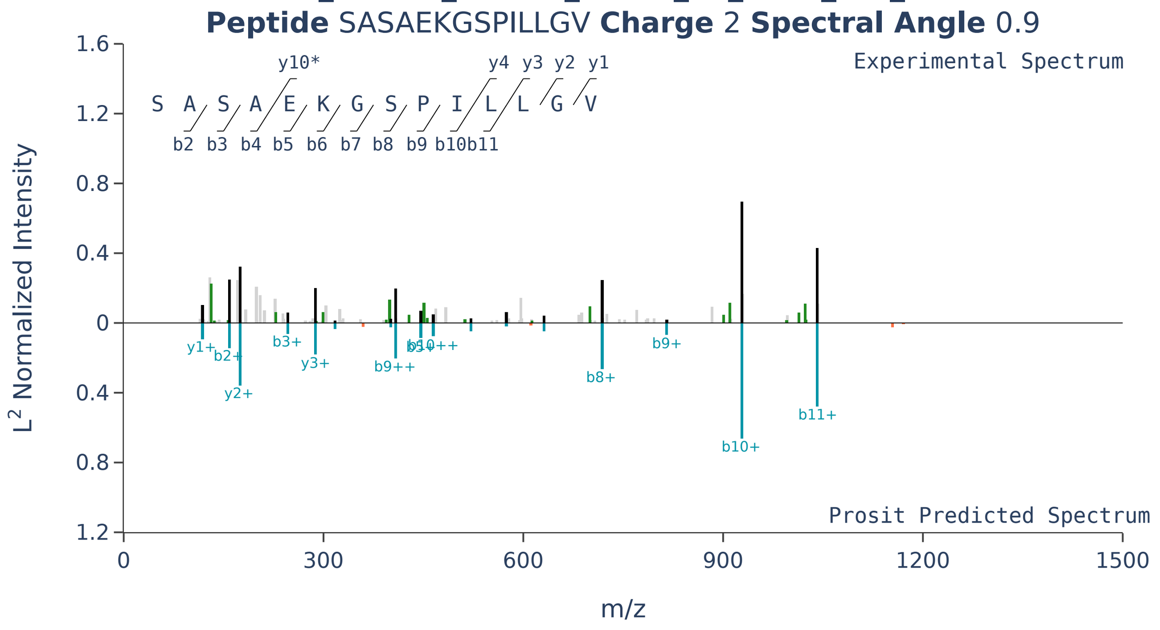

**Source** WSoh_101121_151121_HFGoe_G1_IL37b_4h_R1 **Scan** 13181
**Peptide** HDQDHKVLVLDS **Charge** 2 **Spectral Angle** 0.81

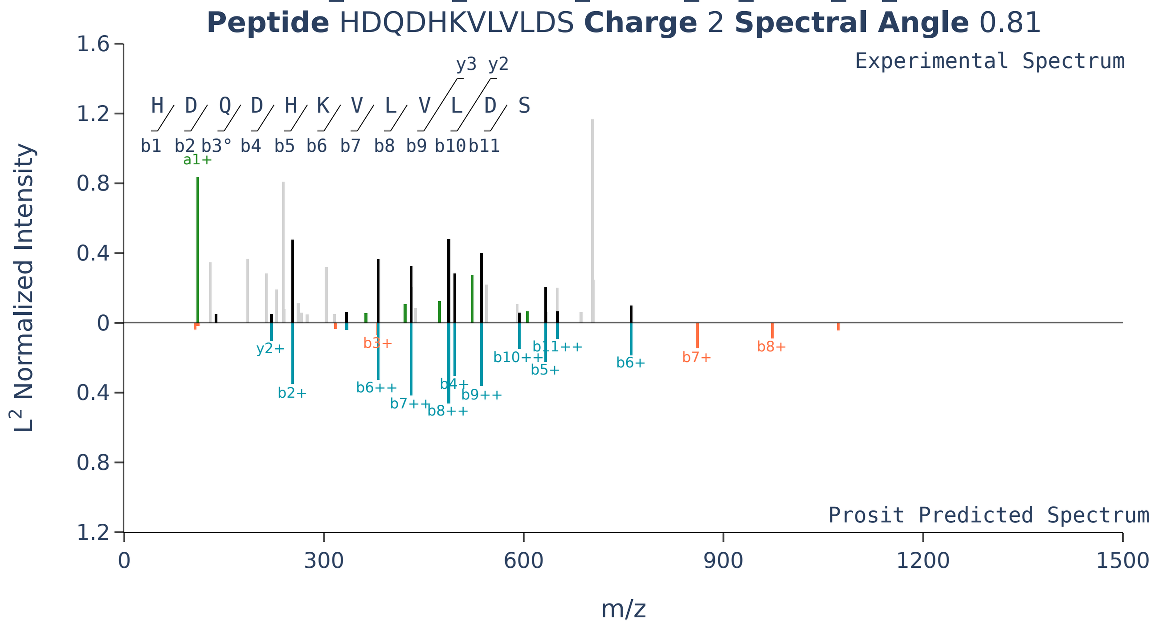

**Source** WSoh_101121_151121_HFGoe_G1_IL37b_1h_R2 **Scan** 6414
**Peptide** KNLNPKKF **Charge** 2 **Spectral Angle** 0.85

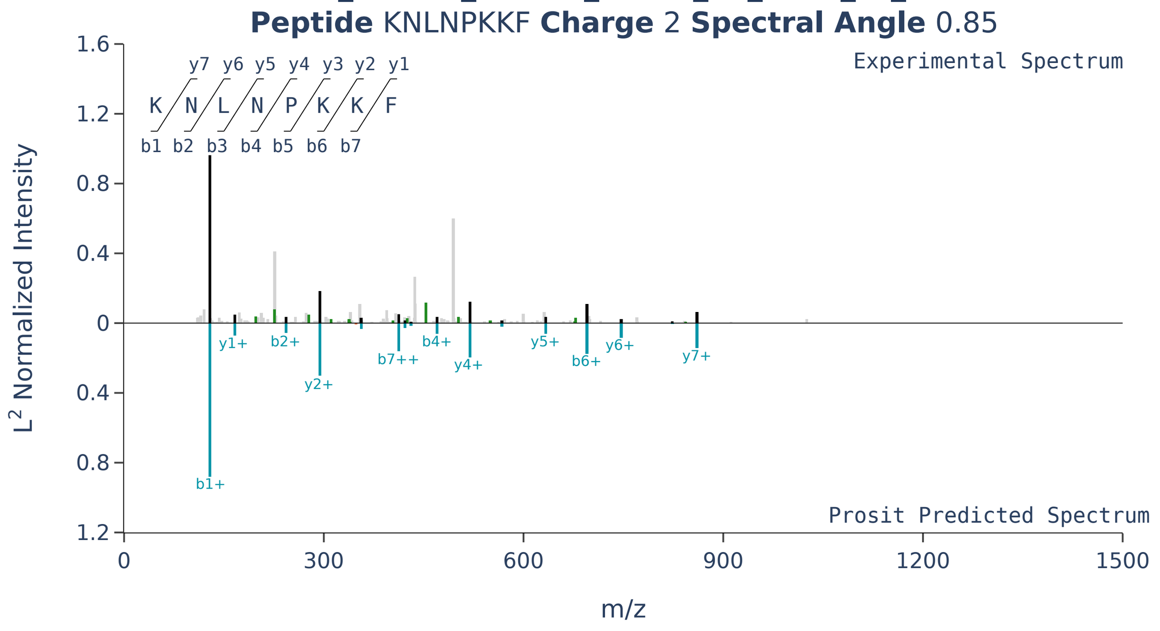

**Source** WSoh_101121_151121_HFGoe_G1_IL37b_24h_R2 **Scan** 7489
**Peptide** NPKKFSIHD **Charge** 2 **Spectral Angle** 0.78

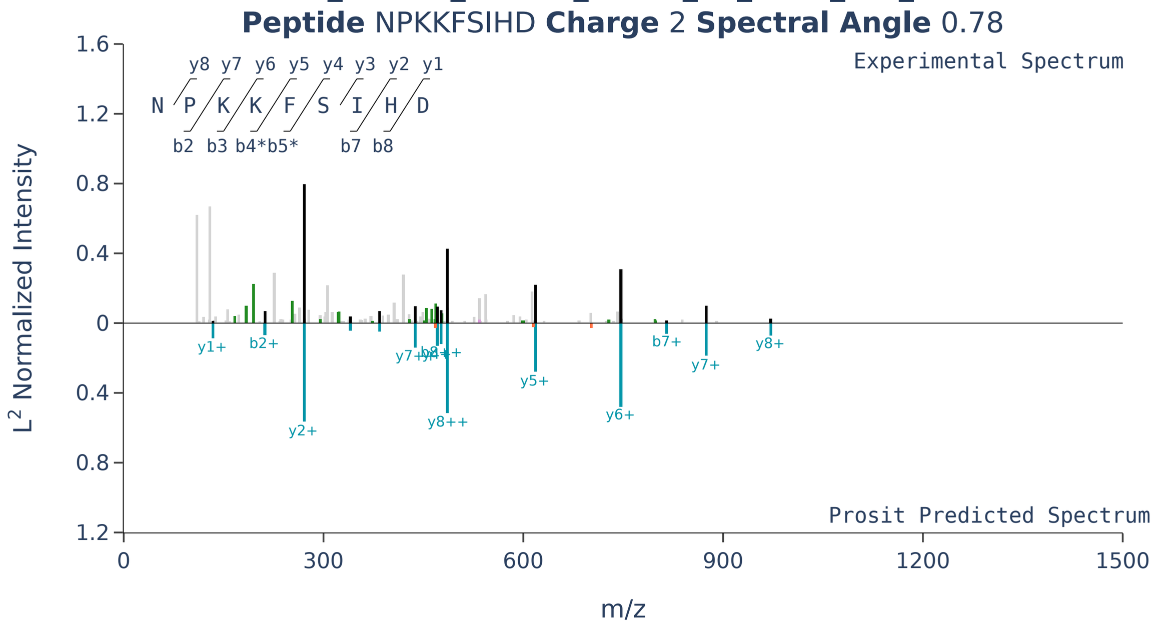

**Source** WSoh_101121_151121_HFGoe_G2_IL37b_24h_R2 **Scan** 15565
**Peptide** SLHDQDHKVLVL **Charge** 4 **Spectral Angle** 0.81

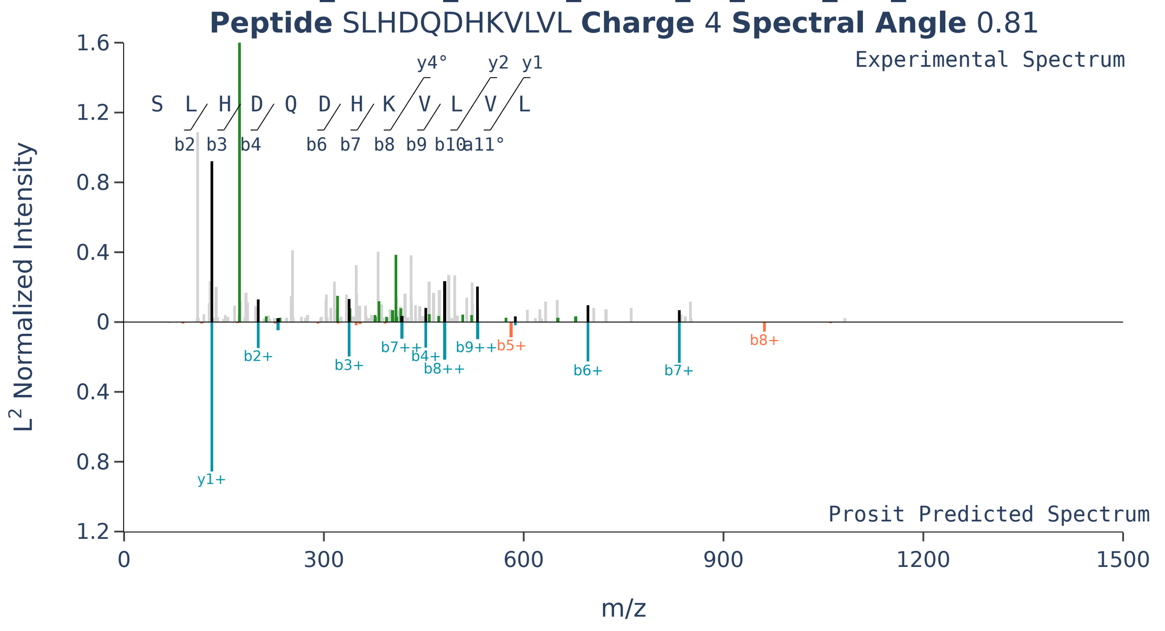

**Source** WSoh_101121_151121_HFGoe_G2_IL37b_4h_R1 **Scan** 11243
**Peptide** NPKKFSI **Charge** 2 **Spectral Angle** 0.84

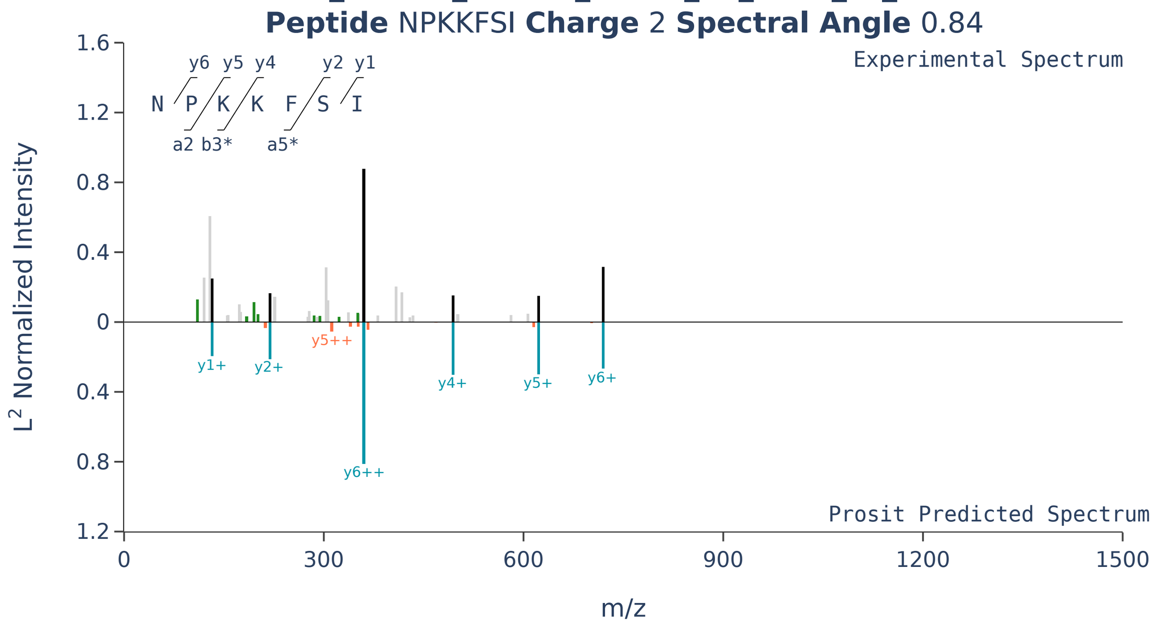

**Source** WSoh_101121_151121_HFGoe_G2_IL37b_24h_R1 **Scan** 16269
**Peptide** AAQKESARRPFIF **Charge** 3 **Spectral Angle** 0.9

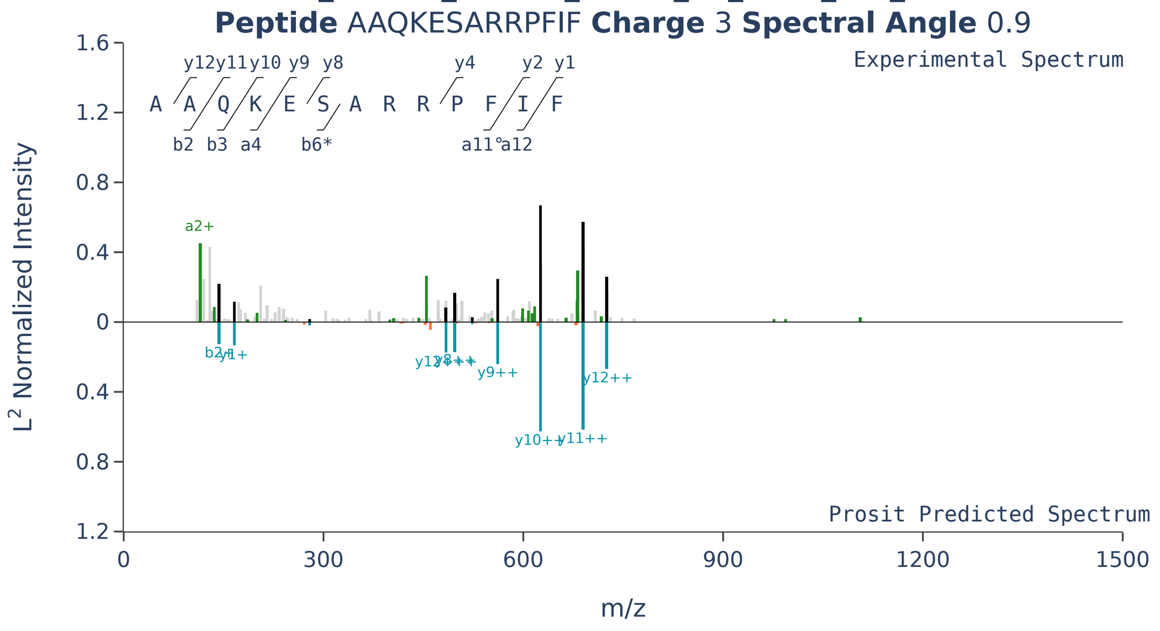

**Source** WSoh_101121_151121_HFGoe_G1_IL37b_24h_R1 **Scan** 11492
**Peptide** TDKFENRKHIEF **Charge** 3 **Spectral Angle** 0.85

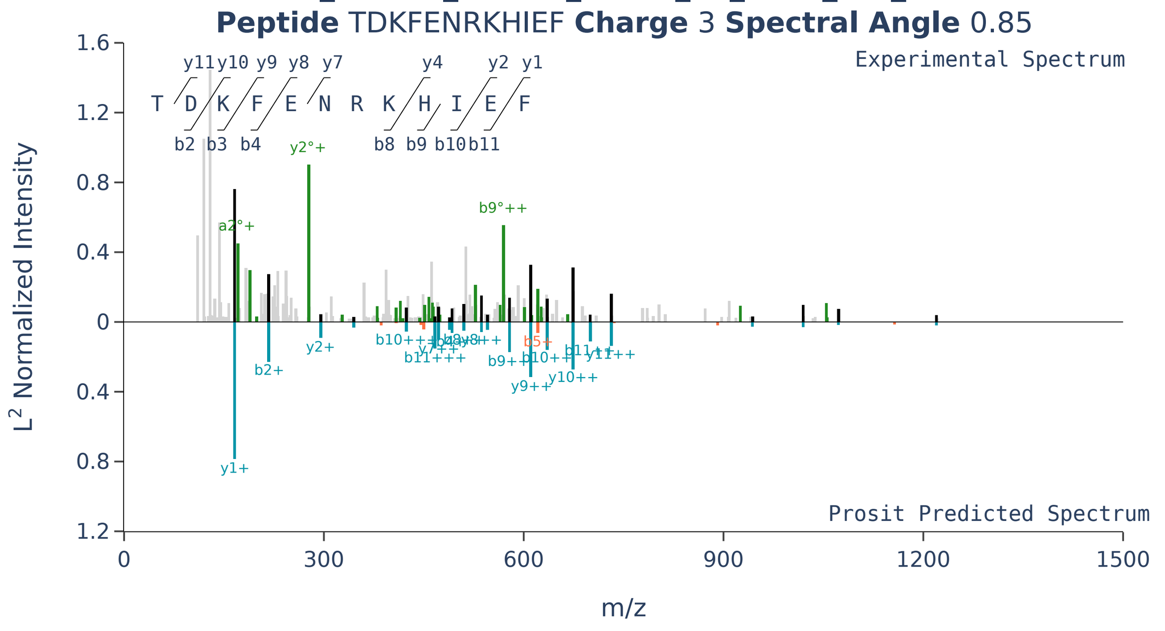

**Source** WSoh_101121_151121_HFGoe_G1_IL37b_2h_R1 **Scan** 14537
**Peptide** SLHDQDHKVLVLD **Charge** 4 **Spectral Angle** 0.83

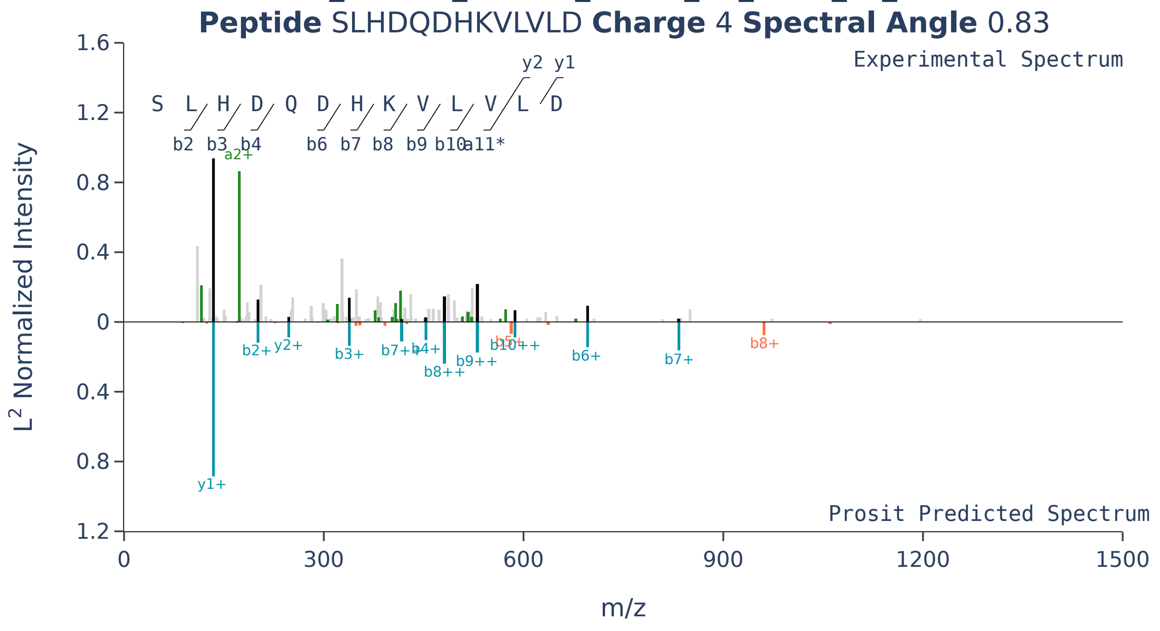

**Source** WSoh_101121_151121_HFGoe_G1_IL37b_24h_R2 **Scan** 21489
**Peptide** VLDSGNLIAVPDKN **Charge** 2 **Spectral Angle** 0.92

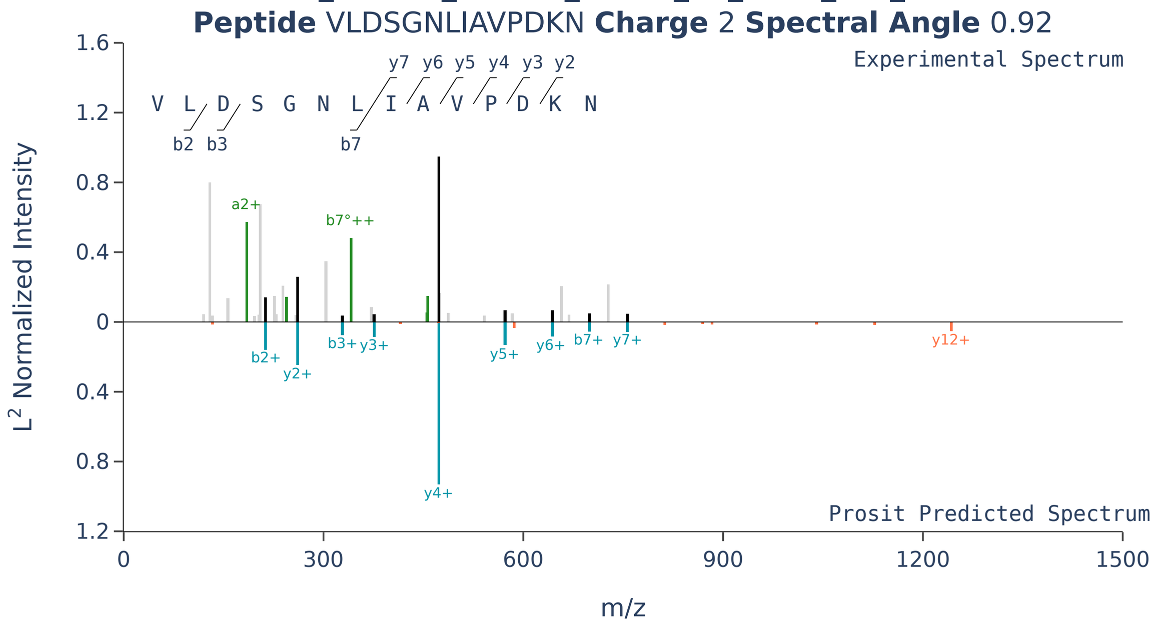

**Source** WSoh_101121_151121_HFGoe_G2_IL37b_24h_R2 **Scan** 13661
**Peptide** EKGSPIL **Charge** 2 **Spectral Angle** 0.93

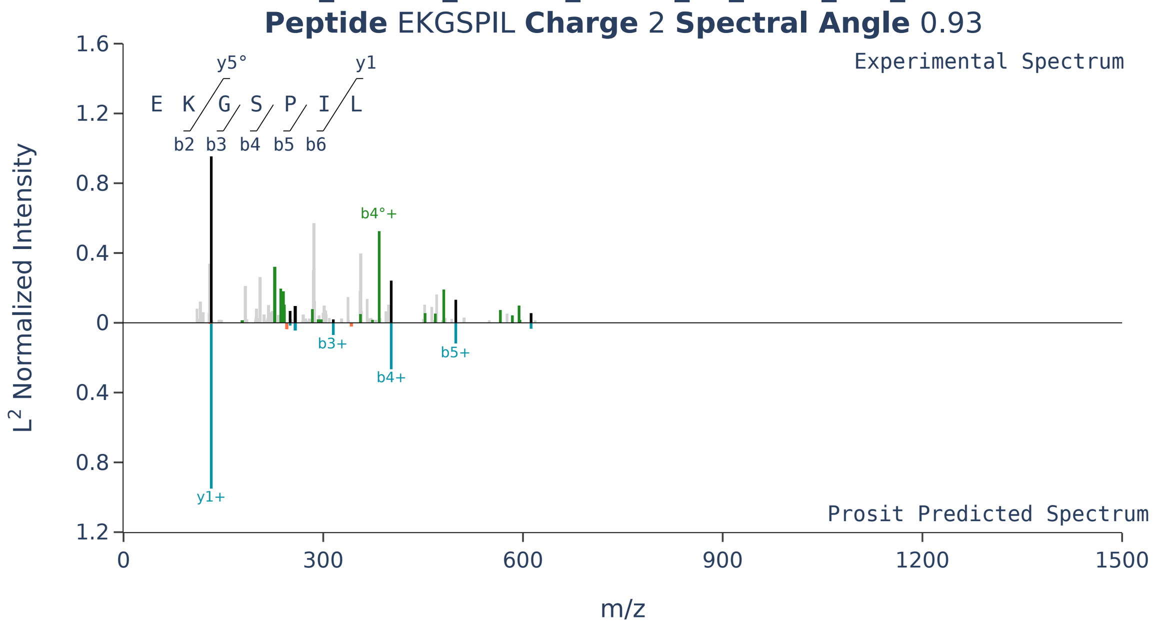


**Source** WSoh_101121_151121_HFGoe_G1_IL37b_24h_R1 **Scan** 18165
**Peptide** SLSSASAEKGSPIL **Charge** 2 **Spectral Angle** 0.86

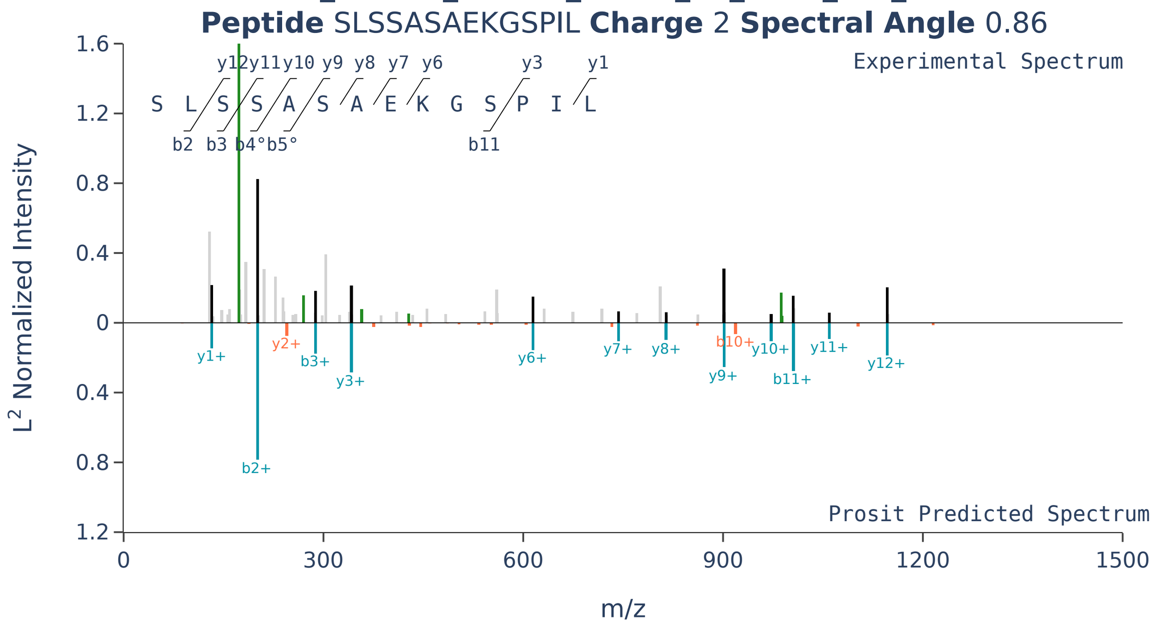


**Source** WSoh_101121_151121_HFGoe_G1_IL37b_24h_R2 **Scan** 11358
**Peptide** GVSKGEFCLYCDKDKGQSH **Charge** 4 **Spectral Angle** 0.83

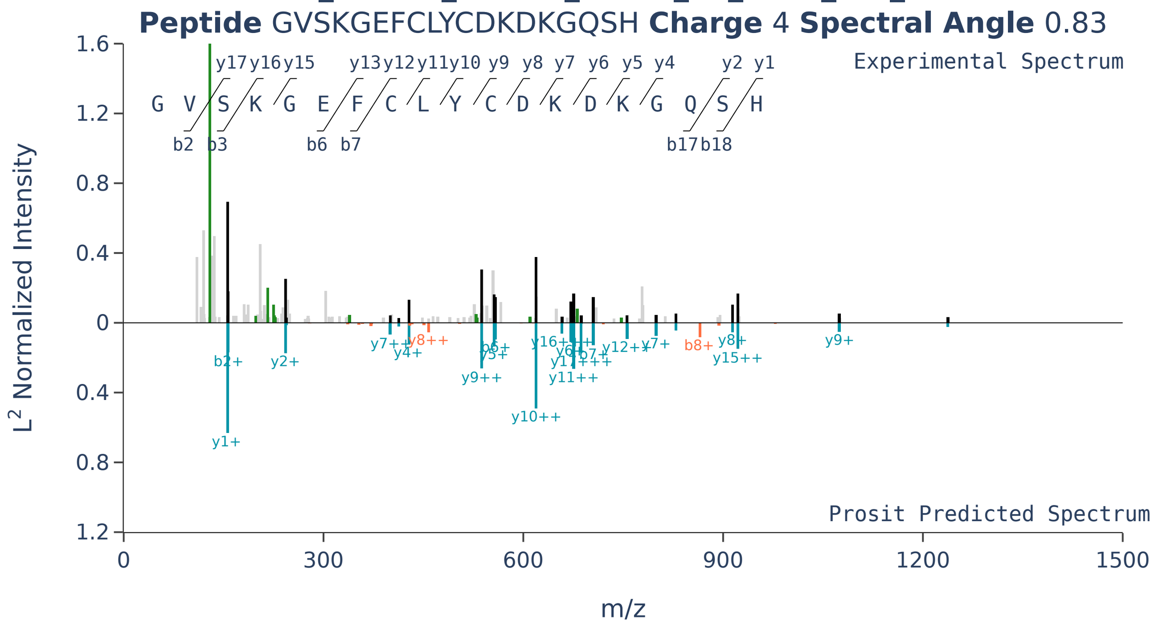


**Source** WSoh_101121_151121_HFGoe_G2_IL37b_24h_R1 **Scan** 15506
**Peptide** ARRPFIFYR **Charge** 3 **Spectral Angle** 0.81

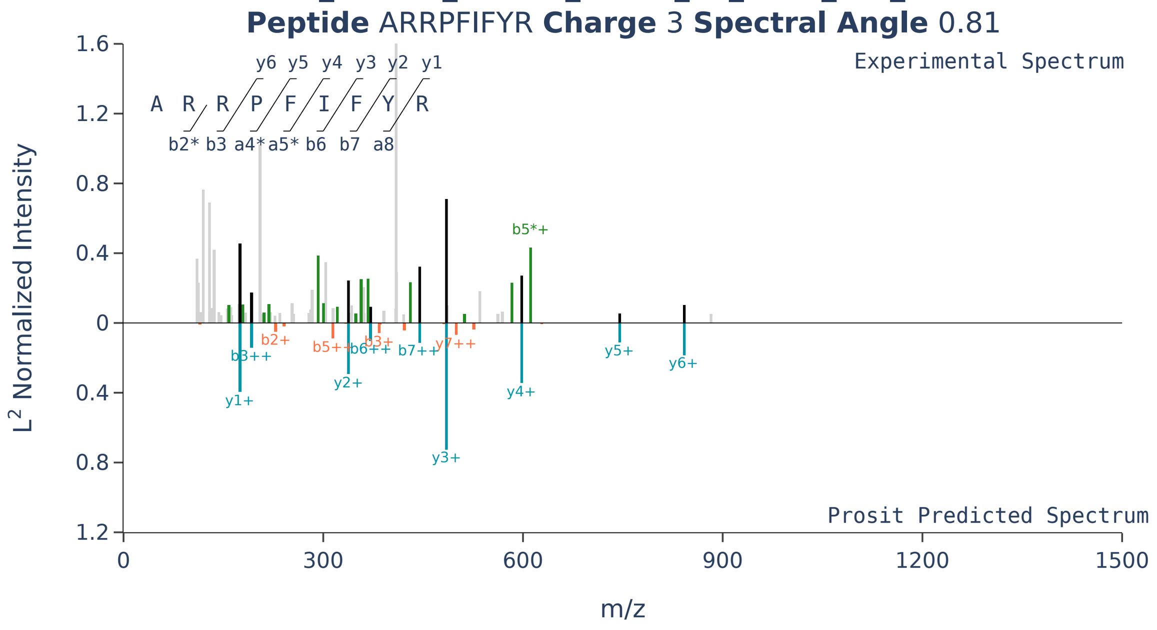


**Source** WSoh_101121_151121_HFGoe_G1_IL37b_4h_R1 **Scan** 6135
**Peptide** KKEKLMKLAAQKESAR **Charge** 4 **Spectral Angle** 0.94

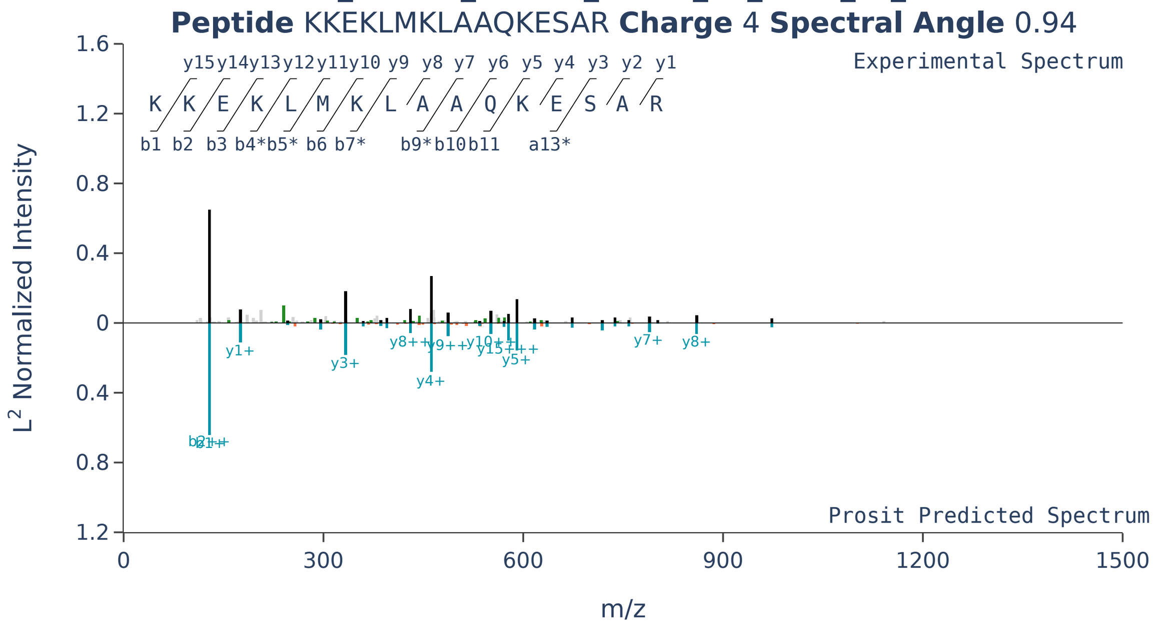


**Source** WSoh_101121_151121_HFGoe_G1_IL37b_4h_R2 **Scan** 9779
**Peptide** PVGVTDKFENRKHLE **Charge** 3 **Spectral Angle** 0.87

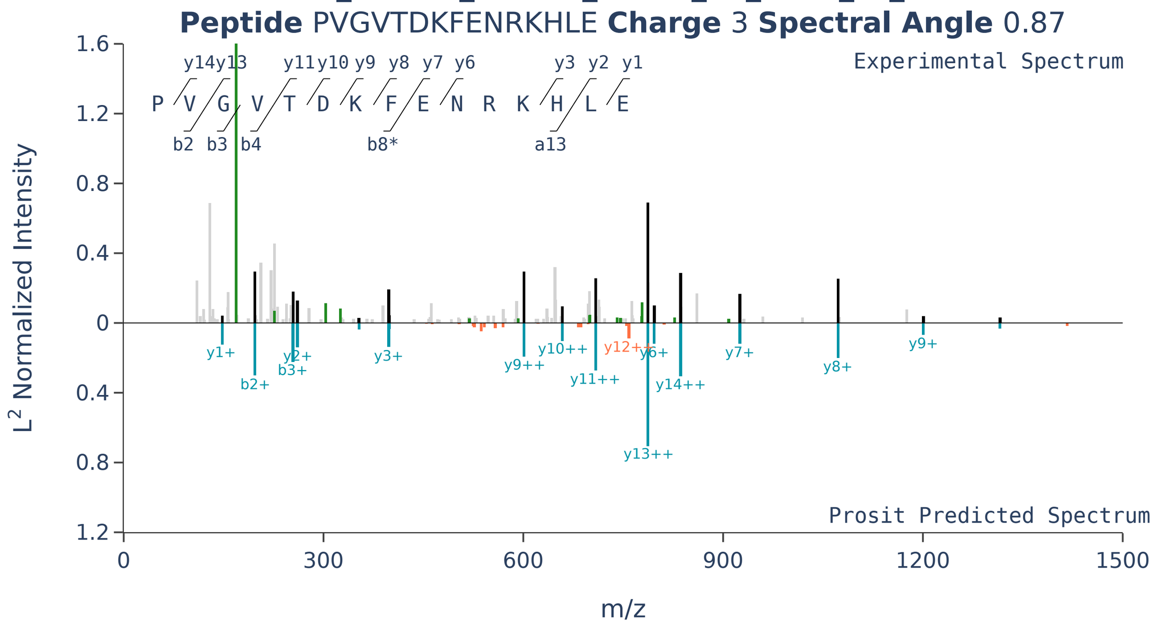


**Source** WSoh_101121_151121_HFGoe_G2_IL37b_24h_R2 **Scan** 5683
**Peptide** DKFENRKHIE **Charge** 3 **Spectral Angle** 0.74

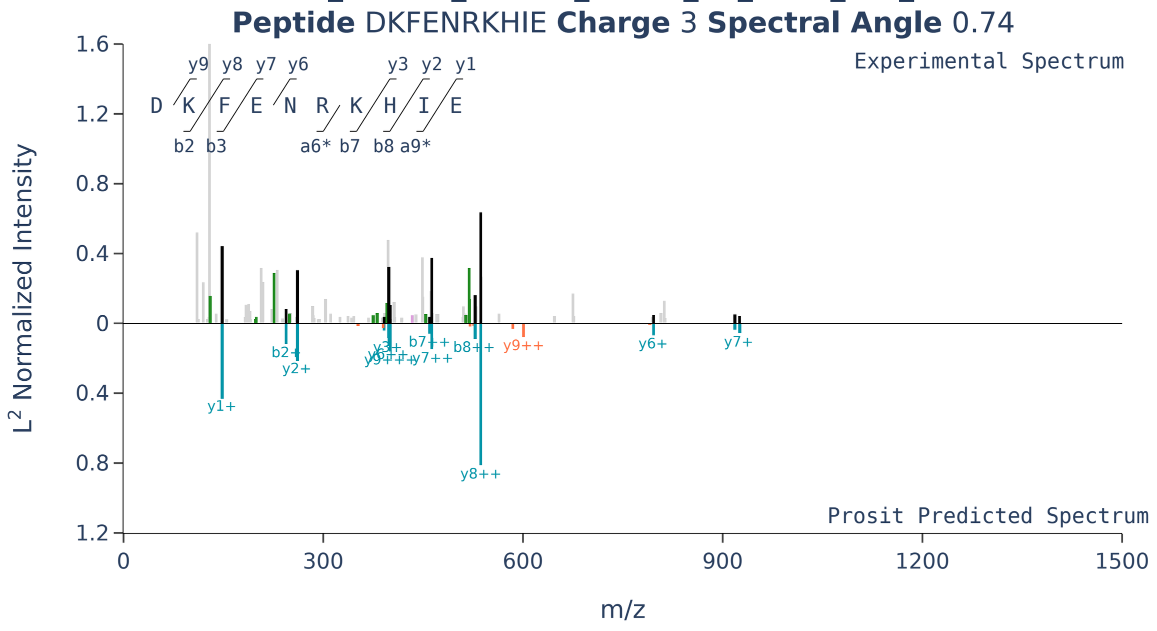


**Source** WSoh_101121_151121_HFGoe_G1_IL37b_24h_R1 **Scan** 19665
**Peptide** NLIAVPDKNY **Charge** 2 **Spectral Angle** 0.87

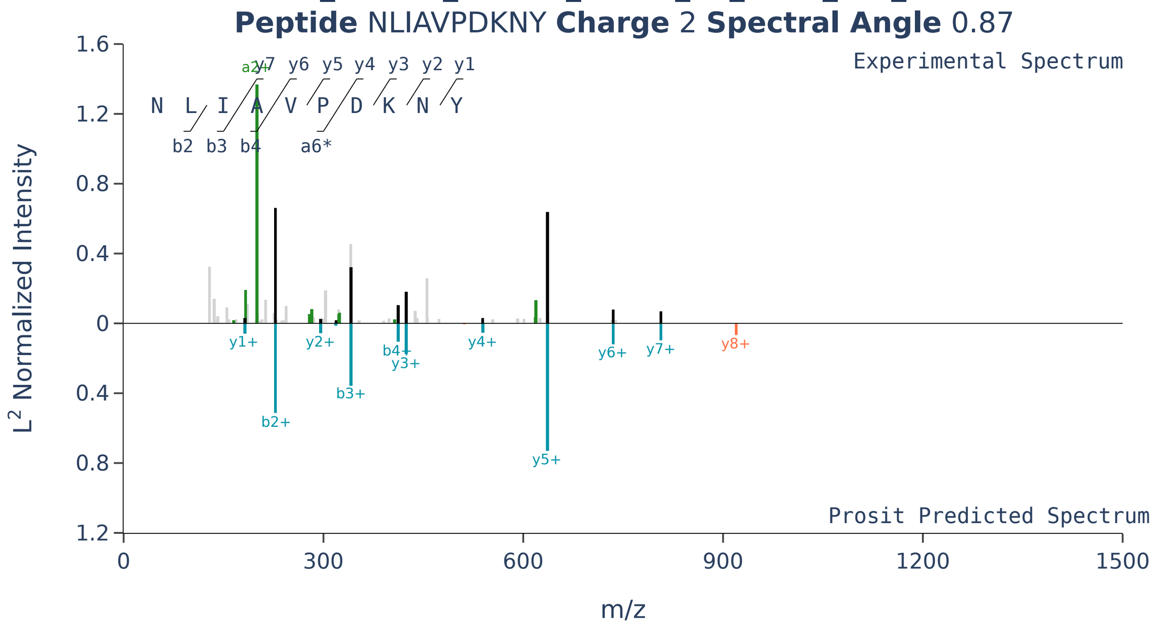


**Source** WSoh_101121_151121_HFGoe_G2_IL37b_4h_R2 **Scan** 21575
**Peptide** AVPDKNYIRPEIF **Charge** 2 **Spectral Angle** 0.79

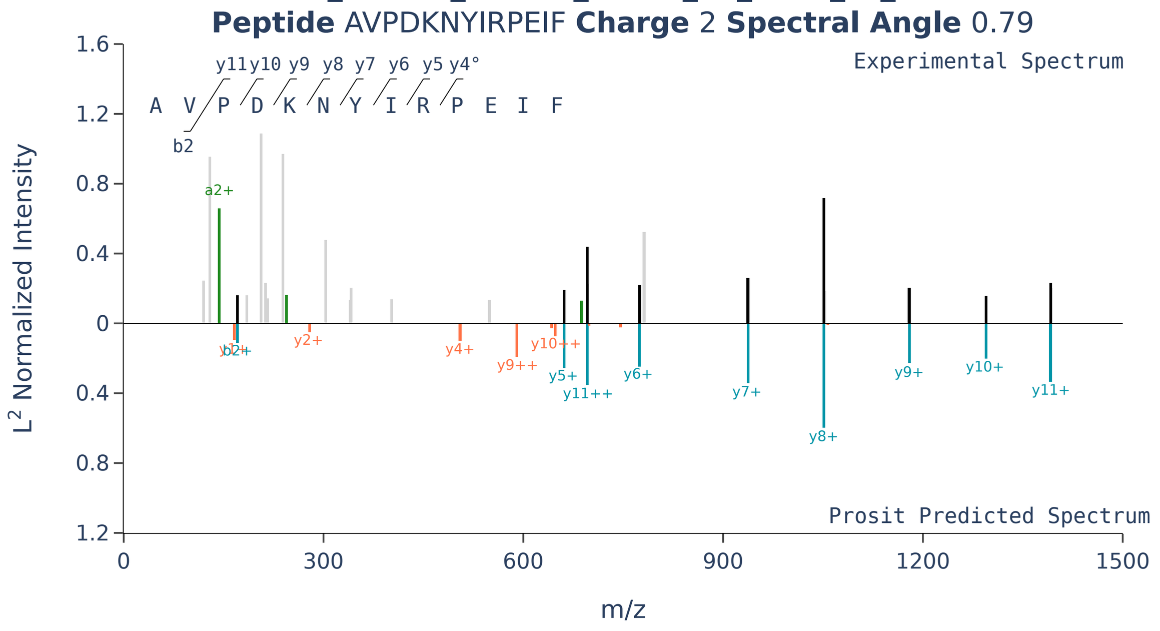


**Source** WSoh_101121_151121_HFGoe_G1_IL37b_4h_R1 **Scan** 6331
**Peptide** CDKDKGQSHPSL **Charge** 2 **Spectral Angle** 0.88

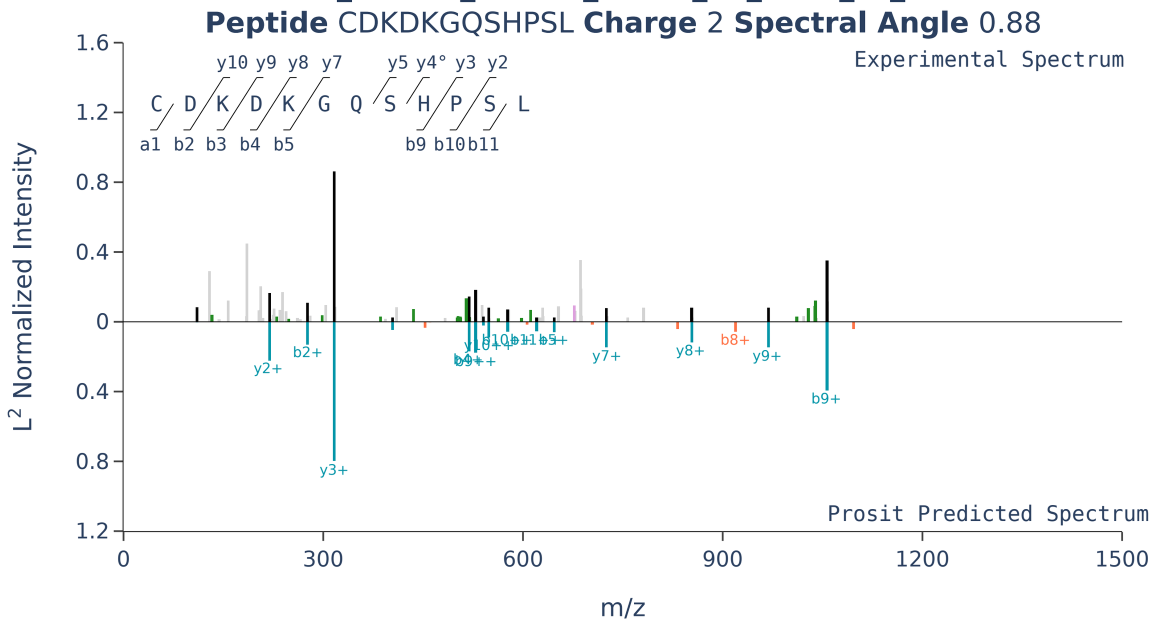


**Source** WSoh_101121_151121_HFGoe_G2_IL37b_2h_R1 **Scan** 22232
**Peptide** AAHPGWFICTS **Charge** 2 **Spectral Angle** 0.79

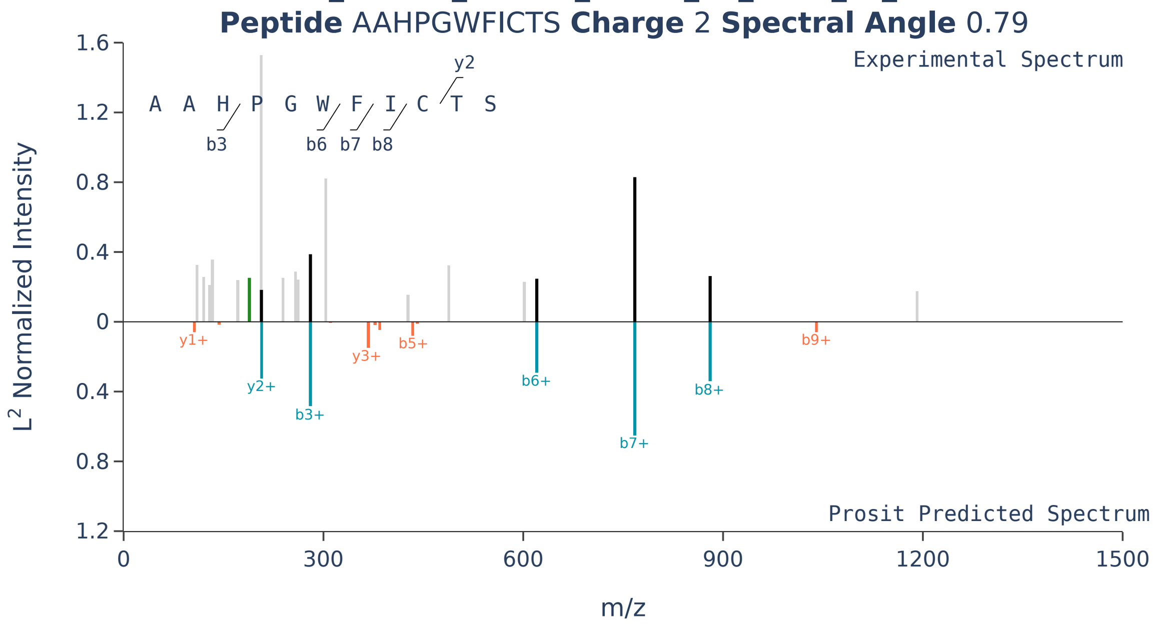


**Source** WSoh_101121_151121_HFGoe_G1_IL37b_4h_R1 **Scan** 11294
**Peptide** SARRPFI **Charge** 2 **Spectral Angle** 0.7

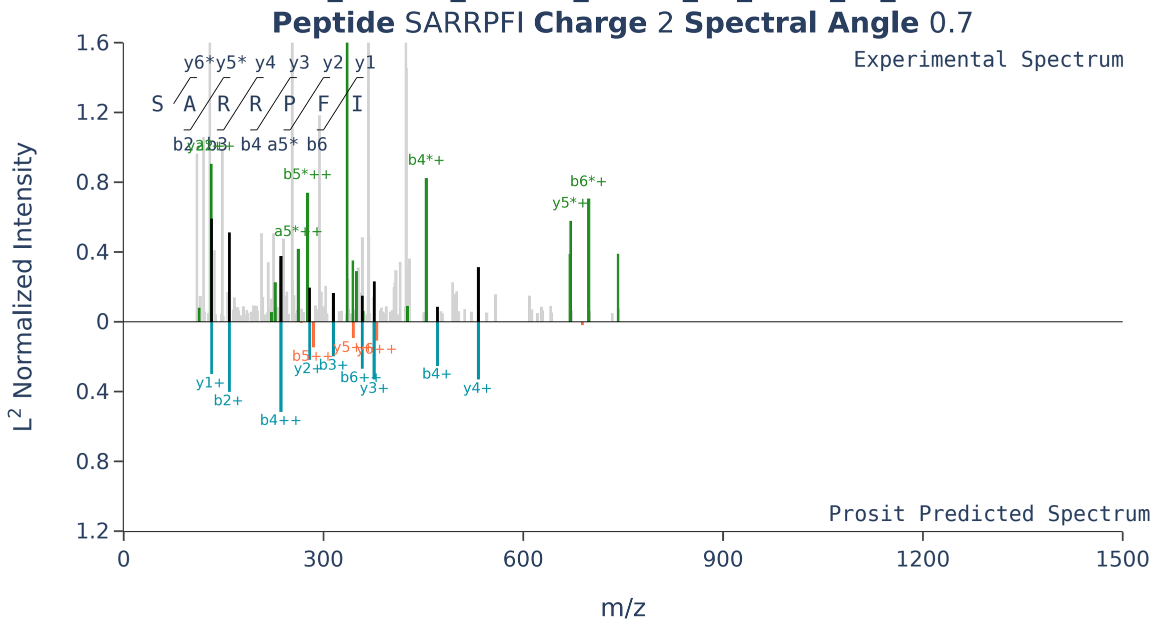


**Source** WSoh_101121_151121_HFGoe_G1_IL37b_24h_R2 **Scan** 8063
**Peptide** ASSLSSASA **Charge** 1 **Spectral Angle** 0.82

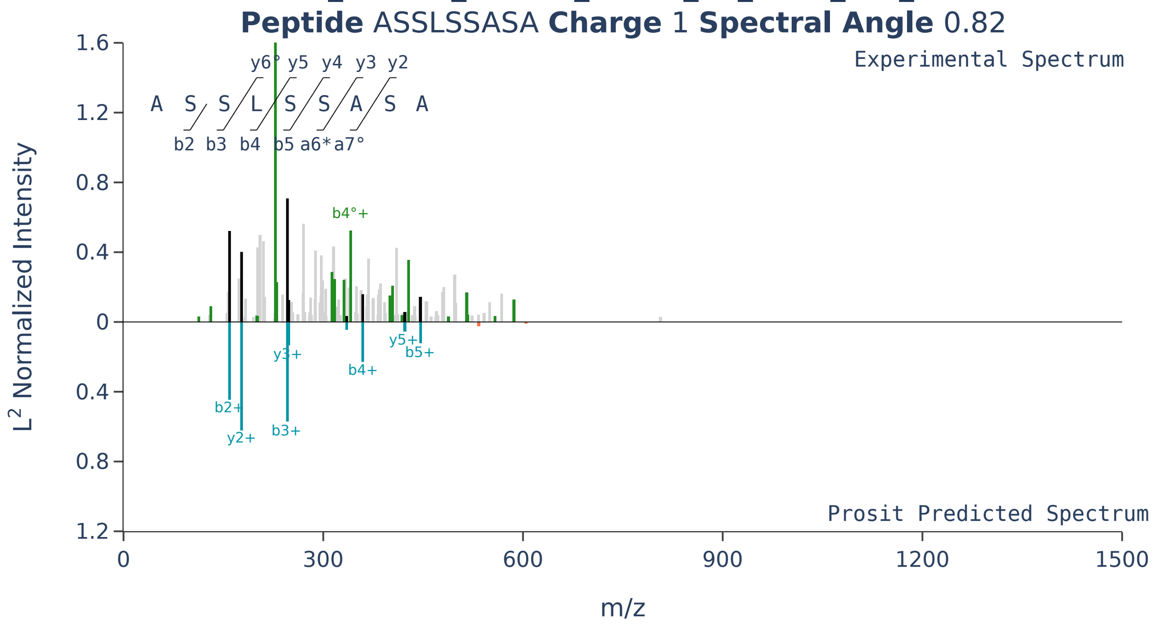


**Source** WSoh_101121_151121_HFGoe_G2_IL37b_24h_R2 **Scan** 6330
**Peptide** LSSASAEKGSP **Charge** 2 **Spectral Angle** 0.77

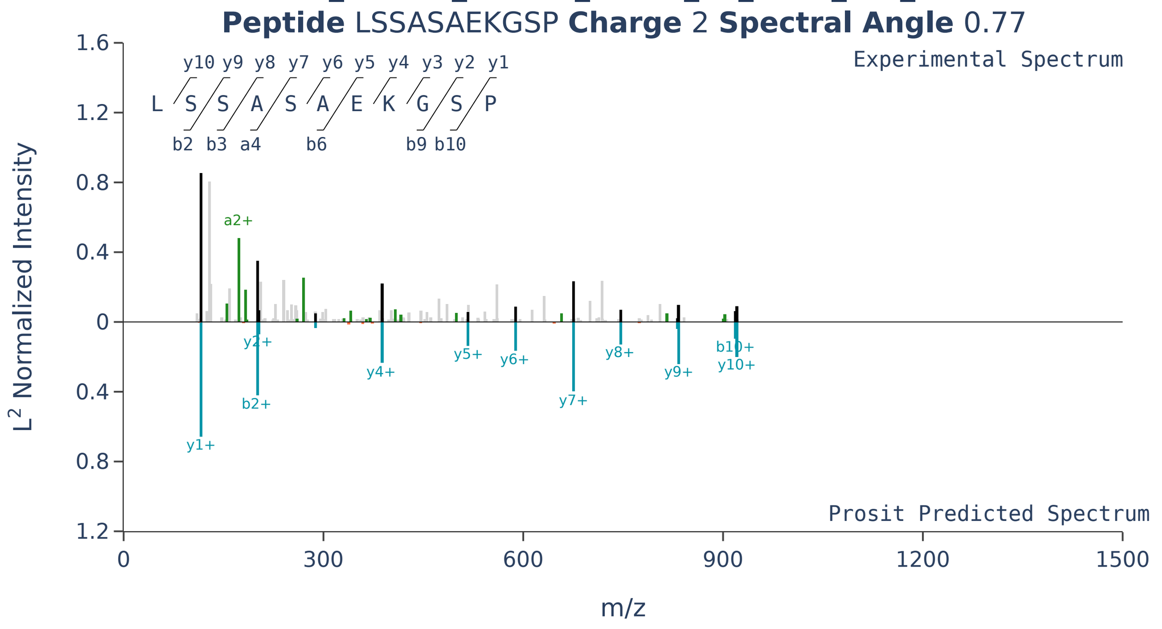


**Source** WSoh_101121_151121_HFGoe_G1_IL37b_1h_R2 **Scan** 6471
**Peptide** TDKFENR **Charge** 2 **Spectral Angle** 0.91

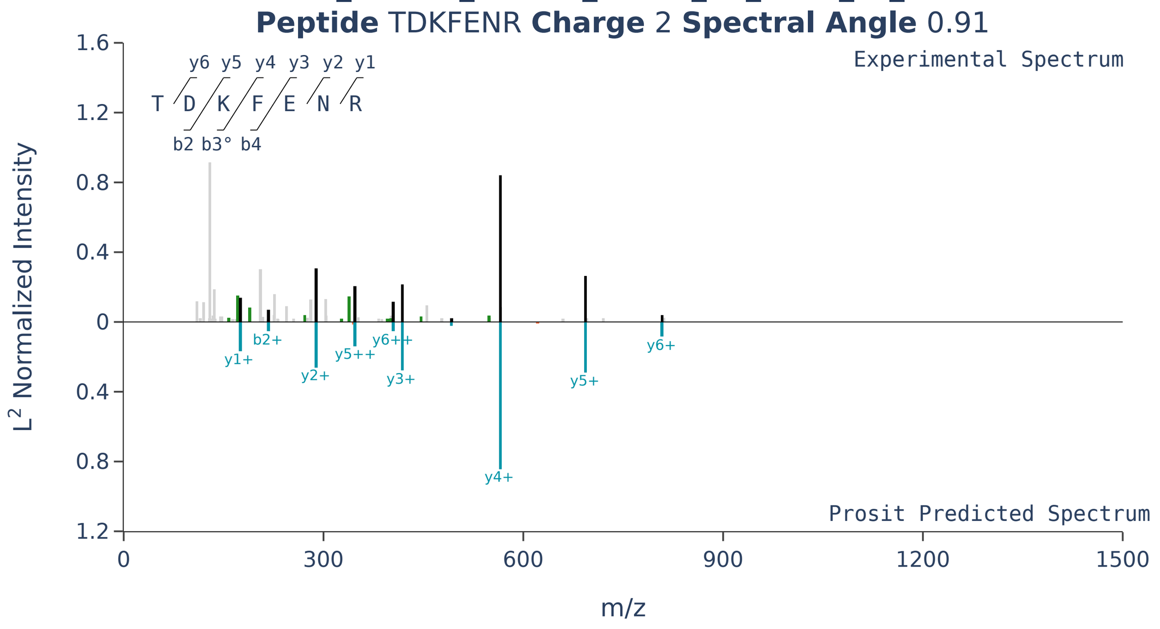

**Source** WSoh_101121_151121_HFGoe_G1_IL37b_4h_R2 **Scan** 14154
**Peptide** AQKESARRPFIFYRAQ **Charge** 4 **Spectral Angle** 0.94

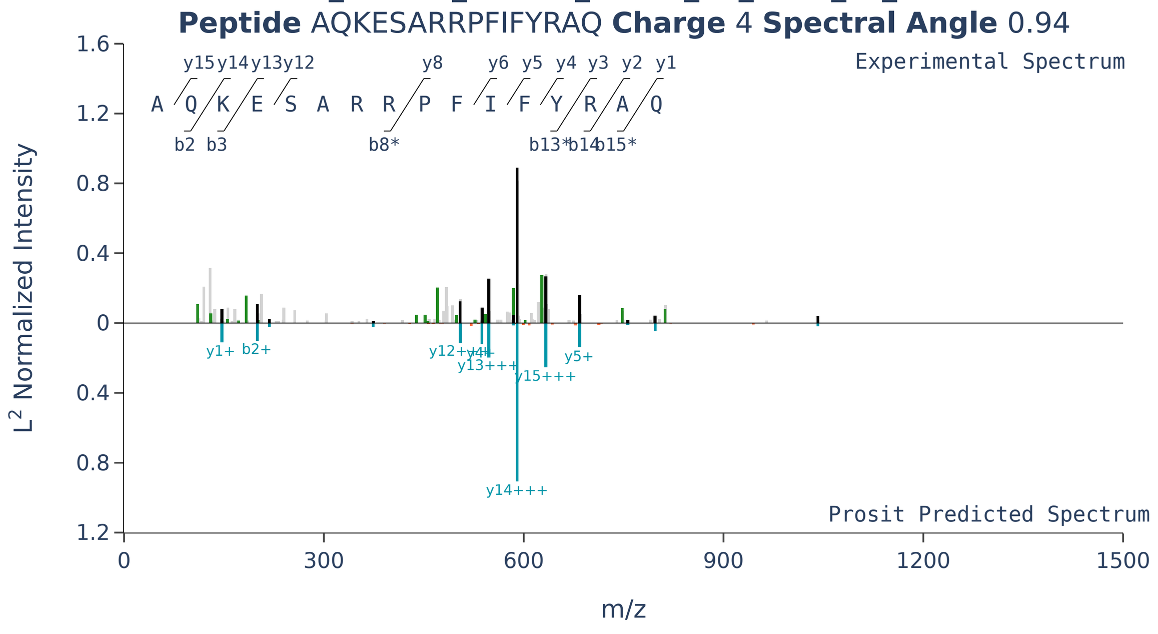


**Source** WSoh_101121_151121_HFGoe_G1_IL37b_24h_R1 **Scan** 8814
**Peptide** KEKLMKLA **Charge** 2 **Spectral Angle** 0.88

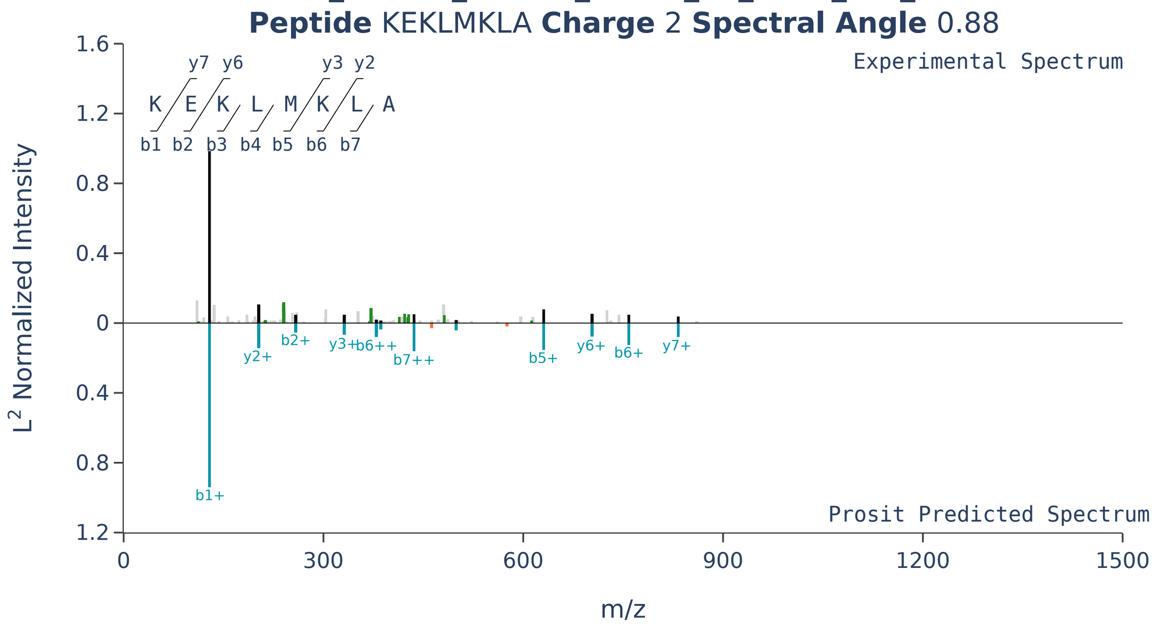


**Source** WSoh_101121_151121_HFGoe_G1_IL37b_4h_R1 **Scan** 14592
**Peptide** ESARRPFIFYRA **Charge** 3 **Spectral Angle** 0.77

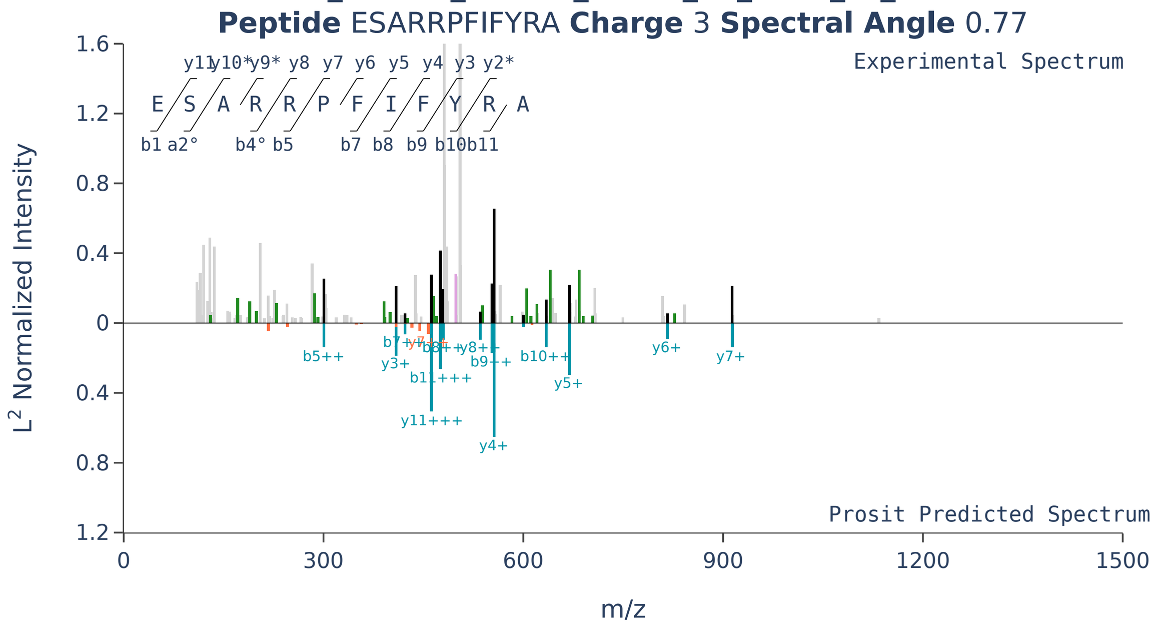


**Source** WSoh_101121_151121_HFGoe_G2_IL37b_24h_R2 **Scan** 5712
**Peptide** QLKKEKLMK **Charge** 2 **Spectral Angle** 0.86

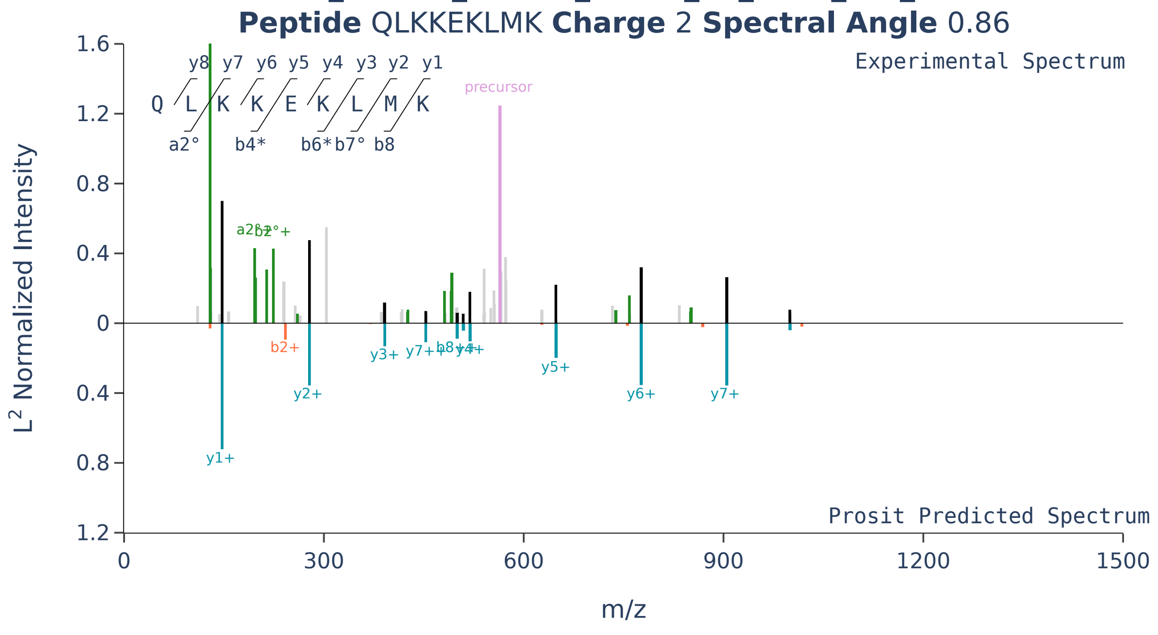


**Source** WSoh_101121_151121_HFGoe_G1_IL37b_24h_R1 **Scan** 21550
**Peptide** DSGNLIAVPDKNYIRPE **Charge** 3 **Spectral Angle** 0.88

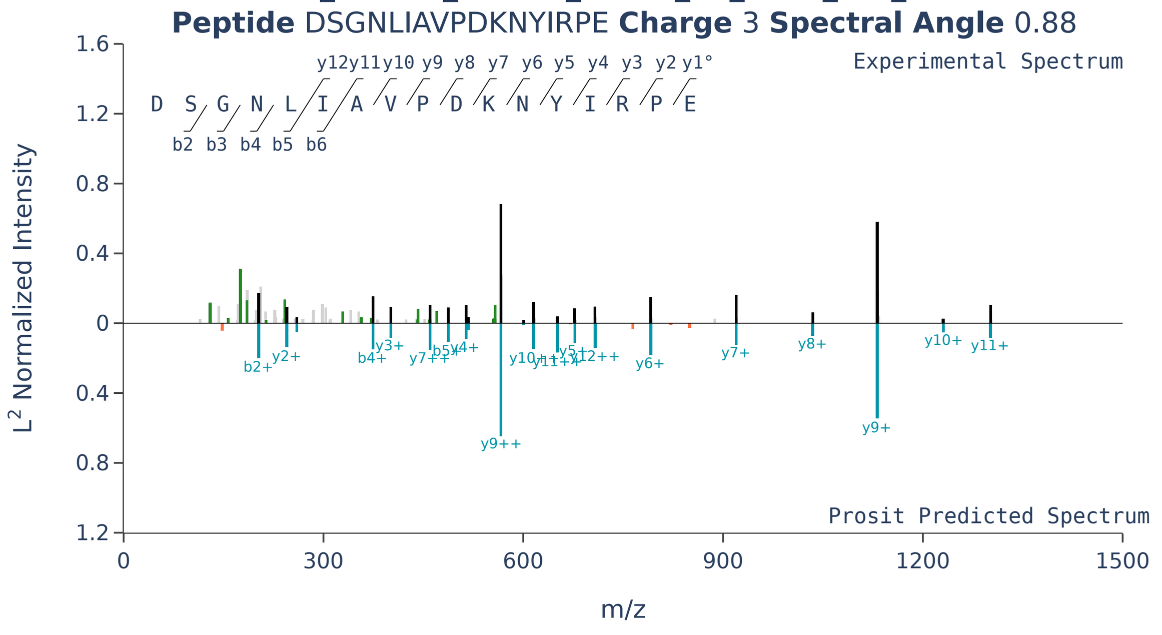


**Source** WSoh_101121_151121_HFGoe_G2_IL37b_24h_R2 **Scan** 10053
**Peptide** PVGVTDKFENRKHI **Charge** 3 **Spectral Angle** 0.81

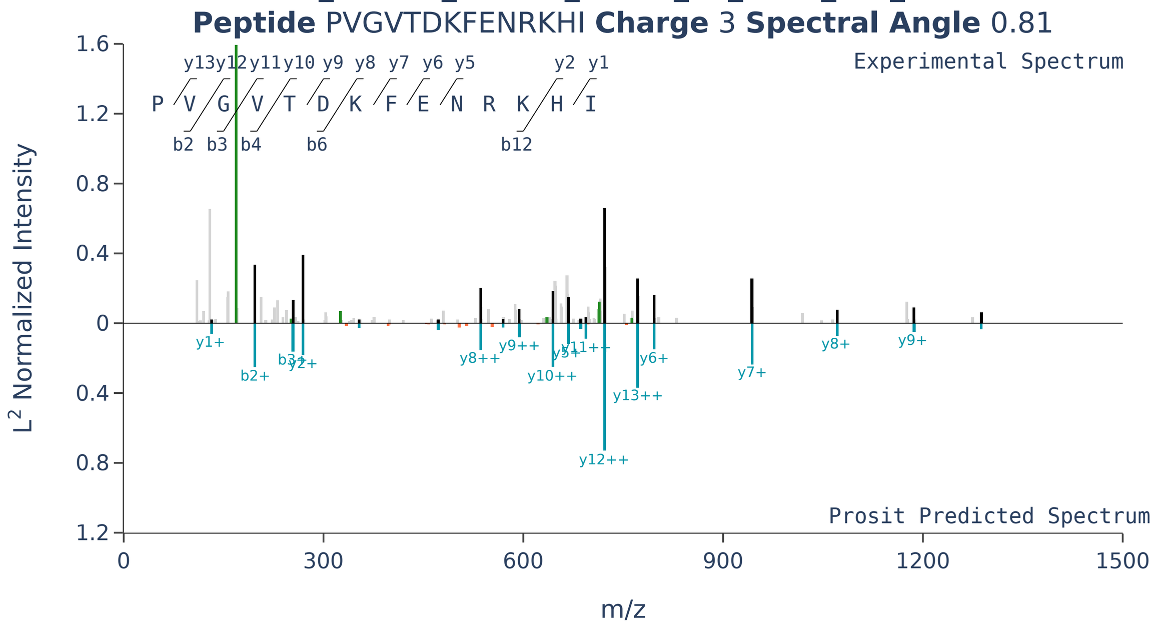


**Source** WSoh_101121_151121_HFGoe_G1_IL37b_1h_R2 **Scan** 20118
**Peptide** GVSKGEFCLY **Charge** 2 **Spectral Angle** 0.79

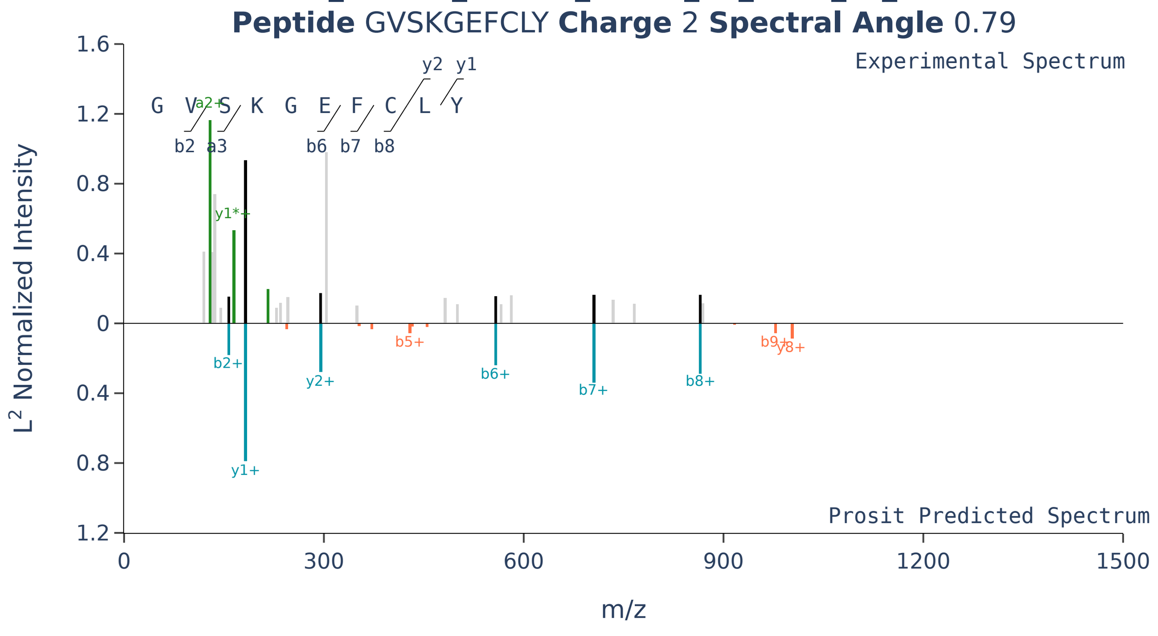


**Source** WSoh_101121_151121_HFGoe_G2_IL37b_2h_R2 **Scan** 10703
**Peptide** CLYCDKDKGQSHPSL **Charge** 3 **Spectral Angle** 0.86

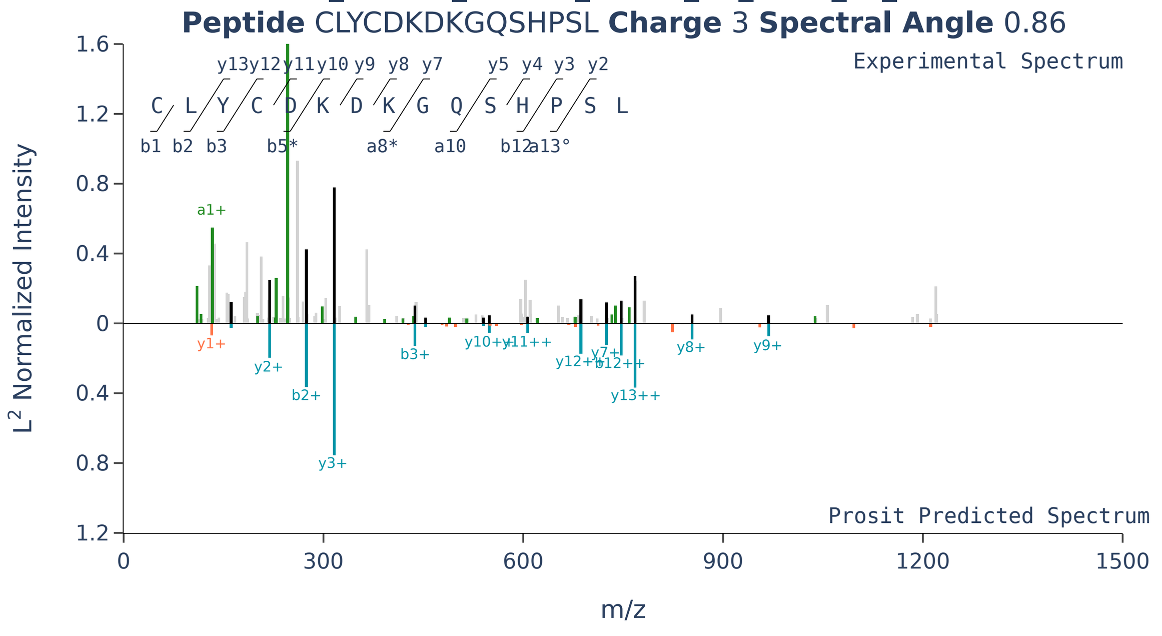


**Source** WSoh_101121_151121_HFGoe_G1_IL37b_24h_R2 **Scan** 14415
**Peptide** SIHDQDHKVLVLDS **Charge** 2 **Spectral Angle** 0.76

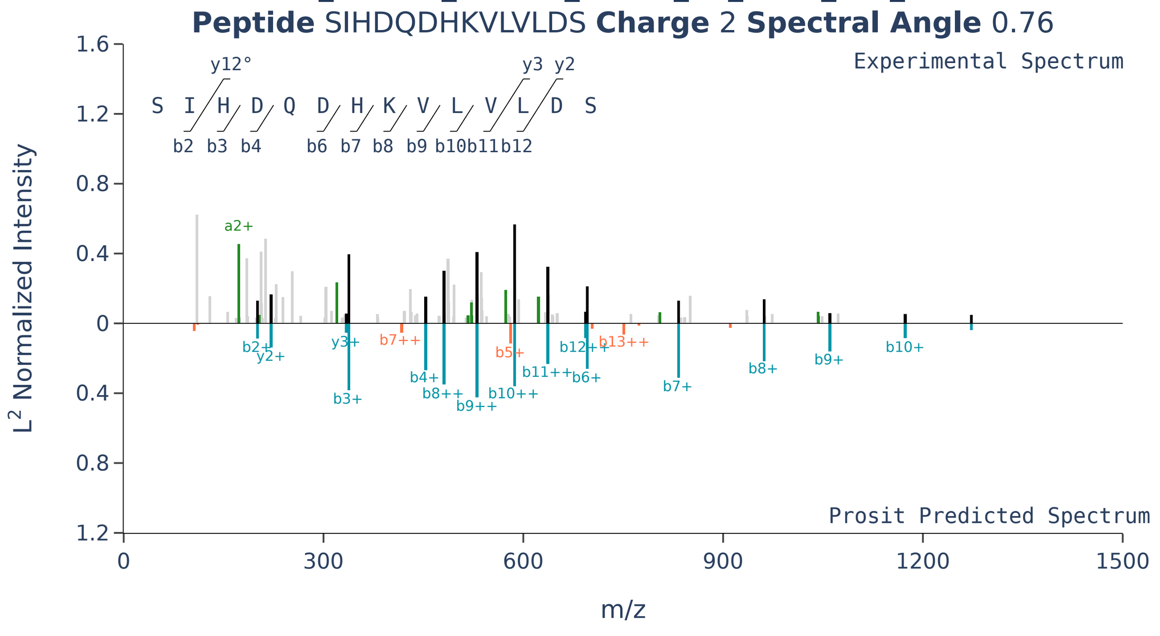


**Source** WSoh_101121_151121_HFGoe_G2_IL37b_24h_R2 **Scan** 6682
**Peptide** KKEKLMKLAAQKESA **Charge** 3 **Spectral Angle** 0.83

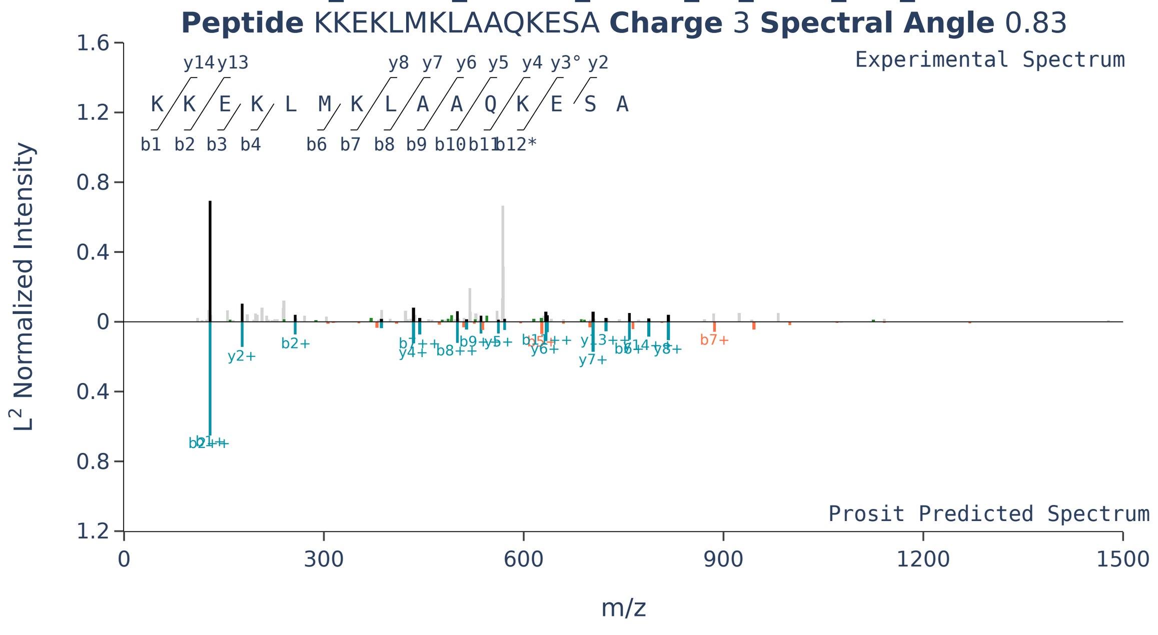


**Source** WSoh_101121_151121_HFGoe_G1_IL37b_24h_R2 **Scan** 7841
**Peptide** KDKGQSHPSLQLKKEKL **Charge** 3 **Spectral Angle** 0.85

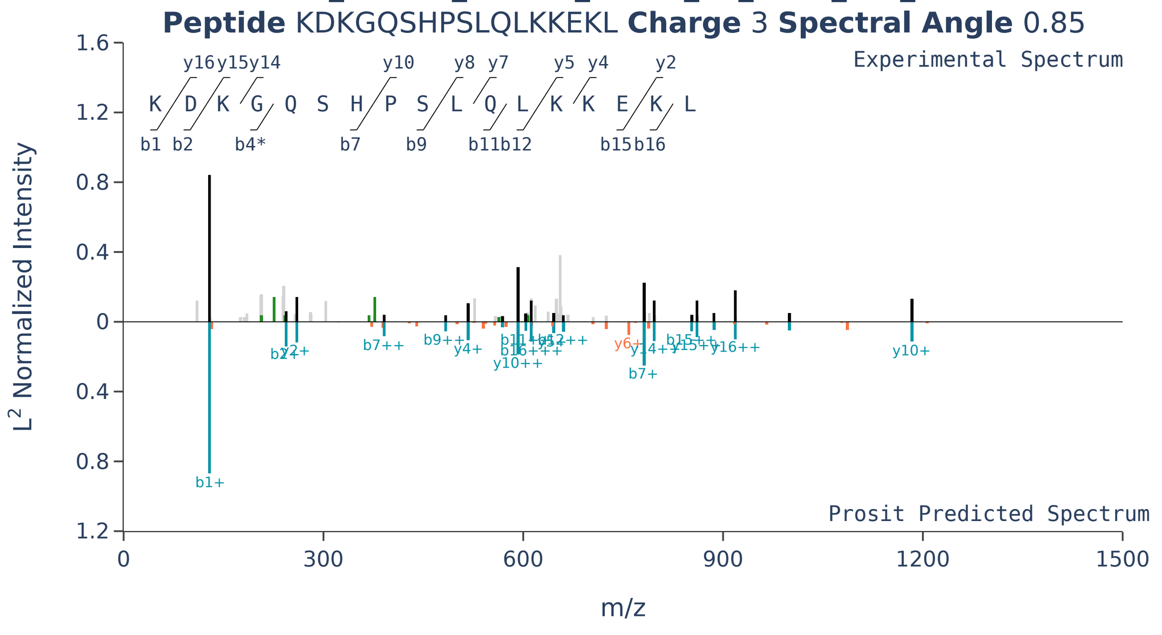


**Source** WSoh_101121_151121_HFGoe_G2_IL37b_4h_R2 **Scan** 21105
**Peptide** ARRPFIFY **Charge** 2 **Spectral Angle** 0.82

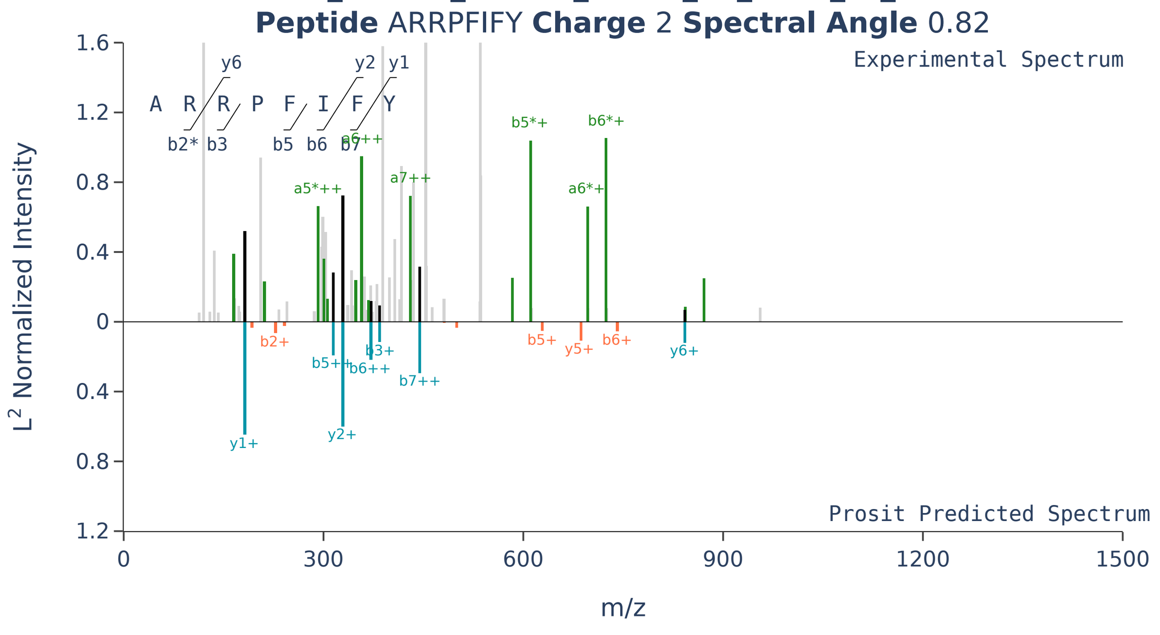


**Source** WSoh_101121_151121_HFGoe_G2_IL37b_24h_R1 **Scan** 9980
**Peptide** TSCNCNEPVGVTDKFENRKH **Charge** 4 **Spectral Angle** 0.81

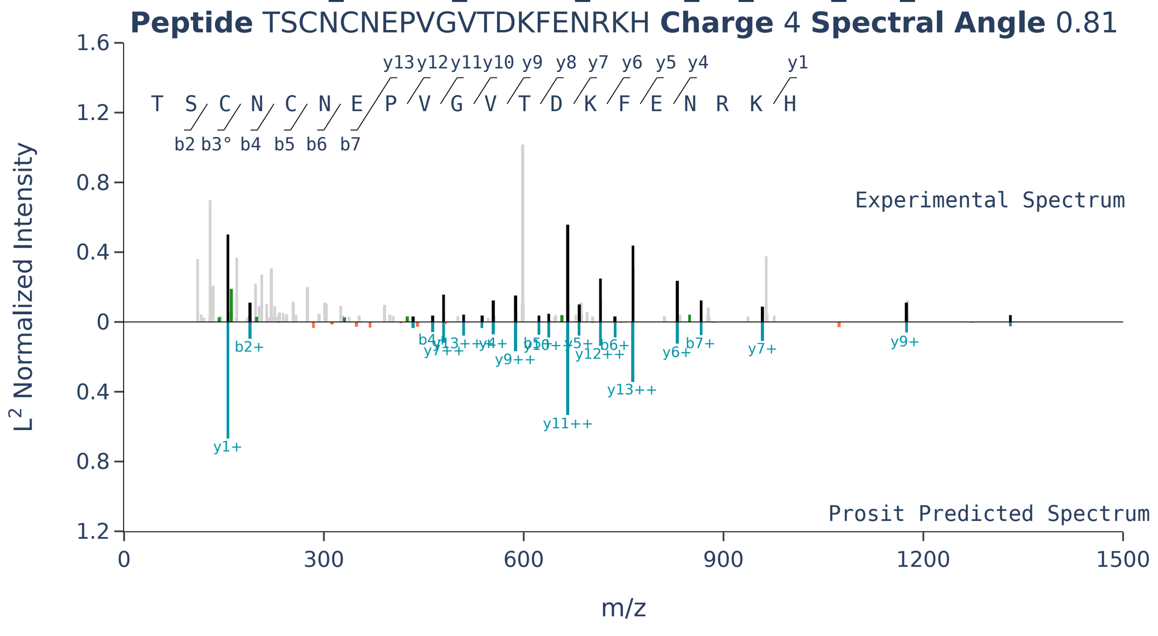


**Source** WSoh_101121_151121_HFGoe_G2_IL37b_2h_R1 **Scan** 14419
**Peptide** AQKESARRPFIFYRA **Charge** 4 **Spectral Angle** 0.93

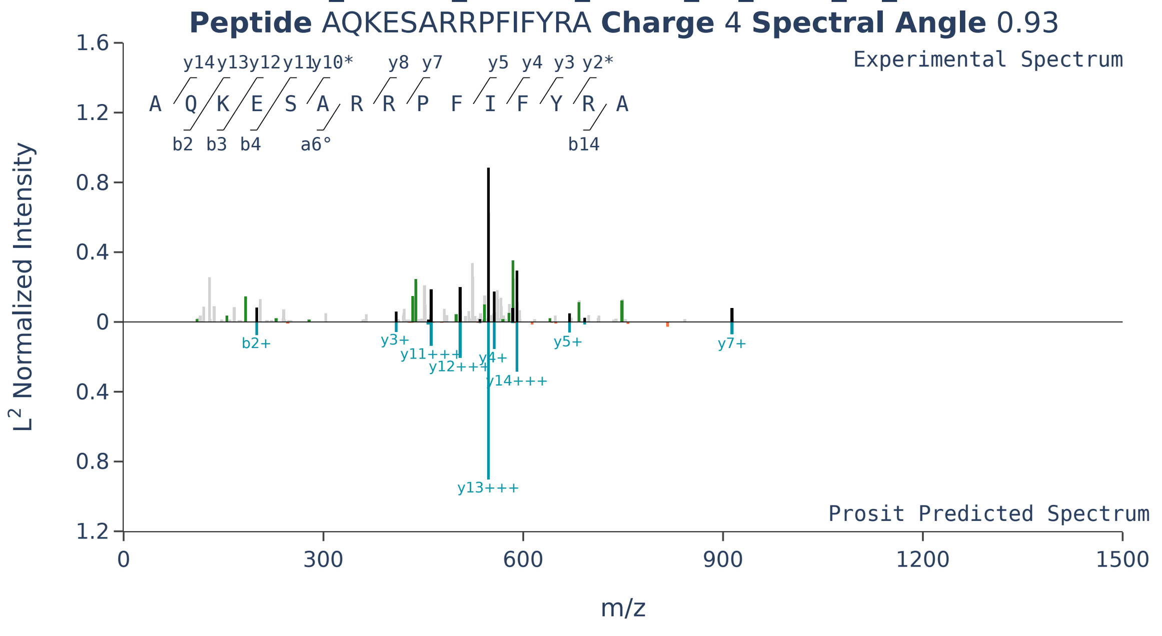


**Source** WSoh_101121_151121_HFGoe_G2_IL37b_24h_R2 **Scan** 5724
**Peptide** NPKKFSIHDQDHK **Charge** 3 **Spectral Angle** 0.82

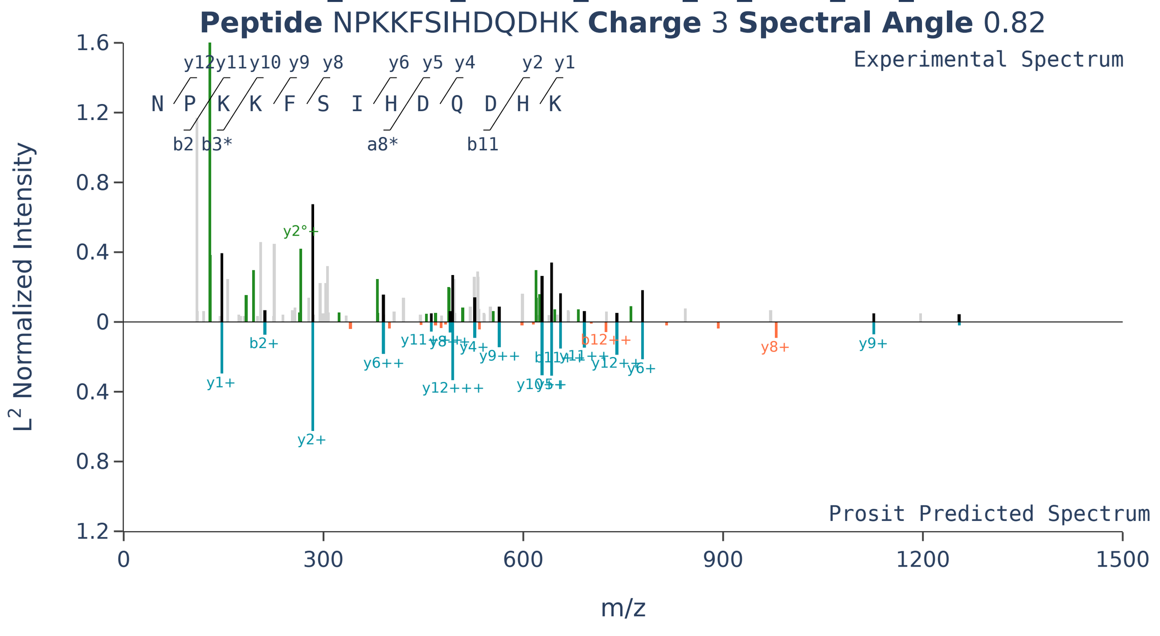

**Source** WSoh_101121_151121_HFGoe_G1_IL37b_24h_R2 **Scan** 24149
**Peptide** EKGSPILLGV **Charge** 2 **Spectral Angle** 0.87

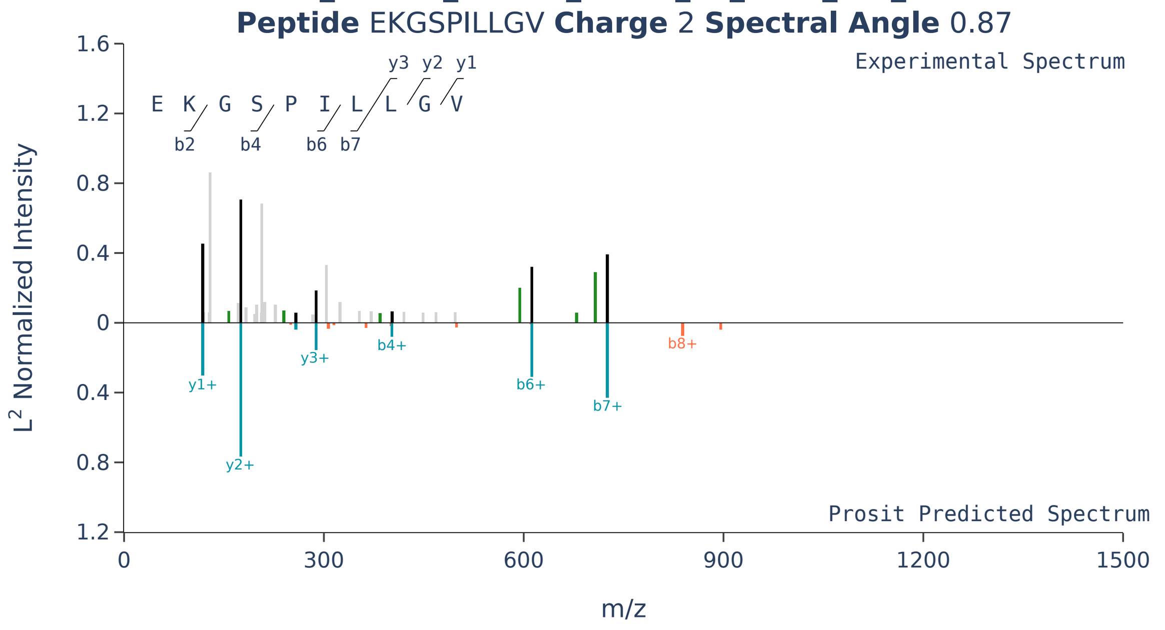


**Source** WSoh_101121_151121_HFGoe_G1_IL37b_24h_R2 **Scan** 8600
**Peptide** NLNPKKFS **Charge** 2 **Spectral Angle** 0.88

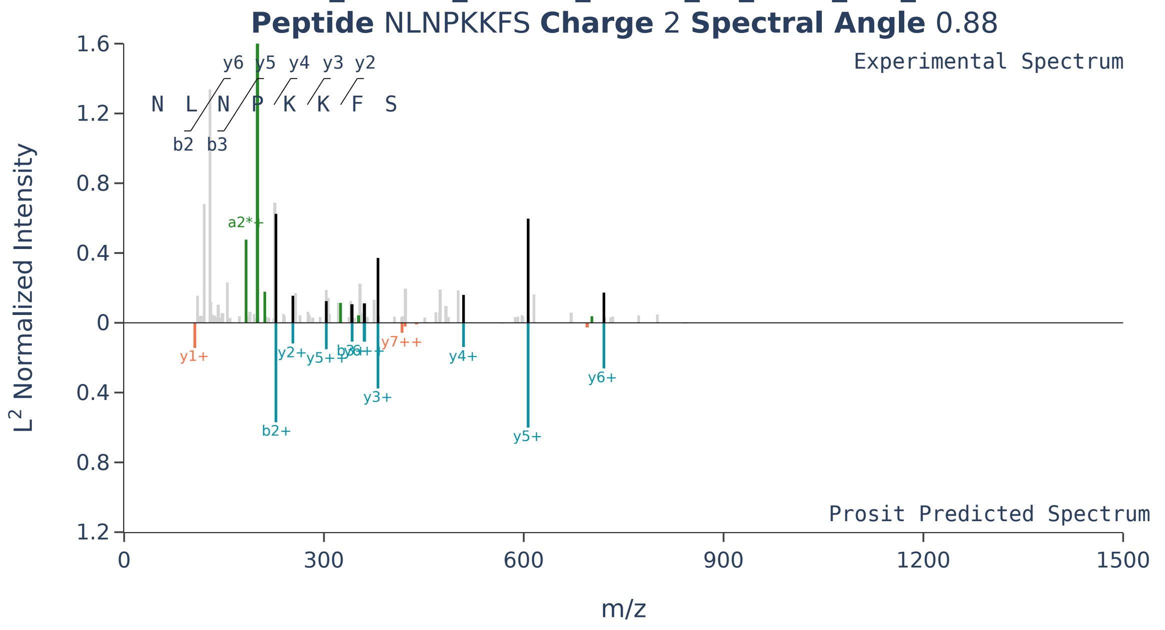


**Source** WSoh_101121_151121_HFGoe_G2_IL37b_4h_R2 **Scan** 16043
**Peptide** GSWNMLESAAH **Charge** 2 **Spectral Angle** 0.76

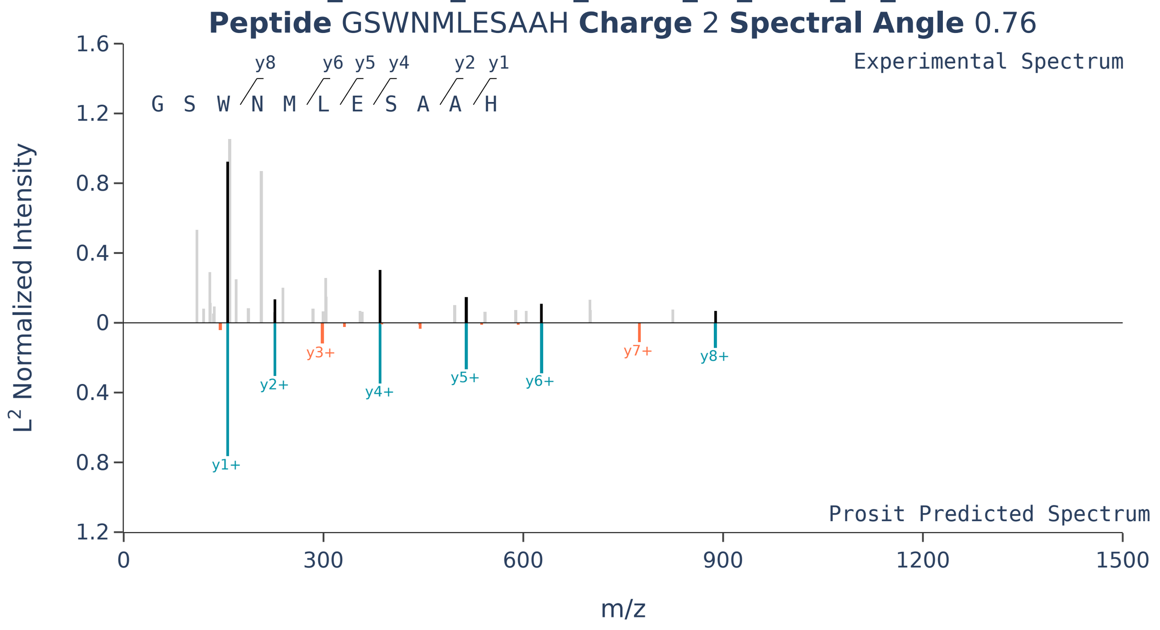


**Source** WSoh_101121_151121_HFGoe_G2_IL37b_24h_R1 **Scan** 18116
**Peptide** DSGNLIAVPDK **Charge** 2 **Spectral Angle** 0.88

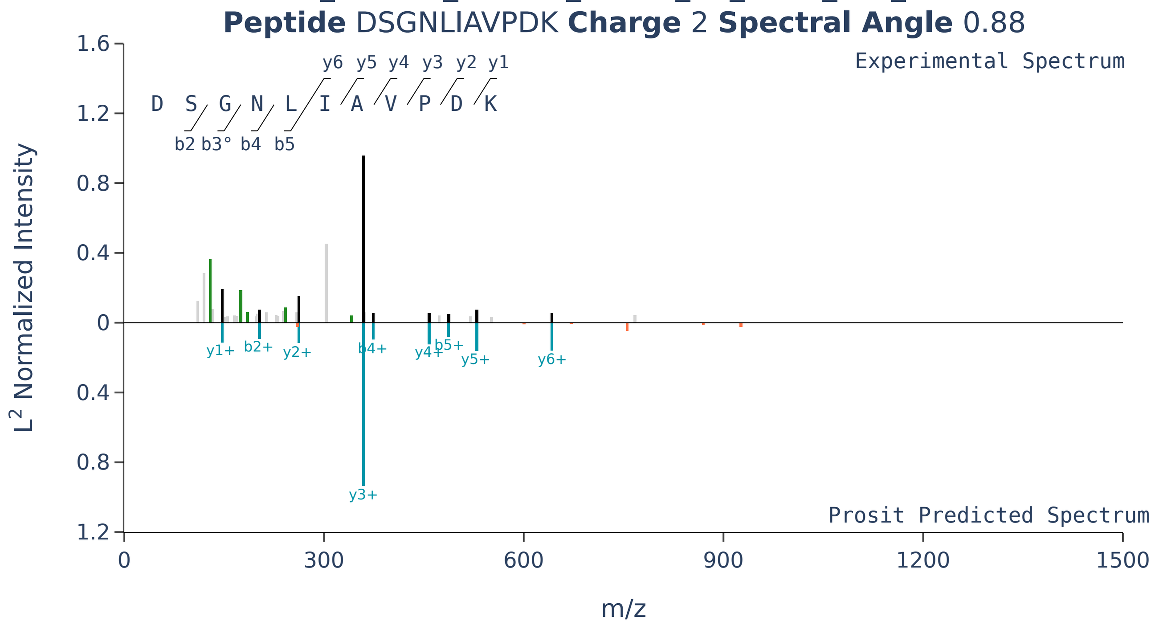


**Source** WSoh_101121_151121_HFGoe_G1_IL37b_24h_R1 **Scan** 16652
**Peptide** NCNEPVGVTDKFENRKHIEF **Charge** 5 **Spectral Angle** 0.79

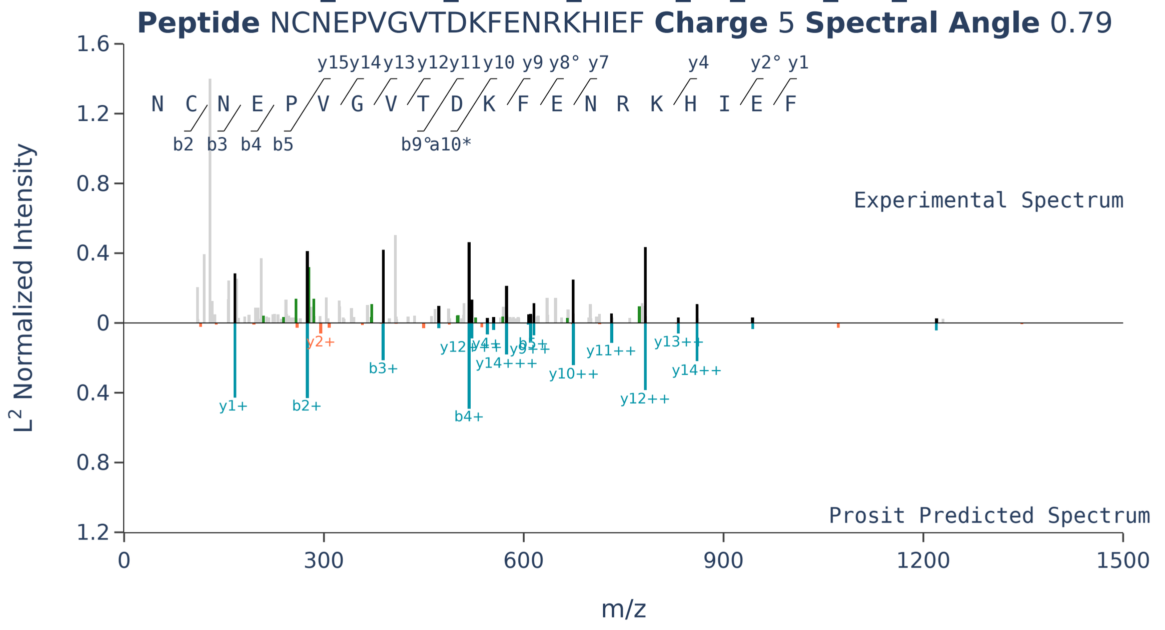


**Source** WSoh_101121_151121_HFGoe_G2_IL37b_4h_R2 **Scan** 12337
**Peptide** LASSLSSA **Charge** 1 **Spectral Angle** 0.93

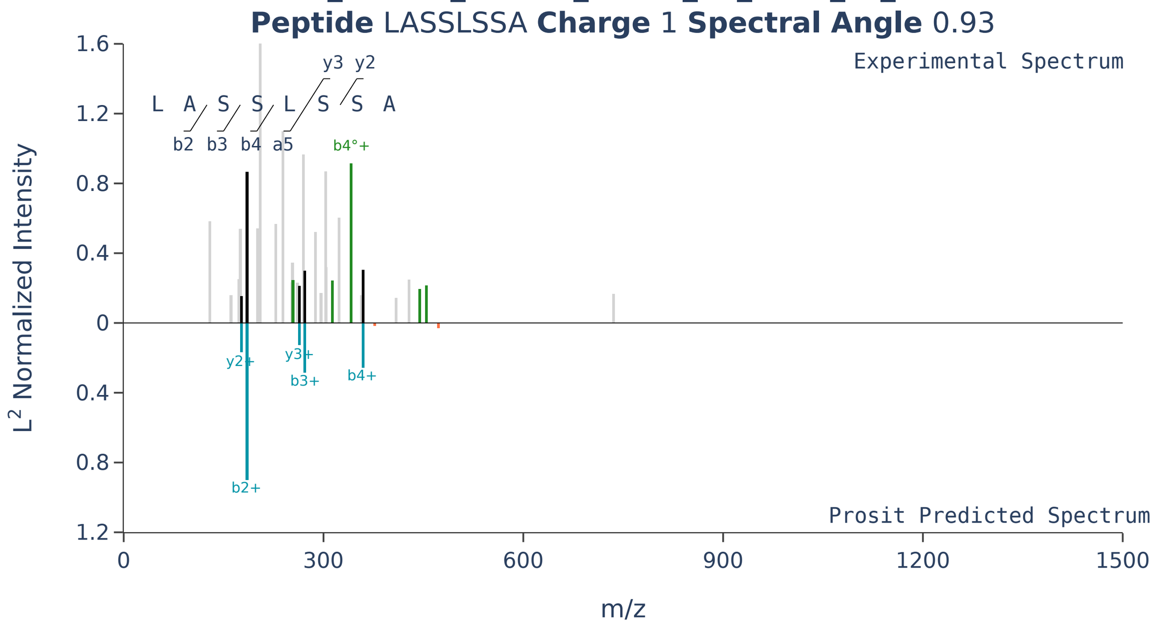


**Source** WSoh_101121_151121_HFGoe_G1_IL37b_24h_R2 **Scan** 10401
**Peptide** PSLQLKKEKLMK **Charge** 3 **Spectral Angle** 0.8

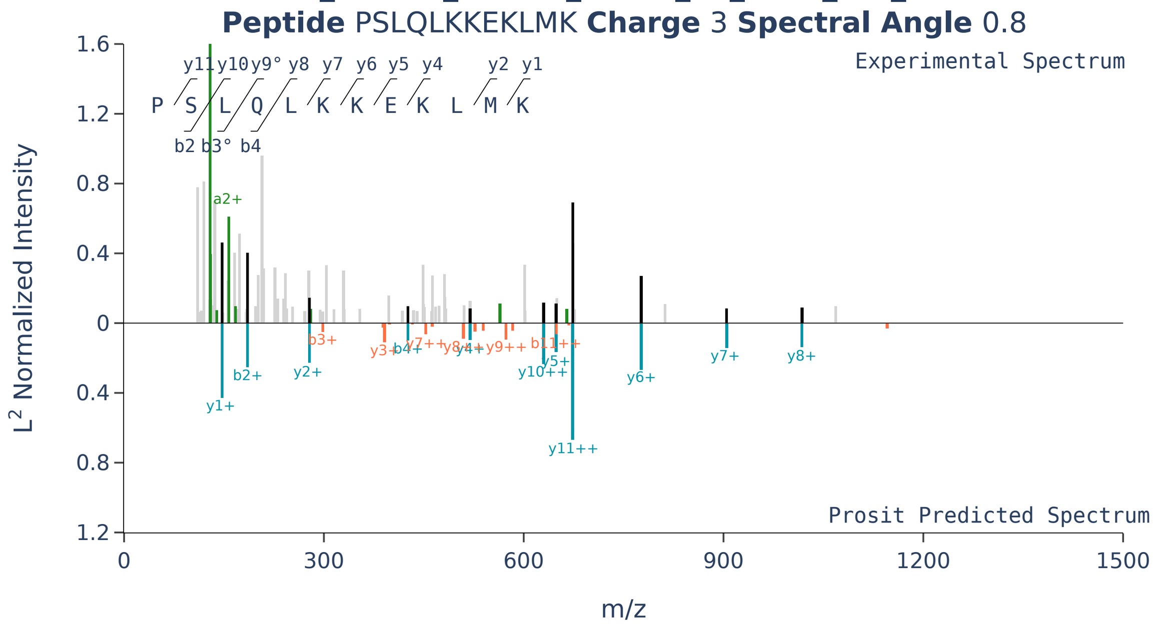


**Source** WSoh_101121_151121_HFGoe_G2_IL37b_24h_R1 **Scan** 19256
**Peptide** SGNLIAVPDKNY **Charge** 2 **Spectral Angle** 0.81

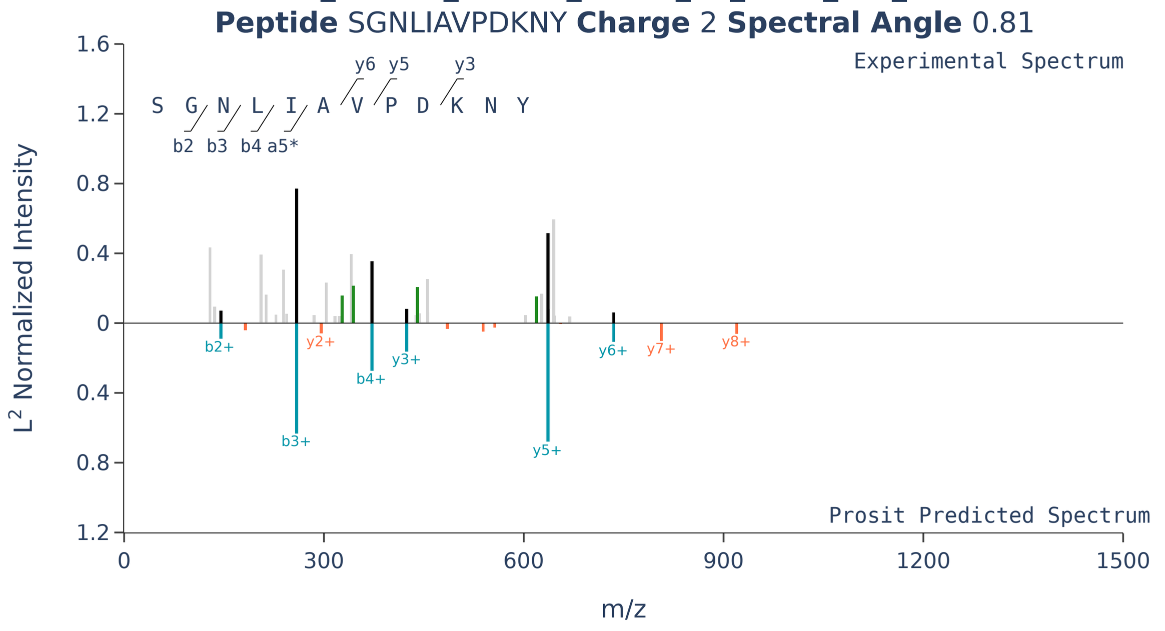


**Source** WSoh_101121_151121_HFGoe_G1_IL37b_4h_R2 **Scan** 17293
**Peptide** GEFCLYCDKDKGQSHPSL **Charge** 3 **Spectral Angle** 0.84

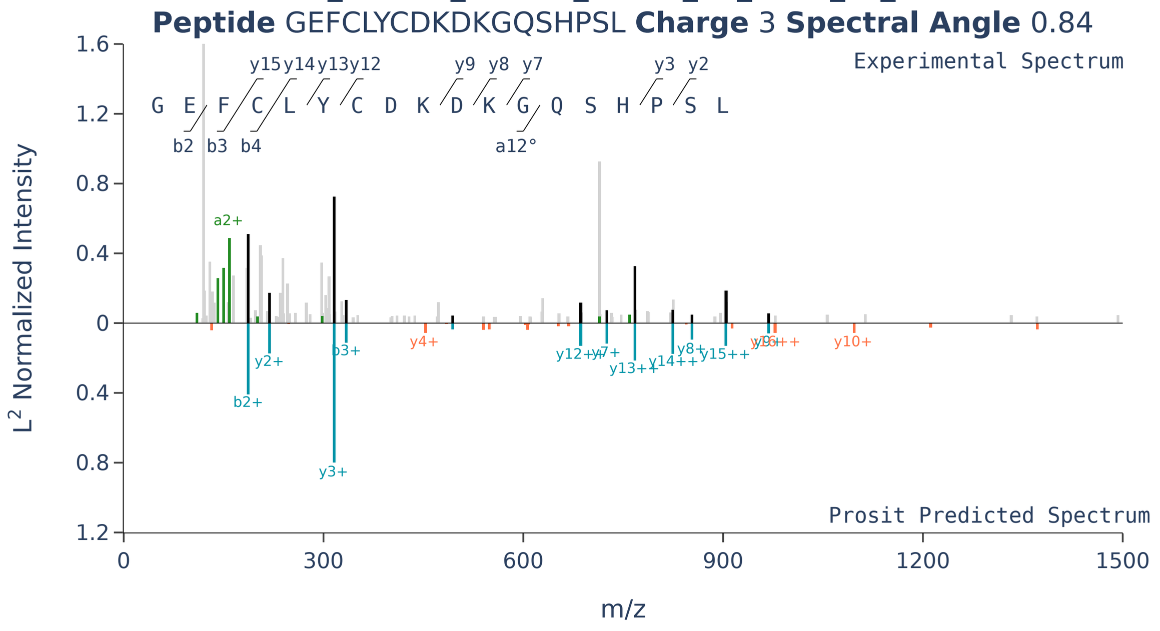


**Source** WSoh_101121_151121_HFGoe_G2_IL37b_24h_R2 **Scan** 17316
**Peptide** TSCNCNEPVGVTDKF **Charge** 2 **Spectral Angle** 0.91

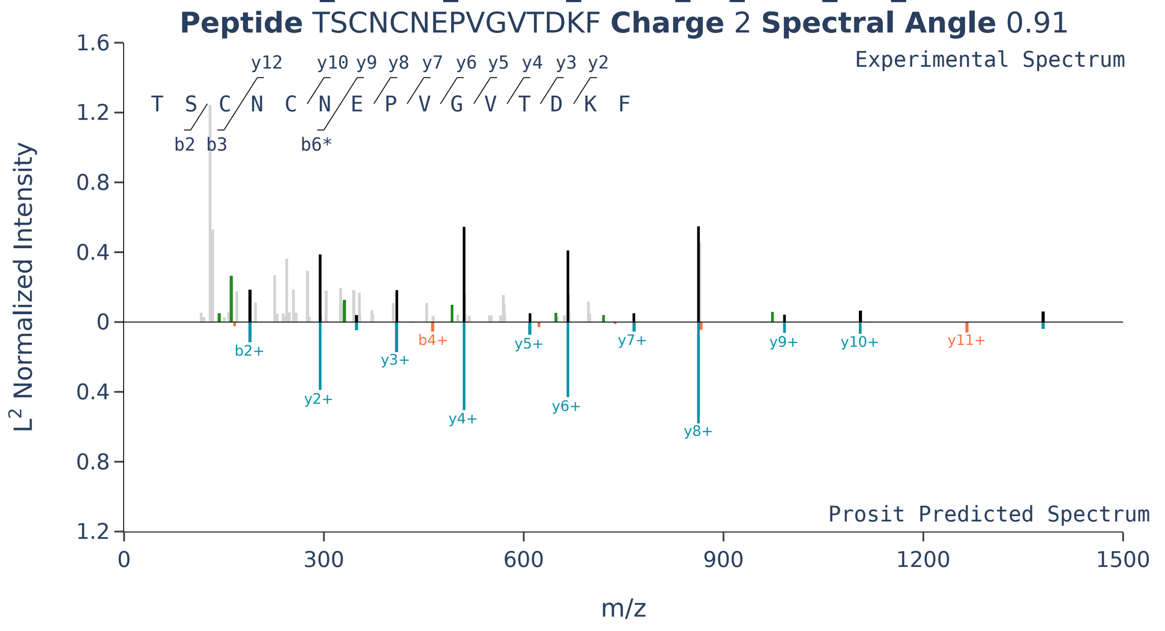


**Source** WSoh_101121_151121_HFGoe_G2_IL37b_24h_R1 **Scan** 17371
**Peptide** LIAVPDKNY **Charge** 2 **Spectral Angle** 0.86

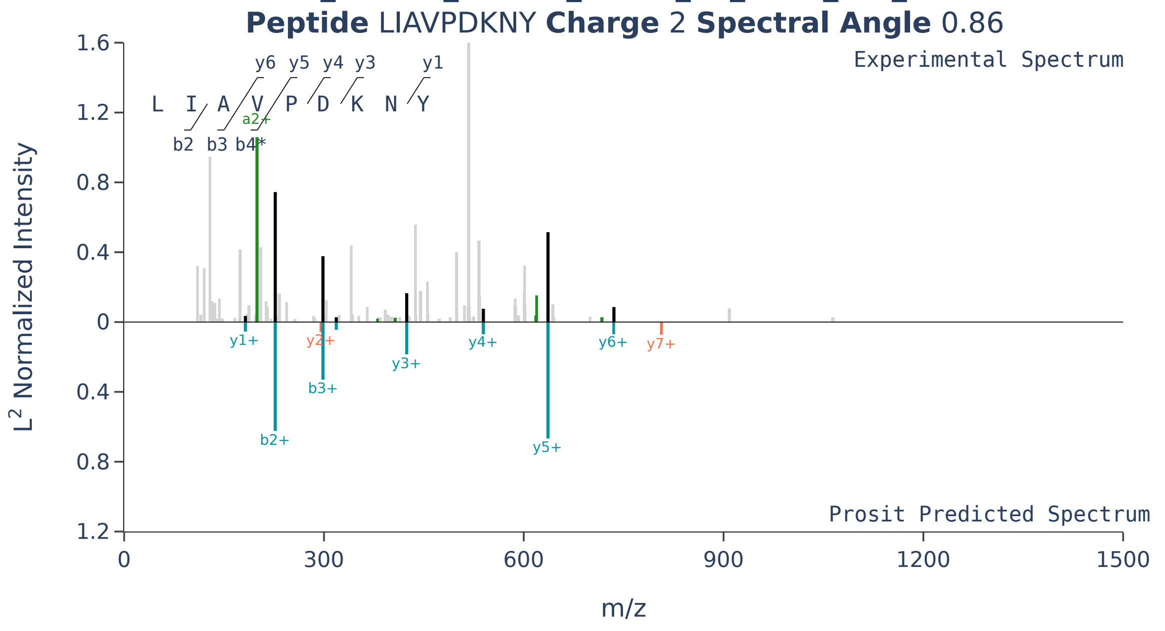


**Source** WSoh_101121_151121_HFGoe_G1_IL37b_24h_R1 **Scan** 7068
**Peptide** YCDKDKGQSHPSLQLKKE **Charge** 3 **Spectral Angle** 0.77

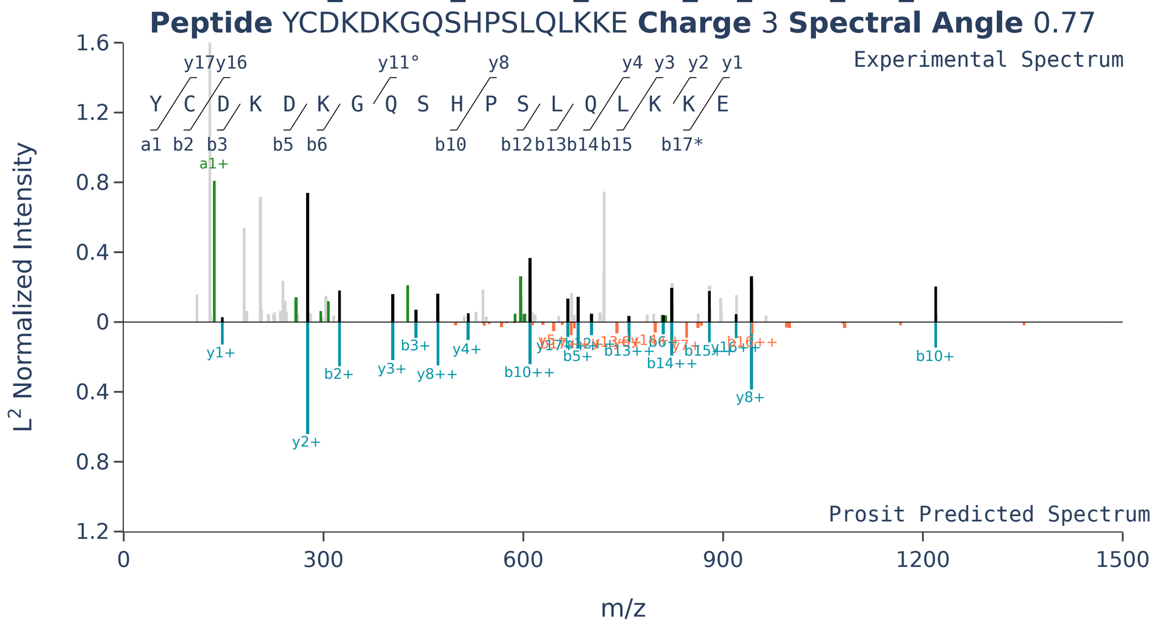


**Source** WSoh_101121_151121_HFGoe_G1_IL37b_4h_R1 **Scan** 16365
**Peptide** NEPVGVTDKFENRKHIEF **Charge** 3 **Spectral Angle** 0.79

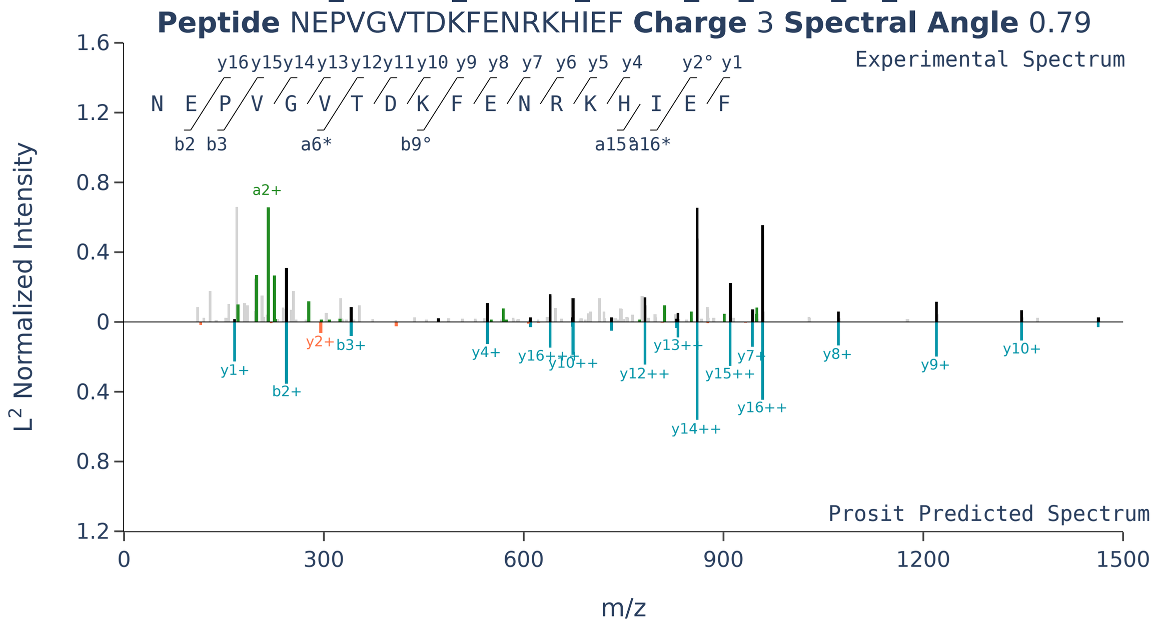


**Source** WSoh_101121_151121_HFGoe_G1_IL37b_24h_R2 **Scan** 7481
**Peptide** NRKHIEF **Charge** 2 **Spectral Angle** 0.87

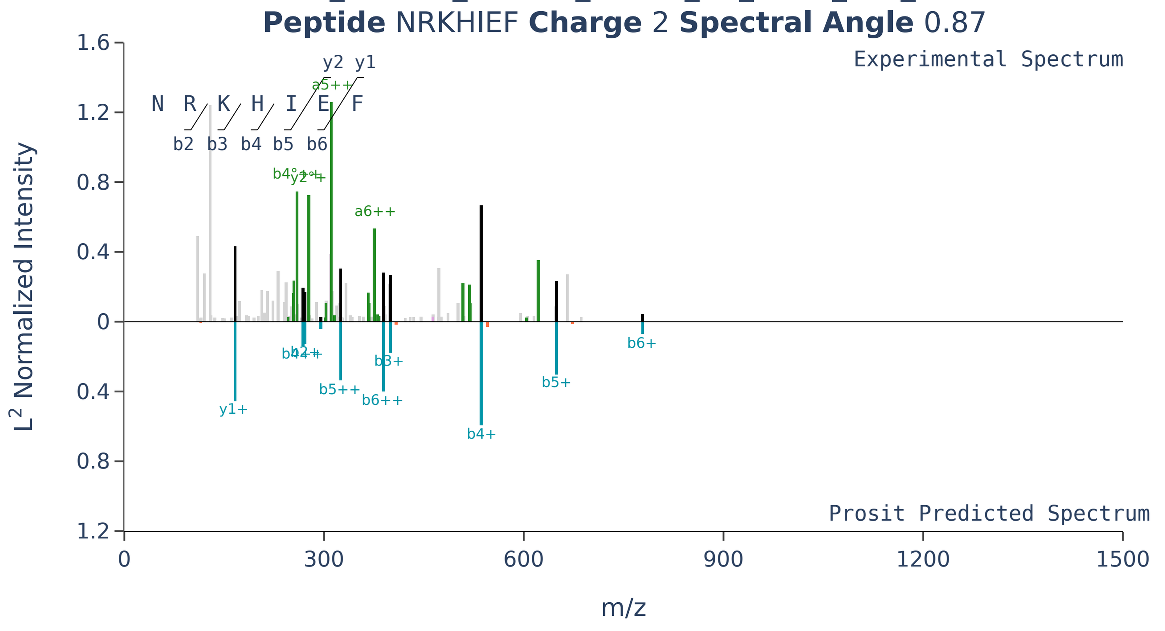


**Source** WSoh_101121_151121_HFGoe_G1_IL37b_24h_R2 **Scan** 14876
**Peptide** SKGEFCLYCDKD **Charge** 2 **Spectral Angle** 0.78

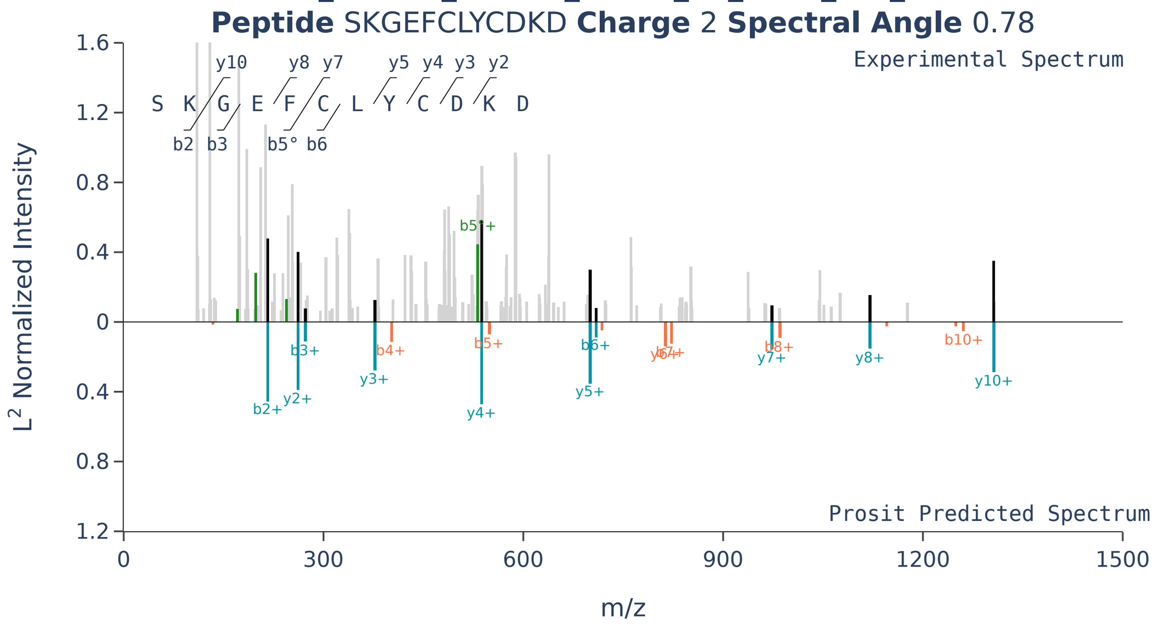

**Source** WSoh_101121_151121_HFGoe_G2_IL37b_24h_R2 **Scan** 5493
**Peptide** LAAQKESARRP **Charge** 3 **Spectral Angle** 0.93

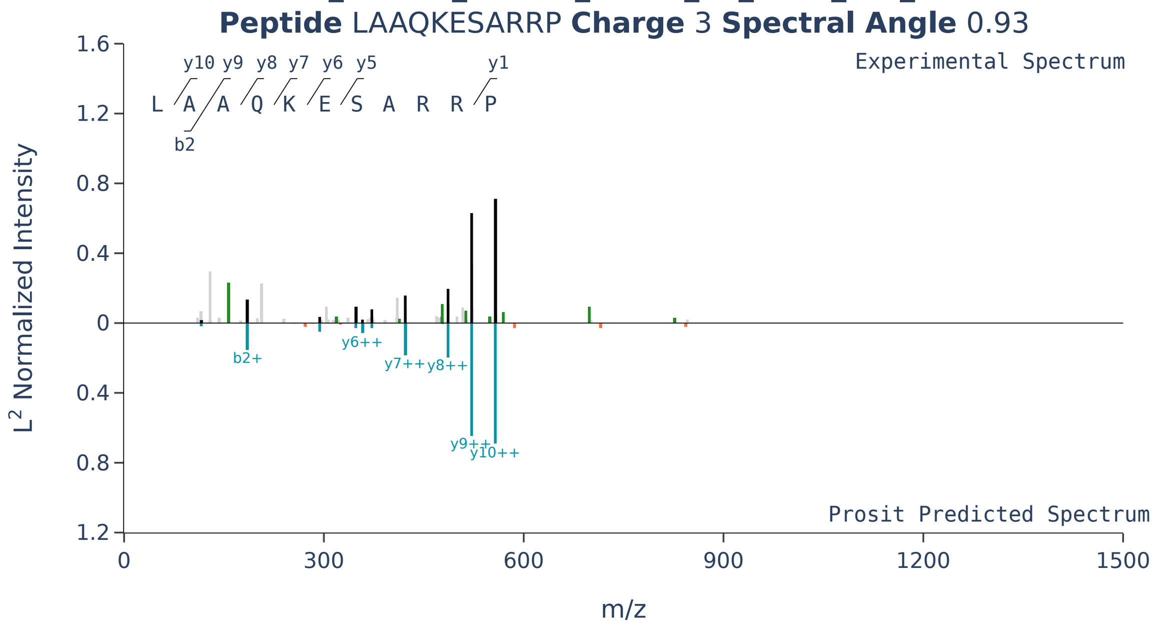


**Source** WSoh_101121_151121_HFGoe_G2_IL37b_24h_R1 **Scan** 23268
**Peptide** NYIRPEIF **Charge** 2 **Spectral Angle** 0.72

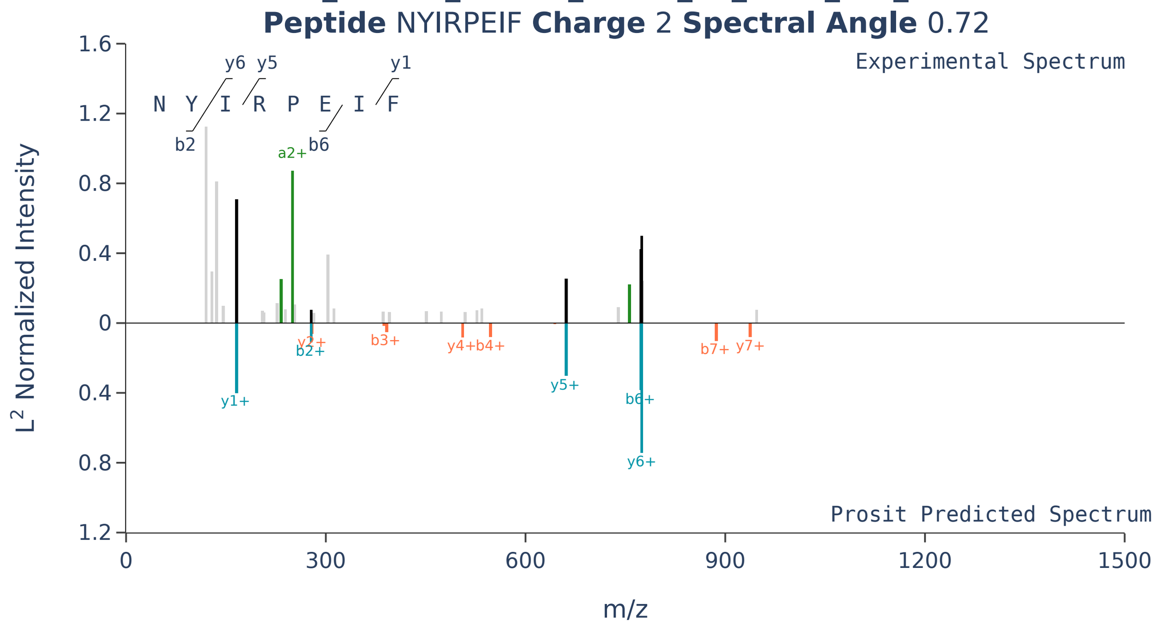


**Source** WSoh_101121_151121_HFGoe_G1_IL37b_2h_R2 **Scan** 13906
**Peptide** AAQKESARRPFIFYR **Charge** 4 **Spectral Angle** 0.85

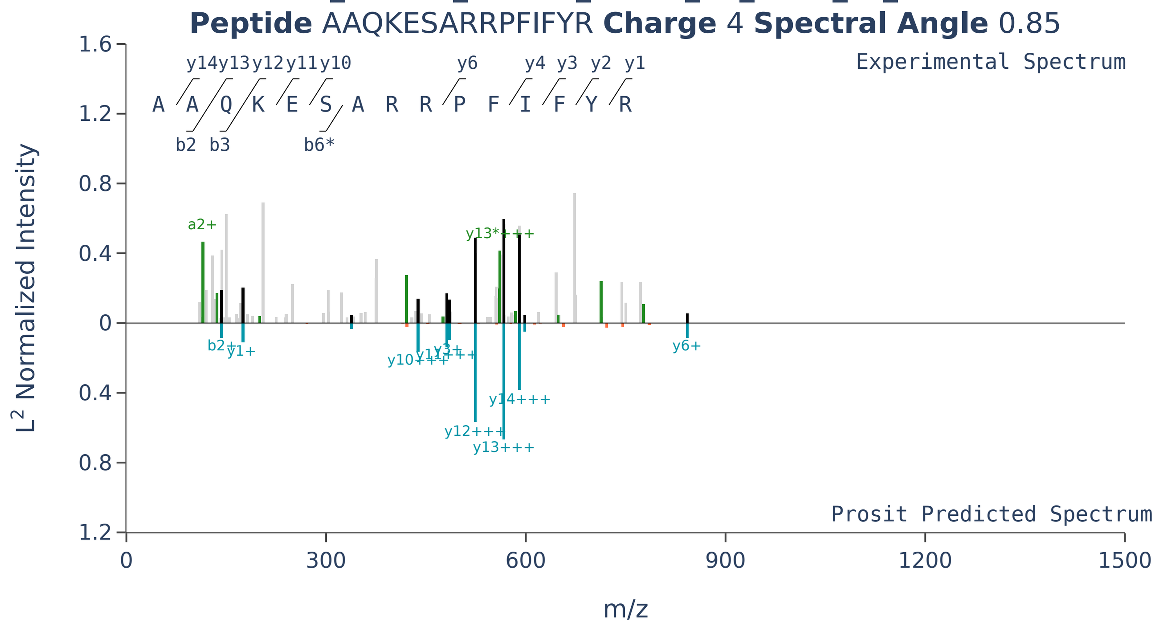


**Source** WSoh_101121_151121_HFGoe_G2_IL37b_2h_R2 **Scan** 22650
**Peptide** SAAHPGWFICTSC **Charge** 2 **Spectral Angle** 0.86

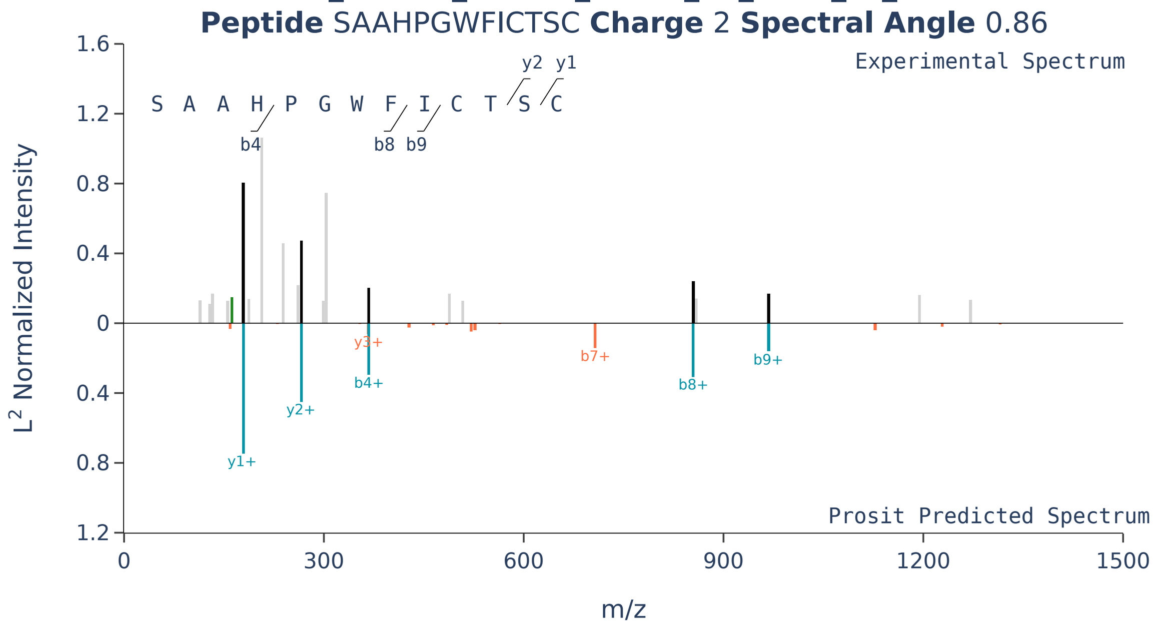


**Source** WSoh_101121_151121_HFGoe_G1_IL37b_24h_R2 **Scan** 11597
**Peptide** SKGEFCLYCDKDKGQ **Charge** 3 **Spectral Angle** 0.79

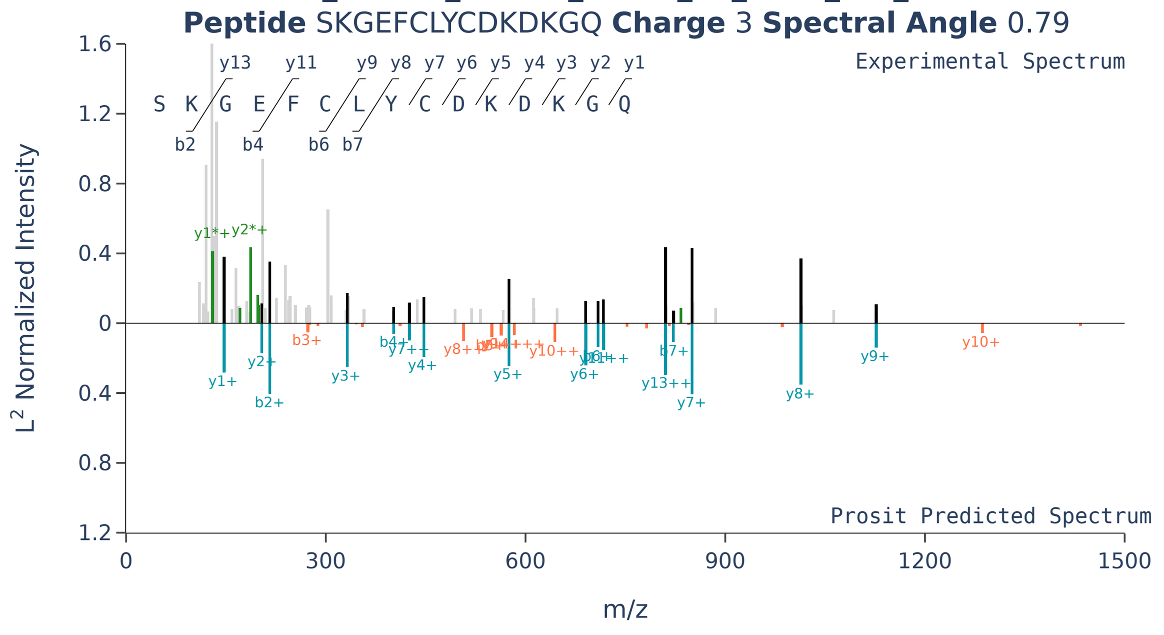


**Source** WSoh_101121_151121_HFGoe_G1_IL37b_4h_R1 **Scan** 11743
**Peptide** SCNCNEPVGVTDKFENRKHLE **Charge** 5 **Spectral Angle** 0.82

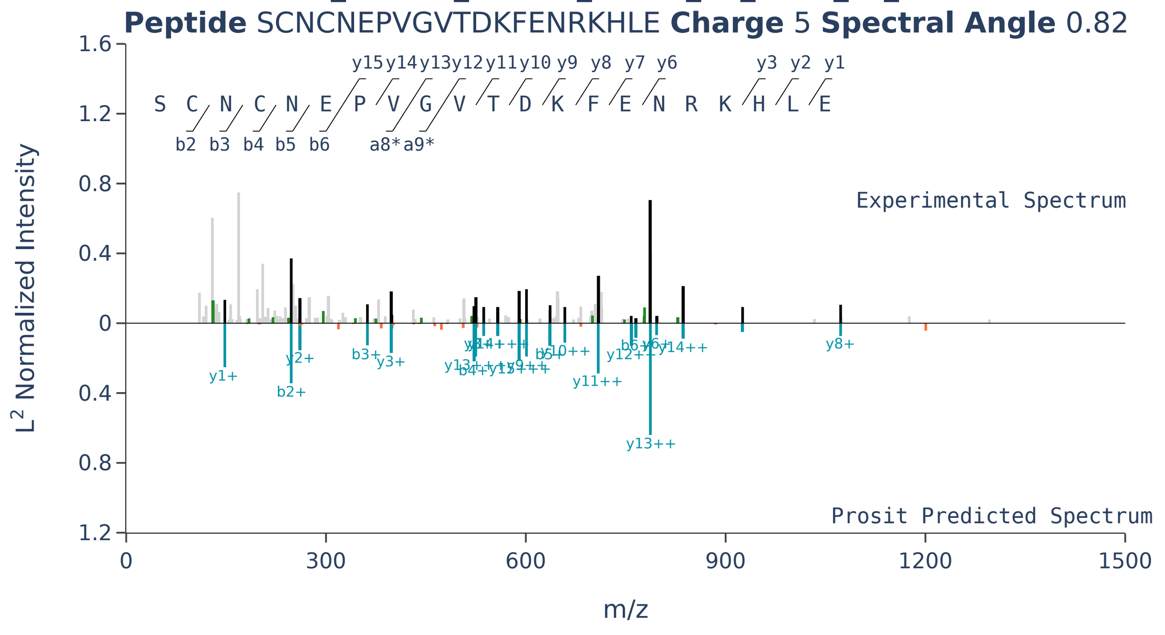


**Source** WSoh_101121_151121_HFGoe_G1_IL37b_4h_R1 **Scan** 5617
**Peptide** VHTSPKVKNLNPKK **Charge** 3 **Spectral Angle** 0.82

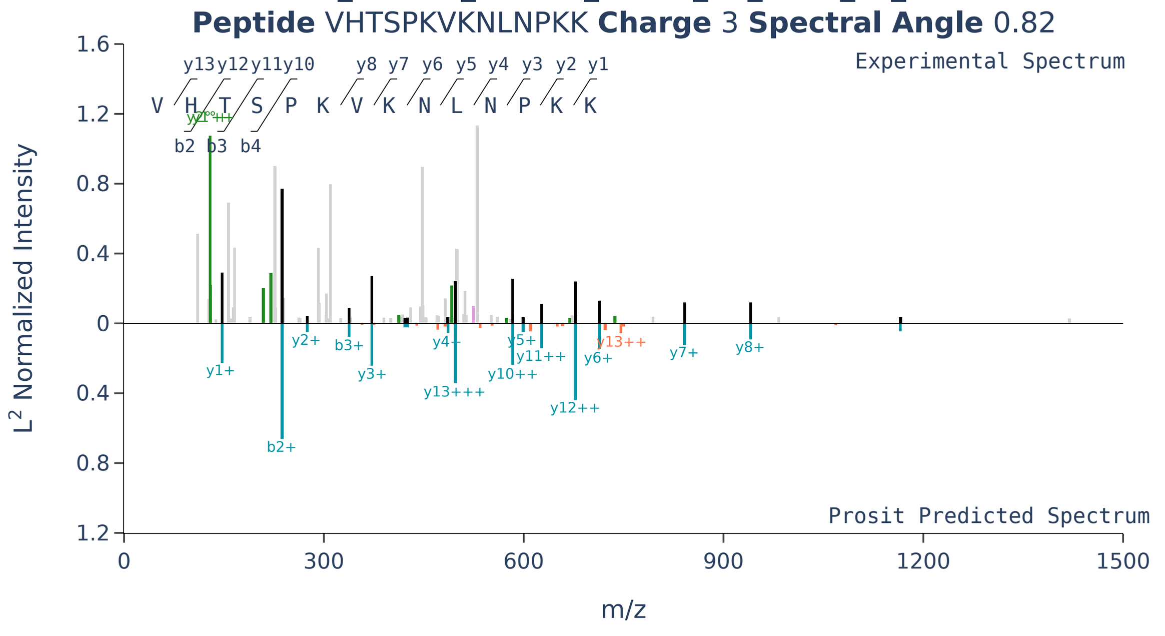


**Source** WSoh_101121_151121_HFGoe_G1_IL37b_24h_R1 **Scan** 23331
**Peptide** SSLSSASAEKGSPILL **Charge** 2 **Spectral Angle** 0.86

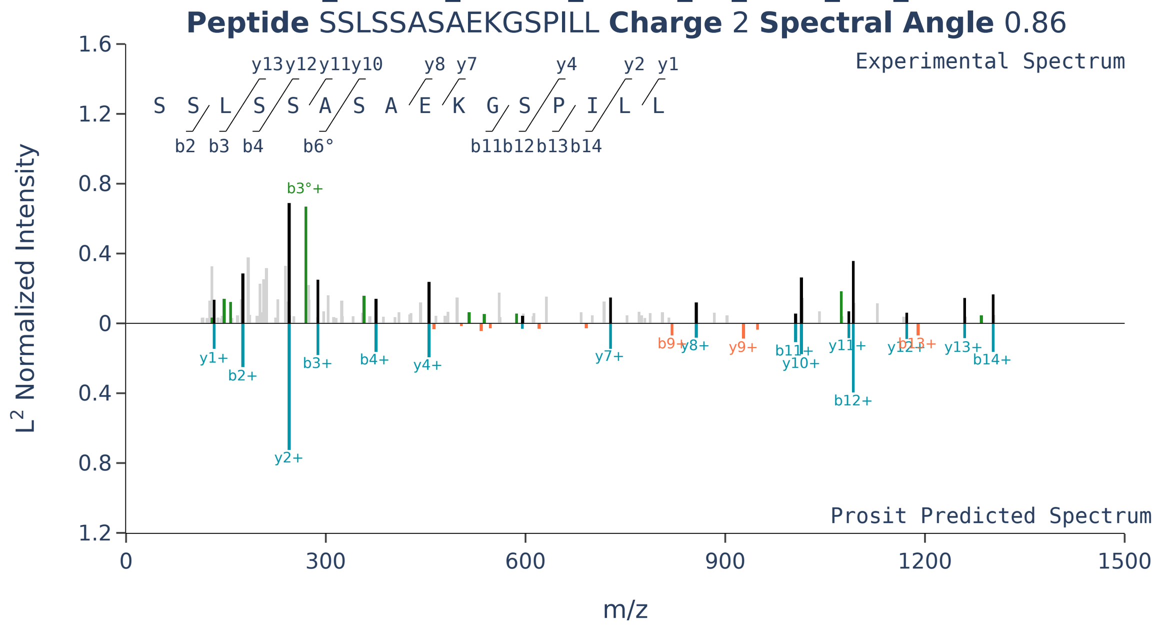


**Source** WSoh_101121_151121_HFGoe_G2_IL37b_2h_R1 **Scan** 19899
**Peptide** DSGNLIAVPDKNYIR **Charge** 2 **Spectral Angle** 0.86

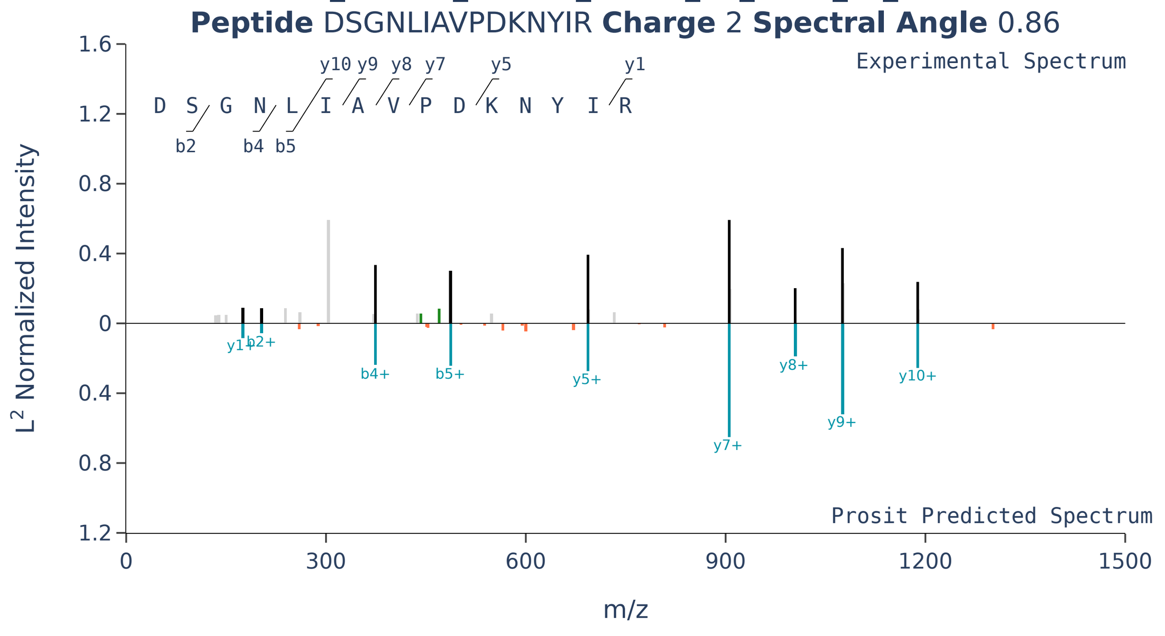


**Source** WSoh_101121_151121_HFGoe_G2_IL37b_2h_R2 **Scan** 9212
**Peptide** KESARRPFI **Charge** 3 **Spectral Angle** 0.78

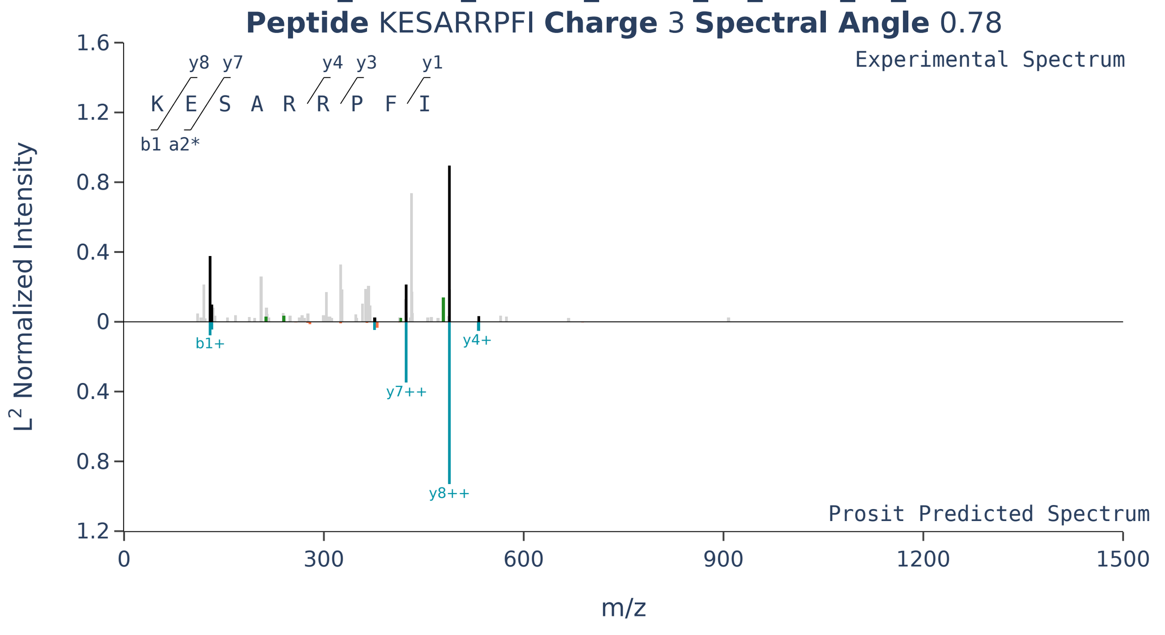


**Source** WSoh_101121_151121_HFGoe_G1_IL37b_4h_R1 **Scan** 8226
**Peptide** DKDKGQSHPSLQLK **Charge** 4 **Spectral Angle** 0.82

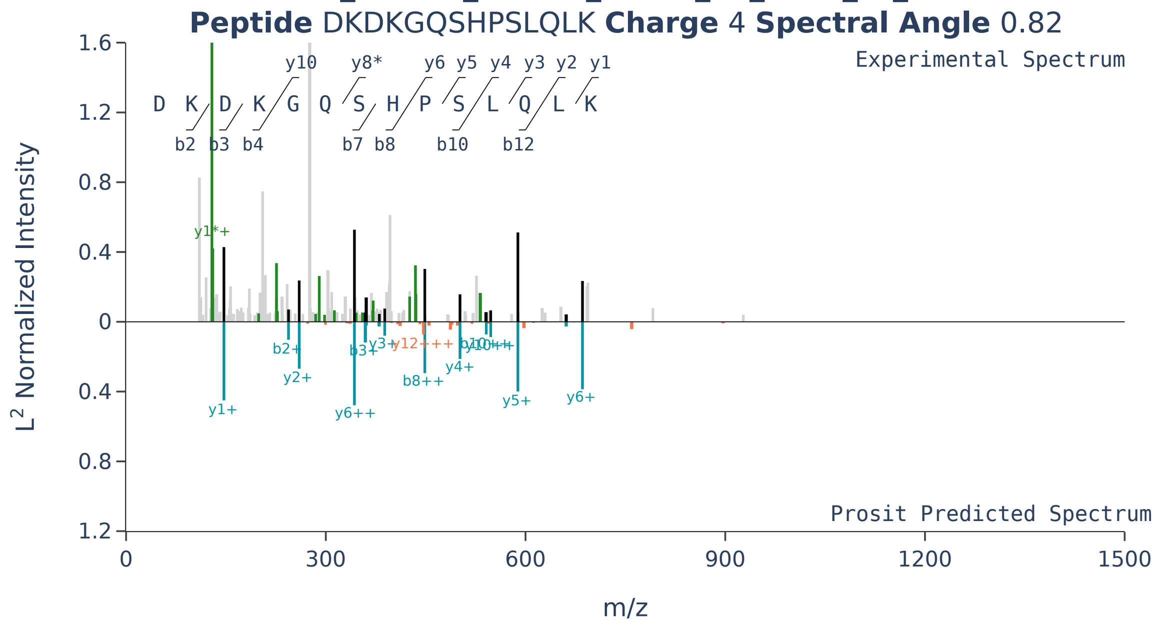


**Source** WSoh_101121_151121_HFGoe_G2_IL37b_24h_R1 **Scan** 18598
**Peptide** KGSPILLG **Charge** 2 **Spectral Angle** 0.65

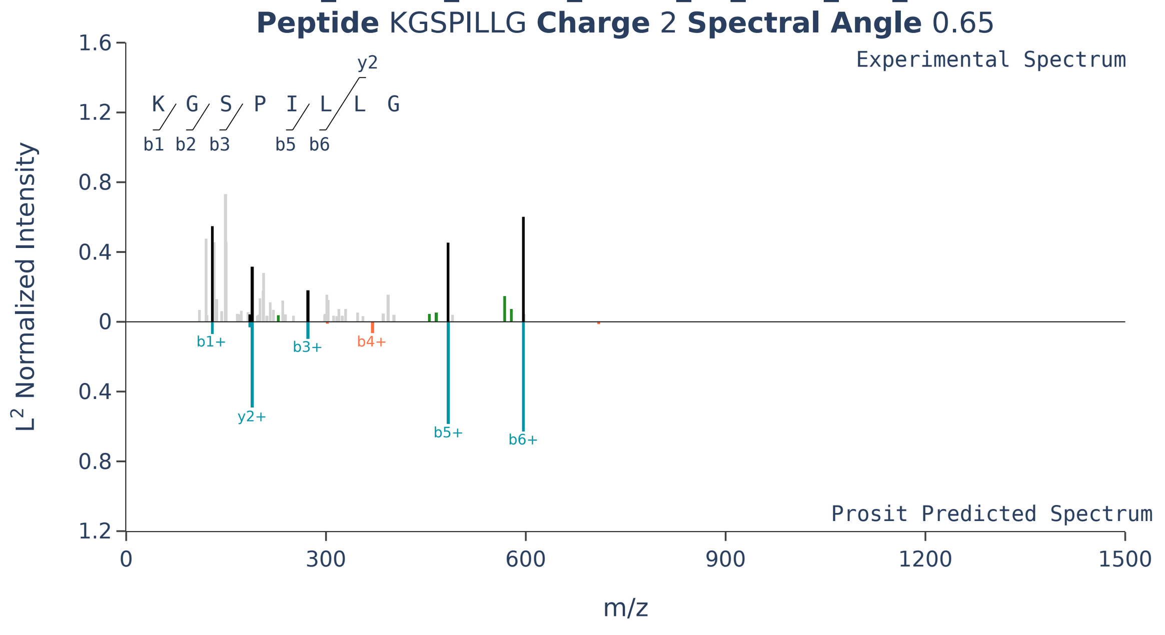


**Source** WSoh_101121_151121_HFGoe_G1_IL37b_24h_R1 **Scan** 11412
**Peptide** SKGEFCLYCDKDKGQS **Charge** 3 **Spectral Angle** 0.81

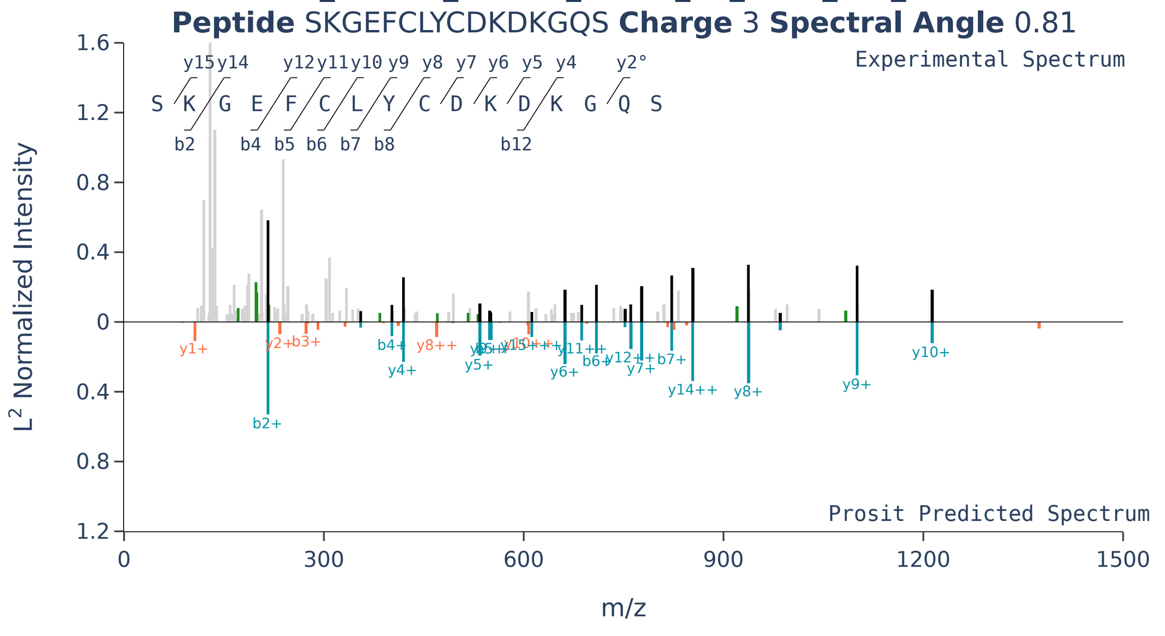


**Source** WSoh_101121_151121_HFGoe_G1_IL37b_24h_R2 **Scan** 23907
**Peptide** GNLIAVPDKNYI **Charge** 2 **Spectral Angle** 0.83

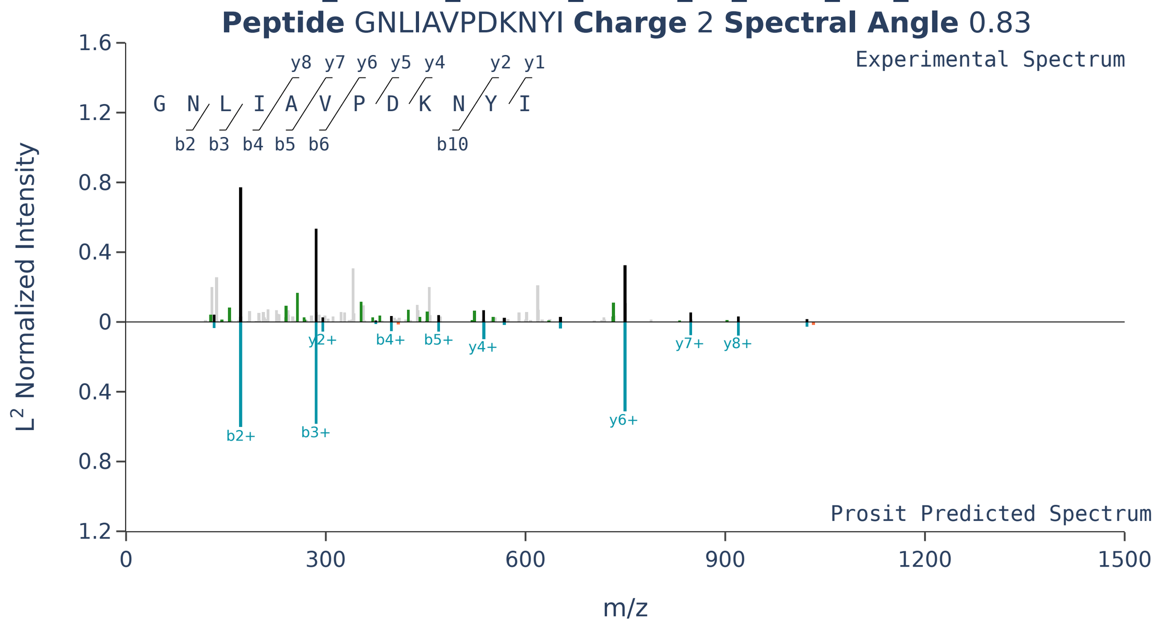


**Source** WSoh_101121_151121_HFGoe_G2_IL37b_24h_R1 **Scan** 10292
**Peptide** PKVKNLNPKKFSIHD **Charge** 3 **Spectral Angle** 0.76

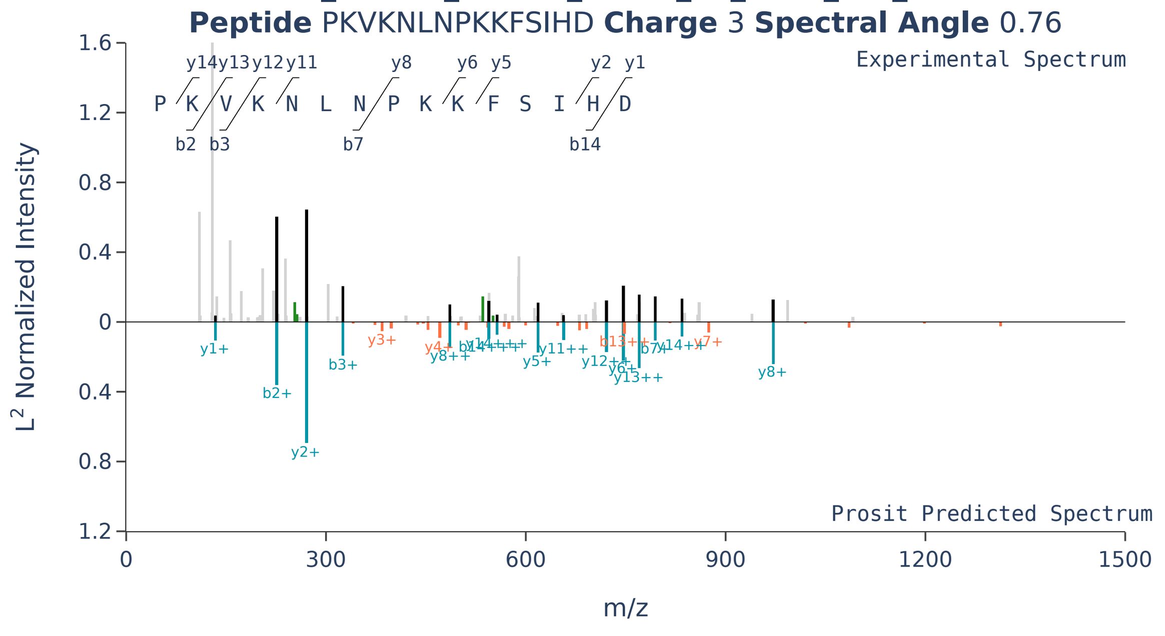

**Source** WSoh_101121_151121_HFGoe_G1_IL37b_4h_R2 **Scan** 11192
**Peptide** TSPKVKNLNPKKFSIHD **Charge** 4 **Spectral Angle** 0.82

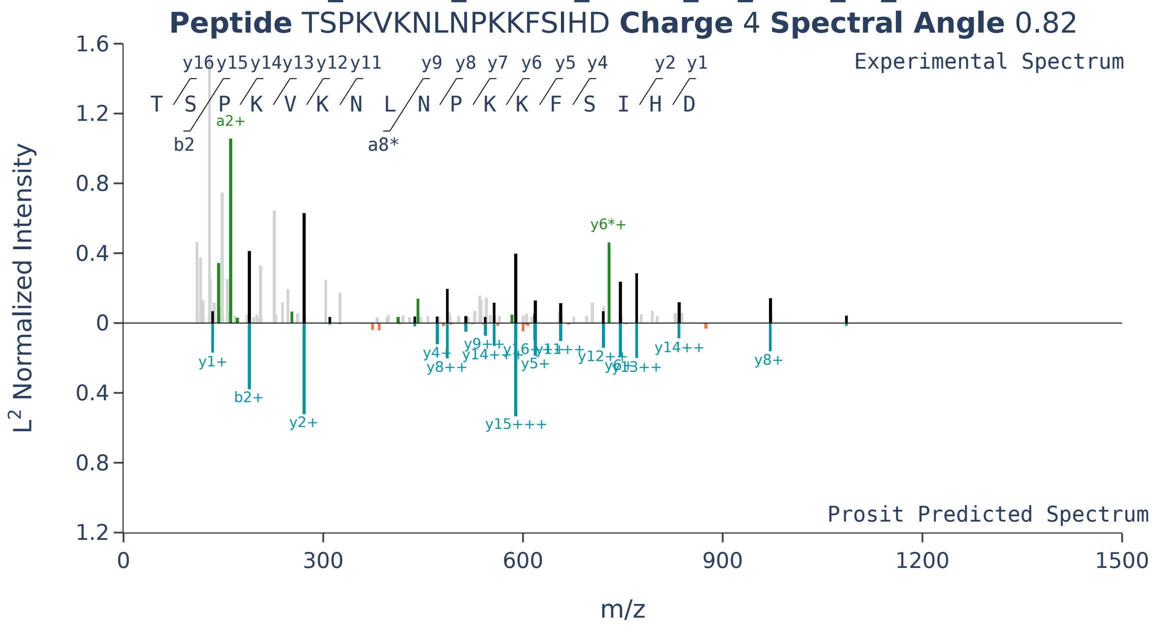

**Source** WSoh_101121_151121_HFGoe_G1_IL37b_24h_R1 **Scan** 8717
**Peptide** YCDKDKGQSHPSLQLKKEKL **Charge** 5 **Spectral Angle** 0.85

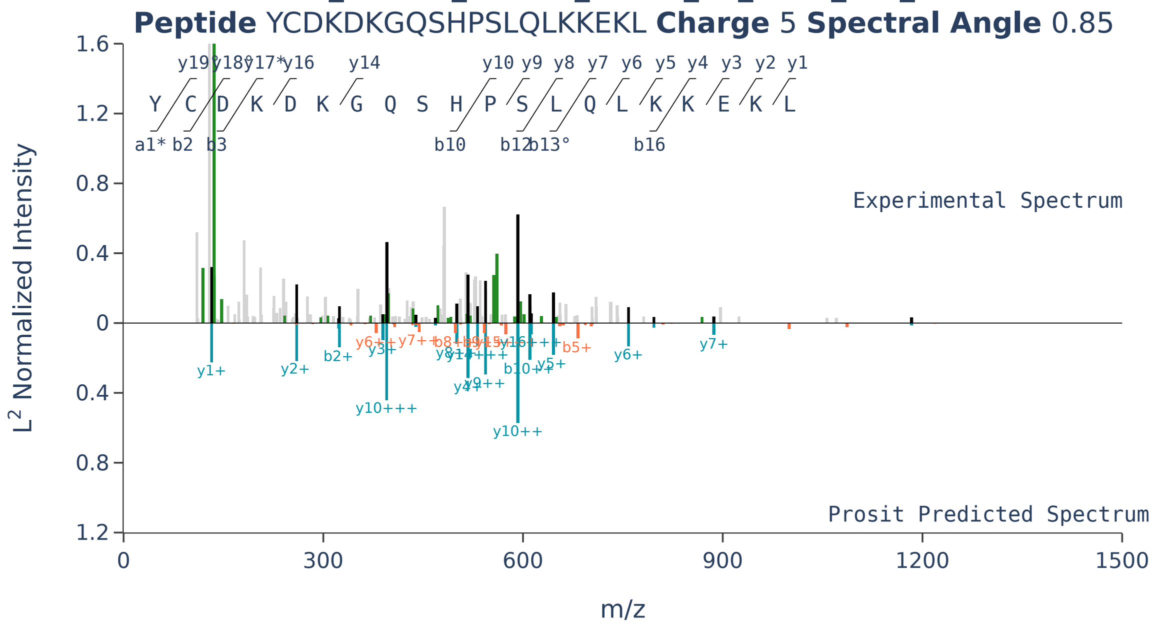

**Source** WSoh_101121_151121_HFGoe_G1_IL37b_24h_R1 **Scan** 10574
**Peptide** VPDKNYIRP **Charge** 3 **Spectral Angle** 0.78

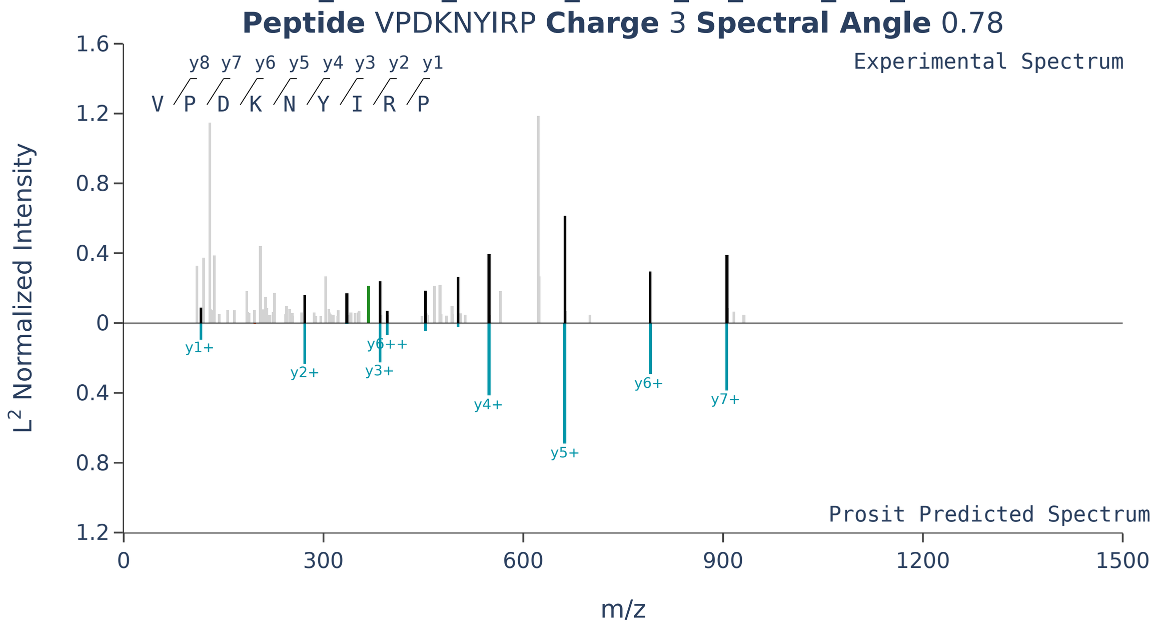

**Source** WSoh_101121_151121_HFGoe_G2_IL37b_24h_R2 **Scan** 5459
**Peptide** LYCDKDKGQSHPS **Charge** 2 **Spectral Angle** 0.82

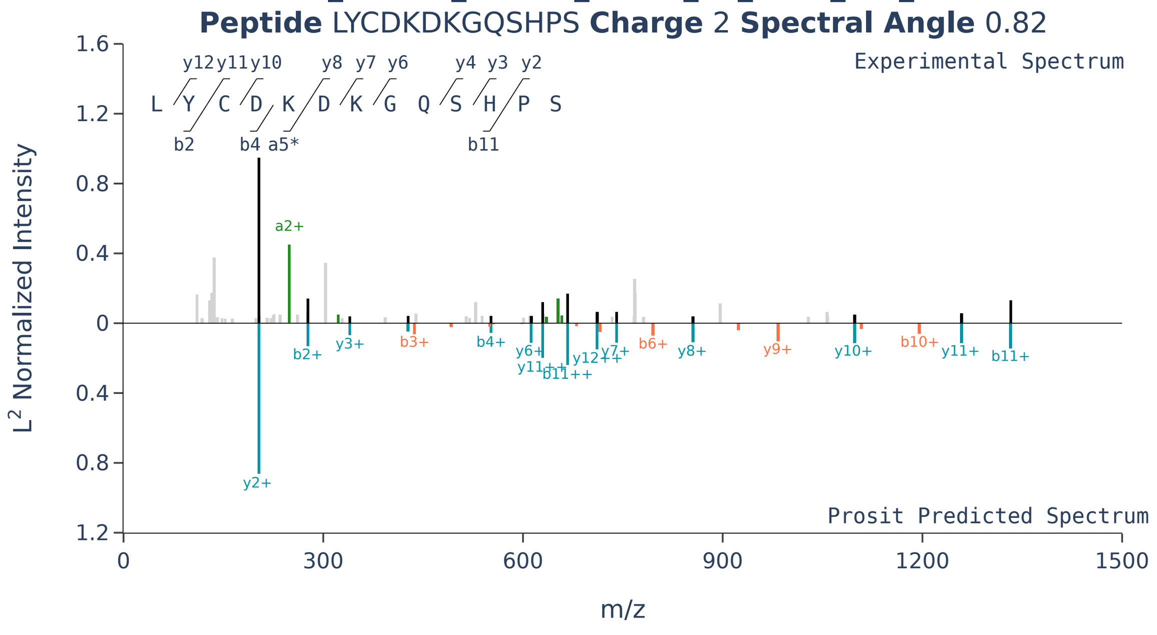

**Source** WSoh_101121_151121_HFGoe_G1_IL37b_4h_R1 **Scan** 10118
**Peptide** DKDKGQSHPSLQLKKEKLMKL **Charge** 3 **Spectral Angle** 0.78

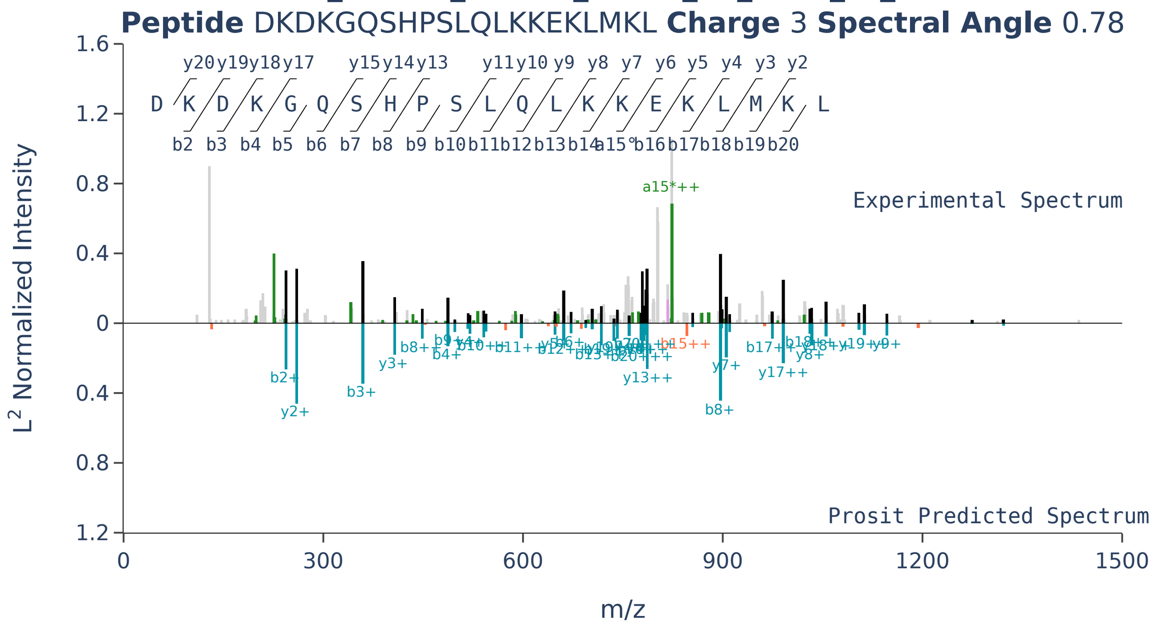

**Source** WSoh_101121_151121_HFGoe_G1_IL37b_4h_R1 **Scan** 7983
**Peptide** KFENRKHIEFS **Charge** 2 **Spectral Angle** 0.8

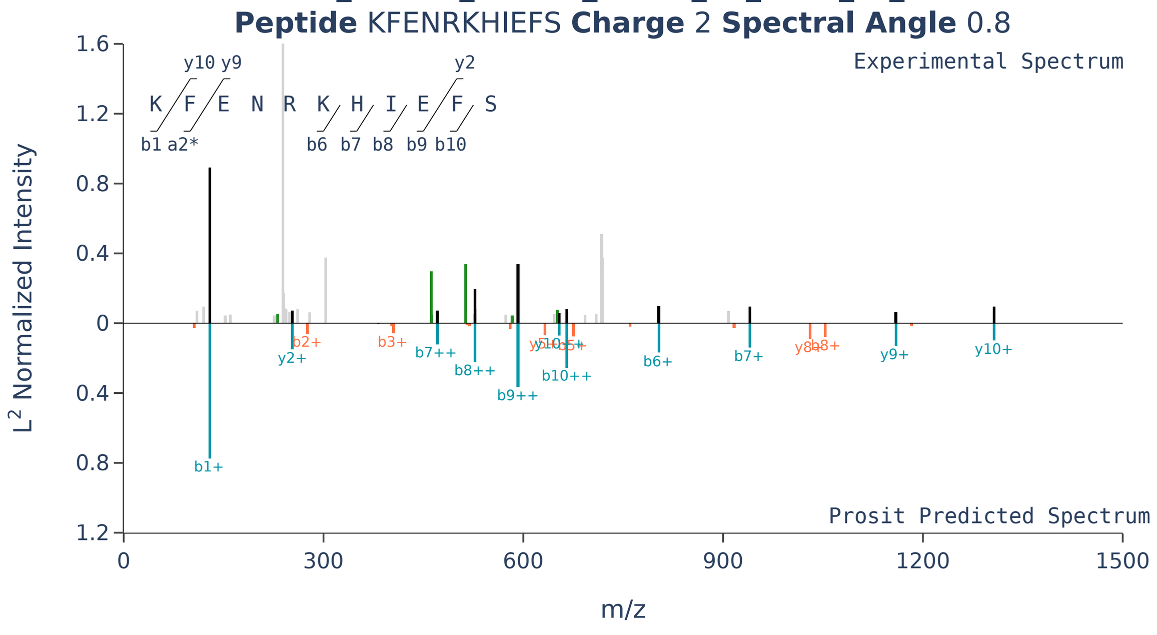

**Source** WSoh_101121_151121_HFGoe_G2_IL37b_24h_R2 **Scan** 19830
**Peptide** SLSSASAEKGSPILLGVSKG **Charge** 3 **Spectral Angle** 0.86

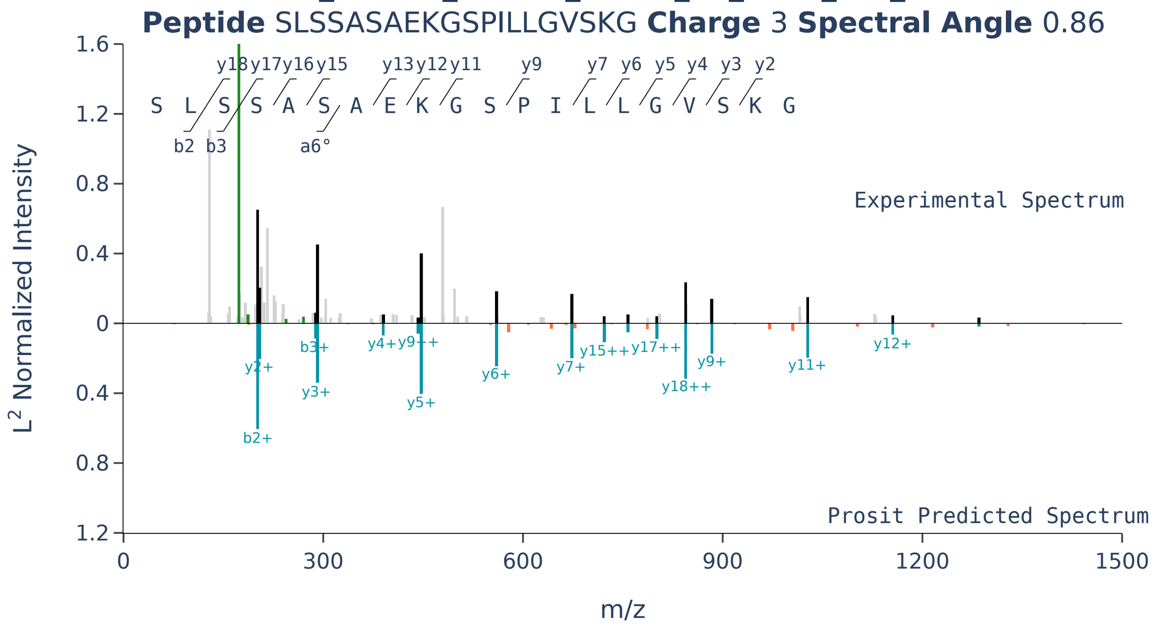

**Source** WSoh_101121_151121_HFGoe_G2_IL37b_24h_R1 **Scan** 12742
**Peptide** PKVKNLNPKKFSI **Charge** 3 **Spectral Angle** 0.74

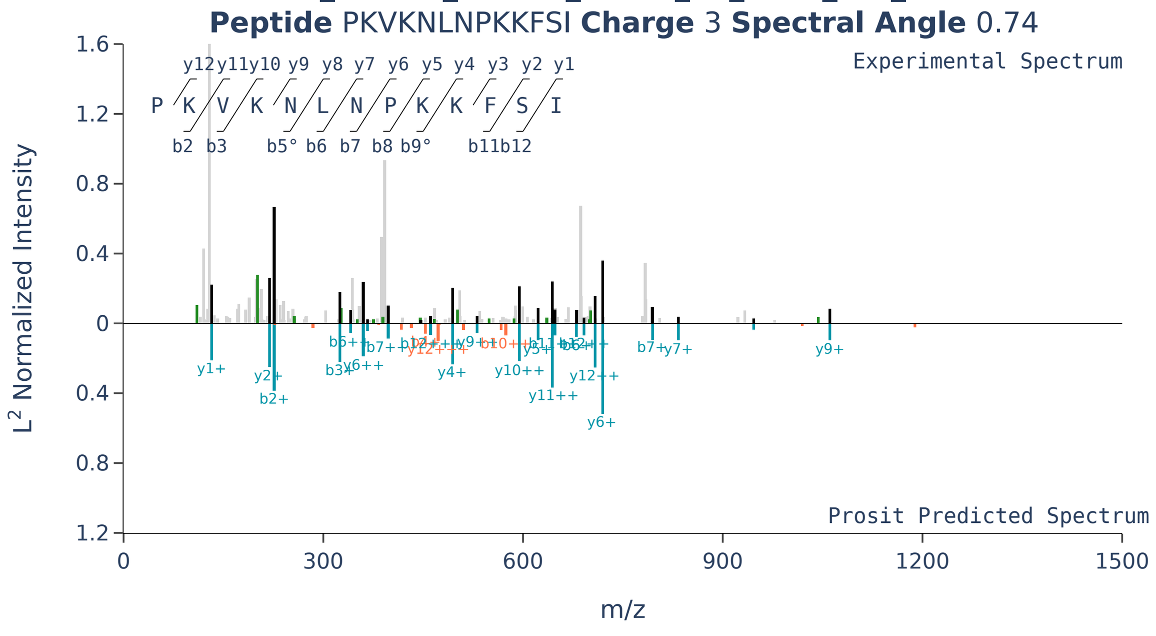

**Source** WSoh_101121_151121_HFGoe_G2_IL37b_24h_R1 **Scan** 14108
**Peptide** AAQKESARRPFIFYRAQ **Charge** 4 **Spectral Angle** 0.89

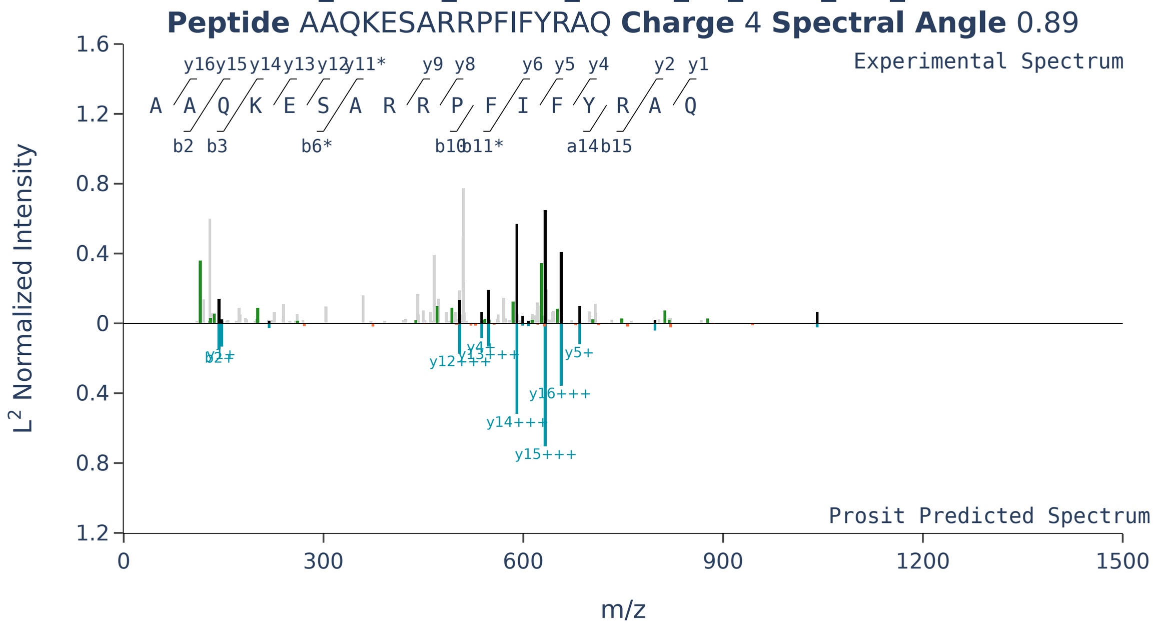

**Source** WSoh_101121_151121_HFGoe_G2_IL37b_24h_R1 **Scan** 14277
**Peptide** SFQPVCKAEMS **Charge** 2 **Spectral Angle** 0.76

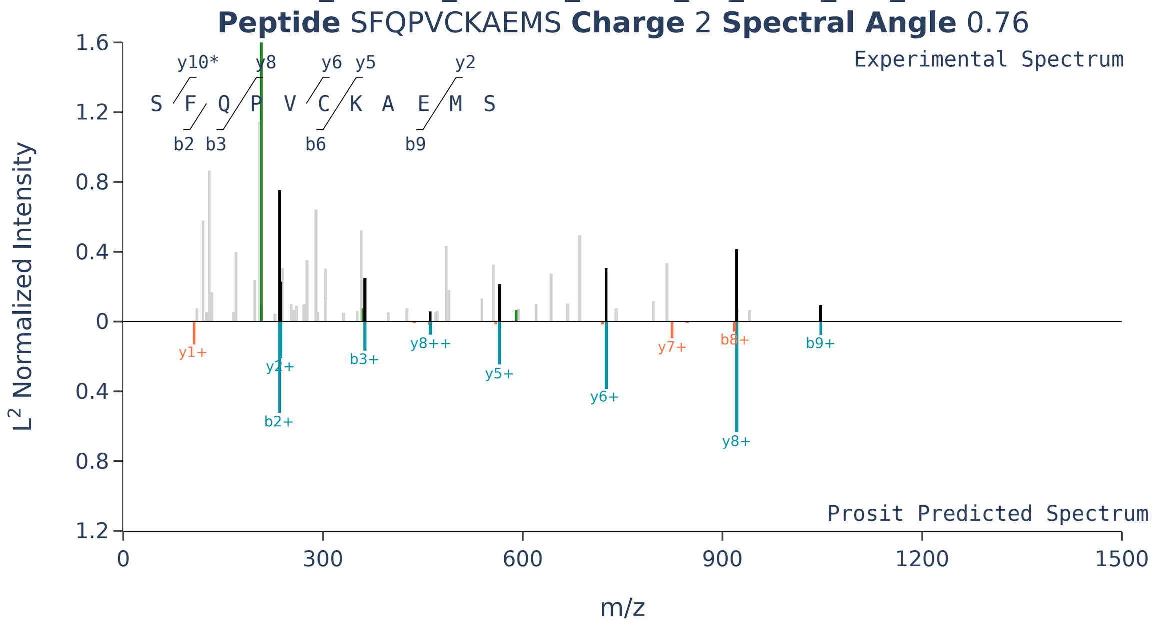

**Source** WSoh_101121_151121_HFGoe_G1_IL37b_4h_R2 **Scan** 15922
**Peptide** SARRPFIFYR **Charge** 3 **Spectral Angle** 0.88

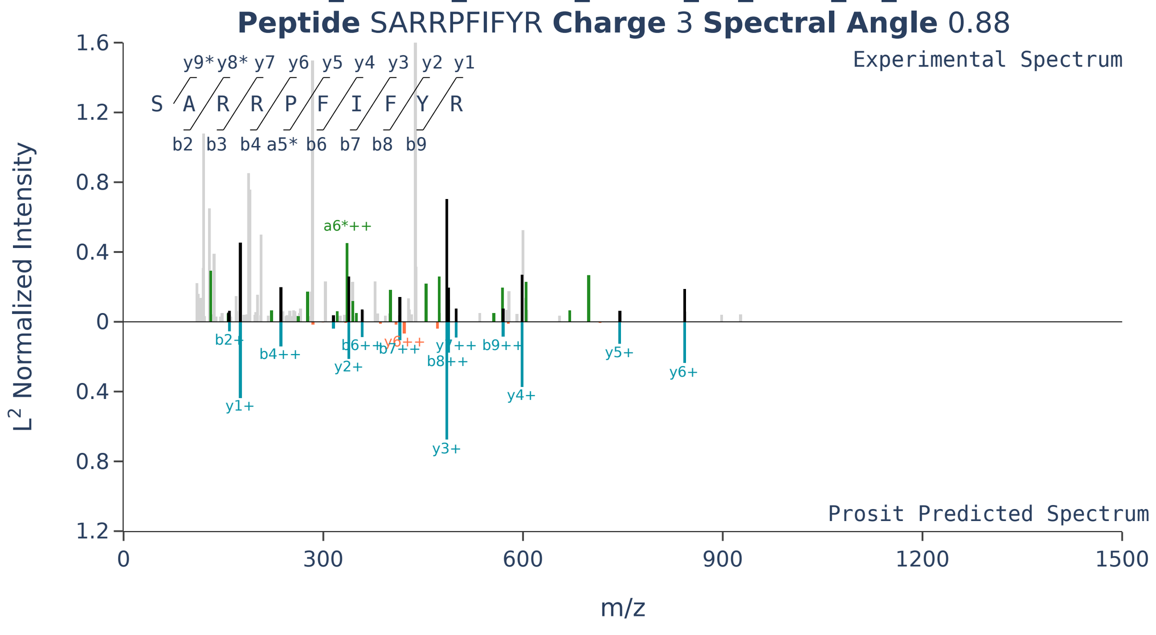

**Source** WSoh_101121_151121_HFGoe_G1_IL37b_24h_R2 **Scan** 9172
**Peptide** QPVCKAEMSPSE **Charge** 2 **Spectral Angle** 0.93

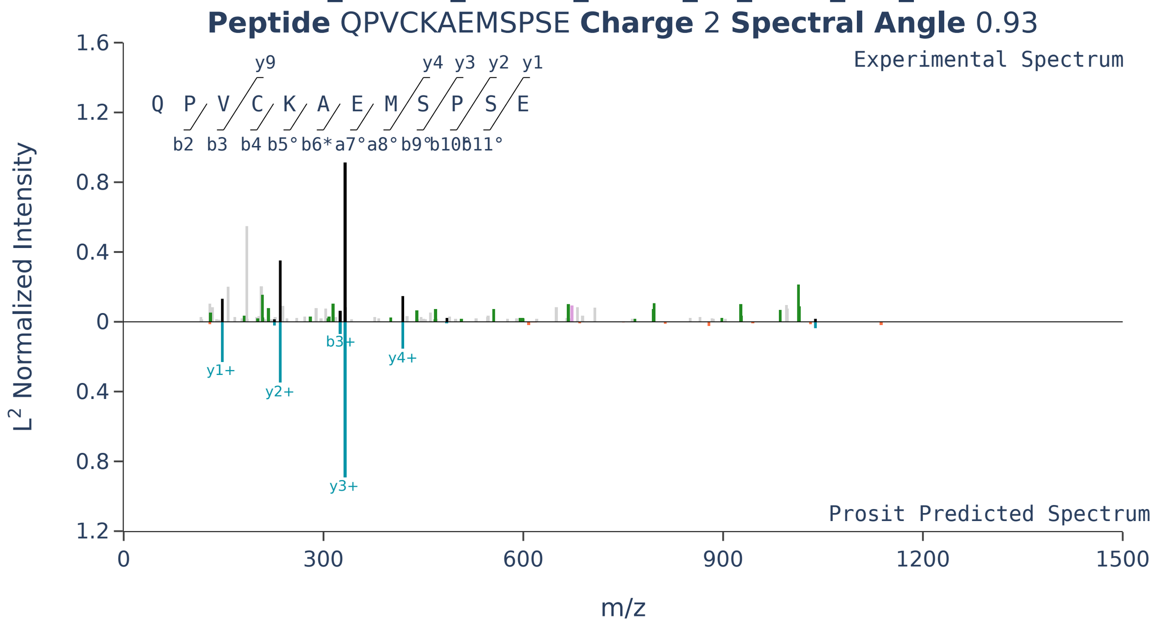

**Source** WSoh_101121_151121_HFGoe_G1_IL37b_4h_R1 **Scan** 9657
**Peptide** CNEPVGVTDKFENRKH **Charge** 4 **Spectral Angle** 0.86

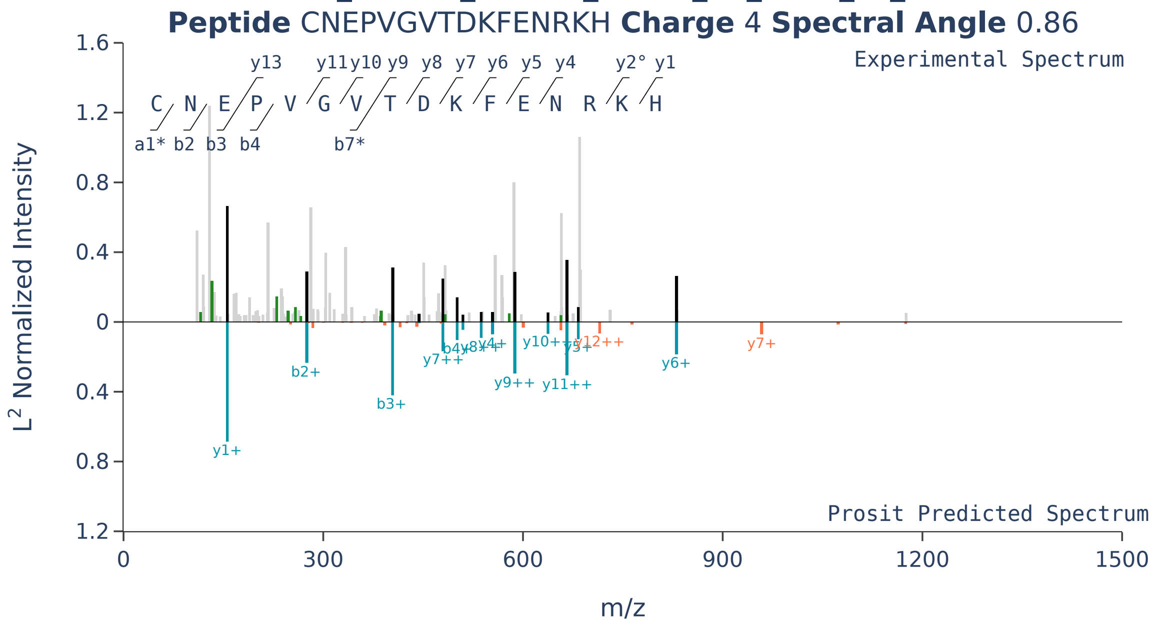

**Source** WSoh_101121_151121_HFGoe_G1_IL37b_24h_R1 **Scan** 7183
**Peptide** VHTSPKVKNLNP **Charge** 3 **Spectral Angle** 0.91

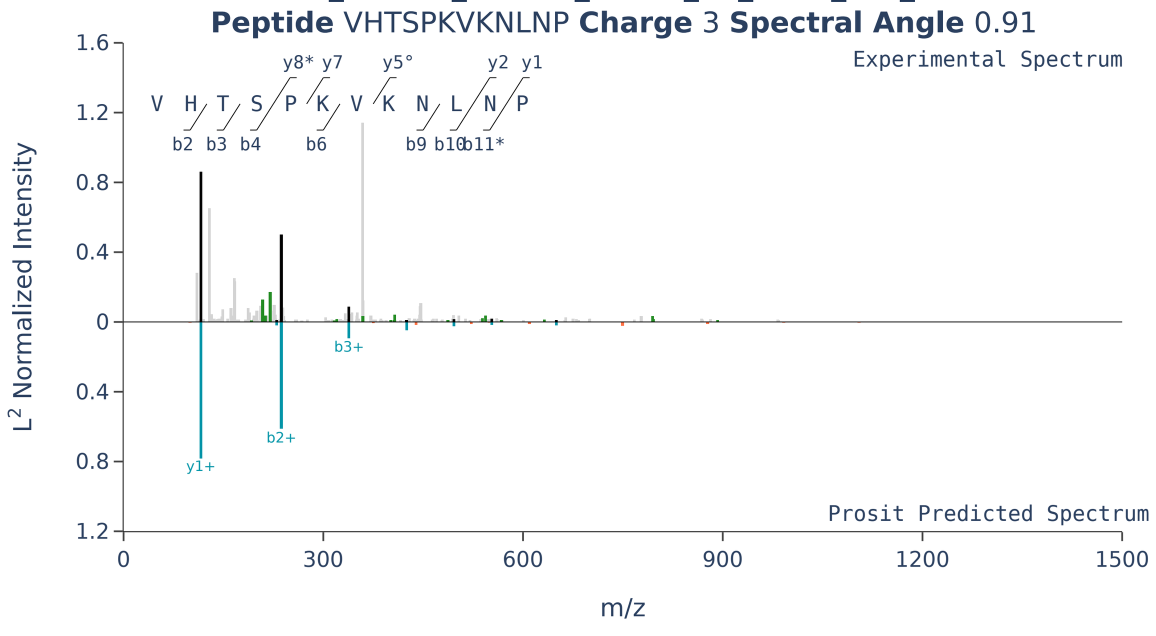

**Source** WSoh_101121_151121_HFGoe_G1_IL37b_24h_R1 **Scan** 11697
**Peptide** SCNCNEPVGVTDKFENRKHIE **Charge** 3 **Spectral Angle** 0.81

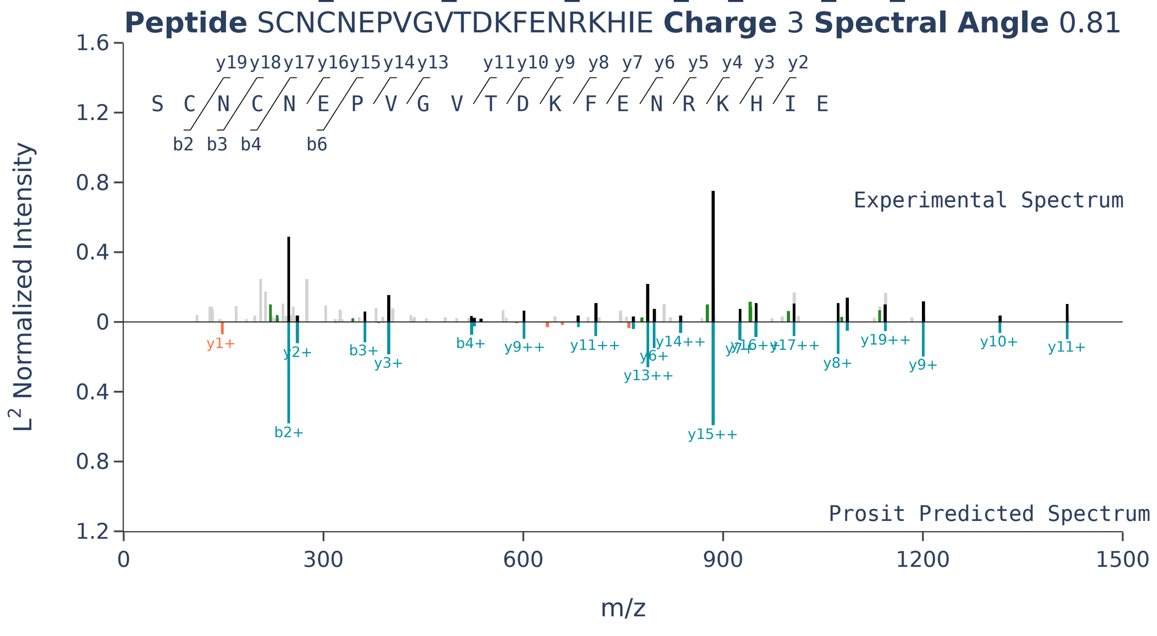

**Source** WSoh_101121_151121_HFGoe_G1_IL37b_2h_R1 **Scan** 6835
**Peptide** SLQLKKEK **Charge** 2 **Spectral Angle** 0.77

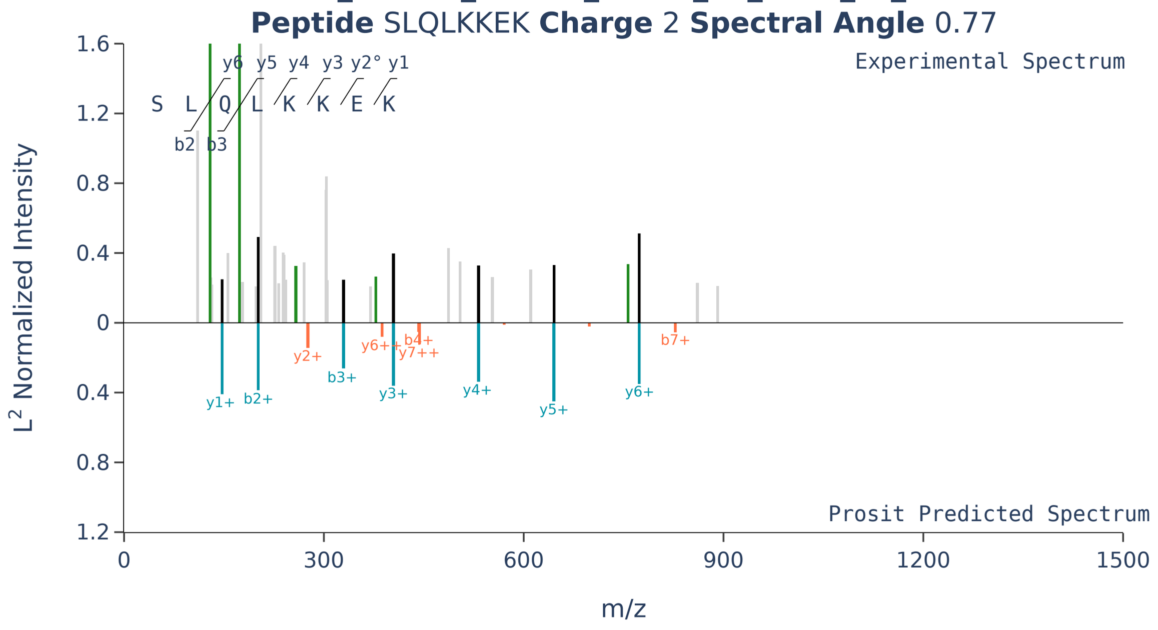

**Source** WSoh_101121_151121_HFGoe_G1_IL37b_24h_R1 **Scan** 20285
**Peptide** KGEFCLY **Charge** 2 **Spectral Angle** 0.83

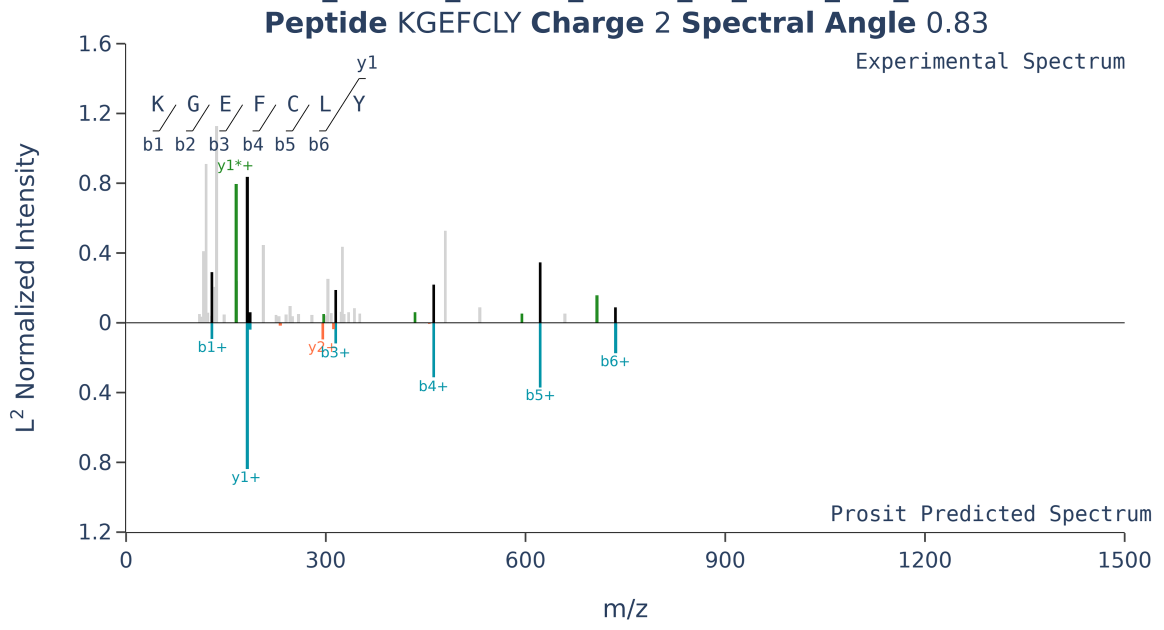

**Source** WSoh_101121_151121_HFGoe_G2_IL37b_1h_R1 **Scan** 18283
**Peptide** SSASAEKGSPILLG **Charge** 2 **Spectral Angle** 0.85

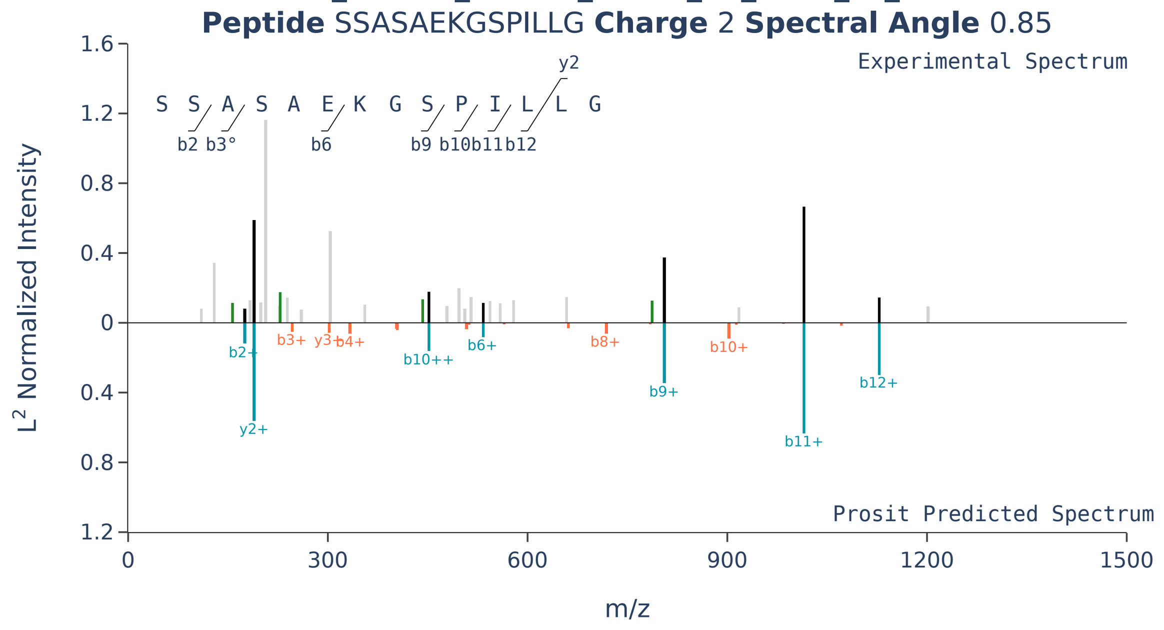

**Source** WSoh_101121_151121_HFGoe_G2_IL37b_24h_R1 **Scan** 21298
**Peptide** AQVGSWNM **Charge** 2 **Spectral Angle** 0.79

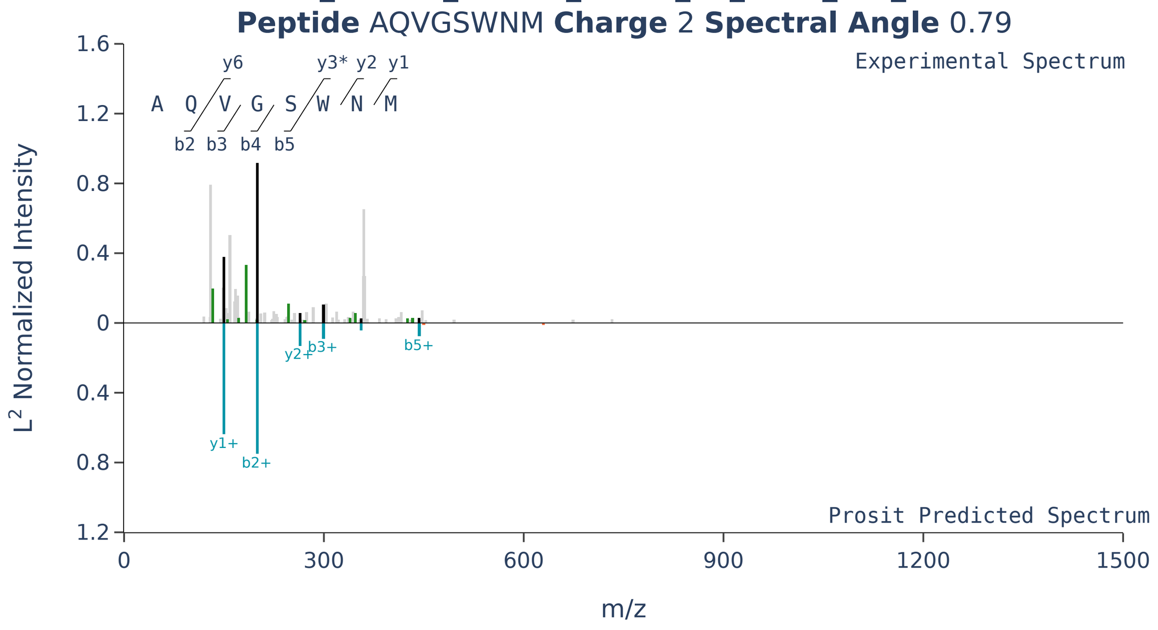

**Source** WSoh_101121_151121_HFGoe_G1_IL37b_4h_R2 **Scan** 19756
**Peptide** SLSSASAEKGSPILLGVSK **Charge** 3 **Spectral Angle** 0.76

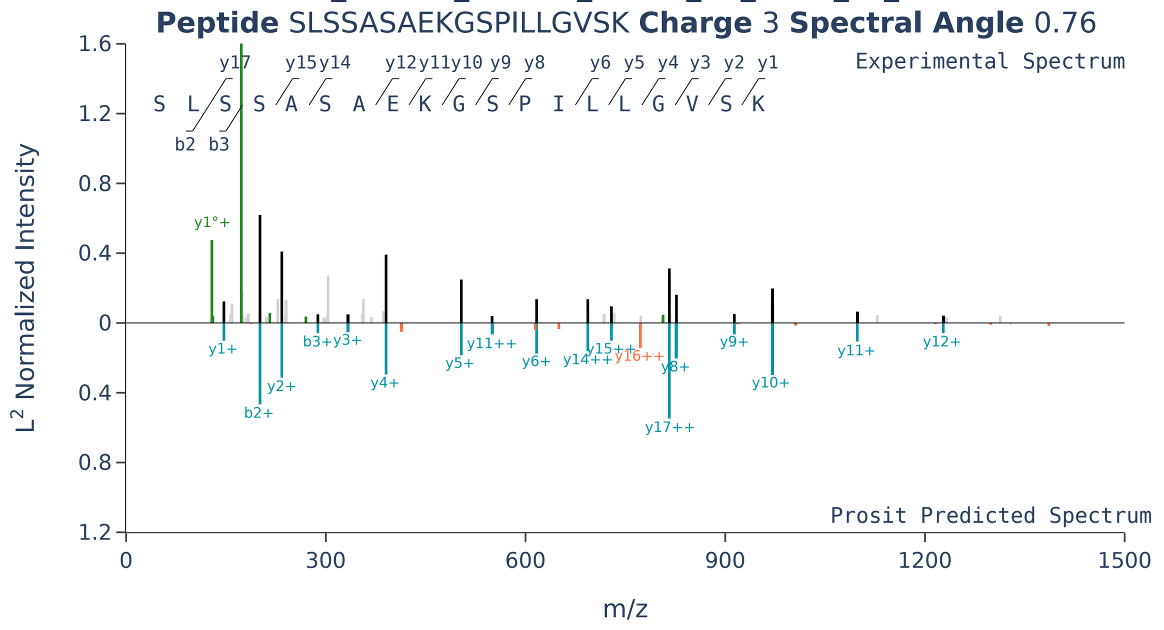

**Source** WSoh_101121_151121_HFGoe_G2_IL37b_24h_R1 **Scan** 16652
**Peptide** NCNEPVGVTDKFE **Charge** 2 **Spectral Angle** 0.85

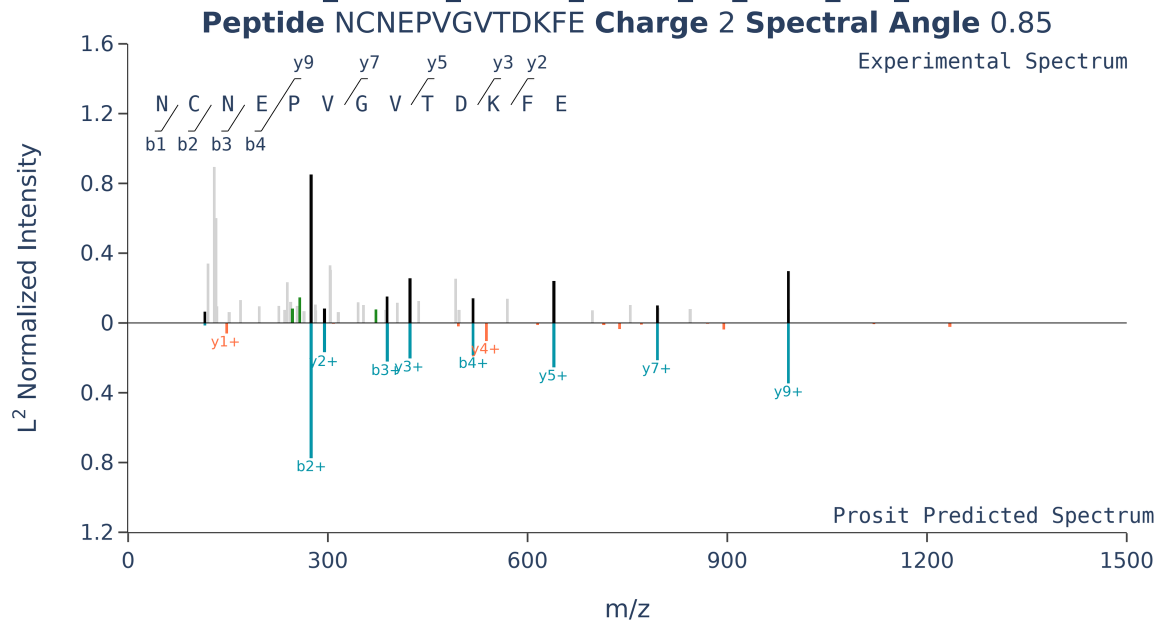

**Source** WSoh_101121_151121_HFGoe_G2_IL37b_4h_R2 **Scan** 18862
**Peptide** LLAVPDKNYIRPE **Charge** 3 **Spectral Angle** 0.79

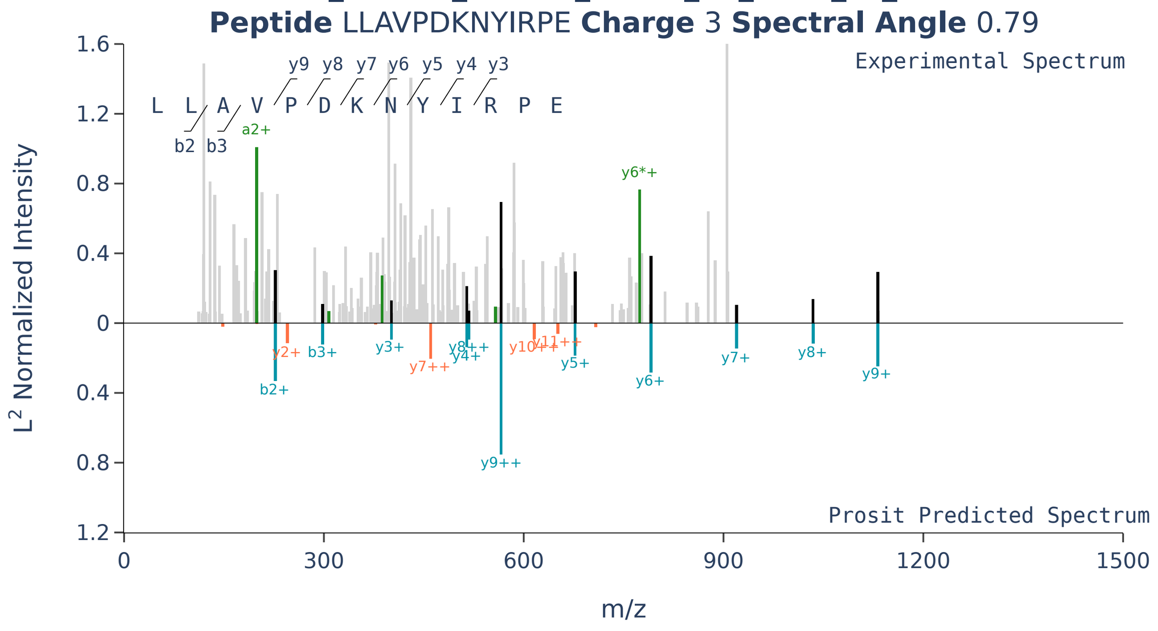

**Source** WSoh_101121_151121_HFGoe_G2_IL37b_24h_R2 **Scan** 7854
**Peptide** KLMKLAAQKESA **Charge** 3 **Spectral Angle** 0.89

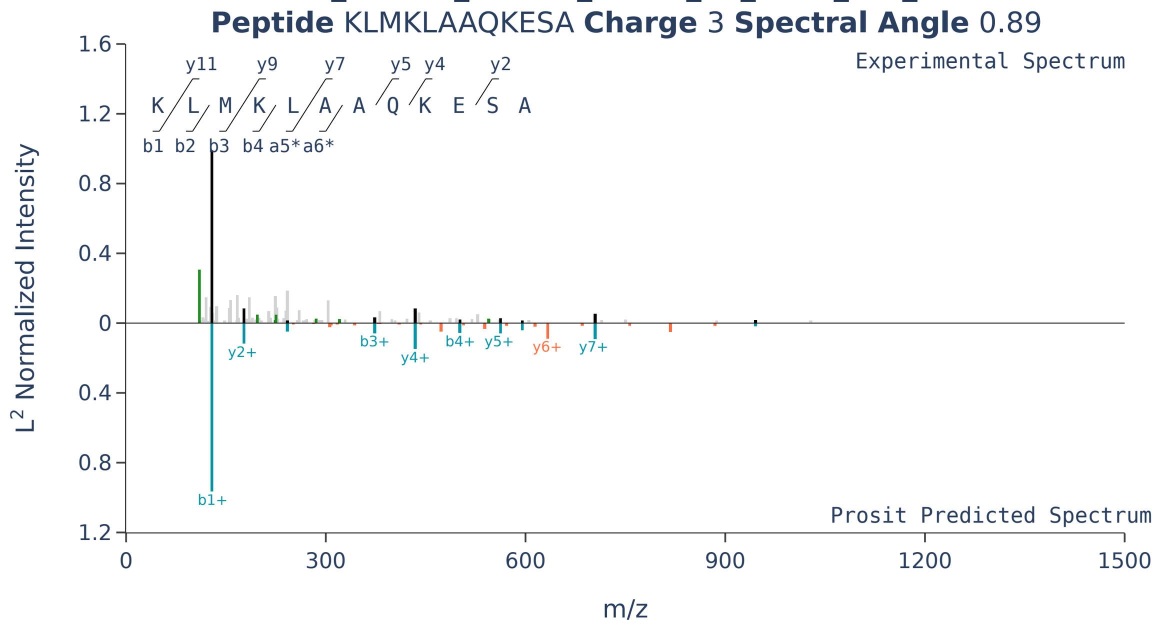

**Source** WSoh_101121_151121_HFGoe_G1_IL37b_24h_R1 **Scan** 11954
**Peptide** NLNPKKFSIHDQD **Charge** 2 **Spectral Angle** 0.81

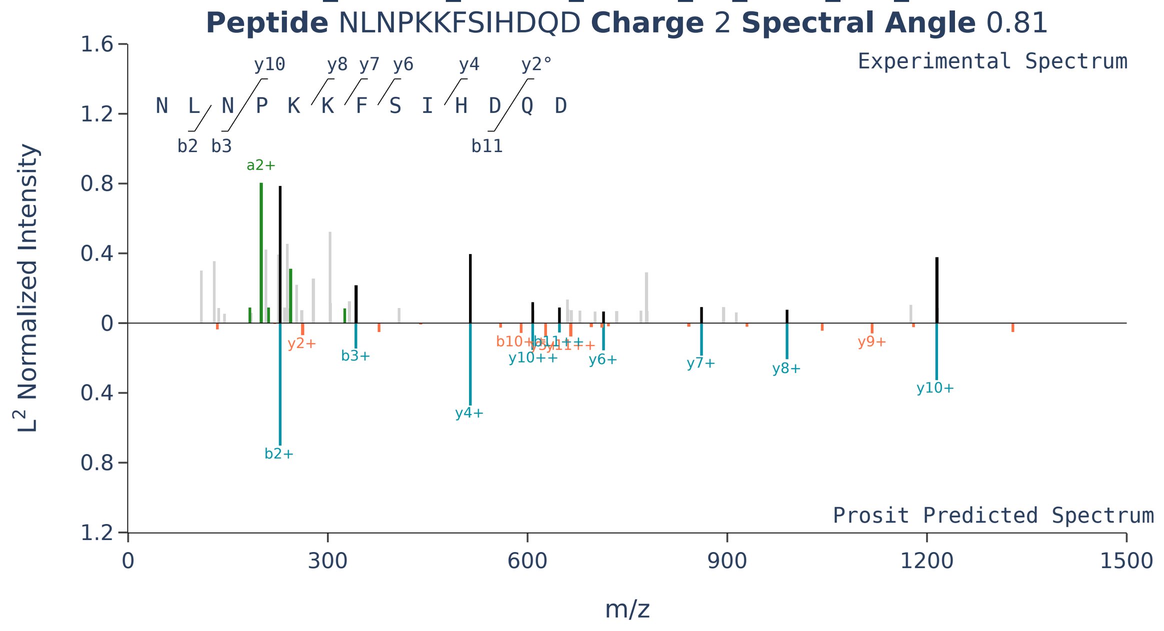

**Source** WSoh_101121_151121_HFGoe_G1_IL37b_24h_R2 **Scan** 10860
**Peptide** NEPVGVTDKFENRKHIE **Charge** 3 **Spectral Angle** 0.85

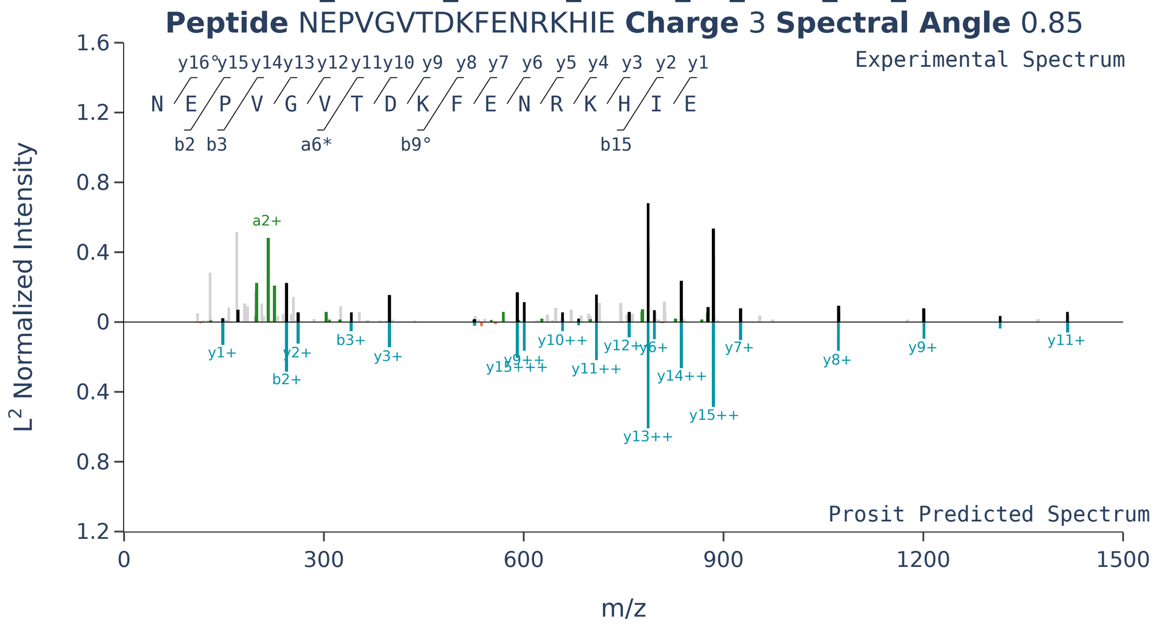

**Source** WSoh_101121_151121_HFGoe_G2_IL37b_24h_R2 **Scan** 5740
**Peptide** LQLKKEK **Charge** 2 **Spectral Angle** 0.64

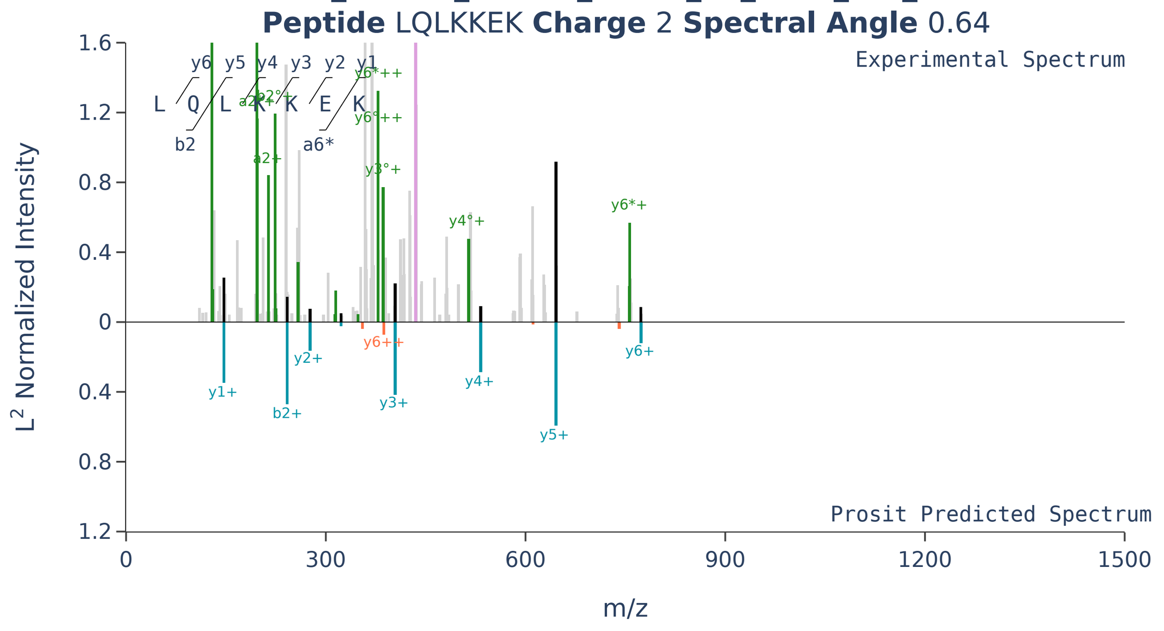

**Source** WSoh_101121_151121_HFGoe_G1_IL37b_24h_R1 **Scan** 7370
**Peptide** DKGQSHPSL **Charge** 2 **Spectral Angle** 0.81

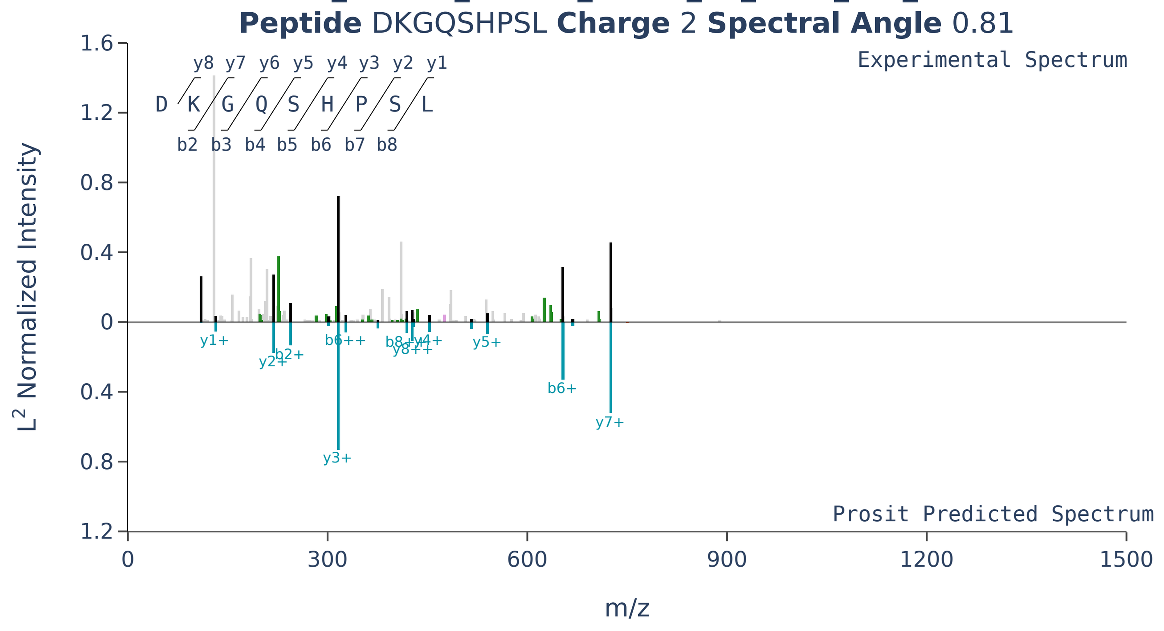

**Source** WSoh_101121_151121_HFGoe_G1_IL37b_24h_R2 **Scan** 9738
**Peptide** QLKKEKLMKLA **Charge** 2 **Spectral Angle** 0.68

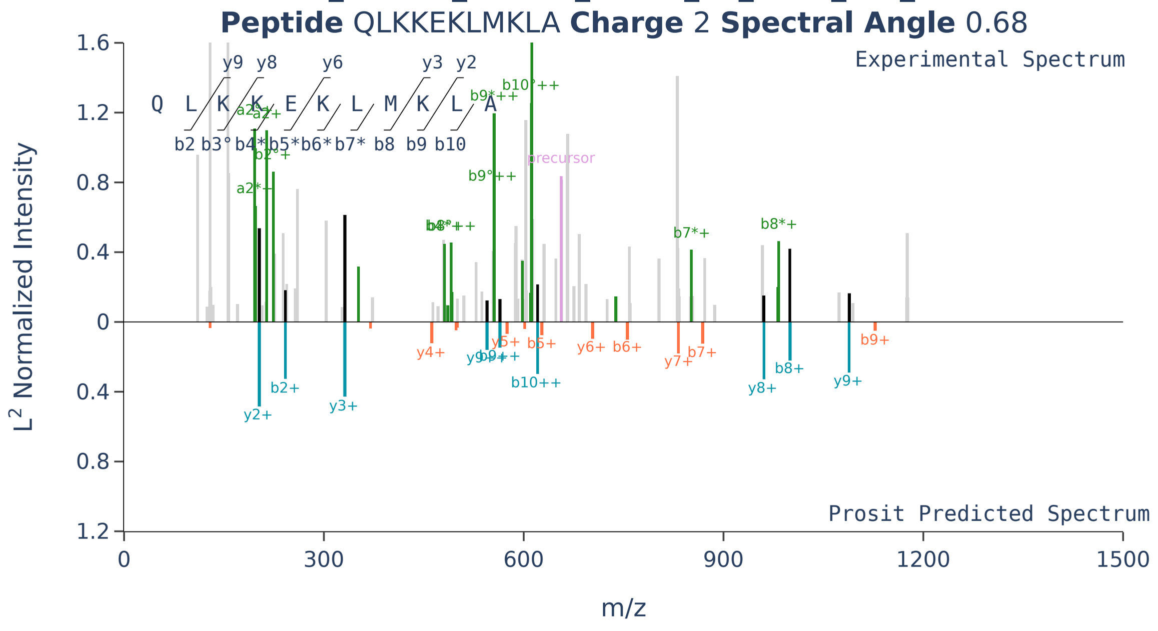

**Source** WSoh_101121_151121_HFGoe_G1_IL37b_4h_R2 **Scan** 15387
**Peptide** NPKKFSIHDQDHKVLVL **Charge** 4 **Spectral Angle** 0.89

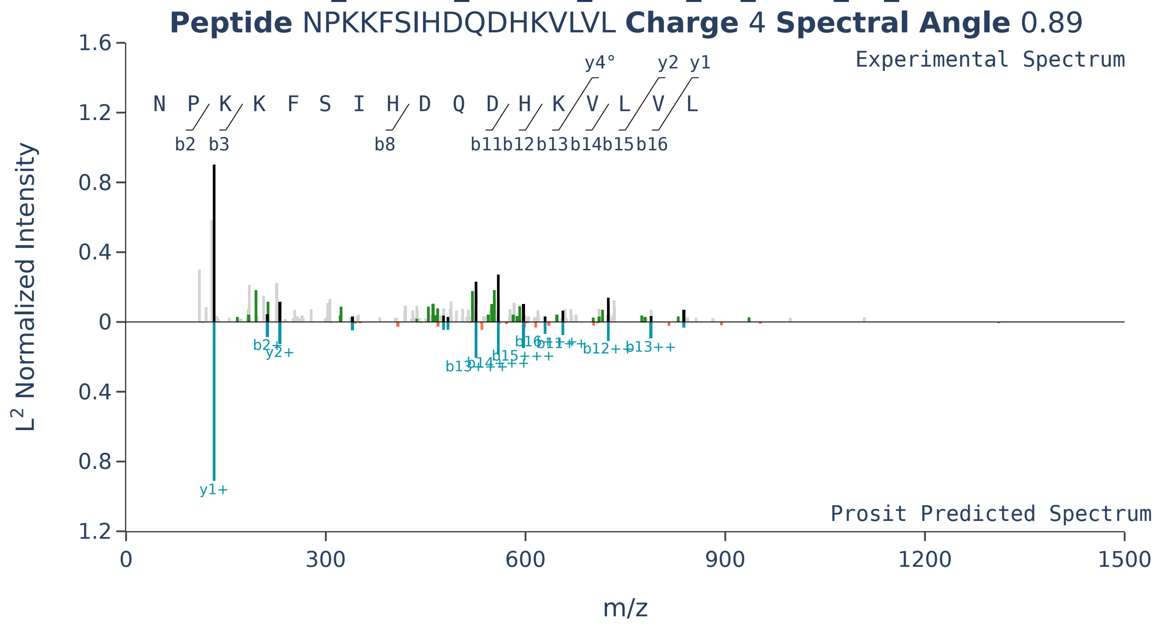

**Source** WSoh_101121_151121_HFGoe_G1_IL37b_4h_R2 **Scan** 9602
**Peptide** SLHDQDHKVLV **Charge** 3 **Spectral Angle** 0.71

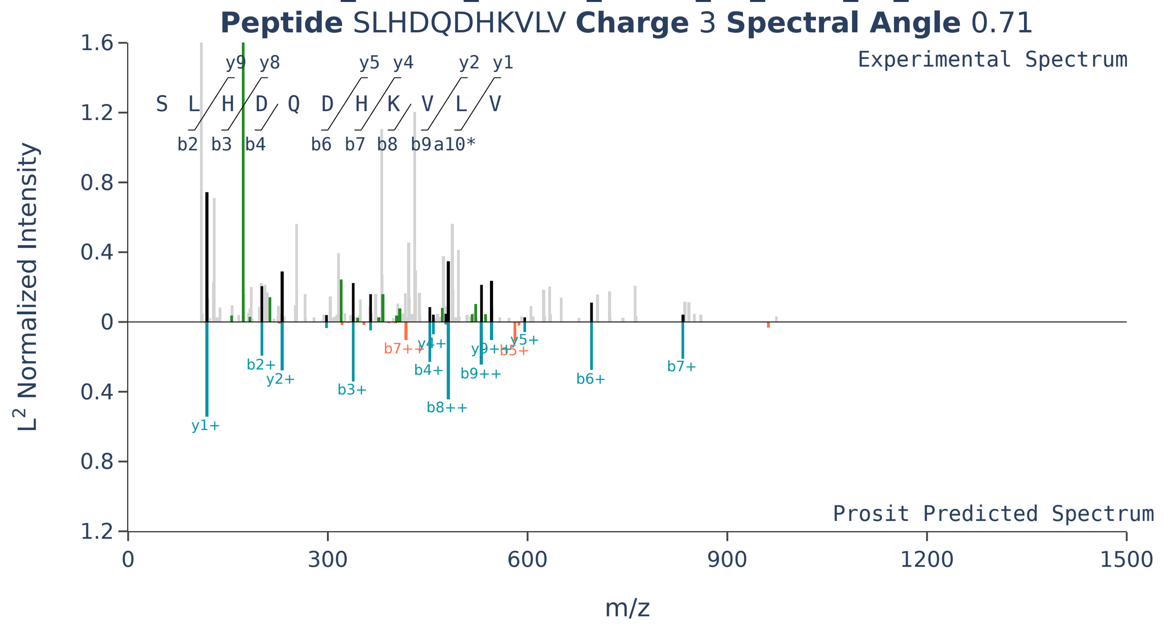

**Source** WSoh_101121_151121_HFGoe_G1_IL37b_2h_R2 **Scan** 22602
**Peptide** KGSPILLGV **Charge** 2 **Spectral Angle** 0.71

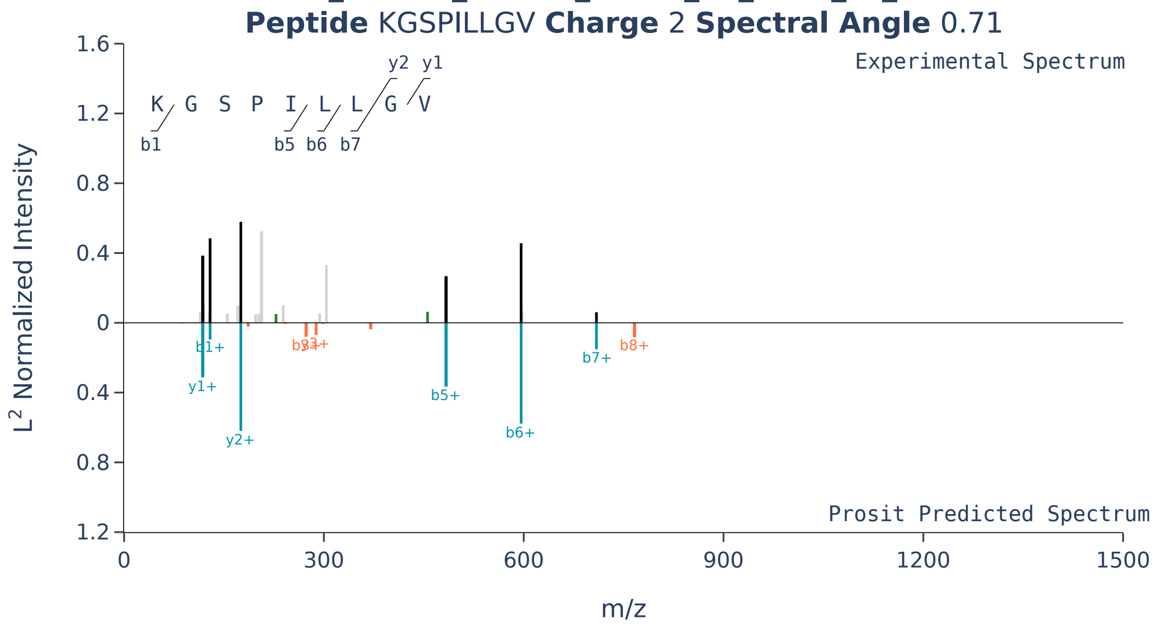


**Source** WSoh_101121_151121_HFGoe_G2_IL37b_24h_R2 **Scan** 15682
**Peptide** SCNCNEPVGVTDKFEN **Charge** 2 **Spectral Angle** 0.8

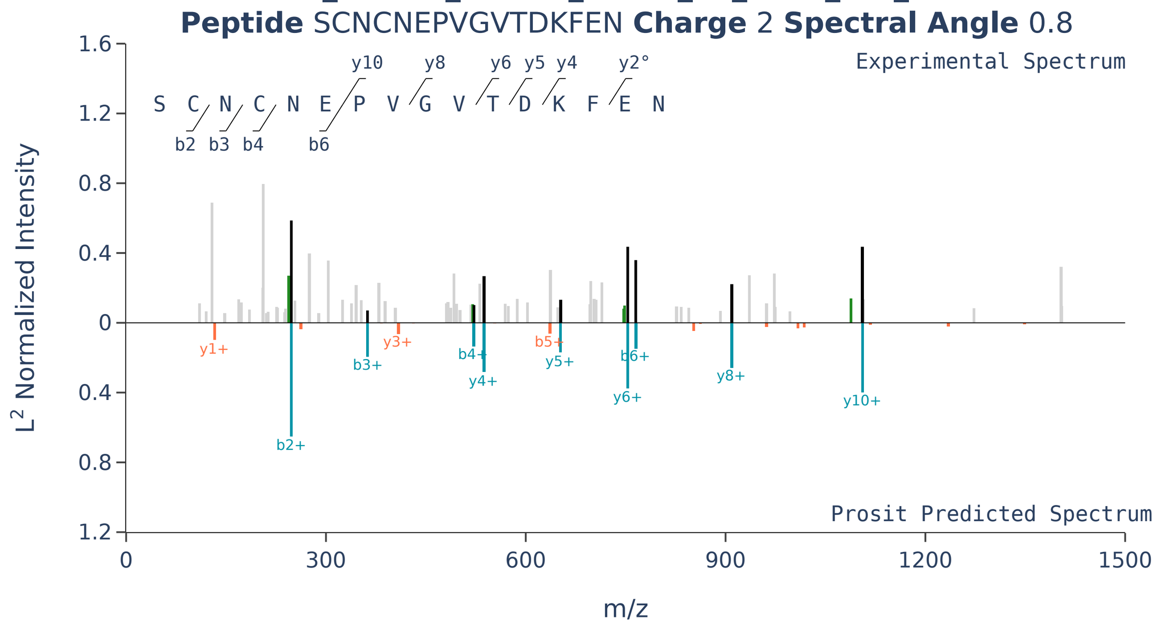


**Source** WSoh_101121_151121_HFGoe_G1_IL37b_24h_R2 **Scan** 16939
**Peptide** LSSASAEKGSPIL **Charge** 2 **Spectral Angle** 0.8

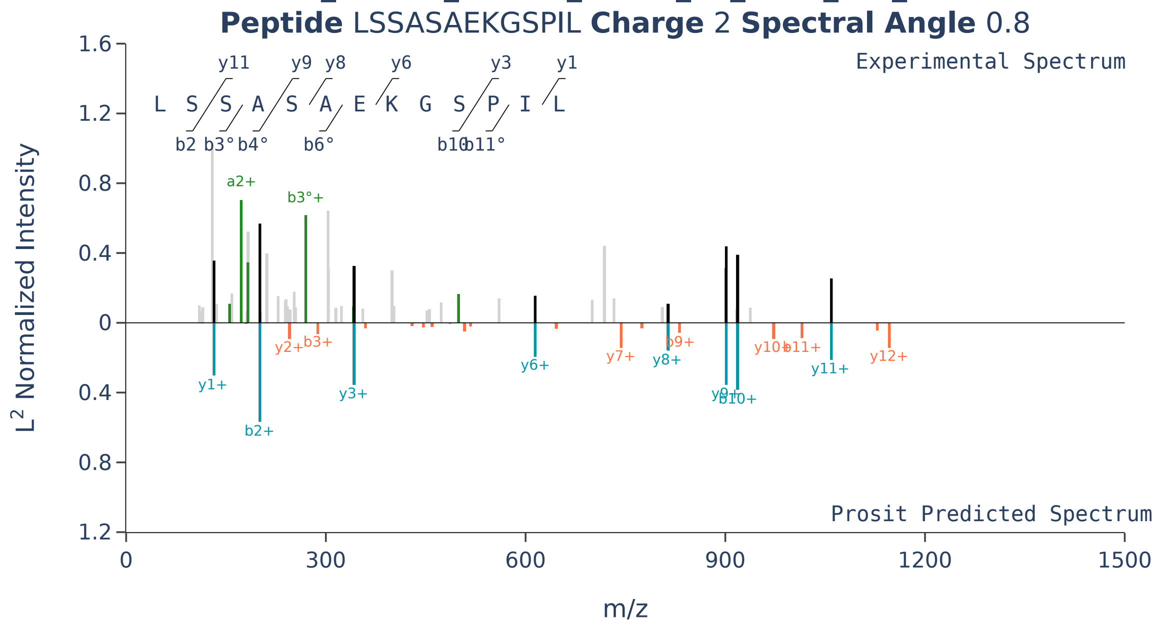


**Source** WSoh_101121_151121_HFGoe_G1_IL37b_24h_R2 **Scan** 14771
**Peptide** EPVGVTDKFEN **Charge** 2 **Spectral Angle** 0.76

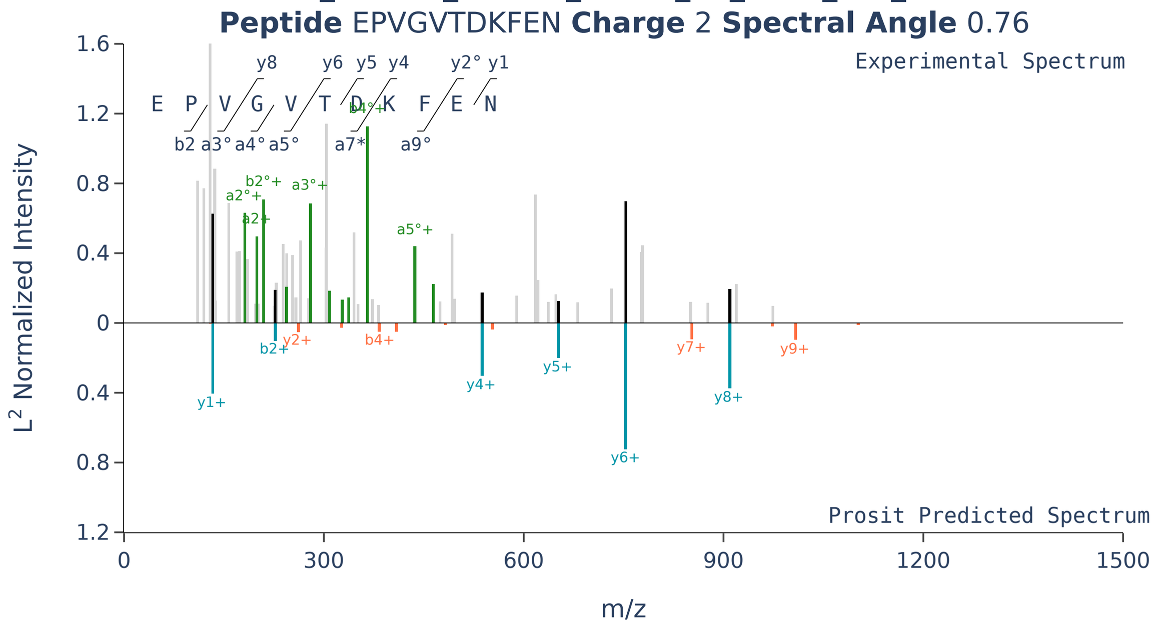


**Source** WSoh_101121_151121_HFGoe_G2_IL37b_24h_R2 **Scan** 14967
**Peptide** NEPVGVTDKFEN **Charge** 2 **Spectral Angle** 0.79

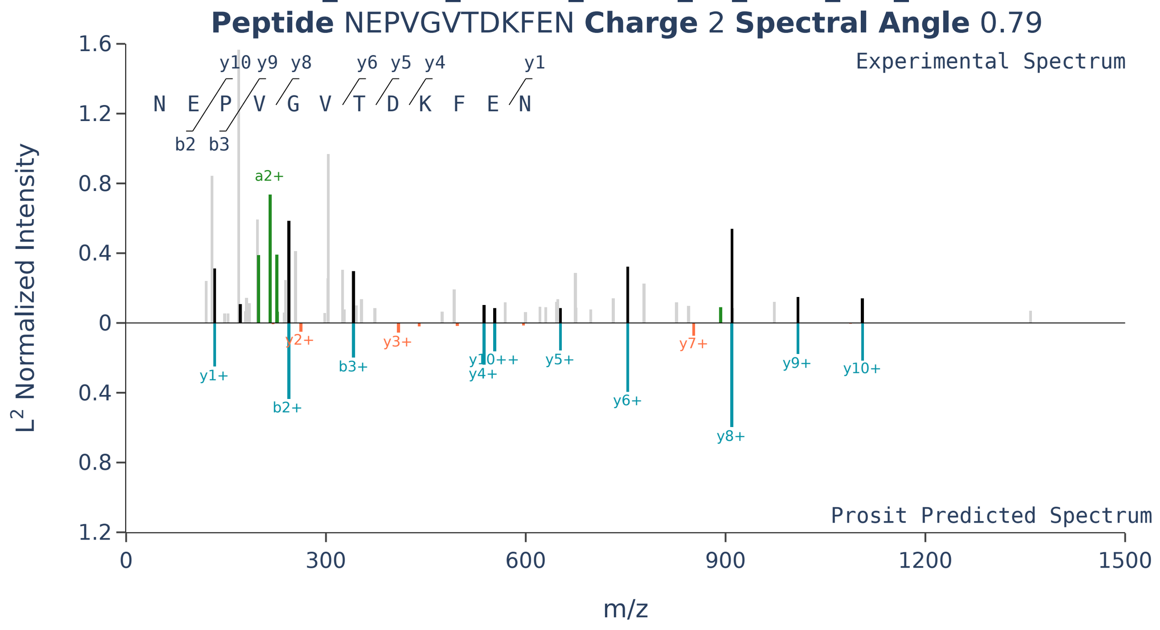


**Source** WSoh_101121_151121_HFGoe_G1_IL37b_24h_R1 **Scan** 25240
**Peptide** AHPGWFI **Charge** 2 **Spectral Angle** 0.84

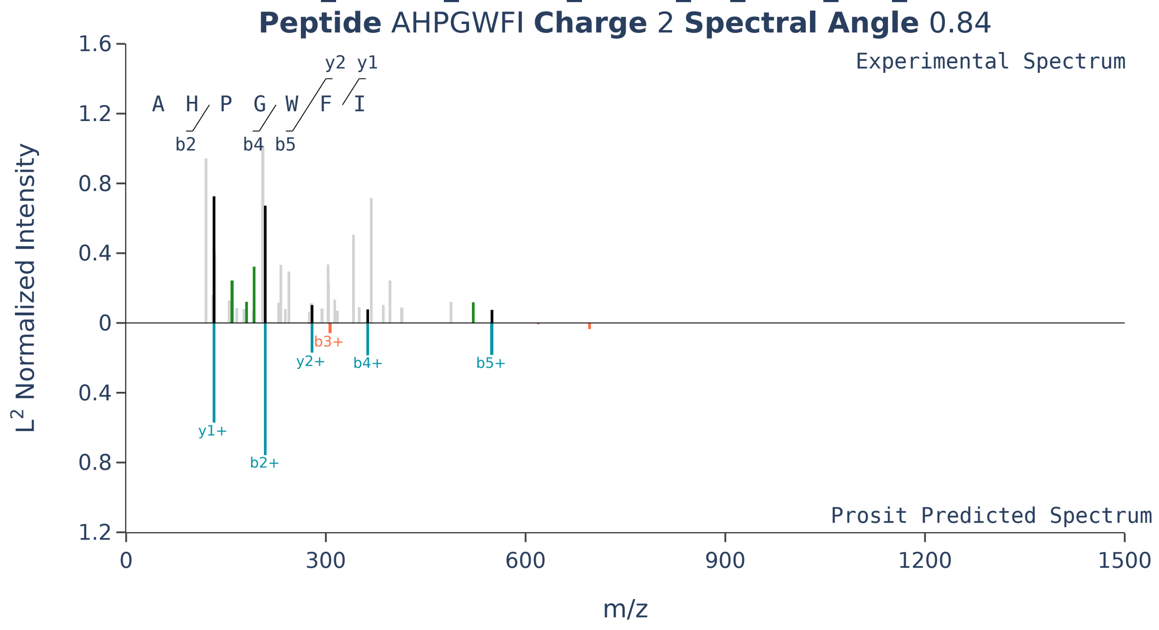


**Source** WSoh_101121_151121_HFGoe_G1_IL37b_24h_R2 **Scan** 22054
**Peptide** DSGNLIAVPDKNYIRP **Charge** 3 **Spectral Angle** 0.87

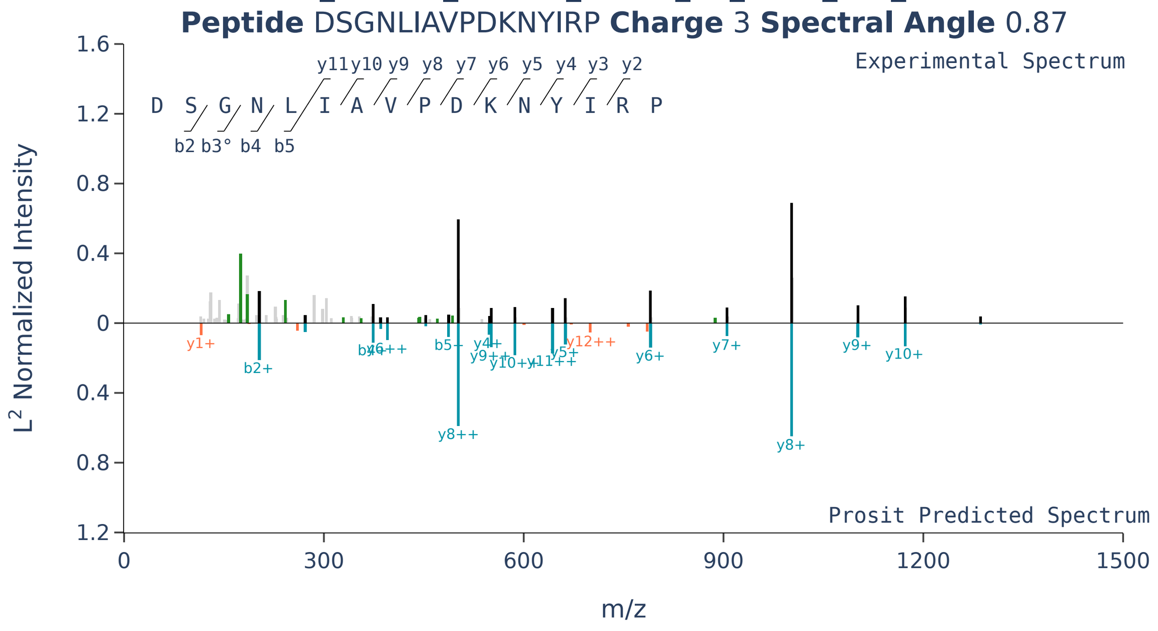


**Source** WSoh_101121_151121_HFGoe_G1_IL37b_4h_R1 **Scan** 15723
**Peptide** ARRPFIFYRAQ **Charge** 3 **Spectral Angle** 0.85

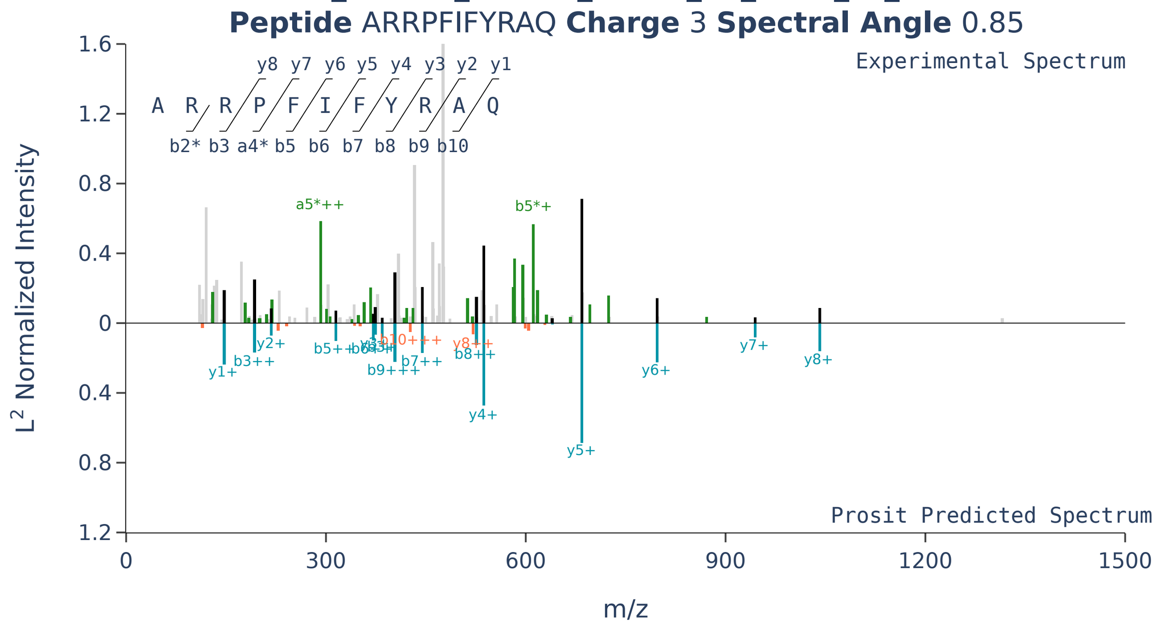


**Source** WSoh_101121_151121_HFGoe_G1_IL37b_1h_R1 **Scan** 6929
**Peptide** KKEKLMKL **Charge** 2 **Spectral Angle** 0.91

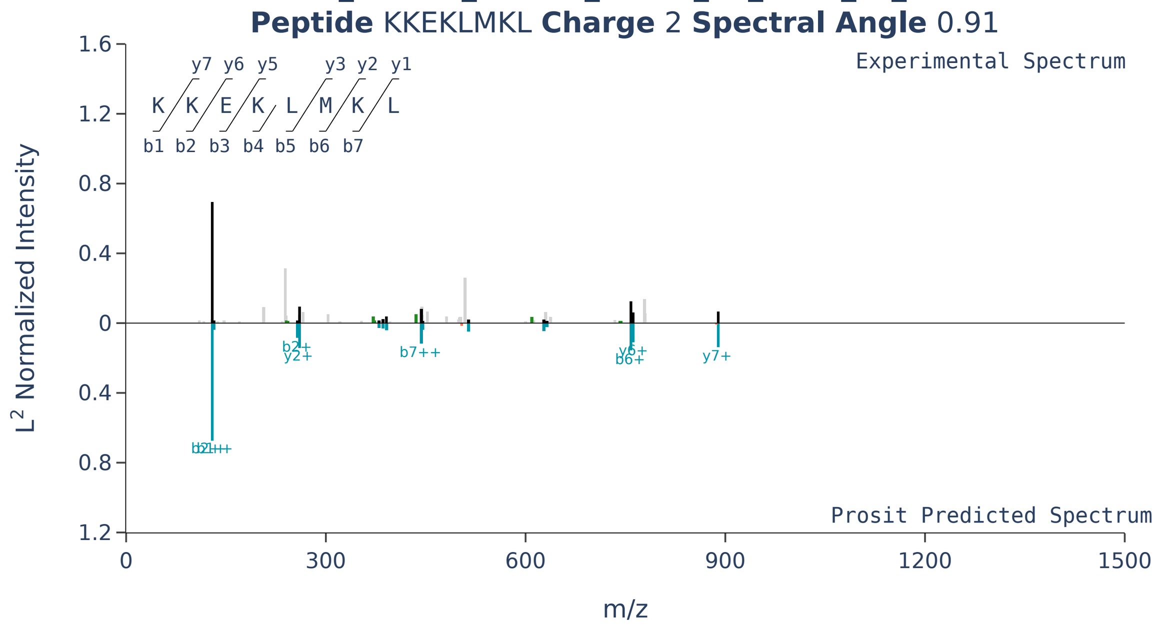


**Source** WSoh_101121_151121_HFGoe_G1_IL37b_4h_R2 **Scan** 16370
**Peptide** ENRKHIEFSFQPVCKA **Charge** 4 **Spectral Angle** 0.83

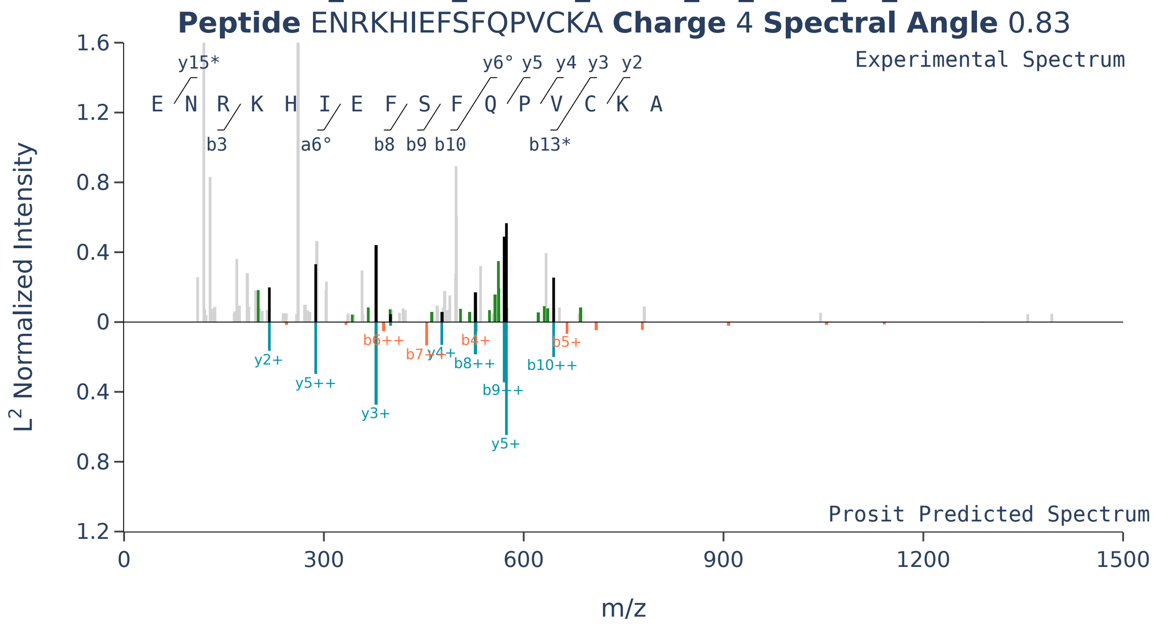


**Source** WSoh_101121_151121_HFGoe_G1_IL37b_24h_R1 **Scan** 7510
**Peptide** VHTSPKVKNLNPKKFS **Charge** 3 **Spectral Angle** 0.85

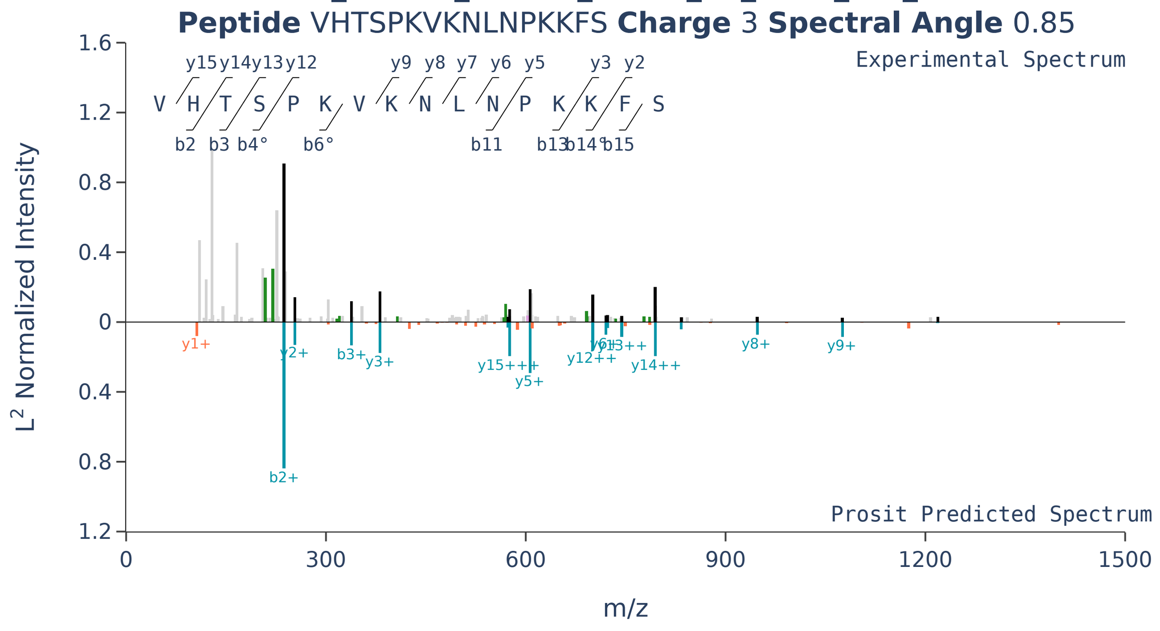


**Source** WSoh_101121_151121_HFGoe_G2_IL37b_24h_R2 **Scan** 6959
**Peptide** NPKKFSIHDQ **Charge** 3 **Spectral Angle** 0.75

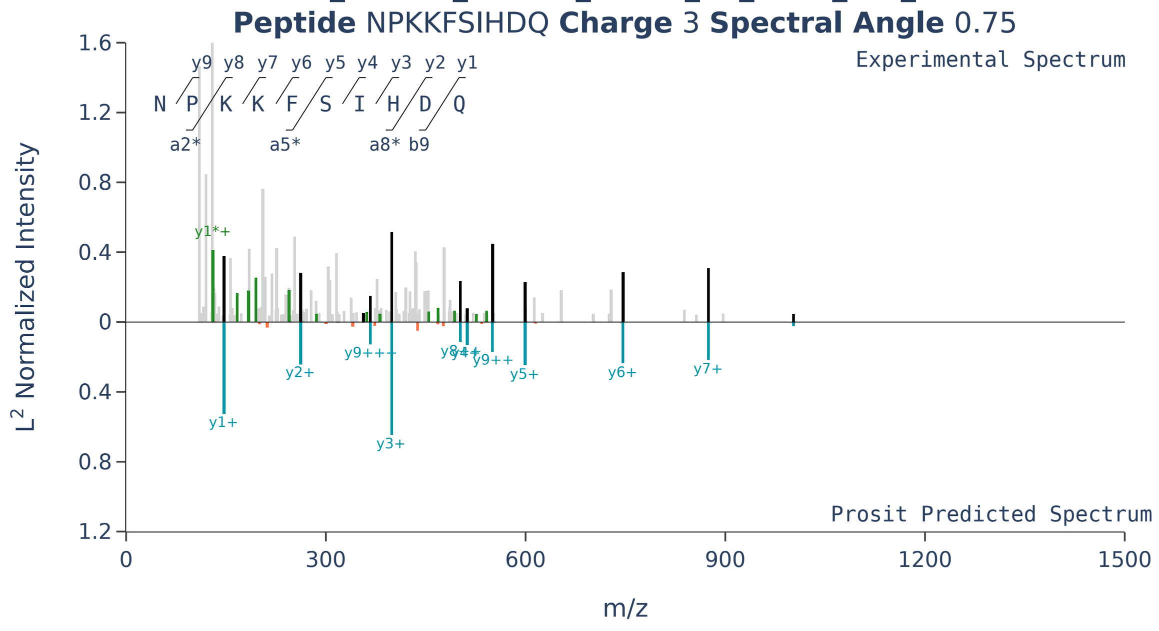


**Source** WSoh_101121_151121_HFGoe_G1_IL37b_4h_R1 **Scan** 16435
**Peptide** SARRPFIFYRA **Charge** 3 **Spectral Angle** 0.81

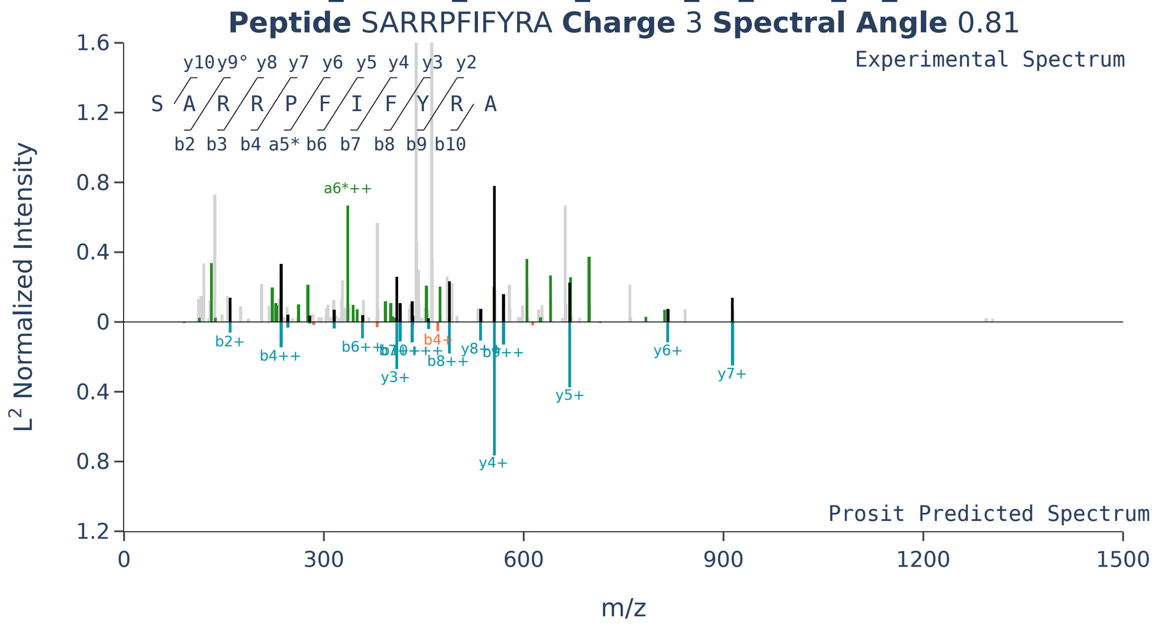


**Source** WSoh_101121_151121_HFGoe_G2_IL37b_24h_R2 **Scan** 13220
**Peptide** SPKVKNLNPKKFSI **Charge** 4 **Spectral Angle** 0.75

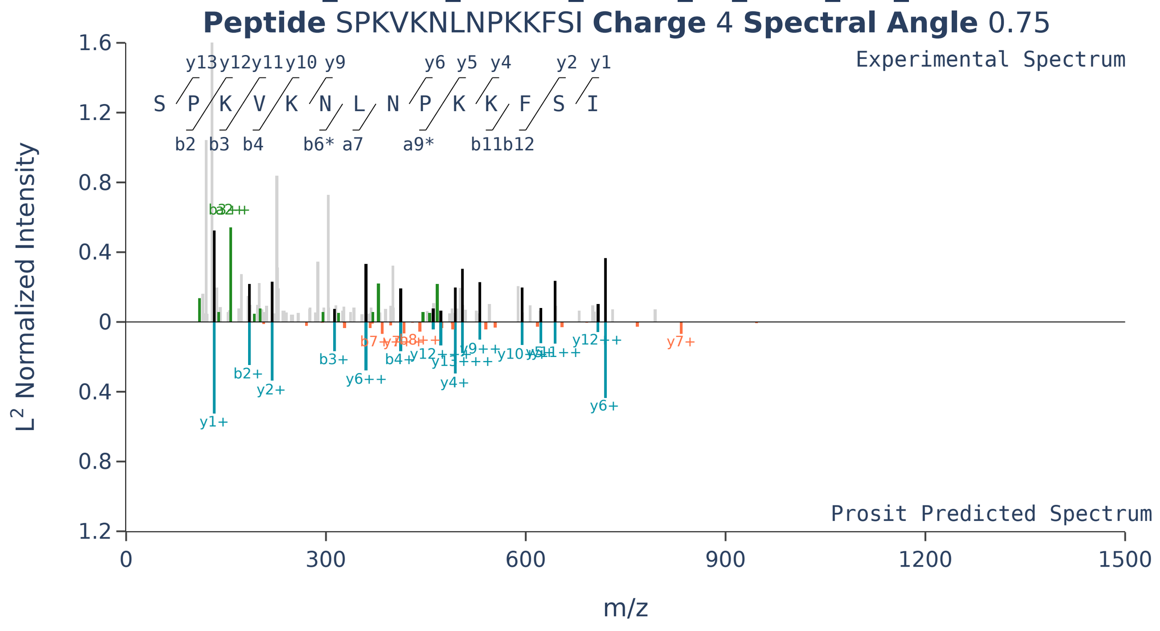


**Source** WSoh_101121_151121_HFGoe_G1_IL37b_4h_R2 **Scan** 16030
**Peptide** SARRPFIFYRAQ **Charge** 3 **Spectral Angle** 0.85

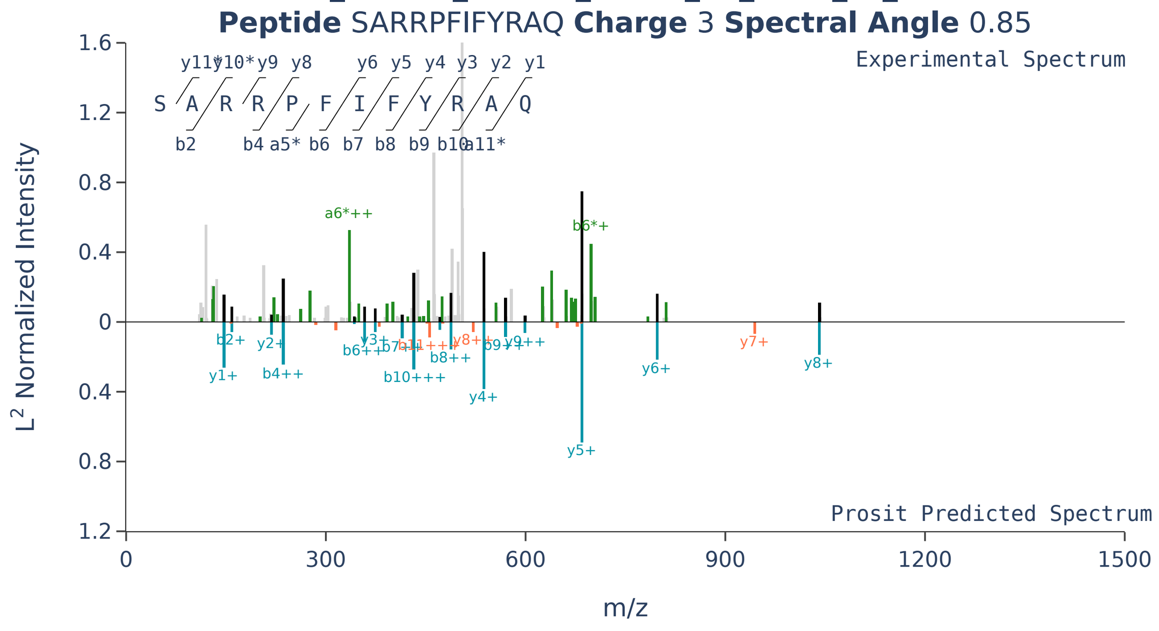

**Source** WSoh_101121_151121_HFGoe_G1_IL37b_24h_R2 **Scan** 9812
**Peptide** SKGEFCLYCDKDKGQSHPS **Charge** 4 **Spectral Angle** 0.89

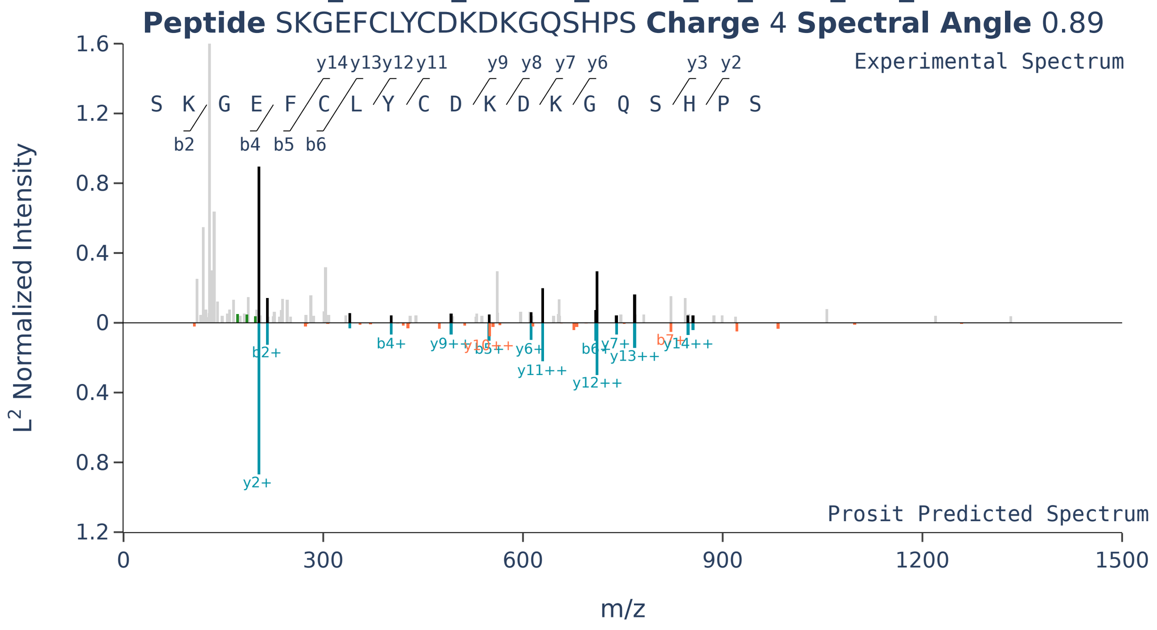


**Source** WSoh_101121_151121_HFGoe_G1_IL37b_24h_R1 **Scan** 20751
**Peptide** SGNLIAVPDKNYIRP **Charge** 3 **Spectral Angle** 0.85

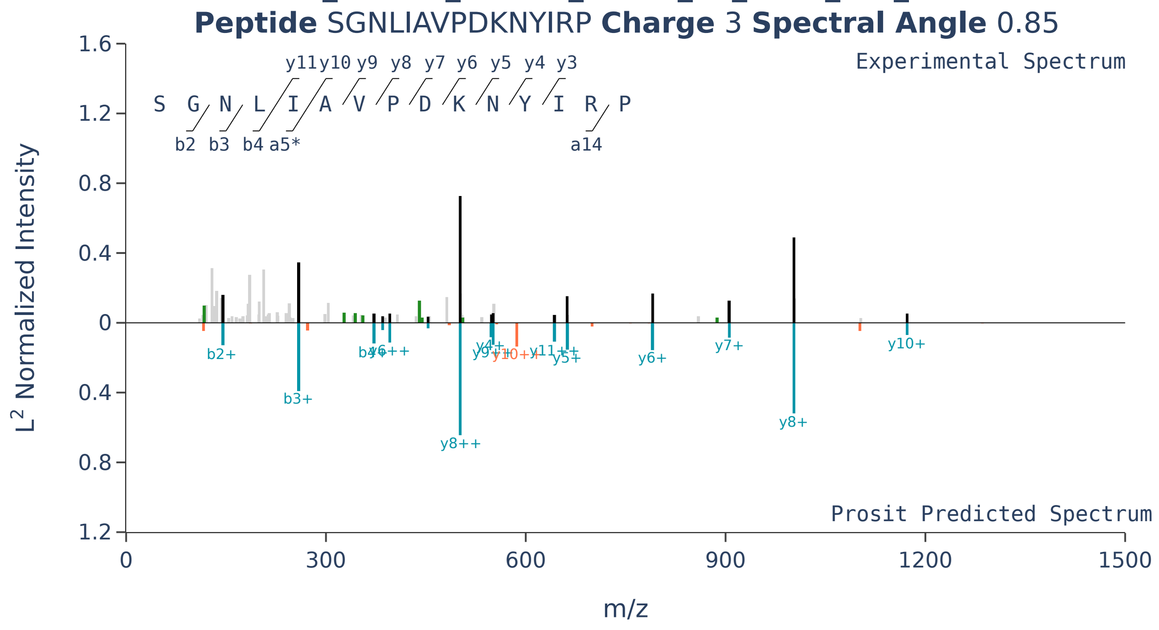


**Source** WSoh_101121_151121_HFGoe_G1_IL37b_24h_R1 **Scan** 19281
**Peptide** SASAEKGSPILLG **Charge** 2 **Spectral Angle** 0.81

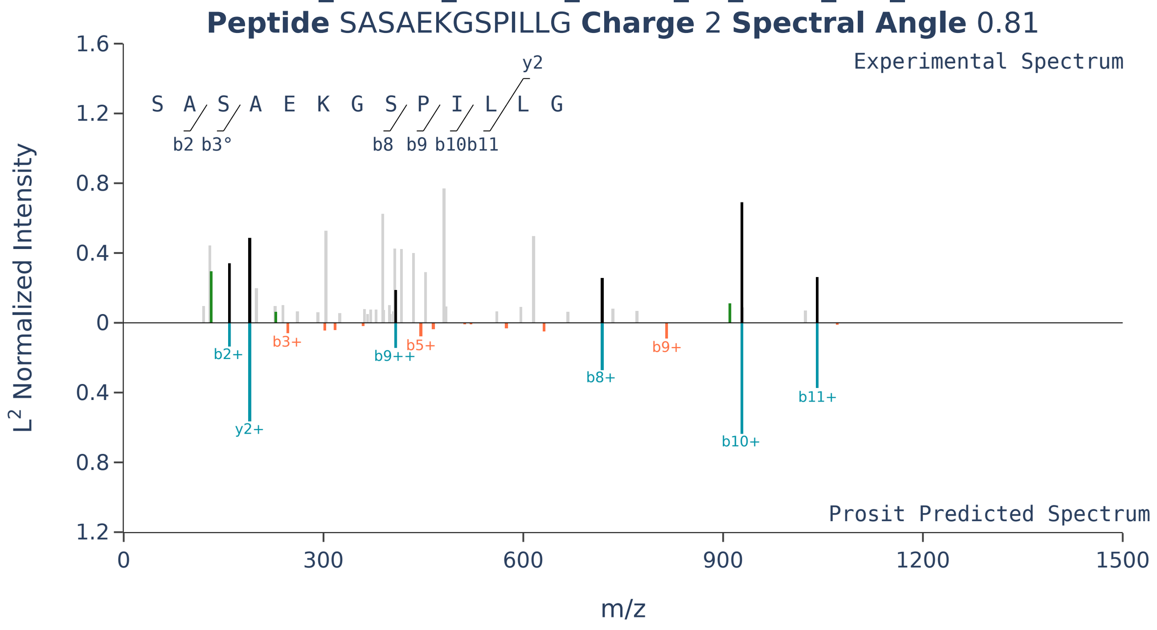


**Source** WSoh_101121_151121_HFGoe_G2_IL37b_24h_R1 **Scan** 19188
**Peptide** ARRPFIF **Charge** 2 **Spectral Angle** 0.77

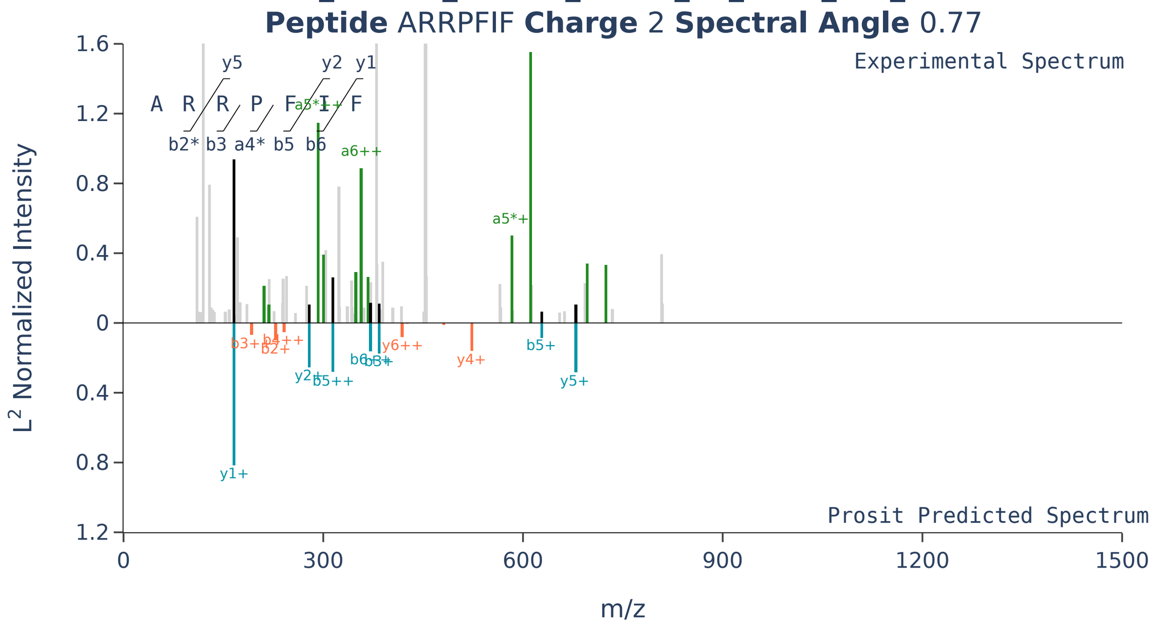


**Source** WSoh_101121_151121_HFGoe_G1_IL37b_4h_R2 **Scan** 21567
**Peptide** SARRPFIFY **Charge** 3 **Spectral Angle** 0.85

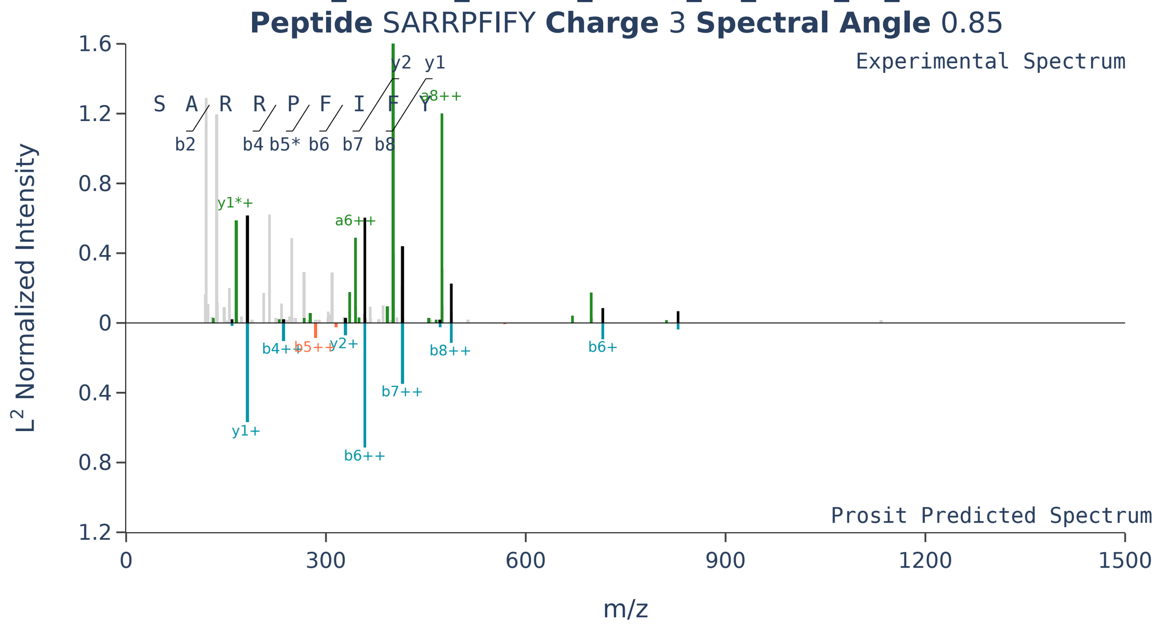


**Source** WSoh_101121_151121_HFGoe_G1_IL37b_24h_R2 **Scan** 10589
**Peptide** FENRKHIEF **Charge** 3 **Spectral Angle** 0.71

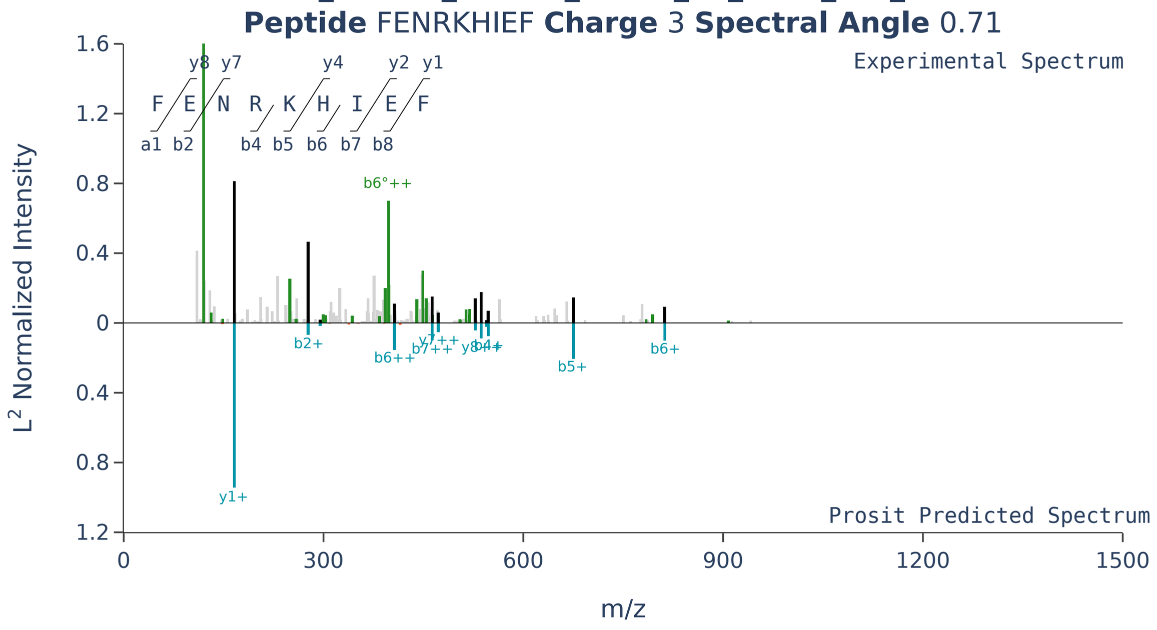


**Source** WSoh_101121_151121_HFGoe_G1_IL37b_4h_R2 **Scan** 10701
**Peptide** DKFENRKHIEF **Charge** 3 **Spectral Angle** 0.83

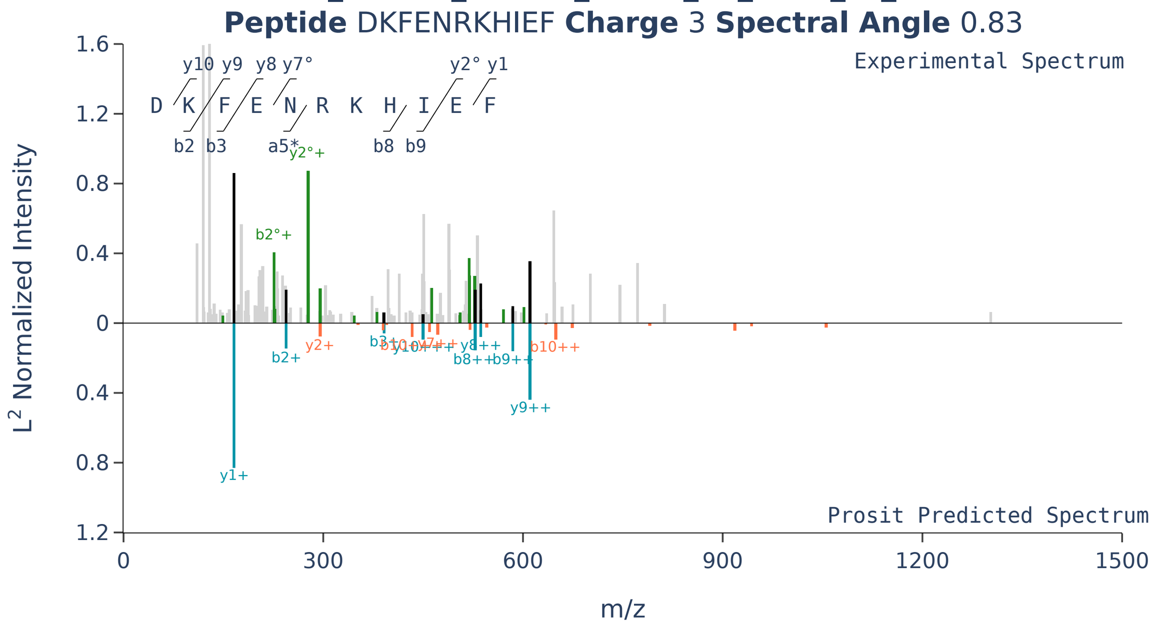


**Source** WSoh_101121_151121_HFGoe_G1_IL37b_4h_R2 **Scan** 6747
**Peptide** SPKVKNL **Charge** 2 **Spectral Angle** 0.83

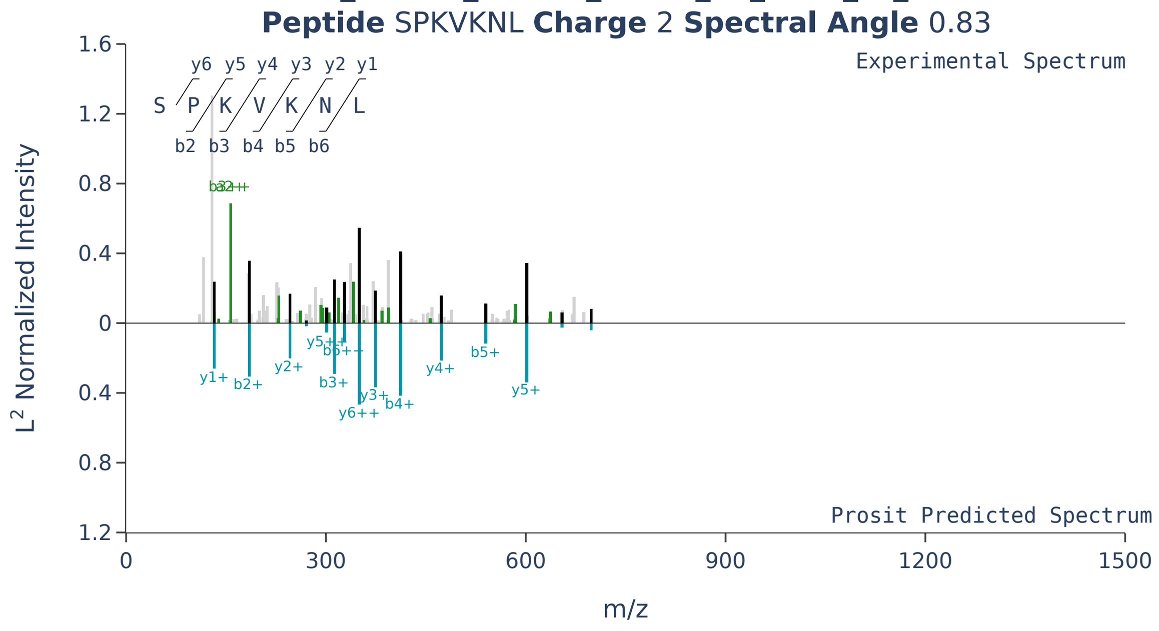


**Source** WSoh_101121_151121_HFGoe_G1_IL37b_4h_R1 **Scan** 10057
**Peptide** KDKGQSHPSLQLKKEKLM **Charge** 4 **Spectral Angle** 0.88

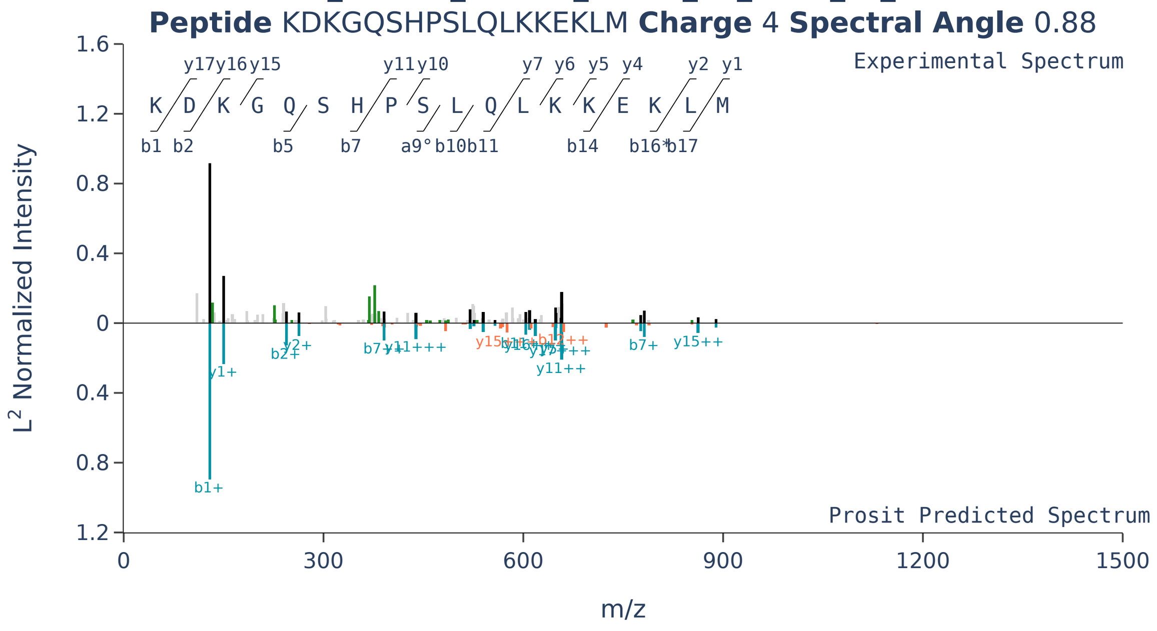


**Source** WSoh_101121_151121_HFGoe_G1_IL37b_1h_R1 **Scan** 9453
**Peptide** NPKKFSIHDQDHKVL **Charge** 3 **Spectral Angle** 0.73

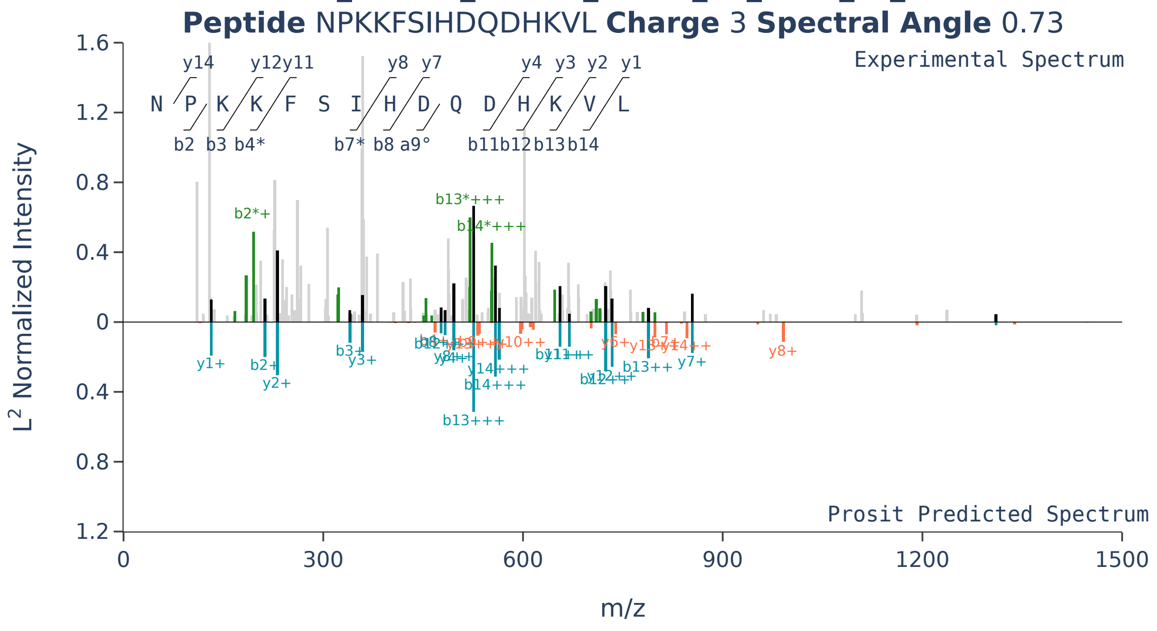


**Source** WSoh_101121_151121_HFGoe_G1_IL37b_24h_R1 **Scan** 10767
**Peptide** SPKVKNLNPKKFSIHD **Charge** 3 **Spectral Angle** 0.8

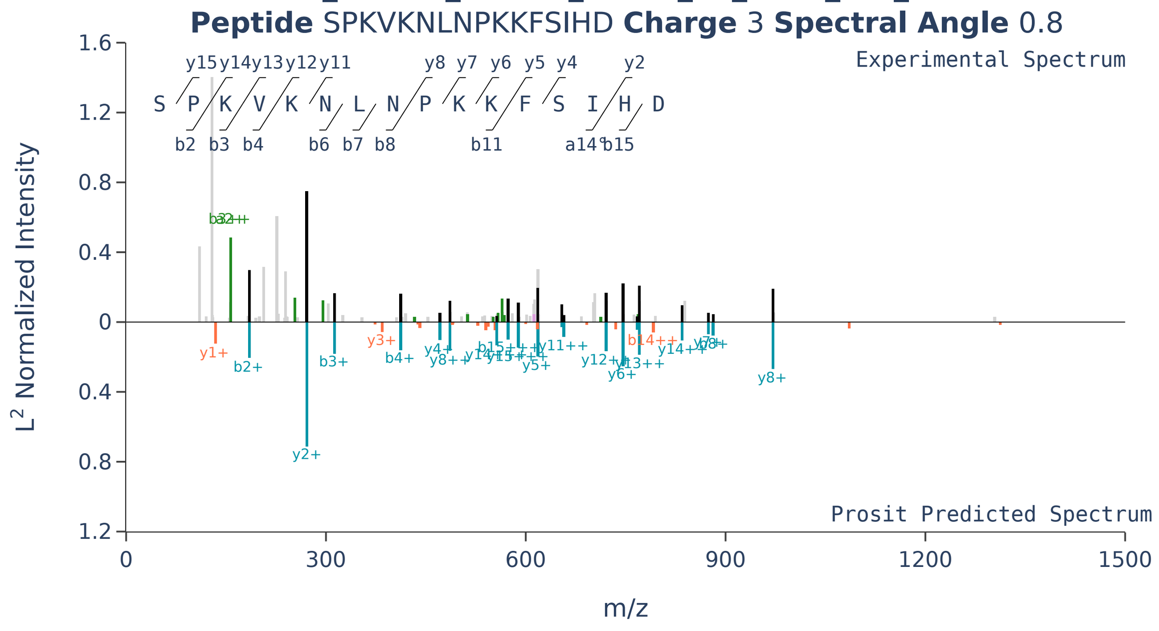


**Source** WSoh_101121_151121_HFGoe_G1_IL37b_24h_R1 **Scan** 20604
**Peptide** EFSFQPVCKA **Charge** 2 **Spectral Angle** 0.82

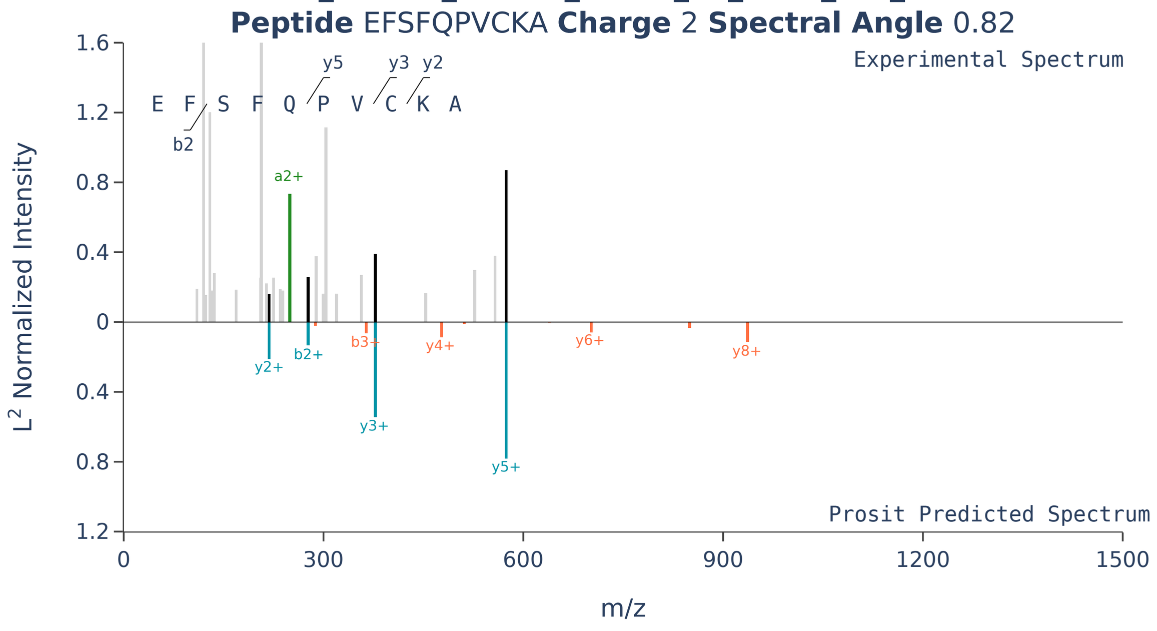


**Source** WSoh_101121_151121_HFGoe_G1_IL37b_24h_R1 **Scan** 16715
**Peptide** DQDHKVLVLD **Charge** 3 **Spectral Angle** 0.84

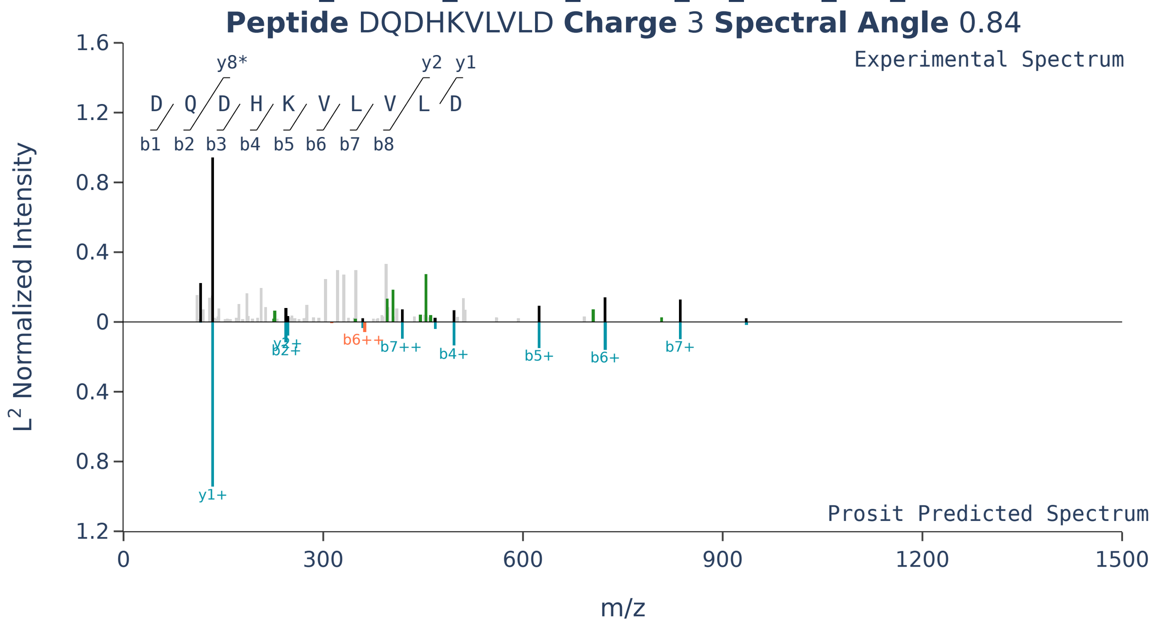


**Source** WSoh_101121_151121_HFGoe_G2_IL37b_24h_R2 **Scan** 10779
**Peptide** VSKGEFCLYCDKDKGQSH **Charge** 4 **Spectral Angle** 0.75

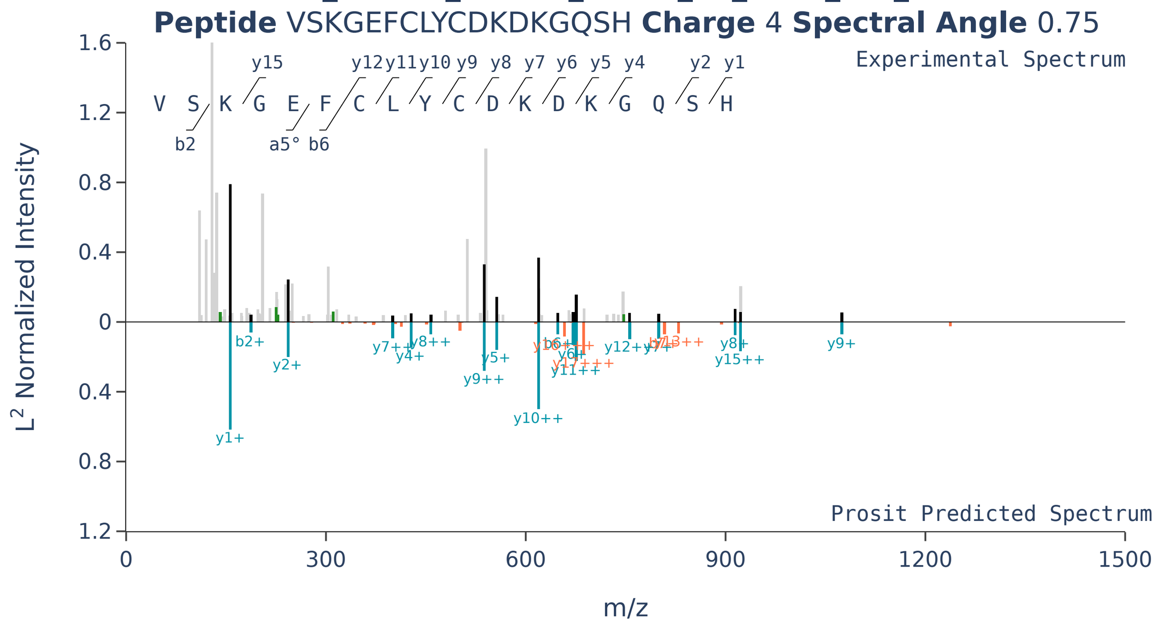


**Source** WSoh_101121_151121_HFGoe_G2_IL37b_24h_R2 **Scan** 8065
**Peptide** TSPKVKNLNPKKFS **Charge** 3 **Spectral Angle** 0.79

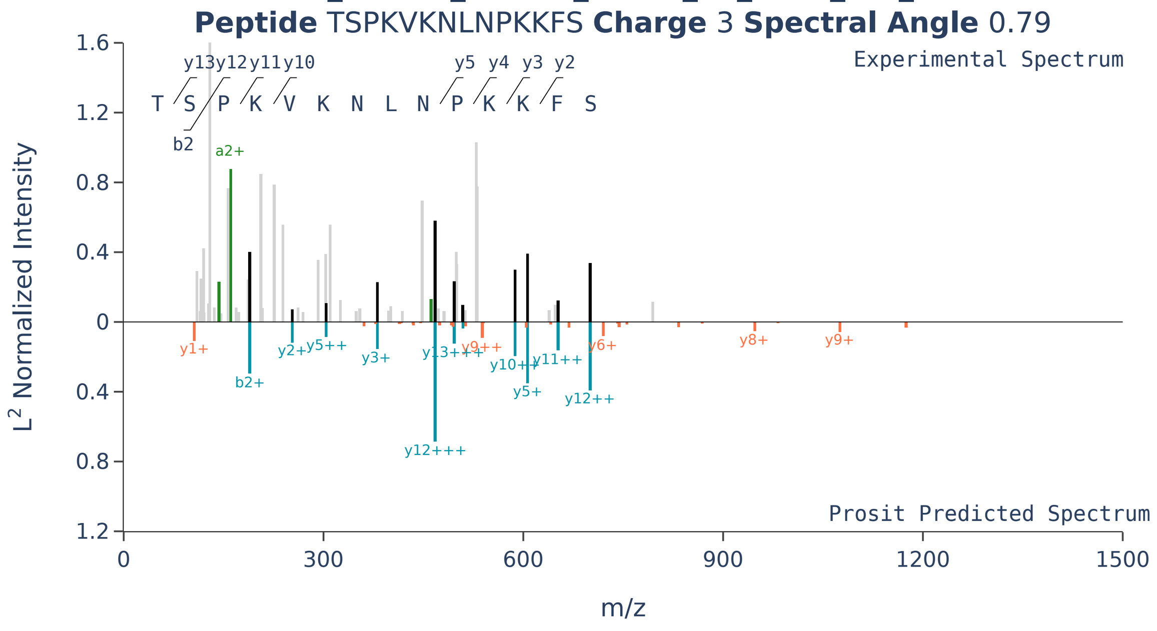

**Source** WSoh_101121_151121_HFGoe_G2_IL37b_2h_R1 **Scan** 22781
**Peptide** ESAAHPGWFICTSC **Charge** 2 **Spectral Angle** 0.84

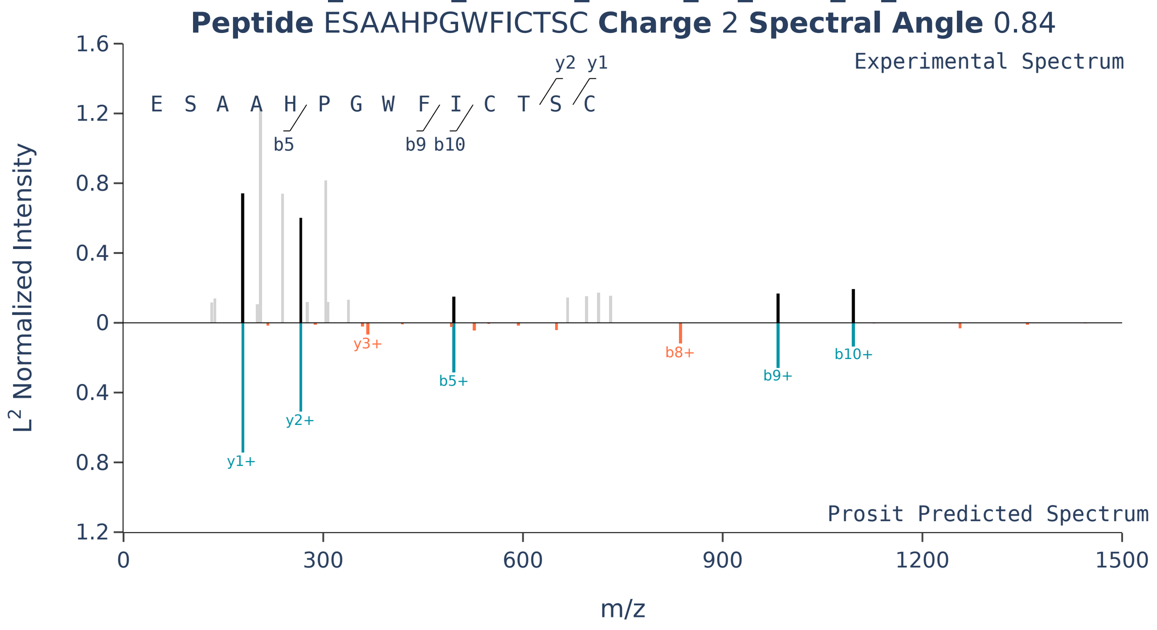


**Source** WSoh_101121_151121_HFGoe_G2_IL37b_24h_R2 **Scan** 19547
**Peptide** GNLIAVPDKNY **Charge** 2 **Spectral Angle** 0.84

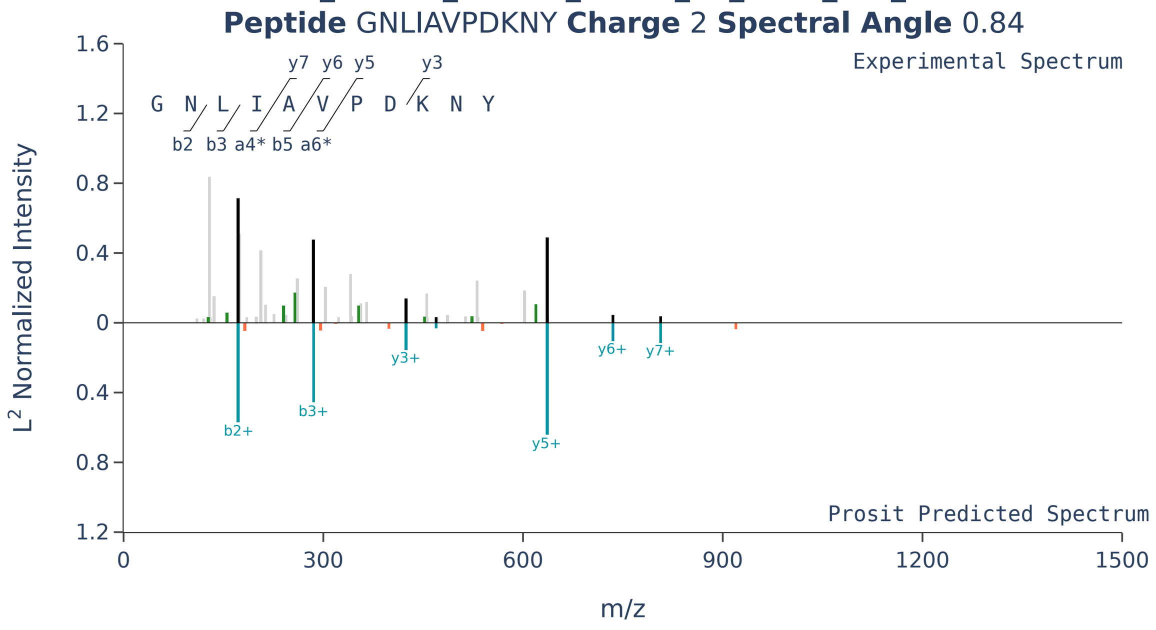


**Source** WSoh_101121_151121_HFGoe_G1_IL37b_24h_R1 **Scan** 13728
**Peptide** IHDQDHKVLVLDSG **Charge** 3 **Spectral Angle** 0.83

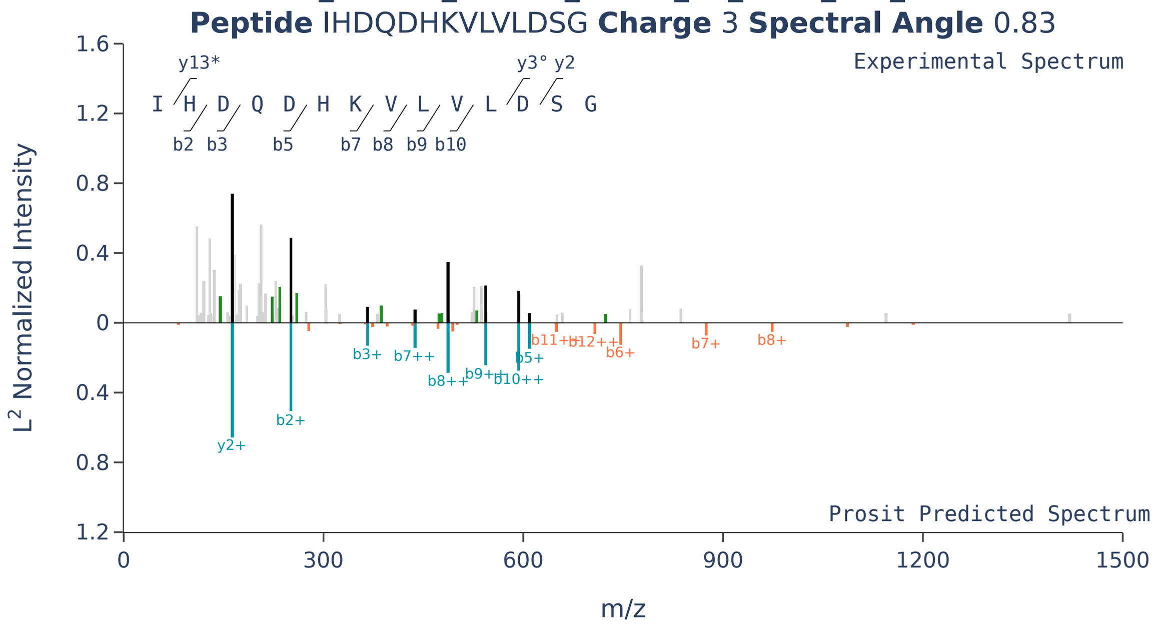


**Source** WSoh_101121_151121_HFGoe_G1_IL37b_4h_R1 **Scan** 13235
**Peptide** PSLQLKKEKLMKLAA **Charge** 4 **Spectral Angle** 0.76

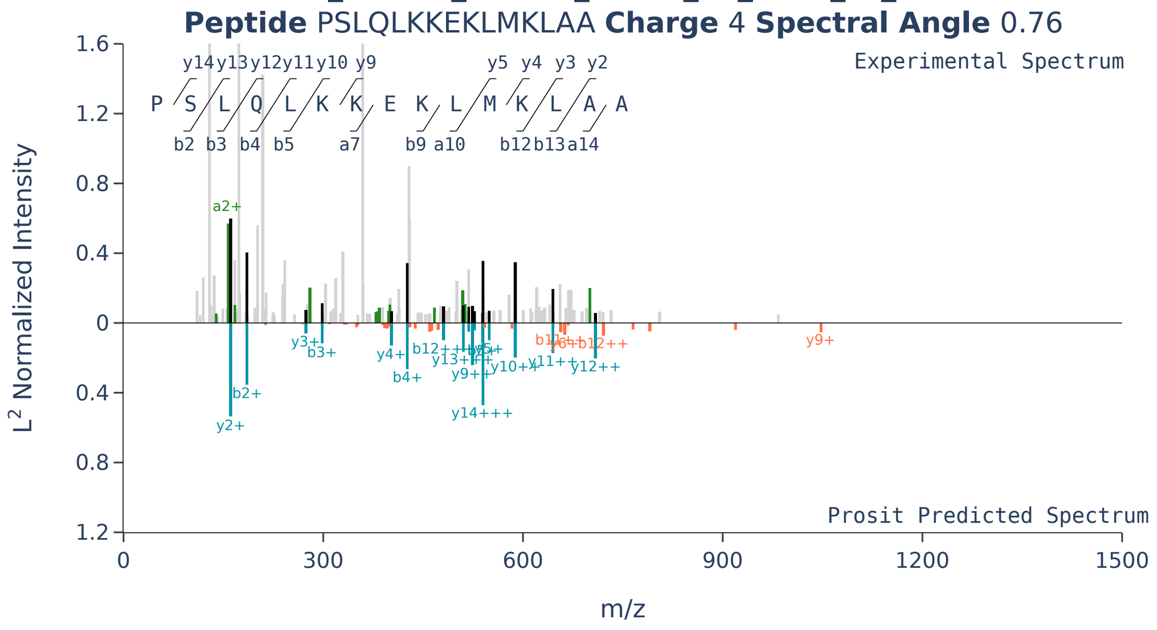


**Source** WSoh_101121_151121_HFGoe_G1_IL37b_1h_R2 **Scan** 24629
**Peptide** QVGSWNMLE **Charge** 2 **Spectral Angle** 0.81

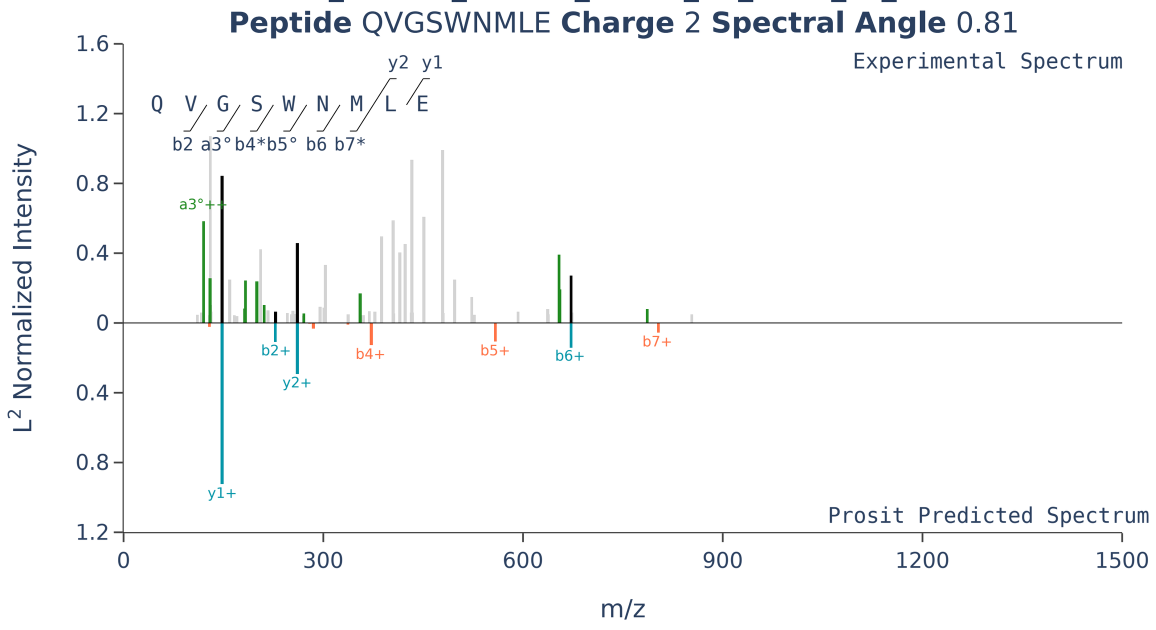


**Source** WSoh_101121_151121_HFGoe_G2_IL37b_24h_R2 **Scan** 15699
**Peptide** ALASSLSSASAE **Charge** 2 **Spectral Angle** 0.92

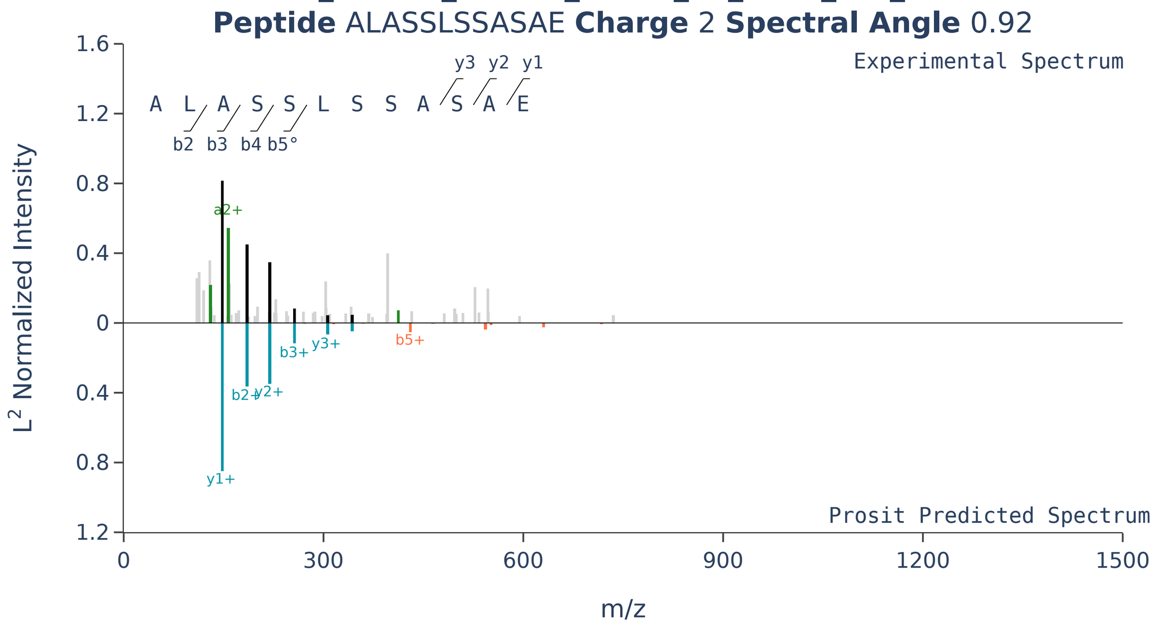


**Source** WSoh_101121_151121_HFGoe_G2_IL37b_2h_R2 **Scan** 7262
**Peptide** SLHDQDHKVL **Charge** 3 **Spectral Angle** 0.73

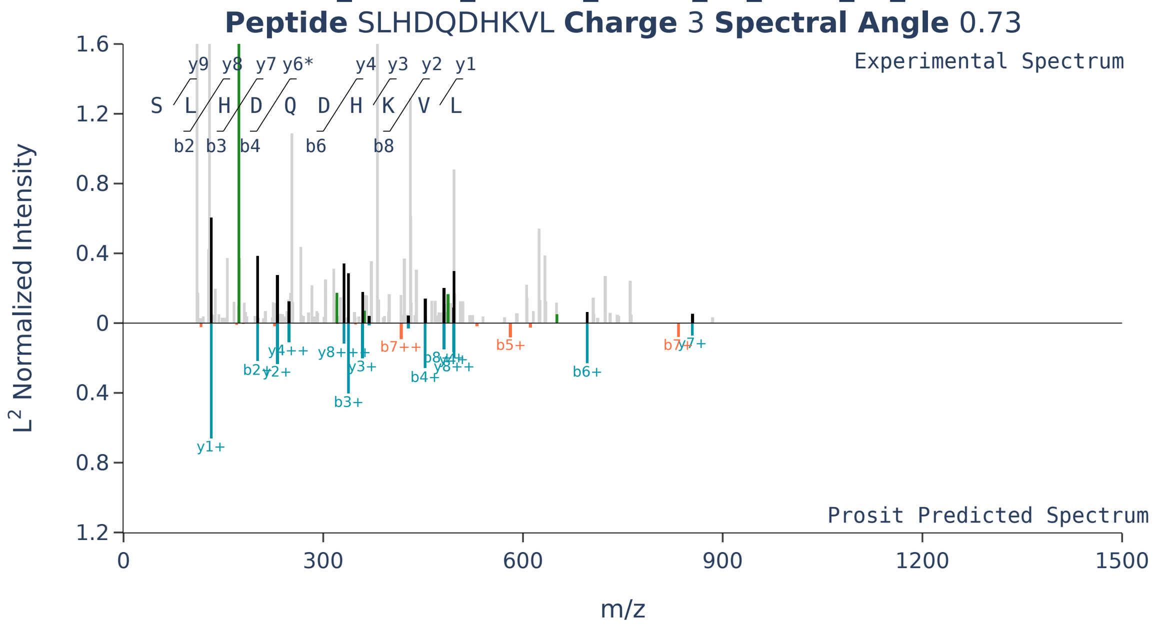


**Source** WSoh_101121_151121_HFGoe_G2_IL37b_24h_R1 **Scan** 18706
**Peptide** QKESARRPFIFY **Charge** 3 **Spectral Angle** 0.76

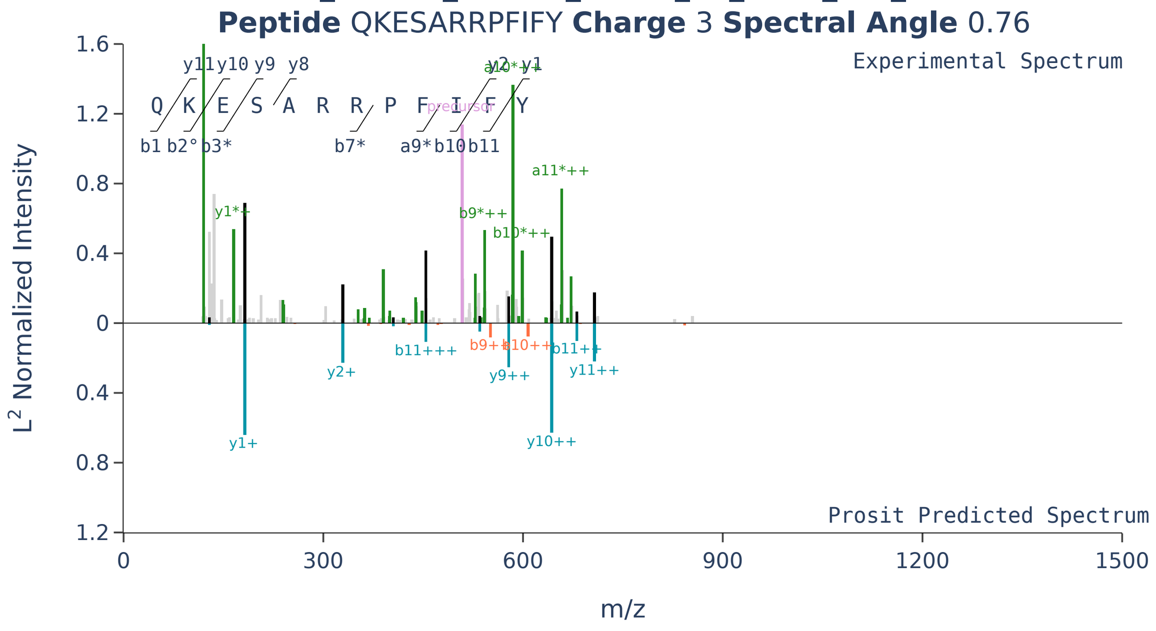


**Source** WSoh_101121_151121_HFGoe_G2_IL37b_24h_R1 **Scan** 15545
**Peptide** PVGVTDKFENRKHIEF **Charge** 4 **Spectral Angle** 0.83

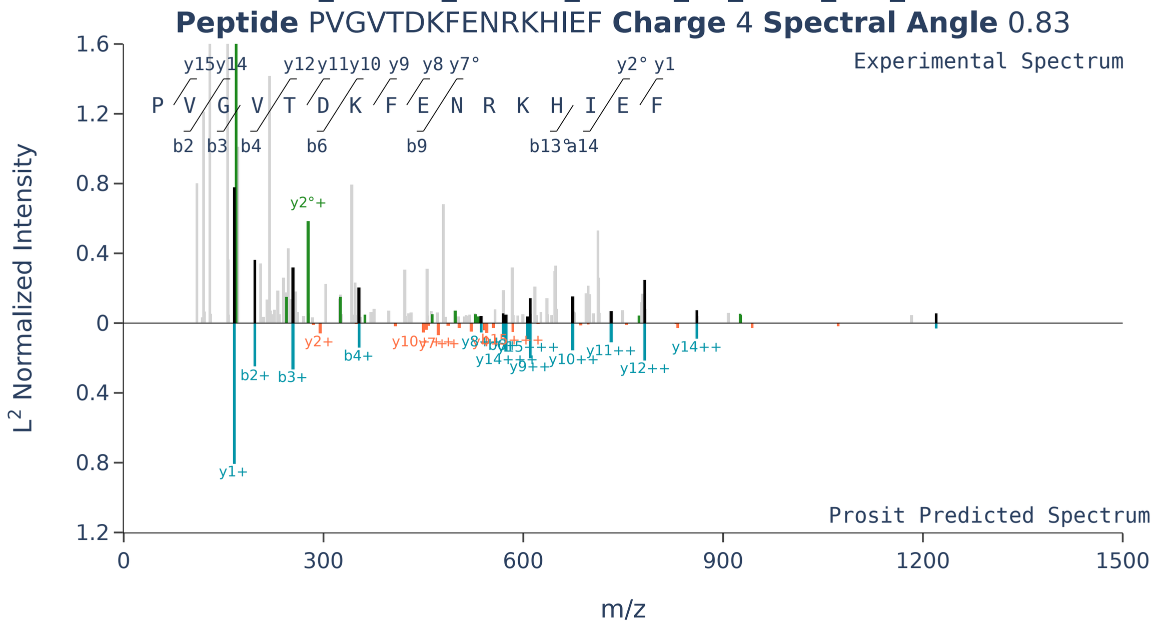


**Source** WSoh_101121_151121_HFGoe_G2_IL37b_24h_R1 **Scan** 14760
**Peptide** KGEFCLYCDKDKGQSHPSL **Charge** 4 **Spectral Angle** 0.78

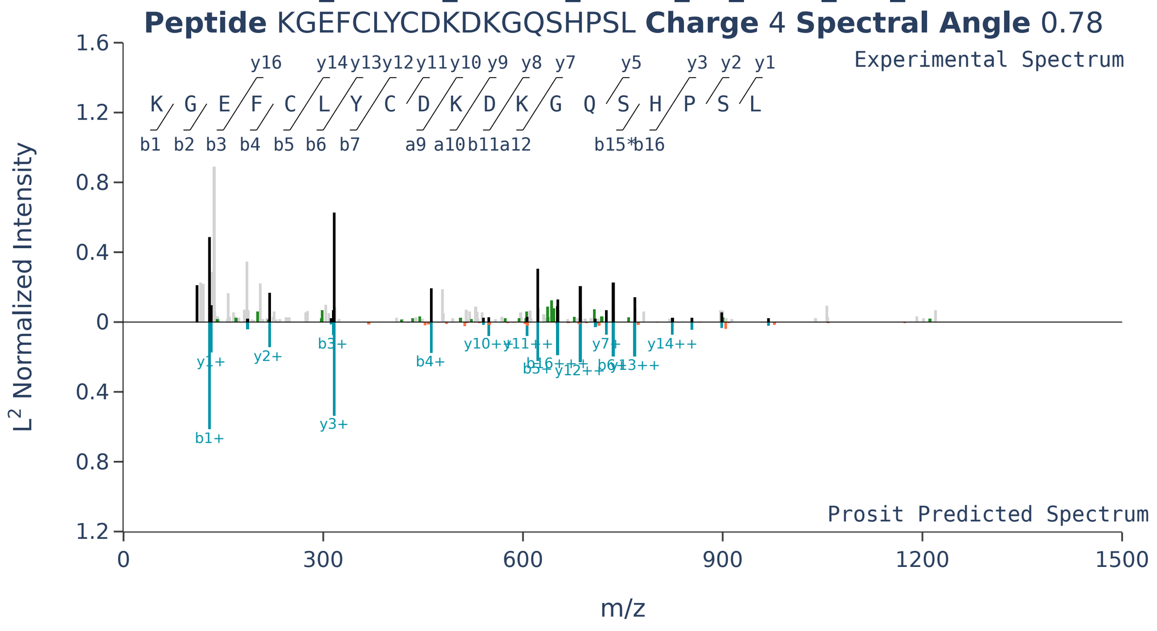


**Source** WSoh_101121_151121_HFGoe_G2_IL37b_24h_R2 **Scan** 22119
**Peptide** SAEKGSPILLGVS **Charge** 2 **Spectral Angle** 0.85

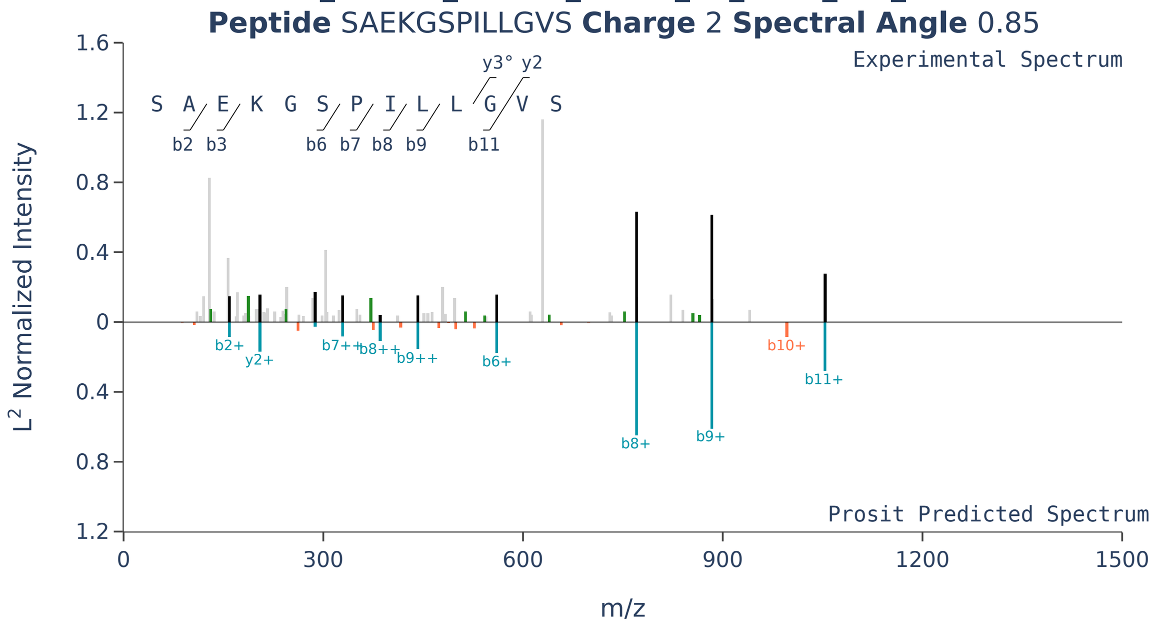


**Source** WSoh_101121_151121_HFGoe_G2_IL37b_24h_R1 **Scan** 11757
**Peptide** NLNPKKFSIHDQ **Charge** 3 **Spectral Angle** 0.7

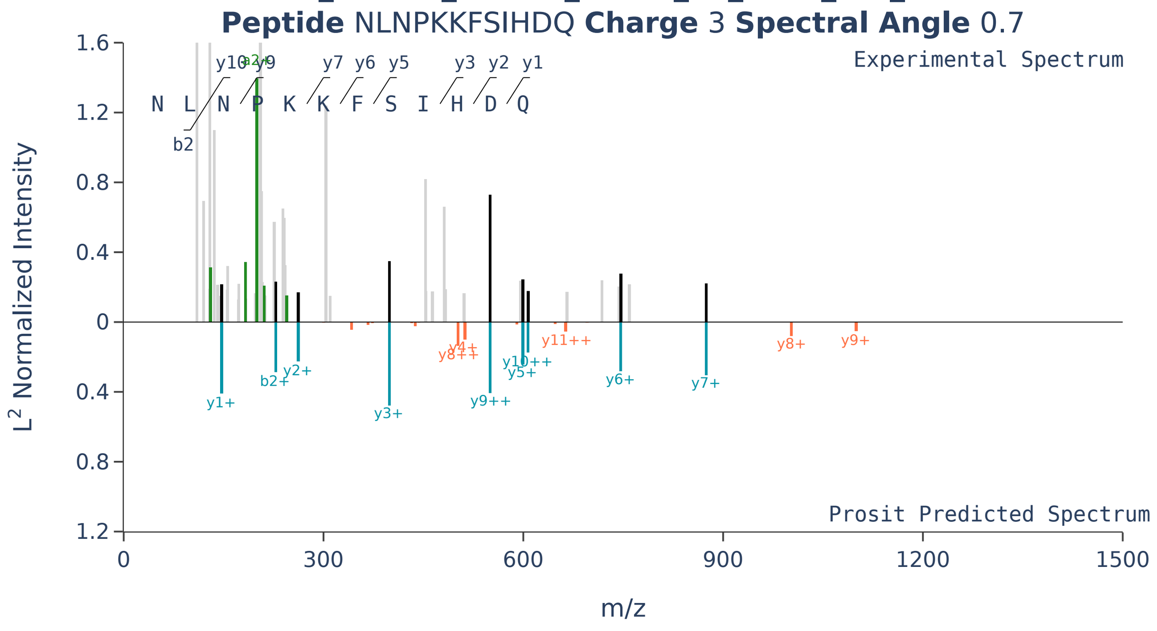


**Source** WSoh_101121_151121_HFGoe_G1_IL37b_1h_R2 **Scan** 20819
**Peptide** SGNLIAV **Charge** 1 **Spectral Angle** 0.92

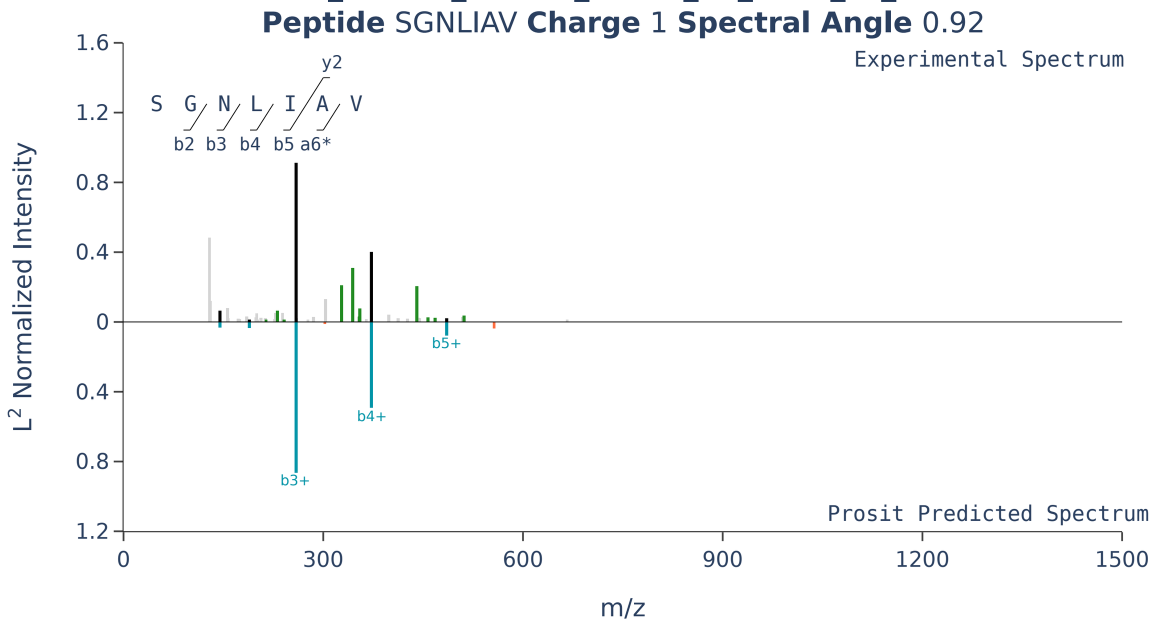


**Source** WSoh_101121_151121_HFGoe_G2_IL37b_24h_R1 **Scan** 8562
**Peptide** LYCDKDKGQSHPSLQLKKE **Charge** 5 **Spectral Angle** 0.8

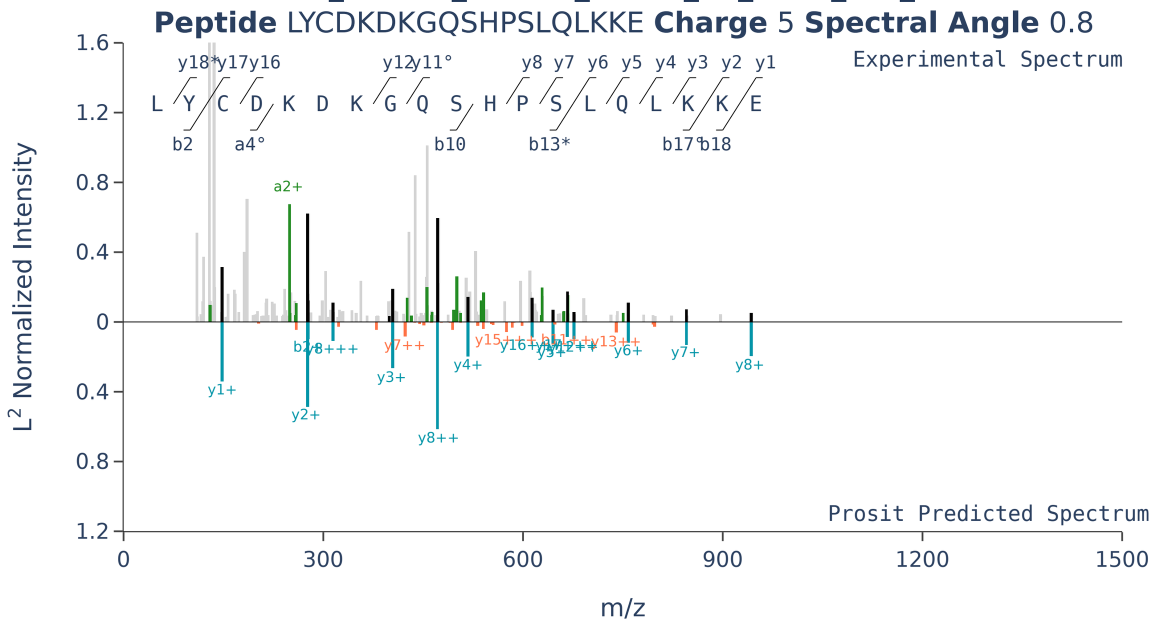


**Source** WSoh_101121_151121_HFGoe_G2_IL37b_24h_R2 **Scan** 7877
**Peptide** KNLNPKKFSIHDQDHKV **Charge** 3 **Spectral Angle** 0.87

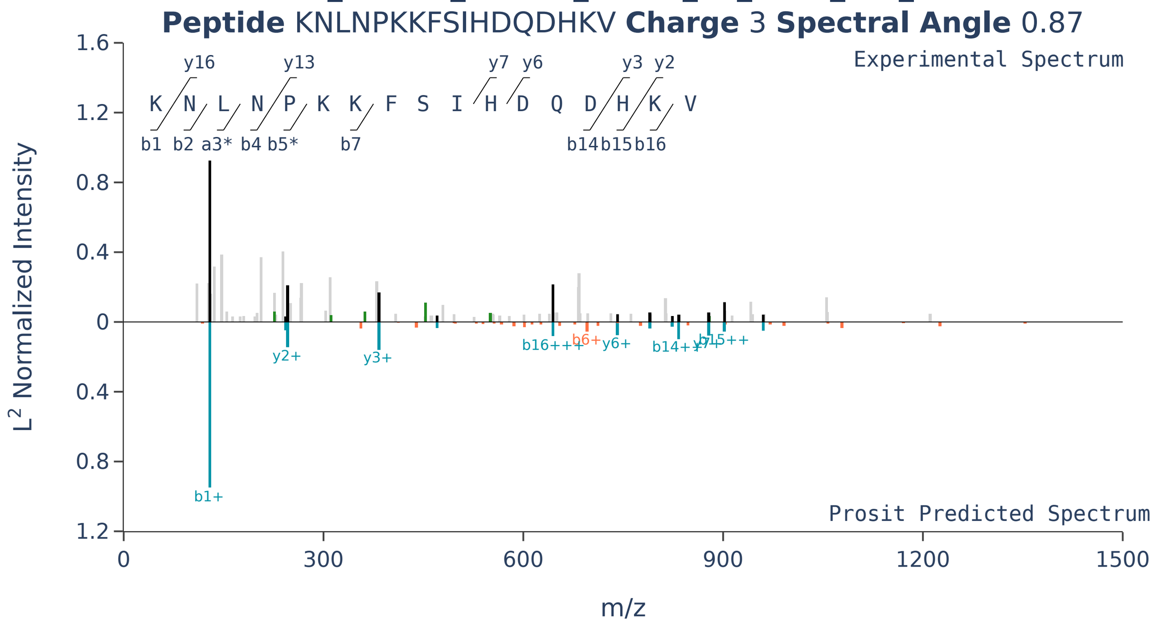

**Source** WSoh_101121_151121_HFGoe_G2_IL37b_24h_R2 **Scan** 5371
**Peptide** KLAAQKESARRP **Charge** 3 **Spectral Angle** 0.93

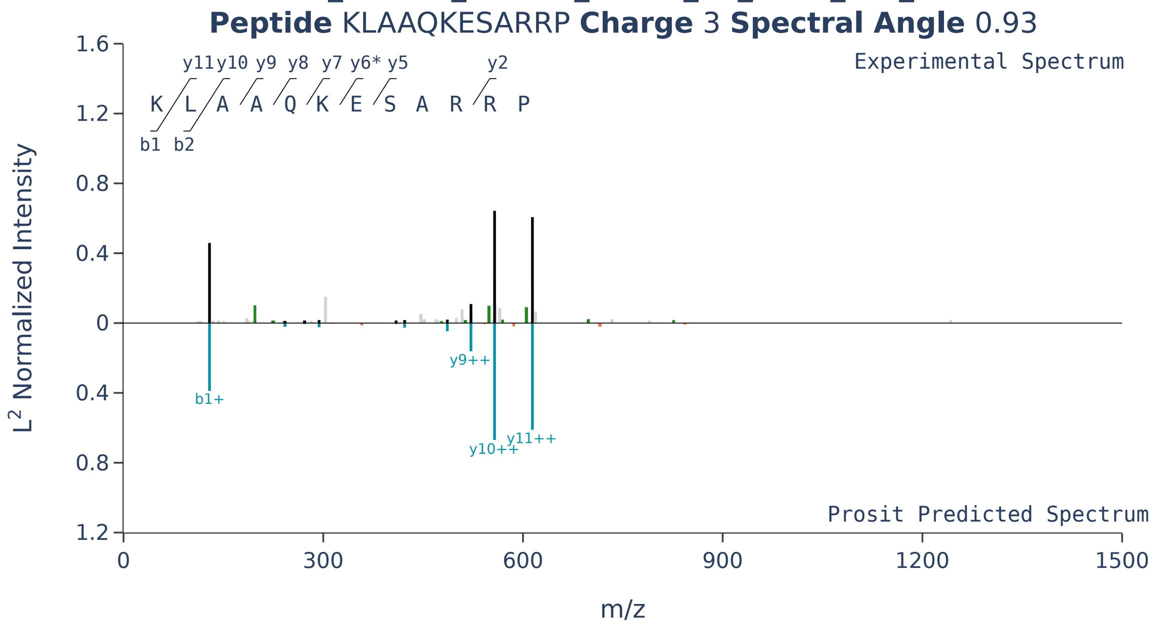


**Source** WSoh_101121_151121_HFGoe_G1_IL37b_4h_R1 **Scan** 10202
**Peptide** VHTSPKVKNLNPKKFSIHDQD **Charge** 4 **Spectral Angle** 0.82

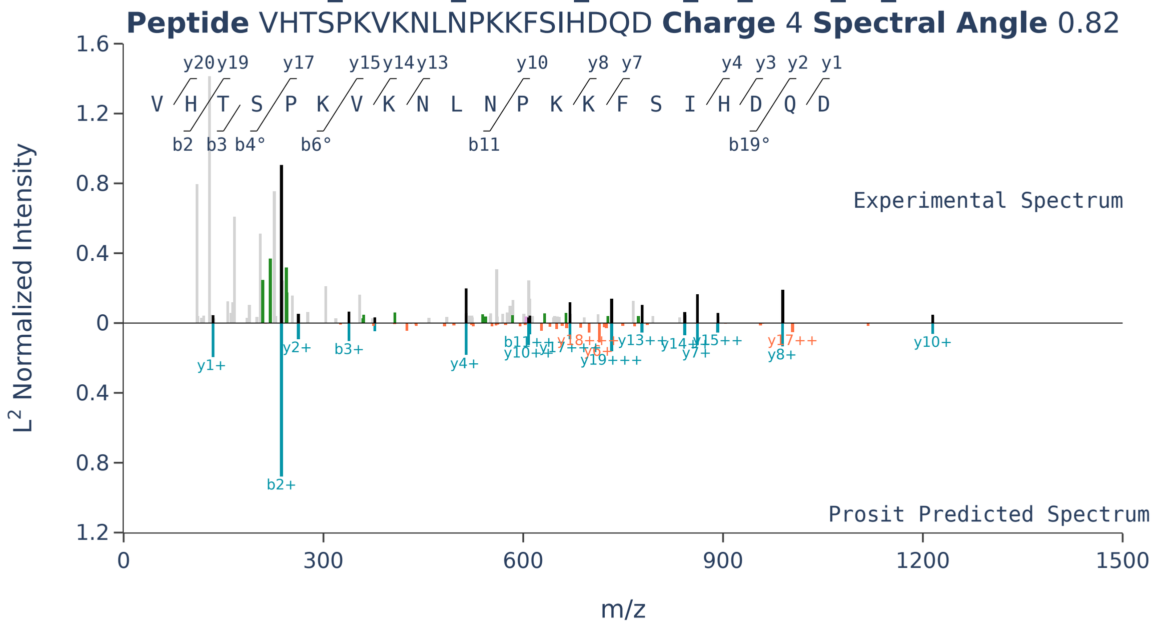


**Source** WSoh_101121_151121_HFGoe_G1_IL37b_4h_R1 **Scan** 14112
**Peptide** QKESARRPFIFYRAQ **Charge** 4 **Spectral Angle** 0.82

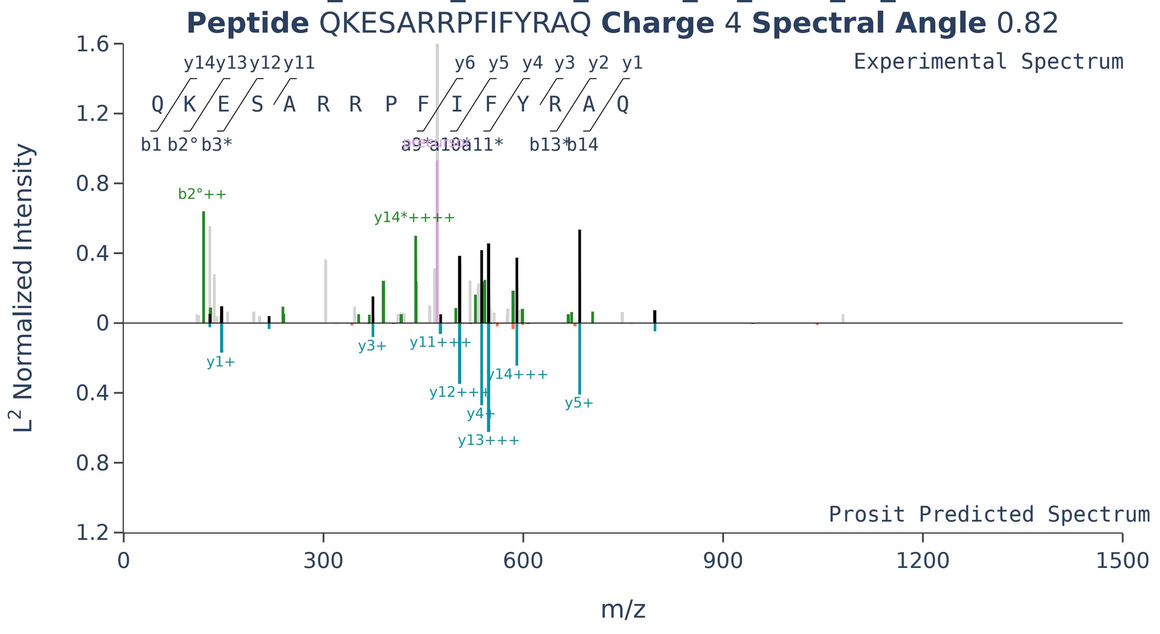


**Source** WSoh_101121_151121_HFGoe_G2_IL37b_24h_R1 **Scan** 14768
**Peptide** FQPVCKAEMSPSE **Charge** 2 **Spectral Angle** 0.85

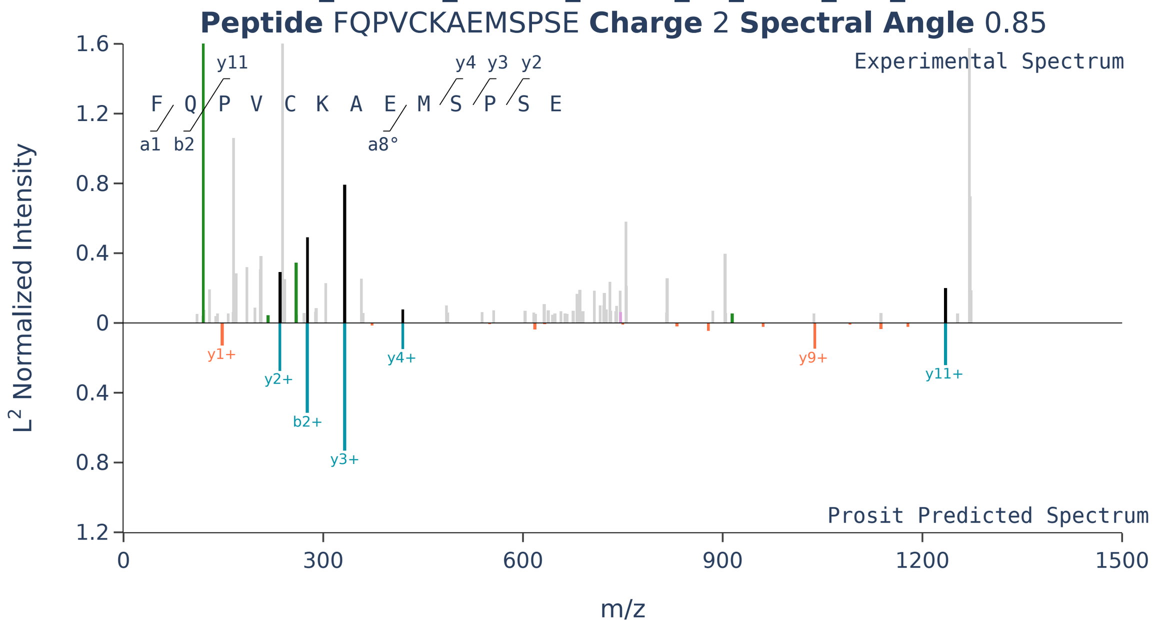


**Source** WSoh_101121_151121_HFGoe_G2_IL37b_24h_R1 **Scan** 18921
**Peptide** KESARRPFIFY **Charge** 3 **Spectral Angle** 0.77

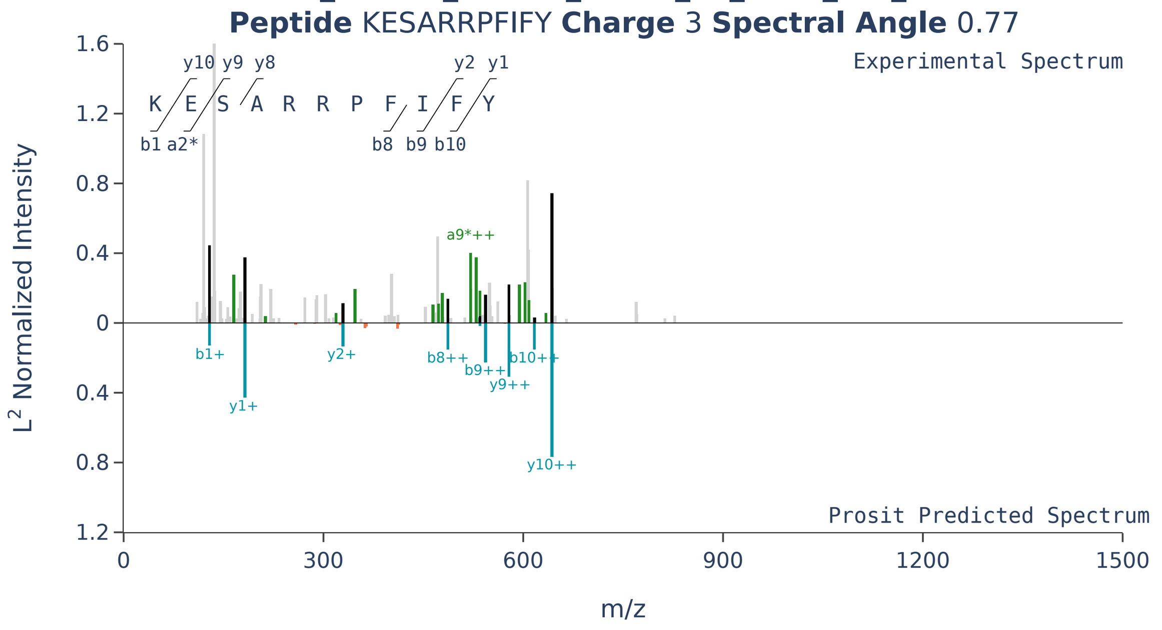


**Source** WSoh_101121_151121_HFGoe_G2_IL37b_24h_R1 **Scan** 14876
**Peptide** NPKKFSIHDQDHKVLVLD **Charge** 5 **Spectral Angle** 0.85

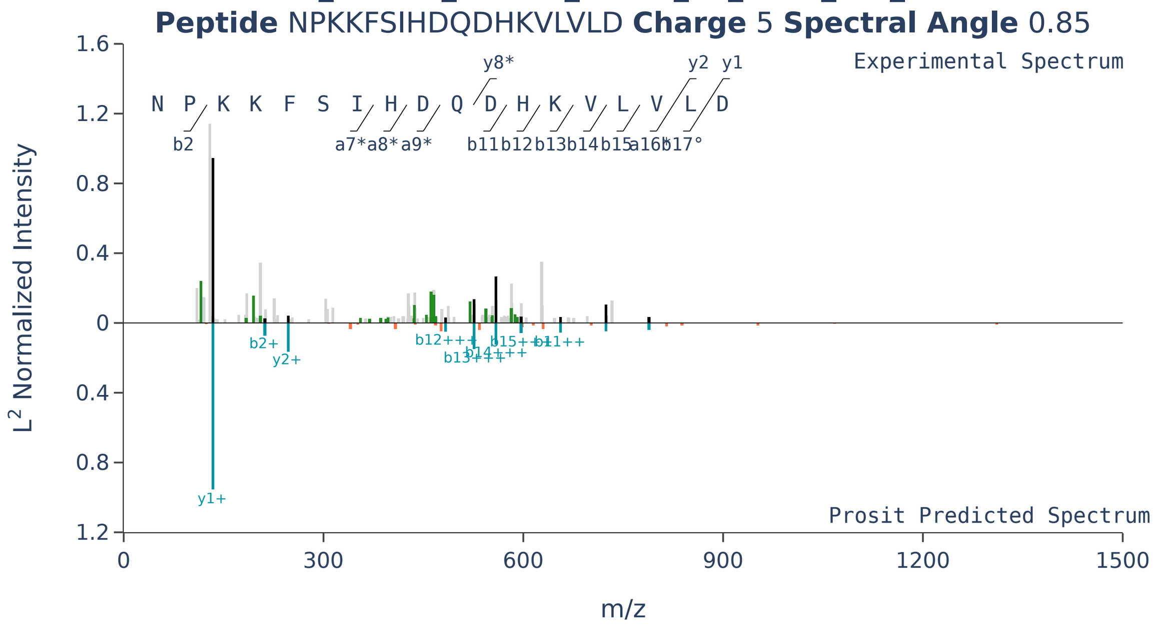


**Source** WSoh_101121_151121_HFGoe_G1_IL37b_4h_R2 **Scan** 22835
**Peptide** YIRPEIF **Charge** 2 **Spectral Angle** 0.79

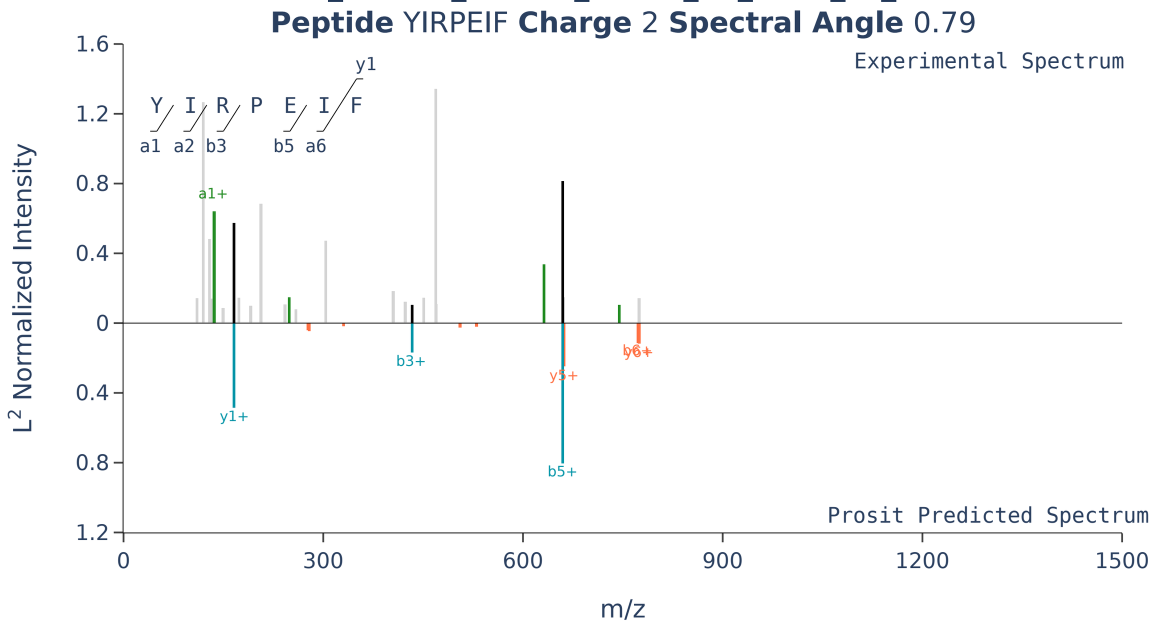


**Source** WSoh_101121_151121_HFGoe_G1_IL37b_24h_R1 **Scan** 12733
**Peptide** PSLQLKKEKLMKLA **Charge** 4 **Spectral Angle** 0.75

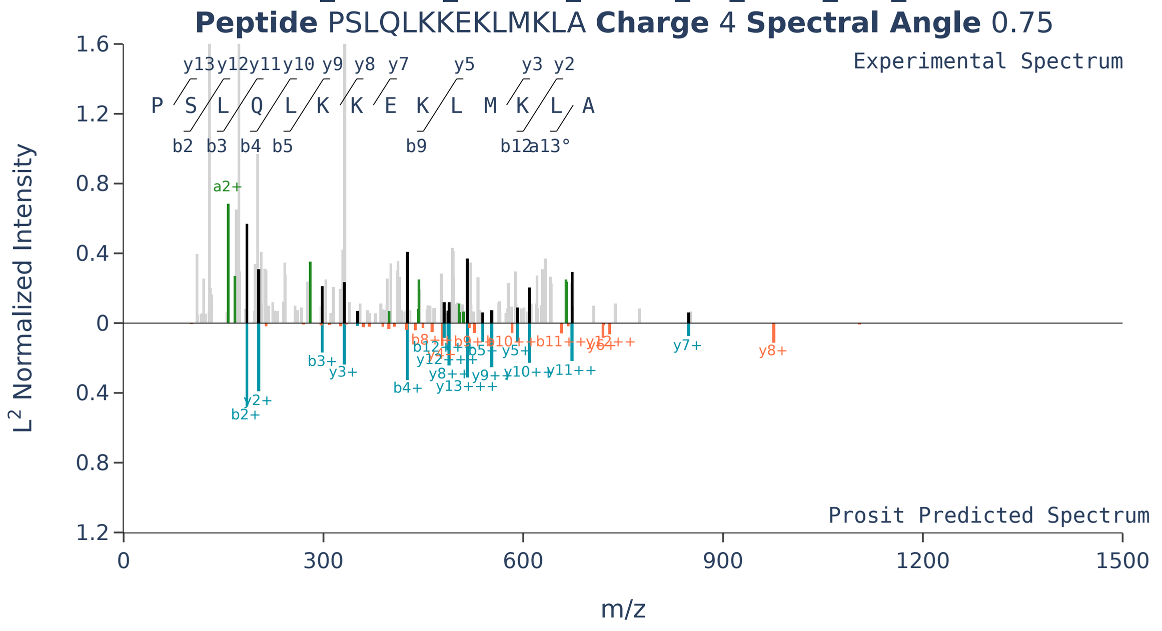


**Source** WSoh_101121_151121_HFGoe_G1_IL37b_1h_R1 **Scan** 20004
**Peptide** FALASSLSSASAEKG **Charge** 2 **Spectral Angle** 0.77

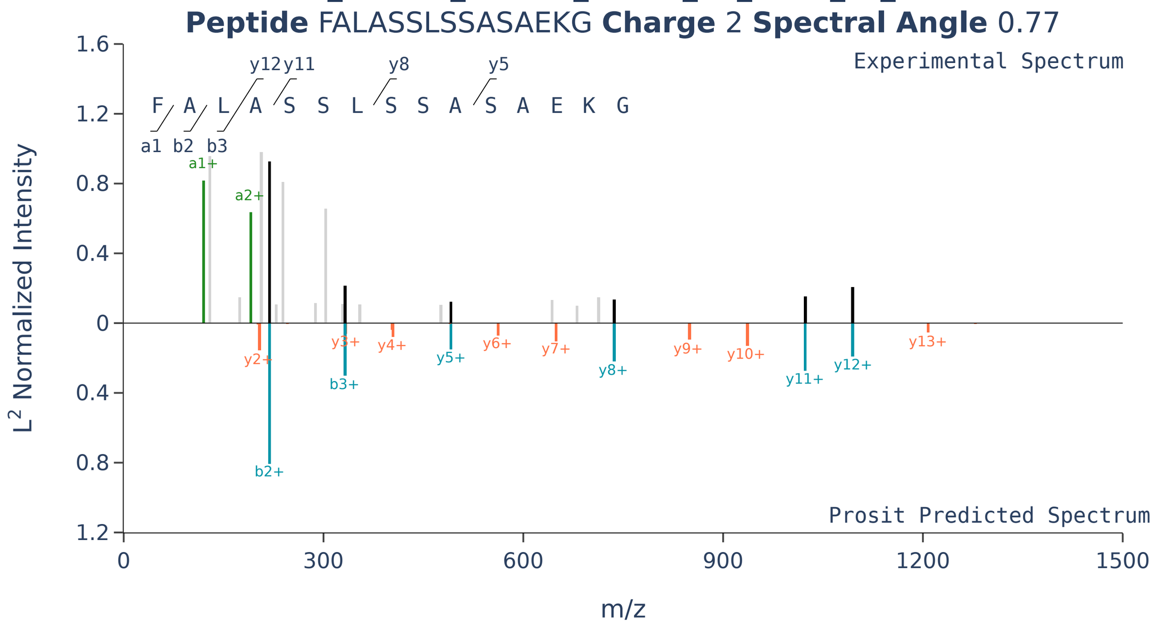


**Source** WSoh_101121_151121_HFGoe_G2_IL37b_24h_R2 **Scan** 13755
**Peptide** TSCNCNEPVGVTDKFENR **Charge** 3 **Spectral Angle** 0.73

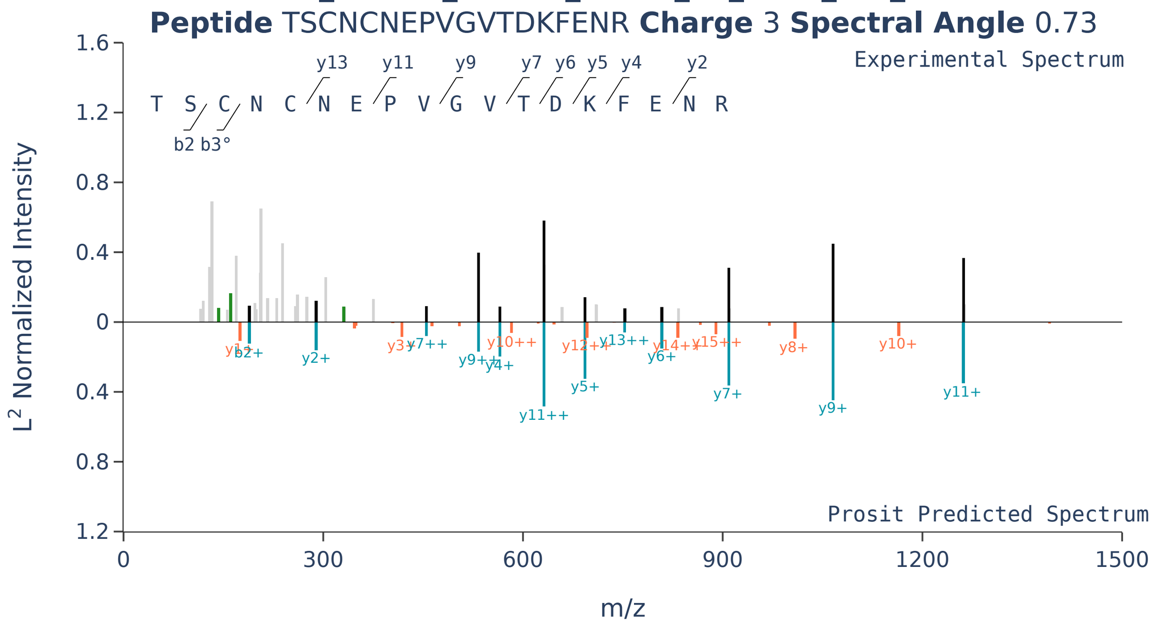


**Source** WSoh_101121_151121_HFGoe_G2_IL37b_24h_R2 **Scan** 6198
**Peptide** KKEKLMKLAAQK **Charge** 4 **Spectral Angle** 0.92

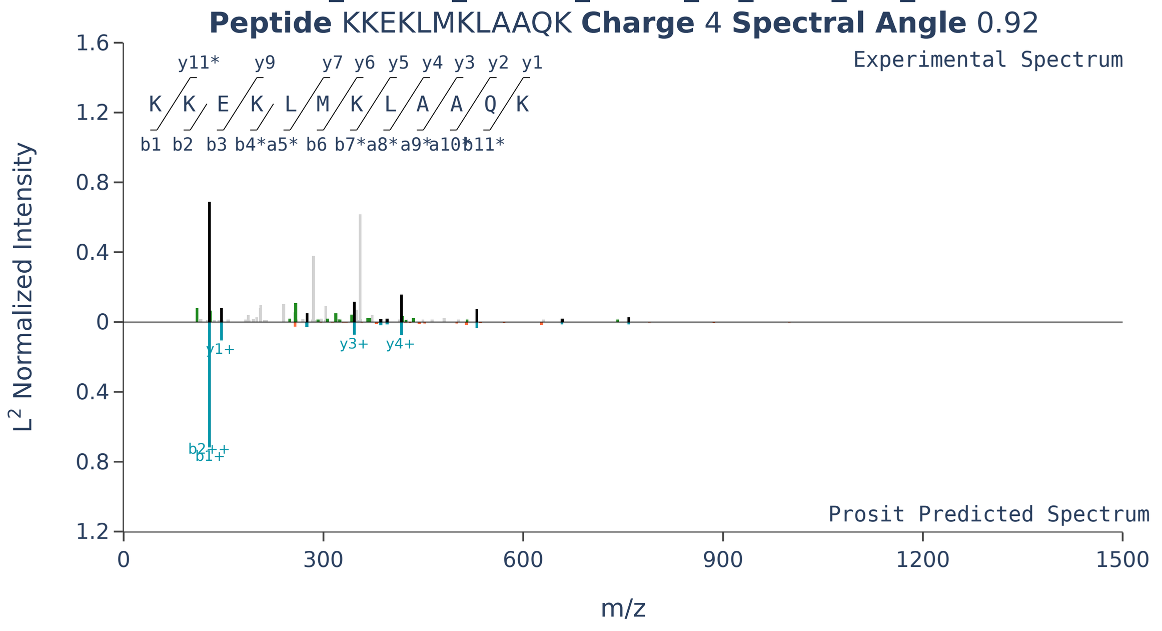


**Source** WSoh_101121_151121_HFGoe_G2_IL37b_24h_R2 **Scan** 11769
**Peptide** TSCNCNEPVGVTDKFENRKHIE **Charge** 4 **Spectral Angle** 0.81

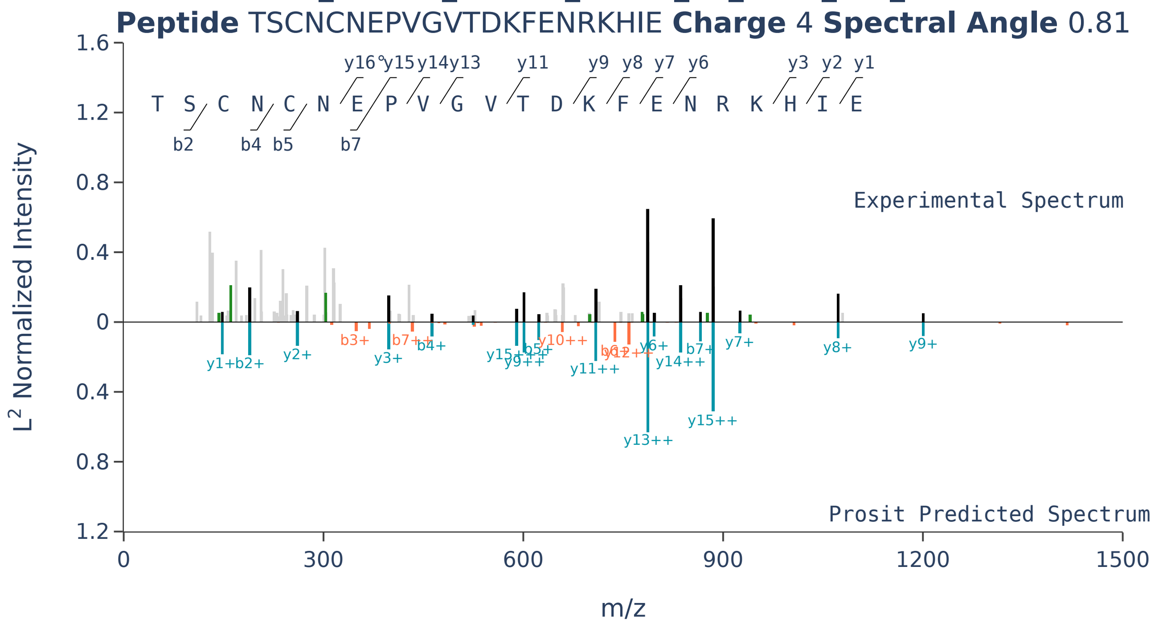


**Source** WSoh_101121_151121_HFGoe_G1_IL37b_4h_R1 **Scan** 10945
**Peptide** YCDKDKGQSHPSLQLKKEKLM **Charge** 4 **Spectral Angle** 0.82

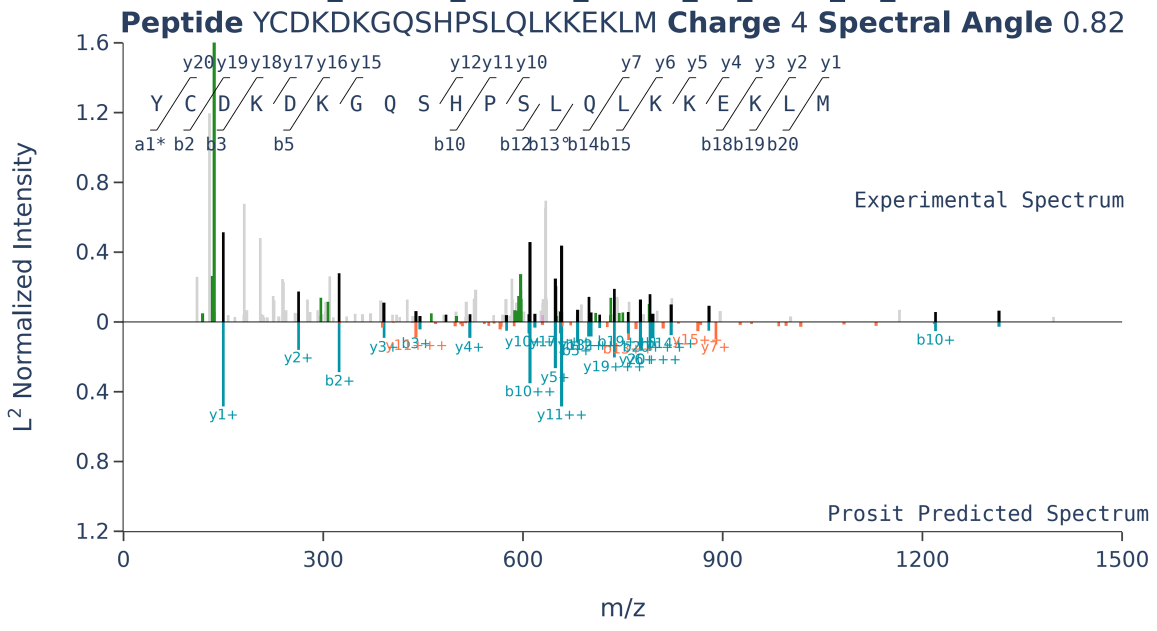


**Source** WSoh_101121_151121_HFGoe_G1_IL37b_24h_R1 **Scan** 20996
**Peptide** NLIAVPDKNYIRP **Charge** 2 **Spectral Angle** 0.82

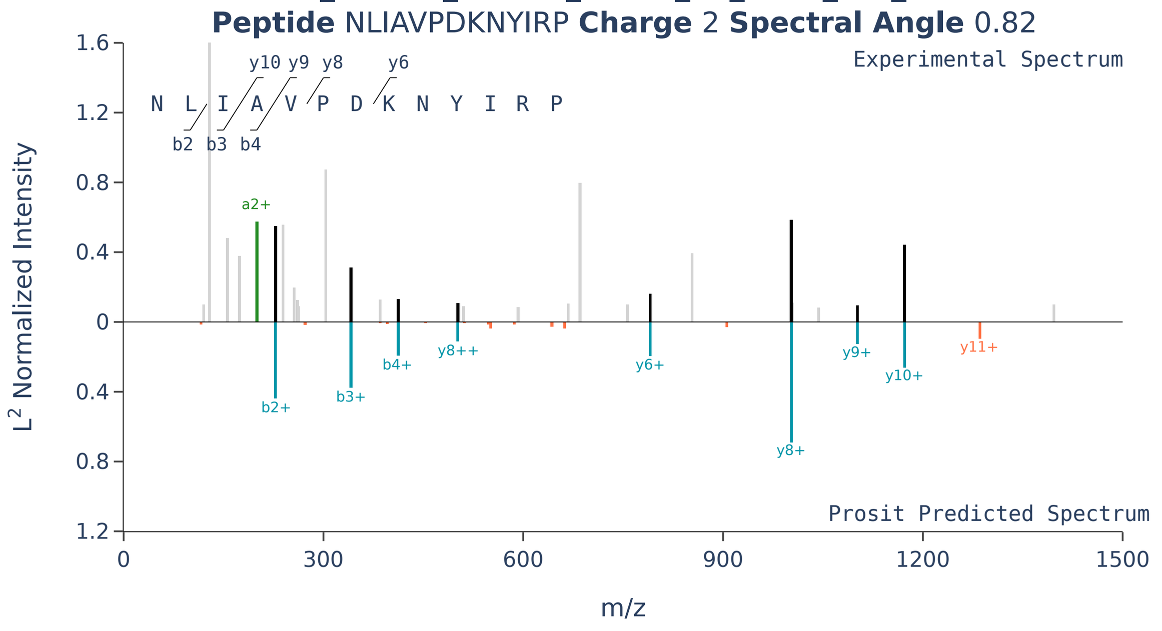


**Source** WSoh_101121_151121_HFGoe_G1_IL37b_4h_R1 **Scan** 15147
**Peptide** LGVSKGEFCLYCDKDKGQSH **Charge** 4 **Spectral Angle** 0.76

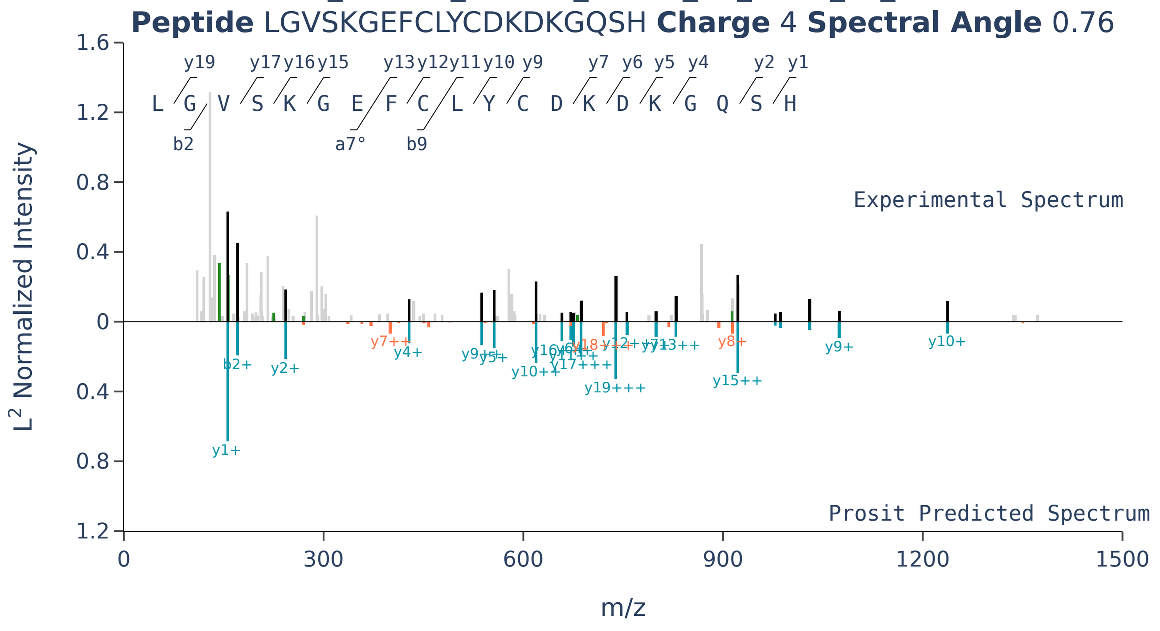

**Source** WSoh_101121_151121_HFGoe_G2_IL37b_2h_R1 **Scan** 22526
**Peptide** AAHPGWFICTSC **Charge** 2 **Spectral Angle** 0.78

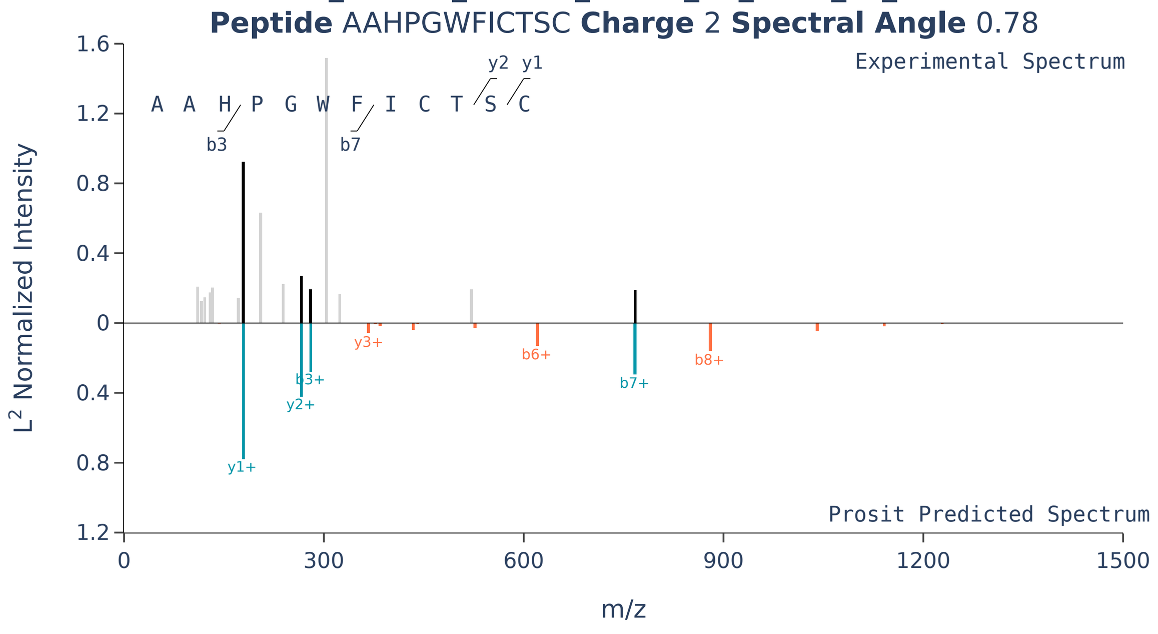


**Source** WSoh_101121_151121_HFGoe_G1_IL37b_24h_R2 **Scan** 8727
**Peptide** IHDQDHKVLV **Charge** 3 **Spectral Angle** 0.73

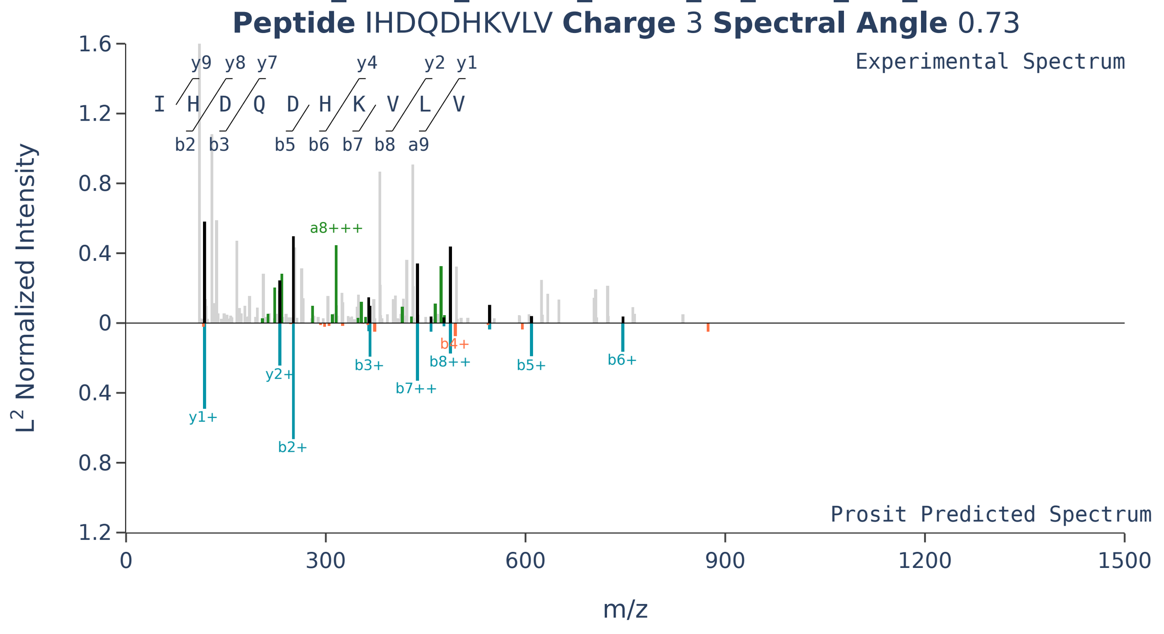


**Source** WSoh_101121_151121_HFGoe_G2_IL37b_24h_R1 **Scan** 23581
**Peptide** SGNLIAVPDKNYI **Charge** 2 **Spectral Angle** 0.86

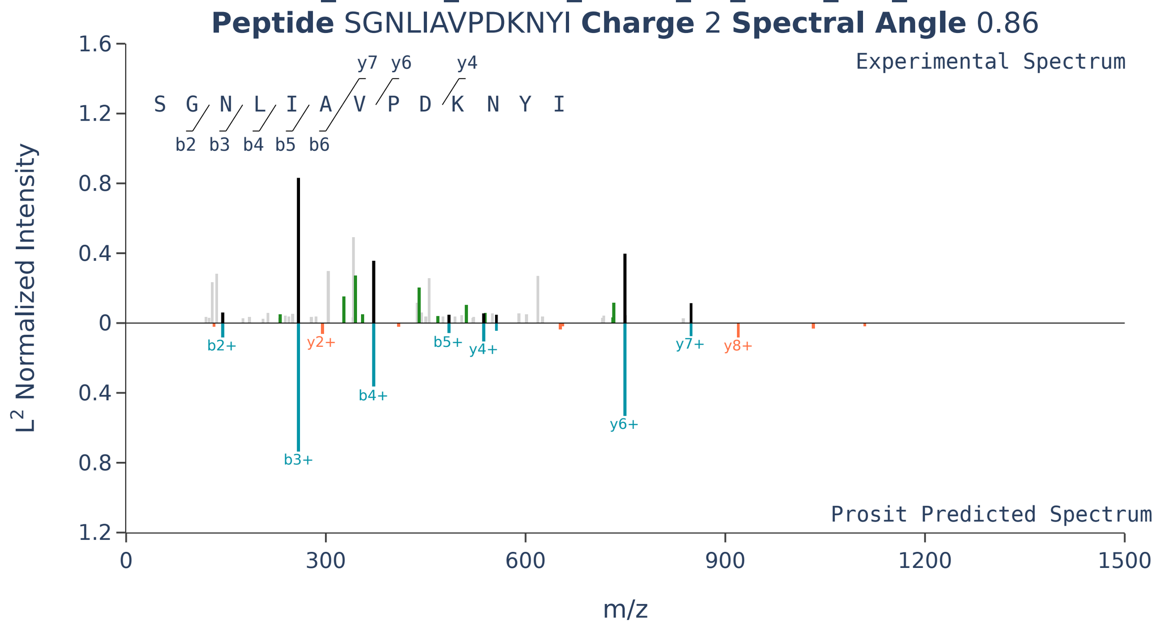


**Source** WSoh_101121_151121_HFGoe_G2_IL37b_24h_R2 **Scan** 9697
**Peptide** MSPSEVSD **Charge** 1 **Spectral Angle** 0.63

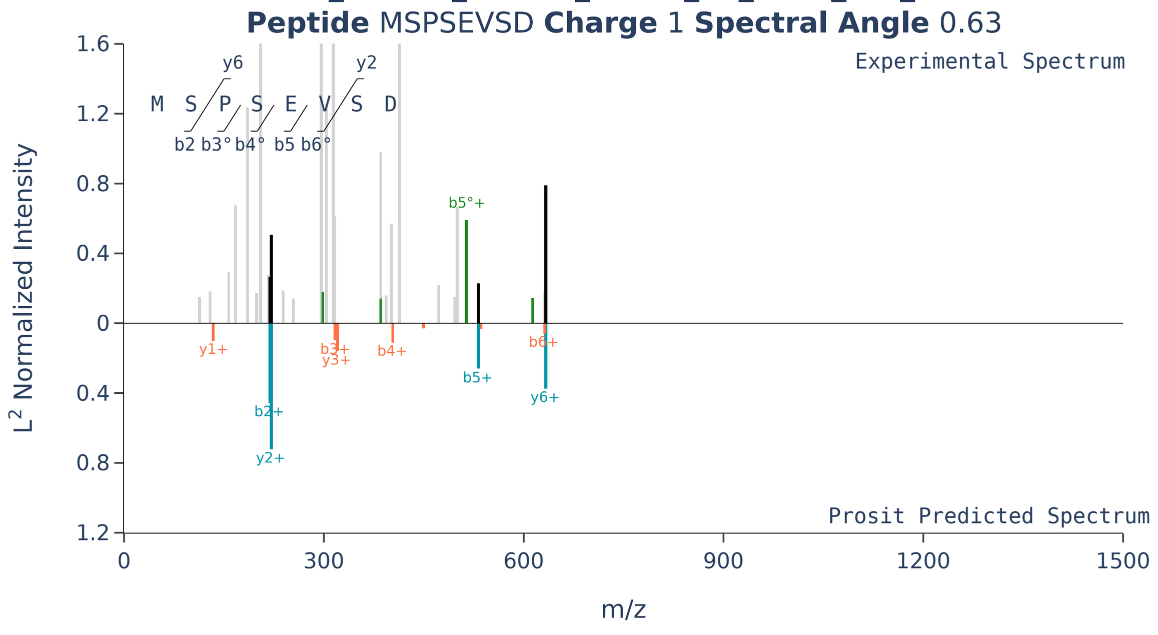


**Source** WSoh_101121_151121_HFGoe_G1_IL37b_1h_R2 **Scan** 22644
**Peptide** AHPGWFICT **Charge** 2 **Spectral Angle** 0.79

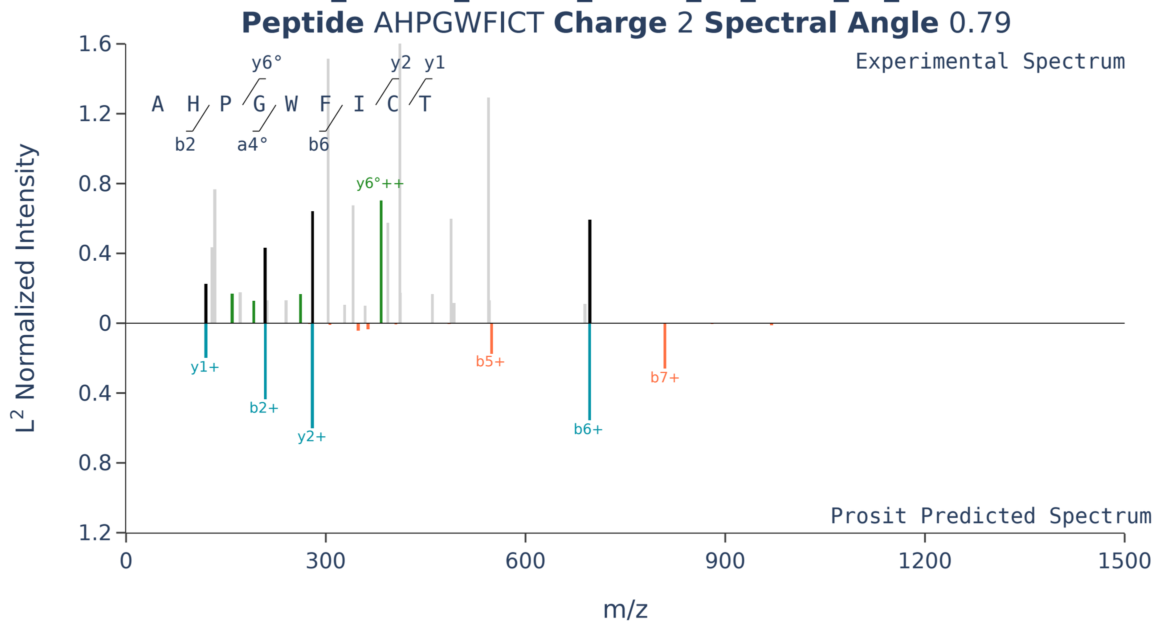


**Source** WSoh_101121_151121_HFGoe_G1_IL37b_4h_R2 **Scan** 12895
**Peptide** PKVKNLNPKKFSL **Charge** 3 **Spectral Angle** 0.68

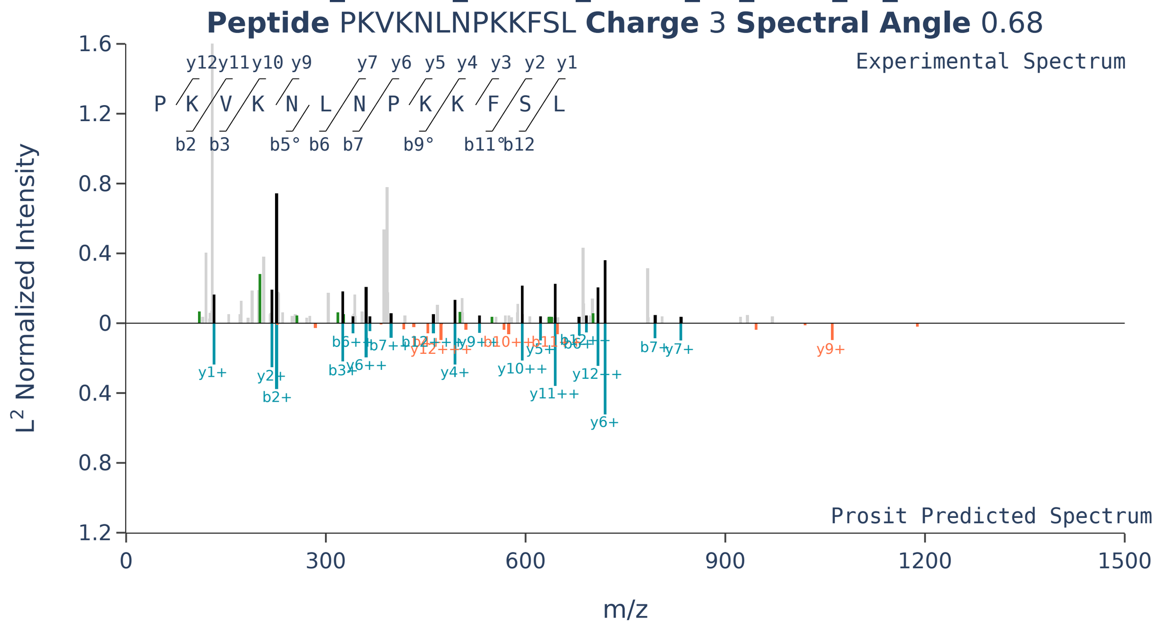


**Source** WSoh_101121_151121_HFGoe_G1_IL37b_24h_R2 **Scan** 23301
**Peptide** SSASAEKGSPILLGVSKGEF **Charge** 3 **Spectral Angle** 0.86

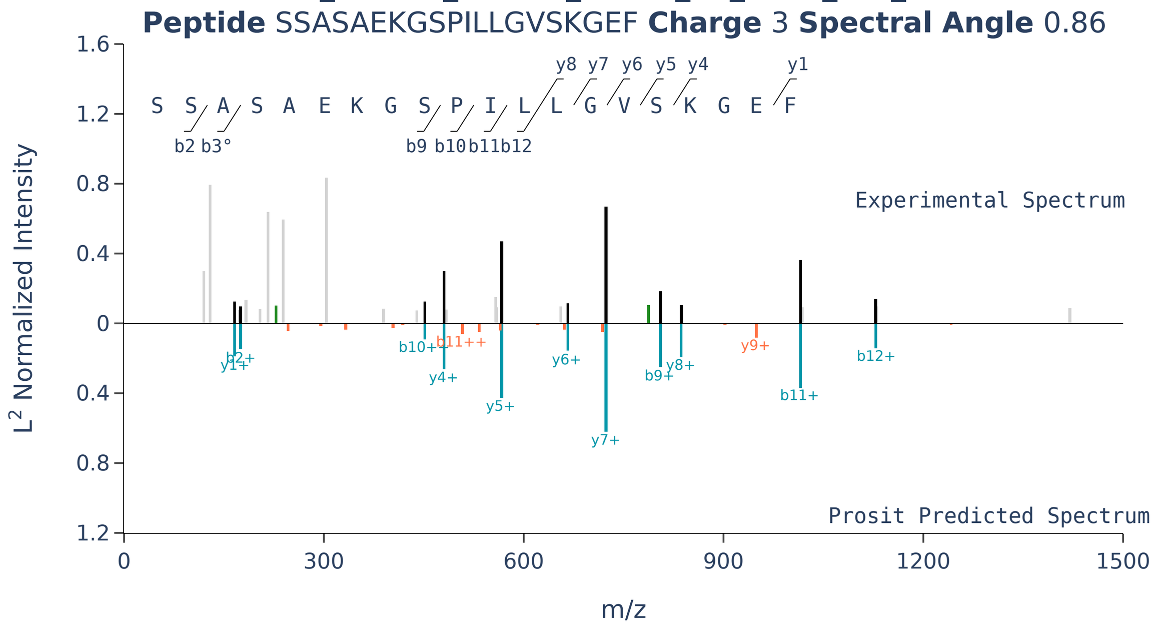


**Source** WSoh_101121_151121_HFGoe_G1_IL37b_4h_R1 **Scan** 16685
**Peptide** ESARRPFIFYR **Charge** 3 **Spectral Angle** 0.82

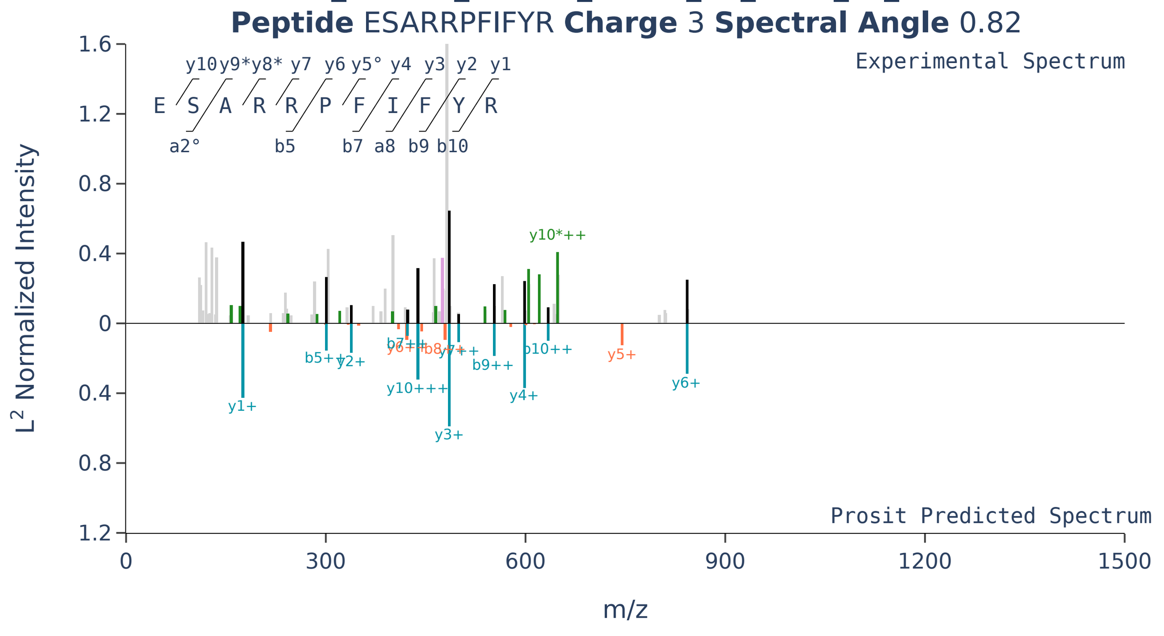


**Source** WSoh_101121_151121_HFGoe_G2_IL37b_24h_R1 **Scan** 8751
**Peptide** KNLNPKKFSIHDQD **Charge** 4 **Spectral Angle** 0.83

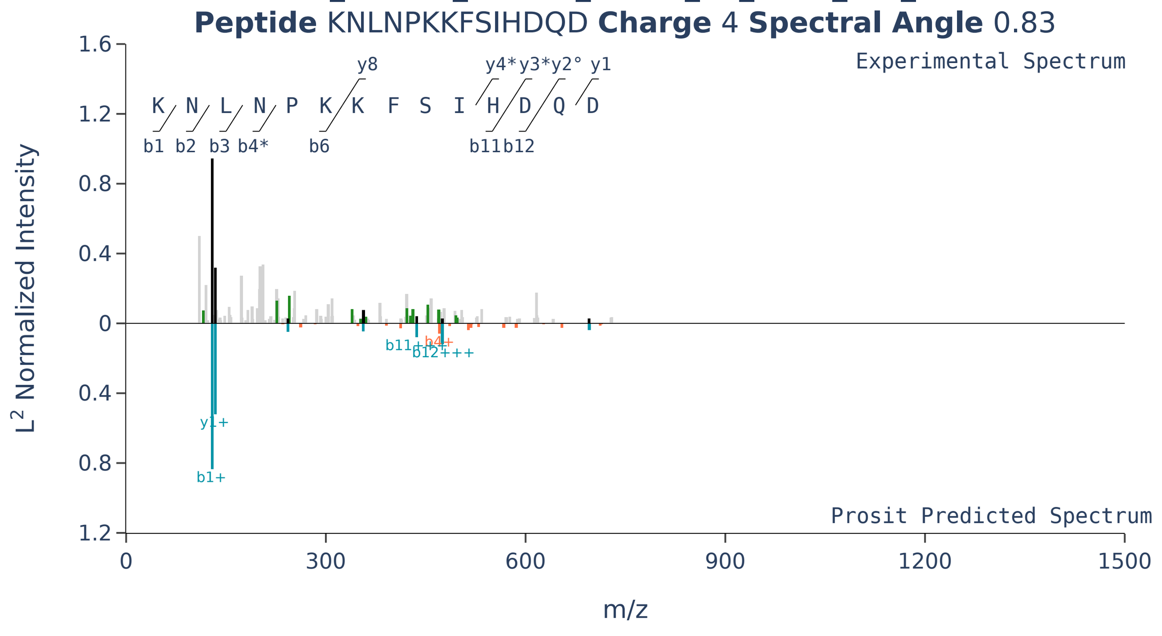


**Source** WSoh_101121_151121_HFGoe_G2_IL37b_2h_R2 **Scan** 13171
**Peptide** TSCNCNEPVGV **Charge** 2 **Spectral Angle** 0.78

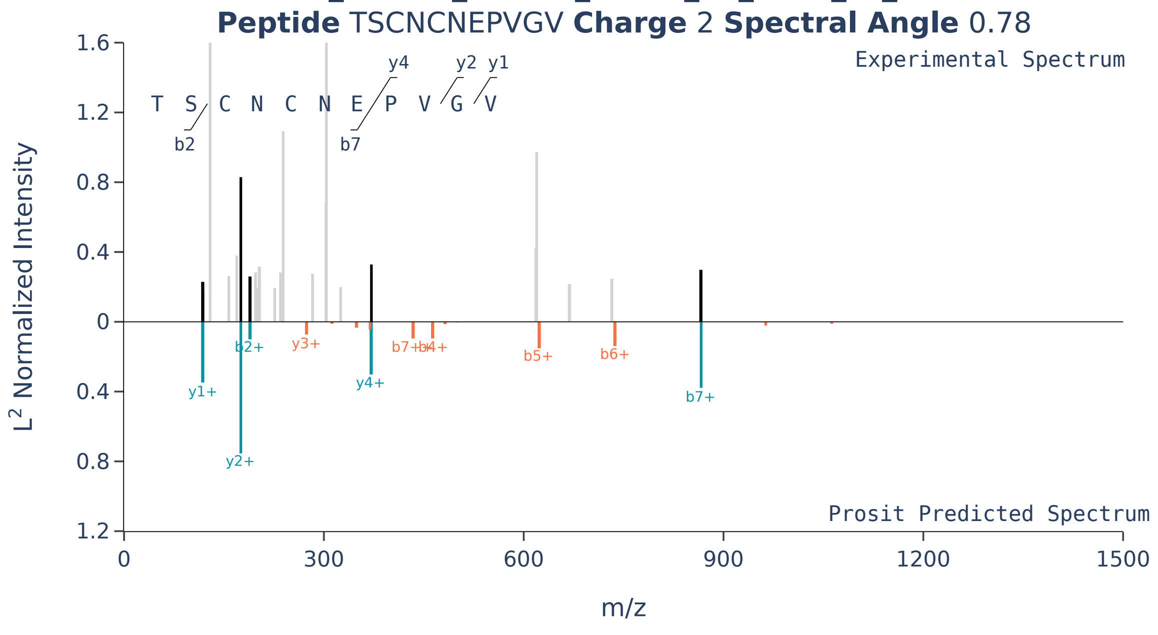


**Source** WSoh_101121_151121_HFGoe_G1_IL37b_24h_R2 **Scan** 23427
**Peptide** SLSSASAEKGSPILL **Charge** 2 **Spectral Angle** 0.82

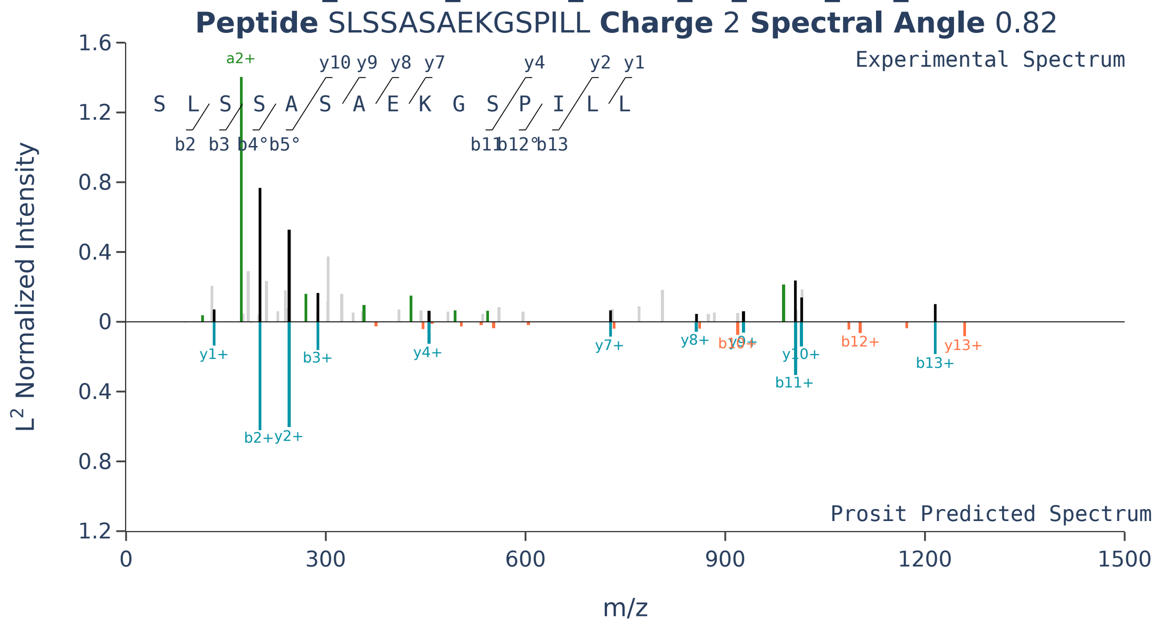


**Source** WSoh_101121_151121_HFGoe_G2_IL37b_24h_R1 **Scan** 21297
**Peptide** RRPFIFY **Charge** 2 **Spectral Angle** 0.82

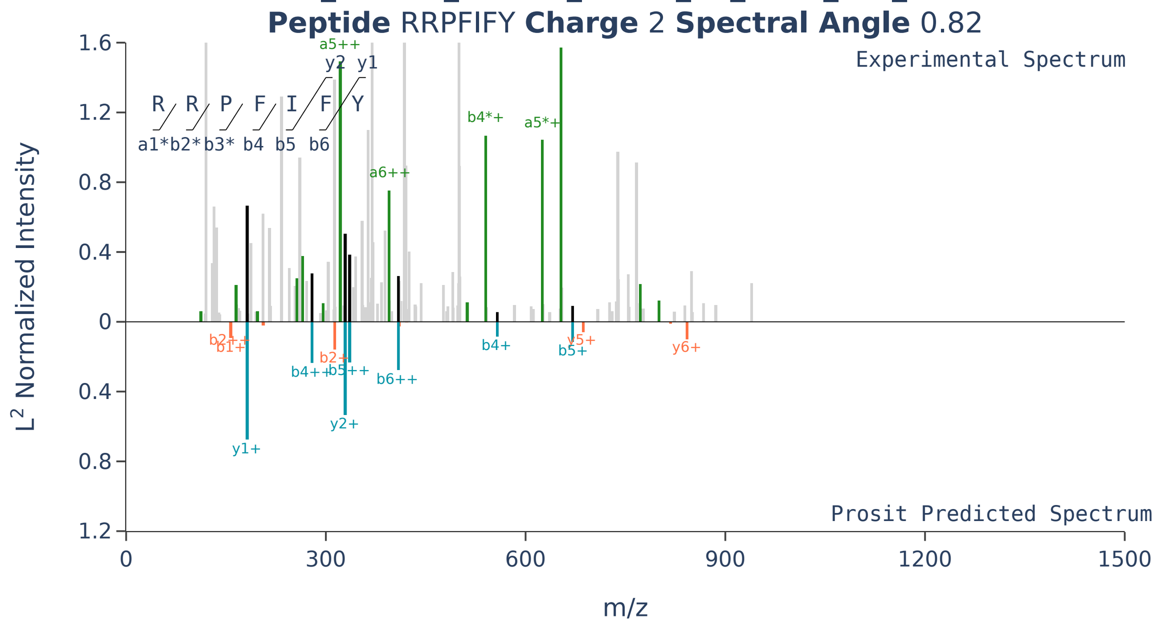


**Source** WSoh_101121_151121_HFGoe_G1_IL37b_4h_R1 **Scan** 11267
**Peptide** NPKKFSIHDQDHKVLV **Charge** 3 **Spectral Angle** 0.72

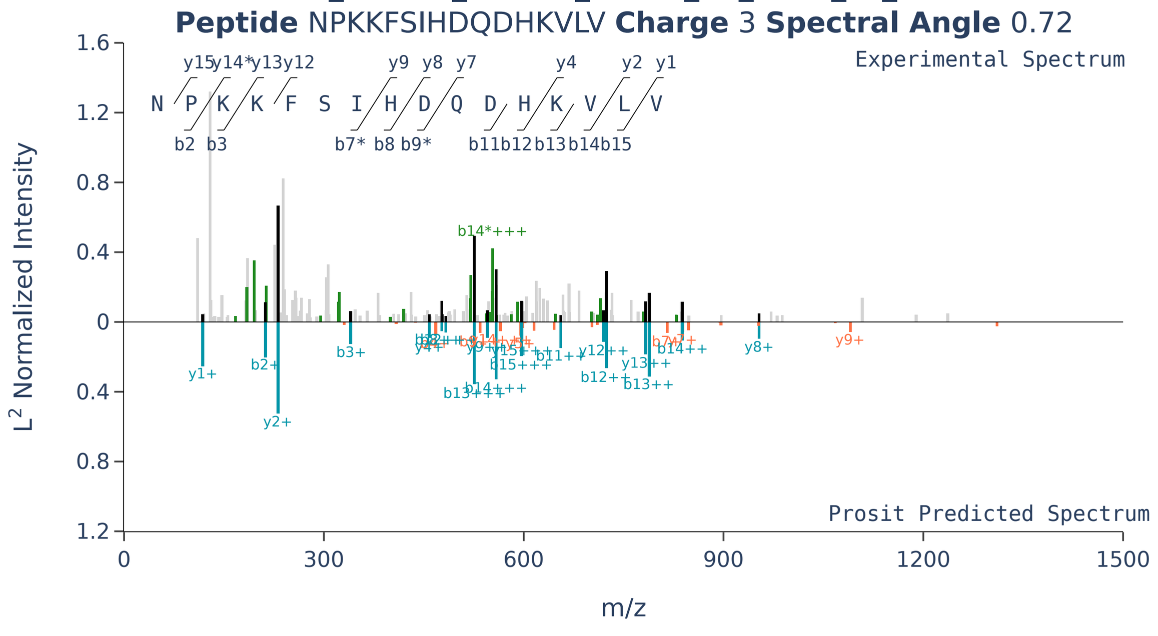


**Source** WSoh_101121_151121_HFGoe_G2_IL37b_24h_R2 **Scan** 7615
**Peptide** GVTDKFENRKH **Charge** 3 **Spectral Angle** 0.83

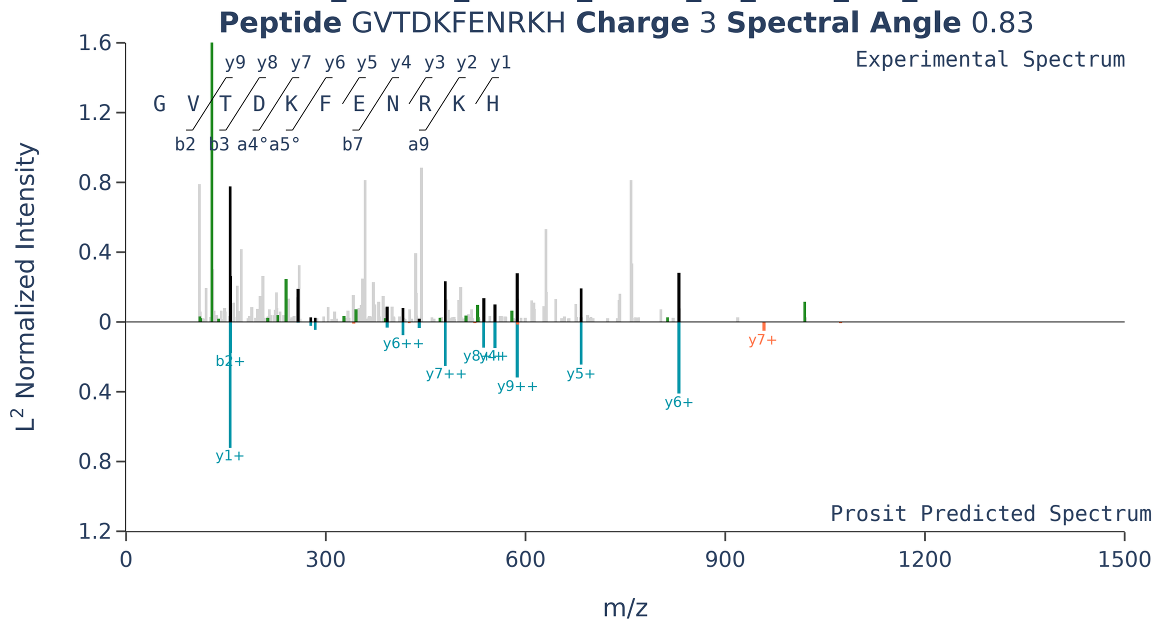


**Source** WSoh_101121_151121_HFGoe_G2_IL37b_24h_R2 **Scan** 6764
**Peptide** DKDKGQSHPSLQLKKEKLMK **Charge** 4 **Spectral Angle** 0.72

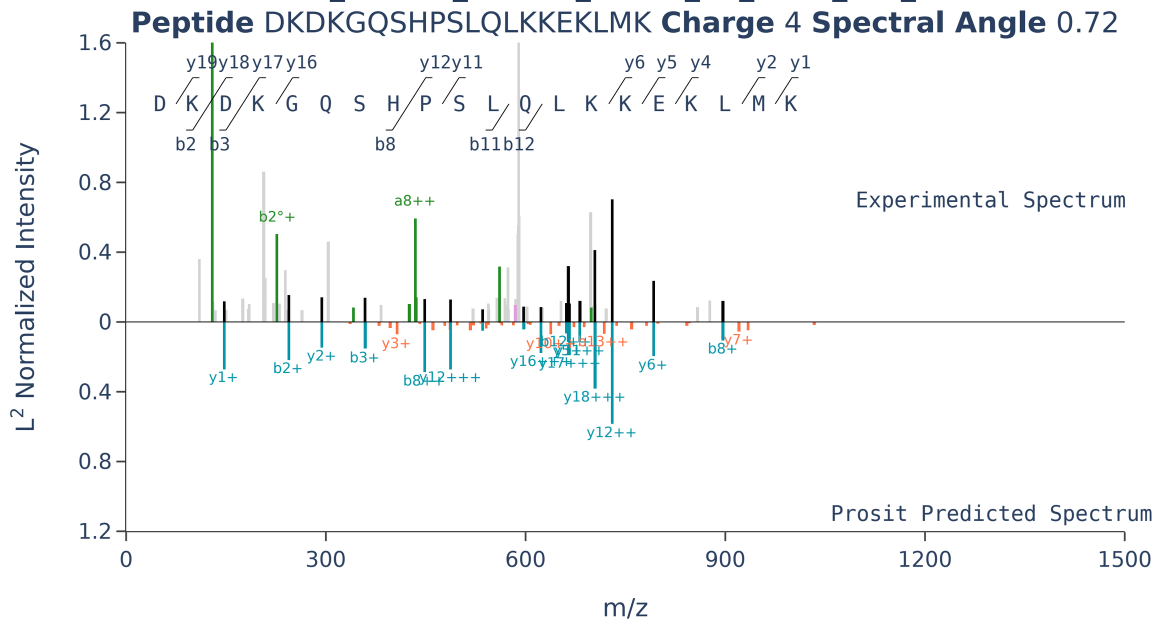

**Source** WSoh_101121_151121_HFGoe_G1_IL37b_4h_R2 **Scan** 7683
**Peptide** VKNLNPKKF **Charge** 3 **Spectral Angle** 0.81

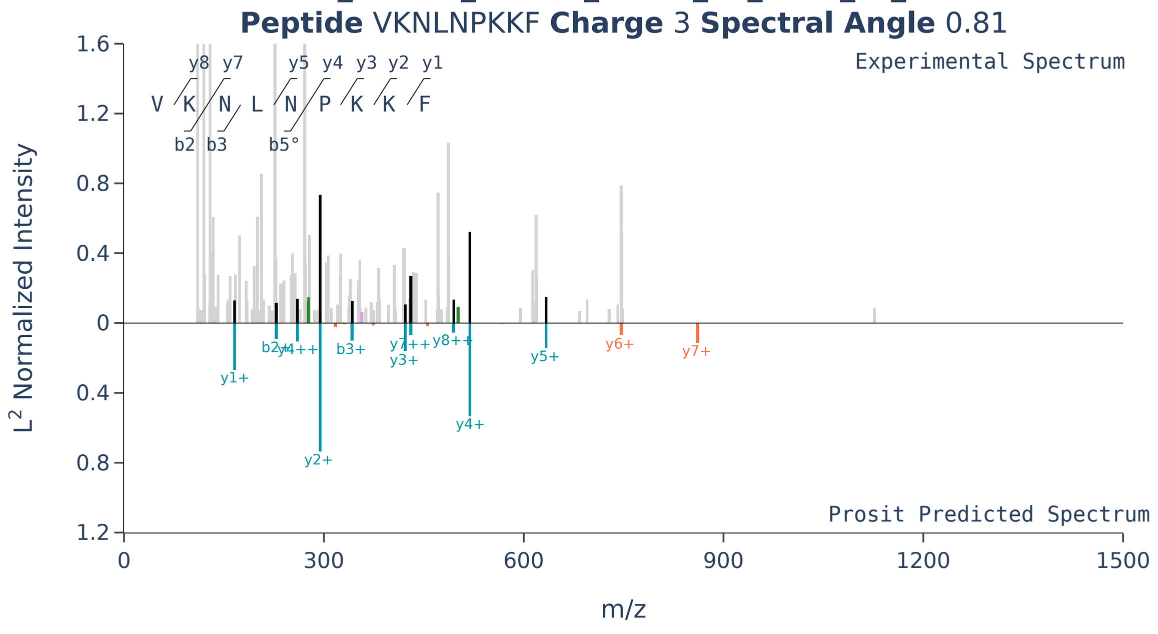


**Source** WSoh_101121_151121_HFGoe_G2_IL37b_2h_R2 **Scan** 24336
**Peptide** SAAHPGWFI **Charge** 2 **Spectral Angle** 0.77

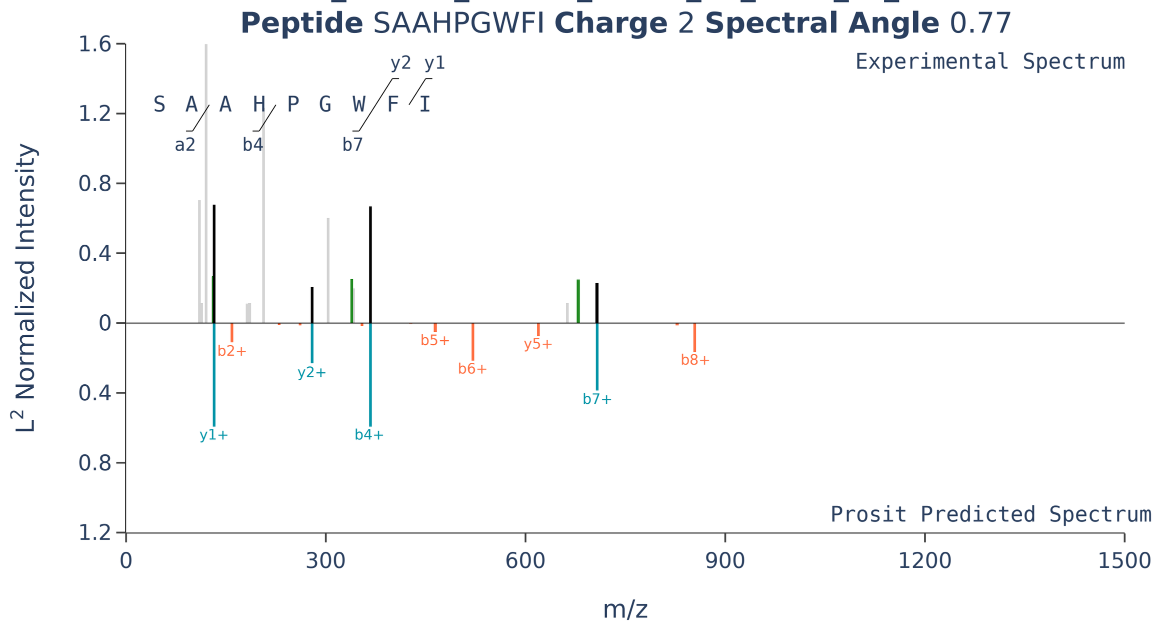


**Source** WSoh_101121_151121_HFGoe_G2_IL37b_24h_R2 **Scan** 9253
**Peptide** LNPKKFSIHDQDHKV **Charge** 4 **Spectral Angle** 0.73

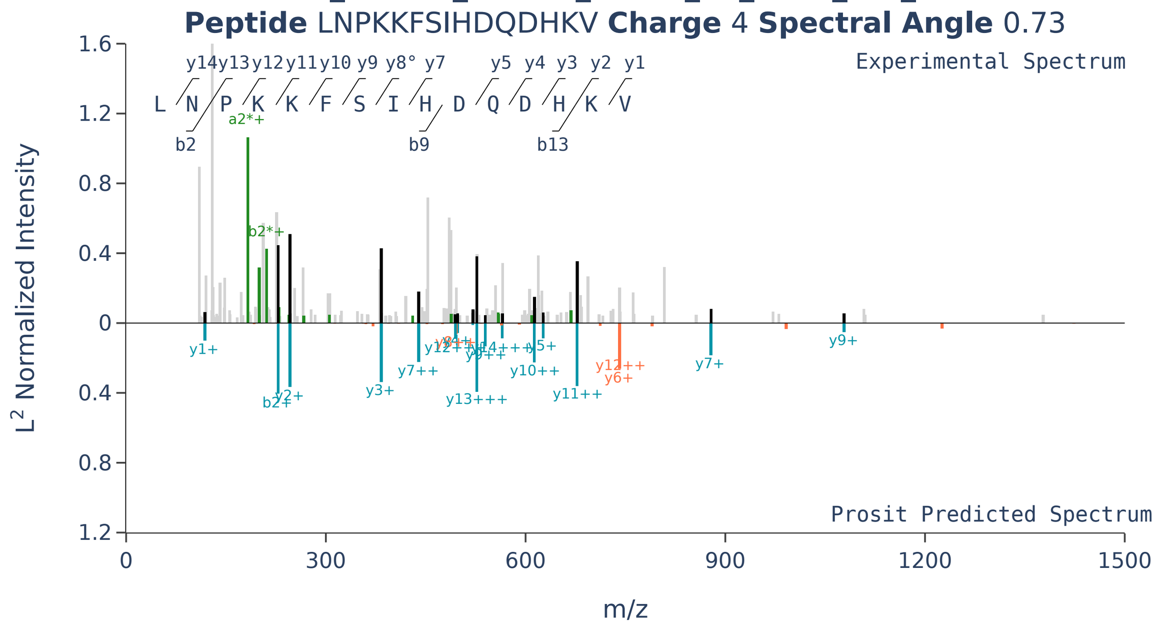


**Source** WSoh_101121_151121_HFGoe_G2_IL37b_1h_R2 **Scan** 6732
**Peptide** MKLAAQKE **Charge** 2 **Spectral Angle** 0.67

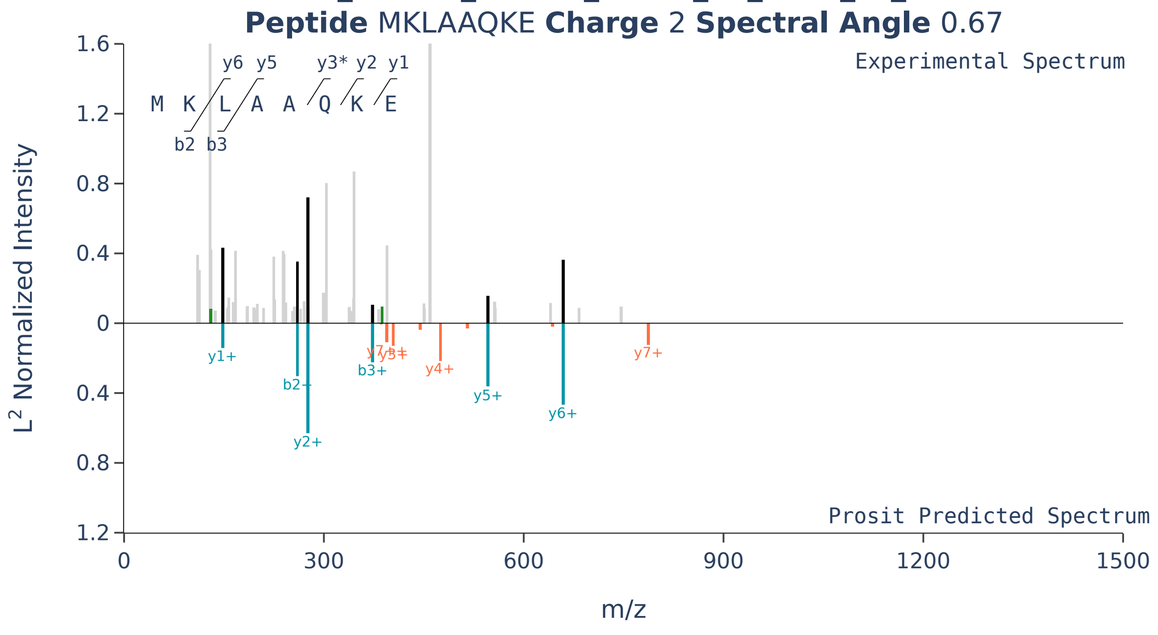


**Source** WSoh_101121_151121_HFGoe_G2_IL37b_2h_R1 **Scan** 25367
**Peptide** AVPDKNYIRPEIFF **Charge** 2 **Spectral Angle** 0.71

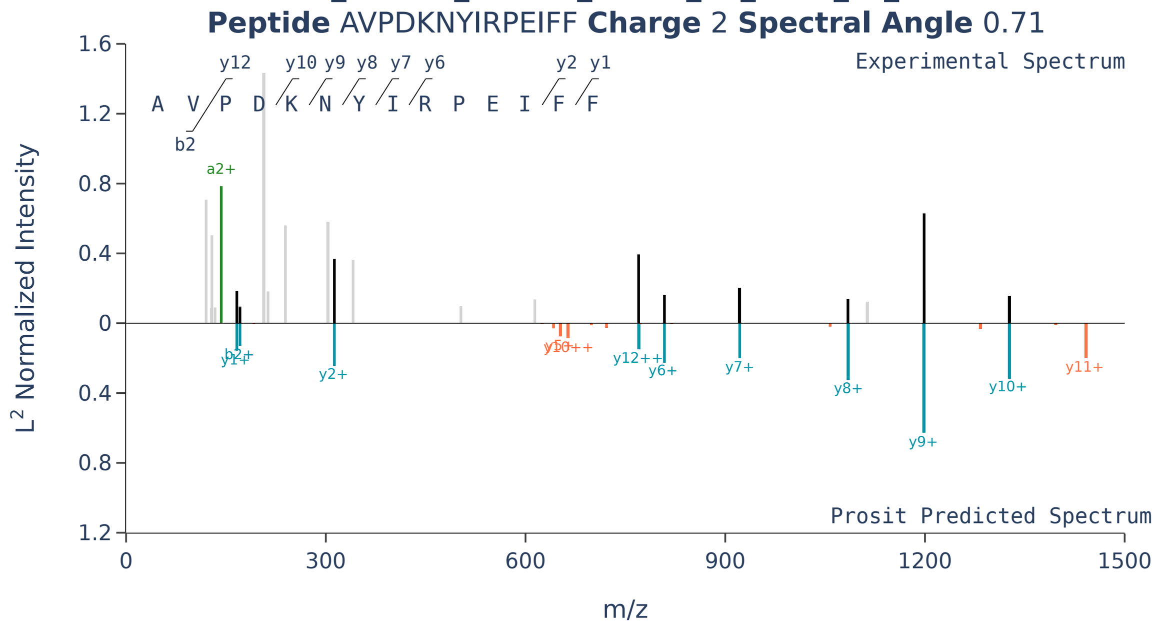


**Source** WSoh_101121_151121_HFGoe_G1_IL37b_2h_R1 **Scan** 22642
**Peptide** ESAAHPGWFICTS **Charge** 2 **Spectral Angle** 0.69

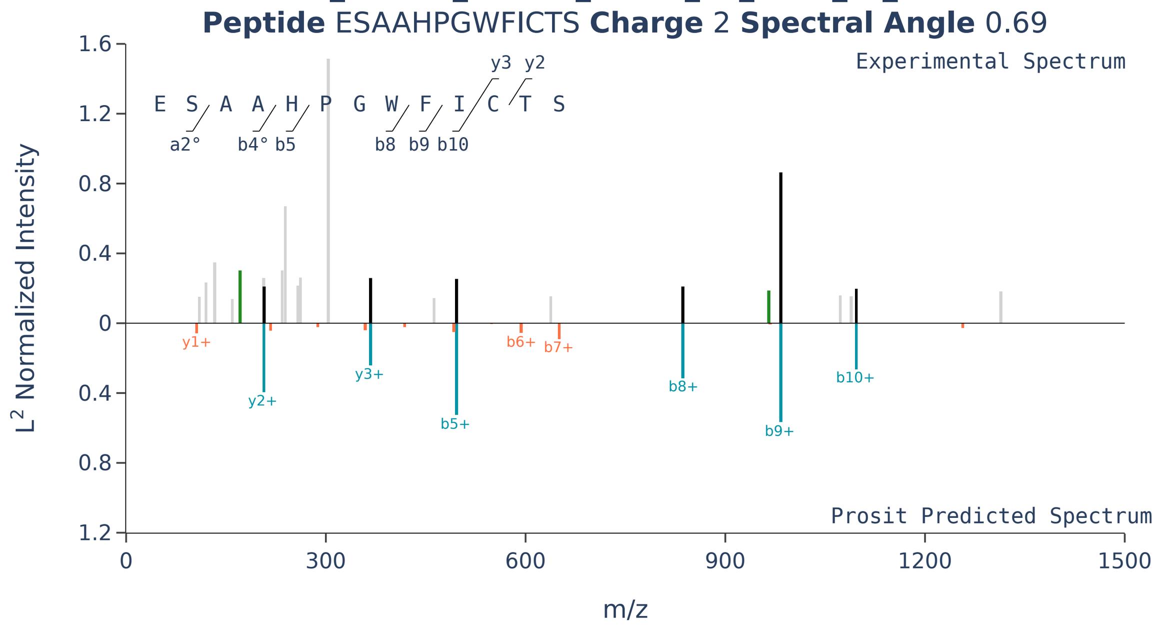


**Source** WSoh_101121_151121_HFGoe_G1_IL37b_4h_R1 **Scan** 11927
**Peptide** PVCKAEMSPSEVSD **Charge** 2 **Spectral Angle** 0.7

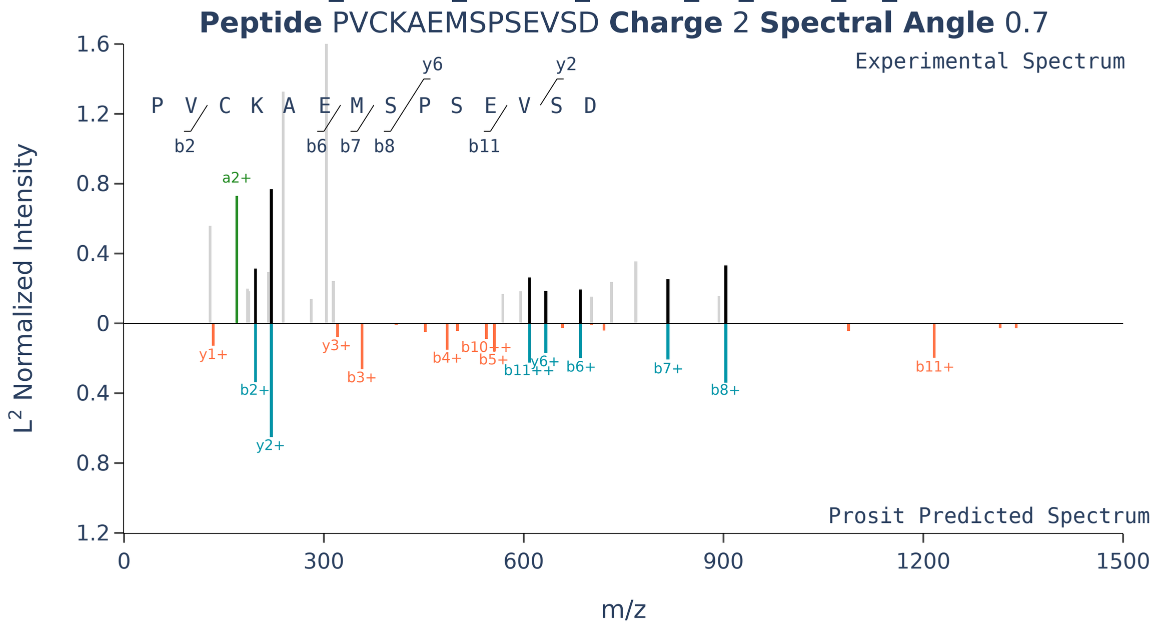


**Source** WSoh_101121_151121_HFGoe_G1_IL37b_4h_R1 **Scan** 10838
**Peptide** KNLNPKKFSIHDQDHKVL **Charge** 3 **Spectral Angle** 0.87

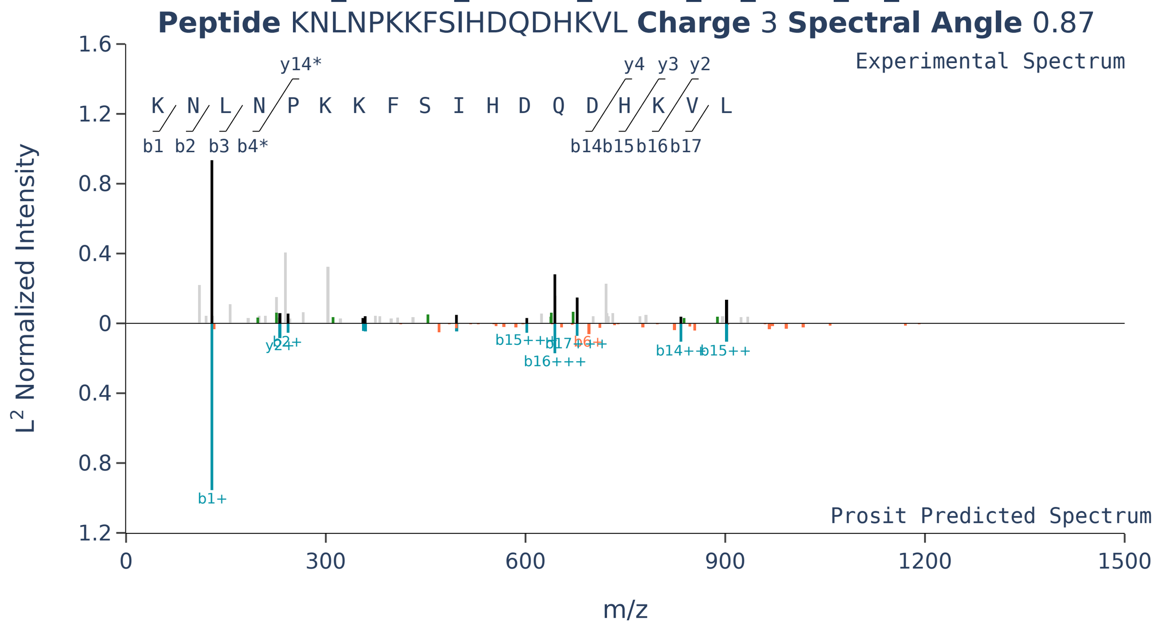


**Source** WSoh_101121_151121_HFGoe_G1_IL37b_4h_R1 **Scan** 17347
**Peptide** KLAAQKESARRPFIFY **Charge** 4 **Spectral Angle** 0.75

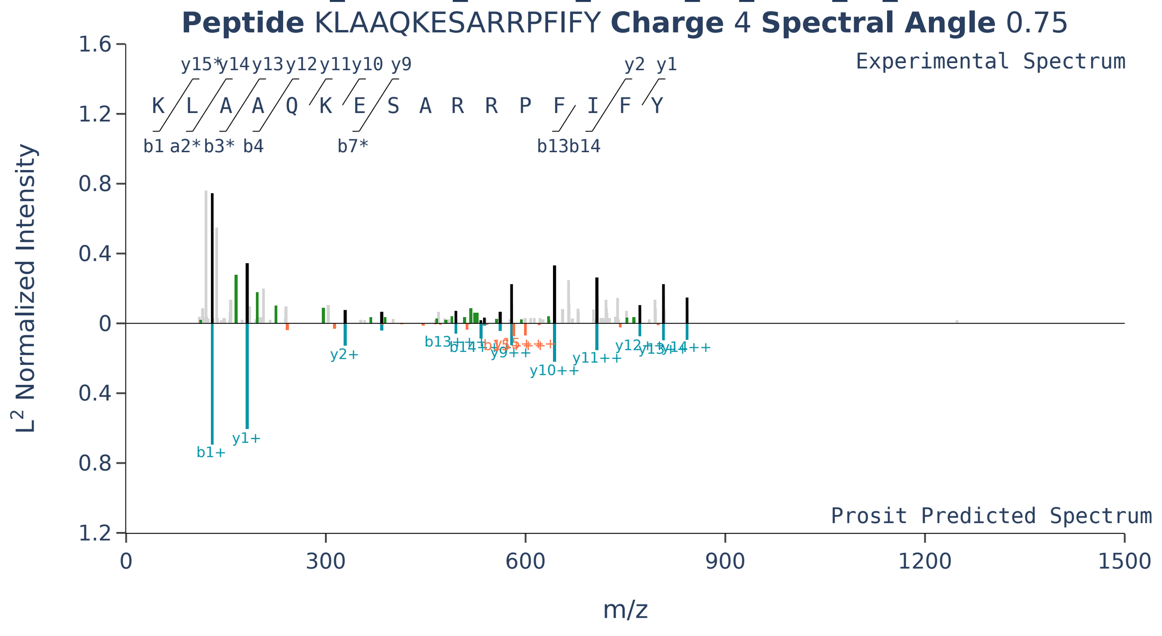


**Source** WSoh_101121_151121_HFGoe_G2_IL37b_24h_R1 **Scan** 10837
**Peptide** NEPVGVTDKFENRKHLE **Charge** 4 **Spectral Angle** 0.77

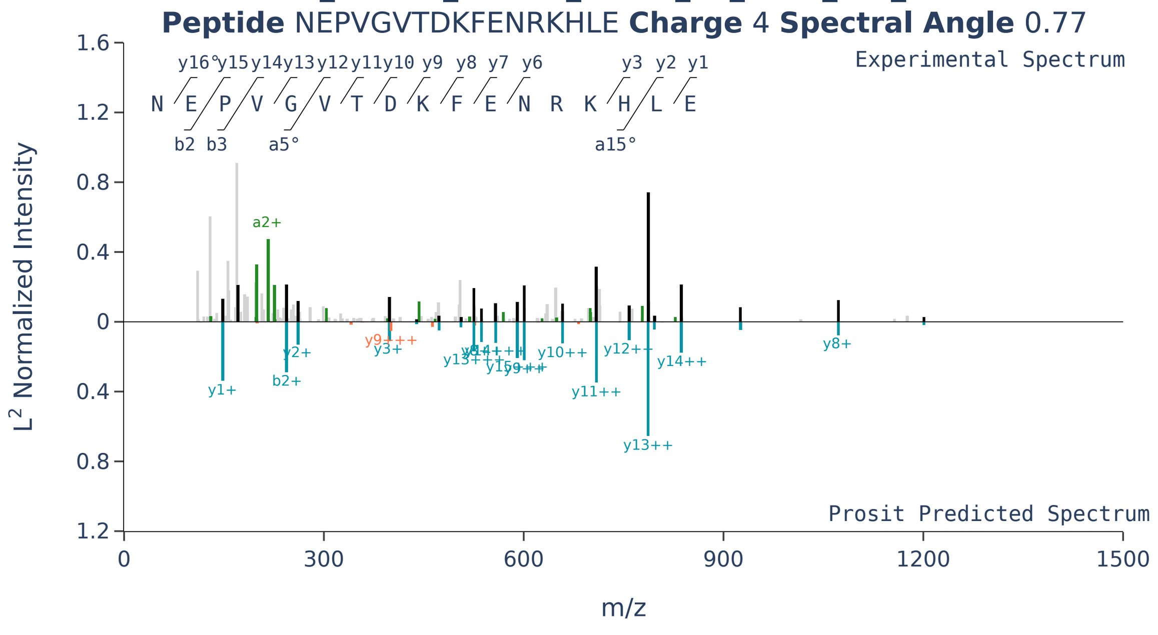


**Source** WSoh_101121_151121_HFGoe_G1_IL37b_24h_R1 **Scan** 11487
**Peptide** GVTDKFENRKHLE **Charge** 3 **Spectral Angle** 0.78

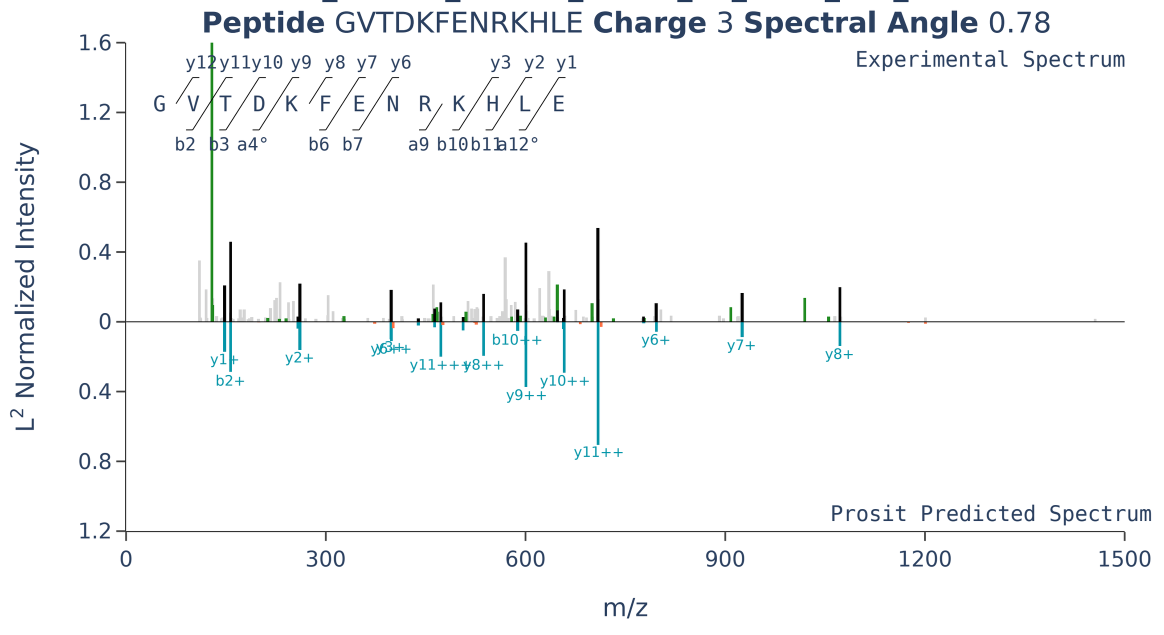


**Source** WSoh_101121_151121_HFGoe_G1_IL37b_4h_R1 **Scan** 20110
**Peptide** SLSSASAEKGSPILLGVSKGE **Charge** 3 **Spectral Angle** 0.92

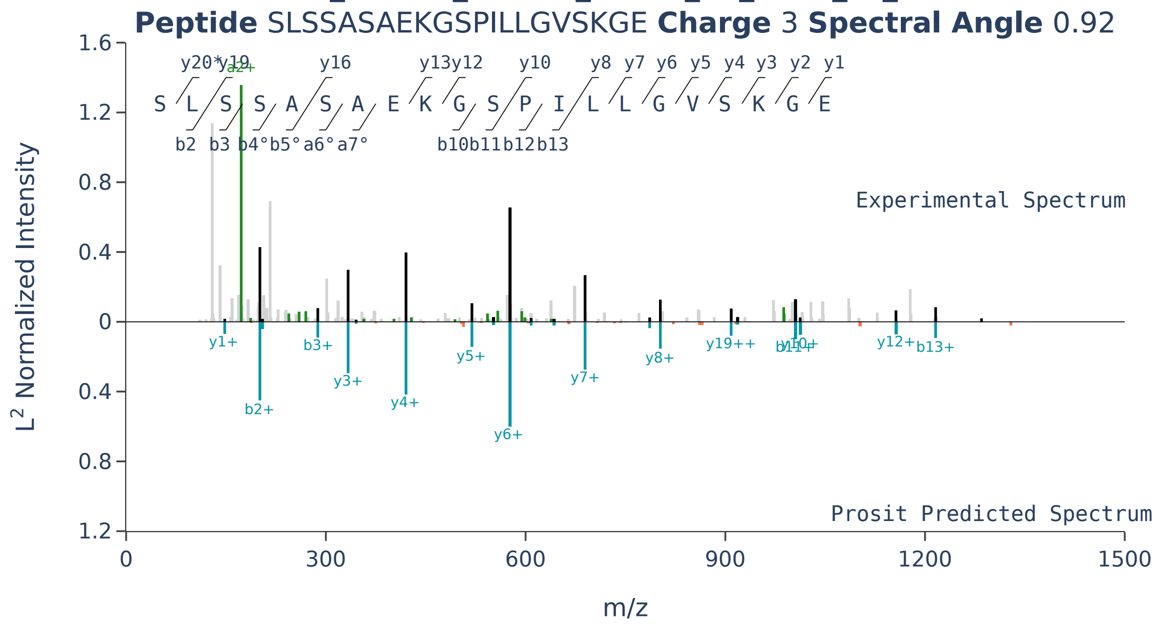


**Source** WSoh_101121_151121_HFGoe_G2_IL37b_24h_R2 **Scan** 13453
**Peptide** NLNPKKFSIHDQDHKVL **Charge** 3 **Spectral Angle** 0.83

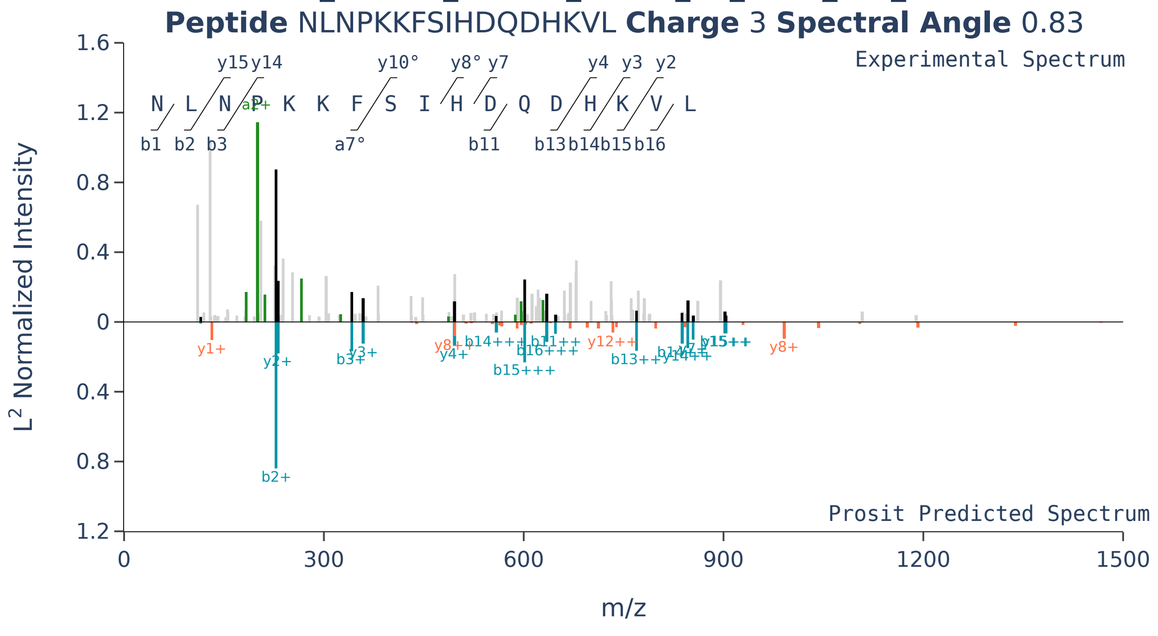


**Source** WSoh_101121_151121_HFGoe_G1_IL37b_24h_R1 **Scan** 16767
**Peptide** SCNCNEPVGVTDKFENRKHIEF **Charge** 4 **Spectral Angle** 0.73

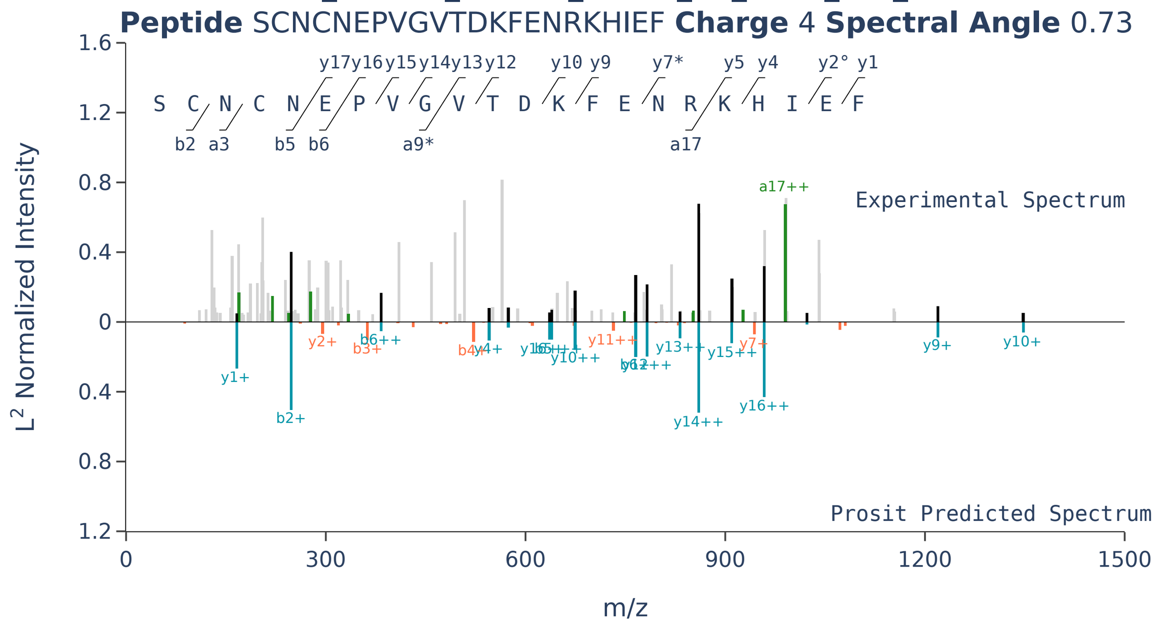


**Source** WSoh_101121_151121_HFGoe_G2_IL37b_2h_R2 **Scan** 21441
**Peptide** SSASAEKGSPILLGVS **Charge** 2 **Spectral Angle** 0.76

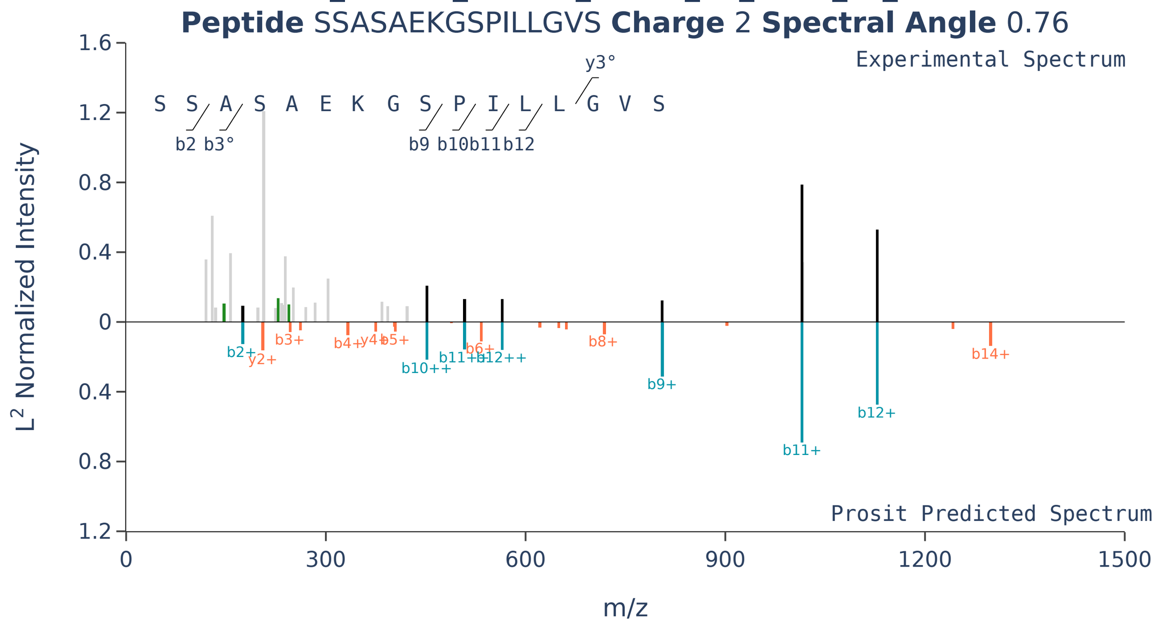

**Source** WSoh_101121_151121_HFGoe_G1_IL37b_24h_R1 **Scan** 10414
**Peptide** NLNPKKFSIHDQDH **Charge** 3 **Spectral Angle** 0.76

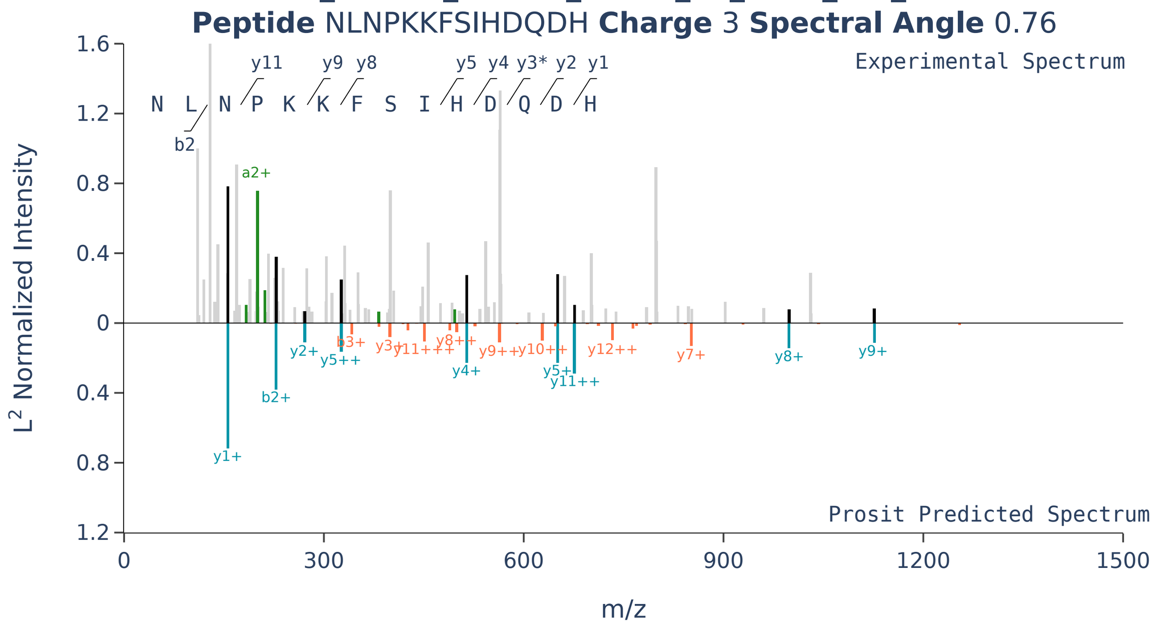


**Source** WSoh_101121_151121_HFGoe_G2_IL37b_4h_R2 **Scan** 5786
**Peptide** KLMKLAAQK **Charge** 2 **Spectral Angle** 0.78

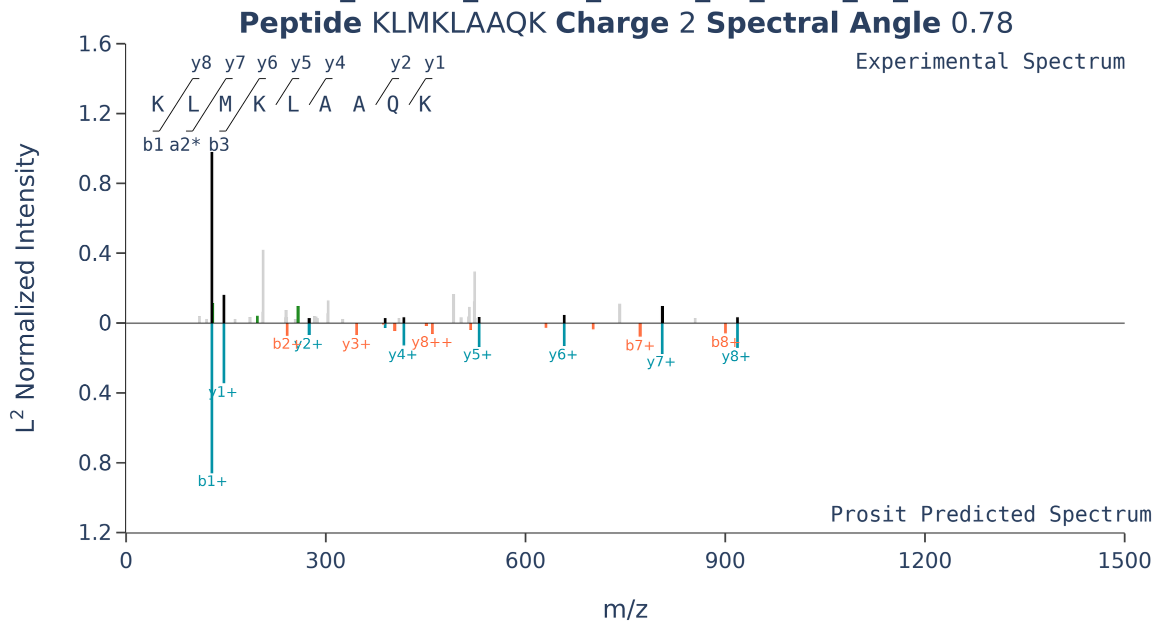


**Source** WSoh_101121_151121_HFGoe_G1_IL37b_2h_R1 **Scan** 21825
**Peptide** QVGSWNML **Charge** 2 **Spectral Angle** 0.79

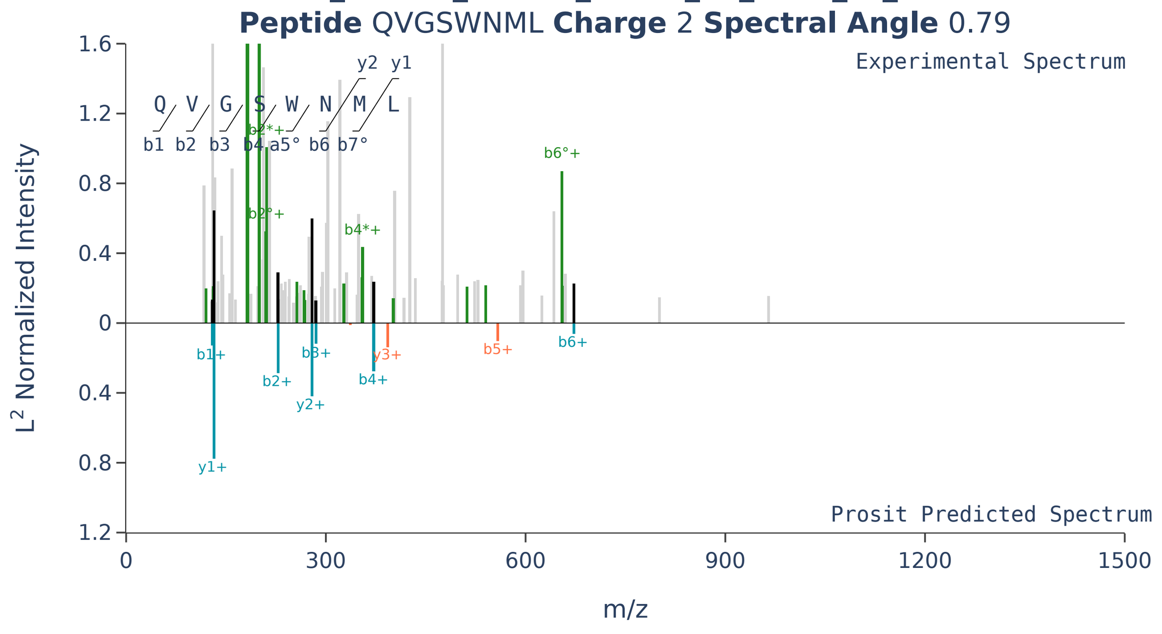


**Source** WSoh_101121_151121_HFGoe_G1_IL37b_2h_R2 **Scan** 5474
**Peptide** FENRKHIE **Charge** 2 **Spectral Angle** 0.72

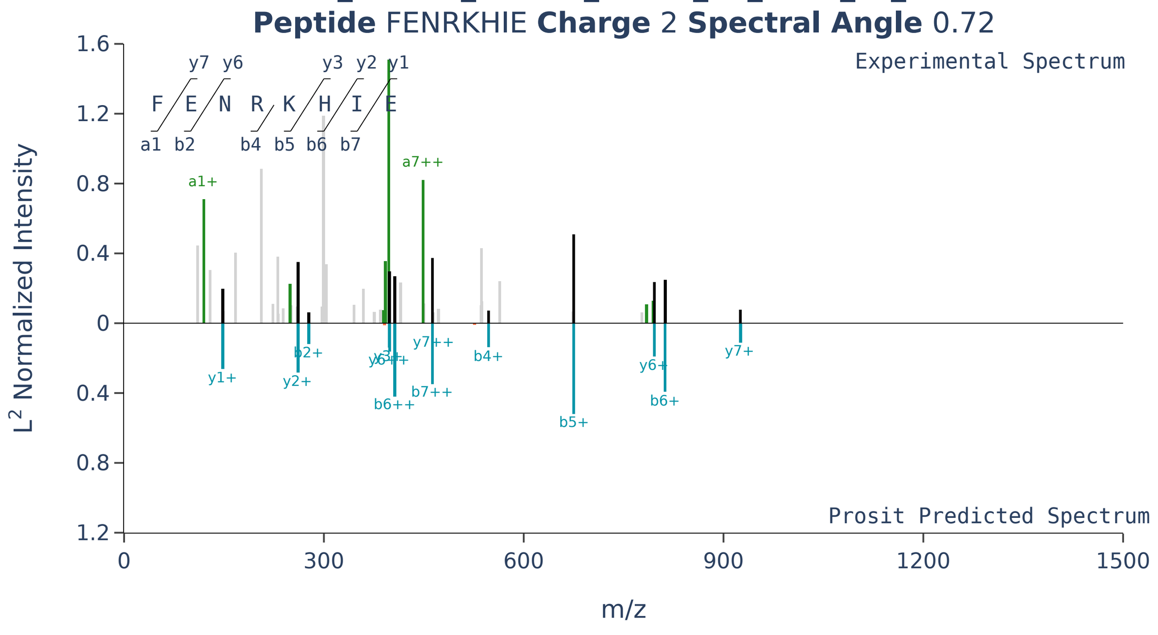


**Source** WSoh_101121_151121_HFGoe_G1_IL37b_1h_R1 **Scan** 22092
**Peptide** AAHPGWFICTSCN **Charge** 2 **Spectral Angle** 0.67

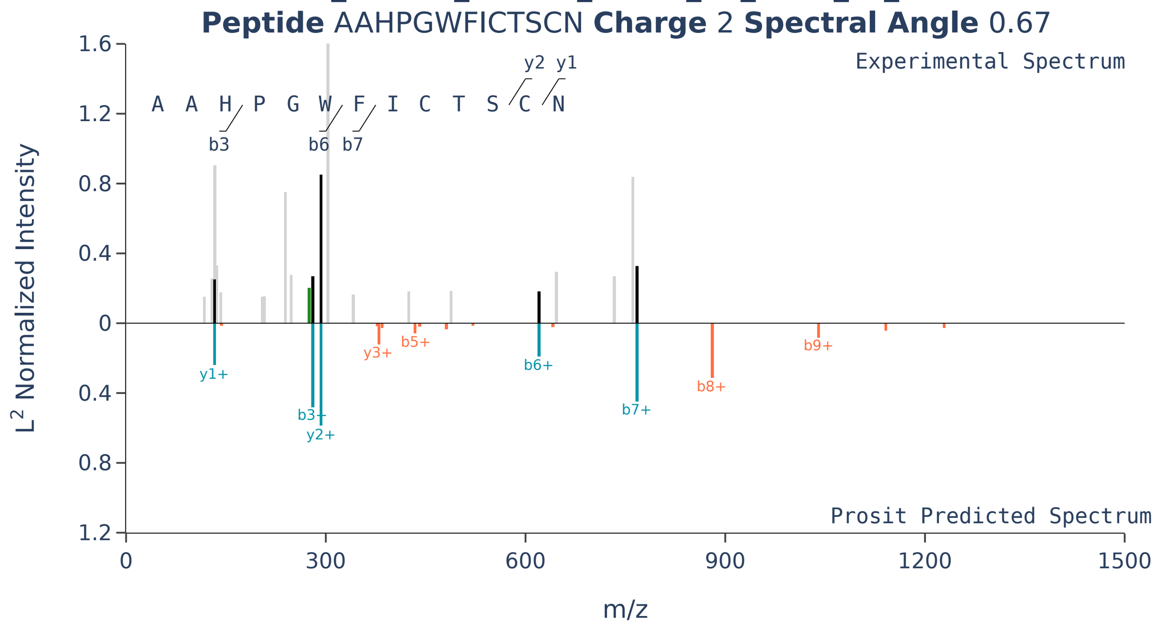


**Source** WSoh_101121_151121_HFGoe_G2_IL37b_24h_R1 **Scan** 22772
**Peptide** AQVGSWNML **Charge** 2 **Spectral Angle** 0.86

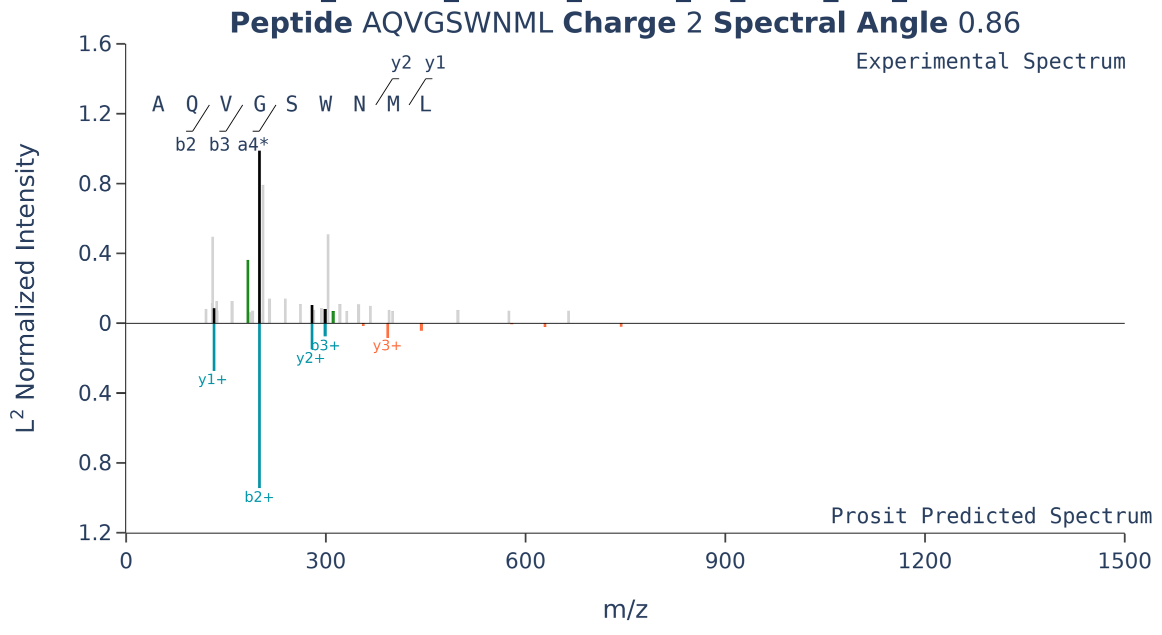


**Source** WSoh_101121_151121_HFGoe_G1_IL37b_1h_R1 **Scan** 10162
**Peptide** CTSCNCNEPVGVTDKFENRKH **Charge** 4 **Spectral Angle** 0.76

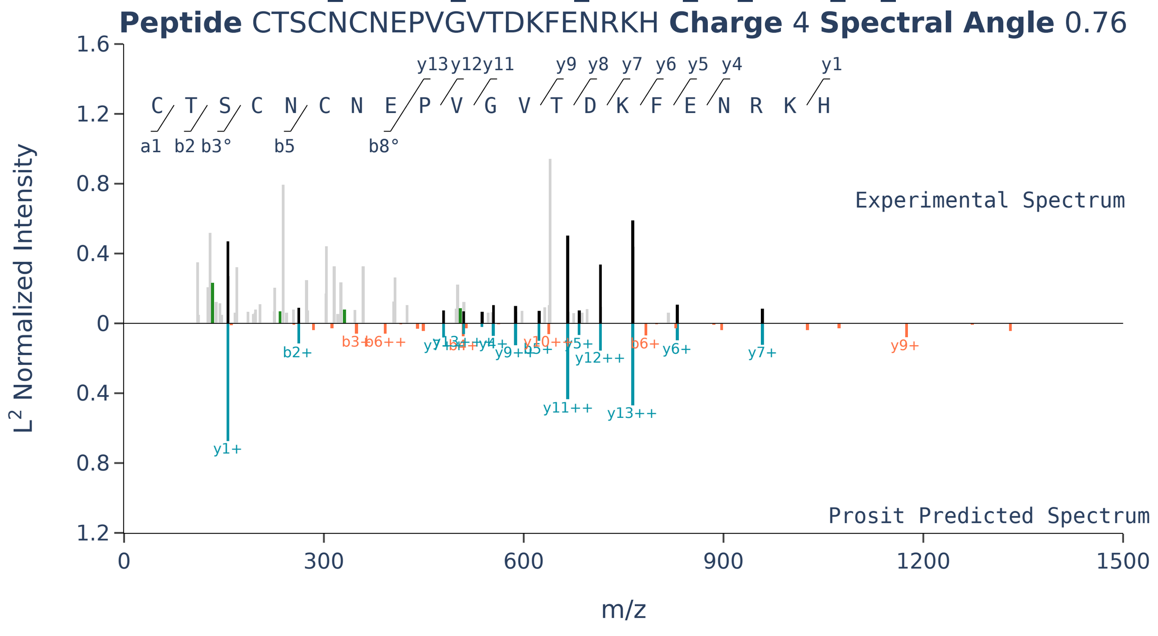


**Source** WSoh_101121_151121_HFGoe_G2_IL37b_1h_R2 **Scan** 19338
**Peptide** GVSKGEFCLYC **Charge** 2 **Spectral Angle** 0.89

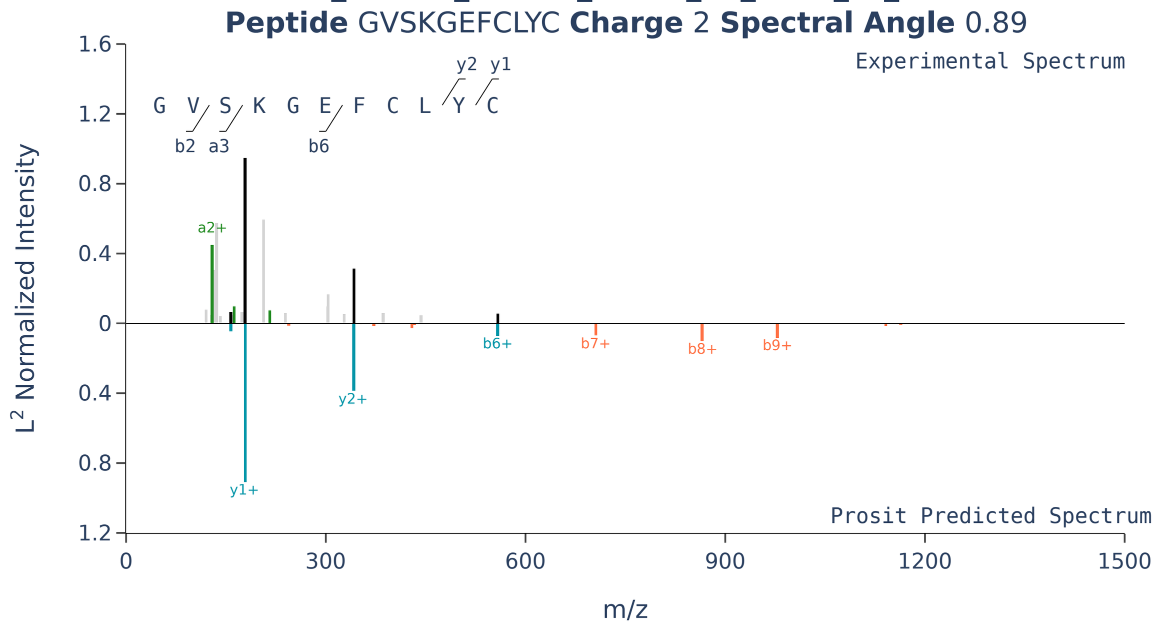


**Source** WSoh_101121_151121_HFGoe_G1_IL37b_2h_R2 **Scan** 21451
**Peptide** ESARRPFIFY **Charge** 3 **Spectral Angle** 0.87

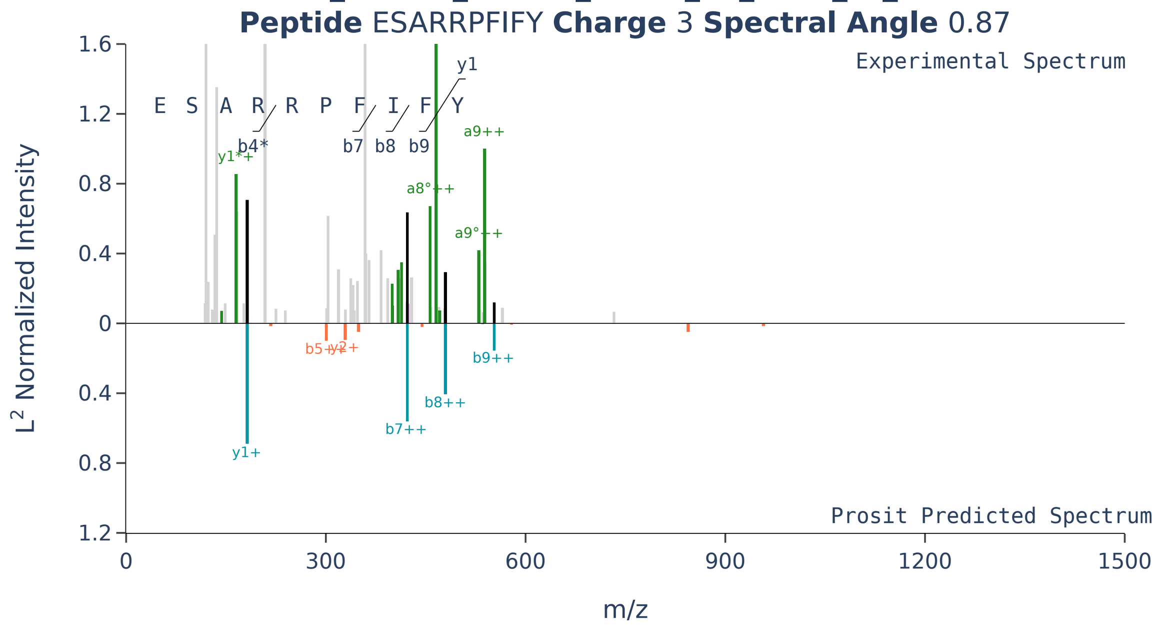


**Source** WSoh_101121_151121_HFGoe_G2_IL37b_24h_R1 **Scan** 25598
**Peptide** SLSSASAEKGSPILLGV **Charge** 2 **Spectral Angle** 0.82

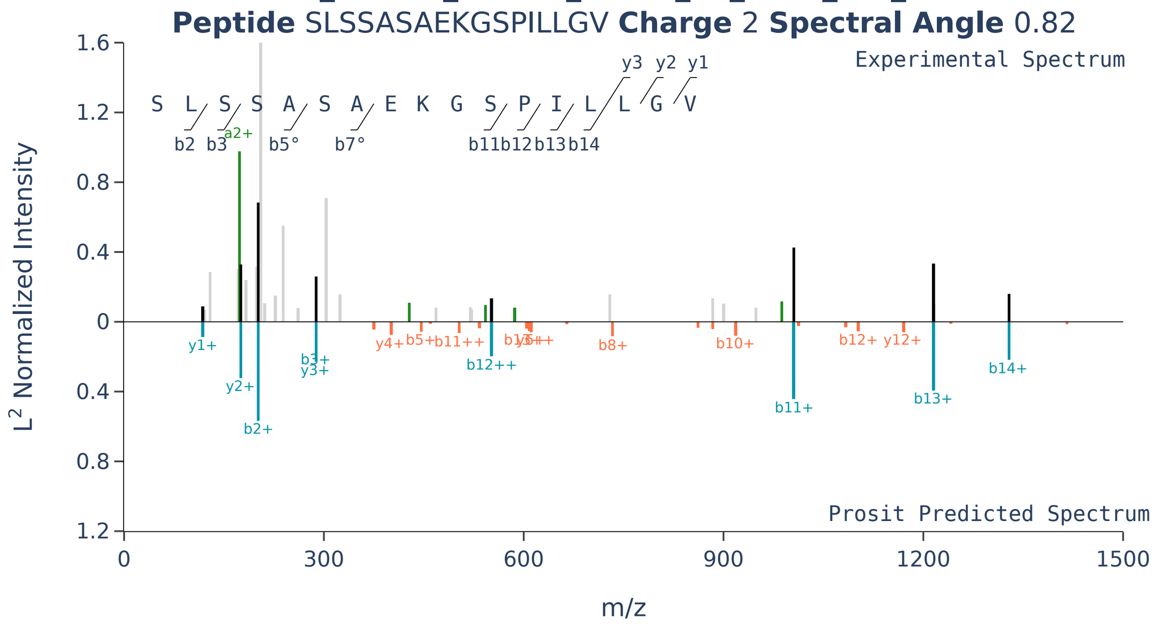


**Source** WSoh_101121_151121_HFGoe_G1_IL37b_1h_R1 **Scan** 16632
**Peptide** KGSPILLGVSKGE **Charge** 3 **Spectral Angle** 0.58

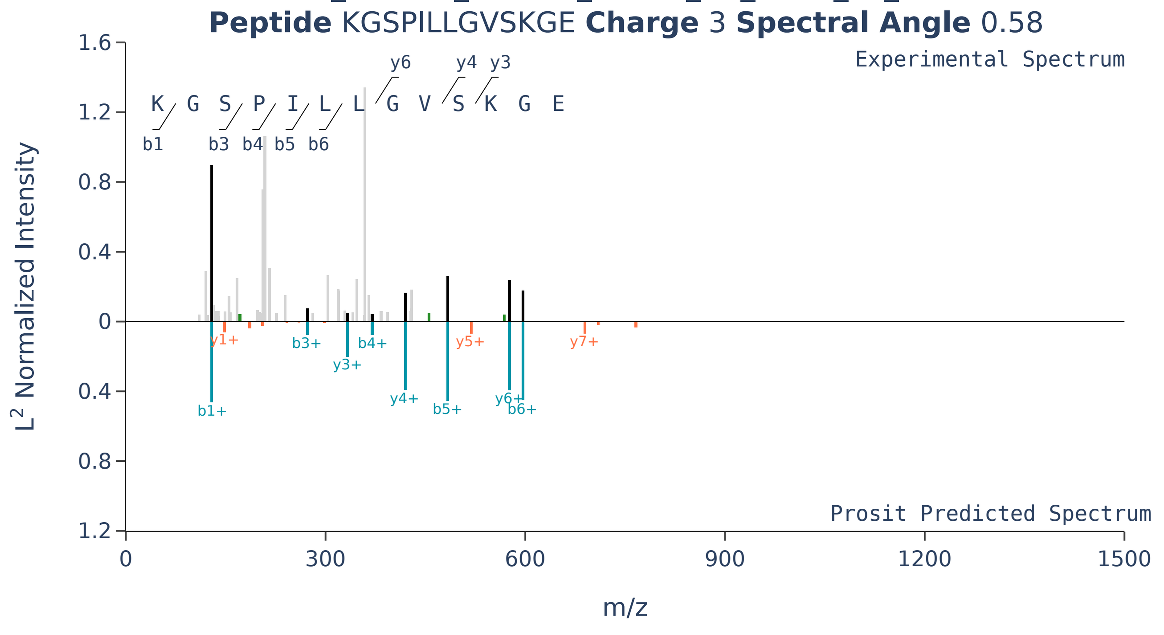


**Source** WSoh_101121_151121_HFGoe_G1_IL37b_4h_R2 **Scan** 11571
**Peptide** CNEPVGVTDKFENRKHIE **Charge** 4 **Spectral Angle** 0.75

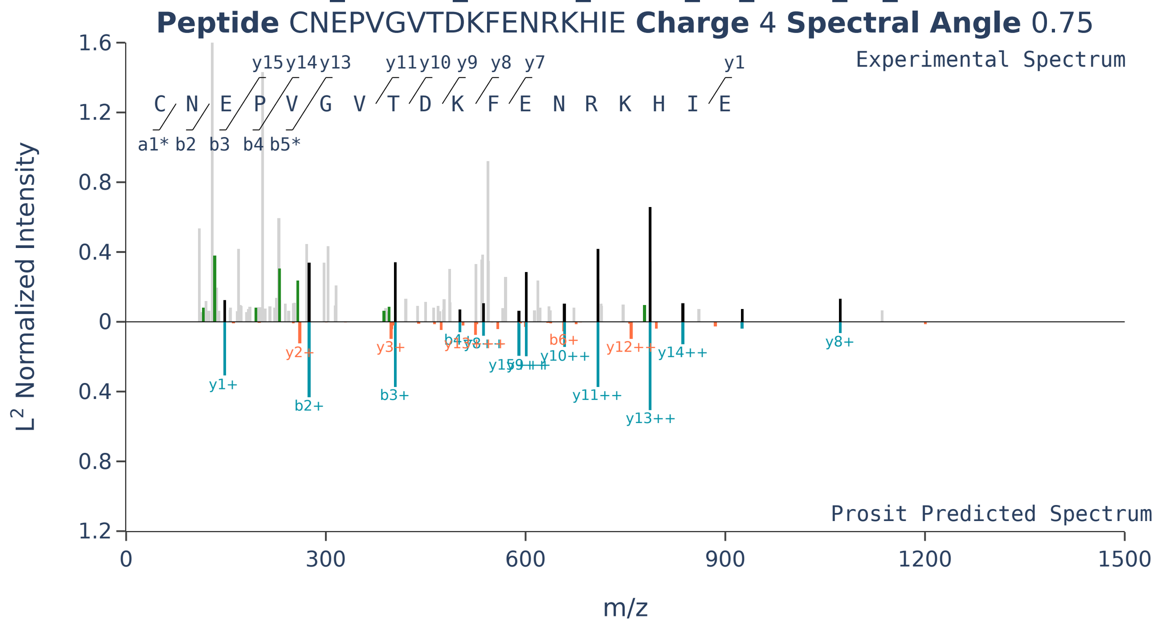


**Source** WSoh_101121_151121_HFGoe_G2_IL37b_24h_R1 **Scan** 7668
**Peptide** SPKVKNLNPKKFS **Charge** 3 **Spectral Angle** 0.67

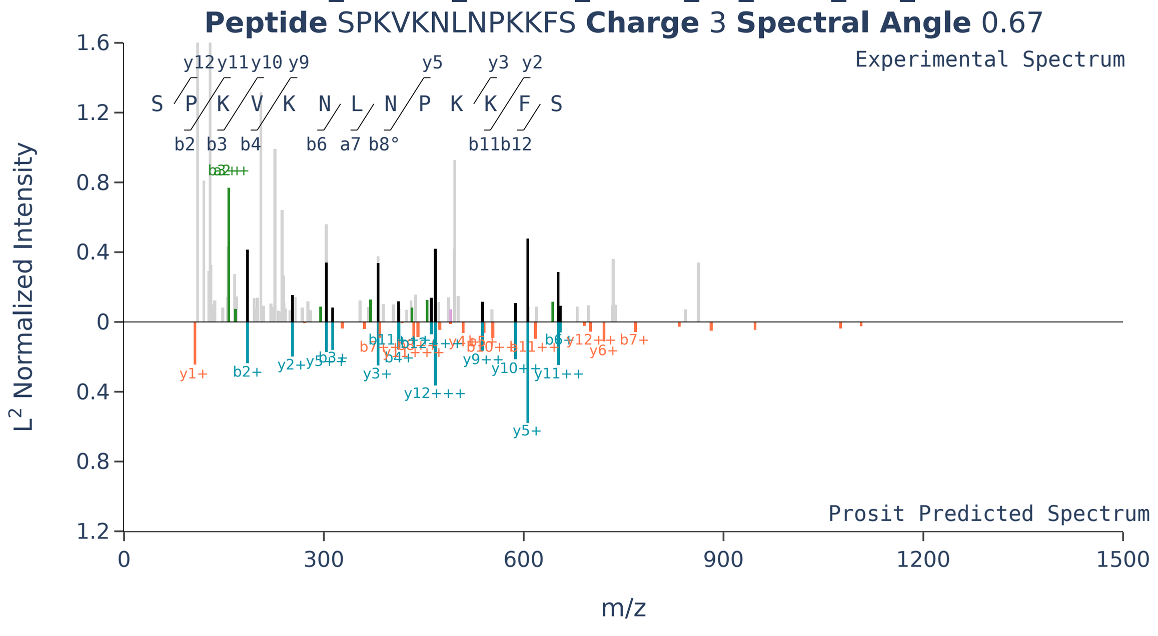


**Source** WSoh_101121_151121_HFGoe_G2_IL37b_24h_R1 **Scan** 21667
**Peptide** DKNYIRPEIF **Charge** 2 **Spectral Angle** 0.73

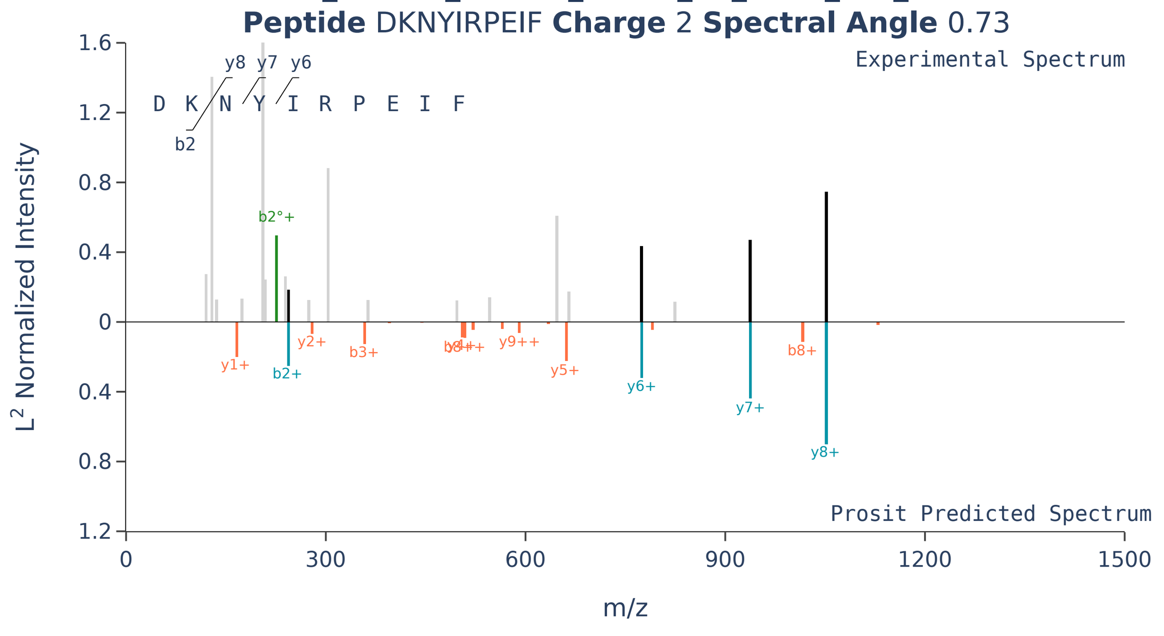


**Source** WSoh_101121_151121_HFGoe_G2_IL37b_24h_R1 **Scan** 23840
**Peptide** SLSSASAEKGSPILLGVS **Charge** 2 **Spectral Angle** 0.76

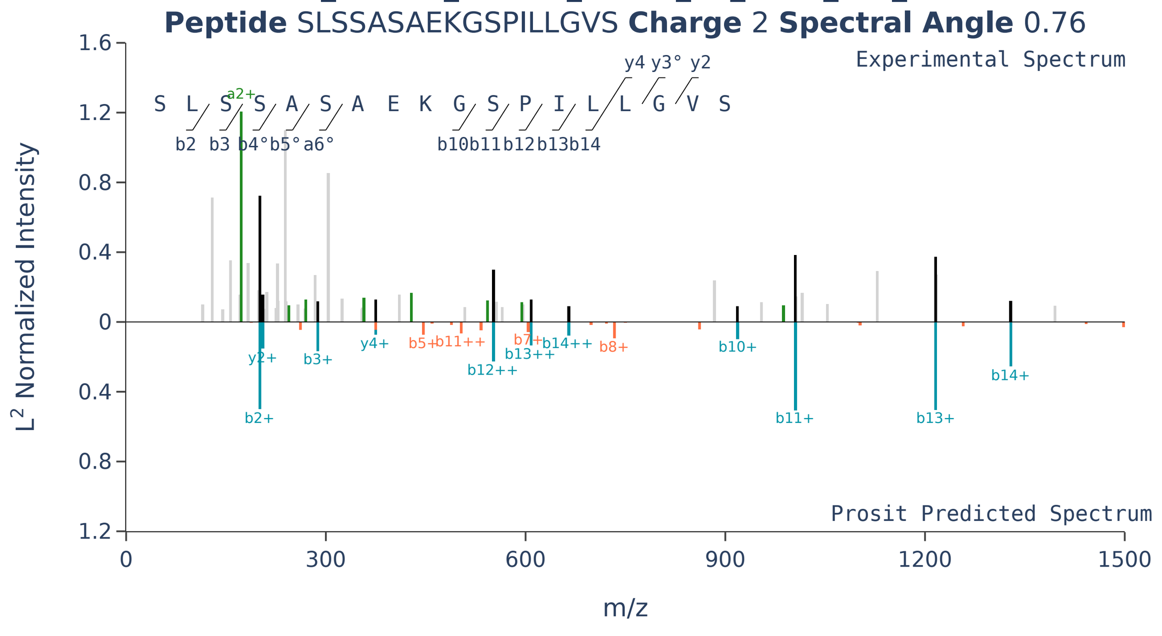

**Source** WSoh_101121_151121_HFGoe_G1_IL37b_24h_R2 **Scan** 17116
**Peptide** EKGSPILLGVSK **Charge** 3 **Spectral Angle** 0.78

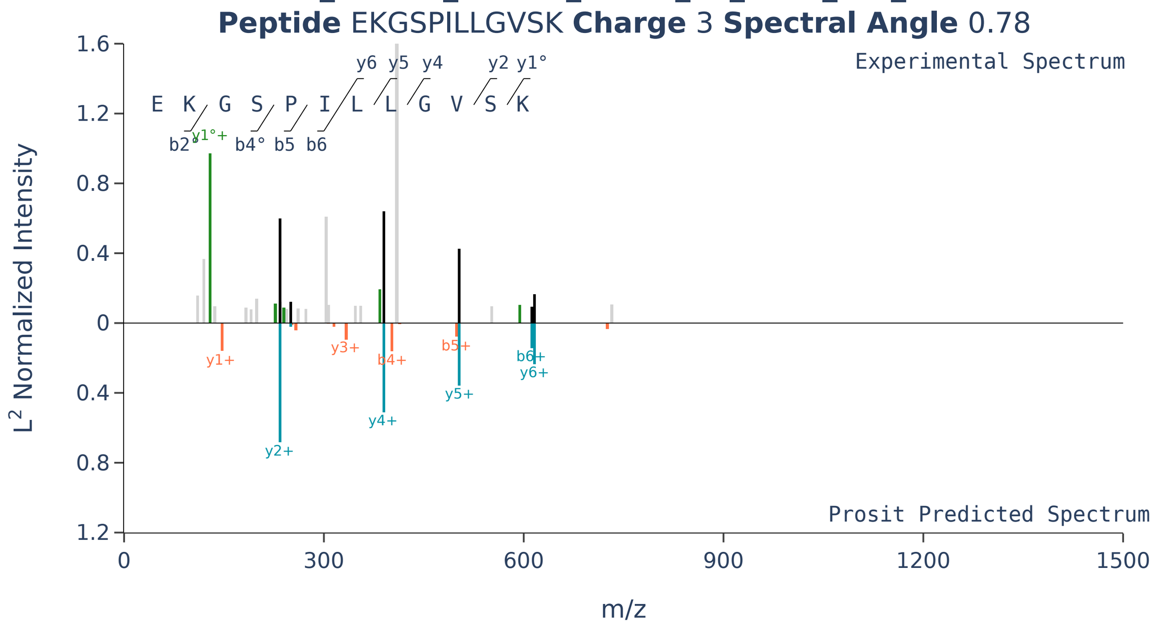


**Source** WSoh_101121_151121_HFGoe_G1_IL37b_4h_R1 **Scan** 19751
**Peptide** LAAQKESARRPFIFY **Charge** 4 **Spectral Angle** 0.77

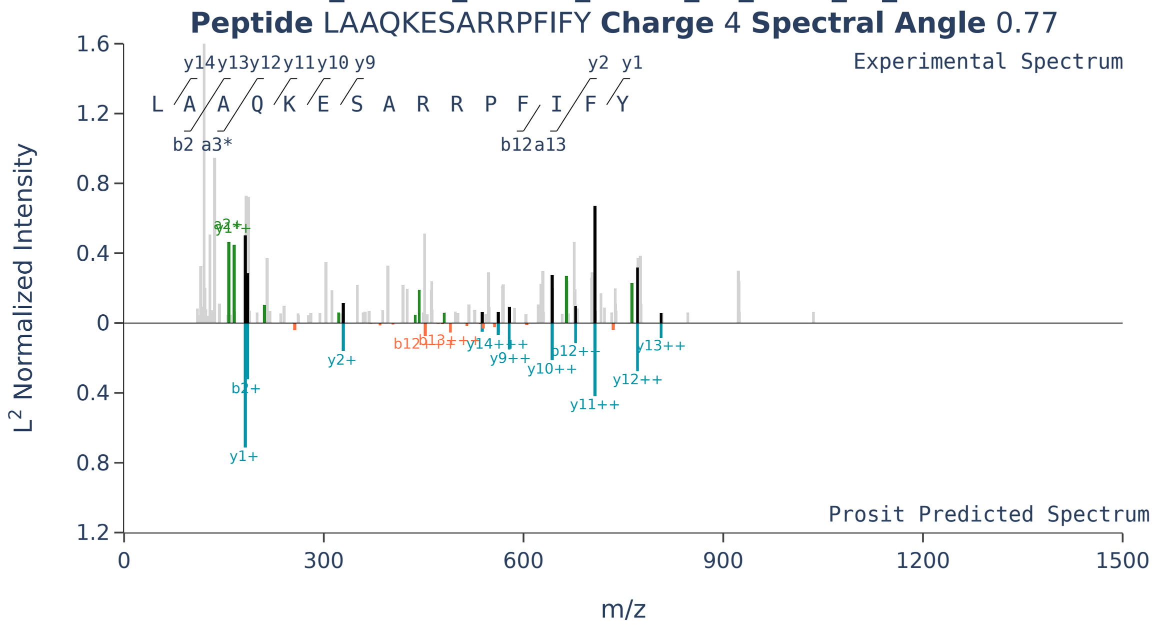


**Source** WSoh_101121_151121_HFGoe_G2_IL37b_24h_R2 **Scan** 5739
**Peptide** KDKGQSHPSLQLKKEK **Charge** 3 **Spectral Angle** 0.82

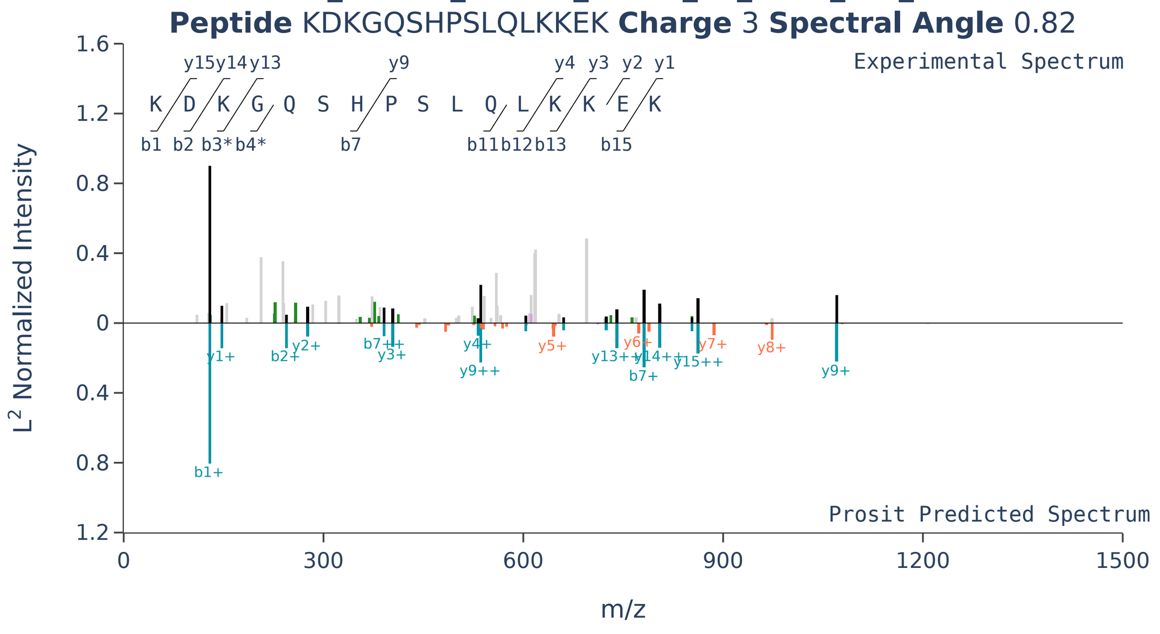


**Source** WSoh_101121_151121_HFGoe_G1_IL37b_4h_R1 **Scan** 5895
**Peptide** KKEKLMKLAAQKESARR **Charge** 3 **Spectral Angle** 0.83

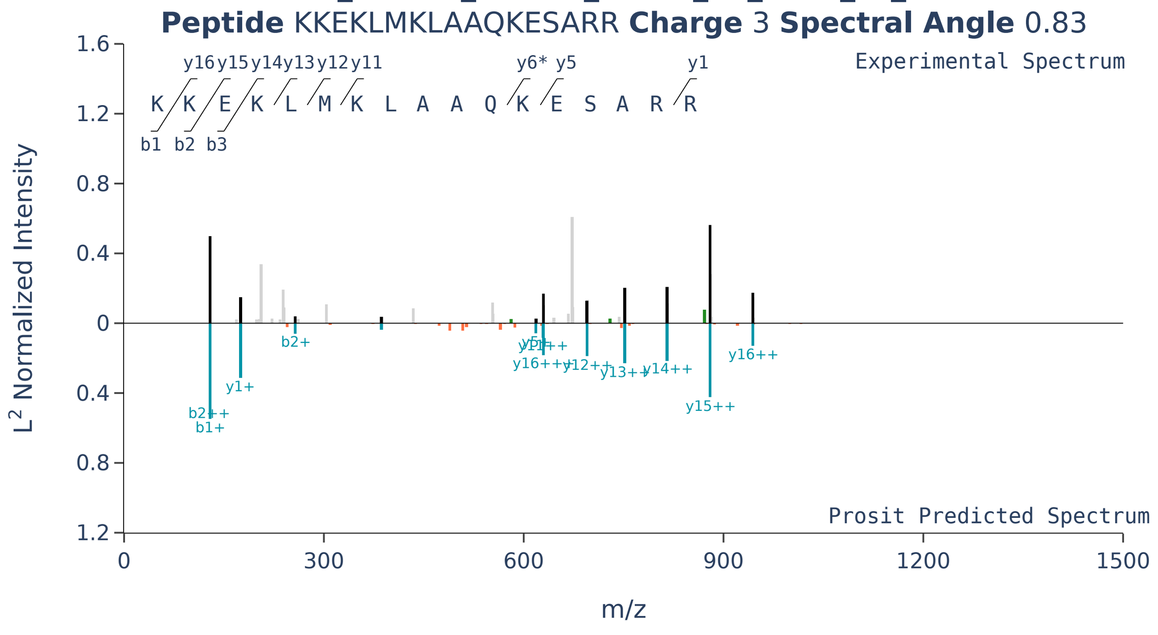


**Source** WSoh_101121_151121_HFGoe_G1_IL37b_24h_R1 **Scan** 28270
**Peptide** YIRPEIFF **Charge** 2 **Spectral Angle** 0.75

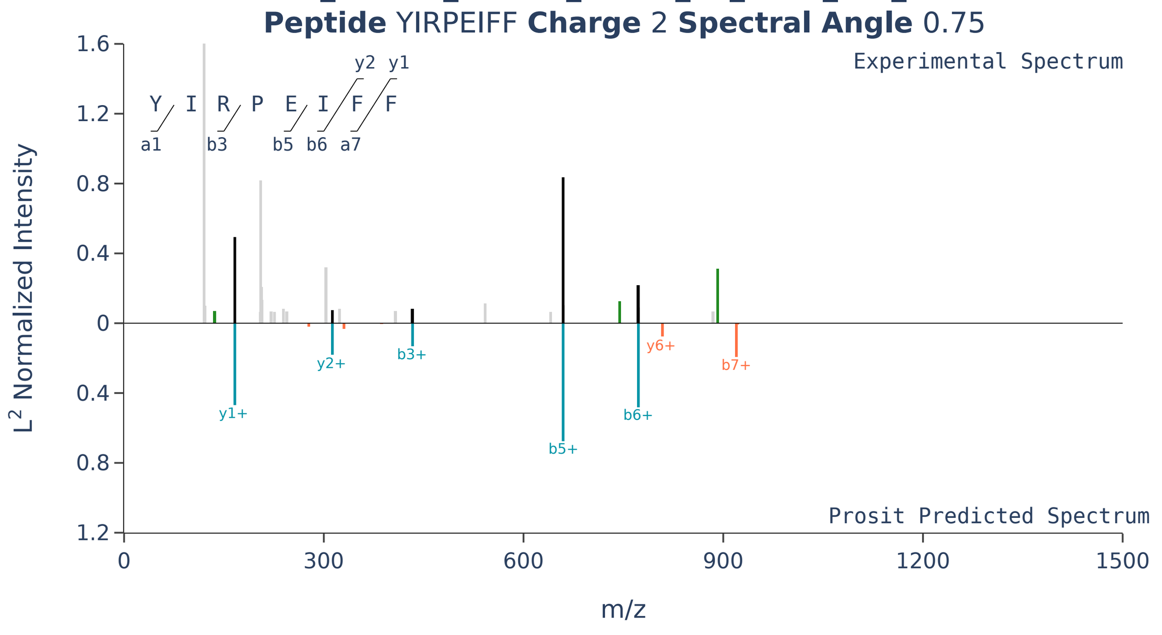


**Source** WSoh_101121_151121_HFGoe_G2_IL37b_24h_R1 **Scan** 25087
**Peptide** DSGNLIAVPDKNYI **Charge** 2 **Spectral Angle** 0.83

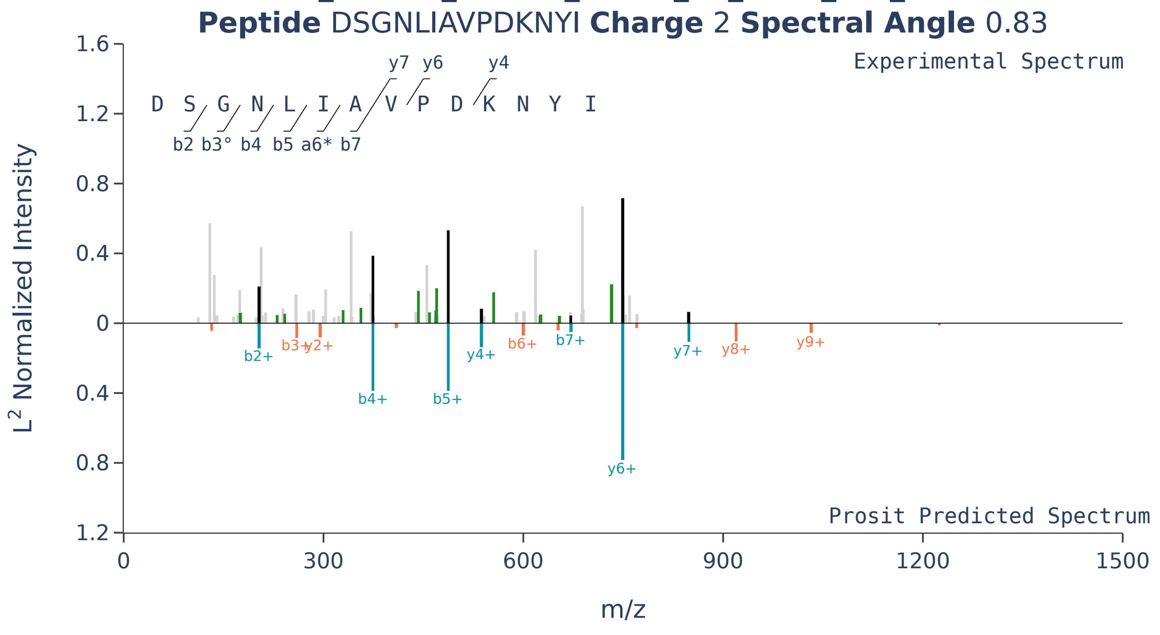


**Source** WSoh_101121_151121_HFGoe_G1_IL37b_4h_R2 **Scan** 19245
**Peptide** SFQPVCKAEMSPSEV **Charge** 2 **Spectral Angle** 0.78

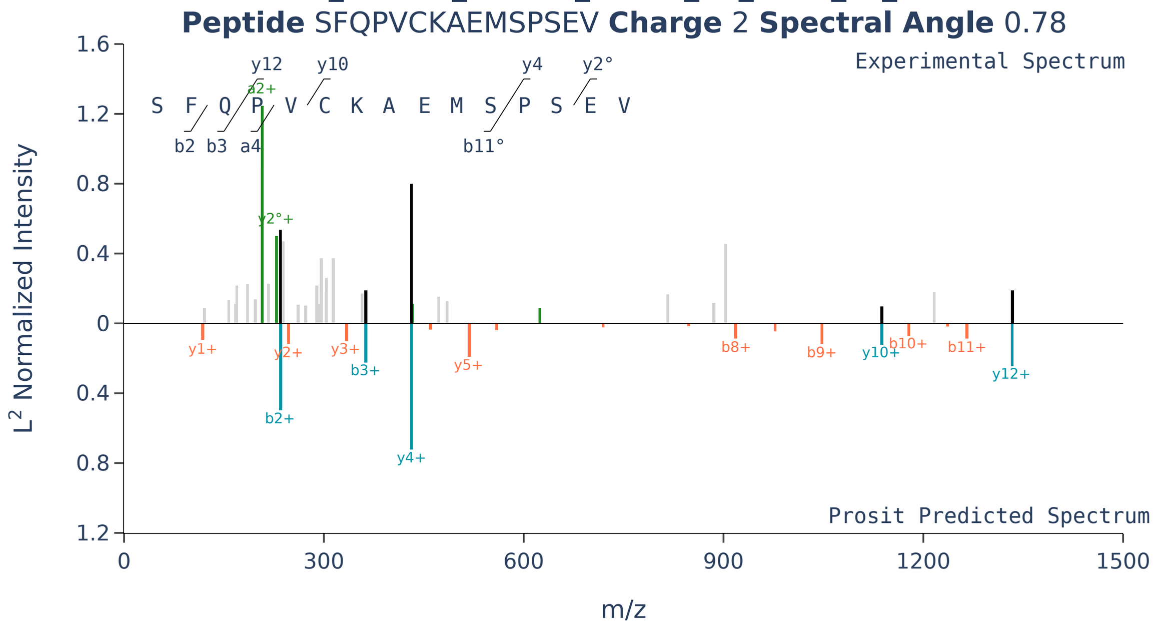


**Source** WSoh_101121_151121_HFGoe_G1_IL37b_4h_R1 **Scan** 11729
**Peptide** CNCNEPVGVTDKFENRKHIE **Charge** 4 **Spectral Angle** 0.71

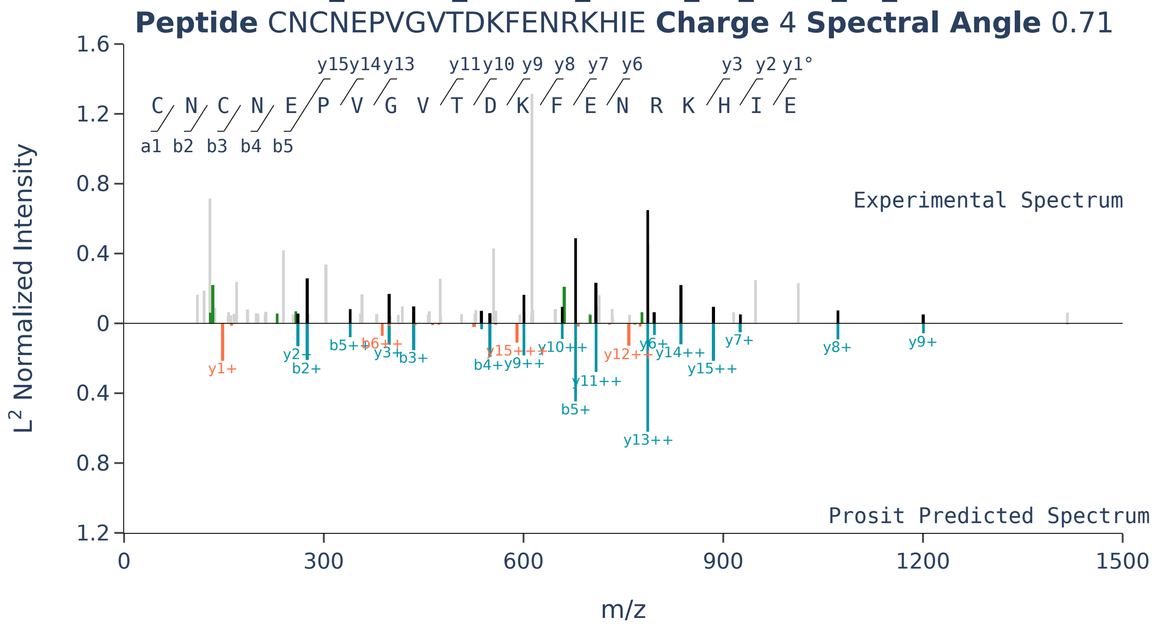


**Source** WSoh_101121_151121_HFGoe_G1_IL37b_4h_R1 **Scan** 9887
**Peptide** HTSPKVKNLNPKKFSIHD **Charge** 5 **Spectral Angle** 0.77

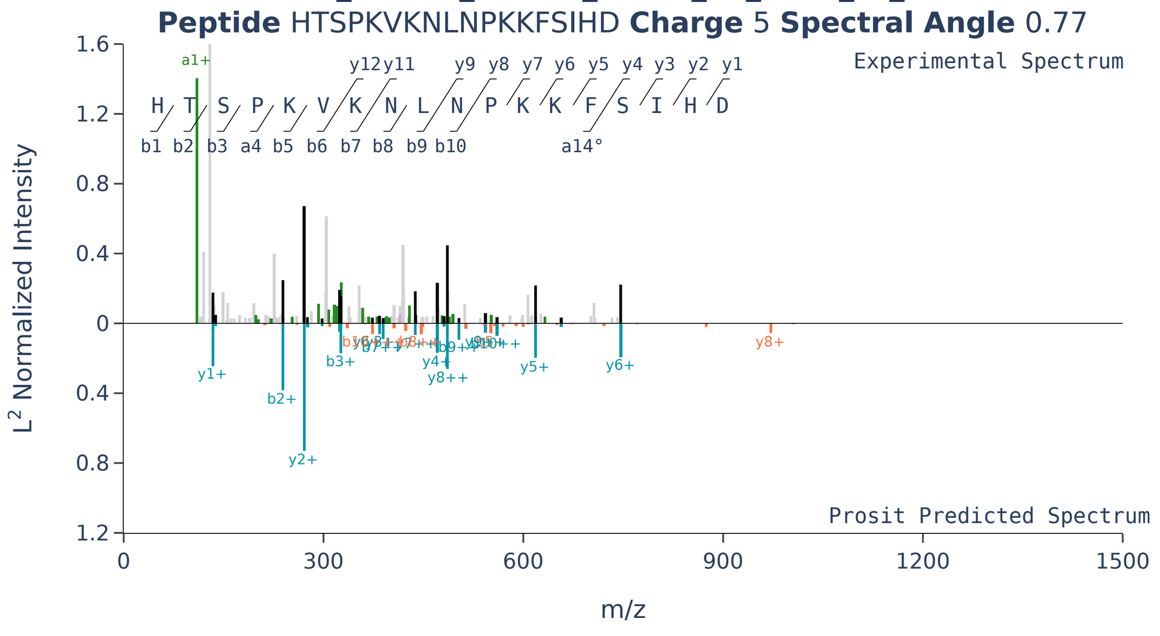


**Source** WSoh_101121_151121_HFGoe_G2_IL37b_24h_R1 **Scan** 6150
**Peptide** YCDKDKGQSHPSLQLKKEK **Charge** 4 **Spectral Angle** 0.78

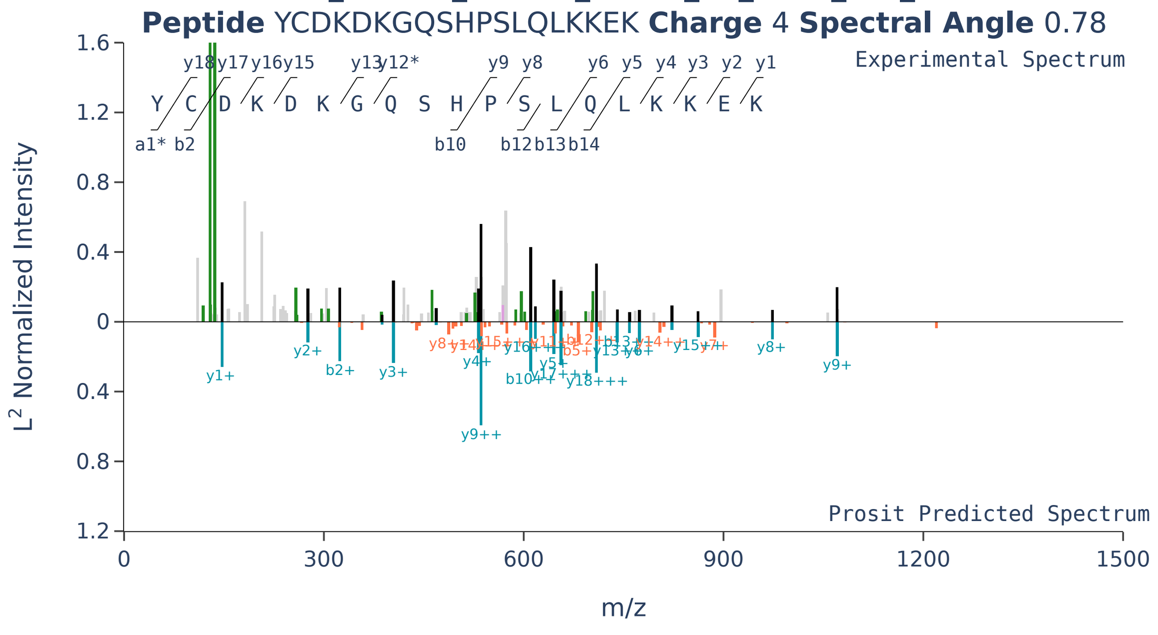


**Source** WSoh_101121_151121_HFGoe_G1_IL37b_2h_R2 **Scan** 17183
**Peptide** CTSCNCNEPVGVTDKF **Charge** 2 **Spectral Angle** 0.79

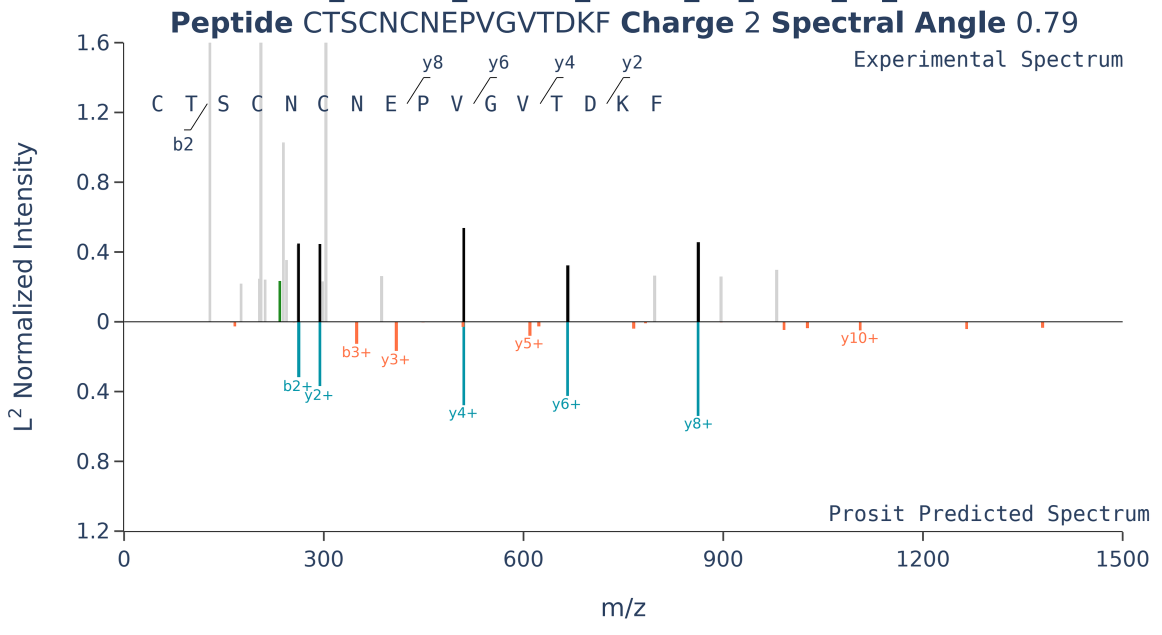


**Source** WSoh_101121_151121_HFGoe_G1_IL37b_4h_R1 **Scan** 22969
**Peptide** ASAEKGSPILLGVSKGEF **Charge** 3 **Spectral Angle** 0.88

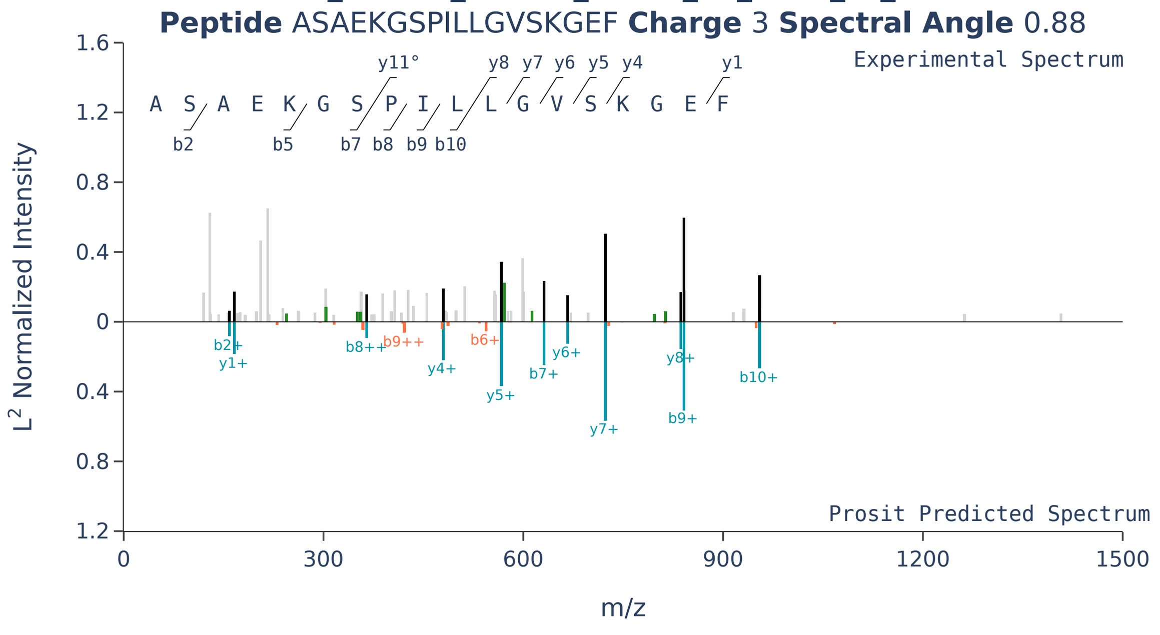


**Source** WSoh_101121_151121_HFGoe_G1_IL37b_24h_R1 **Scan** 18743
**Peptide** SKGEFCLYCDKDKGQSHPSLQL **Charge** 4 **Spectral Angle** 0.78

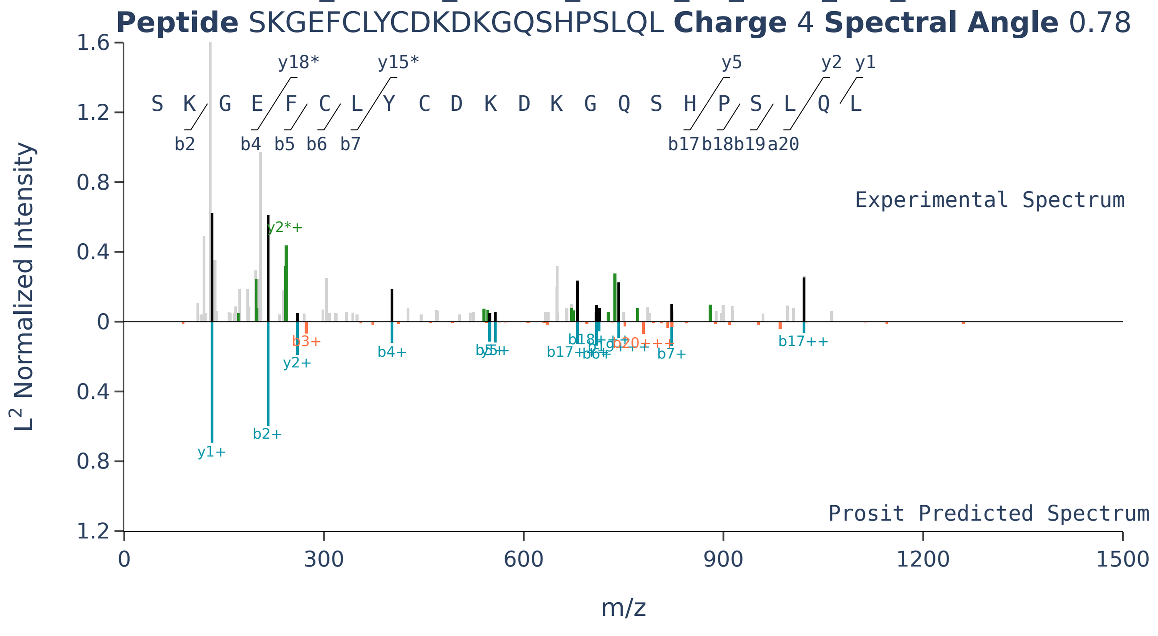


**Source** WSoh_101121_151121_HFGoe_G2_IL37b_24h_R1 **Scan** 9774
**Peptide** GQSHPSLQLKKEKL **Charge** 4 **Spectral Angle** 0.63

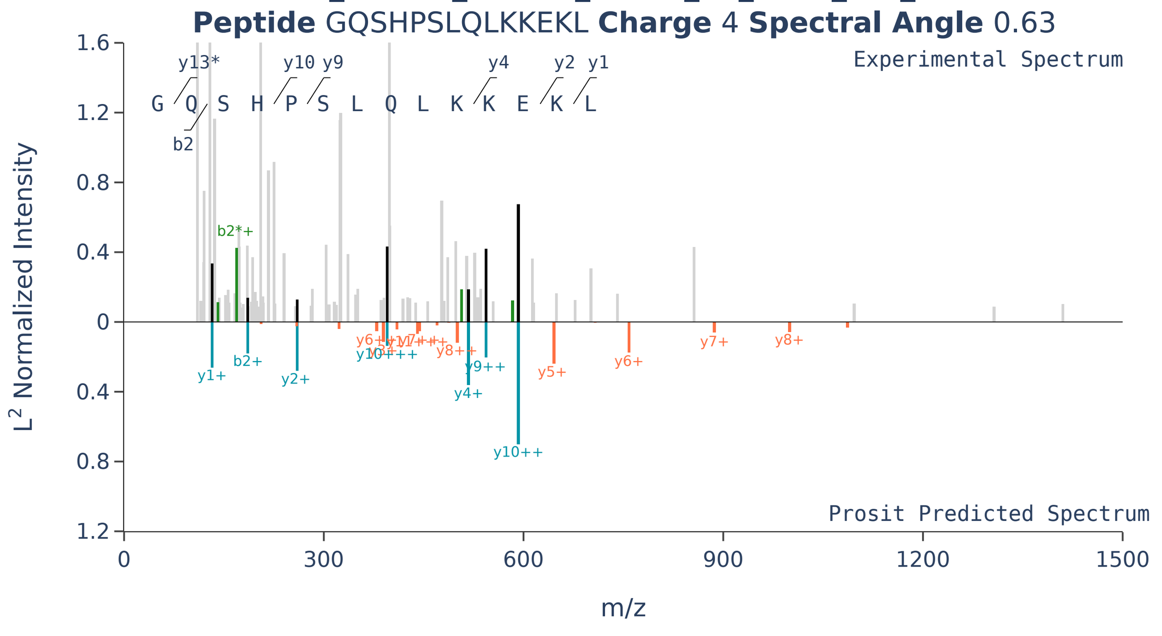


**Source** WSoh_101121_151121_HFGoe_G2_IL37b_24h_R1 **Scan** 23094
**Peptide** SAEKGSPILLGVSKGEF **Charge** 3 **Spectral Angle** 0.76

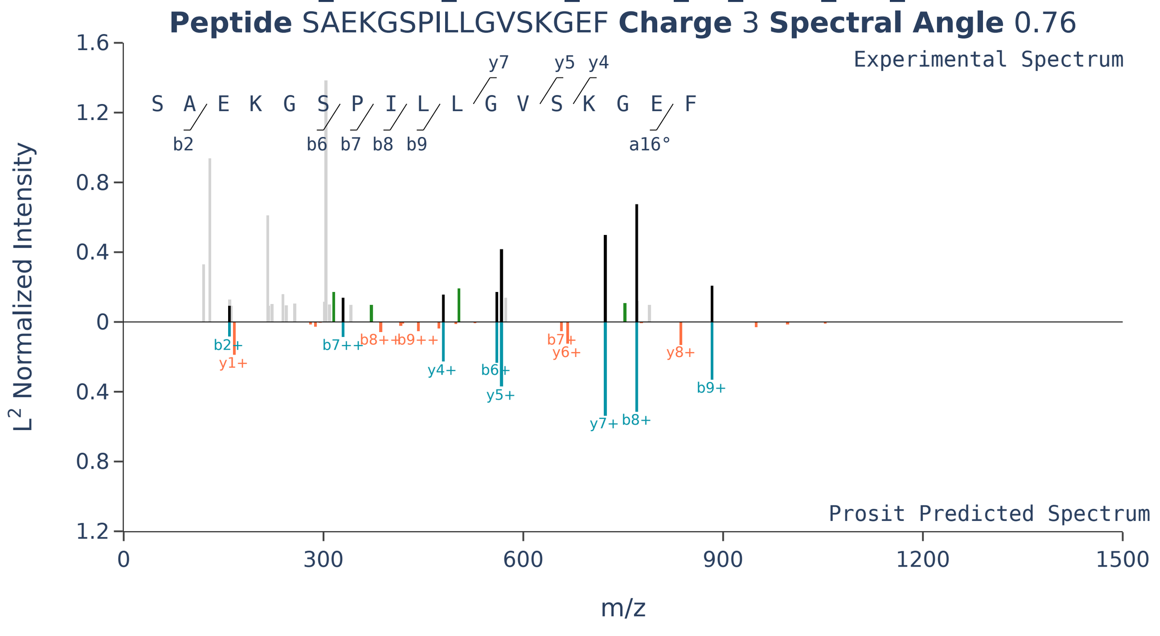

**Source** WSoh_101121_151121_HFGoe_G2_IL37b_24h_R2 **Scan** 17562
**Peptide** EFCLYCDKDKGQSHPSL **Charge** 3 **Spectral Angle** 0.75

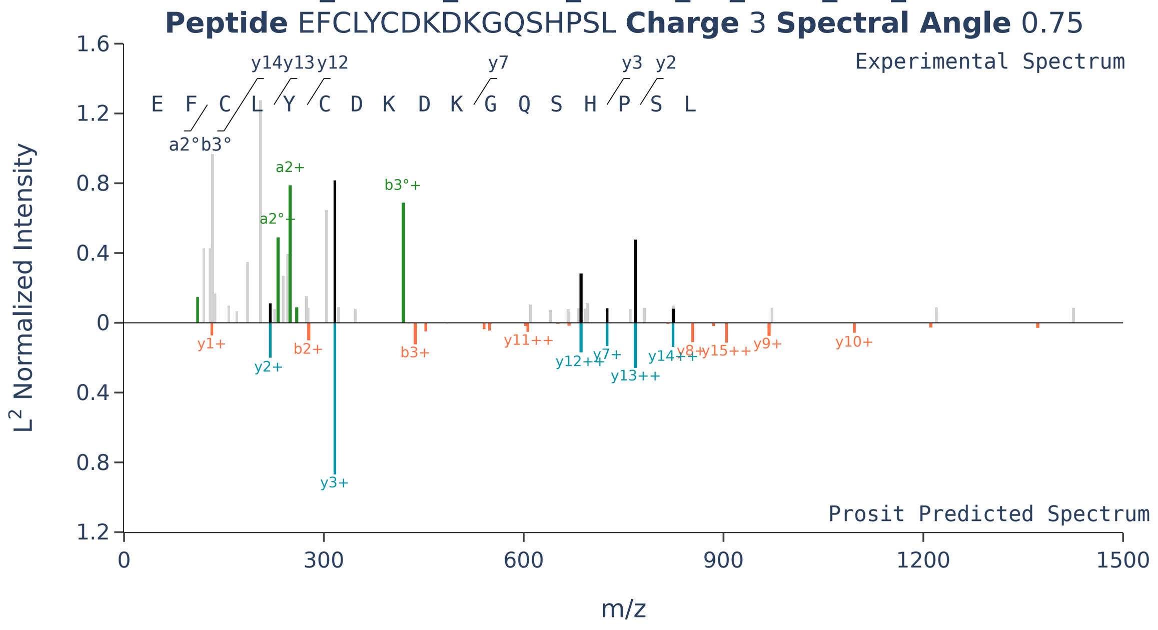


**Source** WSoh_101121_151121_HFGoe_G1_IL37b_24h_R2 **Scan** 20000
**Peptide** ASSLSSASAEKGSPILLGVSK **Charge** 3 **Spectral Angle** 0.71

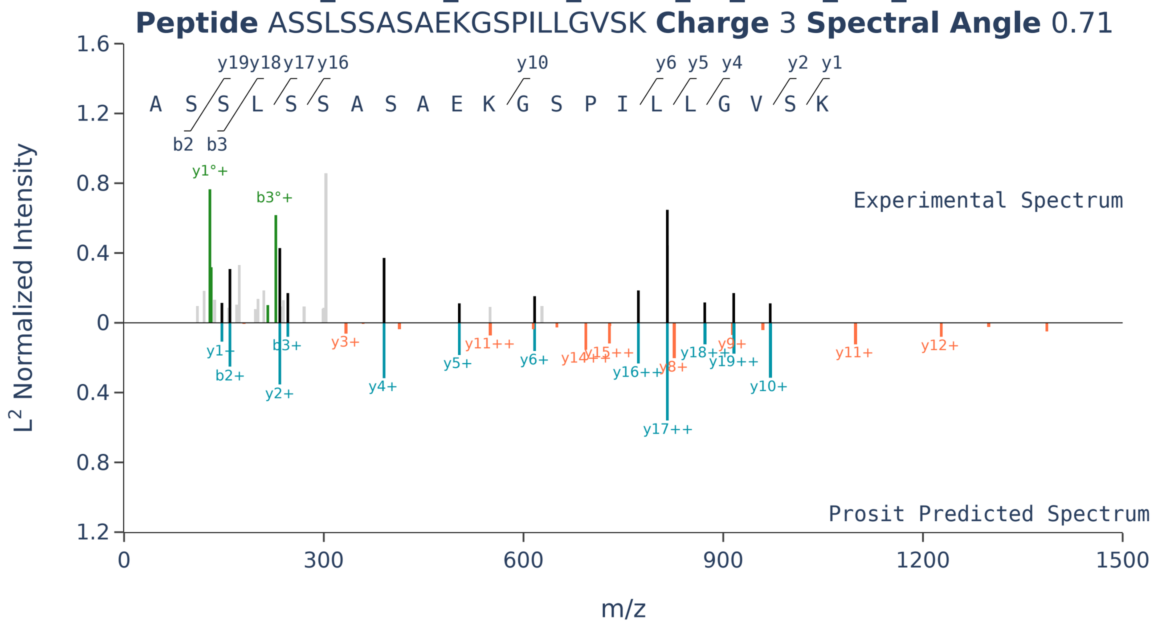


**Source** WSoh_101121_151121_HFGoe_G1_IL37b_24h_R1 **Scan** 18880
**Peptide** SKGEFCLYCD **Charge** 2 **Spectral Angle** 0.84

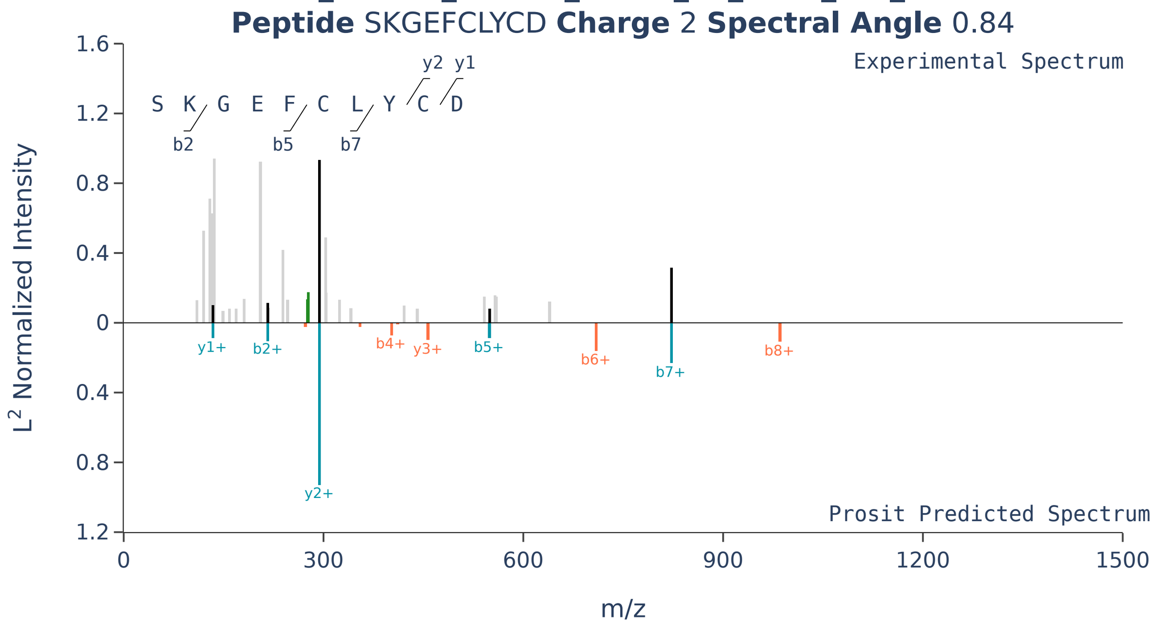


**Source** WSoh_101121_151121_HFGoe_G1_IL37b_1h_R1 **Scan** 6005
**Peptide** KDKGQSHPSL **Charge** 2 **Spectral Angle** 0.79

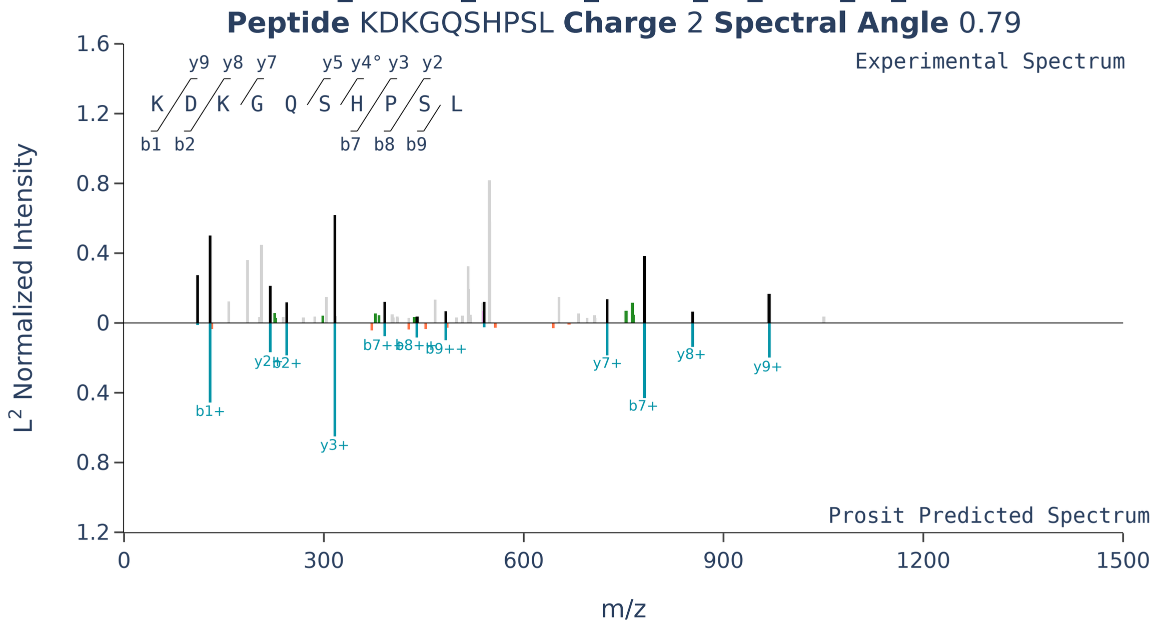


**Source** WSoh_101121_151121_HFGoe_G1_IL37b_2h_R2 **Scan** 19424
**Peptide** EFSFQPVCK **Charge** 2 **Spectral Angle** 0.82

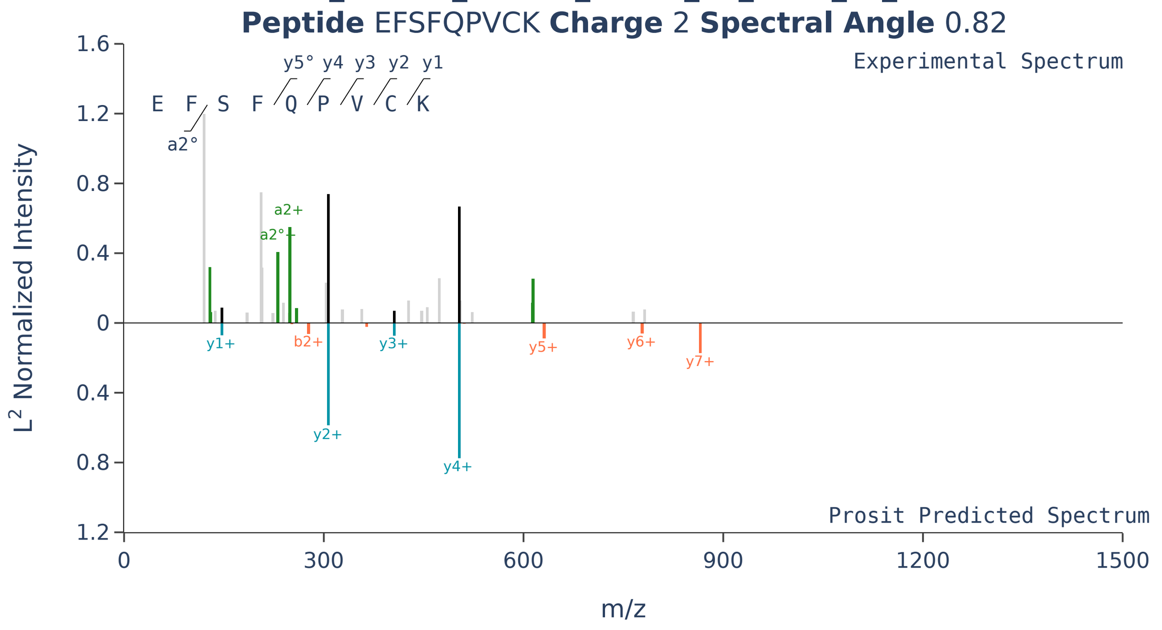


**Source** WSoh_101121_151121_HFGoe_G1_IL37b_24h_R2 **Scan** 20422
**Peptide** GNLIAVPDKNYIRPE **Charge** 2 **Spectral Angle** 0.84

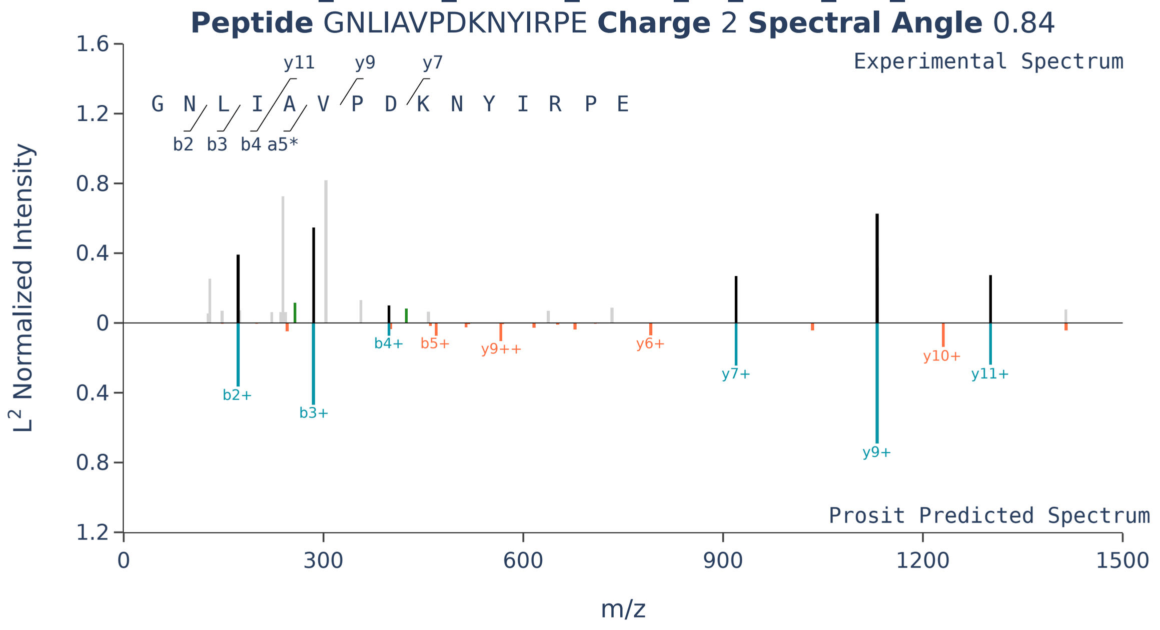


**Source** WSoh_101121_151121_HFGoe_G2_IL37b_2h_R1 **Scan** 22575
**Peptide** AQVGSWNMLESAAH **Charge** 2 **Spectral Angle** 0.74

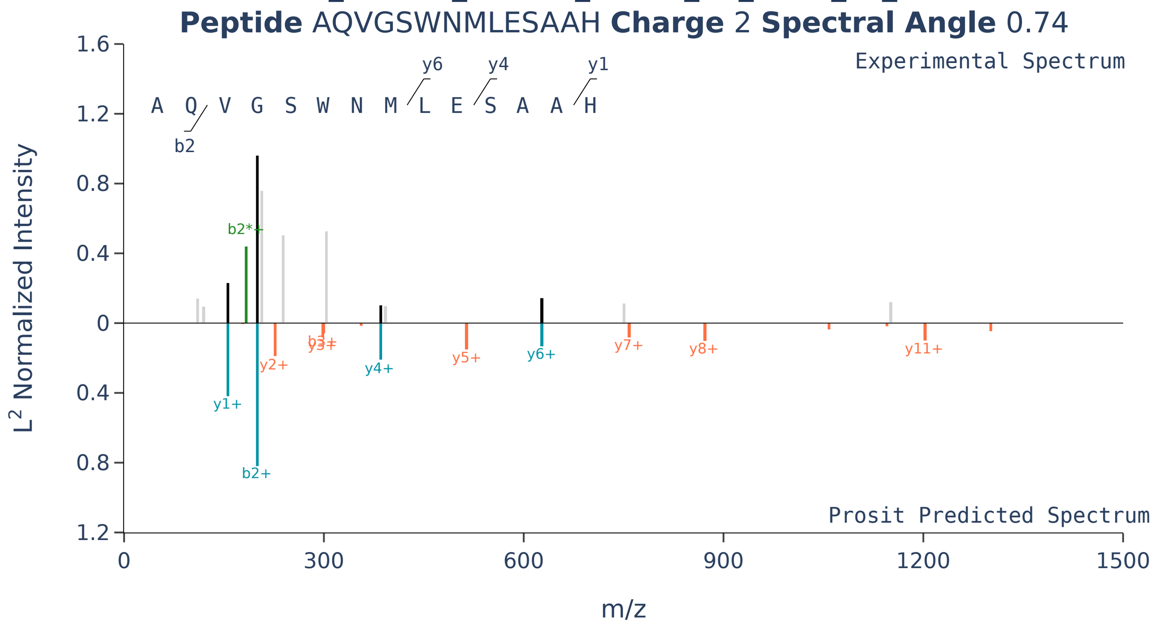


**Source** WSoh_101121_151121_HFGoe_G1_IL37b_4h_R1 **Scan** 13043
**Peptide** DKDKGQSHPSLQLKKEKLMKLA **Charge** 5 **Spectral Angle** 0.75

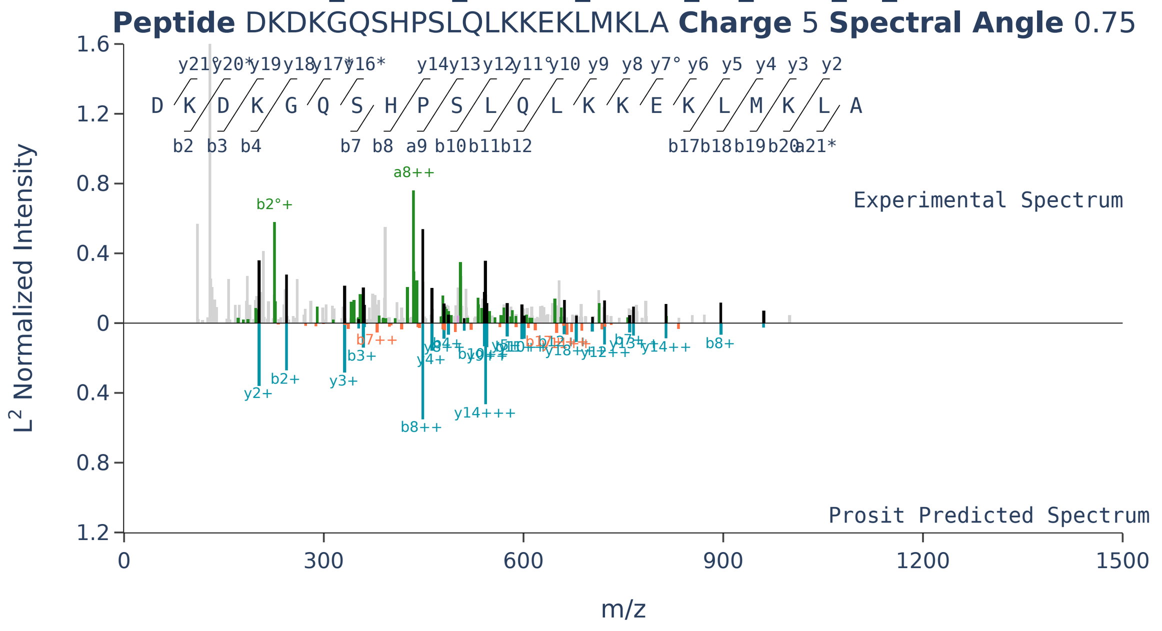


**Source** WSoh_101121_151121_HFGoe_G1_IL37b_24h_R2 **Scan** 7308
**Peptide** QLKKEKLMKLAA **Charge** 3 **Spectral Angle** 0.81

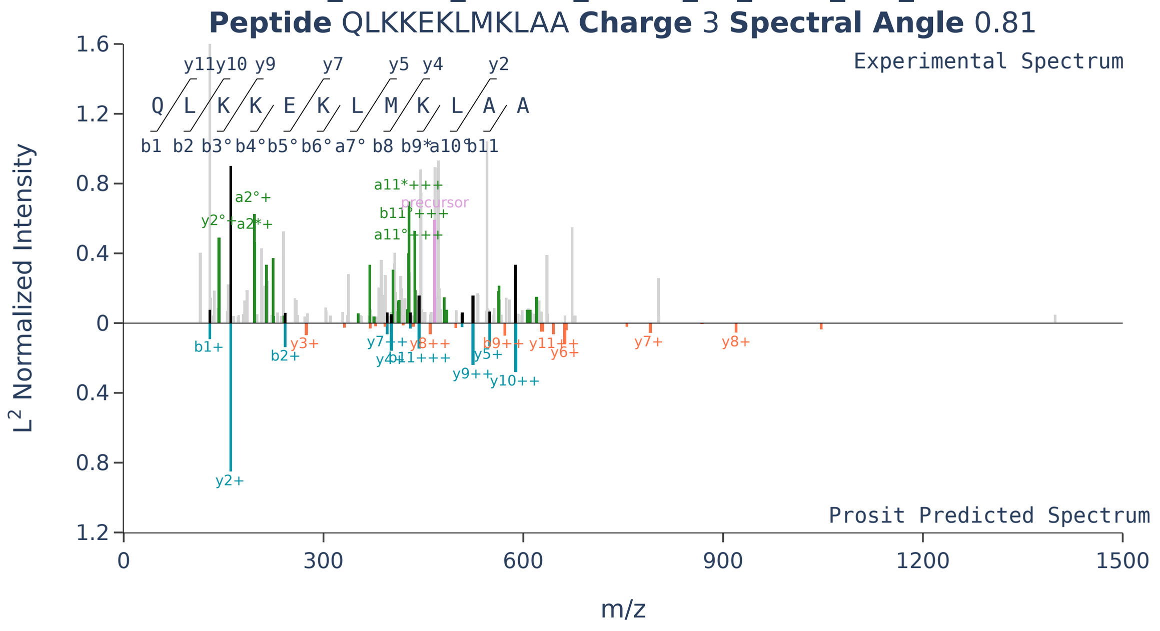


**Source** WSoh_101121_151121_HFGoe_G1_IL37b_24h_R1 **Scan** 10663
**Peptide** DKDKGQSHPSLQLKKEKLMKLAAQ **Charge** 4 **Spectral Angle** 0.81

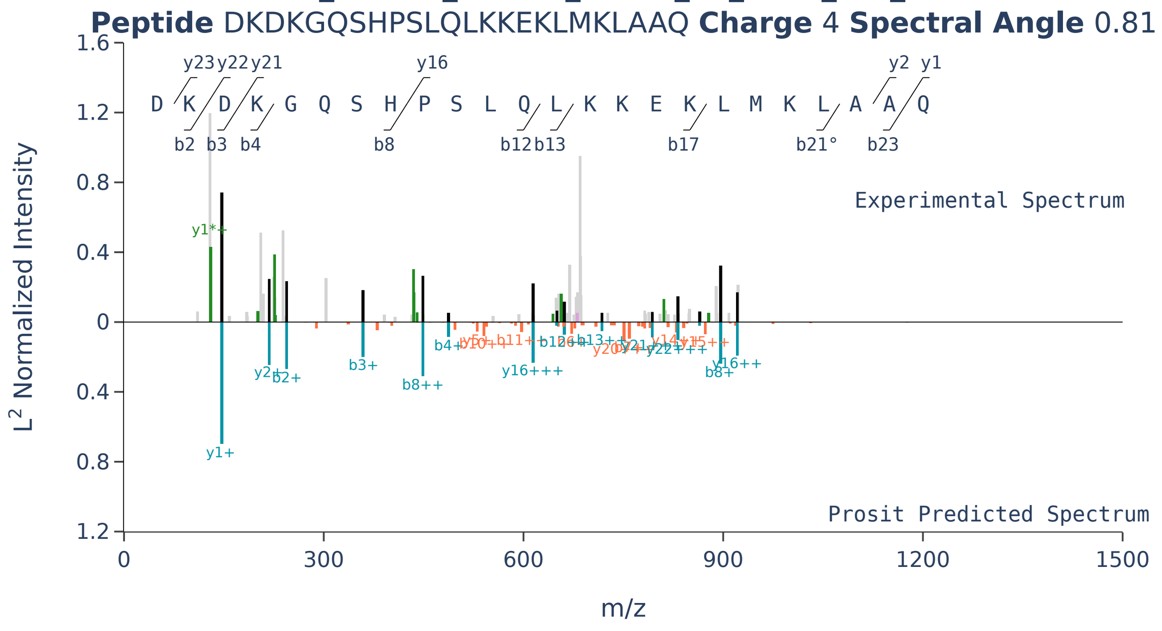


**Source** WSoh_101121_151121_HFGoe_G1_IL37b_24h_R1 **Scan** 25576
**Peptide** ASSLSSASAEKGSPILLGV **Charge** 2 **Spectral Angle** 0.8

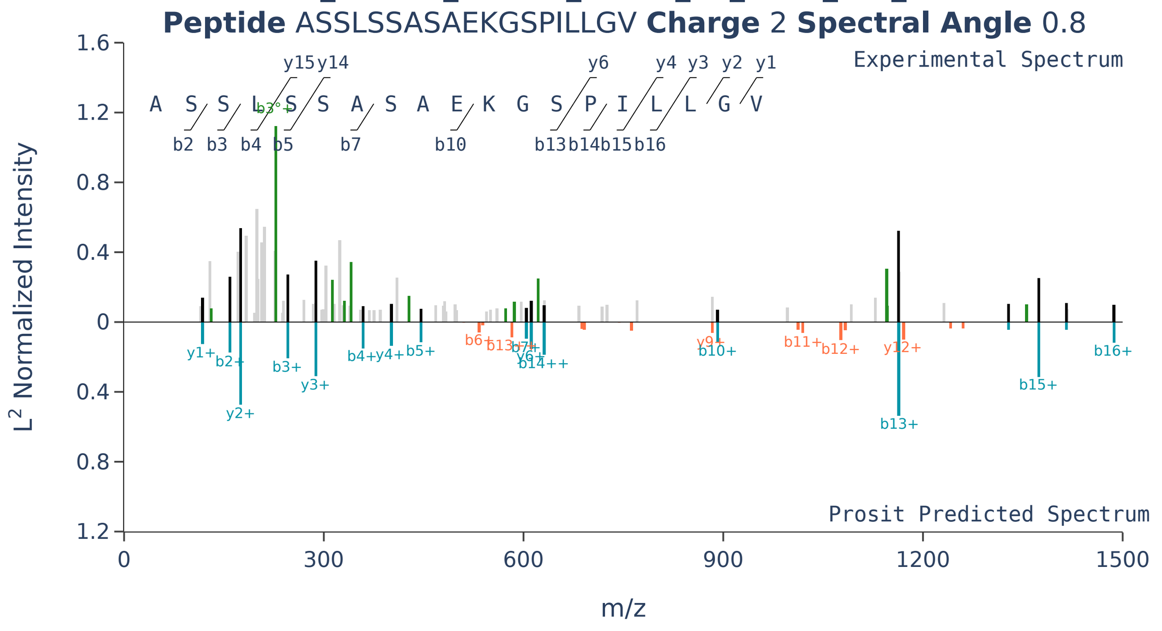


**Source** WSoh_101121_151121_HFGoe_G2_IL37b_24h_R2 **Scan** 5594
**Peptide** LYCDKDKGQSHP **Charge** 3 **Spectral Angle** 0.65

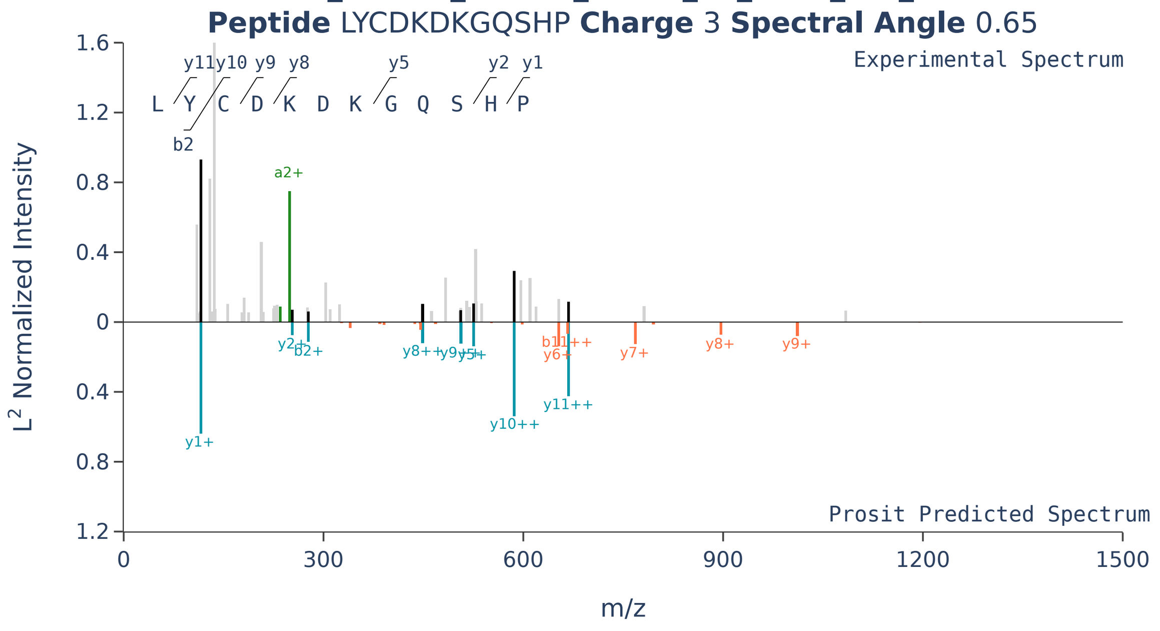


**Source** WSoh_101121_151121_HFGoe_G1_IL37b_4h_R1 **Scan** 16299
**Peptide** CLYCDKDKGQSHPSLQL **Charge** 3 **Spectral Angle** 0.79

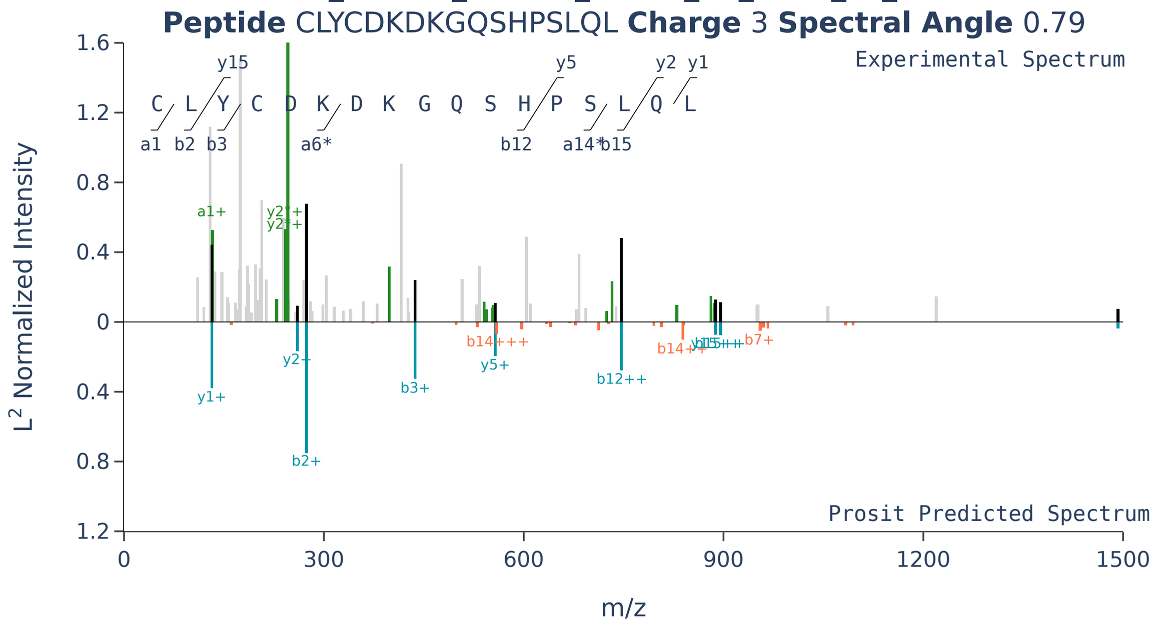


**Source** WSoh_101121_151121_HFGoe_G1_IL37b_4h_R1 **Scan** 10856
**Peptide** DKDKGQSHPSLQLKKEKLMKLAA **Charge** 5 **Spectral Angle** 0.73

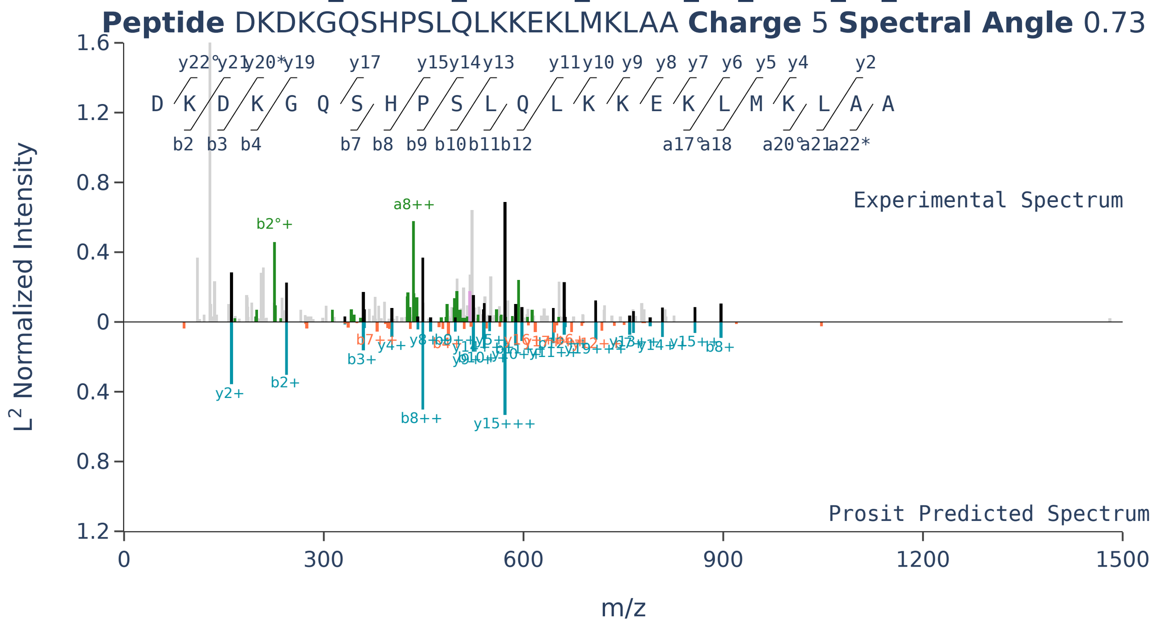


**Source** WSoh_101121_151121_HFGoe_G1_IL37b_4h_R1 **Scan** 10288
**Peptide** LYCDKDKGQSHPSLQLKKEKL **Charge** 4 **Spectral Angle** 0.76

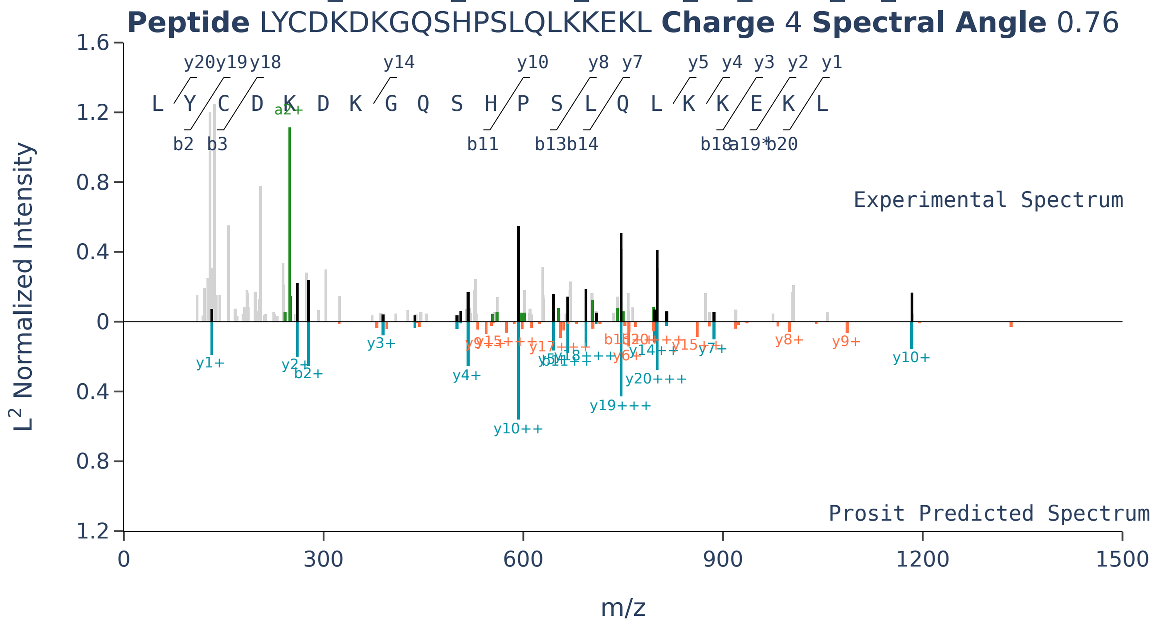

**Source** WSoh_101121_151121_HFGoe_G2_IL37b_24h_R1 **Scan** 21991
**Peptide** ASAEKGSPILLGVS **Charge** 2 **Spectral Angle** 0.76

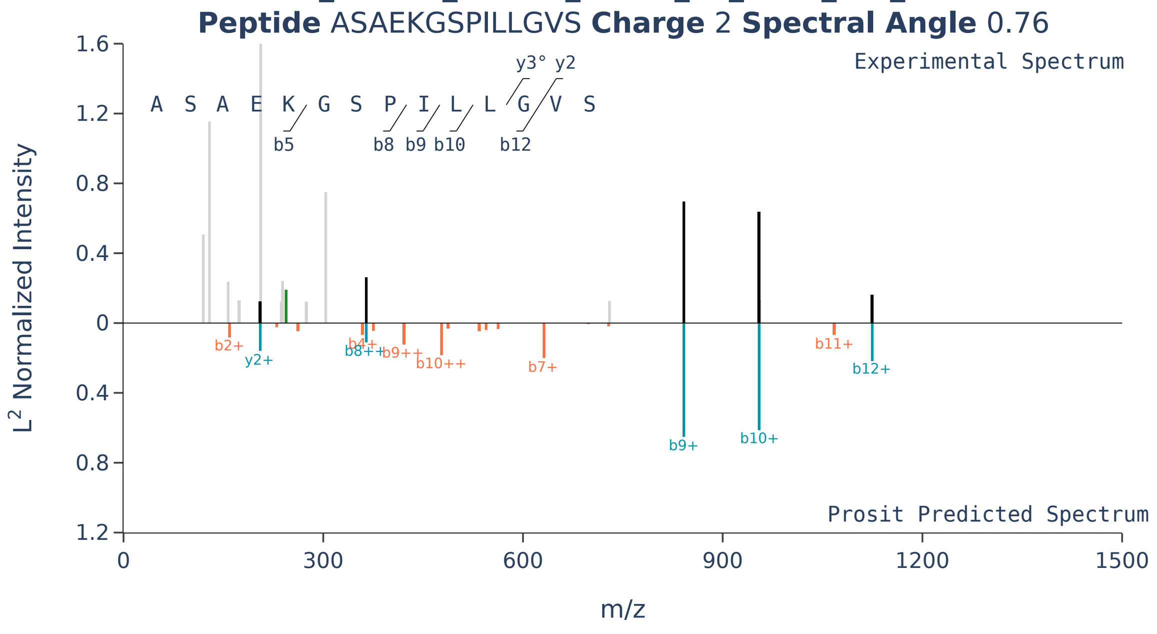


**Source** WSoh_101121_151121_HFGoe_G2_IL37b_2h_R2 **Scan** 13234
**Peptide** CNCNEPVGV **Charge** 2 **Spectral Angle** 0.62

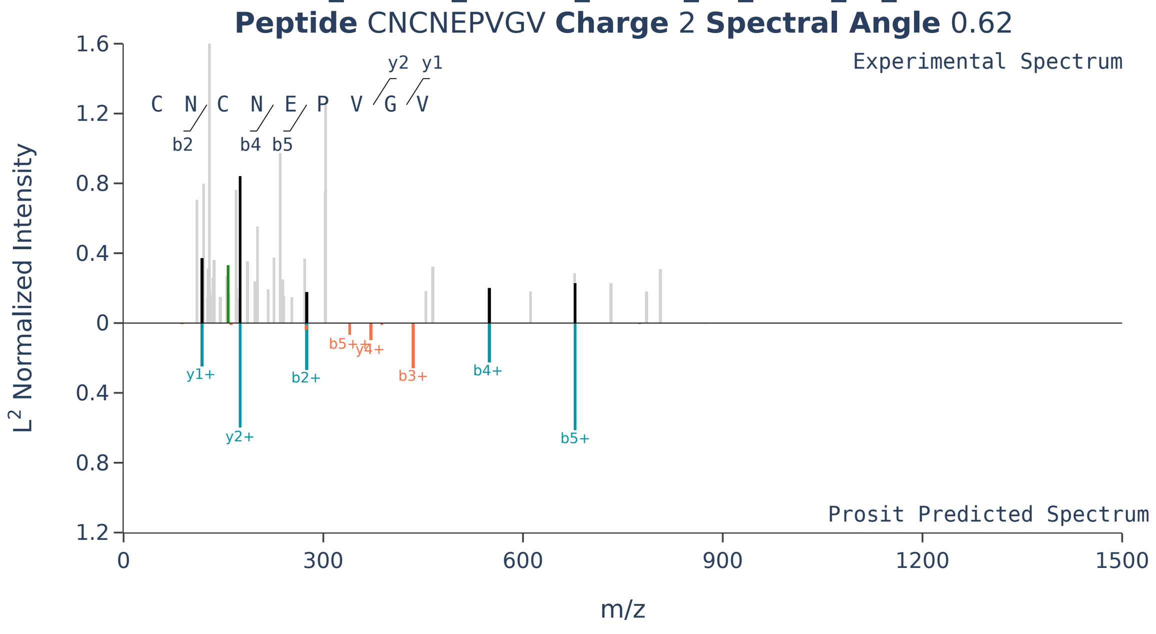


**Source** WSoh_101121_151121_HFGoe_G1_IL37b_4h_R1 **Scan** 6116
**Peptide** PVCKAEMSPSE **Charge** 2 **Spectral Angle** 0.7

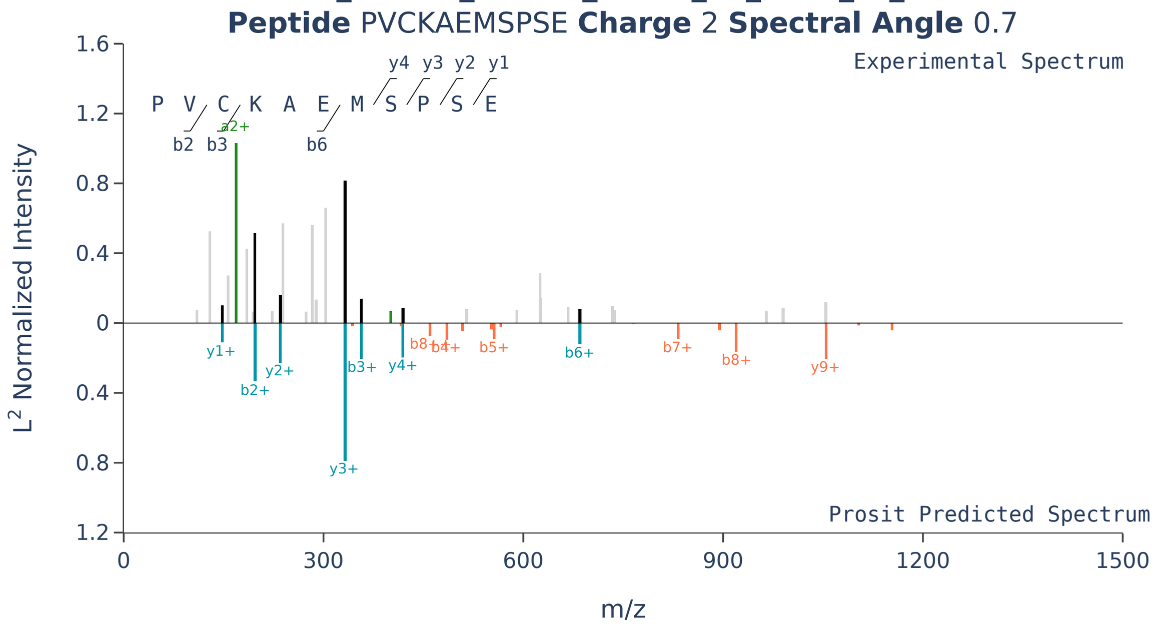


**Source** WSoh_101121_151121_HFGoe_G1_IL37b_24h_R1 **Scan** 23831
**Peptide** SGNLIAVPDKNYIRPEI **Charge** 3 **Spectral Angle** 0.75

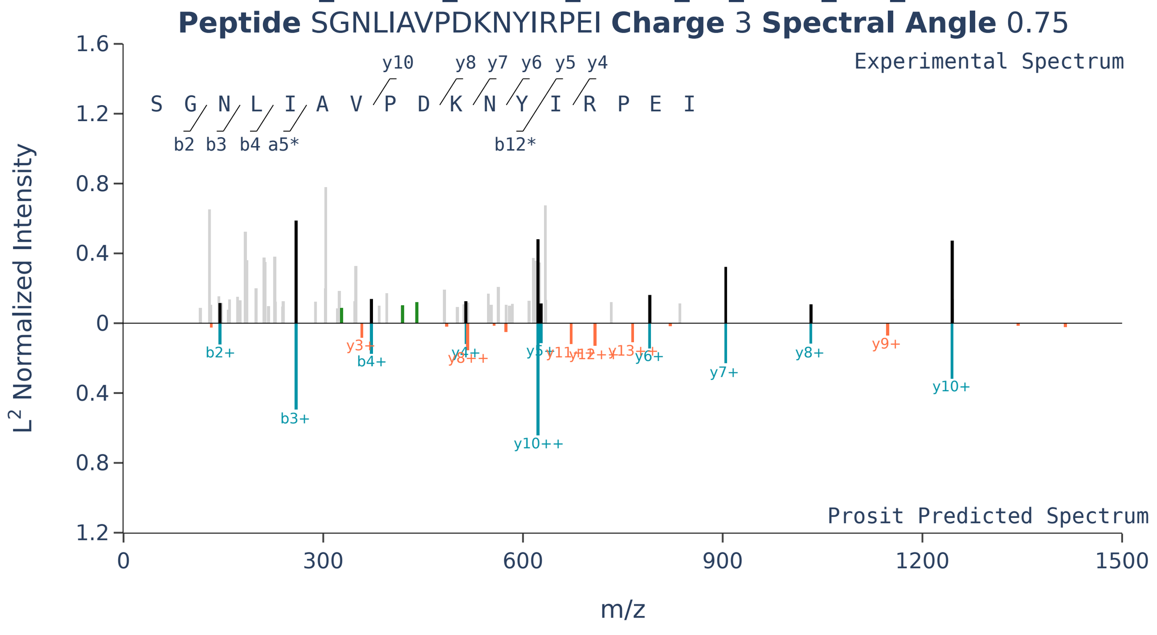


**Source** WSoh_101121_151121_HFGoe_G2_IL37b_24h_R1 **Scan** 14551
**Peptide** QDHKVLVLDS **Charge** 2 **Spectral Angle** 0.68

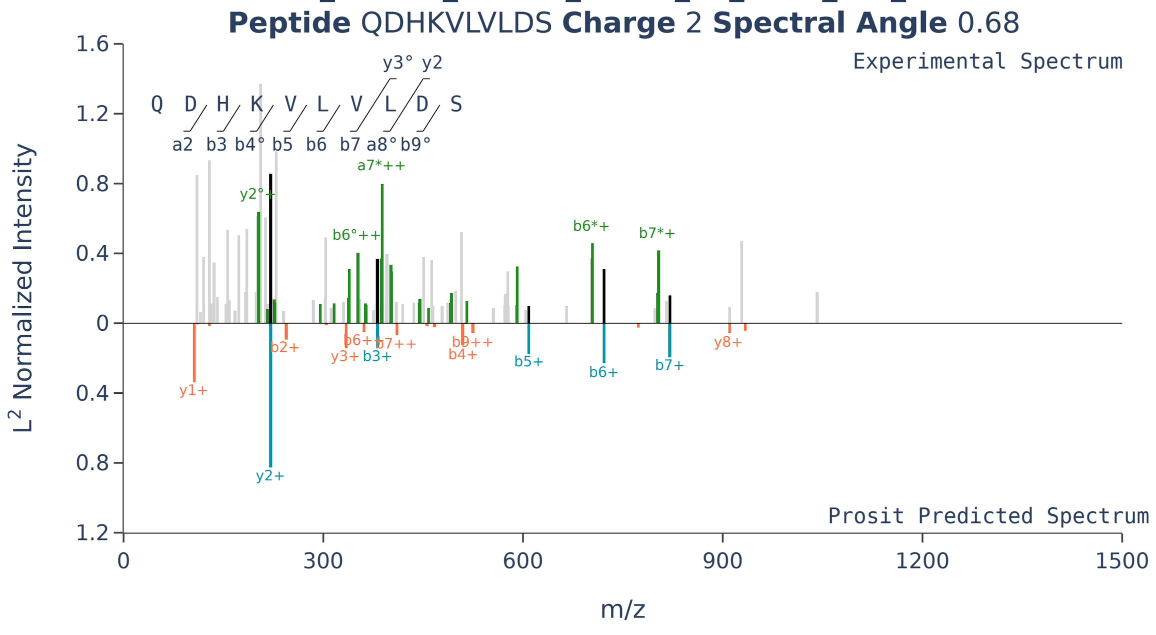


**Source** WSoh_101121_151121_HFGoe_G1_IL37b_4h_R1 **Scan** 8514
**Peptide** QLKKEKLMKLAAQKESAR **Charge** 5 **Spectral Angle** 0.72

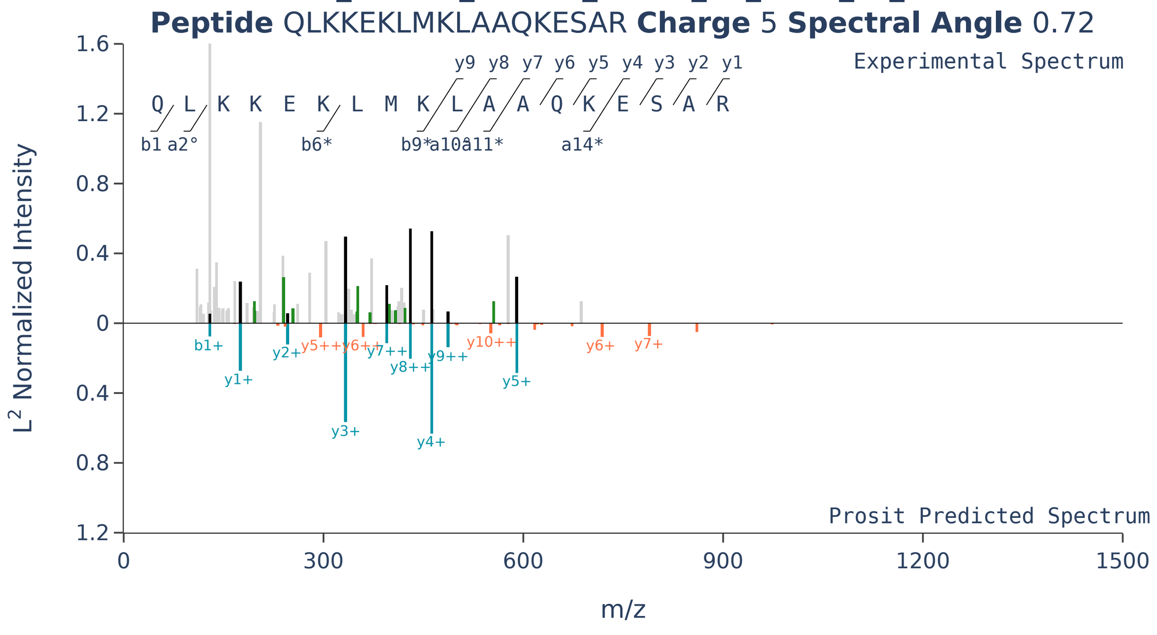


**Source** WSoh_101121_151121_HFGoe_G2_IL37b_24h_R2 **Scan** 22803
**Peptide** ALASSLSSASAEKGSPIL **Charge** 2 **Spectral Angle** 0.78

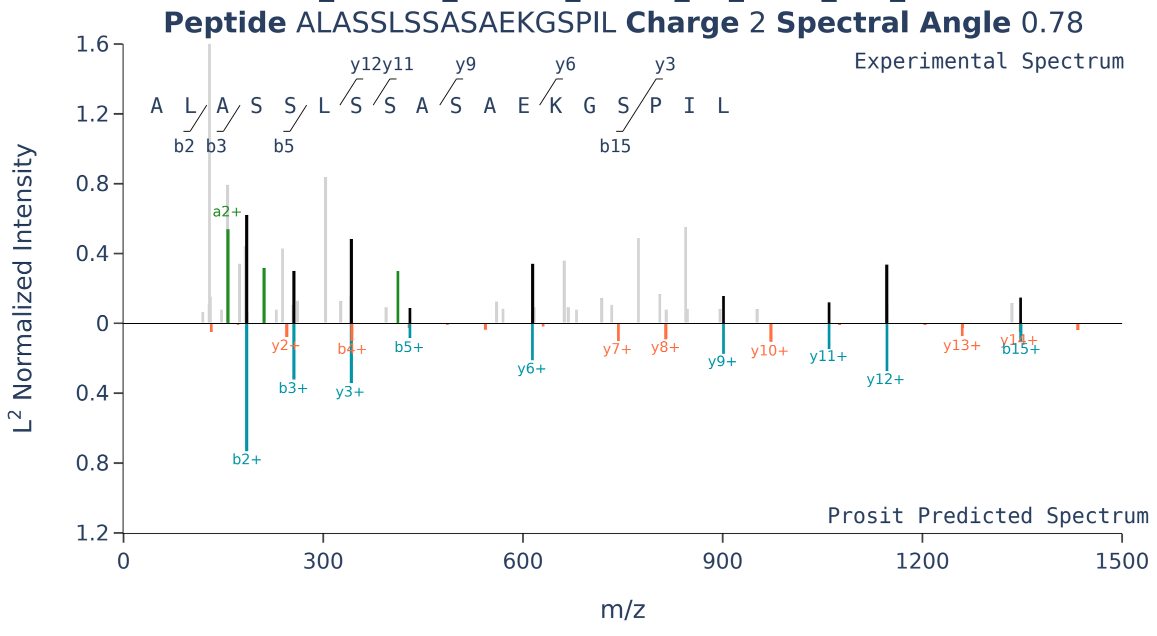


**Source** WSoh_101121_151121_HFGoe_G1_IL37b_4h_R2 **Scan** 6701
**Peptide** RKHIEFS **Charge** 2 **Spectral Angle** 0.74

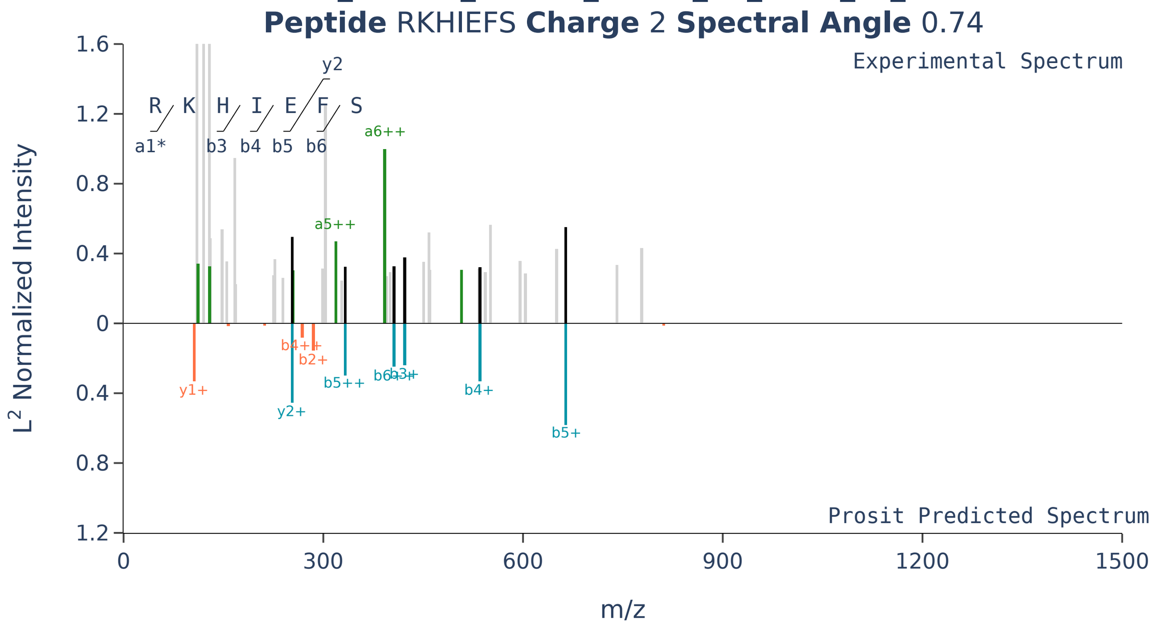


**Source** WSoh_101121_151121_HFGoe_G1_IL37b_24h_R2 **Scan** 23260
**Peptide** ASSLSSASAEKGSPILL **Charge** 2 **Spectral Angle** 0.7

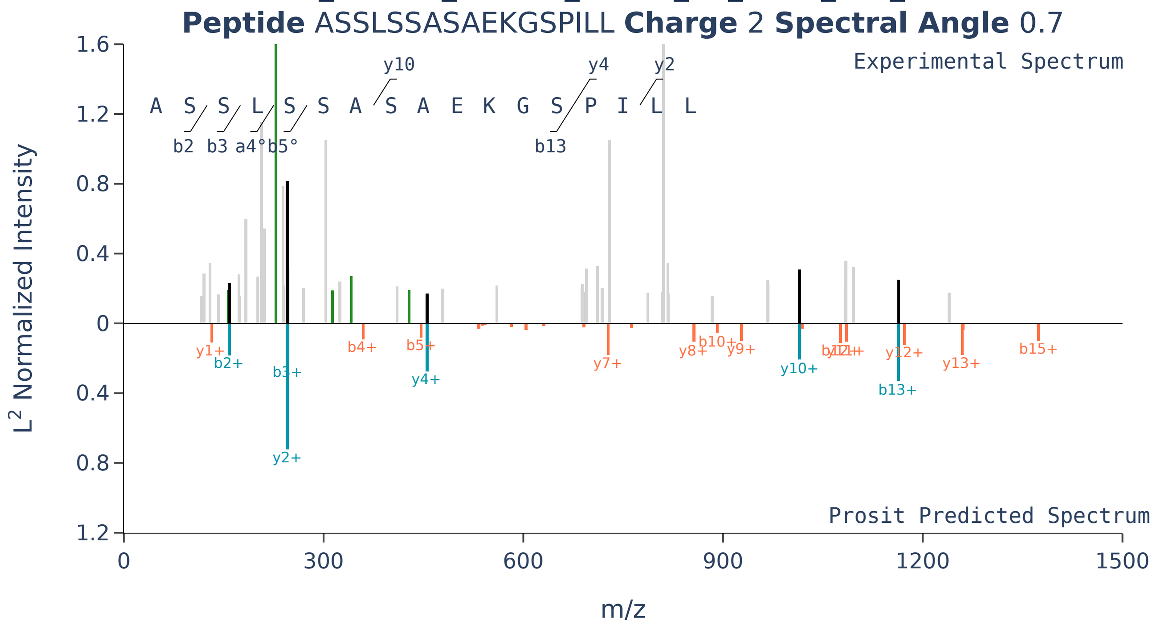


**Source** WSoh_101121_151121_HFGoe_G2_IL37b_24h_R1 **Scan** 19798
**Peptide** SSLSSASAEKGSPILLGVSK **Charge** 3 **Spectral Angle** 0.68

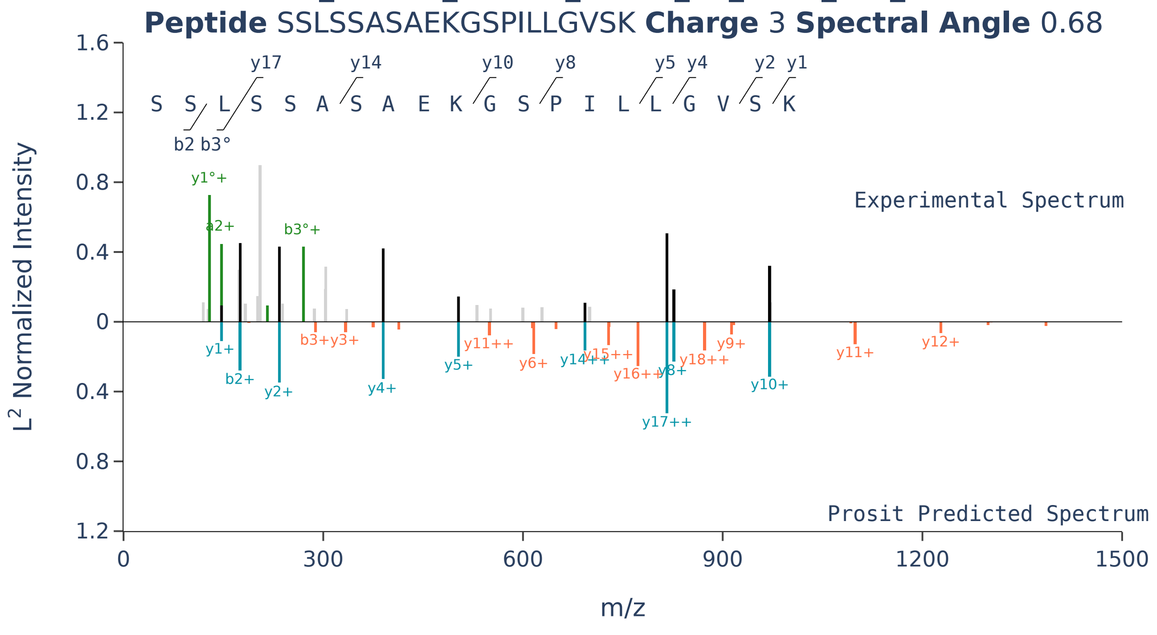


**Source** WSoh_101121_151121_HFGoe_G2_IL37b_4h_R2 **Scan** 5865
**Peptide** VHTSPKV **Charge** 1 **Spectral Angle** 0.86

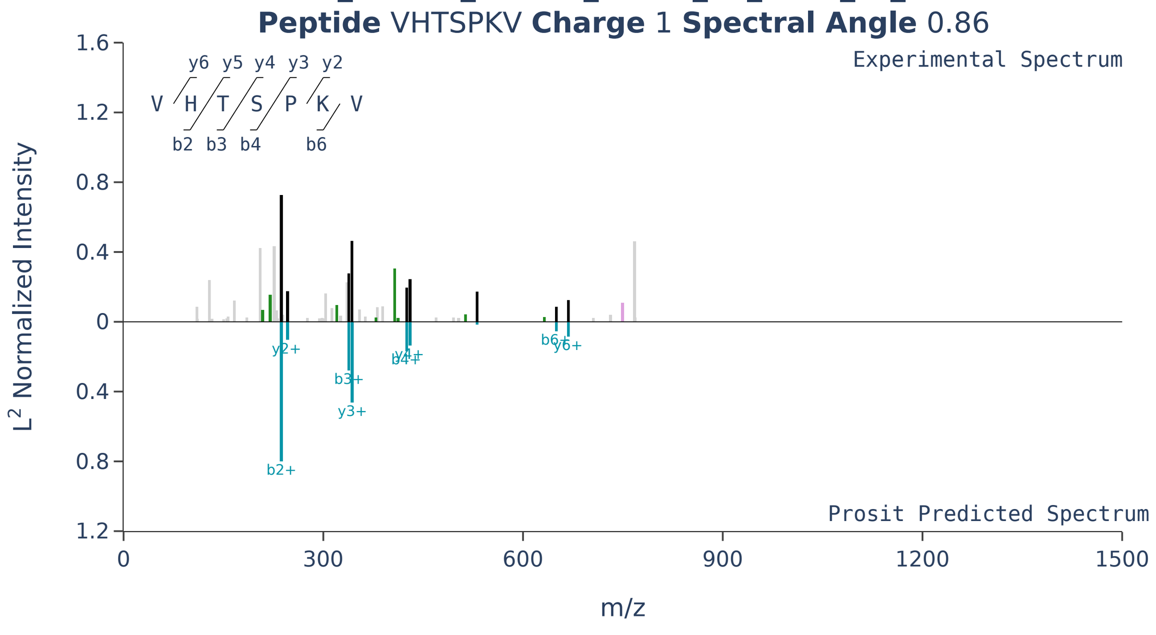


**Source** WSoh_101121_151121_HFGoe_G1_IL37b_24h_R2 **Scan** 7474
**Peptide** SLSSASAE **Charge** 1 **Spectral Angle** 0.9

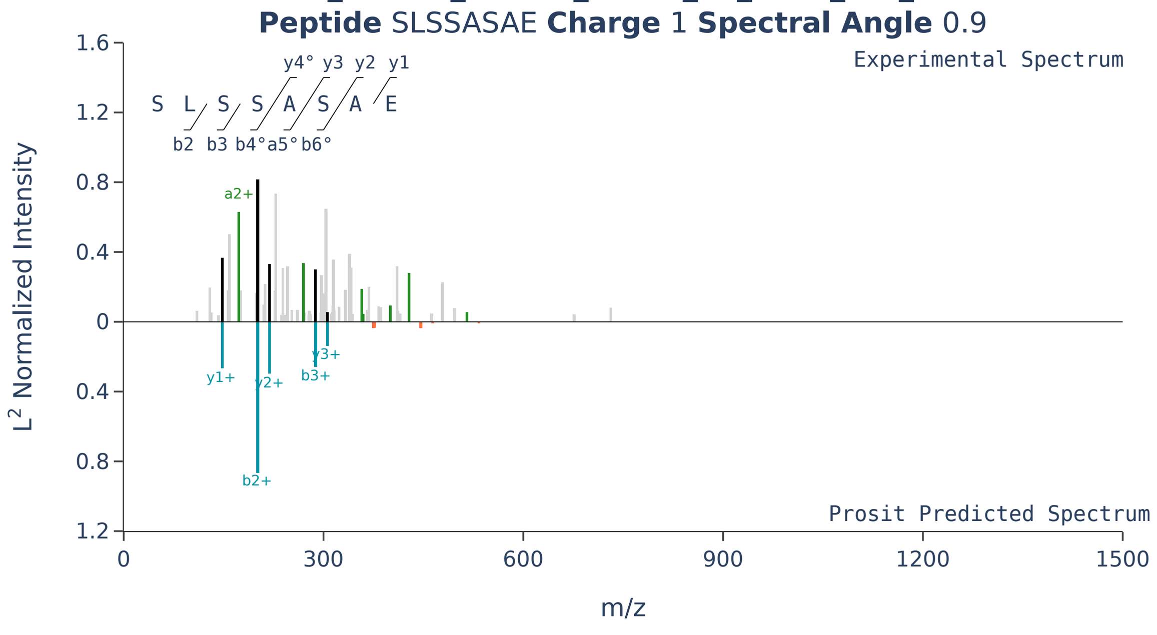


**Source** WSoh_101121_151121_HFGoe_G1_IL37b_4h_R2 **Scan** 21837
**Peptide** SSLSSASAEKGSPILLG **Charge** 2 **Spectral Angle** 0.73

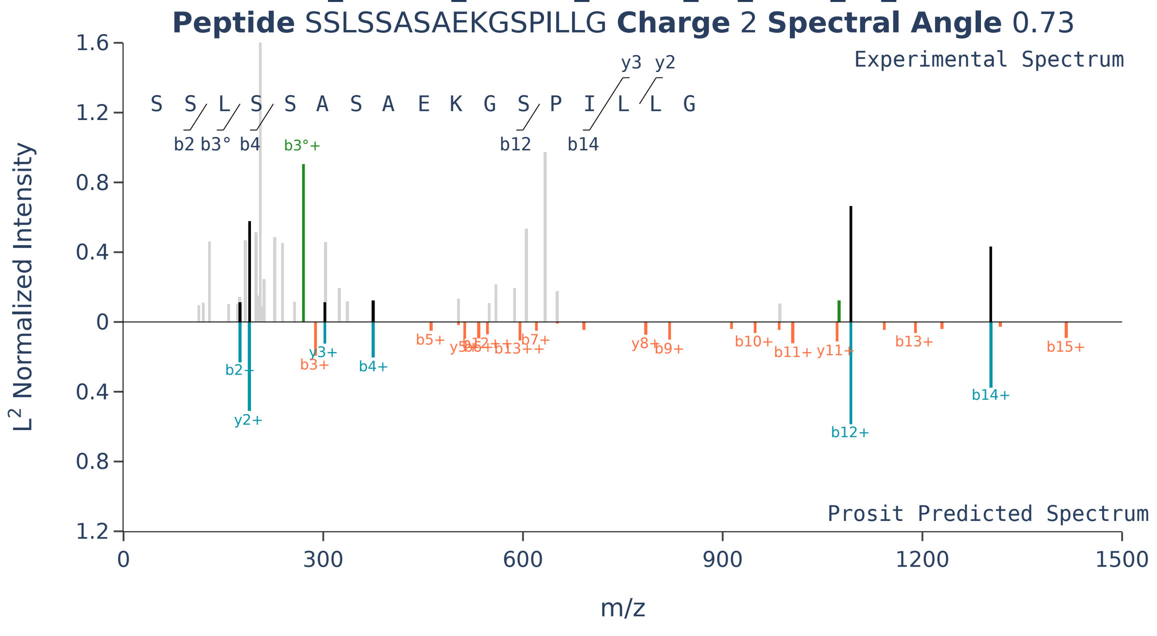


**Source** WSoh_101121_151121_HFGoe_G1_IL37b_4h_R2 **Scan** 22459
**Peptide** SAAHPGWFICTSCN **Charge** 2 **Spectral Angle** 0.64

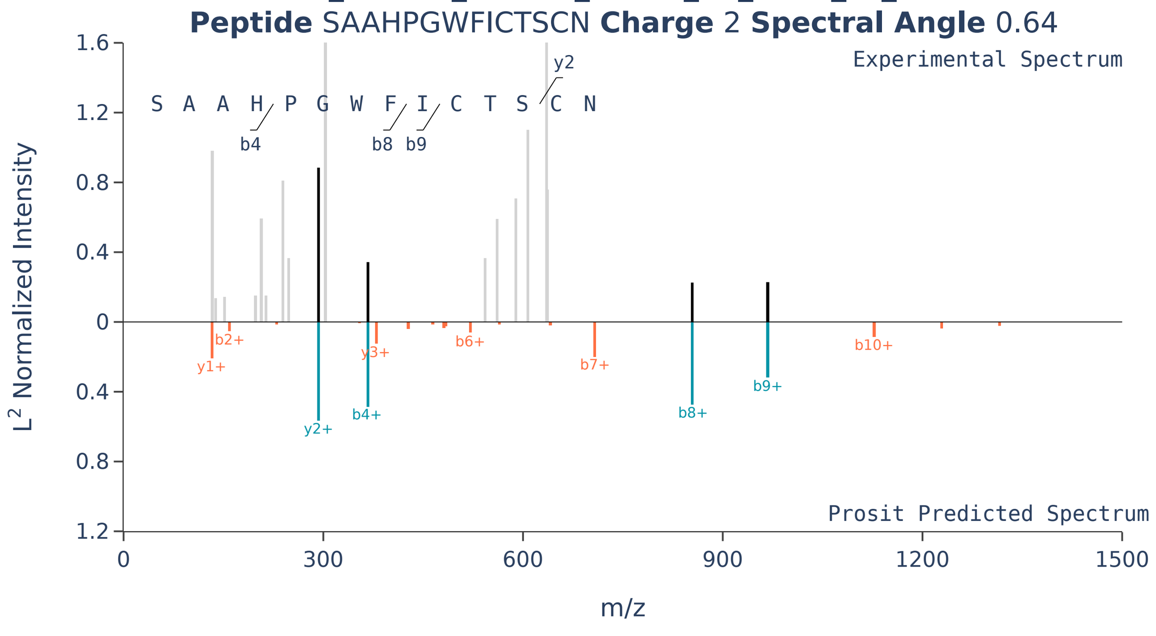


**Source** WSoh_101121_151121_HFGoe_G1_IL37b_4h_R1 **Scan** 14225
**Peptide** KESARRPFIFYRAQ **Charge** 4 **Spectral Angle** 0.81

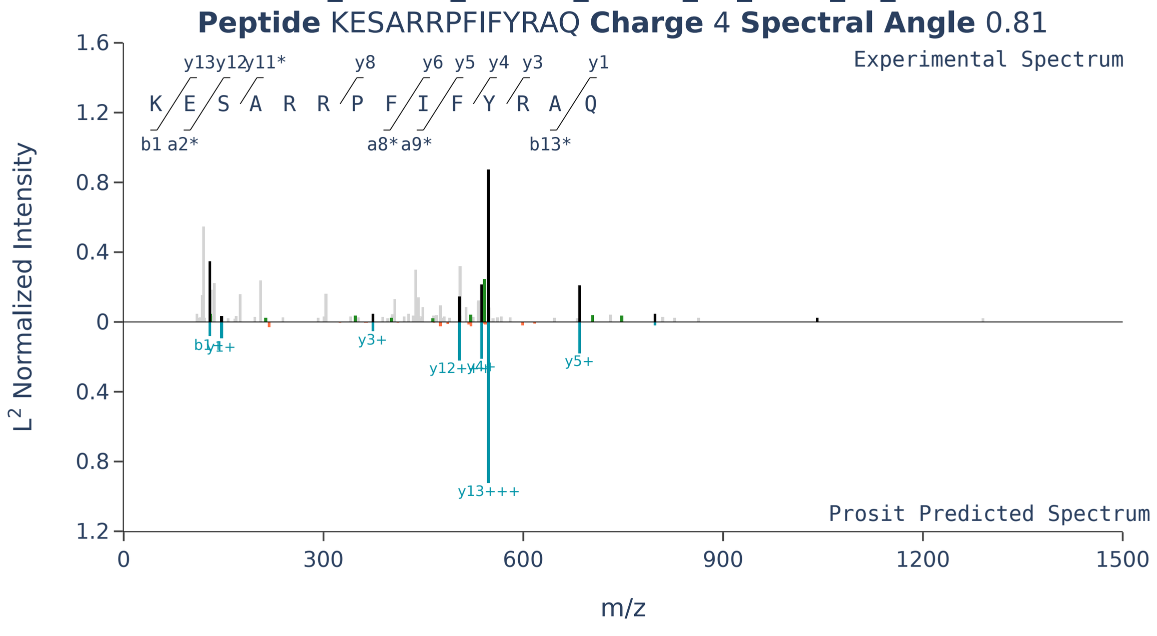

**Source** WSoh_101121_151121_HFGoe_G2_IL37b_24h_R1 **Scan** 8752
**Peptide** KAEMSPSEVS **Charge** 2 **Spectral Angle** 0.45

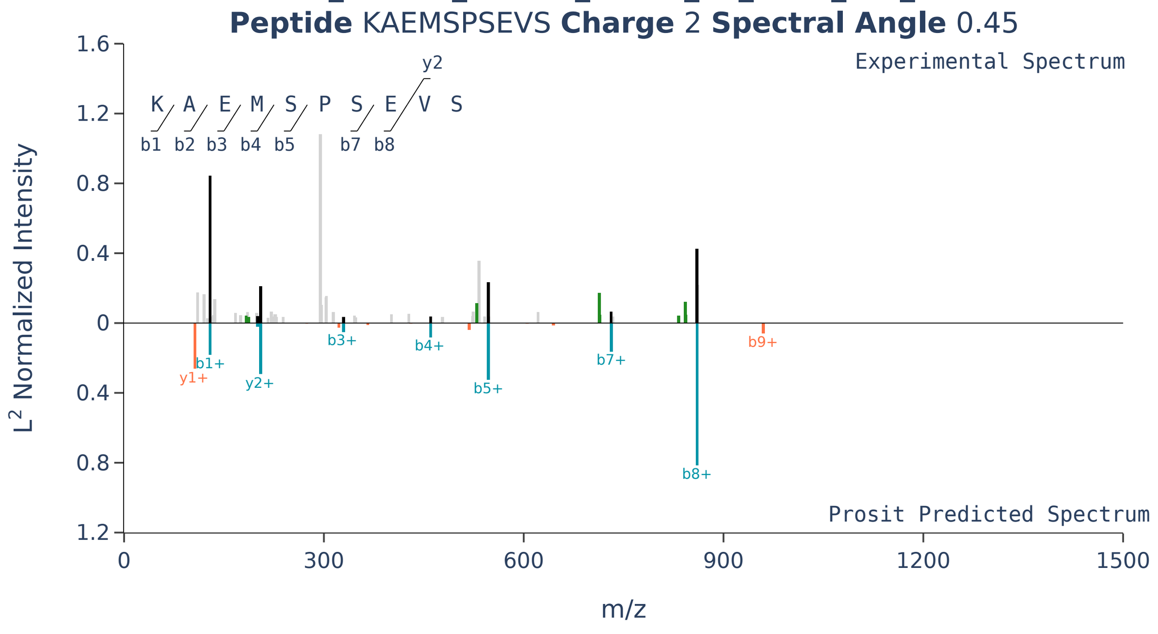

**Source** WSoh_101121_151121_HFGoe_G2_IL37b_2h_R1 **Scan** 16212
**Peptide** FCLYCDKDKGQSHPSL **Charge** 3 **Spectral Angle** 0.79

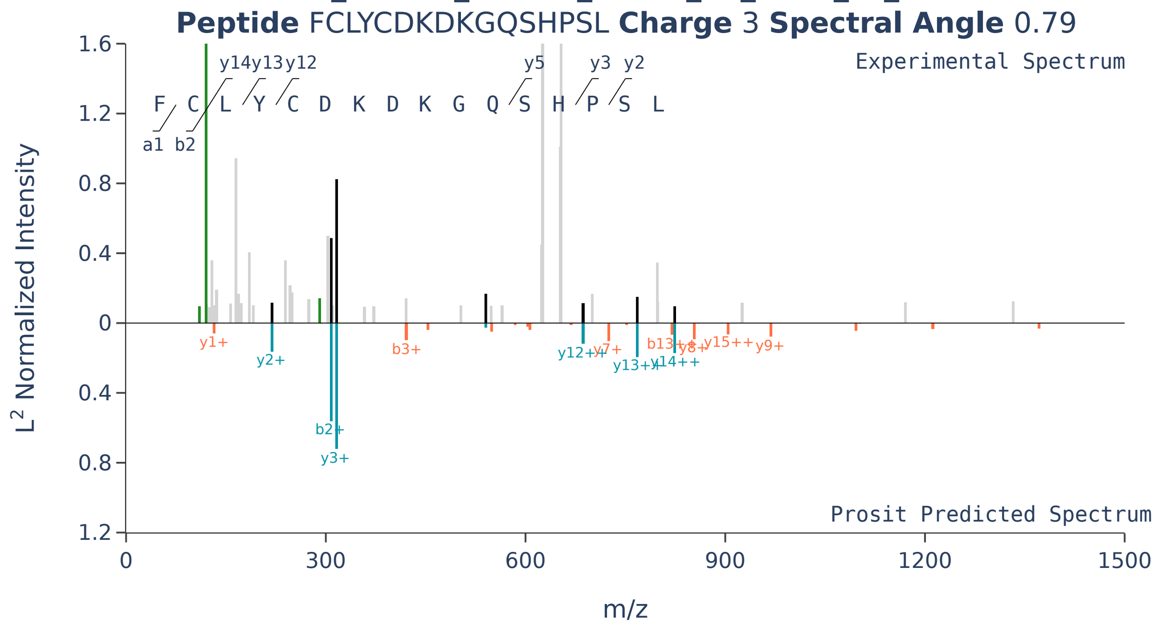

**Source** WSoh_101121_151121_HFGoe_G2_IL37b_24h_R1 **Scan** 20160
**Peptide** SSLSSASAEKGSPILLGVSKGE **Charge** 3 **Spectral Angle** 0.78

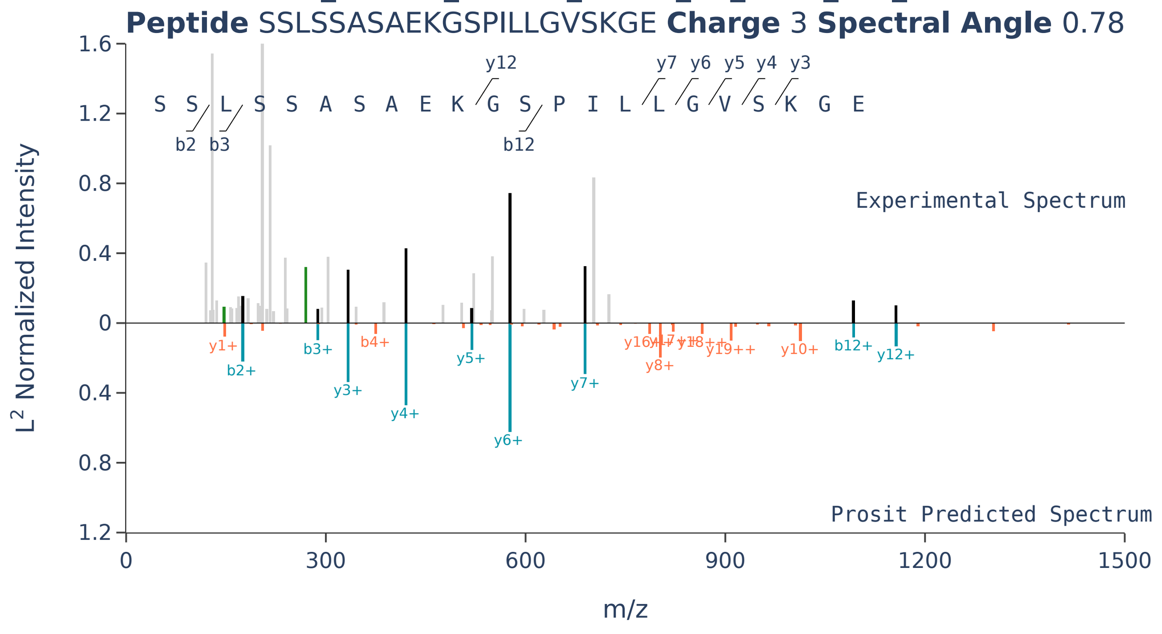

**Source** WSoh_101121_151121_HFGoe_G1_IL37b_4h_R1 **Scan** 13222
**Peptide** SKGEFCLYCDKDKGQSHPSLQLKKE **Charge** 6 **Spectral Angle** 0.7

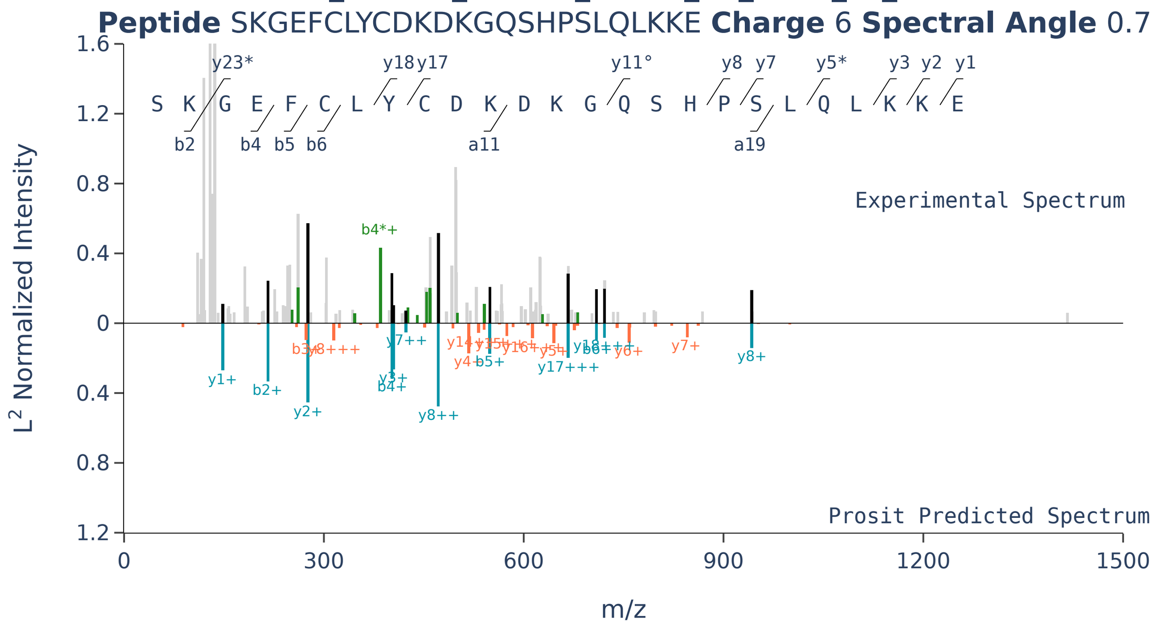

**Source** WSoh_101121_151121_HFGoe_G2_IL37b_24h_R2 **Scan** 5257
**Peptide** HTSPKVK **Charge** 2 **Spectral Angle** 0.62

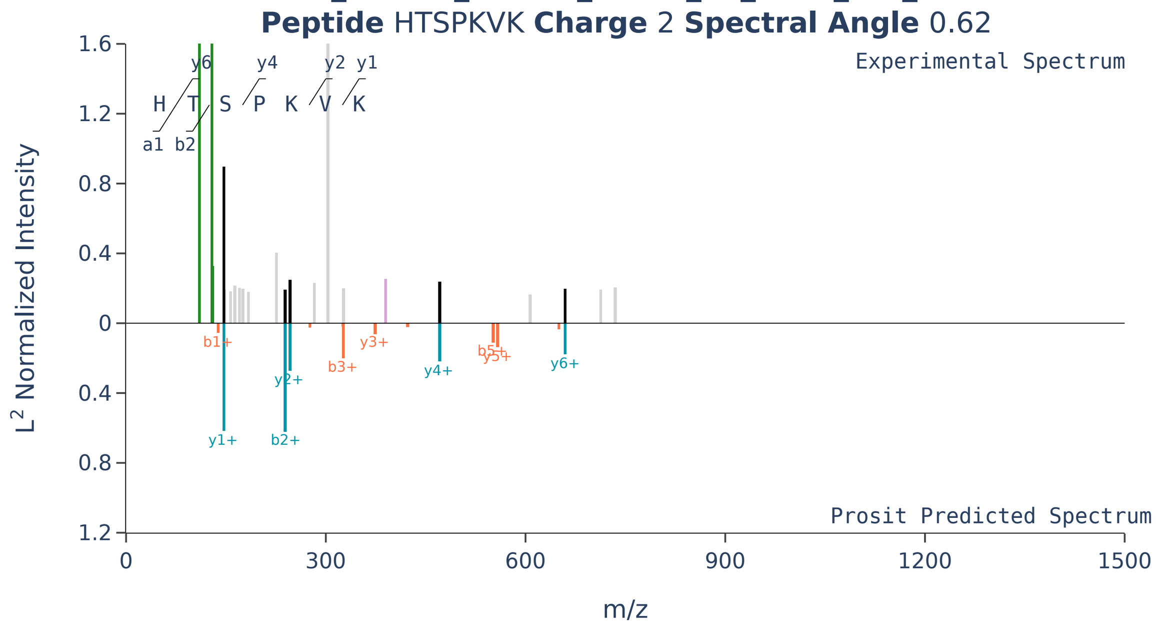

**Source** WSoh_101121_151121_HFGoe_G2_IL37b_24h_R2 **Scan** 5264
**Peptide** KLAAQKESAR **Charge** 2 **Spectral Angle** 0.54

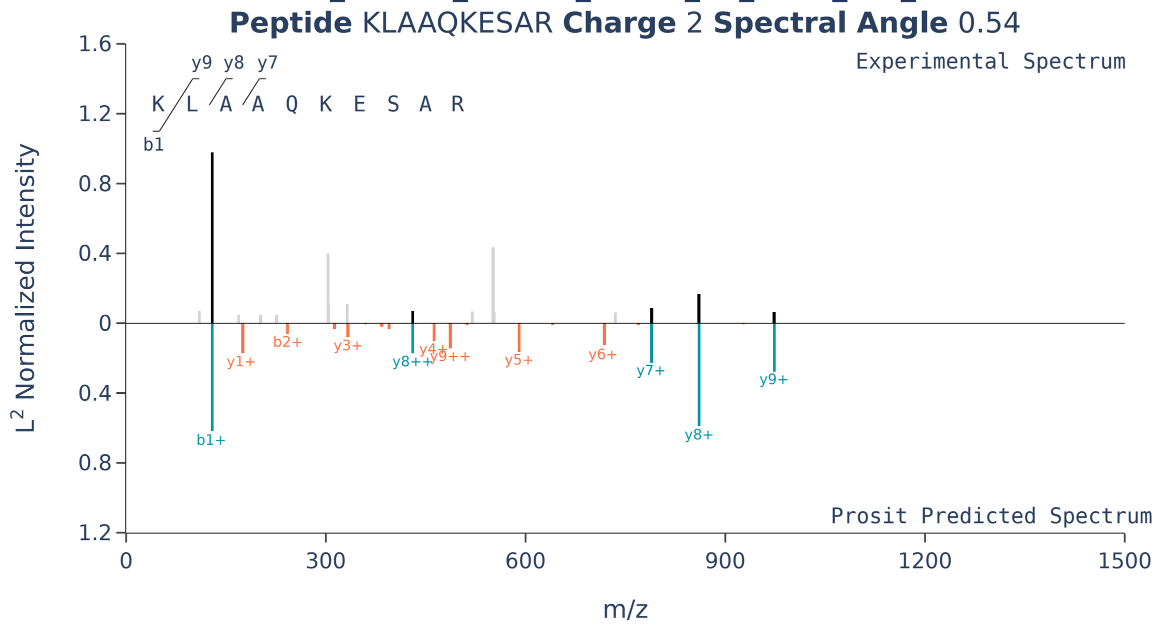

**Source** WSoh_101121_151121_HFGoe_G1_IL37b_1h_R1 **Scan** 26817
**Peptide** DSGNLIAVPDKNYIRPEIF **Charge** 3 **Spectral Angle** 0.73

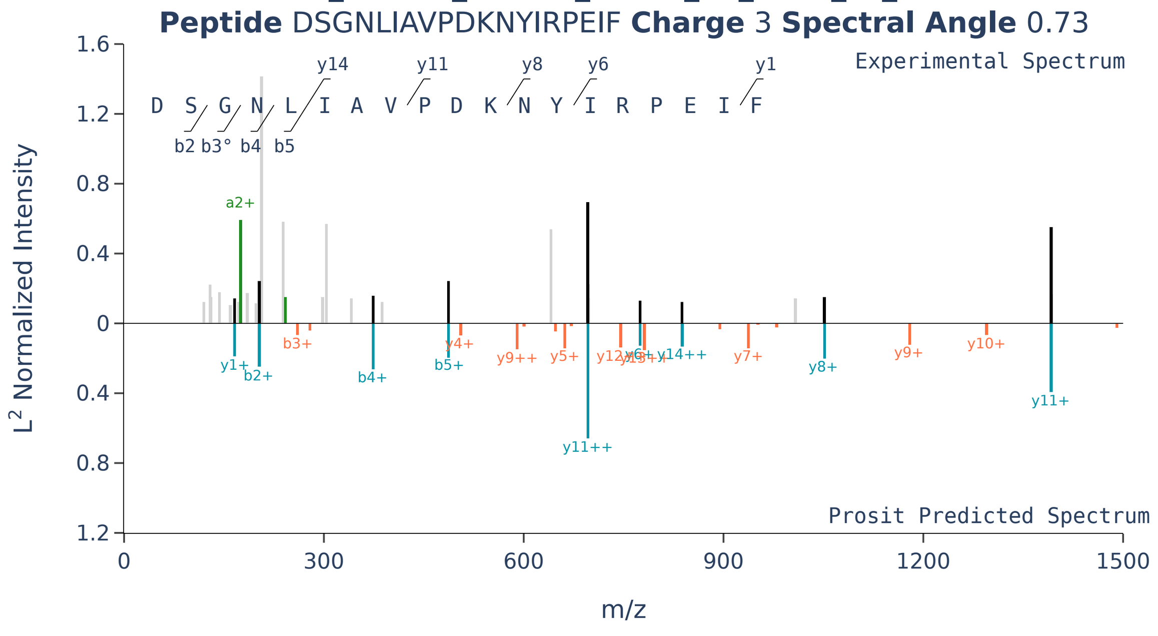

**Source** WSoh_101121_151121_HFGoe_G1_IL37b_4h_R2 **Scan** 8774
**Peptide** PSLQLKK **Charge** 2 **Spectral Angle** 0.9

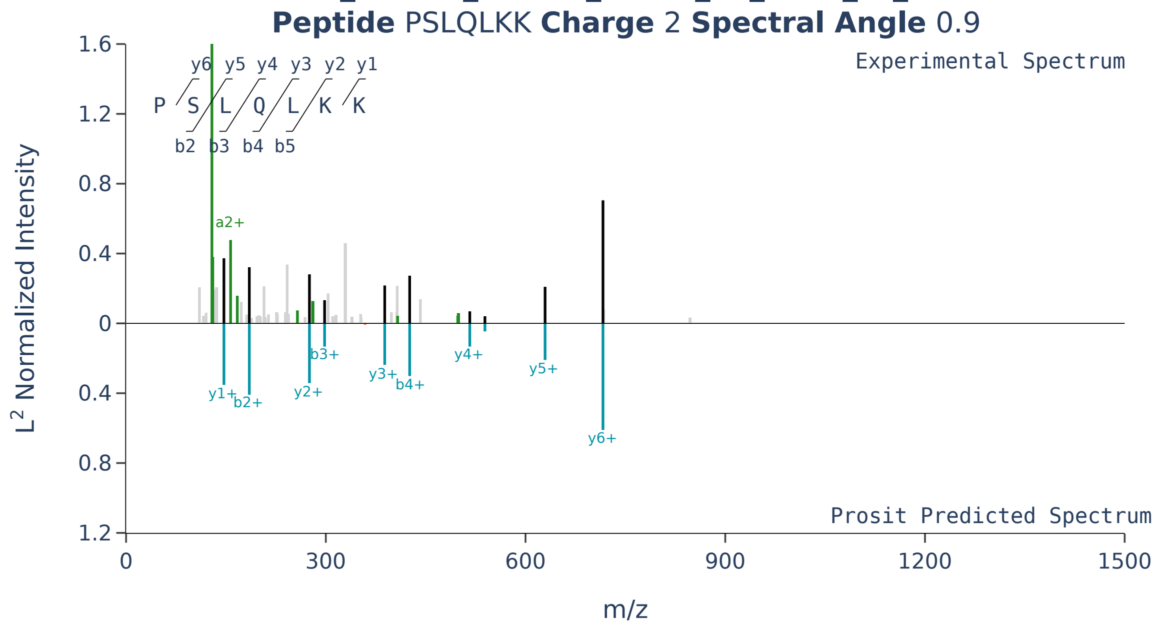

**Source** WSoh_101121_151121_HFGoe_G1_IL37b_24h_R2 **Scan** 5785
**Peptide** QLKKEKL **Charge** 2 **Spectral Angle** 0.87

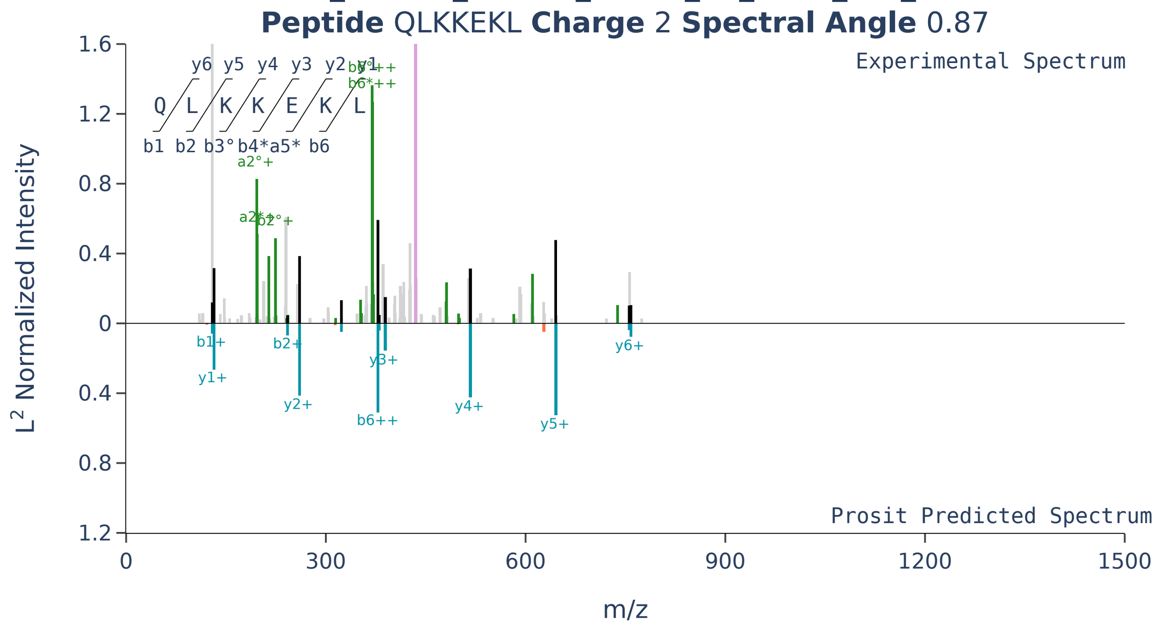

**Source** WSoh_101121_151121_HFGoe_G2_IL37b_24h_R1 **Scan** 7241
**Peptide** PKVKNLNPKKFS **Charge** 3 **Spectral Angle** 0.59

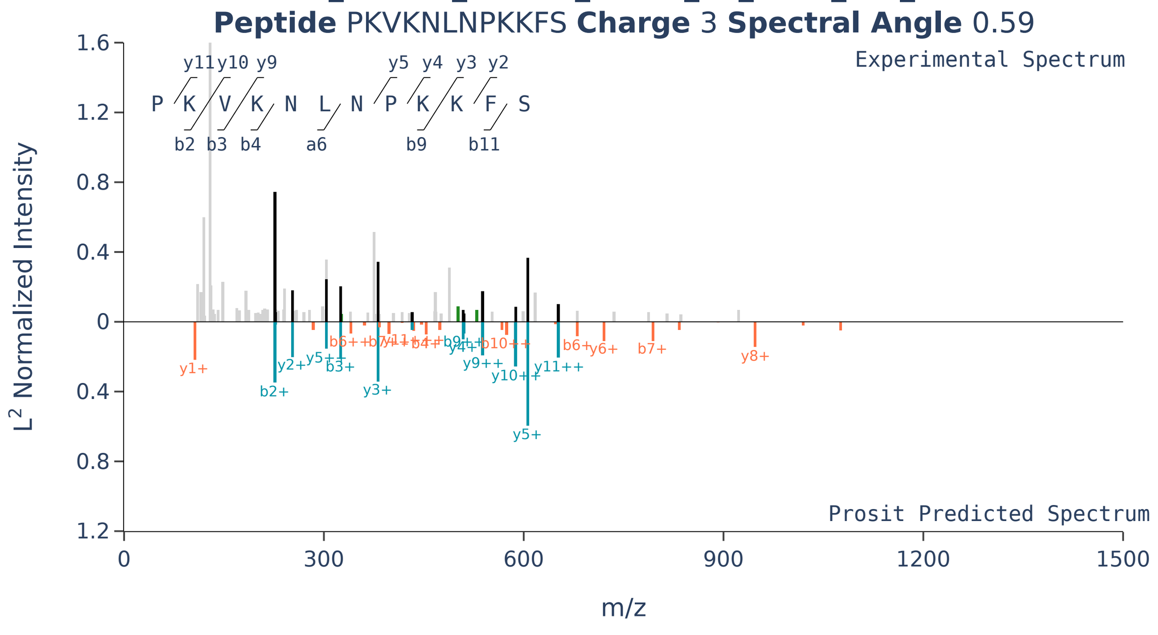

**Source** WSoh_101121_151121_HFGoe_G2_IL37b_2h_R1 **Scan** 25443
**Peptide** SGNLIAVPDKNYIRPEIF **Charge** 3 **Spectral Angle** 0.73

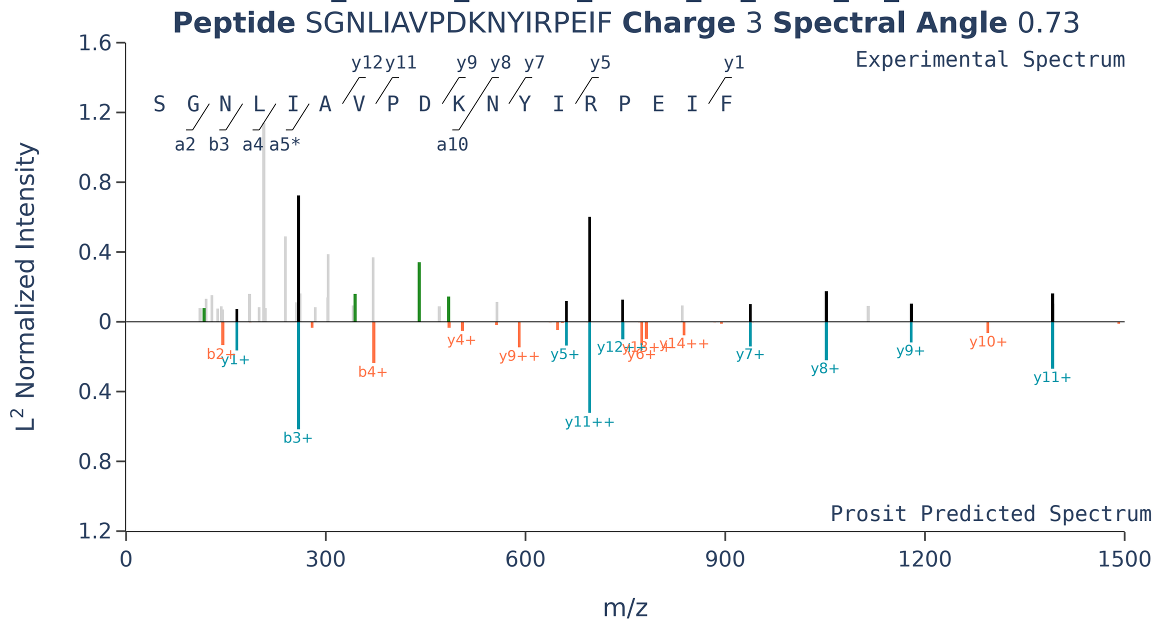

**Source** WSoh_101121_151121_HFGoe_G1_IL37b_4h_R2 **Scan** 16922
**Peptide** ESARRPFIFYRAQ **Charge** 3 **Spectral Angle** 0.73

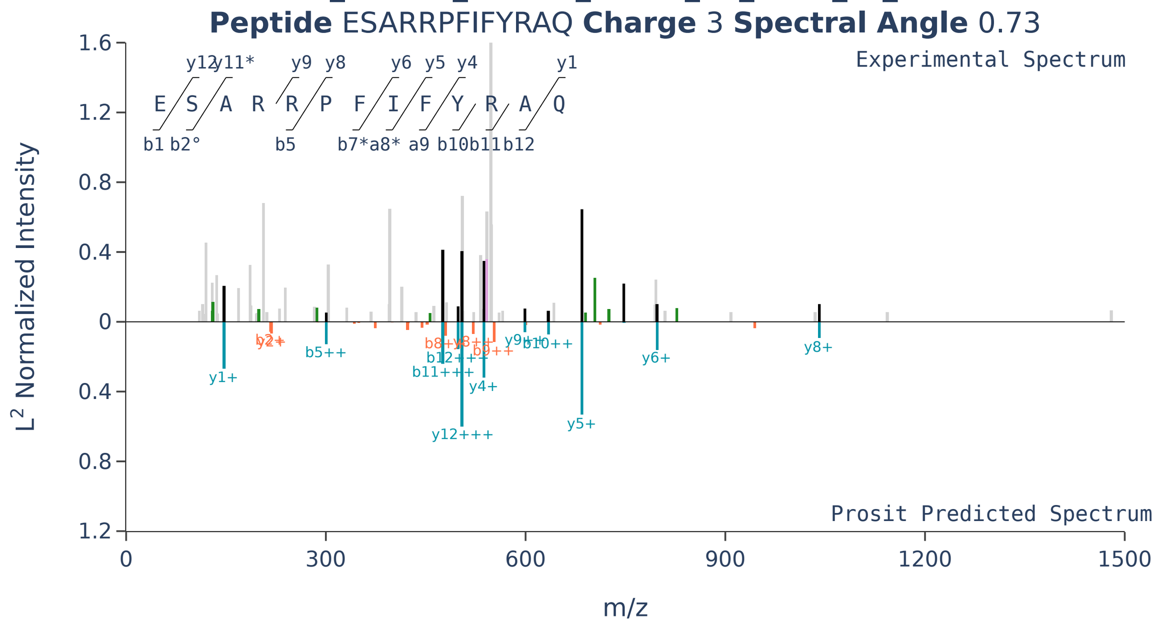

**Source** WSoh_101121_151121_HFGoe_G2_IL37b_4h_R2 **Scan** 6562
**Peptide** AQKESARRPF **Charge** 3 **Spectral Angle** 0.94

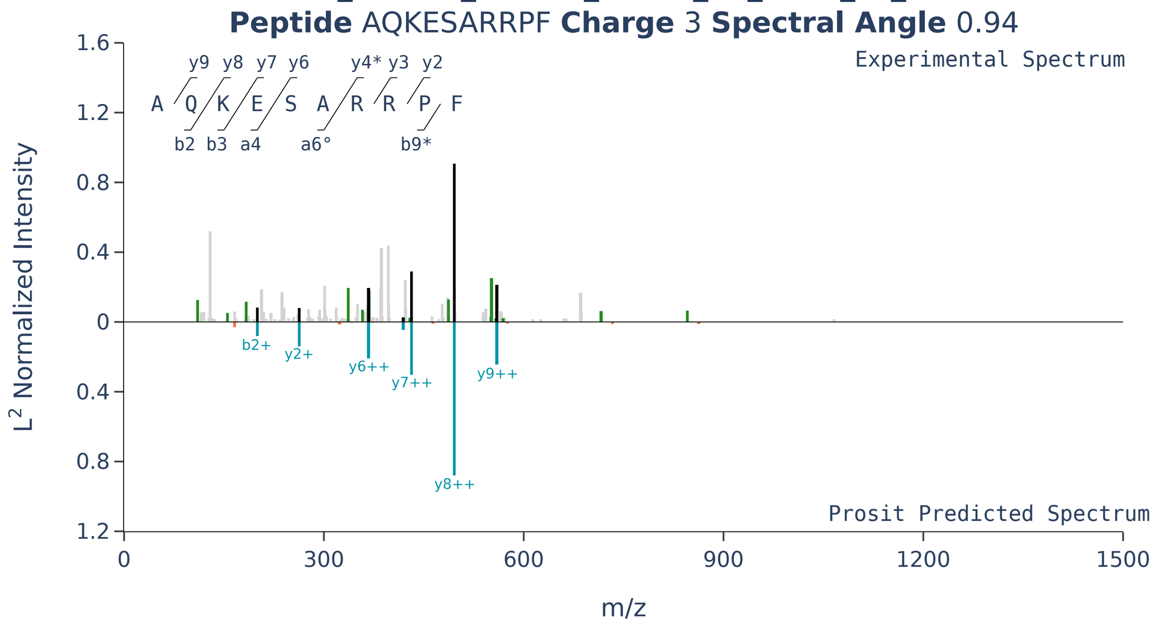

**Source** WSoh_101121_151121_HFGoe_G1_IL37b_2h_R1 **Scan** 21176
**Peptide** SLSSASAEKGSPILLG **Charge** 2 **Spectral Angle** 0.69

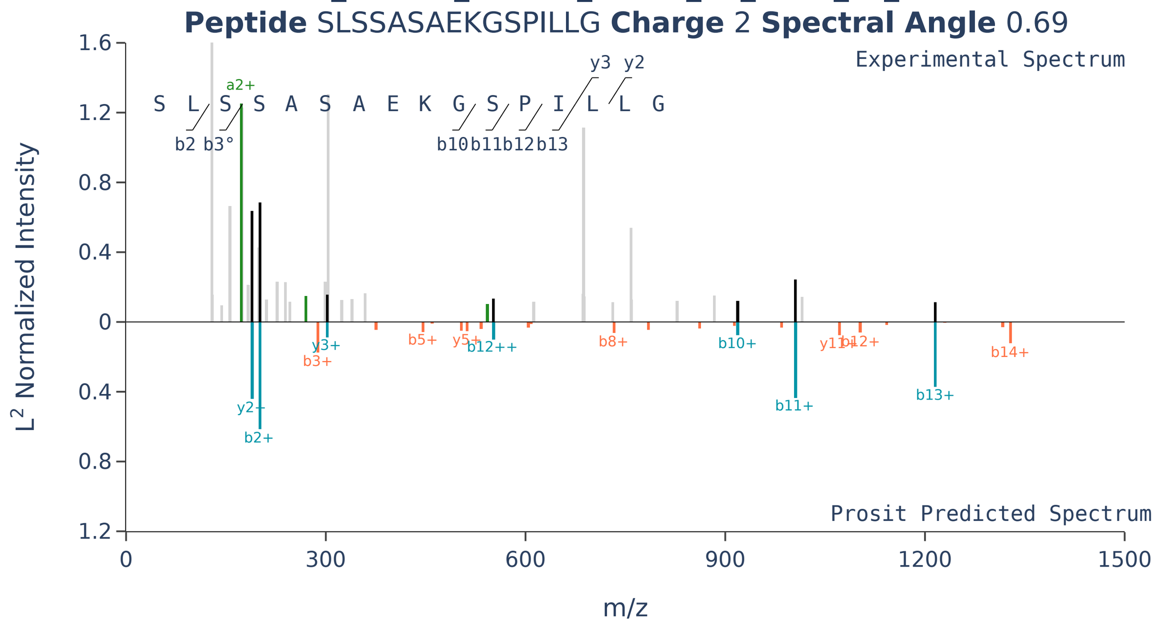

**Source** WSoh_101121_151121_HFGoe_G2_IL37b_2h_R2 **Scan** 5832
**Peptide** CLYCDKDKGQSH **Charge** 3 **Spectral Angle** 0.64

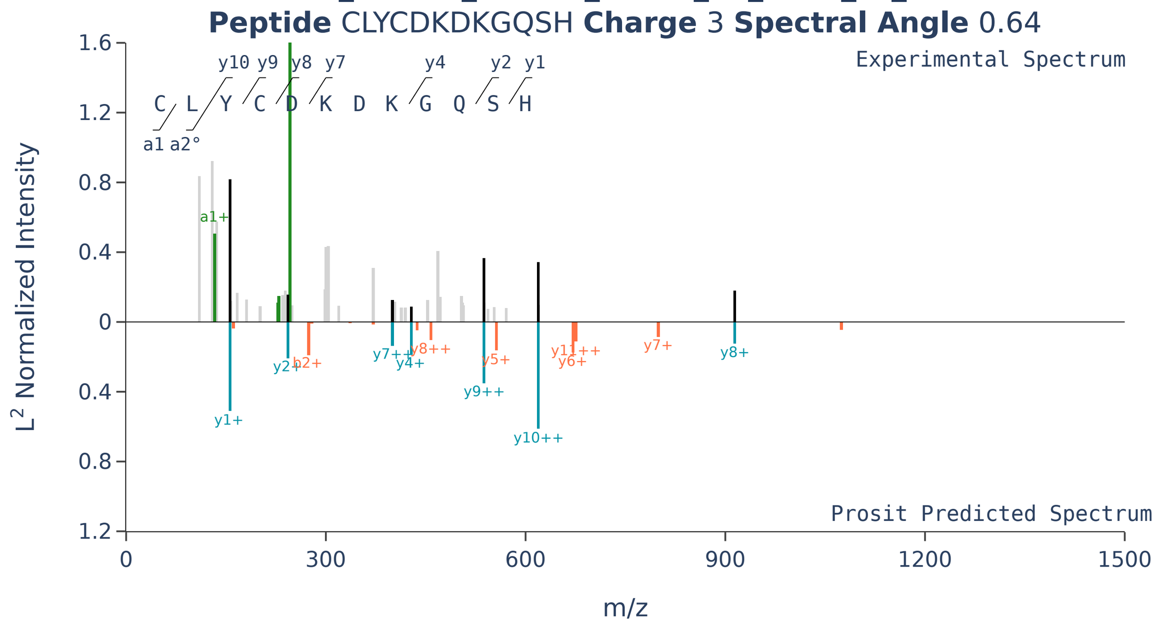

**Source** WSoh_101121_151121_HFGoe_G1_IL37b_24h_R1 **Scan** 17365
**Peptide** CNCNEPVGVTDKF **Charge** 2 **Spectral Angle** 0.58

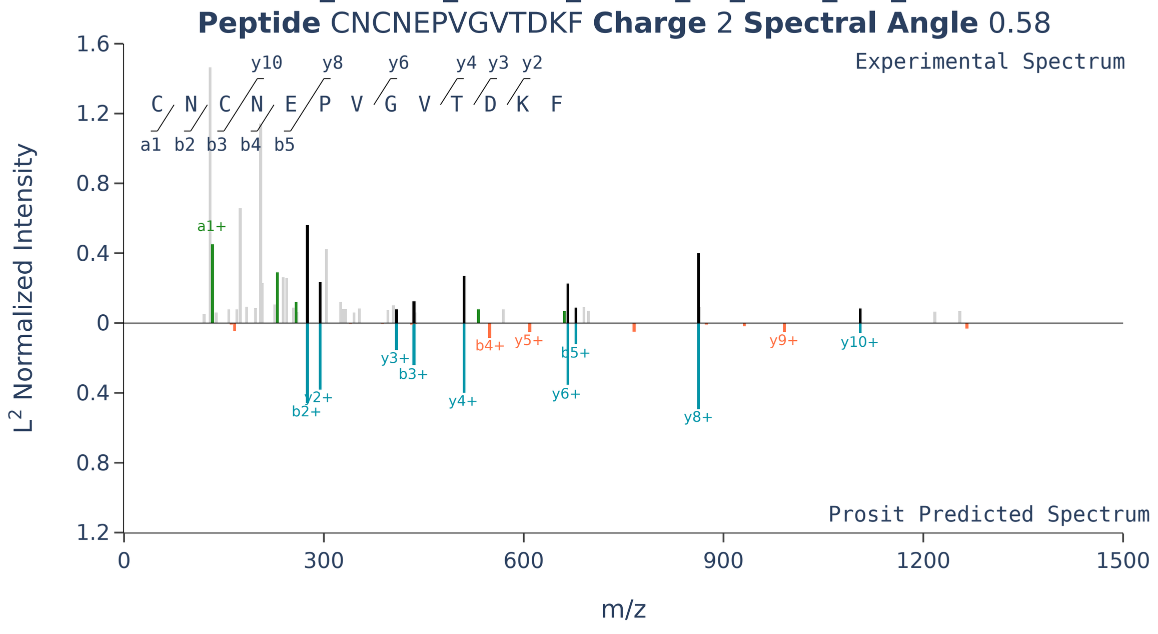


**Source** WSoh_101121_151121_HFGoe_G2_IL37b_24h_R2 **Scan** 10614
**Peptide** KGQSHPSLQLKKEKLM **Charge** 4 **Spectral Angle** 0.82

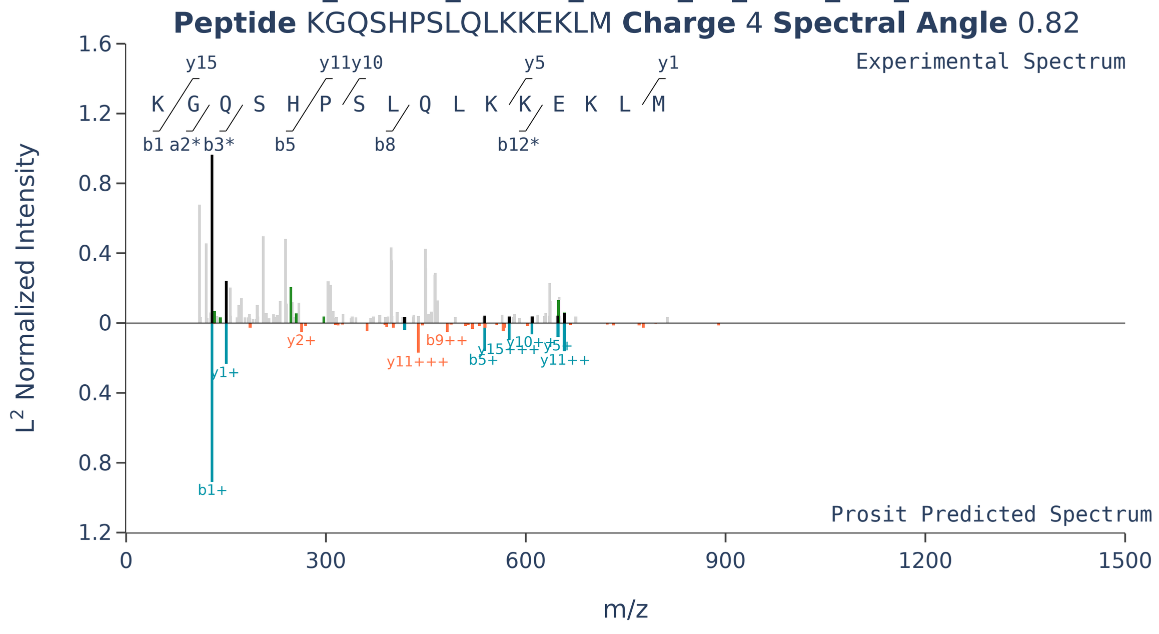


**Source** WSoh_101121_151121_HFGoe_G1_IL37b_24h_R1 **Scan** 26292
**Peptide** VPDKNYIRPEIFF **Charge** 3 **Spectral Angle** 0.85

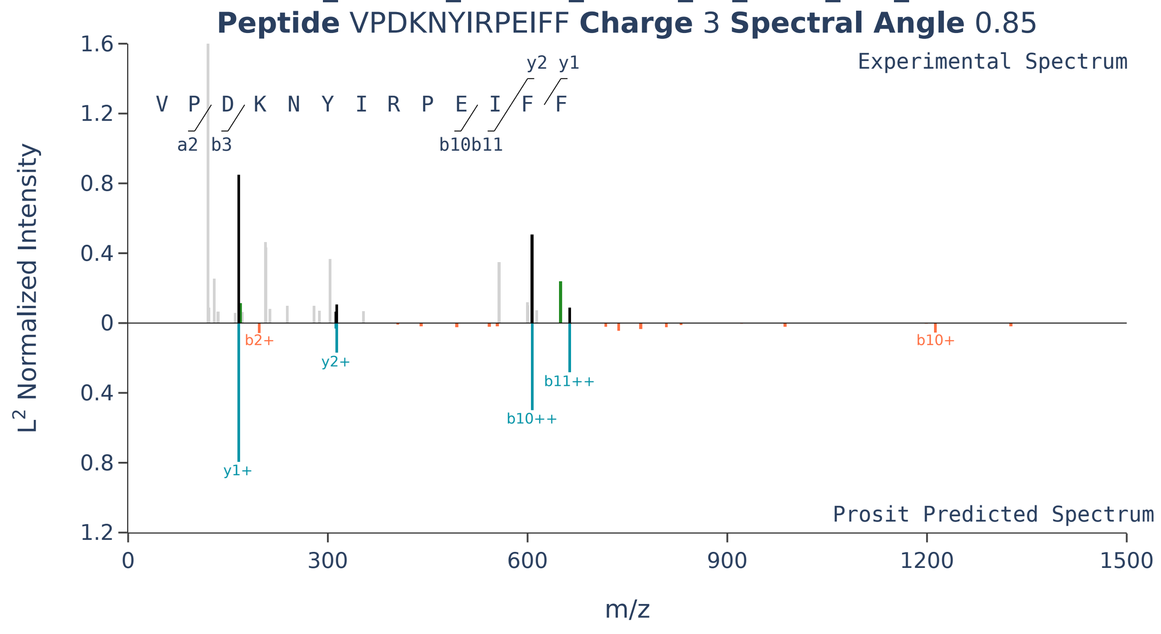


**Source** WSoh_101121_151121_HFGoe_G1_IL37b_4h_R2 **Scan** 20340
**Peptide** ASSLSSASAEKGSPILLGVSKGE **Charge** 3 **Spectral Angle** 0.74

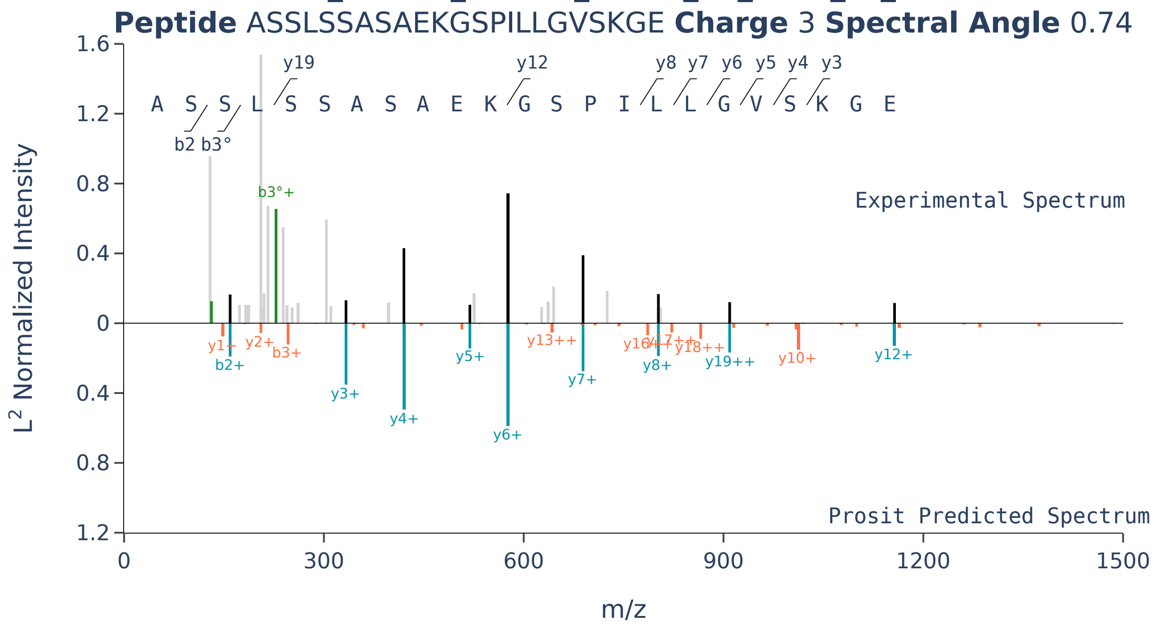


**Source** WSoh_101121_151121_HFGoe_G2_IL37b_2h_R2 **Scan** 20789
**Peptide** LASSLSSASAEKGSPIL **Charge** 2 **Spectral Angle** 0.73

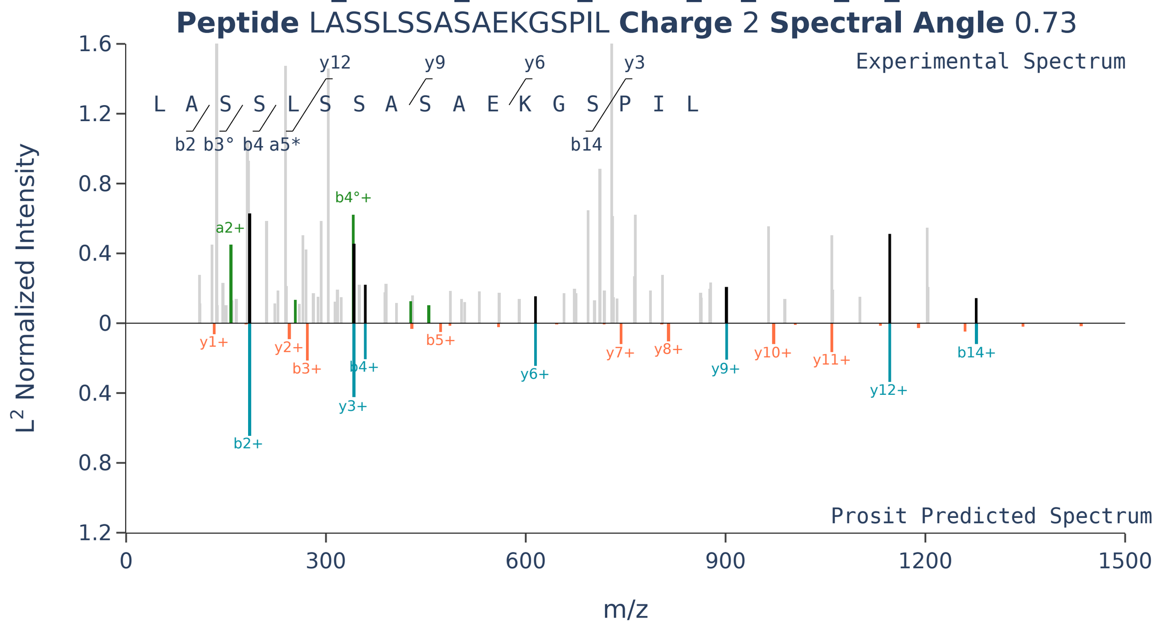


**Source** WSoh_101121_151121_HFGoe_G1_IL37b_24h_R1 **Scan** 16782
**Peptide** ENRKHIEFSFQPVCKAEMSPSE **Charge** 4 **Spectral Angle** 0.78

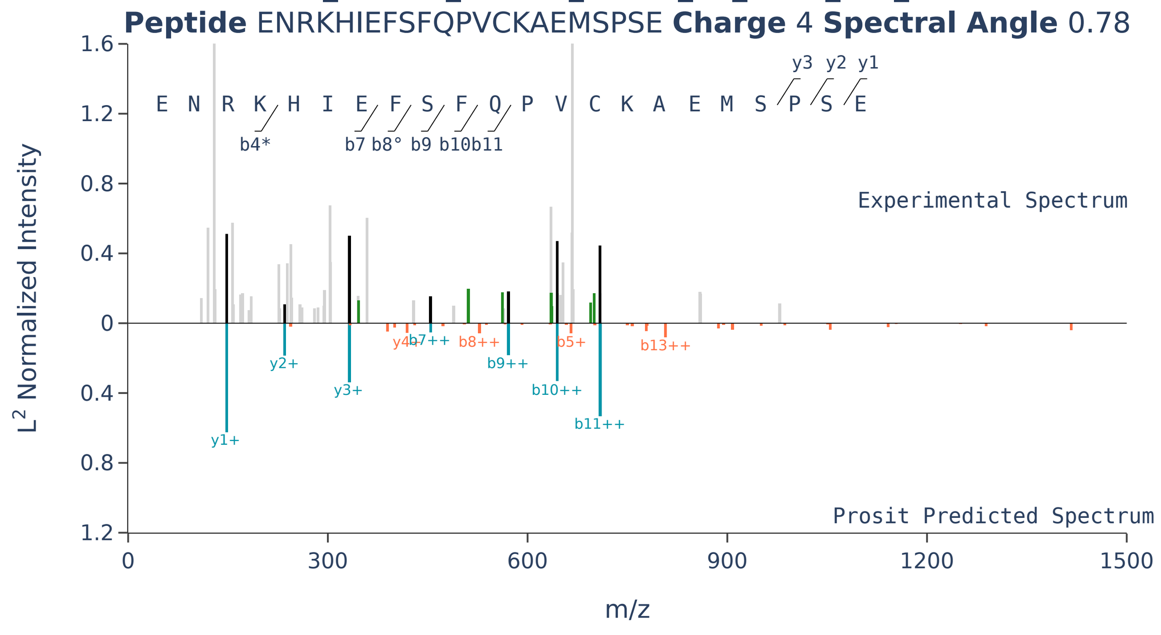


**Source** WSoh_101121_151121_HFGoe_G1_IL37b_24h_R2 **Scan** 24815
**Peptide** LSSASAEKGSPILLGV **Charge** 2 **Spectral Angle** 0.69

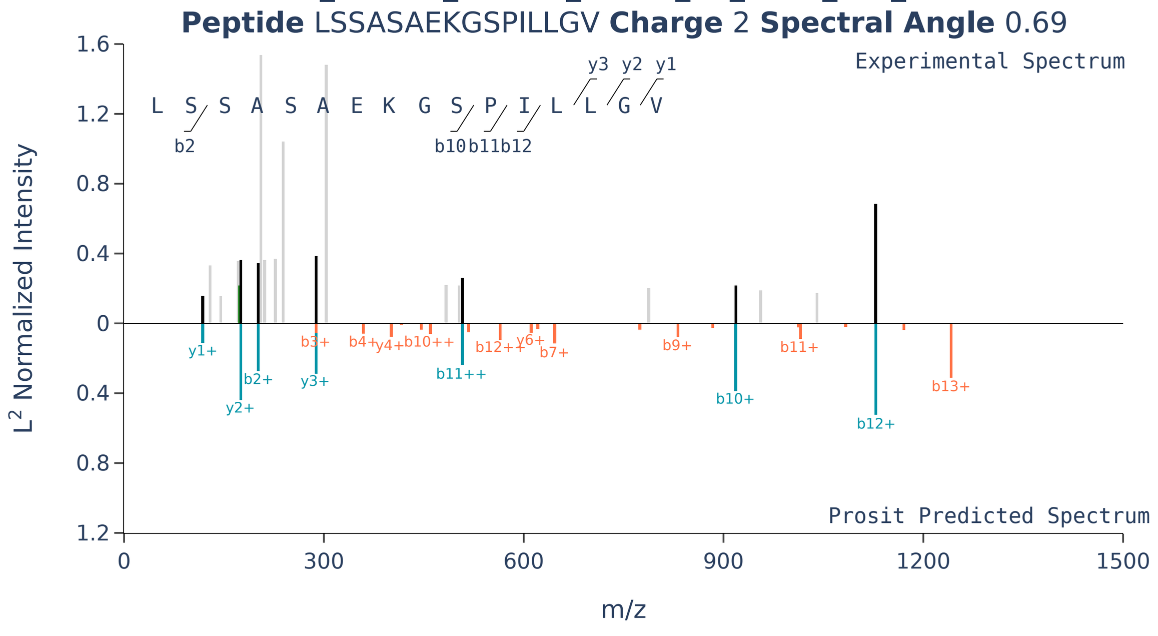


**Source** WSoh_101121_151121_HFGoe_G1_IL37b_4h_R2 **Scan** 15609
**Peptide** GVSKGEFCLYCDKDKGQSHPSL **Charge** 4 **Spectral Angle** 0.78

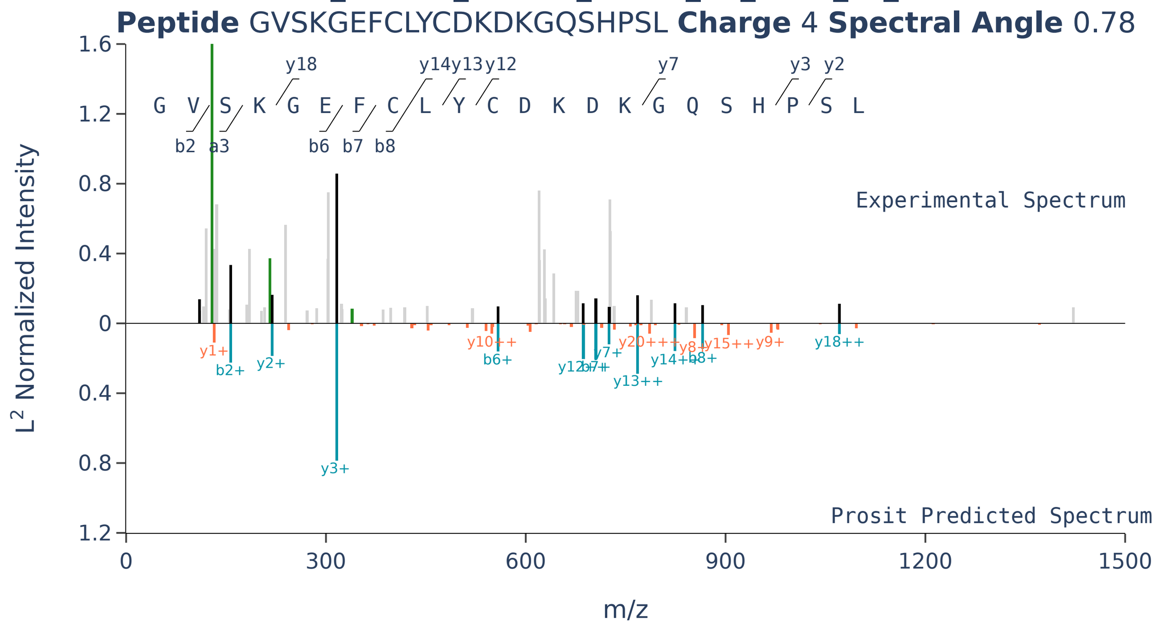


**Source** WSoh_101121_151121_HFGoe_G1_IL37b_24h_R1 **Scan** 9958
**Peptide** DKDKGQSHPSLQLKKEKLMKLAAQKE **Charge** 5 **Spectral Angle** 0.7

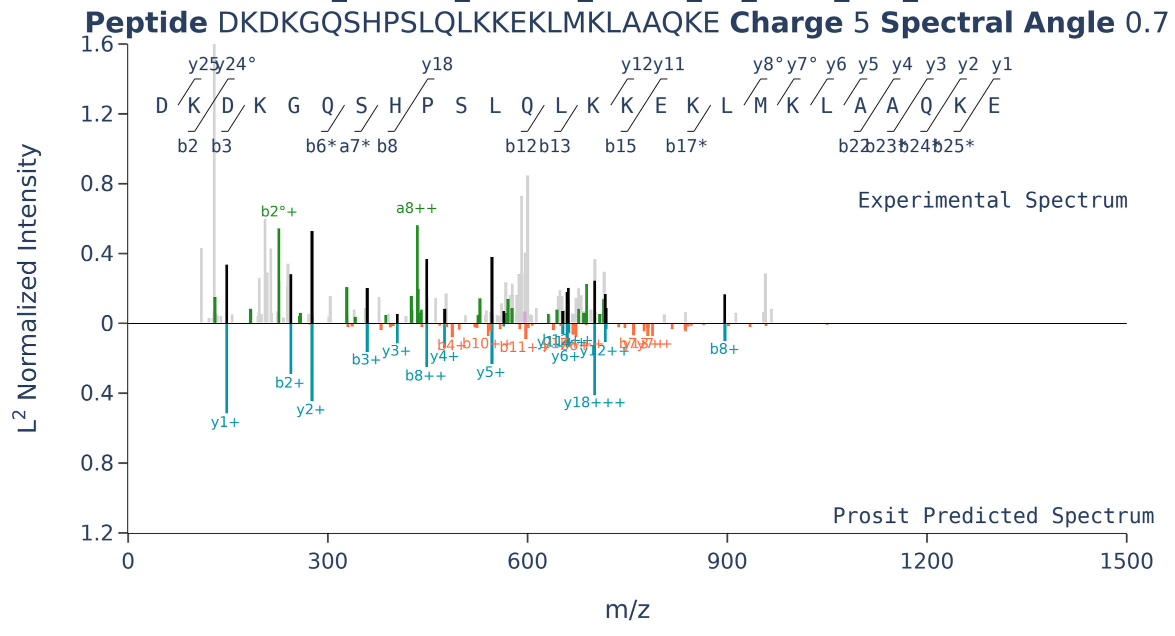


**Source** WSoh_101121_151121_HFGoe_G2_IL37b_24h_R1 **Scan** 6656
**Peptide** QLKKEKLMKLAAQKE **Charge** 4 **Spectral Angle** 0.79

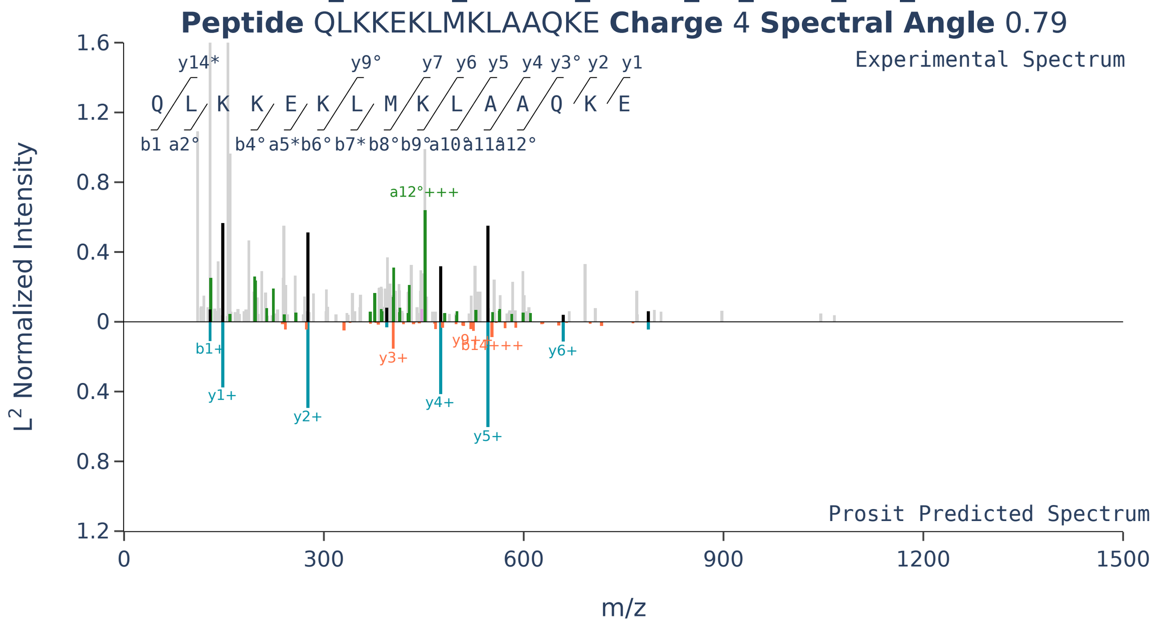


**Source** WSoh_101121_151121_HFGoe_G1_IL37b_24h_R2 **Scan** 7868
**Peptide** QSHPSLQLKKE **Charge** 3 **Spectral Angle** 0.62

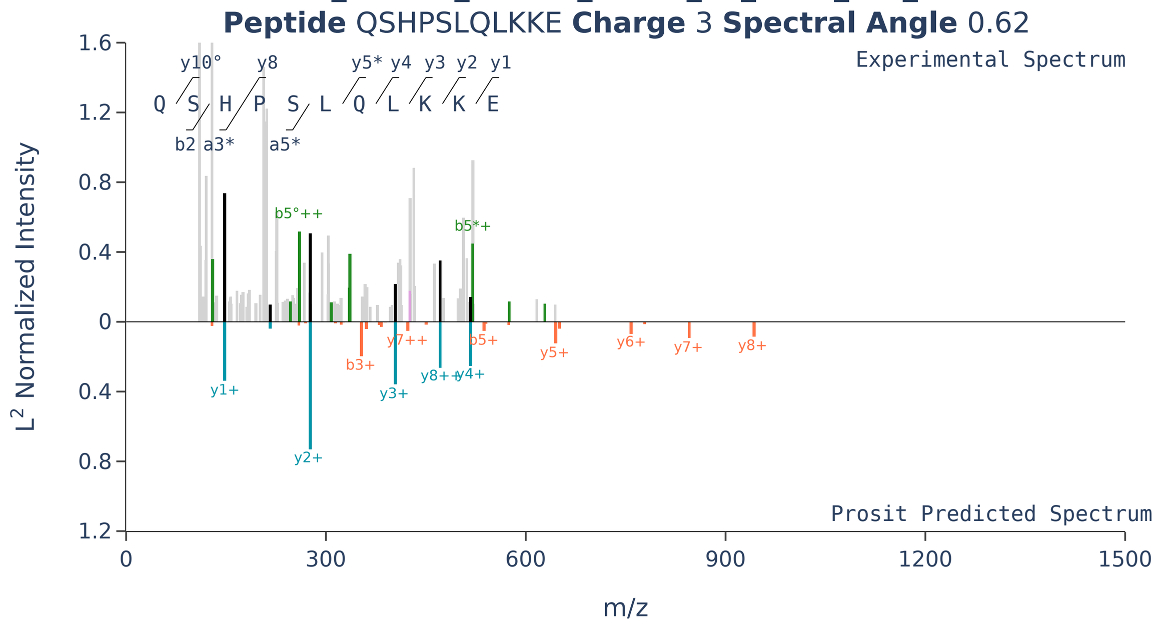


**Source** WSoh_101121_151121_HFGoe_G2_IL37b_4h_R1 **Scan** 14990
**Peptide** KNYIRPEI **Charge** 2 **Spectral Angle** 0.5

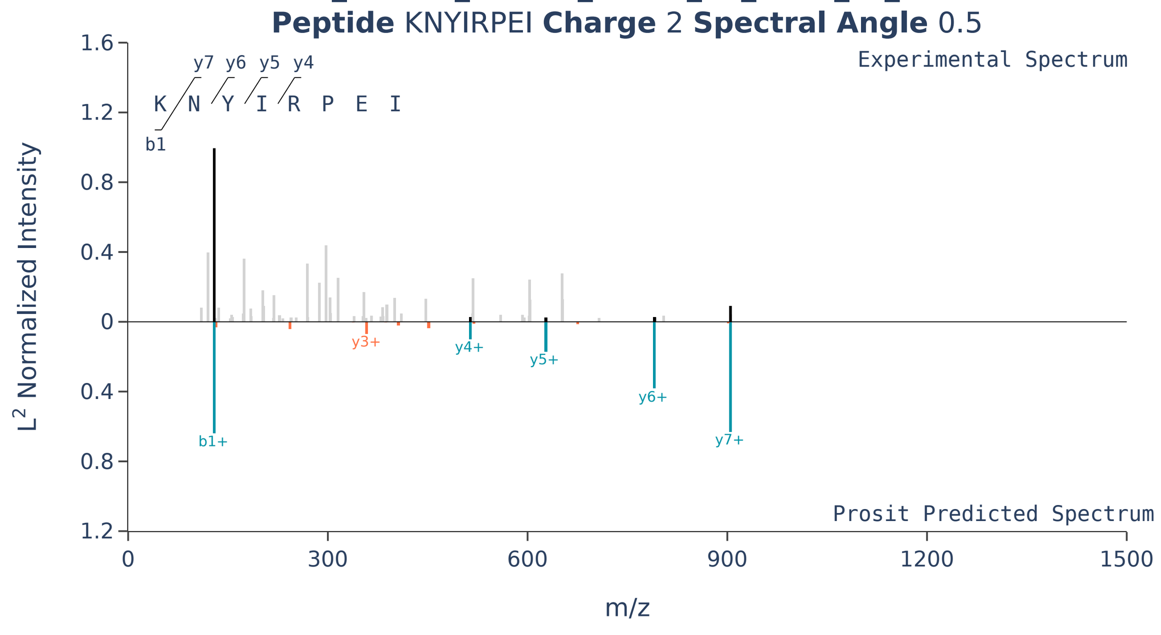


**Source** WSoh_101121_151121_HFGoe_G1_IL37b_24h_R1 **Scan** 21049
**Peptide** LSSASAEKGSPILLG **Charge** 2 **Spectral Angle** 0.76

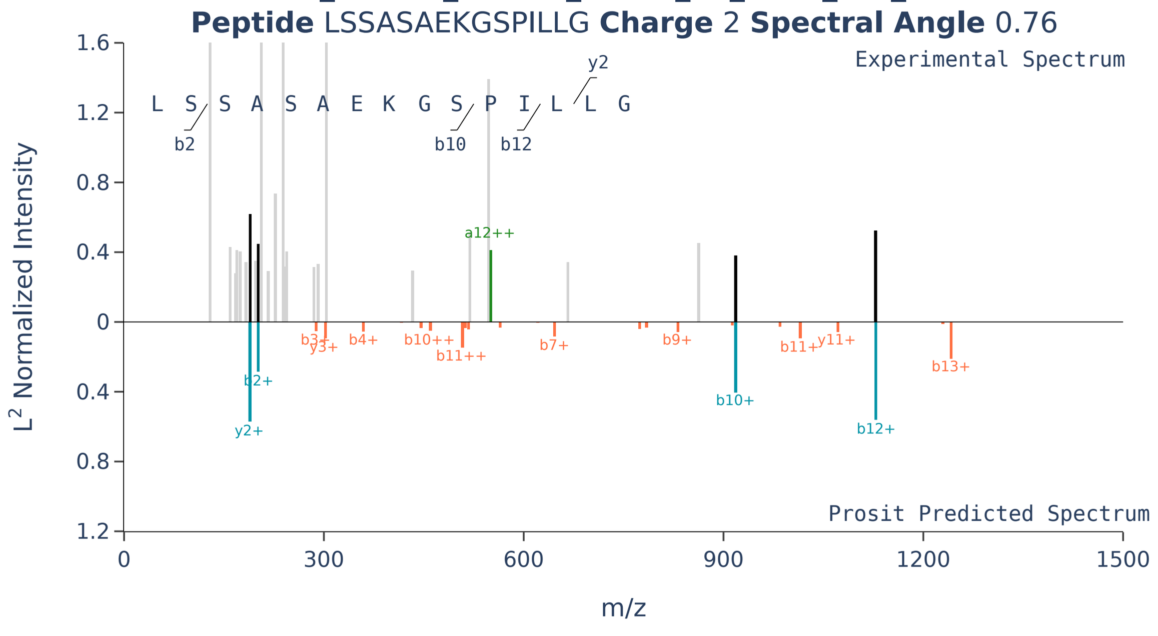


**Source** WSoh_101121_151121_HFGoe_G2_IL37b_24h_R1 **Scan** 24015
**Peptide** IEFSFQPVCKAEMSPSE **Charge** 3 **Spectral Angle** 0.8

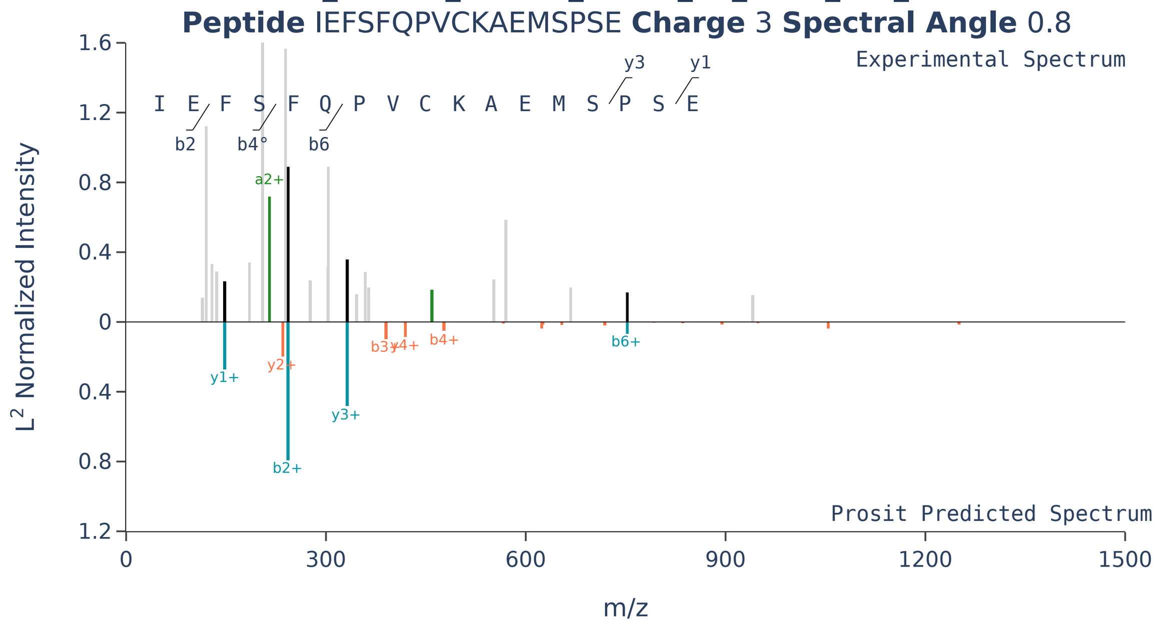


**Source** WSoh_101121_151121_HFGoe_G1_IL37b_4h_R1 **Scan** 11635
**Peptide** SKGEFCLYCDKDKGQSHPSLQLKKEK **Charge** 5 **Spectral Angle** 0.7

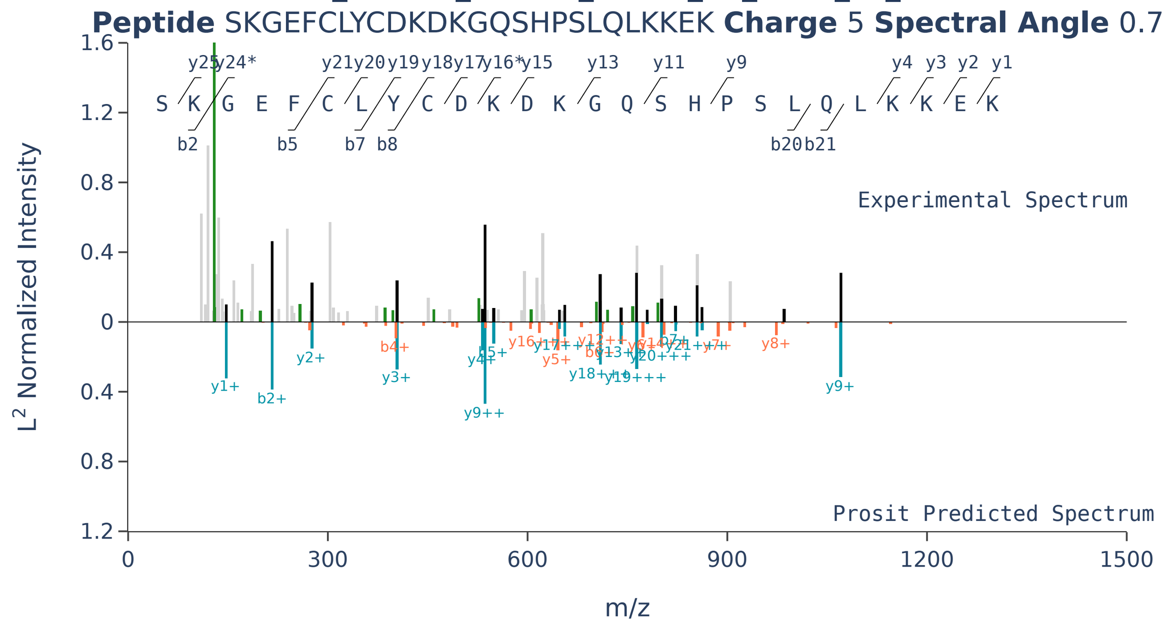

**Source** WSoh_101121_151121_HFGoe_G2_IL37b_24h_R1 **Scan** 13315
**Peptide** SKGEFCLYCDKDKGQSHPSLQ **Charge** 4 **Spectral Angle** 0.78

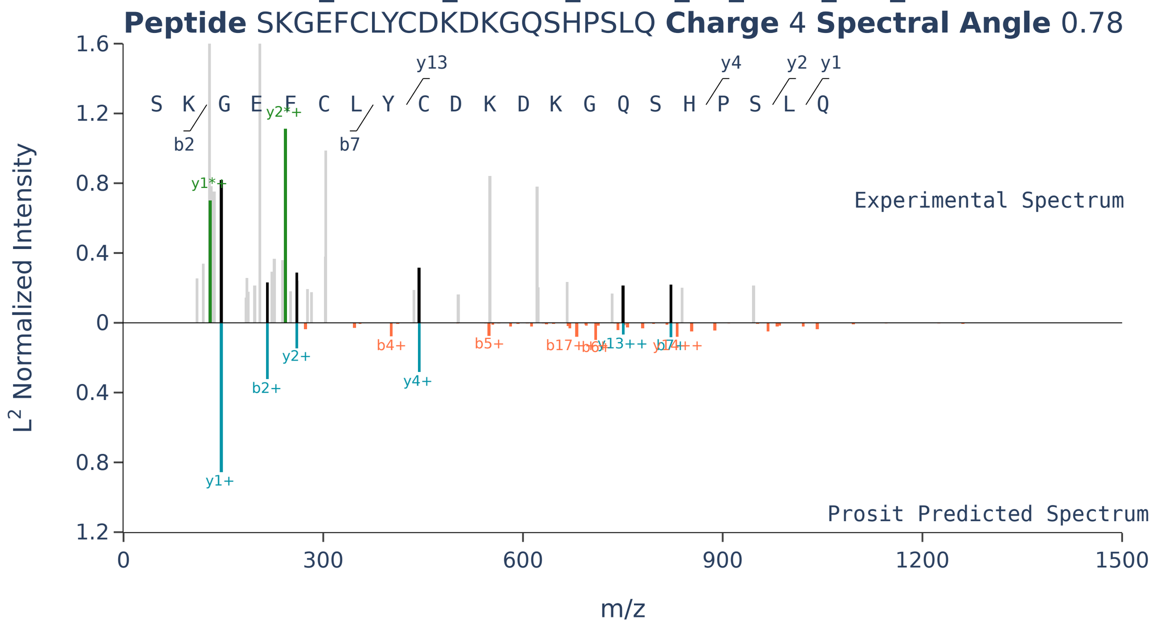


**Source** WSoh_101121_151121_HFGoe_G1_IL37b_4h_R1 **Scan** 19221
**Peptide** ENRKHIEFSFQPVC **Charge** 2 **Spectral Angle** 0.73

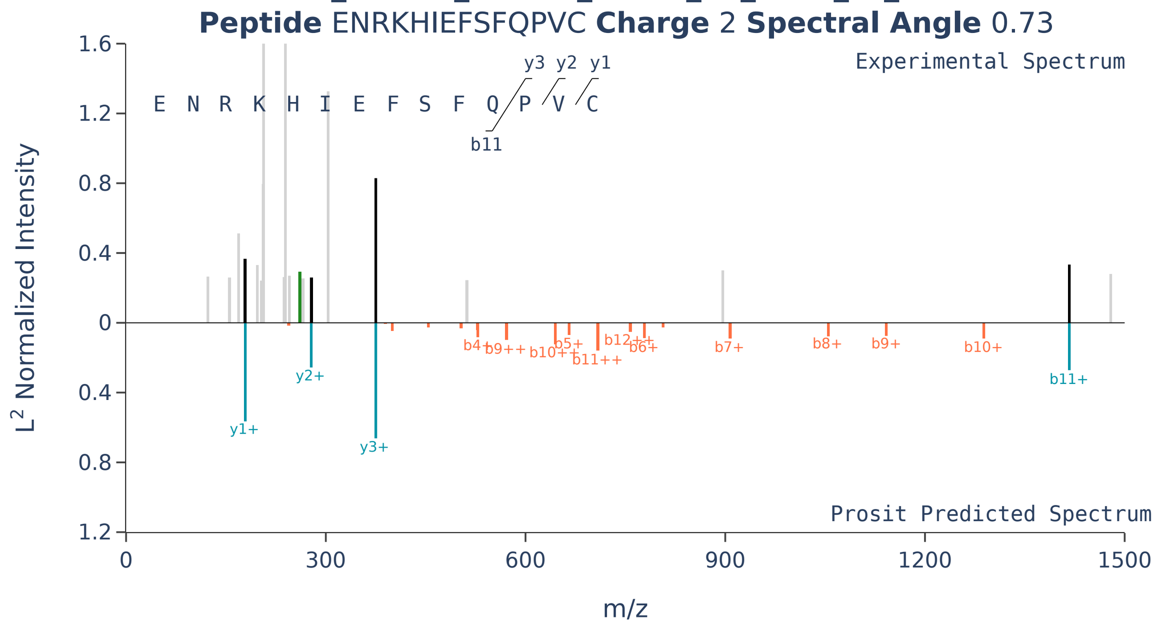


**Source** WSoh_101121_151121_HFGoe_G2_IL37b_2h_R2 **Scan** 21389
**Peptide** FSFQPVCKAEMSPSE **Charge** 2 **Spectral Angle** 0.7

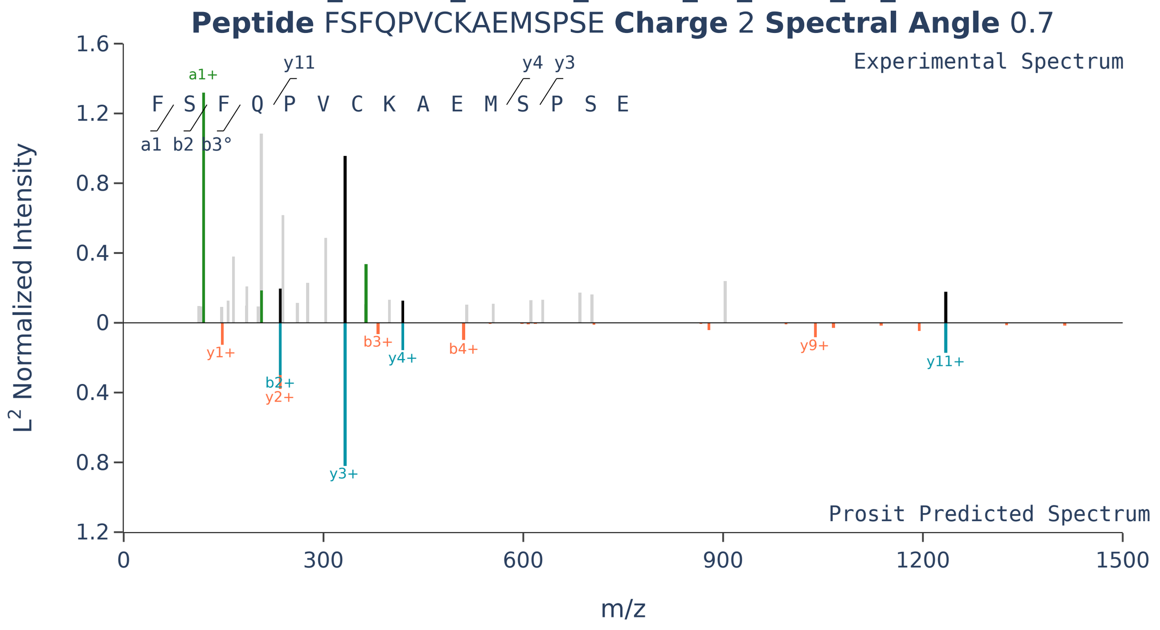


**Source** WSoh_101121_151121_HFGoe_G2_IL37b_24h_R2 **Scan** 7029
**Peptide** HTSPKVKNLNPKKFS **Charge** 4 **Spectral Angle** 0.66

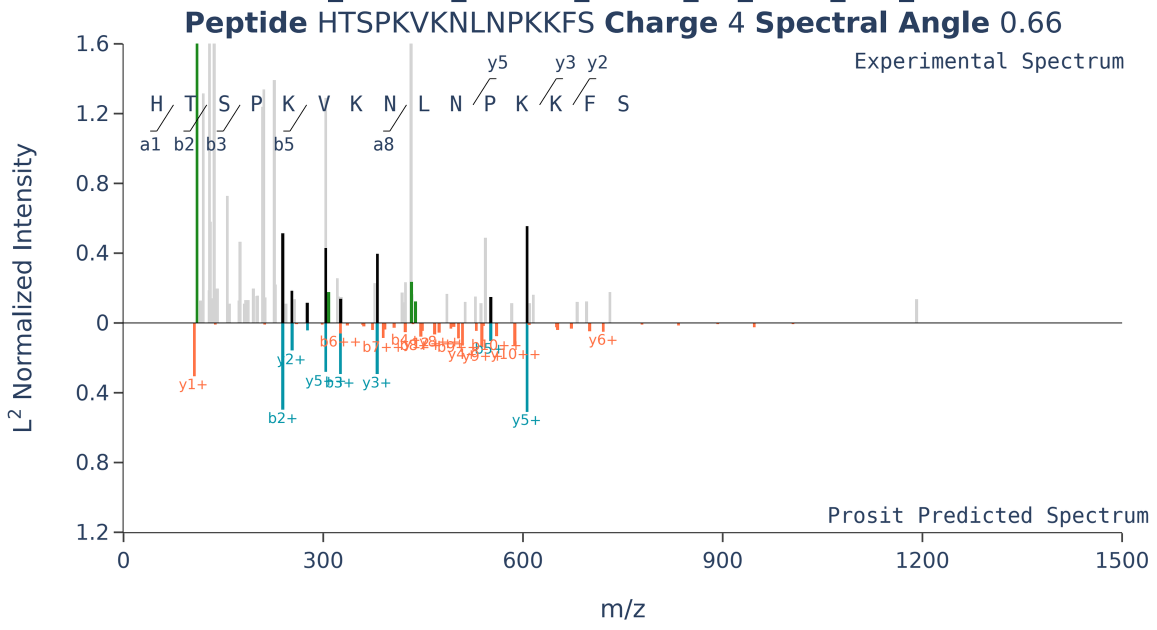


**Source** WSoh_101121_151121_HFGoe_G1_IL37b_24h_R1 **Scan** 25285
**Peptide** LESAAHPGWFICT **Charge** 2 **Spectral Angle** 0.71

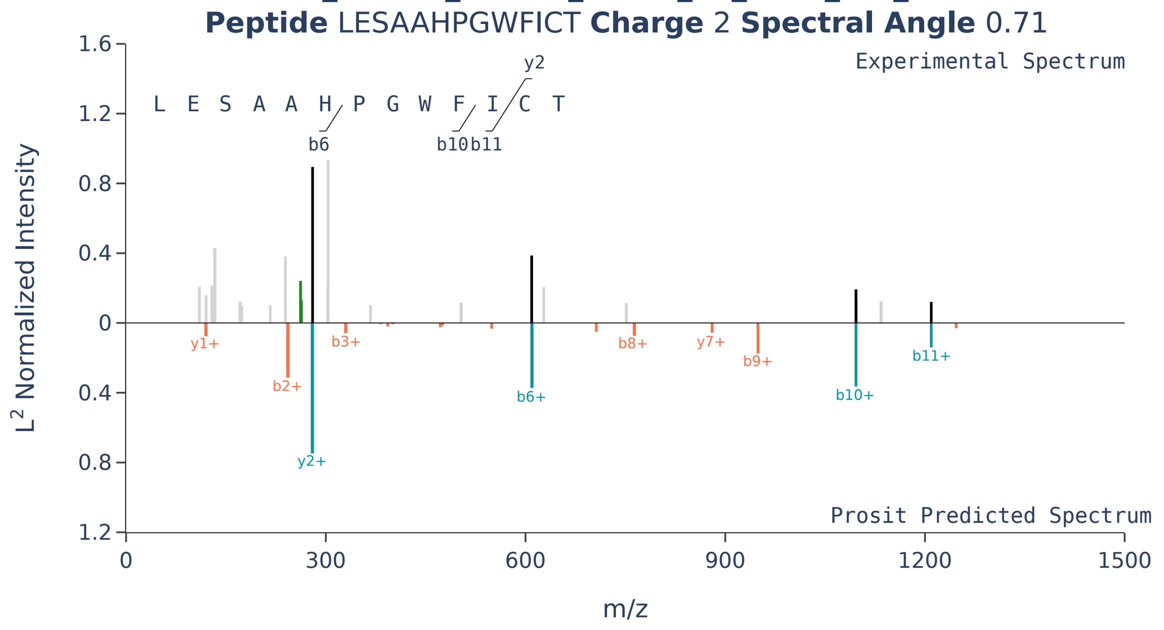


**Source** WSoh_101121_151121_HFGoe_G1_IL37b_1h_R1 **Scan** 6622
**Peptide** CDKDKGQSHPSLQLKKE **Charge** 4 **Spectral Angle** 0.71

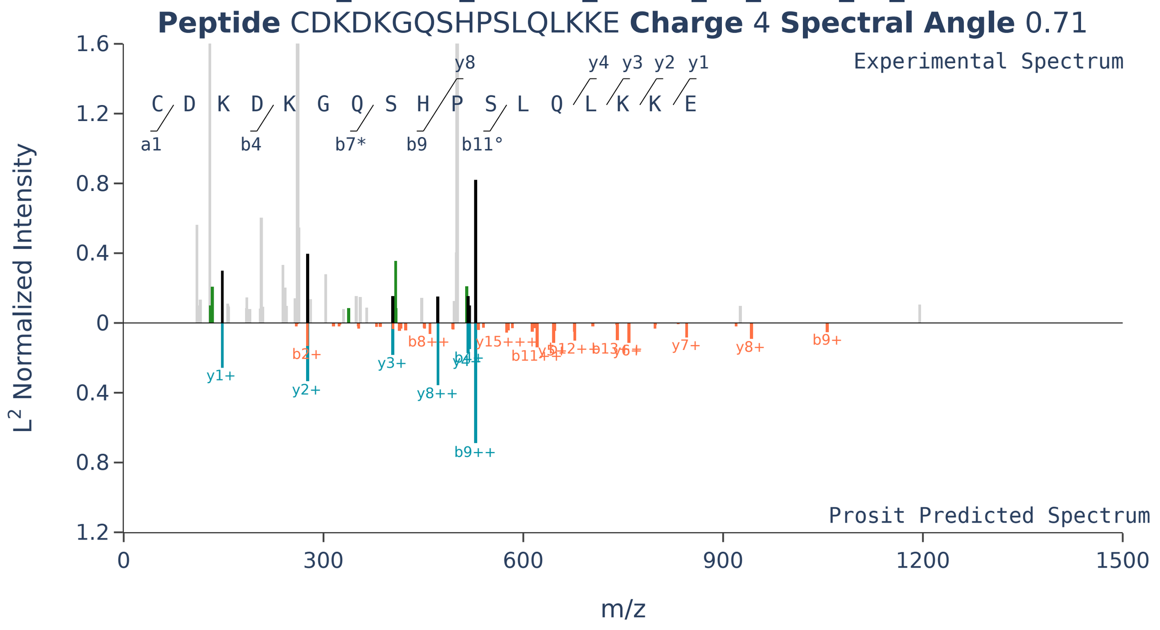


**Source** WSoh_101121_151121_HFGoe_G2_IL37b_24h_R1 **Scan** 9486
**Peptide** PKKFSIHDQDHKVL **Charge** 4 **Spectral Angle** 0.64

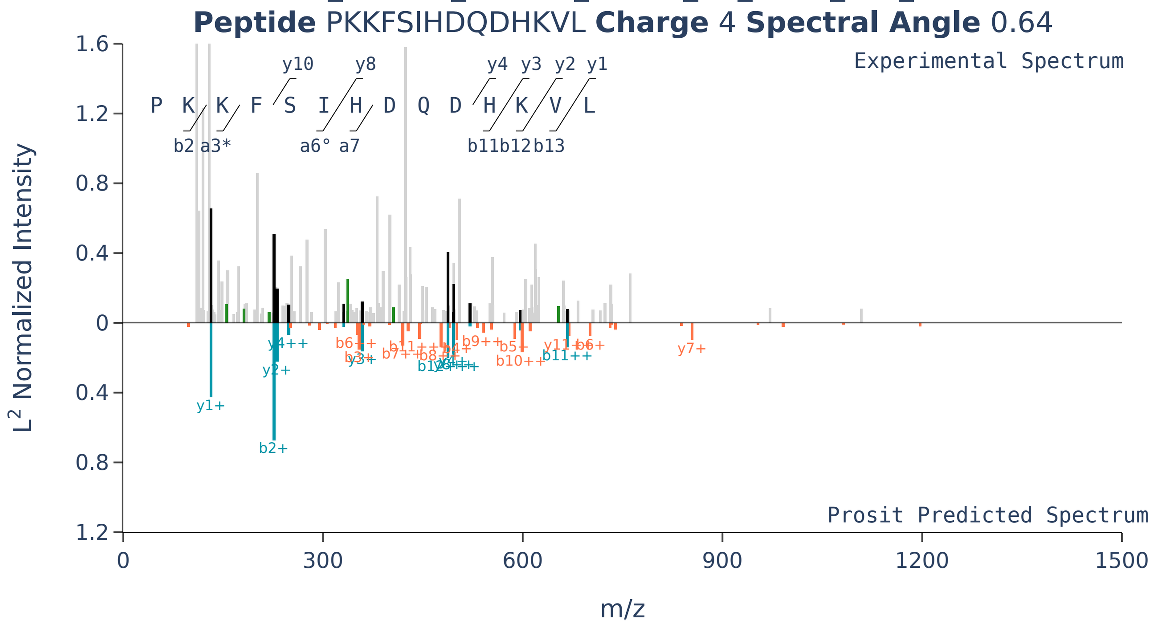


**Source** WSoh_101121_151121_HFGoe_G1_IL37b_24h_R2 **Scan** 11430
**Peptide** LASSLSSASAEKGSP **Charge** 2 **Spectral Angle** 0.62

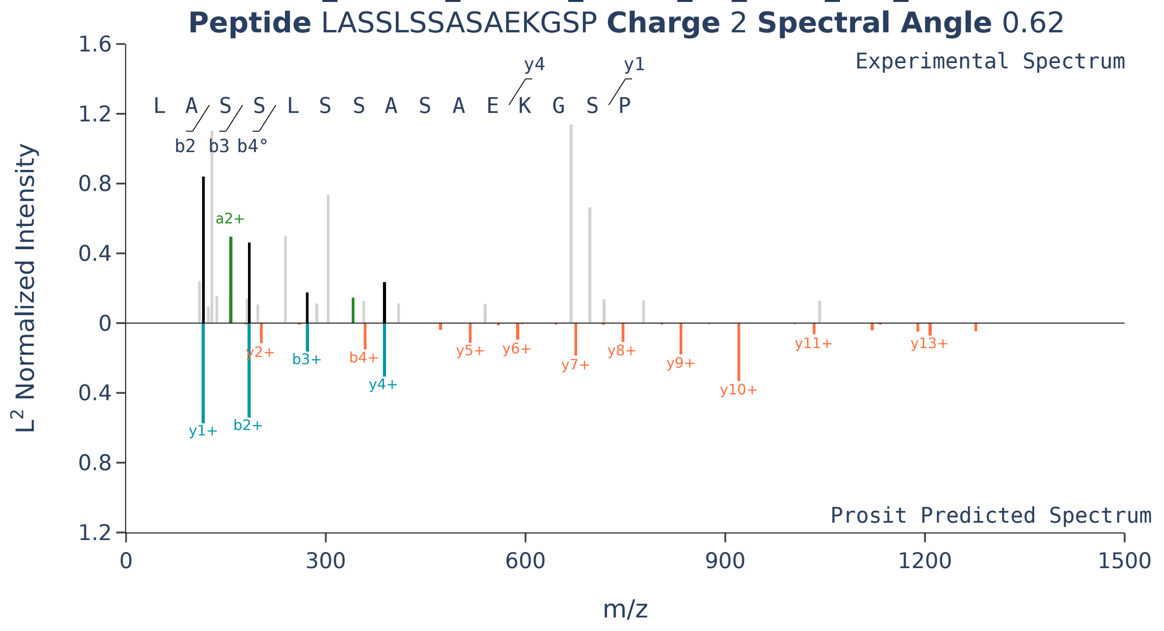


**Source** WSoh_101121_151121_HFGoe_G1_IL37b_24h_R2 **Scan** 18387
**Peptide** VSKGEFCL **Charge** 2 **Spectral Angle** 0.18

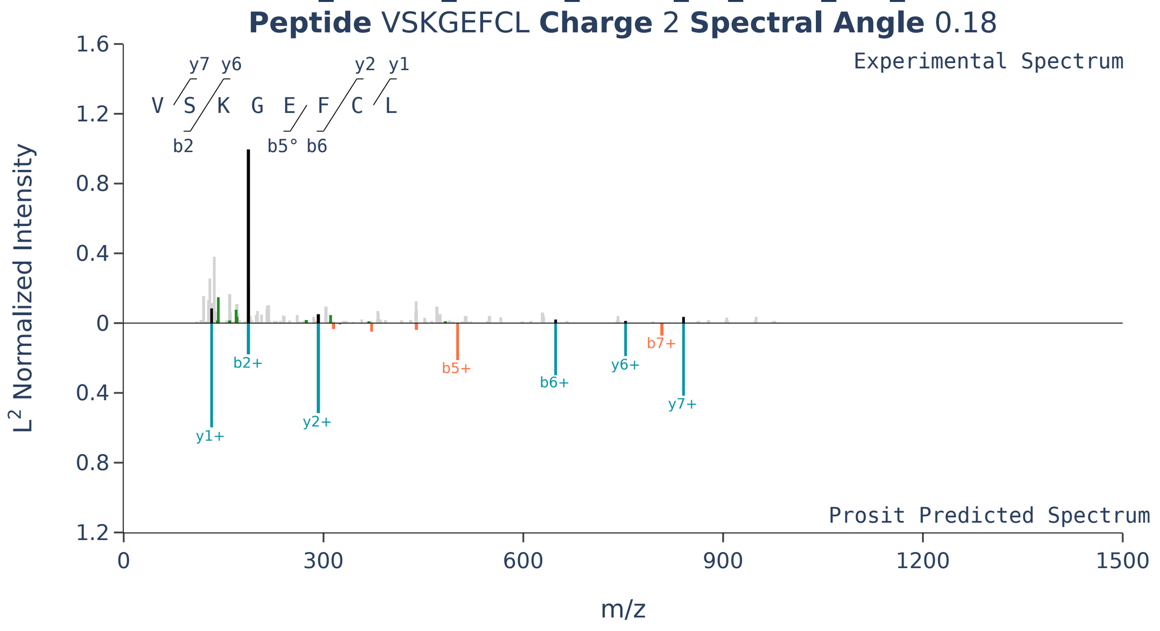


**Source** WSoh_101121_151121_HFGoe_G2_IL37b_2h_R1 **Scan** 28163
**Peptide** SGNLIAVPDKNYIRPEIFF **Charge** 3 **Spectral Angle** 0.74

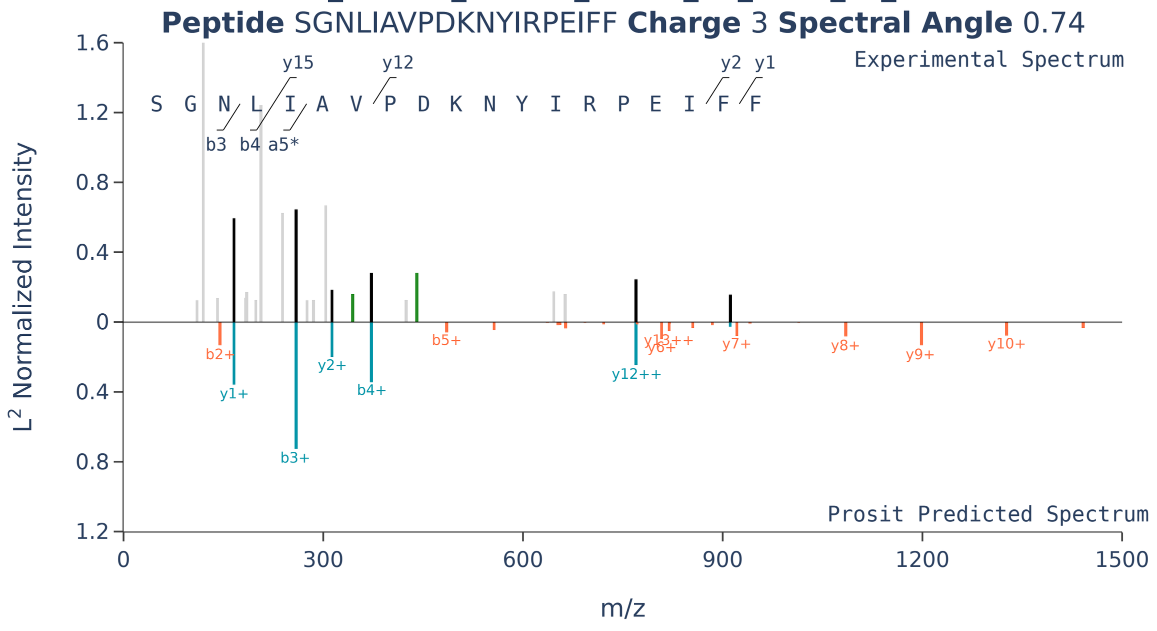


**Source** WSoh_101121_151121_HFGoe_G1_IL37b_24h_R1 **Scan** 25571
**Peptide** SSLSSASAEKGSPILLGV **Charge** 2 **Spectral Angle** 0.66

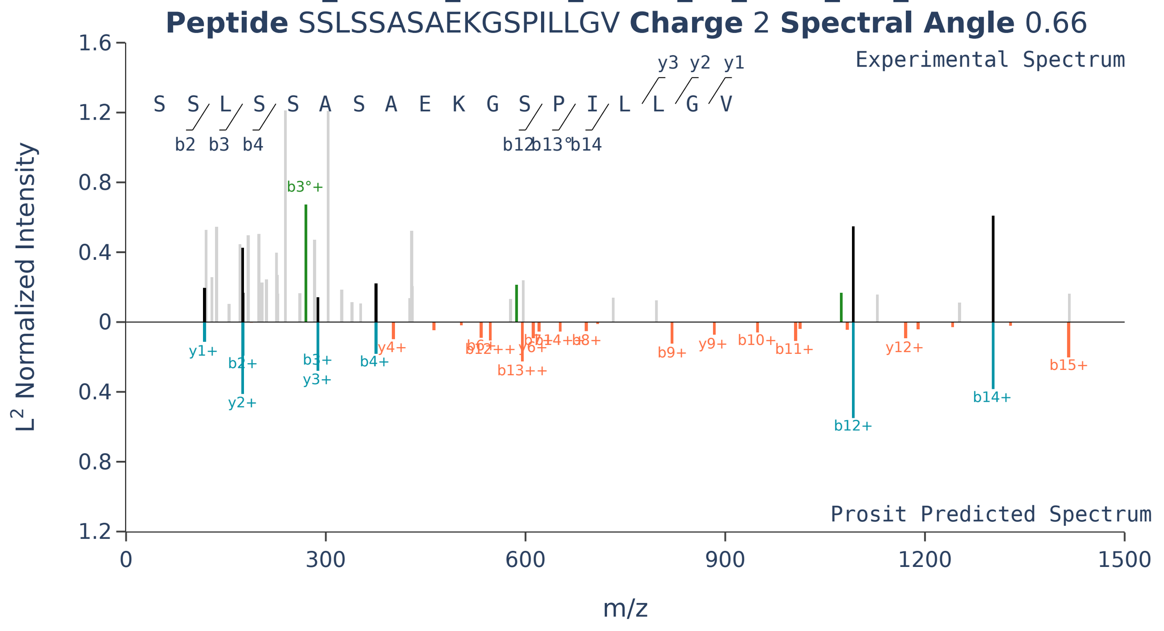


**Source** WSoh_101121_151121_HFGoe_G2_IL37b_2h_R2 **Scan** 12967
**Peptide** DKGQSHPSLQL **Charge** 2 **Spectral Angle** 0.5

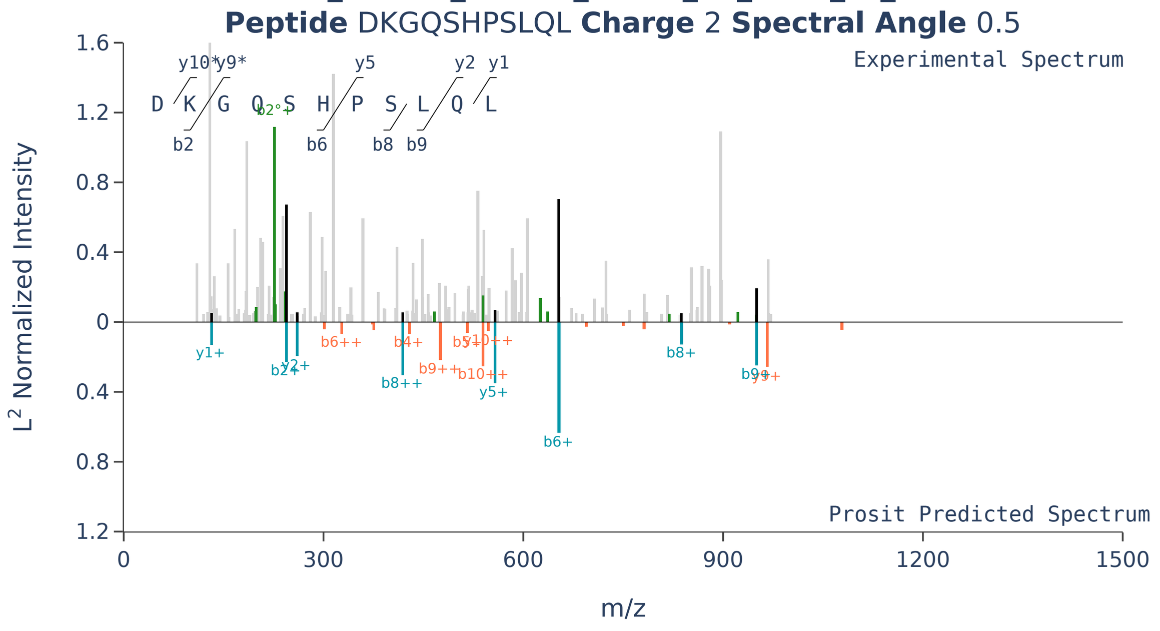


**Source** WSoh_101121_151121_HFGoe_G1_IL37b_4h_R1 **Scan** 8909
**Peptide** QLKKEKLMKLAAQKES **Charge** 4 **Spectral Angle** 0.59

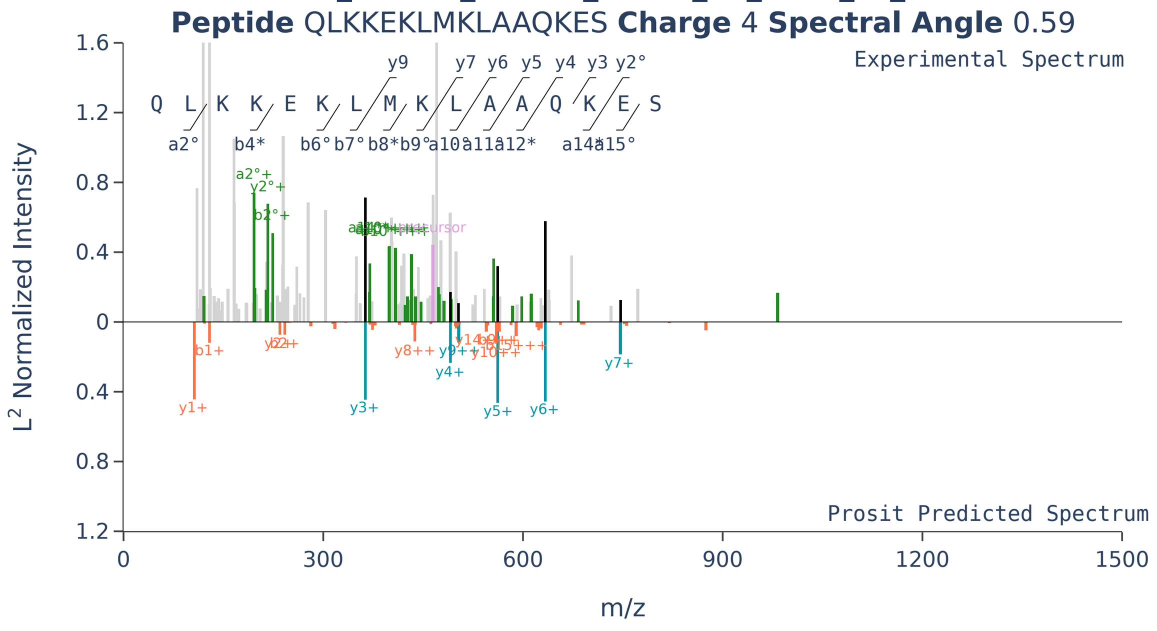


**Source** WSoh_101121_151121_HFGoe_G1_IL37b_24h_R1 **Scan** 19864
**Peptide** SSLSSASAEKGSPILLGVSKG **Charge** 3 **Spectral Angle** 0.64

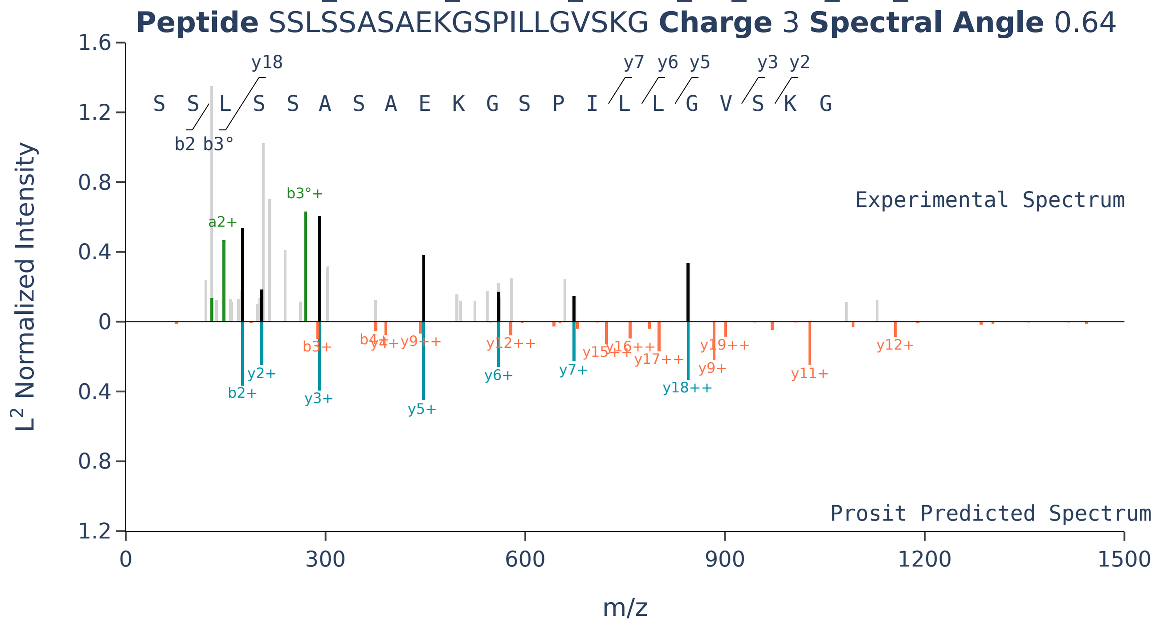


**Source** WSoh_101121_151121_HFGoe_G2_IL37b_4h_R2 **Scan** 18793
**Peptide** LSSASAEKGSPILLGVSK **Charge** 3 **Spectral Angle** 0.52

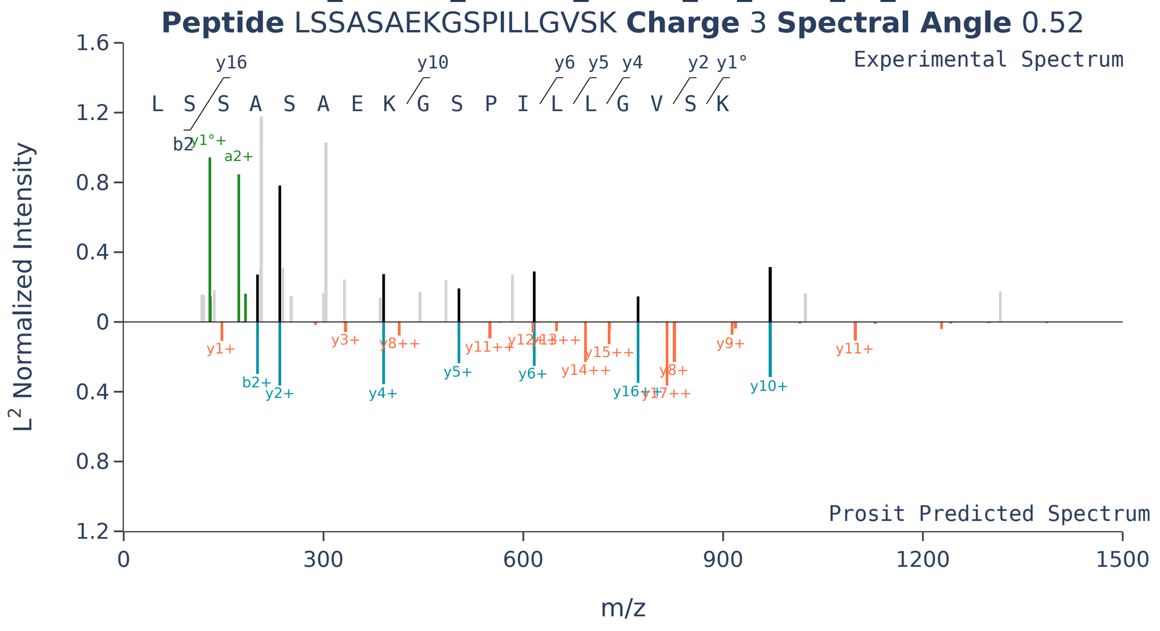

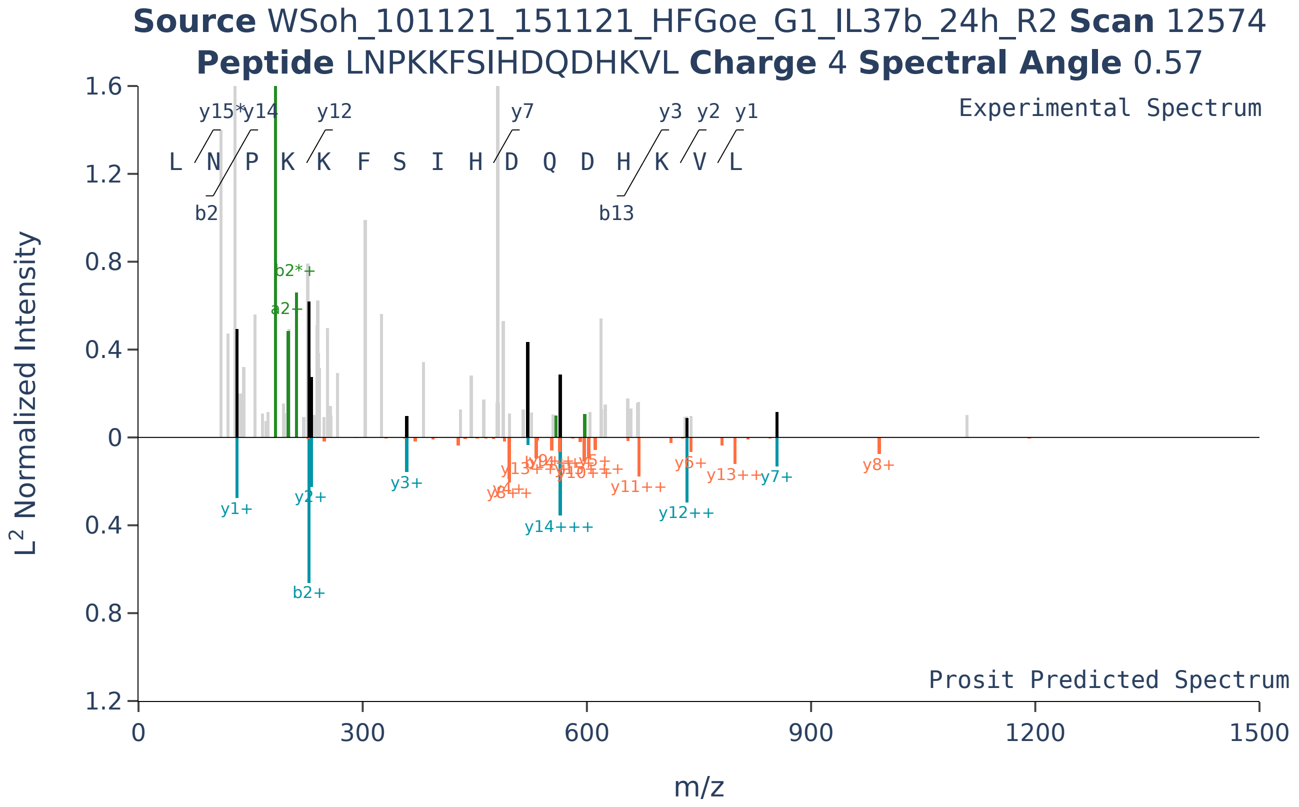
Source WSoh_101121_151121_HFGoe_G1_IL37b_24h_R2 Scan 12574
Peptide LNPKKFSIHDQDHKVL Charge 4 Spectral Angle 0.57
Experimental Spectrum
Prosit Predicted Spectrum
L2 Normalized Intensity
m/z

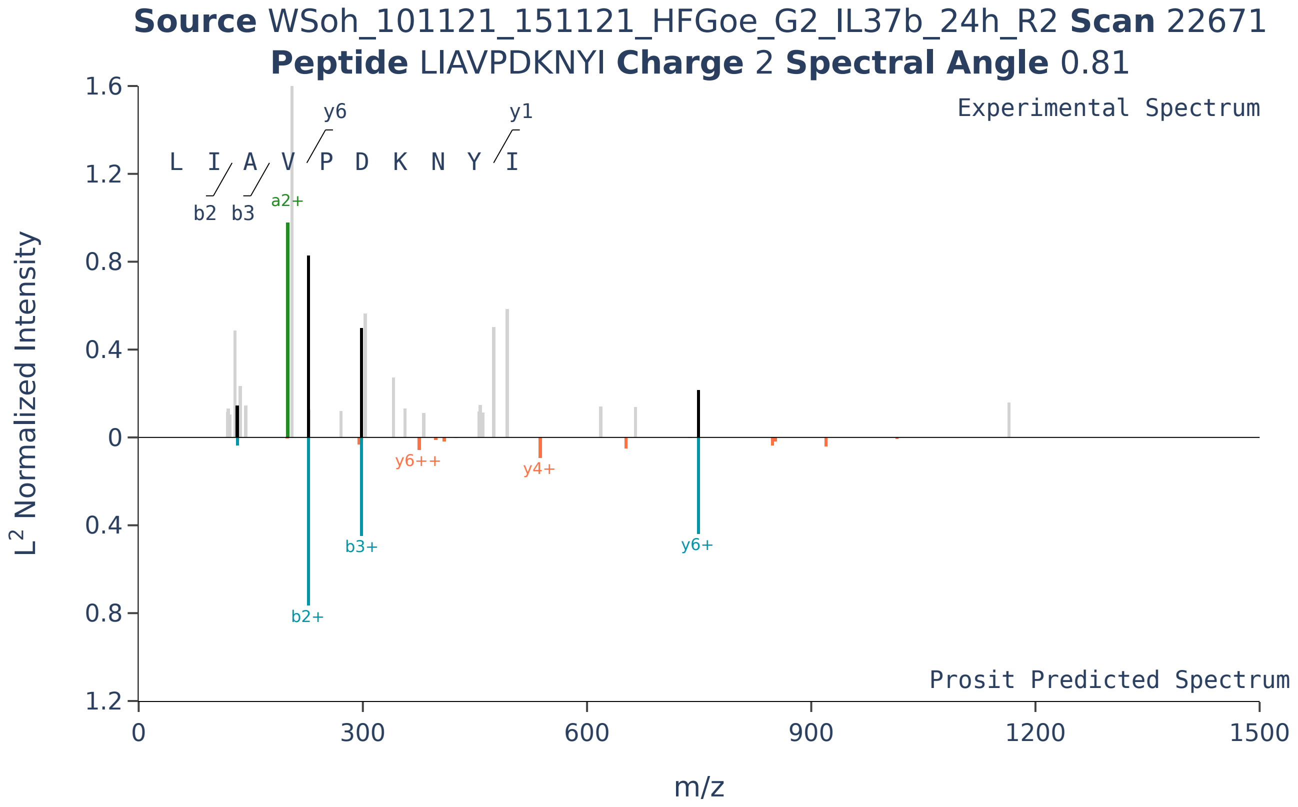
Source WSoh_101121_151121_HFGoe_G2_IL37b_24h_R2 Scan 22671
Peptide LIAVPDKNYI Charge 2 Spectral Angle 0.81
Experimental Spectrum
Prosit Predicted Spectrum
L2 Normalized Intensity
m/z

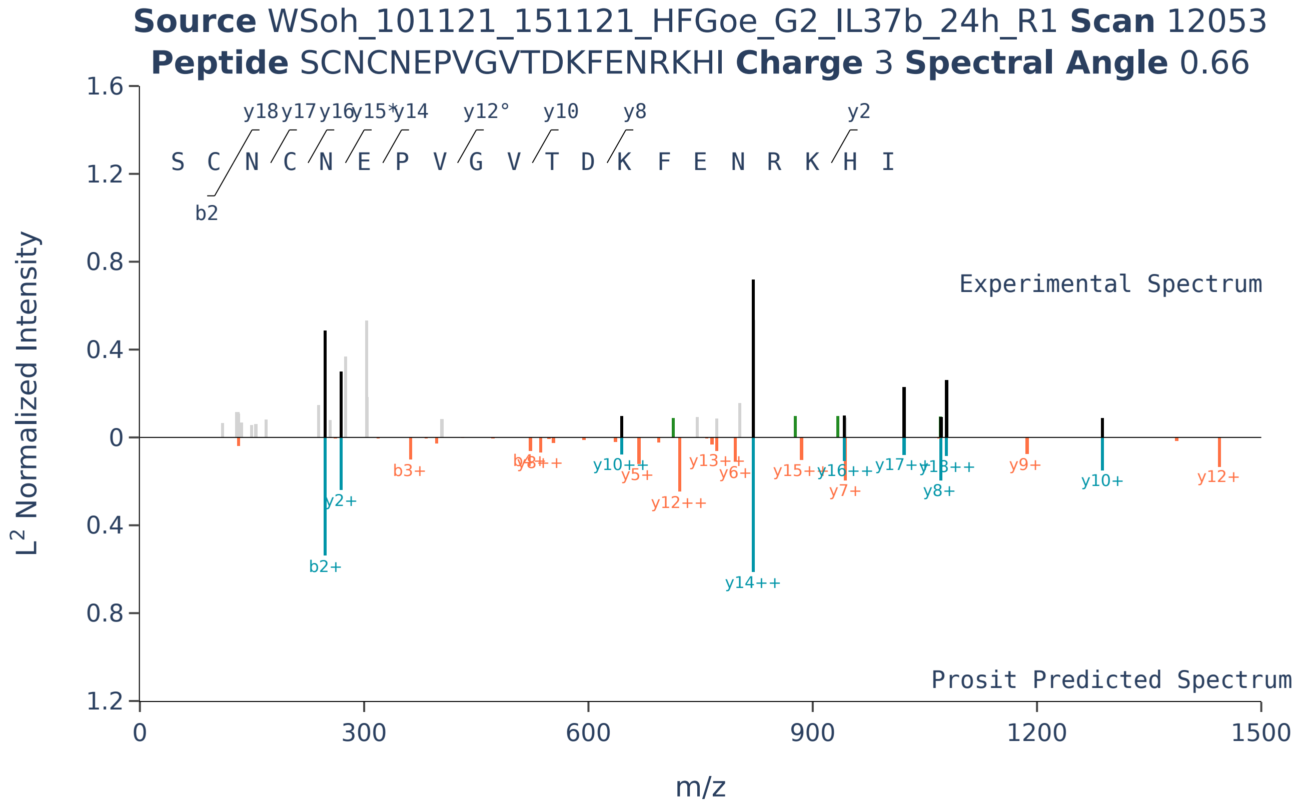
Source WSoh_101121_151121_HFGoe_G2_IL37b_24h_R1 Scan 12053
Peptide SCNCNEPVGVTDKFENRKHI Charge 3 Spectral Angle 0.66
Experimental Spectrum
Prosit Predicted Spectrum
L2 Normalized Intensity
m/z

**Source** HRoetschke_271222_090123_HFGoe_LEDGF_b1R1_2h **Scan** 17973
**Peptide** ASLQVTMQ **Charge** 2 **Spectral Angle** 0.98

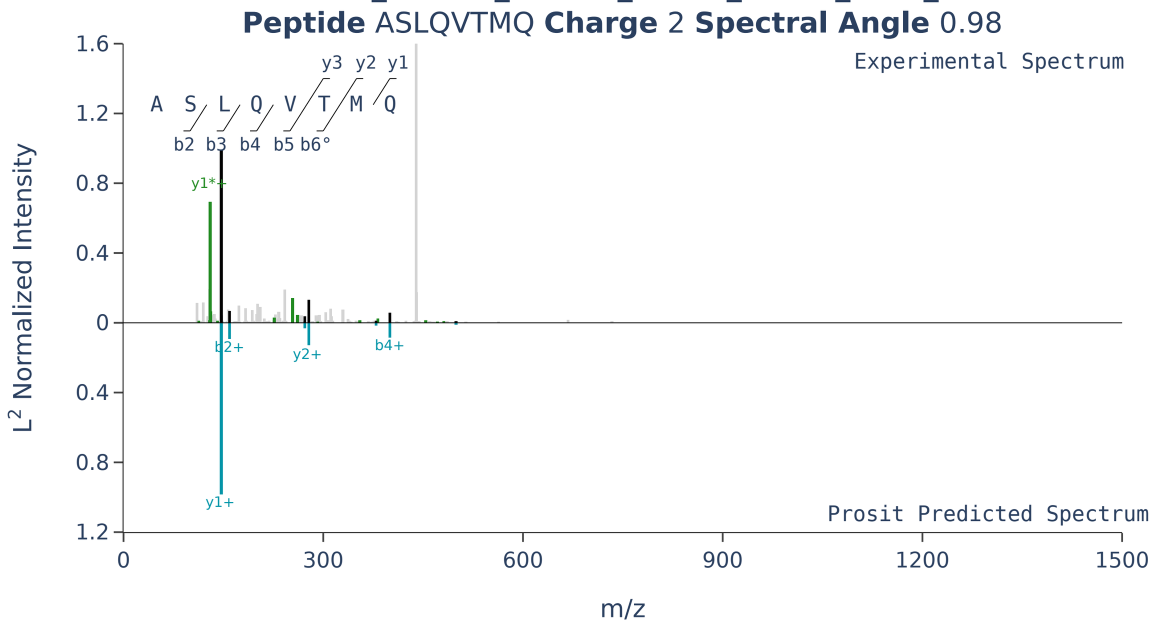


**Source** HRoetschke_271222_090123_HFGoe_LEDGF_b2R2_24h **Scan** 9135
**Peptide** SQVIMEKS **Charge** 1 **Spectral Angle** 0.97

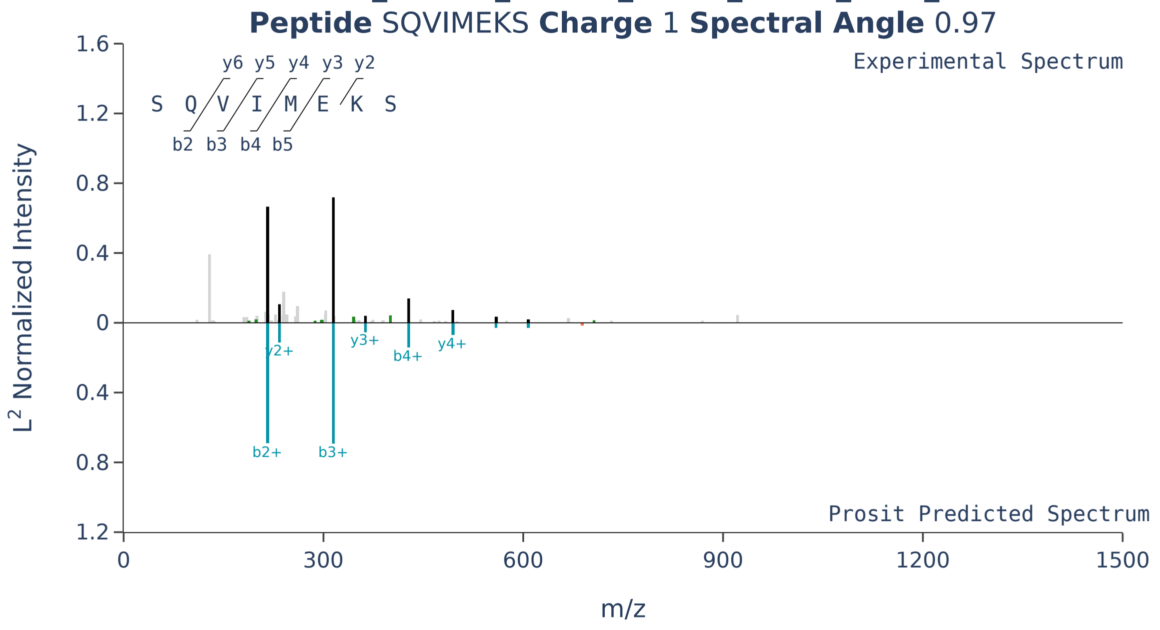


**Source** HRoetschke_271222_090123_HFGoe_LEDGF_b2R2_24h **Scan** 13023
**Peptide** TEISLKD **Charge** 2 **Spectral Angle** 0.94

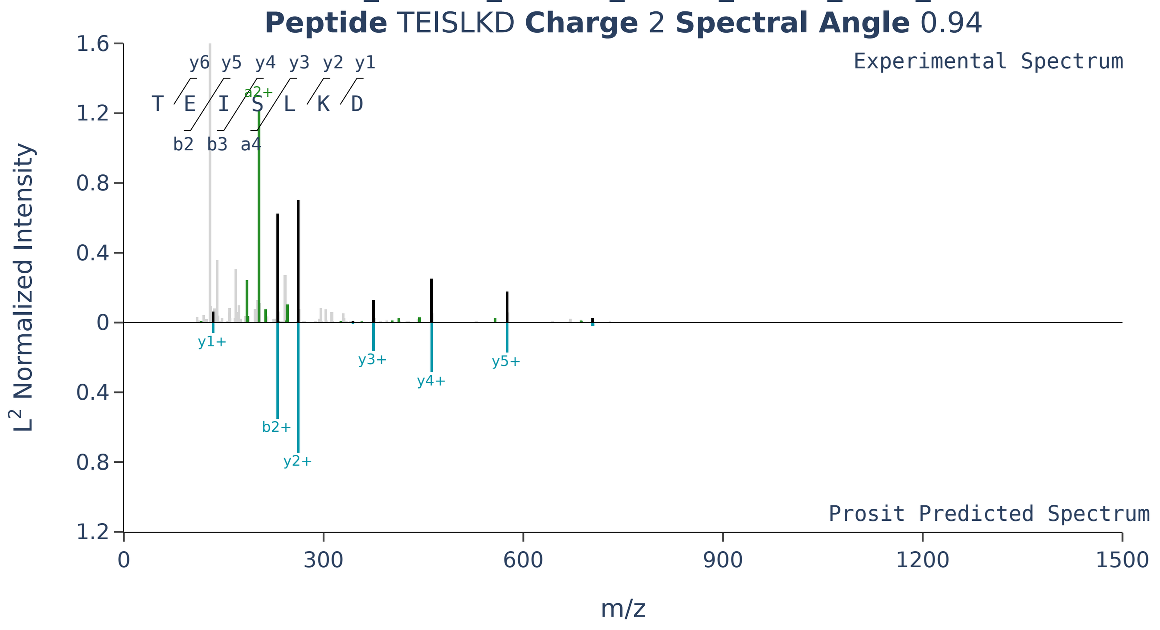


**Source** HRoetschke_271222_090123_HFGoe_LEDGF_b1R2_2h **Scan** 7900
**Peptide** LNKSLAE **Charge** 2 **Spectral Angle** 0.97

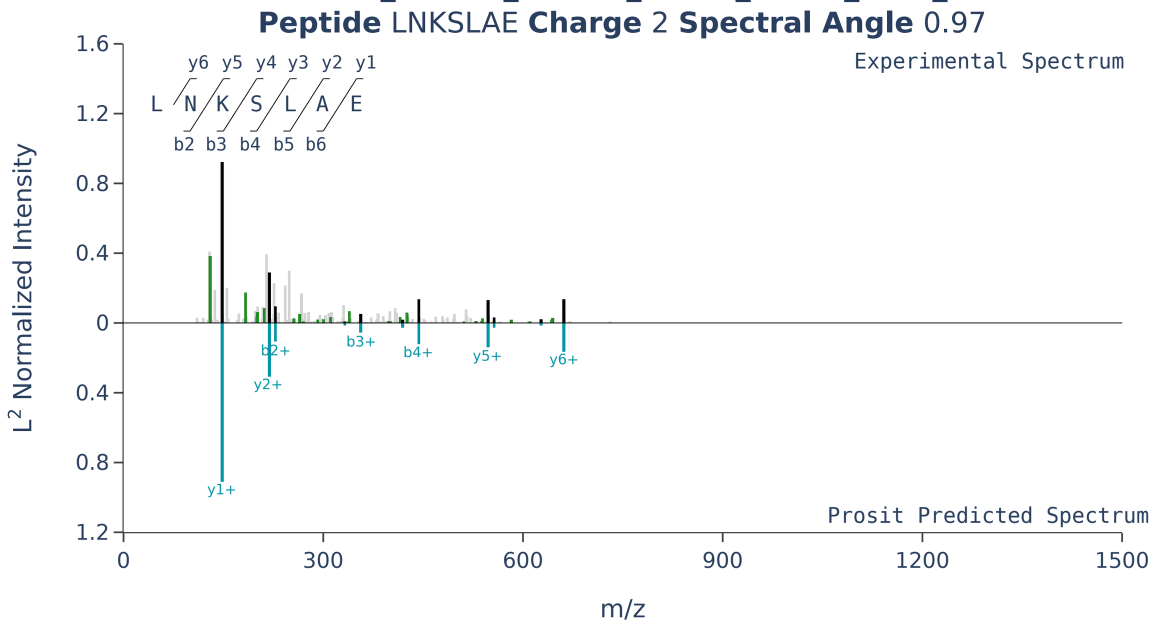


**Source** HRoetschke_271222_090123_HFGoe_LEDGF_b2R1_24h **Scan** 13308
**Peptide** VDITTPKA **Charge** 2 **Spectral Angle** 0.96

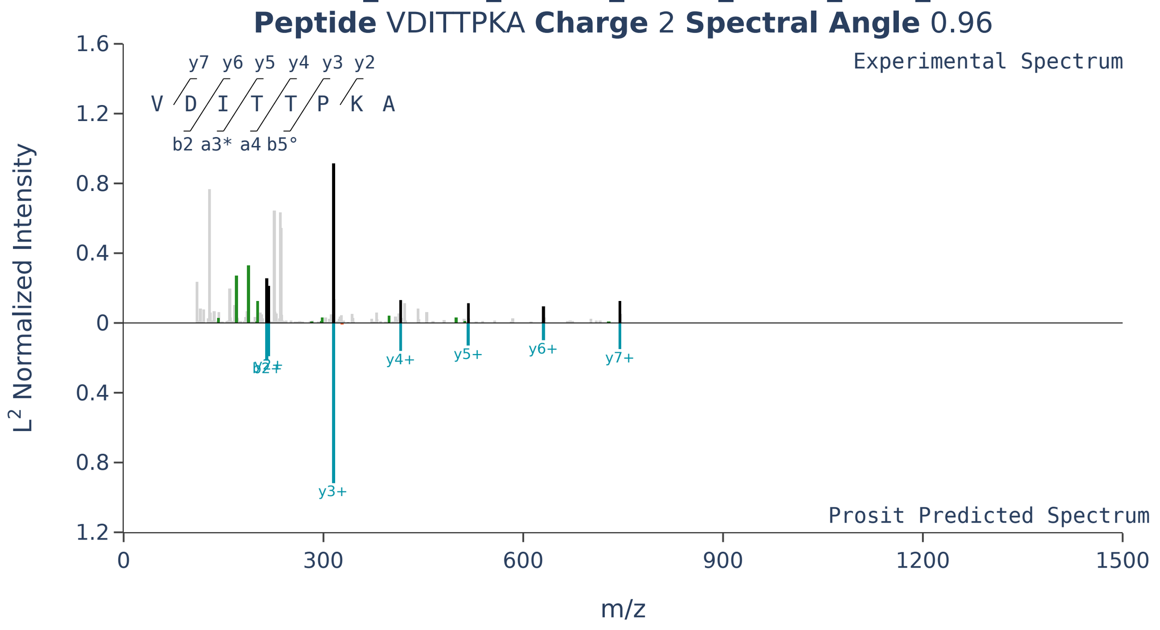


**Source** HRoetschke_271222_090123_HFGoe_LEDGF_b1R2_2h **Scan** 7703
**Peptide** ETSMDSRLQR **Charge** 3 **Spectral Angle** 0.94

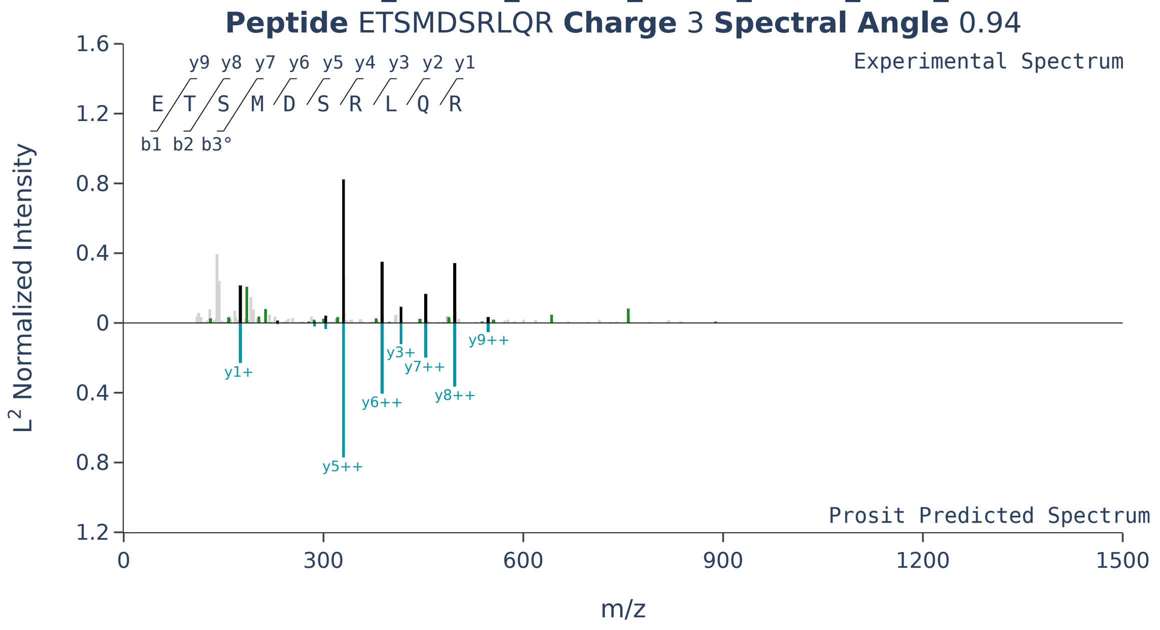


**Source** HRoetschke_271222_090123_HFGoe_LEDGF_b1R2_2h **Scan** 6595
**Peptide** VEVEEKE **Charge** 1 **Spectral Angle** 0.97

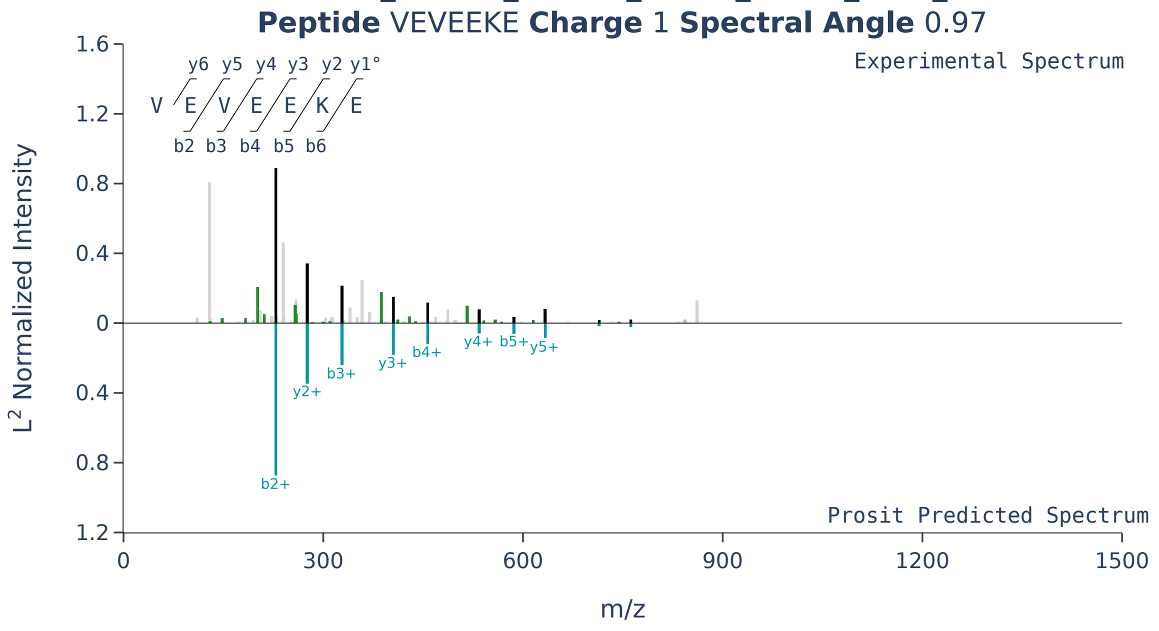


**Source** HRoetschke_271222_090123_HFGoe_LEDGF_b1R1_24h **Scan** 12513
**Peptide** VSQVIME **Charge** 1 **Spectral Angle** 0.94

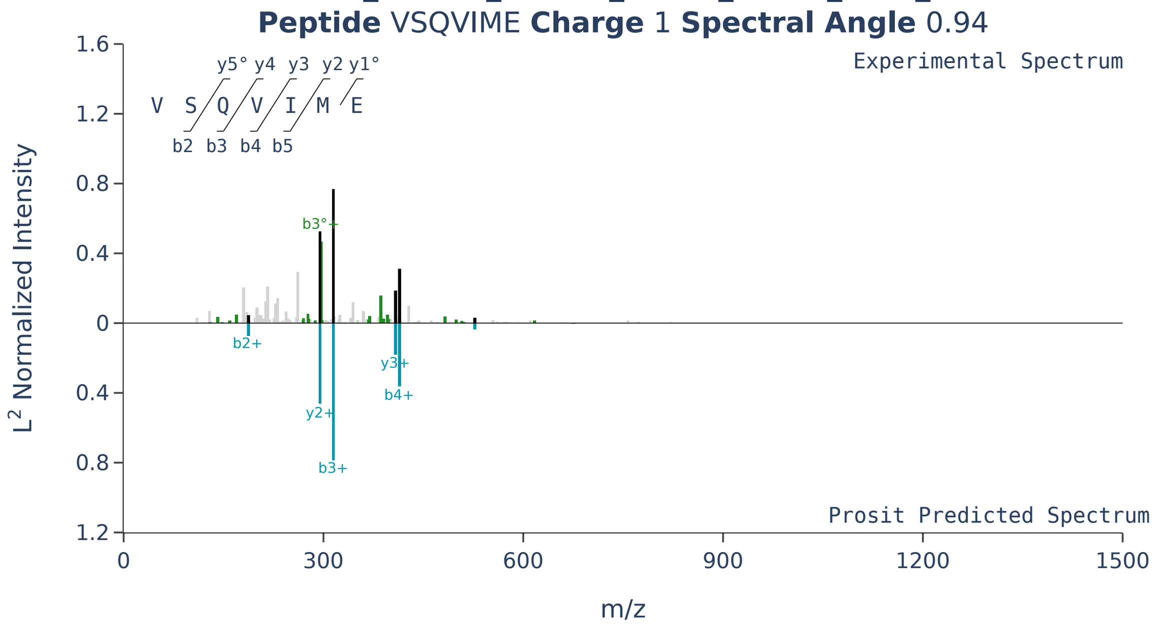


**Source** HRoetschke_271222_090123_HFGoe_LEDGF_b1R2_1h **Scan** 24474
**Peptide** ELASLQVTMQ **Charge** 2 **Spectral Angle** 0.95

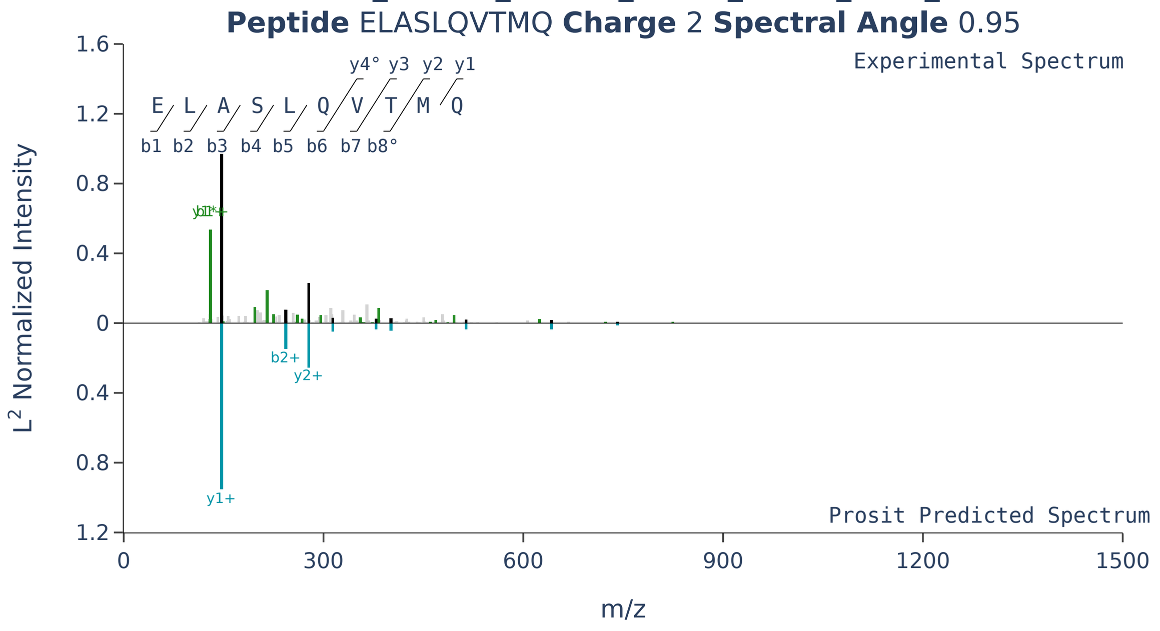


**Source** HRoetschke_271222_090123_HFGoe_LEDGF_b1R2_1h **Scan** 7939
**Peptide** PDGAVKPPTN **Charge** 2 **Spectral Angle** 0.92

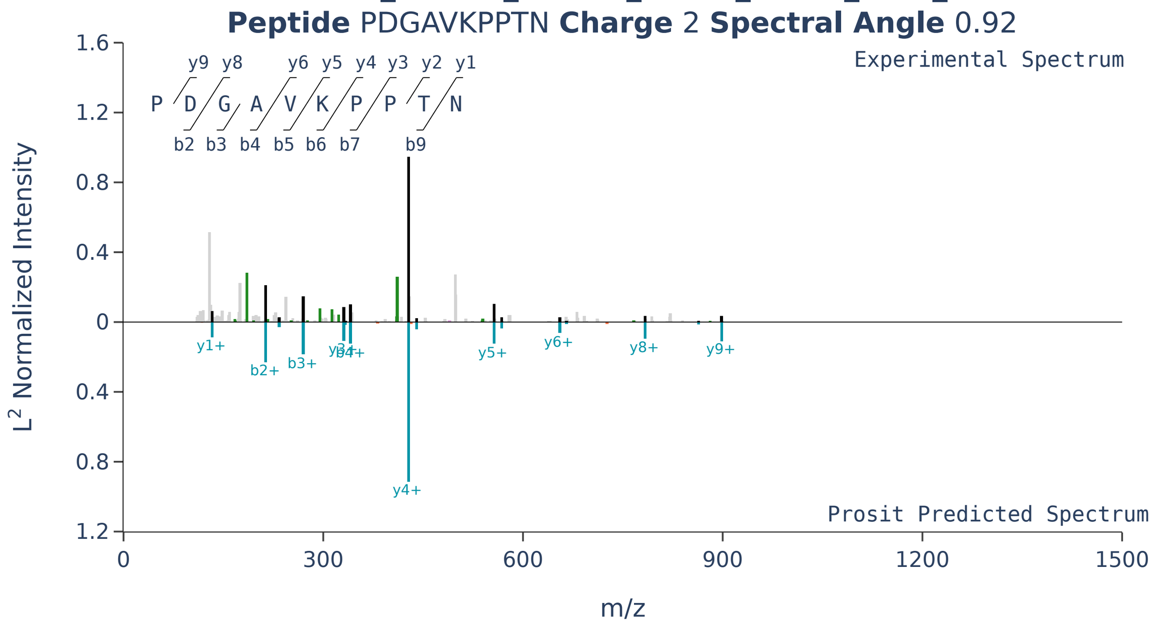


**Source** HRoetschke_271222_090123_HFGoe_LEDGF_b2R1_24h **Scan** 12767
**Peptide** TEEAGVVTTAT **Charge** 2 **Spectral Angle** 0.95

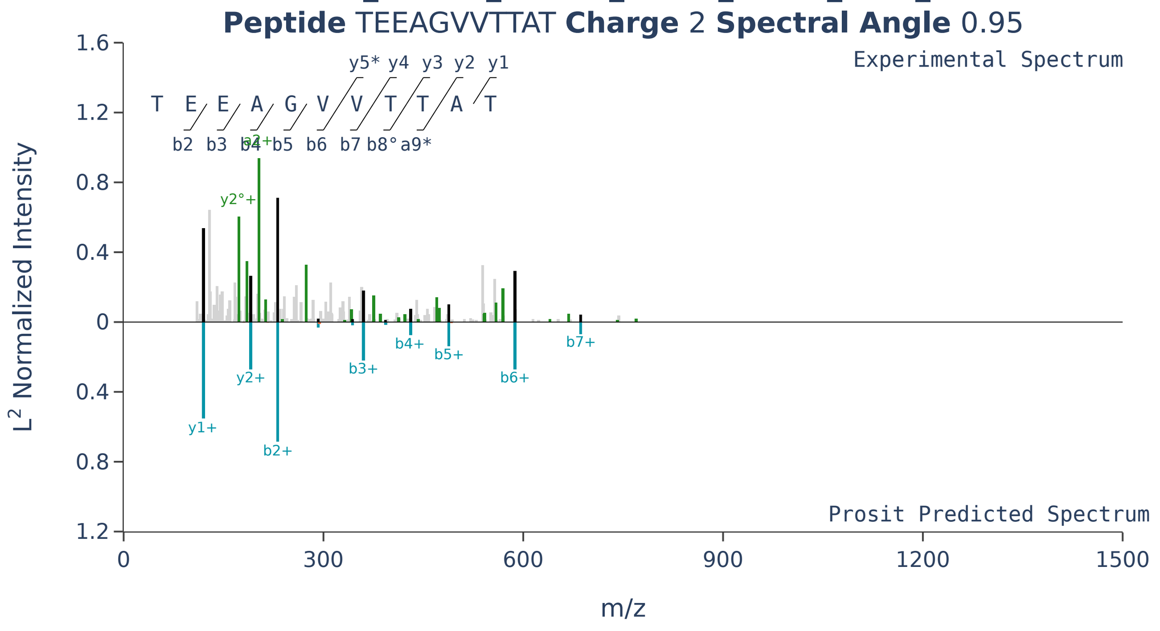


**Source** HRoetschke_271222_090123_HFGoe_LEDGF_b2R2_2h **Scan** 10004
**Peptide** DITTPKAA **Charge** 2 **Spectral Angle** 0.98

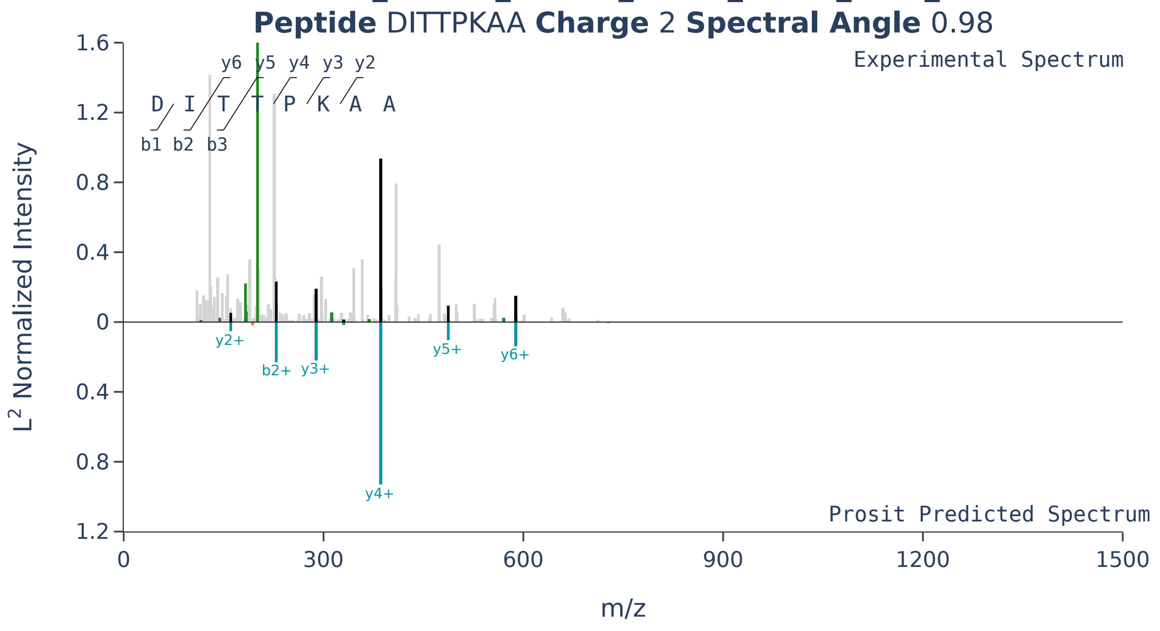


**Source** HRoetschke_271222_090123_HFGoe_LEDGF_b2R1_24h **Scan** 6324
**Peptide** ITTPKAAR **Charge** 2 **Spectral Angle** 0.96

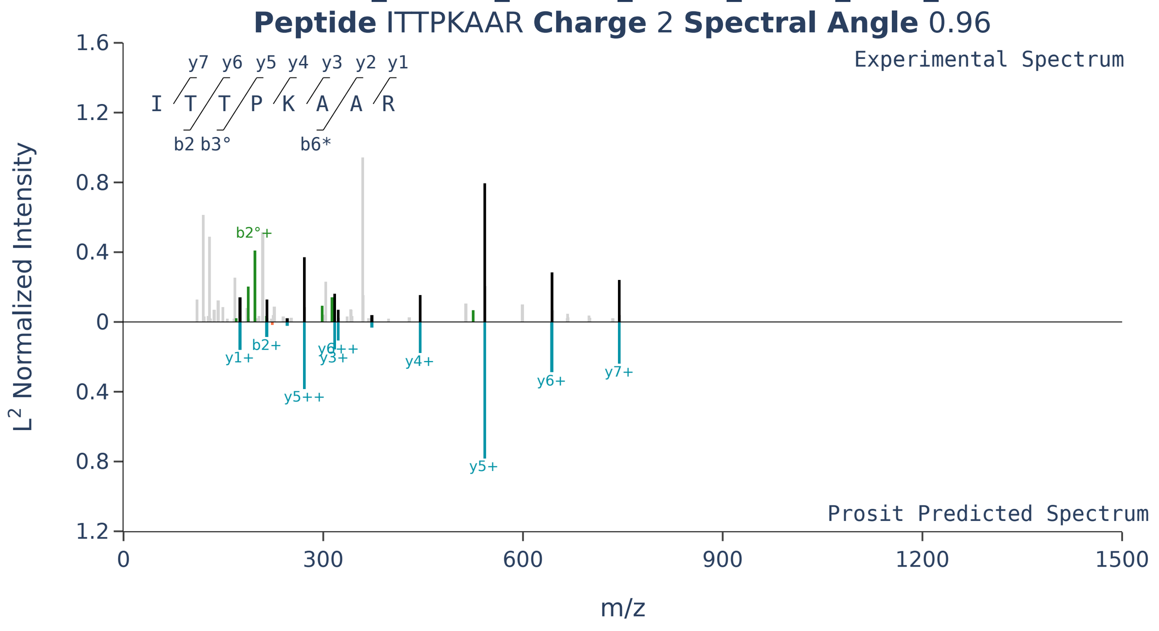


**Source** HRoetschke_271222_090123_HFGoe_LEDGF_b1R1_24h **Scan** 12199
**Peptide** SLQVTMQQA **Charge** 1 **Spectral Angle** 0.95

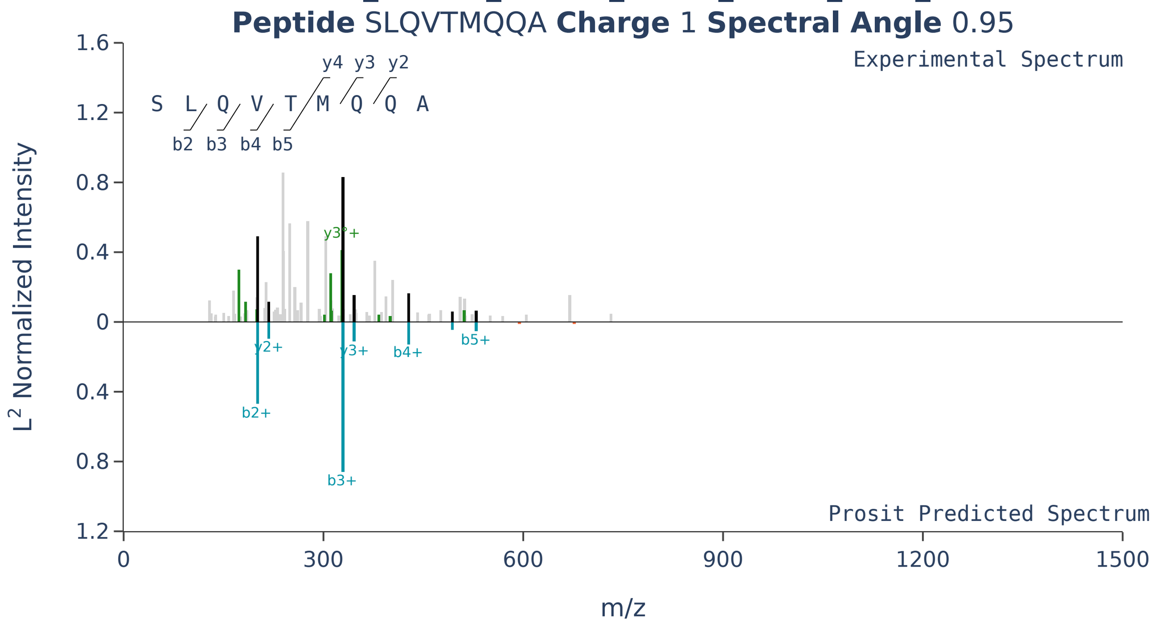


**Source** HRoetschke_271222_090123_HFGoe_LEDGF_b2R1_4h **Scan** 7709
**Peptide** LNKSLAEQ **Charge** 2 **Spectral Angle** 0.97

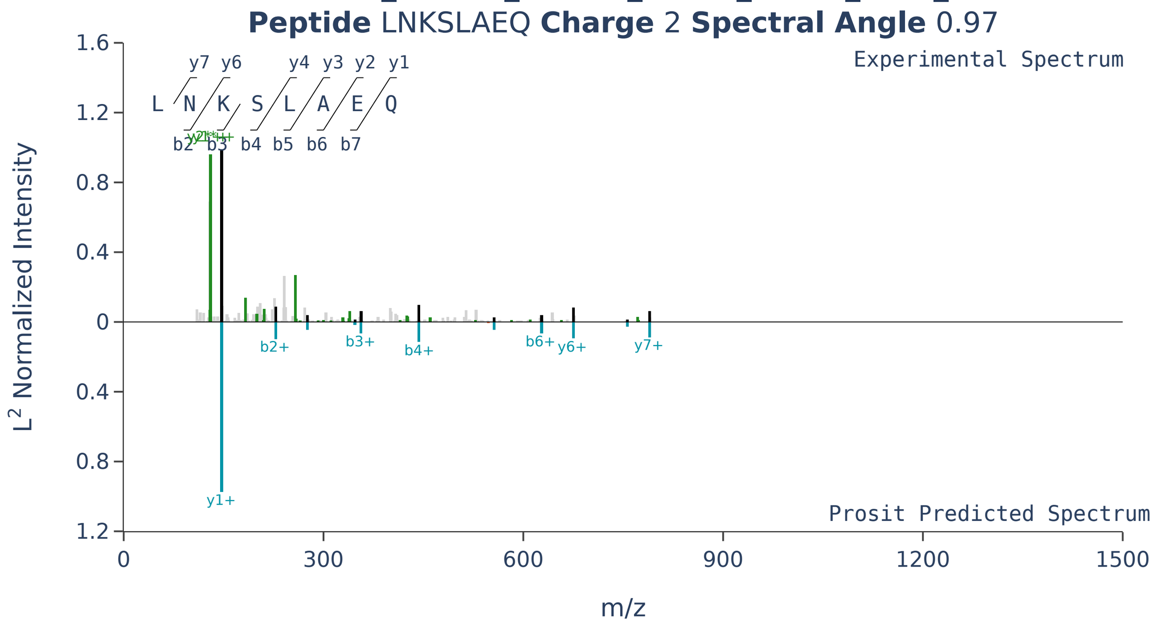

**Source** HRoetschke_271222_090123_HFGoe_LEDGF_b1R1_24h **Scan** 16670
**Peptide** TMLYNKF **Charge** 1 **Spectral Angle** 0.96

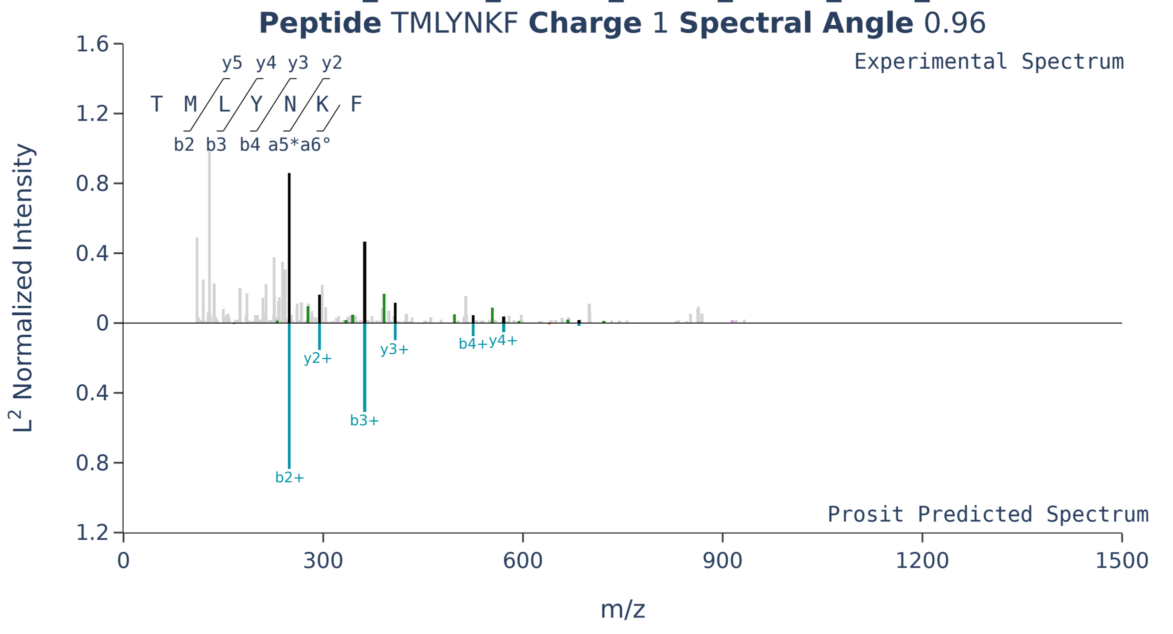


**Source** HRoetschke_271222_090123_HFGoe_LEDGF_b1R1_4h **Scan** 16804
**Peptide** LQVTMQQA **Charge** 1 **Spectral Angle** 0.95

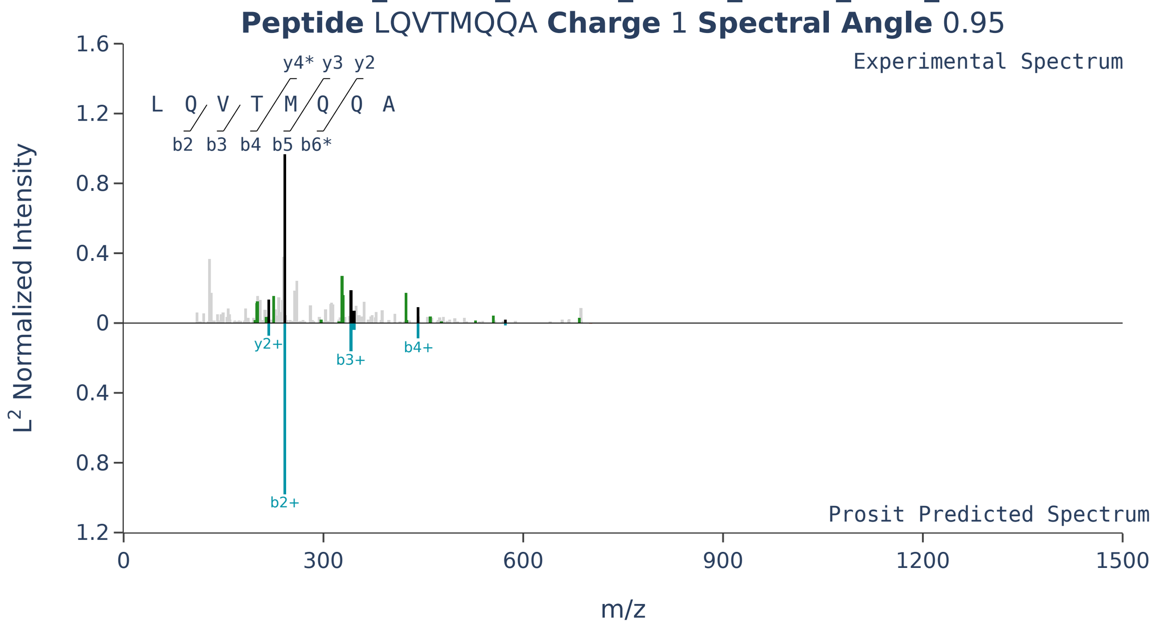


**Source** HRoetschke_271222_090123_HFGoe_LEDGF_b1R1_24h **Scan** 11505
**Peptide** NASSDVEVEE **Charge** 1 **Spectral Angle** 0.91

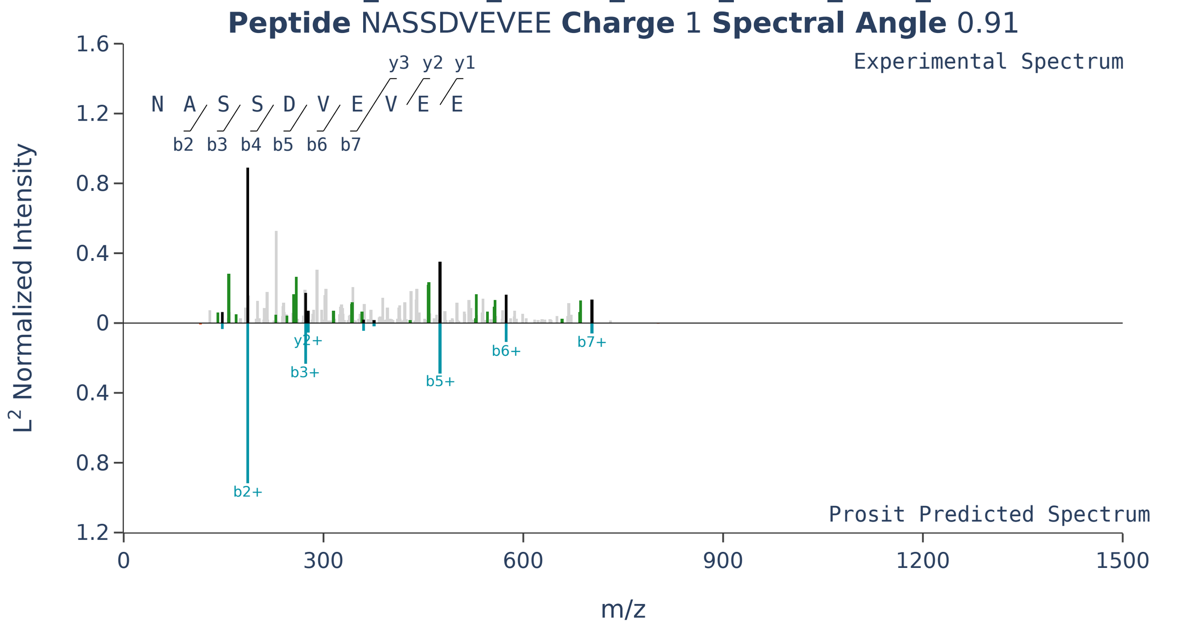


**Source** HRoetschke_271222_090123_HFGoe_LEDGF_b2R2_2h **Scan** 17991
**Peptide** GYPHWPARV **Charge** 3 **Spectral Angle** 0.96

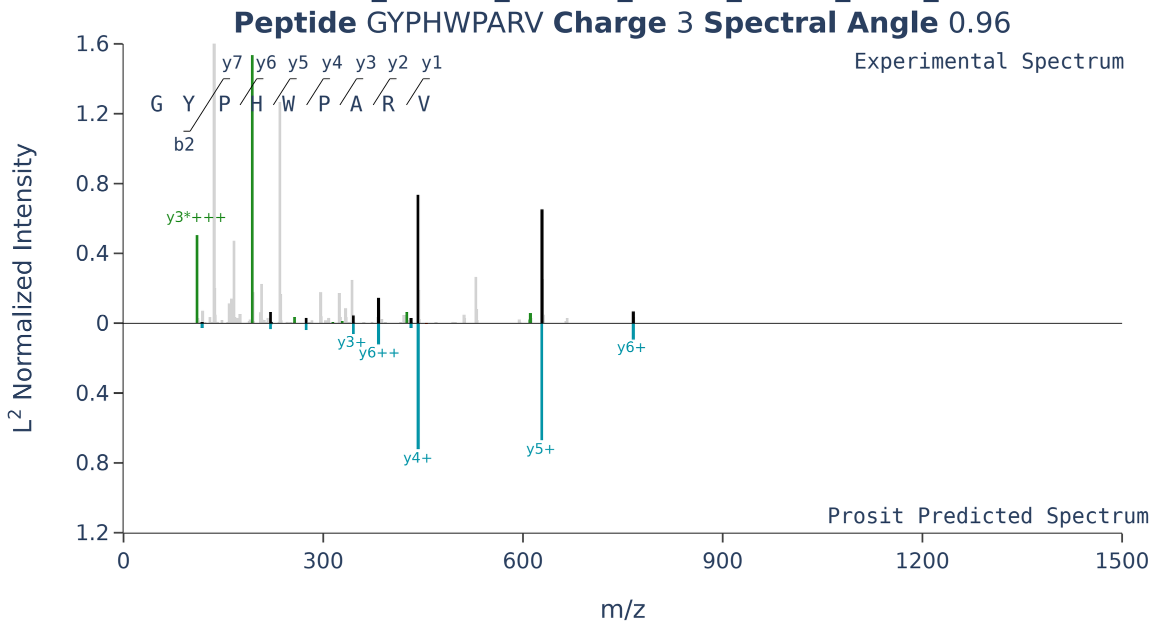


**Source** HRoetschke_271222_090123_HFGoe_LEDGF_b1R1_2h **Scan** 9848
**Peptide** SSDVEVEE **Charge** 2 **Spectral Angle** 0.93

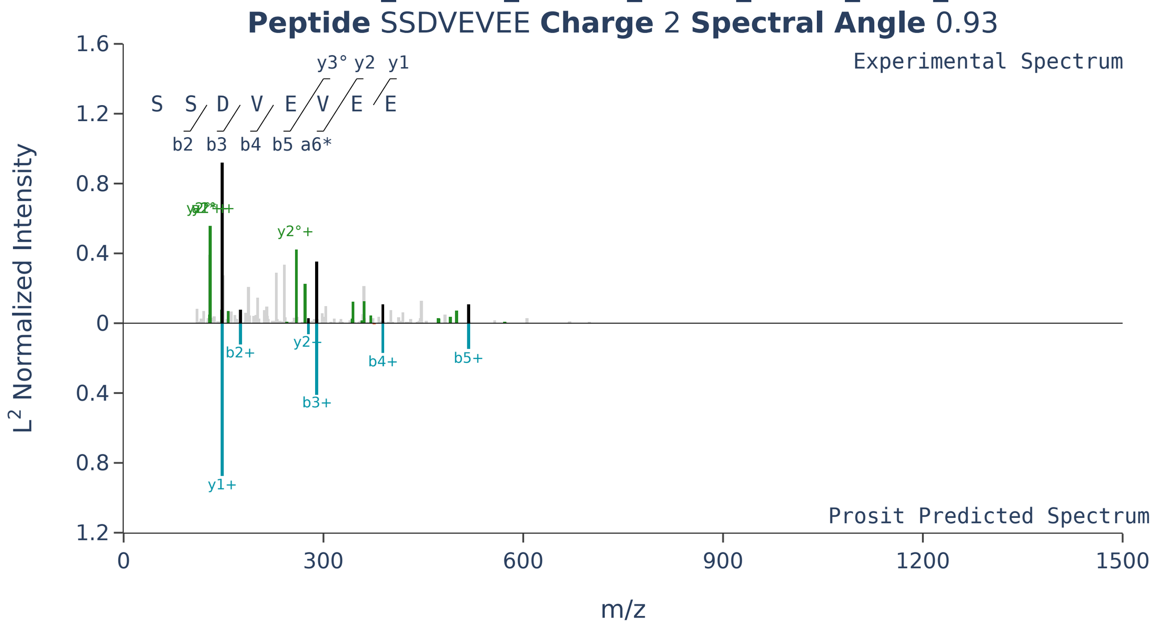


**Source** HRoetschke_271222_090123_HFGoe_LEDGF_b2R1_4h **Scan** 19605
**Peptide** SLKIDNL **Charge** 2 **Spectral Angle** 0.87

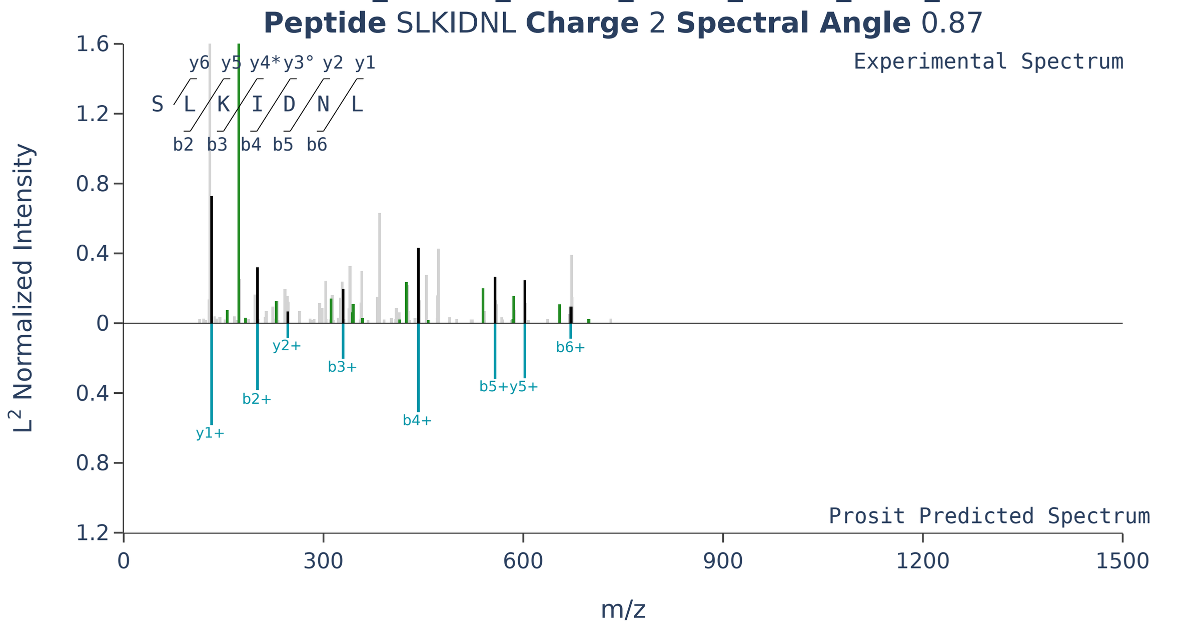


**Source** HRoetschke_271222_090123_HFGoe_LEDGF_b2R1_4h **Scan** 11014
**Peptide** SDVEVEE **Charge** 1 **Spectral Angle** 0.93

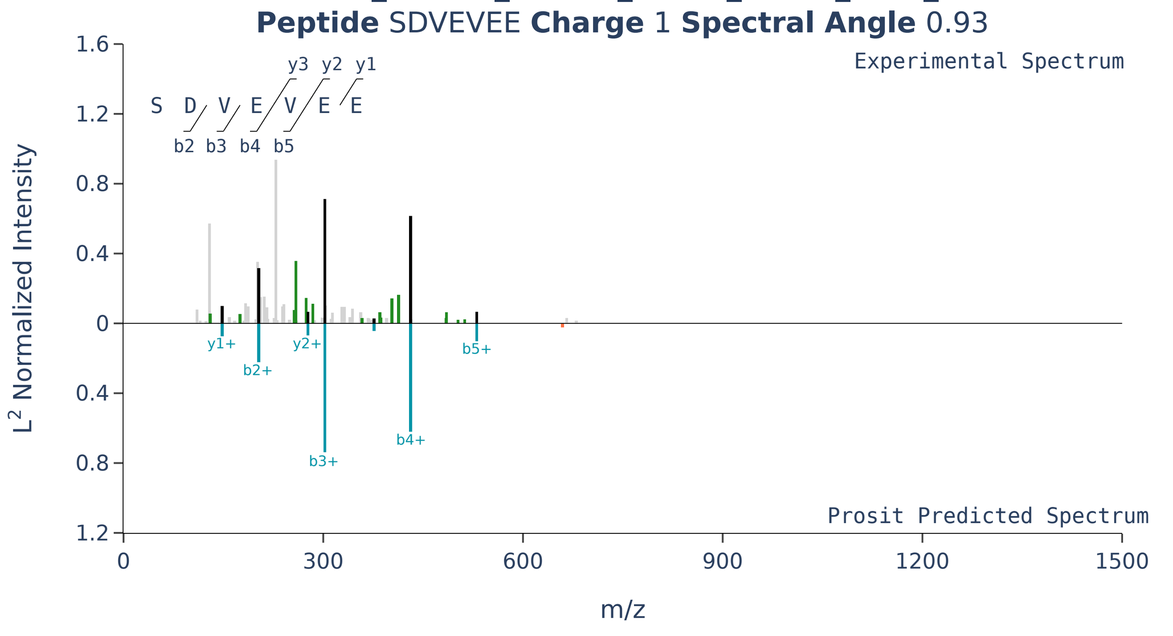


**Source** HRoetschke_271222_090123_HFGoe_LEDGF_b2R1_1h **Scan** 15871
**Peptide** LIFAKMK **Charge** 2 **Spectral Angle** 0.9

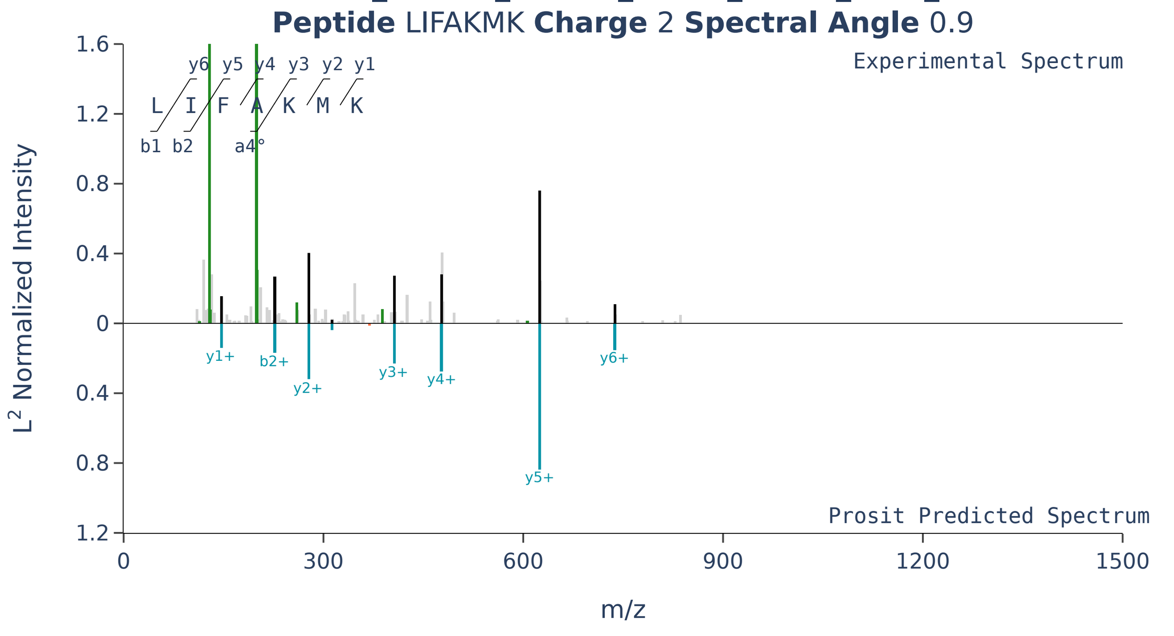


**Source** HRoetschke_271222_090123_HFGoe_LEDGF_b2R1_2h **Scan** 9581
**Peptide** AVKPPTNKL **Charge** 1 **Spectral Angle** 0.95

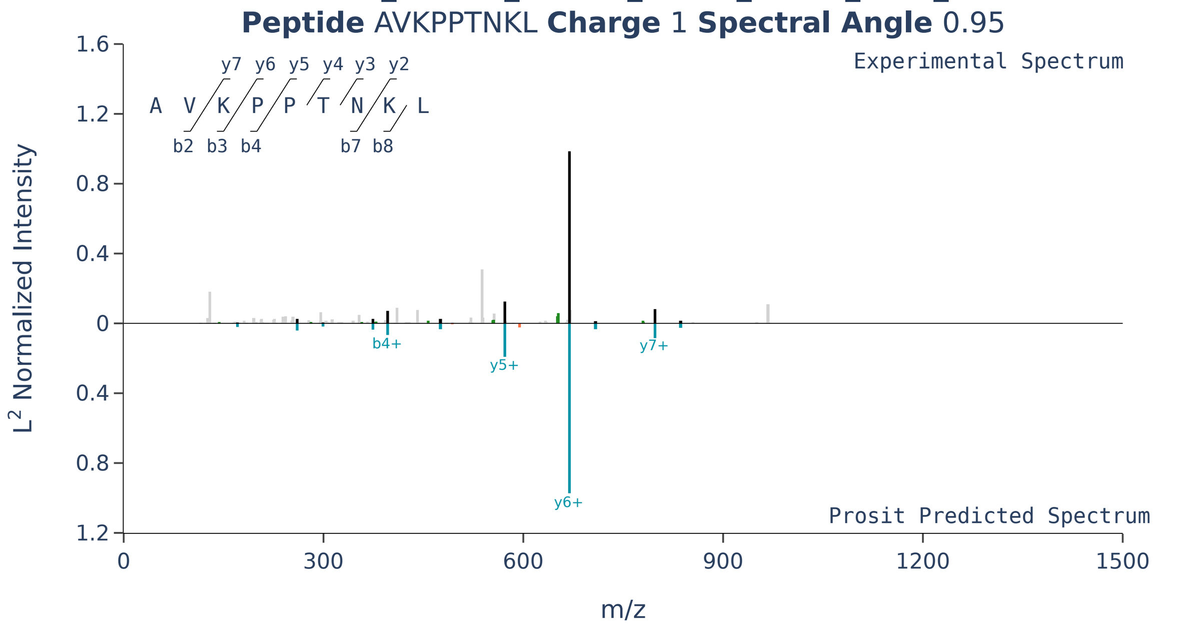


**Source** HRoetschke_271222_090123_HFGoe_LEDGF_b2R1_2h **Scan** 25505
**Peptide** FNEGLWE **Charge** 1 **Spectral Angle** 0.9

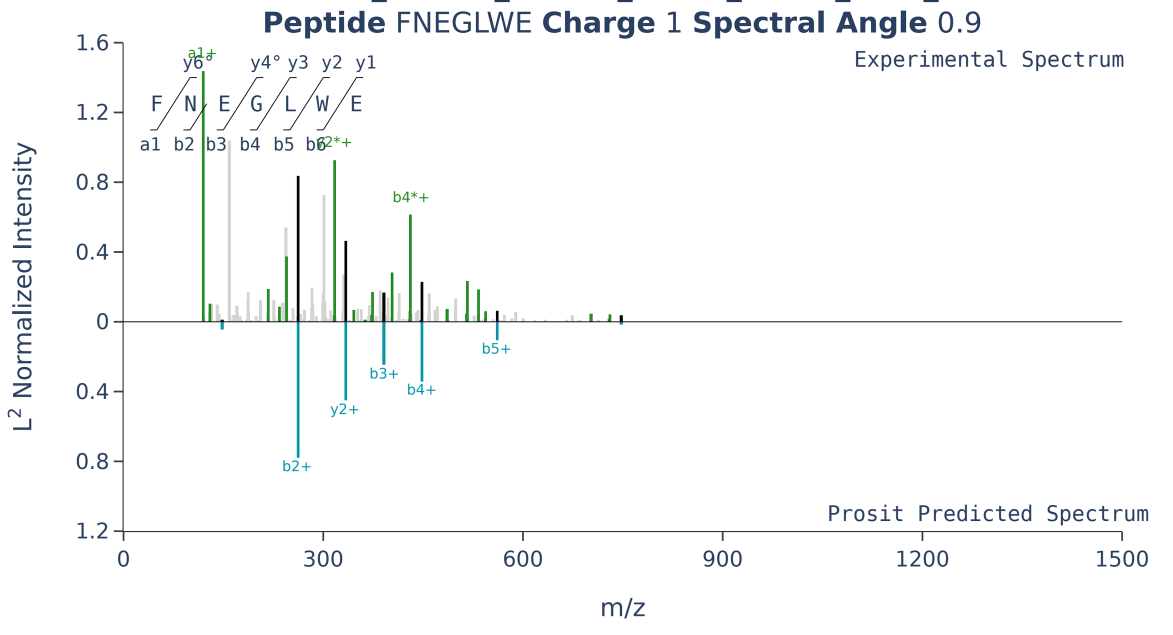


**Source** HRoetschke_271222_090123_HFGoe_LEDGF_b2R2_1h **Scan** 13320
**Peptide** VGEGDSVITQ **Charge** 2 **Spectral Angle** 0.97

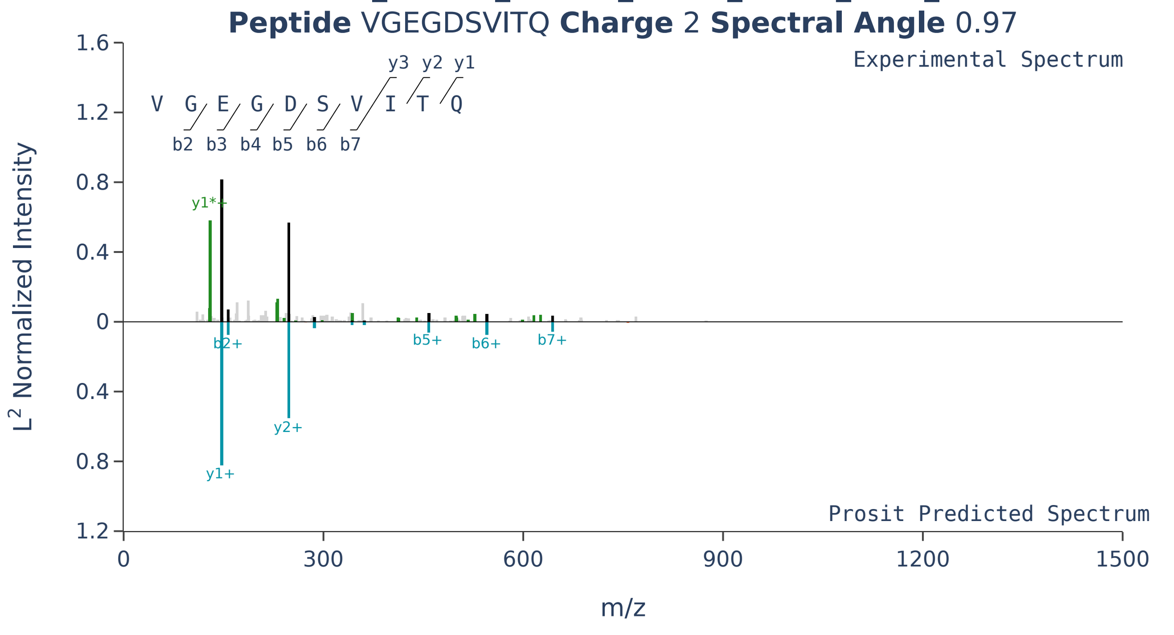


**Source** HRoetschke_271222_090123_HFGoe_LEDGF_b2R1_24h **Scan** 9968
**Peptide** VDITTPKAAR **Charge** 3 **Spectral Angle** 0.94

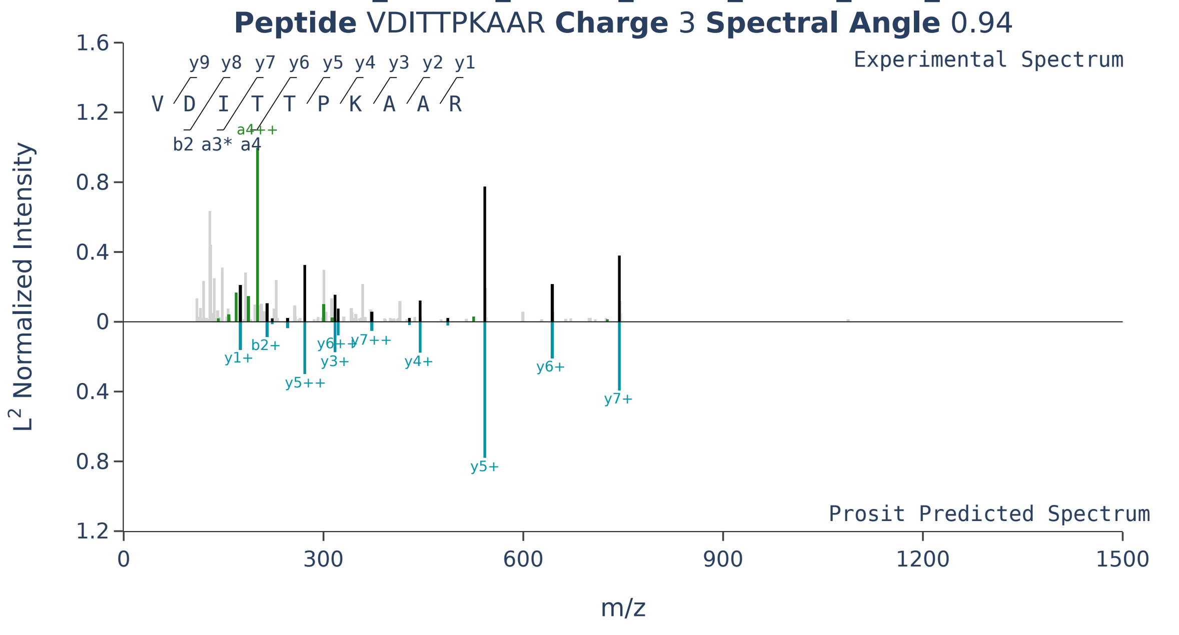


**Source** HRoetschke_271222_090123_HFGoe_LEDGF_b1R2_2h **Scan** 10454
**Peptide** NASSDVEVE **Charge** 1 **Spectral Angle** 0.93

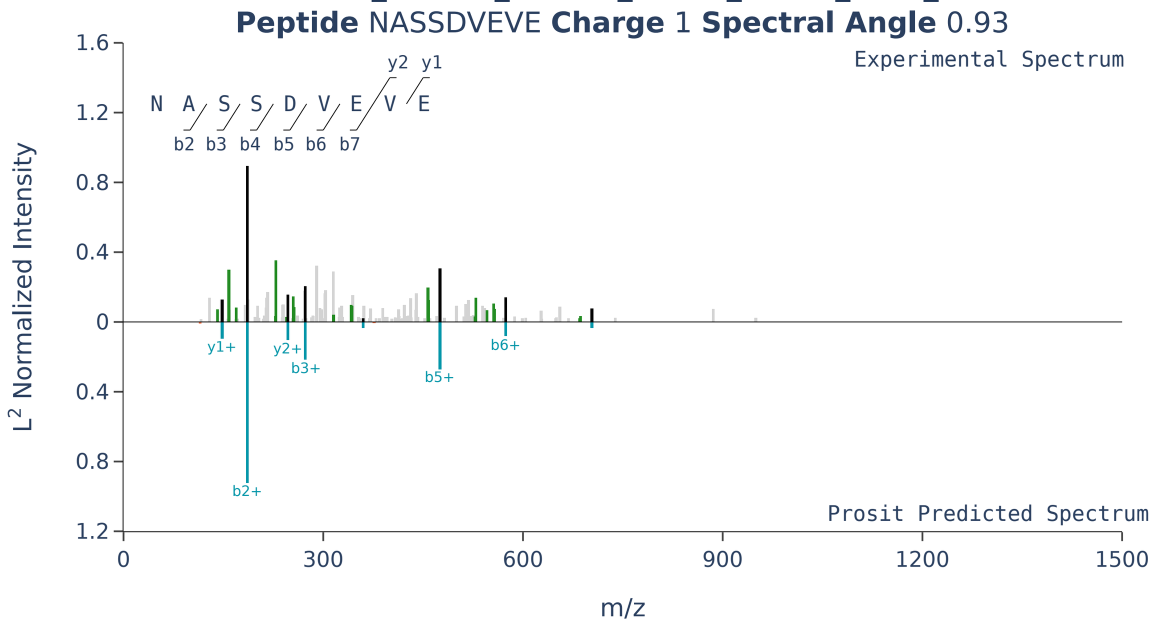


**Source** HRoetschke_271222_090123_HFGoe_LEDGF_b1R2_24h **Scan** 14108
**Peptide** EIDNNPKVKF **Charge** 3 **Spectral Angle** 0.9

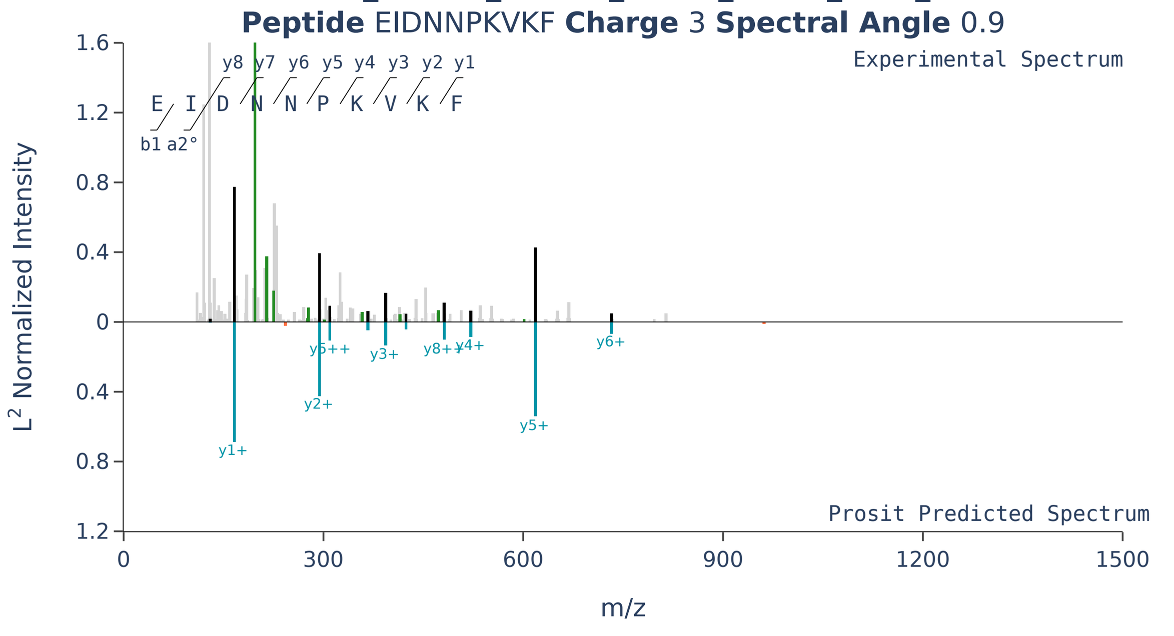


**Source** HRoetschke_271222_090123_HFGoe_LEDGF_b1R1_24h **Scan** 11984
**Peptide** GEGDSVIT **Charge** 1 **Spectral Angle** 0.92

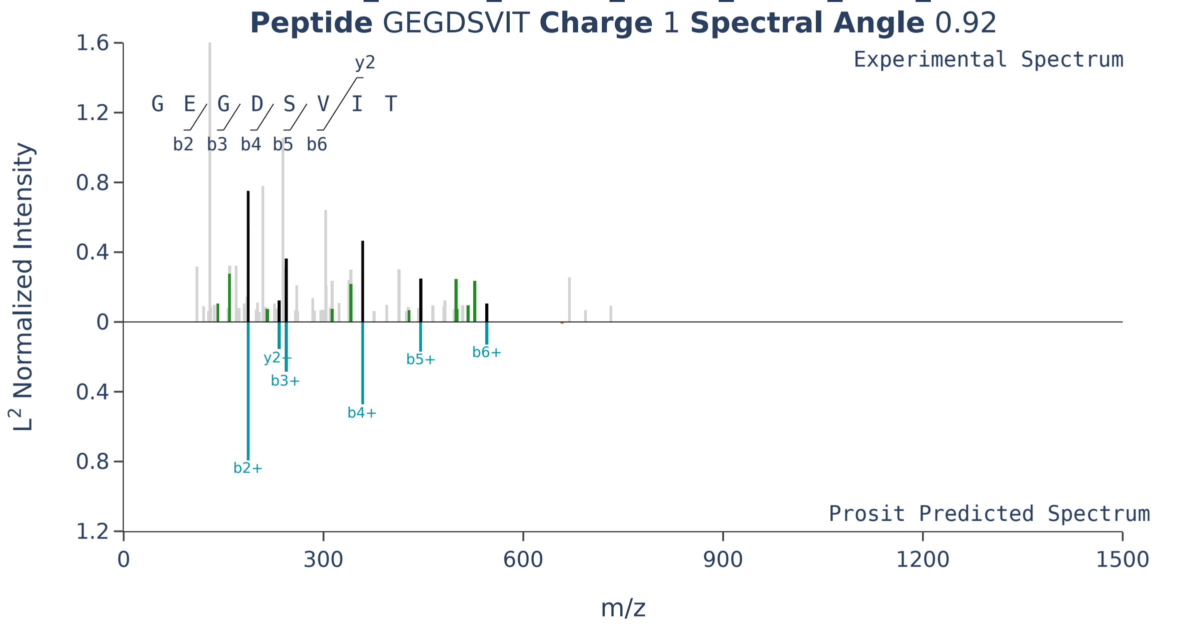

**Source** HRoetschke_271222_090123_HFGoe_LEDGF_b2R1_4h **Scan** 19093
**Peptide** TEMITTLKKI **Charge** 3 **Spectral Angle** 0.88

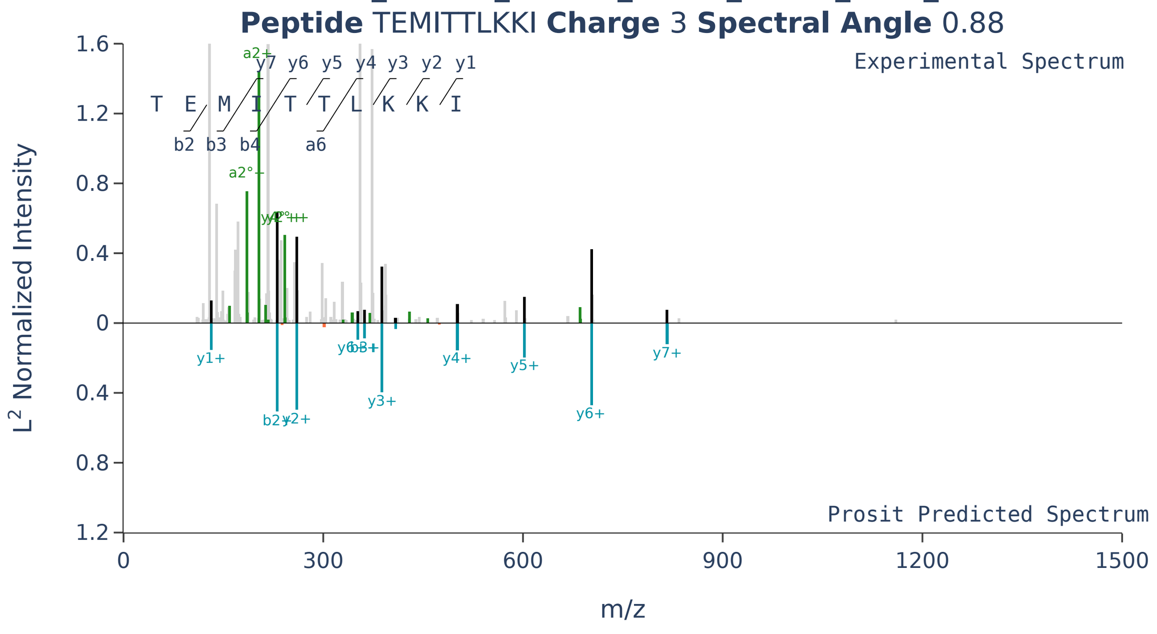


**Source** HRoetschke_271222_090123_HFGoe_LEDGF_b2R1_1h **Scan** 9428
**Peptide** SQVIMEKST **Charge** 2 **Spectral Angle** 0.91

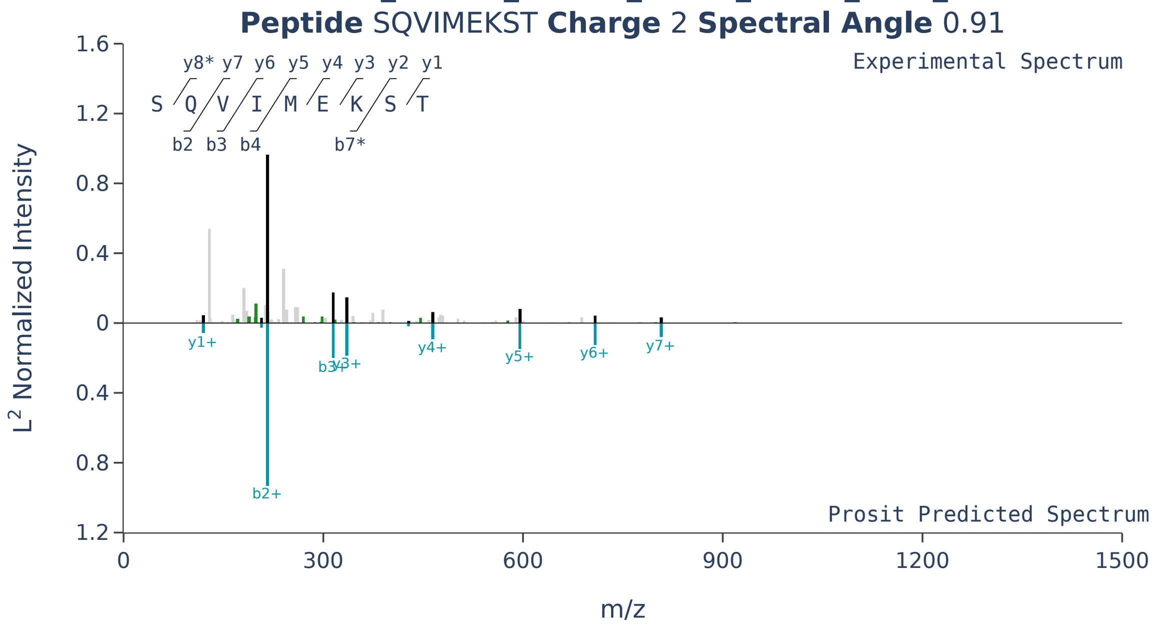


**Source** HRoetschke_271222_090123_HFGoe_LEDGF_b1R2_24h **Scan** 17244
**Peptide** SLKIDNLDVNR **Charge** 3 **Spectral Angle** 0.93

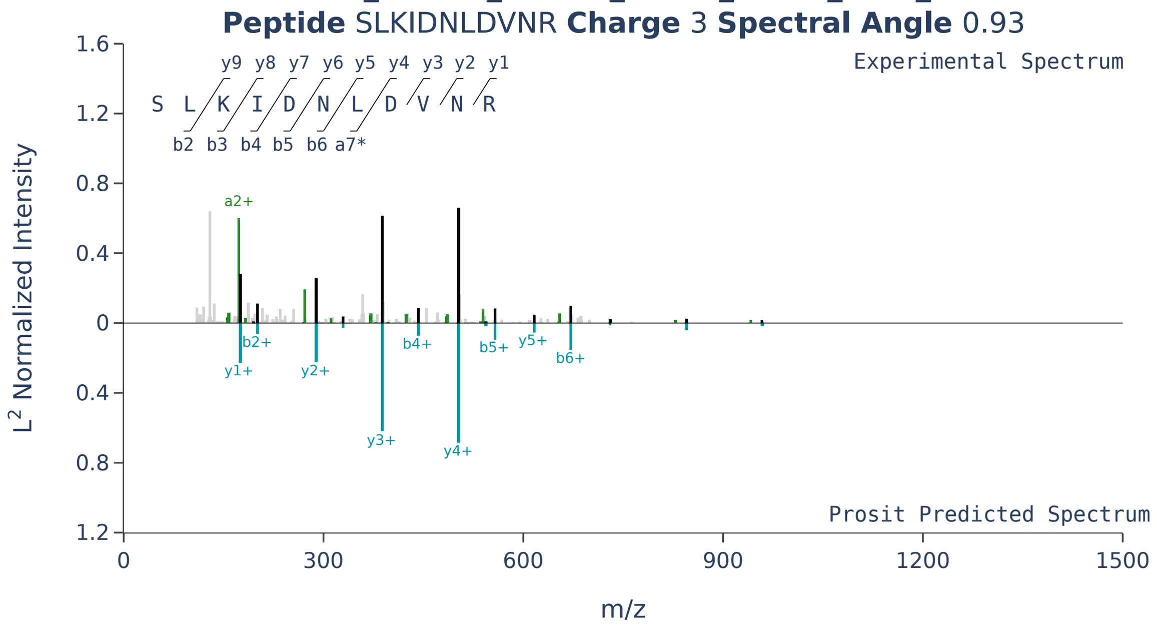


**Source** HRoetschke_271222_090123_HFGoe_LEDGF_b2R1_1h **Scan** 9378
**Peptide** KVSQVIMEK **Charge** 3 **Spectral Angle** 0.84

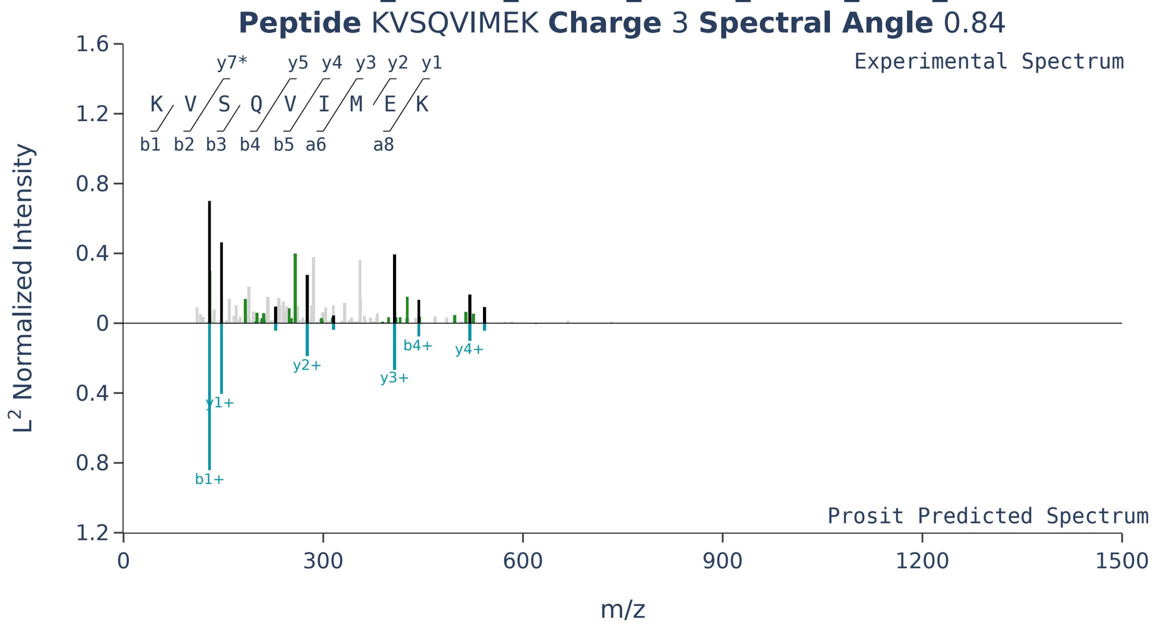


**Source** HRoetschke_271222_090123_HFGoe_LEDGF_b1R1_24h **Scan** 14073
**Peptide** KGFNEGL **Charge** 2 **Spectral Angle** 0.92

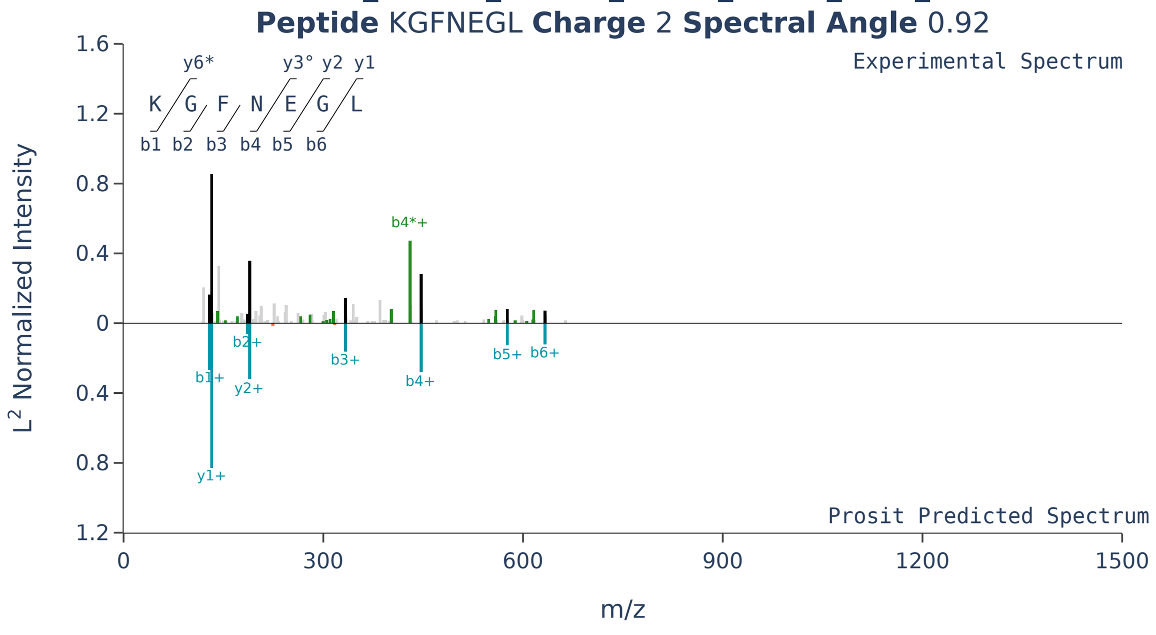


**Source** HRoetschke_271222_090123_HFGoe_LEDGF_b2R1_2h **Scan** 17862
**Peptide** TEISLKDSTLD **Charge** 1 **Spectral Angle** 0.92

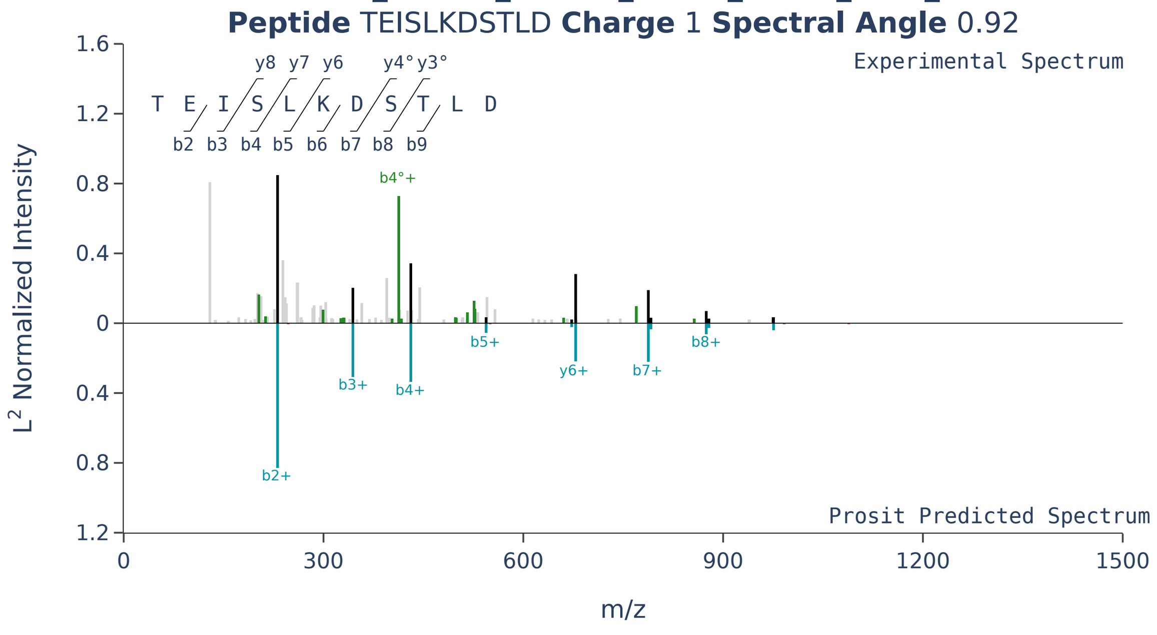


**Source** HRoetschke_271222_090123_HFGoe_LEDGF_b2R2_2h **Scan** 9189
**Peptide** QVIMEKST **Charge** 1 **Spectral Angle** 0.95

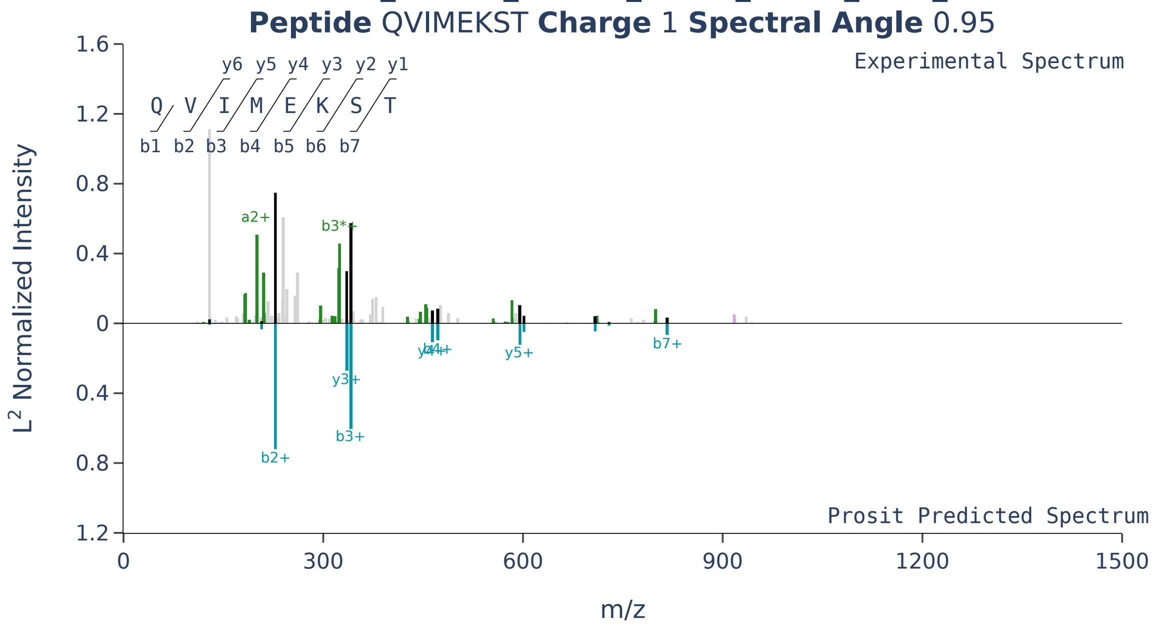


**Source** HRoetschke_271222_090123_HFGoe_LEDGF_b2R1_24h **Scan** 7430
**Peptide** KVSQVIMEKST **Charge** 3 **Spectral Angle** 0.87

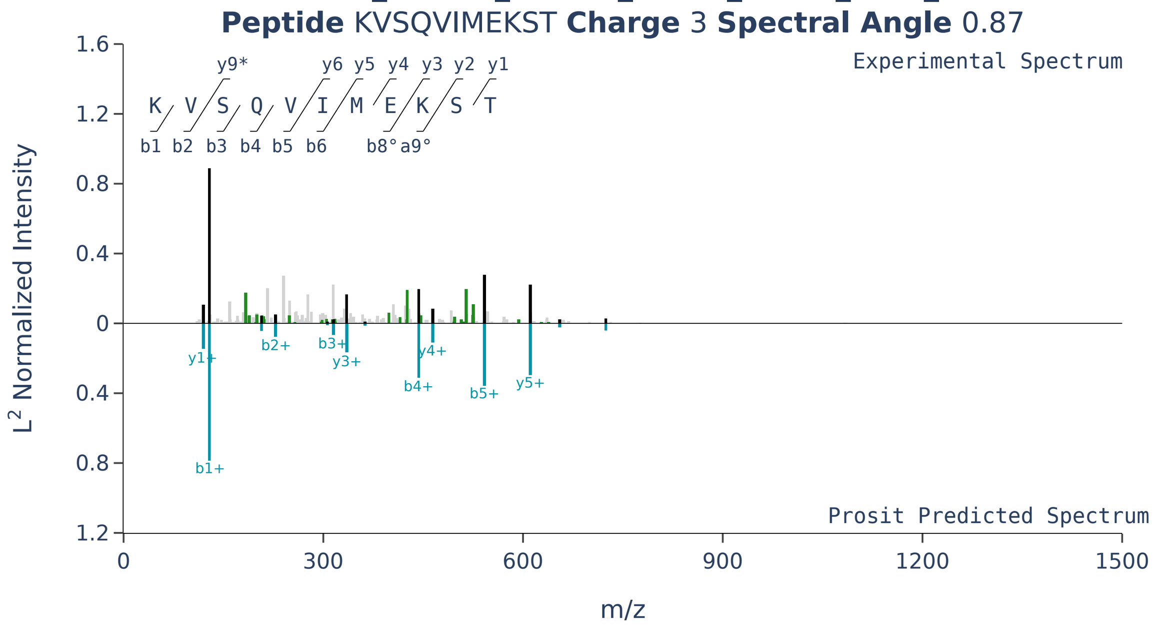


**Source** HRoetschke_271222_090123_HFGoe_LEDGF_b2R2_1h **Scan** 9895
**Peptide** SNASSDVEVE **Charge** 2 **Spectral Angle** 0.95

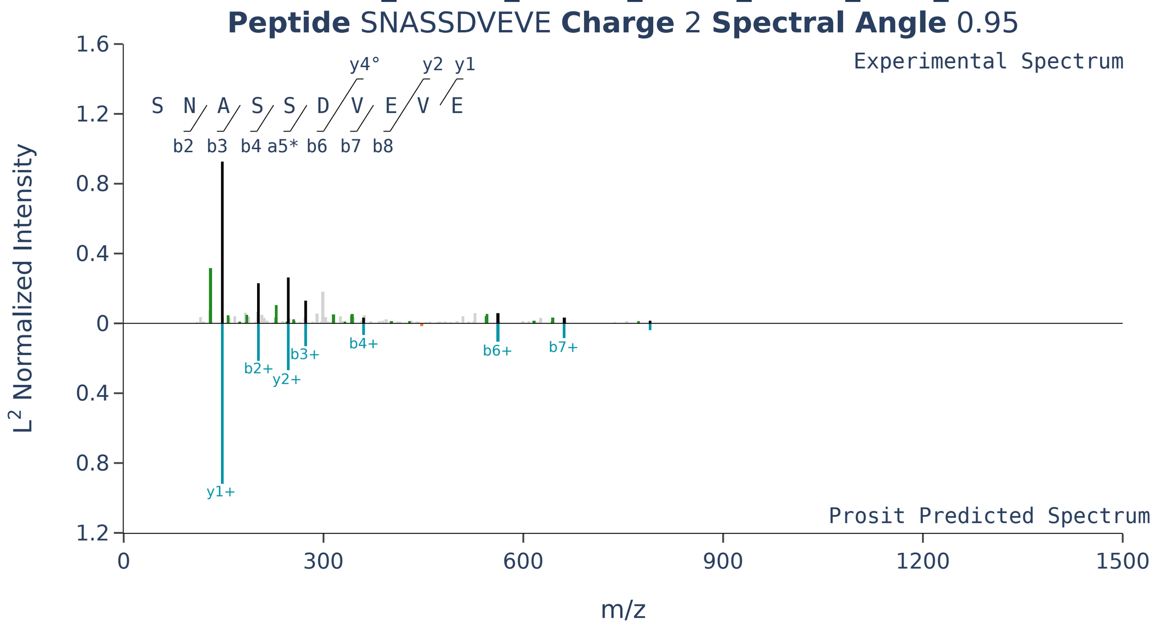


**Source** HRoetschke_271222_090123_HFGoe_LEDGF_b1R2_24h **Scan** 21264
**Peptide** SLKIDNLDVNRCIE **Charge** 2 **Spectral Angle** 0.89

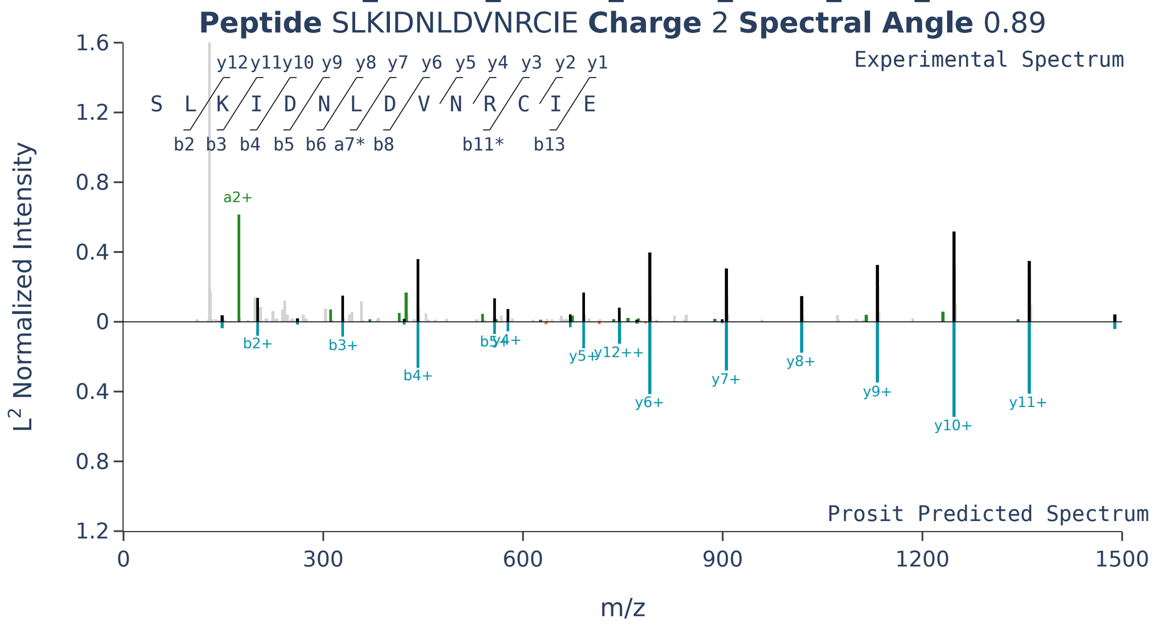


**Source** HRoetschke_271222_090123_HFGoe_LEDGF_b1R1_2h **Scan** 13132
**Peptide** VDITTPKAA **Charge** 2 **Spectral Angle** 0.89

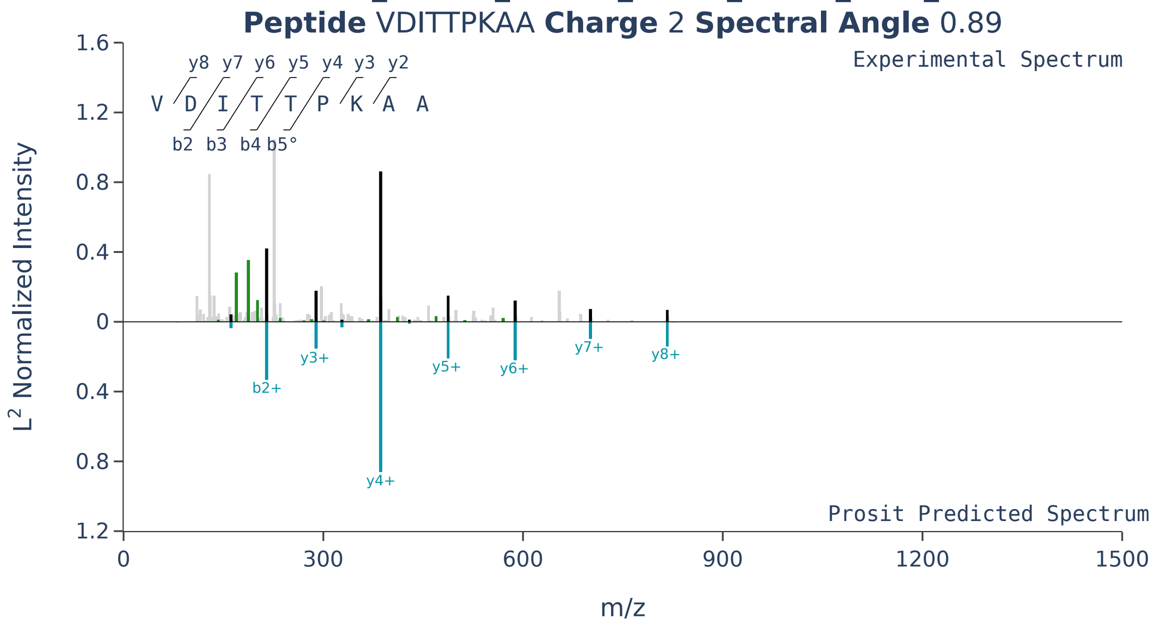


**Source** HRoetschke_271222_090123_HFGoe_LEDGF_b1R2_1h **Scan** 11353
**Peptide** MLYNKFKN **Charge** 3 **Spectral Angle** 0.89

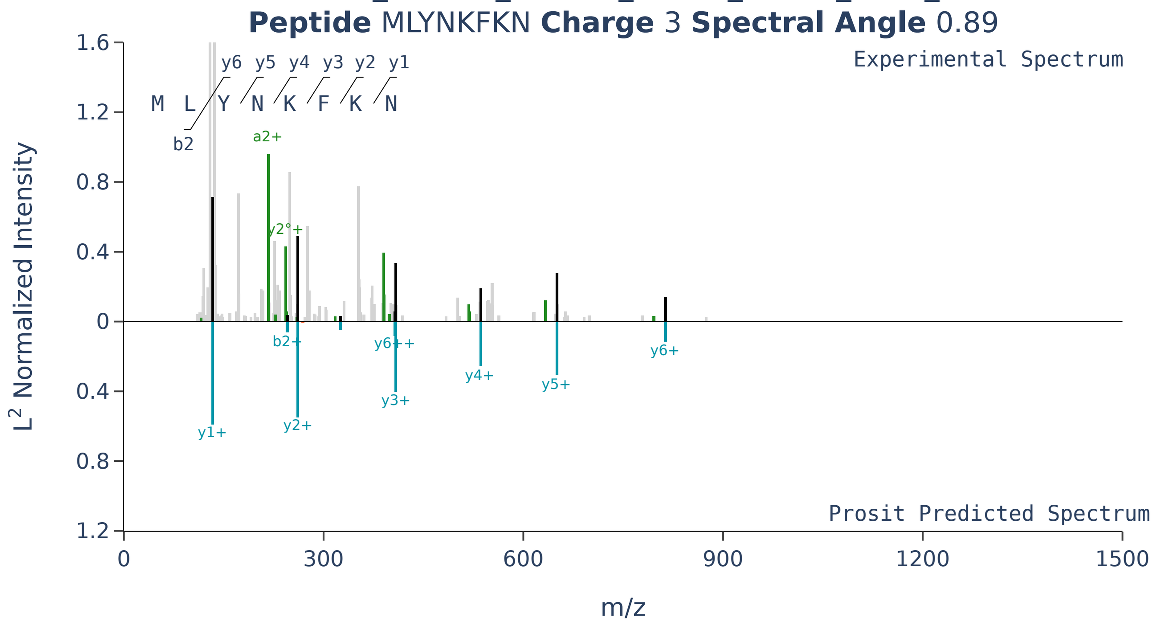


**Source** HRoetschke_271222_090123_HFGoe_LEDGF_b2R1_24h **Scan** 12579
**Peptide** ASLQVTM **Charge** 1 **Spectral Angle** 0.91

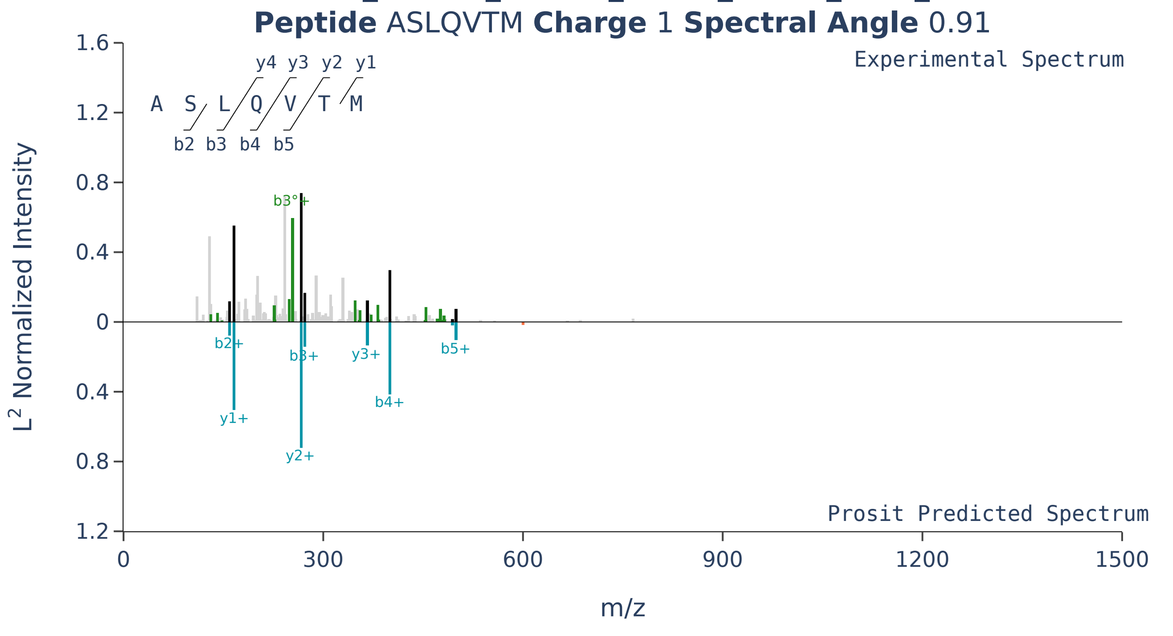


**Source** HRoetschke_271222_090123_HFGoe_LEDGF_b1R1_2h **Scan** 25823
**Peptide** LASLQVTM **Charge** 1 **Spectral Angle** 0.95

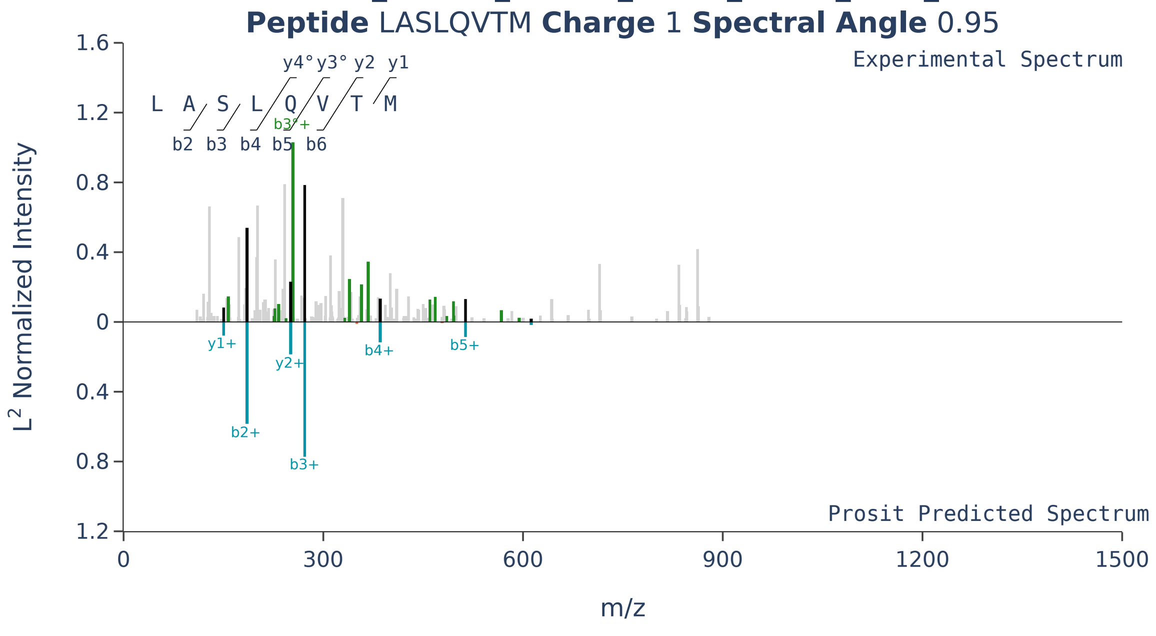


**Source** HRoetschke_271222_090123_HFGoe_LEDGF_b2R1_4h **Scan** 11509
**Peptide** VSQVIMEKS **Charge** 1 **Spectral Angle** 0.95

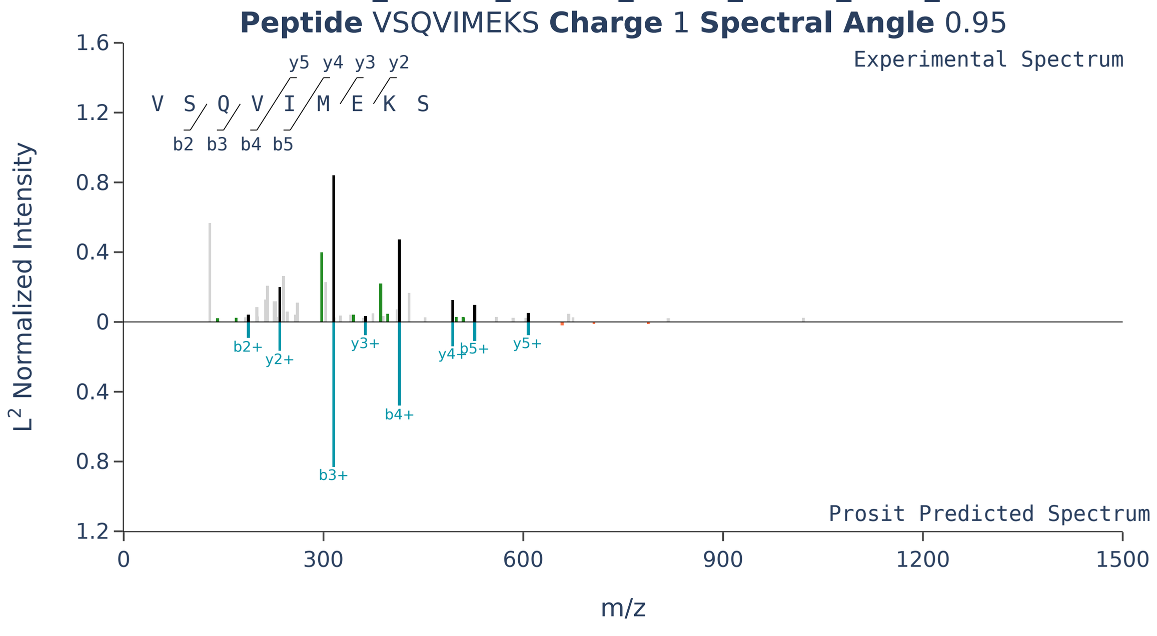

**Source** HRoetschke_271222_090123_HFGoe_LEDGF_b2R1_2h **Scan** 11007
**Peptide** TEEAGVVTT **Charge** 1 **Spectral Angle** 0.91

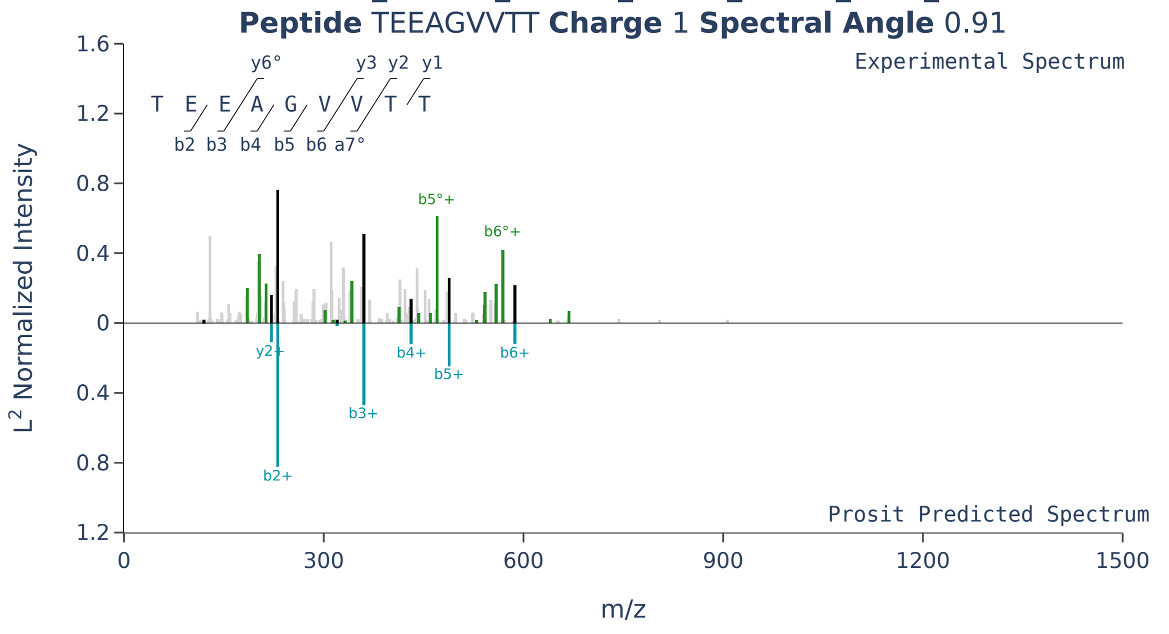


**Source** HRoetschke_271222_090123_HFGoe_LEDGF_b1R2_24h **Scan** 9507
**Peptide** HTEMITTLKK **Charge** 3 **Spectral Angle** 0.92

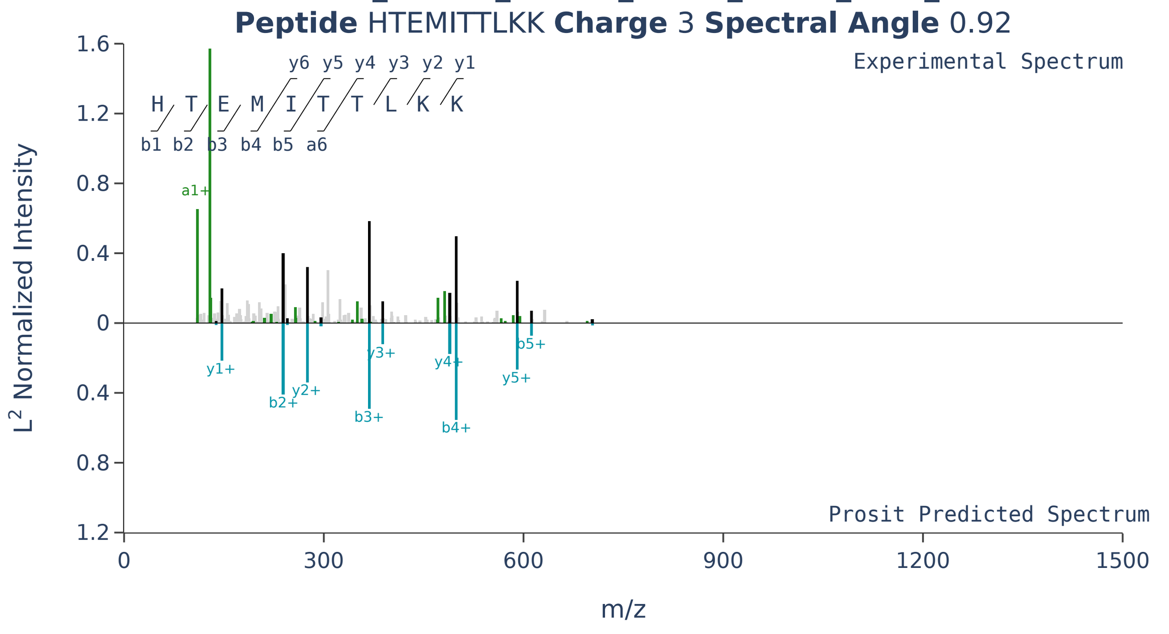


**Source** HRoetschke_271222_090123_HFGoe_LEDGF_b1R2_24h **Scan** 8443
**Peptide** SVNLKVSPKRG **Charge** 3 **Spectral Angle** 0.91

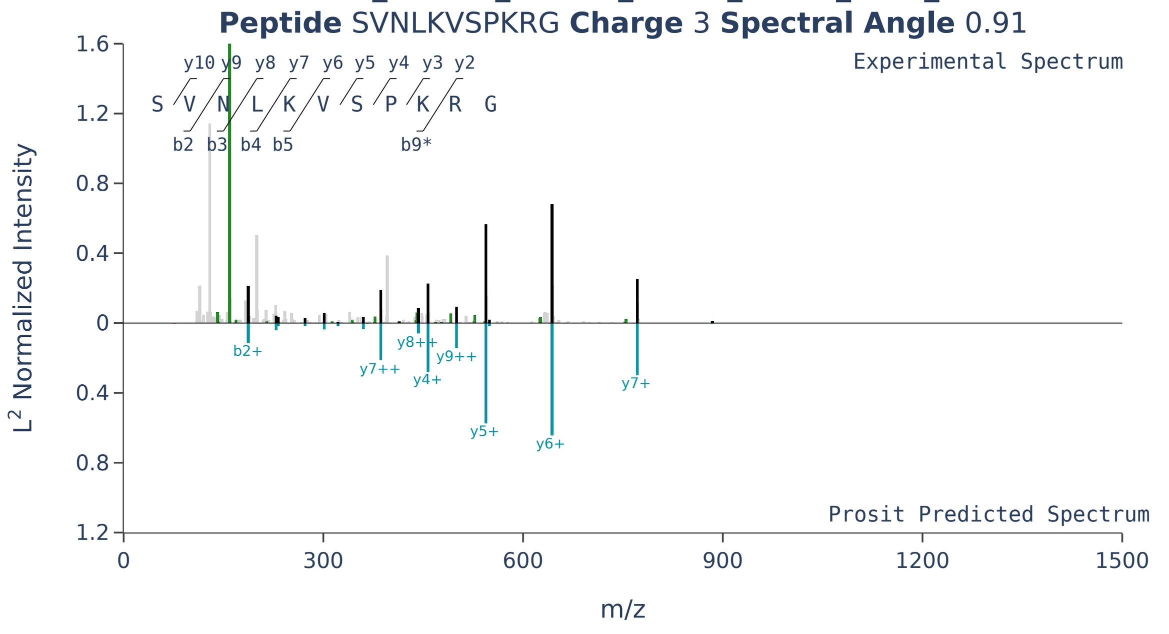


**Source** HRoetschke_271222_090123_HFGoe_LEDGF_b2R2_2h **Scan** 11213
**Peptide** DEVPDGAVKPPTNK **Charge** 2 **Spectral Angle** 0.87

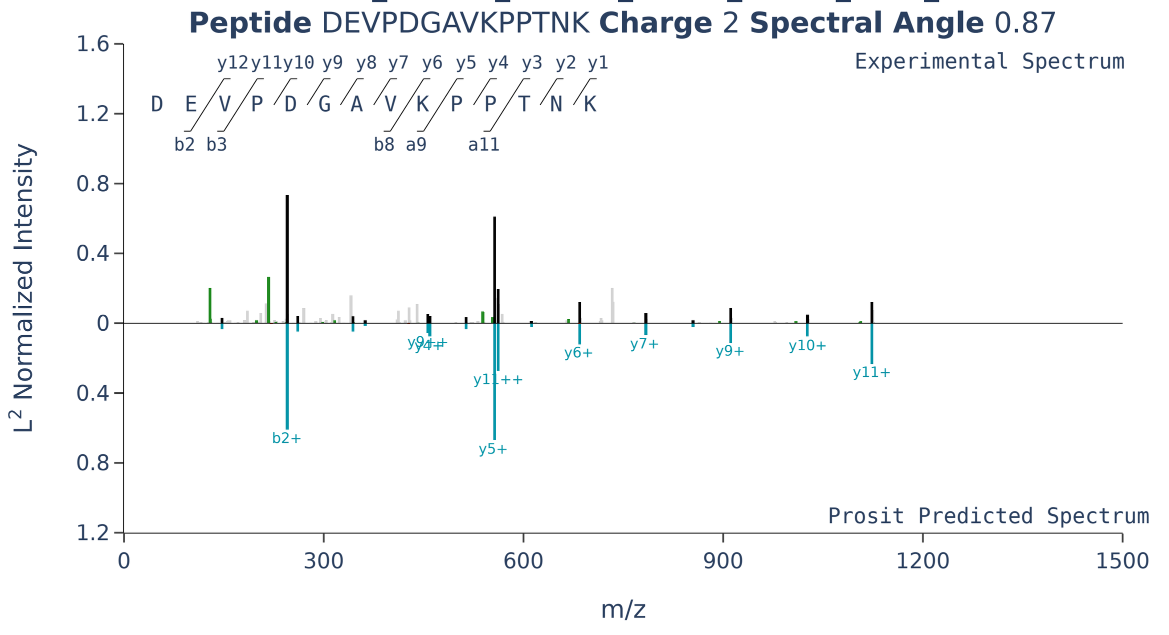


**Source** HRoetschke_271222_090123_HFGoe_LEDGF_b2R1_2h **Scan** 10079
**Peptide** LQVTMQQAQKH **Charge** 3 **Spectral Angle** 0.91

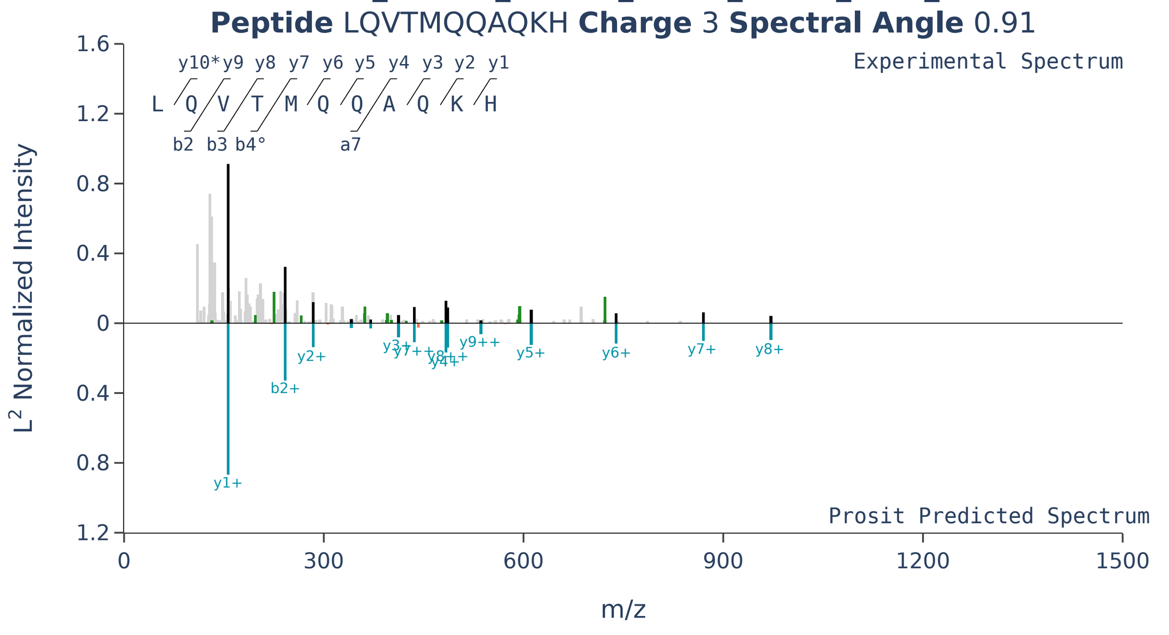


**Source** HRoetschke_271222_090123_HFGoe_LEDGF_b2R1_24h **Scan** 16481
**Peptide** DEVPDGAVKPPTNKL **Charge** 2 **Spectral Angle** 0.87

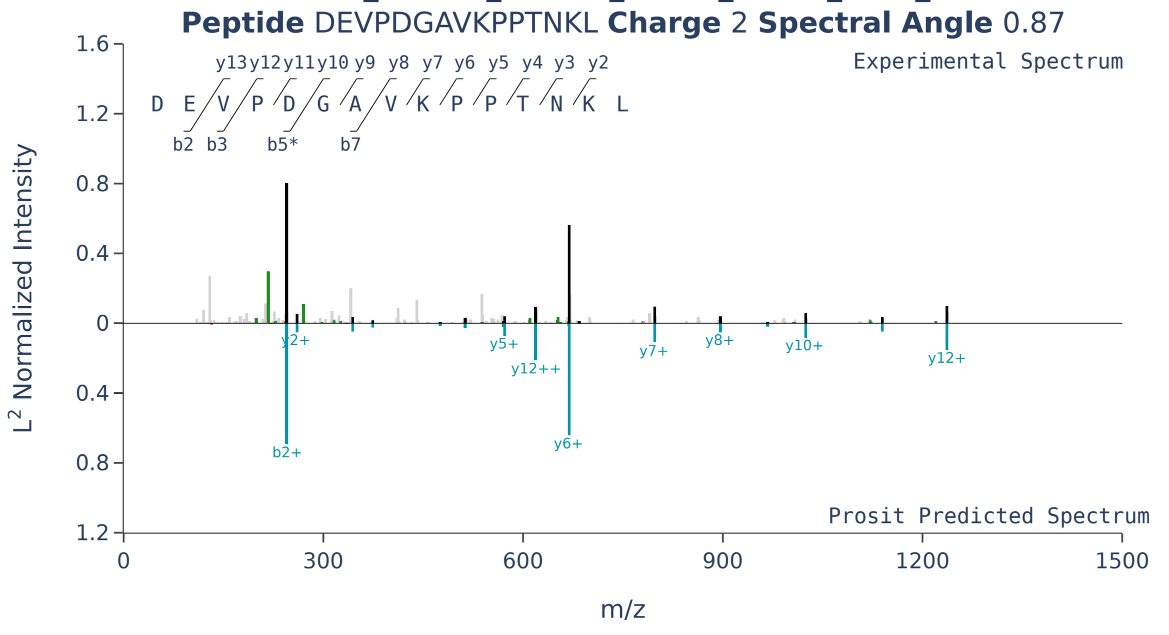


**Source** HRoetschke_271222_090123_HFGoe_LEDGF_b1R2_24h **Scan** 10491
**Peptide** AGVVTTAT **Charge** 1 **Spectral Angle** 0.93

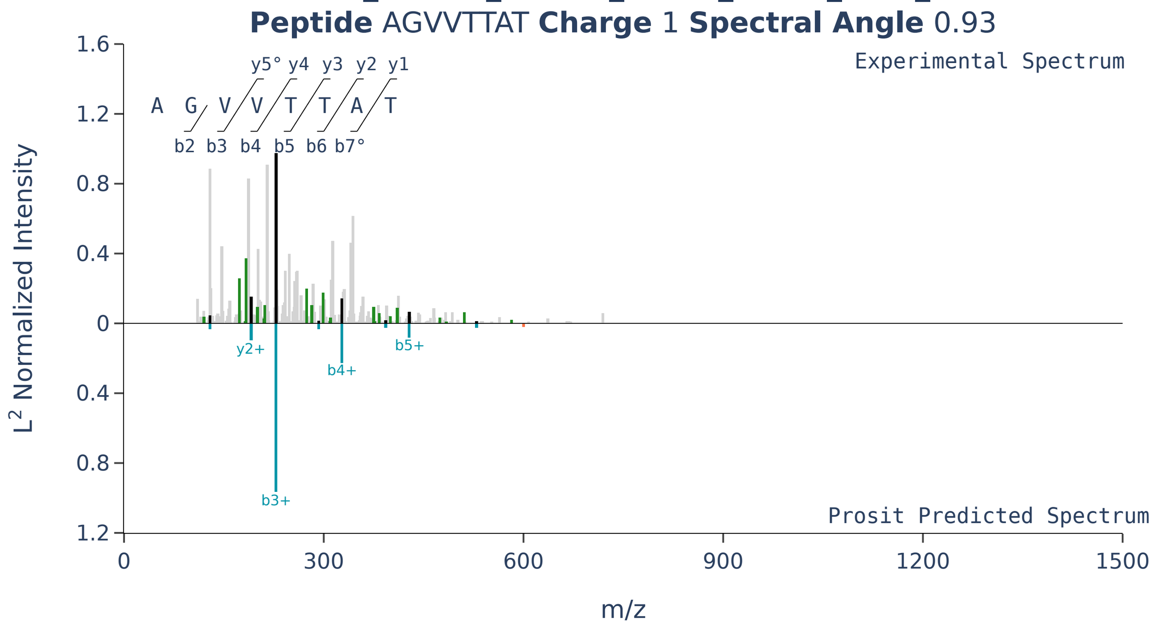


**Source** HRoetschke_271222_090123_HFGoe_LEDGF_b2R2_4h **Scan** 12864
**Peptide** KSTMLYNKF **Charge** 3 **Spectral Angle** 0.93

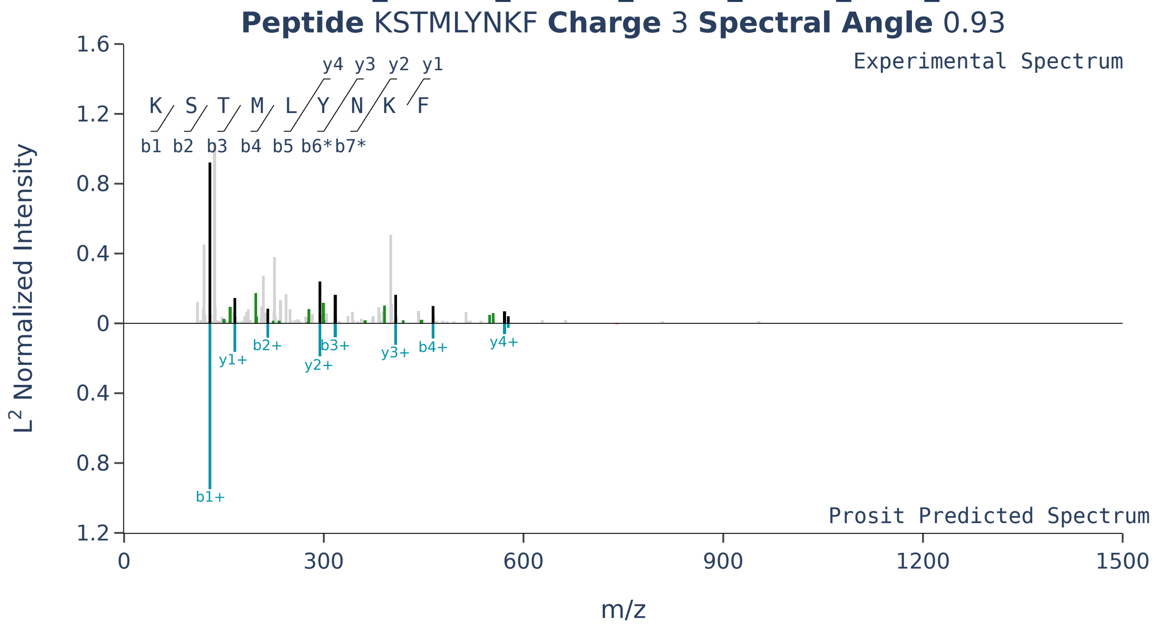


**Source** HRoetschke_271222_090123_HFGoe_LEDGF_b2R1_4h **Scan** 7226
**Peptide** VNLKVSPKRG **Charge** 3 **Spectral Angle** 0.94

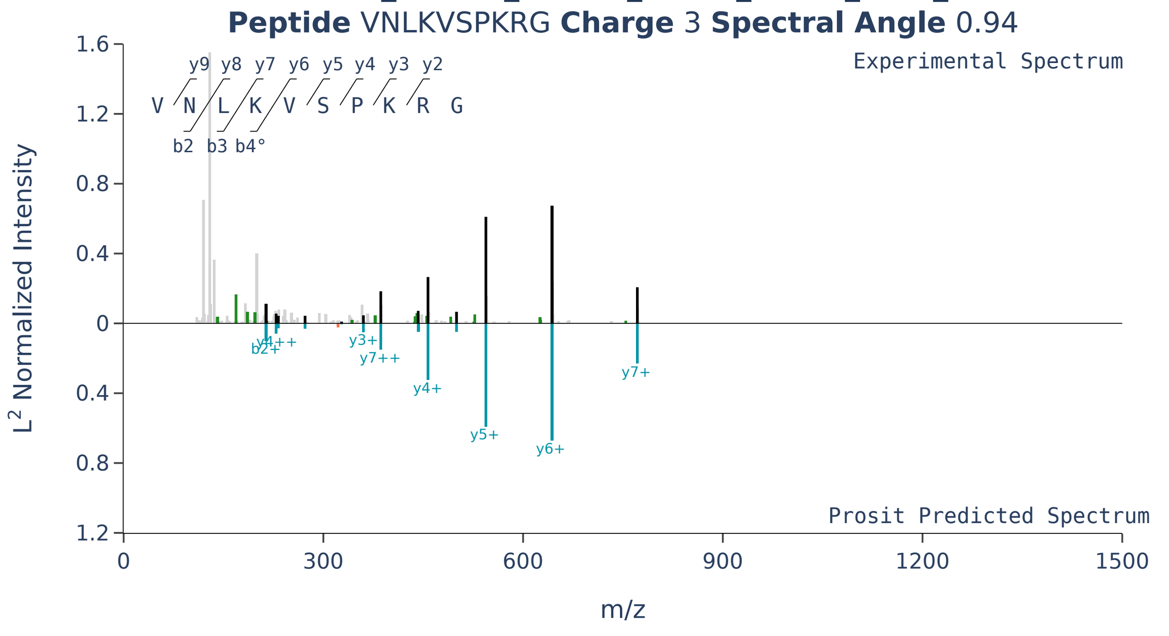


**Source** HRoetschke_271222_090123_HFGoe_LEDGF_b2R1_2h **Scan** 11380
**Peptide** SNASSDVEVEE **Charge** 2 **Spectral Angle** 0.93

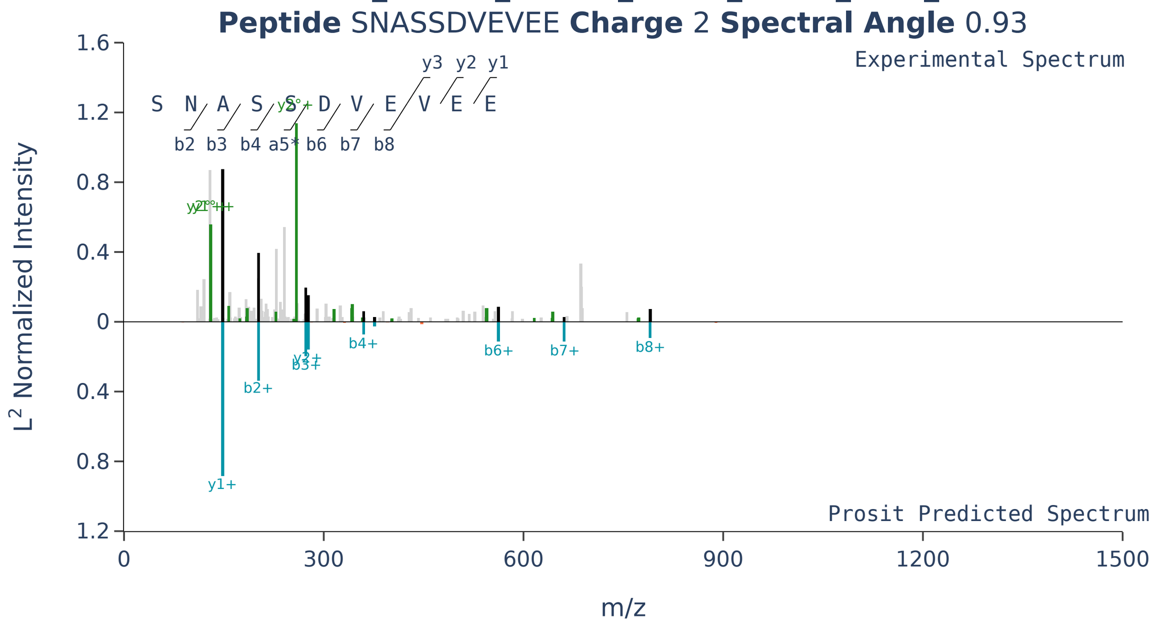


**Source** HRoetschke_271222_090123_HFGoe_LEDGF_b1R2_24h **Scan** 17311
**Peptide** TEISLKDSTLDN **Charge** 1 **Spectral Angle** 0.92

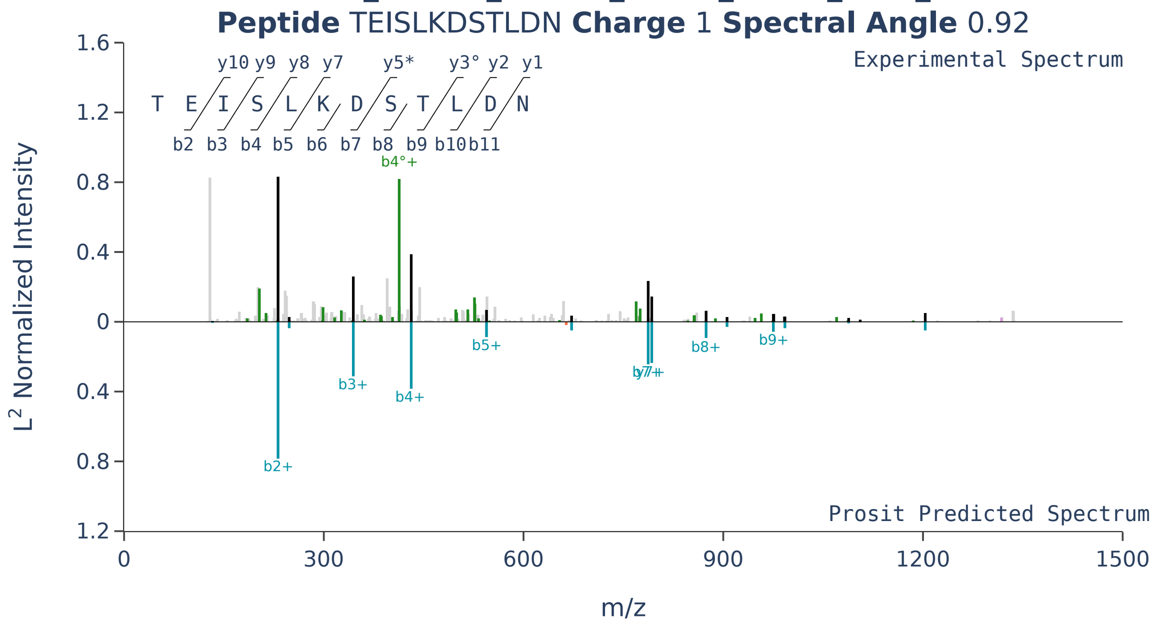


**Source** HRoetschke_271222_090123_HFGoe_LEDGF_b1R1_2h **Scan** 20501
**Peptide** ELASLQVTM **Charge** 2 **Spectral Angle** 0.94

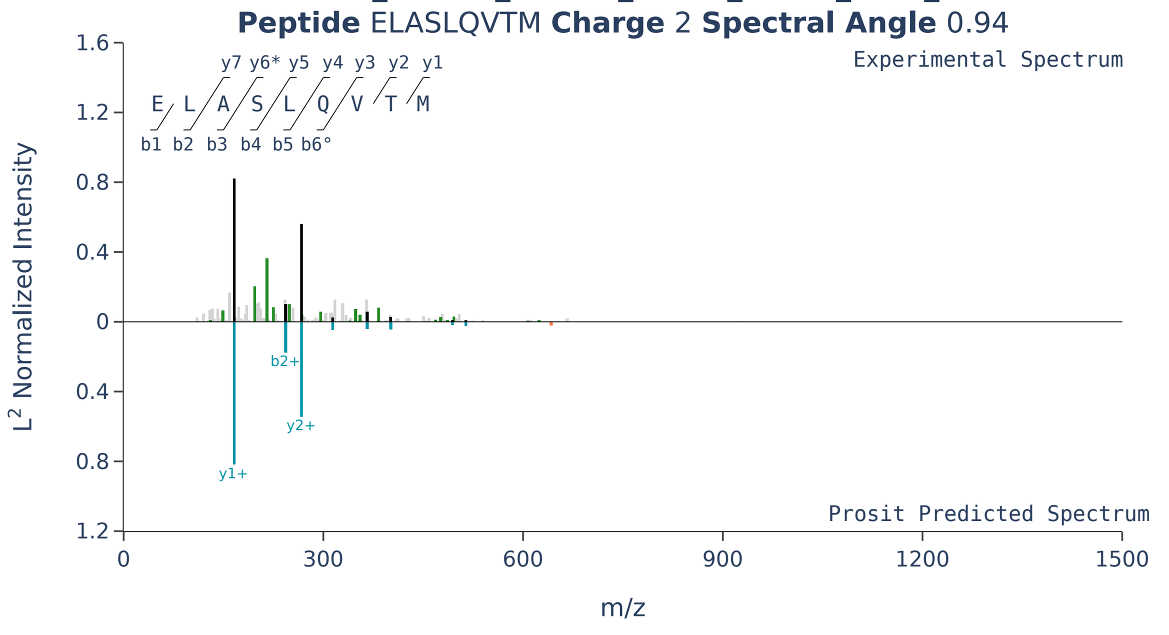


**Source** HRoetschke_271222_090123_HFGoe_LEDGF_b2R1_1h **Scan** 11101
**Peptide** ERETEISLKD **Charge** 3 **Spectral Angle** 0.94

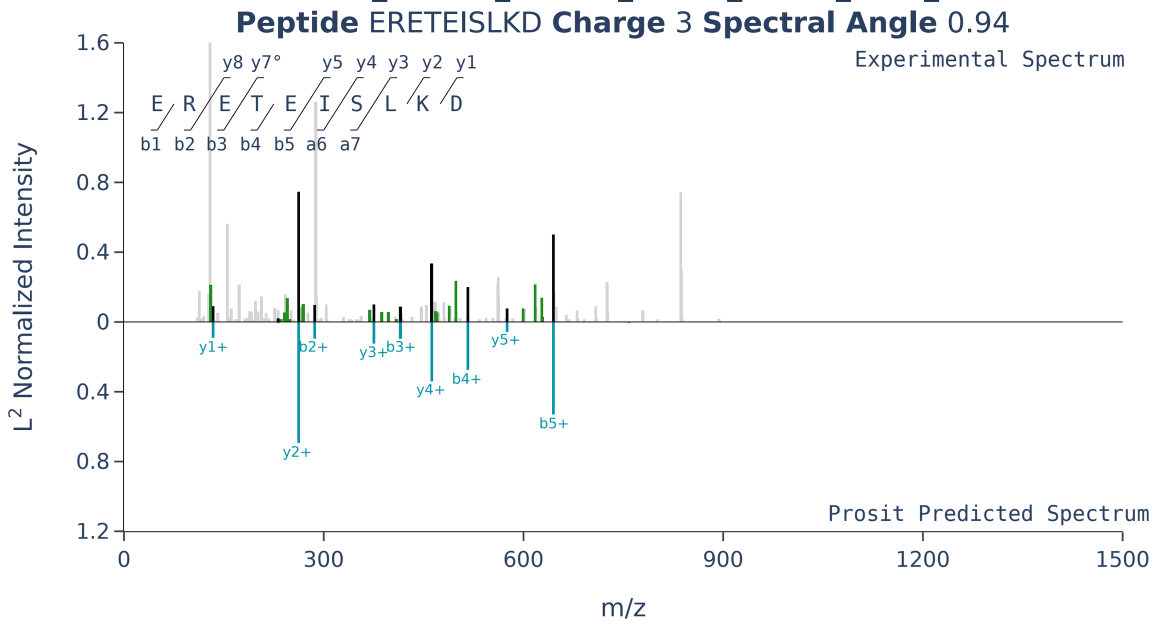


**Source** HRoetschke_271222_090123_HFGoe_LEDGF_b2R1_24h **Scan** 17071
**Peptide** YPHWPARVD **Charge** 3 **Spectral Angle** 0.93

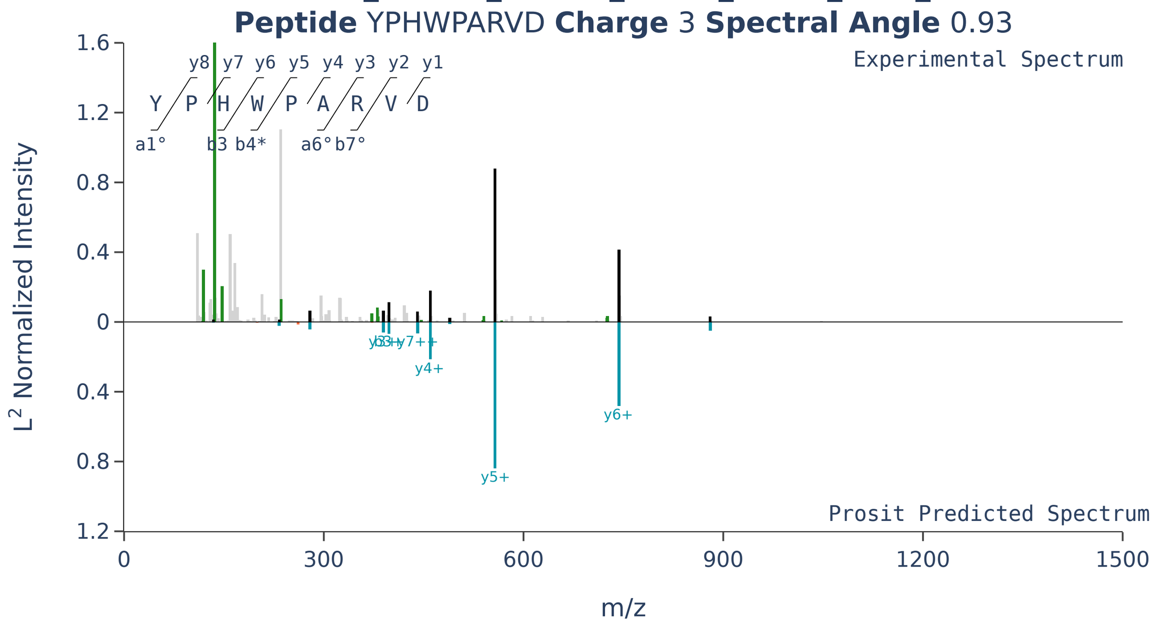


**Source** HRoetschke_271222_090123_HFGoe_LEDGF_b1R1_2h **Scan** 8083
**Peptide** SMDSRLQ **Charge** 2 **Spectral Angle** 0.95

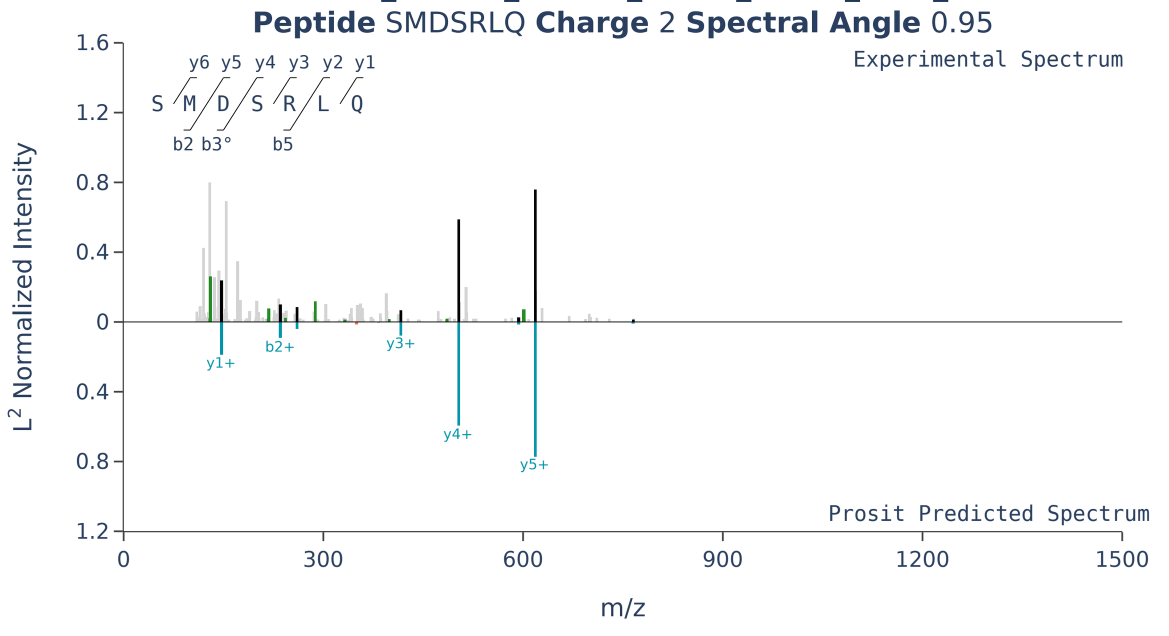

**Source** HRoetschke_271222_090123_HFGoe_LEDGF_b1R1_2h **Scan** 10614
**Peptide** RETEISLKD **Charge** 3 **Spectral Angle** 0.86

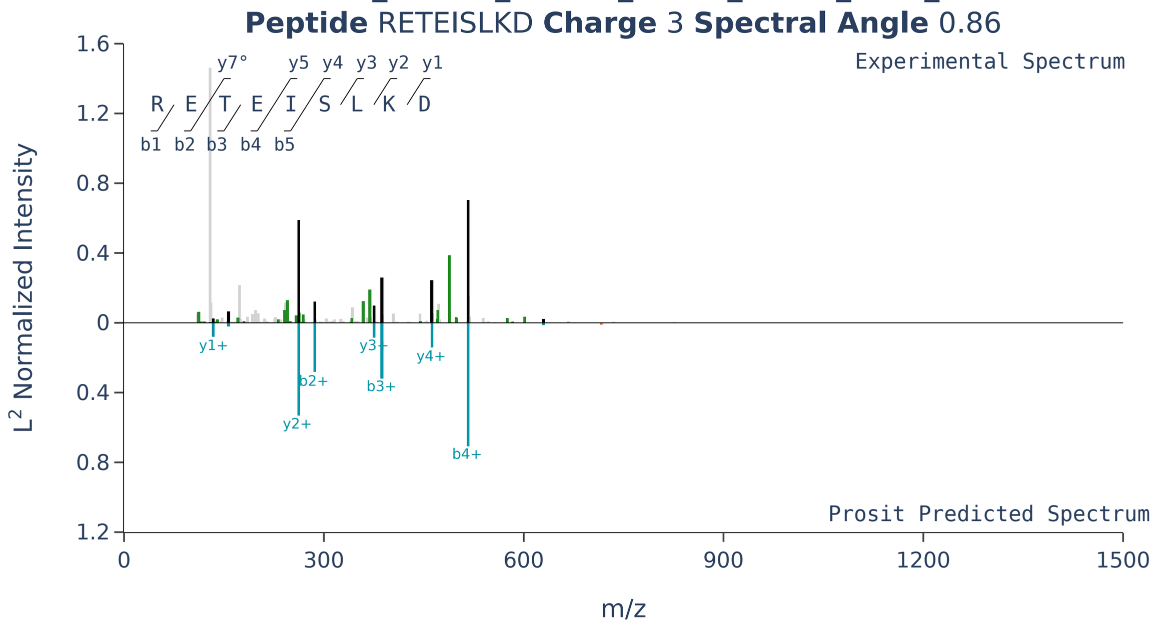


**Source** HRoetschke_271222_090123_HFGoe_LEDGF_b2R2_2h **Scan** 16869
**Peptide** MQQAQKHTEMITTL **Charge** 2 **Spectral Angle** 0.9

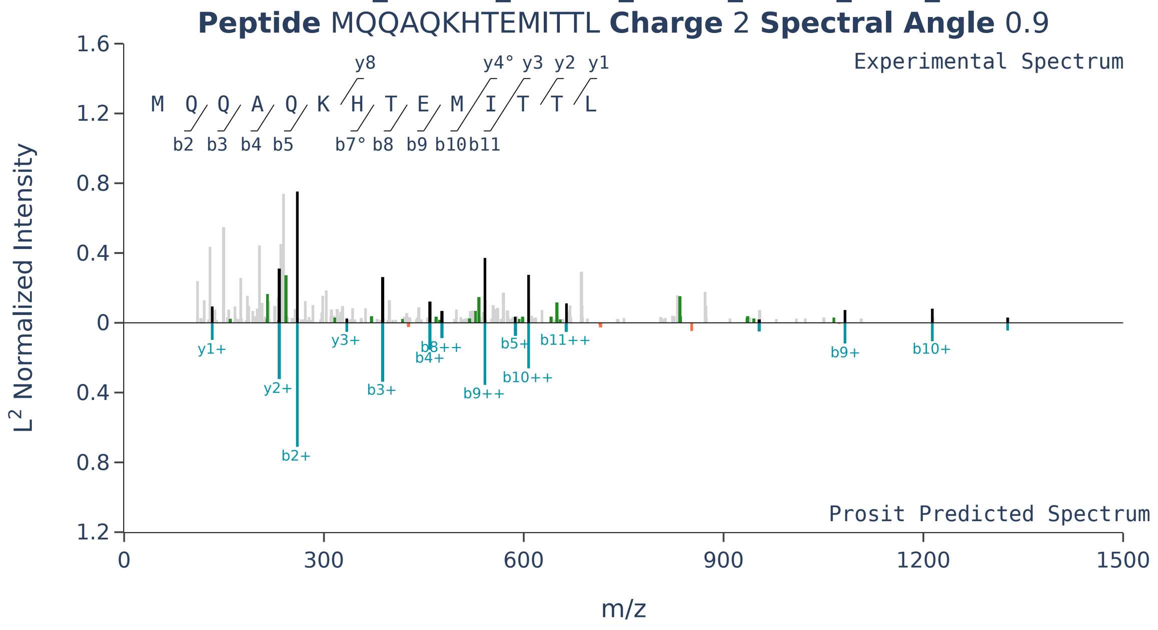


**Source** HRoetschke_271222_090123_HFGoe_LEDGF_b2R2_24h **Scan** 9480
**Peptide** SNEDVTKAV **Charge** 2 **Spectral Angle** 0.89

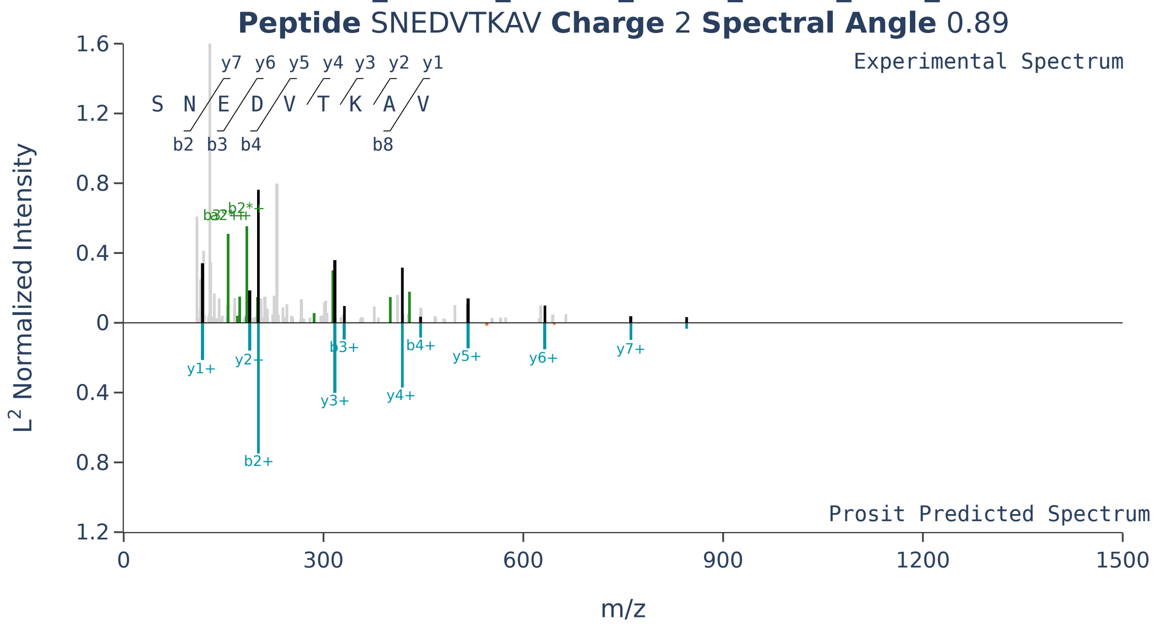


**Source** HRoetschke_271222_090123_HFGoe_LEDGF_b1R1_1h **Scan** 12520
**Peptide** IDNNPKVKF **Charge** 3 **Spectral Angle** 0.88

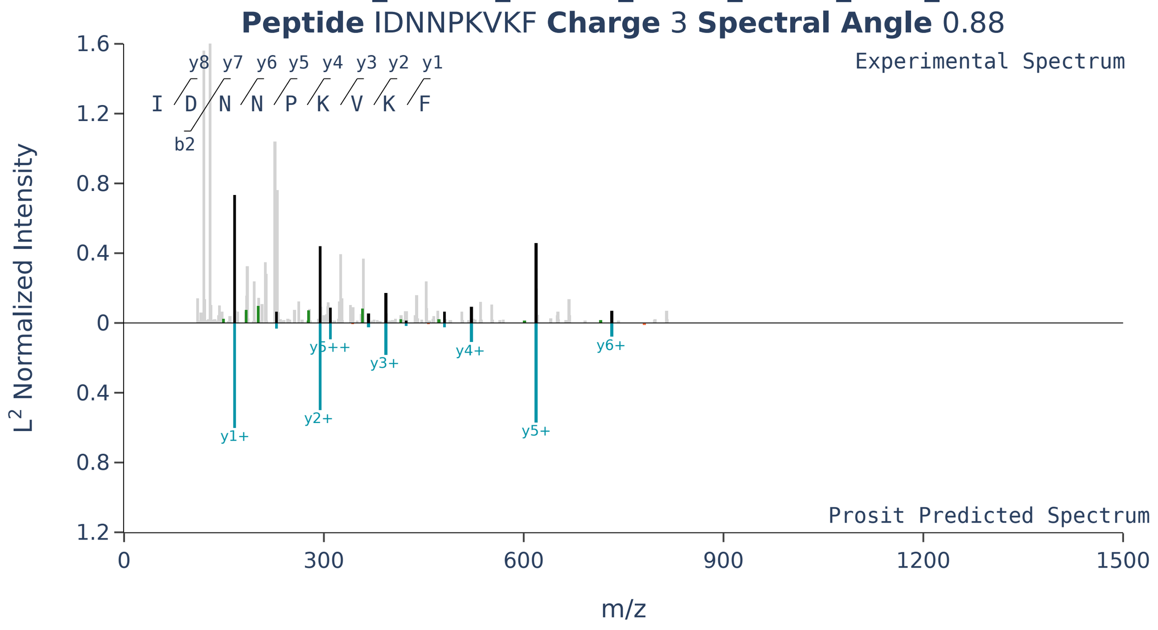


**Source** HRoetschke_271222_090123_HFGoe_LEDGF_b2R1_2h **Scan** 23840
**Peptide** NEGLWEIDNNPKVKFS **Charge** 2 **Spectral Angle** 0.95

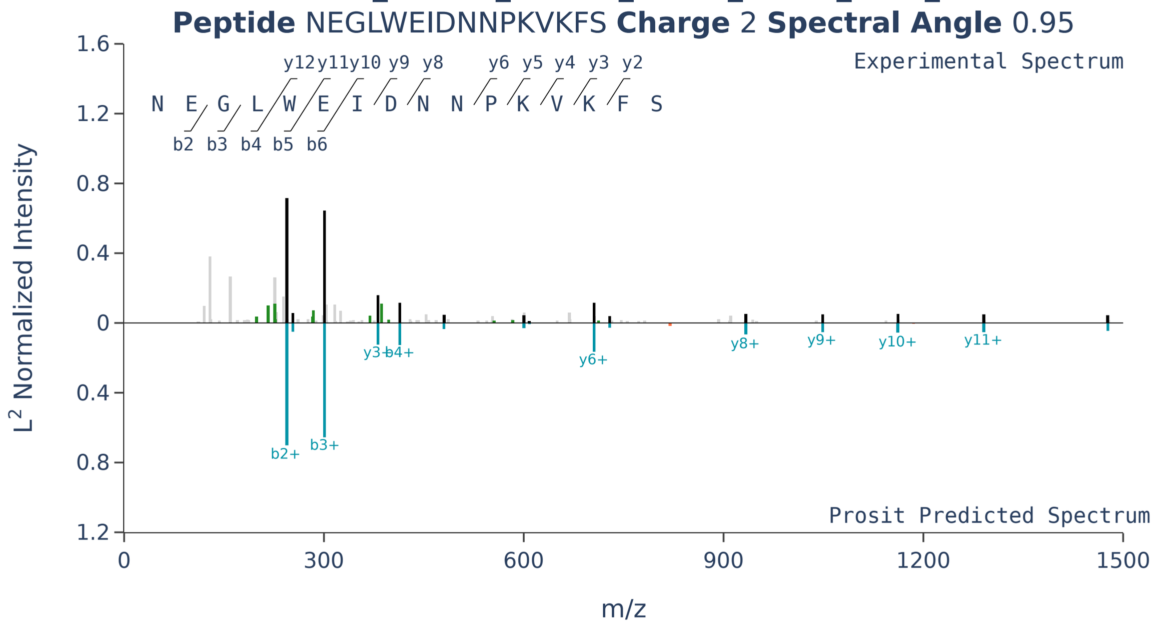


**Source** HRoetschke_271222_090123_HFGoe_LEDGF_b1R1_24h **Scan** 17764
**Peptide** SLKIDNLD **Charge** 1 **Spectral Angle** 0.93

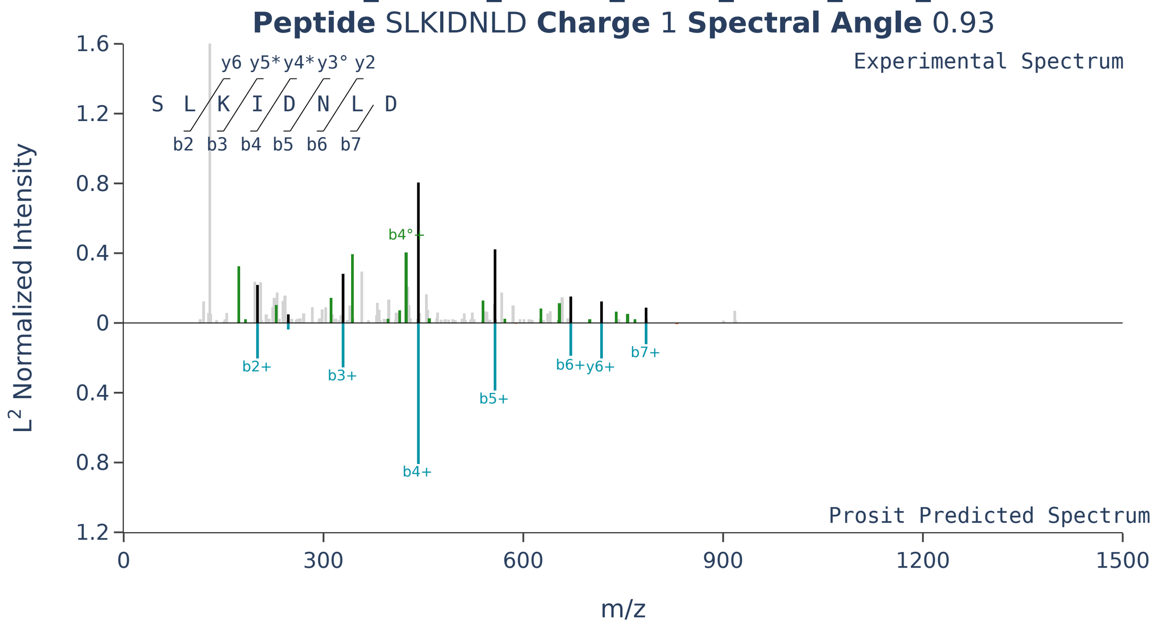


**Source** HRoetschke_271222_090123_HFGoe_LEDGF_b2R2_2h **Scan** 8249
**Peptide** FPYSENKEK **Charge** 3 **Spectral Angle** 0.92

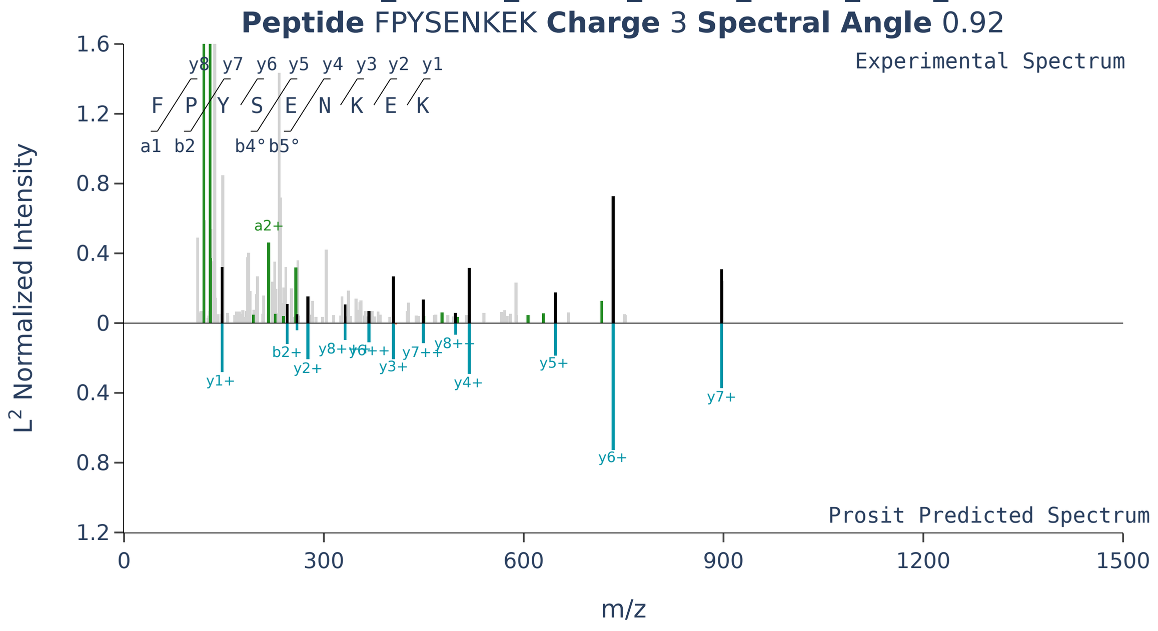


**Source** HRoetschke_271222_090123_HFGoe_LEDGF_b2R2_24h **Scan** 7368
**Peptide** DGAVKPPTN **Charge** 2 **Spectral Angle** 0.9

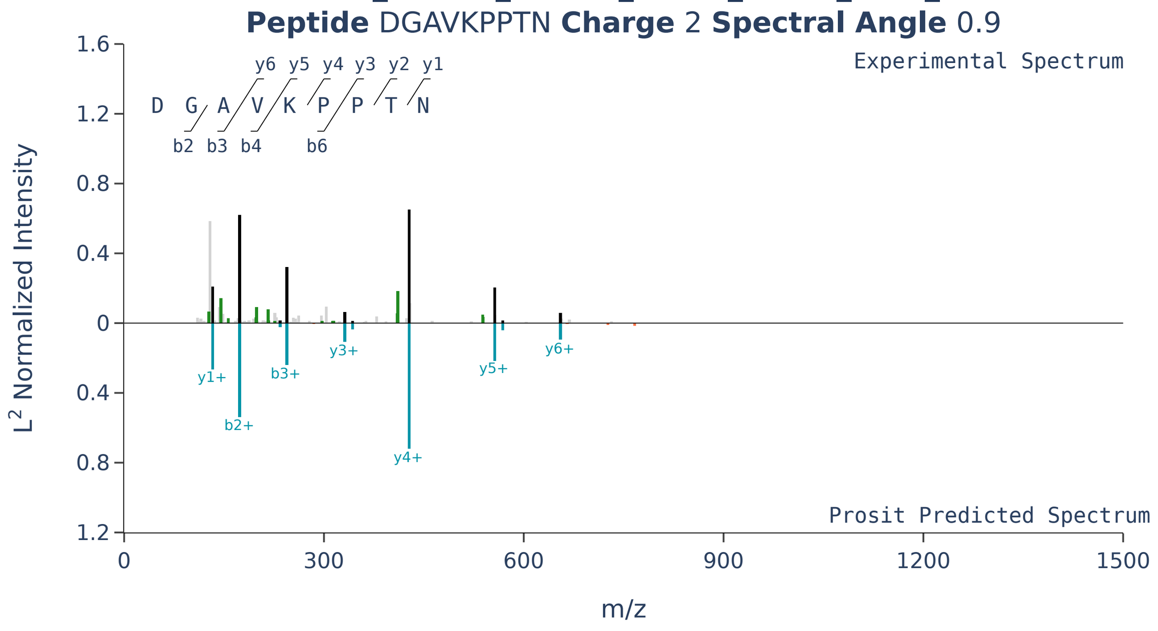


**Source** HRoetschke_271222_090123_HFGoe_LEDGF_b2R1_4h **Scan** 6319
**Peptide** LNKSLAEQRQ **Charge** 3 **Spectral Angle** 0.88

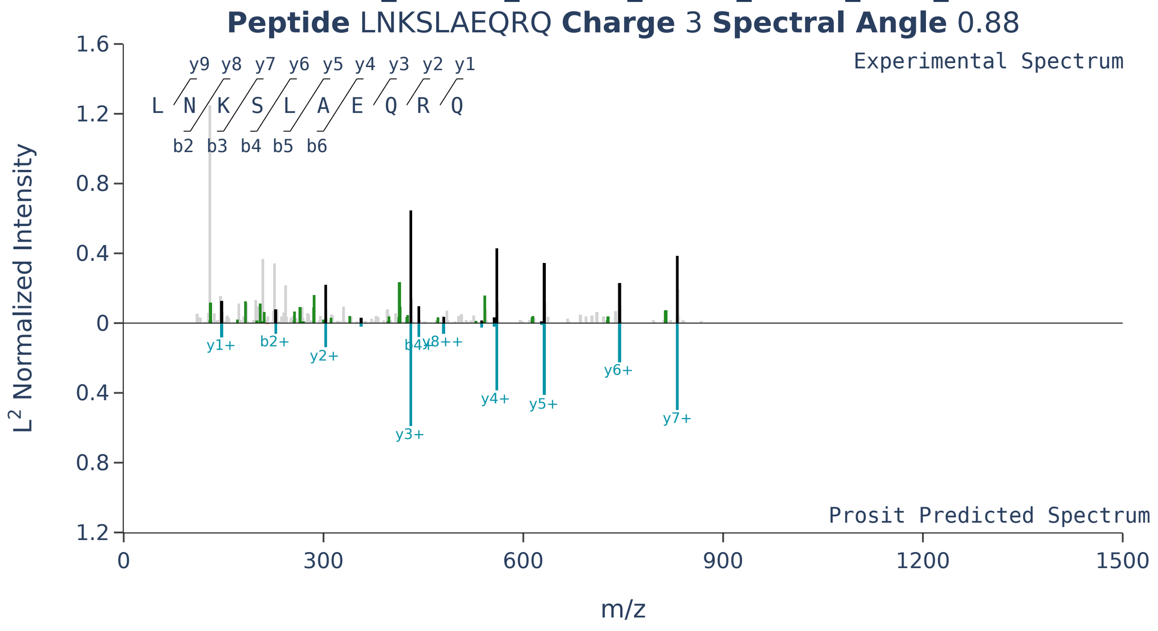


**Source** HRoetschke_271222_090123_HFGoe_LEDGF_b1R1_4h **Scan** 19567
**Peptide** LIFAKMKGY **Charge** 3 **Spectral Angle** 0.89

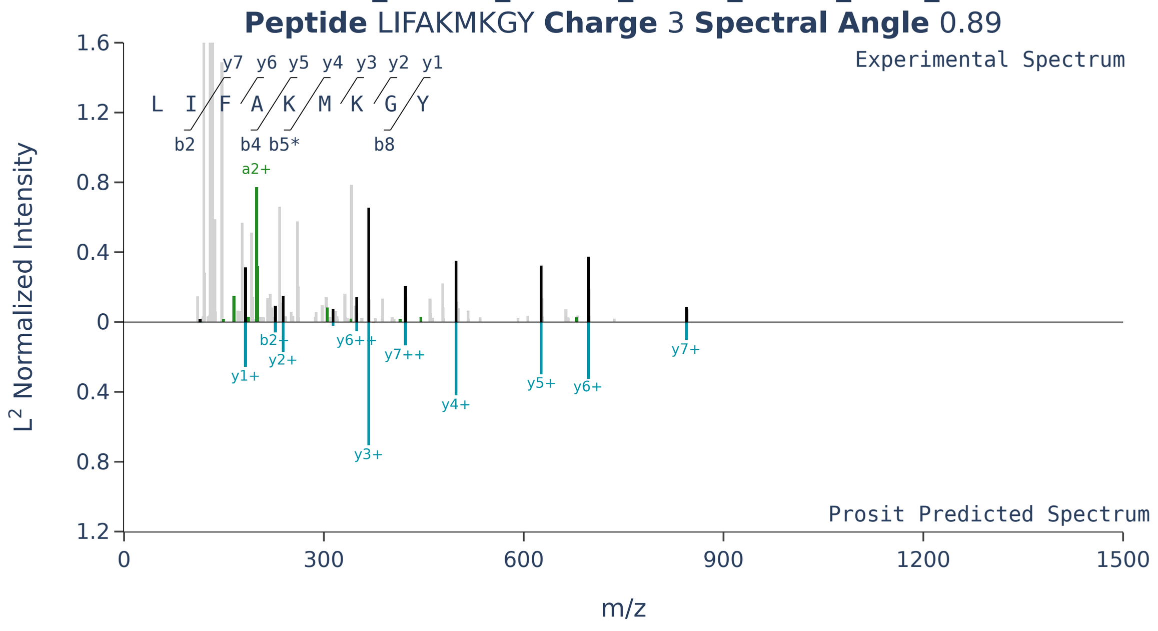


**Source** HRoetschke_271222_090123_HFGoe_LEDGF_b2R2_24h **Scan** 7263
**Peptide** TSMDSRLQR **Charge** 3 **Spectral Angle** 0.92

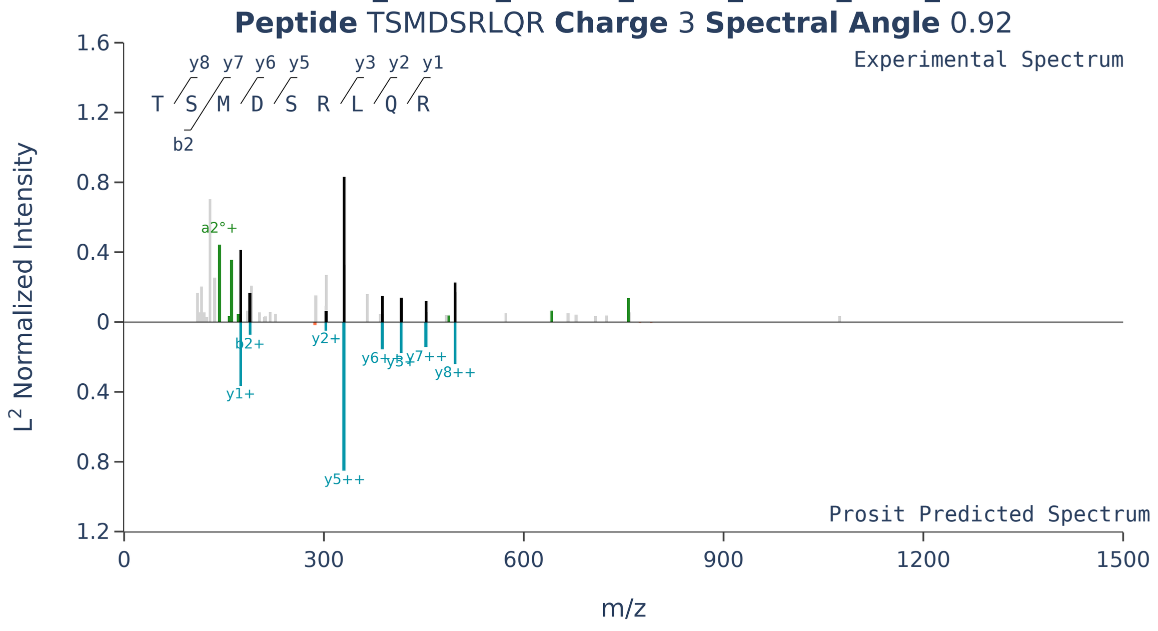


**Source** HRoetschke_271222_090123_HFGoe_LEDGF_b2R1_24h **Scan** 8694
**Peptide** ETSMDSRLQRIHA **Charge** 4 **Spectral Angle** 0.9

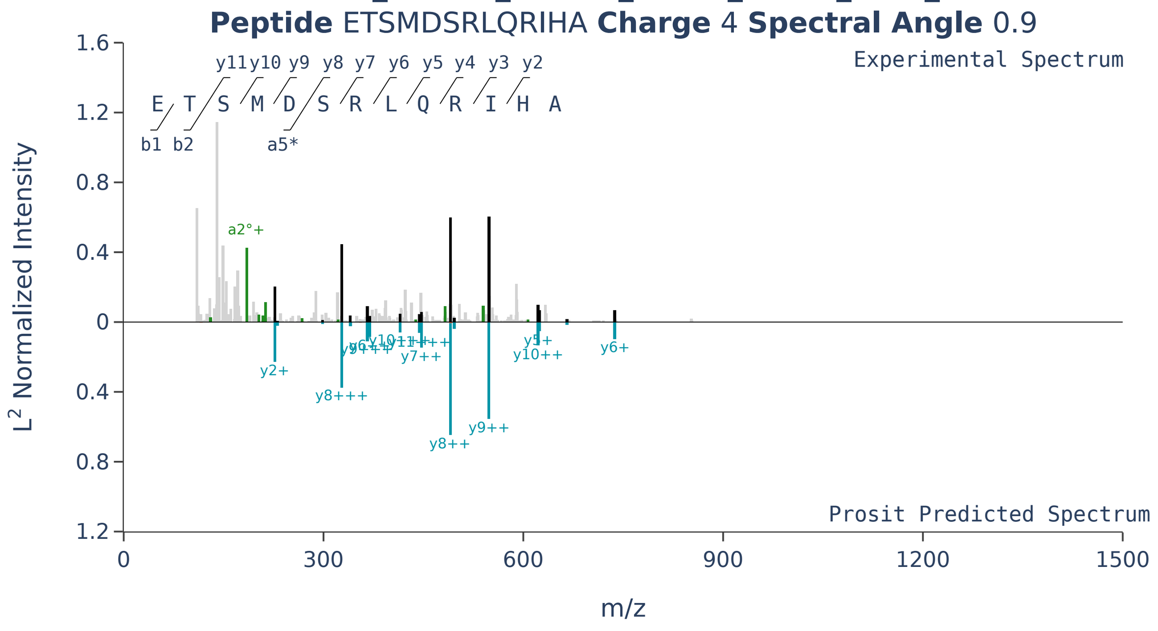


**Source** HRoetschke_271222_090123_HFGoe_LEDGF_b1R2_4h **Scan** 7963
**Peptide** DNLDVNR **Charge** 2 **Spectral Angle** 0.91

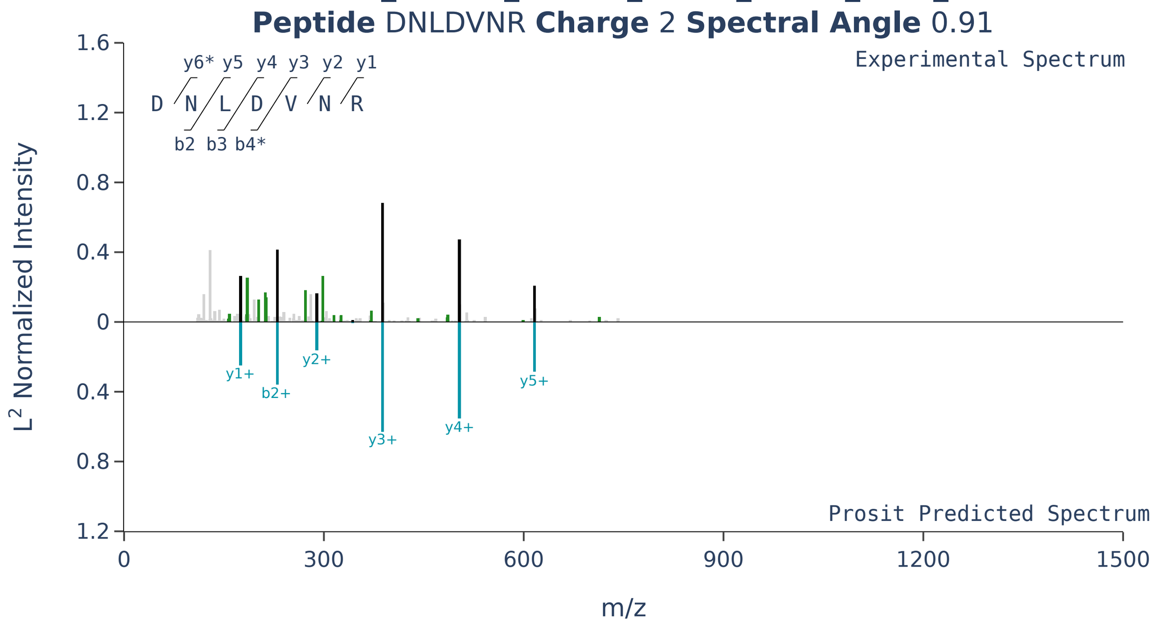


**Source** HRoetschke_271222_090123_HFGoe_LEDGF_b1R1_2h **Scan** 17303
**Peptide** YPHWPARVDE **Charge** 3 **Spectral Angle** 0.92

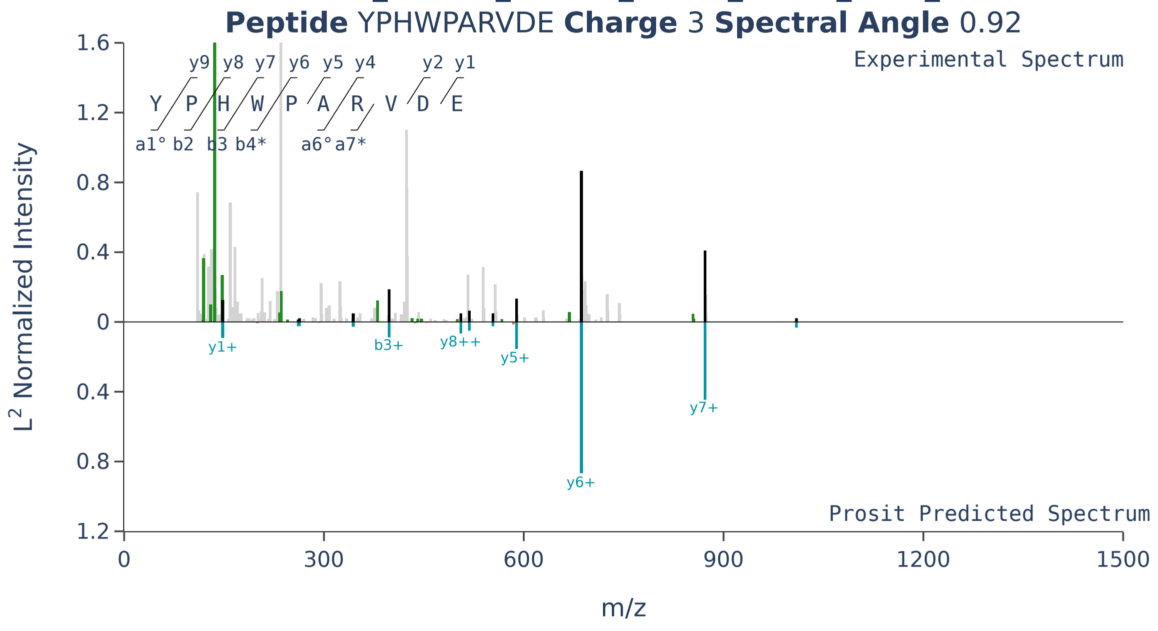


**Source** HRoetschke_271222_090123_HFGoe_LEDGF_b1R1_24h **Scan** 14836
**Peptide** TEMITTLKKIR **Charge** 3 **Spectral Angle** 0.91

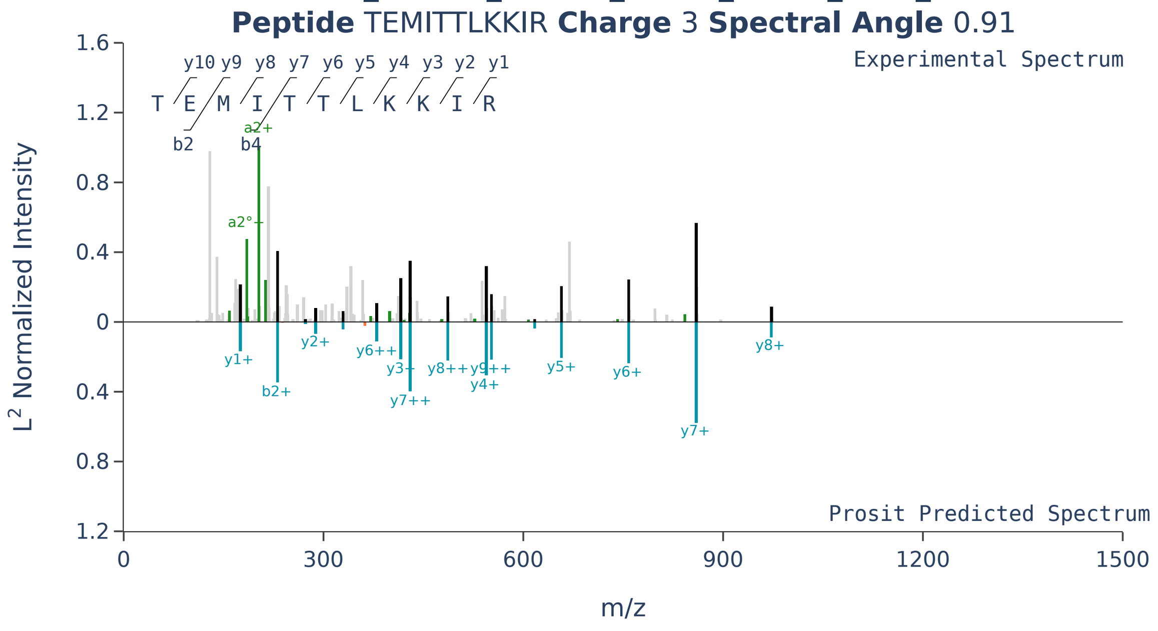

**Source** HRoetschke_271222_090123_HFGoe_LEDGF_b2R2_2h **Scan** 23236
**Peptide** GPKDIFPY **Charge** 2 **Spectral Angle** 0.91

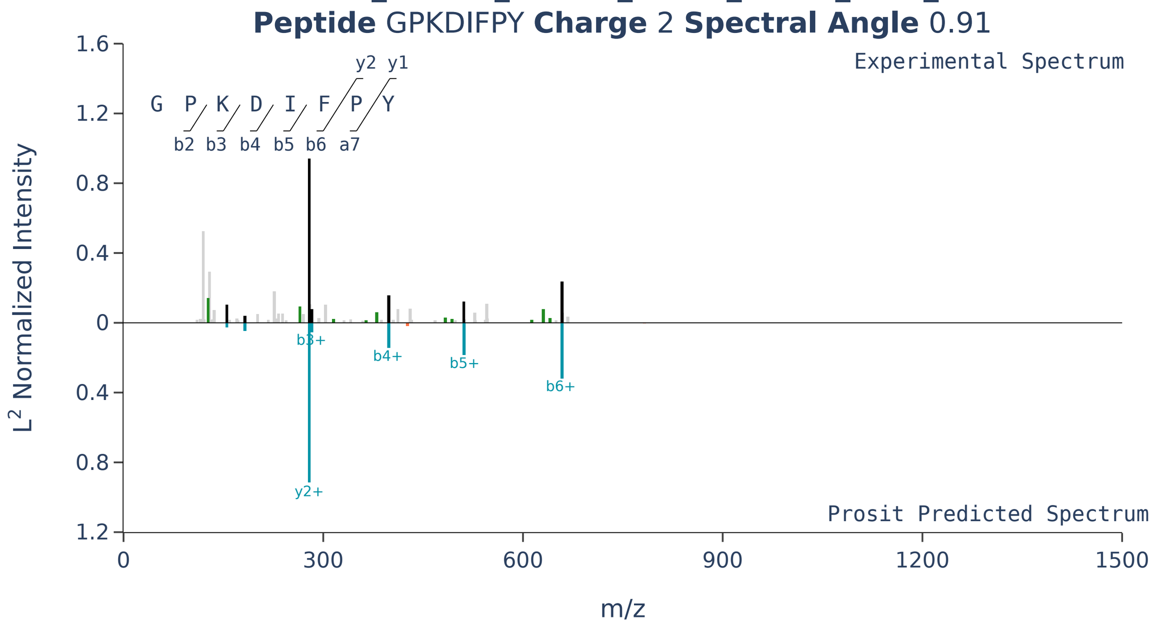


**Source** HRoetschke_271222_090123_HFGoe_LEDGF_b2R2_24h **Scan** 17058
**Peptide** GYPHWPARVD **Charge** 3 **Spectral Angle** 0.94

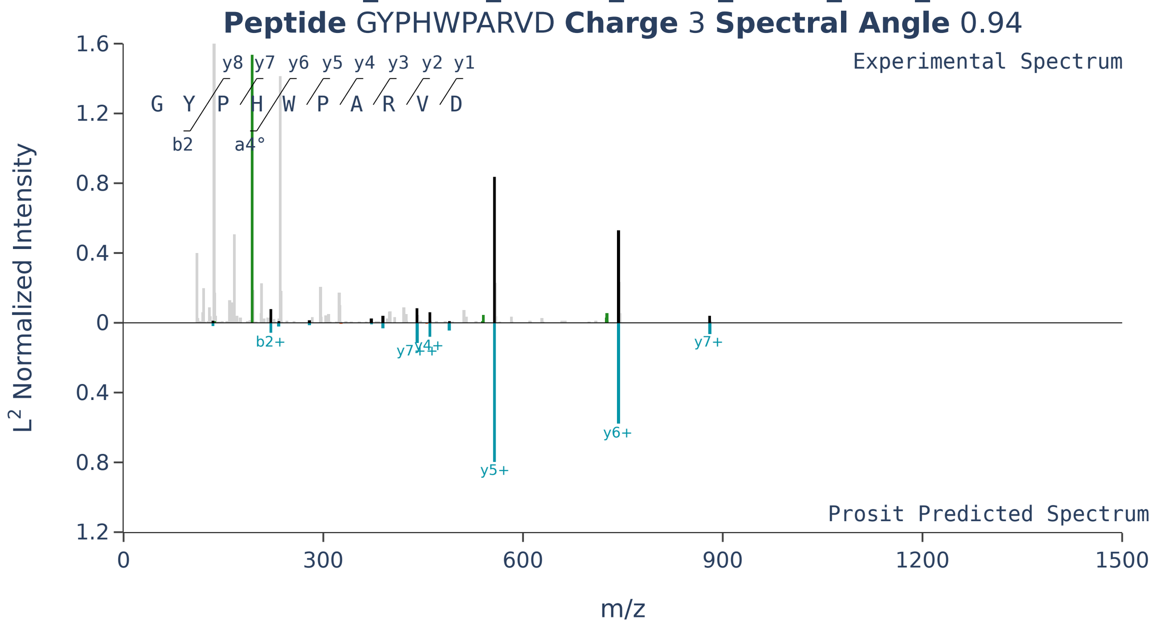


**Source** HRoetschke_271222_090123_HFGoe_LEDGF_b2R2_4h **Scan** 7954
**Peptide** ITTPKAA **Charge** 2 **Spectral Angle** 0.91

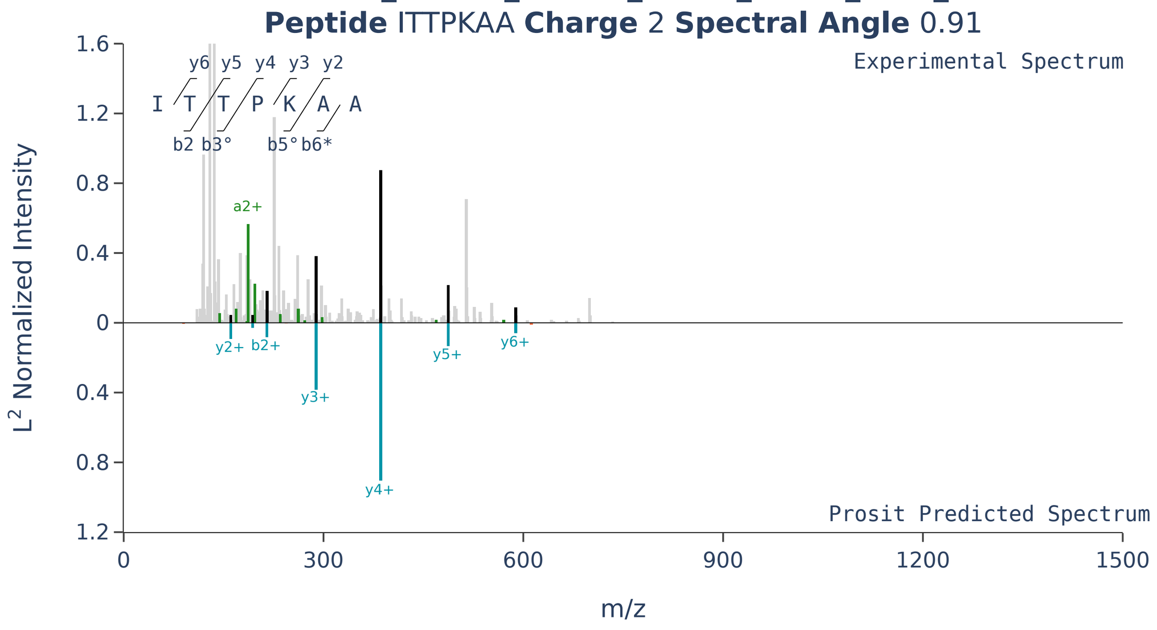


**Source** HRoetschke_271222_090123_HFGoe_LEDGF_b1R1_2h **Scan** 10308
**Peptide** ASSDVEVEE **Charge** 2 **Spectral Angle** 0.94

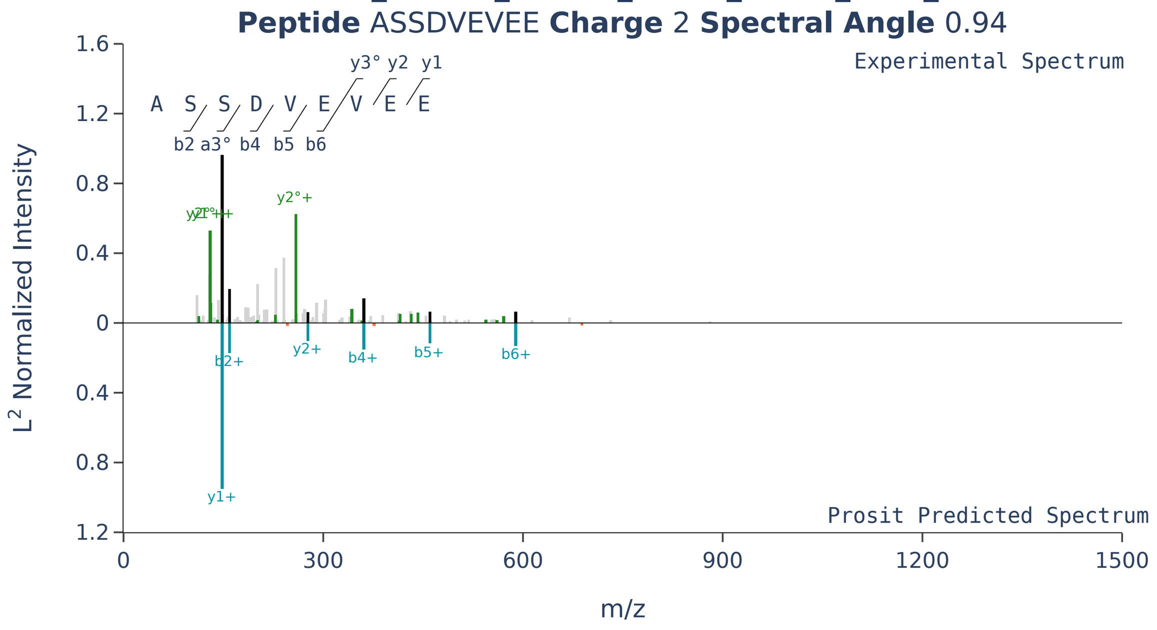


**Source** HRoetschke_271222_090123_HFGoe_LEDGF_b2R1_4h **Scan** 16720
**Peptide** DNLDVNRCIE **Charge** 2 **Spectral Angle** 0.9

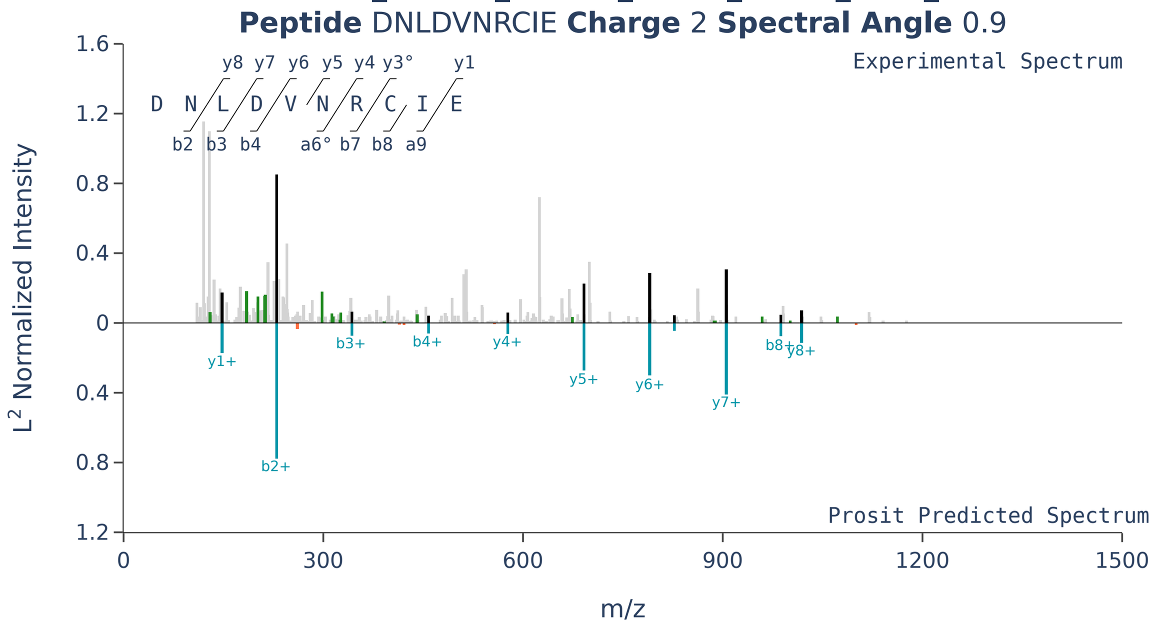


**Source** HRoetschke_271222_090123_HFGoe_LEDGF_b1R2_2h **Scan** 6763
**Peptide** MDSRLQR **Charge** 2 **Spectral Angle** 0.97

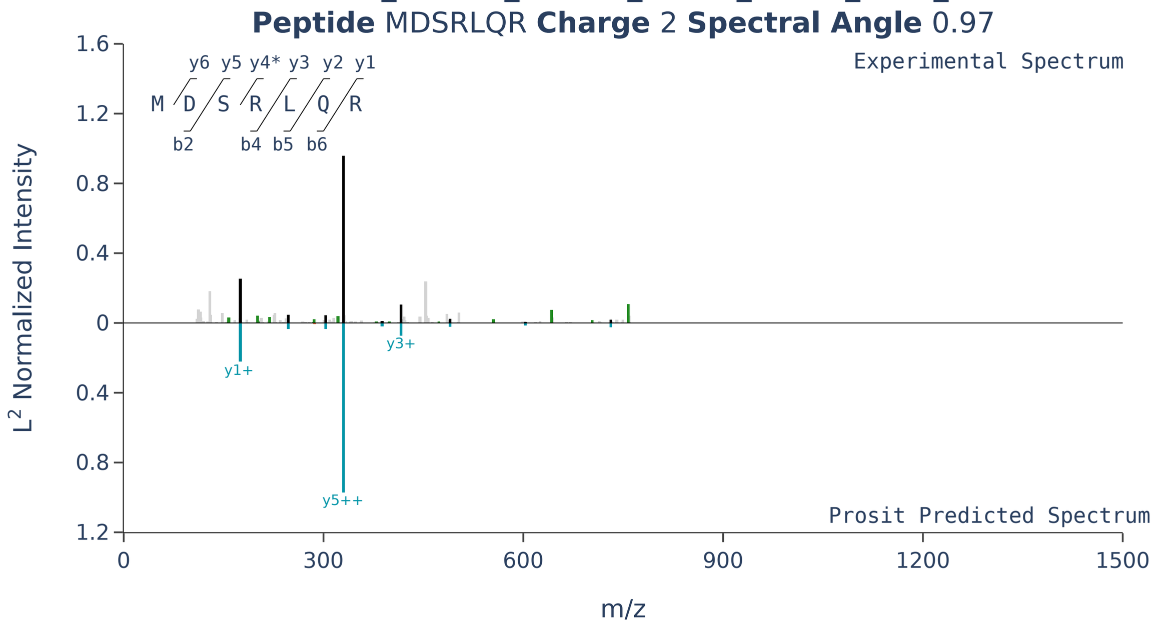


**Source** HRoetschke_271222_090123_HFGoe_LEDGF_b2R1_24h **Scan** 8891
**Peptide** DNNPKVKFSSQ **Charge** 2 **Spectral Angle** 0.9

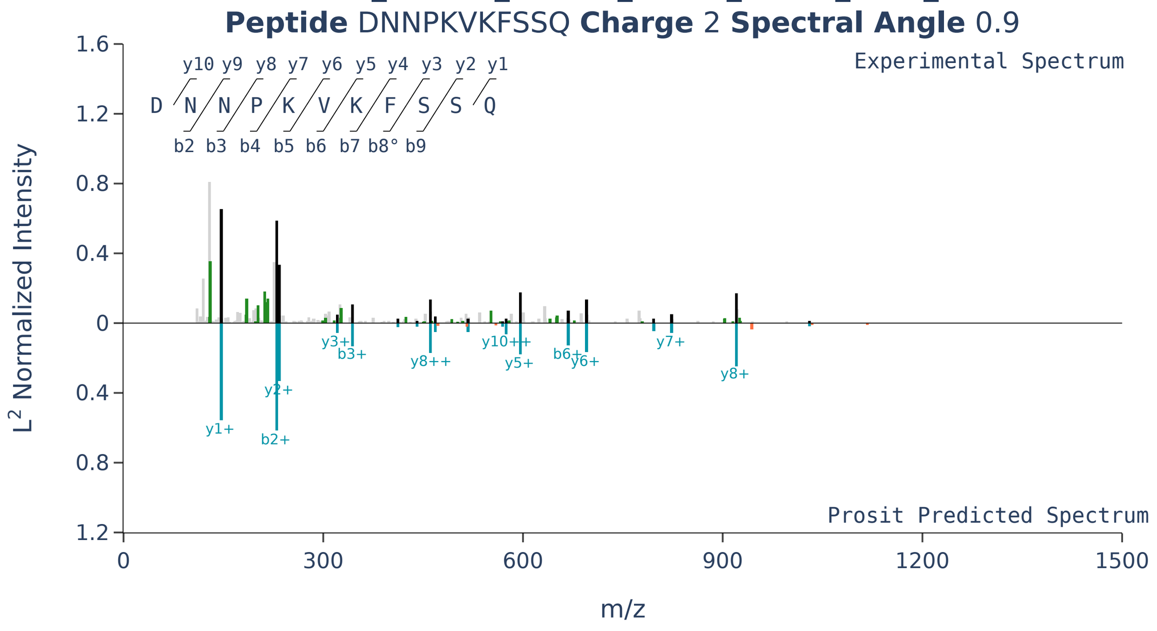


**Source** HRoetschke_271222_090123_HFGoe_LEDGF_b1R1_4h **Scan** 11772
**Peptide** DGAVKPPTNKL **Charge** 3 **Spectral Angle** 0.85

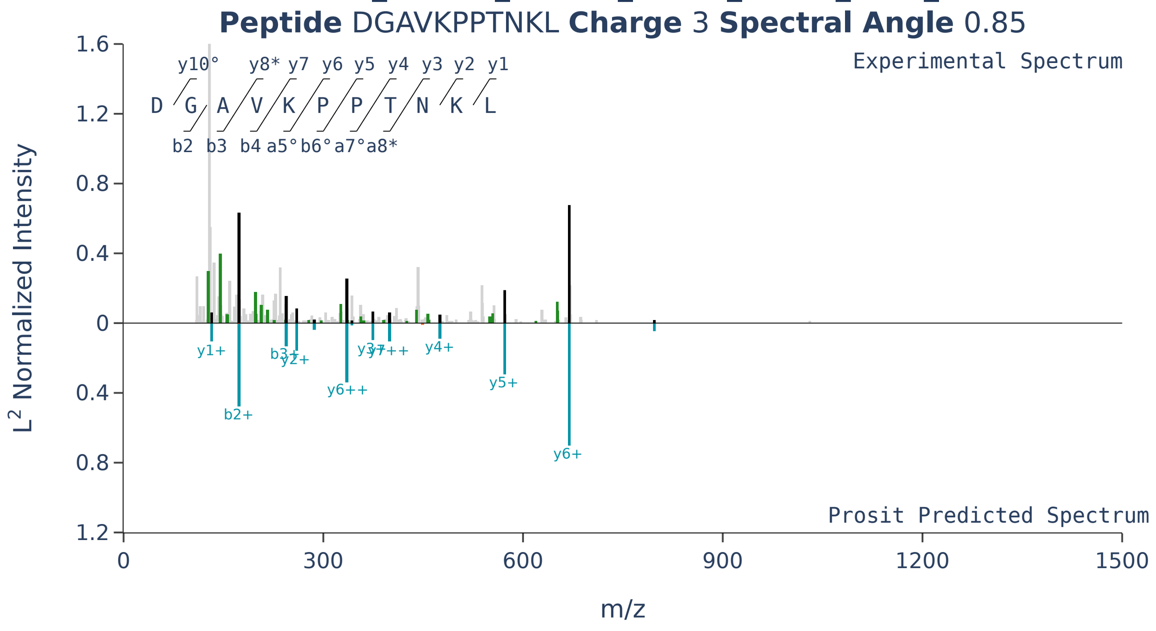


**Source** HRoetschke_271222_090123_HFGoe_LEDGF_b2R2_1h **Scan** 11613
**Peptide** MLYNKFKNM **Charge** 3 **Spectral Angle** 0.87

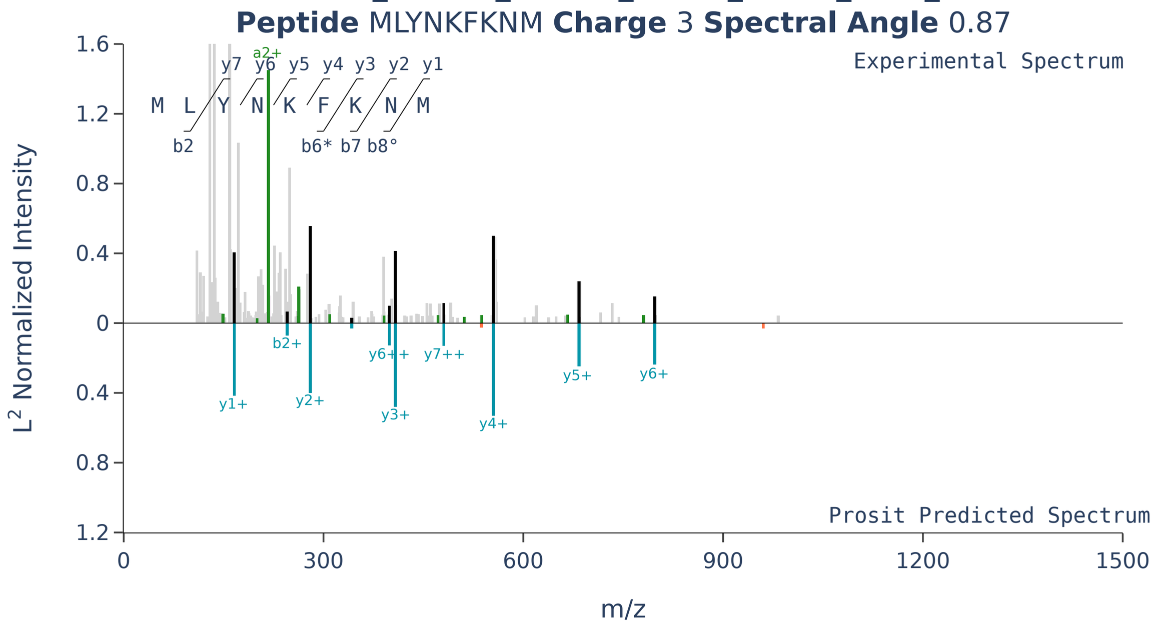


**Source** HRoetschke_271222_090123_HFGoe_LEDGF_b1R2_24h **Scan** 13059
**Peptide** TEEAGVVTTA **Charge** 1 **Spectral Angle** 0.89

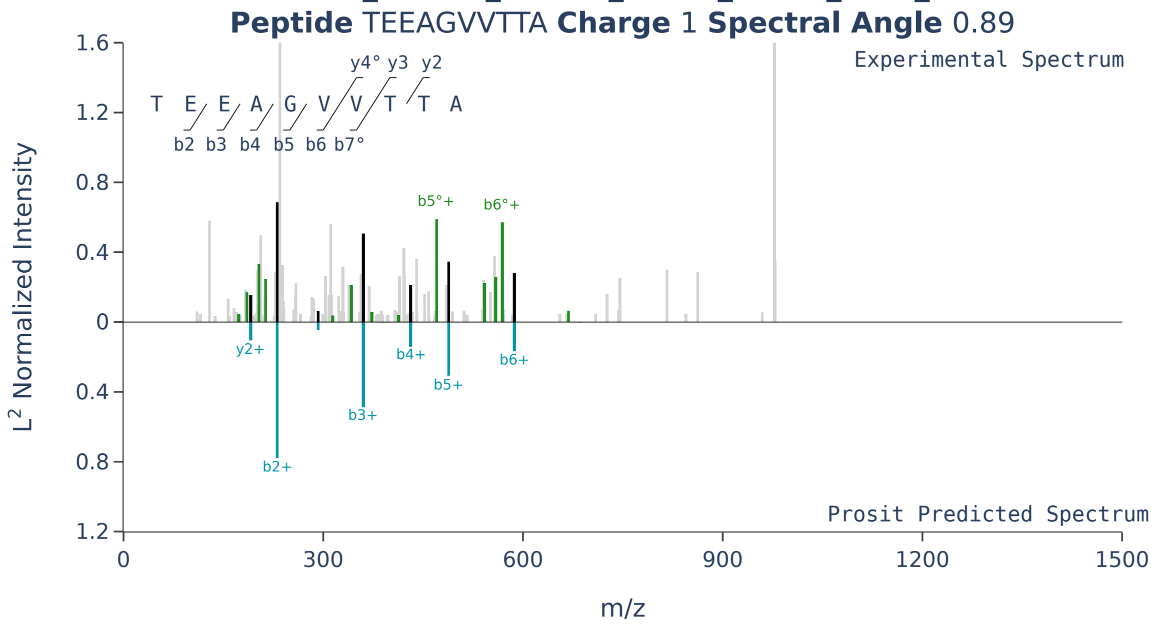


**Source** HRoetschke_271222_090123_HFGoe_LEDGF_b1R2_2h **Scan** 9475
**Peptide** KVSQVIMEKS **Charge** 2 **Spectral Angle** 0.92

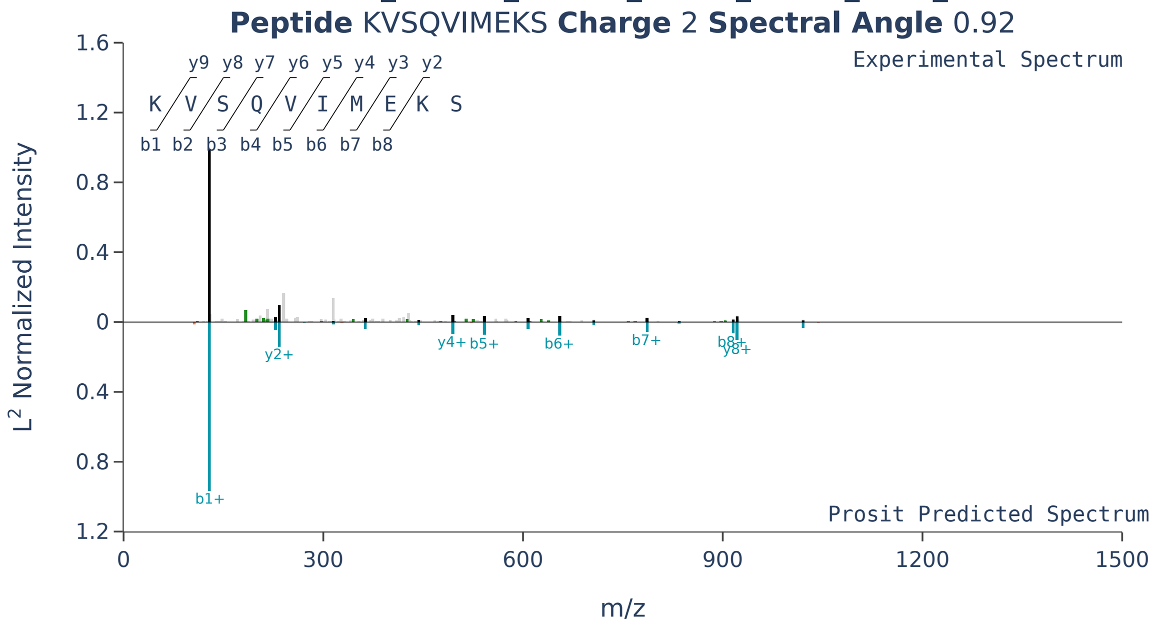


**Source** HRoetschke_271222_090123_HFGoe_LEDGF_b2R2_24h **Scan** 13876
**Peptide** TEEAGVVTTATA **Charge** 2 **Spectral Angle** 0.92

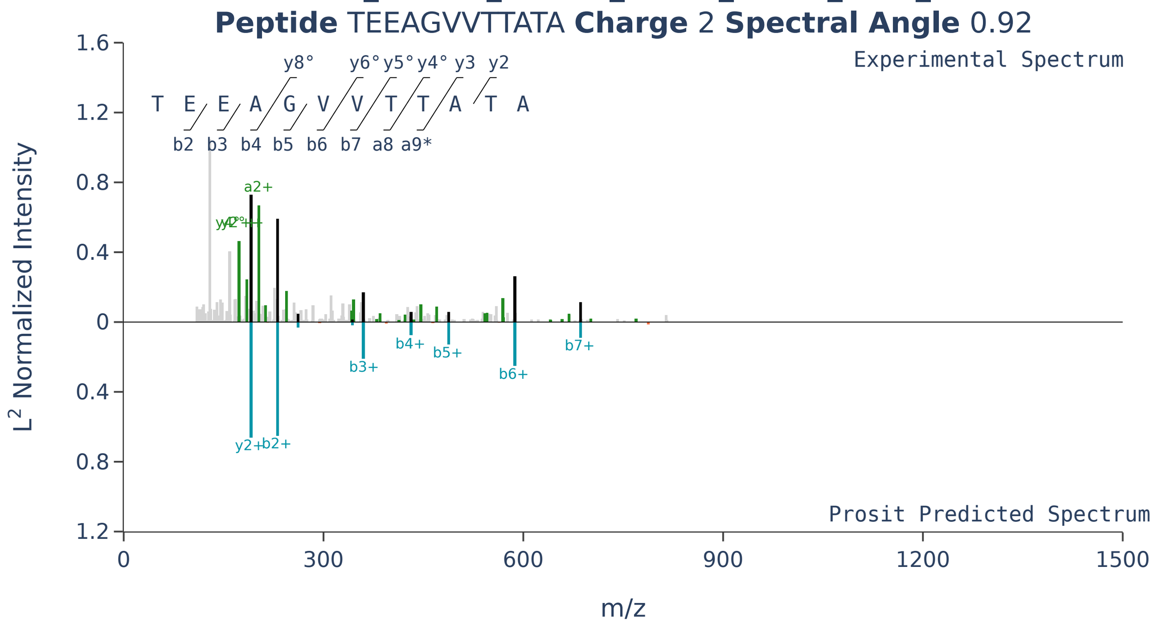


**Source** HRoetschke_271222_090123_HFGoe_LEDGF_b2R2_4h **Scan** 13335
**Peptide** EMITTLKKIR **Charge** 3 **Spectral Angle** 0.9

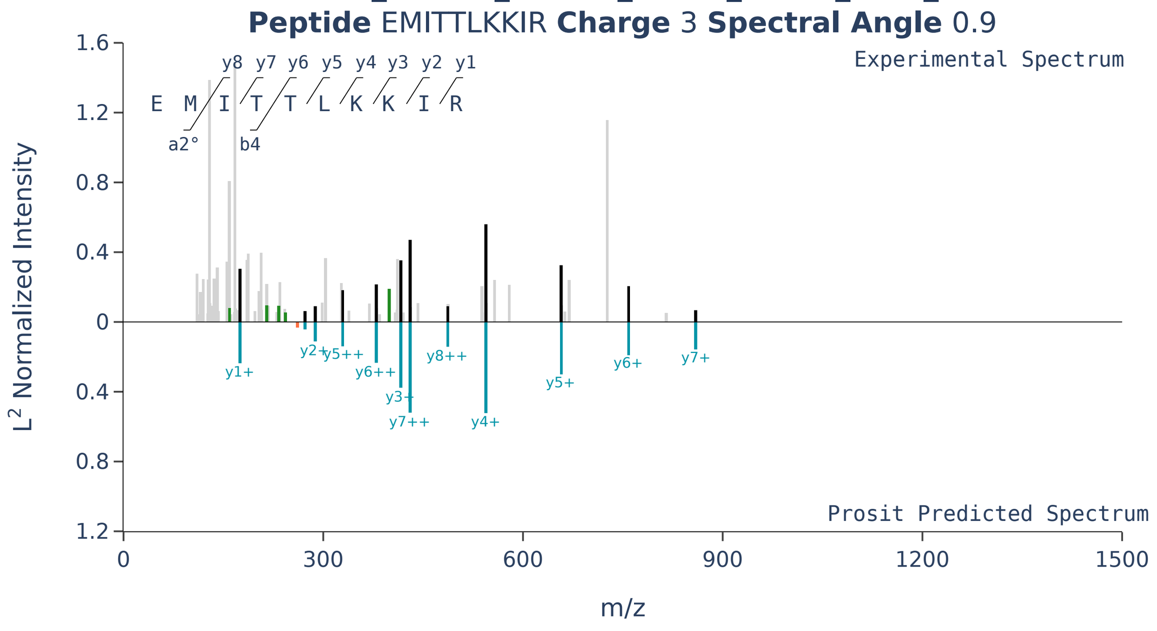


**Source** HRoetschke_271222_090123_HFGoe_LEDGF_b1R2_4h **Scan** 7086
**Peptide** IFPYSENKEKYGKPNKRKG **Charge** 3 **Spectral Angle** 0.89

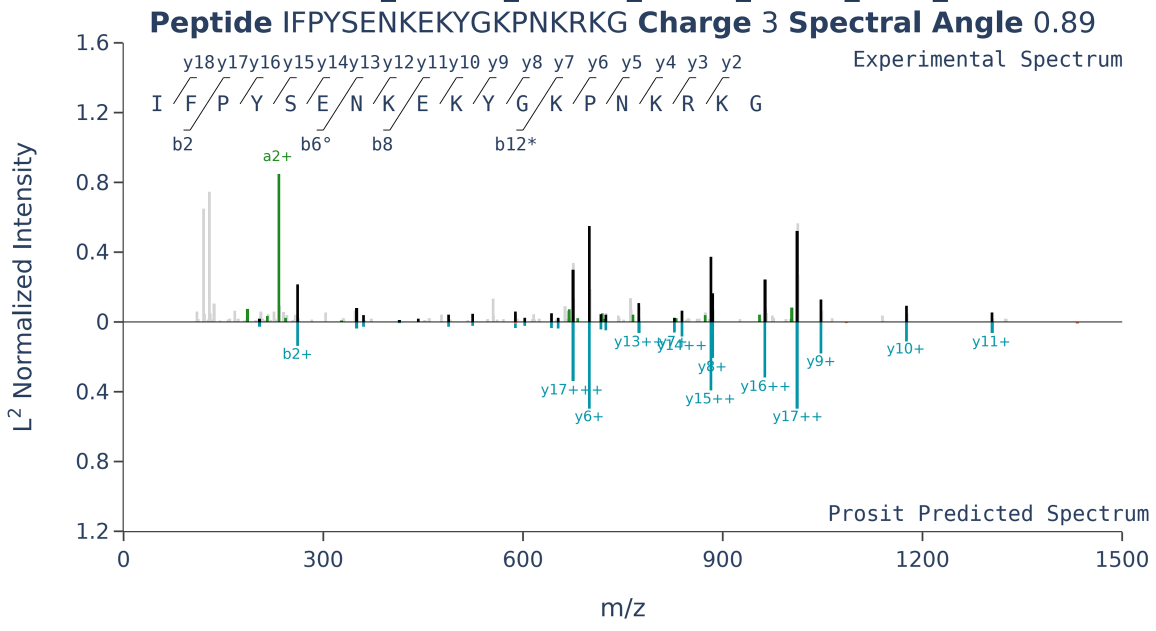


**Source** HRoetschke_271222_090123_HFGoe_LEDGF_b1R2_2h **Scan** 7755
**Peptide** VIMEKST **Charge** 2 **Spectral Angle** 0.92

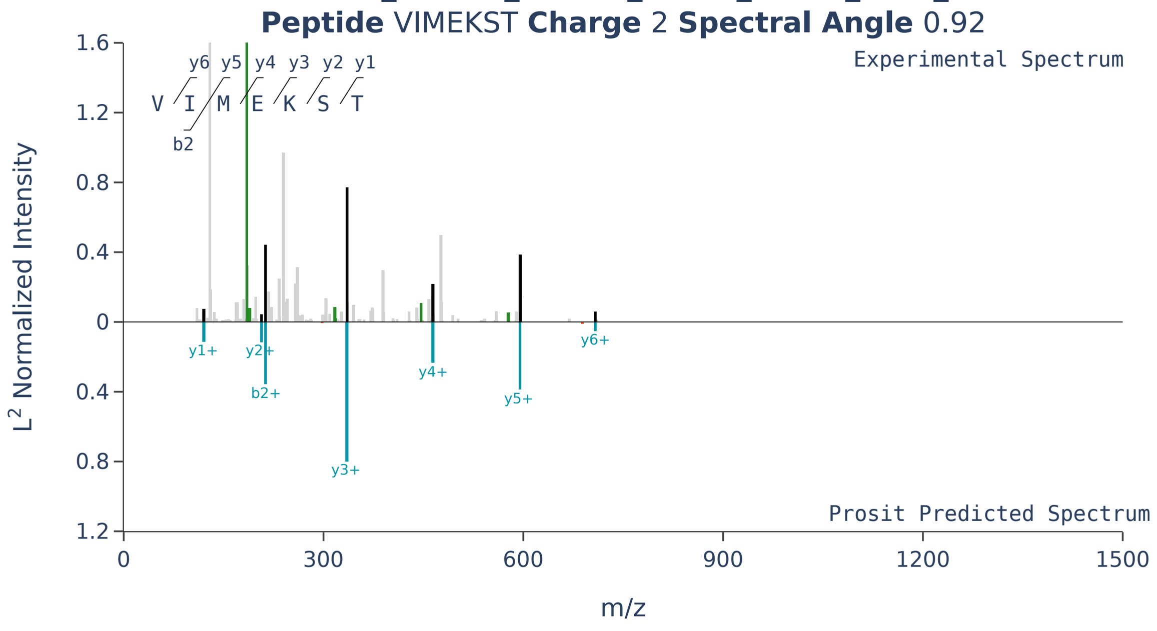

**Source** HRoetschke_271222_090123_HFGoe_LEDGF_b2R1_4h **Scan** 17087
**Peptide** FGTHETAFLGPKD **Charge** 2 **Spectral Angle** 0.87

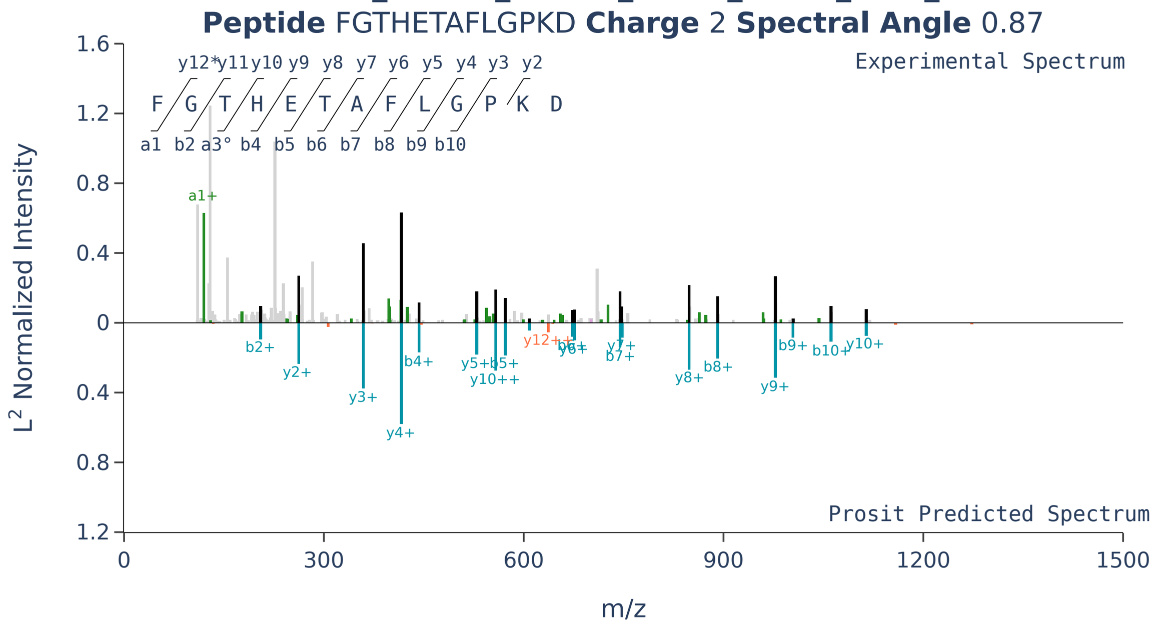


**Source** HRoetschke_271222_090123_HFGoe_LEDGF_b1R1_24h **Scan** 17039
**Peptide** RETEISLKDSTL **Charge** 2 **Spectral Angle** 0.9

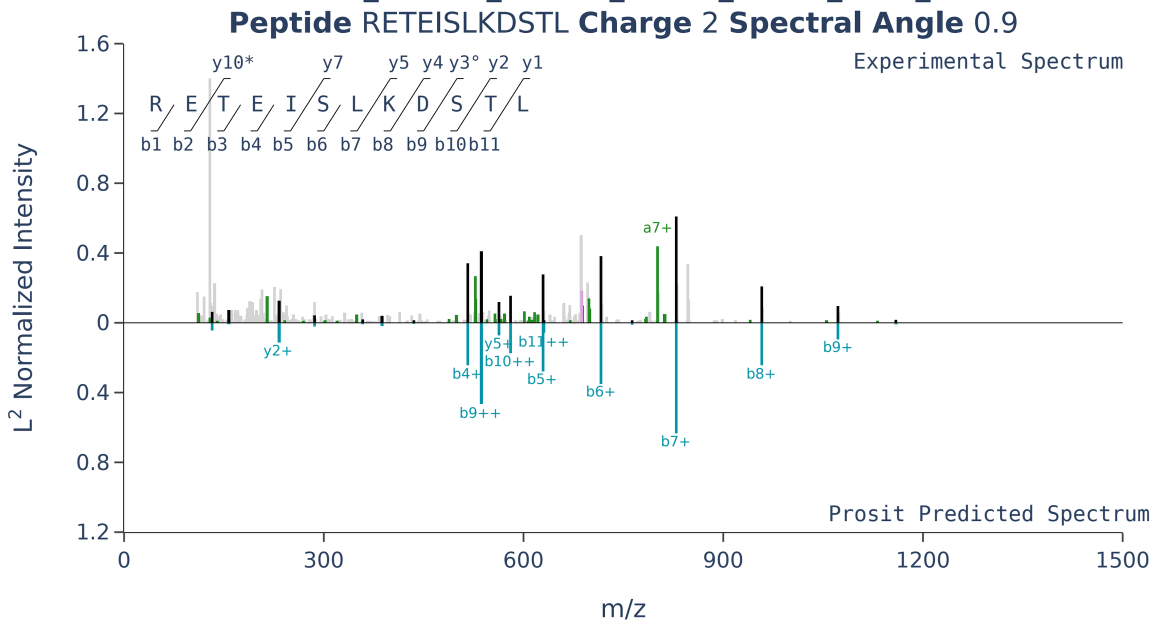


**Source** HRoetschke_271222_090123_HFGoe_LEDGF_b2R1_24h **Scan** 18986
**Peptide** ELASLQVTMQQAQKH **Charge** 2 **Spectral Angle** 0.86

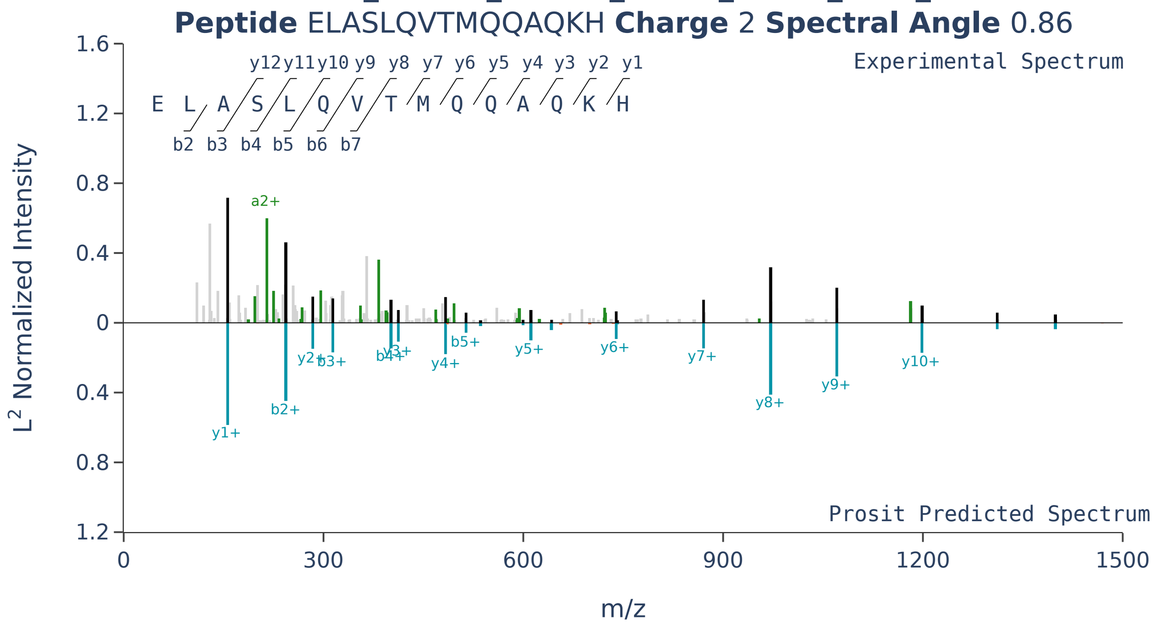


**Source** HRoetschke_271222_090123_HFGoe_LEDGF_b1R1_24h **Scan** 7176
**Peptide** EVKIPKPRGR **Charge** 3 **Spectral Angle** 0.88

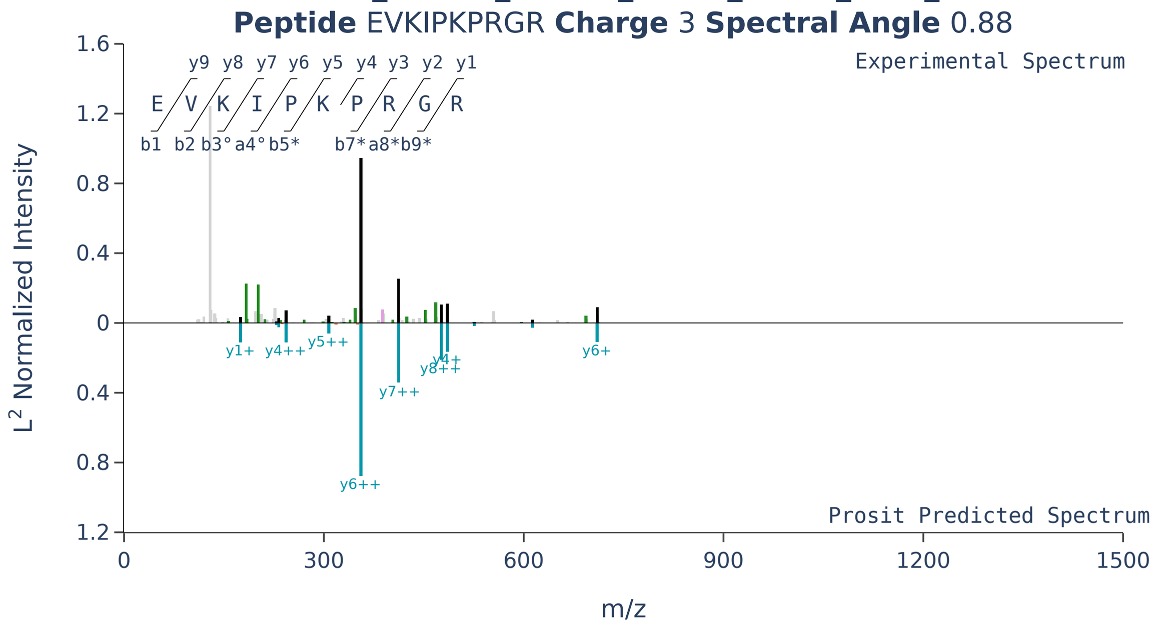


**Source** HRoetschke_271222_090123_HFGoe_LEDGF_b2R2_2h **Scan** 22808
**Peptide** SQVIMEKSTMLY **Charge** 2 **Spectral Angle** 0.94

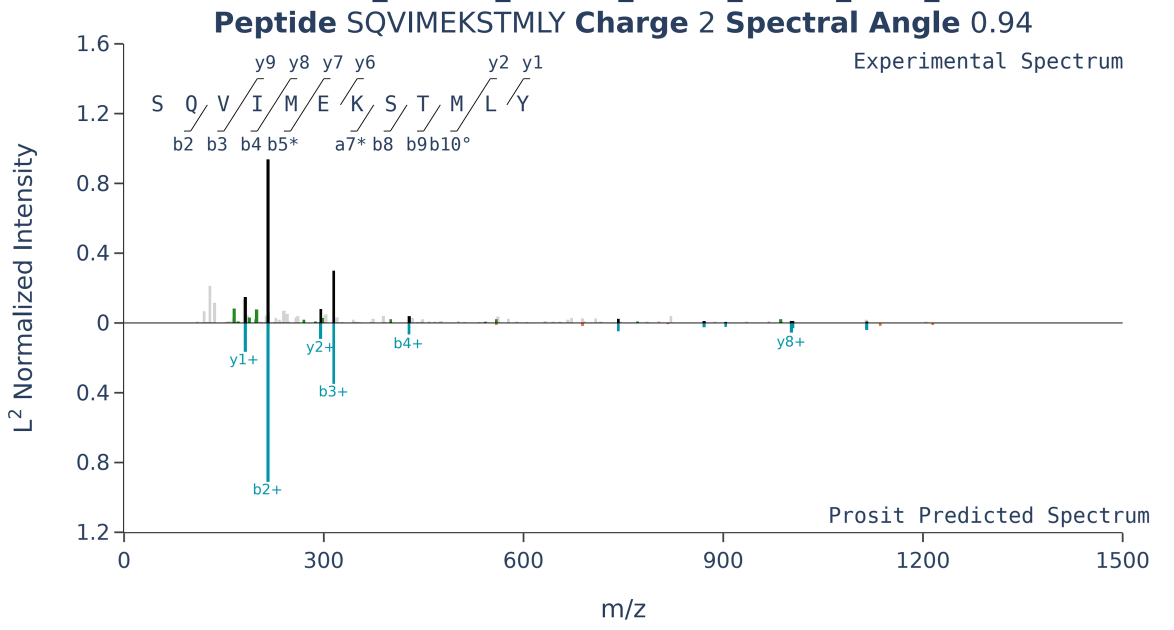


**Source** HRoetschke_271222_090123_HFGoe_LEDGF_b1R2_2h **Scan** 8357
**Peptide** MITTLKKIRR **Charge** 3 **Spectral Angle** 0.85

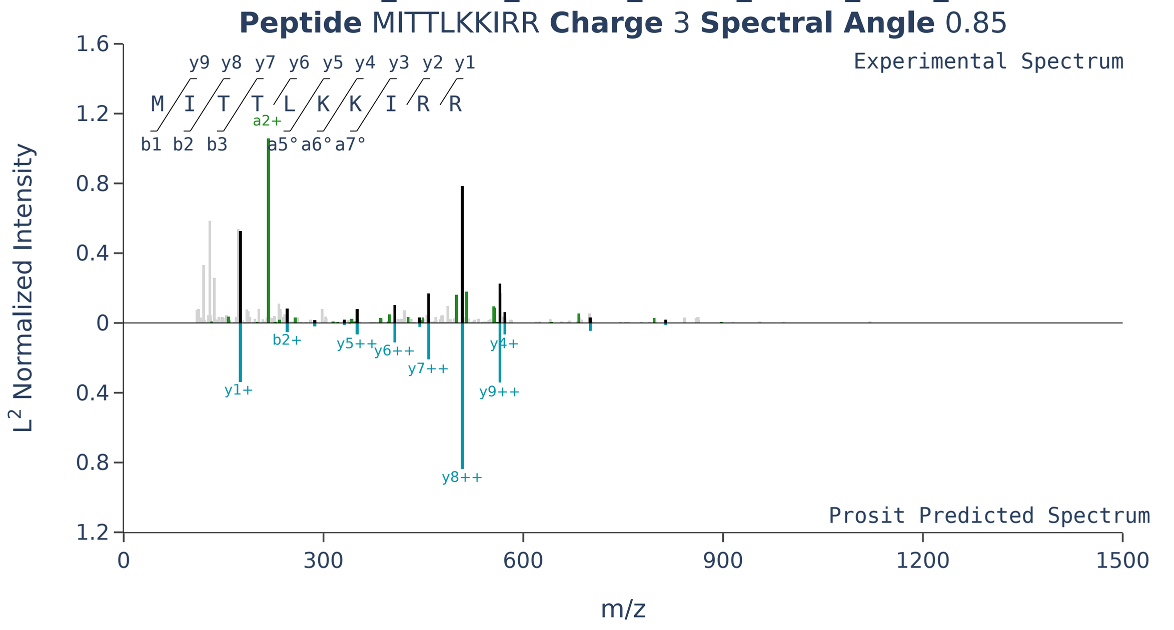


**Source** HRoetschke_271222_090123_HFGoe_LEDGF_b1R2_4h **Scan** 17649
**Peptide** GYPHWPARVDE **Charge** 3 **Spectral Angle** 0.92

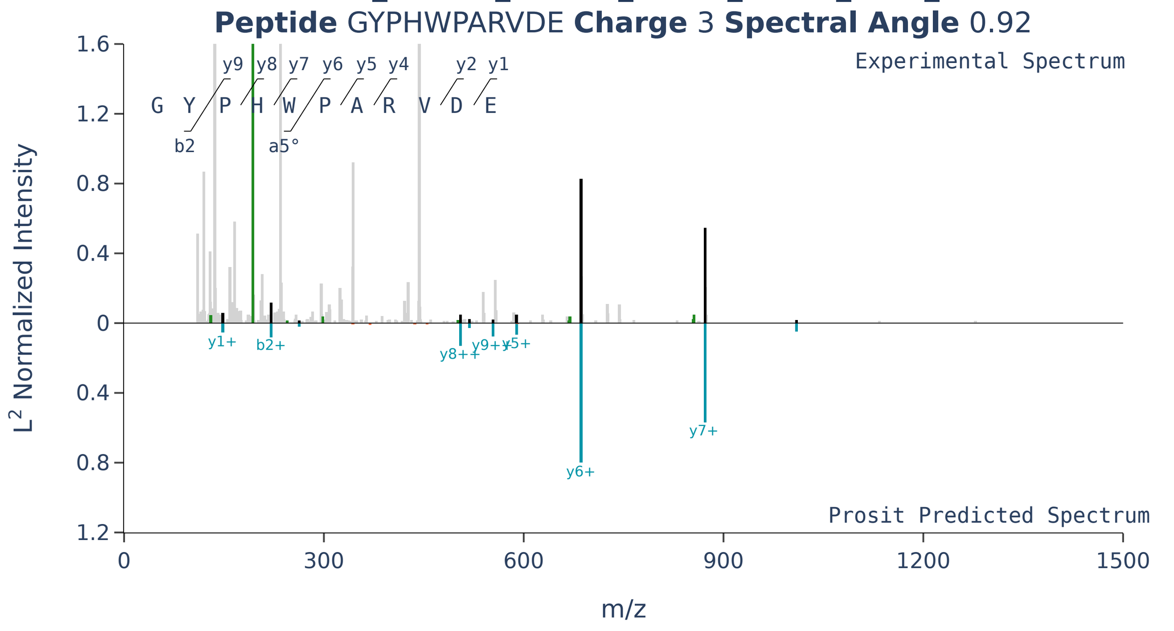


**Source** HRoetschke_271222_090123_HFGoe_LEDGF_b1R1_24h **Scan** 14583
**Peptide** TAFLGPKD **Charge** 2 **Spectral Angle** 0.87

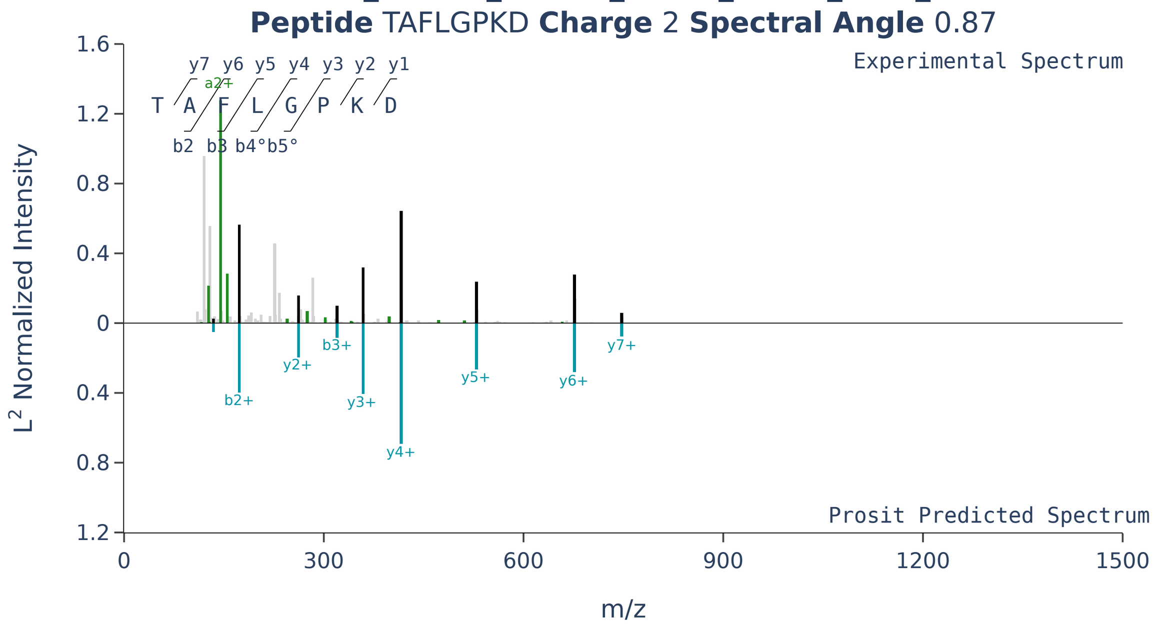


**Source** HRoetschke_271222_090123_HFGoe_LEDGF_b1R2_4h **Scan** 6989
**Peptide** KASNEDVTKAVD **Charge** 3 **Spectral Angle** 0.92

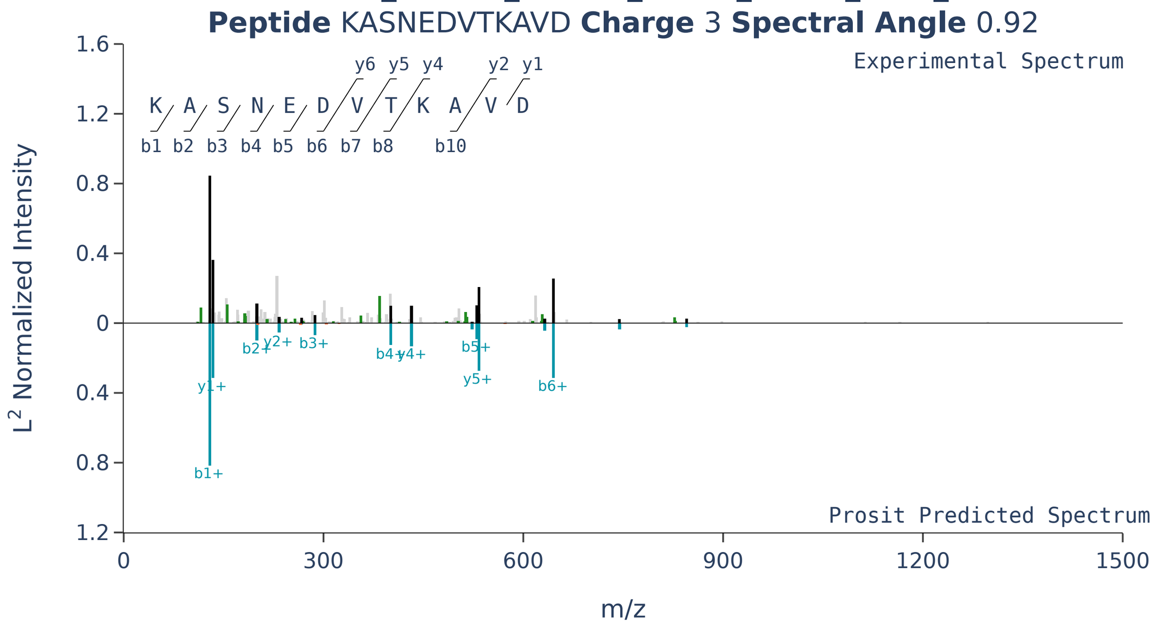


**Source** HRoetschke_271222_090123_HFGoe_LEDGF_b2R2_2h **Scan** 7230
**Peptide** PKMVKQPCPSE **Charge** 2 **Spectral Angle** 0.86

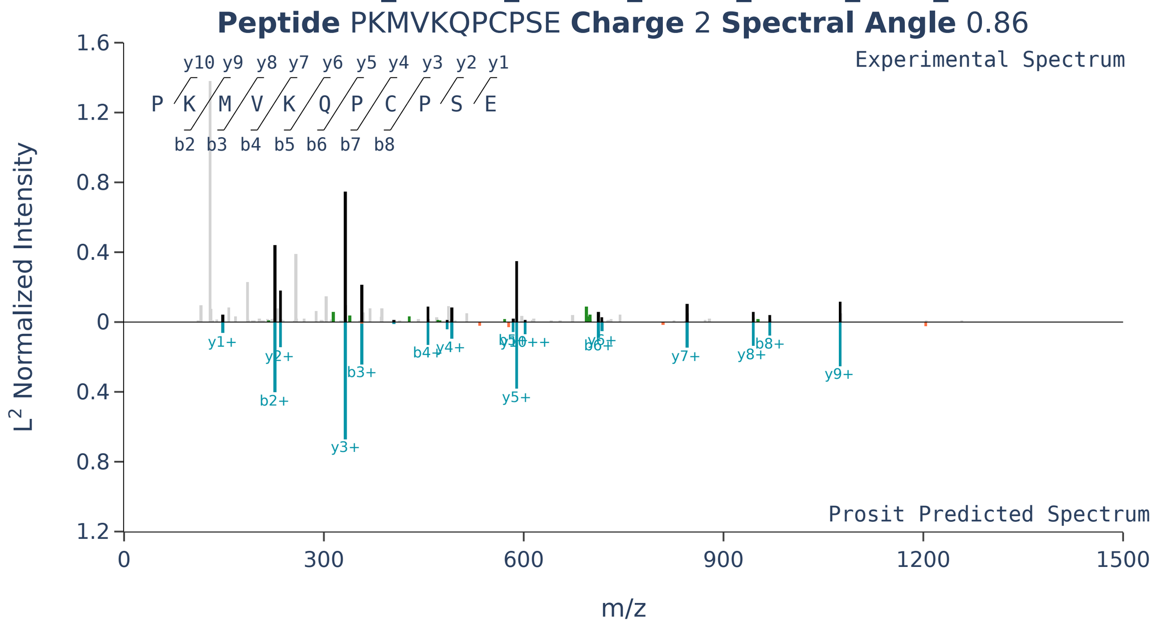


**Source** HRoetschke_271222_090123_HFGoe_LEDGF_b2R2_24h **Scan** 8366
**Peptide** ASVNLKVSPKRG **Charge** 3 **Spectral Angle** 0.89

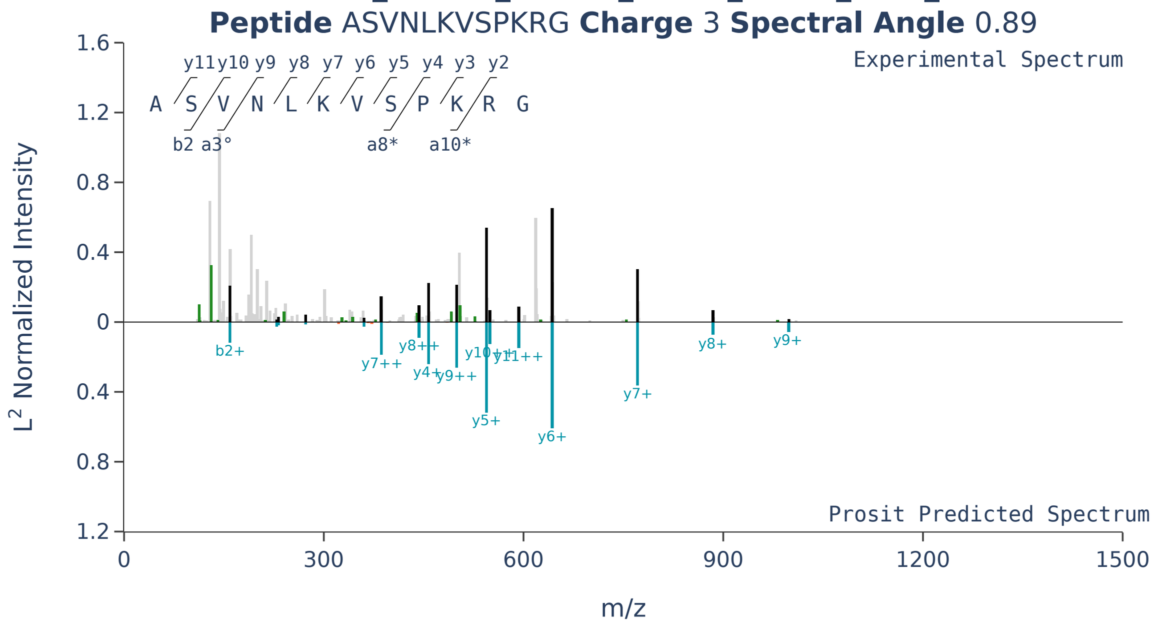


**Source** HRoetschke_271222_090123_HFGoe_LEDGF_b1R2_24h **Scan** 6484
**Peptide** VNLKVSPKRGR **Charge** 3 **Spectral Angle** 0.86

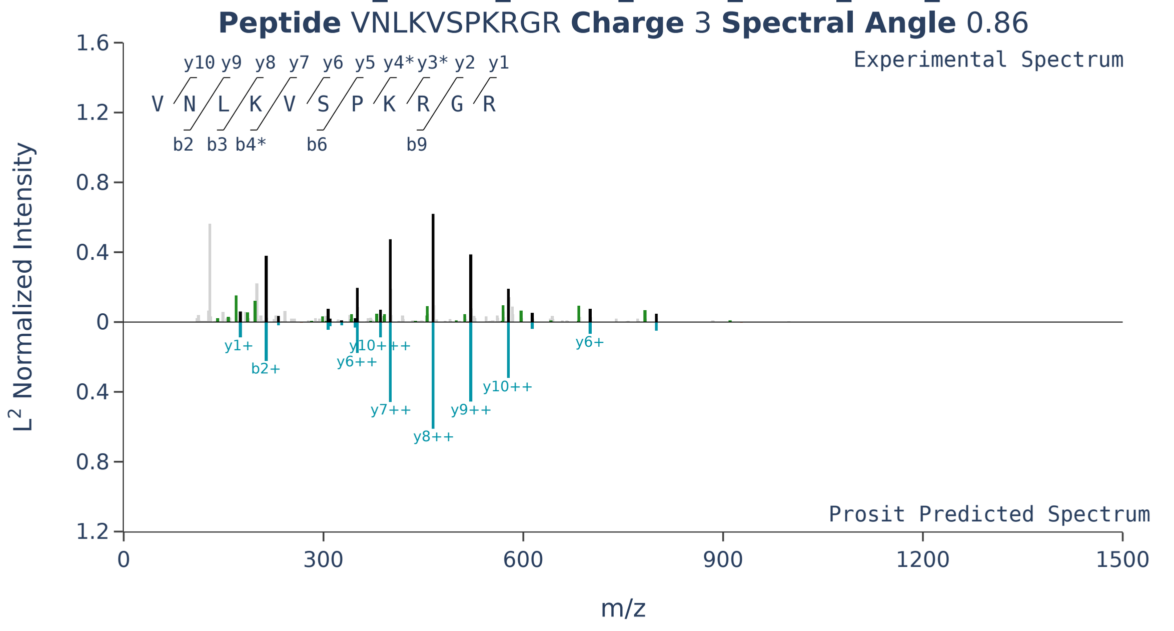


**Source** HRoetschke_271222_090123_HFGoe_LEDGF_b1R2_4h **Scan** 6642
**Peptide** SMDSRLQRIH **Charge** 3 **Spectral Angle** 0.96

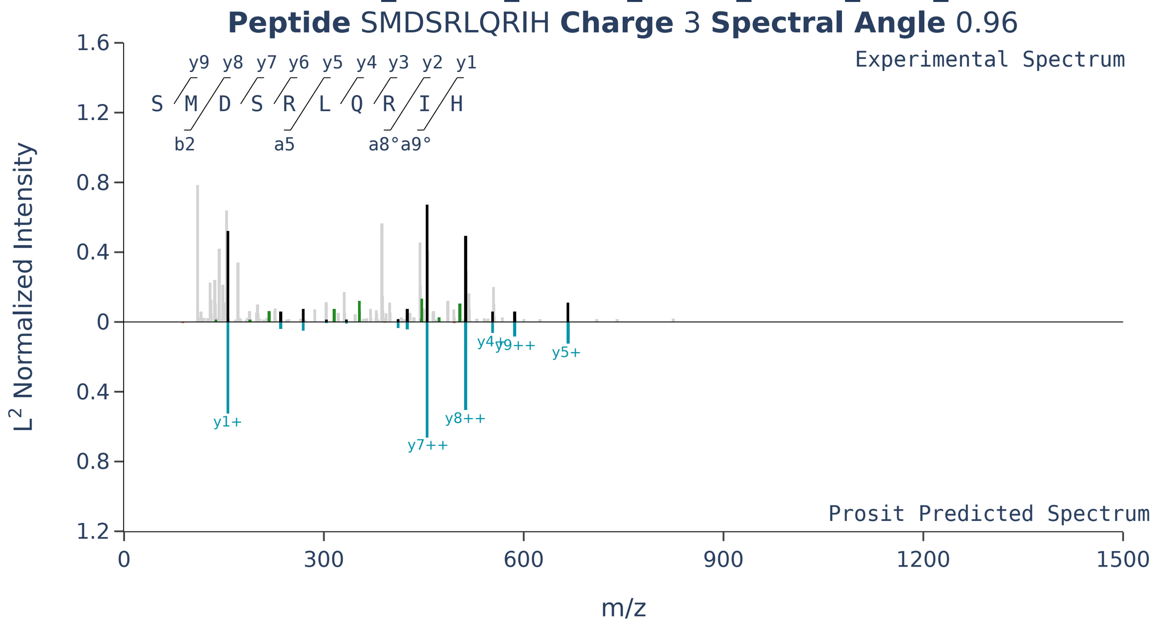


**Source** HRoetschke_271222_090123_HFGoe_LEDGF_b2R1_1h **Scan** 18111
**Peptide** GTHETAFL **Charge** 1 **Spectral Angle** 0.88

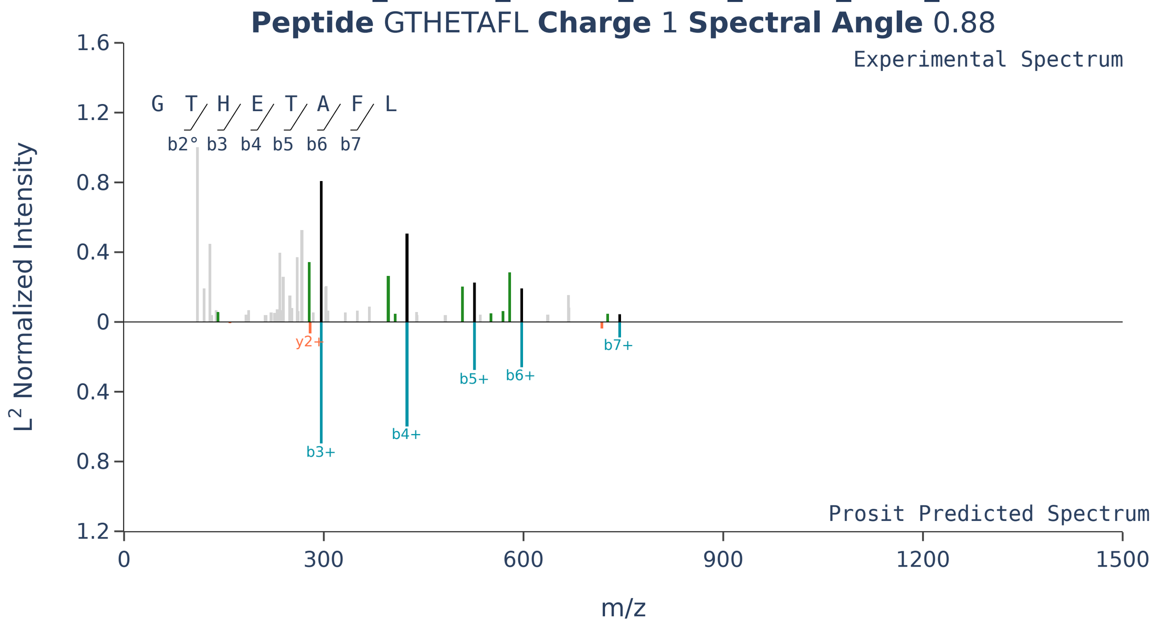


**Source** HRoetschke_271222_090123_HFGoe_LEDGF_b2R1_1h **Scan** 8269
**Peptide** SNEDVTKAVD **Charge** 1 **Spectral Angle** 0.91

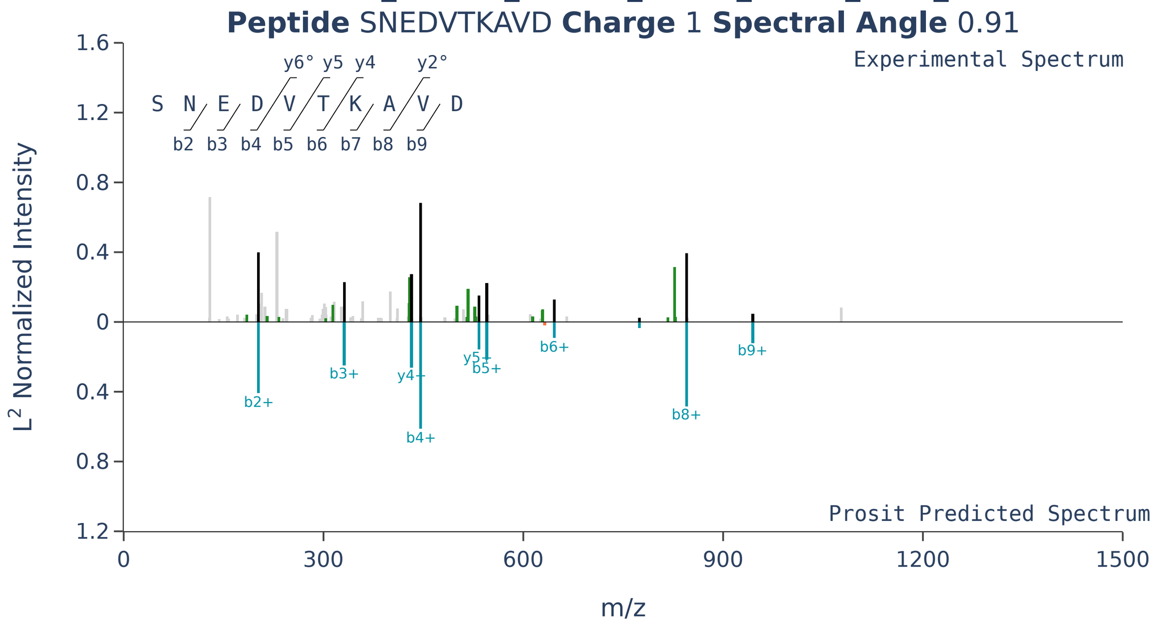

**Source** HRoetschke_271222_090123_HFGoe_LEDGF_b1R1_2h **Scan** 24613
**Peptide** SVITQVLN **Charge** 1 **Spectral Angle** 0.91

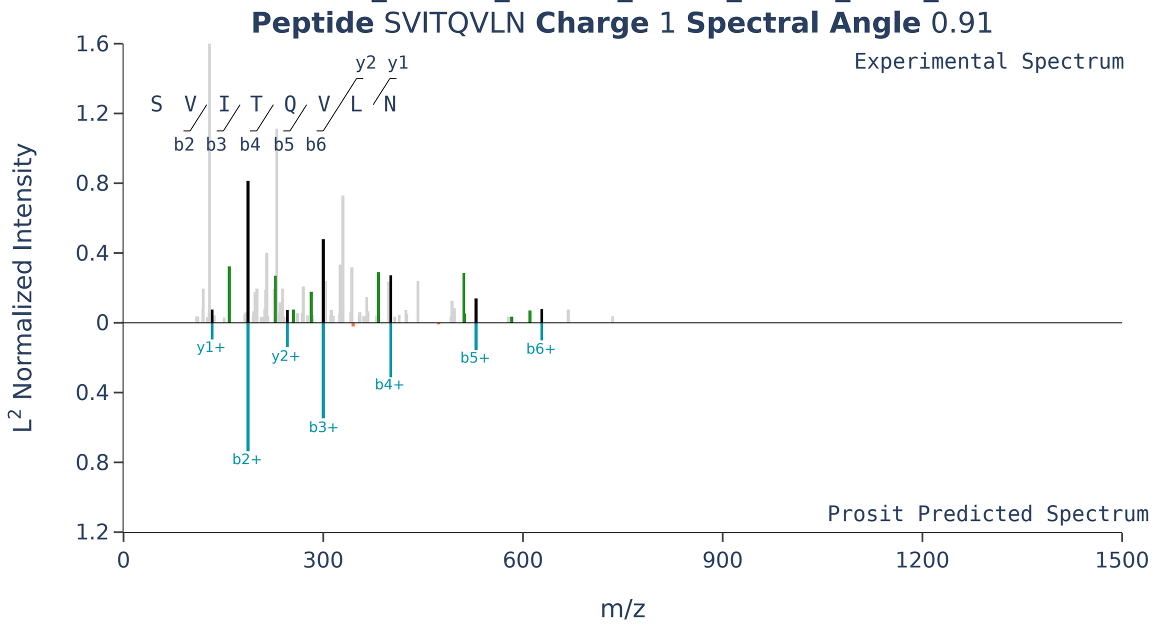


**Source** HRoetschke_271222_090123_HFGoe_LEDGF_b1R1_24h **Scan** 6851
**Peptide** FPYSENKEKYGKPNKR **Charge** 5 **Spectral Angle** 0.9

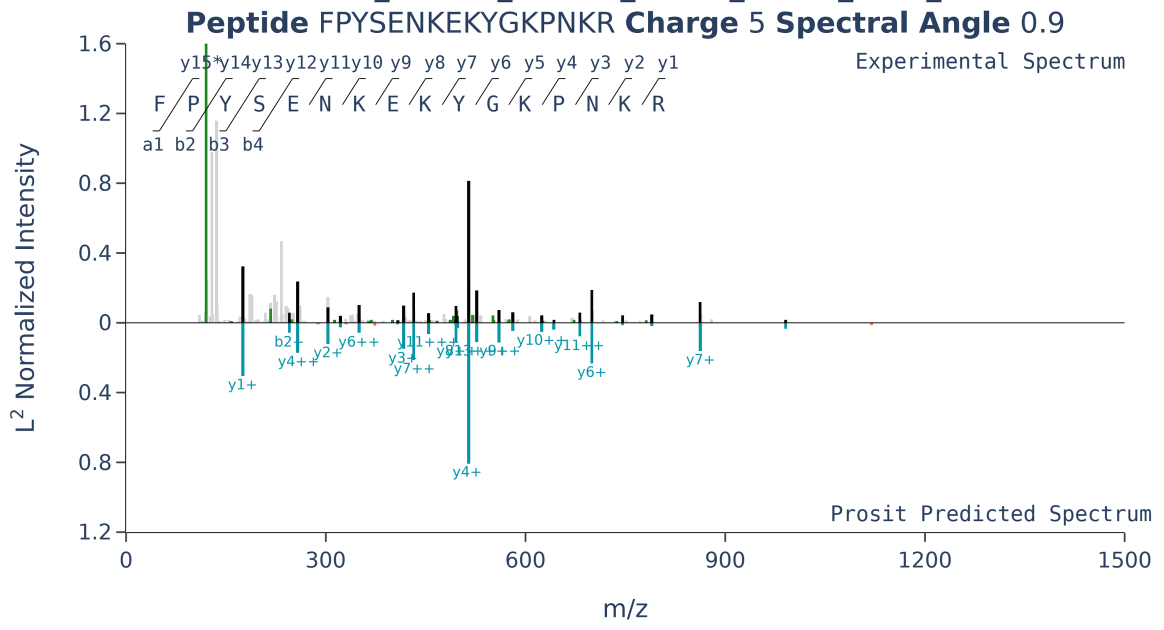


**Source** HRoetschke_271222_090123_HFGoe_LEDGF_b2R1_2h **Scan** 21203
**Peptide** AFLGPKDI **Charge** 2 **Spectral Angle** 0.87

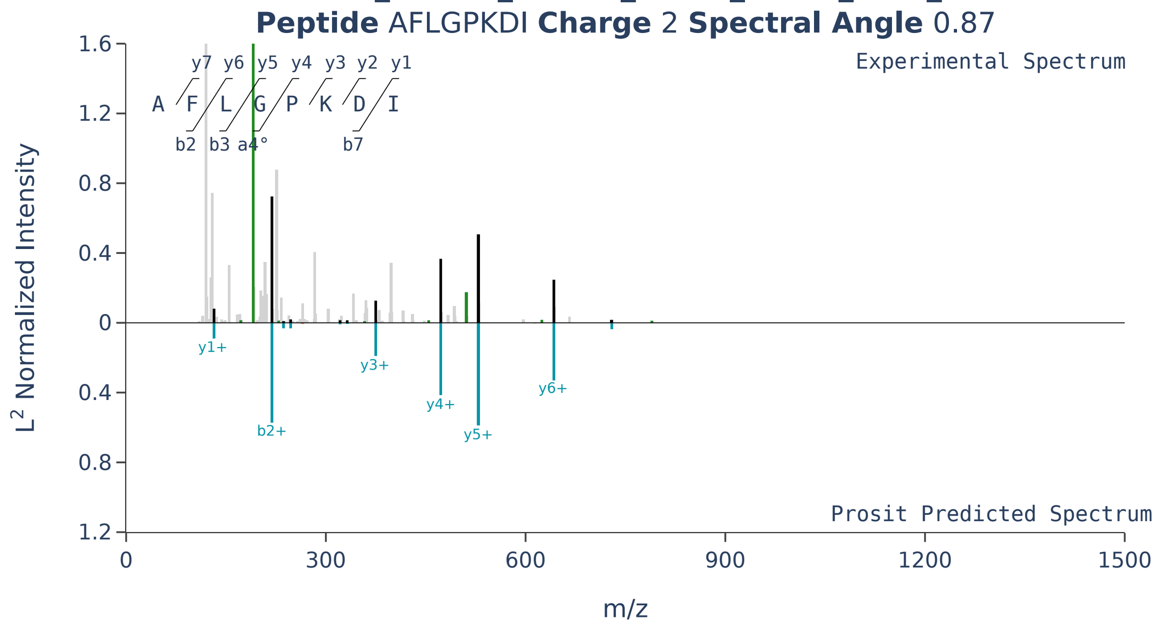


**Source** HRoetschke_271222_090123_HFGoe_LEDGF_b2R1_24h **Scan** 5930
**Peptide** VKQPCPSE **Charge** 2 **Spectral Angle** 0.87

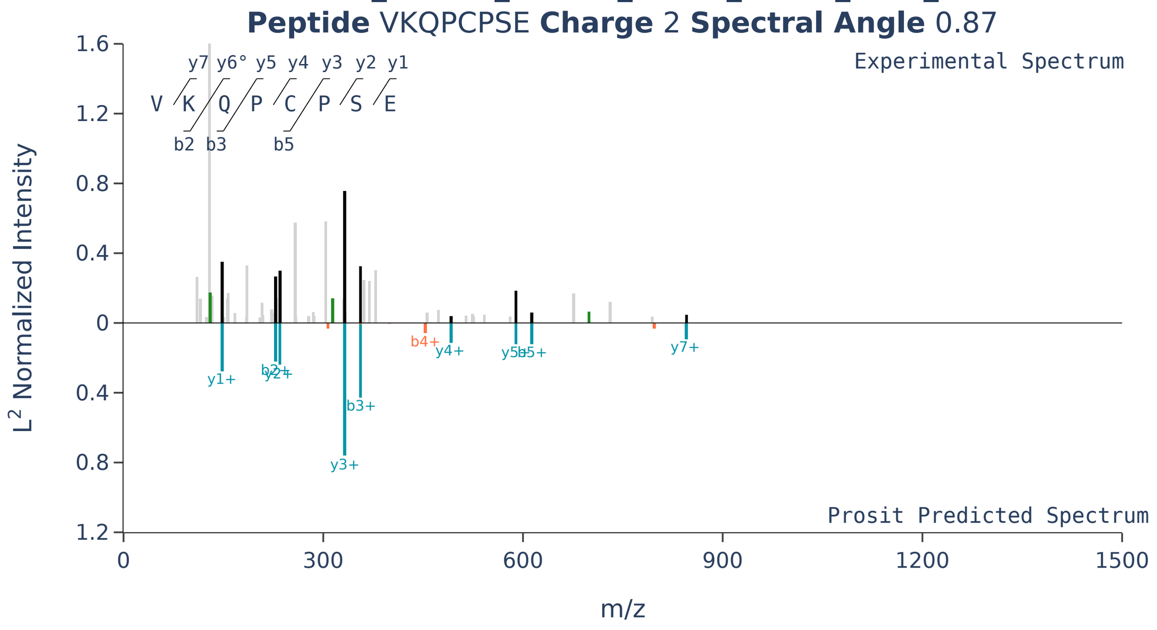


**Source** HRoetschke_271222_090123_HFGoe_LEDGF_b1R1_2h **Scan** 9372
**Peptide** SMDSRLQRI **Charge** 3 **Spectral Angle** 0.88

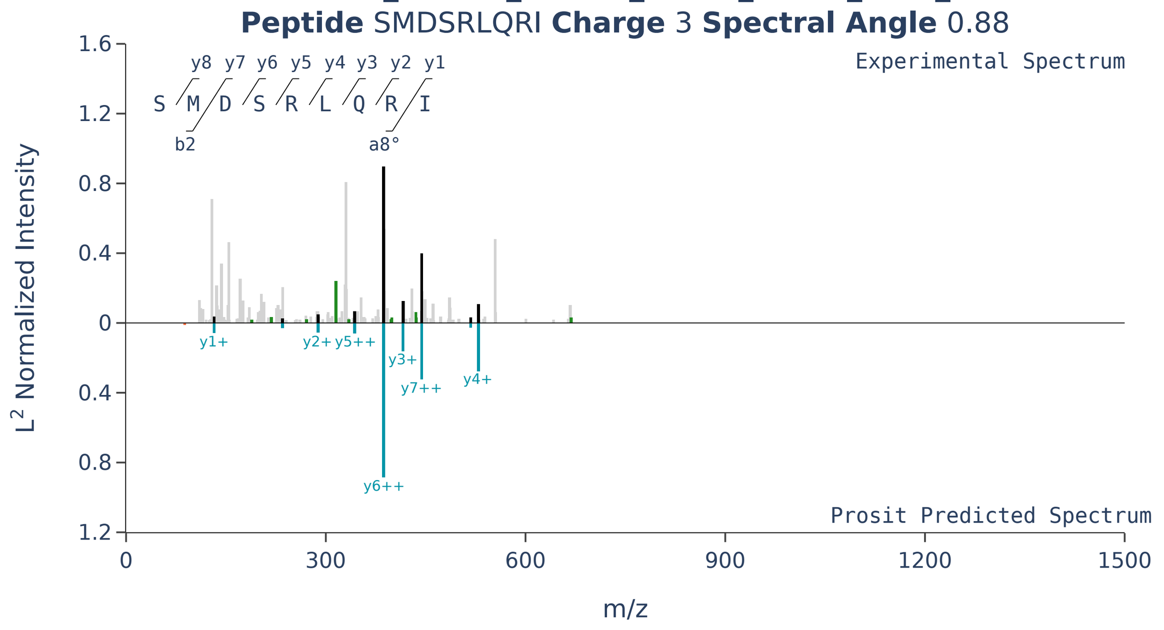


**Source** HRoetschke_271222_090123_HFGoe_LEDGF_b1R2_24h **Scan** 18572
**Peptide** PCPSESDIITE **Charge** 2 **Spectral Angle** 0.88

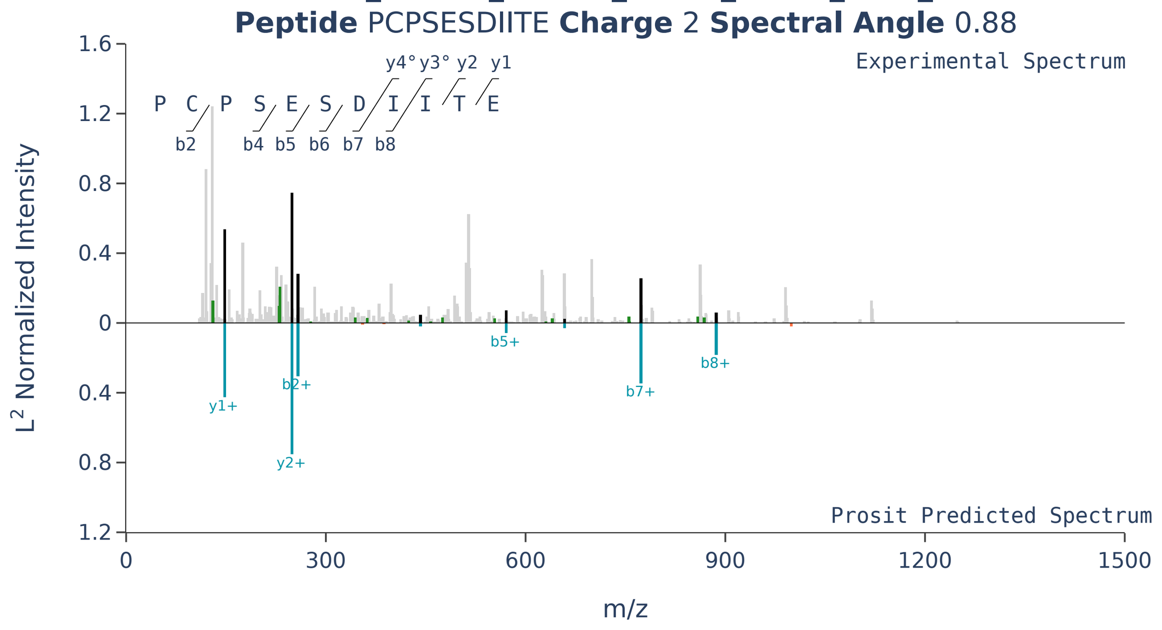


**Source** HRoetschke_271222_090123_HFGoe_LEDGF_b2R1_24h **Scan** 24979
**Peptide** TEMITTL **Charge** 2 **Spectral Angle** 0.96

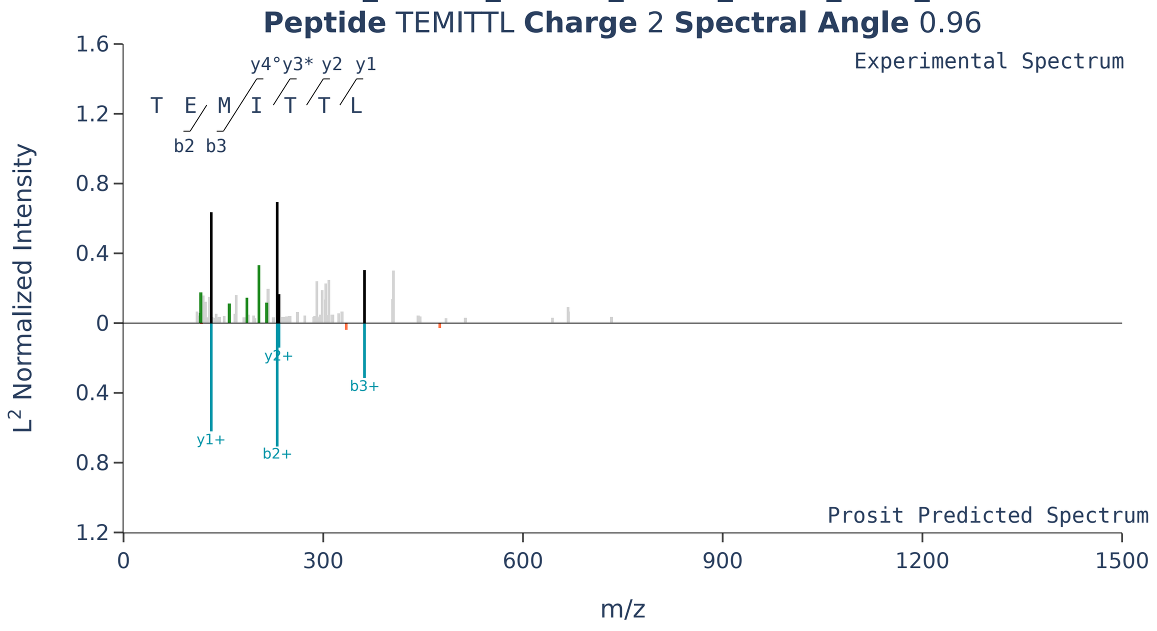


**Source** HRoetschke_271222_090123_HFGoe_LEDGF_b1R2_2h **Scan** 21191
**Peptide** LWEIDNNPKVKFSSQQ **Charge** 3 **Spectral Angle** 0.88

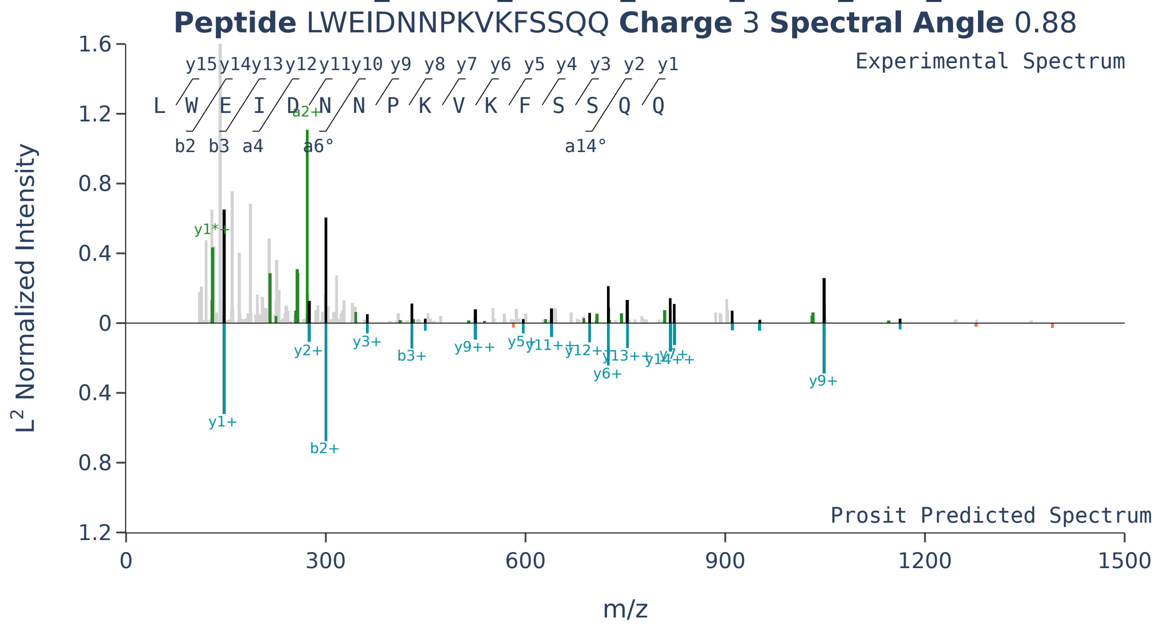


**Source** HRoetschke_271222_090123_HFGoe_LEDGF_b1R1_24h **Scan** 7980
**Peptide** SVNLKVSPKR **Charge** 3 **Spectral Angle** 0.89

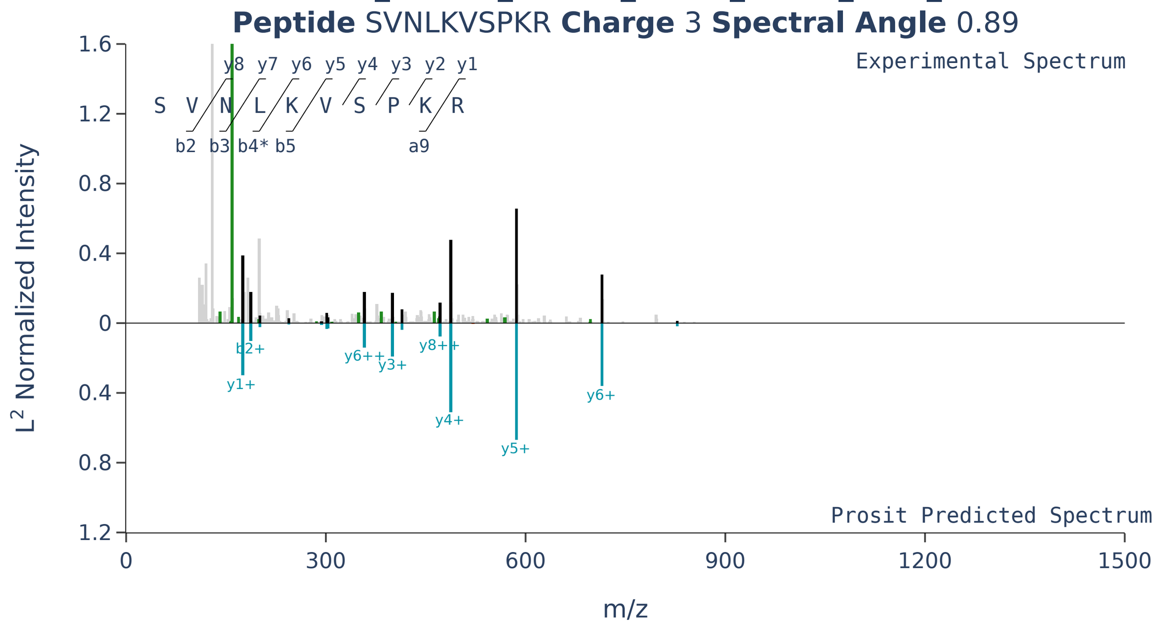


**Source** HRoetschke_271222_090123_HFGoe_LEDGF_b1R1_2h **Scan** 11225
**Peptide** HAEIKNSLKID **Charge** 3 **Spectral Angle** 0.91

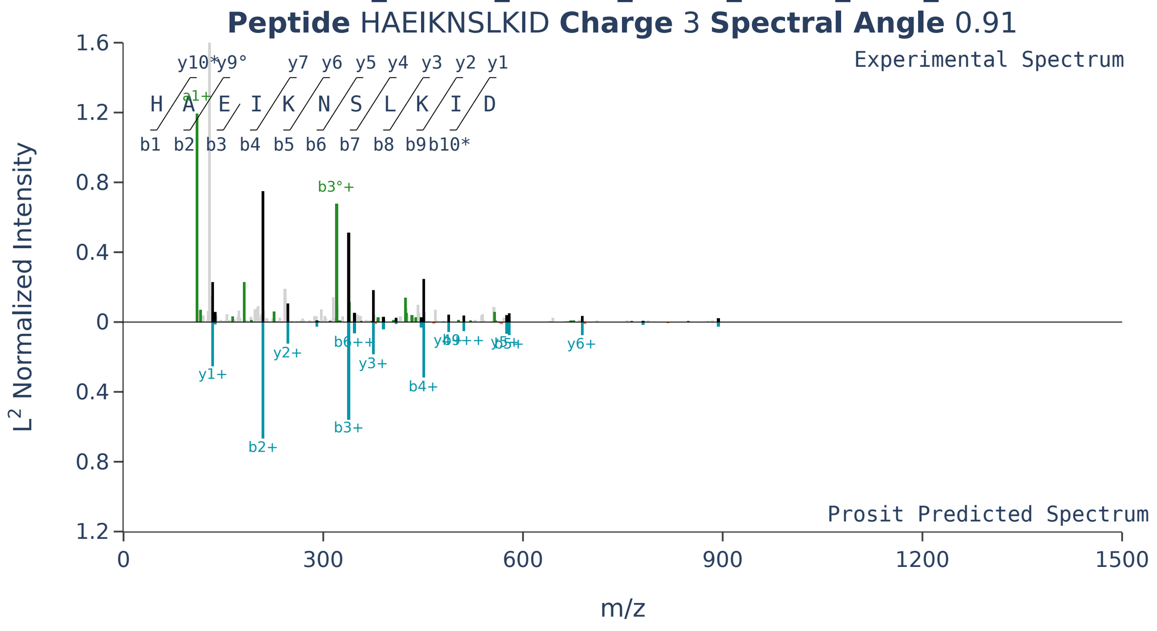


**Source** HRoetschke_271222_090123_HFGoe_LEDGF_b2R2_4h **Scan** 7593
**Peptide** IDNNPKV **Charge** 2 **Spectral Angle** 0.89

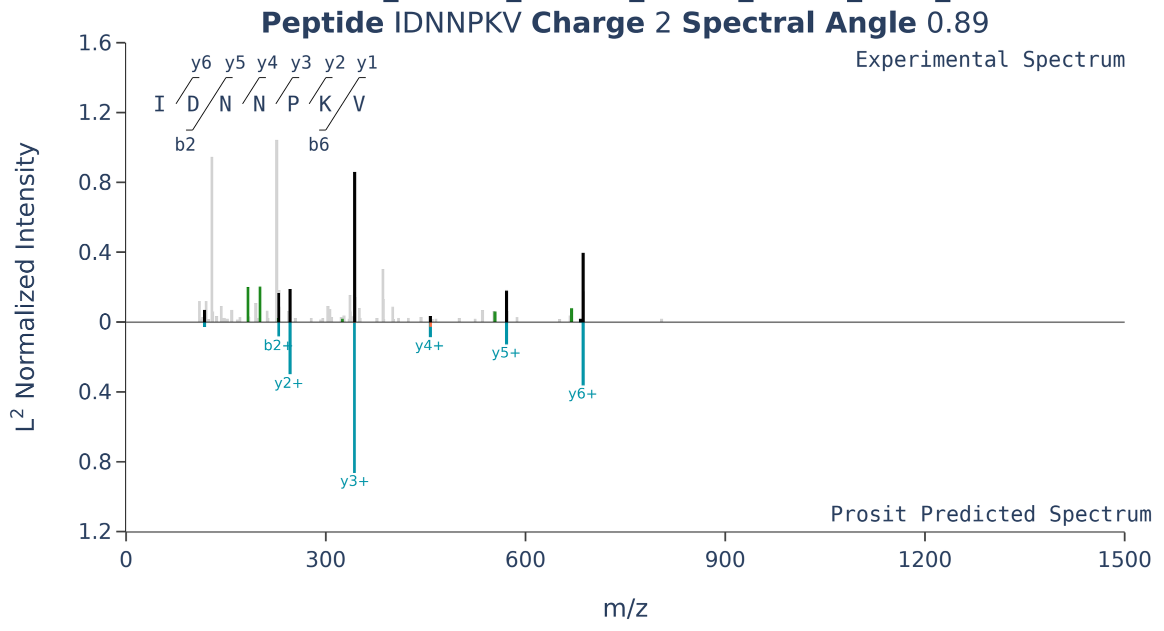


**Source** HRoetschke_271222_090123_HFGoe_LEDGF_b1R2_4h **Scan** 9477
**Peptide** GTHETAF **Charge** 1 **Spectral Angle** 0.9

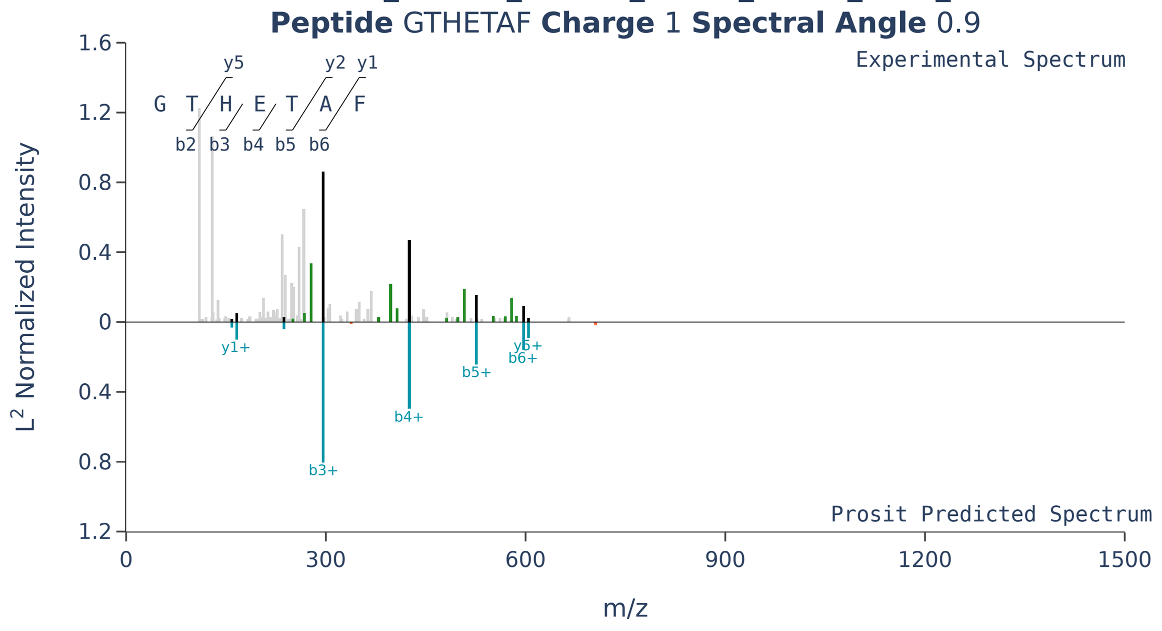


**Source** HRoetschke_271222_090123_HFGoe_LEDGF_b2R1_2h **Scan** 22866
**Peptide** NEGLWEIDNNPKVKFSSQ **Charge** 3 **Spectral Angle** 0.89

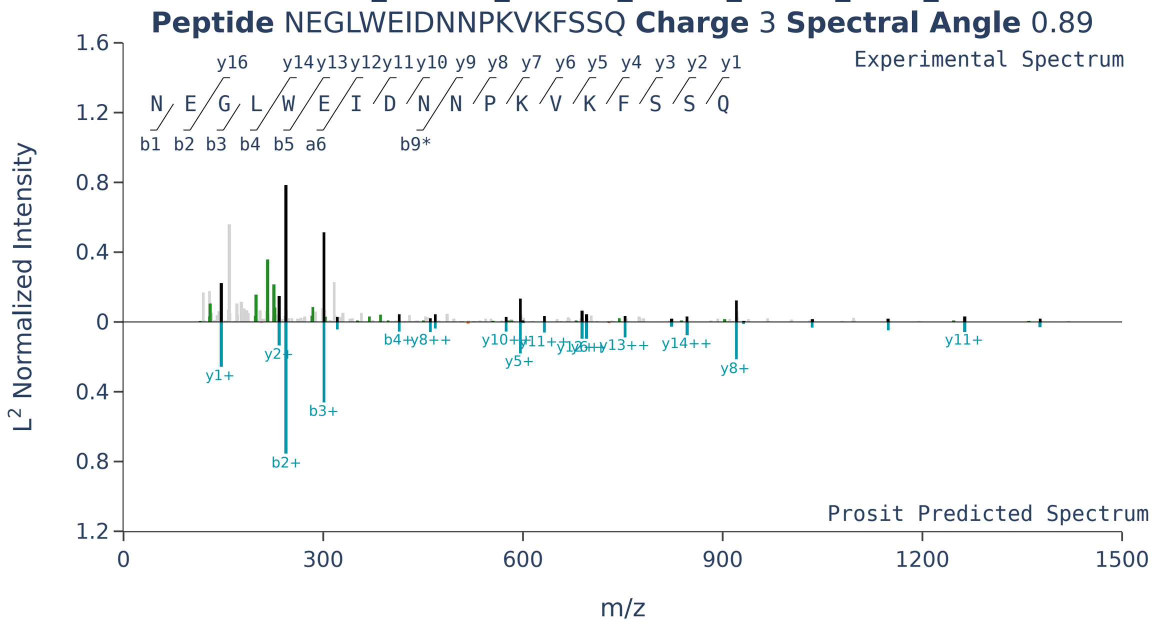


**Source** HRoetschke_271222_090123_HFGoe_LEDGF_b1R2_2h **Scan** 17631
**Peptide** LASLQVTMQQAQKHTE **Charge** 2 **Spectral Angle** 0.9

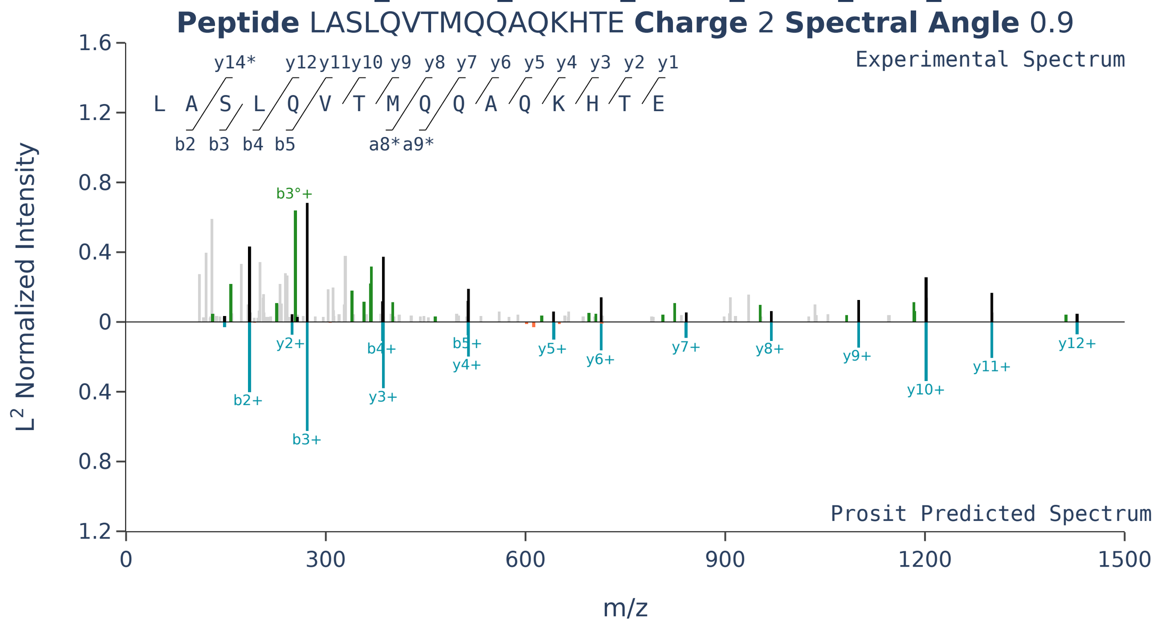


**Source** HRoetschke_271222_090123_HFGoe_LEDGF_b1R2_24h **Scan** 8839
**Peptide** MDSRLQRIH **Charge** 3 **Spectral Angle** 0.87

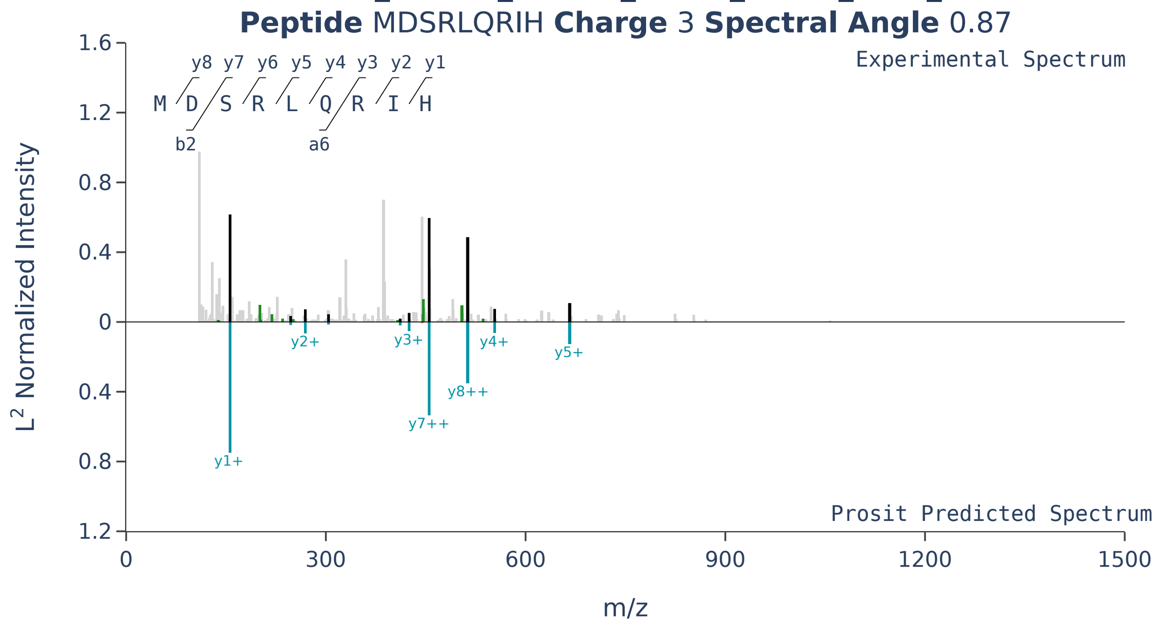

**Source** HRoetschke_271222_090123_HFGoe_LEDGF_b2R2_2h **Scan** 9553
**Peptide** DEVPDGA **Charge** 1 **Spectral Angle** 0.89

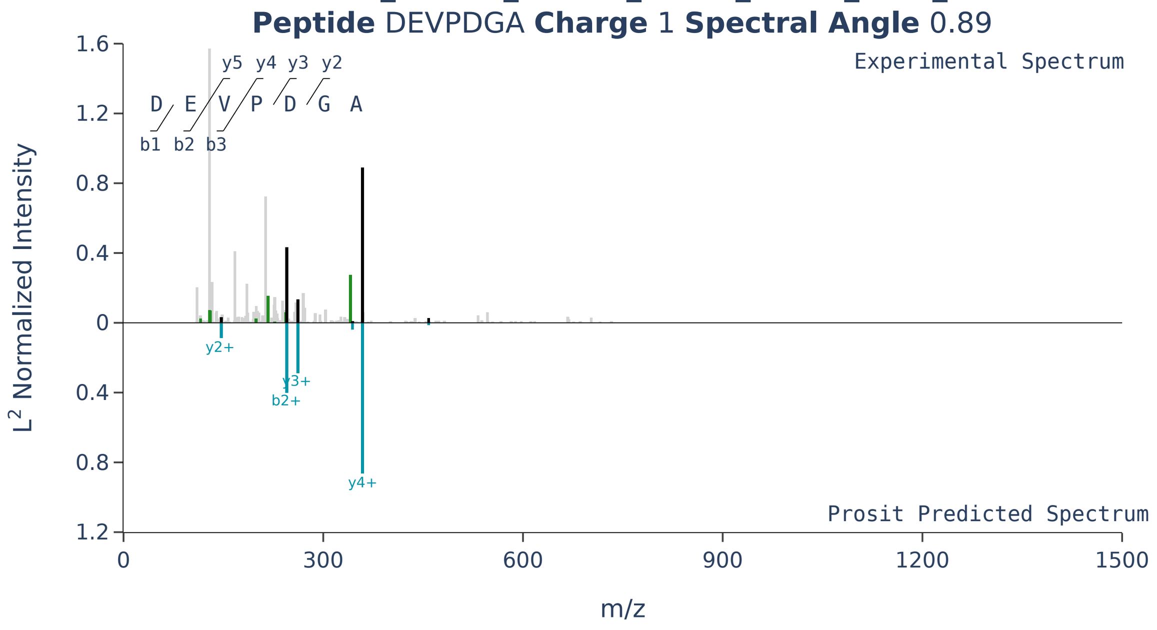


**Source** HRoetschke_271222_090123_HFGoe_LEDGF_b1R1_4h **Scan** 26323
**Peptide** AFLGPKDIFPYSENKE **Charge** 3 **Spectral Angle** 0.86

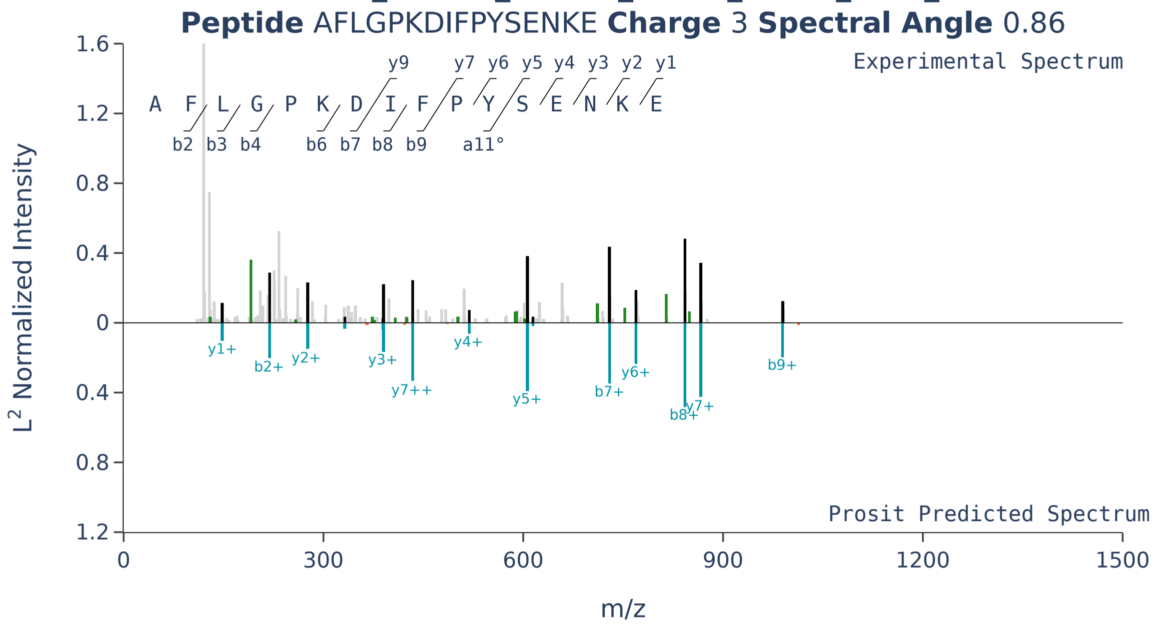


**Source** HRoetschke_271222_090123_HFGoe_LEDGF_b2R1_1h **Scan** 27274
**Peptide** ALDELASLQVTM **Charge** 2 **Spectral Angle** 0.94

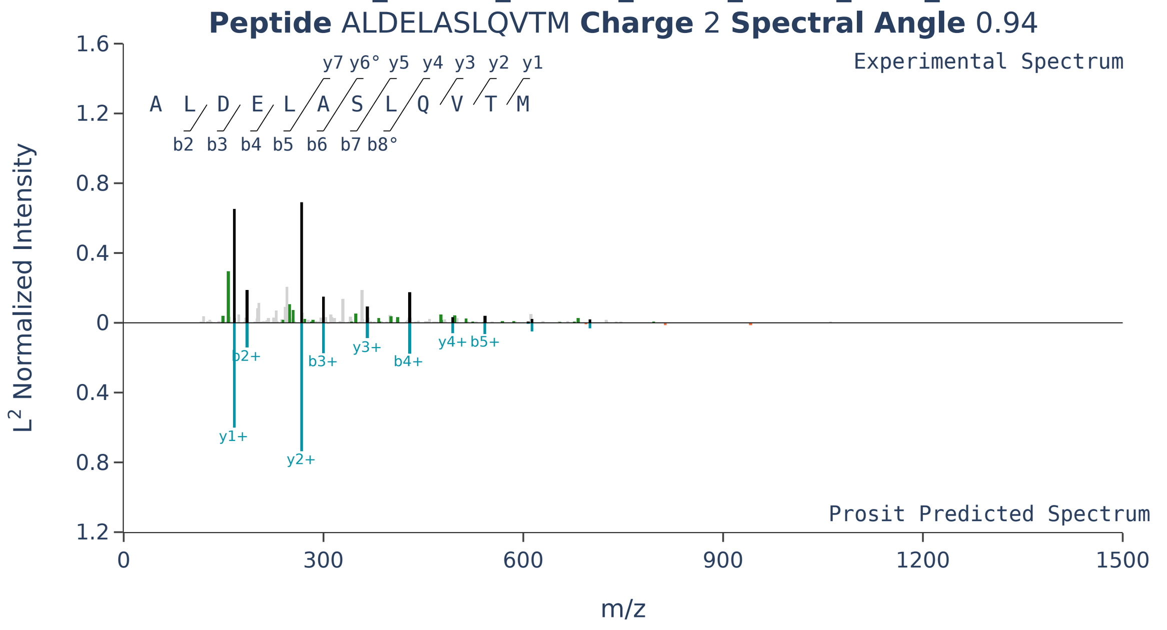


**Source** HRoetschke_271222_090123_HFGoe_LEDGF_b2R1_24h **Scan** 16806
**Peptide** STMLYNKF **Charge** 1 **Spectral Angle** 0.88

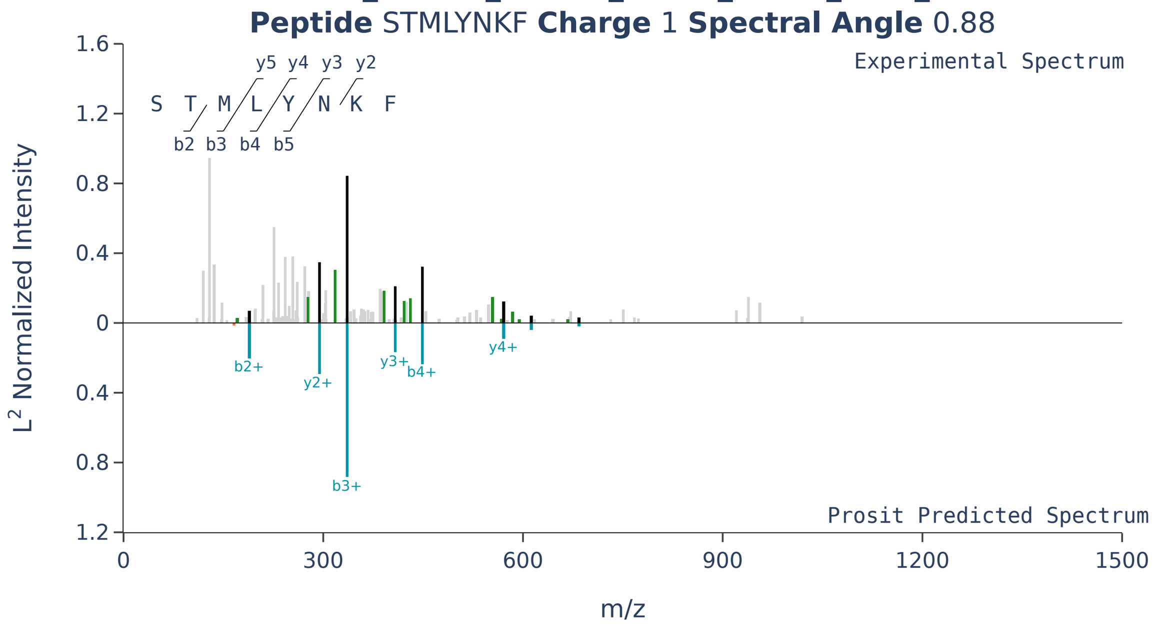


**Source** HRoetschke_271222_090123_HFGoe_LEDGF_b1R1_2h **Scan** 18659
**Peptide** LWEIDNNPKVKFSSQQAATKQ **Charge** 3 **Spectral Angle** 0.94

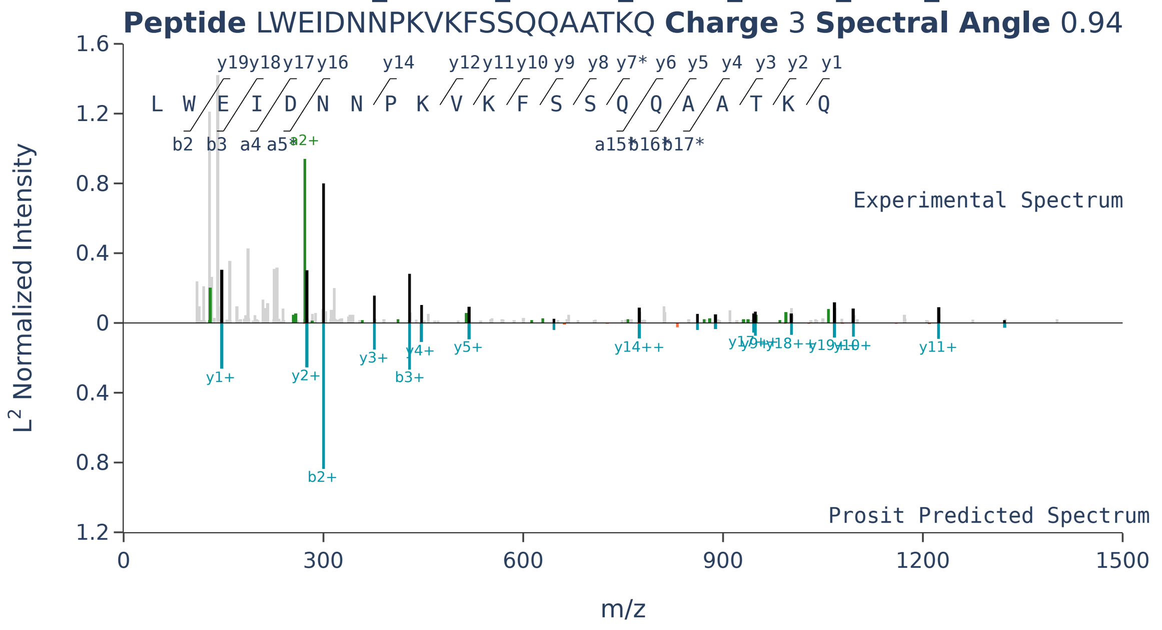


**Source** HRoetschke_271222_090123_HFGoe_LEDGF_b2R1_4h **Scan** 7018
**Peptide** ASVNLKVSPKRGR **Charge** 4 **Spectral Angle** 0.93

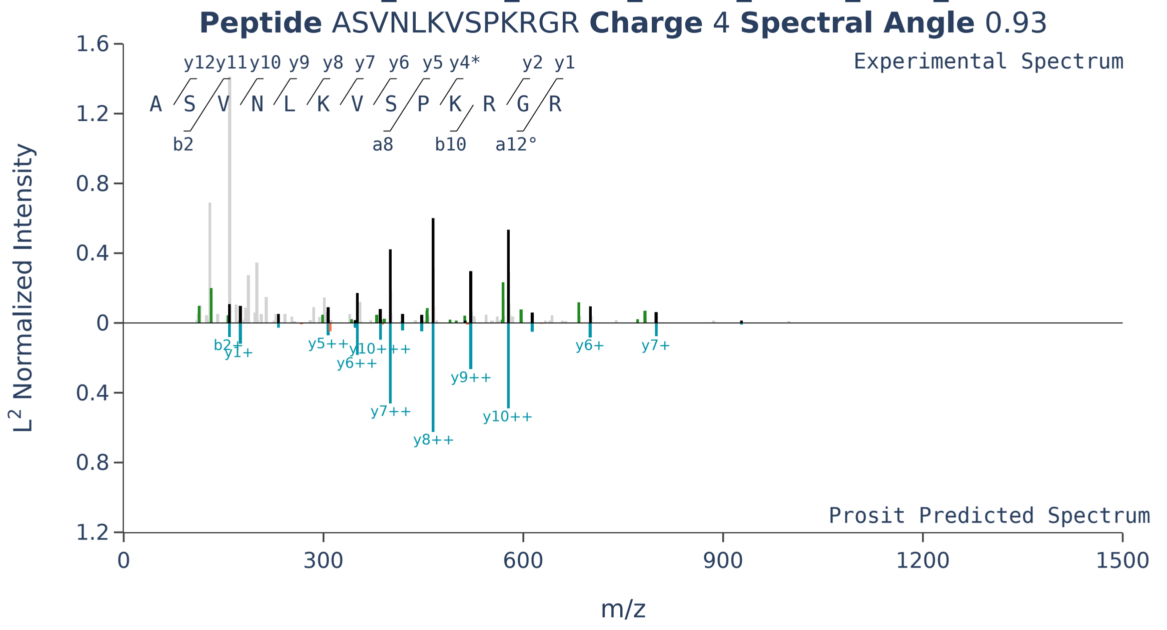


**Source** HRoetschke_271222_090123_HFGoe_LEDGF_b1R2_24h **Scan** 6007
**Peptide** LNKSLAEQRQHEE **Charge** 2 **Spectral Angle** 0.87

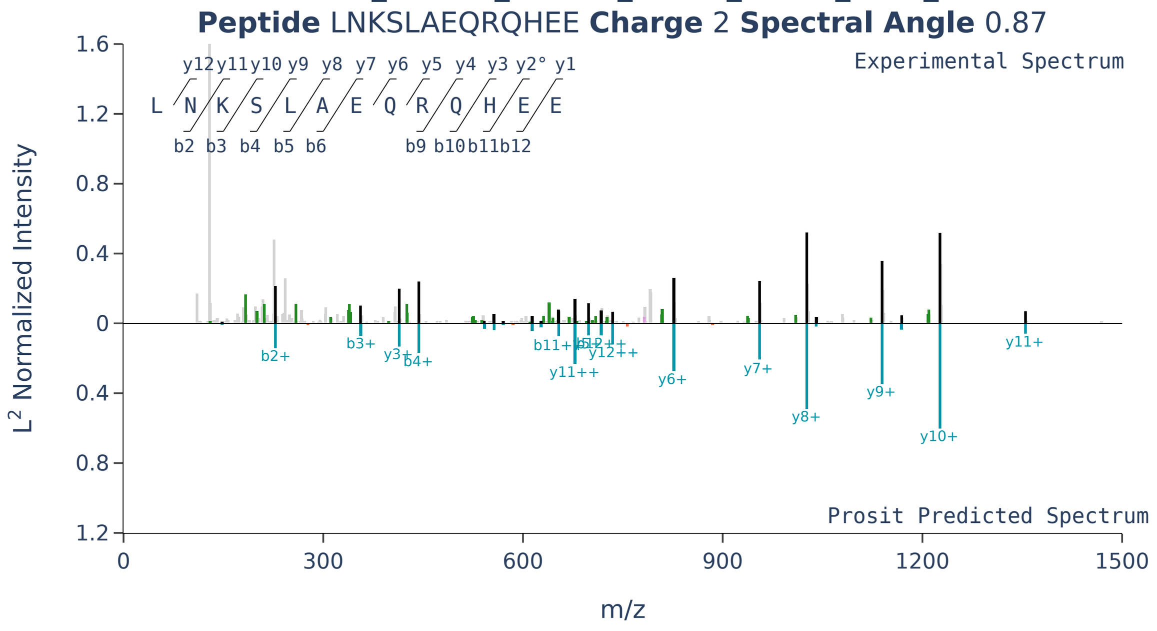


**Source** HRoetschke_271222_090123_HFGoe_LEDGF_b2R1_24h **Scan** 7743
**Peptide** TASVNLKVSPKRGR **Charge** 4 **Spectral Angle** 0.9

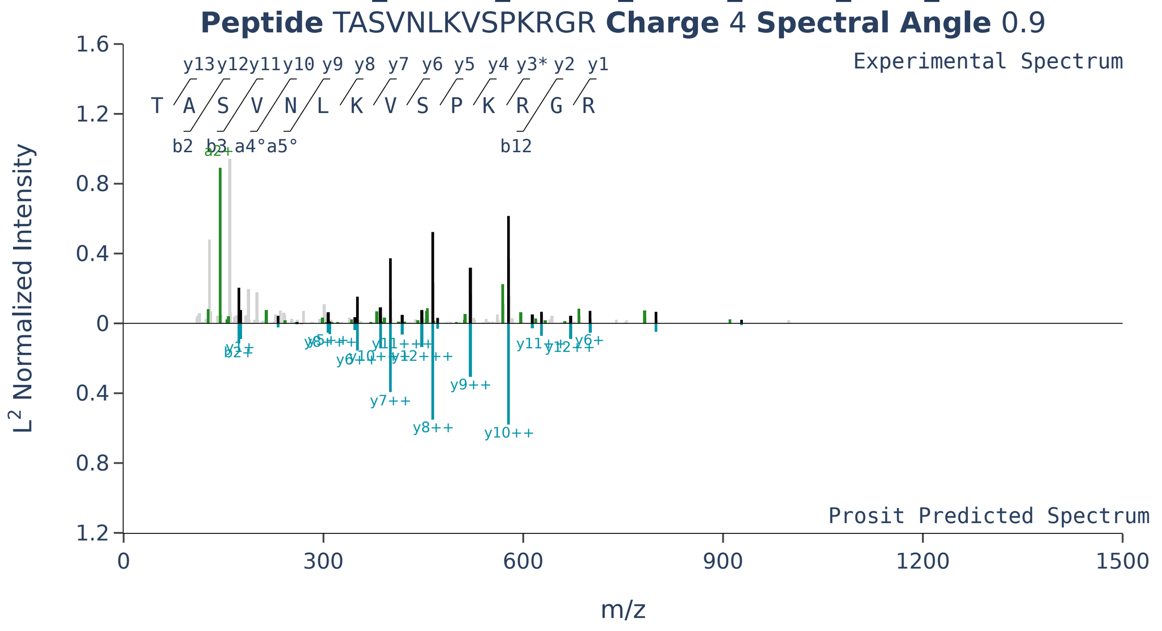


**Source** HRoetschke_271222_090123_HFGoe_LEDGF_b1R2_24h **Scan** 6899
**Peptide** VLNKSLAEQRQHE **Charge** 4 **Spectral Angle** 0.86

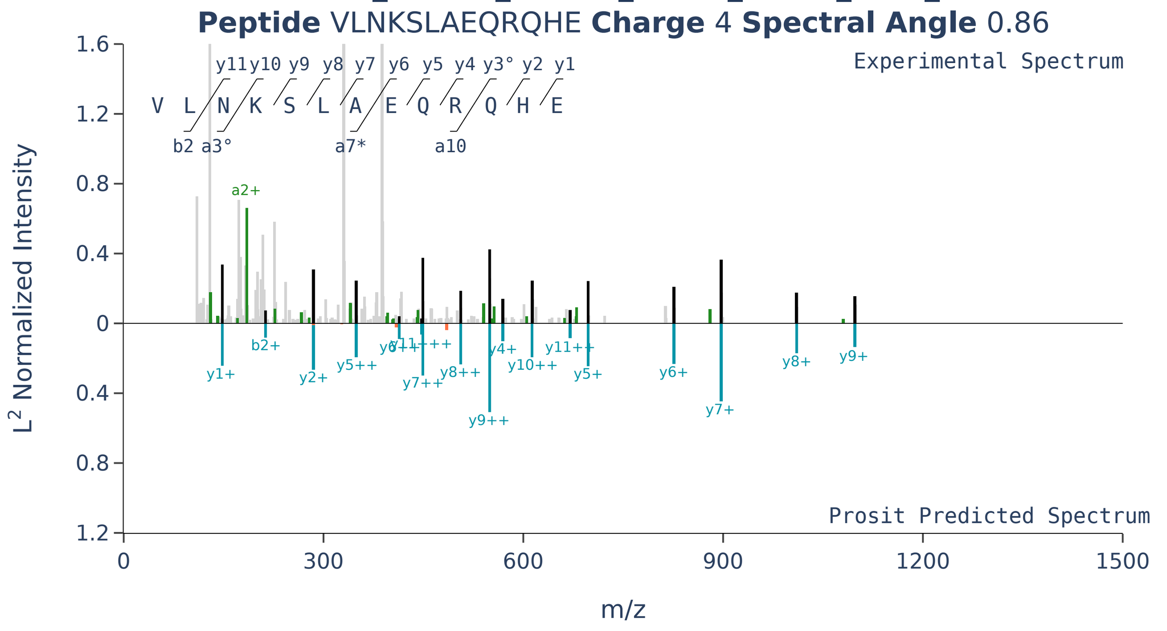


**Source** HRoetschke_271222_090123_HFGoe_LEDGF_b2R2_4h **Scan** 15946
**Peptide** KIDNLDV **Charge** 2 **Spectral Angle** 0.86

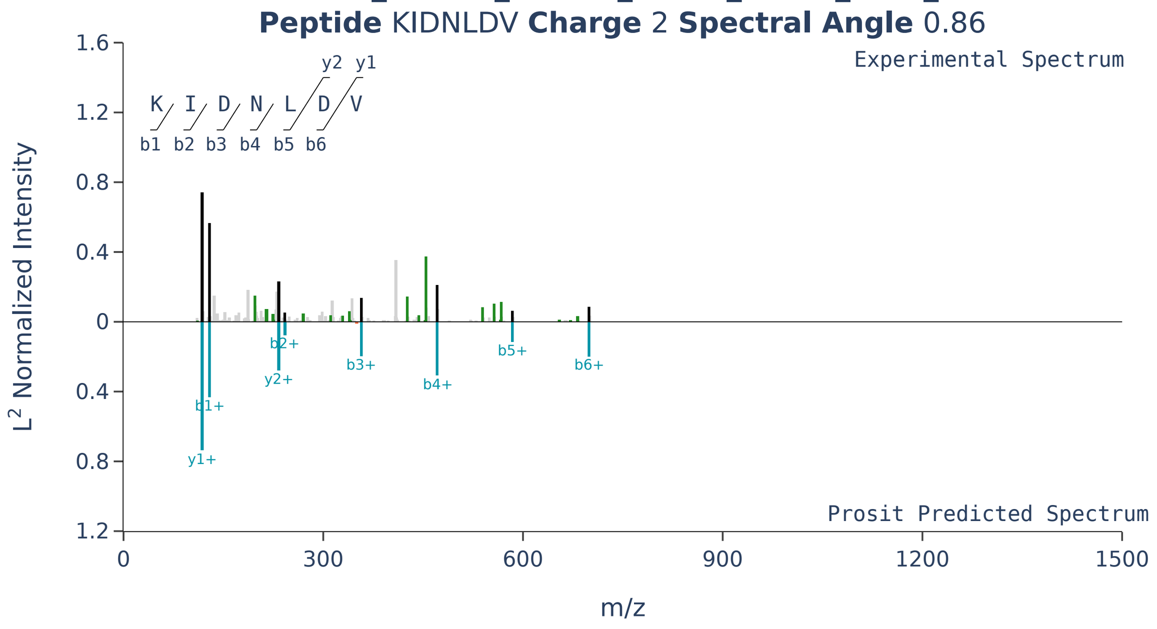


**Source** HRoetschke_271222_090123_HFGoe_LEDGF_b1R2_2h **Scan** 7783
**Peptide** ATASVNLKVSPKRGR **Charge** 4 **Spectral Angle** 0.84

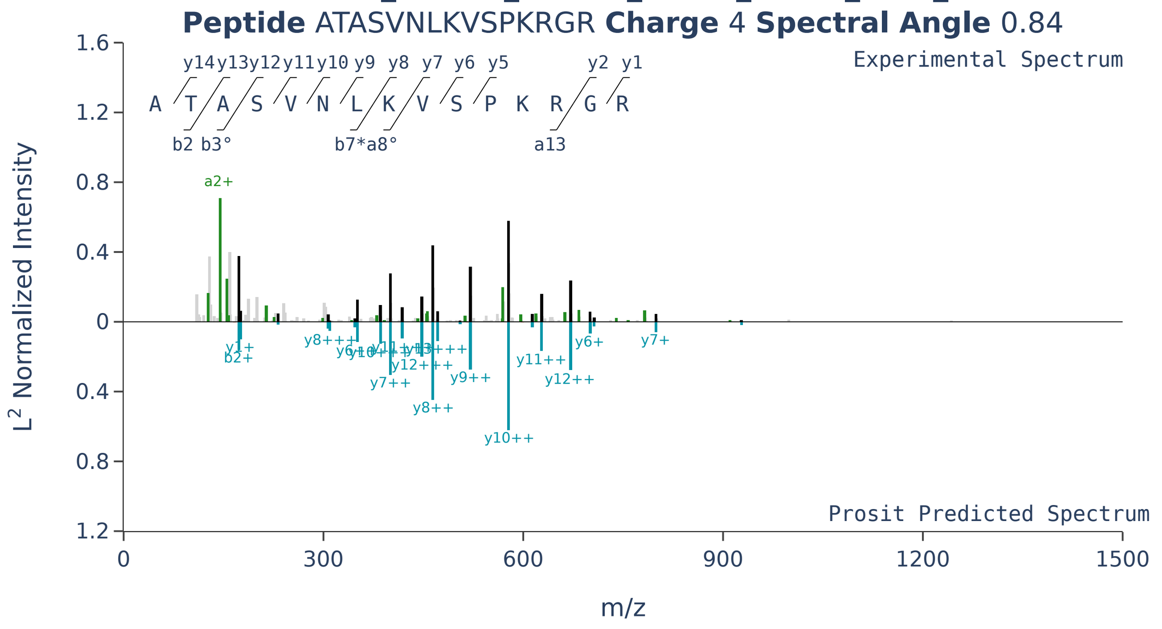


**Source** HRoetschke_271222_090123_HFGoe_LEDGF_b2R1_4h **Scan** 6776
**Peptide** VLNKSLAEQRQHEE **Charge** 2 **Spectral Angle** 0.88

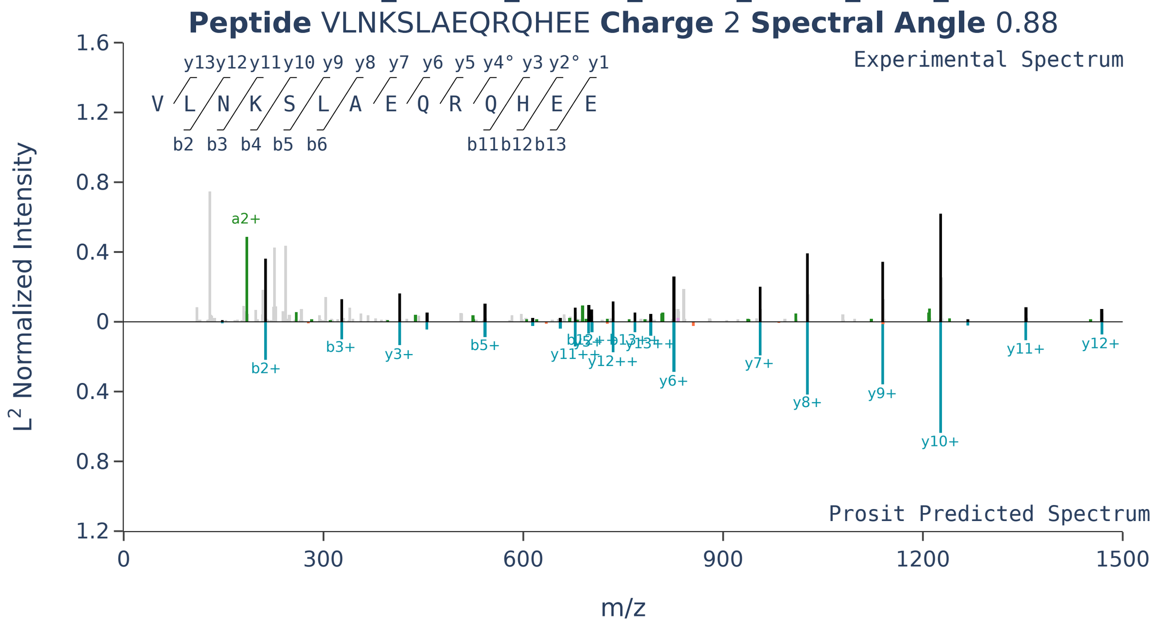


**Source** HRoetschke_271222_090123_HFGoe_LEDGF_b1R2_1h **Scan** 12000
**Peptide** VSQVIMEKST **Charge** 2 **Spectral Angle** 0.85

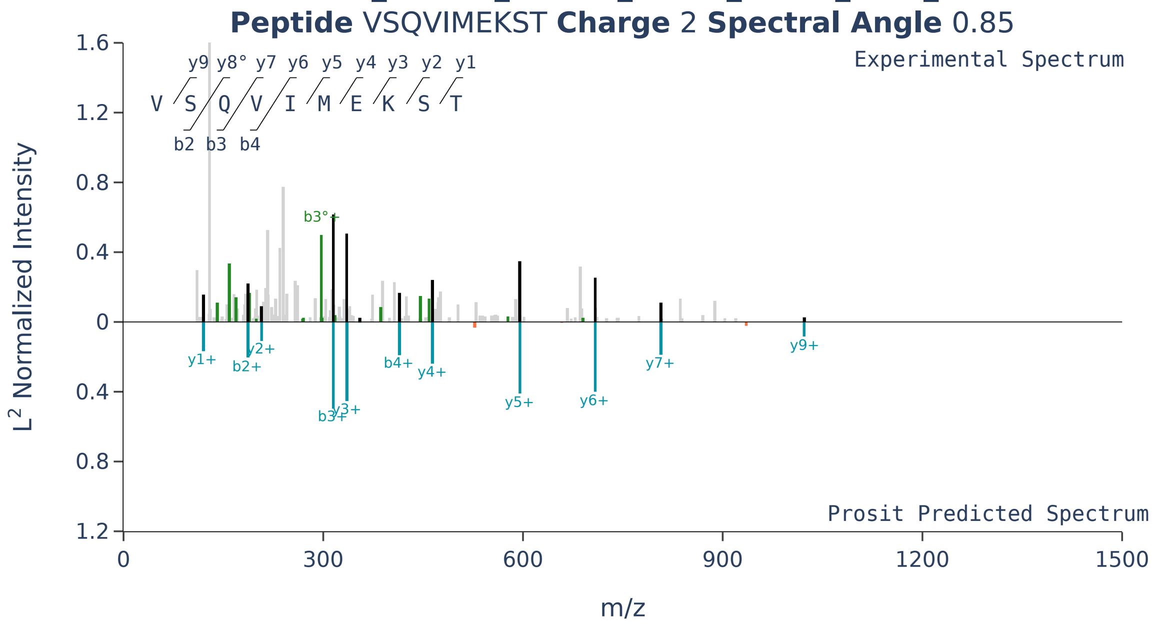


**Source** HRoetschke_271222_090123_HFGoe_LEDGF_b2R1_1h **Scan** 24915
**Peptide** DELASLQV **Charge** 1 **Spectral Angle** 0.95

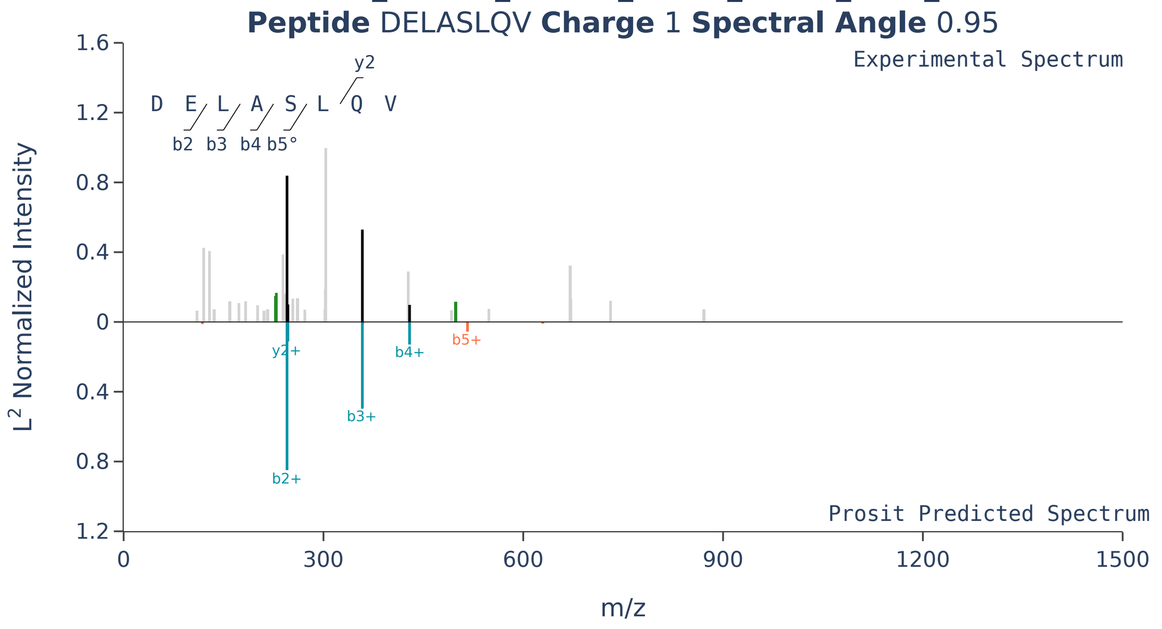


**Source** HRoetschke_271222_090123_HFGoe_LEDGF_b1R1_4h **Scan** 11591
**Peptide** EGDSVIT **Charge** 1 **Spectral Angle** 0.85

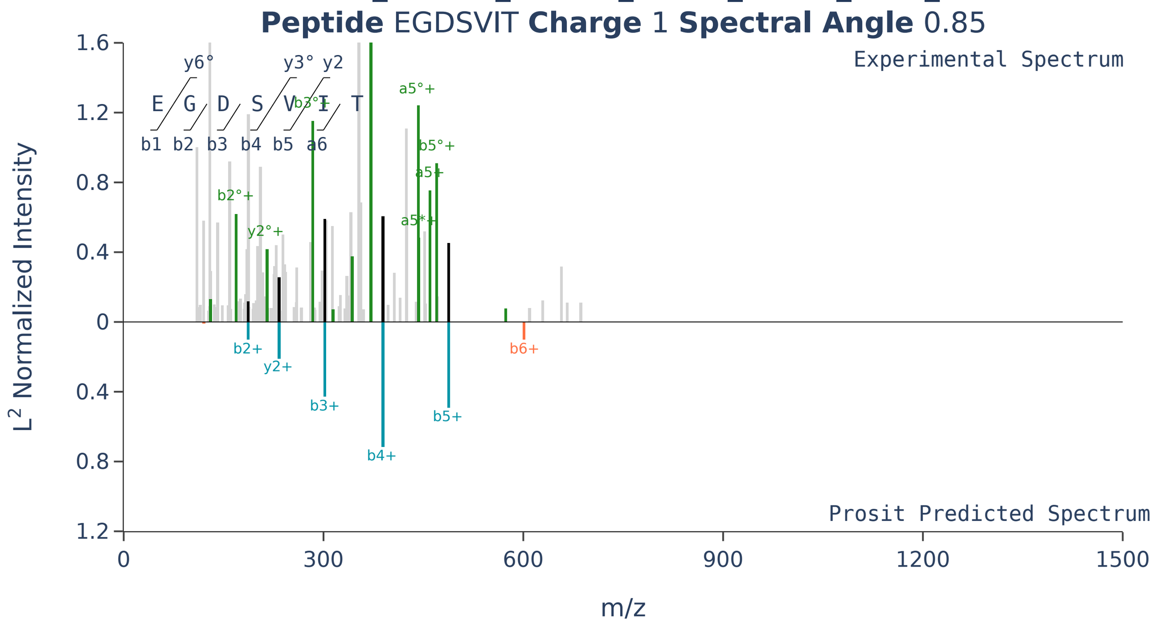

**Source** HRoetschke_271222_090123_HFGoe_LEDGF_b2R1_2h **Scan** 19501
**Peptide** TEISLKDSTL **Charge** 2 **Spectral Angle** 0.86

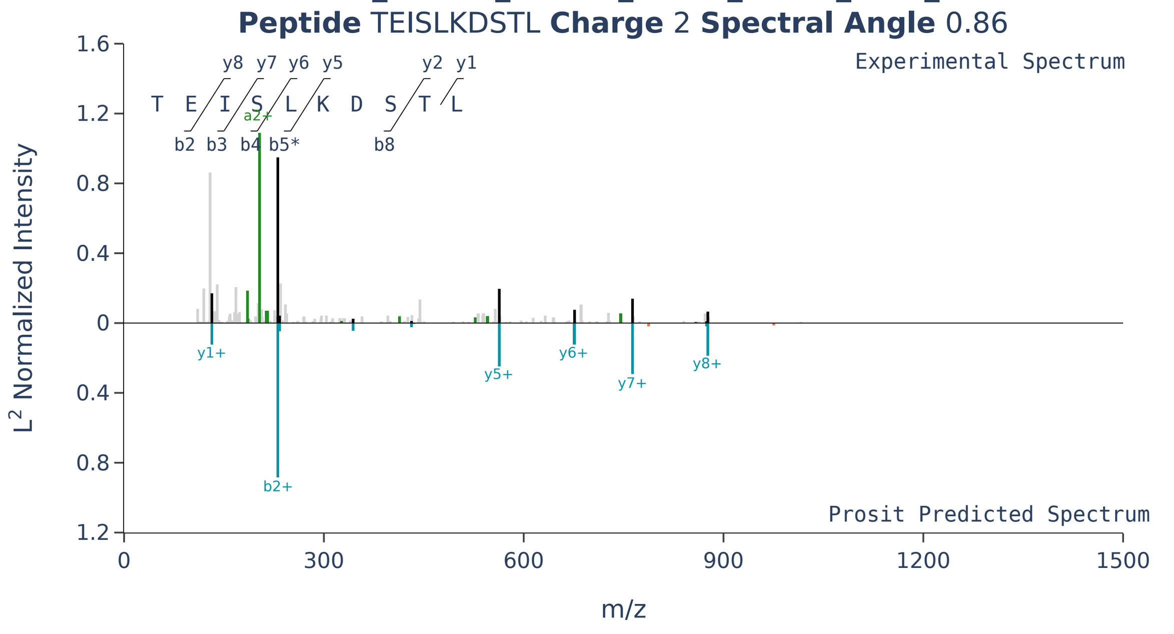


**Source** HRoetschke_271222_090123_HFGoe_LEDGF_b2R2_4h **Scan** 7718
**Peptide** VLNKSLAEQRQ **Charge** 2 **Spectral Angle** 0.88

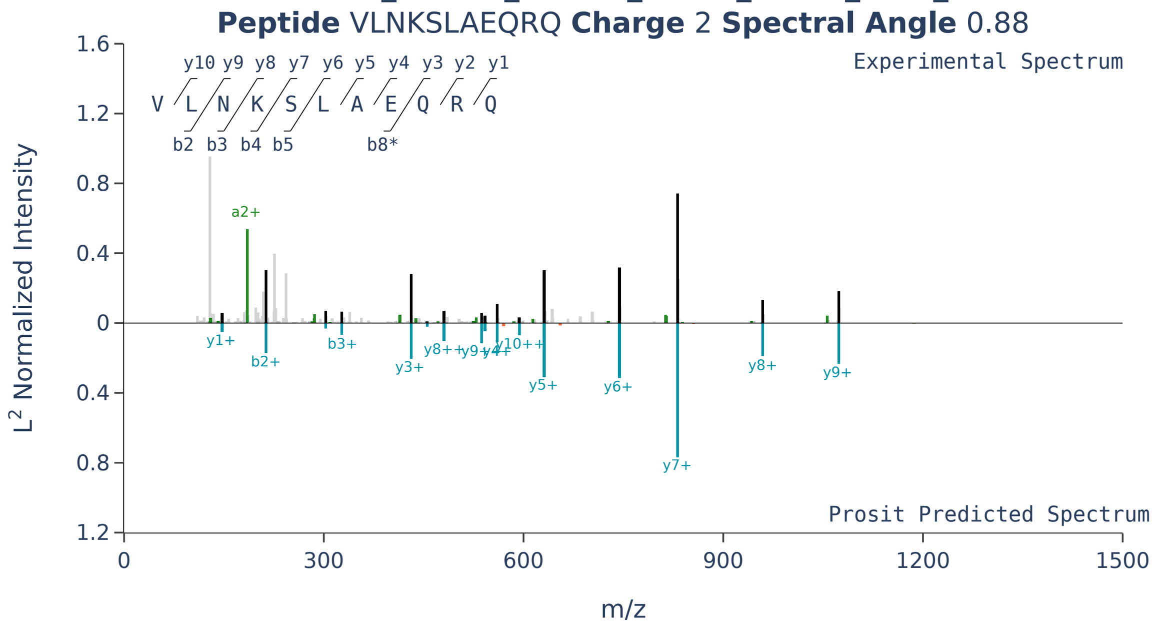


**Source** HRoetschke_271222_090123_HFGoe_LEDGF_b2R1_24h **Scan** 18633
**Peptide** GDSVITQV **Charge** 1 **Spectral Angle** 0.85

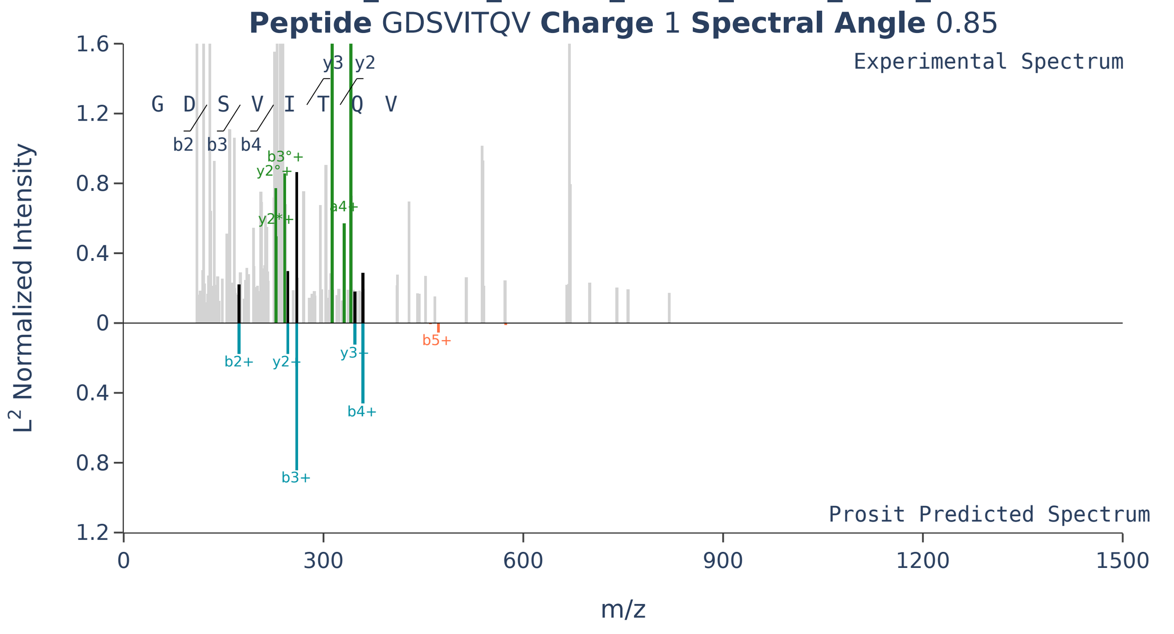


**Source** HRoetschke_271222_090123_HFGoe_LEDGF_b1R1_24h **Scan** 11506
**Peptide** AGVVTTATA **Charge** 1 **Spectral Angle** 0.95

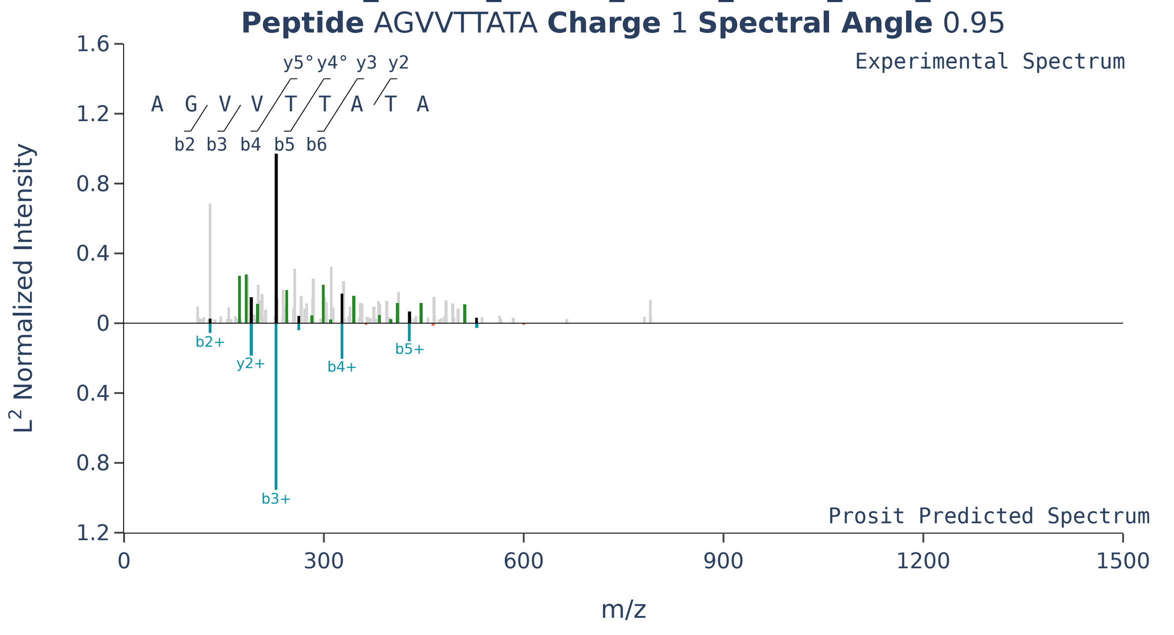


**Source** HRoetschke_271222_090123_HFGoe_LEDGF_b2R1_24h **Scan** 10642
**Peptide** EEAGVVTT **Charge** 1 **Spectral Angle** 0.87

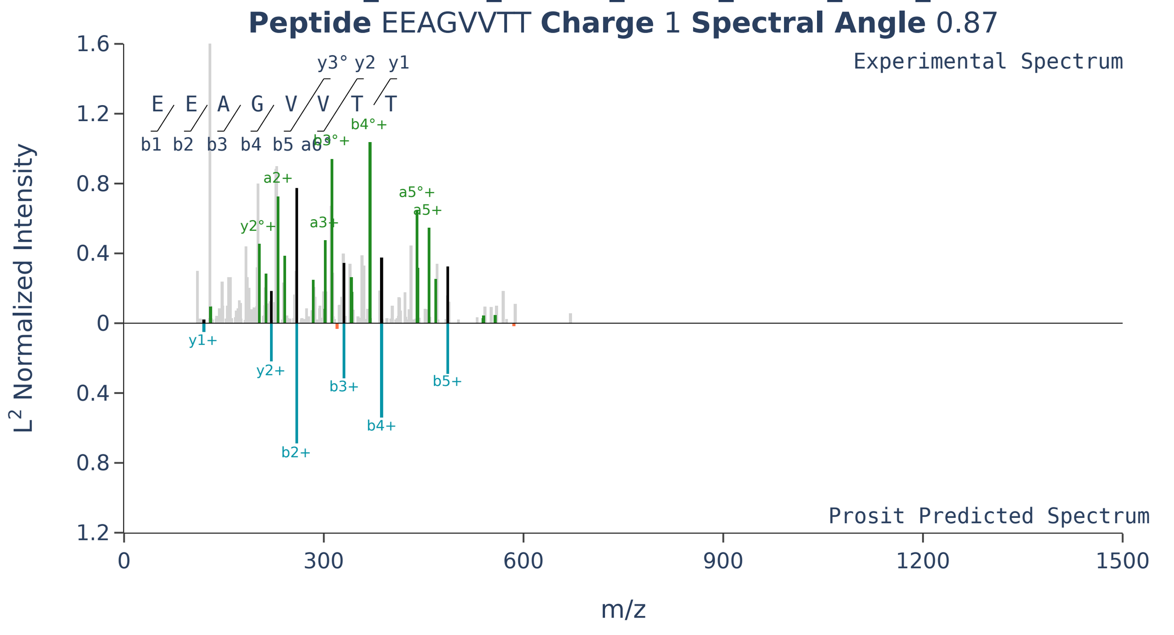


**Source** HRoetschke_271222_090123_HFGoe_LEDGF_b1R1_4h **Scan** 27872
**Peptide** SVITQVL **Charge** 1 **Spectral Angle** 0.9

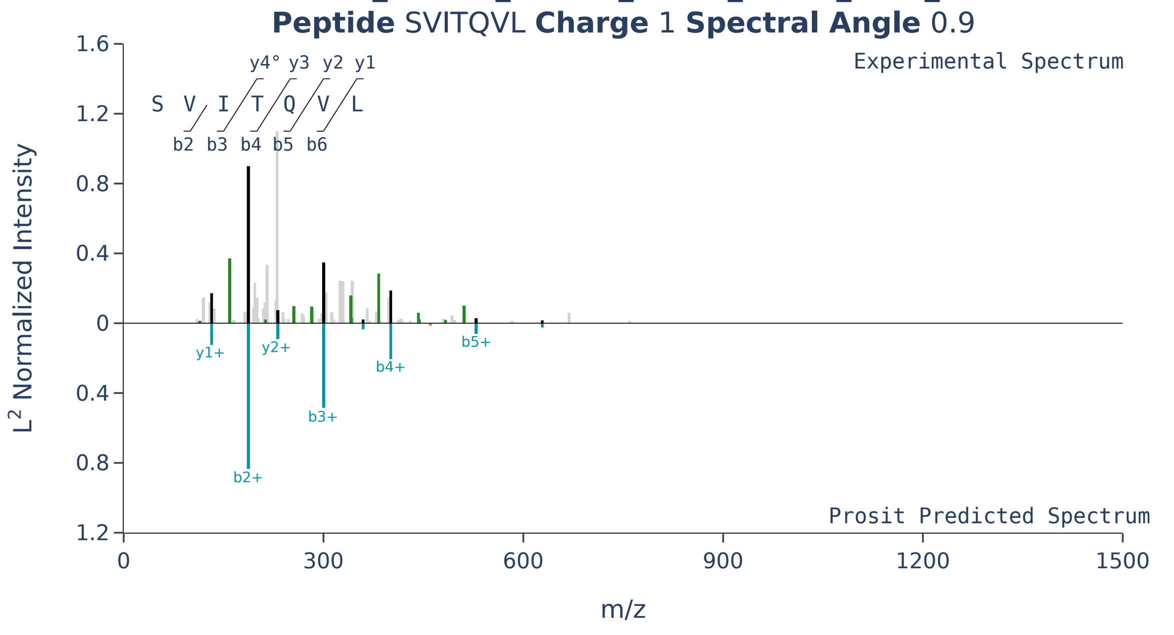


**Source** HRoetschke_271222_090123_HFGoe_LEDGF_b2R2_2h **Scan** 11950
**Peptide** ETEEAGVVTT **Charge** 2 **Spectral Angle** 0.85

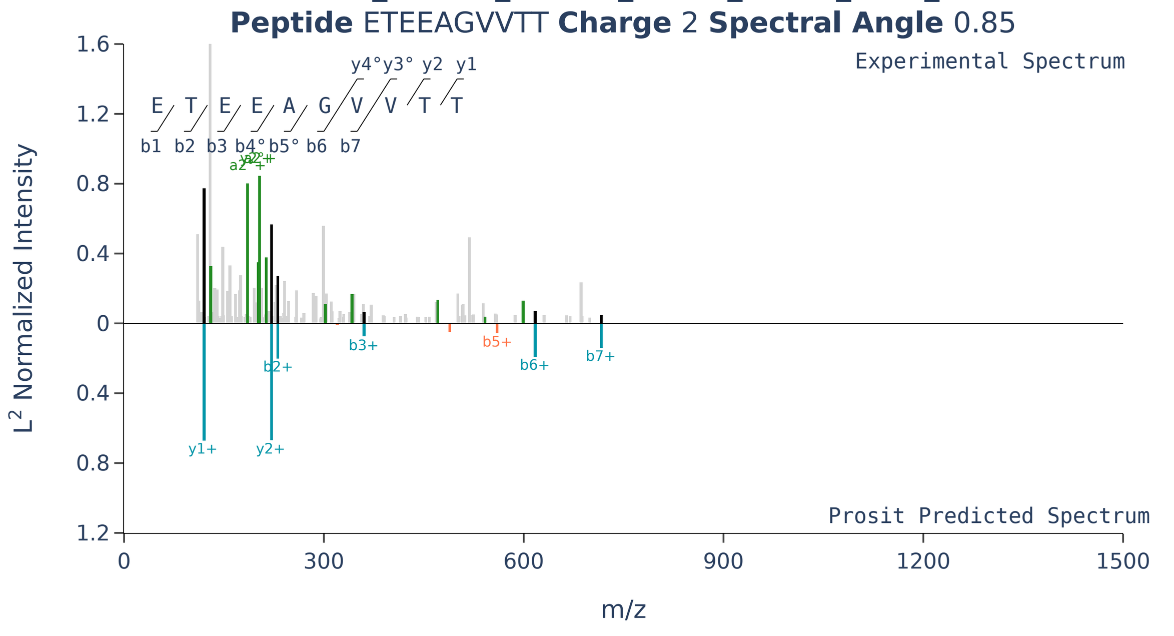


**Source** HRoetschke_271222_090123_HFGoe_LEDGF_b1R1_2h **Scan** 10385
**Peptide** KSTMLYNKFKN **Charge** 3 **Spectral Angle** 0.88

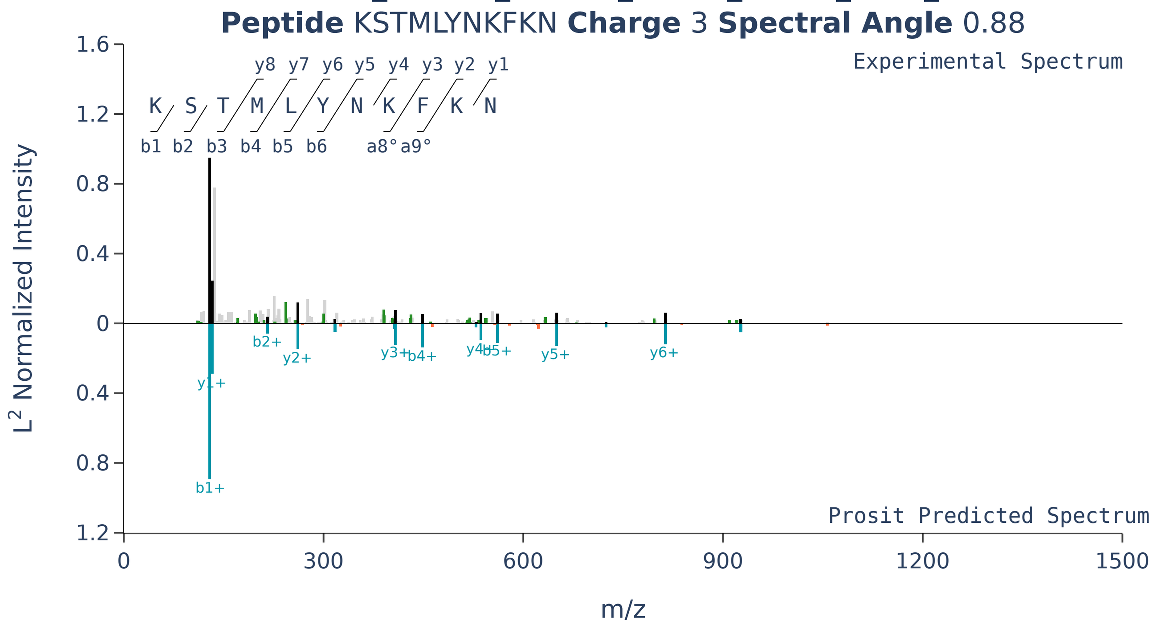


**Source** HRoetschke_271222_090123_HFGoe_LEDGF_b1R2_0h **Scan** 7557
**Peptide** RVDEVPD **Charge** 2 **Spectral Angle** 0.91

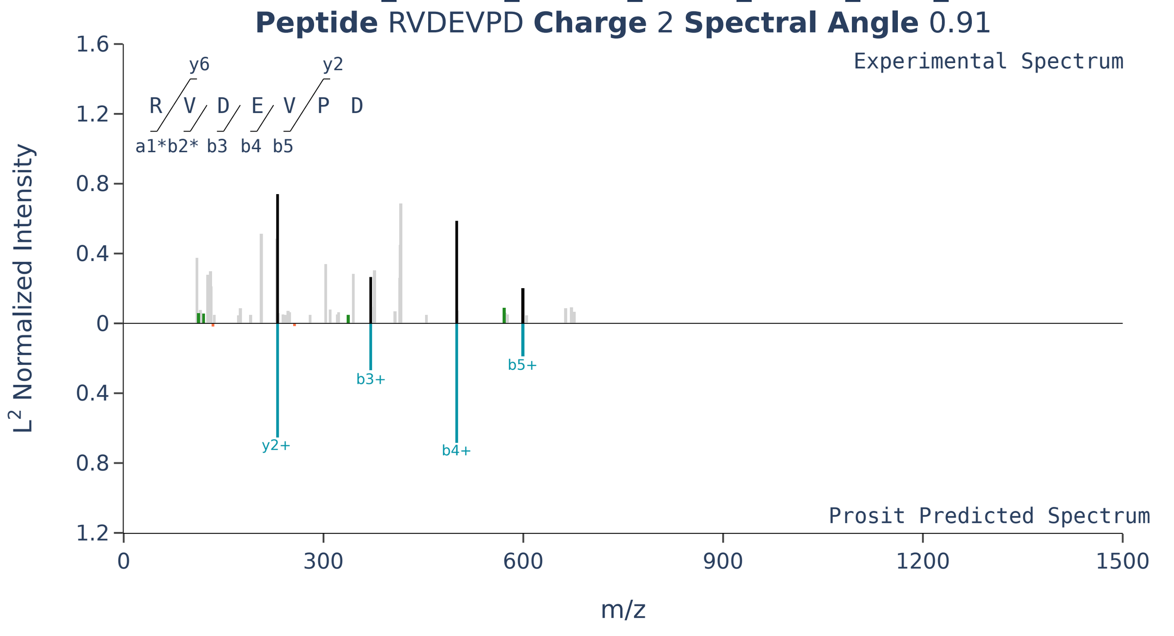


**Source** HRoetschke_271222_090123_HFGoe_LEDGF_b1R1_24h **Scan** 13177
**Peptide** DEVPDGAVKPPTN **Charge** 2 **Spectral Angle** 0.9

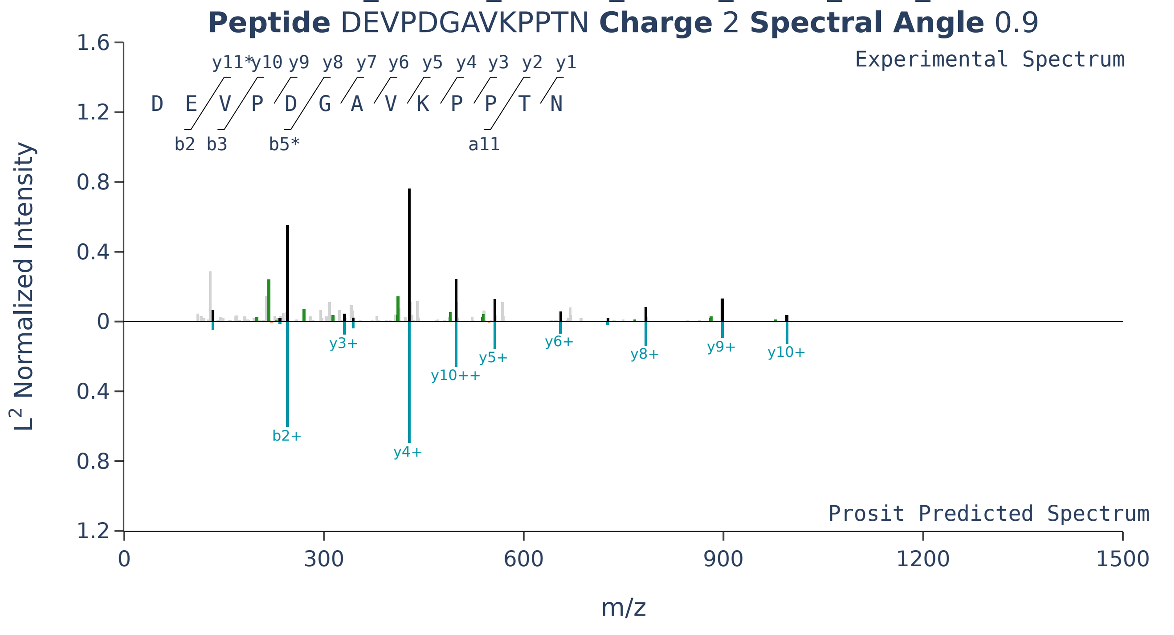


**Source** HRoetschke_271222_090123_HFGoe_LEDGF_b2R1_4h **Scan** 17343
**Peptide** SLKIDNLDVNRC **Charge** 3 **Spectral Angle** 0.94

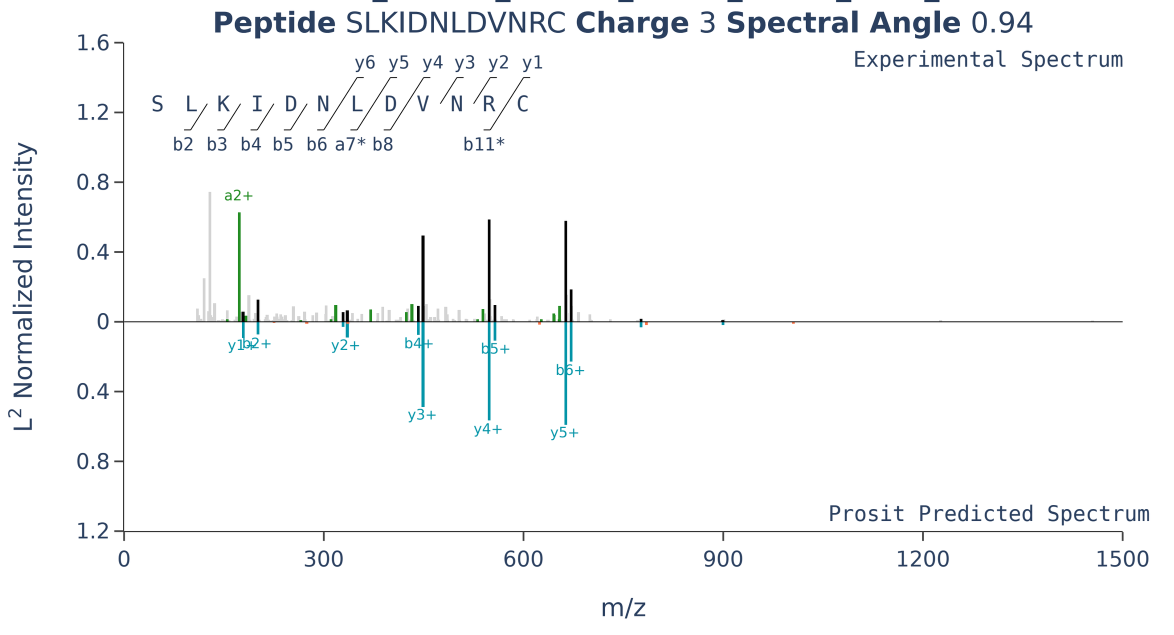


**Source** HRoetschke_271222_090123_HFGoe_LEDGF_b2R1_24h **Scan** 11609
**Peptide** TSMDSRLQRIHA **Charge** 4 **Spectral Angle** 0.94

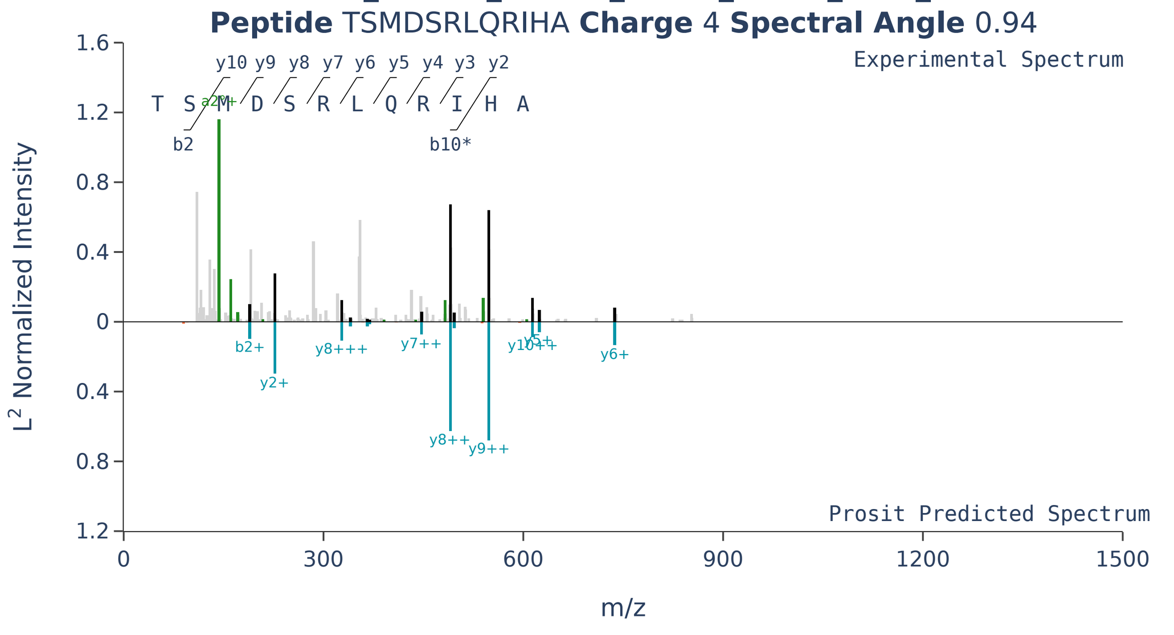


**Source** HRoetschke_271222_090123_HFGoe_LEDGF_b2R1_1h **Scan** 8711
**Peptide** ATKQSNASSDVEVEE **Charge** 2 **Spectral Angle** 0.86

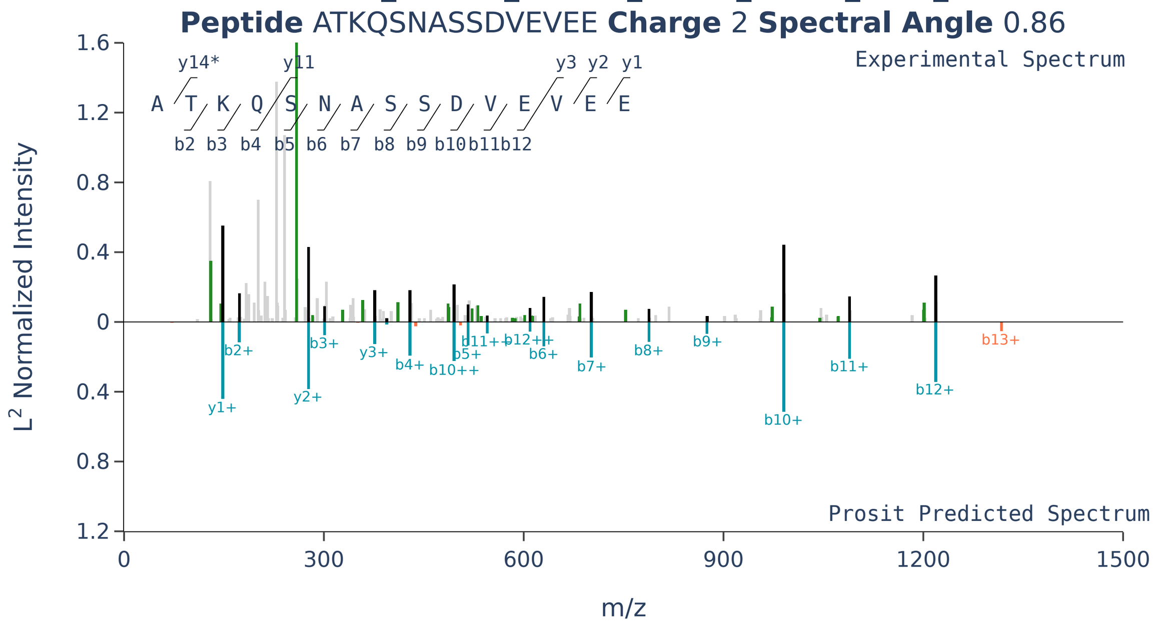


**Source** HRoetschke_271222_090123_HFGoe_LEDGF_b2R1_1h **Scan** 12746
**Peptide** GTHETAFLGPKD **Charge** 3 **Spectral Angle** 0.94

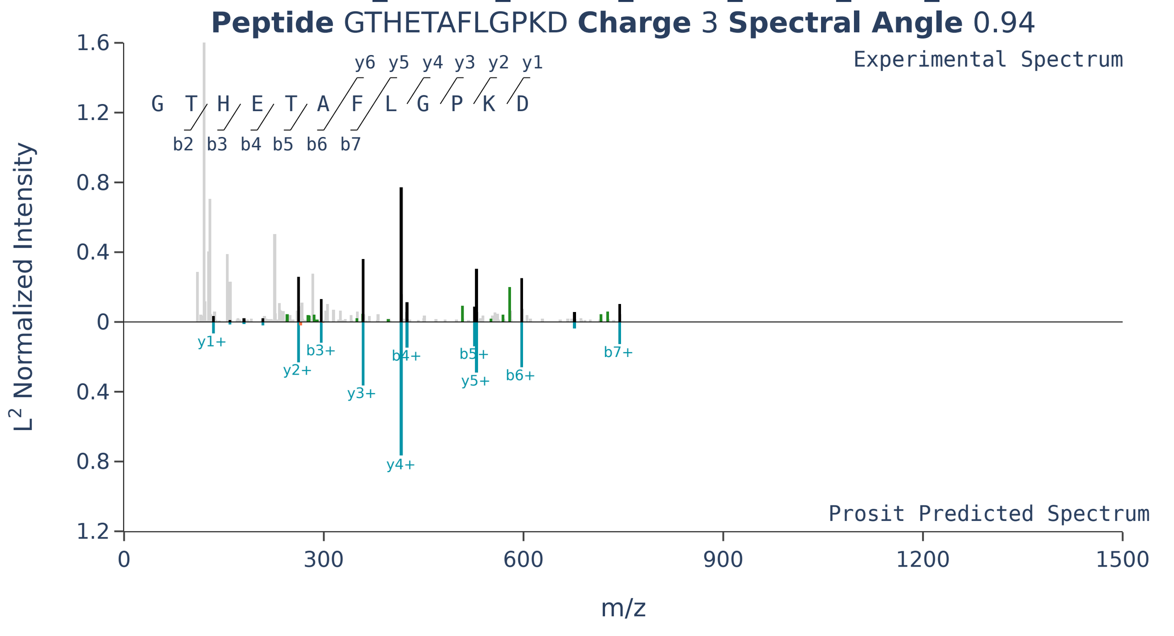


**Source** HRoetschke_271222_090123_HFGoe_LEDGF_b2R1_2h **Scan** 9448
**Peptide** ASSDVEVE **Charge** 1 **Spectral Angle** 0.9

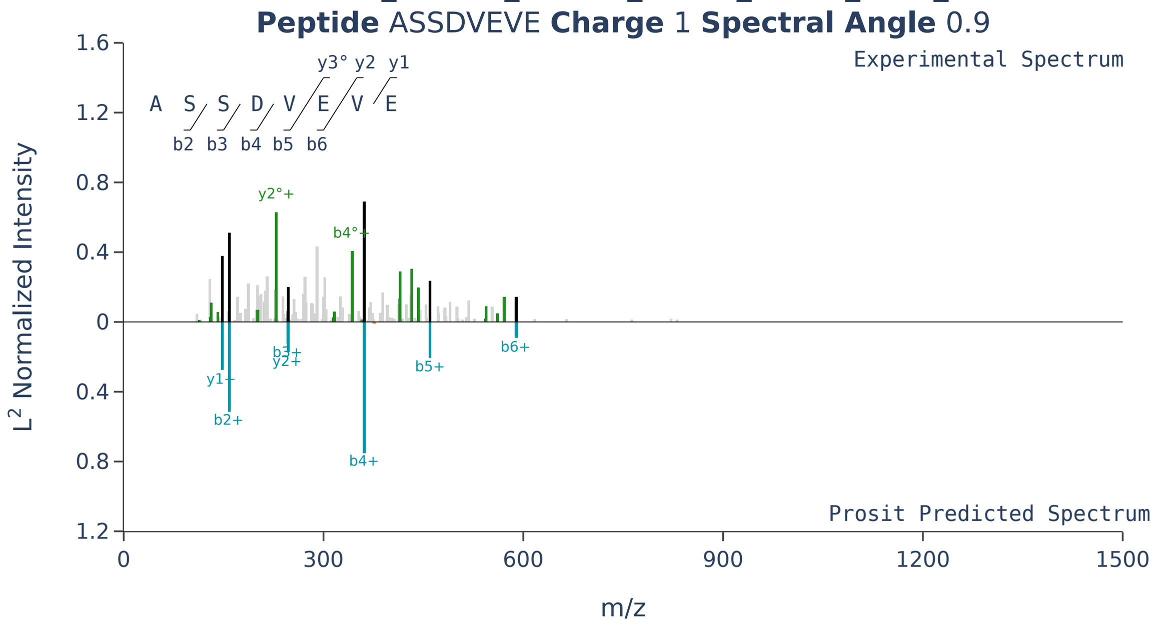

**Source** HRoetschke_271222_090123_HFGoe_LEDGF_b1R2_24h **Scan** 6752
**Peptide** LNKSLAEQR **Charge** 2 **Spectral Angle** 0.88

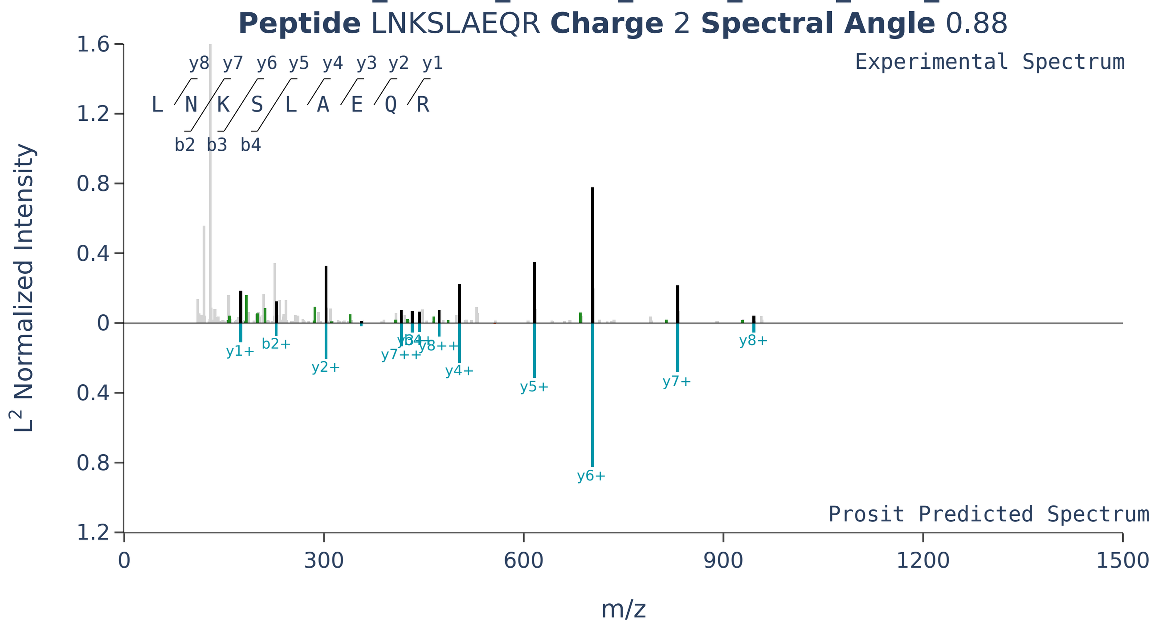


**Source** HRoetschke_271222_090123_HFGoe_LEDGF_b2R1_4h **Scan** 5746
**Peptide** FPYSENKEKYGKPNKRKG **Charge** 3 **Spectral Angle** 0.84

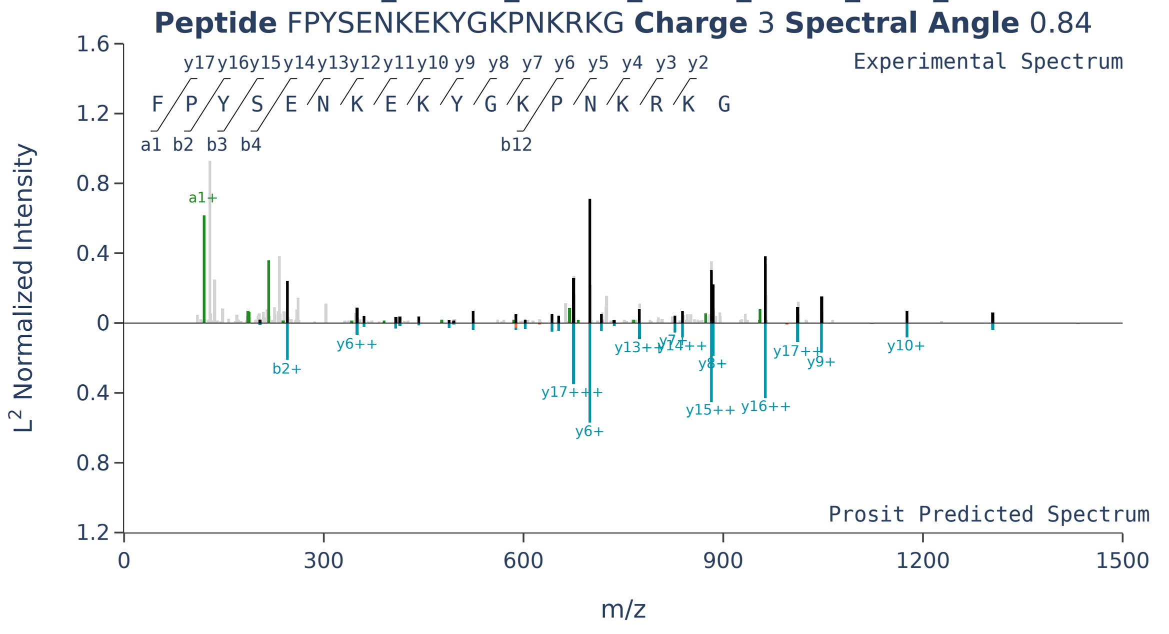


**Source** HRoetschke_271222_090123_HFGoe_LEDGF_b2R1_4h **Scan** 6614
**Peptide** SVNLKVSPKRGR **Charge** 3 **Spectral Angle** 0.88

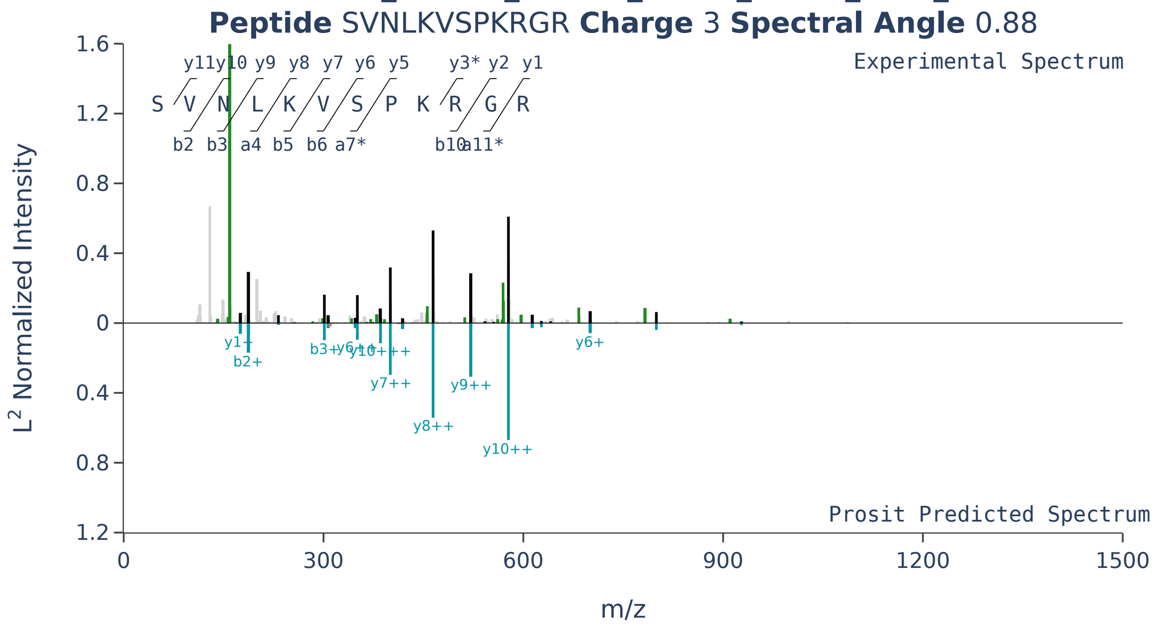


**Source** HRoetschke_271222_090123_HFGoe_LEDGF_b2R1_4h **Scan** 17542
**Peptide** ERETEISL **Charge** 1 **Spectral Angle** 0.87

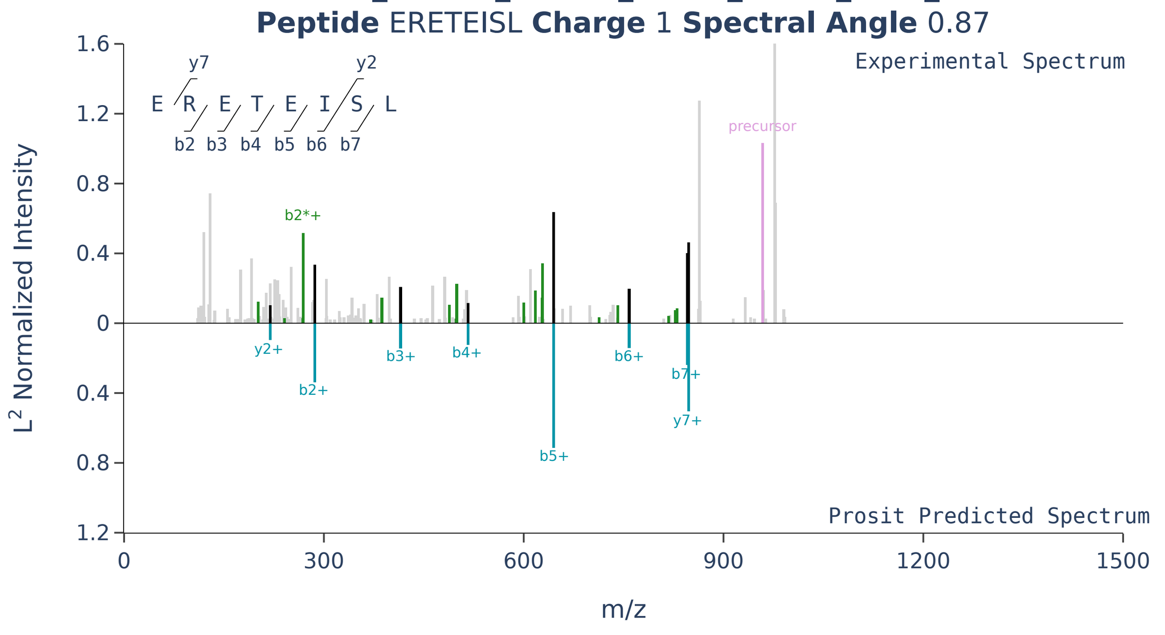


**Source** HRoetschke_271222_090123_HFGoe_LEDGF_b1R2_24h **Scan** 15922
**Peptide** FGTHETAF **Charge** 2 **Spectral Angle** 0.93

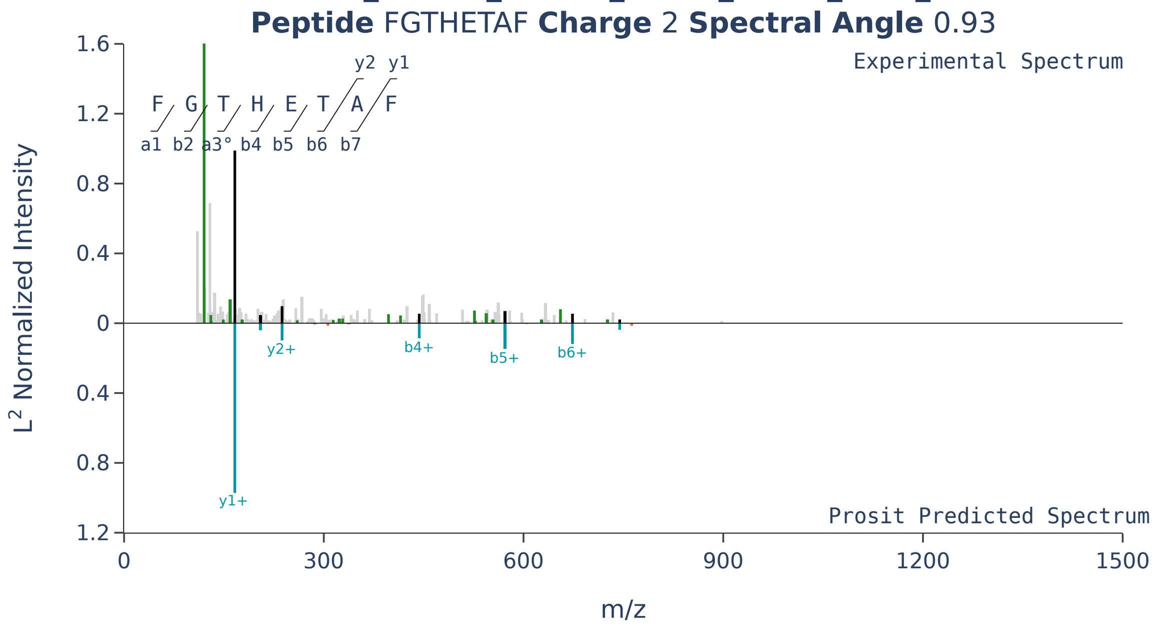


**Source** HRoetschke_271222_090123_HFGoe_LEDGF_b2R2_2h **Scan** 20972
**Peptide** ELASLQVTMQQAQK **Charge** 2 **Spectral Angle** 0.84

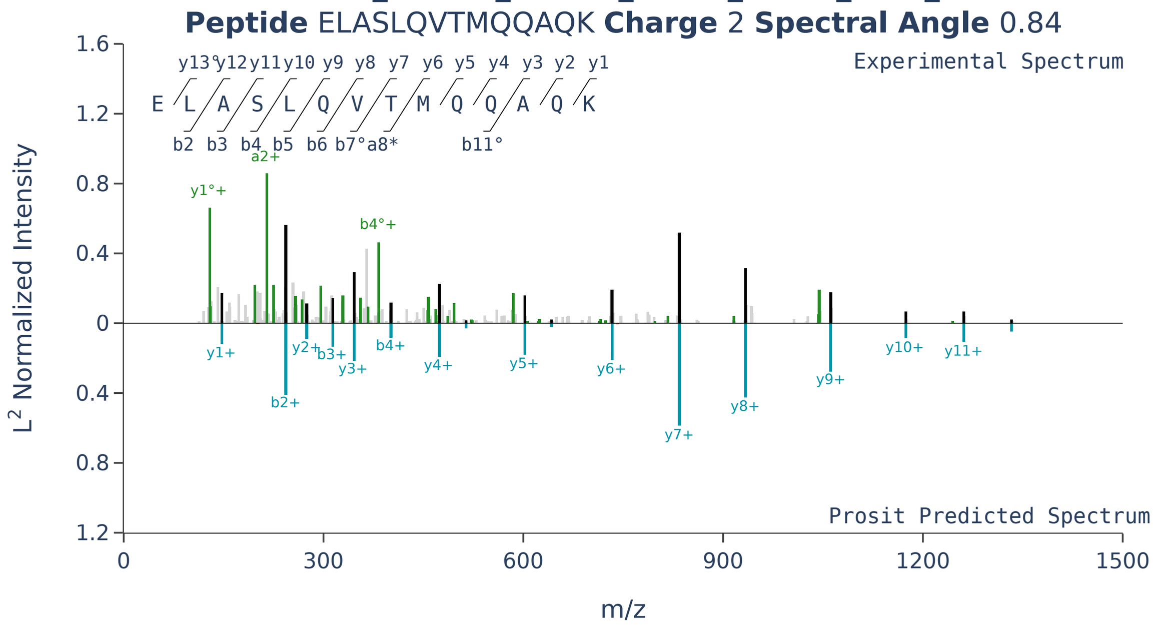


**Source** HRoetschke_271222_090123_HFGoe_LEDGF_b1R1_2h **Scan** 10369
**Peptide** VLNKSLAE **Charge** 1 **Spectral Angle** 0.85

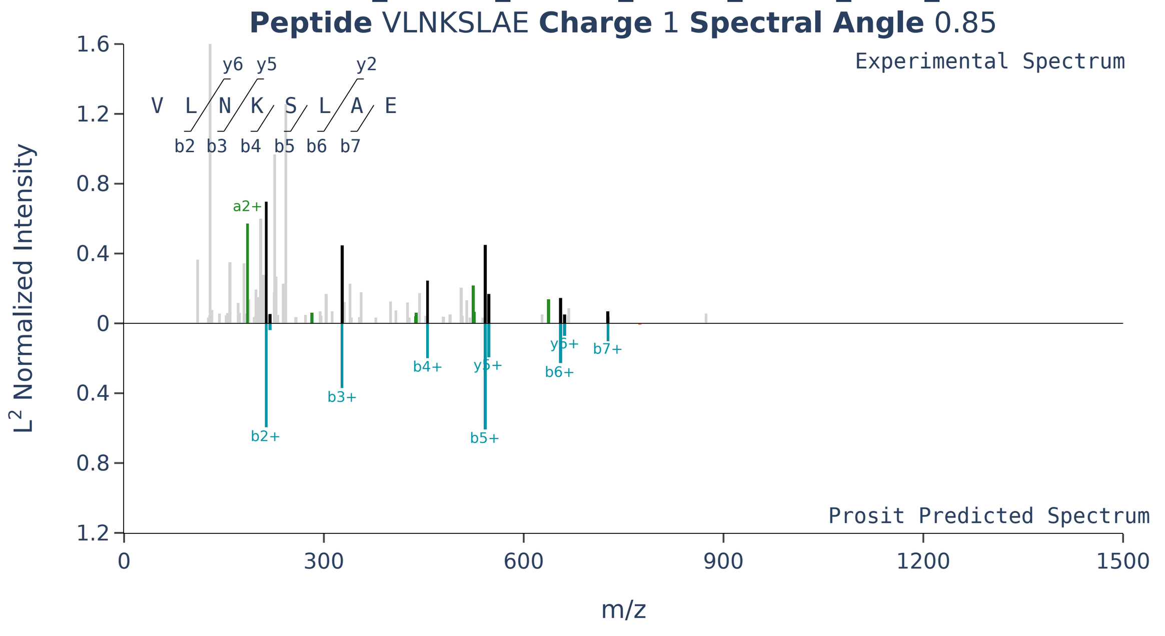


**Source** HRoetschke_271222_090123_HFGoe_LEDGF_b1R2_24h **Scan** 16162
**Peptide** RETEISLKDSTLD **Charge** 2 **Spectral Angle** 0.89

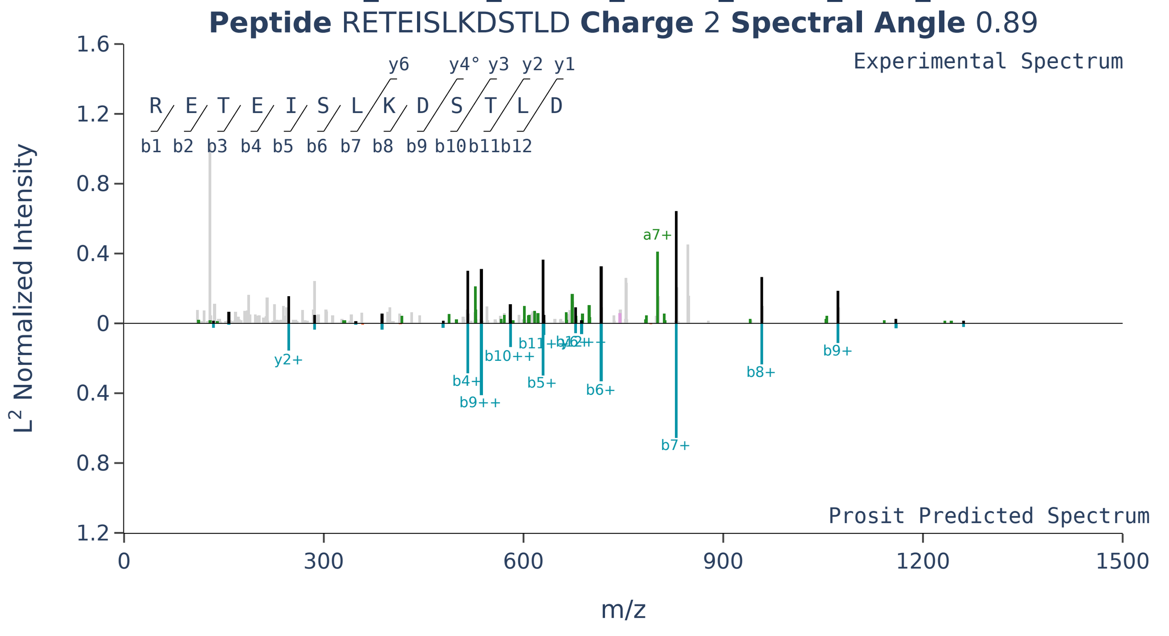


**Source** HRoetschke_271222_090123_HFGoe_LEDGF_b1R2_4h **Scan** 10616
**Peptide** KSTMLYNKFK **Charge** 3 **Spectral Angle** 0.85

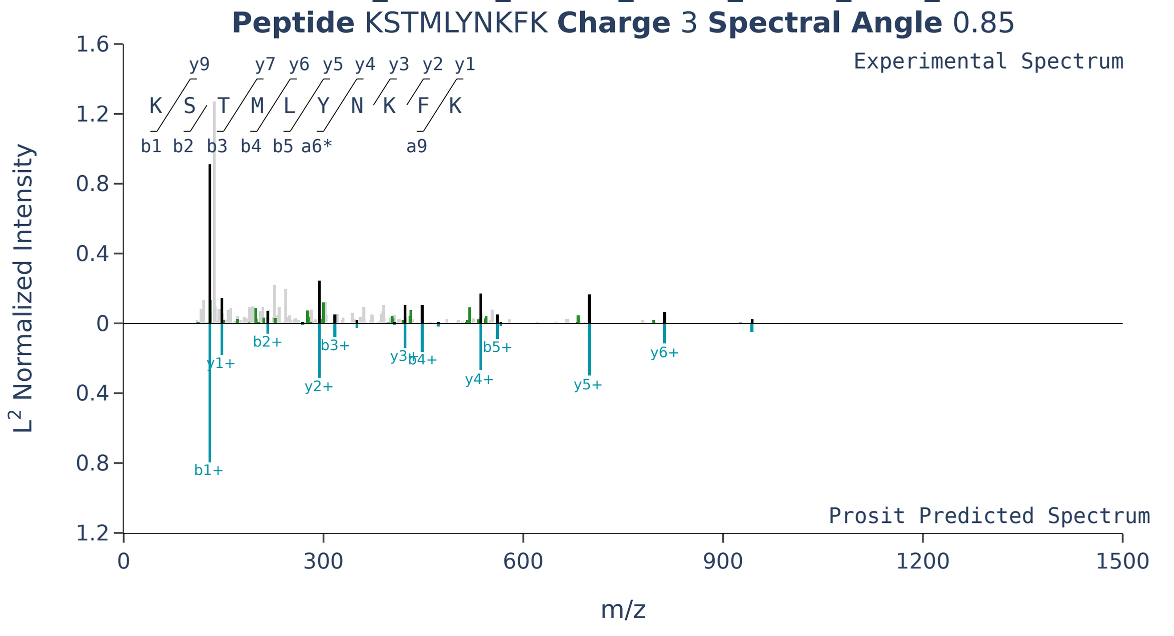


**Source** HRoetschke_271222_090123_HFGoe_LEDGF_b2R1_4h **Scan** 19179
**Peptide** GTHETAFLGPKDI **Charge** 3 **Spectral Angle** 0.91

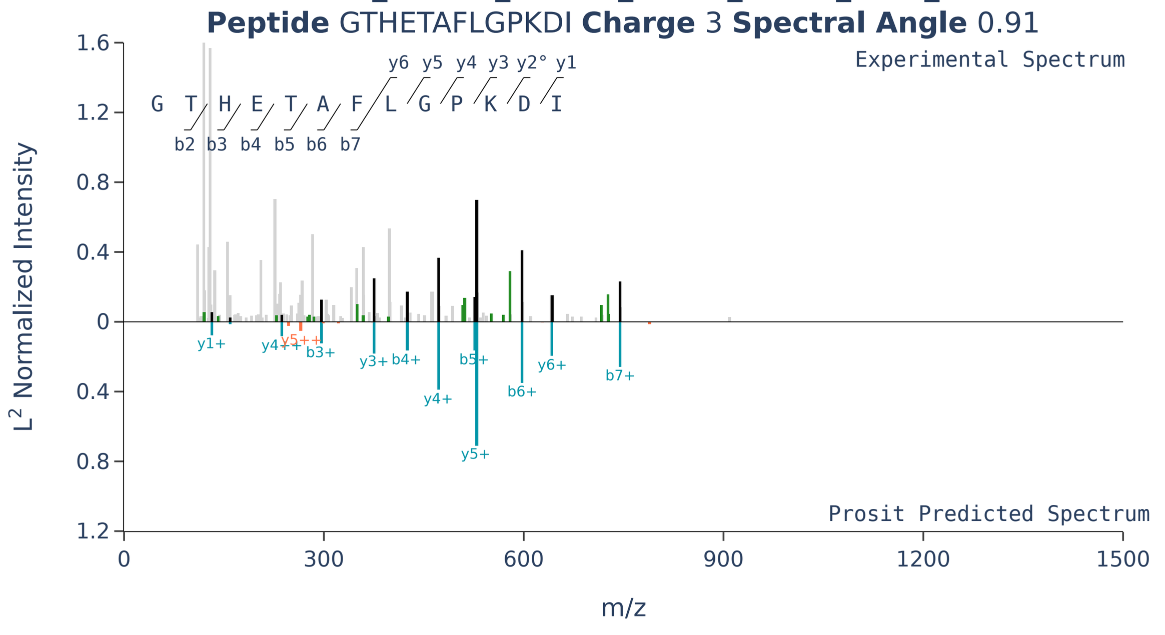


**Source** HRoetschke_271222_090123_HFGoe_LEDGF_b2R1_24h **Scan** 8593
**Peptide** MITTLKKIR **Charge** 3 **Spectral Angle** 0.9

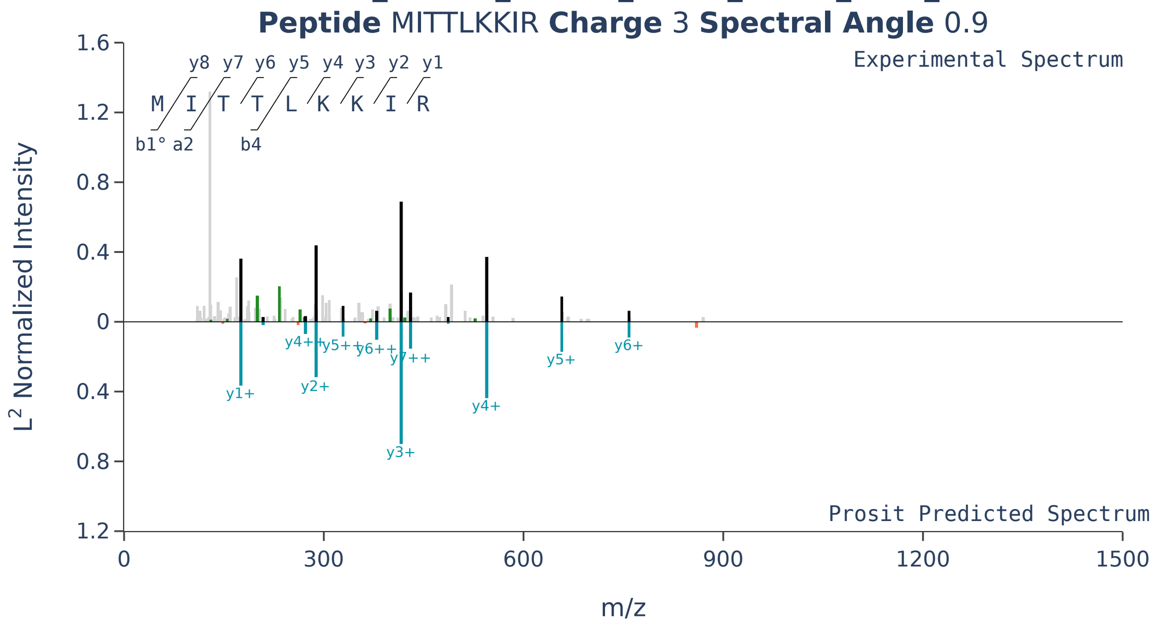


**Source** HRoetschke_271222_090123_HFGoe_LEDGF_b1R2_24h **Scan** 15211
**Peptide** RFKVSQVIMEK **Charge** 3 **Spectral Angle** 0.87

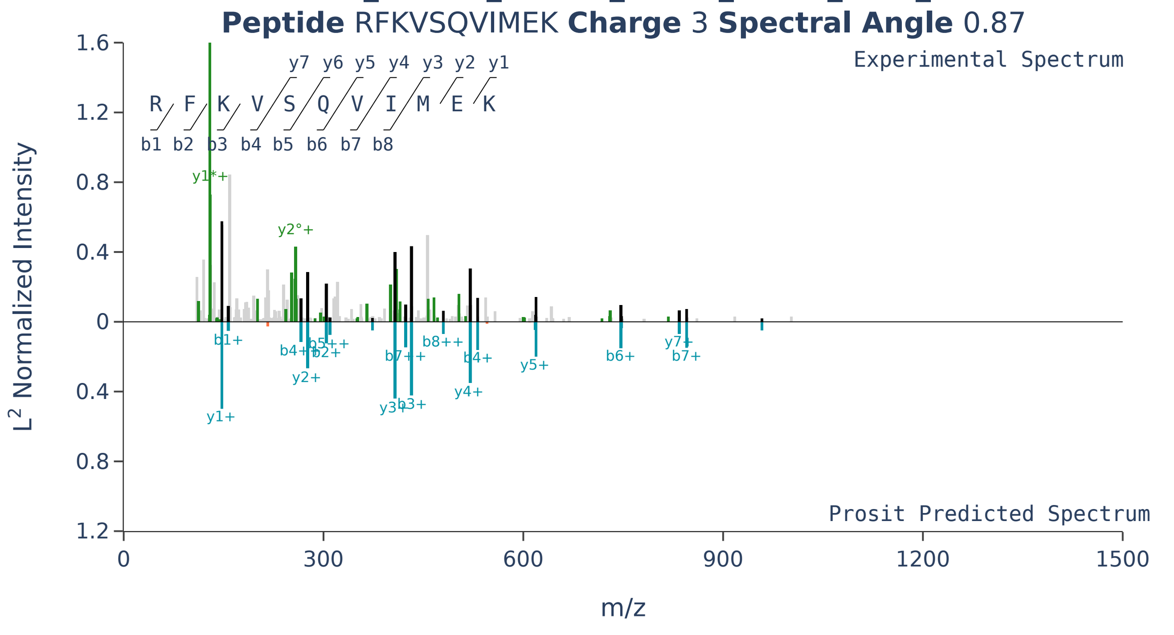


**Source** HRoetschke_271222_090123_HFGoe_LEDGF_b1R1_1h **Scan** 14793
**Peptide** ELASLQVTMQQAQKHTE **Charge** 3 **Spectral Angle** 0.85

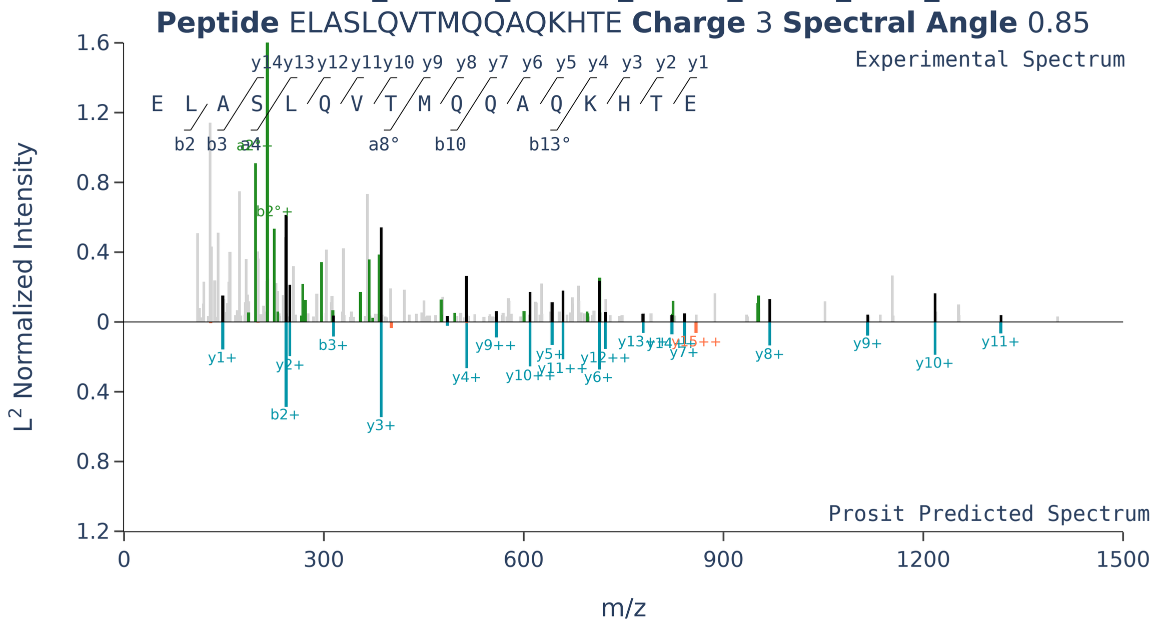


**Source** HRoetschke_271222_090123_HFGoe_LEDGF_b1R2_24h **Scan** 16765
**Peptide** EMITTLKKIRRF **Charge** 3 **Spectral Angle** 0.93

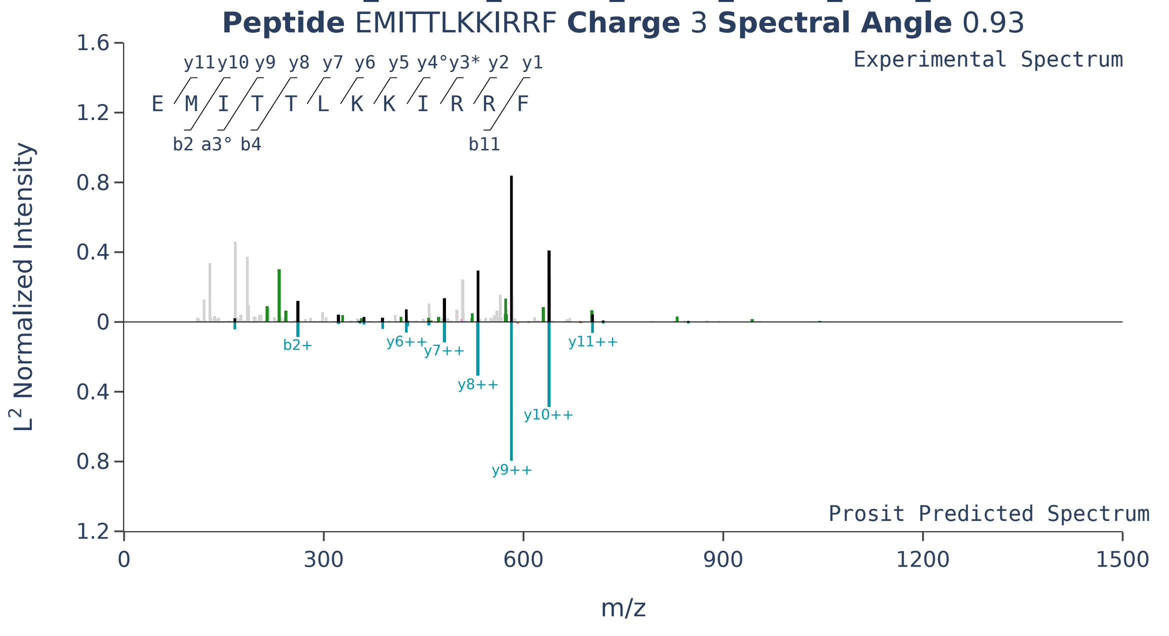


**Source** HRoetschke_271222_090123_HFGoe_LEDGF_b1R1_24h **Scan** 13685
**Peptide** PHWPARV **Charge** 2 **Spectral Angle** 0.89

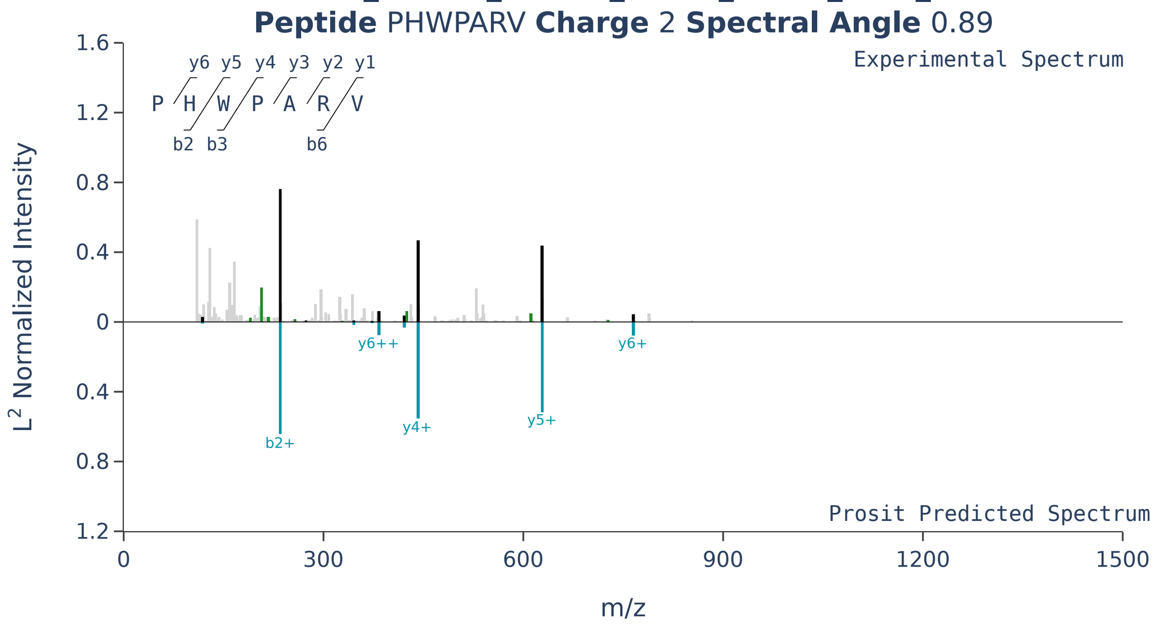

**Source** HRoetschke_271222_090123_HFGoe_LEDGF_b1R1_24h **Scan** 15156
**Peptide** RETEISLKDSTLDN **Charge** 2 **Spectral Angle** 0.89

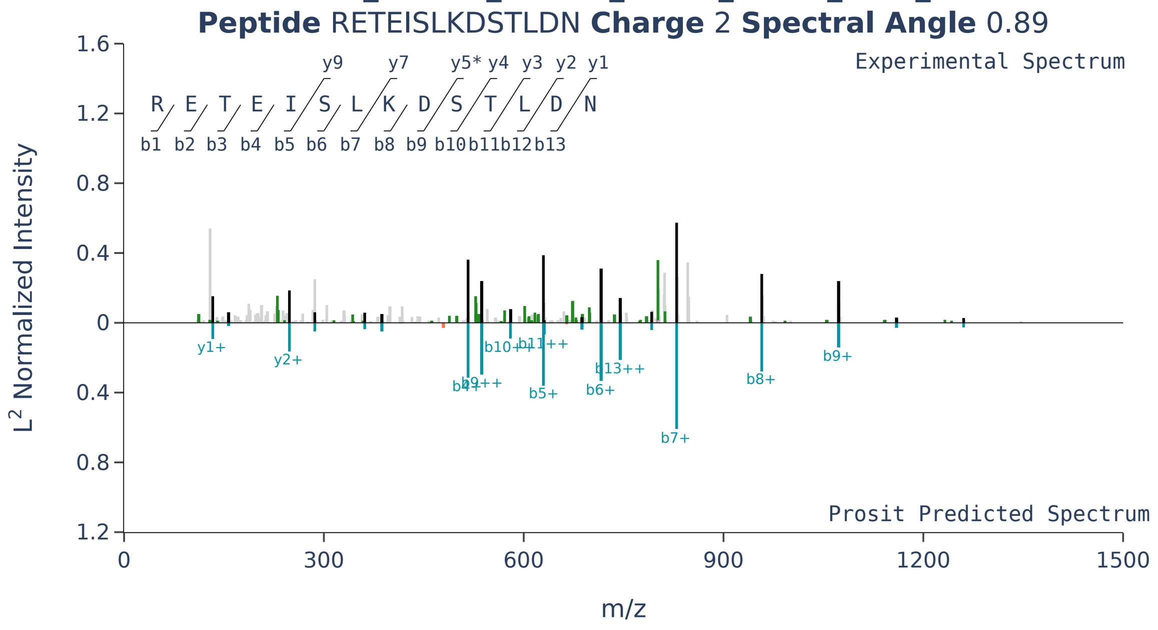


**Source** HRoetschke_271222_090123_HFGoe_LEDGF_b1R2_24h **Scan** 6459
**Peptide** VKIPKPRGR **Charge** 3 **Spectral Angle** 0.89

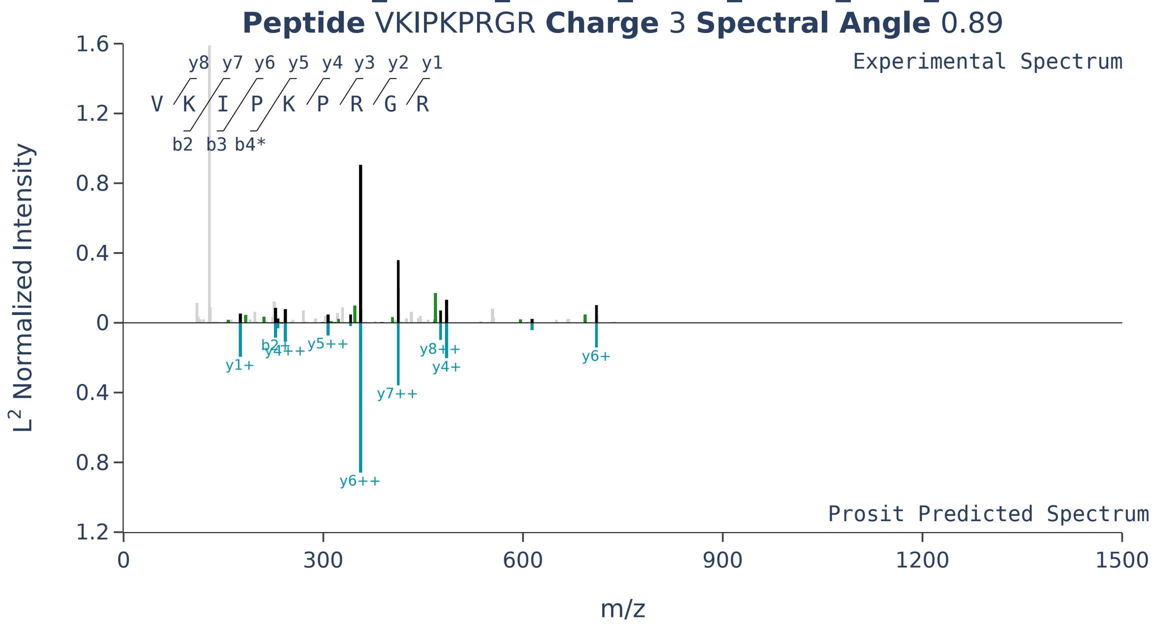


**Source** HRoetschke_271222_090123_HFGoe_LEDGF_b2R2_2h **Scan** 20655
**Peptide** GYPHWPARVDEV **Charge** 2 **Spectral Angle** 0.91

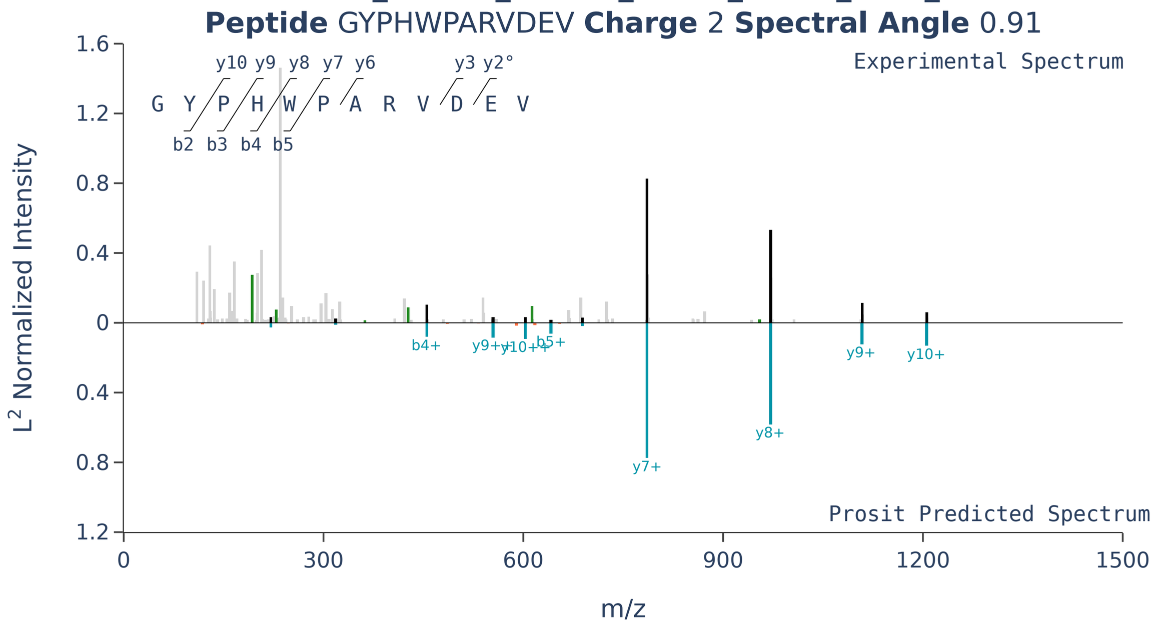


**Source** HRoetschke_271222_090123_HFGoe_LEDGF_b2R1_24h **Scan** 12429
**Peptide** HWPARVDE **Charge** 2 **Spectral Angle** 0.9

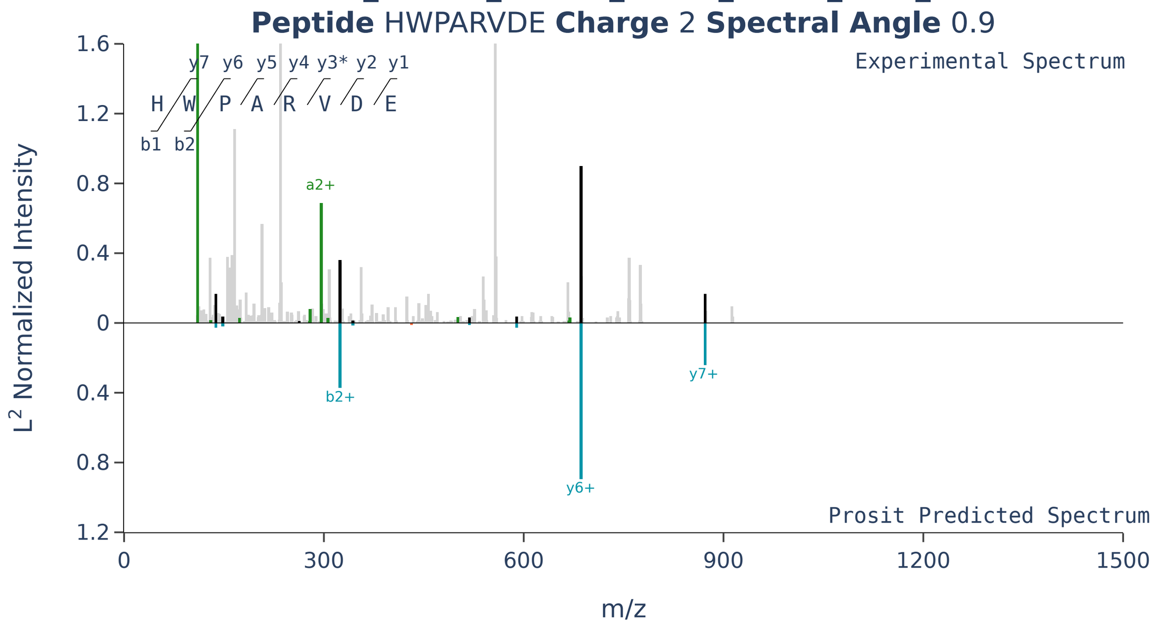


**Source** HRoetschke_271222_090123_HFGoe_LEDGF_b1R1_4h **Scan** 27422
**Peptide** FNEGLWEIDNNPKV **Charge** 2 **Spectral Angle** 0.81

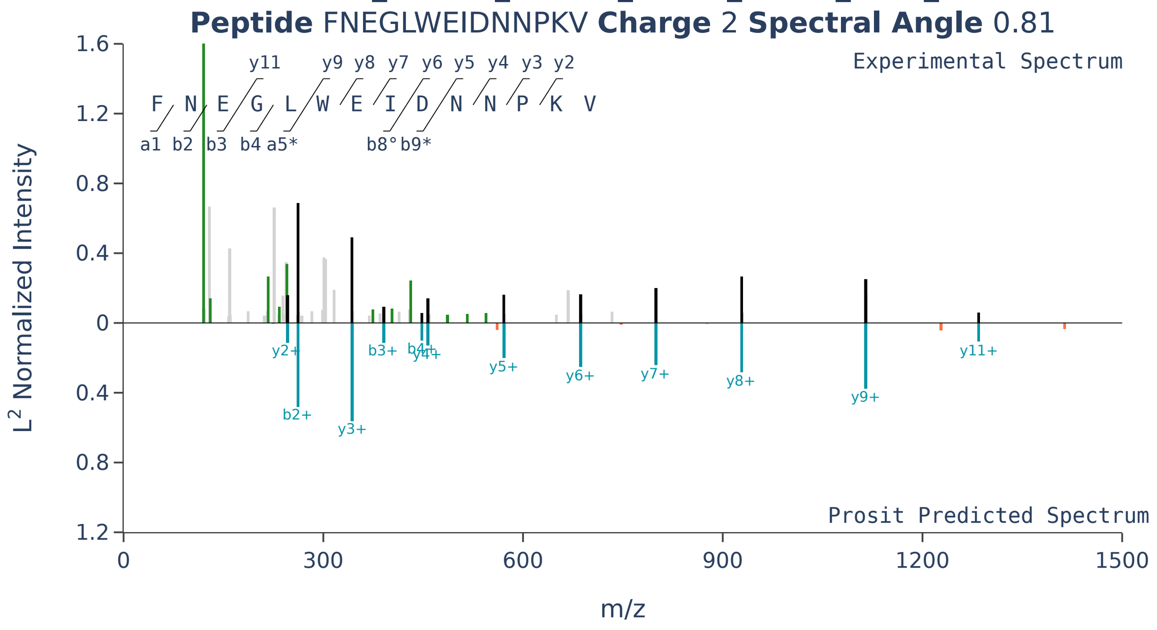


**Source** HRoetschke_271222_090123_HFGoe_LEDGF_b1R2_2h **Scan** 24541
**Peptide** NEGLWEIDNNPKV **Charge** 2 **Spectral Angle** 0.86

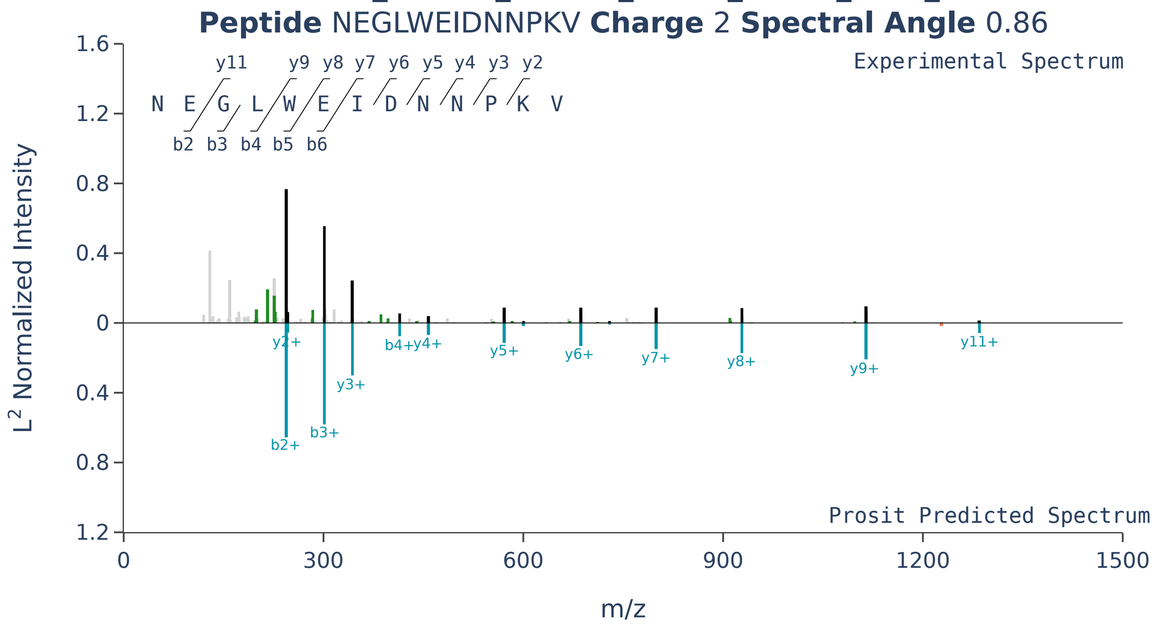


**Source** HRoetschke_271222_090123_HFGoe_LEDGF_b1R1_4h **Scan** 22438
**Peptide** FNEGLWEIDNNPKVKFSSQQAATK **Charge** 3 **Spectral Angle** 0.87

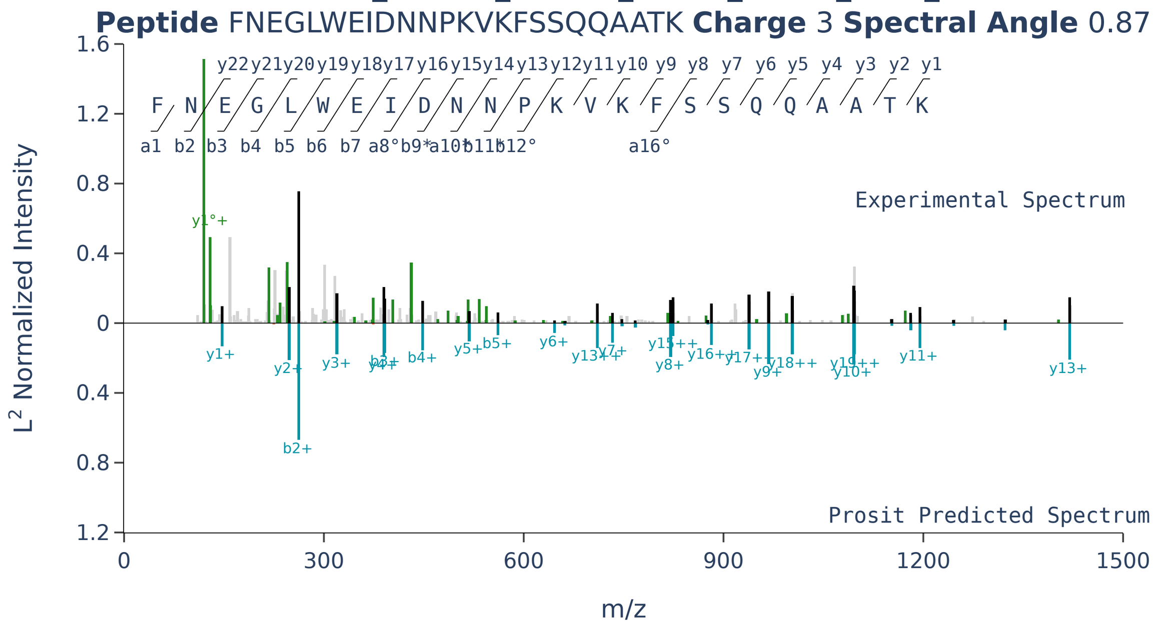


**Source** HRoetschke_271222_090123_HFGoe_LEDGF_b1R2_4h **Scan** 23351
**Peptide** GLWEIDNNPKVKFSSQ **Charge** 2 **Spectral Angle** 0.84

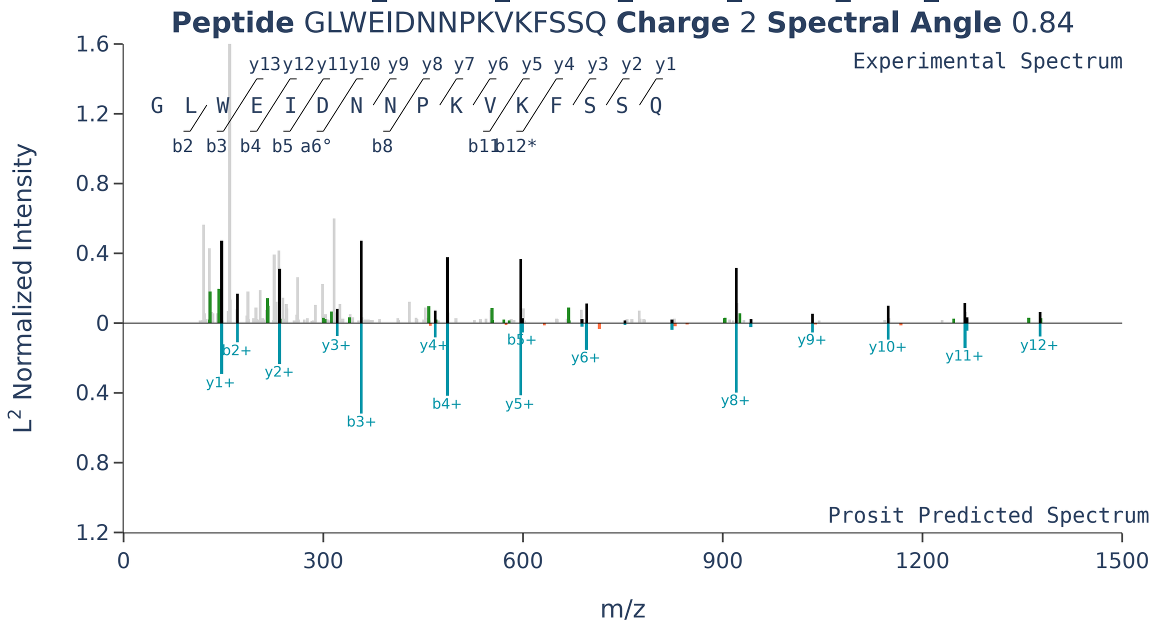


**Source** HRoetschke_271222_090123_HFGoe_LEDGF_b2R1_0h **Scan** 13621
**Peptide** RETEISL **Charge** 2 **Spectral Angle** 0.87

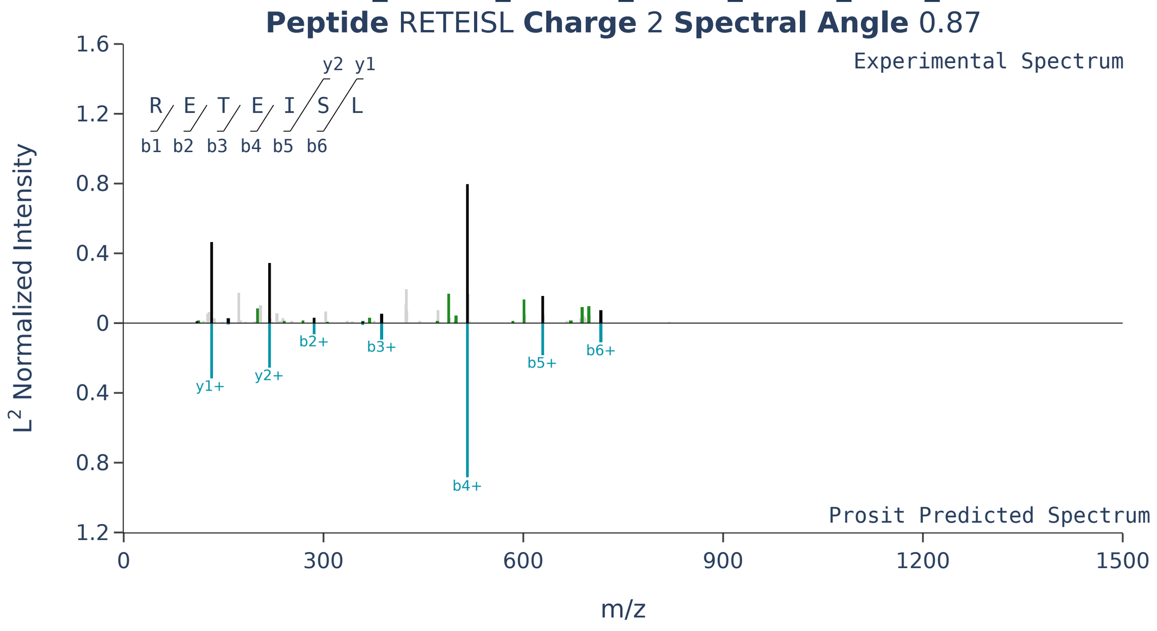


**Source** HRoetschke_271222_090123_HFGoe_LEDGF_b2R2_4h **Scan** 6714
**Peptide** GAVKPPTN **Charge** 2 **Spectral Angle** 0.89

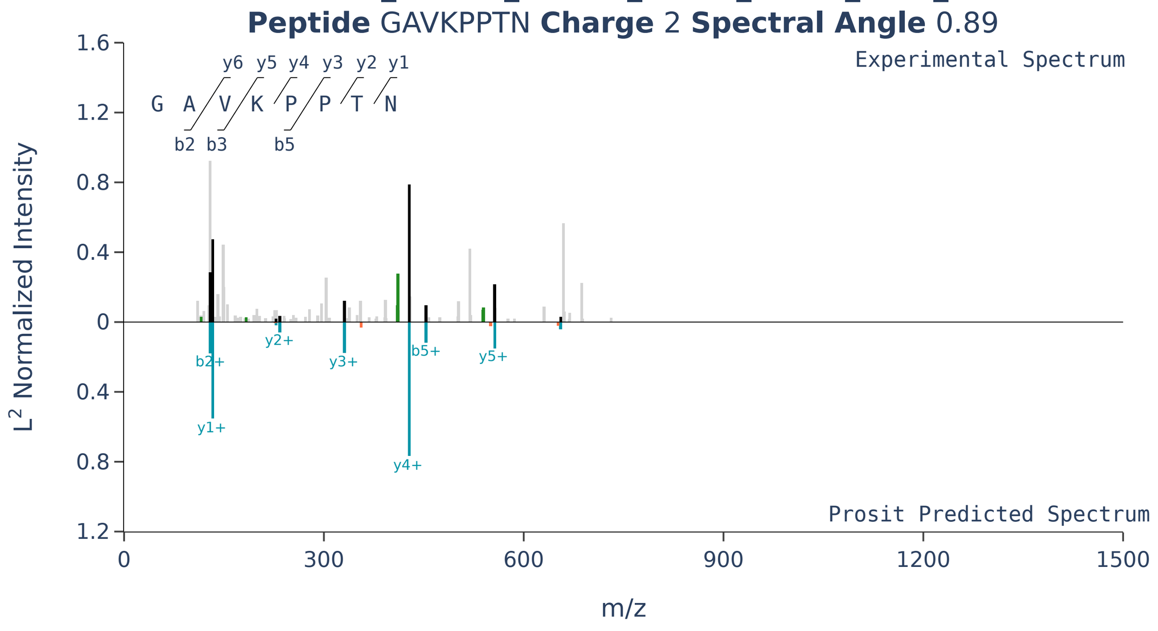


**Source** HRoetschke_271222_090123_HFGoe_LEDGF_b2R2_24h **Scan** 12170
**Peptide** HTEMITTLKKIR **Charge** 4 **Spectral Angle** 0.89

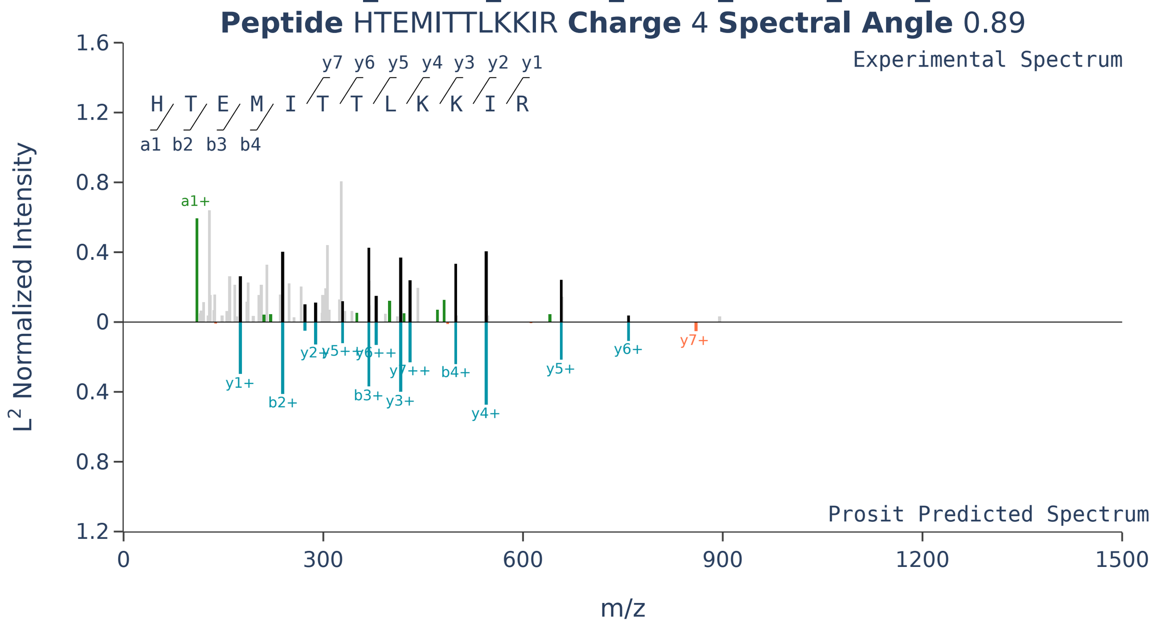


**Source** HRoetschke_271222_090123_HFGoe_LEDGF_b1R2_24h **Scan** 15468
**Peptide** IFPYSENKEKY **Charge** 2 **Spectral Angle** 0.83

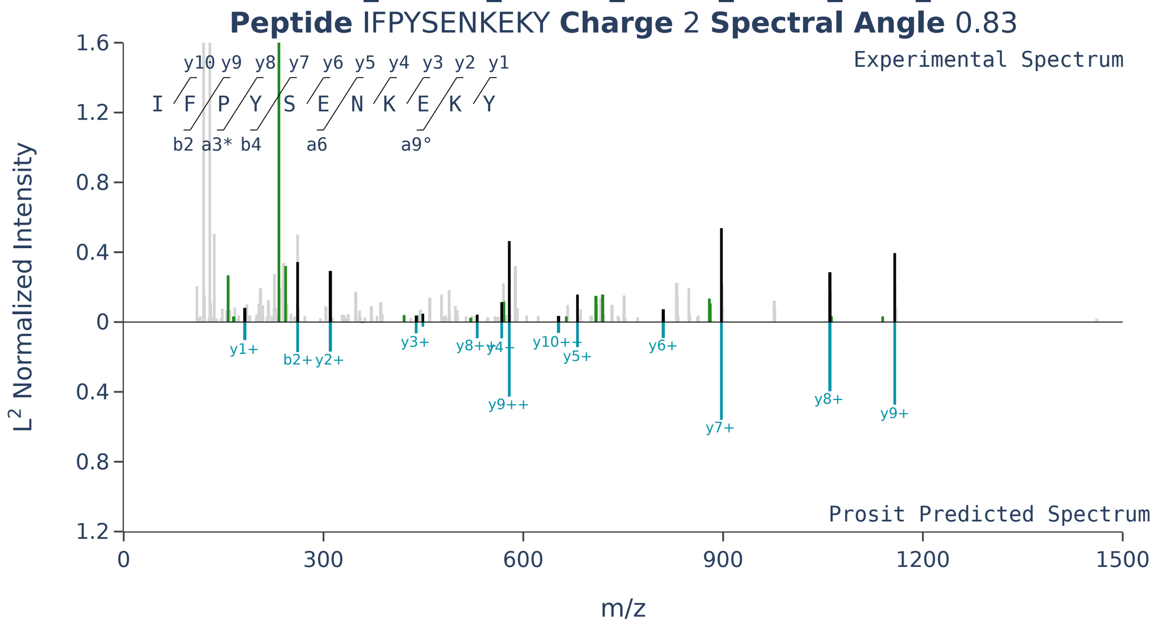


**Source** HRoetschke_271222_090123_HFGoe_LEDGF_b1R2_1h **Scan** 8164
**Peptide** NLDVNRC **Charge** 2 **Spectral Angle** 0.94

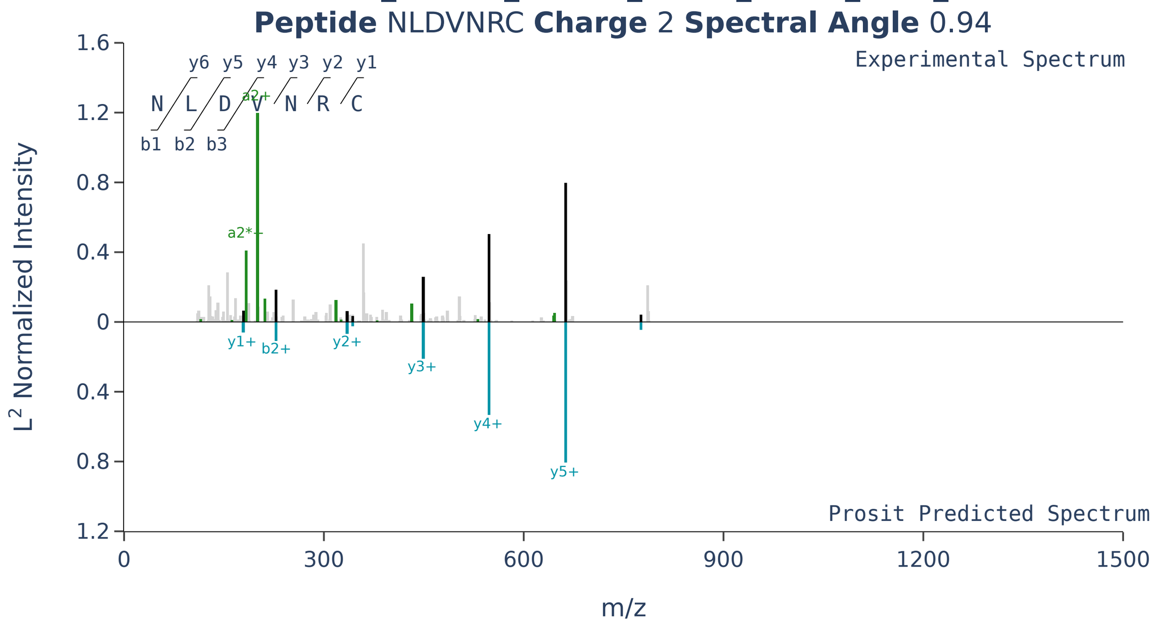


**Source** HRoetschke_271222_090123_HFGoe_LEDGF_b1R2_2h **Scan** 19920
**Peptide** HTEMITTL **Charge** 2 **Spectral Angle** 0.91

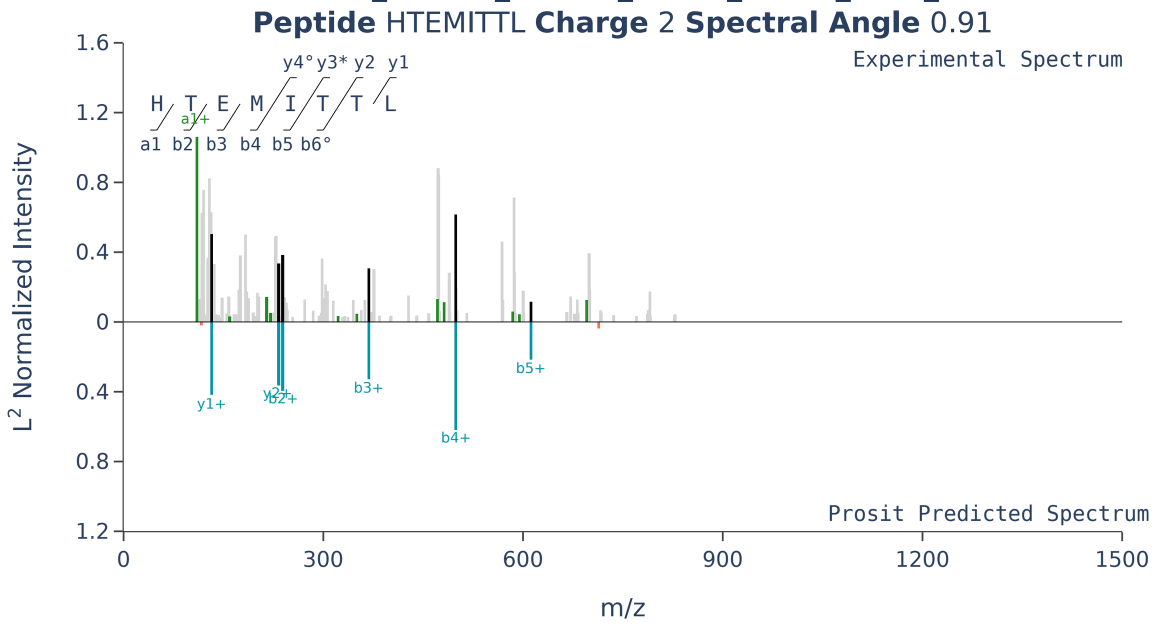


**Source** HRoetschke_271222_090123_HFGoe_LEDGF_b2R1_1h **Scan** 10239
**Peptide** KVSQVIME **Charge** 2 **Spectral Angle** 0.85

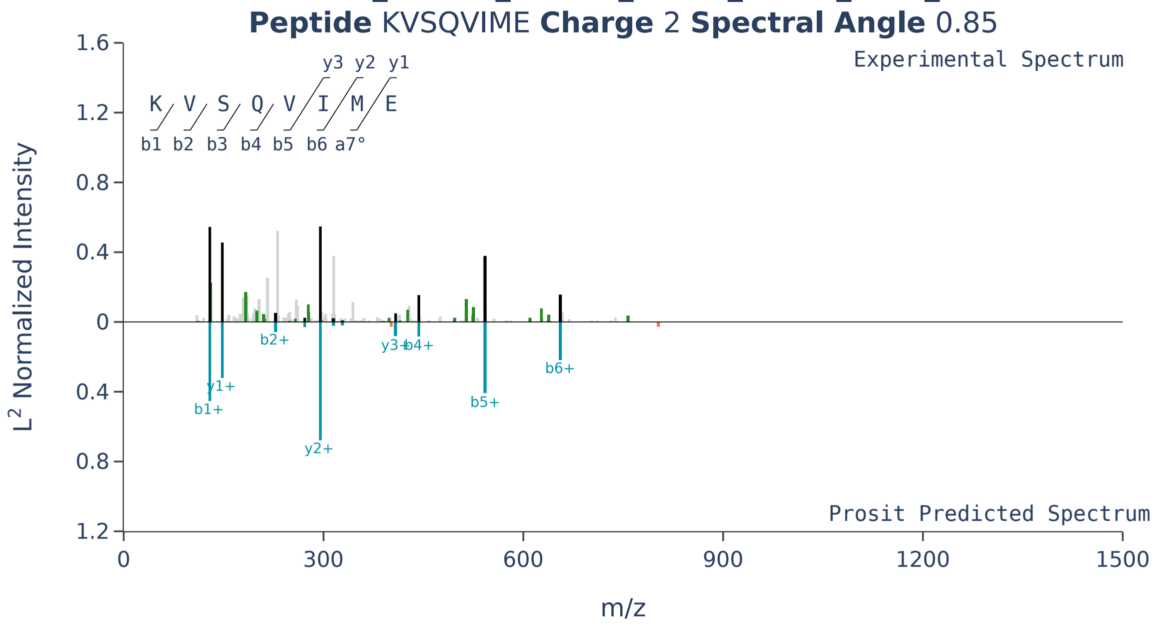

**Source** HRoetschke_271222_090123_HFGoe_LEDGF_b1R1_4h **Scan** 7064
**Peptide** TDHEEKASNEDVTKAVD **Charge** 2 **Spectral Angle** 0.86

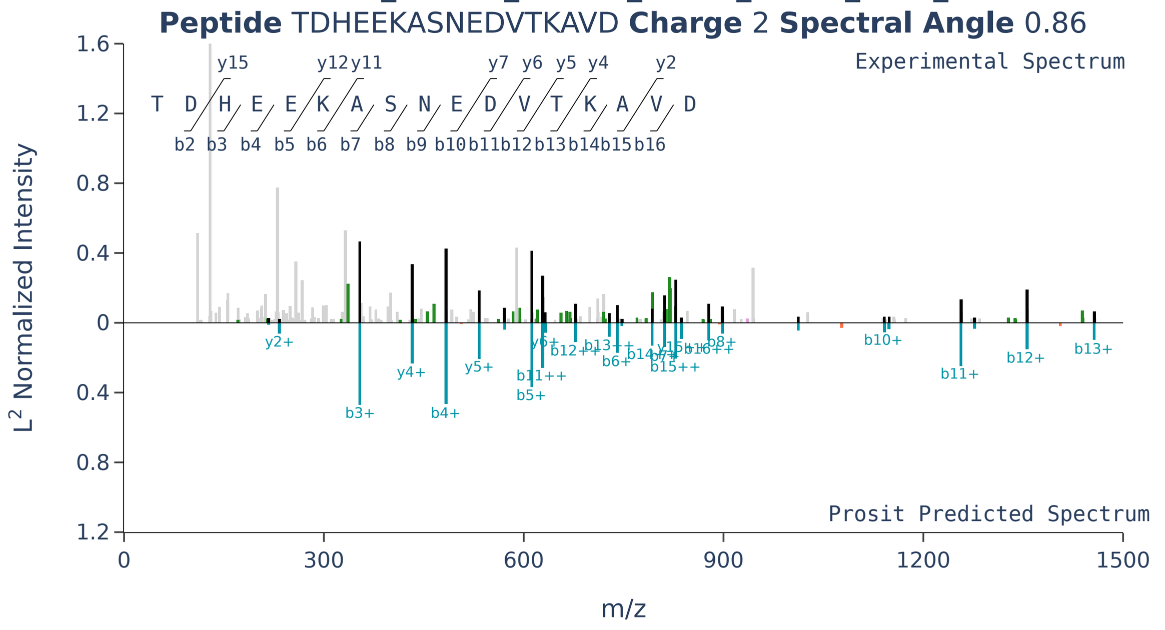


**Source** HRoetschke_271222_090123_HFGoe_LEDGF_b1R2_24h **Scan** 13610
**Peptide** YPHWPAR **Charge** 2 **Spectral Angle** 0.88

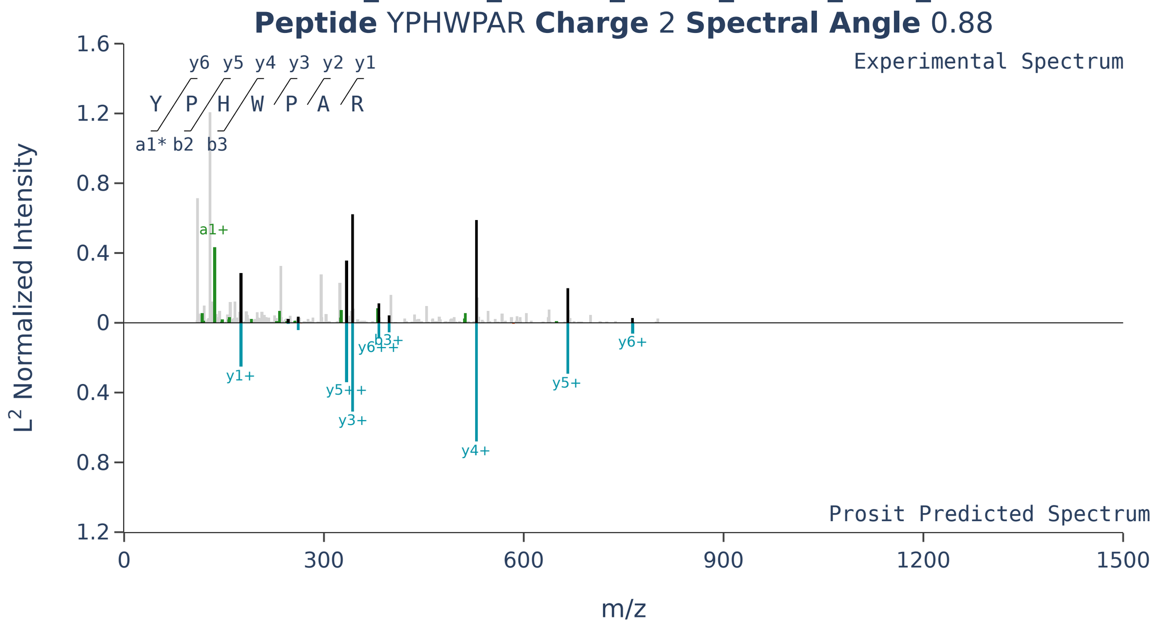


**Source** HRoetschke_271222_090123_HFGoe_LEDGF_b2R2_2h **Scan** 19513
**Peptide** KVSQVIMEKSTML **Charge** 2 **Spectral Angle** 0.89

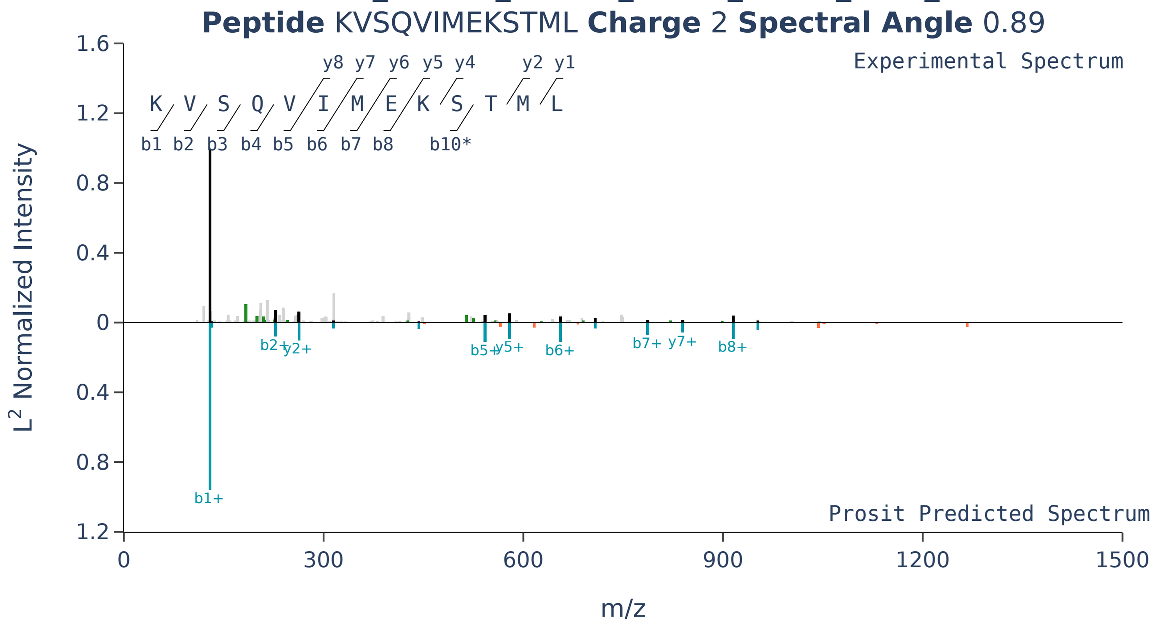


**Source** HRoetschke_271222_090123_HFGoe_LEDGF_b1R1_4h **Scan** 17494
**Peptide** FLGPKDIFPYSENKEKYKGKPNKR **Charge** 5 **Spectral Angle** 0.81

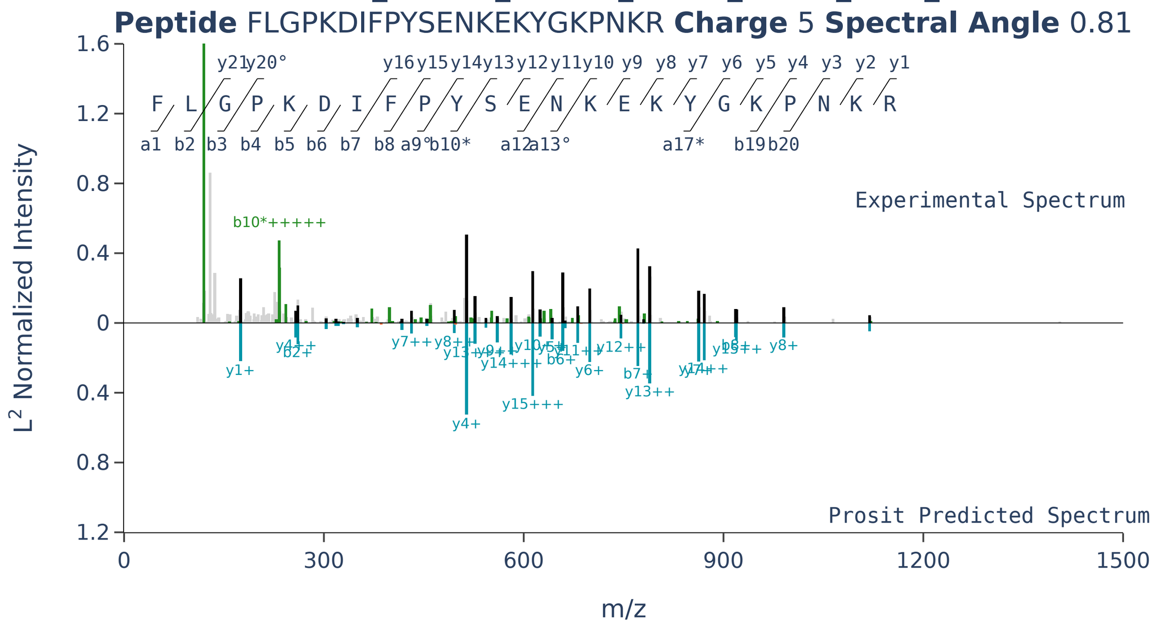


**Source** HRoetschke_271222_090123_HFGoe_LEDGF_b2R2_24h **Scan** 18517
**Peptide** ASLQVTMQQA **Charge** 2 **Spectral Angle** 0.88

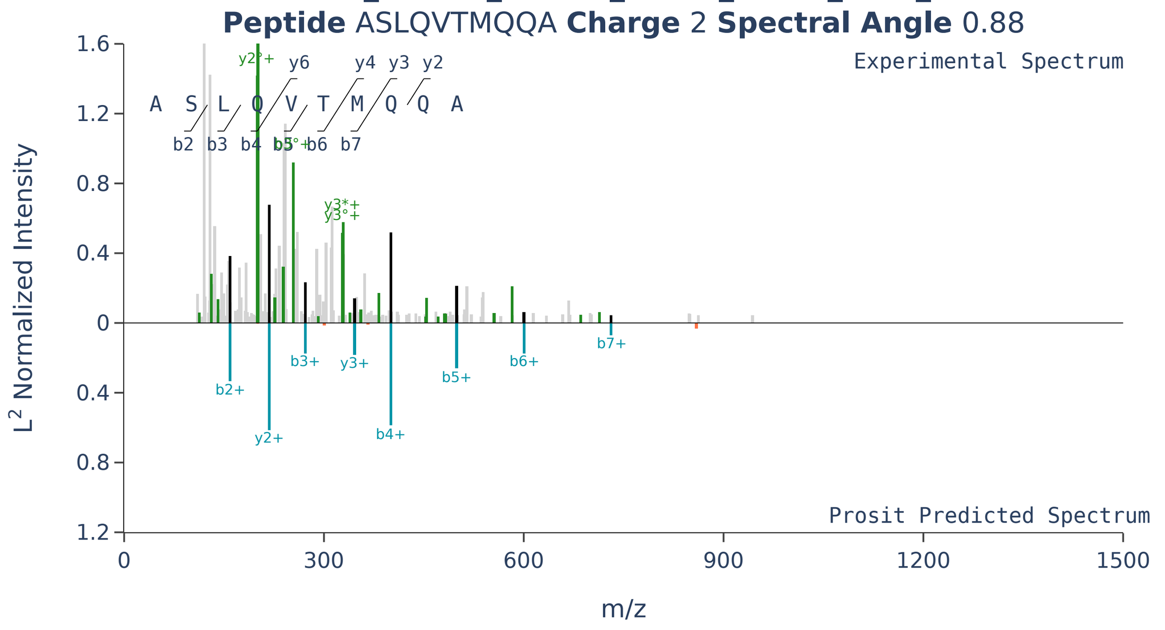


**Source** HRoetschke_271222_090123_HFGoe_LEDGF_b1R2_2h **Scan** 20885
**Peptide** SVITQVLNK **Charge** 1 **Spectral Angle** 0.86

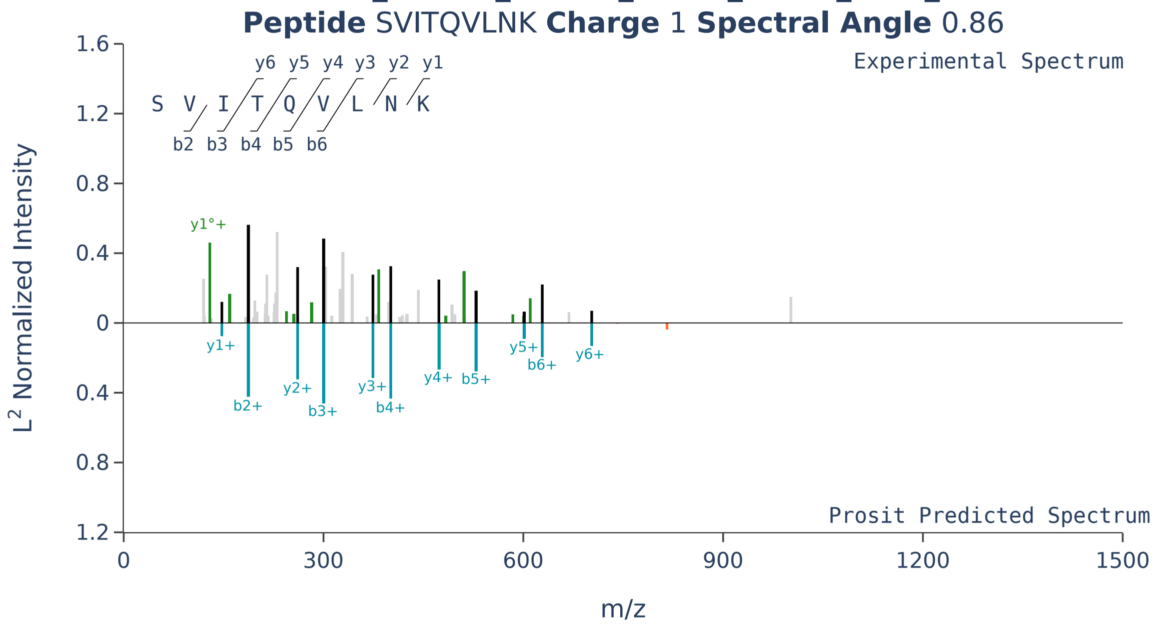


**Source** HRoetschke_271222_090123_HFGoe_LEDGF_b1R1_24h **Scan** 22640
**Peptide** PKDIFPY **Charge** 2 **Spectral Angle** 0.91

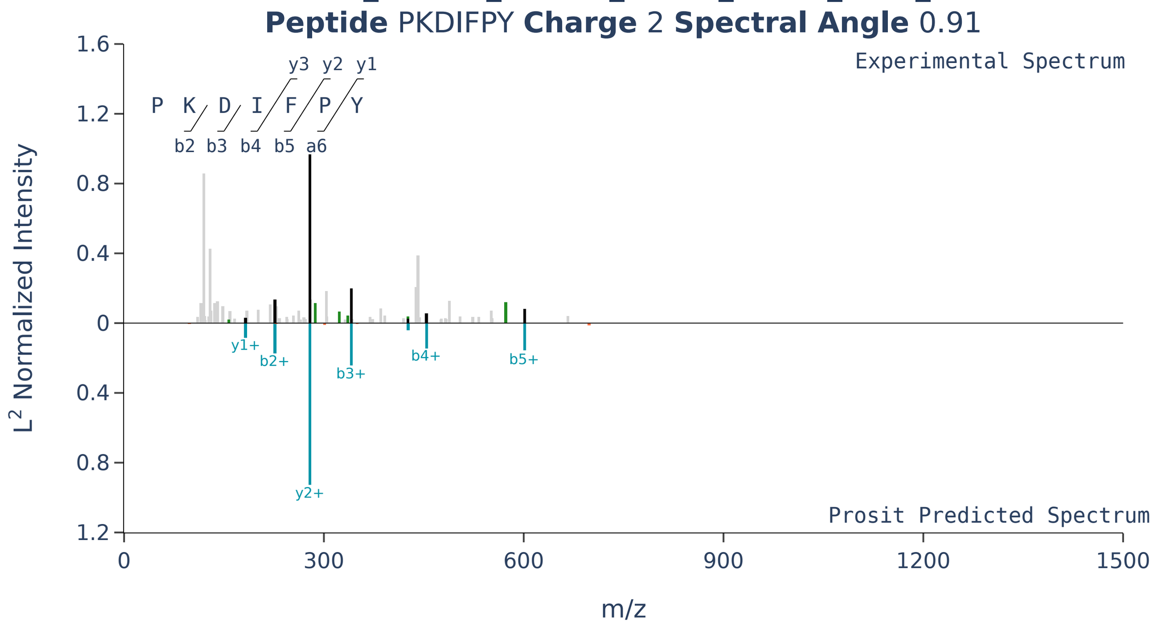


**Source** HRoetschke_271222_090123_HFGoe_LEDGF_b2R2_24h **Scan** 9737
**Peptide** DSRLQRI **Charge** 2 **Spectral Angle** 0.89

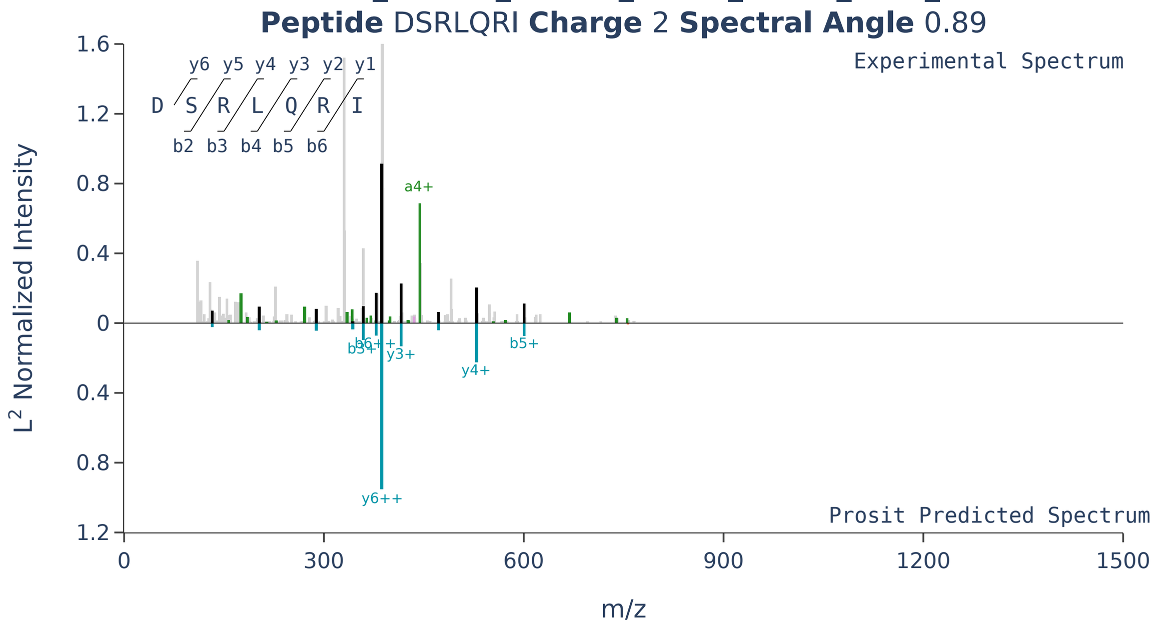


**Source** HRoetschke_271222_090123_HFGoe_LEDGF_b1R2_24h **Scan** 12624
**Peptide** HWPARVD **Charge** 2 **Spectral Angle** 0.95

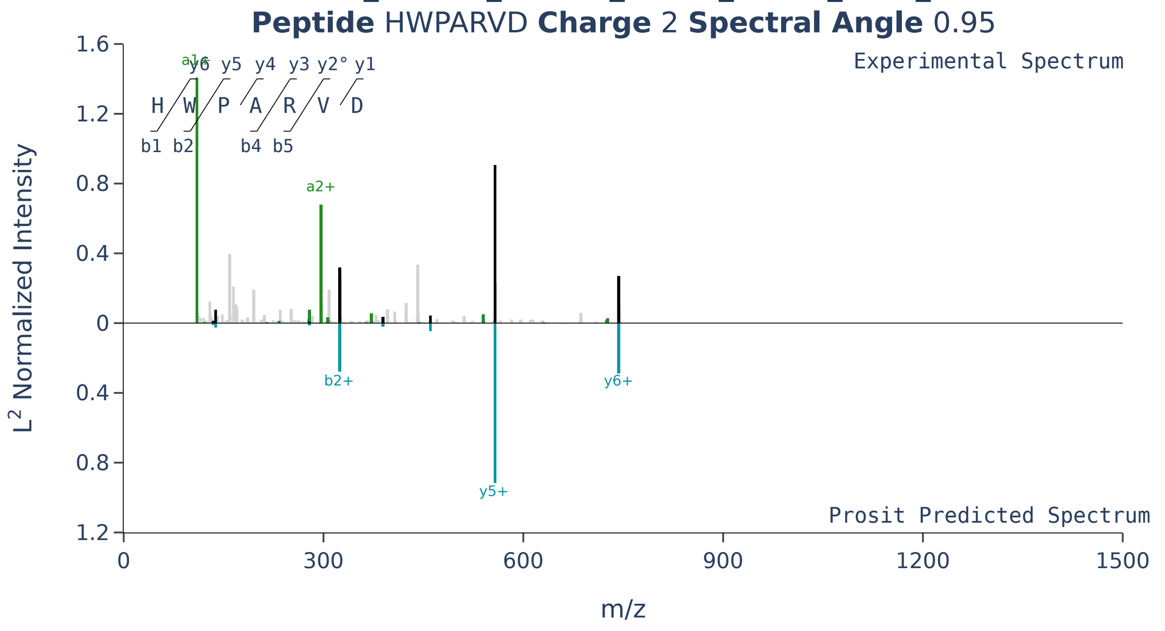


**Source** HRoetschke_271222_090123_HFGoe_LEDGF_b1R2_24h **Scan** 6810
**Peptide** VLNKSLAEQRQH **Charge** 4 **Spectral Angle** 0.85

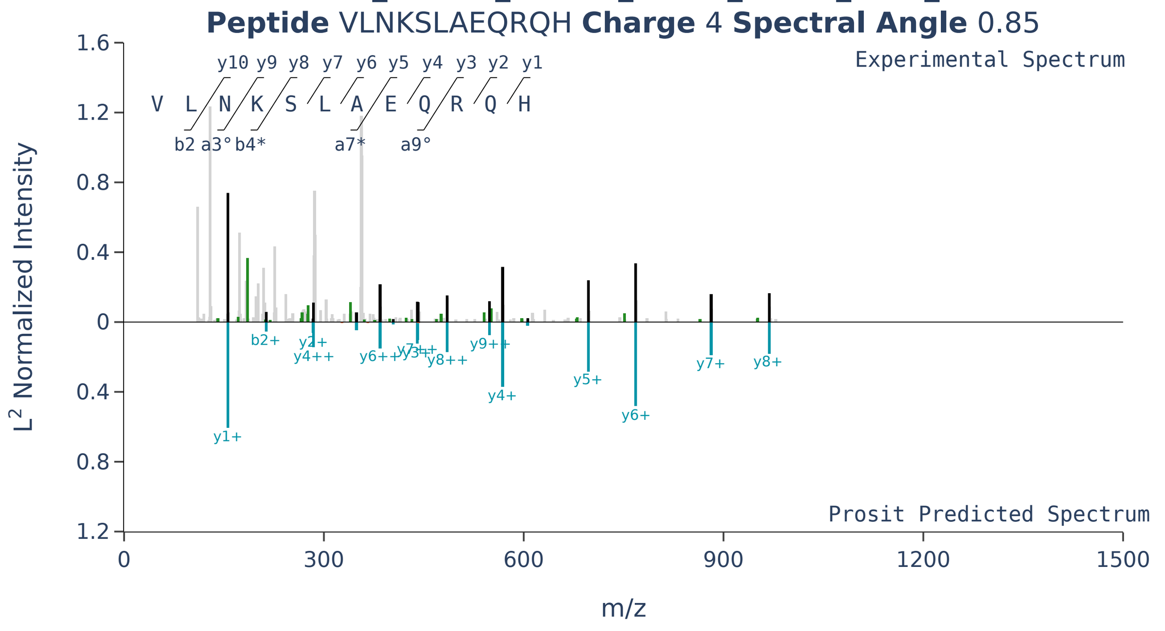


**Source** HRoetschke_271222_090123_HFGoe_LEDGF_b1R1_24h **Scan** 11228
**Peptide** EMITTLKKIRR **Charge** 3 **Spectral Angle** 0.86

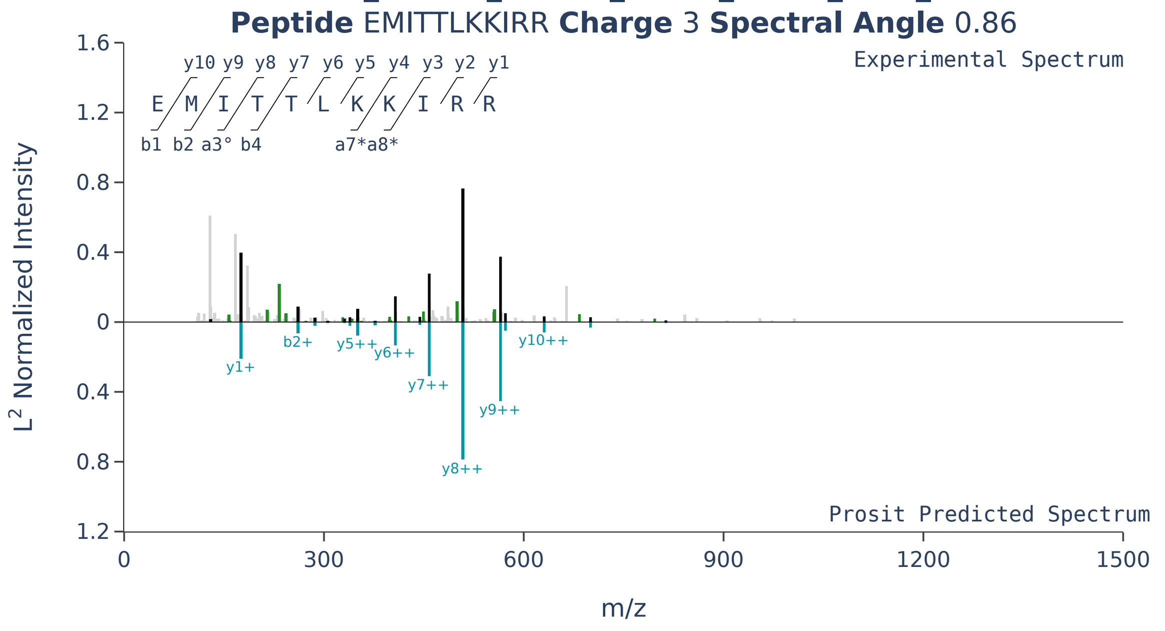


**Source** HRoetschke_271222_090123_HFGoe_LEDGF_b1R1_4h **Scan** 9343
**Peptide** ATASVNLKVSPKRG **Charge** 4 **Spectral Angle** 0.85

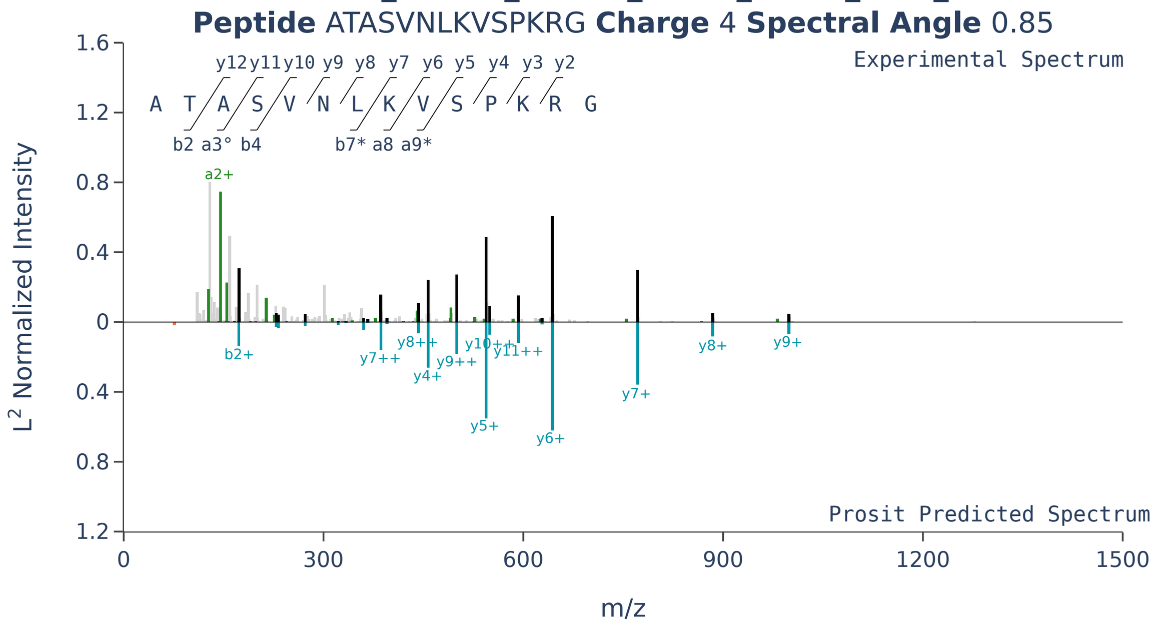


**Source** HRoetschke_271222_090123_HFGoe_LEDGF_b1R2_24h **Scan** 13924
**Peptide** KVSQVIMEKSTM **Charge** 2 **Spectral Angle** 0.91

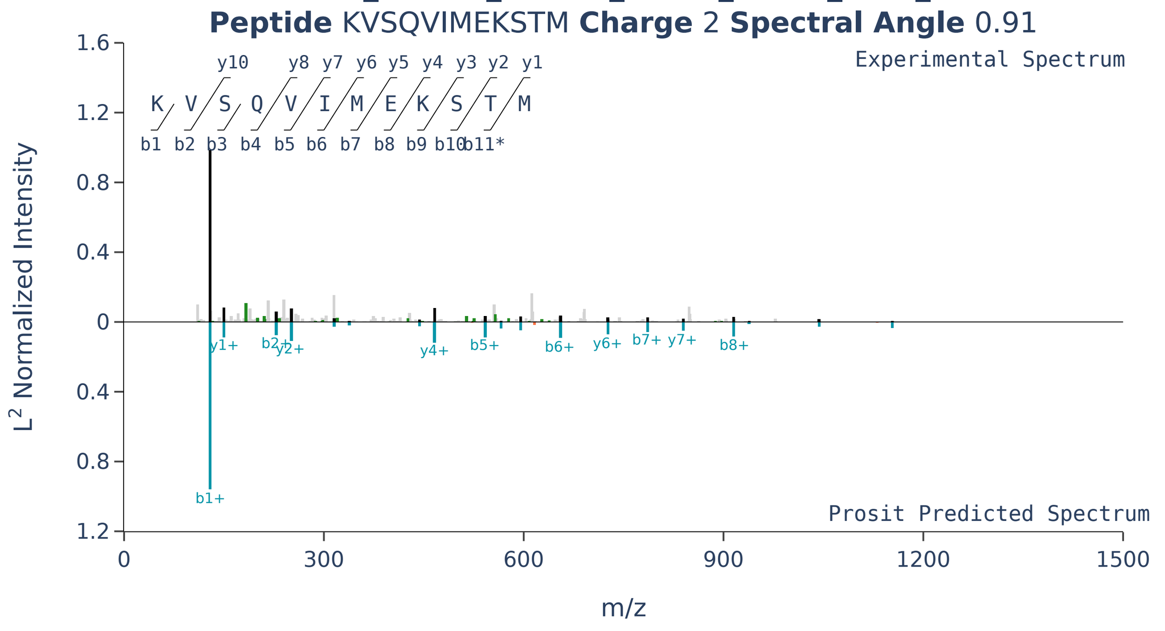


**Source** HRoetschke_271222_090123_HFGoe_LEDGF_b2R1_4h **Scan** 6322
**Peptide** PDGAVKPPTNK **Charge** 3 **Spectral Angle** 0.82

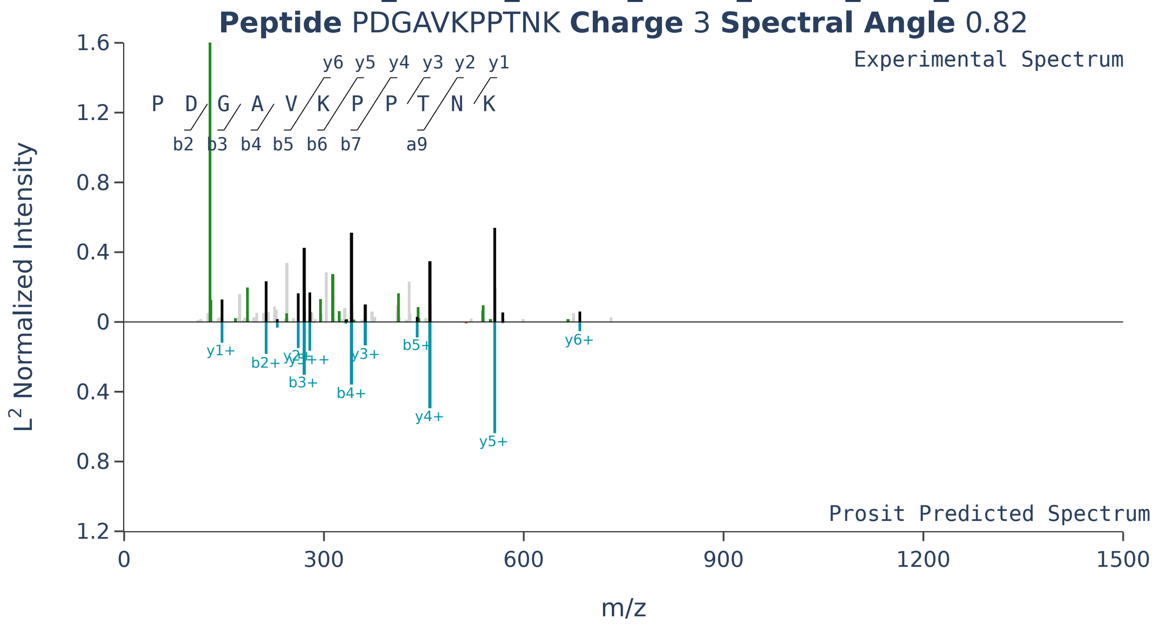


**Source** HRoetschke_271222_090123_HFGoe_LEDGF_b1R1_2h **Scan** 23368
**Peptide** KGFNEGLWEIDNNPKVKFSSQ **Charge** 3 **Spectral Angle** 0.9

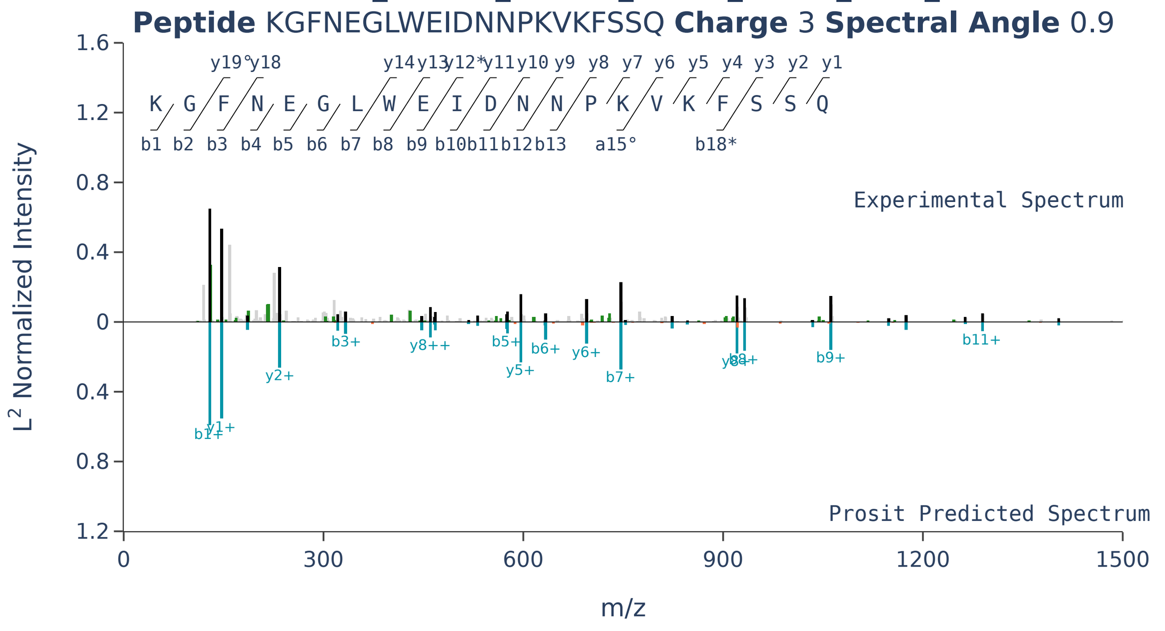

**Source** HRoetschke_271222_090123_HFGoe_LEDGF_b1R2_1h **Scan** 10631
**Peptide** AGVVTTATAS **Charge** 1 **Spectral Angle** 0.88

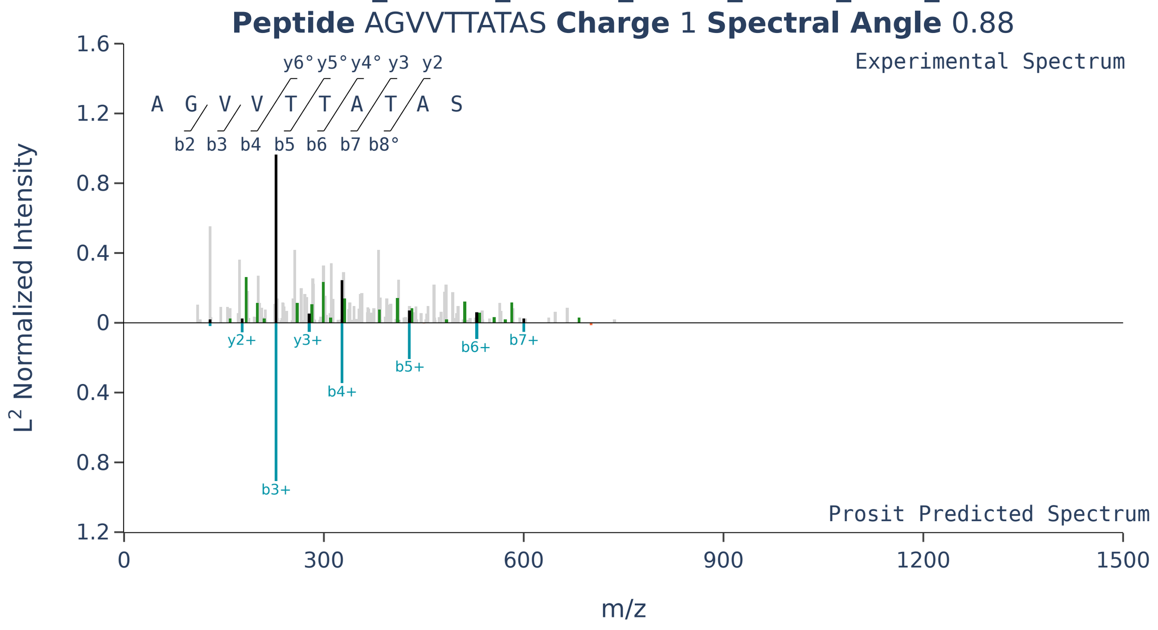


**Source** HRoetschke_271222_090123_HFGoe_LEDGF_b1R2_24h **Scan** 17353
**Peptide** LKIDNLD **Charge** 2 **Spectral Angle** 0.85

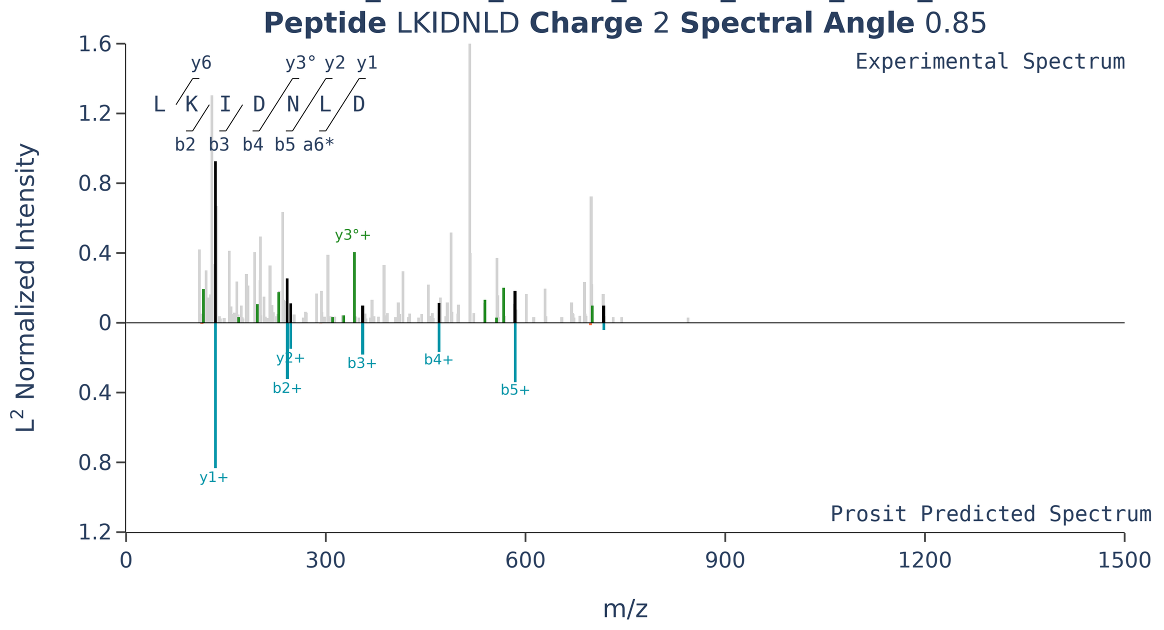


**Source** HRoetschke_271222_090123_HFGoe_LEDGF_b1R2_2h **Scan** 18529
**Peptide** FKVSQVIMEKST **Charge** 3 **Spectral Angle** 0.81

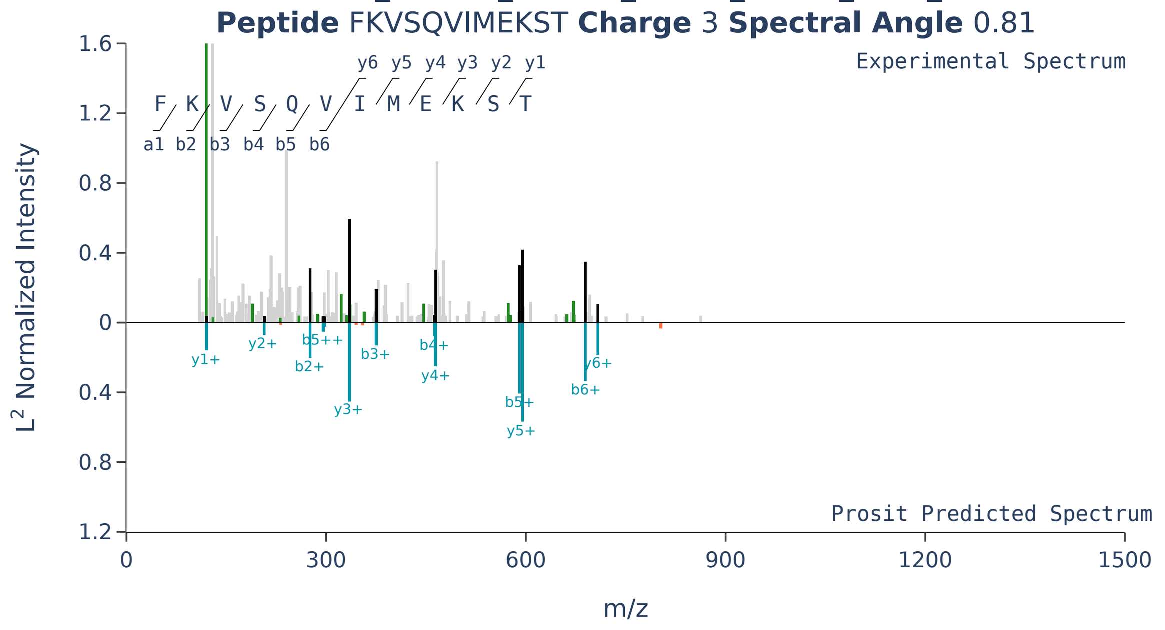


**Source** HRoetschke_271222_090123_HFGoe_LEDGF_b1R1_2h **Scan** 7947
**Peptide** IFPYSENKEKYGKPNKR **Charge** 5 **Spectral Angle** 0.86

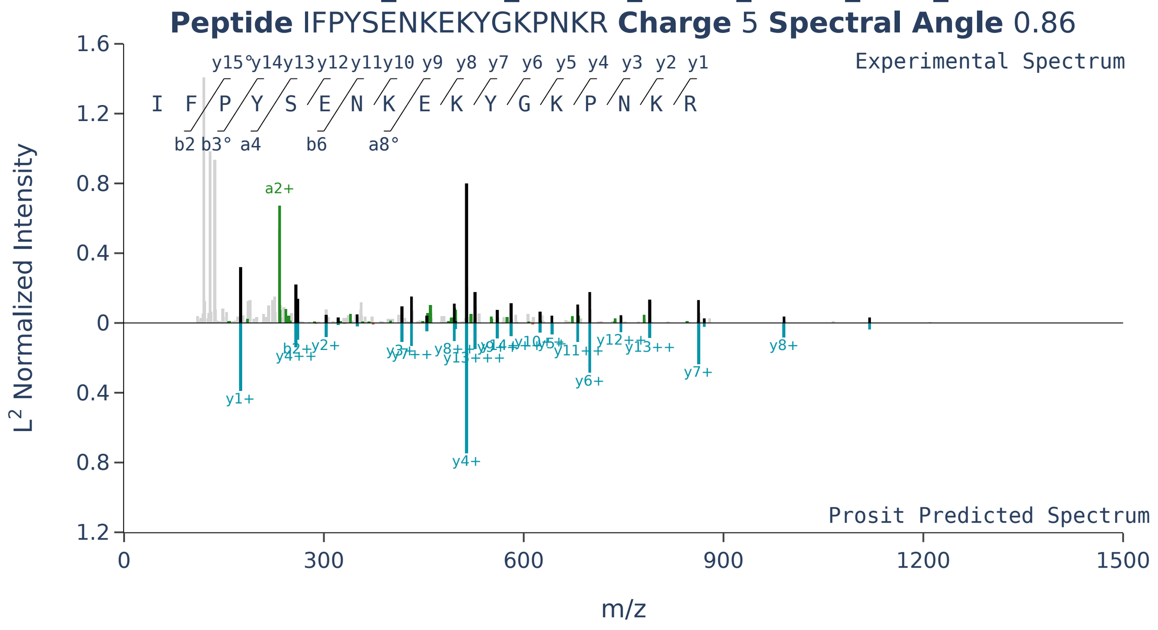


**Source** HRoetschke_271222_090123_HFGoe_LEDGF_b1R1_24h **Scan** 15128
**Peptide** EVPDGAVKPPTNKL **Charge** 2 **Spectral Angle** 0.91

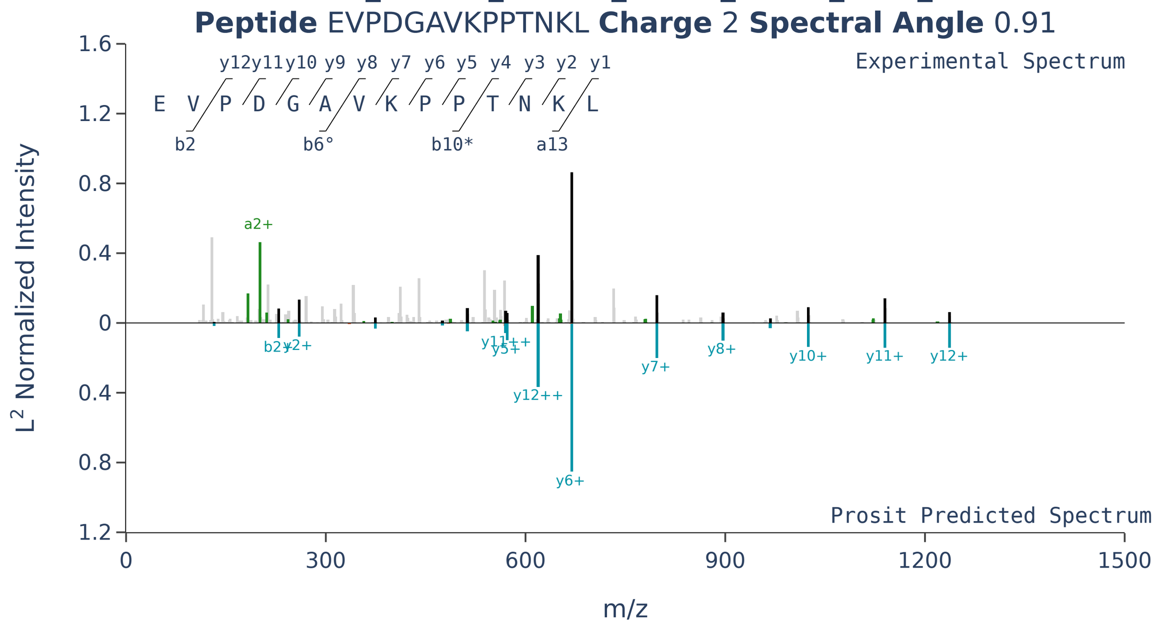


**Source** HRoetschke_271222_090123_HFGoe_LEDGF_b2R1_24h **Scan** 7060
**Peptide** VNLKVSPKRGRPAAT **Charge** 4 **Spectral Angle** 0.93

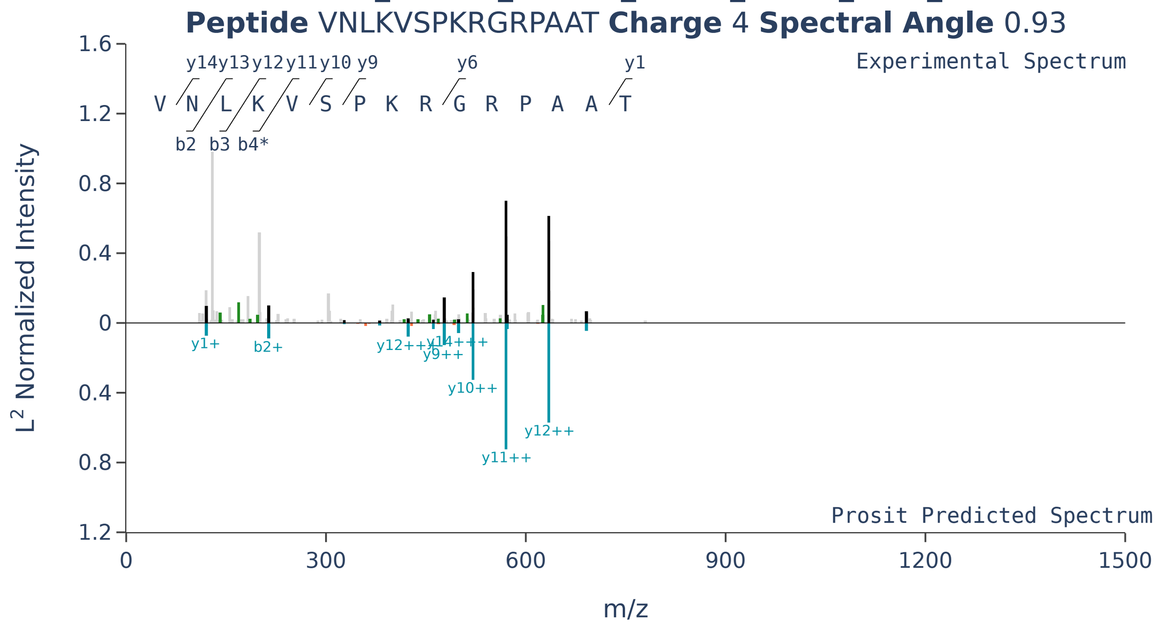


**Source** HRoetschke_271222_090123_HFGoe_LEDGF_b2R2_2h **Scan** 10705
**Peptide** QSNASSDVEVEE **Charge** 2 **Spectral Angle** 0.91

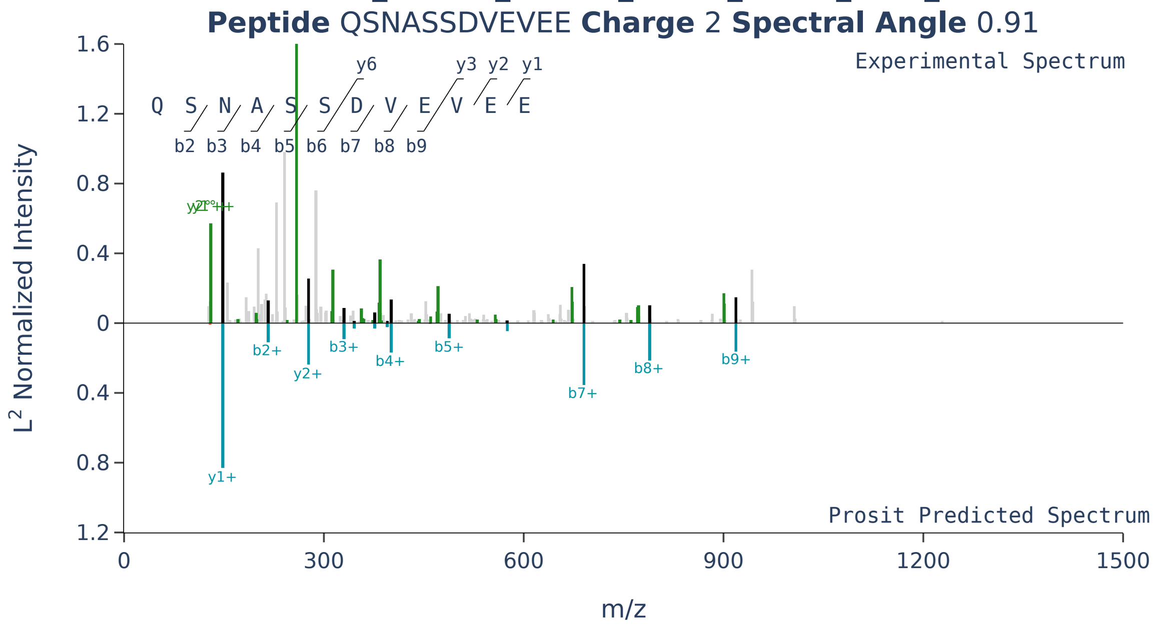


**Source** HRoetschke_271222_090123_HFGoe_LEDGF_b1R2_1h **Scan** 10131
**Peptide** QSNASSDVEVE **Charge** 2 **Spectral Angle** 0.91

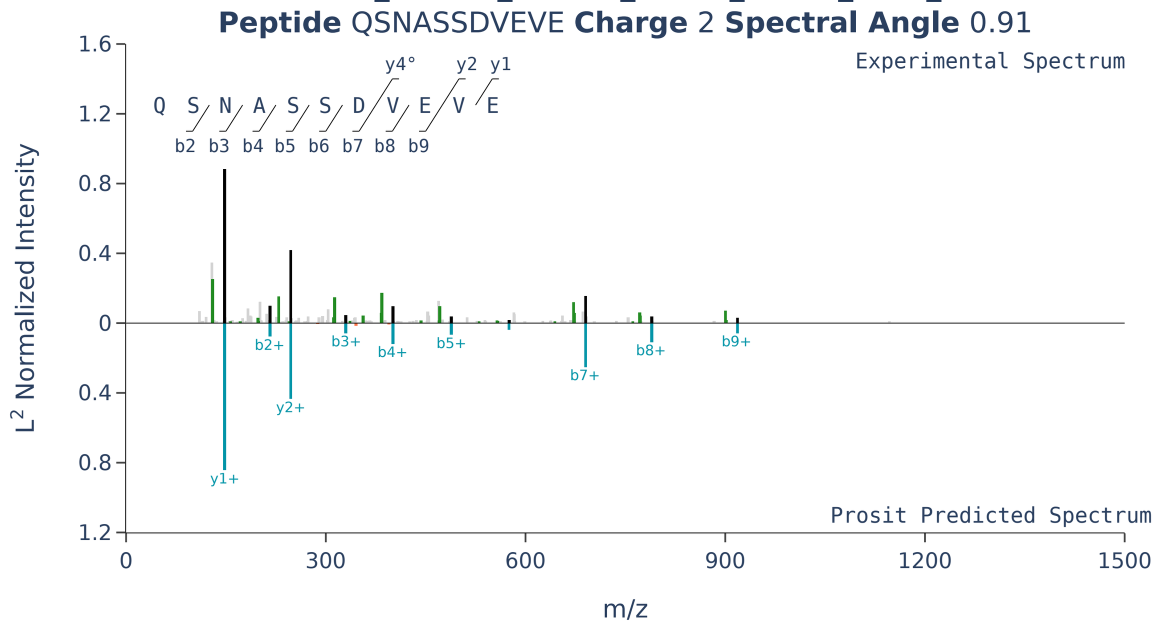


**Source** HRoetschke_271222_090123_HFGoe_LEDGF_b2R1_1h **Scan** 8601
**Peptide** IMEKSTM **Charge** 2 **Spectral Angle** 0.84

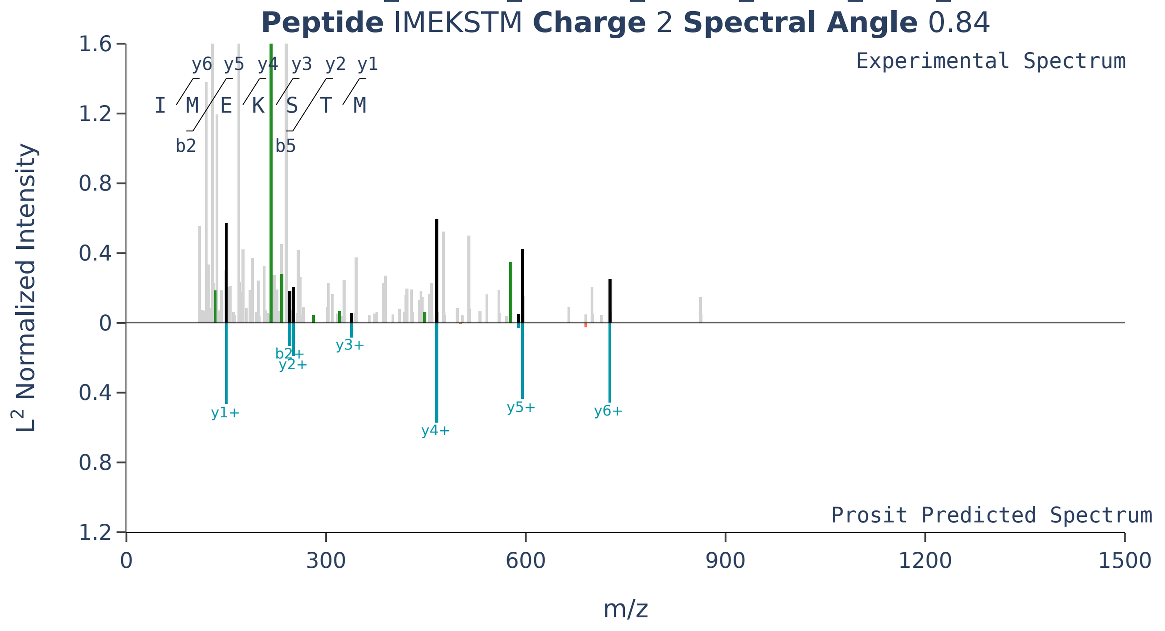


**Source** HRoetschke_271222_090123_HFGoe_LEDGF_b1R1_24h **Scan** 14718
**Peptide** AFLGPKD **Charge** 1 **Spectral Angle** 0.87

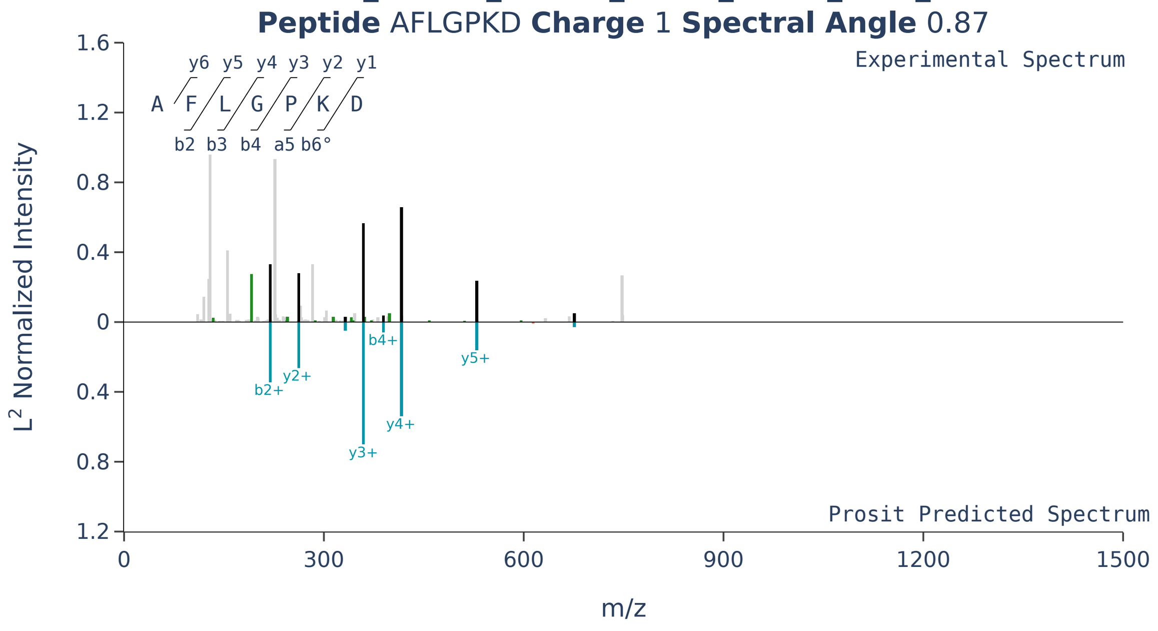


**Source** HRoetschke_271222_090123_HFGoe_LEDGF_b2R2_4h **Scan** 11979
**Peptide** PDGAVKPPTNKL **Charge** 3 **Spectral Angle** 0.86

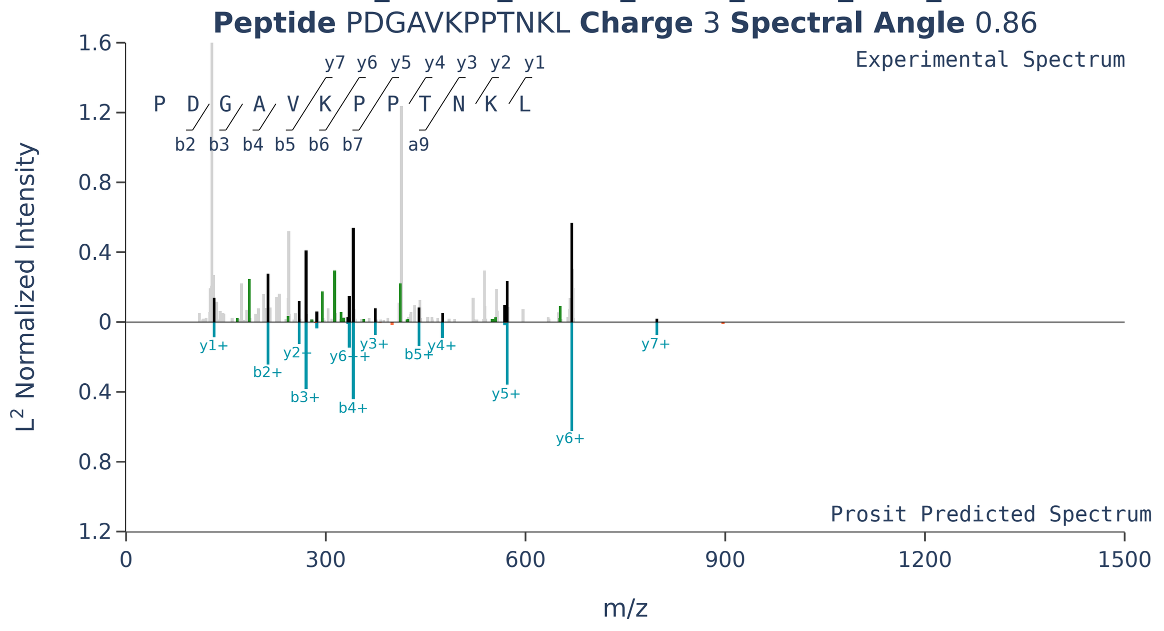


**Source** HRoetschke_271222_090123_HFGoe_LEDGF_b2R1_24h **Scan** 10449
**Peptide** TMLYNKFKN **Charge** 3 **Spectral Angle** 0.87

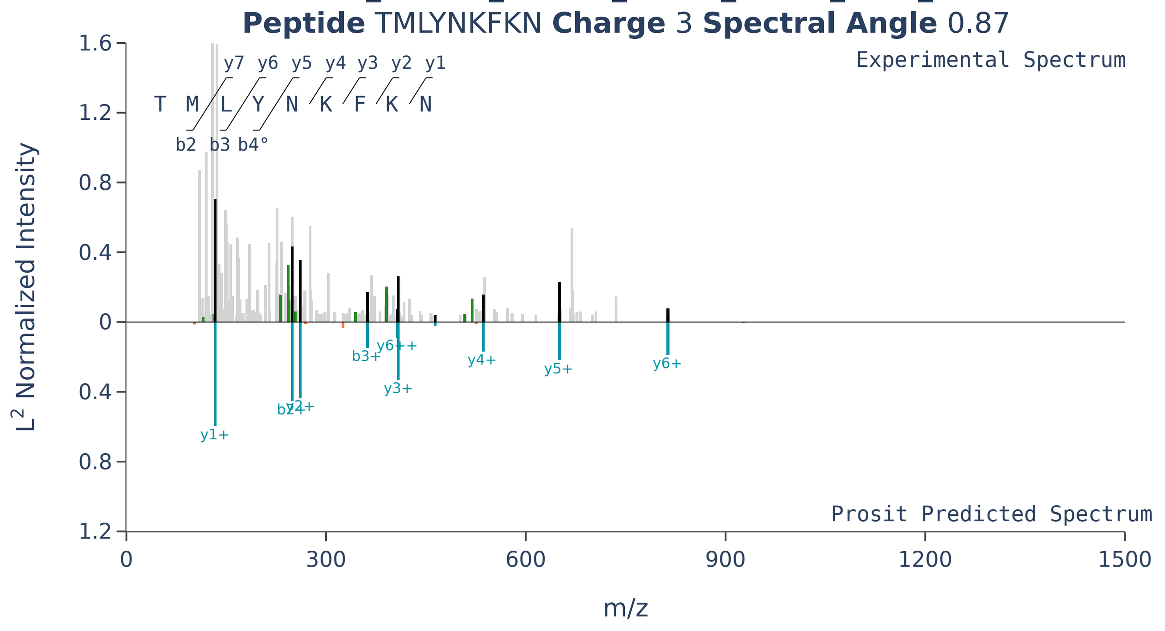


**Source** HRoetschke_271222_090123_HFGoe_LEDGF_b2R2_24h **Scan** 19152
**Peptide** KNSLKIDNLDV **Charge** 3 **Spectral Angle** 0.88

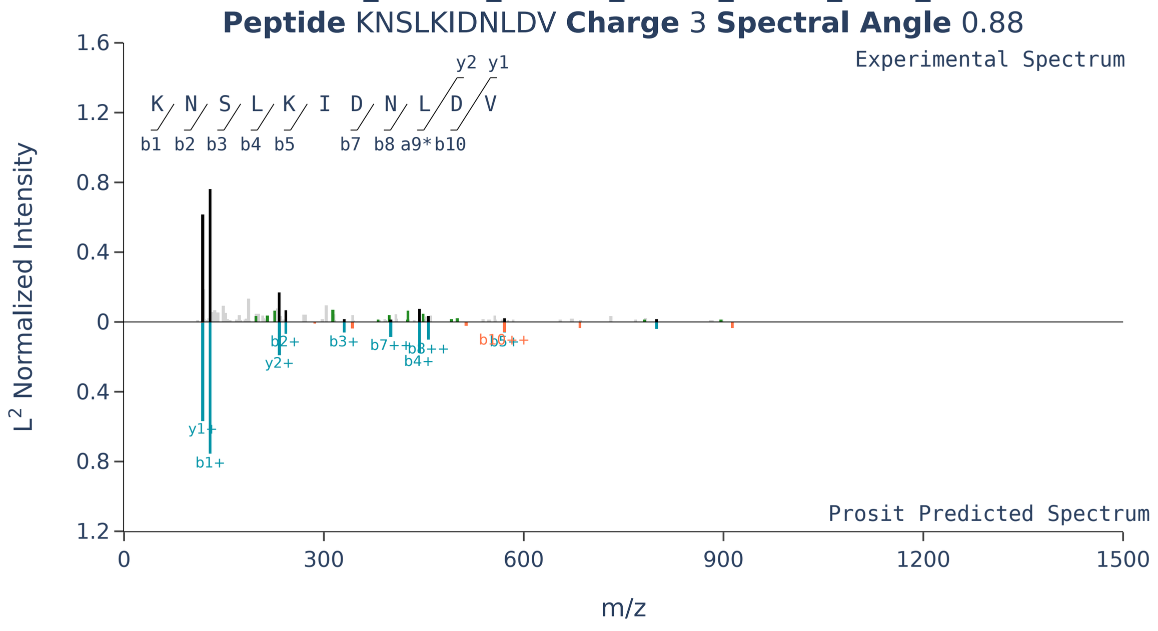


**Source** HRoetschke_271222_090123_HFGoe_LEDGF_b1R1_4h **Scan** 7767
**Peptide** DTDHEEKASNEDVTKAVD **Charge** 2 **Spectral Angle** 0.89

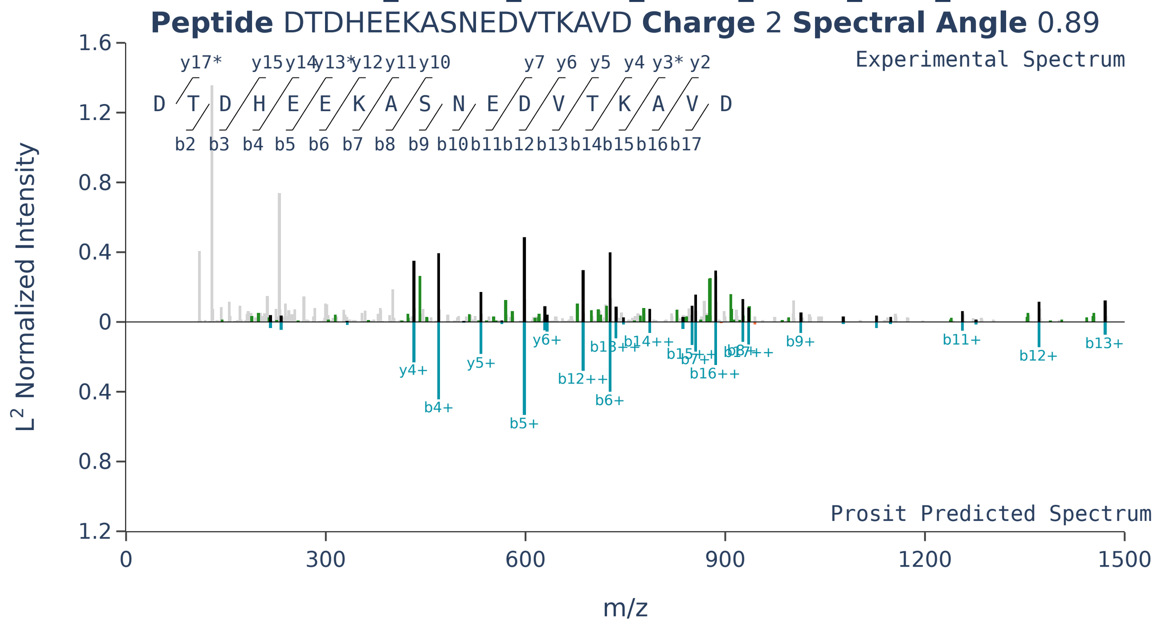


**Source** HRoetschke_271222_090123_HFGoe_LEDGF_b1R1_4h **Scan** 27995
**Peptide** KGFNEGLWEID **Charge** 2 **Spectral Angle** 0.78

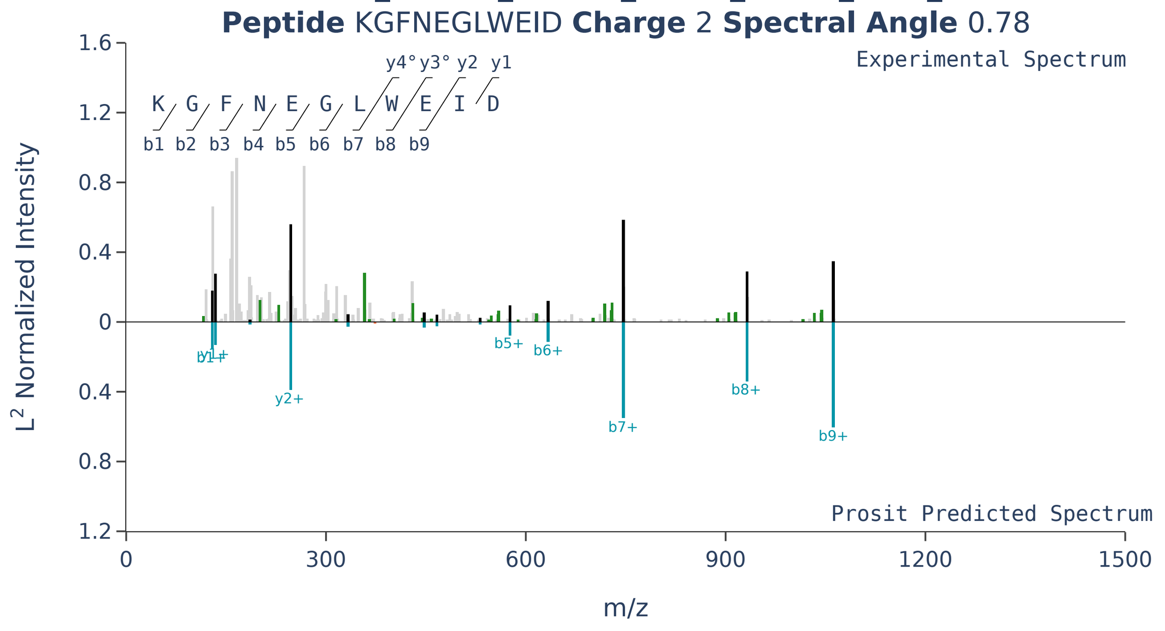

**Source** HRoetschke_271222_090123_HFGoe_LEDGF_b2R1_24h **Scan** 17346
**Peptide** TMQQAQKHTEMITTL **Charge** 2 **Spectral Angle** 0.87

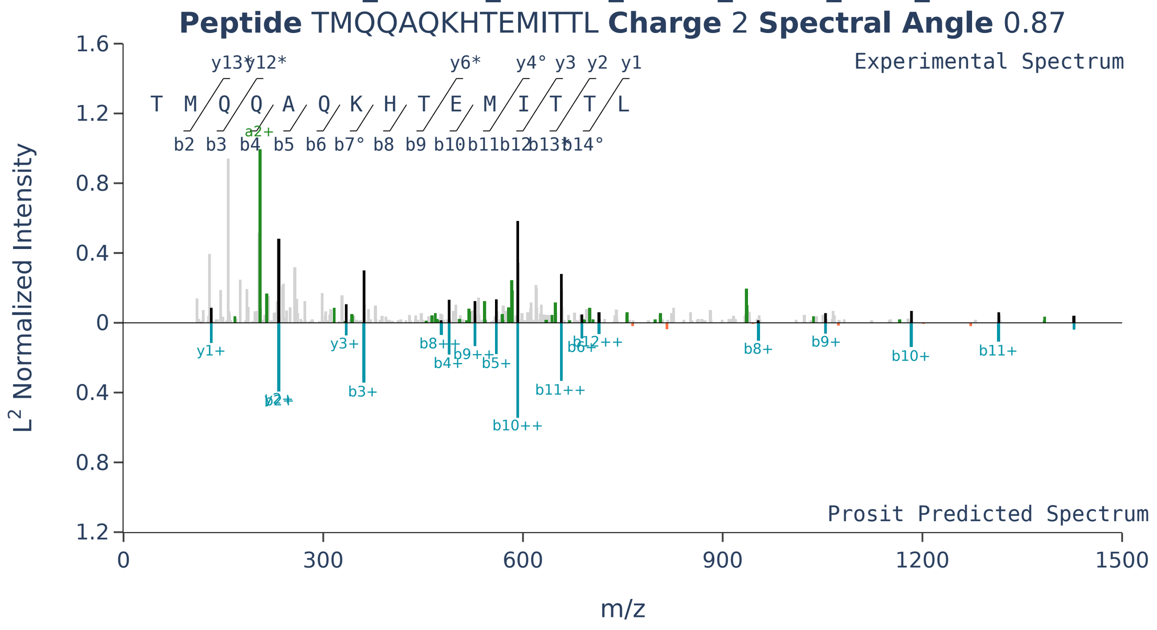


**Source** HRoetschke_271222_090123_HFGoe_LEDGF_b1R1_4h **Scan** 11032
**Peptide** SRLQRIHAEI **Charge** 3 **Spectral Angle** 0.89

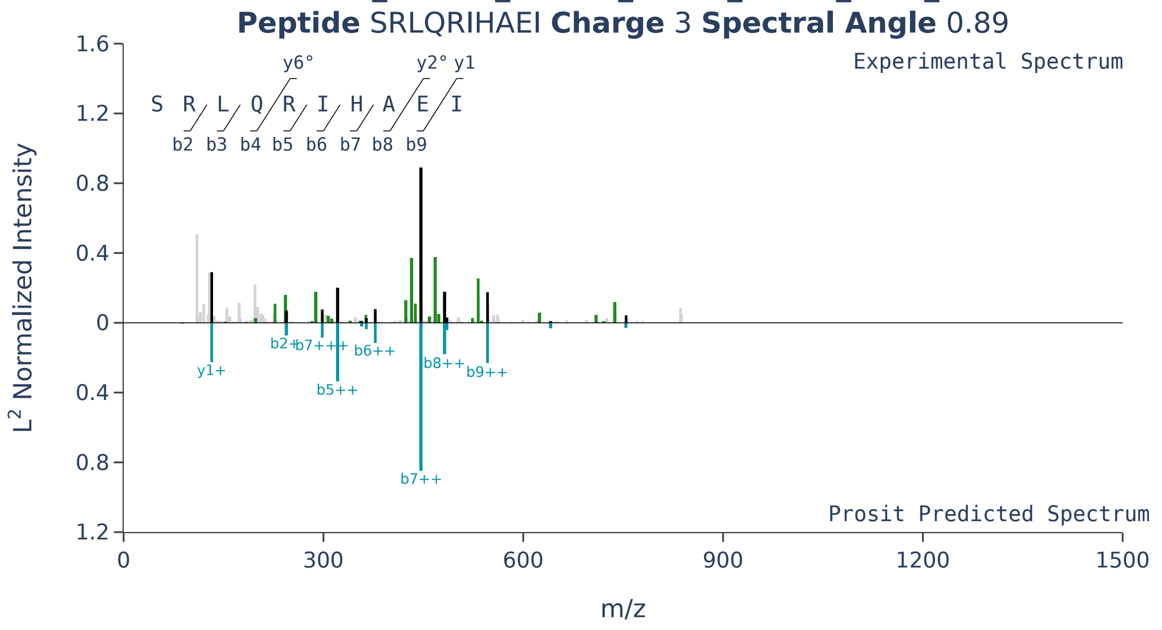


**Source** HRoetschke_271222_090123_HFGoe_LEDGF_b2R2_24h **Scan** 13593
**Peptide** STMLYNKFKN **Charge** 3 **Spectral Angle** 0.81

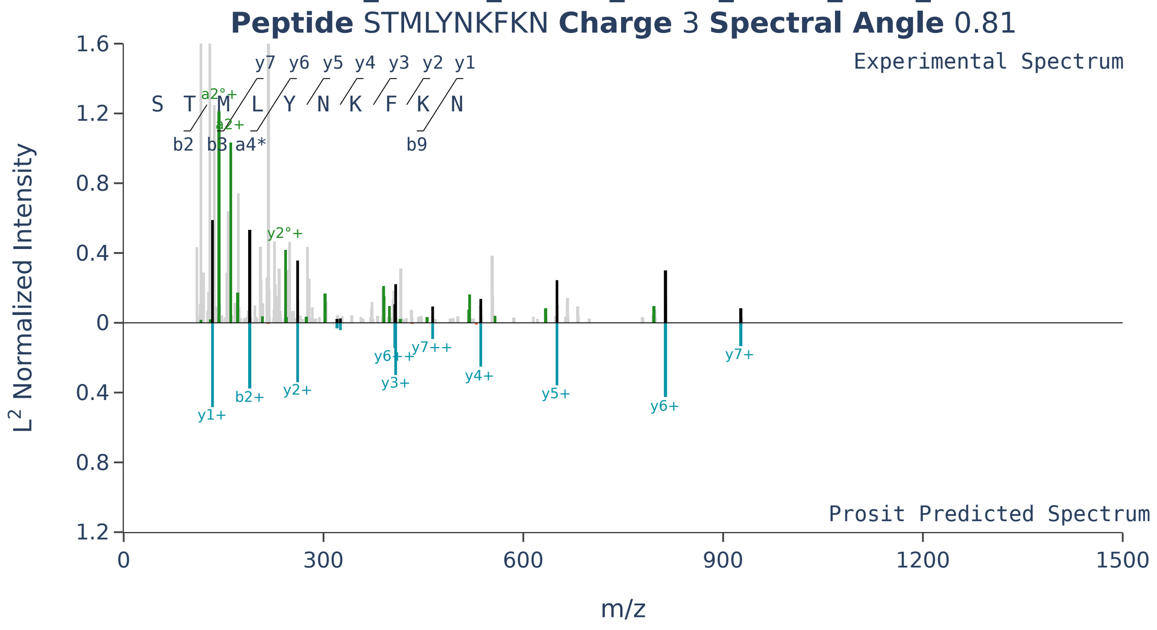


**Source** HRoetschke_271222_090123_HFGoe_LEDGF_b1R1_24h **Scan** 16845
**Peptide** GPKDIFPYSENKE **Charge** 2 **Spectral Angle** 0.79

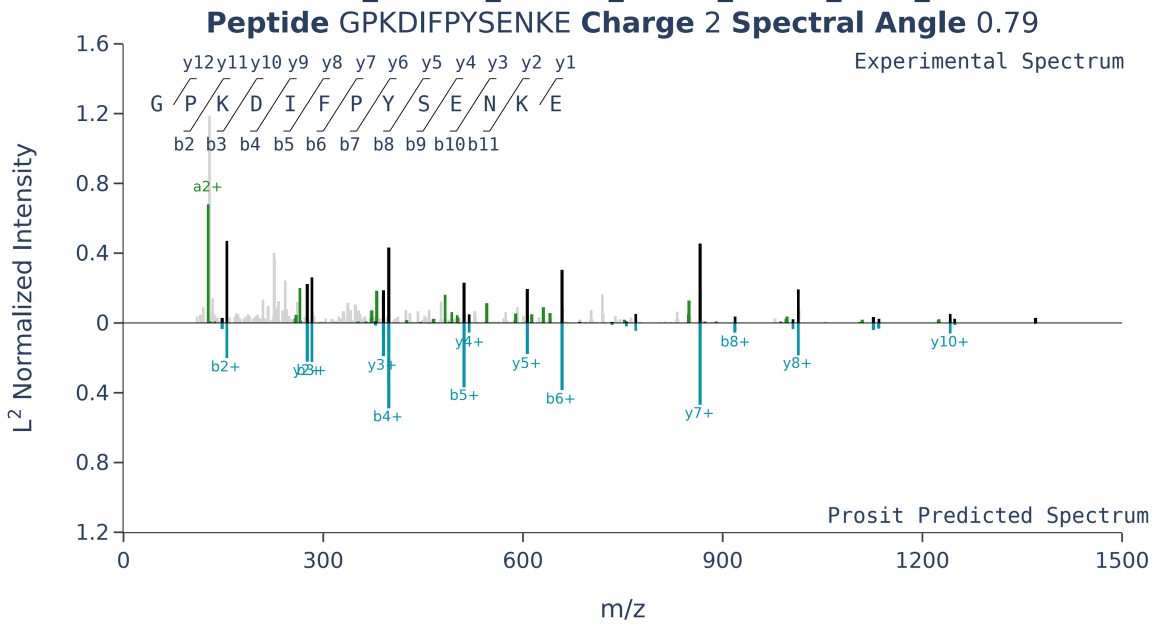


**Source** HRoetschke_271222_090123_HFGoe_LEDGF_b2R2_2h **Scan** 24112
**Peptide** EGDSVITQVLNKS **Charge** 2 **Spectral Angle** 0.88

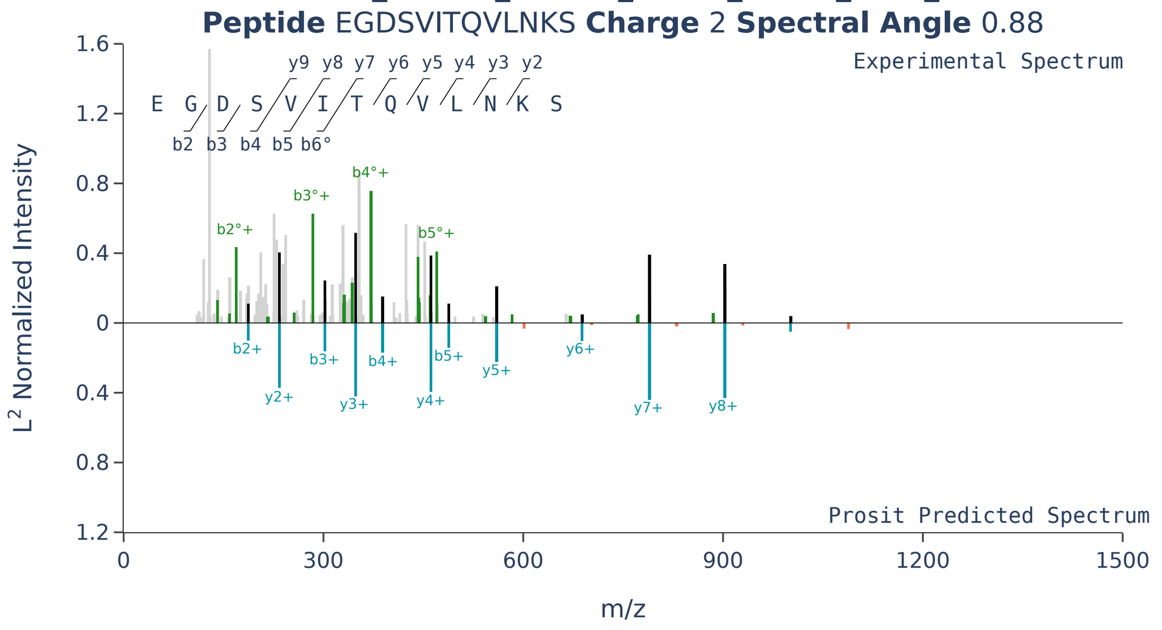


**Source** HRoetschke_271222_090123_HFGoe_LEDGF_b1R1_2h **Scan** 7071
**Peptide** LFPYSENKEKYGKPNKRKG **Charge** 3 **Spectral Angle** 0.88

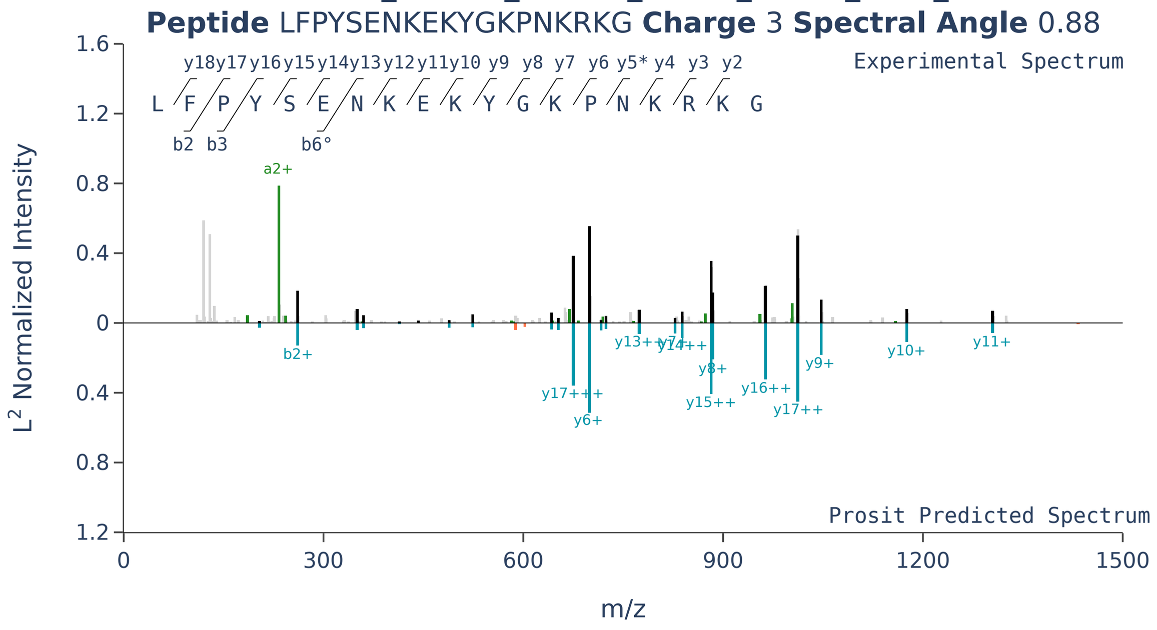


**Source** HRoetschke_271222_090123_HFGoe_LEDGF_b1R1_4h **Scan** 18873
**Peptide** LWEIDNNPKVKFSSQQAATK **Charge** 3 **Spectral Angle** 0.86

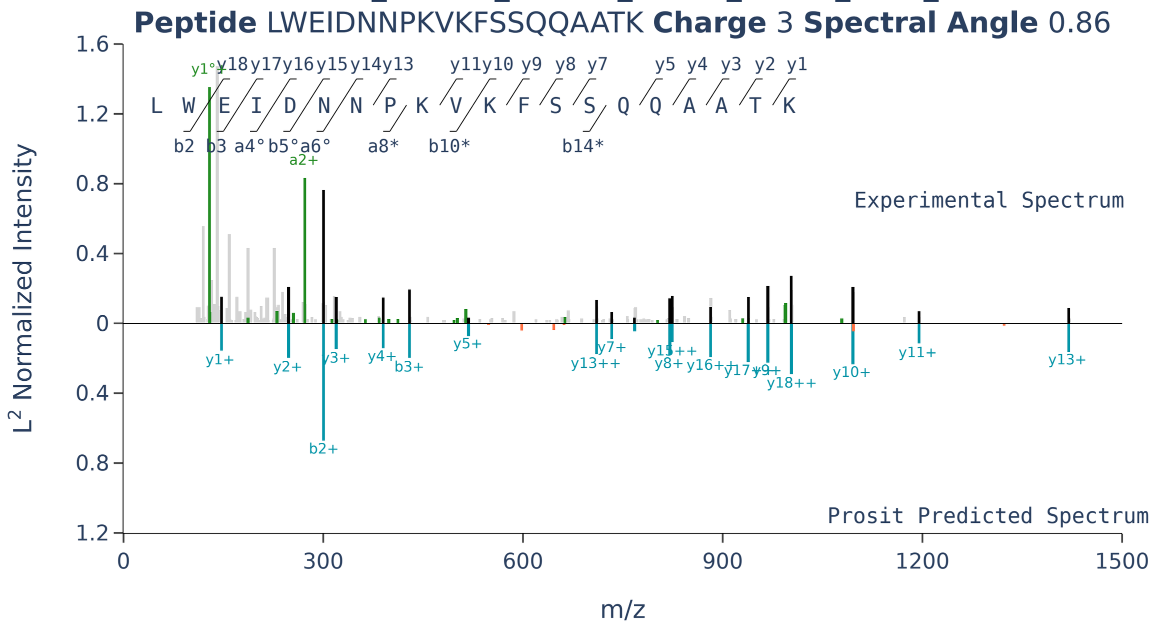


**Source** HRoetschke_271222_090123_HFGoe_LEDGF_b2R1_24h **Scan** 8686
**Peptide** KSTMLYNKFKNM **Charge** 4 **Spectral Angle** 0.93

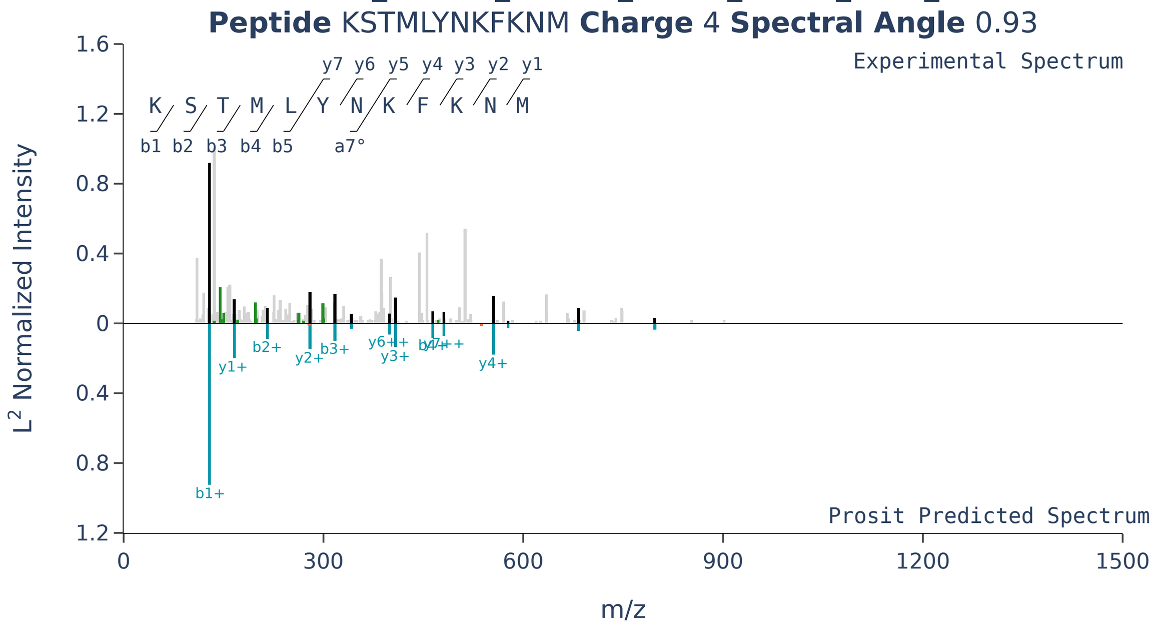


**Source** HRoetschke_271222_090123_HFGoe_LEDGF_b2R2_24h **Scan** 21755
**Peptide** SLKIDNLDVNRCI **Charge** 3 **Spectral Angle** 0.91

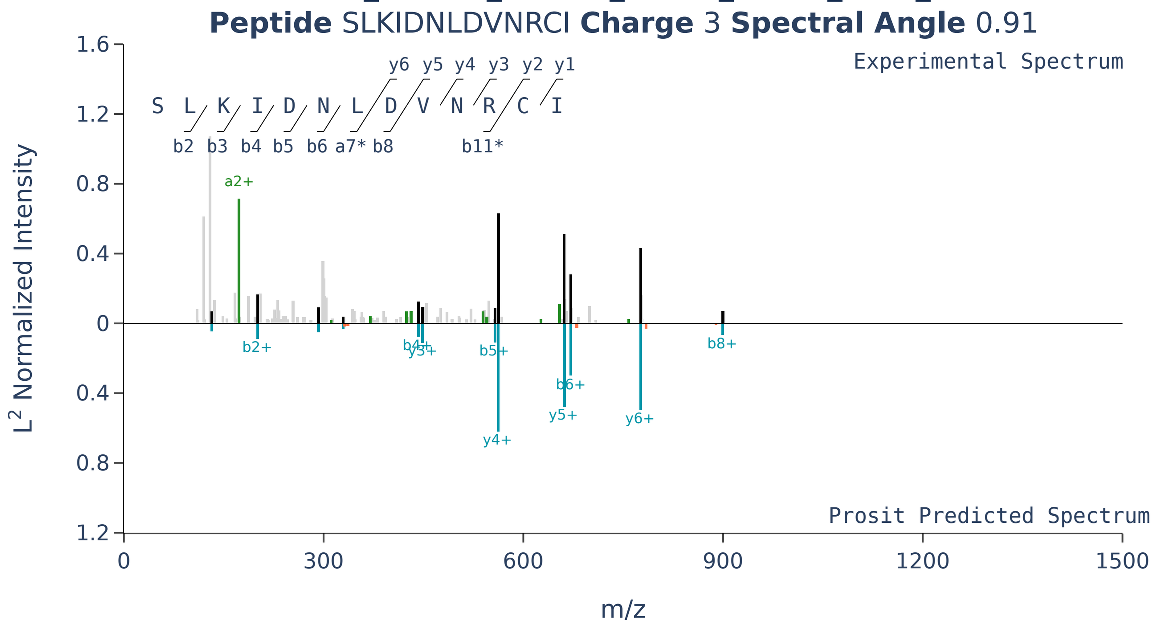


**Source** HRoetschke_271222_090123_HFGoe_LEDGF_b1R1_4h **Scan** 12244
**Peptide** HAEIKNSLKL **Charge** 3 **Spectral Angle** 0.9

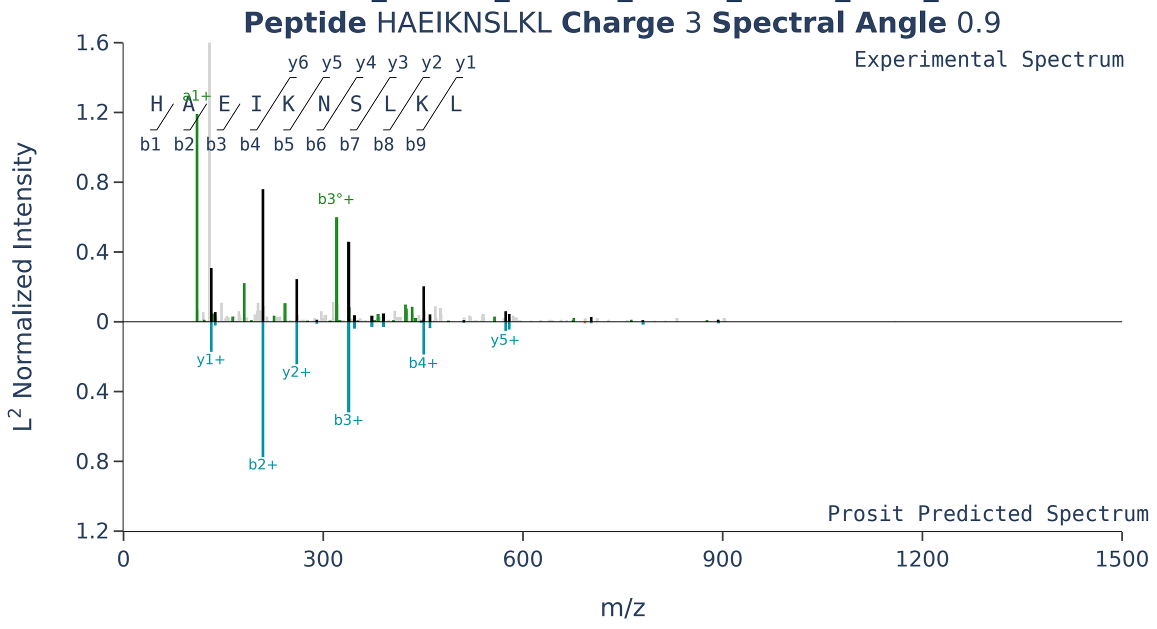


**Source** HRoetschke_271222_090123_HFGoe_LEDGF_b2R1_2h **Scan** 8177
**Peptide** NEDVTKAVD **Charge** 2 **Spectral Angle** 0.85

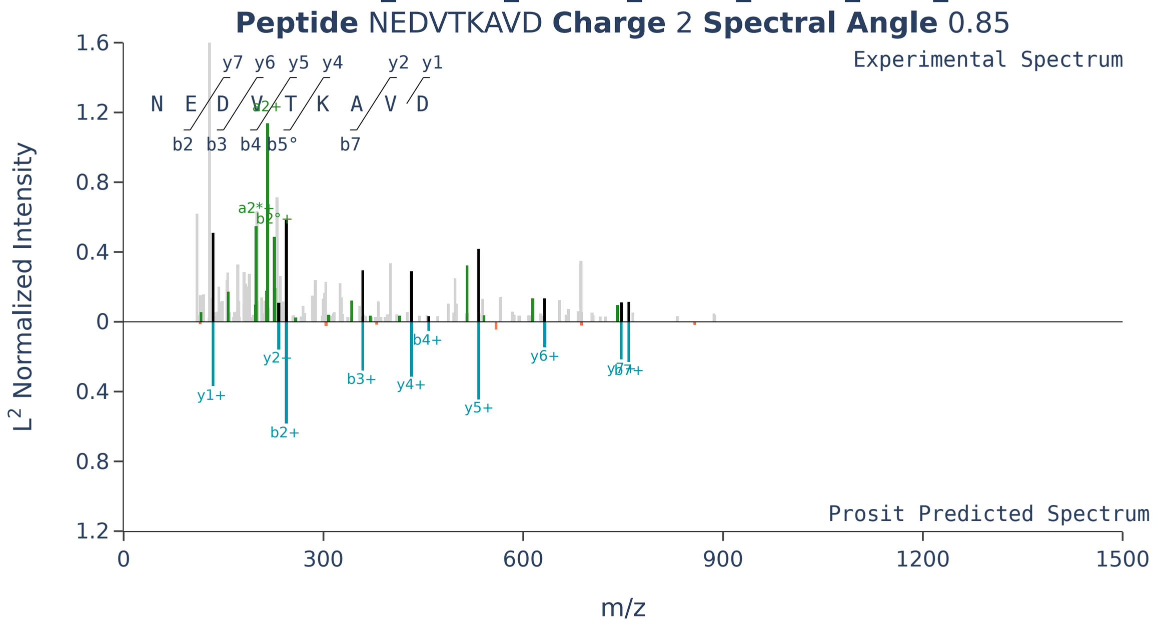


**Source** HRoetschke_271222_090123_HFGoe_LEDGF_b2R1_4h **Scan** 5681
**Peptide** NLKVSPKRGRPAA **Charge** 3 **Spectral Angle** 0.95

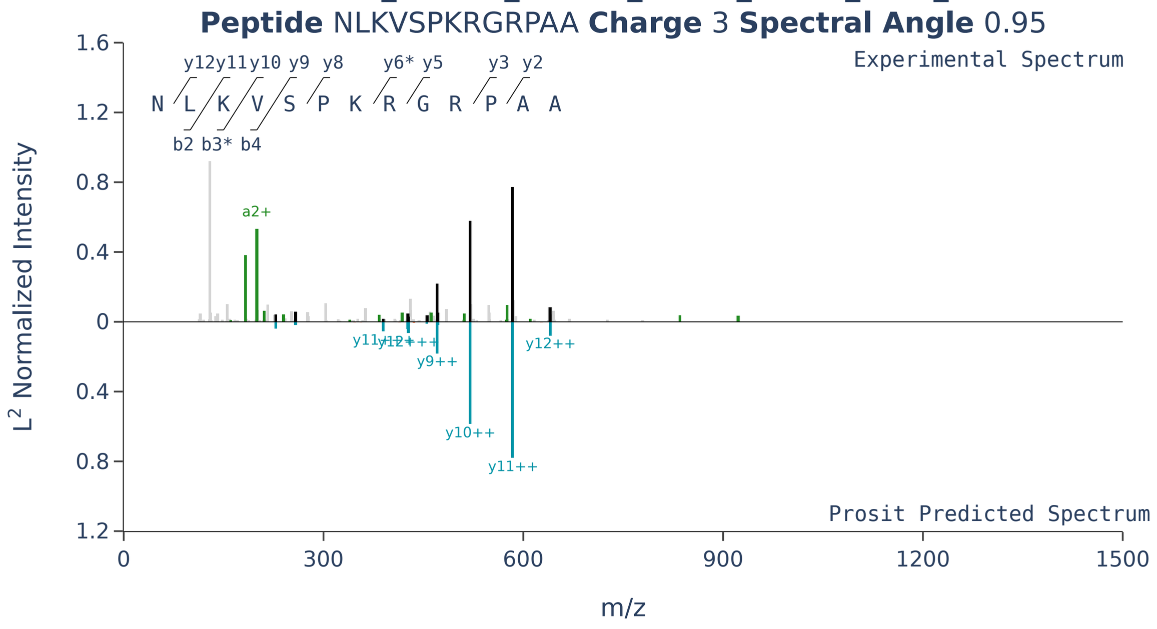


**Source** HRoetschke_271222_090123_HFGoe_LEDGF_b2R1_1h **Scan** 16545
**Peptide** EKSTMLYNKF **Charge** 2 **Spectral Angle** 0.88

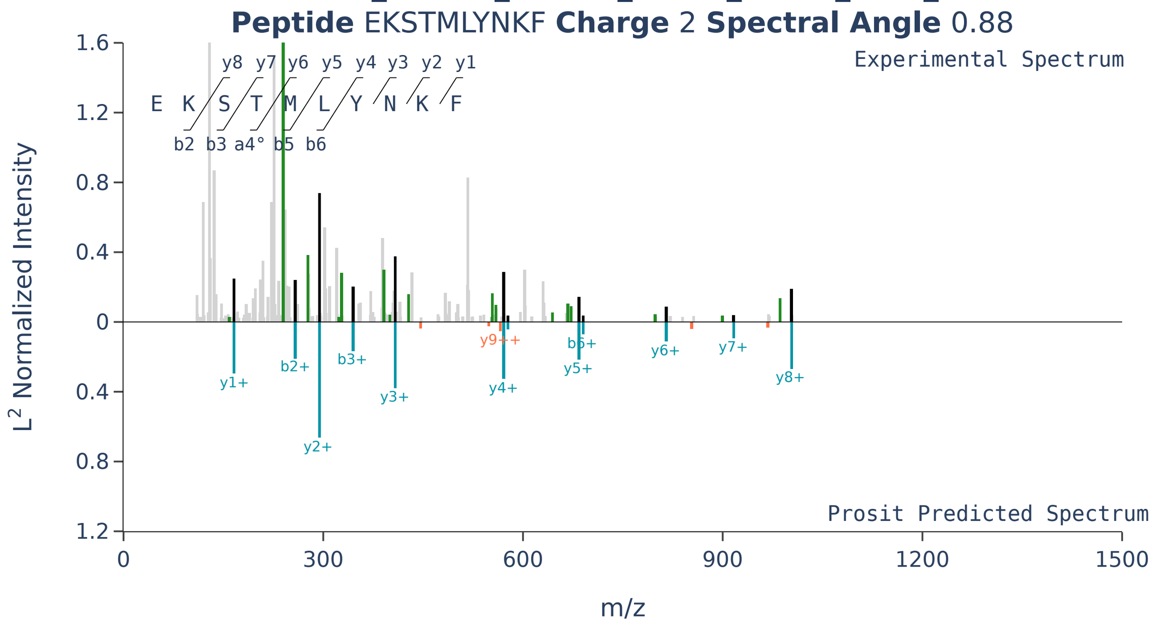


**Source** HRoetschke_271222_090123_HFGoe_LEDGF_b2R2_4h **Scan** 15095
**Peptide** MKGYPHWPARVD **Charge** 3 **Spectral Angle** 0.9

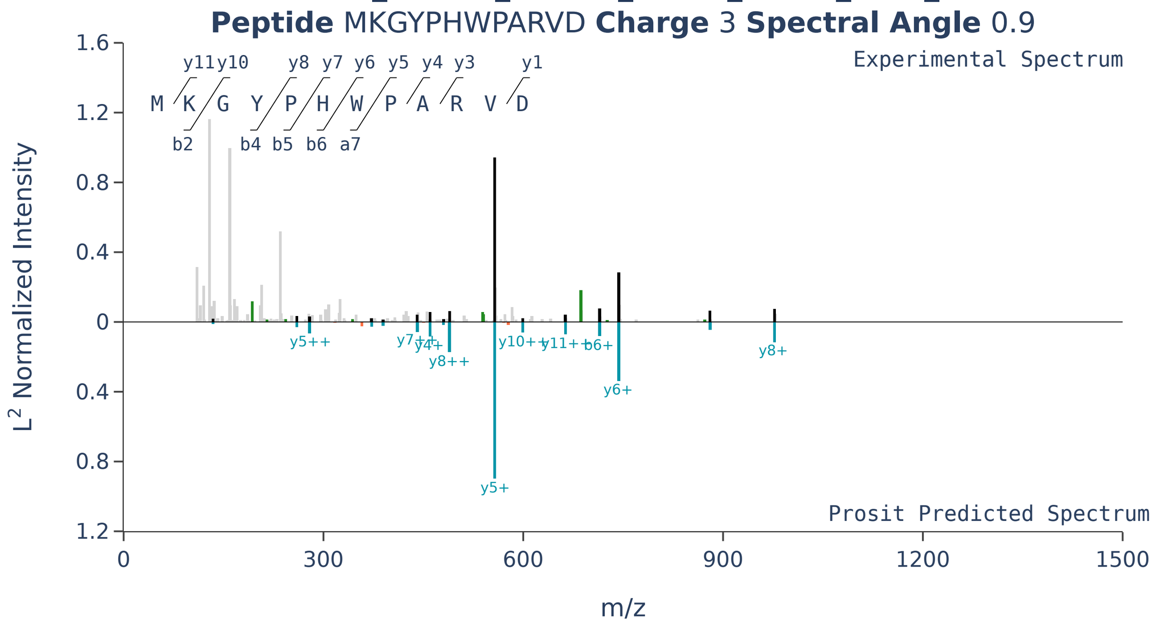


**Source** HRoetschke_271222_090123_HFGoe_LEDGF_b2R2_2h **Scan** 8110
**Peptide** VDITTPKAARRG **Charge** 2 **Spectral Angle** 0.93

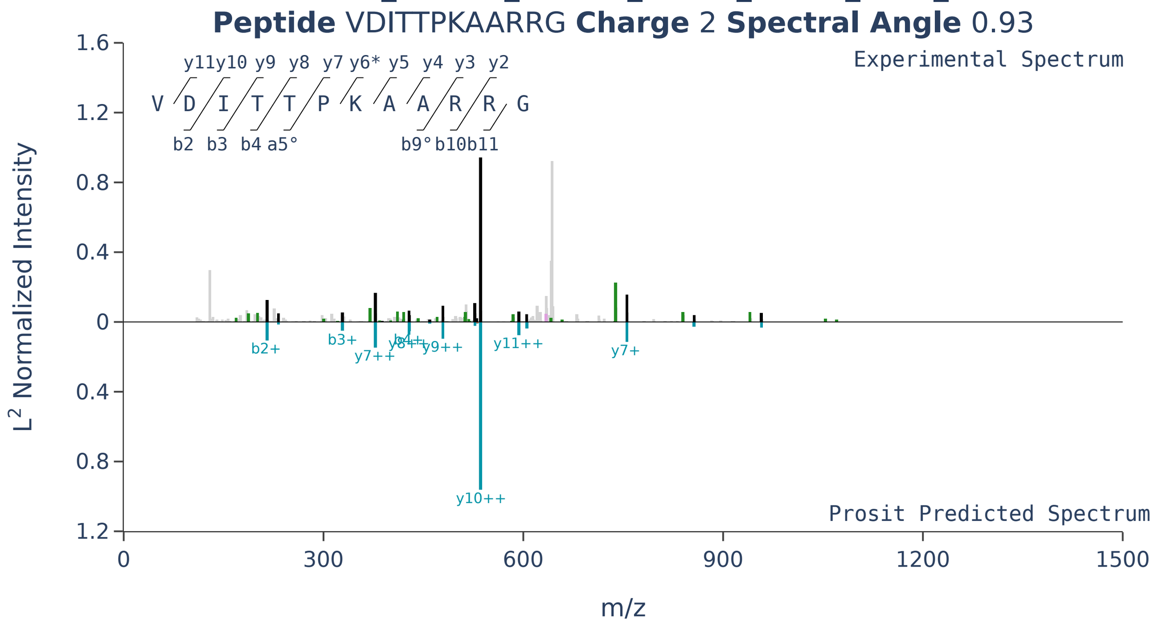

**Source** HRoetschke_271222_090123_HFGoe_LEDGF_b2R1_2h **Scan** 17837
**Peptide** THETAFL **Charge** 2 **Spectral Angle** 0.86

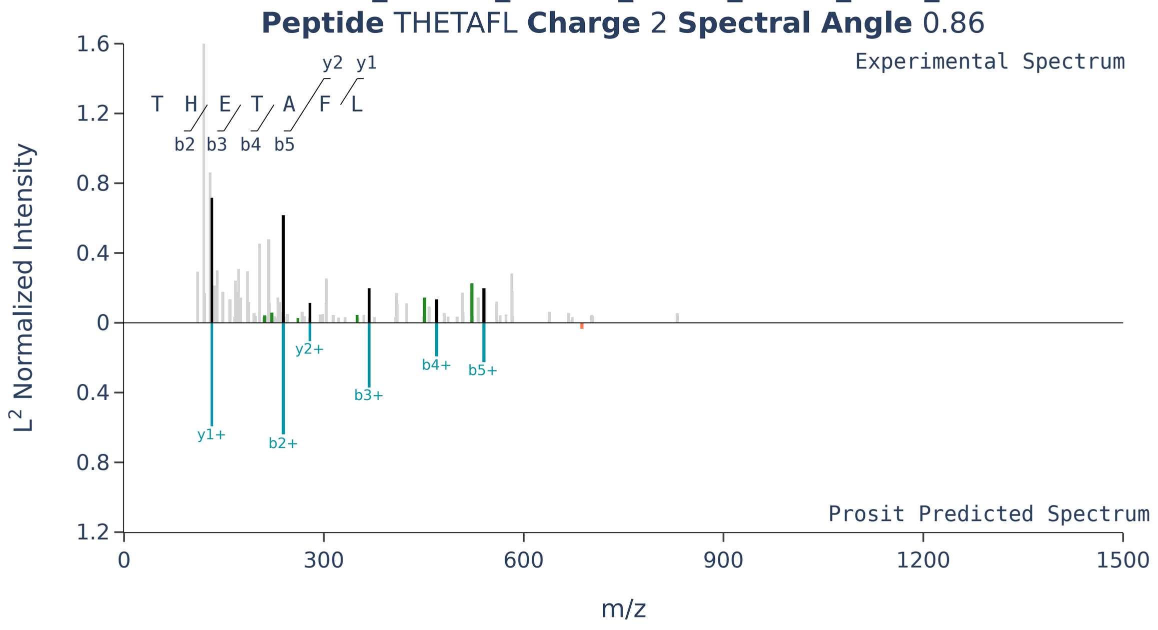


**Source** HRoetschke_271222_090123_HFGoe_LEDGF_b1R2_24h **Scan** 13879
**Peptide** TMLYNKFKNM **Charge** 2 **Spectral Angle** 0.83

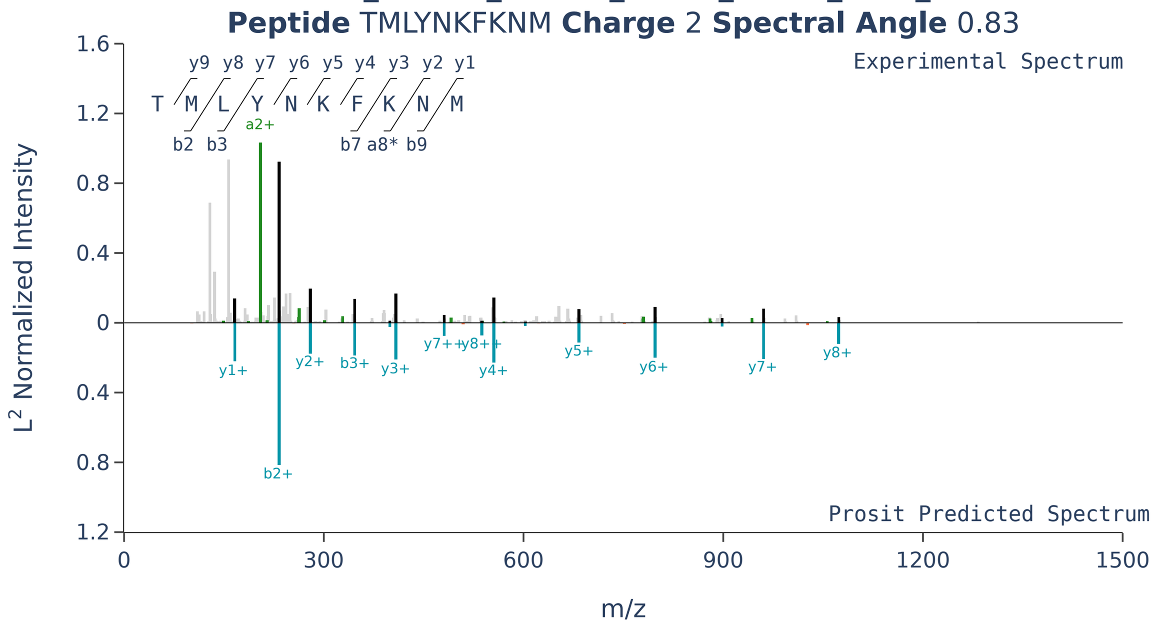


**Source** HRoetschke_271222_090123_HFGoe_LEDGF_b1R1_2h **Scan** 11144
**Peptide** KSTMLYN **Charge** 2 **Spectral Angle** 0.88

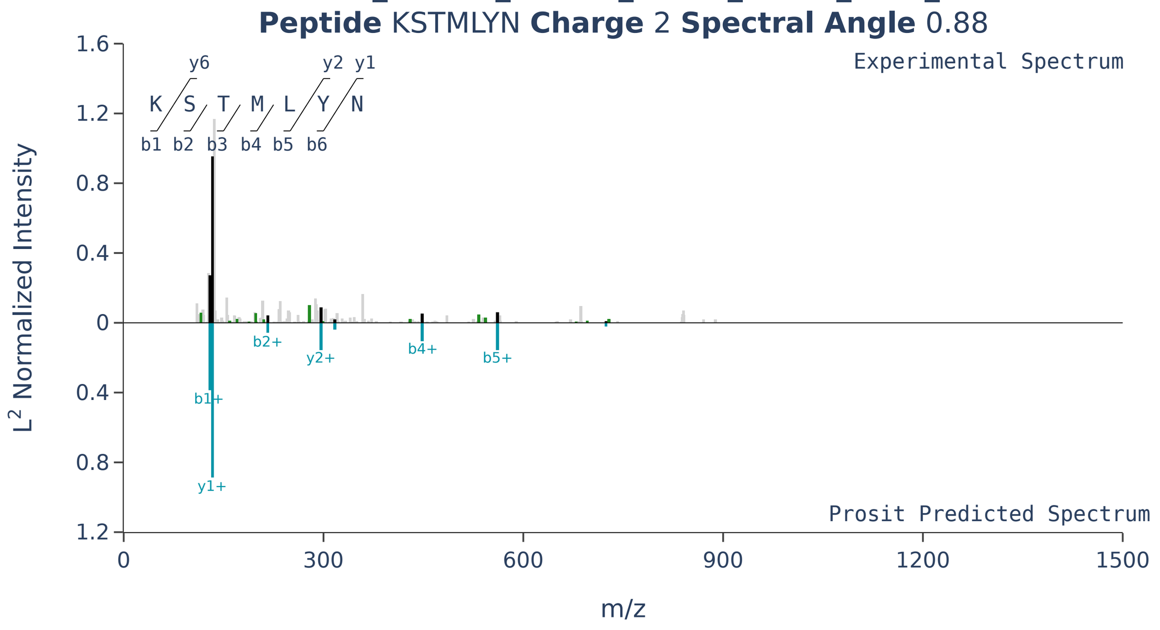


**Source** HRoetschke_271222_090123_HFGoe_LEDGF_b2R1_1h **Scan** 24419
**Peptide** KPGDLIF **Charge** 2 **Spectral Angle** 0.88

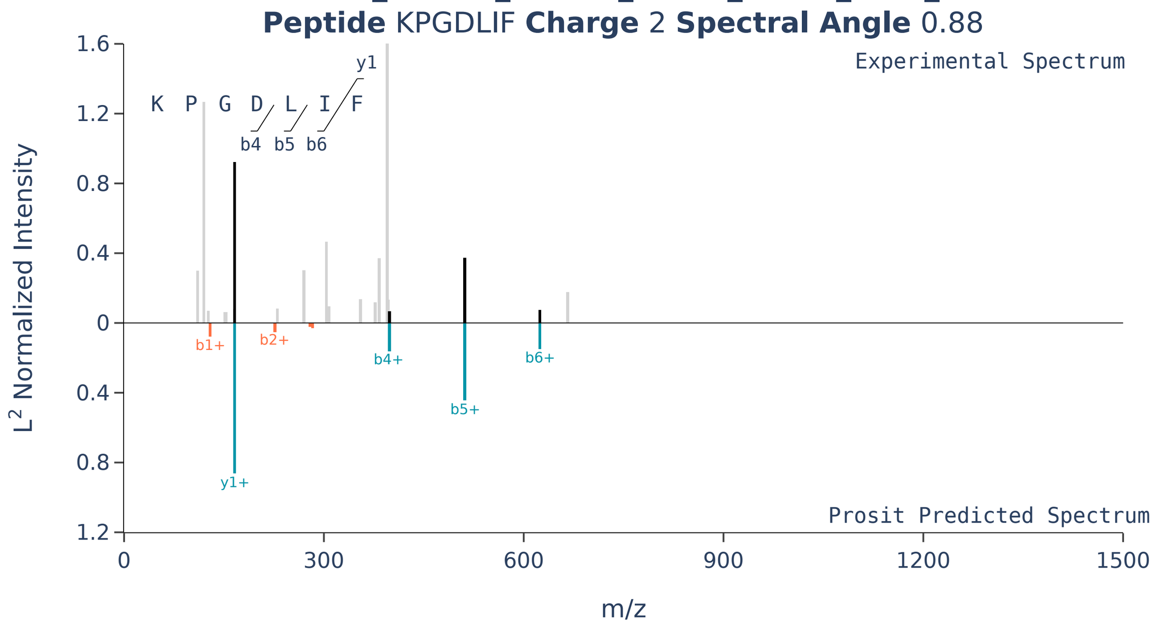


**Source** HRoetschke_271222_090123_HFGoe_LEDGF_b1R2_24h **Scan** 6260
**Peptide** LEKEQTGSKTLNGGSD **Charge** 2 **Spectral Angle** 0.85

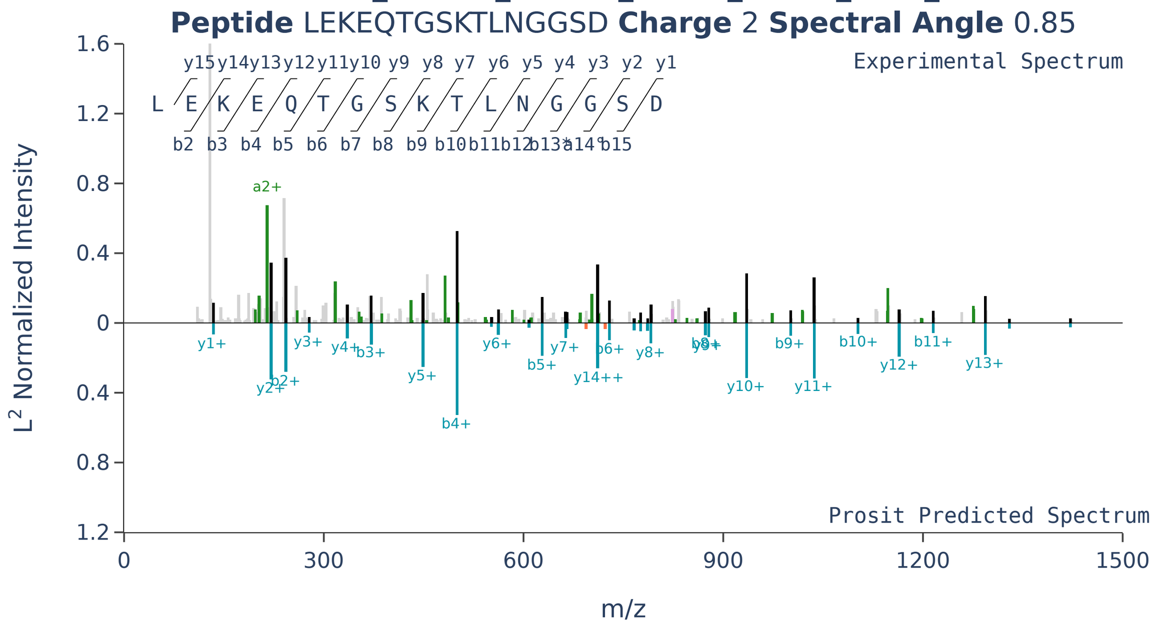


**Source** HRoetschke_271222_090123_HFGoe_LEDGF_b1R2_24h **Scan** 15099
**Peptide** SDIITEED **Charge** 1 **Spectral Angle** 0.9

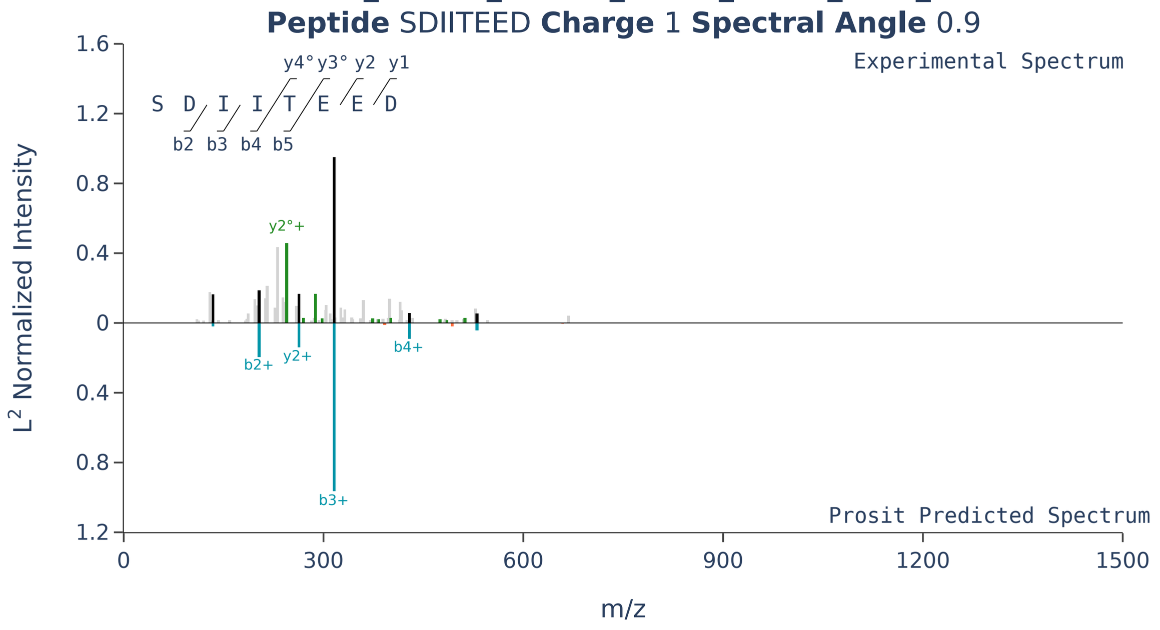


**Source** HRoetschke_271222_090123_HFGoe_LEDGF_b1R2_1h **Scan** 15920
**Peptide** LIFAKMKG **Charge** 2 **Spectral Angle** 0.85

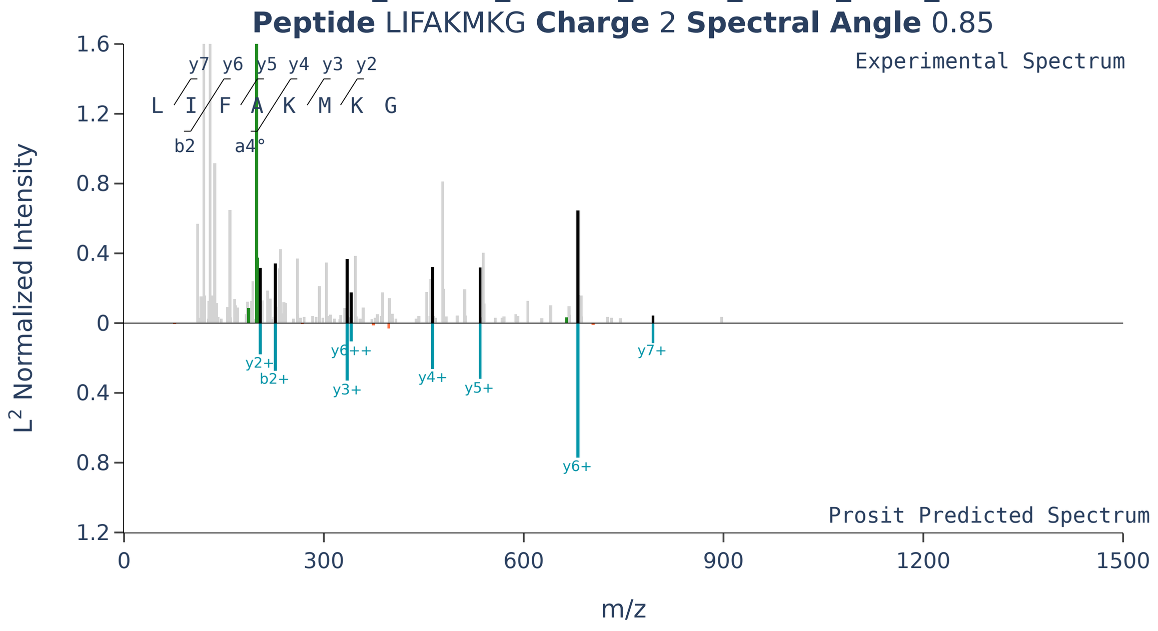


**Source** HRoetschke_271222_090123_HFGoe_LEDGF_b1R2_1h **Scan** 7985
**Peptide** QKHTEMITT **Charge** 3 **Spectral Angle** 0.88

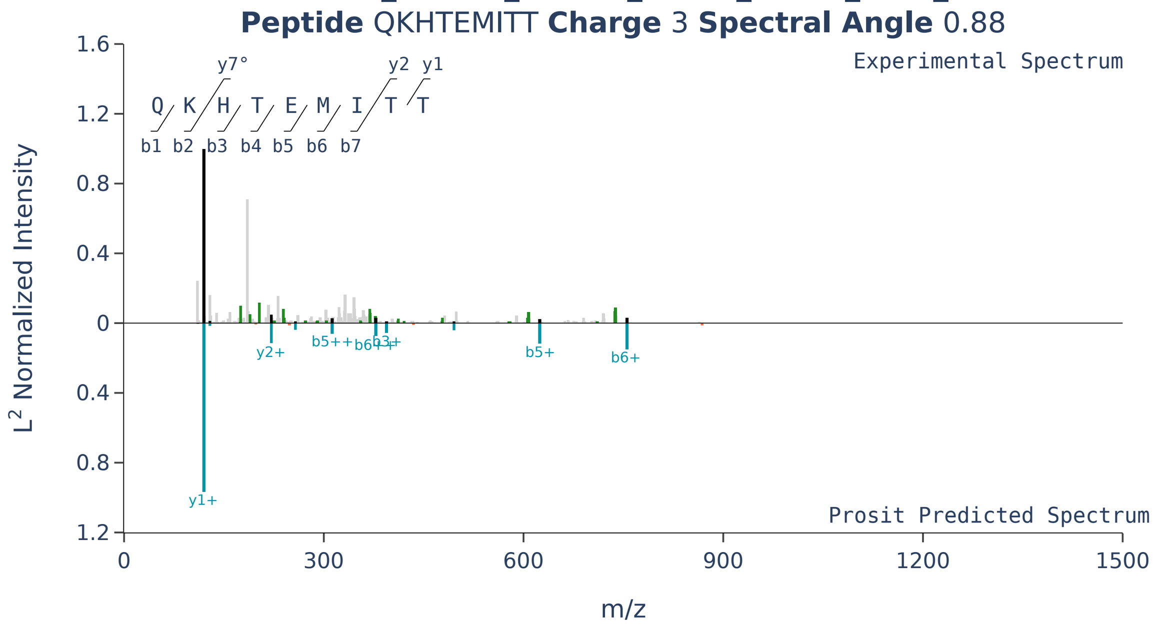


**Source** HRoetschke_271222_090123_HFGoe_LEDGF_b2R1_4h **Scan** 6289
**Peptide** SDIITEEDKSKKKGQEE **Charge** 2 **Spectral Angle** 0.9

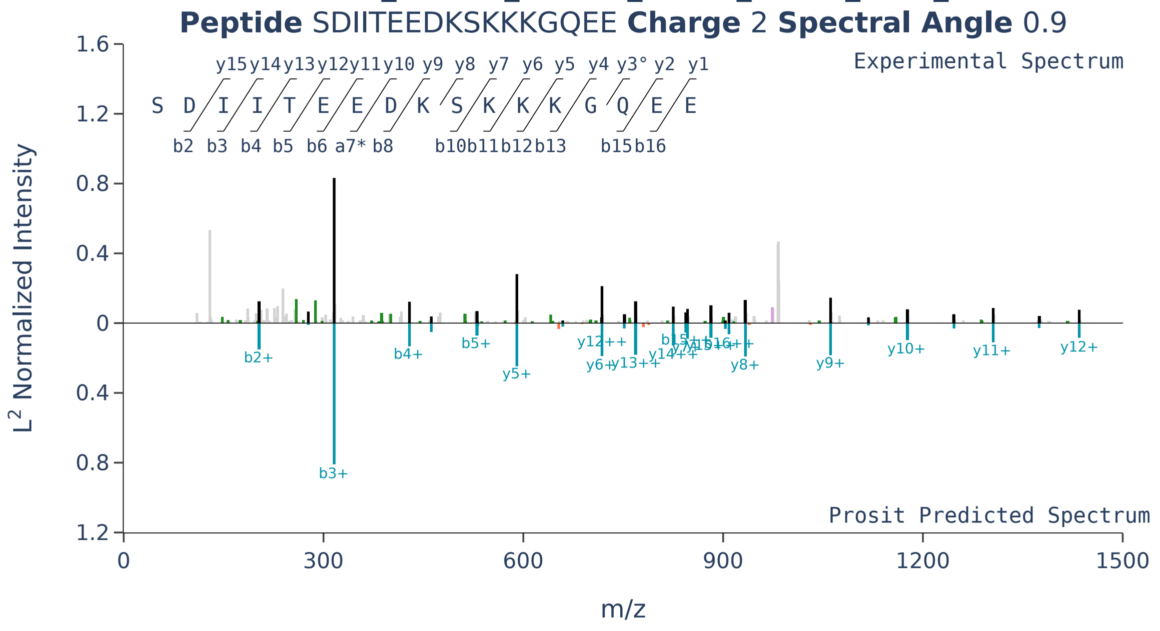


**Source** HRoetschke_271222_090123_HFGoe_LEDGF_b2R2_24h **Scan** 12624
**Peptide** PHWPARVD **Charge** 2 **Spectral Angle** 0.87

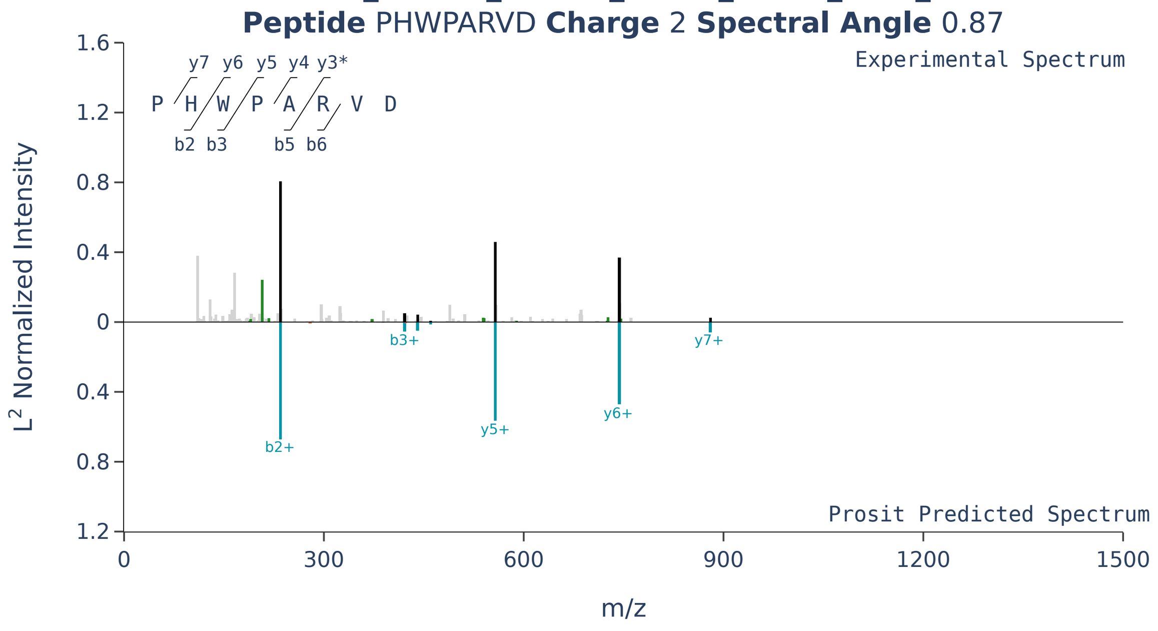


**Source** HRoetschke_271222_090123_HFGoe_LEDGF_b2R1_24h **Scan** 26125
**Peptide** NEGLWEIDNNPKVKF **Charge** 2 **Spectral Angle** 0.93

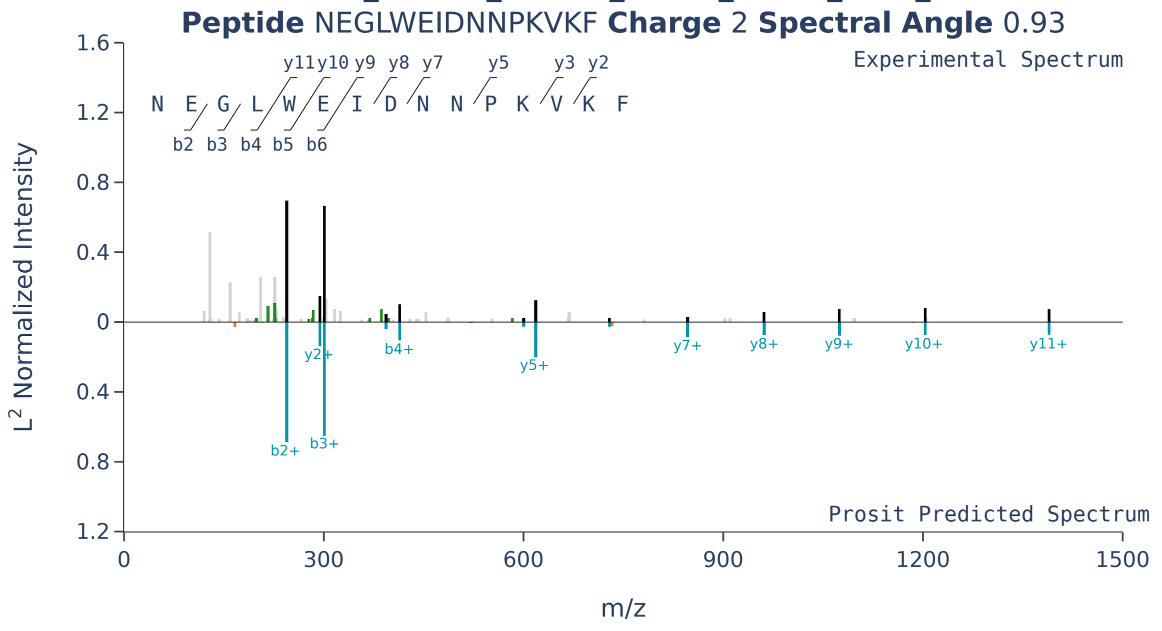


**Source** HRoetschke_271222_090123_HFGoe_LEDGF_b1R1_1h **Scan** 7821
**Peptide** TKQSNASSDVEVE **Charge** 2 **Spectral Angle** 0.84

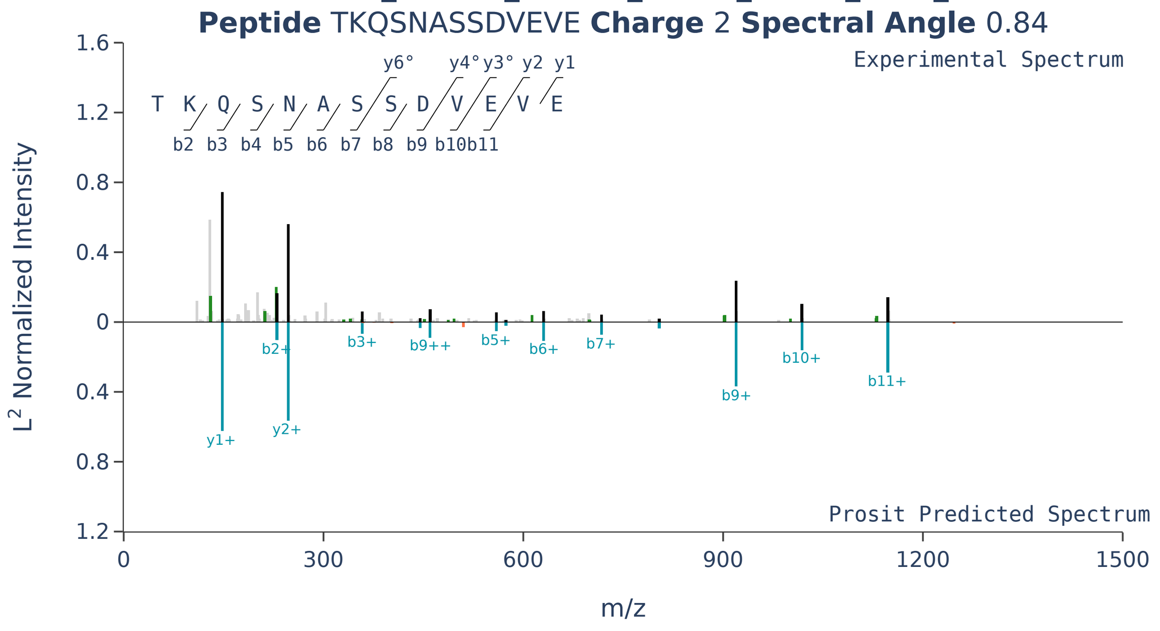


**Source** HRoetschke_271222_090123_HFGoe_LEDGF_b2R2_4h **Scan** 12641
**Peptide** KIDNLDVN **Charge** 2 **Spectral Angle** 0.85

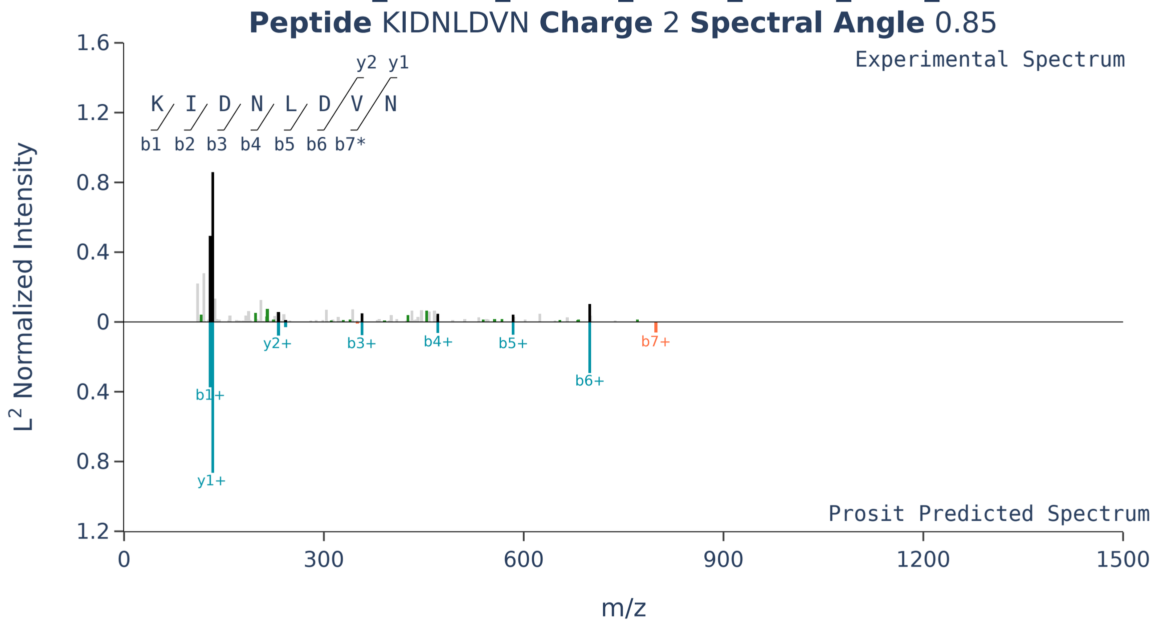


**Source** HRoetschke_271222_090123_HFGoe_LEDGF_b1R1_24h **Scan** 16878
**Peptide** YNKFKNMF **Charge** 3 **Spectral Angle** 0.95

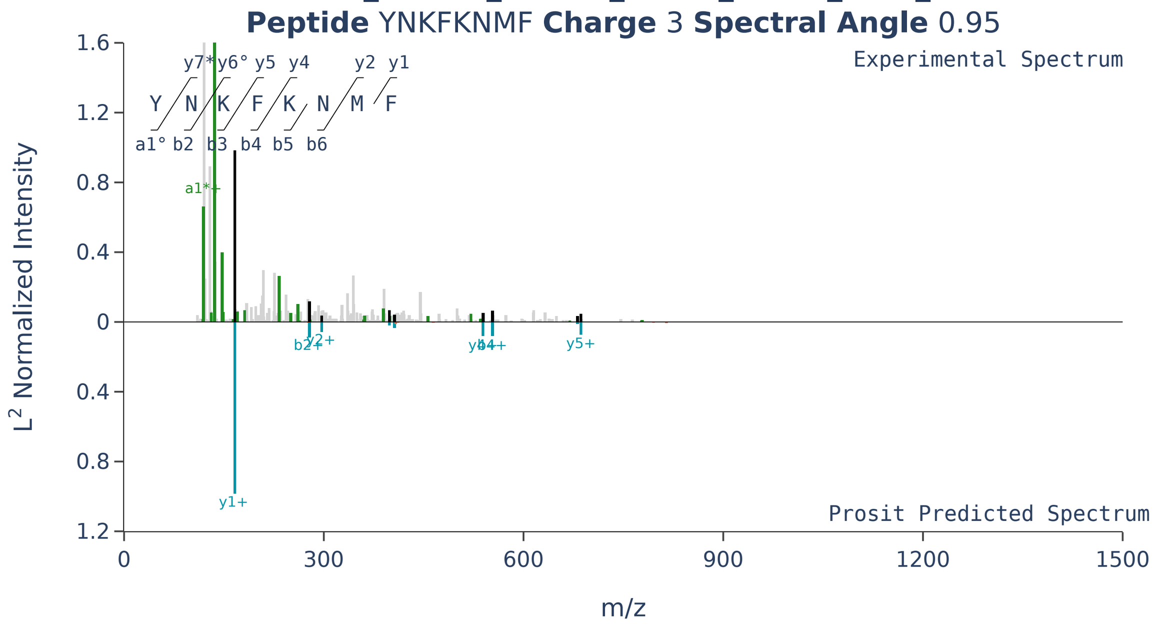


**Source** HRoetschke_271222_090123_HFGoe_LEDGF_b1R1_24h **Scan** 8576
**Peptide** VPDGAVKPPTNK **Charge** 3 **Spectral Angle** 0.88

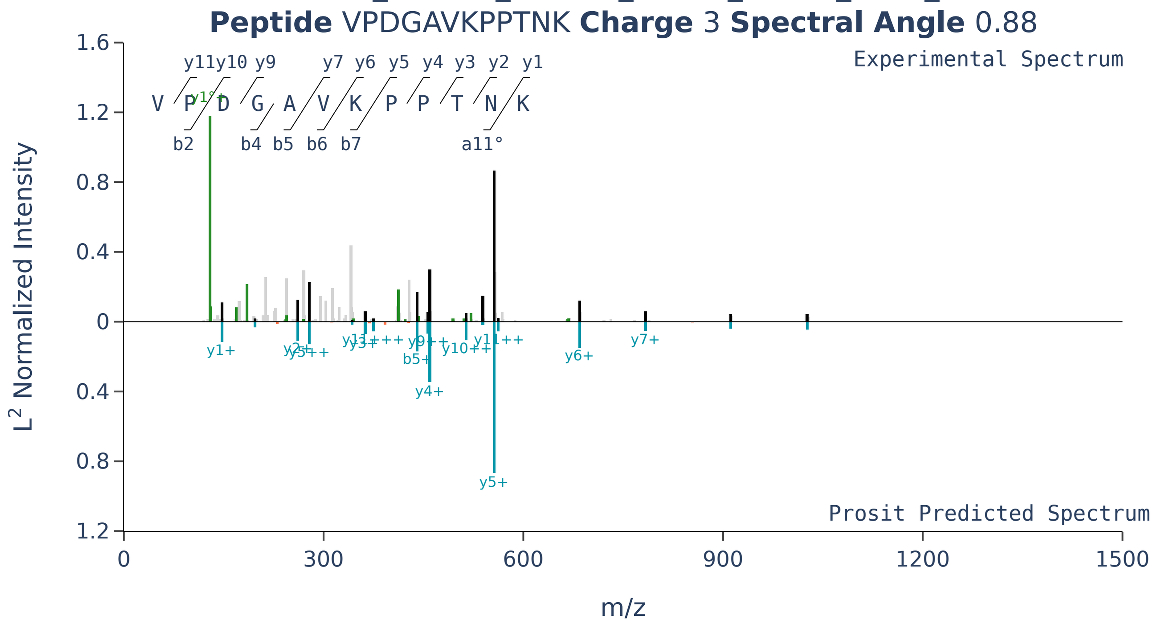

**Source** HRoetschke_271222_090123_HFGoe_LEDGF_b2R2_24h **Scan** 9341
**Peptide** ETSMDSRLQ **Charge** 2 **Spectral Angle** 0.86

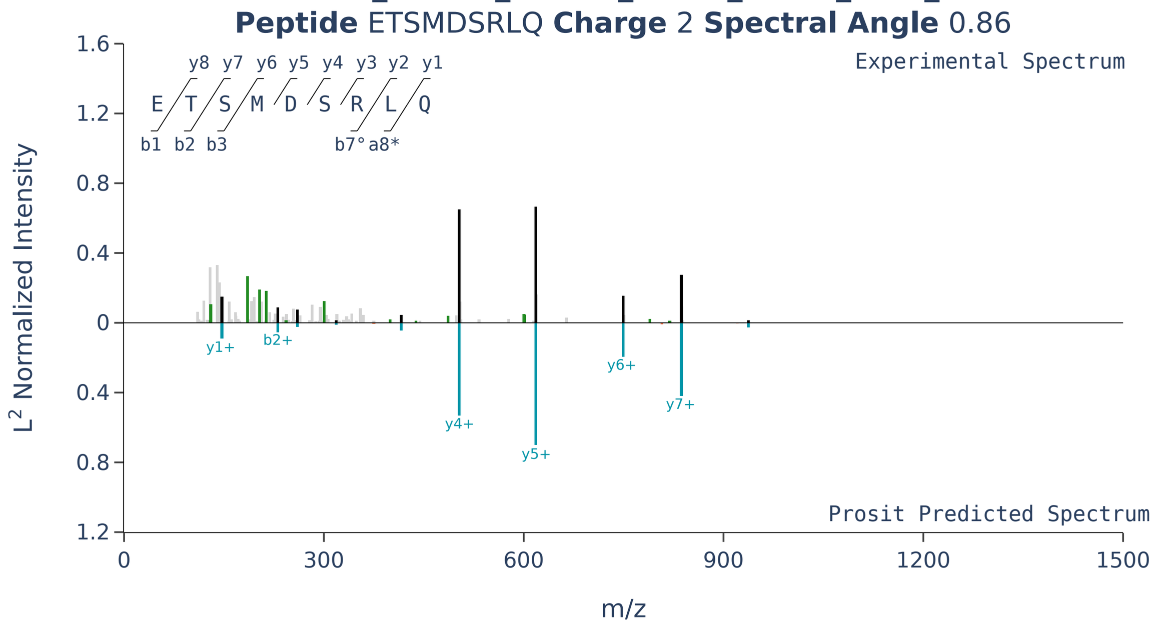


**Source** HRoetschke_271222_090123_HFGoe_LEDGF_b1R2_24h **Scan** 13135
**Peptide** MEKSTML **Charge** 2 **Spectral Angle** 0.94

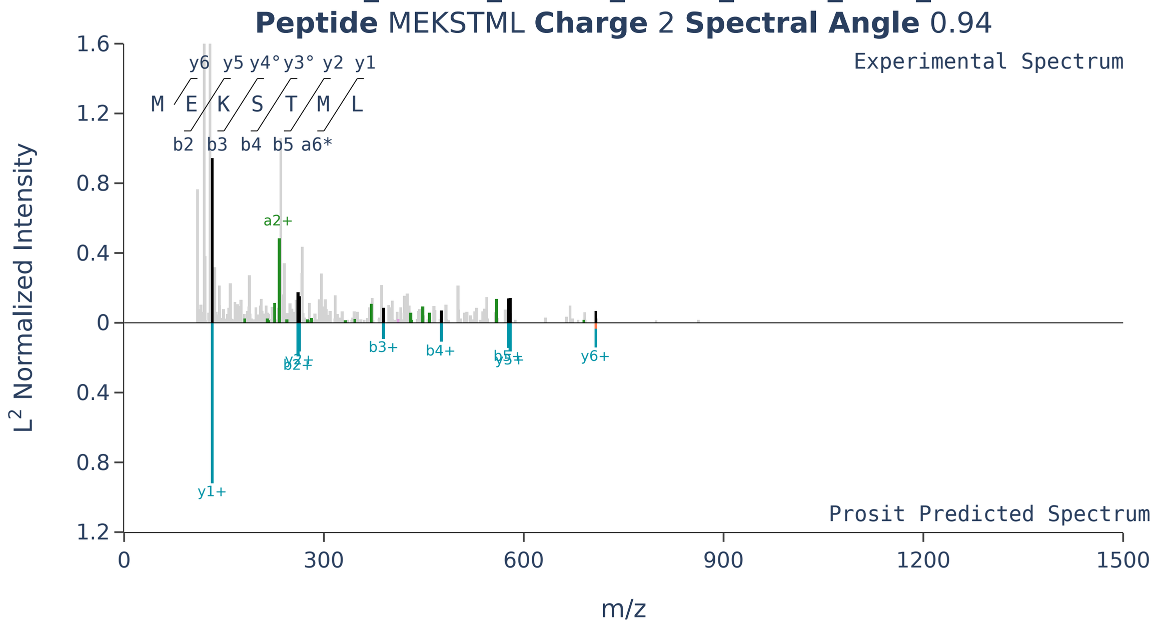


**Source** HRoetschke_271222_090123_HFGoe_LEDGF_b1R2_1h **Scan** 14170
**Peptide** KGYPHWPARV **Charge** 3 **Spectral Angle** 0.91

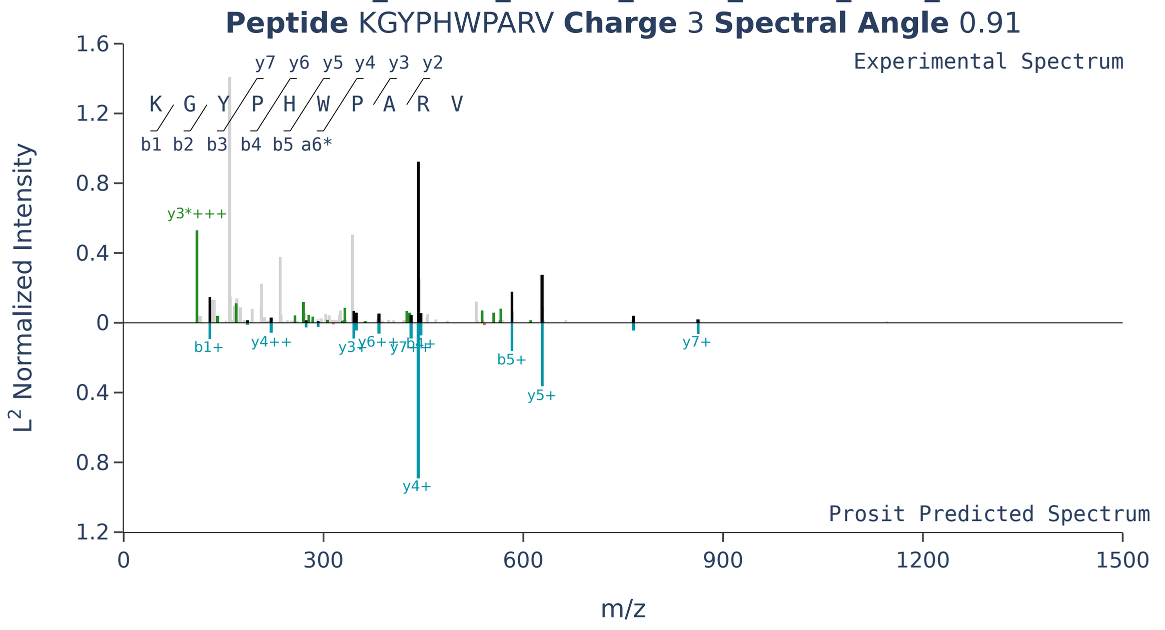


**Source** HRoetschke_271222_090123_HFGoe_LEDGF_b2R2_2h **Scan** 10552
**Peptide** LQVTMQQAQKHTE **Charge** 2 **Spectral Angle** 0.89

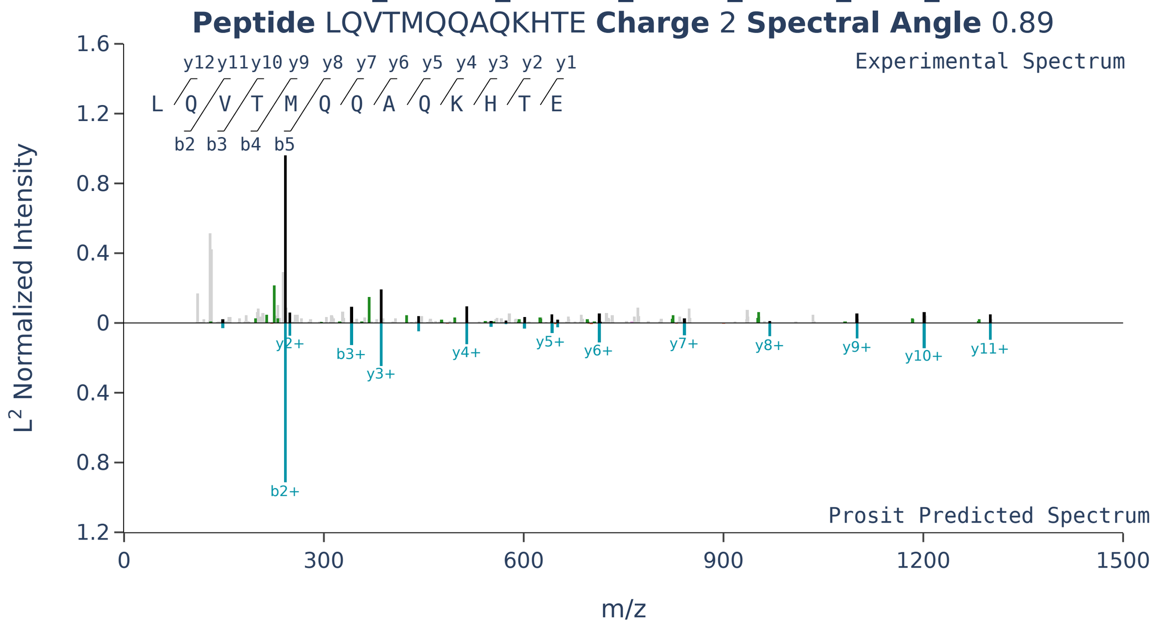


**Source** HRoetschke_271222_090123_HFGoe_LEDGF_b1R1_2h **Scan** 26272
**Peptide** MFLVGEGD **Charge** 1 **Spectral Angle** 0.92

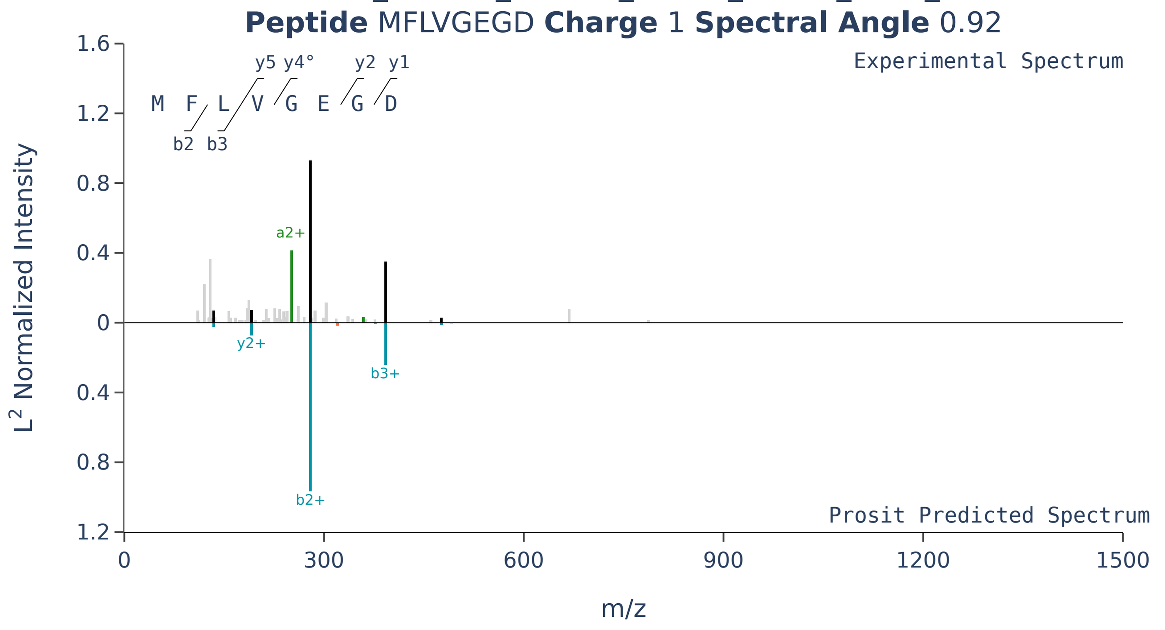


**Source** HRoetschke_271222_090123_HFGoe_LEDGF_b1R2_4h **Scan** 13678
**Peptide** RDFKPGDL **Charge** 2 **Spectral Angle** 0.84

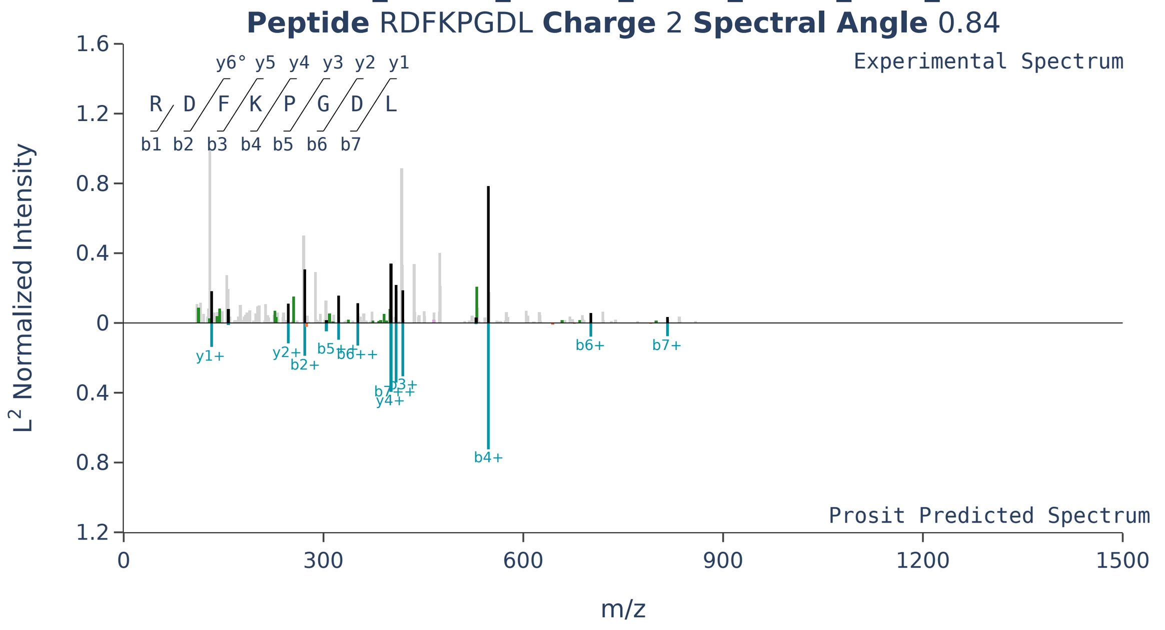


**Source** HRoetschke_271222_090123_HFGoe_LEDGF_b2R1_1h **Scan** 12078
**Peptide** EVPDGAVKPPTN **Charge** 2 **Spectral Angle** 0.81

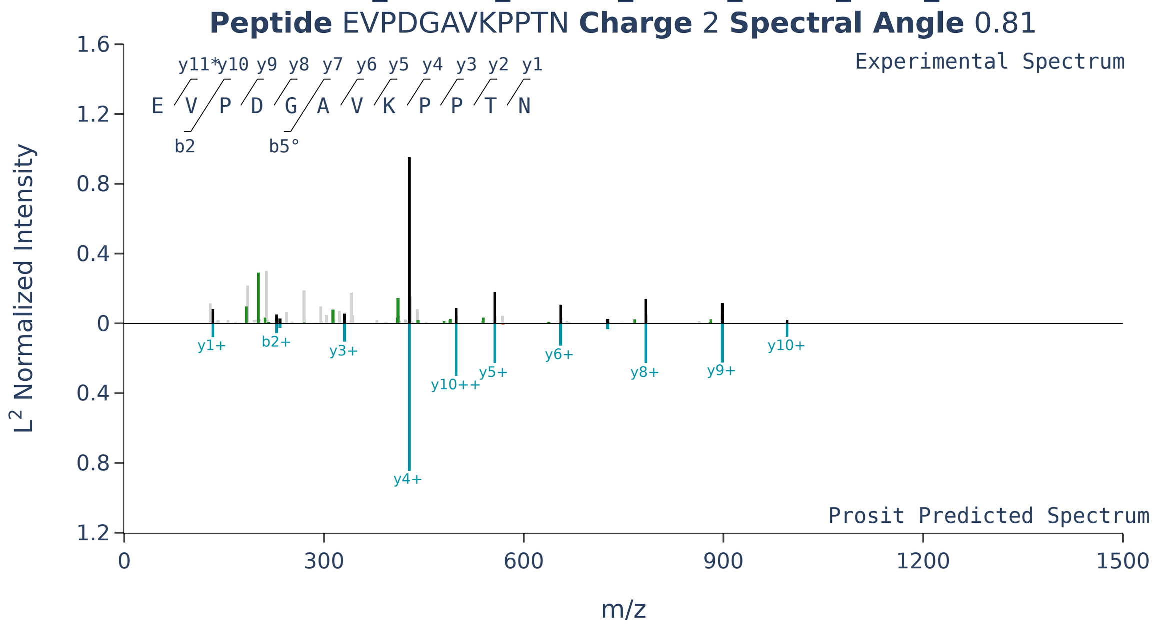


**Source** HRoetschke_271222_090123_HFGoe_LEDGF_b2R2_4h **Scan** 5529
**Peptide** NHEASTKKKPSSEERETEI **Charge** 3 **Spectral Angle** 0.84

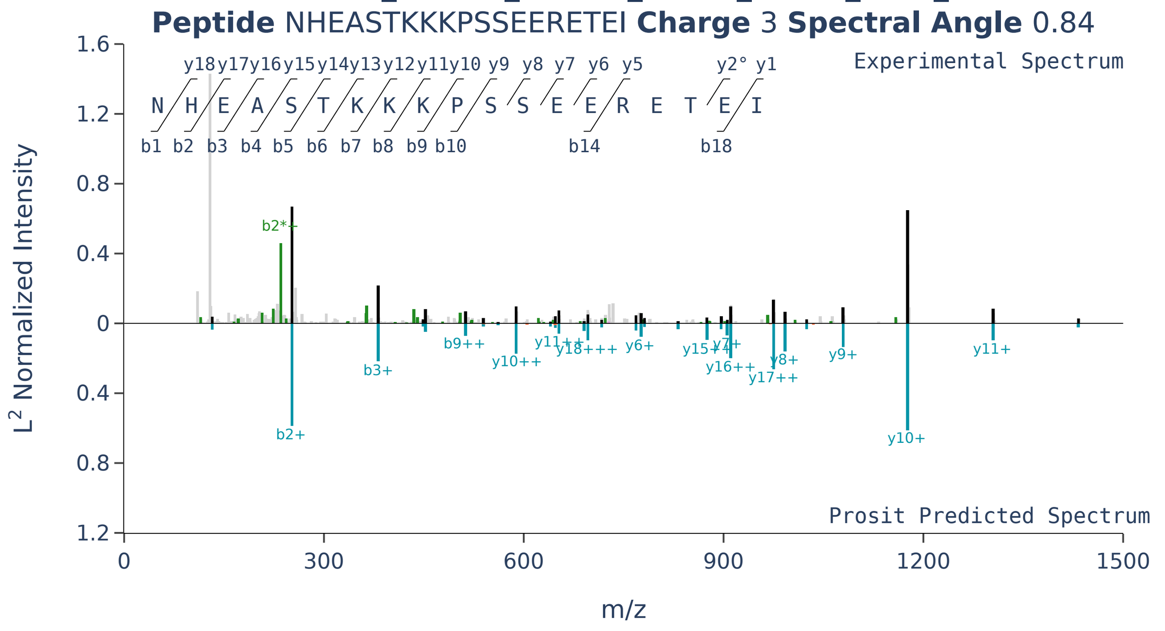


**Source** HRoetschke_271222_090123_HFGoe_LEDGF_b1R1_24h **Scan** 15787
**Peptide** AQKHTEMITTL **Charge** 2 **Spectral Angle** 0.78

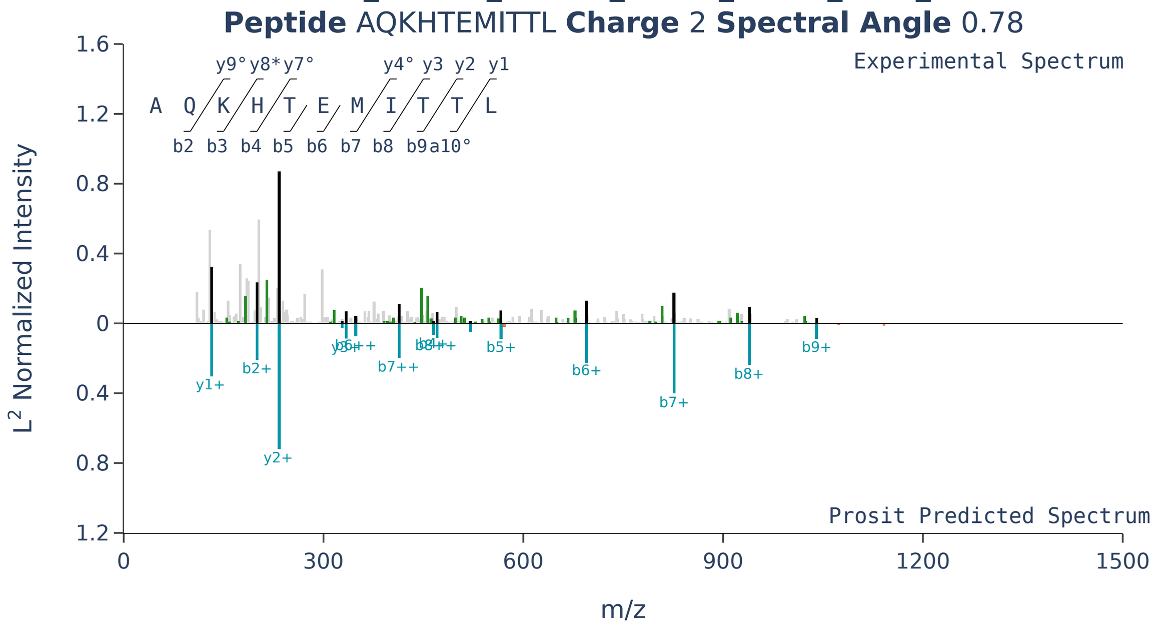


**Source** HRoetschke_271222_090123_HFGoe_LEDGF_b2R2_2h **Scan** 6923
**Peptide** LAKTGVTSTSD **Charge** 1 **Spectral Angle** 0.89

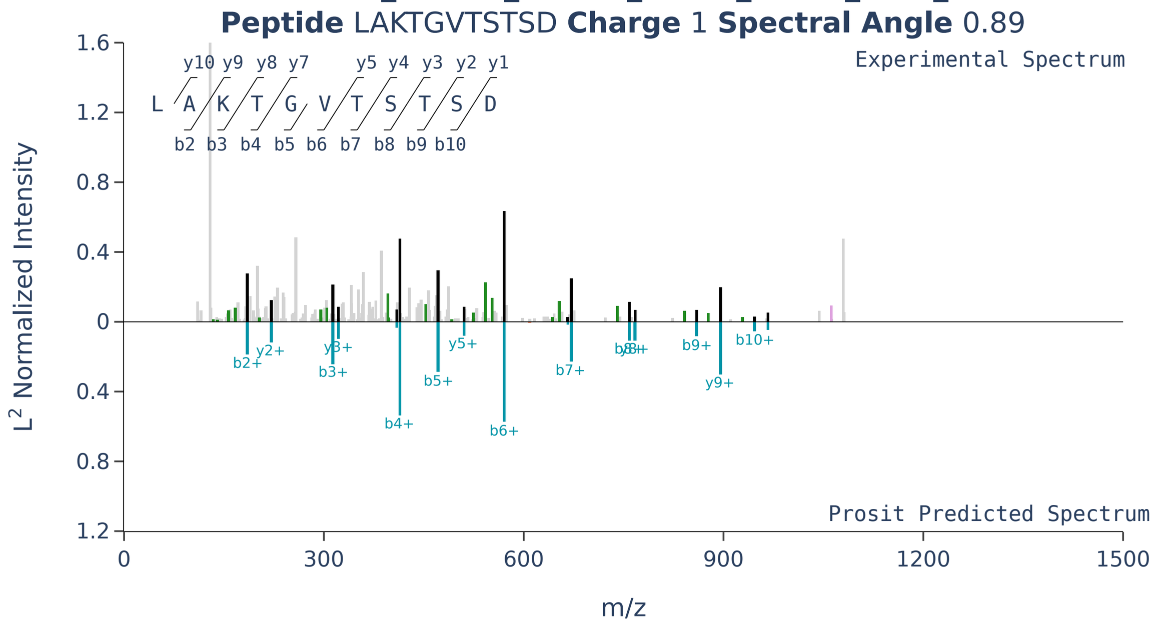


**Source** HRoetschke_271222_090123_HFGoe_LEDGF_b2R1_24h **Scan** 17817
**Peptide** YPHWPARV **Charge** 2 **Spectral Angle** 0.89

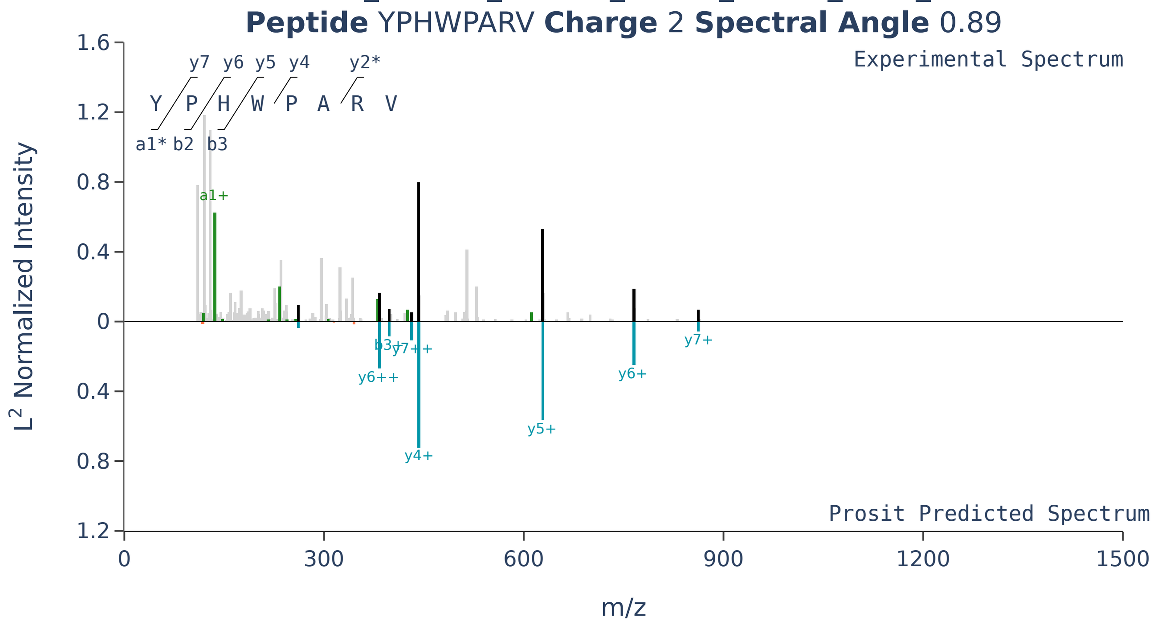


**Source** HRoetschke_271222_090123_HFGoe_LEDGF_b2R1_24h **Scan** 25060
**Peptide** FNEGLWEIDNNPKVKFSSQQ **Charge** 3 **Spectral Angle** 0.87

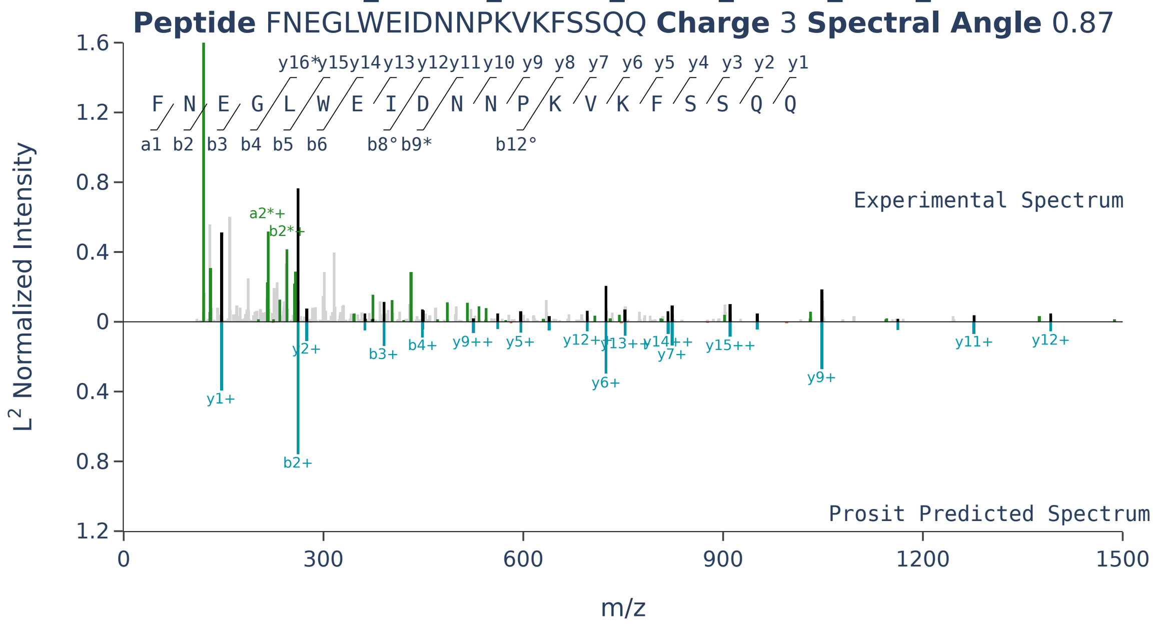


**Source** HRoetschke_271222_090123_HFGoe_LEDGF_b1R2_4h **Scan** 22517
**Peptide** FGTHETAFLGPKDI **Charge** 3 **Spectral Angle** 0.89

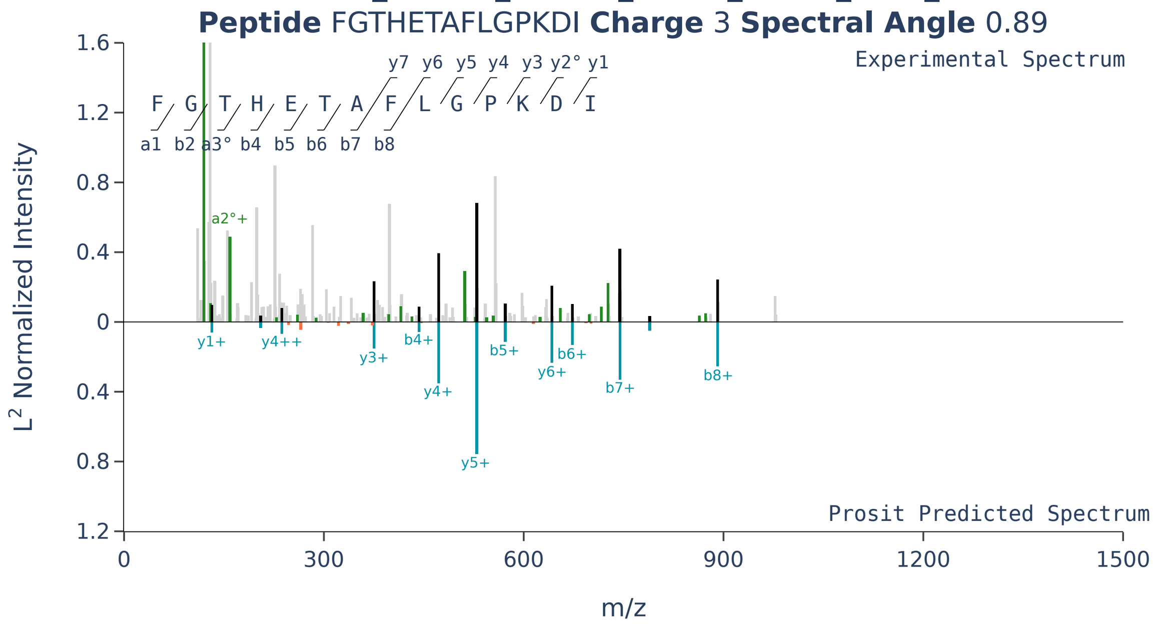


**Source** HRoetschke_271222_090123_HFGoe_LEDGF_b2R1_4h **Scan** 14312
**Peptide** STMLYNKFK **Charge** 3 **Spectral Angle** 0.88

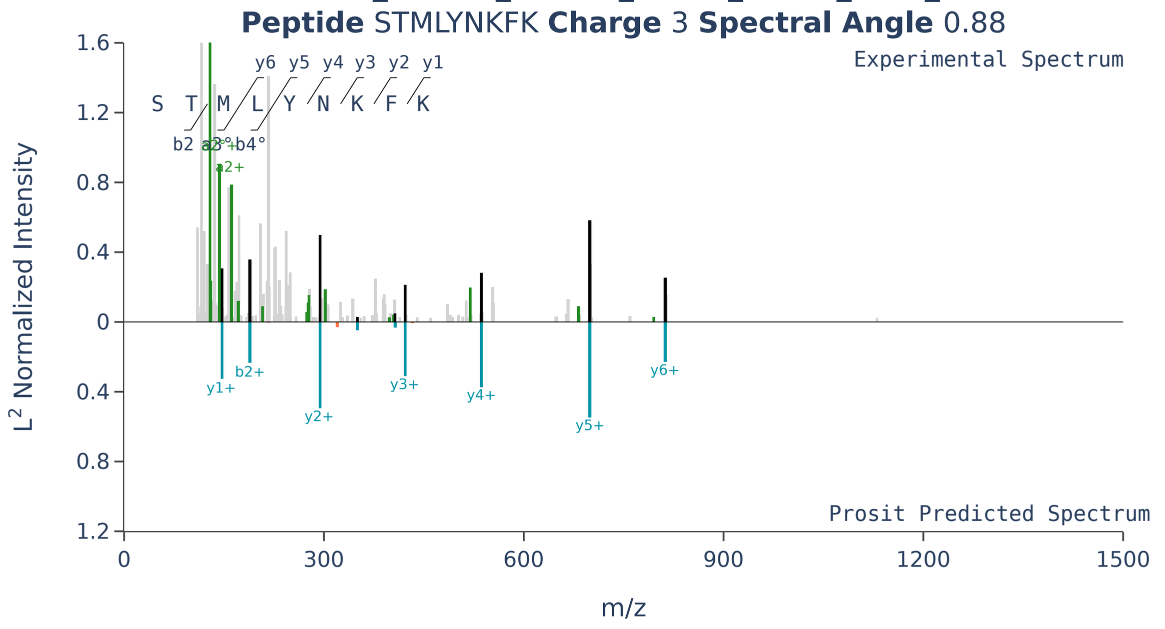


**Source** HRoetschke_271222_090123_HFGoe_LEDGF_b1R1_24h **Scan** 10787
**Peptide** VPDGAVKPPTN **Charge** 1 **Spectral Angle** 0.85

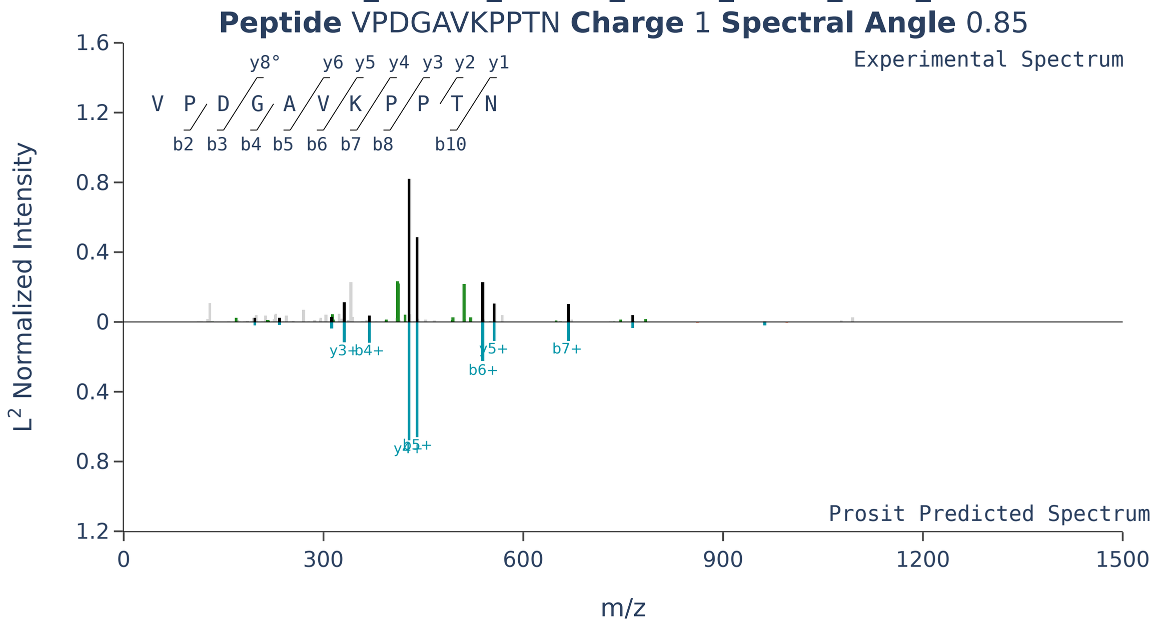

**Source** HRoetschke_271222_090123_HFGoe_LEDGF_b1R2_1h **Scan** 12630
**Peptide** LGPKDIFPYSENKEKYGKPNKRKG **Charge** 5 **Spectral Angle** 0.83

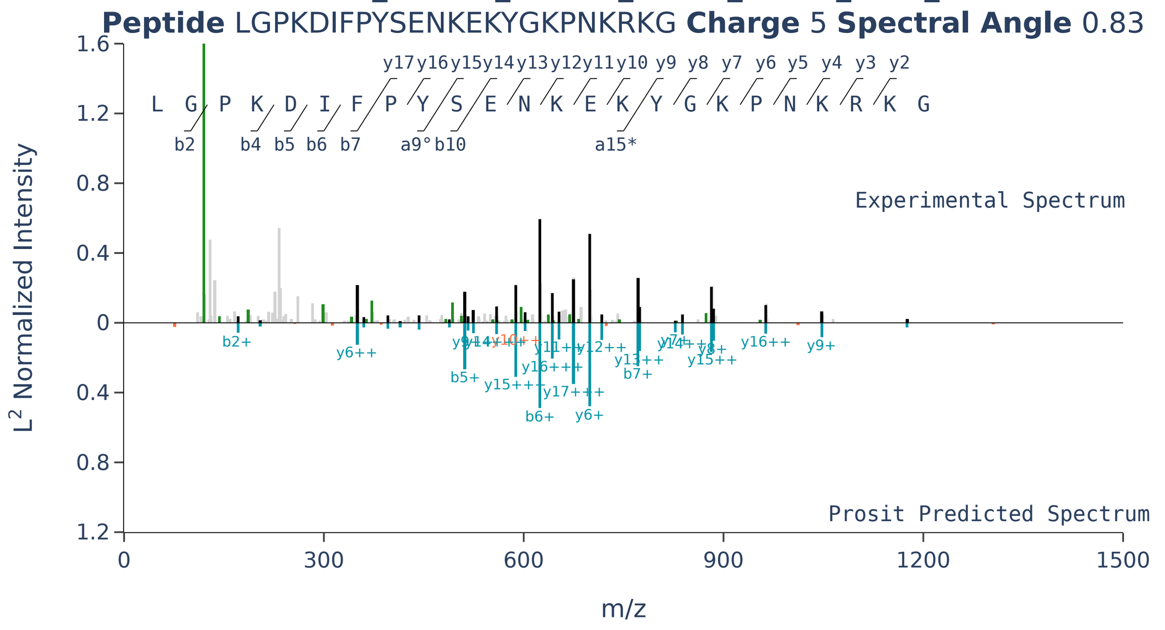


**Source** HRoetschke_271222_090123_HFGoe_LEDGF_b1R1_24h **Scan** 16556
**Peptide** DVTKAVDITTPKAA **Charge** 3 **Spectral Angle** 0.86

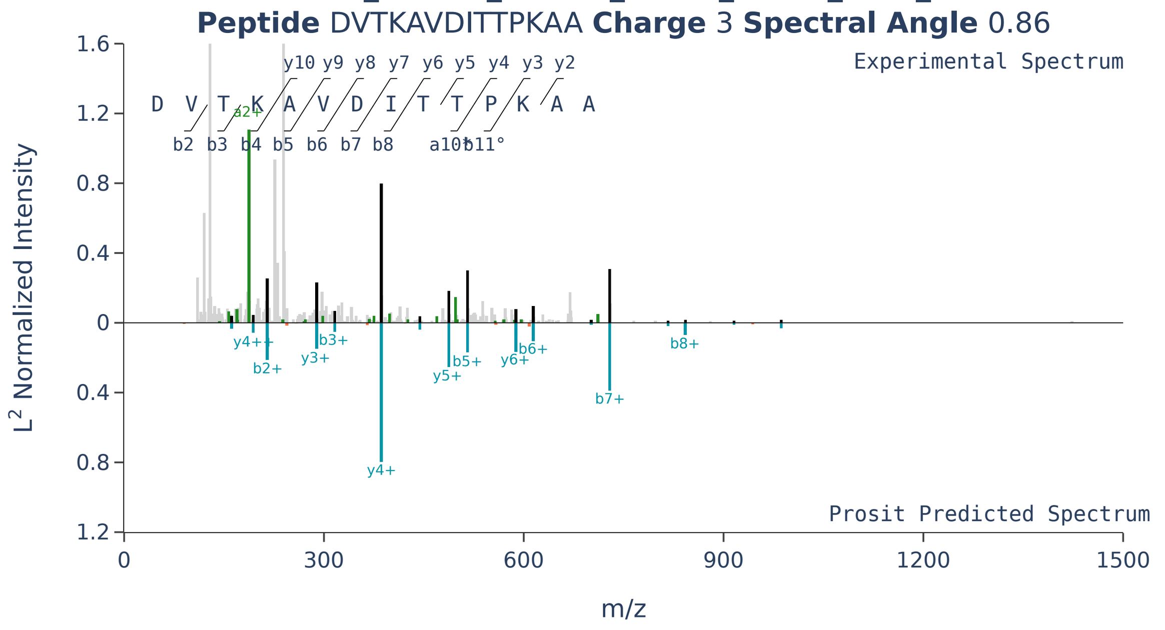


**Source** HRoetschke_271222_090123_HFGoe_LEDGF_b1R2_4h **Scan** 27319
**Peptide** THETAFLGPKDIFPYSEN **Charge** 3 **Spectral Angle** 0.82

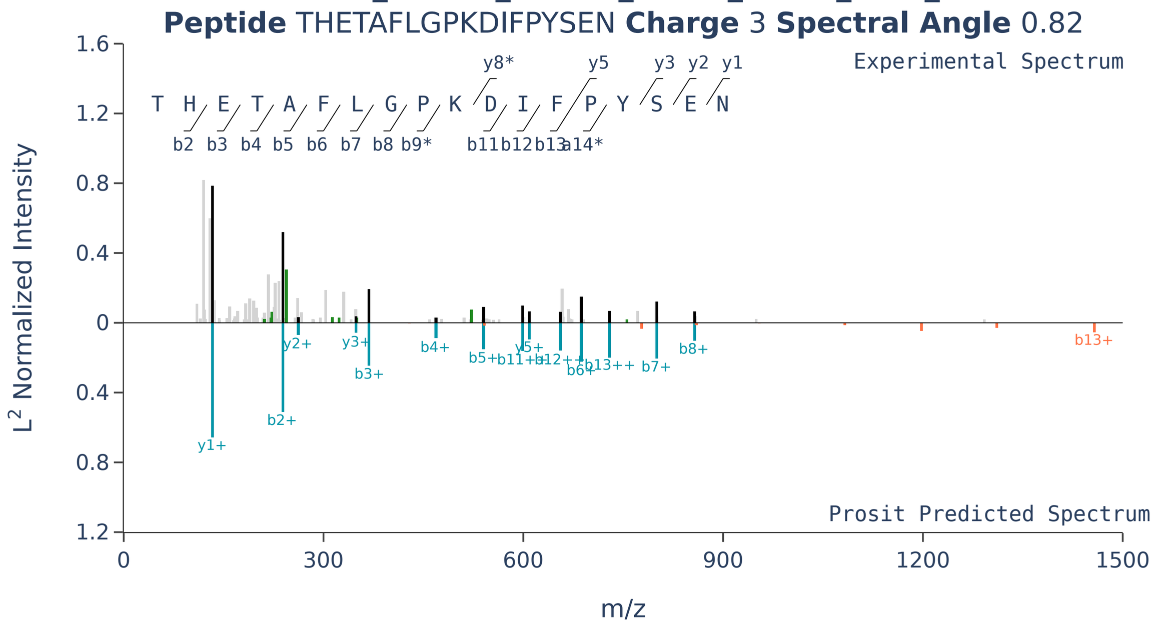


**Source** HRoetschke_271222_090123_HFGoe_LEDGF_b2R1_4h **Scan** 5640
**Peptide** ASTKKKPSSEERETEI **Charge** 2 **Spectral Angle** 0.89

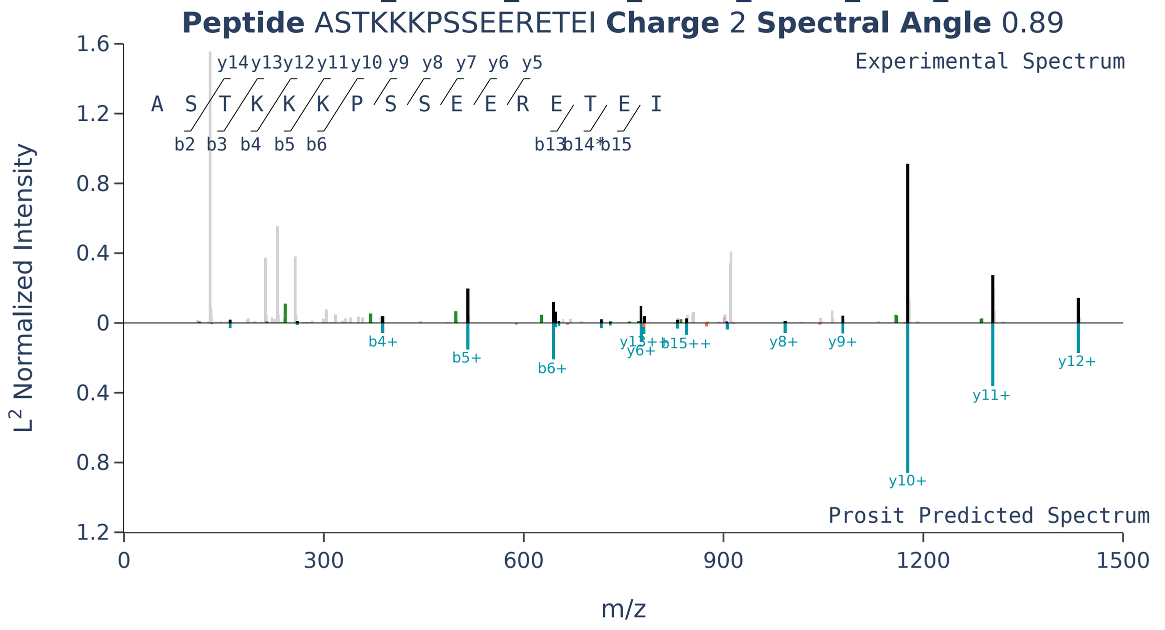


**Source** HRoetschke_271222_090123_HFGoe_LEDGF_b2R1_24h **Scan** 7550
**Peptide** TEVKIPKPRGR **Charge** 3 **Spectral Angle** 0.81

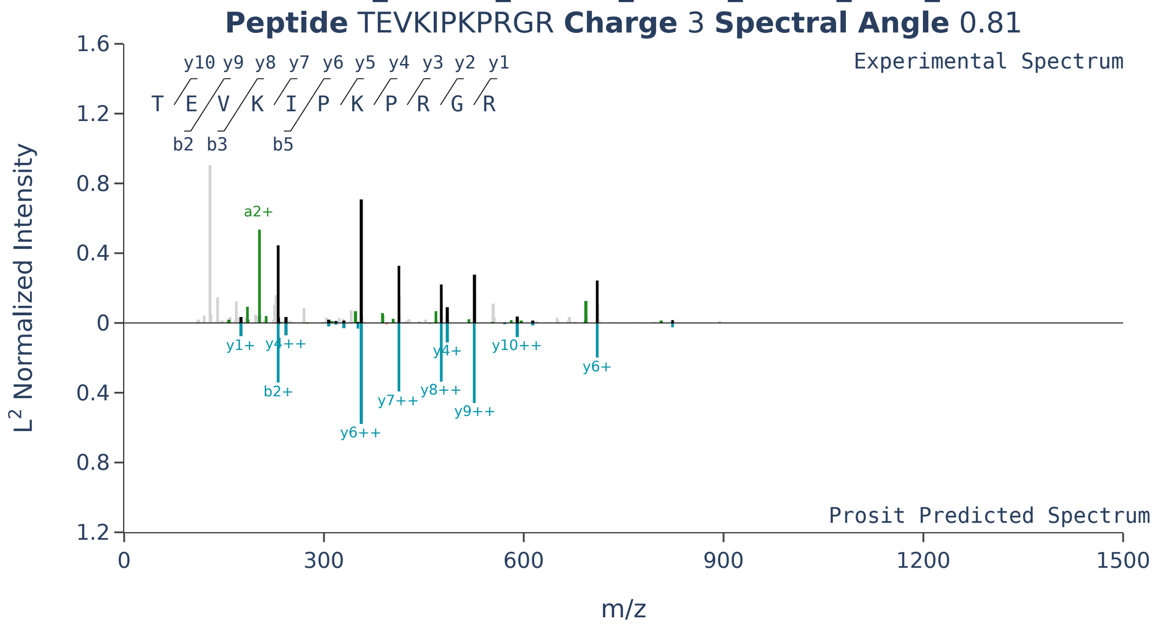


**Source** HRoetschke_271222_090123_HFGoe_LEDGF_b2R2_2h **Scan** 17397
**Peptide** SLQVTMQ **Charge** 1 **Spectral Angle** 0.83

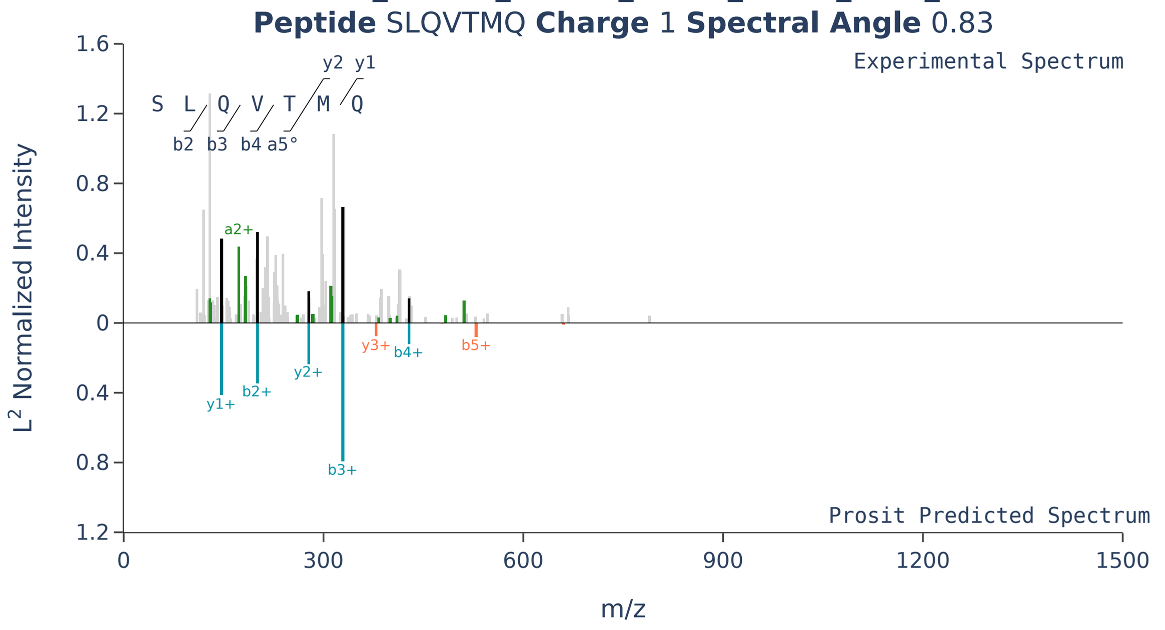


**Source** HRoetschke_271222_090123_HFGoe_LEDGF_b1R1_4h **Scan** 25657
**Peptide** FNEGLWEIDNNPKVKFSSQ **Charge** 3 **Spectral Angle** 0.88

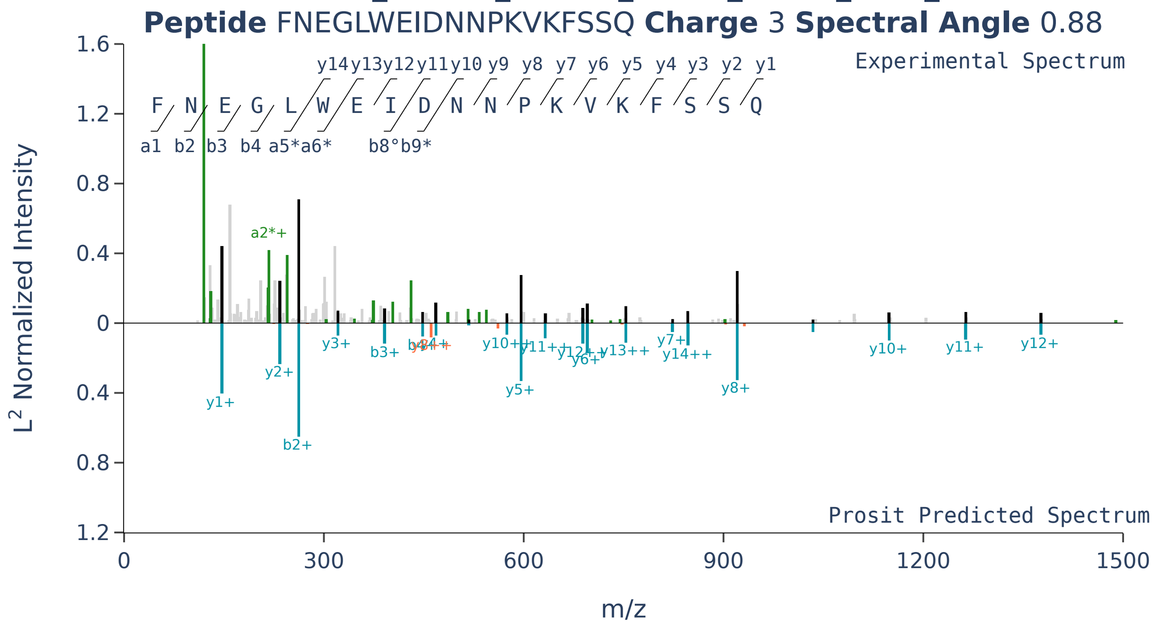


**Source** HRoetschke_271222_090123_HFGoe_LEDGF_b1R1_4h **Scan** 7974
**Peptide** ATKQSNASSDVEVE **Charge** 1 **Spectral Angle** 0.84

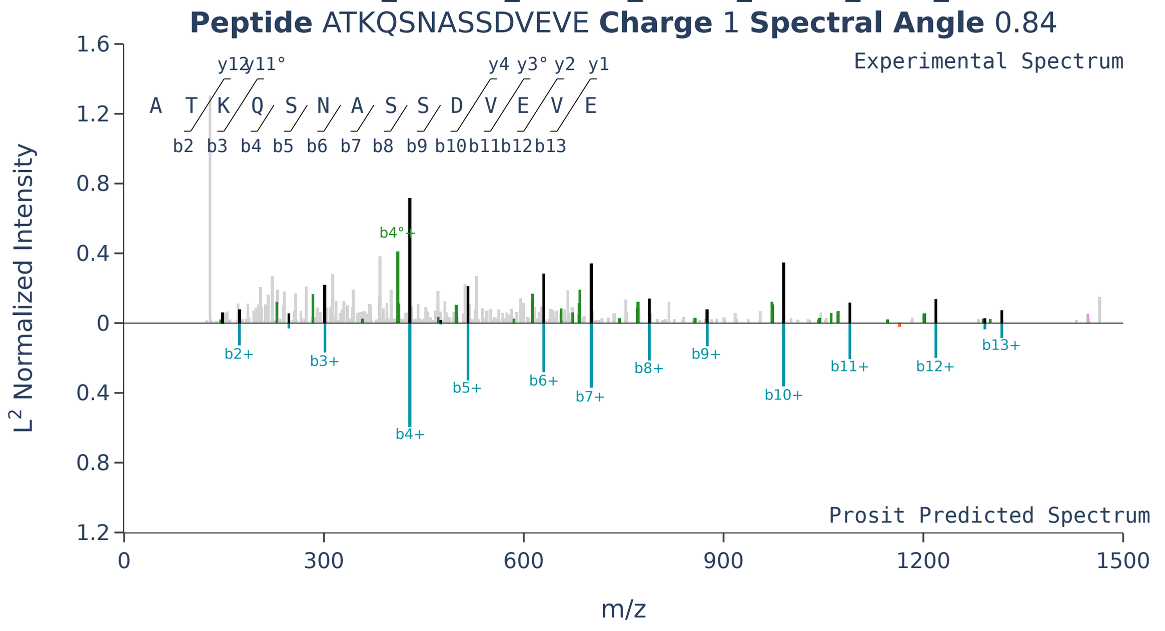


**Source** HRoetschke_271222_090123_HFGoe_LEDGF_b1R1_24h **Scan** 13762
**Peptide** AEIKNSLKID **Charge** 3 **Spectral Angle** 0.84

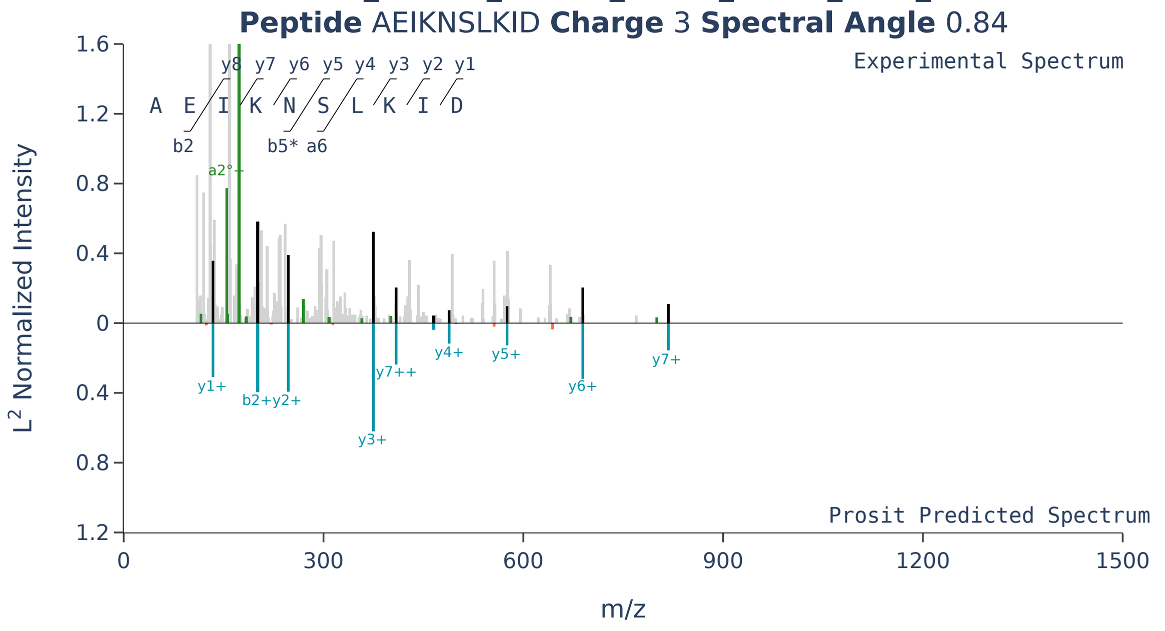


**Source** HRoetschke_271222_090123_HFGoe_LEDGF_b1R1_2h **Scan** 19363
**Peptide** EGDSVITQV **Charge** 1 **Spectral Angle** 0.8

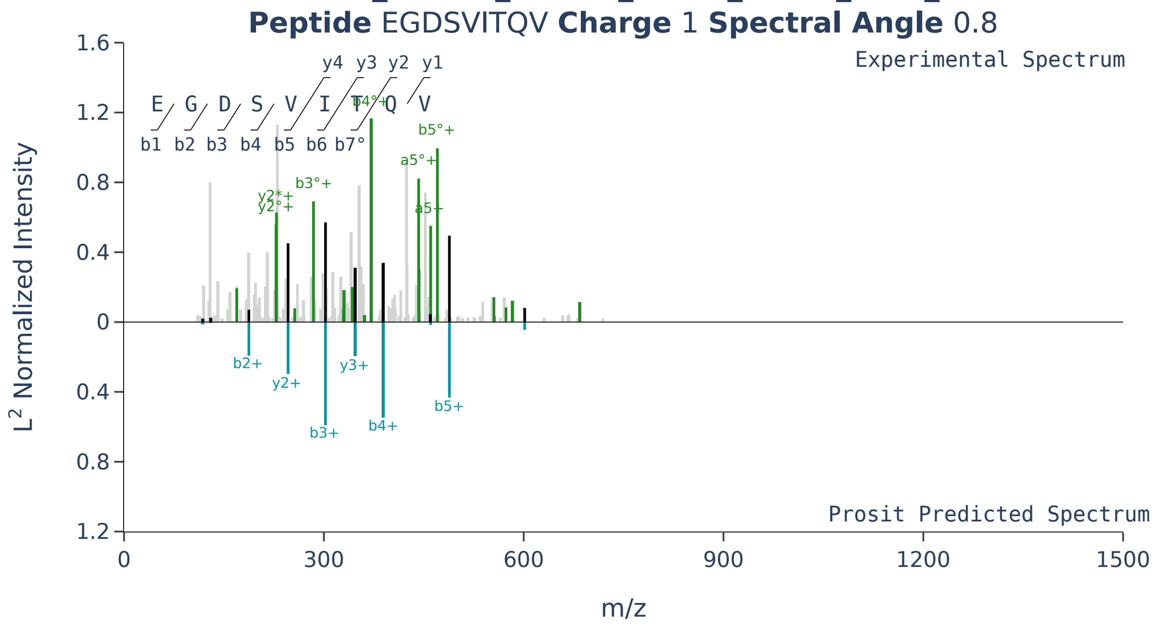


**Source** HRoetschke_271222_090123_HFGoe_LEDGF_b2R1_2h **Scan** 17048
**Peptide** KGYPHWPARVDEVPDG **Charge** 2 **Spectral Angle** 0.84

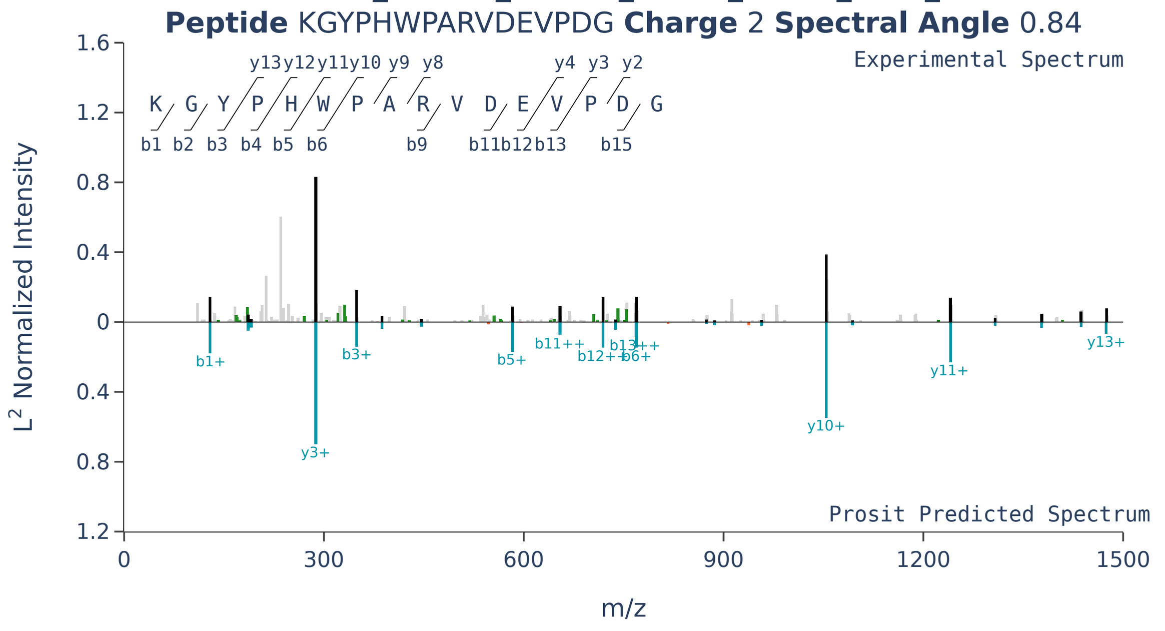


**Source** HRoetschke_271222_090123_HFGoe_LEDGF_b1R2_24h **Scan** 10587
**Peptide** STMLYNK **Charge** 2 **Spectral Angle** 0.9

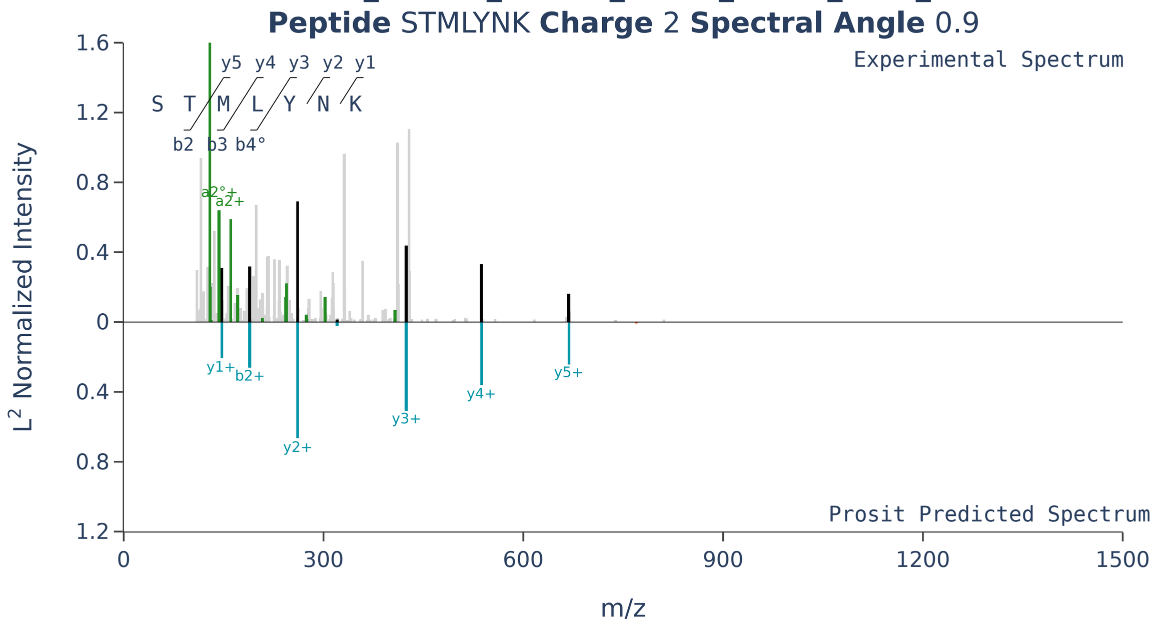


**Source** HRoetschke_271222_090123_HFGoe_LEDGF_b1R1_2h **Scan** 30583
**Peptide** ALDELASLQV **Charge** 1 **Spectral Angle** 0.79

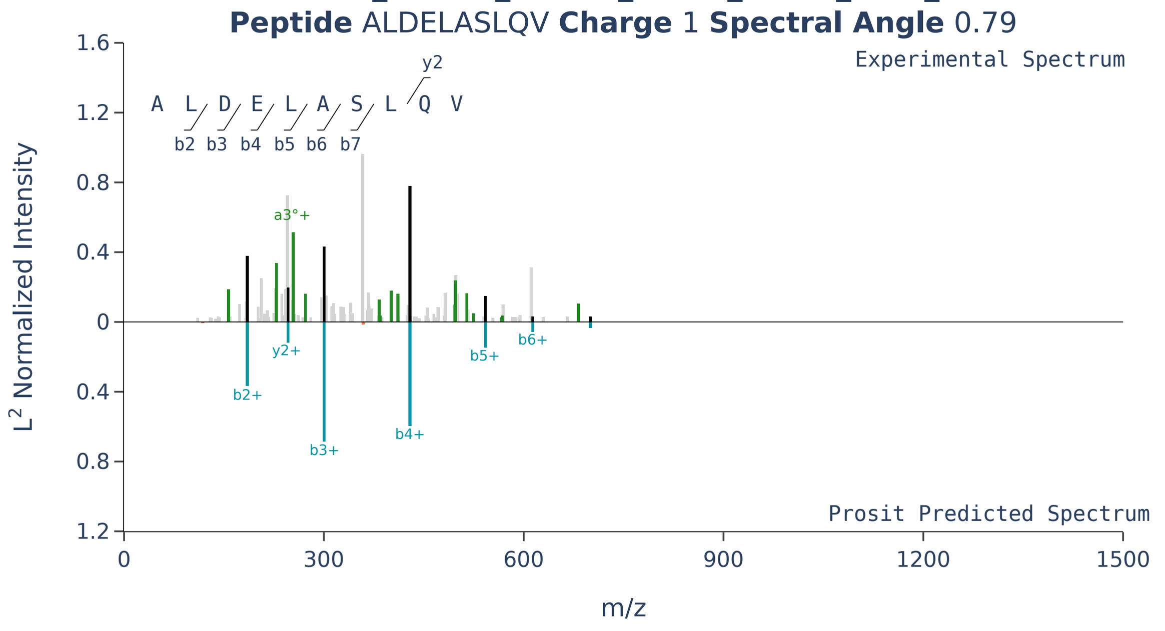


**Source** HRoetschke_271222_090123_HFGoe_LEDGF_b2R2_1h **Scan** 8503
**Peptide** QVIMEKS **Charge** 2 **Spectral Angle** 0.88

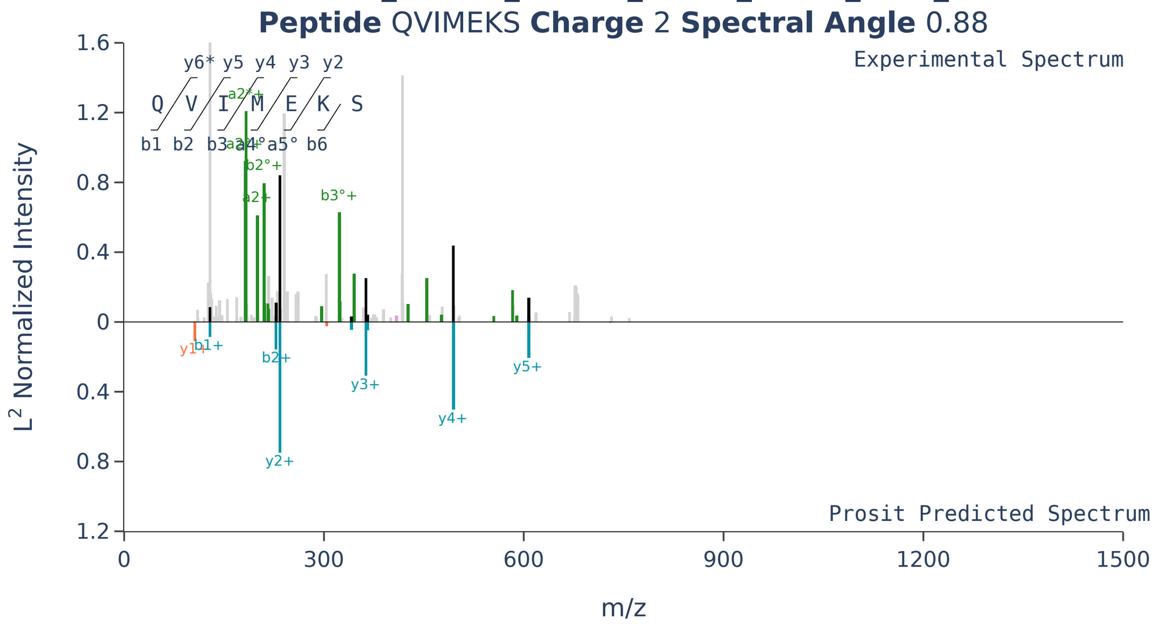


**Source** HRoetschke_271222_090123_HFGoe_LEDGF_b1R2_2h **Scan** 9357
**Peptide** DEVPDGAVK **Charge** 2 **Spectral Angle** 0.87

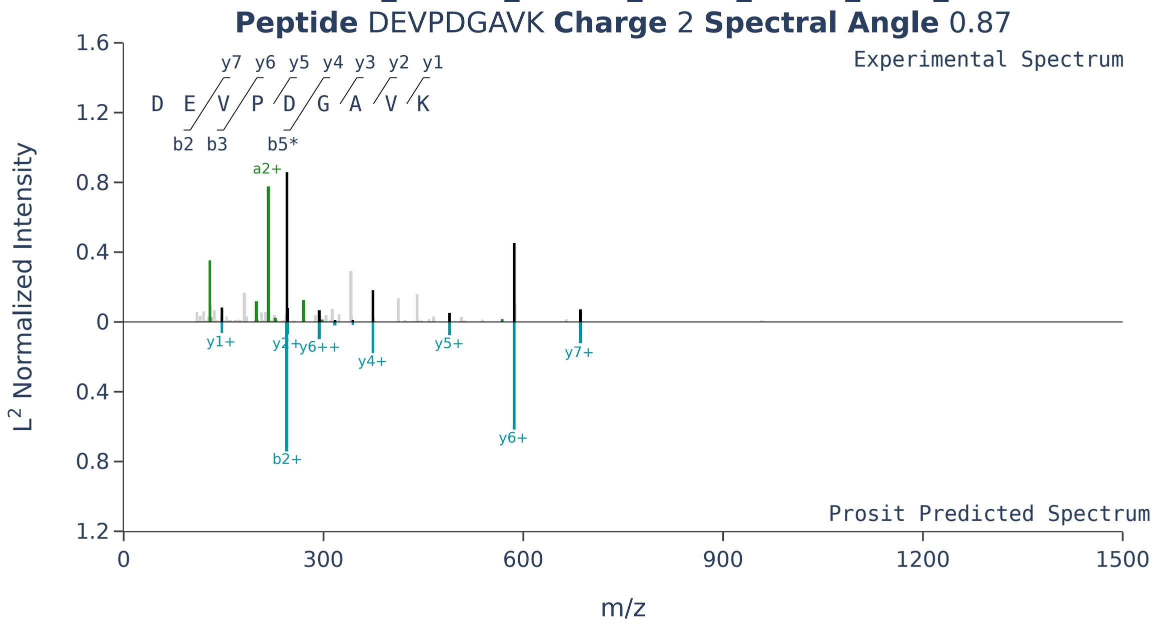

**Source** HRoetschke_271222_090123_HFGoe_LEDGF_b1R1_2h **Scan** 25339
**Peptide** EGDSVITQVLNKSLAEQRQHE **Charge** 3 **Spectral Angle** 0.89

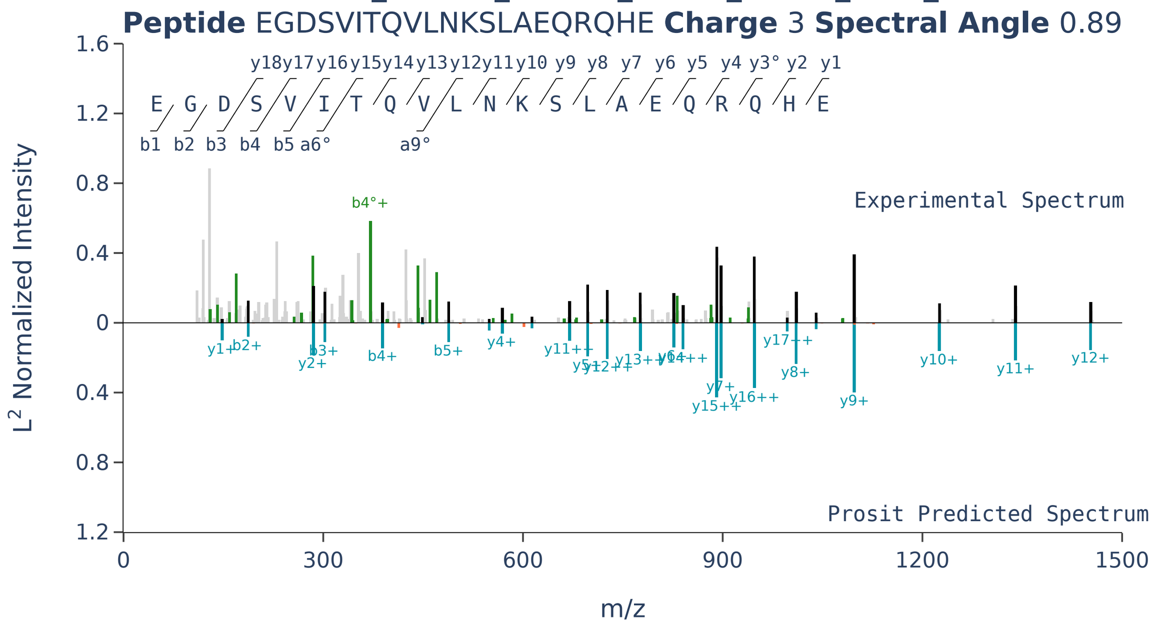


**Source** HRoetschke_271222_090123_HFGoe_LEDGF_b1R1_4h **Scan** 23546
**Peptide** ELASLQV **Charge** 2 **Spectral Angle** 0.79

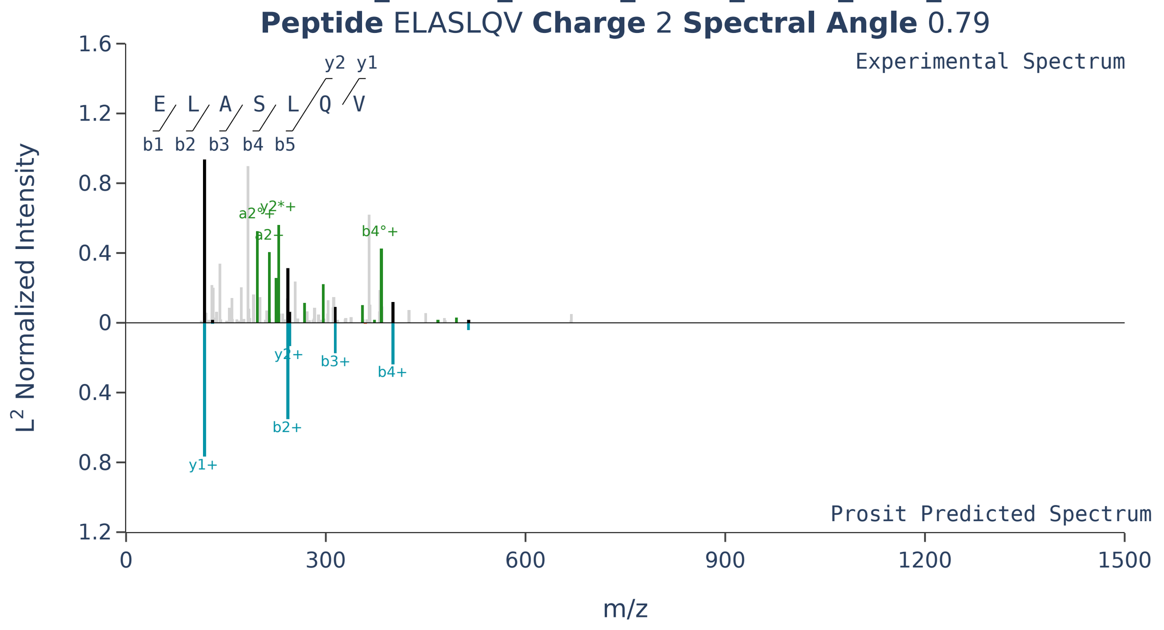


**Source** HRoetschke_271222_090123_HFGoe_LEDGF_b2R1_2h **Scan** 13169
**Peptide** PHWPARVDE **Charge** 2 **Spectral Angle** 0.82

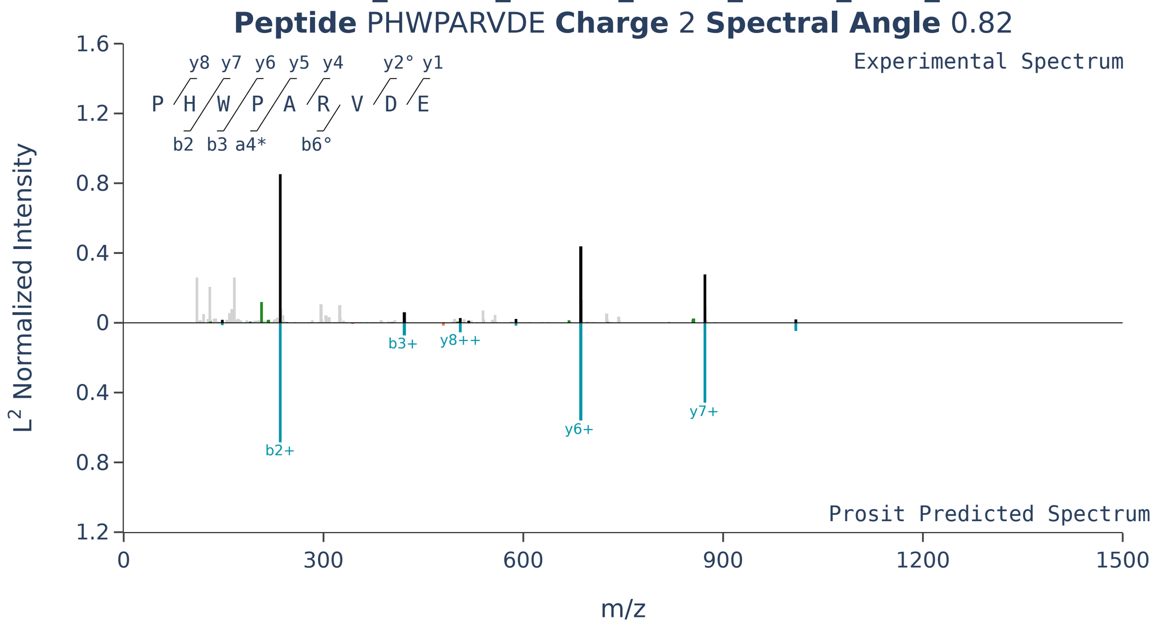


**Source** HRoetschke_271222_090123_HFGoe_LEDGF_b1R1_24h **Scan** 13785
**Peptide** GPGMTRDF **Charge** 2 **Spectral Angle** 0.83

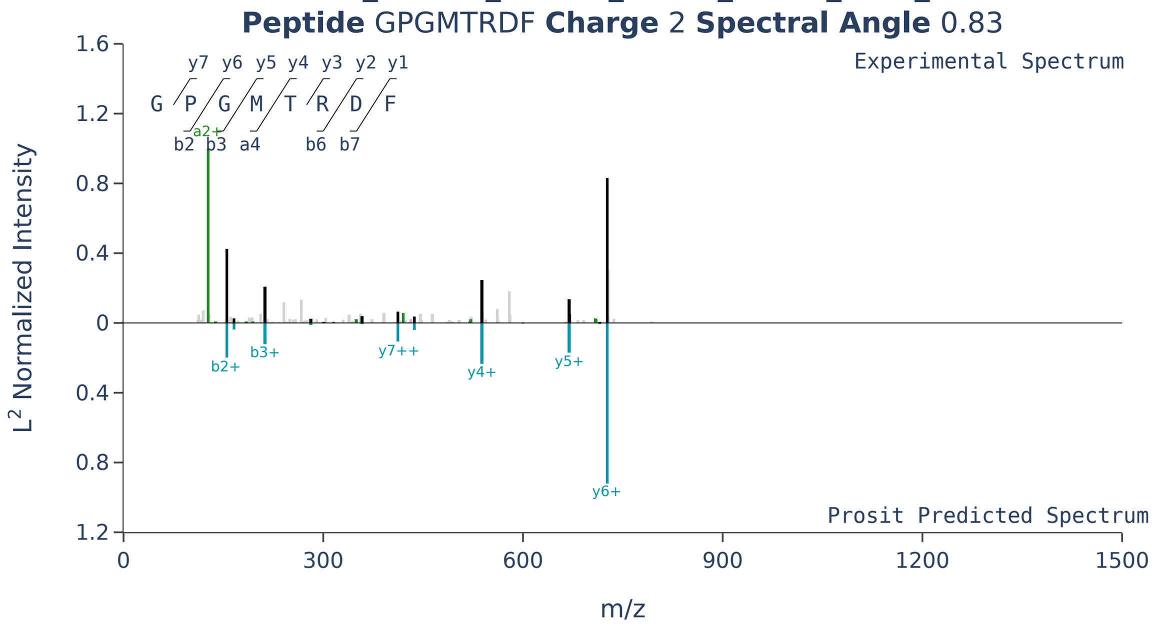


**Source** HRoetschke_271222_090123_HFGoe_LEDGF_b1R1_4h **Scan** 7461
**Peptide** QKHTEMITTLKKIR **Charge** 4 **Spectral Angle** 0.9

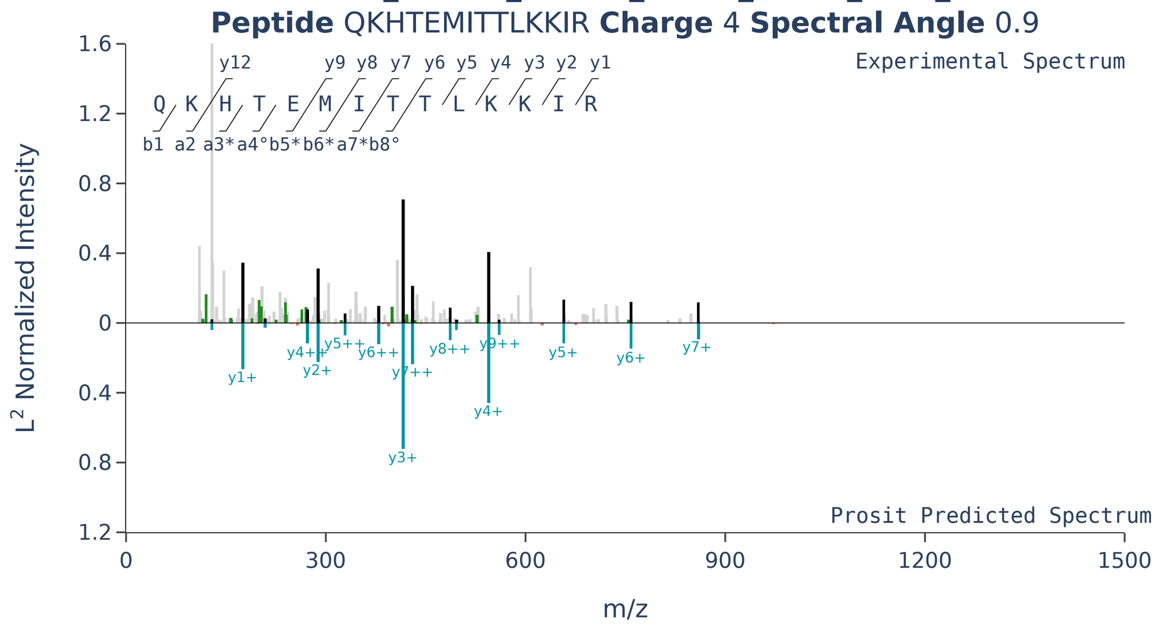


**Source** HRoetschke_271222_090123_HFGoe_LEDGF_b2R2_2h **Scan** 7548
**Peptide** SVNLKVSPKRGRPAAT **Charge** 3 **Spectral Angle** 0.82

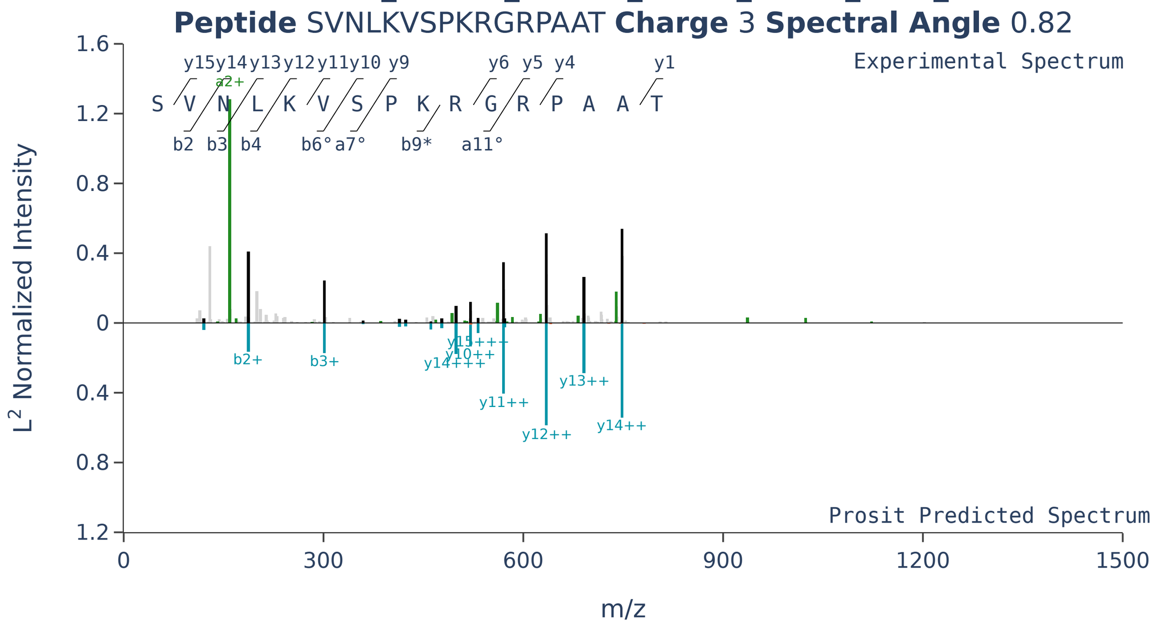


**Source** HRoetschke_271222_090123_HFGoe_LEDGF_b1R2_4h **Scan** 15322
**Peptide** MKGYPHWPARVDE **Charge** 2 **Spectral Angle** 0.9

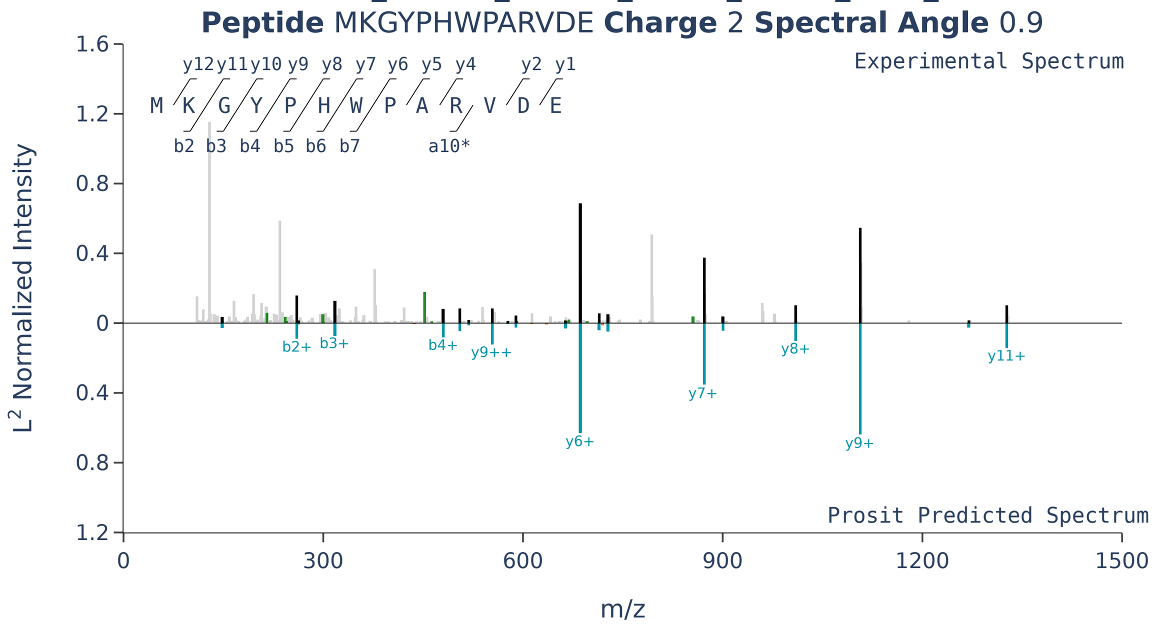


**Source** HRoetschke_271222_090123_HFGoe_LEDGF_b1R2_4h **Scan** 16078
**Peptide** EKSTMLYNKFKNM **Charge** 4 **Spectral Angle** 0.79

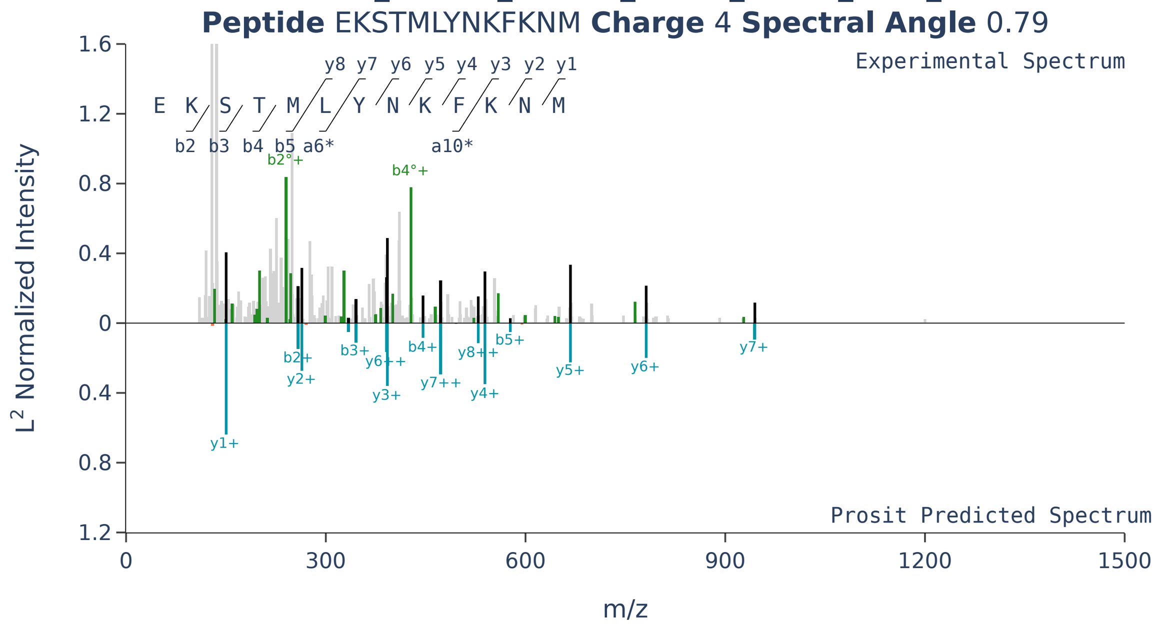


**Source** HRoetschke_271222_090123_HFGoe_LEDGF_b1R1_1h **Scan** 11566
**Peptide** EERETEISLKD **Charge** 2 **Spectral Angle** 0.89

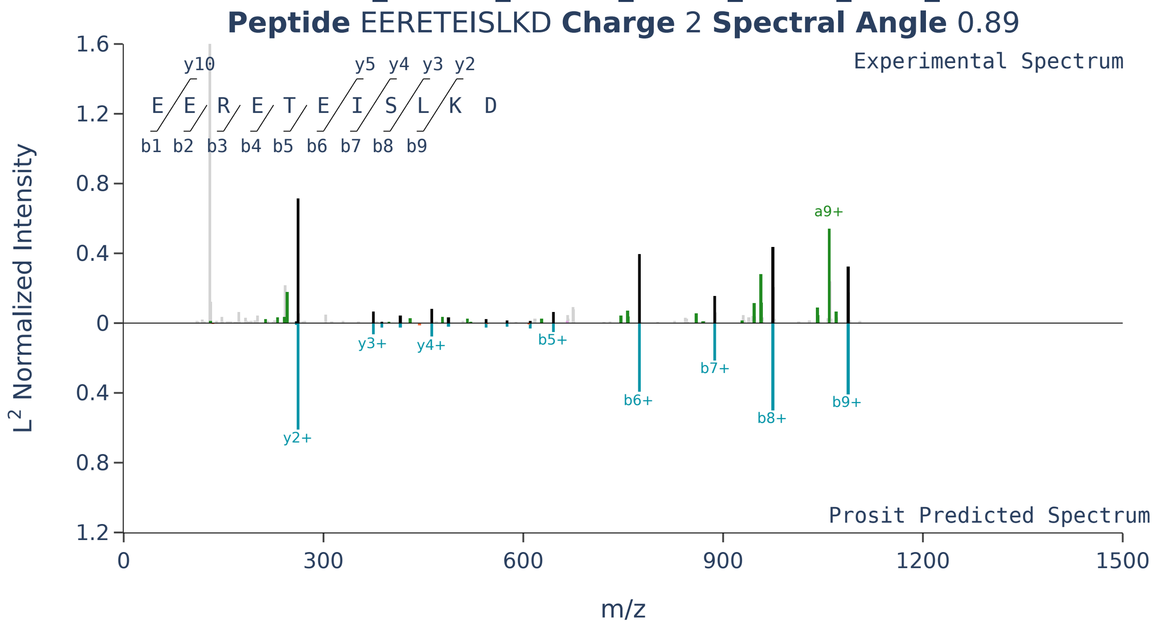


**Source** HRoetschke_271222_090123_HFGoe_LEDGF_b1R2_4h **Scan** 19974
**Peptide** GLWEIDNNPKVKFSSQQAATKQ **Charge** 3 **Spectral Angle** 0.87

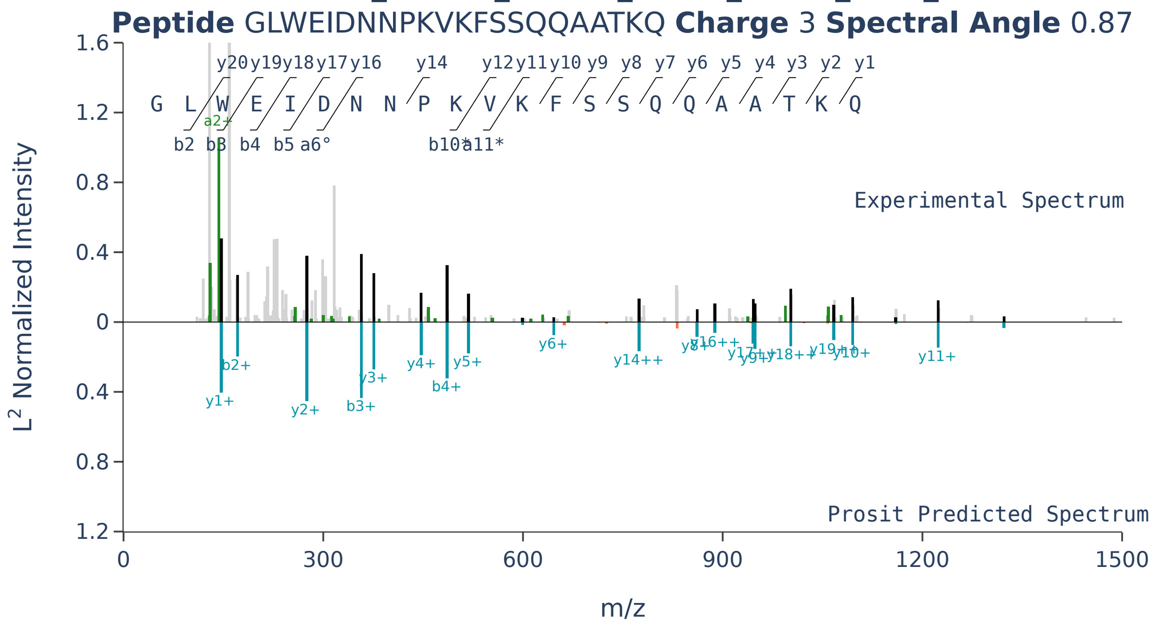


**Source** HRoetschke_271222_090123_HFGoe_LEDGF_b1R2_24h **Scan** 10829
**Peptide** TEEAGVVT **Charge** 1 **Spectral Angle** 0.84

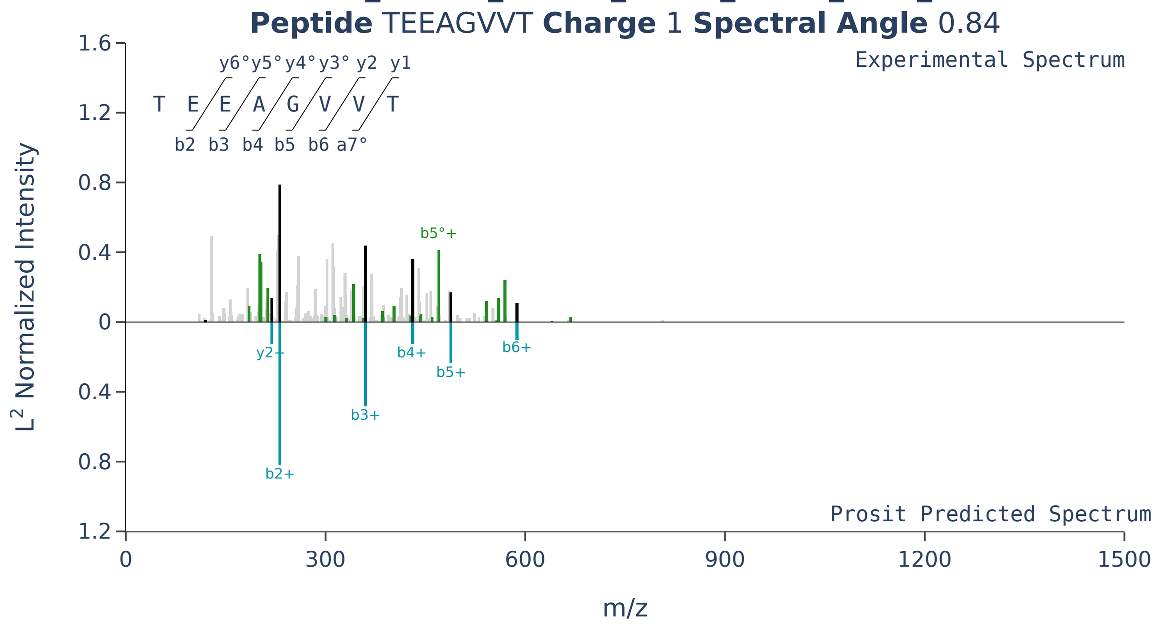


**Source** HRoetschke_271222_090123_HFGoe_LEDGF_b1R1_24h **Scan** 5477
**Peptide** NMLKGQHEKEAADRKRKQEEQME **Charge** 4 **Spectral Angle** 0.86

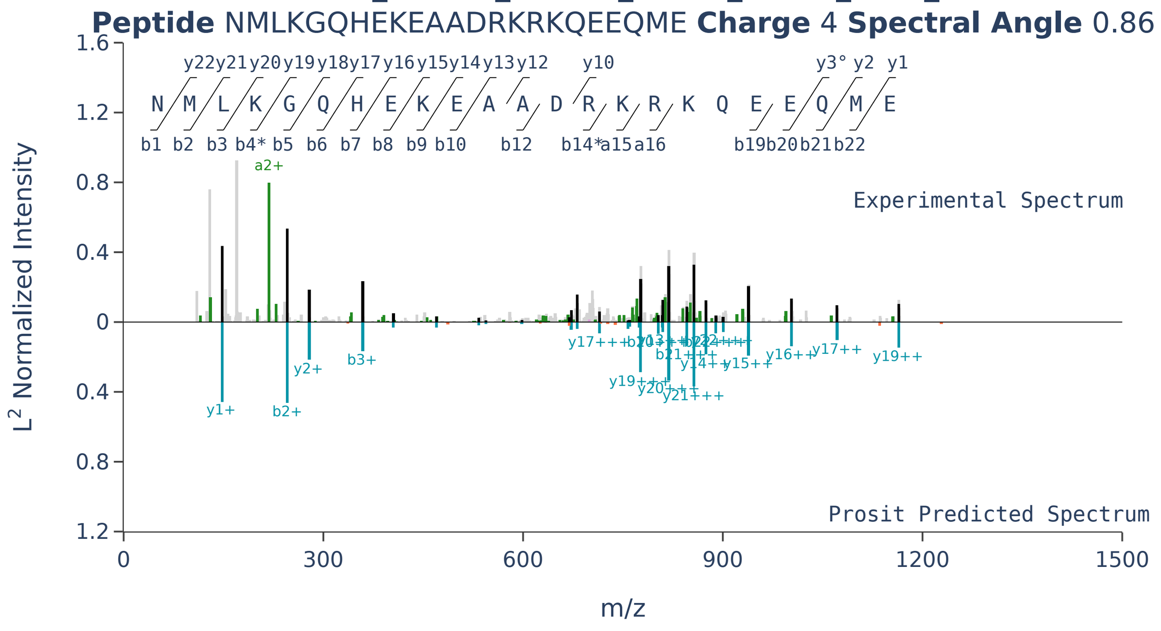


**Source** HRoetschke_271222_090123_HFGoe_LEDGF_b1R1_4h **Scan** 23554
**Peptide** LASLQVTMQQA **Charge** 1 **Spectral Angle** 0.81

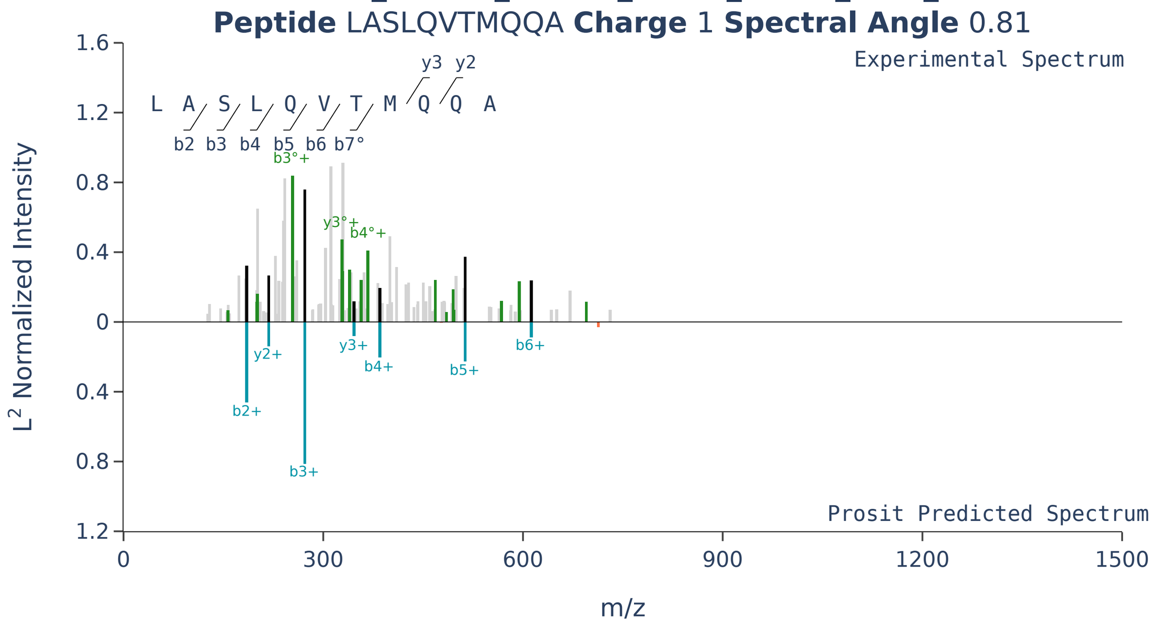


**Source** HRoetschke_271222_090123_HFGoe_LEDGF_b1R2_4h **Scan** 10320
**Peptide** HTEMITTLKKIRR **Charge** 4 **Spectral Angle** 0.86

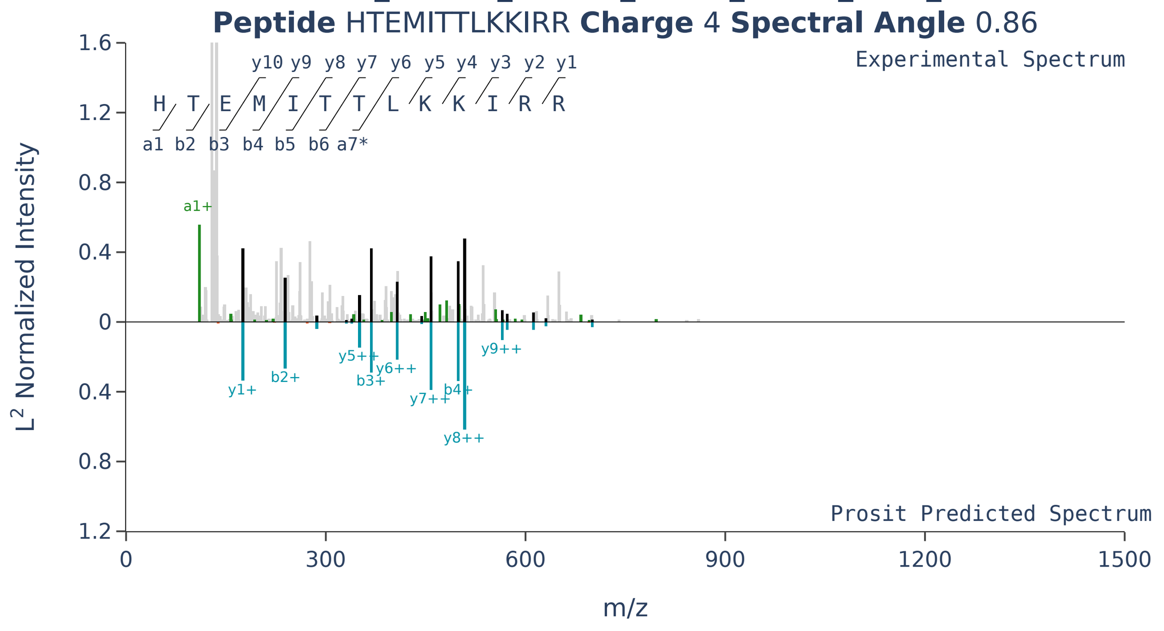


**Source** HRoetschke_271222_090123_HFGoe_LEDGF_b1R1_24h **Scan** 7131
**Peptide** VNLKVSPKRGRPAA **Charge** 4 **Spectral Angle** 0.91

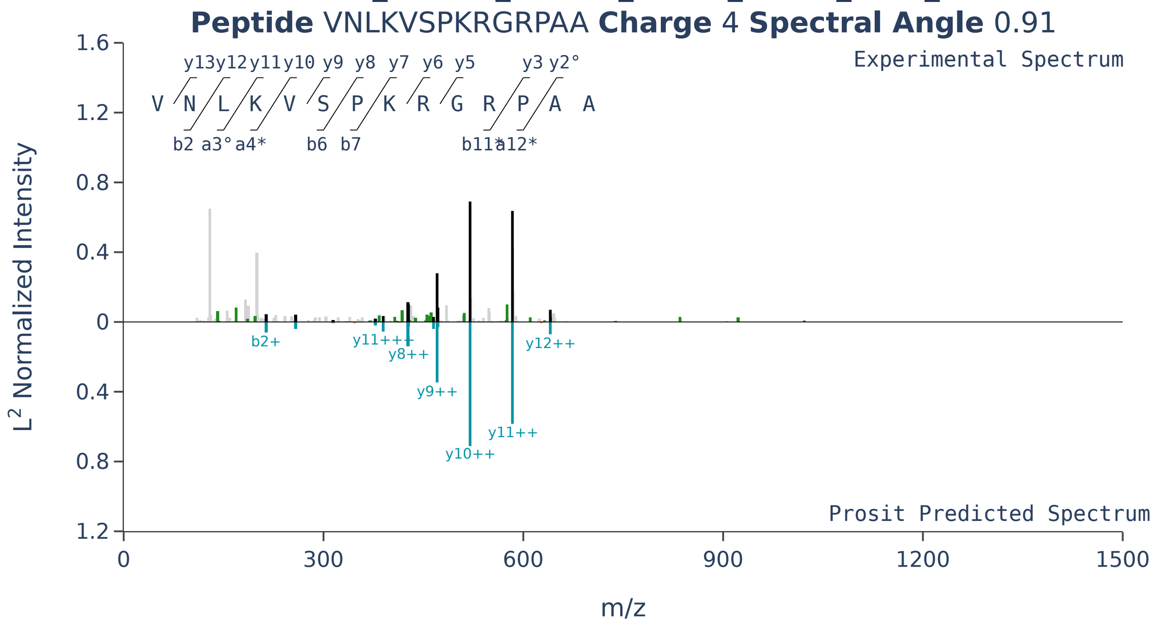

**Source** HRoetschke_271222_090123_HFGoe_LEDGF_b2R2_24h **Scan** 10446
**Peptide** RLQRIHAEI **Charge** 3 **Spectral Angle** 0.85

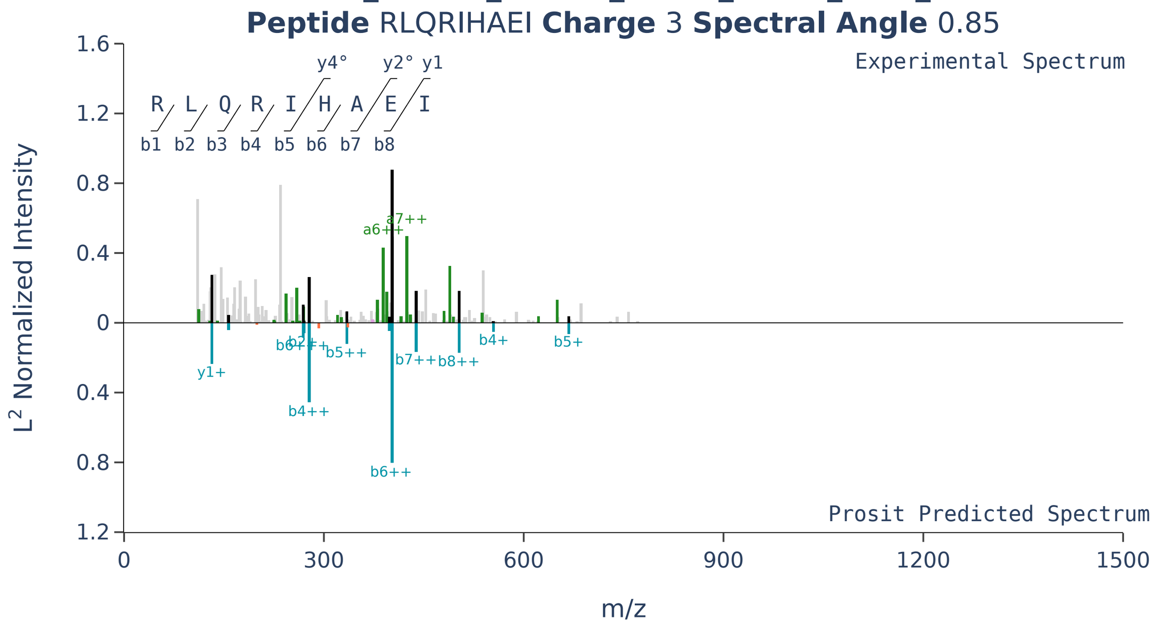


**Source** HRoetschke_271222_090123_HFGoe_LEDGF_b2R1_4h **Scan** 5708
**Peptide** LNKSLAEQRQHE **Charge** 2 **Spectral Angle** 0.88

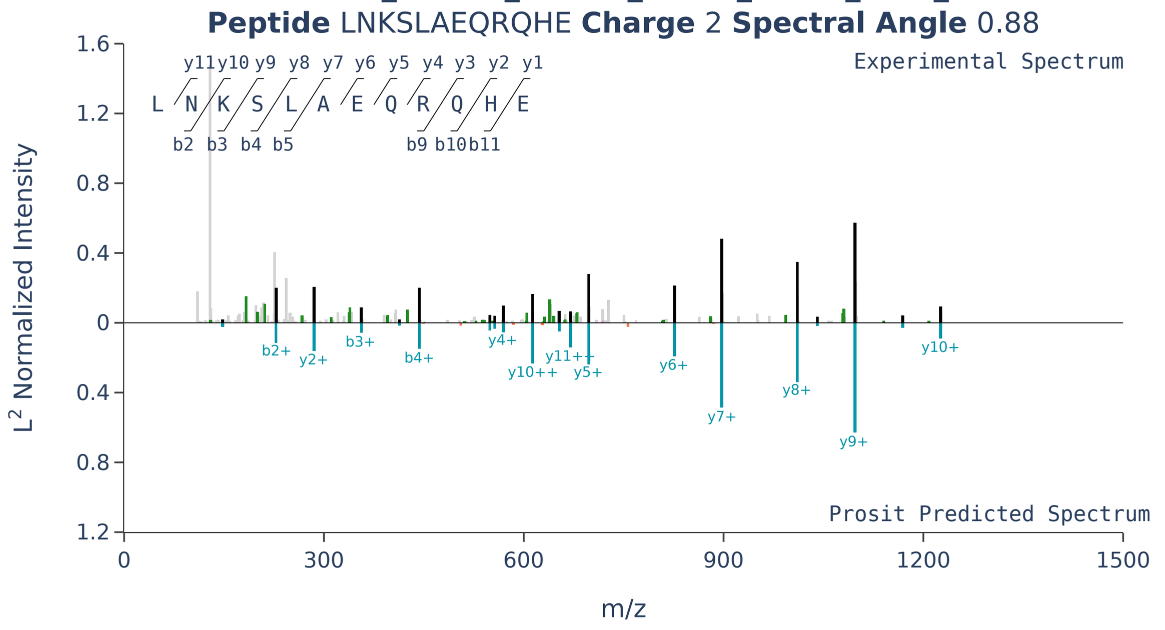


**Source** HRoetschke_271222_090123_HFGoe_LEDGF_b1R2_2h **Scan** 13808
**Peptide** VPDGAVKPPTNKL **Charge** 2 **Spectral Angle** 0.81

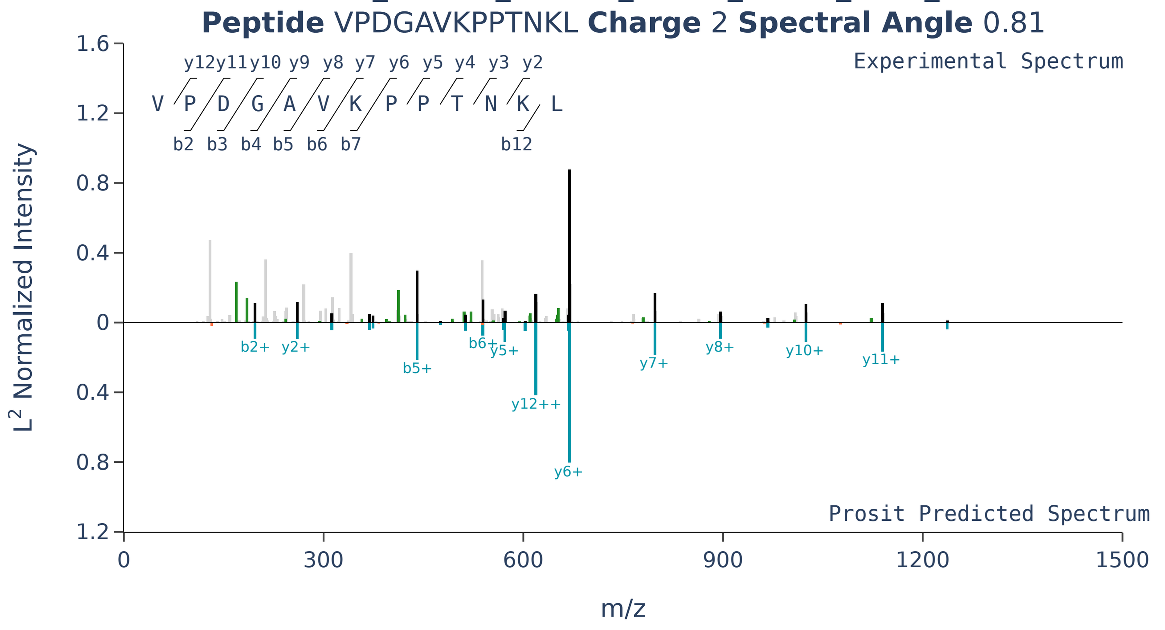


**Source** HRoetschke_271222_090123_HFGoe_LEDGF_b1R2_4h **Scan** 15890
**Peptide** IFPYSENKE **Charge** 2 **Spectral Angle** 0.77

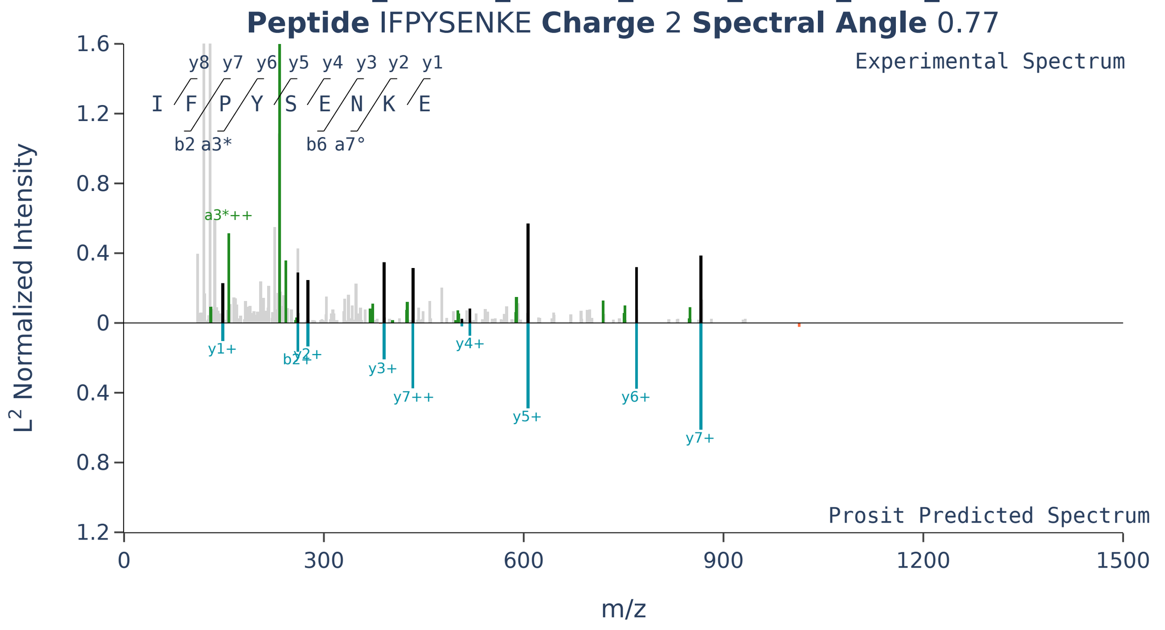


**Source** HRoetschke_271222_090123_HFGoe_LEDGF_b2R1_4h **Scan** 6571
**Peptide** QVTMQQAQK **Charge** 2 **Spectral Angle** 0.77

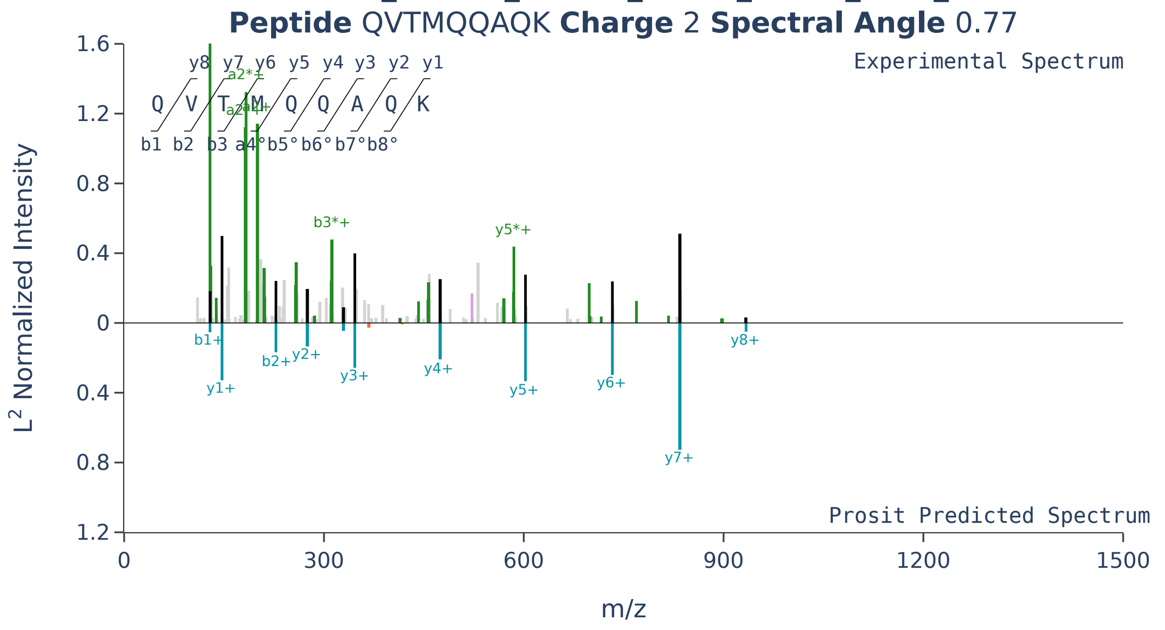


**Source** HRoetschke_271222_090123_HFGoe_LEDGF_b1R1_4h **Scan** 15172
**Peptide** ISLKDSTLDN **Charge** 1 **Spectral Angle** 0.91

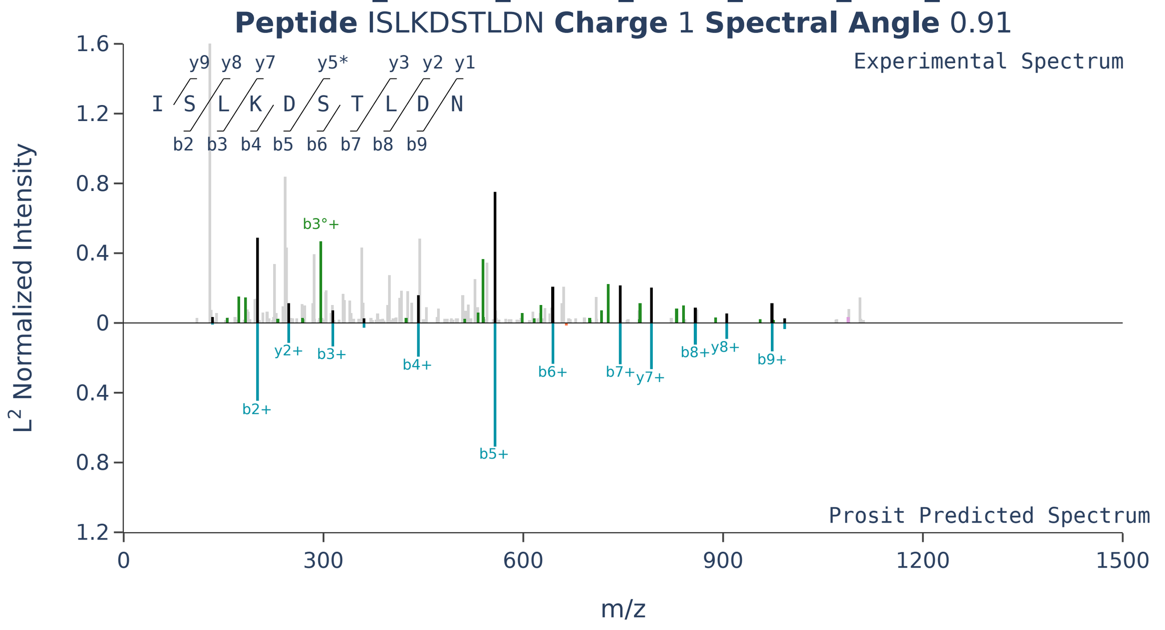


**Source** HRoetschke_271222_090123_HFGoe_LEDGF_b2R1_1h **Scan** 7739
**Peptide** ASSDVEVEEKE **Charge** 2 **Spectral Angle** 0.8

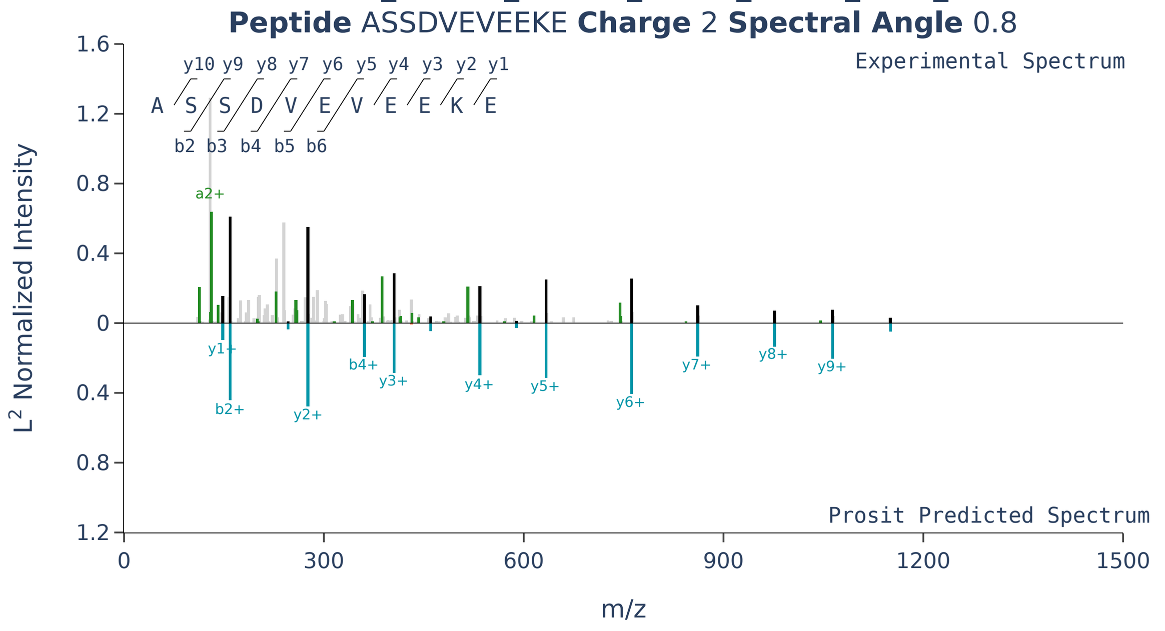


**Source** HRoetschke_271222_090123_HFGoe_LEDGF_b2R2_2h **Scan** 16077
**Peptide** PKDIFPYSENKE **Charge** 2 **Spectral Angle** 0.84

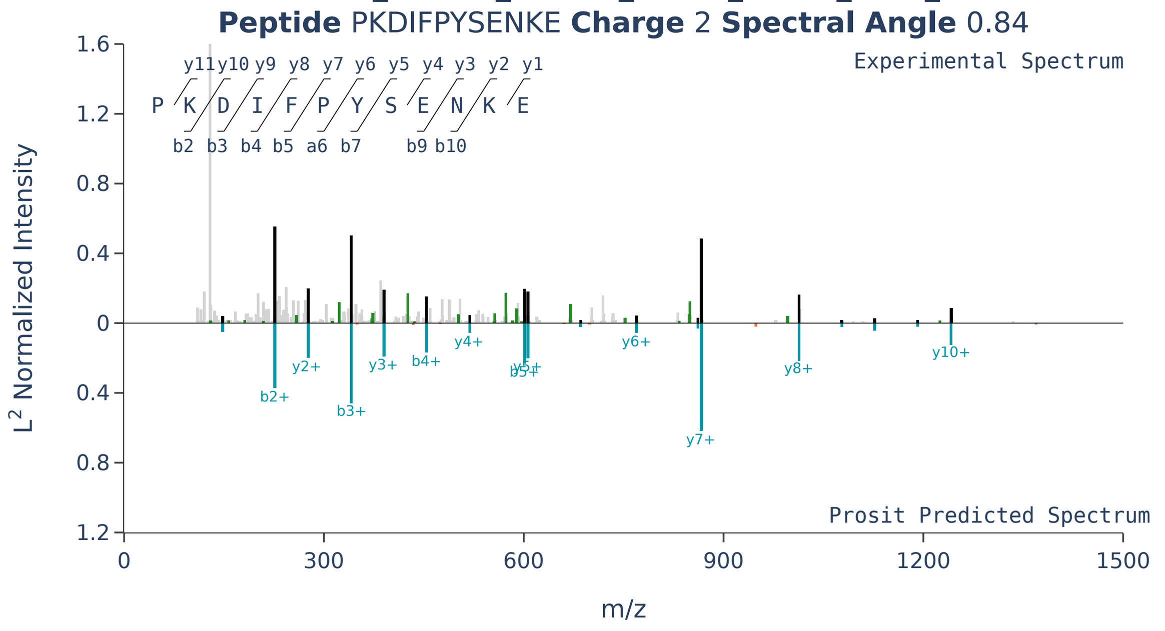


**Source** HRoetschke_271222_090123_HFGoe_LEDGF_b1R2_24h **Scan** 10359
**Peptide** GAVKPPTNKL **Charge** 2 **Spectral Angle** 0.89

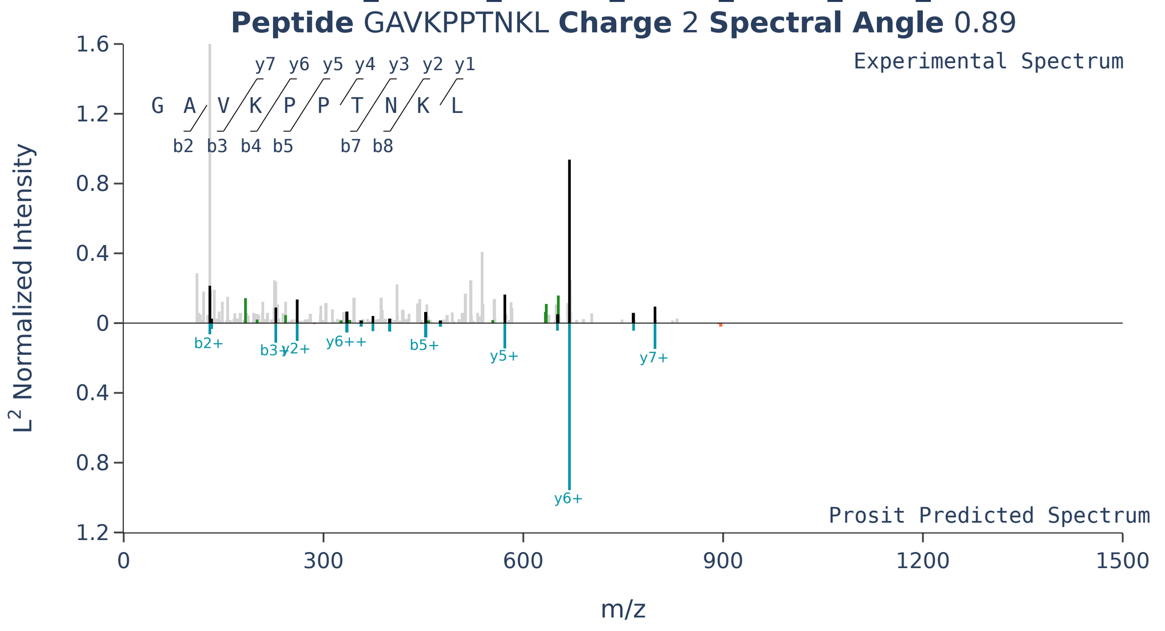


**Source** HRoetschke_271222_090123_HFGoe_LEDGF_b1R1_2h **Scan** 31084
**Peptide** KLPIFFFGTHE **Charge** 3 **Spectral Angle** 0.79

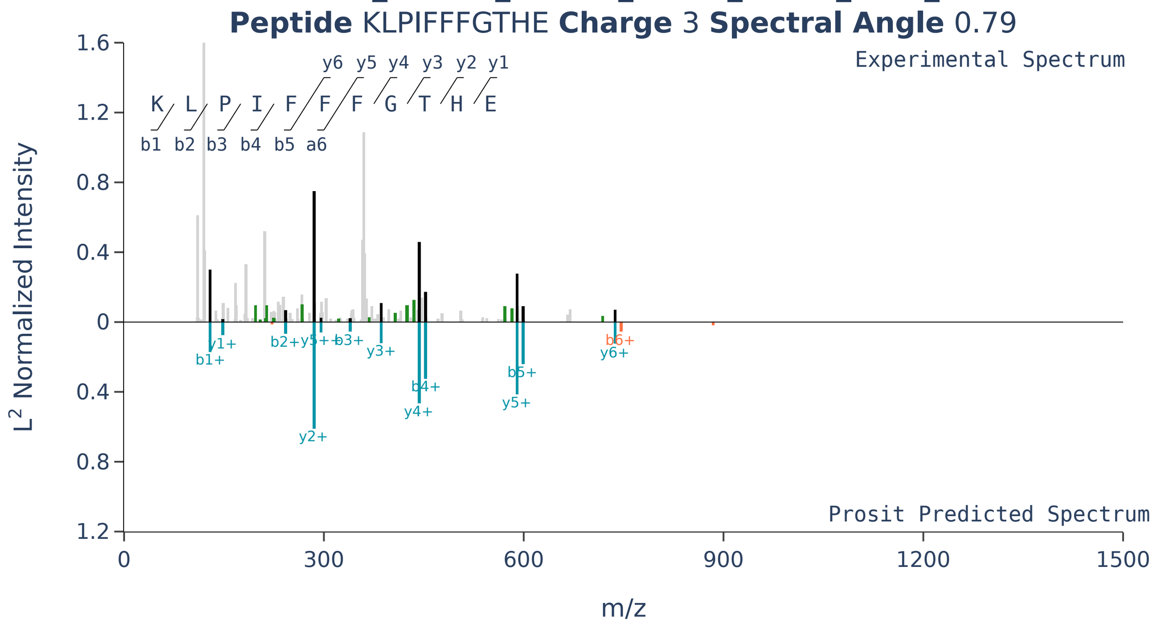


**Source** HRoetschke_271222_090123_HFGoe_LEDGF_b1R1_2h **Scan** 12730
**Peptide** IMEKSTMLYNK **Charge** 3 **Spectral Angle** 0.84

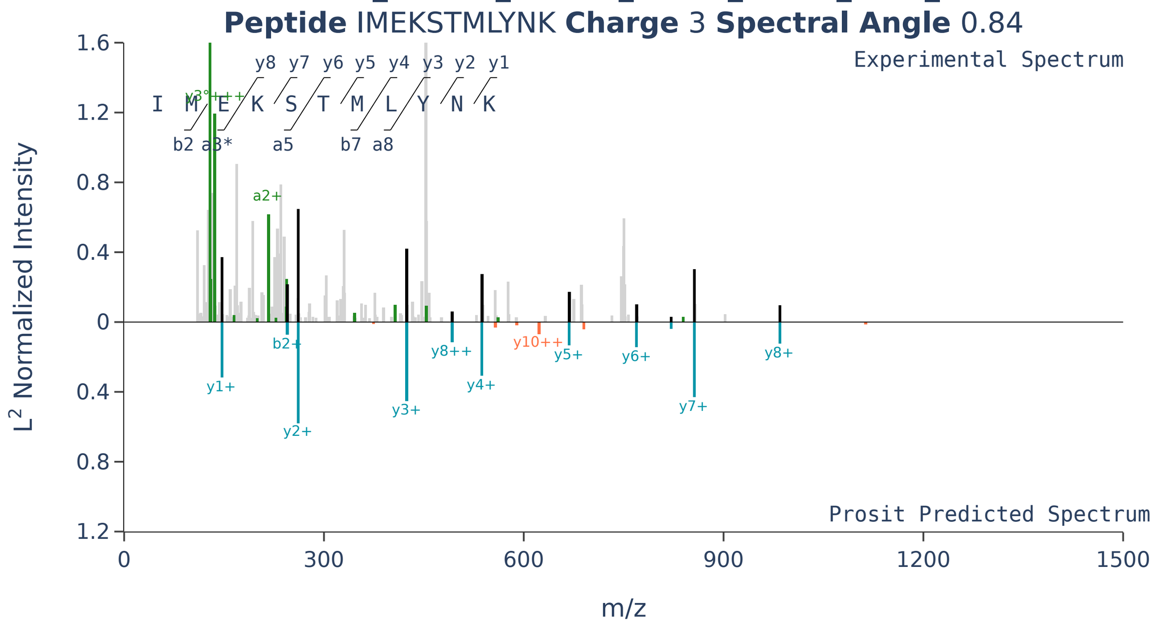


**Source** HRoetschke_271222_090123_HFGoe_LEDGF_b1R2_4h **Scan** 7700
**Peptide** SVNLKVSPKRGRPAATE **Charge** 3 **Spectral Angle** 0.86

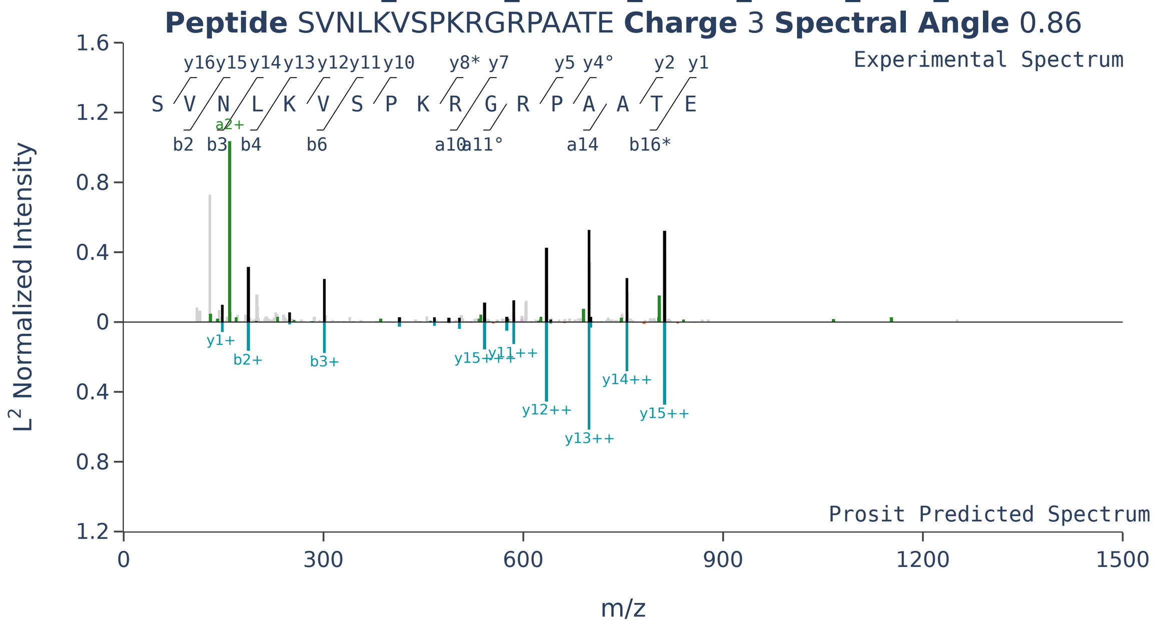


**Source** HRoetschke_271222_090123_HFGoe_LEDGF_b2R1_4h **Scan** 17992
**Peptide** EERETEISL **Charge** 1 **Spectral Angle** 0.88

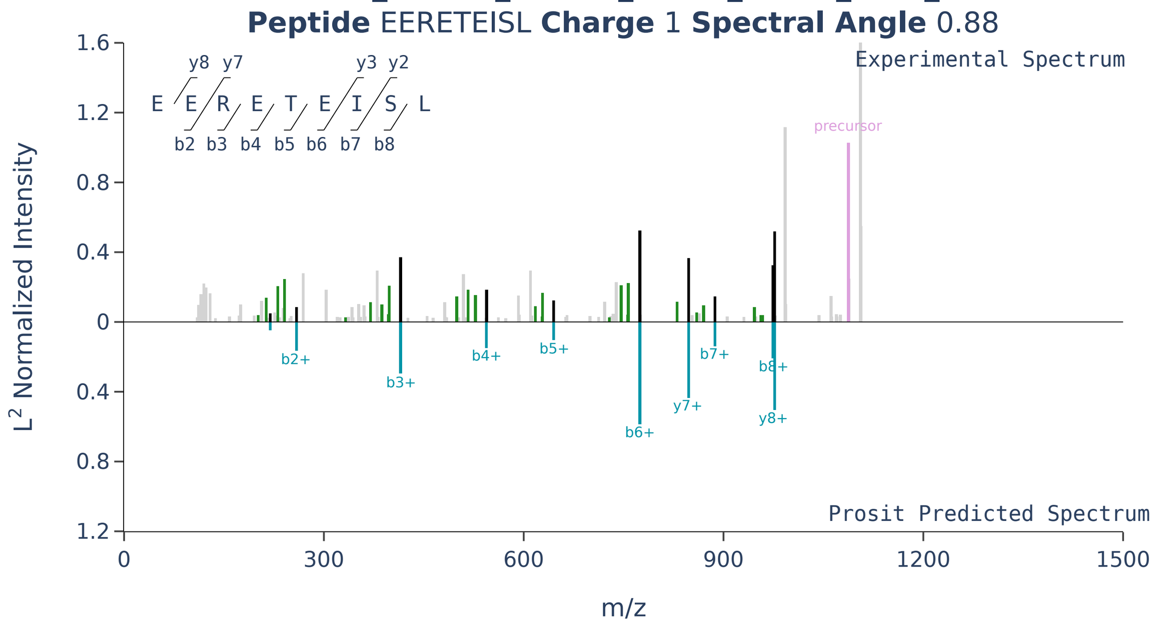


**Source** HRoetschke_271222_090123_HFGoe_LEDGF_b1R1_4h **Scan** 19519
**Peptide** GLWEIDNNPKVK **Charge** 3 **Spectral Angle** 0.84

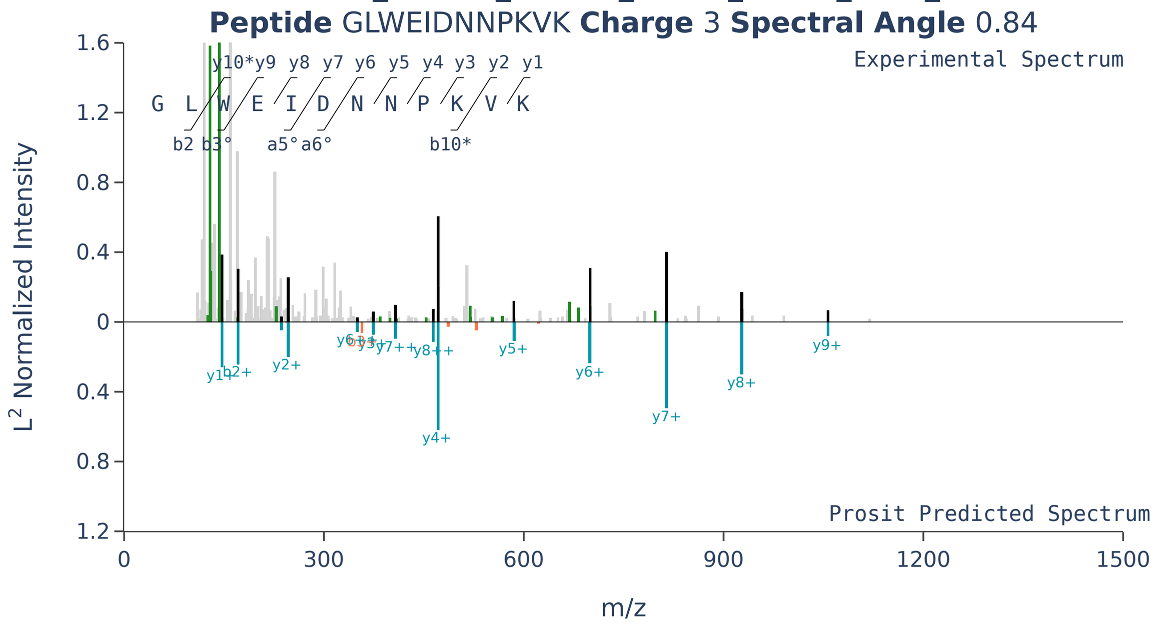


**Source** HRoetschke_271222_090123_HFGoe_LEDGF_b1R2_24h **Scan** 12358
**Peptide** EEAGVVTTAT **Charge** 2 **Spectral Angle** 0.84

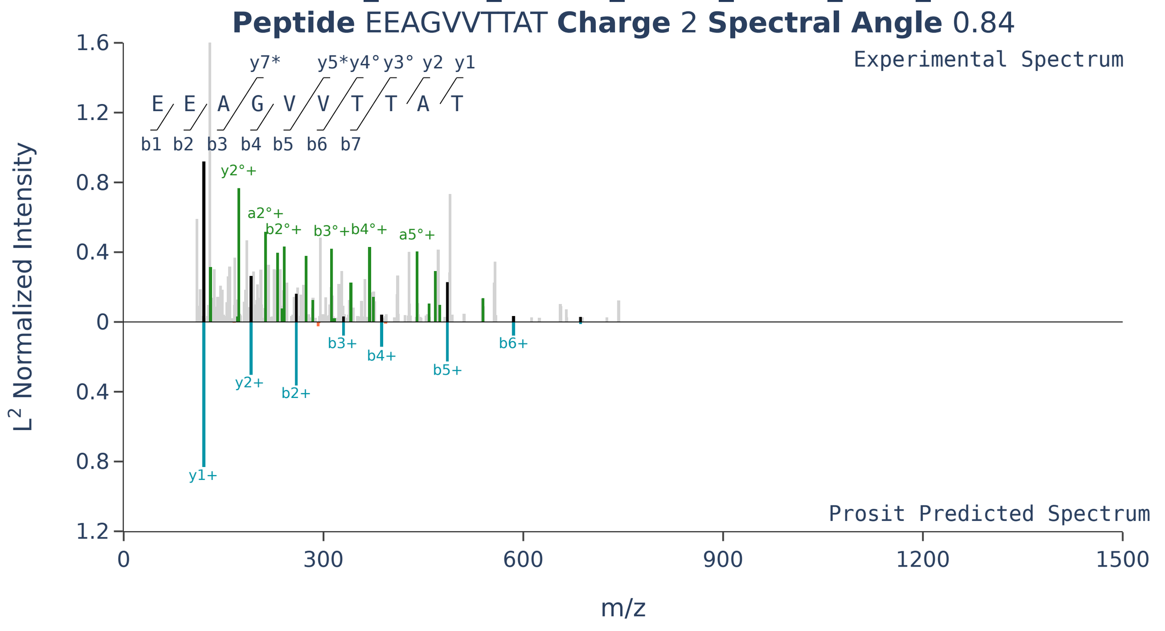

**Source** HRoetschke_271222_090123_HFGoe_LEDGF_b2R1_24h **Scan** 9488
**Peptide** PAATEVKIPKPRGR **Charge** 4 **Spectral Angle** 0.85

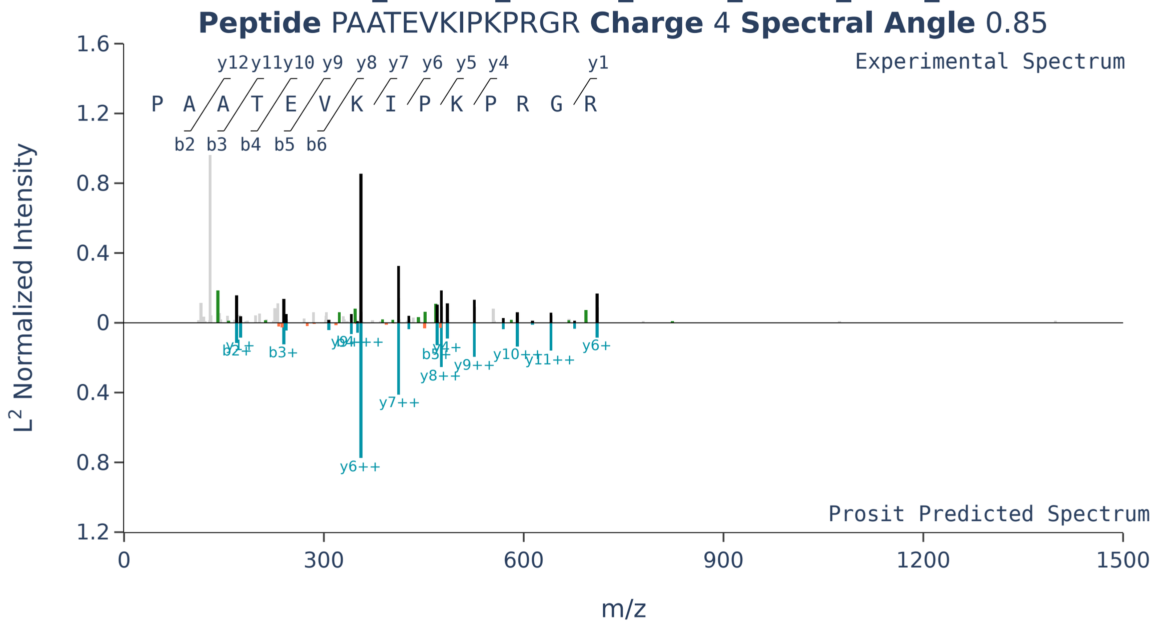


**Source** HRoetschke_271222_090123_HFGoe_LEDGF_b2R1_1h **Scan** 12219
**Peptide** HAEIKNSLKI **Charge** 3 **Spectral Angle** 0.89

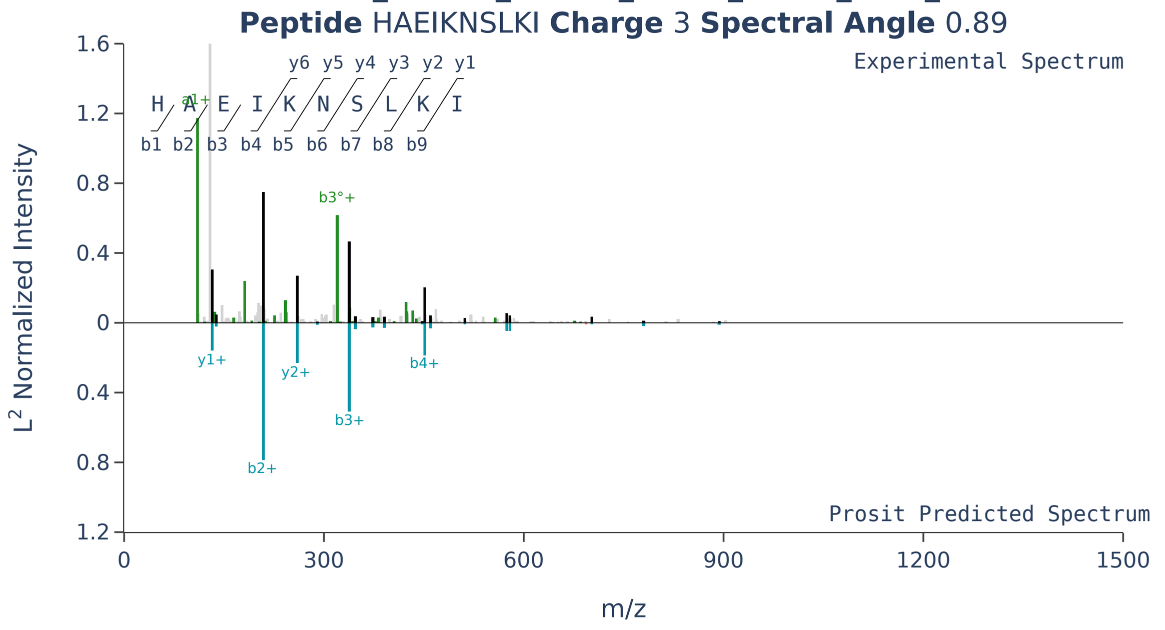


**Source** HRoetschke_271222_090123_HFGoe_LEDGF_b2R1_24h **Scan** 14922
**Peptide** VDEVPDGAVKPPTN **Charge** 2 **Spectral Angle** 0.88

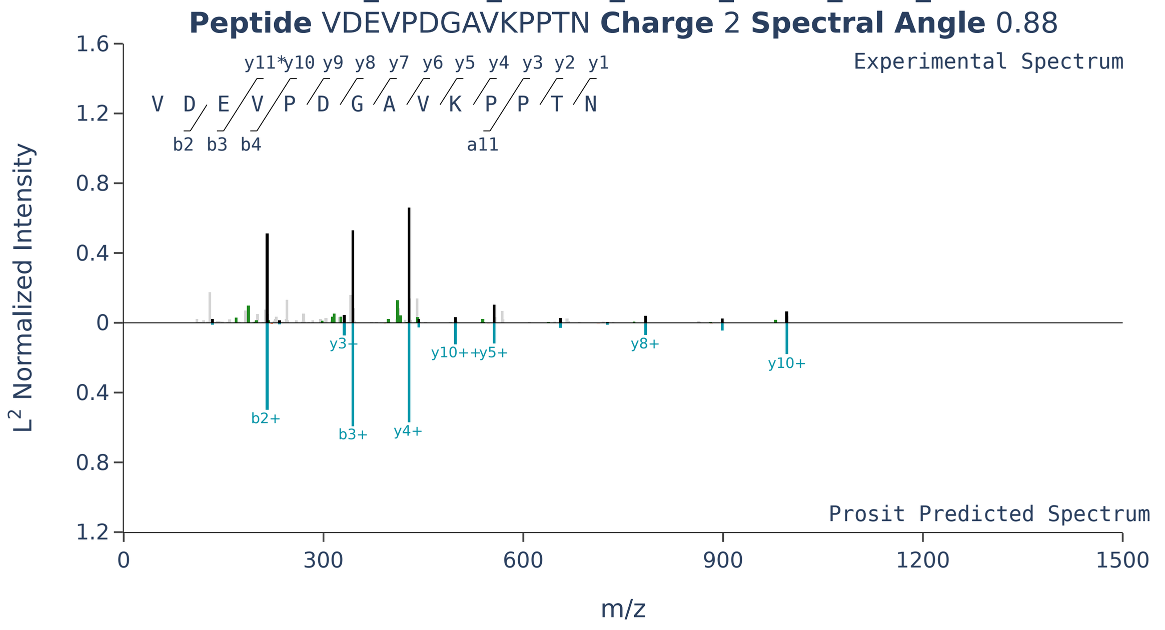


**Source** HRoetschke_271222_090123_HFGoe_LEDGF_b1R1_4h **Scan** 15812
**Peptide** MKGYPHWPARV **Charge** 3 **Spectral Angle** 0.93

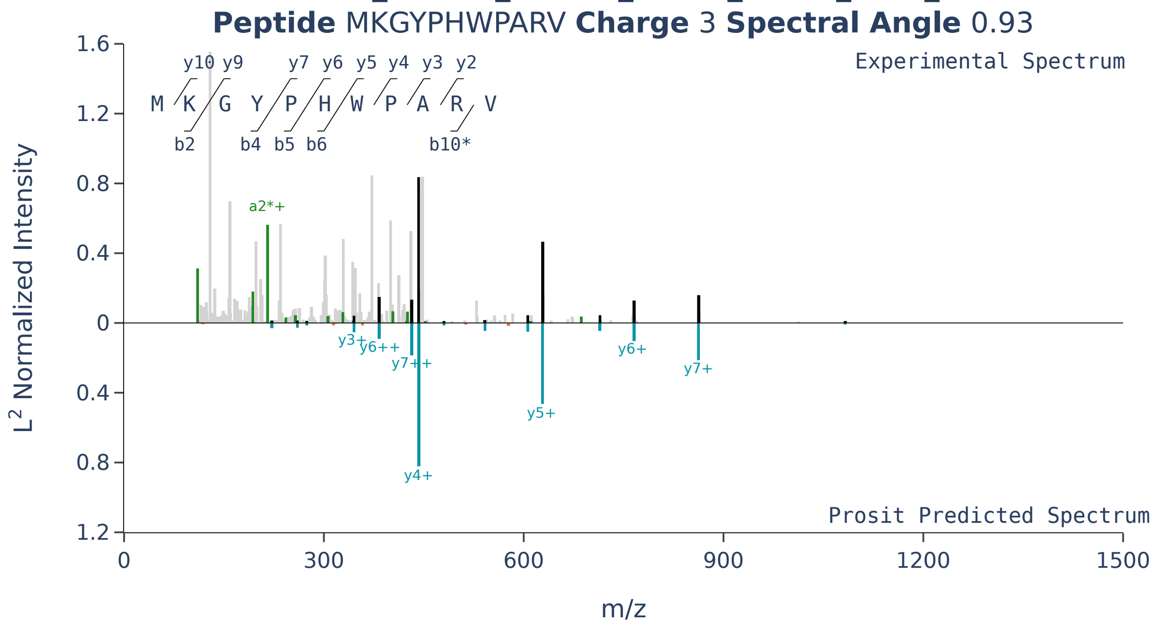


**Source** HRoetschke_271222_090123_HFGoe_LEDGF_b1R2_24h **Scan** 16348
**Peptide** QKHTEMITTL **Charge** 2 **Spectral Angle** 0.82

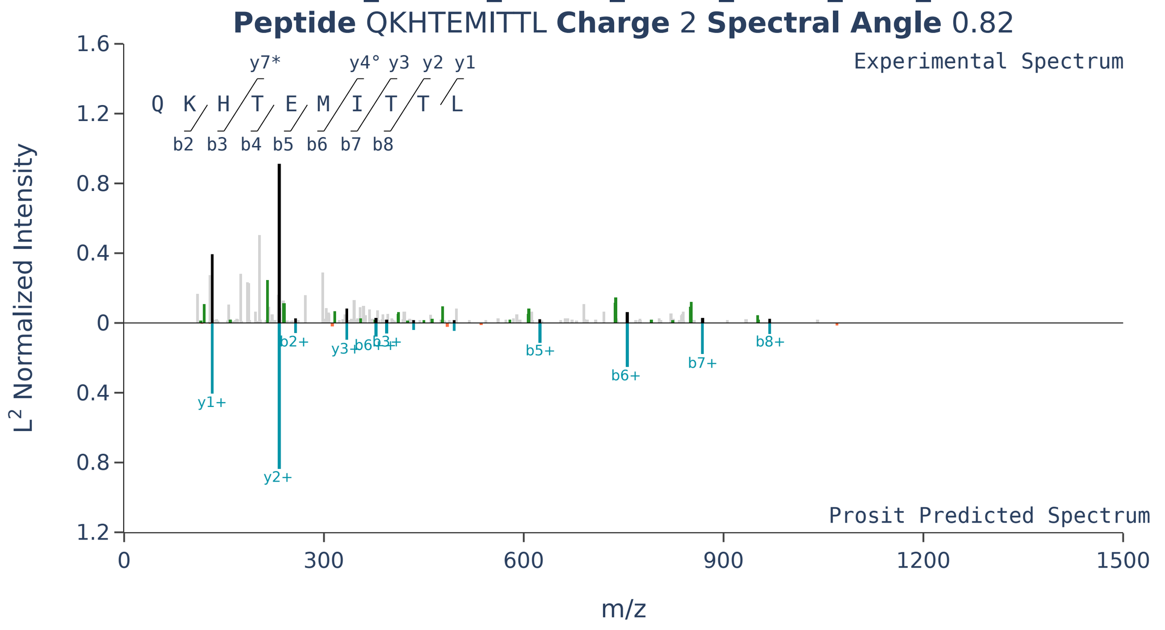


**Source** HRoetschke_271222_090123_HFGoe_LEDGF_b1R1_4h **Scan** 12401
**Peptide** TEMITTLKKIRR **Charge** 2 **Spectral Angle** 0.87

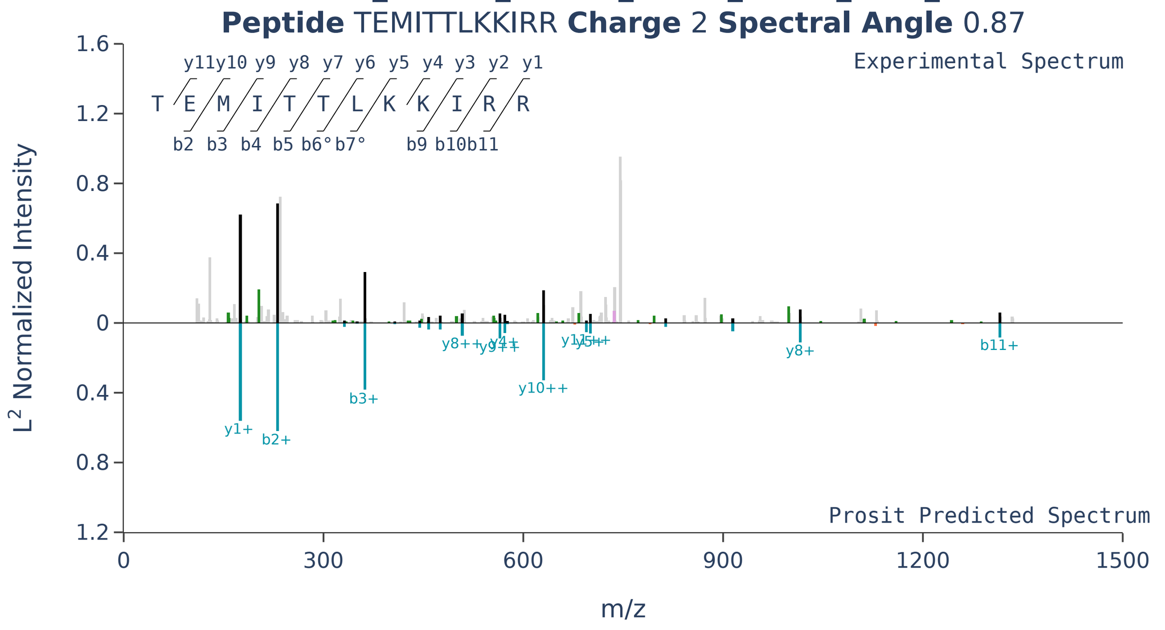


**Source** HRoetschke_271222_090123_HFGoe_LEDGF_b2R2_2h **Scan** 7008
**Peptide** KQSNASSDVEVEEKE **Charge** 2 **Spectral Angle** 0.88

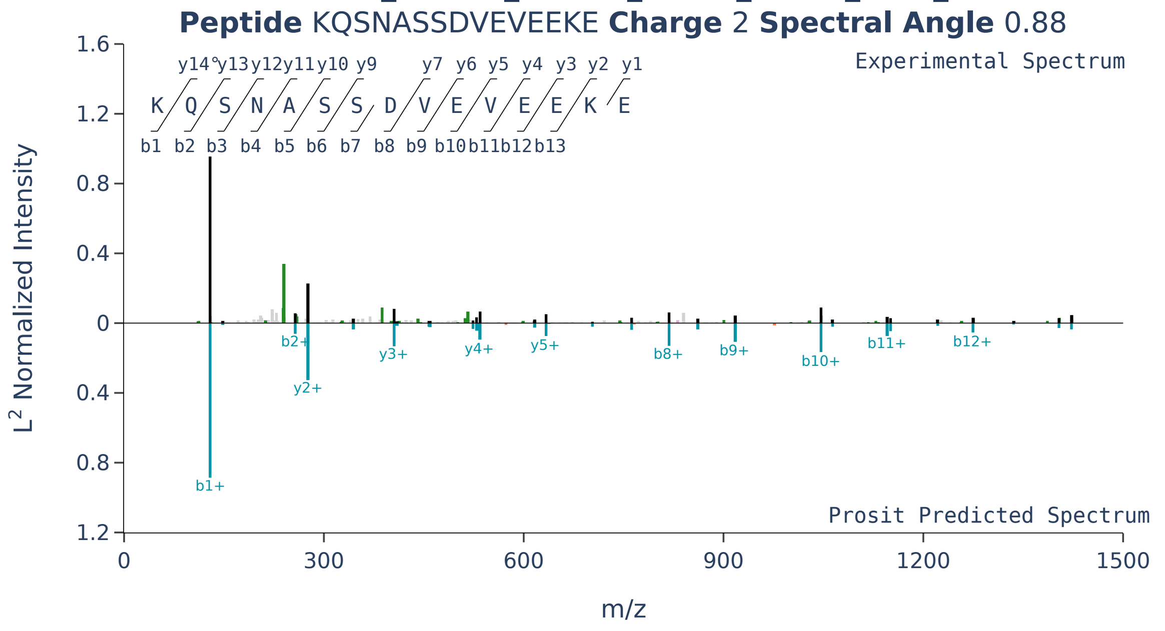


**Source** HRoetschke_271222_090123_HFGoe_LEDGF_b1R2_4h **Scan** 7417
**Peptide** MVKQPCPSE **Charge** 2 **Spectral Angle** 0.84

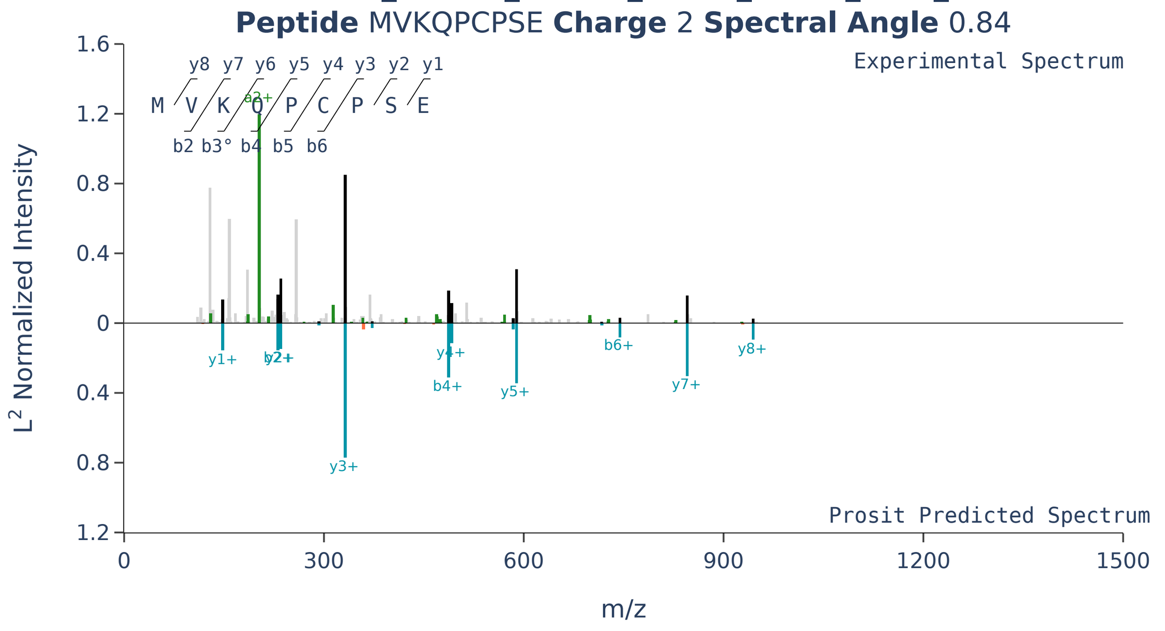


**Source** HRoetschke_271222_090123_HFGoe_LEDGF_b2R1_4h **Scan** 6328
**Peptide** KRETSMDSRLQ **Charge** 3 **Spectral Angle** 0.9

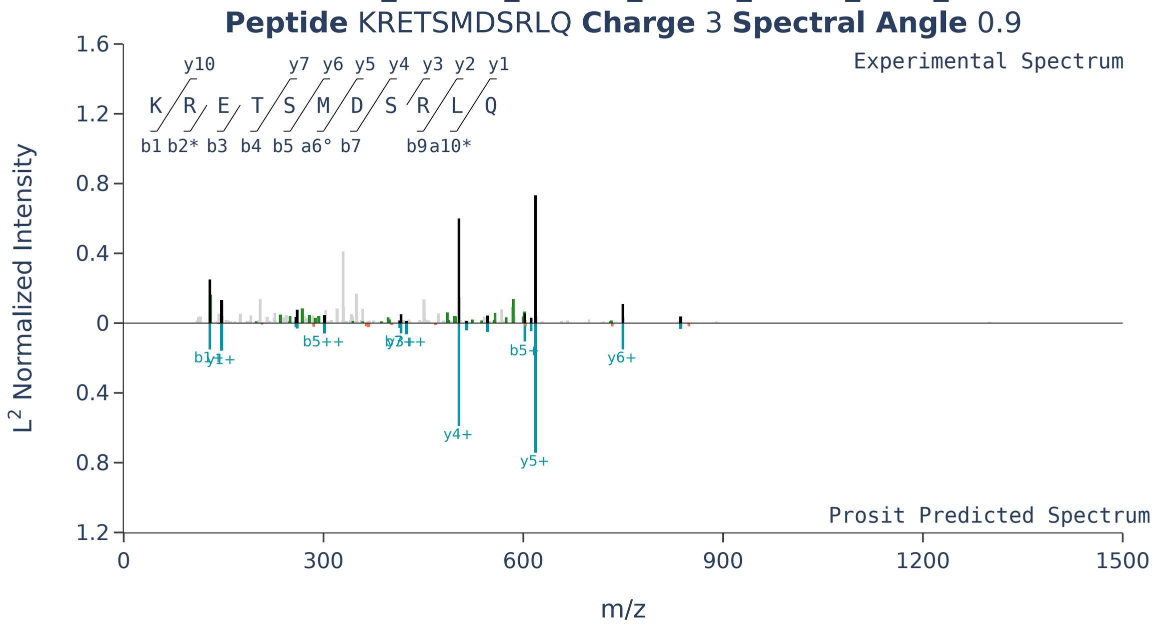


**Source** HRoetschke_271222_090123_HFGoe_LEDGF_b1R1_2h **Scan** 24395
**Peptide** GDSVITQVLNKSLAEQRQHE **Charge** 3 **Spectral Angle** 0.86

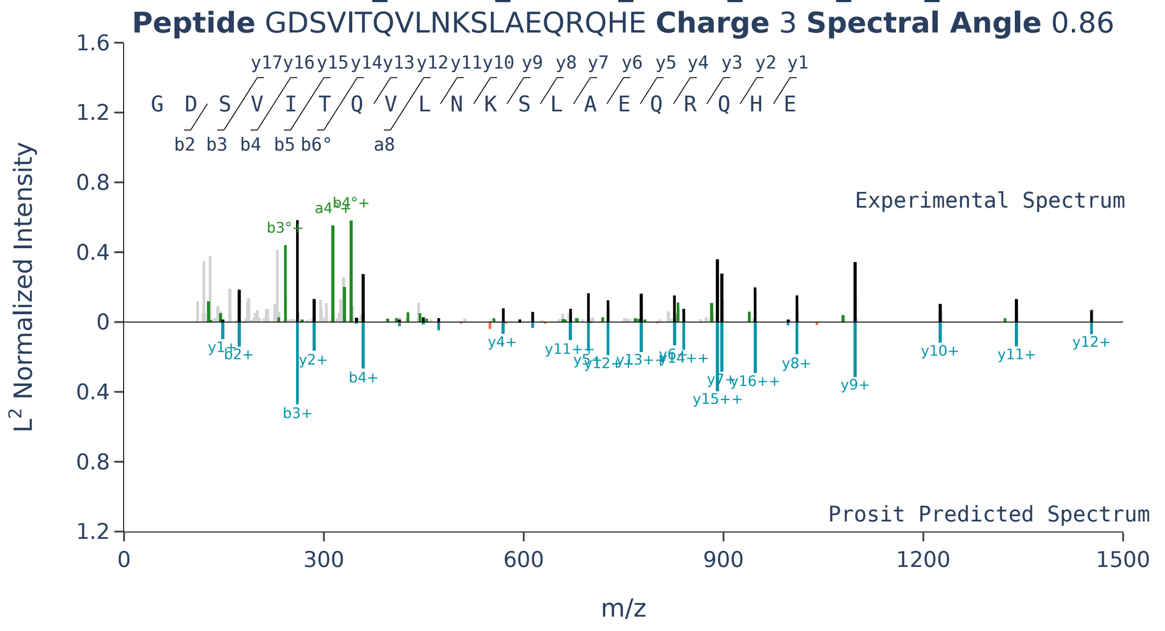


**Source** HRoetschke_271222_090123_HFGoe_LEDGF_b2R2_4h **Scan** 7462
**Peptide** SVNLKVSPKRGRPAA **Charge** 4 **Spectral Angle** 0.91

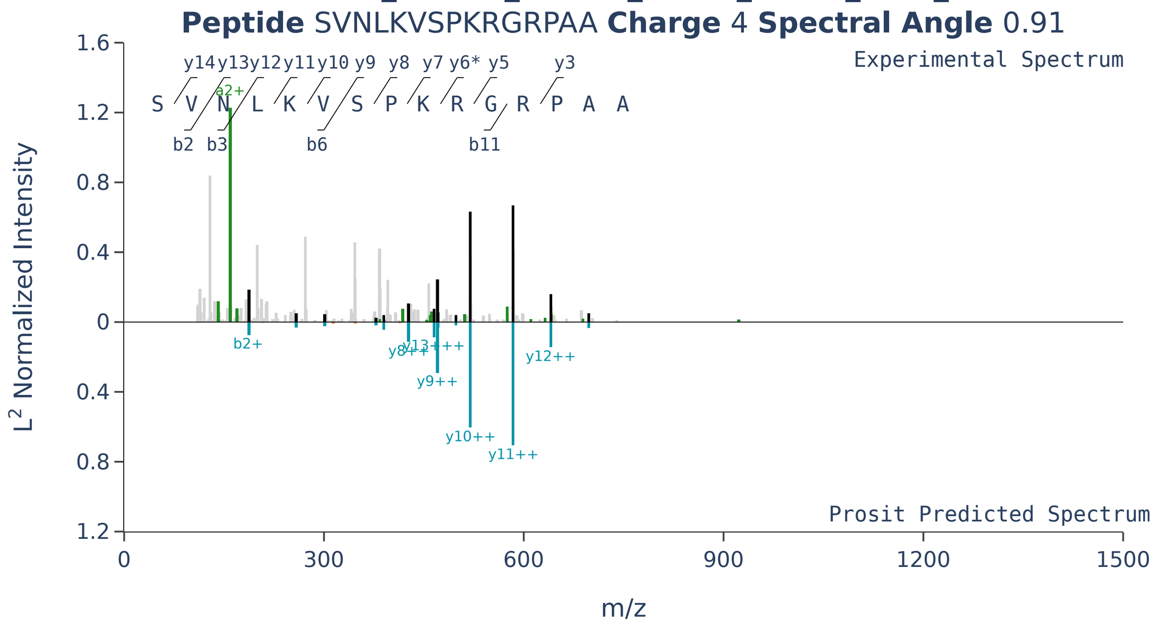


**Source** HRoetschke_271222_090123_HFGoe_LEDGF_b2R2_2h **Scan** 19863
**Peptide** QVTMQQAQKHTEMITTL **Charge** 3 **Spectral Angle** 0.94

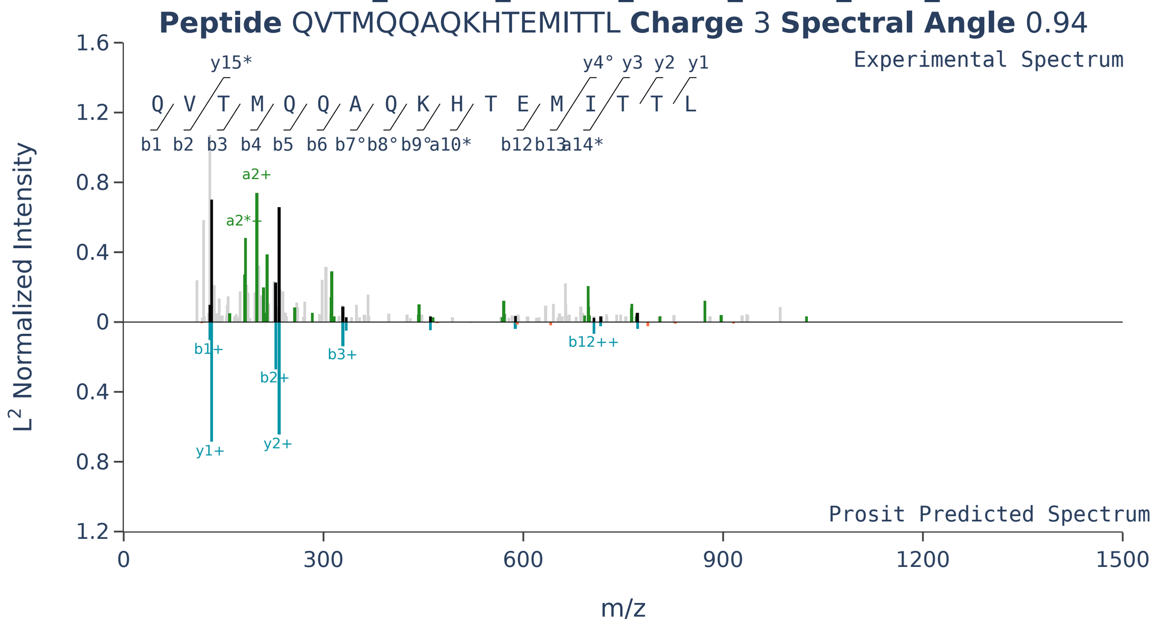


**Source** HRoetschke_271222_090123_HFGoe_LEDGF_b2R2_24h **Scan** 10240
**Peptide** PARVDEVPDG **Charge** 2 **Spectral Angle** 0.79

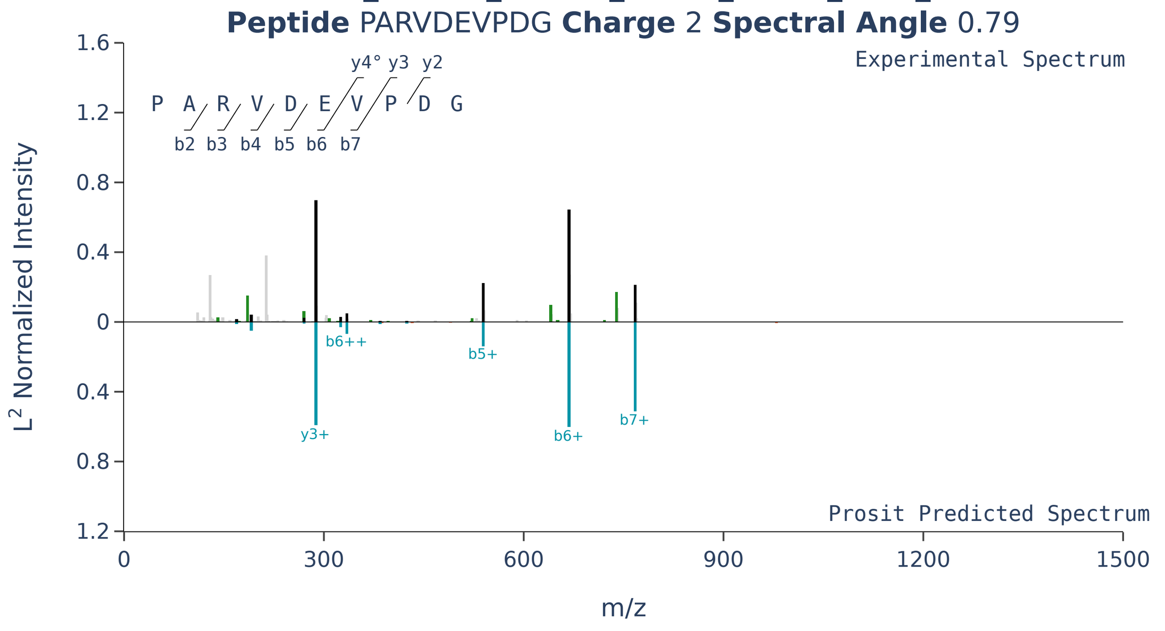


**Source** HRoetschke_271222_090123_HFGoe_LEDGF_b1R1_1h **Scan** 7993
**Peptide** VDITTPKAARR **Charge** 3 **Spectral Angle** 0.82

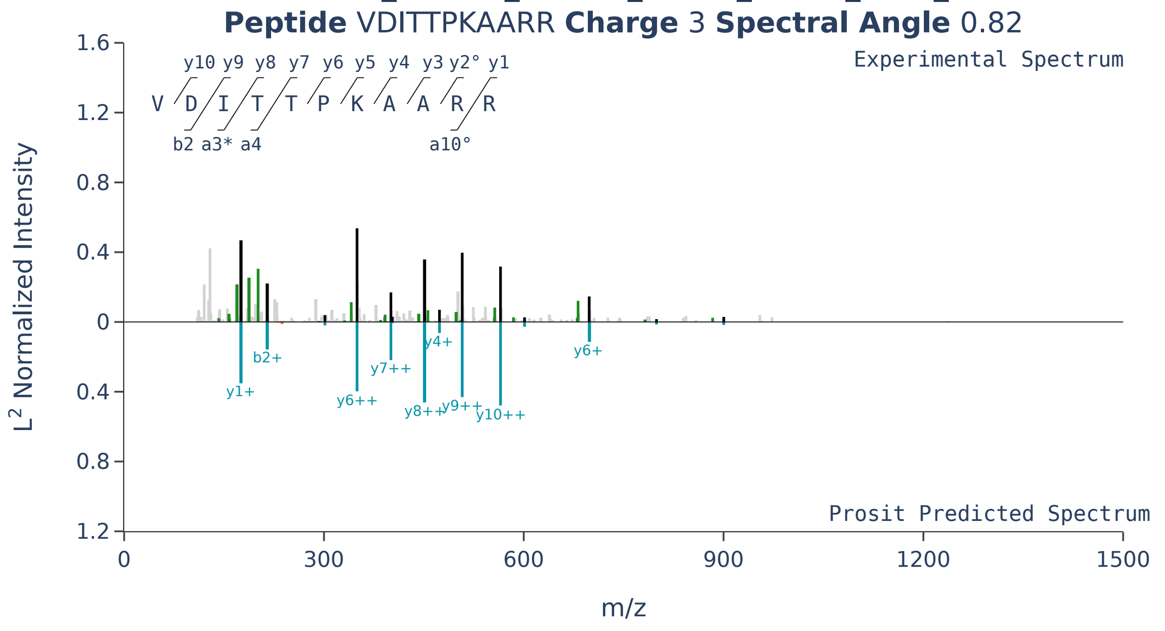


**Source** HRoetschke_271222_090123_HFGoe_LEDGF_b2R1_2h **Scan** 23925
**Peptide** TAFLGPKDIFPYSENKEKY **Charge** 3 **Spectral Angle** 0.86

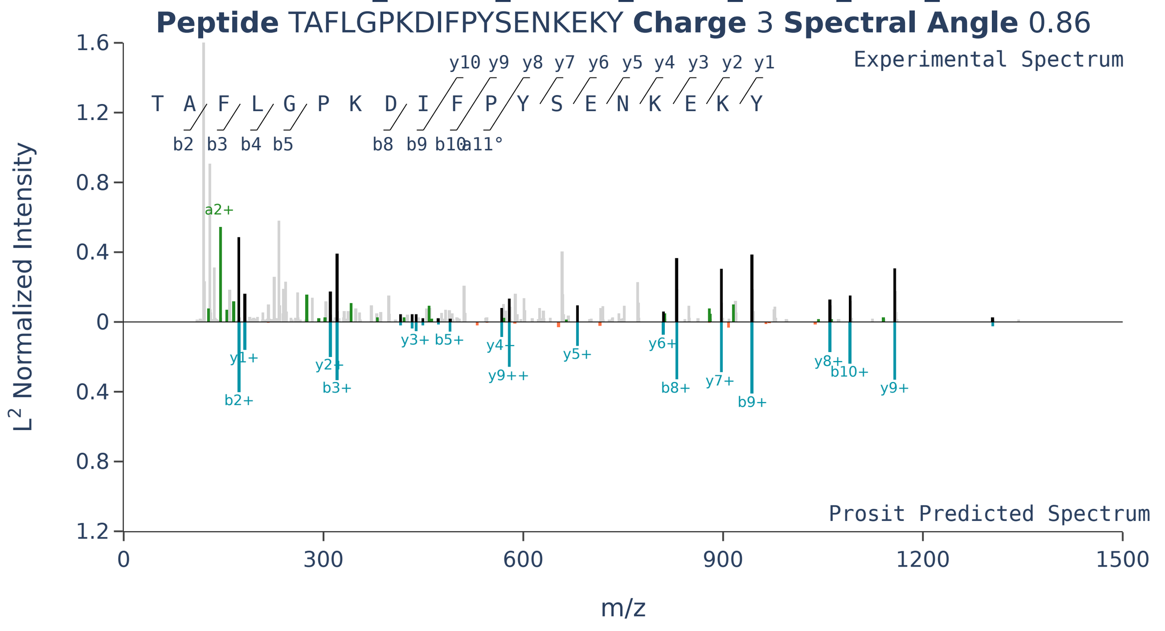

**Source** HRoetschke_271222_090123_HFGoe_LEDGF_b1R2_24h **Scan** 22689
**Peptide** YNKFKNMFL **Charge** 3 **Spectral Angle** 0.93

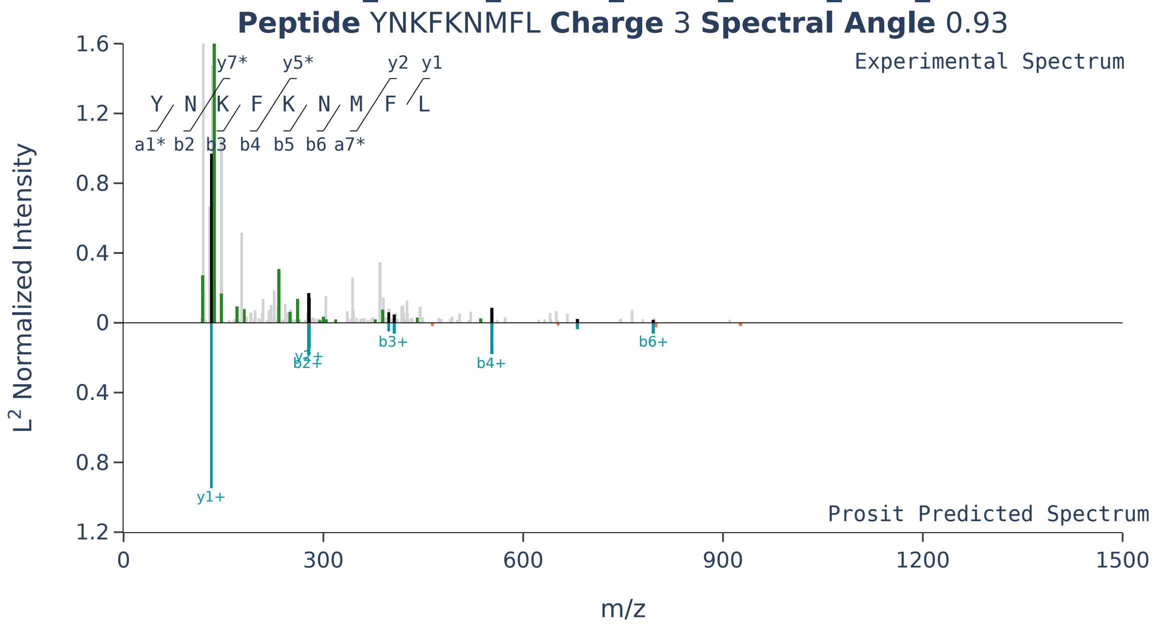


**Source** HRoetschke_271222_090123_HFGoe_LEDGF_b2R1_24h **Scan** 21190
**Peptide** NMFLVGEGD **Charge** 1 **Spectral Angle** 0.97

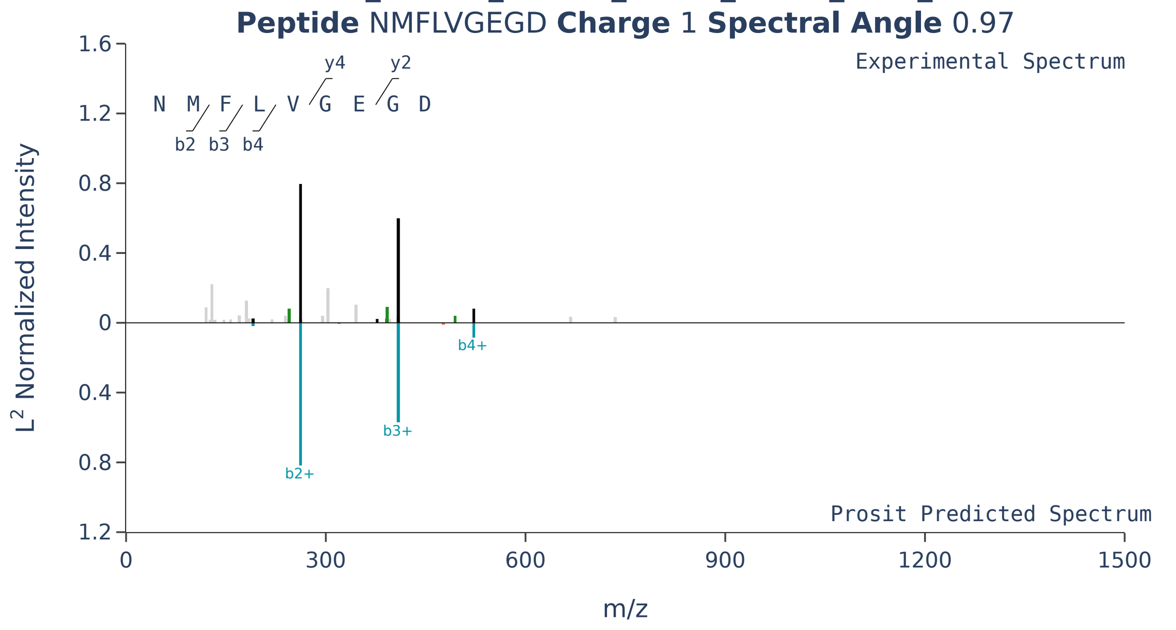


**Source** HRoetschke_271222_090123_HFGoe_LEDGF_b1R1_4h **Scan** 8522
**Peptide** ATASVNLKVSPKRGRPAA **Charge** 3 **Spectral Angle** 0.79

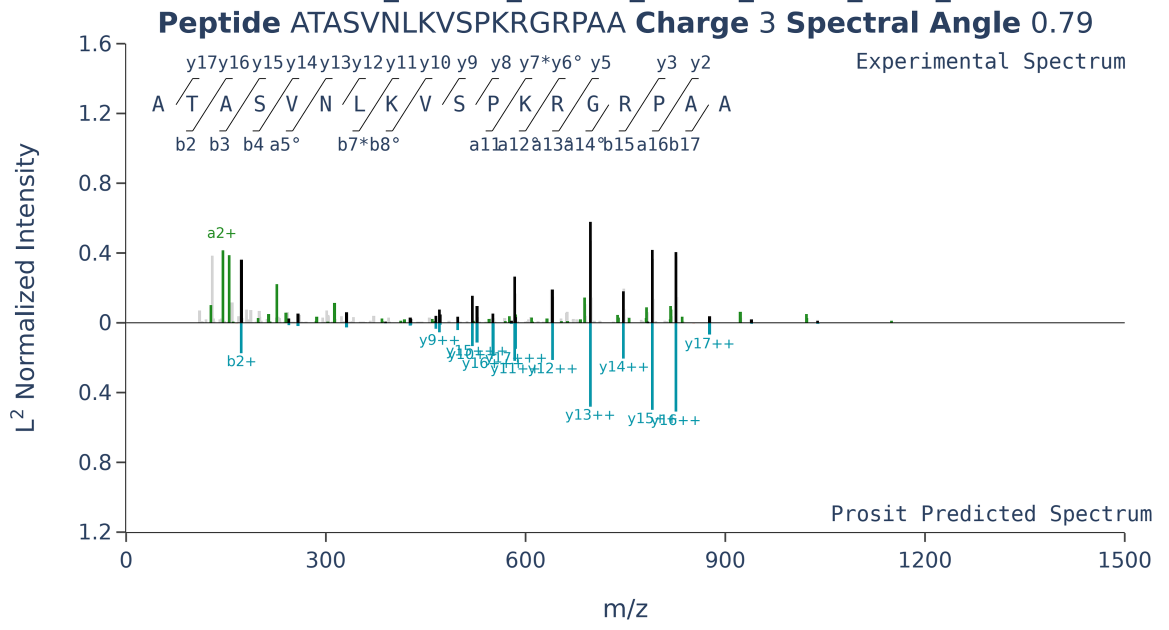


**Source** HRoetschke_271222_090123_HFGoe_LEDGF_b2R2_1h **Scan** 24724
**Peptide** FLGPKDIFPYSENKE **Charge** 3 **Spectral Angle** 0.86

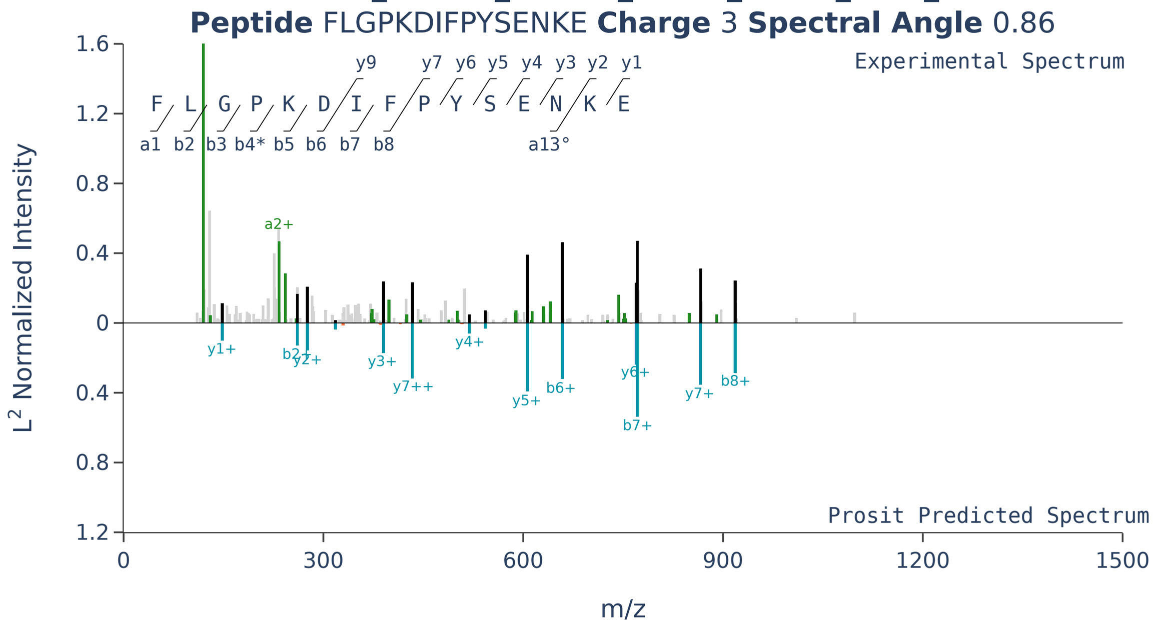


**Source** HRoetschke_271222_090123_HFGoe_LEDGF_b2R1_4h **Scan** 18076
**Peptide** PSSEERETEISL **Charge** 2 **Spectral Angle** 0.79

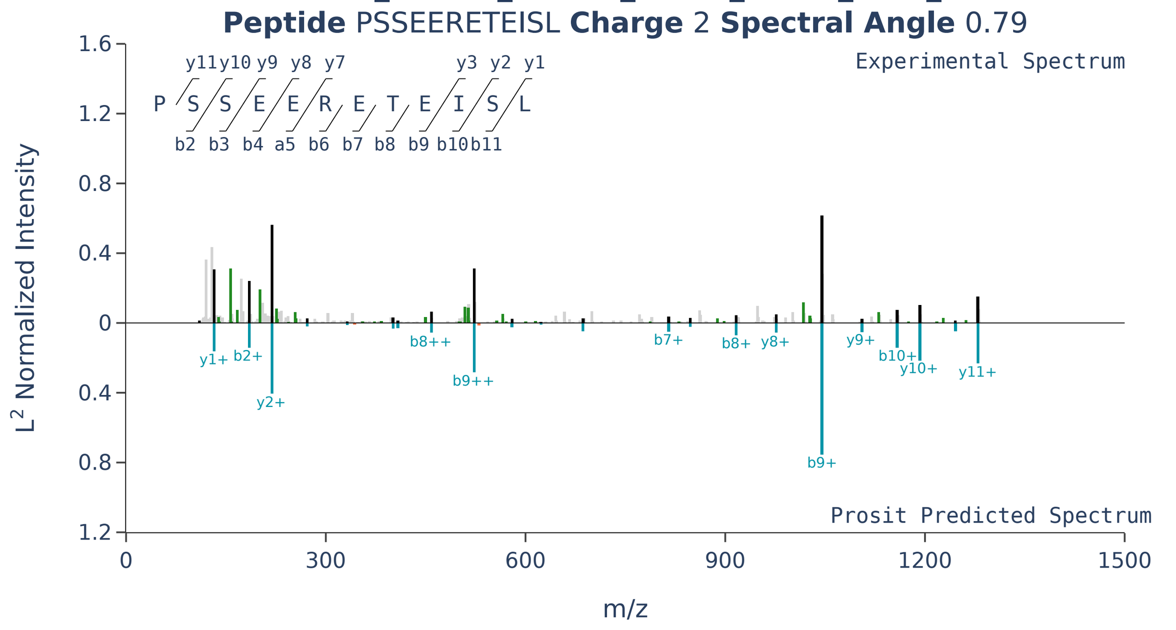


**Source** HRoetschke_271222_090123_HFGoe_LEDGF_b1R1_2h **Scan** 10113
**Peptide** GPKDIFPYSENKEKYGKPNKR **Charge** 5 **Spectral Angle** 0.82

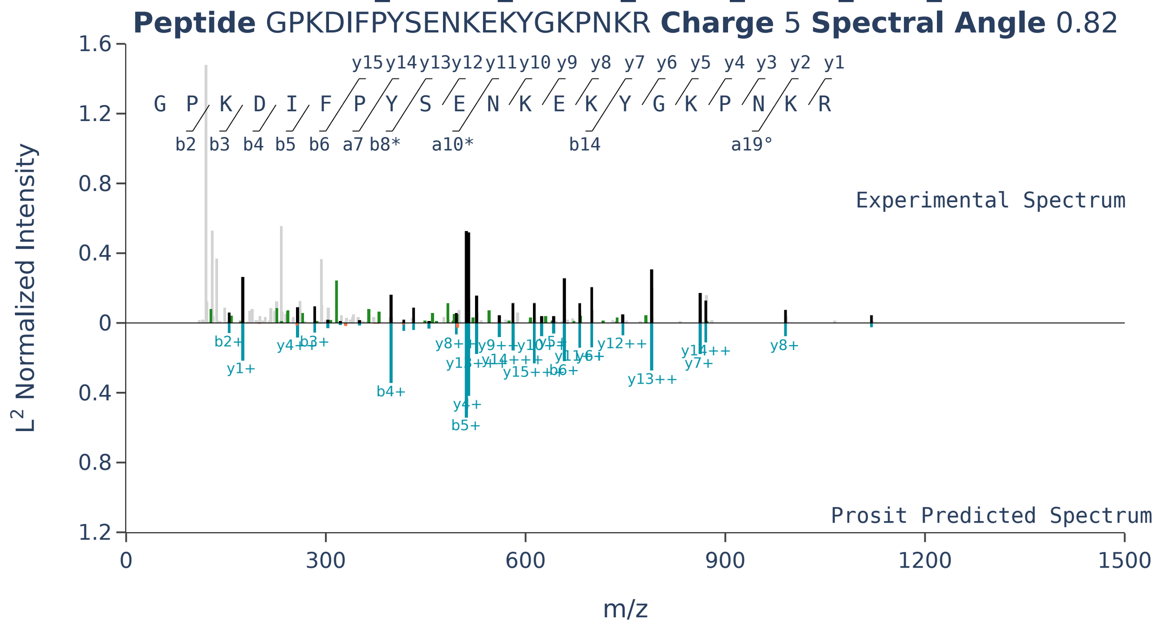


**Source** HRoetschke_271222_090123_HFGoe_LEDGF_b1R2_24h **Scan** 11633
**Peptide** PAATEVKIPKPRG **Charge** 2 **Spectral Angle** 0.87

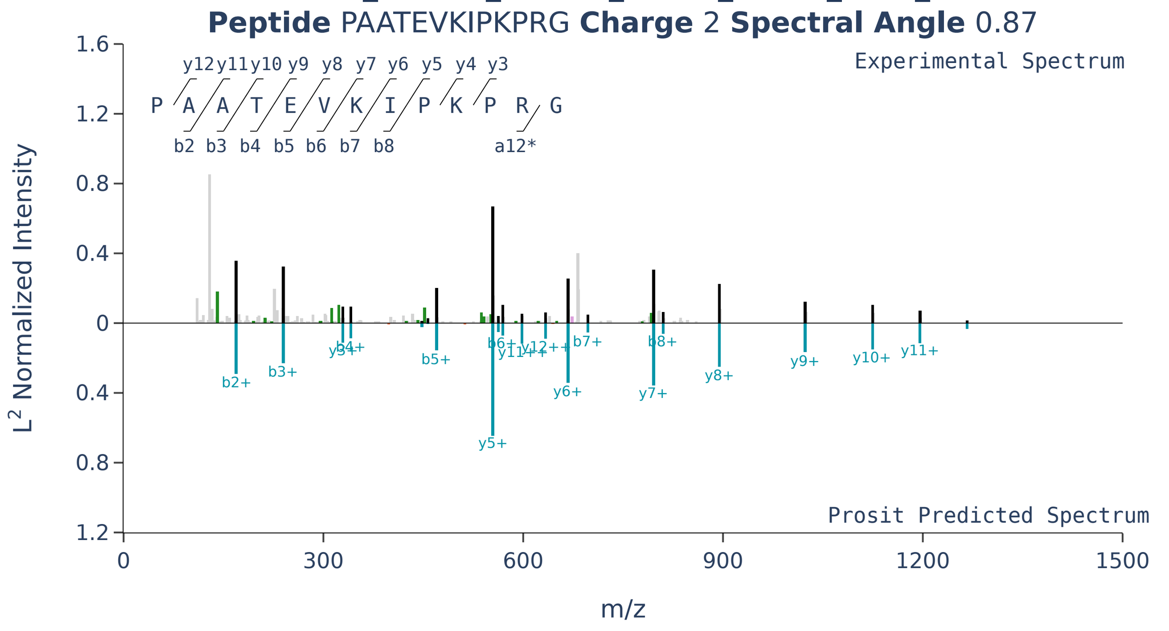


**Source** HRoetschke_271222_090123_HFGoe_LEDGF_b1R2_24h **Scan** 6463
**Peptide** DITTPKAARR **Charge** 3 **Spectral Angle** 0.84

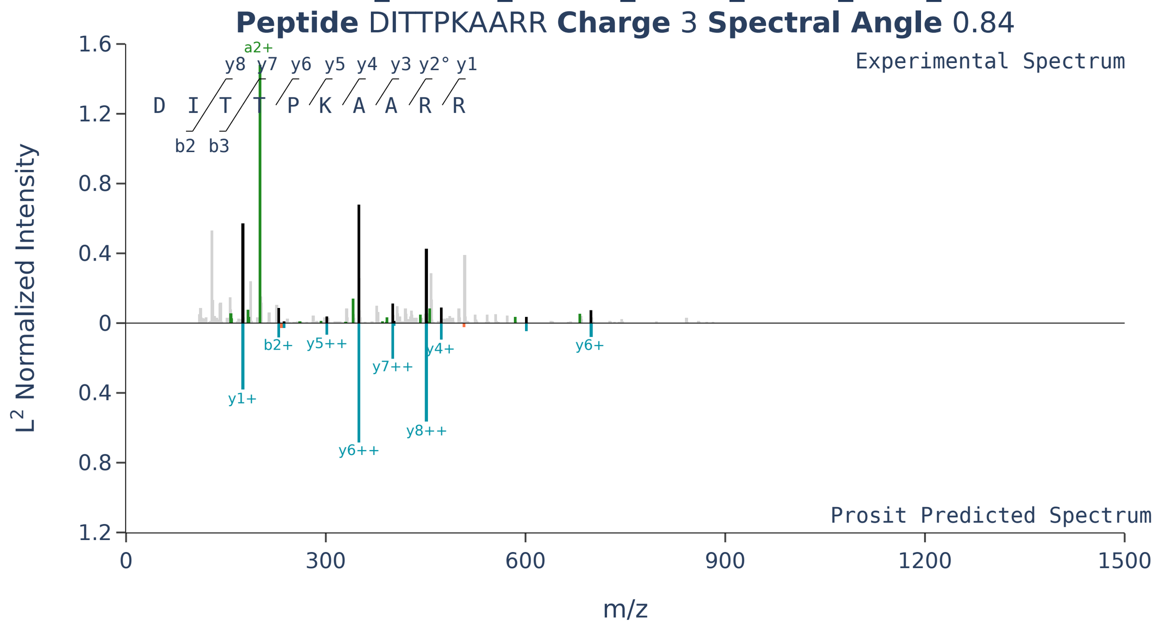


**Source** HRoetschke_271222_090123_HFGoe_LEDGF_b2R2_2h **Scan** 7129
**Peptide** PKMVKQPCPSESD **Charge** 2 **Spectral Angle** 0.85

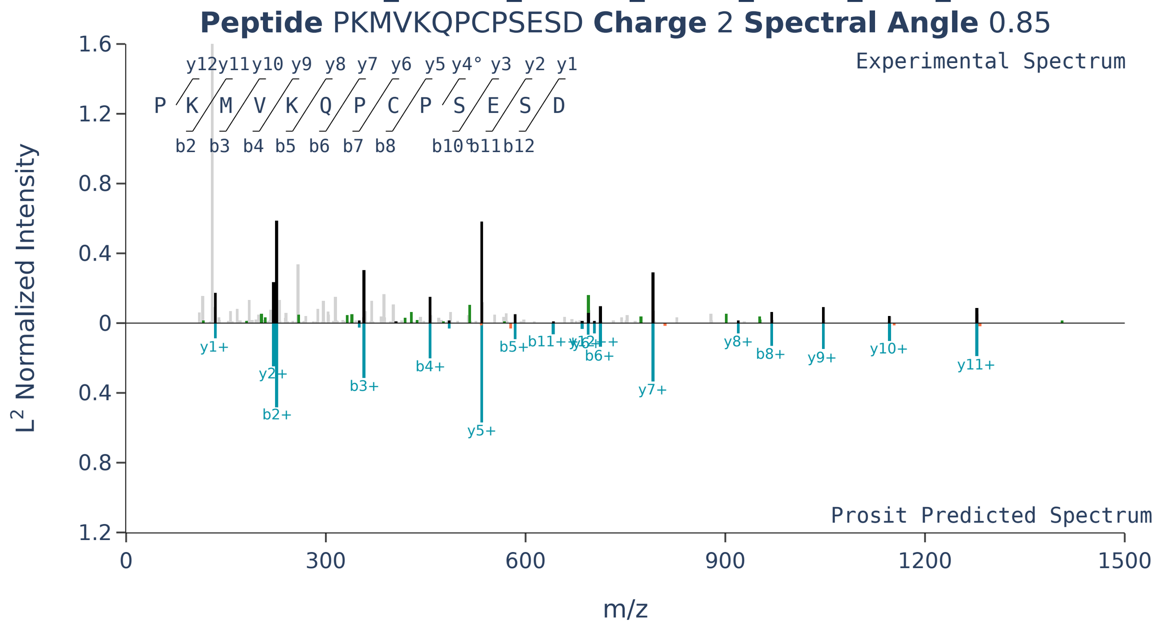


**Source** HRoetschke_271222_090123_HFGoe_LEDGF_b1R1_24h **Scan** 16124
**Peptide** HTEMITTLKKI **Charge** 3 **Spectral Angle** 0.87

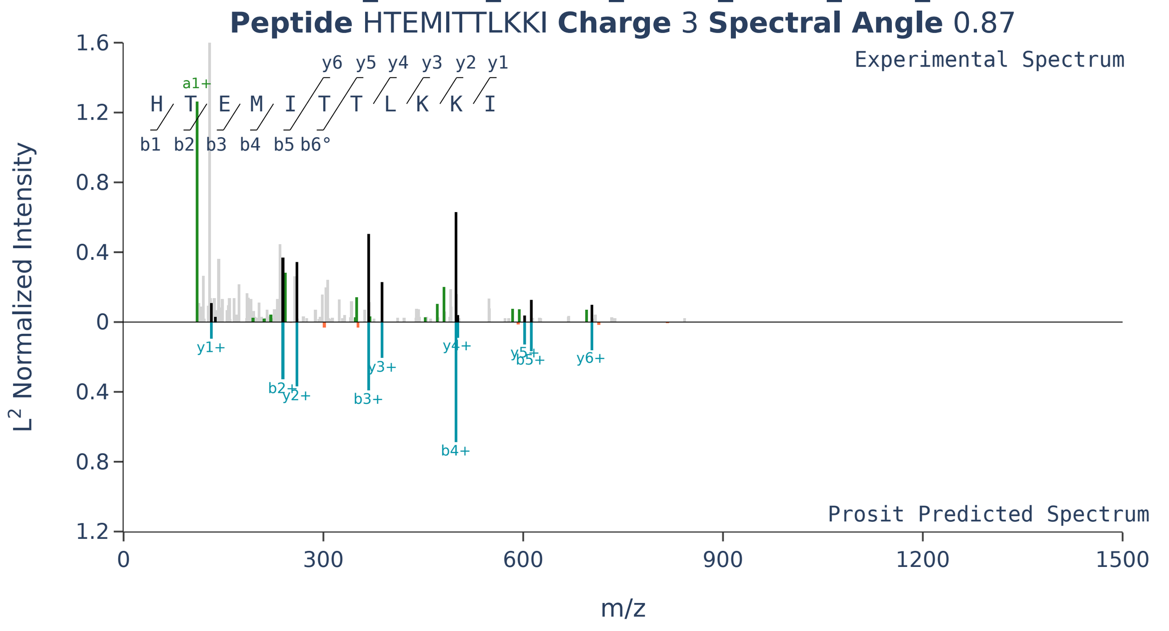


**Source** HRoetschke_271222_090123_HFGoe_LEDGF_b1R2_2h **Scan** 17101
**Peptide** PHWPARVDEVPDG **Charge** 2 **Spectral Angle** 0.81

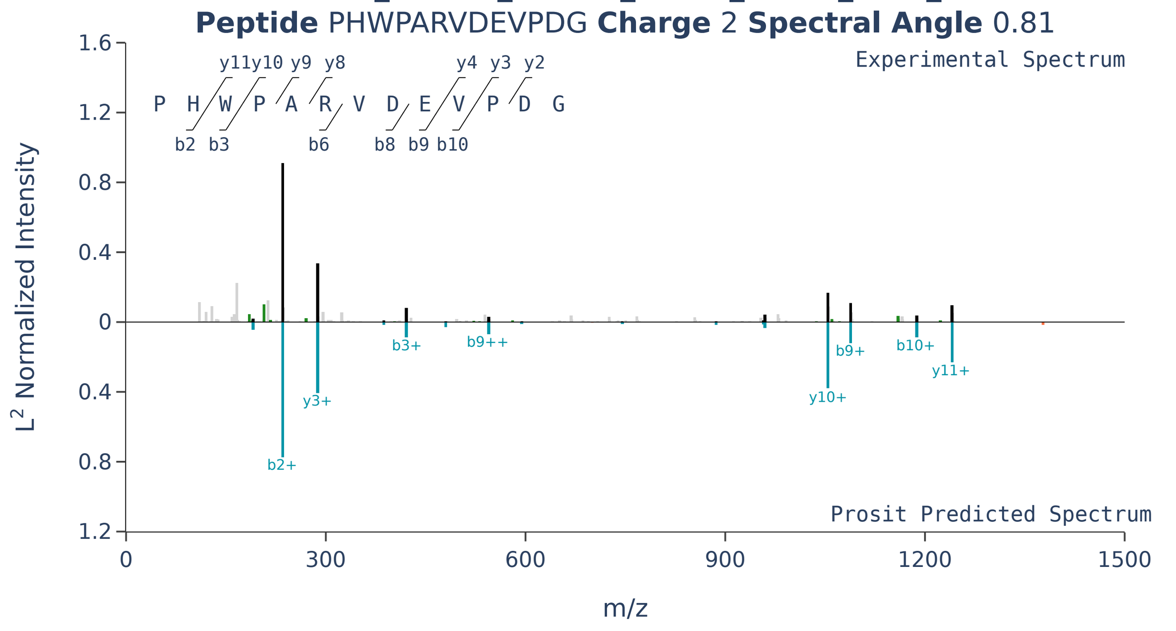


**Source** HRoetschke_271222_090123_HFGoe_LEDGF_b1R1_2h **Scan** 25487
**Peptide** TEEAGVVTTATASVNL **Charge** 2 **Spectral Angle** 0.88

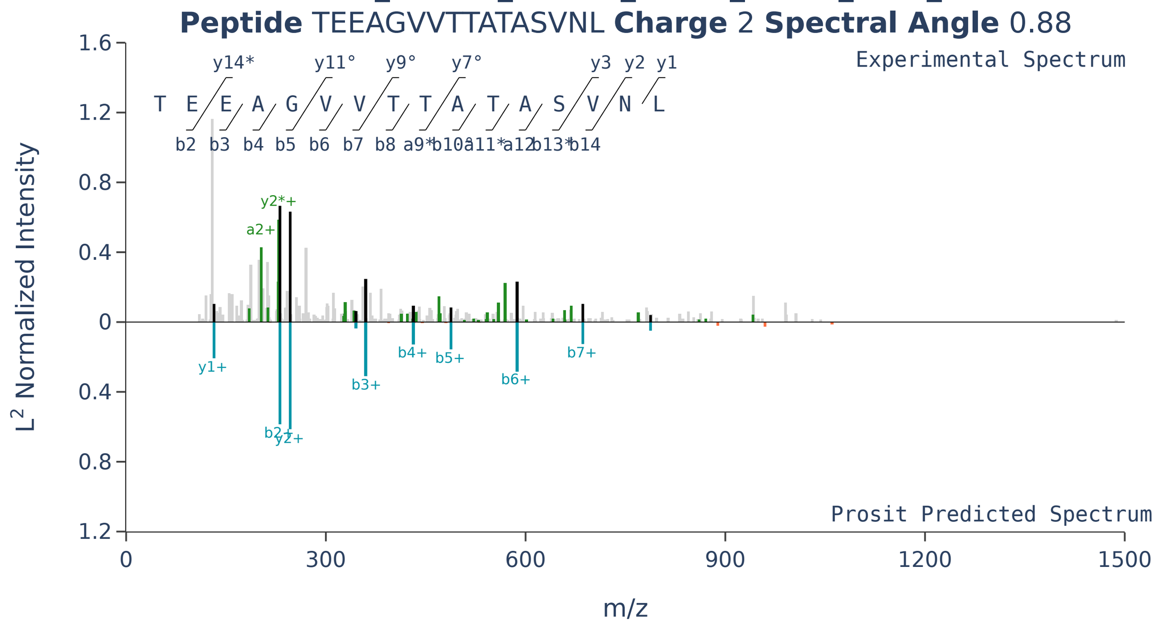


**Source** HRoetschke_271222_090123_HFGoe_LEDGF_b1R1_4h **Scan** 23369
**Peptide** KGFNEGLWEIDNNPKVKFSSQQA **Charge** 4 **Spectral Angle** 0.93

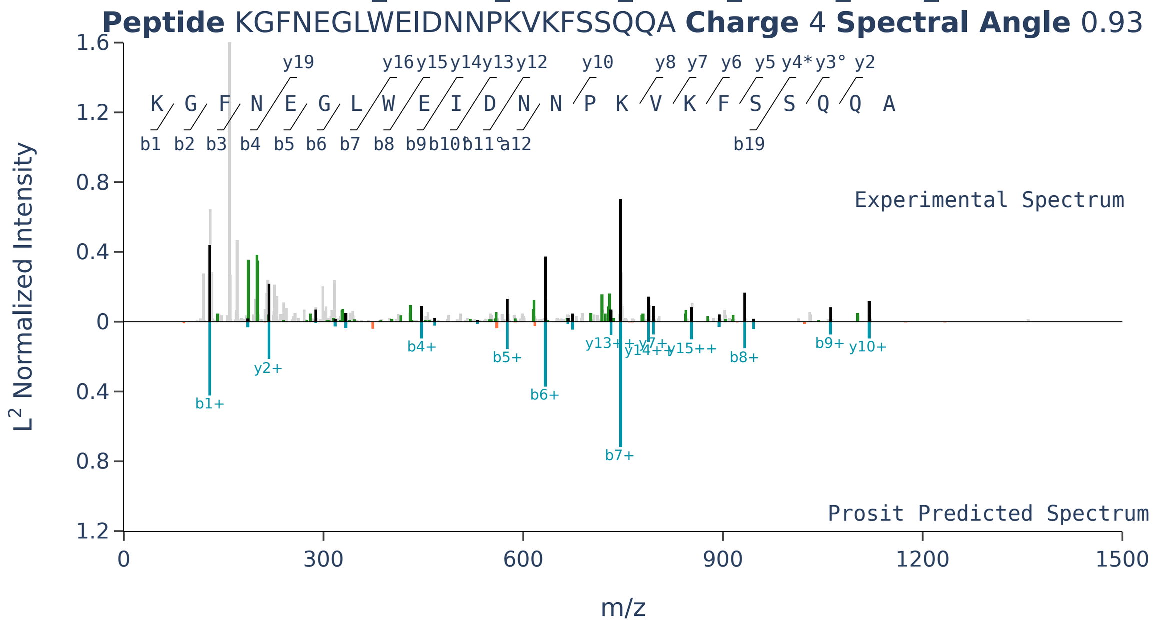


**Source** HRoetschke_271222_090123_HFGoe_LEDGF_b1R2_24h **Scan** 17833
**Peptide** KAEKQVETEEAGVVTTATASV **Charge** 3 **Spectral Angle** 0.89

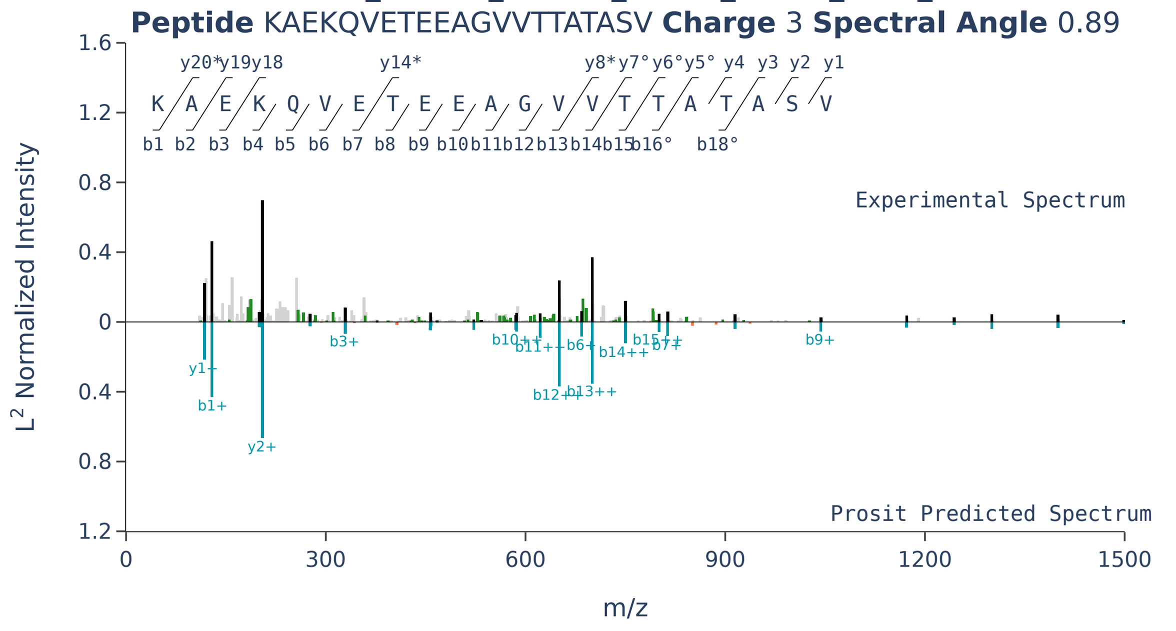


**Source** HRoetschke_271222_090123_HFGoe_LEDGF_b2R1_2h **Scan** 21756
**Peptide** TAFLGPKDI **Charge** 2 **Spectral Angle** 0.81

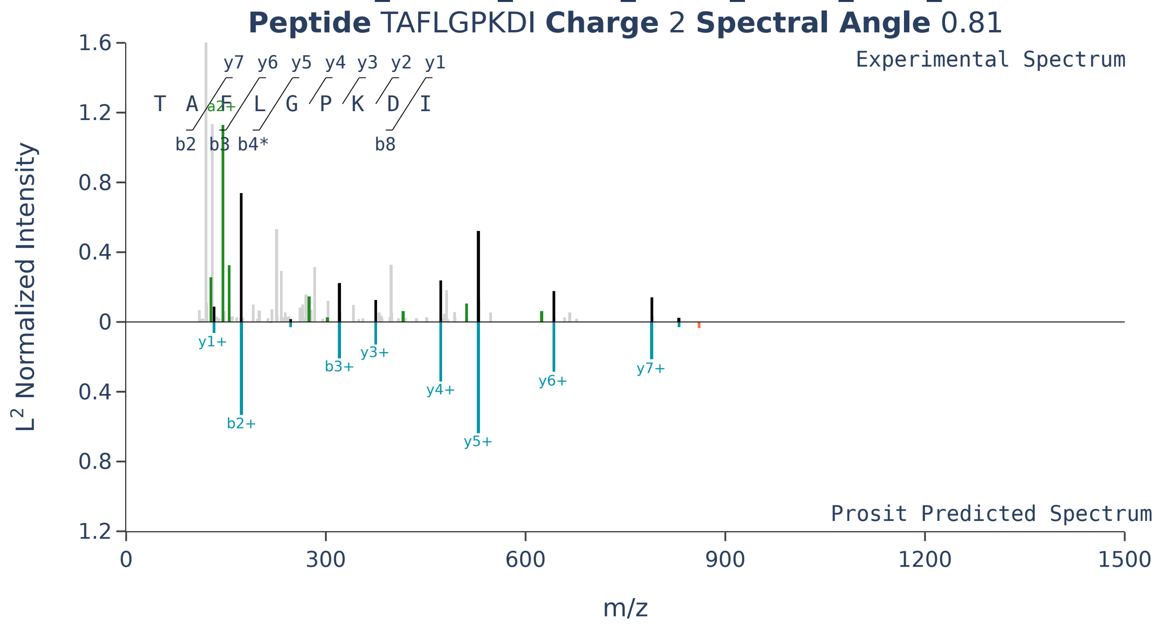

**Source** HRoetschke_271222_090123_HFGoe_LEDGF_b1R2_4h **Scan** 10135
**Peptide** IDNNPKVKFSSQ **Charge** 2 **Spectral Angle** 0.8

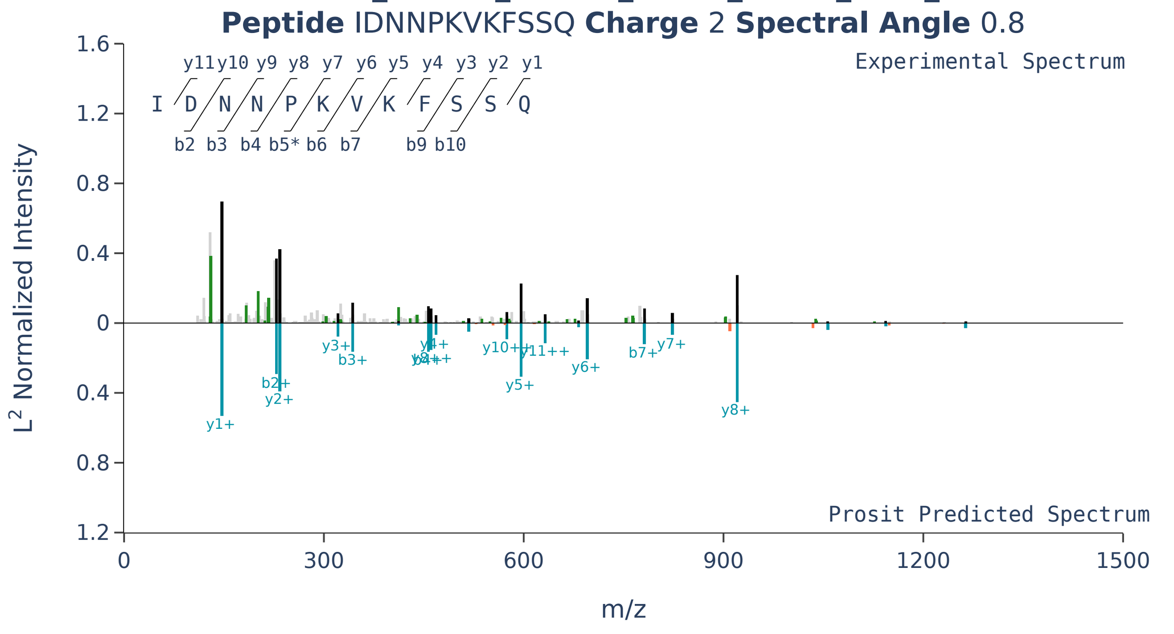


**Source** HRoetschke_271222_090123_HFGoe_LEDGF_b1R2_24h **Scan** 15965
**Peptide** KIDNLDVNRCIE **Charge** 2 **Spectral Angle** 0.87

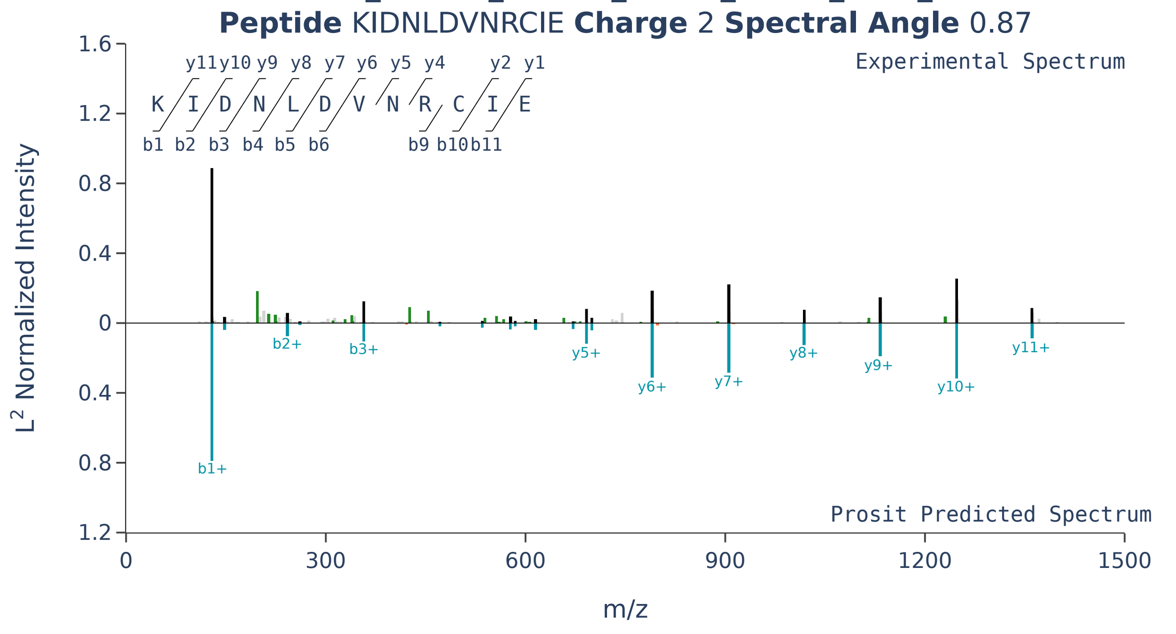


**Source** HRoetschke_271222_090123_HFGoe_LEDGF_b1R1_2h **Scan** 24356
**Peptide** SVITQVLNKSLAEQRQ **Charge** 2 **Spectral Angle** 0.82

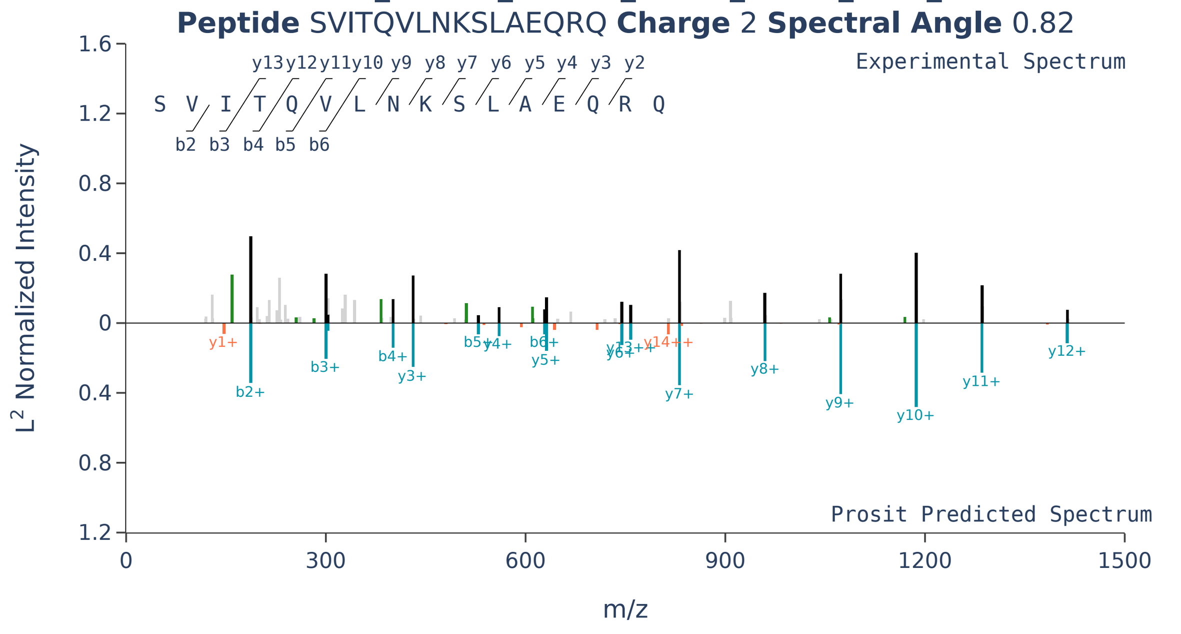


**Source** HRoetschke_271222_090123_HFGoe_LEDGF_b1R2_2h **Scan** 8759
**Peptide** VKPPTNKL **Charge** 2 **Spectral Angle** 0.93

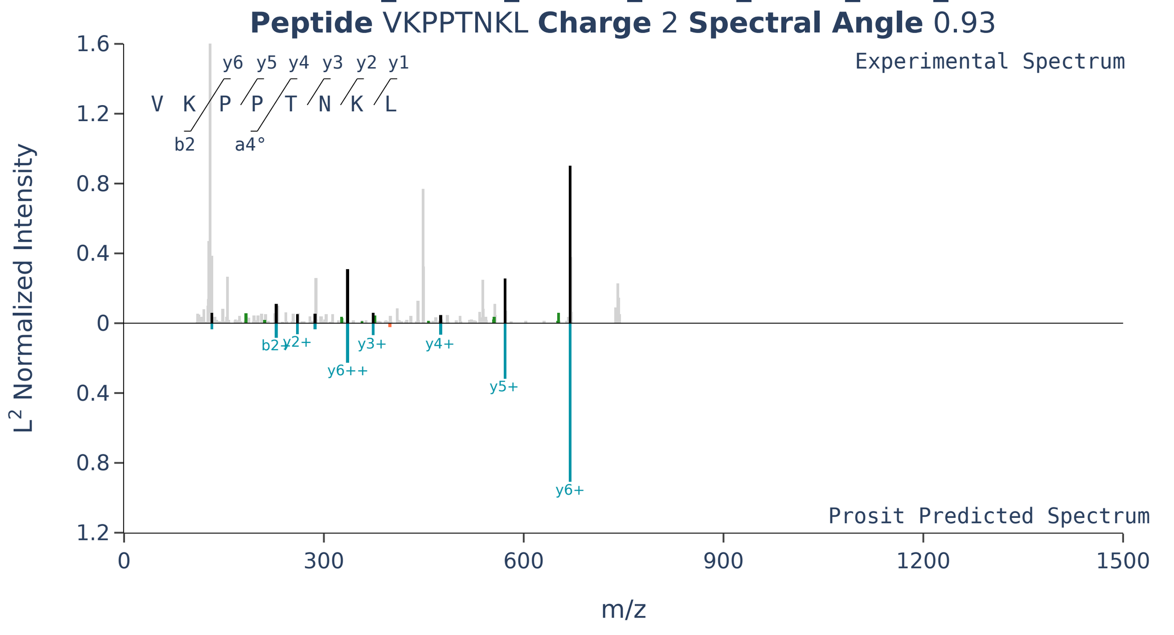


**Source** HRoetschke_271222_090123_HFGoe_LEDGF_b2R2_24h **Scan** 20324
**Peptide** FLGPKDI **Charge** 2 **Spectral Angle** 0.84

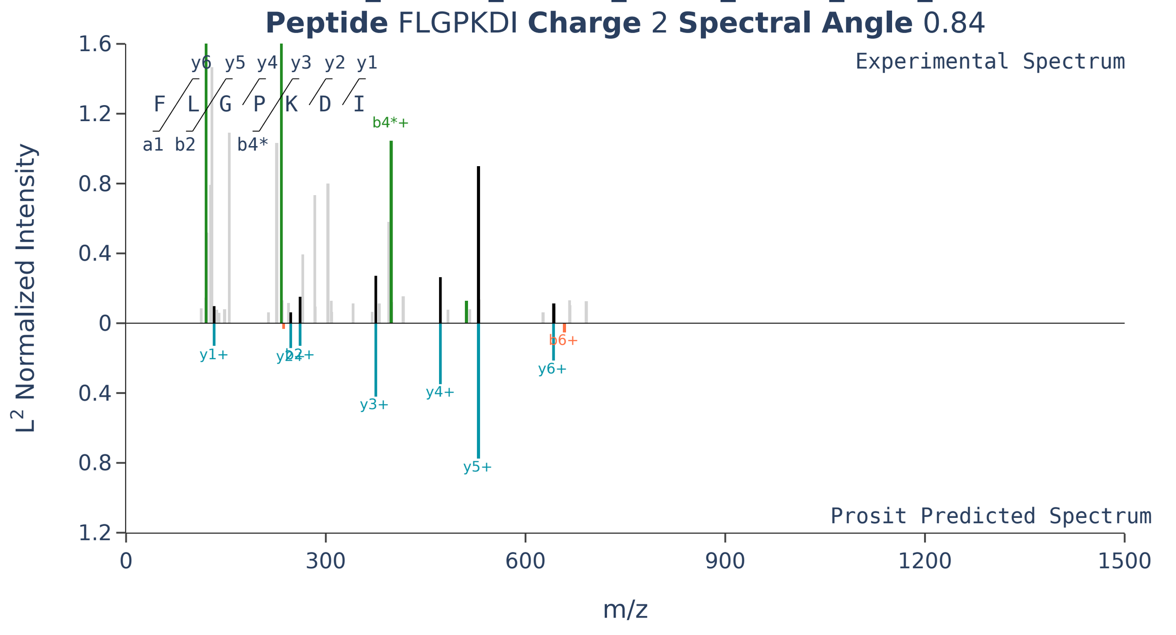


**Source** HRoetschke_271222_090123_HFGoe_LEDGF_b2R2_24h **Scan** 8359
**Peptide** VSQVIMEK **Charge** 2 **Spectral Angle** 0.82

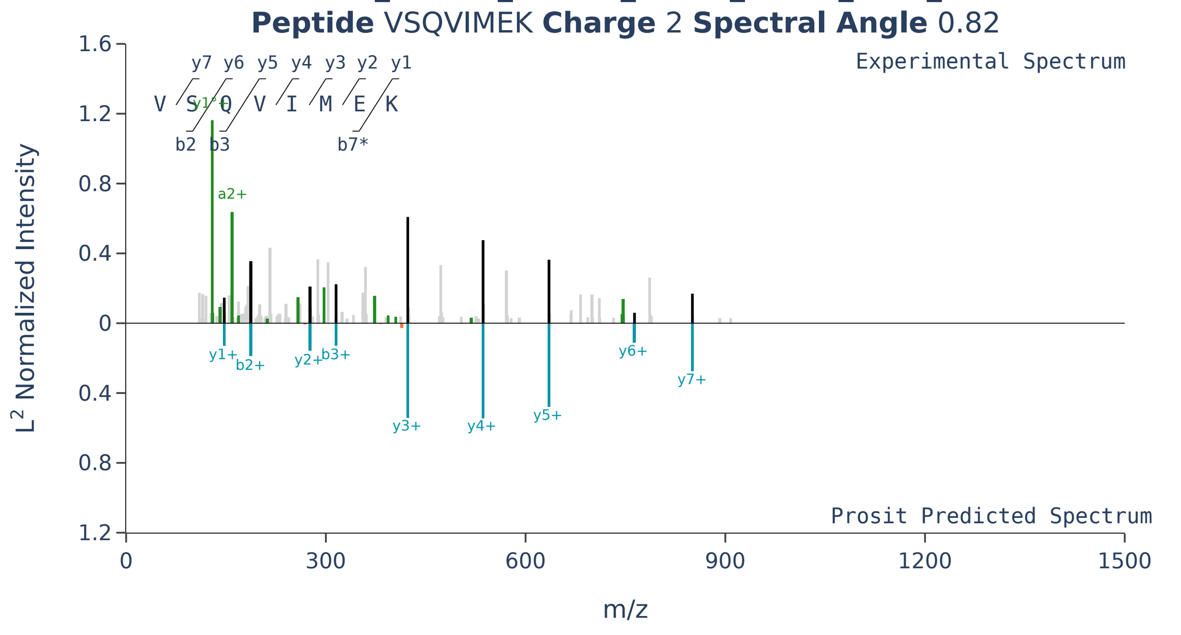


**Source** HRoetschke_271222_090123_HFGoe_LEDGF_b2R1_24h **Scan** 20406
**Peptide** NEGLWEIDNNPKVKFSSQQAATK **Charge** 3 **Spectral Angle** 0.89

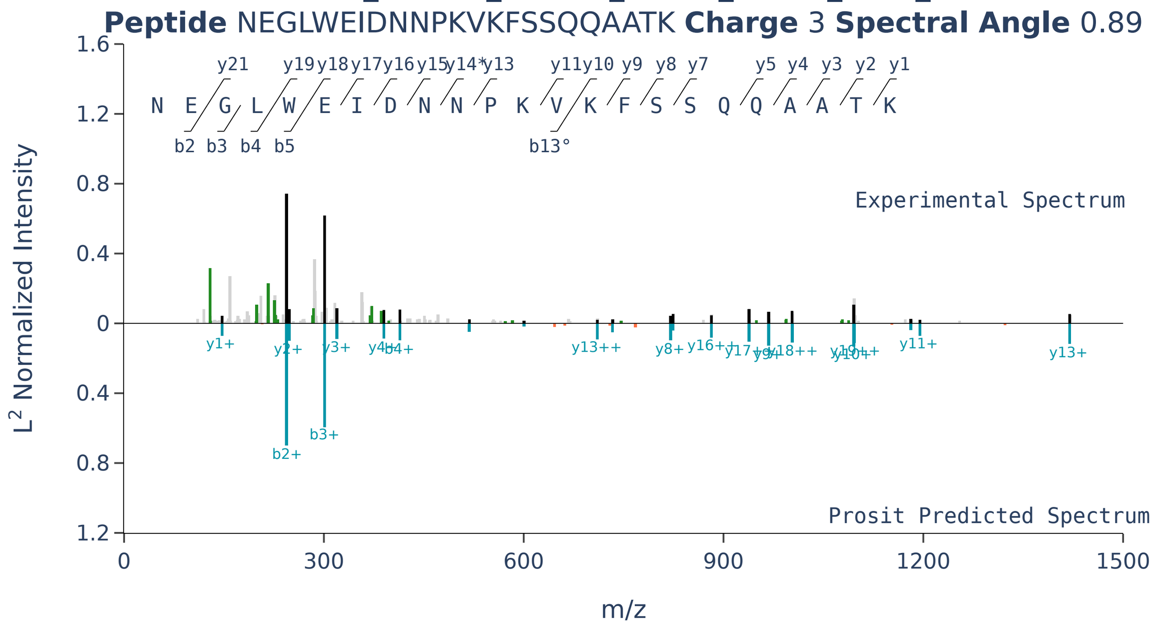


**Source** HRoetschke_271222_090123_HFGoe_LEDGF_b2R2_24h **Scan** 16028
**Peptide** FKVSQVIMEKS **Charge** 3 **Spectral Angle** 0.86

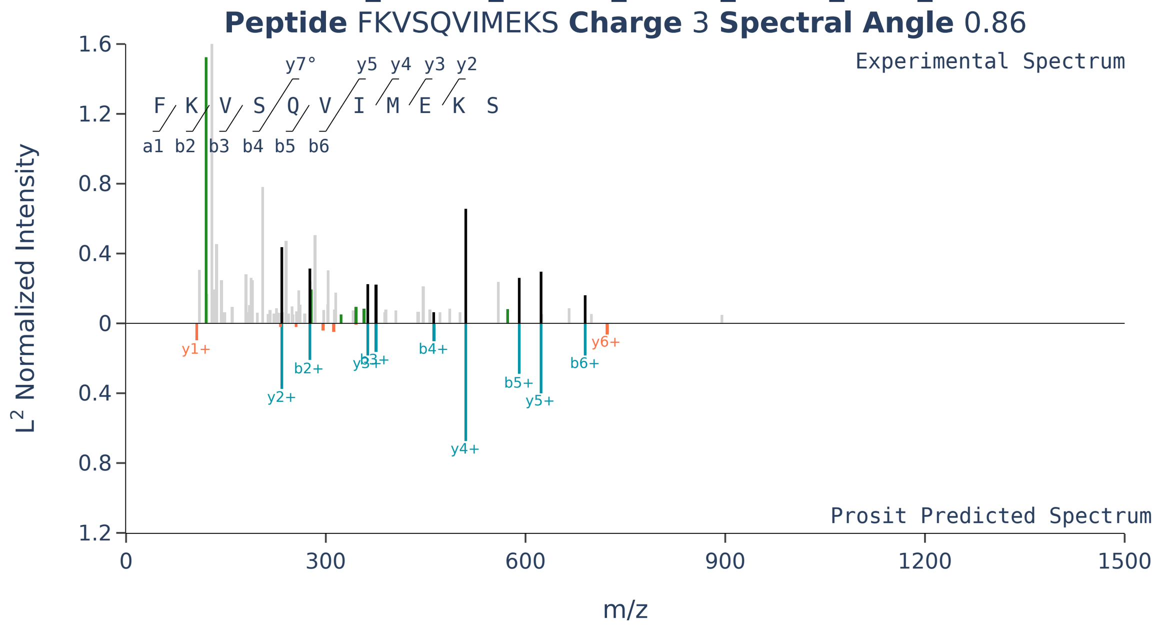


**Source** HRoetschke_271222_090123_HFGoe_LEDGF_b1R2_24h **Scan** 15703
**Peptide** ERETEISLKDSTLDN **Charge** 2 **Spectral Angle** 0.87

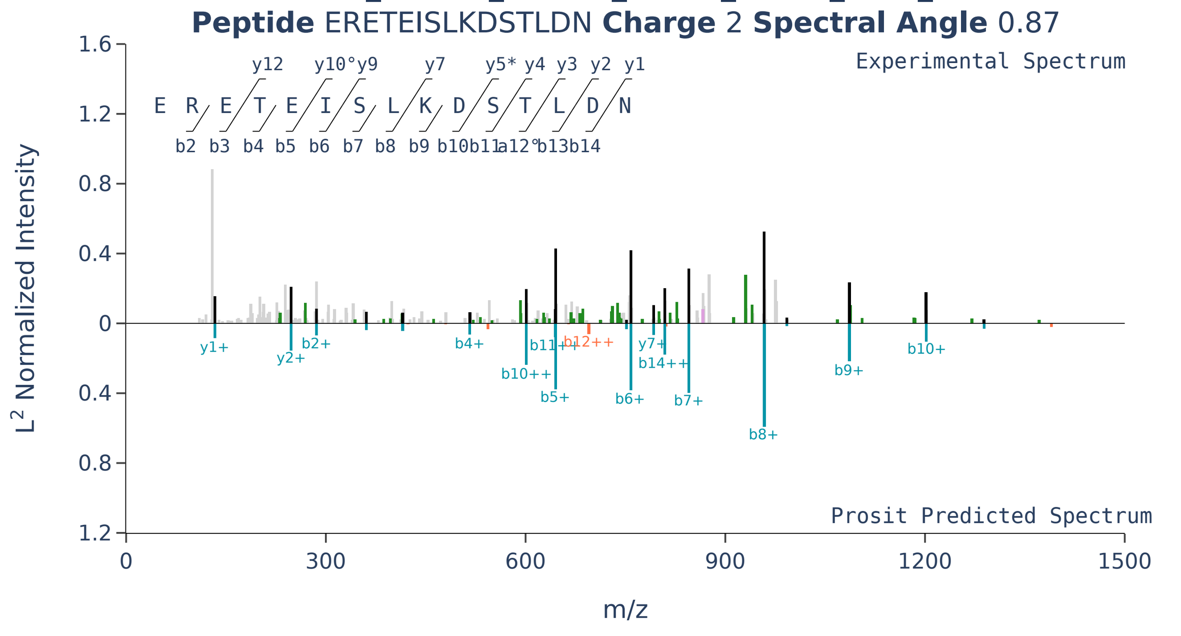


**Source** HRoetschke_271222_090123_HFGoe_LEDGF_b2R1_24h **Scan** 17523
**Peptide** LWEIDNNPKVK **Charge** 3 **Spectral Angle** 0.8

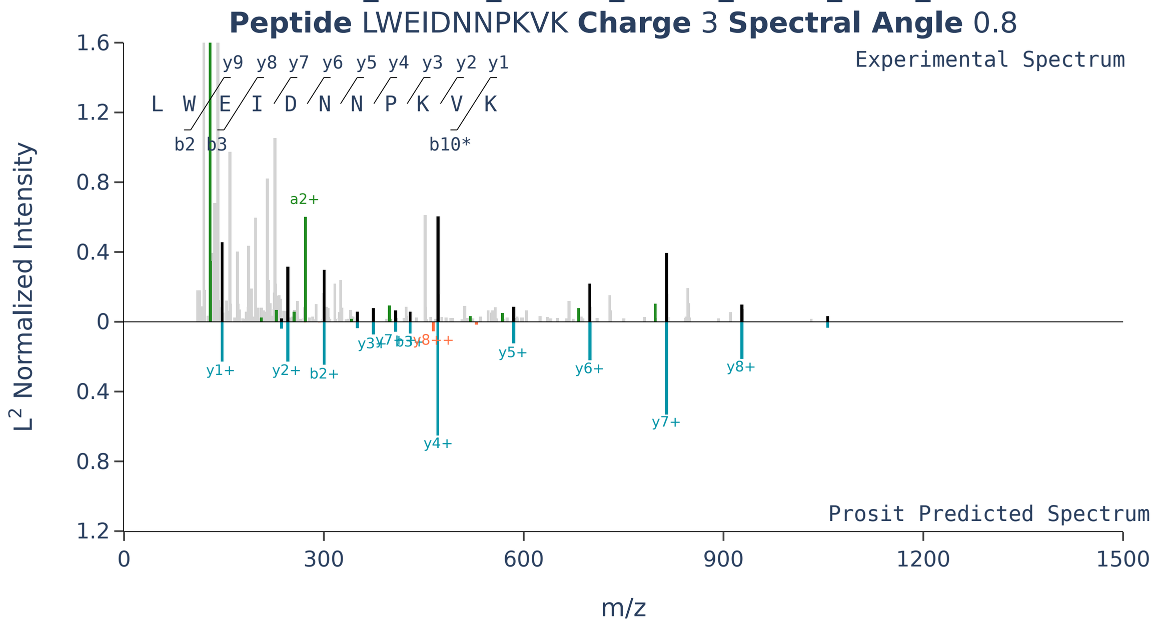


**Source** HRoetschke_271222_090123_HFGoe_LEDGF_b2R1_24h **Scan** 24046
**Peptide** SLKIDNLDV **Charge** 1 **Spectral Angle** 0.84

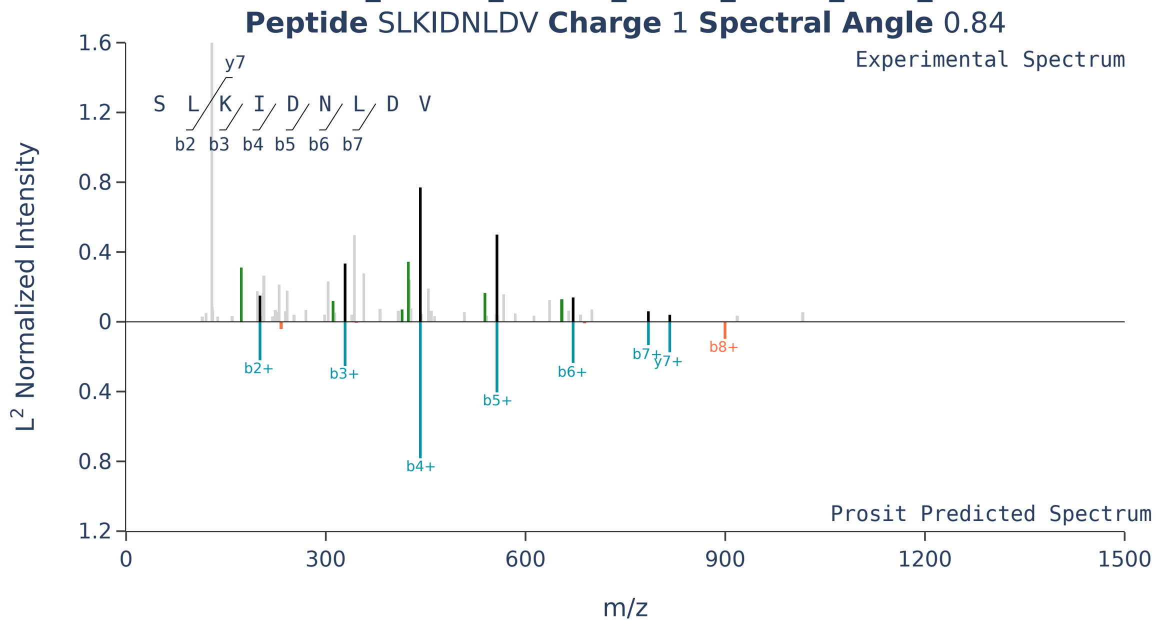


**Source** HRoetschke_271222_090123_HFGoe_LEDGF_b1R1_4h **Scan** 23143
**Peptide** NKFKNMFLVGE **Charge** 3 **Spectral Angle** 0.92

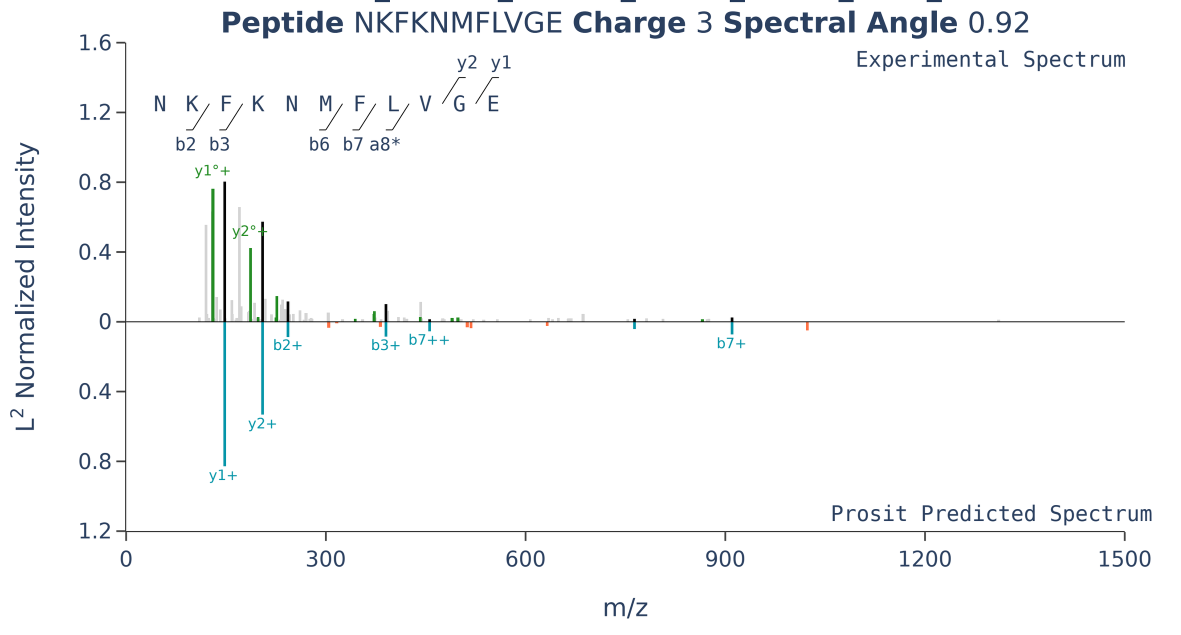


**Source** HRoetschke_271222_090123_HFGoe_LEDGF_b1R1_4h **Scan** 8055
**Peptide** AATKQSNASSDVEVE **Charge** 2 **Spectral Angle** 0.81

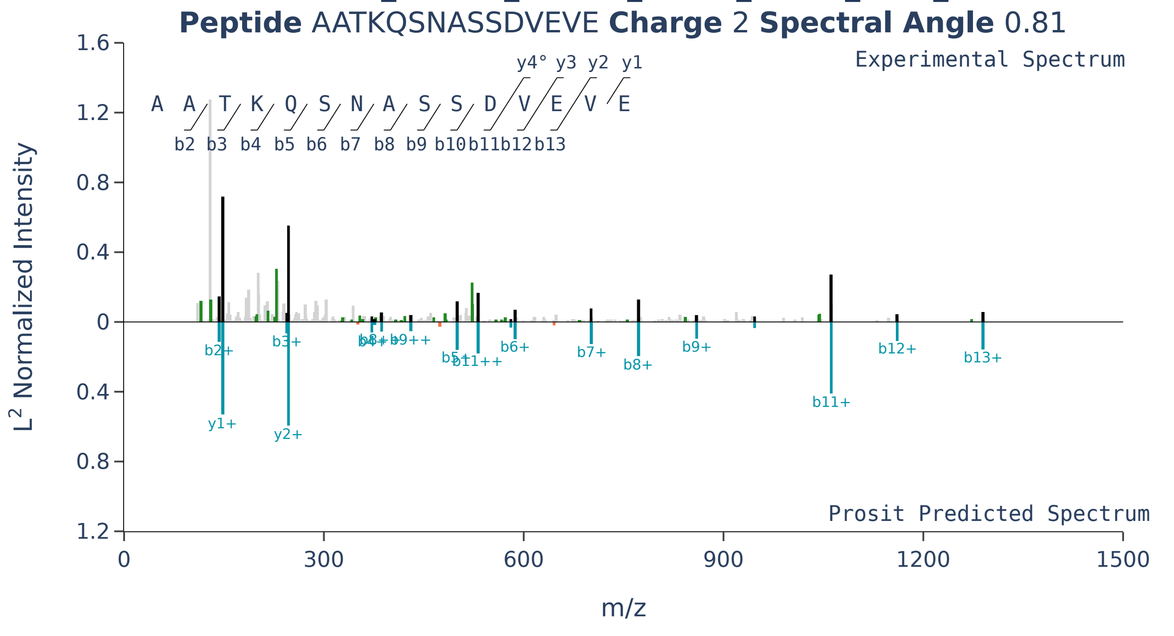


**Source** HRoetschke_271222_090123_HFGoe_LEDGF_b2R1_4h **Scan** 7967
**Peptide** SMDSRLQRIHA **Charge** 2 **Spectral Angle** 0.92

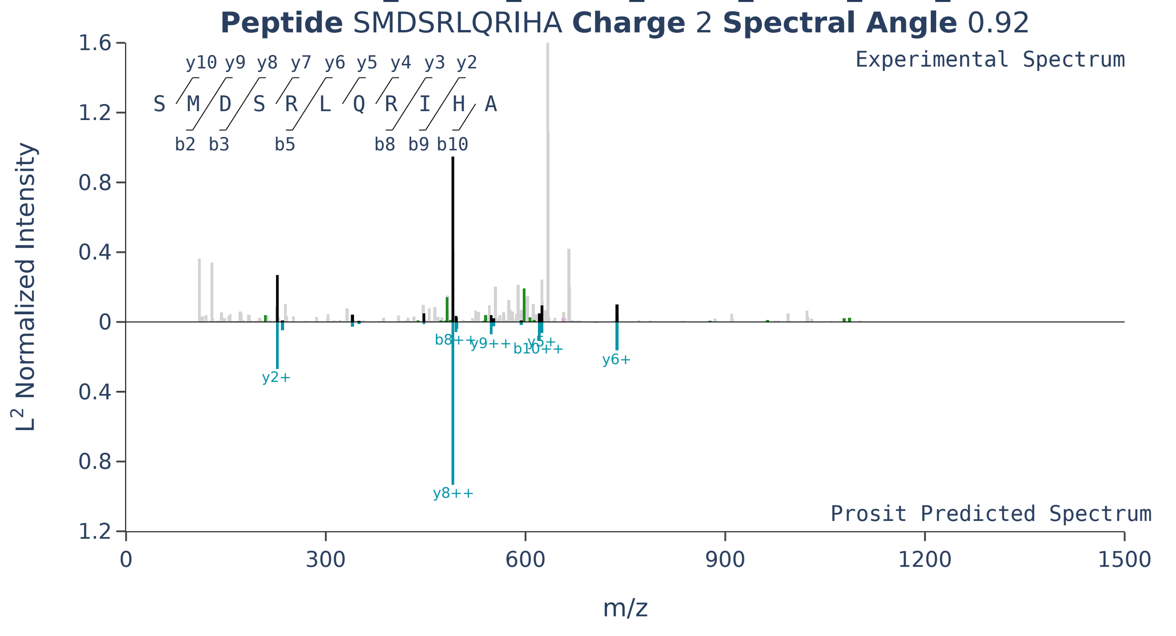


**Source** HRoetschke_271222_090123_HFGoe_LEDGF_b1R1_4h **Scan** 20903
**Peptide** SQVIMEKSTML **Charge** 2 **Spectral Angle** 0.91

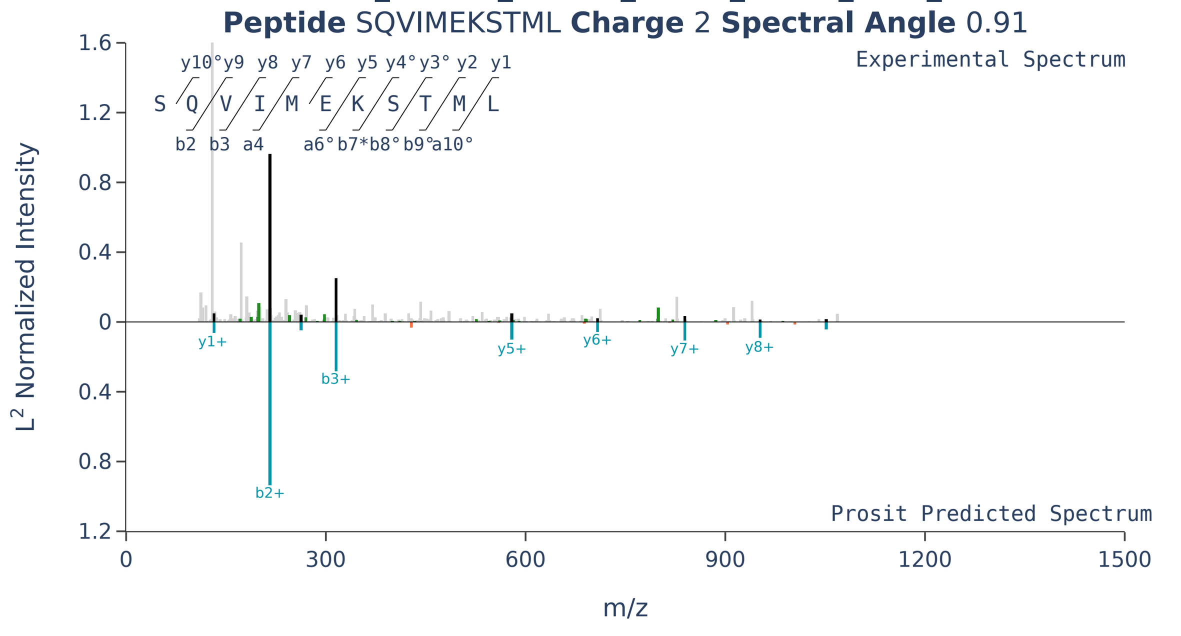

**Source** HRoetschke_271222_090123_HFGoe_LEDGF_b1R1_4h **Scan** 22408
**Peptide** LIFAKMKGYPHWPARVD **Charge** 5 **Spectral Angle** 0.91

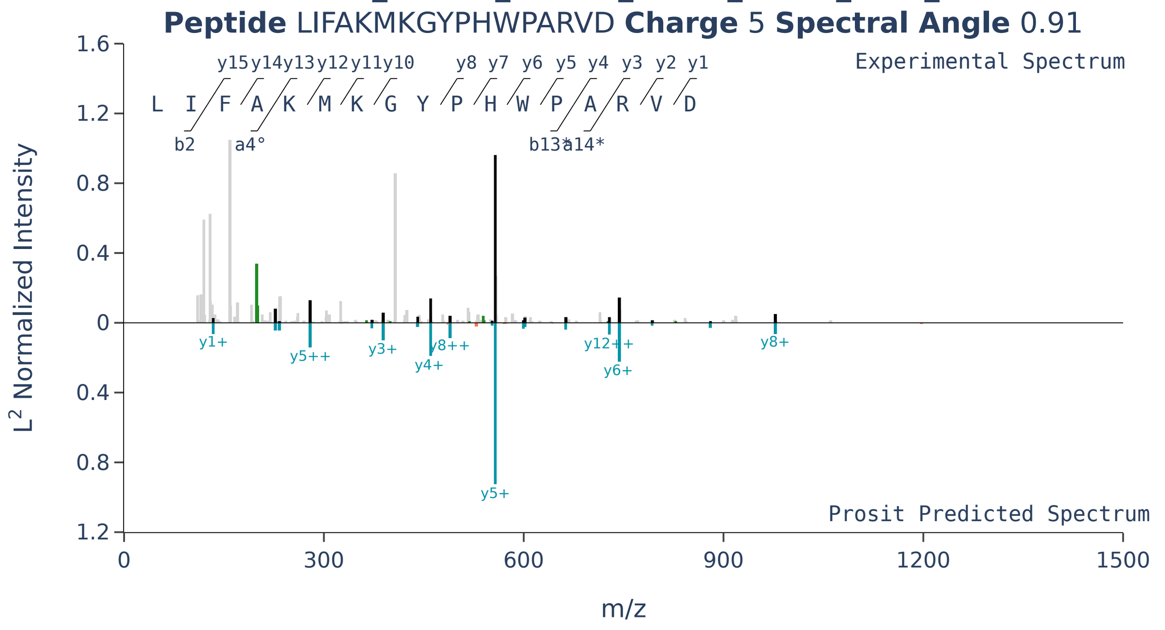


**Source** HRoetschke_271222_090123_HFGoe_LEDGF_b1R1_24h **Scan** 15826
**Peptide** ASVNLKV **Charge** 2 **Spectral Angle** 0.88

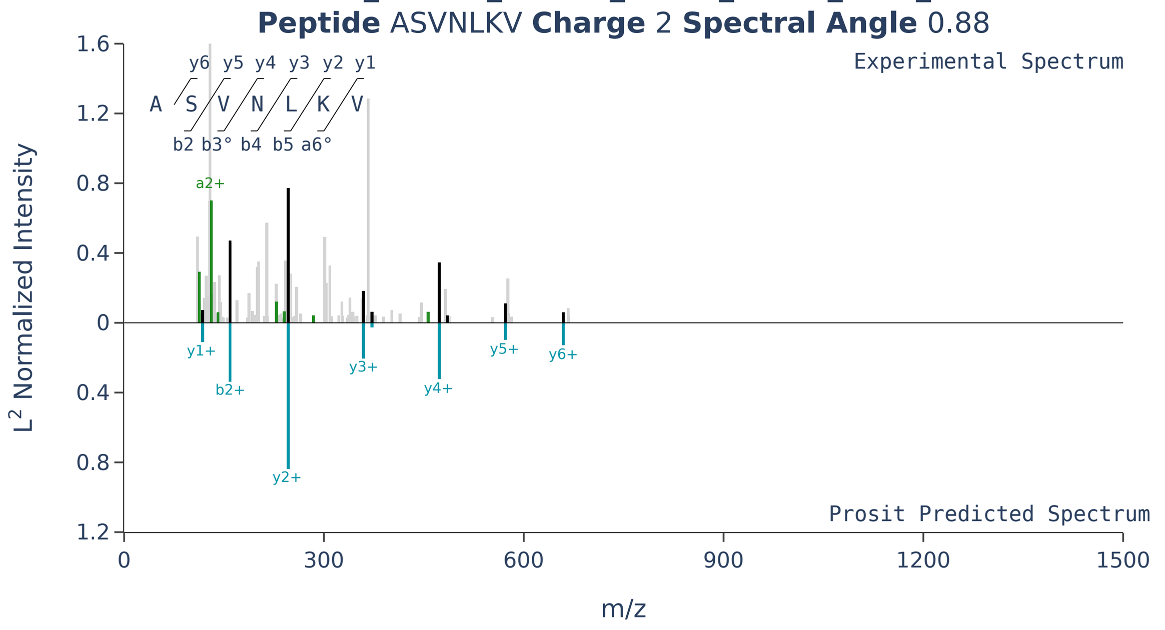


**Source** HRoetschke_271222_090123_HFGoe_LEDGF_b1R1_4h **Scan** 21915
**Peptide** ALDELASLQVTMQQAQKH **Charge** 3 **Spectral Angle** 0.83

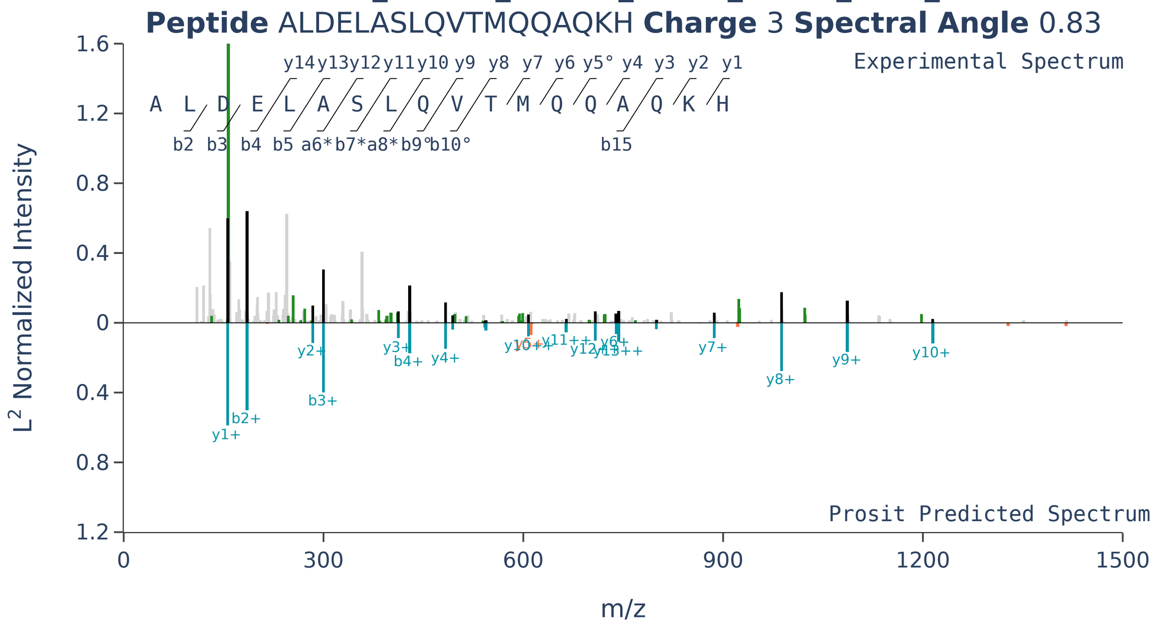


**Source** HRoetschke_271222_090123_HFGoe_LEDGF_b1R2_24h **Scan** 6868
**Peptide** SMDSRLQR **Charge** 2 **Spectral Angle** 0.93

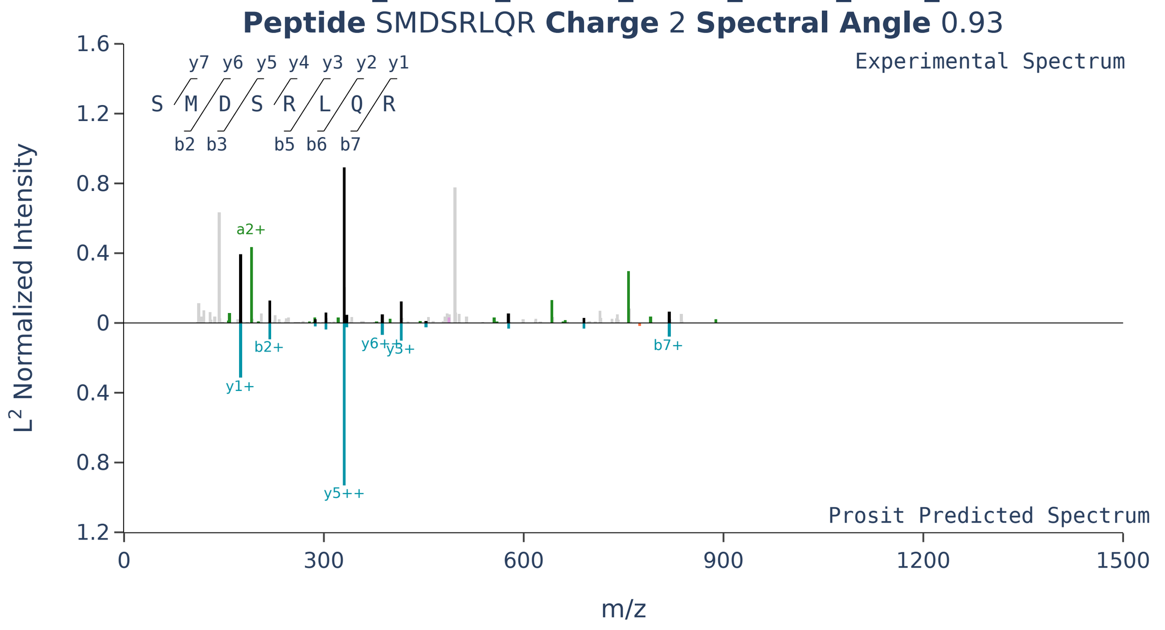


**Source** HRoetschke_271222_090123_HFGoe_LEDGF_b2R2_4h **Scan** 11862
**Peptide** TEMITTLKK **Charge** 3 **Spectral Angle** 0.84

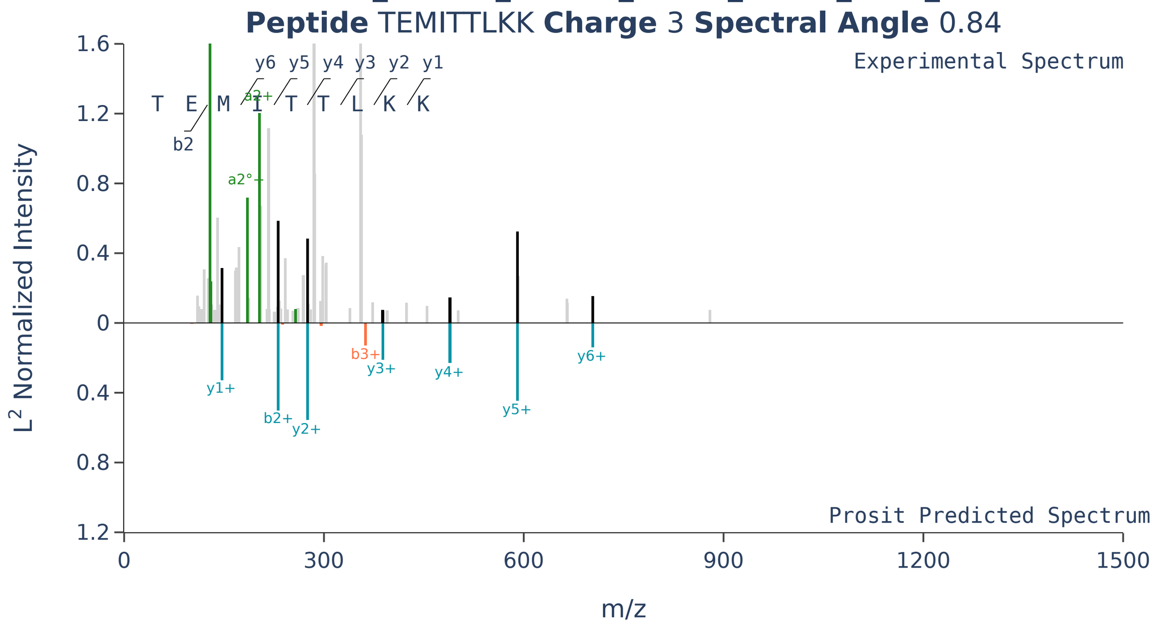


**Source** HRoetschke_271222_090123_HFGoe_LEDGF_b2R2_24h **Scan** 16107
**Peptide** AGVVTTATASVNLKVSPKRGRPAATEV **Charge** 4 **Spectral Angle** 0.82

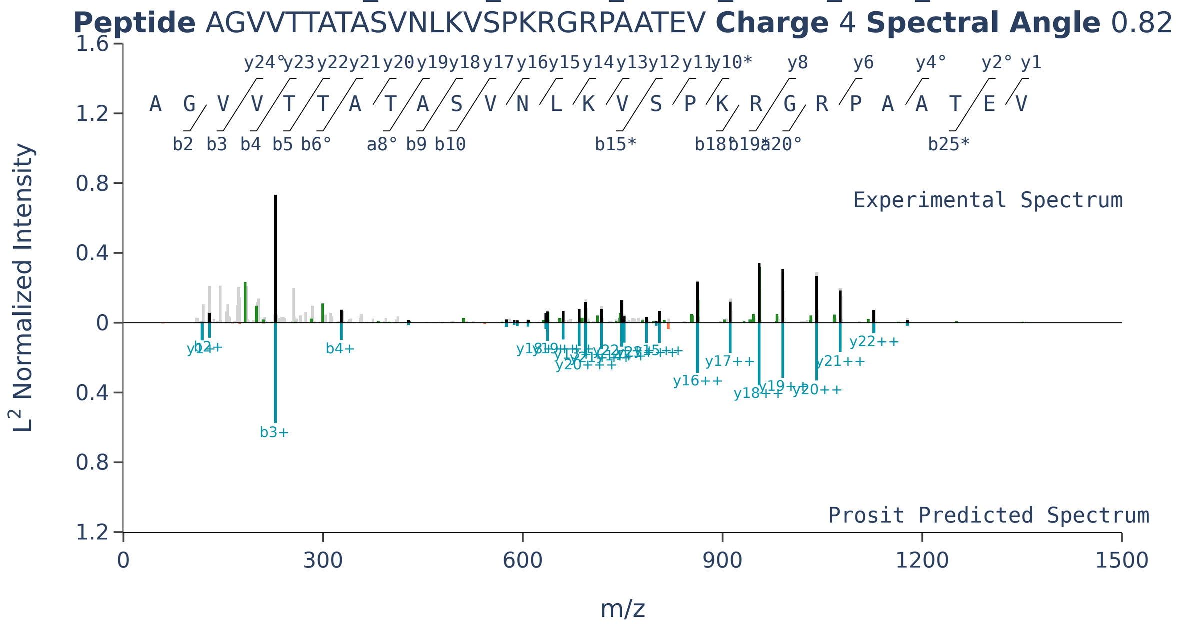


**Source** HRoetschke_271222_090123_HFGoe_LEDGF_b1R2_1h **Scan** 26144
**Peptide** TAFLGPKDIFPYSENKE **Charge** 3 **Spectral Angle** 0.85

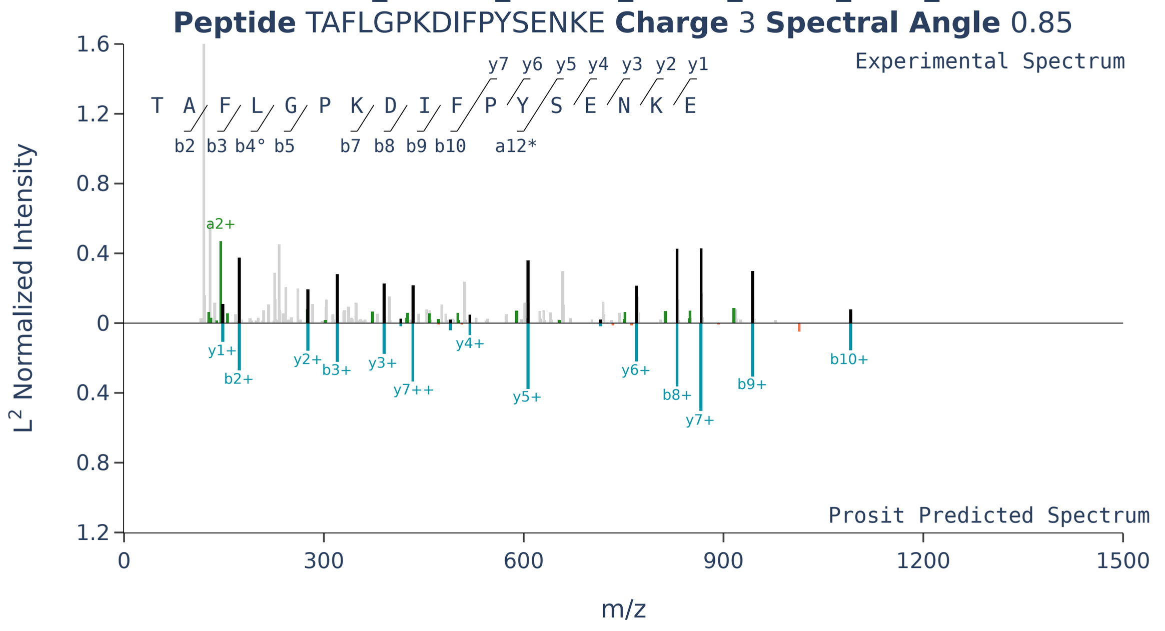


**Source** HRoetschke_271222_090123_HFGoe_LEDGF_b2R1_2h **Scan** 9105
**Peptide** SQQAATKQSNASSDVEVEE **Charge** 2 **Spectral Angle** 0.89

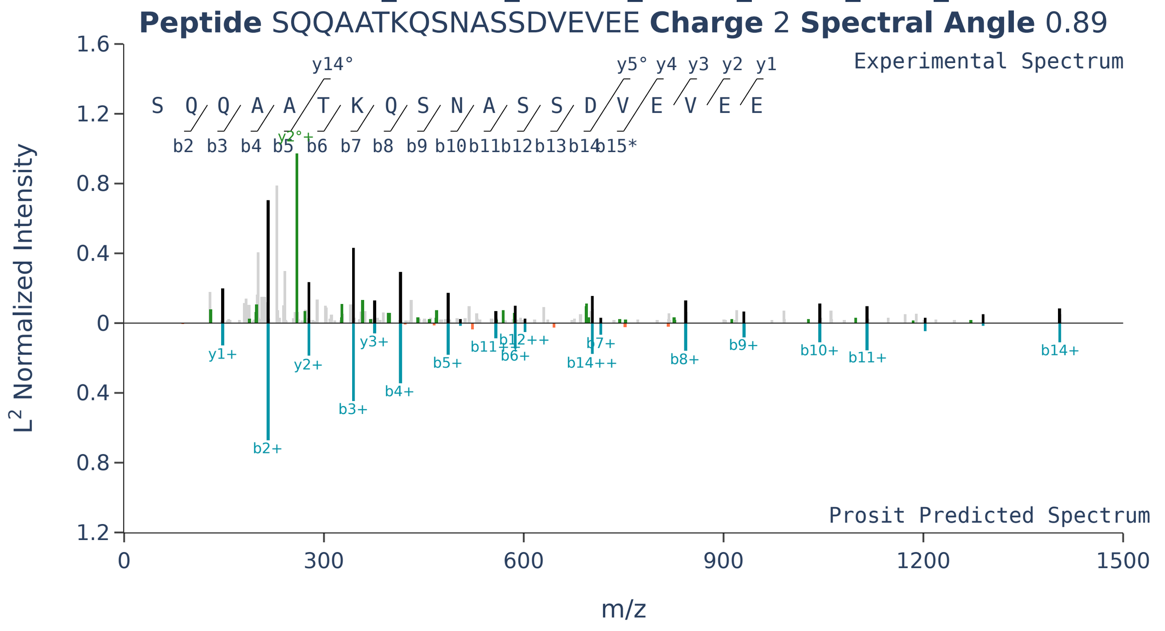


**Source** HRoetschke_271222_090123_HFGoe_LEDGF_b1R1_24h **Scan** 18274
**Peptide** KNSLKIDNLDVNRCIE **Charge** 3 **Spectral Angle** 0.89

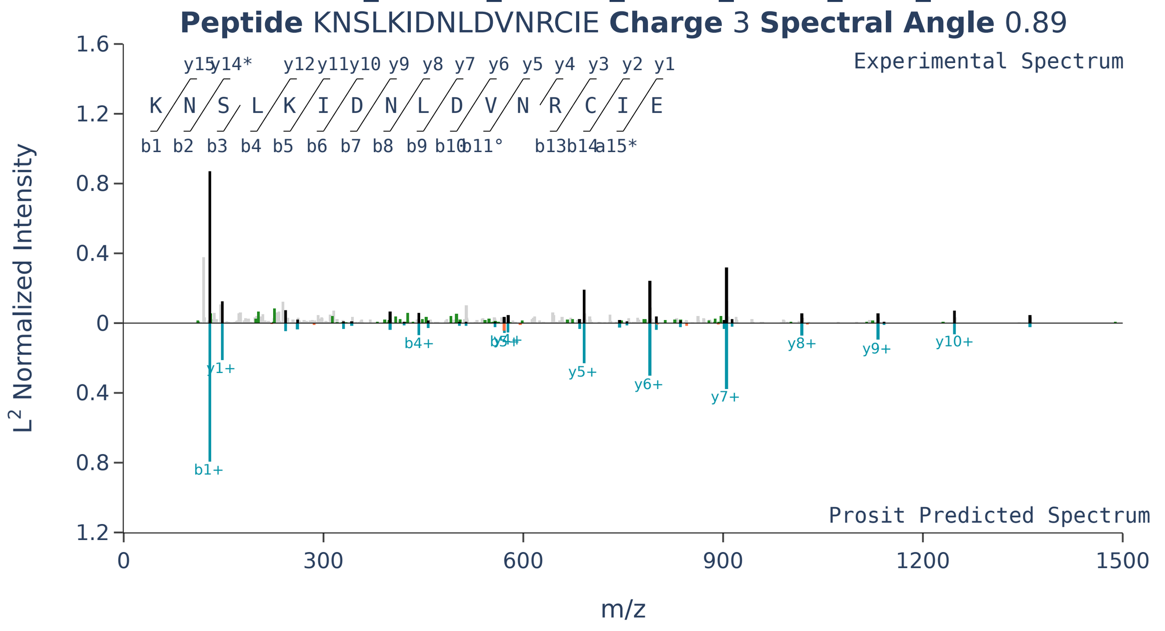


**Source** HRoetschke_271222_090123_HFGoe_LEDGF_b2R2_2h **Scan** 12633
**Peptide** THETAFLGPKD **Charge** 3 **Spectral Angle** 0.9

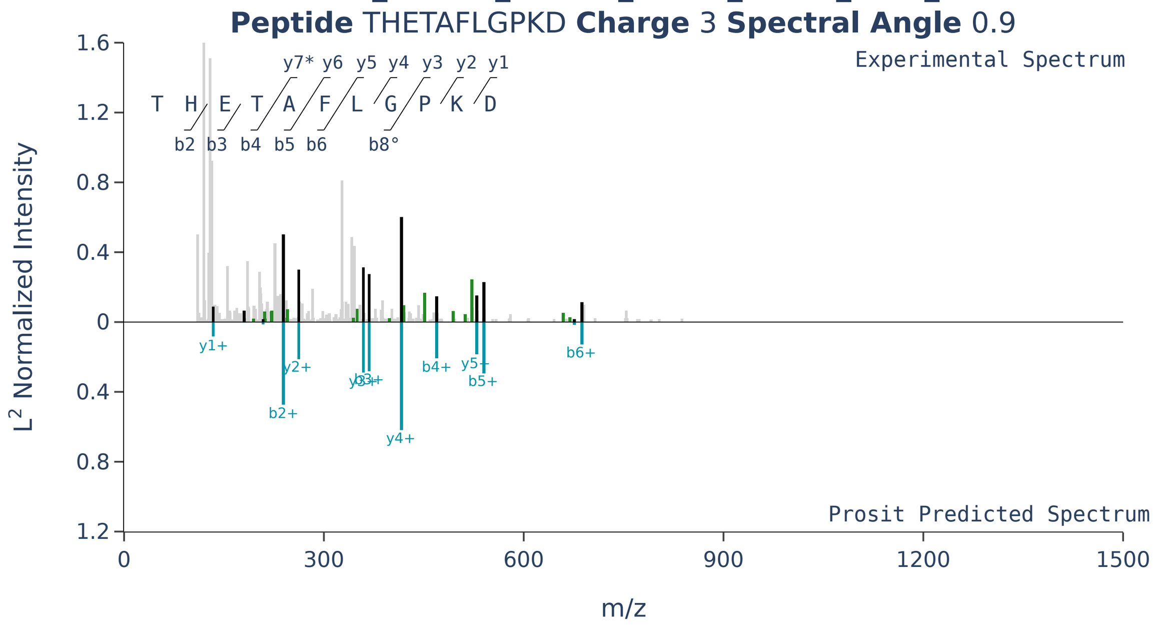


**Source** HRoetschke_271222_090123_HFGoe_LEDGF_b1R1_4h **Scan** 10988
**Peptide** TMLYNKFK **Charge** 3 **Spectral Angle** 0.82

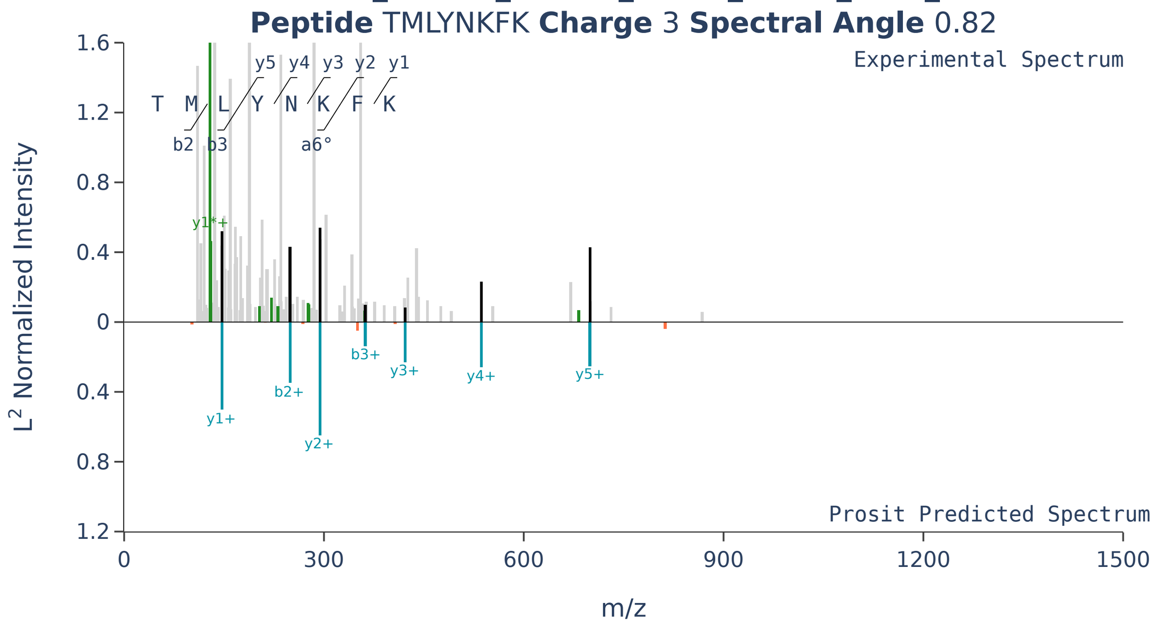


**Source** HRoetschke_271222_090123_HFGoe_LEDGF_b2R1_24h **Scan** 26862
**Peptide** DELASLQVTMQ **Charge** 2 **Spectral Angle** 0.91

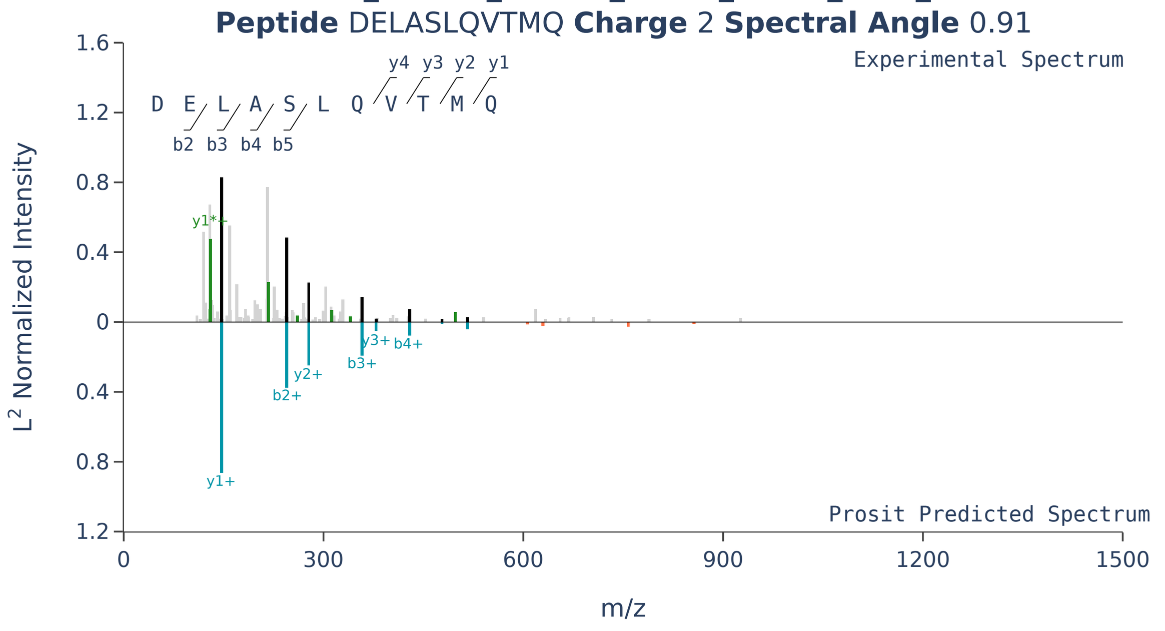


**Source** HRoetschke_271222_090123_HFGoe_LEDGF_b1R2_2h **Scan** 30051
**Peptide** PIFFFGTHE **Charge** 1 **Spectral Angle** 0.88

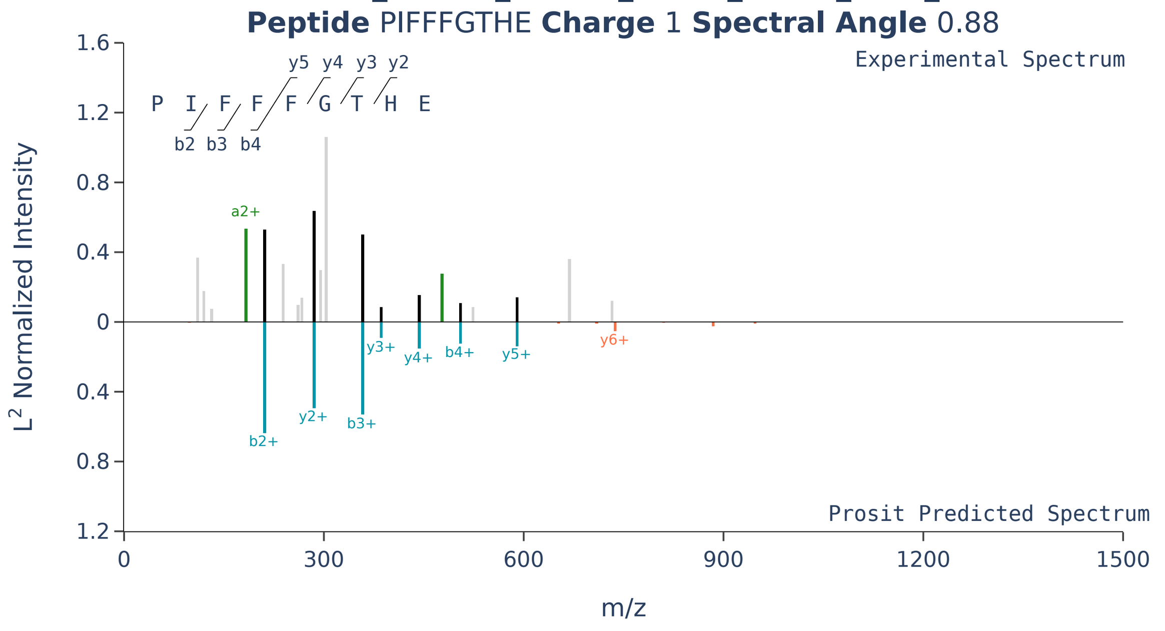


**Source** HRoetschke_271222_090123_HFGoe_LEDGF_b2R2_24h **Scan** 8267
**Peptide** TASVNLKVSPKRGRPAAT **Charge** 3 **Spectral Angle** 0.81

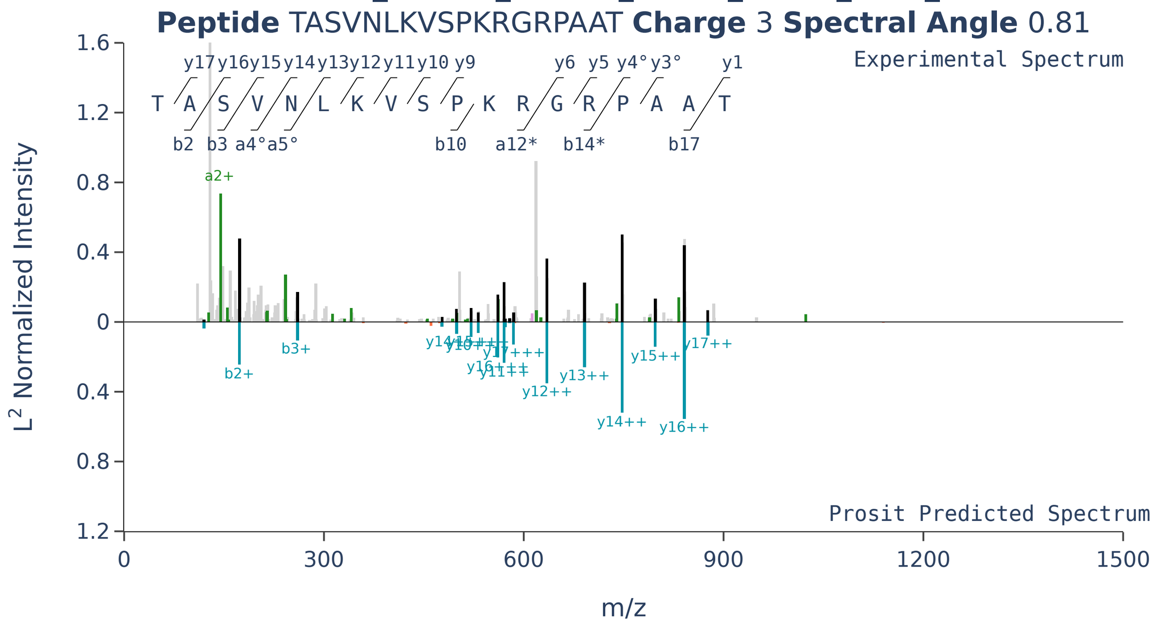


**Source** HRoetschke_271222_090123_HFGoe_LEDGF_b2R1_24h **Scan** 25086
**Peptide** VGEGDSVITQVLNK **Charge** 2 **Spectral Angle** 0.81

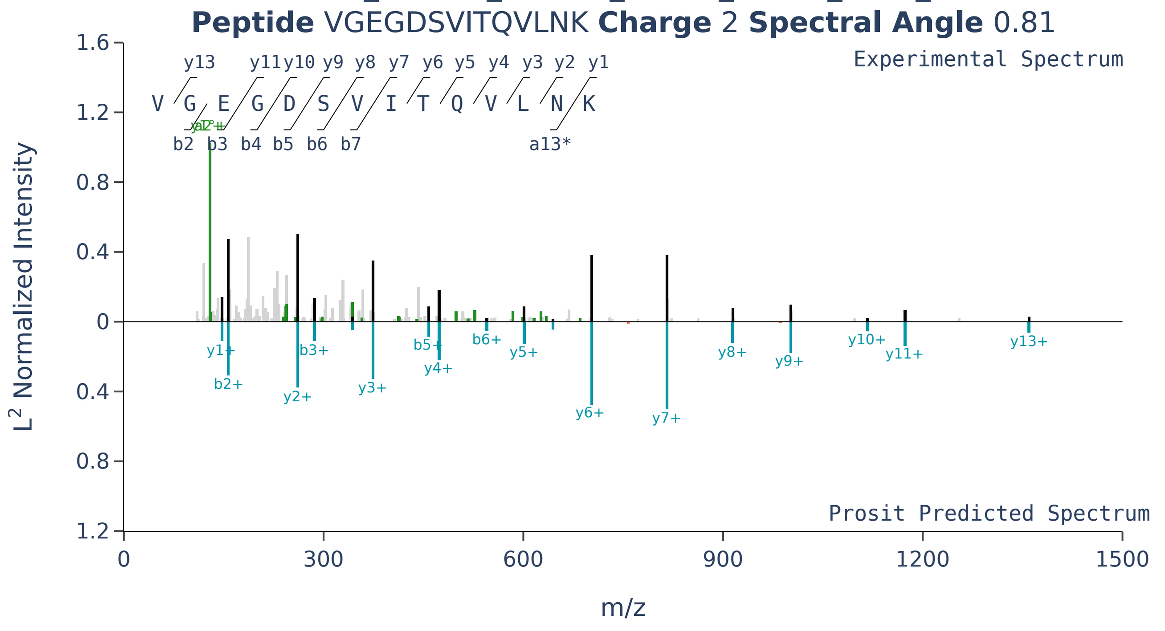

**Source** HRoetschke_271222_090123_HFGoe_LEDGF_b1R2_1h **Scan** 15660
**Peptide** IHAEIKNSLKI **Charge** 3 **Spectral Angle** 0.89

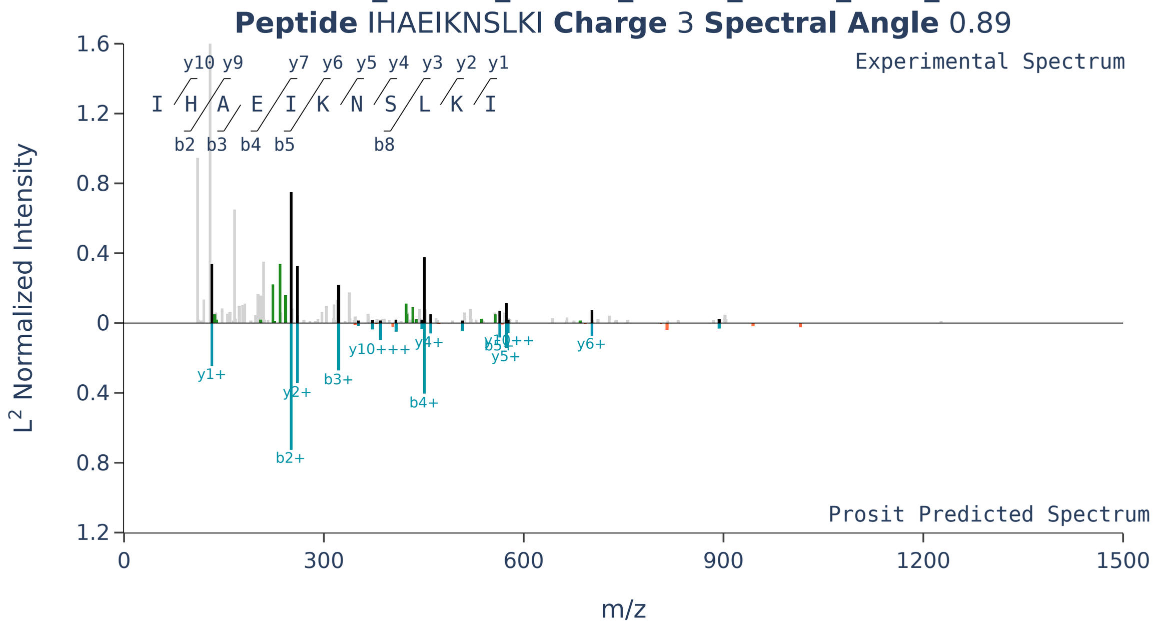


**Source** HRoetschke_271222_090123_HFGoe_LEDGF_b1R1_1h **Scan** 22250
**Peptide** KGFNEGLWEIDNNPKVKFSSQQ **Charge** 4 **Spectral Angle** 0.85

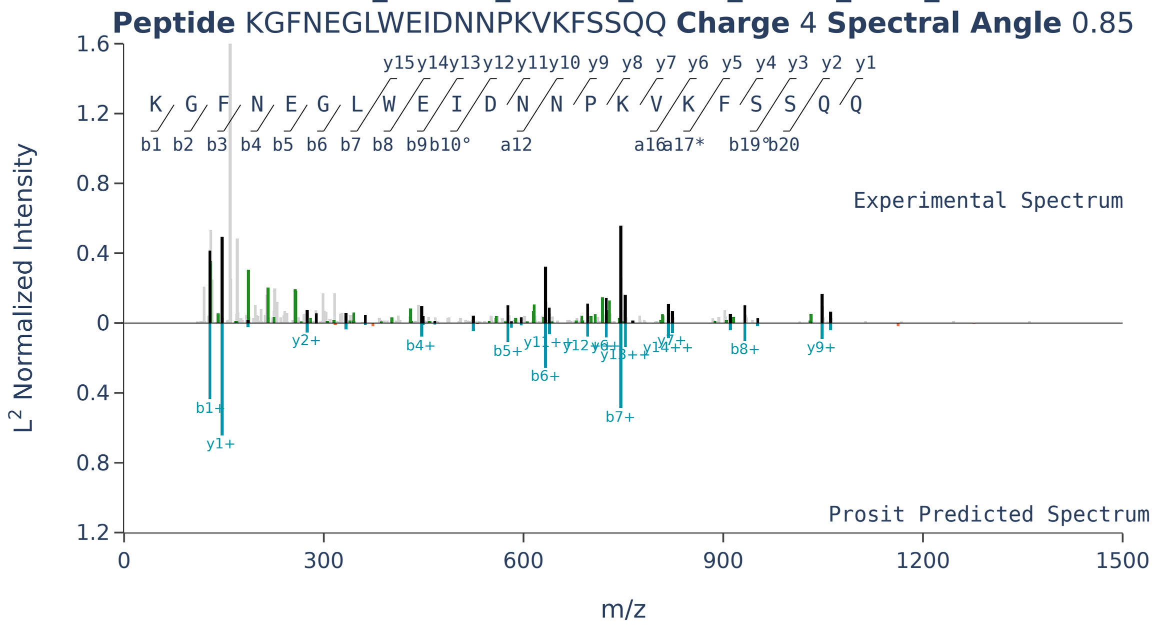


**Source** HRoetschke_271222_090123_HFGoe_LEDGF_b2R1_24h **Scan** 23094
**Peptide** GFNEGLWEIDNNPKVKFSSQQAATKQS **Charge** 3 **Spectral Angle** 0.83

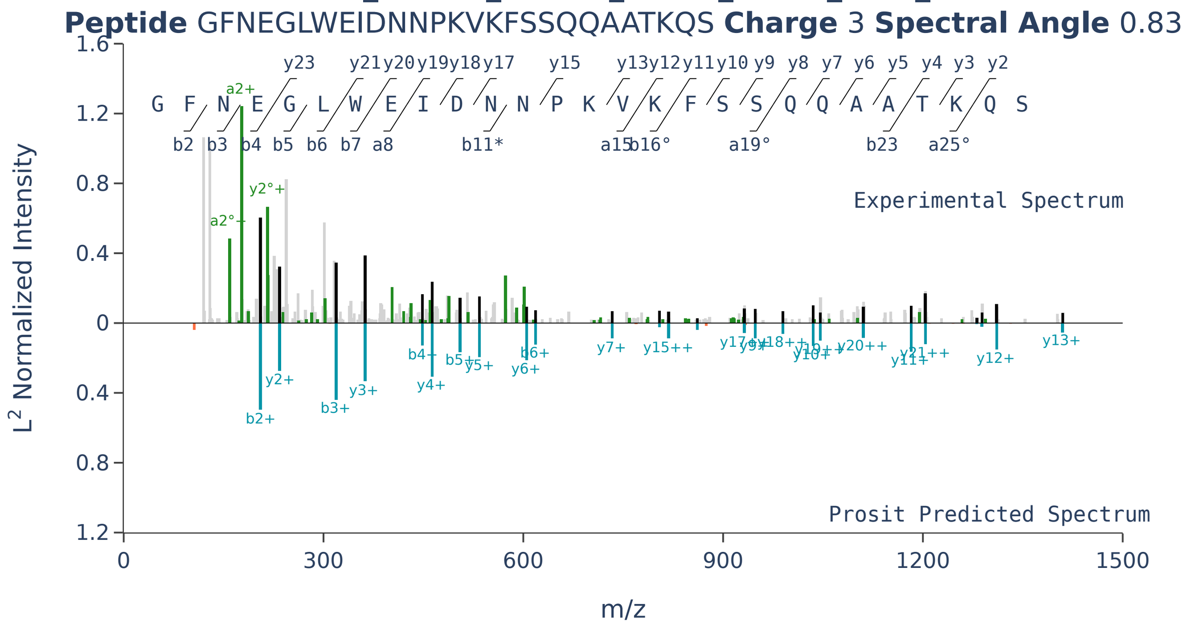


**Source** HRoetschke_271222_090123_HFGoe_LEDGF_b2R1_4h **Scan** 14298
**Peptide** ETSMDSRLQRI **Charge** 2 **Spectral Angle** 0.89

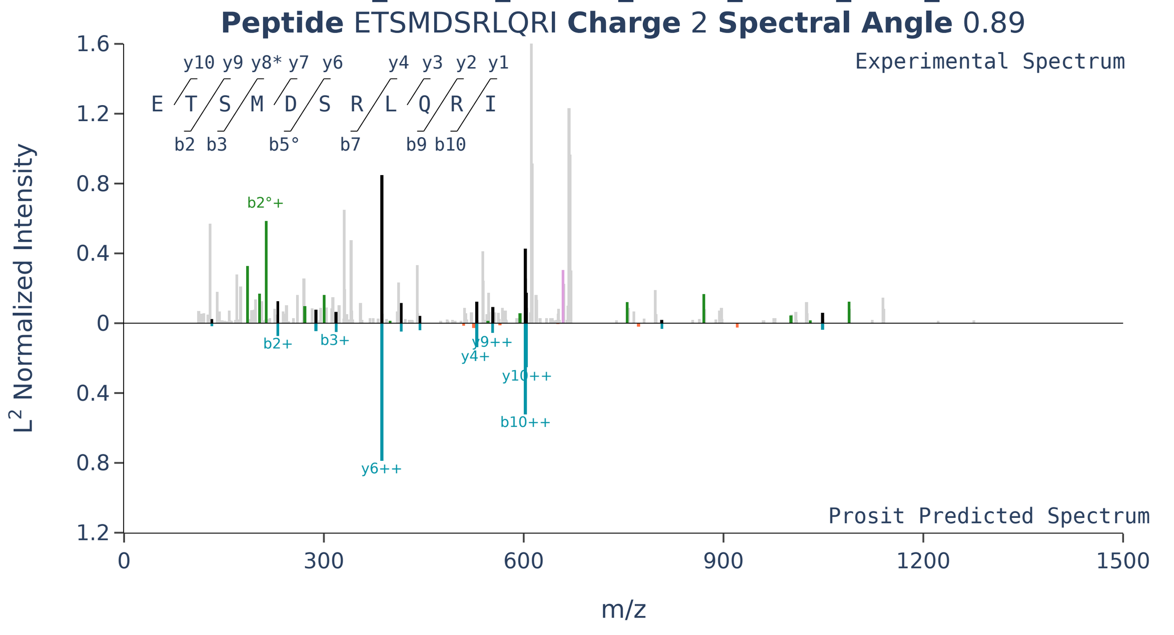


**Source** HRoetschke_271222_090123_HFGoe_LEDGF_b2R1_2h **Scan** 22307
**Peptide** FNEGLWEIDNNPKVK **Charge** 2 **Spectral Angle** 0.84

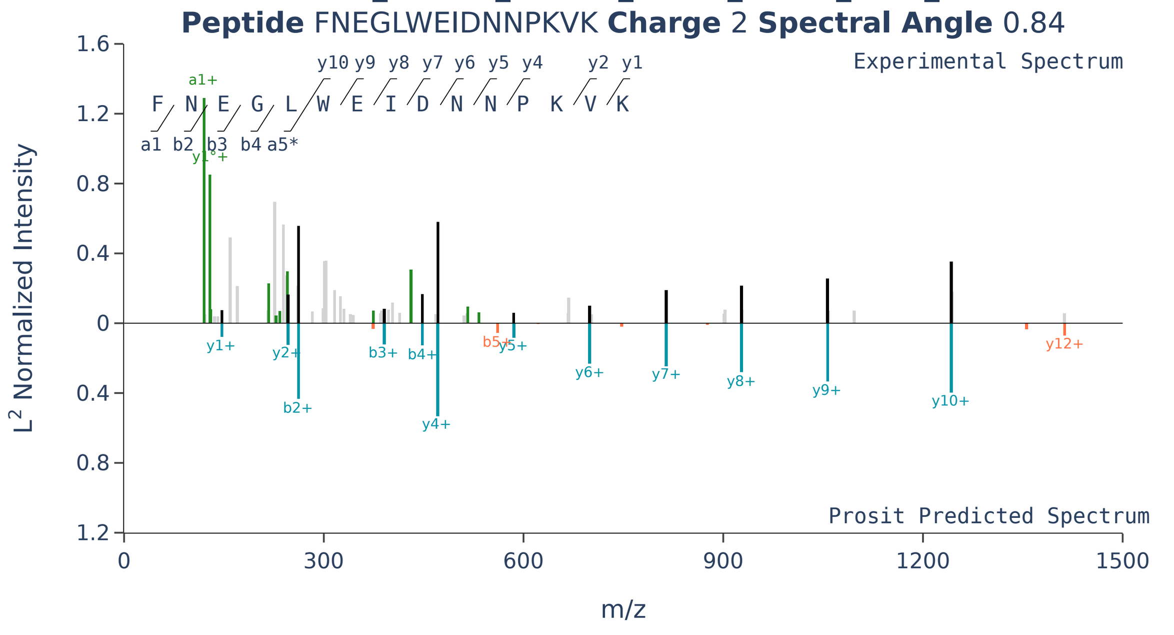


**Source** HRoetschke_271222_090123_HFGoe_LEDGF_b1R2_4h **Scan** 8718
**Peptide** IDNNPKVKFSSQQAATKQ **Charge** 2 **Spectral Angle** 0.85

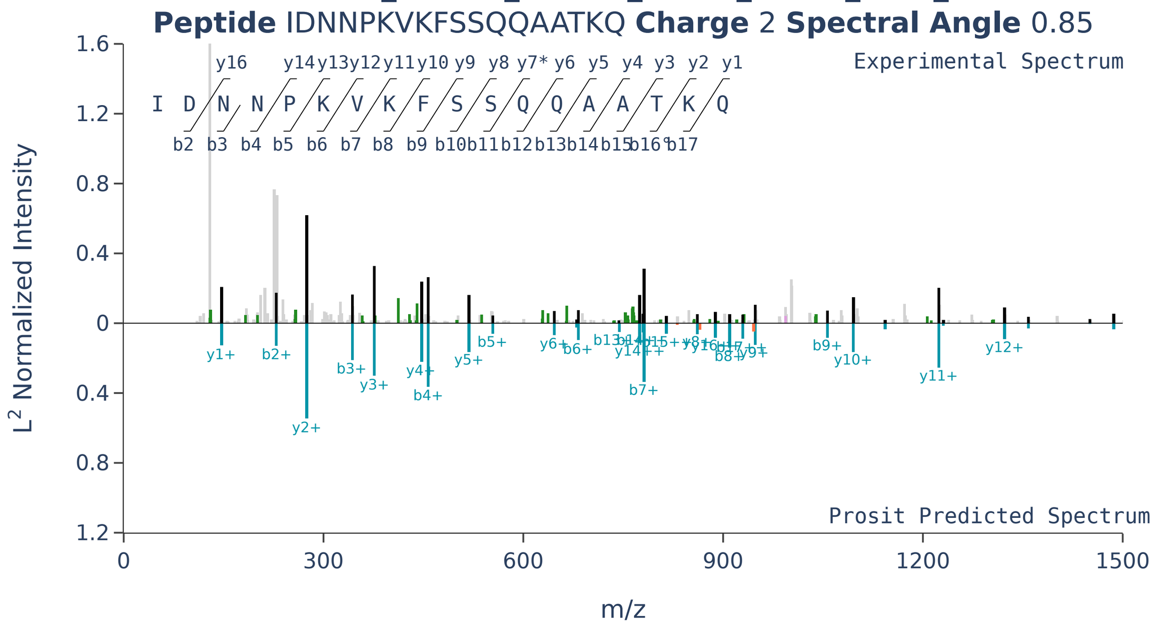


**Source** HRoetschke_271222_090123_HFGoe_LEDGF_b1R2_1h **Scan** 18350
**Peptide** TAFLGPKDIFPYSENKEKYGKPNKR **Charge** 5 **Spectral Angle** 0.79

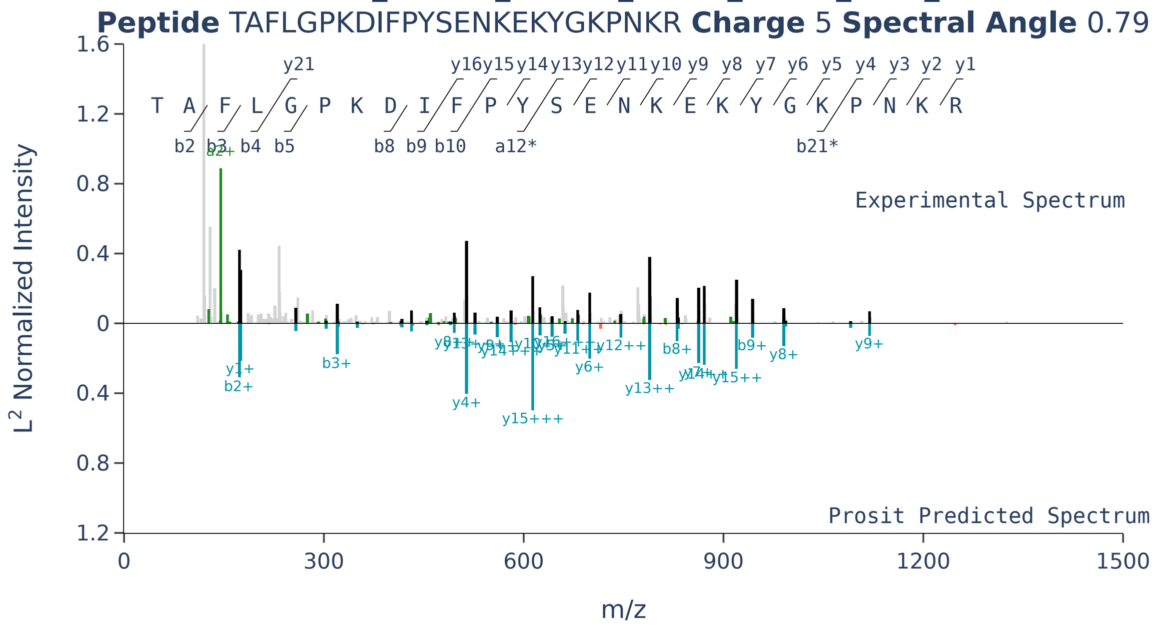


**Source** HRoetschke_271222_090123_HFGoe_LEDGF_b2R2_4h **Scan** 8308
**Peptide** SQQAATKQSNASSDVEVE **Charge** 3 **Spectral Angle** 0.86

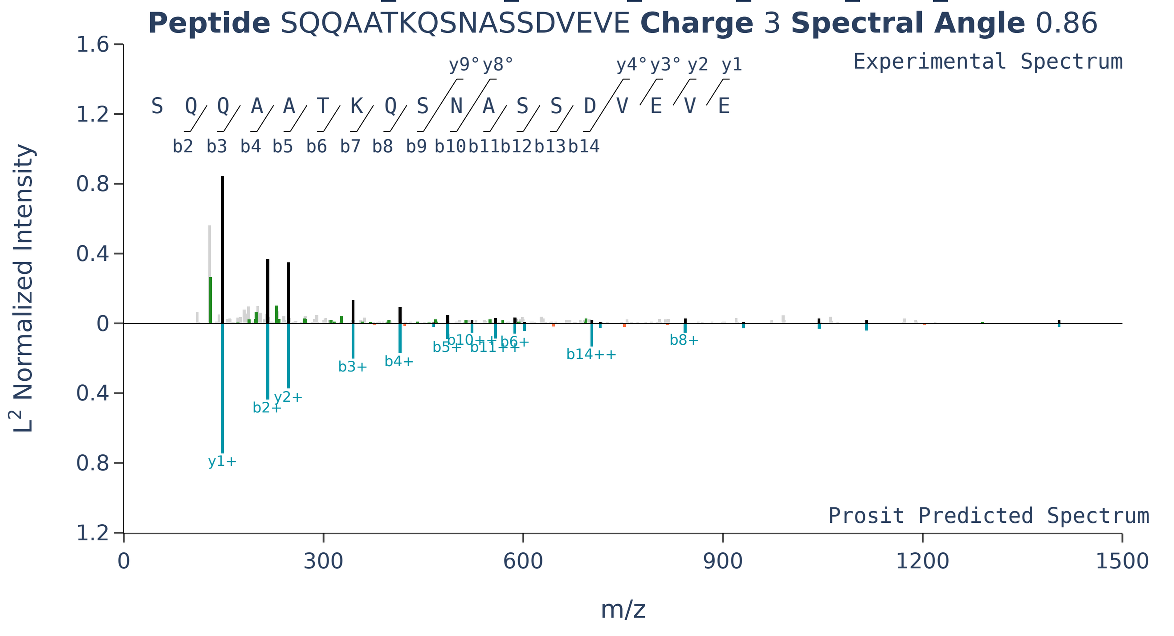


**Source** HRoetschke_271222_090123_HFGoe_LEDGF_b1R1_24h **Scan** 6412
**Peptide** AVKPPTN **Charge** 2 **Spectral Angle** 0.83

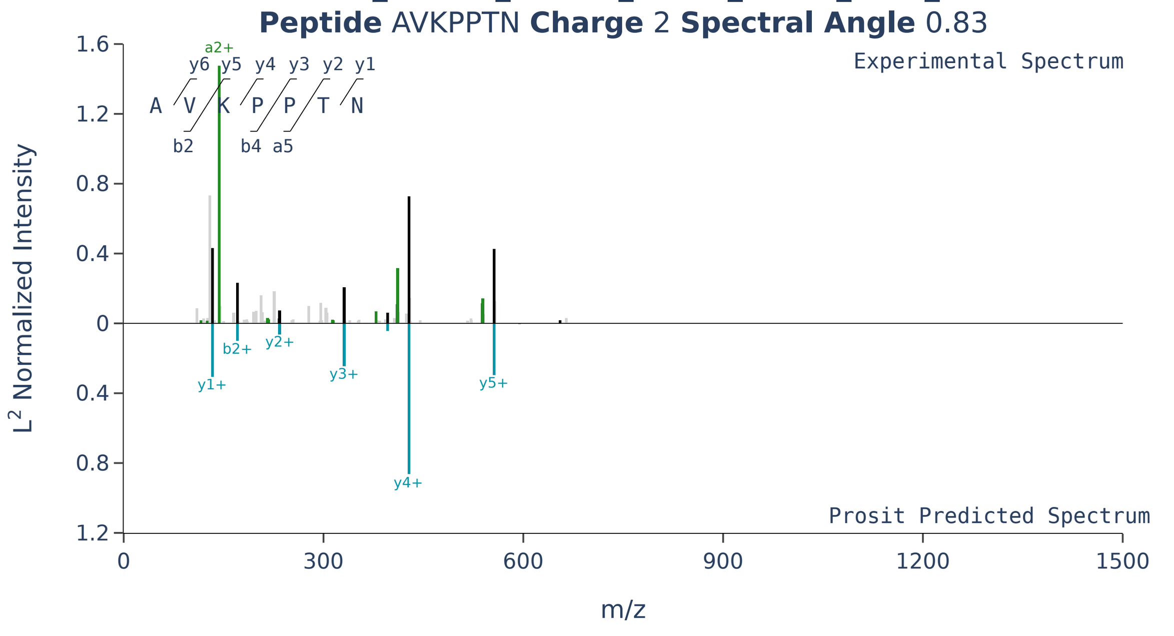


**Source** HRoetschke_271222_090123_HFGoe_LEDGF_b1R1_2h **Scan** 11495
**Peptide** DVTKAVDITTPKAARRG **Charge** 3 **Spectral Angle** 0.89

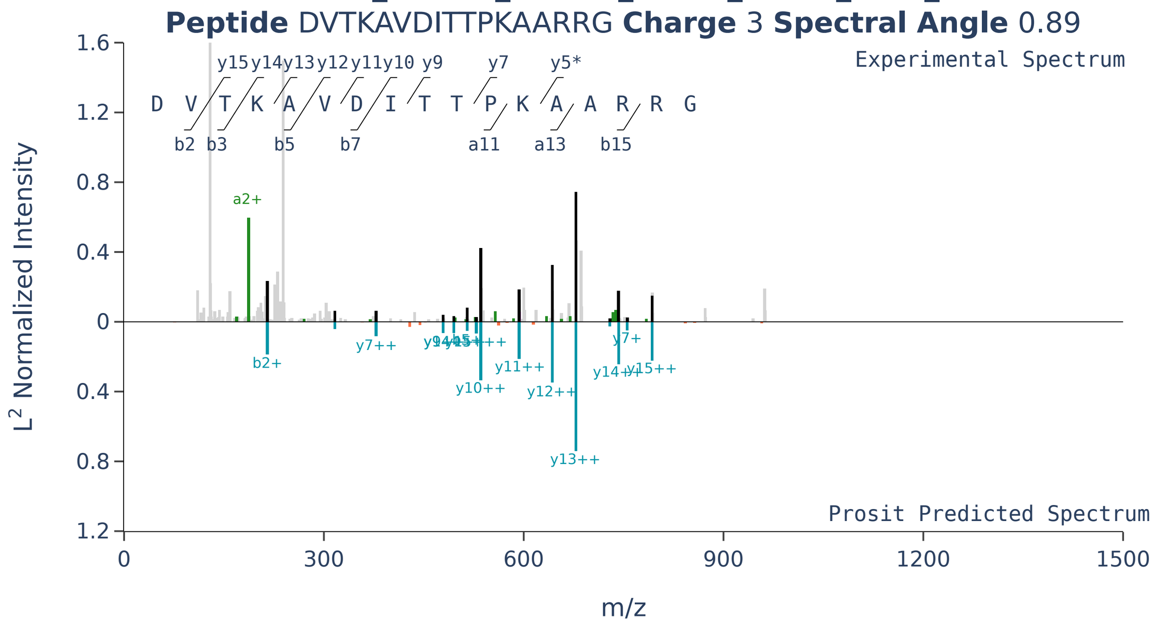


**Source** HRoetschke_271222_090123_HFGoe_LEDGF_b1R1_2h **Scan** 9425
**Peptide** KFSSQQAATKQSNASSDVEVE **Charge** 3 **Spectral Angle** 0.9

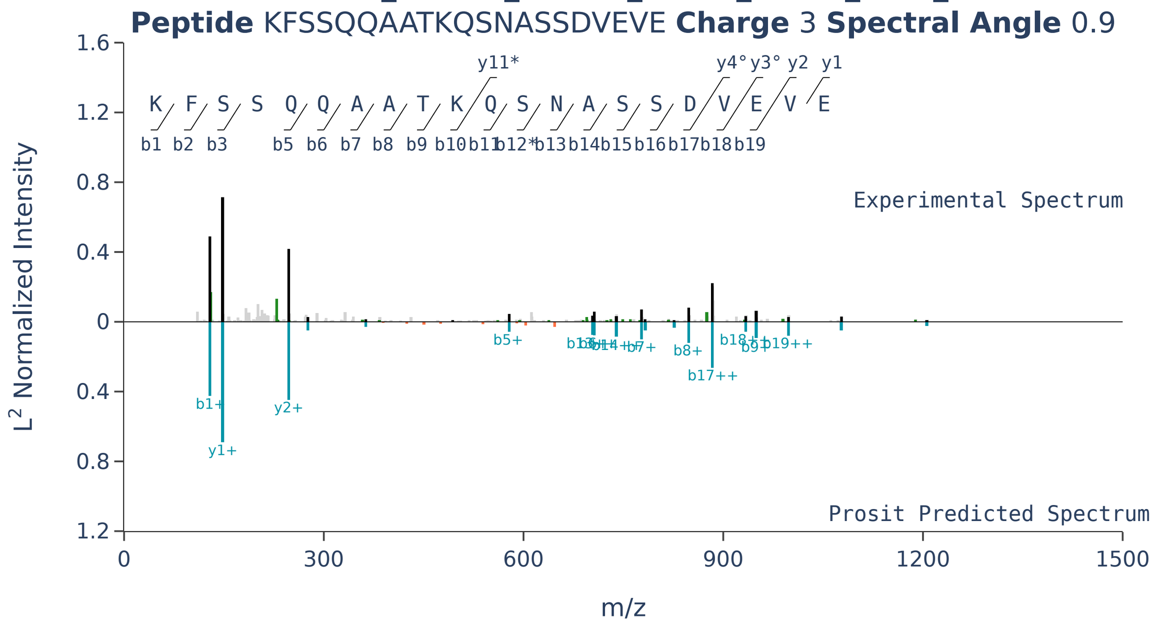


**Source** HRoetschke_271222_090123_HFGoe_LEDGF_b2R1_24h **Scan** 8542
**Peptide** ATASVNLKVSPKRGRPAAT **Charge** 3 **Spectral Angle** 0.84

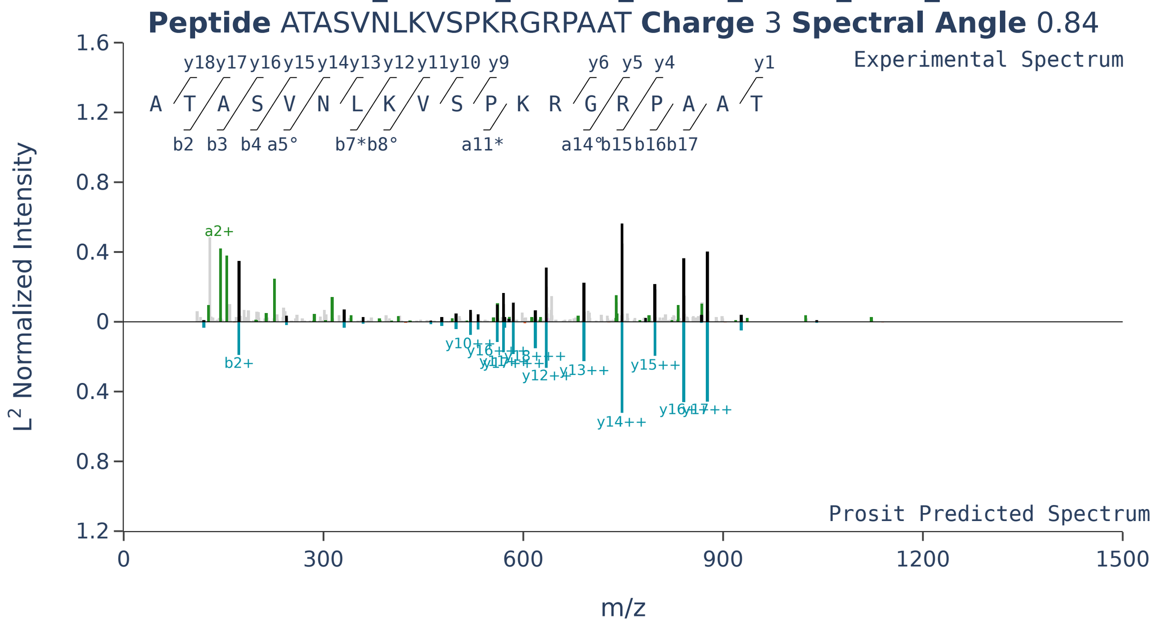


**Source** HRoetschke_271222_090123_HFGoe_LEDGF_b1R2_2h **Scan** 8217
**Peptide** TASVNLKVSPKRGRPAA **Charge** 4 **Spectral Angle** 0.83

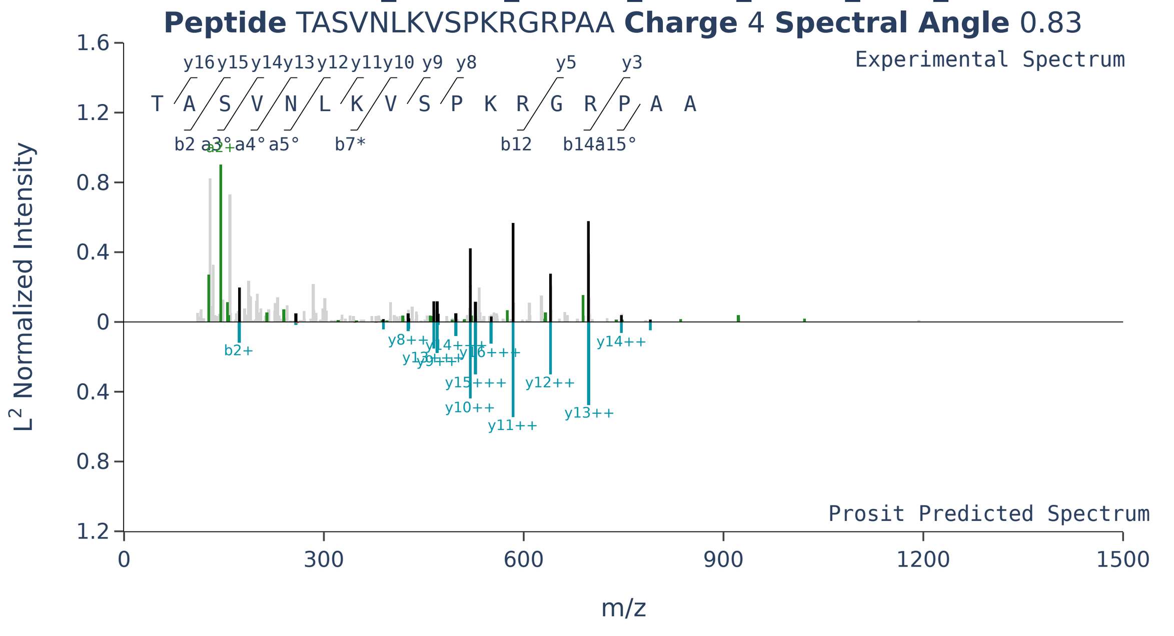


**Source** HRoetschke_271222_090123_HFGoe_LEDGF_b2R2_24h **Scan** 13662
**Peptide** GYPHWPAR **Charge** 2 **Spectral Angle** 0.85

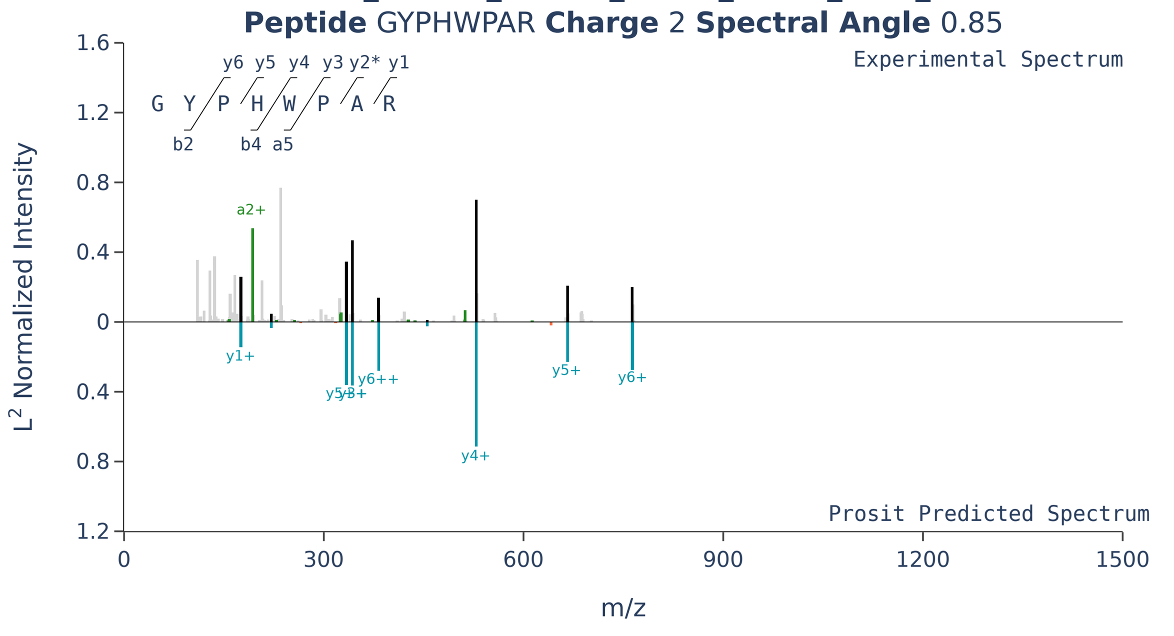


**Source** HRoetschke_271222_090123_HFGoe_LEDGF_b2R1_2h **Scan** 6719
**Peptide** HEEKASNEDVTKAVD **Charge** 2 **Spectral Angle** 0.82

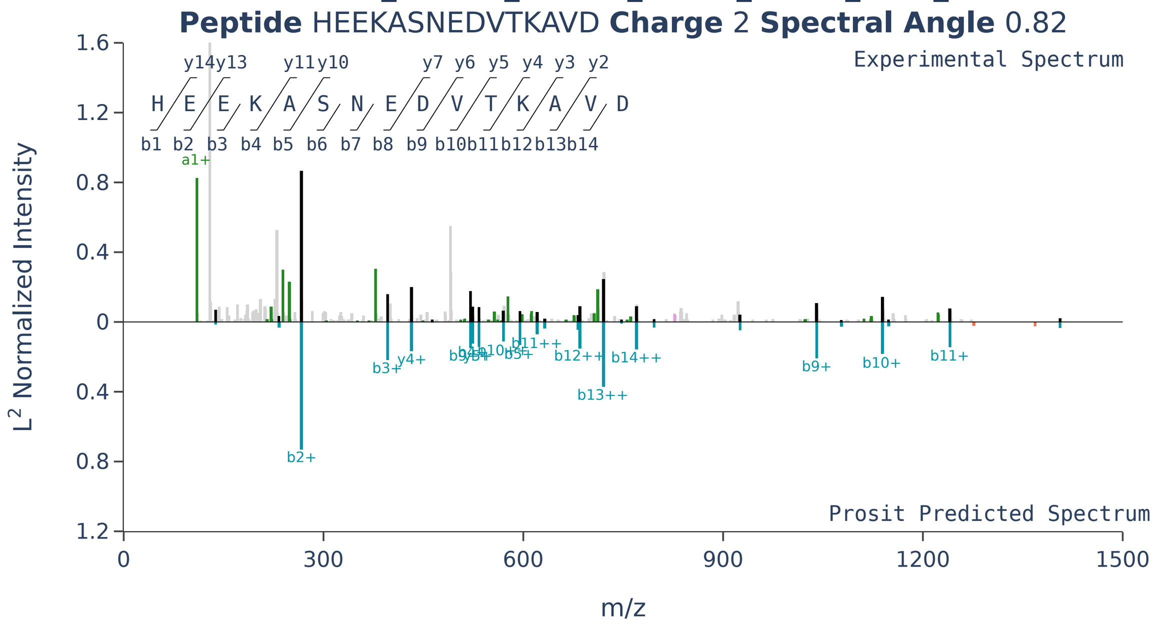

**Source** HRoetschke_271222_090123_HFGoe_LEDGF_b2R1_2h **Scan** 9739
**Peptide** DNNPKVKFSSQQAA **Charge** 2 **Spectral Angle** 0.88

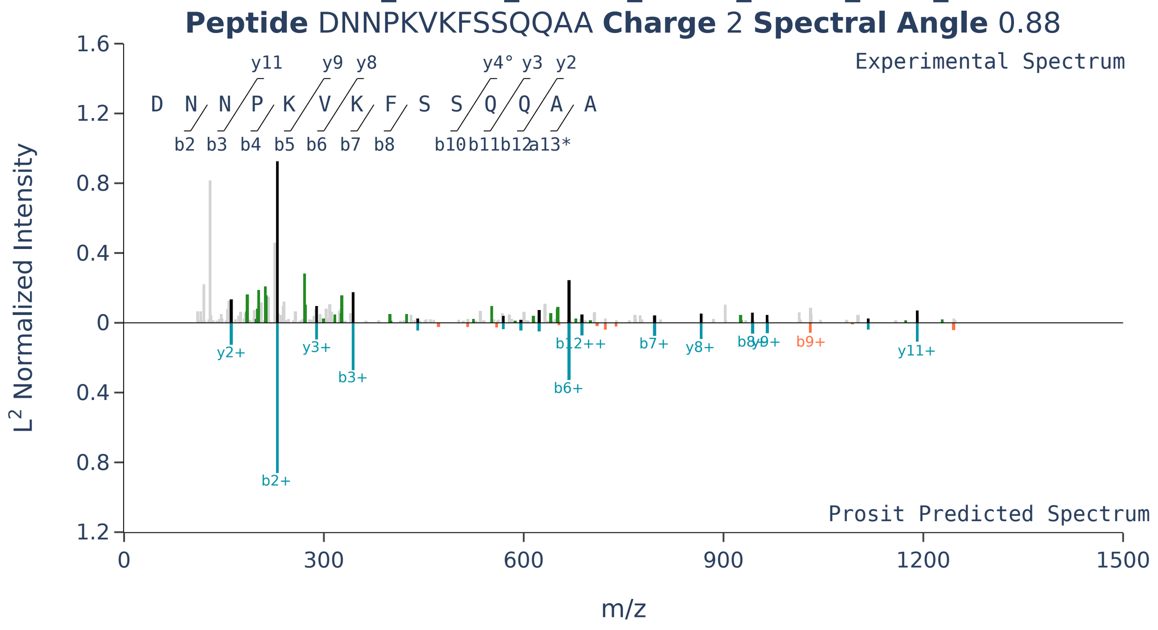


**Source** HRoetschke_271222_090123_HFGoe_LEDGF_b1R2_2h **Scan** 9358
**Peptide** TEVKIPKPRG **Charge** 3 **Spectral Angle** 0.94

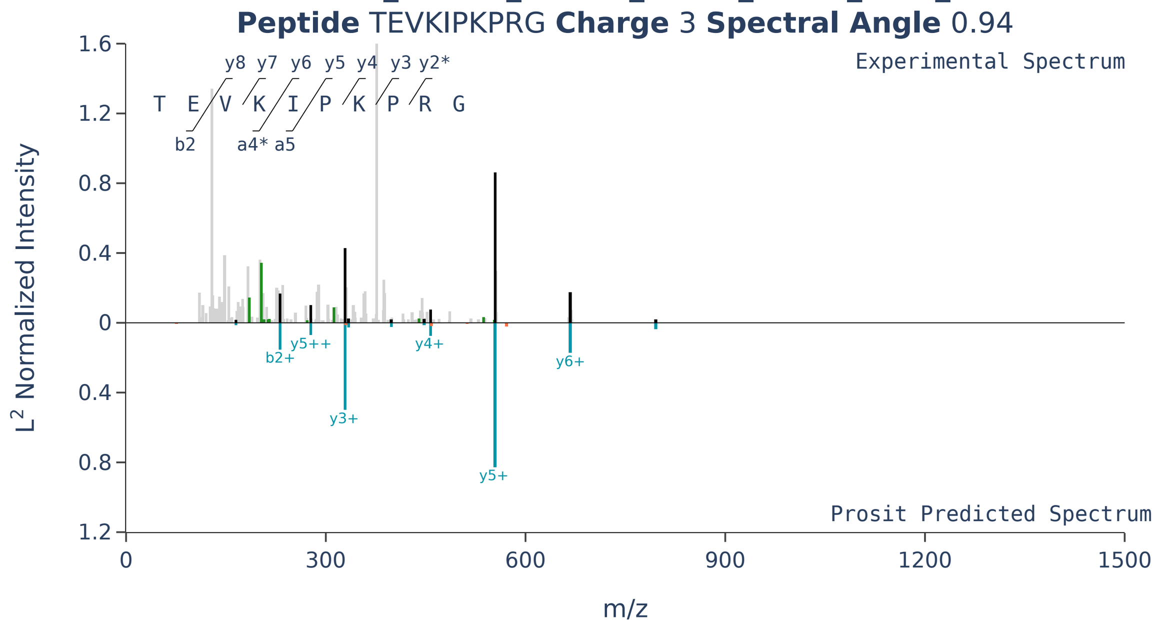


**Source** HRoetschke_271222_090123_HFGoe_LEDGF_b1R2_4h **Scan** 8303
**Peptide** AQKHTEMI **Charge** 2 **Spectral Angle** 0.76

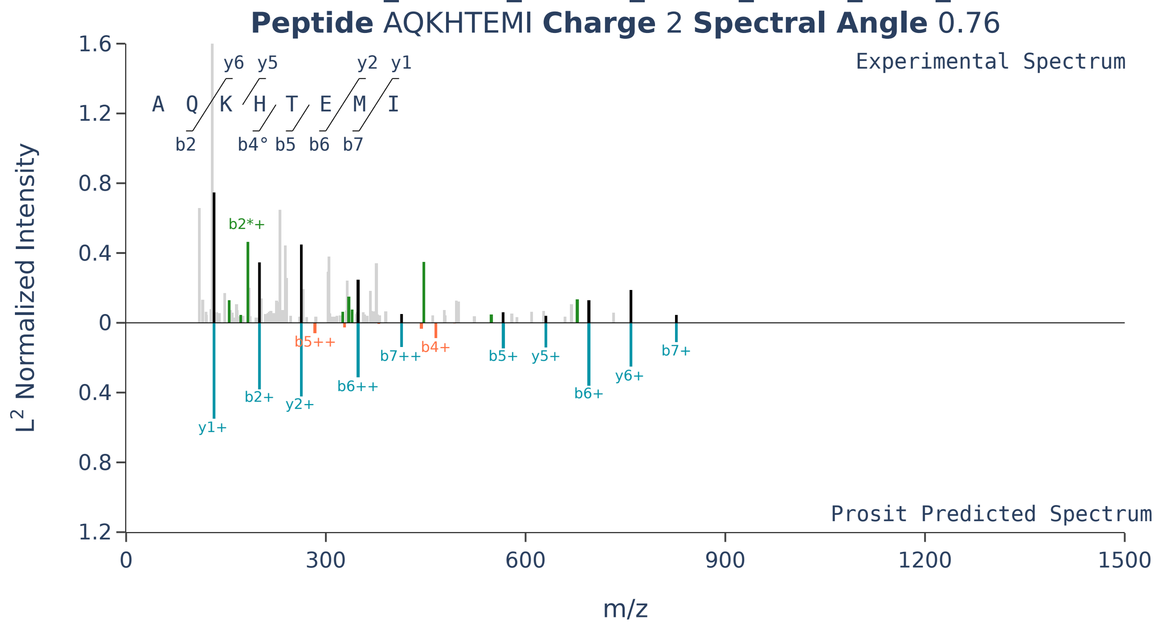


**Source** HRoetschke_271222_090123_HFGoe_LEDGF_b1R1_4h **Scan** 14298
**Peptide** AGVVTTATASVNLKVSPKRGR **Charge** 5 **Spectral Angle** 0.84

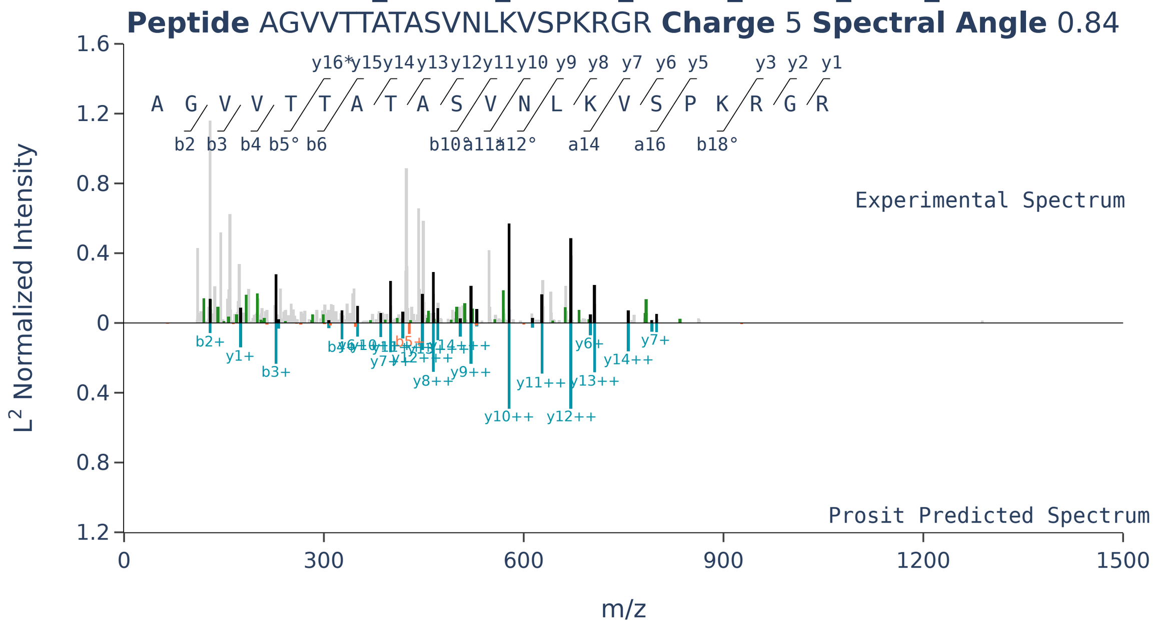


**Source** HRoetschke_271222_090123_HFGoe_LEDGF_b1R2_1h **Scan** 10696
**Peptide** EAGVVTTA **Charge** 1 **Spectral Angle** 0.84

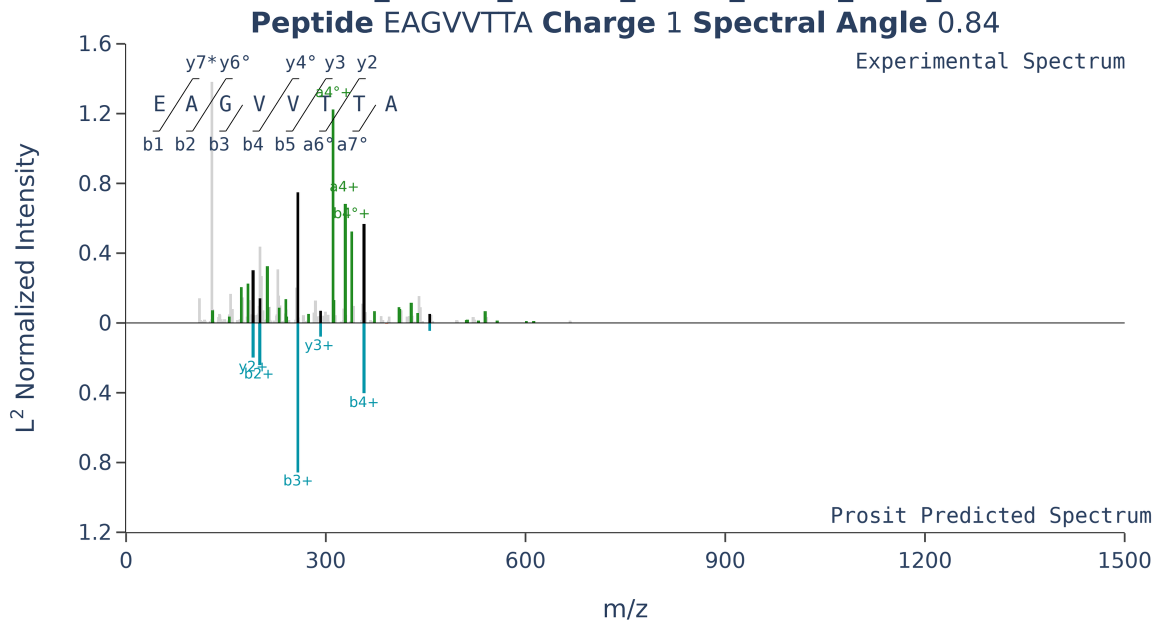


**Source** HRoetschke_271222_090123_HFGoe_LEDGF_b1R1_2h **Scan** 33131
**Peptide** DFKPGDLIFA **Charge** 2 **Spectral Angle** 0.83

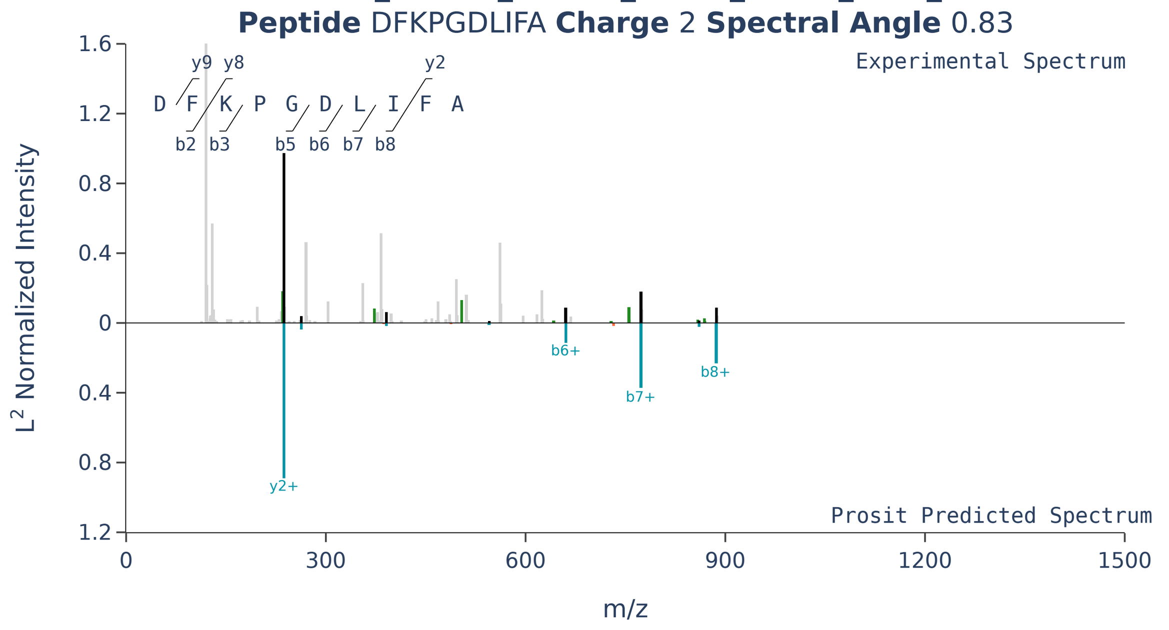


**Source** HRoetschke_271222_090123_HFGoe_LEDGF_b2R1_24h **Scan** 10903
**Peptide** IDNNPKVKFSSQQAA **Charge** 2 **Spectral Angle** 0.82

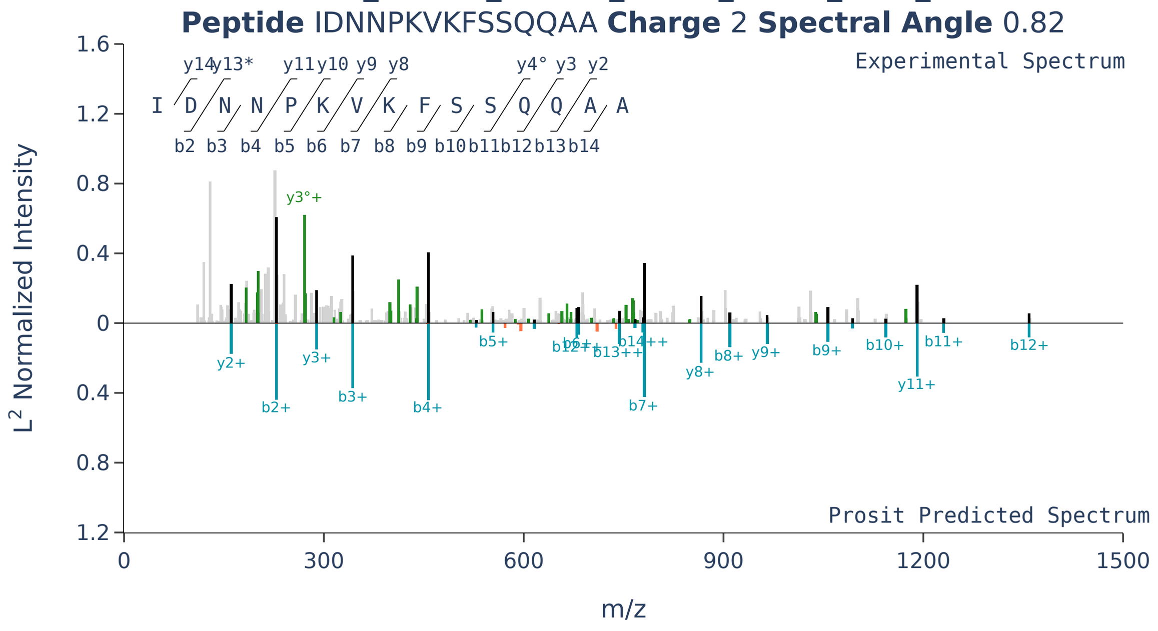


**Source** HRoetschke_271222_090123_HFGoe_LEDGF_b1R2_24h **Scan** 18005
**Peptide** IDNLDVNRCIE **Charge** 2 **Spectral Angle** 0.85

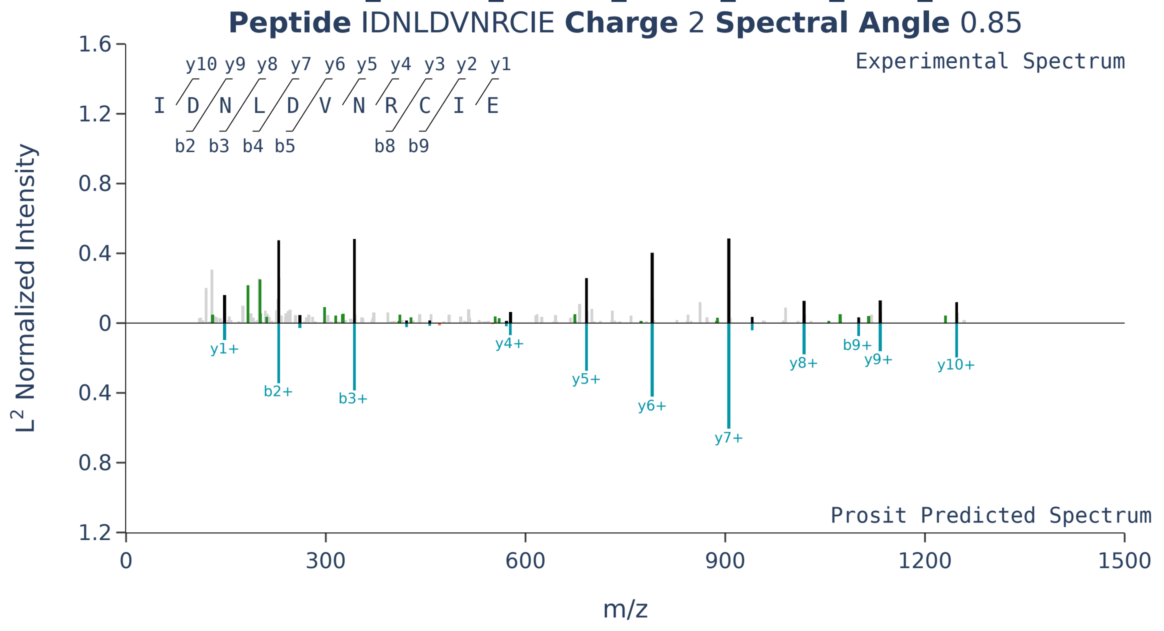


**Source** HRoetschke_271222_090123_HFGoe_LEDGF_b2R2_24h **Scan** 8110
**Peptide** NLAKTGVTSTSD **Charge** 2 **Spectral Angle** 0.84

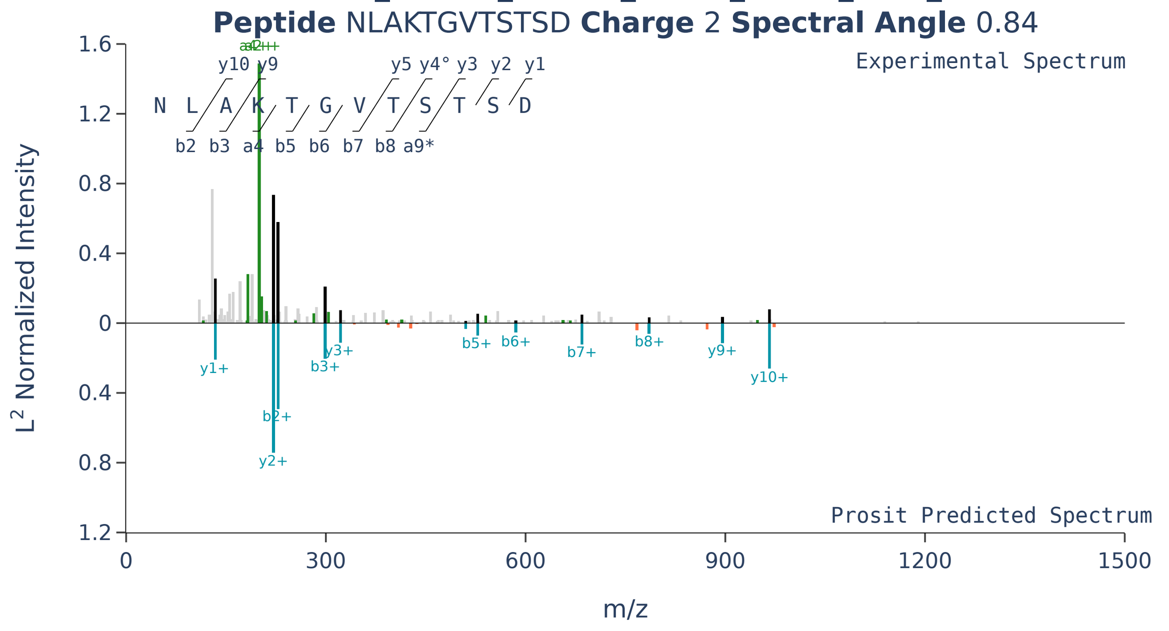


**Source** HRoetschke_271222_090123_HFGoe_LEDGF_b2R1_24h **Scan** 21928
**Peptide** LWEIDNNPKVKFSSQ **Charge** 3 **Spectral Angle** 0.8

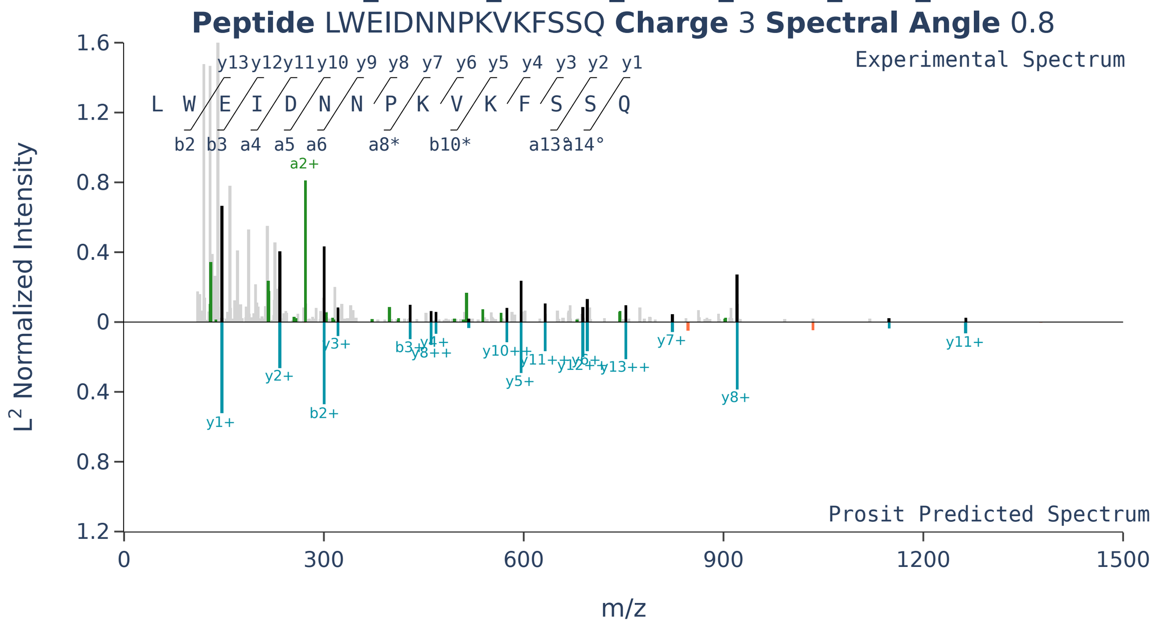


**Source** HRoetschke_271222_090123_HFGoe_LEDGF_b2R2_2h **Scan** 22567
**Peptide** DELASLQVTMQQAQKH **Charge** 2 **Spectral Angle** 0.86

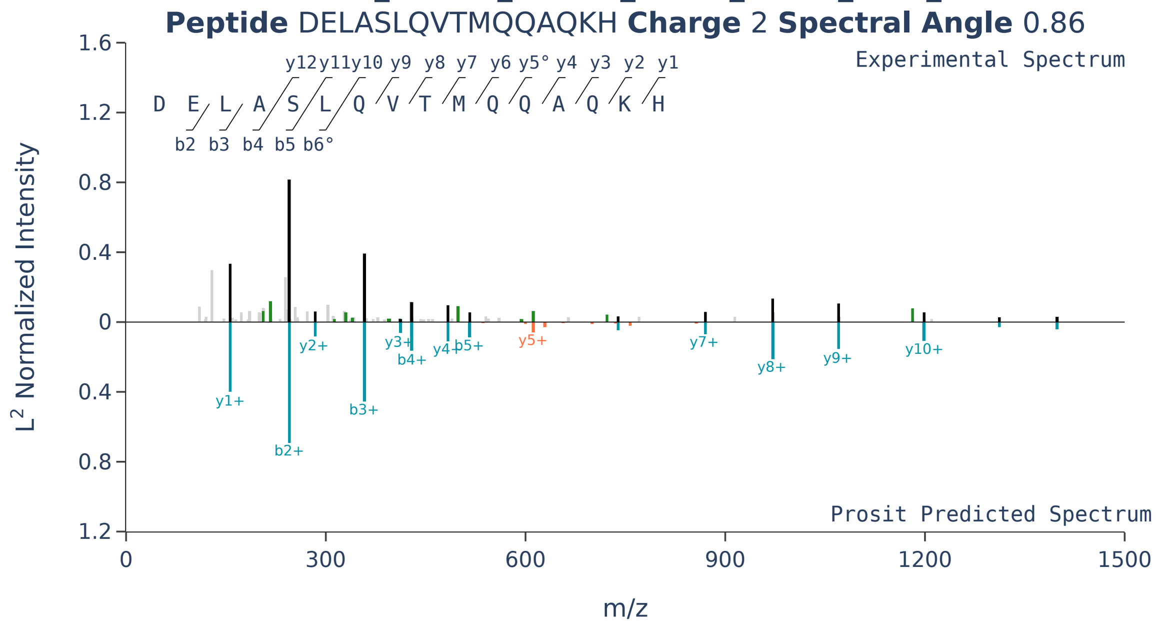


**Source** HRoetschke_271222_090123_HFGoe_LEDGF_b1R1_24h **Scan** 9984
**Peptide** KIDNLDVNR **Charge** 2 **Spectral Angle** 0.76

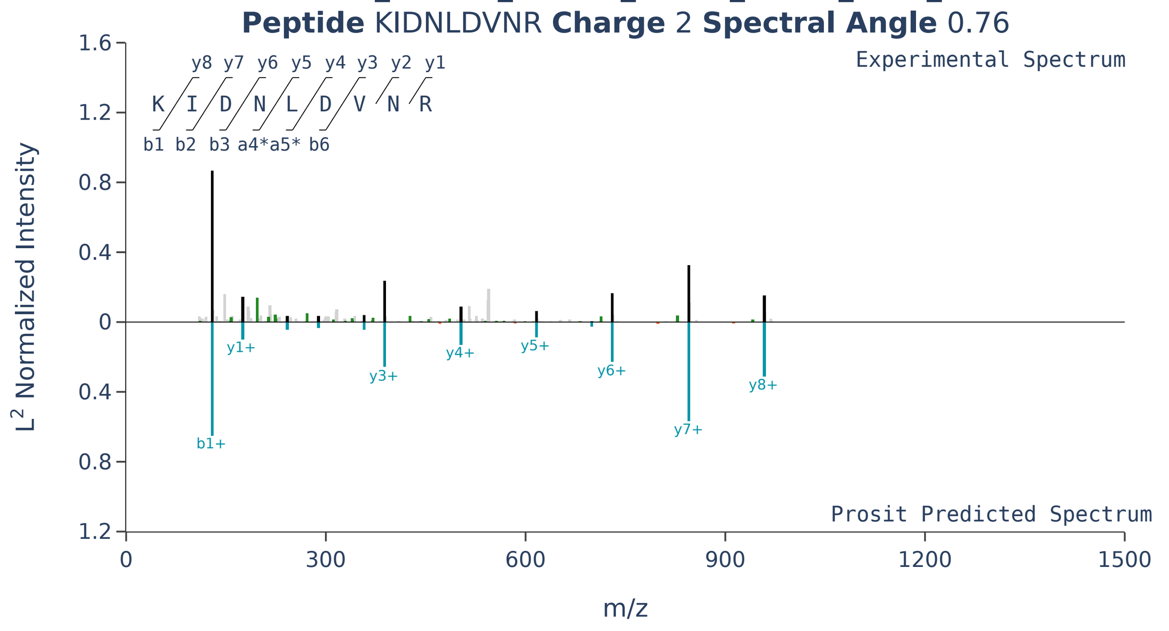


**Source** HRoetschke_271222_090123_HFGoe_LEDGF_b1R2_24h **Scan** 9635
**Peptide** IMEKSTML **Charge** 1 **Spectral Angle** 0.94

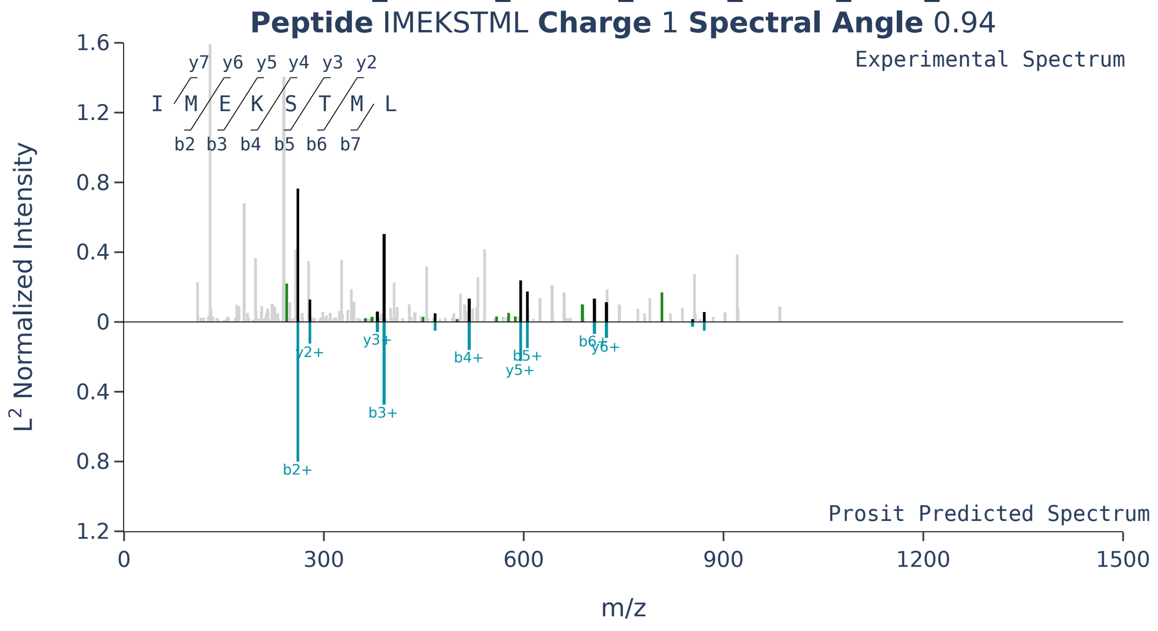


**Source** HRoetschke_271222_090123_HFGoe_LEDGF_b2R1_1h **Scan** 7425
**Peptide** SDVEVEEKE **Charge** 2 **Spectral Angle** 0.78

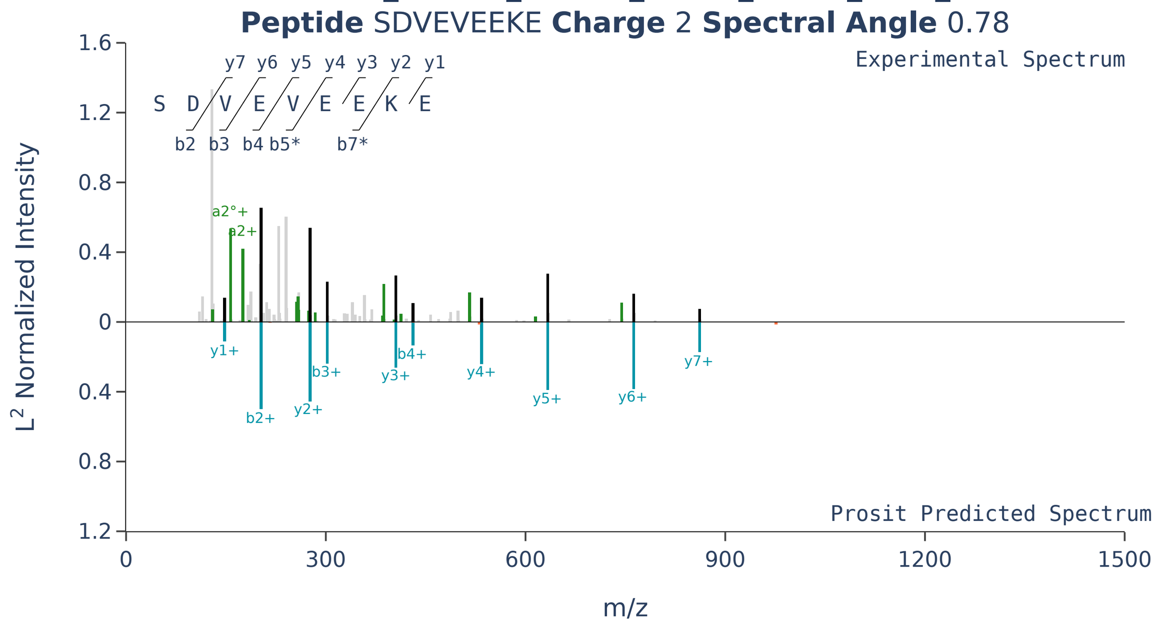


**Source** HRoetschke_271222_090123_HFGoe_LEDGF_b1R1_2h **Scan** 20486
**Peptide** YPHWPARVDEVPDGAVKPPTN **Charge** 3 **Spectral Angle** 0.91

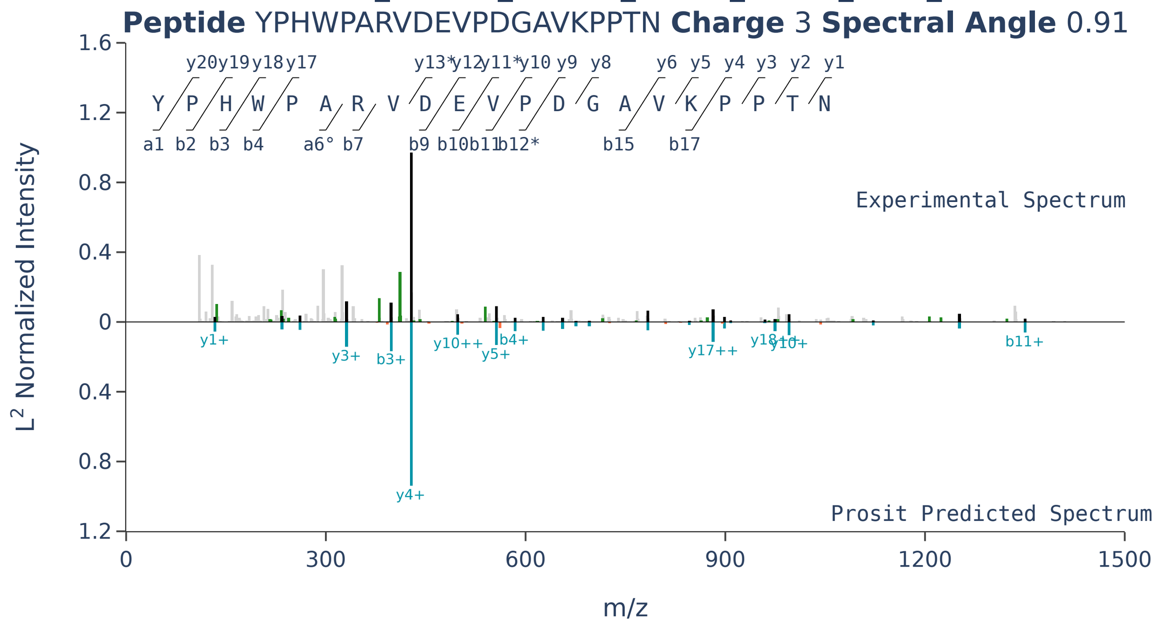

**Source** HRoetschke_271222_090123_HFGoe_LEDGF_b1R2_24h **Scan** 17110
**Peptide** PHWPARVDEV **Charge** 3 **Spectral Angle** 0.94

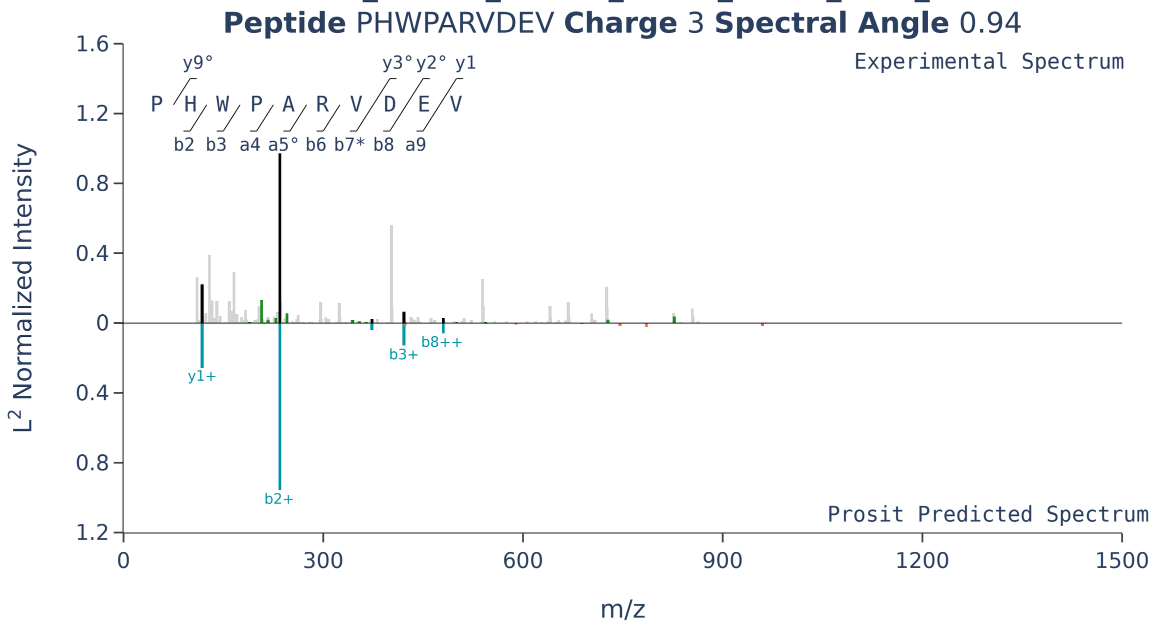


**Source** HRoetschke_271222_090123_HFGoe_LEDGF_b2R2_4h **Scan** 13839
**Peptide** STMLYNKFKNM **Charge** 3 **Spectral Angle** 0.78

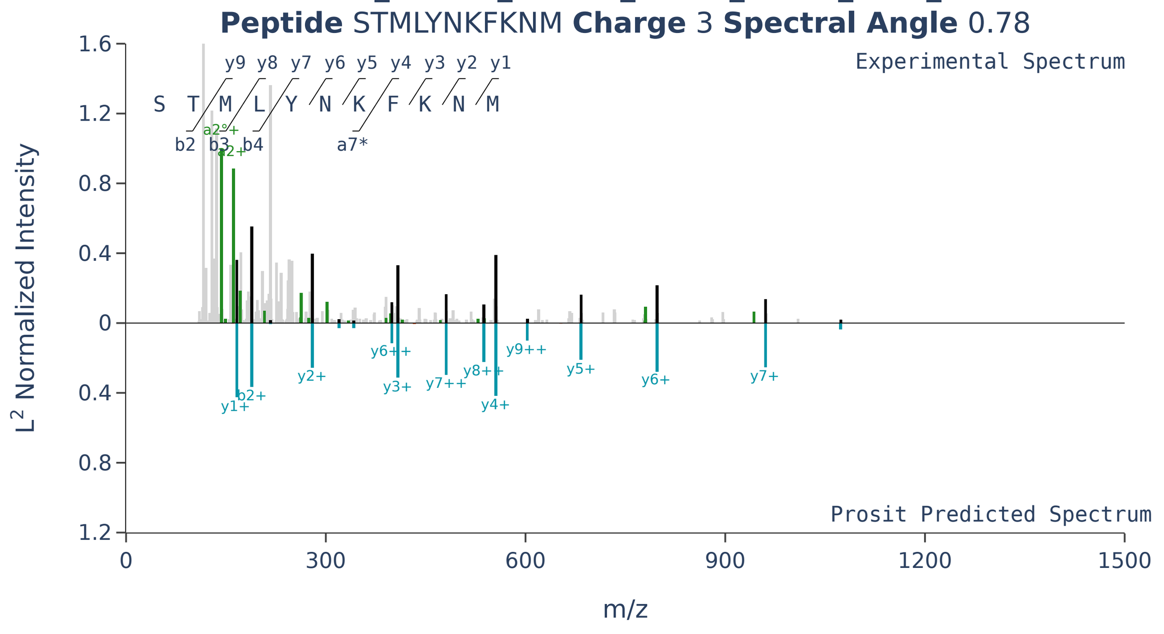


**Source** HRoetschke_271222_090123_HFGoe_LEDGF_b1R2_24h **Scan** 15204
**Peptide** KASNEDVTKAVDI **Charge** 2 **Spectral Angle** 0.78

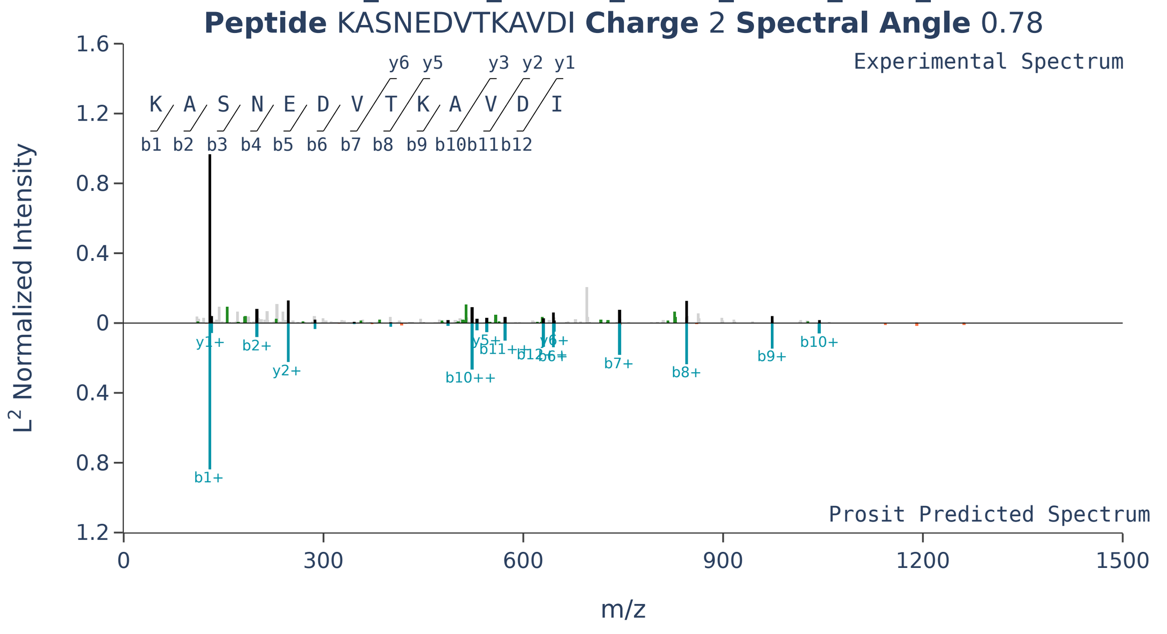


**Source** HRoetschke_271222_090123_HFGoe_LEDGF_b1R1_2h **Scan** 21066
**Peptide** KVSQVIMEKSTMLY **Charge** 2 **Spectral Angle** 0.88

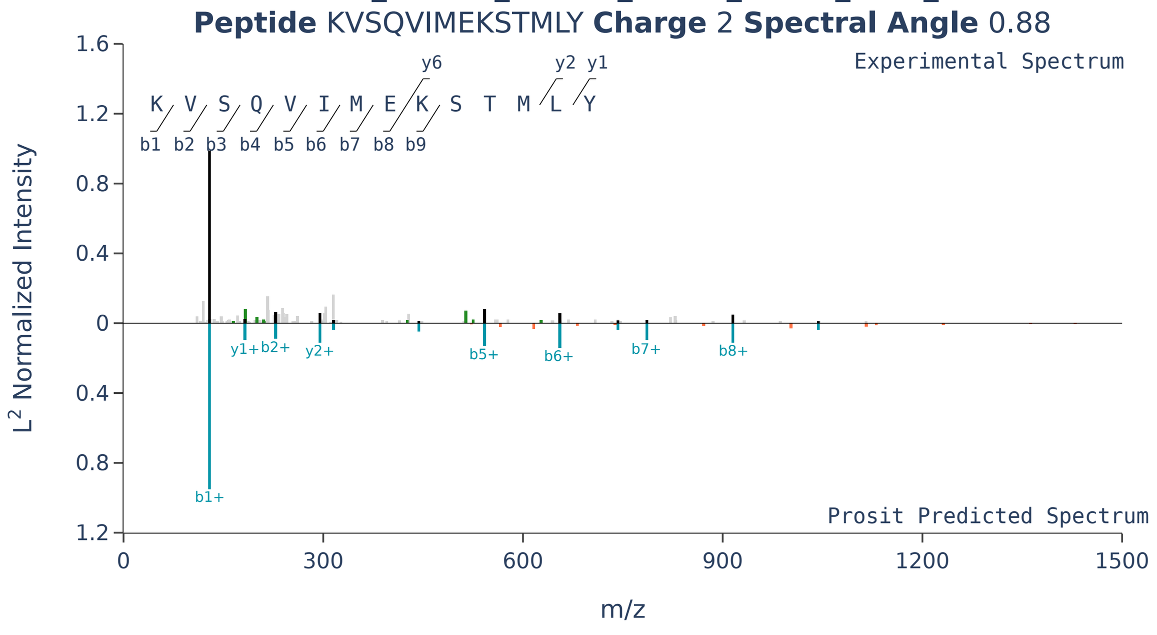


**Source** HRoetschke_271222_090123_HFGoe_LEDGF_b1R2_4h **Scan** 18354
**Peptide** PHWPARVDEVPDGAVKPPTN **Charge** 3 **Spectral Angle** 0.87

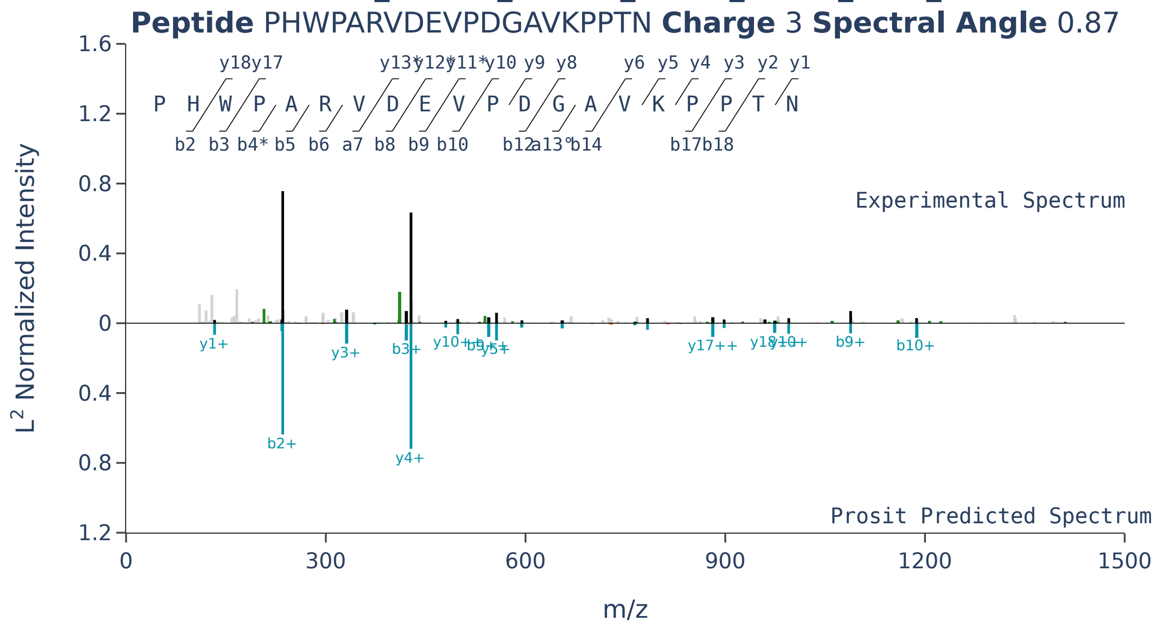


**Source** HRoetschke_271222_090123_HFGoe_LEDGF_b1R2_24h **Scan** 6453
**Peptide** KNLAKTGVTSTSD **Charge** 3 **Spectral Angle** 0.86

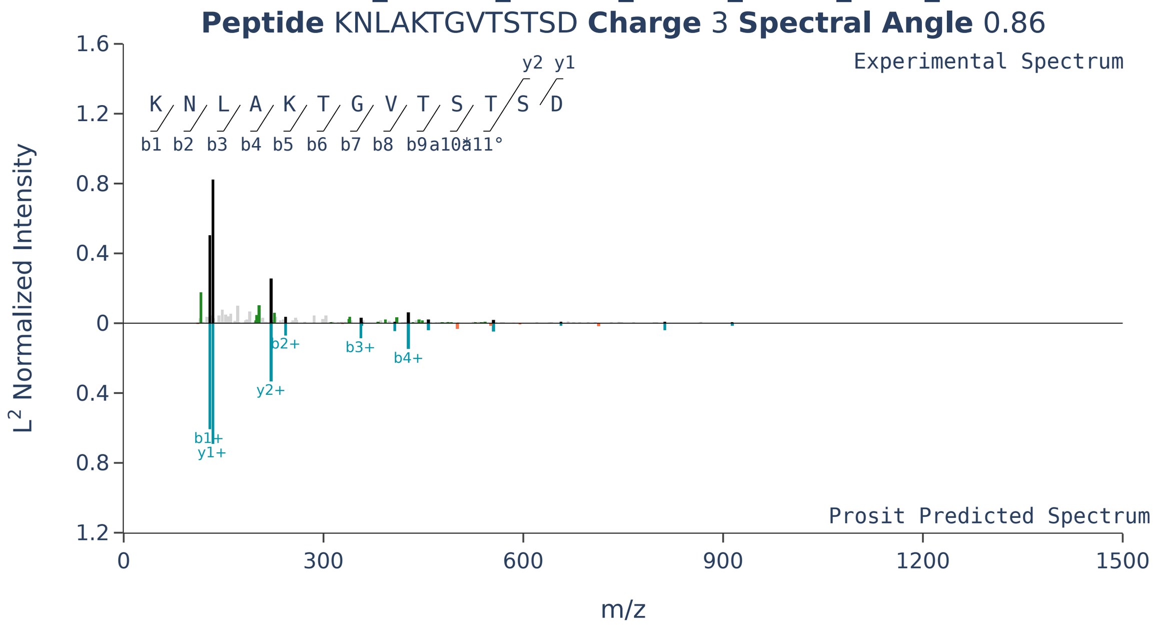


**Source** HRoetschke_271222_090123_HFGoe_LEDGF_b1R2_24h **Scan** 5756
**Peptide** DTDHEEKASNEDVTKA **Charge** 2 **Spectral Angle** 0.83

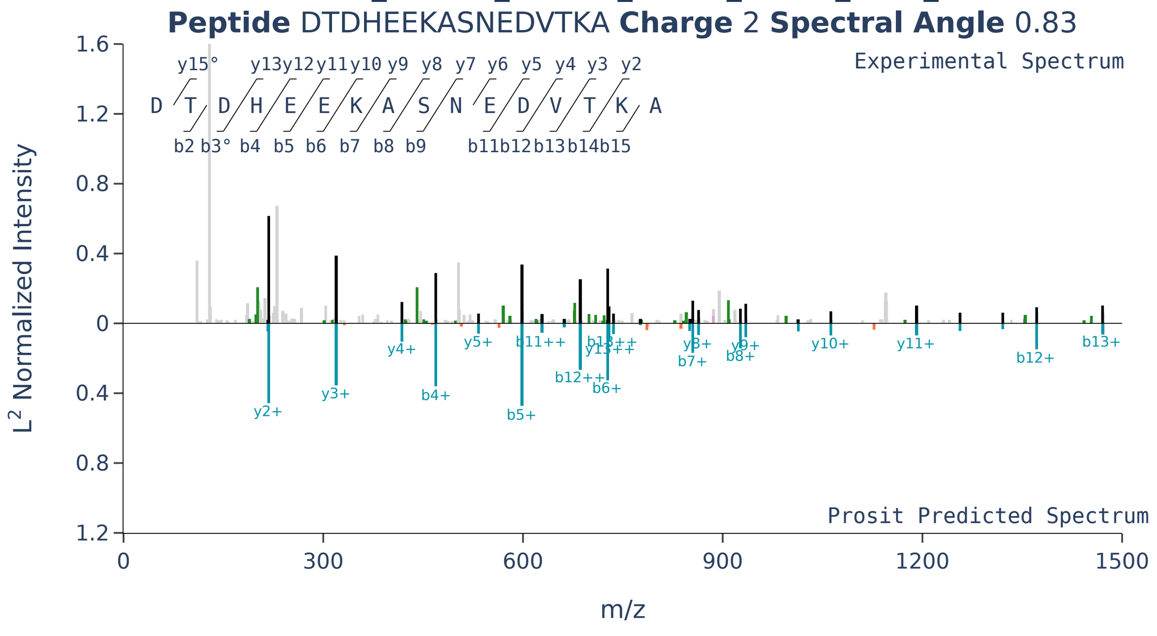


**Source** HRoetschke_271222_090123_HFGoe_LEDGF_b1R2_1h **Scan** 26153
**Peptide** TRDFKPGDLIFA **Charge** 2 **Spectral Angle** 0.85

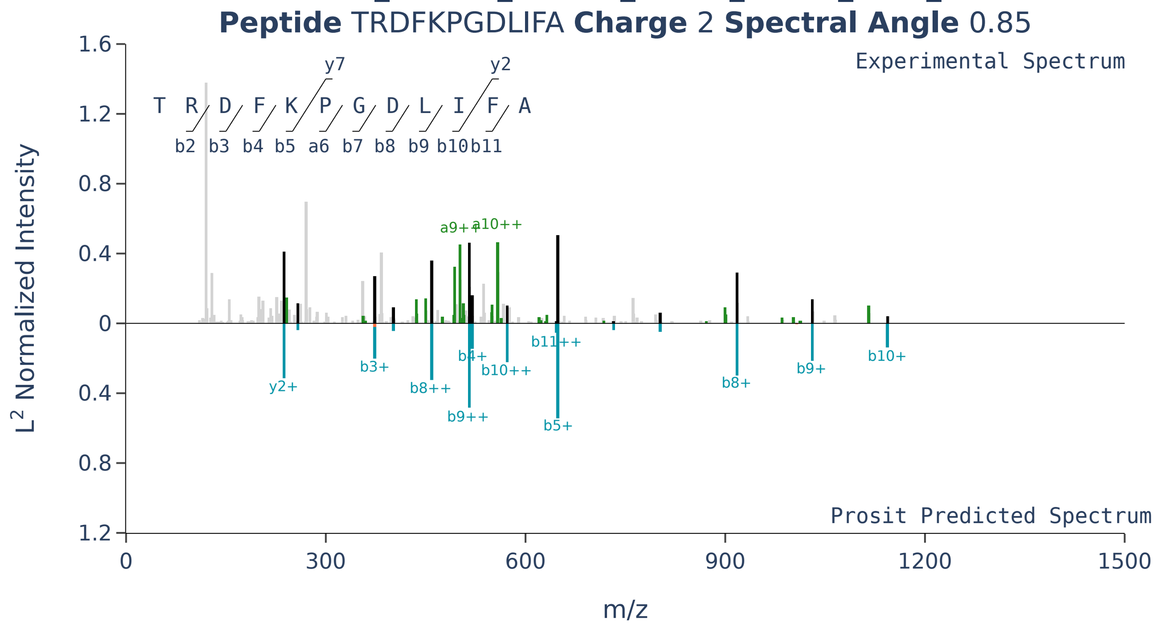


**Source** HRoetschke_271222_090123_HFGoe_LEDGF_b1R2_4h **Scan** 25254
**Peptide** ELASLQVTMQQA **Charge** 2 **Spectral Angle** 0.89

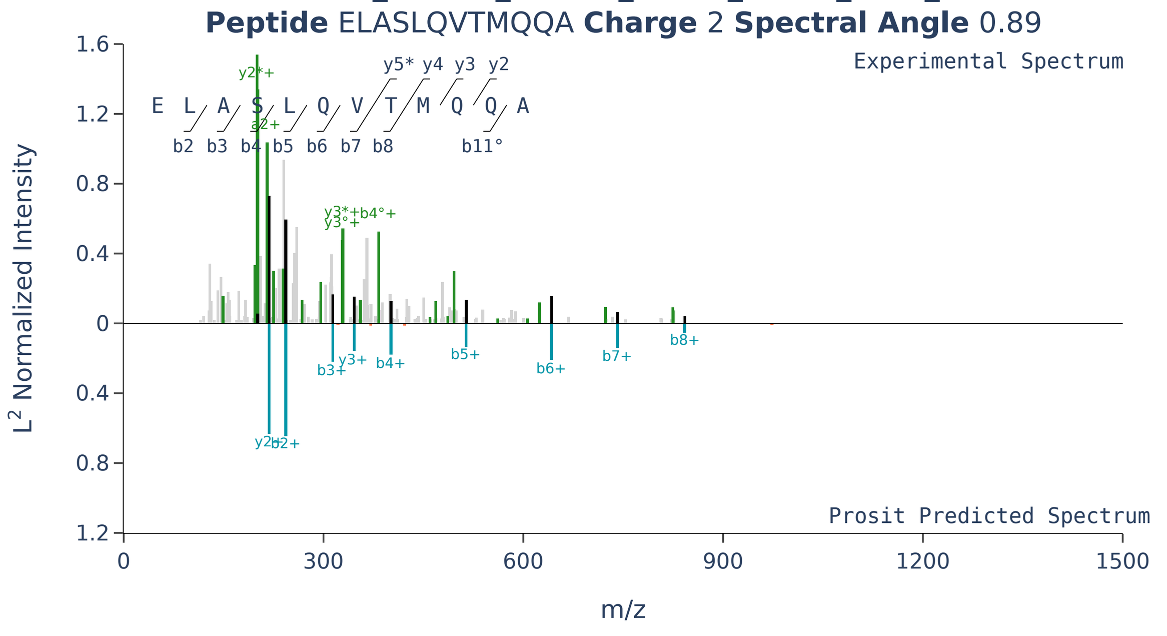


**Source** HRoetschke_271222_090123_HFGoe_LEDGF_b1R1_2h **Scan** 27031
**Peptide** GFNEGLWE **Charge** 1 **Spectral Angle** 0.87

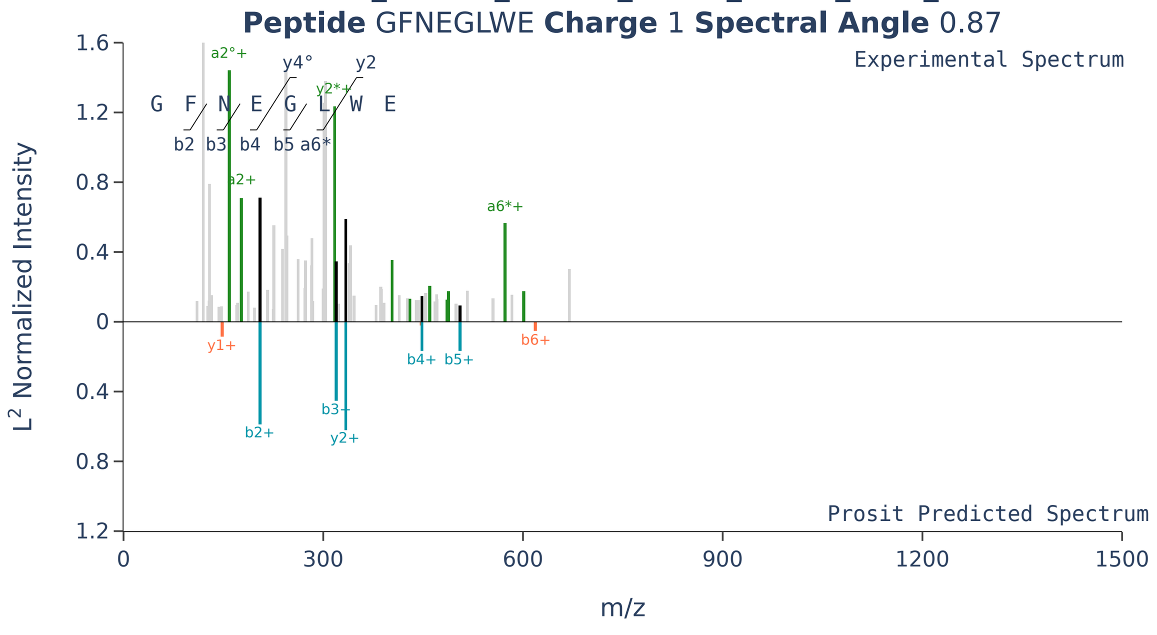


**Source** HRoetschke_271222_090123_HFGoe_LEDGF_b2R1_2h **Scan** 23121
**Peptide** EGDSVITQVLNK **Charge** 2 **Spectral Angle** 0.76

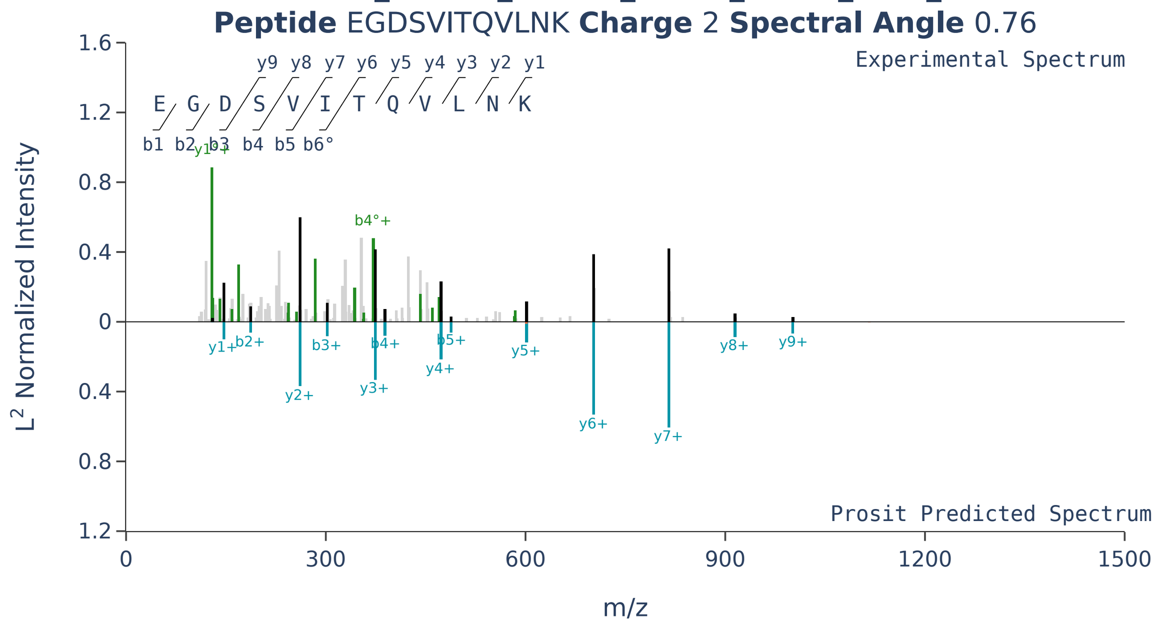


**Source** HRoetschke_271222_090123_HFGoe_LEDGF_b1R2_24h **Scan** 13718
**Peptide** EEAGVVTTATA **Charge** 1 **Spectral Angle** 0.85

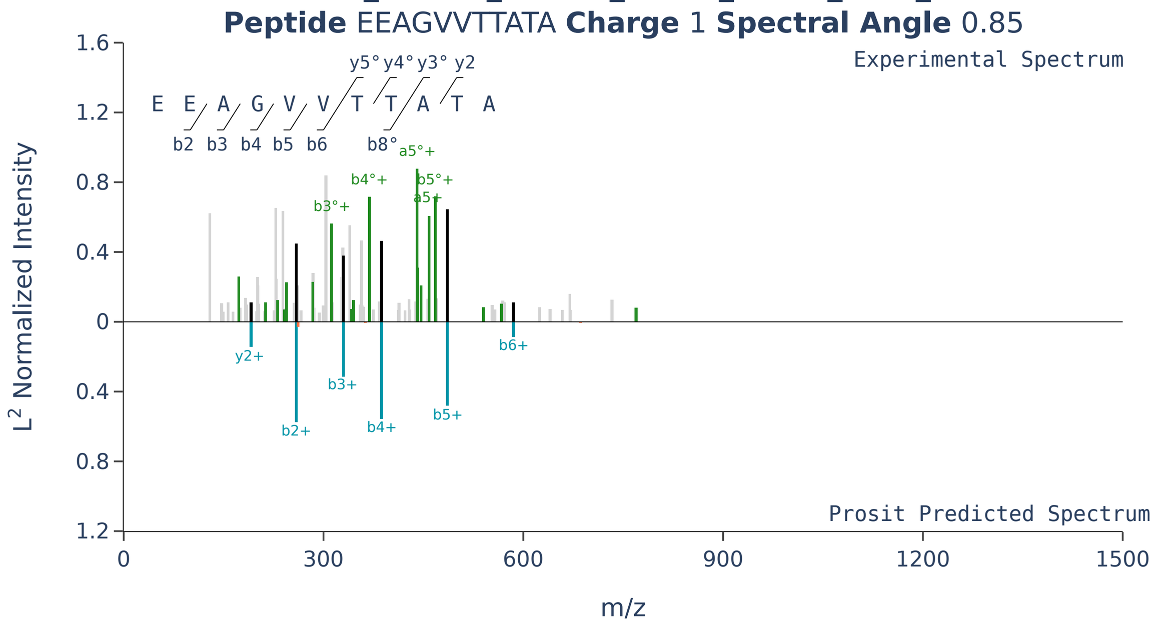


**Source** HRoetschke_271222_090123_HFGoe_LEDGF_b1R1_4h **Scan** 15085
**Peptide** RRFKVSQVIME **Charge** 2 **Spectral Angle** 0.85

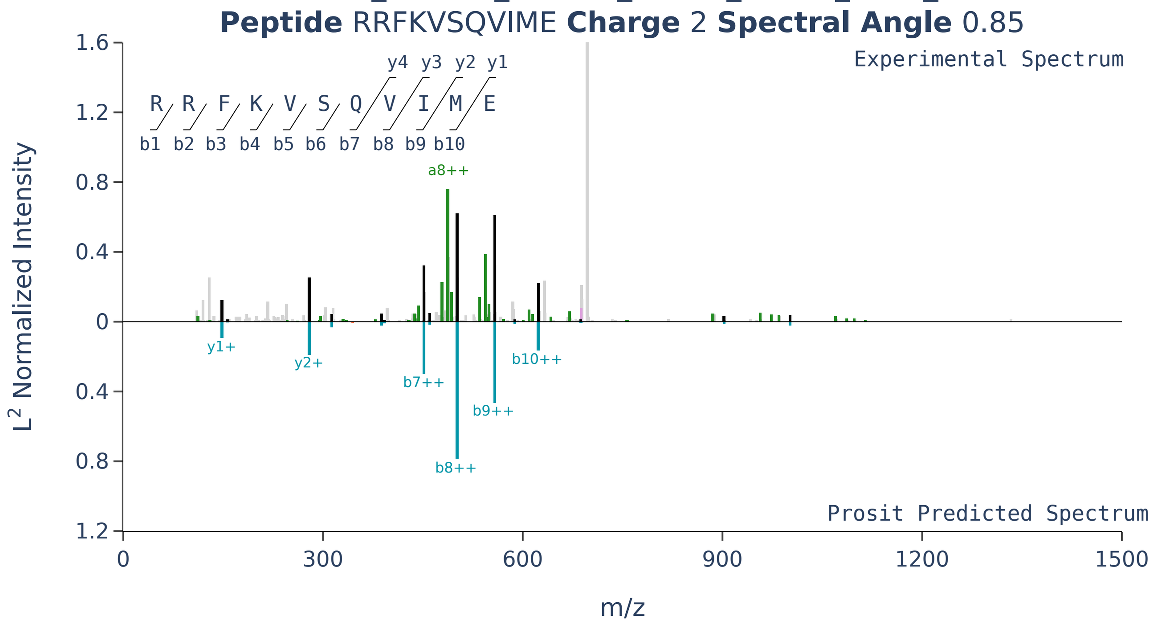


**Source** HRoetschke_271222_090123_HFGoe_LEDGF_b2R2_2h **Scan** 8433
**Peptide** NASSDVEVEEKE **Charge** 1 **Spectral Angle** 0.86

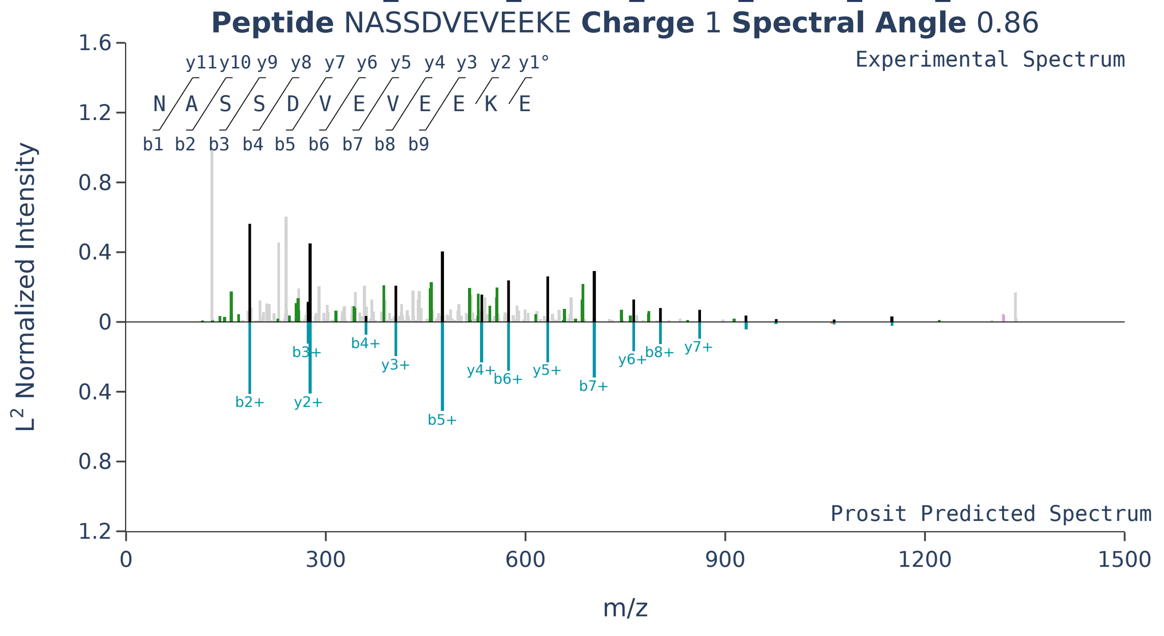


**Source** HRoetschke_271222_090123_HFGoe_LEDGF_b1R1_2h **Scan** 28331
**Peptide** NEGLWEID **Charge** 1 **Spectral Angle** 0.89

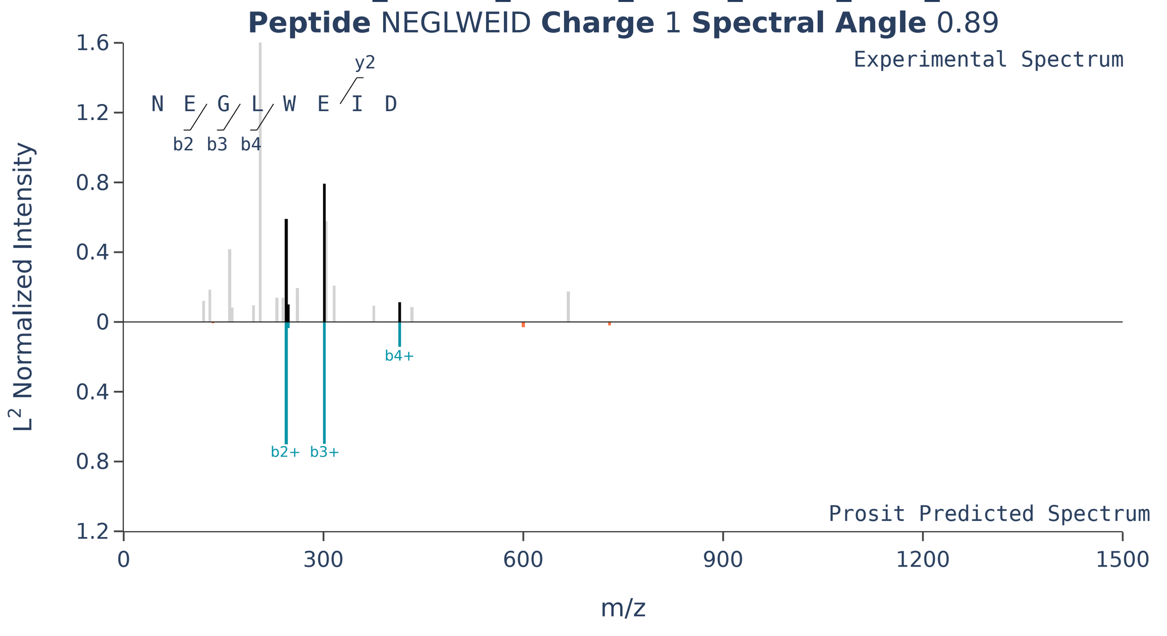

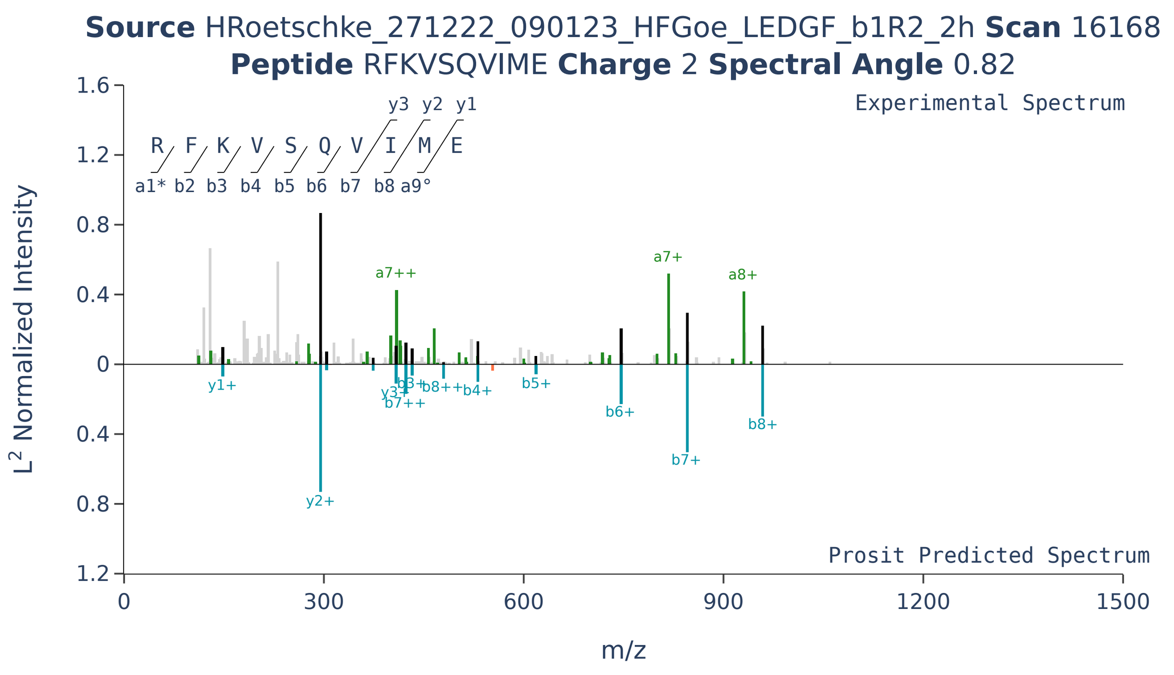
Source HRoetschke_271222_090123_HFGoe_LEDGF_b1R2_2h Scan 16168
Peptide RFKVSQVIME Charge 2 Spectral Angle 0.82
Experimental Spectrum
Prosit Predicted Spectrum
L² Normalized Intensity
m/z

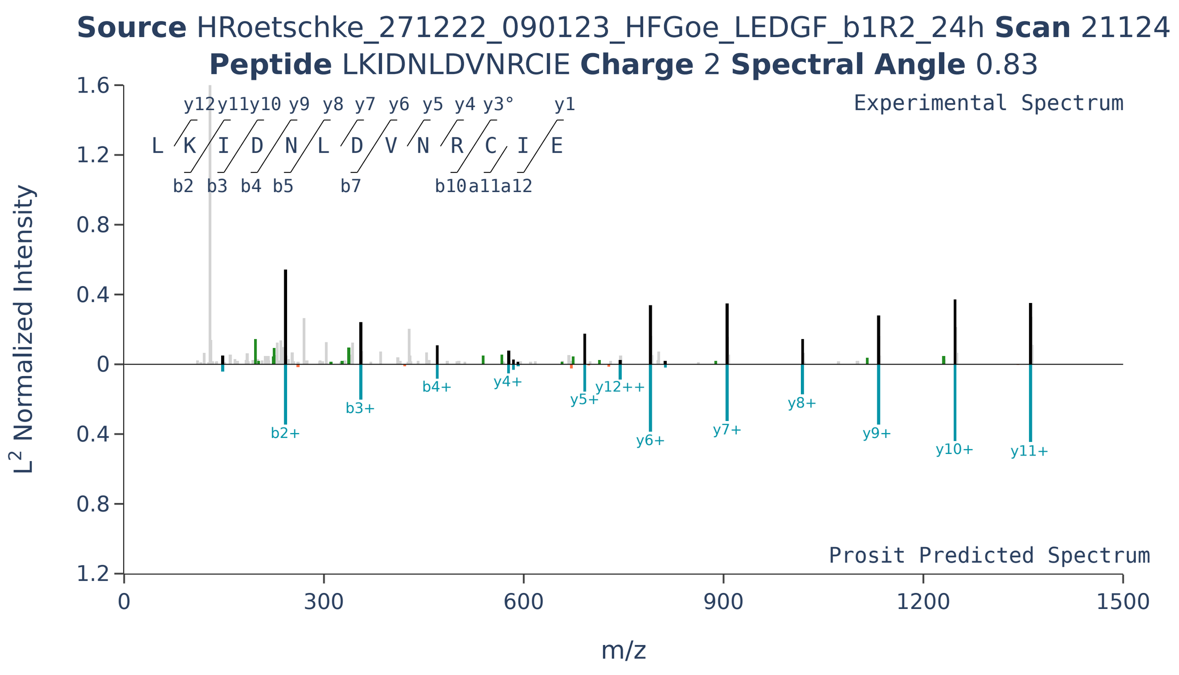
Source HRoetschke_271222_090123_HFGoe_LEDGF_b1R2_24h Scan 21124
Peptide LKIDNLDVNRCIE Charge 2 Spectral Angle 0.83
Experimental Spectrum
Prosit Predicted Spectrum
L² Normalized Intensity
m/z

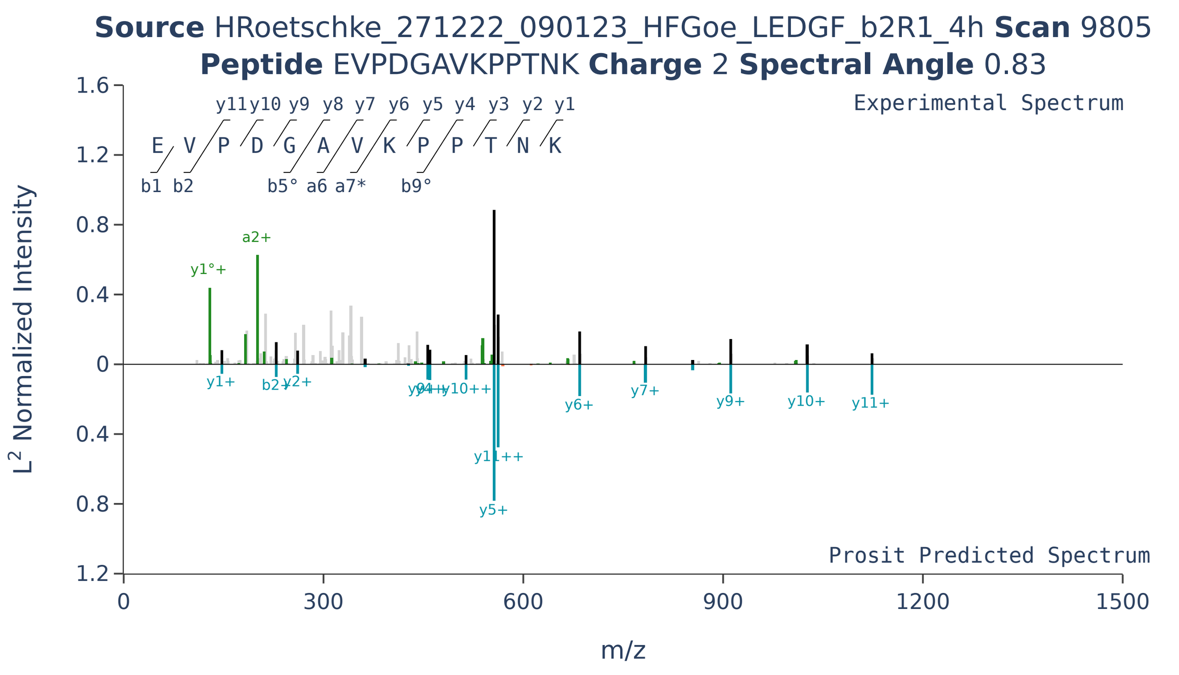
Source HRoetschke_271222_090123_HFGoe_LEDGF_b2R1_4h Scan 9805
Peptide EVPDGAVKPPTNK Charge 2 Spectral Angle 0.83
Experimental Spectrum
Prosit Predicted Spectrum
L² Normalized Intensity
m/z

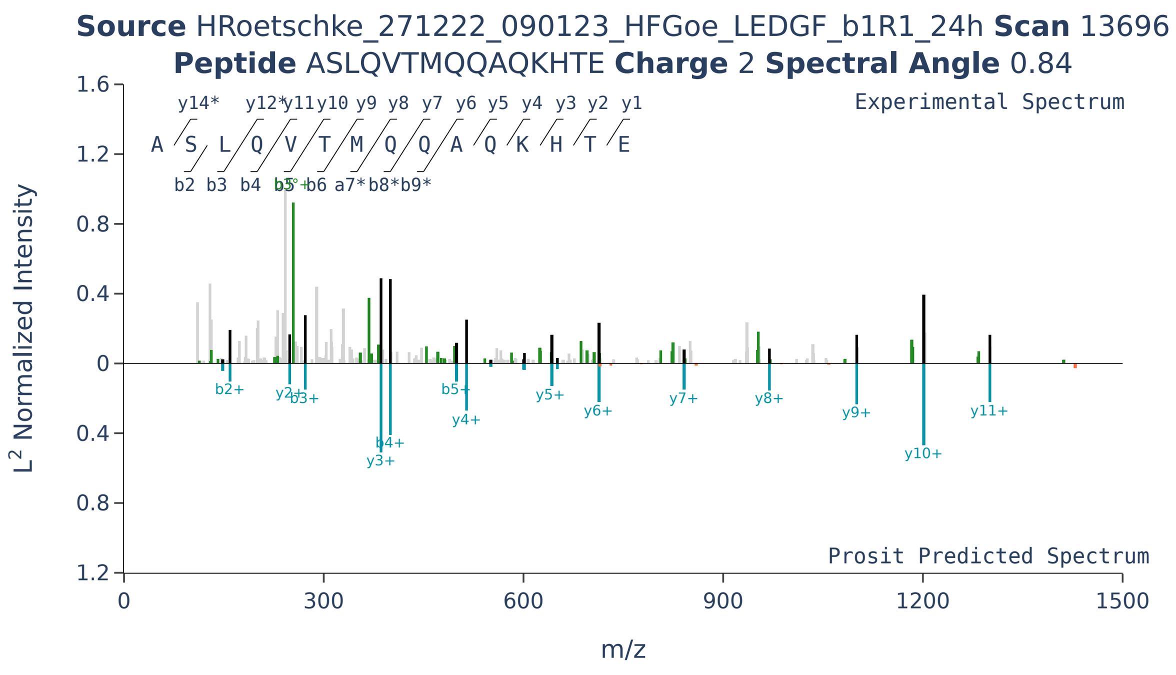
Source HRoetschke_271222_090123_HFGoe_LEDGF_b1R1_24h Scan 13696
Peptide ASLQVTMQQAQKHTE Charge 2 Spectral Angle 0.84
Experimental Spectrum
Prosit Predicted Spectrum
L² Normalized Intensity
m/z

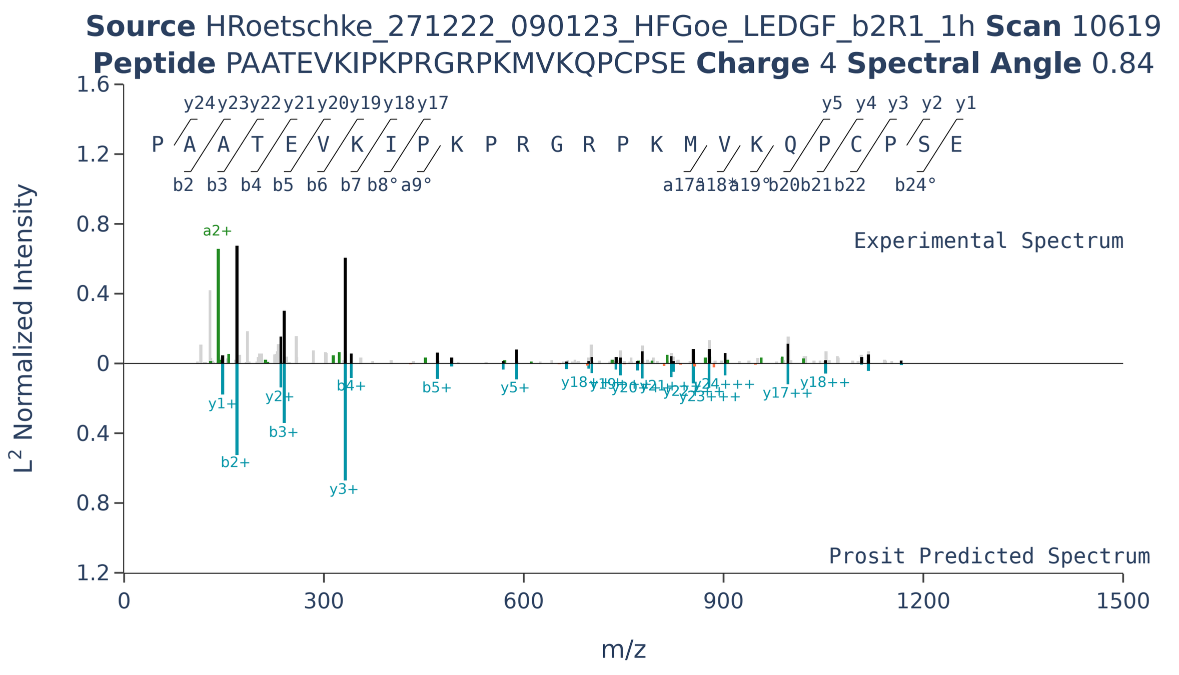
Source HRoetschke_271222_090123_HFGoe_LEDGF_b2R1_1h Scan 10619
Peptide PAATEVKIPKPRGRPKMVKQPCPSE Charge 4 Spectral Angle 0.84
Experimental Spectrum
Prosit Predicted Spectrum
L² Normalized Intensity
m/z

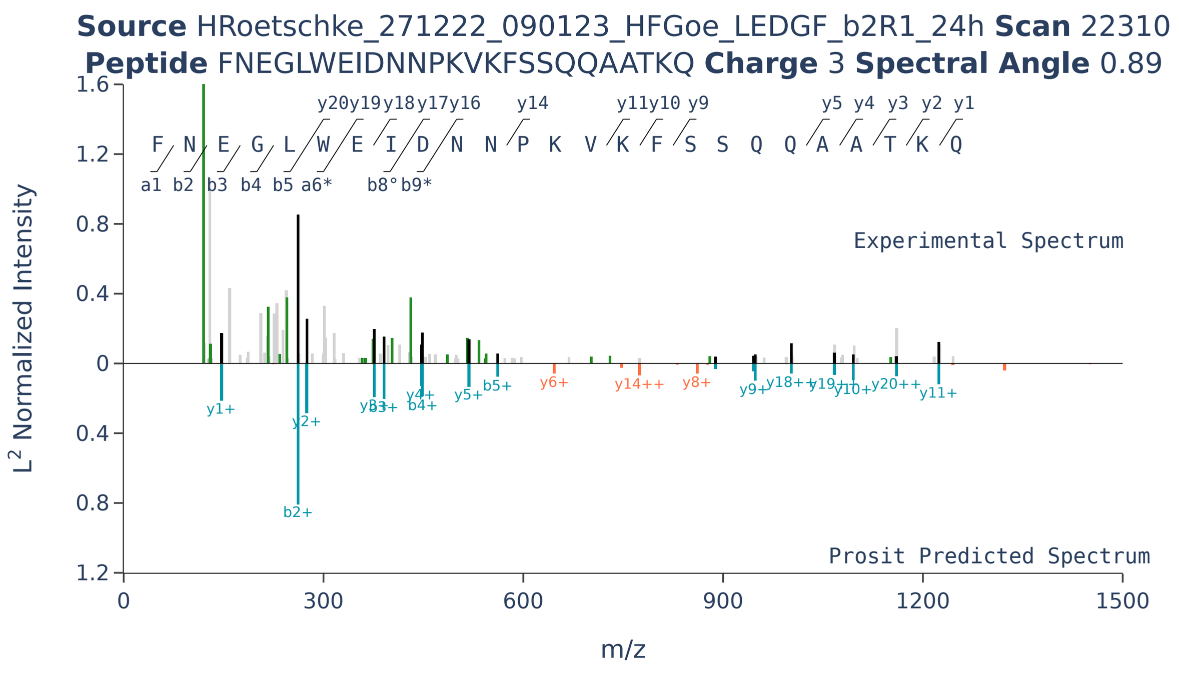
Source HRoetschke_271222_090123_HFGoe_LEDGF_b2R1_24h Scan 22310
Peptide FNEGLWEIDNNPKVKFSSQQAATKQ Charge 3 Spectral Angle 0.89
Experimental Spectrum
Prosit Predicted Spectrum
L² Normalized Intensity
m/z

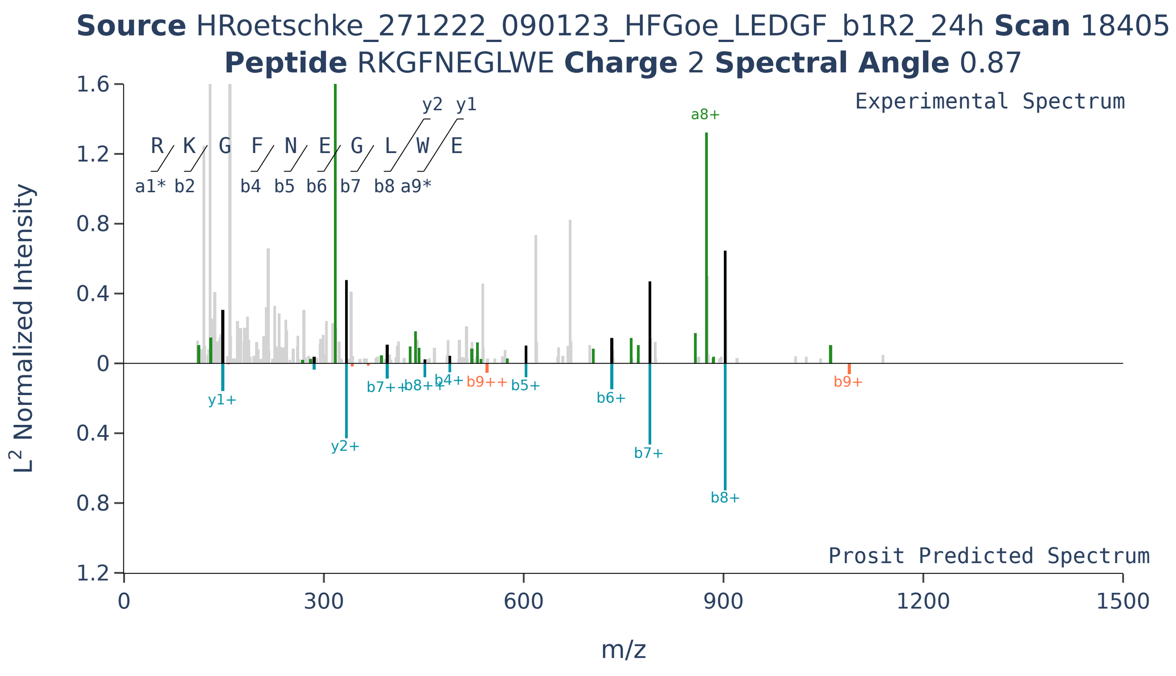
Source HRoetschke_271222_090123_HFGoe_LEDGF_b1R2_24h Scan 18405
Peptide RKGFNEGLWE Charge 2 Spectral Angle 0.87
Experimental Spectrum
Prosit Predicted Spectrum
L² Normalized Intensity
m/z

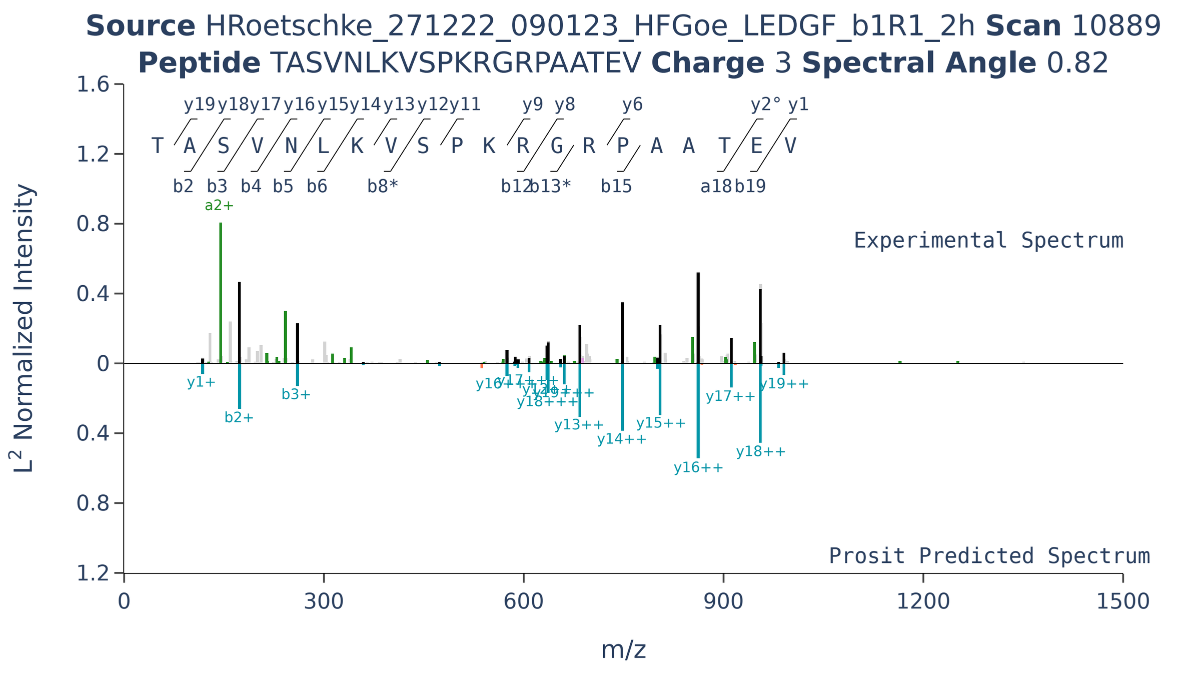
Source HRoetschke_271222_090123_HFGoe_LEDGF_b1R1_2h Scan 10889
Peptide TASVNLKVSPKRGRPAATEV Charge 3 Spectral Angle 0.82
Experimental Spectrum
Prosit Predicted Spectrum
L² Normalized Intensity
m/z

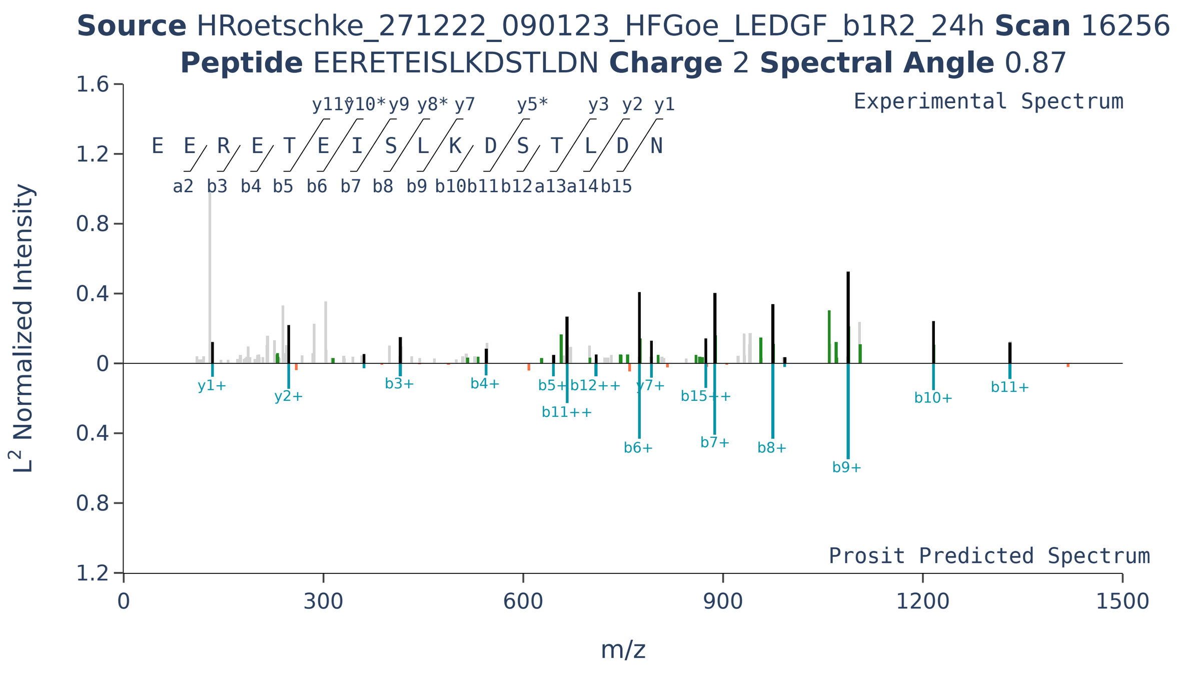
Source HRoetschke_271222_090123_HFGoe_LEDGF_b1R2_24h Scan 16256
Peptide EERETEISLKDSTLDN Charge 2 Spectral Angle 0.87
Experimental Spectrum
Prosit Predicted Spectrum
L² Normalized Intensity
m/z

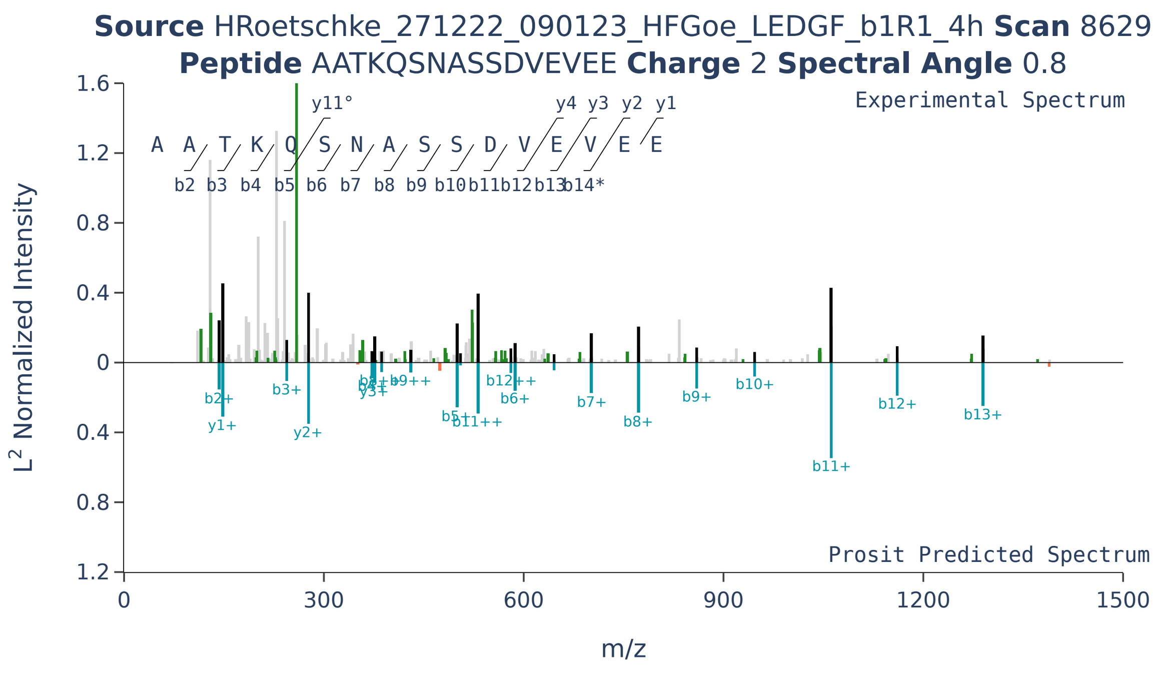
Source HRoetschke_271222_090123_HFGoe_LEDGF_b1R1_4h Scan 8629
Peptide AATKQSNASSDVEVEE Charge 2 Spectral Angle 0.8
Experimental Spectrum
Prosit Predicted Spectrum
L² Normalized Intensity
m/z

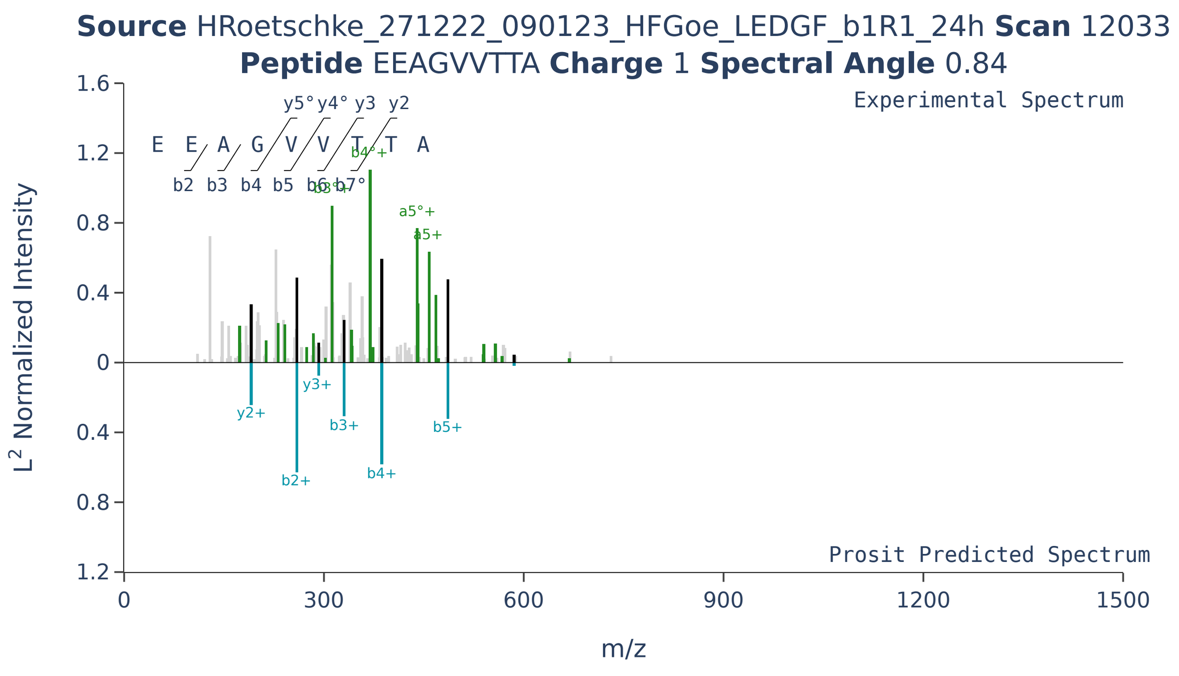
Source HRoetschke_271222_090123_HFGoe_LEDGF_b1R1_24h Scan 12033
Peptide EEAGVVTTA Charge 1 Spectral Angle 0.84
Experimental Spectrum
Prosit Predicted Spectrum
L² Normalized Intensity
m/z

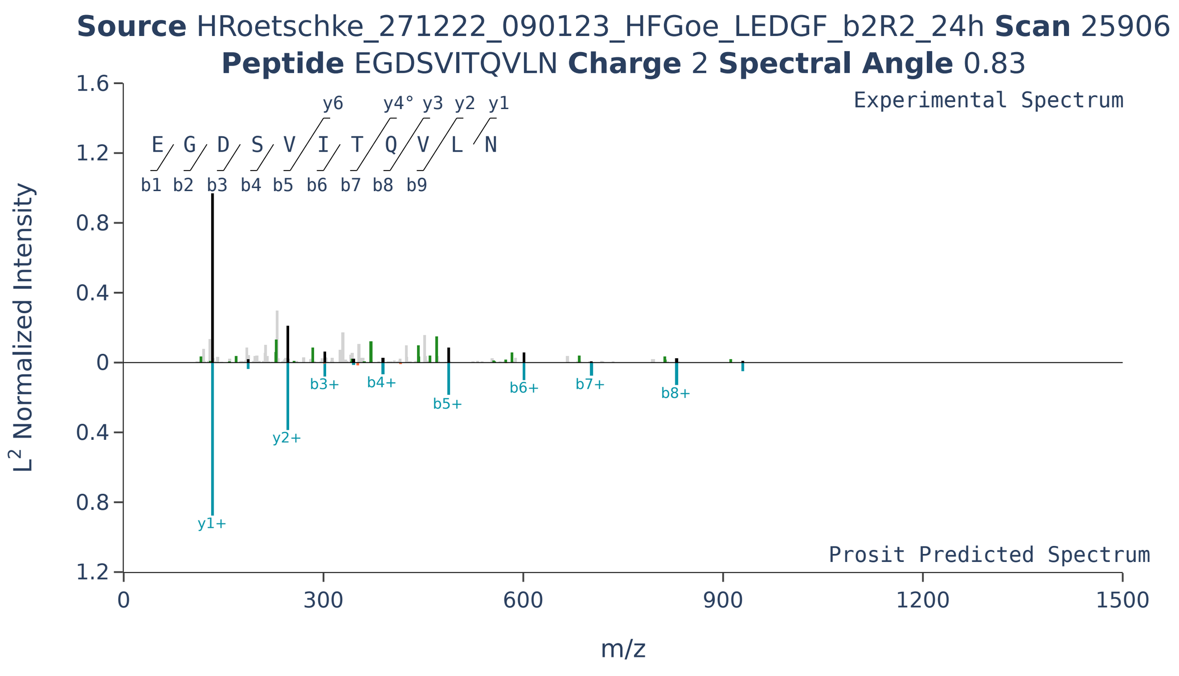
Source HRoetschke_271222_090123_HFGoe_LEDGF_b2R2_24h Scan 25906
Peptide EGDSVITQVLN Charge 2 Spectral Angle 0.83
Experimental Spectrum
Prosit Predicted Spectrum
L² Normalized Intensity
m/z

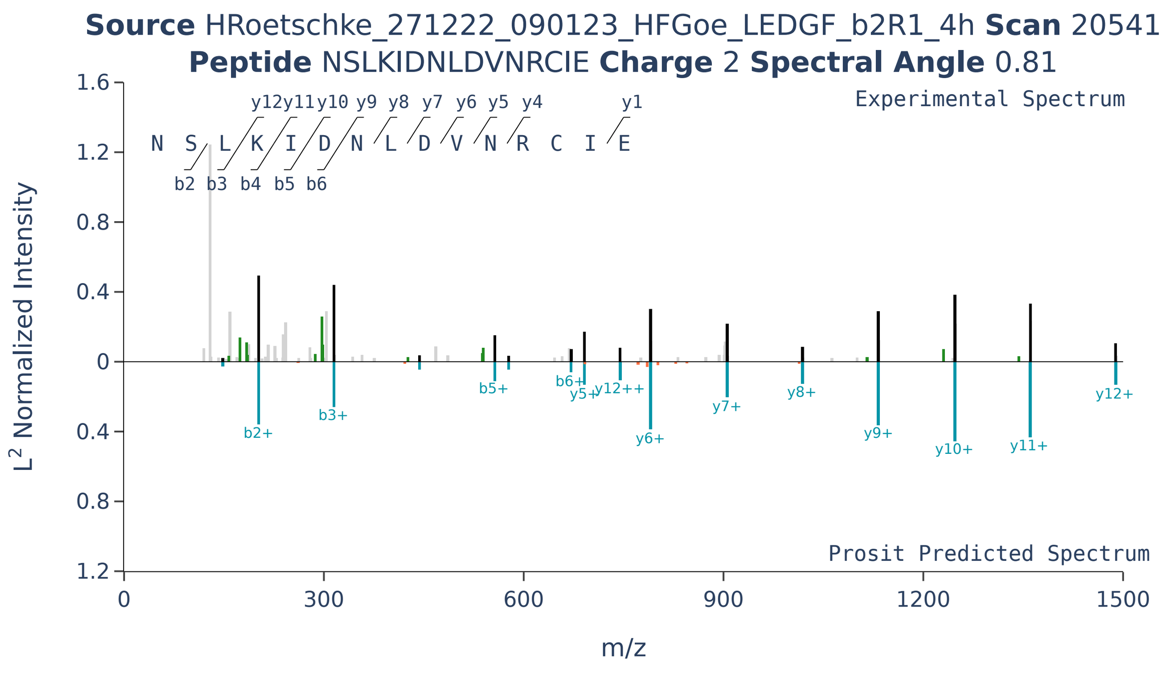
Source HRoetschke_271222_090123_HFGoe_LEDGF_b2R1_4h Scan 20541
Peptide NSLKIDNLDVNRCIE Charge 2 Spectral Angle 0.81
Experimental Spectrum
Prosit Predicted Spectrum
L² Normalized Intensity
m/z

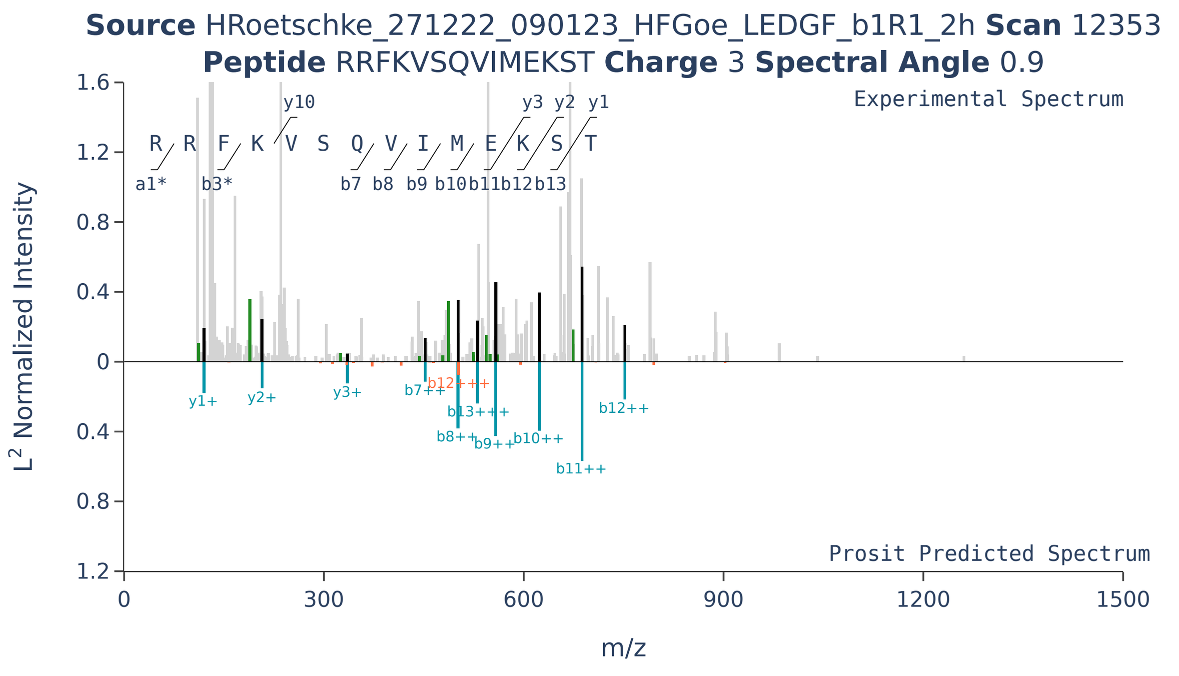
Source HRoetschke_271222_090123_HFGoe_LEDGF_b1R1_2h Scan 12353
Peptide RRFKVSQVIMEKST Charge 3 Spectral Angle 0.9
Experimental Spectrum
Prosit Predicted Spectrum
L² Normalized Intensity
m/z

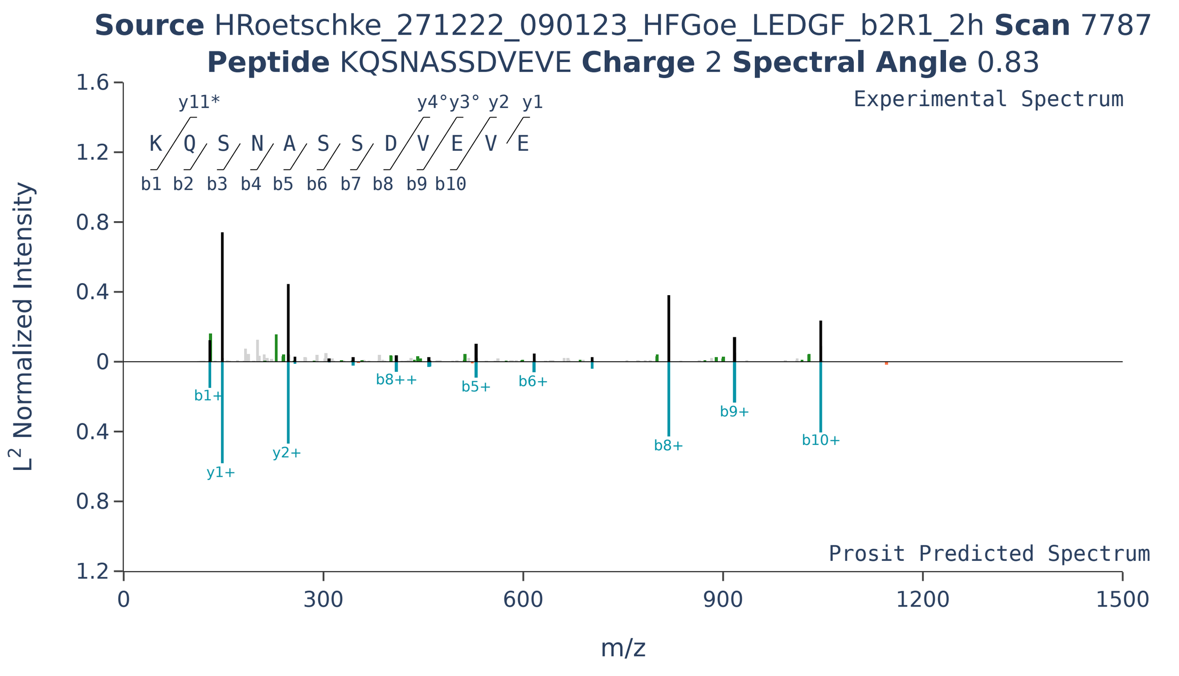
Source HRoetschke_271222_090123_HFGoe_LEDGF_b2R1_2h Scan 7787
Peptide KQSNASSDVEVE Charge 2 Spectral Angle 0.83
Experimental Spectrum
Prosit Predicted Spectrum
L² Normalized Intensity
m/z

**Source** HRoetschke_271222_090123_HFGoe_LEDGF_b2R1_24h **Scan** 16227
**Peptide** ATASVNL **Charge** 1 **Spectral Angle** 0.91

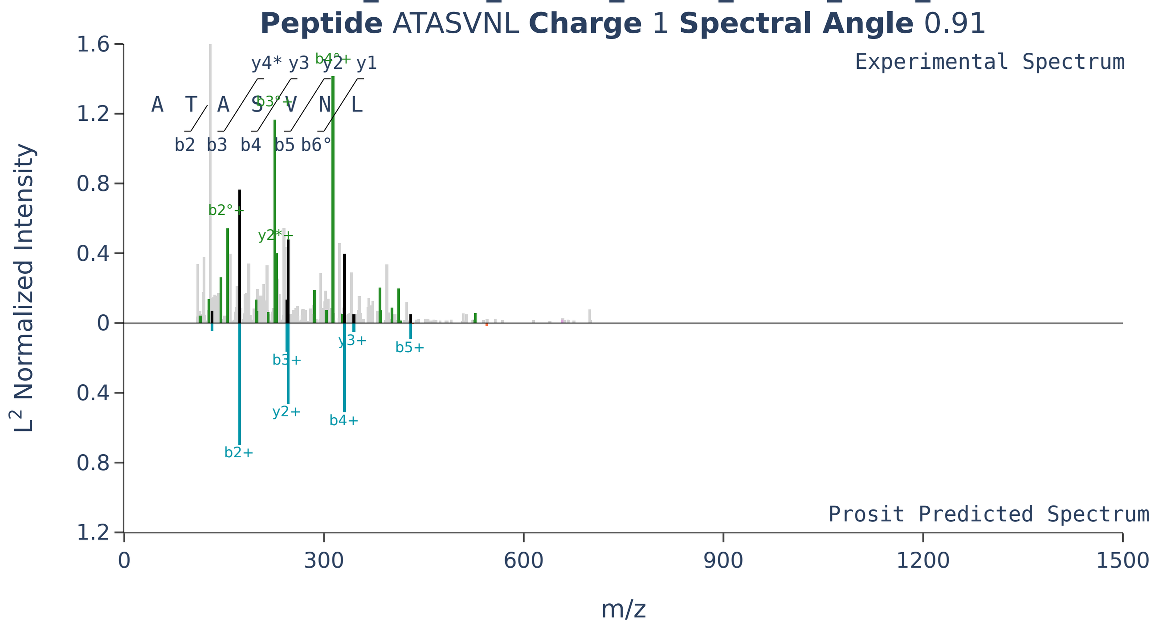


**Source** HRoetschke_271222_090123_HFGoe_LEDGF_b1R2_2h **Scan** 27253
**Peptide** VGEGDSVITQVLN **Charge** 2 **Spectral Angle** 0.79

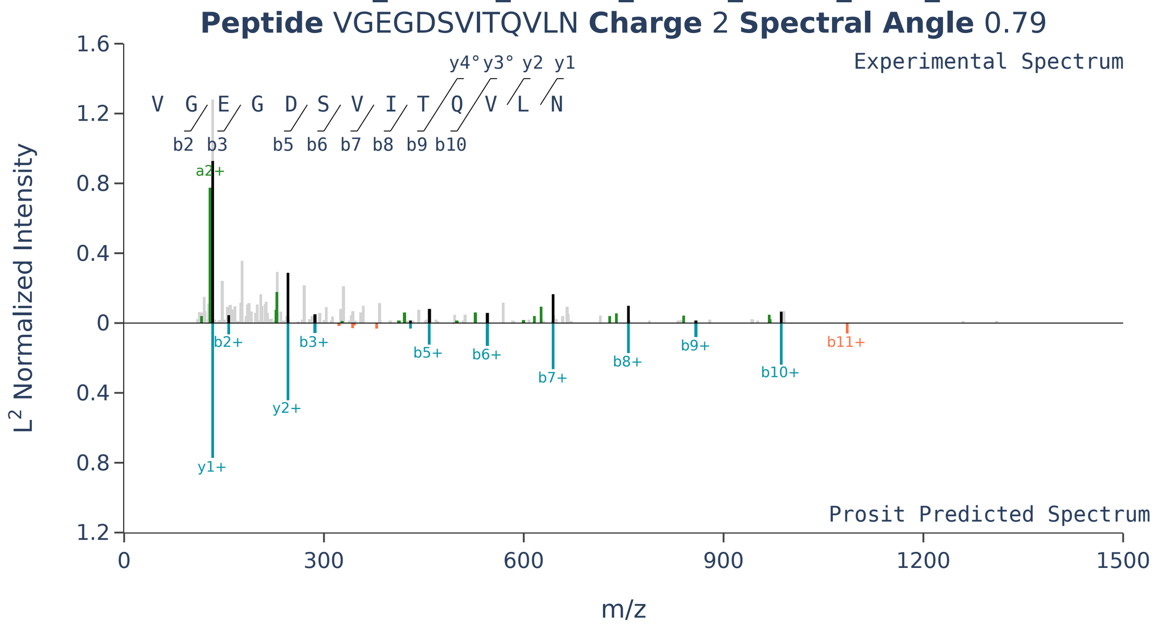


**Source** HRoetschke_271222_090123_HFGoe_LEDGF_b2R1_24h **Scan** 27123
**Peptide** RDFKPGDLIF **Charge** 2 **Spectral Angle** 0.86

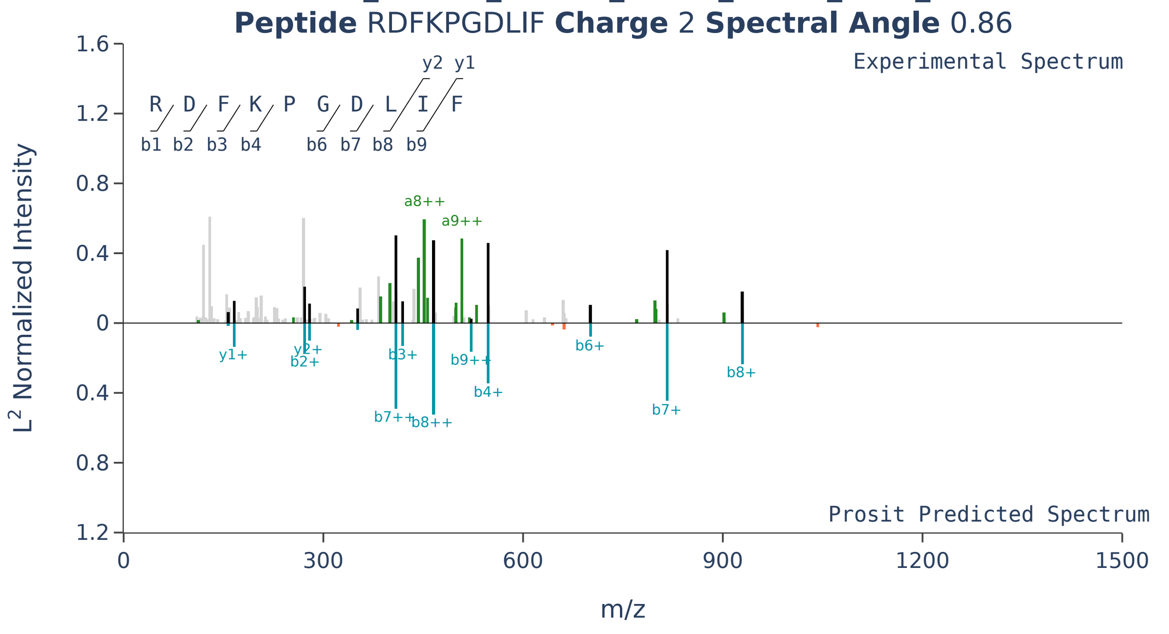


**Source** HRoetschke_271222_090123_HFGoe_LEDGF_b1R2_24h **Scan** 13349
**Peptide** HTEMITTLK **Charge** 2 **Spectral Angle** 0.79

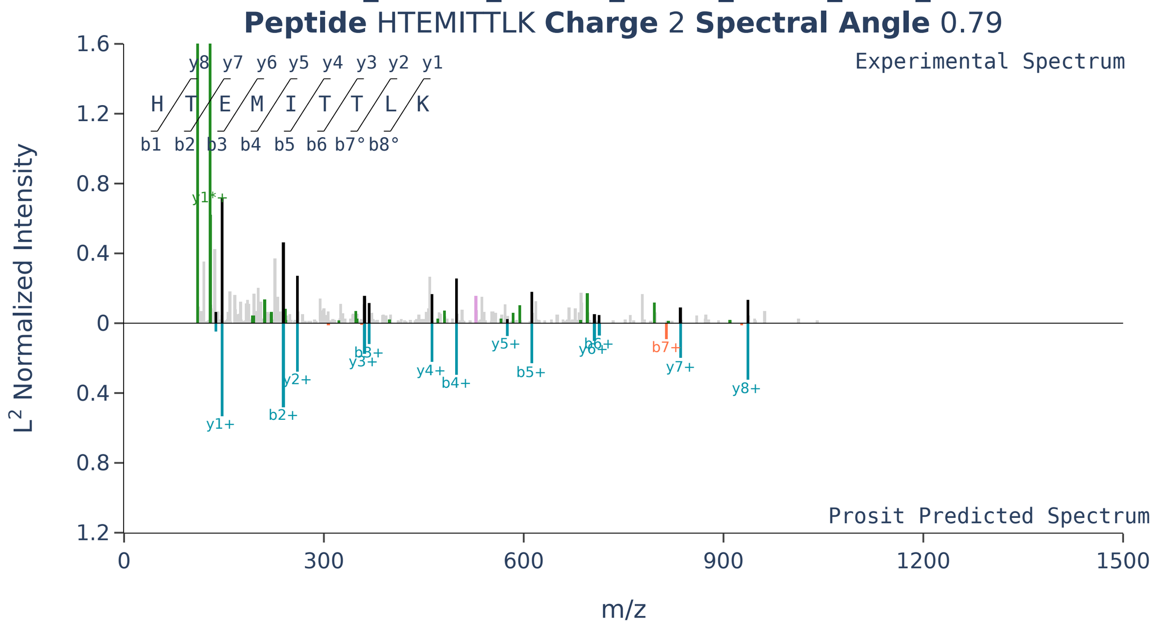


**Source** HRoetschke_271222_090123_HFGoe_LEDGF_b1R2_4h **Scan** 12205
**Peptide** HETAFLGPKD **Charge** 2 **Spectral Angle** 0.87

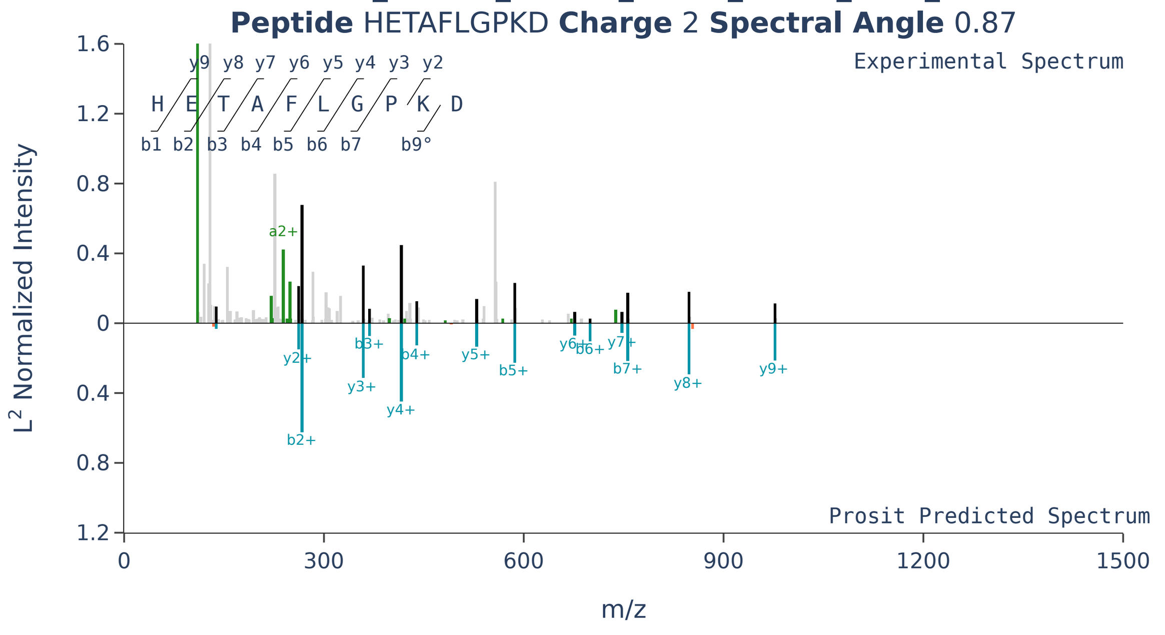


**Source** HRoetschke_271222_090123_HFGoe_LEDGF_b2R1_4h **Scan** 17152
**Peptide** KGYPHWPARVDEV **Charge** 2 **Spectral Angle** 0.85

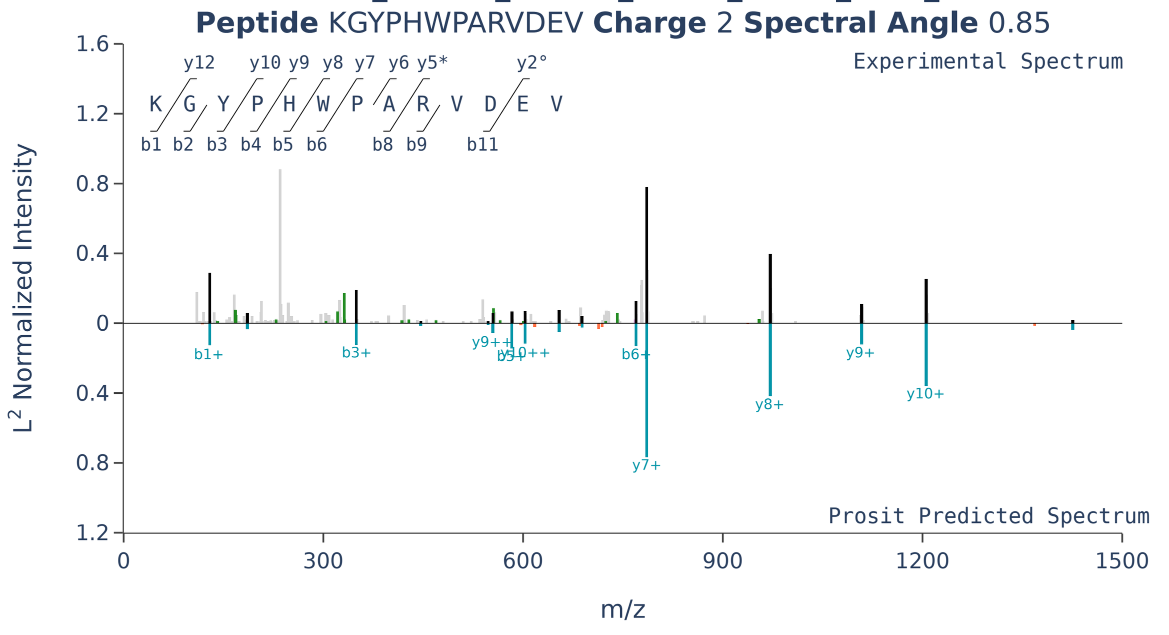


**Source** HRoetschke_271222_090123_HFGoe_LEDGF_b1R1_4h **Scan** 5811
**Peptide** VDITTPKAARRGRKRKAEKQVETEE **Charge** 4 **Spectral Angle** 0.81

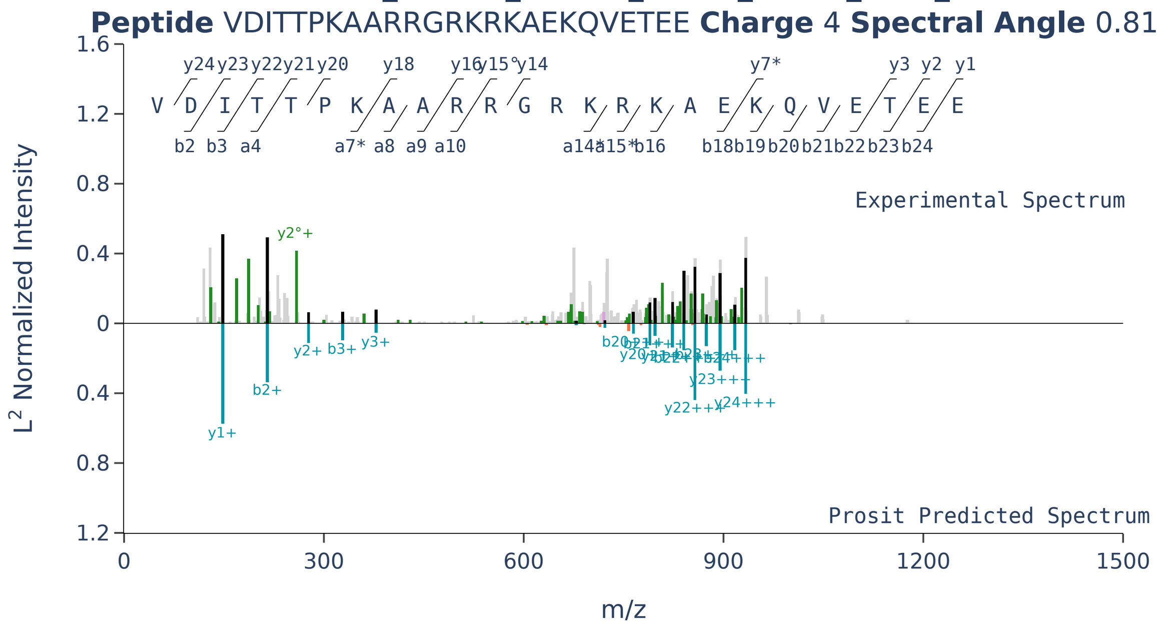


**Source** HRoetschke_271222_090123_HFGoe_LEDGF_b1R2_1h **Scan** 9537
**Peptide** TEVKIPKPRGRPKMVKQPCPSE **Charge** 4 **Spectral Angle** 0.83

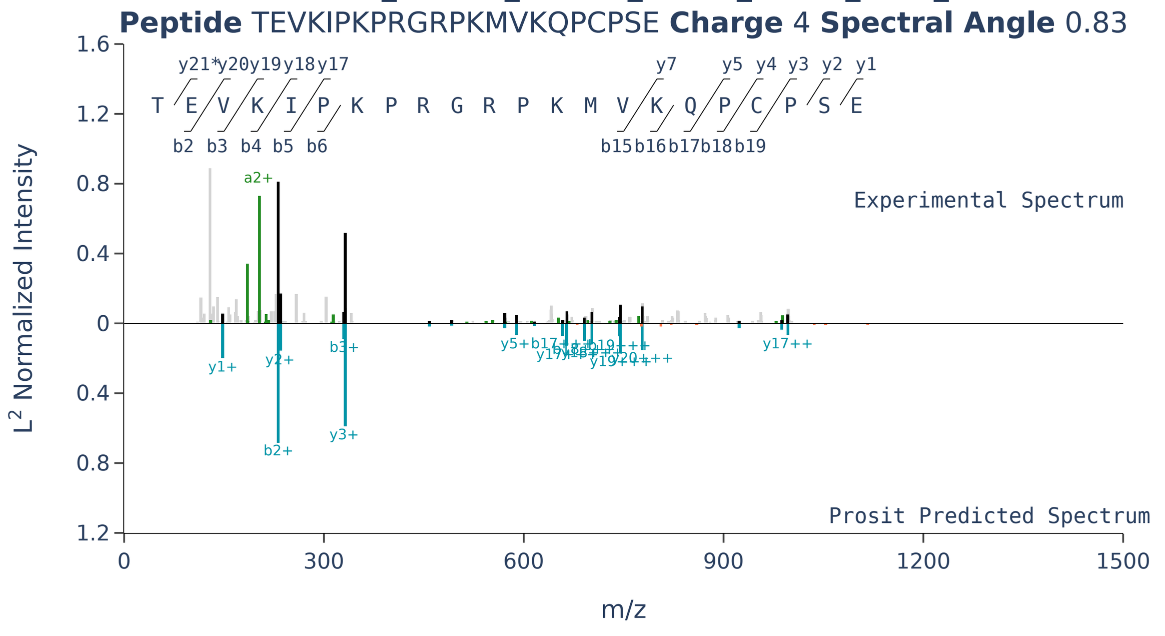


**Source** HRoetschke_271222_090123_HFGoe_LEDGF_b1R1_2h **Scan** 24808
**Peptide** GLWEIDNNPKV **Charge** 2 **Spectral Angle** 0.82

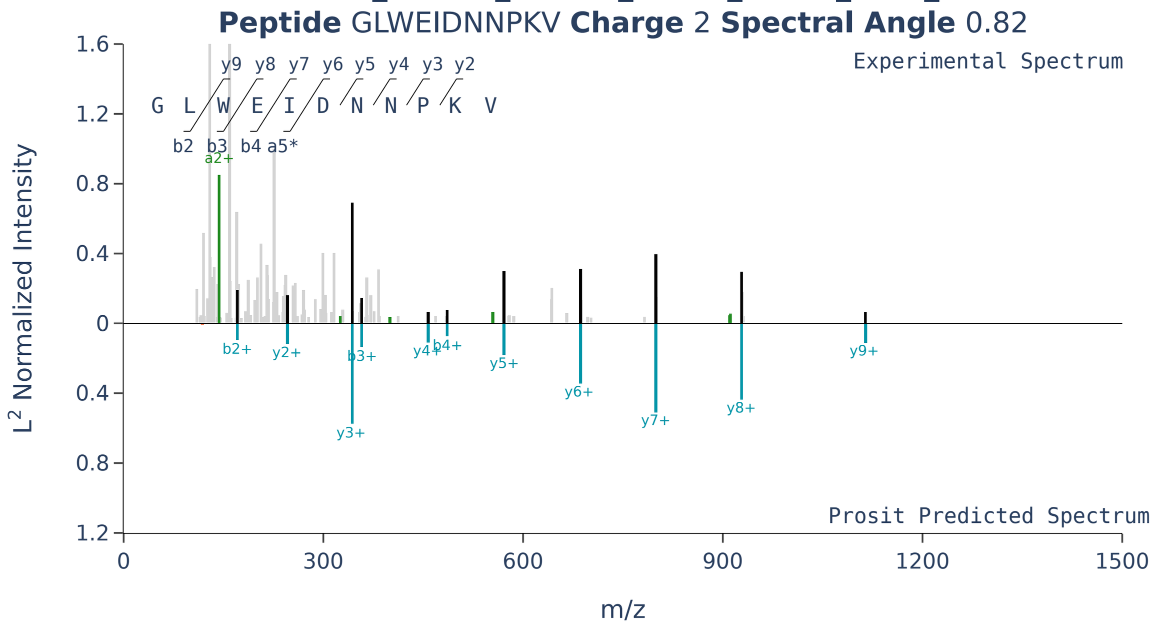


**Source** HRoetschke_271222_090123_HFGoe_LEDGF_b1R1_24h **Scan** 16232
**Peptide** GPKDIFPYSENKEKY **Charge** 2 **Spectral Angle** 0.78

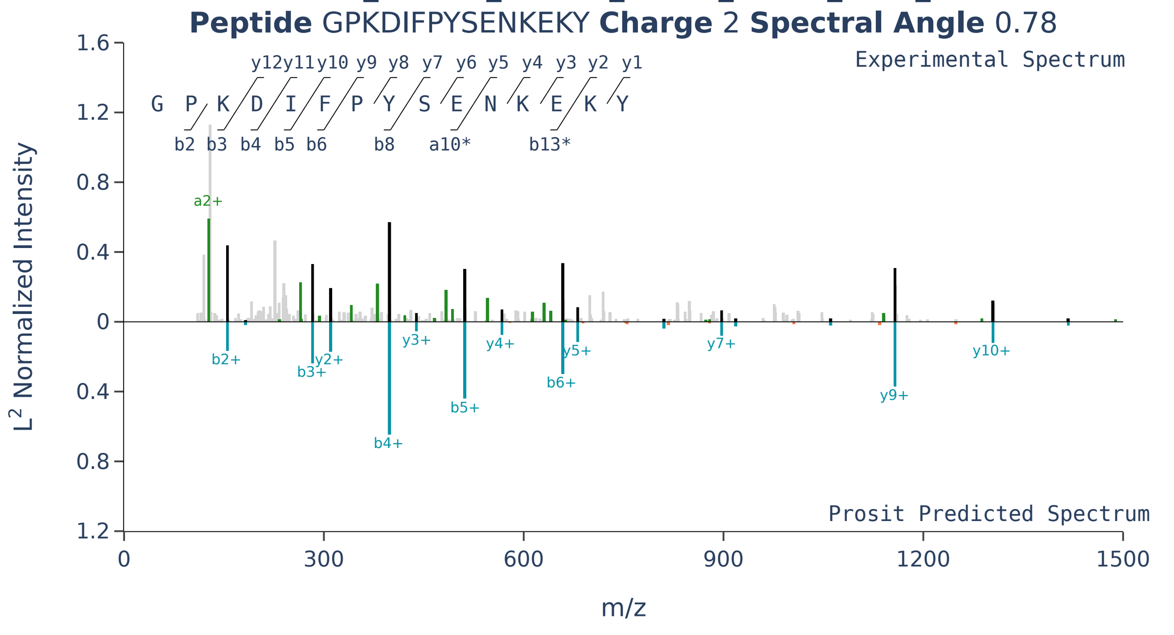


**Source** HRoetschke_271222_090123_HFGoe_LEDGF_b2R2_24h **Scan** 9383
**Peptide** TASVNLKVSPKRG **Charge** 3 **Spectral Angle** 0.73

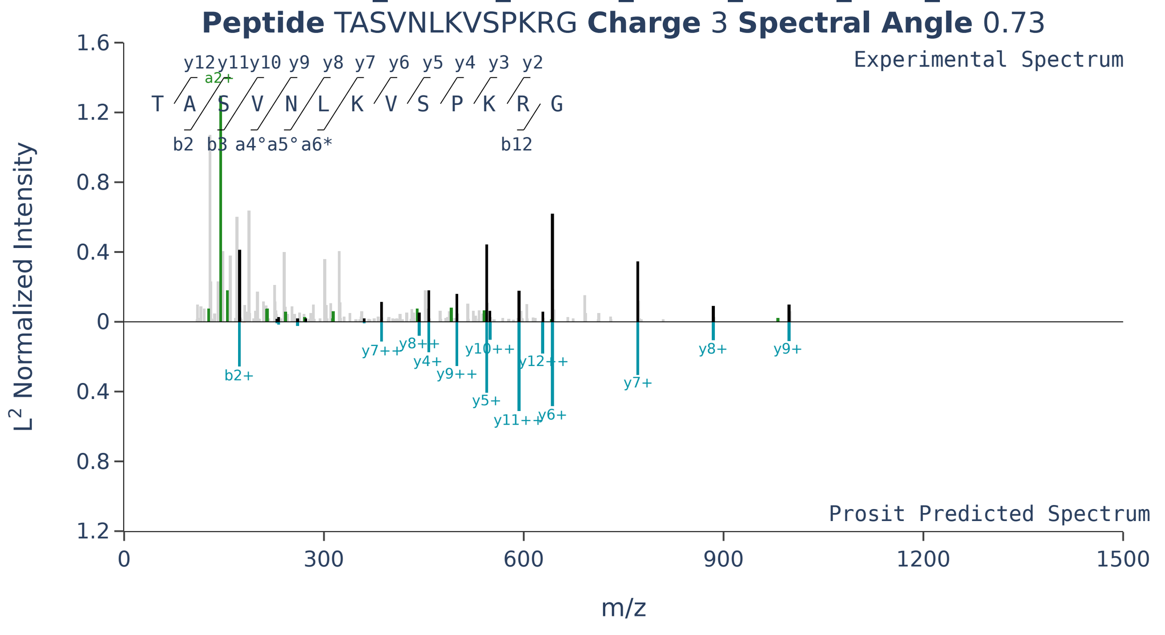


**Source** HRoetschke_271222_090123_HFGoe_LEDGF_b1R1_4h **Scan** 21301
**Peptide** GYPHWPARVDEVPDGAVKPPTNKL **Charge** 5 **Spectral Angle** 0.86

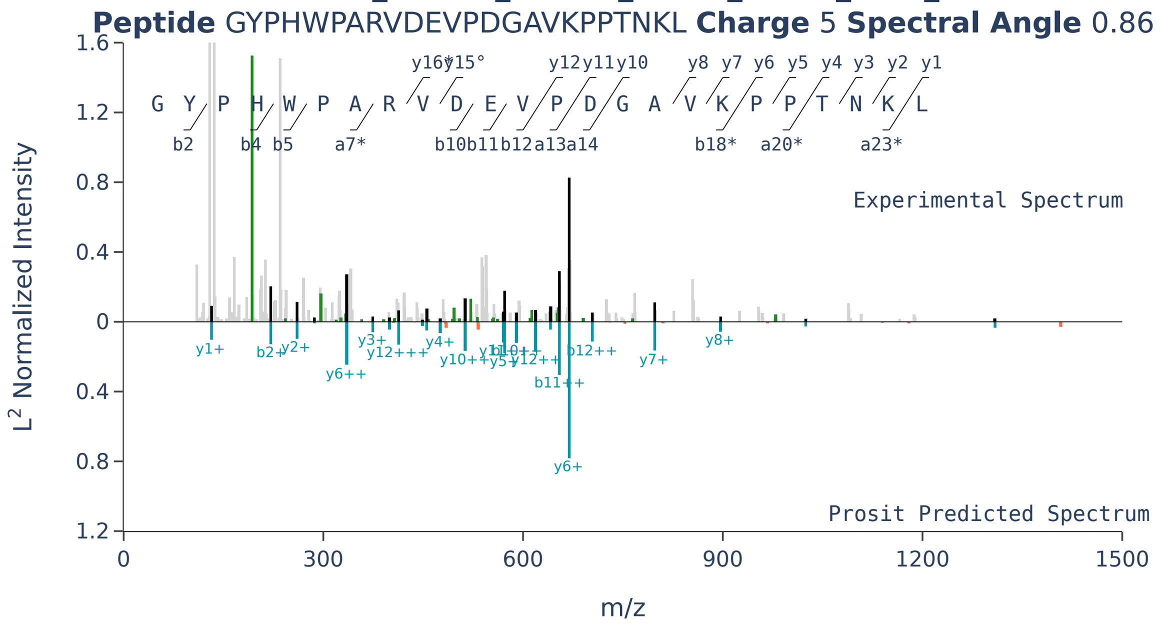


**Source** HRoetschke_271222_090123_HFGoe_LEDGF_b2R2_2h **Scan** 30173
**Peptide** EGDSVITQVLNKSL **Charge** 2 **Spectral Angle** 0.83

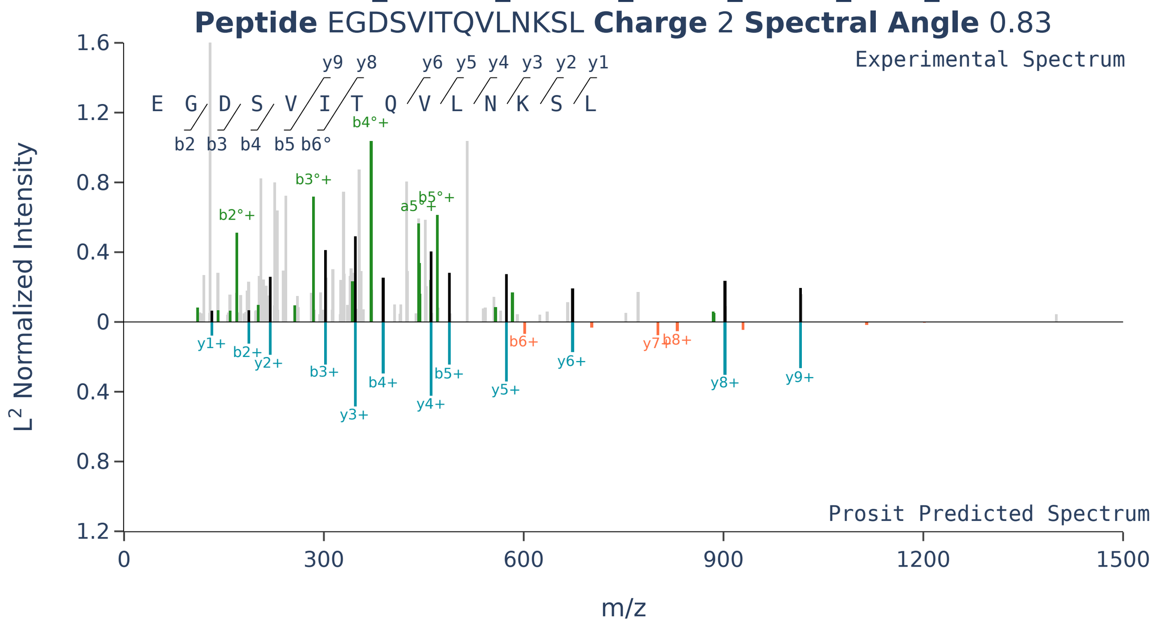


**Source** HRoetschke_271222_090123_HFGoe_LEDGF_b2R2_2h **Scan** 19679
**Peptide** GEGDSVITQV **Charge** 1 **Spectral Angle** 0.83

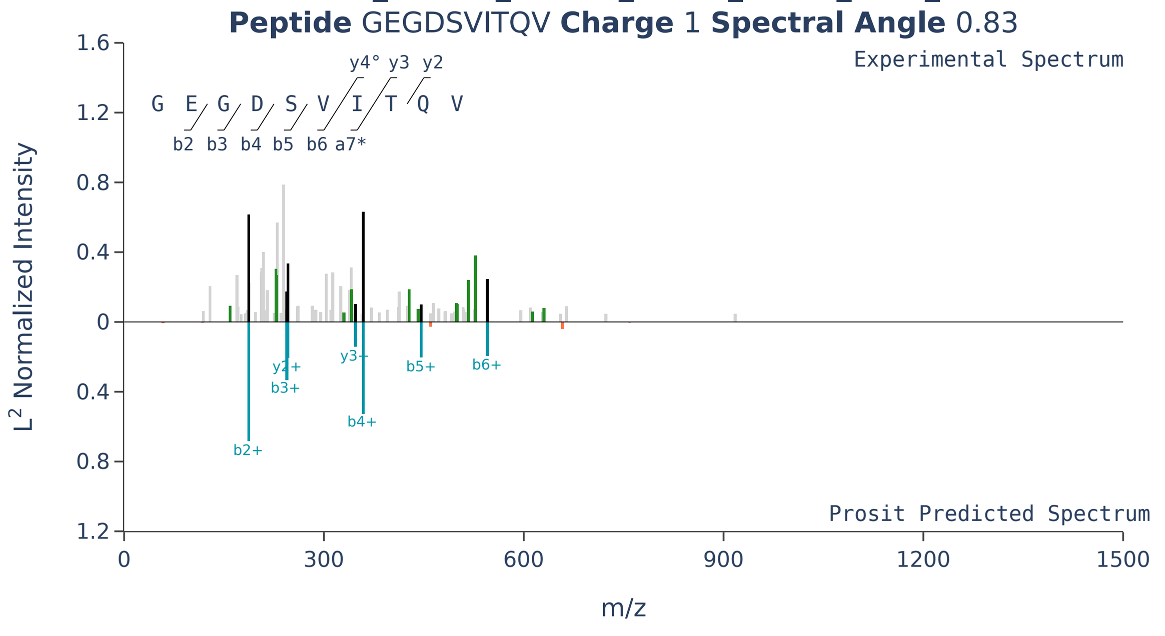


**Source** HRoetschke_271222_090123_HFGoe_LEDGF_b1R1_1h **Scan** 10677
**Peptide** IDNNPKVKFSSQQAAT **Charge** 2 **Spectral Angle** 0.82

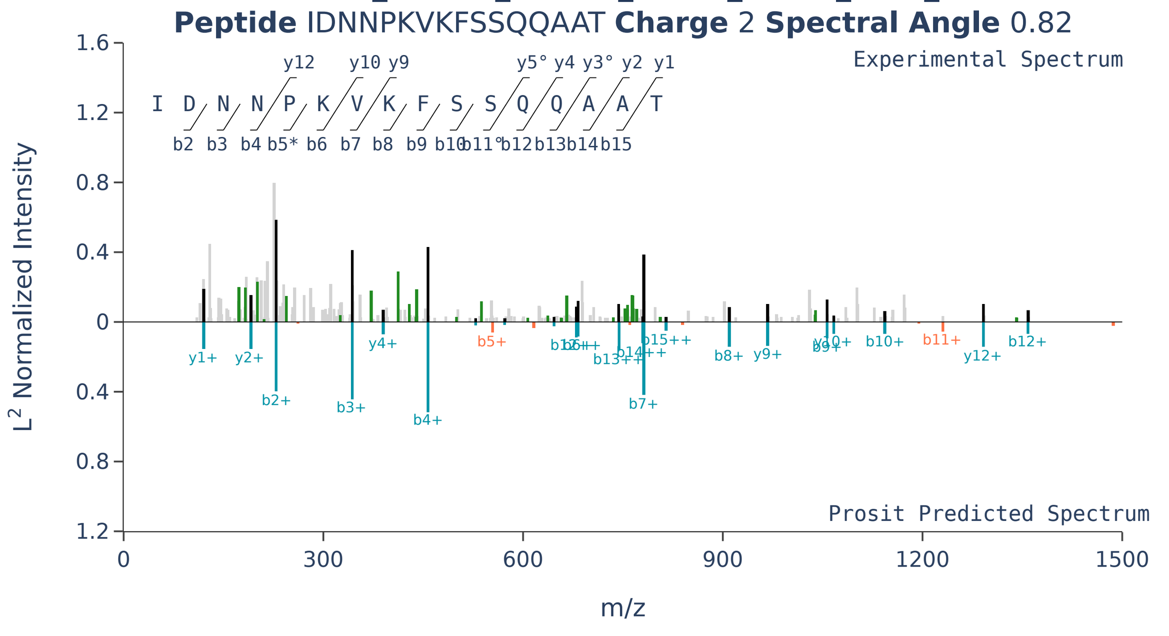

**Source** HRoetschke_271222_090123_HFGoe_LEDGF_b2R1_2h **Scan** 9503
**Peptide** DNNPKVKFSSQQA **Charge** 3 **Spectral Angle** 0.89

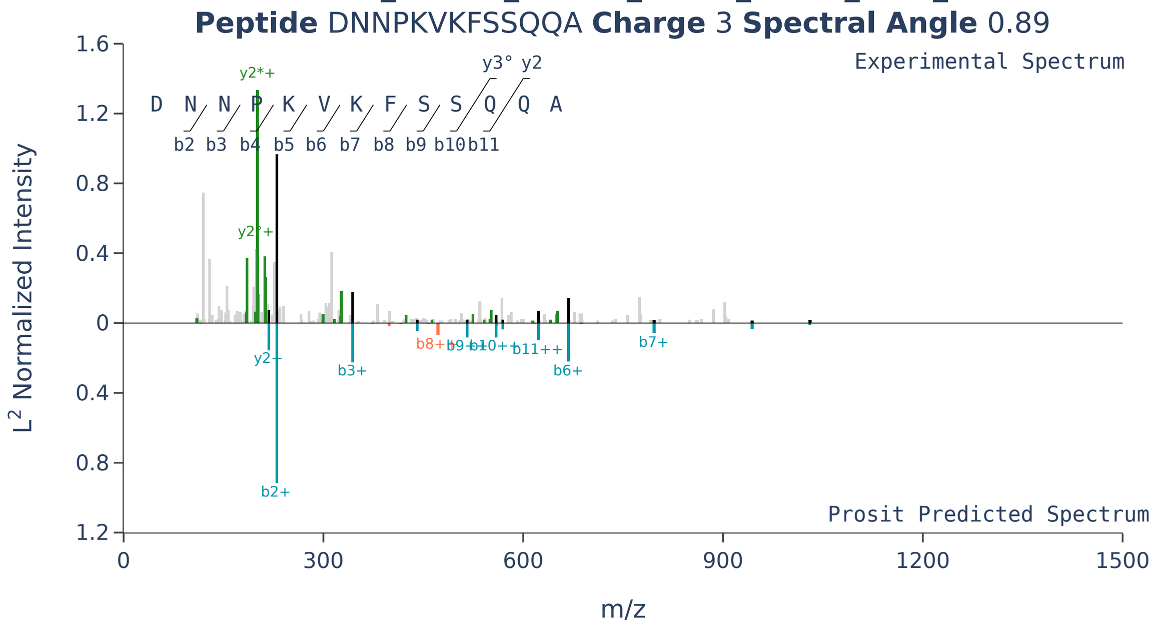


**Source** HRoetschke_271222_090123_HFGoe_LEDGF_b2R1_2h **Scan** 9363
**Peptide** GMTRDFKPGD **Charge** 3 **Spectral Angle** 0.92

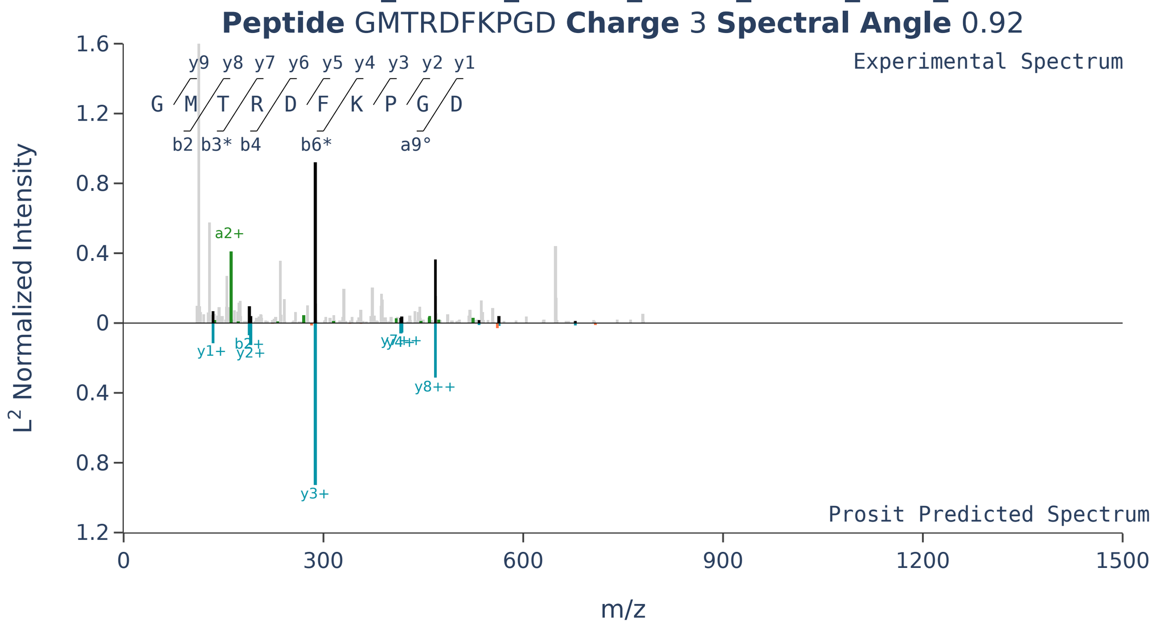


**Source** HRoetschke_271222_090123_HFGoe_LEDGF_b1R1_1h **Scan** 20059
**Peptide** GYPHWPARVDEVPDGAVKPPTN **Charge** 3 **Spectral Angle** 0.93

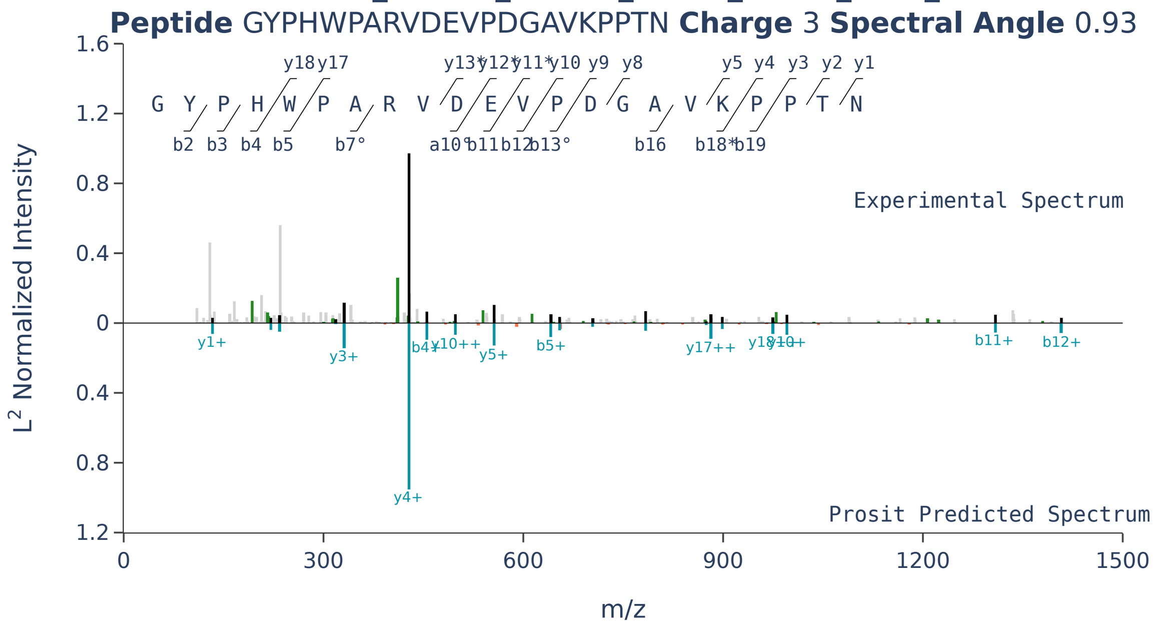


**Source** HRoetschke_271222_090123_HFGoe_LEDGF_b2R2_24h **Scan** 22268
**Peptide** KGFNEGLWE **Charge** 2 **Spectral Angle** 0.8

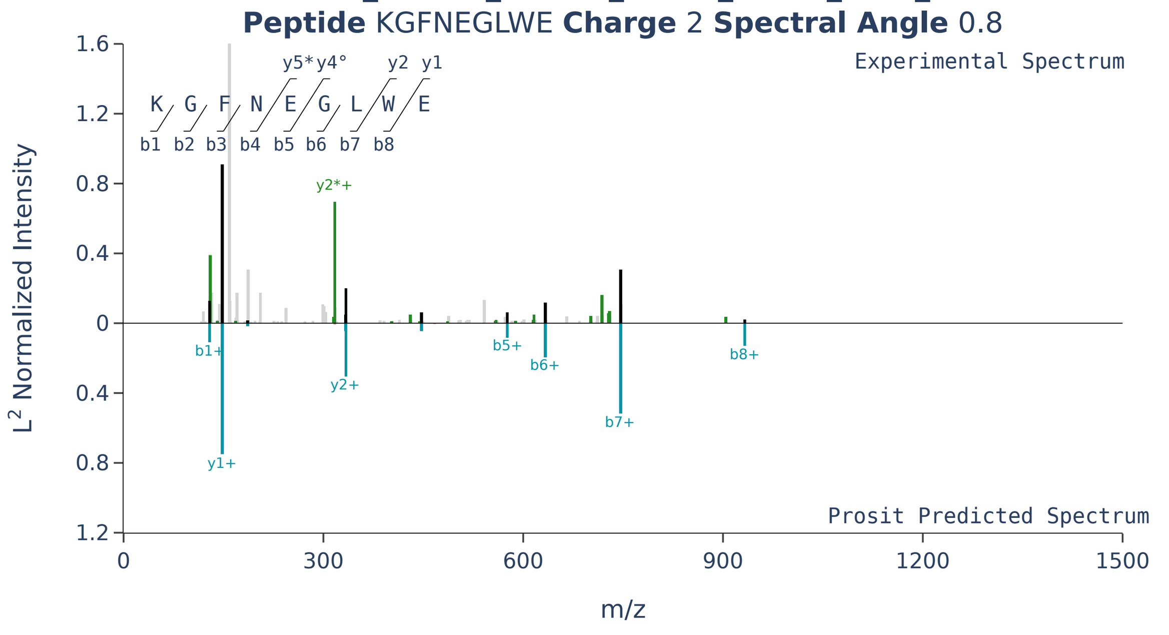


**Source** HRoetschke_271222_090123_HFGoe_LEDGF_b2R1_2h **Scan** 9830
**Peptide** SSDVEVE **Charge** 1 **Spectral Angle** 0.89

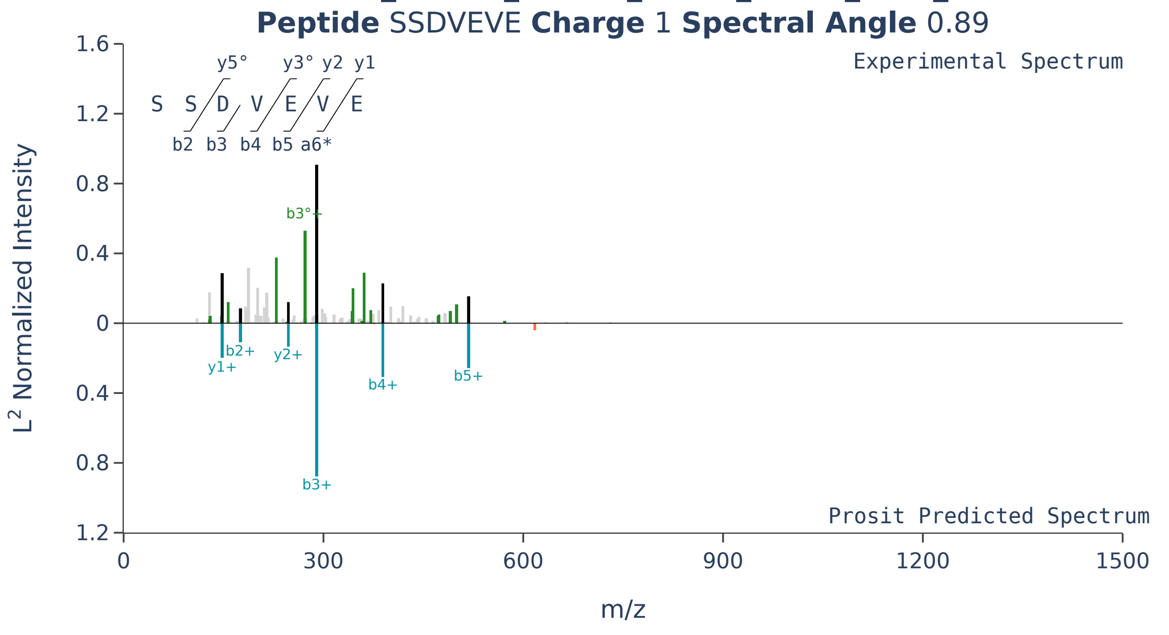


**Source** HRoetschke_271222_090123_HFGoe_LEDGF_b1R1_24h **Scan** 13924
**Peptide** KNSLKIDNLDVNR **Charge** 3 **Spectral Angle** 0.82

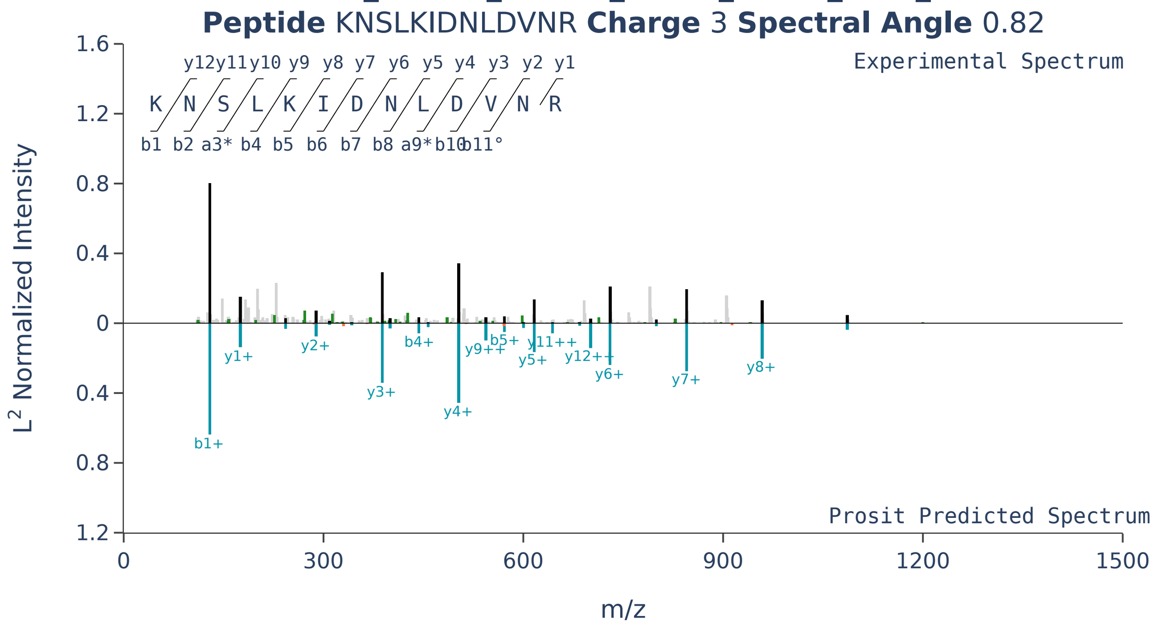


**Source** HRoetschke_271222_090123_HFGoe_LEDGF_b2R1_24h **Scan** 28434
**Peptide** ALDELASL **Charge** 1 **Spectral Angle** 0.84

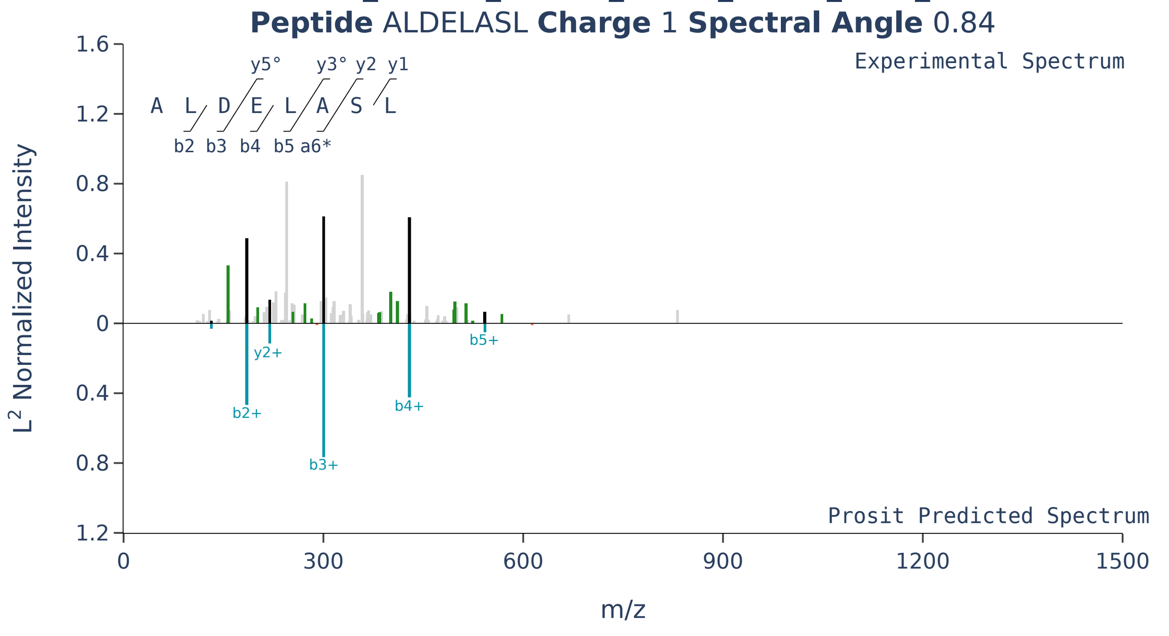


**Source** HRoetschke_271222_090123_HFGoe_LEDGF_b2R2_2h **Scan** 12902
**Peptide** ASLQVTMQQAQKH **Charge** 2 **Spectral Angle** 0.74

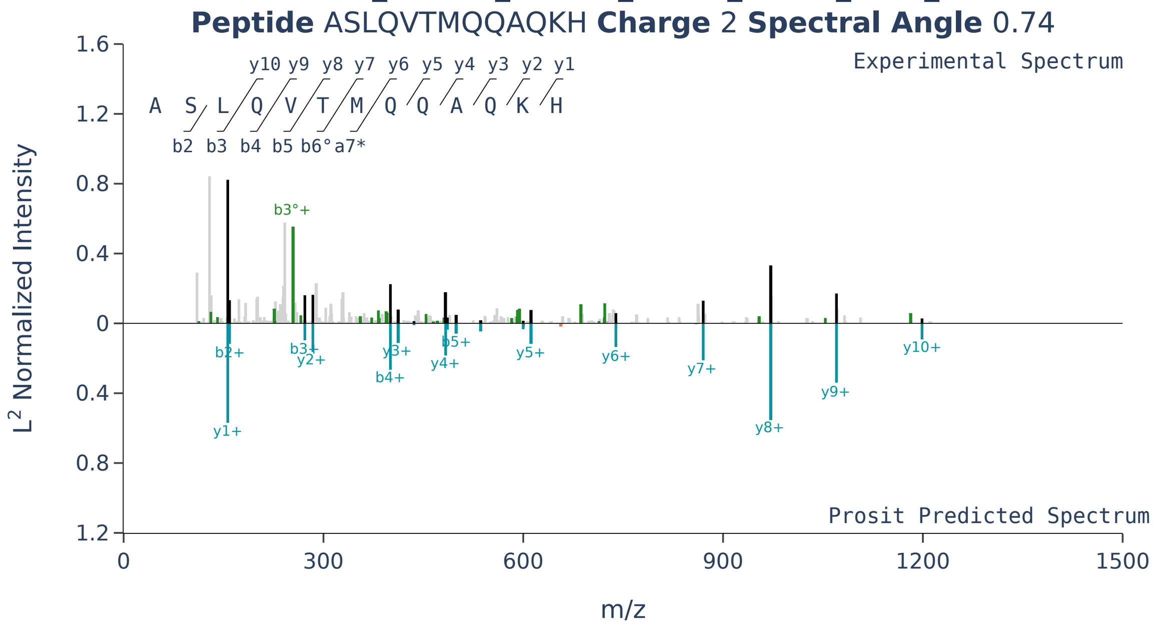


**Source** HRoetschke_271222_090123_HFGoe_LEDGF_b2R2_24h **Scan** 10220
**Peptide** KGYPHWPAR **Charge** 3 **Spectral Angle** 0.86

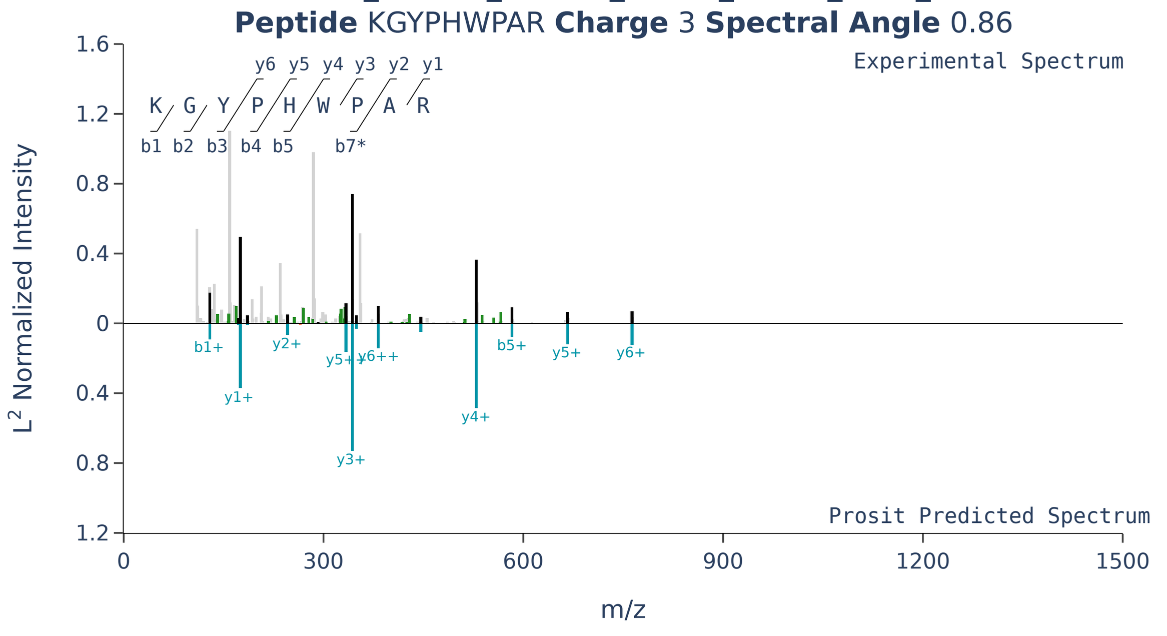


**Source** HRoetschke_271222_090123_HFGoe_LEDGF_b1R1_4h **Scan** 13340
**Peptide** RIHAEIKNSLKI **Charge** 3 **Spectral Angle** 0.86

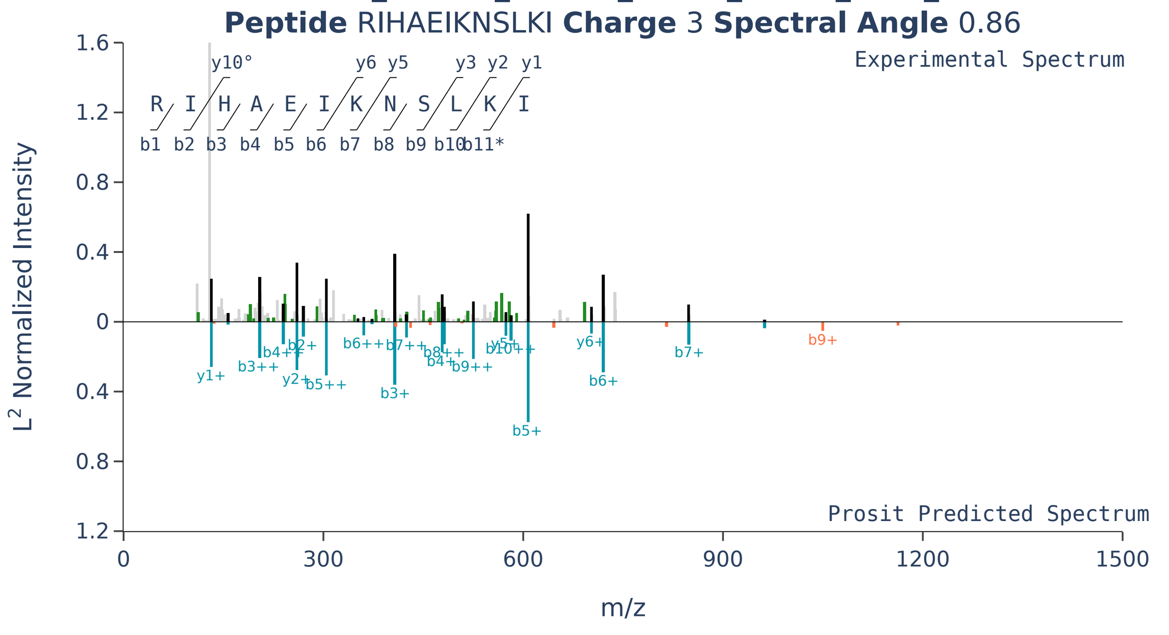


**Source** HRoetschke_271222_090123_HFGoe_LEDGF_b2R1_1h **Scan** 13131
**Peptide** KMKGYPHWPARV **Charge** 4 **Spectral Angle** 0.94

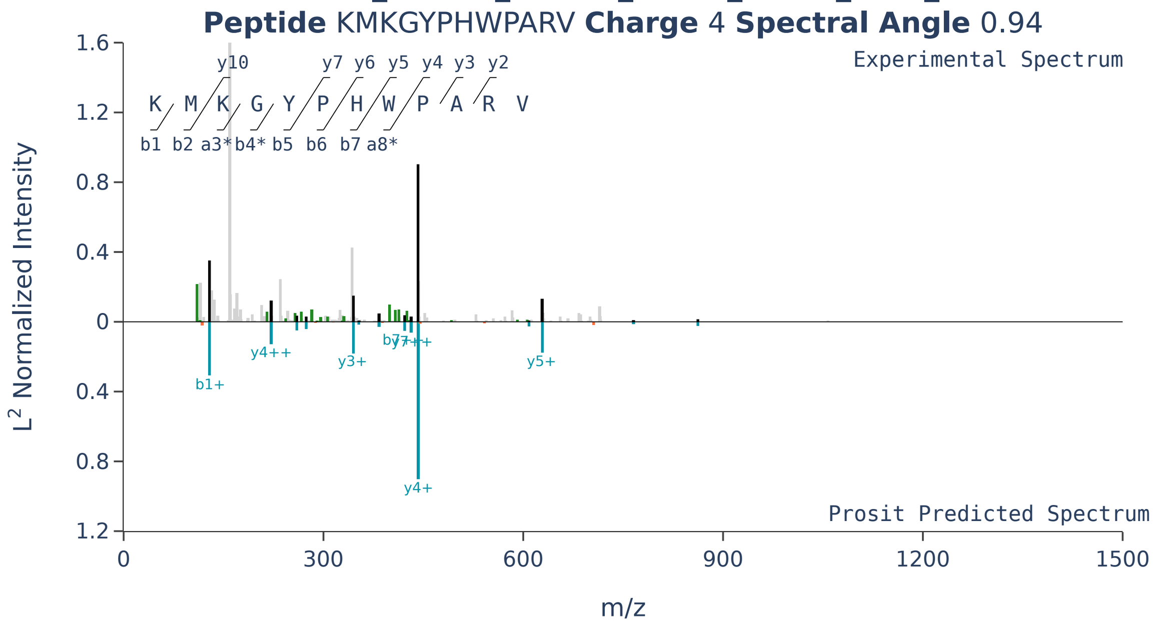


**Source** HRoetschke_271222_090123_HFGoe_LEDGF_b2R2_2h **Scan** 10029
**Peptide** IDNNPKVKFSSQQ **Charge** 2 **Spectral Angle** 0.79

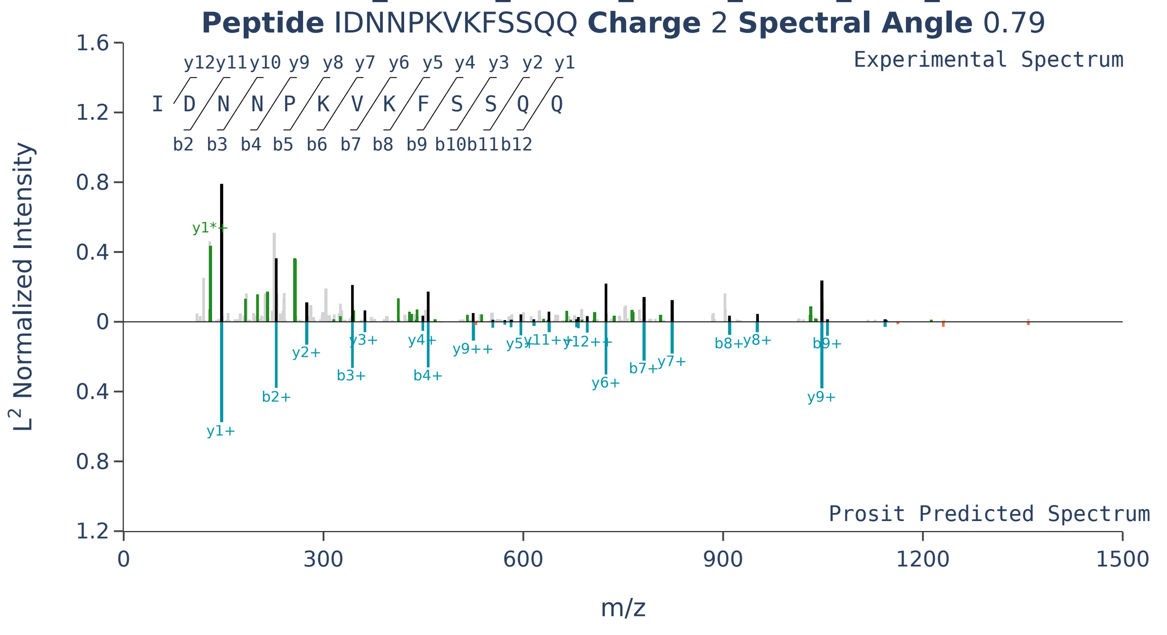


**Source** HRoetschke_271222_090123_HFGoe_LEDGF_b2R1_4h **Scan** 20868
**Peptide** KFKNMFL **Charge** 2 **Spectral Angle** 0.83

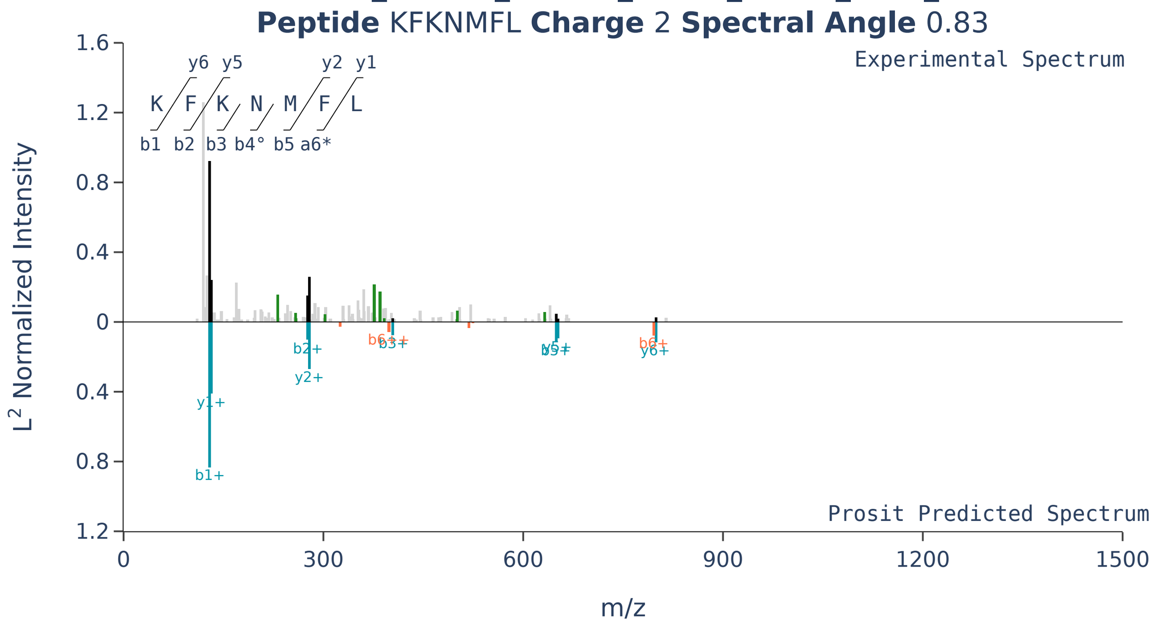


**Source** HRoetschke_271222_090123_HFGoe_LEDGF_b2R2_2h **Scan** 22324
**Peptide** KVSQVIMEKSTMLYNKF **Charge** 3 **Spectral Angle** 0.93

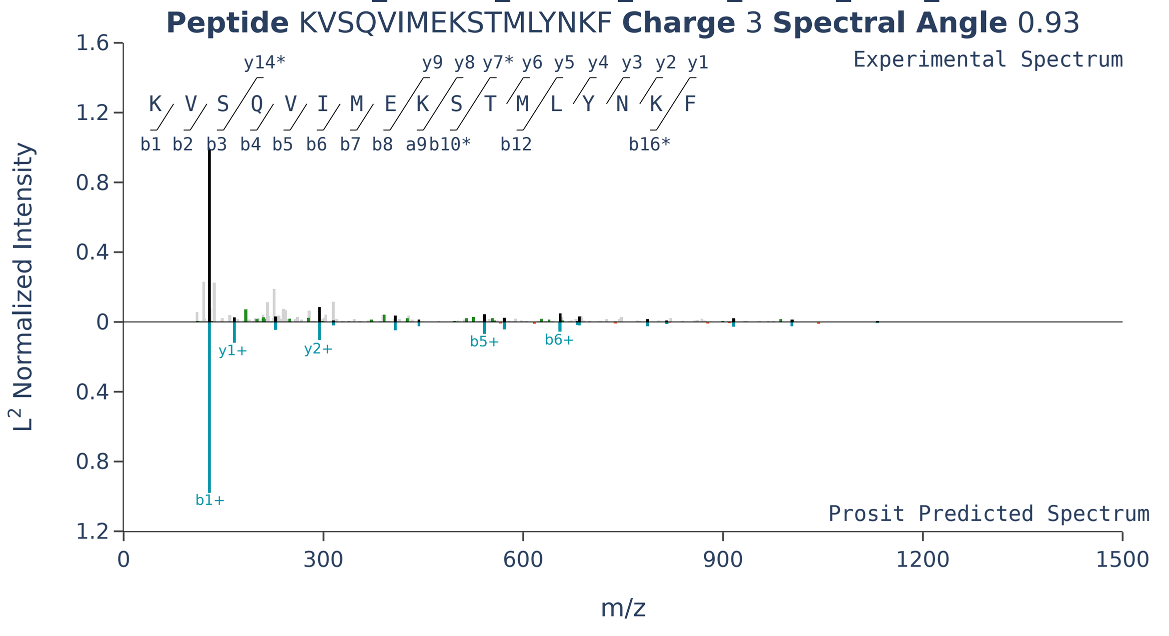


**Source** HRoetschke_271222_090123_HFGoe_LEDGF_b1R2_2h **Scan** 7544
**Peptide** SQQAATKQSNASSDVEVEEKE **Charge** 2 **Spectral Angle** 0.9

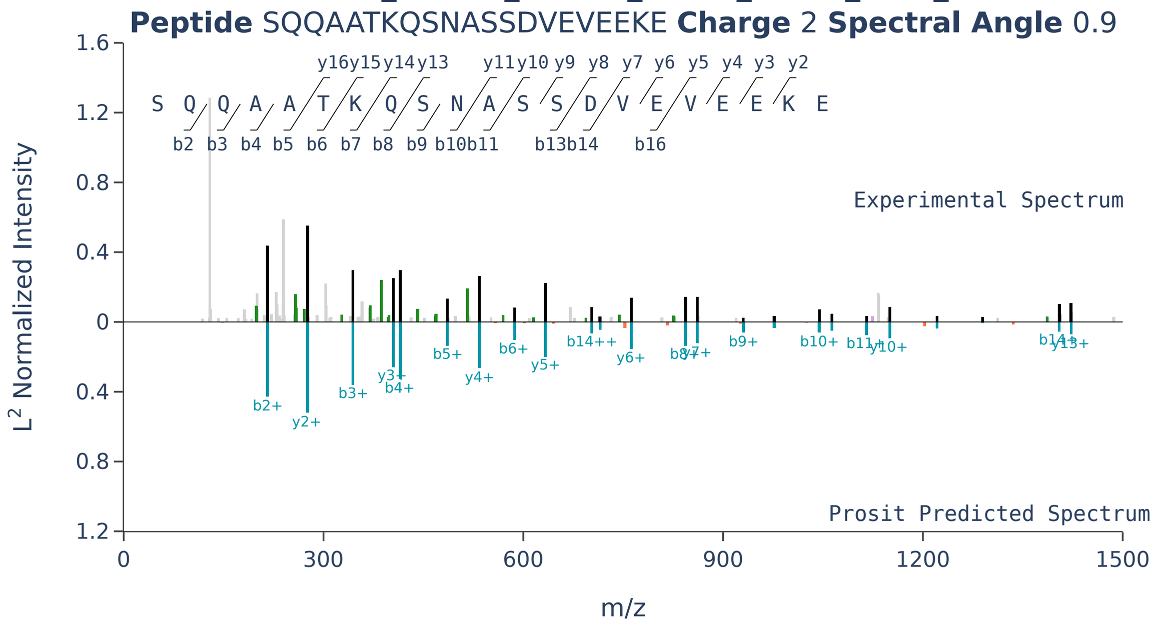

**Source** HRoetschke_271222_090123_HFGoe_LEDGF_b1R1_2h **Scan** 23523
**Peptide** KPGDLIFAKM **Charge** 3 **Spectral Angle** 0.9

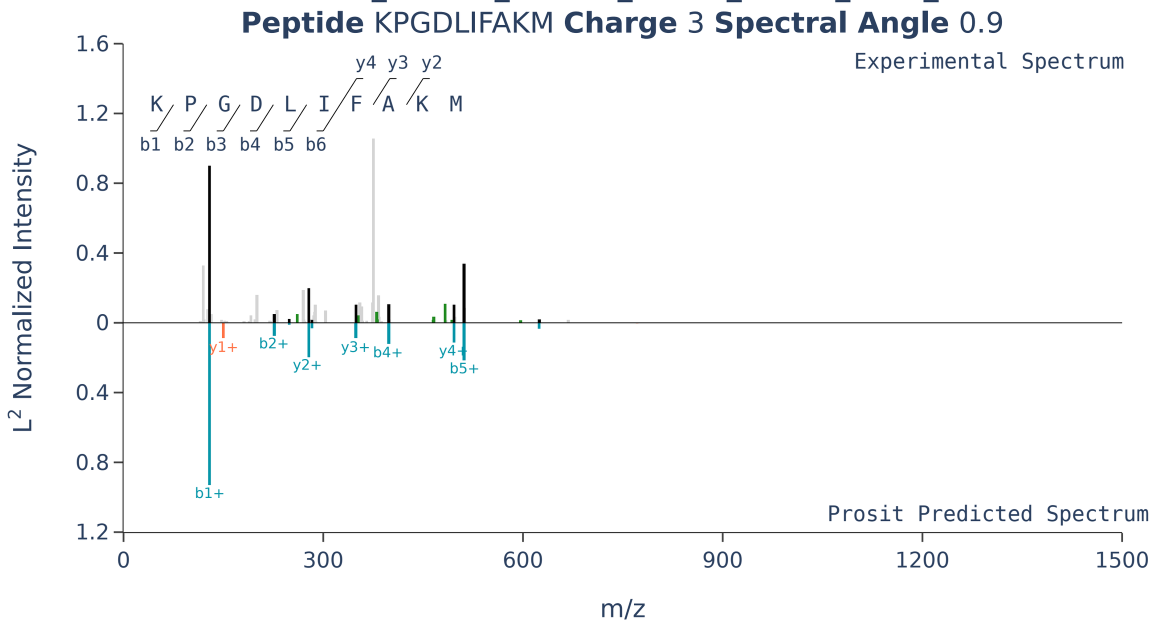


**Source** HRoetschke_271222_090123_HFGoe_LEDGF_b1R1_2h **Scan** 22061
**Peptide** RDFKPGDLIFAKMK **Charge** 4 **Spectral Angle** 0.82

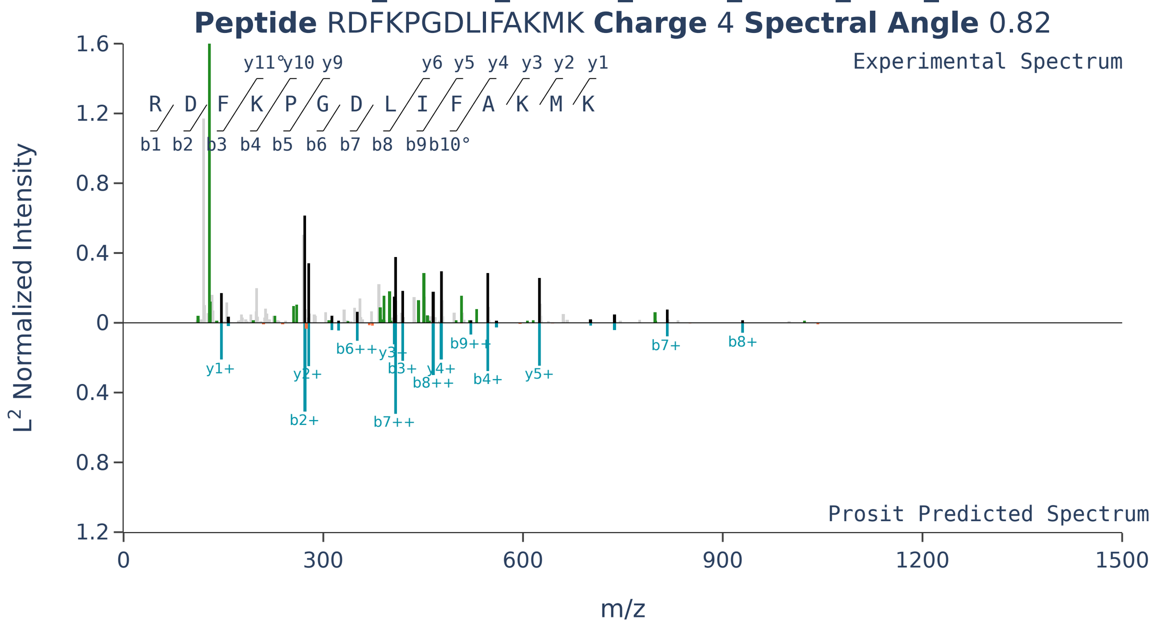


**Source** HRoetschke_271222_090123_HFGoe_LEDGF_b2R1_2h **Scan** 15371
**Peptide** FLGPKDIFPYSENKEKYGKPNKRKG **Charge** 5 **Spectral Angle** 0.84

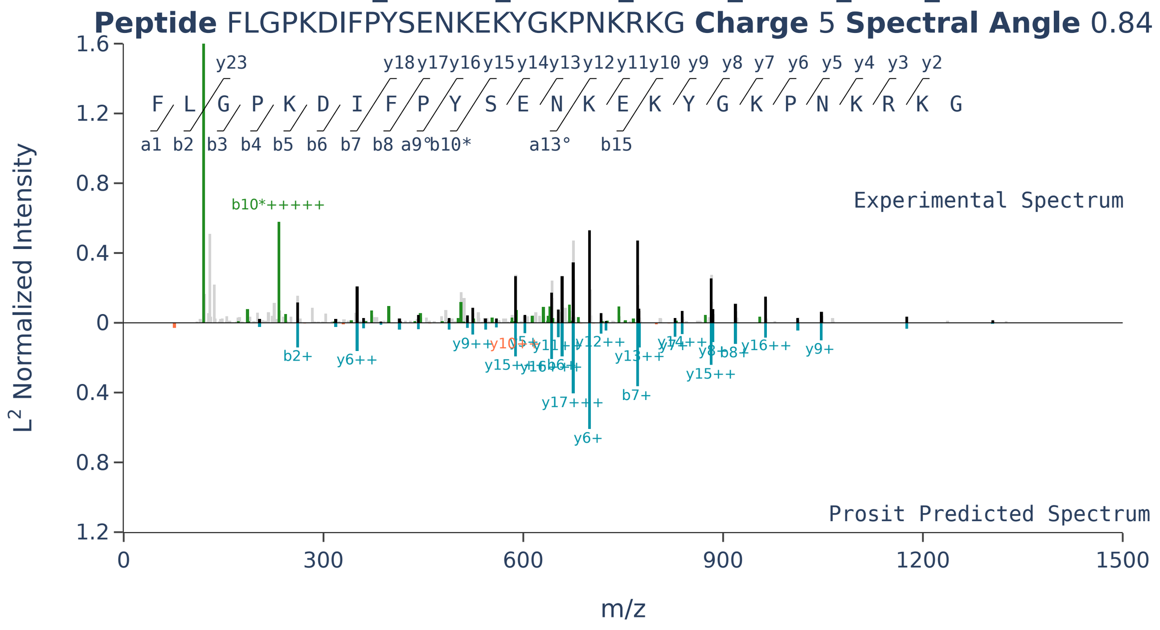


**Source** HRoetschke_271222_090123_HFGoe_LEDGF_b1R1_4h **Scan** 8674
**Peptide** QKHTEMITTLKKIRR **Charge** 5 **Spectral Angle** 0.89

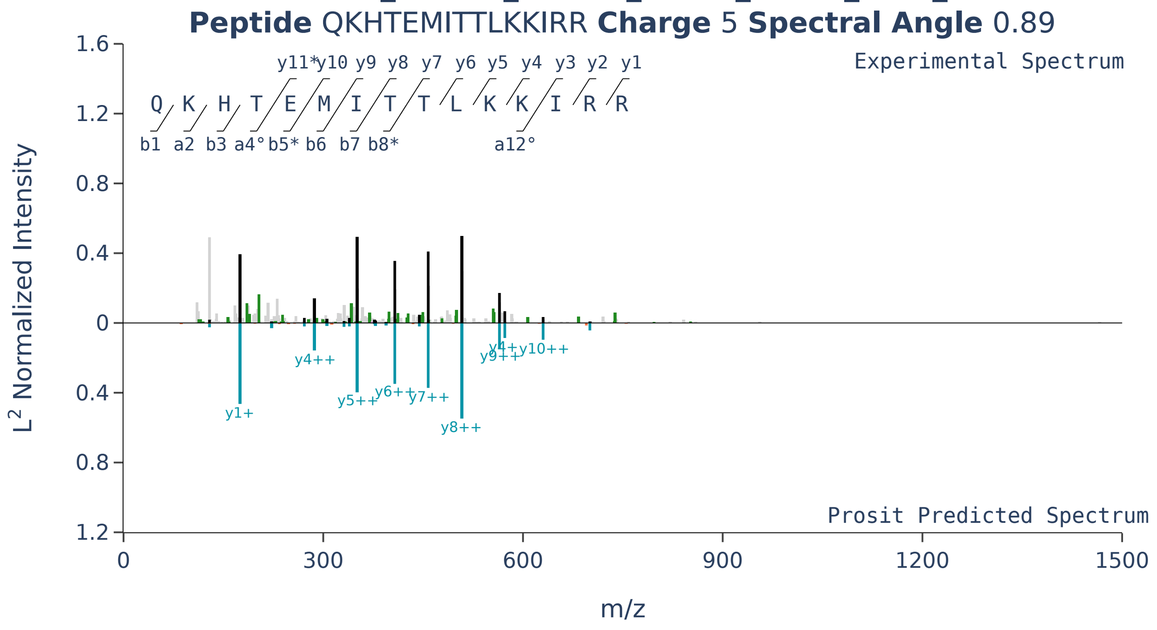


**Source** HRoetschke_271222_090123_HFGoe_LEDGF_b1R2_2h **Scan** 29414
**Peptide** PIFFFGTHET **Charge** 2 **Spectral Angle** 0.81

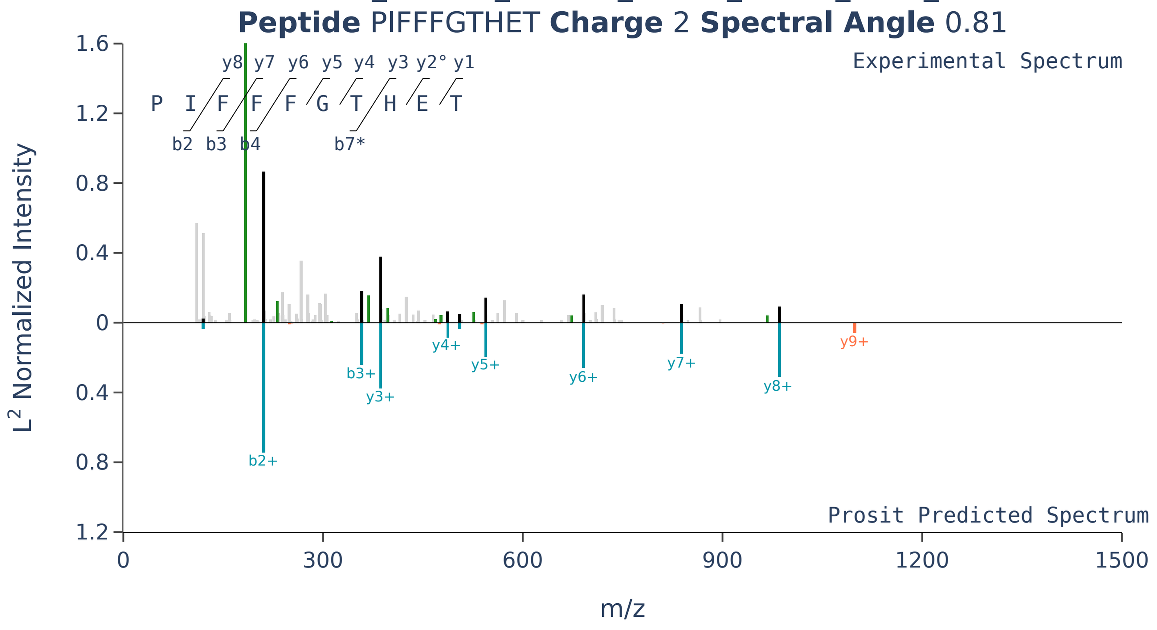


**Source** HRoetschke_271222_090123_HFGoe_LEDGF_b2R1_2h **Scan** 23609
**Peptide** AFLGPKDIFPYSENKEKY **Charge** 3 **Spectral Angle** 0.86

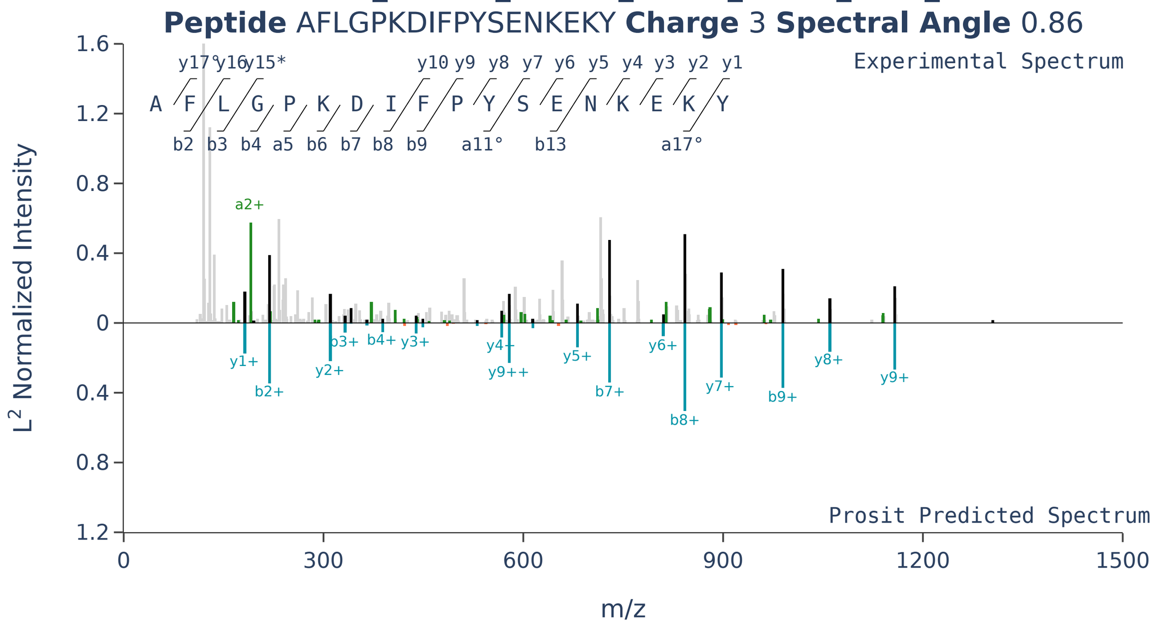


**Source** HRoetschke_271222_090123_HFGoe_LEDGF_b1R1_4h **Scan** 6649
**Peptide** DSRLQRIH **Charge** 2 **Spectral Angle** 0.87

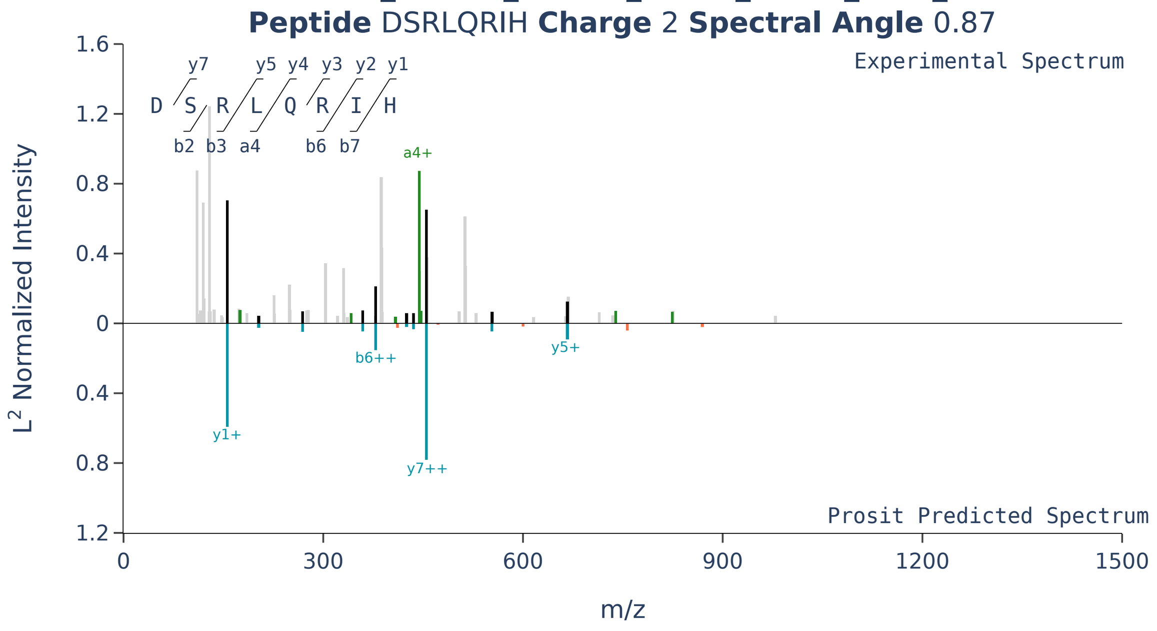


**Source** HRoetschke_271222_090123_HFGoe_LEDGF_b2R1_4h **Scan** 13310
**Peptide** WEIDNNPKVK **Charge** 3 **Spectral Angle** 0.86

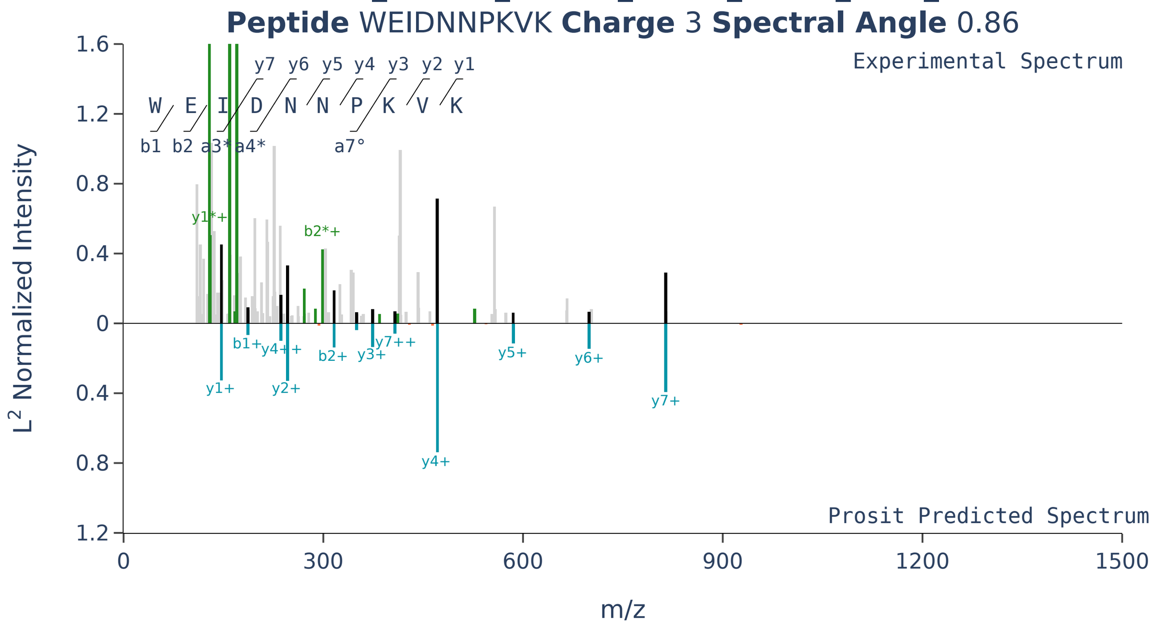


**Source** HRoetschke_271222_090123_HFGoe_LEDGF_b2R1_4h **Scan** 6692
**Peptide** NNPKVKFSSQQAATK **Charge** 2 **Spectral Angle** 0.8

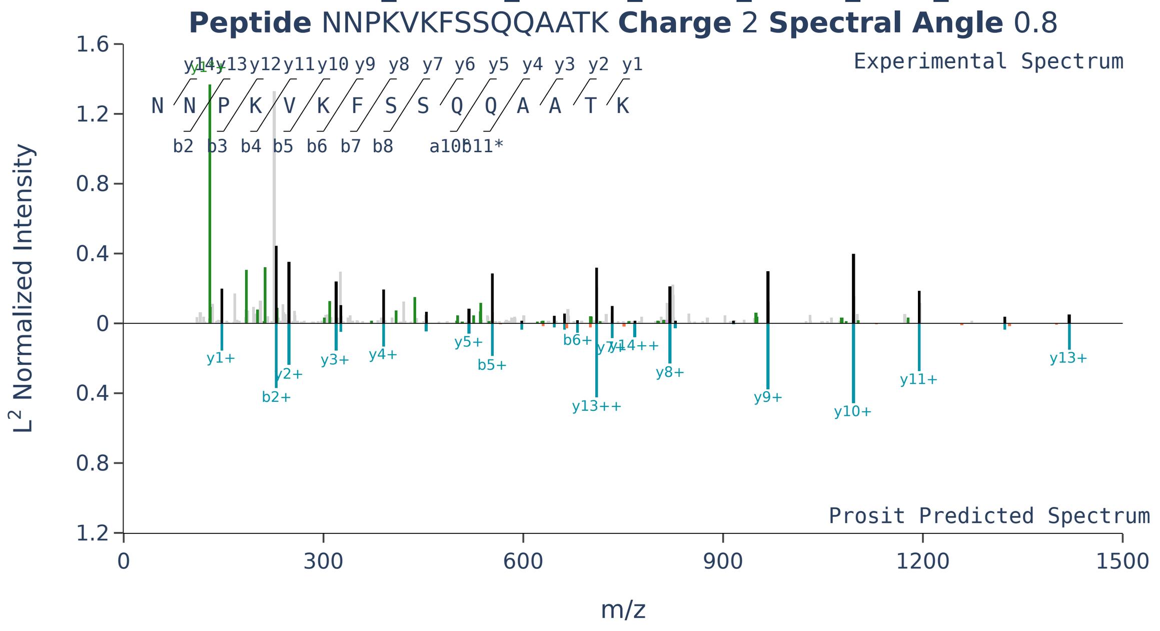


**Source** HRoetschke_271222_090123_HFGoe_LEDGF_b1R1_2h **Scan** 22896
**Peptide** NEGLWEIDNNPKVKFSSQQA **Charge** 3 **Spectral Angle** 0.94

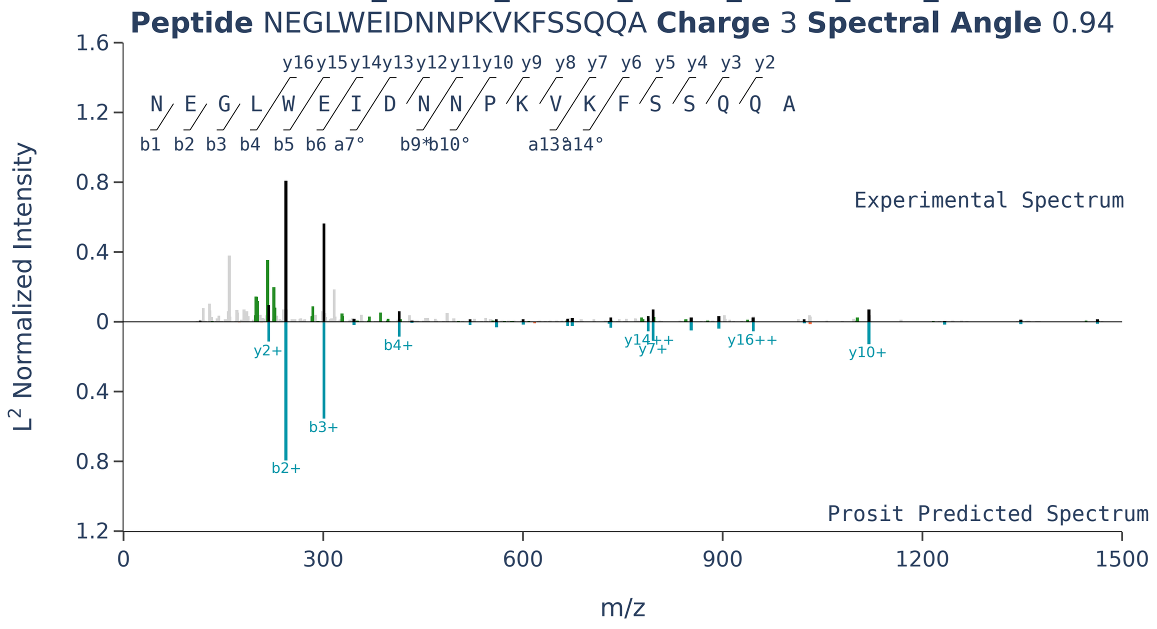


**Source** HRoetschke_271222_090123_HFGoe_LEDGF_b2R1_4h **Scan** 5544
**Peptide** PNKKLEKEQTGSKTL **Charge** 2 **Spectral Angle** 0.79

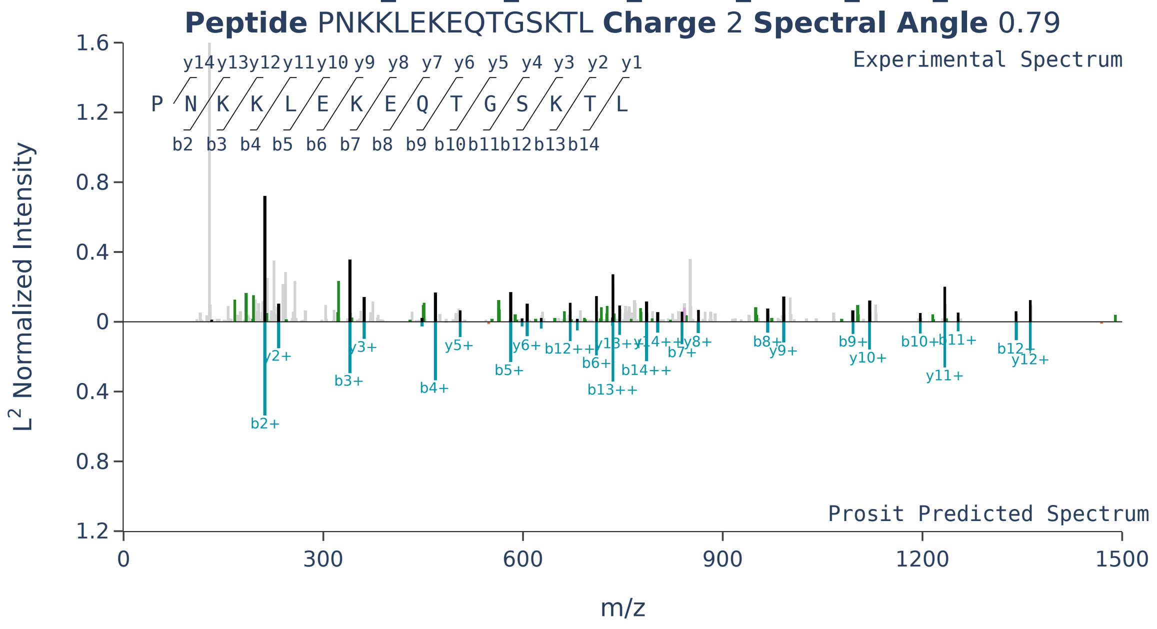


**Source** HRoetschke_271222_090123_HFGoe_LEDGF_b2R1_4h **Scan** 22218
**Peptide** GDSVITQVLNK **Charge** 2 **Spectral Angle** 0.75

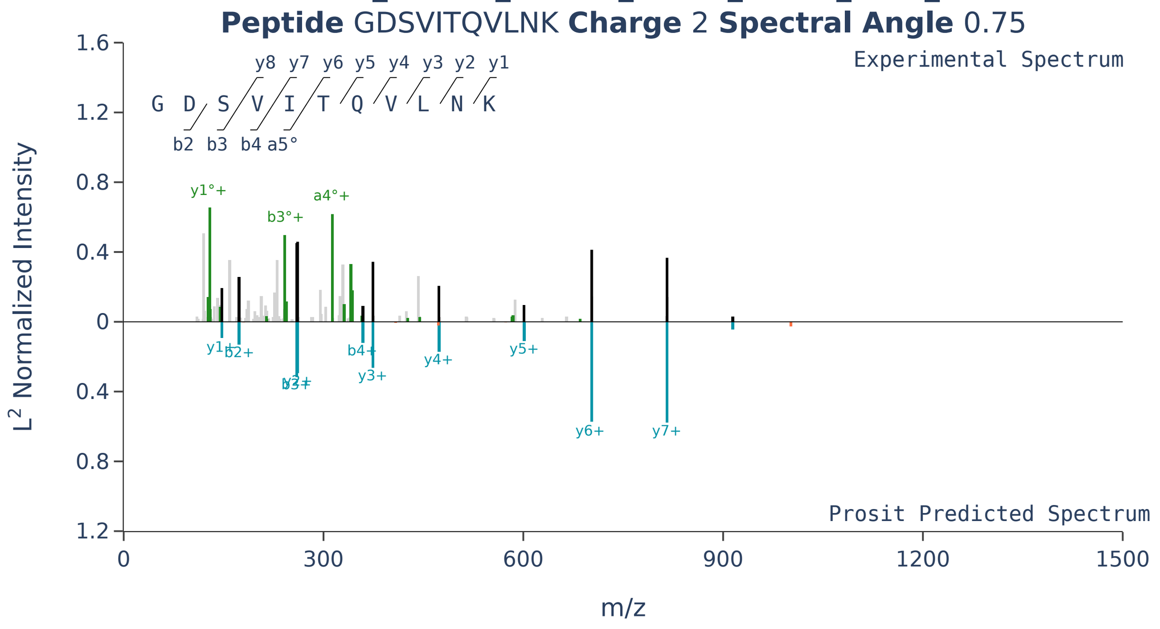


**Source** HRoetschke_271222_090123_HFGoe_LEDGF_b1R2_2h **Scan** 29393
**Peptide** KGFNEGLWEI **Charge** 2 **Spectral Angle** 0.74

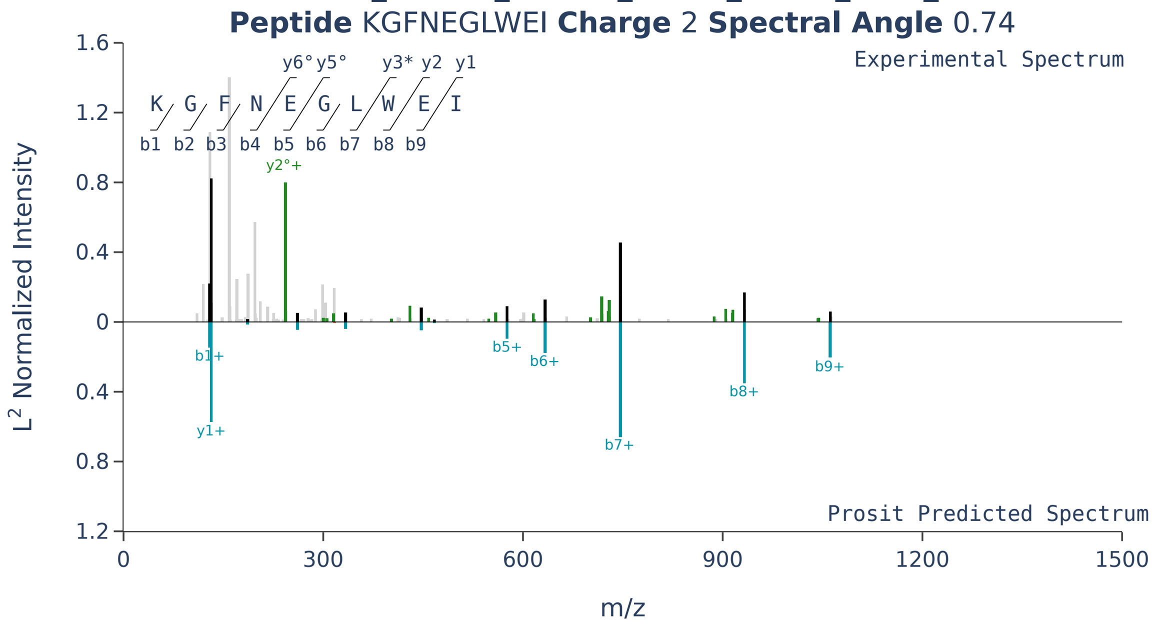


**Source** HRoetschke_271222_090123_HFGoe_LEDGF_b1R1_1h **Scan** 20355
**Peptide** SLKIDNLDVN **Charge** 2 **Spectral Angle** 0.8

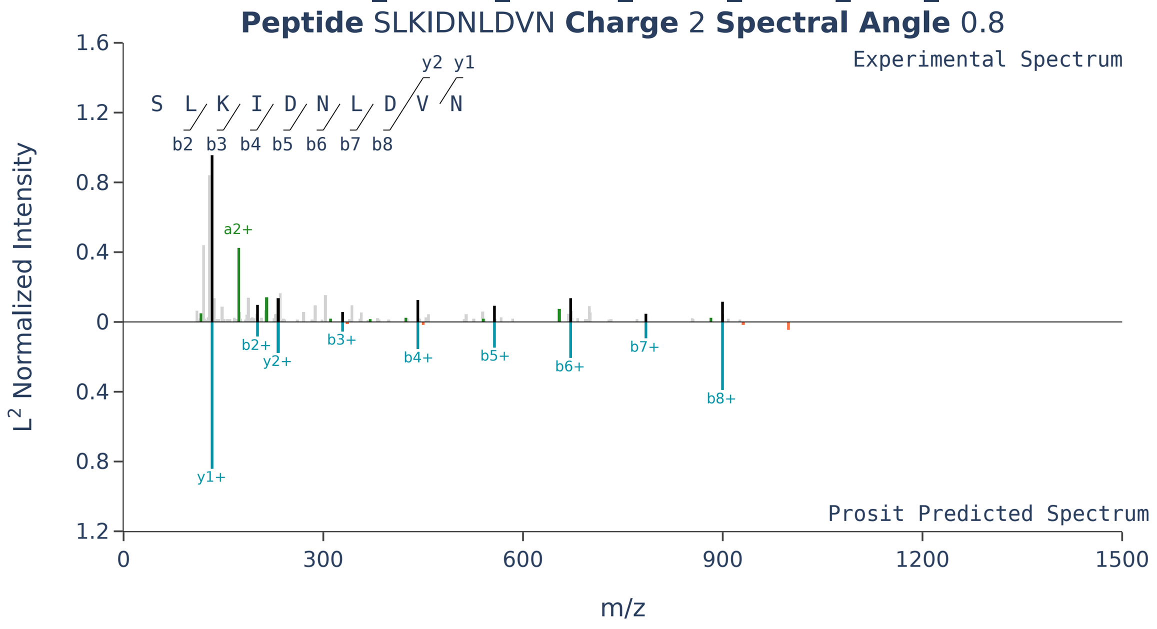


**Source** HRoetschke_271222_090123_HFGoe_LEDGF_b2R1_4h **Scan** 19500
**Peptide** KVSQVIMEKSTMLYN **Charge** 2 **Spectral Angle** 0.91

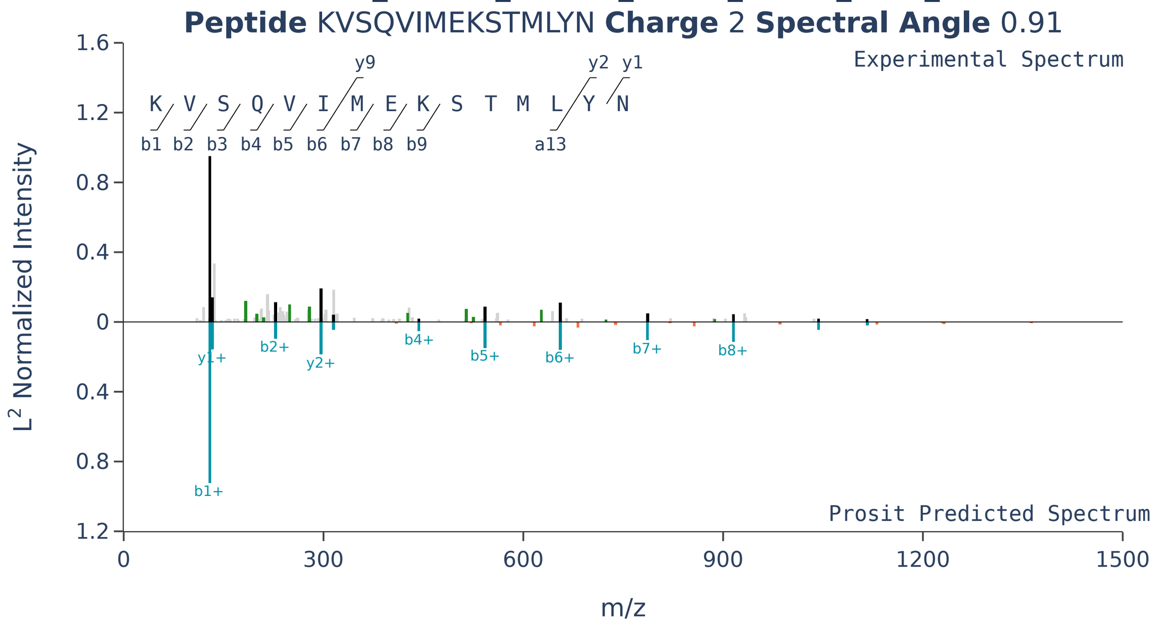

**Source** HRoetschke_271222_090123_HFGoe_LEDGF_b1R1_2h **Scan** 26922
**Peptide** ALDELASLQVTMQ **Charge** 2 **Spectral Angle** 0.87

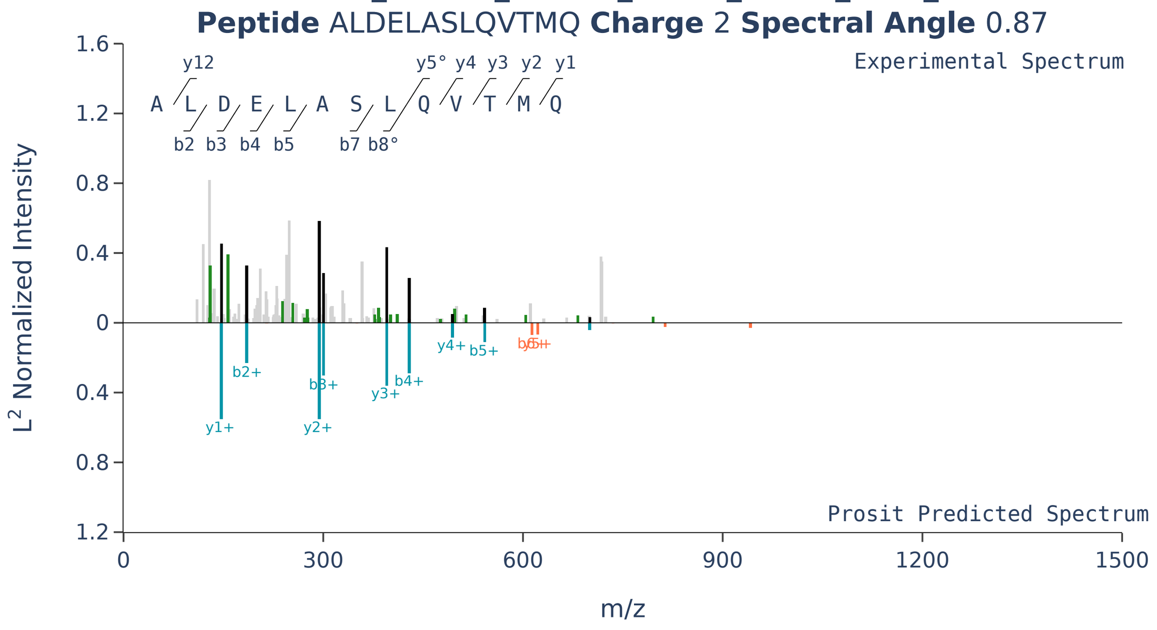


**Source** HRoetschke_271222_090123_HFGoe_LEDGF_b2R2_4h **Scan** 11343
**Peptide** SLQVTMQQAQKH **Charge** 3 **Spectral Angle** 0.85

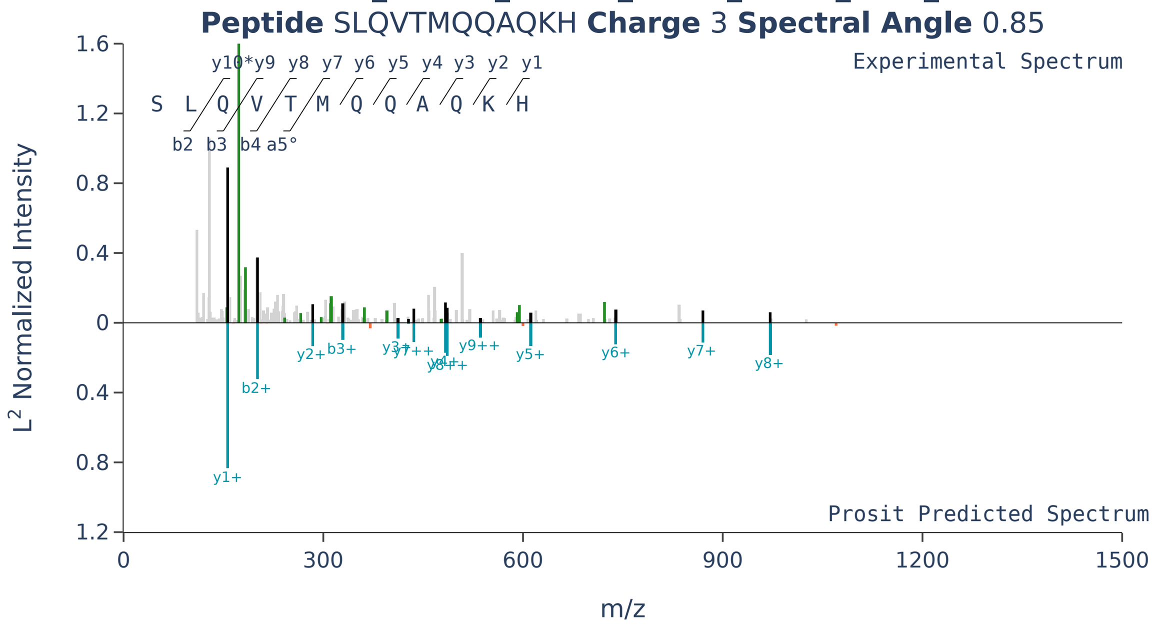


**Source** HRoetschke_271222_090123_HFGoe_LEDGF_b1R2_24h **Scan** 5529
**Peptide** SKDNHEASTKKKPSSEERETEI **Charge** 3 **Spectral Angle** 0.85

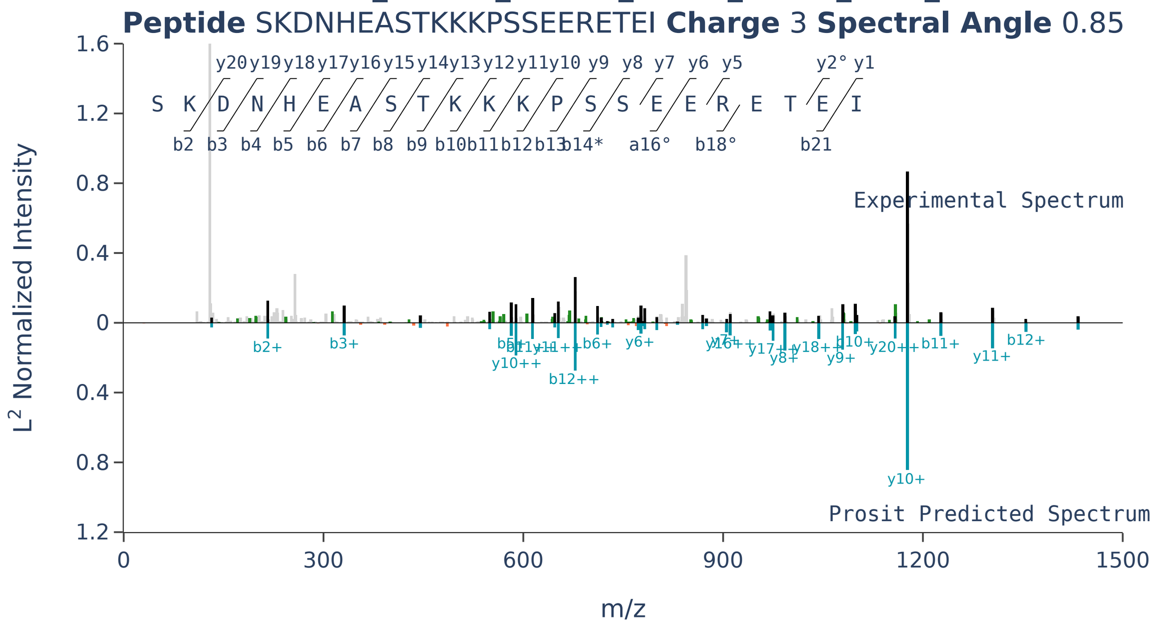


**Source** HRoetschke_271222_090123_HFGoe_LEDGF_b1R1_4h **Scan** 10584
**Peptide** IDNNPKVKFSSQQA **Charge** 2 **Spectral Angle** 0.81

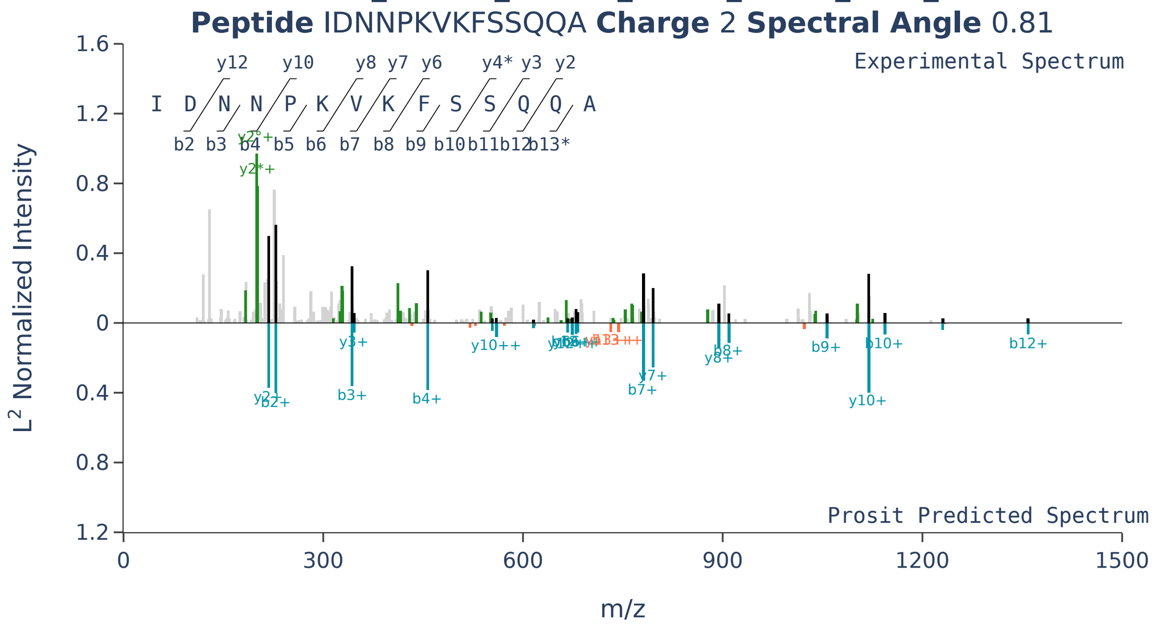


**Source** HRoetschke_271222_090123_HFGoe_LEDGF_b1R1_4h **Scan** 11355
**Peptide** DNNPKVKF **Charge** 1 **Spectral Angle** 0.89

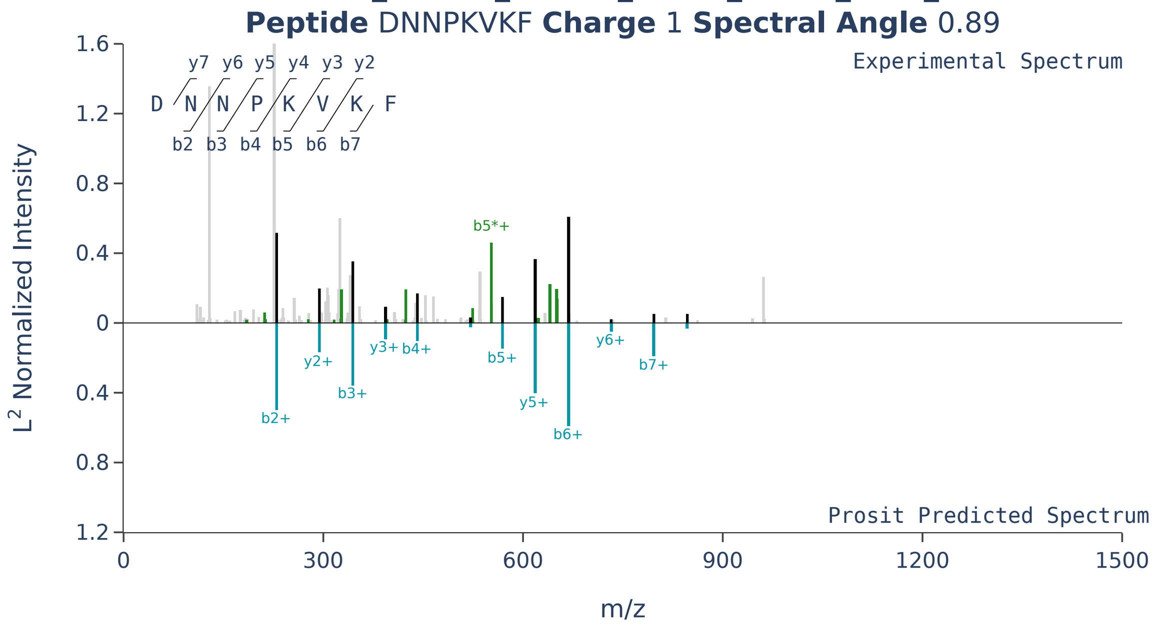


**Source** HRoetschke_271222_090123_HFGoe_LEDGF_b1R1_4h **Scan** 8468
**Peptide** TKQSNASSDVEVEE **Charge** 2 **Spectral Angle** 0.77

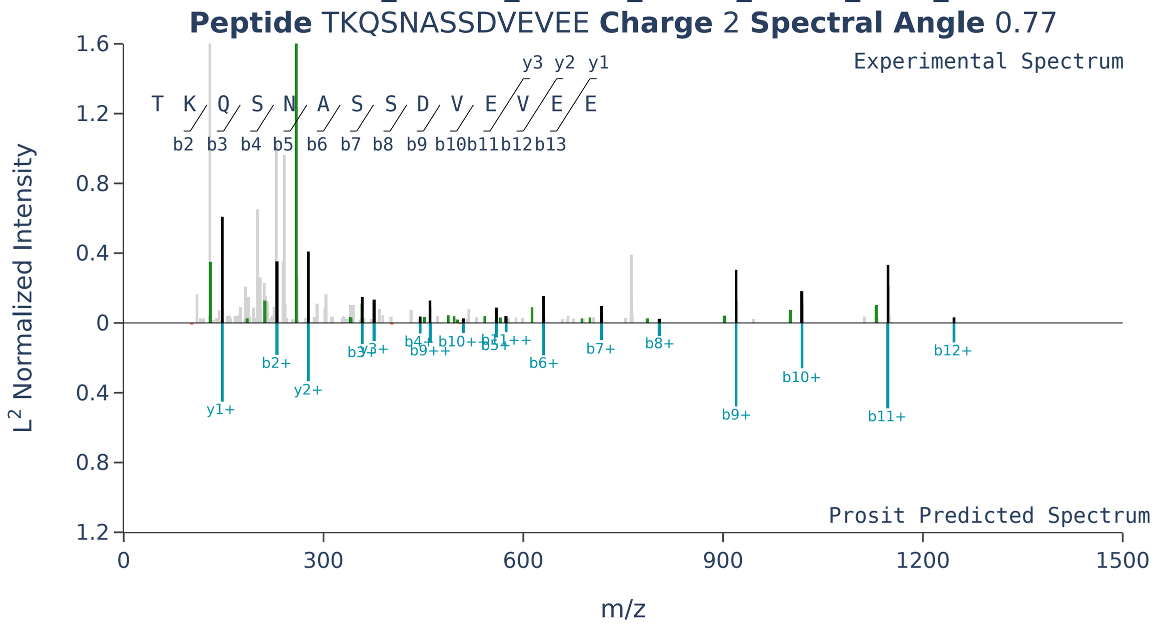


**Source** HRoetschke_271222_090123_HFGoe_LEDGF_b1R1_4h **Scan** 26824
**Peptide** RDFKPGDLIFA **Charge** 2 **Spectral Angle** 0.82

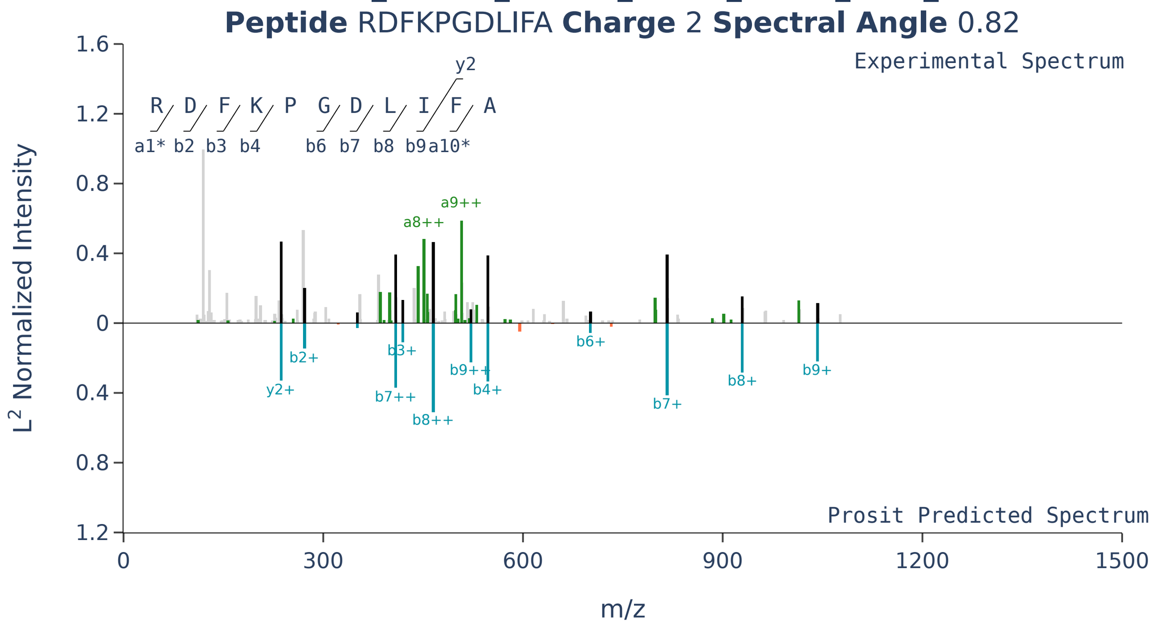


**Source** HRoetschke_271222_090123_HFGoe_LEDGF_b1R2_4h **Scan** 30504
**Peptide** GEGDSVITQVLNKSLAE **Charge** 2 **Spectral Angle** 0.82

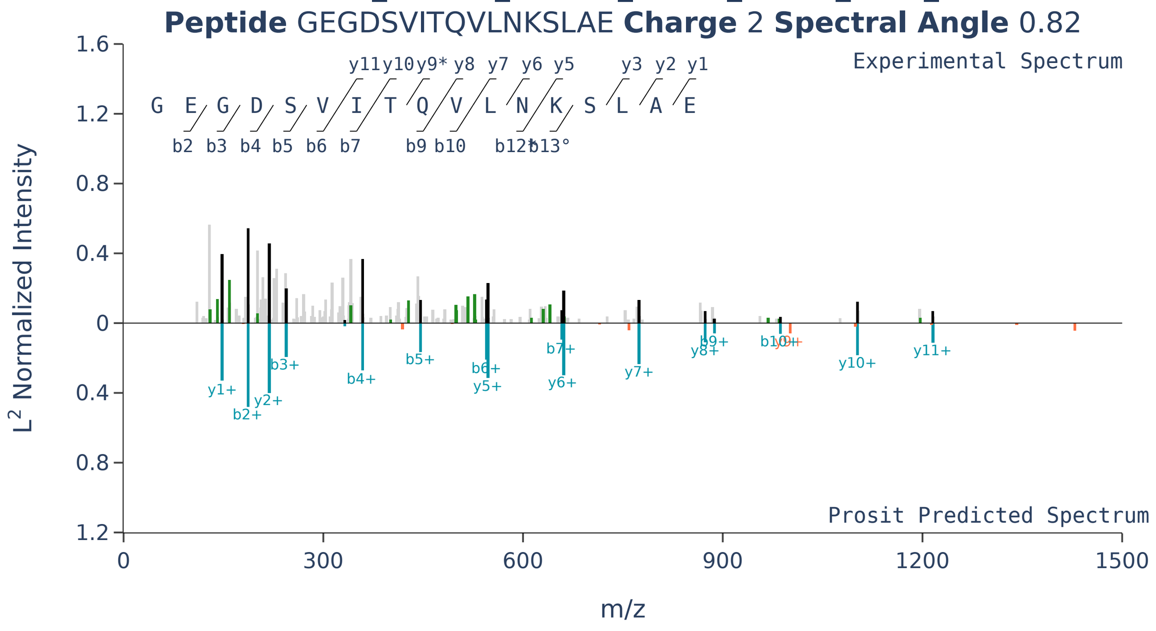


**Source** HRoetschke_271222_090123_HFGoe_LEDGF_b1R1_24h **Scan** 7741
**Peptide** KASNEDVTKAV **Charge** 2 **Spectral Angle** 0.82

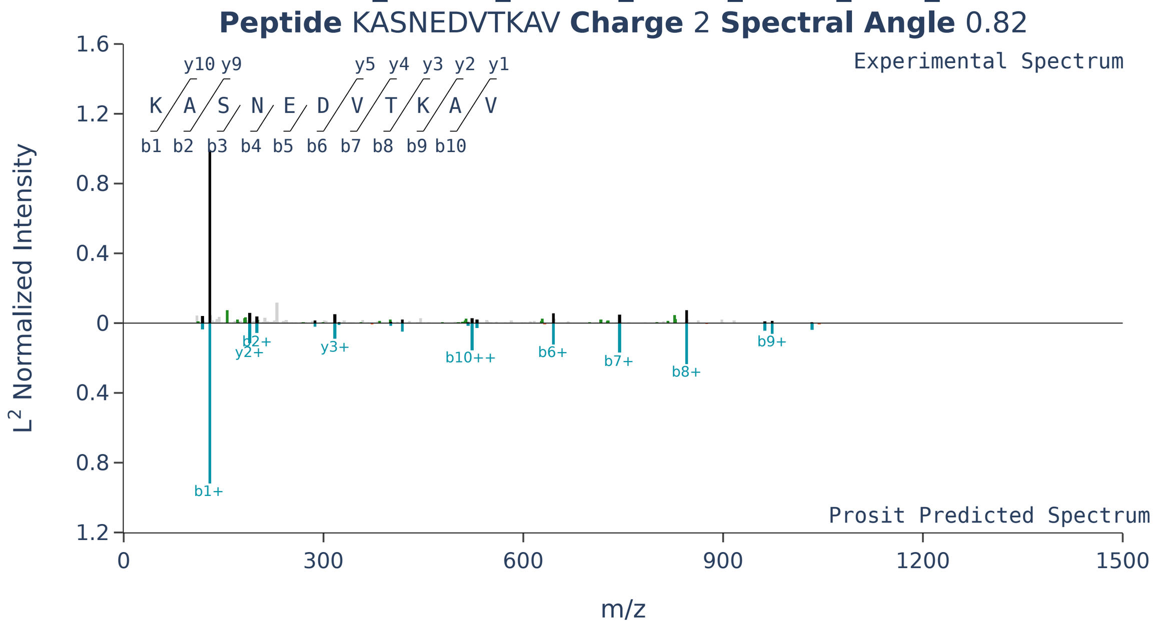


**Source** HRoetschke_271222_090123_HFGoe_LEDGF_b1R1_4h **Scan** 8220
**Peptide** NNPKVKFSSQQAAT **Charge** 3 **Spectral Angle** 0.88

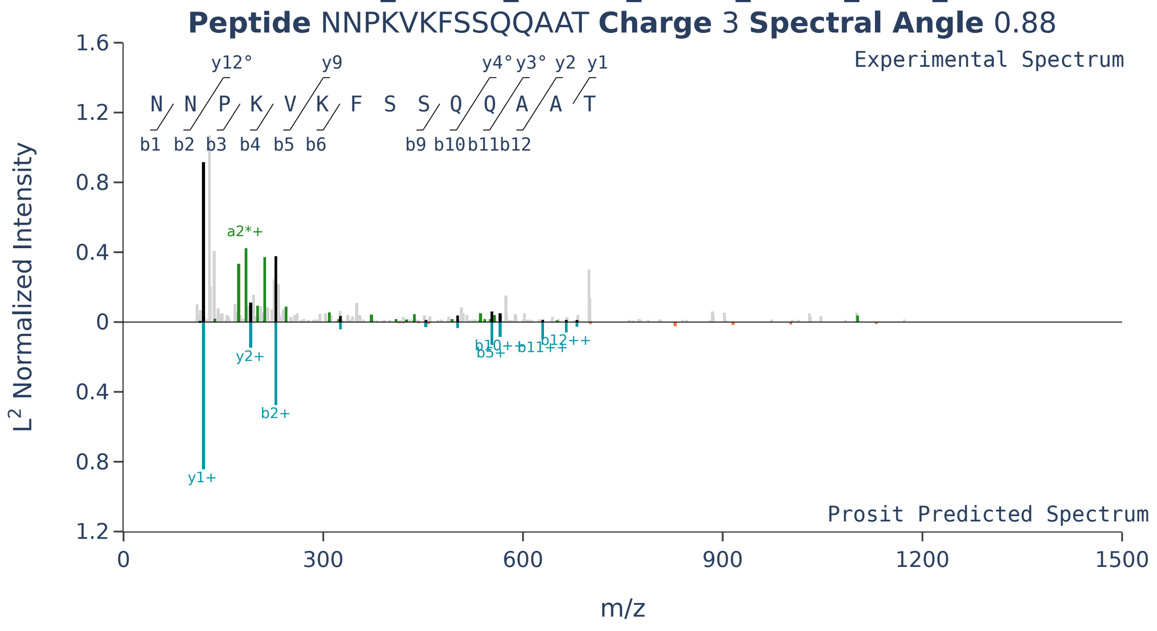


**Source** HRoetschke_271222_090123_HFGoe_LEDGF_b1R2_24h **Scan** 13031
**Peptide** TEISLKDST **Charge** 2 **Spectral Angle** 0.84

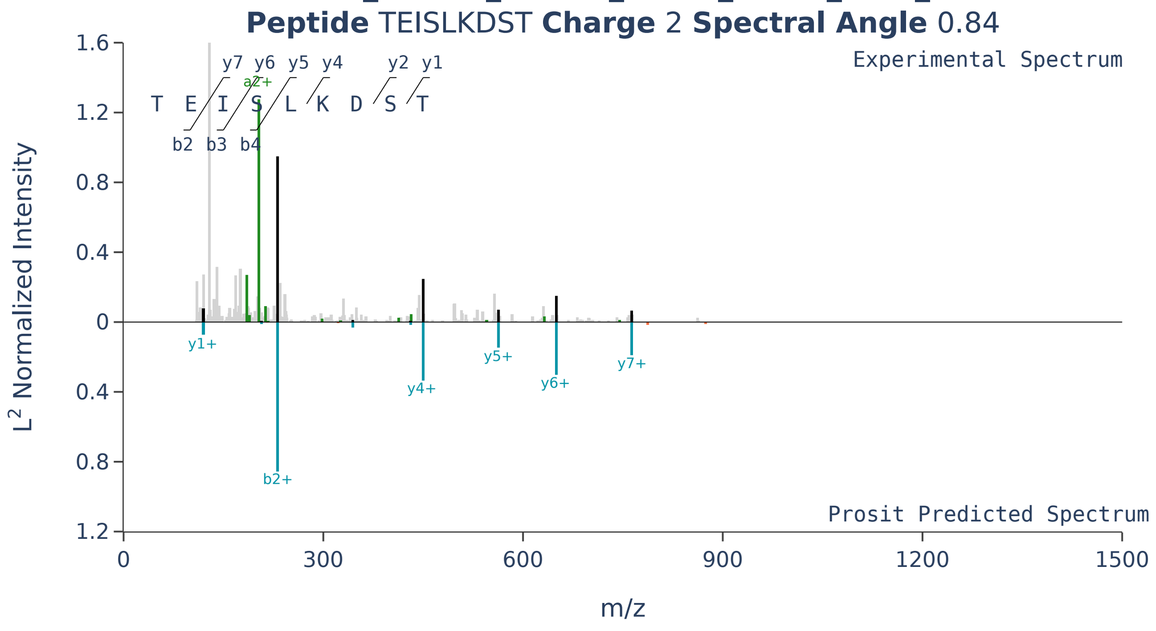


**Source** HRoetschke_271222_090123_HFGoe_LEDGF_b1R1_2h **Scan** 13773
**Peptide** KGYPHWPARVD **Charge** 3 **Spectral Angle** 0.91

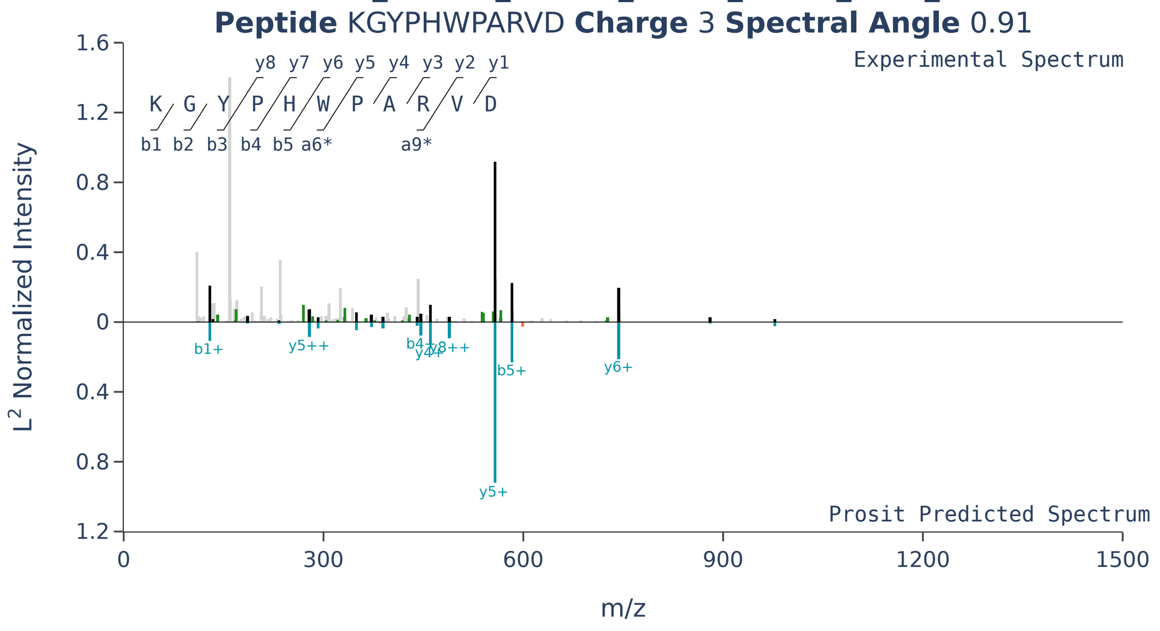


**Source** HRoetschke_271222_090123_HFGoe_LEDGF_b1R1_24h **Scan** 8152
**Peptide** VTKAVDITTPKAARRG **Charge** 3 **Spectral Angle** 0.83

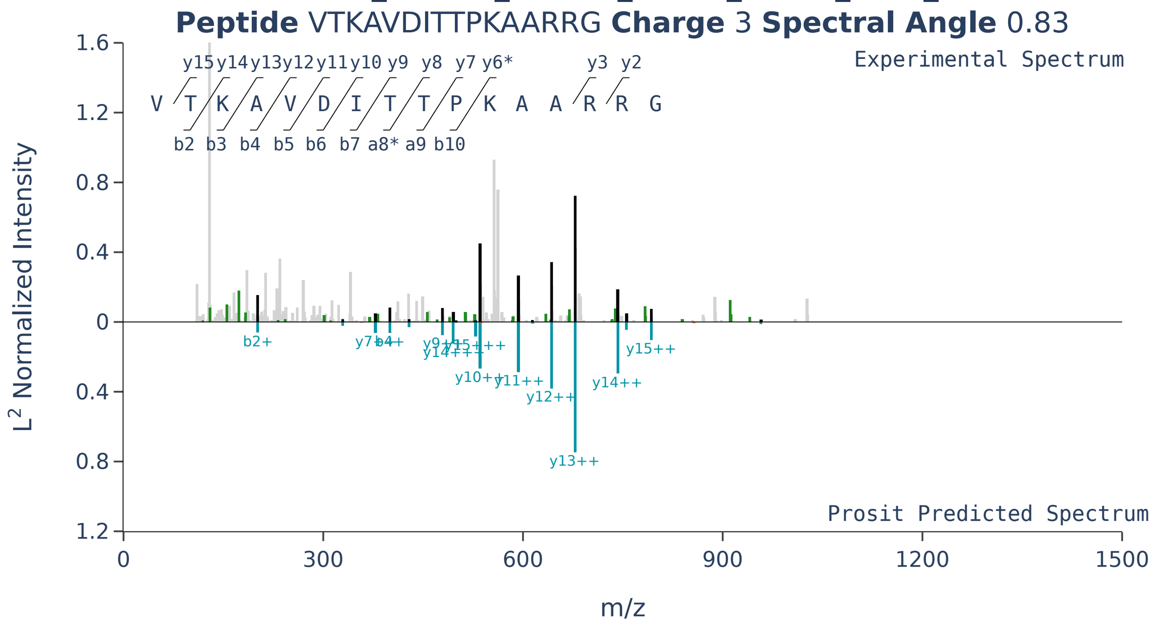


**Source** HRoetschke_271222_090123_HFGoe_LEDGF_b2R1_2h **Scan** 18822
**Peptide** SQVIMEKSTMLYNKFKN **Charge** 3 **Spectral Angle** 0.88

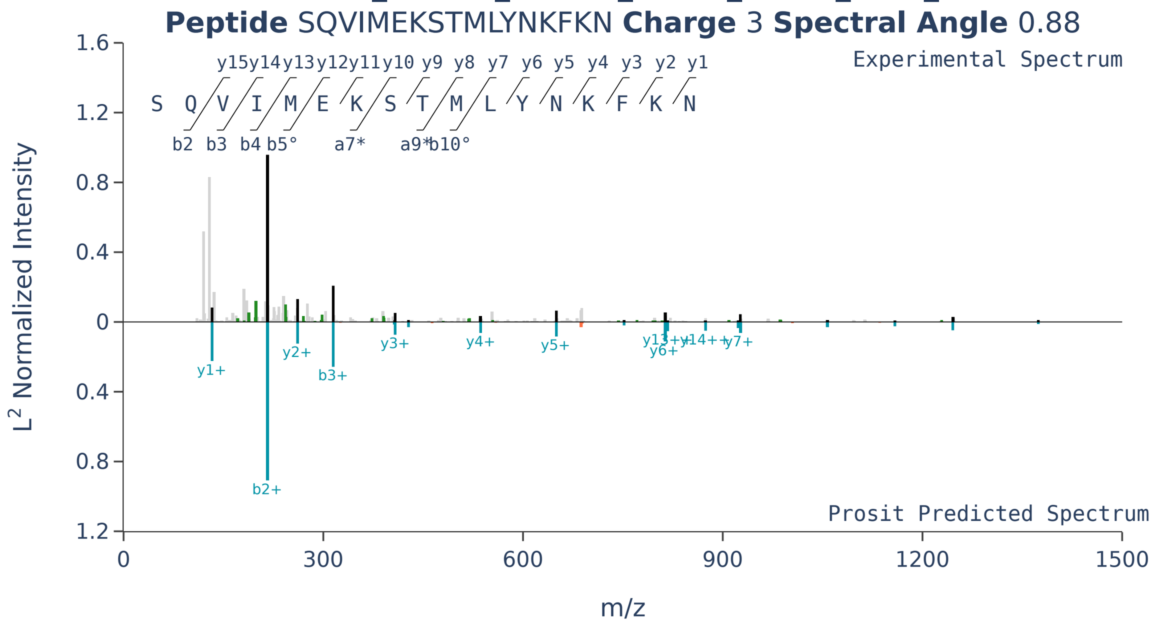


**Source** HRoetschke_271222_090123_HFGoe_LEDGF_b1R2_4h **Scan** 7694
**Peptide** KSTMLYNK **Charge** 2 **Spectral Angle** 0.82

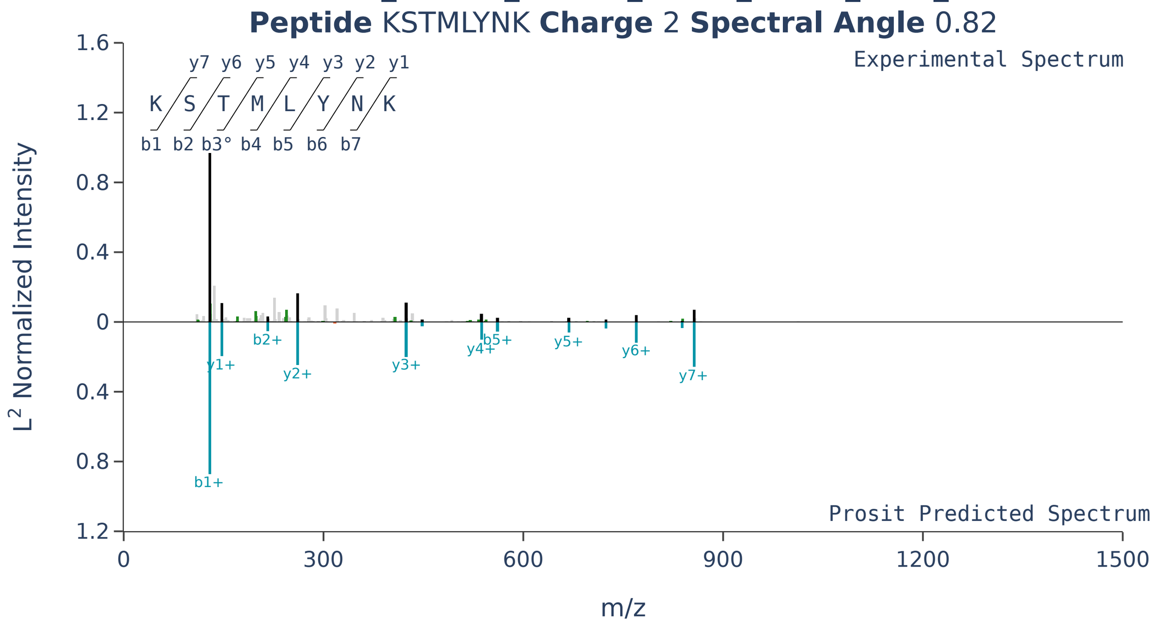

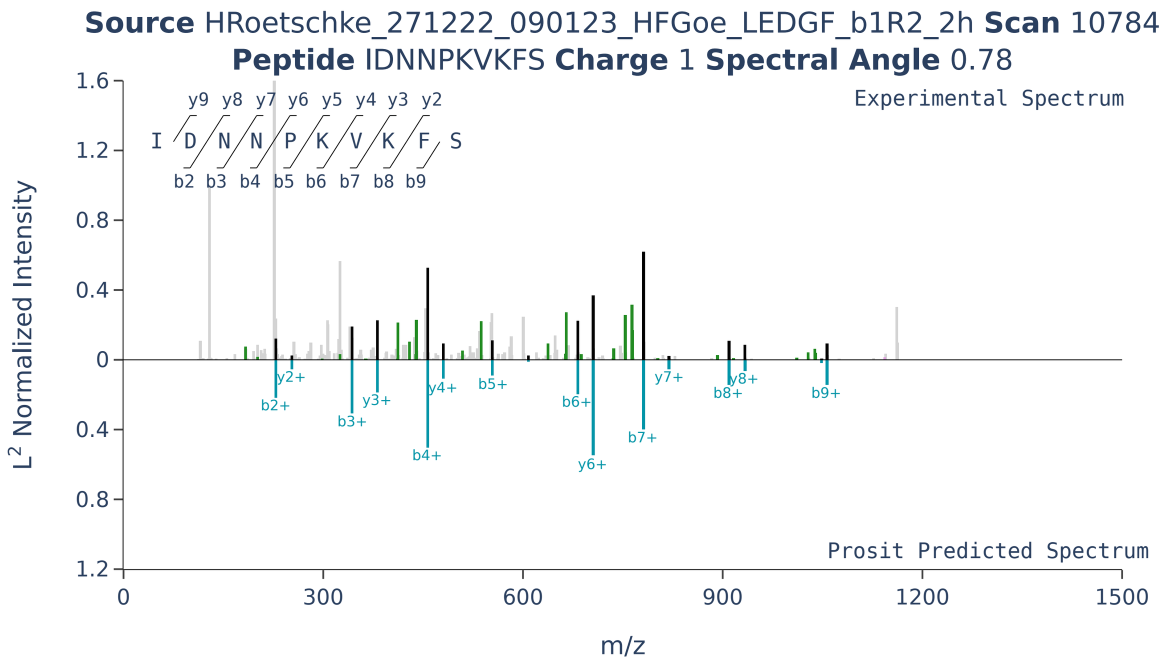
**Source** HRoetschke_271222_090123_HFGoe_LEDGF_b1R2_2h **Scan** 10784
**Peptide** IDNNPKVKFS **Charge** 1 **Spectral Angle** 0.78
Experimental Spectrum
Prosit Predicted Spectrum
L² Normalized Intensity
m/z

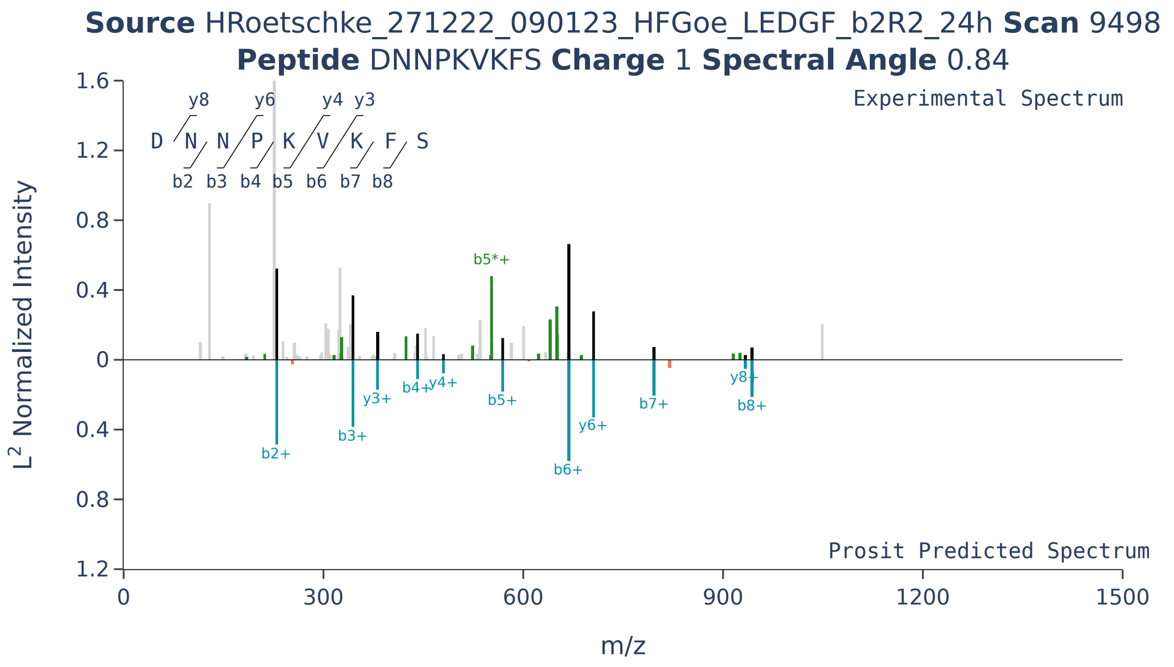
**Source** HRoetschke_271222_090123_HFGoe_LEDGF_b2R2_24h **Scan** 9498
**Peptide** DNNPKVKFS **Charge** 1 **Spectral Angle** 0.84
Experimental Spectrum
Prosit Predicted Spectrum
L² Normalized Intensity
m/z

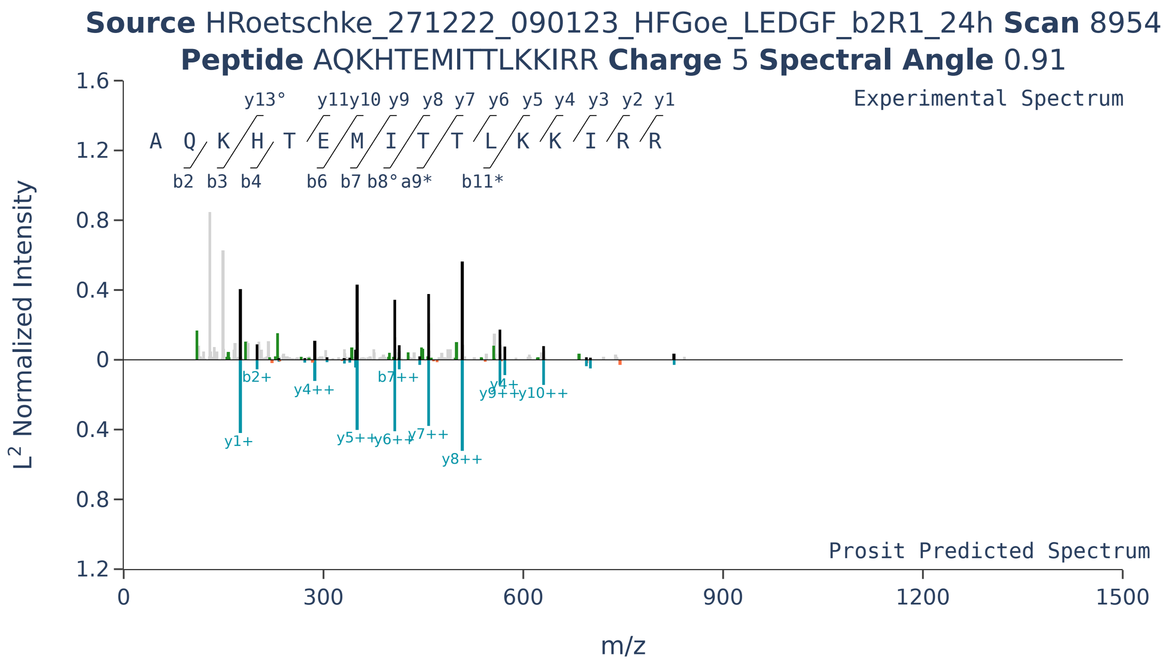
**Source** HRoetschke_271222_090123_HFGoe_LEDGF_b2R1_24h **Scan** 8954
**Peptide** AQKHTEMITTLKKIRR **Charge** 5 **Spectral Angle** 0.91
Experimental Spectrum
Prosit Predicted Spectrum
L² Normalized Intensity
m/z

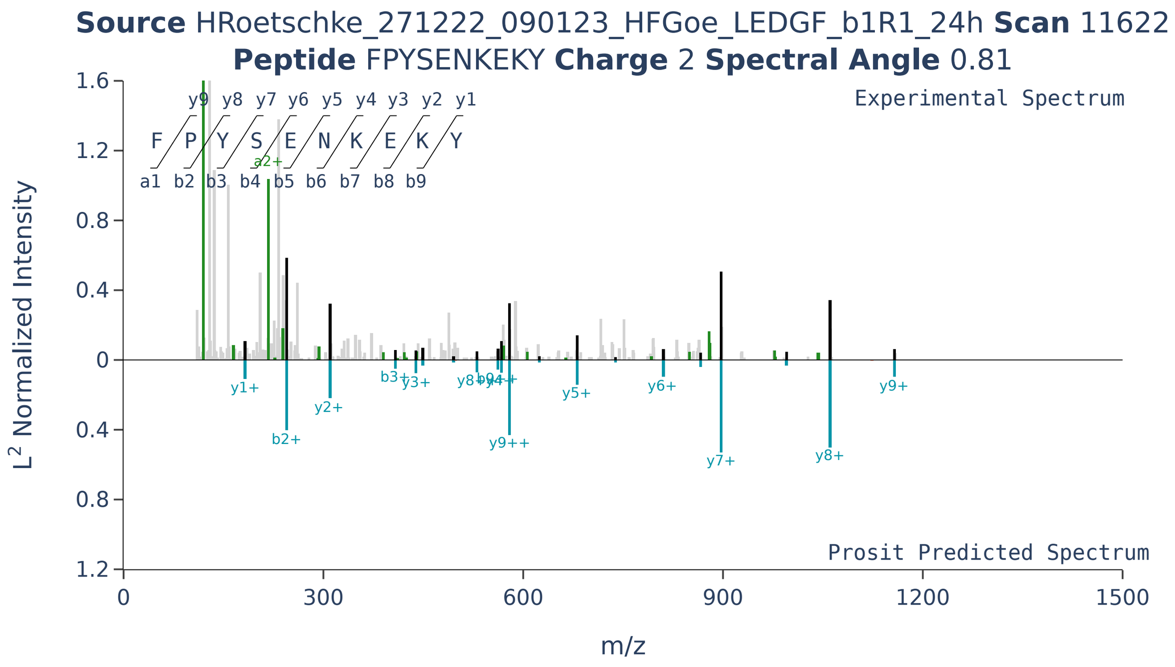
**Source** HRoetschke_271222_090123_HFGoe_LEDGF_b1R1_24h **Scan** 11622
**Peptide** FPYSENKEKY **Charge** 2 **Spectral Angle** 0.81
Experimental Spectrum
Prosit Predicted Spectrum
L² Normalized Intensity
m/z

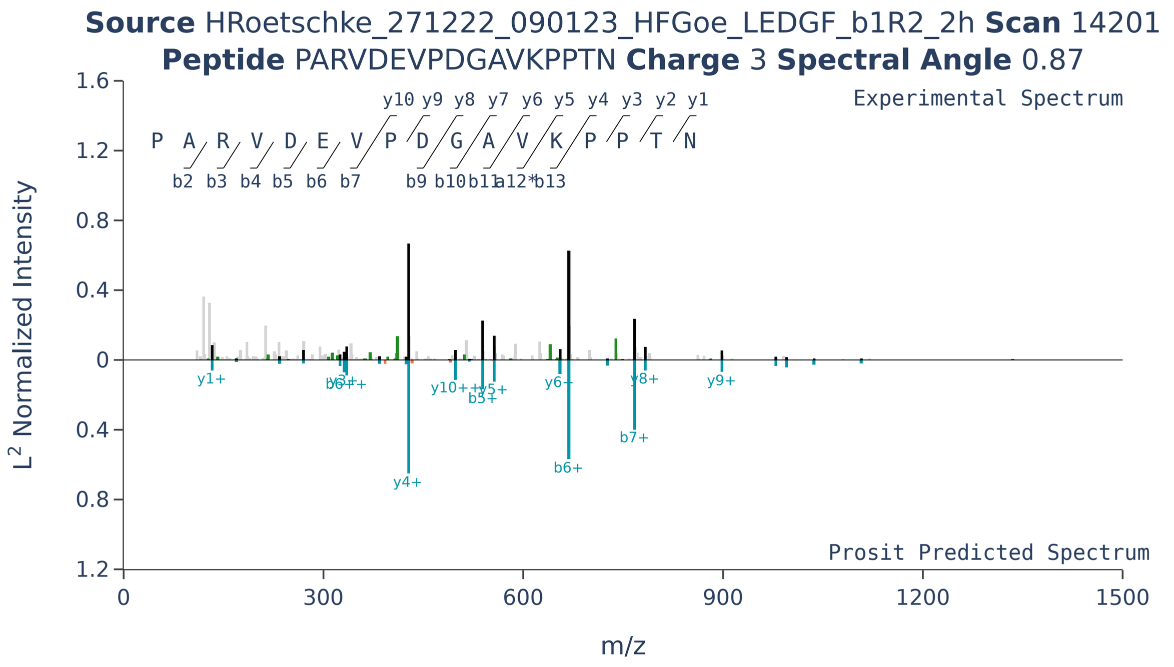
**Source** HRoetschke_271222_090123_HFGoe_LEDGF_b1R2_2h **Scan** 14201
**Peptide** PARVDEVPDGAVKPPTN **Charge** 3 **Spectral Angle** 0.87
Experimental Spectrum
Prosit Predicted Spectrum
L² Normalized Intensity
m/z

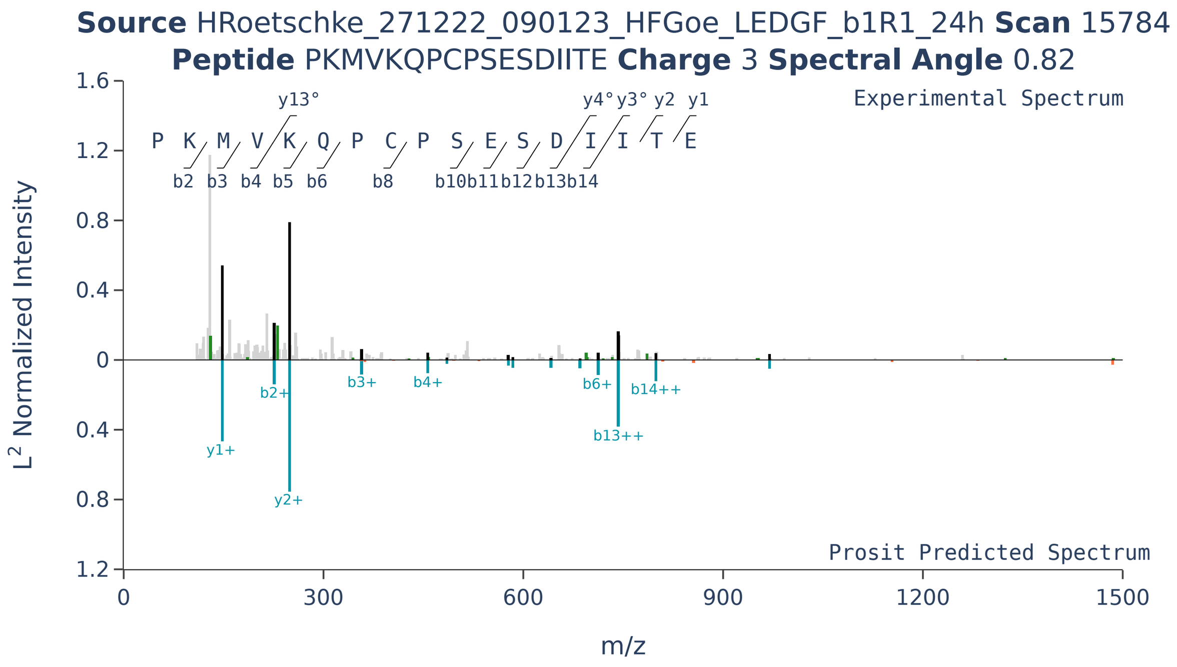
**Source** HRoetschke_271222_090123_HFGoe_LEDGF_b1R1_24h **Scan** 15784
**Peptide** PKMVKQPCPSESDIITE **Charge** 3 **Spectral Angle** 0.82
Experimental Spectrum
Prosit Predicted Spectrum
L² Normalized Intensity
m/z

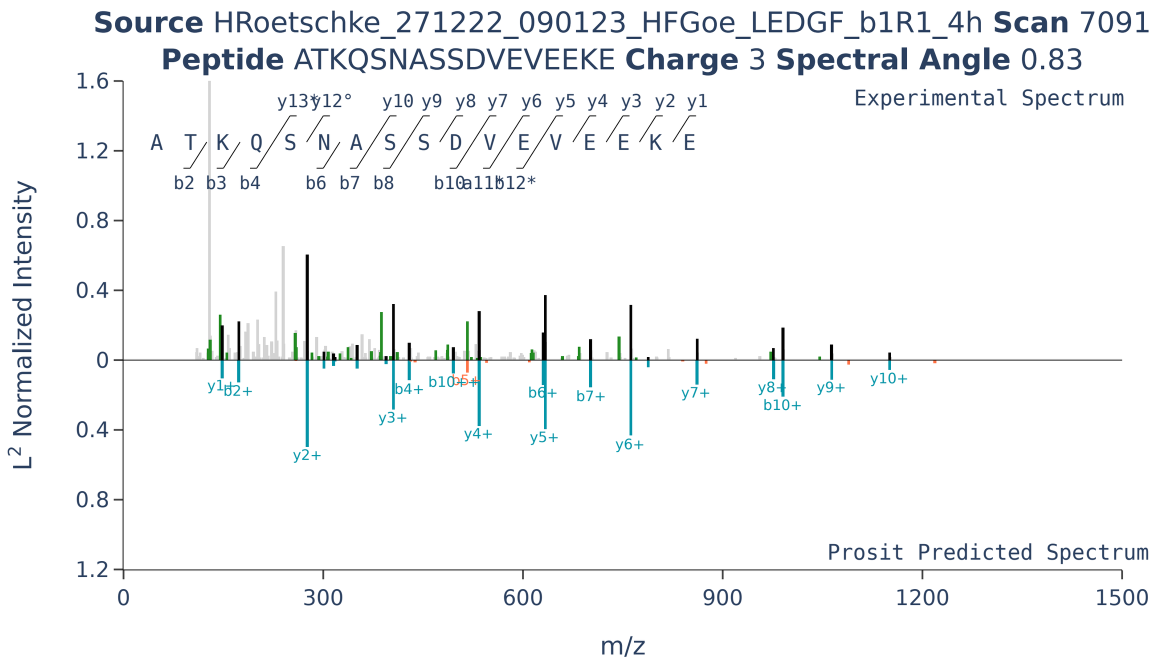
**Source** HRoetschke_271222_090123_HFGoe_LEDGF_b1R1_4h **Scan** 7091
**Peptide** ATKQSNASSDVEVEEKE **Charge** 3 **Spectral Angle** 0.83
Experimental Spectrum
Prosit Predicted Spectrum
L² Normalized Intensity
m/z

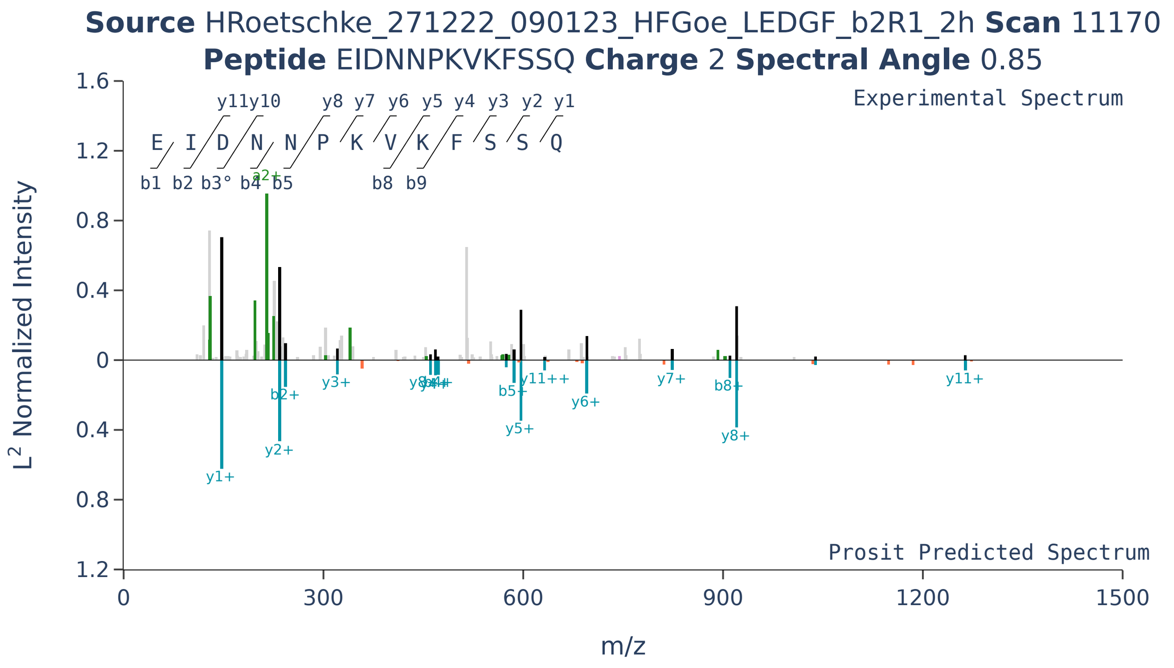
**Source** HRoetschke_271222_090123_HFGoe_LEDGF_b2R1_2h **Scan** 11170
**Peptide** EIDNNPKVKFSSQ **Charge** 2 **Spectral Angle** 0.85
Experimental Spectrum
Prosit Predicted Spectrum
L² Normalized Intensity
m/z

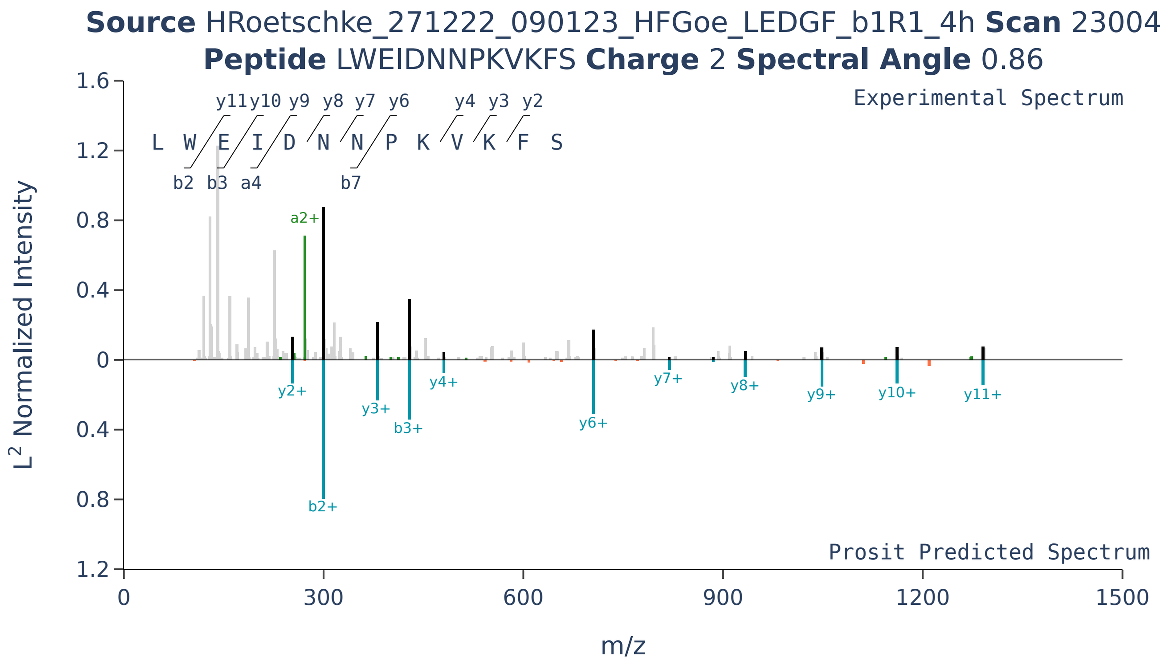
**Source** HRoetschke_271222_090123_HFGoe_LEDGF_b1R1_4h **Scan** 23004
**Peptide** LWEIDNNPKVKFS **Charge** 2 **Spectral Angle** 0.86
Experimental Spectrum
Prosit Predicted Spectrum
L² Normalized Intensity
m/z

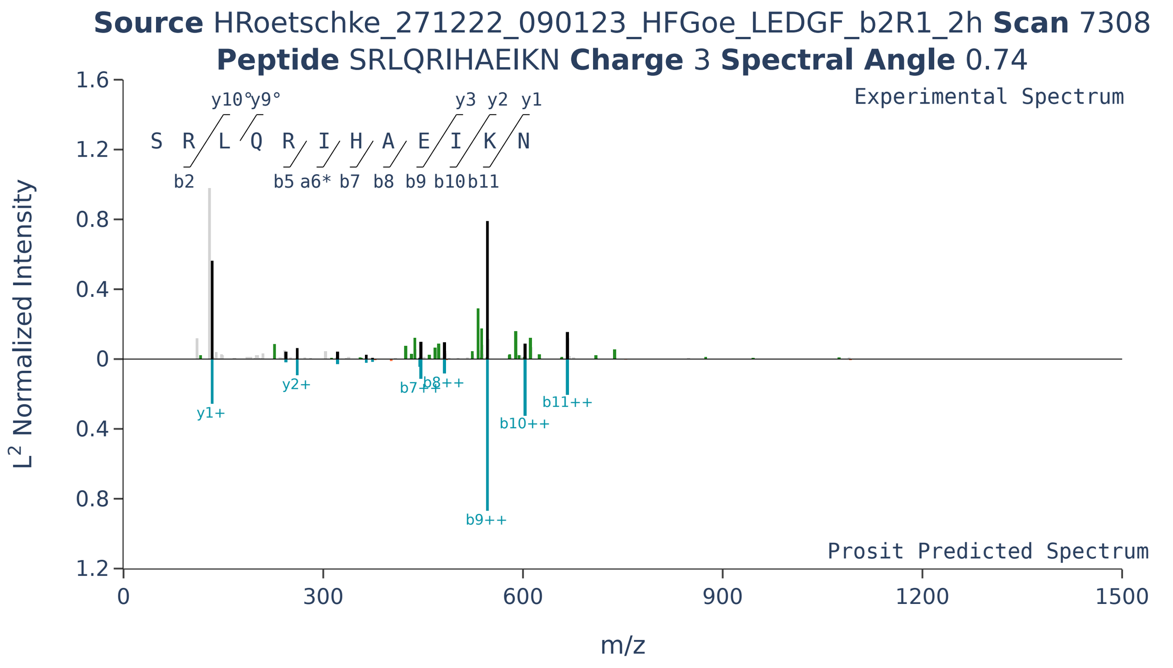
**Source** HRoetschke_271222_090123_HFGoe_LEDGF_b2R1_2h **Scan** 7308
**Peptide** SRLQRIHAEIKN **Charge** 3 **Spectral Angle** 0.74
Experimental Spectrum
Prosit Predicted Spectrum
L² Normalized Intensity
m/z

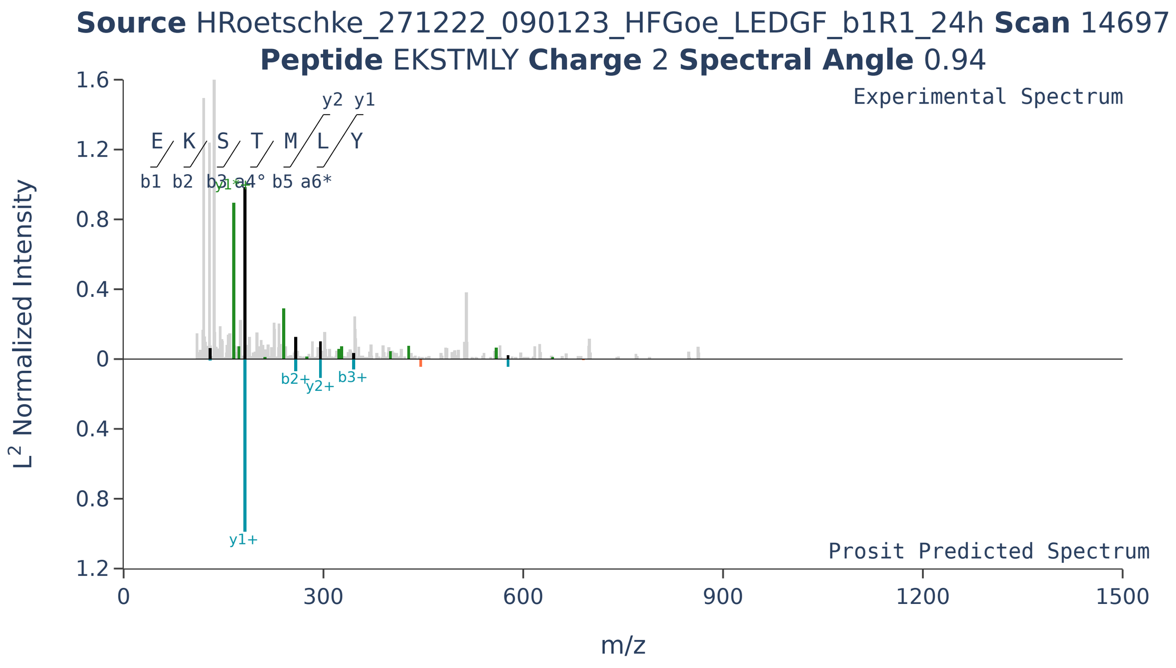
**Source** HRoetschke_271222_090123_HFGoe_LEDGF_b1R1_24h **Scan** 14697
**Peptide** EKSTMLY **Charge** 2 **Spectral Angle** 0.94
Experimental Spectrum
Prosit Predicted Spectrum
L² Normalized Intensity
m/z

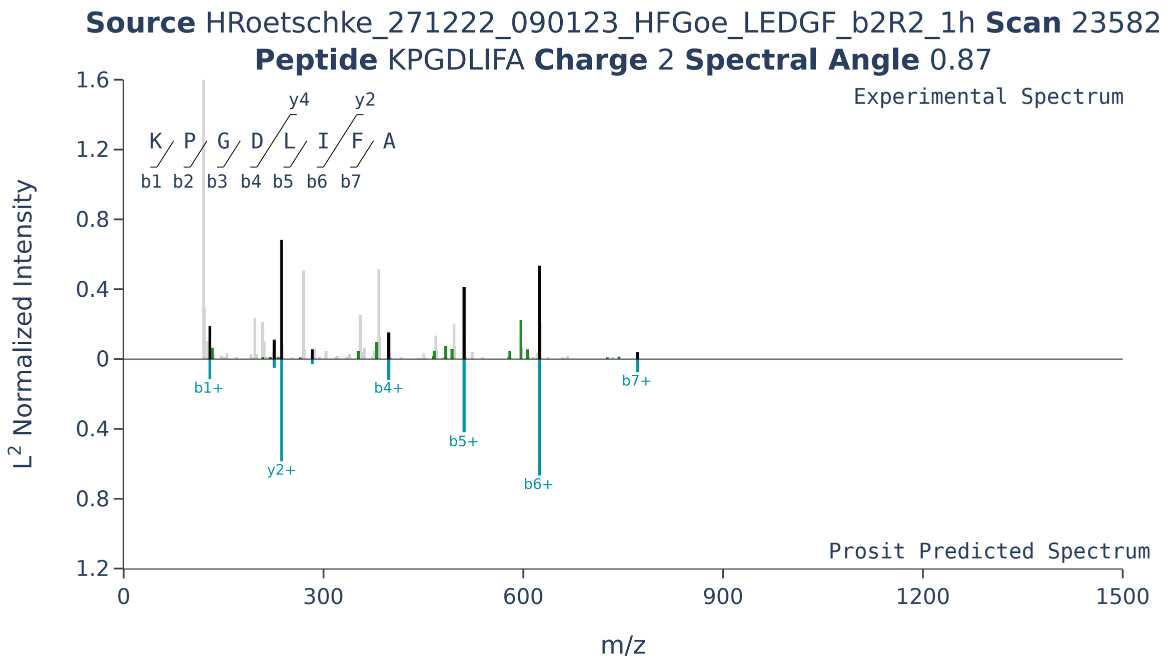
**Source** HRoetschke_271222_090123_HFGoe_LEDGF_b2R2_1h **Scan** 23582
**Peptide** KPGDLIFA **Charge** 2 **Spectral Angle** 0.87
Experimental Spectrum
Prosit Predicted Spectrum
L² Normalized Intensity
m/z

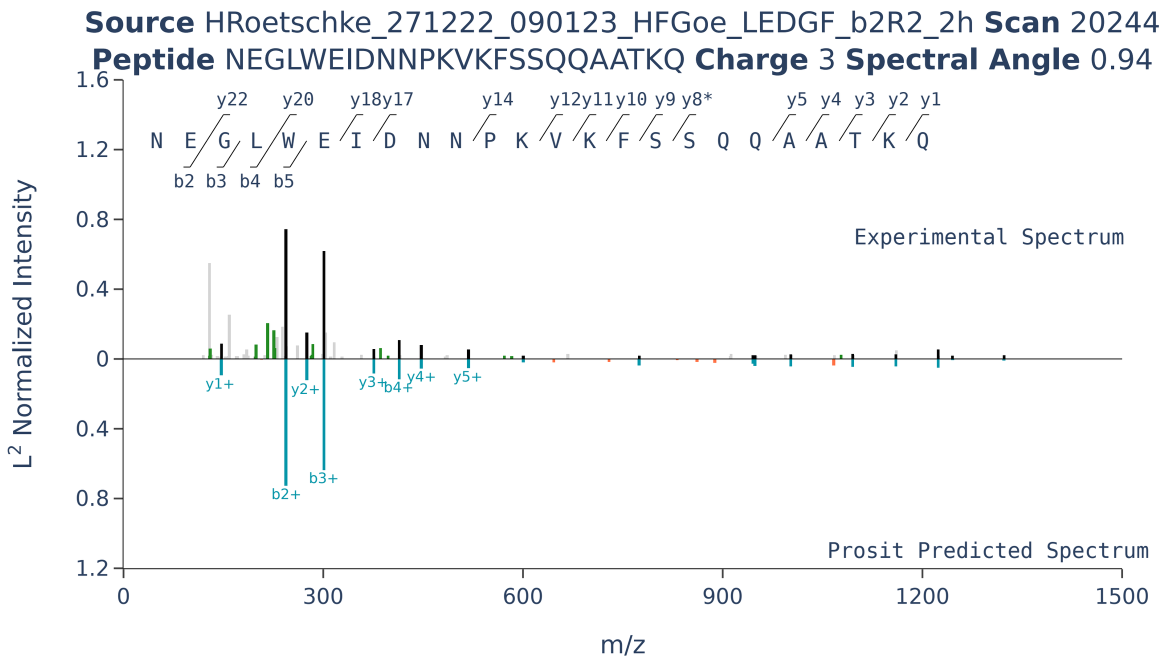
**Source** HRoetschke_271222_090123_HFGoe_LEDGF_b2R2_2h **Scan** 20244
**Peptide** NEGLWEIDNNPKVKFSSQQAATKQ **Charge** 3 **Spectral Angle** 0.94
Experimental Spectrum
Prosit Predicted Spectrum
L² Normalized Intensity
m/z

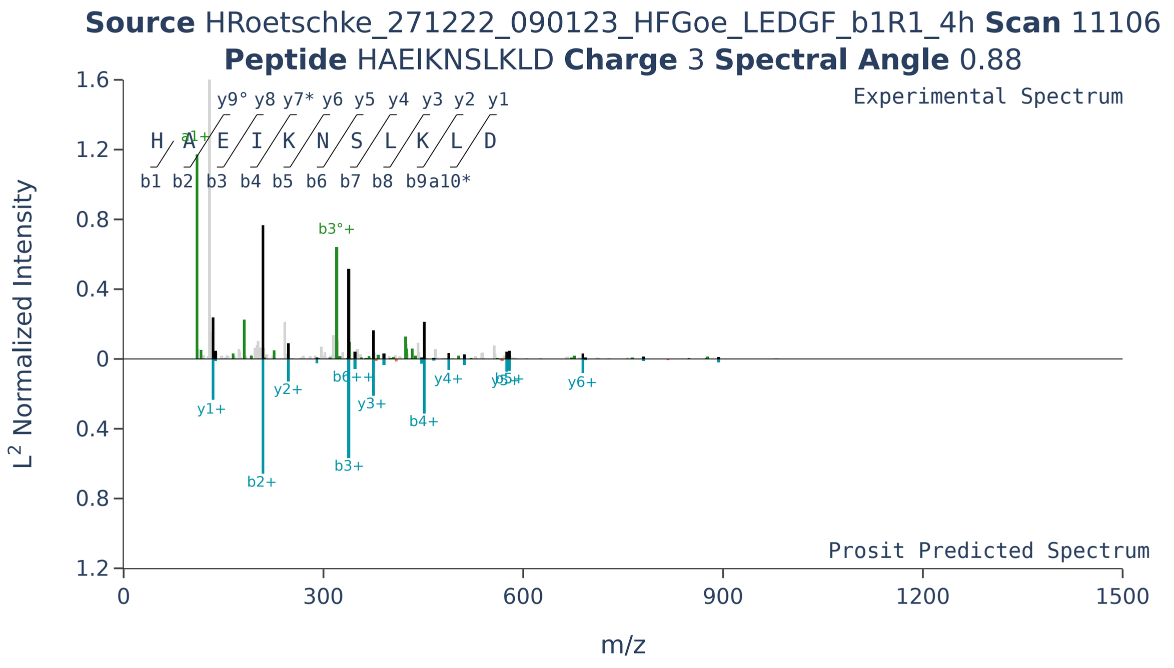
**Source** HRoetschke_271222_090123_HFGoe_LEDGF_b1R1_4h **Scan** 11106
**Peptide** HAEIKNSLKLD **Charge** 3 **Spectral Angle** 0.88
Experimental Spectrum
Prosit Predicted Spectrum
L² Normalized Intensity
m/z

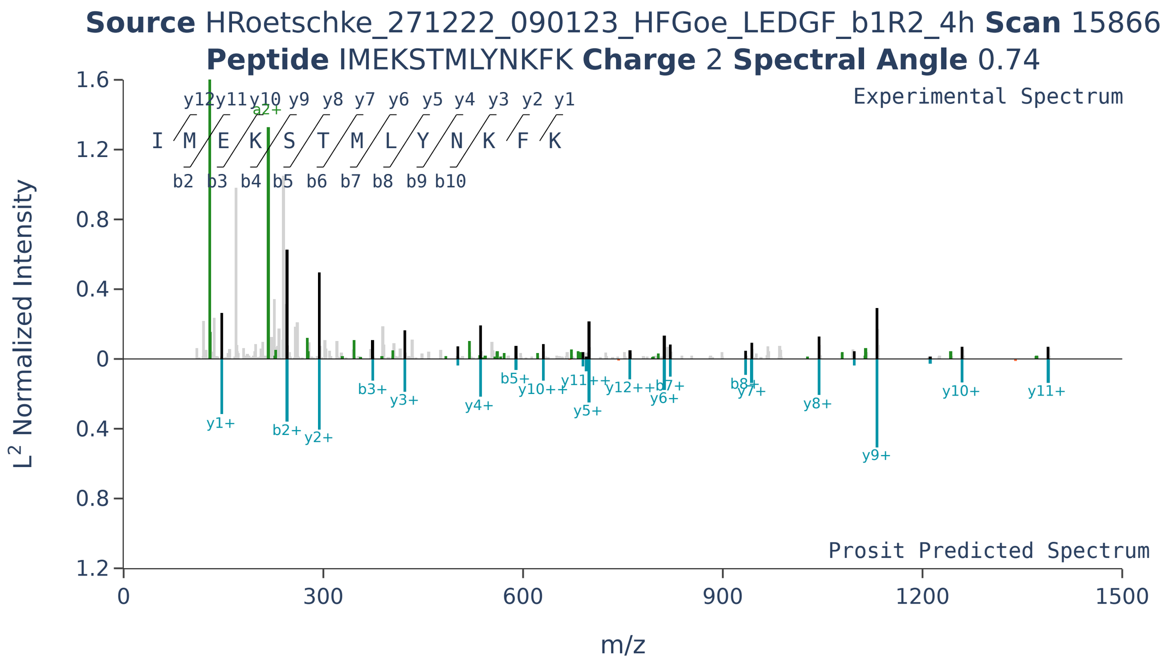
**Source** HRoetschke_271222_090123_HFGoe_LEDGF_b1R2_4h **Scan** 15866
**Peptide** IMEKSTMLYNKFK **Charge** 2 **Spectral Angle** 0.74
Experimental Spectrum
Prosit Predicted Spectrum
L² Normalized Intensity
m/z

**Source** HRoetschke_271222_090123_HFGoe_LEDGF_b1R2_2h **Scan** 7876
**Peptide** DNNPKVKFSSQQAATKQSNASSD **Charge** 3 **Spectral Angle** 0.87

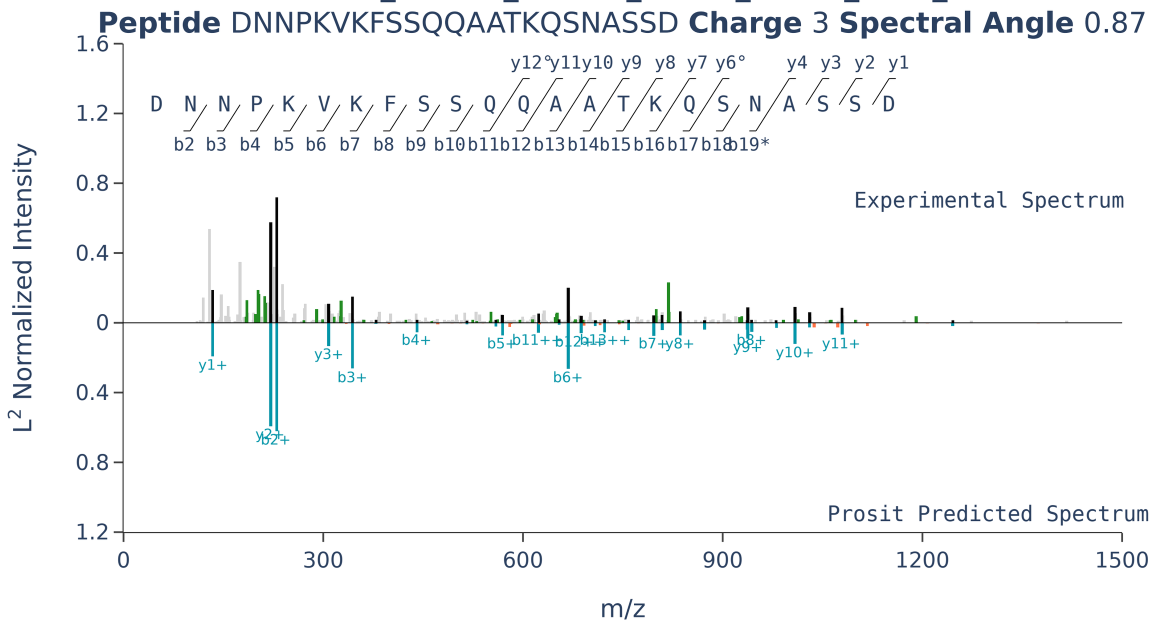


**Source** HRoetschke_271222_090123_HFGoe_LEDGF_b1R2_24h **Scan** 8484
**Peptide** FPYSENKEKYGK **Charge** 4 **Spectral Angle** 0.91

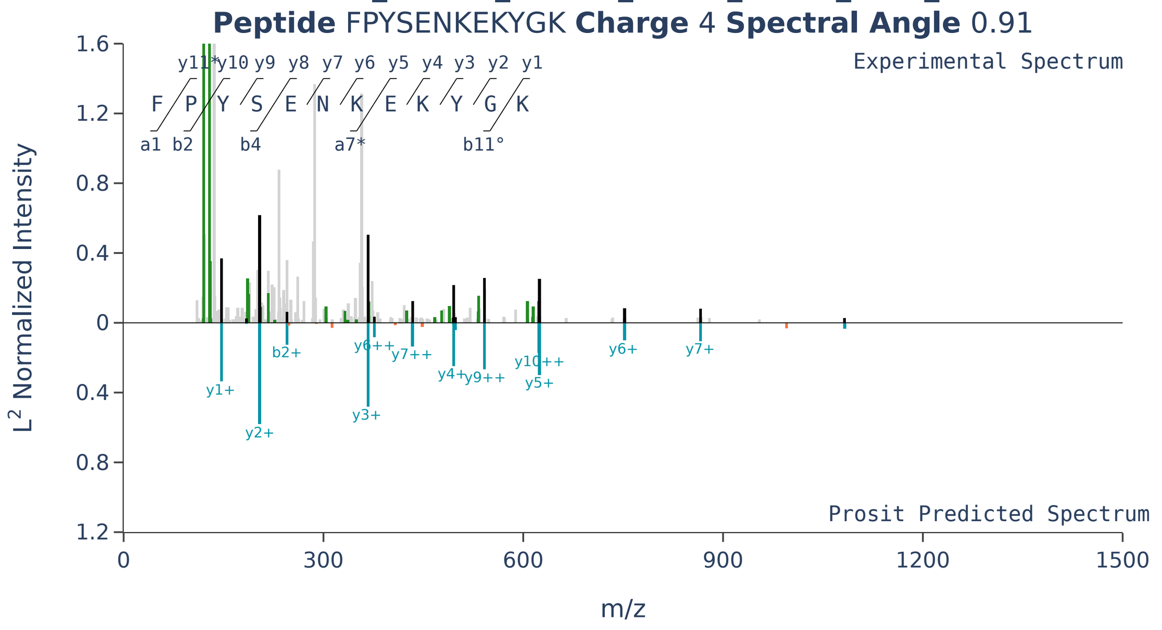


**Source** HRoetschke_271222_090123_HFGoe_LEDGF_b1R1_4h **Scan** 8968
**Peptide** ATASVNLKVSPKRGRPAATE **Charge** 3 **Spectral Angle** 0.8

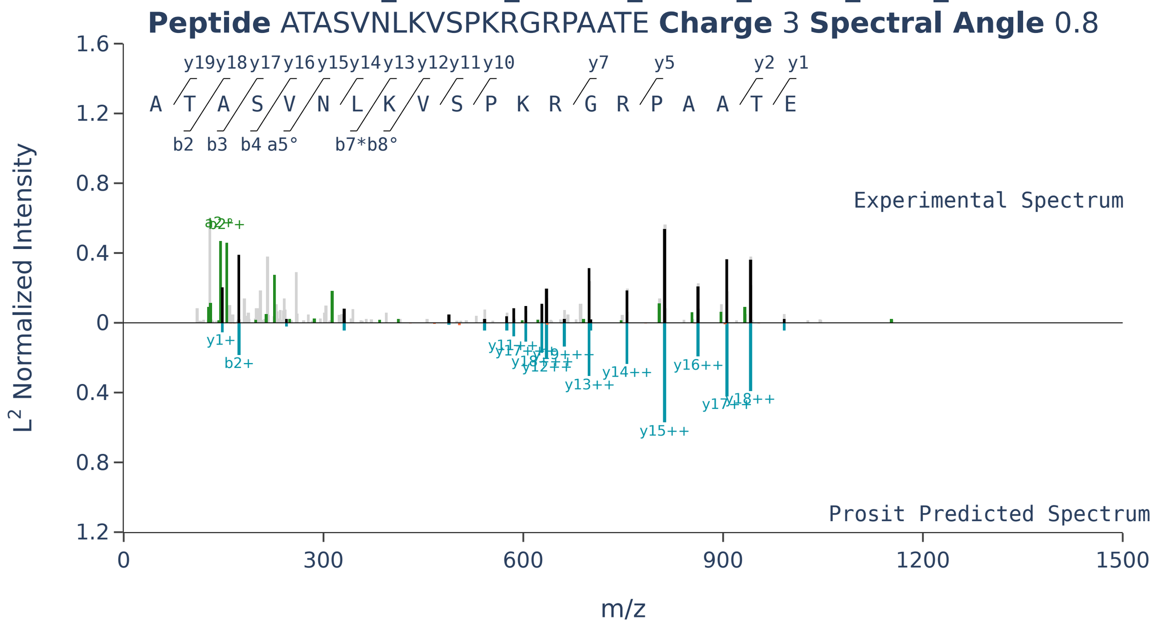


**Source** HRoetschke_271222_090123_HFGoe_LEDGF_b2R1_24h **Scan** 19662
**Peptide** TEEAGVVTTATASV **Charge** 2 **Spectral Angle** 0.85

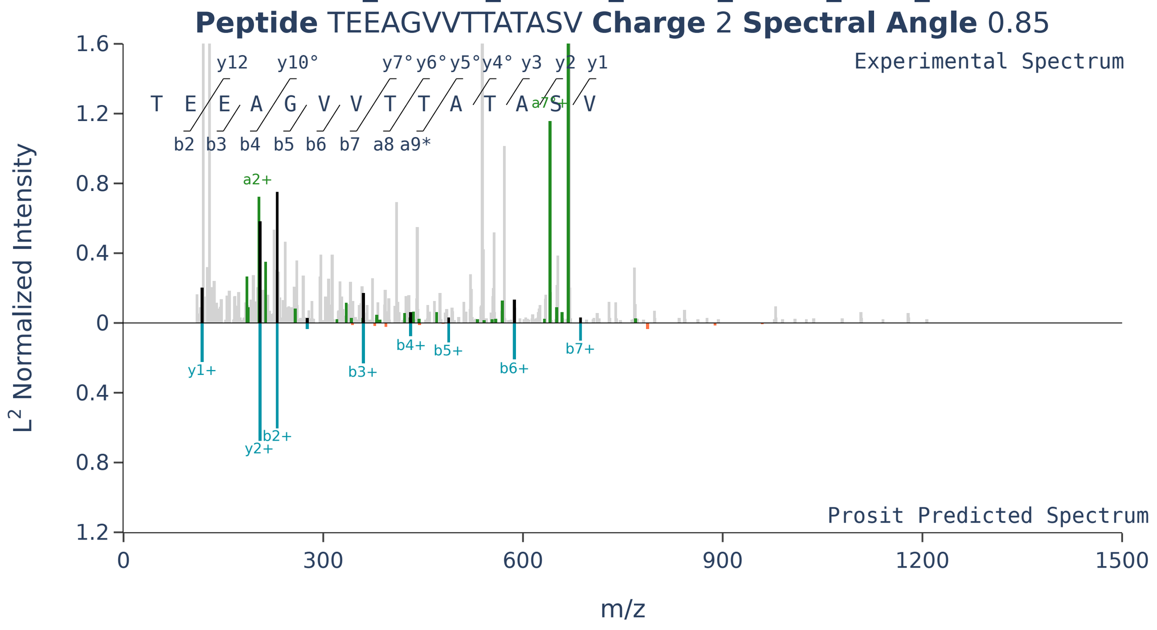


**Source** HRoetschke_271222_090123_HFGoe_LEDGF_b1R1_4h **Scan** 13210
**Peptide** AKMKGYPHWPARV **Charge** 4 **Spectral Angle** 0.93

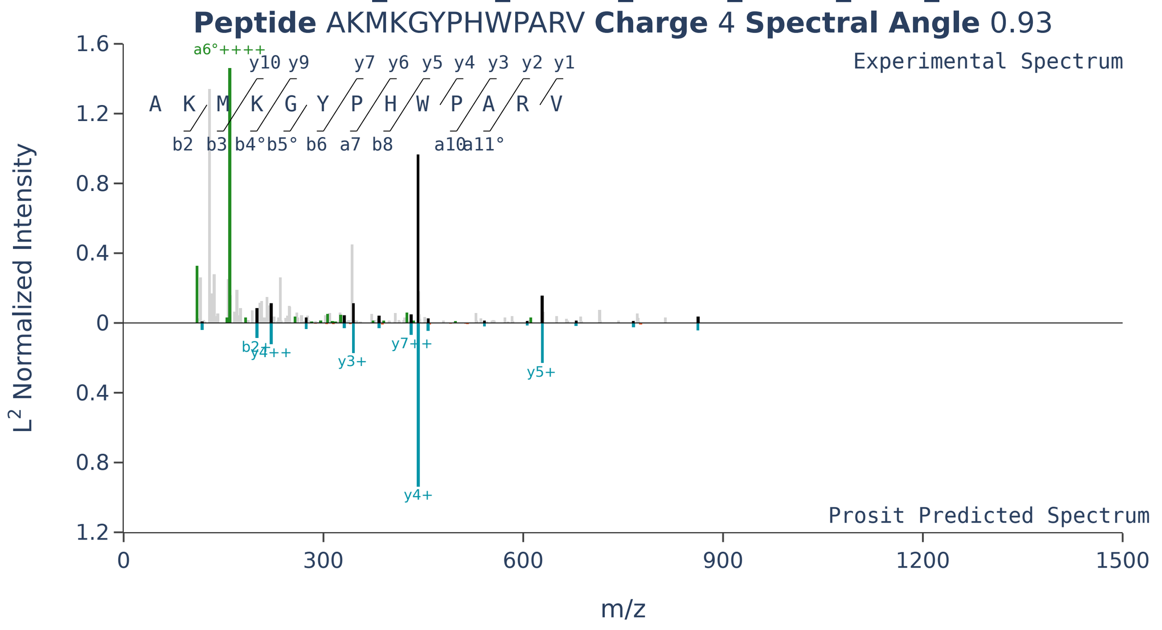


**Source** HRoetschke_271222_090123_HFGoe_LEDGF_b1R2_2h **Scan** 11104
**Peptide** FPYSENKE **Charge** 2 **Spectral Angle** 0.78

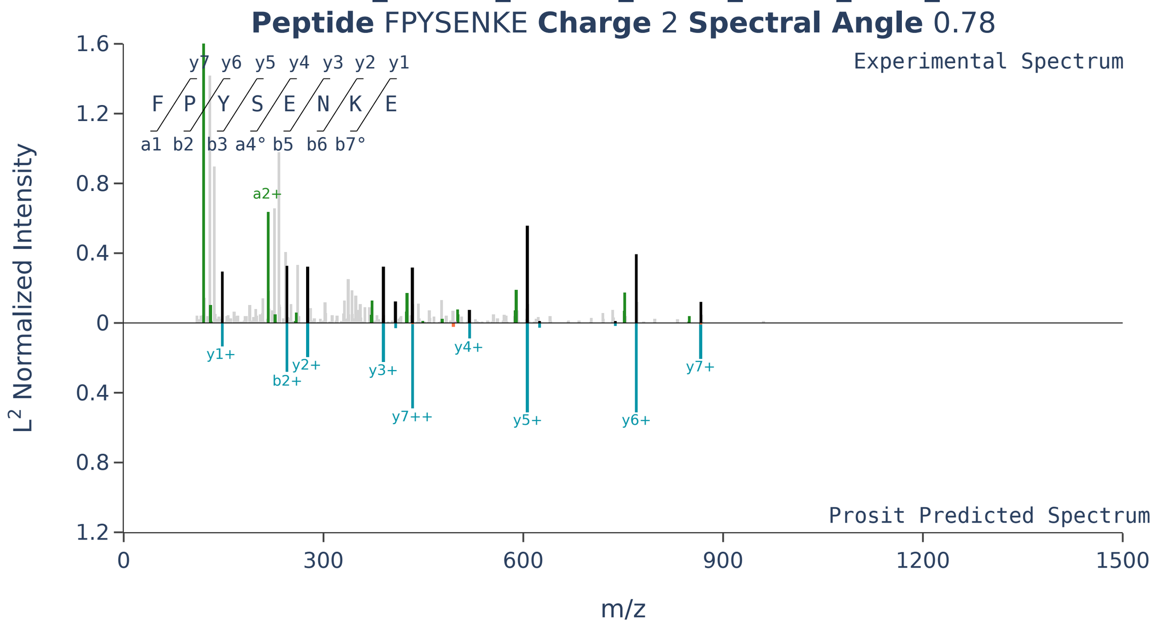


**Source** HRoetschke_271222_090123_HFGoe_LEDGF_b1R2_4h **Scan** 9504
**Peptide** QRIHAEI **Charge** 2 **Spectral Angle** 0.8

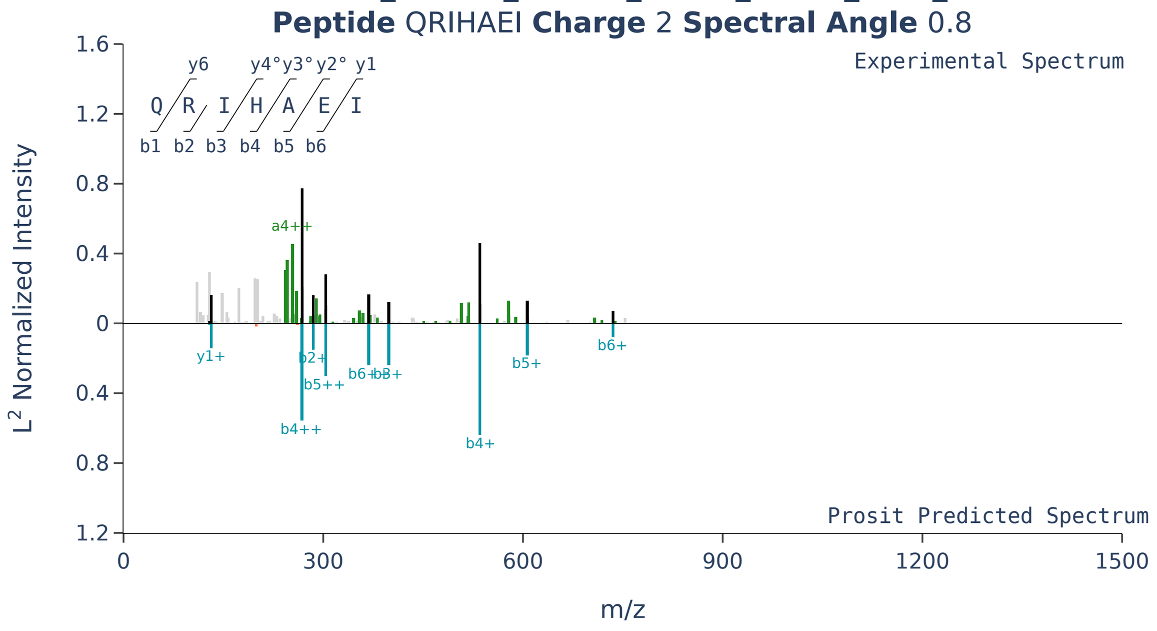


**Source** HRoetschke_271222_090123_HFGoe_LEDGF_b2R2_2h **Scan** 23950
**Peptide** KGFNEGLW **Charge** 2 **Spectral Angle** 0.77

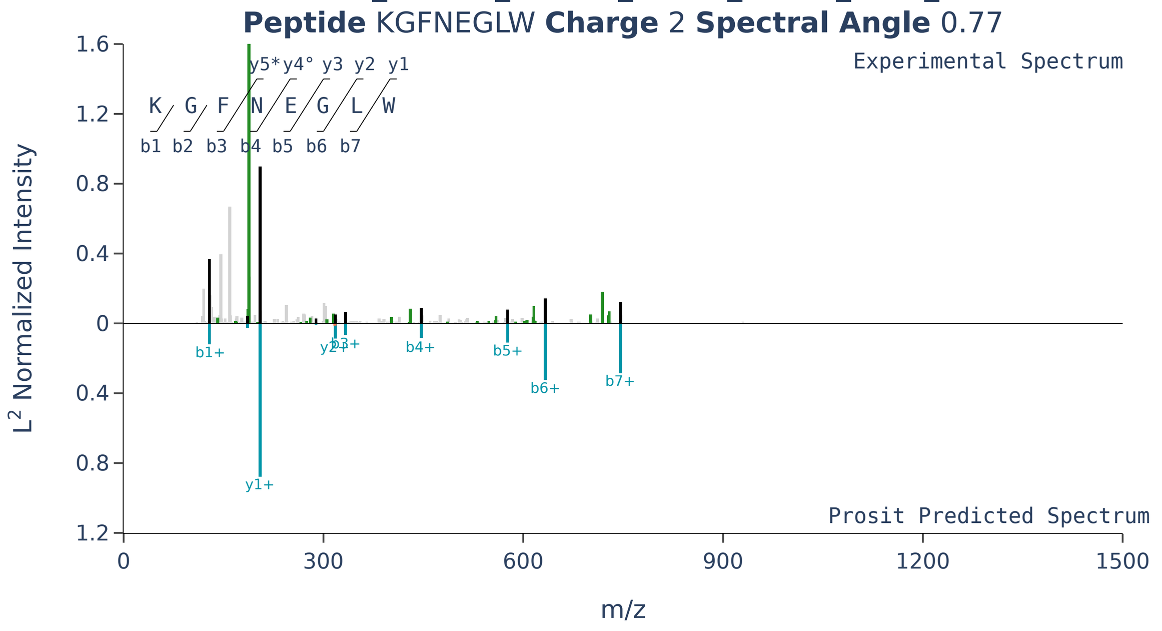


**Source** HRoetschke_271222_090123_HFGoe_LEDGF_b1R1_2h **Scan** 10482
**Peptide** QQAQKHTEMITTLKKIR **Charge** 5 **Spectral Angle** 0.85

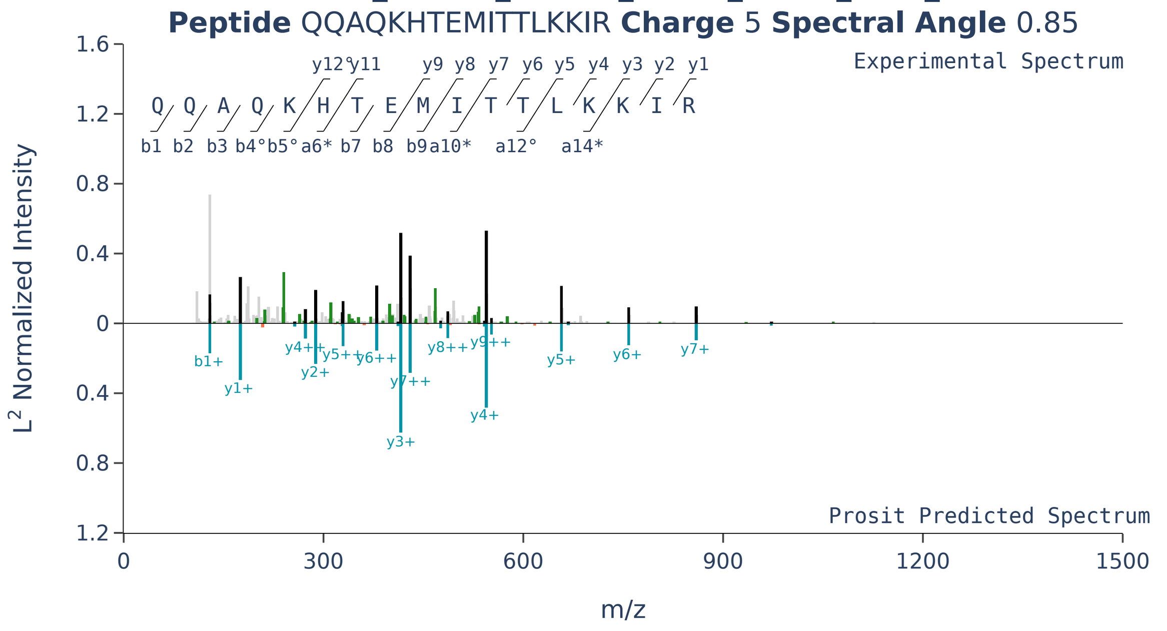


**Source** HRoetschke_271222_090123_HFGoe_LEDGF_b2R1_24h **Scan** 8725
**Peptide** IDNNPKVKFSSQQAATK **Charge** 2 **Spectral Angle** 0.8

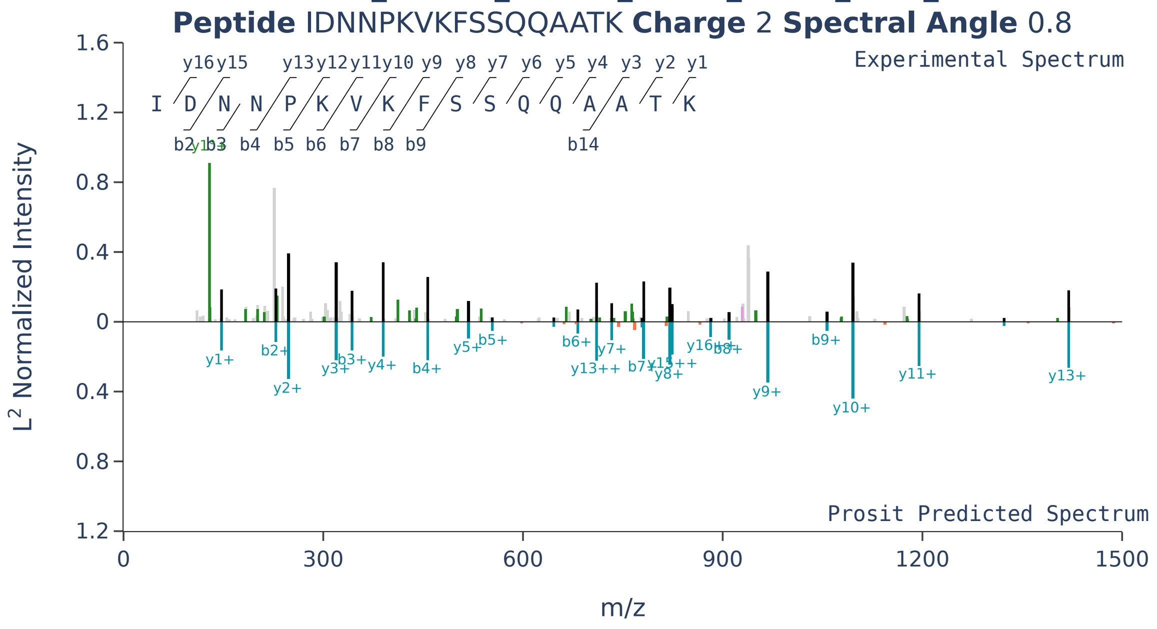


**Source** HRoetschke_271222_090123_HFGoe_LEDGF_b1R2_24h **Scan** 6359
**Peptide** RPKMVKQPCPSE **Charge** 2 **Spectral Angle** 0.83

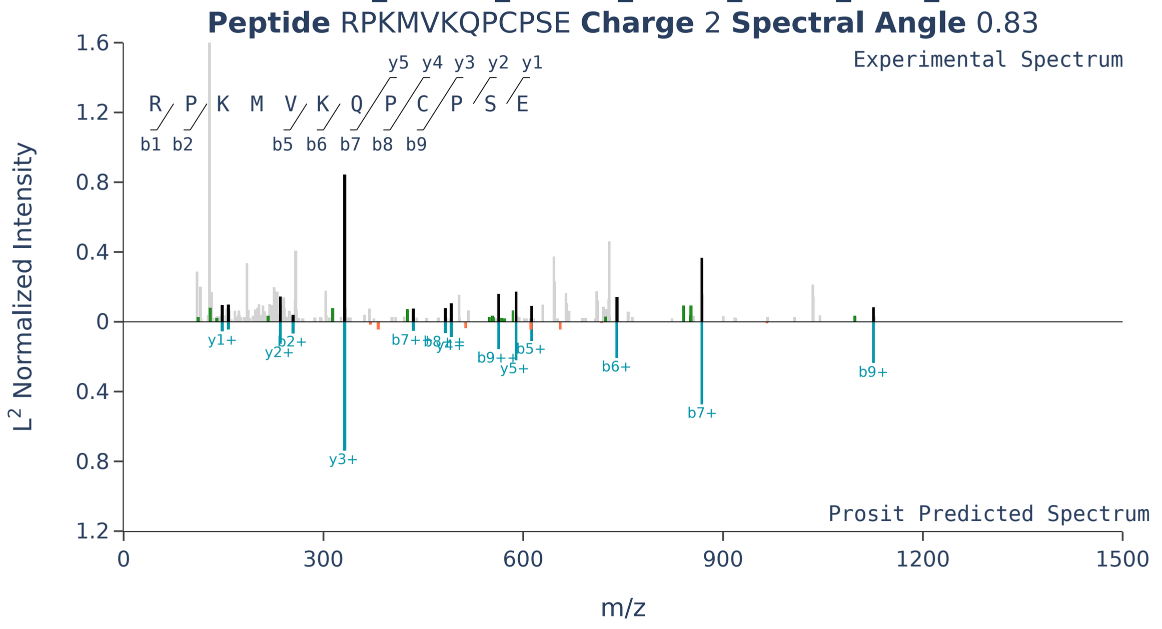


**Source** HRoetschke_271222_090123_HFGoe_LEDGF_b2R1_24h **Scan** 24706
**Peptide** GLWEIDNNPKVKFS **Charge** 2 **Spectral Angle** 0.85

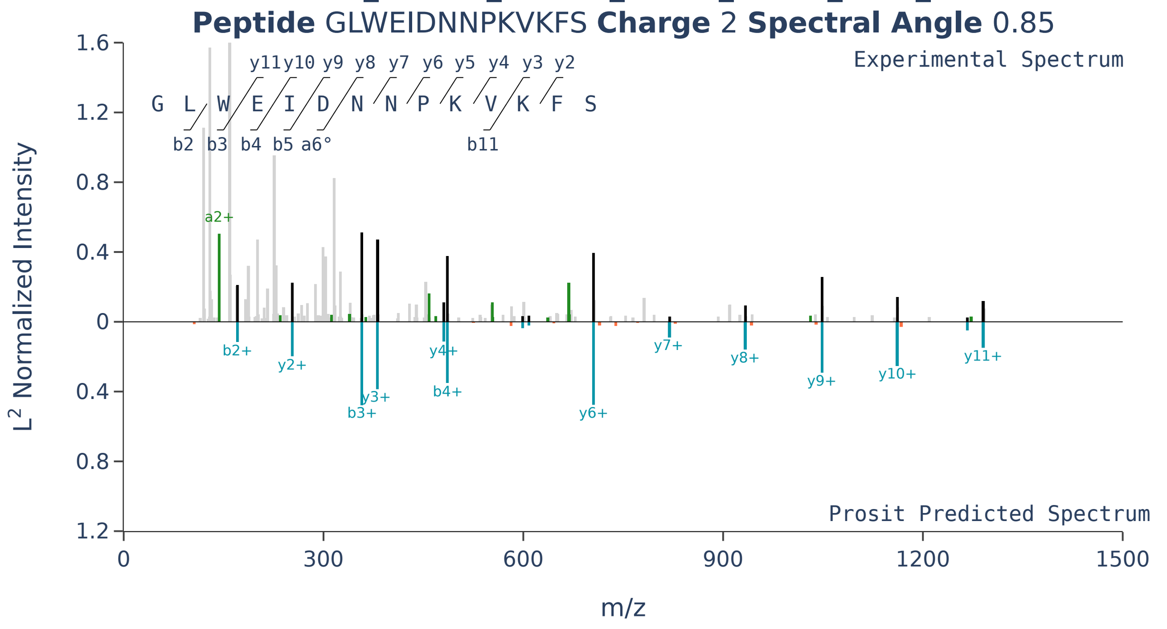


**Source** HRoetschke_271222_090123_HFGoe_LEDGF_b2R1_24h **Scan** 23013
**Peptide** KNMFLVGEGDSVITQ **Charge** 2 **Spectral Angle** 0.88

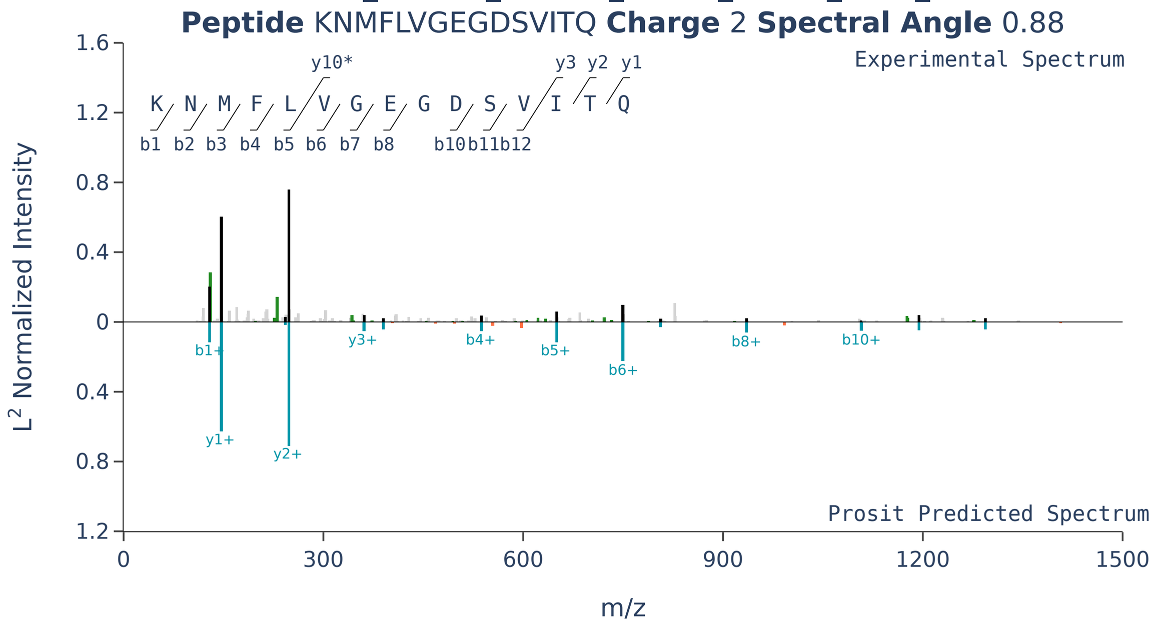


**Source** HRoetschke_271222_090123_HFGoe_LEDGF_b1R2_2h **Scan** 11070
**Peptide** EGDSVITQ **Charge** 1 **Spectral Angle** 0.82

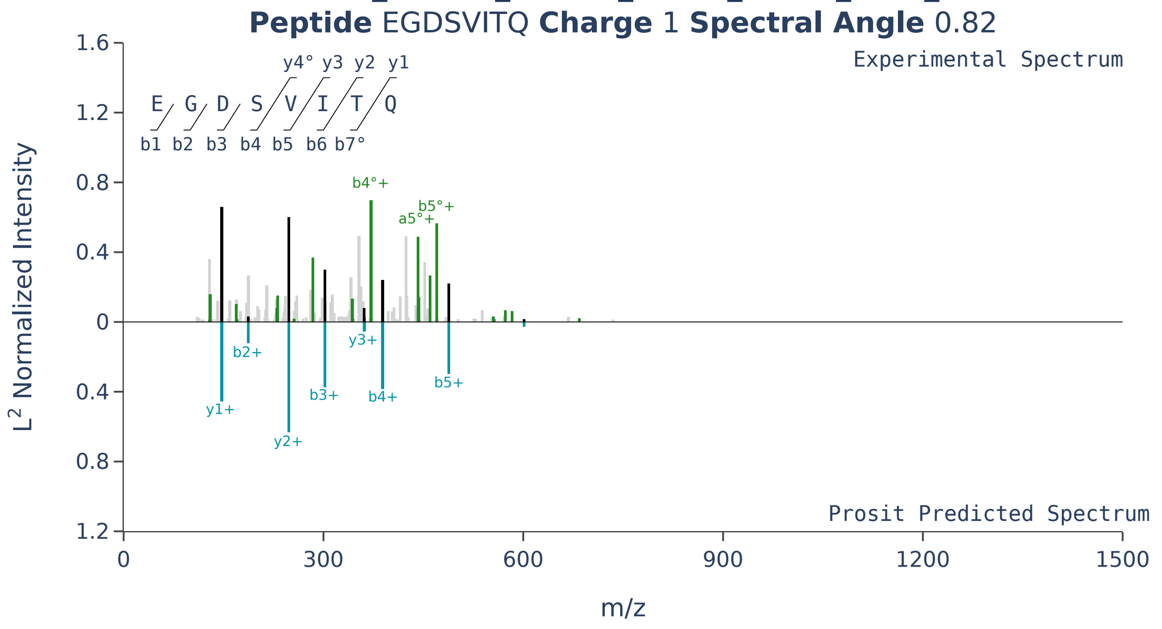


**Source** HRoetschke_271222_090123_HFGoe_LEDGF_b1R2_24h **Scan** 14989
**Peptide** SDIITEE **Charge** 1 **Spectral Angle** 0.9

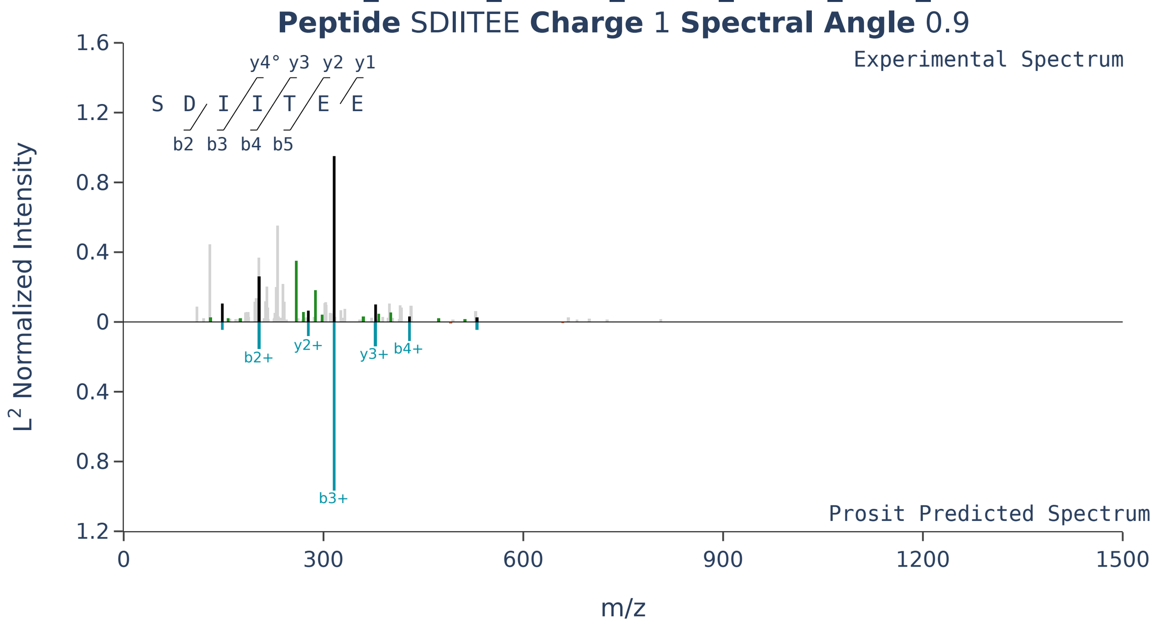

**Source** HRoetschke_271222_090123_HFGoe_LEDGF_b1R1_2h **Scan** 22491
**Peptide** LIFAKMKGYPHWPARV **Charge** 3 **Spectral Angle** 0.79

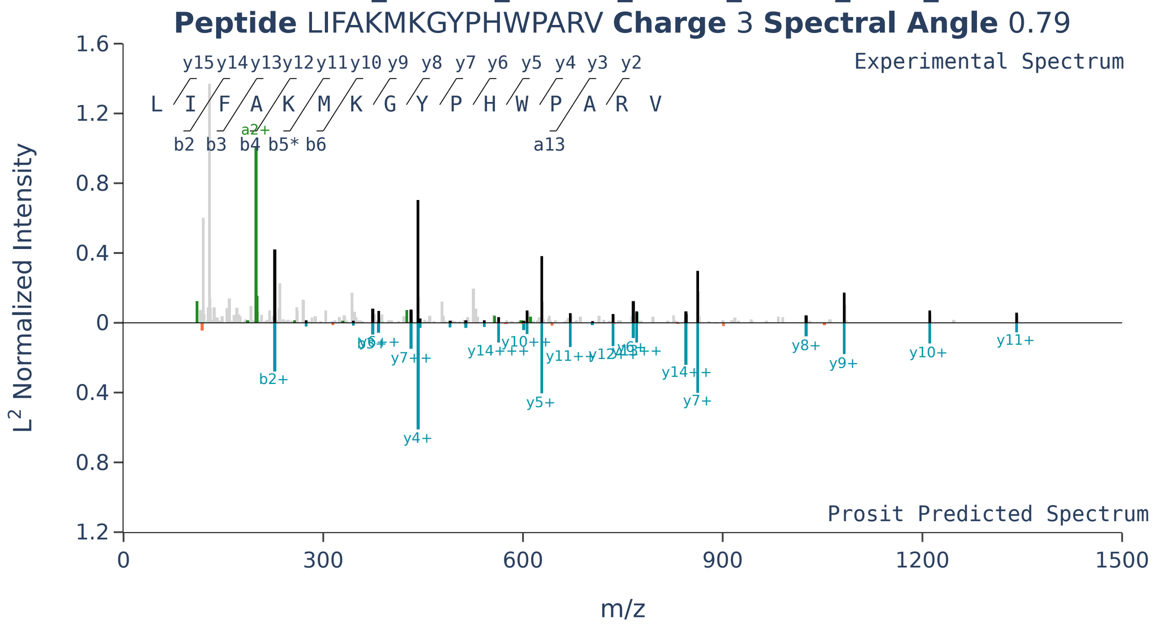


**Source** HRoetschke_271222_090123_HFGoe_LEDGF_b1R2_4h **Scan** 20117
**Peptide** KVSQVIMEKSTMLYNKFKN **Charge** 3 **Spectral Angle** 0.93

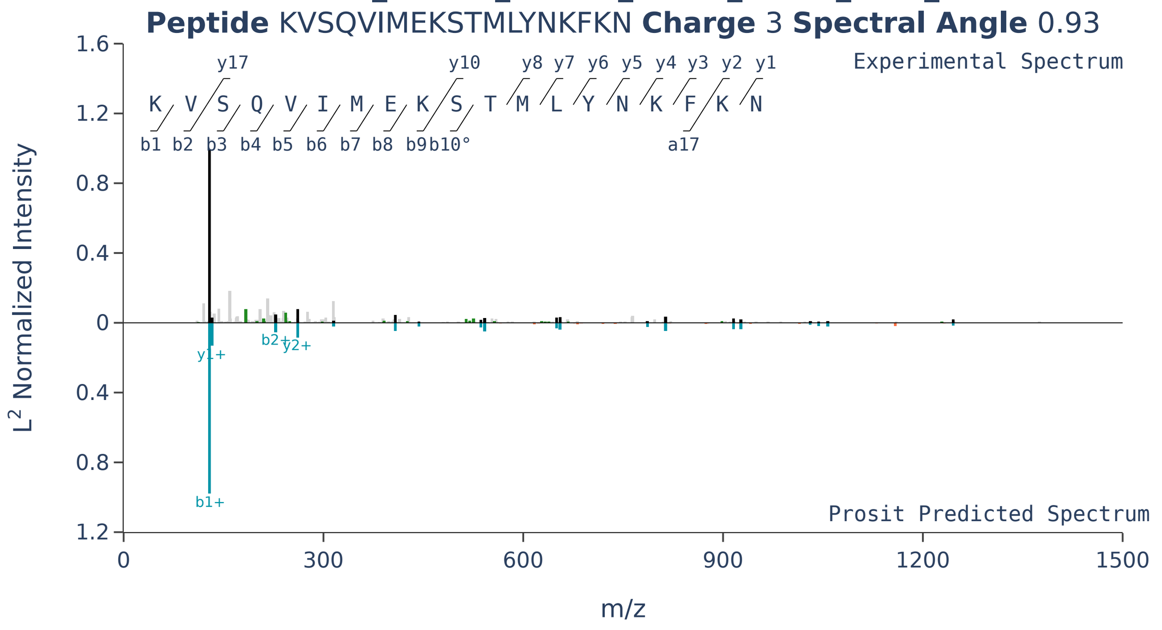


**Source** HRoetschke_271222_090123_HFGoe_LEDGF_b2R1_4h **Scan** 20477
**Peptide** AEIKNSLKIDNLDVNRCIE **Charge** 3 **Spectral Angle** 0.88

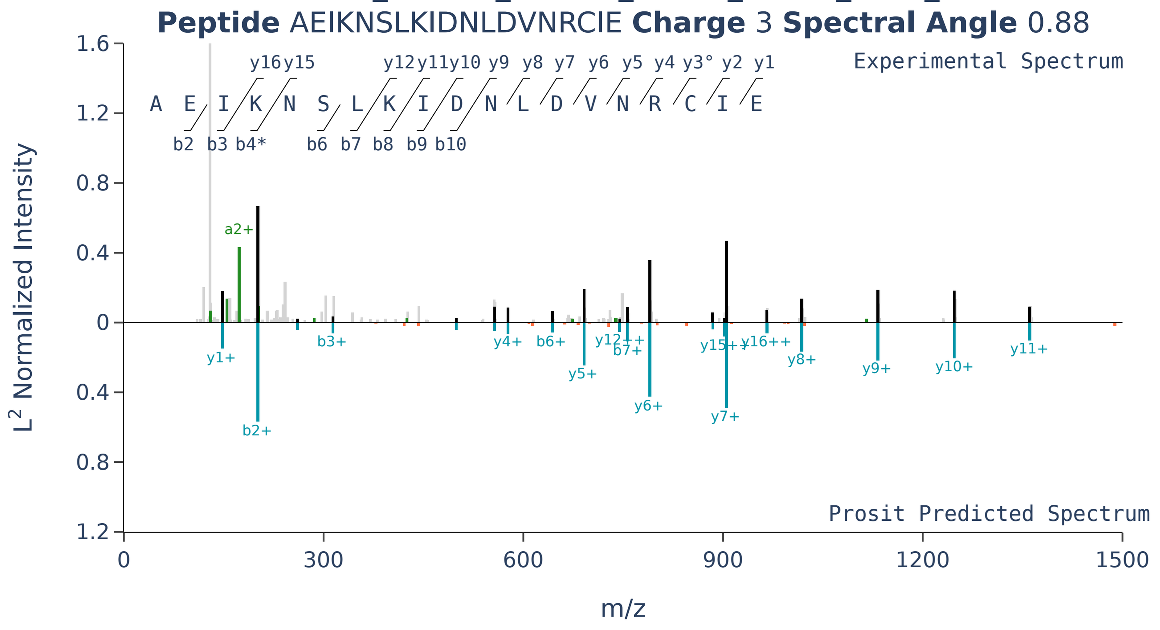


**Source** HRoetschke_271222_090123_HFGoe_LEDGF_b1R1_4h **Scan** 14484
**Peptide** DSRLQRIHAEI **Charge** 3 **Spectral Angle** 0.8

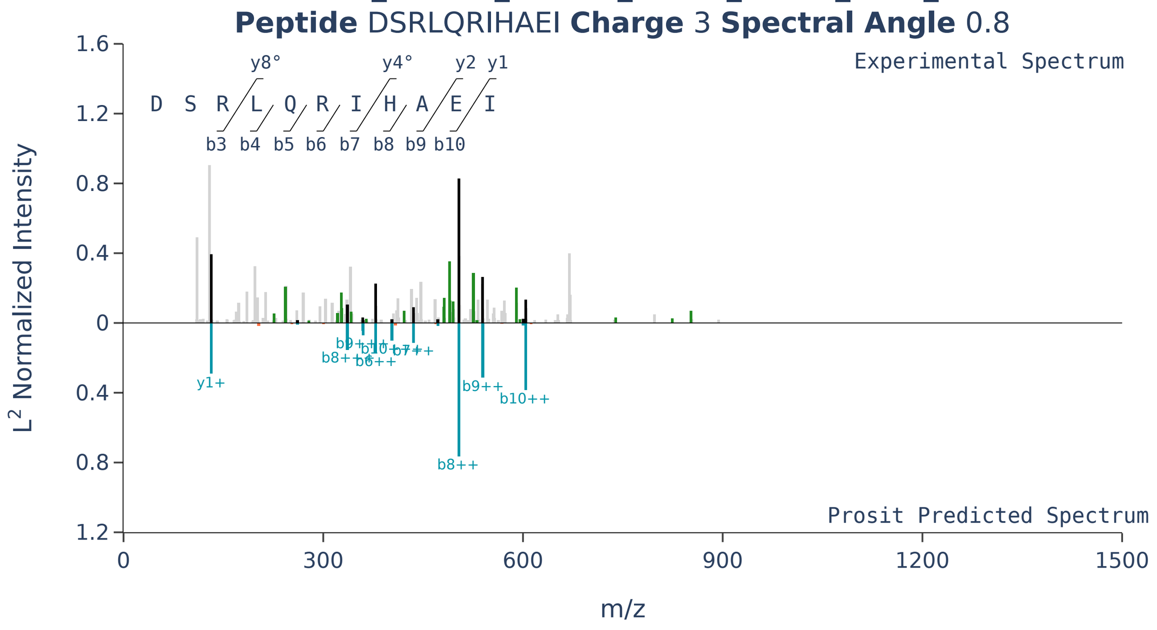


**Source** HRoetschke_271222_090123_HFGoe_LEDGF_b1R1_2h **Scan** 20161
**Peptide** GLWEIDNNPKVKFSSQQAATK **Charge** 3 **Spectral Angle** 0.84

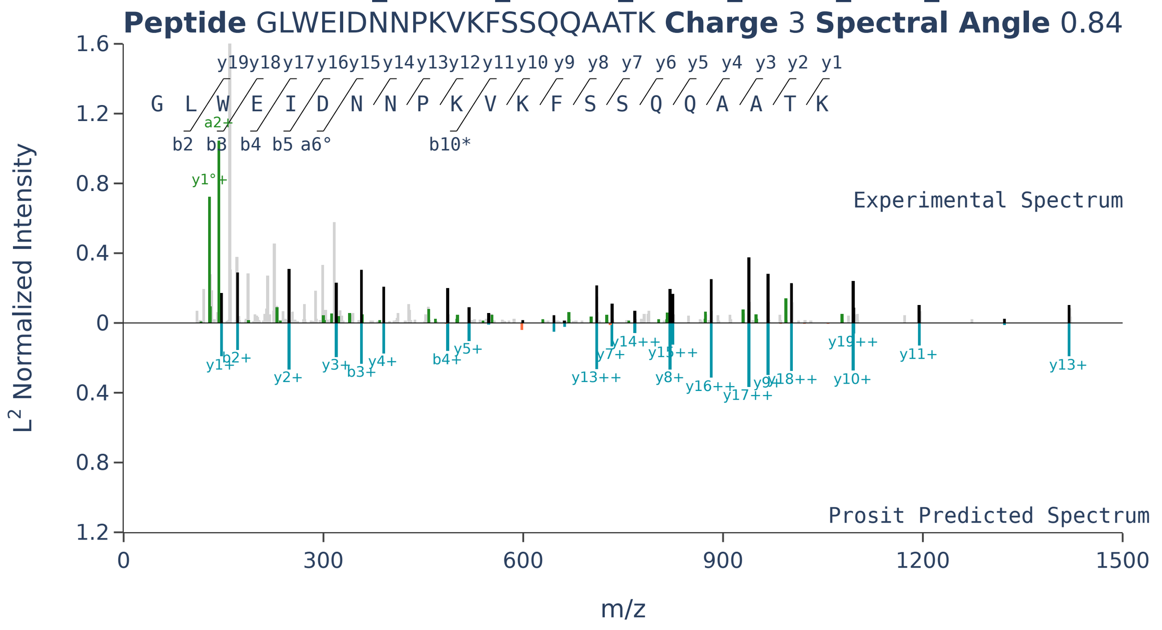


**Source** HRoetschke_271222_090123_HFGoe_LEDGF_b2R2_2h **Scan** 14165
**Peptide** MITTLKKIRRFKV **Charge** 4 **Spectral Angle** 0.9

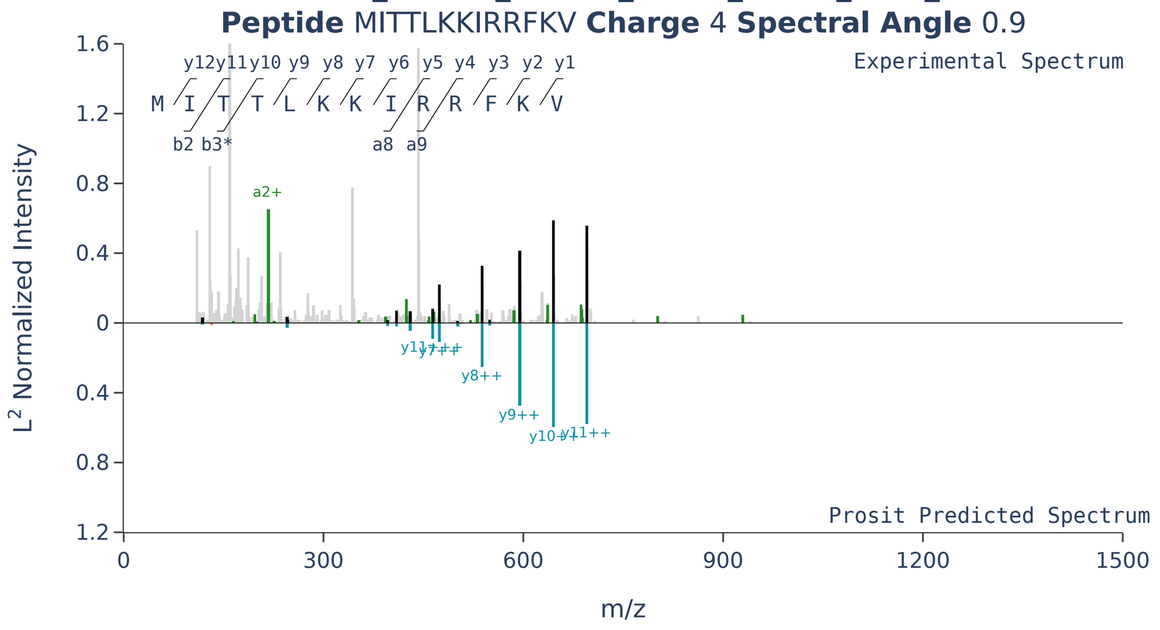


**Source** HRoetschke_271222_090123_HFGoe_LEDGF_b1R2_1h **Scan** 29193
**Peptide** EGDSVITQVLNKSLAE **Charge** 2 **Spectral Angle** 0.73

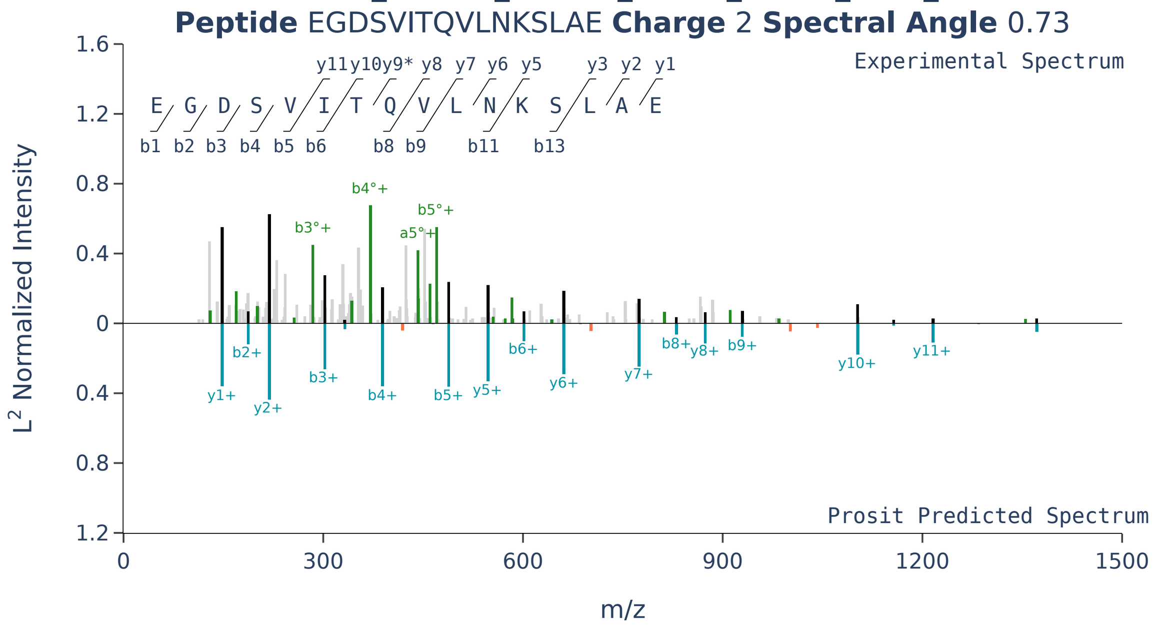


**Source** HRoetschke_271222_090123_HFGoe_LEDGF_b2R2_2h **Scan** 7947
**Peptide** DNNPKVKFSSQQAATK **Charge** 3 **Spectral Angle** 0.81

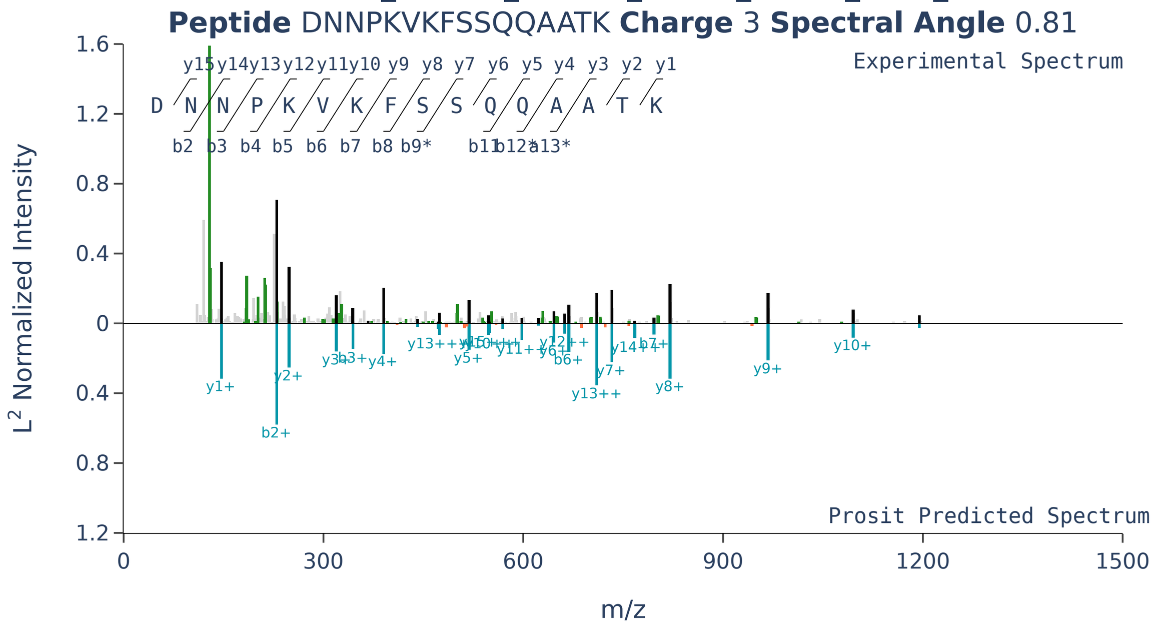


**Source** HRoetschke_271222_090123_HFGoe_LEDGF_b1R2_2h **Scan** 21117
**Peptide** ELASLQVT **Charge** 1 **Spectral Angle** 0.87

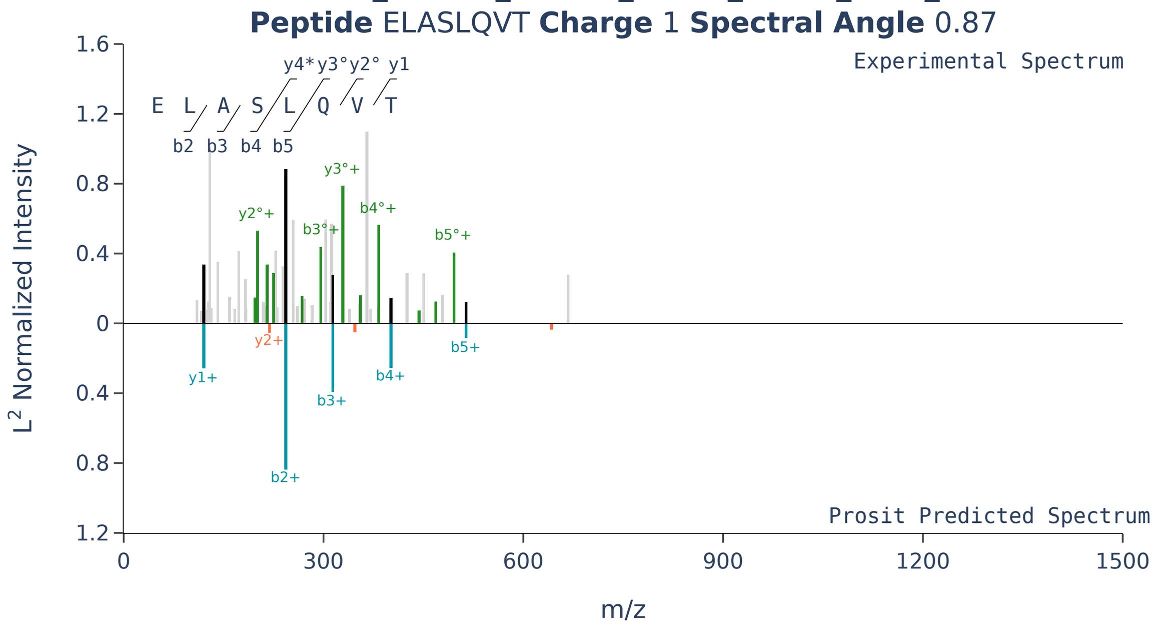


**Source** HRoetschke_271222_090123_HFGoe_LEDGF_b2R2_X **Scan** 8110
**Peptide** ATASVNLK **Charge** 2 **Spectral Angle** 0.84

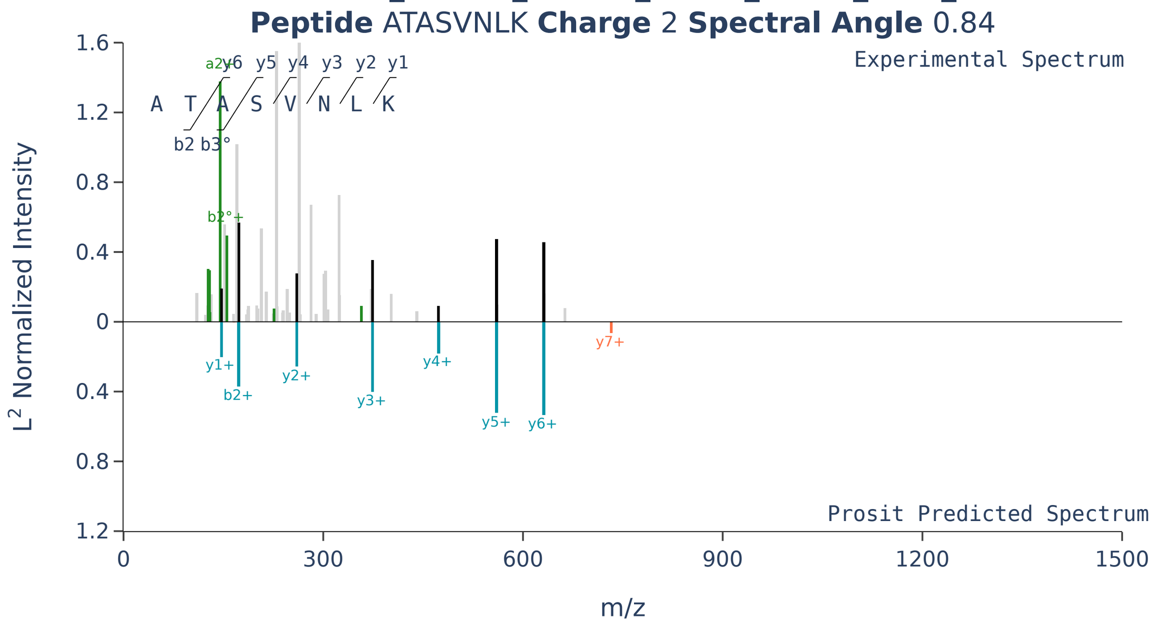


**Source** HRoetschke_271222_090123_HFGoe_LEDGF_b1R2_4h **Scan** 12134
**Peptide** AEKQVETEEAGVVTT **Charge** 2 **Spectral Angle** 0.88

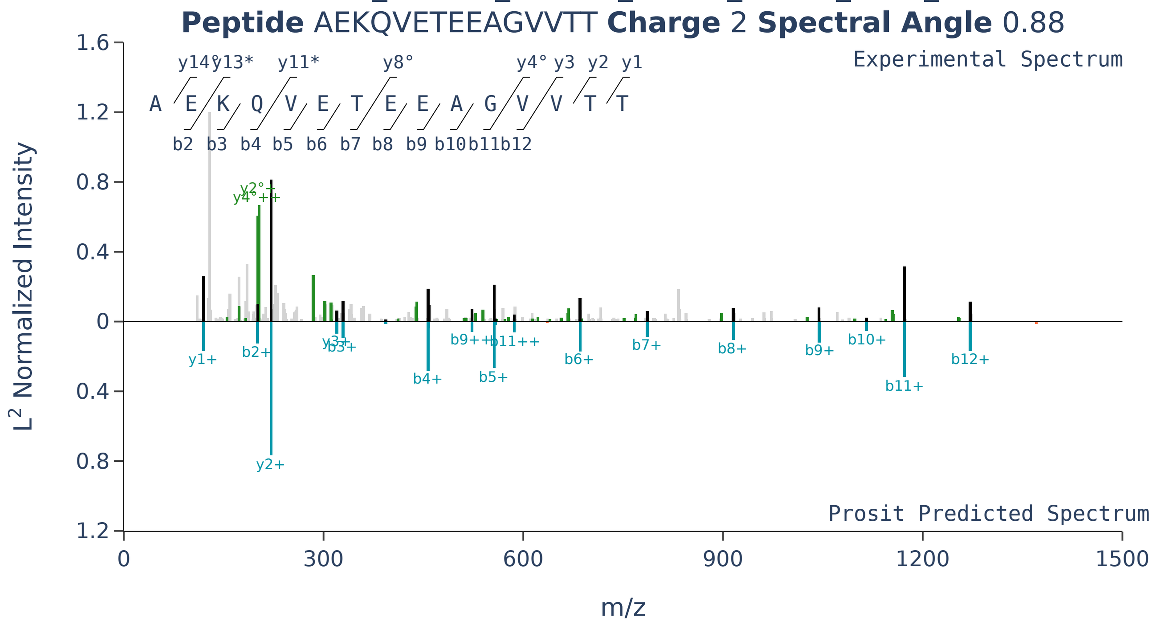


**Source** HRoetschke_271222_090123_HFGoe_LEDGF_b1R1_4h **Scan** 14494
**Peptide** ASLQVTMQQAQK **Charge** 2 **Spectral Angle** 0.82

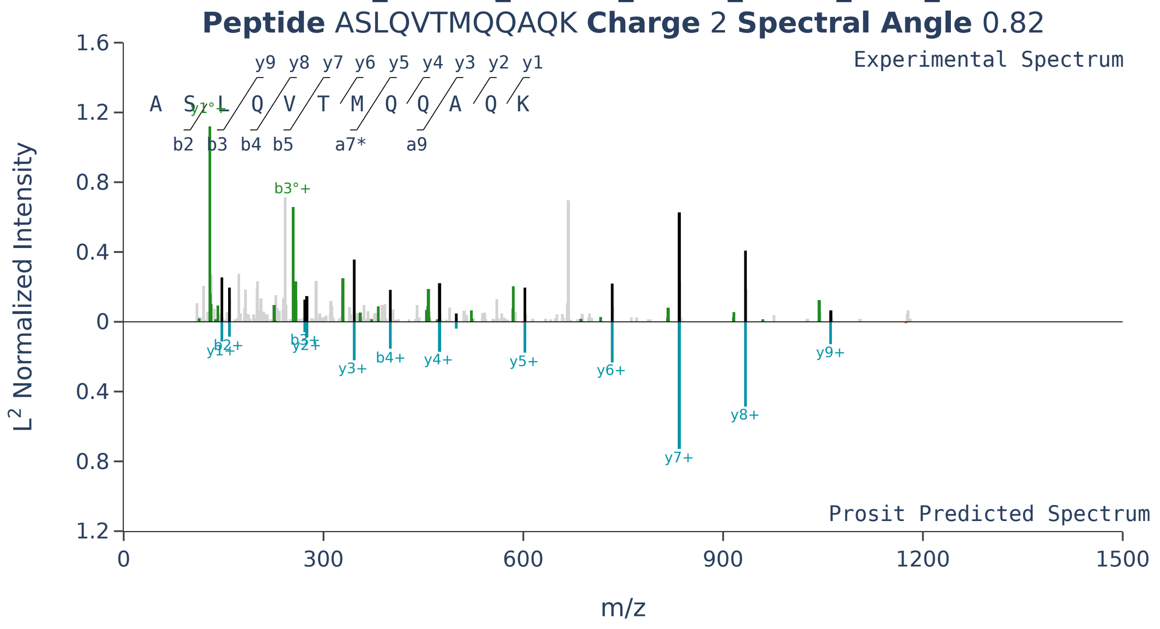


**Source** HRoetschke_271222_090123_HFGoe_LEDGF_b2R1_4h **Scan** 13557
**Peptide** MEKSTMLYN **Charge** 2 **Spectral Angle** 0.84

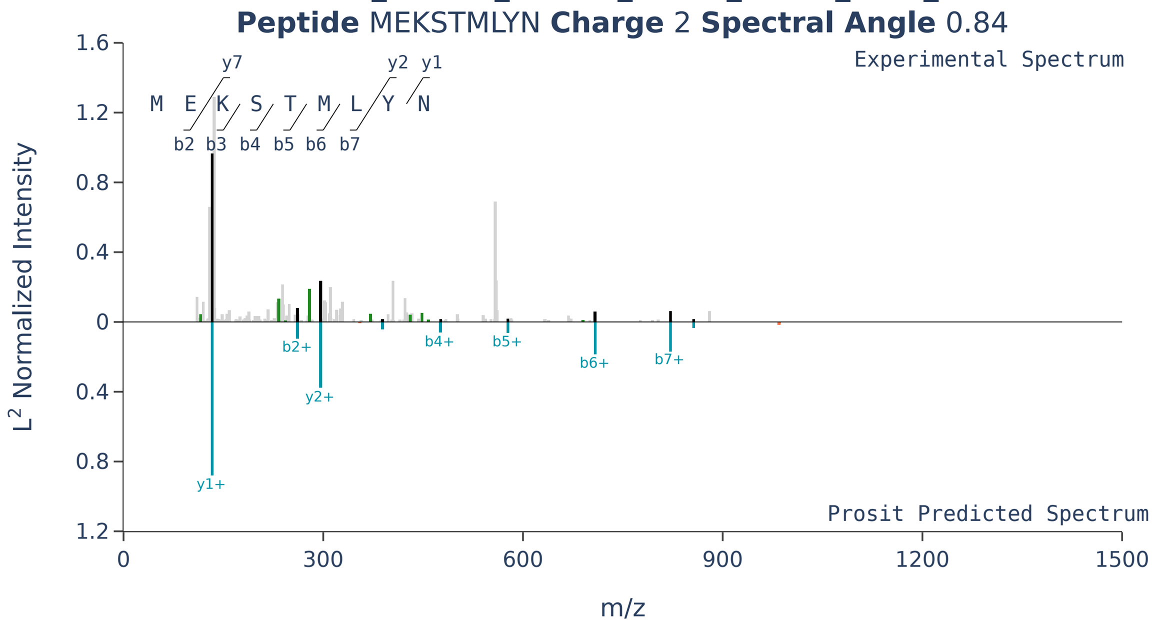


**Source** HRoetschke_271222_090123_HFGoe_LEDGF_b2R2_2h **Scan** 12168
**Peptide** EKSTMLYNKFK **Charge** 2 **Spectral Angle** 0.81

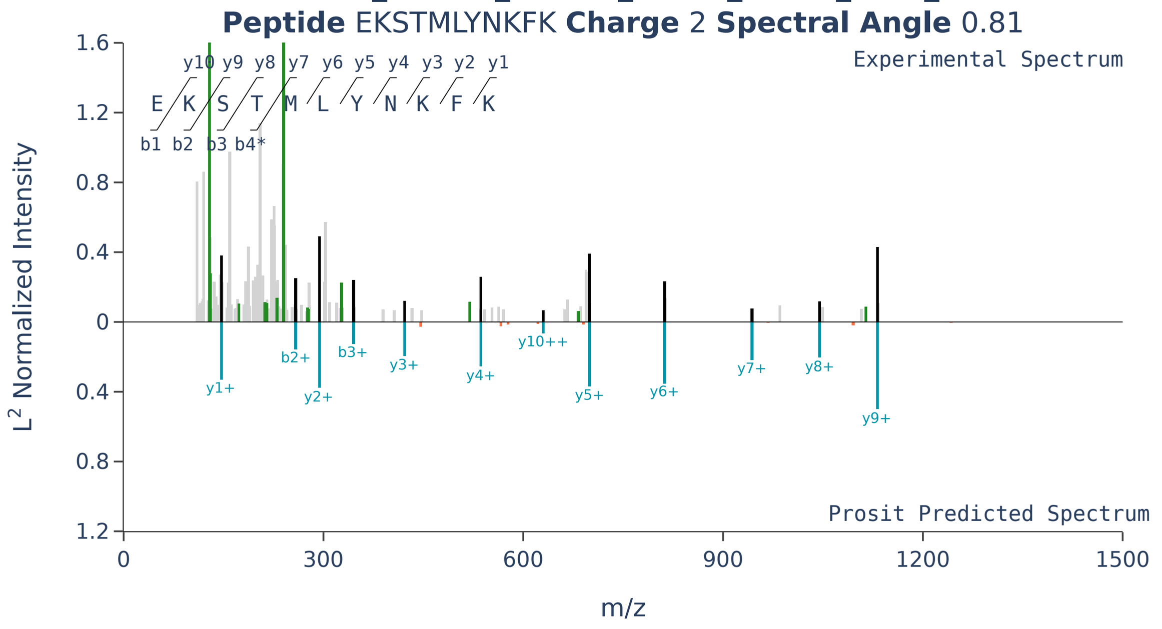


**Source** HRoetschke_271222_090123_HFGoe_LEDGF_b1R1_2h **Scan** 25152
**Peptide** FNEGLWEIDNNPKVKFSSQQA **Charge** 3 **Spectral Angle** 0.88

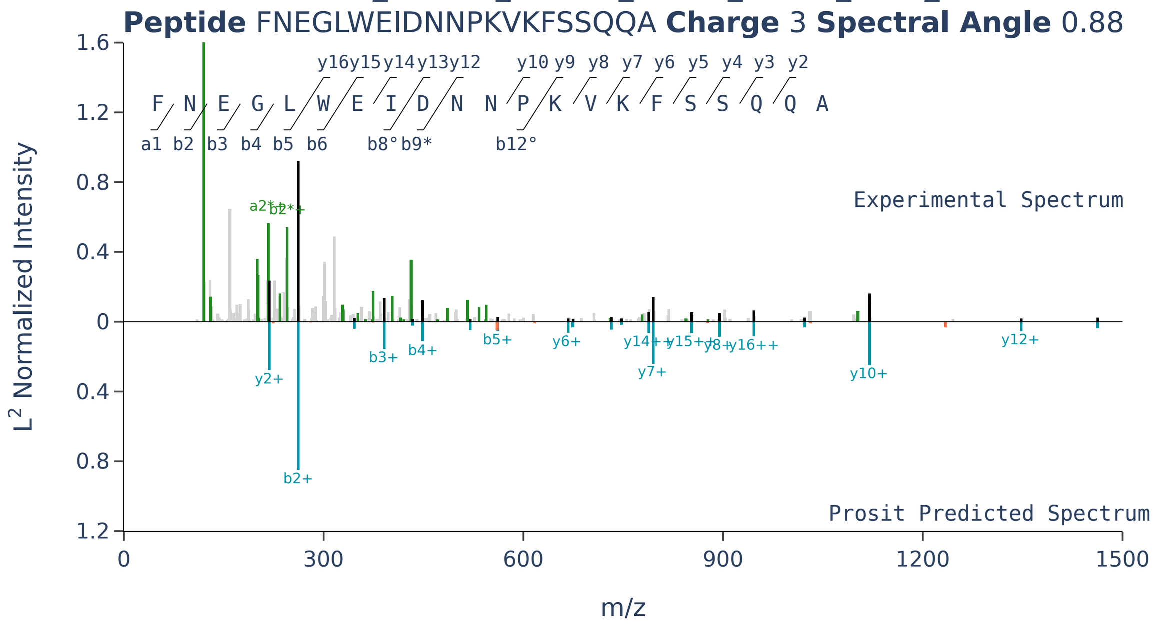

**Source** HRoetschke_271222_090123_HFGoe_LEDGF_b1R1_2h **Scan** 13996
**Peptide** LGPKDIFPYSENKEKYGKPNKR **Charge** 4 **Spectral Angle** 0.76

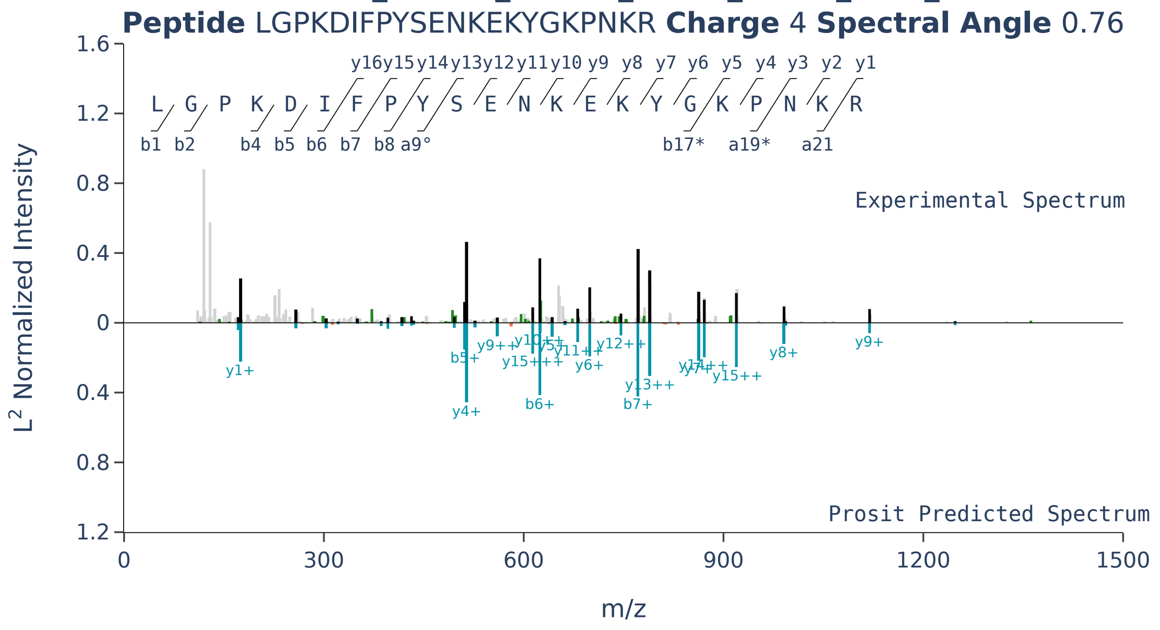


**Source** HRoetschke_271222_090123_HFGoe_LEDGF_b2R1_1h **Scan** 8343
**Peptide** MLTTLKKIRR **Charge** 3 **Spectral Angle** 0.78

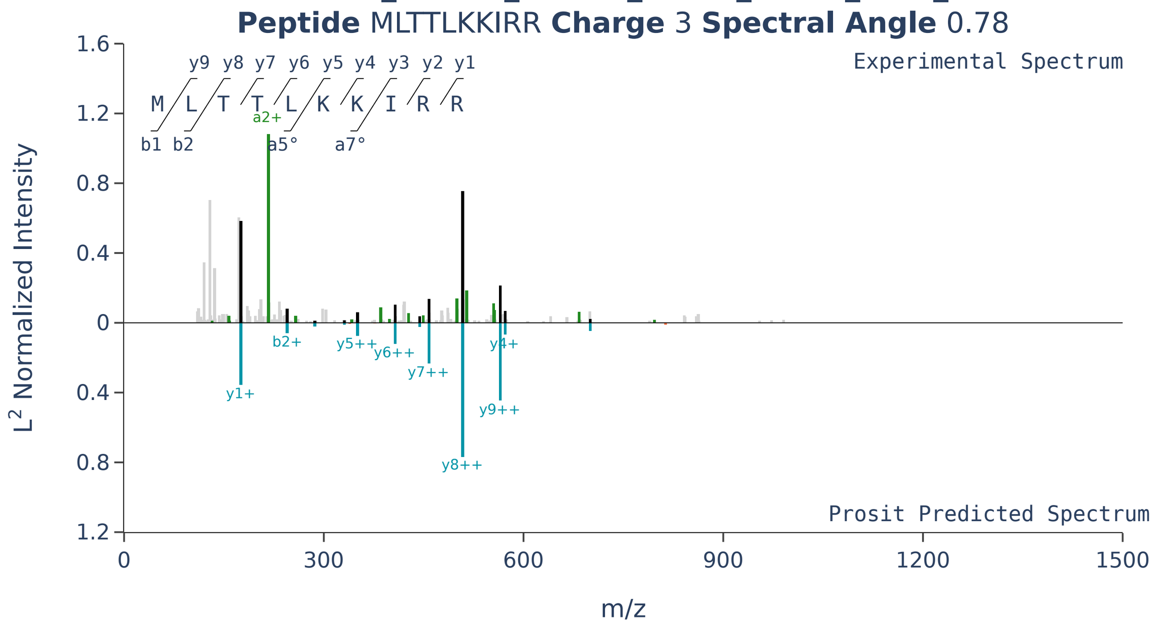


**Source** HRoetschke_271222_090123_HFGoe_LEDGF_b2R1_4h **Scan** 12258
**Peptide** PSSEERETEISLKD **Charge** 2 **Spectral Angle** 0.81

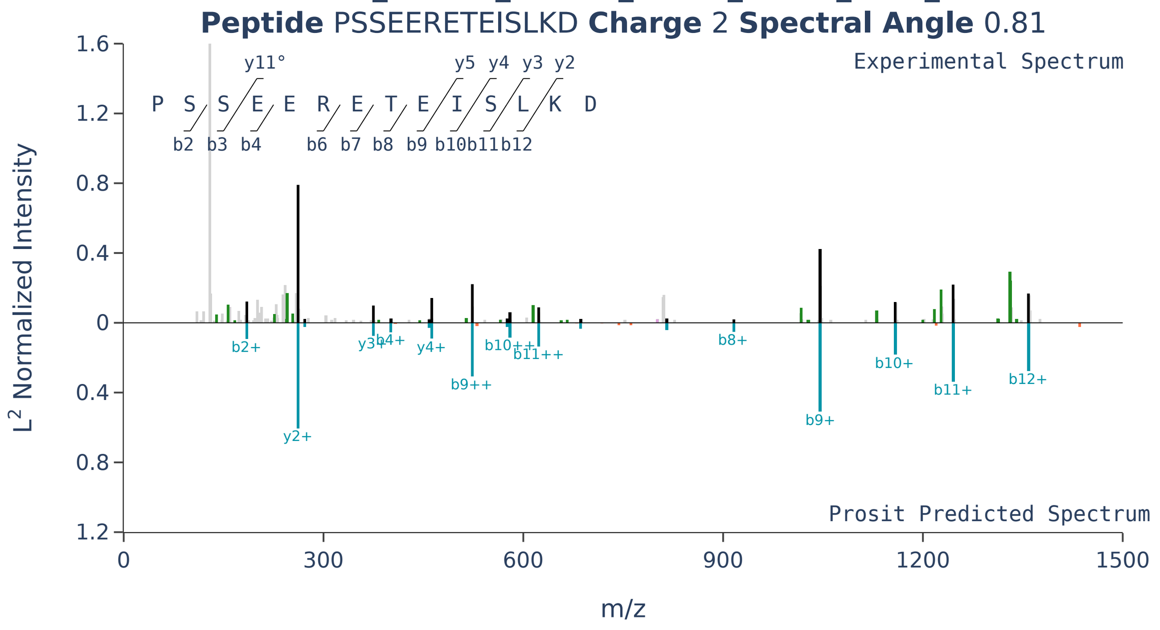


**Source** HRoetschke_271222_090123_HFGoe_LEDGF_b2R1_2h **Scan** 9255
**Peptide** QVTMQQA **Charge** 1 **Spectral Angle** 0.82

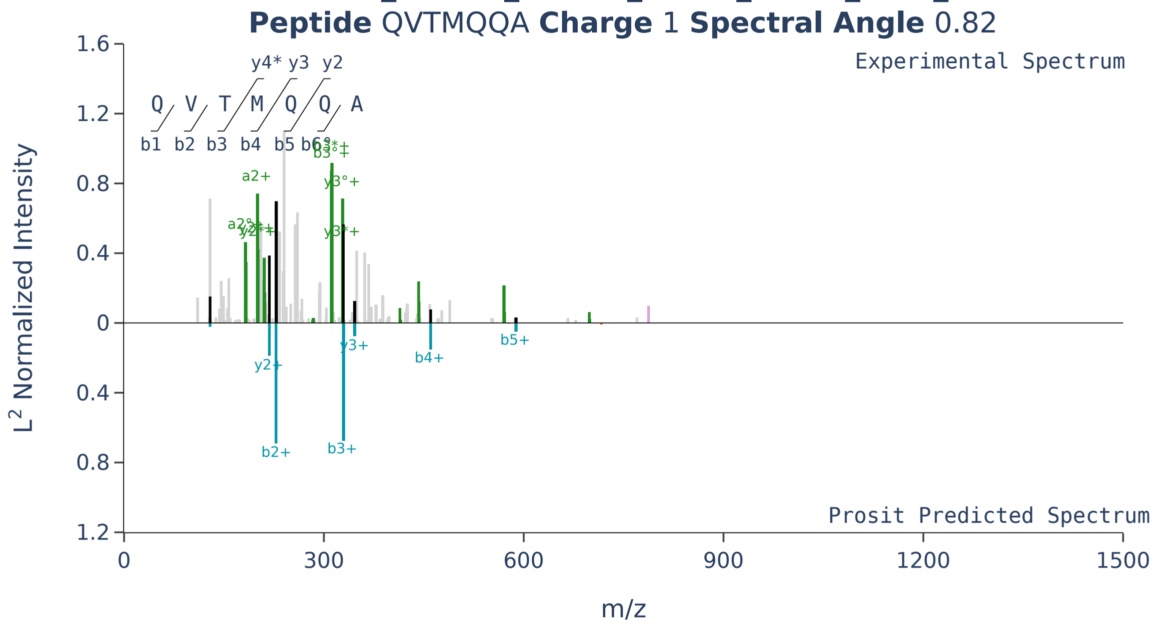


**Source** HRoetschke_271222_090123_HFGoe_LEDGF_b1R2_4h **Scan** 19697
**Peptide** ELASLQVTMQQAQKHT **Charge** 2 **Spectral Angle** 0.83

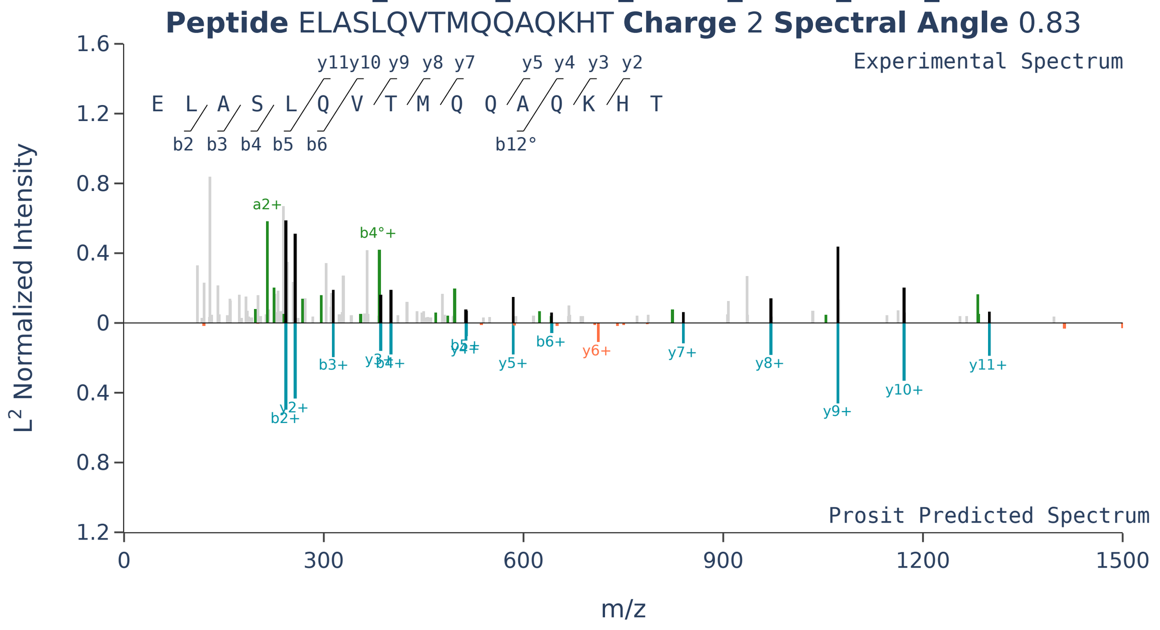


**Source** HRoetschke_271222_090123_HFGoe_LEDGF_b1R1_4h **Scan** 20858
**Peptide** KGFNEGLWEIDNNPKVKFSSQQAATK **Charge** 3 **Spectral Angle** 0.83

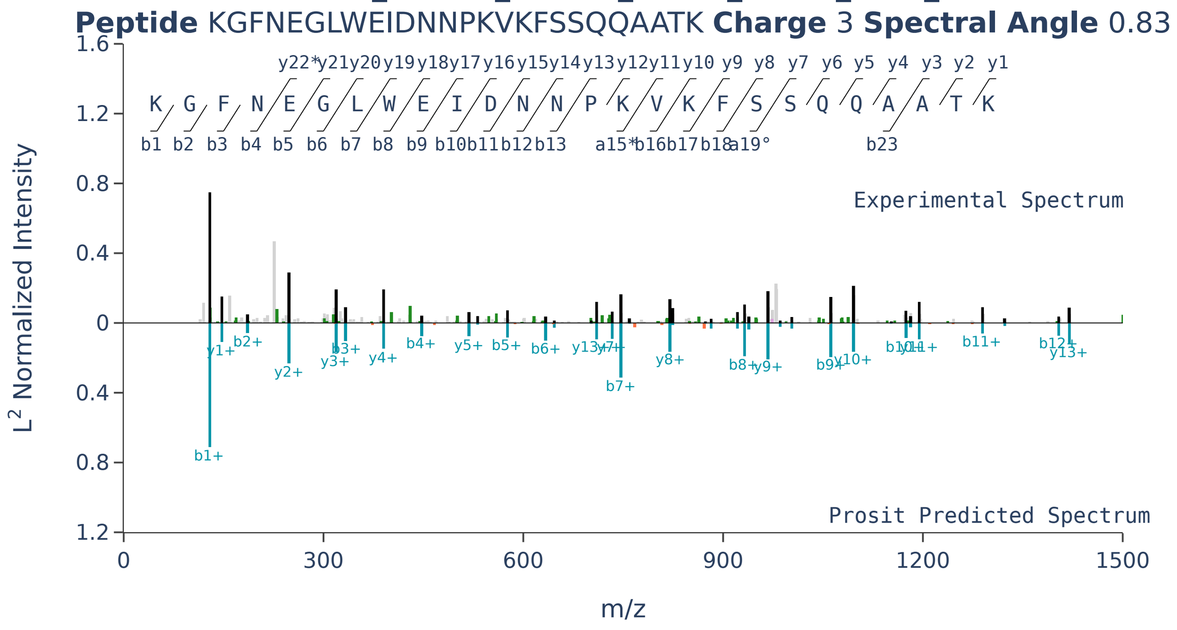


**Source** HRoetschke_271222_090123_HFGoe_LEDGF_b1R2_2h **Scan** 8672
**Peptide** IDNNPKVKFSSQQAATKQSNASSD **Charge** 3 **Spectral Angle** 0.88

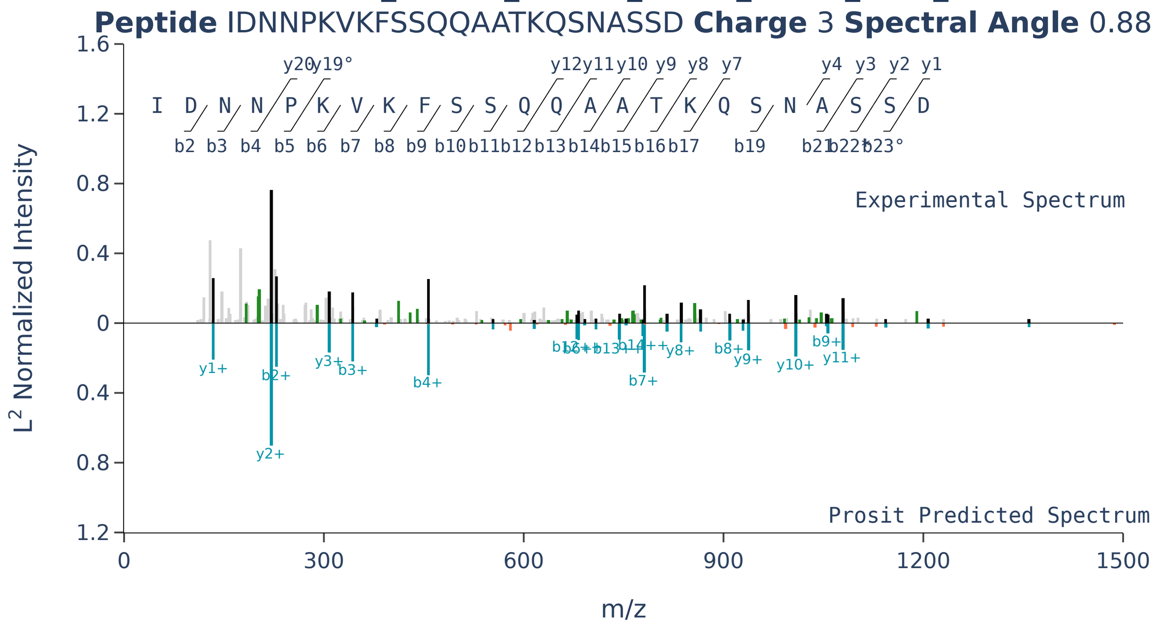


**Source** HRoetschke_271222_090123_HFGoe_LEDGF_b2R2_24h **Scan** 18056
**Peptide** AGVVTTATASVNLK **Charge** 2 **Spectral Angle** 0.76

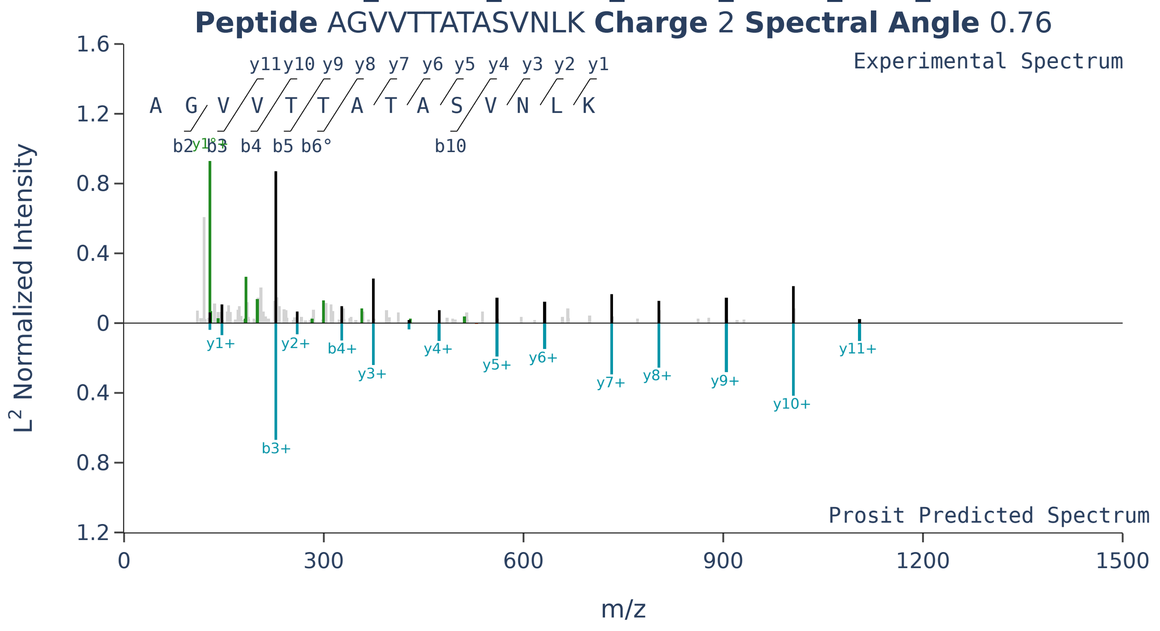


**Source** HRoetschke_271222_090123_HFGoe_LEDGF_b1R2_24h **Scan** 15666
**Peptide** RFKVSQVIMEKST **Charge** 2 **Spectral Angle** 0.8

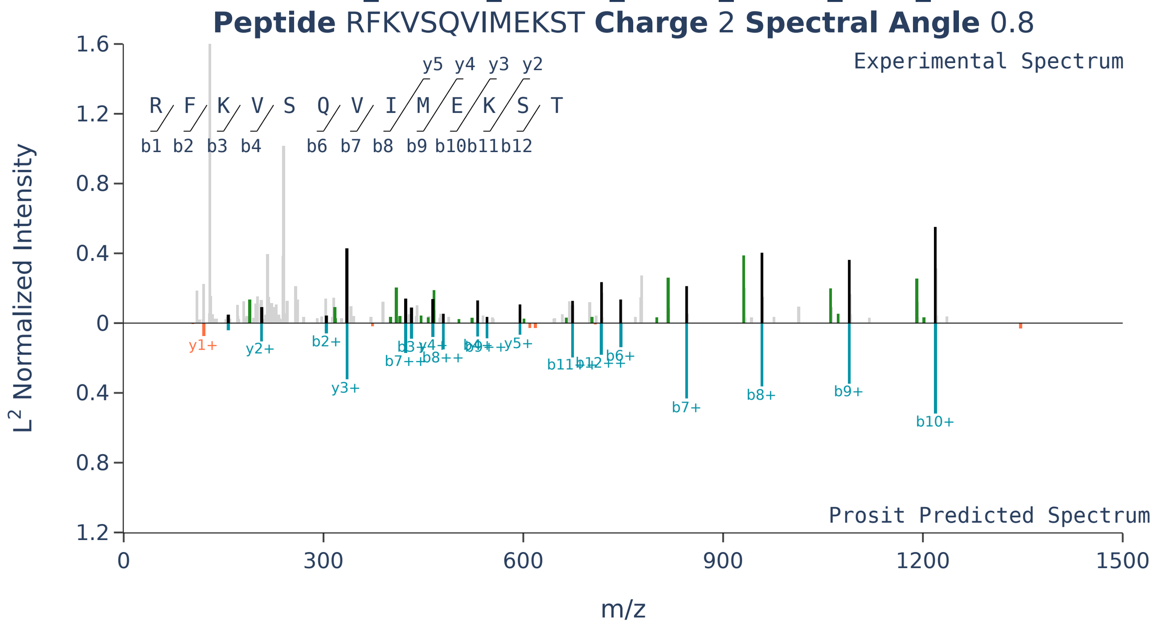


**Source** HRoetschke_271222_090123_HFGoe_LEDGF_b2R1_24h **Scan** 16205
**Peptide** NKFKNMFL **Charge** 3 **Spectral Angle** 0.9

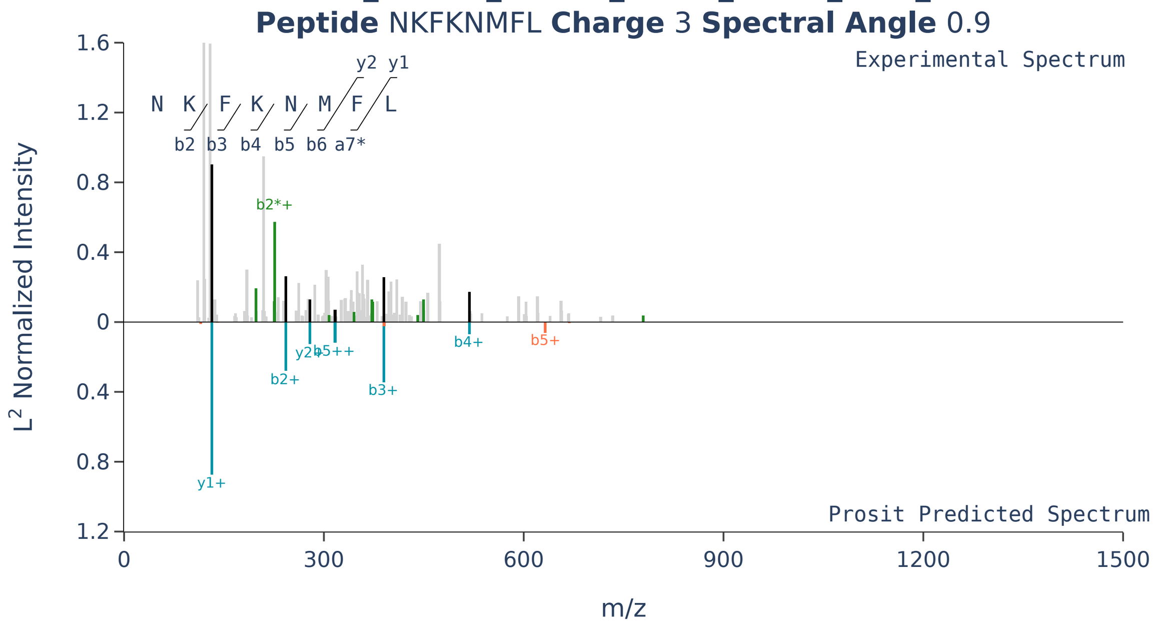


**Source** HRoetschke_271222_090123_HFGoe_LEDGF_b2R1_4h **Scan** 6163
**Peptide** SDIITEEDKSKKKGQE **Charge** 3 **Spectral Angle** 0.83

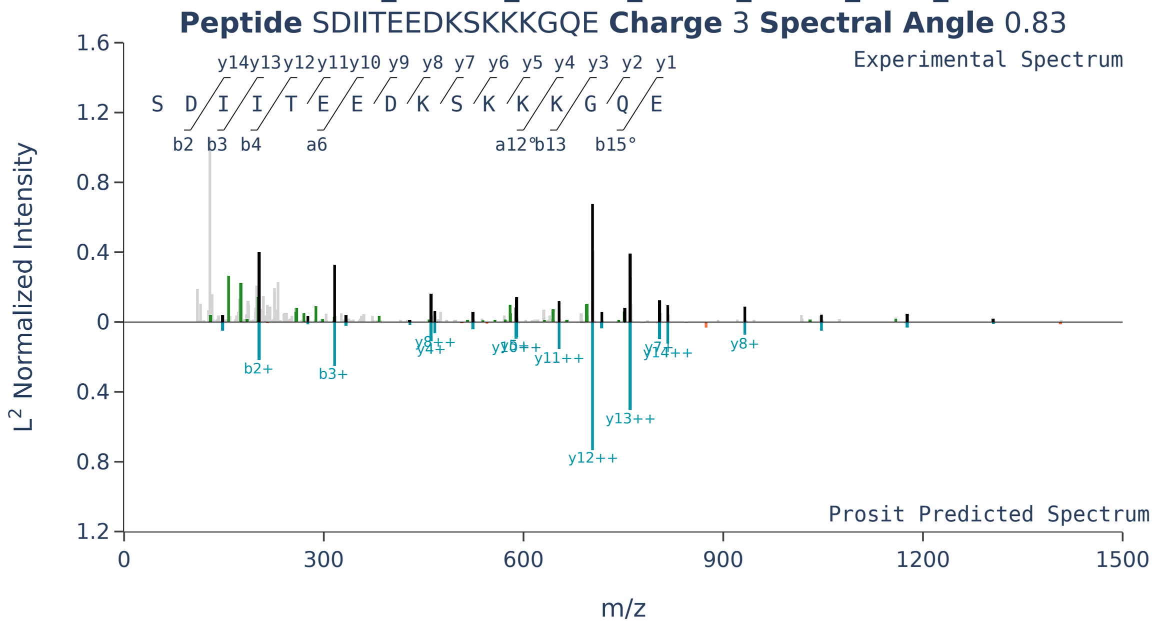


**Source** HRoetschke_271222_090123_HFGoe_LEDGF_b2R1_4h **Scan** 16530
**Peptide** IMEKSTMLYN **Charge** 2 **Spectral Angle** 0.8

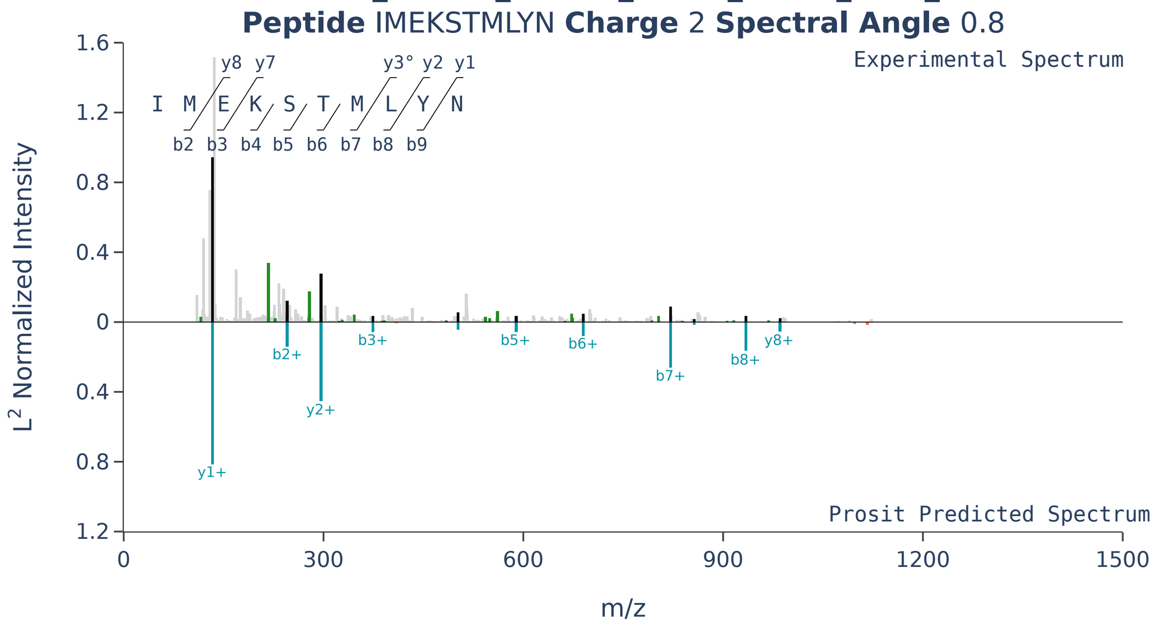


**Source** HRoetschke_271222_090123_HFGoe_LEDGF_b1R1_2h **Scan** 27124
**Peptide** EGDSVITQVLNKSLAEQRQ **Charge** 3 **Spectral Angle** 0.81

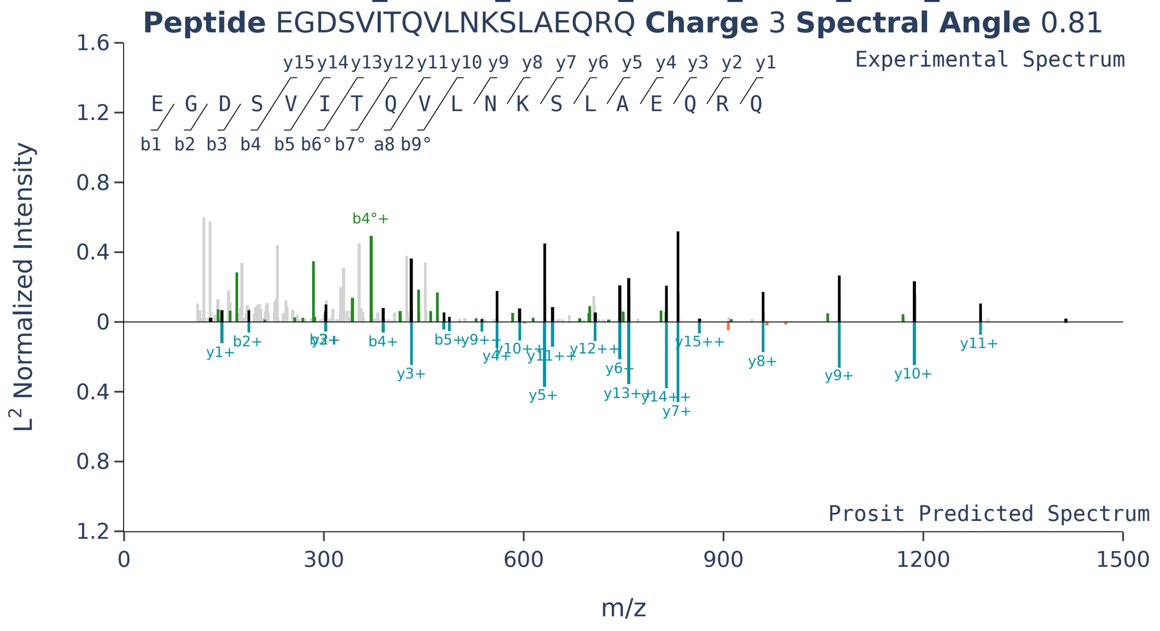


**Source** HRoetschke_271222_090123_HFGoe_LEDGF_b1R1_1h **Scan** 23834
**Peptide** KGFNEGLWEIDNNPKV **Charge** 3 **Spectral Angle** 0.71

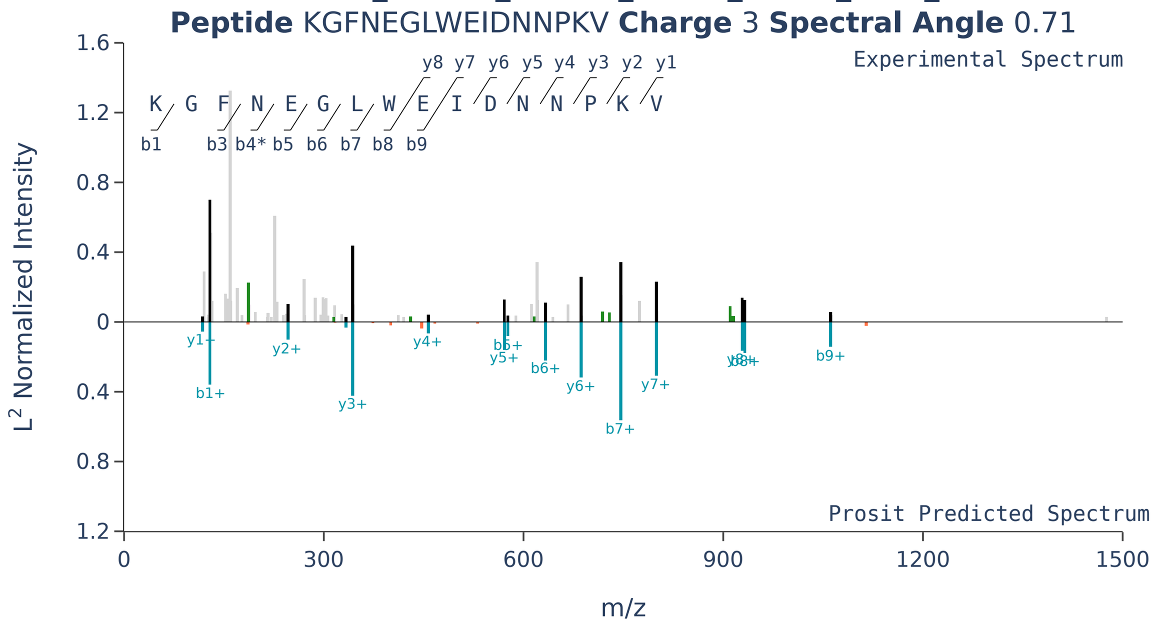


**Source** HRoetschke_271222_090123_HFGoe_LEDGF_b1R2_2h **Scan** 21027
**Peptide** YPHWPARVDEVPDGAVKPPTNKL **Charge** 3 **Spectral Angle** 0.88

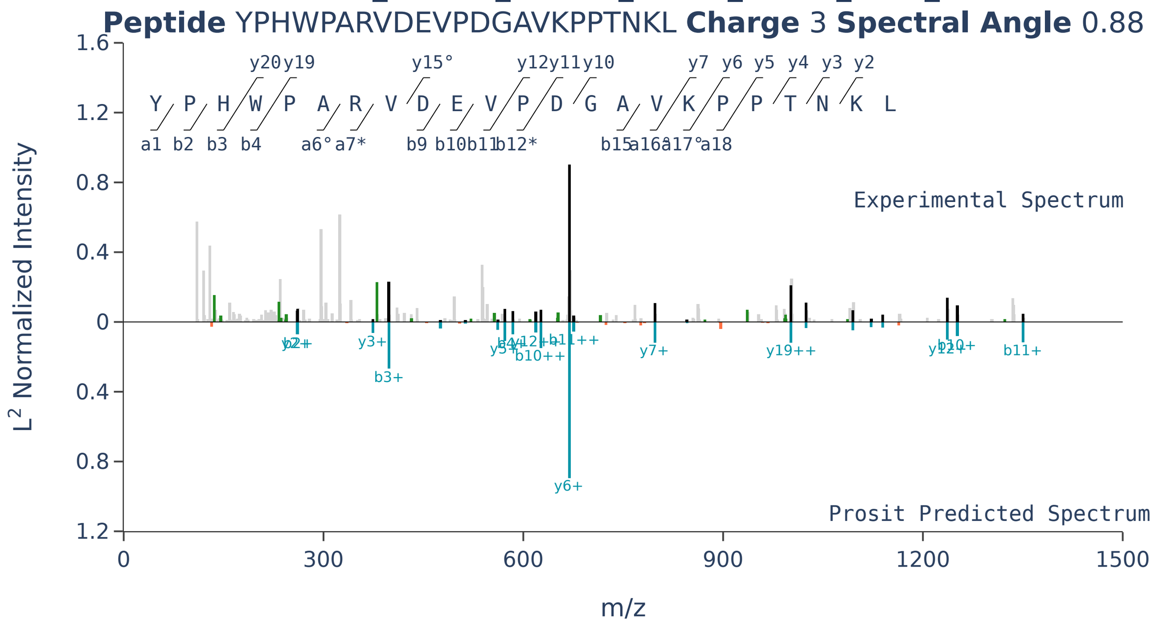

**Source** HRoetschke_271222_090123_HFGoe_LEDGF_b2R1_24h **Scan** 12991
**Peptide** SMDSRLQRIHAEIK **Charge** 3 **Spectral Angle** 0.86

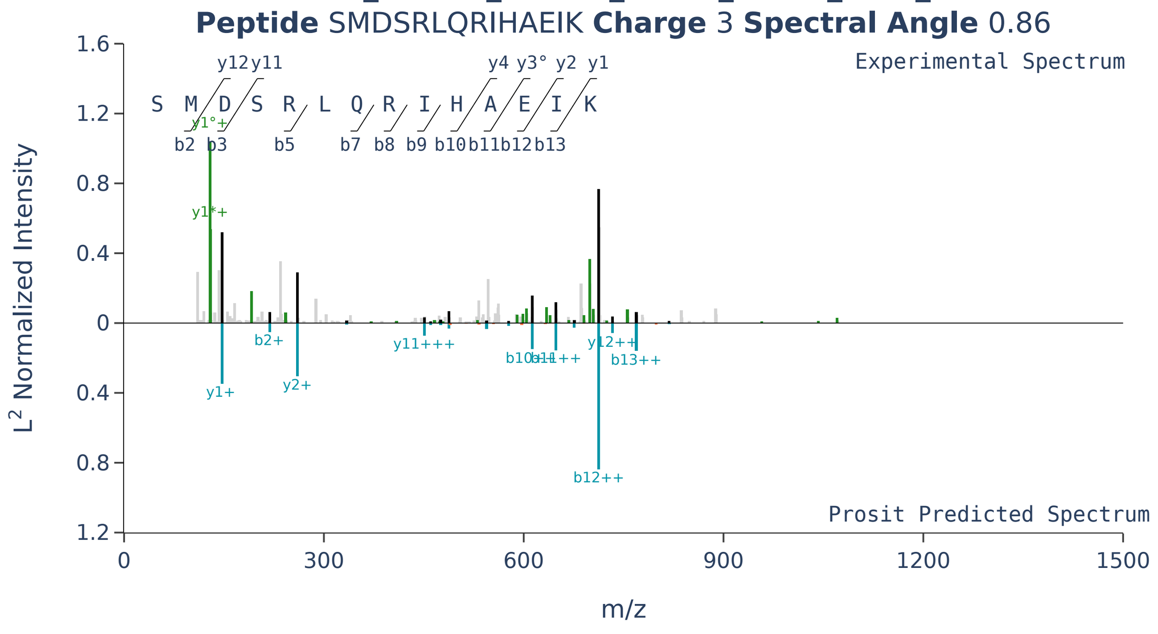


**Source** HRoetschke_271222_090123_HFGoe_LEDGF_b1R2_24h **Scan** 7552
**Peptide** TKAVDITTPKAARRG **Charge** 4 **Spectral Angle** 0.91

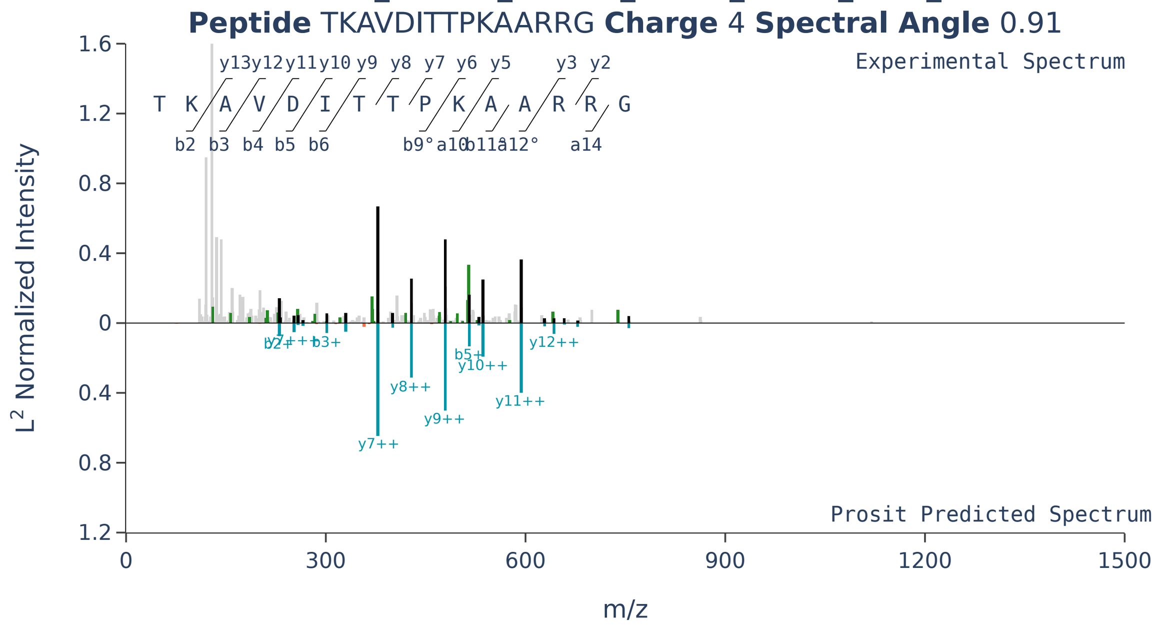


**Source** HRoetschke_271222_090123_HFGoe_LEDGF_b2R2_4h **Scan** 12807
**Peptide** EKQVETEEAGVVTTA **Charge** 2 **Spectral Angle** 0.79

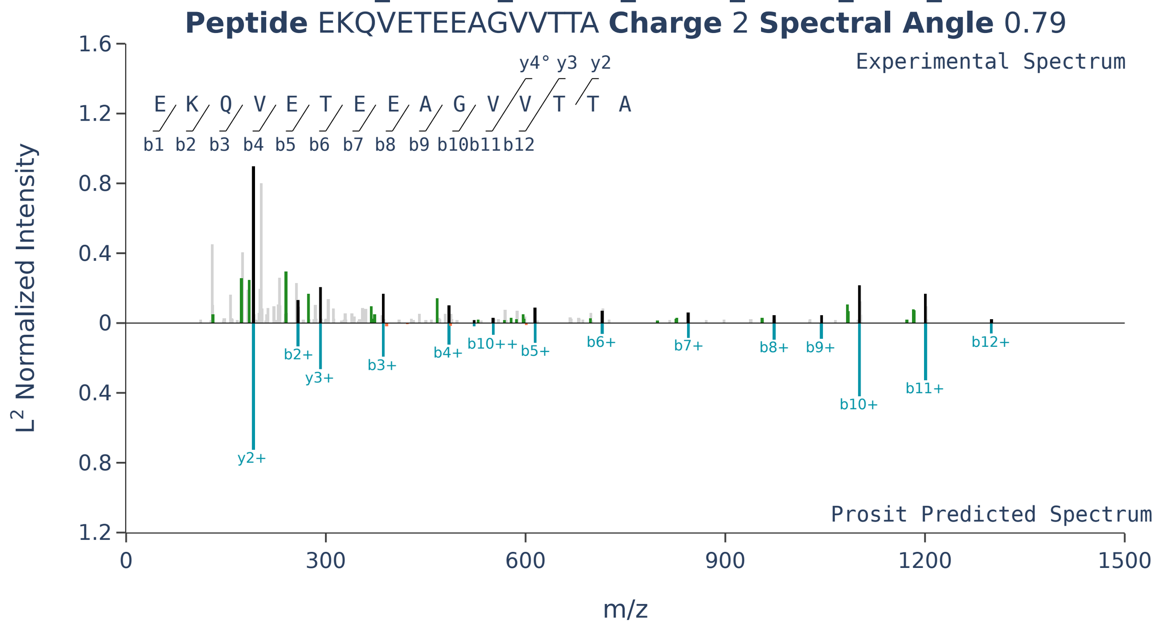


**Source** HRoetschke_271222_090123_HFGoe_LEDGF_b2R2_4h **Scan** 5904
**Peptide** SQQAATKQSNASSDV **Charge** 2 **Spectral Angle** 0.86

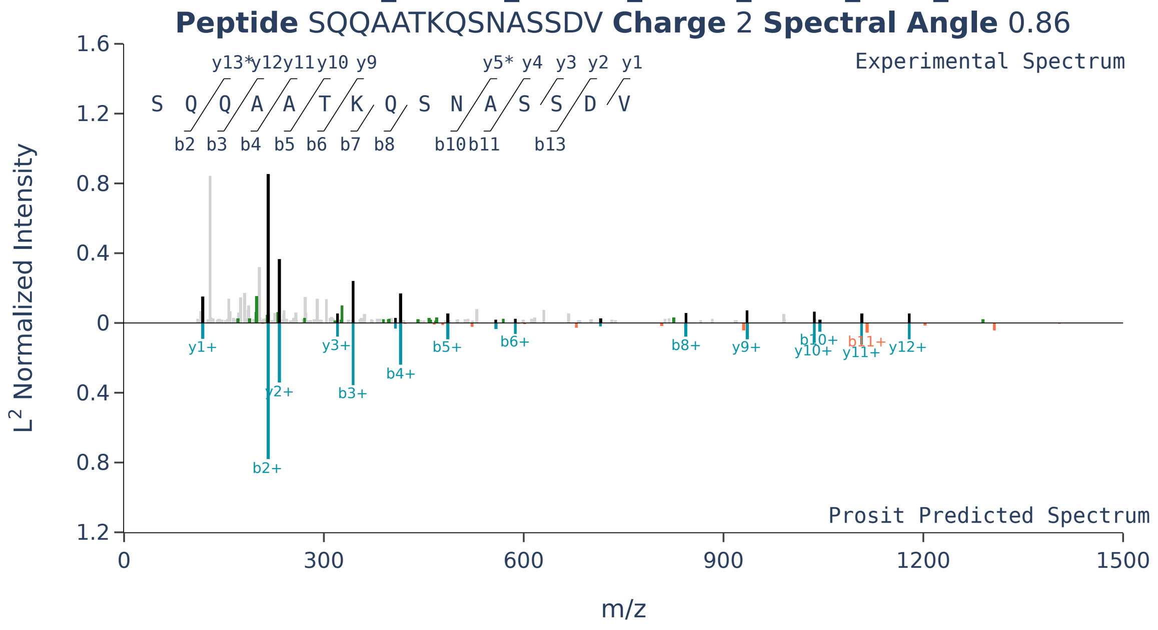


**Source** HRoetschke_271222_090123_HFGoe_LEDGF_b1R1_4h **Scan** 20271
**Peptide** YPHWPARVDEVPDG **Charge** 2 **Spectral Angle** 0.84

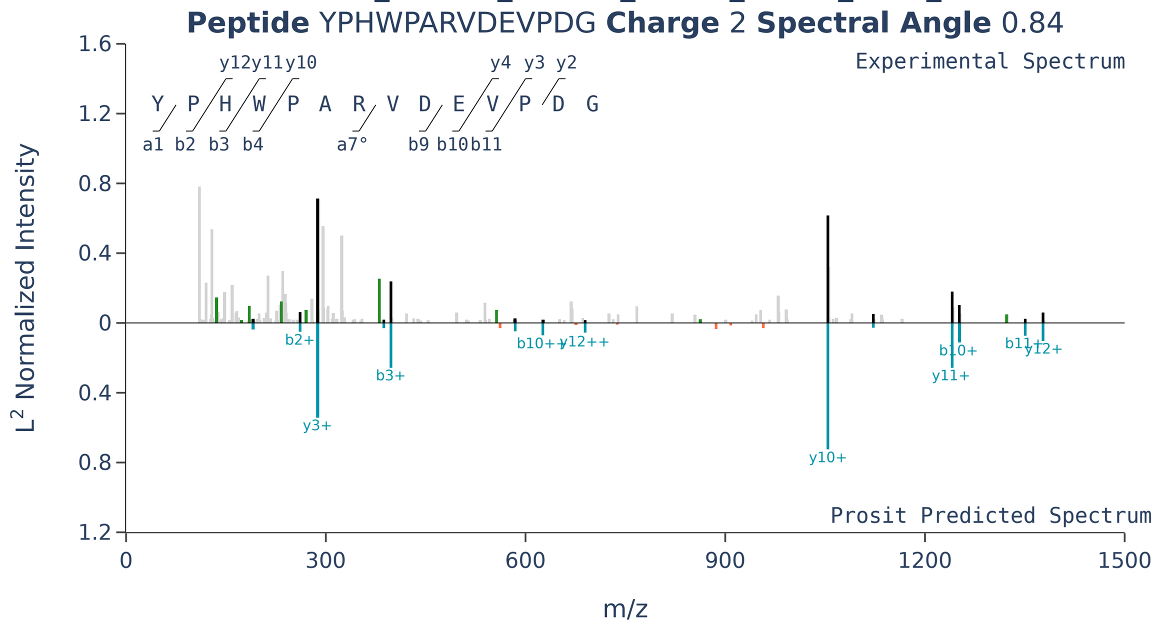


**Source** HRoetschke_271222_090123_HFGoe_LEDGF_b1R2_24h **Scan** 6028
**Peptide** FPYSENKEKYGKPNKRK **Charge** 5 **Spectral Angle** 0.86

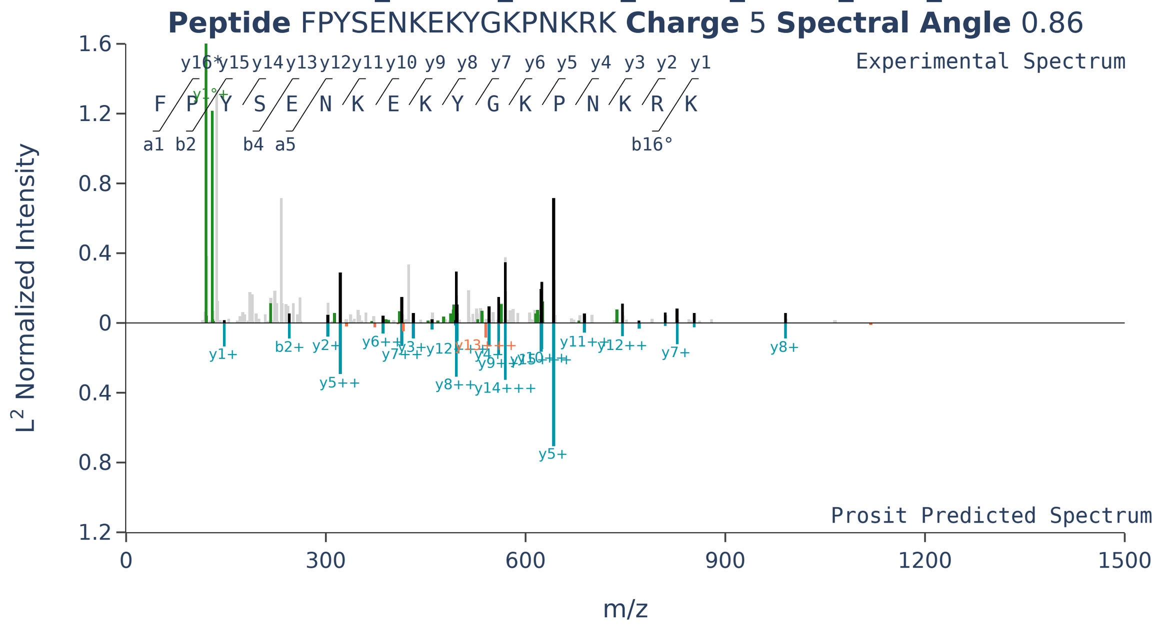


**Source** HRoetschke_271222_090123_HFGoe_LEDGF_b2R2_2h **Scan** 20943
**Peptide** RDFKPGDLI **Charge** 2 **Spectral Angle** 0.84

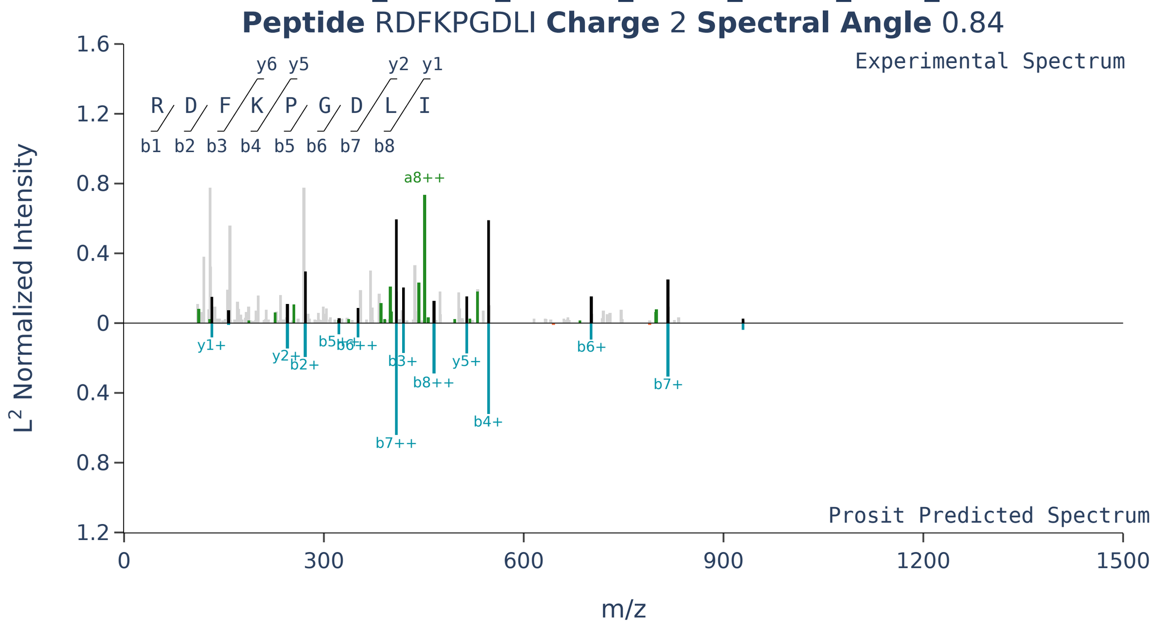


**Source** HRoetschke_271222_090123_HFGoe_LEDGF_b1R1_2h **Scan** 23137
**Peptide** LASLQVTMQ **Charge** 2 **Spectral Angle** 0.95

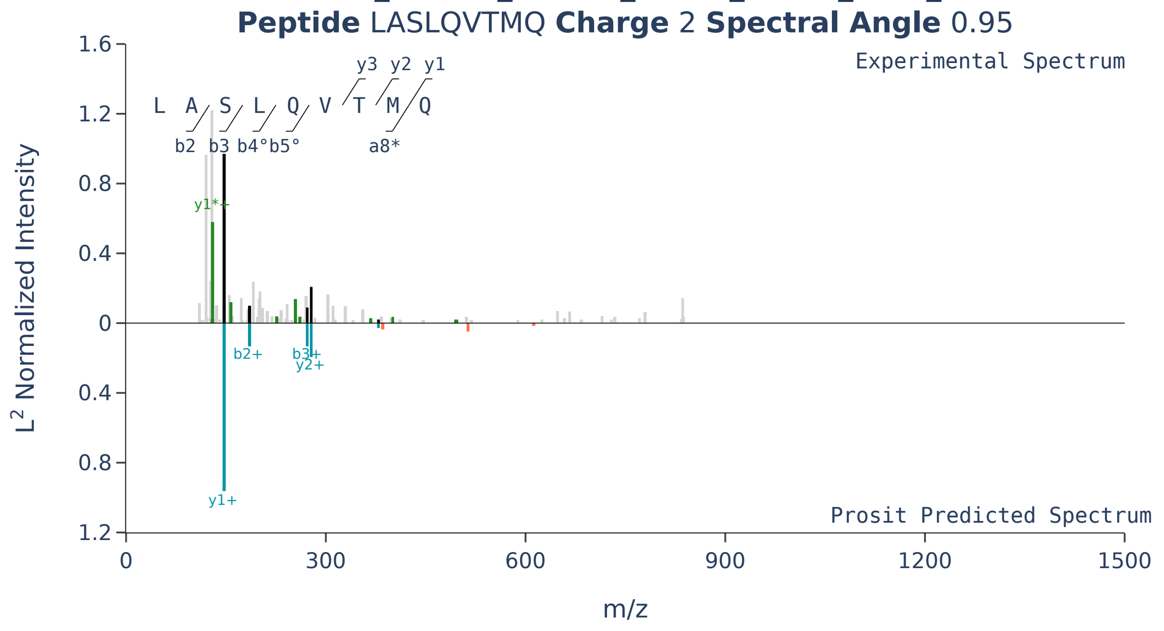


**Source** HRoetschke_271222_090123_HFGoe_LEDGF_b1R1_4h **Scan** 19587
**Peptide** IMEKSTMLYNKFKNM **Charge** 2 **Spectral Angle** 0.78

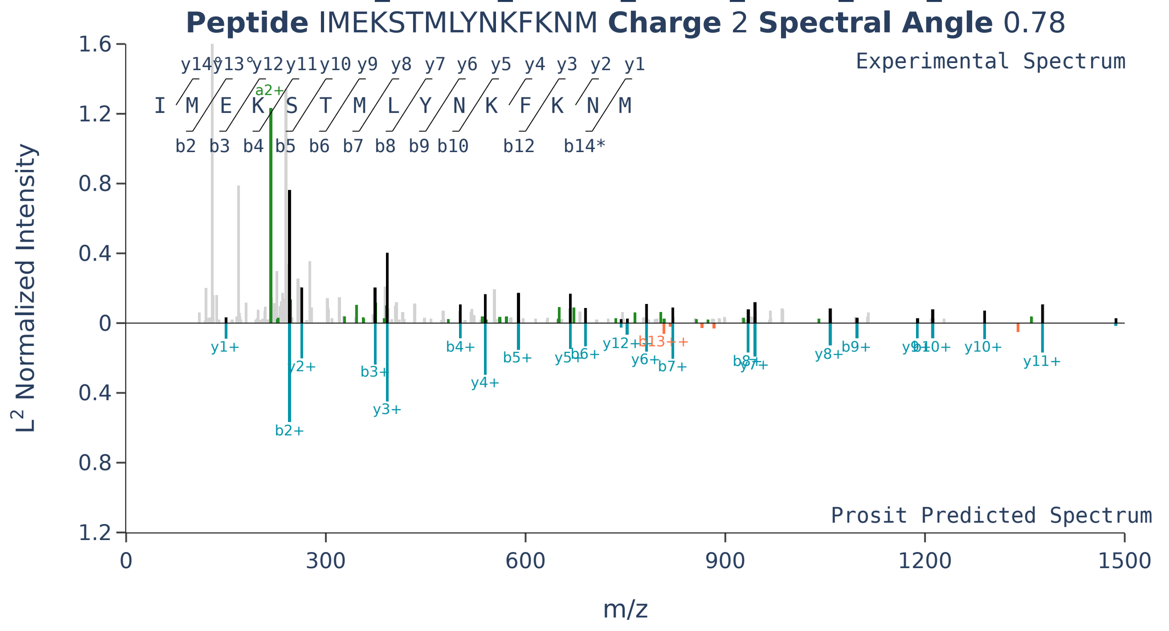


**Source** HRoetschke_271222_090123_HFGoe_LEDGF_b2R2_2h **Scan** 6658
**Peptide** VKIPKPRGRPKM **Charge** 4 **Spectral Angle** 0.95

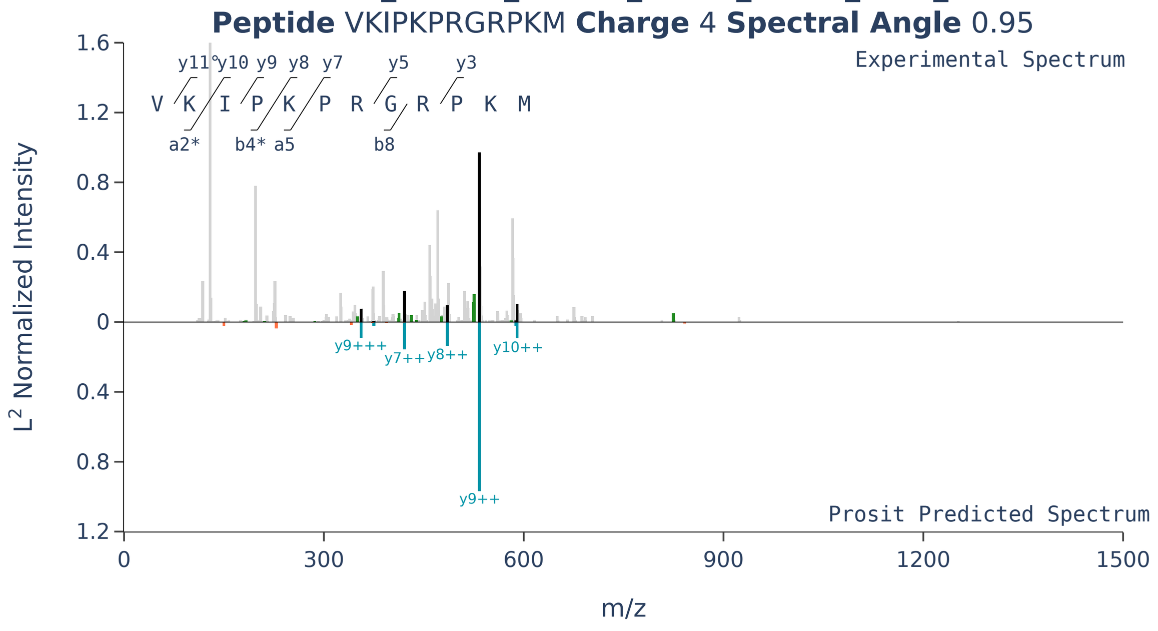


**Source** HRoetschke_271222_090123_HFGoe_LEDGF_b1R2_4h **Scan** 18945
**Peptide** QVIMEKSTMLYNKFK **Charge** 3 **Spectral Angle** 0.8

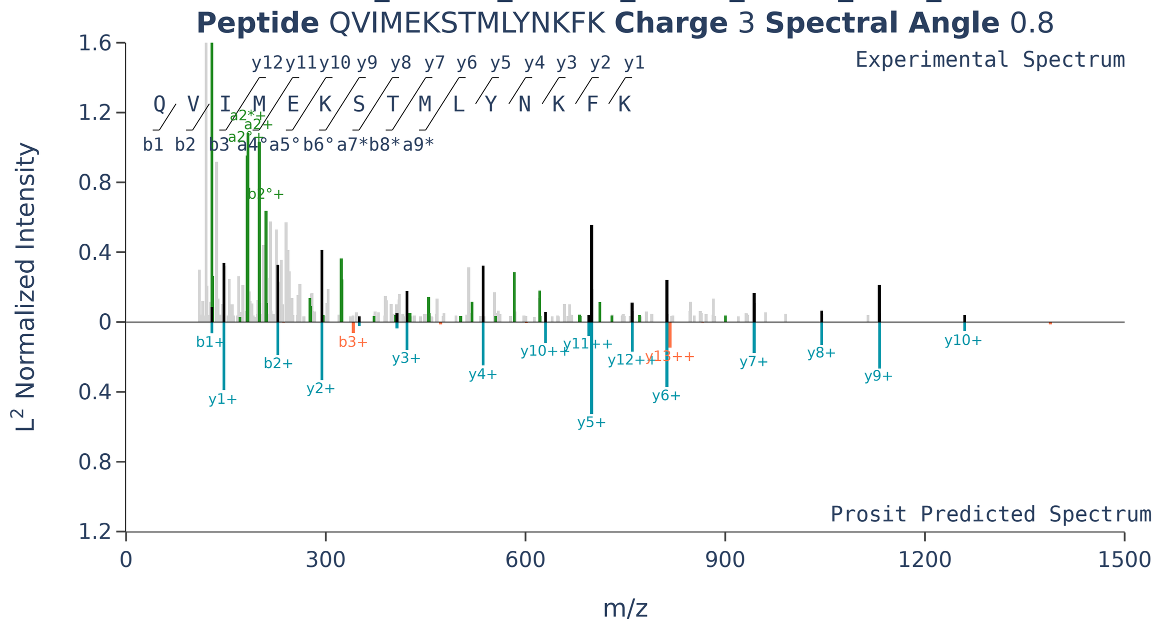


**Source** HRoetschke_271222_090123_HFGoe_LEDGF_b1R2_2h **Scan** 8949
**Peptide** PKDIFPYSENKEKYGKPNKR **Charge** 3 **Spectral Angle** 0.86

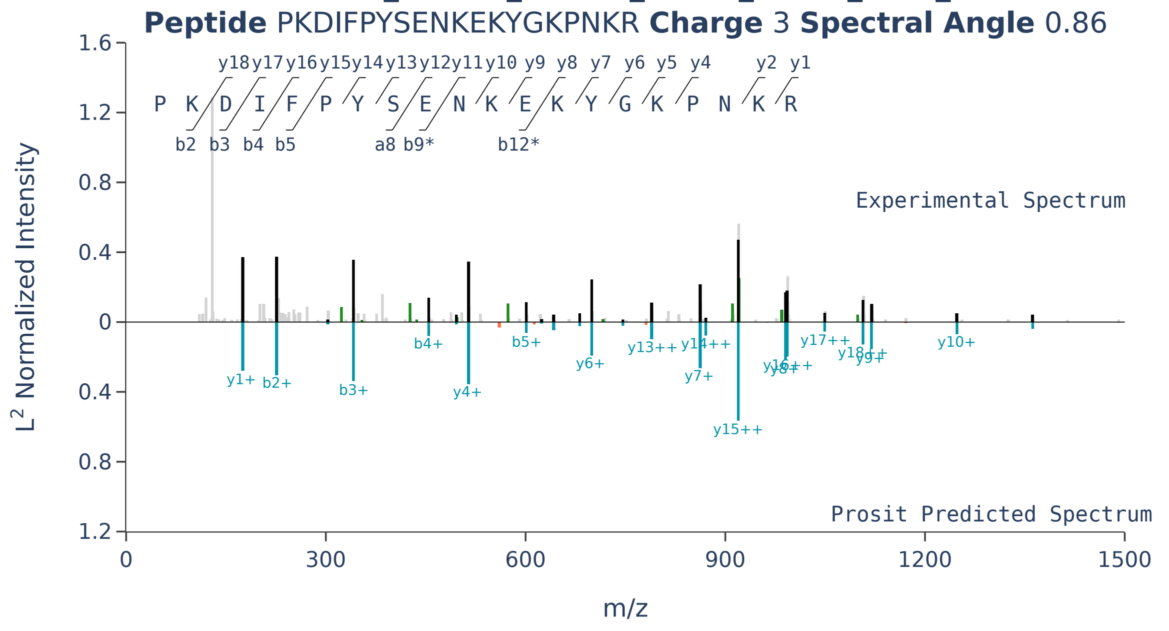


**Source** HRoetschke_271222_090123_HFGoe_LEDGF_b1R2_2h **Scan** 5913
**Peptide** AKTGVTSTSDSEEEGD **Charge** 2 **Spectral Angle** 0.72

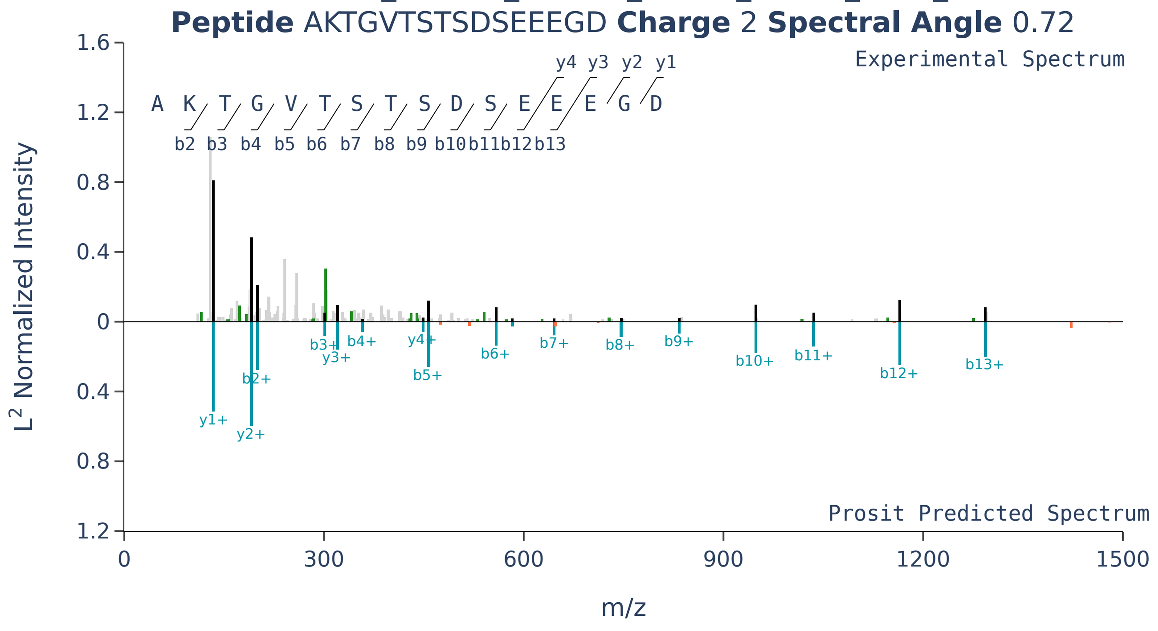


**Source** HRoetschke_271222_090123_HFGoe_LEDGF_b2R2_2h **Scan** 22615
**Peptide** KNMFLVG **Charge** 2 **Spectral Angle** 0.82

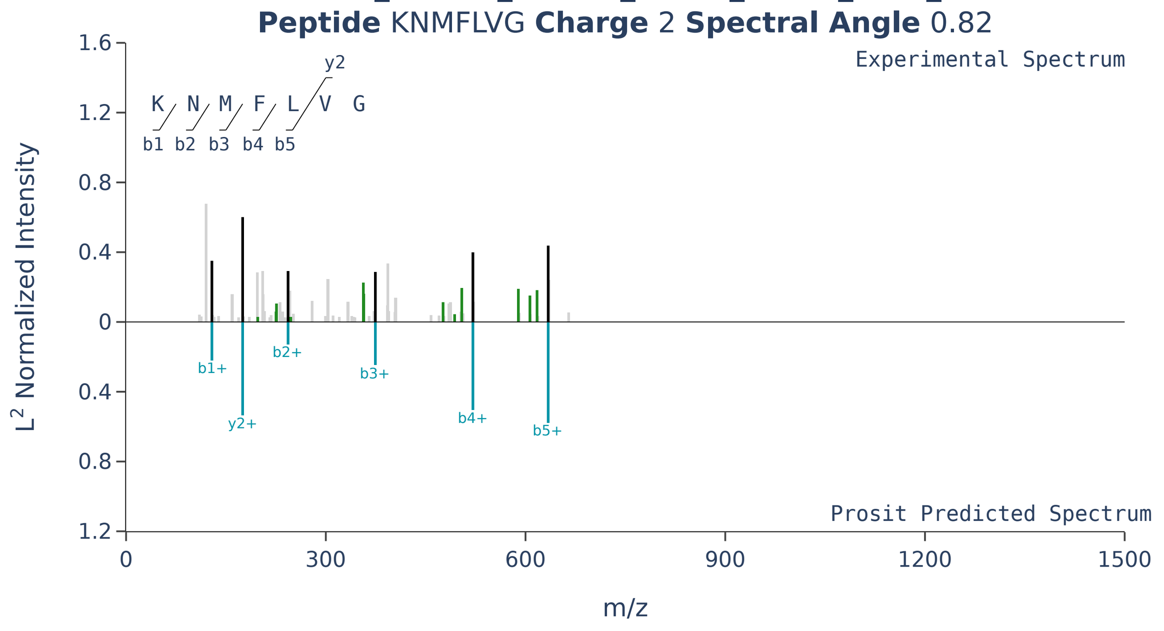


**Source** HRoetschke_271222_090123_HFGoe_LEDGF_b2R2_4h **Scan** 21199
**Peptide** SVITQVLNKS **Charge** 2 **Spectral Angle** 0.77

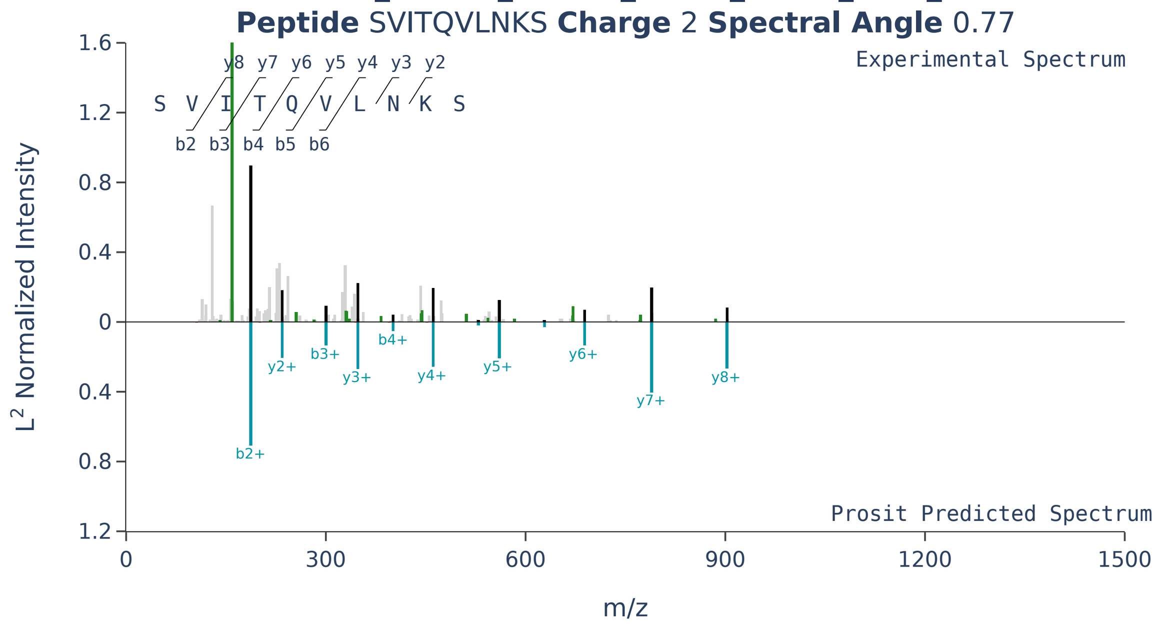

**Source** HRoetschke_271222_090123_HFGoe_LEDGF_b1R1_2h **Scan** 9771
**Peptide** DSRLQRIHAEIKN **Charge** 4 **Spectral Angle** 0.82

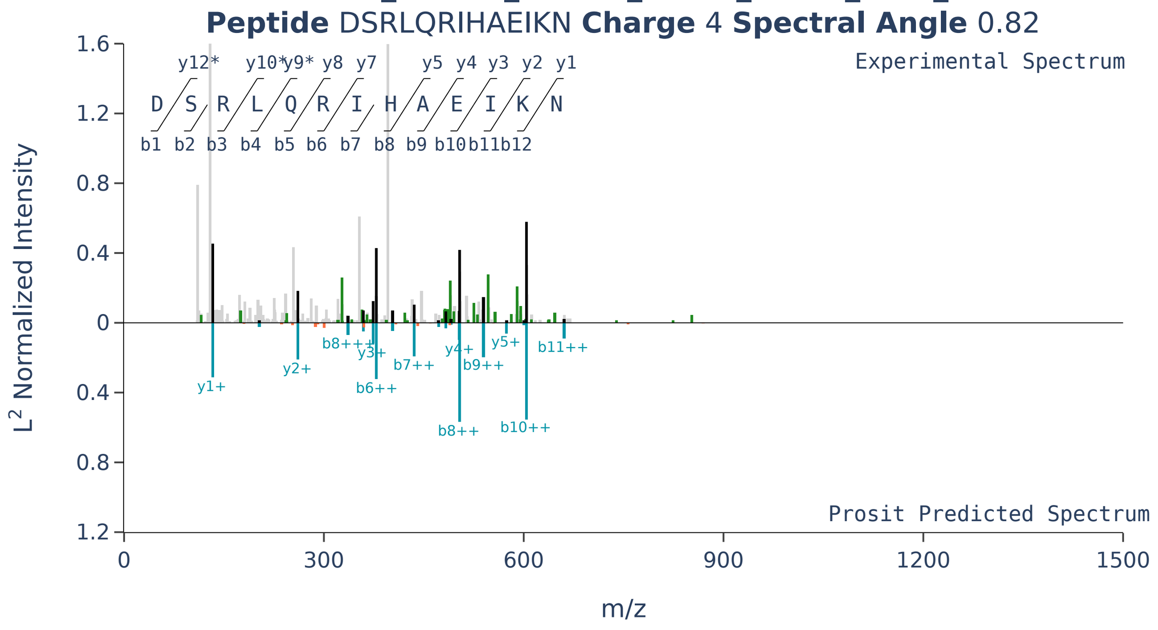


**Source** HRoetschke_271222_090123_HFGoe_LEDGF_b1R1_2h **Scan** 20545
**Peptide** LGPKDIFPYSENKEKY **Charge** 3 **Spectral Angle** 0.85

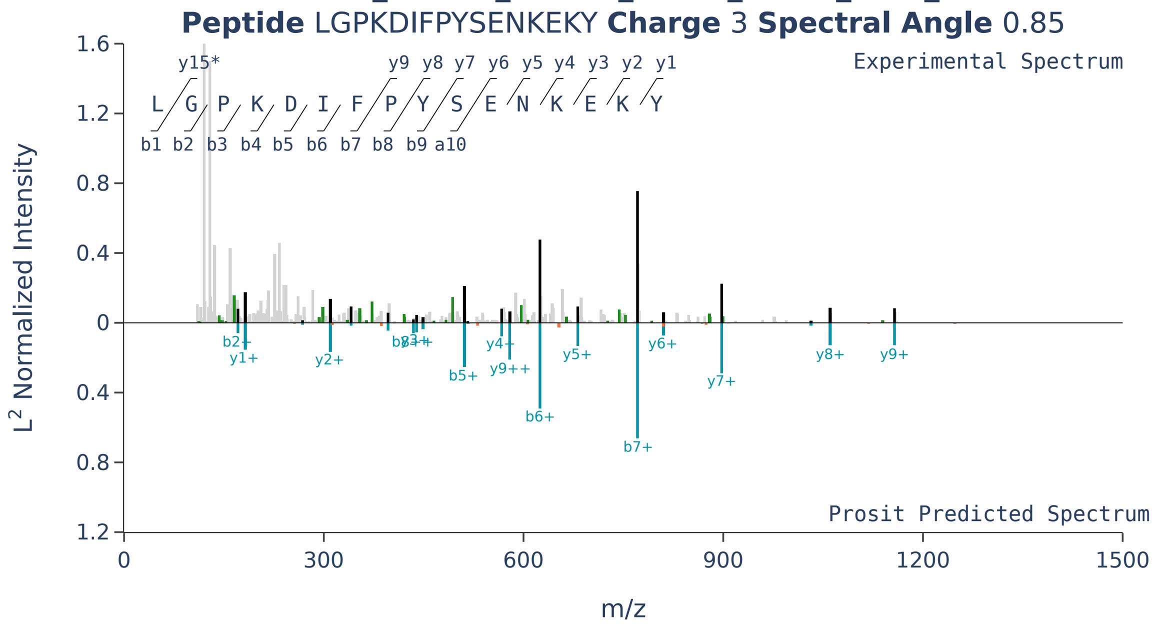


**Source** HRoetschke_271222_090123_HFGoe_LEDGF_b1R2_4h **Scan** 29953
**Peptide** PIFFFGTHETA **Charge** 2 **Spectral Angle** 0.79

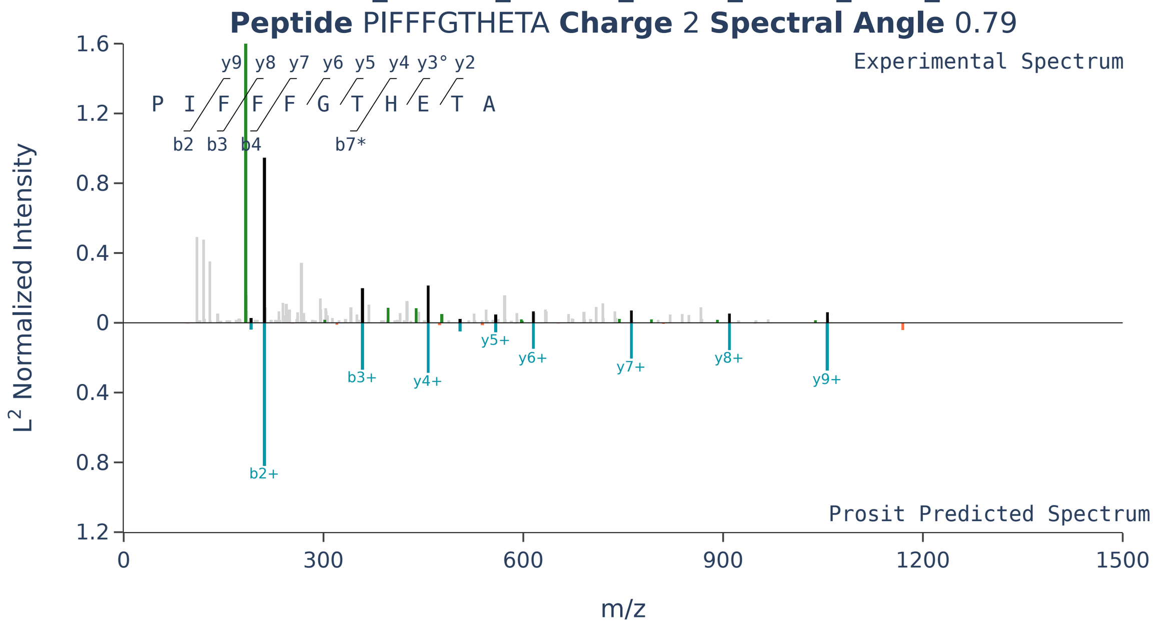


**Source** HRoetschke_271222_090123_HFGoe_LEDGF_b1R2_2h **Scan** 9787
**Peptide** KFSSQQAATKQSNASSDVEVEE **Charge** 3 **Spectral Angle** 0.88

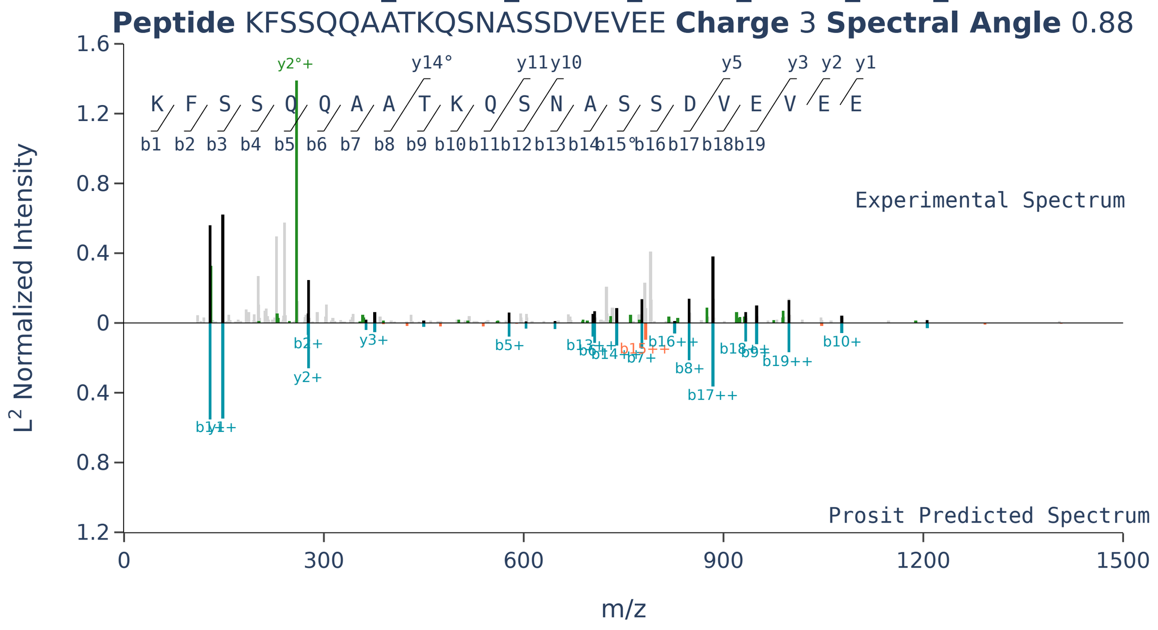


**Source** HRoetschke_271222_090123_HFGoe_LEDGF_b1R1_1h **Scan** 21567
**Peptide** FNEGLWEIDNNPKVKFSSQQAATKQS **Charge** 3 **Spectral Angle** 0.9

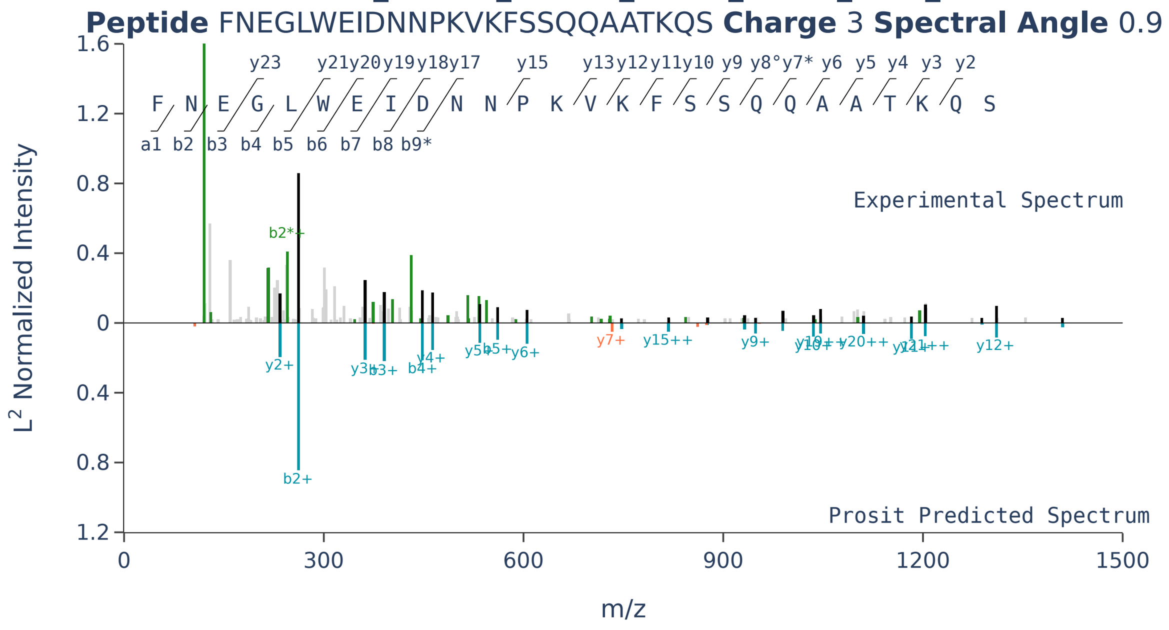


**Source** HRoetschke_271222_090123_HFGoe_LEDGF_b1R1_2h **Scan** 21326
**Peptide** WEIDNNPKVKF **Charge** 3 **Spectral Angle** 0.83

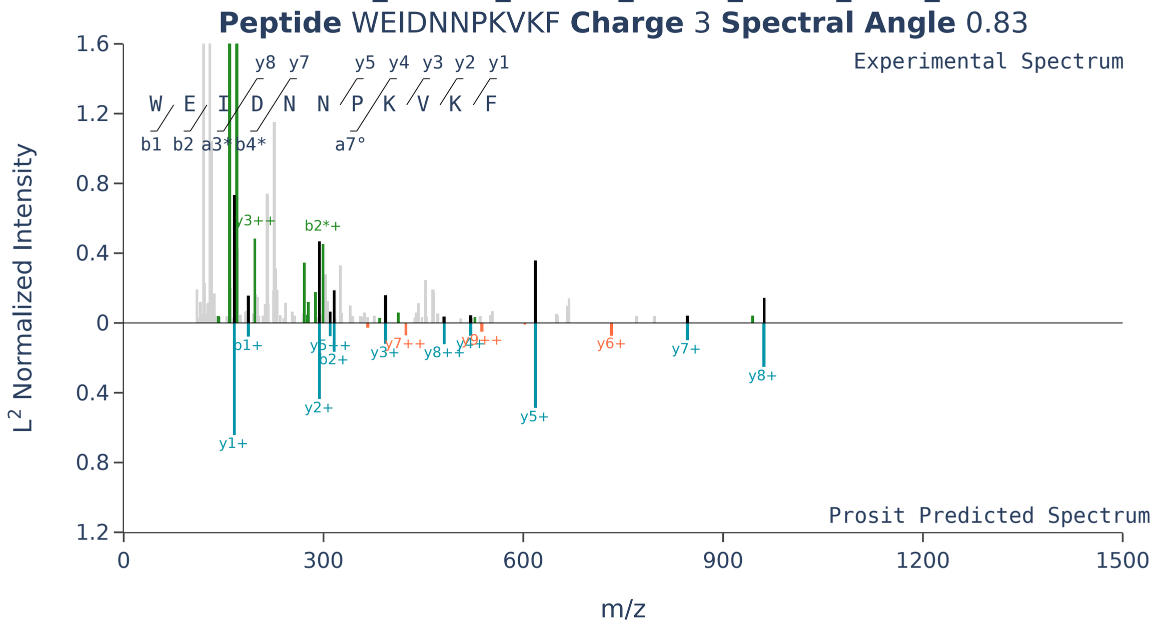


**Source** HRoetschke_271222_090123_HFGoe_LEDGF_b1R2_24h **Scan** 5834
**Peptide** LNKSLAEQRQH **Charge** 3 **Spectral Angle** 0.83

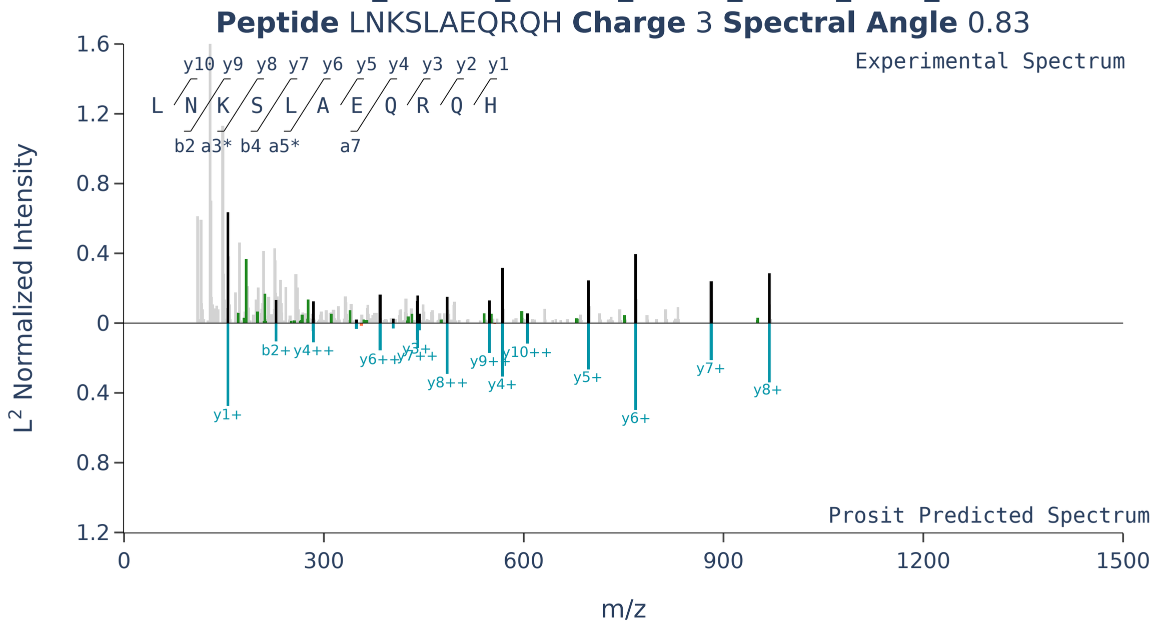


**Source** HRoetschke_271222_090123_HFGoe_LEDGF_b1R1_4h **Scan** 16220
**Peptide** RIHAEIKNSLKIDNLD **Charge** 4 **Spectral Angle** 0.86

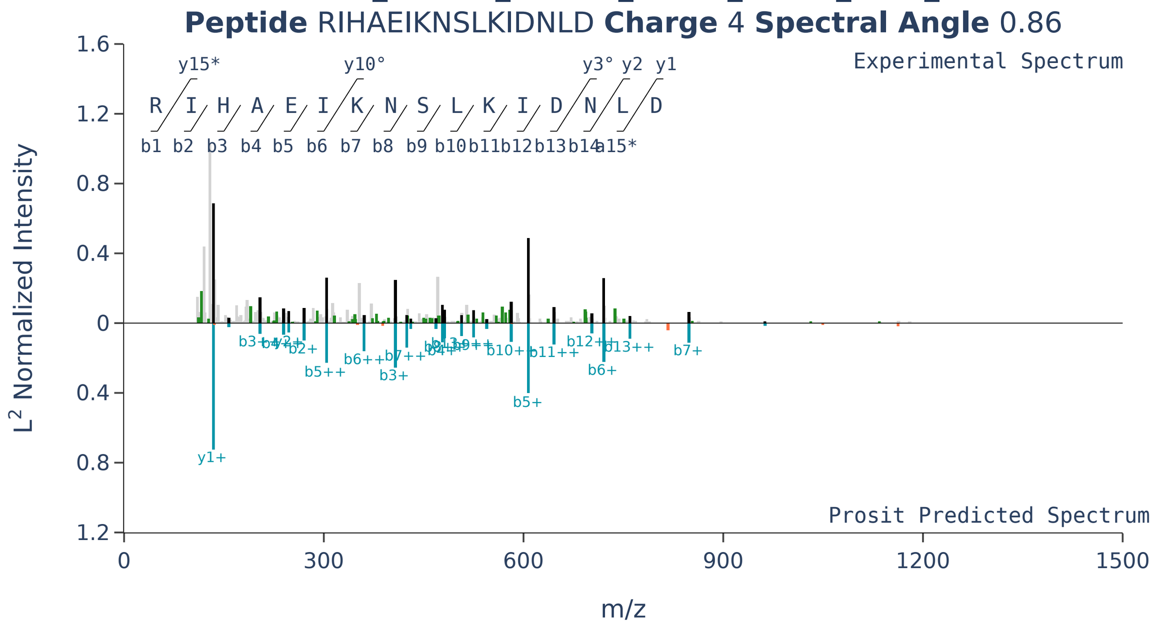


**Source** HRoetschke_271222_090123_HFGoe_LEDGF_b1R1_4h **Scan** 6733
**Peptide** RKRKAEKQVETEEAGVVTT **Charge** 3 **Spectral Angle** 0.82

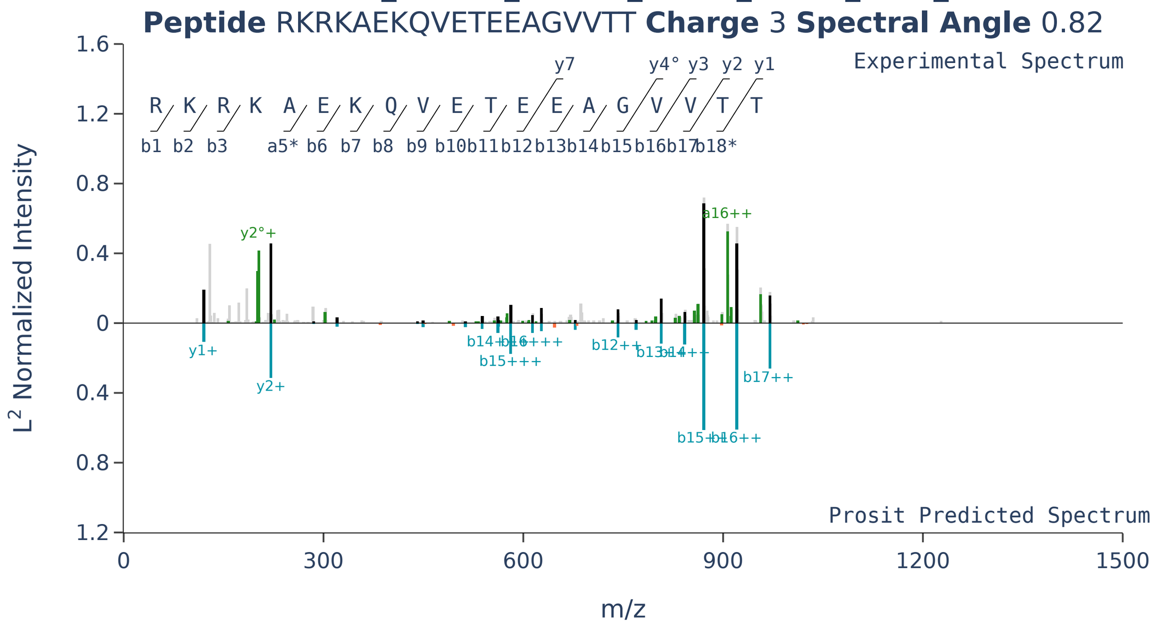


**Source** HRoetschke_271222_090123_HFGoe_LEDGF_b2R2_2h **Scan** 7345
**Peptide** AATKQSNASSDVEVEEKE **Charge** 2 **Spectral Angle** 0.84

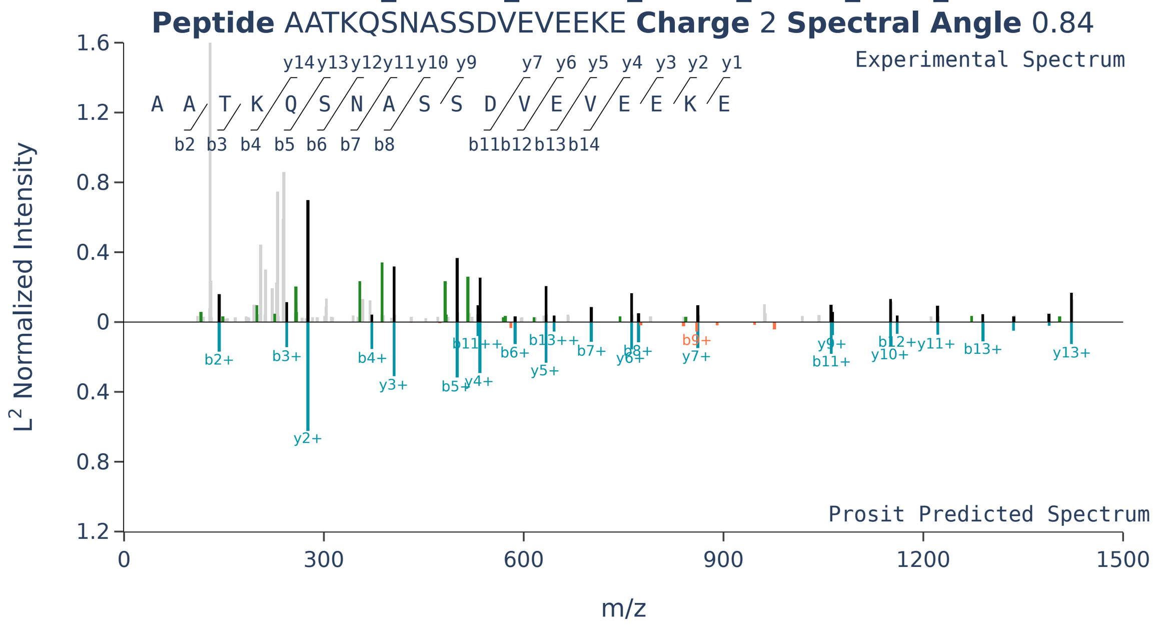


**Source** HRoetschke_271222_090123_HFGoe_LEDGF_b1R2_24h **Scan** 7226
**Peptide** KAVDITTPKAARR **Charge** 4 **Spectral Angle** 0.69

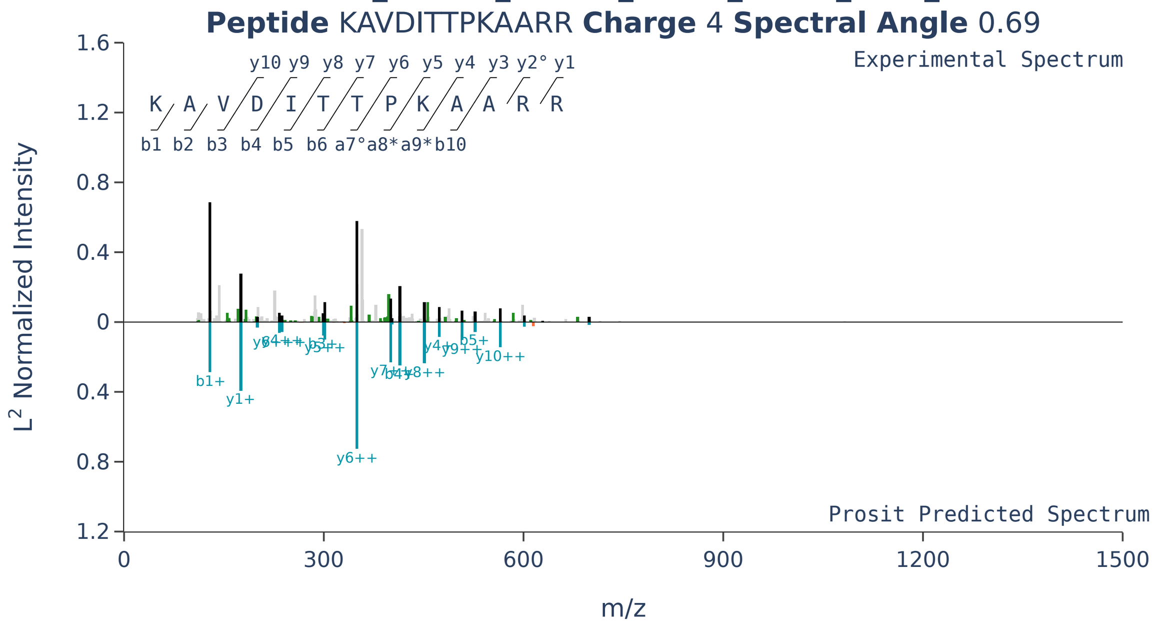


**Source** HRoetschke_271222_090123_HFGoe_LEDGF_b2R1_24h **Scan** 24086
**Peptide** ELASLQVTMQQ **Charge** 1 **Spectral Angle** 0.76

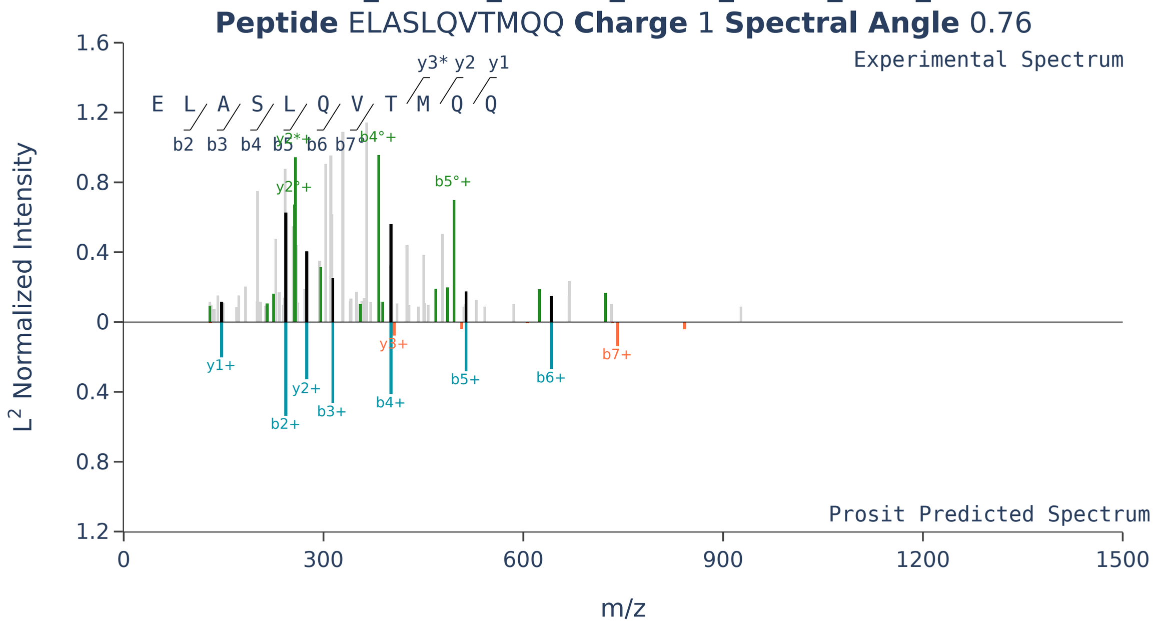


**Source** HRoetschke_271222_090123_HFGoe_LEDGF_b1R1_24h **Scan** 16223
**Peptide** WEIDNNPKVKFSSQQAATK **Charge** 3 **Spectral Angle** 0.81

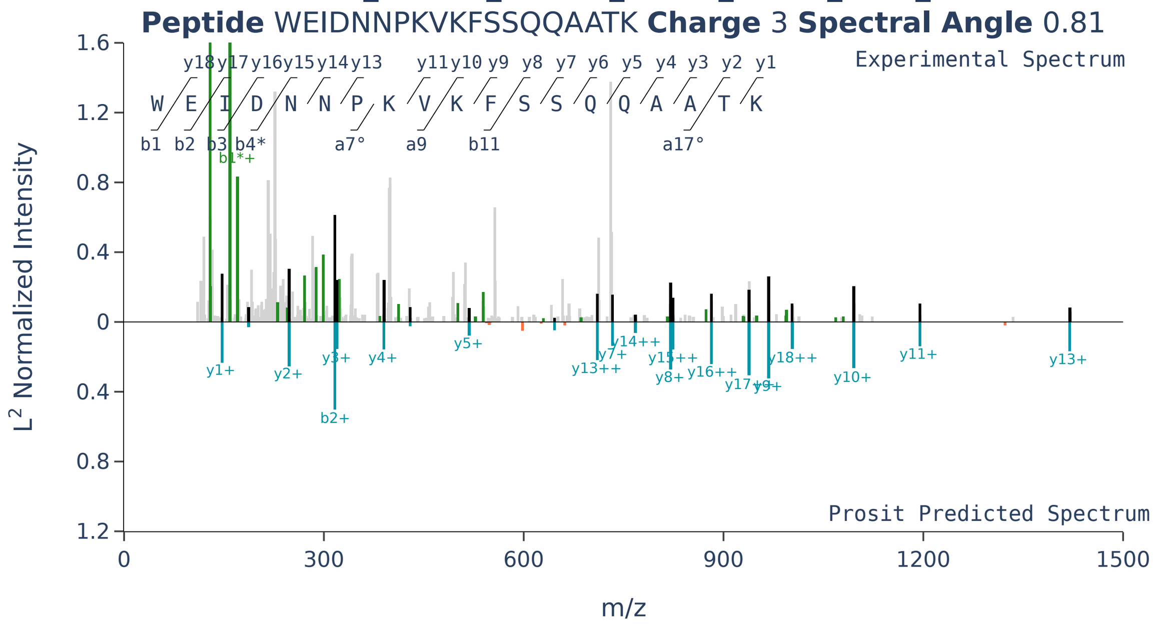


**Source** HRoetschke_271222_090123_HFGoe_LEDGF_b1R2_4h **Scan** 18783
**Peptide** GYPHWPARVDEVPDGAVKPPTNK **Charge** 3 **Spectral Angle** 0.9

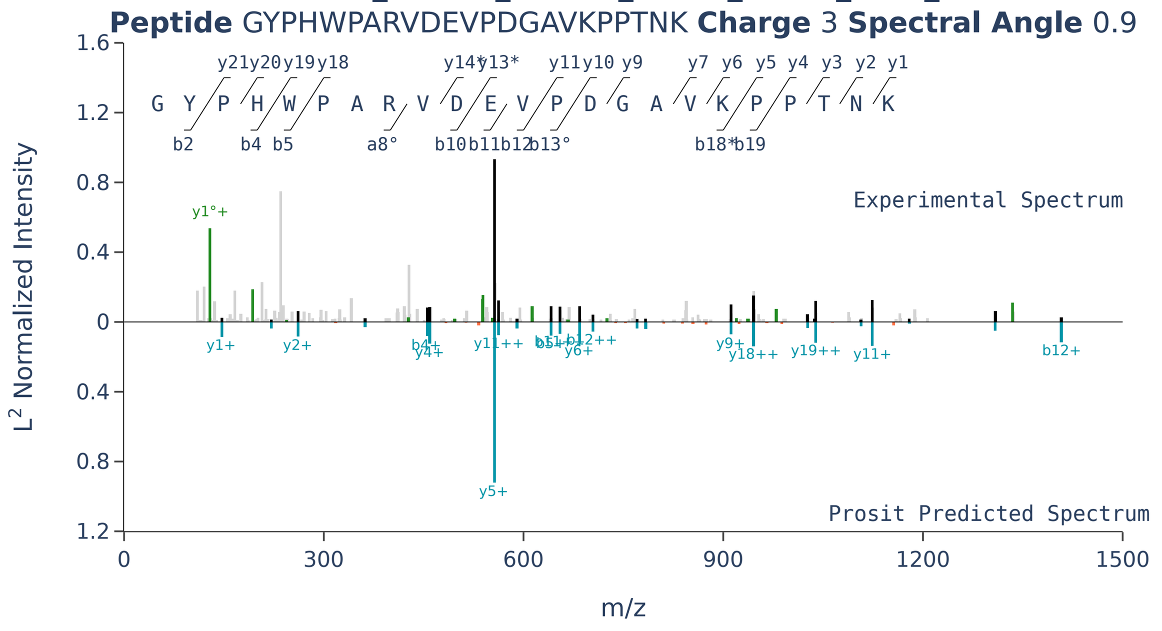


**Source** HRoetschke_271222_090123_HFGoe_LEDGF_b2R1_2h **Scan** 6739
**Peptide** NNPKVKFSSQQAATKQ **Charge** 2 **Spectral Angle** 0.85

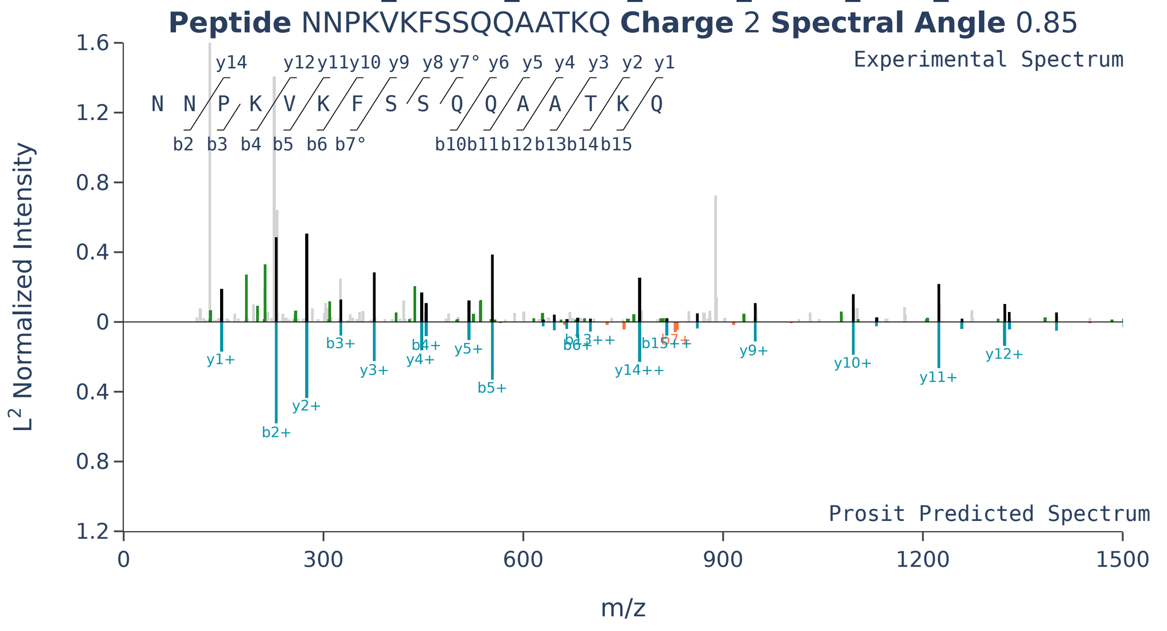

**Source** HRoetschke_271222_090123_HFGoe_LEDGF_b2R1_24h **Scan** 11798
**Peptide** GPGMTRDFKPGD **Charge** 2 **Spectral Angle** 0.77

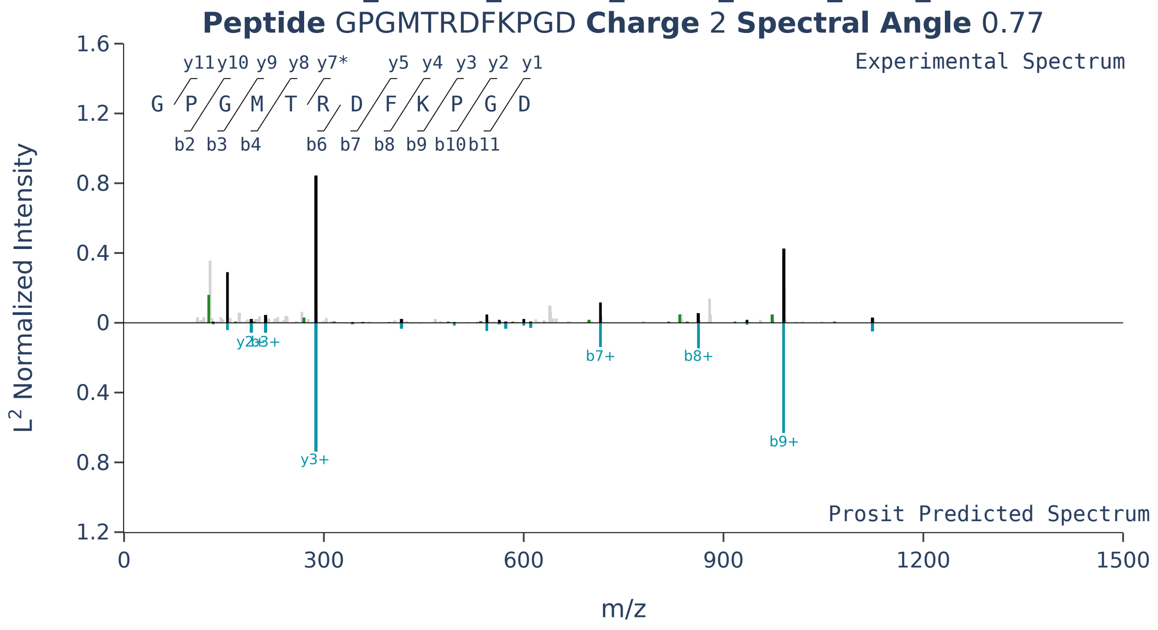


**Source** HRoetschke_271222_090123_HFGoe_LEDGF_b2R1_4h **Scan** 12773
**Peptide** MDSRLQRI **Charge** 2 **Spectral Angle** 0.94

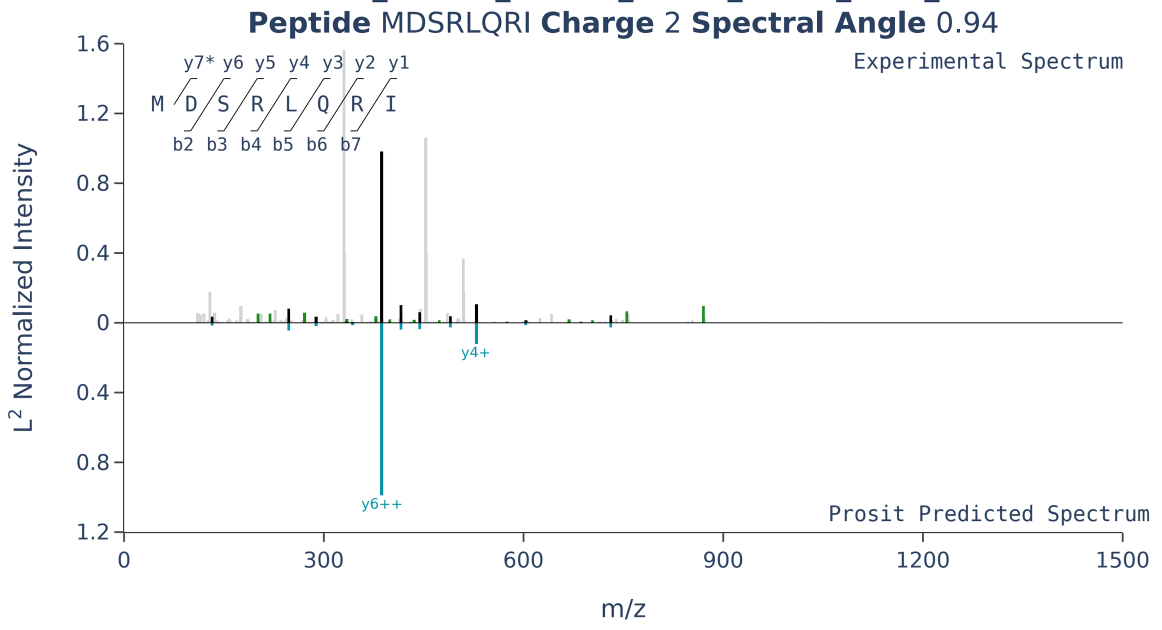


**Source** HRoetschke_271222_090123_HFGoe_LEDGF_b1R1_24h **Scan** 12484
**Peptide** QVLNKSL **Charge** 1 **Spectral Angle** 0.77

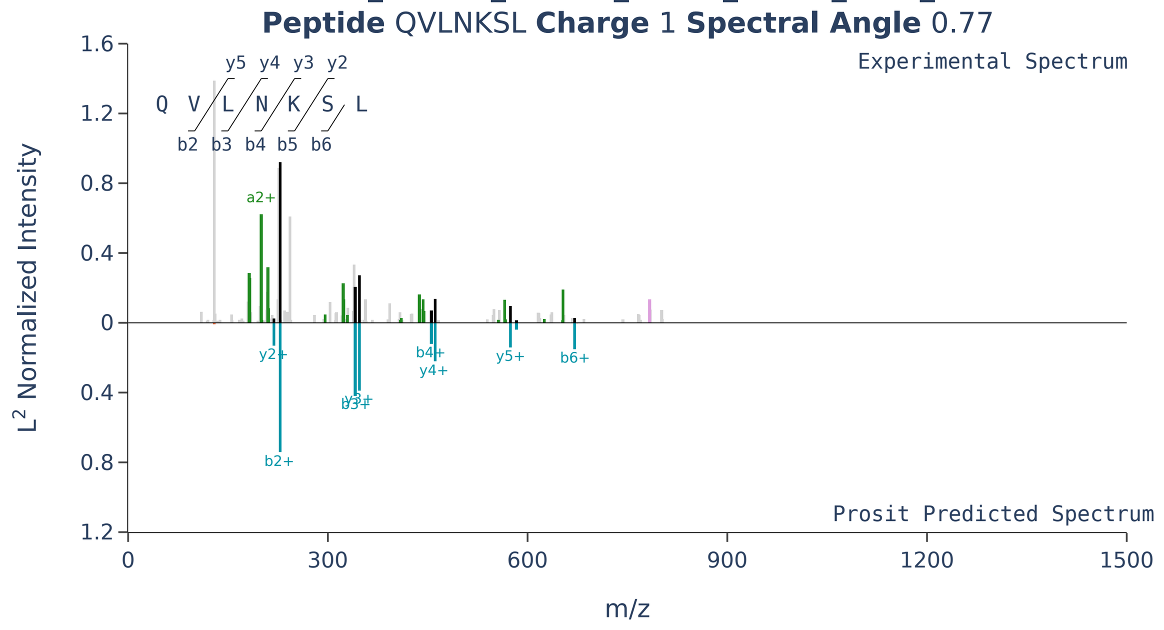


**Source** HRoetschke_271222_090123_HFGoe_LEDGF_b1R2_24h **Scan** 12418
**Peptide** SVNLKVS **Charge** 2 **Spectral Angle** 0.92

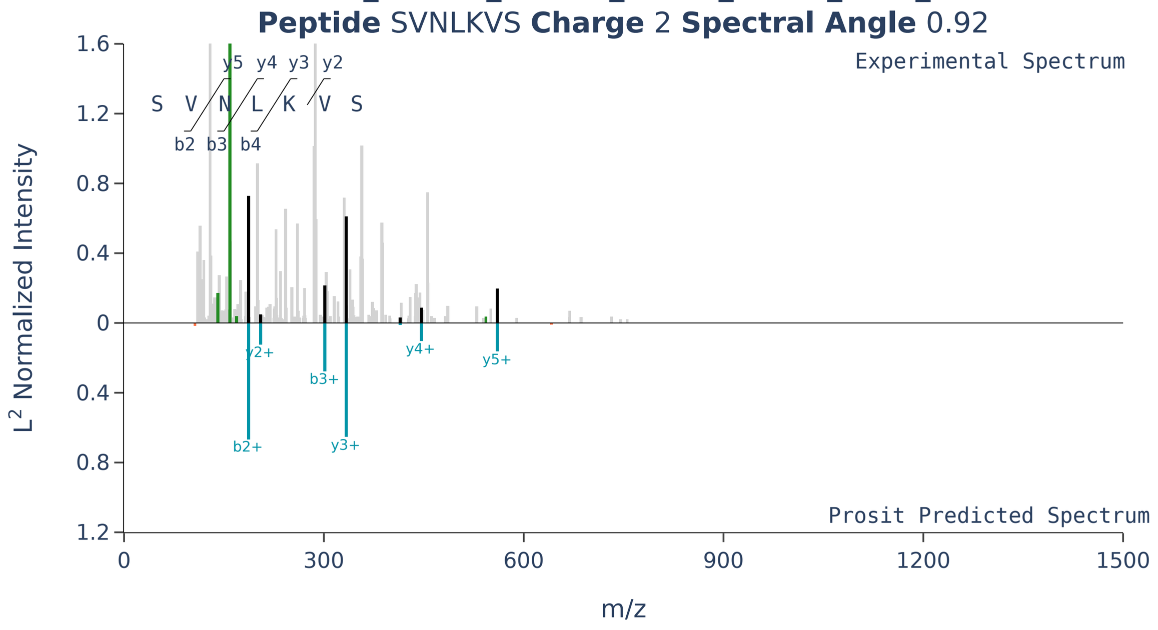


**Source** HRoetschke_271222_090123_HFGoe_LEDGF_b2R1_2h **Scan** 16083
**Peptide** KMKGYPHWPARVDEVPDG **Charge** 3 **Spectral Angle** 0.85

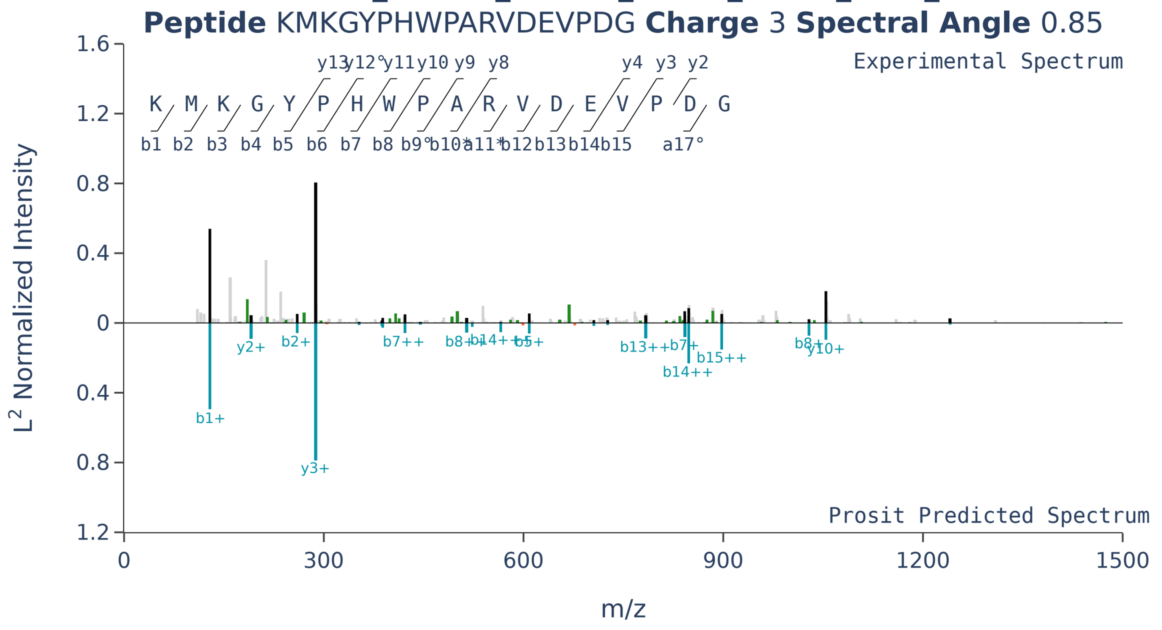


**Source** HRoetschke_271222_090123_HFGoe_LEDGF_b1R2_24h **Scan** 14876
**Peptide** LGPKDIFPYSENKEKYGKPNKRKGF **Charge** 5 **Spectral Angle** 0.85

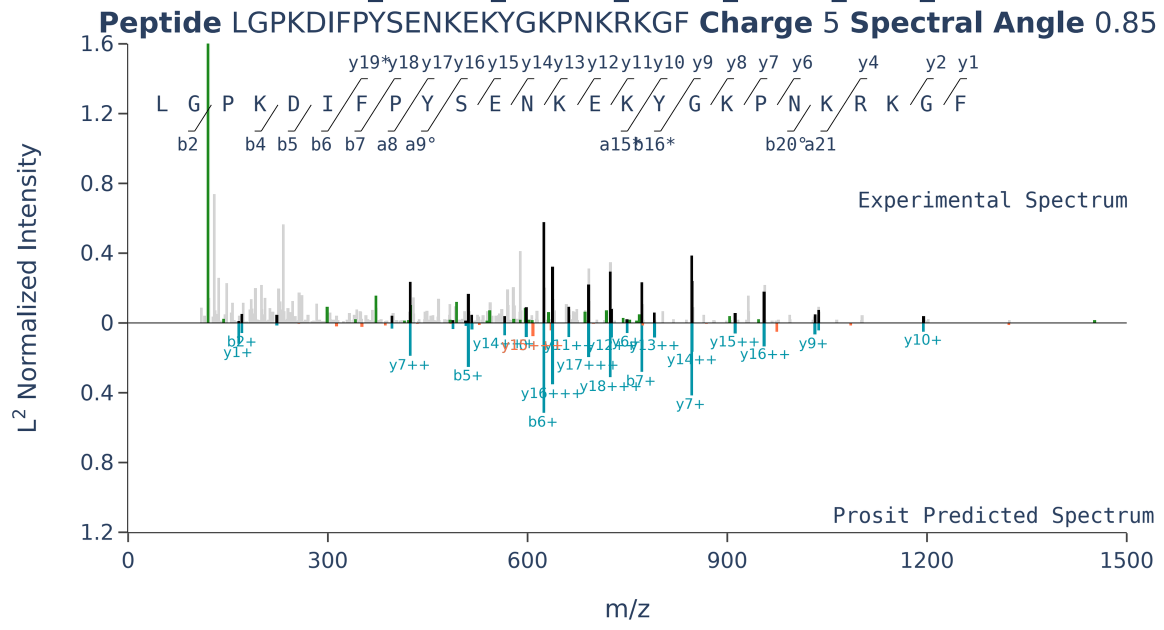


**Source** HRoetschke_271222_090123_HFGoe_LEDGF_b1R1_24h **Scan** 10883
**Peptide** MDSRLQRIHA **Charge** 2 **Spectral Angle** 0.9

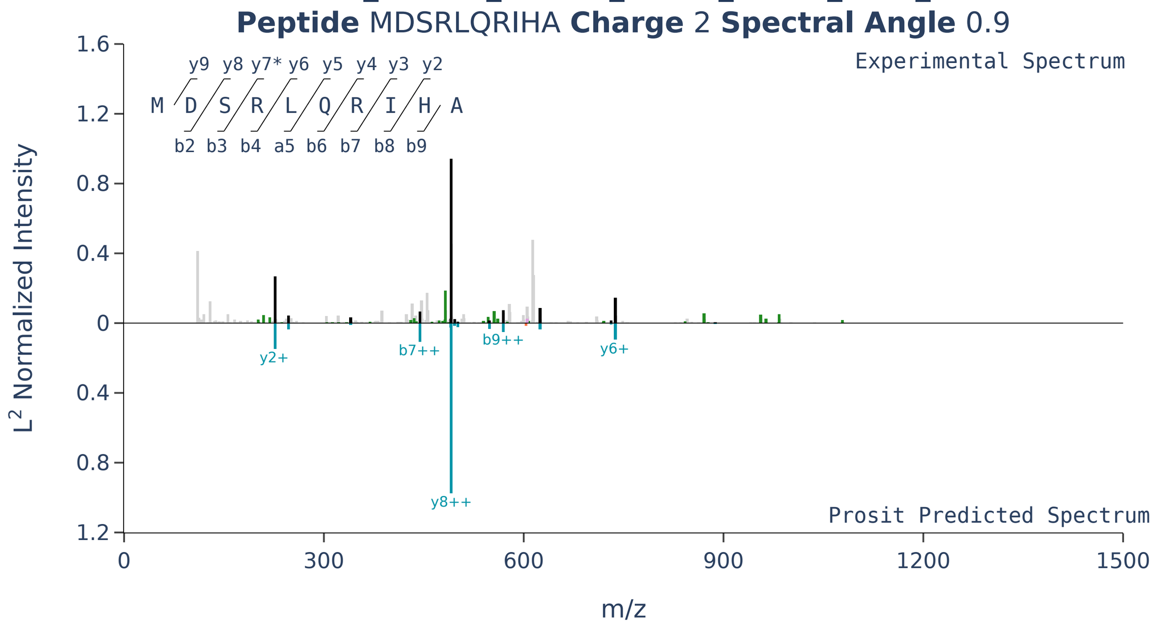


**Source** HRoetschke_271222_090123_HFGoe_LEDGF_b1R1_24h **Scan** 12003
**Peptide** ASLQVTMQQ **Charge** 2 **Spectral Angle** 0.81

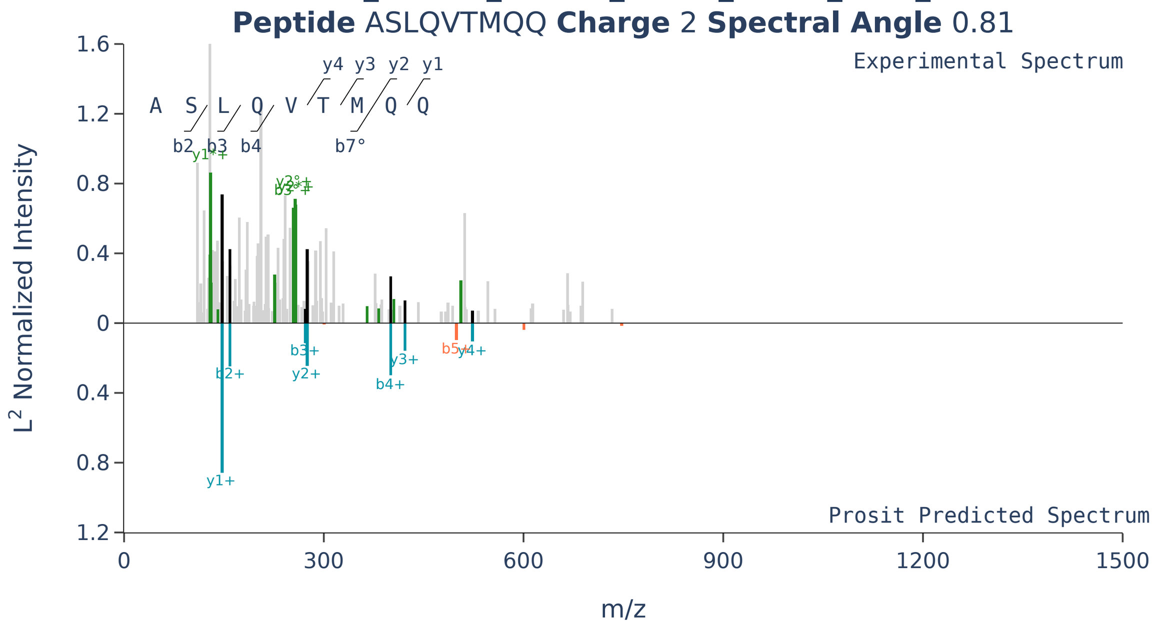


**Source** HRoetschke_271222_090123_HFGoe_LEDGF_b2R1_24h **Scan** 24036
**Peptide** GEGDSVITQVLNK **Charge** 2 **Spectral Angle** 0.74

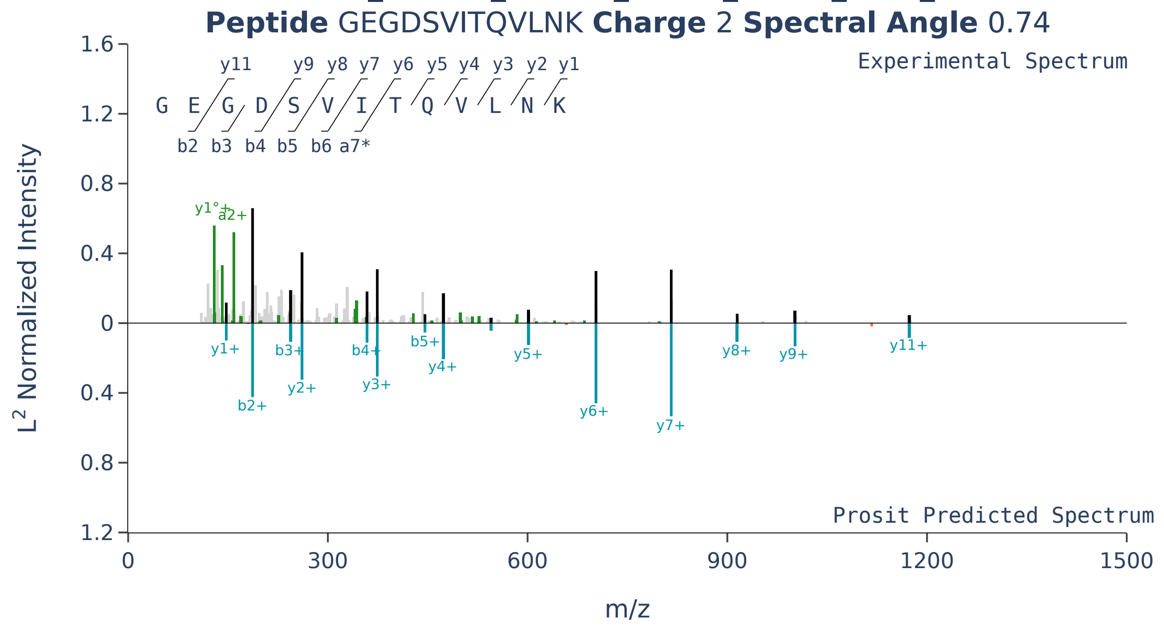


**Source** HRoetschke_271222_090123_HFGoe_LEDGF_b2R1_24h **Scan** 18097
**Peptide** AEIKNSLKIDNLD **Charge** 3 **Spectral Angle** 0.86

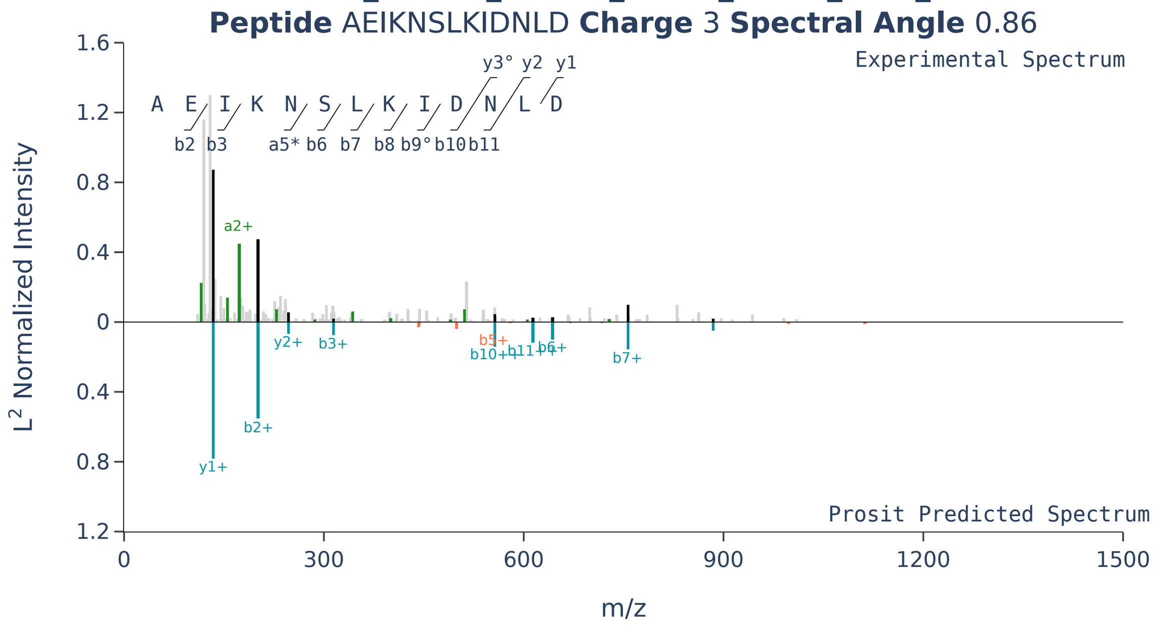


**Source** HRoetschke_271222_090123_HFGoe_LEDGF_b1R2_24h **Scan** 6288
**Peptide** TTLKKIRRFK **Charge** 3 **Spectral Angle** 0.78

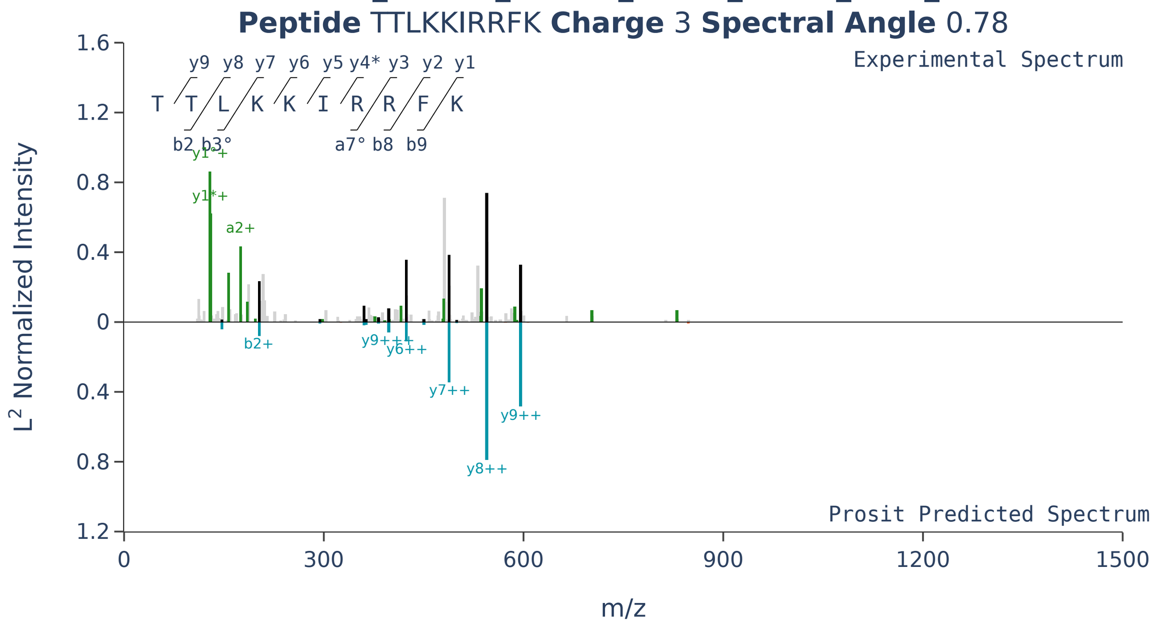


**Source** HRoetschke_271222_090123_HFGoe_LEDGF_b2R1_2h **Scan** 22741
**Peptide** GFNEGLWEIDNNPKVKFSSQQAATK **Charge** 4 **Spectral Angle** 0.86

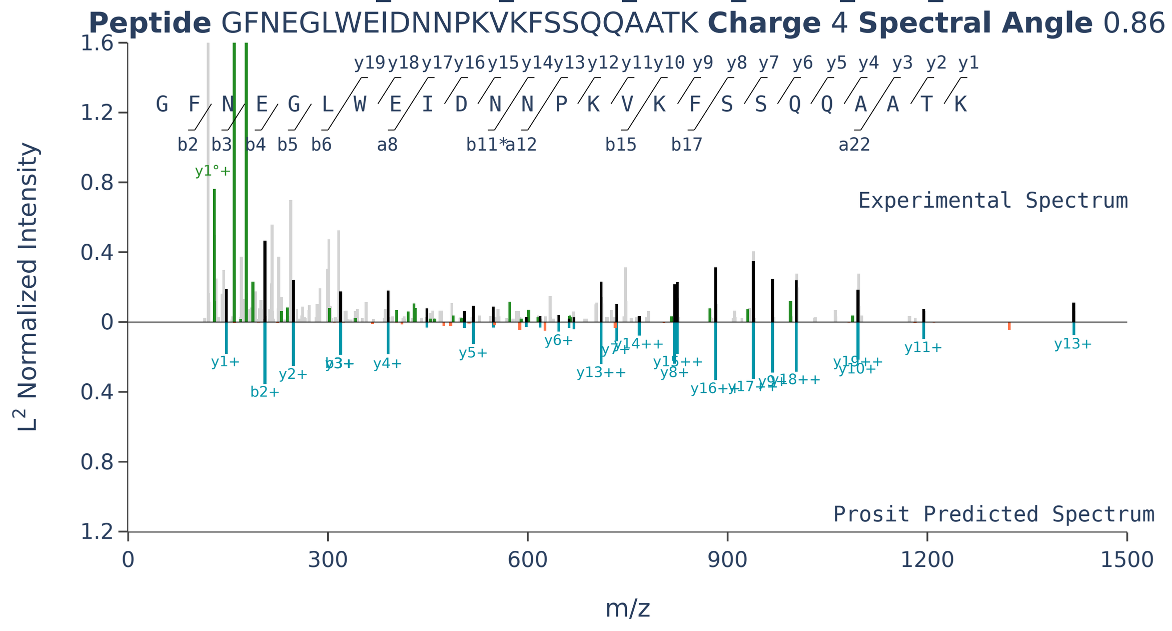


**Source** HRoetschke_271222_090123_HFGoe_LEDGF_b2R2_2h **Scan** 12710
**Peptide** EDVTKAVDITTPKAARR **Charge** 3 **Spectral Angle** 0.82

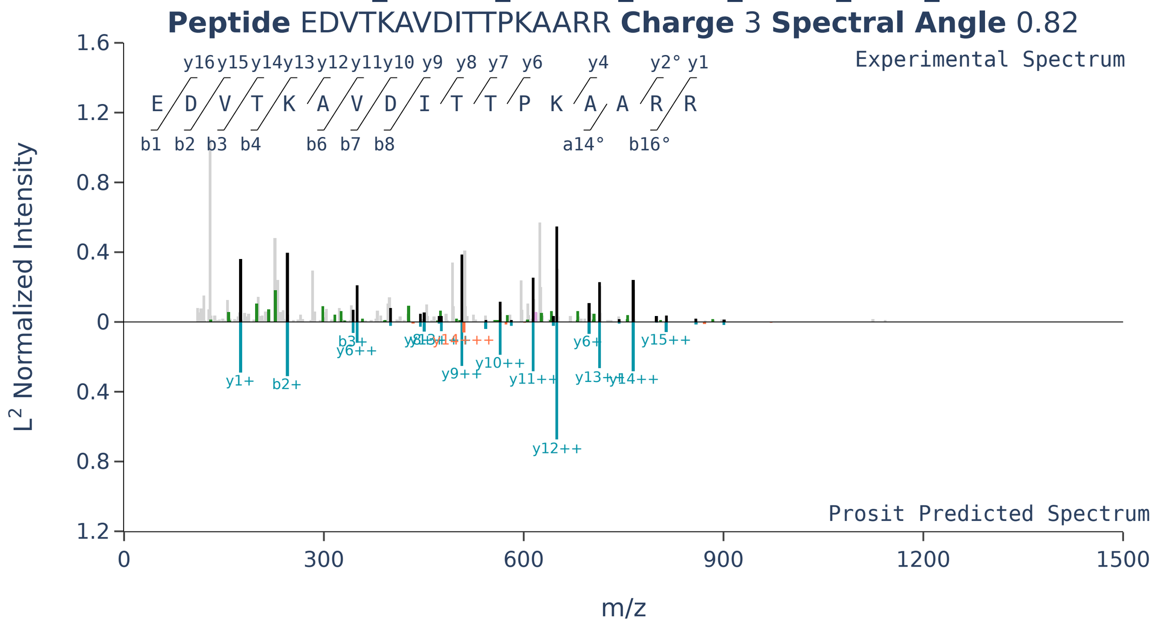


**Source** HRoetschke_271222_090123_HFGoe_LEDGF_b1R2_2h **Scan** 11430
**Peptide** ETSMDSRL **Charge** 2 **Spectral Angle** 0.87

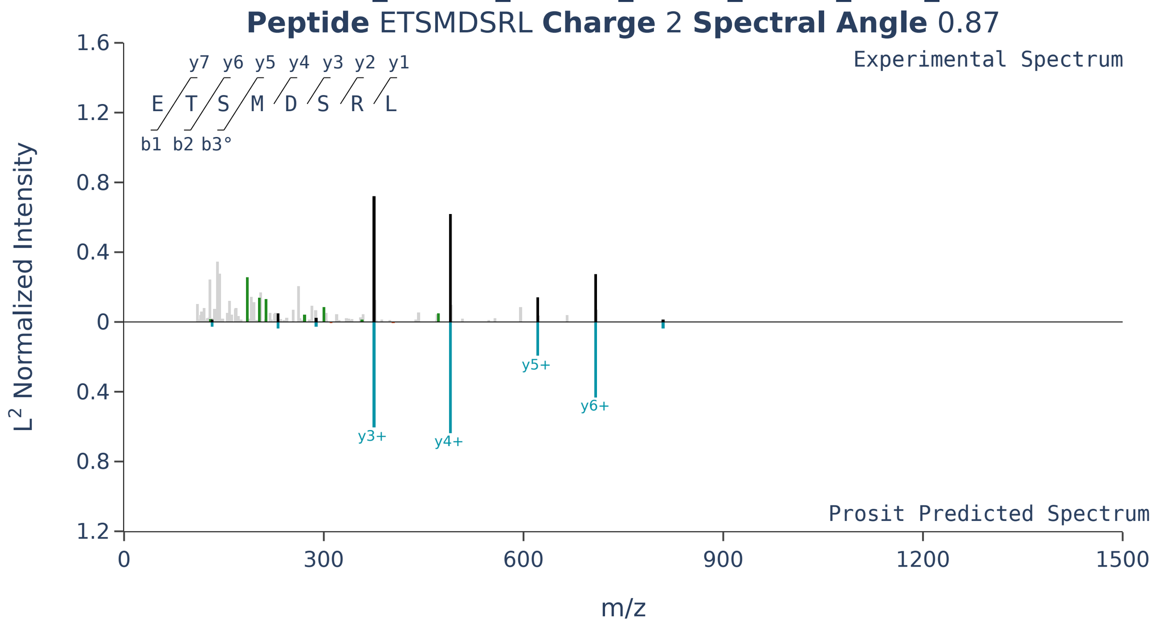


**Source** HRoetschke_271222_090123_HFGoe_LEDGF_b1R1_2h **Scan** 28732
**Peptide** ALDELASLQVT **Charge** 2 **Spectral Angle** 0.82

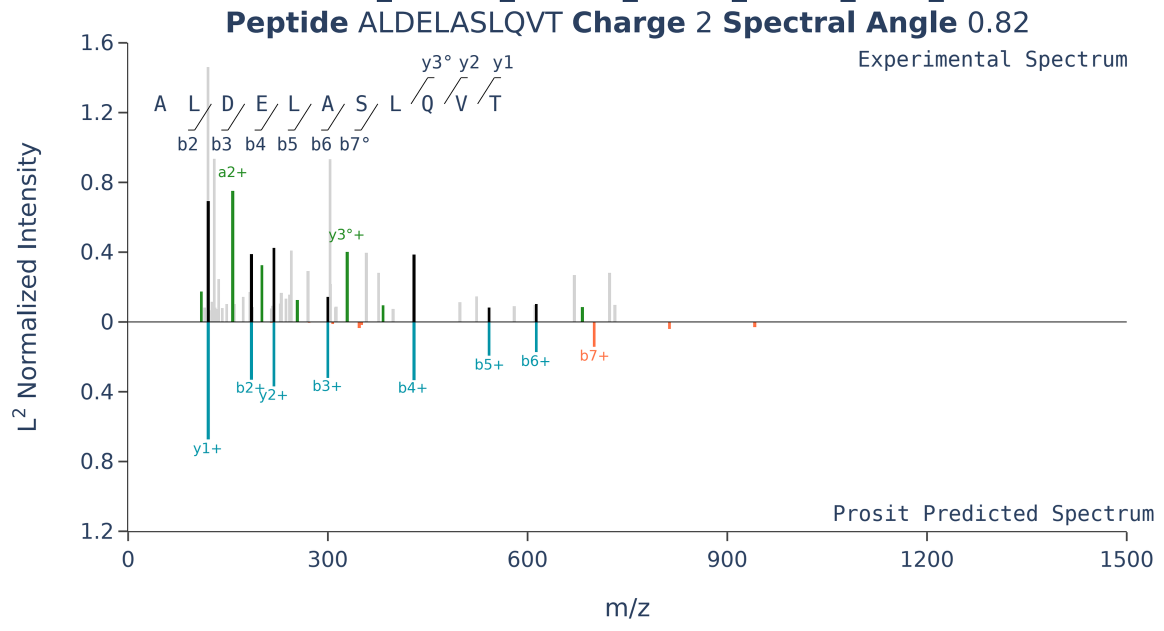

**Source** HRoetschke_271222_090123_HFGoe_LEDGF_b2R1_4h **Scan** 7351
**Peptide** QQAQKHTEMIT **Charge** 3 **Spectral Angle** 0.94

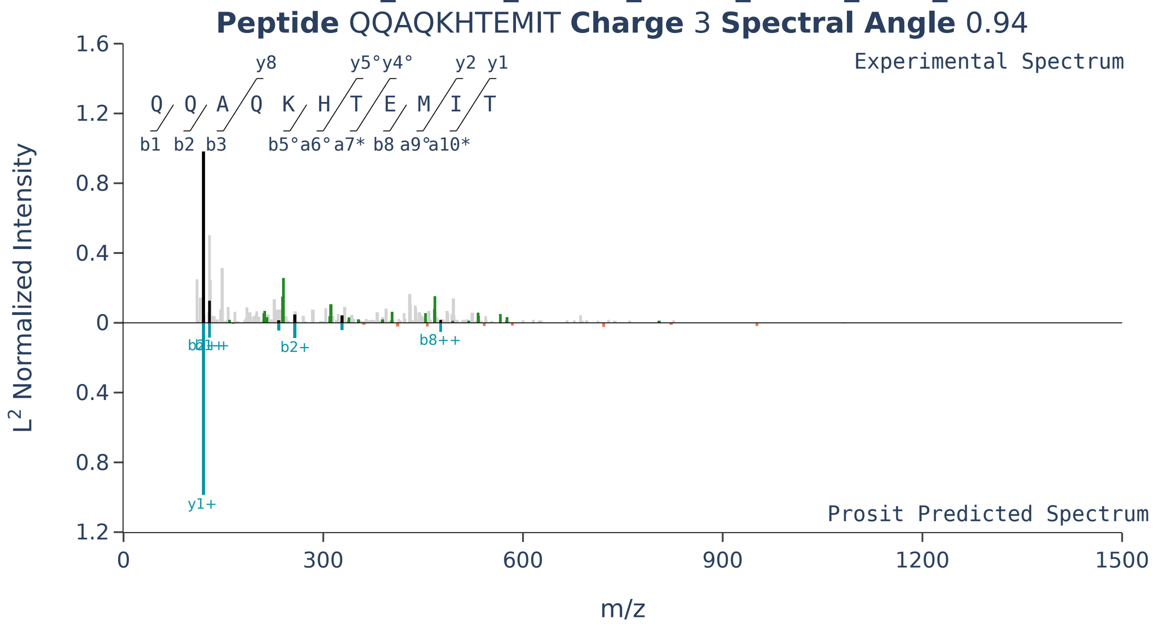


**Source** HRoetschke_271222_090123_HFGoe_LEDGF_b1R1_1h **Scan** 9753
**Peptide** AGVVTTA **Charge** 1 **Spectral Angle** 0.91

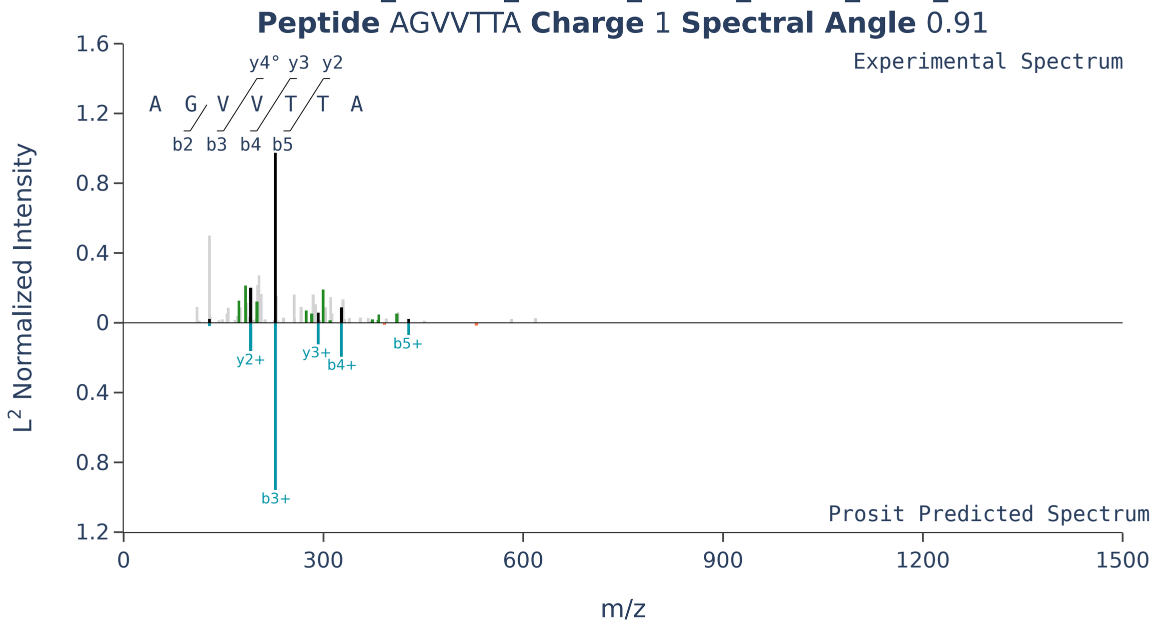


**Source** HRoetschke_271222_090123_HFGoe_LEDGF_b2R2_24h **Scan** 7820
**Peptide** QVLNKSLAEQRQHE **Charge** 4 **Spectral Angle** 0.82

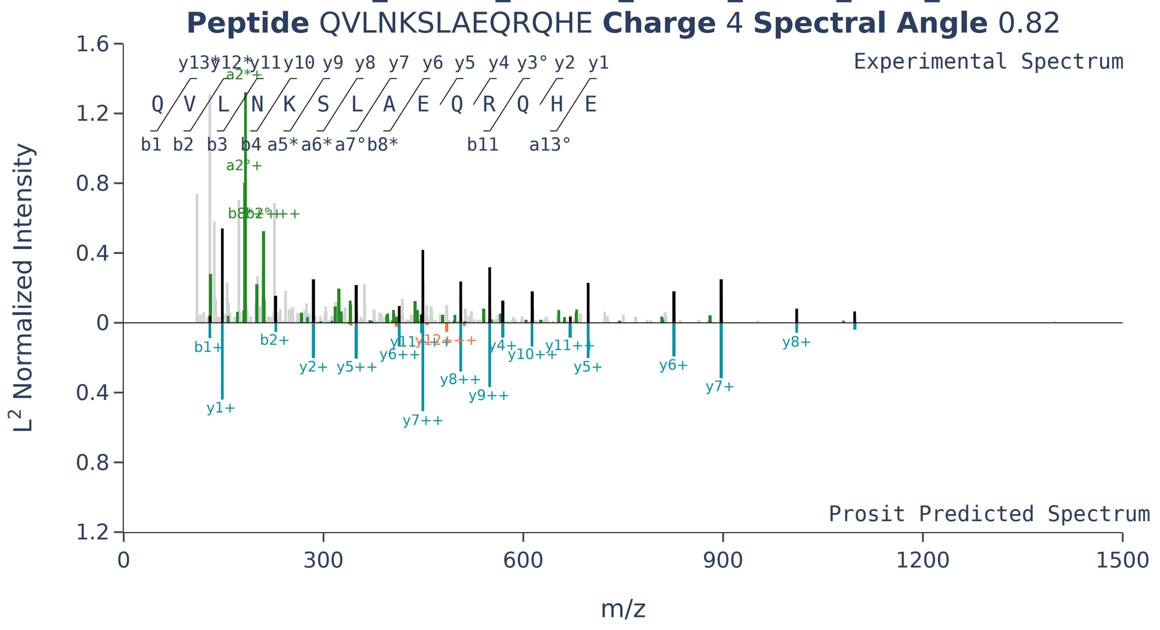


**Source** HRoetschke_271222_090123_HFGoe_LEDGF_b1R1_4h **Scan** 8445
**Peptide** DNLDVNRC **Charge** 2 **Spectral Angle** 0.87

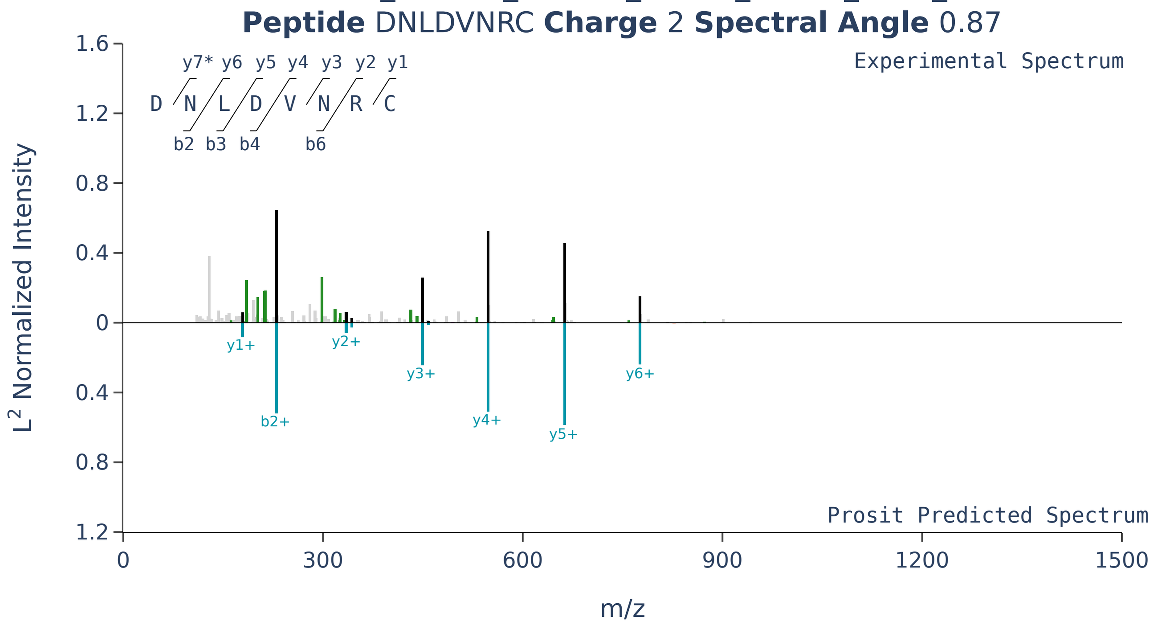


**Source** HRoetschke_271222_090123_HFGoe_LEDGF_b1R1_4h **Scan** 8475
**Peptide** TASVNLKVSPKRGRPAATE **Charge** 3 **Spectral Angle** 0.85

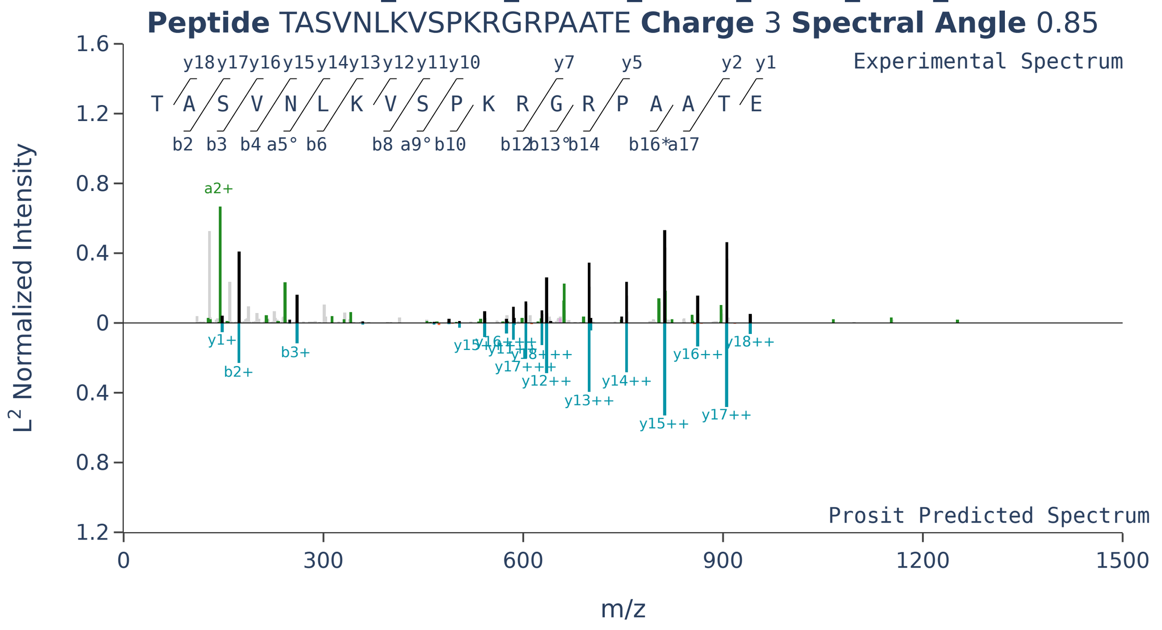


**Source** HRoetschke_271222_090123_HFGoe_LEDGF_b1R2_1h **Scan** 12198
**Peptide** QVLNKSLAE **Charge** 2 **Spectral Angle** 0.84

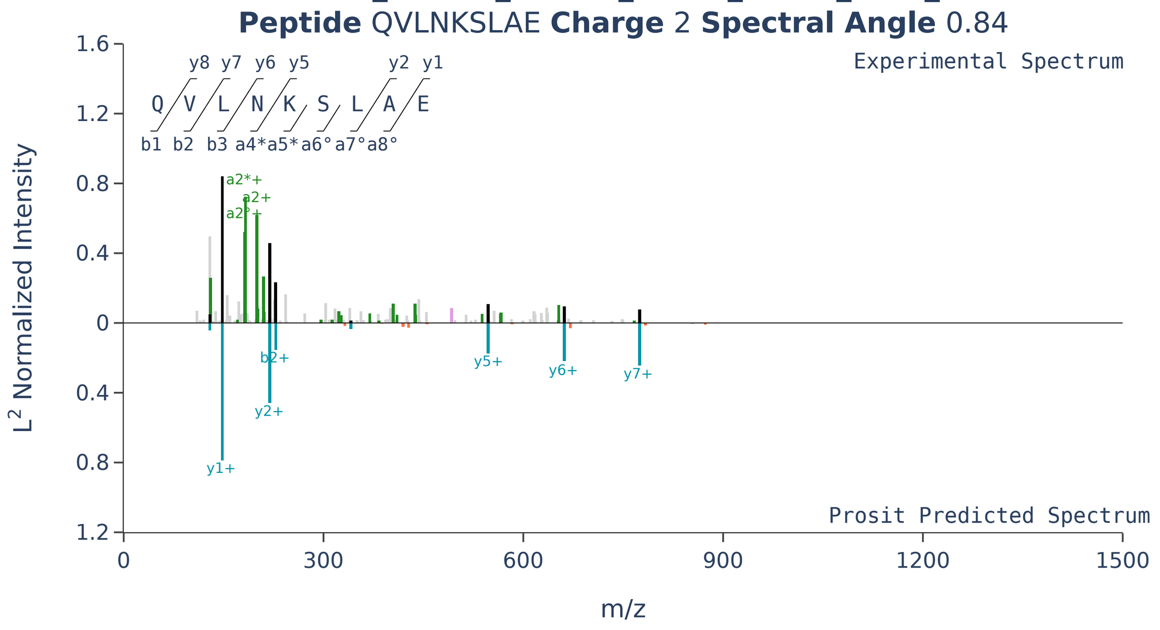


**Source** HRoetschke_271222_090123_HFGoe_LEDGF_b2R2_24h **Scan** 8283
**Peptide** LFPYSENKEKYGKPNKR **Charge** 5 **Spectral Angle** 0.85

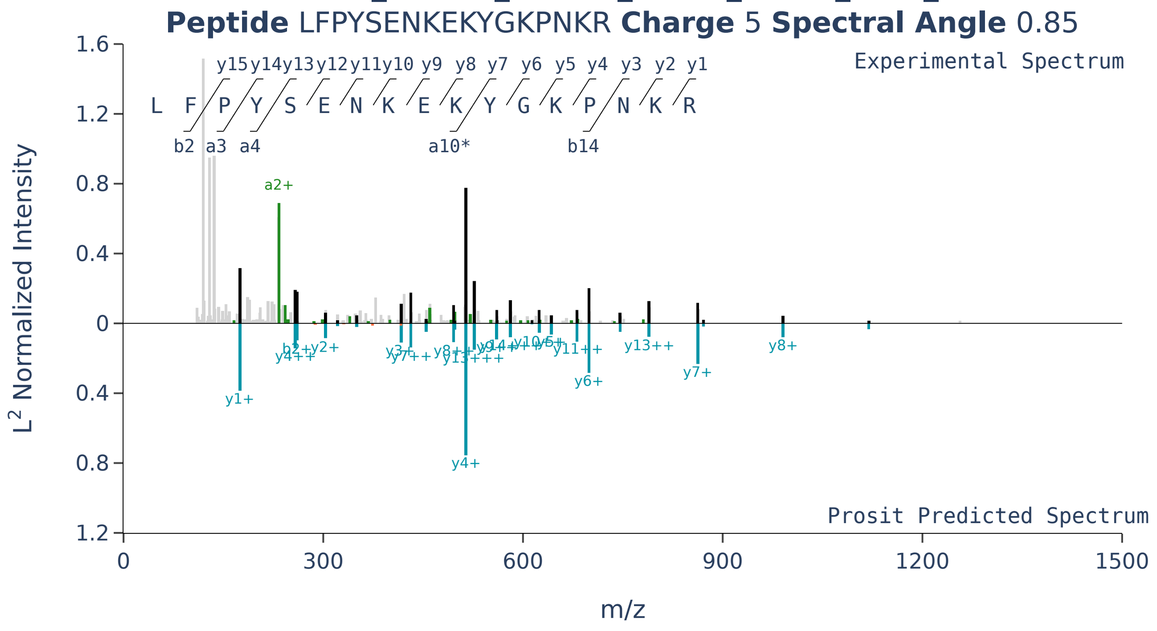


**Source** HRoetschke_271222_090123_HFGoe_LEDGF_b1R1_24h **Scan** 12479
**Peptide** EDVTKAVDITTPKAARRG **Charge** 3 **Spectral Angle** 0.85

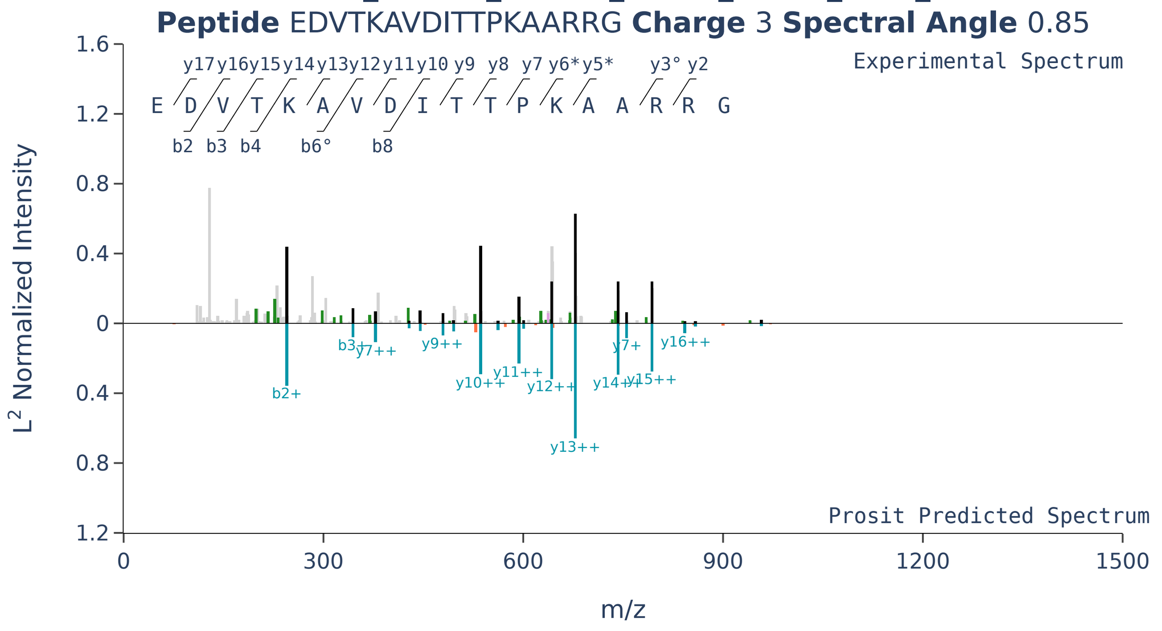


**Source** HRoetschke_271222_090123_HFGoe_LEDGF_b1R2_4h **Scan** 22741
**Peptide** GLWEIDNNPKVKFSSQQ **Charge** 3 **Spectral Angle** 0.78

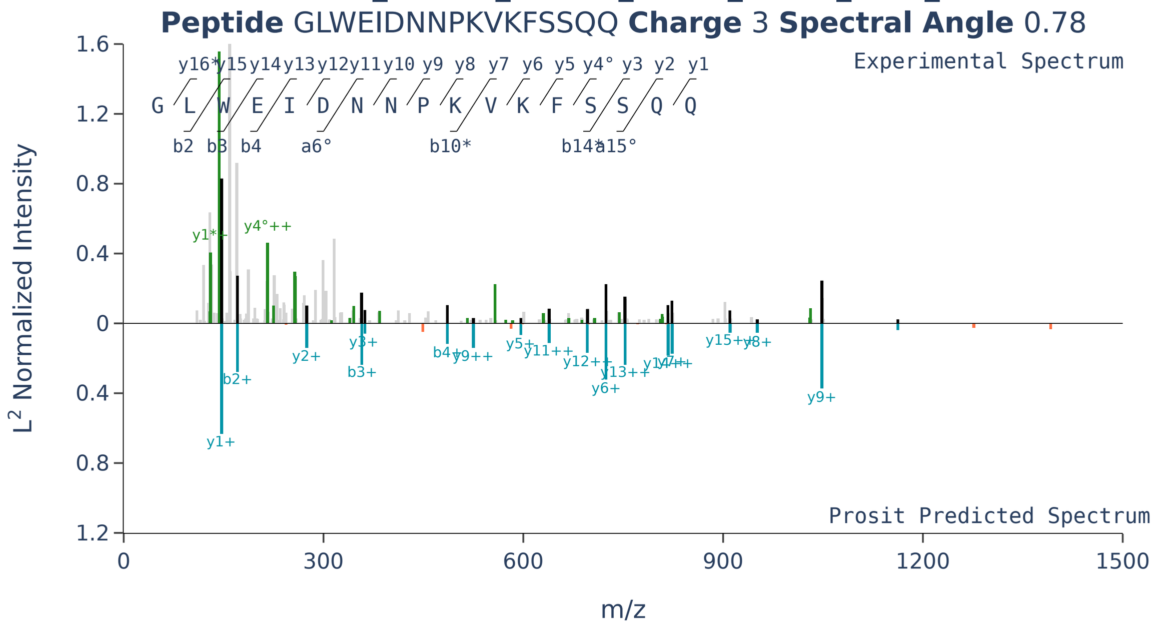


**Source** HRoetschke_271222_090123_HFGoe_LEDGF_b2R1_24h **Scan** 6742
**Peptide** NNPKVKFSSQQAATKQS **Charge** 2 **Spectral Angle** 0.82

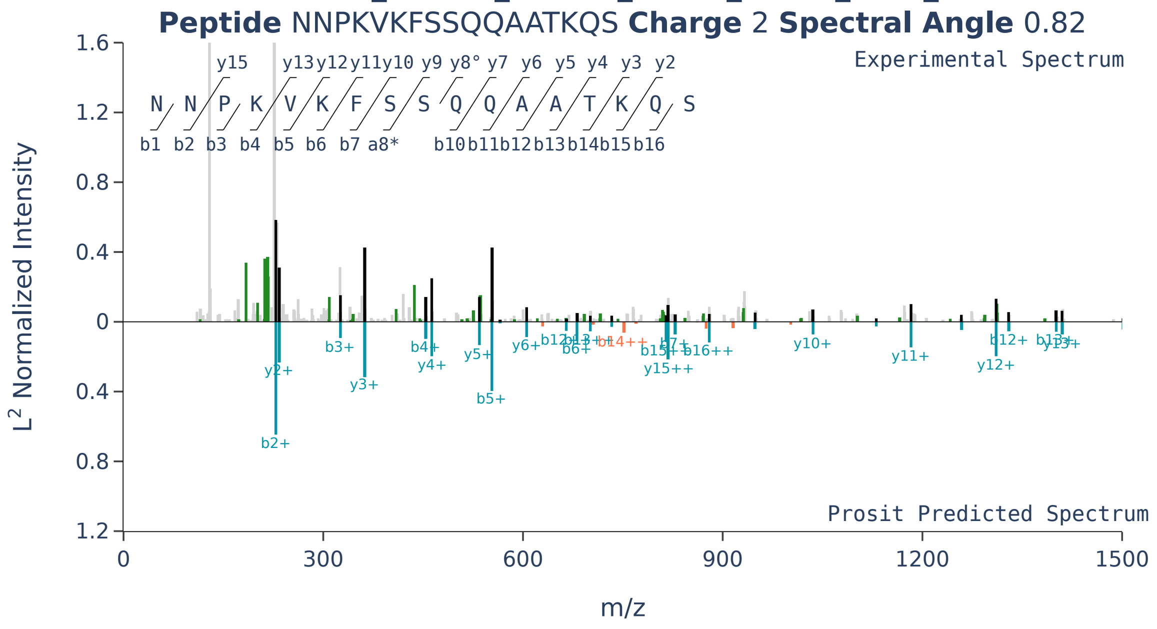


**Source** HRoetschke_271222_090123_HFGoe_LEDGF_b2R2_4h **Scan** 19258
**Peptide** PKDIFPYSEN **Charge** 2 **Spectral Angle** 0.75

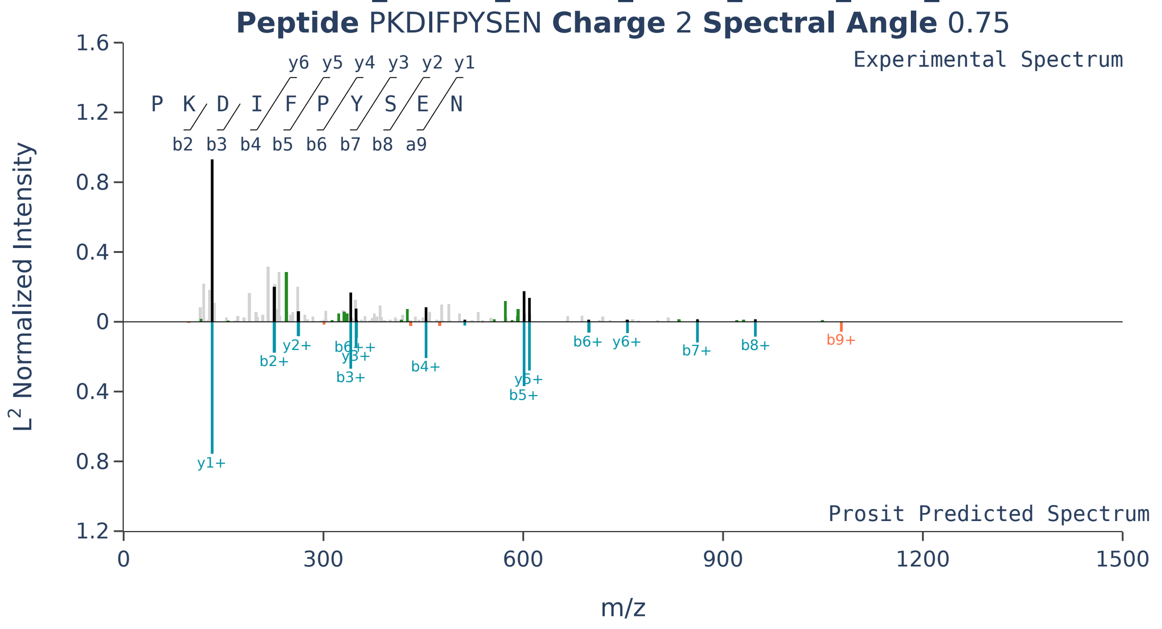


**Source** HRoetschke_271222_090123_HFGoe_LEDGF_b1R2_2h **Scan** 14322
**Peptide** NLDVNRCIE **Charge** 1 **Spectral Angle** 0.89

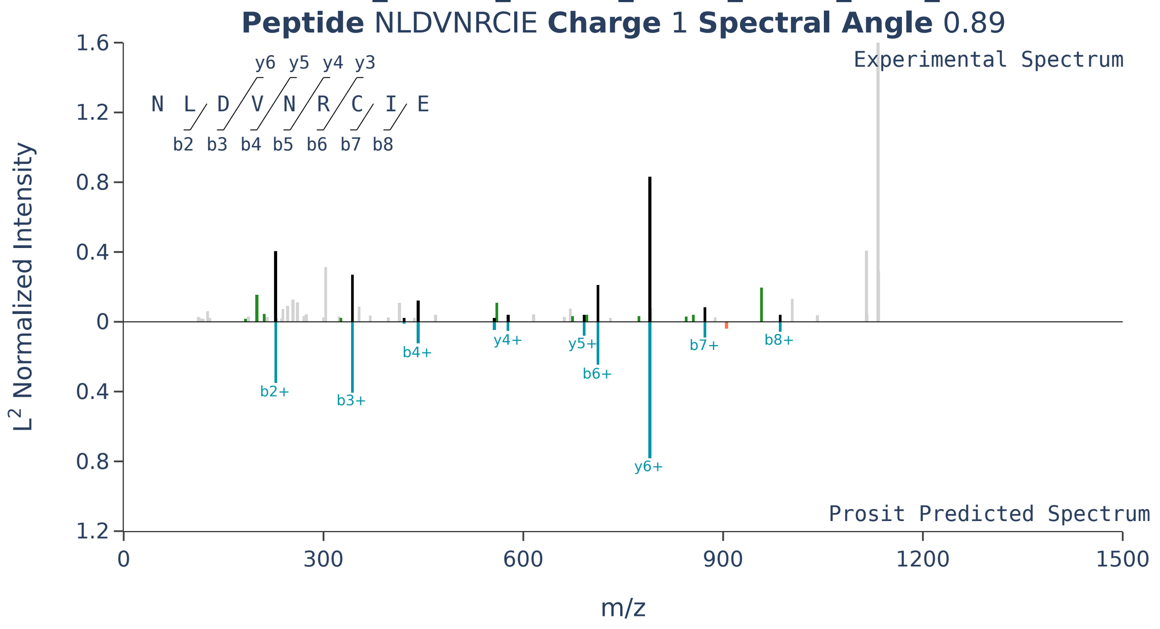


**Source** HRoetschke_271222_090123_HFGoe_LEDGF_b2R1_4h **Scan** 5511
**Peptide** KVSPKRGRPAATEV **Charge** 3 **Spectral Angle** 0.88

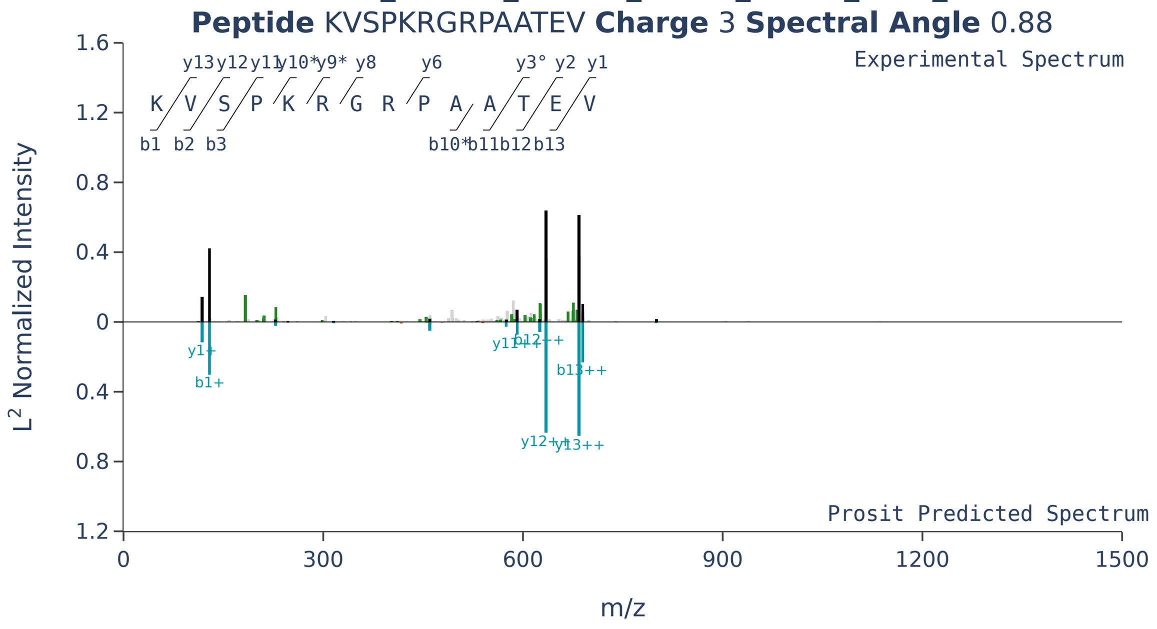


**Source** HRoetschke_271222_090123_HFGoe_LEDGF_b2R1_24h **Scan** 13652
**Peptide** KPGDLIFAKMK **Charge** 3 **Spectral Angle** 0.78

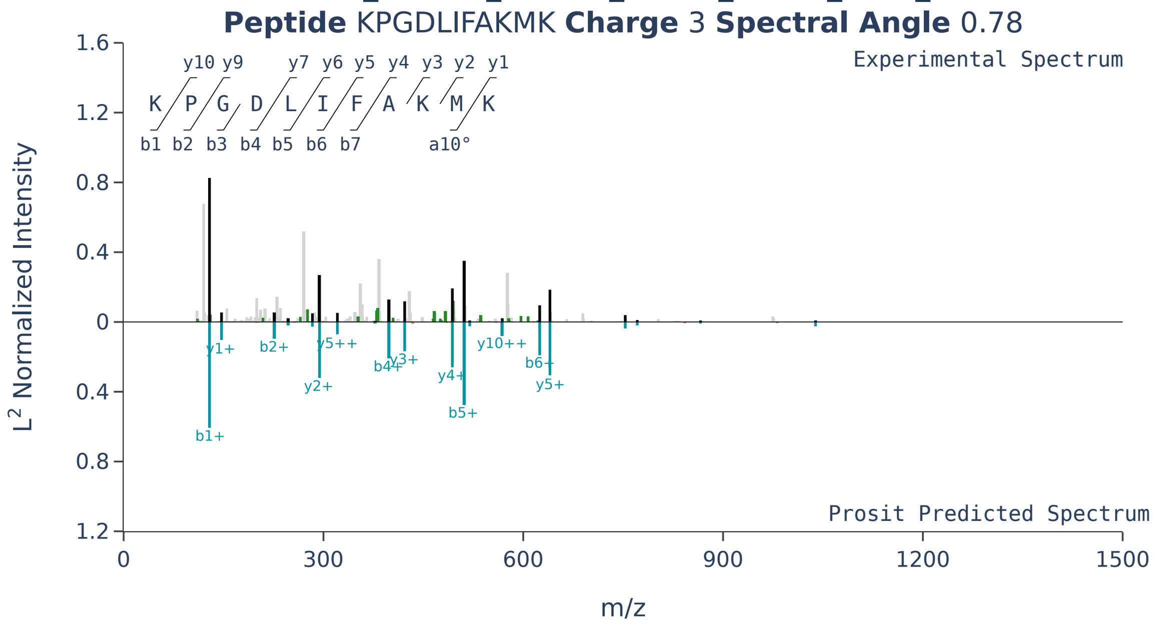


**Source** HRoetschke_271222_090123_HFGoe_LEDGF_b2R1_24h **Scan** 23126
**Peptide** GFNEGLWEIDNNPKVKFSSQQAATKQ **Charge** 3 **Spectral Angle** 0.82

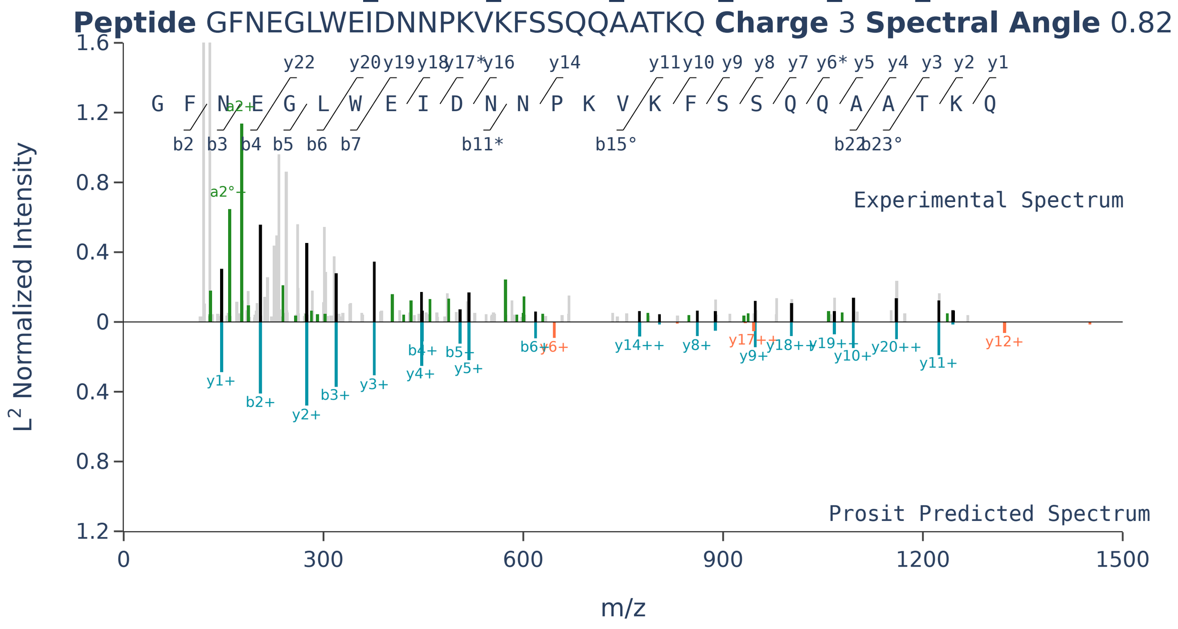

**Source** HRoetschke_271222_090123_HFGoe_LEDGF_b2R1_4h **Scan** 10294
**Peptide** AQKHTEMITTLKKIR **Charge** 4 **Spectral Angle** 0.88

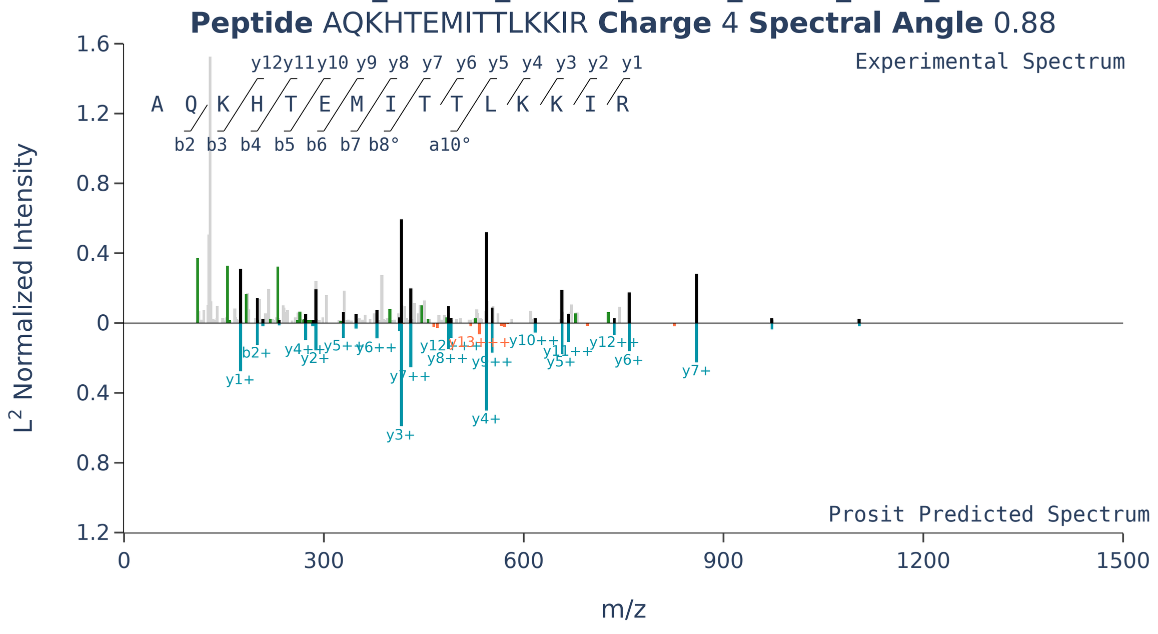


**Source** HRoetschke_271222_090123_HFGoe_LEDGF_b1R1_2h **Scan** 28041
**Peptide** ALDELASLQVTMQQAQK **Charge** 3 **Spectral Angle** 0.86

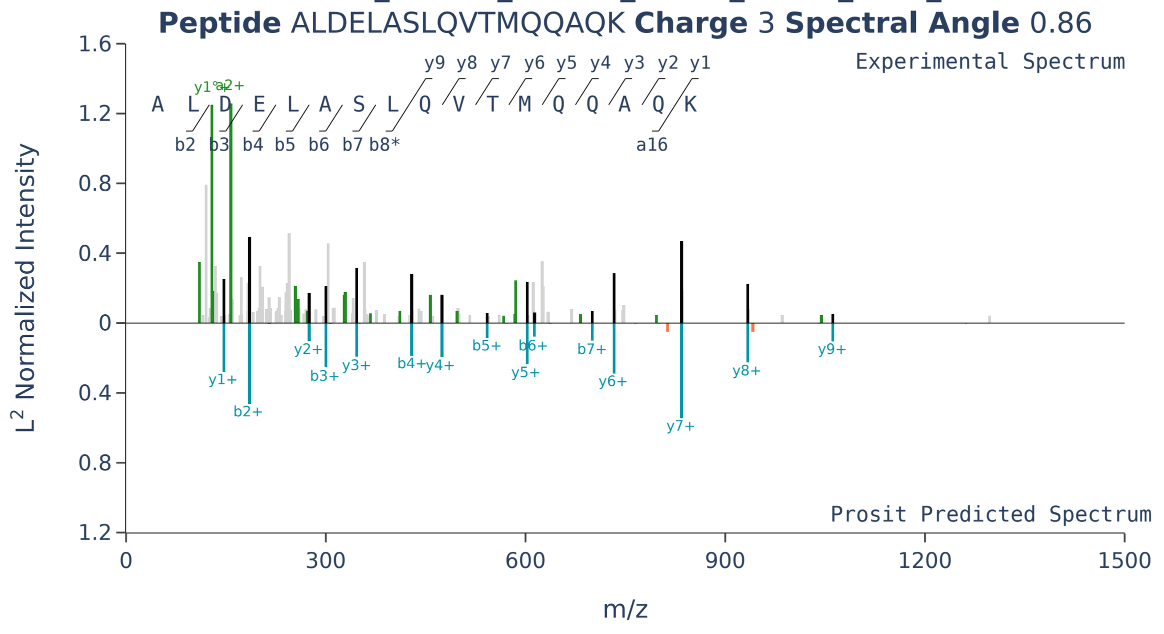


**Source** HRoetschke_271222_090123_HFGoe_LEDGF_b2R1_24h **Scan** 10082
**Peptide** RIHAEIKNSL **Charge** 2 **Spectral Angle** 0.85

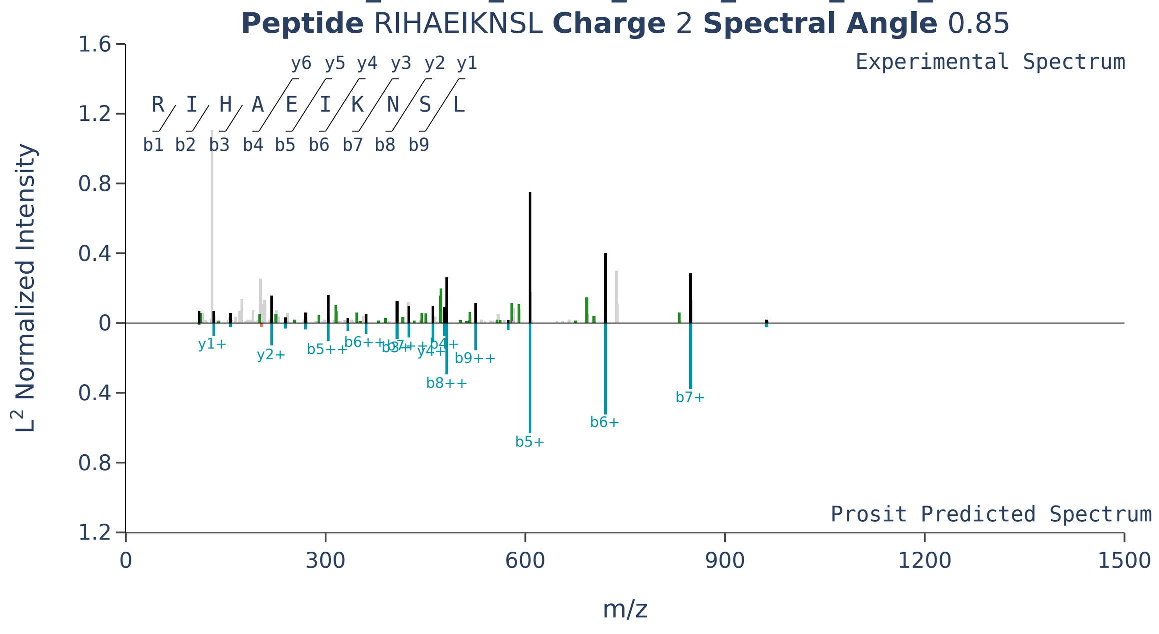


**Source** HRoetschke_271222_090123_HFGoe_LEDGF_b1R2_24h **Scan** 6217
**Peptide** VDITTPKAARRGRK **Charge** 4 **Spectral Angle** 0.84

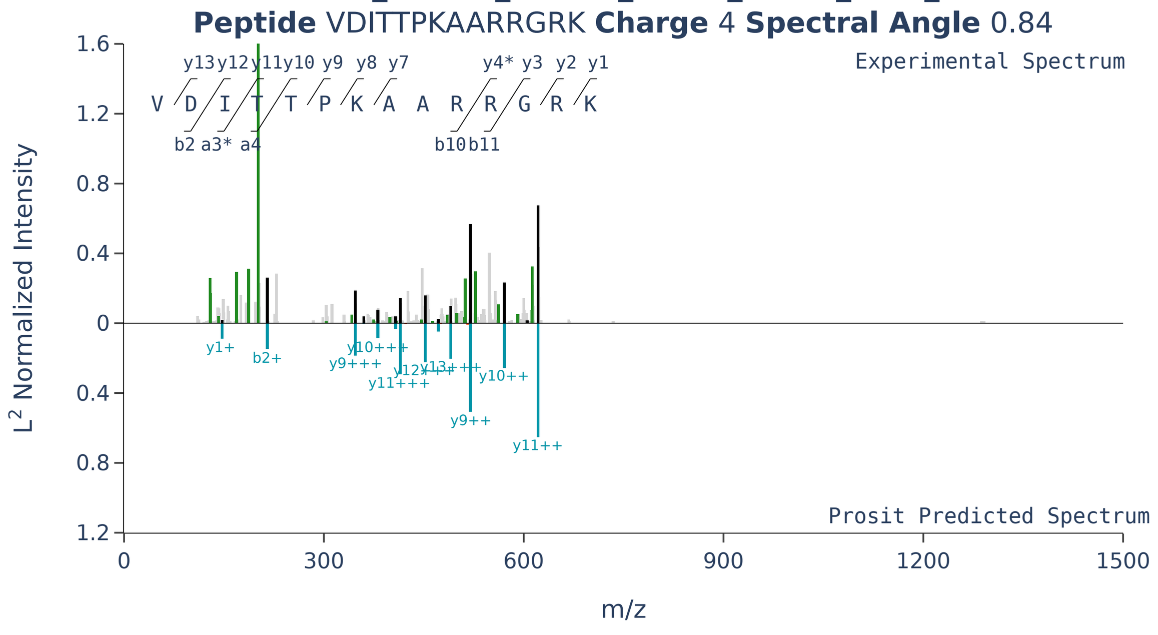


**Source** HRoetschke_271222_090123_HFGoe_LEDGF_b2R1_4h **Scan** 6234
**Peptide** QVTMQQAQKHTE **Charge** 2 **Spectral Angle** 0.8

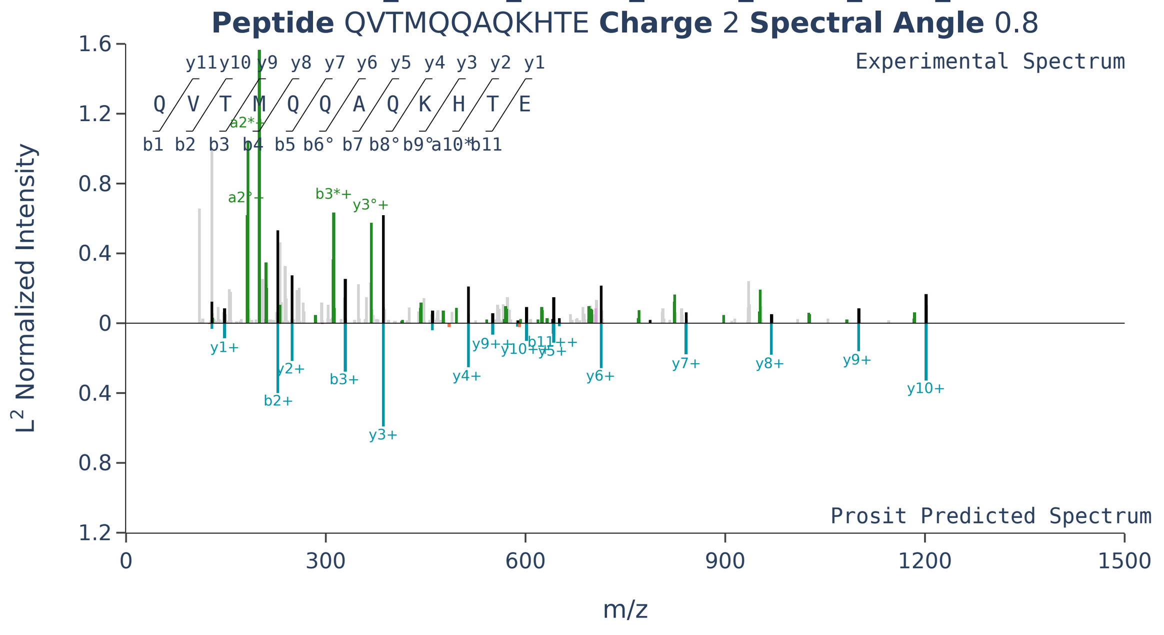


**Source** HRoetschke_271222_090123_HFGoe_LEDGF_b1R2_24h **Scan** 10434
**Peptide** DSRLQRIHAEIK **Charge** 3 **Spectral Angle** 0.89

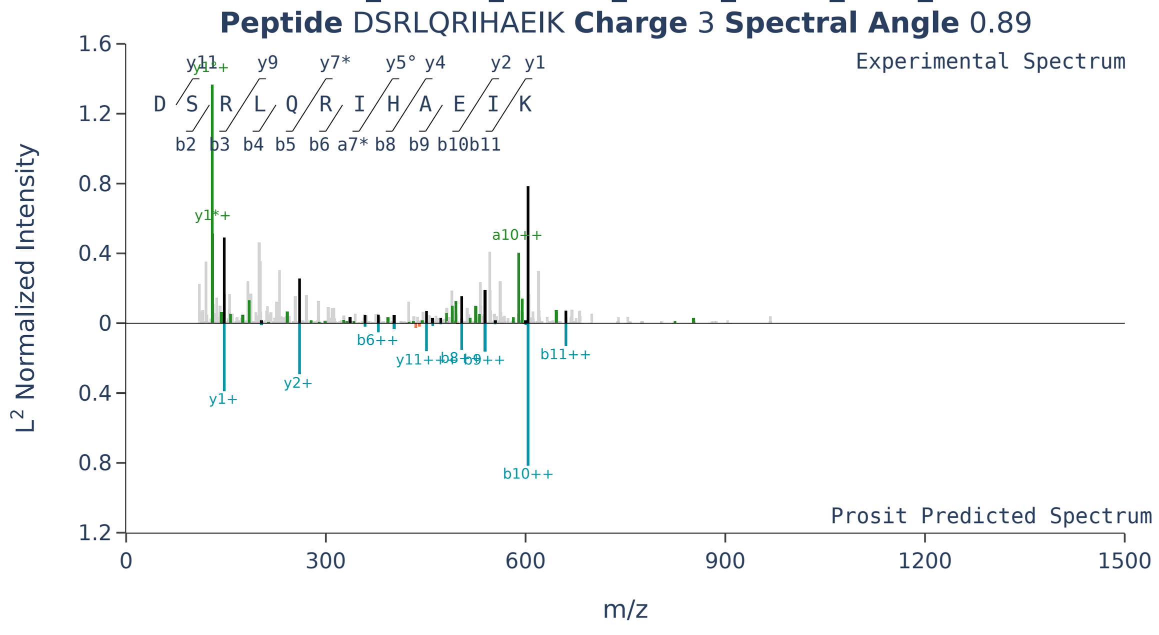


**Source** HRoetschke_271222_090123_HFGoe_LEDGF_b1R1_24h **Scan** 8440
**Peptide** HAEIKNSL **Charge** 1 **Spectral Angle** 0.88

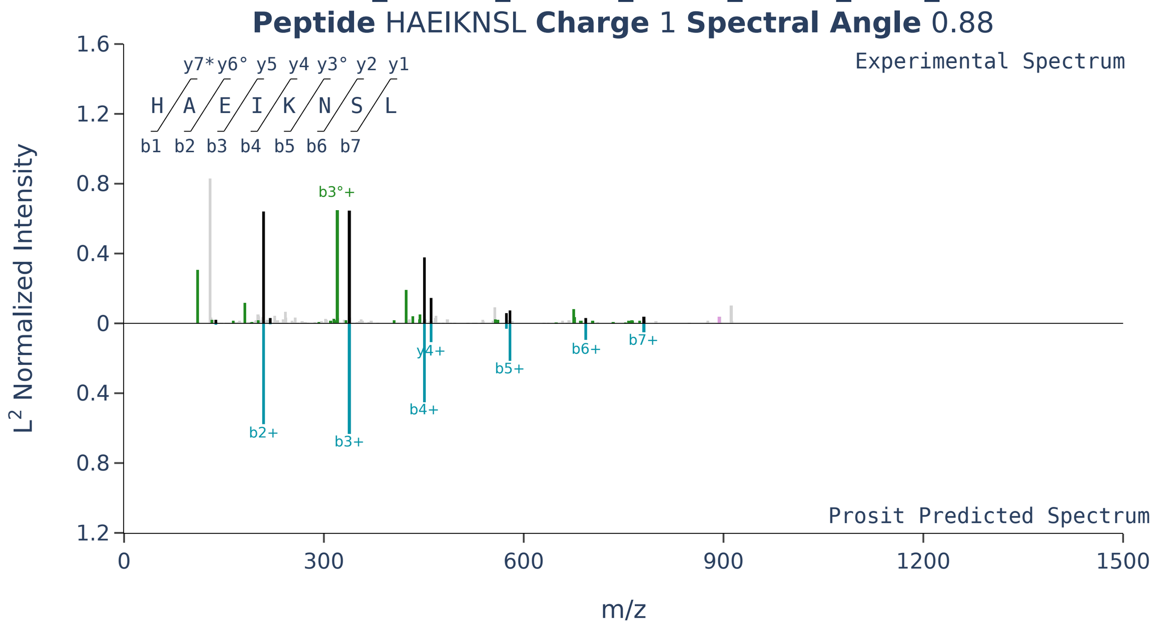


**Source** HRoetschke_271222_090123_HFGoe_LEDGF_b2R1_24h **Scan** 31330
**Peptide** VGEGDSVITQVLNKSLAE **Charge** 2 **Spectral Angle** 0.86

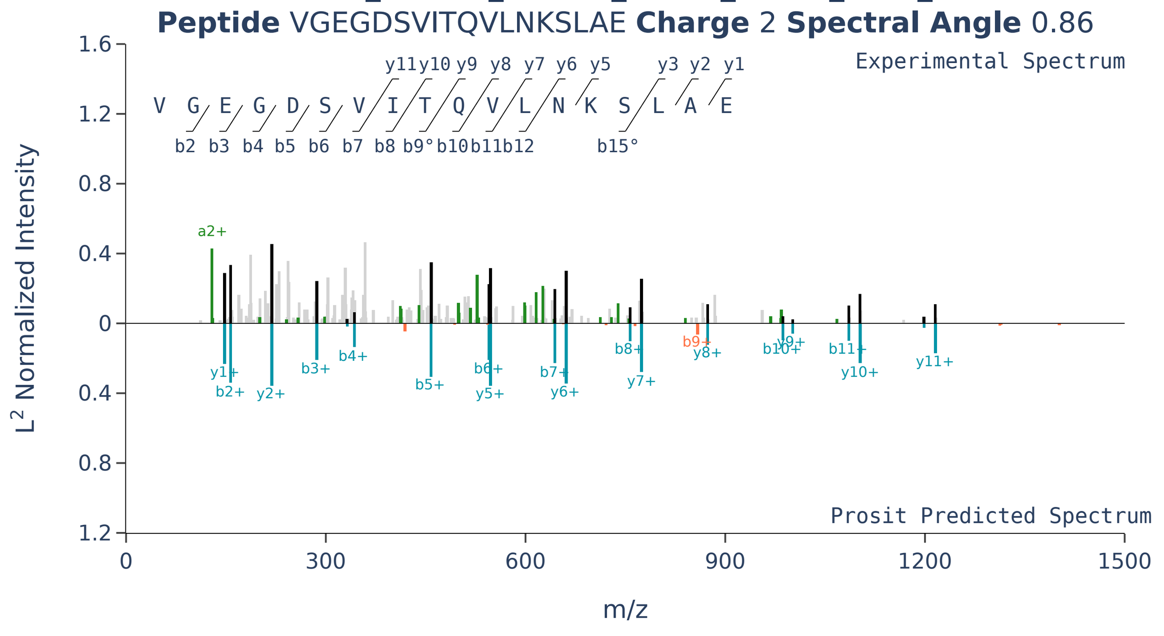


**Source** HRoetschke_271222_090123_HFGoe_LEDGF_b1R1_4h **Scan** 29055
**Peptide** DELASLQVTM **Charge** 1 **Spectral Angle** 0.86

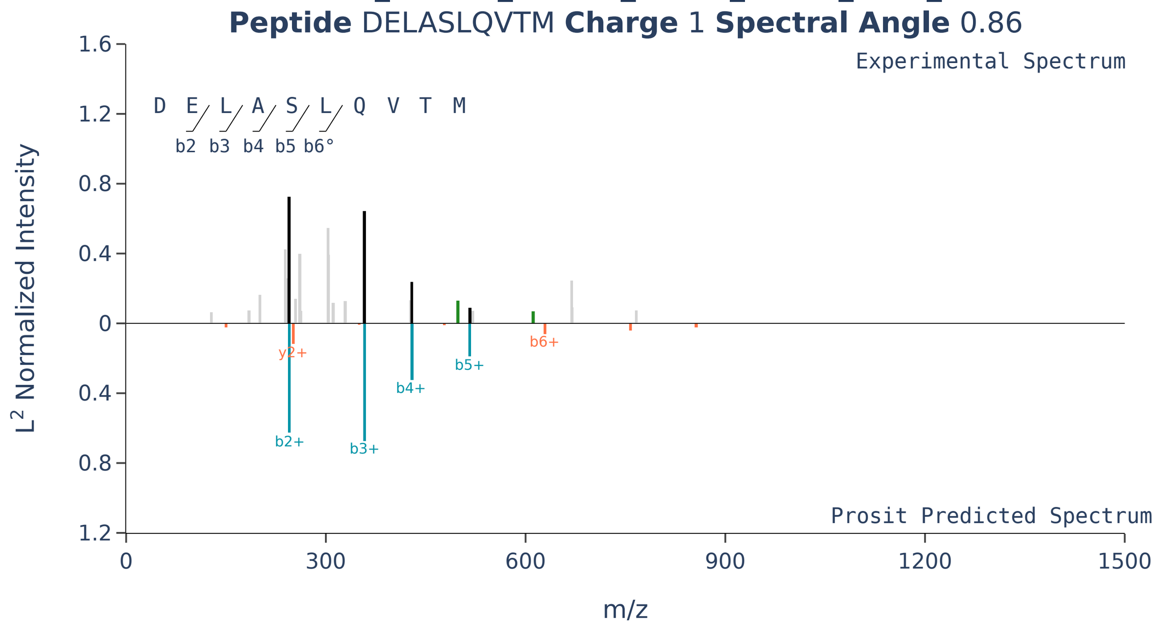


**Source** HRoetschke_271222_090123_HFGoe_LEDGF_b1R2_4h **Scan** 24356
**Peptide** VSQVIMEKSTMLYNKF **Charge** 2 **Spectral Angle** 0.9

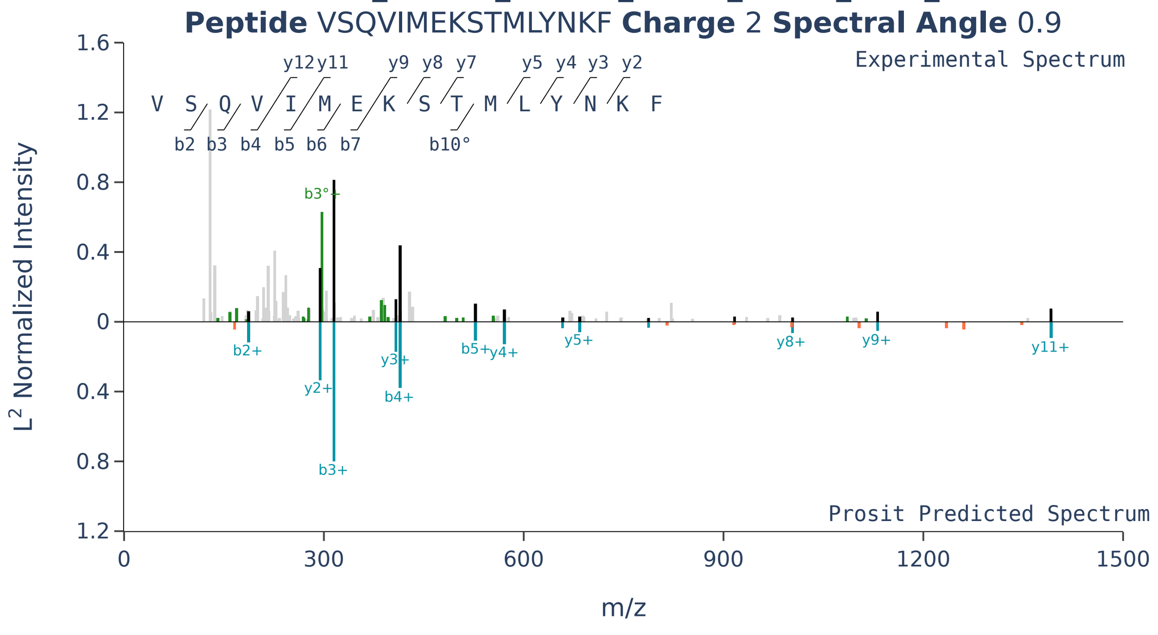


**Source** HRoetschke_271222_090123_HFGoe_LEDGF_b1R1_1h **Scan** 7258
**Peptide** FPYSENKEKYGKPNK **Charge** 4 **Spectral Angle** 0.85

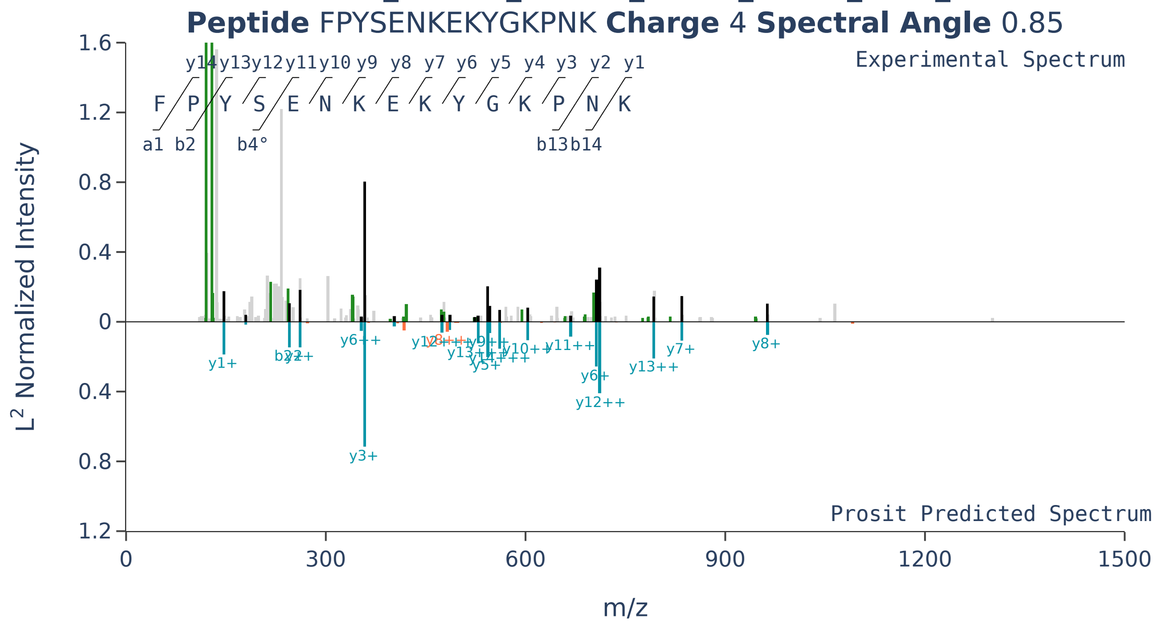


**Source** HRoetschke_271222_090123_HFGoe_LEDGF_b2R2_2h **Scan** 16006
**Peptide** AFLGPKDIFPYSENKEKYGKPNKRKG **Charge** 4 **Spectral Angle** 0.87

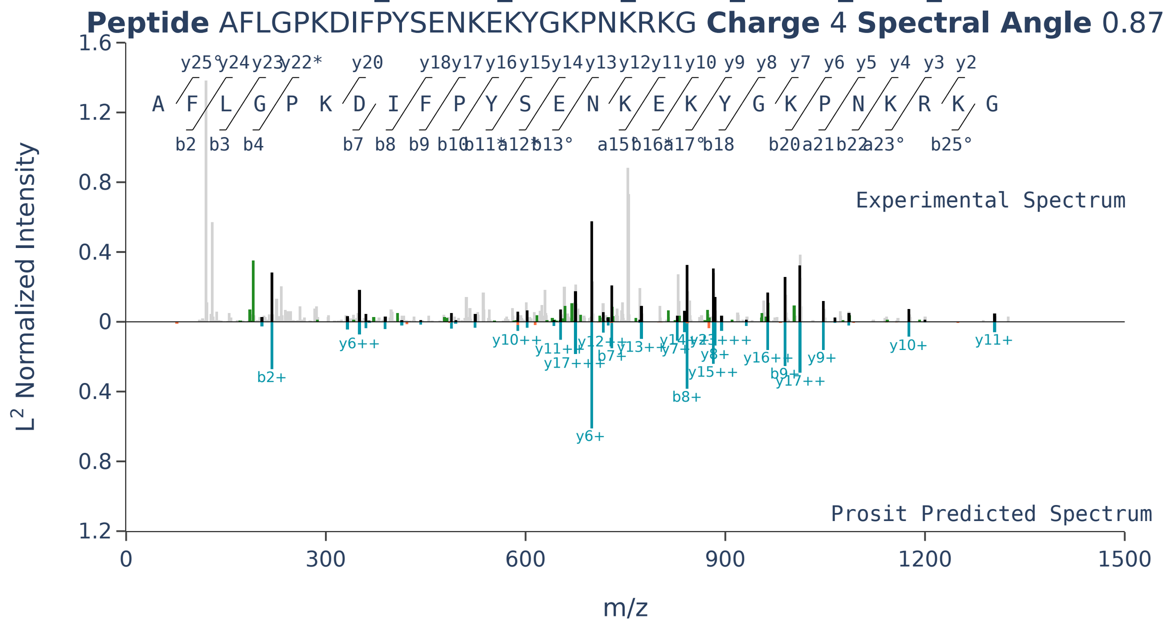


**Source** HRoetschke_271222_090123_HFGoe_LEDGF_b2R2_2h **Scan** 7350
**Peptide** FPYSENKEKYGKPNKRKGFNEG **Charge** 3 **Spectral Angle** 0.85

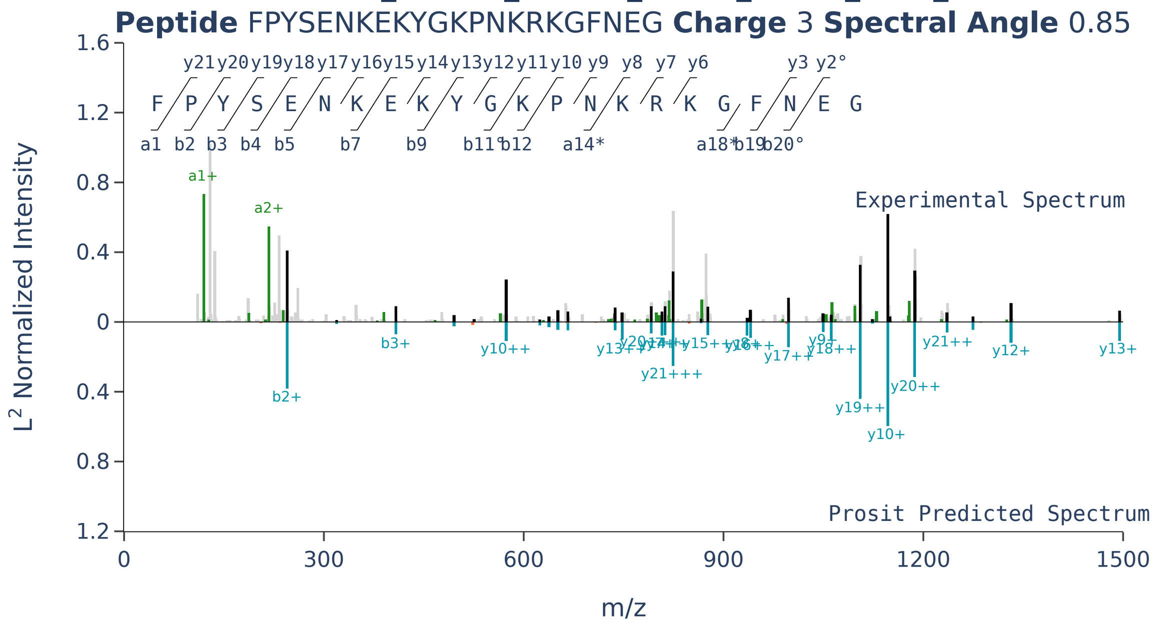


**Source** HRoetschke_271222_090123_HFGoe_LEDGF_b1R2_4h **Scan** 30041
**Peptide** LVGEGDSVITQVLN **Charge** 2 **Spectral Angle** 0.76

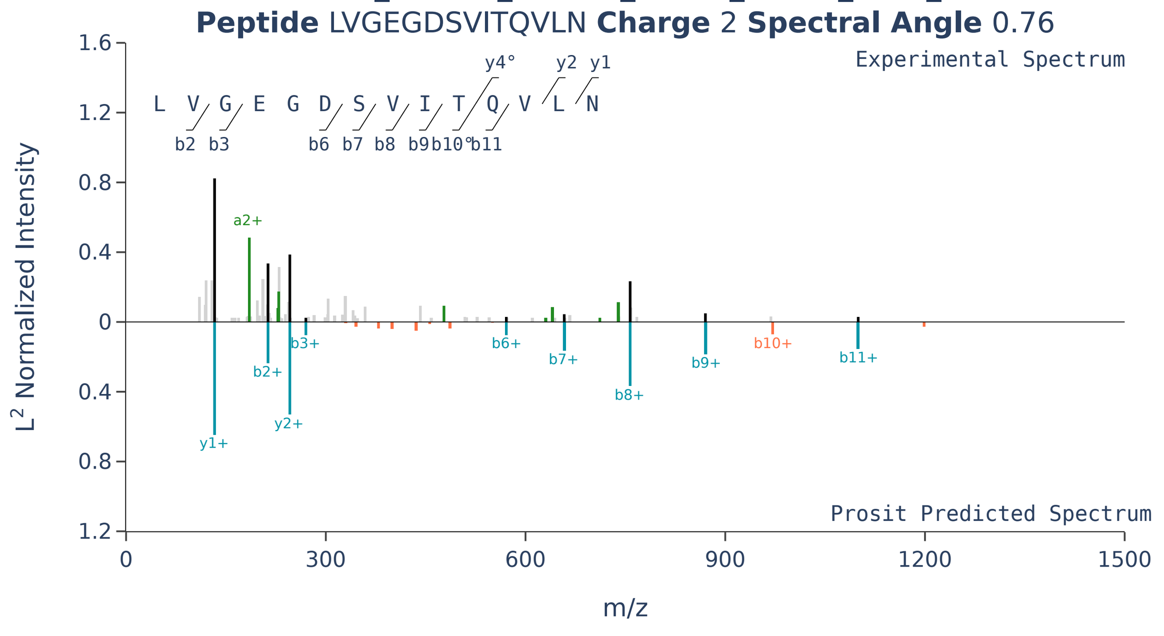


**Source** HRoetschke_271222_090123_HFGoe_LEDGF_b1R2_24h **Scan** 7908
**Peptide** ASVNLKVSPKRGRPAAT **Charge** 5 **Spectral Angle** 0.84

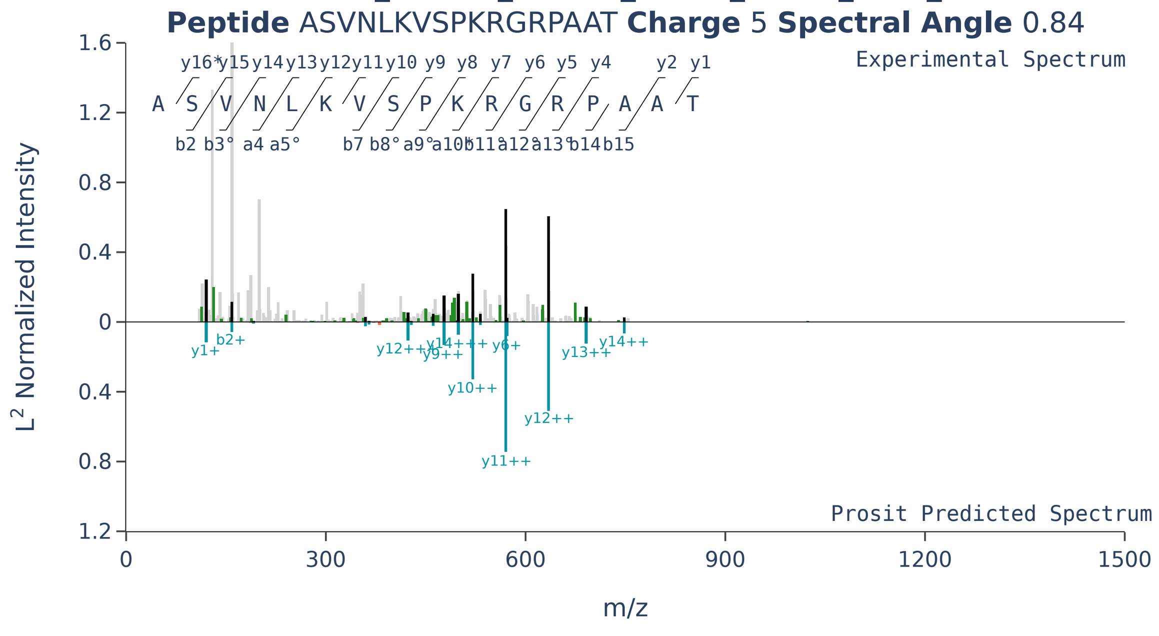

**Source** HRoetschke_271222_090123_HFGoe_LEDGF_b1R2_4h **Scan** 28304
**Peptide** SVITQVLNKSLAE **Charge** 2 **Spectral Angle** 0.85

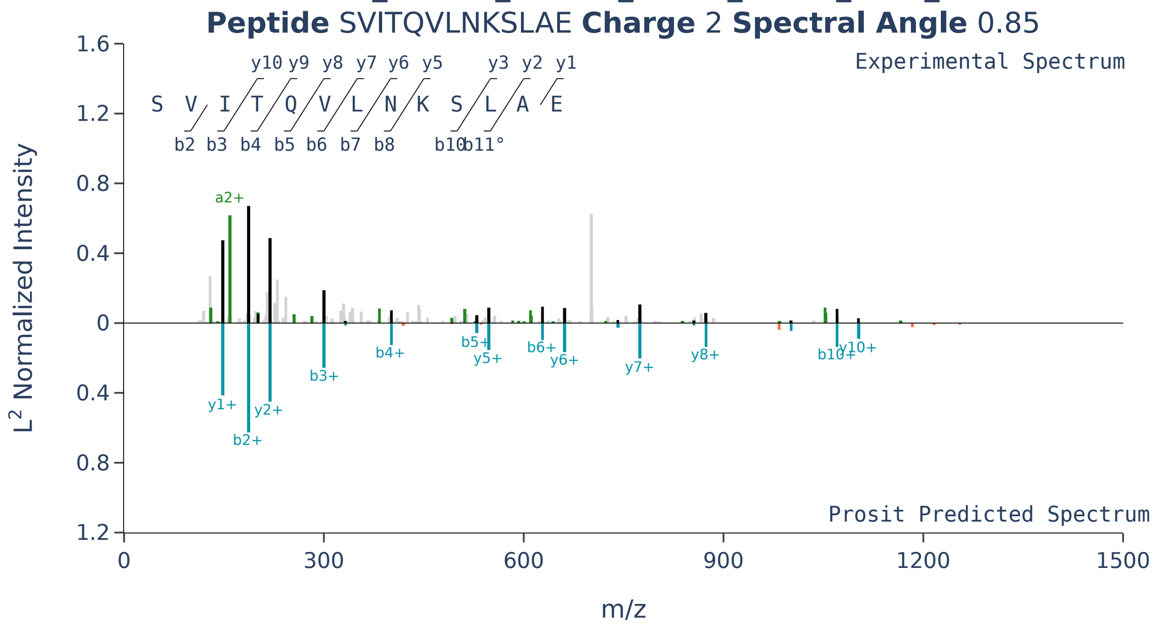


**Source** HRoetschke_271222_090123_HFGoe_LEDGF_b1R1_24h **Scan** 8245
**Peptide** DSRLQRIHA **Charge** 2 **Spectral Angle** 0.87

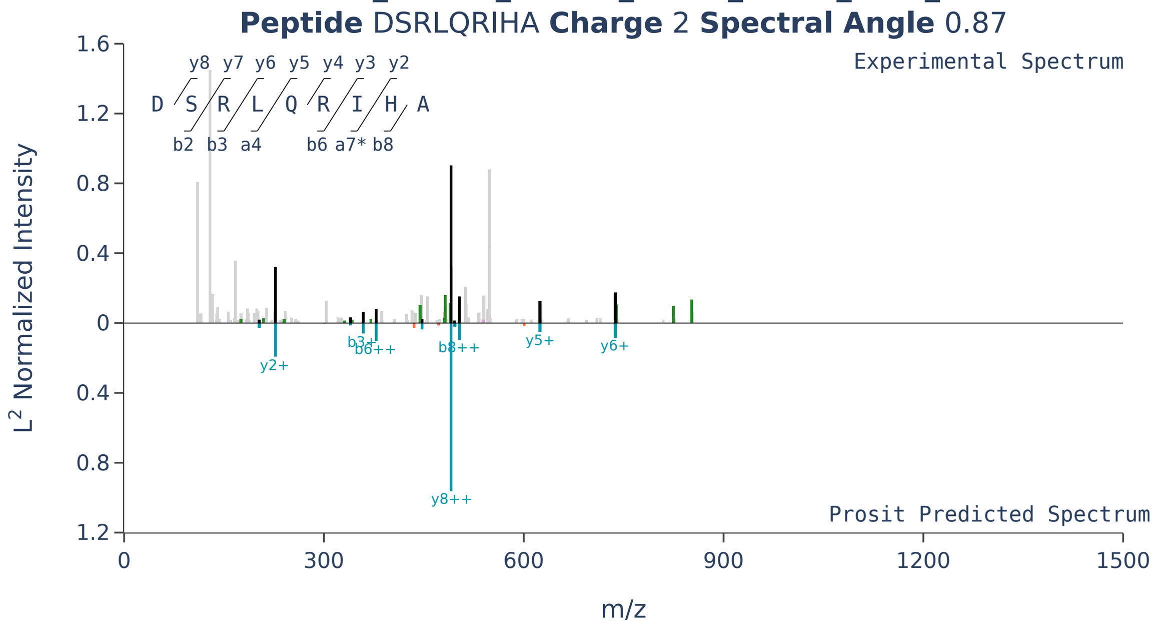


**Source** HRoetschke_271222_090123_HFGoe_LEDGF_b1R2_2h **Scan** 30077
**Peptide** FLVGEGDSVITQV **Charge** 2 **Spectral Angle** 0.88

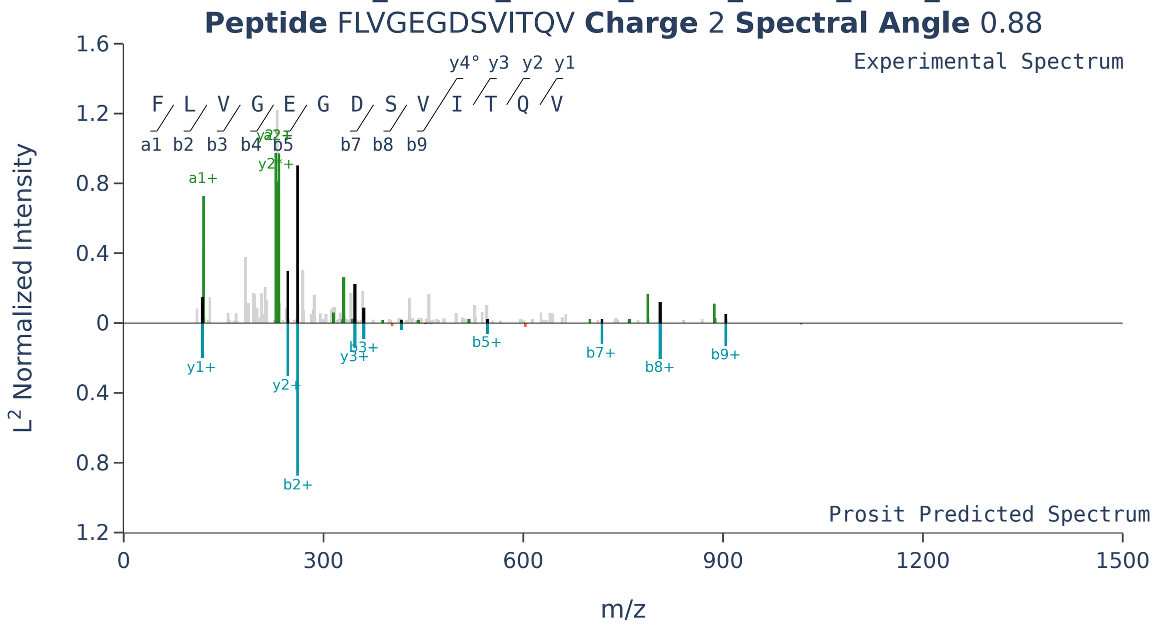


**Source** HRoetschke_271222_090123_HFGoe_LEDGF_b2R2_2h **Scan** 13123
**Peptide** IDNNPKVKFSSQQAATKQSNASSDVEVE **Charge** 4 **Spectral Angle** 0.84

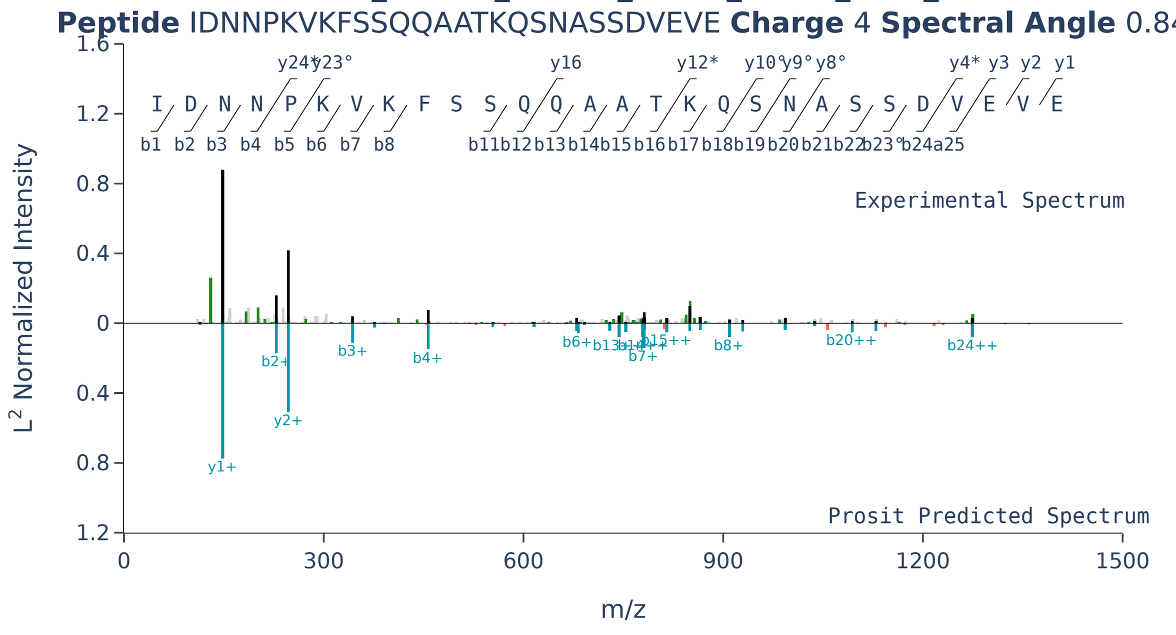


**Source** HRoetschke_271222_090123_HFGoe_LEDGF_b1R1_4h **Scan** 21696
**Peptide** KPGDLIFAKMKGY **Charge** 4 **Spectral Angle** 0.82

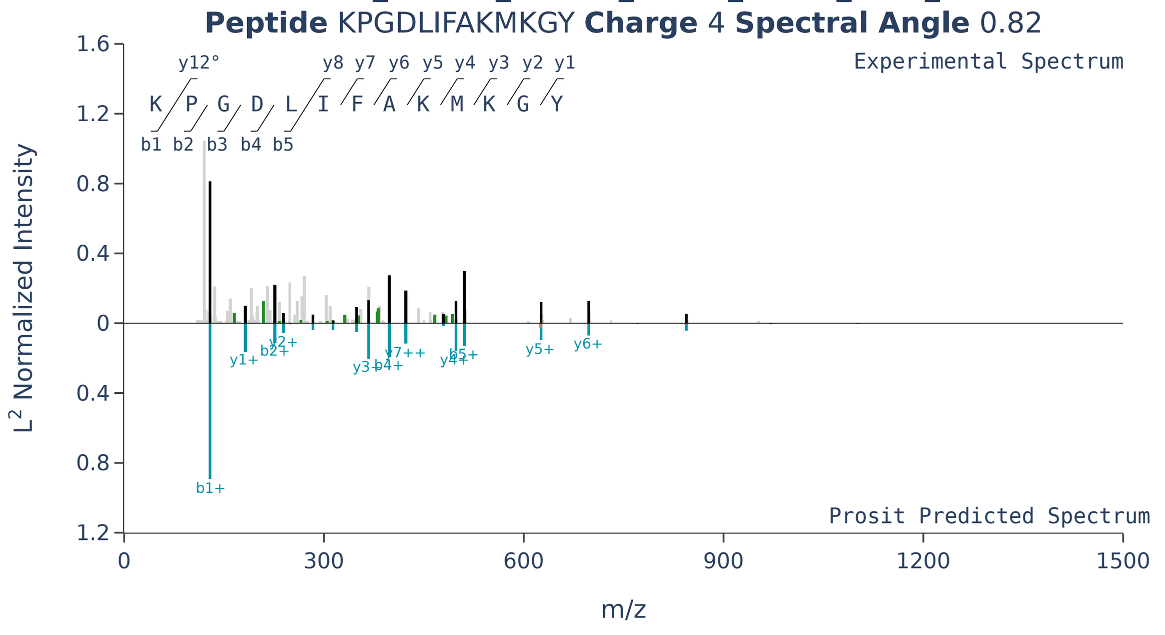


**Source** HRoetschke_271222_090123_HFGoe_LEDGF_b2R1_24h **Scan** 23771
**Peptide** FLGPKDIFPYSENKEKYG **Charge** 3 **Spectral Angle** 0.78

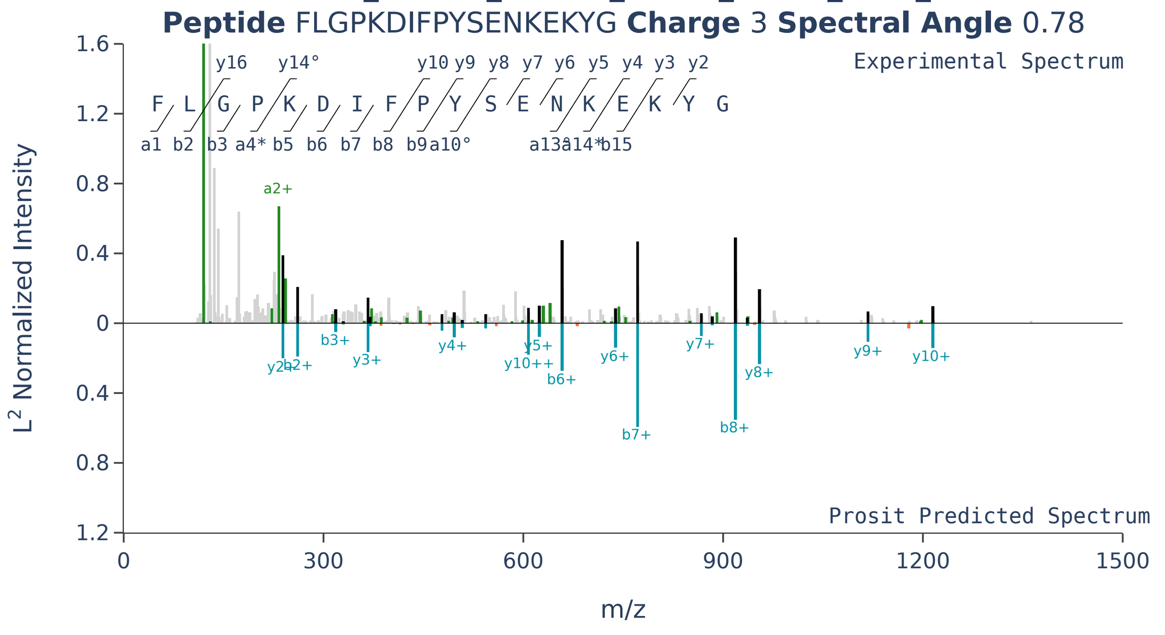


**Source** HRoetschke_271222_090123_HFGoe_LEDGF_b2R1_24h **Scan** 18494
**Peptide** FKVSQVIMEK **Charge** 3 **Spectral Angle** 0.89

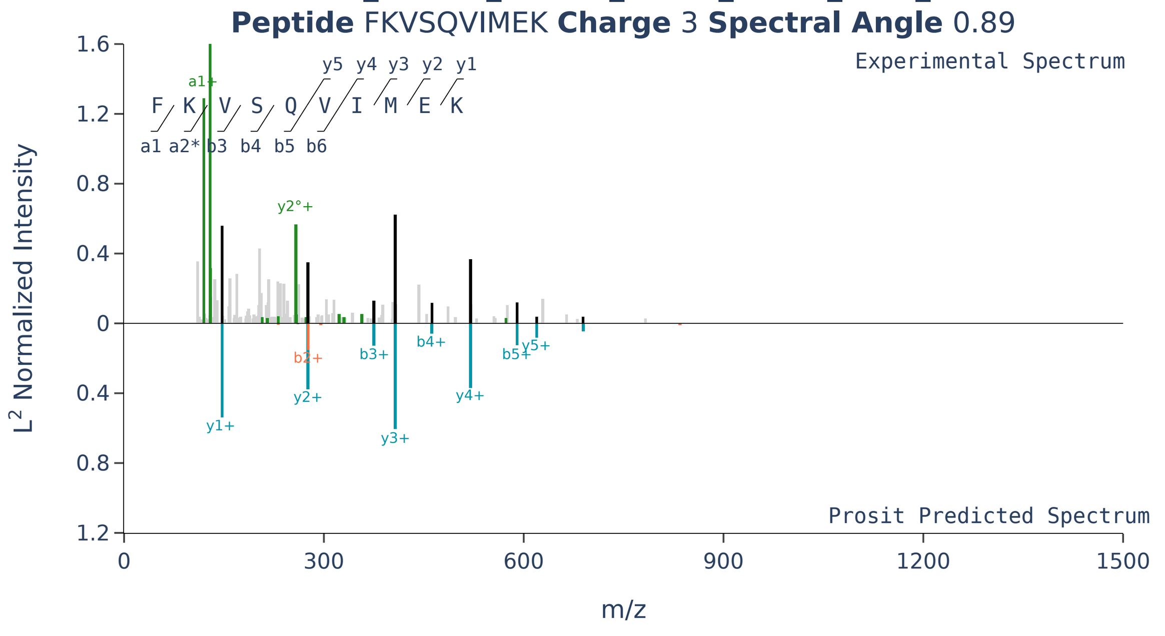


**Source** HRoetschke_271222_090123_HFGoe_LEDGF_b1R2_24h **Scan** 13621
**Peptide** AEKQVETEEAGVVTTA **Charge** 2 **Spectral Angle** 0.82

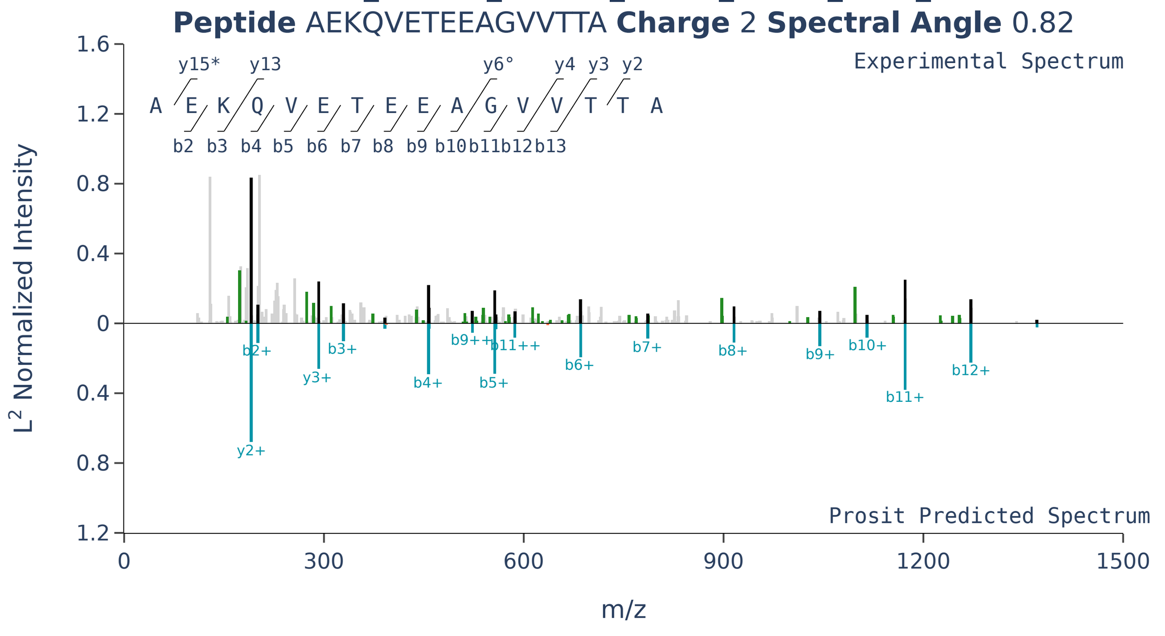


**Source** HRoetschke_271222_090123_HFGoe_LEDGF_b1R1_4h **Scan** 20611
**Peptide** KGFNEGLWEIDNNPKVK **Charge** 2 **Spectral Angle** 0.67

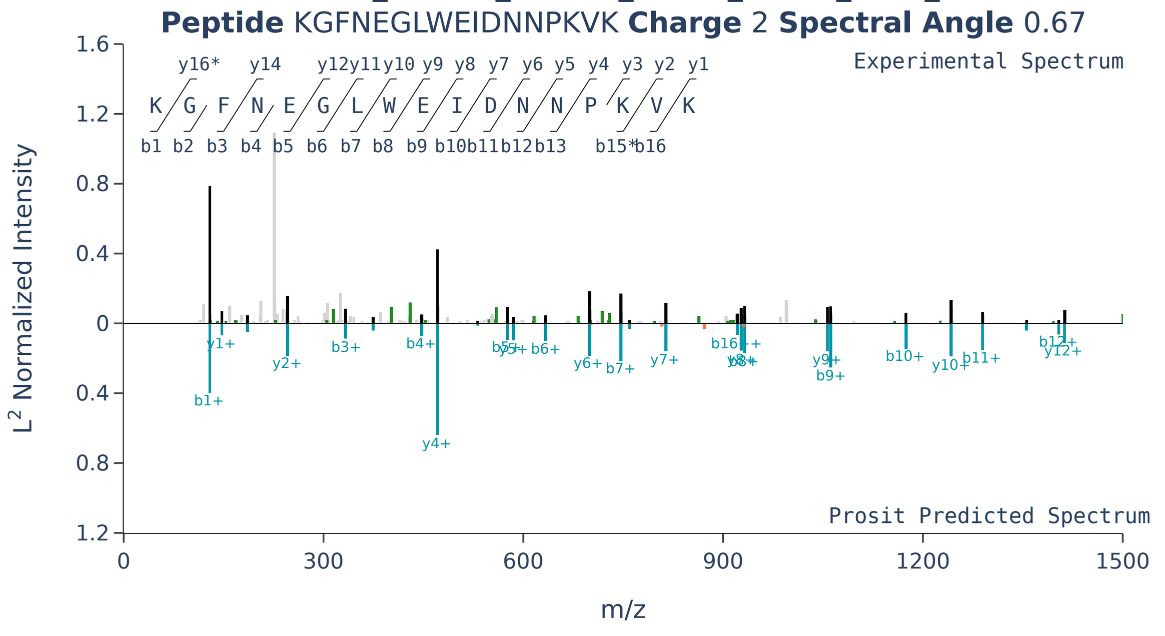


**Source** HRoetschke_271222_090123_HFGoe_LEDGF_b1R1_2h **Scan** 9000
**Peptide** SSQQAATKQSNASSDVEVEE **Charge** 2 **Spectral Angle** 0.75

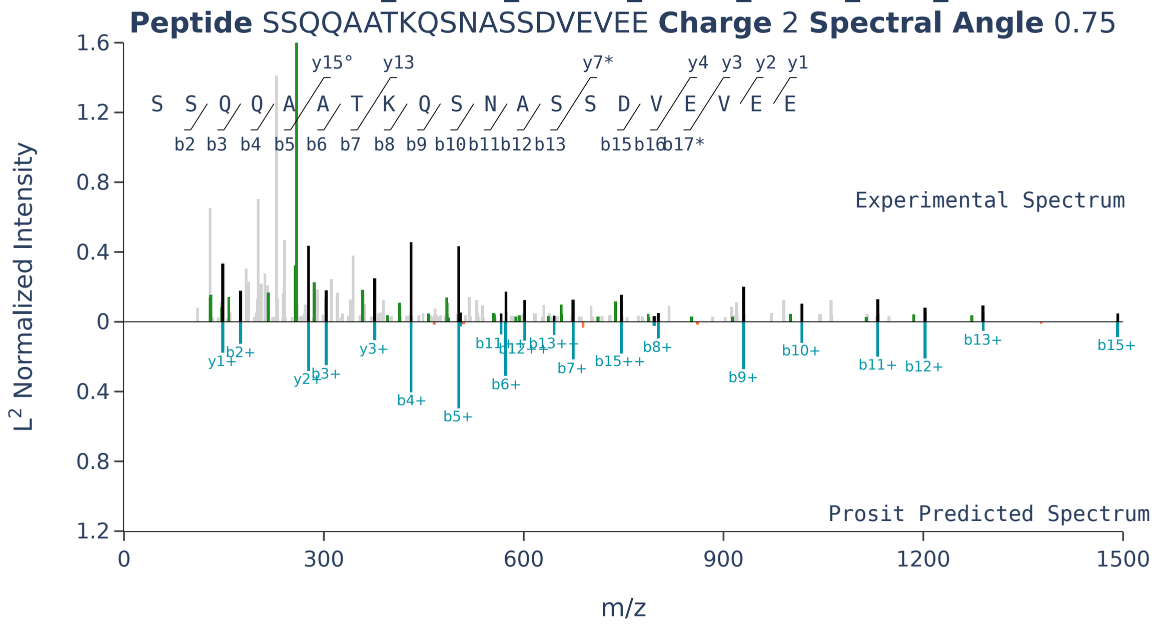


**Source** HRoetschke_271222_090123_HFGoe_LEDGF_b2R1_24h **Scan** 21654
**Peptide** GPKDIFPYSE **Charge** 2 **Spectral Angle** 0.78

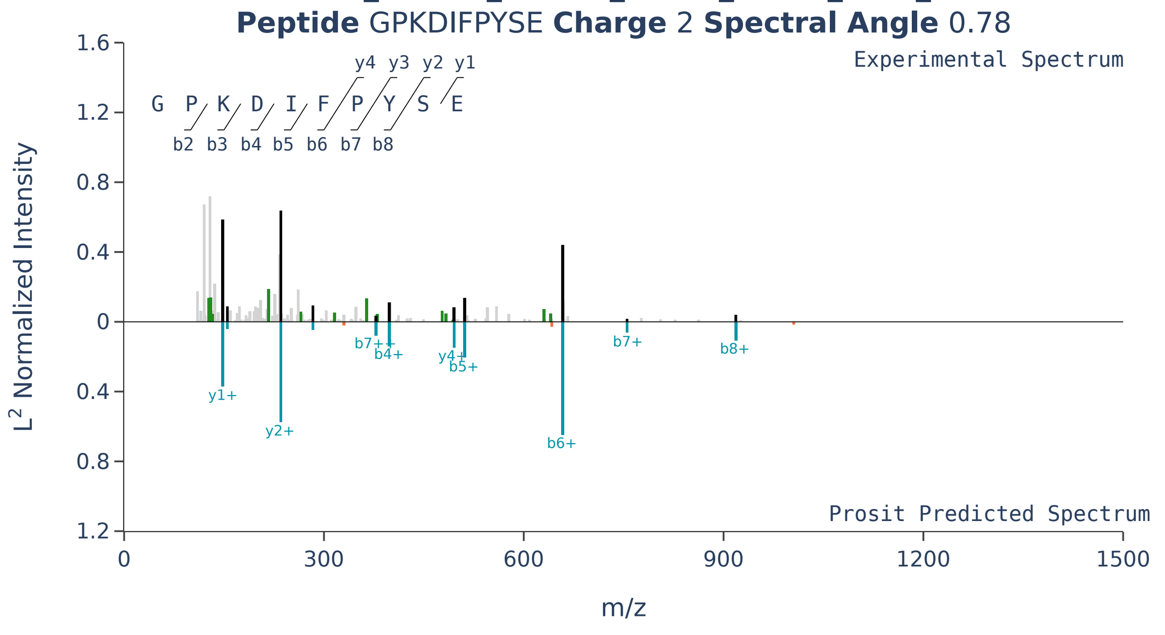


**Source** HRoetschke_271222_090123_HFGoe_LEDGF_b2R1_1h **Scan** 17284
**Peptide** KMKGYPHWPARVDEVPDGAVKPPTN **Charge** 4 **Spectral Angle** 0.84

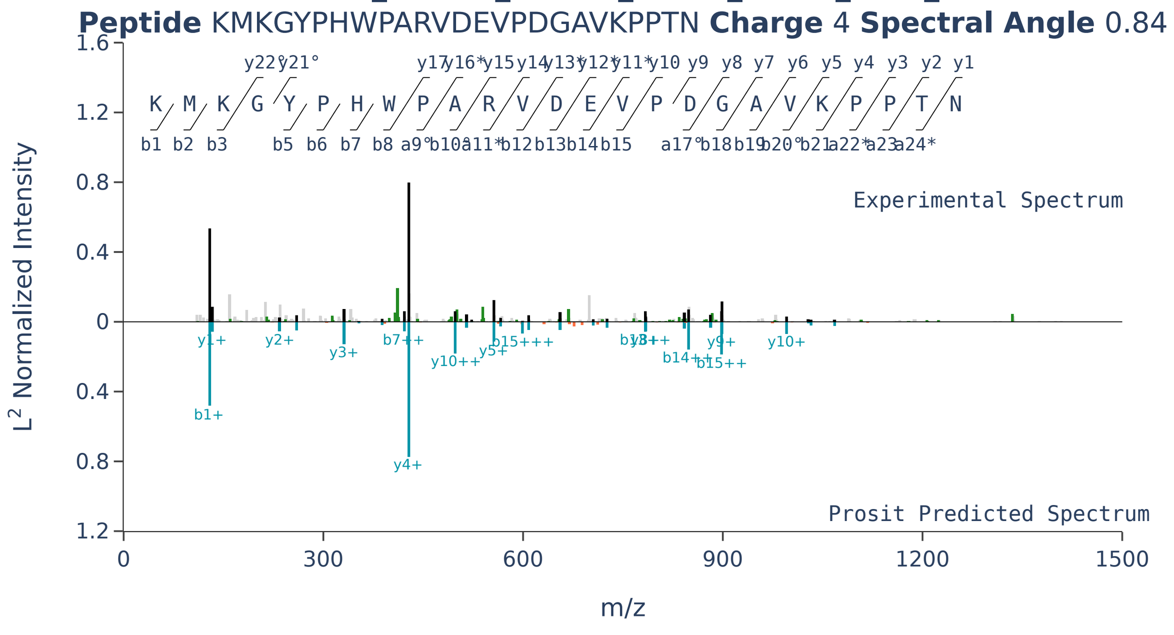


**Source** HRoetschke_271222_090123_HFGoe_LEDGF_b2R1_24h **Scan** 23585
**Peptide** TAFLGPKDIFPYSENKEK **Charge** 3 **Spectral Angle** 0.83

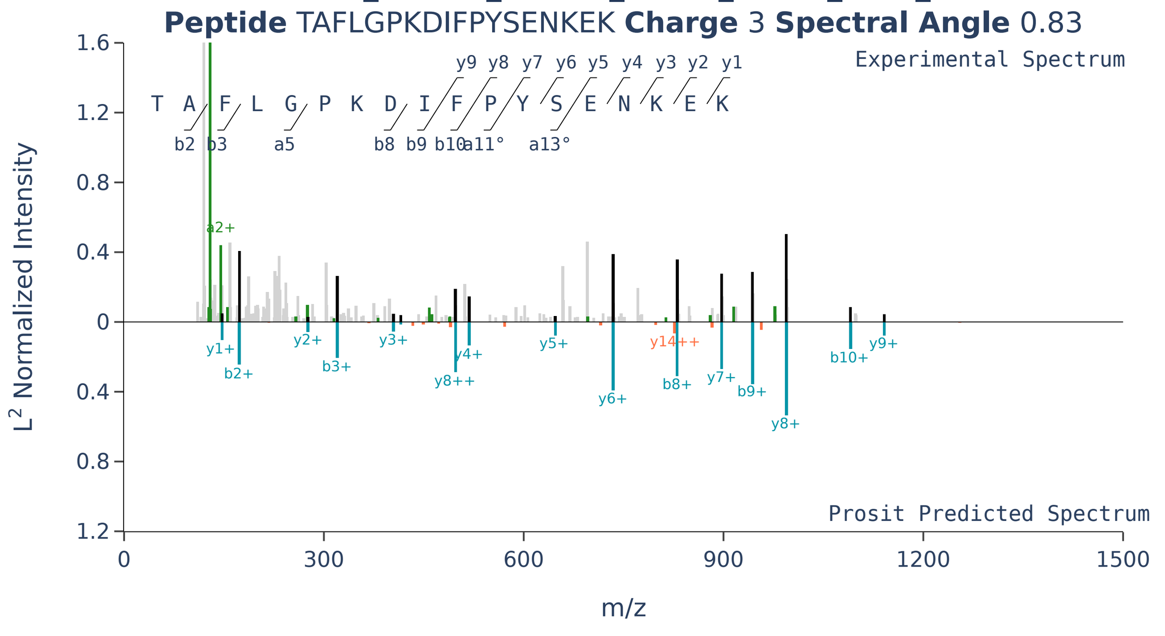


**Source** HRoetschke_271222_090123_HFGoe_LEDGF_b2R2_2h **Scan** 11294
**Peptide** ATASVNLKVSPKRGRPAATEV **Charge** 3 **Spectral Angle** 0.84

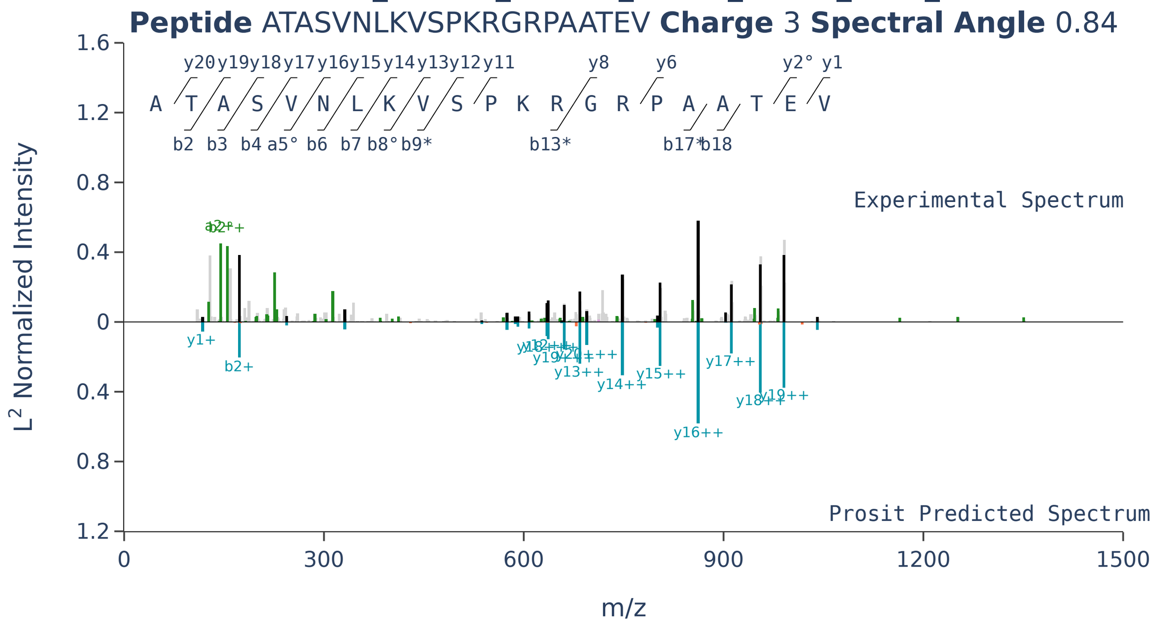


**Source** HRoetschke_271222_090123_HFGoe_LEDGF_b1R2_4h **Scan** 9587
**Peptide** KPPTNKL **Charge** 2 **Spectral Angle** 0.87

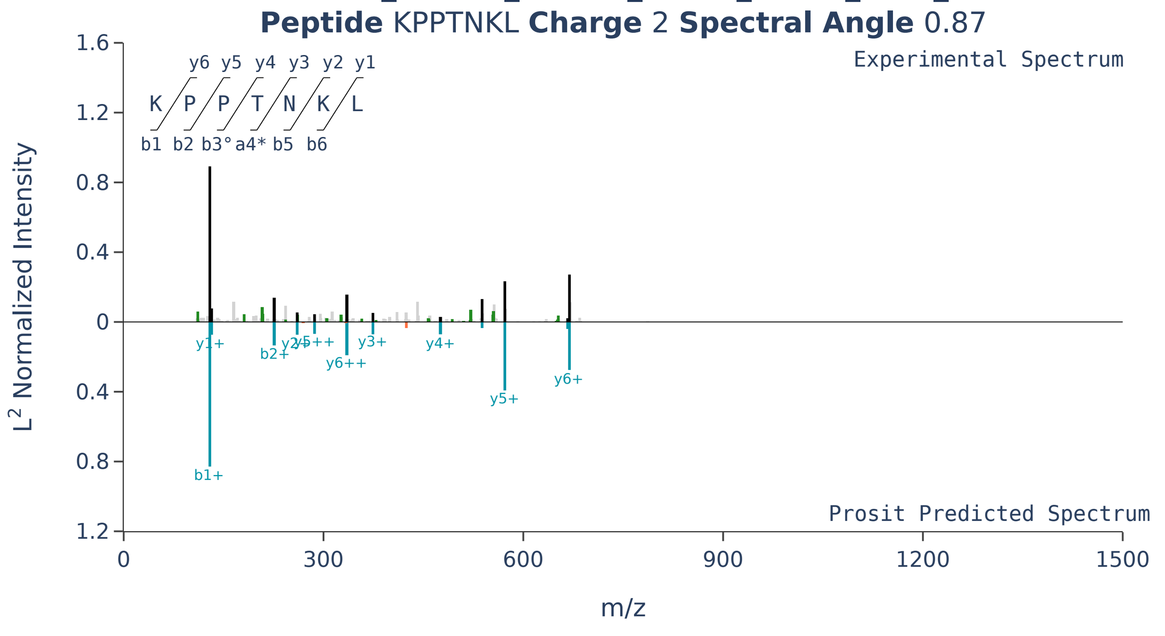

**Source** HRoetschke_271222_090123_HFGoe_LEDGF_b1R2_2h **Scan** 26287
**Peptide** NMFLVGEGDSVITQ **Charge** 2 **Spectral Angle** 0.91

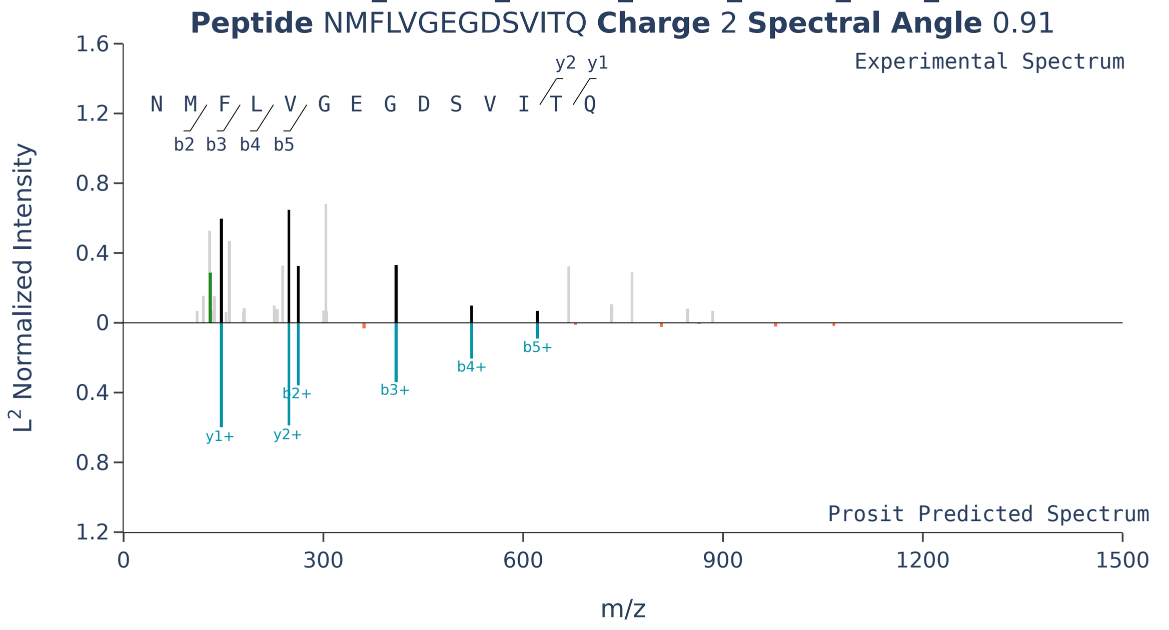


**Source** HRoetschke_271222_090123_HFGoe_LEDGF_b2R1_2h **Scan** 11721
**Peptide** EKQVETEEAGVVTT **Charge** 2 **Spectral Angle** 0.85

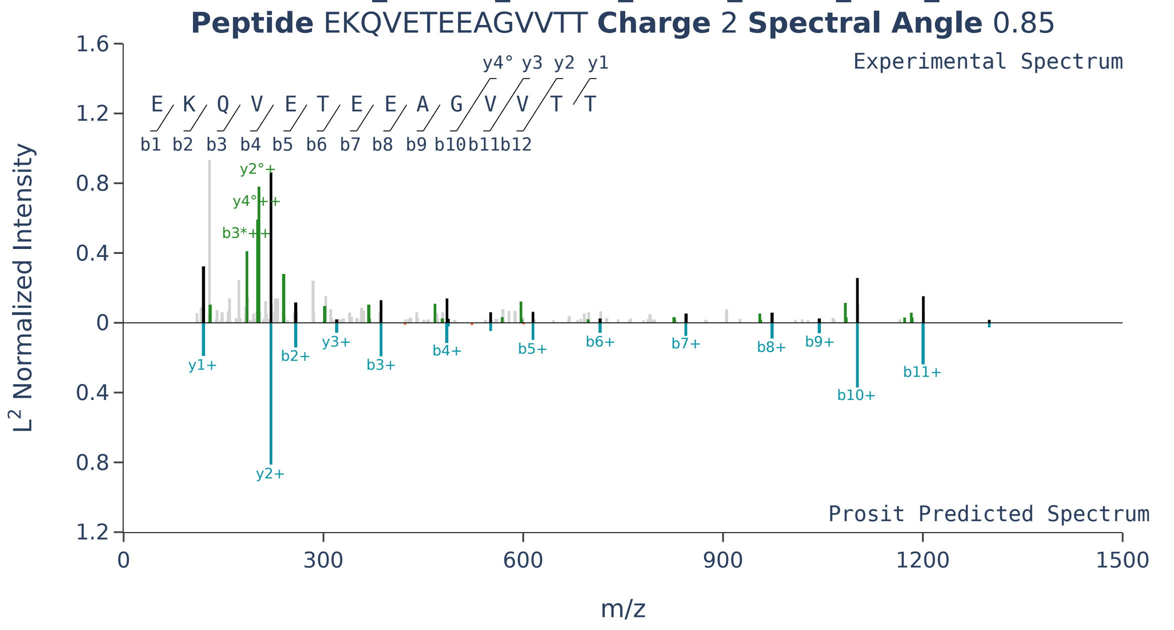


**Source** HRoetschke_271222_090123_HFGoe_LEDGF_b1R2_4h **Scan** 13425
**Peptide** EKQVETEEAGVVTTAT **Charge** 2 **Spectral Angle** 0.8

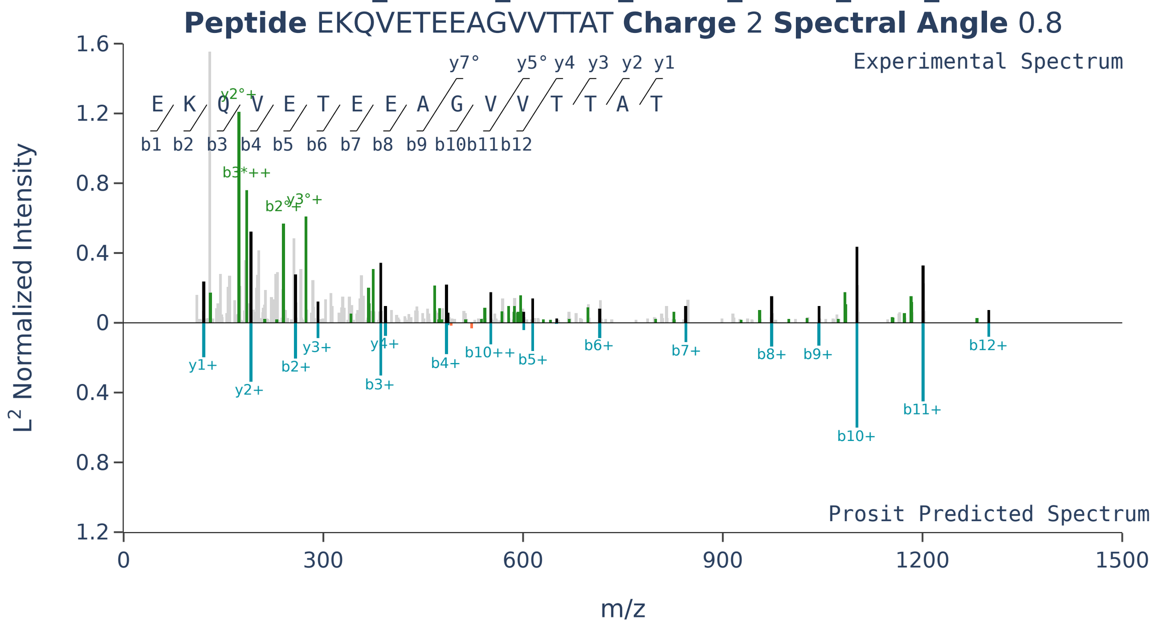


**Source** HRoetschke_271222_090123_HFGoe_LEDGF_b2R1_4h **Scan** 16334
**Peptide** TAFLGPKDIFPYSENKEKYGKPNKRKG **Charge** 4 **Spectral Angle** 0.82

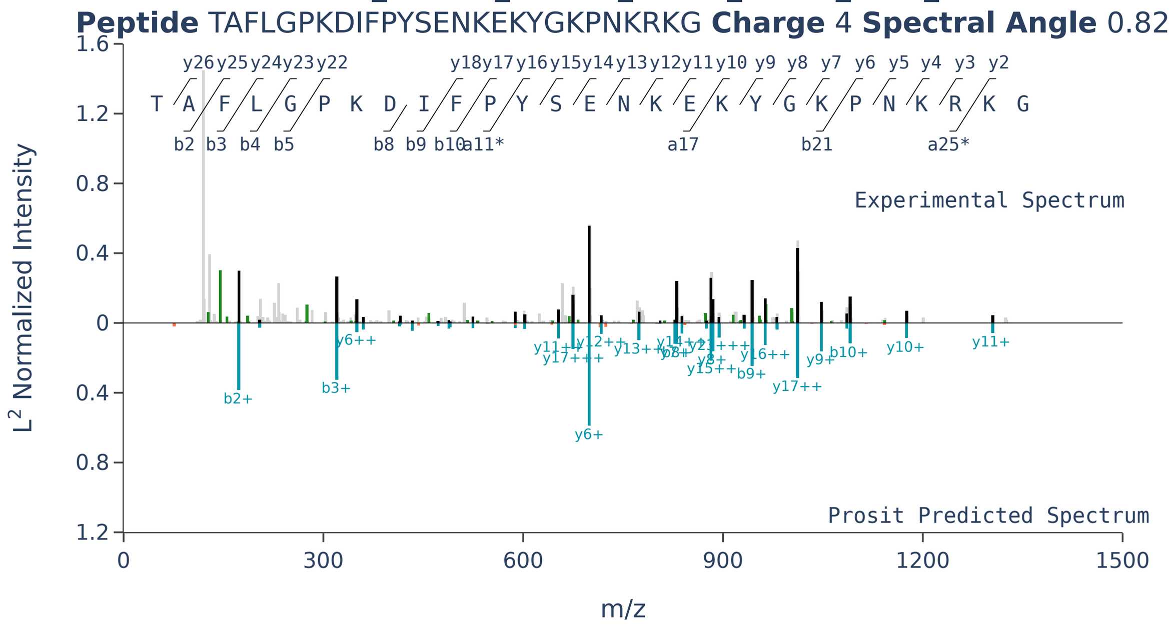


**Source** HRoetschke_271222_090123_HFGoe_LEDGF_b2R1_4h **Scan** 17818
**Peptide** TEMITTLKKIRRF **Charge** 4 **Spectral Angle** 0.92

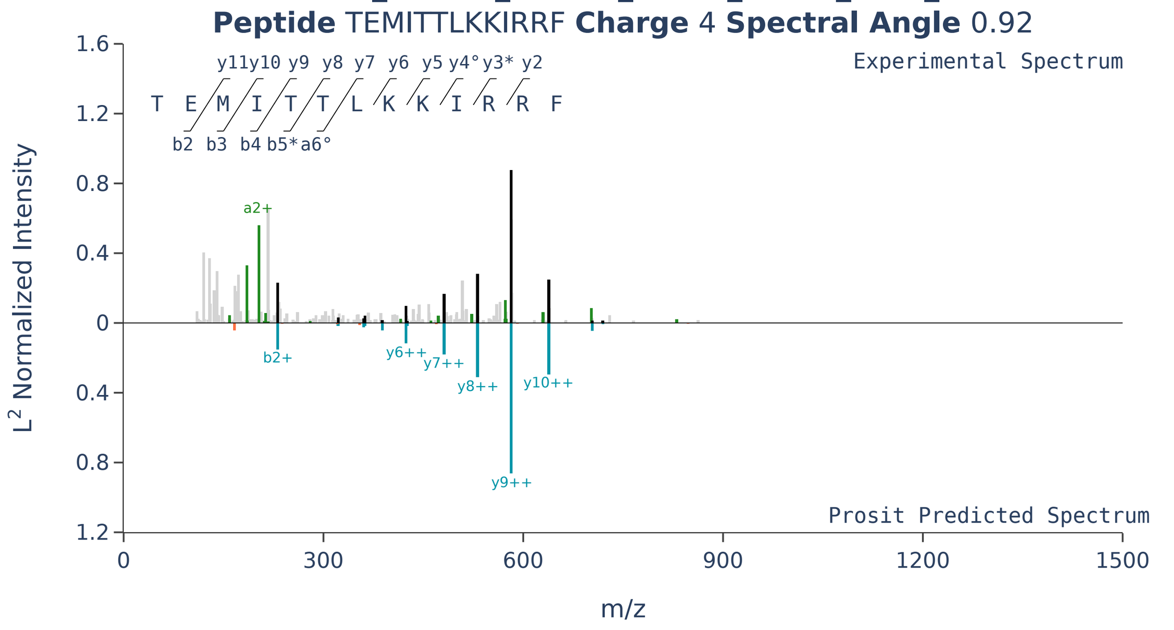


**Source** HRoetschke_271222_090123_HFGoe_LEDGF_b1R2_2h **Scan** 7407
**Peptide** QAATKQSNASSDVEVEEKE **Charge** 3 **Spectral Angle** 0.83

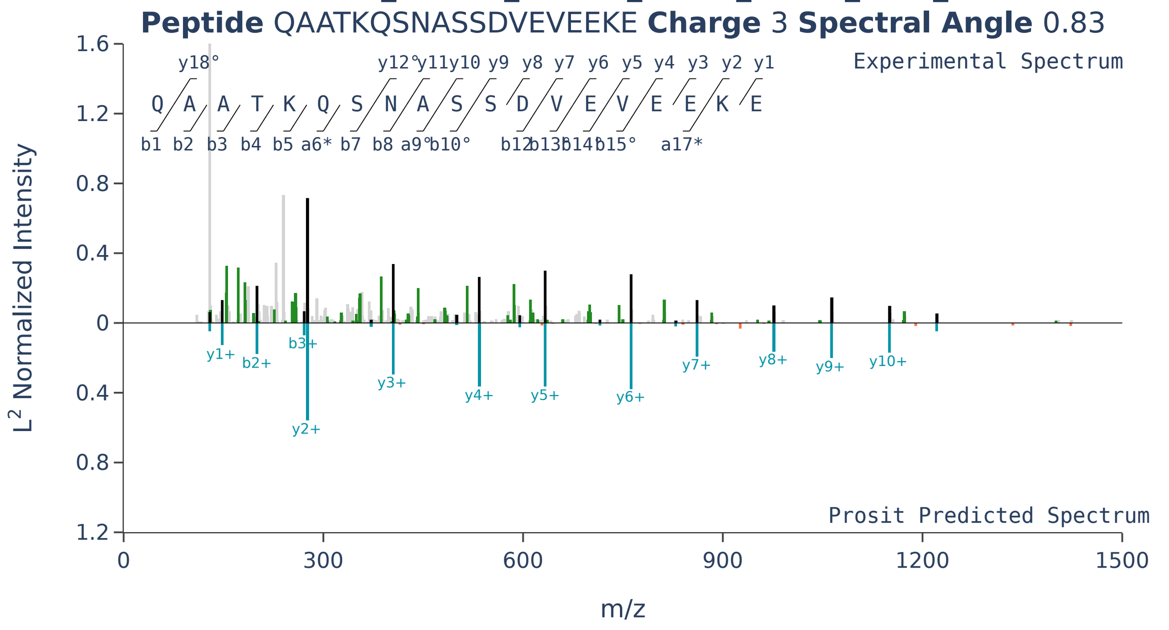


**Source** HRoetschke_271222_090123_HFGoe_LEDGF_b2R2_4h **Scan** 12983
**Peptide** RRFKVSQVI **Charge** 3 **Spectral Angle** 0.88

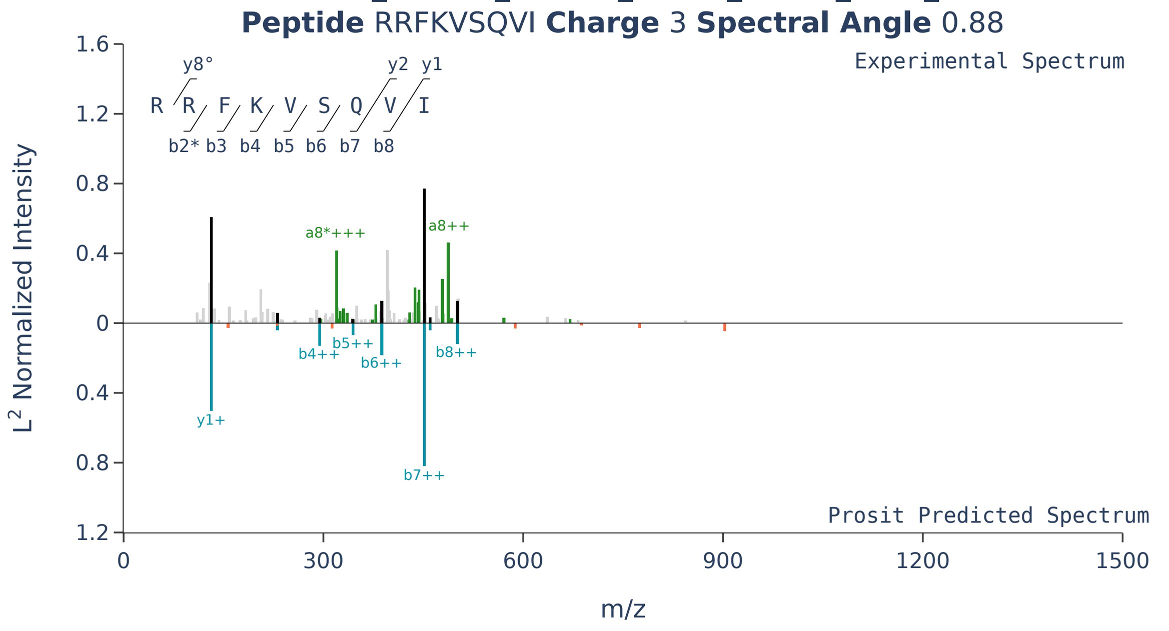


**Source** HRoetschke_271222_090123_HFGoe_LEDGF_b1R2_24h **Scan** 14904
**Peptide** NKFKNMF **Charge** 1 **Spectral Angle** 0.82

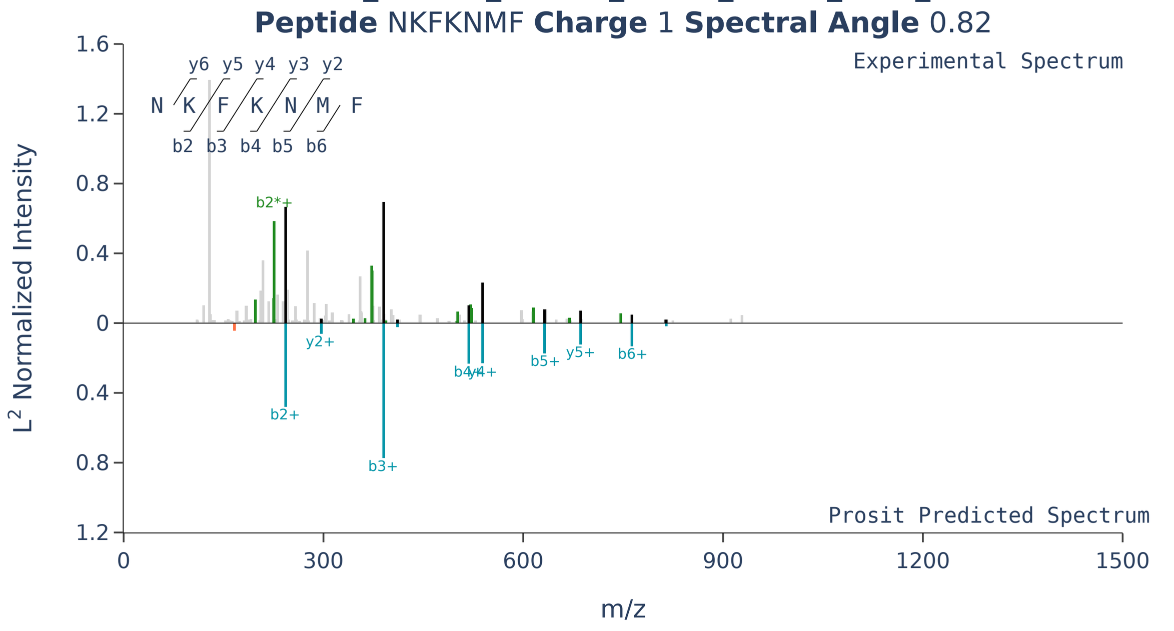


**Source** HRoetschke_271222_090123_HFGoe_LEDGF_b1R1_2h **Scan** 26898
**Peptide** DFKPGDLI **Charge** 2 **Spectral Angle** 0.79

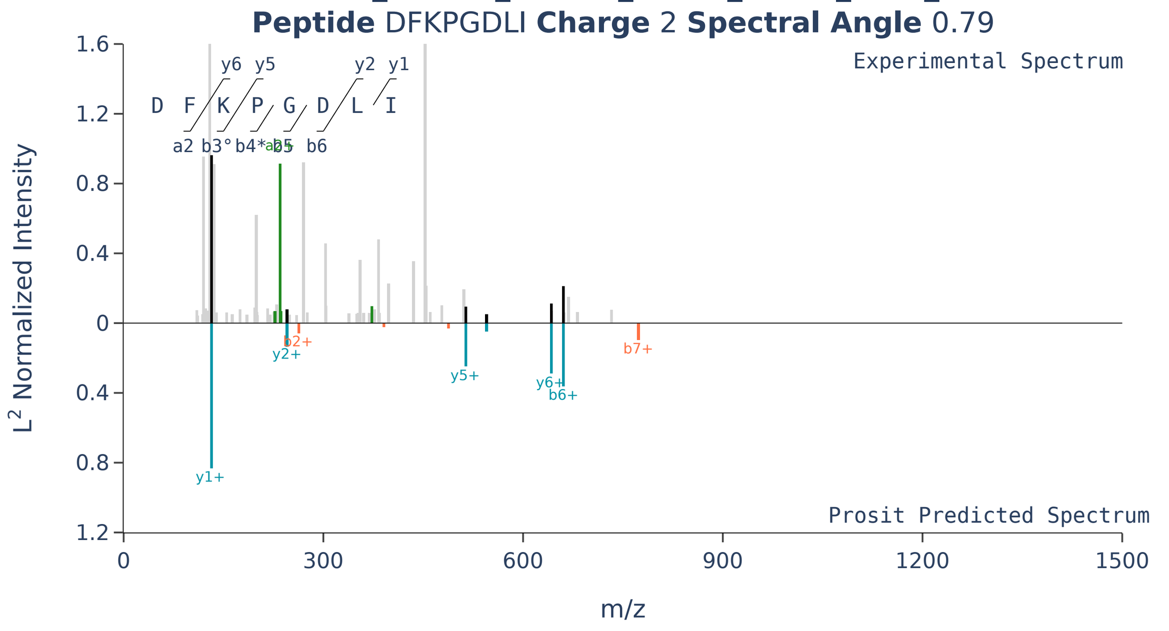


**Source** HRoetschke_271222_090123_HFGoe_LEDGF_b1R2_24h **Scan** 6318
**Peptide** VSPKRGRPAATEV **Charge** 3 **Spectral Angle** 0.86

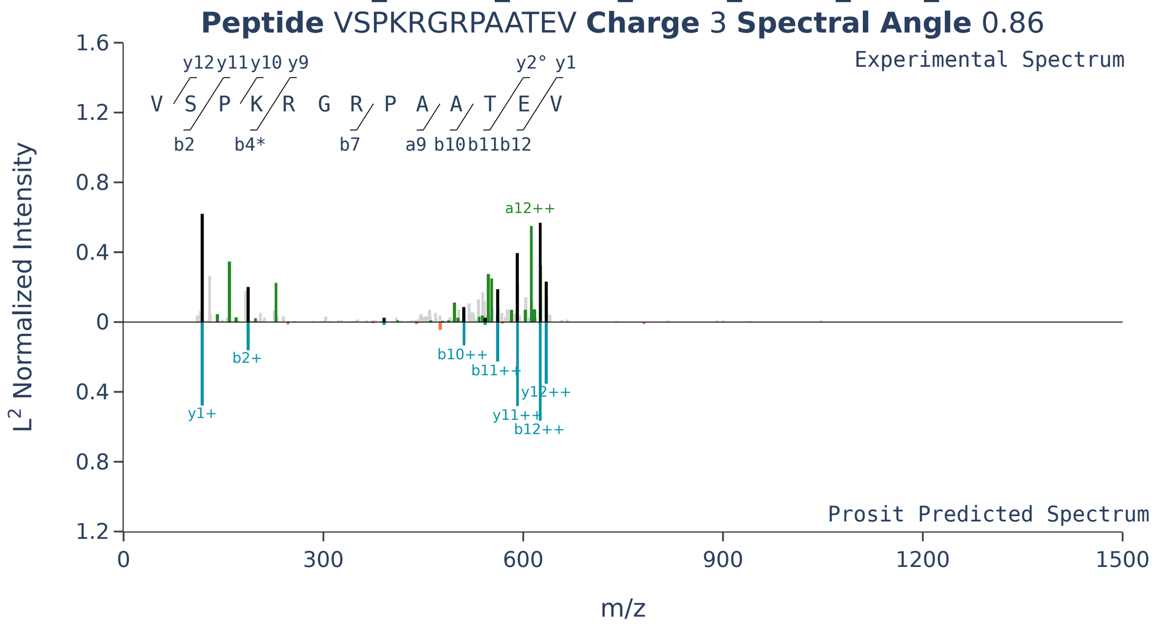


**Source** HRoetschke_271222_090123_HFGoe_LEDGF_b1R1_2h **Scan** 23031
**Peptide** NKFKNMFLVGEGD **Charge** 2 **Spectral Angle** 0.77

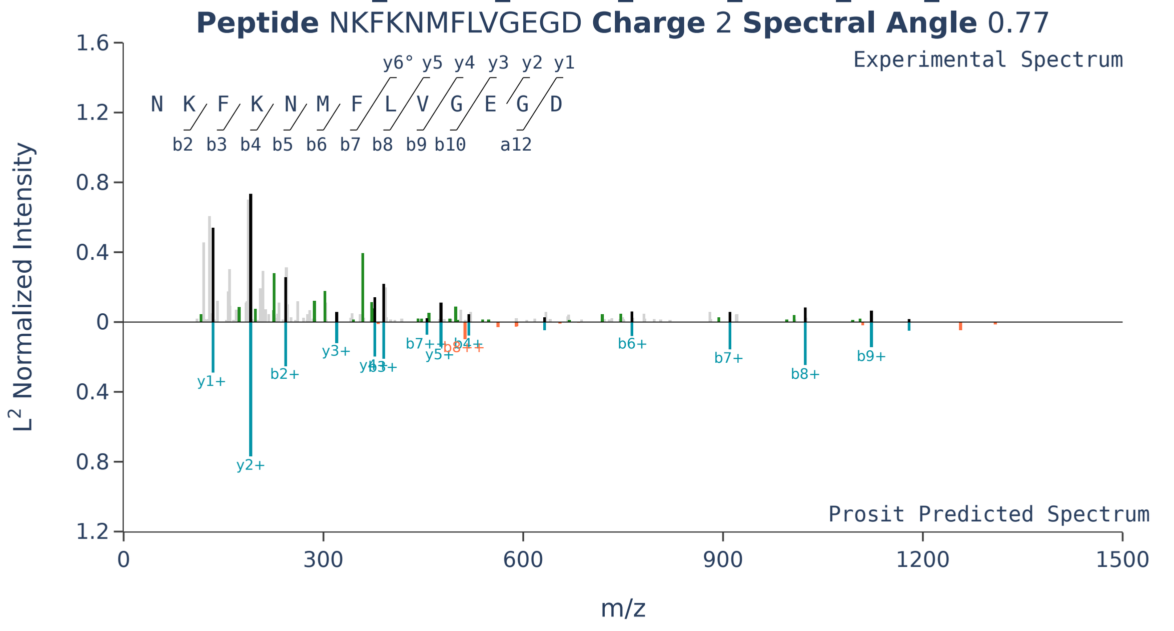


**Source** HRoetschke_271222_090123_HFGoe_LEDGF_b1R2_4h **Scan** 13726
**Peptide** KNSLKIDNLD **Charge** 3 **Spectral Angle** 0.89

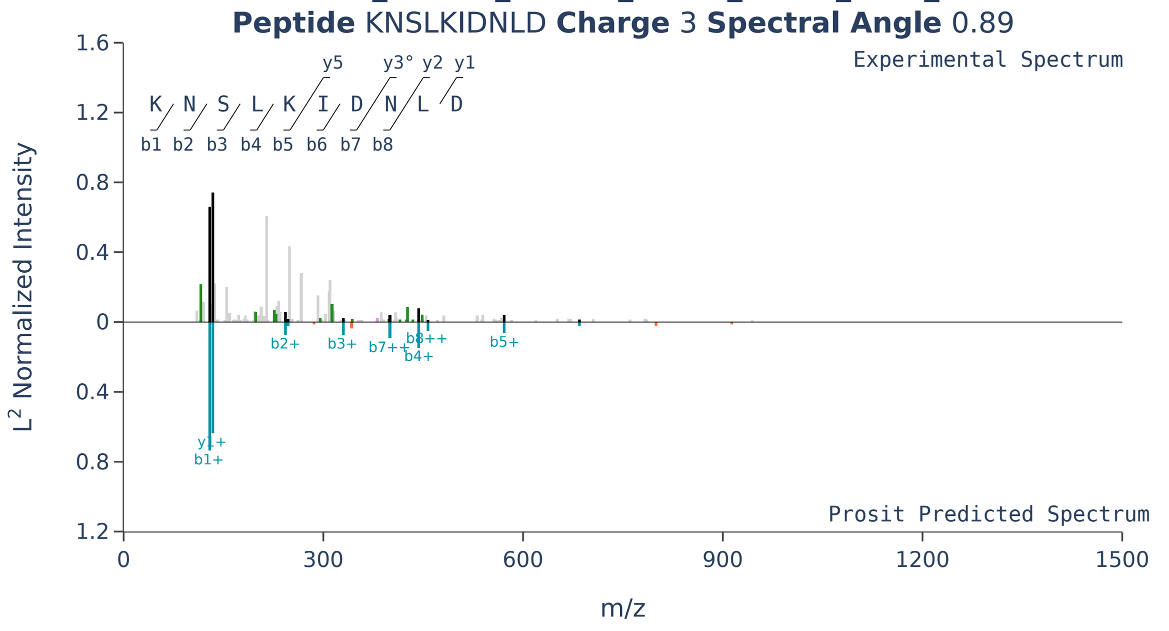


**Source** HRoetschke_271222_090123_HFGoe_LEDGF_b1R1_4h **Scan** 11560
**Peptide** QVLNKSLAEQ **Charge** 2 **Spectral Angle** 0.84

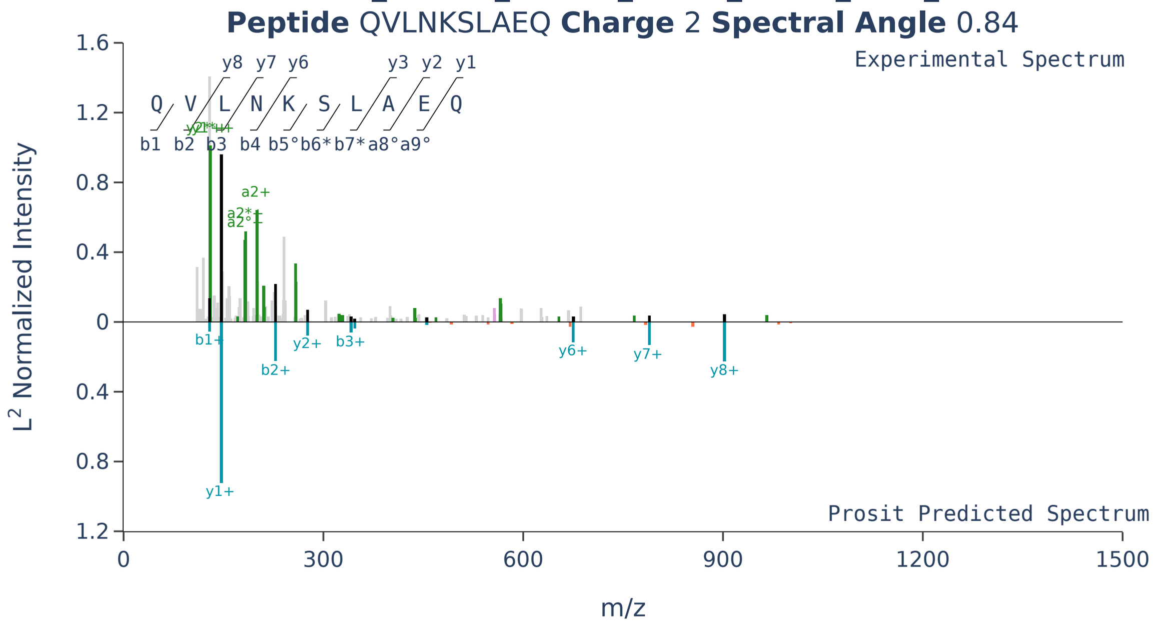


**Source** HRoetschke_271222_090123_HFGoe_LEDGF_b2R1_24h **Scan** 9609
**Peptide** ATASVNLKVSPKR **Charge** 3 **Spectral Angle** 0.74

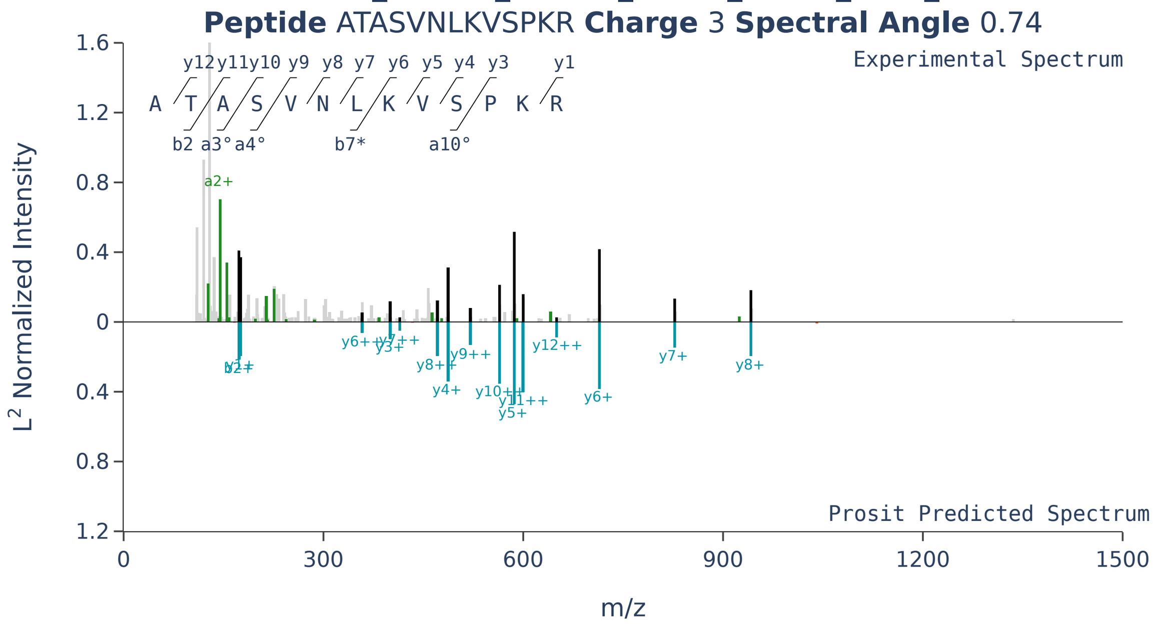


**Source** HRoetschke_271222_090123_HFGoe_LEDGF_b1R1_24h **Scan** 14807
**Peptide** PKDIFPYSENKEKYG **Charge** 4 **Spectral Angle** 0.79

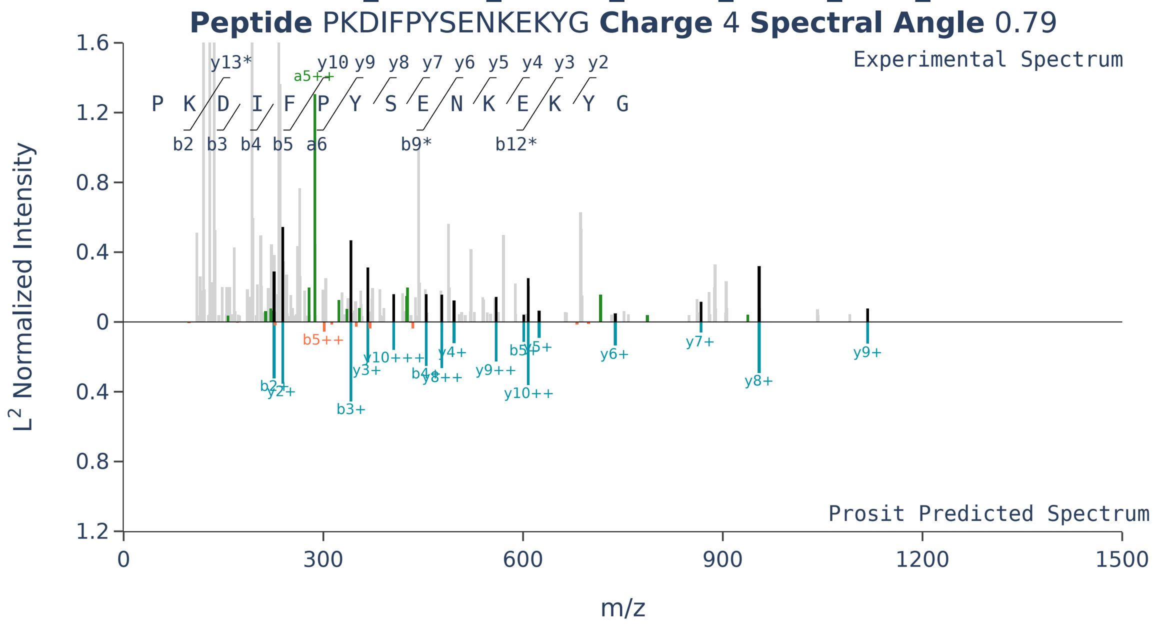

**Source** HRoetschke_271222_090123_HFGoe_LEDGF_b1R1_24h **Scan** 10441
**Peptide** KIDNLDVNRC **Charge** 2 **Spectral Angle** 0.81

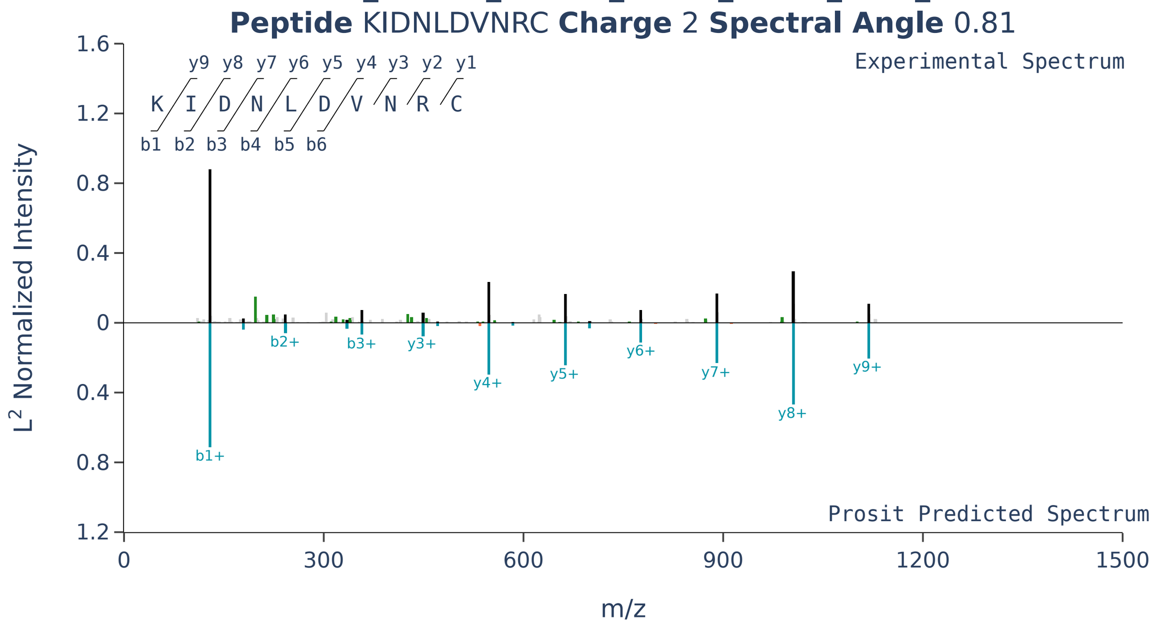


**Source** HRoetschke_271222_090123_HFGoe_LEDGF_b1R1_4h **Scan** 8034
**Peptide** ASVNLKVSPKRGRPAATE **Charge** 4 **Spectral Angle** 0.73

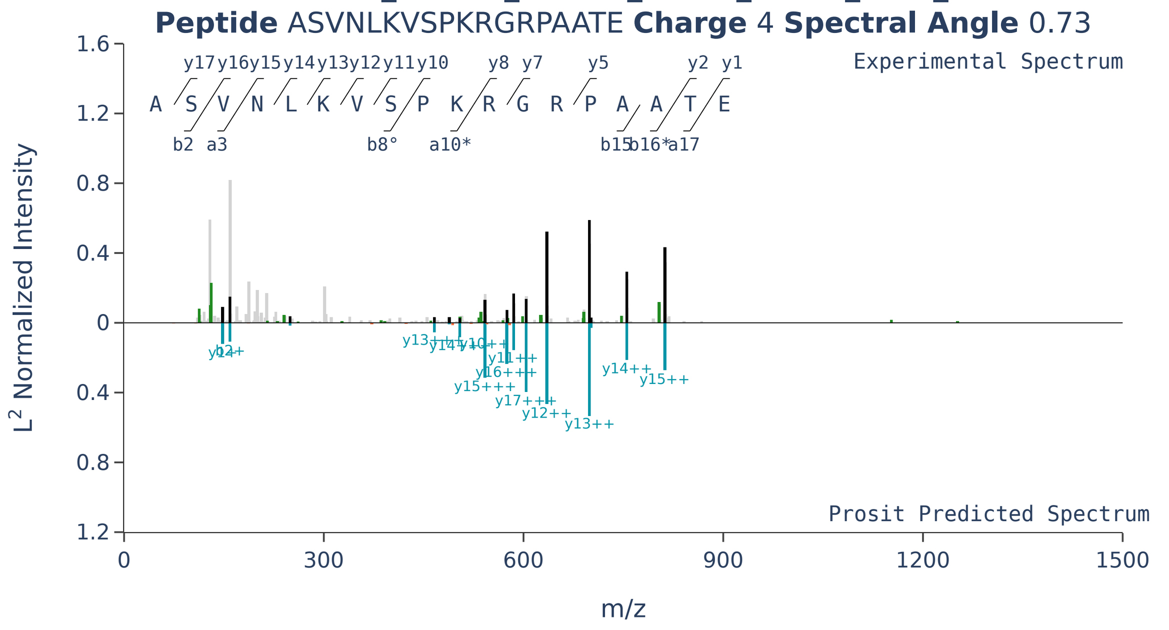


**Source** HRoetschke_271222_090123_HFGoe_LEDGF_b2R1_24h **Scan** 19939
**Peptide** GLWEIDNNPKVKFSSQQAATKQS **Charge** 3 **Spectral Angle** 0.87

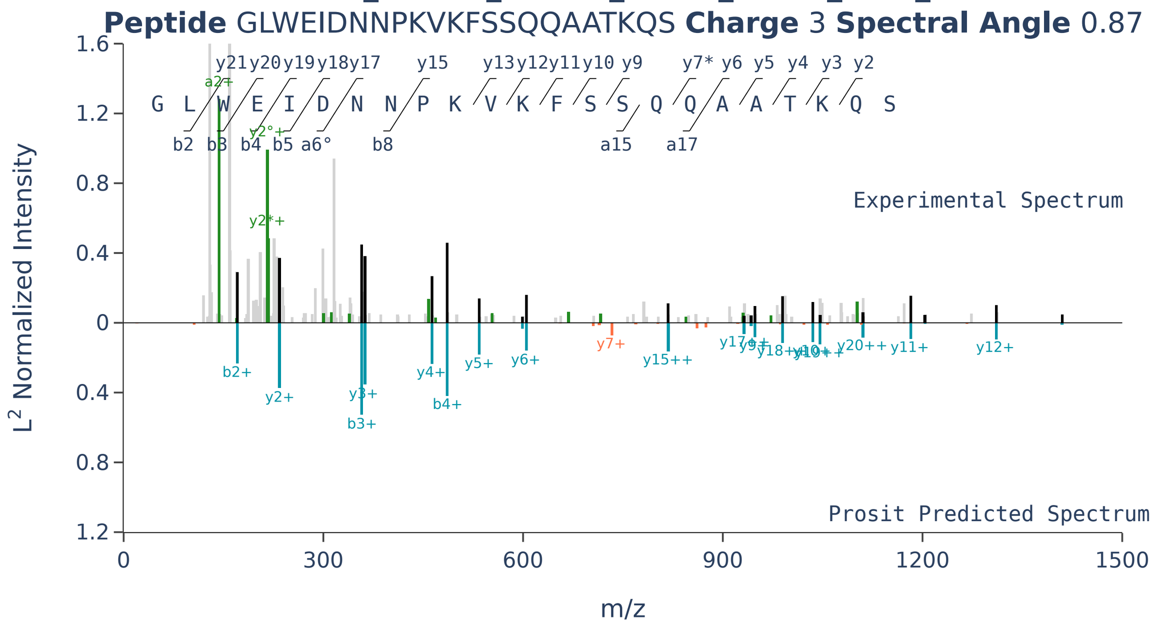


**Source** HRoetschke_271222_090123_HFGoe_LEDGF_b1R2_4h **Scan** 22048
**Peptide** DELASLQVTMQQA **Charge** 2 **Spectral Angle** 0.91

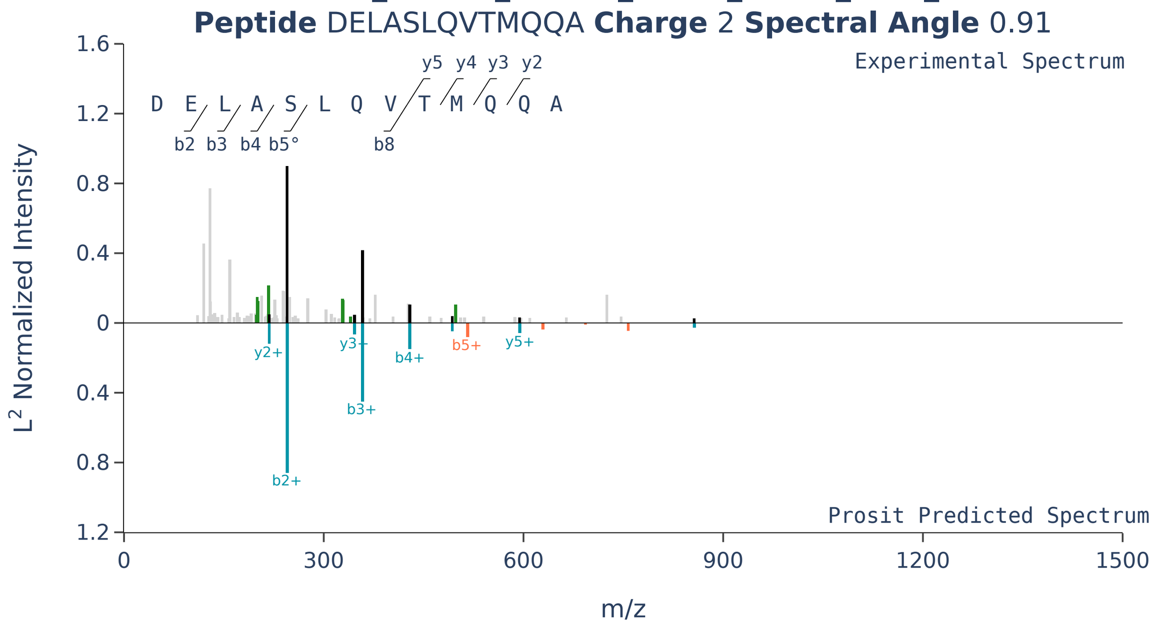


**Source** HRoetschke_271222_090123_HFGoe_LEDGF_b2R2_4h **Scan** 13603
**Peptide** TRDFKPGDL **Charge** 2 **Spectral Angle** 0.85

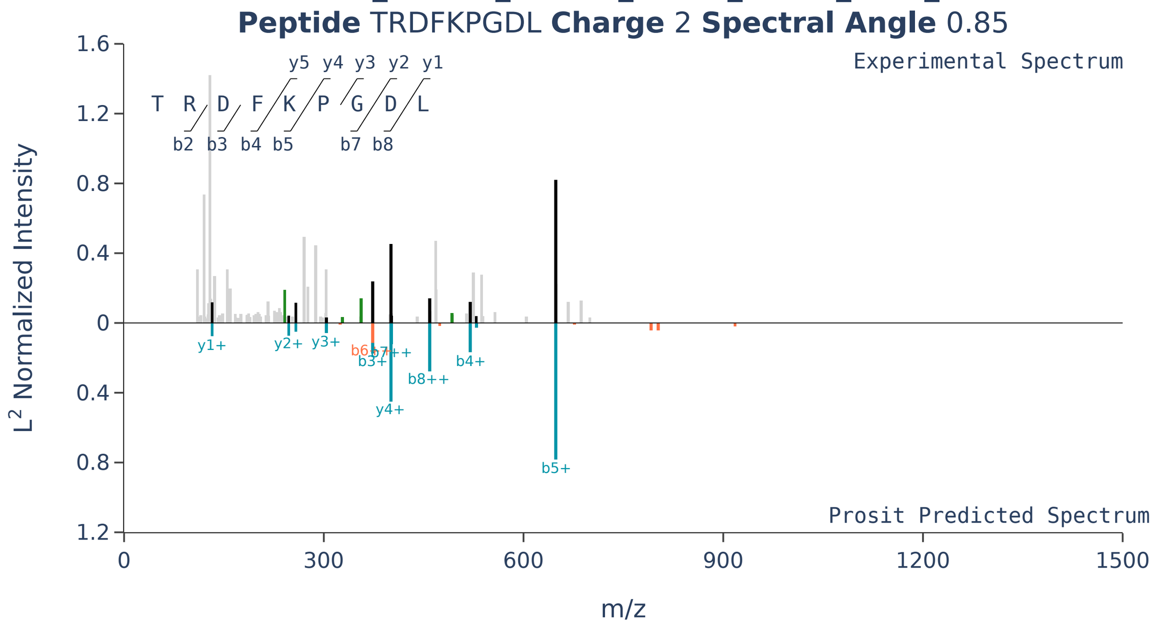


**Source** HRoetschke_271222_090123_HFGoe_LEDGF_b1R2_4h **Scan** 23275
**Peptide** FGTHETAFL **Charge** 2 **Spectral Angle** 0.76

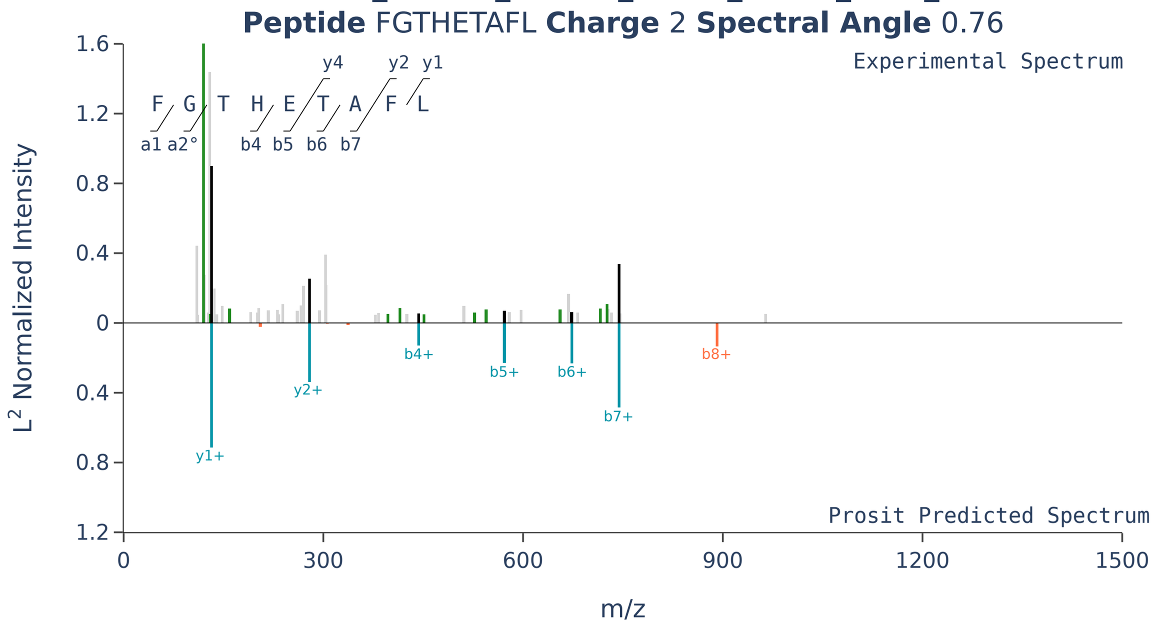


**Source** HRoetschke_271222_090123_HFGoe_LEDGF_b1R2_2h **Scan** 17610
**Peptide** AFLGPKDIFPYSENKEKYGKPNKR **Charge** 6 **Spectral Angle** 0.74

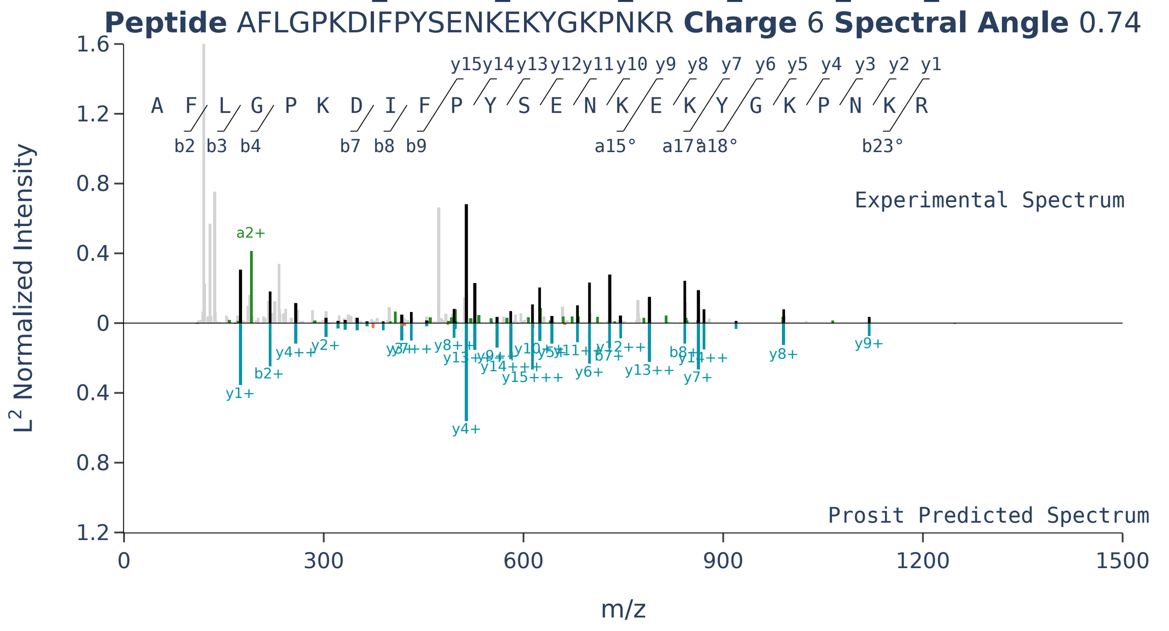


**Source** HRoetschke_271222_090123_HFGoe_LEDGF_b1R1_1h **Scan** 18889
**Peptide** FLVGEGD **Charge** 1 **Spectral Angle** 0.9

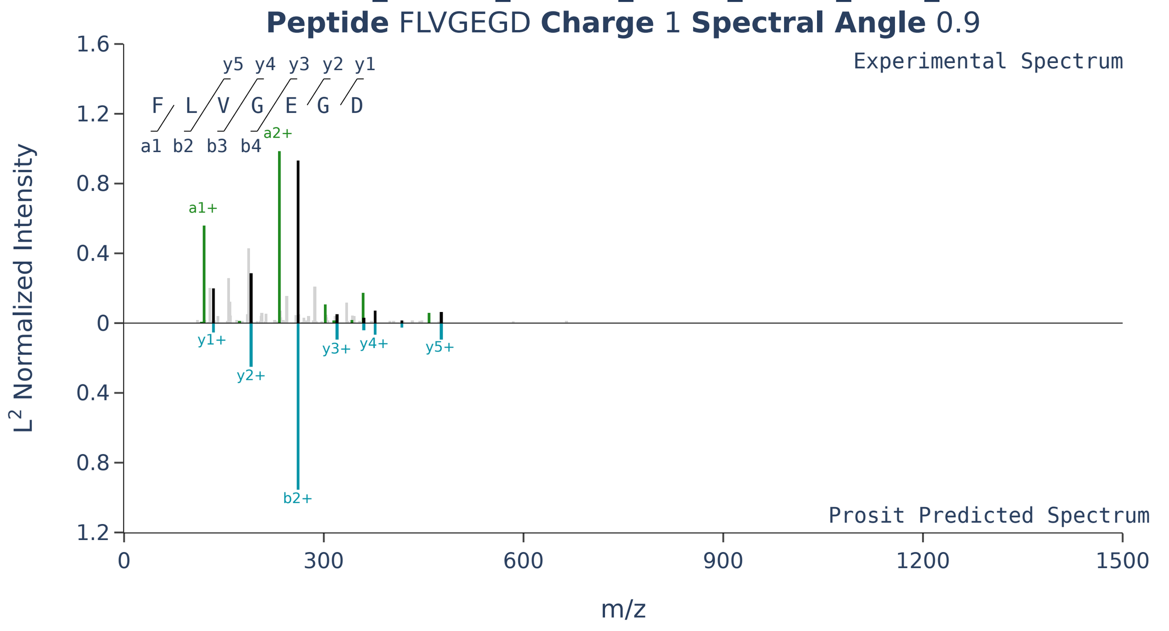


**Source** HRoetschke_271222_090123_HFGoe_LEDGF_b1R1_2h **Scan** 23090
**Peptide** LWEIDNNPKV **Charge** 2 **Spectral Angle** 0.76

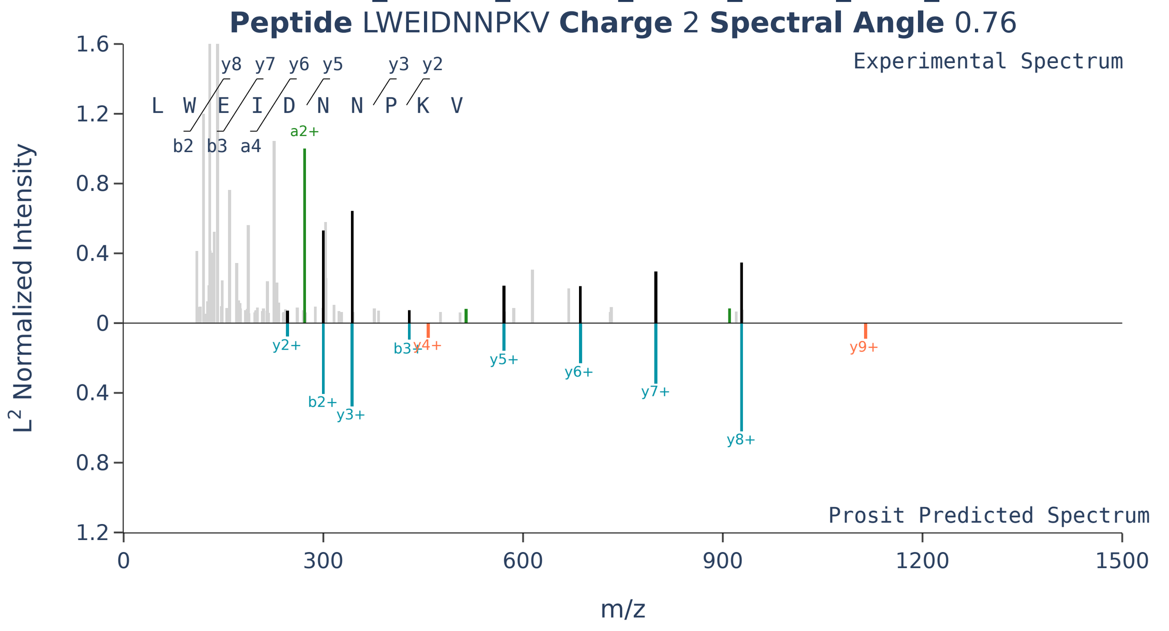


**Source** HRoetschke_271222_090123_HFGoe_LEDGF_b1R2_4h **Scan** 18664
**Peptide** YPHWPARVDEVPDGAVKPPTNK **Charge** 3 **Spectral Angle** 0.87

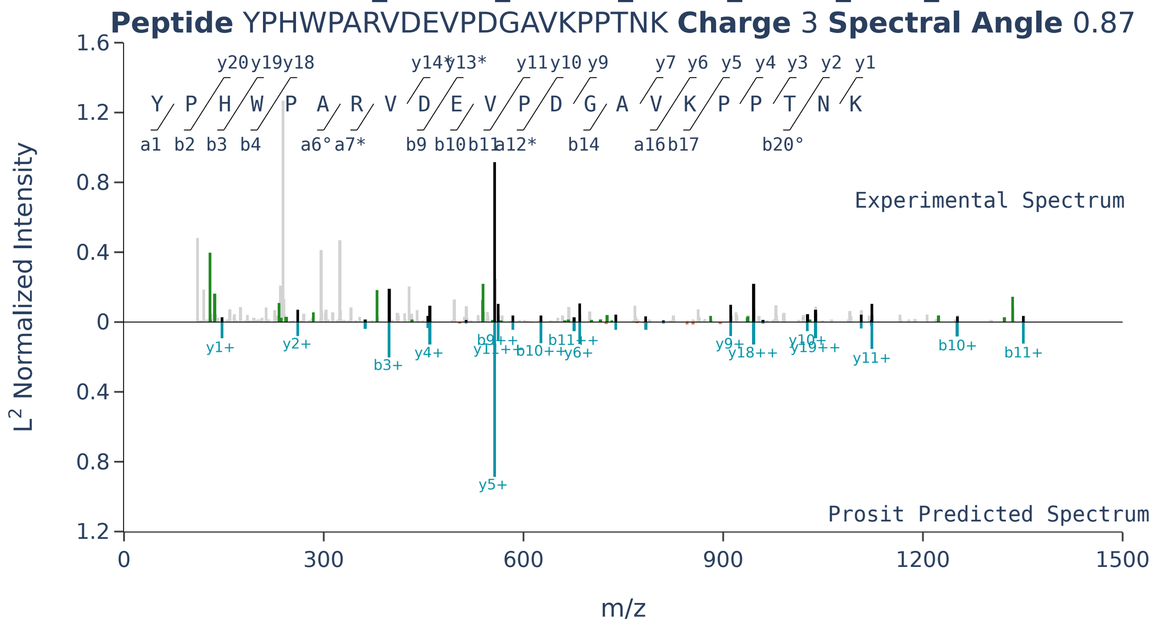


**Source** HRoetschke_271222_090123_HFGoe_LEDGF_b2R1_24h **Scan** 17111
**Peptide** RIHAEIKNSLKIDNL **Charge** 4 **Spectral Angle** 0.82

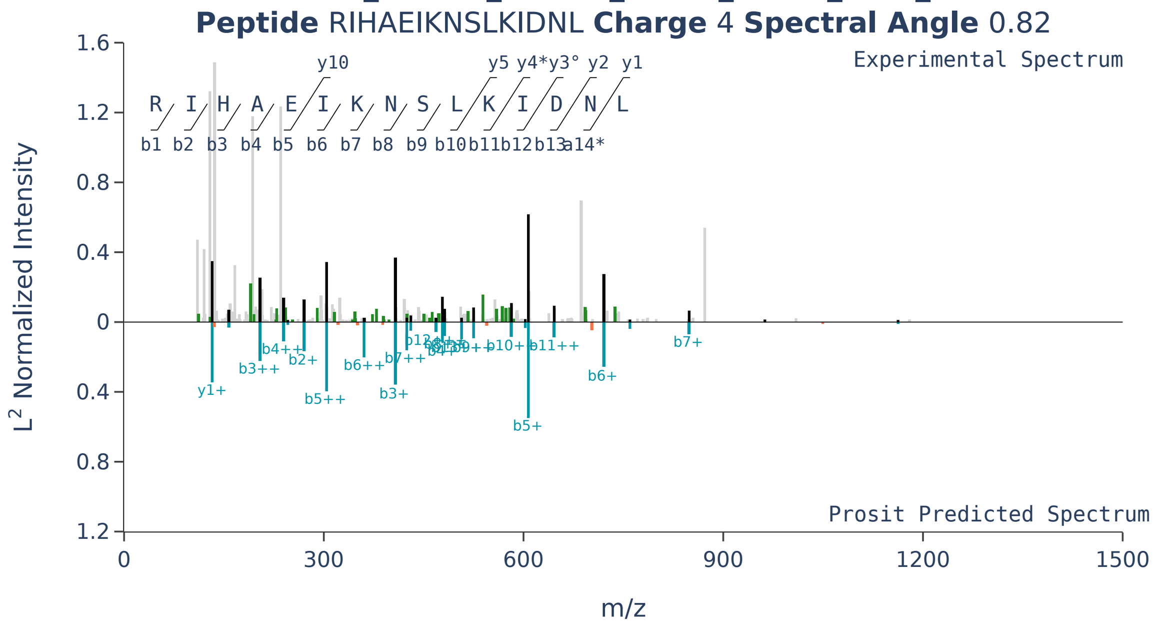


**Source** HRoetschke_271222_090123_HFGoe_LEDGF_b2R2_24h **Scan** 22752
**Peptide** NKFKNMFLVG **Charge** 2 **Spectral Angle** 0.76

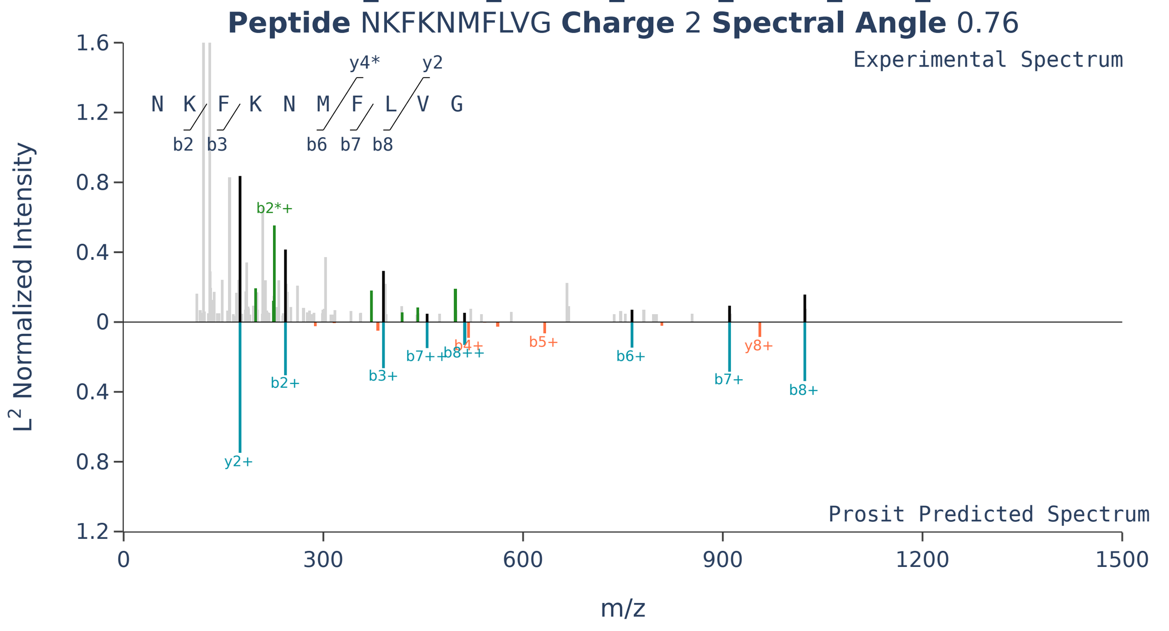


**Source** HRoetschke_271222_090123_HFGoe_LEDGF_b1R2_1h **Scan** 10199
**Peptide** ETSMDSRLQRIH **Charge** 4 **Spectral Angle** 0.87

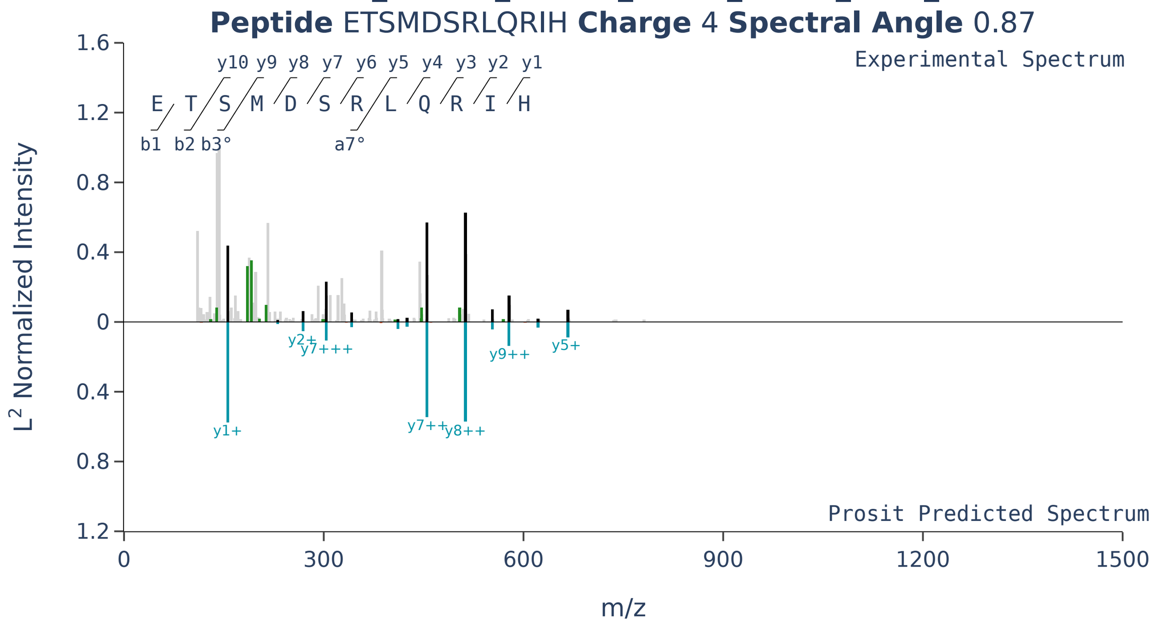


**Source** HRoetschke_271222_090123_HFGoe_LEDGF_b1R2_4h **Scan** 19213
**Peptide** AFLGPKDIFPYSENKEKYGKPNK **Charge** 4 **Spectral Angle** 0.82

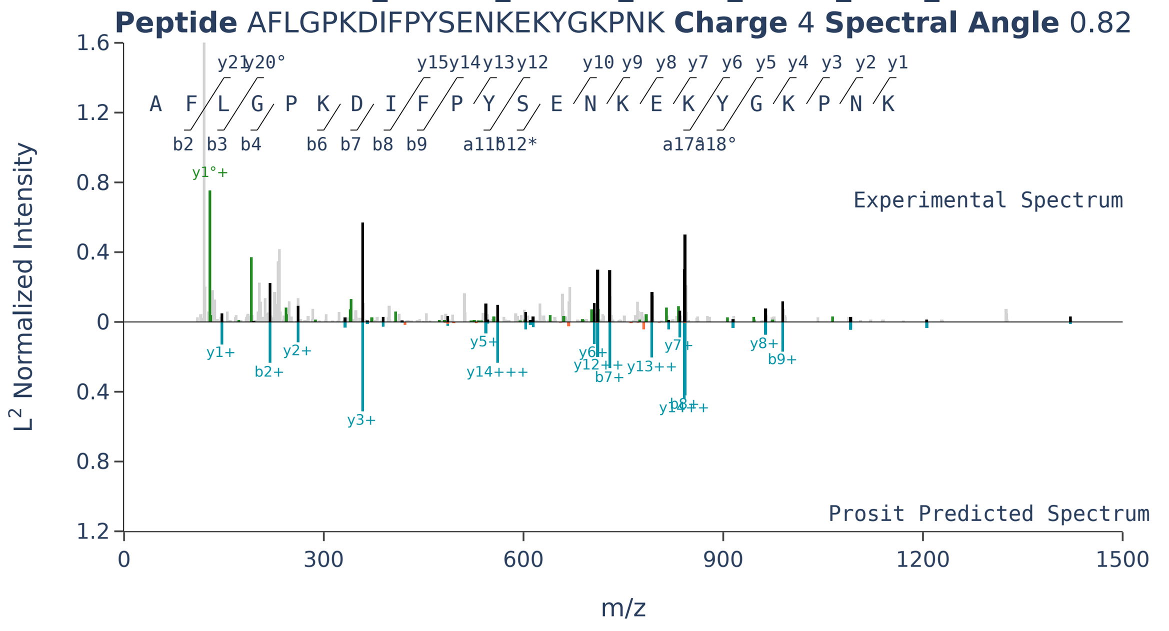


**Source** HRoetschke_271222_090123_HFGoe_LEDGF_b1R1_4h **Scan** 12139
**Peptide** TMQQAQKHTEMITTLKKIR **Charge** 5 **Spectral Angle** 0.87

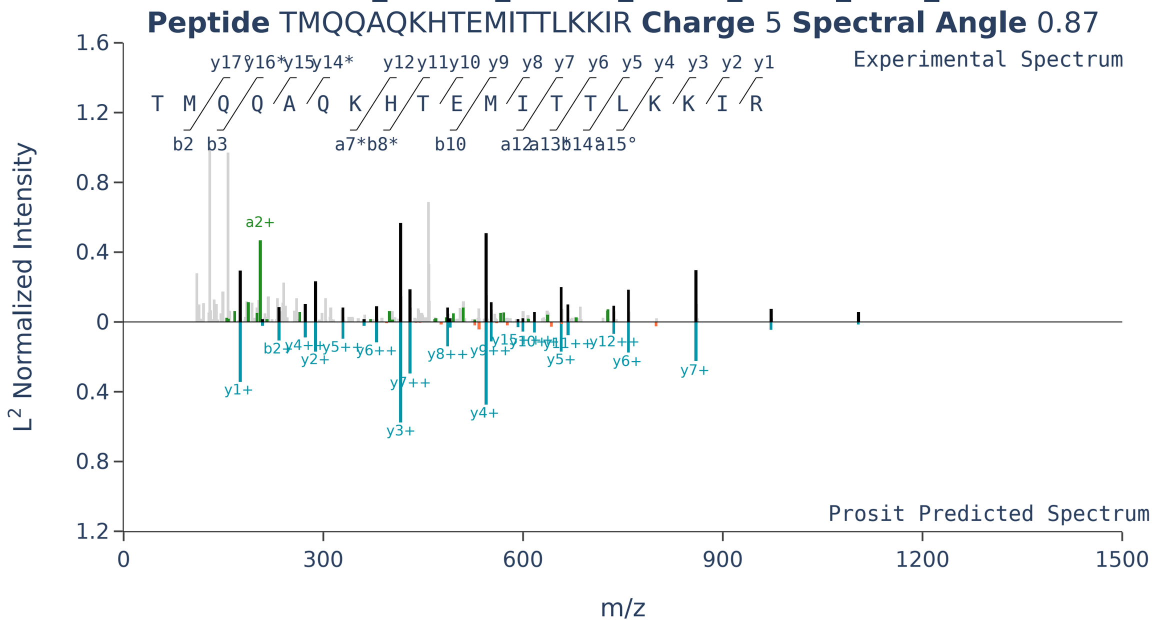

**Source** HRoetschke_271222_090123_HFGoe_LEDGF_b2R1_24h **Scan** 9114
**Peptide** TASVNLKVSPKR **Charge** 3 **Spectral Angle** 0.8

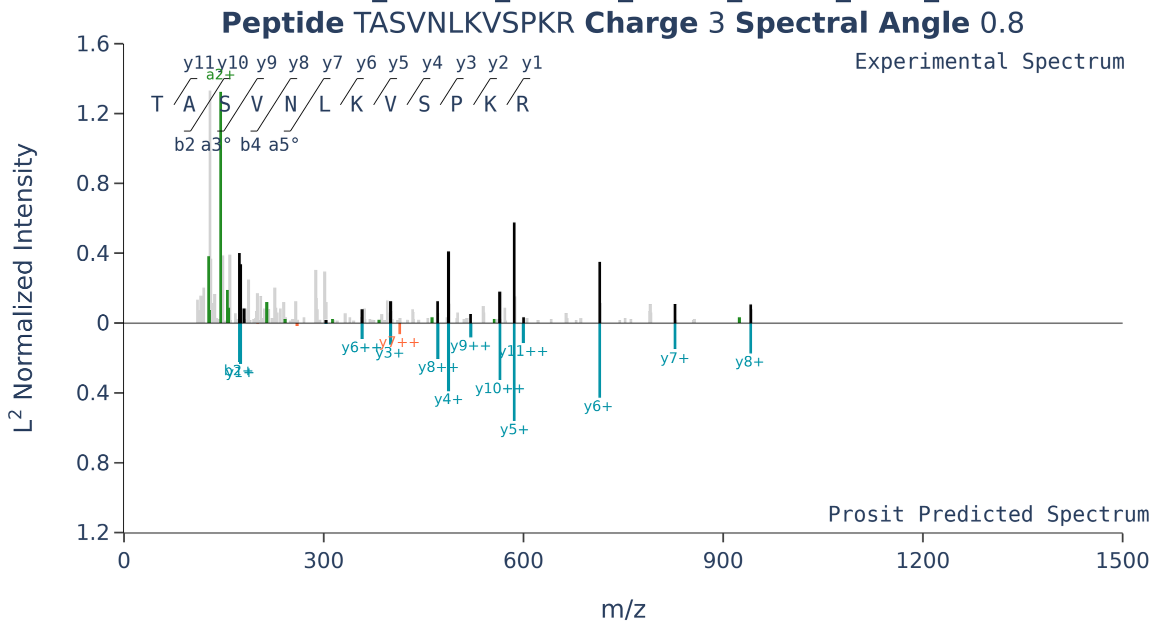


**Source** HRoetschke_271222_090123_HFGoe_LEDGF_b2R1_2h **Scan** 11339
**Peptide** EKQVETEEAGVVT **Charge** 2 **Spectral Angle** 0.86

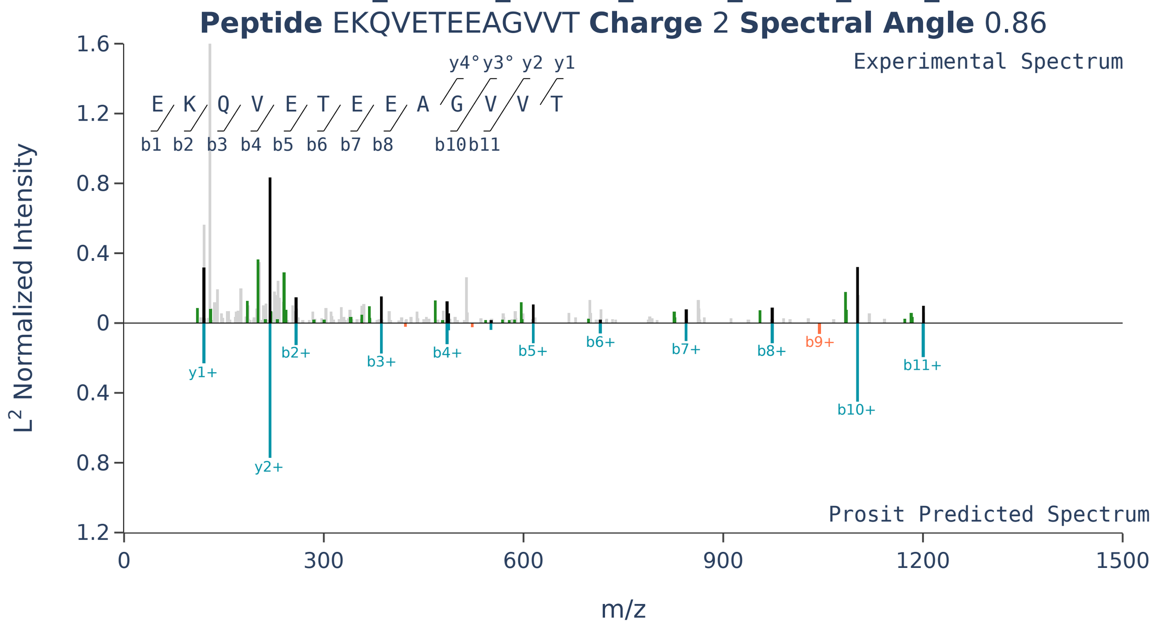


**Source** HRoetschke_271222_090123_HFGoe_LEDGF_b2R1_24h **Scan** 24462
**Peptide** EKQVETEEAGVVTTATASVNL **Charge** 2 **Spectral Angle** 0.81

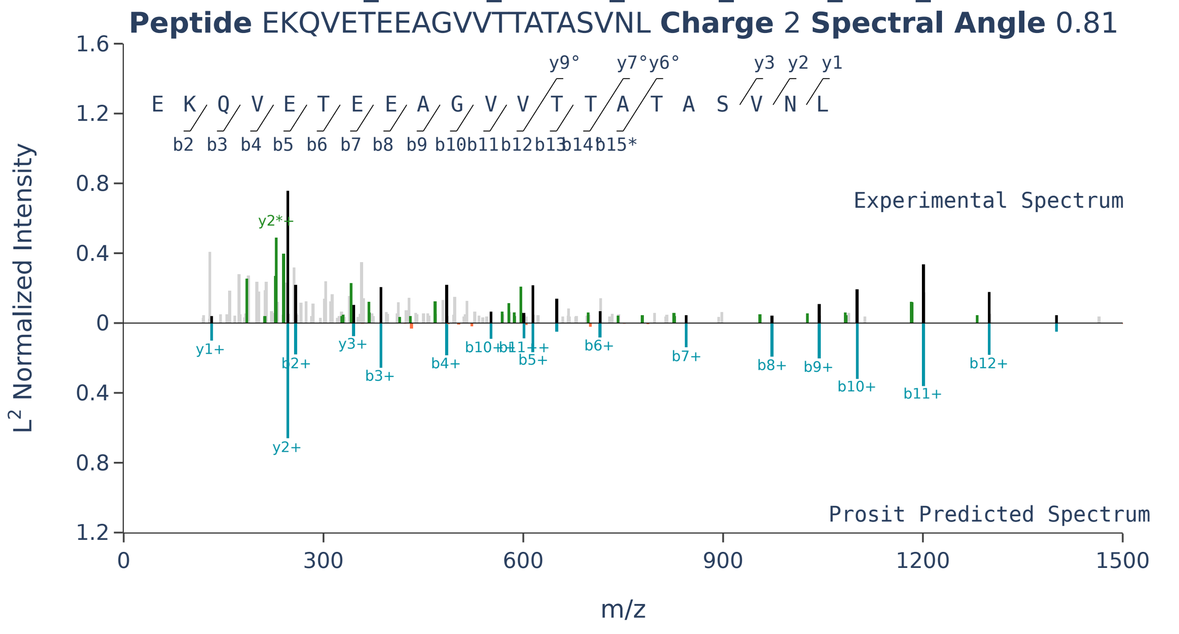


**Source** HRoetschke_271222_090123_HFGoe_LEDGF_b2R1_24h **Scan** 15225
**Peptide** PKDIFPYSENKEKY **Charge** 2 **Spectral Angle** 0.87

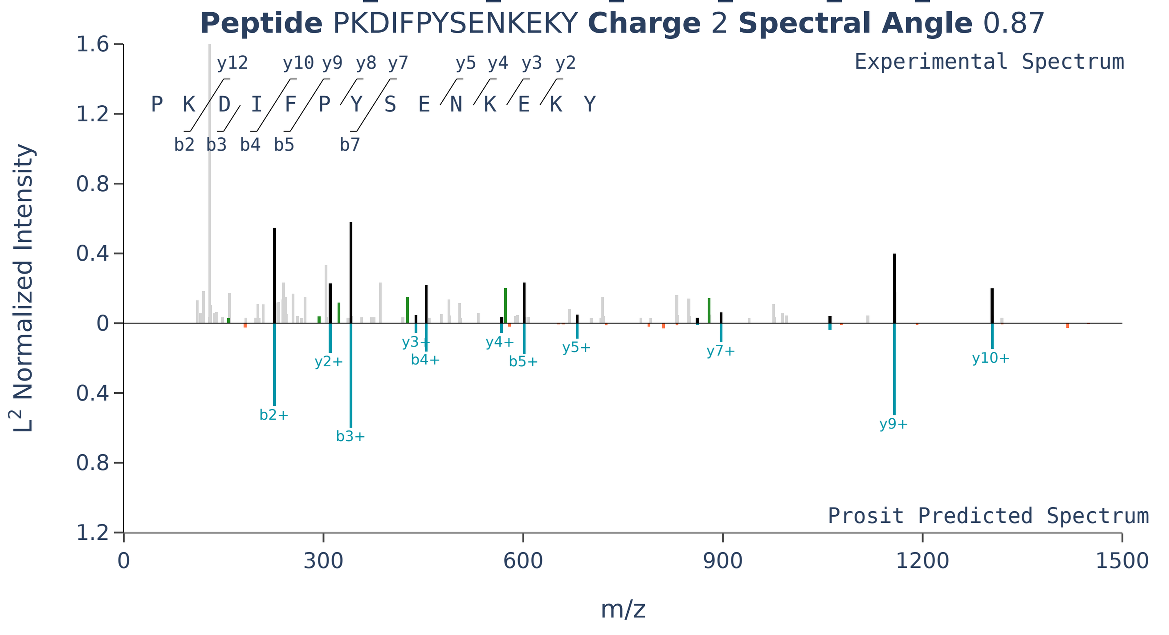


**Source** HRoetschke_271222_090123_HFGoe_LEDGF_b1R2_4h **Scan** 16668
**Peptide** HAEIKNSLKIDNL **Charge** 2 **Spectral Angle** 0.85

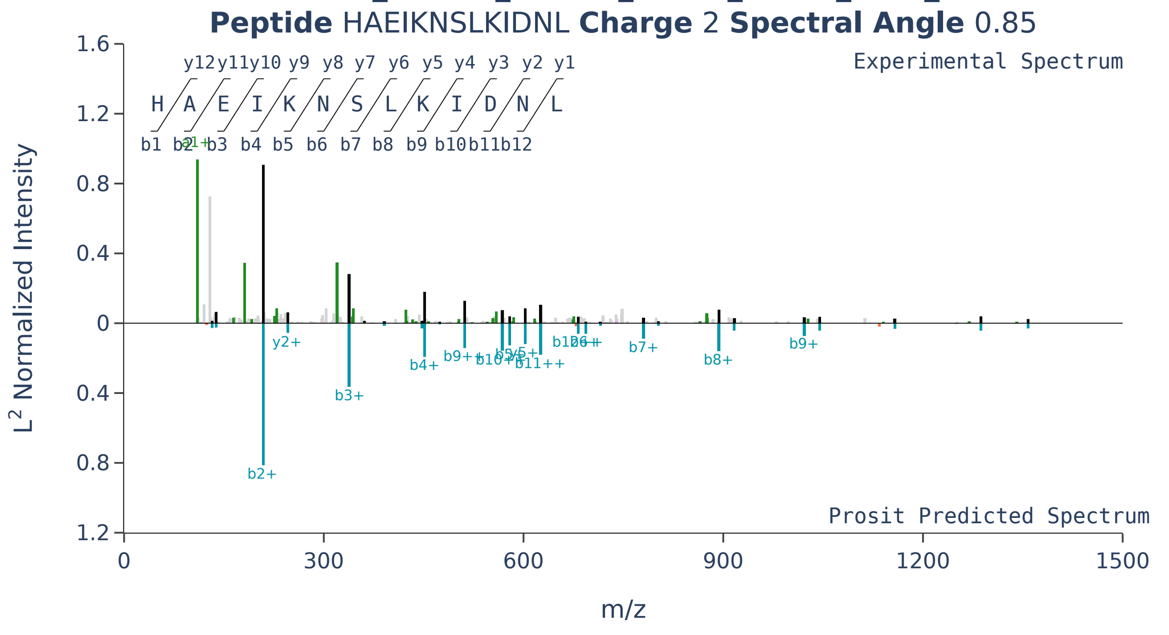


**Source** HRoetschke_271222_090123_HFGoe_LEDGF_b1R2_4h **Scan** 21336
**Peptide** VGEGDSVITQV **Charge** 2 **Spectral Angle** 0.81

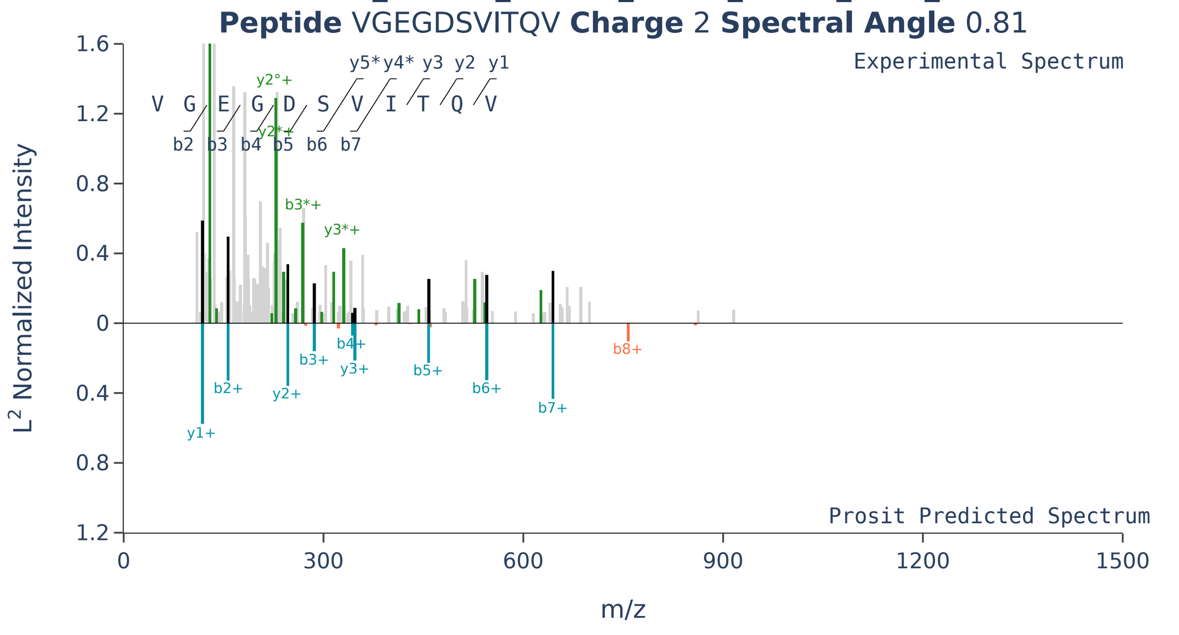


**Source** HRoetschke_271222_090123_HFGoe_LEDGF_b1R1_4h **Scan** 20412
**Peptide** GYPHWPARVDEVPDG **Charge** 2 **Spectral Angle** 0.81

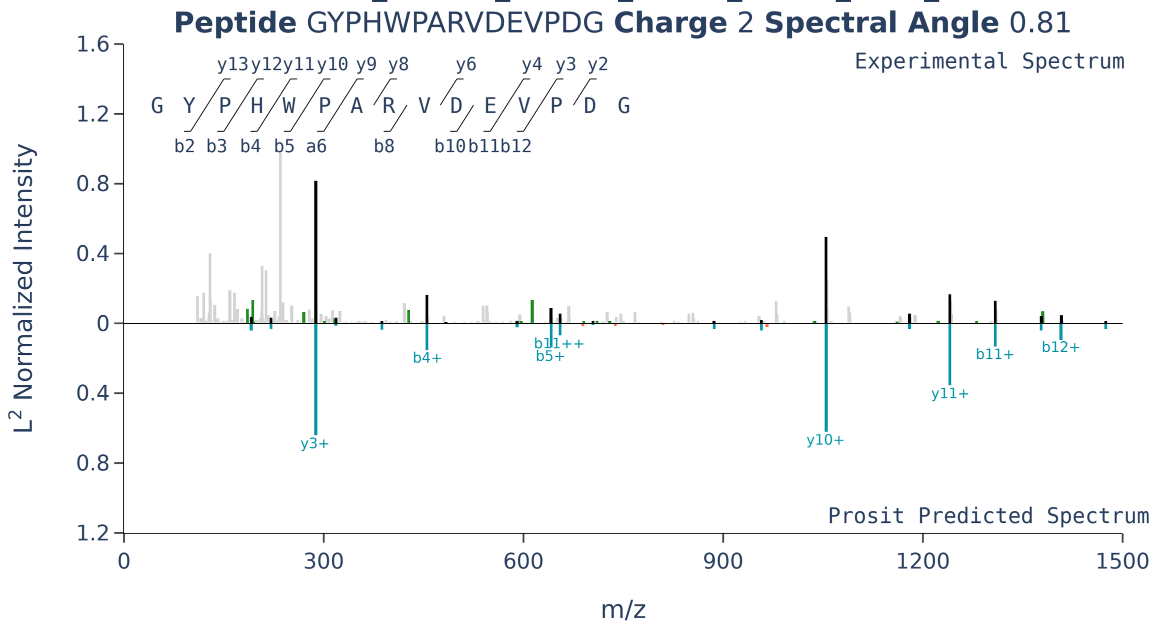


**Source** HRoetschke_271222_090123_HFGoe_LEDGF_b2R1_24h **Scan** 20525
**Peptide** EIKNSLKIDNLDVNRCIE **Charge** 2 **Spectral Angle** 0.87

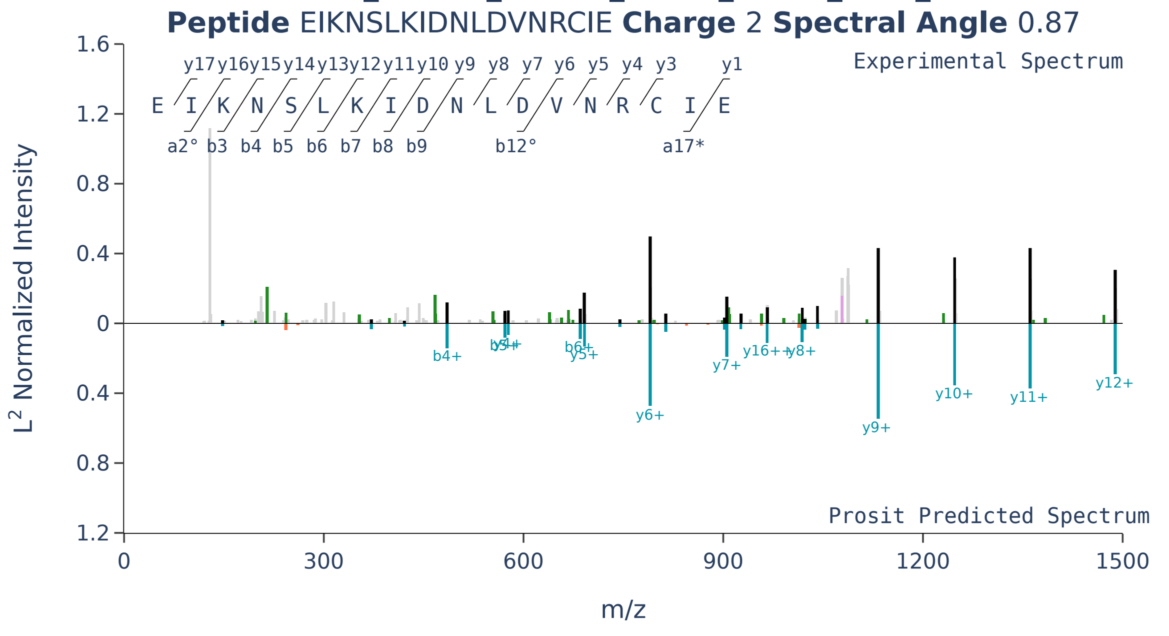


**Source** HRoetschke_271222_090123_HFGoe_LEDGF_b1R2_24h **Scan** 18938
**Peptide** TEMITTLKKIRRFKV **Charge** 4 **Spectral Angle** 0.91

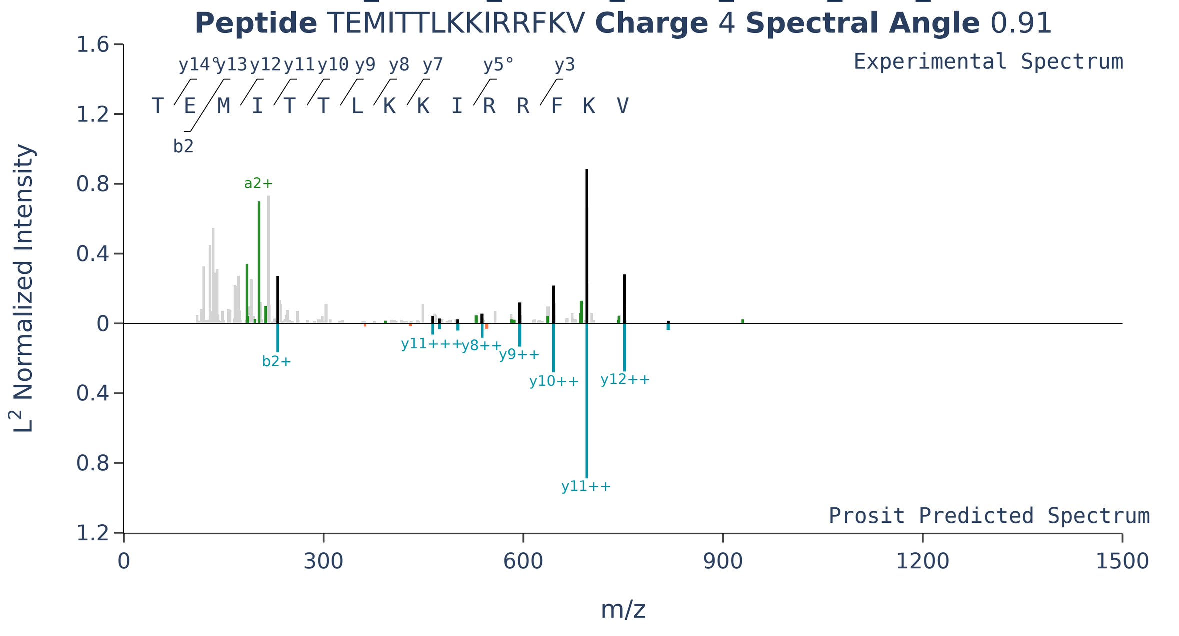


**Source** HRoetschke_271222_090123_HFGoe_LEDGF_b1R1_4h **Scan** 17972
**Peptide** PHWPARVDEVPDGA **Charge** 2 **Spectral Angle** 0.77

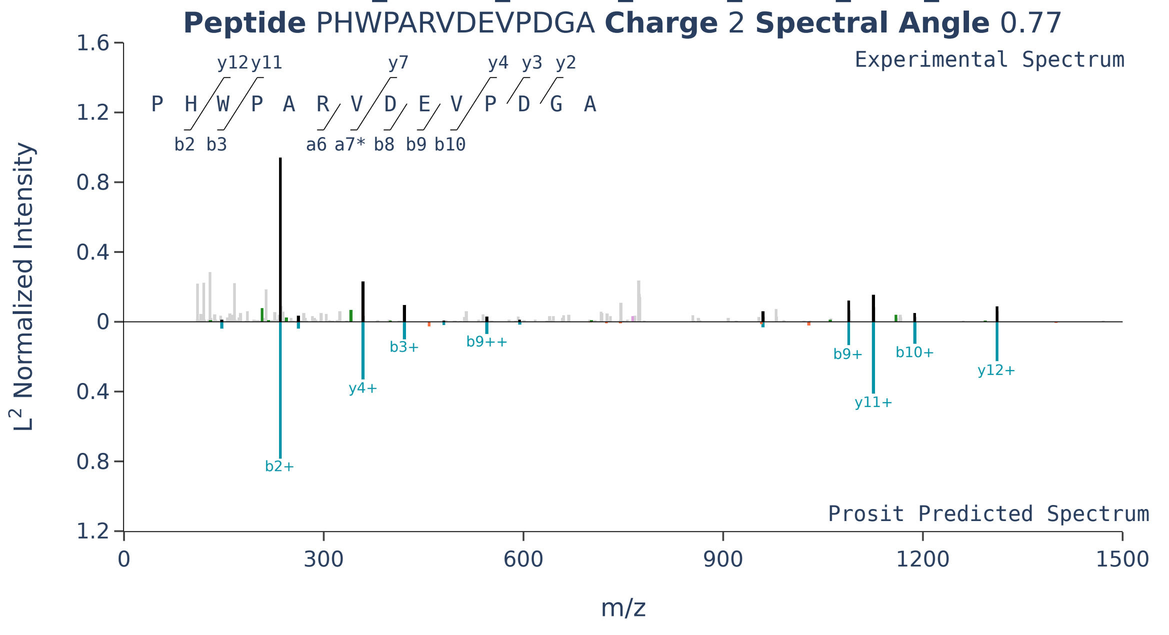


**Source** HRoetschke_271222_090123_HFGoe_LEDGF_b1R2_4h **Scan** 12709
**Peptide** SNEDVTKAVDITTPKAARRG **Charge** 3 **Spectral Angle** 0.84

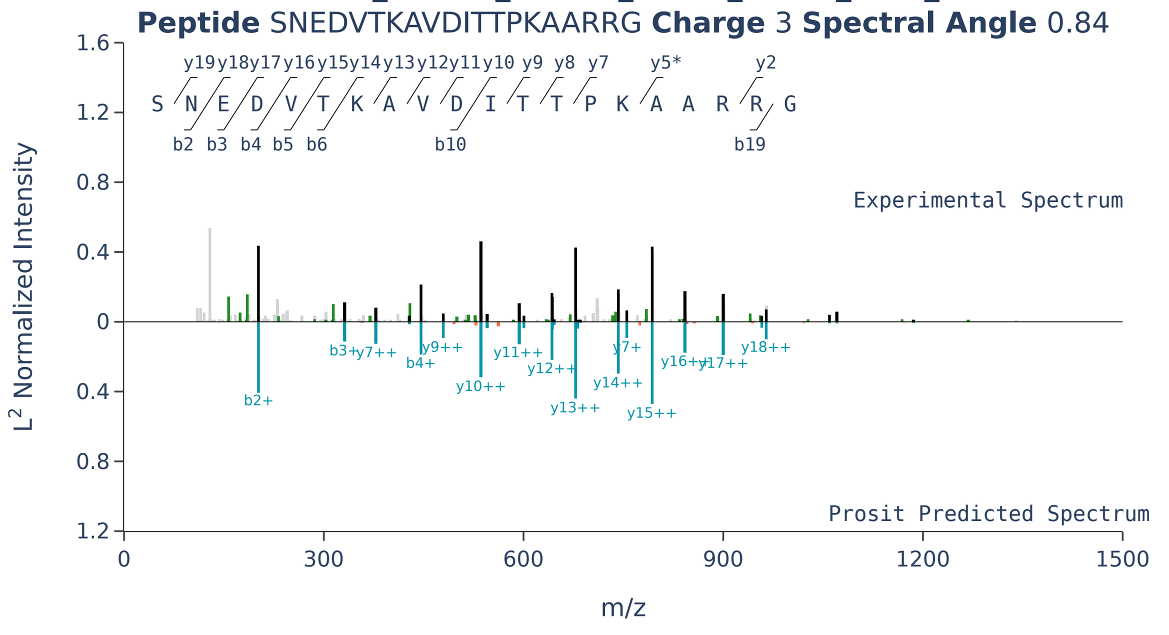


**Source** HRoetschke_271222_090123_HFGoe_LEDGF_b1R2_4h **Scan** 15085
**Peptide** MITTLKKIRRFKVSQV **Charge** 3 **Spectral Angle** 0.92

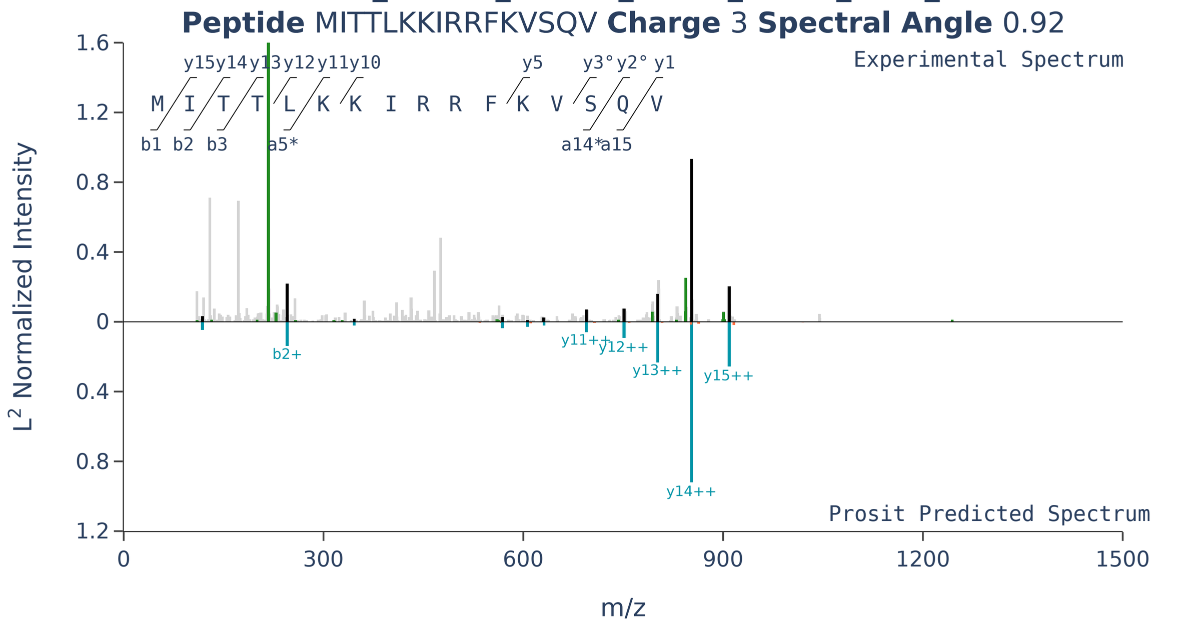


**Source** HRoetschke_271222_090123_HFGoe_LEDGF_b2R2_1h **Scan** 25970
**Peptide** GTHETAFLGPKDIFPYSEN **Charge** 3 **Spectral Angle** 0.75

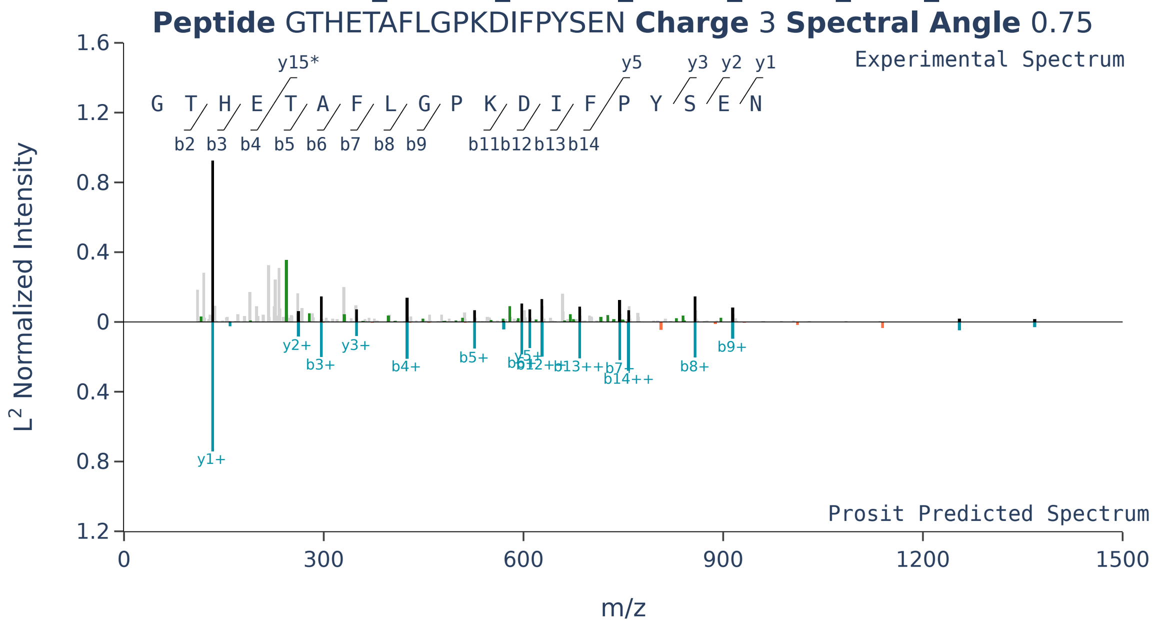


**Source** HRoetschke_271222_090123_HFGoe_LEDGF_b2R1_4h **Scan** 10130
**Peptide** QKHTEMITTLK **Charge** 3 **Spectral Angle** 0.87

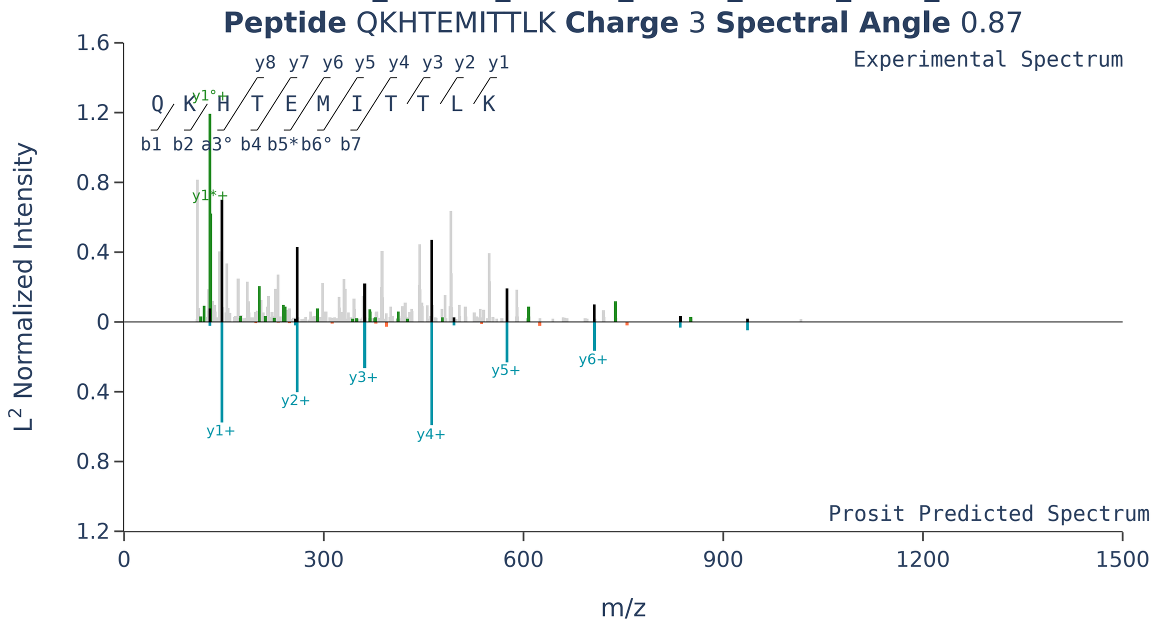


**Source** HRoetschke_271222_090123_HFGoe_LEDGF_b2R1_24h **Scan** 29463
**Peptide** KNMFLVGEGDSVITQVLNK **Charge** 3 **Spectral Angle** 0.78

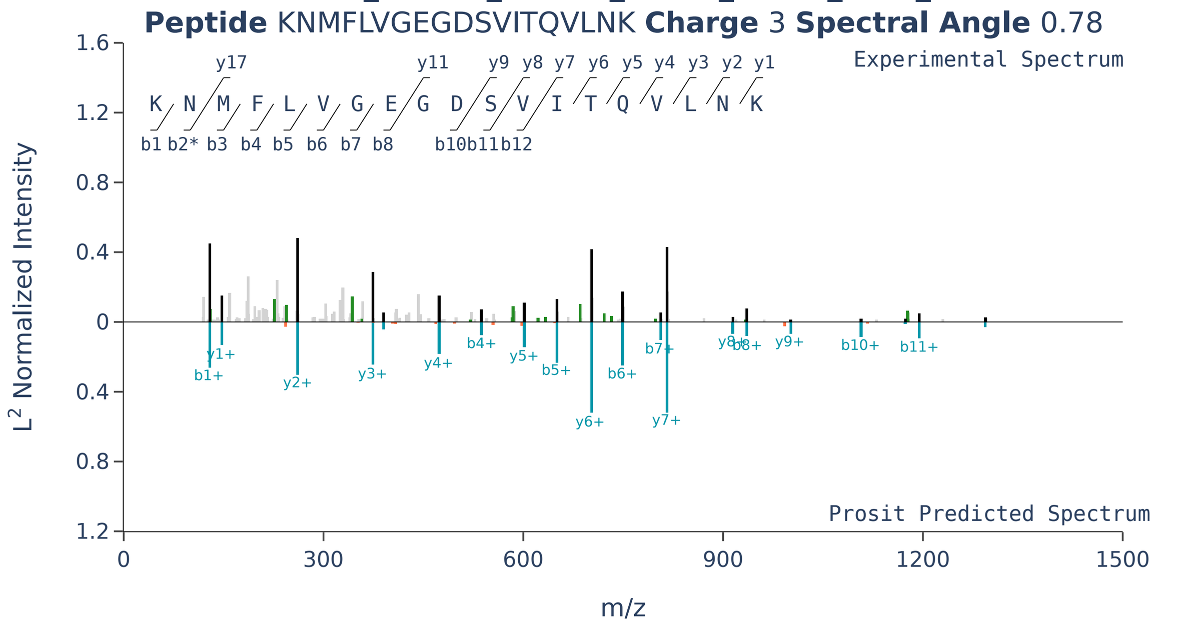

**Source** HRoetschke_271222_090123_HFGoe_LEDGF_b1R2_2h **Scan** 18644
**Peptide** HAEIKNSLKIDNLDVNRCIE **Charge** 3 **Spectral Angle** 0.86

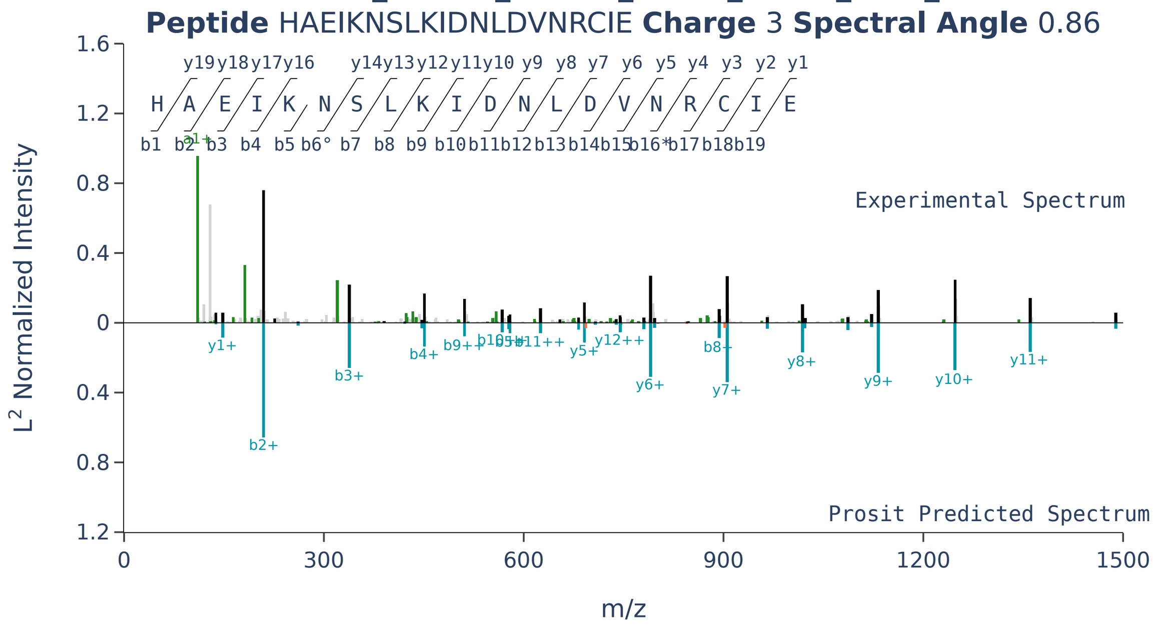


**Source** HRoetschke_271222_090123_HFGoe_LEDGF_b1R2_4h **Scan** 8596
**Peptide** IDNNPKVKFSSQQAATKQS **Charge** 2 **Spectral Angle** 0.81

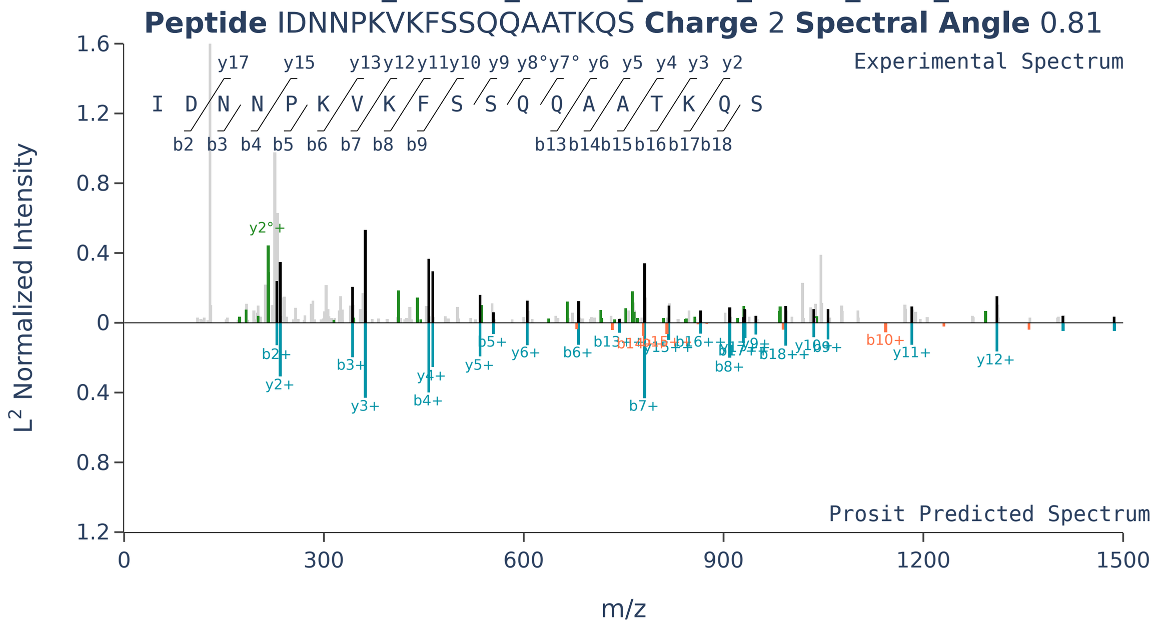


**Source** HRoetschke_271222_090123_HFGoe_LEDGF_b1R1_4h **Scan** 12590
**Peptide** RIHAEIKNSLKID **Charge** 2 **Spectral Angle** 0.76

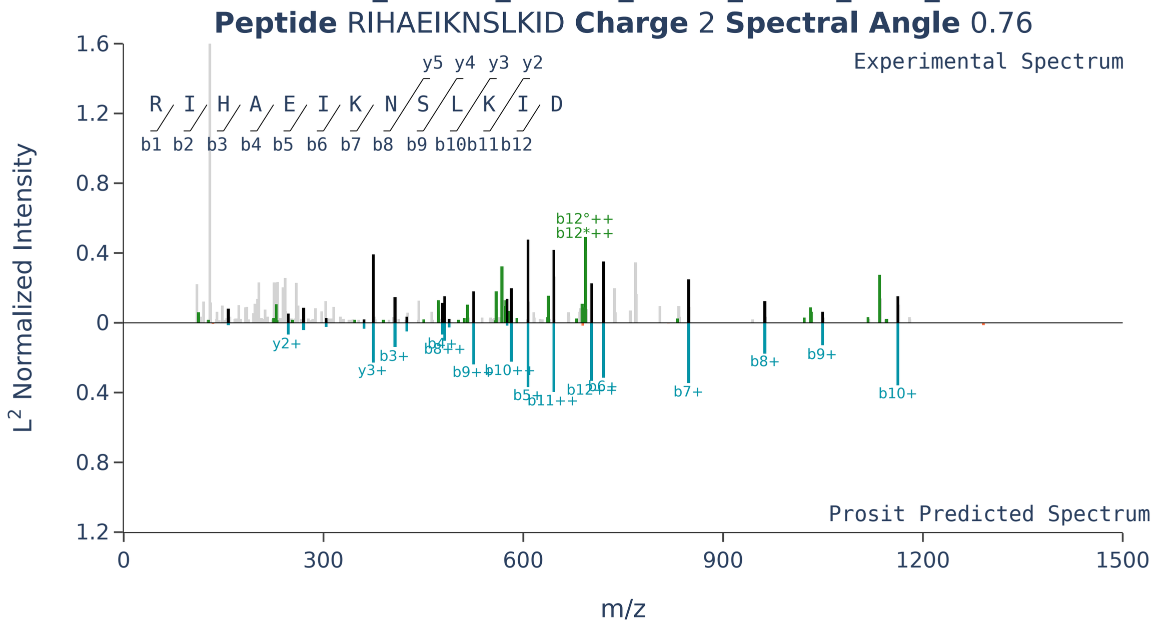


**Source** HRoetschke_271222_090123_HFGoe_LEDGF_b2R1_2h **Scan** 15534
**Peptide** WEIDNNPKVKFSSQQAATKQ **Charge** 3 **Spectral Angle** 0.86

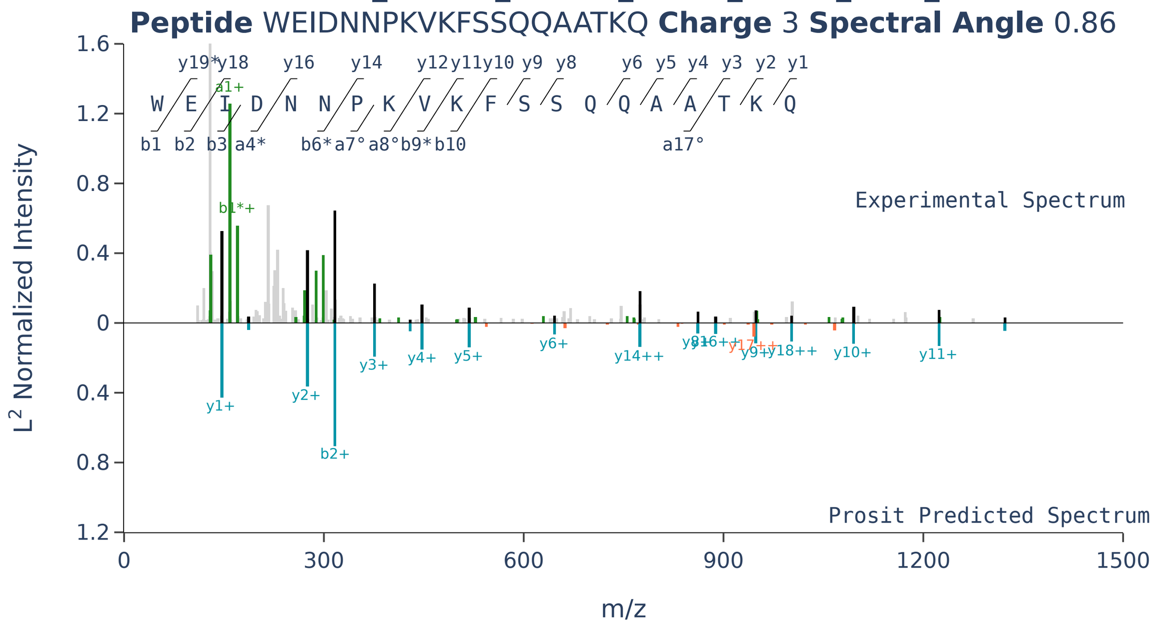


**Source** HRoetschke_271222_090123_HFGoe_LEDGF_b1R1_2h **Scan** 25987
**Peptide** GFNEGLWEIDNNPKVKFSSQ **Charge** 3 **Spectral Angle** 0.79

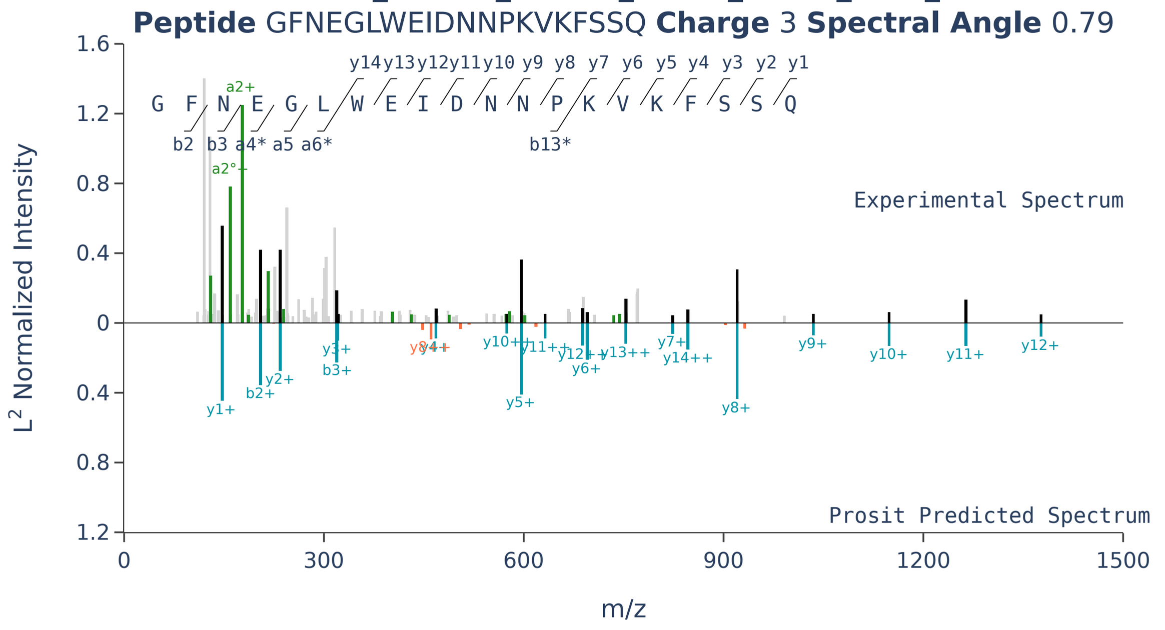


**Source** HRoetschke_271222_090123_HFGoe_LEDGF_b2R1_4h **Scan** 8051
**Peptide** AQKHTEMITT **Charge** 2 **Spectral Angle** 0.76

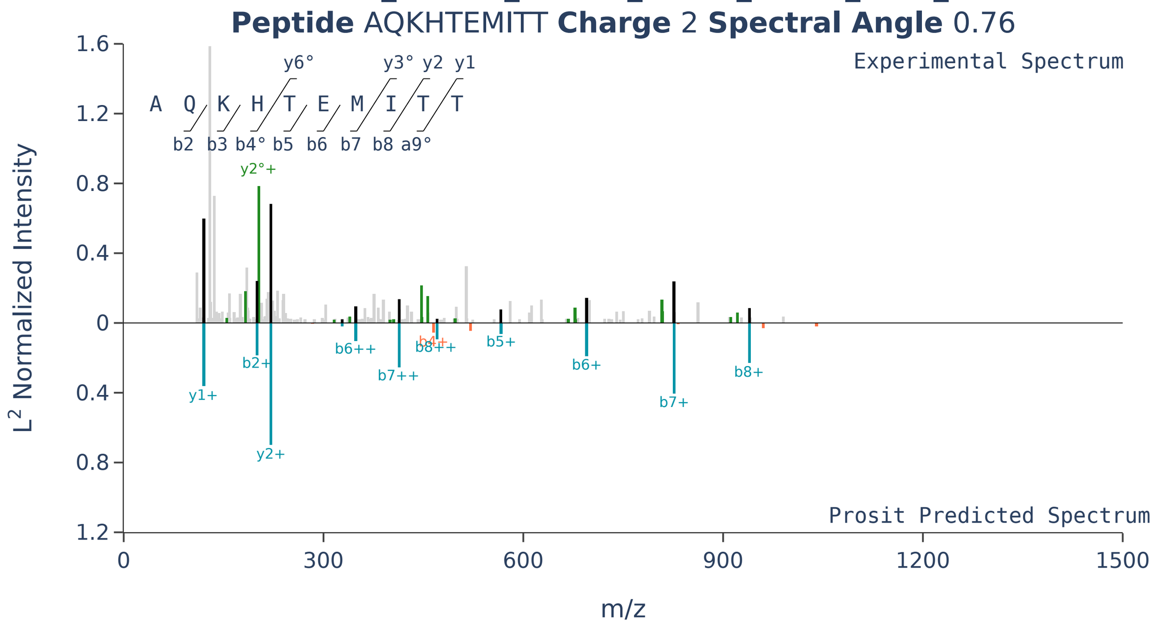


**Source** HRoetschke_271222_090123_HFGoe_LEDGF_b1R1_4h **Scan** 18616
**Peptide** LWEIDNNPKVKFSSQQAATKQS **Charge** 2 **Spectral Angle** 0.88

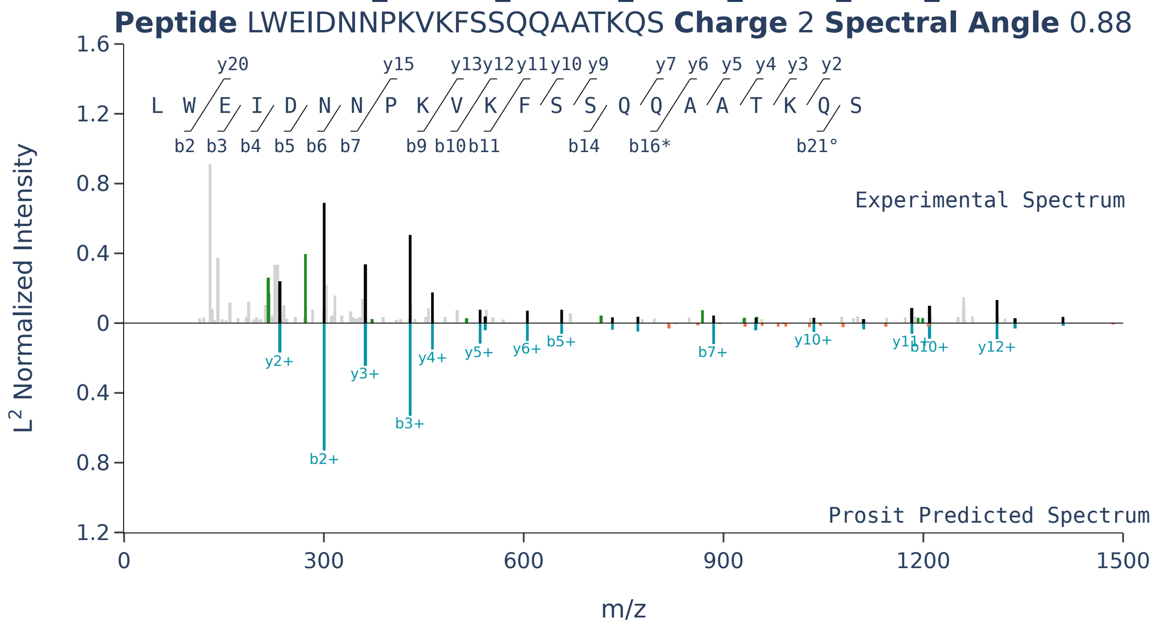


**Source** HRoetschke_271222_090123_HFGoe_LEDGF_b1R1_2h **Scan** 25865
**Peptide** GEGDSVITQVLNKSLAEQRQHE **Charge** 3 **Spectral Angle** 0.84

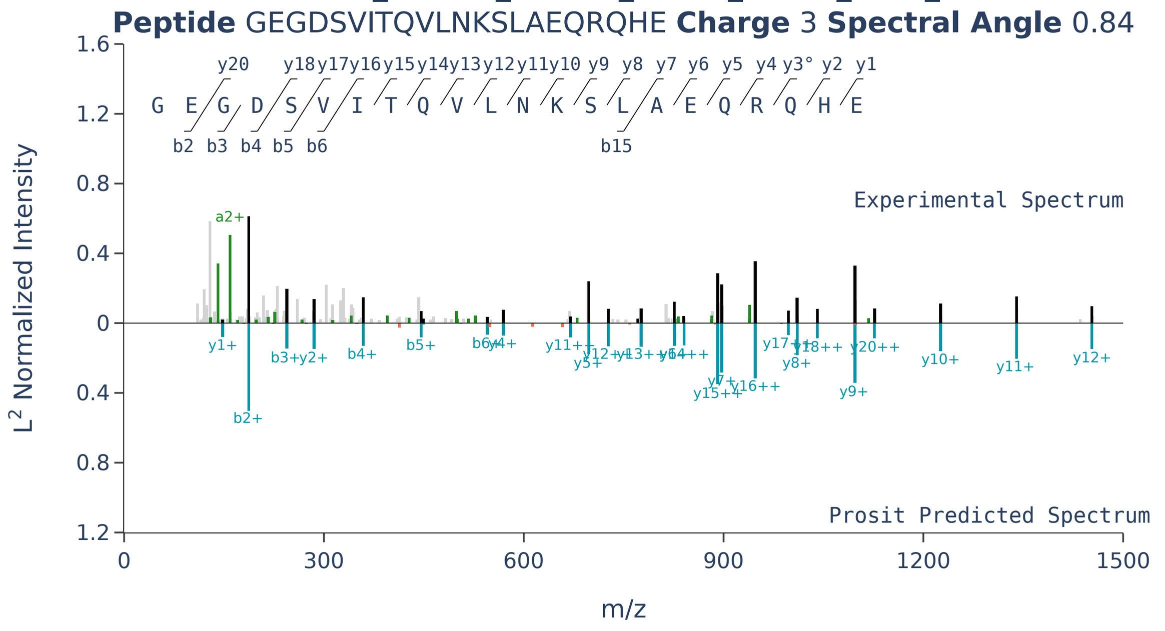


**Source** HRoetschke_271222_090123_HFGoe_LEDGF_b2R2_24h **Scan** 26618
**Peptide** ETAFLGPKDIFPYSENKE **Charge** 3 **Spectral Angle** 0.86

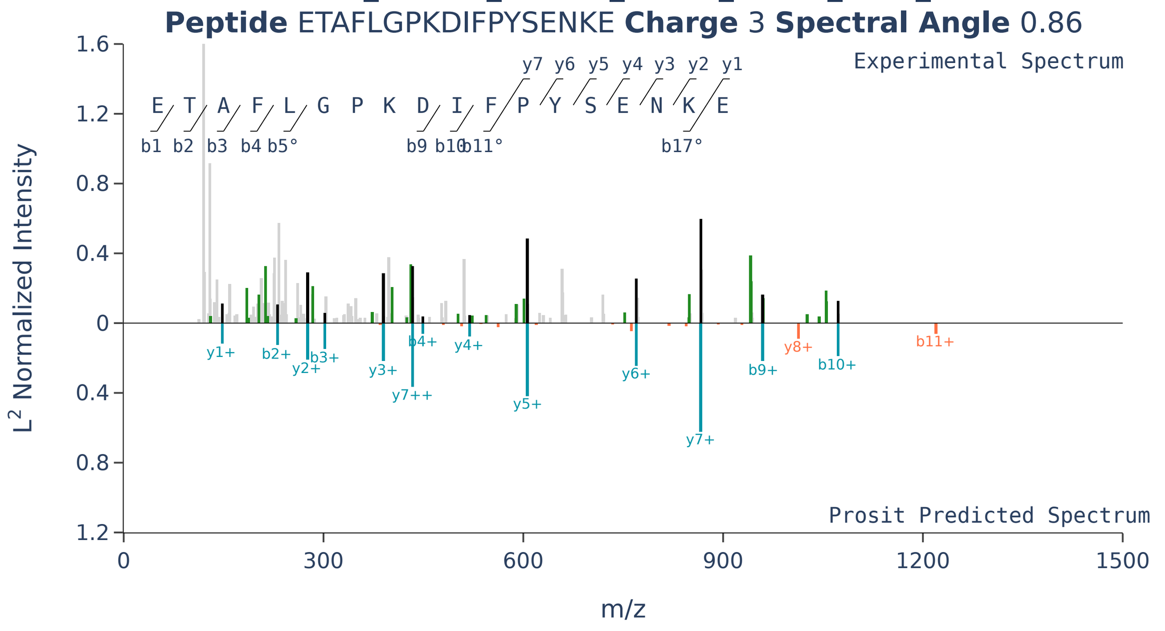


**Source** HRoetschke_271222_090123_HFGoe_LEDGF_b2R1_2h **Scan** 6843
**Peptide** LEKEQTGSKTL **Charge** 3 **Spectral Angle** 0.83

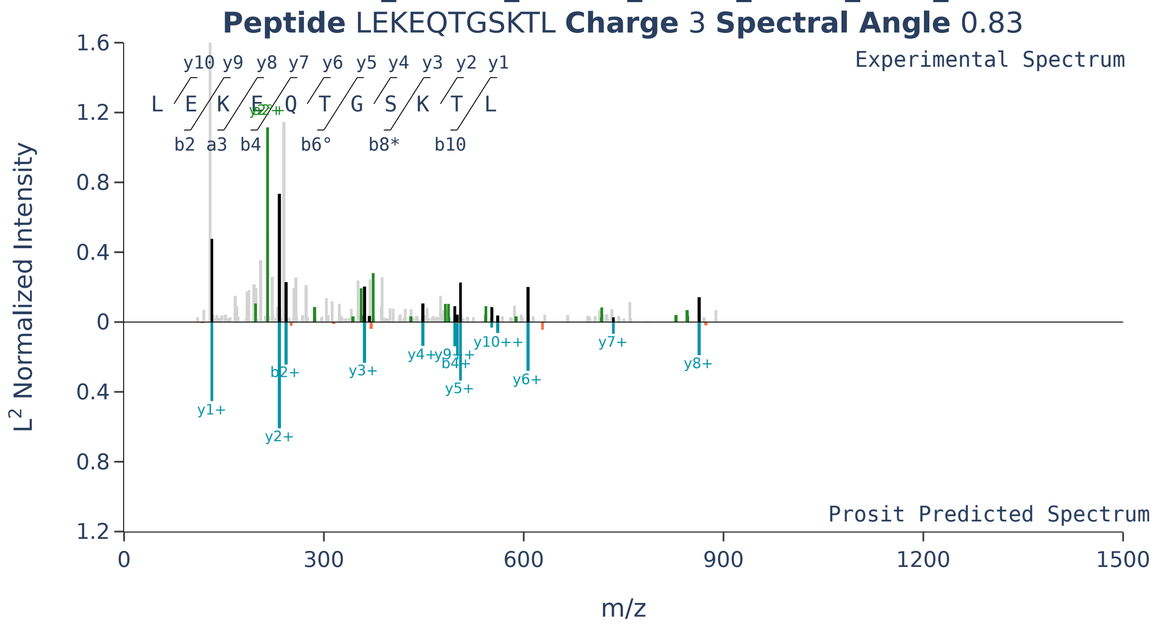


**Source** HRoetschke_271222_090123_HFGoe_LEDGF_b1R2_24h **Scan** 17610
**Peptide** SNEDVTKAVDITTPKA **Charge** 2 **Spectral Angle** 0.91

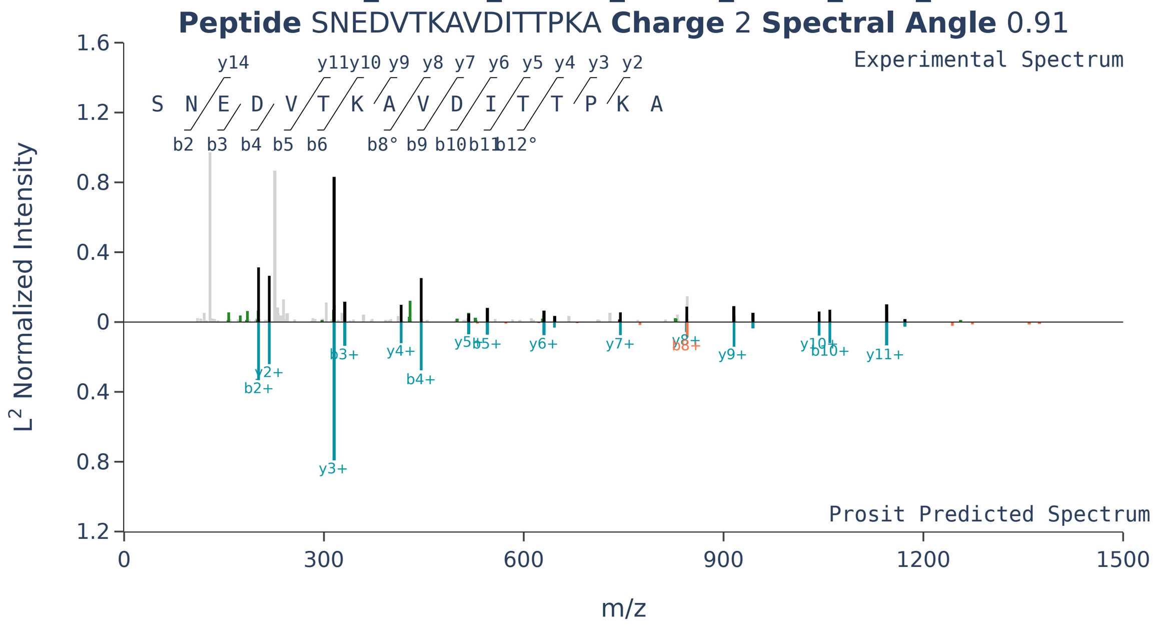


**Source** HRoetschke_271222_090123_HFGoe_LEDGF_b2R1_2h **Scan** 26321
**Peptide** TRDFKPGDLIF **Charge** 3 **Spectral Angle** 0.77

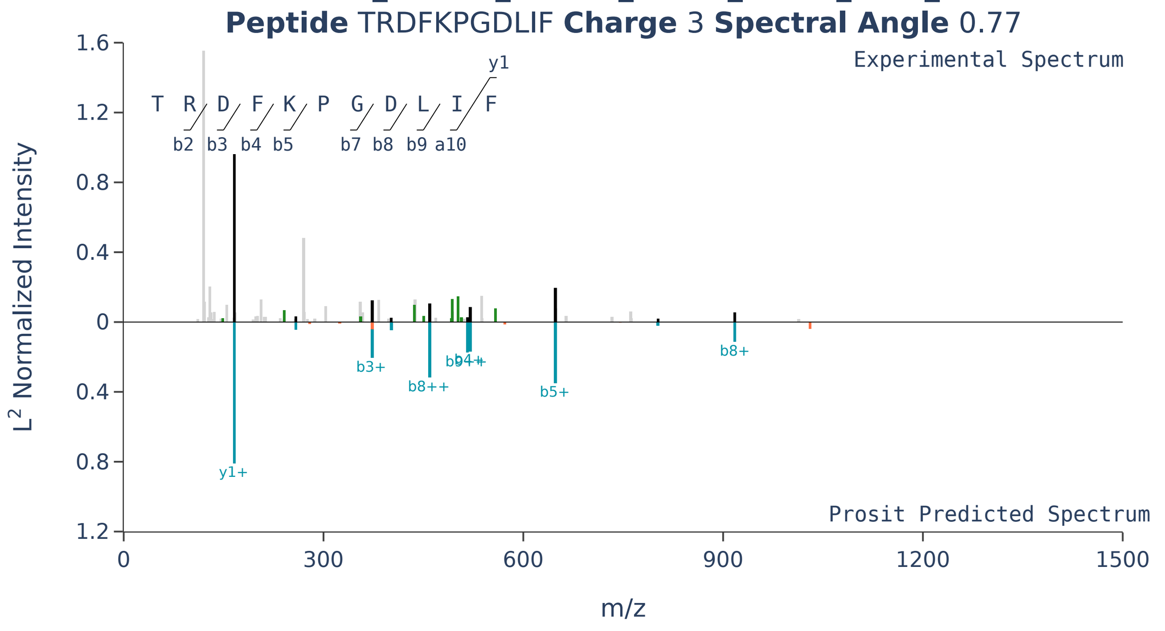


**Source** HRoetschke_271222_090123_HFGoe_LEDGF_b1R1_2h **Scan** 11105
**Peptide** KASNEDVTKAVDITTPKAARR **Charge** 4 **Spectral Angle** 0.85

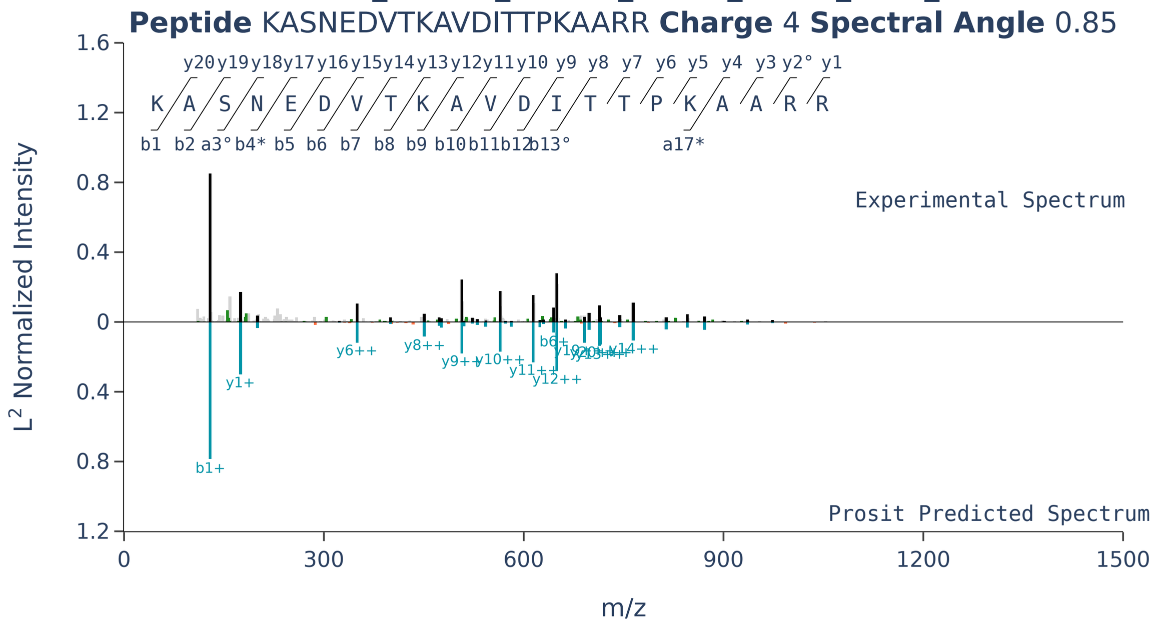


**Source** HRoetschke_271222_090123_HFGoe_LEDGF_b2R2_24h **Scan** 12701
**Peptide** SRLQRIHAEIKNSL **Charge** 4 **Spectral Angle** 0.87

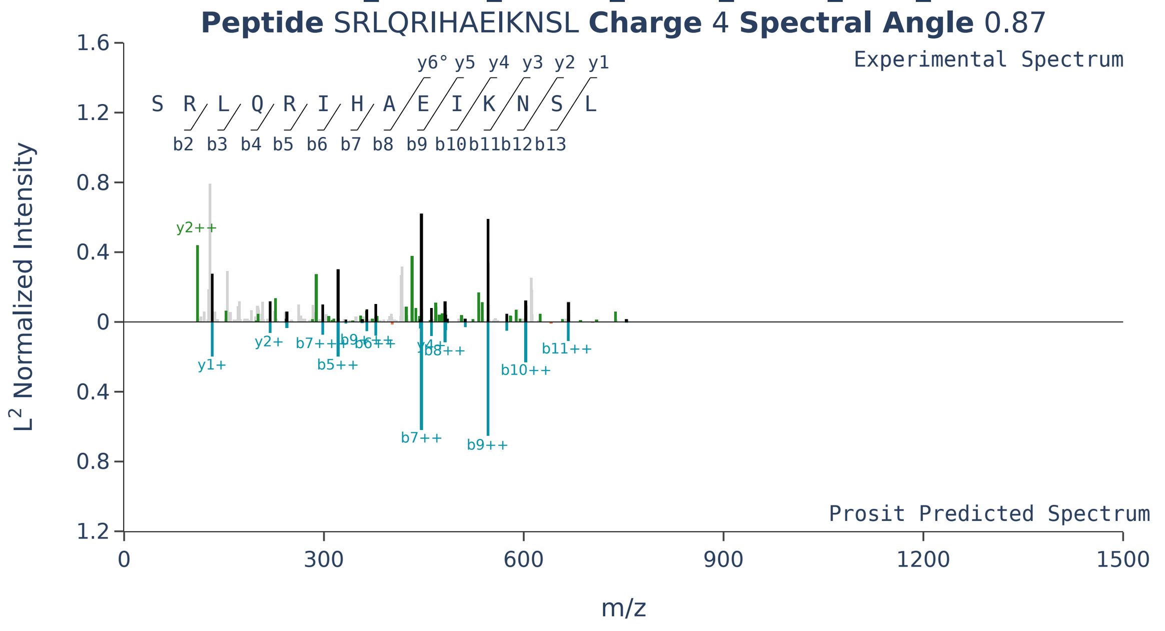


**Source** HRoetschke_271222_090123_HFGoe_LEDGF_b2R1_24h **Scan** 27594
**Peptide** LVGEGDSVITQVLNK **Charge** 2 **Spectral Angle** 0.77

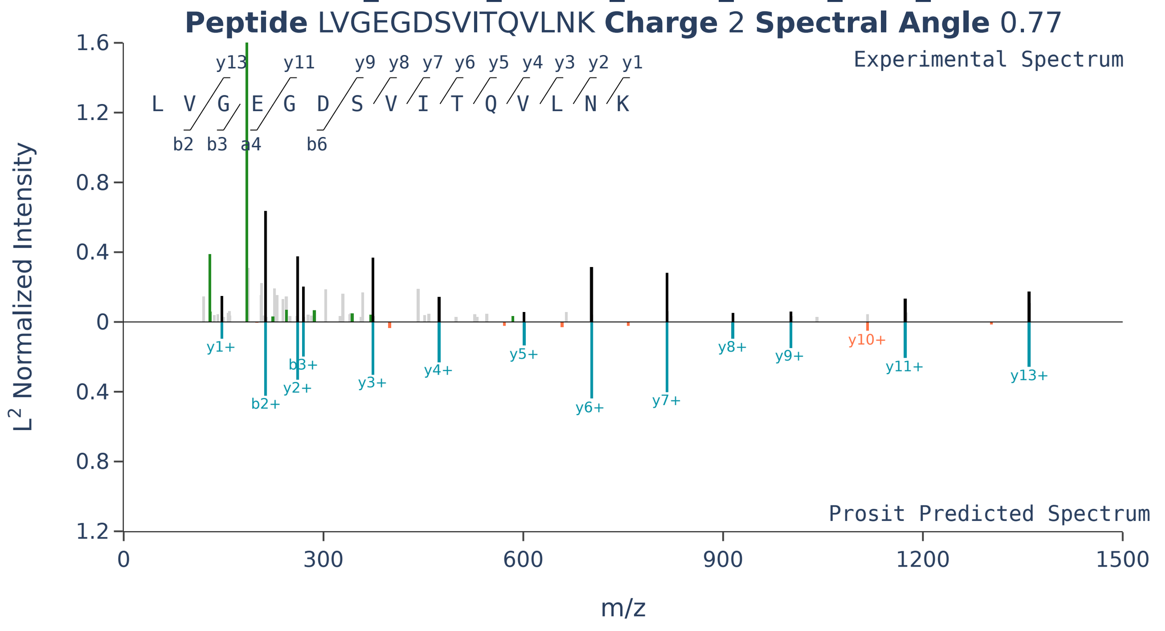

**Source** HRoetschke_271222_090123_HFGoe_LEDGF_b2R2_4h **Scan** 13383
**Peptide** KGYPHWPARVDE **Charge** 2 **Spectral Angle** 0.83

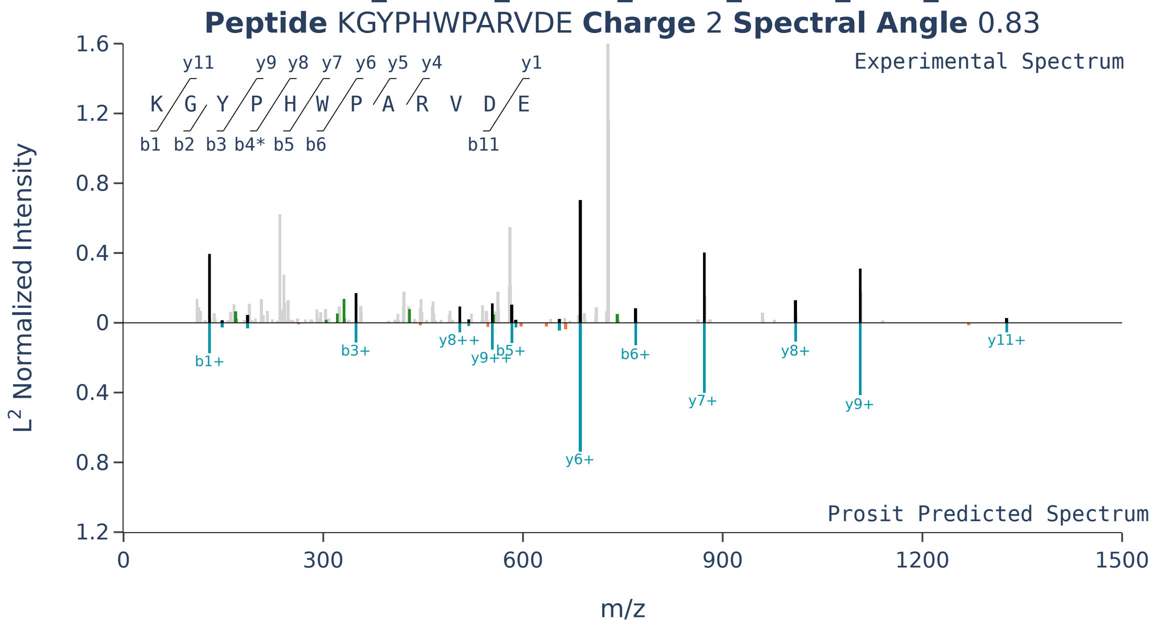


**Source** HRoetschke_271222_090123_HFGoe_LEDGF_b1R1_2h **Scan** 12913
**Peptide** AKMKGYPHWPARVD **Charge** 4 **Spectral Angle** 0.91

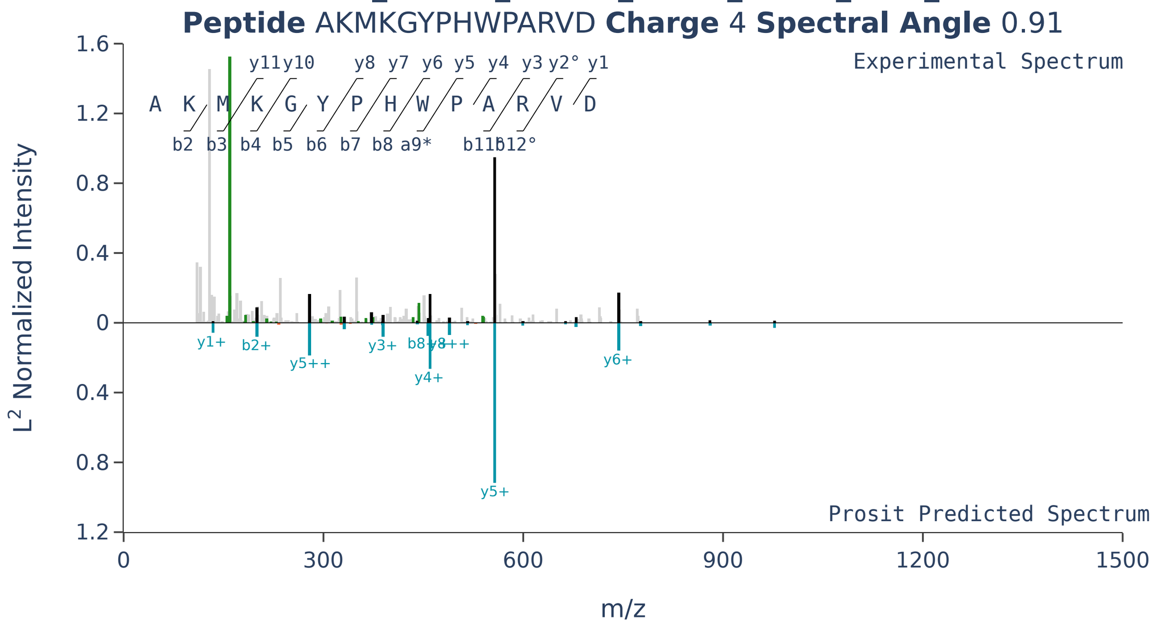


**Source** HRoetschke_271222_090123_HFGoe_LEDGF_b1R1_4h **Scan** 23871
**Peptide** FLGPKDIFPYSENKEKY **Charge** 3 **Spectral Angle** 0.81

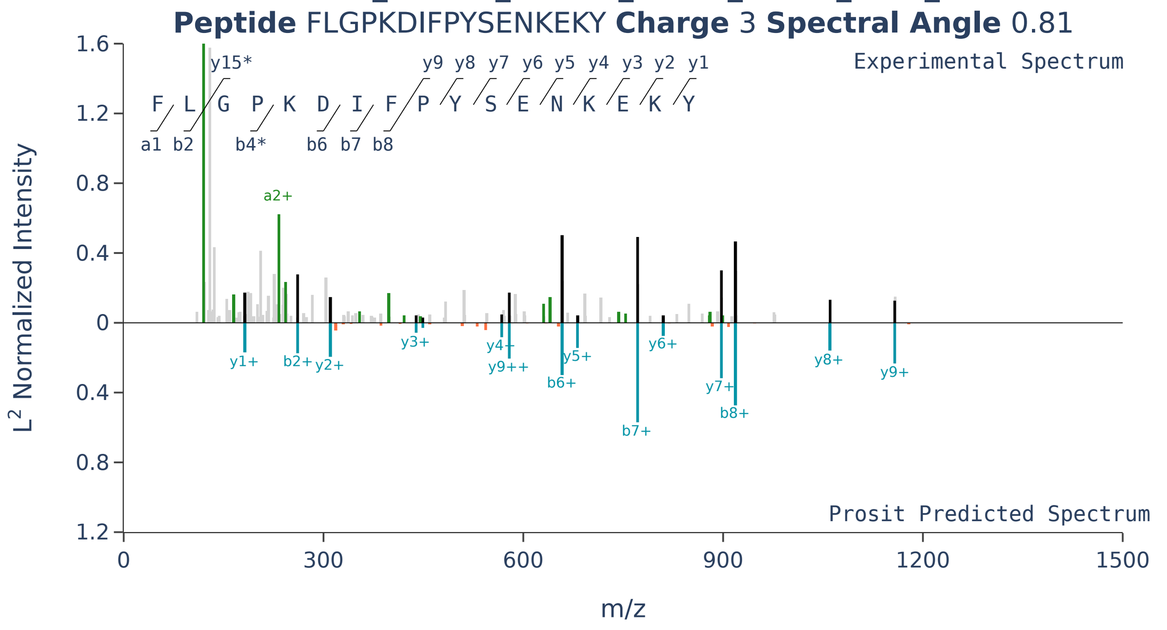


**Source** HRoetschke_271222_090123_HFGoe_LEDGF_b1R1_2h **Scan** 24011
**Peptide** YNKFKNMFLVGEGD **Charge** 2 **Spectral Angle** 0.78

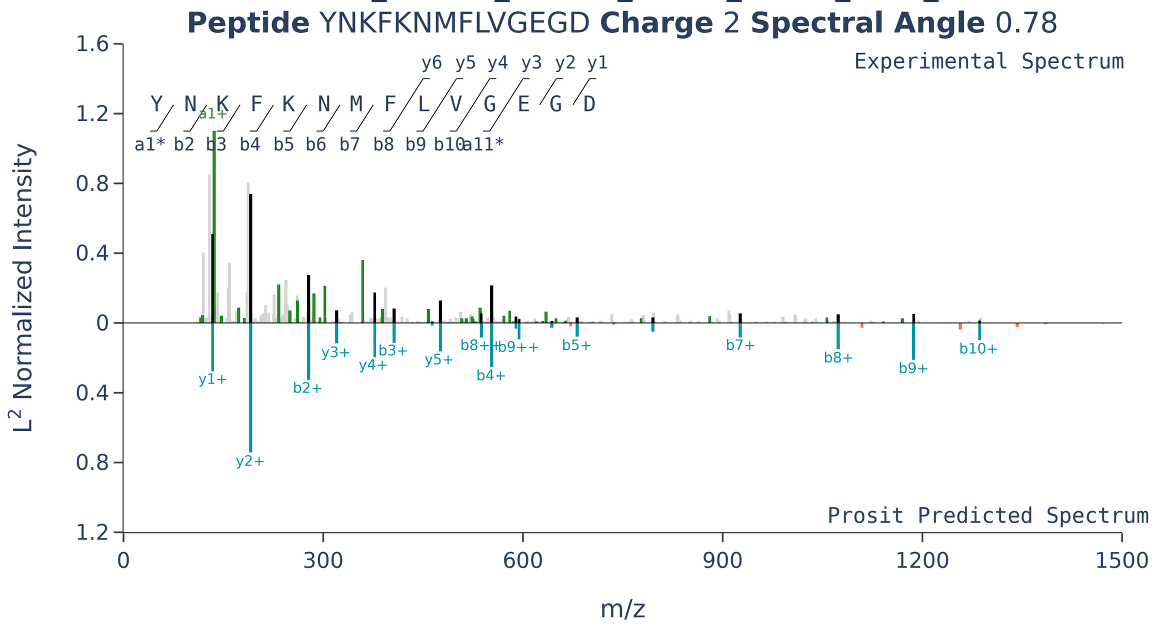


**Source** HRoetschke_271222_090123_HFGoe_LEDGF_b1R2_24h **Scan** 6253
**Peptide** DITTPKAARRGRK **Charge** 4 **Spectral Angle** 0.89

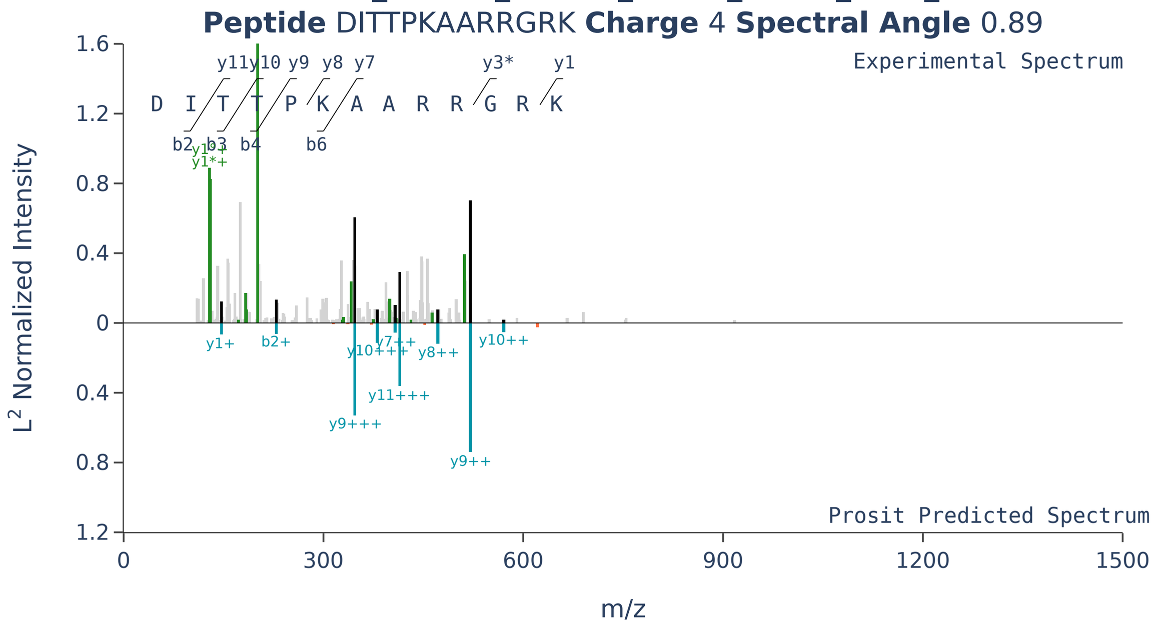


**Source** HRoetschke_271222_090123_HFGoe_LEDGF_b1R1_2h **Scan** 14531
**Peptide** AGVVTTATASVNLKVSPKRGRPAA **Charge** 4 **Spectral Angle** 0.76

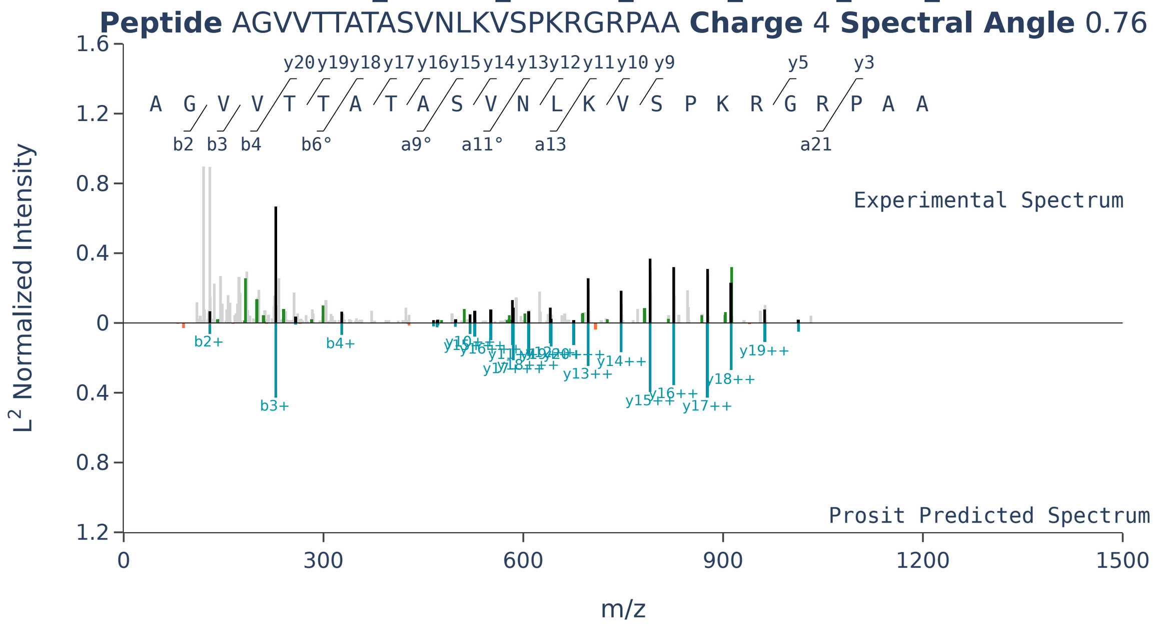


**Source** HRoetschke_271222_090123_HFGoe_LEDGF_b2R2_24h **Scan** 8886
**Peptide** DNNPKVKFSSQQ **Charge** 2 **Spectral Angle** 0.83

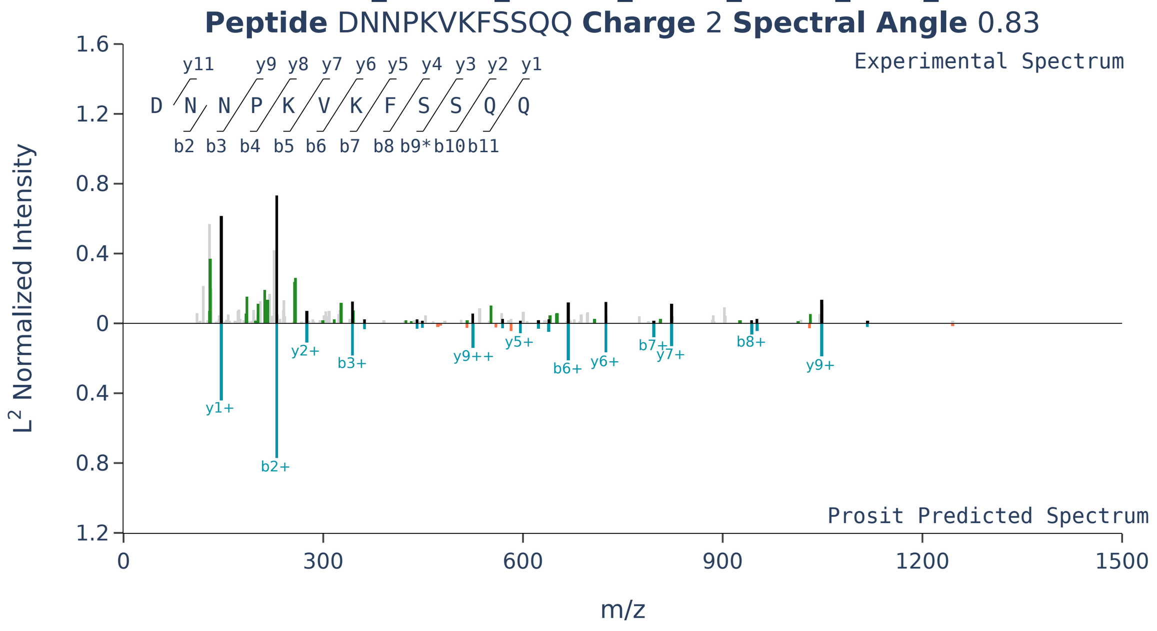


**Source** HRoetschke_271222_090123_HFGoe_LEDGF_b2R1_1h **Scan** 11050
**Peptide** SRLQRIHAEL **Charge** 3 **Spectral Angle** 0.91

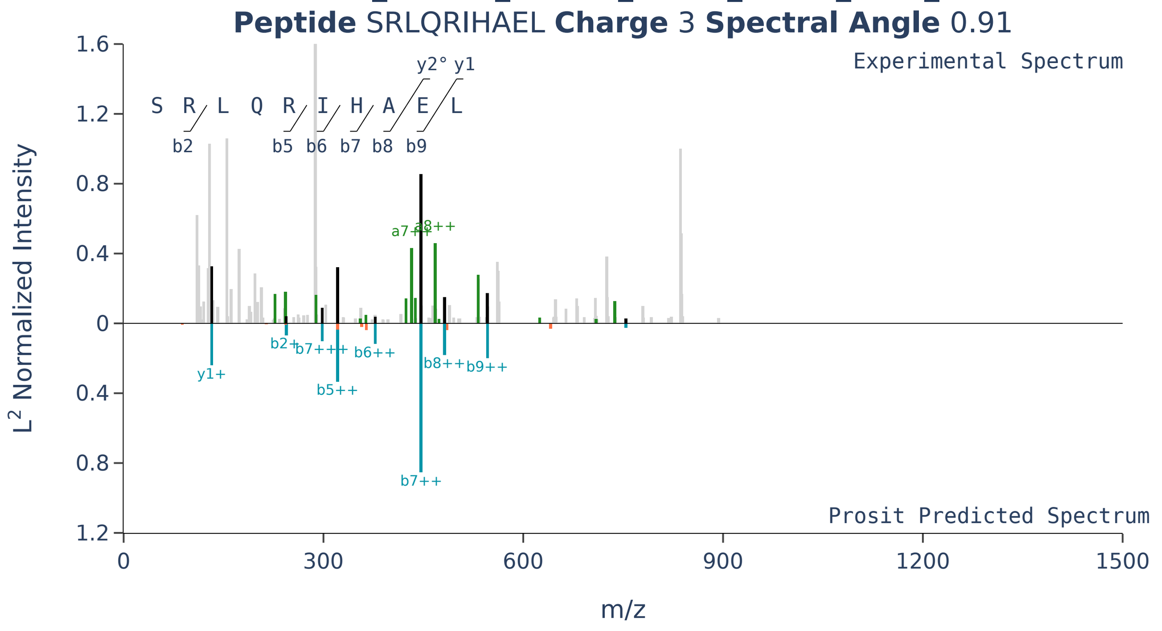


**Source** HRoetschke_271222_090123_HFGoe_LEDGF_b1R2_4h **Scan** 19744
**Peptide** NEGLWEIDNNPKVK **Charge** 3 **Spectral Angle** 0.75

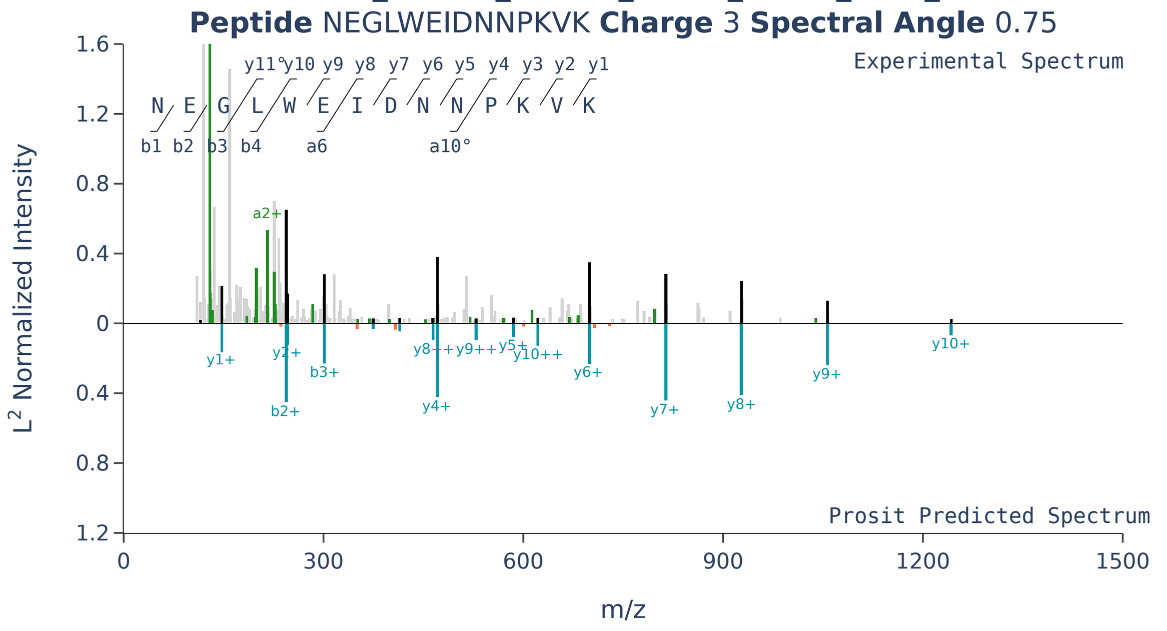


**Source** HRoetschke_271222_090123_HFGoe_LEDGF_b1R2_2h **Scan** 22990
**Peptide** FKVSQVIME **Charge** 2 **Spectral Angle** 0.69

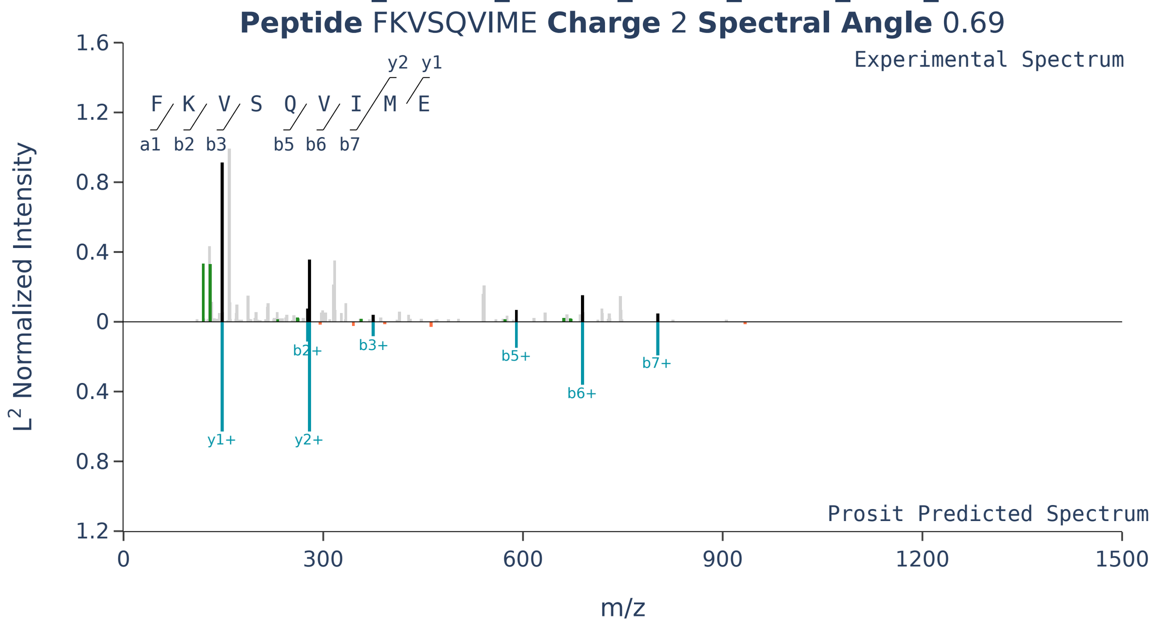


**Source** HRoetschke_271222_090123_HFGoe_LEDGF_b1R2_2h **Scan** 23877
**Peptide** GTHETAFLGPKDIFPYSENKE **Charge** 4 **Spectral Angle** 0.81

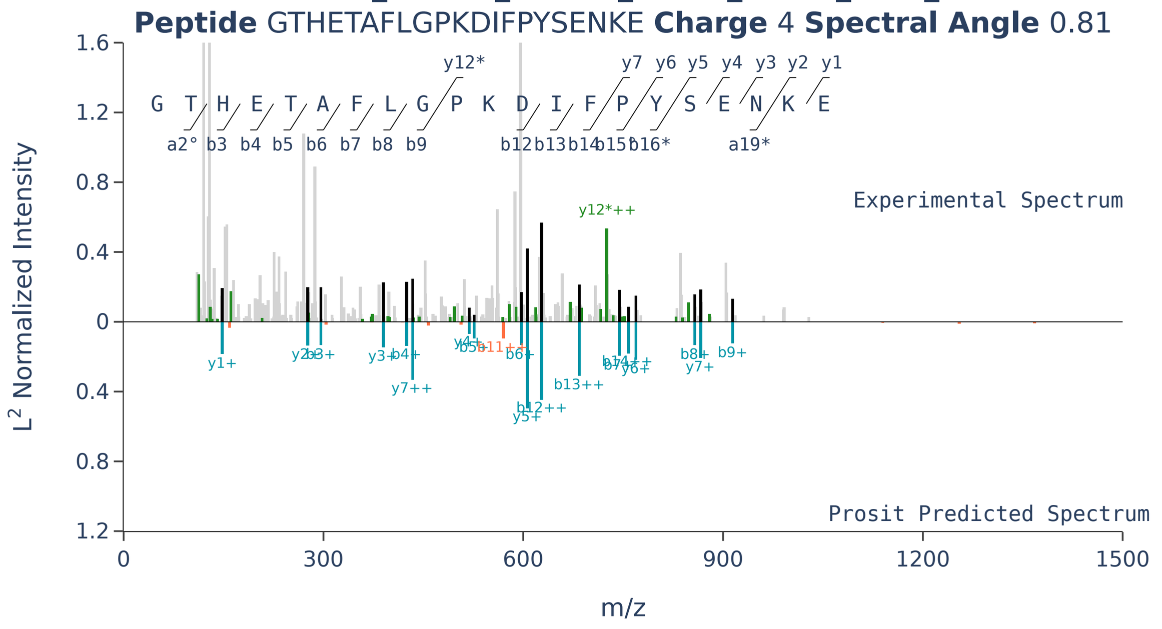


**Source** HRoetschke_271222_090123_HFGoe_LEDGF_b1R1_4h **Scan** 16653
**Peptide** KGYPHWPARVDEVPDGAVKPPTNK **Charge** 3 **Spectral Angle** 0.88

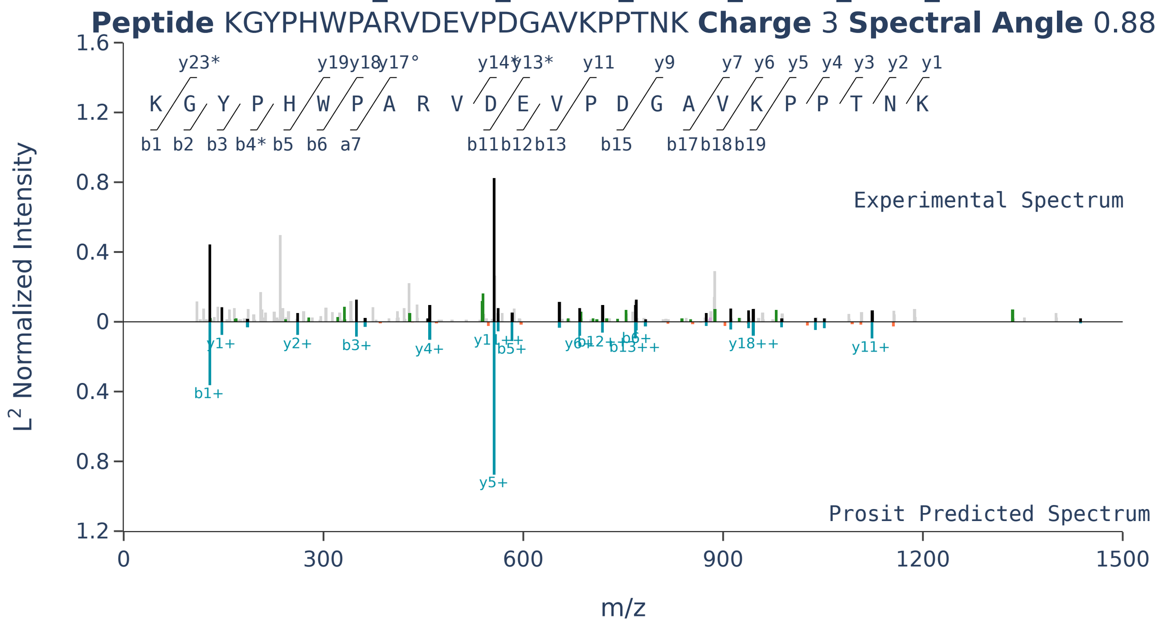


**Source** HRoetschke_271222_090123_HFGoe_LEDGF_b1R1_4h **Scan** 18511
**Peptide** QVIMEKSTMLYNKFKN **Charge** 4 **Spectral Angle** 0.81

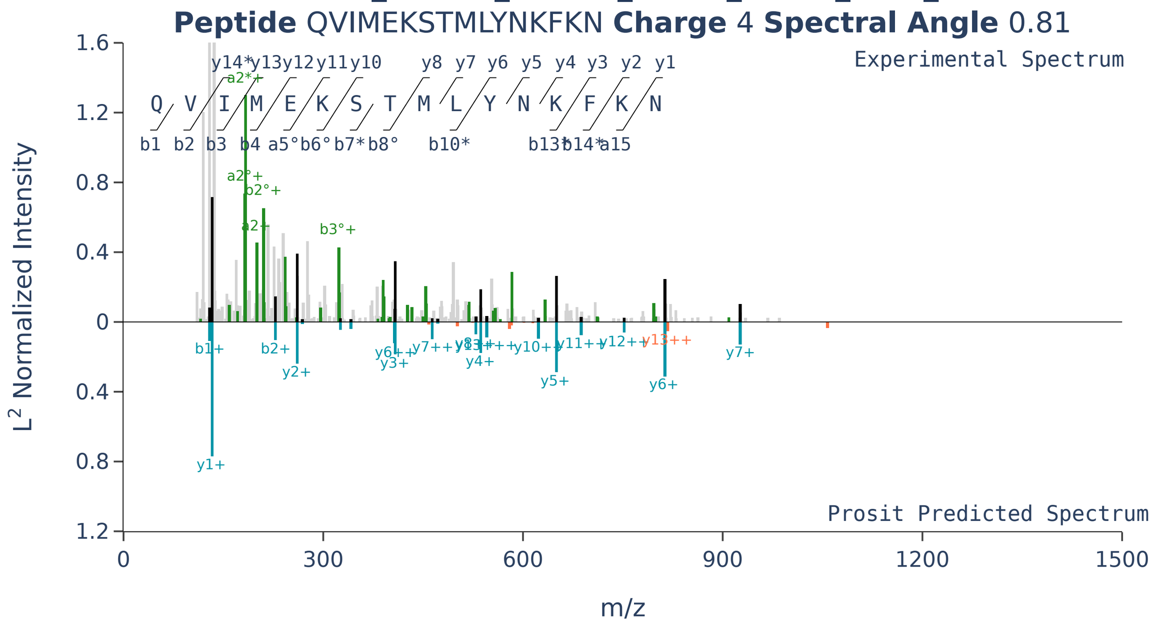


**Source** HRoetschke_271222_090123_HFGoe_LEDGF_b1R2_4h **Scan** 19088
**Peptide** IMEKSTMLY **Charge** 2 **Spectral Angle** 0.84

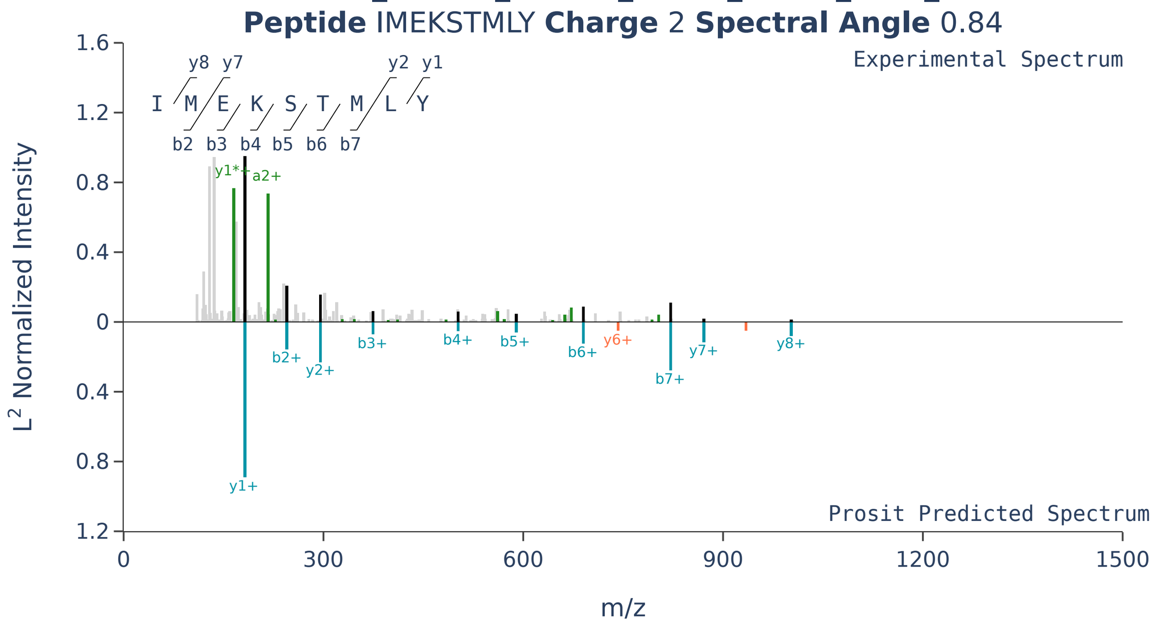


**Source** HRoetschke_271222_090123_HFGoe_LEDGF_b1R1_24h **Scan** 6061
**Peptide** NLKVSPKRGRPAATE **Charge** 3 **Spectral Angle** 0.93

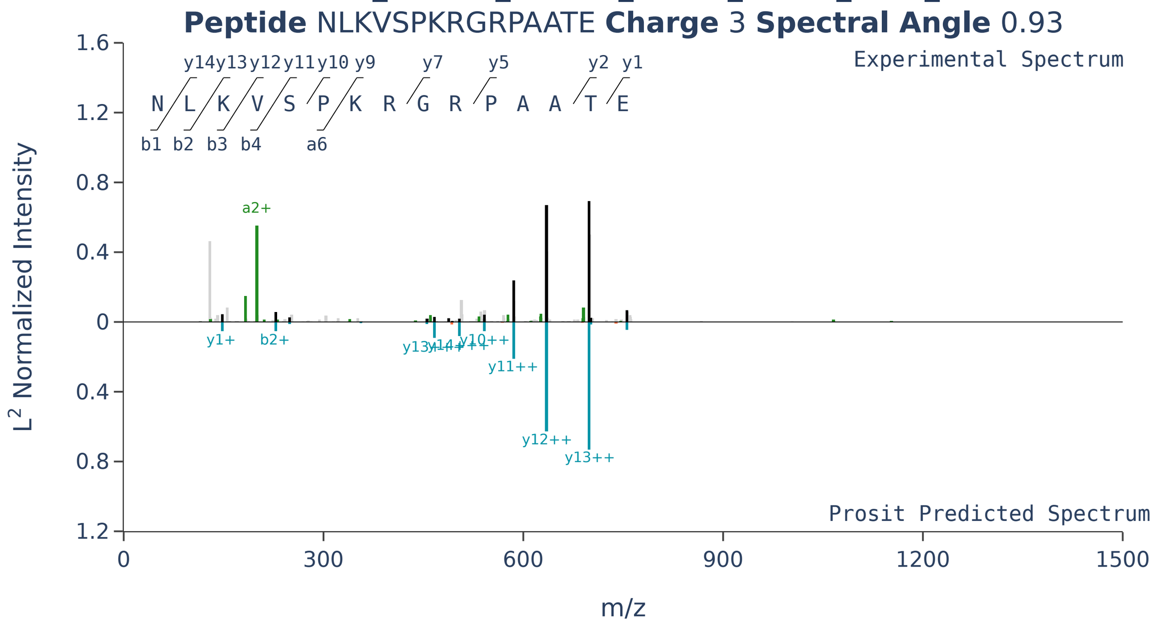

**Source** HRoetschke_271222_090123_HFGoe_LEDGF_b2R1_24h **Scan** 14354
**Peptide** LGPKDIFPYSENKEKYGKPNKRKGFNE **Charge** 6 **Spectral Angle** 0.78

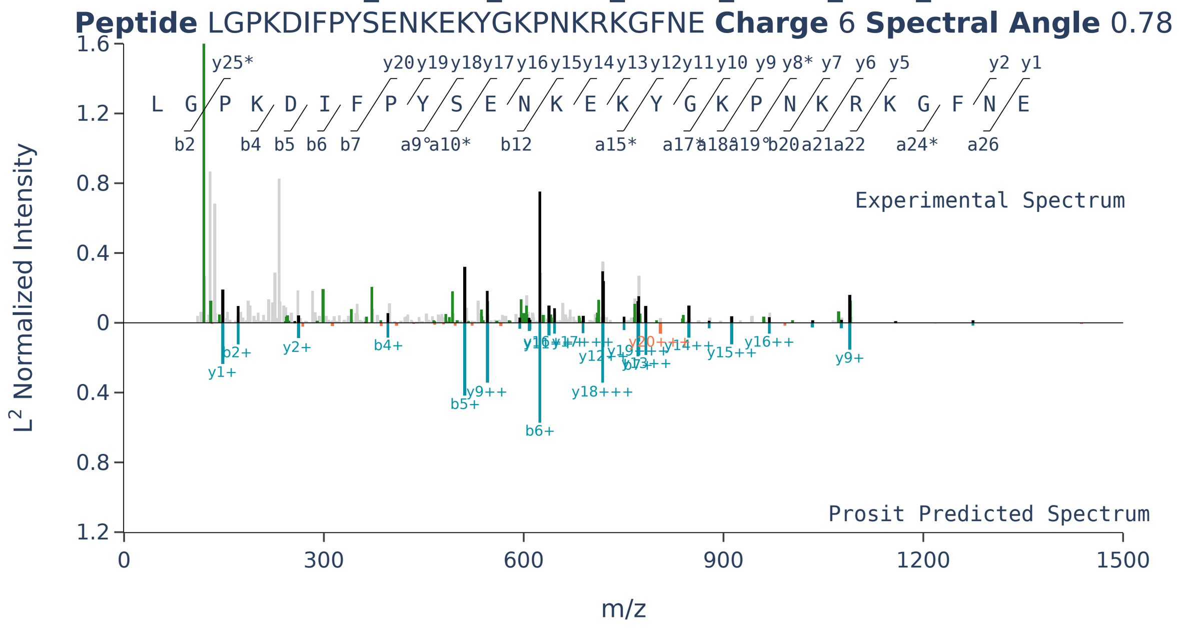


**Source** HRoetschke_271222_090123_HFGoe_LEDGF_b1R2_4h **Scan** 18756
**Peptide** WEIDNNPKVKFSSQ **Charge** 2 **Spectral Angle** 0.85

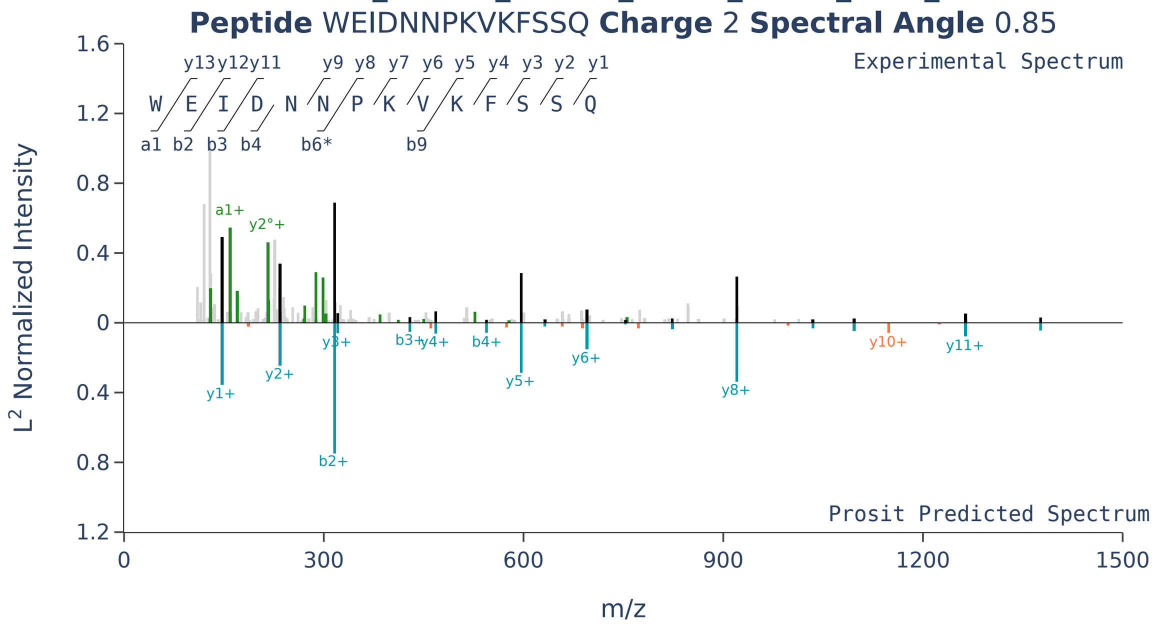


**Source** HRoetschke_271222_090123_HFGoe_LEDGF_b1R1_2h **Scan** 21976
**Peptide** LGPKDIFPYSENKE **Charge** 2 **Spectral Angle** 0.82

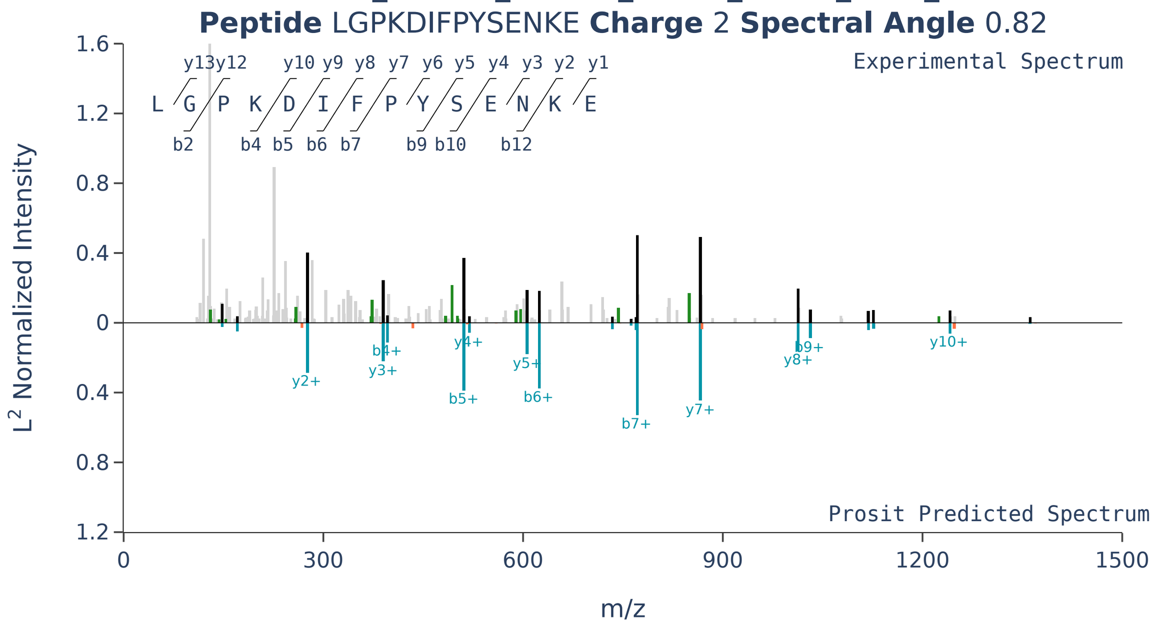


**Source** HRoetschke_271222_090123_HFGoe_LEDGF_b2R1_24h **Scan** 7104
**Peptide** LKVSPKRGRPAATEV **Charge** 3 **Spectral Angle** 0.87

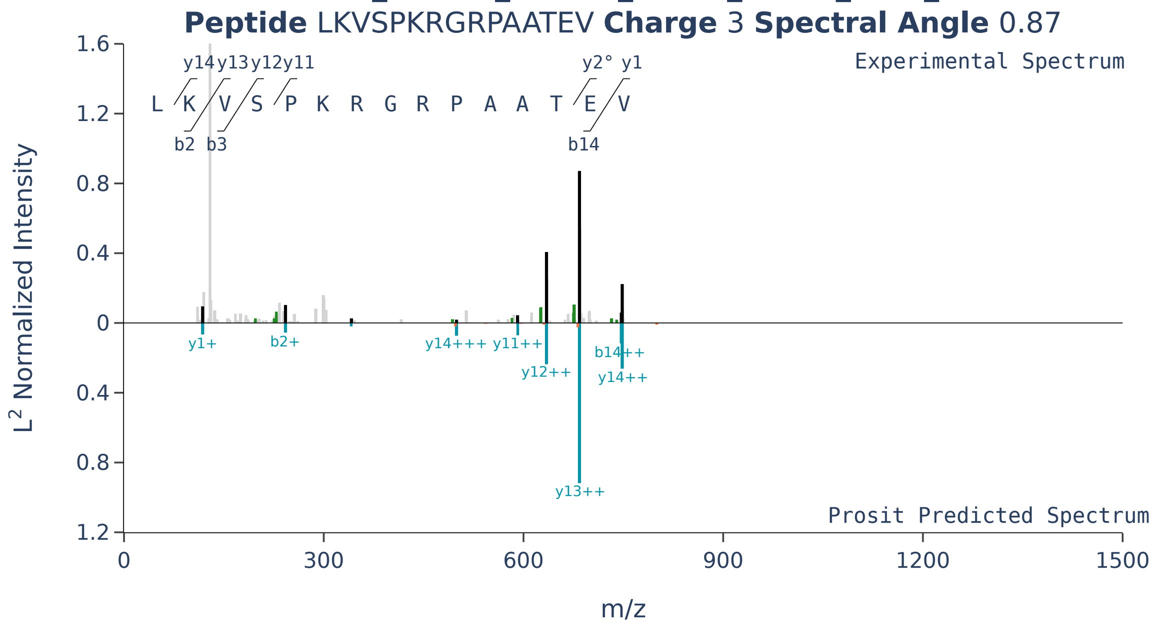


**Source** HRoetschke_271222_090123_HFGoe_LEDGF_b2R2_1h **Scan** 7445
**Peptide** PKVKFSSQ **Charge** 2 **Spectral Angle** 0.82

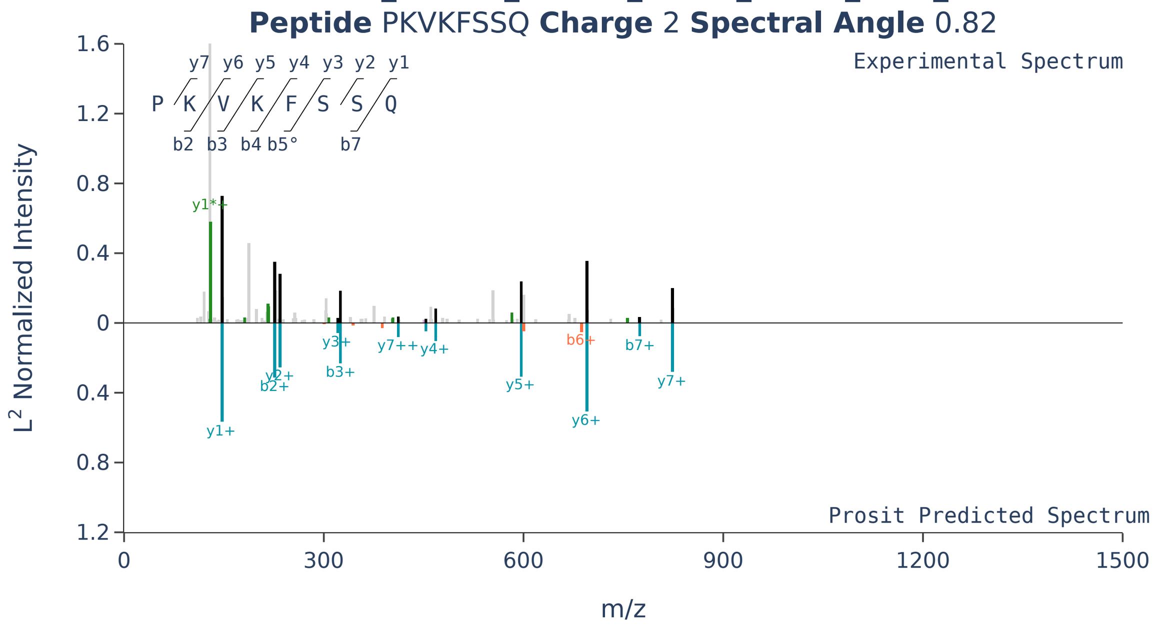


**Source** HRoetschke_271222_090123_HFGoe_LEDGF_b2R1_24h **Scan** 26559
**Peptide** FNEGLWEIDNNPKVKFS **Charge** 3 **Spectral Angle** 0.85

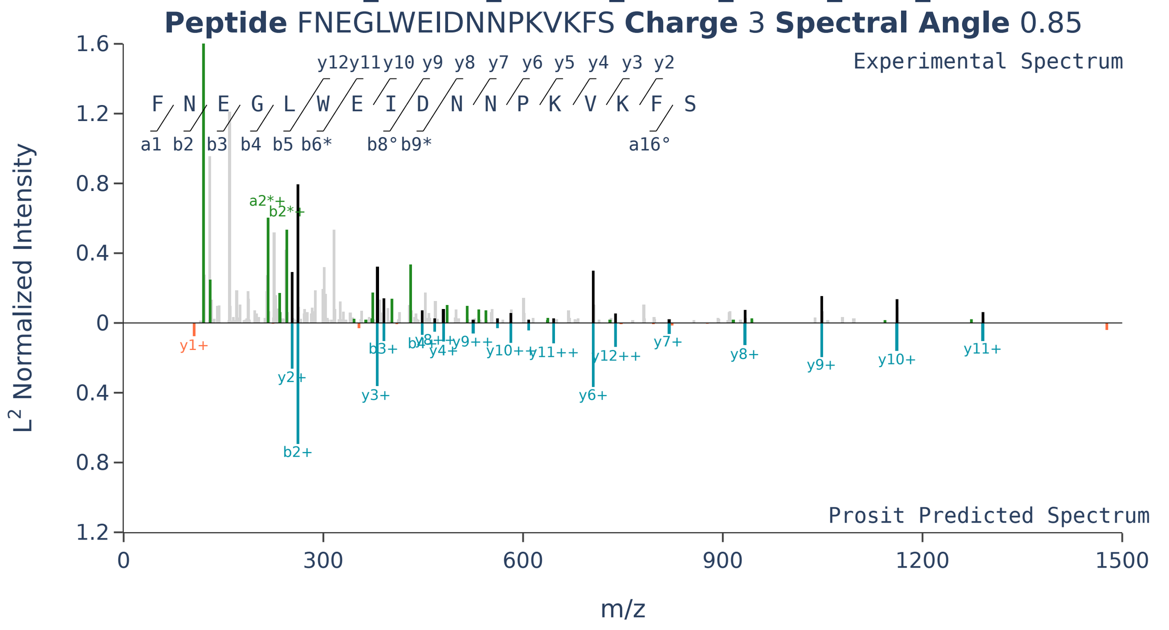


**Source** HRoetschke_271222_090123_HFGoe_LEDGF_b1R2_24h **Scan** 9835
**Peptide** TTLKKIRRF **Charge** 3 **Spectral Angle** 0.88

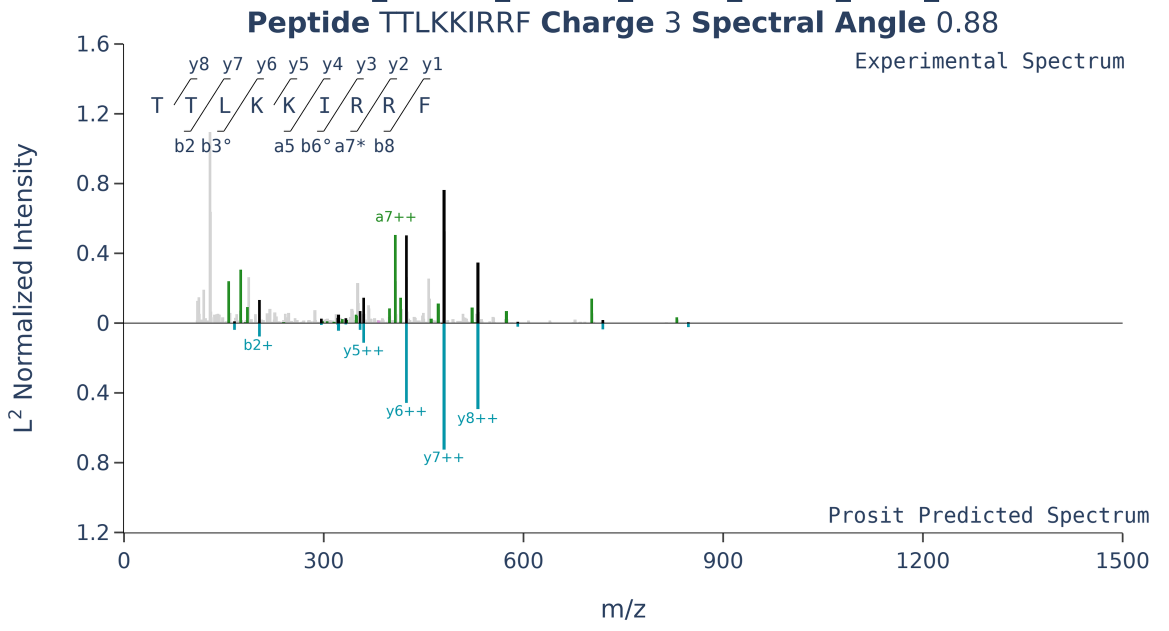


**Source** HRoetschke_271222_090123_HFGoe_LEDGF_b1R1_2h **Scan** 8982
**Peptide** IFPYSENKEKYGKPNKRKGFNEG **Charge** 4 **Spectral Angle** 0.82

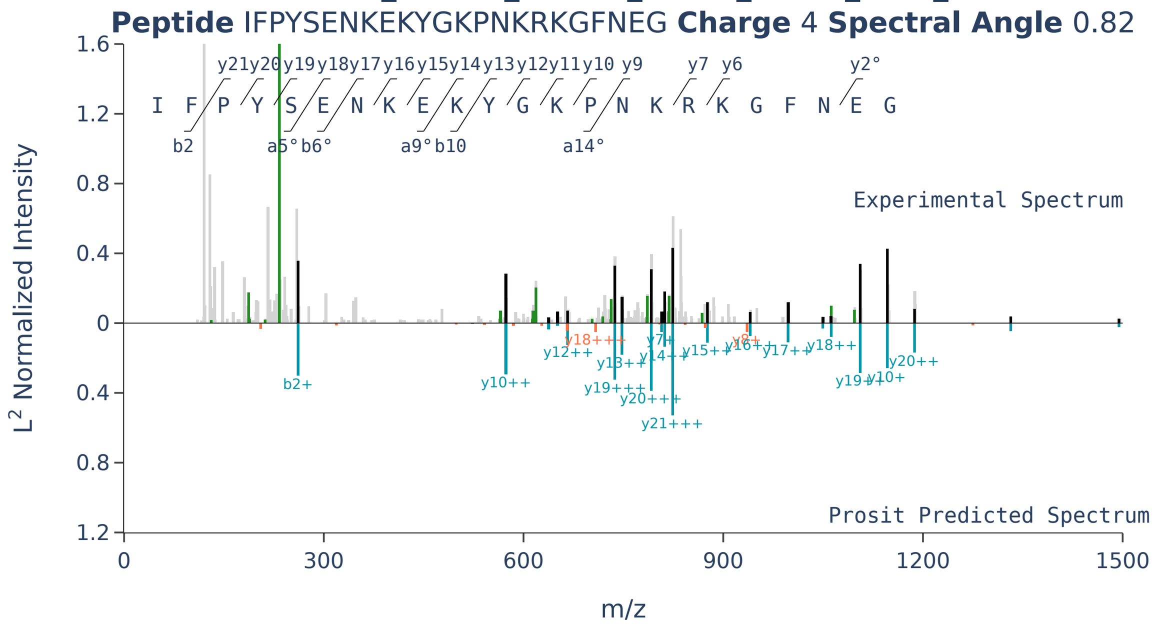


**Source** HRoetschke_271222_090123_HFGoe_LEDGF_b1R2_2h **Scan** 9245
**Peptide** PSSEERETEI **Charge** 2 **Spectral Angle** 0.84

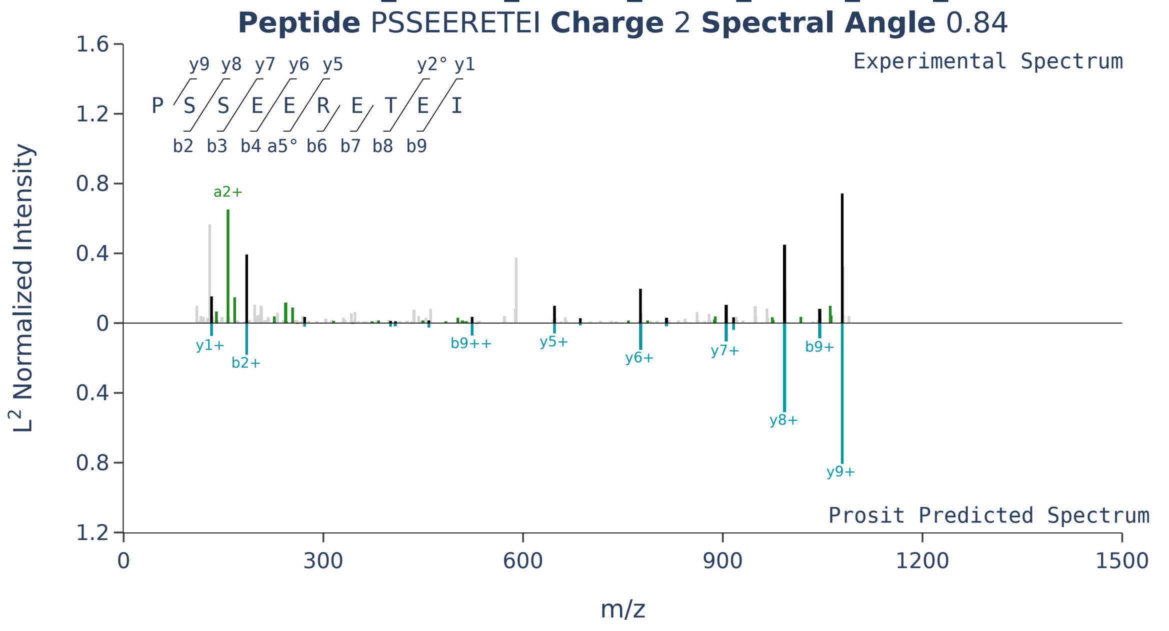


**Source** HRoetschke_271222_090123_HFGoe_LEDGF_b2R1_4h **Scan** 17071
**Peptide** SNEDVTKAVDITTPKAA **Charge** 3 **Spectral Angle** 0.86

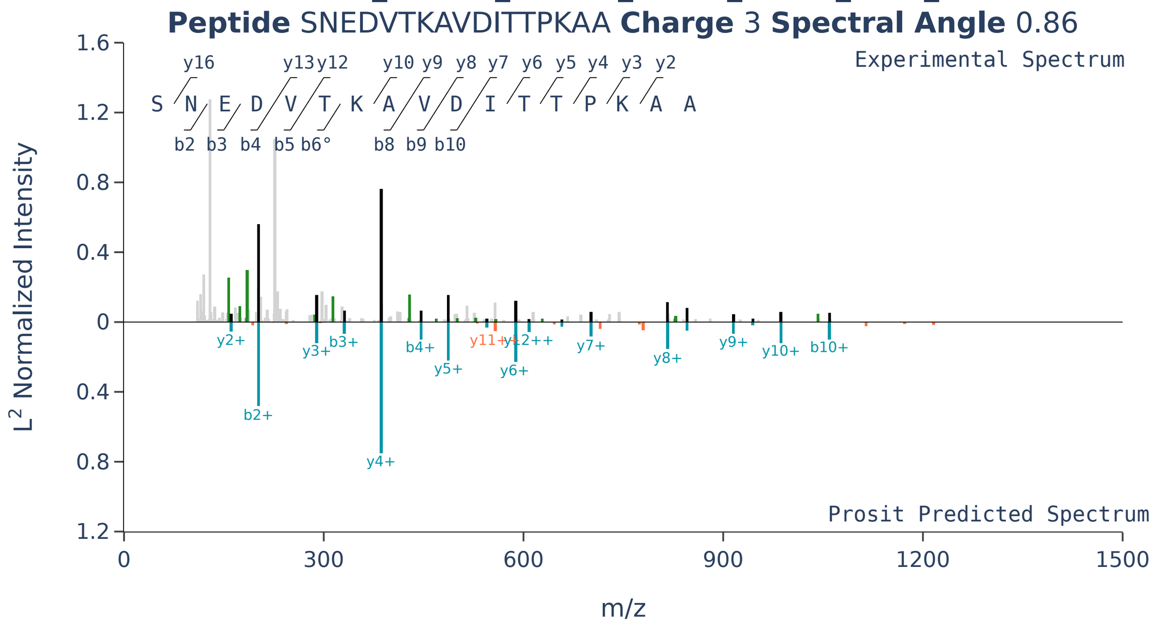


**Source** HRoetschke_271222_090123_HFGoe_LEDGF_b2R2_2h **Scan** 19351
**Peptide** IKNSLKIDNLDVNRCIE **Charge** 3 **Spectral Angle** 0.82

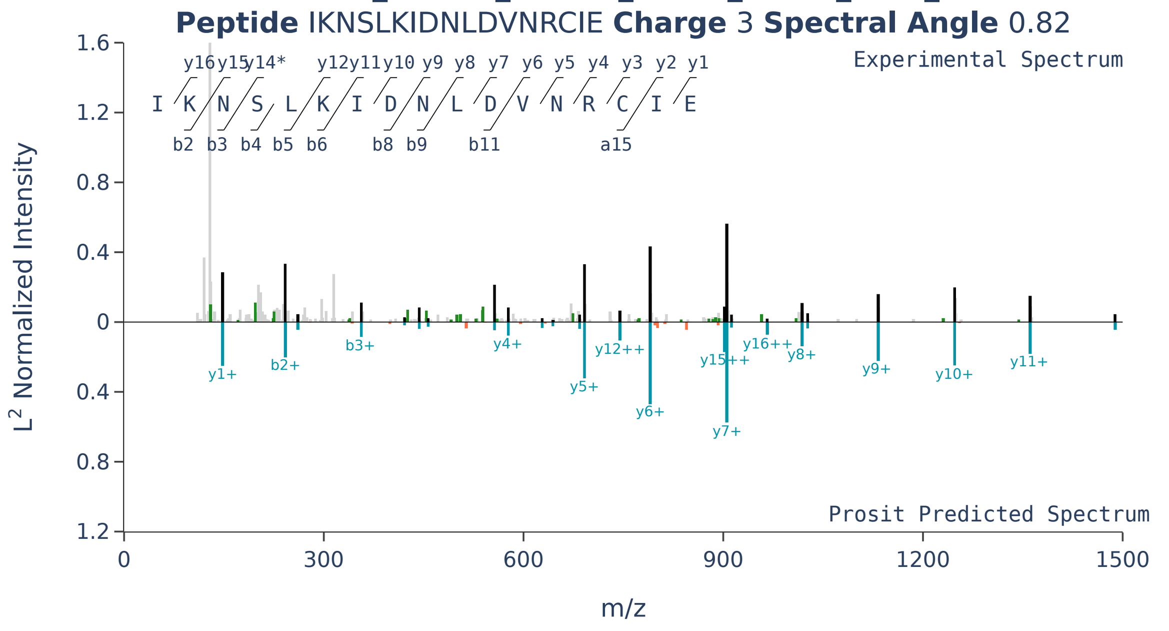


**Source** HRoetschke_271222_090123_HFGoe_LEDGF_b2R2_4h **Scan** 14583
**Peptide** KASNEDVTKAVDITTPKAA **Charge** 3 **Spectral Angle** 0.87

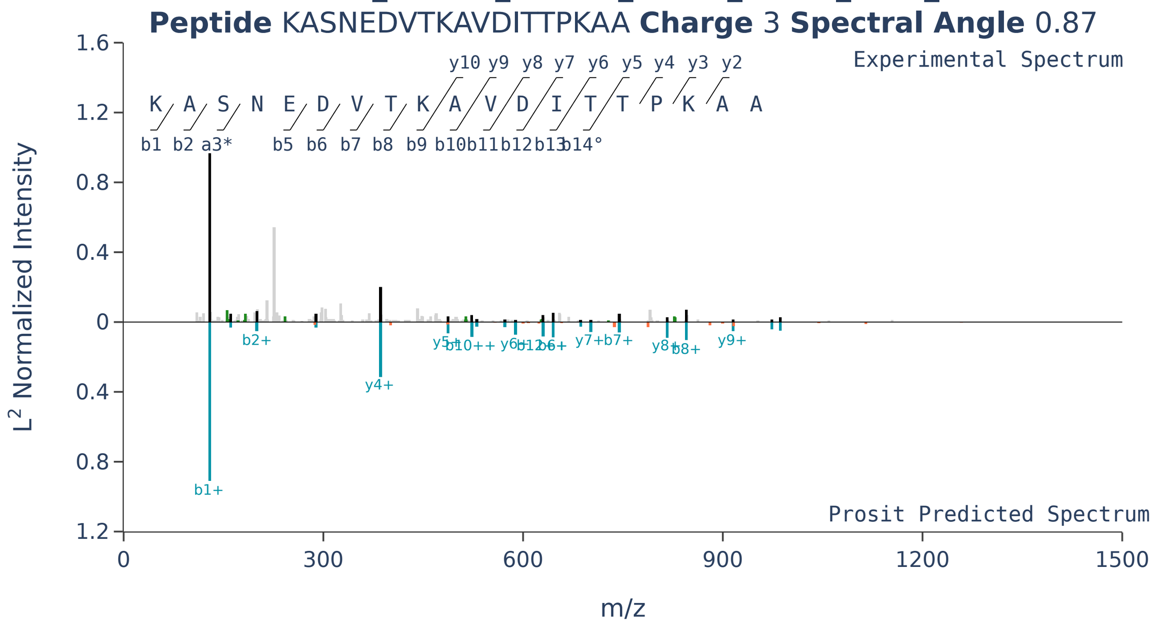


**Source** HRoetschke_271222_090123_HFGoe_LEDGF_b1R1_2h **Scan** 24315
**Peptide** RDFKPGDLIFAKMKGY **Charge** 3 **Spectral Angle** 0.81

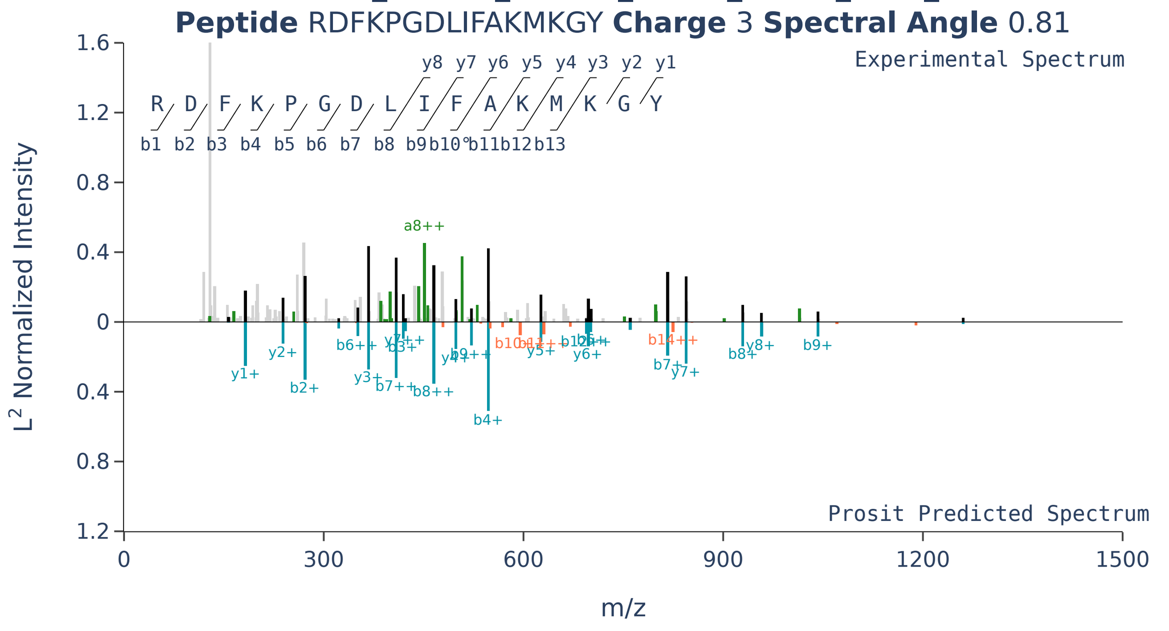


**Source** HRoetschke_271222_090123_HFGoe_LEDGF_b2R1_2h **Scan** 21459
**Peptide** VIMEKSTMLYNKF **Charge** 3 **Spectral Angle** 0.79

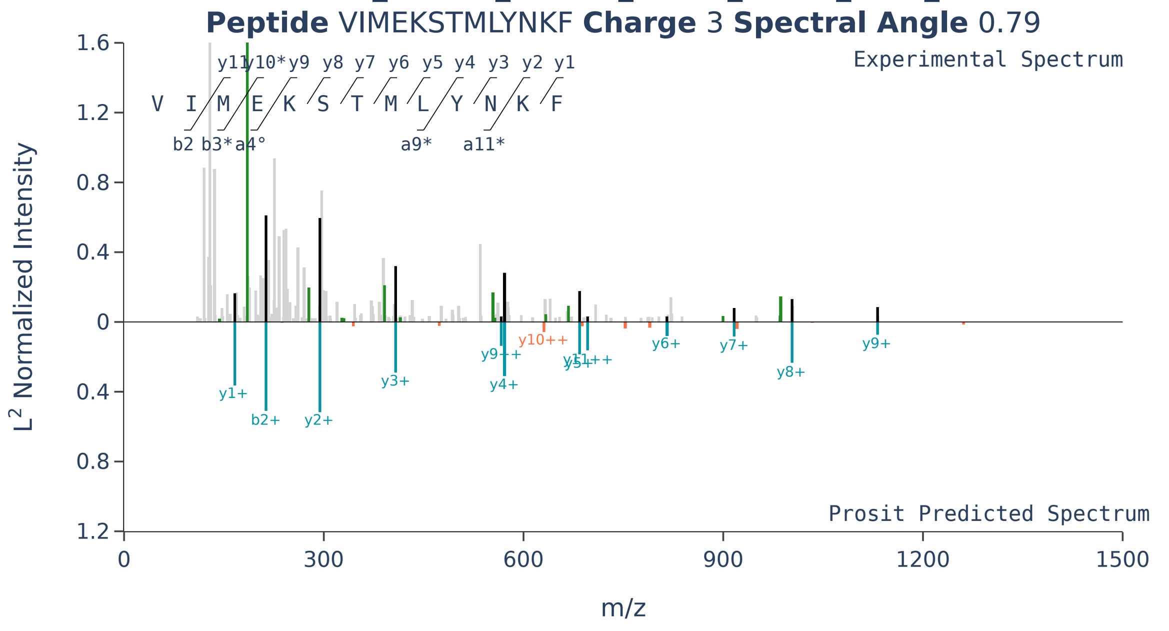


**Source** HRoetschke_271222_090123_HFGoe_LEDGF_b1R1_24h **Scan** 13761
**Peptide** QRIHAEIKNSLKI **Charge** 3 **Spectral Angle** 0.79

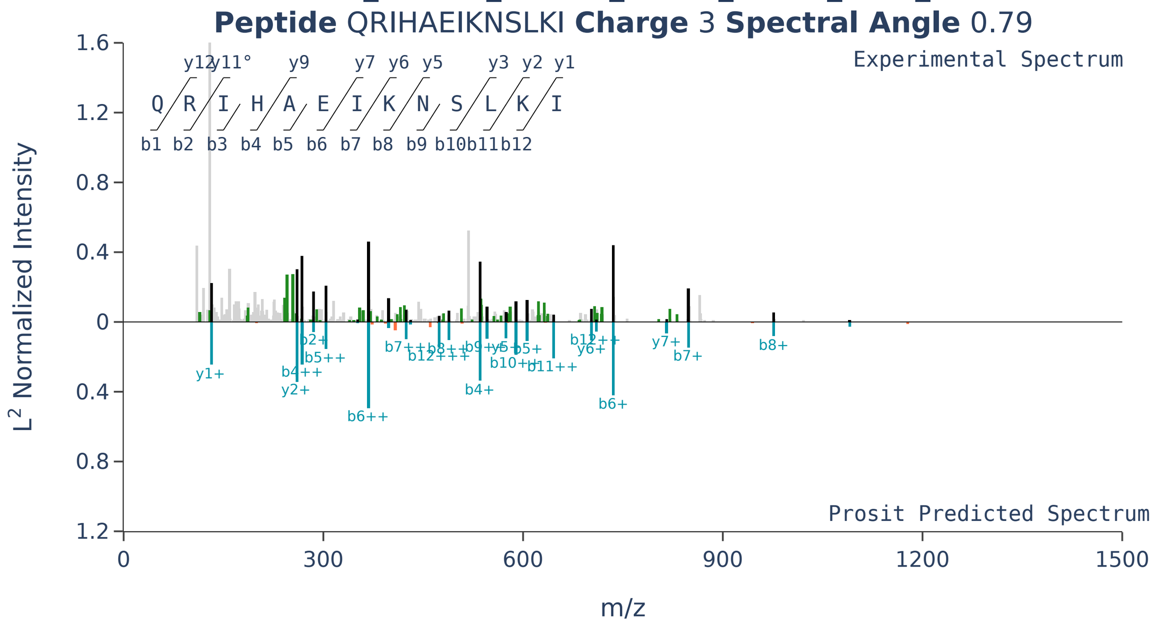

**Source** HRoetschke_271222_090123_HFGoe_LEDGF_b1R1_2h **Scan** 24101
**Peptide** YNKFKNMFLVGE **Charge** 2 **Spectral Angle** 0.81

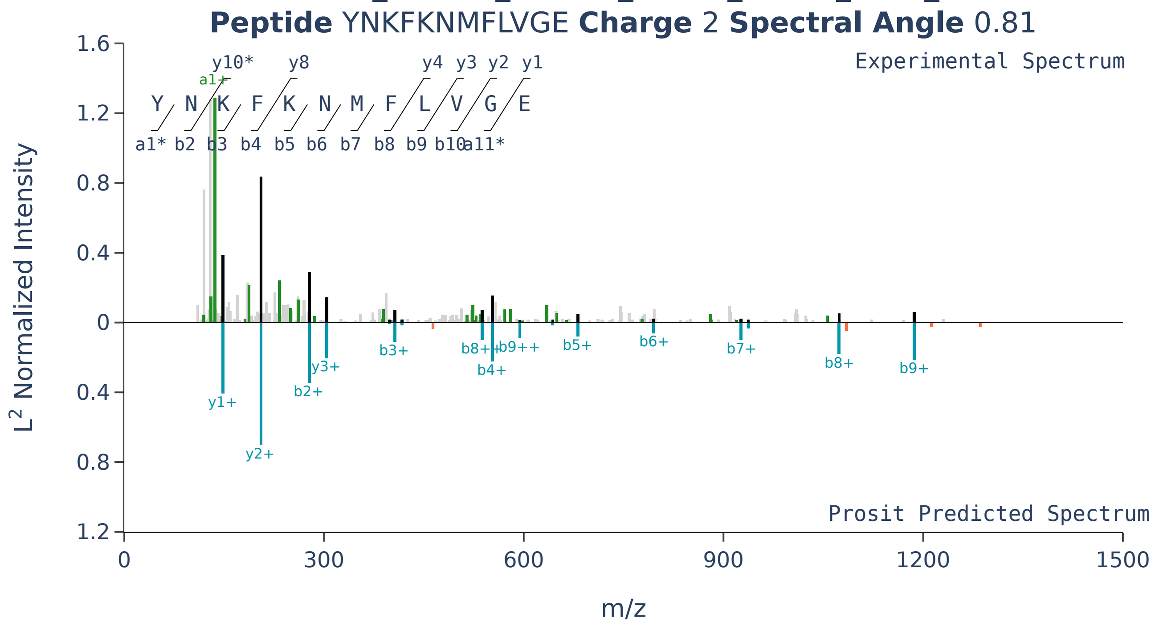


**Source** HRoetschke_271222_090123_HFGoe_LEDGF_b2R2_4h **Scan** 12030
**Peptide** LQVTMQQAQK **Charge** 2 **Spectral Angle** 0.75

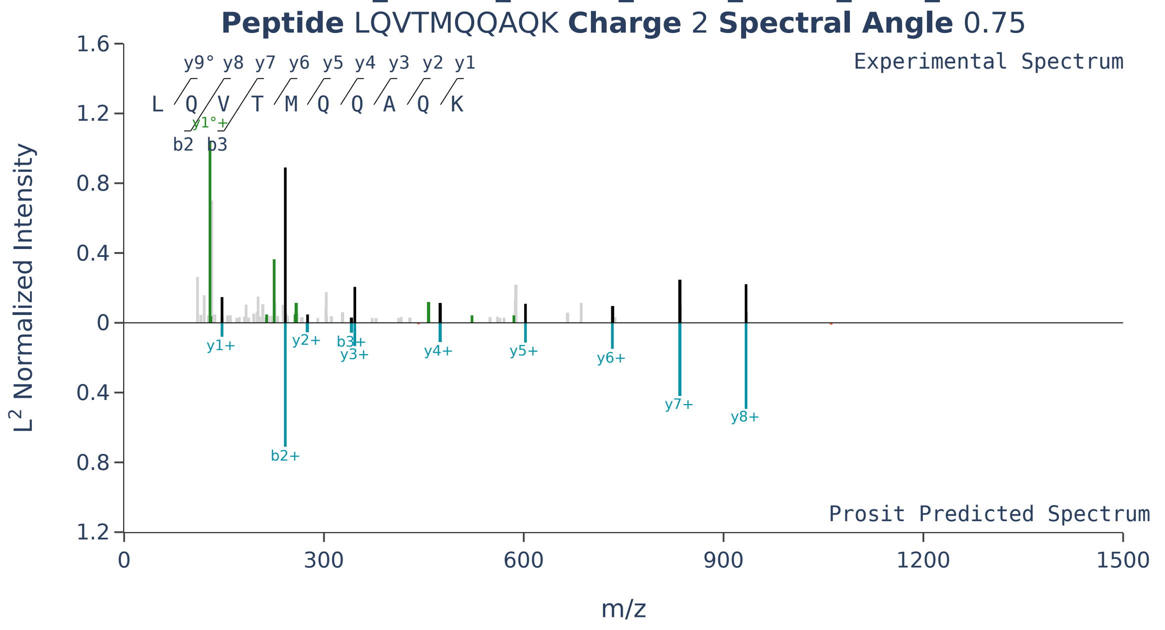


**Source** HRoetschke_271222_090123_HFGoe_LEDGF_b2R1_4h **Scan** 11803
**Peptide** LYNKFKNM **Charge** 3 **Spectral Angle** 0.82

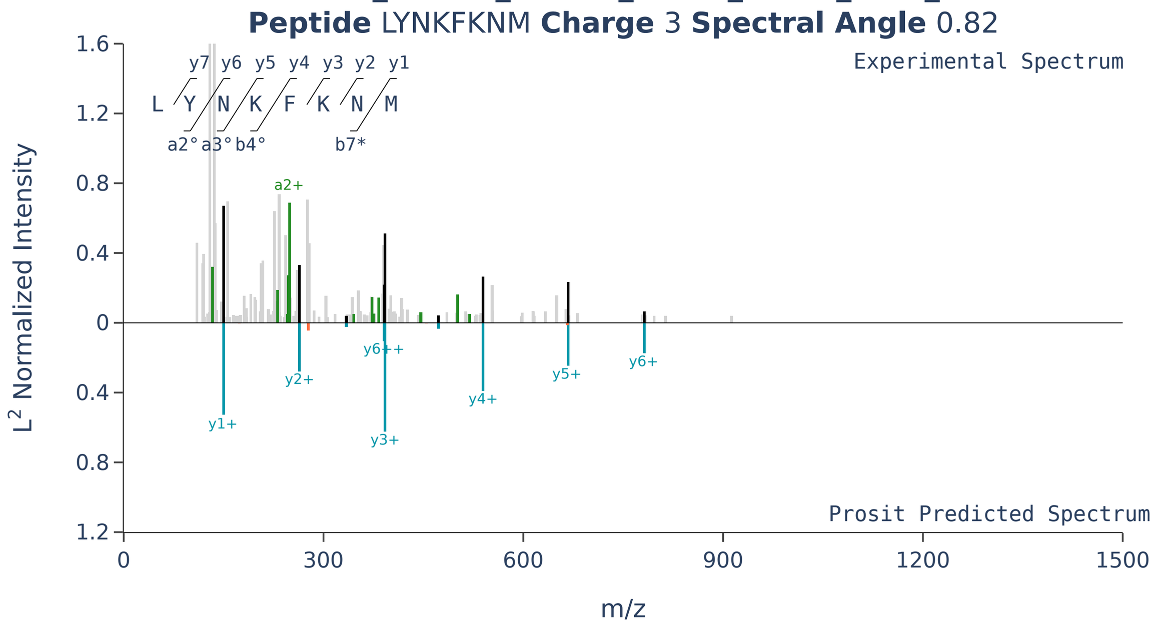


**Source** HRoetschke_271222_090123_HFGoe_LEDGF_b1R1_2h **Scan** 22692
**Peptide** SVITQVLNKSLAEQRQHE **Charge** 2 **Spectral Angle** 0.84

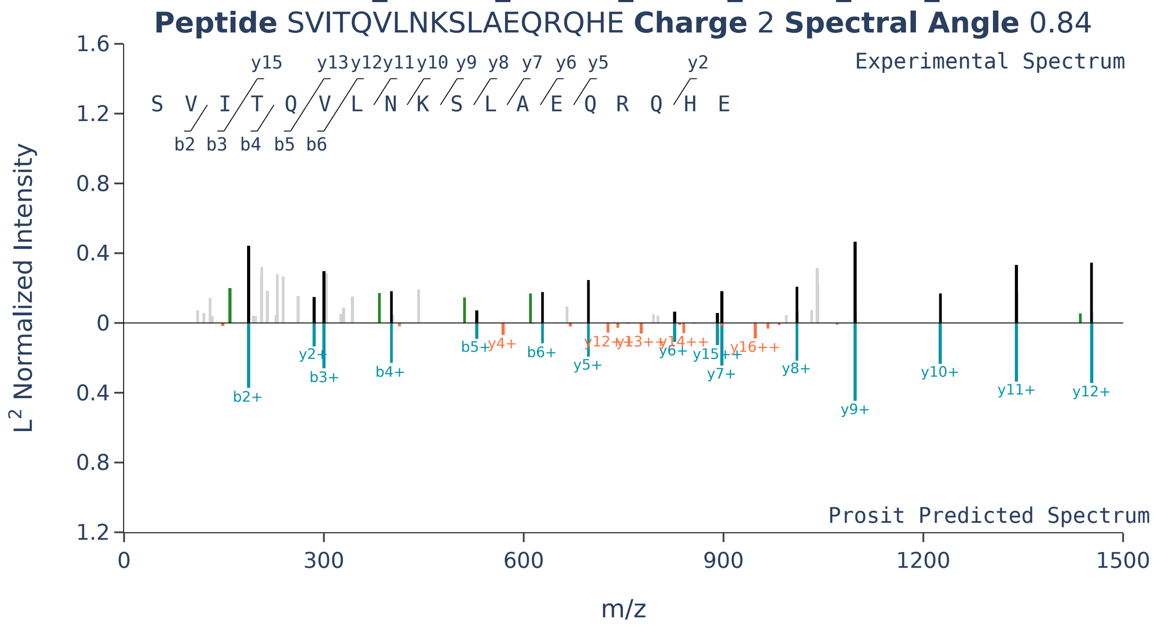


**Source** HRoetschke_271222_090123_HFGoe_LEDGF_b2R1_24h **Scan** 15736
**Peptide** LGPKDIFPYSENKEKYGKPNK **Charge** 5 **Spectral Angle** 0.76

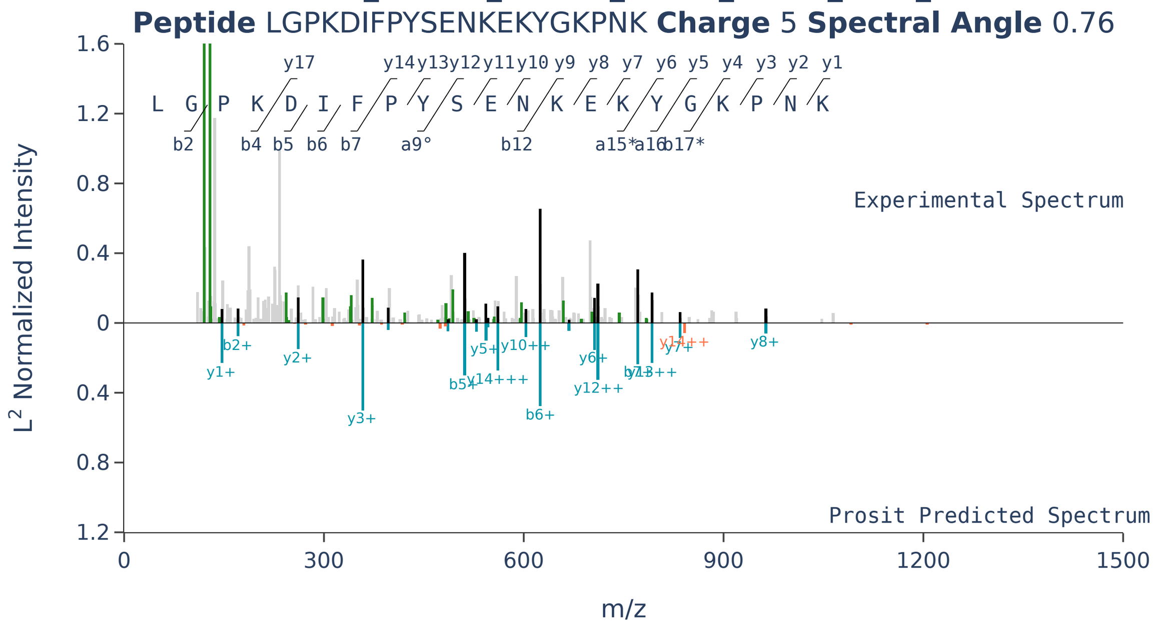


**Source** HRoetschke_271222_090123_HFGoe_LEDGF_b1R1_24h **Scan** 5853
**Peptide** NLKVSPKRGRPAAT **Charge** 3 **Spectral Angle** 0.94

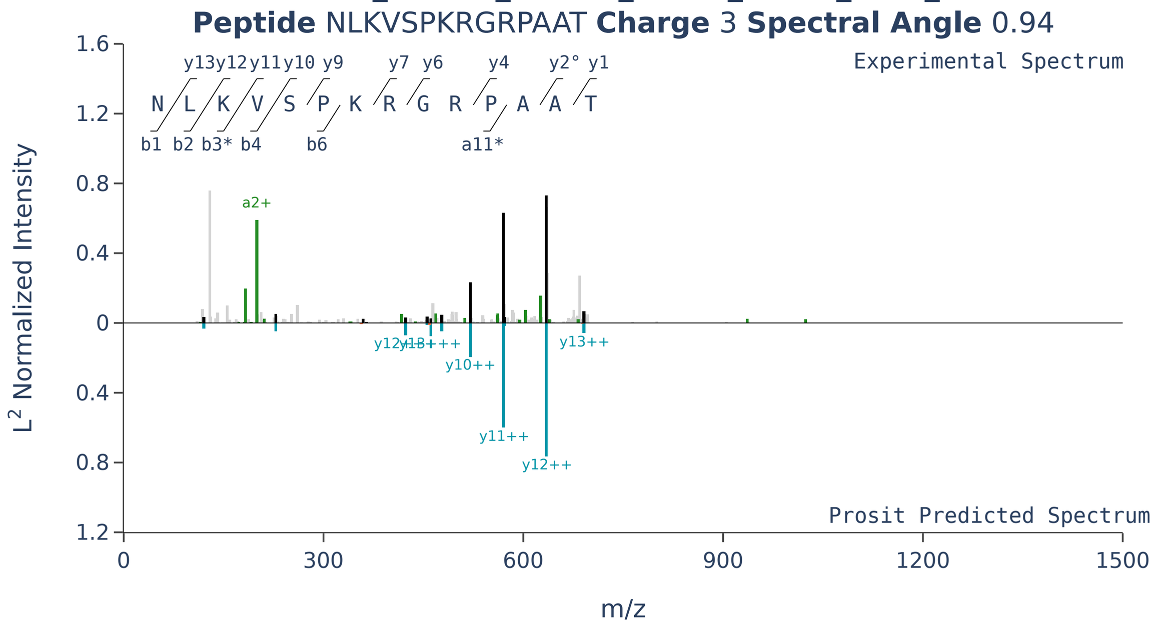


**Source** HRoetschke_271222_090123_HFGoe_LEDGF_b1R1_2h **Scan** 25663
**Peptide** GFNEGLWEIDNNPKVKFSSQQ **Charge** 3 **Spectral Angle** 0.83

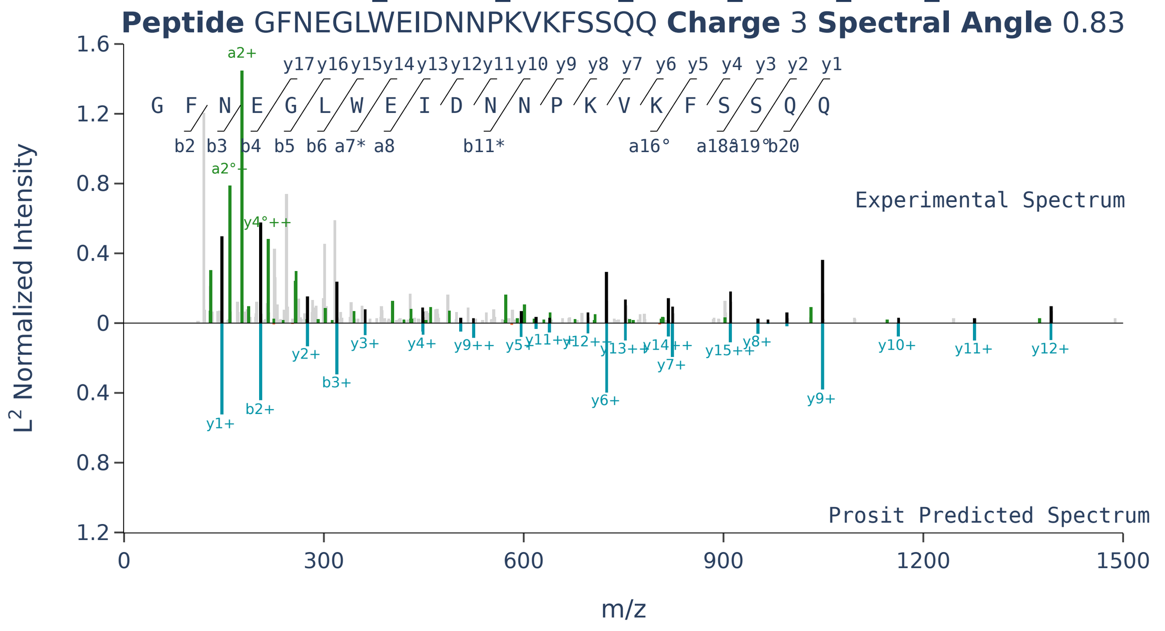


**Source** HRoetschke_271222_090123_HFGoe_LEDGF_b1R1_2h **Scan** 19622
**Peptide** PHWPARVDEVPDGAVKPPTNKL **Charge** 3 **Spectral Angle** 0.82

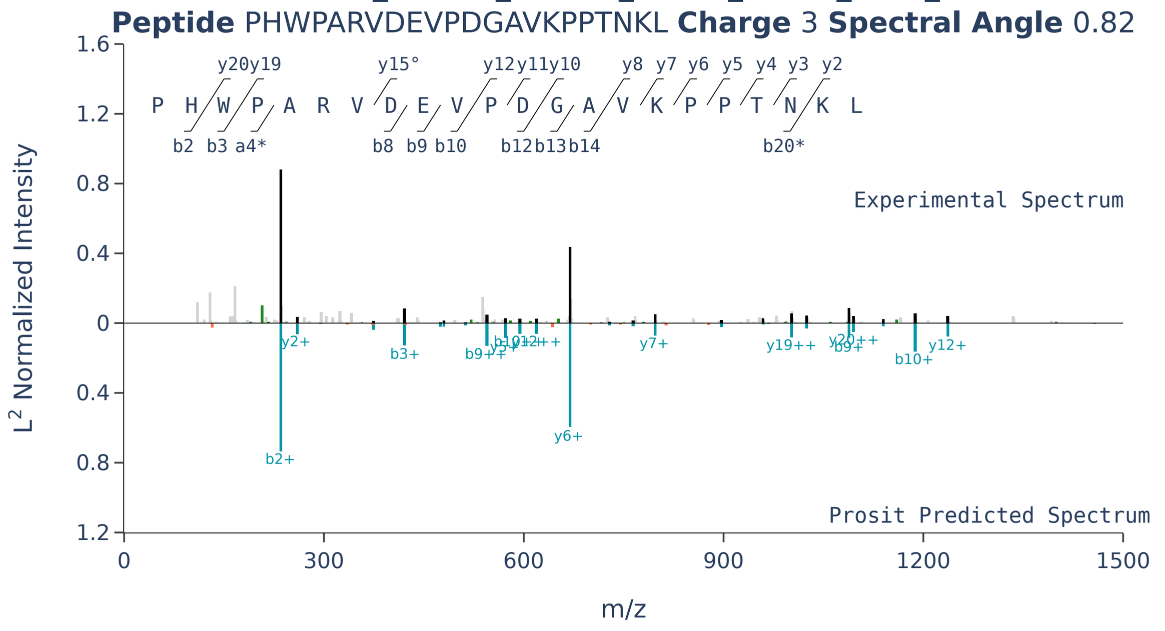


**Source** HRoetschke_271222_090123_HFGoe_LEDGF_b2R2_1h **Scan** 8774
**Peptide** SLKDSTLDN **Charge** 2 **Spectral Angle** 0.85

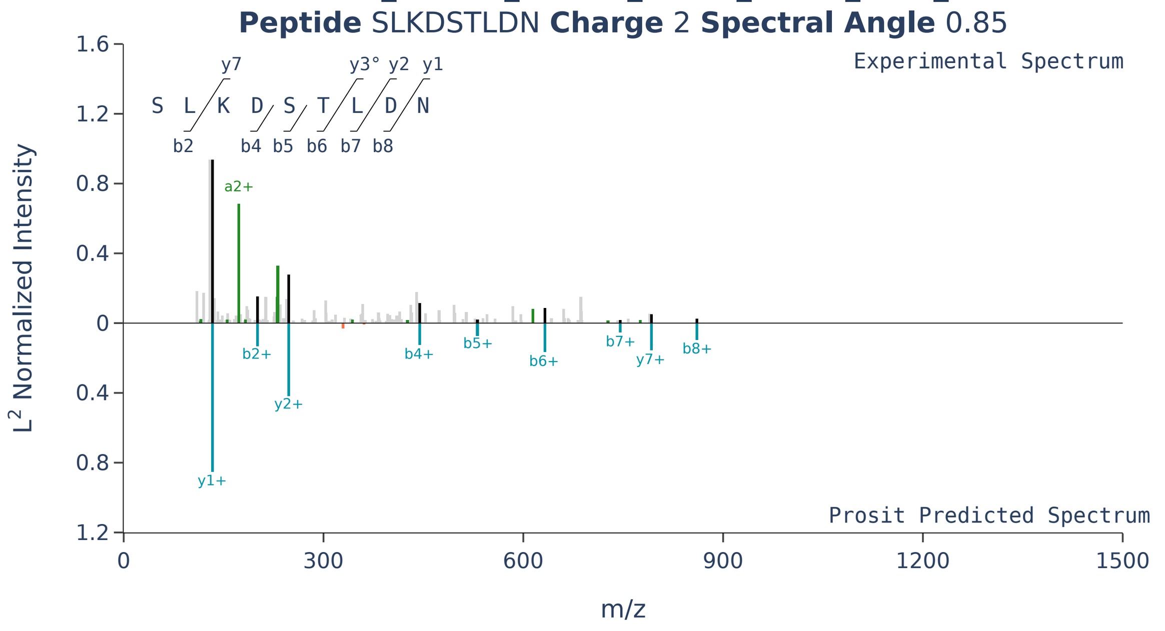


**Source** HRoetschke_271222_090123_HFGoe_LEDGF_b2R1_24h **Scan** 12980
**Peptide** WPARVDE **Charge** 2 **Spectral Angle** 0.94

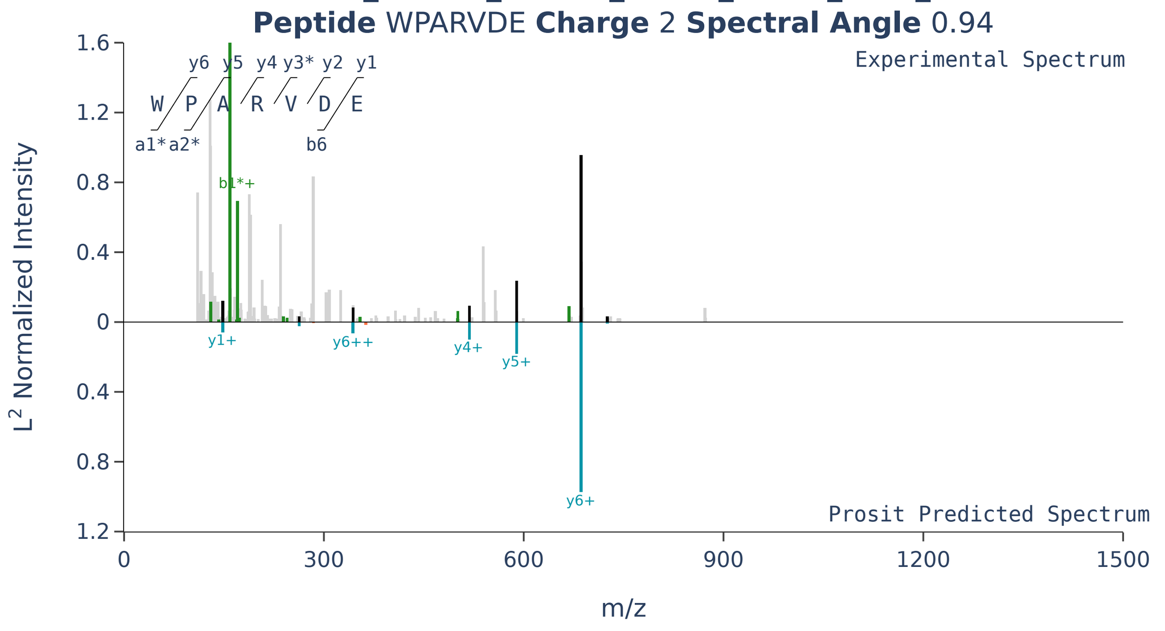


**Source** HRoetschke_271222_090123_HFGoe_LEDGF_b2R1_1h **Scan** 28983
**Peptide** AFLGPKDIFPYSEN **Charge** 2 **Spectral Angle** 0.65

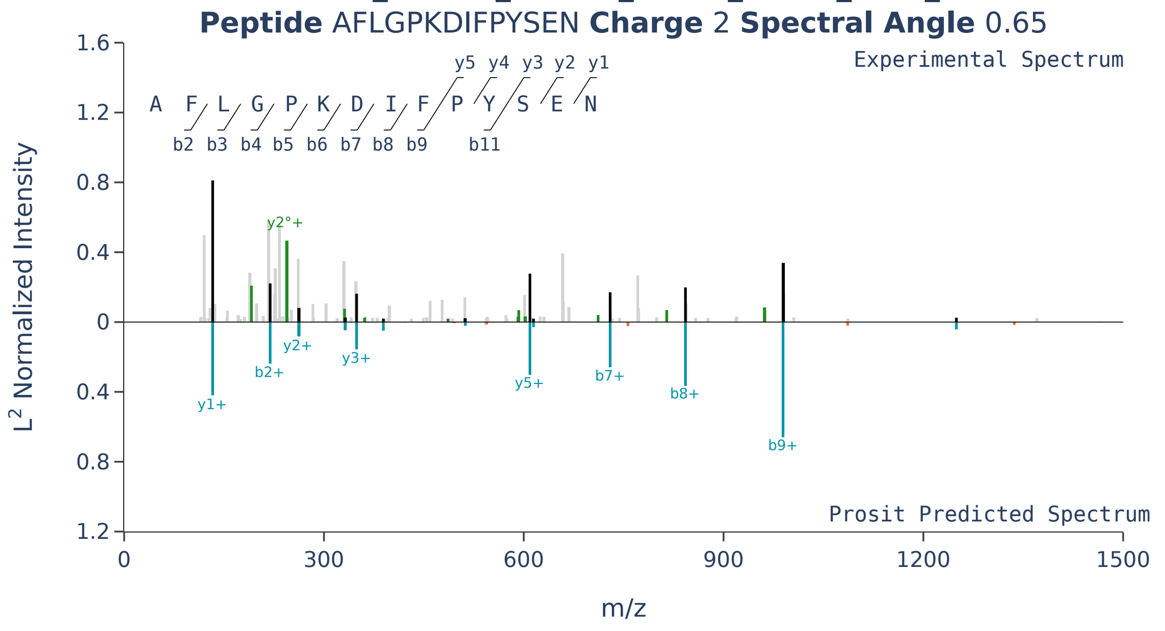


**Source** HRoetschke_271222_090123_HFGoe_LEDGF_b2R1_24h **Scan** 14437
**Peptide** EKQVETEEAGVVTTATA **Charge** 2 **Spectral Angle** 0.79

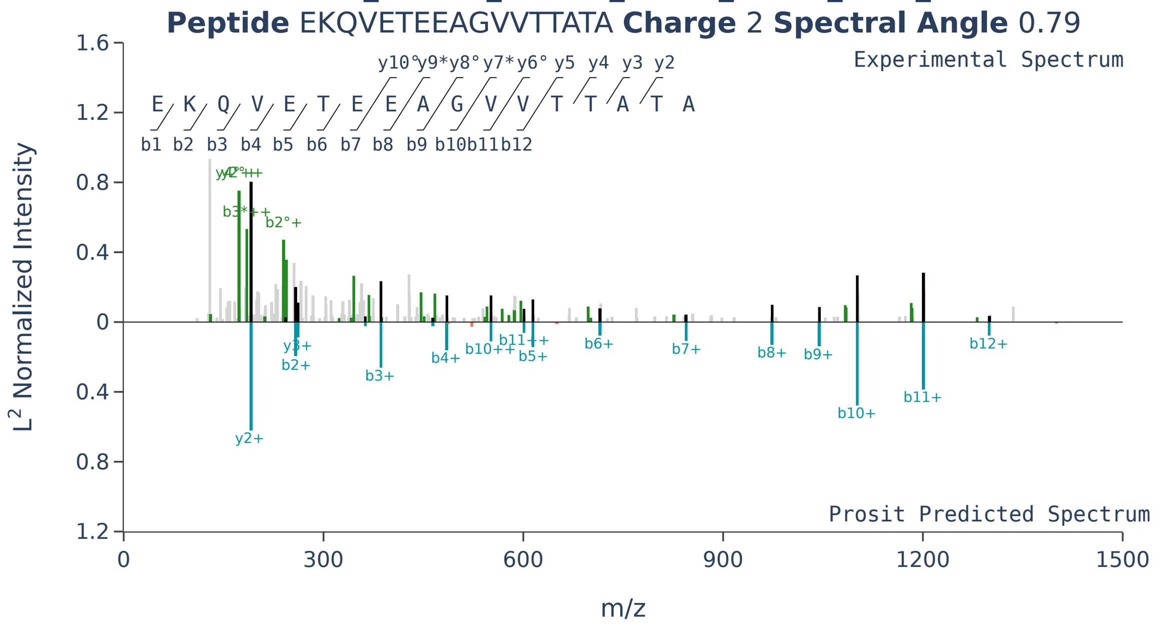


**Source** HRoetschke_271222_090123_HFGoe_LEDGF_b1R2_2h **Scan** 13275
**Peptide** KMKGYPHWPARVD **Charge** 4 **Spectral Angle** 0.87

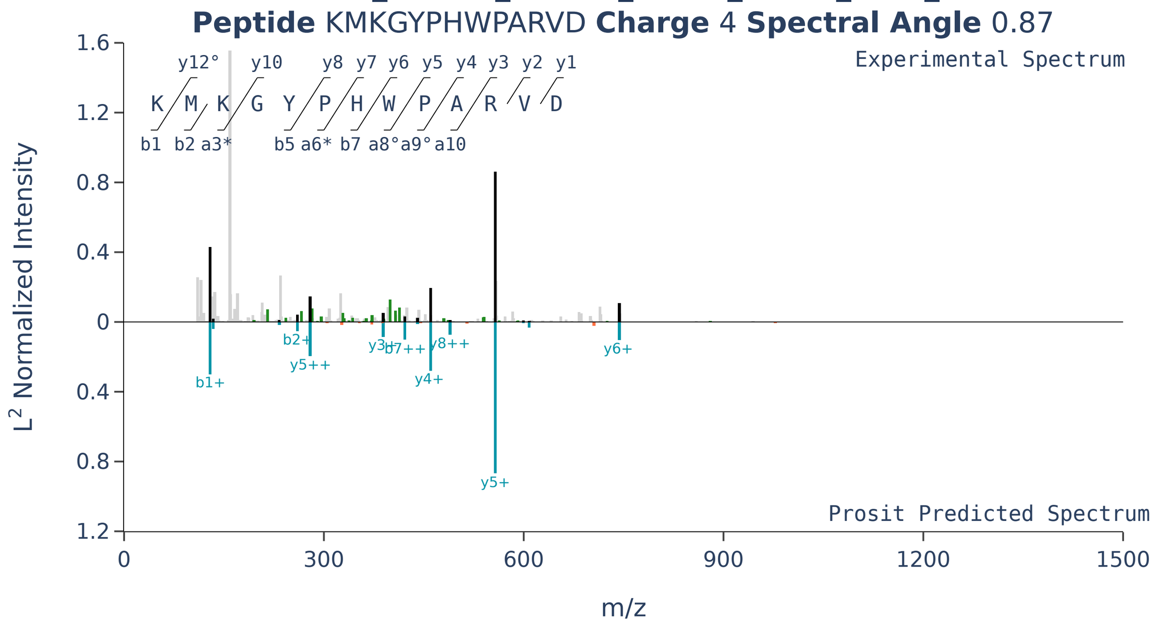


**Source** HRoetschke_271222_090123_HFGoe_LEDGF_b1R1_24h **Scan** 15099
**Peptide** AEIKNSLKI **Charge** 1 **Spectral Angle** 0.75

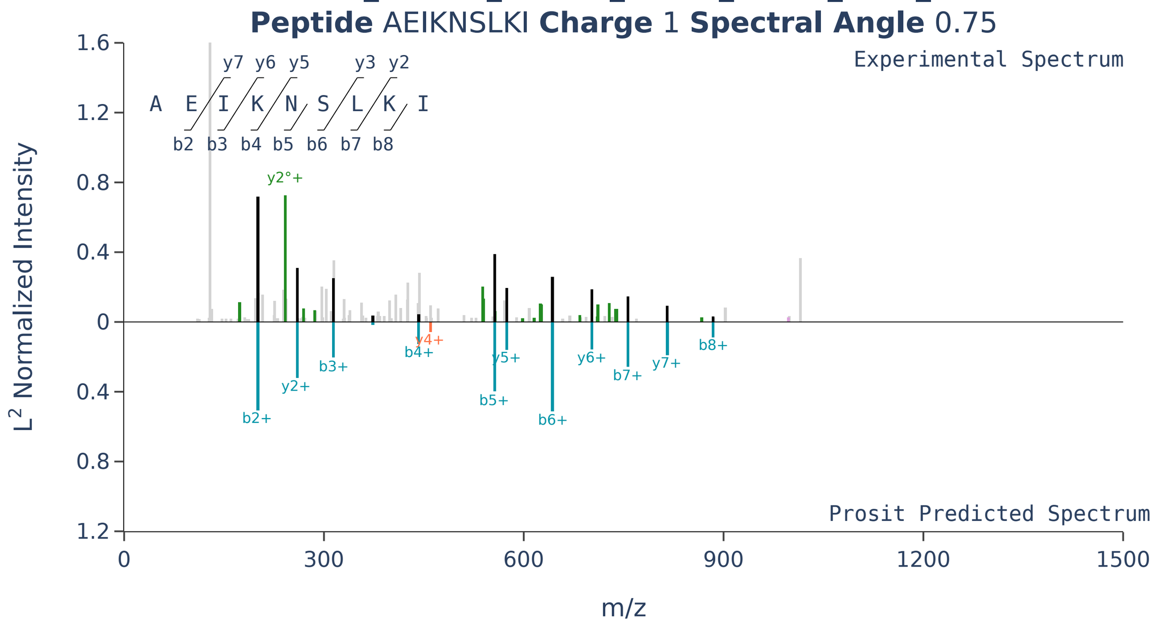


**Source** HRoetschke_271222_090123_HFGoe_LEDGF_b1R2_4h **Scan** 18587
**Peptide** KPGDLIFAKMKG **Charge** 2 **Spectral Angle** 0.87

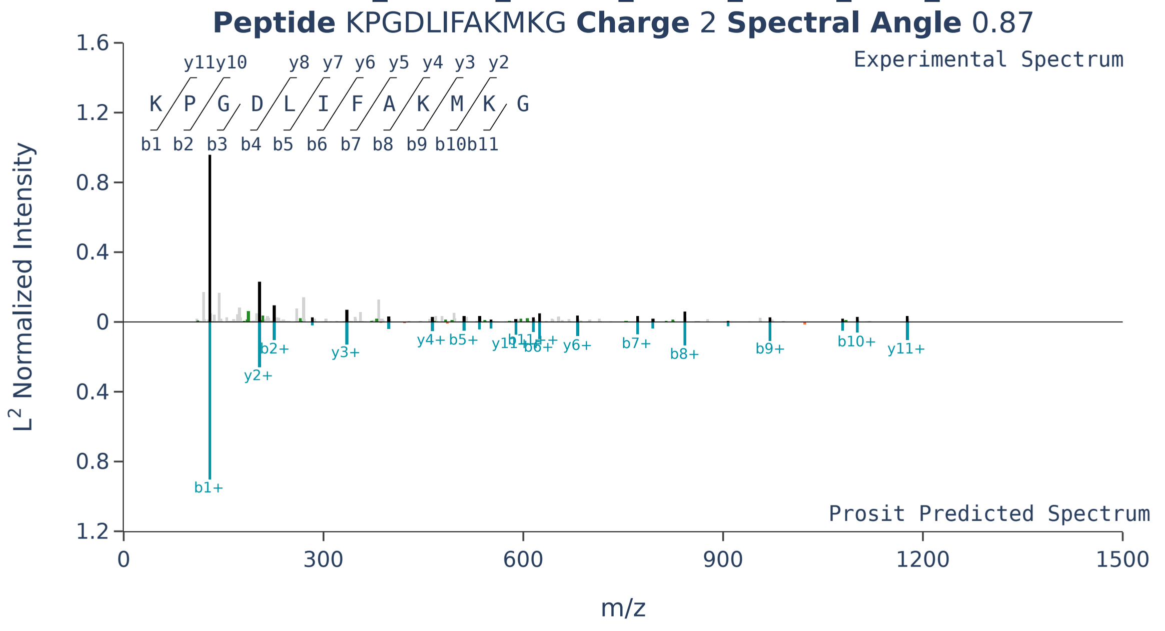

**Source** HRoetschke_271222_090123_HFGoe_LEDGF_b2R1_4h **Scan** 13457
**Peptide** ETEISLKD **Charge** 2 **Spectral Angle** 0.9

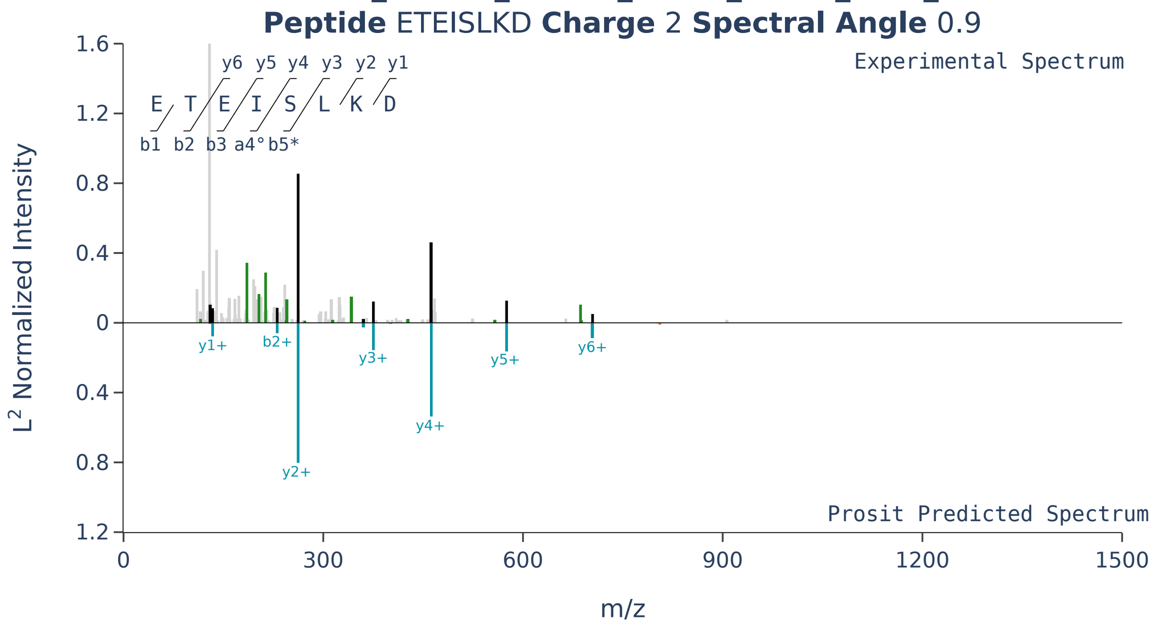


**Source** HRoetschke_271222_090123_HFGoe_LEDGF_b1R1_24h **Scan** 10241
**Peptide** AQKHTEMITTLK **Charge** 3 **Spectral Angle** 0.75

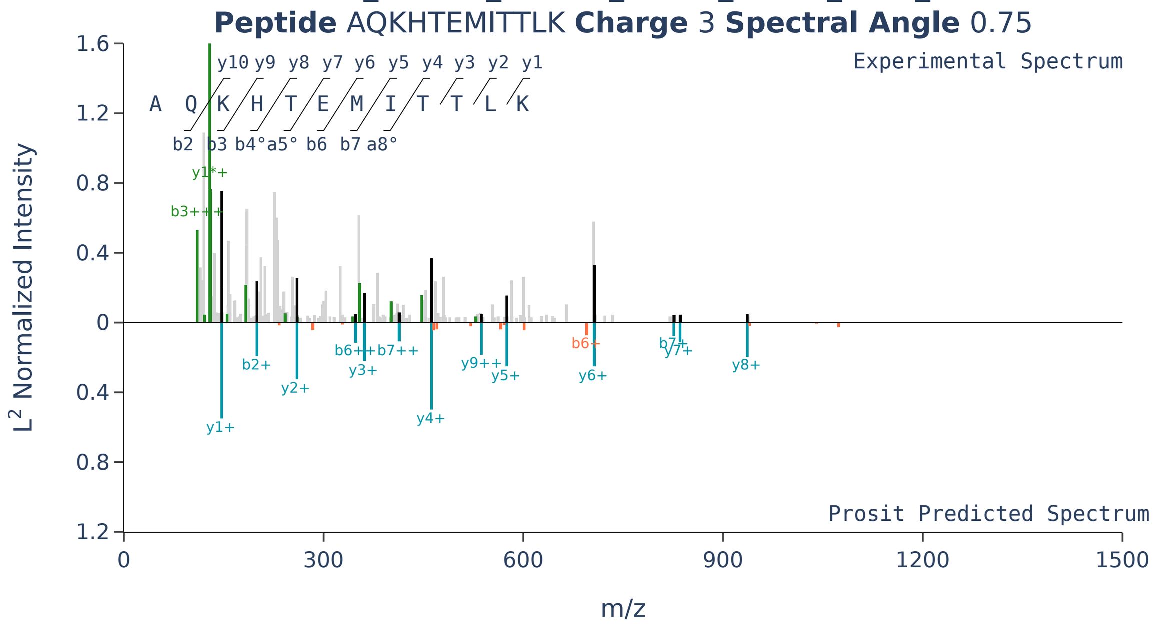


**Source** HRoetschke_271222_090123_HFGoe_LEDGF_b2R2_4h **Scan** 6499
**Peptide** RDFKPGD **Charge** 2 **Spectral Angle** 0.87

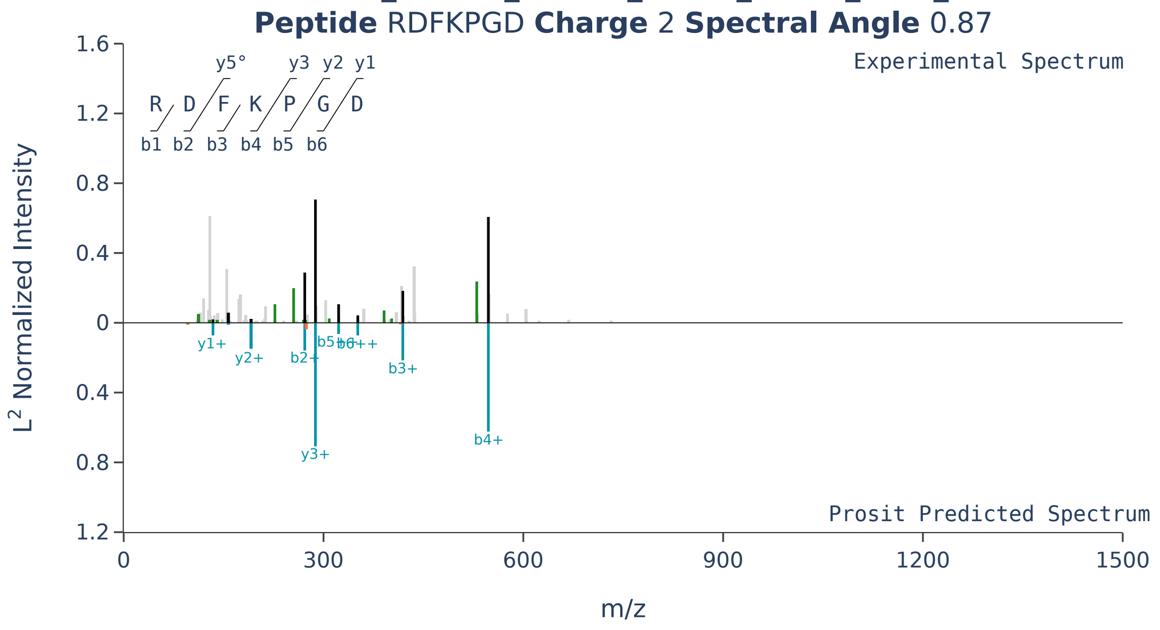


**Source** HRoetschke_271222_090123_HFGoe_LEDGF_b1R1_4h **Scan** 23088
**Peptide** DFKPGDLIFAKMKG **Charge** 2 **Spectral Angle** 0.82

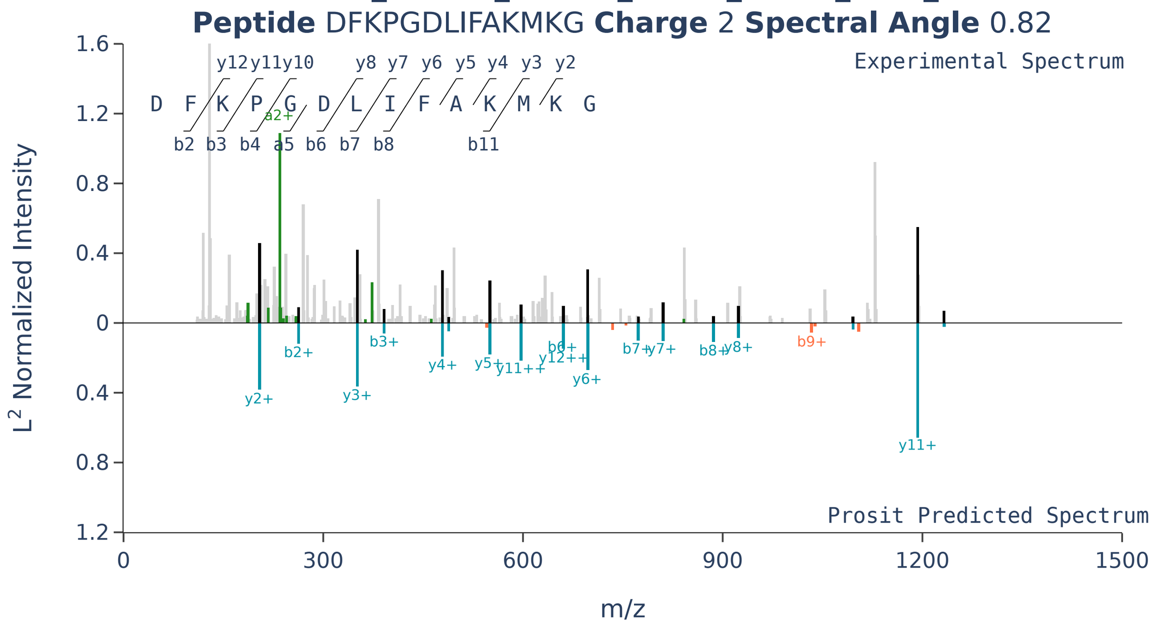


**Source** HRoetschke_271222_090123_HFGoe_LEDGF_b1R1_24h **Scan** 9366
**Peptide** KVSQVIM **Charge** 2 **Spectral Angle** 0.8

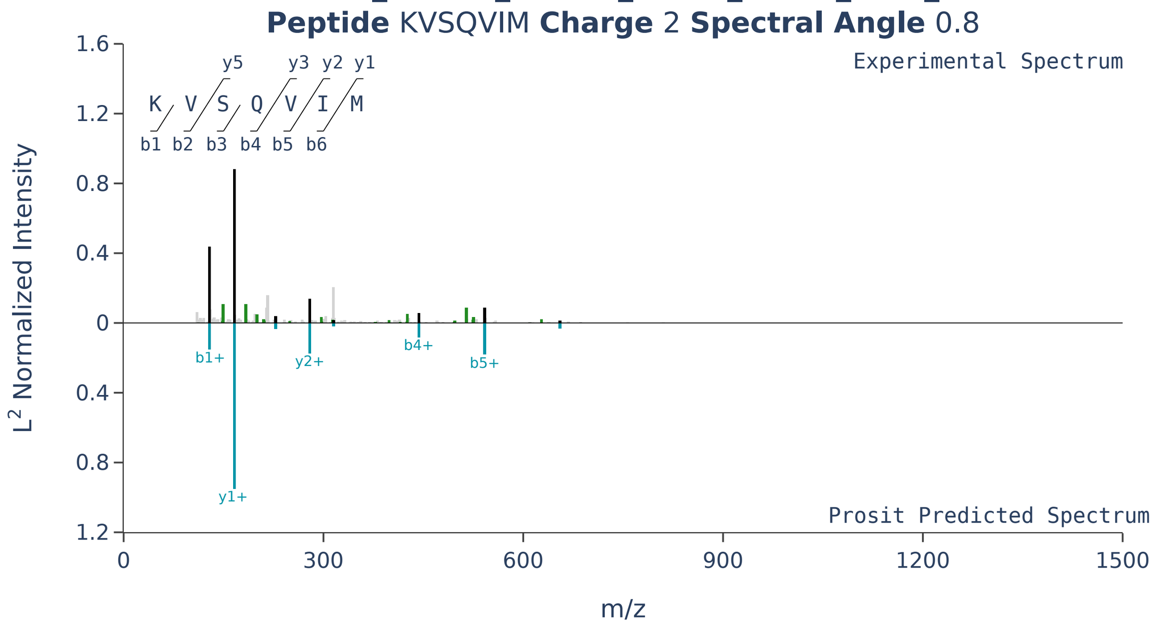


**Source** HRoetschke_271222_090123_HFGoe_LEDGF_b2R1_4h **Scan** 15791
**Peptide** KHTEMITTL **Charge** 3 **Spectral Angle** 0.76

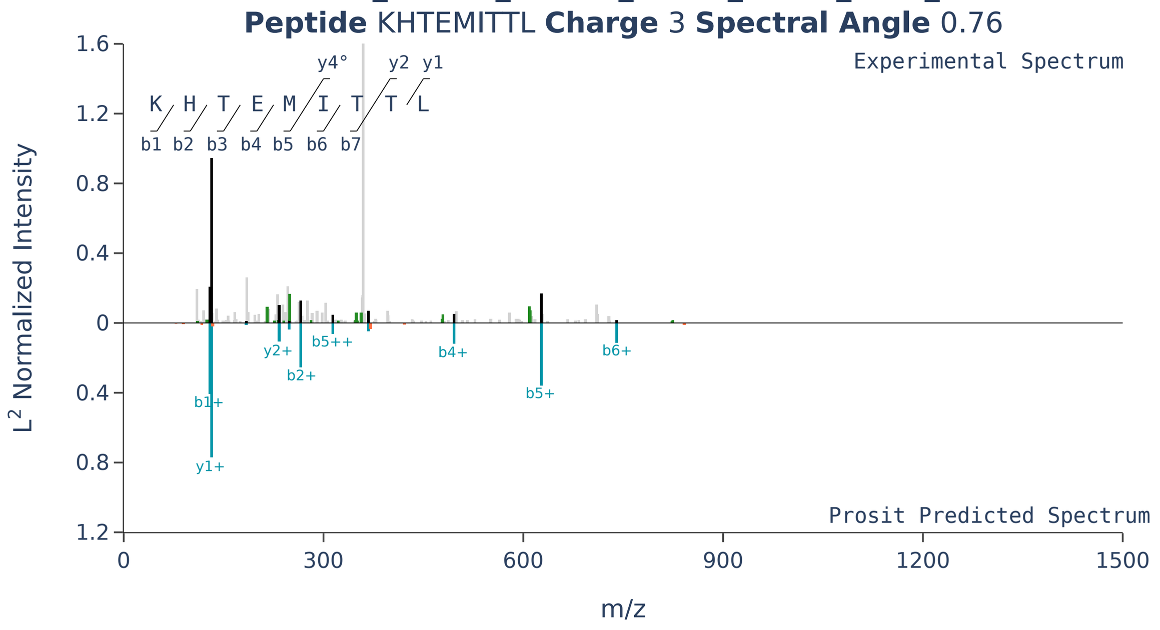


**Source** HRoetschke_271222_090123_HFGoe_LEDGF_b1R1_24h **Scan** 7645
**Peptide** QKHTEMITTLKK **Charge** 2 **Spectral Angle** 0.77

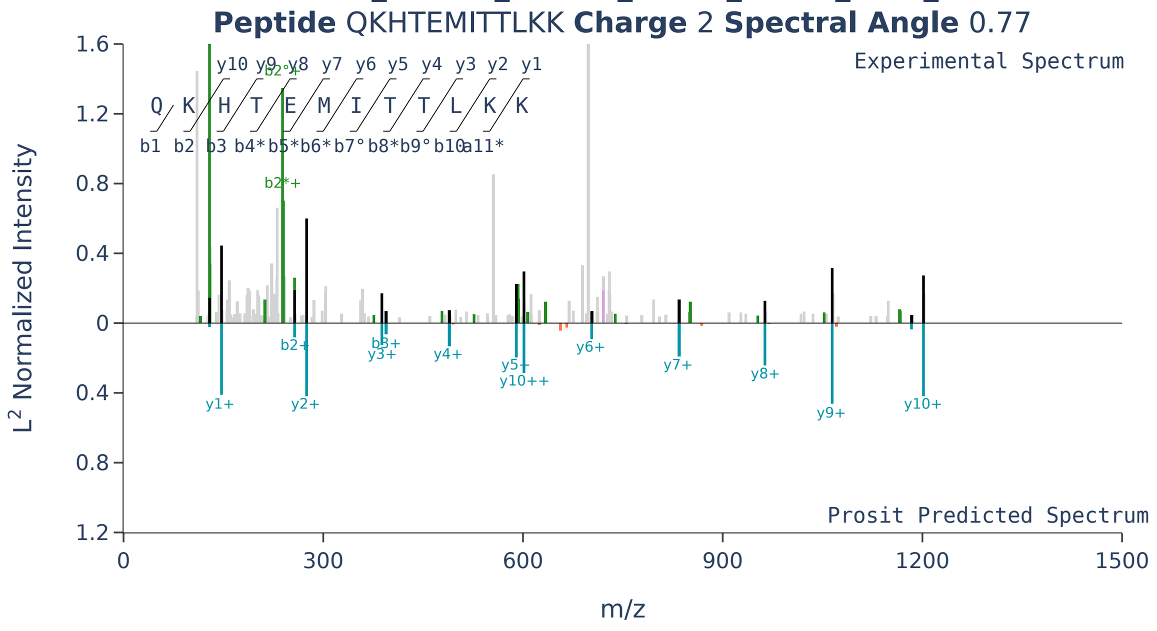


**Source** HRoetschke_271222_090123_HFGoe_LEDGF_b2R1_1h **Scan** 8306
**Peptide** KQSNASSDVEVEE **Charge** 2 **Spectral Angle** 0.78

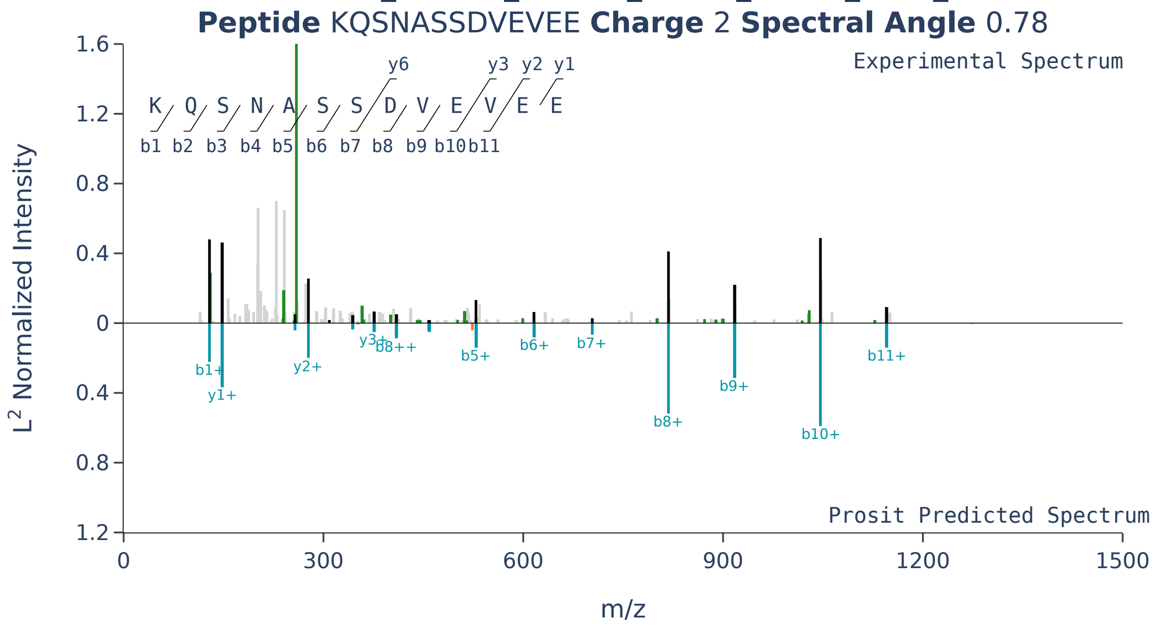


**Source** HRoetschke_271222_090123_HFGoe_LEDGF_b2R2_2h **Scan** 8774
**Peptide** TQVLNKSLAEQRQHE **Charge** 4 **Spectral Angle** 0.77

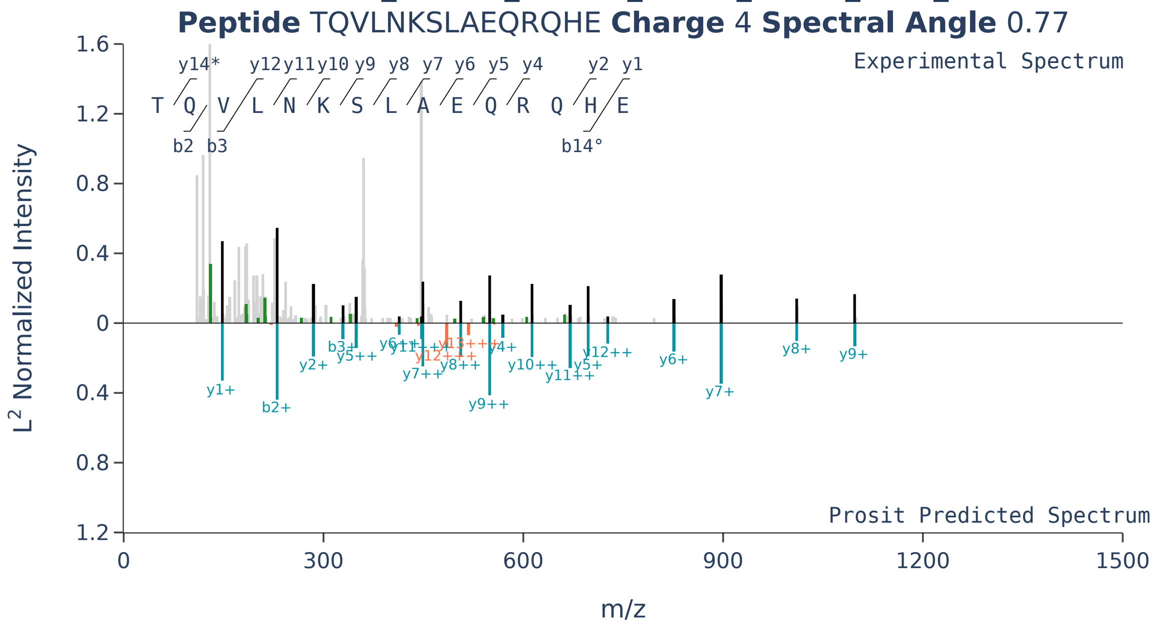


**Source** HRoetschke_271222_090123_HFGoe_LEDGF_b1R2_24h **Scan** 11904
**Peptide** DVTKAVDITTPKAARR **Charge** 2 **Spectral Angle** 0.83

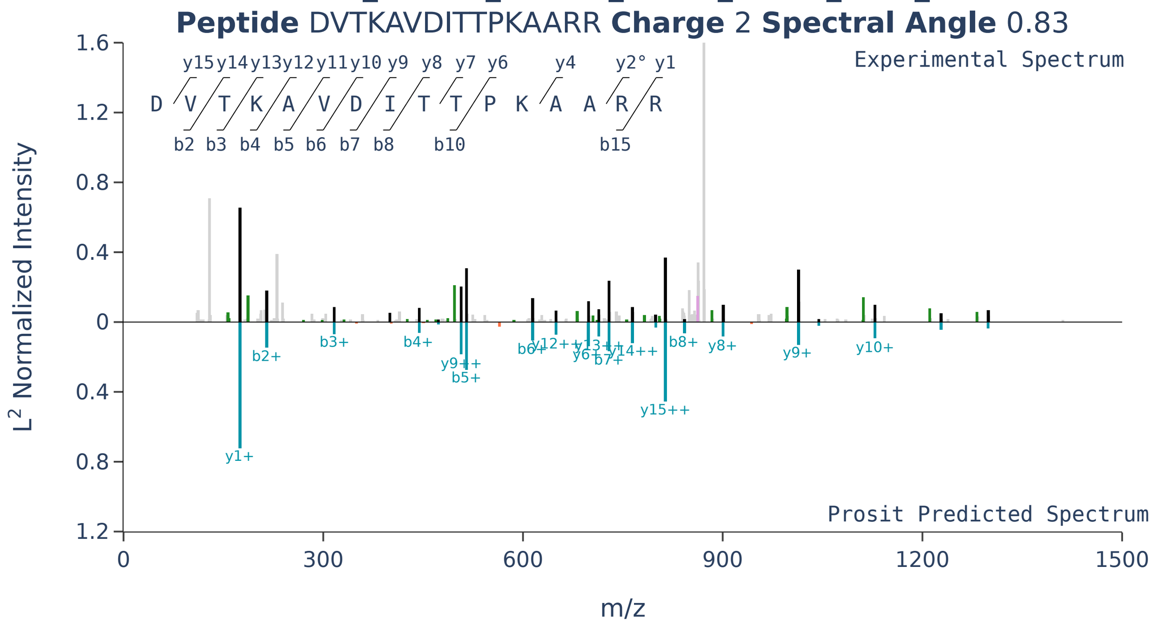


**Source** HRoetschke_271222_090123_HFGoe_LEDGF_b1R2_24h **Scan** 12656
**Peptide** EAGVVTTATA **Charge** 1 **Spectral Angle** 0.72

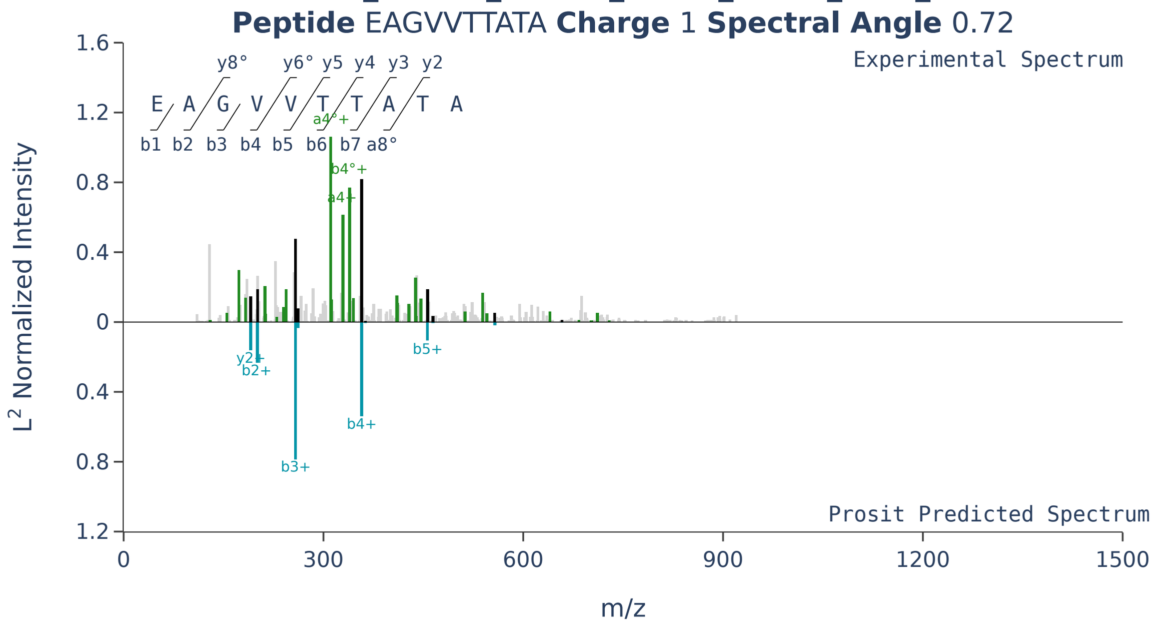


**Source** HRoetschke_271222_090123_HFGoe_LEDGF_b1R2_24h **Scan** 10578
**Peptide** QQAQKHTEMITTLK **Charge** 4 **Spectral Angle** 0.89

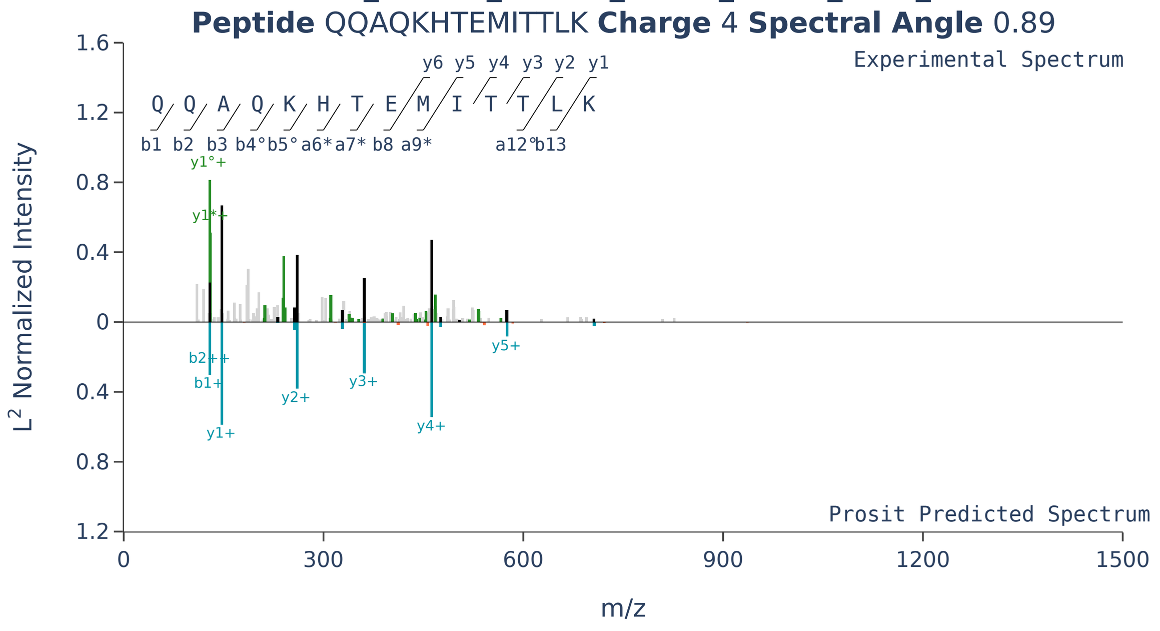


**Source** HRoetschke_271222_090123_HFGoe_LEDGF_b1R1_24h **Scan** 7092
**Peptide** KRETSMDSRL **Charge** 3 **Spectral Angle** 0.88

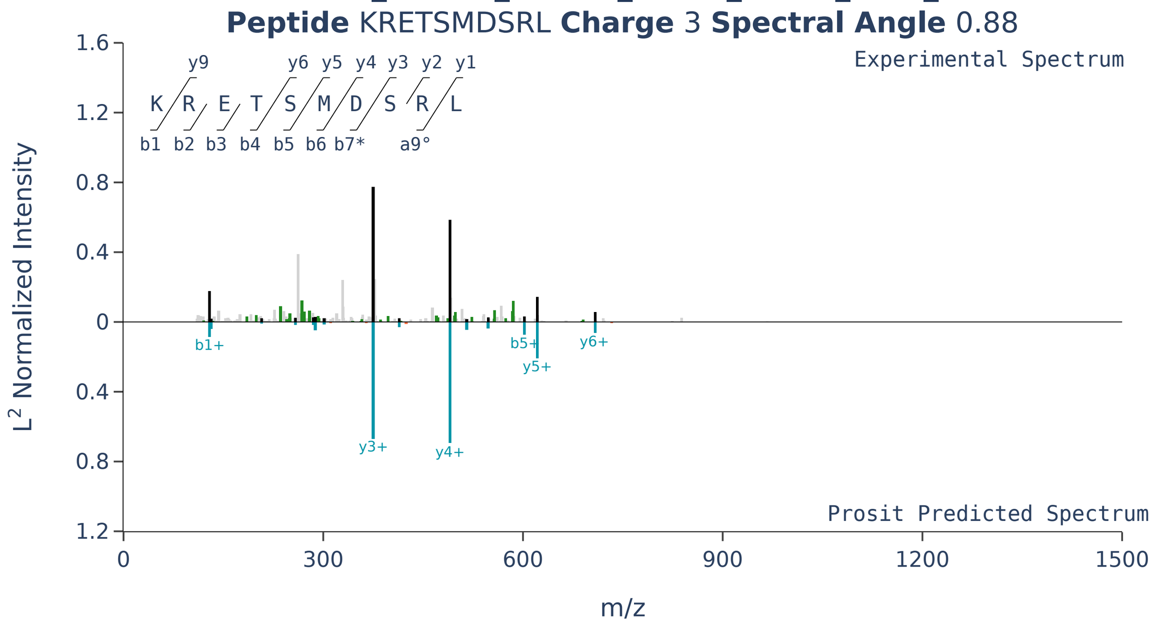


**Source** HRoetschke_271222_090123_HFGoe_LEDGF_b2R1_24h **Scan** 13653
**Peptide** QRIHAEIKNSLKL **Charge** 3 **Spectral Angle** 0.79

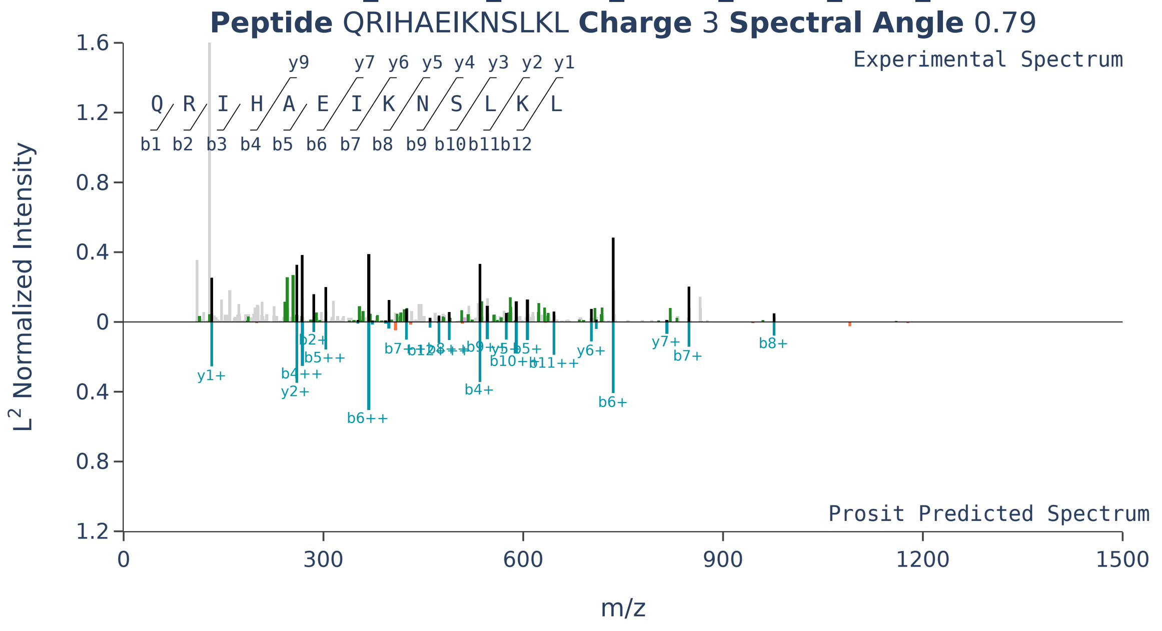


**Source** HRoetschke_271222_090123_HFGoe_LEDGF_b1R1_4h **Scan** 14606
**Peptide** IFPYSENKEKYG **Charge** 3 **Spectral Angle** 0.73

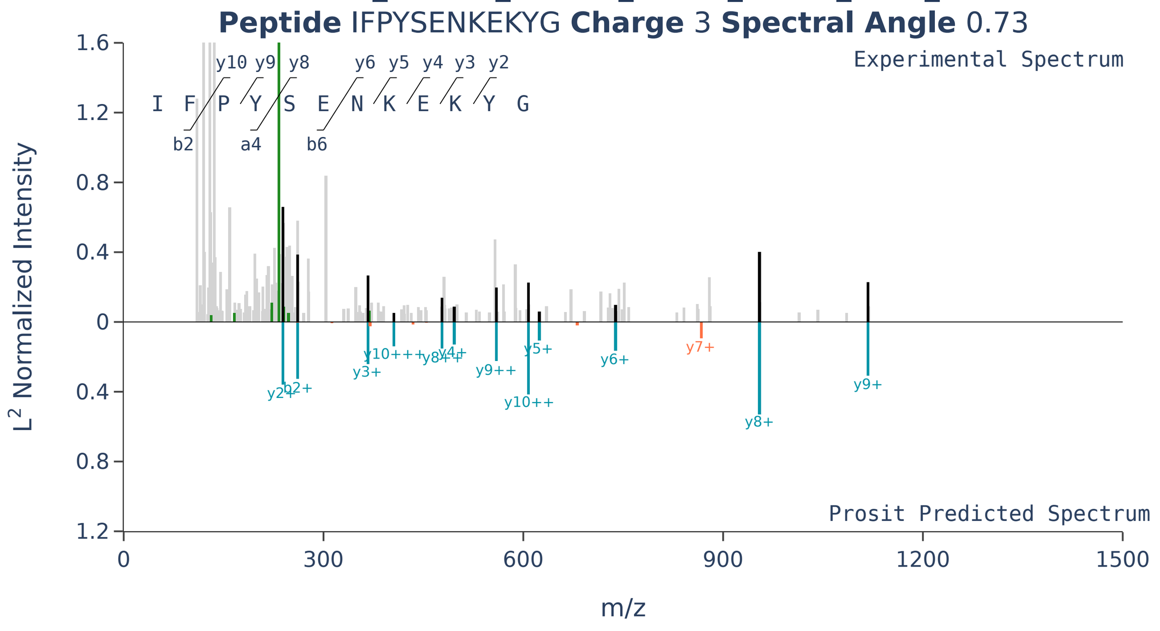

**Source** HRoetschke_271222_090123_HFGoe_LEDGF_b2R1_1h **Scan** 16231
**Peptide** PHWPARVDEVPDGAVKPPTNK **Charge** 3 **Spectral Angle** 0.78

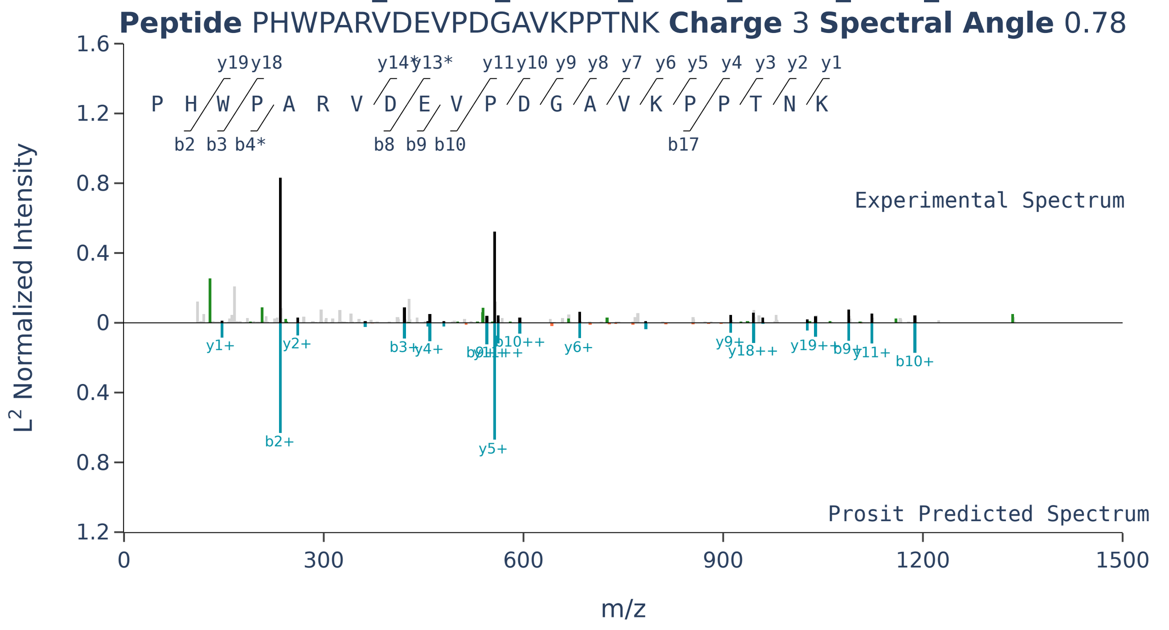

**Source** HRoetschke_271222_090123_HFGoe_LEDGF_b1R2_4h **Scan** 11266
**Peptide** GPKDIFPYSENKEKYGKPNK **Charge** 4 **Spectral Angle** 0.83

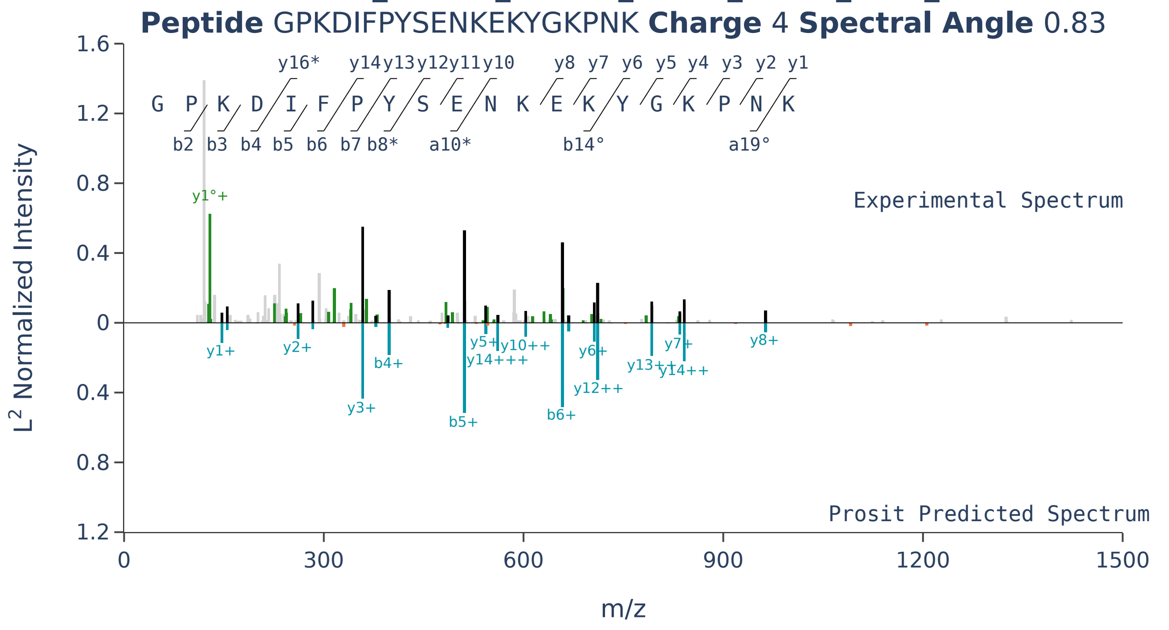

**Source** HRoetschke_271222_090123_HFGoe_LEDGF_b1R2_24h **Scan** 13914
**Peptide** ASLQVTMQQAQKHT **Charge** 3 **Spectral Angle** 0.7

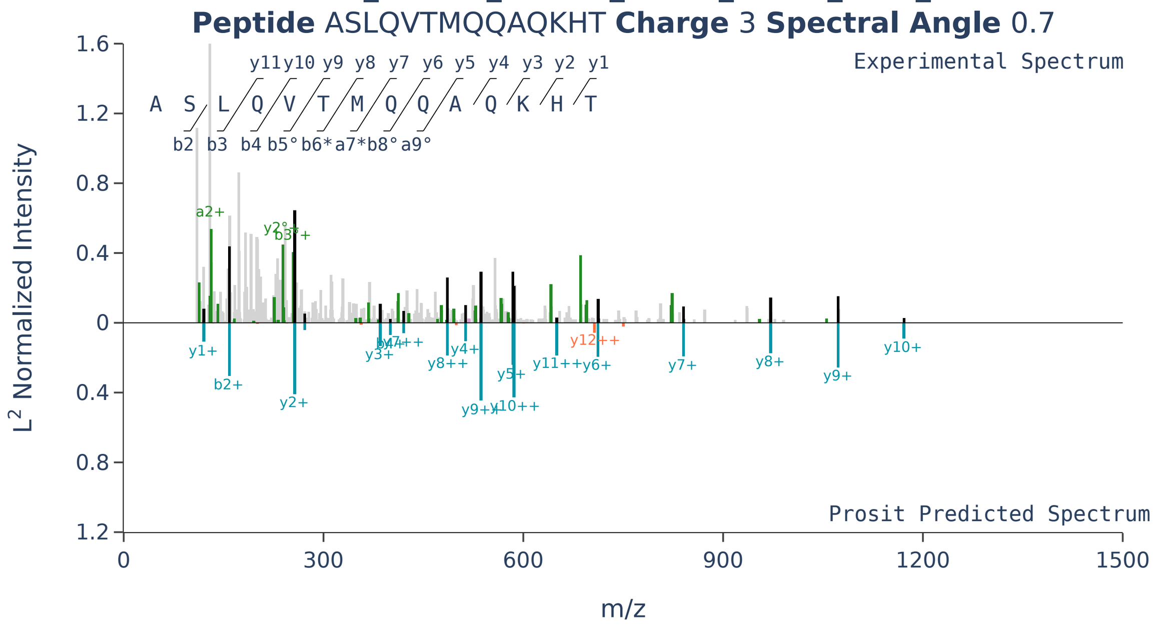

**Source** HRoetschke_271222_090123_HFGoe_LEDGF_b1R1_2h **Scan** 12185
**Peptide** DNNPKVKFSSQQAATKQSNASSDVEVE **Charge** 4 **Spectral Angle** 0.87

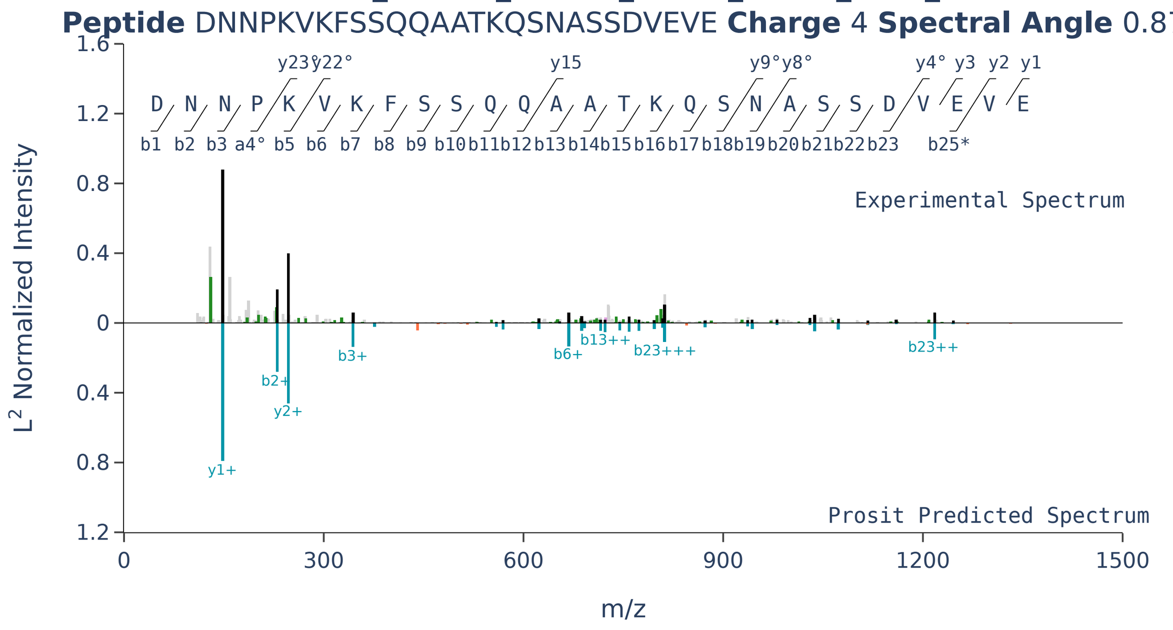

**Source** HRoetschke_271222_090123_HFGoe_LEDGF_b1R1_24h **Scan** 10383
**Peptide** ASVNLKVSPKRGRPAATEV **Charge** 3 **Spectral Angle** 0.85

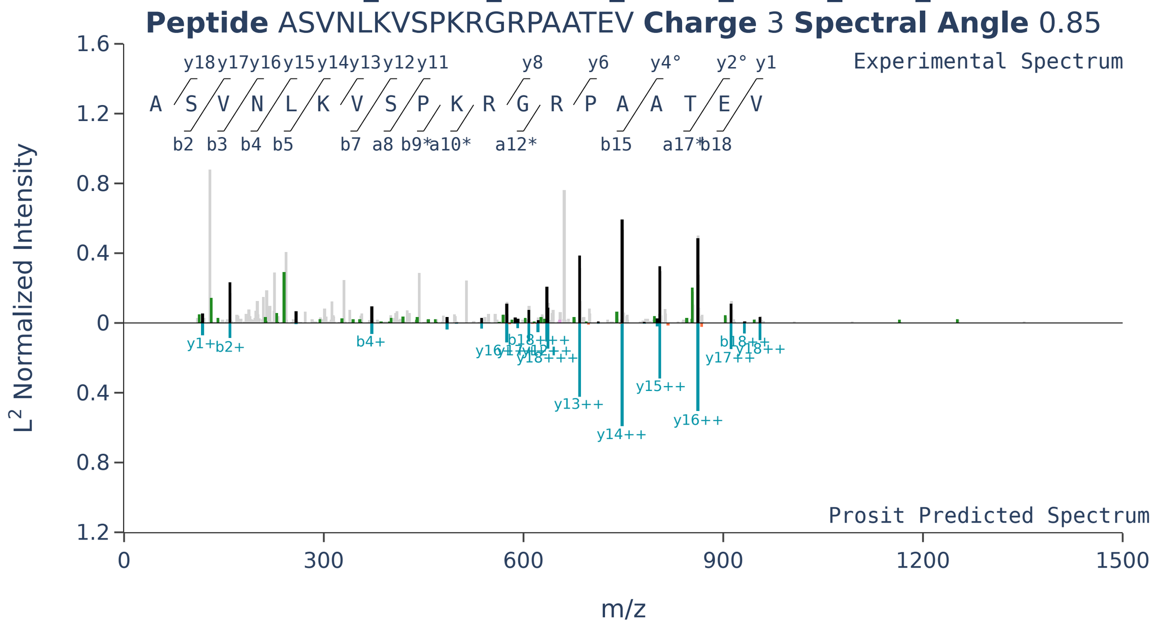

**Source** HRoetschke_271222_090123_HFGoe_LEDGF_b2R1_4h **Scan** 20030
**Peptide** NEGLWEIDNNPKVKFSSQQAATKQS **Charge** 4 **Spectral Angle** 0.92

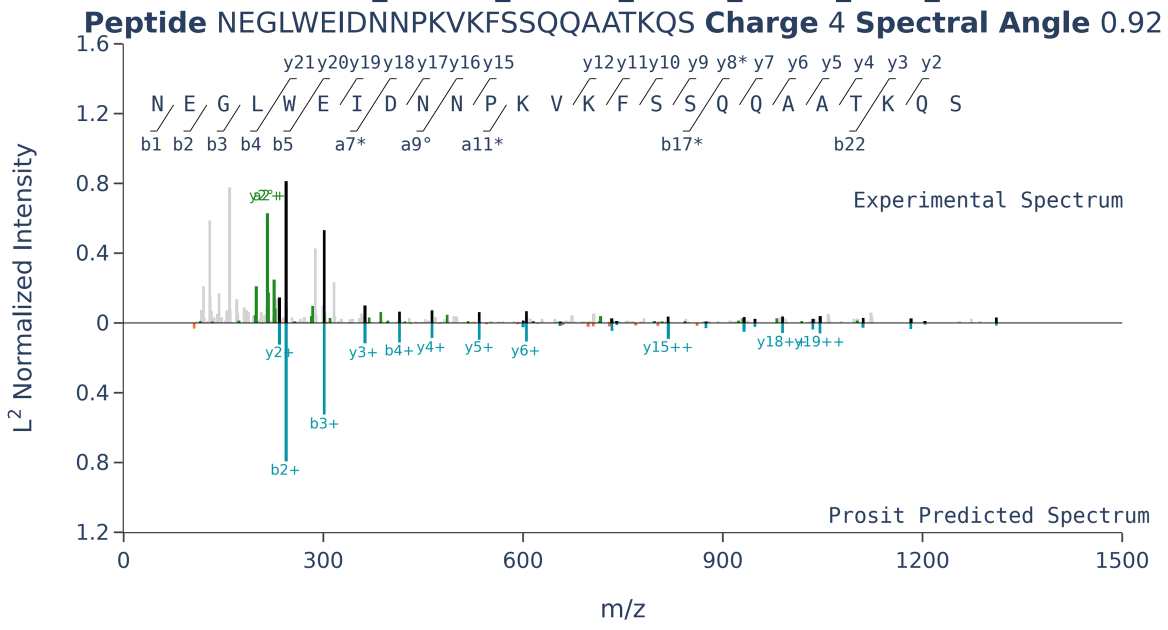

**Source** HRoetschke_271222_090123_HFGoe_LEDGF_b2R1_2h **Scan** 7564
**Peptide** SSQQAATKQSNASSDVEVEEKE **Charge** 3 **Spectral Angle** 0.85

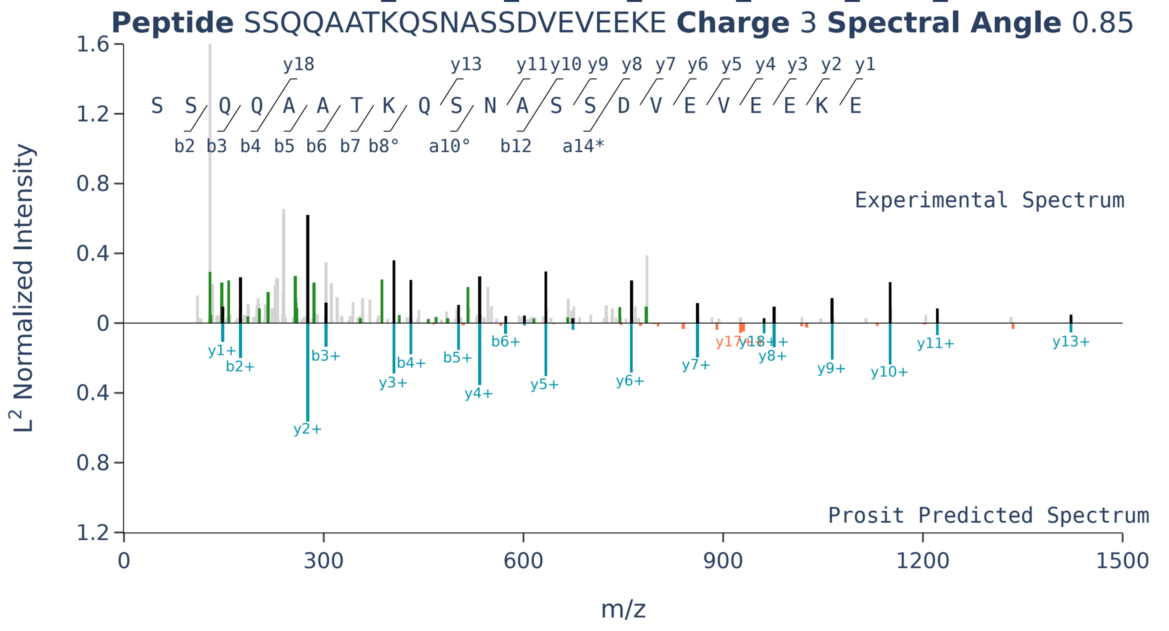

**Source** HRoetschke_271222_090123_HFGoe_LEDGF_b1R1_2h **Scan** 26491
**Peptide** ALDELASLQVTMQQ **Charge** 2 **Spectral Angle** 0.85

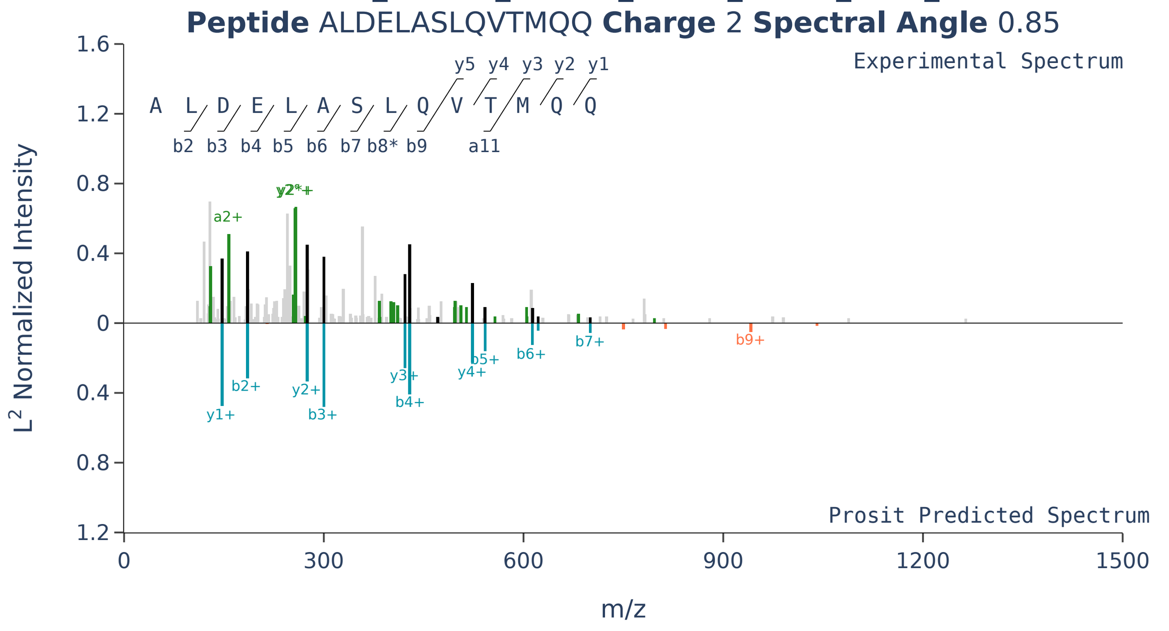

**Source** HRoetschke_271222_090123_HFGoe_LEDGF_b1R1_24h **Scan** 16071
**Peptide** HAEIKNSLKIDNLD **Charge** 2 **Spectral Angle** 0.85

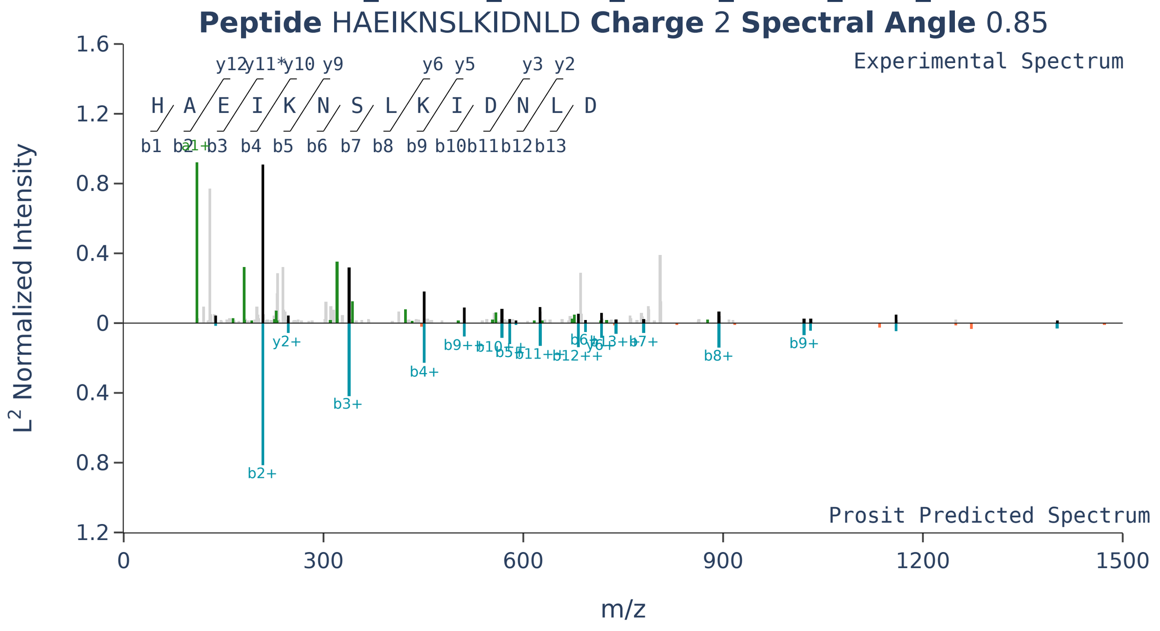

**Source** HRoetschke_271222_090123_HFGoe_LEDGF_b2R1_2h **Scan** 14843
**Peptide** RRFKVSQVIM **Charge** 3 **Spectral Angle** 0.77

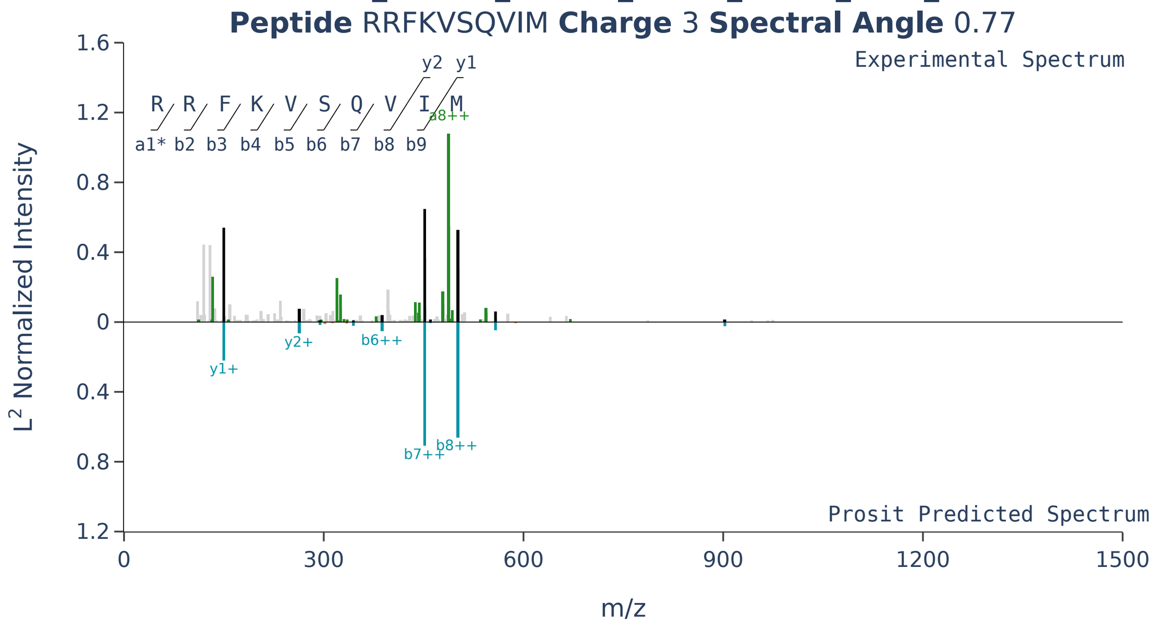

**Source** HRoetschke_271222_090123_HFGoe_LEDGF_b2R2_2h **Scan** 10479
**Peptide** GDSVITQ **Charge** 2 **Spectral Angle** 0.8

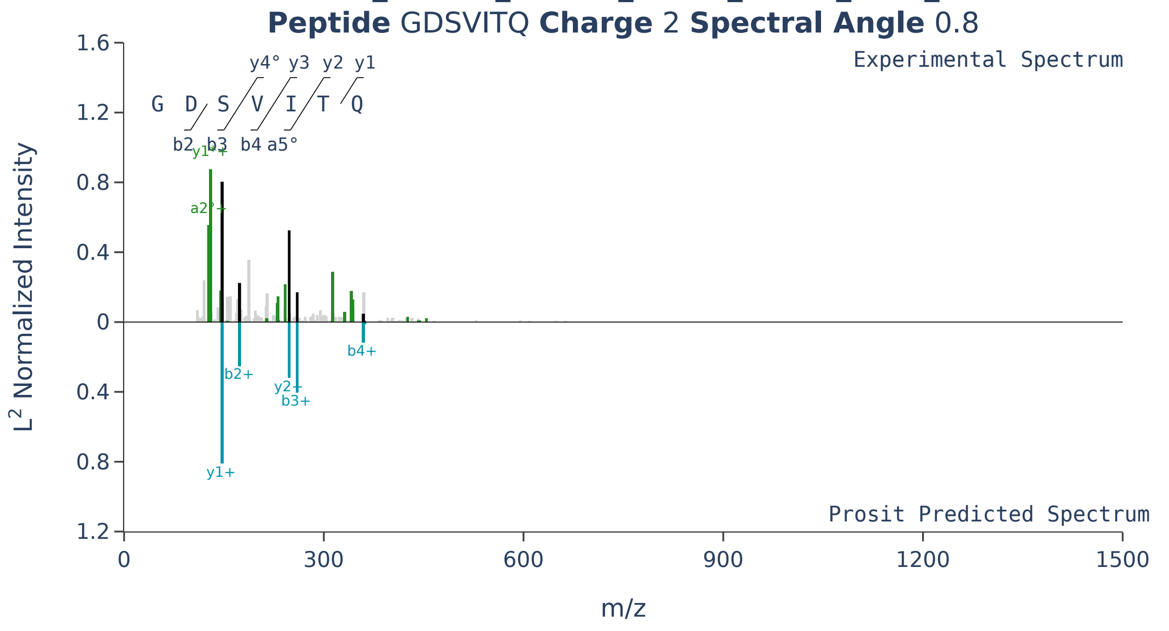

**Source** HRoetschke_271222_090123_HFGoe_LEDGF_b1R1_2h **Scan** 26685
**Peptide** DFKPGDLIFAKMK **Charge** 4 **Spectral Angle** 0.88

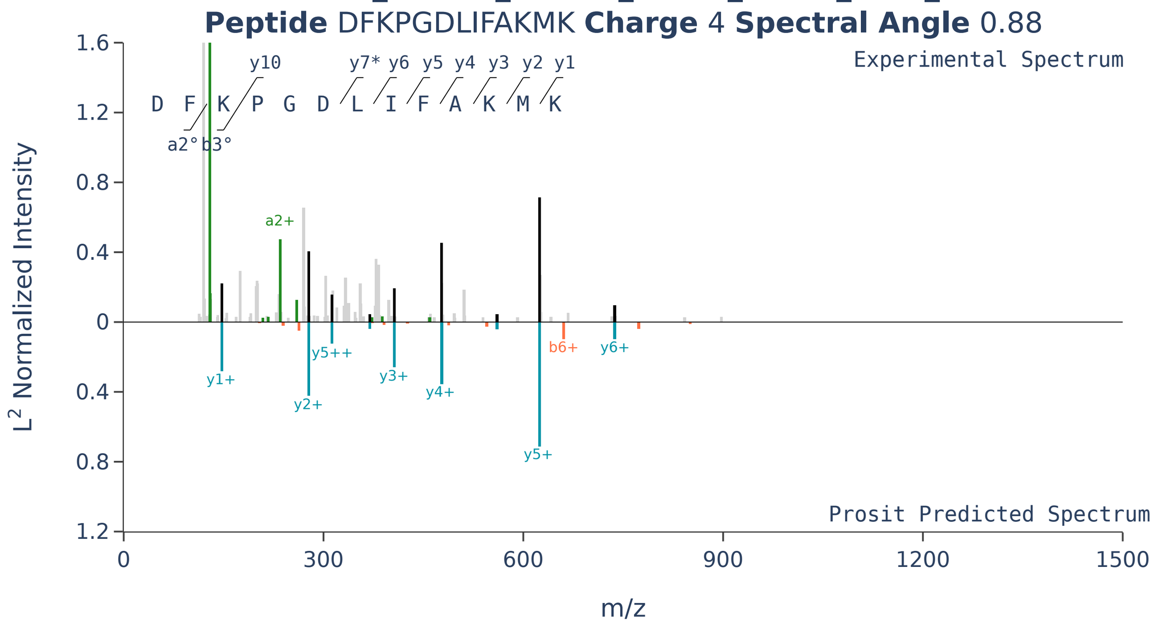

**Source** HRoetschke_271222_090123_HFGoe_LEDGF_b1R2_24h **Scan** 6130
**Peptide** FSSQQAATKQSNASSD **Charge** 2 **Spectral Angle** 0.83

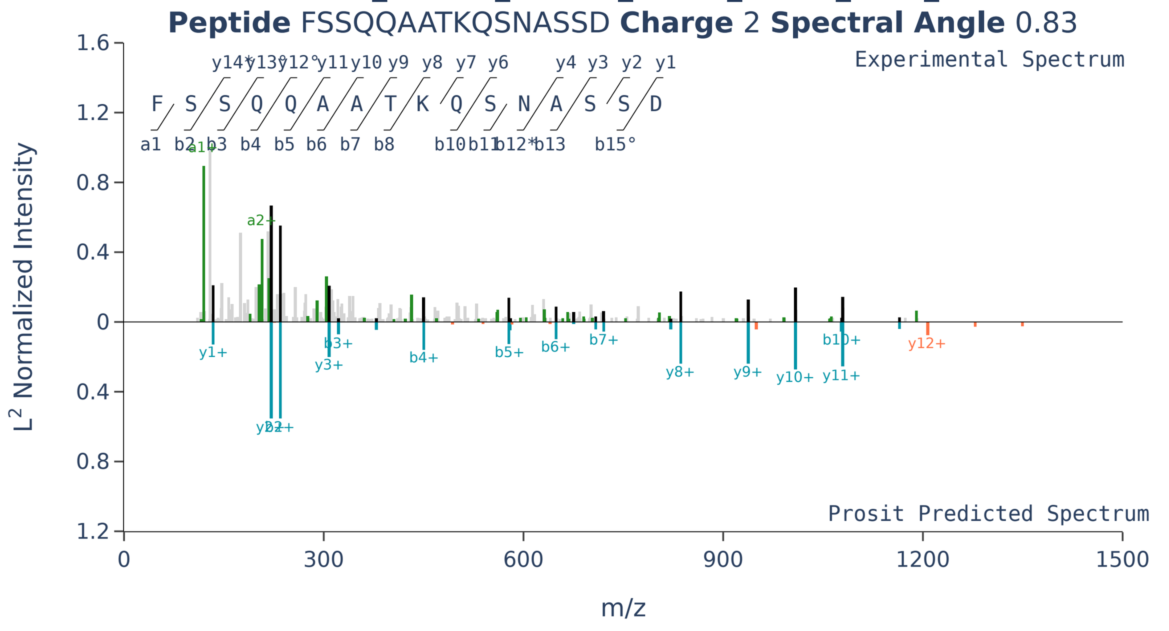

**Source** HRoetschke_271222_090123_HFGoe_LEDGF_b2R2_4h **Scan** 15853
**Peptide** AFLGPKDIFPYSENKEKYGKPNKRK **Charge** 5 **Spectral Angle** 0.73

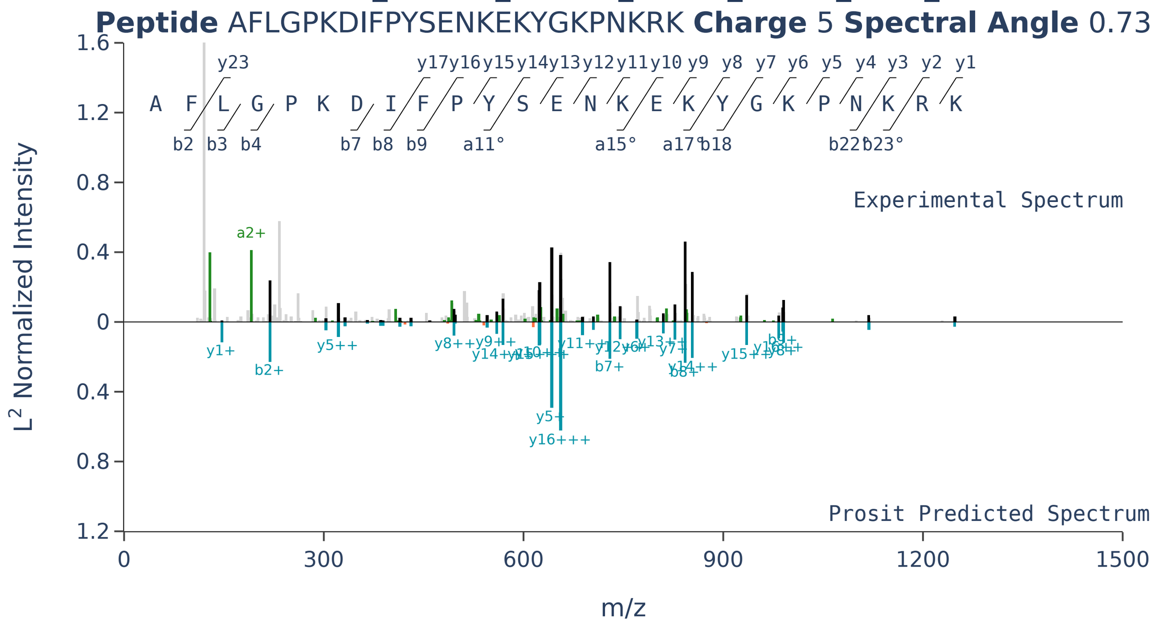

**Source** HRoetschke_271222_090123_HFGoe_LEDGF_b1R2_4h **Scan** 19680
**Peptide** SQVIMEKSTMLYNKFK **Charge** 4 **Spectral Angle** 0.78

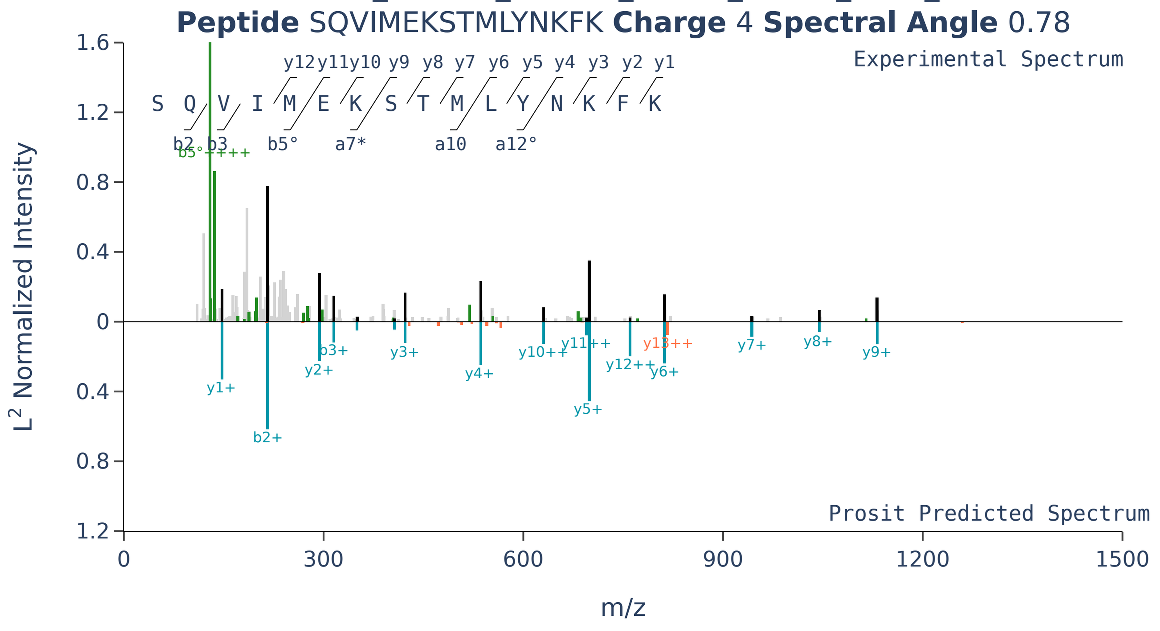

**Source** HRoetschke_271222_090123_HFGoe_LEDGF_b1R2_24h **Scan** 12350
**Peptide** SLQVTMQQAQKHTE **Charge** 2 **Spectral Angle** 0.81

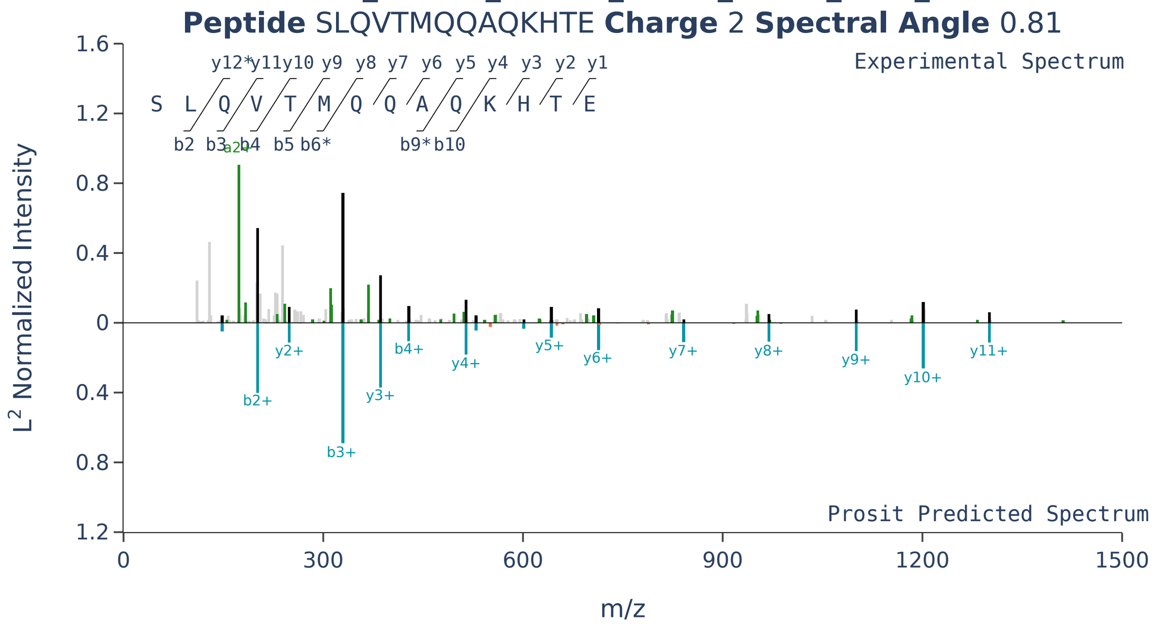


**Source** HRoetschke_271222_090123_HFGoe_LEDGF_b2R1_2h **Scan** 13986
**Peptide** VGEGDSVIT **Charge** 1 **Spectral Angle** 0.8

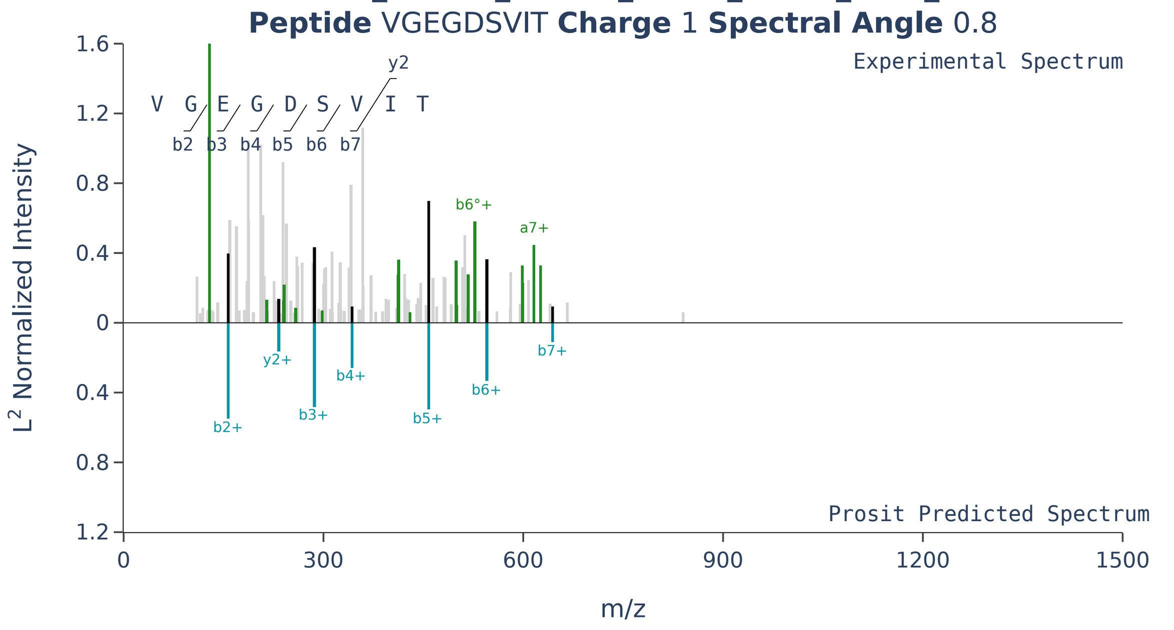


**Source** HRoetschke_271222_090123_HFGoe_LEDGF_b2R1_1h **Scan** 27813
**Peptide** GPGMTRDFKPGDLIFA **Charge** 2 **Spectral Angle** 0.78

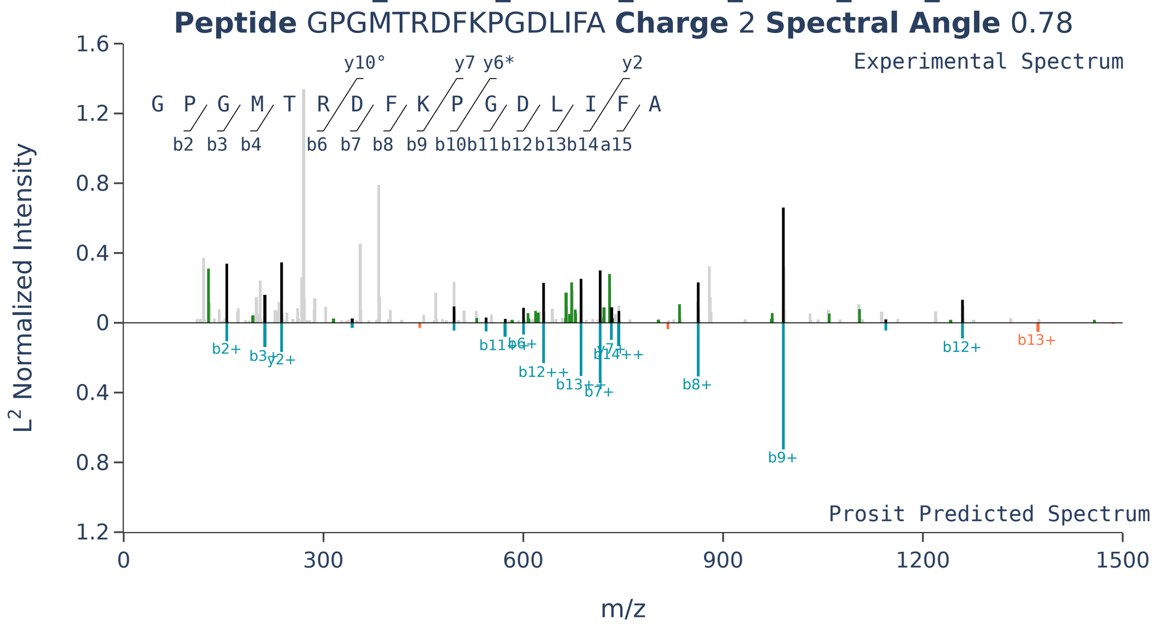


**Source** HRoetschke_271222_090123_HFGoe_LEDGF_b2R2_2h **Scan** 28562
**Peptide** GTHETAFLGPKDIFPY **Charge** 3 **Spectral Angle** 0.88

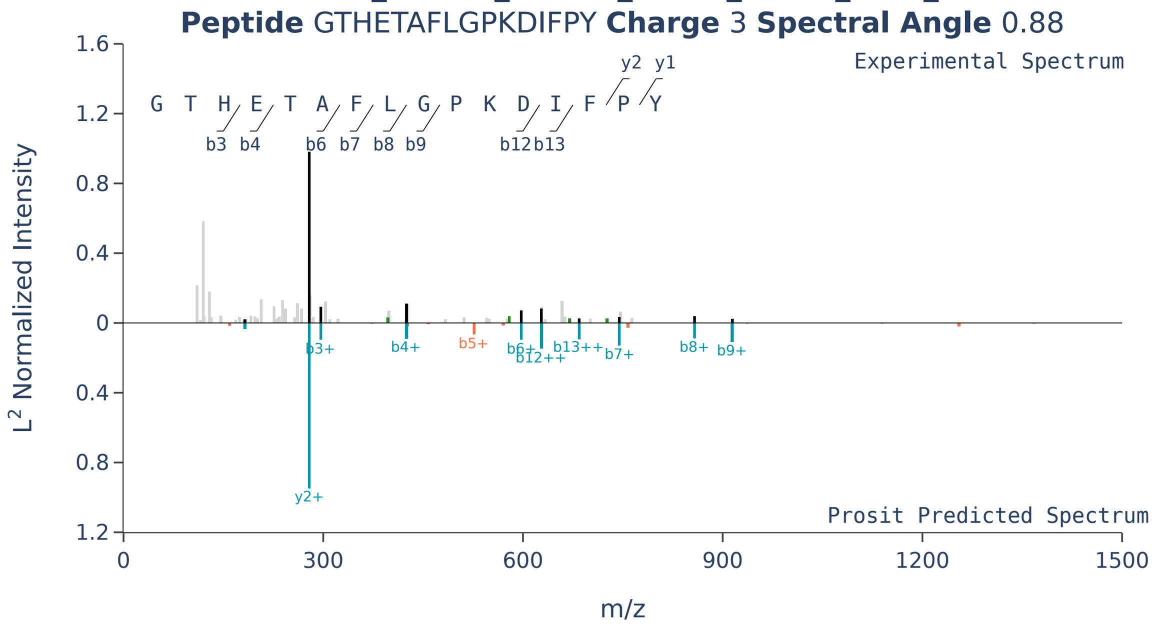


**Source** HRoetschke_271222_090123_HFGoe_LEDGF_b1R1_4h **Scan** 6811
**Peptide** SKEDTDHEEKASNEDVTKAVD **Charge** 3 **Spectral Angle** 0.8

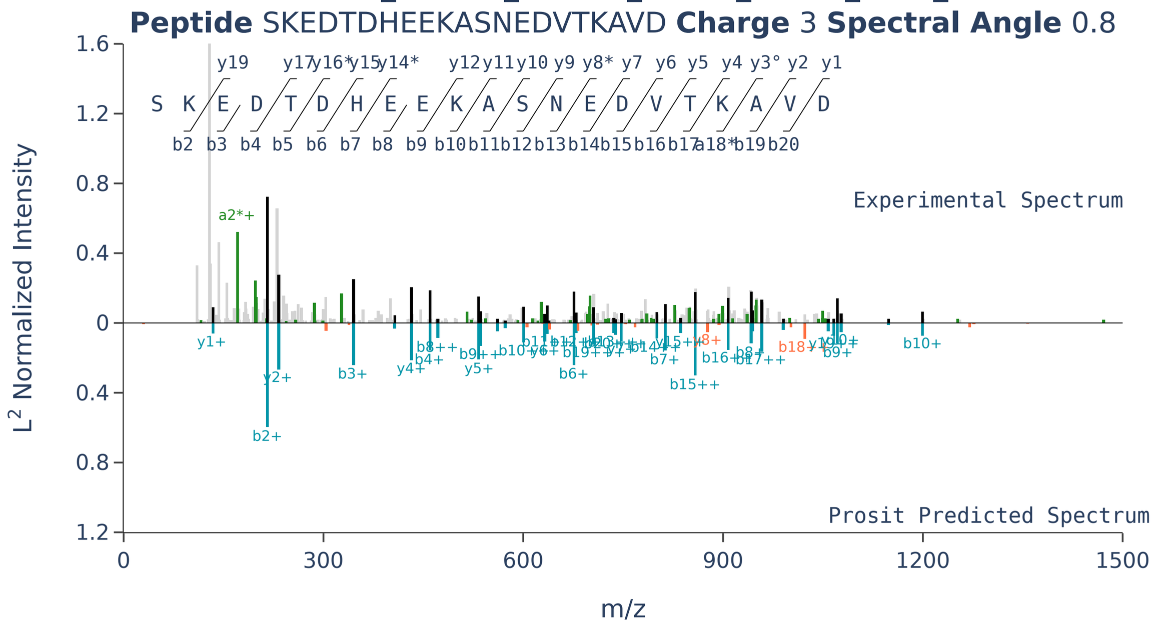


**Source** HRoetschke_271222_090123_HFGoe_LEDGF_b2R1_4h **Scan** 19347
**Peptide** DFKPGDL **Charge** 2 **Spectral Angle** 0.83

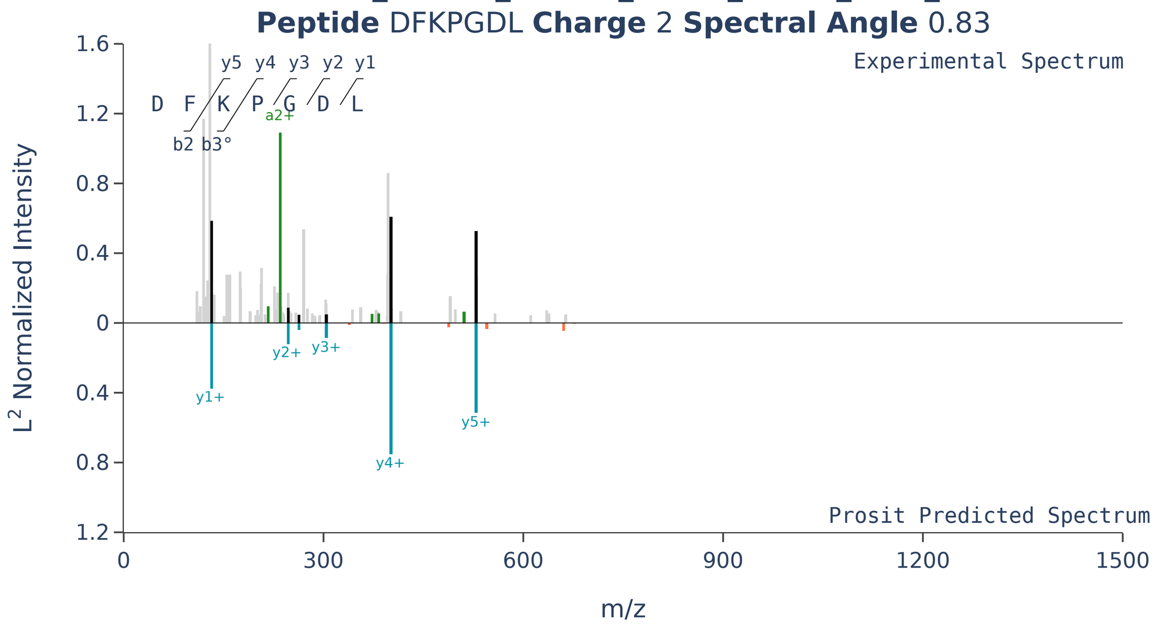


**Source** HRoetschke_271222_090123_HFGoe_LEDGF_b2R2_24h **Scan** 16241
**Peptide** TEEAGVVTTATASVNLKVSPKRGR **Charge** 5 **Spectral Angle** 0.84

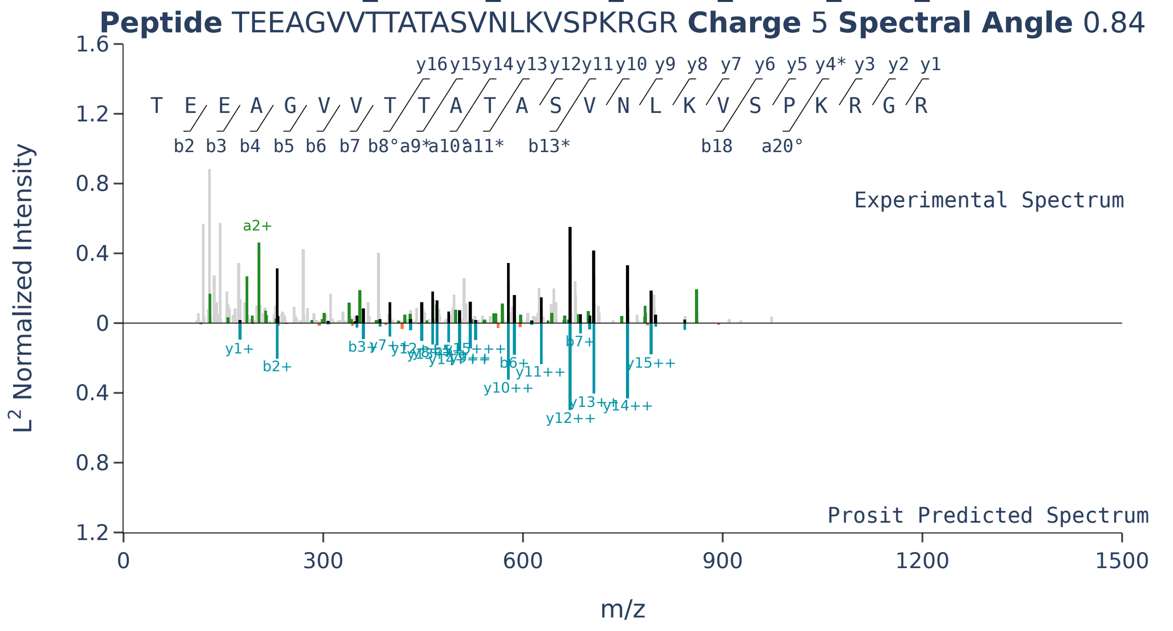


**Source** HRoetschke_271222_090123_HFGoe_LEDGF_b1R1_2h **Scan** 12851
**Peptide** KMKGYPHWPARVDE **Charge** 3 **Spectral Angle** 0.83

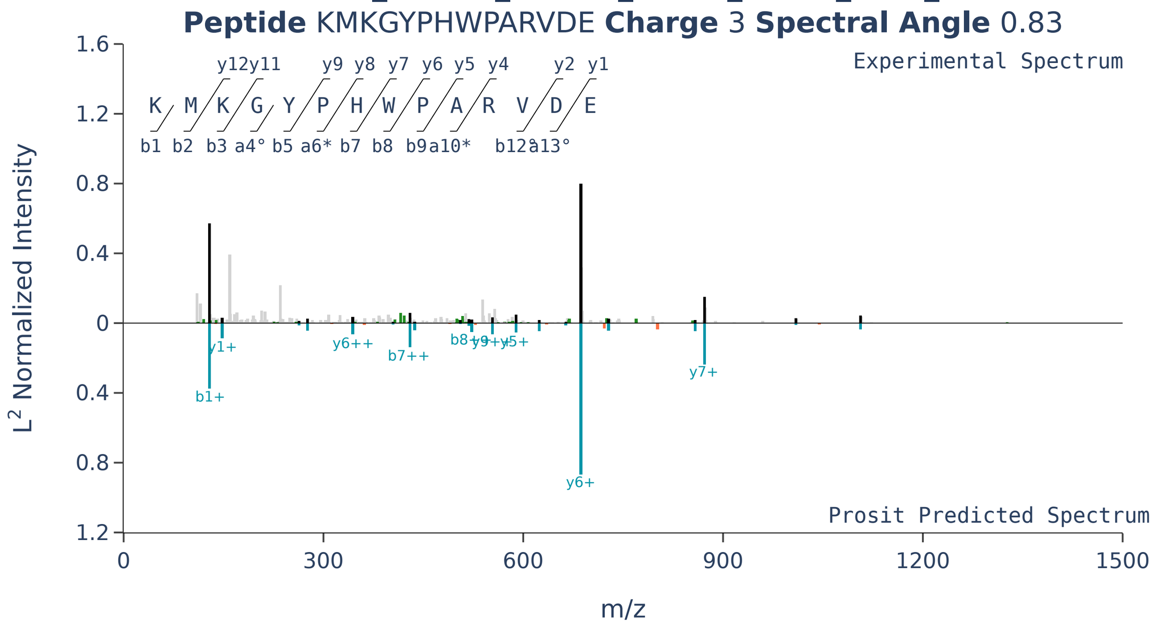


**Source** HRoetschke_271222_090123_HFGoe_LEDGF_b1R1_24h **Scan** 12409
**Peptide** IHAEIKNSL **Charge** 1 **Spectral Angle** 0.9

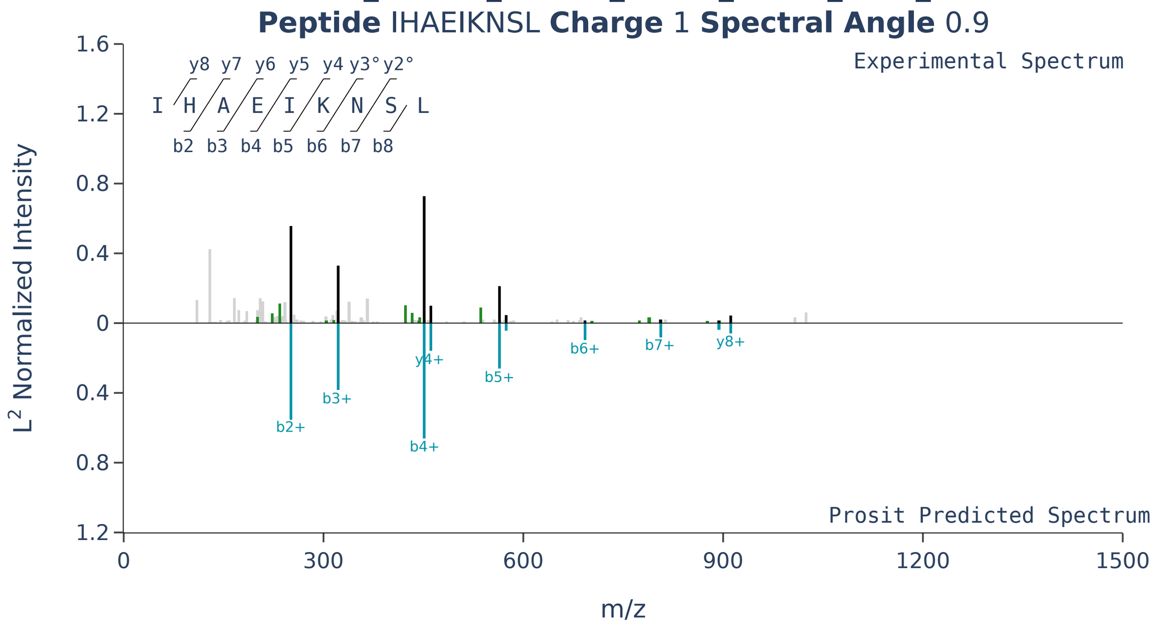


**Source** HRoetschke_271222_090123_HFGoe_LEDGF_b1R2_24h **Scan** 10161
**Peptide** ASTKKKPSSEERETEISL **Charge** 2 **Spectral Angle** 0.87

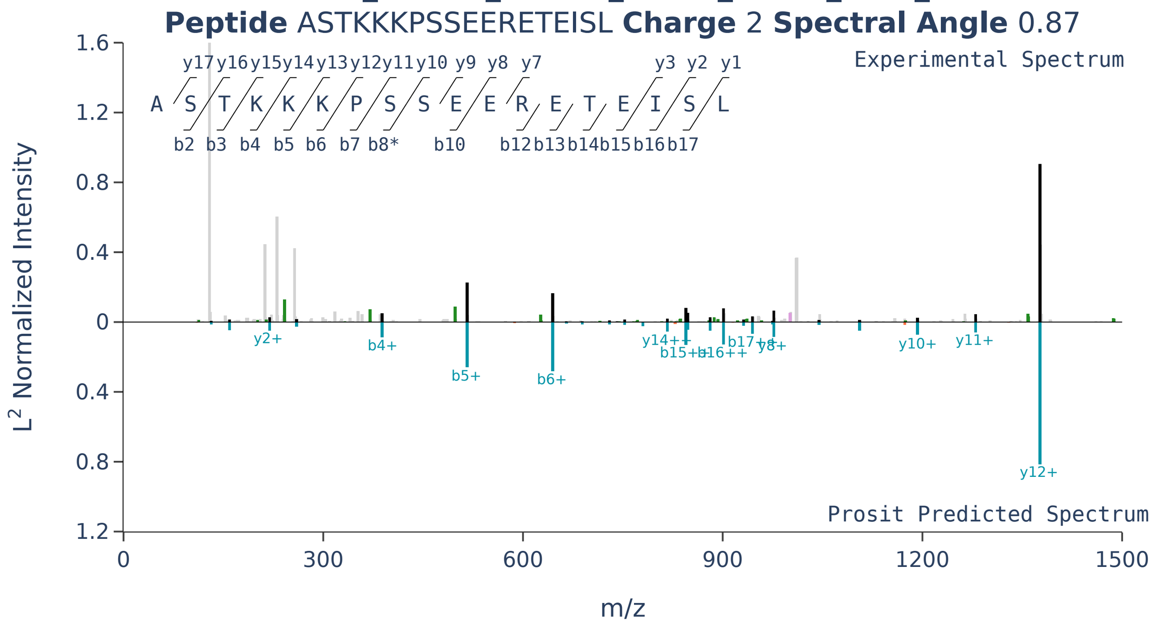


**Source** HRoetschke_271222_090123_HFGoe_LEDGF_b2R1_2h **Scan** 8253
**Peptide** DVNRCIE **Charge** 2 **Spectral Angle** 0.86

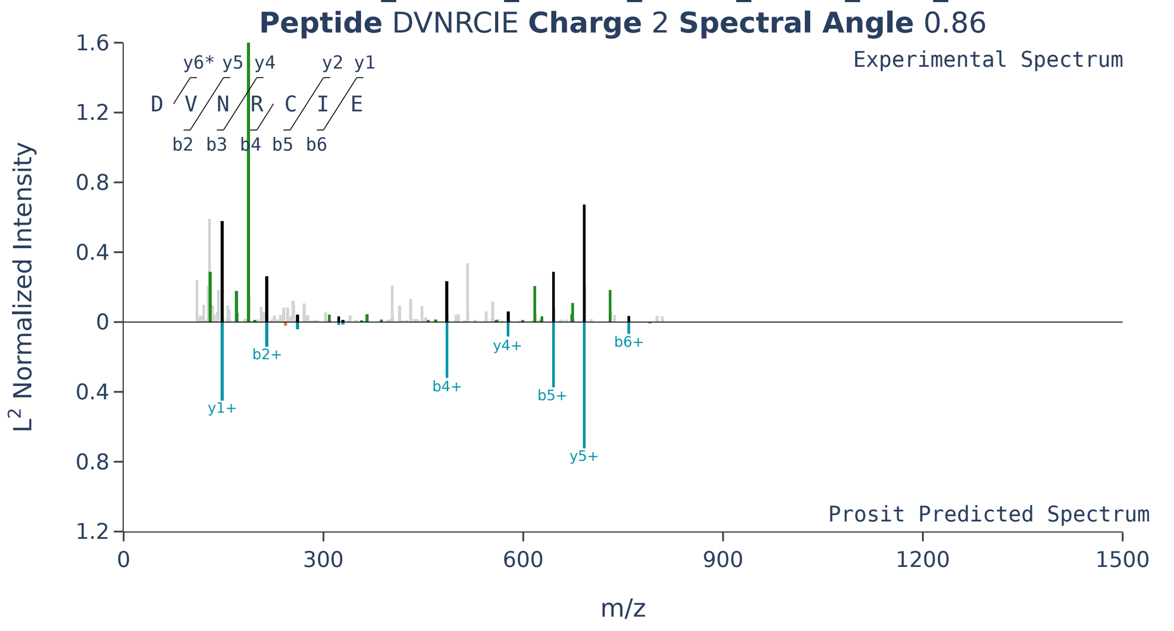


**Source** HRoetschke_271222_090123_HFGoe_LEDGF_b2R1_24h **Scan** 26214
**Peptide** GLWEIDNNPKVKF **Charge** 2 **Spectral Angle** 0.78

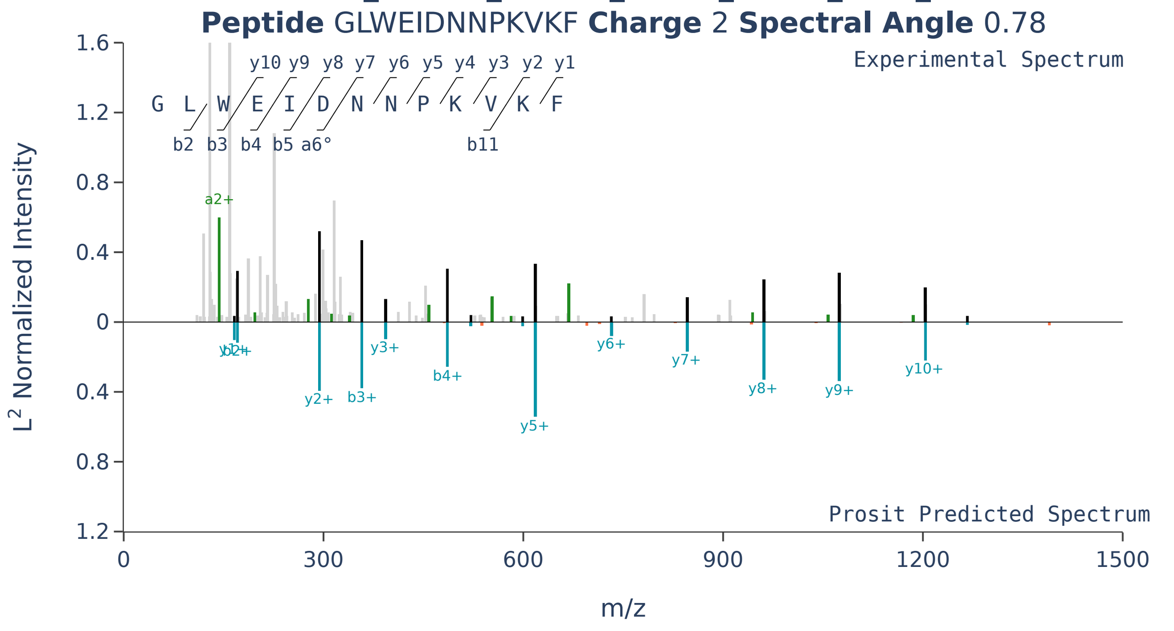


**Source** HRoetschke_271222_090123_HFGoe_LEDGF_b1R1_2h **Scan** 32857
**Peptide** DEVPDGAVKPPTNKLPIFF **Charge** 3 **Spectral Angle** 0.9

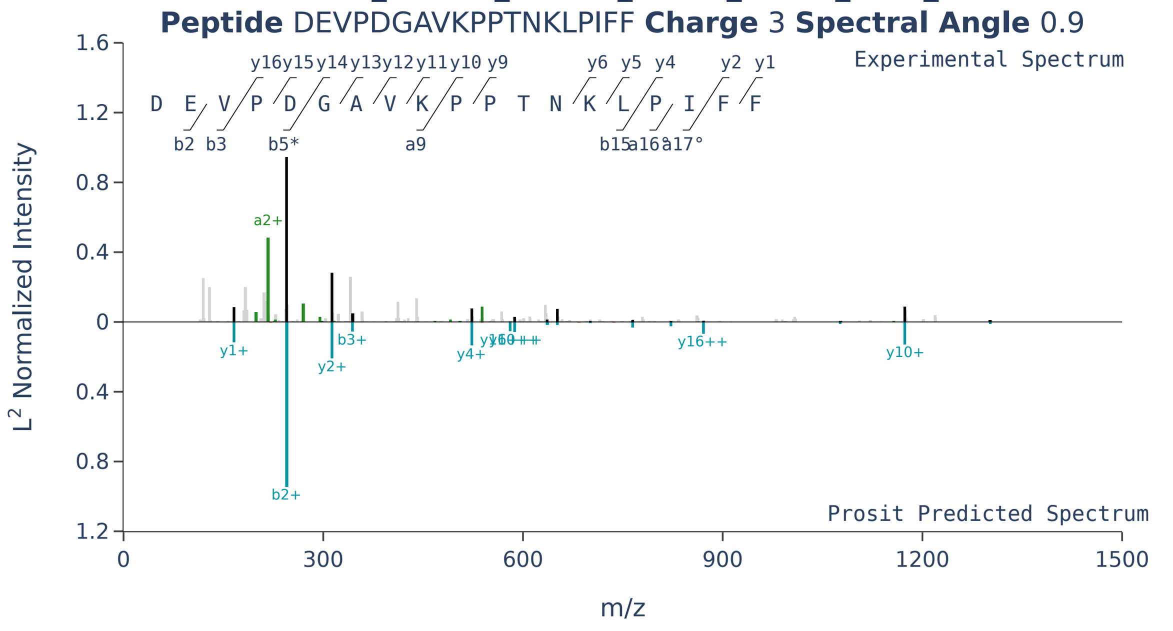


**Source** HRoetschke_271222_090123_HFGoe_LEDGF_b1R2_1h **Scan** 8412
**Peptide** QSNASSDVEVEEKE **Charge** 2 **Spectral Angle** 0.82

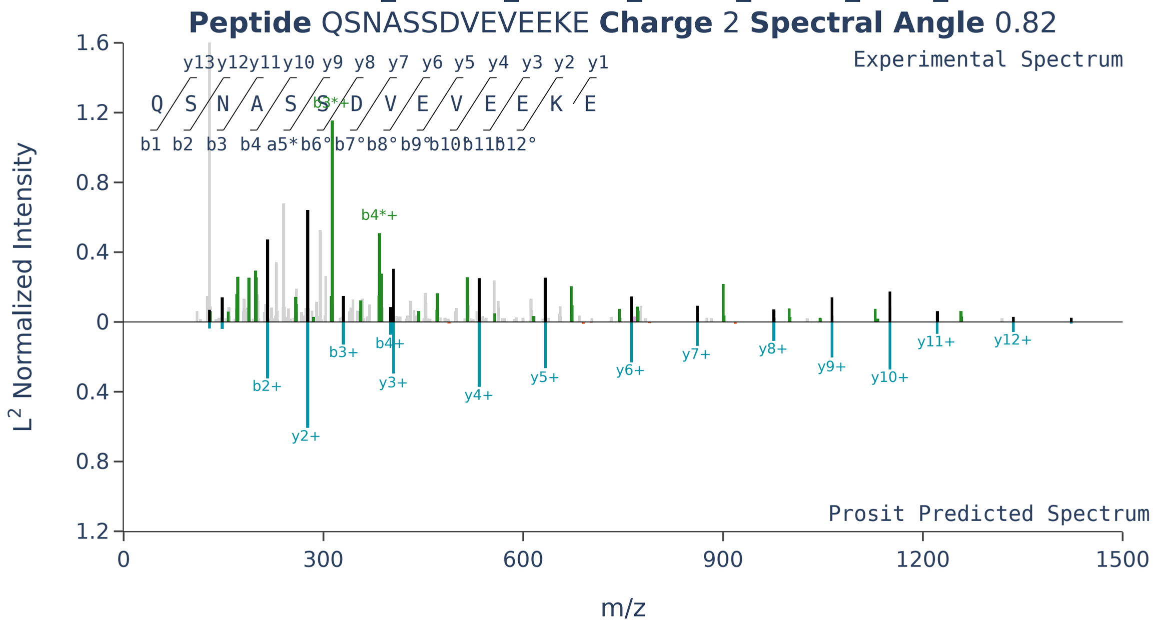


**Source** HRoetschke_271222_090123_HFGoe_LEDGF_b2R2_4h **Scan** 5903
**Peptide** FSSQQAATKQS **Charge** 2 **Spectral Angle** 0.79

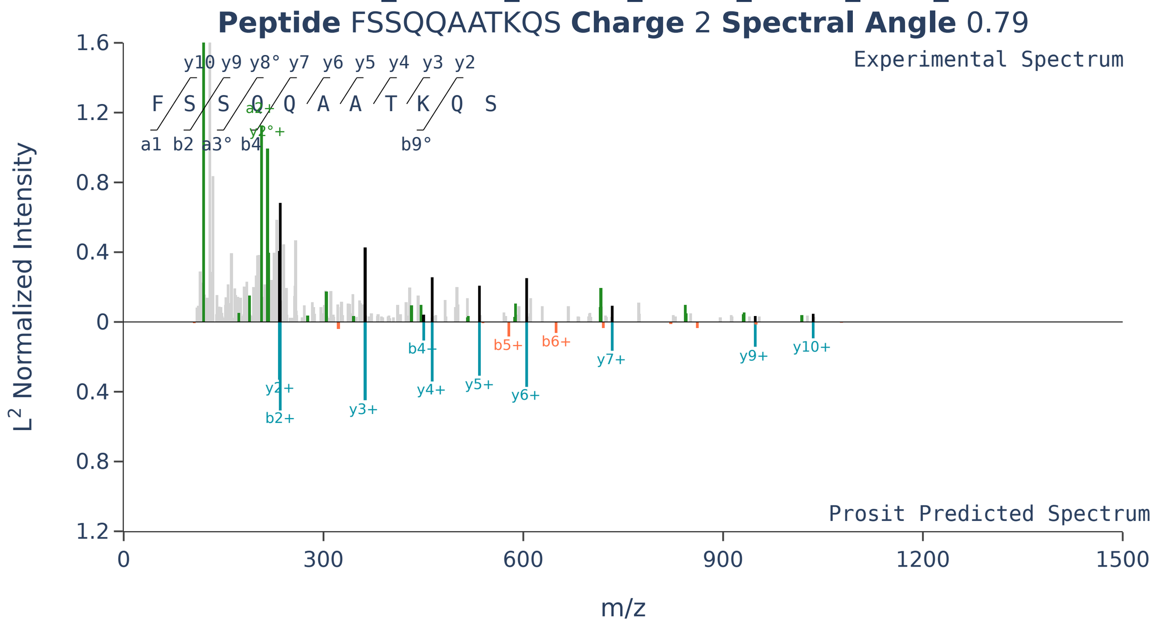

**Source** HRoetschke_271222_090123_HFGoe_LEDGF_b2R1_24h **Scan** 23085
**Peptide** SQVIMEKSTMLYNKF **Charge** 2 **Spectral Angle** 0.96

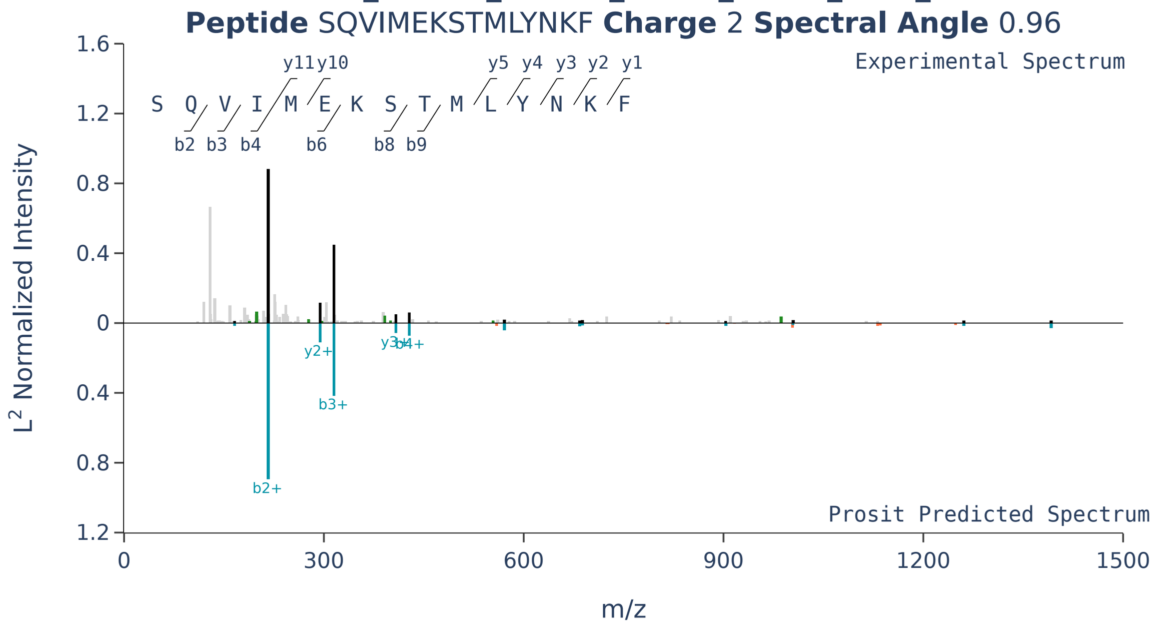


**Source** HRoetschke_271222_090123_HFGoe_LEDGF_b2R1_24h **Scan** 7485
**Peptide** QAQKHTEMITTLKKIR **Charge** 4 **Spectral Angle** 0.85

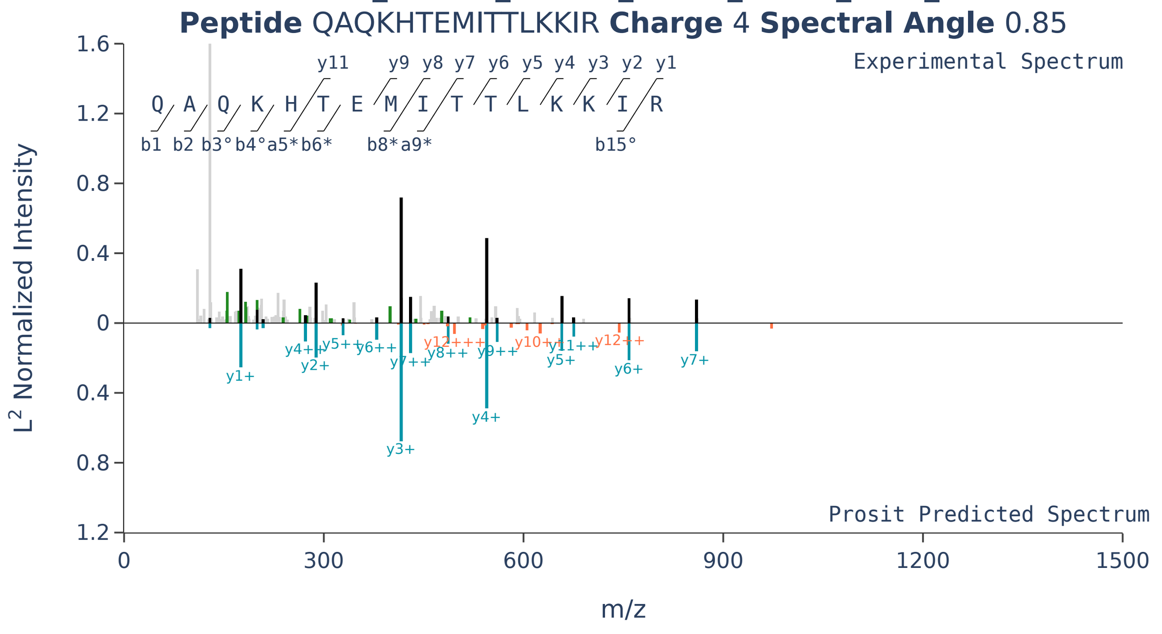


**Source** HRoetschke_271222_090123_HFGoe_LEDGF_b2R1_24h **Scan** 20481
**Peptide** SQVIMEKSTMLYN **Charge** 2 **Spectral Angle** 0.94

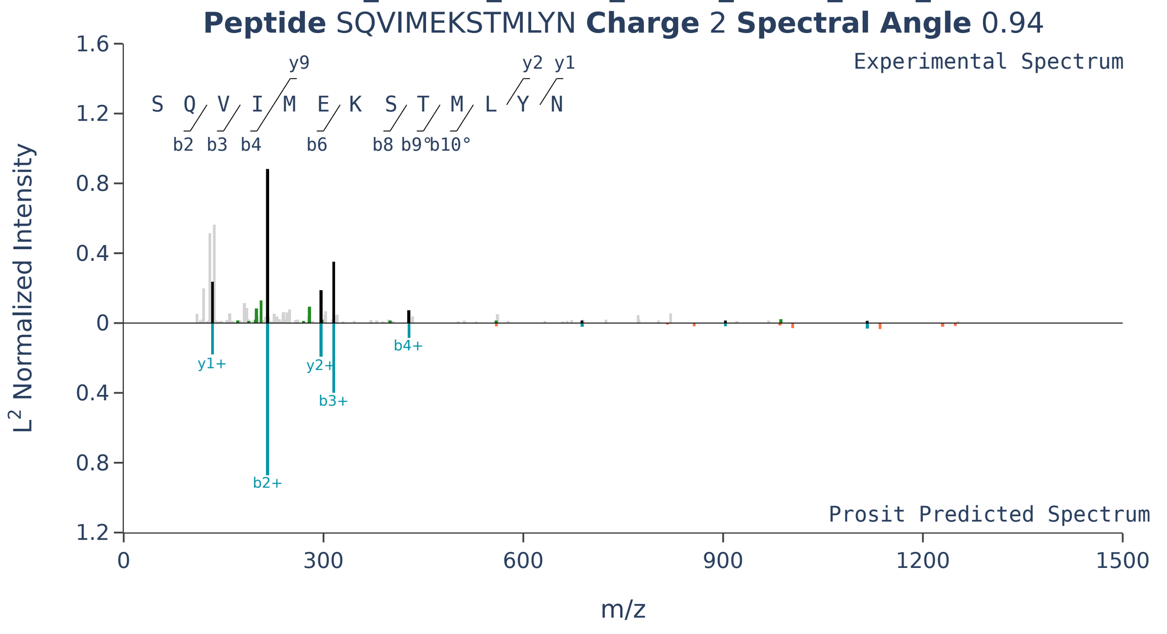


**Source** HRoetschke_271222_090123_HFGoe_LEDGF_b1R2_4h **Scan** 8288
**Peptide** GPKDIFPYSENKEKYGKPNKRKG **Charge** 4 **Spectral Angle** 0.82

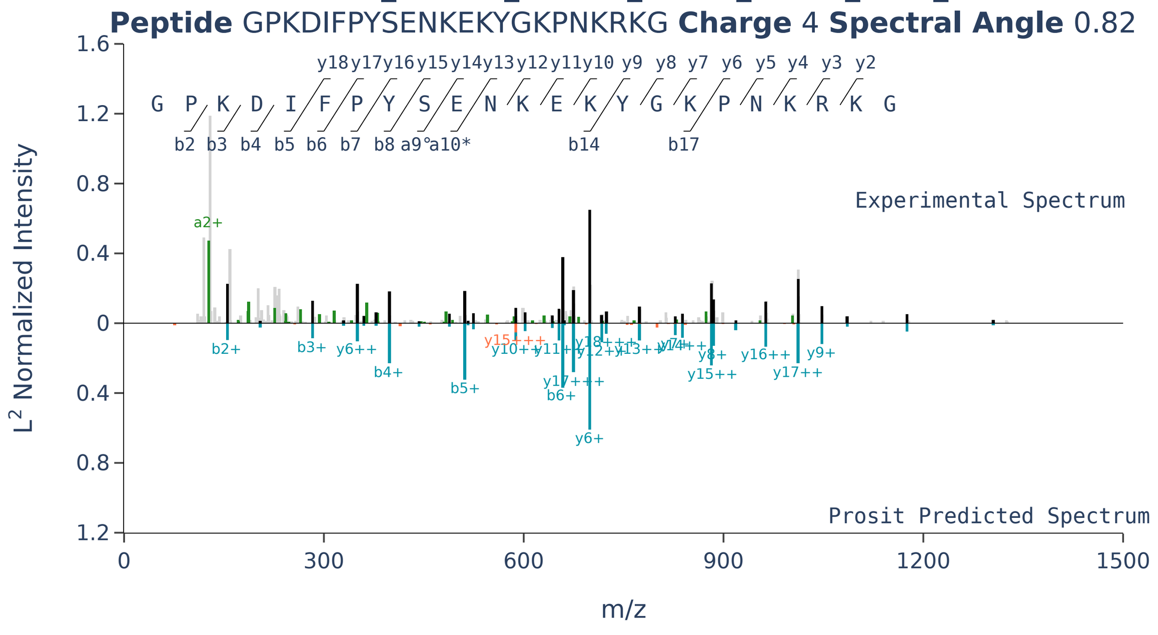


**Source** HRoetschke_271222_090123_HFGoe_LEDGF_b1R2_4h **Scan** 17285
**Peptide** VDEVPDGAVKPPTNKL **Charge** 3 **Spectral Angle** 0.76

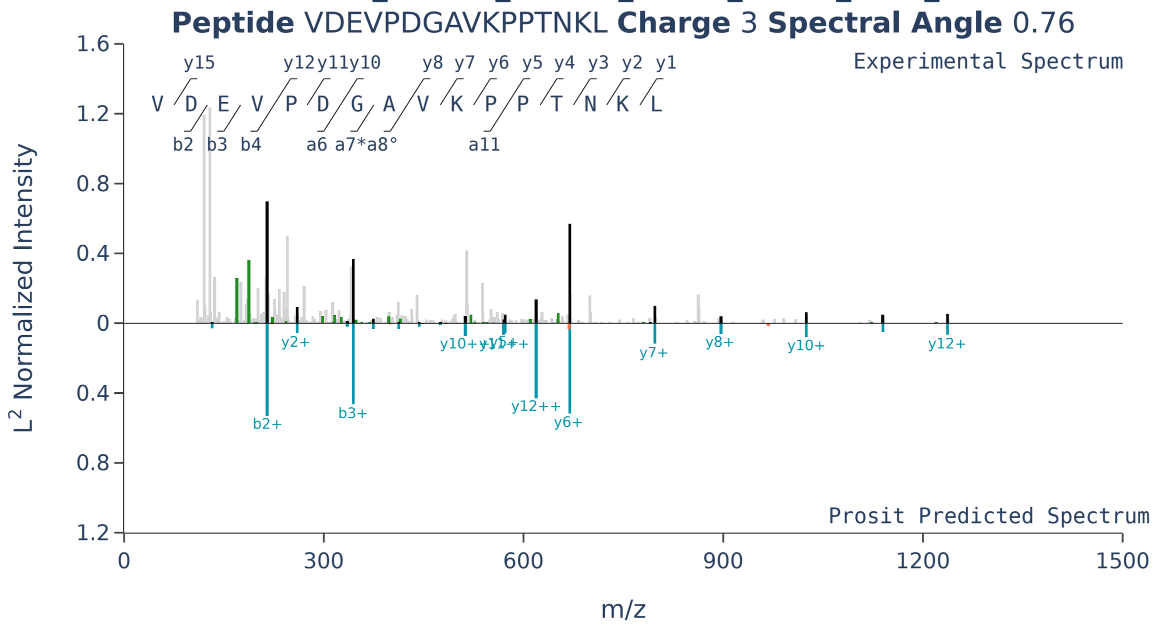


**Source** HRoetschke_271222_090123_HFGoe_LEDGF_b1R1_4h **Scan** 27233
**Peptide** DFKPGDLIFAK **Charge** 3 **Spectral Angle** 0.84

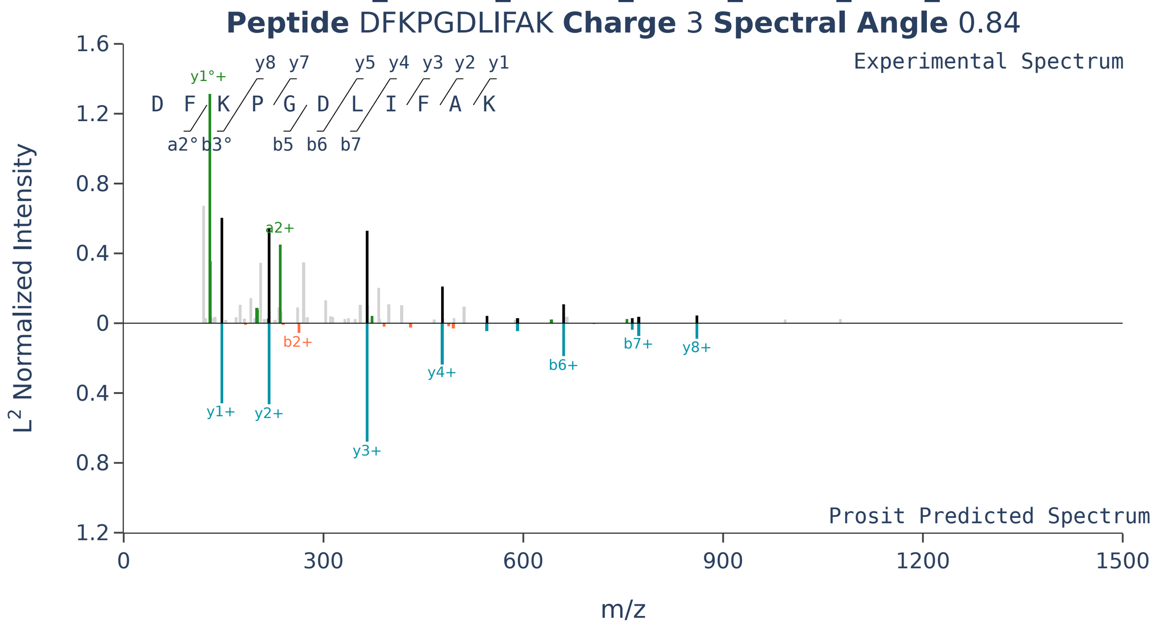


**Source** HRoetschke_271222_090123_HFGoe_LEDGF_b2R1_2h **Scan** 19536
**Peptide** NEGLWEIDNNPKVKFSSQQAATKQSN **Charge** 4 **Spectral Angle** 0.93

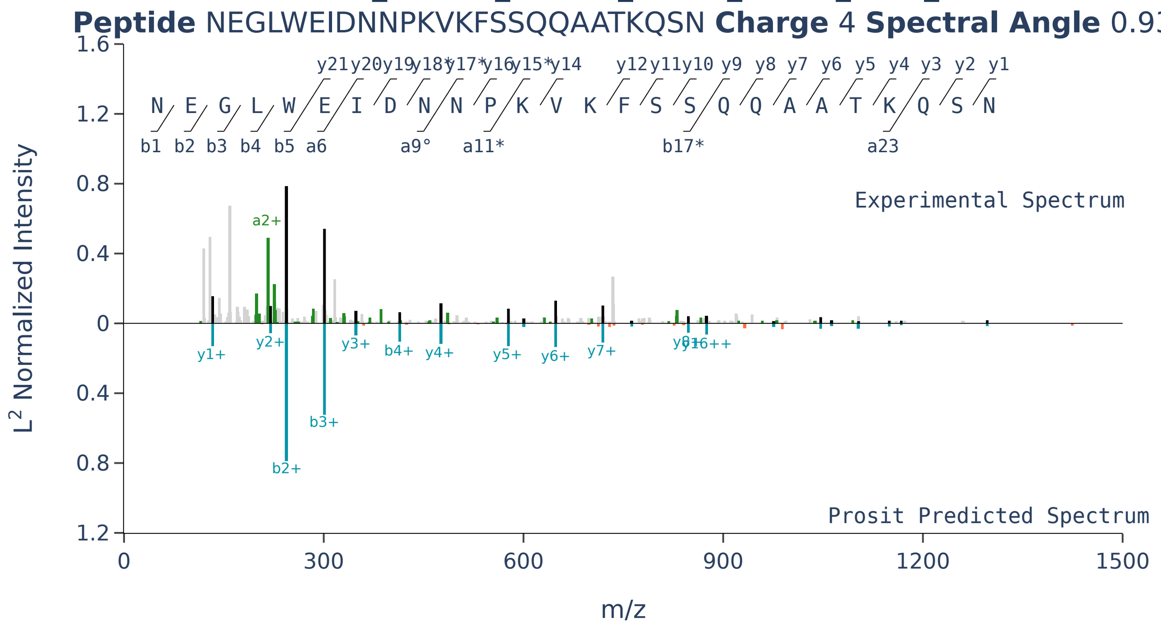


**Source** HRoetschke_271222_090123_HFGoe_LEDGF_b1R1_4h **Scan** 11823
**Peptide** PAATEVKIPKPRGRPKMV **Charge** 5 **Spectral Angle** 0.86

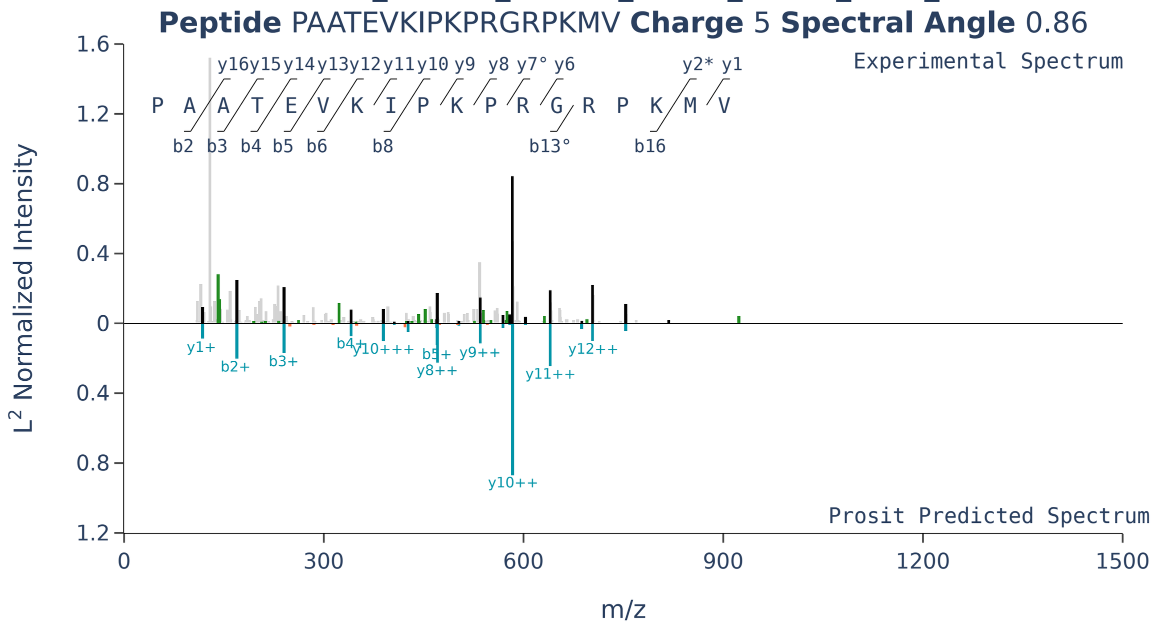


**Source** HRoetschke_271222_090123_HFGoe_LEDGF_b2R1_4h **Scan** 6570
**Peptide** KKIRRFKVSQV **Charge** 3 **Spectral Angle** 0.69

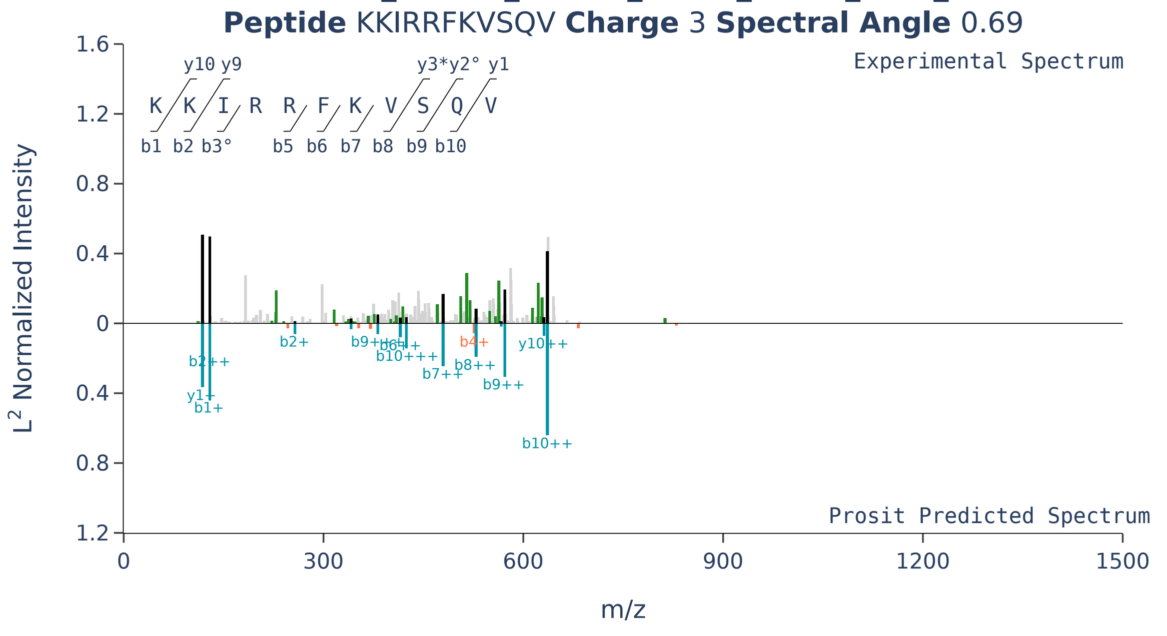


**Source** HRoetschke_271222_090123_HFGoe_LEDGF_b1R1_24h **Scan** 6905
**Peptide** RLQRIHAEIKN **Charge** 3 **Spectral Angle** 0.78

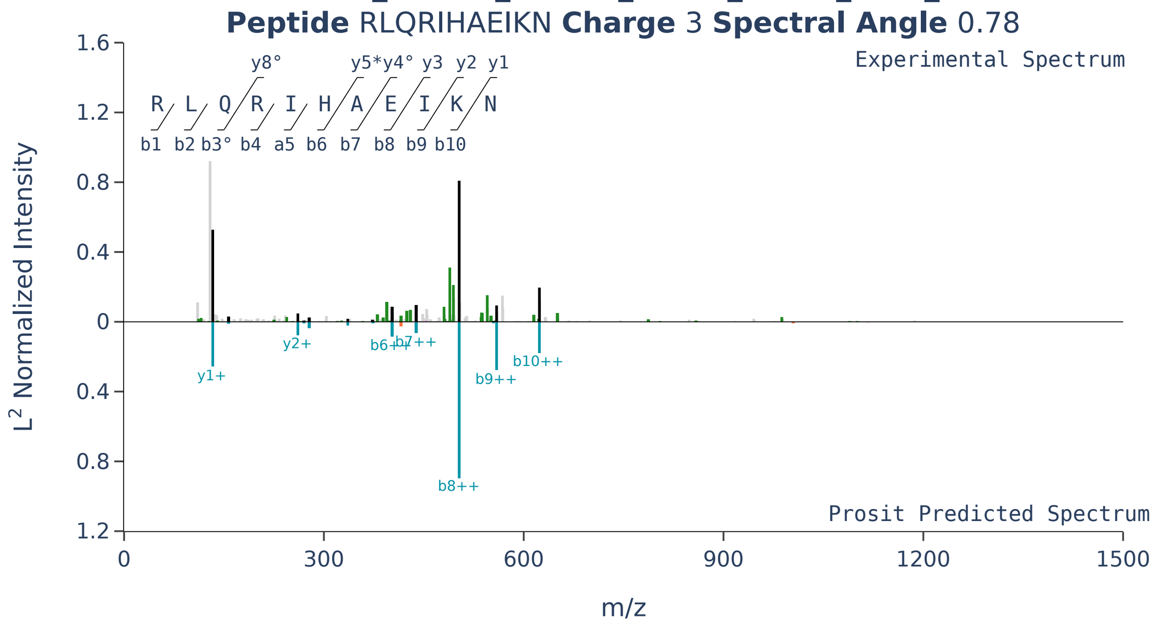


**Source** HRoetschke_271222_090123_HFGoe_LEDGF_b2R1_24h **Scan** 27209
**Peptide** ALDELASLQVTMQQA **Charge** 2 **Spectral Angle** 0.86

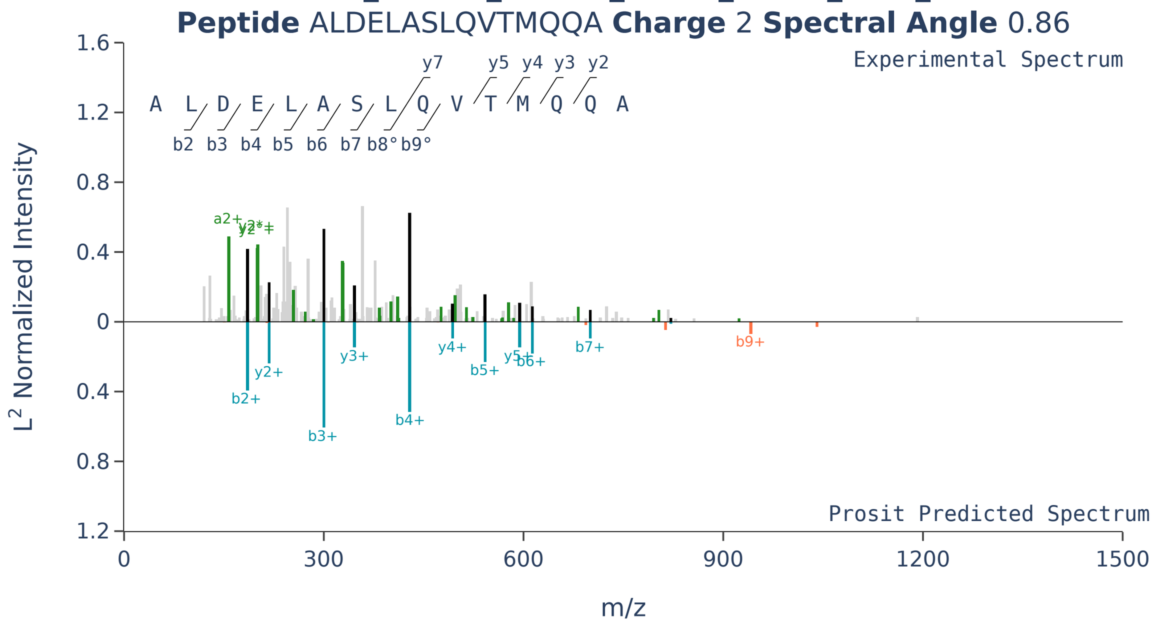


**Source** HRoetschke_271222_090123_HFGoe_LEDGF_b2R1_2h **Scan** 6763
**Peptide** NNPKVKFSSQQAATKQSNASSD **Charge** 3 **Spectral Angle** 0.89

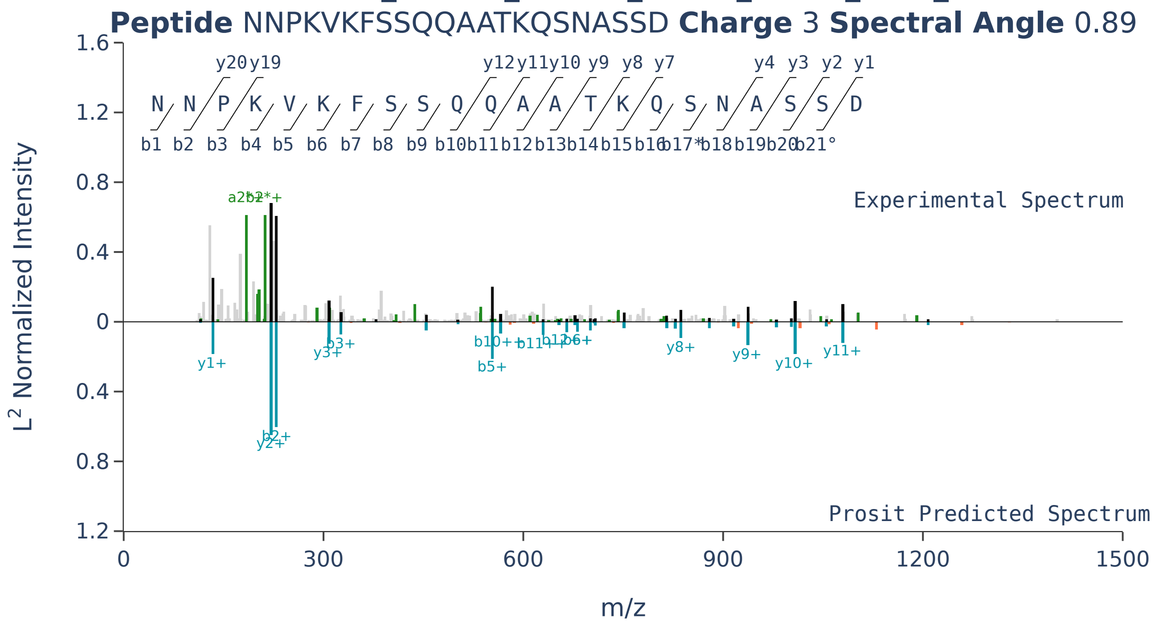


**Source** HRoetschke_271222_090123_HFGoe_LEDGF_b2R2_4h **Scan** 18166
**Peptide** ETAFLGPKDIFPYSENKEKYGKPNKR **Charge** 5 **Spectral Angle** 0.79

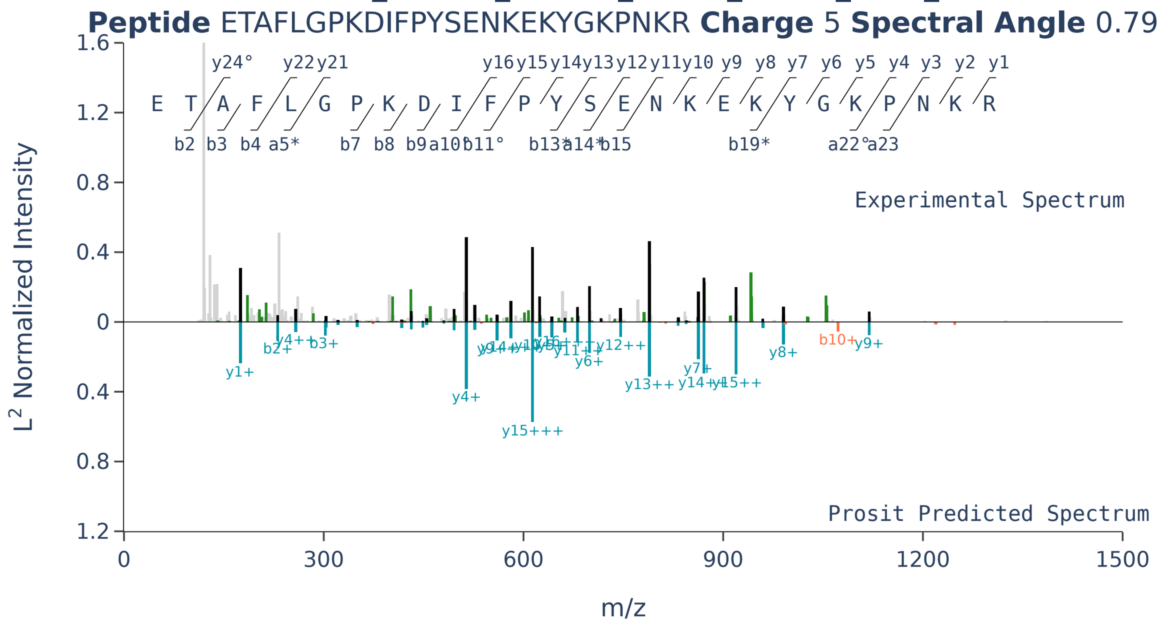


**Source** HRoetschke_271222_090123_HFGoe_LEDGF_b1R1_2h **Scan** 26727
**Peptide** DFKPGDLIFAKM **Charge** 3 **Spectral Angle** 0.77

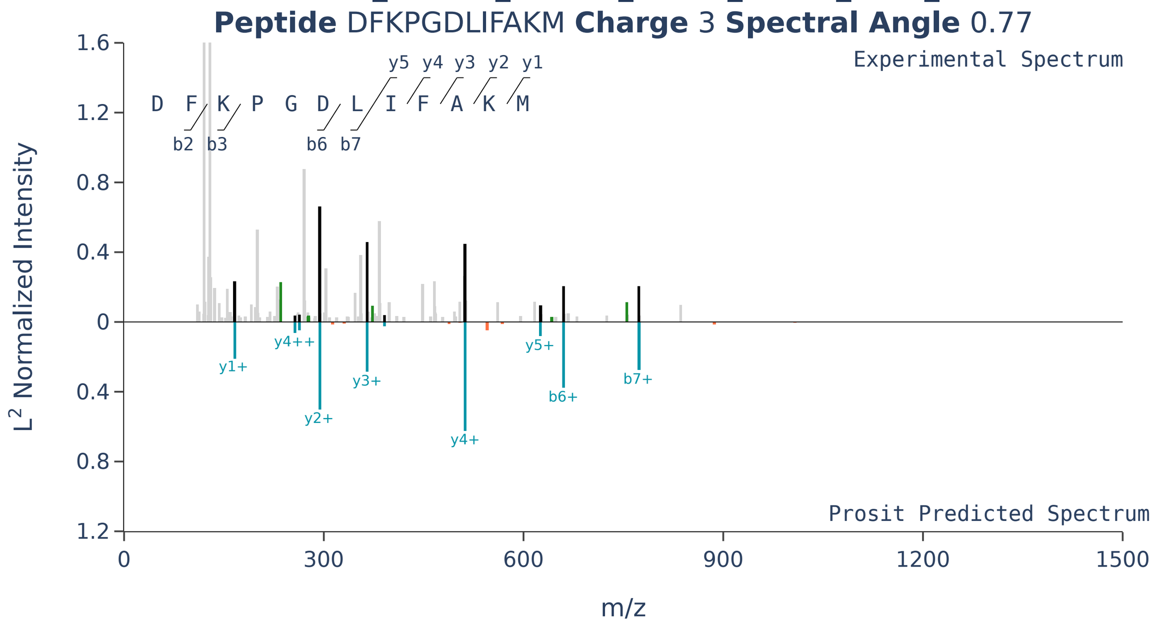


**Source** HRoetschke_271222_090123_HFGoe_LEDGF_b1R2_4h **Scan** 15048
**Peptide** EAGVVTTATASVNLKVSPKRGR **Charge** 4 **Spectral Angle** 0.89

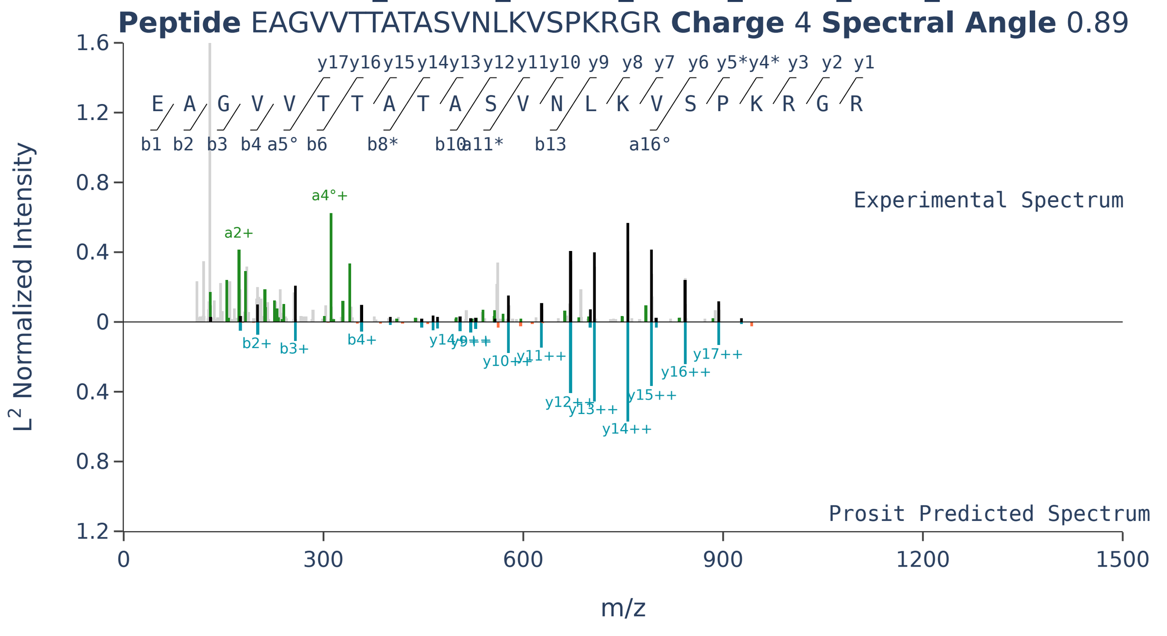

**Source** HRoetschke_271222_090123_HFGoe_LEDGF_b1R1_2h **Scan** 30579
**Peptide** PDGAVKPPTNKLPIFFFGTHE **Charge** 3 **Spectral Angle** 0.82

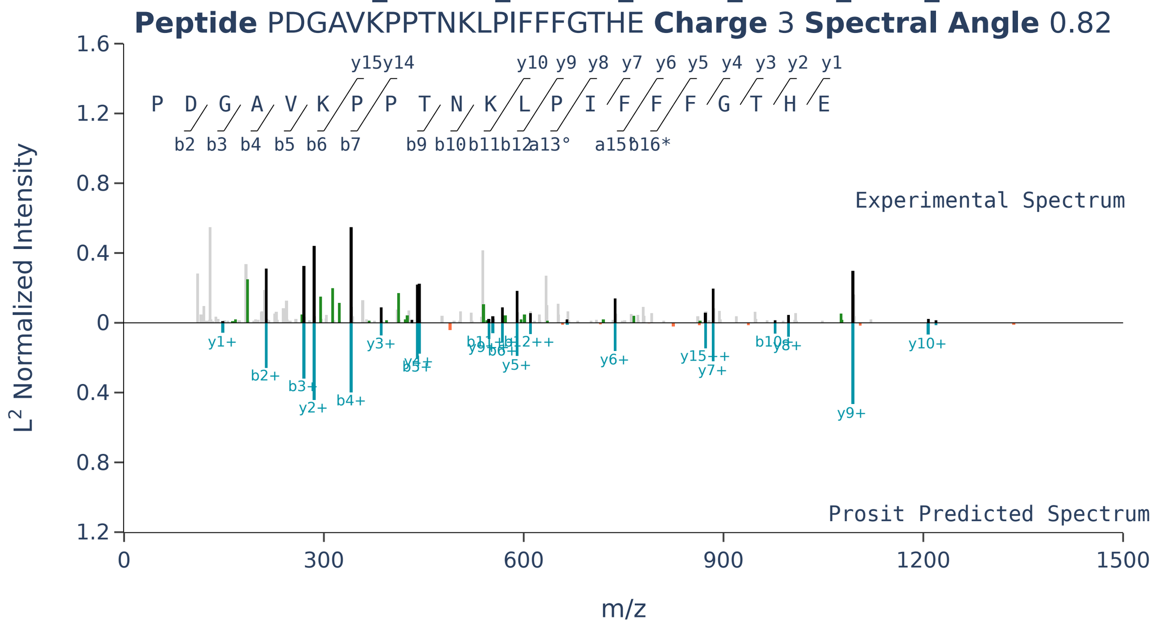


**Source** HRoetschke_271222_090123_HFGoe_LEDGF_b2R2_24h **Scan** 6430
**Peptide** TTPKAAR **Charge** 2 **Spectral Angle** 0.85

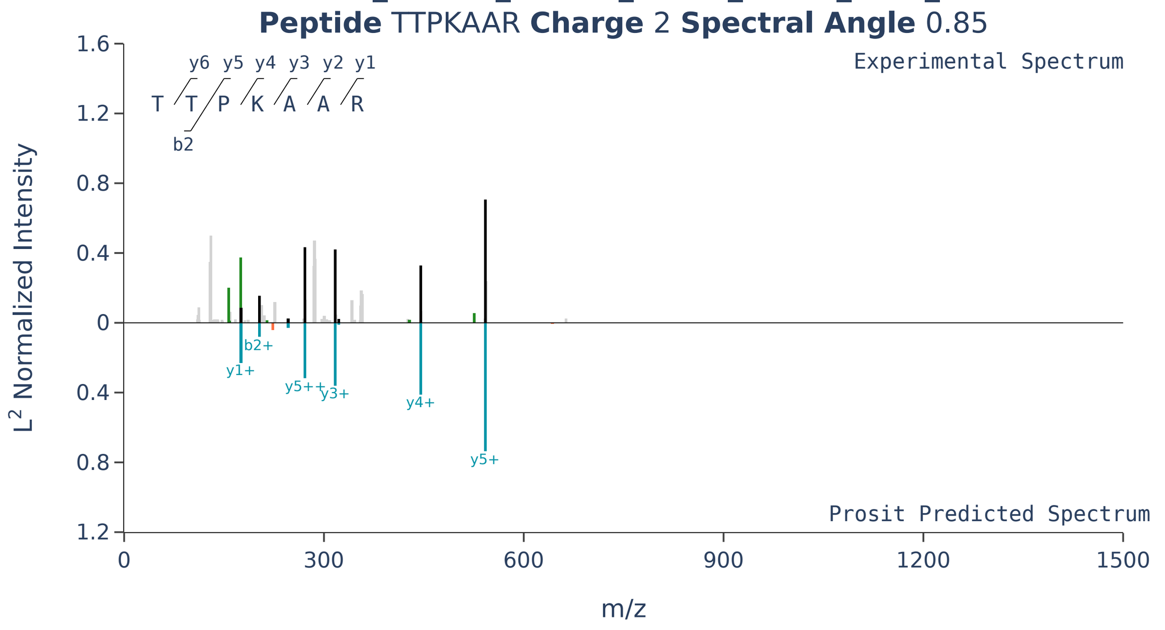


**Source** HRoetschke_271222_090123_HFGoe_LEDGF_b1R2_24h **Scan** 15990
**Peptide** QAQKHTEMITTL **Charge** 2 **Spectral Angle** 0.79

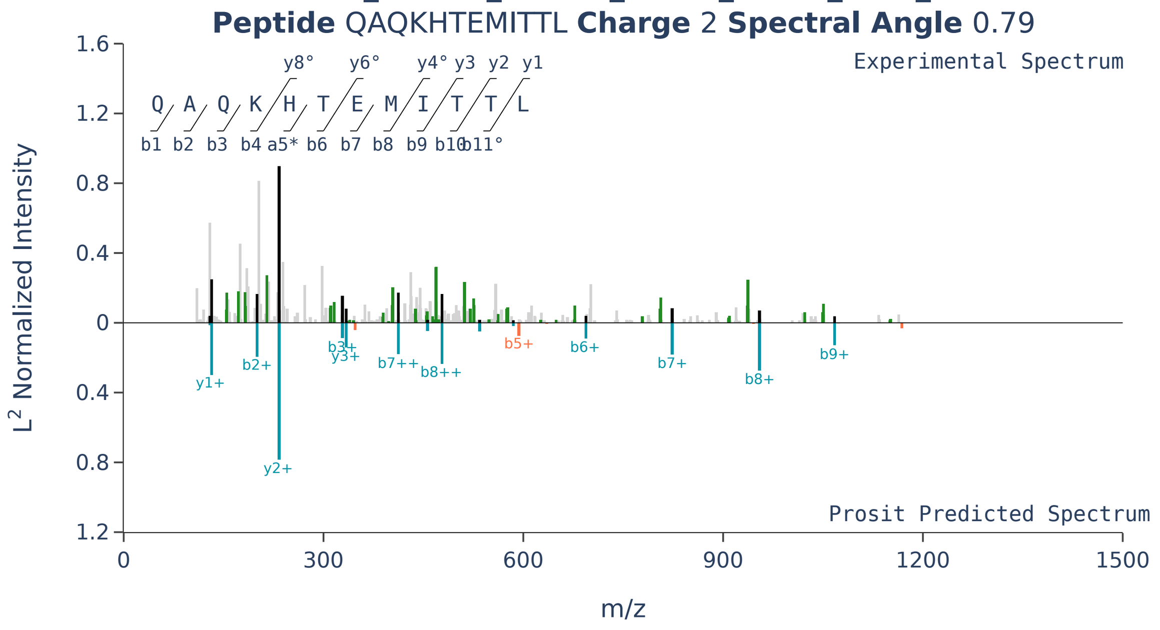


**Source** HRoetschke_271222_090123_HFGoe_LEDGF_b2R1_2h **Scan** 15634
**Peptide** KMKGYPHWPARVDEV **Charge** 3 **Spectral Angle** 0.8

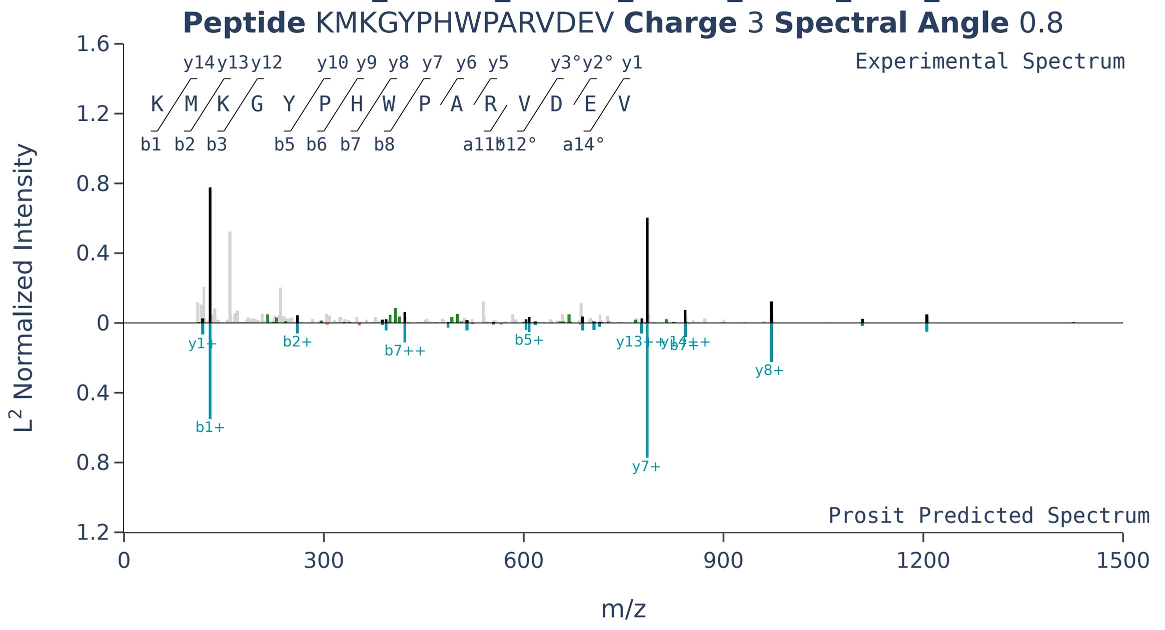


**Source** HRoetschke_271222_090123_HFGoe_LEDGF_b1R2_24h **Scan** 7976
**Peptide** LQRIHAEIKN **Charge** 3 **Spectral Angle** 0.7

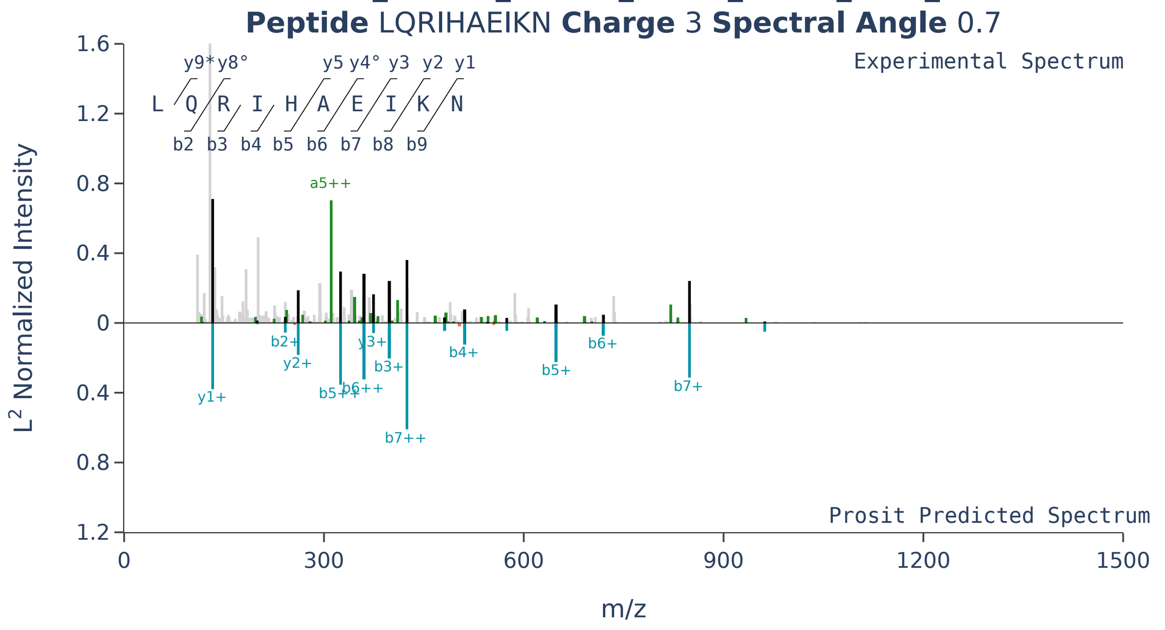


**Source** HRoetschke_271222_090123_HFGoe_LEDGF_b2R1_24h **Scan** 14786
**Peptide** AGVVTTATASVNLKVSPKRGRPAATE **Charge** 5 **Spectral Angle** 0.75

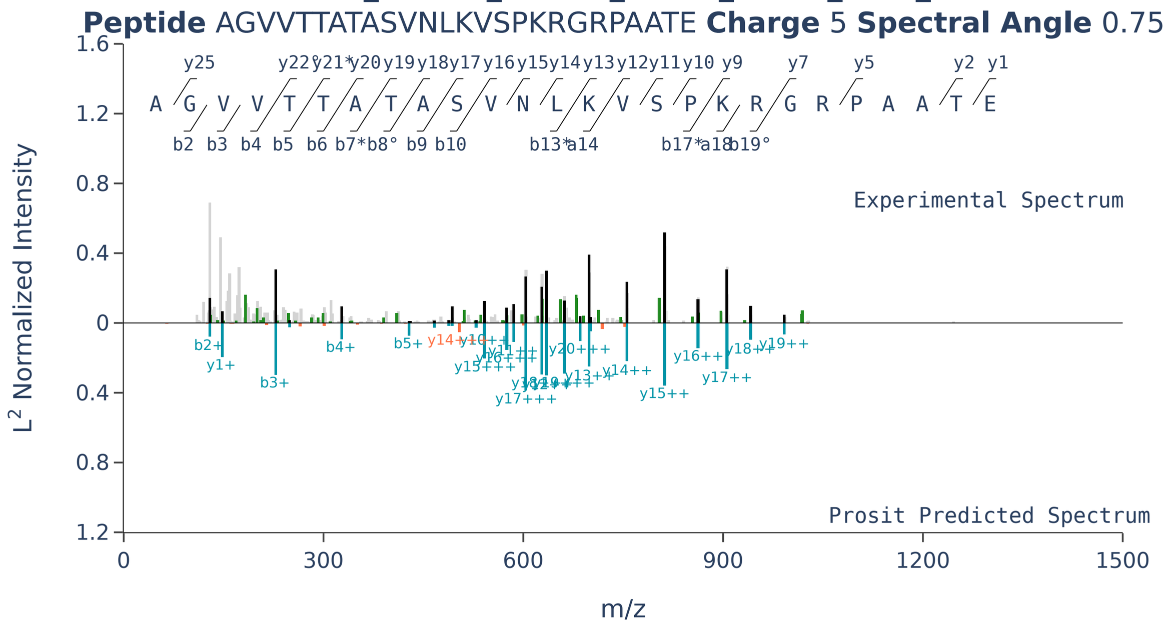


**Source** HRoetschke_271222_090123_HFGoe_LEDGF_b2R1_24h **Scan** 24791
**Peptide** EAGVVTTATASVNL **Charge** 2 **Spectral Angle** 0.88

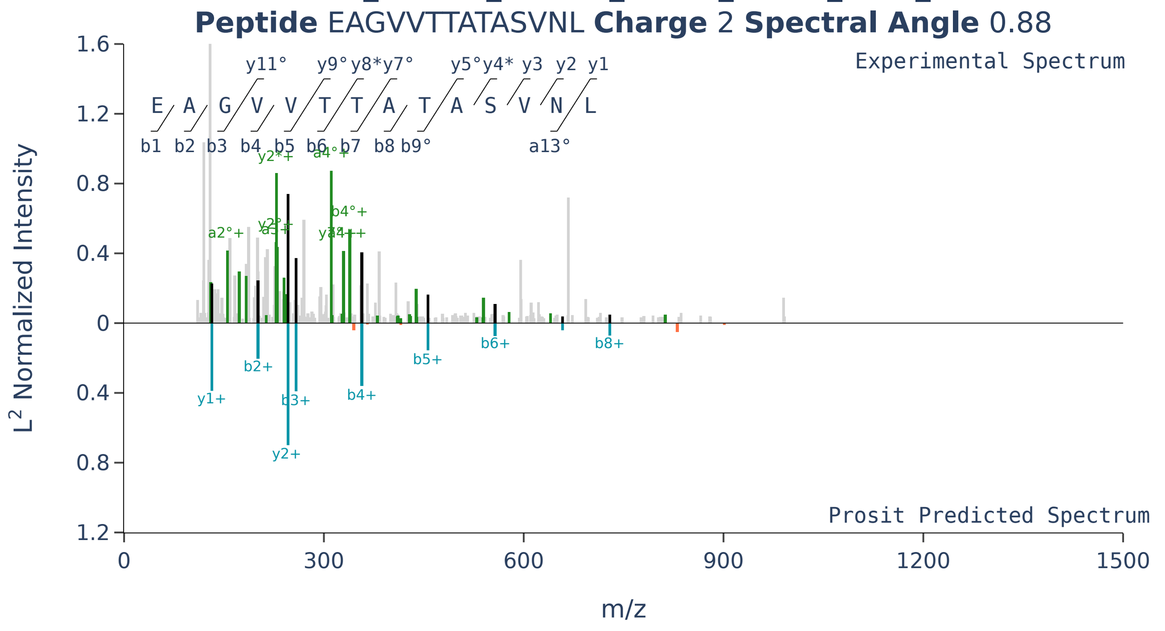


**Source** HRoetschke_271222_090123_HFGoe_LEDGF_b1R1_4h **Scan** 14479
**Peptide** AGVVTTATASVNLKVSPKRGRPAAT **Charge** 4 **Spectral Angle** 0.8

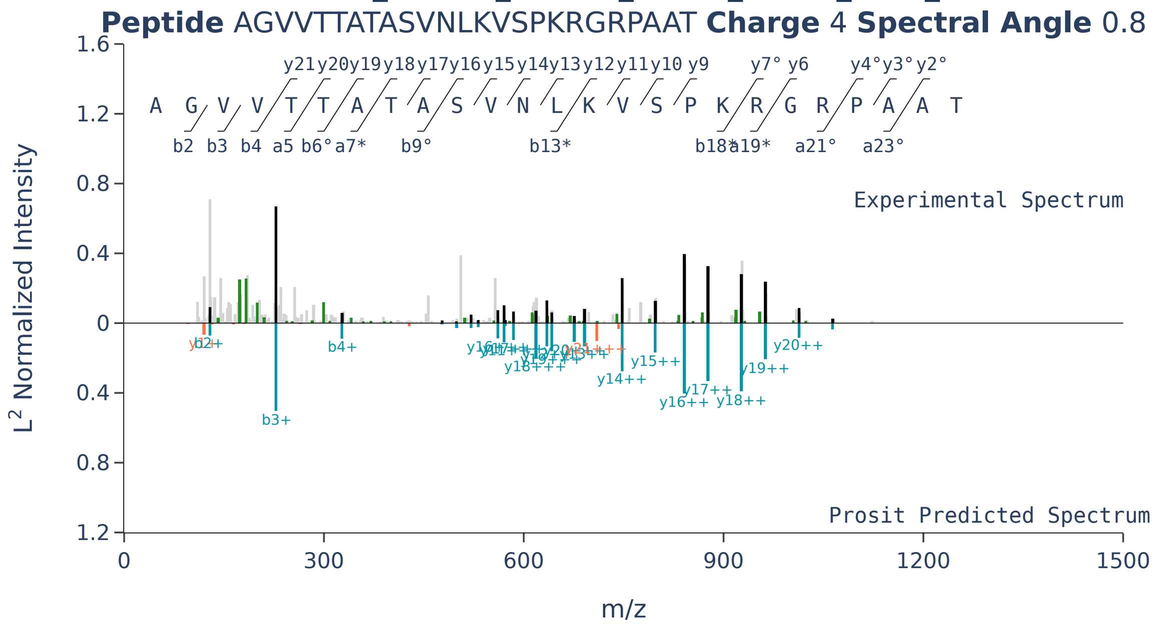


**Source** HRoetschke_271222_090123_HFGoe_LEDGF_b1R2_24h **Scan** 6301
**Peptide** KLEKEQTGSKTL **Charge** 3 **Spectral Angle** 0.9

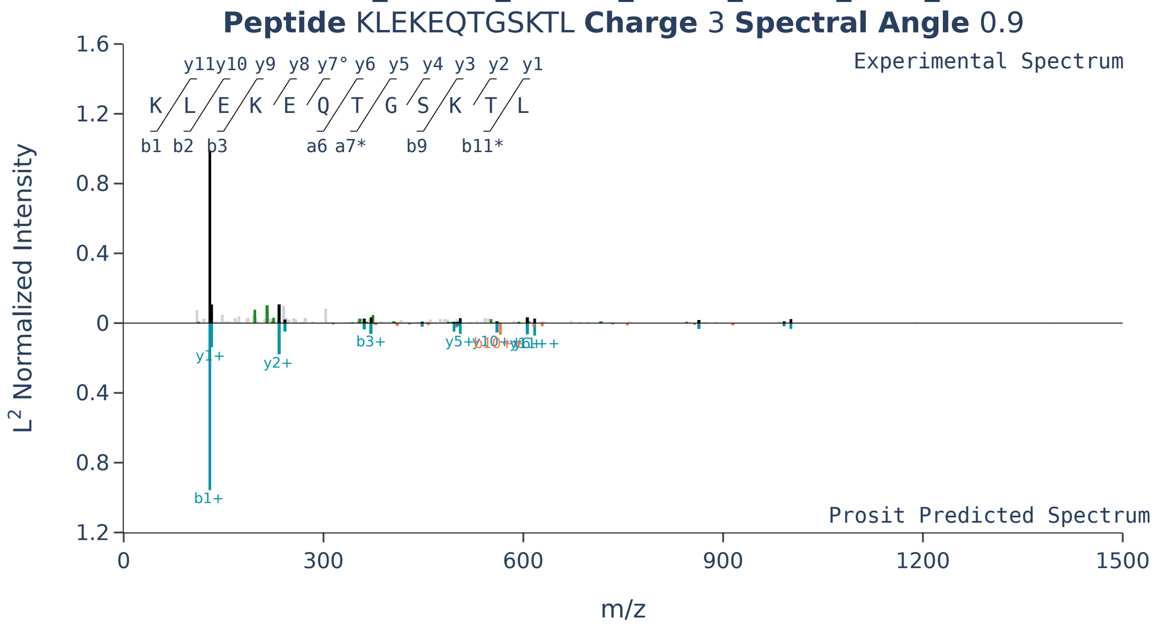


**Source** HRoetschke_271222_090123_HFGoe_LEDGF_b1R1_2h **Scan** 25974
**Peptide** GDSVITQVLNKSLAEQRQ **Charge** 3 **Spectral Angle** 0.76

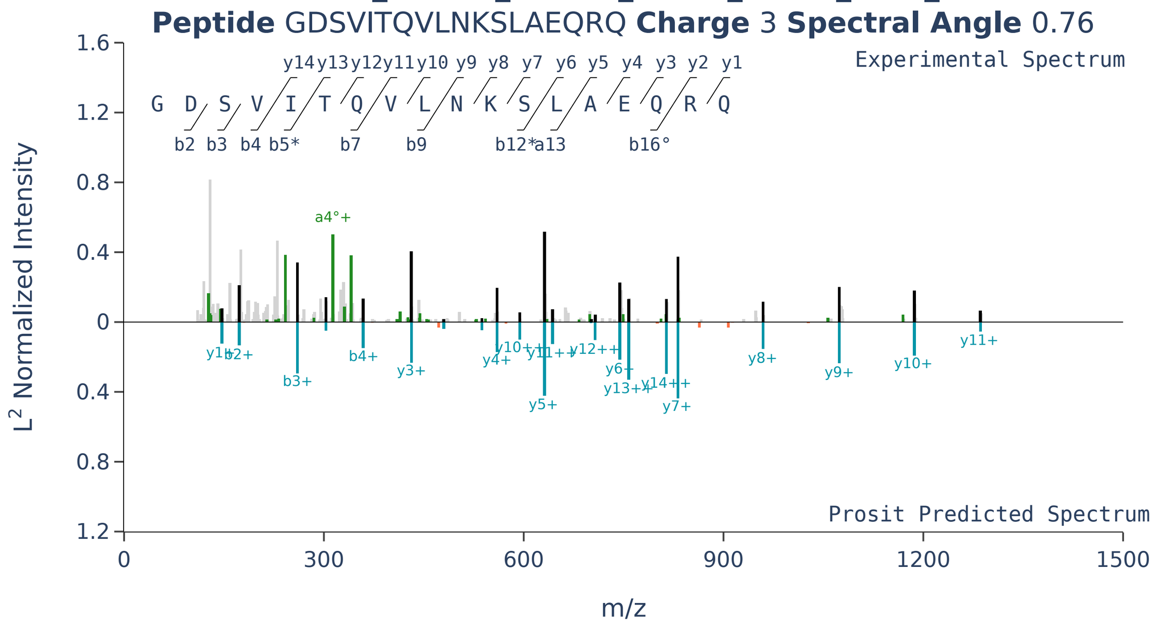


**Source** HRoetschke_271222_090123_HFGoe_LEDGF_b2R1_2h **Scan** 10846
**Peptide** KMKGYPHW **Charge** 2 **Spectral Angle** 0.81

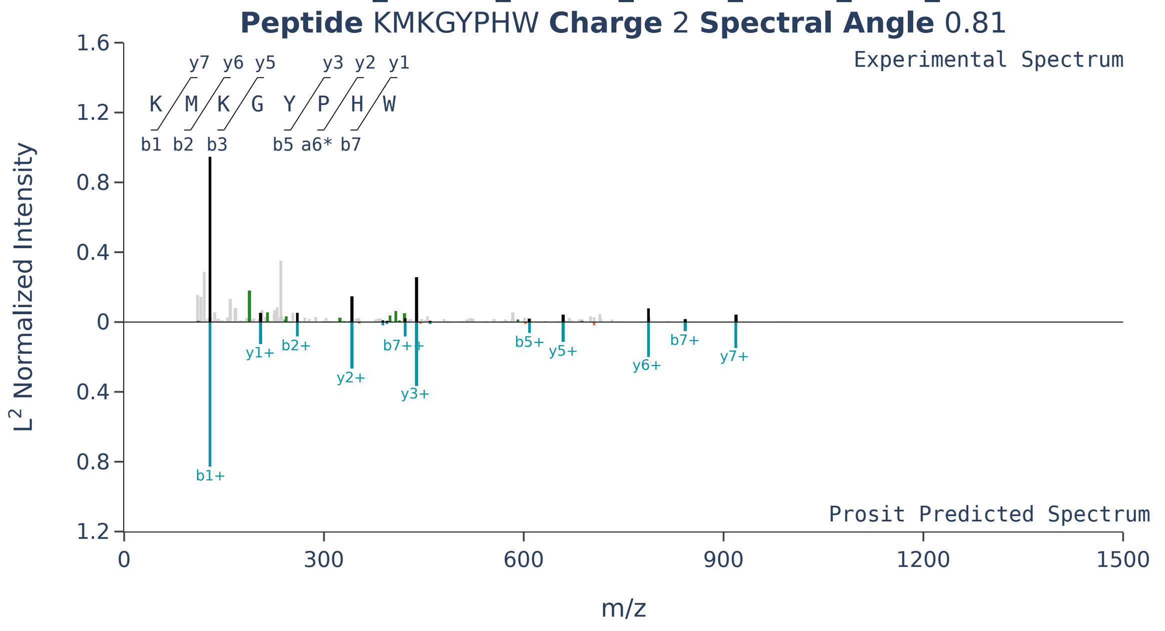


**Source** HRoetschke_271222_090123_HFGoe_LEDGF_b1R1_2h **Scan** 18407
**Peptide** SRLQRIHAEIKNSLKIDNLD **Charge** 4 **Spectral Angle** 0.81

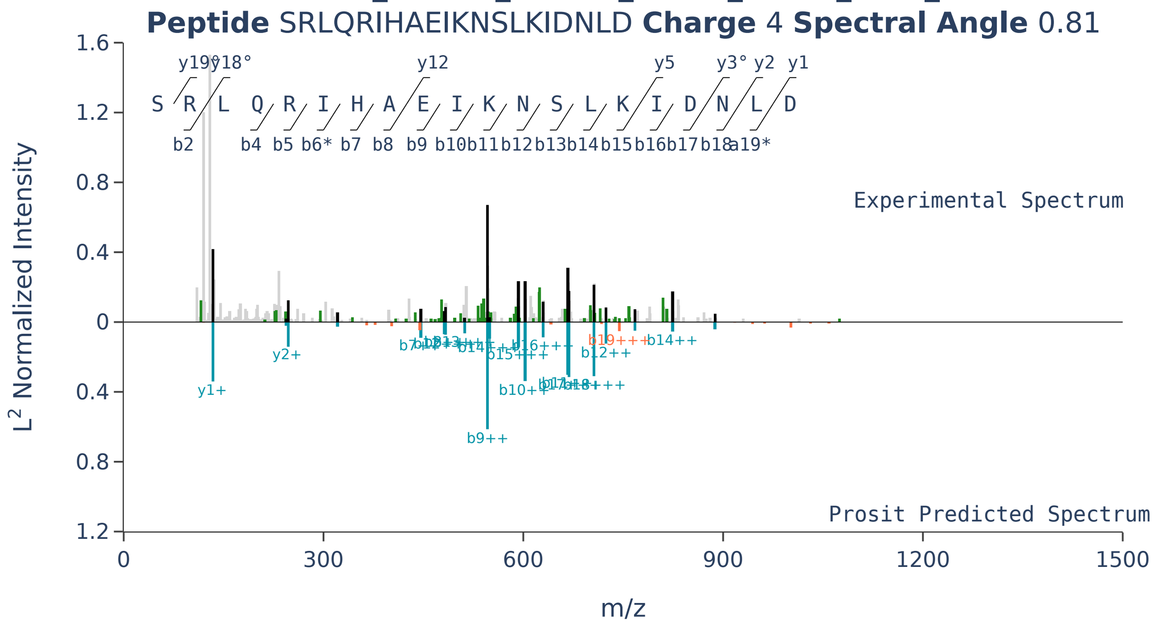


**Source** HRoetschke_271222_090123_HFGoe_LEDGF_b1R1_4h **Scan** 10246
**Peptide** RFKVSQV **Charge** 2 **Spectral Angle** 0.84

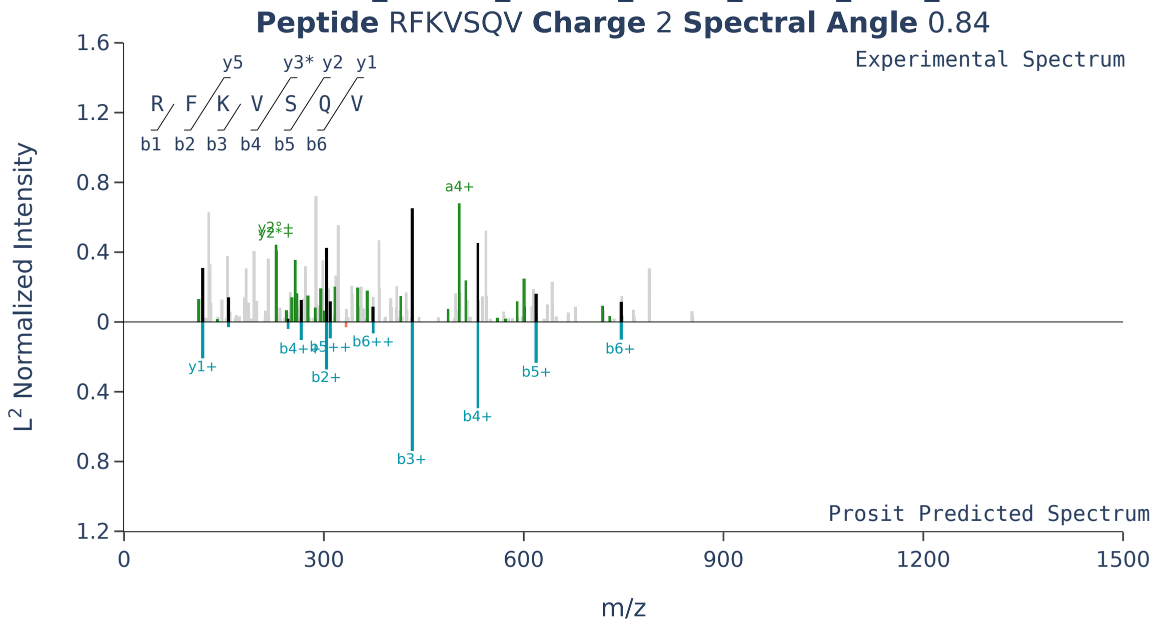


**Source** HRoetschke_271222_090123_HFGoe_LEDGF_b1R2_24h **Scan** 5142
**Peptide** SDIITEEDKSKKKGQEEKQPKKQPKKD **Charge** 5 **Spectral Angle** 0.84

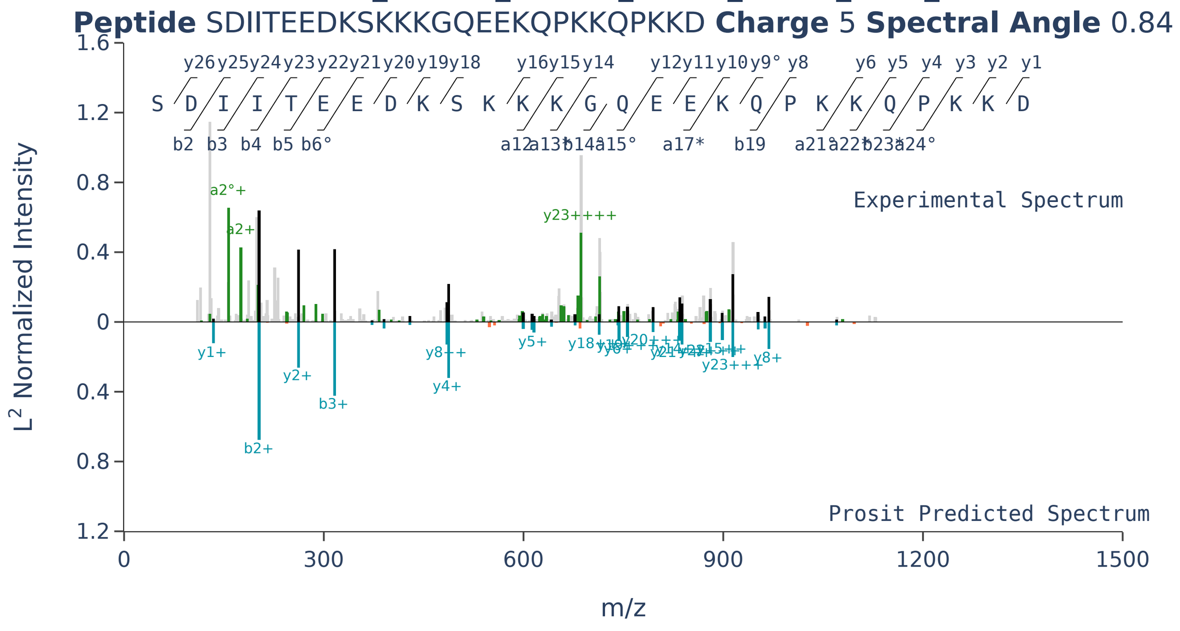


**Source** HRoetschke_271222_090123_HFGoe_LEDGF_b1R2_24h **Scan** 5582
**Peptide** KFSSQQAATKQSNASSD **Charge** 2 **Spectral Angle** 0.68

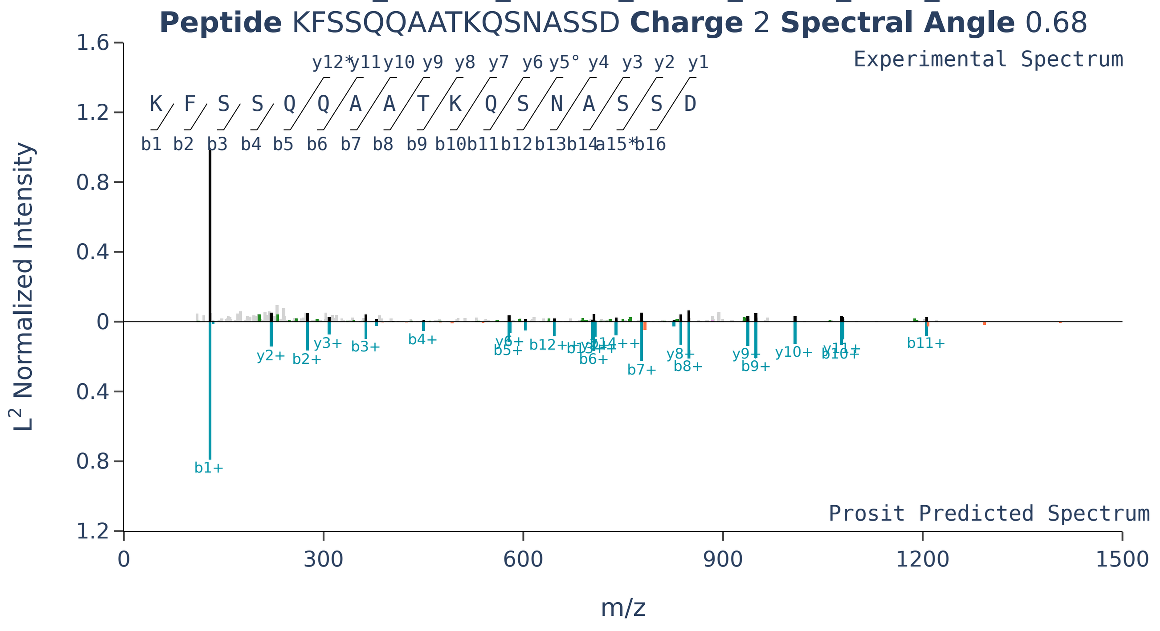

**Source** HRoetschke_271222_090123_HFGoe_LEDGF_b2R2_2h **Scan** 20661
**Peptide** AEIKNSLKIDNLDVNRCLE **Charge** 3 **Spectral Angle** 0.88

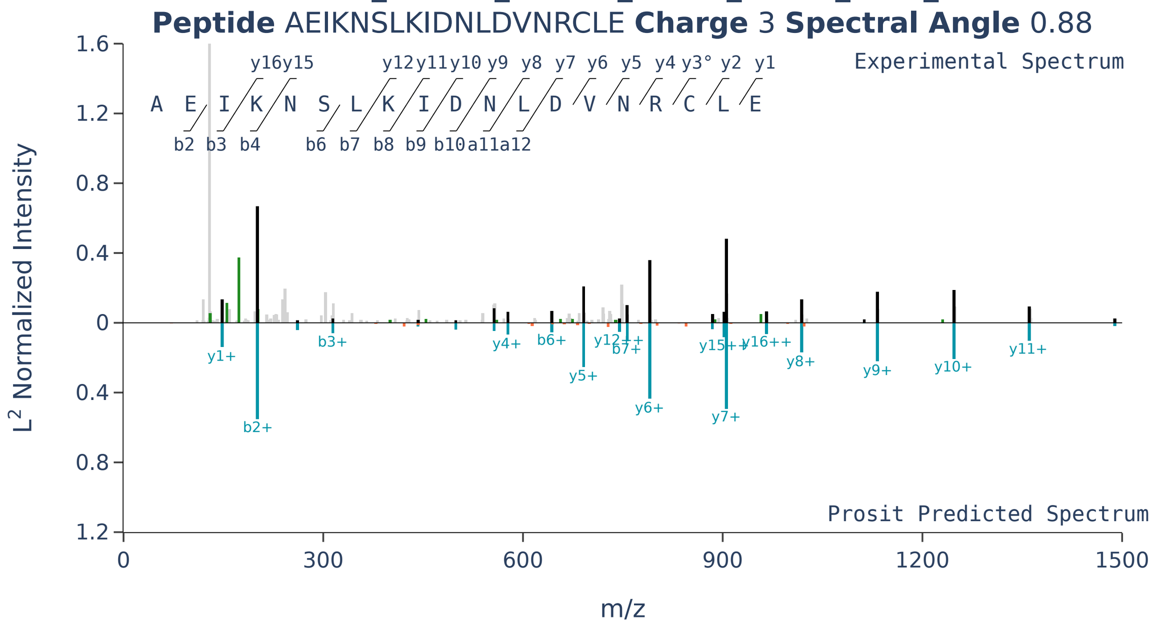


**Source** HRoetschke_271222_090123_HFGoe_LEDGF_b2R2_2h **Scan** 9893
**Peptide** KMKGYPHWPAR **Charge** 3 **Spectral Angle** 0.79

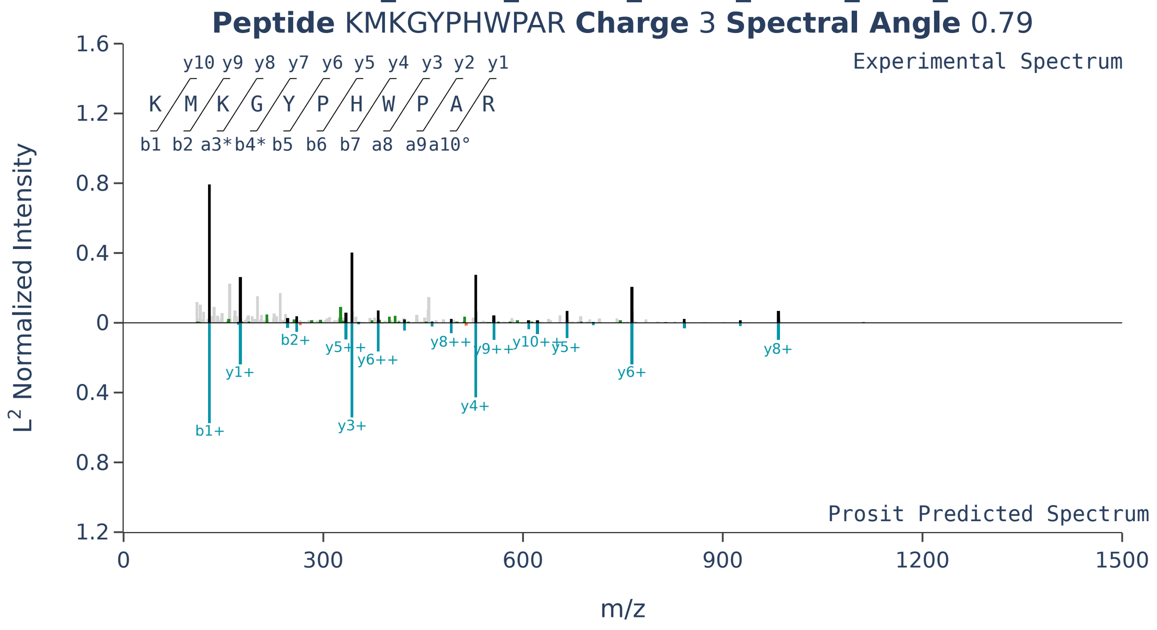


**Source** HRoetschke_271222_090123_HFGoe_LEDGF_b2R1_24h **Scan** 6505
**Peptide** KIPKPRGRPKMV **Charge** 3 **Spectral Angle** 0.74

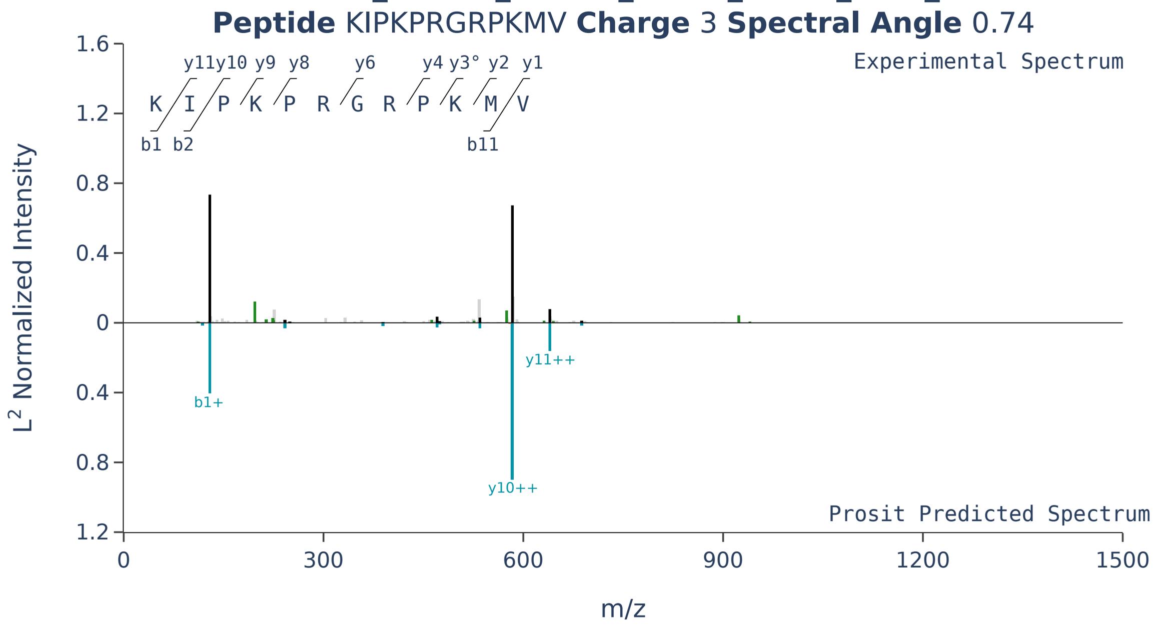


**Source** HRoetschke_271222_090123_HFGoe_LEDGF_b1R1_2h **Scan** 24278
**Peptide** TRDFKPGDLIFAKMKGY **Charge** 3 **Spectral Angle** 0.78

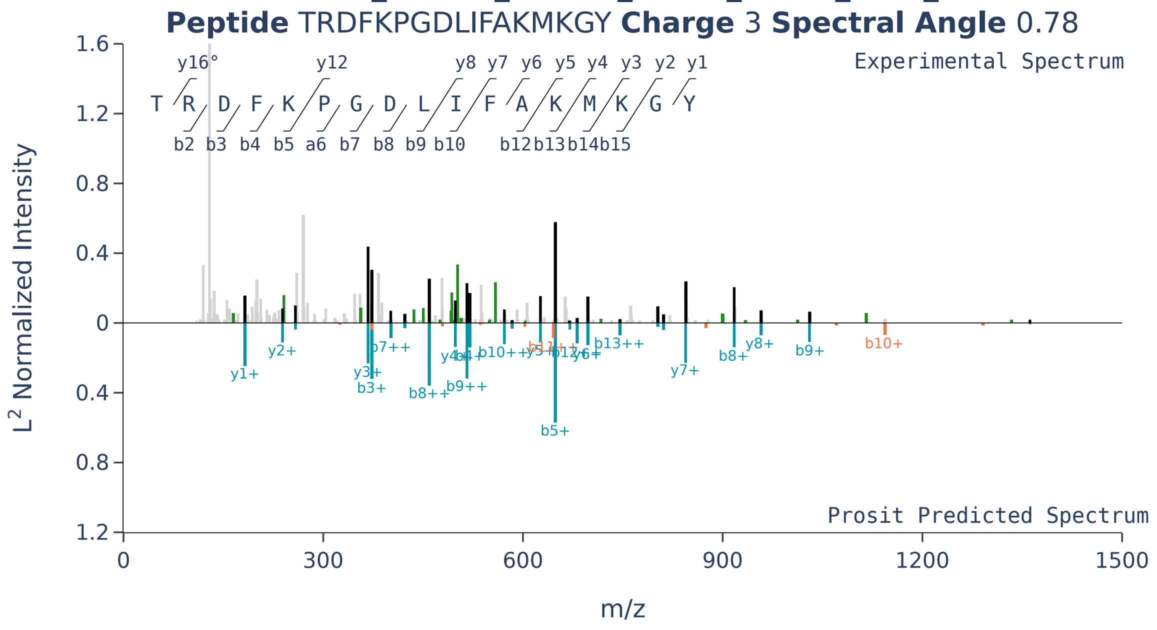


**Source** HRoetschke_271222_090123_HFGoe_LEDGF_b1R1_4h **Scan** 15522
**Peptide** IMEKSTMLYNKFKN **Charge** 3 **Spectral Angle** 0.83

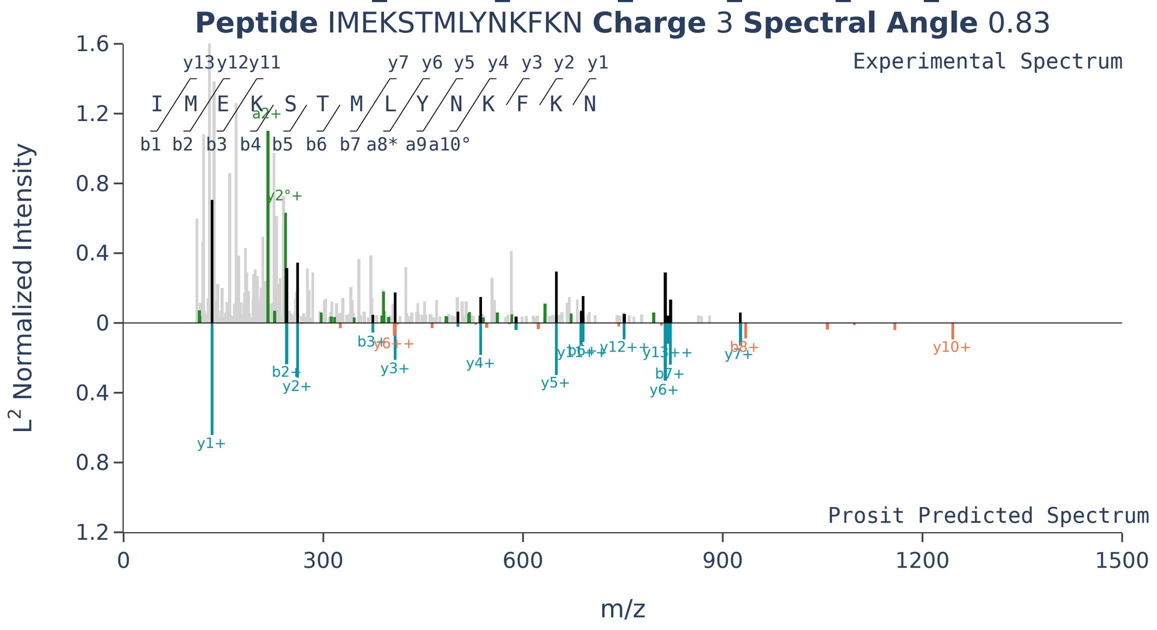


**Source** HRoetschke_271222_090123_HFGoe_LEDGF_b2R1_24h **Scan** 18187
**Peptide** ETSMDSRLQRIHAEI **Charge** 2 **Spectral Angle** 0.83

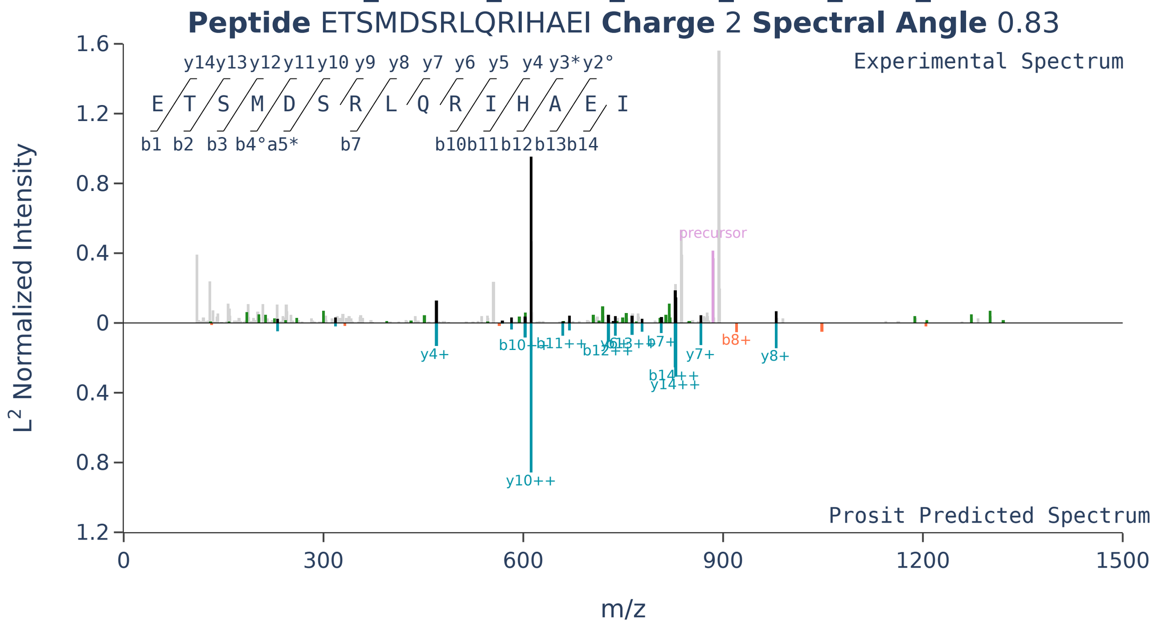


**Source** HRoetschke_271222_090123_HFGoe_LEDGF_b1R1_24h **Scan** 15068
**Peptide** EIKNSLKI **Charge** 2 **Spectral Angle** 0.8

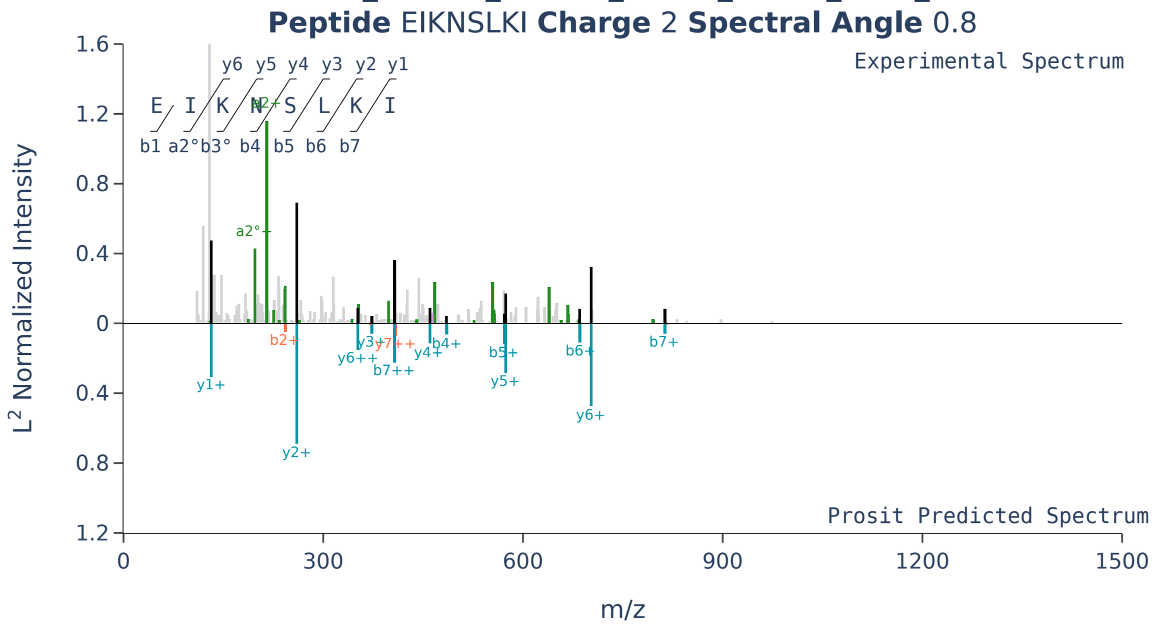


**Source** HRoetschke_271222_090123_HFGoe_LEDGF_b1R1_4h **Scan** 6686
**Peptide** GRKRKAEKQVETEEAGVVTT **Charge** 3 **Spectral Angle** 0.81

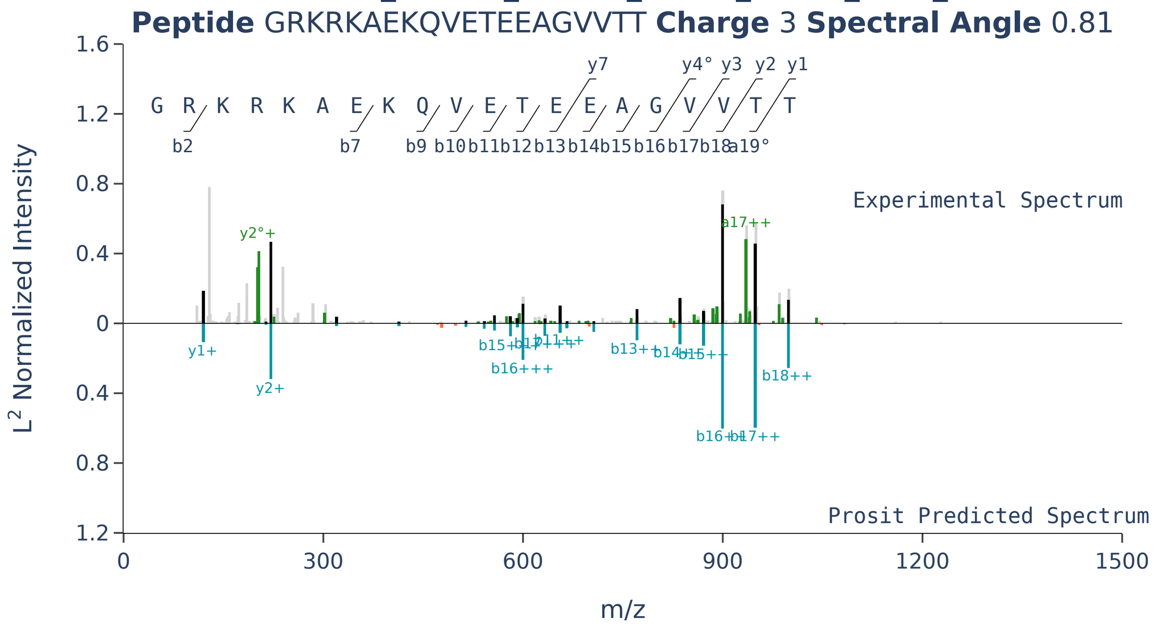


**Source** HRoetschke_271222_090123_HFGoe_LEDGF_b1R2_1h **Scan** 22809
**Peptide** AFLGPKDIFPYSENKEK **Charge** 4 **Spectral Angle** 0.8

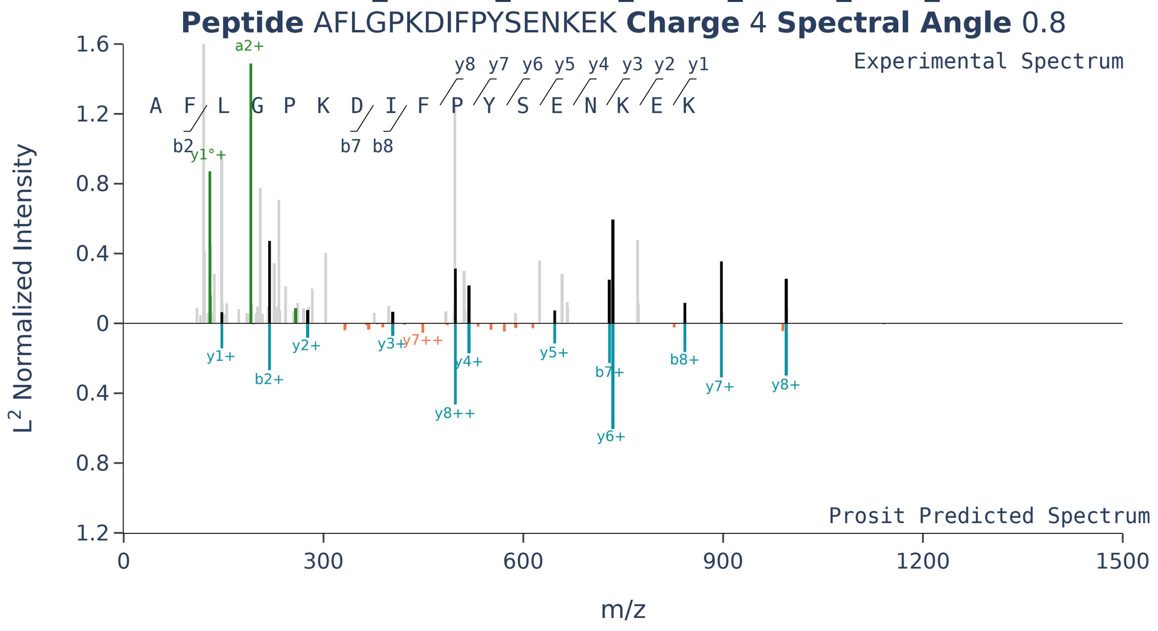


**Source** HRoetschke_271222_090123_HFGoe_LEDGF_b2R2_2h **Scan** 5912
**Peptide** AKTGVTSTSDSEEE **Charge** 2 **Spectral Angle** 0.78

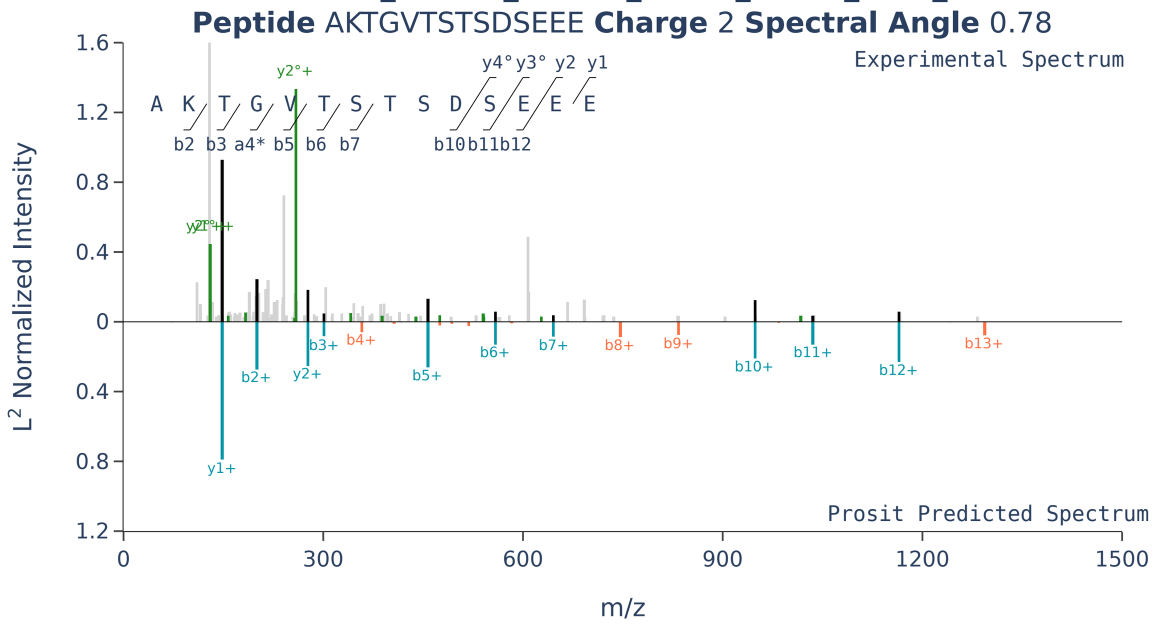


**Source** HRoetschke_271222_090123_HFGoe_LEDGF_b1R1_2h **Scan** 14779
**Peptide** QVTMQQAQKHTEMITTLKKIR **Charge** 5 **Spectral Angle** 0.82

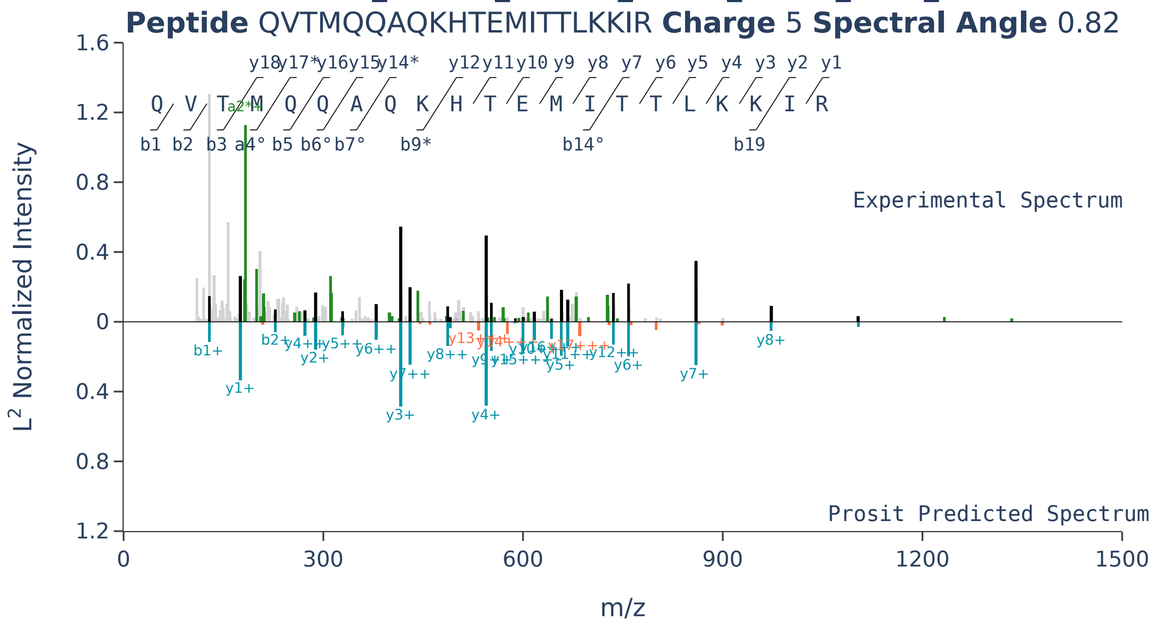


**Source** HRoetschke_271222_090123_HFGoe_LEDGF_b1R2_24h **Scan** 13117
**Peptide** SNEDVTKAVDITTPKAARR **Charge** 3 **Spectral Angle** 0.81

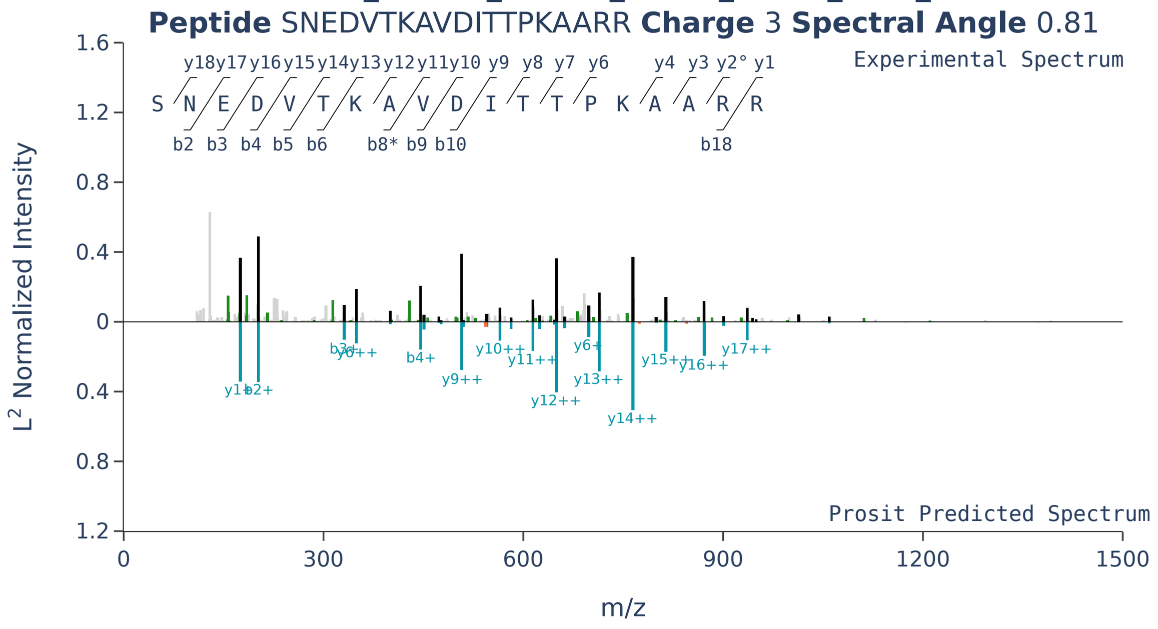


**Source** HRoetschke_271222_090123_HFGoe_LEDGF_b2R1_24h **Scan** 15855
**Peptide** FLGPKDIFPYSENKEKYGKPNKRK **Charge** 5 **Spectral Angle** 0.79

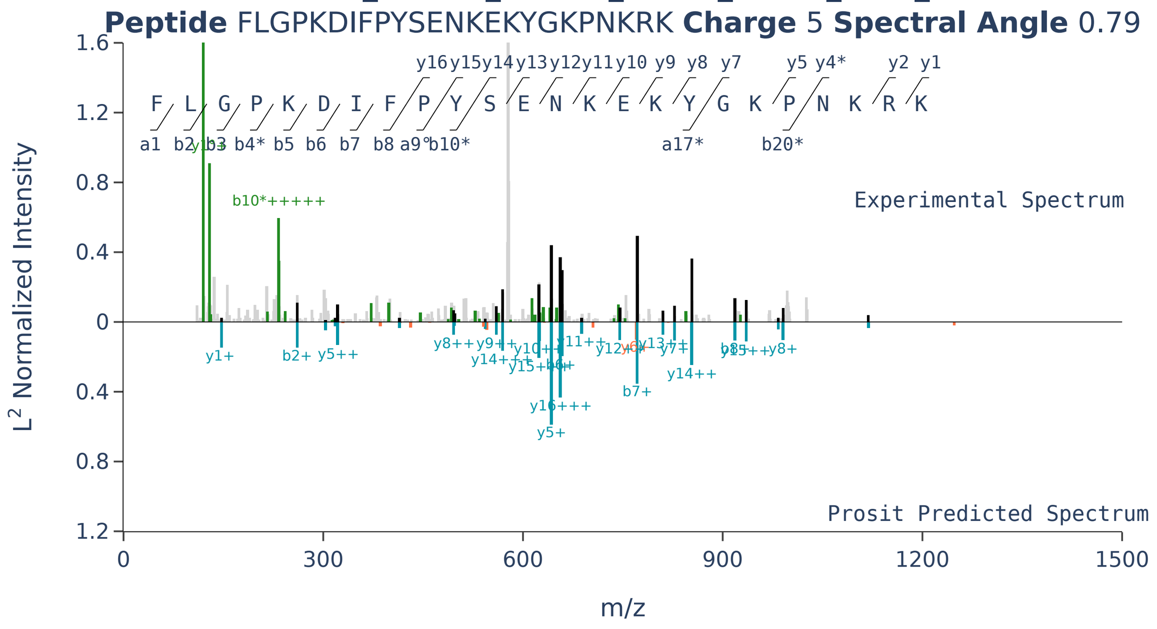


**Source** HRoetschke_271222_090123_HFGoe_LEDGF_b1R2_2h **Scan** 9130
**Peptide** IFPYSENKEKYGKPNKRKGFNE **Charge** 4 **Spectral Angle** 0.72

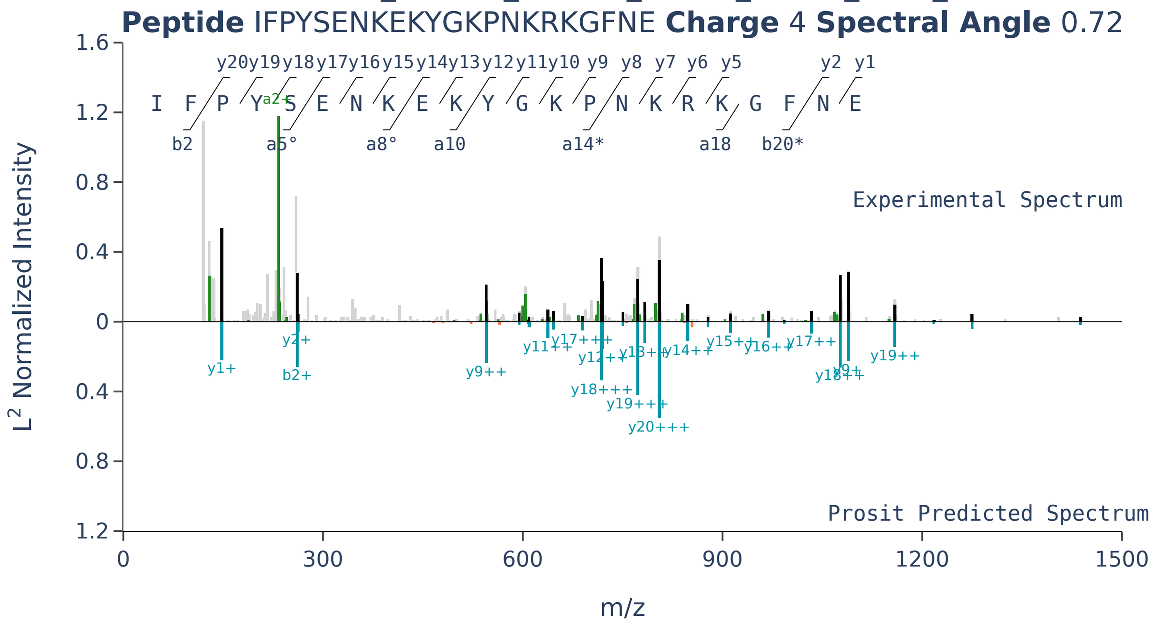


**Source** HRoetschke_271222_090123_HFGoe_LEDGF_b1R2_4h **Scan** 24687
**Peptide** GEGDSVITQVLNKS **Charge** 2 **Spectral Angle** 0.8

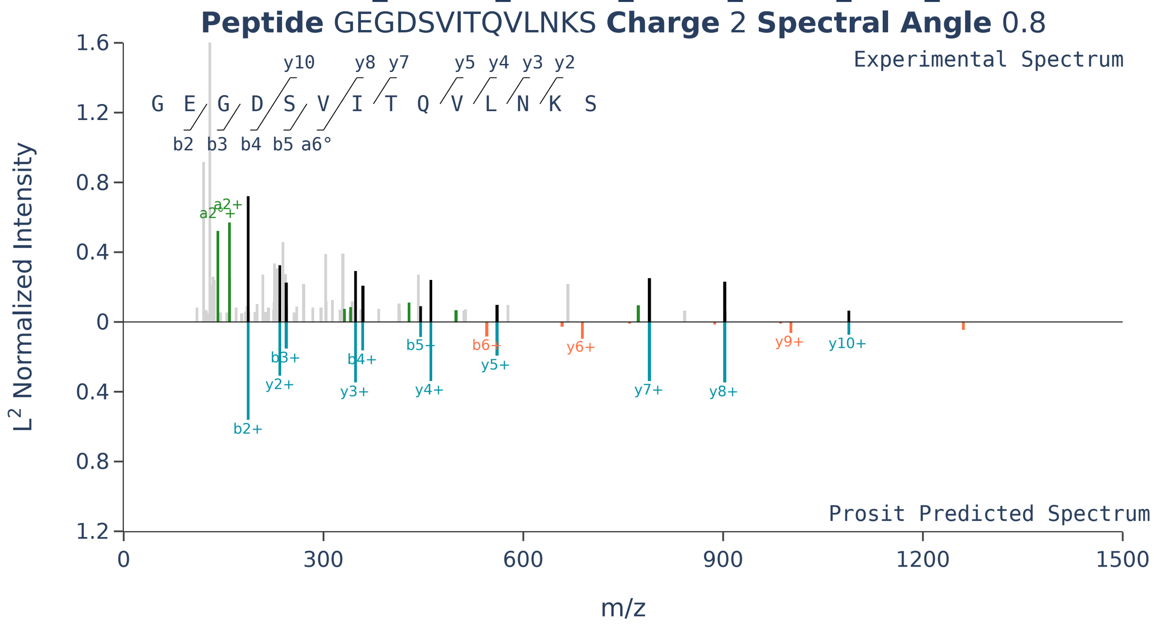

**Source** HRoetschke_271222_090123_HFGoe_LEDGF_b1R2_24h **Scan** 6421
**Peptide** DITTPKAARRG **Charge** 3 **Spectral Angle** 0.91

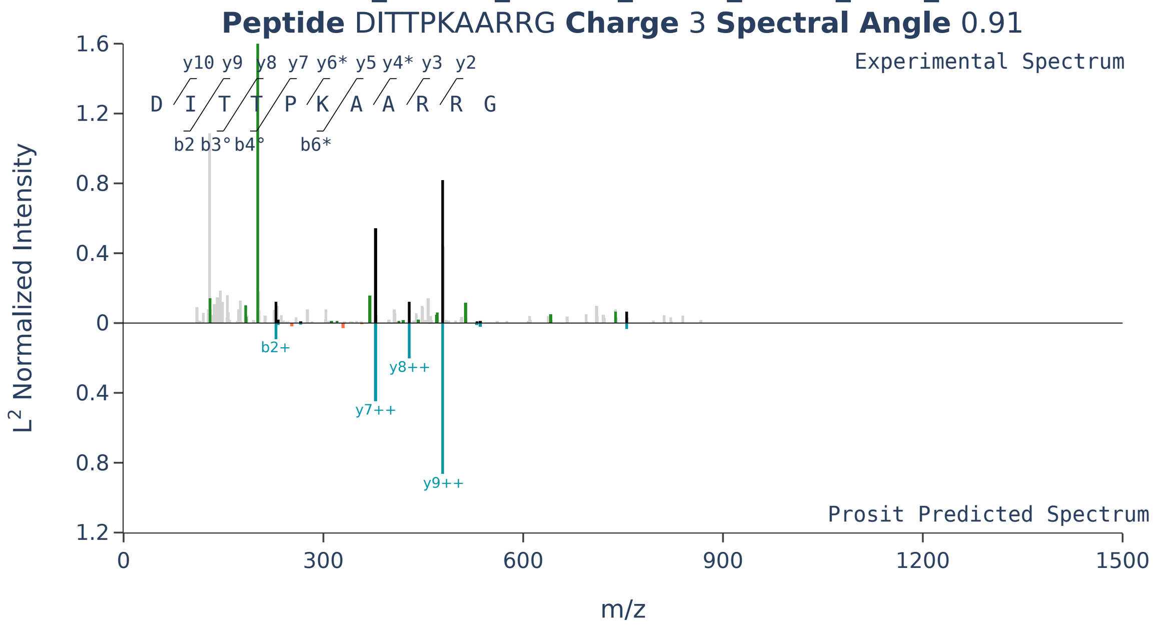


**Source** HRoetschke_271222_090123_HFGoe_LEDGF_b1R1_2h **Scan** 11104
**Peptide** KASNEDVTKAVDITTPKAARRG **Charge** 4 **Spectral Angle** 0.82

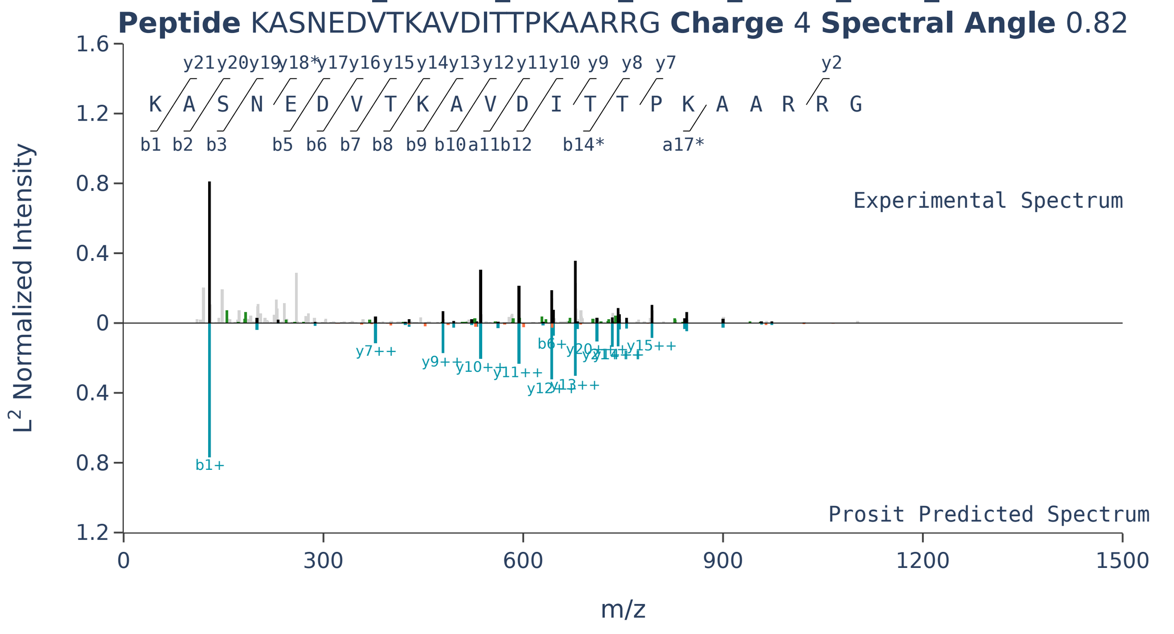


**Source** HRoetschke_271222_090123_HFGoe_LEDGF_b2R1_2h **Scan** 19340
**Peptide** WEIDNNPKVKFS **Charge** 2 **Spectral Angle** 0.81

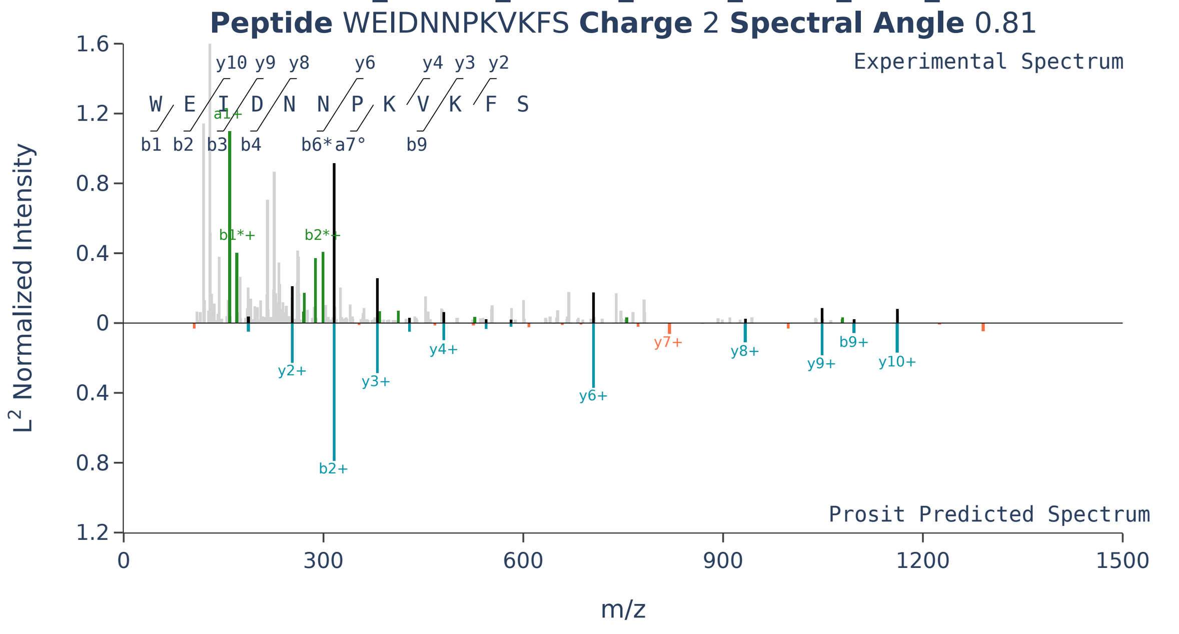


**Source** HRoetschke_271222_090123_HFGoe_LEDGF_b2R1_24h **Scan** 9703
**Peptide** EAGVVTT **Charge** 1 **Spectral Angle** 0.89

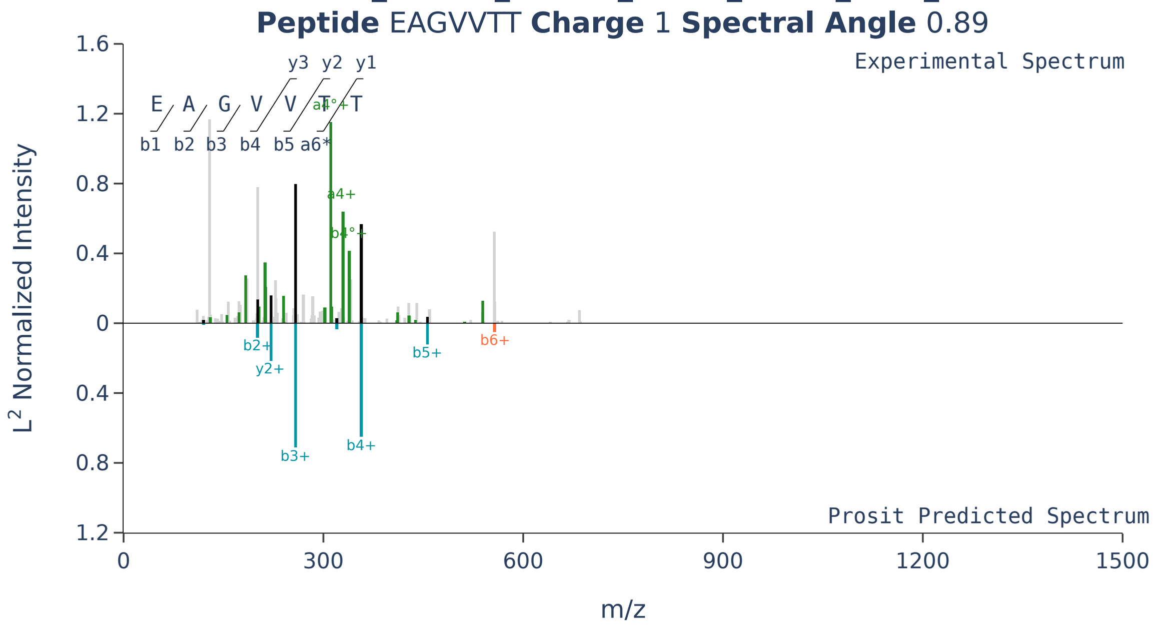


**Source** HRoetschke_271222_090123_HFGoe_LEDGF_b2R1_2h **Scan** 12401
**Peptide** LDNNPKVKFSSQQAATKQSNASSDVEVE **Charge** 4 **Spectral Angle** 0.84

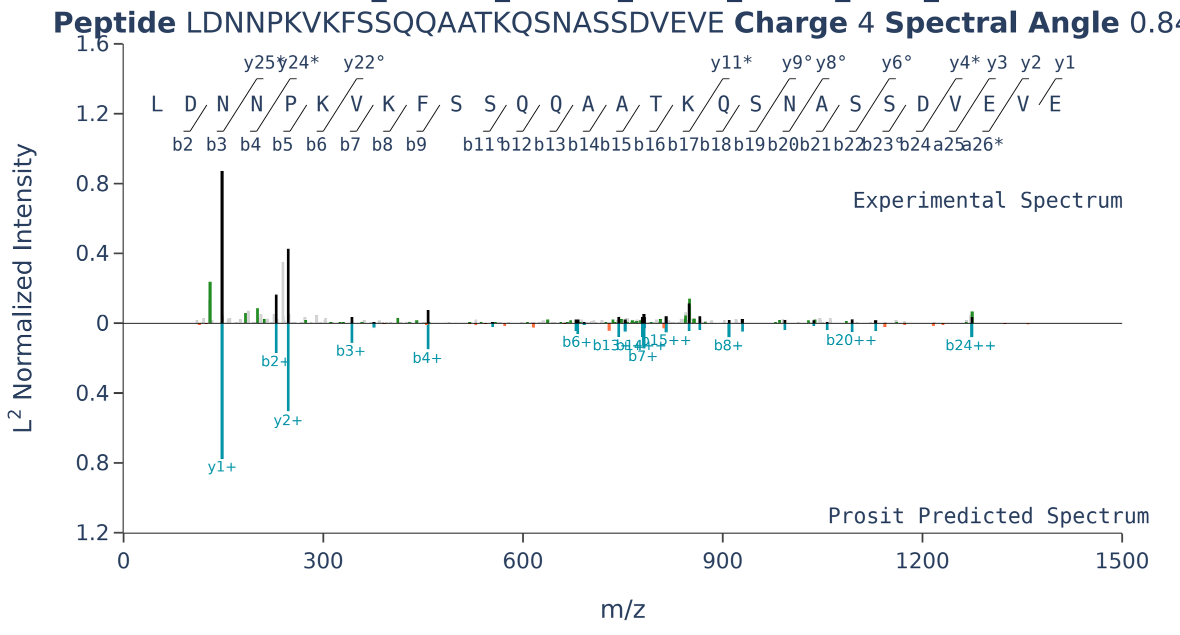


**Source** HRoetschke_271222_090123_HFGoe_LEDGF_b2R1_24h **Scan** 24459
**Peptide** AGVVTTATASVNL **Charge** 1 **Spectral Angle** 0.87

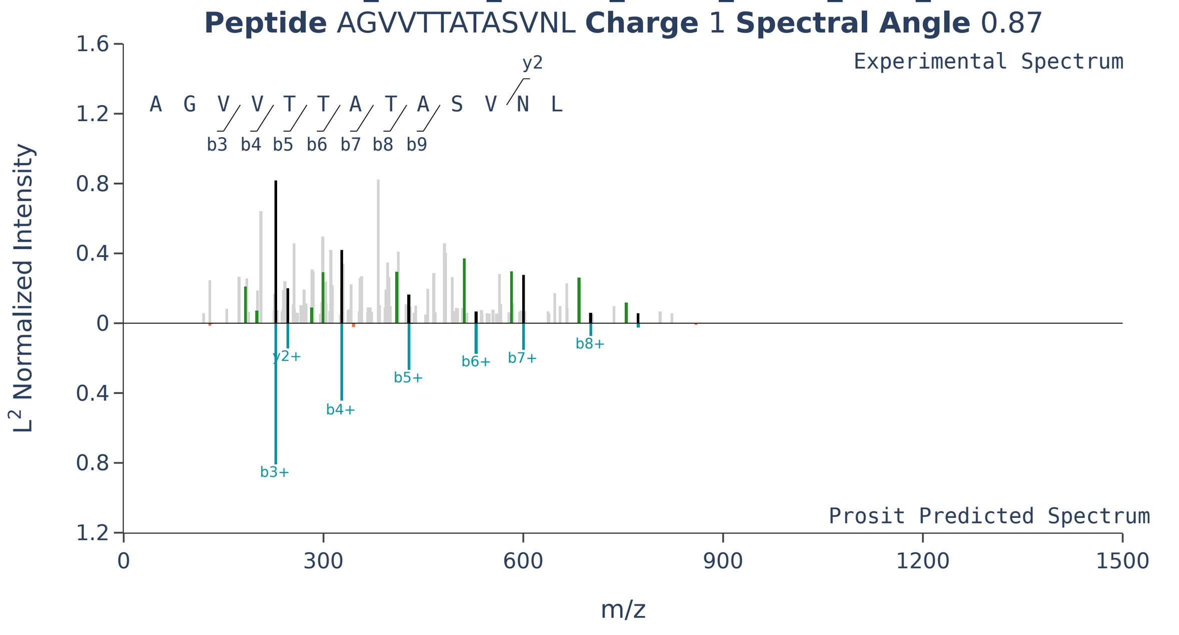


**Source** HRoetschke_271222_090123_HFGoe_LEDGF_b1R2_2h **Scan** 12104
**Peptide** EIDNNPKVKFS **Charge** 3 **Spectral Angle** 0.79

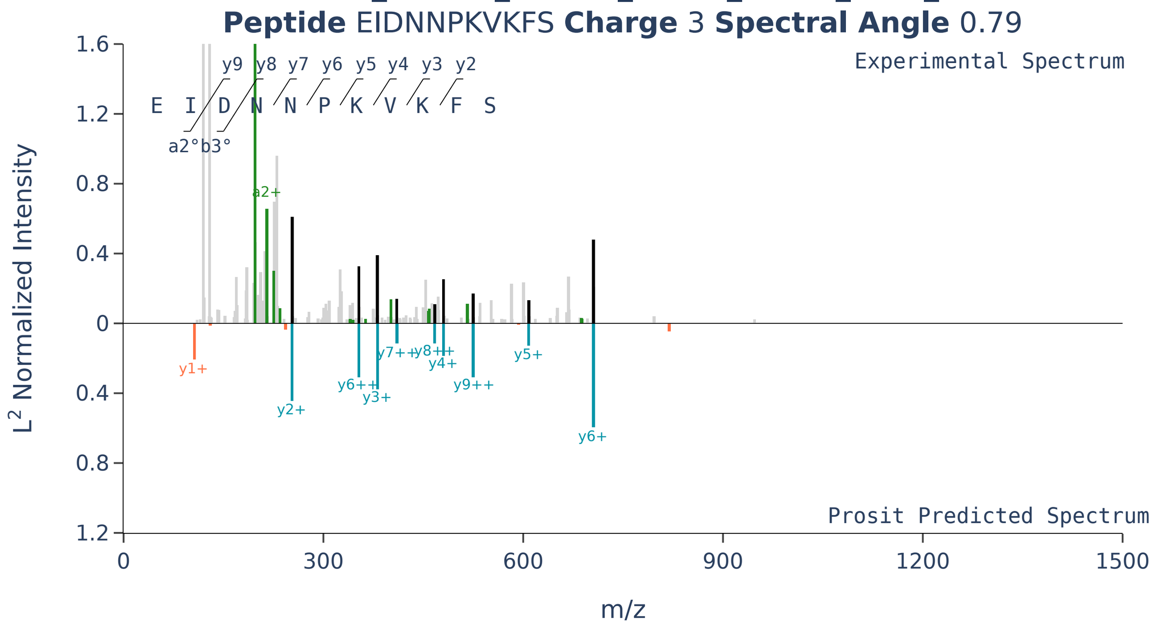


**Source** HRoetschke_271222_090123_HFGoe_LEDGF_b2R1_24h **Scan** 31245
**Peptide** NEGLWEI **Charge** 1 **Spectral Angle** 0.93

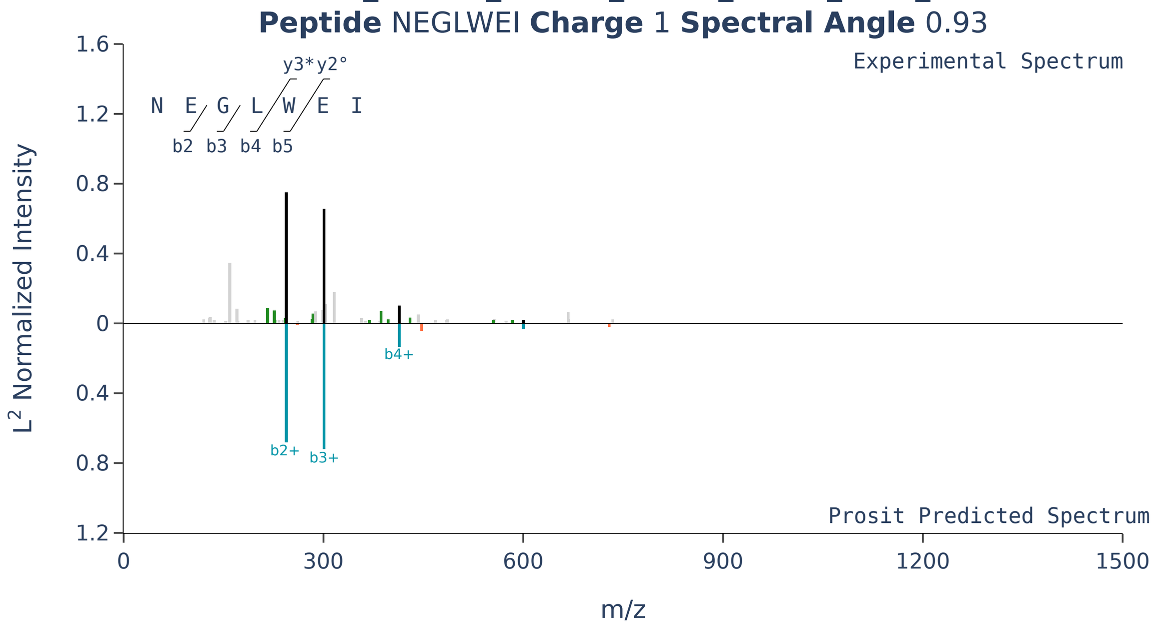


**Source** HRoetschke_271222_090123_HFGoe_LEDGF_b2R2_24h **Scan** 12391
**Peptide** KAEKQVETEEAGVVTTATA **Charge** 3 **Spectral Angle** 0.81

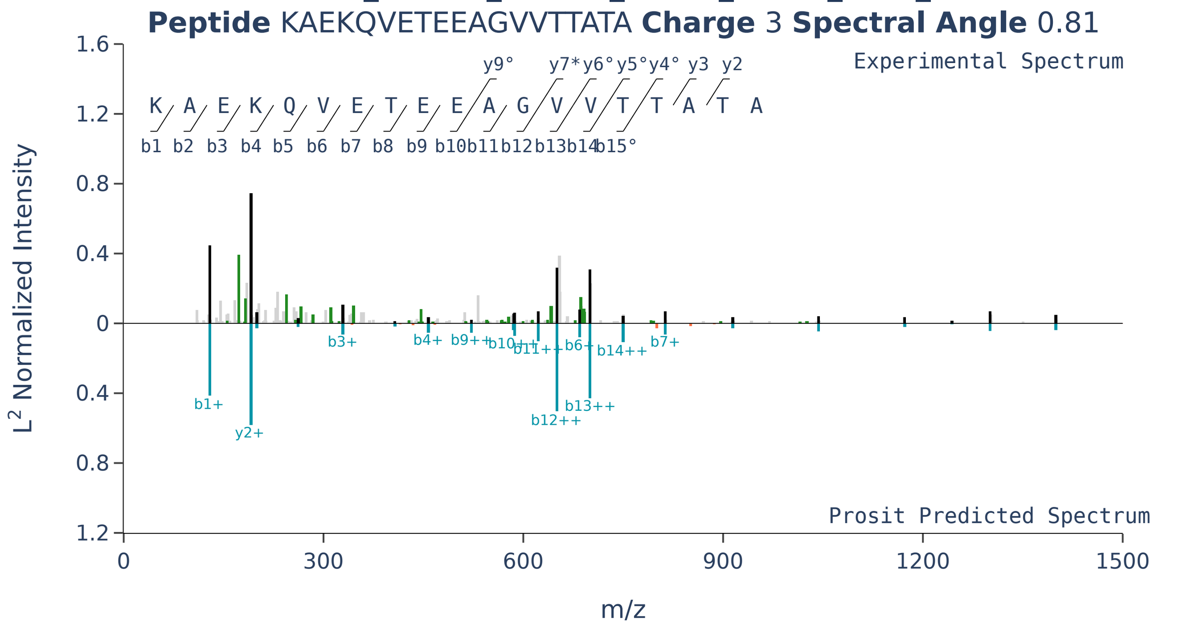


**Source** HRoetschke_271222_090123_HFGoe_LEDGF_b1R1_2h **Scan** 22026
**Peptide** TRDFKPGDLIFAKMK **Charge** 4 **Spectral Angle** 0.81

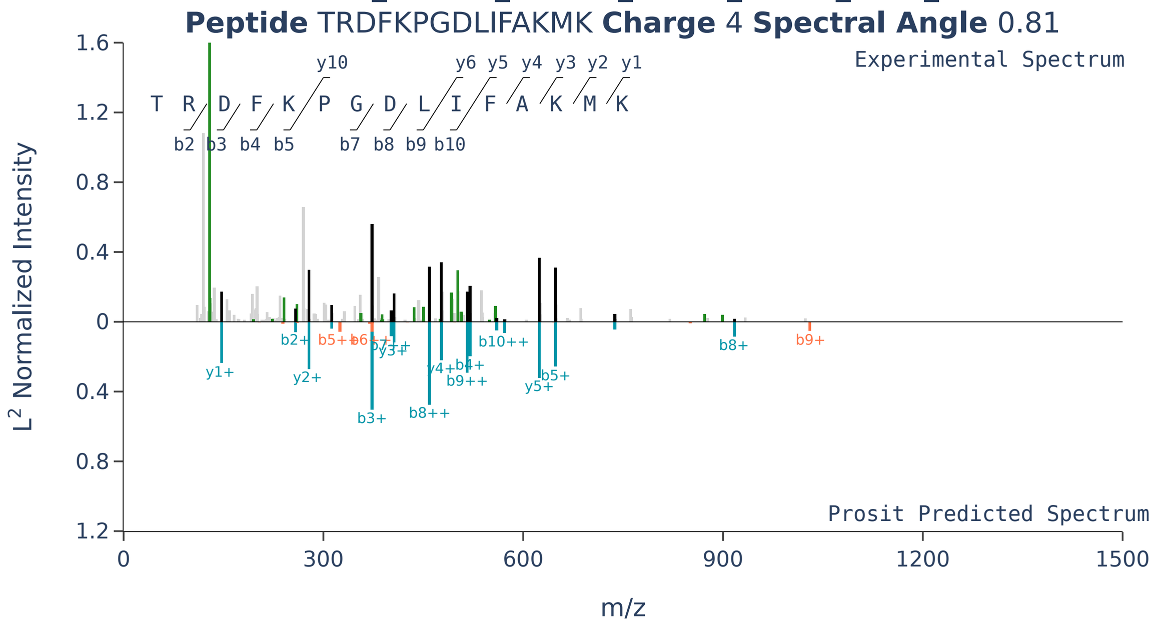


**Source** HRoetschke_271222_090123_HFGoe_LEDGF_b1R1_24h **Scan** 23277
**Peptide** GDSVITQVLNKS **Charge** 2 **Spectral Angle** 0.81

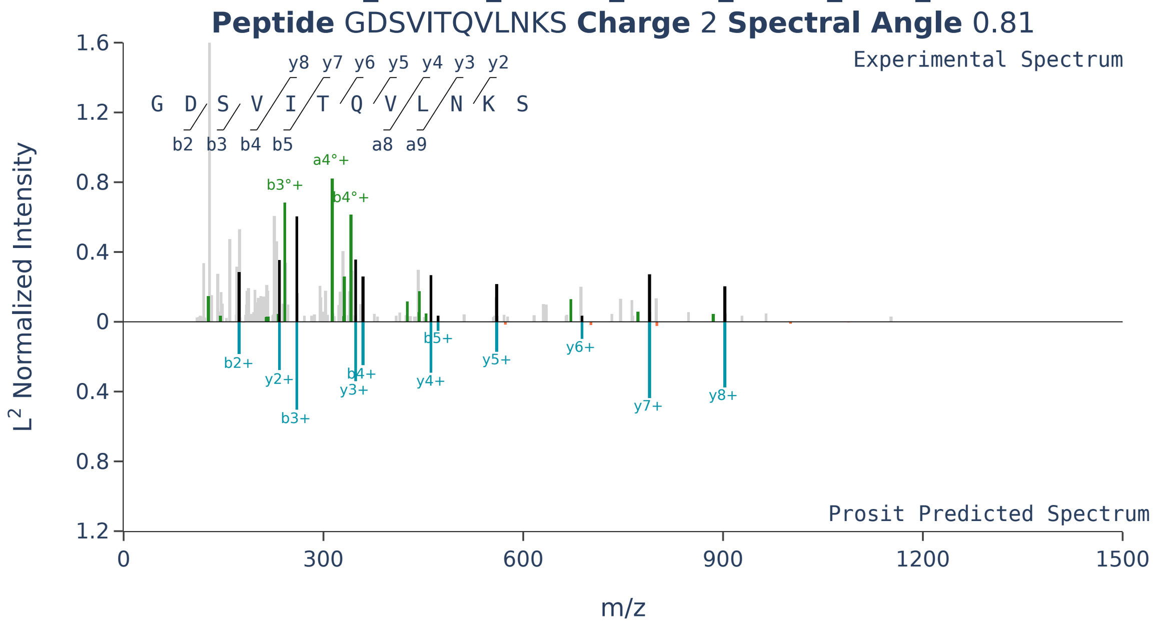


**Source** HRoetschke_271222_090123_HFGoe_LEDGF_b2R2_2h **Scan** 7176
**Peptide** IFPYSENKEKYGKPNKRK **Charge** 3 **Spectral Angle** 0.83

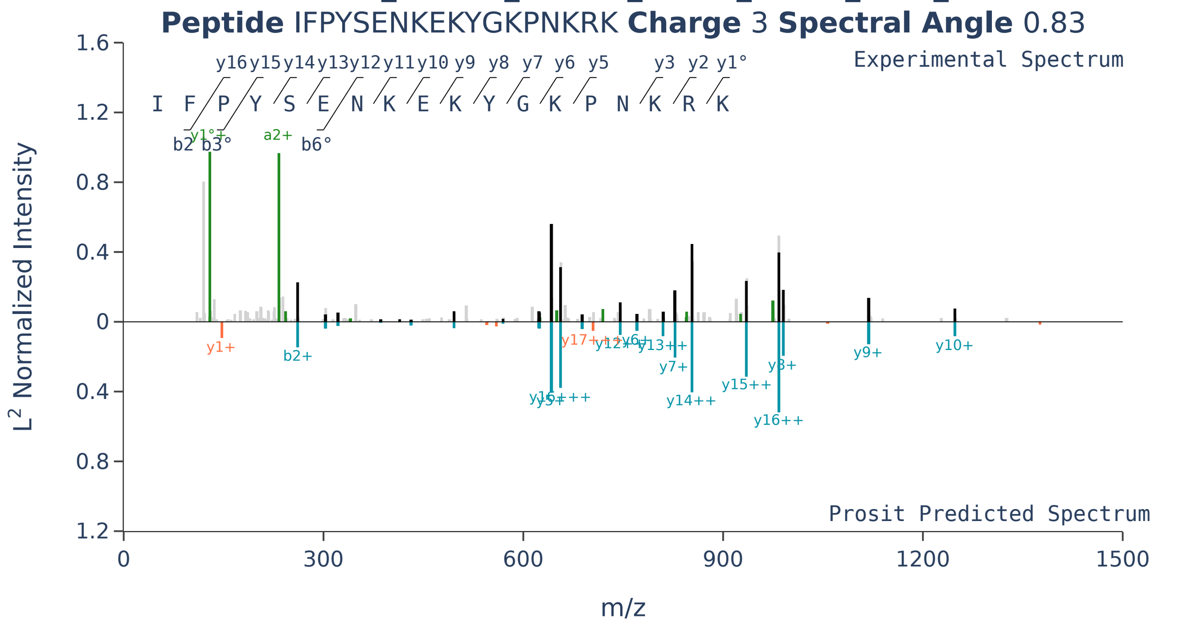


**Source** HRoetschke_271222_090123_HFGoe_LEDGF_b2R1_1h **Scan** 21954
**Peptide** RDFKPGDLIFAKMKG **Charge** 4 **Spectral Angle** 0.81

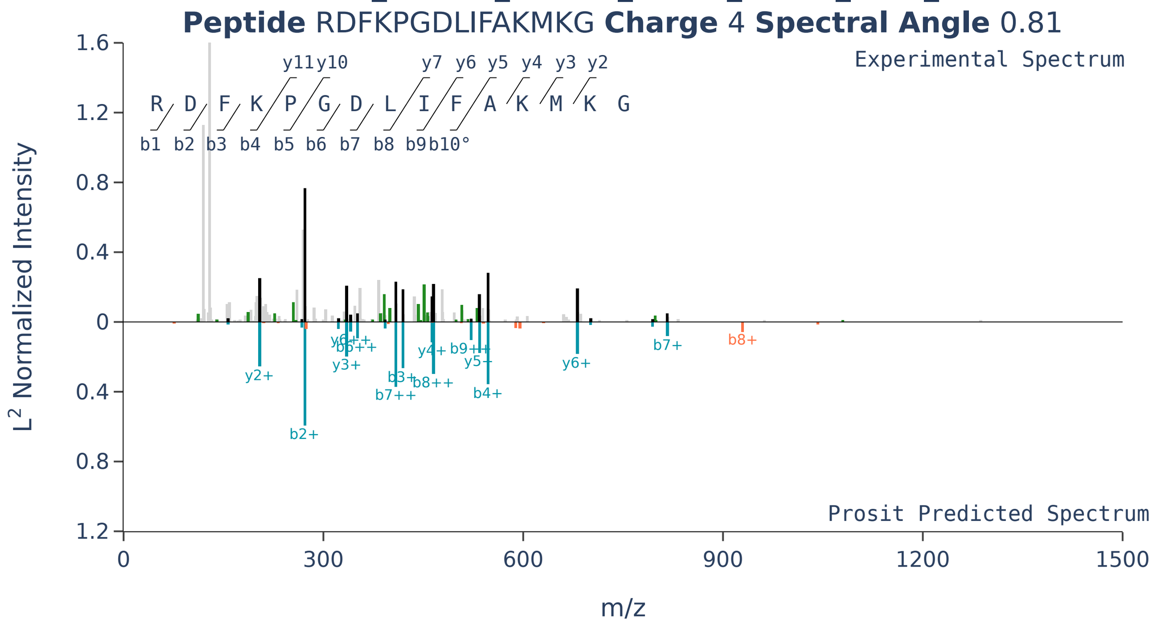


**Source** HRoetschke_271222_090123_HFGoe_LEDGF_b1R1_4h **Scan** 26333
**Peptide** FLVGEGDSVITQ **Charge** 1 **Spectral Angle** 0.88

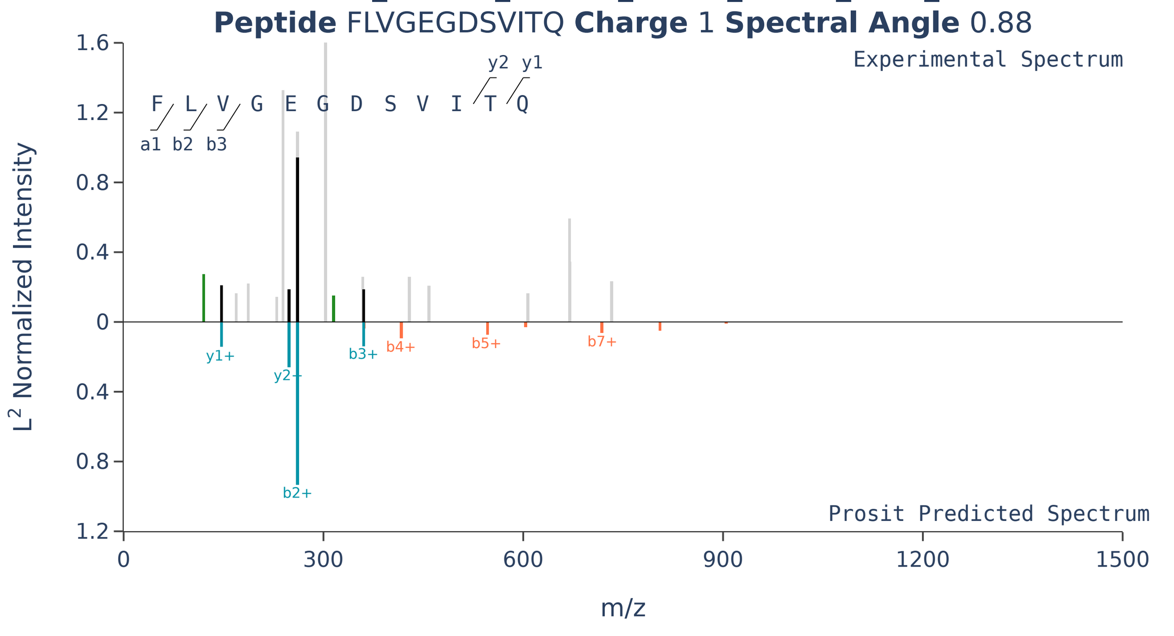


**Source** HRoetschke_271222_090123_HFGoe_LEDGF_b1R1_24h **Scan** 7501
**Peptide** TKAVDITTPKAARR **Charge** 4 **Spectral Angle** 0.81

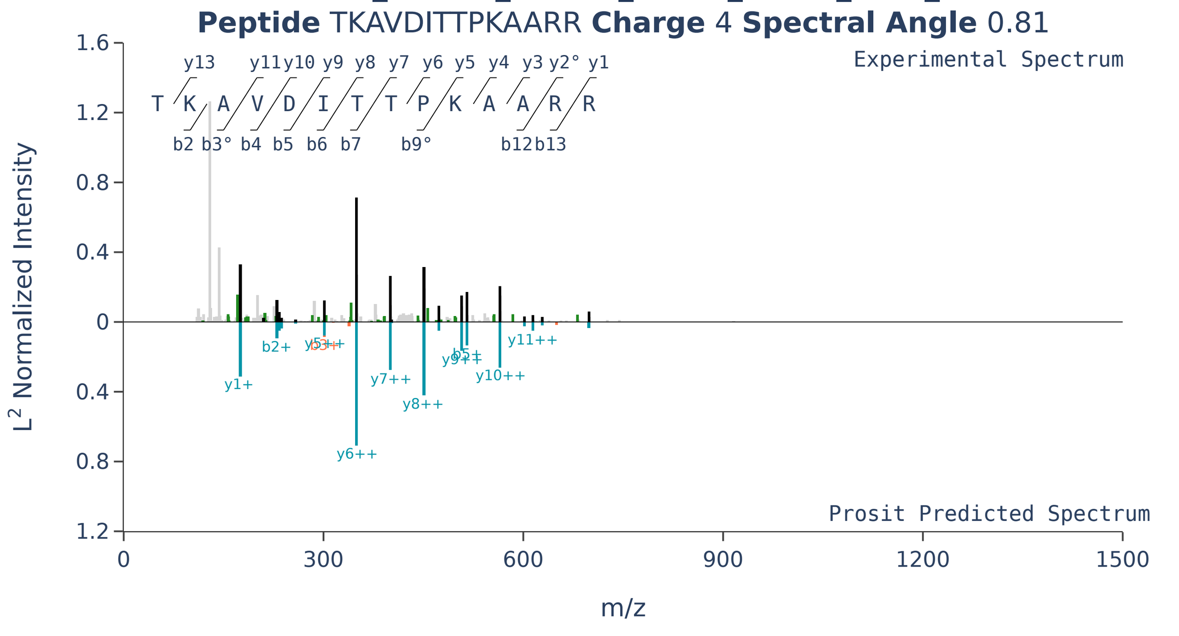

**Source** HRoetschke_271222_090123_HFGoe_LEDGF_b1R2_4h **Scan** 9890
**Peptide** SVNLKVSPKRGRPAATEV **Charge** 3 **Spectral Angle** 0.82

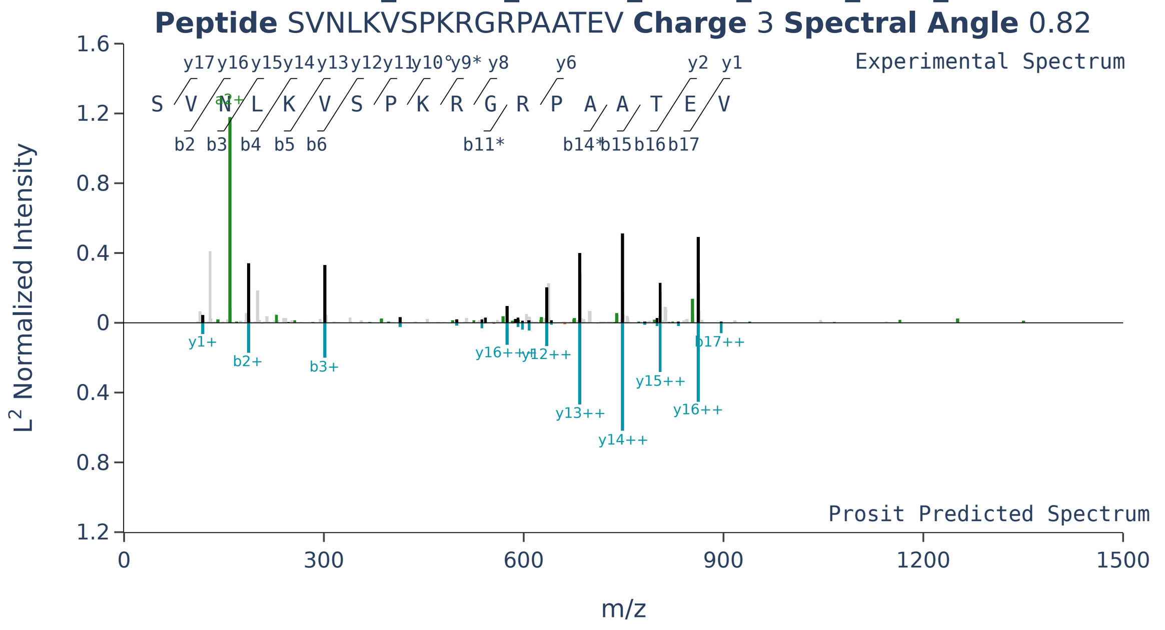


**Source** HRoetschke_271222_090123_HFGoe_LEDGF_b1R2_24h **Scan** 9269
**Peptide** QAQKHTEMITTLKKIRR **Charge** 3 **Spectral Angle** 0.86

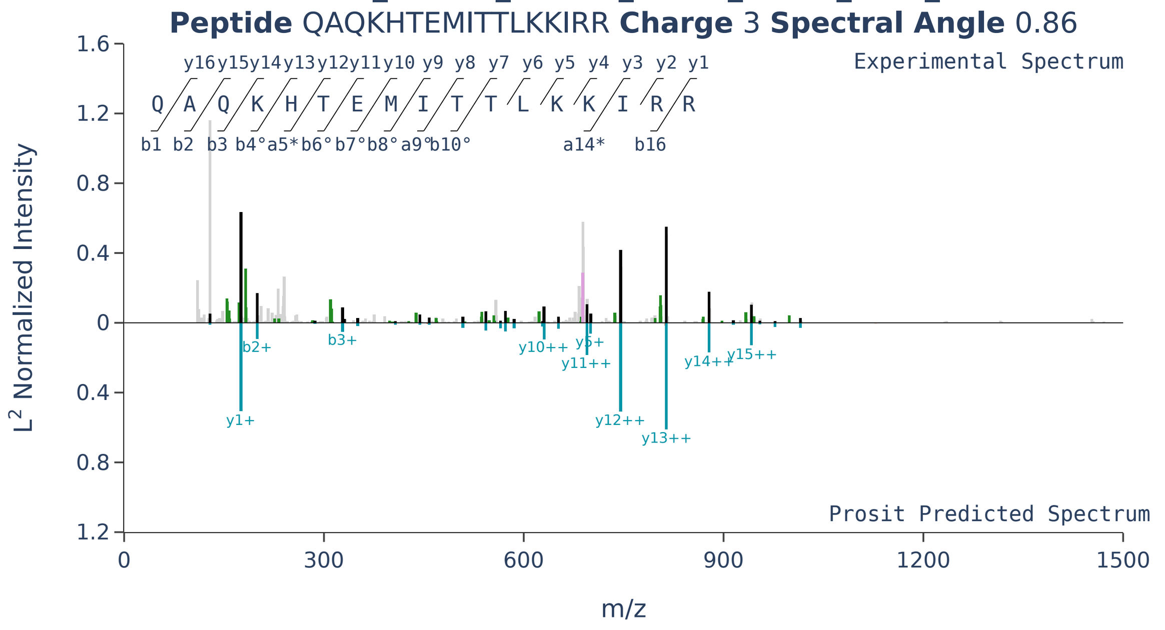


**Source** HRoetschke_271222_090123_HFGoe_LEDGF_b1R1_2h **Scan** 25907
**Peptide** DELASLQVTMQQ **Charge** 2 **Spectral Angle** 0.81

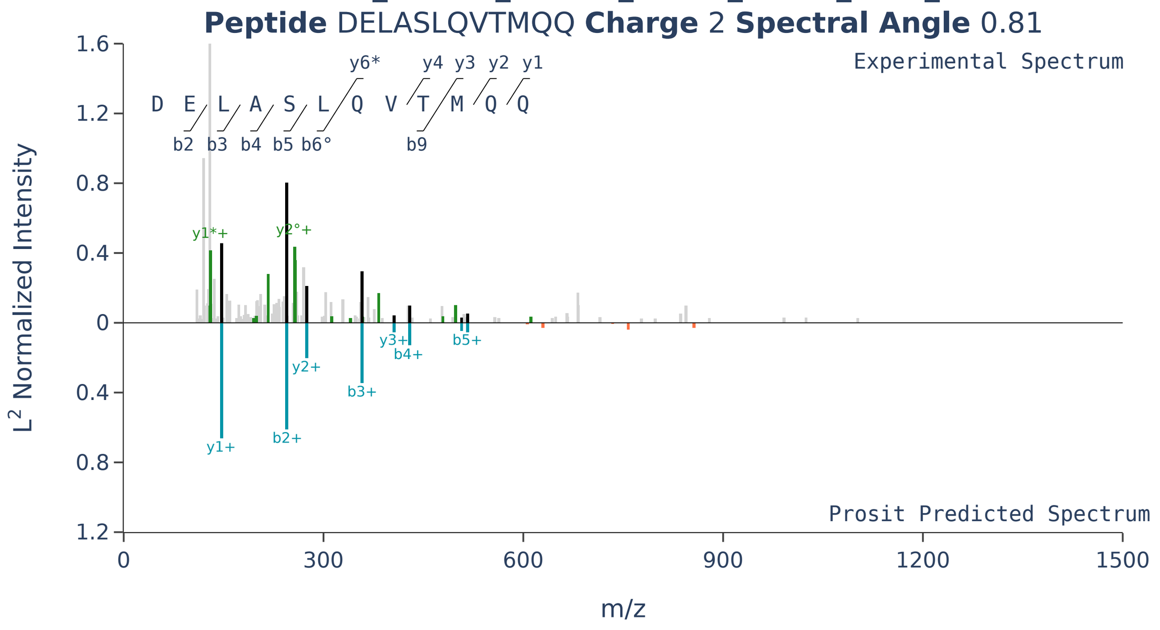


**Source** HRoetschke_271222_090123_HFGoe_LEDGF_b1R2_24h **Scan** 5590
**Peptide** PNKKLEKEQTGSKTLNGGSDAQD **Charge** 3 **Spectral Angle** 0.71

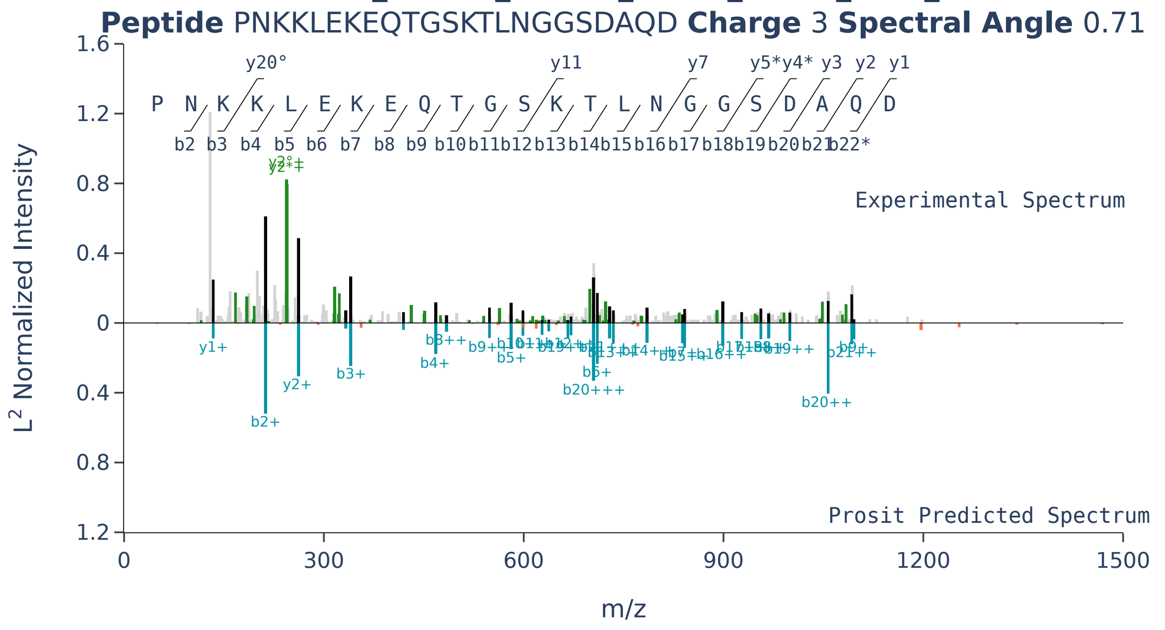


**Source** HRoetschke_271222_090123_HFGoe_LEDGF_b2R2_24h **Scan** 16936
**Peptide** RLHAEIKNSLKIDNL **Charge** 4 **Spectral Angle** 0.82

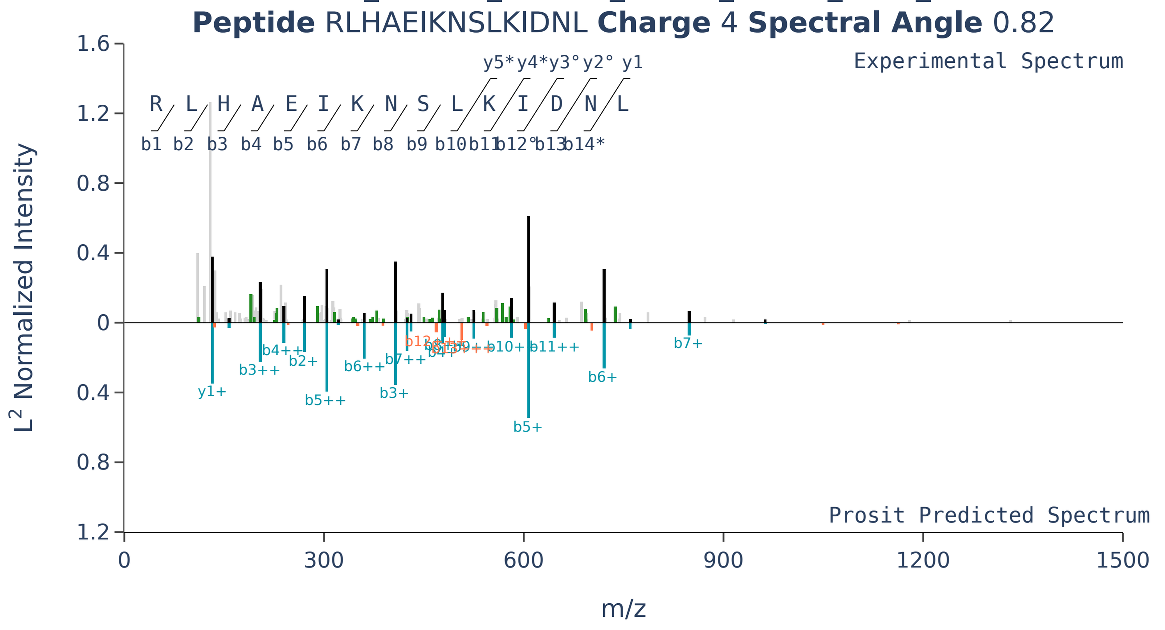


**Source** HRoetschke_271222_090123_HFGoe_LEDGF_b2R1_2h **Scan** 20962
**Peptide** AFLGPKDIFPYSENKEKYGKPN **Charge** 4 **Spectral Angle** 0.81

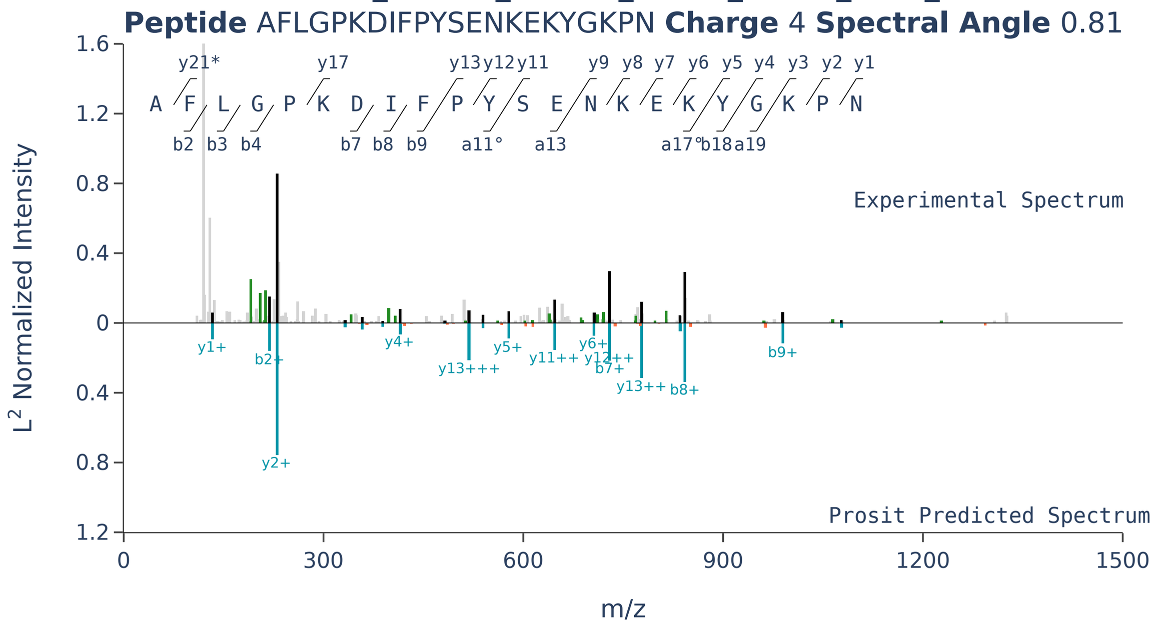


**Source** HRoetschke_271222_090123_HFGoe_LEDGF_b2R2_24h **Scan** 17794
**Peptide** TEEAGVVTTATASVNLKVSPKRGRPAATEV **Charge** 4 **Spectral Angle** 0.79

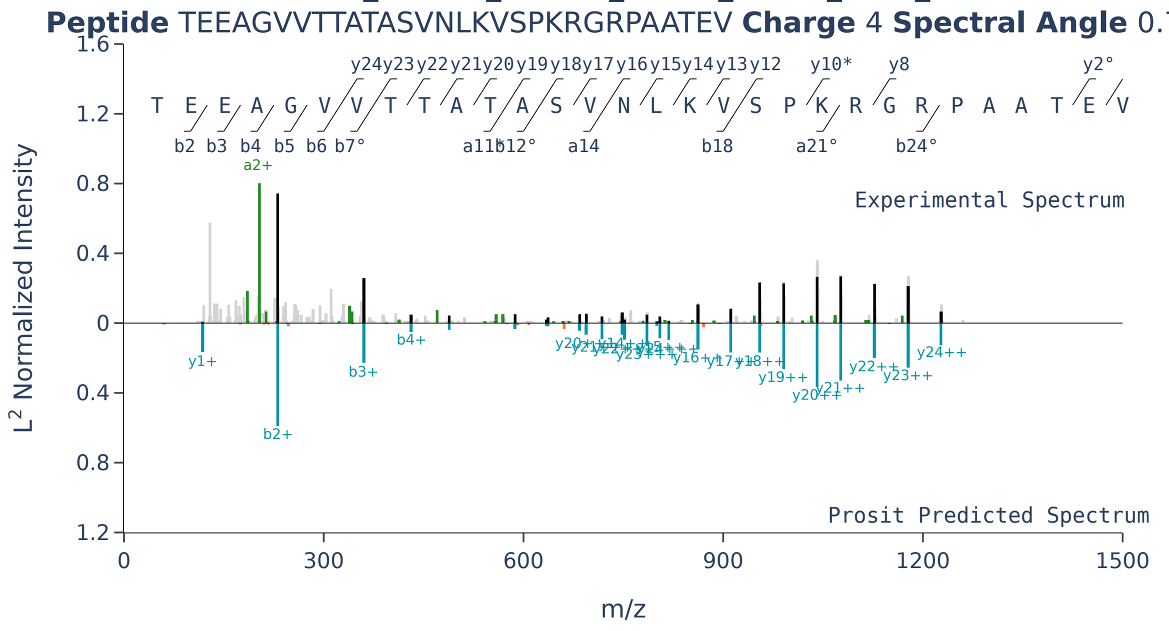


**Source** HRoetschke_271222_090123_HFGoe_LEDGF_b2R1_24h **Scan** 12560
**Peptide** DNNPKVKFSSQQAATKQSNASSDVEVEE **Charge** 4 **Spectral Angle** 0.84

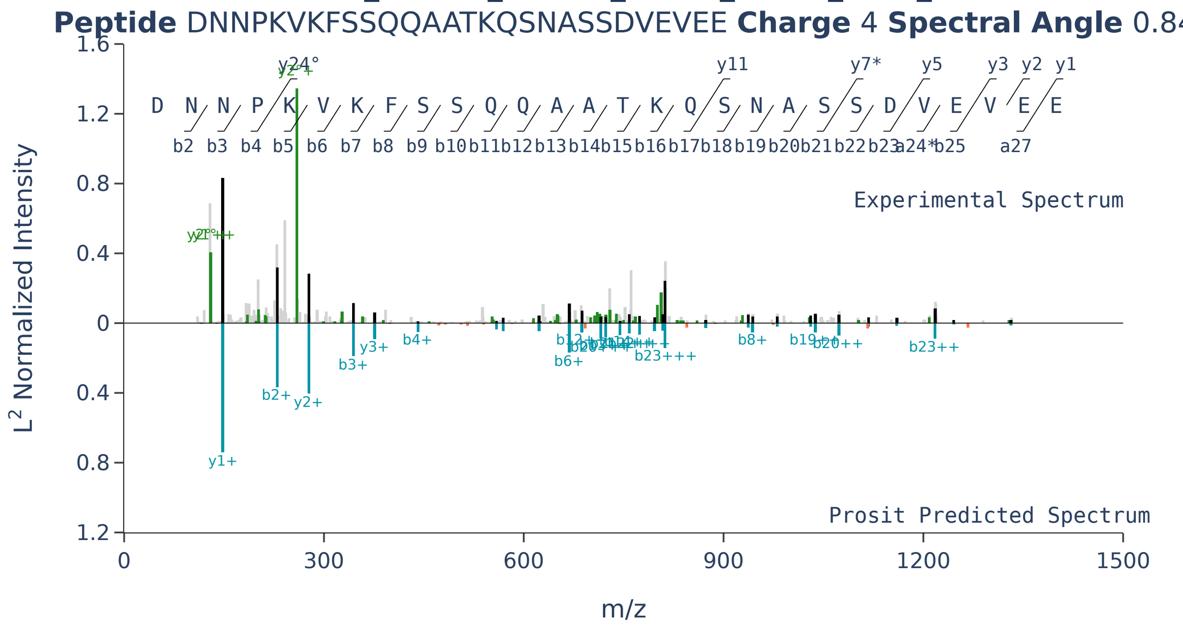


**Source** HRoetschke_271222_090123_HFGoe_LEDGF_b1R1_4h **Scan** 8347
**Peptide** GPKDIFPYSENKEKYGKPNKRK **Charge** 4 **Spectral Angle** 0.78

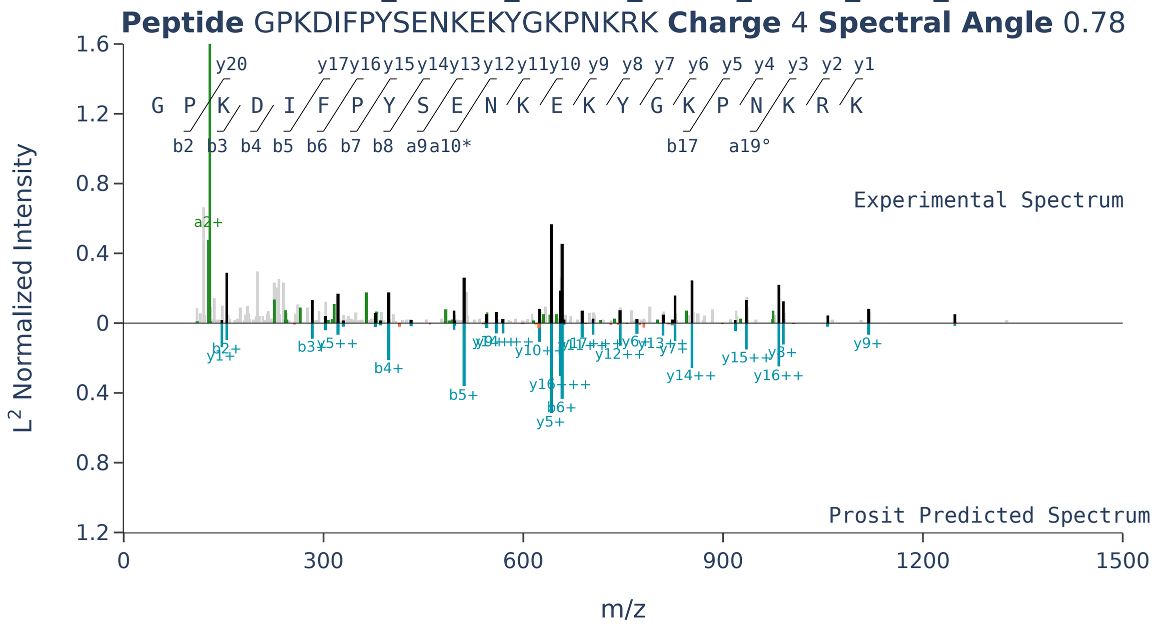


**Source** HRoetschke_271222_090123_HFGoe_LEDGF_b1R2_24h **Scan** 11191
**Peptide** EAGVVTTAT **Charge** 1 **Spectral Angle** 0.69

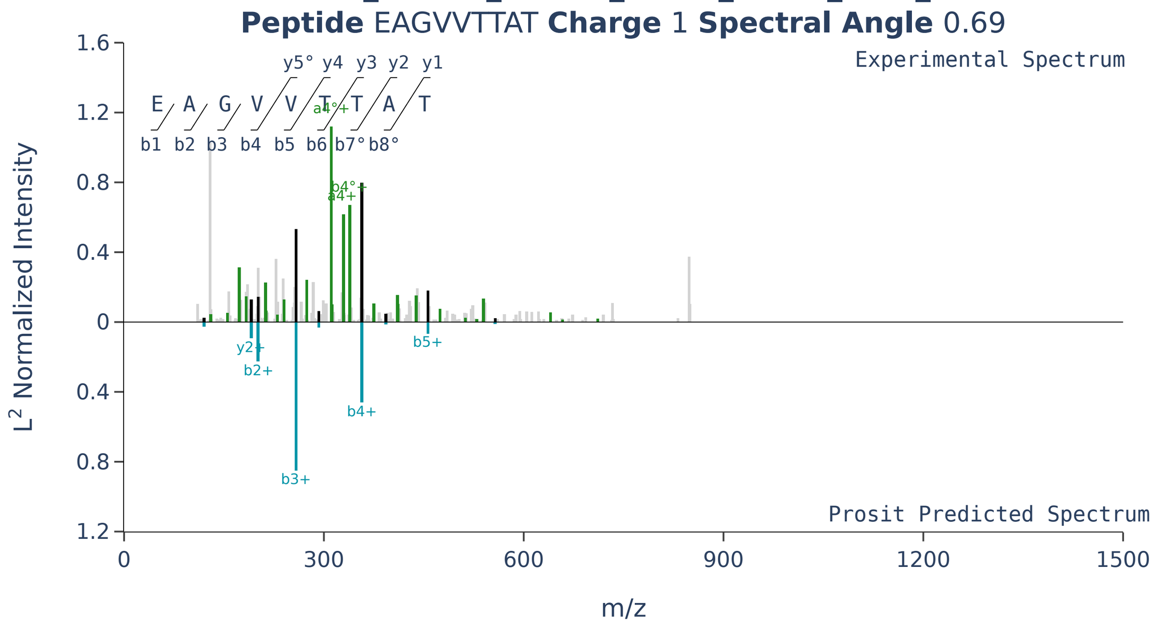


**Source** HRoetschke_271222_090123_HFGoe_LEDGF_b1R1_4h **Scan** 21558
**Peptide** LWEIDNNPKVKFSSQQA **Charge** 3 **Spectral Angle** 0.86

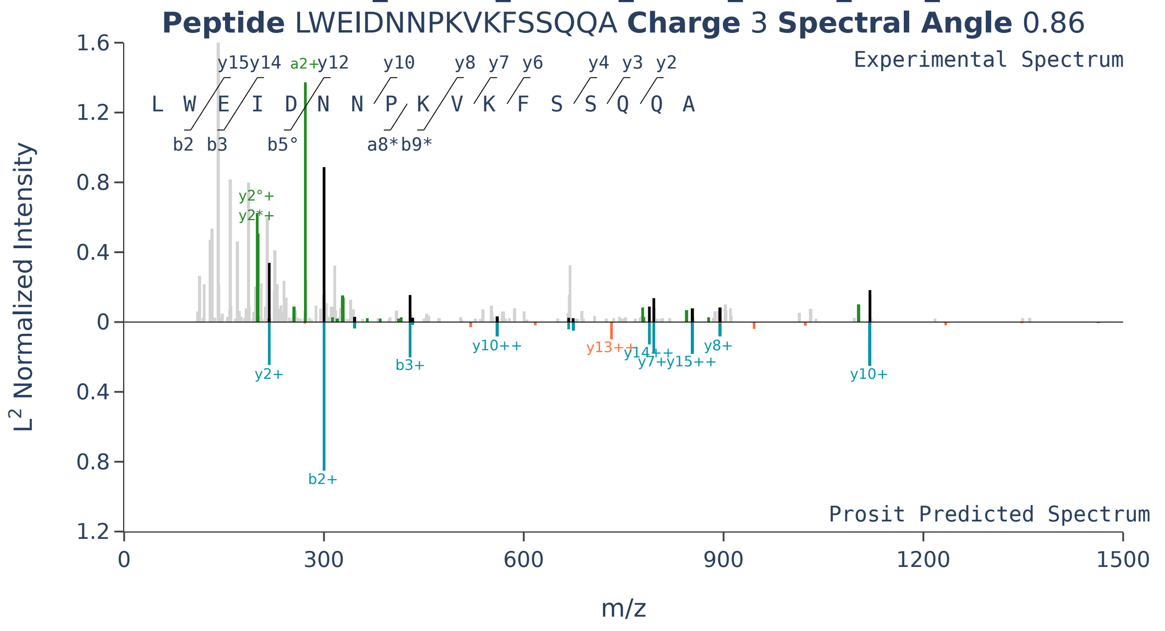


**Source** HRoetschke_271222_090123_HFGoe_LEDGF_b2R1_24h **Scan** 21378
**Peptide** GTHETAFLGPKDIFPYSENKEK **Charge** 4 **Spectral Angle** 0.8

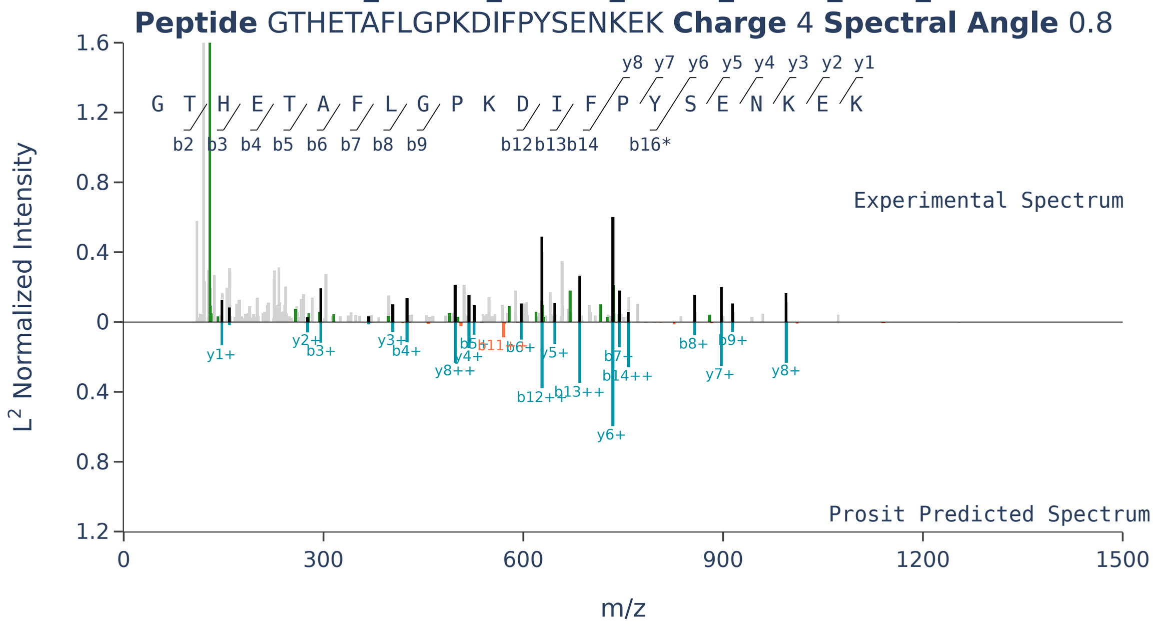


**Source** HRoetschke_271222_090123_HFGoe_LEDGF_b1R1_2h **Scan** 28792
**Peptide** AVKPPTNKLPIFFFGTHET **Charge** 4 **Spectral Angle** 0.78

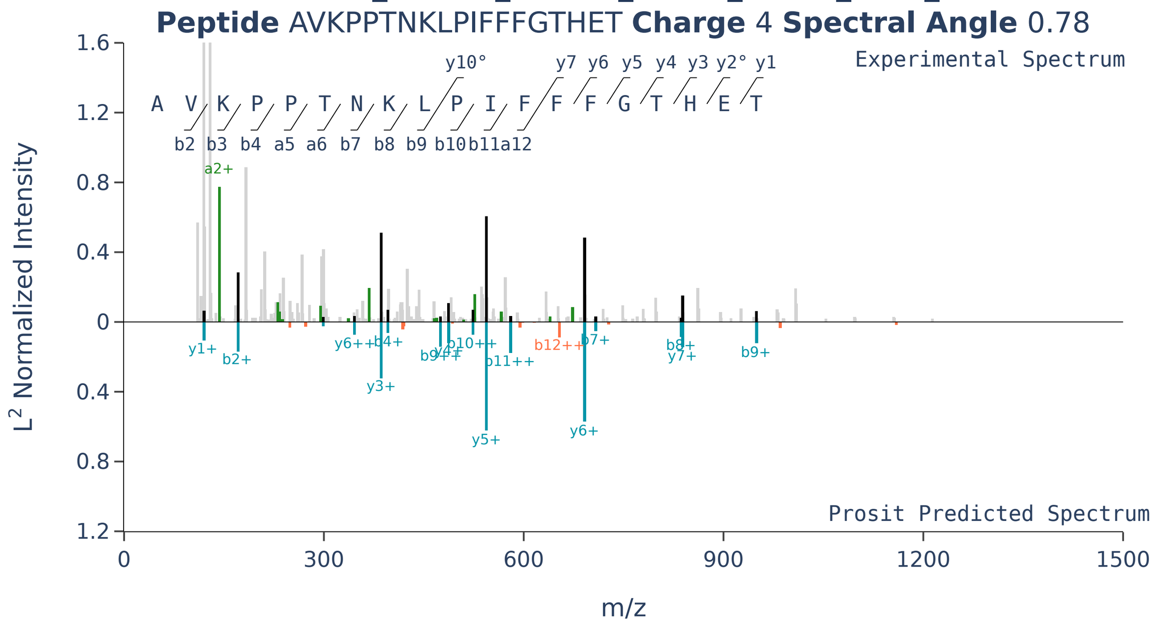


**Source** HRoetschke_271222_090123_HFGoe_LEDGF_b1R1_2h **Scan** 26562
**Peptide** VGEGDSVITQVLNKSLAEQRQHE **Charge** 3 **Spectral Angle** 0.79

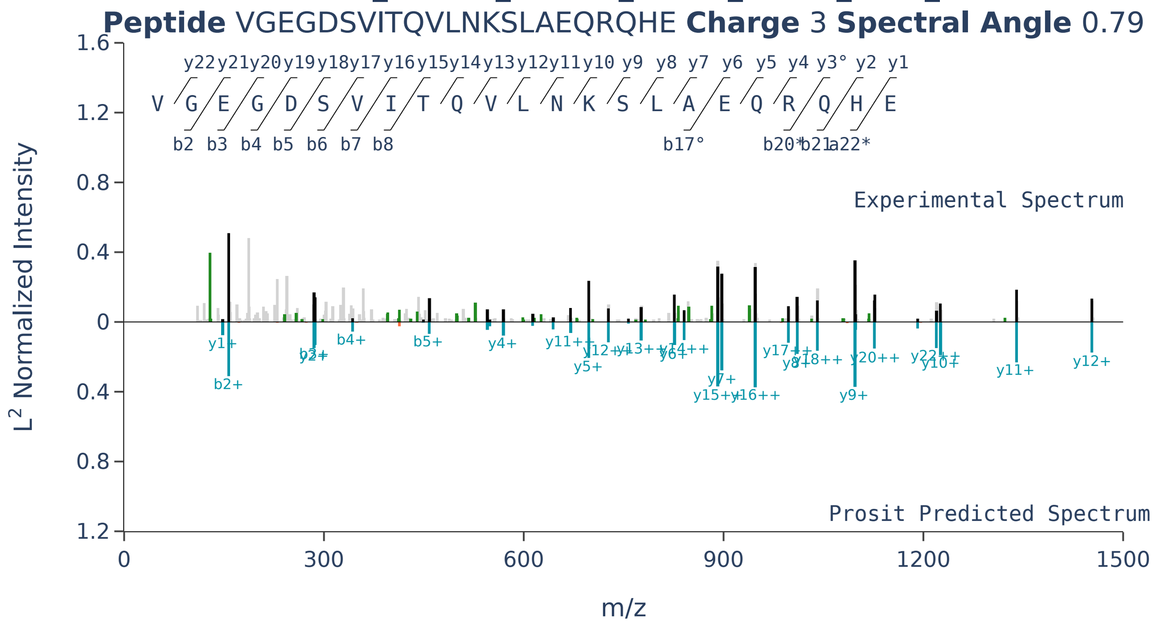


**Source** HRoetschke_271222_090123_HFGoe_LEDGF_b1R1_2h **Scan** 18117
**Peptide** GPGMTRDFKPGDL **Charge** 2 **Spectral Angle** 0.78

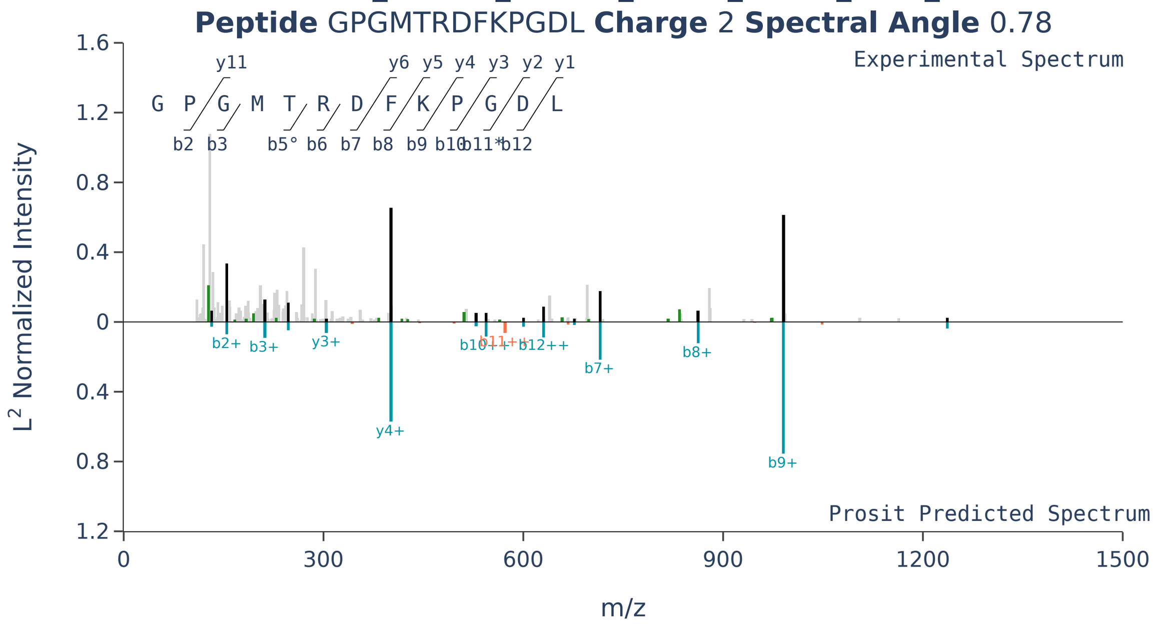

**Source** HRoetschke_271222_090123_HFGoe_LEDGF_b2R1_24h **Scan** 13729
**Peptide** AEKQVETEEAGVVTTAT **Charge** 2 **Spectral Angle** 0.8

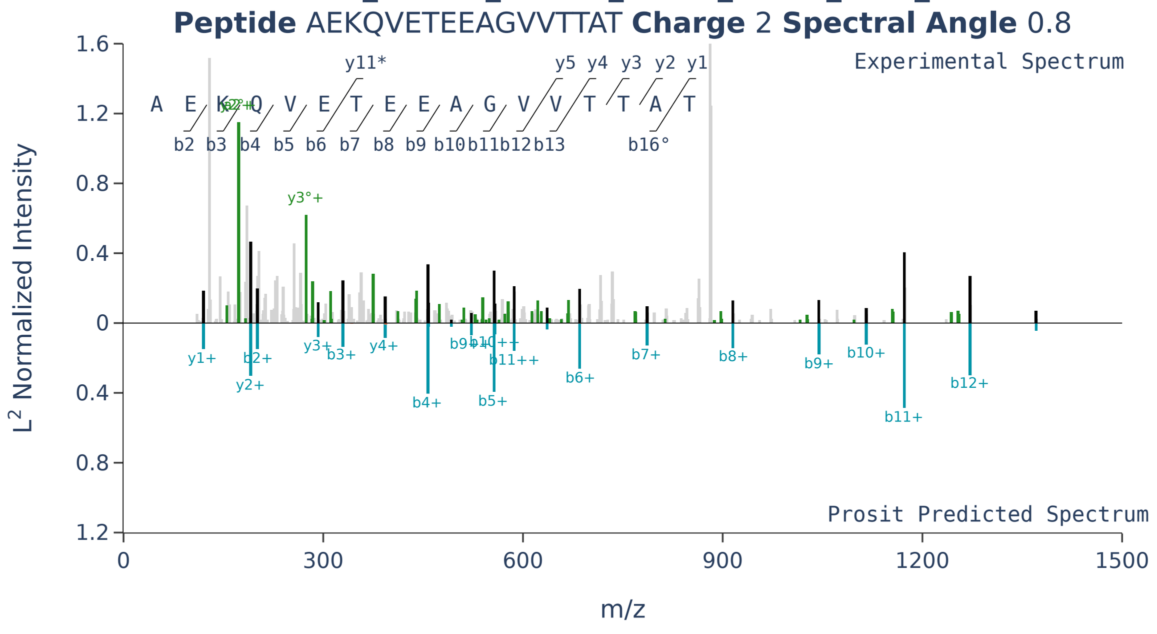

**Source** HRoetschke_271222_090123_HFGoe_LEDGF_b2R1_24h **Scan** 18201
**Peptide** TEEAGVVTTATASVNLKVSPKRG **Charge** 3 **Spectral Angle** 0.78

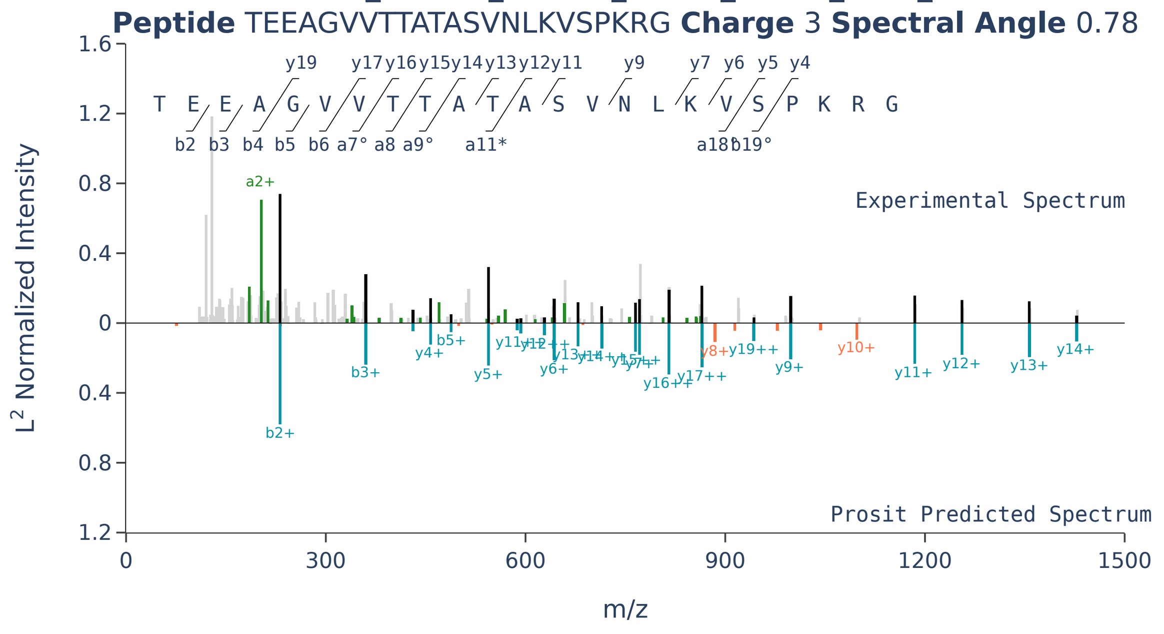

**Source** HRoetschke_271222_090123_HFGoe_LEDGF_b2R1_4h **Scan** 5759
**Peptide** TSVSKEDTDHEEKASNEDVTKA **Charge** 3 **Spectral Angle** 0.84

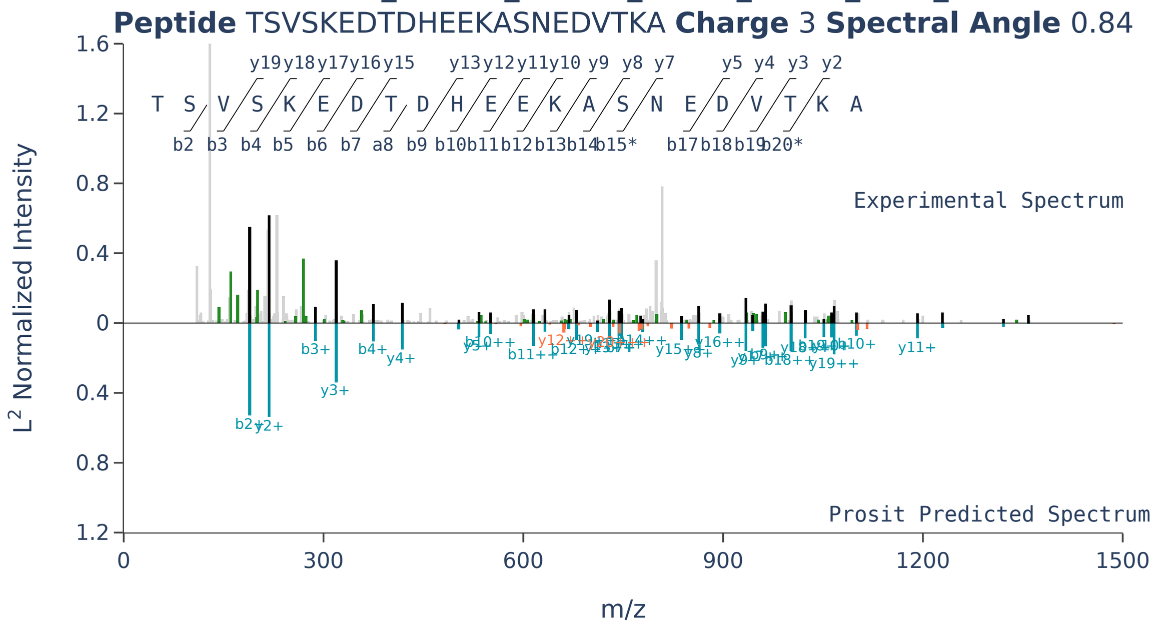

**Source** HRoetschke_271222_090123_HFGoe_LEDGF_b1R1_4h **Scan** 20310
**Peptide** LGPKDIFPYSENKEKYG **Charge** 4 **Spectral Angle** 0.72

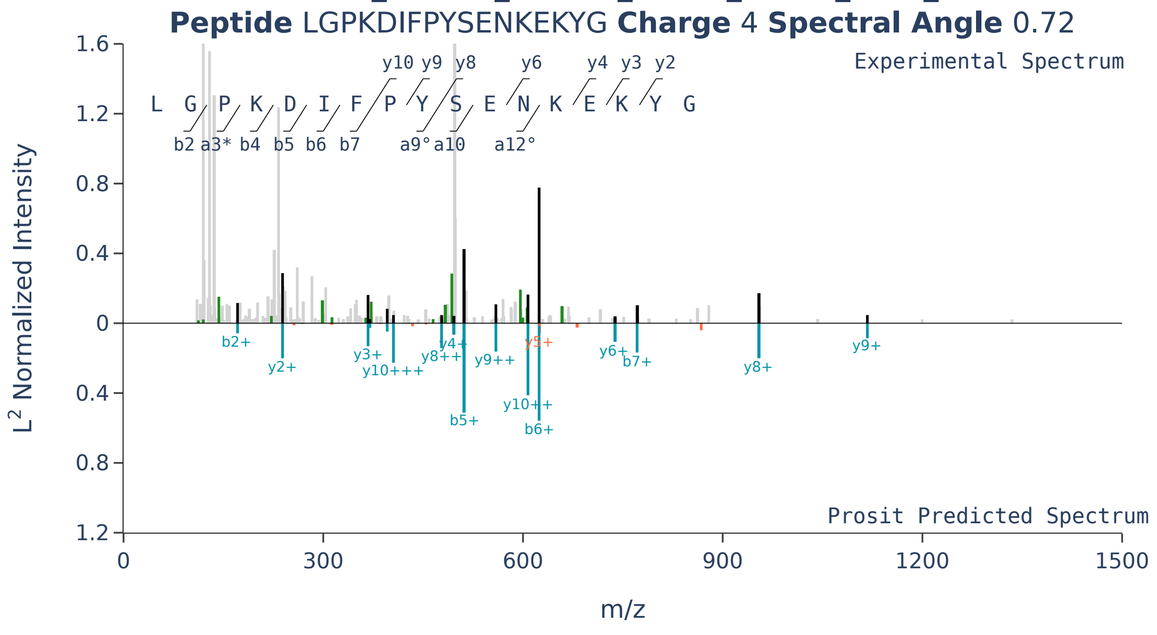

**Source** HRoetschke_271222_090123_HFGoe_LEDGF_b1R1_24h **Scan** 17604
**Peptide** DVTKAVDI **Charge** 2 **Spectral Angle** 0.83

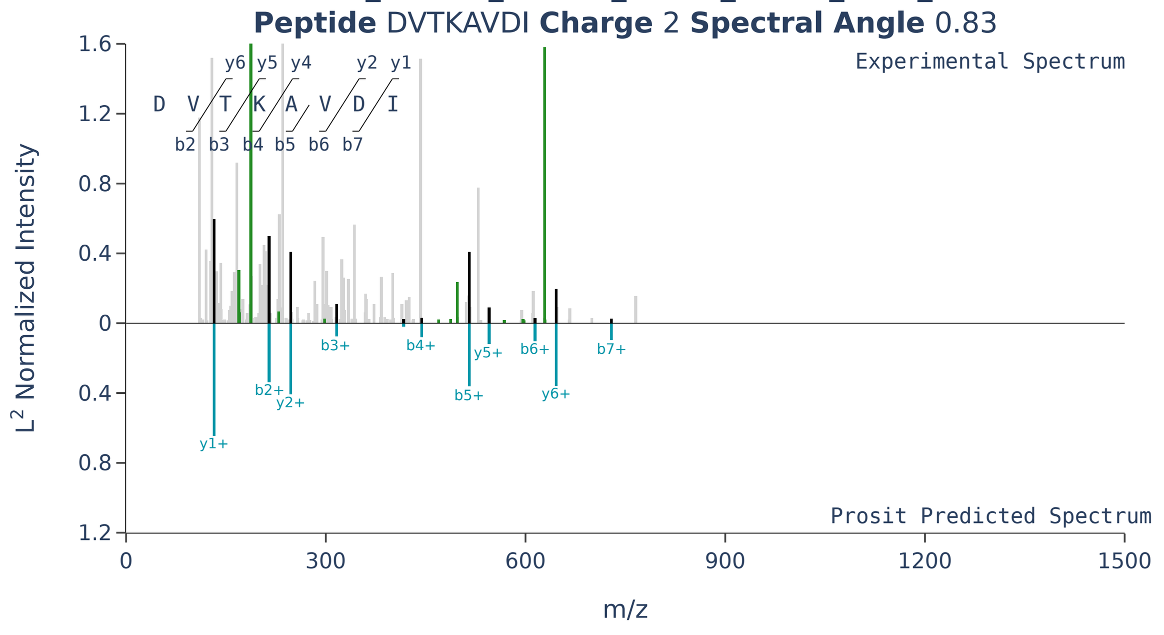

**Source** HRoetschke_271222_090123_HFGoe_LEDGF_b1R1_2h **Scan** 8340
**Peptide** SKDNHEASTKKKPSSEERETEISL **Charge** 3 **Spectral Angle** 0.82

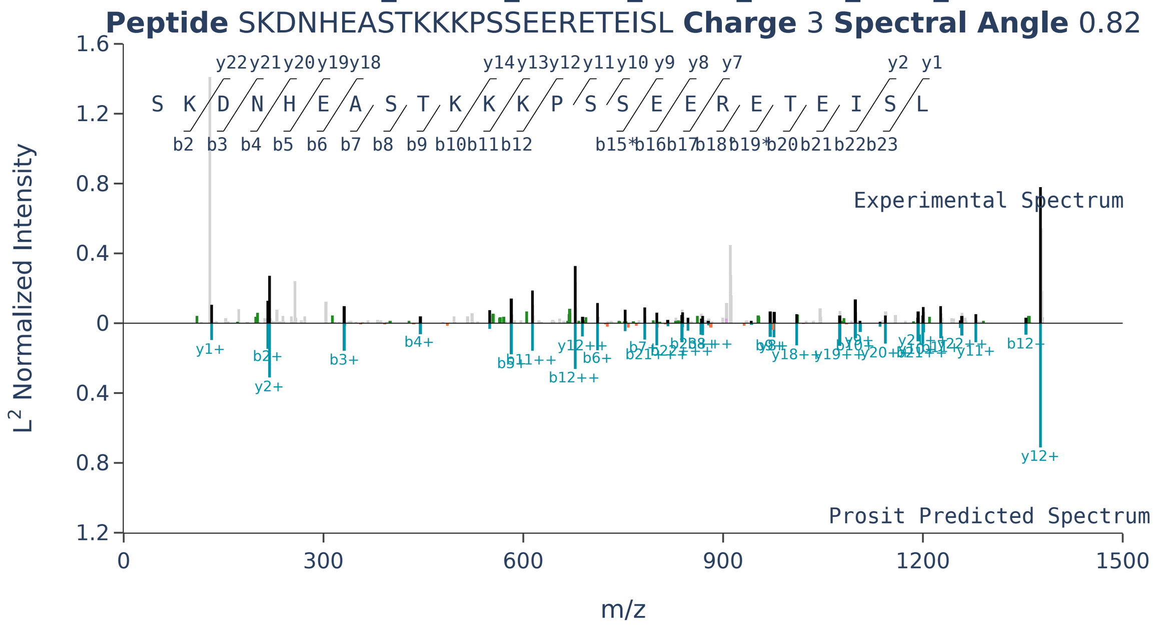

**Source** HRoetschke_271222_090123_HFGoe_LEDGF_b1R1_2h **Scan** 31231
**Peptide** EVPDGAVKPPTNKLPIFFFGTHE **Charge** 3 **Spectral Angle** 0.86

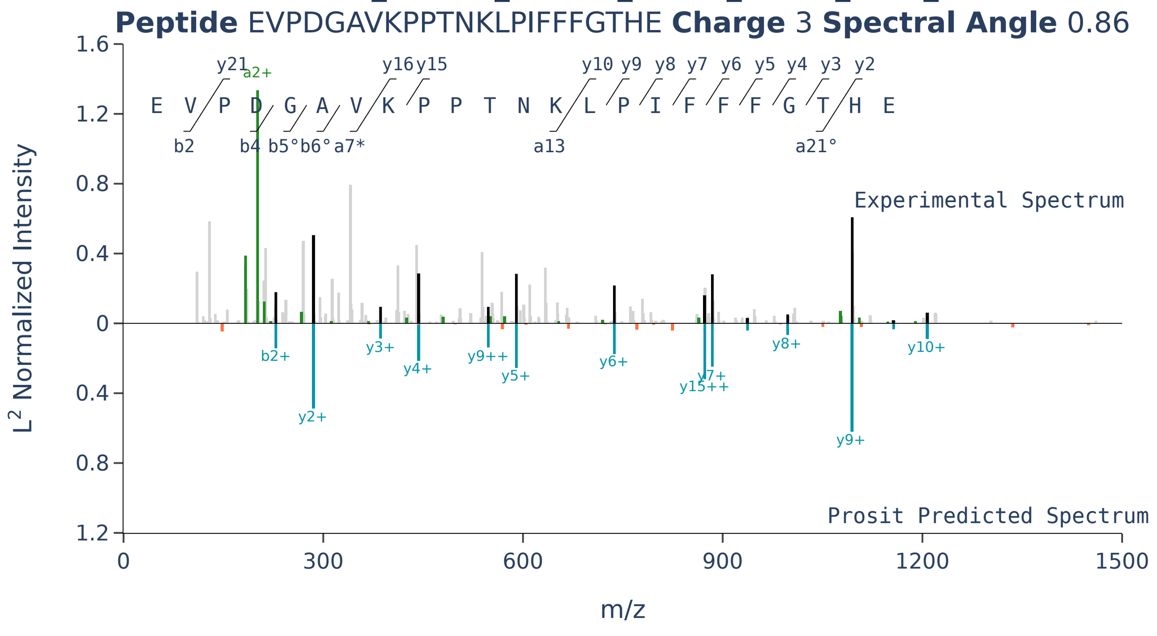

**Source** HRoetschke_271222_090123_HFGoe_LEDGF_b2R1_4h **Scan** 6432
**Peptide** EKKRETSMDSRL **Charge** 3 **Spectral Angle** 0.82

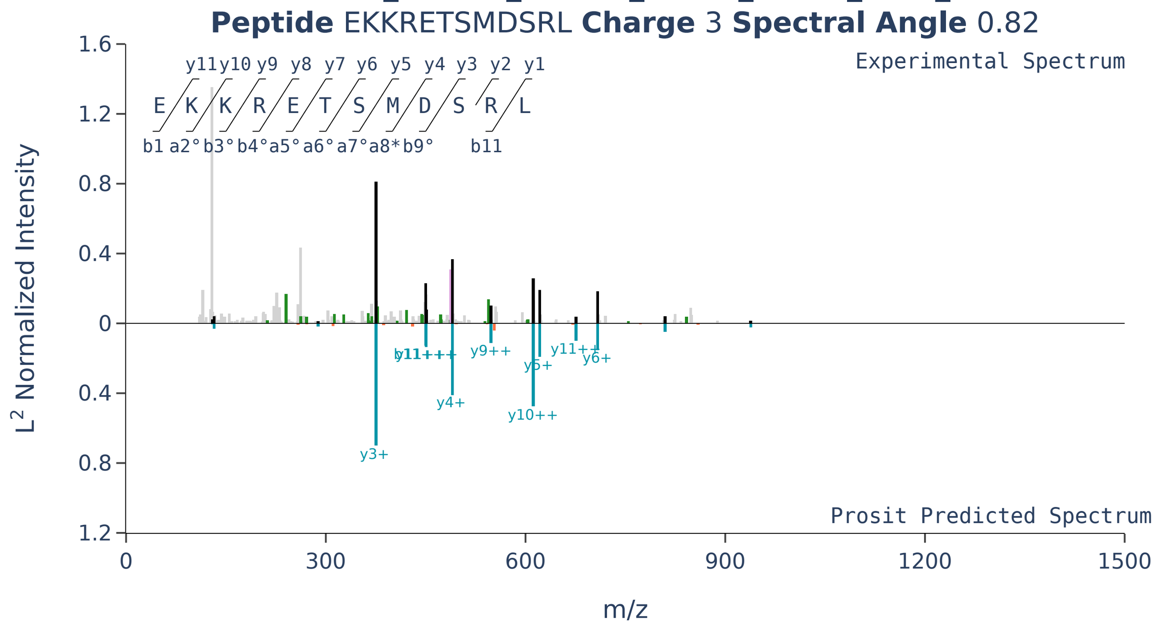

**Source** HRoetschke_271222_090123_HFGoe_LEDGF_b1R1_2h **Scan** 25901
**Peptide** GPGMTRDFKPGDLIFAKMKGY **Charge** 3 **Spectral Angle** 0.73

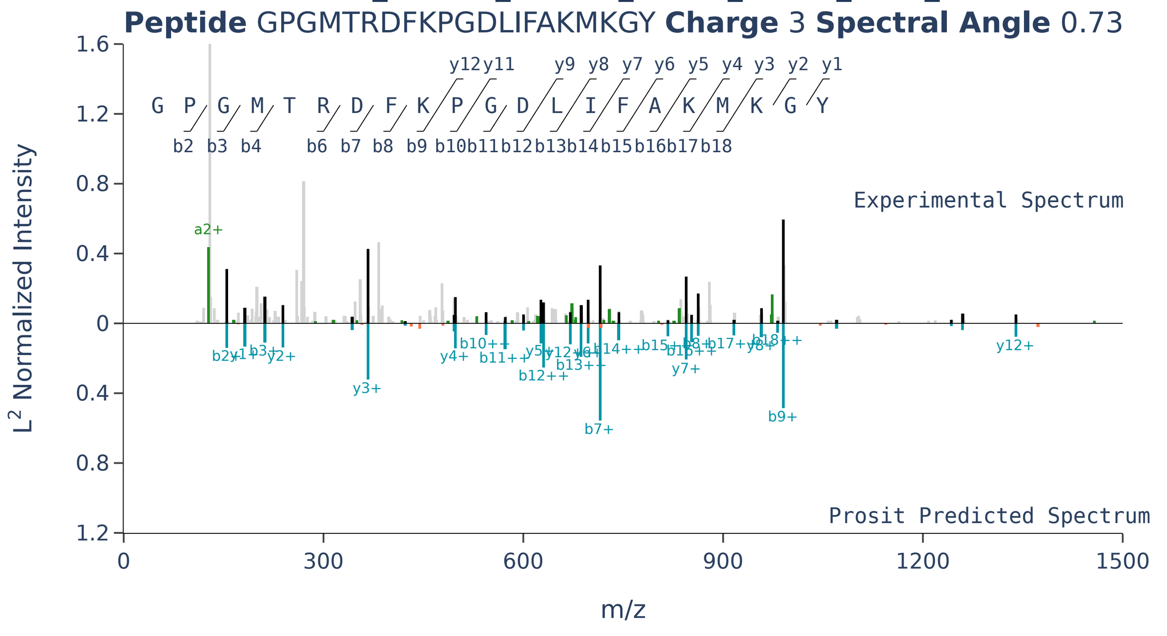

**Source** HRoetschke_271222_090123_HFGoe_LEDGF_b2R2_4h **Scan** 11805
**Peptide** RRFKVSQVIMEK **Charge** 4 **Spectral Angle** 0.85

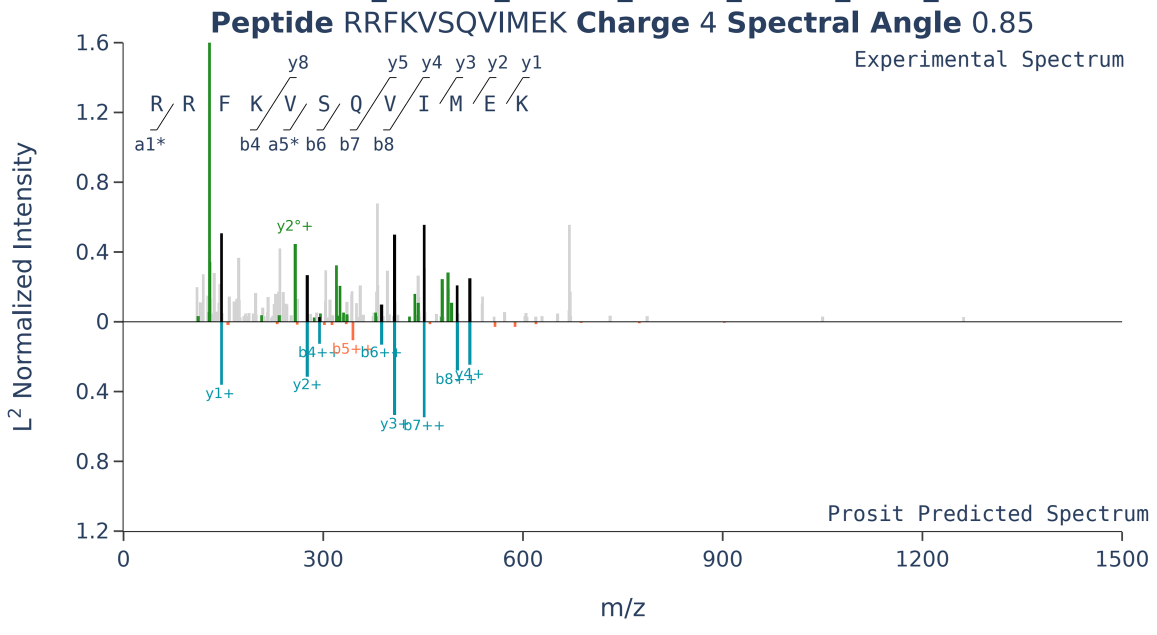

**Source** HRoetschke_271222_090123_HFGoe_LEDGF_b1R1_4h **Scan** 10208
**Peptide** SMDSRLQRIHAE **Charge** 2 **Spectral Angle** 0.89

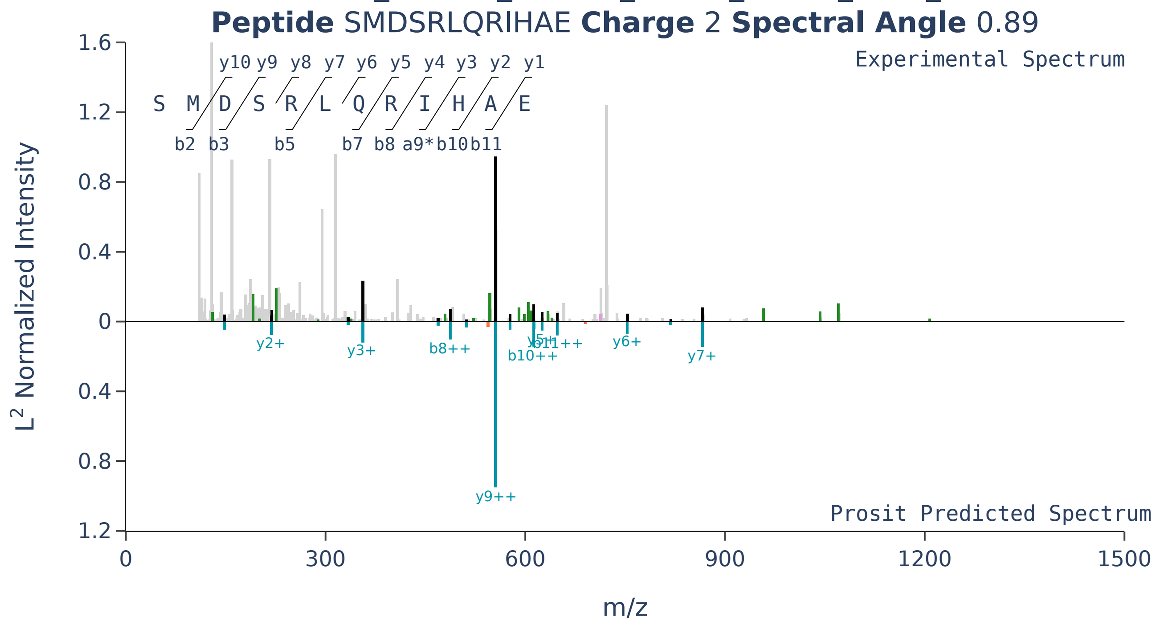

**Source** HRoetschke_271222_090123_HFGoe_LEDGF_b2R1_2h **Scan** 8539
**Peptide** LDNNPKVKFSSQQAATKQSNASSD **Charge** 3 **Spectral Angle** 0.85

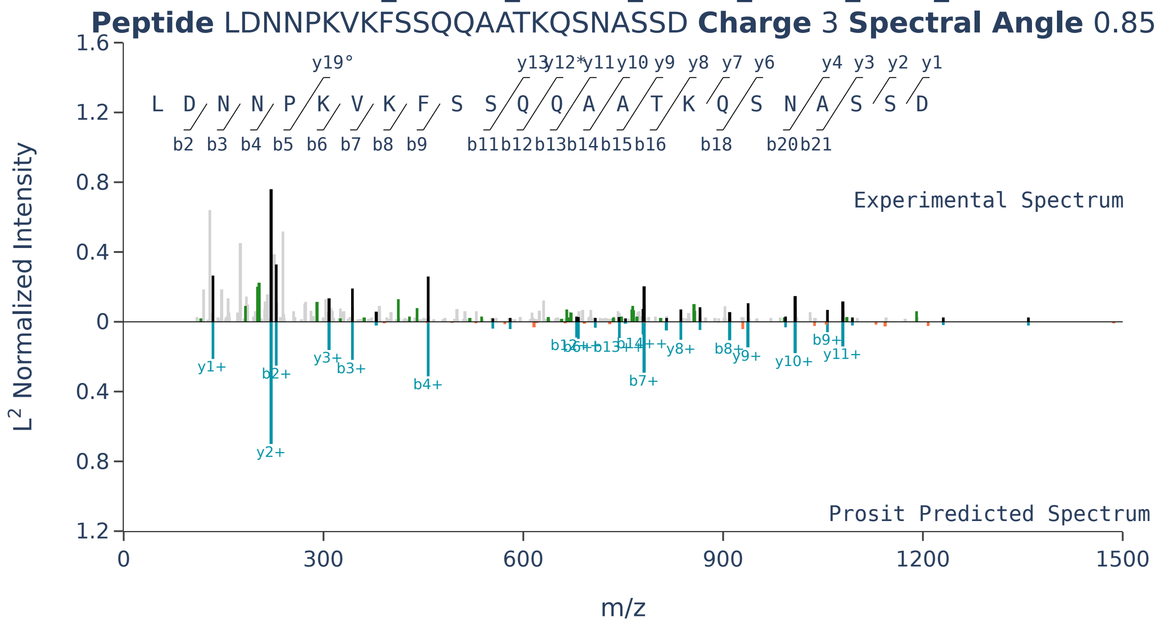

**Source** HRoetschke_271222_090123_HFGoe_LEDGF_b1R2_4h **Scan** 18506
**Peptide** WEIDNNPKVKFSSQQA **Charge** 2 **Spectral Angle** 0.92

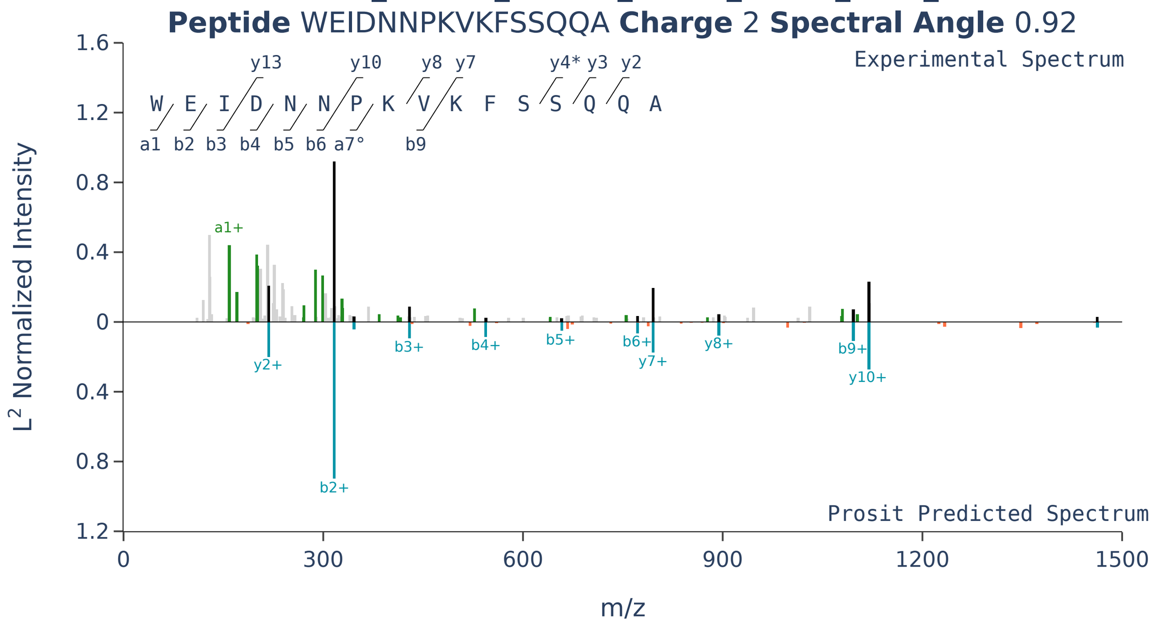

**Source** HRoetschke_271222_090123_HFGoe_LEDGF_b1R1_1h **Scan** 22614
**Peptide** KFKNMFLVGEGD **Charge** 2 **Spectral Angle** 0.77

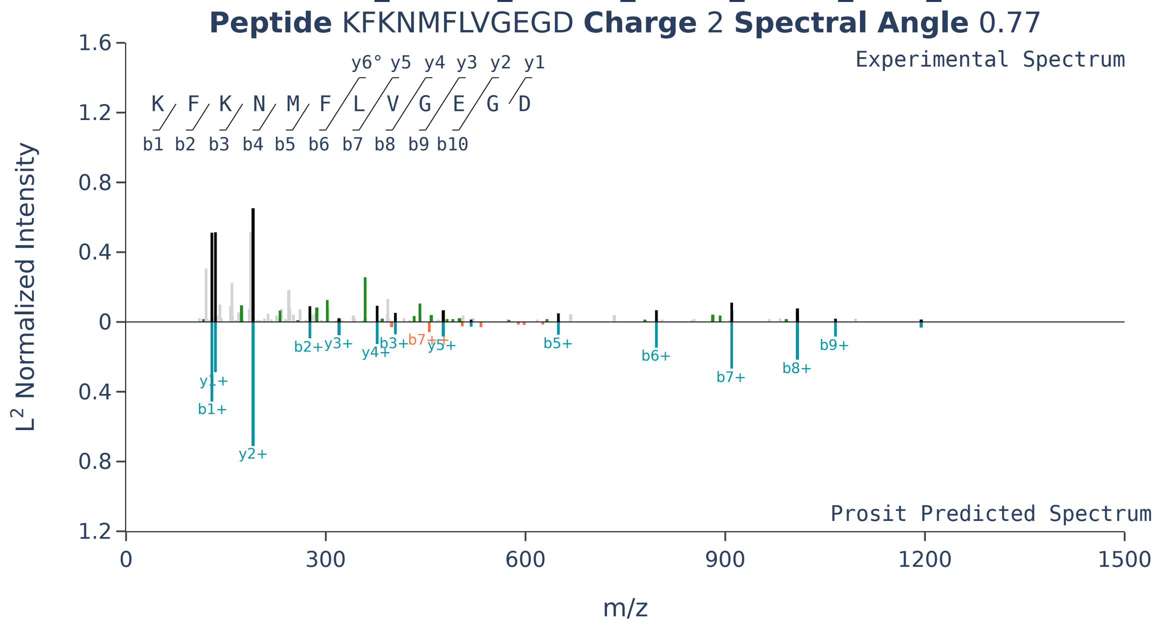

**Source** HRoetschke_271222_090123_HFGoe_LEDGF_b1R1_4h **Scan** 24207
**Peptide** YNKFKNMFLVG **Charge** 3 **Spectral Angle** 0.8

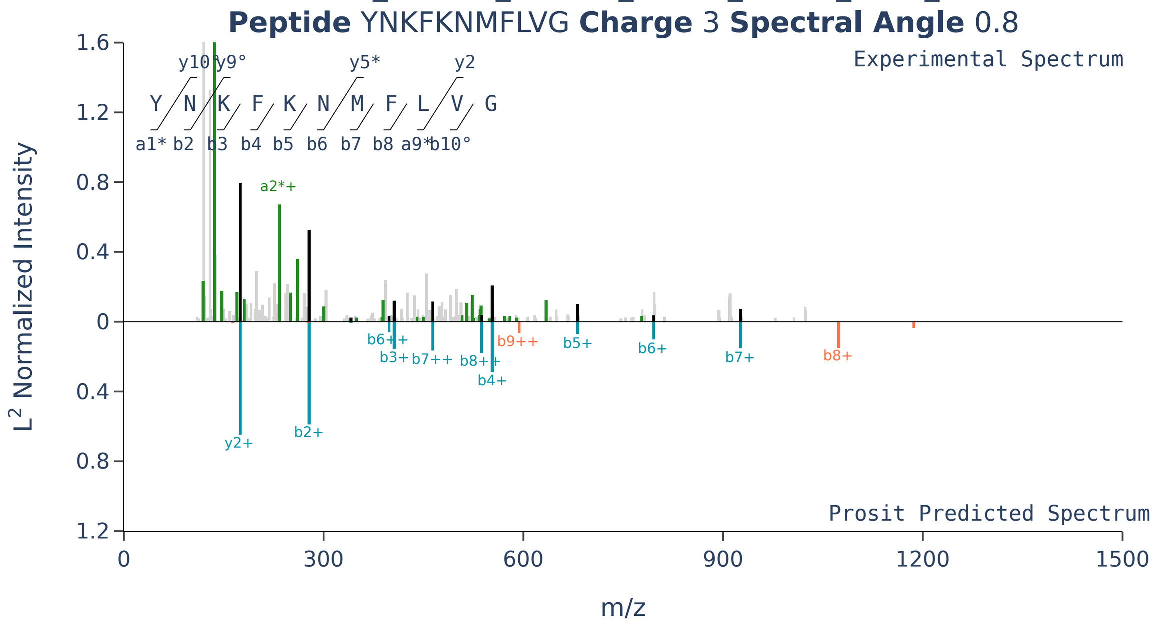

**Source** HRoetschke_271222_090123_HFGoe_LEDGF_b1R1_24h **Scan** 6496
**Peptide** LEKEQTGSKTLNGGSDAQD **Charge** 3 **Spectral Angle** 0.69

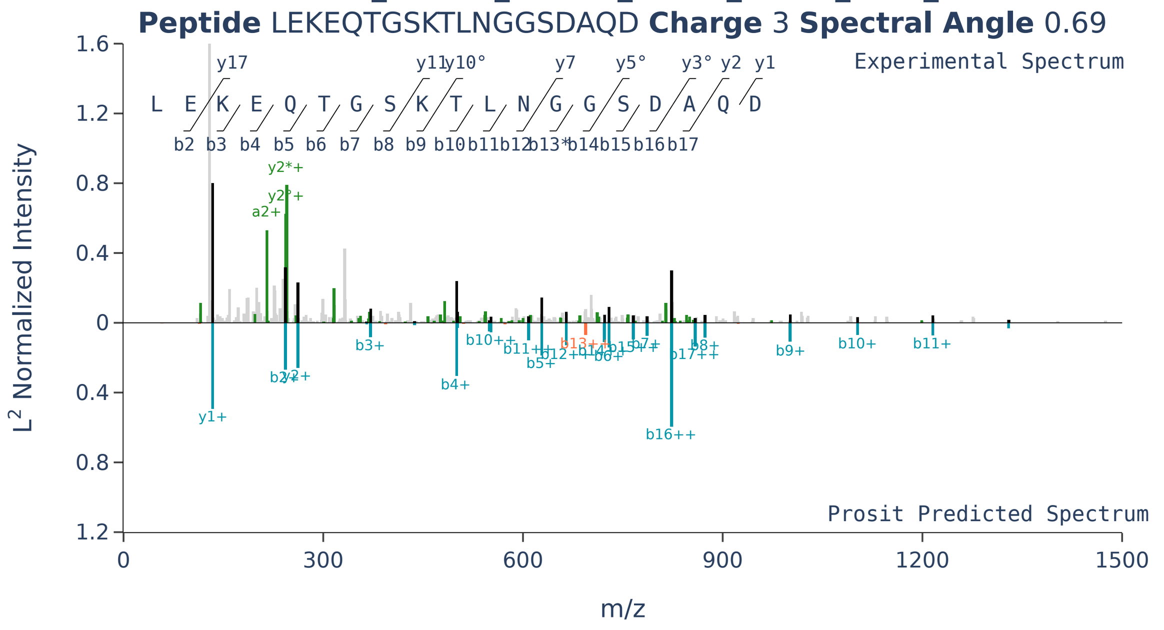


**Source** HRoetschke_271222_090123_HFGoe_LEDGF_b2R1_24h **Scan** 16923
**Peptide** EAGVVTTATASVNLKVSPKRGRPAATEV **Charge** 4 **Spectral Angle** 0.83

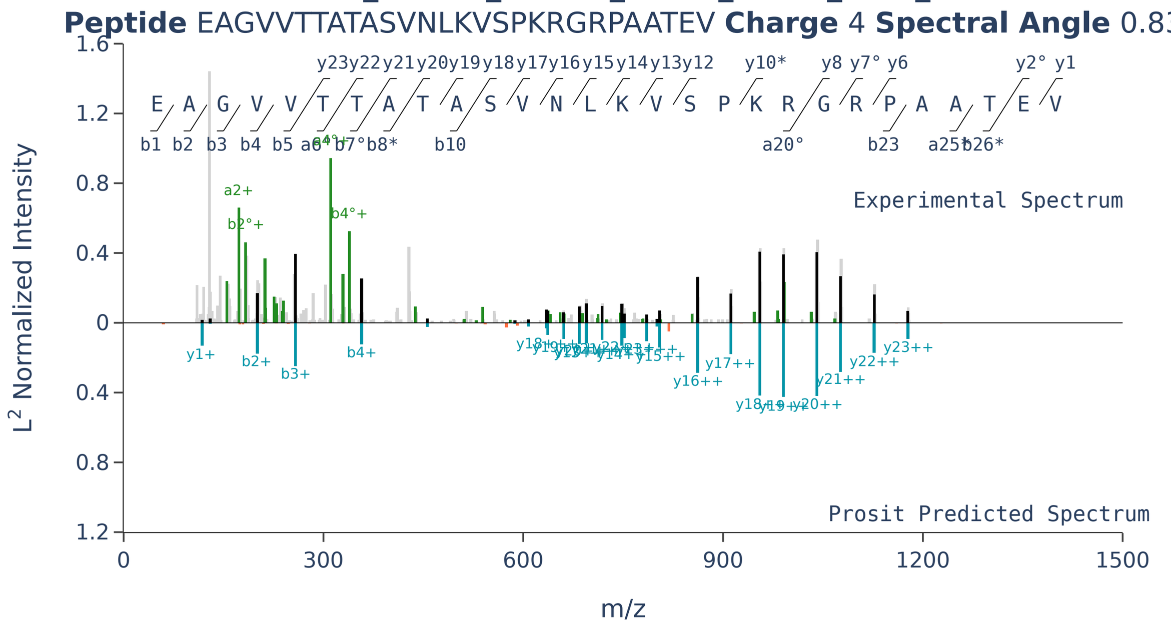


**Source** HRoetschke_271222_090123_HFGoe_LEDGF_b1R2_24h **Scan** 12984
**Peptide** LGPKDIFPYSENKEKYGKPNKRK **Charge** 4 **Spectral Angle** 0.76

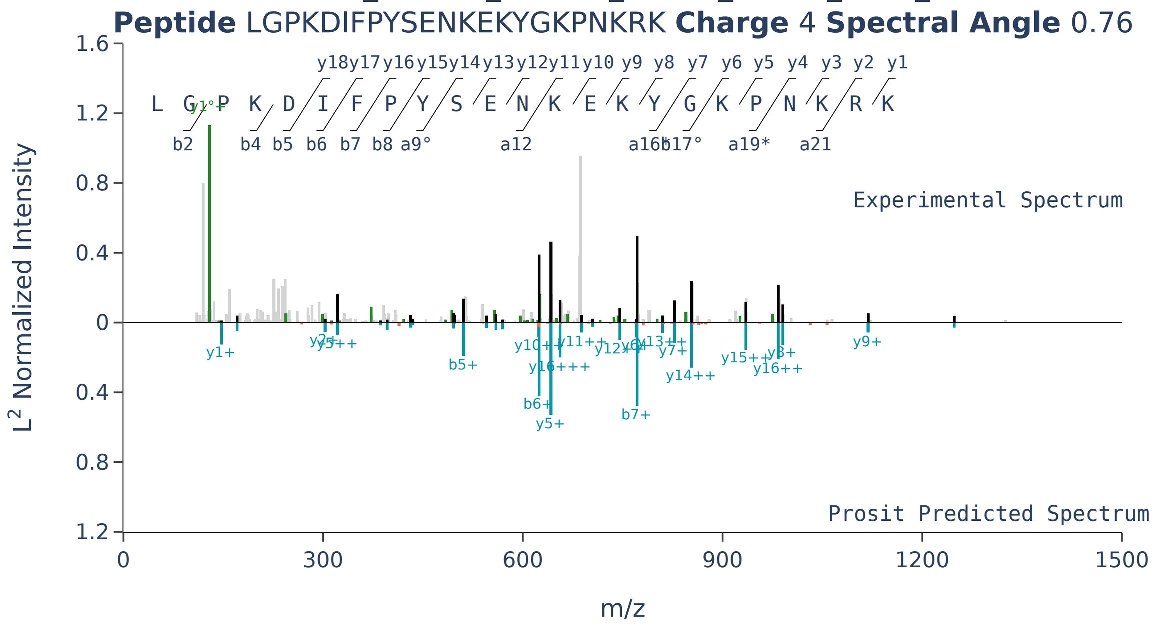


**Source** HRoetschke_271222_090123_HFGoe_LEDGF_b1R1_4h **Scan** 7262
**Peptide** ASTKKKPSSEERETEISLKD **Charge** 5 **Spectral Angle** 0.82

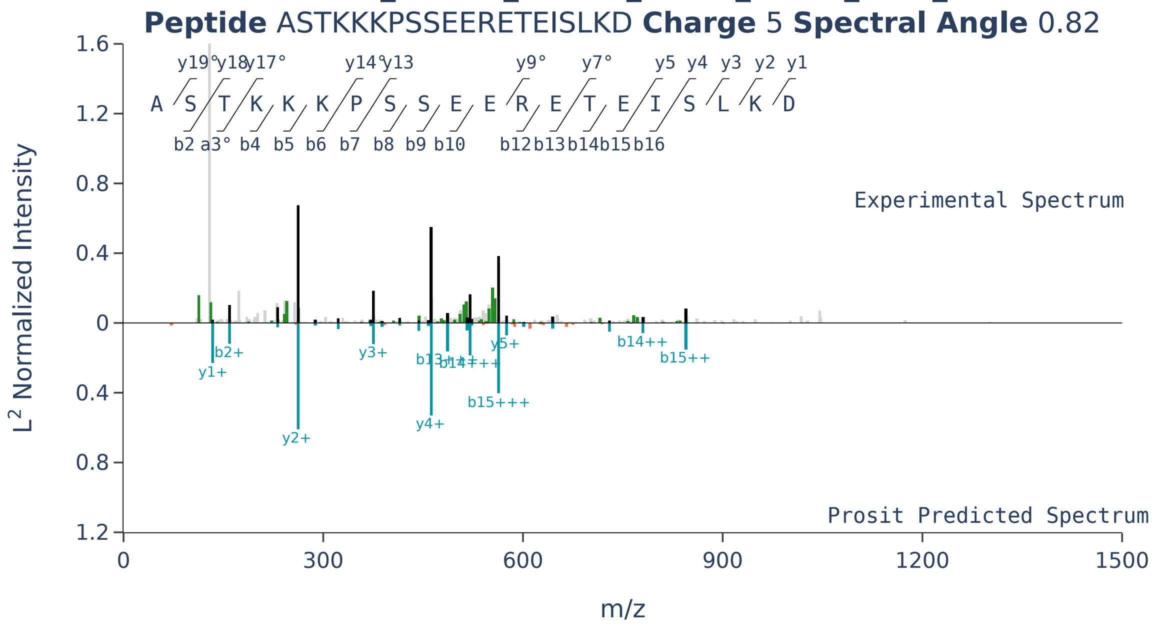


**Source** HRoetschke_271222_090123_HFGoe_LEDGF_b2R1_24h **Scan** 15401
**Peptide** EAGVVTTATASVNLKVSPKRGRPAATE **Charge** 4 **Spectral Angle** 0.84

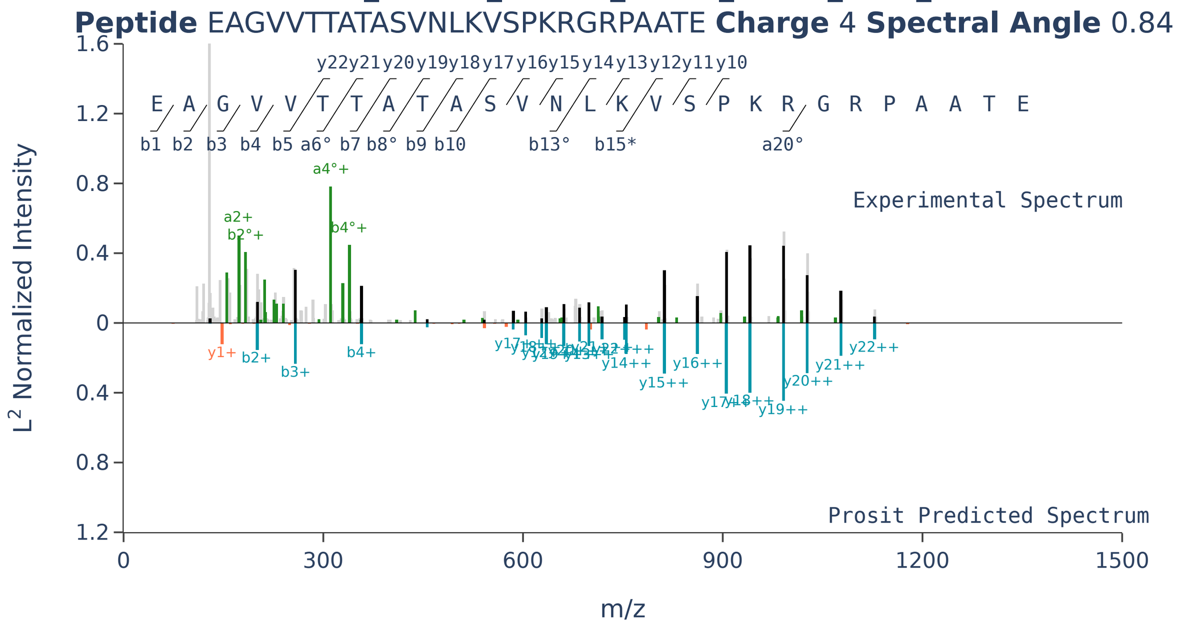


**Source** HRoetschke_271222_090123_HFGoe_LEDGF_b1R1_4h **Scan** 7926
**Peptide** DNNPKVKFSSQQAATKQ **Charge** 2 **Spectral Angle** 0.87

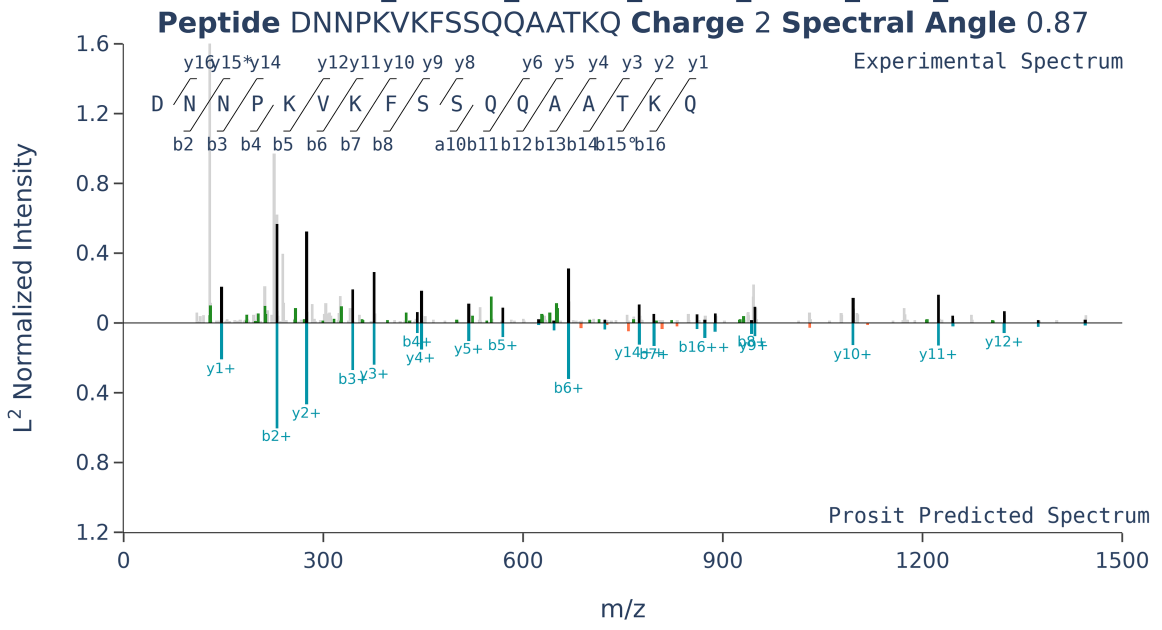


**Source** HRoetschke_271222_090123_HFGoe_LEDGF_b2R2_2h **Scan** 24522
**Peptide** LWEIDNNPKVKF **Charge** 3 **Spectral Angle** 0.79

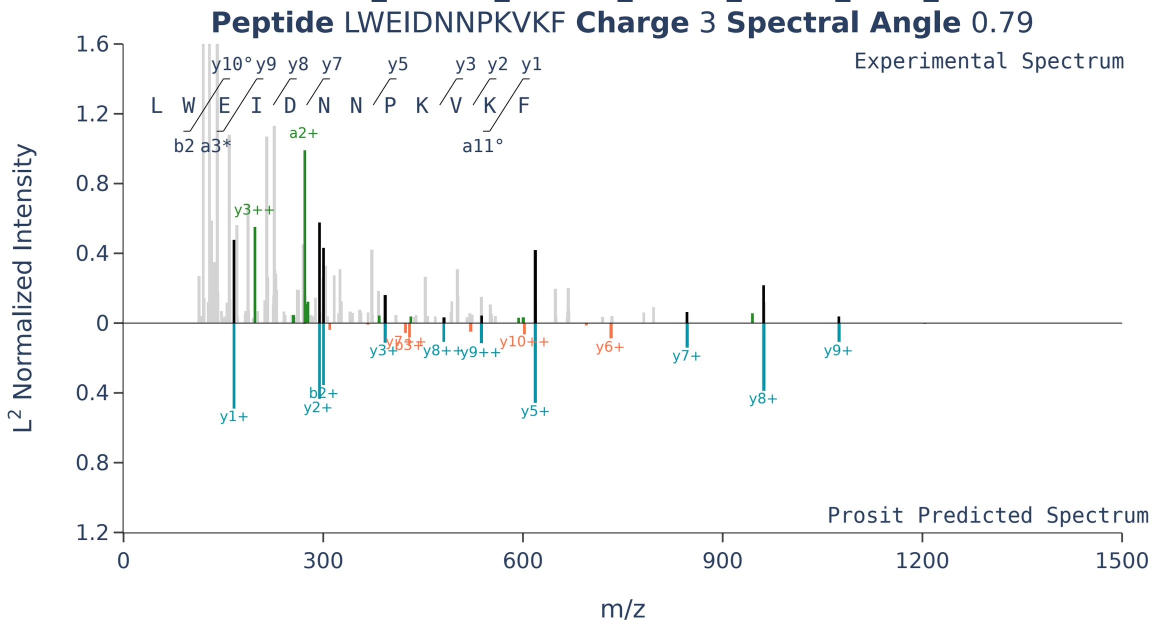


**Source** HRoetschke_271222_090123_HFGoe_LEDGF_b2R1_1h **Scan** 26480
**Peptide** KSTMLYNKFKNMFLVG **Charge** 3 **Spectral Angle** 0.78

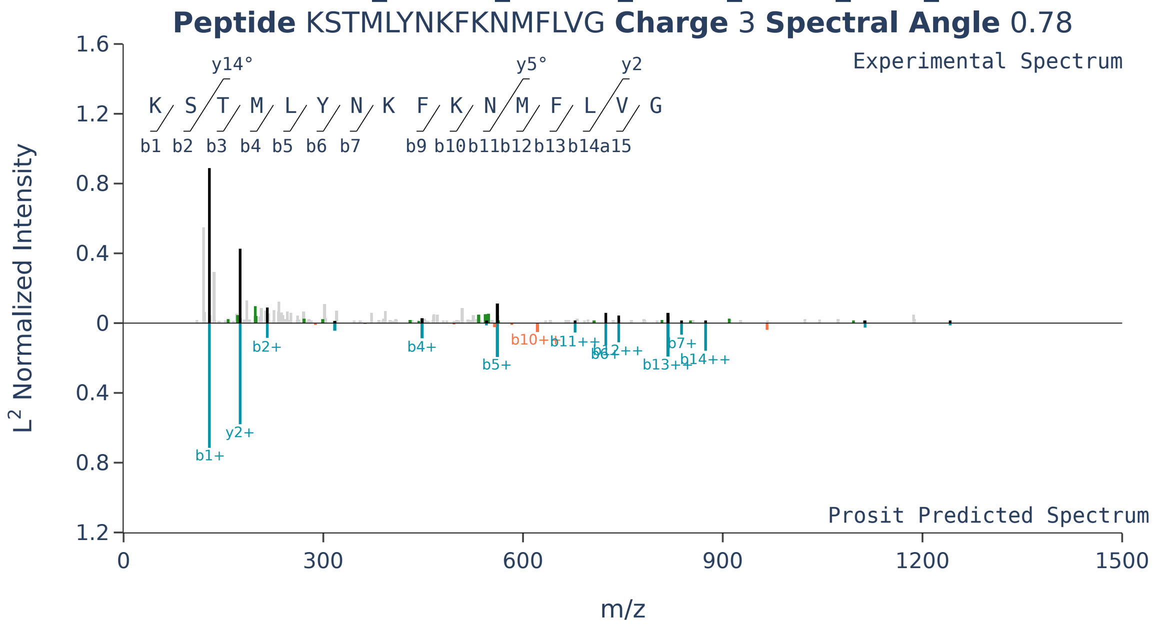


**Source** HRoetschke_271222_090123_HFGoe_LEDGF_b1R1_24h **Scan** 17291
**Peptide** TATASVNL **Charge** 1 **Spectral Angle** 0.85

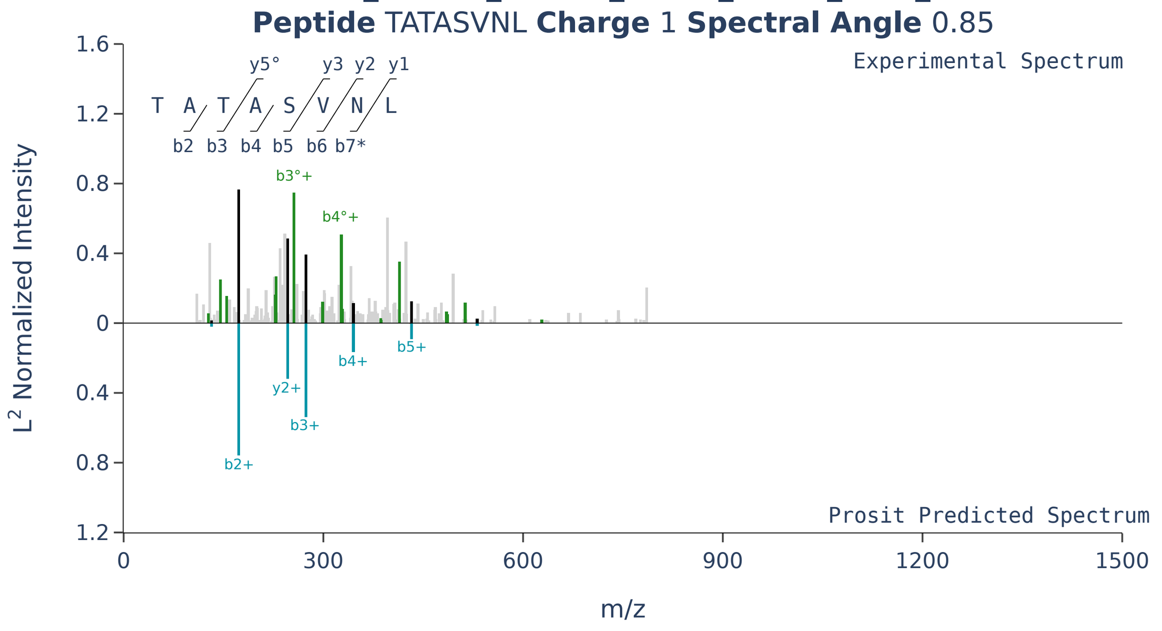


**Source** HRoetschke_271222_090123_HFGoe_LEDGF_b1R1_2h **Scan** 9446
**Peptide** VNLKVSPKRGRPAATEV **Charge** 3 **Spectral Angle** 0.8

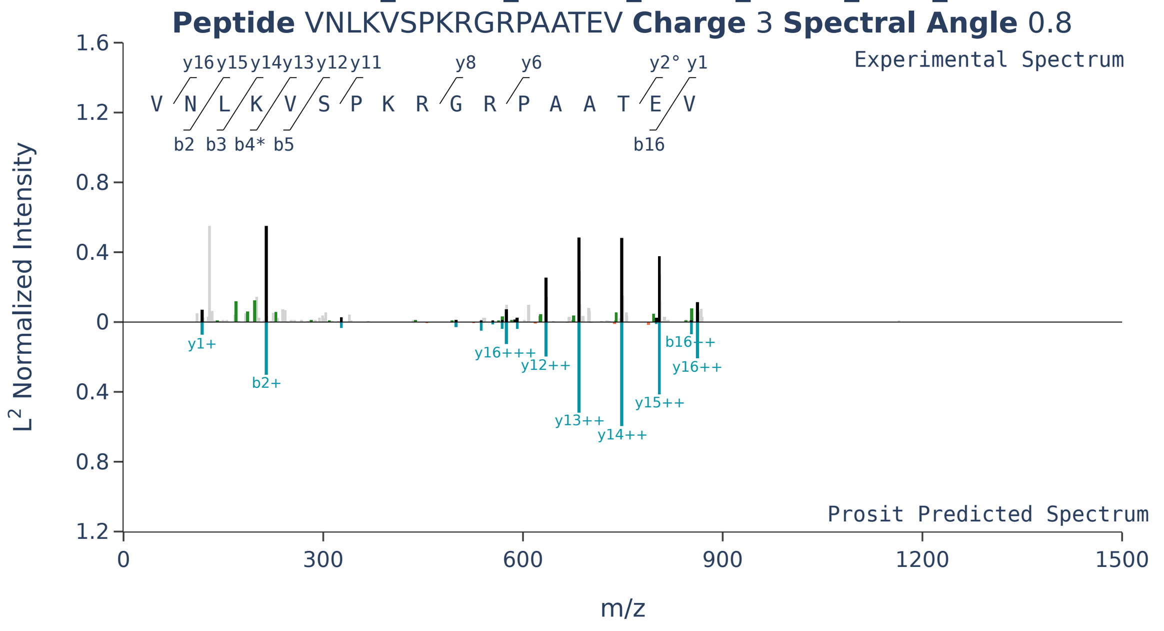


**Source** HRoetschke_271222_090123_HFGoe_LEDGF_b1R1_4h **Scan** 16935
**Peptide** LASLQVTMQQAQKH **Charge** 3 **Spectral Angle** 0.72

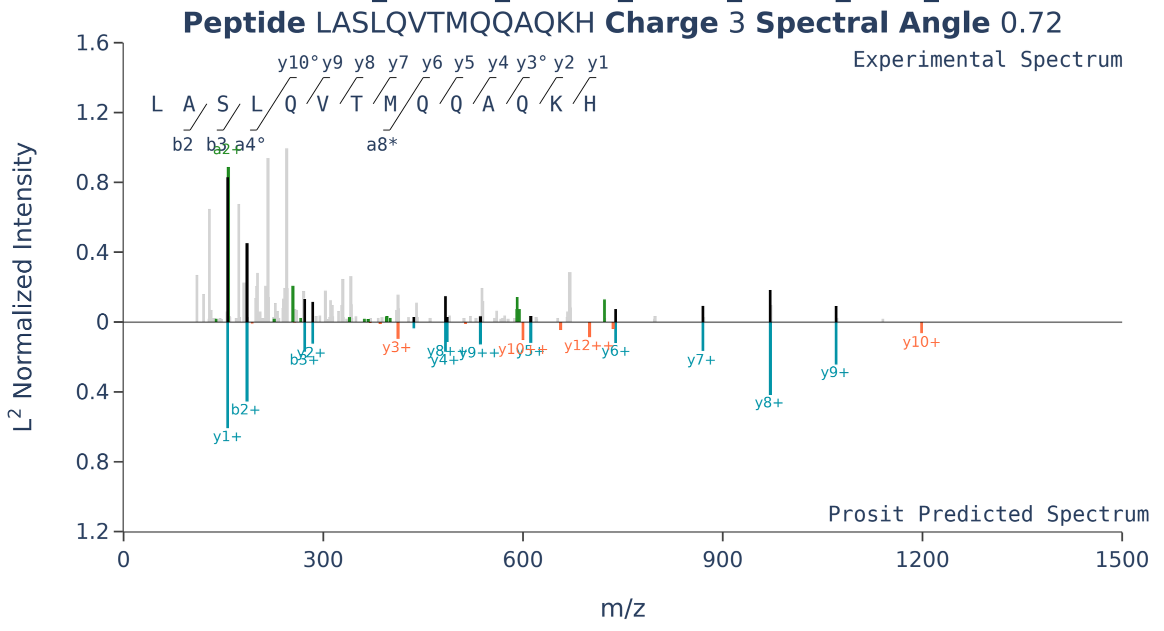


**Source** HRoetschke_271222_090123_HFGoe_LEDGF_b2R2_24h **Scan** 17545
**Peptide** EIKNSLKIDNLD **Charge** 3 **Spectral Angle** 0.92

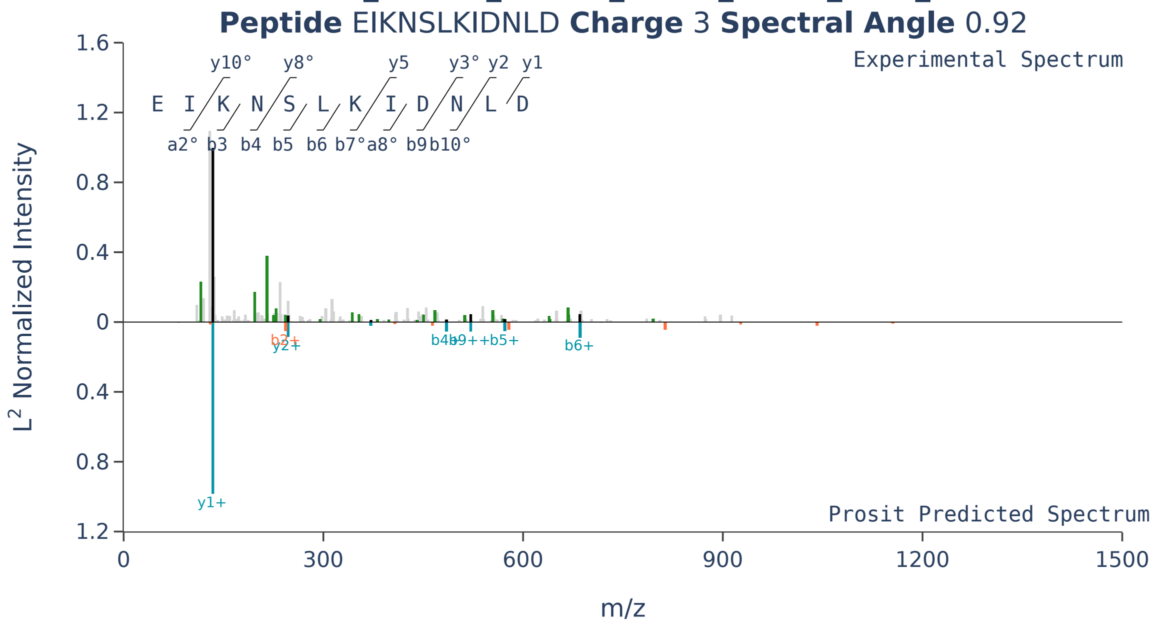


**Source** HRoetschke_271222_090123_HFGoe_LEDGF_b1R2_2h **Scan** 8920
**Peptide** NHEASTKKKPSSEERETEISL **Charge** 3 **Spectral Angle** 0.85

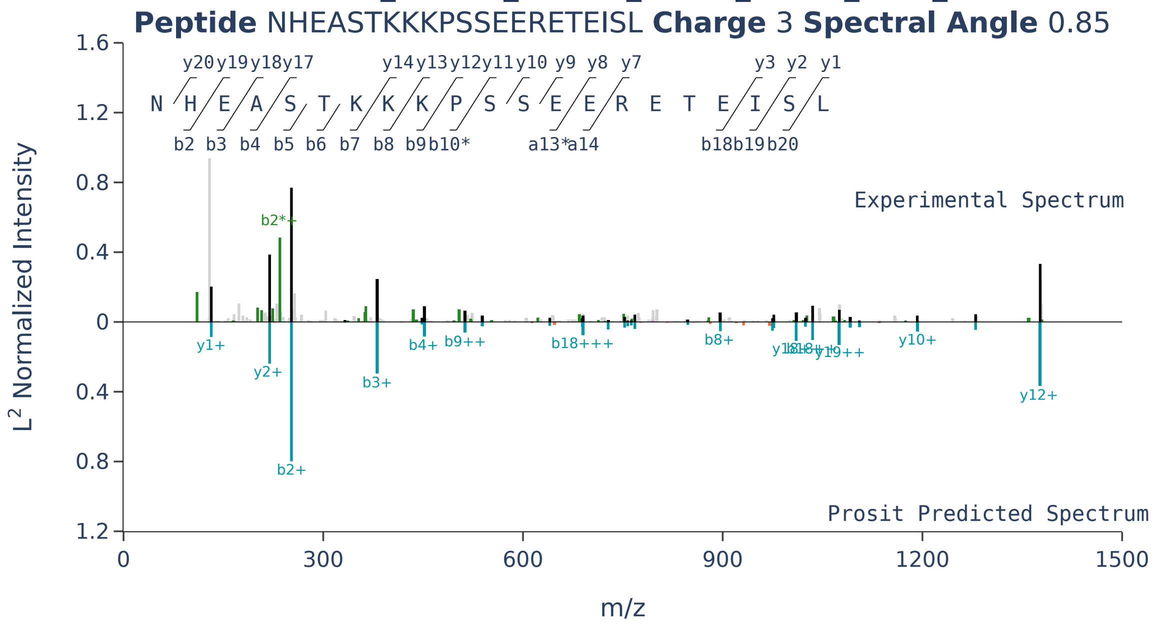


**Source** HRoetschke_271222_090123_HFGoe_LEDGF_b1R2_24h **Scan** 14532
**Peptide** ATASVNLKVS **Charge** 2 **Spectral Angle** 0.77

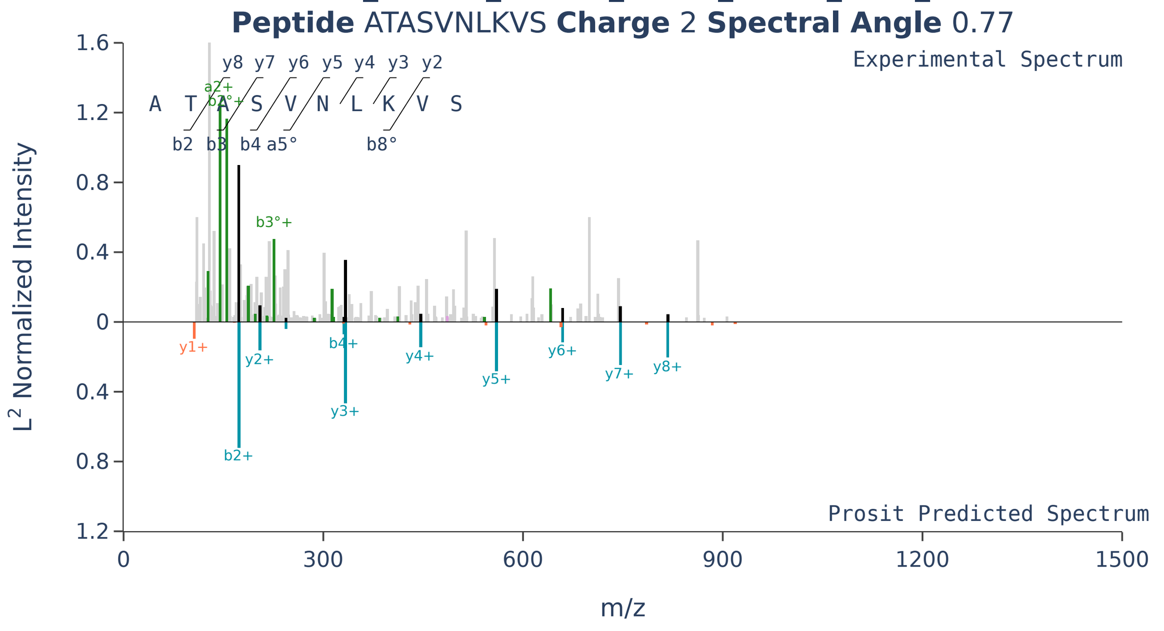


**Source** HRoetschke_271222_090123_HFGoe_LEDGF_b1R1_1h **Scan** 21138
**Peptide** TRDFKPGDLIFAK **Charge** 3 **Spectral Angle** 0.76

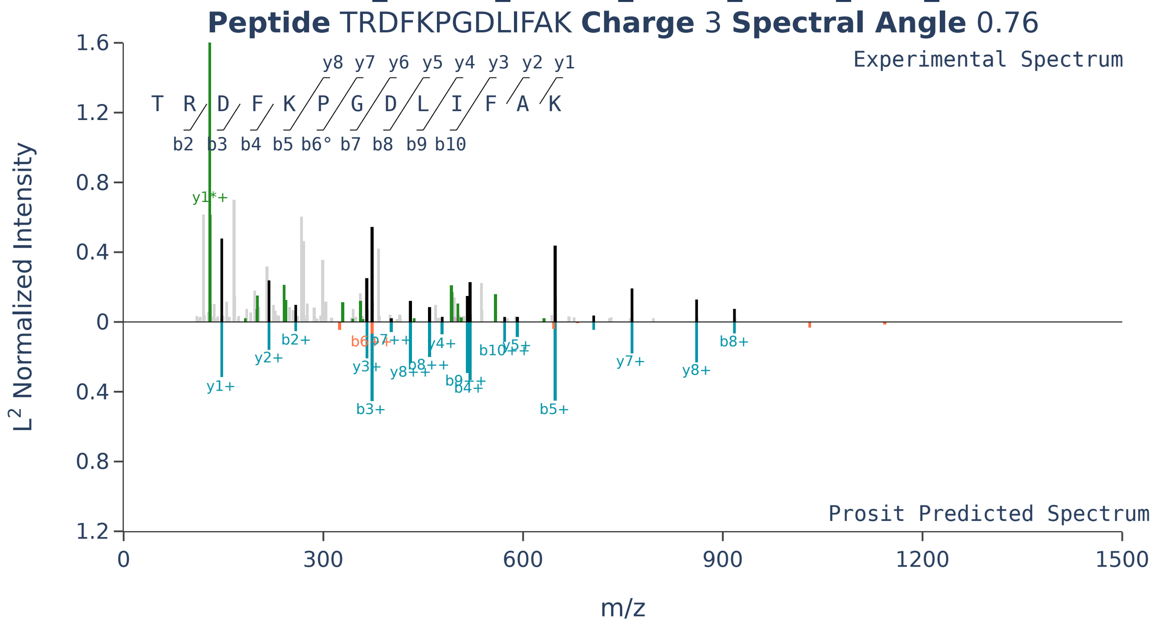

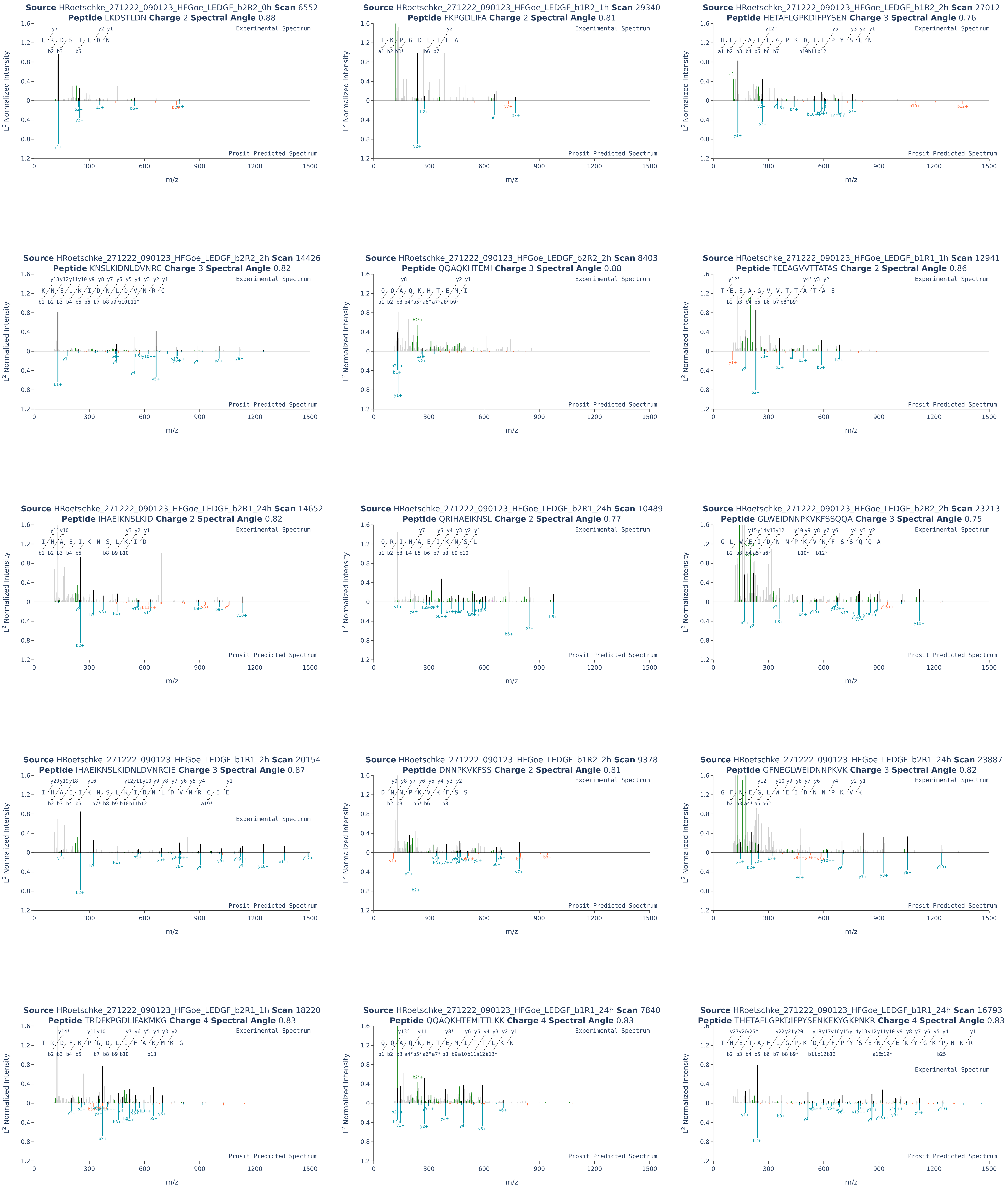
Source HRoetschke_271222_090123_HFGoe_LEDGF_b2R2_0h Scan 6552
Peptide LKDSTLDN Charge 2 Spectral Angle 0.88
Source HRoetschke_271222_090123_HFGoe_LEDGF_b1R2_1h Scan 29340
Peptide FKPGDLIFA Charge 2 Spectral Angle 0.81
Source HRoetschke_271222_090123_HFGoe_LEDGF_b1R2_2h Scan 27012
Peptide HETAFLGPKDIFPYSEN Charge 3 Spectral Angle 0.76
Source HRoetschke_271222_090123_HFGoe_LEDGF_b2R2_2h Scan 14426
Peptide KNSLKIDNLDVNRC Charge 3 Spectral Angle 0.82
Source HRoetschke_271222_090123_HFGoe_LEDGF_b2R2_2h Scan 8403
Peptide QQAQKHTEMI Charge 3 Spectral Angle 0.88
Source HRoetschke_271222_090123_HFGoe_LEDGF_b1R1_1h Scan 12941
Peptide TEEAGVVTTATAS Charge 2 Spectral Angle 0.86
Source HRoetschke_271222_090123_HFGoe_LEDGF_b2R1_24h Scan 14652
Peptide IHAEIKNSLKID Charge 2 Spectral Angle 0.82
Source HRoetschke_271222_090123_HFGoe_LEDGF_b2R1_24h Scan 10489
Peptide QRIHAEIKNSL Charge 2 Spectral Angle 0.77
Source HRoetschke_271222_090123_HFGoe_LEDGF_b2R2_2h Scan 23213
Peptide GLWEIDNNPKVKFSSQQA Charge 3 Spectral Angle 0.75
Source HRoetschke_271222_090123_HFGoe_LEDGF_b1R1_2h Scan 20154
Peptide IHAEIKNSLKIDNLDVNRCIE Charge 3 Spectral Angle 0.87
Source HRoetschke_271222_090123_HFGoe_LEDGF_b1R2_2h Scan 9378
Peptide DNNPKVKFSS Charge 2 Spectral Angle 0.81
Source HRoetschke_271222_090123_HFGoe_LEDGF_b2R1_24h Scan 23887
Peptide GFNEGLWEIDNNPKVK Charge 3 Spectral Angle 0.82
Source HRoetschke_271222_090123_HFGoe_LEDGF_b2R1_1h Scan 18220
Peptide TRDFKPGDLIFAKMKG Charge 4 Spectral Angle 0.83
Source HRoetschke_271222_090123_HFGoe_LEDGF_b1R1_24h Scan 7840
Peptide QQAQKHTEMITTLKK Charge 4 Spectral Angle 0.83
Source HRoetschke_271222_090123_HFGoe_LEDGF_b1R1_24h Scan 16793
Peptide THETAFLGPKDIFPYSENKEKYGKPNKR Charge 4 Spectral Angle 0.83
Experimental Spectrum
Prosit Predicted Spectrum
L² Normalized Intensity
m/z

**Source** HRoetschke_271222_090123_HFGoe_LEDGF_b1R1_4h **Scan** 30295
**Peptide** VGEGDSVITQVL **Charge** 2 **Spectral Angle** 0.85

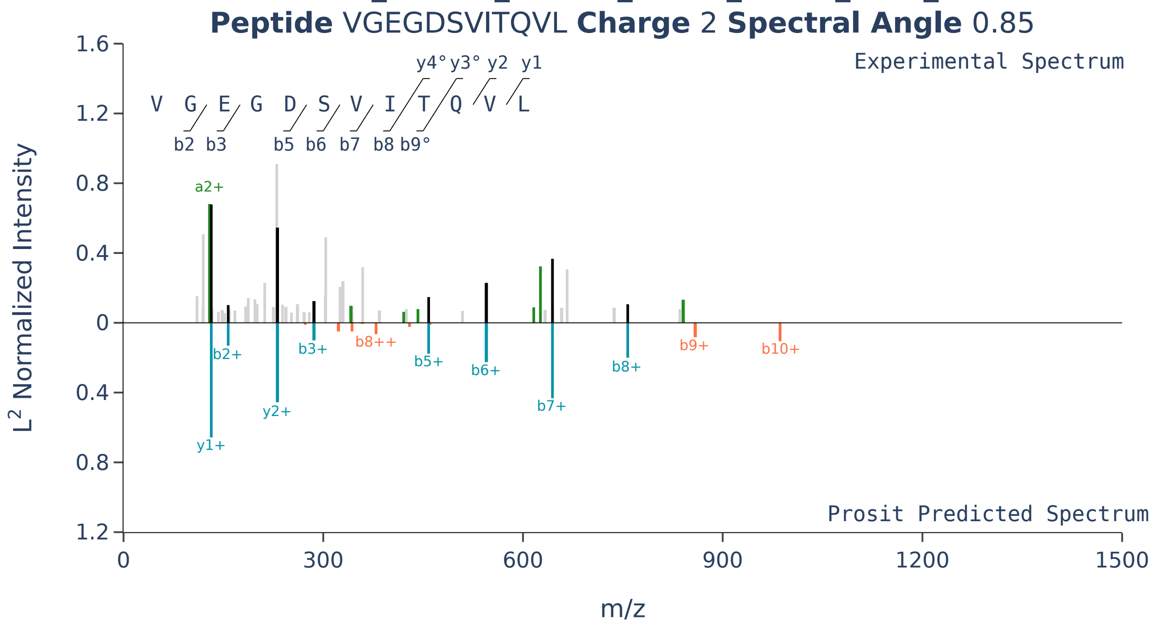


**Source** HRoetschke_271222_090123_HFGoe_LEDGF_b1R2_1h **Scan** 12455
**Peptide** NRCIEALD **Charge** 2 **Spectral Angle** 0.8

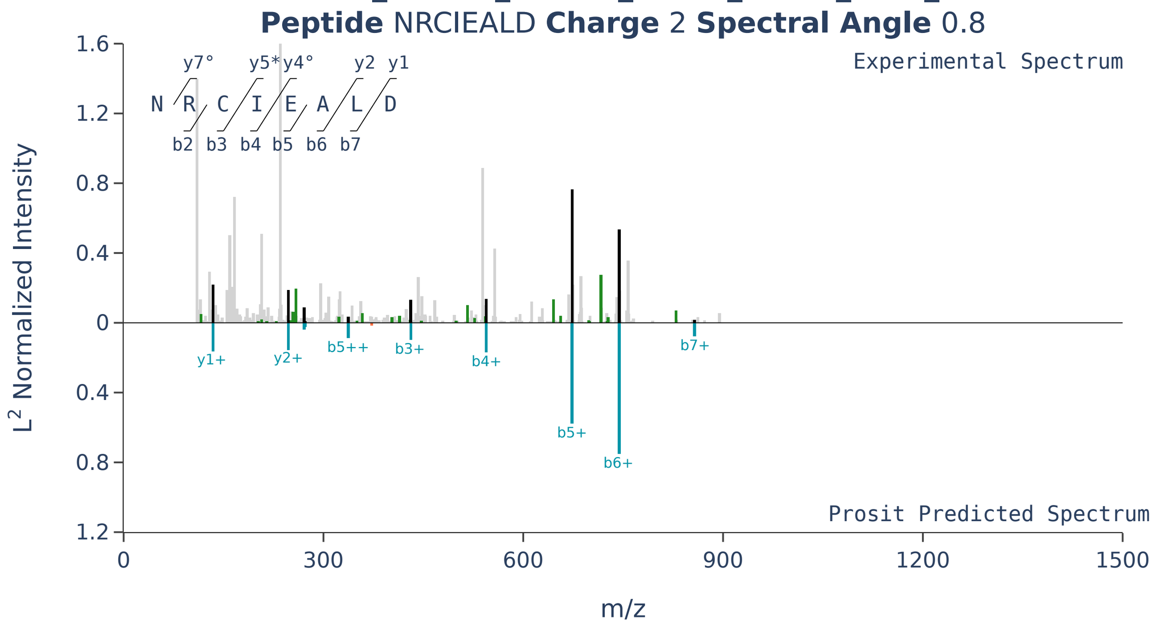


**Source** HRoetschke_271222_090123_HFGoe_LEDGF_b1R1_4h **Scan** 13071
**Peptide** AKMKGYPHWPARVDE **Charge** 3 **Spectral Angle** 0.88

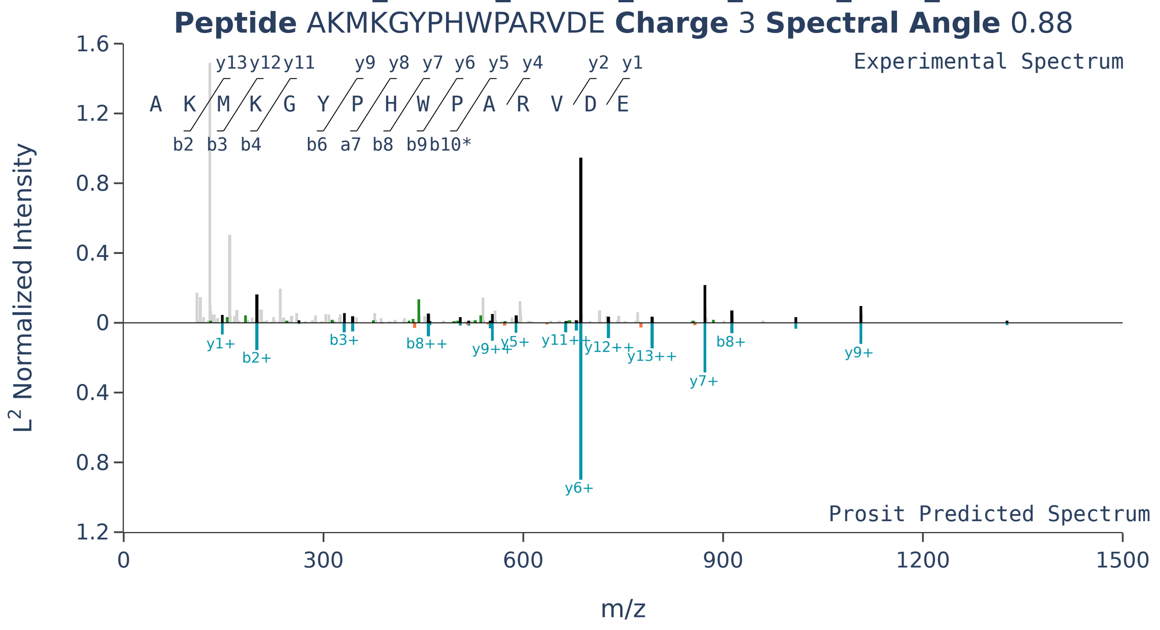


**Source** HRoetschke_271222_090123_HFGoe_LEDGF_b2R1_2h **Scan** 16588
**Peptide** TEMITTLKKIRRFKVSQ **Charge** 4 **Spectral Angle** 0.84

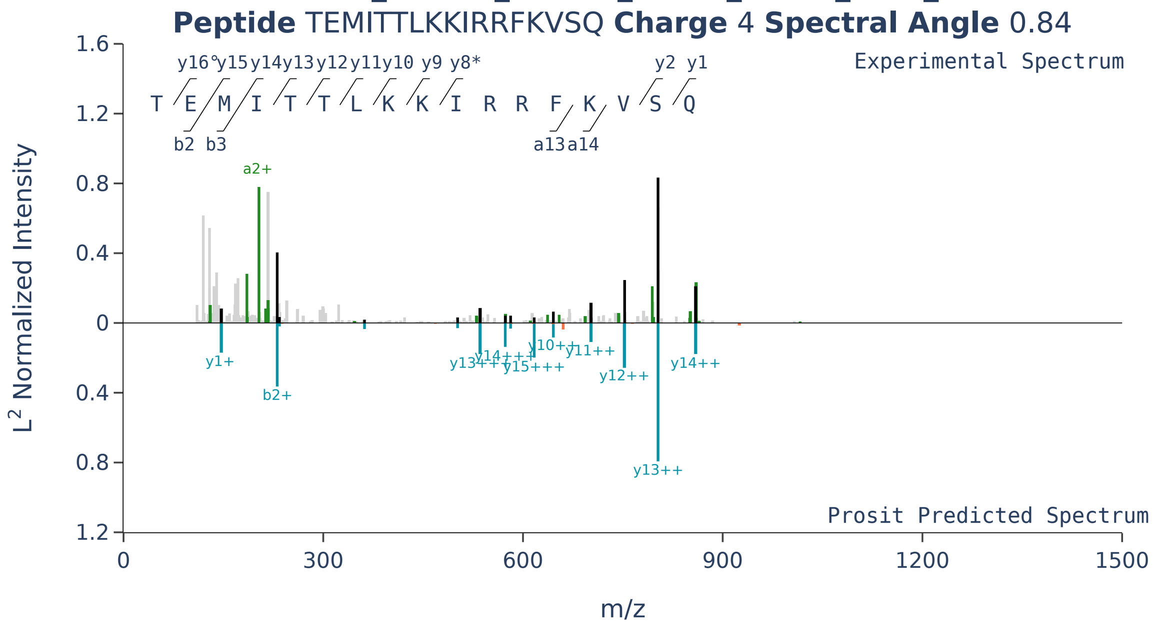


**Source** HRoetschke_271222_090123_HFGoe_LEDGF_b1R1_24h **Scan** 9295
**Peptide** ATEVKIPKPRGR **Charge** 3 **Spectral Angle** 0.76

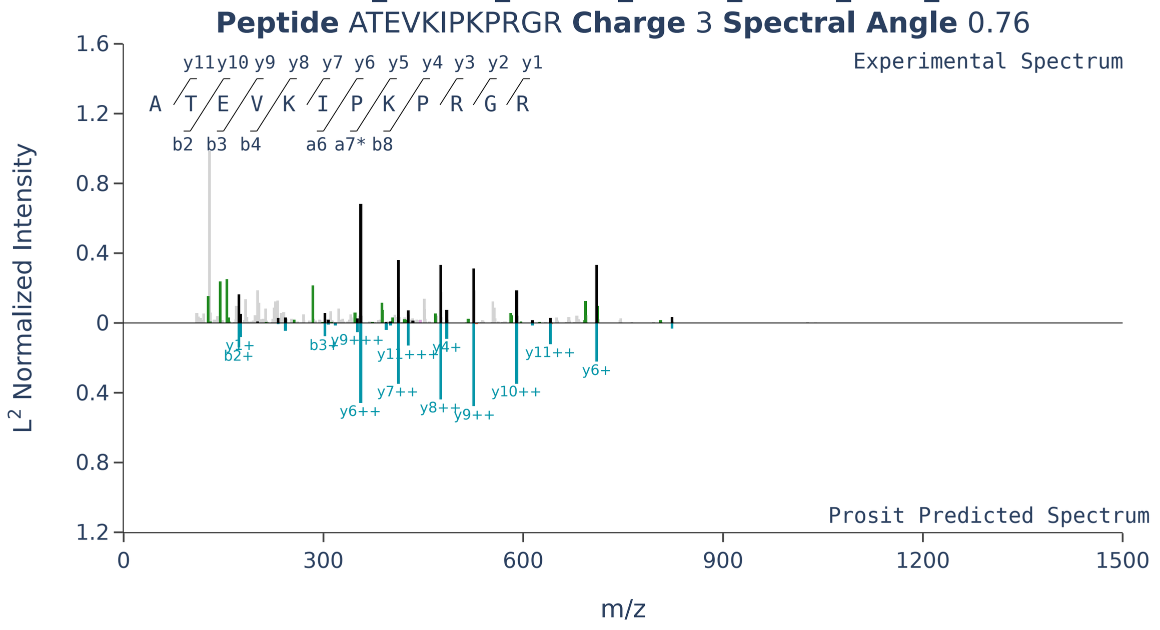


**Source** HRoetschke_271222_090123_HFGoe_LEDGF_b1R2_24h **Scan** 6031
**Peptide** VTMQQAQKHTE **Charge** 2 **Spectral Angle** 0.69

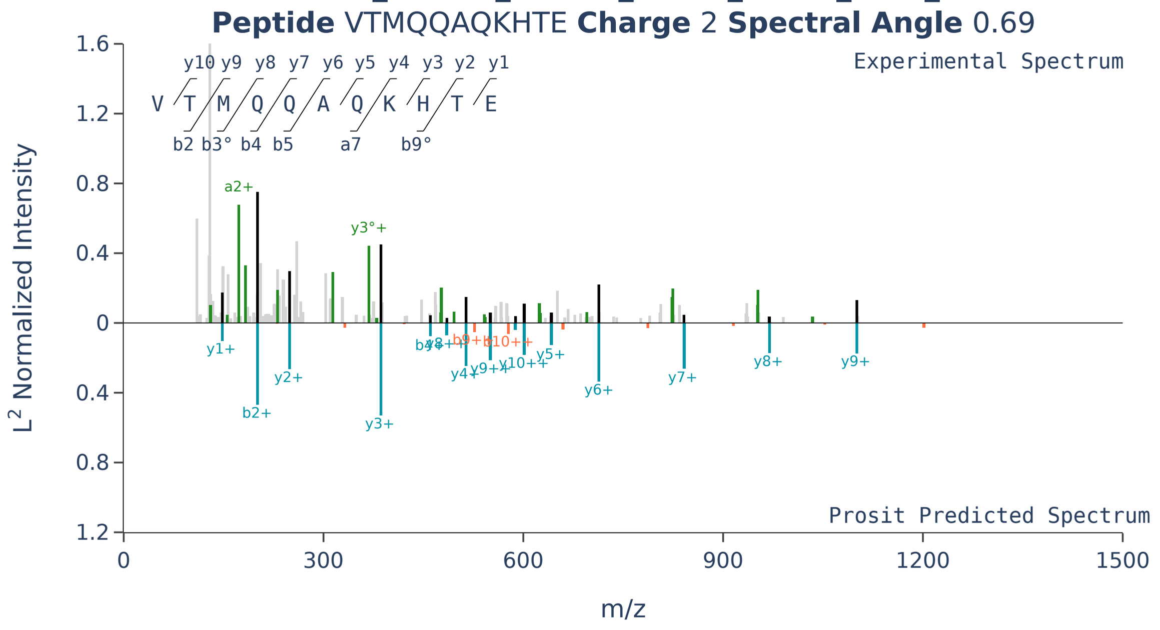


**Source** HRoetschke_271222_090123_HFGoe_LEDGF_b1R1_4h **Scan** 24380
**Peptide** GDSVITQVLNKSLAEQRQHEE **Charge** 3 **Spectral Angle** 0.8

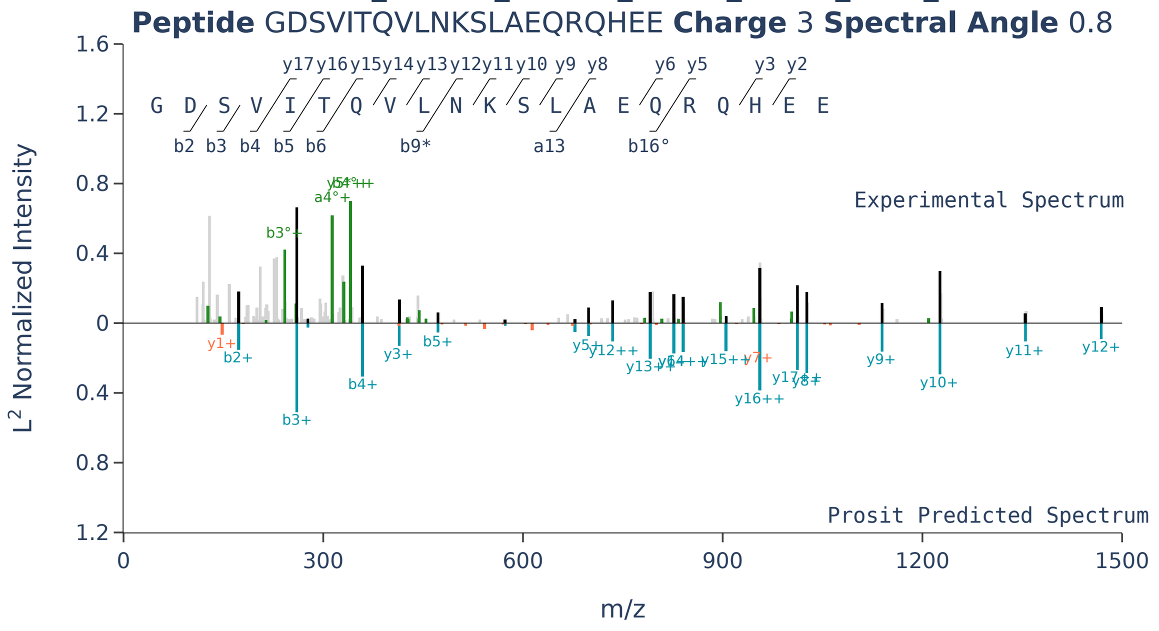


**Source** HRoetschke_271222_090123_HFGoe_LEDGF_b1R1_24h **Scan** 12872
**Peptide** KHTEMITTLKKI **Charge** 4 **Spectral Angle** 0.93

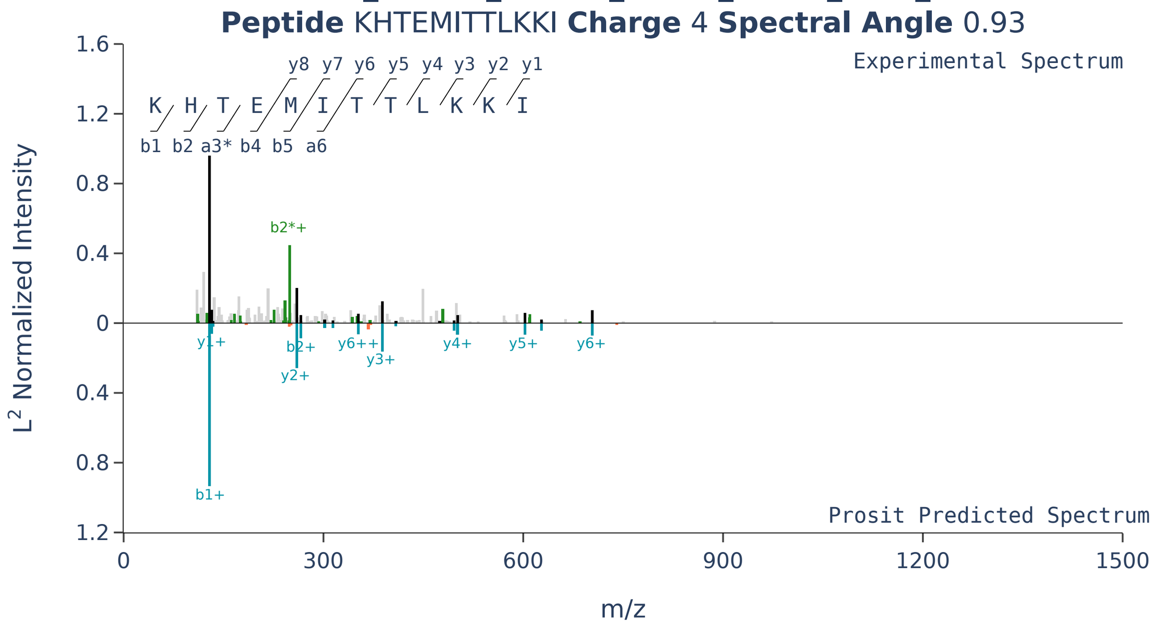


**Source** HRoetschke_271222_090123_HFGoe_LEDGF_b2R1_4h **Scan** 20135
**Peptide** IMEKSTMLYNKF **Charge** 2 **Spectral Angle** 0.77

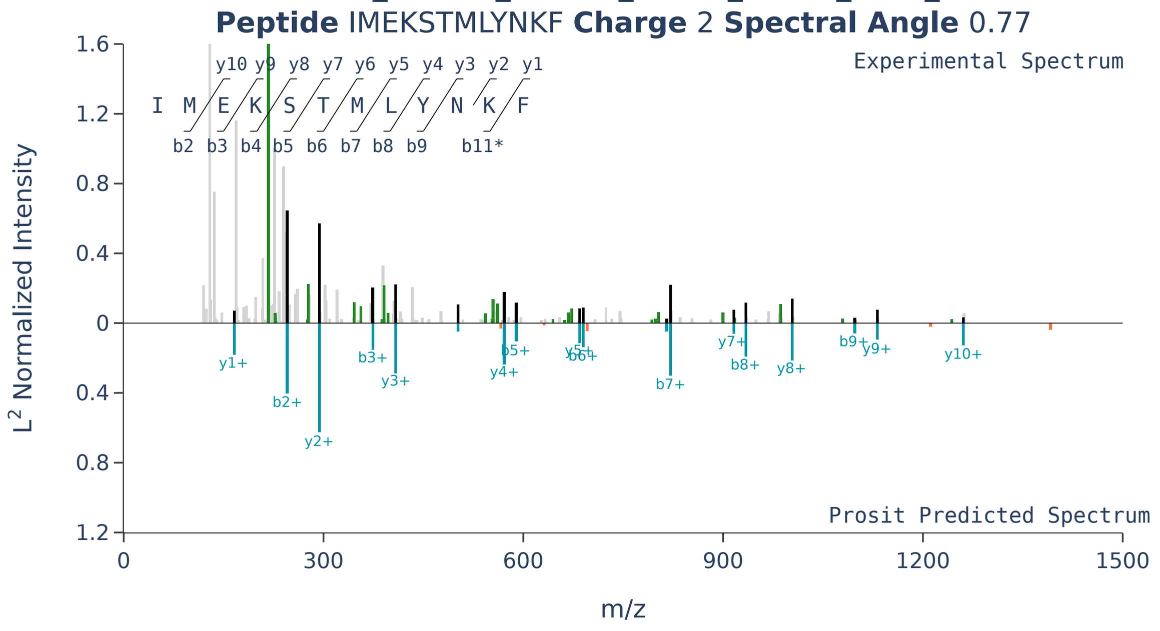


**Source** HRoetschke_271222_090123_HFGoe_LEDGF_b1R1_2h **Scan** 12259
**Peptide** IFPYSENKEKYGKPNKRKGFNEGL **Charge** 4 **Spectral Angle** 0.82

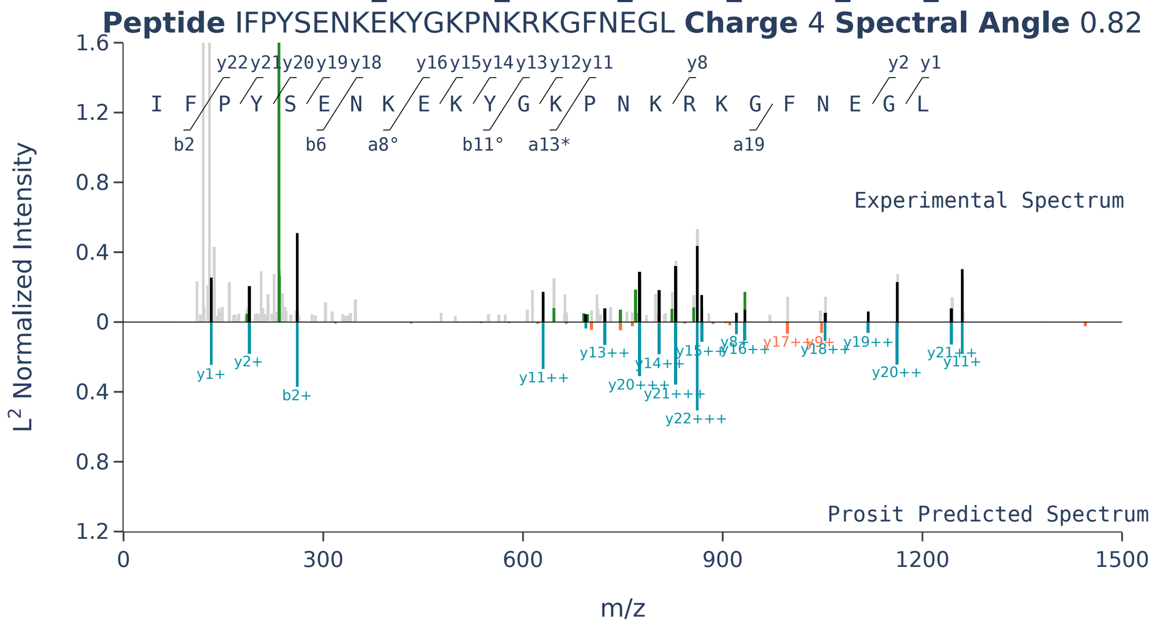


**Source** HRoetschke_271222_090123_HFGoe_LEDGF_b1R1_1h **Scan** 28824
**Peptide** KLPIFFFGTHET **Charge** 2 **Spectral Angle** 0.87

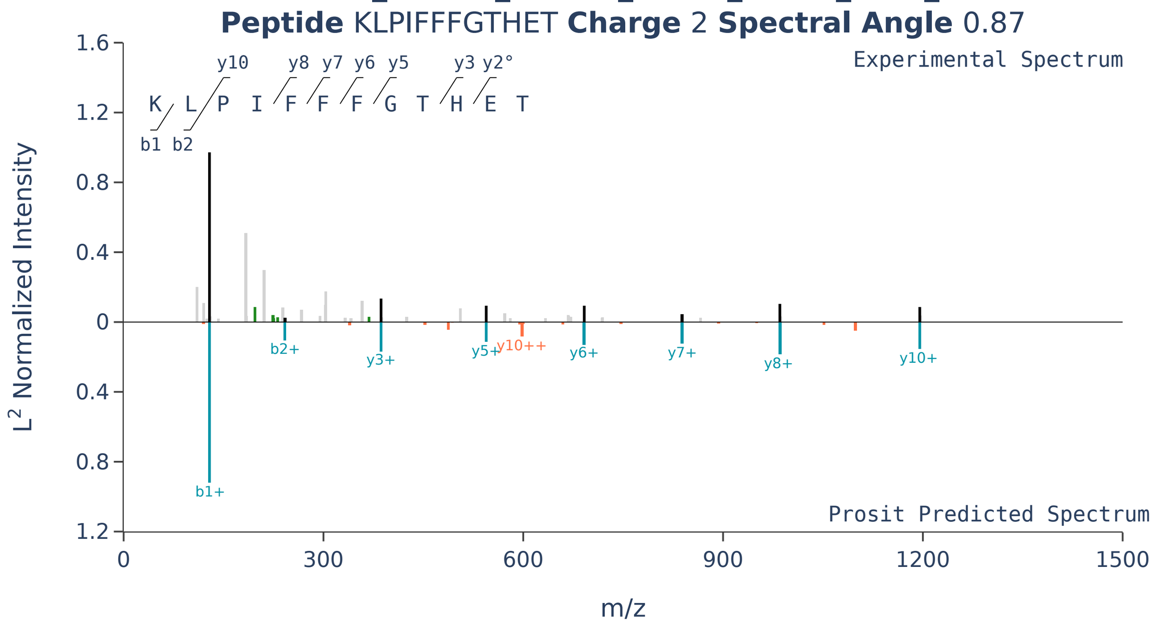


**Source** HRoetschke_271222_090123_HFGoe_LEDGF_b1R2_1h **Scan** 14884
**Peptide** KQPCPSESDIITE **Charge** 2 **Spectral Angle** 0.82

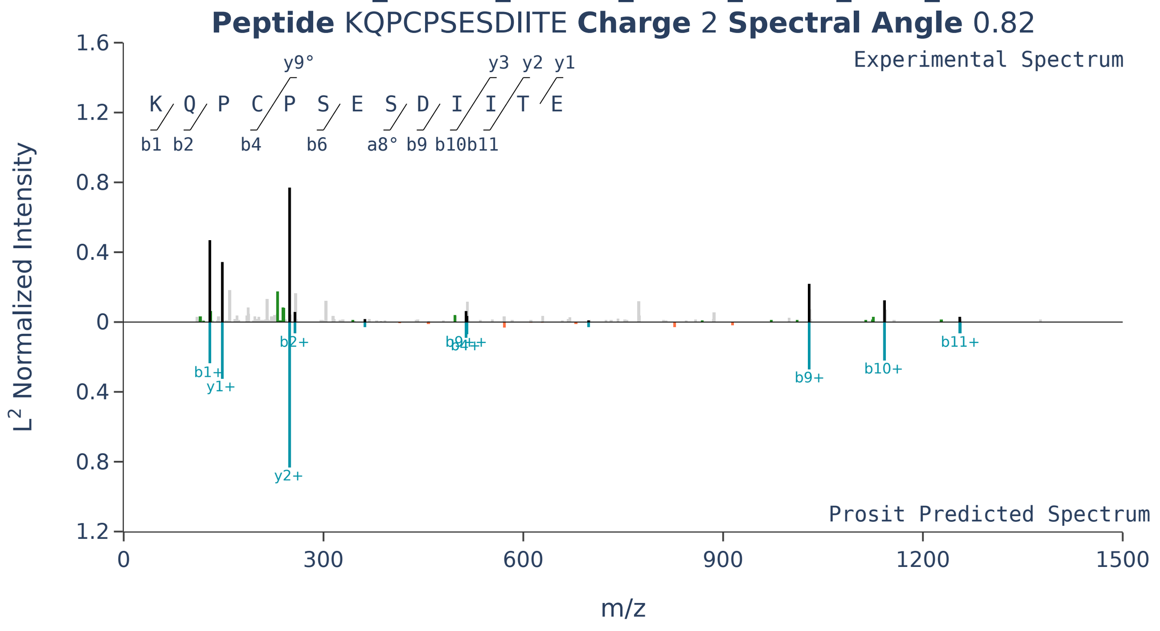


**Source** HRoetschke_271222_090123_HFGoe_LEDGF_b1R2_1h **Scan** 18025
**Peptide** ETSMDSRLQRIHAEL **Charge** 3 **Spectral Angle** 0.84

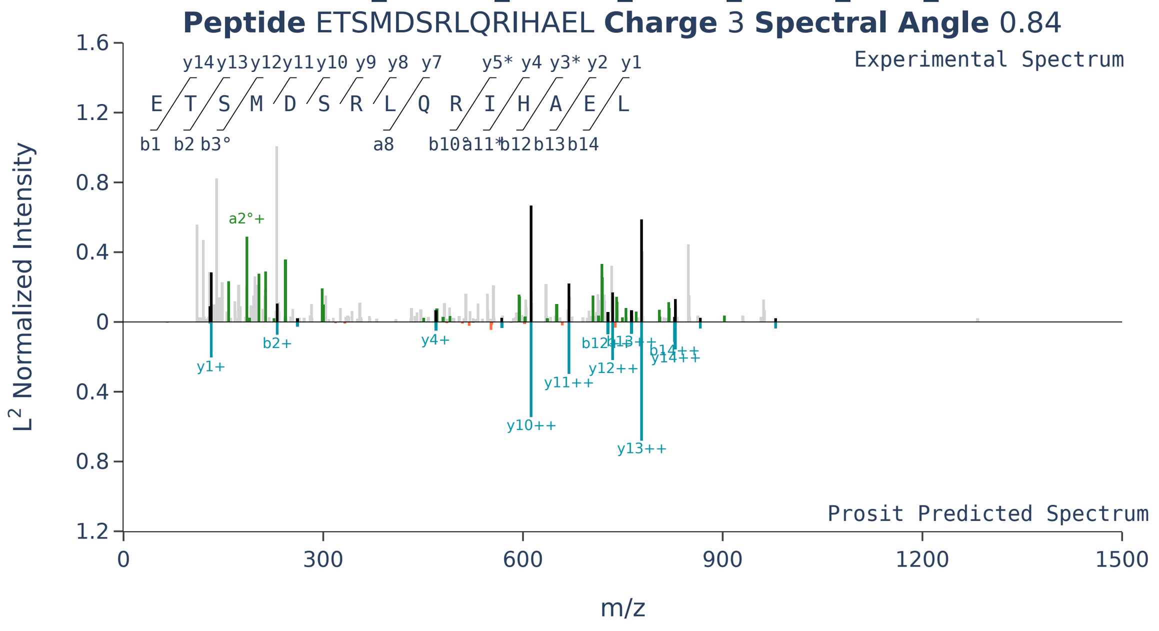


**Source** HRoetschke_271222_090123_HFGoe_LEDGF_b1R1_4h **Scan** 20774
**Peptide** TRDFKPGDLI **Charge** 3 **Spectral Angle** 0.64

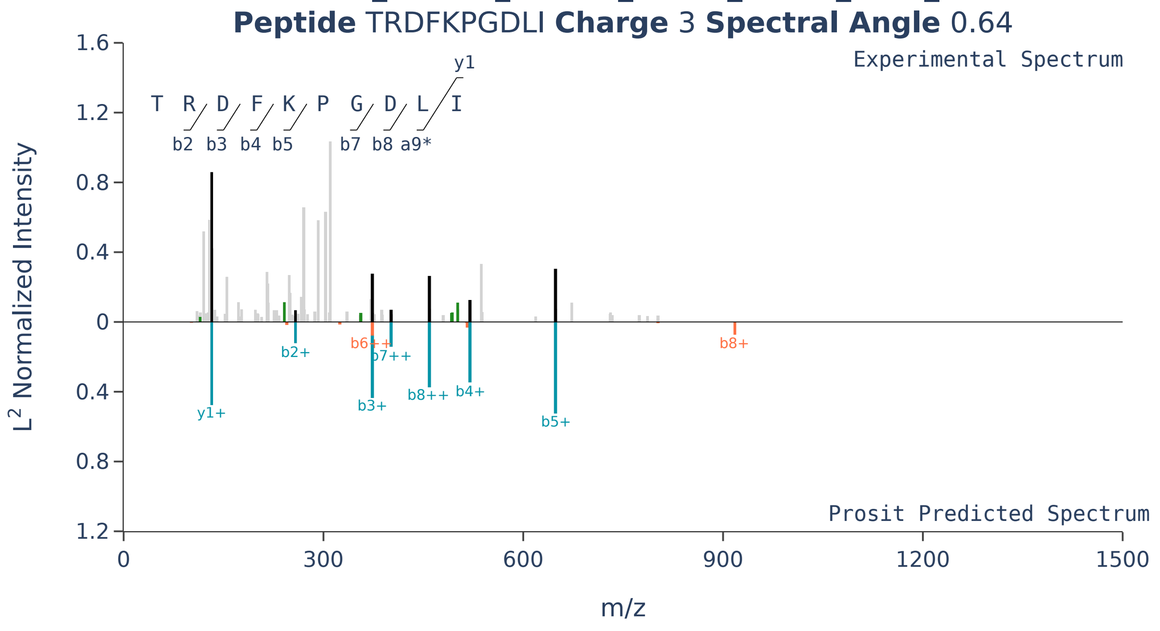


**Source** HRoetschke_271222_090123_HFGoe_LEDGF_b1R1_2h **Scan** 19736
**Peptide** GLWEIDNNPKVKFSSQQAATKQSN **Charge** 3 **Spectral Angle** 0.82

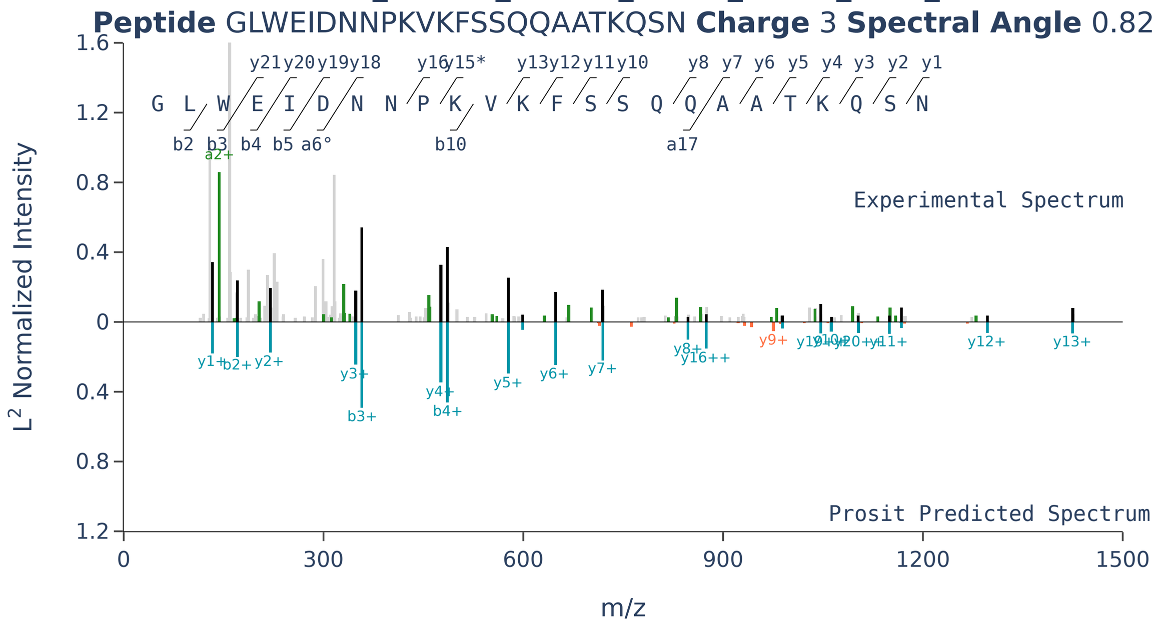

**Source** HRoetschke_271222_090123_HFGoe_LEDGF_b2R1_24h **Scan** 23015
**Peptide** NEGLWEIDNNPKVKFSSQQ **Charge** 2 **Spectral Angle** 0.92

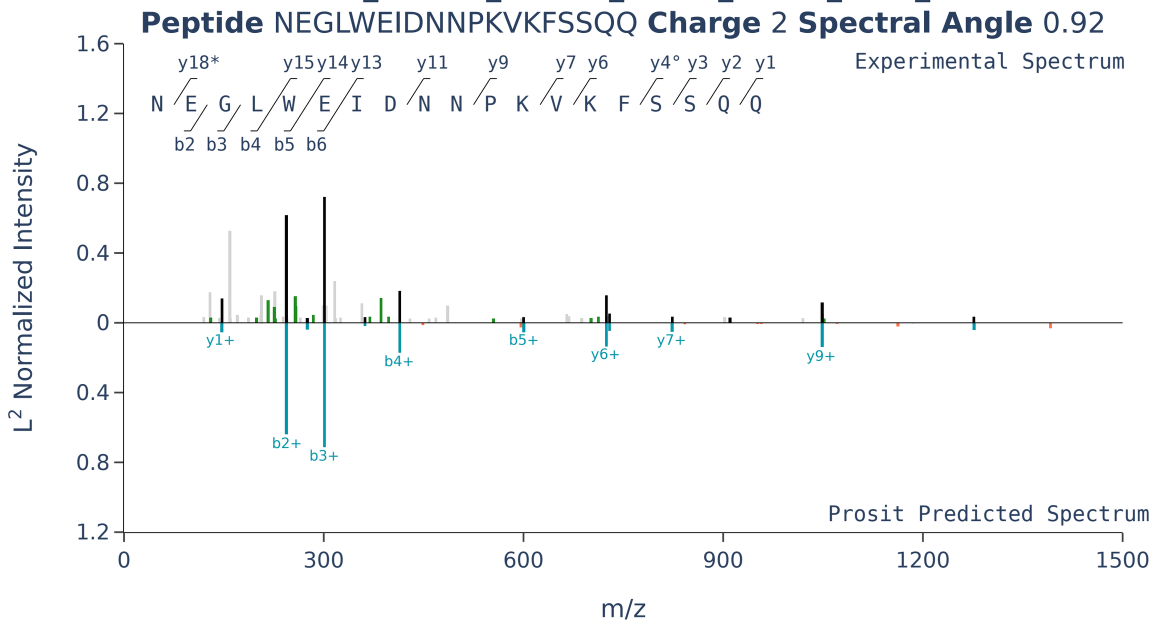


**Source** HRoetschke_271222_090123_HFGoe_LEDGF_b1R1_2h **Scan** 23285
**Peptide** SQVIMEKSTMLYNKFKNM **Charge** 3 **Spectral Angle** 0.93

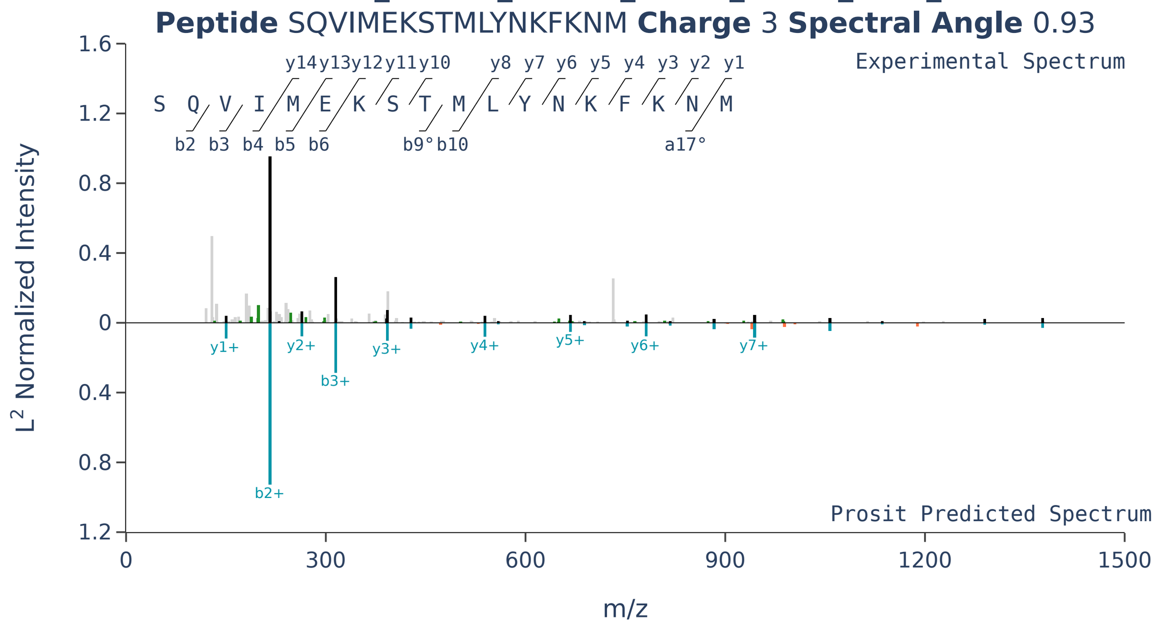


**Source** HRoetschke_271222_090123_HFGoe_LEDGF_b1R1_24h **Scan** 5668
**Peptide** SVSKEDTDHEEKASNEDVTKA **Charge** 3 **Spectral Angle** 0.8

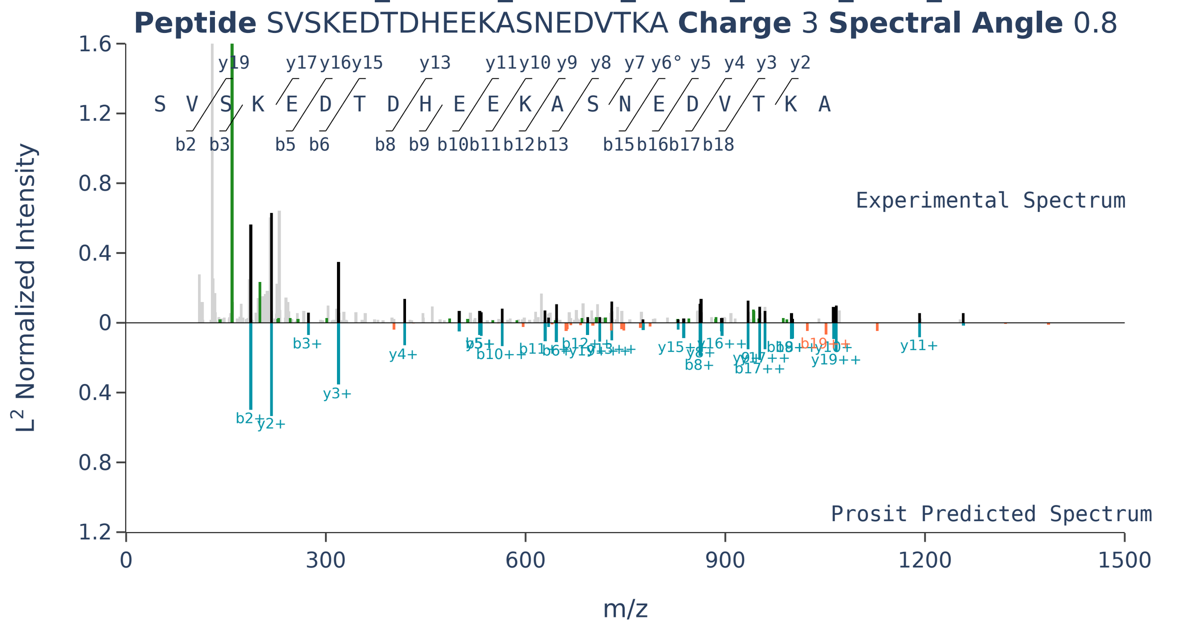


**Source** HRoetschke_271222_090123_HFGoe_LEDGF_b1R1_2h **Scan** 18521
**Peptide** RIHAEIKNSLKIDNLDVNRCIE **Charge** 3 **Spectral Angle** 0.81

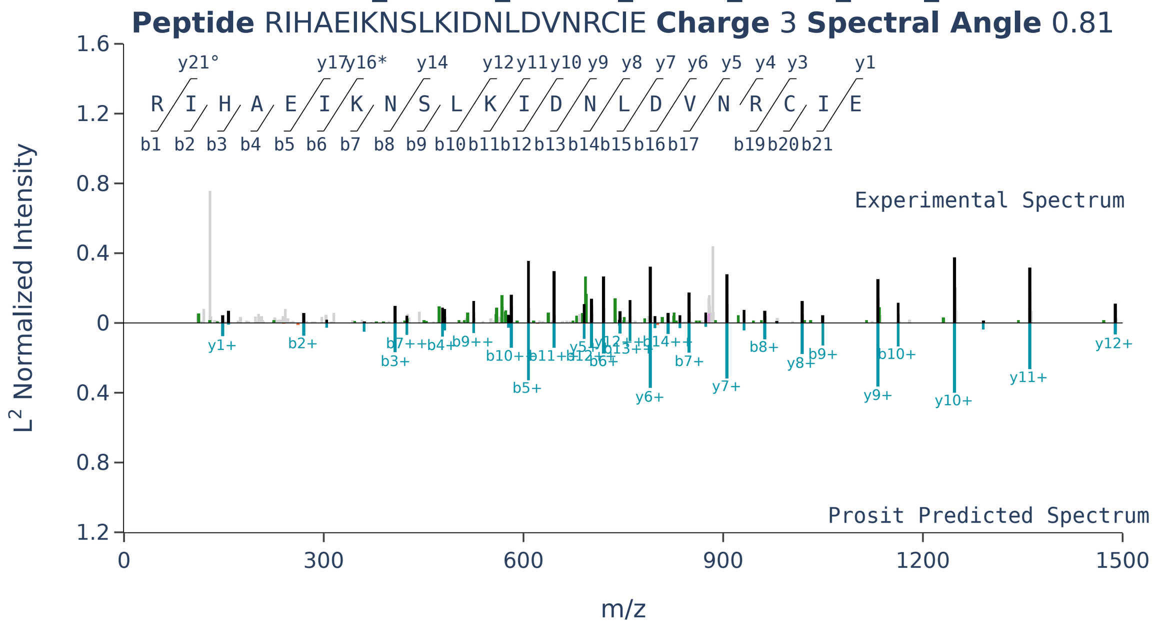


**Source** HRoetschke_271222_090123_HFGoe_LEDGF_b1R2_1h **Scan** 9595
**Peptide** SLKDSTLD **Charge** 2 **Spectral Angle** 0.92

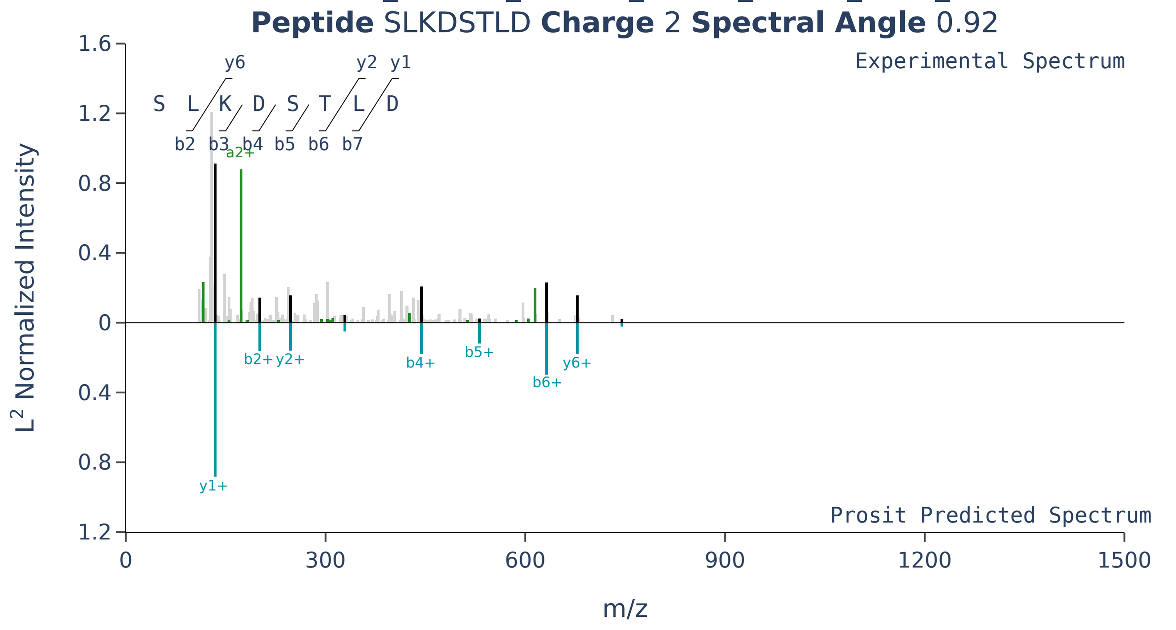


**Source** HRoetschke_271222_090123_HFGoe_LEDGF_b2R2_2h **Scan** 15750
**Peptide** GPKDIFPYSENKEKYG **Charge** 4 **Spectral Angle** 0.77

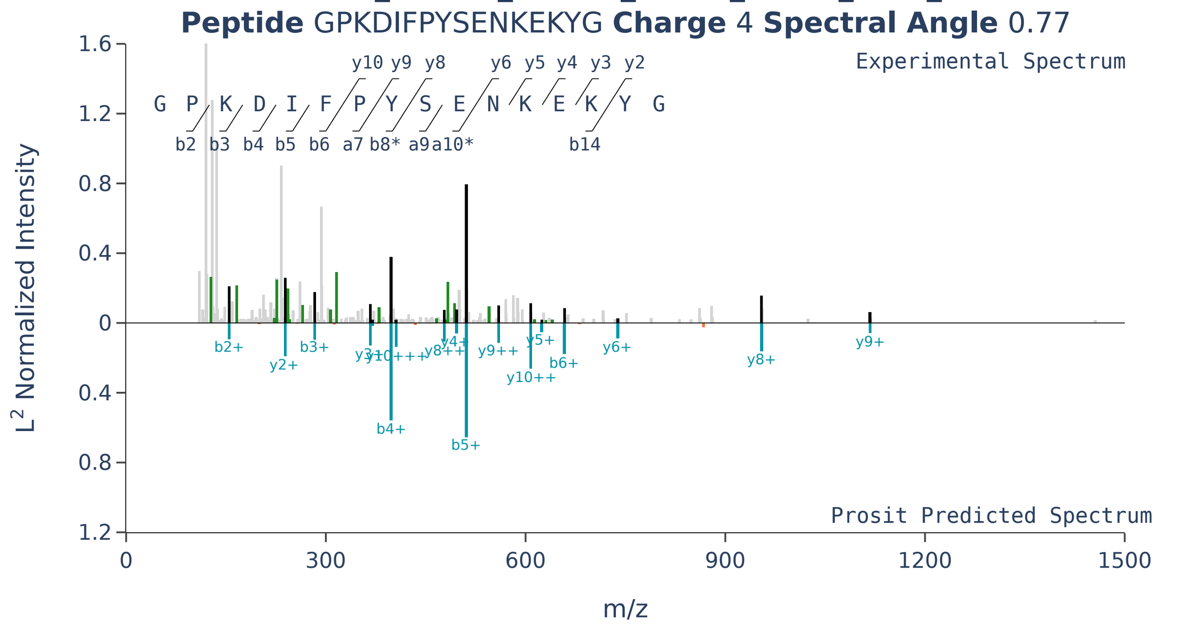


**Source** HRoetschke_271222_090123_HFGoe_LEDGF_b1R2_24h **Scan** 17053
**Peptide** ISLKDSTLD **Charge** 2 **Spectral Angle** 0.75

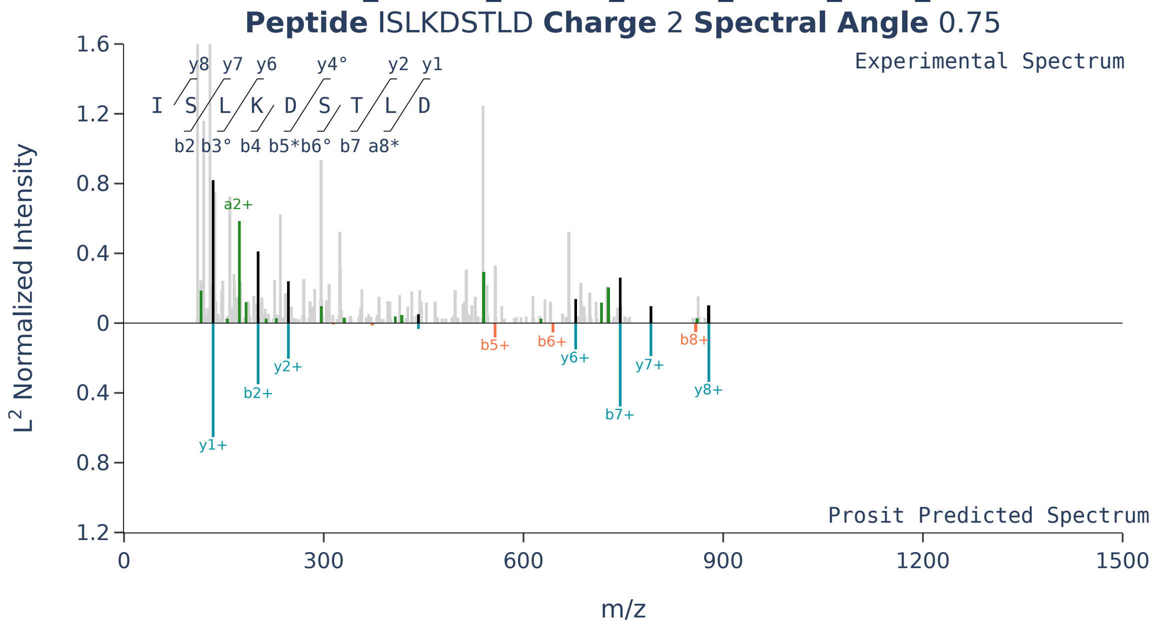


**Source** HRoetschke_271222_090123_HFGoe_LEDGF_b2R1_24h **Scan** 18292
**Peptide** MDSRLQRIHAEIKNSL **Charge** 3 **Spectral Angle** 0.78

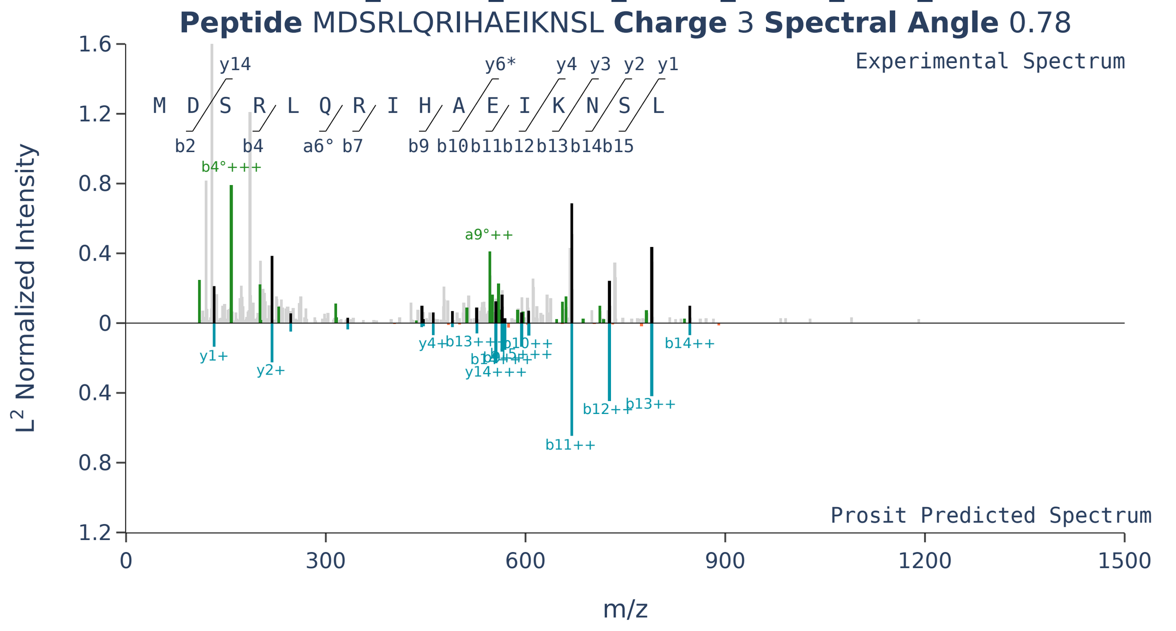


**Source** HRoetschke_271222_090123_HFGoe_LEDGF_b2R1_24h **Scan** 25637
**Peptide** GDSVITQVLN **Charge** 1 **Spectral Angle** 0.85

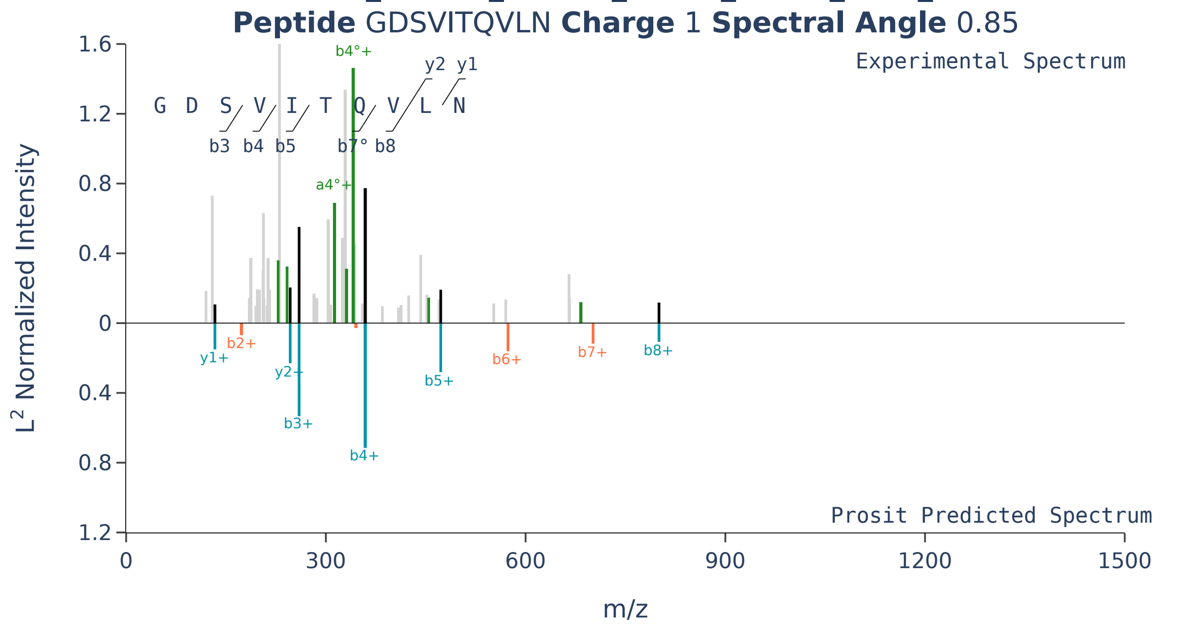


**Source** HRoetschke_271222_090123_HFGoe_LEDGF_b1R1_2h **Scan** 23496
**Peptide** SMDSRLQRIHAEIKNSLKIDNLD **Charge** 5 **Spectral Angle** 0.87

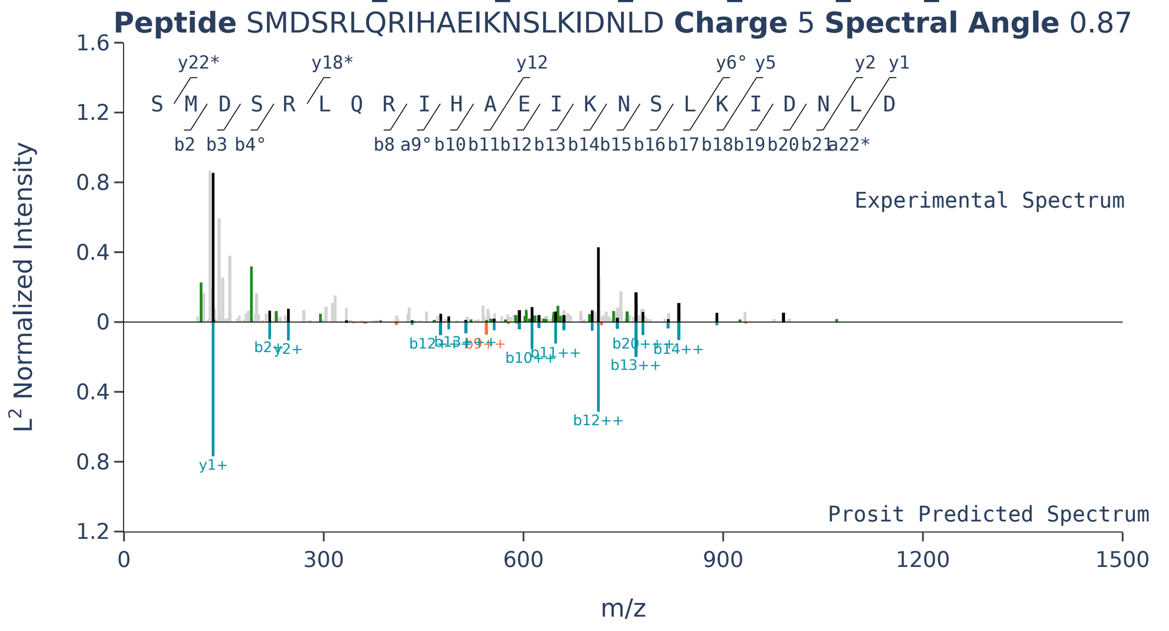


**Source** HRoetschke_271222_090123_HFGoe_LEDGF_b2R1_24h **Scan** 8514
**Peptide** AVDITTPKAARRG **Charge** 3 **Spectral Angle** 0.84

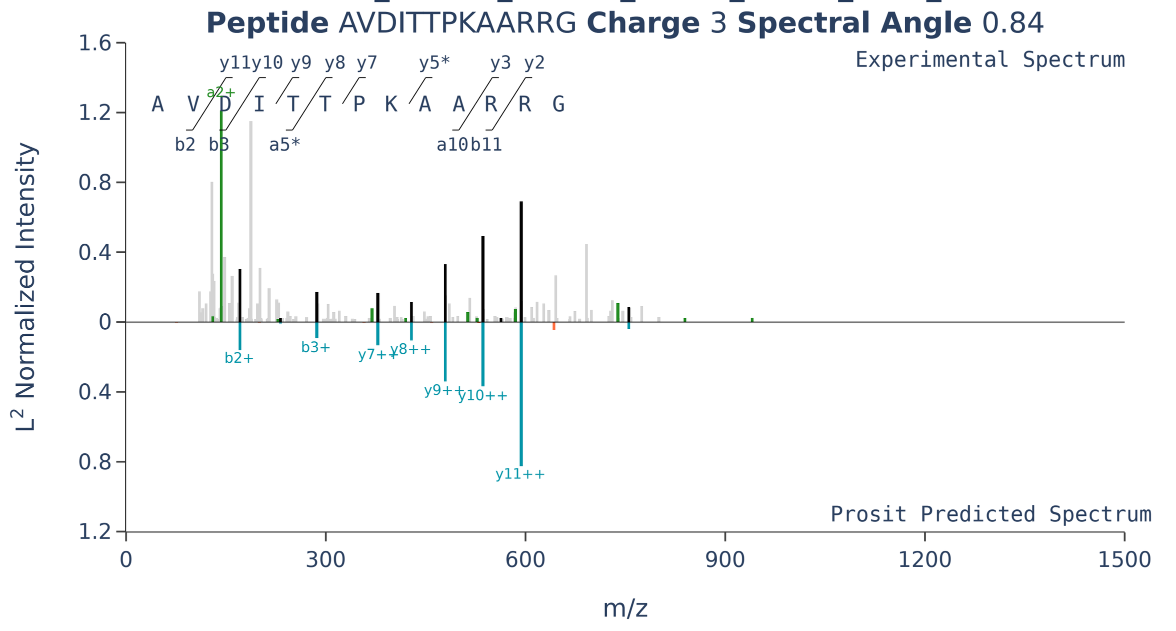


**Source** HRoetschke_271222_090123_HFGoe_LEDGF_b2R2_24h **Scan** 15667
**Peptide** GVVTTATASVNLKVSPKRGRPAATEV **Charge** 4 **Spectral Angle** 0.72

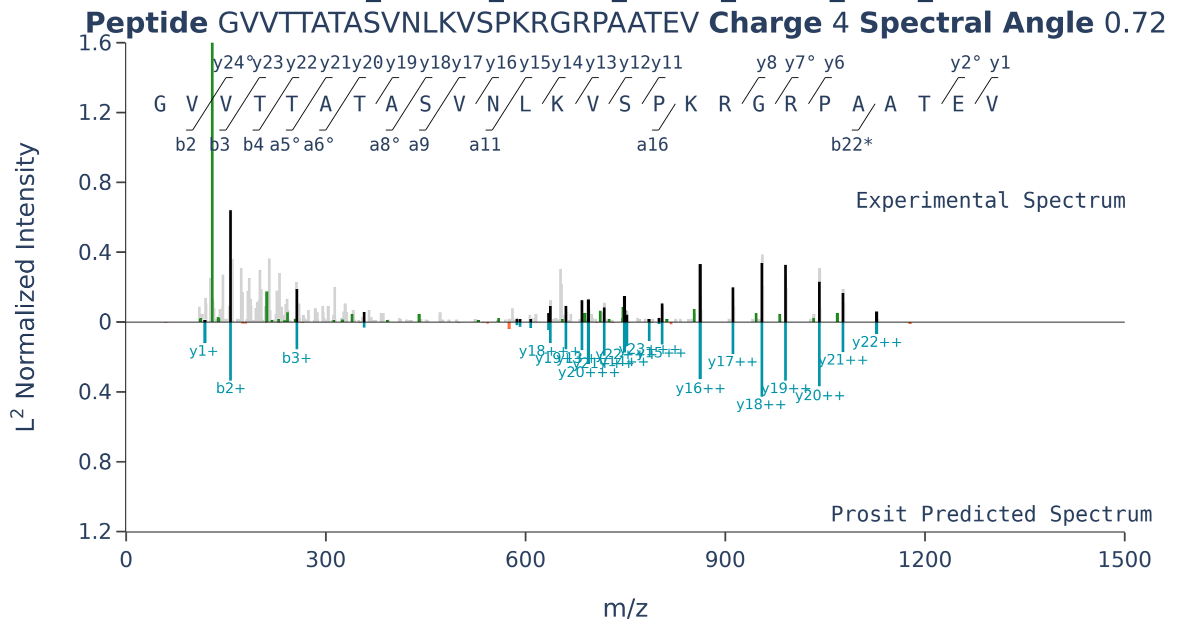


**Source** HRoetschke_271222_090123_HFGoe_LEDGF_b2R1_4h **Scan** 6414
**Peptide** QMETEQQNKDEGKKPEV **Charge** 3 **Spectral Angle** 0.83

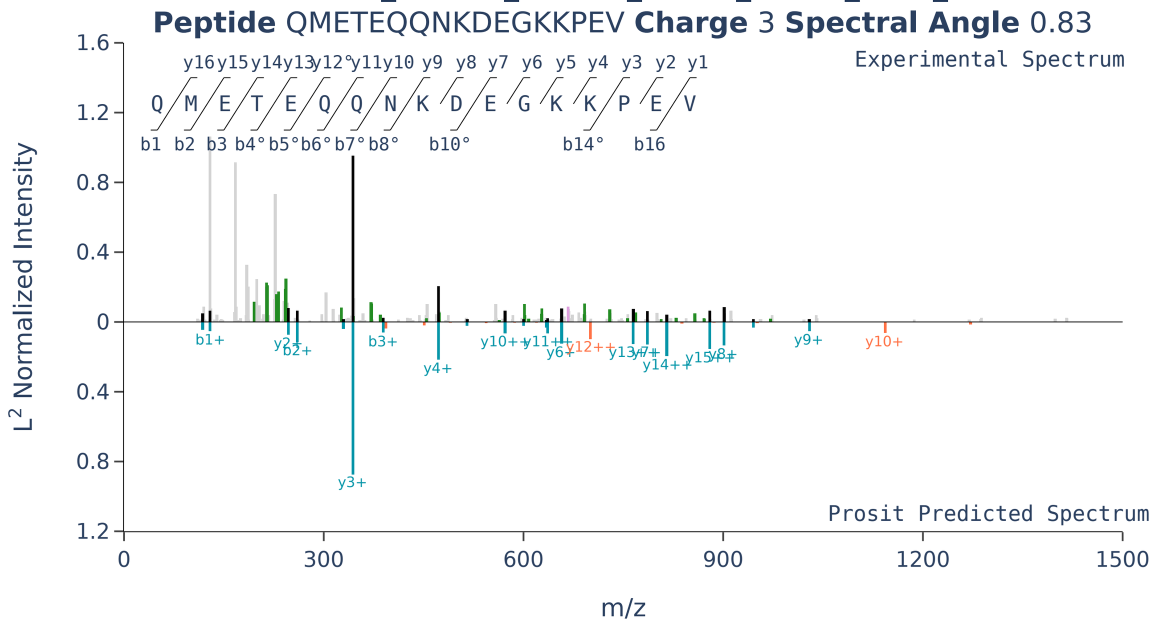


**Source** HRoetschke_271222_090123_HFGoe_LEDGF_b2R2_2h **Scan** 26521
**Peptide** GEGDSVITQVLN **Charge** 2 **Spectral Angle** 0.82

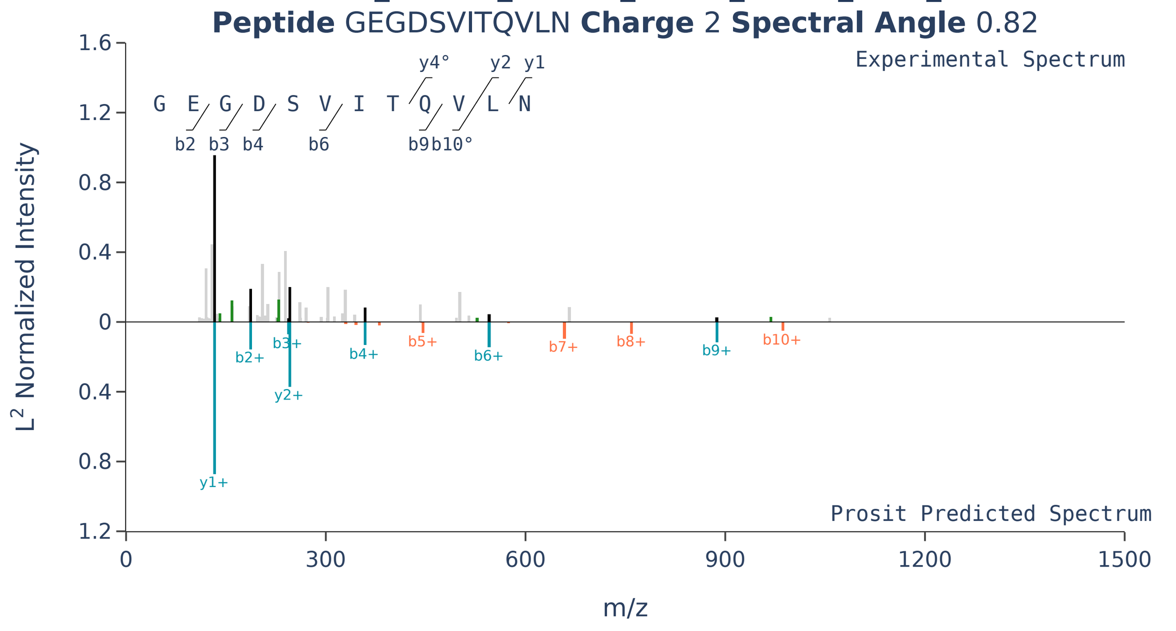


**Source** HRoetschke_271222_090123_HFGoe_LEDGF_b1R1_4h **Scan** 13134
**Peptide** IDNNPKVKFSSQQAATKQSNASSDVEVEE **Charge** 4 **Spectral Angle** 0.71

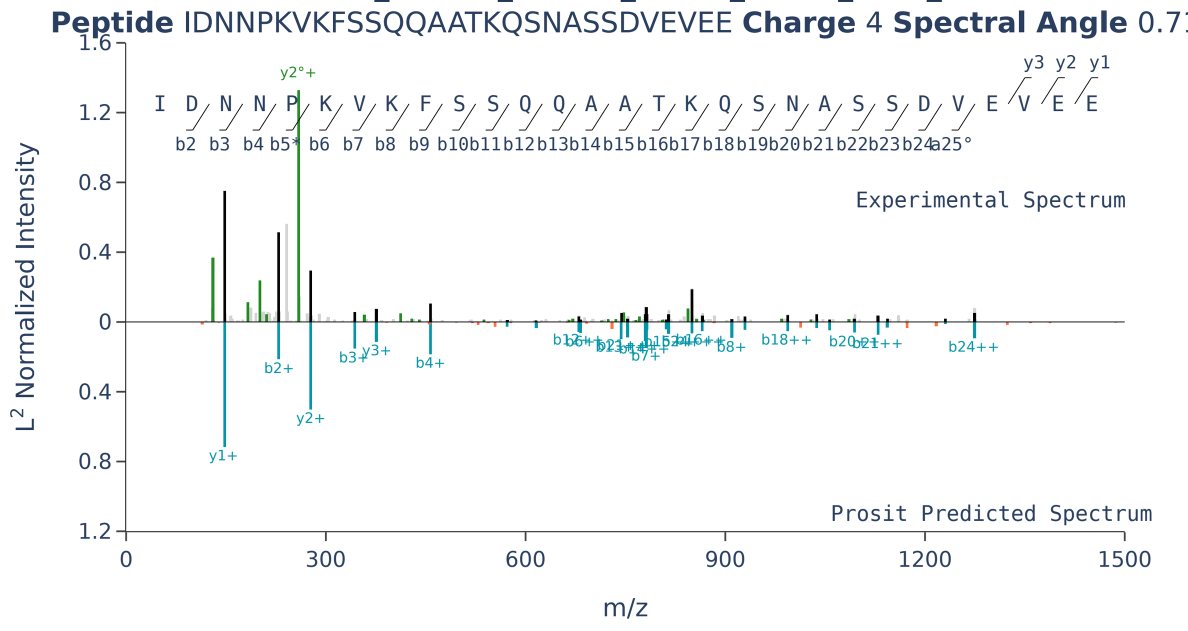

**Source** HRoetschke_271222_090123_HFGoe_LEDGF_b1R1_1h **Scan** 11902
**Peptide** QQAQKHTEMITTL **Charge** 3 **Spectral Angle** 0.8

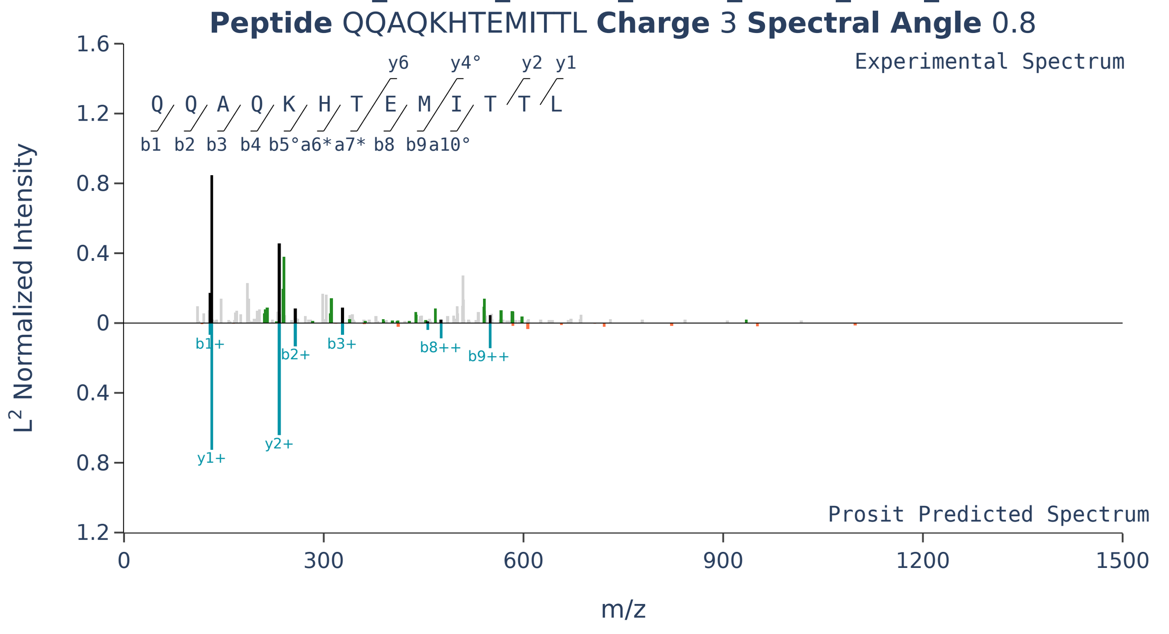


**Source** HRoetschke_271222_090123_HFGoe_LEDGF_b1R1_2h **Scan** 16531
**Peptide** PHWPARVDEVPDGAVK **Charge** 3 **Spectral Angle** 0.79

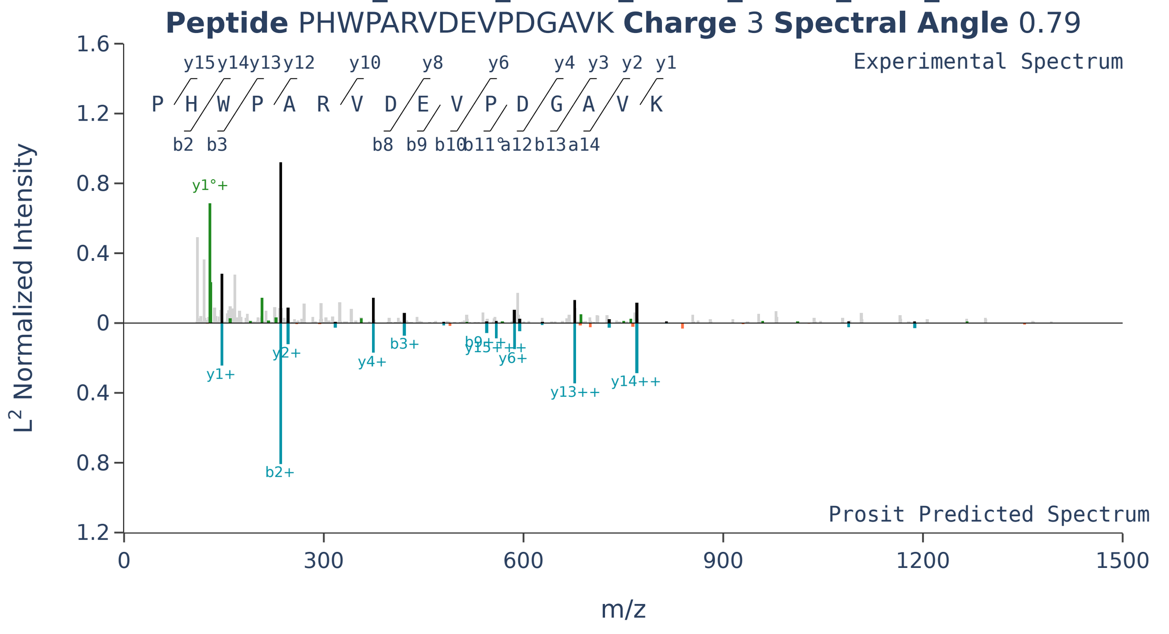


**Source** HRoetschke_271222_090123_HFGoe_LEDGF_b1R1_1h **Scan** 12390
**Peptide** RLHAEIKNSLKID **Charge** 4 **Spectral Angle** 0.76

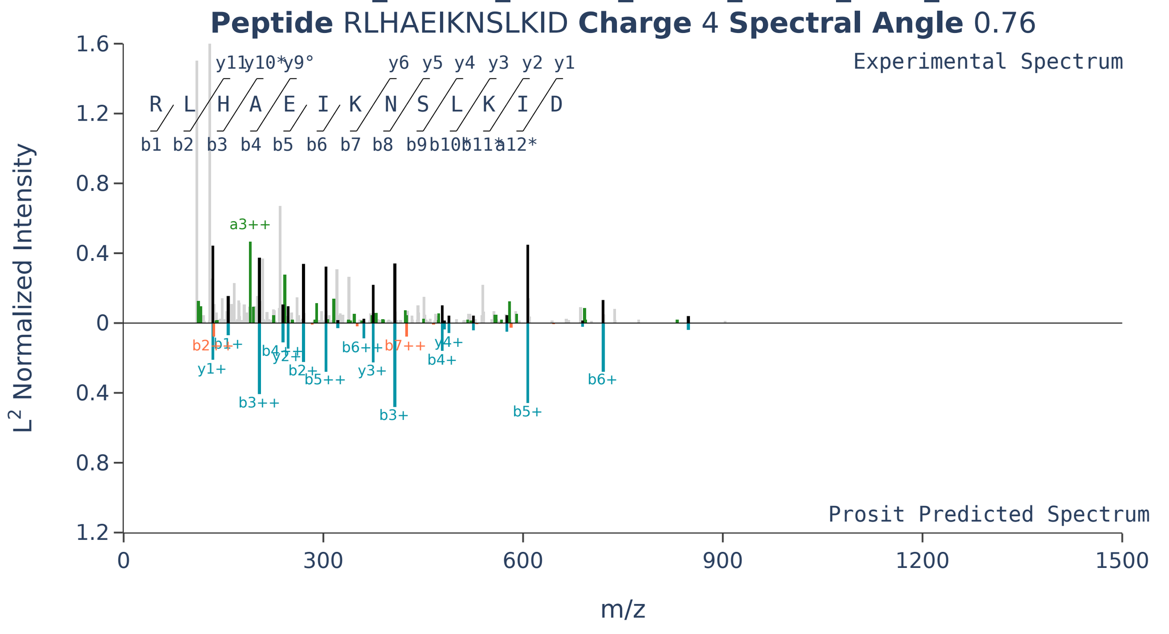


**Source** HRoetschke_271222_090123_HFGoe_LEDGF_b2R1_24h **Scan** 7841
**Peptide** RPAATEVKIPKPRGR **Charge** 4 **Spectral Angle** 0.81

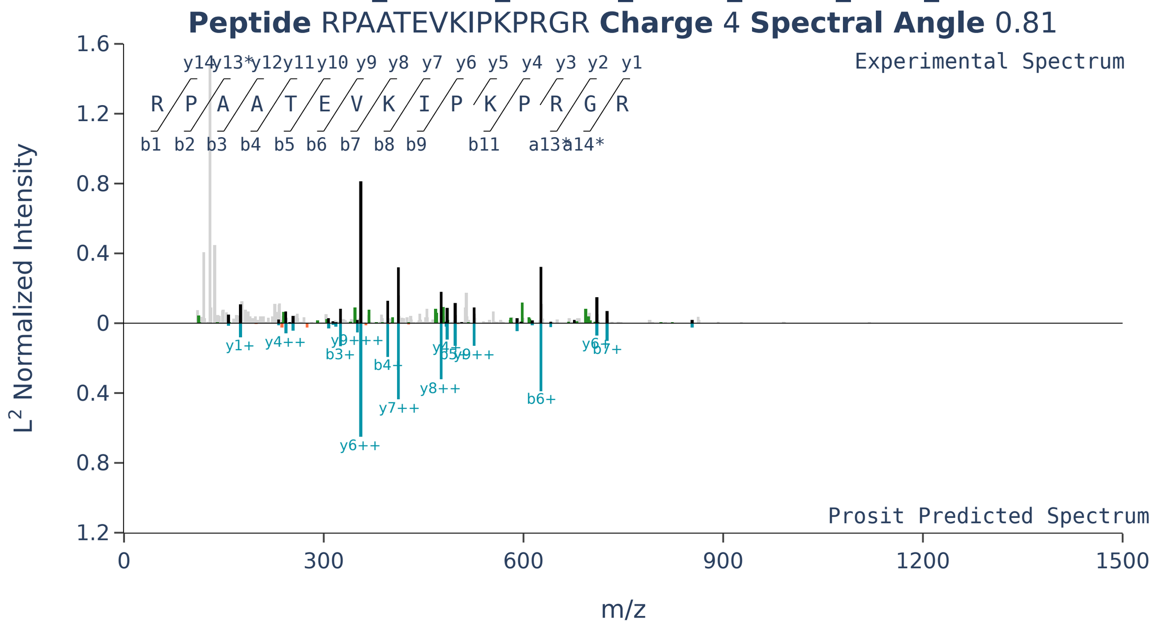


**Source** HRoetschke_271222_090123_HFGoe_LEDGF_b1R2_24h **Scan** 9744
**Peptide** EIDNNPKVKFSSQQAATKQS **Charge** 2 **Spectral Angle** 0.84

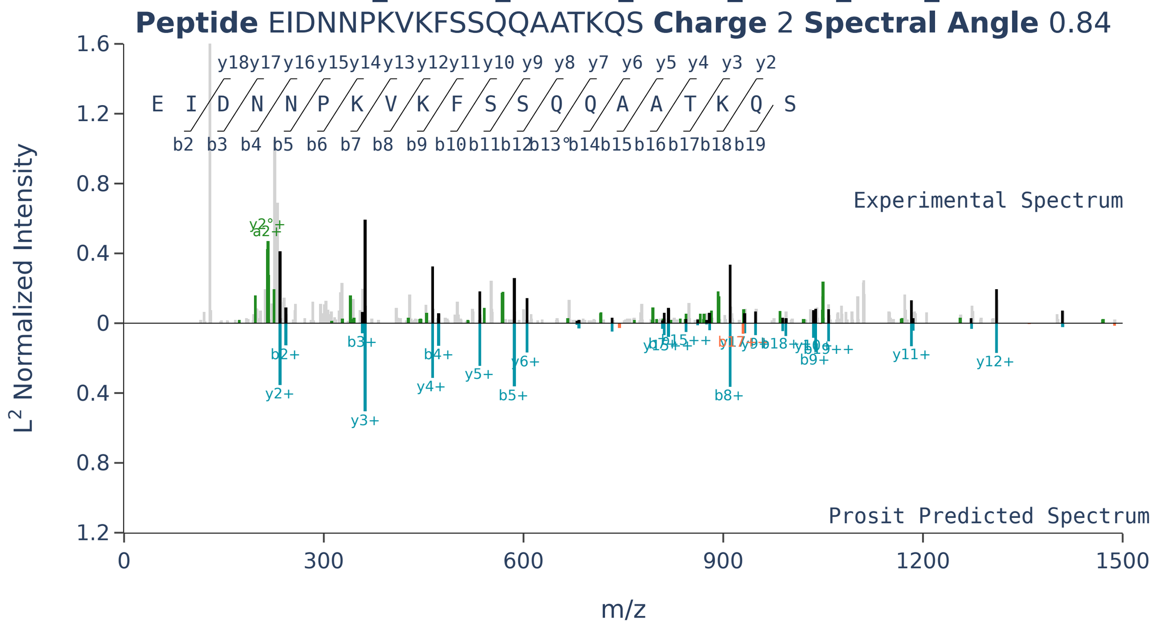


**Source** HRoetschke_271222_090123_HFGoe_LEDGF_b2R1_24h **Scan** 17162
**Peptide** QRIHAEIKNSLKIDNL **Charge** 4 **Spectral Angle** 0.81

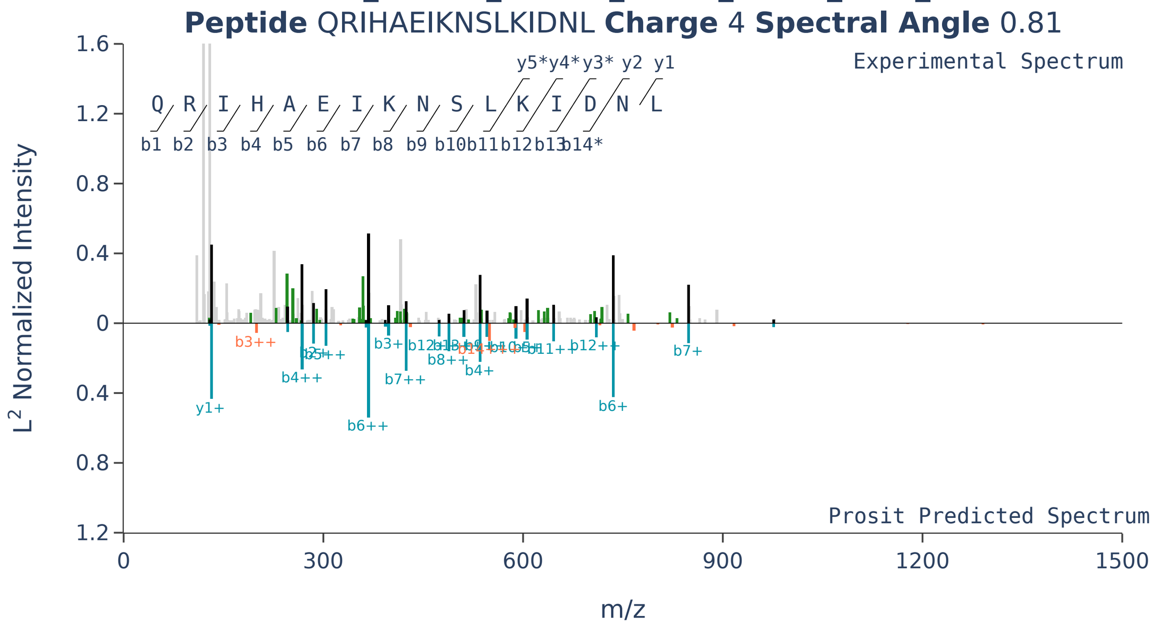


**Source** HRoetschke_271222_090123_HFGoe_LEDGF_b1R2_24h **Scan** 16254
**Peptide** WEIDNNPKVKFSSQQAATKQS **Charge** 2 **Spectral Angle** 0.81

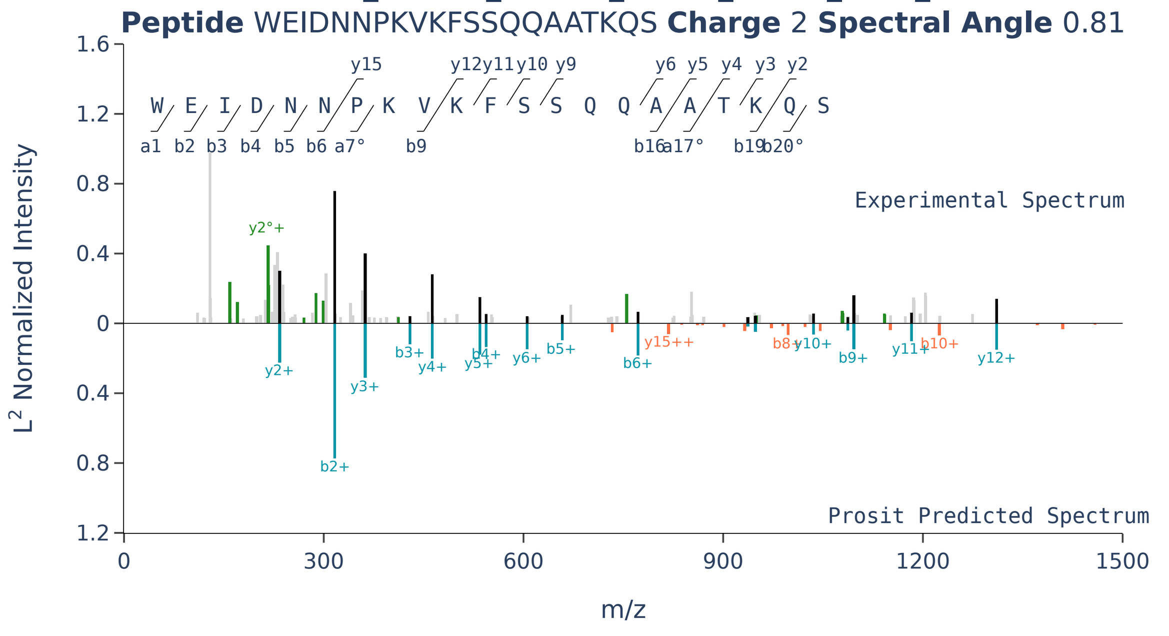


**Source** HRoetschke_271222_090123_HFGoe_LEDGF_b2R1_2h **Scan** 5573
**Peptide** DSKDNHEASTKKKPSSEERETEI **Charge** 3 **Spectral Angle** 0.86

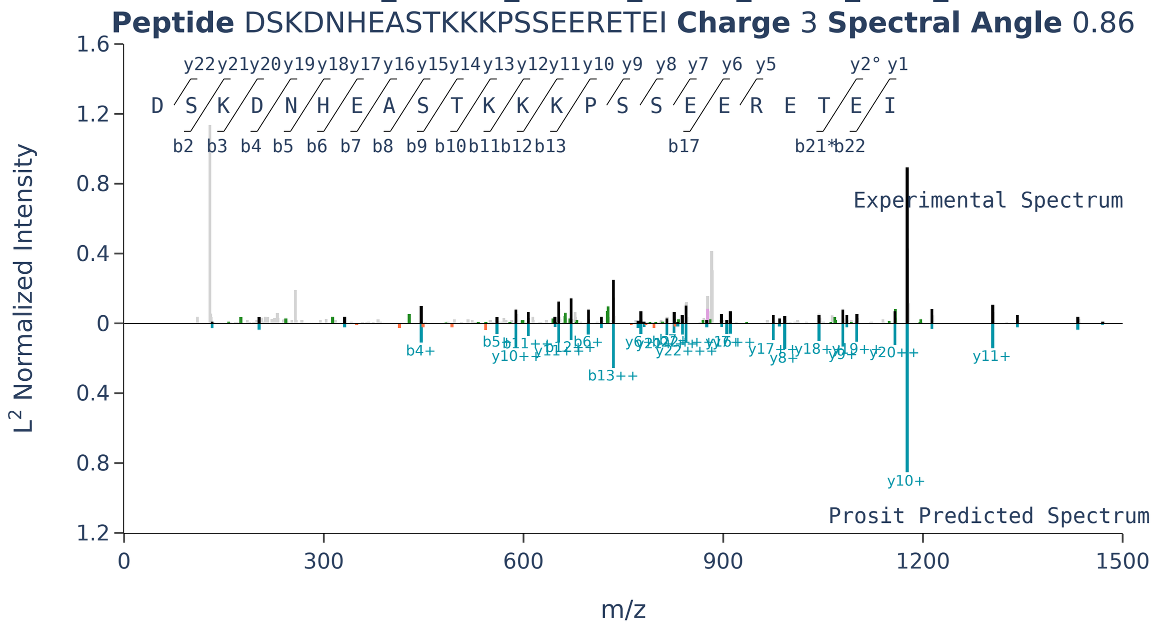


**Source** HRoetschke_271222_090123_HFGoe_LEDGF_b2R1_24h **Scan** 22872
**Peptide** FLGPKDIFPYSENKEK **Charge** 4 **Spectral Angle** 0.73

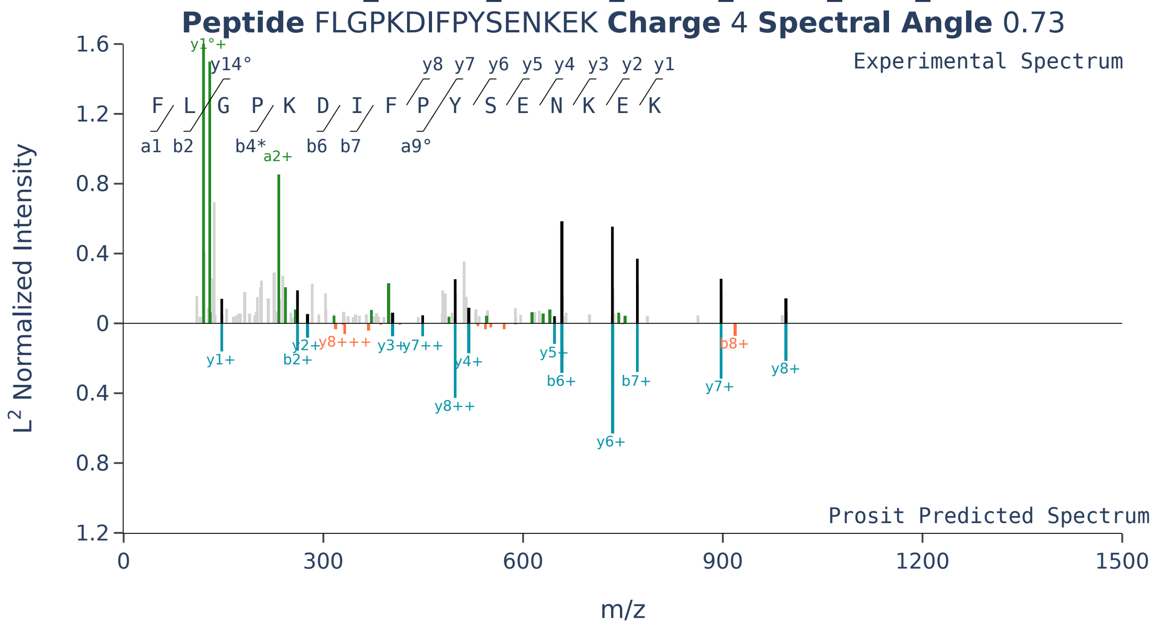


**Source** HRoetschke_271222_090123_HFGoe_LEDGF_b2R2_2h **Scan** 11220
**Peptide** NNPKVKFSSQQAATKQSNASSDVEVEE **Charge** 4 **Spectral Angle** 0.85

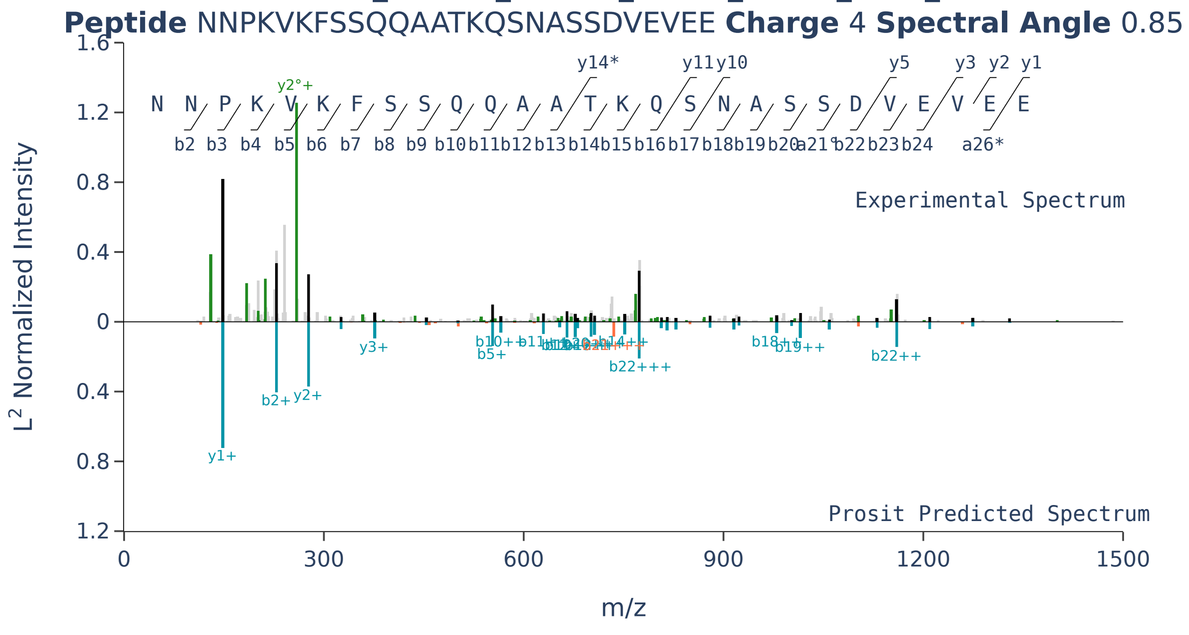


**Source** HRoetschke_271222_090123_HFGoe_LEDGF_b1R1_1h **Scan** 21382
**Peptide** FNEGLWEIDNNPKVKFSSQQAATKQSN **Charge** 4 **Spectral Angle** 0.89

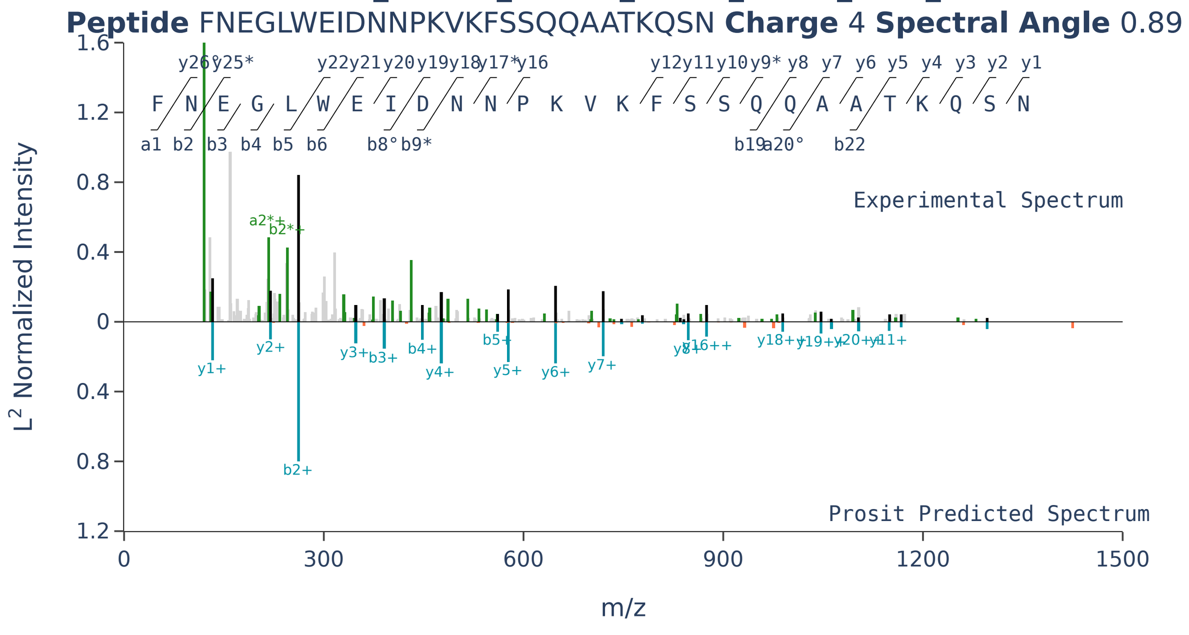


**Source** HRoetschke_271222_090123_HFGoe_LEDGF_b2R1_24h **Scan** 20719
**Peptide** KGFNEGLWEIDNNPKVKFSSQQAATKQ **Charge** 4 **Spectral Angle** 0.84

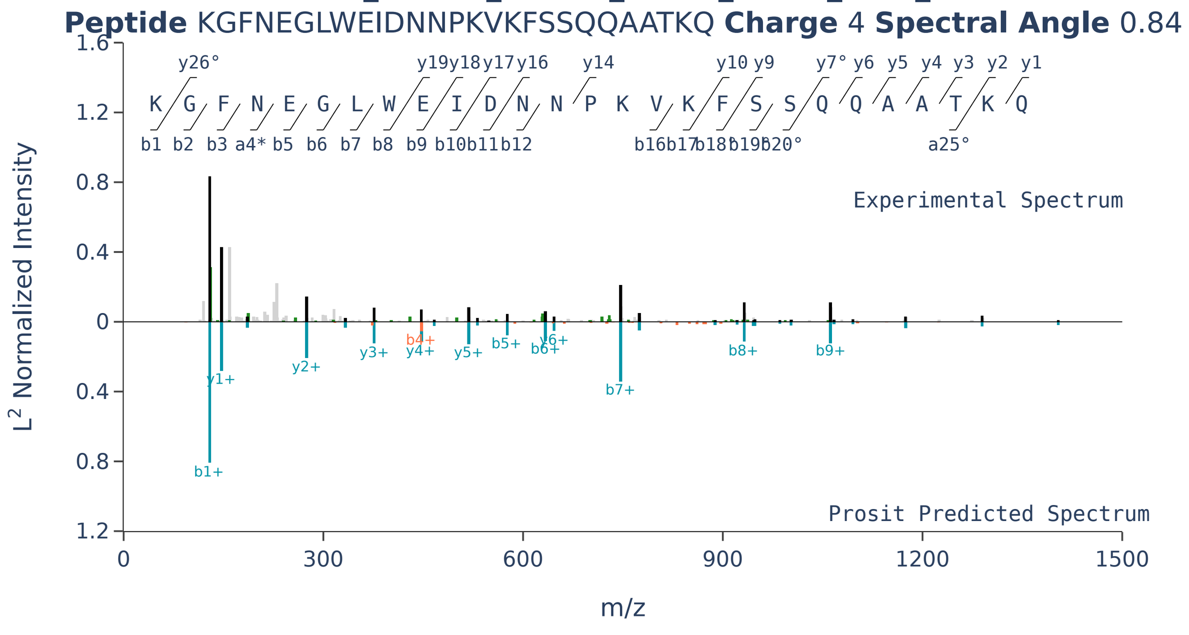


**Source** HRoetschke_271222_090123_HFGoe_LEDGF_b1R1_4h **Scan** 20735
**Peptide** KGFNEGLWEIDNNPKVKFSSQQAATKQS **Charge** 4 **Spectral Angle** 0.87

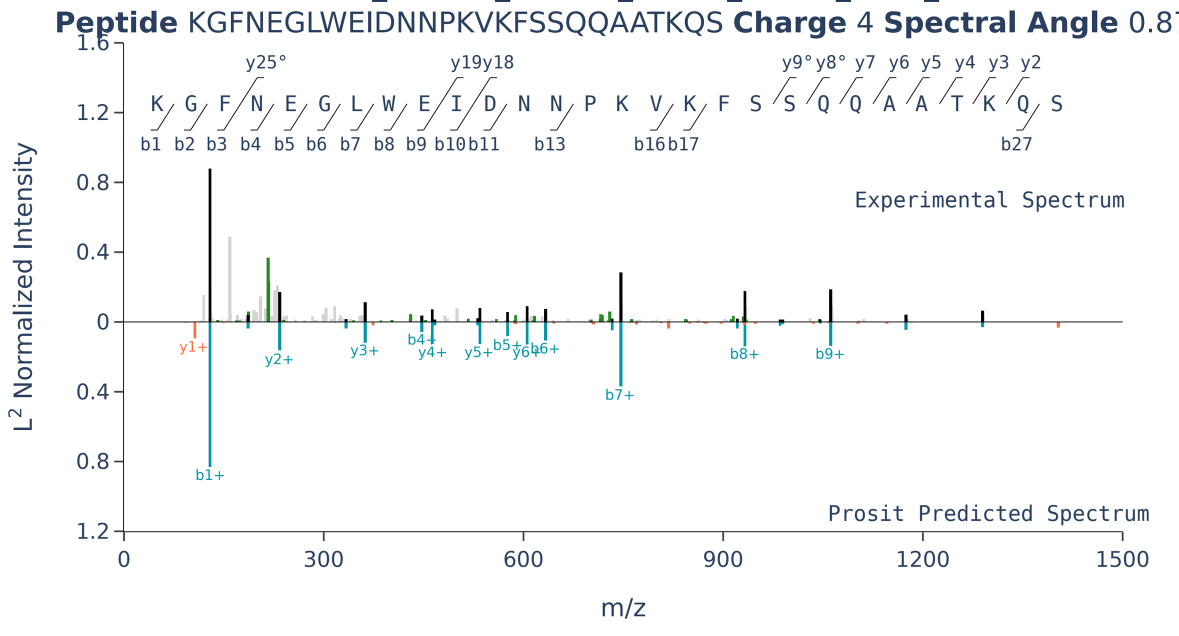


**Source** HRoetschke_271222_090123_HFGoe_LEDGF_b2R1_4h **Scan** 5724
**Peptide** VLNKSLAEQRQHEEANKTKD **Charge** 3 **Spectral Angle** 0.75

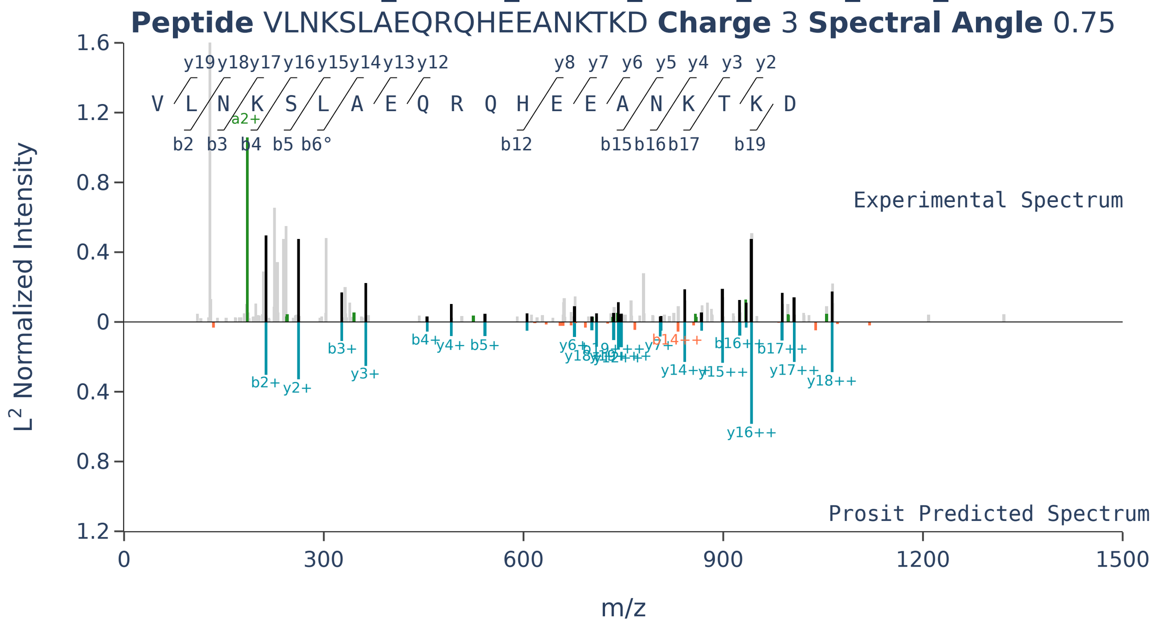


**Source** HRoetschke_271222_090123_HFGoe_LEDGF_b1R1_4h **Scan** 22919
**Peptide** KGFNEGLWEIDNNPKVKFSSQQAAT **Charge** 3 **Spectral Angle** 0.86

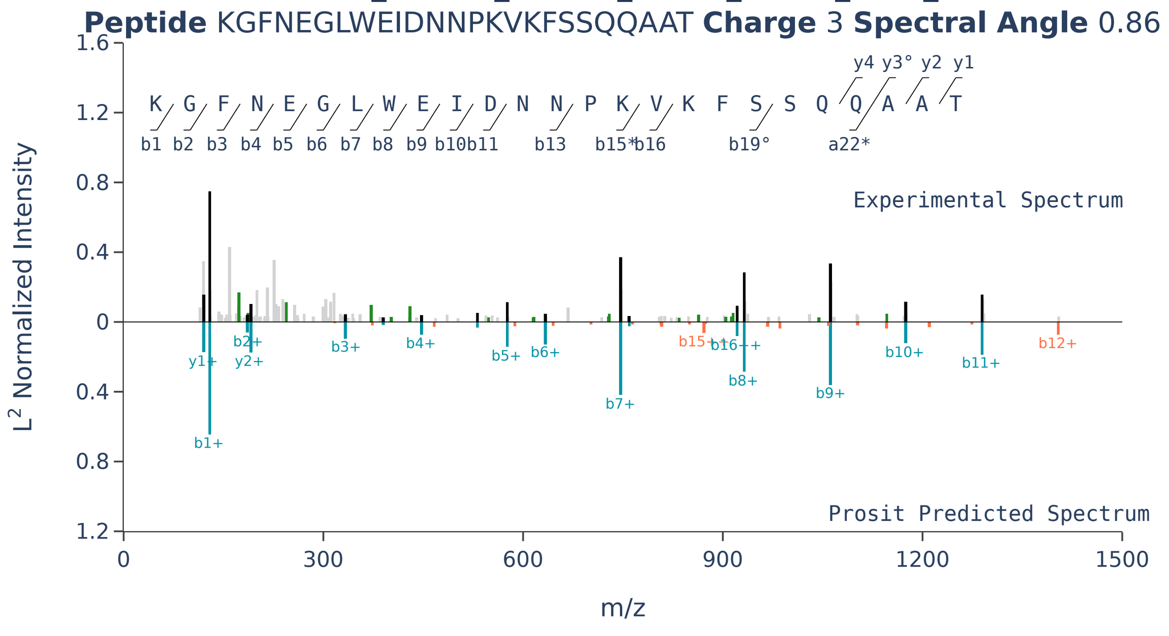

**Source** HRoetschke_271222_090123_HFGoe_LEDGF_b2R1_1h **Scan** 14981
**Peptide** KNSLKIDNL **Charge** 2 **Spectral Angle** 0.85

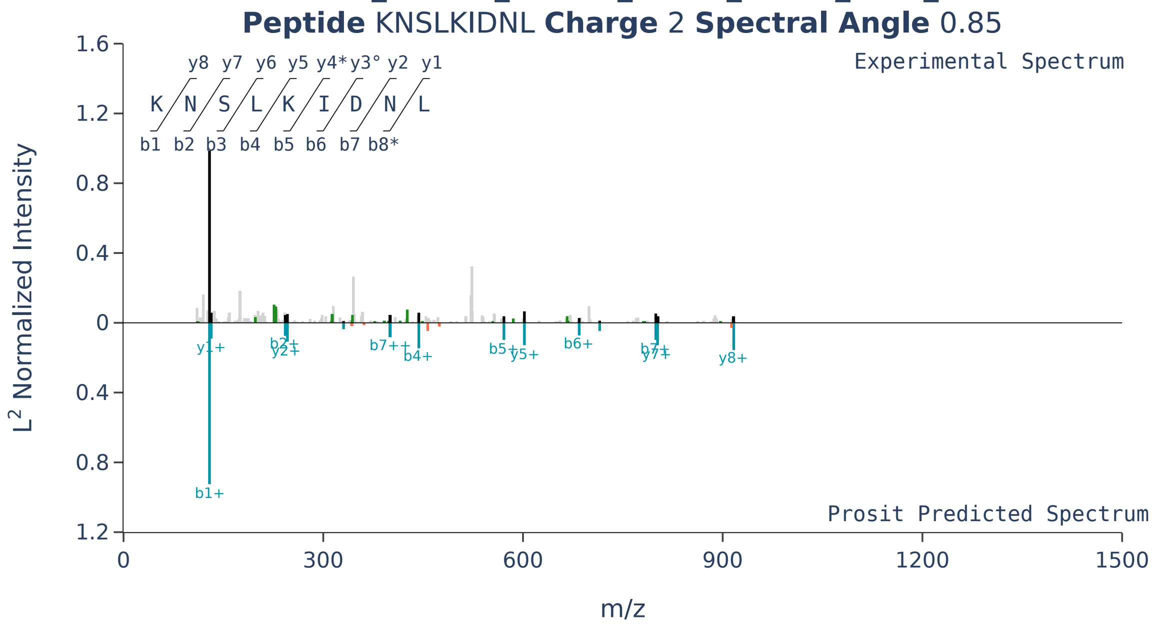


**Source** HRoetschke_271222_090123_HFGoe_LEDGF_b2R1_24h **Scan** 22217
**Peptide** GTHETAFLGPKDIFPYSENKEKYG **Charge** 3 **Spectral Angle** 0.79

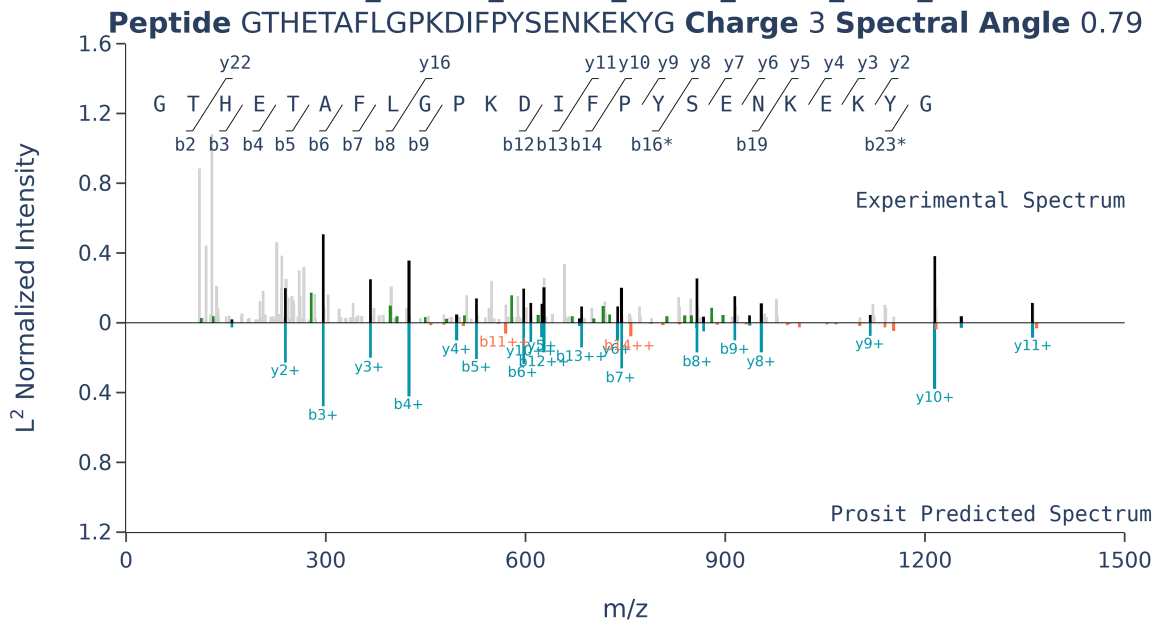


**Source** HRoetschke_271222_090123_HFGoe_LEDGF_b1R1_2h **Scan** 19116
**Peptide** LIFAKMKGYPHWPARVDE **Charge** 3 **Spectral Angle** 0.86

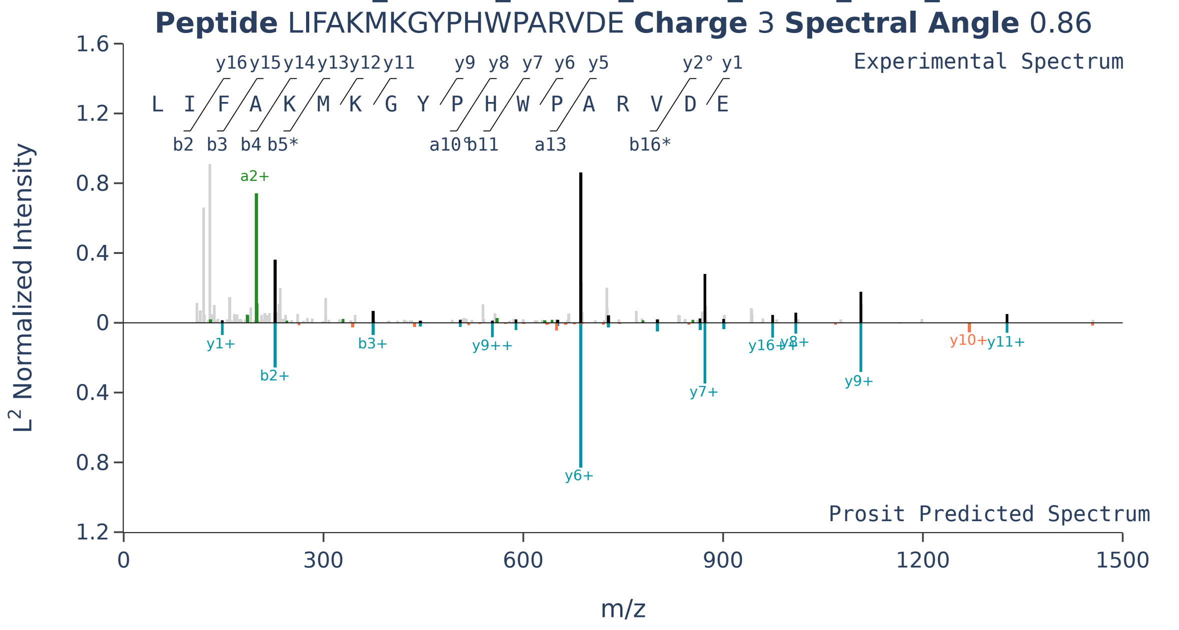


**Source** HRoetschke_271222_090123_HFGoe_LEDGF_b2R2_24h **Scan** 26720
**Peptide** ALDELASLQVTMQQAQKHTE **Charge** 3 **Spectral Angle** 0.79

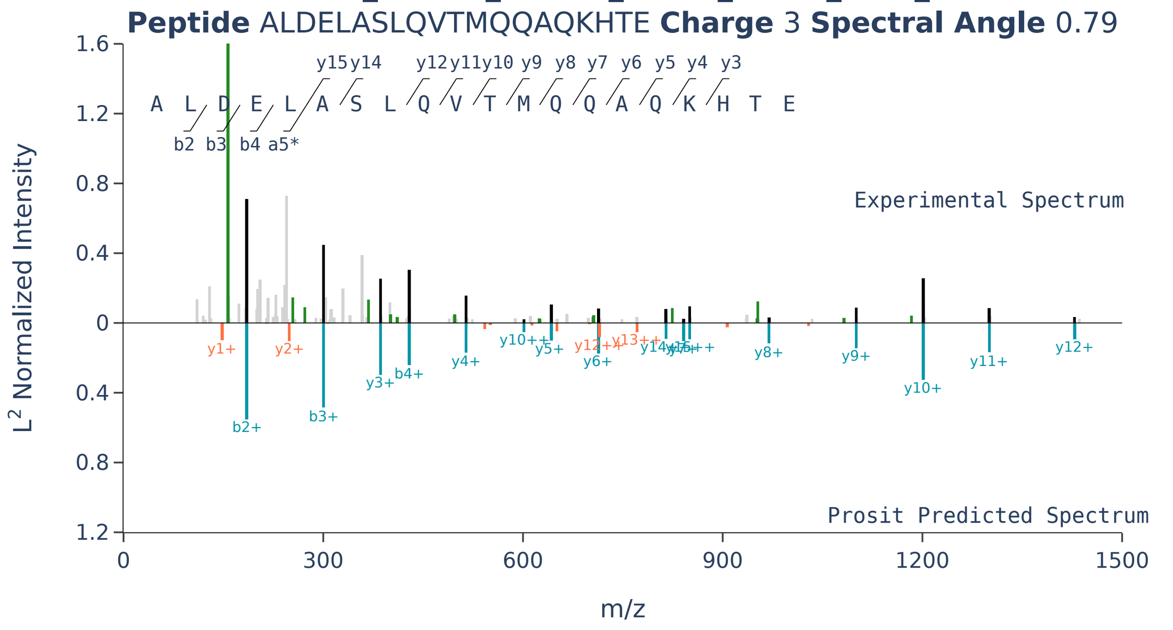


**Source** HRoetschke_271222_090123_HFGoe_LEDGF_b2R1_24h **Scan** 17508
**Peptide** EIKNSLKIDNLDVNRC **Charge** 3 **Spectral Angle** 0.88

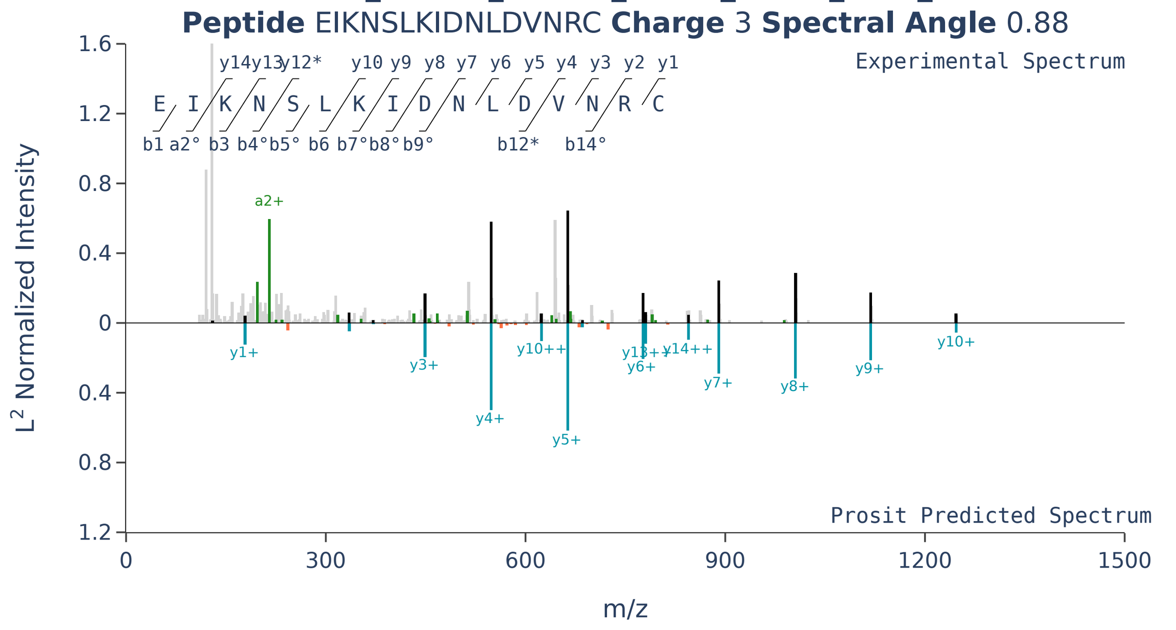


**Source** HRoetschke_271222_090123_HFGoe_LEDGF_b2R1_24h **Scan** 15543
**Peptide** DTDHEEKASNEDVTKAVDITTPKAA **Charge** 3 **Spectral Angle** 0.83

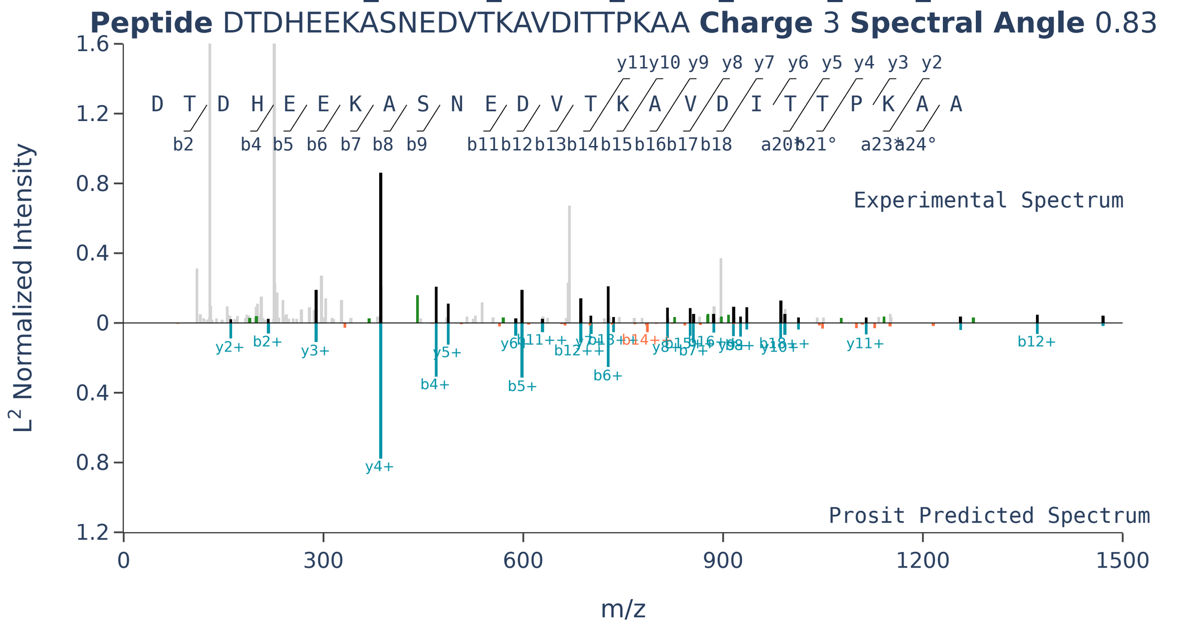


**Source** HRoetschke_271222_090123_HFGoe_LEDGF_b2R2_24h **Scan** 25770
**Peptide** NKFKNMFLVGEGDSVITQ **Charge** 3 **Spectral Angle** 0.94

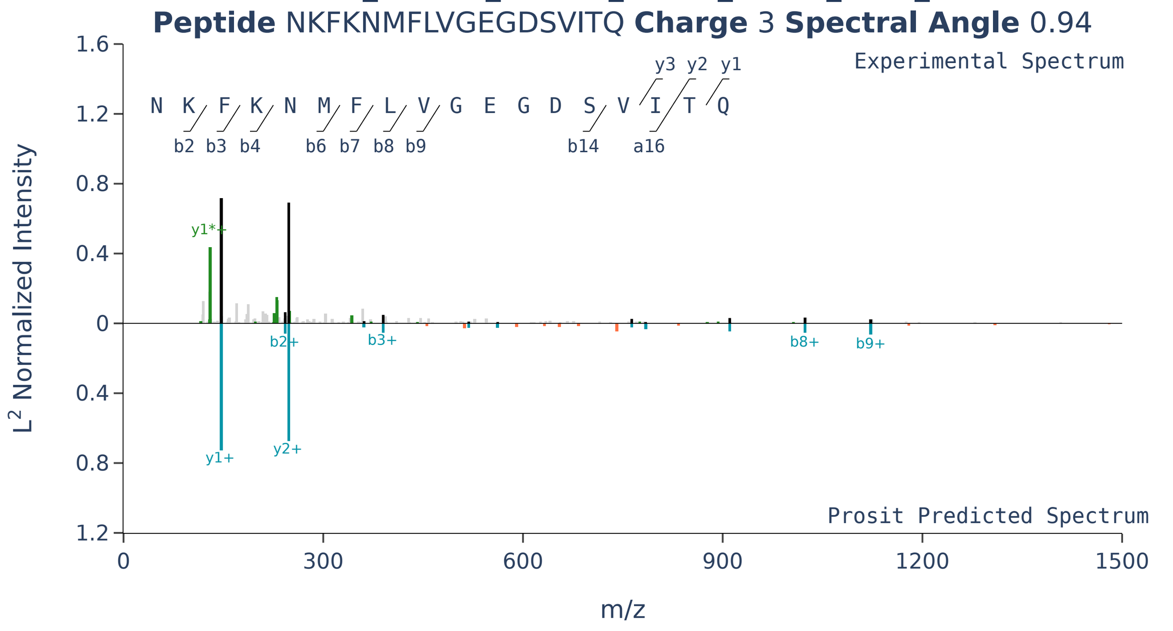


**Source** HRoetschke_271222_090123_HFGoe_LEDGF_b2R2_4h **Scan** 6692
**Peptide** NNPKVKFSSQQAATKQSN **Charge** 4 **Spectral Angle** 0.82

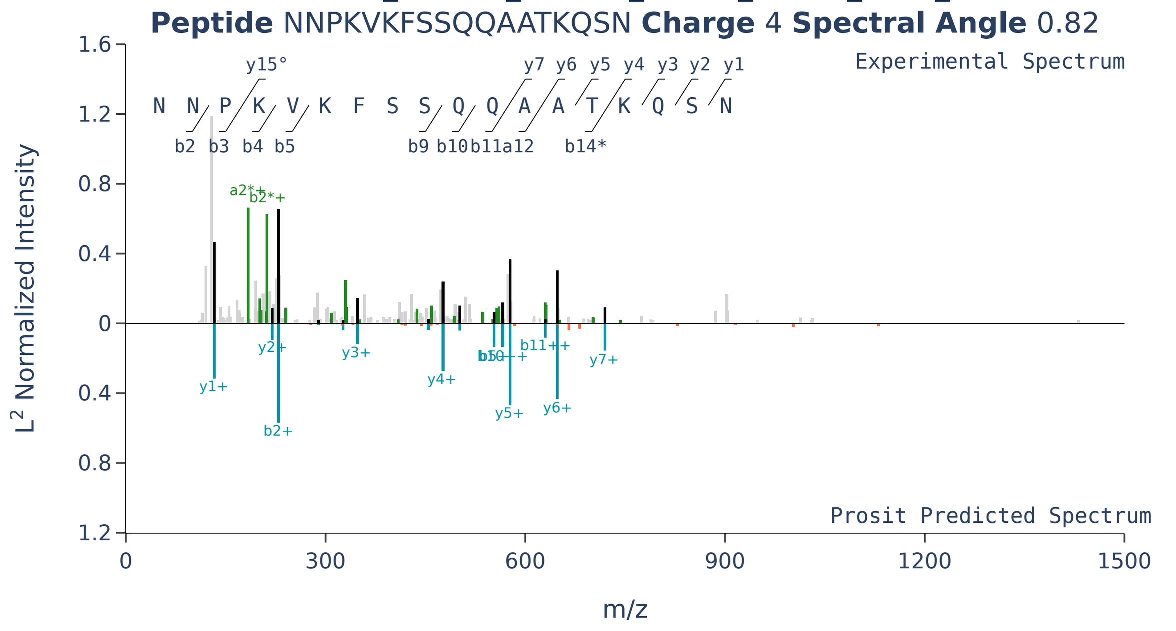


**Source** HRoetschke_271222_090123_HFGoe_LEDGF_b1R1_4h **Scan** 10955
**Peptide** TMQQAQKHTEMITTLKKIRR **Charge** 4 **Spectral Angle** 0.77

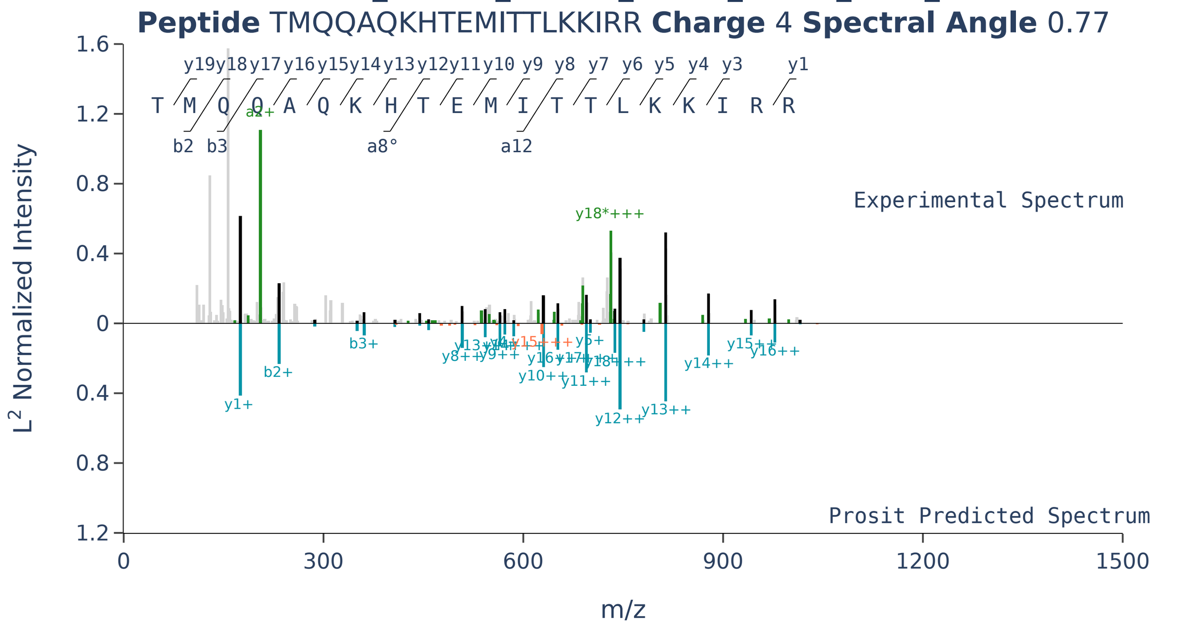


**Source** HRoetschke_271222_090123_HFGoe_LEDGF_b2R1_4h **Scan** 5876
**Peptide** LEKEQTGSKTLN **Charge** 2 **Spectral Angle** 0.71

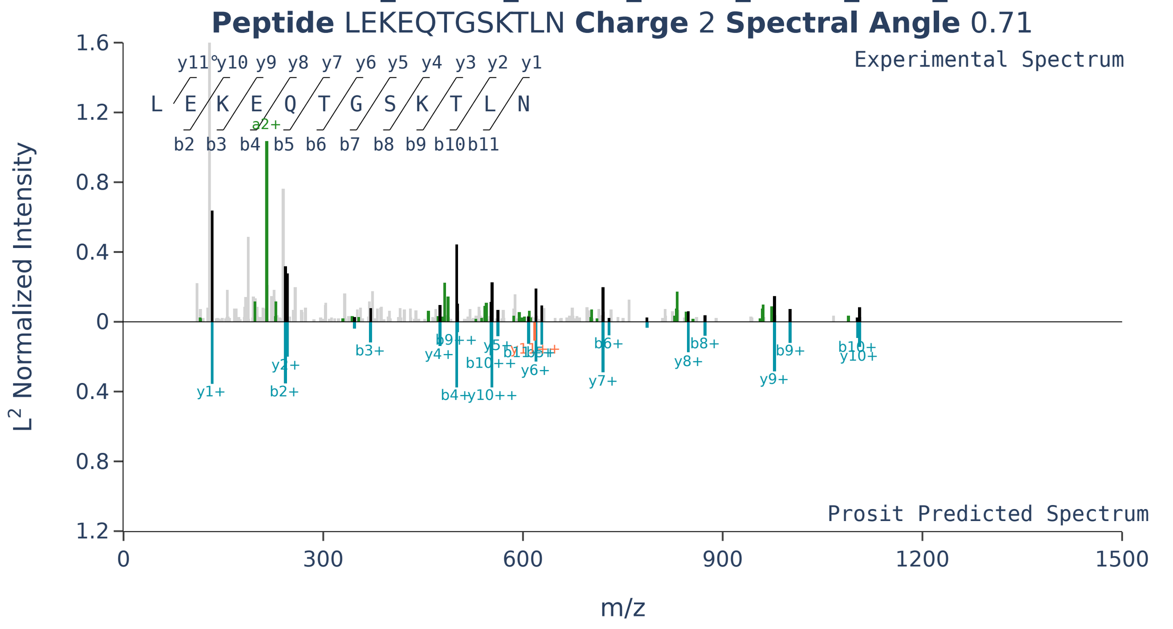


**Source** HRoetschke_271222_090123_HFGoe_LEDGF_b1R1_2h **Scan** 24171
**Peptide** ELASLQVTMQQAQKHTEMITTLKKIR **Charge** 5 **Spectral Angle** 0.71

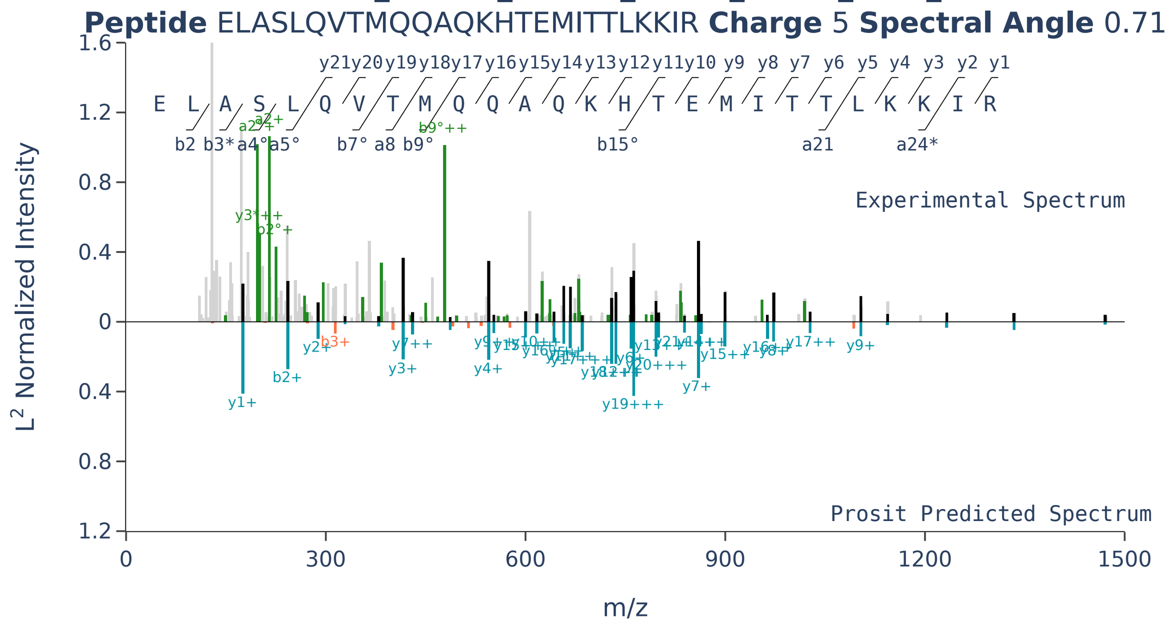


**Source** HRoetschke_271222_090123_HFGoe_LEDGF_b1R1_1h **Scan** 23720
**Peptide** KGFNEGLWEIDNNPKVKFS **Charge** 3 **Spectral Angle** 0.87

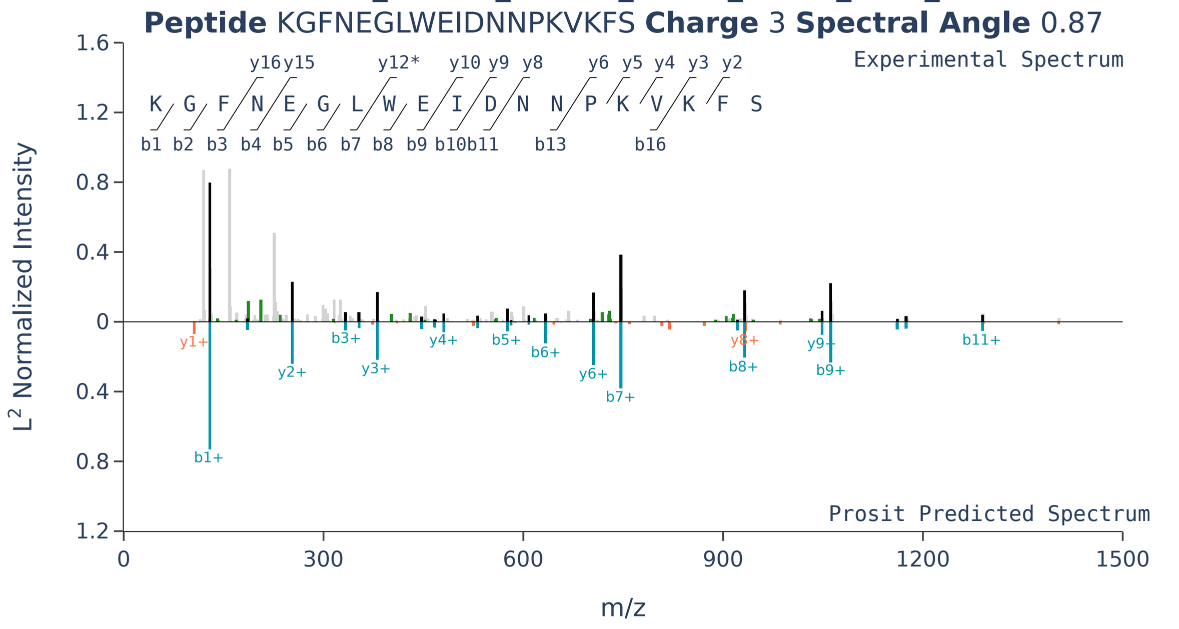


**Source** HRoetschke_271222_090123_HFGoe_LEDGF_b1R1_24h **Scan** 9998
**Peptide** DNNPKVKFSSQQAAT **Charge** 2 **Spectral Angle** 0.84

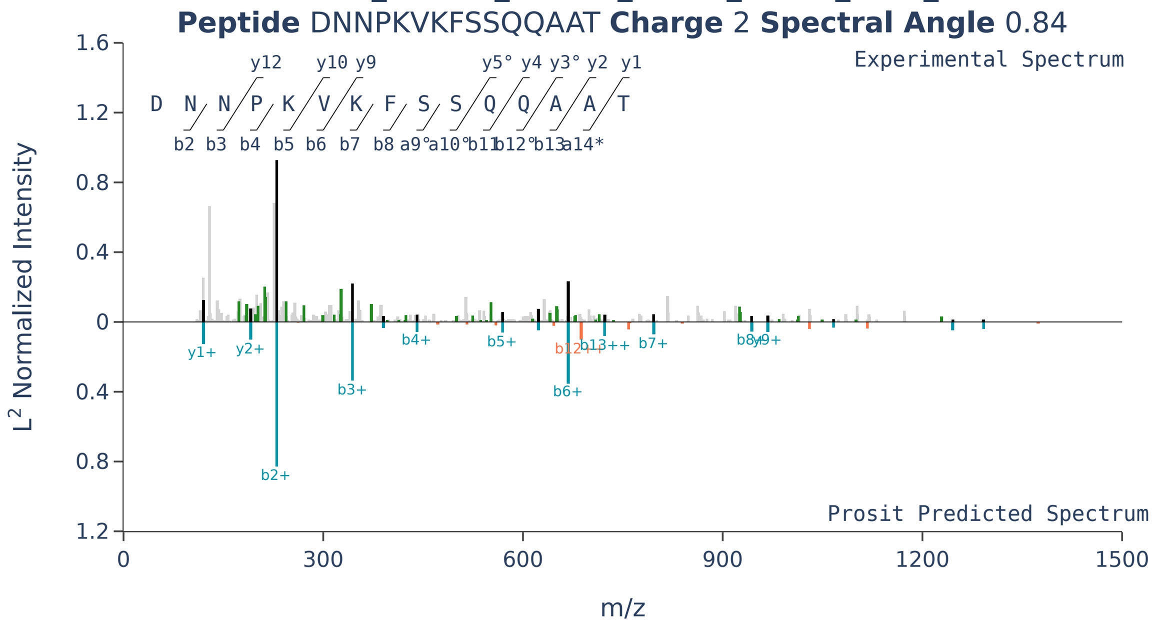


**Source** HRoetschke_271222_090123_HFGoe_LEDGF_b1R1_2h **Scan** 18938
**Peptide** ASLQVTMQQAQKHTEMITTLKKIRR **Charge** 6 **Spectral Angle** 0.78

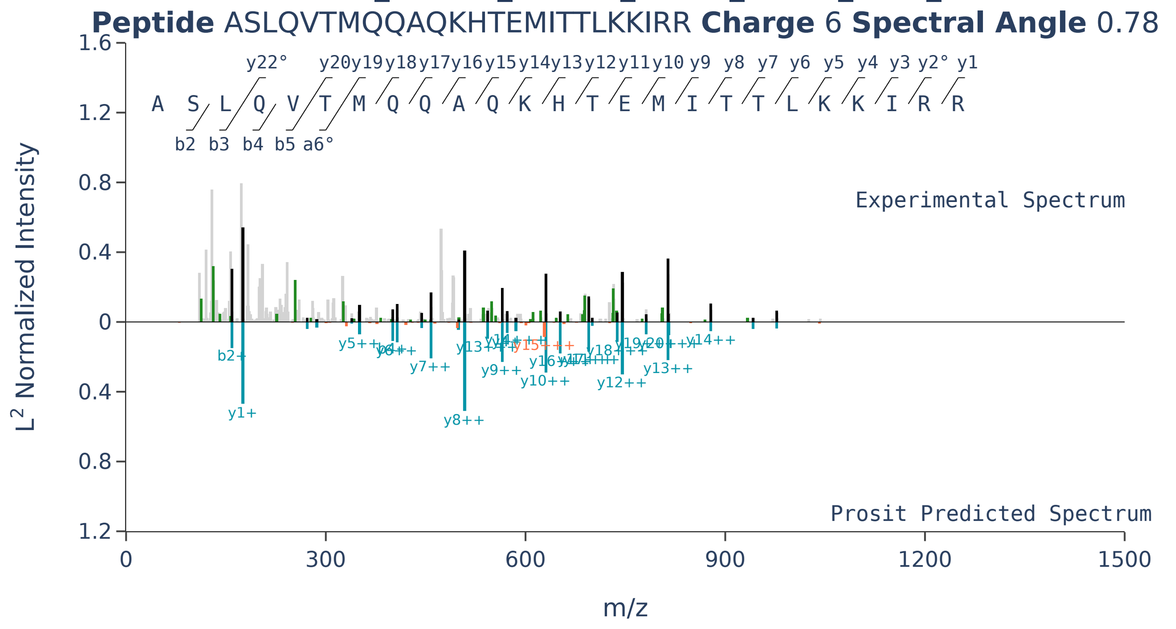


**Source** HRoetschke_271222_090123_HFGoe_LEDGF_b2R2_2h **Scan** 14786
**Peptide** TEMITTLKKIRRFK **Charge** 4 **Spectral Angle** 0.86

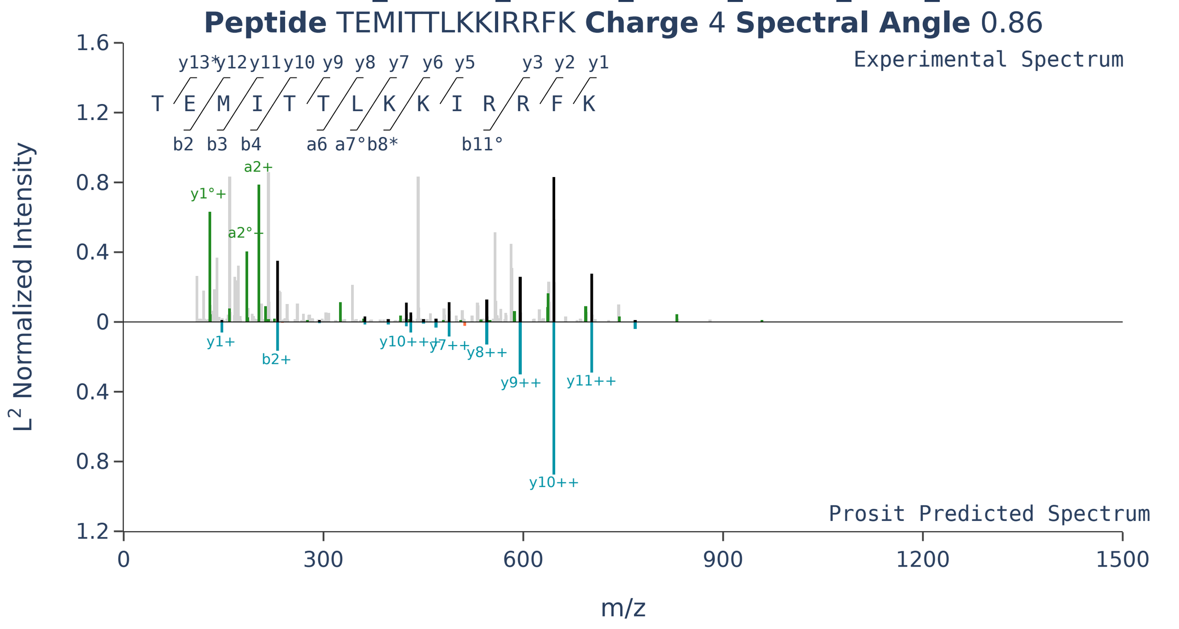

**Source** HRoetschke_271222_090123_HFGoe_LEDGF_b1R2_2h **Scan** 5973
**Peptide** FSSQQAATKQ **Charge** 2 **Spectral Angle** 0.75

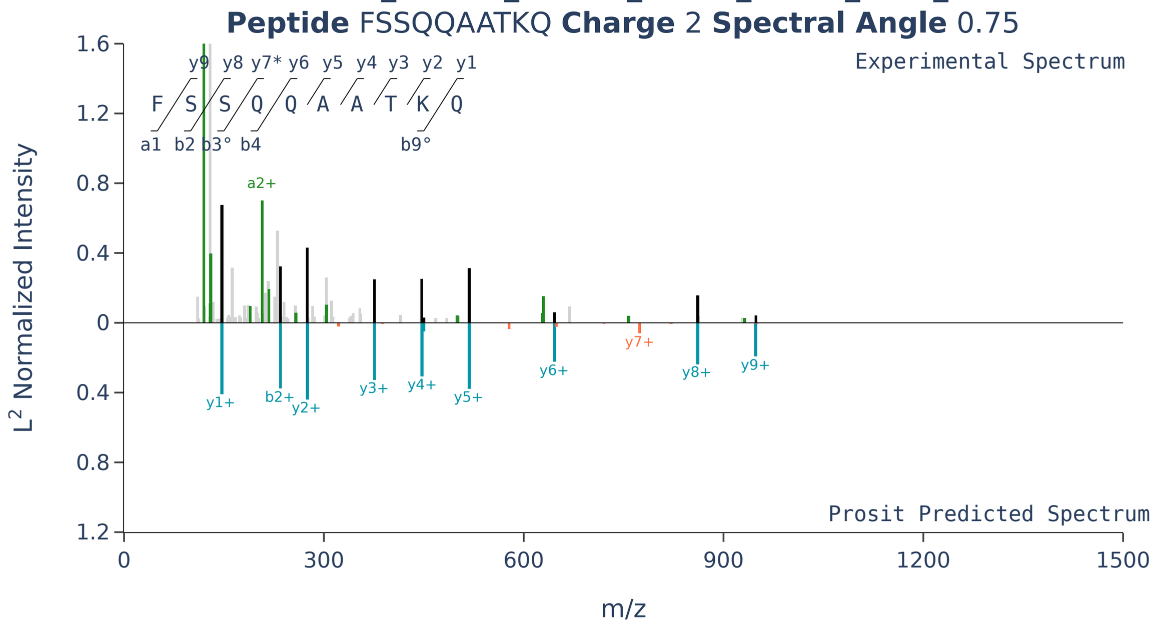


**Source** HRoetschke_271222_090123_HFGoe_LEDGF_b1R1_4h **Scan** 20474
**Peptide** KPGDLIFAKMKGYPHWPARV **Charge** 5 **Spectral Angle** 0.86

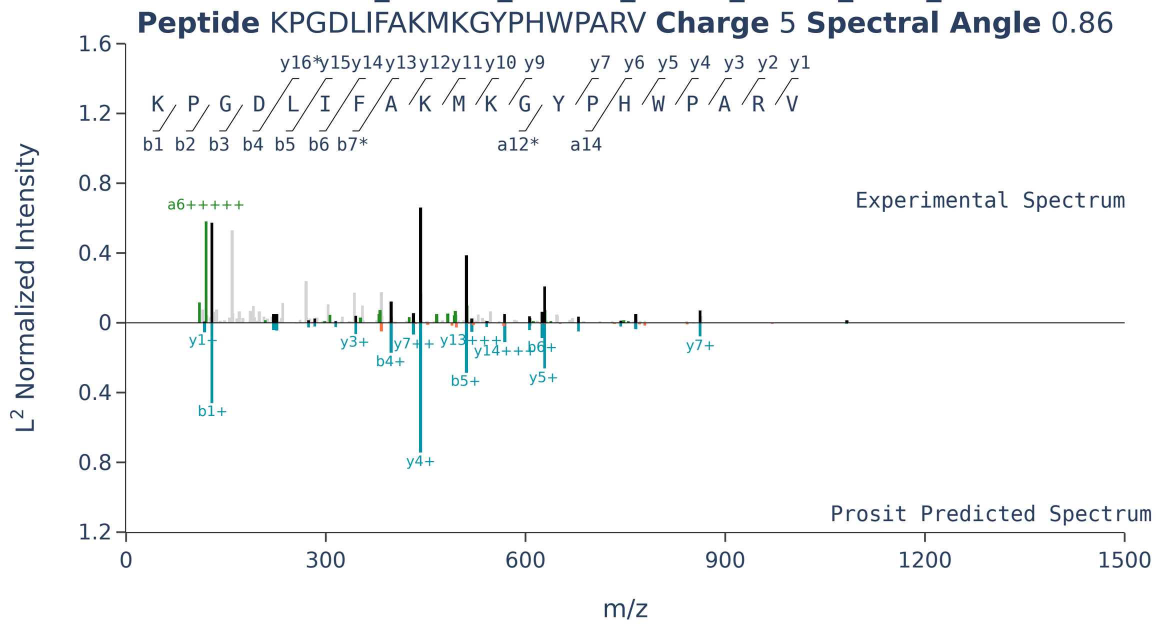


**Source** HRoetschke_271222_090123_HFGoe_LEDGF_b1R1_2h **Scan** 22604
**Peptide** SVITQVLNKSLAEQRQHEE **Charge** 2 **Spectral Angle** 0.8

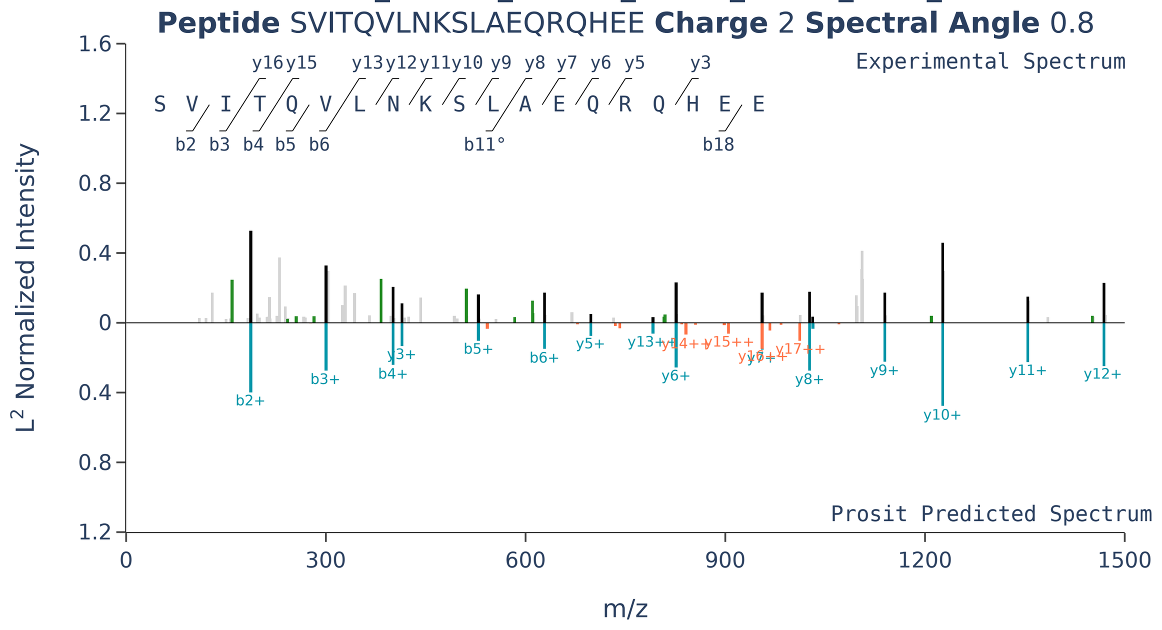


**Source** HRoetschke_271222_090123_HFGoe_LEDGF_b2R1_2h **Scan** 9058
**Peptide** QQAQKHTEMITTLKKIRR **Charge** 5 **Spectral Angle** 0.83

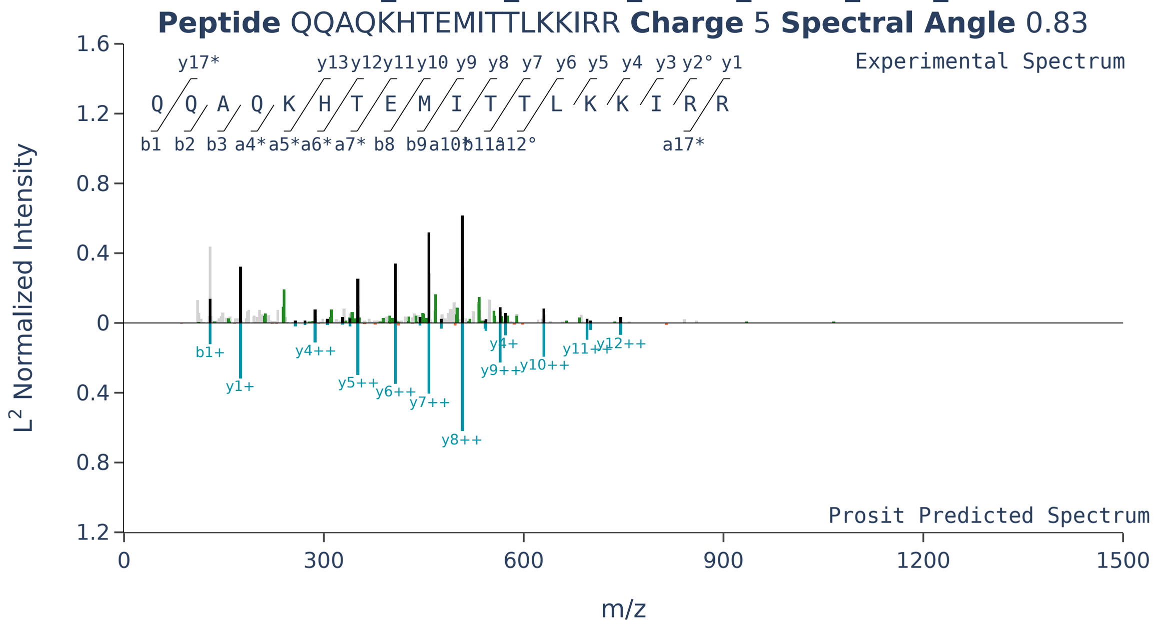


**Source** HRoetschke_271222_090123_HFGoe_LEDGF_b1R1_2h **Scan** 27472
**Peptide** FLVGEGDSVIT **Charge** 2 **Spectral Angle** 0.83

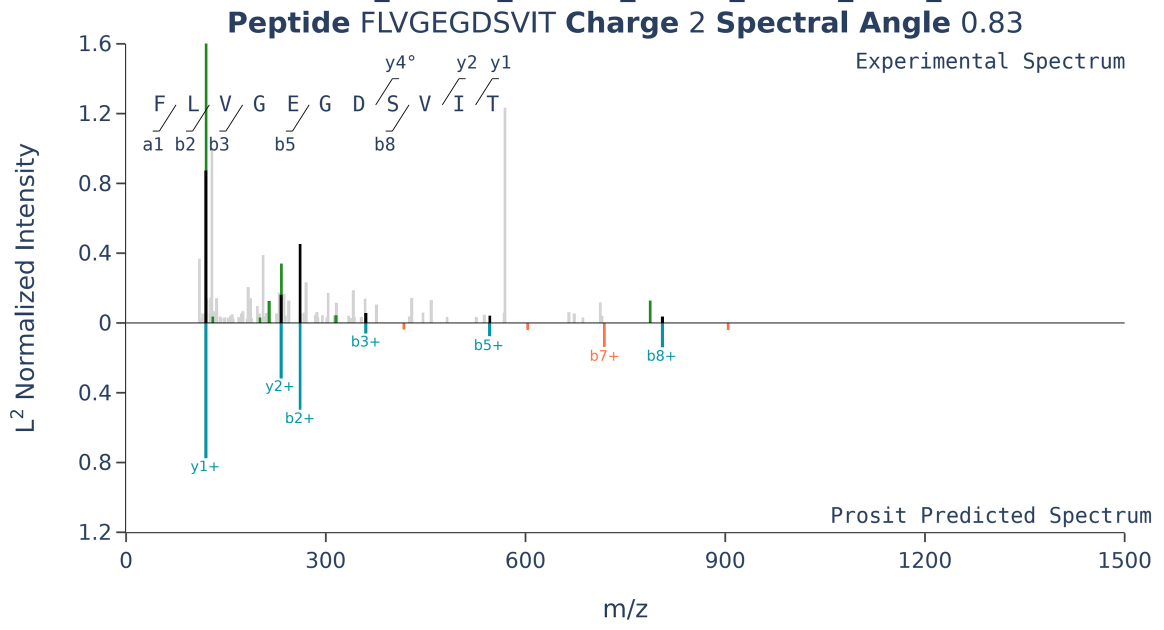


**Source** HRoetschke_271222_090123_HFGoe_LEDGF_b2R1_1h **Scan** 10667
**Peptide** LDNNPKVKFSSQ **Charge** 3 **Spectral Angle** 0.72

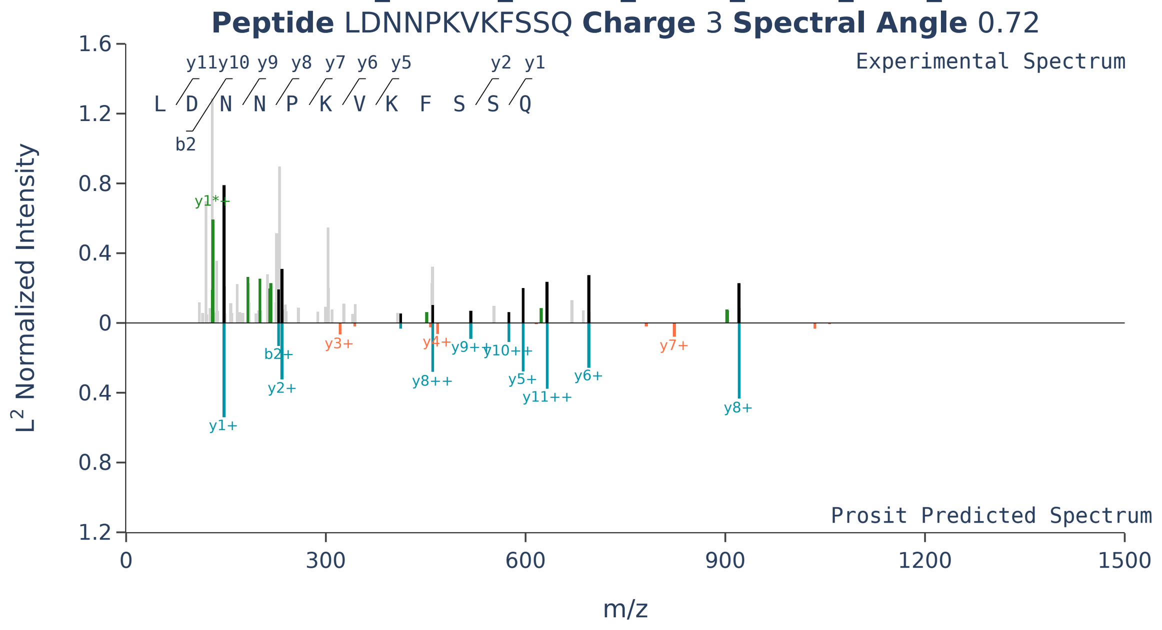


**Source** HRoetschke_271222_090123_HFGoe_LEDGF_b1R1_2h **Scan** 19144
**Peptide** KGYPHWPARVDEVPDGAVKPPTNKL **Charge** 3 **Spectral Angle** 0.84

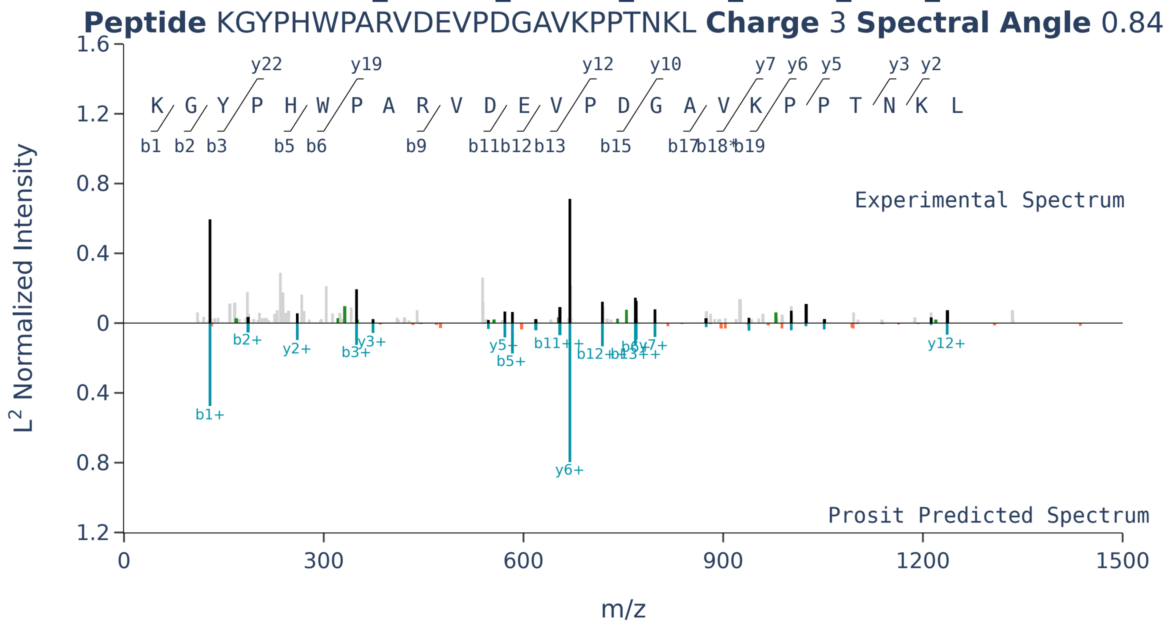


**Source** HRoetschke_271222_090123_HFGoe_LEDGF_b1R1_4h **Scan** 15548
**Peptide** SRLQRIHAEIKNSLKID **Charge** 3 **Spectral Angle** 0.81

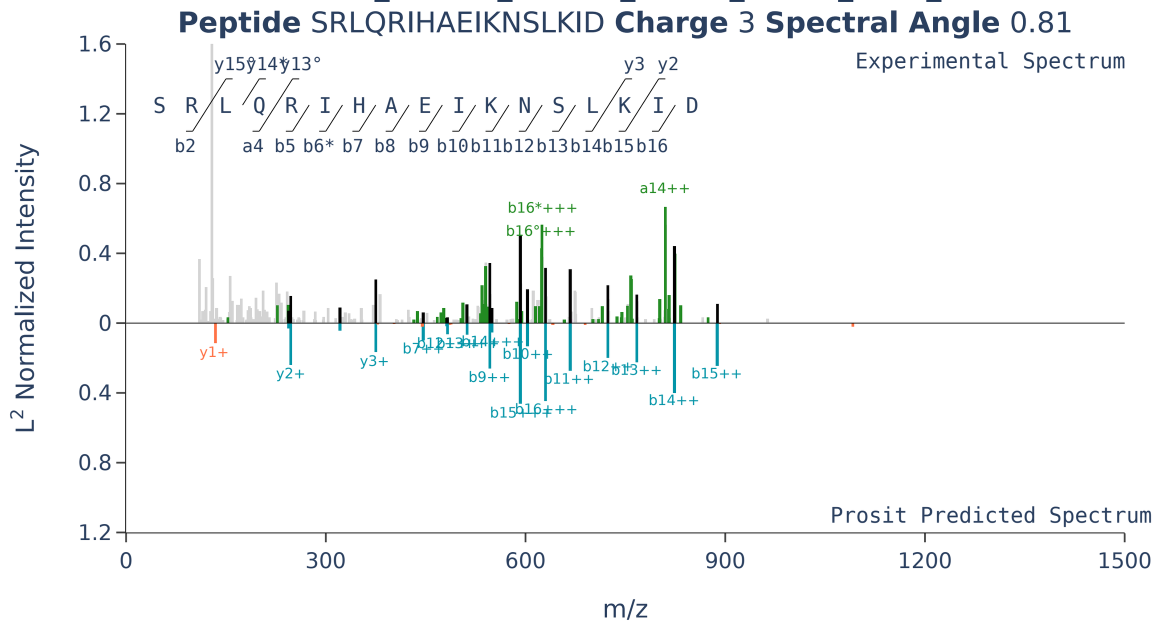


**Source** HRoetschke_271222_090123_HFGoe_LEDGF_b1R1_4h **Scan** 31609
**Peptide** FLGPKDIFPY **Charge** 2 **Spectral Angle** 0.85

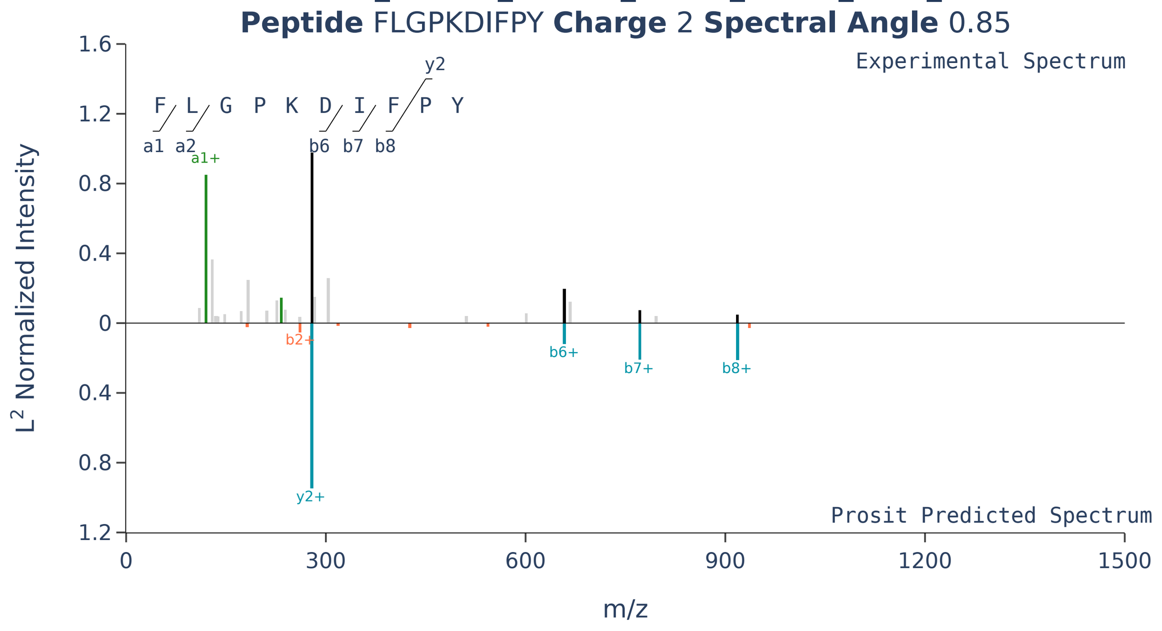


**Source** HRoetschke_271222_090123_HFGoe_LEDGF_b1R1_24h **Scan** 17507
**Peptide** TSMDSRLQRIHAEI **Charge** 2 **Spectral Angle** 0.87

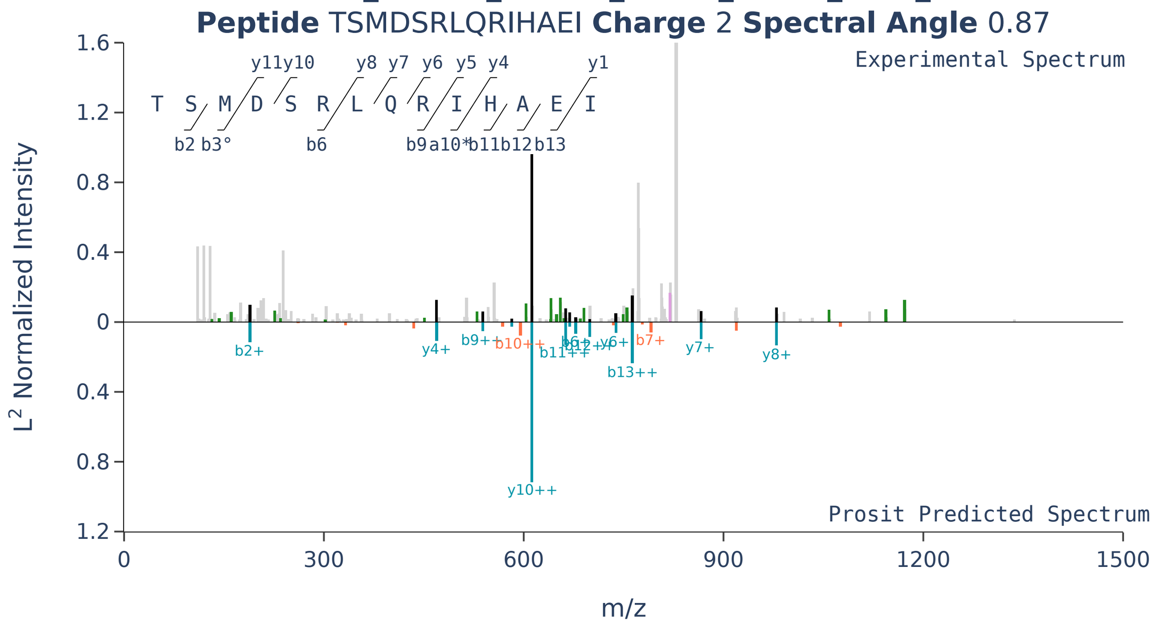


**Source** HRoetschke_271222_090123_HFGoe_LEDGF_b2R1_2h **Scan** 17514
**Peptide** MEKSTMLYNKF **Charge** 2 **Spectral Angle** 0.72

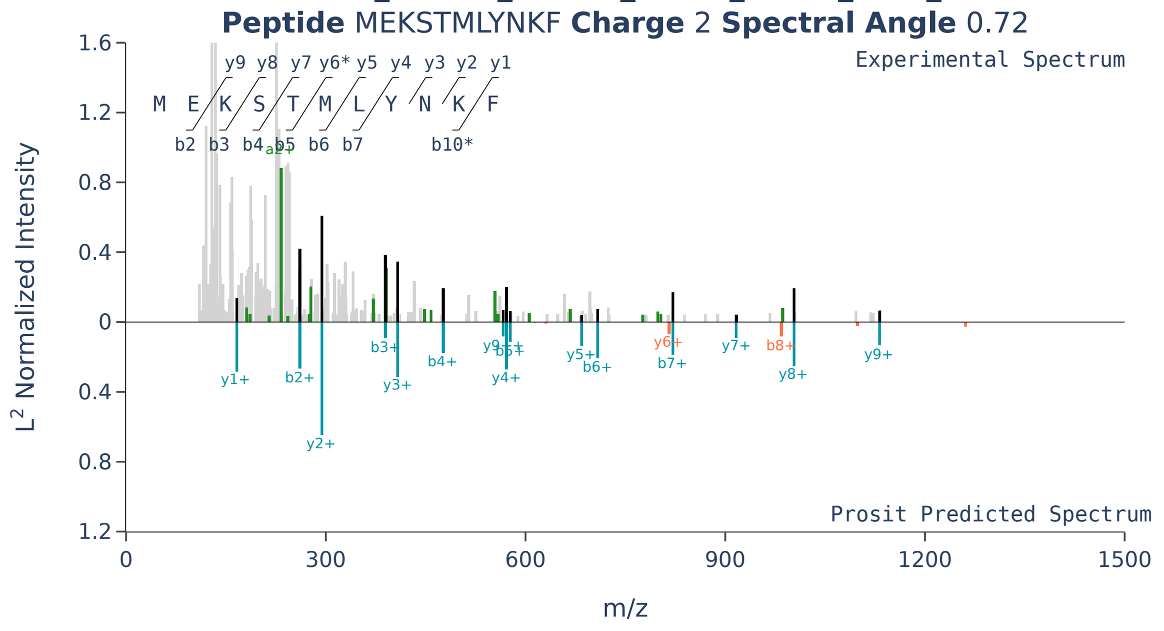


**Source** HRoetschke_271222_090123_HFGoe_LEDGF_b1R1_2h **Scan** 25743
**Peptide** GMTRDFKPGDLIFAKMKGY **Charge** 4 **Spectral Angle** 0.75

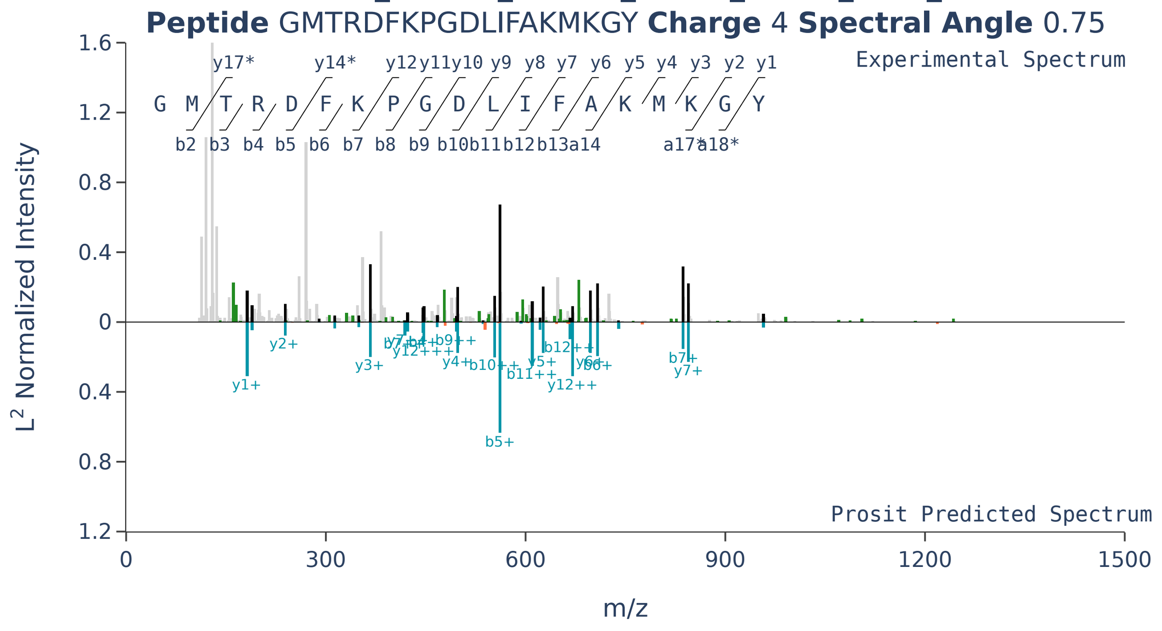


**Source** HRoetschke_271222_090123_HFGoe_LEDGF_b1R1_4h **Scan** 11299
**Peptide** SNEDVTKAVDITTPKAARRGR **Charge** 4 **Spectral Angle** 0.8

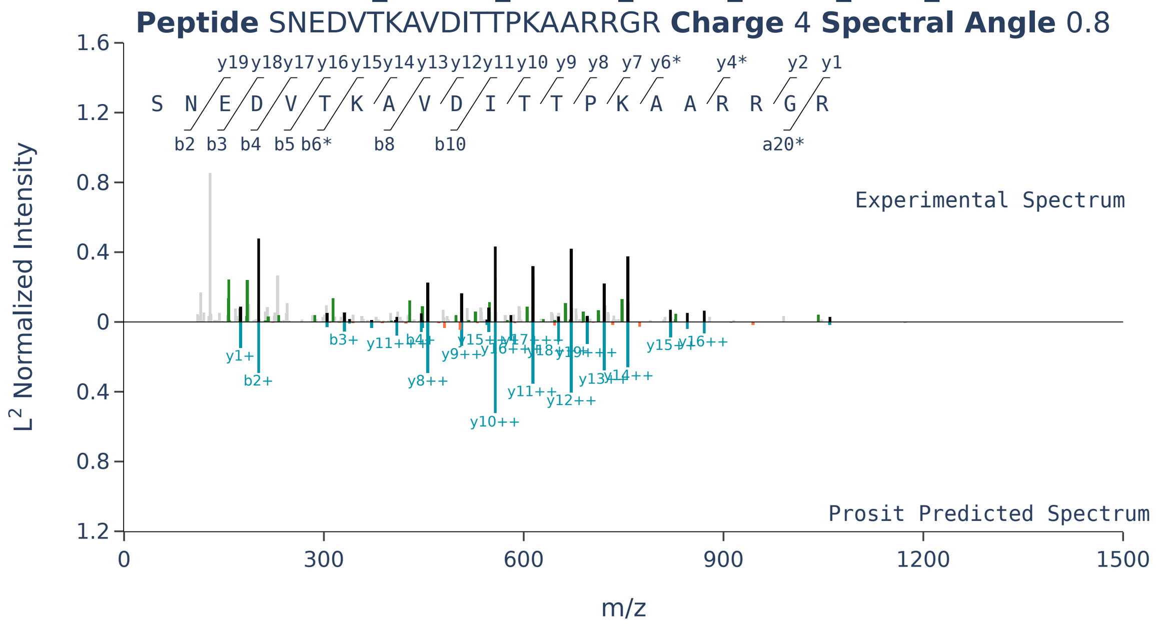


**Source** HRoetschke_271222_090123_HFGoe_LEDGF_b1R1_2h **Scan** 7773
**Peptide** YNKFKNM **Charge** 2 **Spectral Angle** 0.77

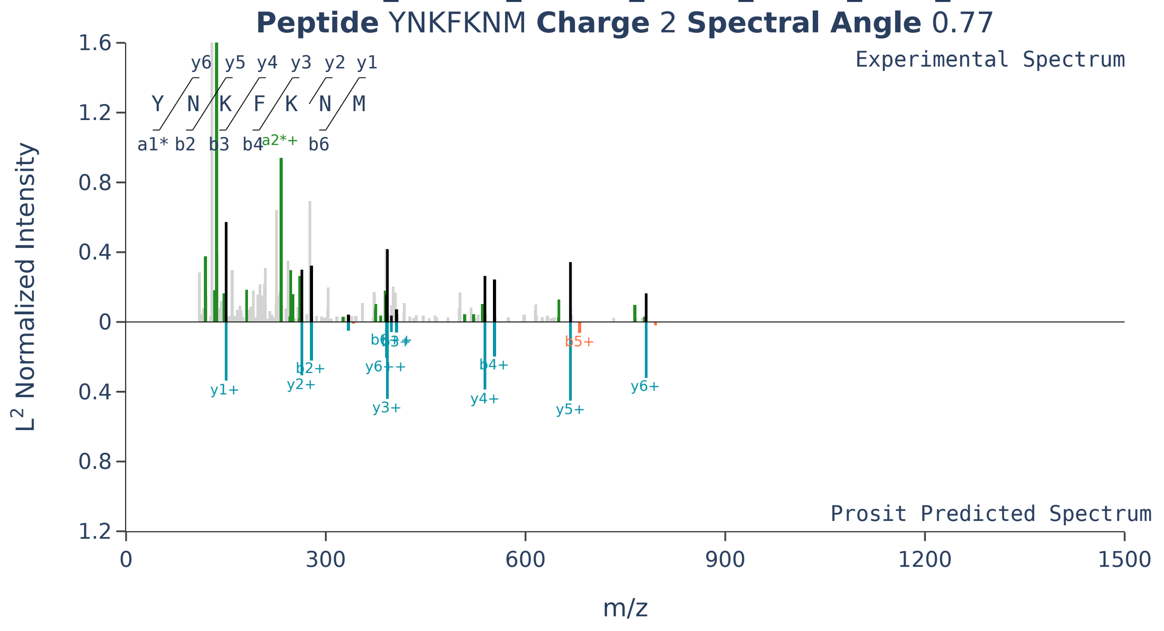


**Source** HRoetschke_271222_090123_HFGoe_LEDGF_b2R1_24h **Scan** 24173
**Peptide** THETAFLGPKDIFPYSENKE **Charge** 4 **Spectral Angle** 0.69

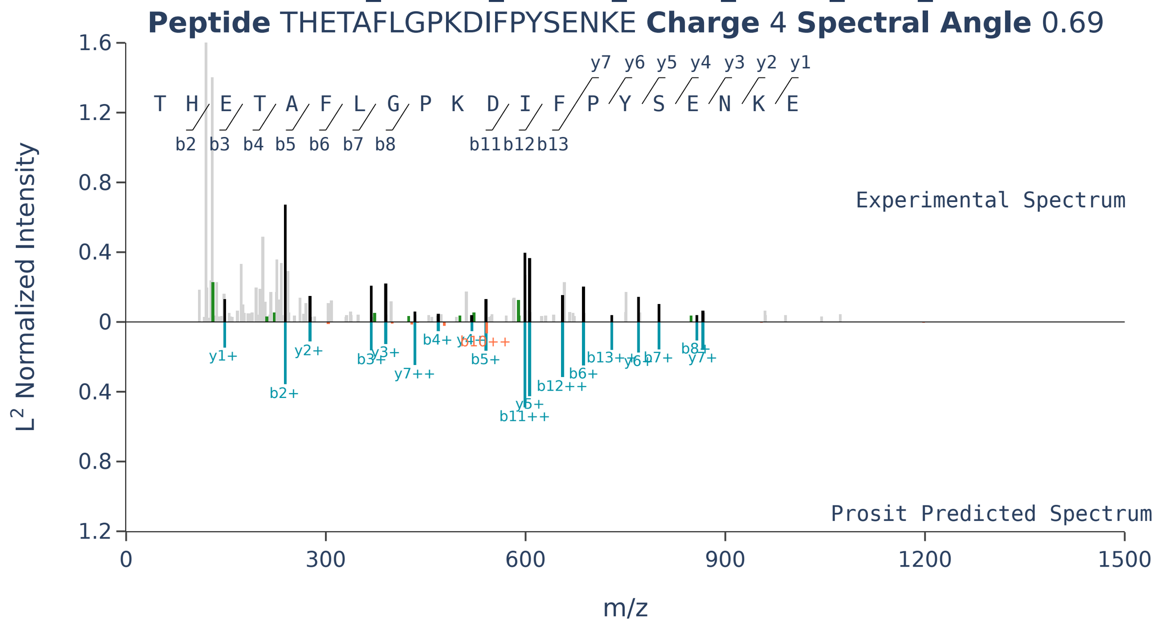

**Source** HRoetschke_271222_090123_HFGoe_LEDGF_b1R2_24h **Scan** 7097
**Peptide** VDITTPKAARRGR **Charge** 4 **Spectral Angle** 0.85

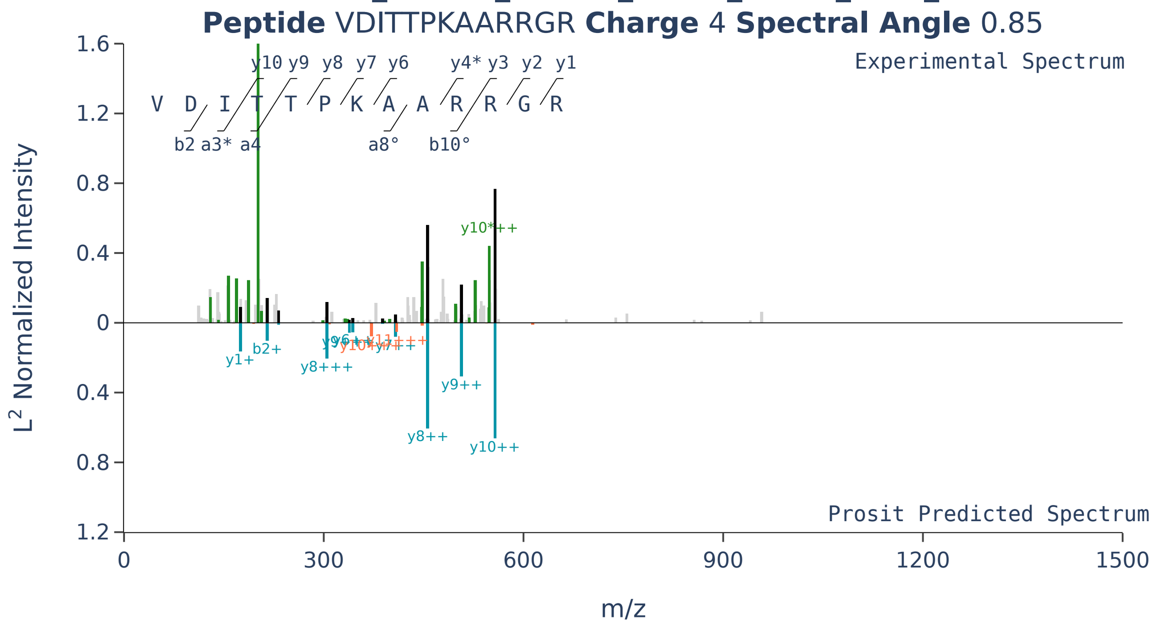

**Source** HRoetschke_271222_090123_HFGoe_LEDGF_b1R1_2h **Scan** 21893
**Peptide** RDFKPGDLIFAK **Charge** 3 **Spectral Angle** 0.75

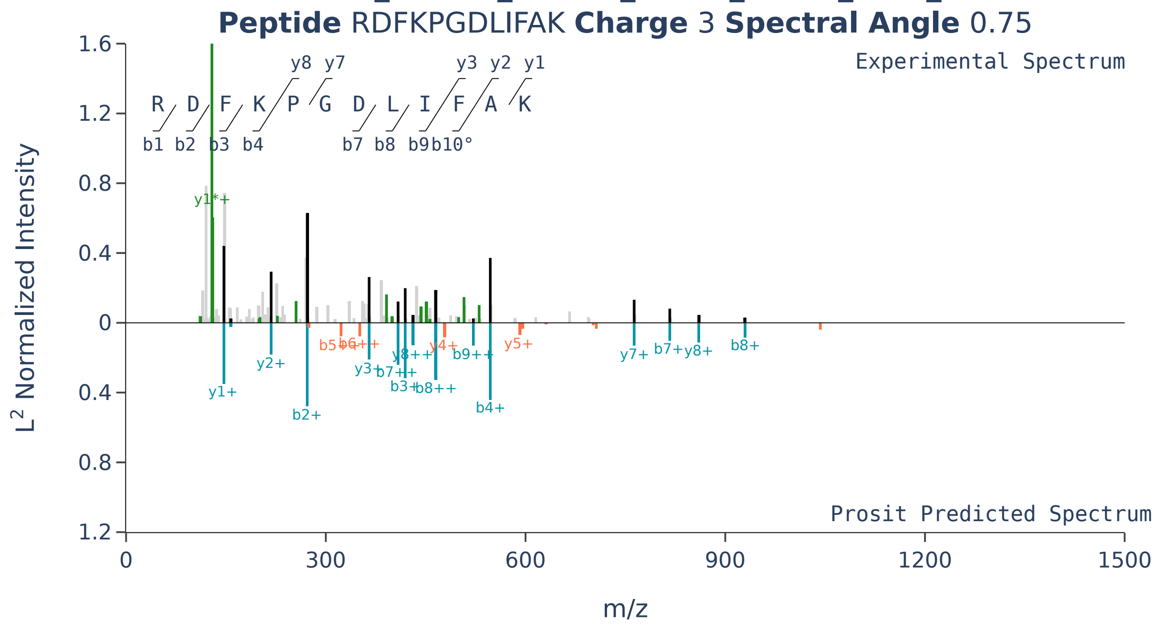

**Source** HRoetschke_271222_090123_HFGoe_LEDGF_b2R1_2h **Scan** 15638
**Peptide** KMKGYPHWPARVDEVPDGAVKPPTNK **Charge** 6 **Spectral Angle** 0.8

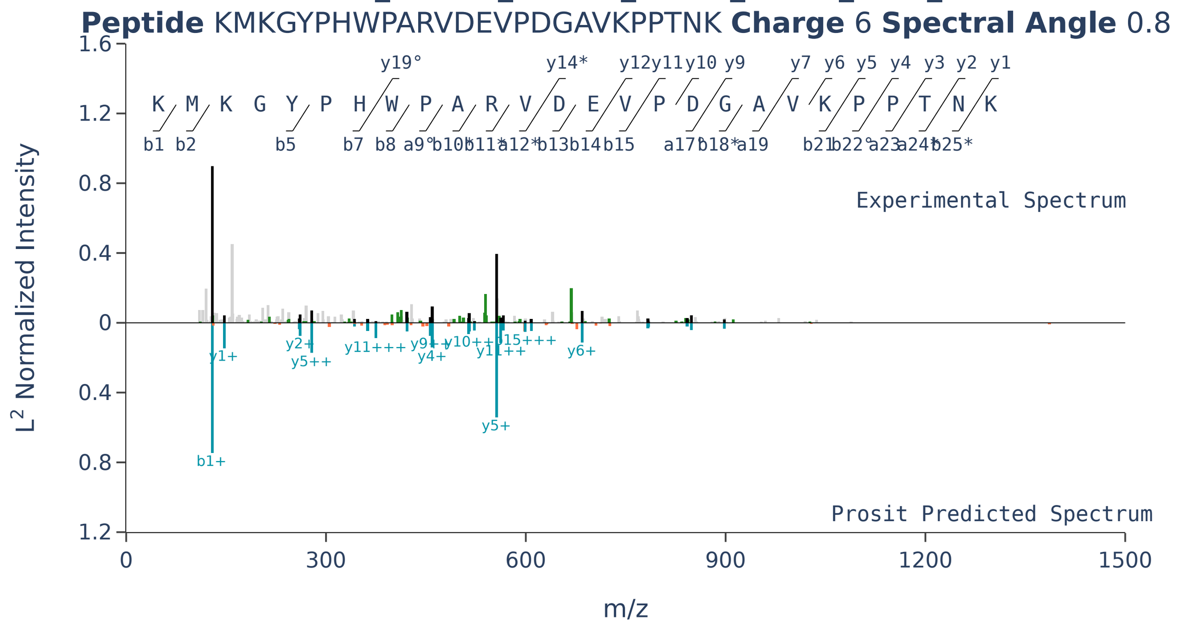

**Source** HRoetschke_271222_090123_HFGoe_LEDGF_b1R2_24h **Scan** 14499
**Peptide** SMDSRLQRIHAEIKN **Charge** 3 **Spectral Angle** 0.81

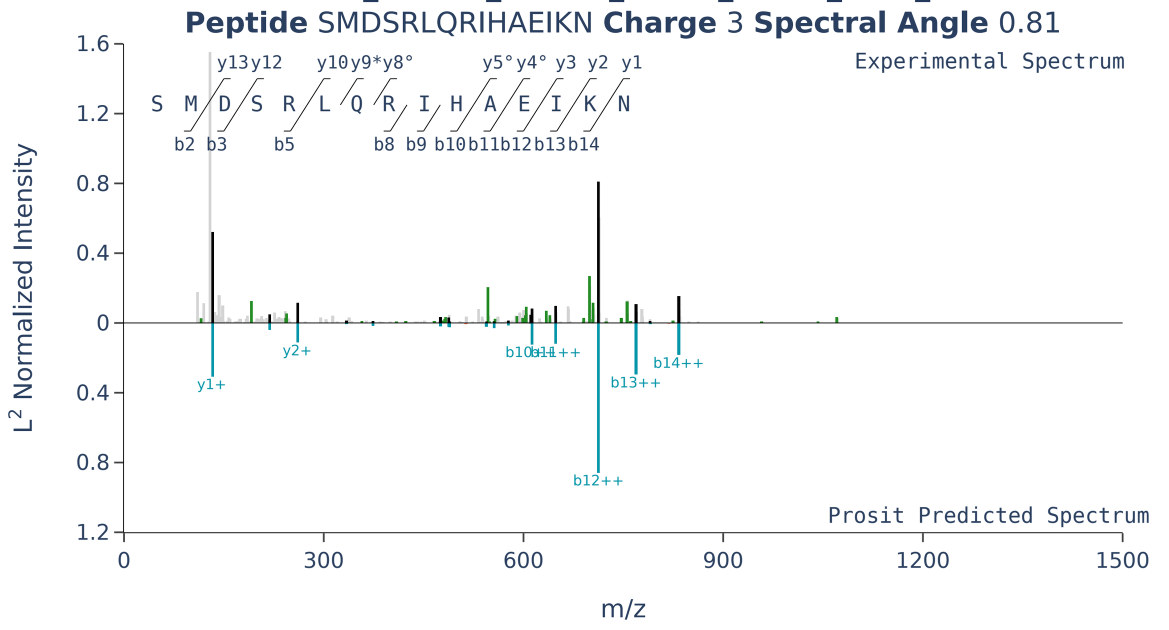

**Source** HRoetschke_271222_090123_HFGoe_LEDGF_b1R2_24h **Scan** 18011
**Peptide** IKNSLKIDNL **Charge** 3 **Spectral Angle** 0.81

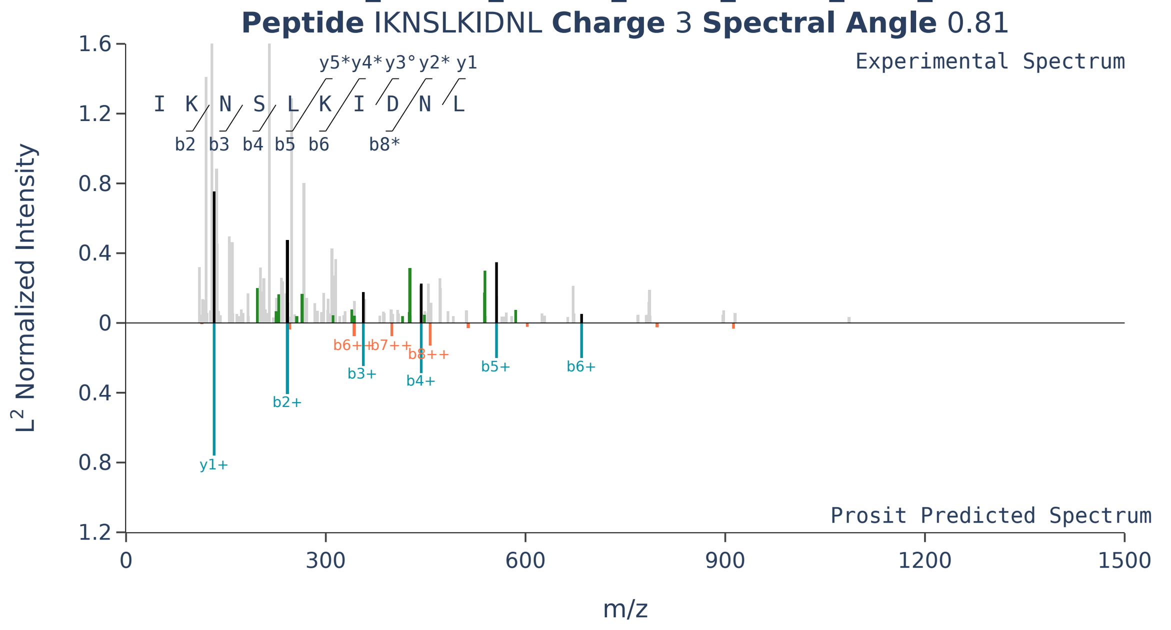

**Source** HRoetschke_271222_090123_HFGoe_LEDGF_b1R2_4h **Scan** 7556
**Peptide** NNPKVKFSSQQ **Charge** 1 **Spectral Angle** 0.77

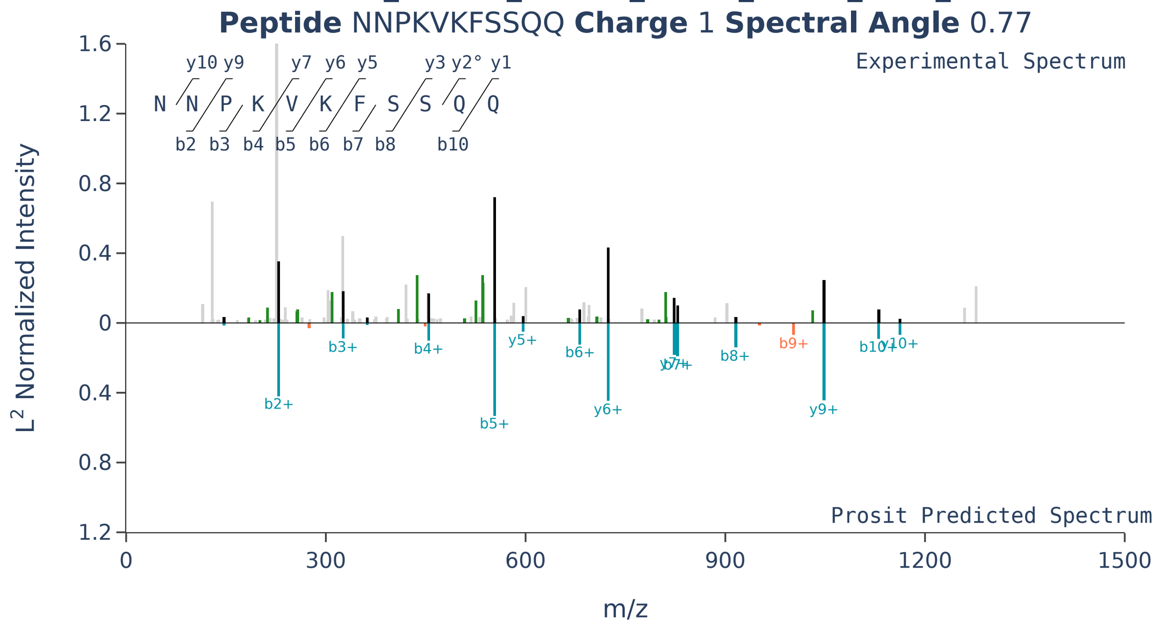

**Source** HRoetschke_271222_090123_HFGoe_LEDGF_b1R2_4h **Scan** 19949
**Peptide** GTHETAFLGPKDIFPYSENKEKYGK **Charge** 4 **Spectral Angle** 0.79

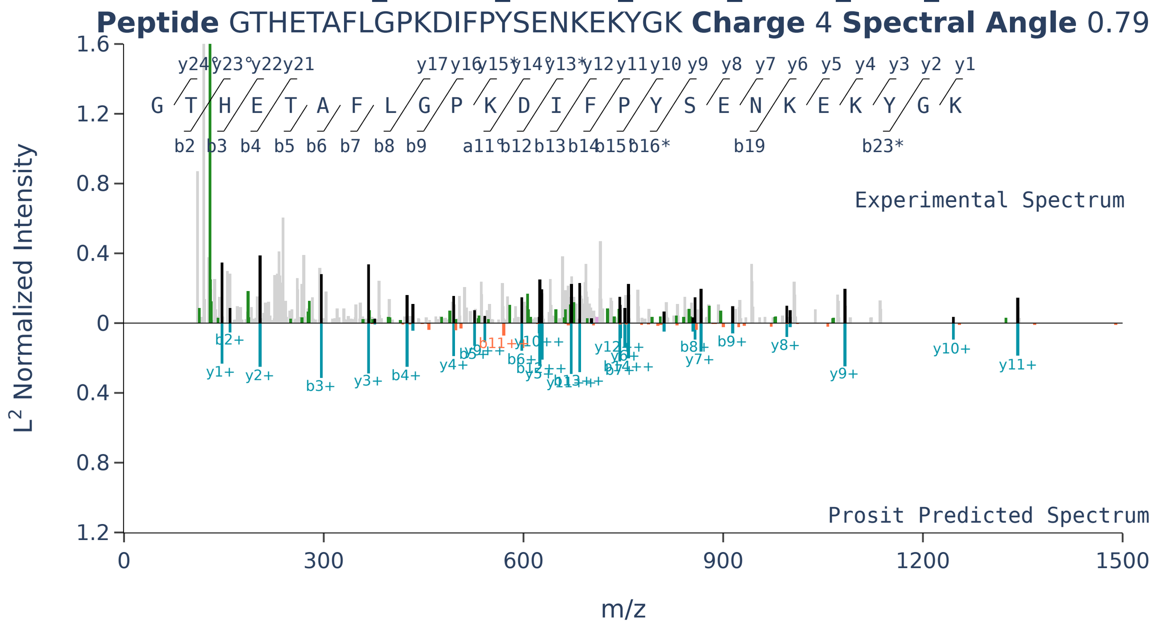

**Source** HRoetschke_271222_090123_HFGoe_LEDGF_b1R1_2h **Scan** 30614
**Peptide** ALDELASLQVTMQQAQ **Charge** 2 **Spectral Angle** 0.85

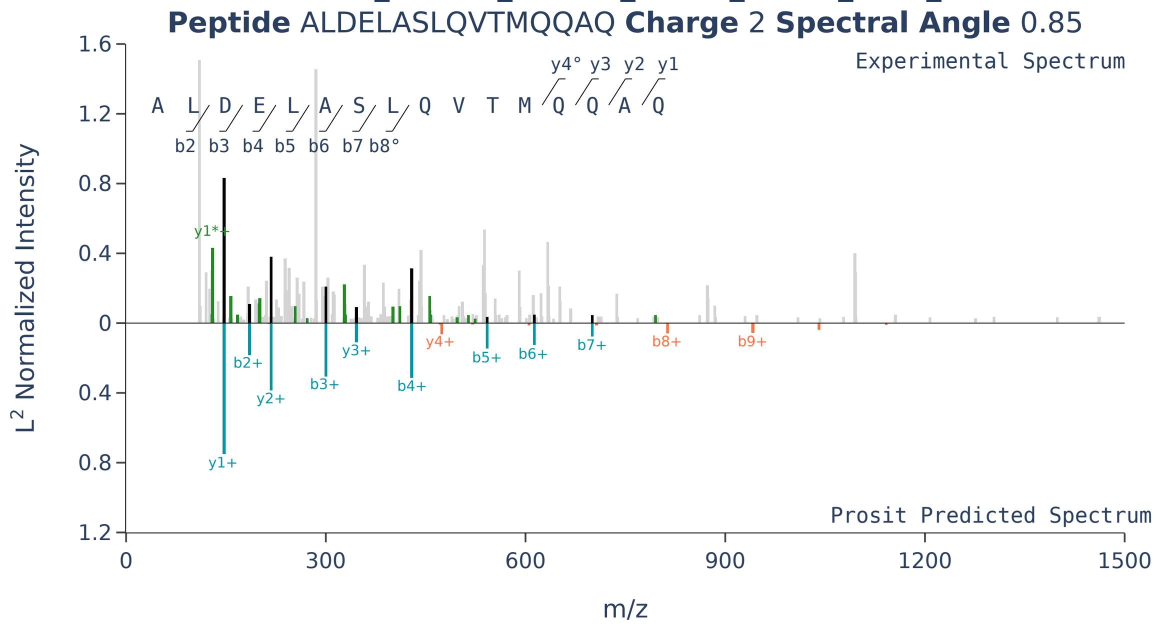

**Source** HRoetschke_271222_090123_HFGoe_LEDGF_b1R1_4h **Scan** 7968
**Peptide** DNNPKVKFSSQQAATKQSNA **Charge** 3 **Spectral Angle** 0.84

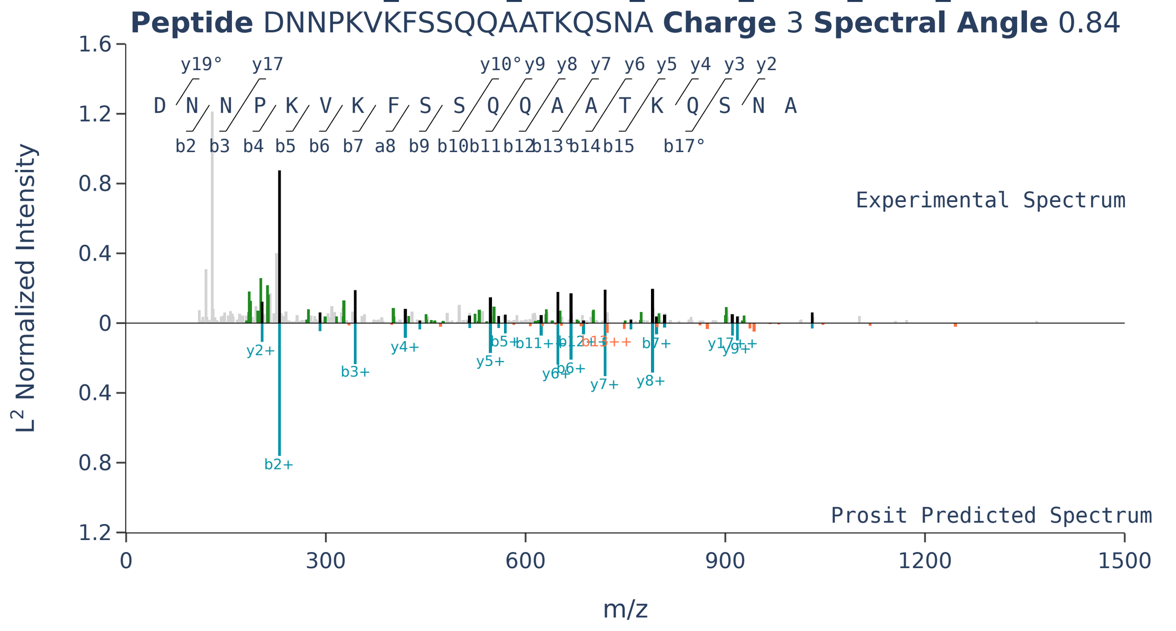

**Source** HRoetschke_271222_090123_HFGoe_LEDGF_b1R1_2h **Scan** 21891
**Peptide** KNMFLVGEGD **Charge** 2 **Spectral Angle** 0.74

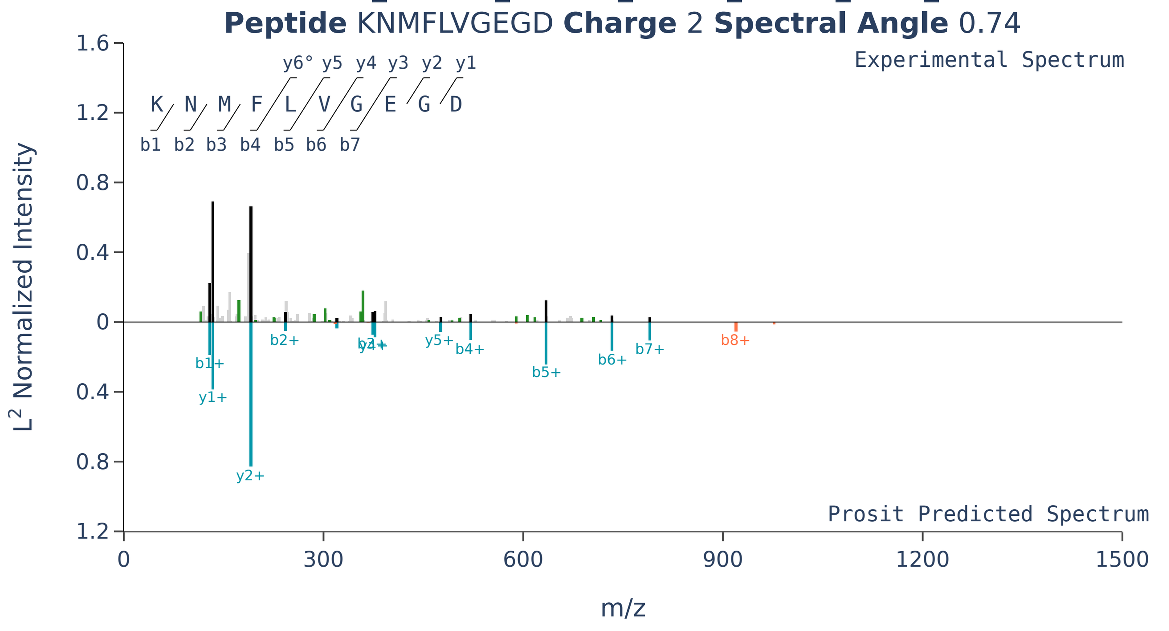

**Source** HRoetschke_271222_090123_HFGoe_LEDGF_b2R1_4h **Scan** 20899
**Peptide** PKDIFPYSE **Charge** 2 **Spectral Angle** 0.69

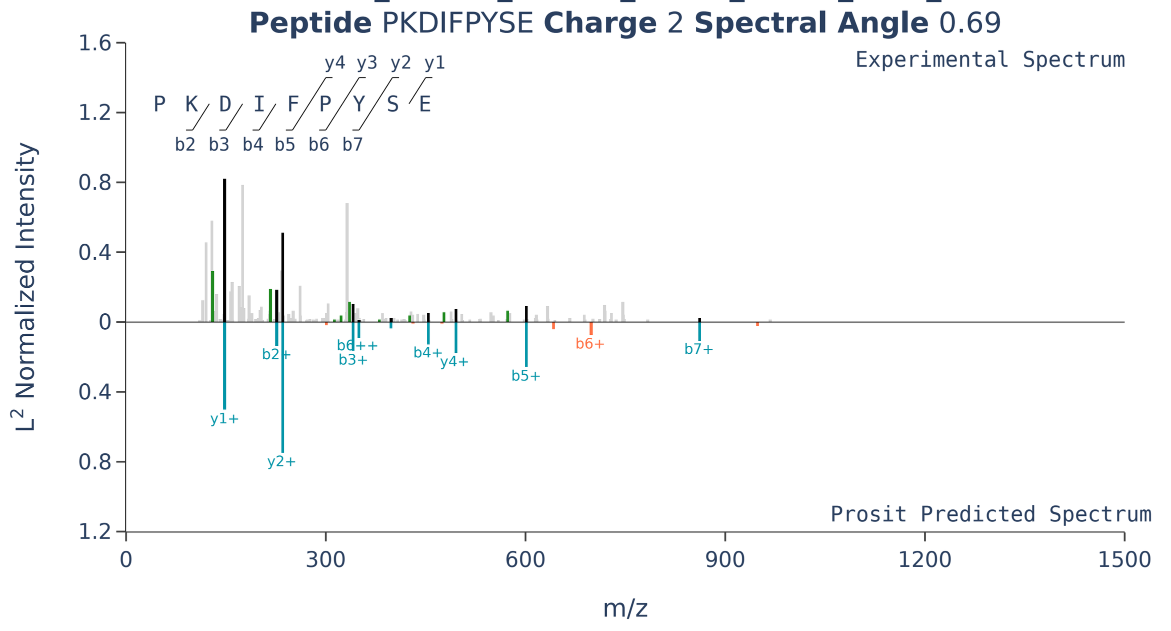

**Source** HRoetschke_271222_090123_HFGoe_LEDGF_b2R1_24h **Scan** 19673
**Peptide** TSMDSRLQRIHAEIKNSL **Charge** 3 **Spectral Angle** 0.86

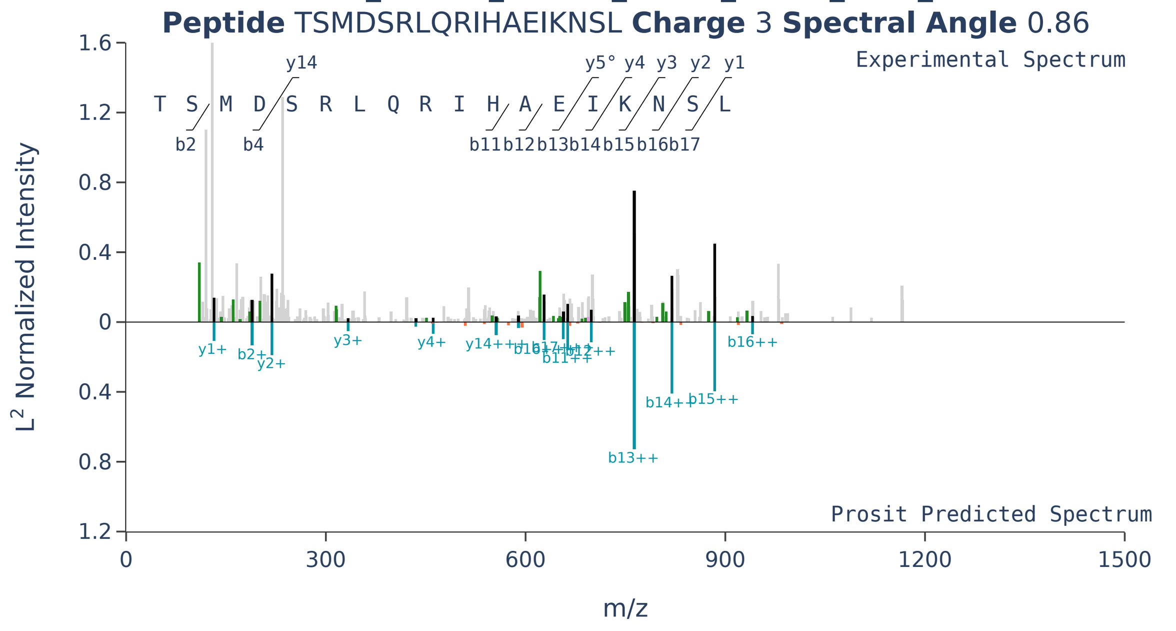

**Source** HRoetschke_271222_090123_HFGoe_LEDGF_b2R1_1h **Scan** 29199
**Peptide** VPDGAVKPPTNKLPIFFFGTHET **Charge** 3 **Spectral Angle** 0.73

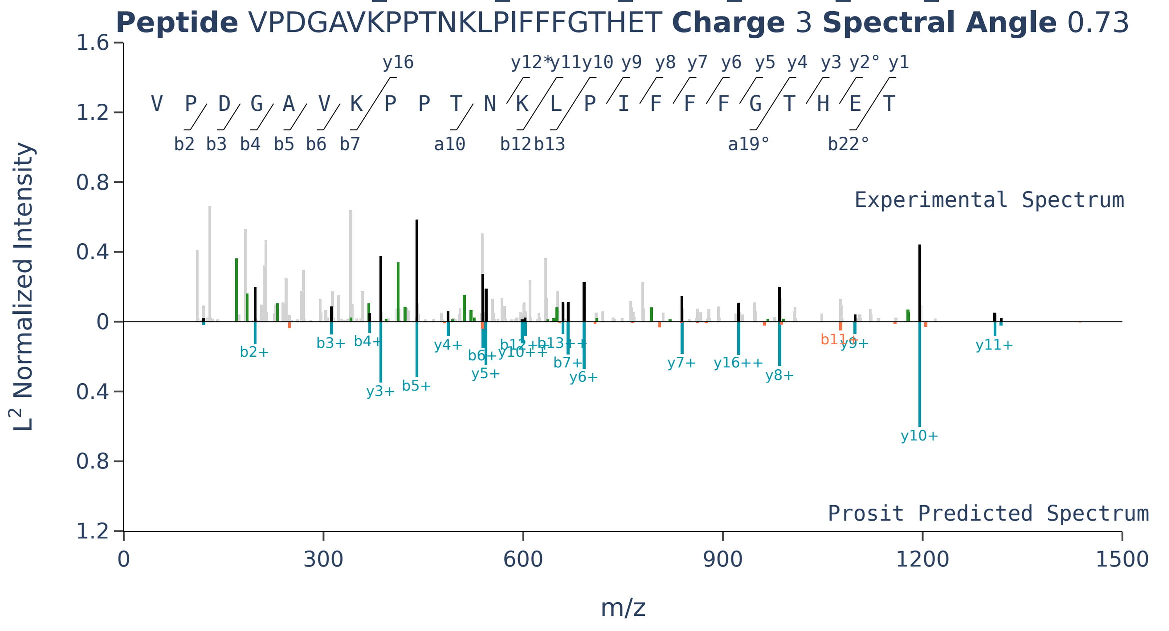

**Source** HRoetschke_271222_090123_HFGoe_LEDGF_b1R2_1h **Scan** 12083
**Peptide** RRFKVSQVIMEKS **Charge** 4 **Spectral Angle** 0.85

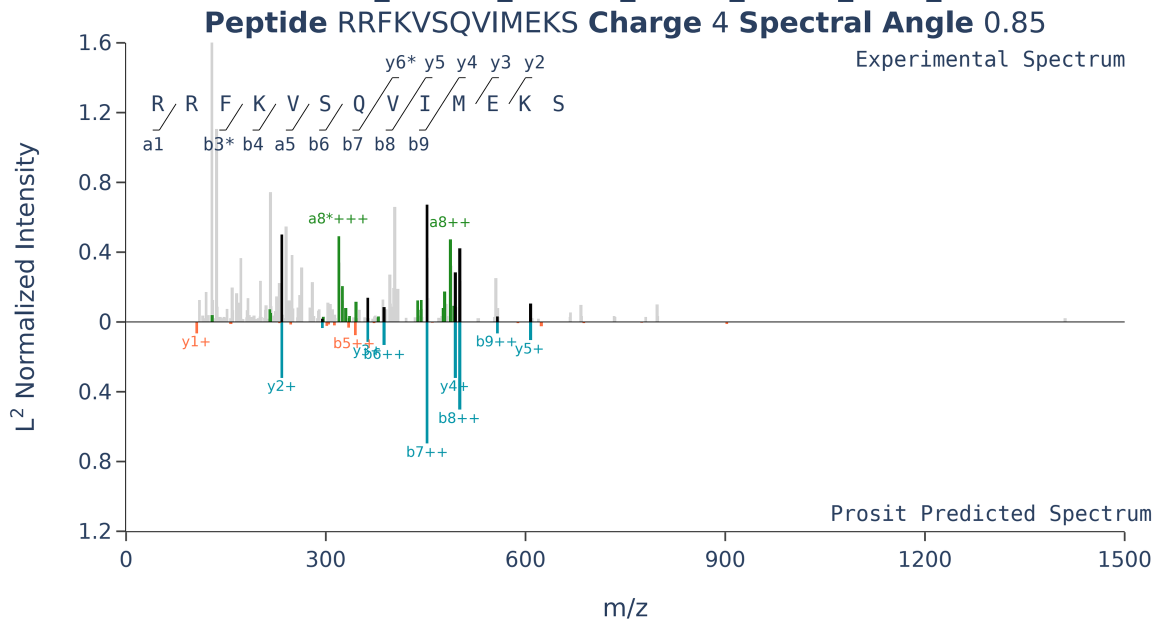

**Source** HRoetschke_271222_090123_HFGoe_LEDGF_b1R1_2h **Scan** 24195
**Peptide** KVSQVIMEKSTMLYNKFKNM **Charge** 3 **Spectral Angle** 0.95

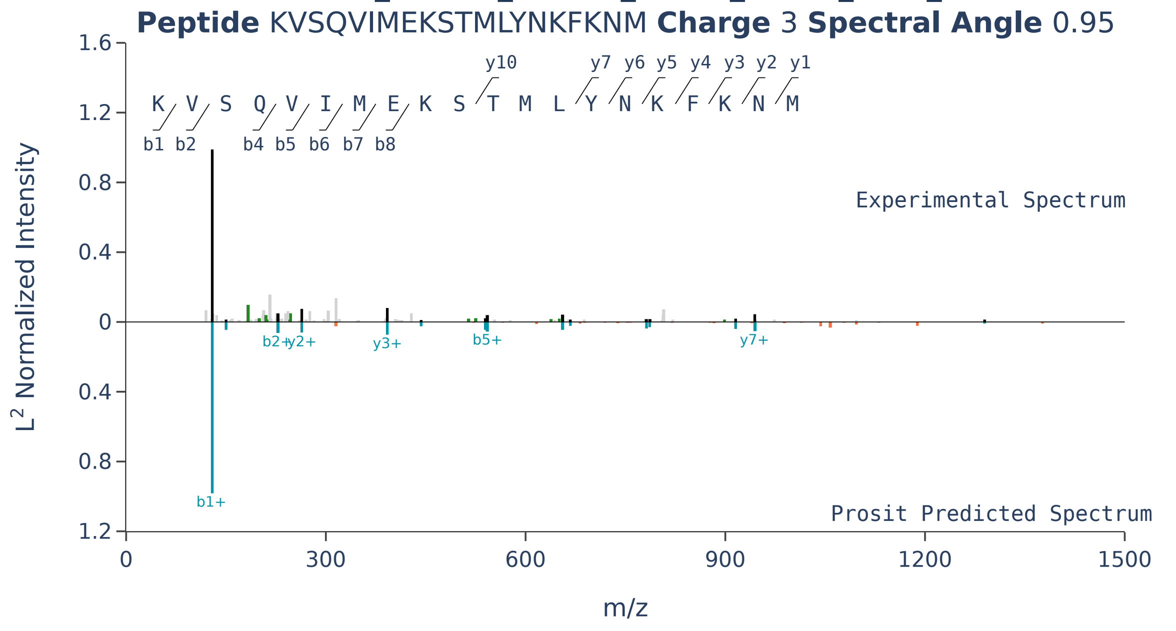

**Source** HRoetschke_271222_090123_HFGoe_LEDGF_b1R1_2h **Scan** 13391
**Peptide** QVTMQQAQKHTEMITTLKKIRR **Charge** 6 **Spectral Angle** 0.87

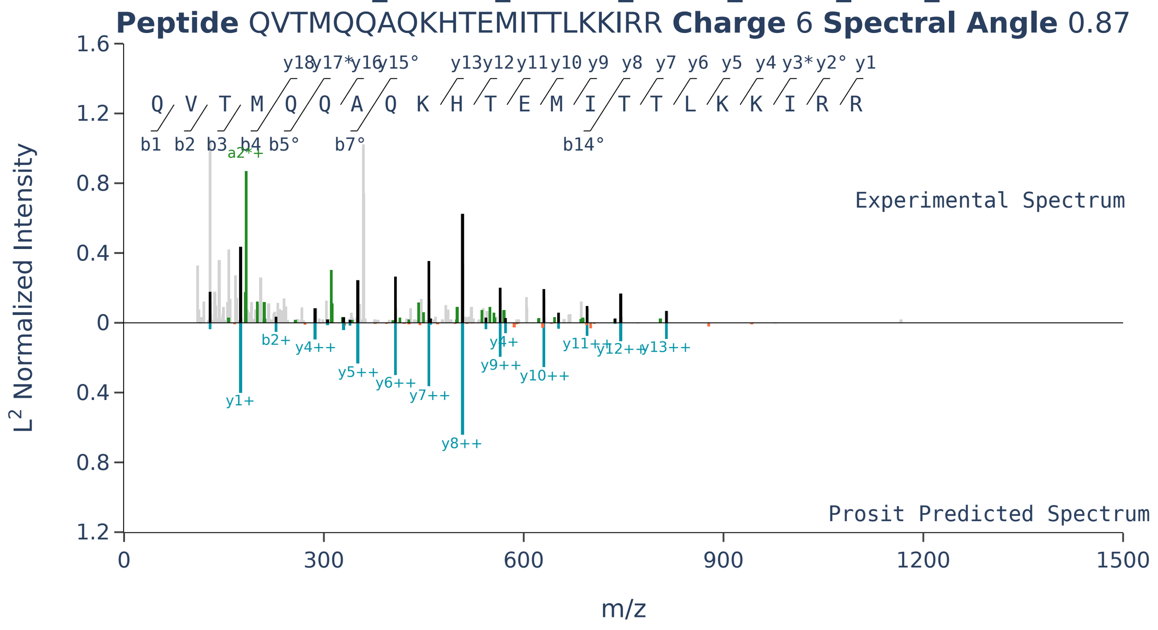


**Source** HRoetschke_271222_090123_HFGoe_LEDGF_b2R1_2h **Scan** 21966
**Peptide** GTHETAFLGPKDIFPYSENKEKY **Charge** 3 **Spectral Angle** 0.79

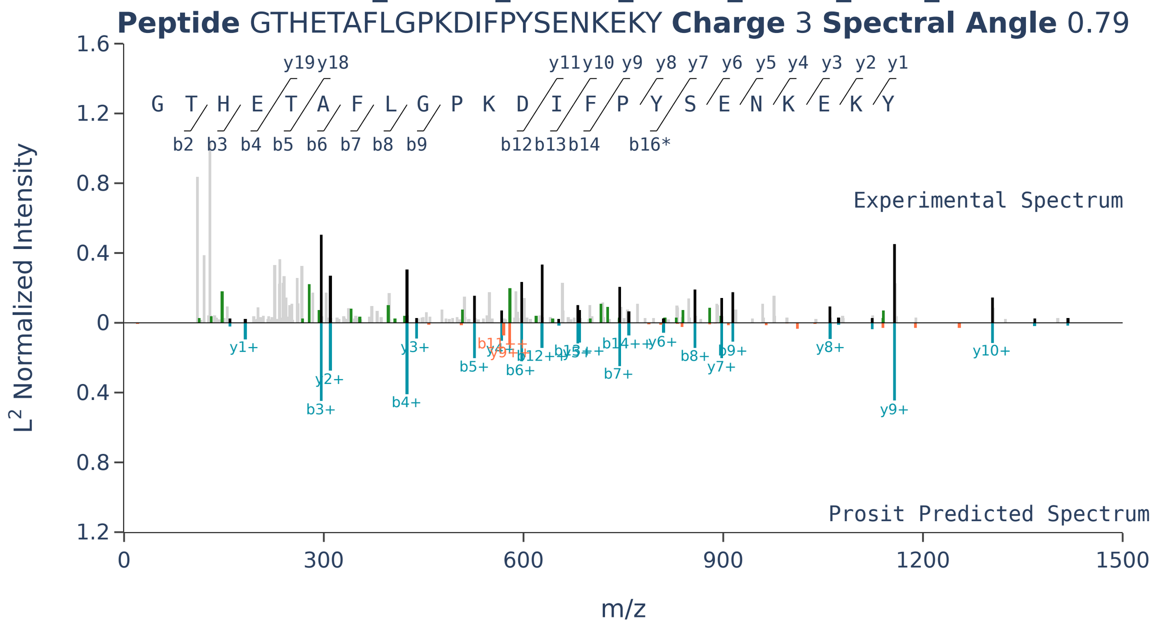


**Source** HRoetschke_271222_090123_HFGoe_LEDGF_b1R2_24h **Scan** 5767
**Peptide** VDITTPKAARRGRKR **Charge** 3 **Spectral Angle** 0.74

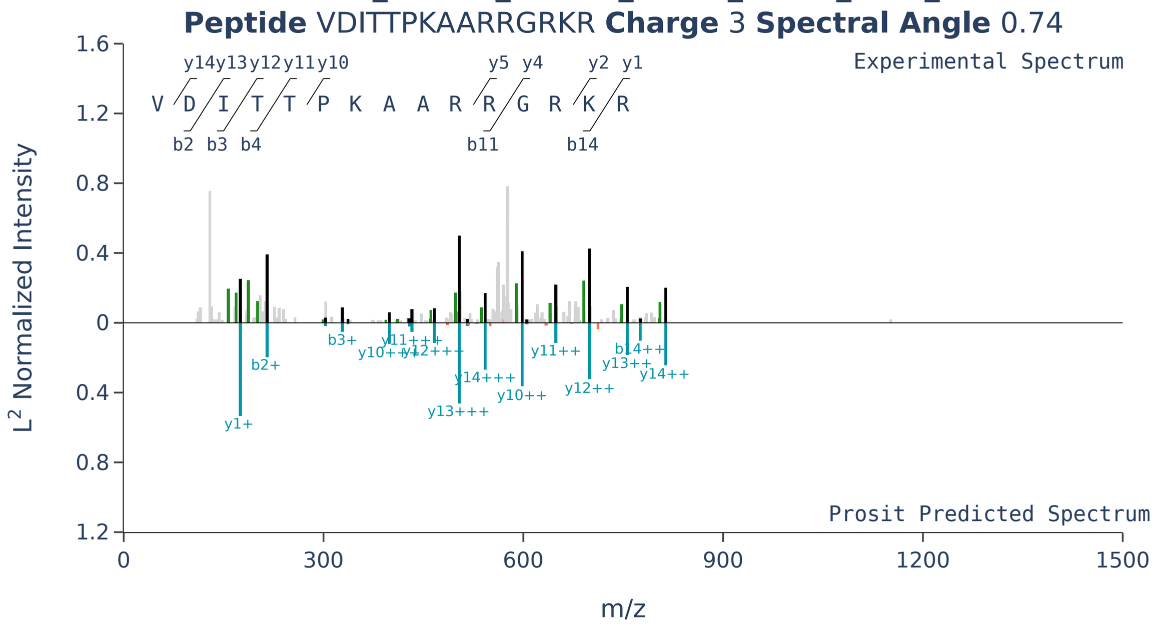


**Source** HRoetschke_271222_090123_HFGoe_LEDGF_b2R1_24h **Scan** 28617
**Peptide** VGEGDSVITQVLNKSLAEQRQ **Charge** 3 **Spectral Angle** 0.81

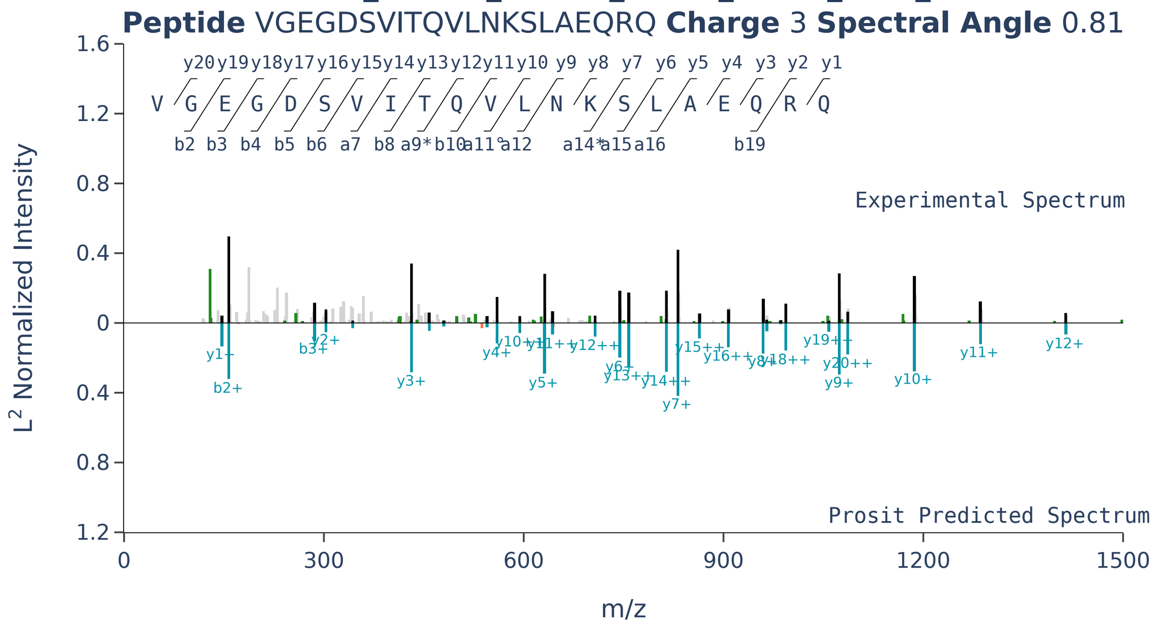


**Source** HRoetschke_271222_090123_HFGoe_LEDGF_b2R2_2h **Scan** 8658
**Peptide** EERETEI **Charge** 1 **Spectral Angle** 0.85

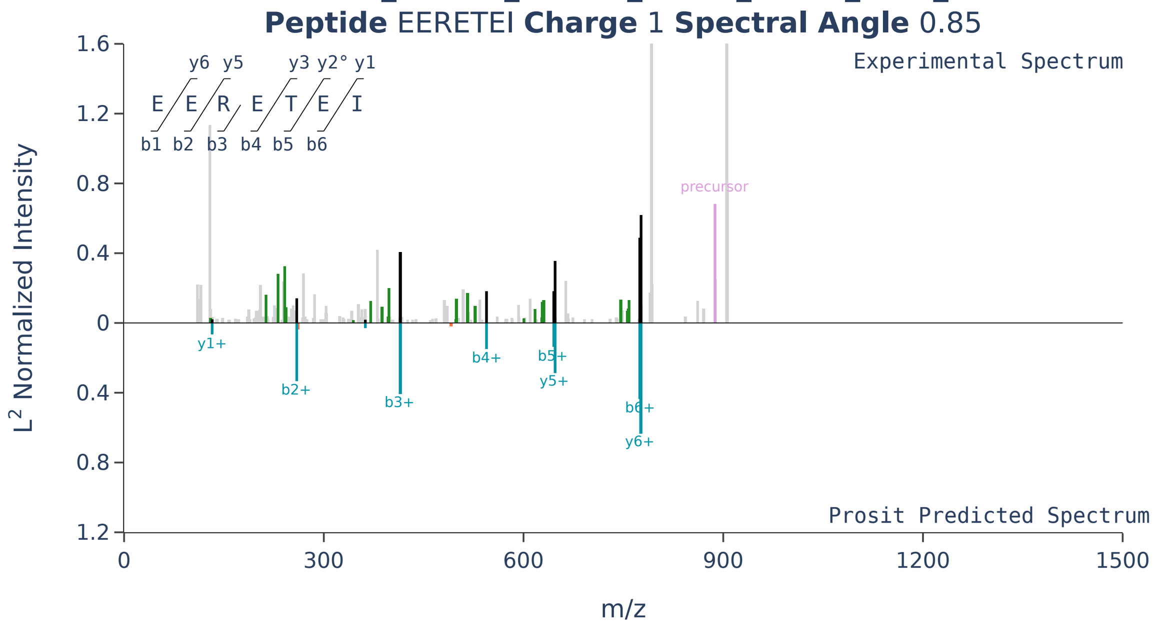


**Source** HRoetschke_271222_090123_HFGoe_LEDGF_b2R2_2h **Scan** 25756
**Peptide** KSTMLYNKFKNMFL **Charge** 3 **Spectral Angle** 0.87

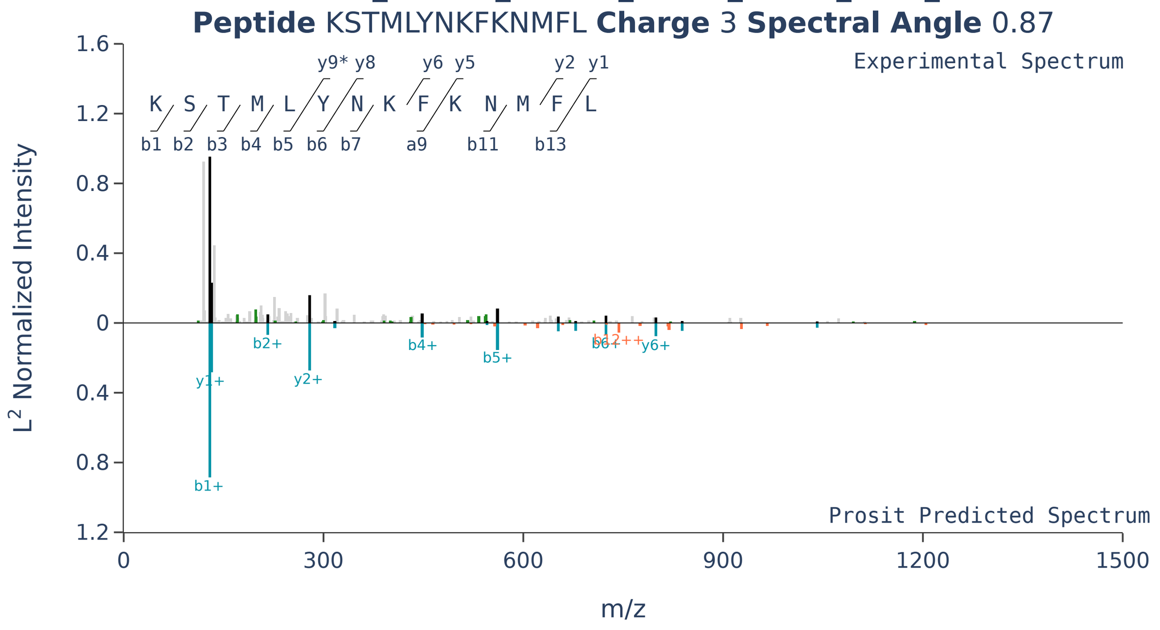


**Source** HRoetschke_271222_090123_HFGoe_LEDGF_b1R1_4h **Scan** 22660
**Peptide** QVIMEKSTMLYNKF **Charge** 3 **Spectral Angle** 0.77

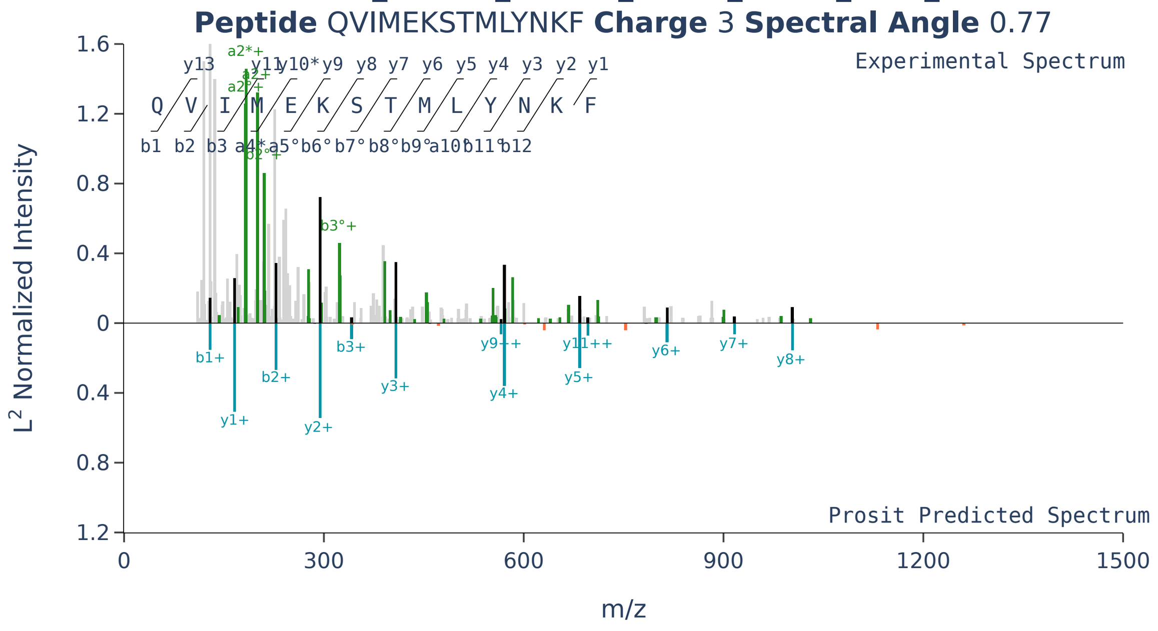


**Source** HRoetschke_271222_090123_HFGoe_LEDGF_b1R1_4h **Scan** 6786
**Peptide** NHEASTKKKPSSEERETEISLKD **Charge** 4 **Spectral Angle** 0.81

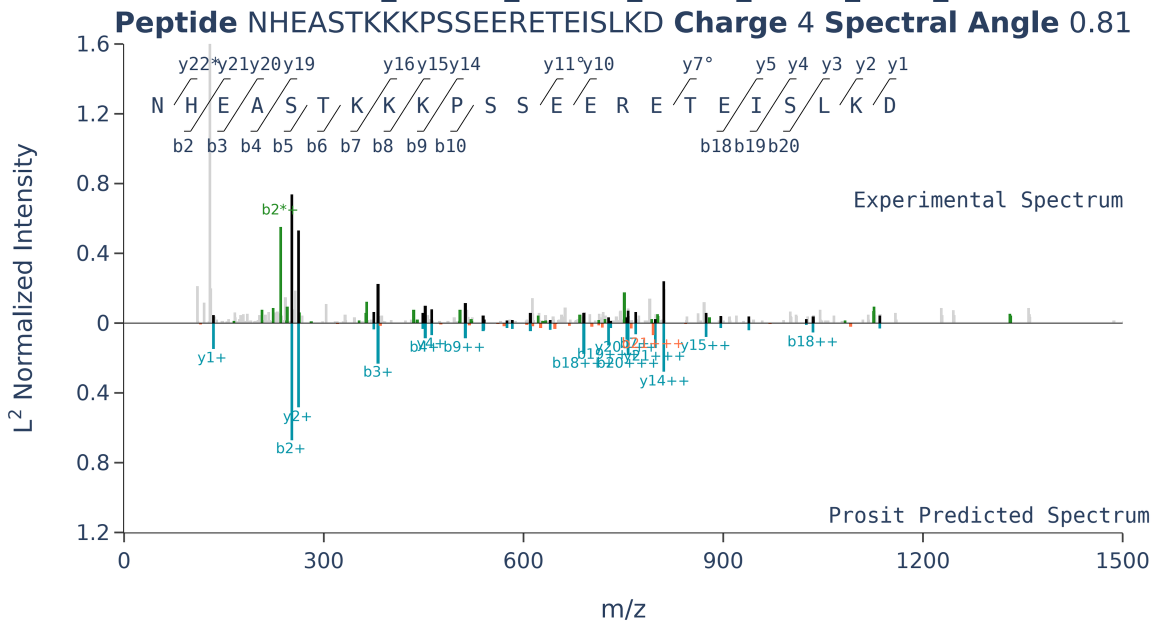


**Source** HRoetschke_271222_090123_HFGoe_LEDGF_b1R2_4h **Scan** 25366
**Peptide** EGDSVITQVLNKSLAEQRQHEE **Charge** 3 **Spectral Angle** 0.83

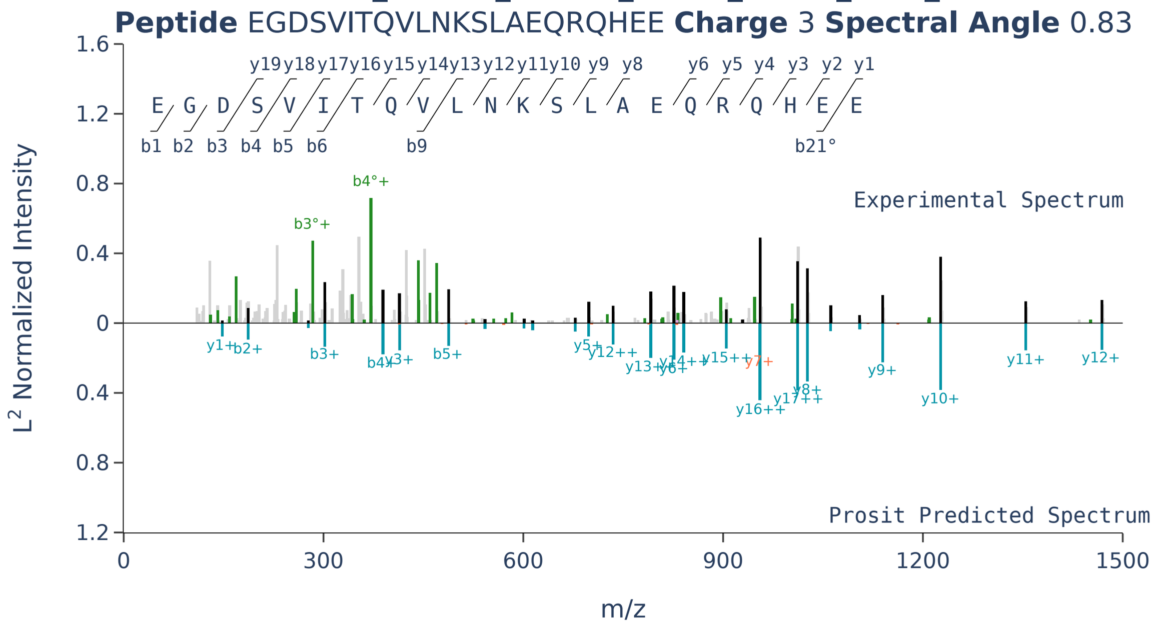


**Source** HRoetschke_271222_090123_HFGoe_LEDGF_b2R2_24h **Scan** 16386
**Peptide** SMDSRLQRIHAEI **Charge** 3 **Spectral Angle** 0.75

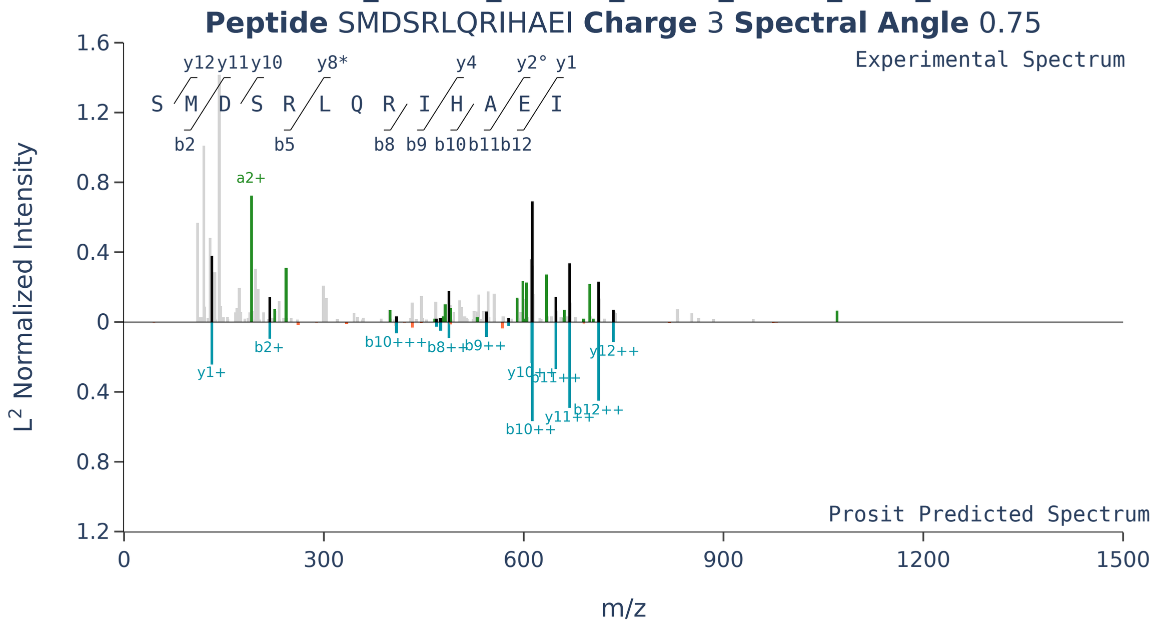


**Source** HRoetschke_271222_090123_HFGoe_LEDGF_b1R2_24h **Scan** 10468
**Peptide** RETEISLKDS **Charge** 2 **Spectral Angle** 0.84

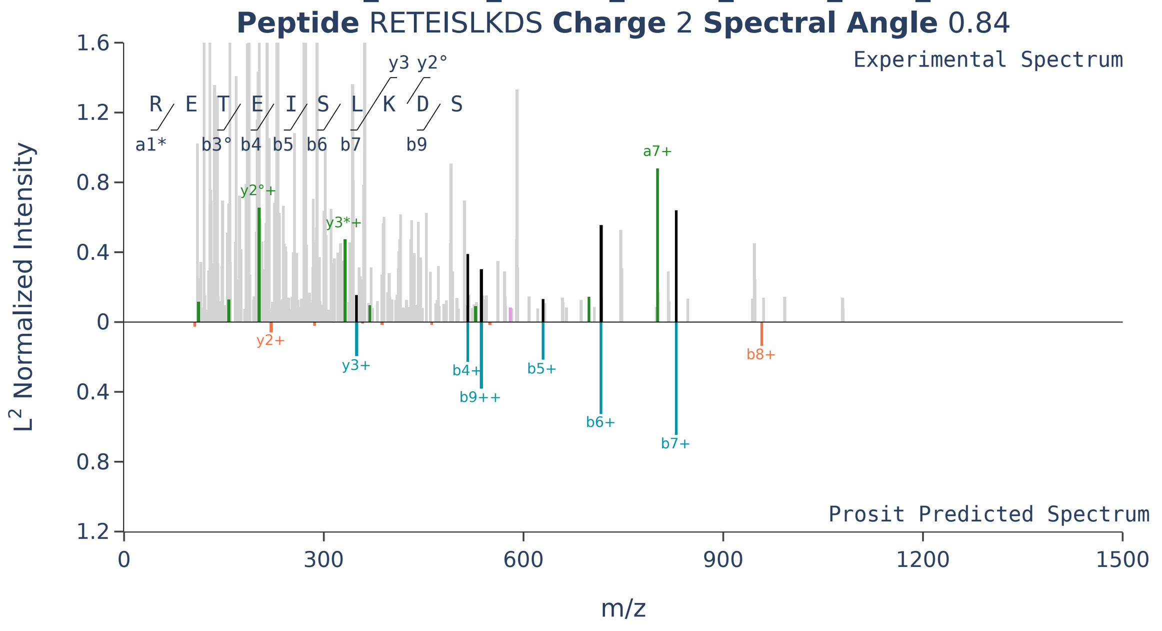


**Source** HRoetschke_271222_090123_HFGoe_LEDGF_b2R1_2h **Scan** 22978
**Peptide** DELASLQVTMQQAQKHTE **Charge** 3 **Spectral Angle** 0.89

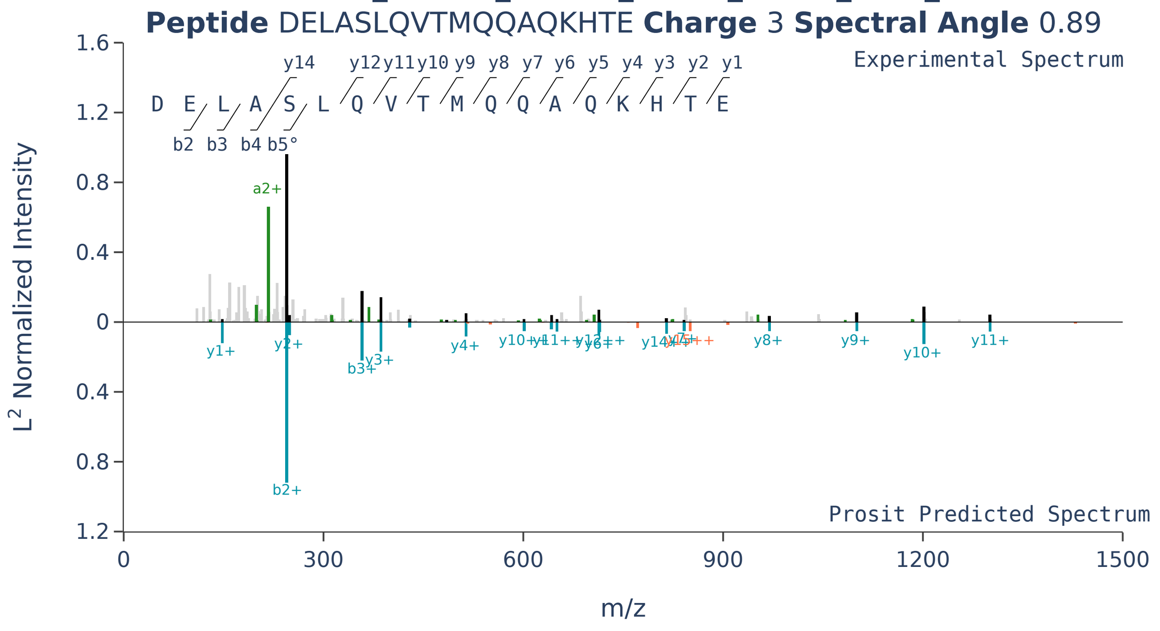


**Source** HRoetschke_271222_090123_HFGoe_LEDGF_b1R2_4h **Scan** 15032
**Peptide** RFKVSQVIMEKS **Charge** 2 **Spectral Angle** 0.78

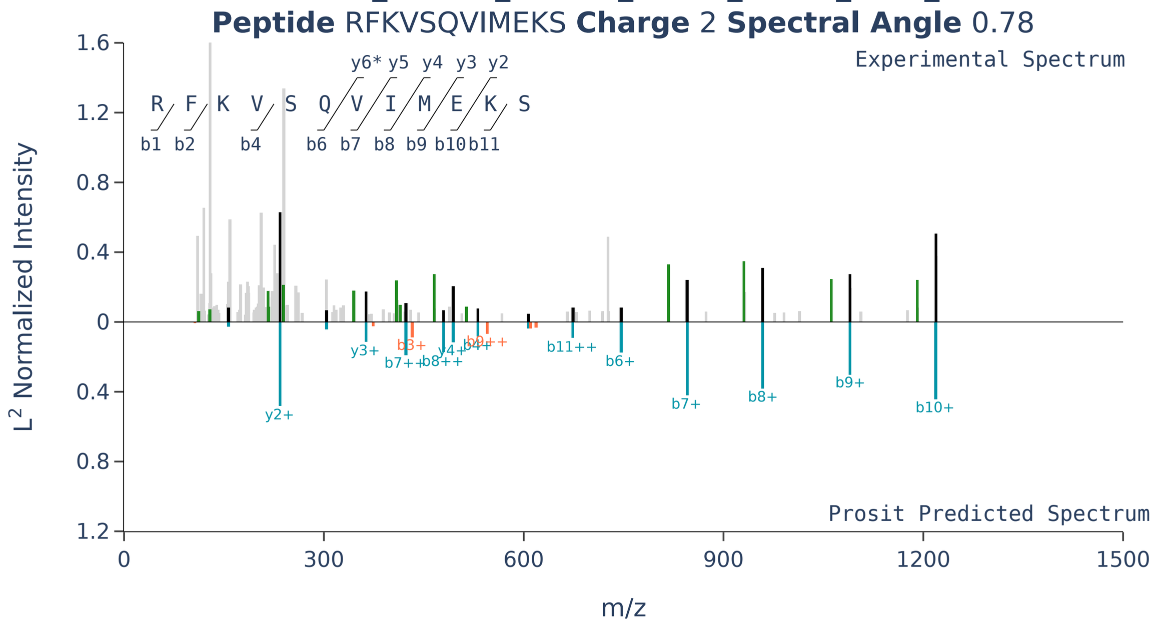


**Source** HRoetschke_271222_090123_HFGoe_LEDGF_b2R1_24h **Scan** 13088
**Peptide** GPKDIFPYSENKEKYGK **Charge** 4 **Spectral Angle** 0.76

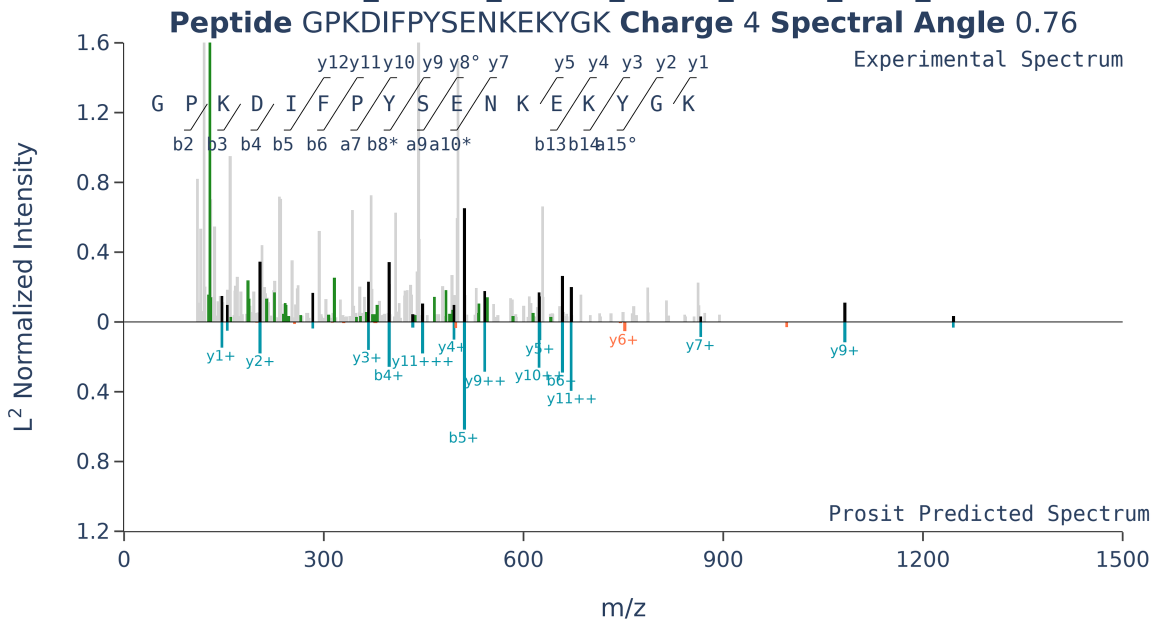


**Source** HRoetschke_271222_090123_HFGoe_LEDGF_b2R1_2h **Scan** 5506
**Peptide** SNEDSKDNHEASTKKKPSSEERETEI **Charge** 4 **Spectral Angle** 0.86

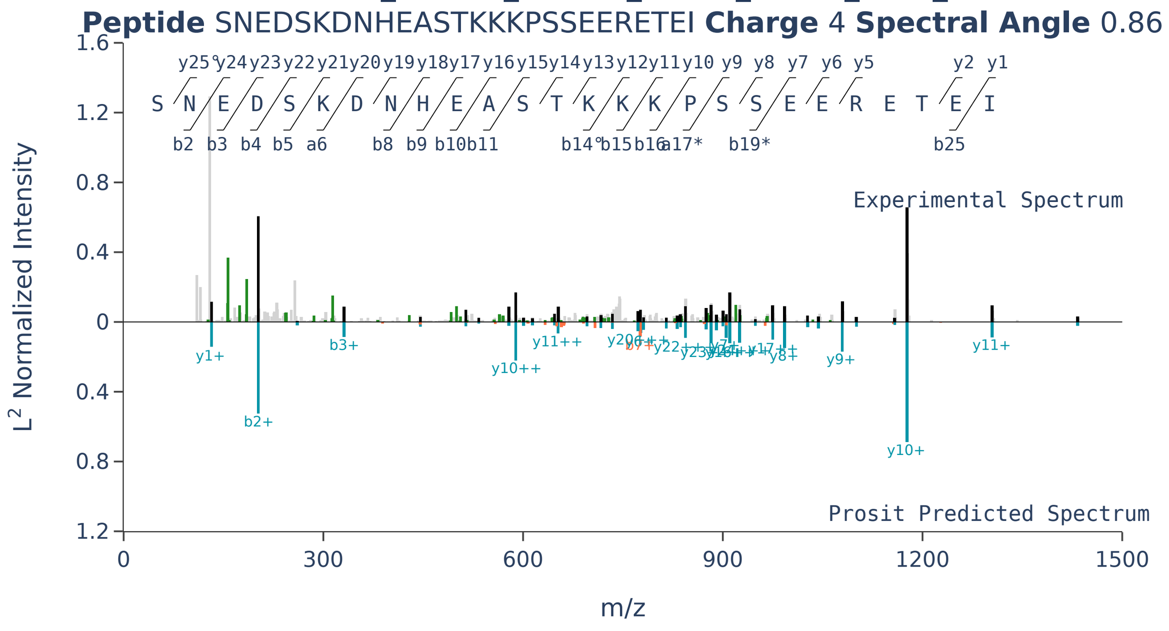

**Source** HRoetschke_271222_090123_HFGoe_LEDGF_b1R2_24h **Scan** 19337
**Peptide** KNSLKIDNLDVNRCI **Charge** 3 **Spectral Angle** 0.77

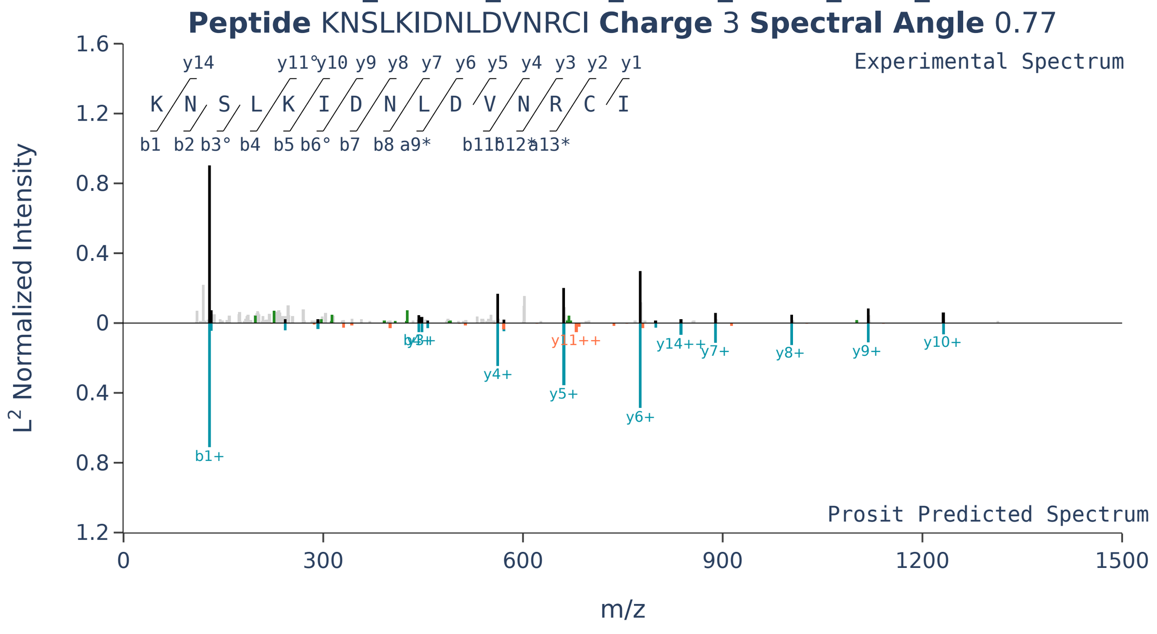


**Source** HRoetschke_271222_090123_HFGoe_LEDGF_b1R1_1h **Scan** 17680
**Peptide** WEIDNNPKVKFSSQQ **Charge** 3 **Spectral Angle** 0.78

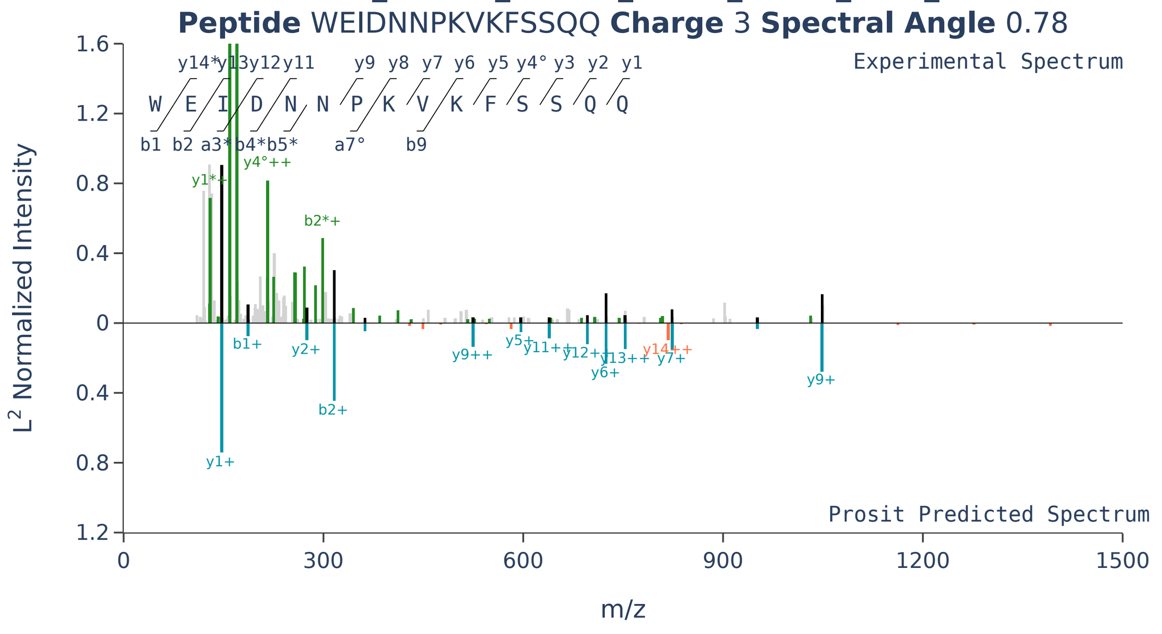


**Source** HRoetschke_271222_090123_HFGoe_LEDGF_b1R1_2h **Scan** 26130
**Peptide** RDFKPGDLIFAKM **Charge** 4 **Spectral Angle** 0.71

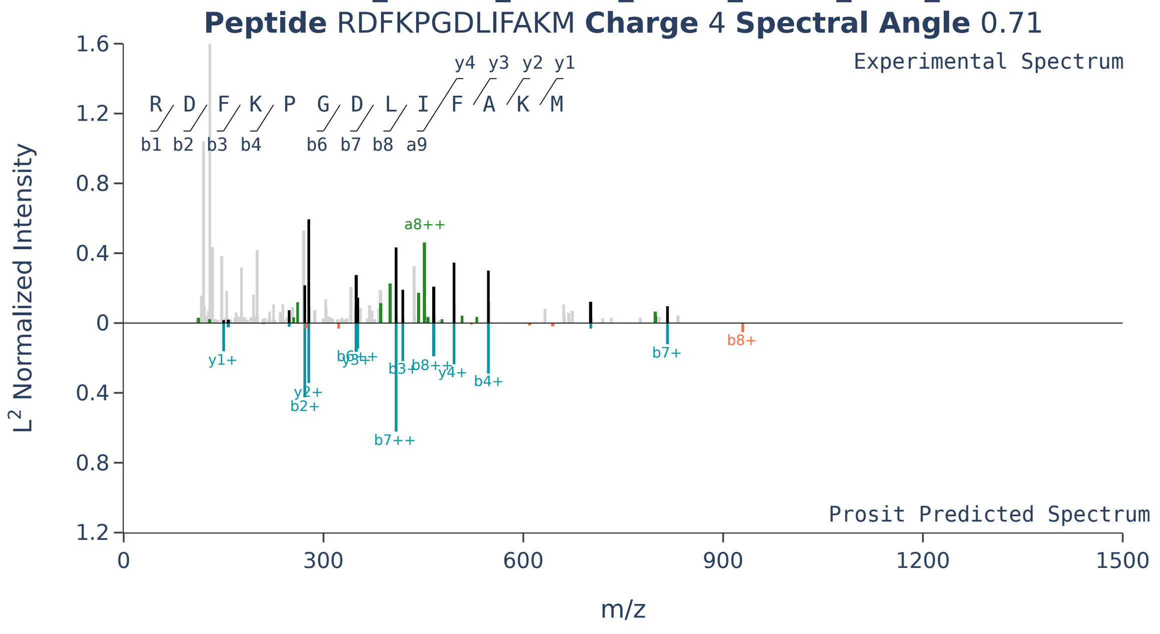


**Source** HRoetschke_271222_090123_HFGoe_LEDGF_b1R1_2h **Scan** 19246
**Peptide** MKGYPHWPARVDEVPDGAVKPPTN **Charge** 4 **Spectral Angle** 0.79

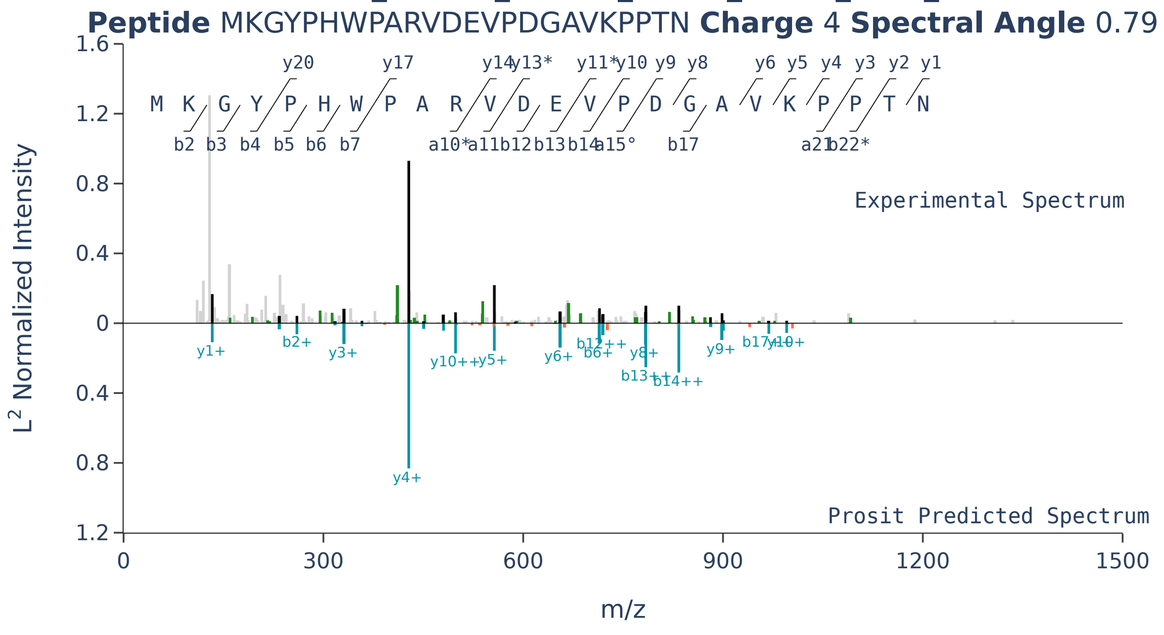


**Source** HRoetschke_271222_090123_HFGoe_LEDGF_b2R1_24h **Scan** 10103
**Peptide** TMQQAQKHTEMITT **Charge** 3 **Spectral Angle** 0.79

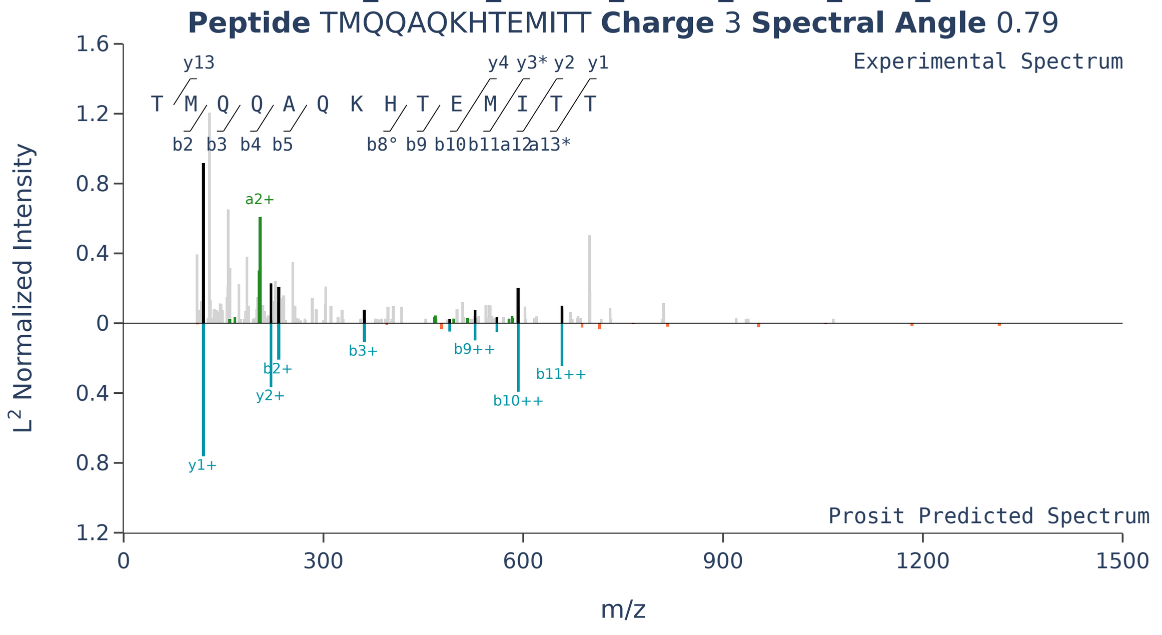


**Source** HRoetschke_271222_090123_HFGoe_LEDGF_b1R2_24h **Scan** 19155
**Peptide** PGDLIFAKMKG **Charge** 2 **Spectral Angle** 0.8

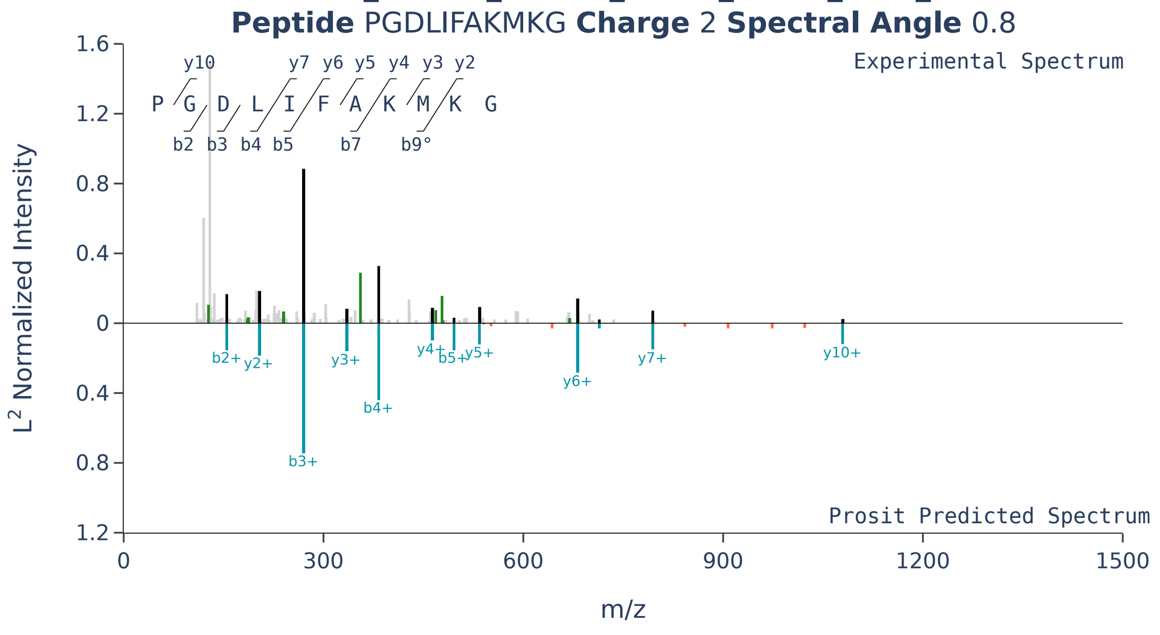


**Source** HRoetschke_271222_090123_HFGoe_LEDGF_b1R1_4h **Scan** 24460
**Peptide** GDSVITQVLNKSLAEQRQH **Charge** 3 **Spectral Angle** 0.75

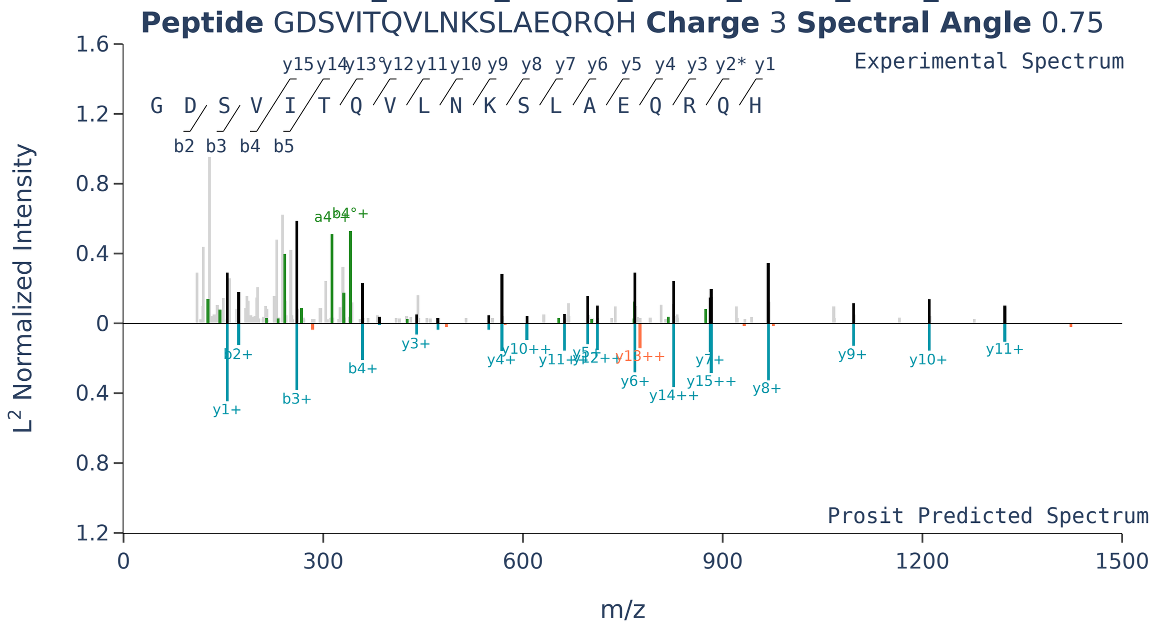


**Source** HRoetschke_271222_090123_HFGoe_LEDGF_b2R1_24h **Scan** 19652
**Peptide** AEIKNSLKIDNL **Charge** 2 **Spectral Angle** 0.8

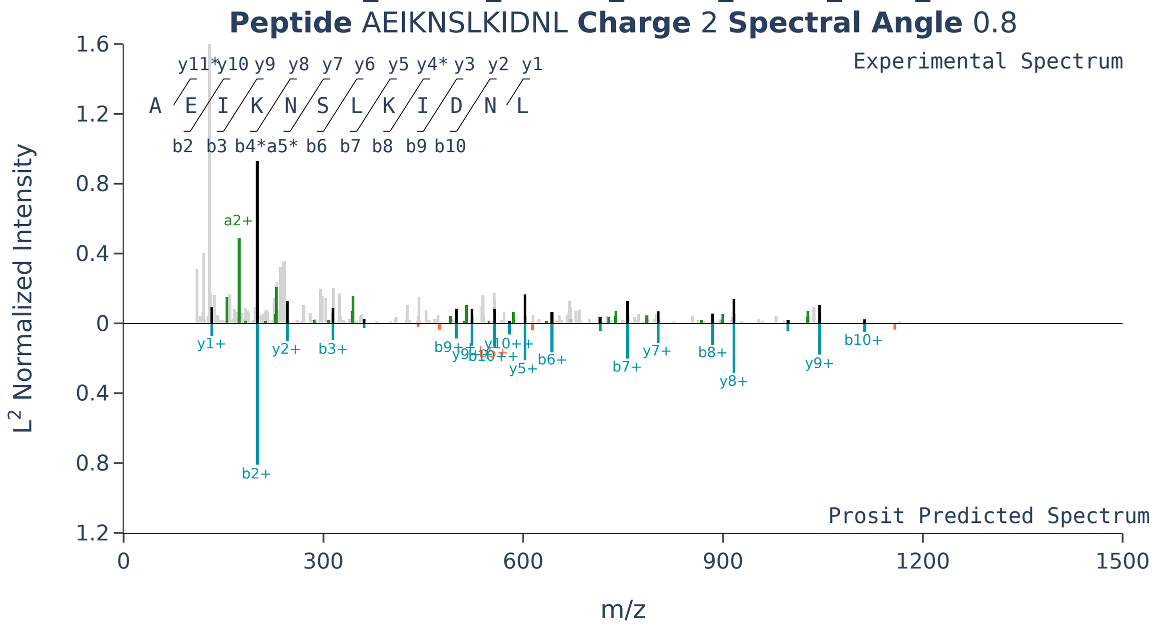


**Source** HRoetschke_271222_090123_HFGoe_LEDGF_b2R2_24h **Scan** 12927
**Peptide** QKHTEMITTLKKI **Charge** 4 **Spectral Angle** 0.9

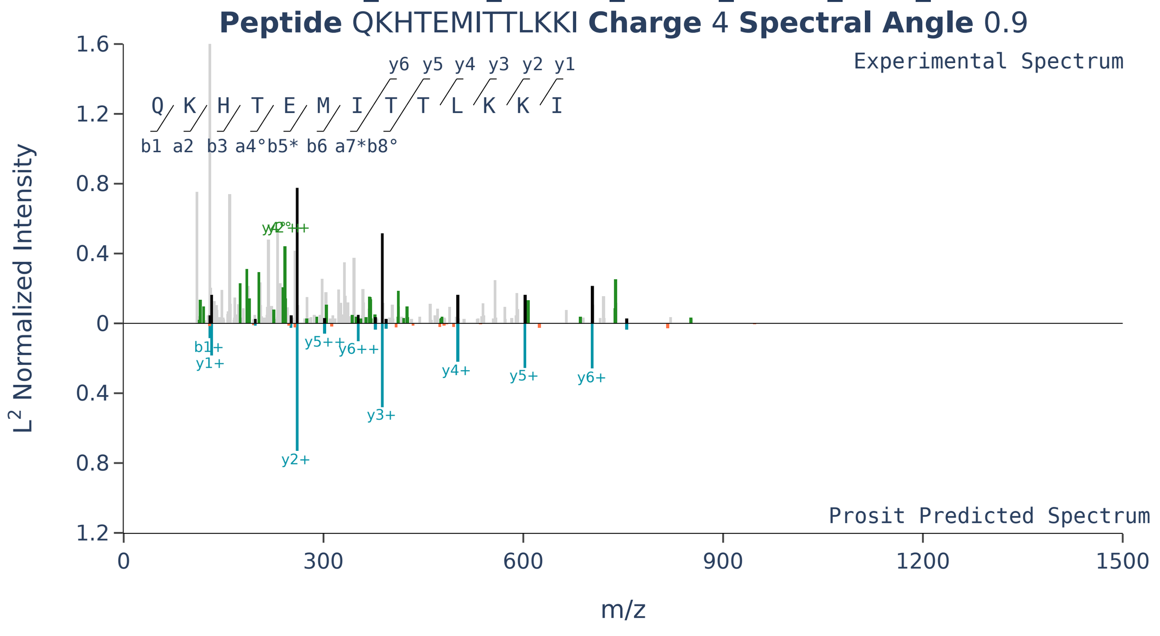


**Source** HRoetschke_271222_090123_HFGoe_LEDGF_b1R1_4h **Scan** 21820
**Peptide** TAFLGPKDIFPYSENKEKYGK **Charge** 4 **Spectral Angle** 0.71

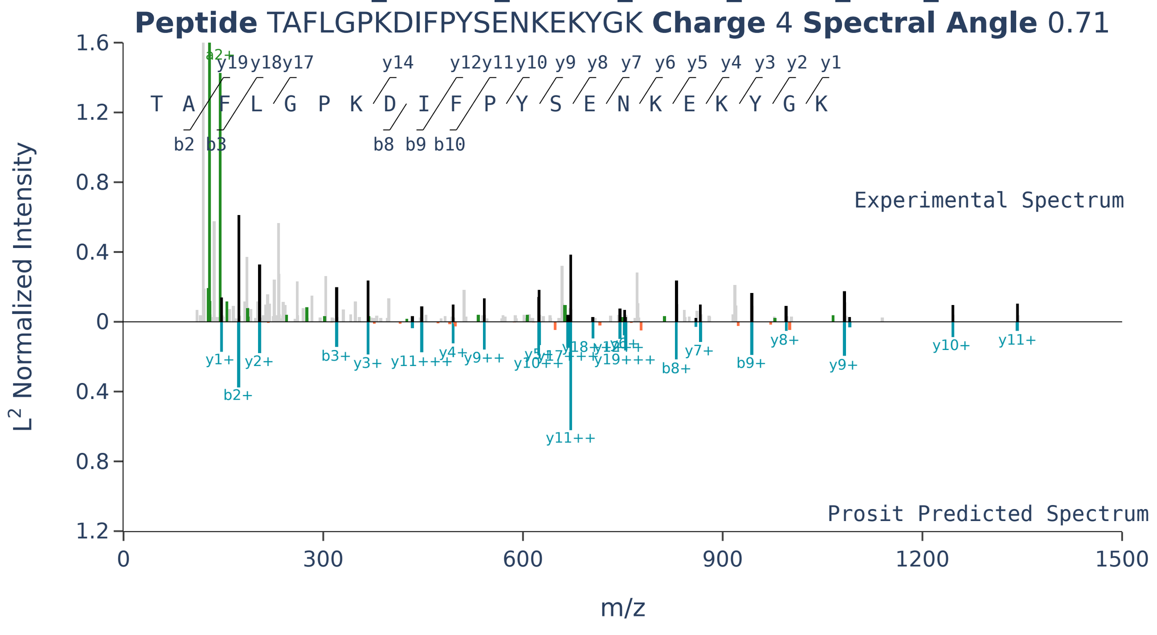


**Source** HRoetschke_271222_090123_HFGoe_LEDGF_b2R2_24h **Scan** 16219
**Peptide** TAFLGPKDIFPYSENKEKYGKPNKRK **Charge** 5 **Spectral Angle** 0.77

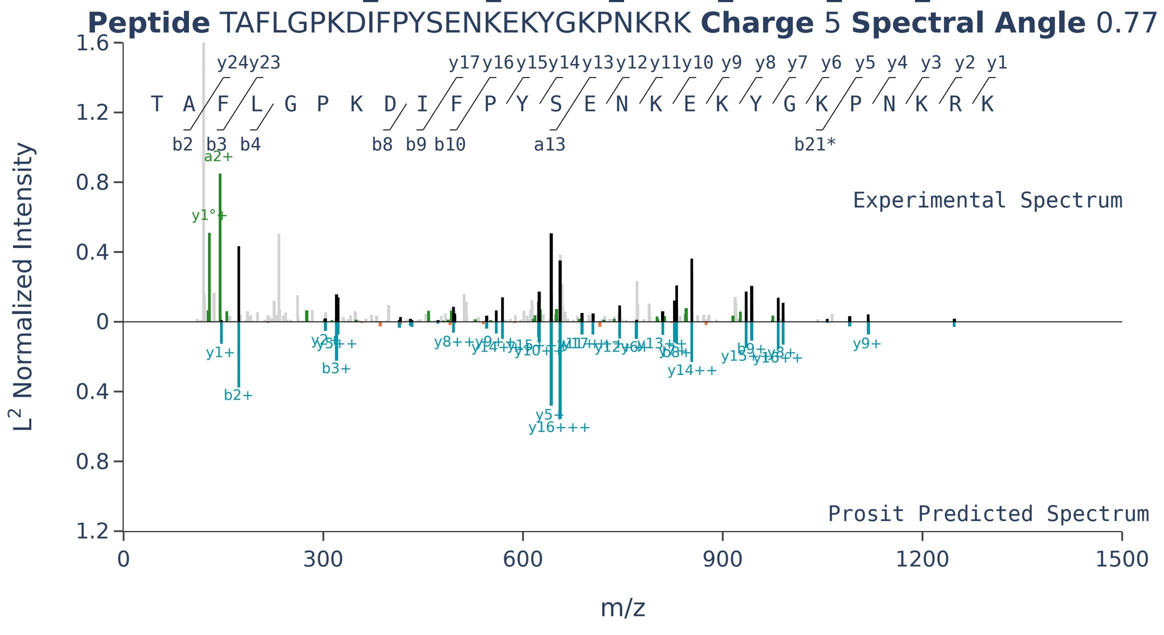


**Source** HRoetschke_271222_090123_HFGoe_LEDGF_b1R2_4h **Scan** 18944
**Peptide** FLGPKDIFPYSENKEKYGKPNK **Charge** 4 **Spectral Angle** 0.8

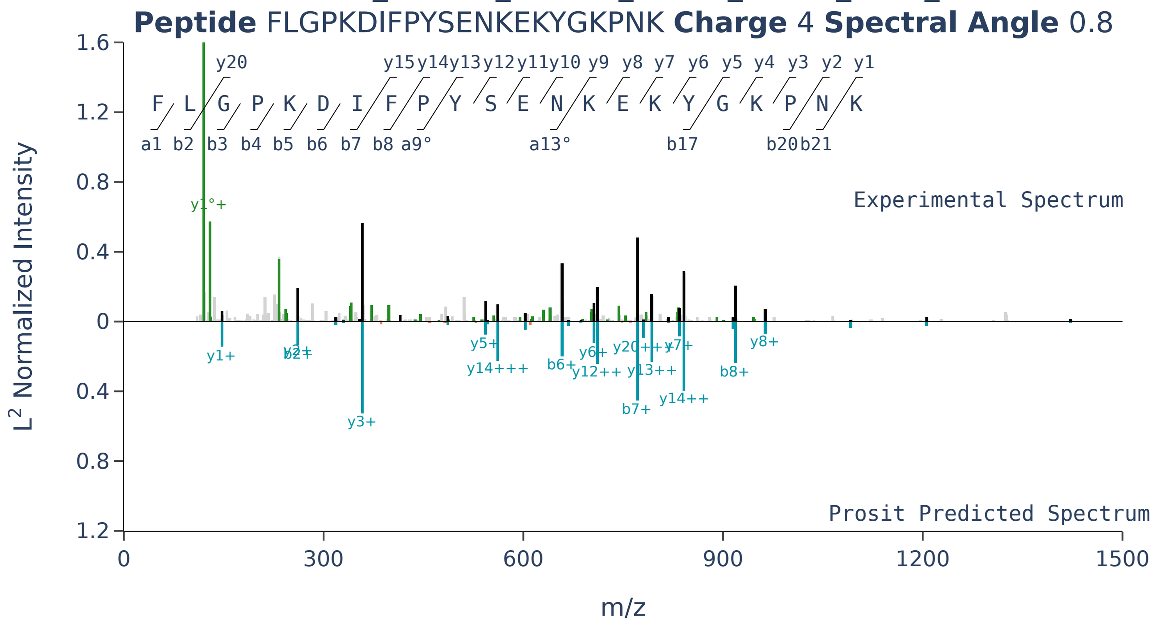


**Source** HRoetschke_271222_090123_HFGoe_LEDGF_b2R1_2h **Scan** 9180
**Peptide** ASSDVEVEEKETSVSKE **Charge** 3 **Spectral Angle** 0.78

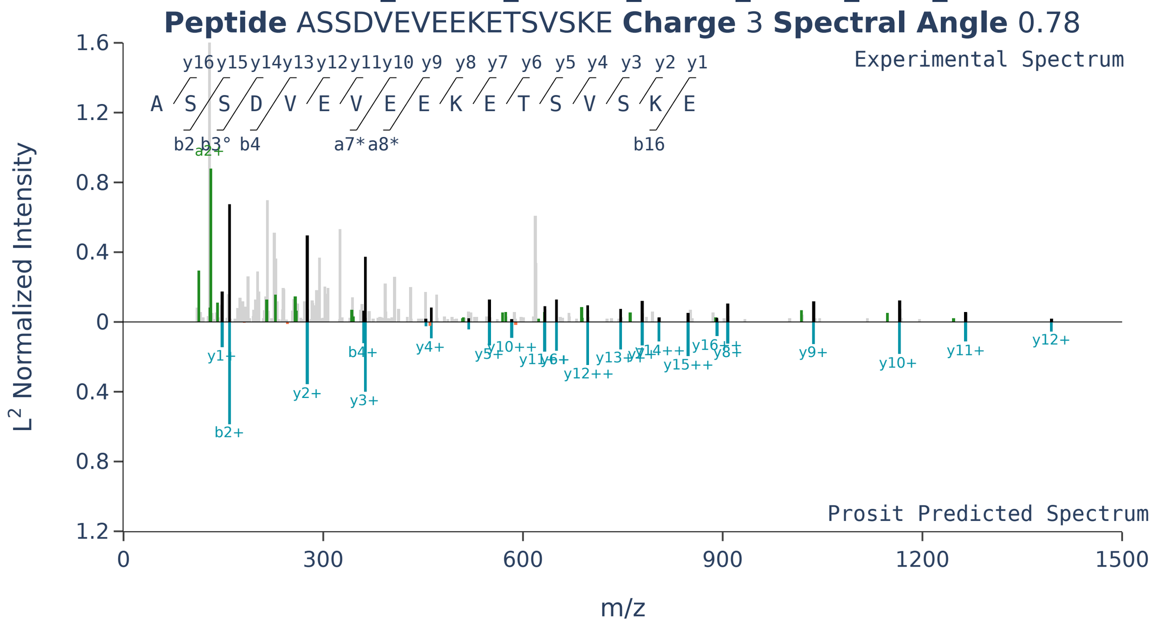


**Source** HRoetschke_271222_090123_HFGoe_LEDGF_b1R1_4h **Scan** 12042
**Peptide** PKDIFPYSENKEKYGK **Charge** 4 **Spectral Angle** 0.73

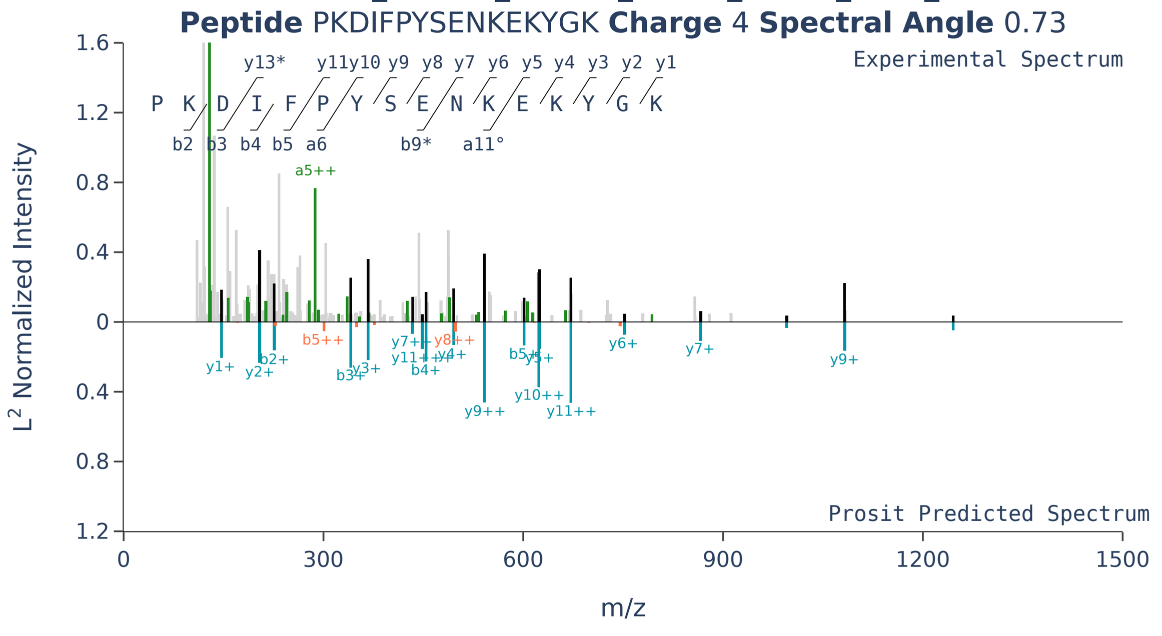


**Source** HRoetschke_271222_090123_HFGoe_LEDGF_b1R1_4h **Scan** 8086
**Peptide** EDVTKAVDITTPKAARRGRKR **Charge** 4 **Spectral Angle** 0.67

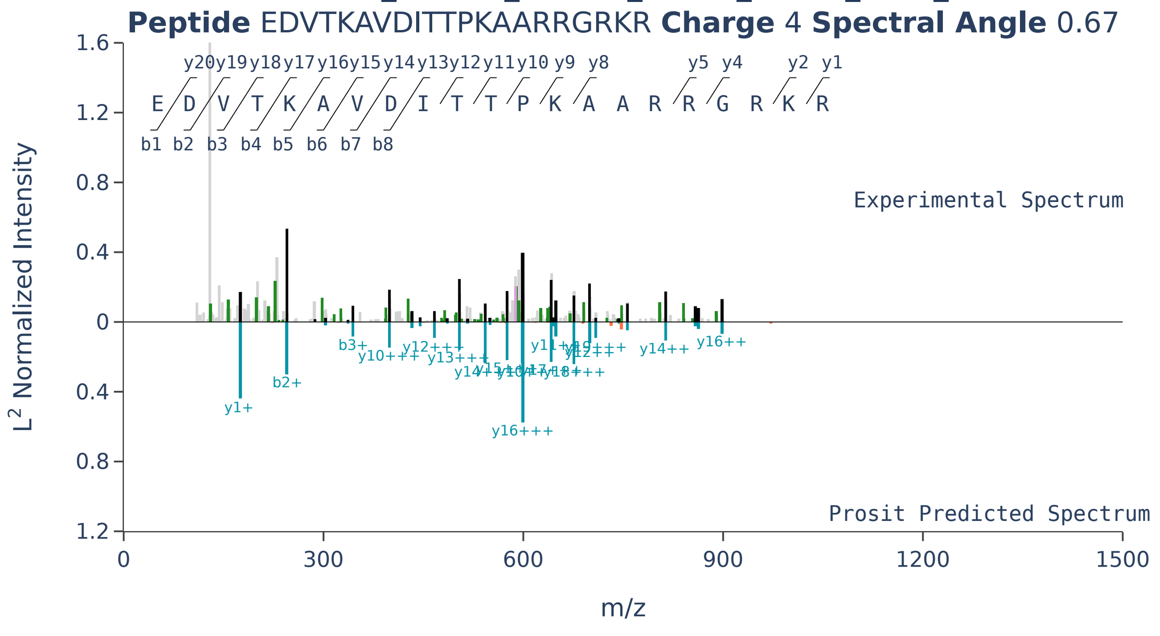

**Source** HRoetschke_271222_090123_HFGoe_LEDGF_b1R1_2h **Scan** 29144
**Peptide** EGDSVITQVL **Charge** 2 **Spectral Angle** 0.87

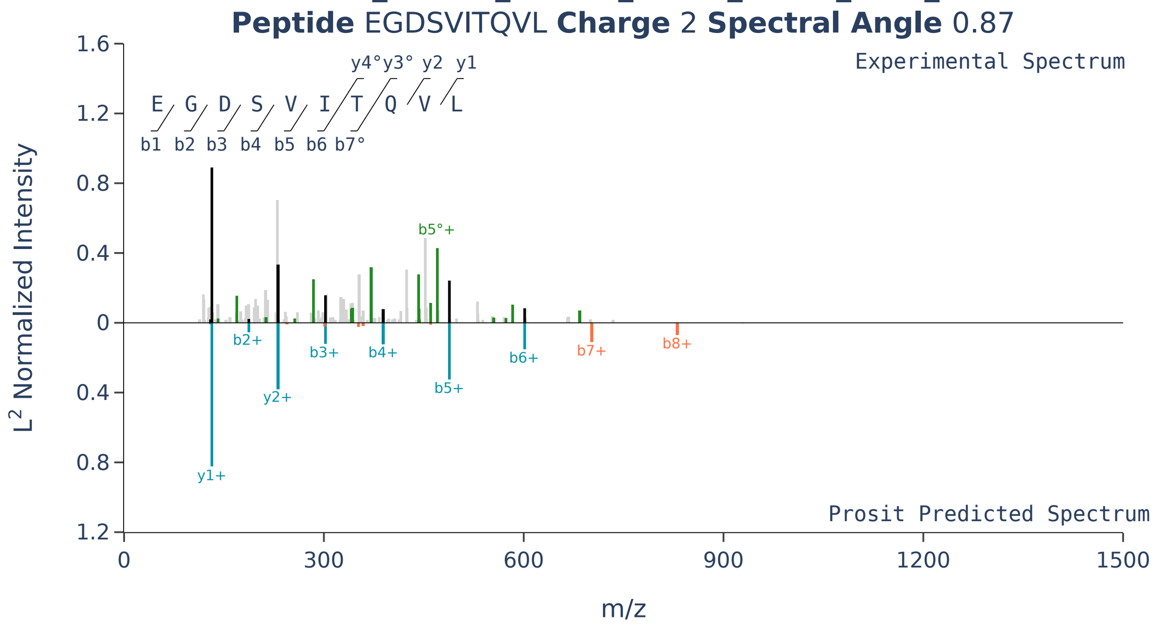


**Source** HRoetschke_271222_090123_HFGoe_LEDGF_b1R1_4h **Scan** 22121
**Peptide** VSQVIMEKSTMLYN **Charge** 2 **Spectral Angle** 0.82

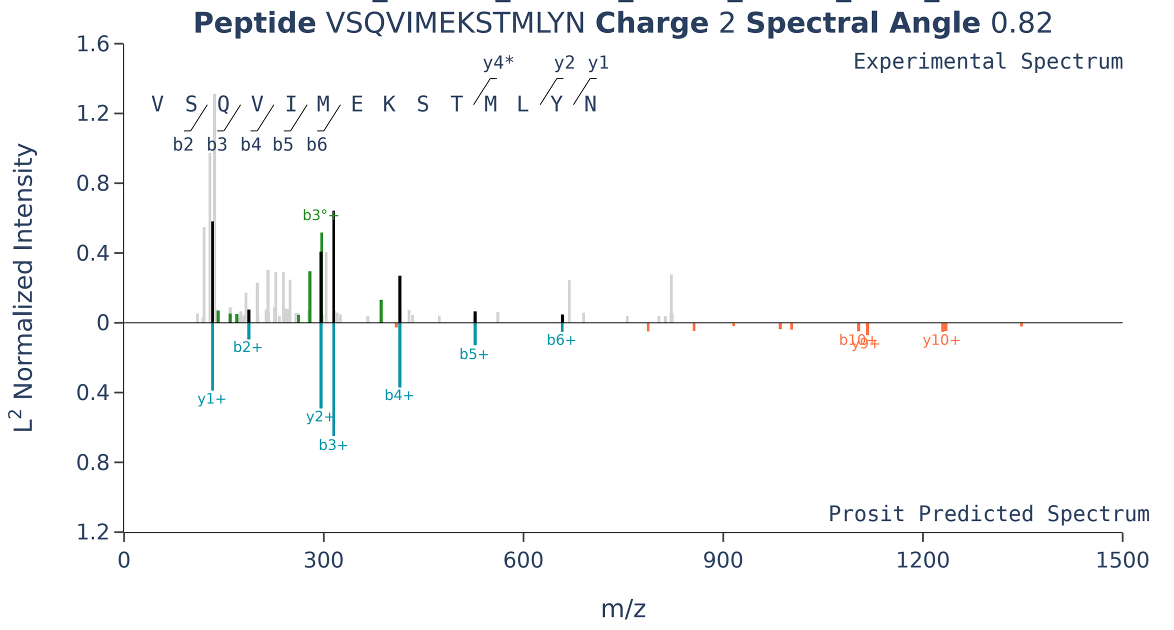


**Source** HRoetschke_271222_090123_HFGoe_LEDGF_b2R1_2h **Scan** 10903
**Peptide** NNPKVKFSSQQAATKQSNASSDVEVE **Charge** 4 **Spectral Angle** 0.89

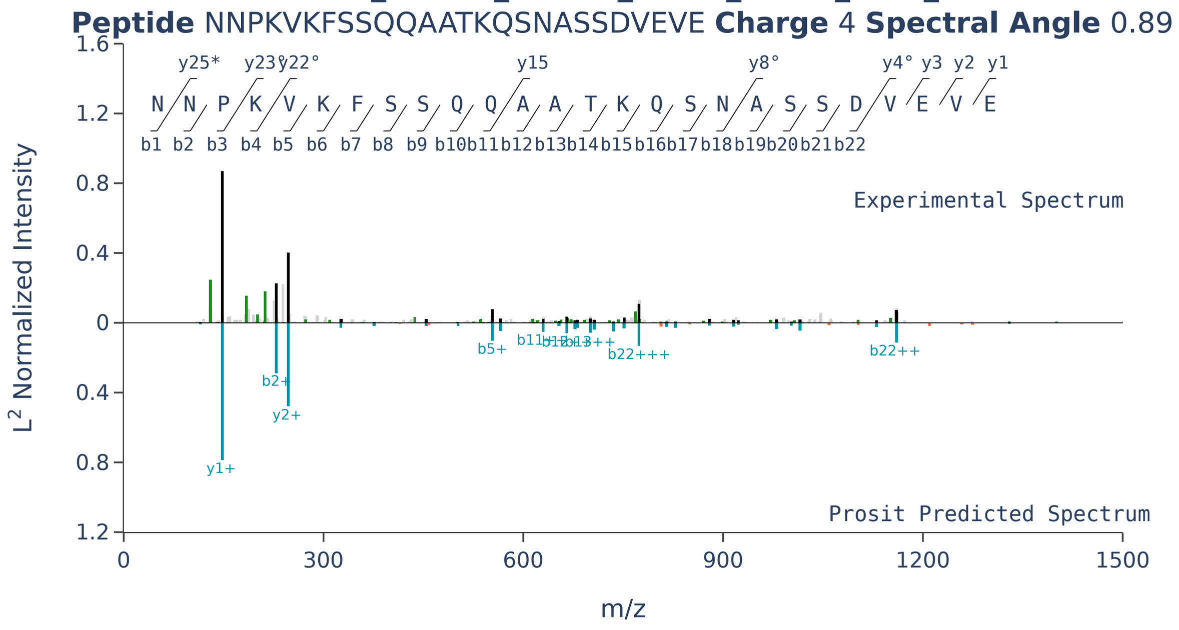


**Source** HRoetschke_271222_090123_HFGoe_LEDGF_b2R1_2h **Scan** 22593
**Peptide** DNLDVNRCIEALD **Charge** 2 **Spectral Angle** 0.88

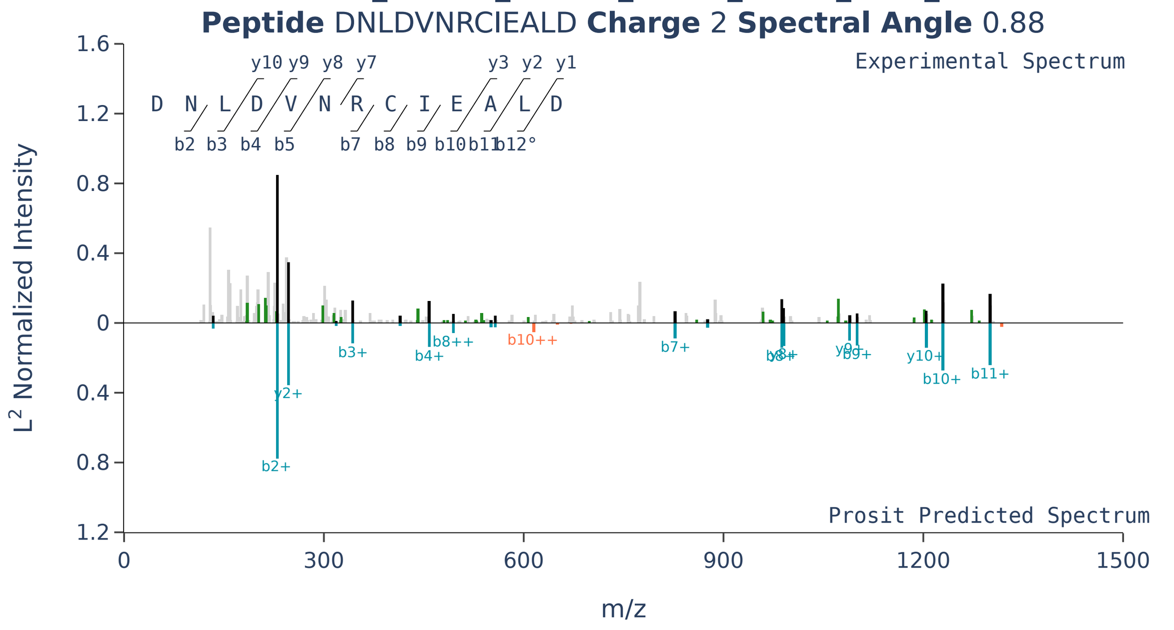


**Source** HRoetschke_271222_090123_HFGoe_LEDGF_b2R2_24h **Scan** 11320
**Peptide** LDNNPKVKFS **Charge** 3 **Spectral Angle** 0.79

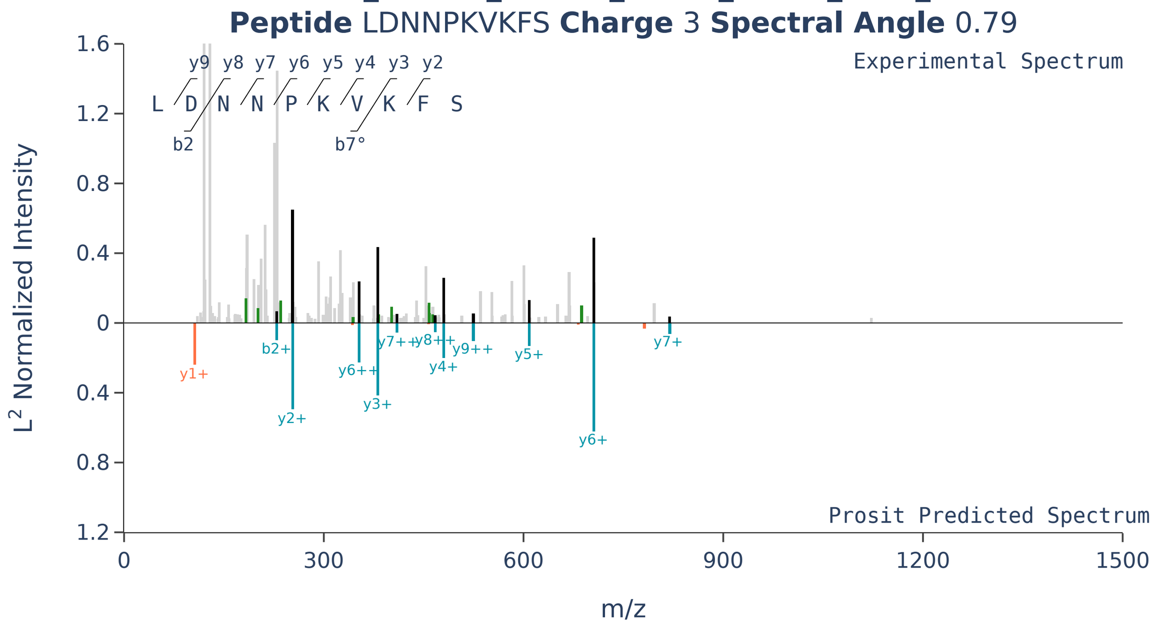


**Source** HRoetschke_271222_090123_HFGoe_LEDGF_b1R2_2h **Scan** 11823
**Peptide** FSSQQAATKQSNASSDVEVEE **Charge** 3 **Spectral Angle** 0.79

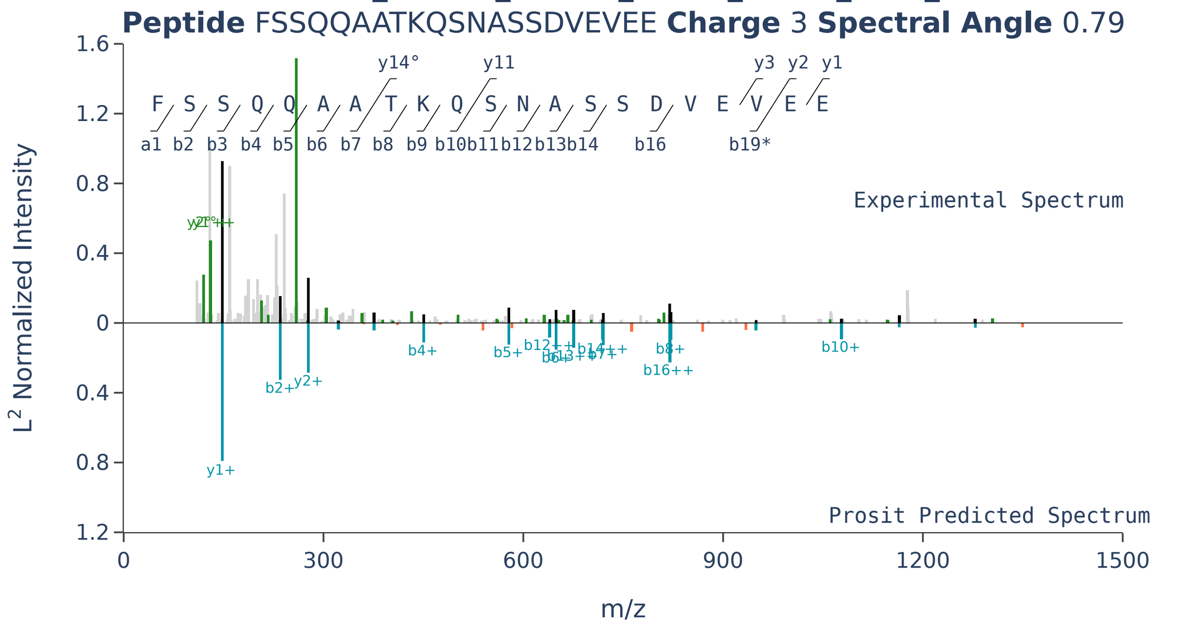


**Source** HRoetschke_271222_090123_HFGoe_LEDGF_b1R2_24h **Scan** 16854
**Peptide** DVTKAVDITTPKA **Charge** 3 **Spectral Angle** 0.85

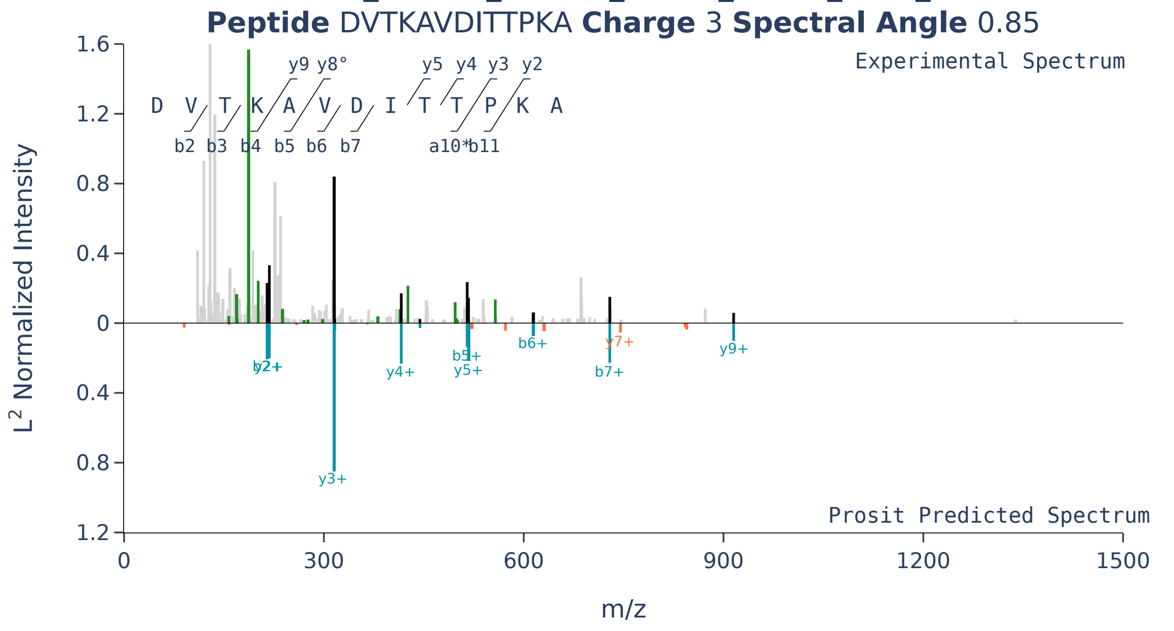


**Source** HRoetschke_271222_090123_HFGoe_LEDGF_b1R2_4h **Scan** 7490
**Peptide** NLKVSPKRGRPAATEV **Charge** 3 **Spectral Angle** 0.9

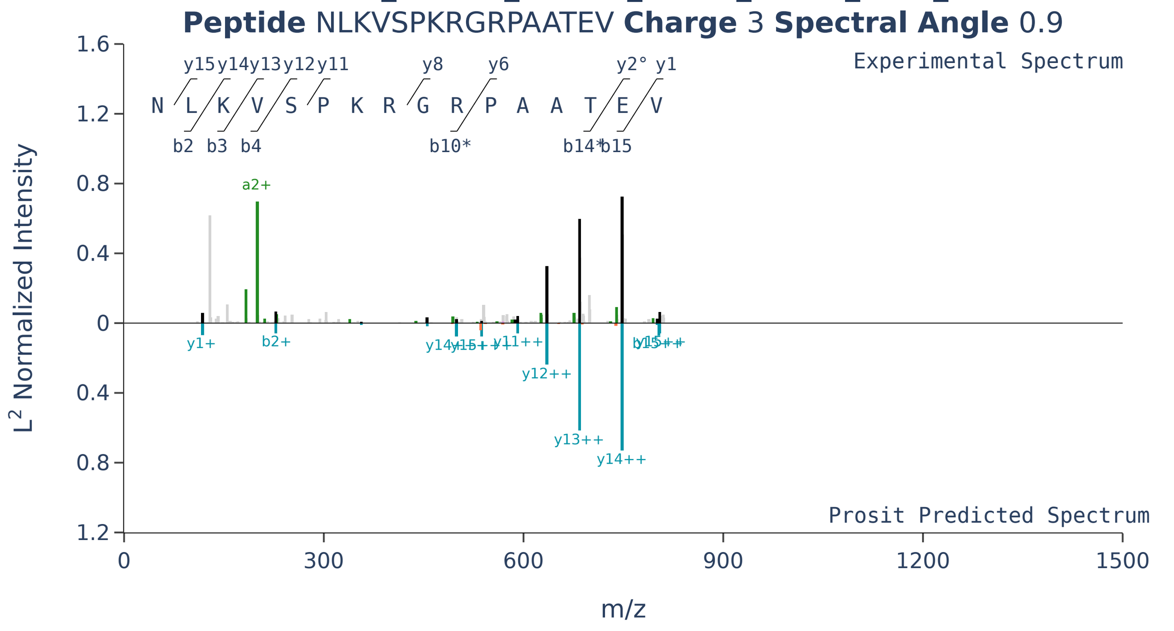


**Source** HRoetschke_271222_090123_HFGoe_LEDGF_b2R1_24h **Scan** 20705
**Peptide** GPKDIFPYSEN **Charge** 1 **Spectral Angle** 0.79

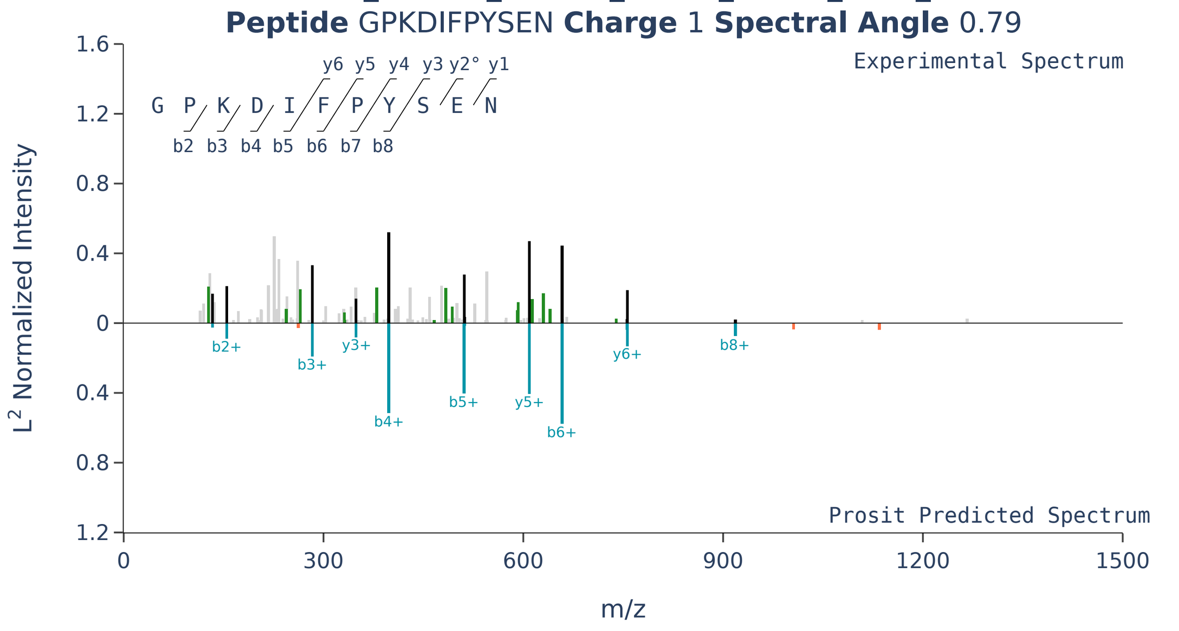


**Source** HRoetschke_271222_090123_HFGoe_LEDGF_b1R2_1h **Scan** 27335
**Peptide** LGPKDIFPY **Charge** 2 **Spectral Angle** 0.84

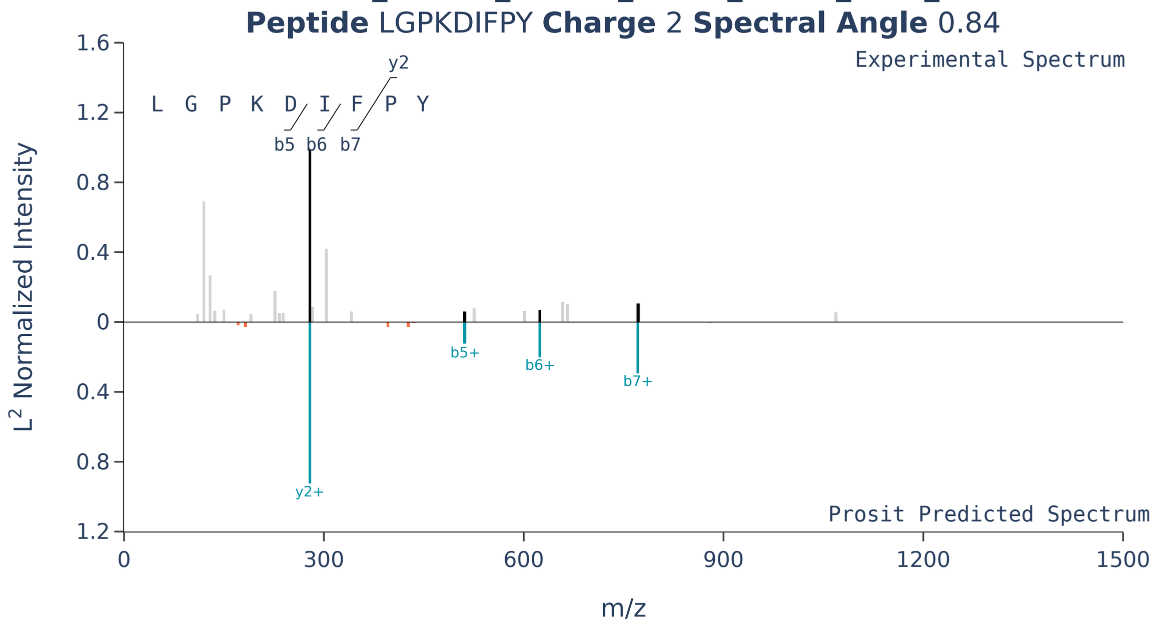


**Source** HRoetschke_271222_090123_HFGoe_LEDGF_b2R1_1h **Scan** 9967
**Peptide** SNASSDVEVEEKETSVSKE **Charge** 3 **Spectral Angle** 0.9

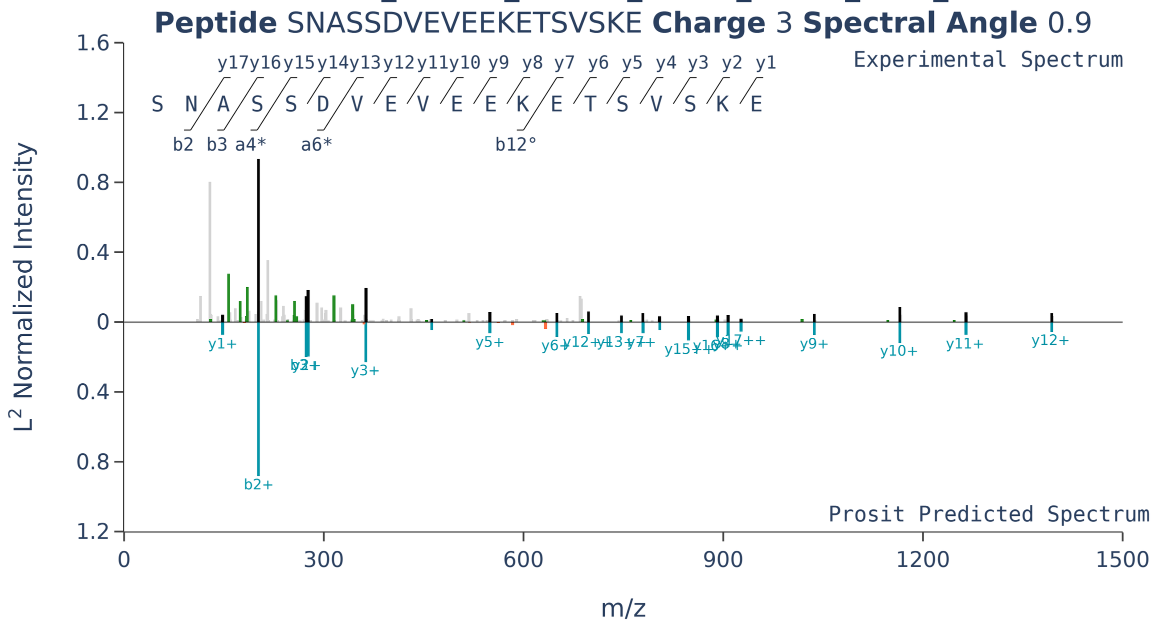


**Source** HRoetschke_271222_090123_HFGoe_LEDGF_b2R2_2h **Scan** 14210
**Peptide** LGPKDIFPYSENKEKYGKPNKRKGFNEG **Charge** 5 **Spectral Angle** 0.85

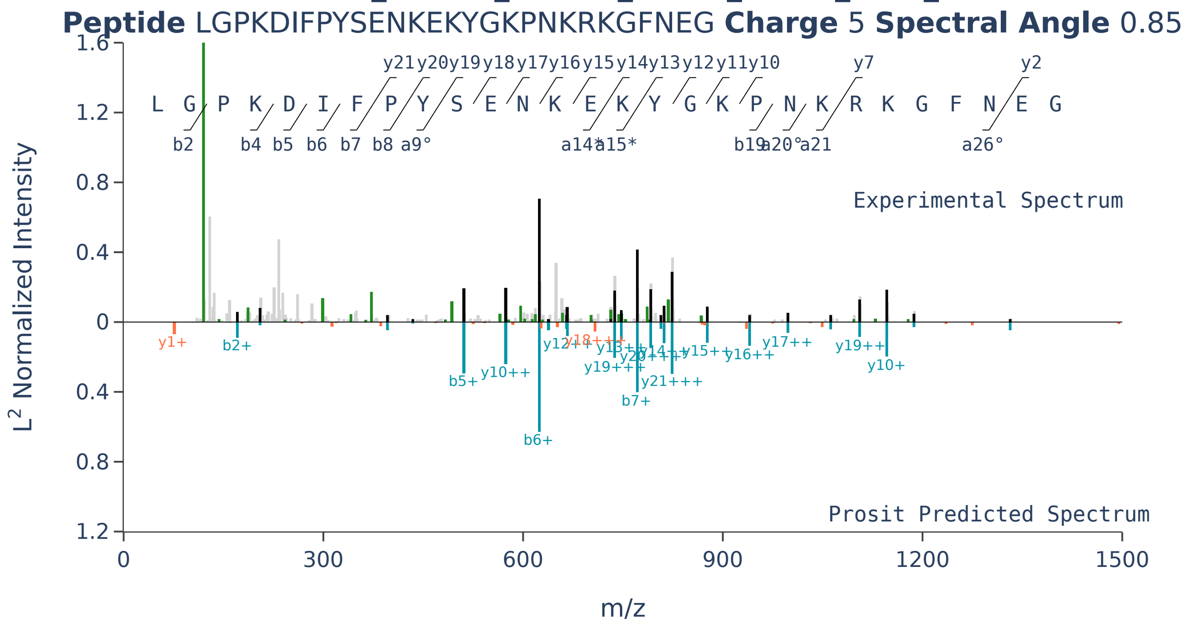


**Source** HRoetschke_271222_090123_HFGoe_LEDGF_b2R2_1h **Scan** 16418
**Peptide** GTHETAFLGPKDIFPYSENKEKYGKPNKR **Charge** 4 **Spectral Angle** 0.8

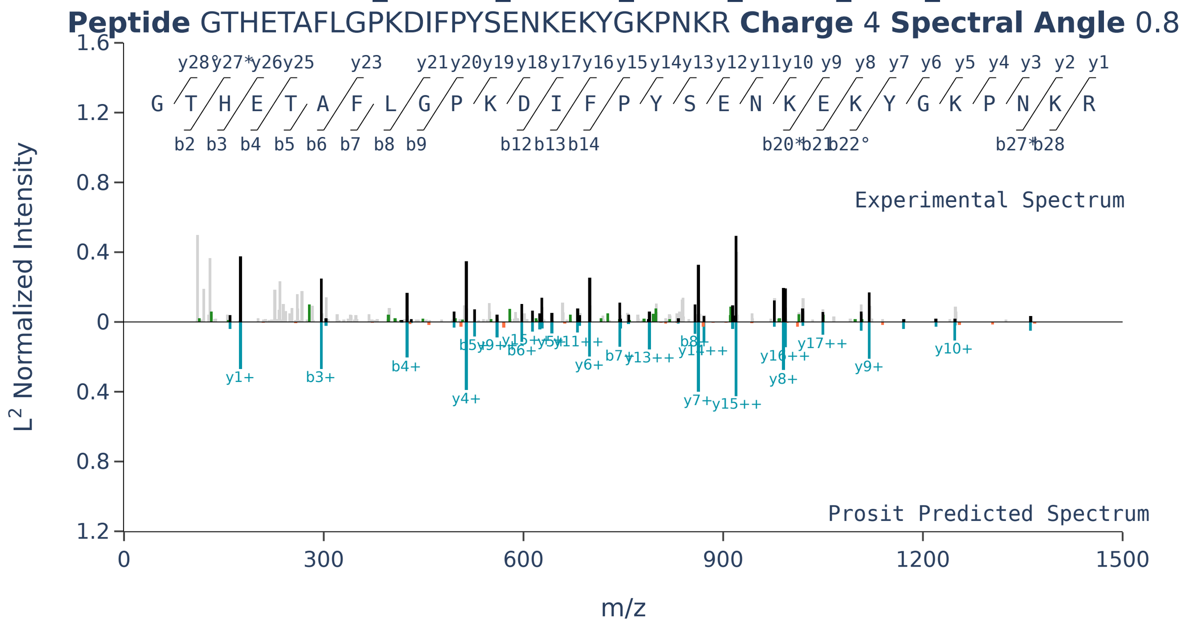


**Source** HRoetschke_271222_090123_HFGoe_LEDGF_b1R2_24h **Scan** 5072
**Peptide** QMETEQQNKDEGKKPEVKKVEKKRET **Charge** 3 **Spectral Angle** 0.74

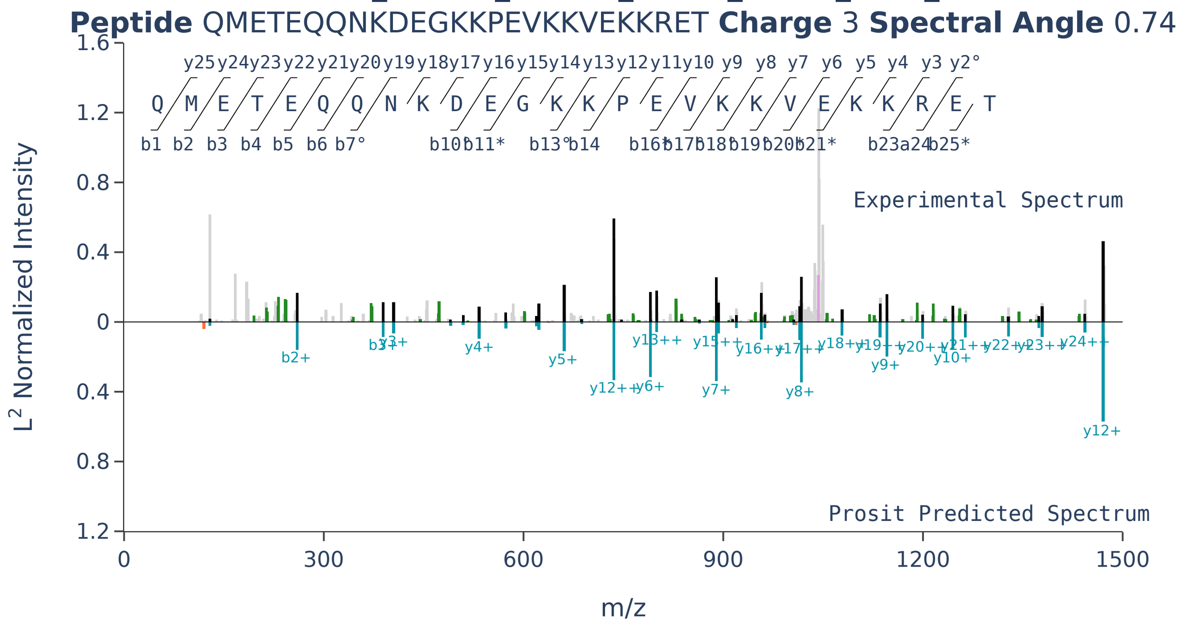


**Source** HRoetschke_271222_090123_HFGoe_LEDGF_b1R2_1h **Scan** 7469
**Peptide** PKVKFSSQQ **Charge** 2 **Spectral Angle** 0.74

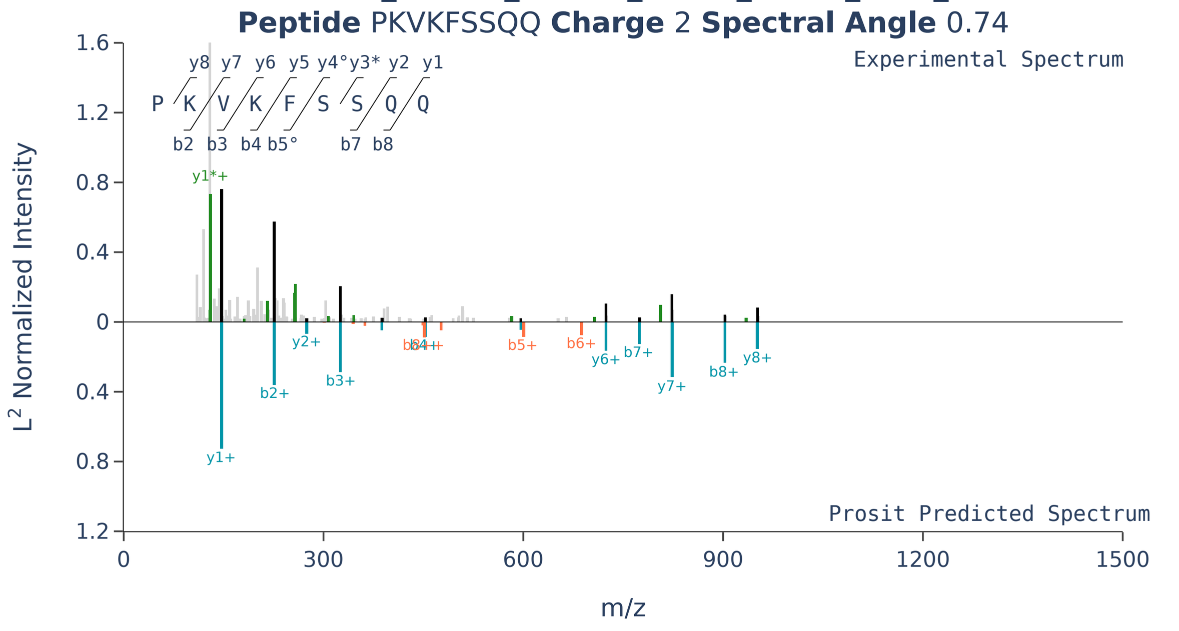

**Source** HRoetschke_271222_090123_HFGoe_LEDGF_b2R1_4h **Scan** 13578
**Peptide** LDVNRCIE **Charge** 2 **Spectral Angle** 0.82

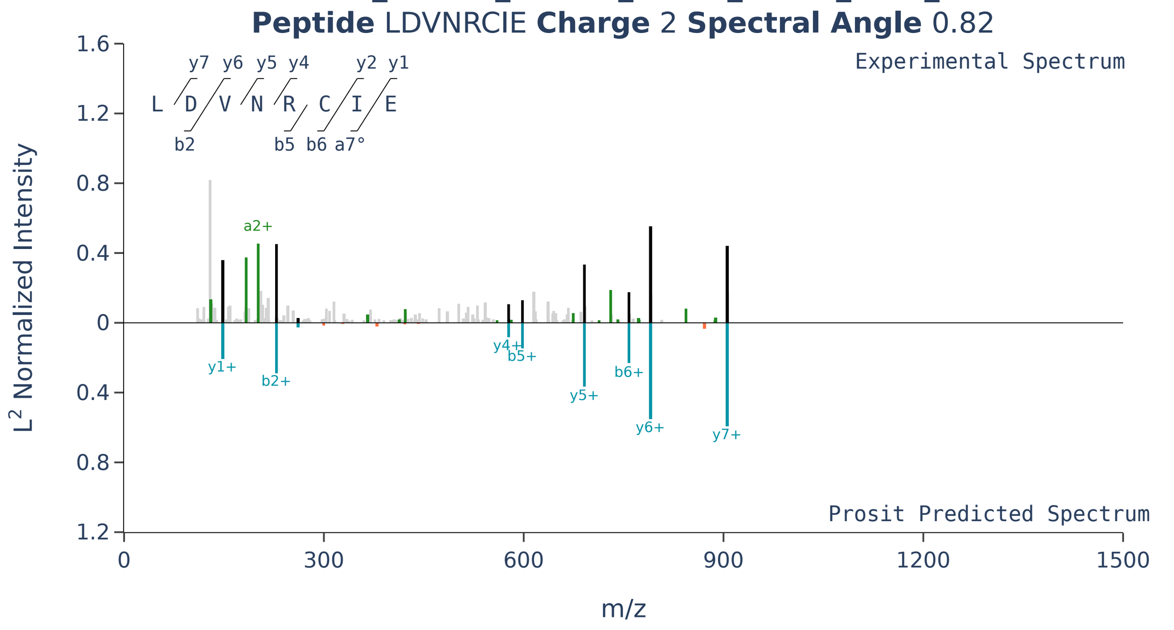


**Source** HRoetschke_271222_090123_HFGoe_LEDGF_b2R1_24h **Scan** 21209
**Peptide** FLGPKDIFPYSENKEKYGK **Charge** 3 **Spectral Angle** 0.75

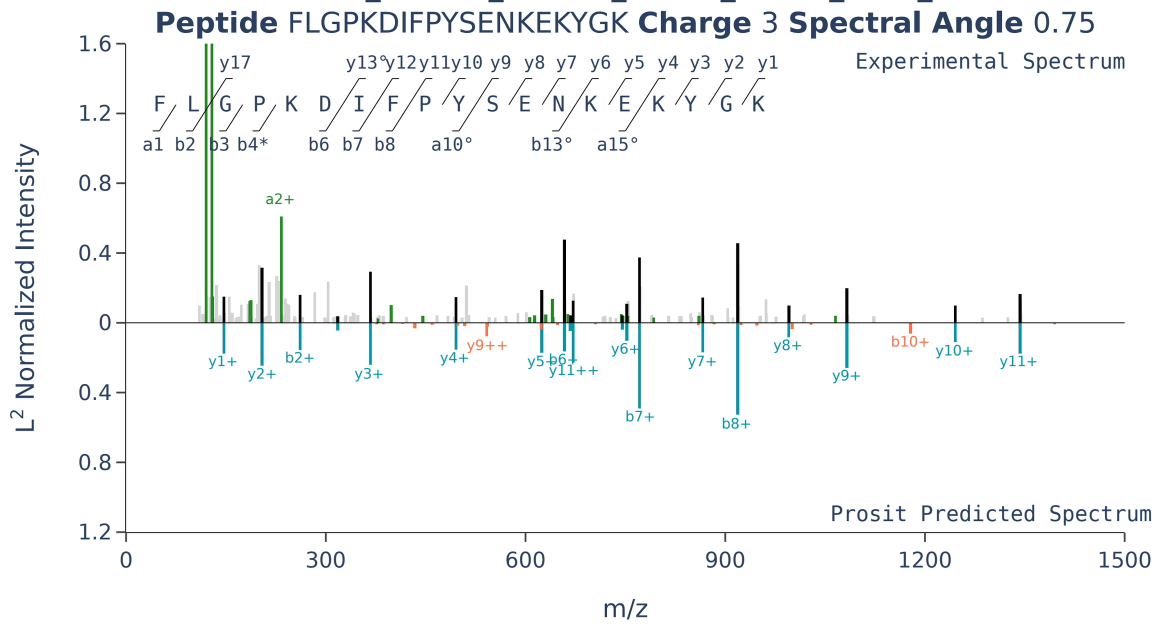


**Source** HRoetschke_271222_090123_HFGoe_LEDGF_b2R2_2h **Scan** 8303
**Peptide** VTKAVDITTPKAARR **Charge** 3 **Spectral Angle** 0.7

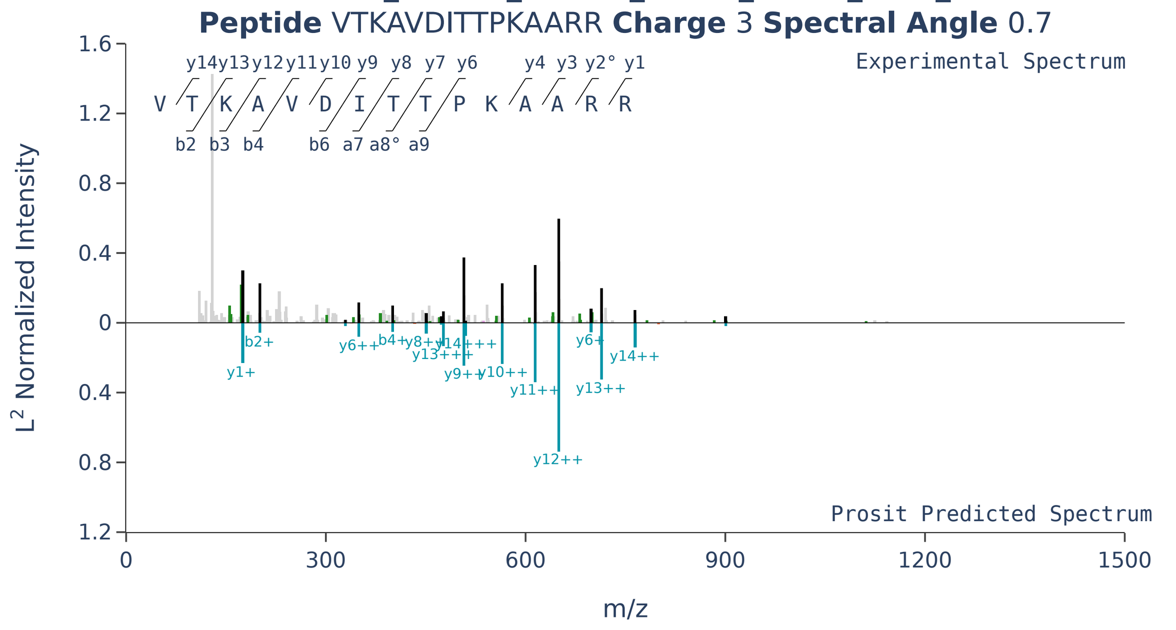


**Source** HRoetschke_271222_090123_HFGoe_LEDGF_b1R2_1h **Scan** 16244
**Peptide** PARVDEVPDGAVKPPTNKL **Charge** 4 **Spectral Angle** 0.78

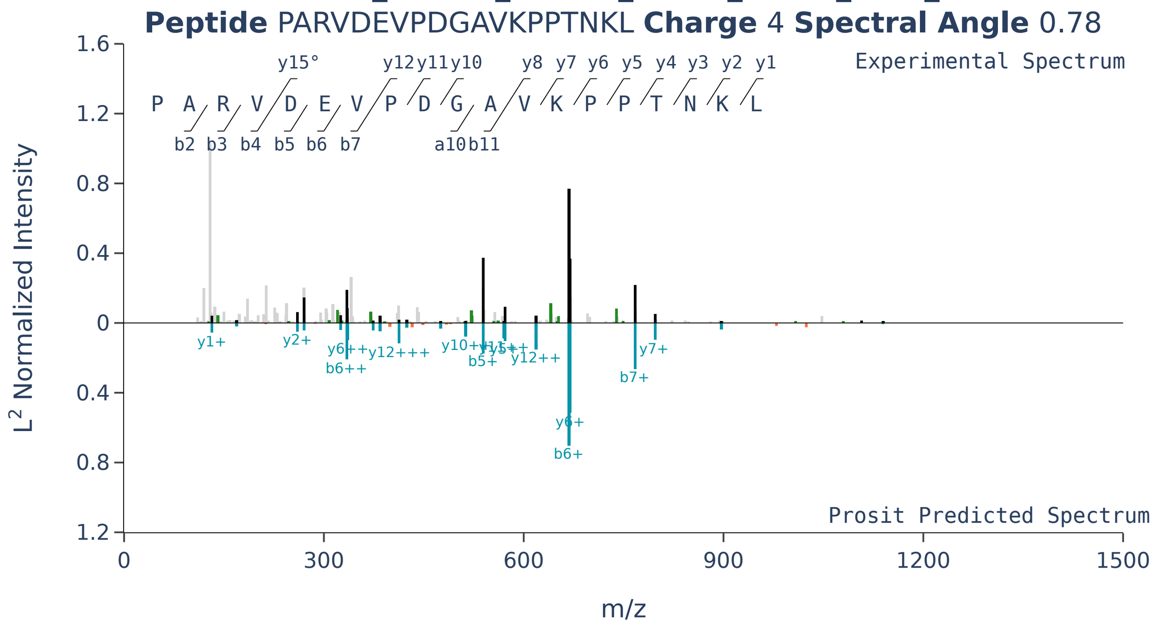


**Source** HRoetschke_271222_090123_HFGoe_LEDGF_b2R2_24h **Scan** 26931
**Peptide** KNMFLVGEGDSVITQV **Charge** 2 **Spectral Angle** 0.81

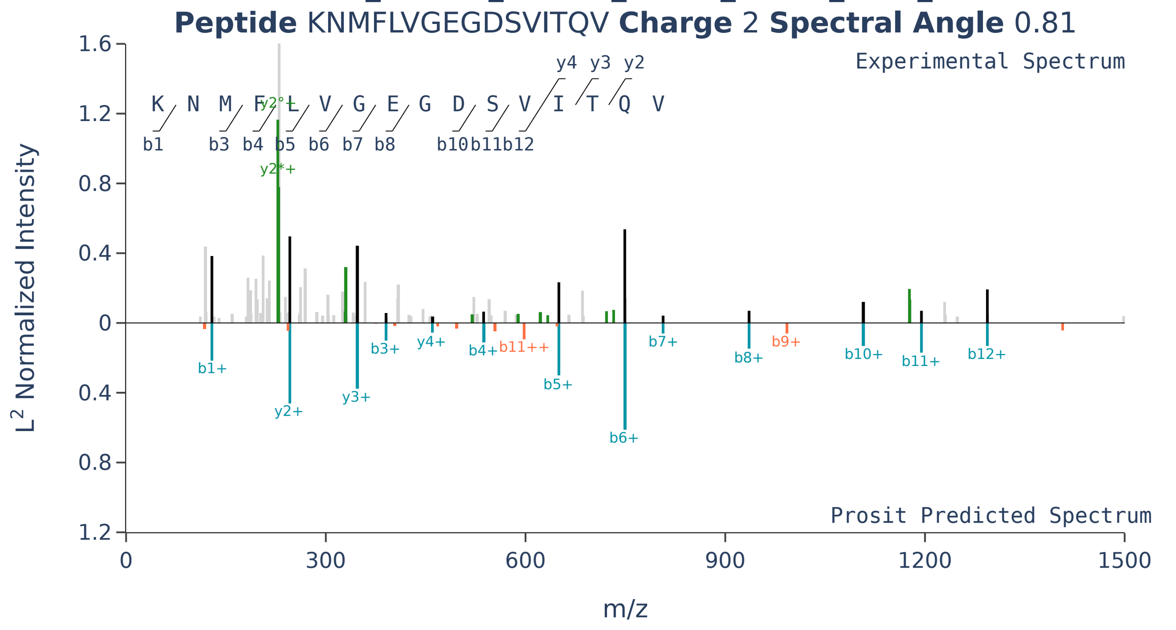


**Source** HRoetschke_271222_090123_HFGoe_LEDGF_b2R2_4h **Scan** 10444
**Peptide** IDNNPKVKFSS **Charge** 3 **Spectral Angle** 0.71

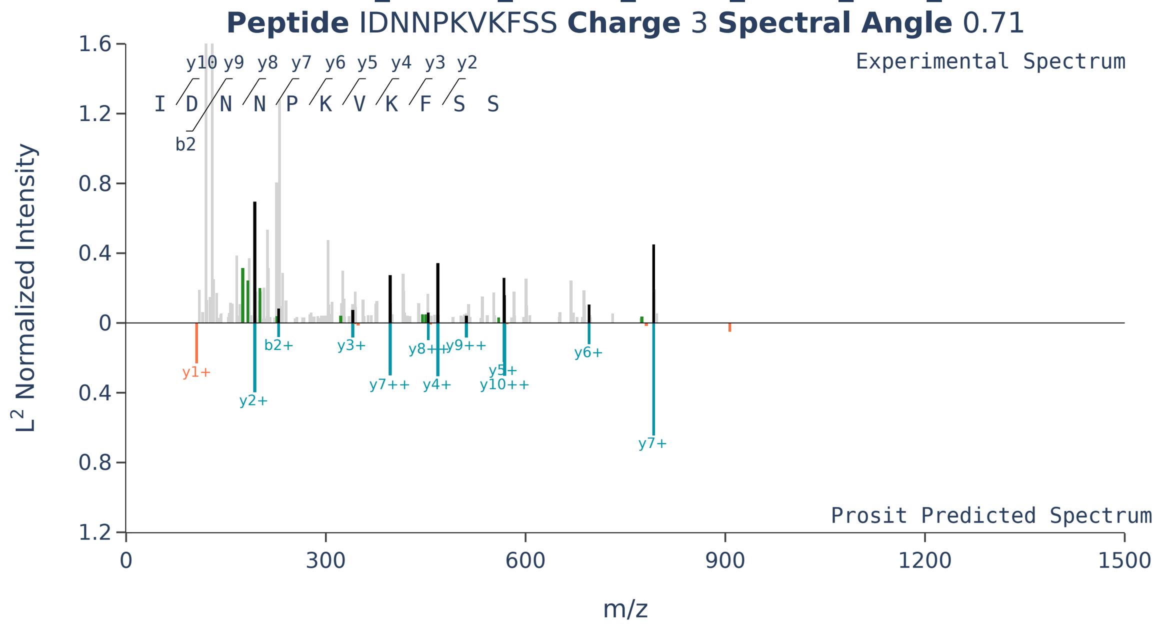


**Source** HRoetschke_271222_090123_HFGoe_LEDGF_b1R1_4h **Scan** 9991
**Peptide** DVTKAVDITTPKAARRGR **Charge** 3 **Spectral Angle** 0.75

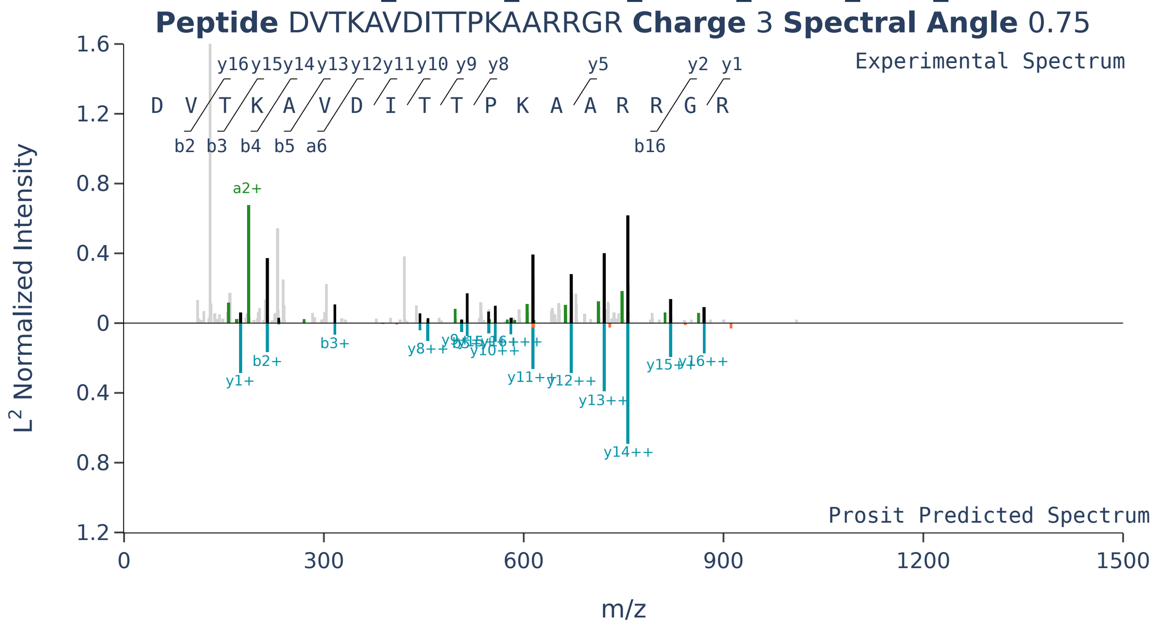


**Source** HRoetschke_271222_090123_HFGoe_LEDGF_b2R1_24h **Scan** 20392
**Peptide** NSLKIDNLDVN **Charge** 2 **Spectral Angle** 0.68

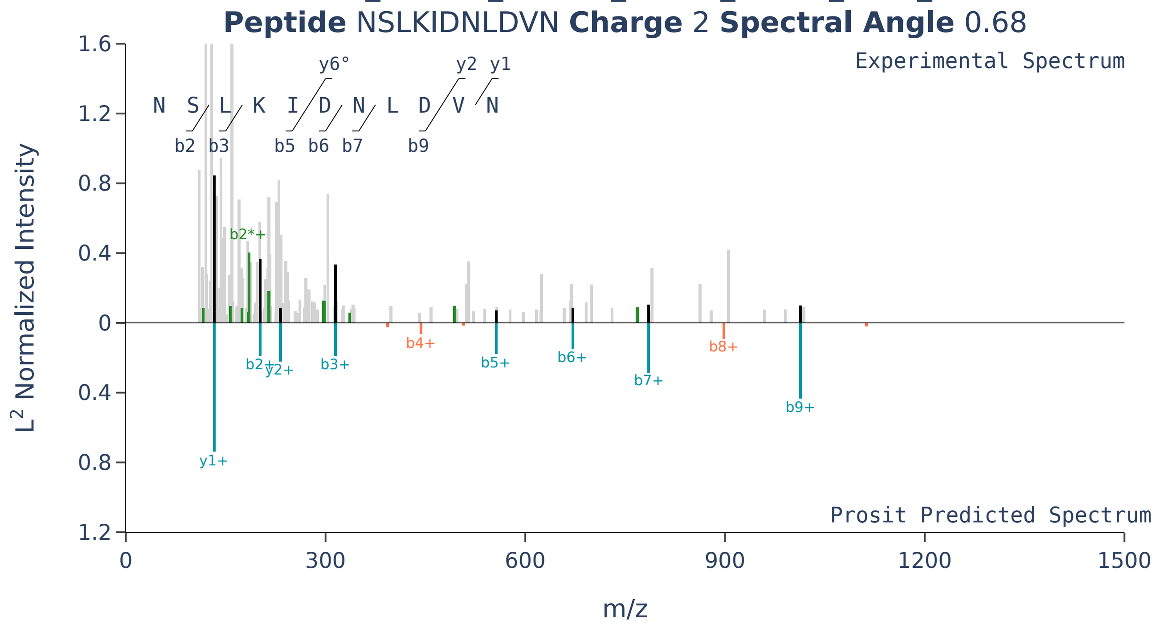


**Source** HRoetschke_271222_090123_HFGoe_LEDGF_b1R1_24h **Scan** 13123
**Peptide** EIKNSLKID **Charge** 3 **Spectral Angle** 0.78

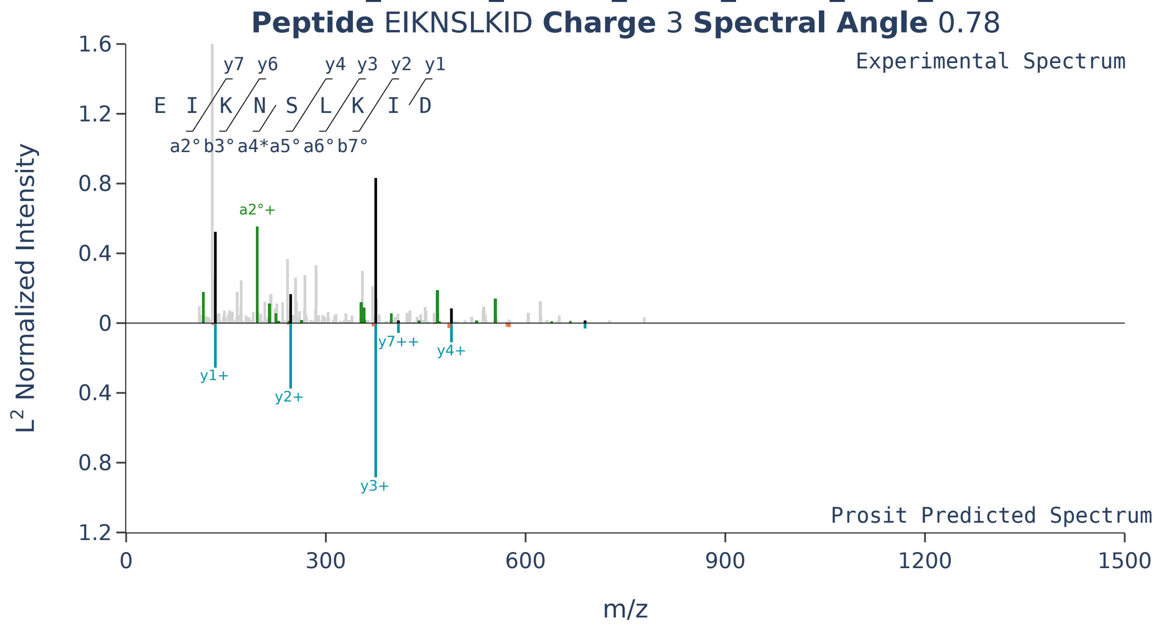


**Source** HRoetschke_271222_090123_HFGoe_LEDGF_b2R1_2h **Scan** 13625
**Peptide** VNRCIEALD **Charge** 2 **Spectral Angle** 0.82

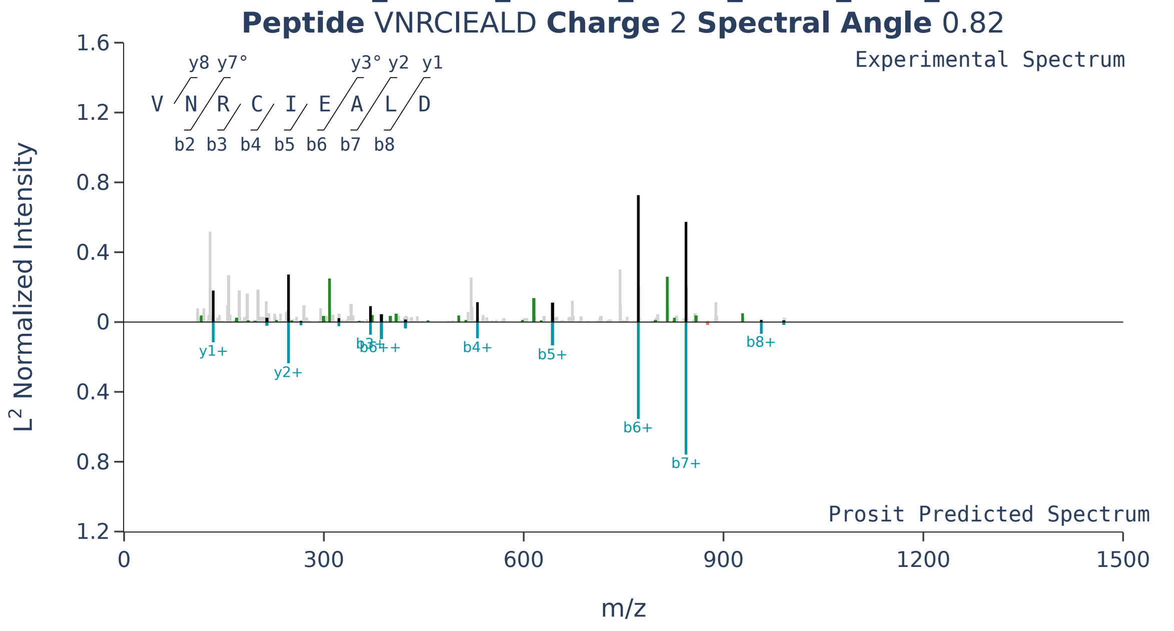


**Source** HRoetschke_271222_090123_HFGoe_LEDGF_b1R2_24h **Scan** 13860
**Peptide** DVTKAVDITTPKAAR **Charge** 3 **Spectral Angle** 0.78

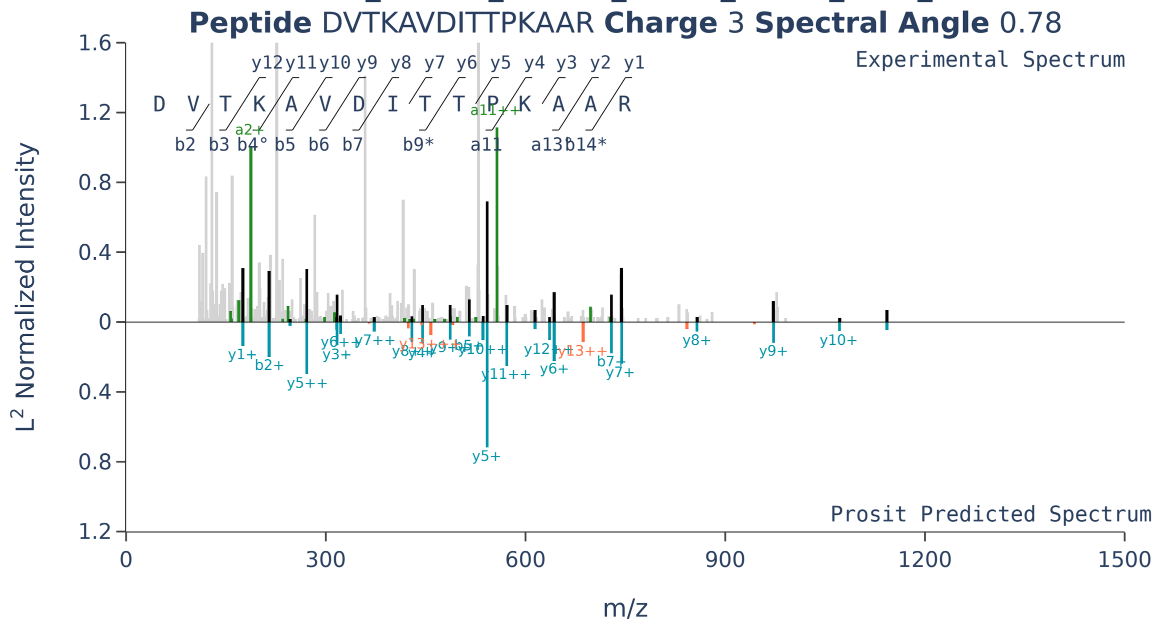


**Source** HRoetschke_271222_090123_HFGoe_LEDGF_b2R1_24h **Scan** 16456
**Peptide** TEEAGVVTTATASVNLKVSPKRGRPAAT **Charge** 4 **Spectral Angle** 0.79

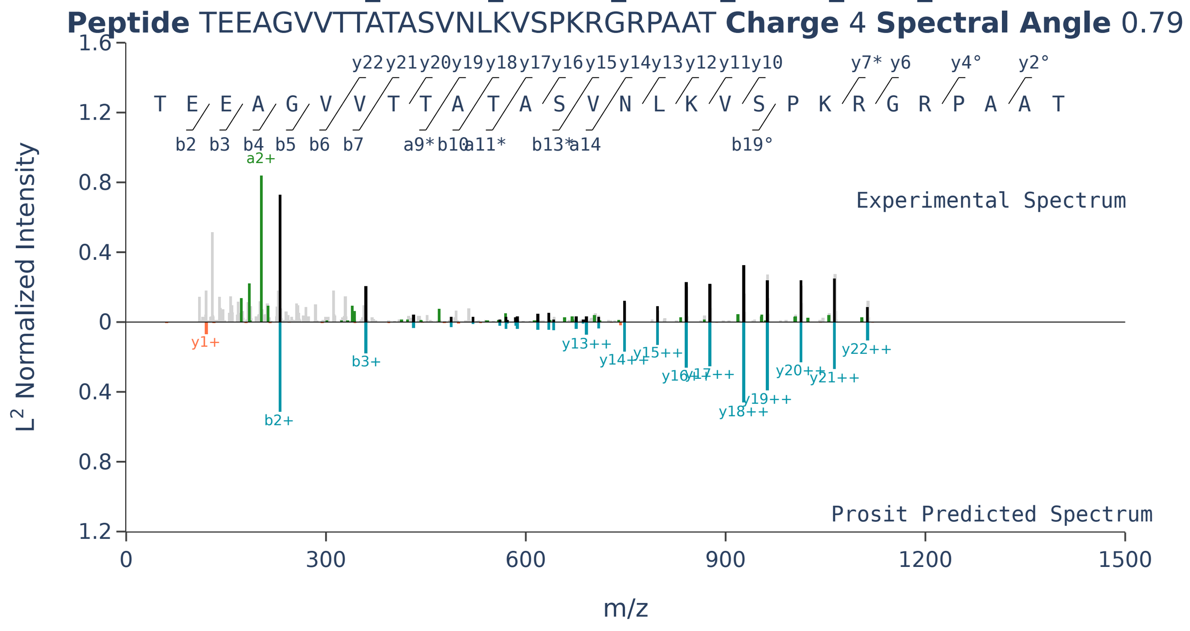


**Source** HRoetschke_271222_090123_HFGoe_LEDGF_b2R1_1h **Scan** 28681
**Peptide** FLGPKDIFPYSEN **Charge** 2 **Spectral Angle** 0.72

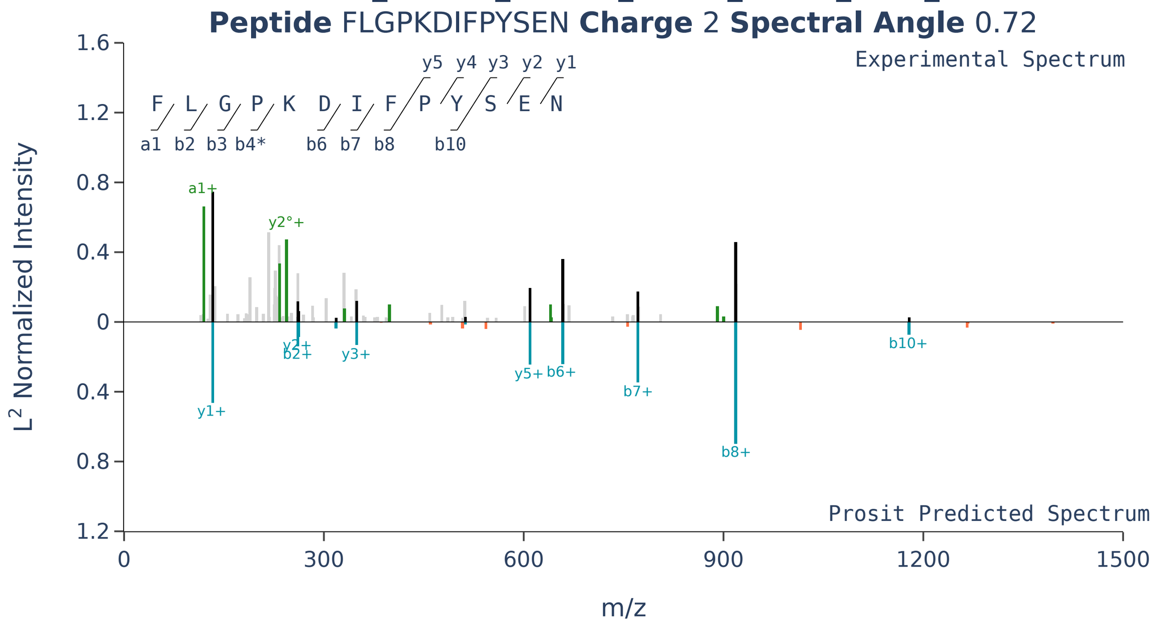


**Source** HRoetschke_271222_090123_HFGoe_LEDGF_b1R2_2h **Scan** 16058
**Peptide** SRLQRIHAEIKNSLKI **Charge** 3 **Spectral Angle** 0.74

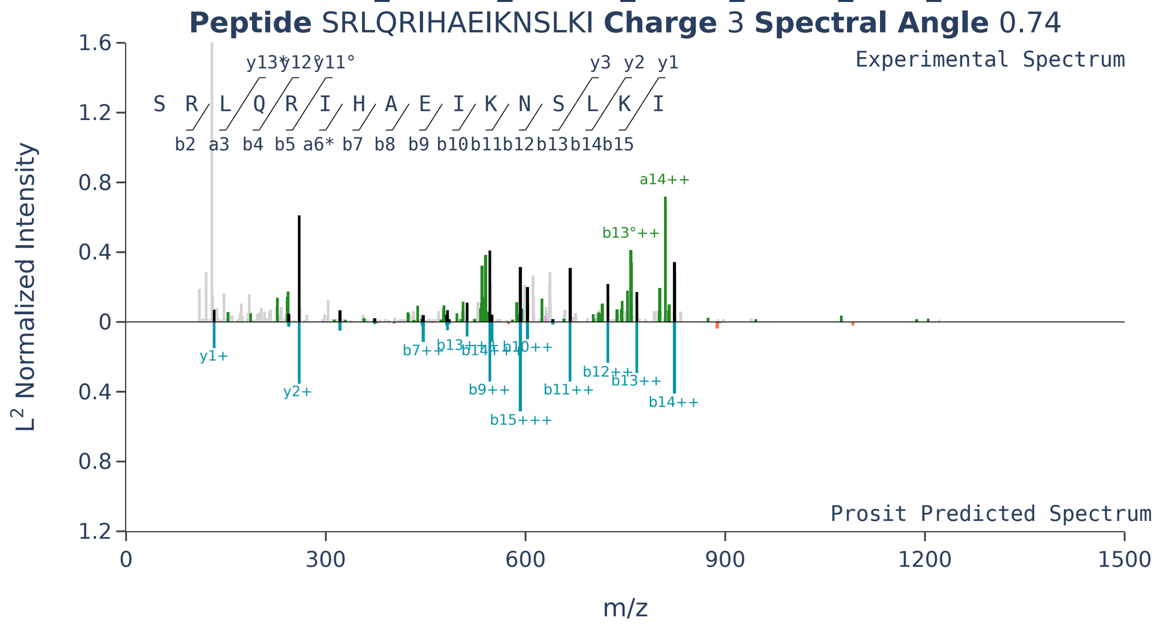


**Source** HRoetschke_271222_090123_HFGoe_LEDGF_b1R2_4h **Scan** 8327
**Peptide** KASNEDVTKAVDITTPKAARRGRK **Charge** 5 **Spectral Angle** 0.8

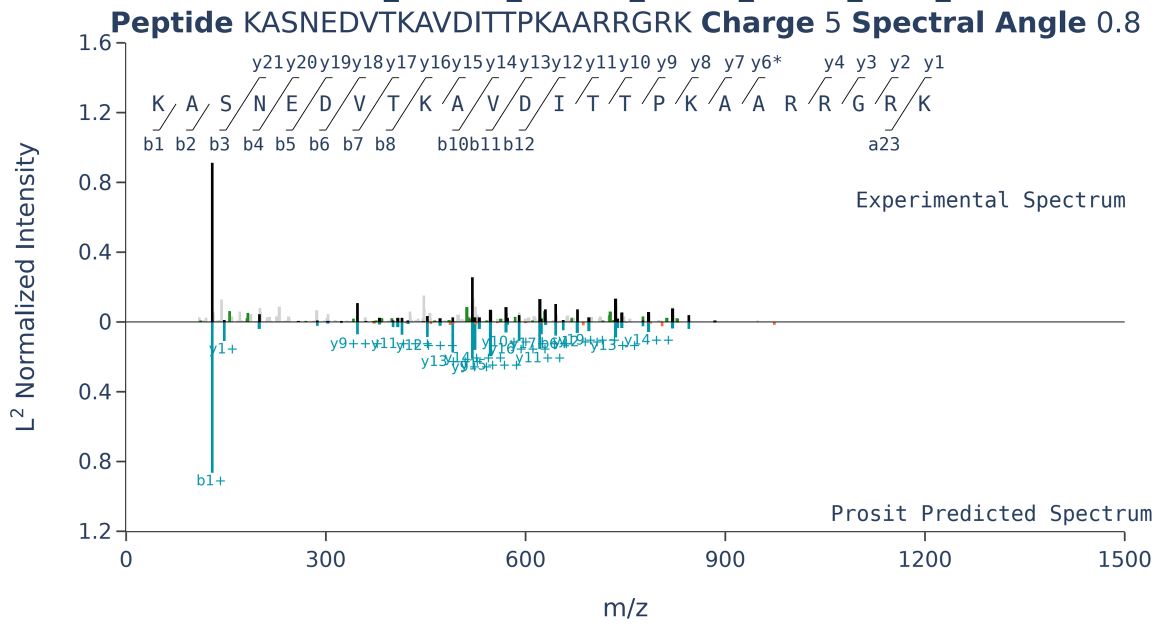

**Source** HRoetschke_271222_090123_HFGoe_LEDGF_b1R1_4h **Scan** 20677
**Peptide** SVITQVLNKSLAEQRQHEEANKT **Charge** 3 **Spectral Angle** 0.87

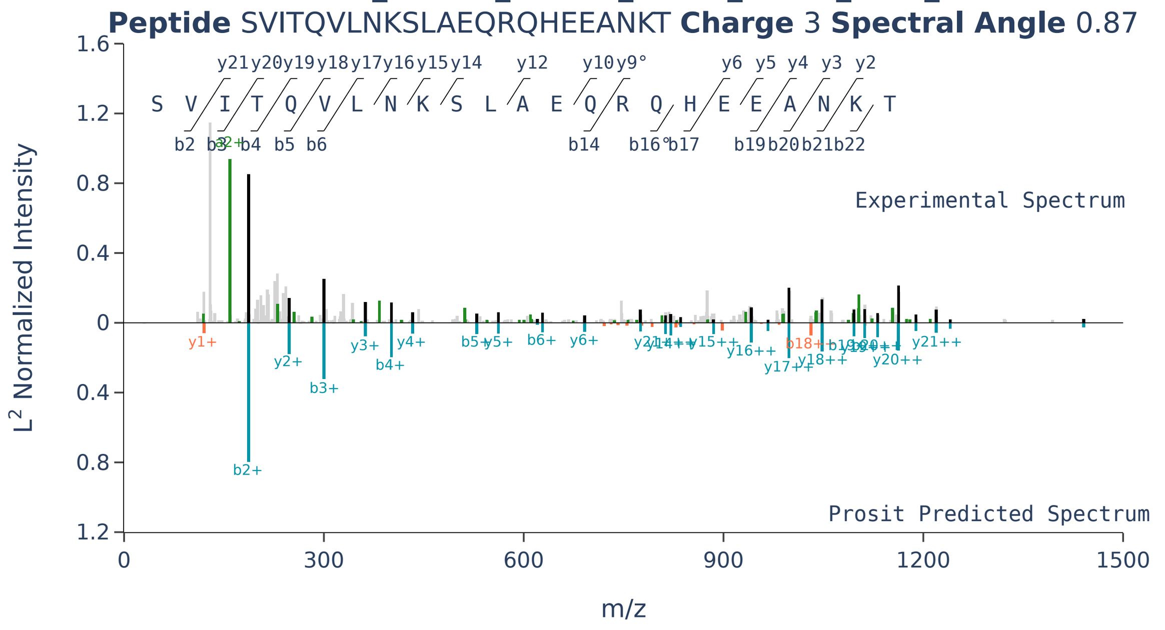


**Source** HRoetschke_271222_090123_HFGoe_LEDGF_b1R1_24h **Scan** 16695
**Peptide** DSRLQRIHAEIKNSL **Charge** 3 **Spectral Angle** 0.7

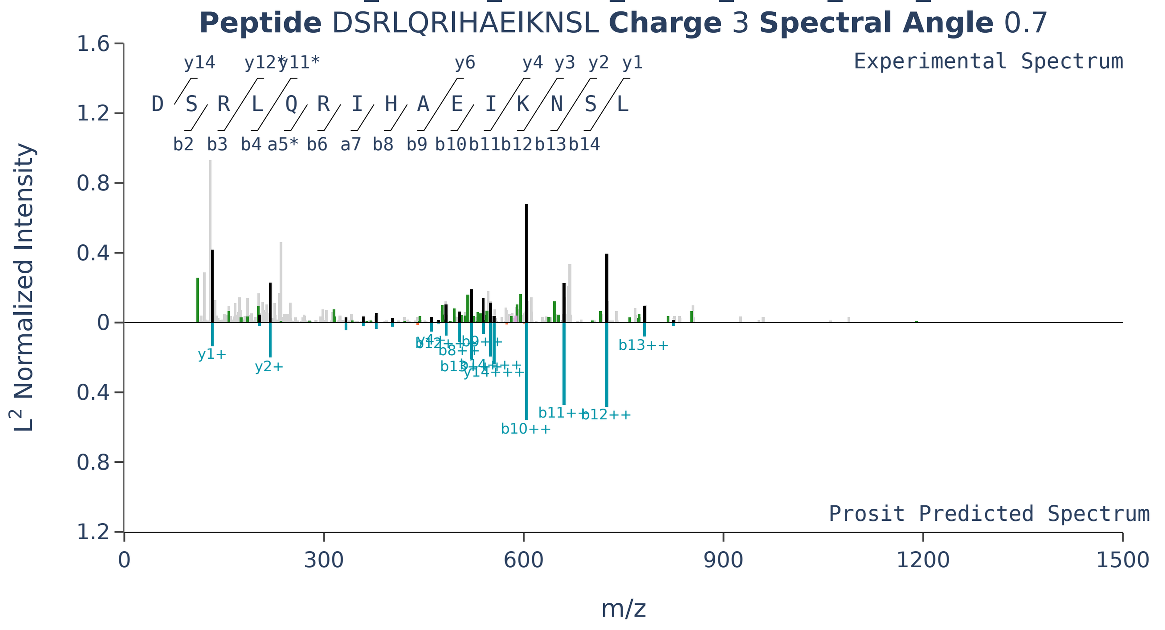


**Source** HRoetschke_271222_090123_HFGoe_LEDGF_b1R2_24h **Scan** 5510
**Peptide** NMLKGQHEKEAADRKRKQEEQM **Charge** 4 **Spectral Angle** 0.76

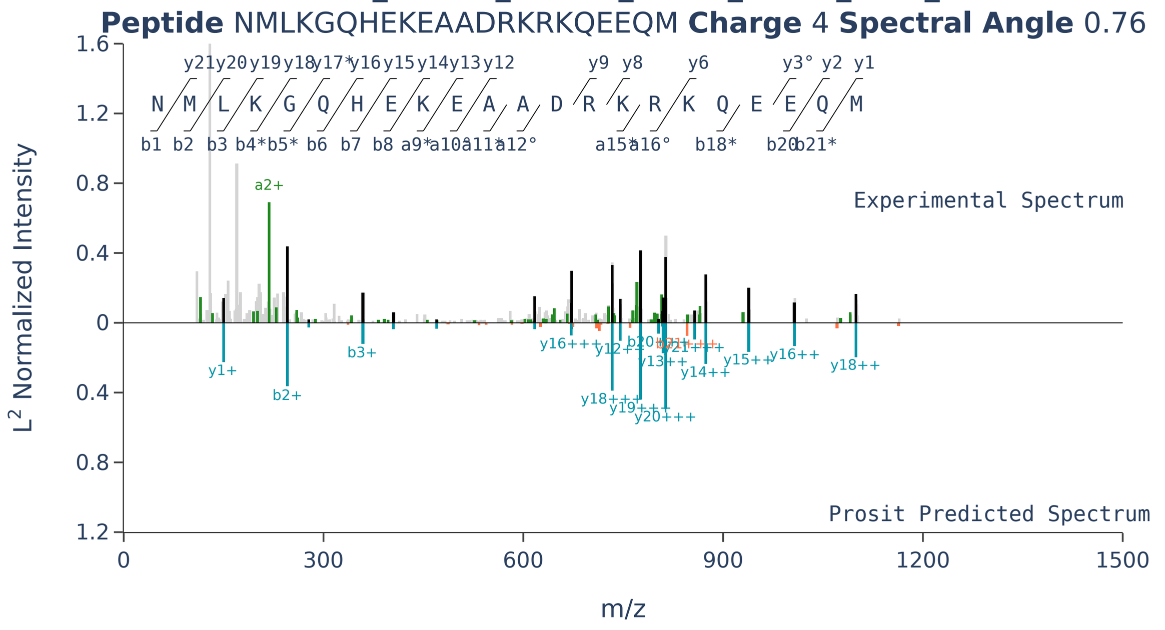


**Source** HRoetschke_271222_090123_HFGoe_LEDGF_b2R1_24h **Scan** 26291
**Peptide** AFLGPKDIFPYSENK **Charge** 3 **Spectral Angle** 0.8

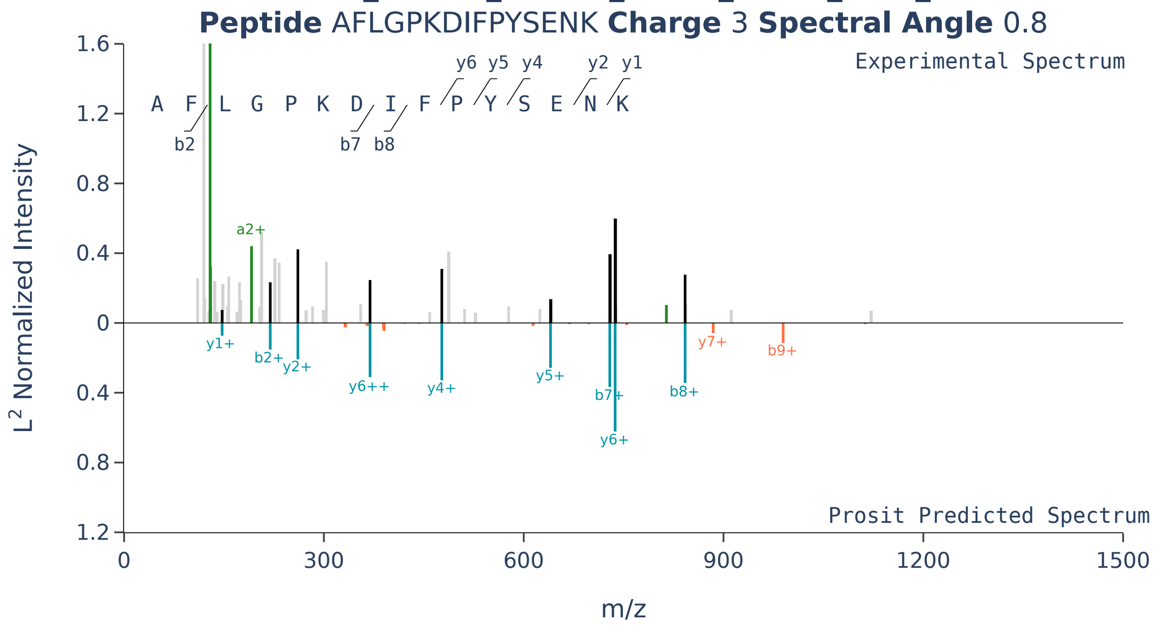


**Source** HRoetschke_271222_090123_HFGoe_LEDGF_b2R1_2h **Scan** 18619
**Peptide** TEMITTLKKIRRFKVSQV **Charge** 3 **Spectral Angle** 0.76

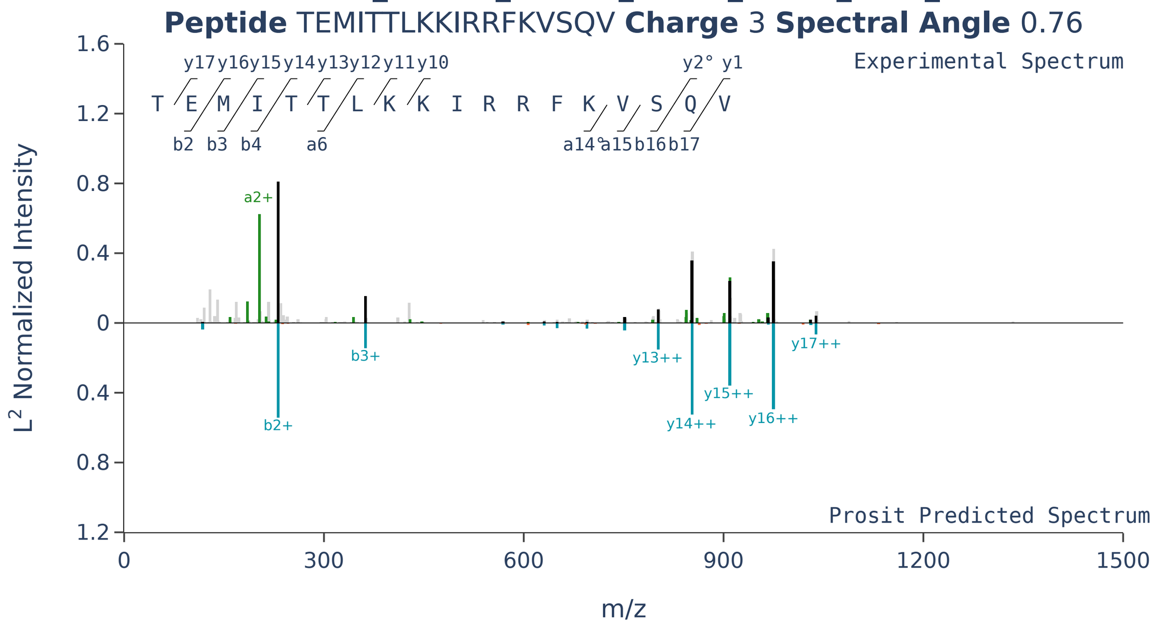


**Source** HRoetschke_271222_090123_HFGoe_LEDGF_b1R1_2h **Scan** 22630
**Peptide** SVITQVLNKSLAEQRQH **Charge** 3 **Spectral Angle** 0.77

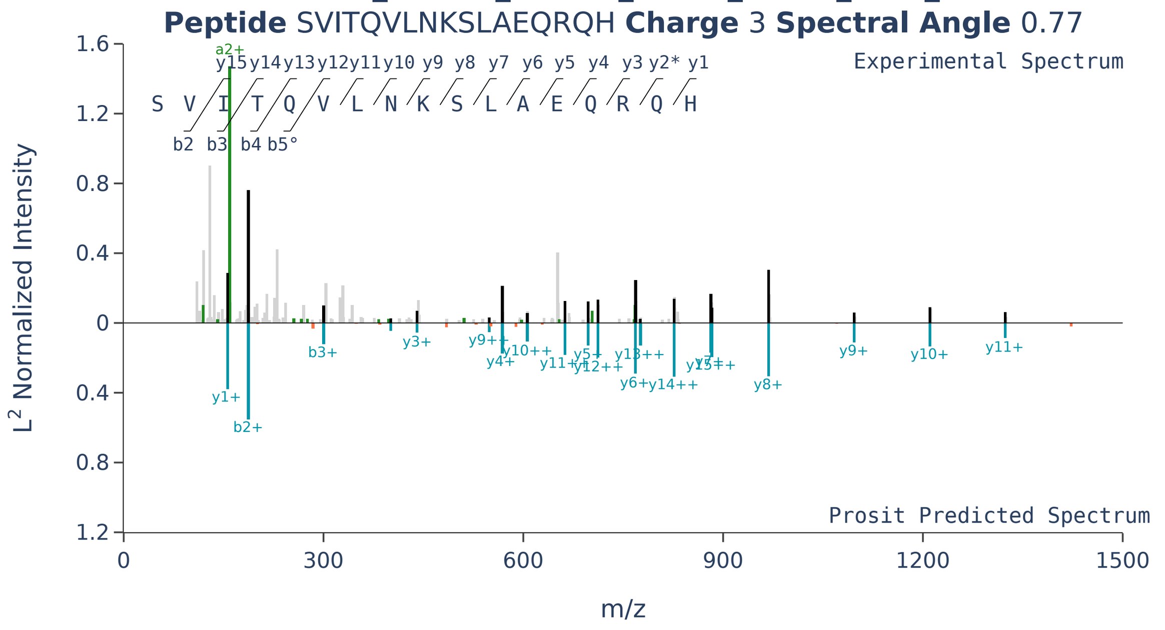


**Source** HRoetschke_271222_090123_HFGoe_LEDGF_b2R1_2h **Scan** 18630
**Peptide** SMDSRLQRIHAEIKNSL **Charge** 3 **Spectral Angle** 0.83

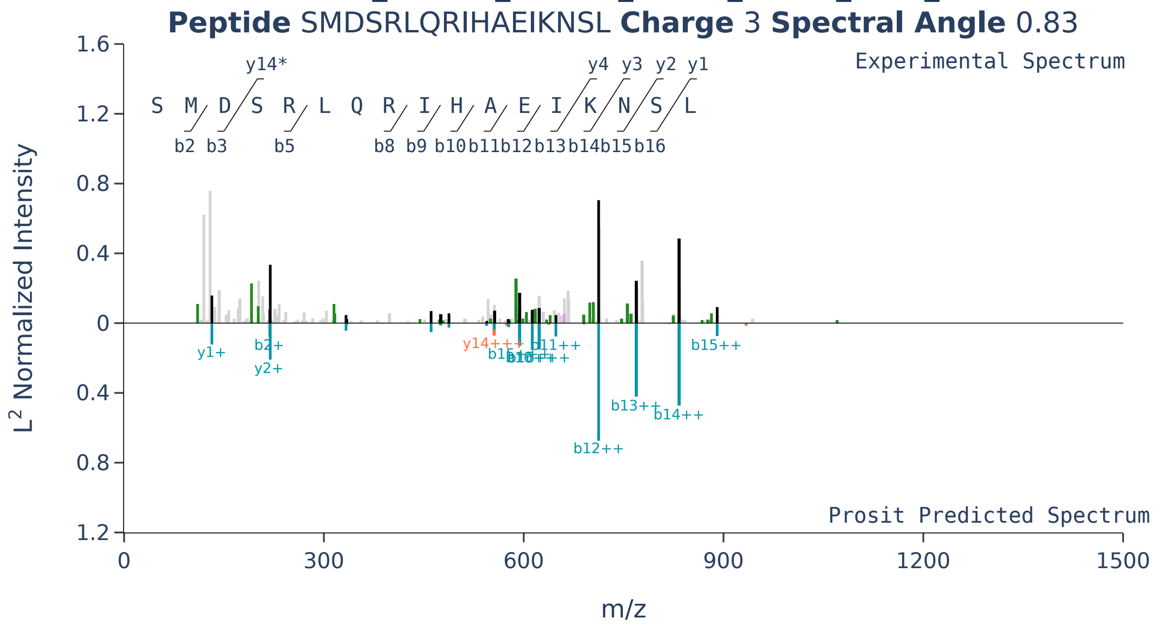


**Source** HRoetschke_271222_090123_HFGoe_LEDGF_b1R2_4h **Scan** 19827
**Peptide** NEGLWEIDNNPKVKFSSQQAATKQSNASSD **Charge** 4 **Spectral Angle** 0.91

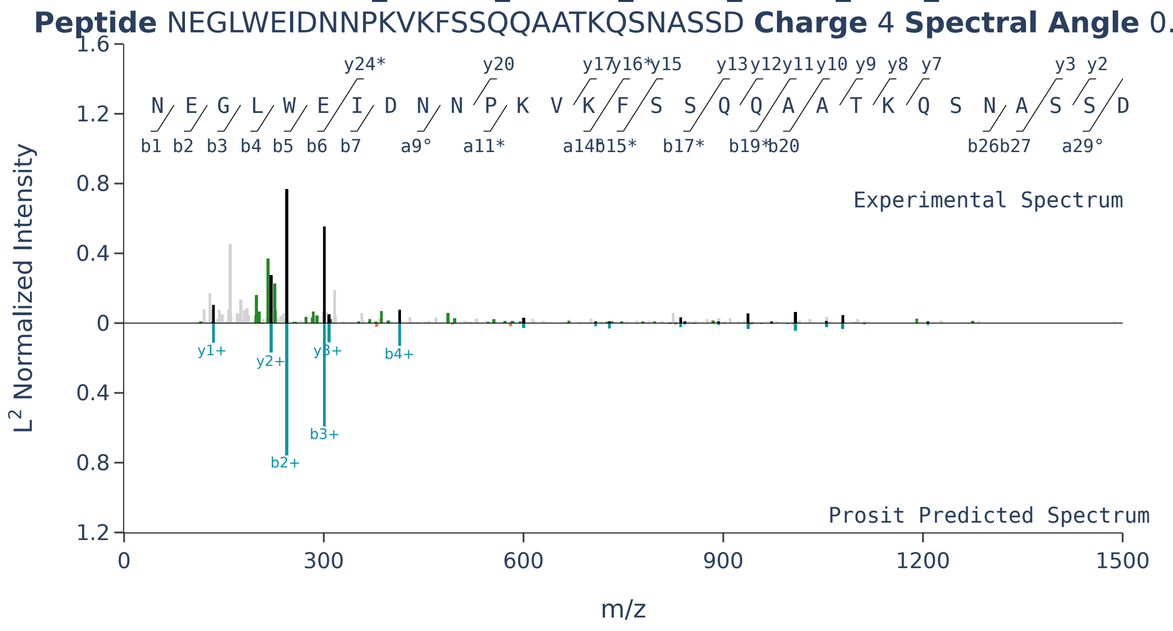


**Source** HRoetschke_271222_090123_HFGoe_LEDGF_b2R1_1h **Scan** 29669
**Peptide** VPDGAVKPPTNKLPIFFFGTHE **Charge** 3 **Spectral Angle** 0.81

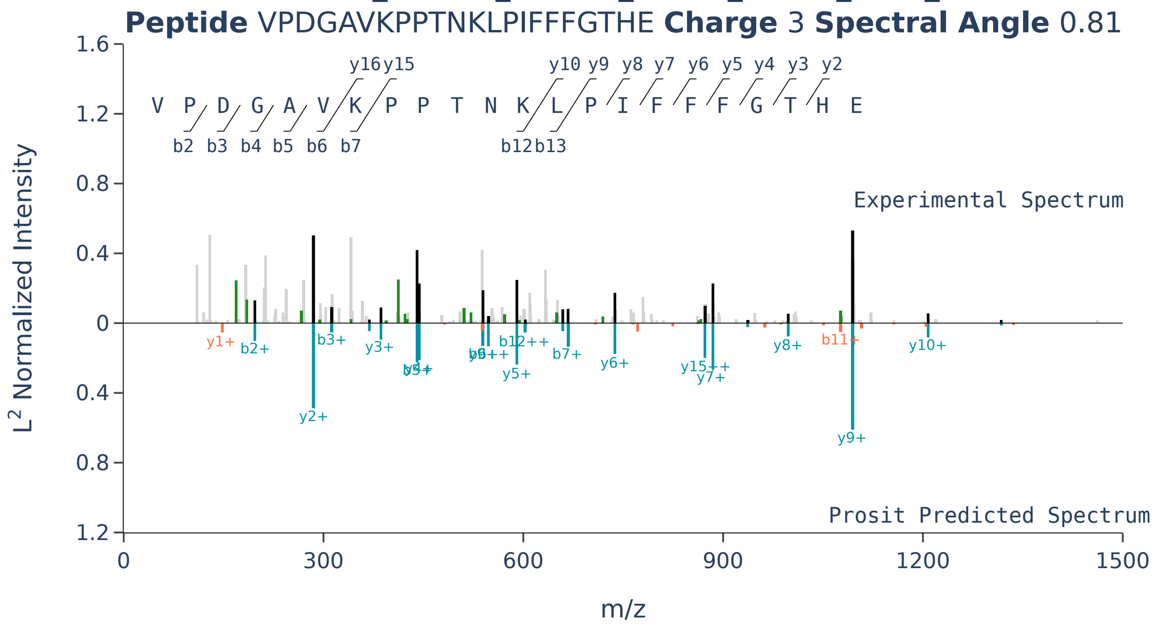


**Source** HRoetschke_271222_090123_HFGoe_LEDGF_b2R2_24h **Scan** 31682
**Peptide** KNMFLVGEGDSVITQVL **Charge** 2 **Spectral Angle** 0.82

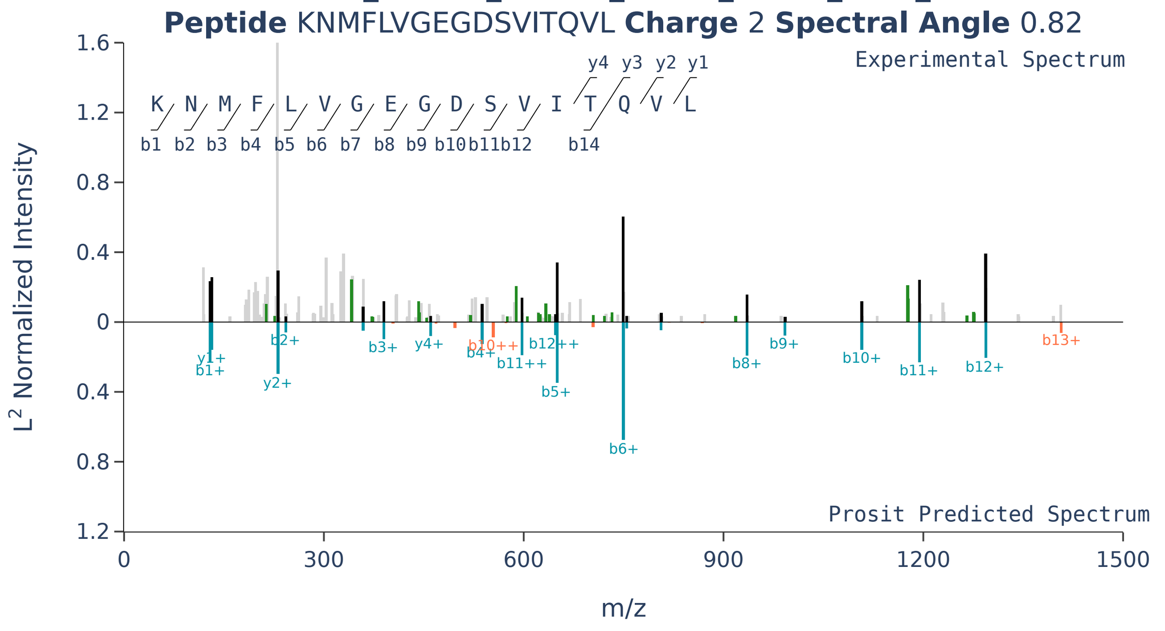


**Source** HRoetschke_271222_090123_HFGoe_LEDGF_b1R2_1h **Scan** 8388
**Peptide** SNASSDVEVEEKE **Charge** 1 **Spectral Angle** 0.79

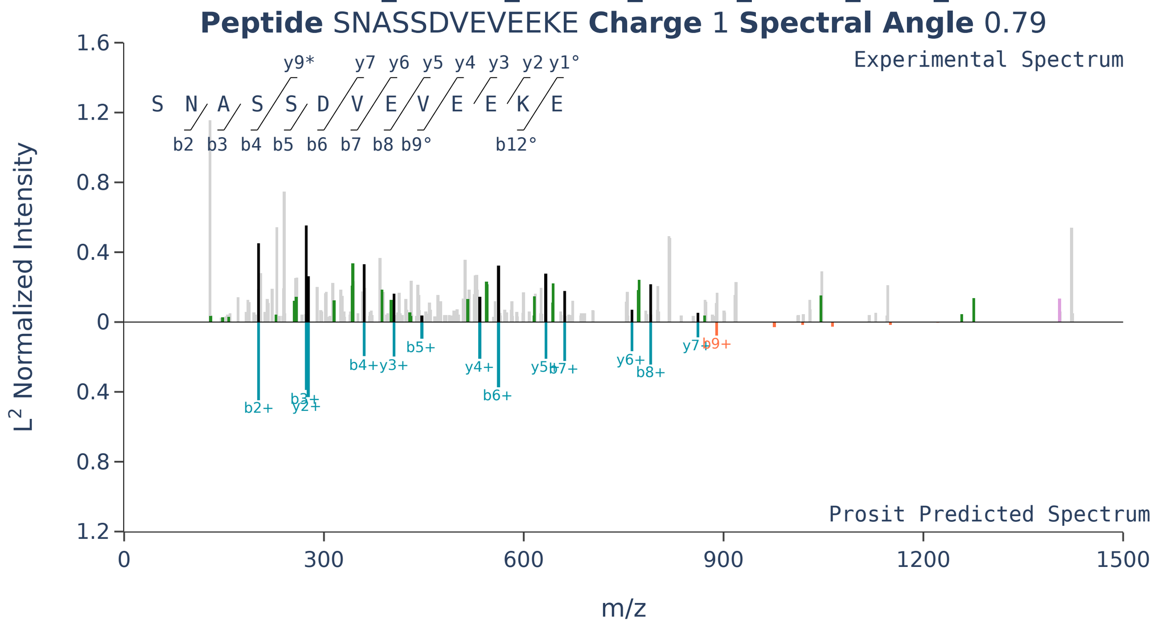


**Source** HRoetschke_271222_090123_HFGoe_LEDGF_b2R1_1h **Scan** 30386
**Peptide** LDELASLQVTM **Charge** 2 **Spectral Angle** 0.88

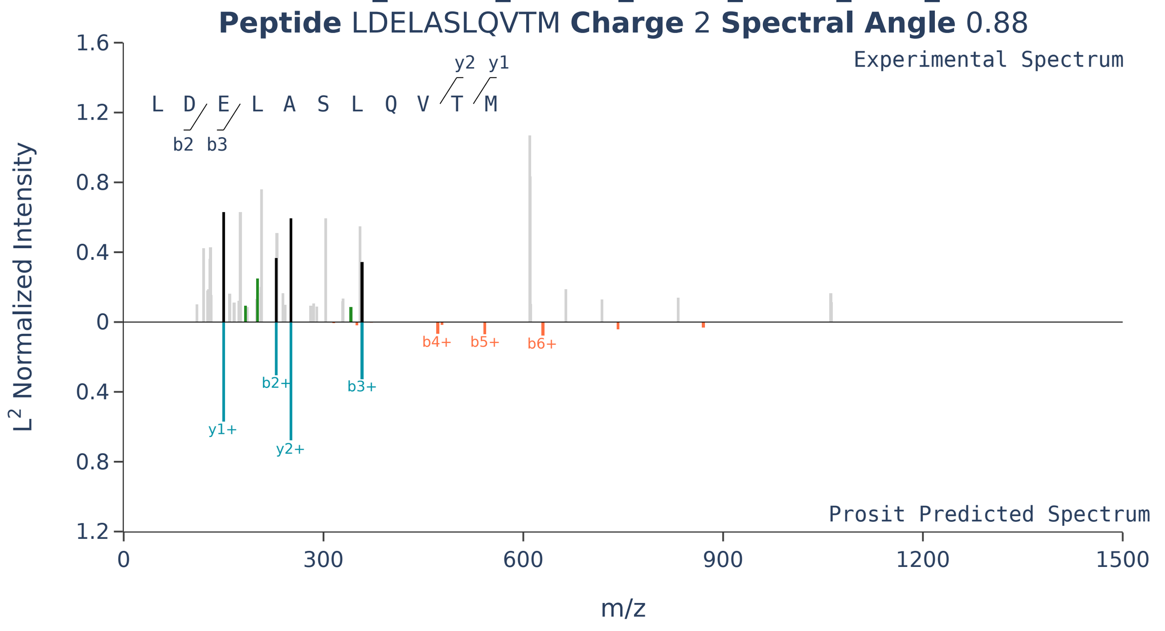


**Source** HRoetschke_271222_090123_HFGoe_LEDGF_b1R2_24h **Scan** 14954
**Peptide** SNEDVTKAVDITTPKAAR **Charge** 4 **Spectral Angle** 0.79

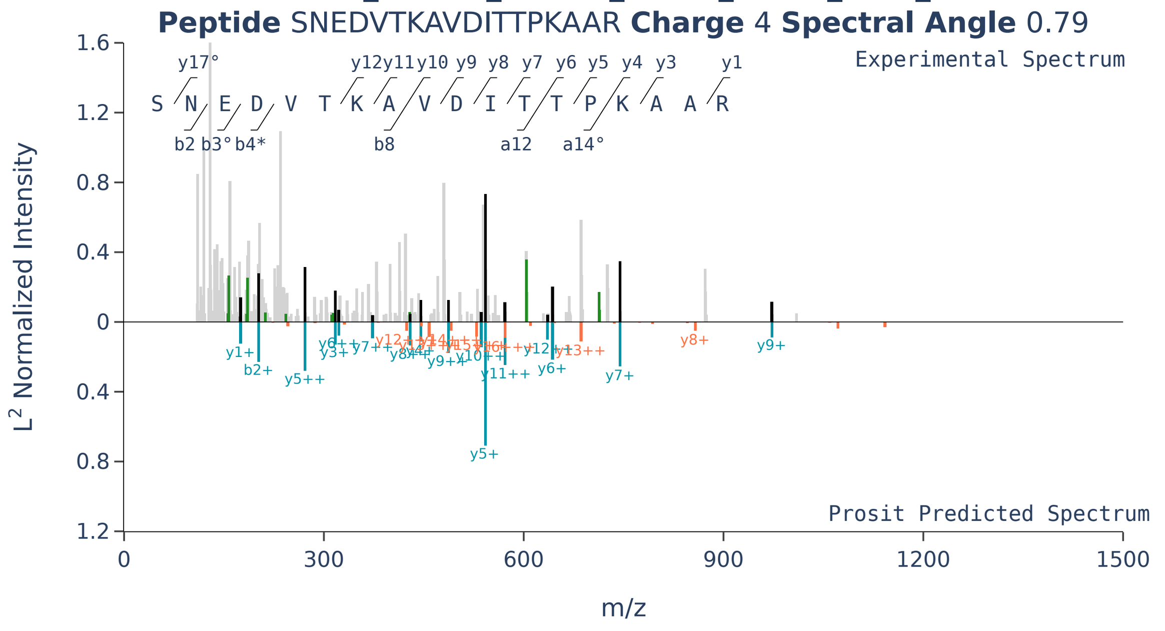


**Source** HRoetschke_271222_090123_HFGoe_LEDGF_b1R1_4h **Scan** 18349
**Peptide** KGYPHWPARVDEVPDGAVKPPTN **Charge** 3 **Spectral Angle** 0.89

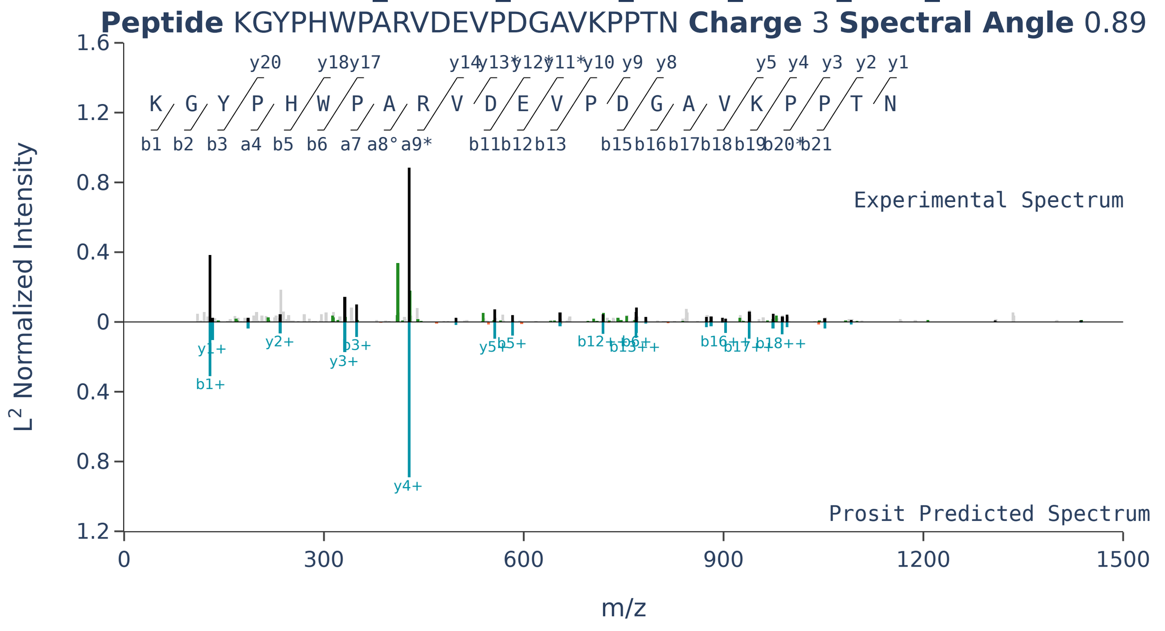


**Source** HRoetschke_271222_090123_HFGoe_LEDGF_b2R2_2h **Scan** 9199
**Peptide** QAATKQSNASSDVEVEE **Charge** 2 **Spectral Angle** 0.78

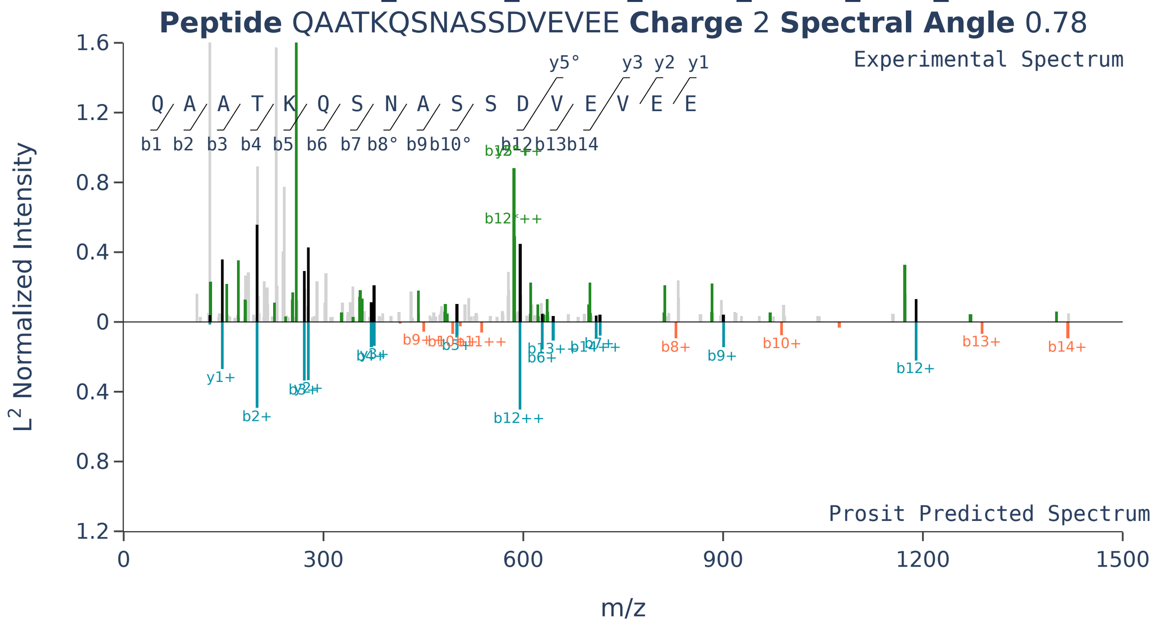

**Source** HRoetschke_271222_090123_HFGoe_LEDGF_b1R1_2h **Scan** 9829
**Peptide** PKDIFPYSENKEKYGKPNKRKGFNE **Charge** 4 **Spectral Angle** 0.84

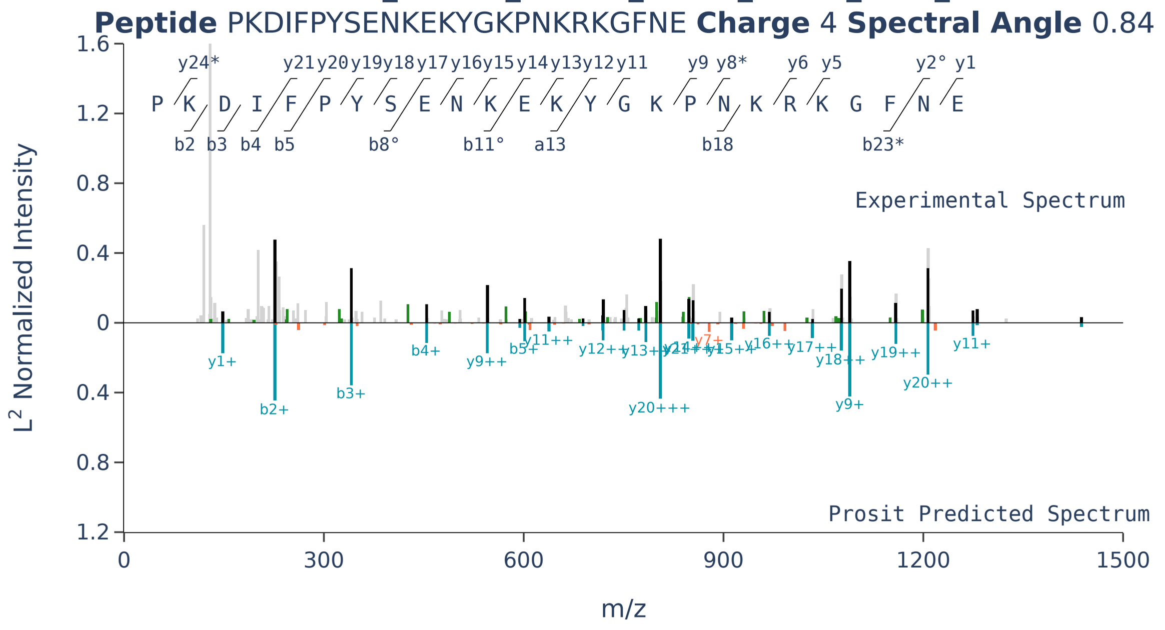


**Source** HRoetschke_271222_090123_HFGoe_LEDGF_b1R2_24h **Scan** 10754
**Peptide** RPAATEVKIPKPRGRPKMV **Charge** 4 **Spectral Angle** 0.86

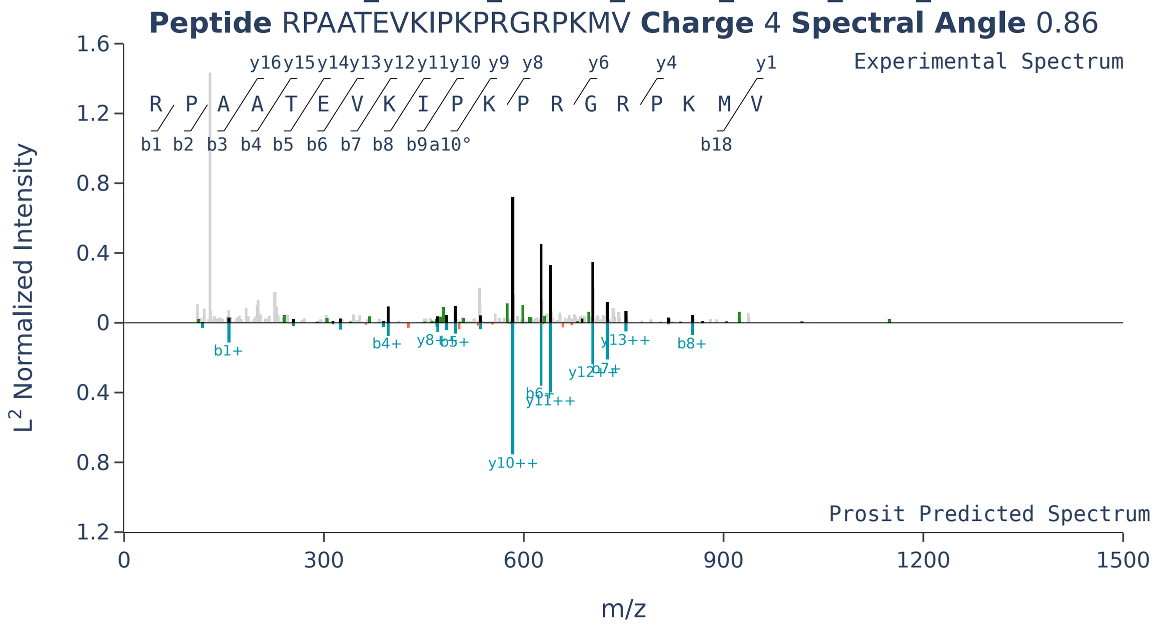


**Source** HRoetschke_271222_090123_HFGoe_LEDGF_b1R1_4h **Scan** 25573
**Peptide** LGPKDIFPYSEN **Charge** 2 **Spectral Angle** 0.73

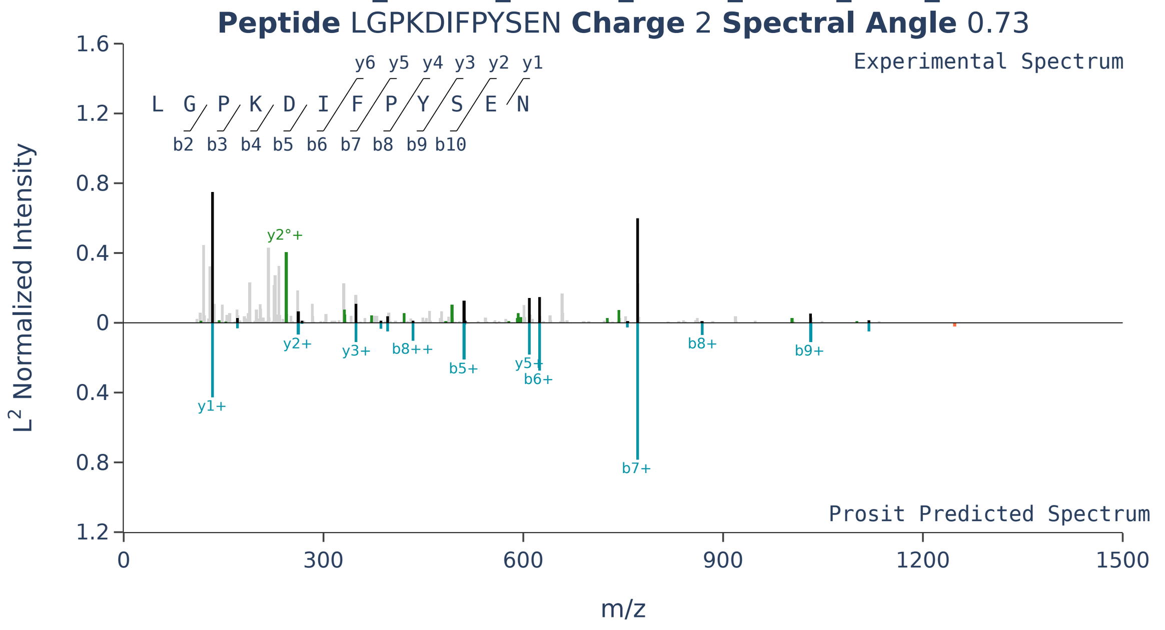


**Source** HRoetschke_271222_090123_HFGoe_LEDGF_b2R1_2h **Scan** 16803
**Peptide** IRRFKVSQVIME **Charge** 3 **Spectral Angle** 0.78

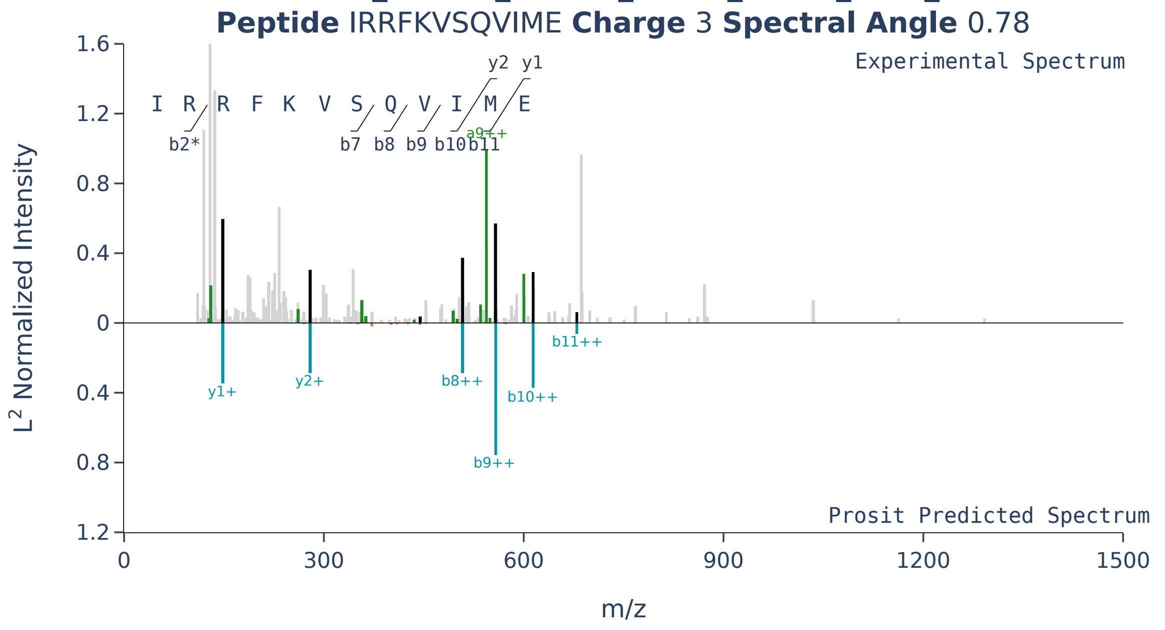


**Source** HRoetschke_271222_090123_HFGoe_LEDGF_b1R2_2h **Scan** 7343
**Peptide** FPYSENKEKYGKPNKRKGFNE **Charge** 3 **Spectral Angle** 0.73

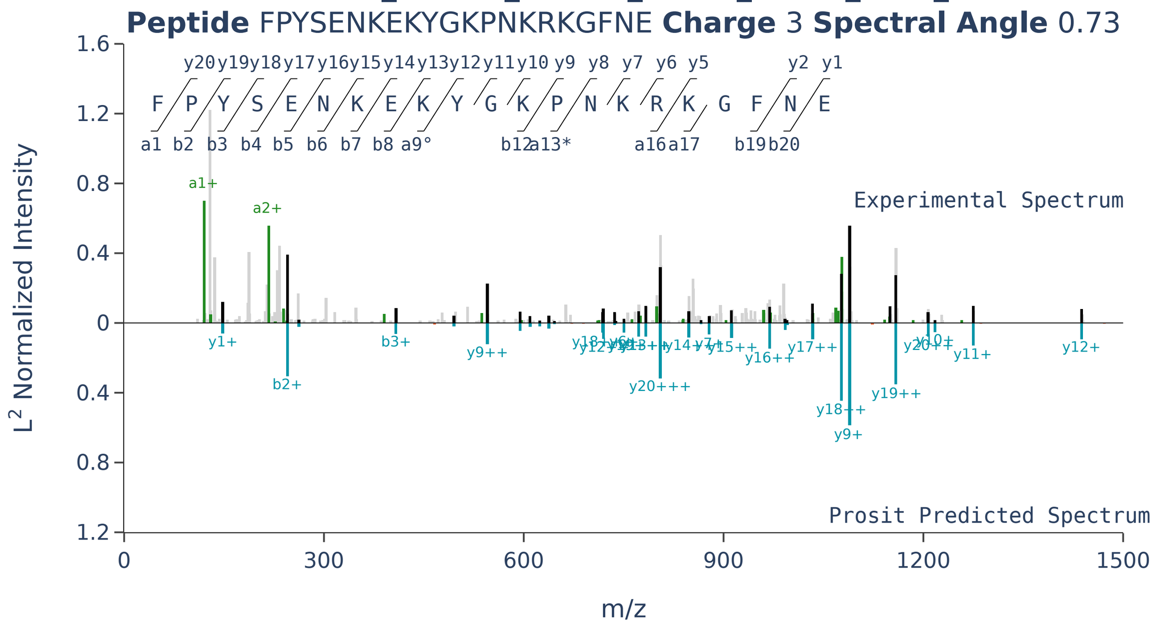


**Source** HRoetschke_271222_090123_HFGoe_LEDGF_b1R1_2h **Scan** 24042
**Peptide** GPGMTRDFKPGDLIFAKMKG **Charge** 3 **Spectral Angle** 0.69

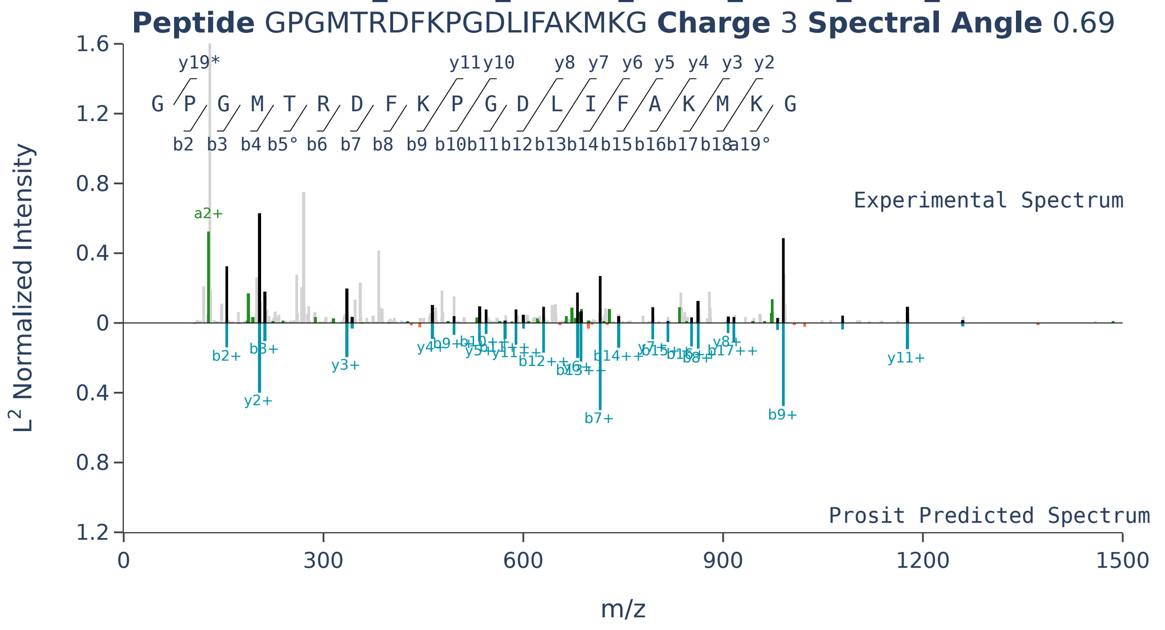


**Source** HRoetschke_271222_090123_HFGoe_LEDGF_b2R1_24h **Scan** 24203
**Peptide** HETAFLGPKDIFPYSENKE **Charge** 3 **Spectral Angle** 0.8

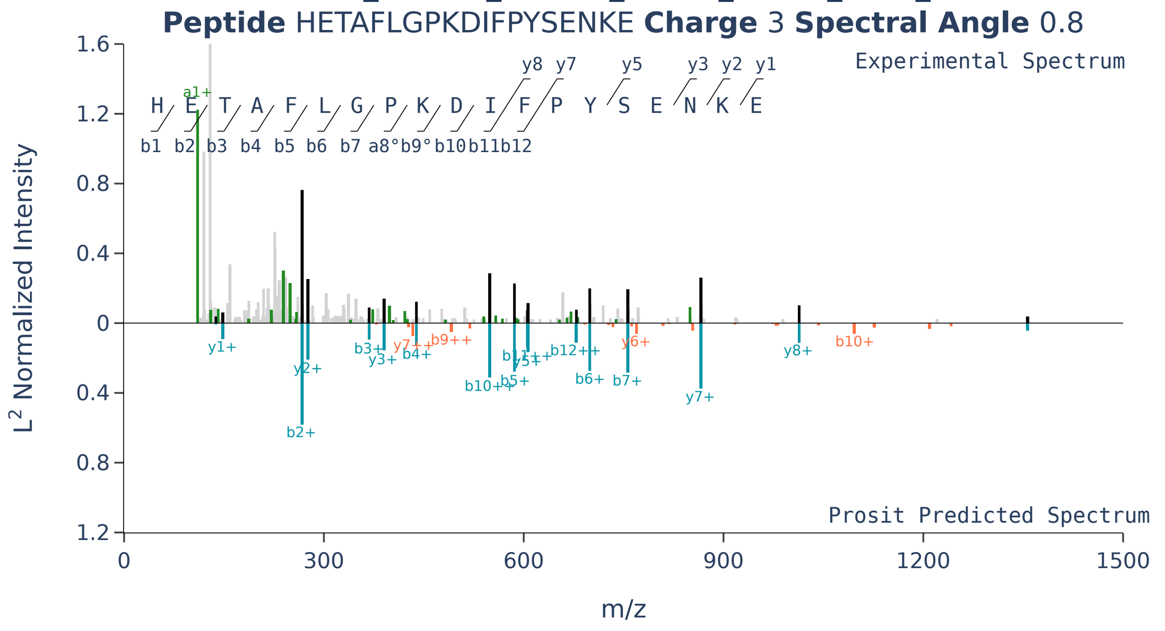


**Source** HRoetschke_271222_090123_HFGoe_LEDGF_b1R1_24h **Scan** 8664
**Peptide** ASNEDVTKAVD **Charge** 2 **Spectral Angle** 0.74

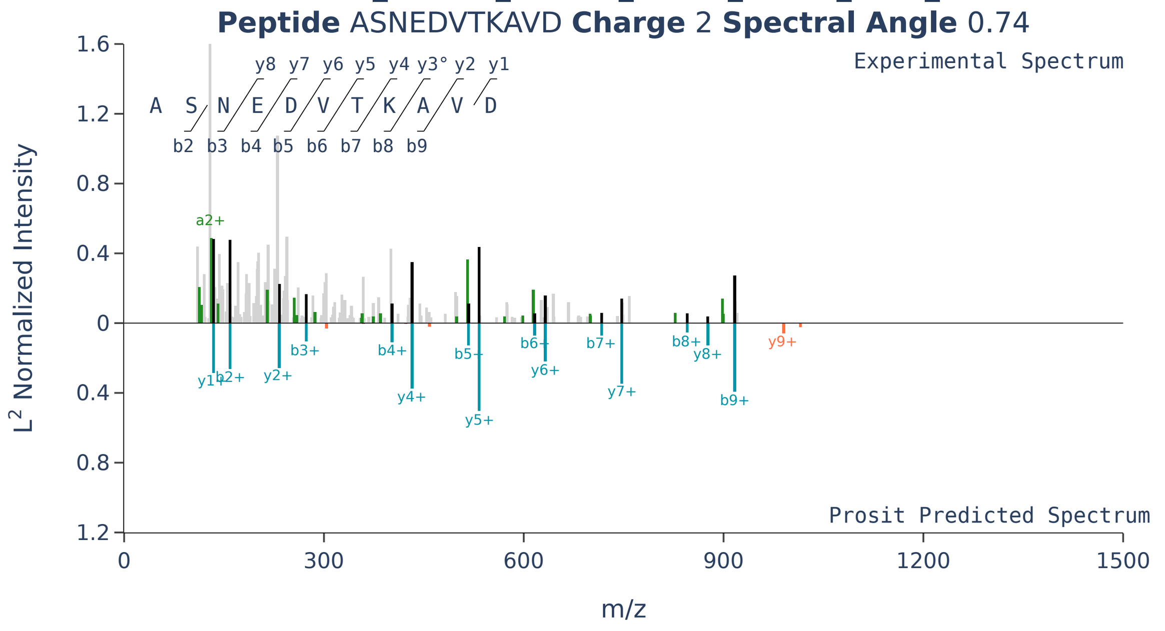


**Source** HRoetschke_271222_090123_HFGoe_LEDGF_b2R1_2h **Scan** 25201
**Peptide** VGEGDSVITQVLNKS **Charge** 2 **Spectral Angle** 0.78

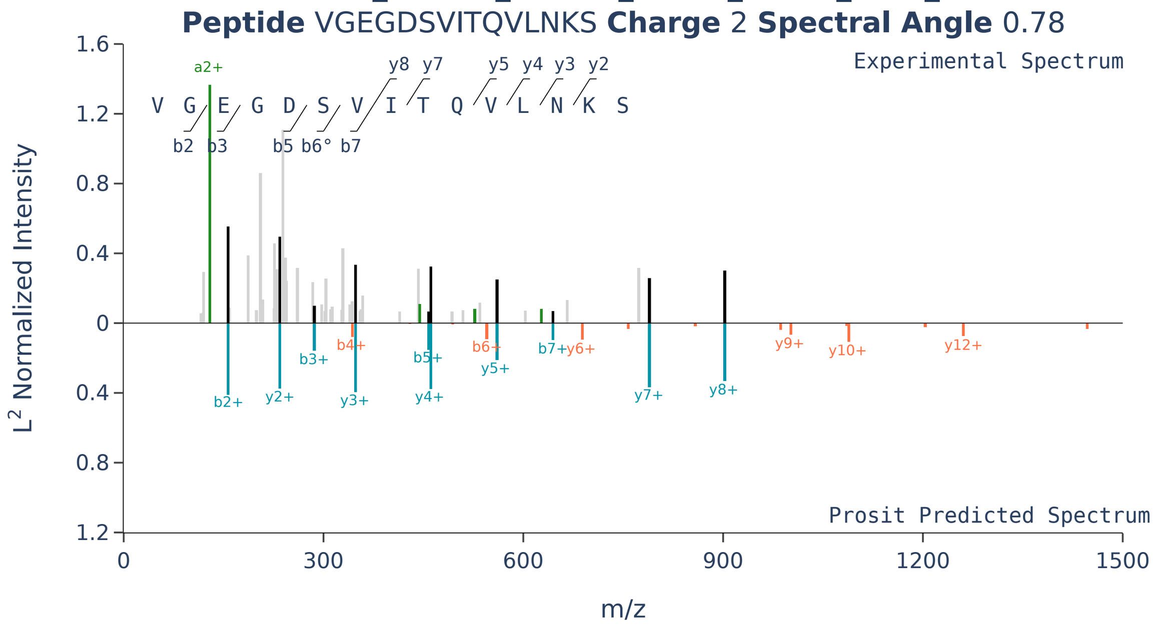


**Source** HRoetschke_271222_090123_HFGoe_LEDGF_b2R2_4h **Scan** 21706
**Peptide** KNMFLVGE **Charge** 2 **Spectral Angle** 0.81

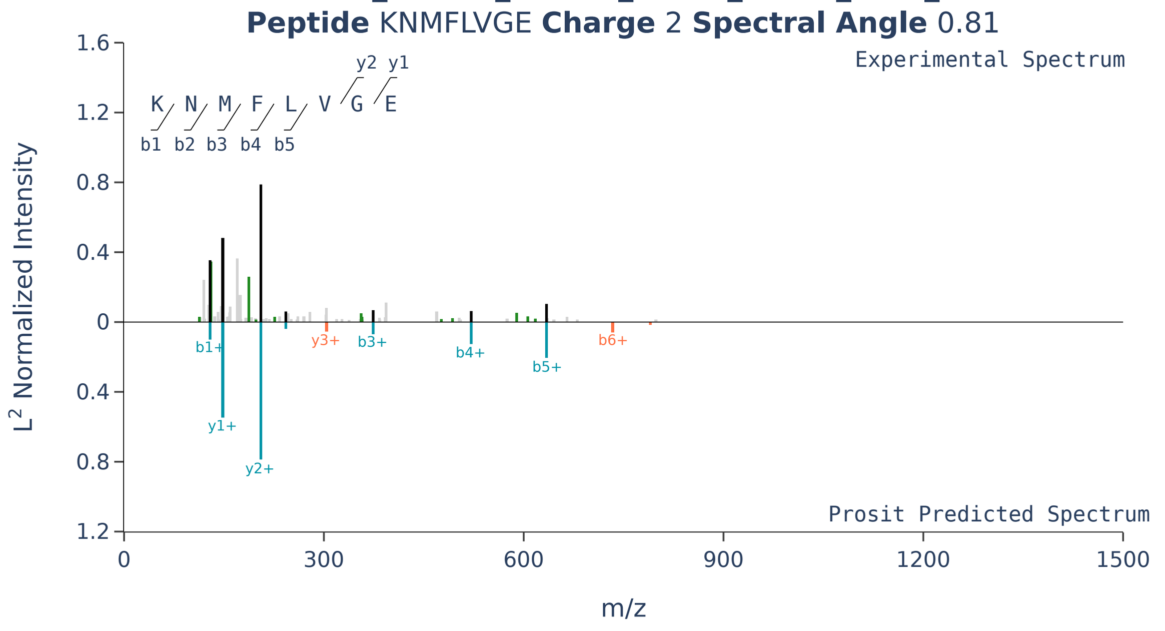


**Source** HRoetschke_271222_090123_HFGoe_LEDGF_b2R1_24h **Scan** 9821
**Peptide** KASNEDVTKAVDITTPKAARRGR **Charge** 4 **Spectral Angle** 0.8

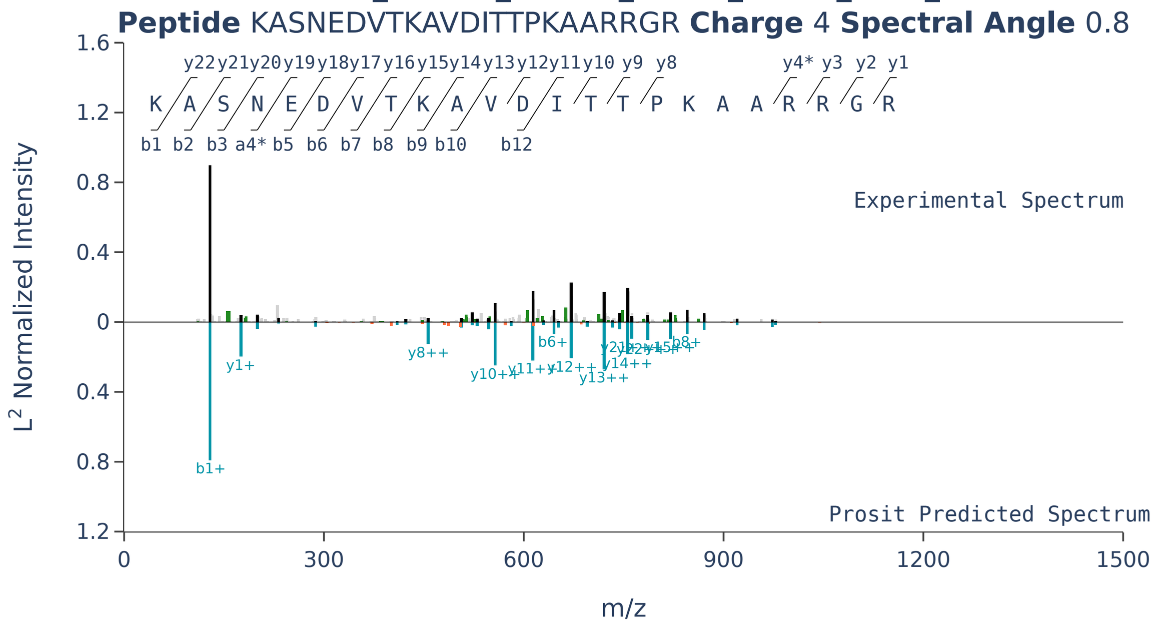


**Source** HRoetschke_271222_090123_HFGoe_LEDGF_b2R1_24h **Scan** 16675
**Peptide** TEEAGVVTTATASVNLKVSPKRGRPAATE **Charge** 4 **Spectral Angle** 0.78

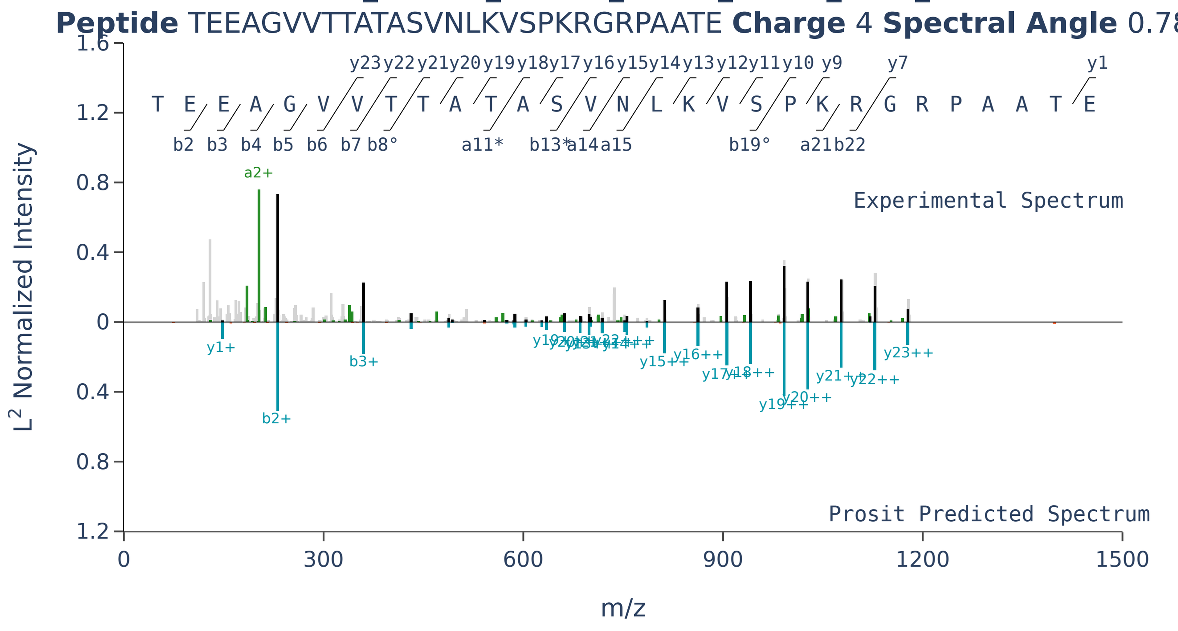


**Source** HRoetschke_271222_090123_HFGoe_LEDGF_b1R2_1h **Scan** 22387
**Peptide** GMTRDFKPGDLIFAKMKG **Charge** 4 **Spectral Angle** 0.78

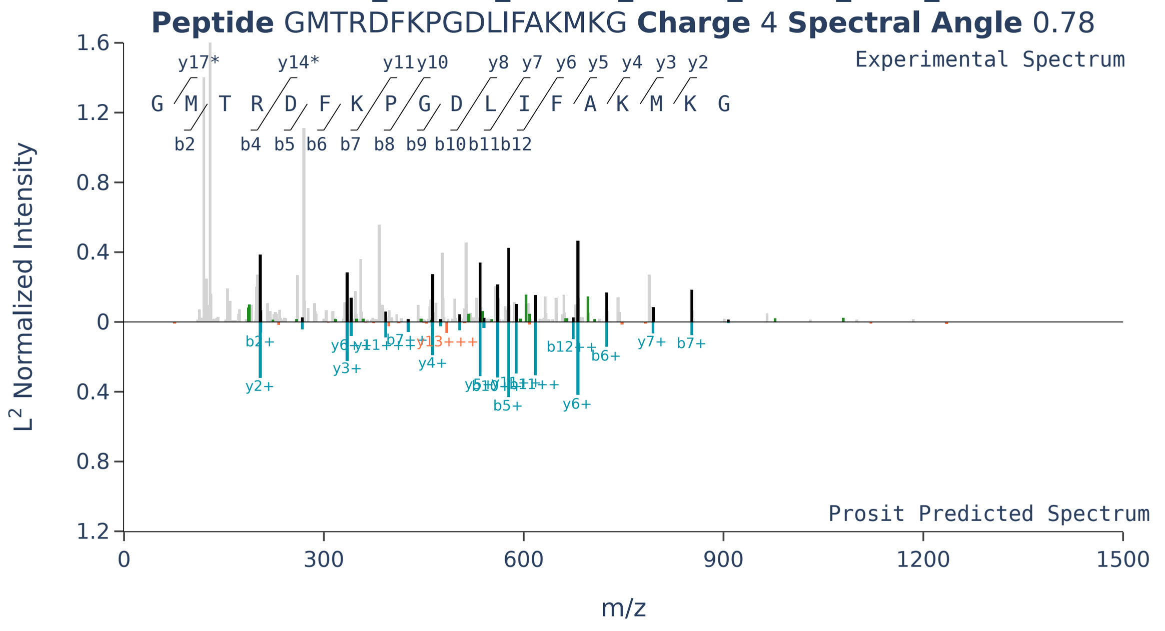


**Source** HRoetschke_271222_090123_HFGoe_LEDGF_b2R1_4h **Scan** 16485
**Peptide** HETAFLGPKDIFPYSENKEKYGKPNKR **Charge** 5 **Spectral Angle** 0.74

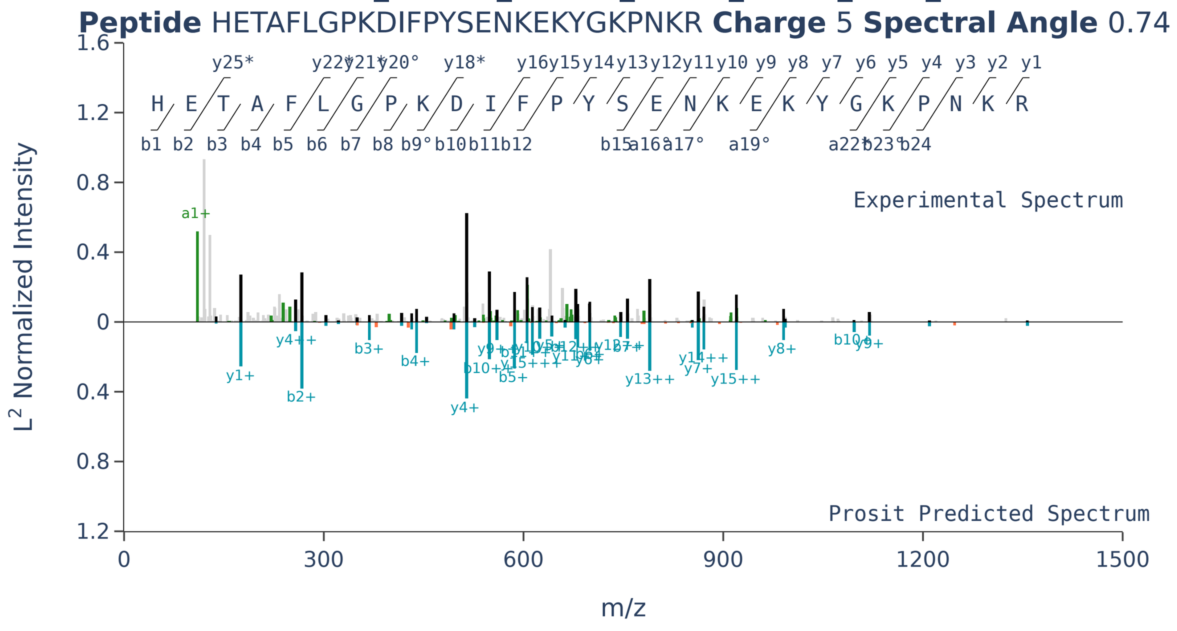


**Source** HRoetschke_271222_090123_HFGoe_LEDGF_b1R2_4h **Scan** 28787
**Peptide** DFKPGDLIFAKMKGY **Charge** 4 **Spectral Angle** 0.8

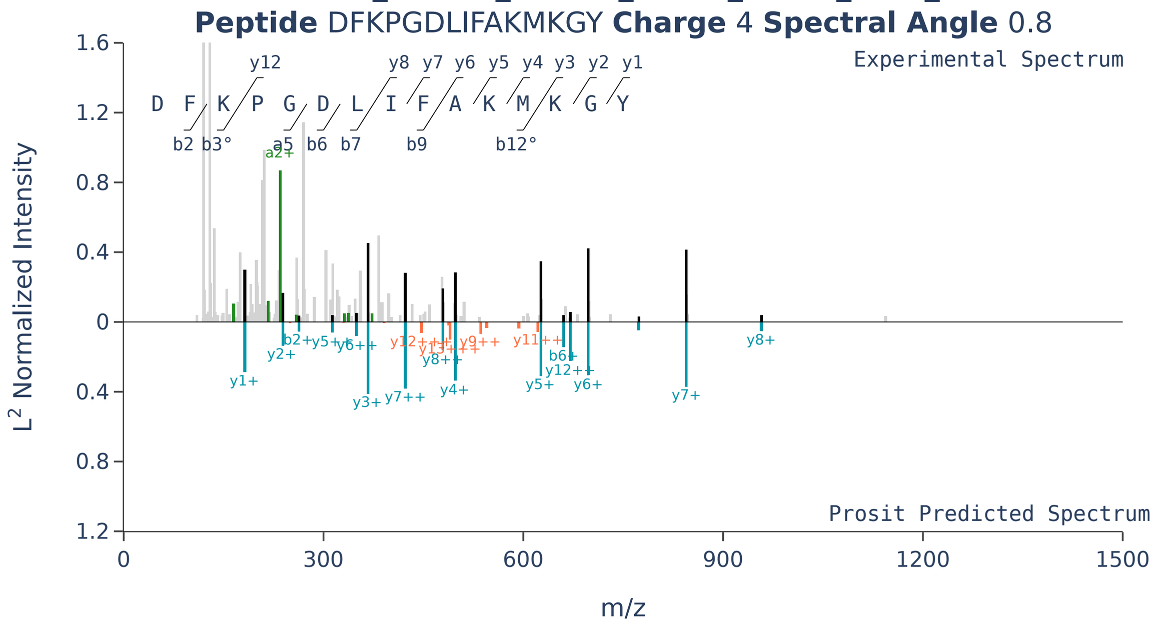

**Source** HRoetschke_271222_090123_HFGoe_LEDGF_b2R1_4h **Scan** 10393
**Peptide** TMQQAQKHTEMI **Charge** 2 **Spectral Angle** 0.71

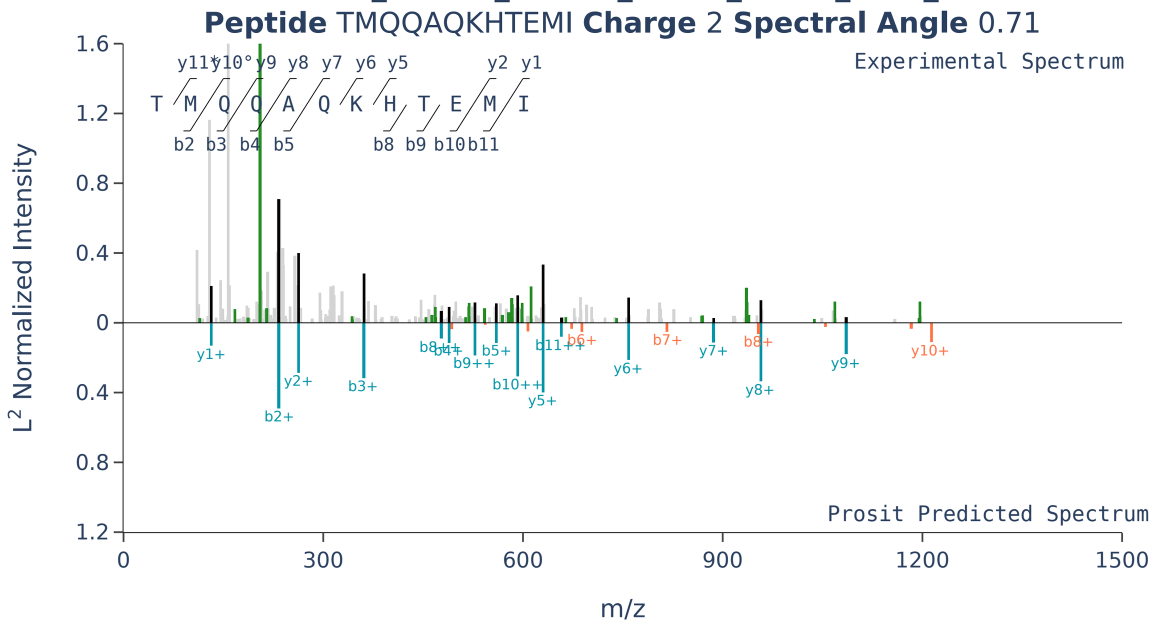


**Source** HRoetschke_271222_090123_HFGoe_LEDGF_b2R1_2h **Scan** 9318
**Peptide** AATEVKIPKPRGR **Charge** 4 **Spectral Angle** 0.86

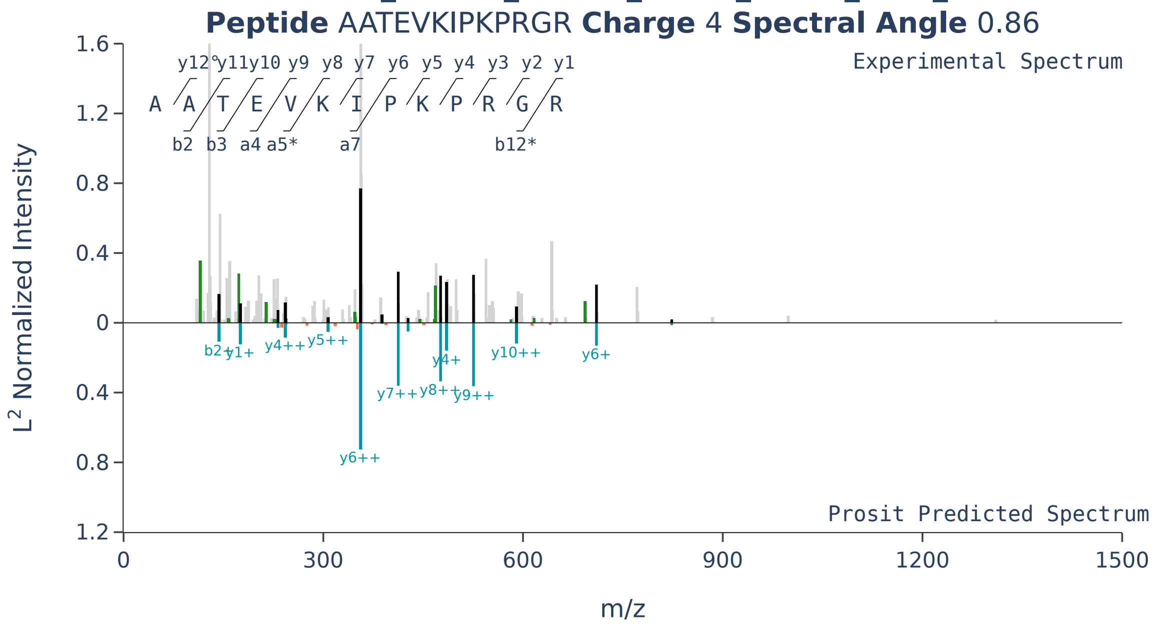


**Source** HRoetschke_271222_090123_HFGoe_LEDGF_b1R2_1h **Scan** 12283
**Peptide** ASSDVEVEEKETSV **Charge** 2 **Spectral Angle** 0.81

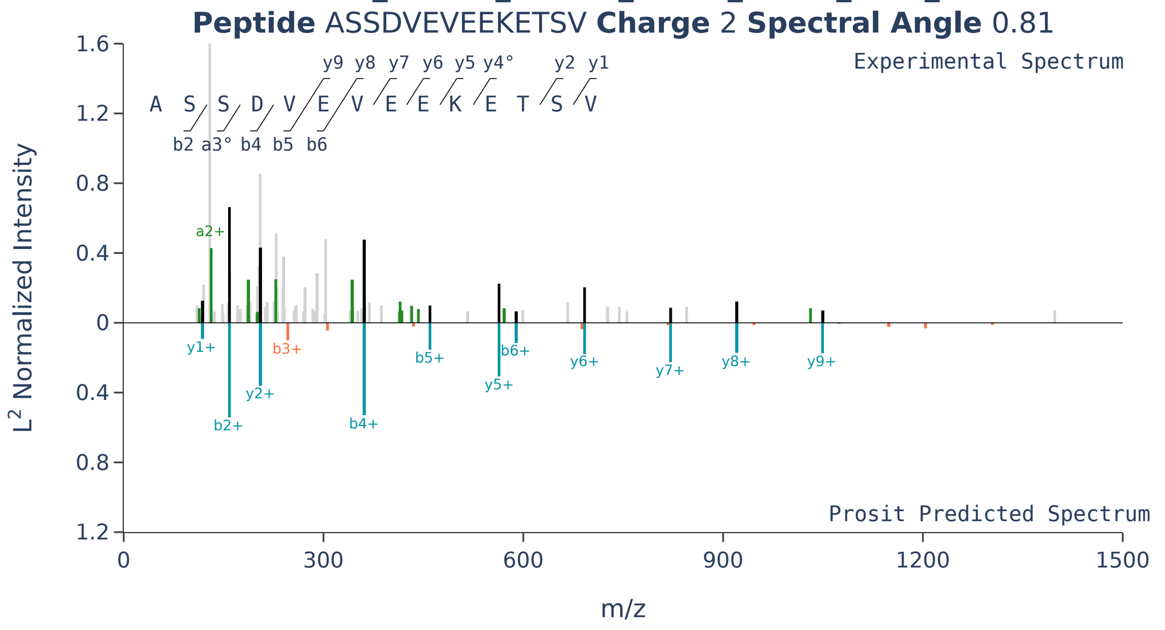


**Source** HRoetschke_271222_090123_HFGoe_LEDGF_b1R2_4h **Scan** 7952
**Peptide** NNPKVKFSSQQA **Charge** 3 **Spectral Angle** 0.89

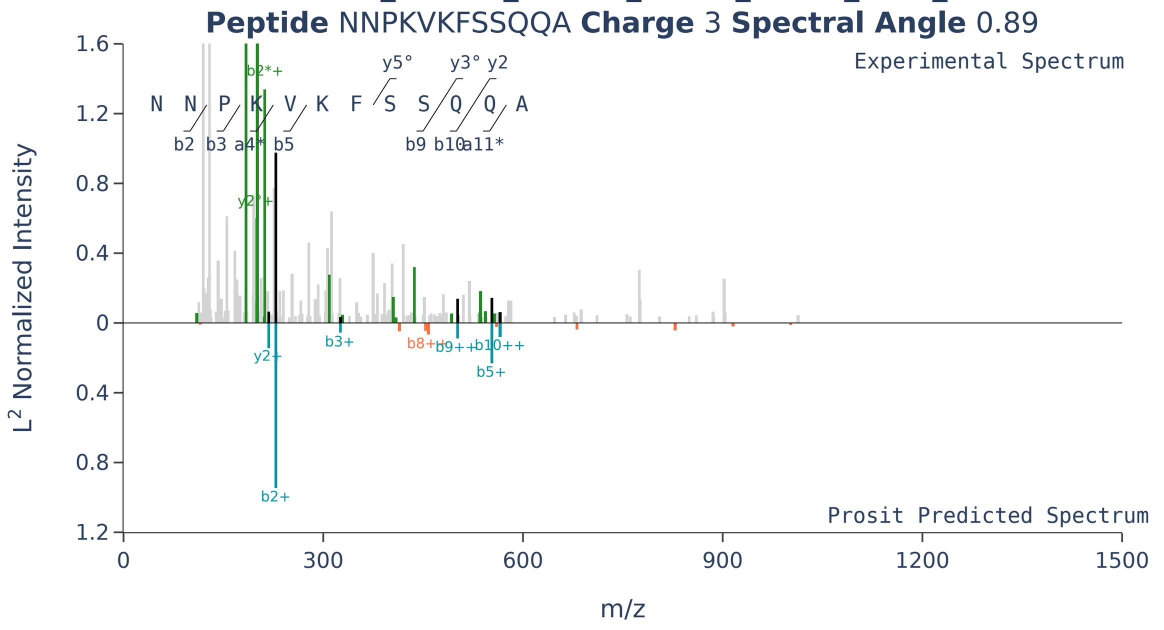


**Source** HRoetschke_271222_090123_HFGoe_LEDGF_b2R1_2h **Scan** 11301
**Peptide** FSSQQAATKQSNASSDVEVE **Charge** 3 **Spectral Angle** 0.85

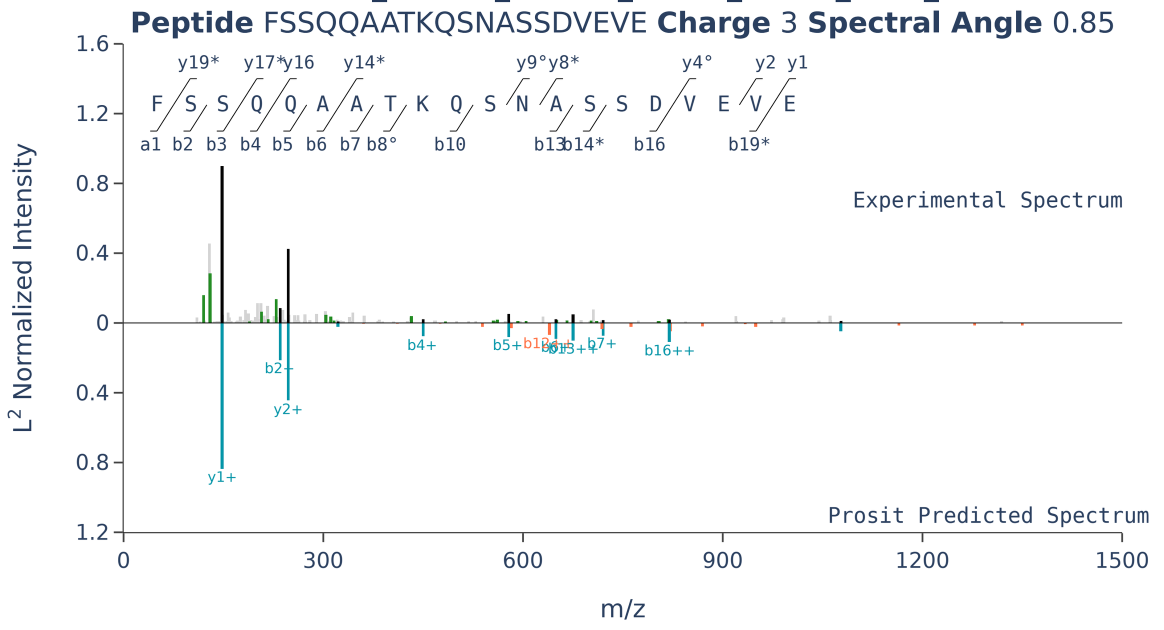


**Source** HRoetschke_271222_090123_HFGoe_LEDGF_b1R1_24h **Scan** 10924
**Peptide** FPYSENKEKYG **Charge** 3 **Spectral Angle** 0.7

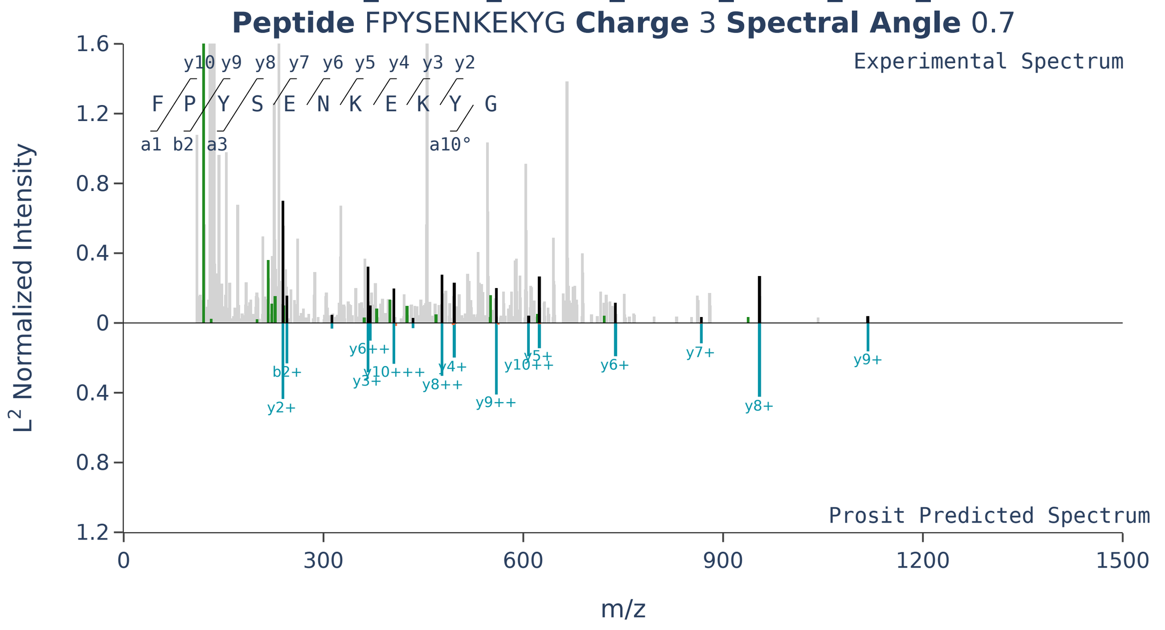


**Source** HRoetschke_271222_090123_HFGoe_LEDGF_b2R1_2h **Scan** 26736
**Peptide** GFNEGLWEIDNNPKVKFS **Charge** 2 **Spectral Angle** 0.76

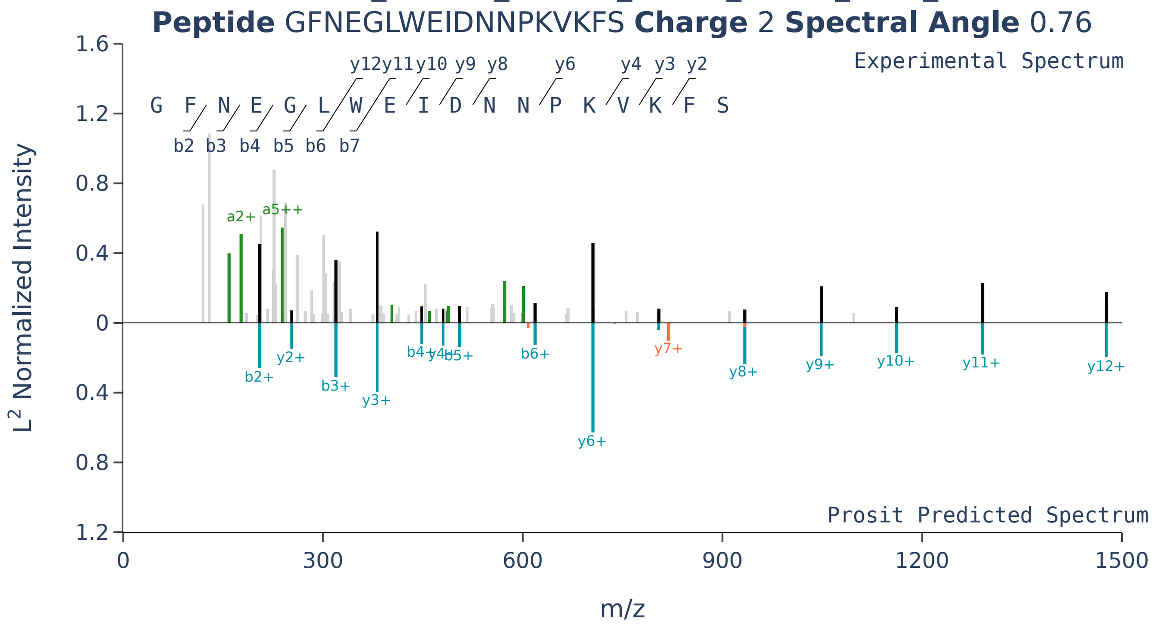


**Source** HRoetschke_271222_090123_HFGoe_LEDGF_b2R2_24h **Scan** 8207
**Peptide** QQAQKHTEMITT **Charge** 3 **Spectral Angle** 0.81

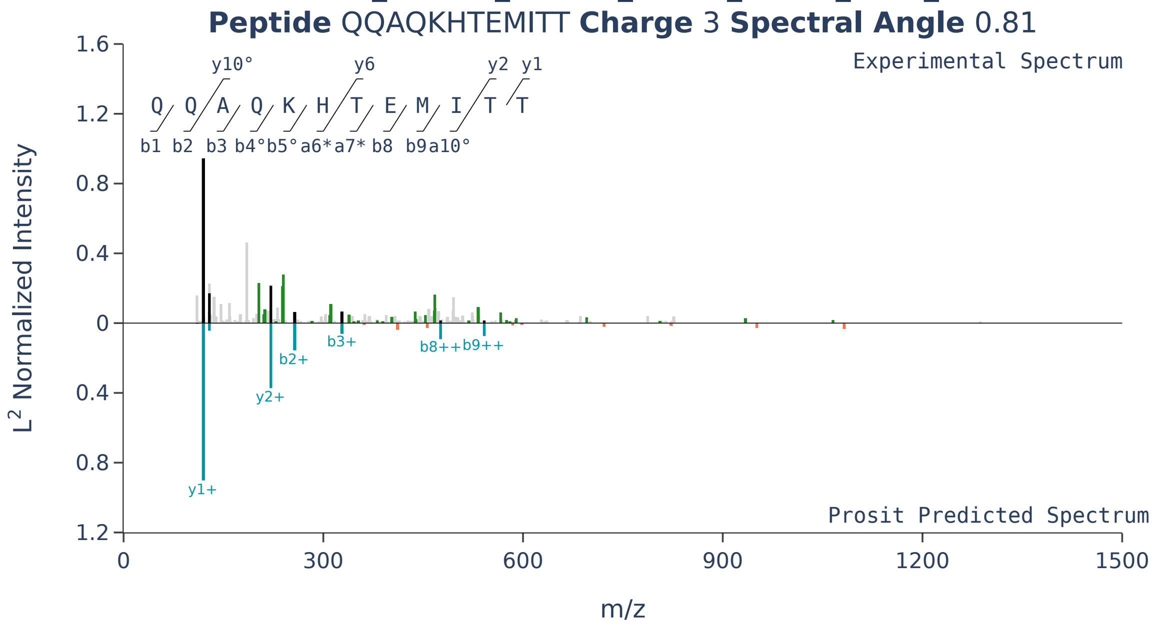


**Source** HRoetschke_271222_090123_HFGoe_LEDGF_b1R2_24h **Scan** 14875
**Peptide** QVETEEAGVVTT **Charge** 2 **Spectral Angle** 0.86

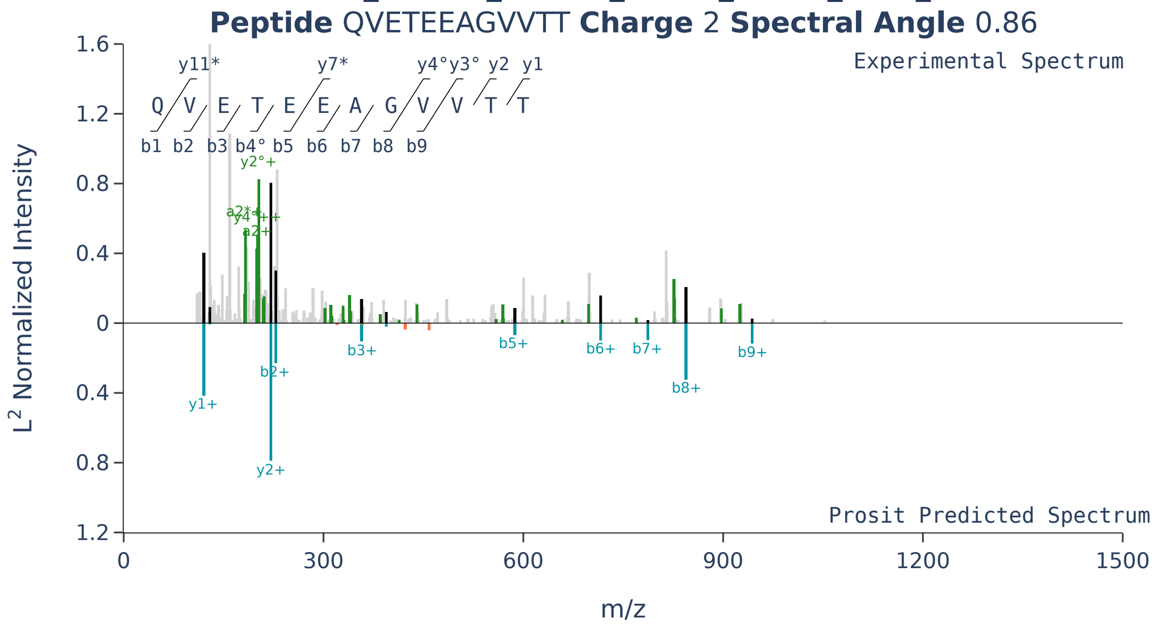


**Source** HRoetschke_271222_090123_HFGoe_LEDGF_b2R2_24h **Scan** 10899
**Peptide** AEIKNSL **Charge** 1 **Spectral Angle** 0.81

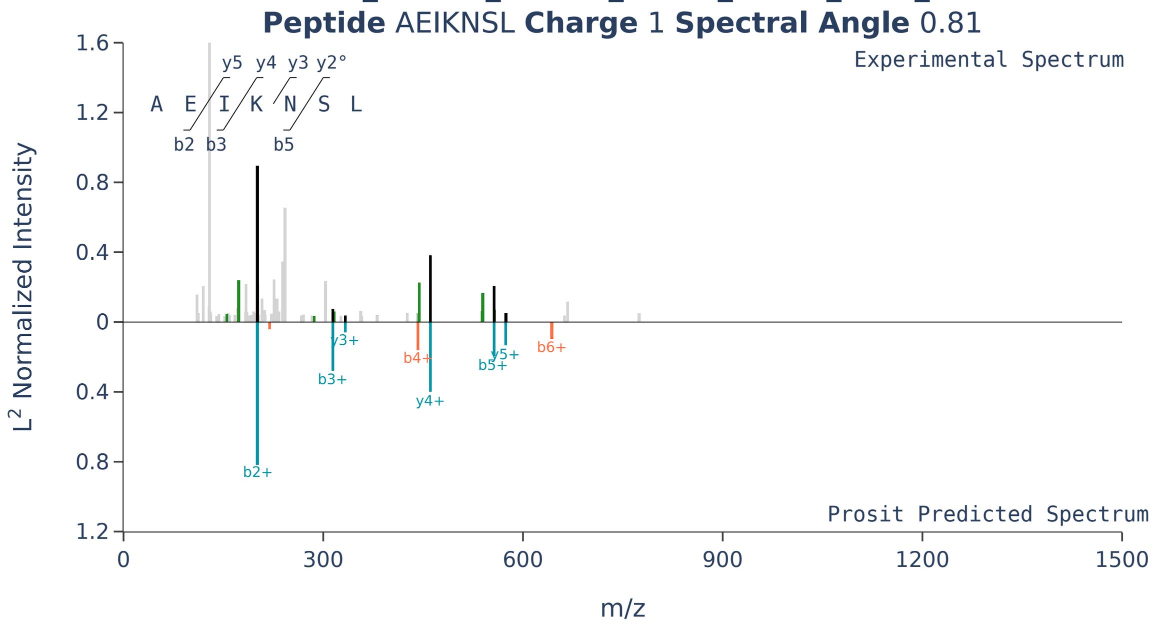


**Source** HRoetschke_271222_090123_HFGoe_LEDGF_b1R2_24h **Scan** 5527
**Peptide** PNKKLEKEQTGSKTLNGGSD **Charge** 2 **Spectral Angle** 0.78

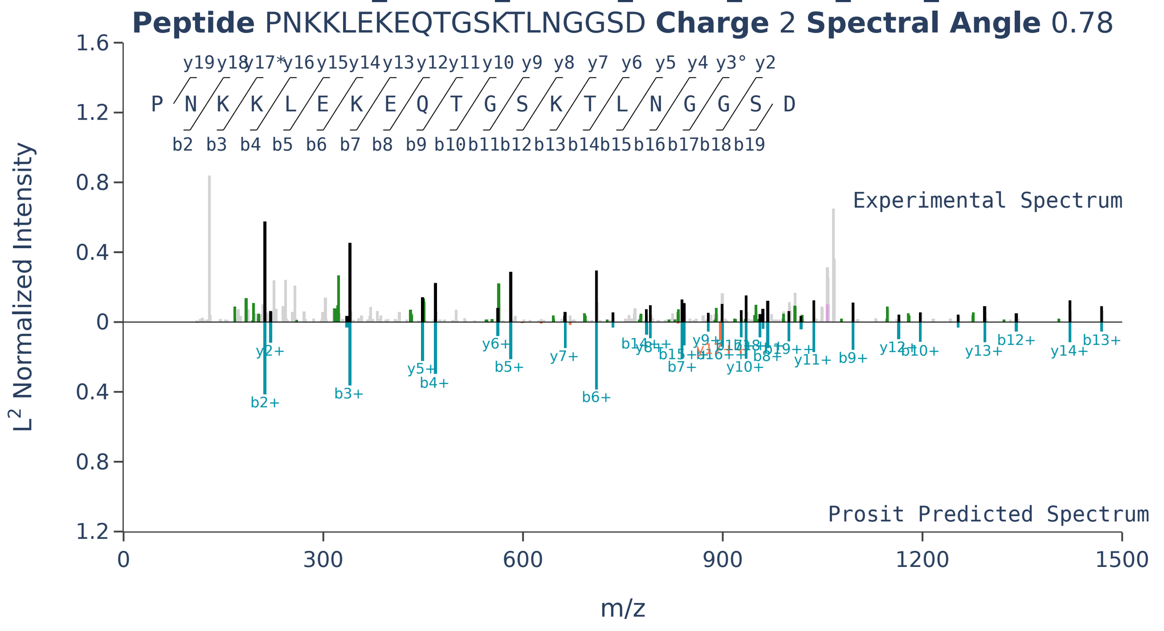


**Source** HRoetschke_271222_090123_HFGoe_LEDGF_b1R2_4h **Scan** 13128
**Peptide** AQKHTEMITTLKKI **Charge** 4 **Spectral Angle** 0.88

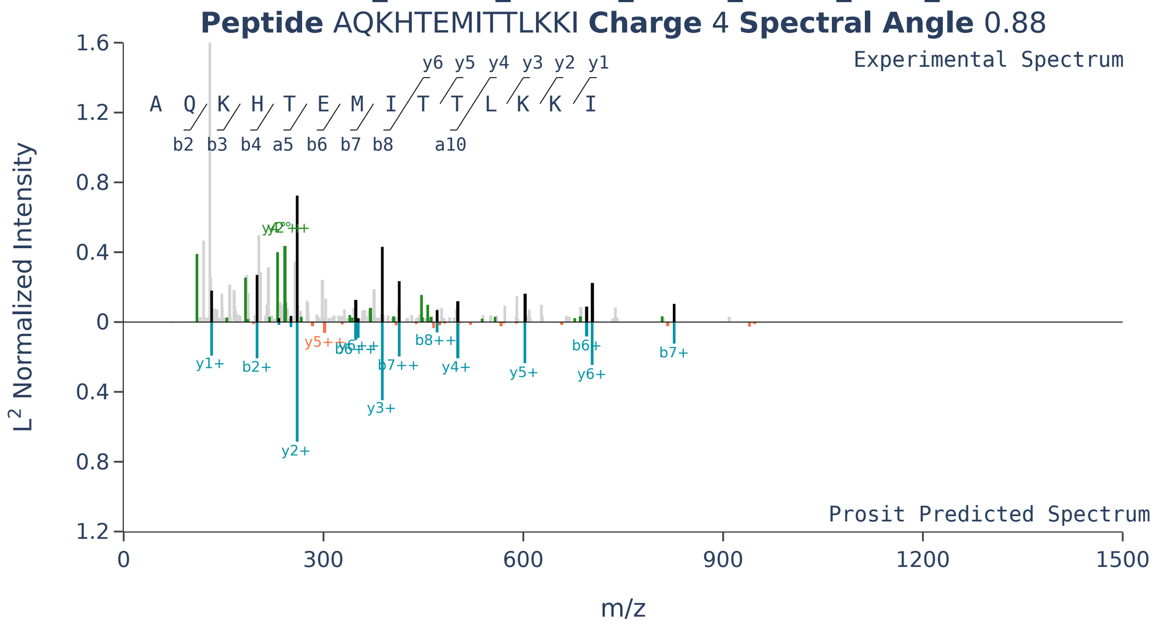


**Source** HRoetschke_271222_090123_HFGoe_LEDGF_b2R2_2h **Scan** 7392
**Peptide** STKKKPSSEERETEISLKD **Charge** 3 **Spectral Angle** 0.68

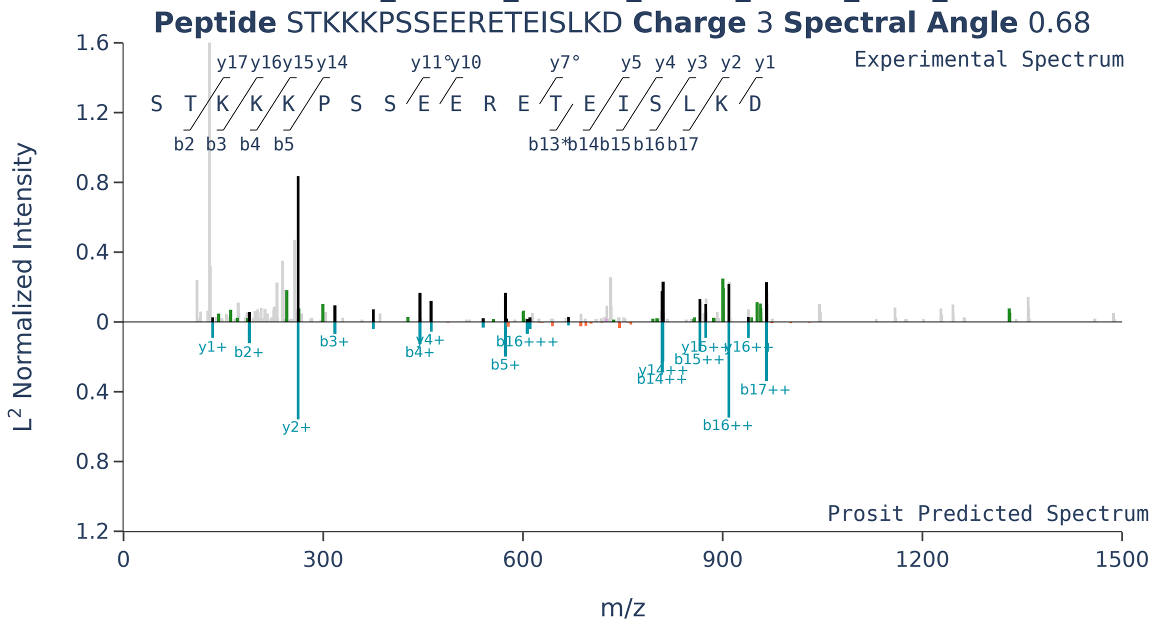


**Source** HRoetschke_271222_090123_HFGoe_LEDGF_b1R1_24h **Scan** 7256
**Peptide** GRKRKAEKQVETEEAGVVTTA **Charge** 3 **Spectral Angle** 0.86

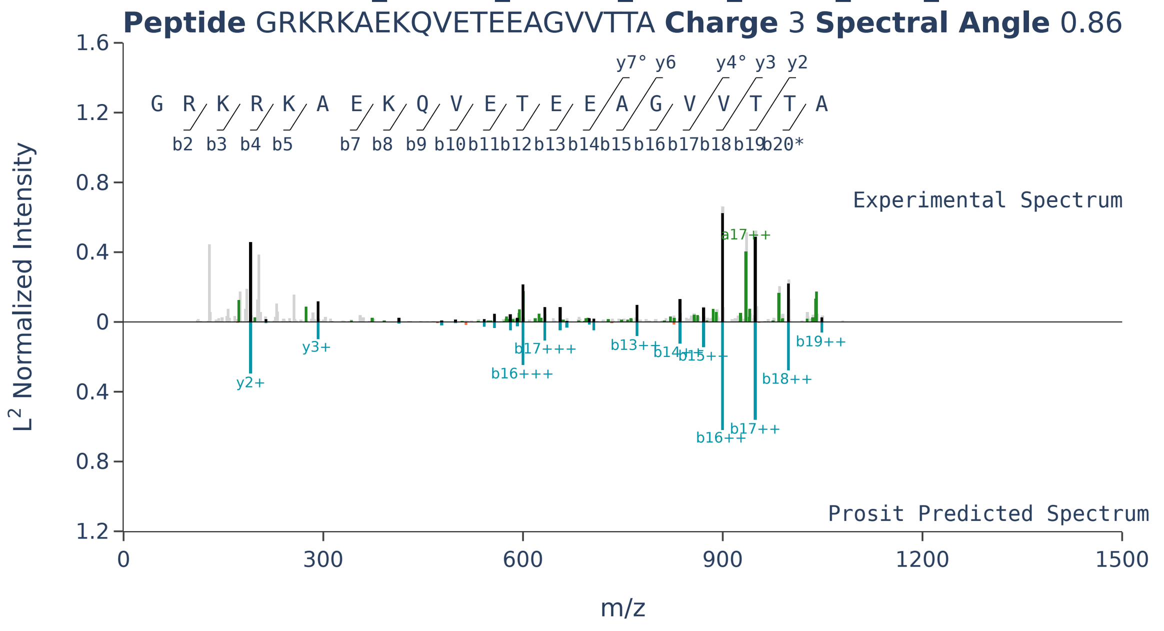


**Source** HRoetschke_271222_090123_HFGoe_LEDGF_b2R1_1h **Scan** 8563
**Peptide** LDNNPKVKFSSQQAATKQS **Charge** 3 **Spectral Angle** 0.81

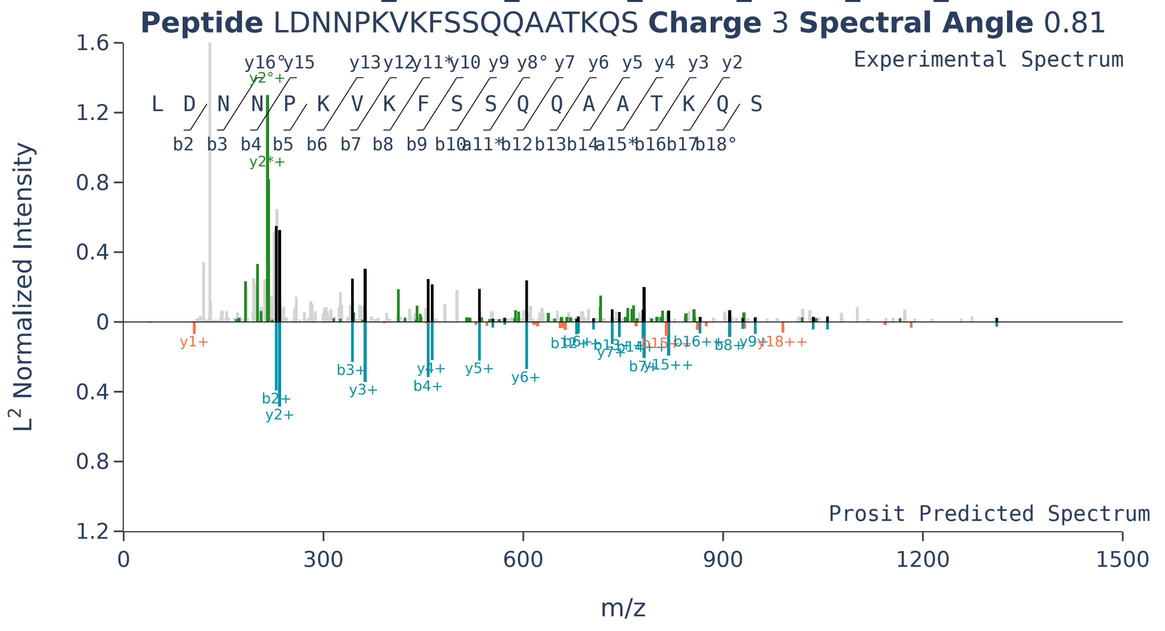

**Source** HRoetschke_271222_090123_HFGoe_LEDGF_b2R1_24h **Scan** 17717
**Peptide** EEAGVVTTATASVNLKVSPKRGRPAATEV **Charge** 3 **Spectral Angle** 0.86

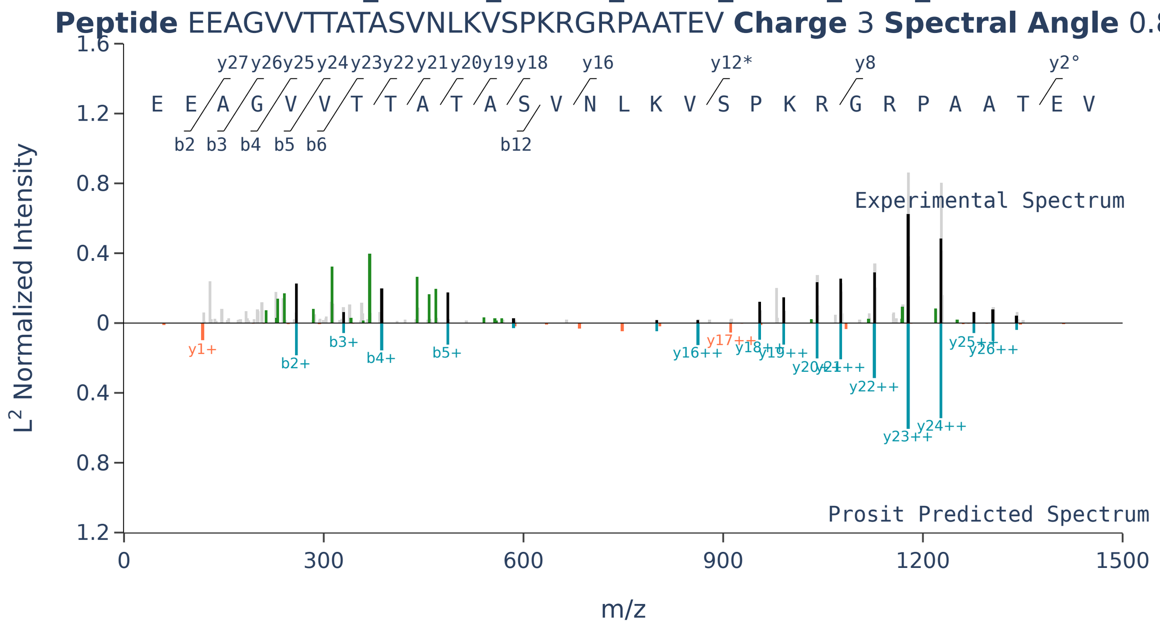


**Source** HRoetschke_271222_090123_HFGoe_LEDGF_b2R1_2h **Scan** 10989
**Peptide** PGMTRDFKPGD **Charge** 2 **Spectral Angle** 0.76

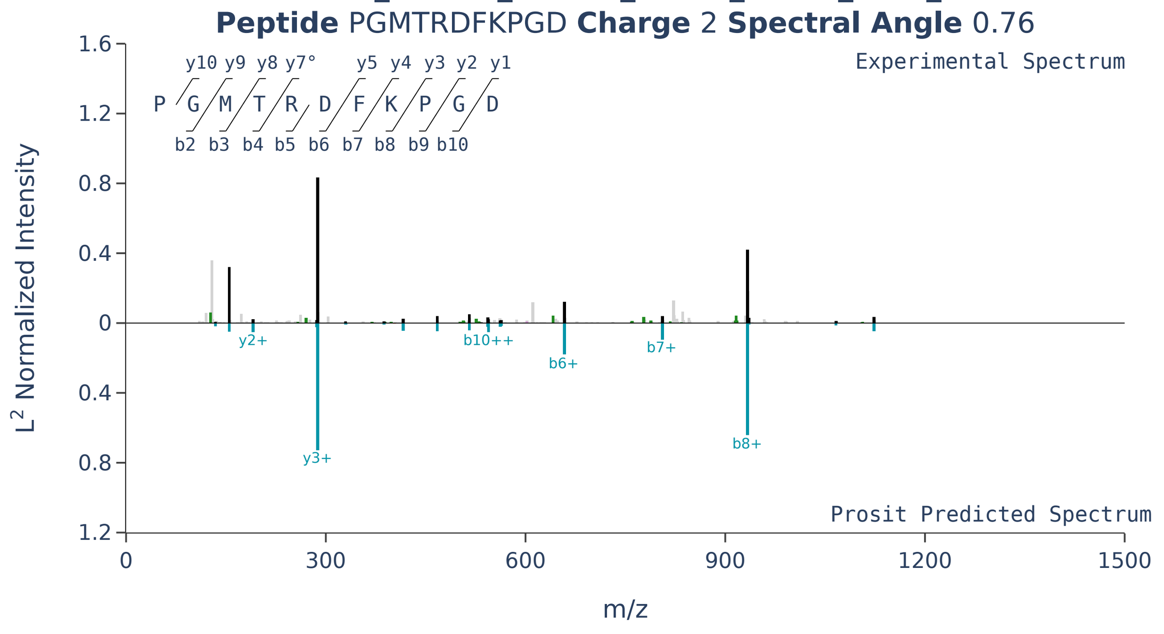


**Source** HRoetschke_271222_090123_HFGoe_LEDGF_b2R1_2h **Scan** 13084
**Peptide** SLQVTMQQAQK **Charge** 2 **Spectral Angle** 0.69

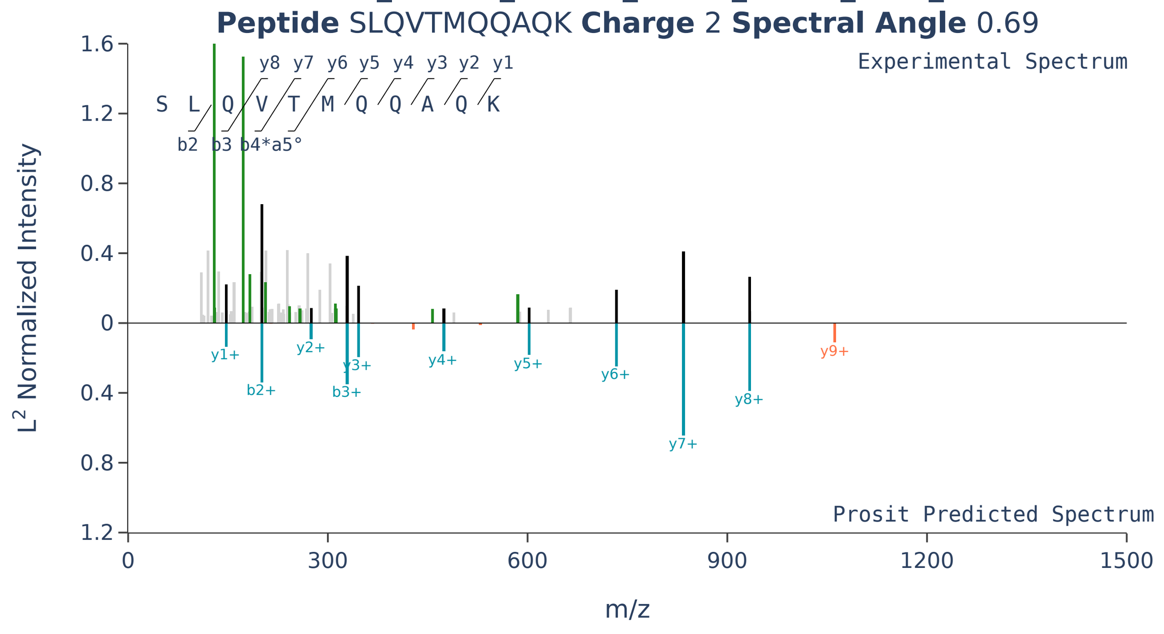


**Source** HRoetschke_271222_090123_HFGoe_LEDGF_b1R2_24h **Scan** 13489
**Peptide** MITTLKKIRRF **Charge** 4 **Spectral Angle** 0.89

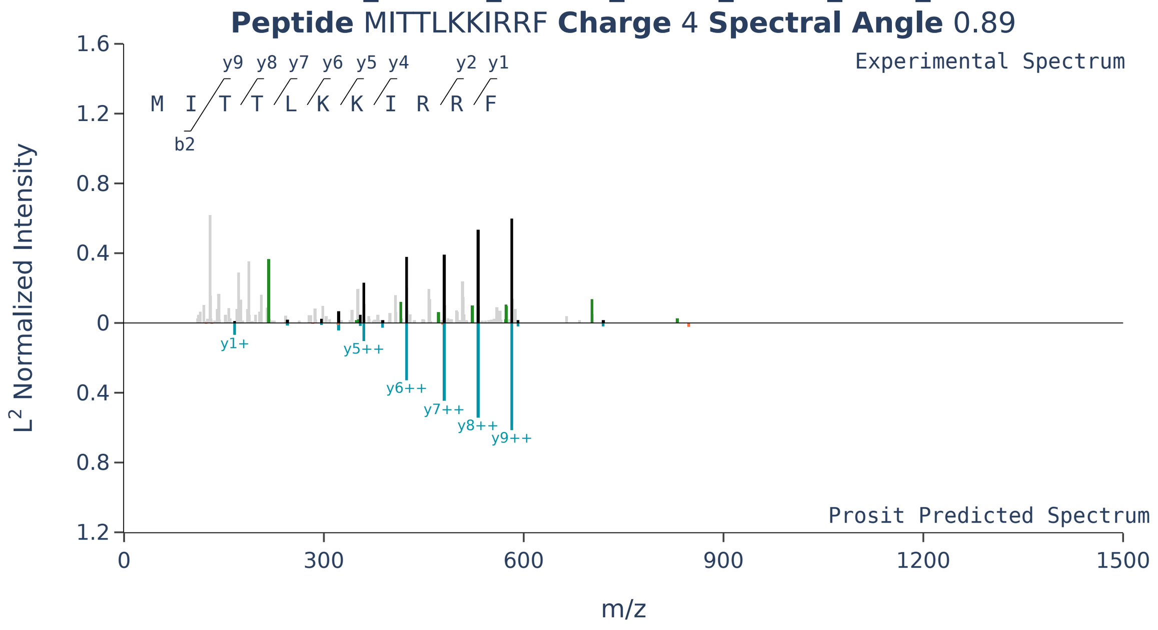


**Source** HRoetschke_271222_090123_HFGoe_LEDGF_b2R1_24h **Scan** 7844
**Peptide** PKVKFSSQQA **Charge** 3 **Spectral Angle** 0.73

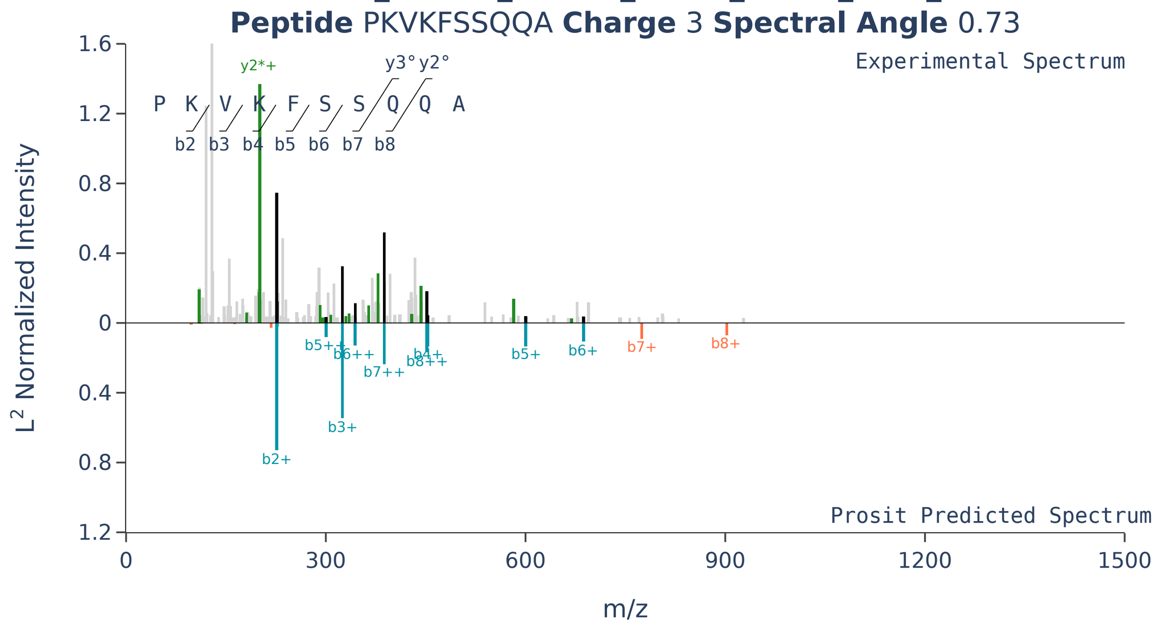


**Source** HRoetschke_271222_090123_HFGoe_LEDGF_b1R2_24h **Scan** 10451
**Peptide** PKDIFPYSENKEKYGKPNK **Charge** 5 **Spectral Angle** 0.81

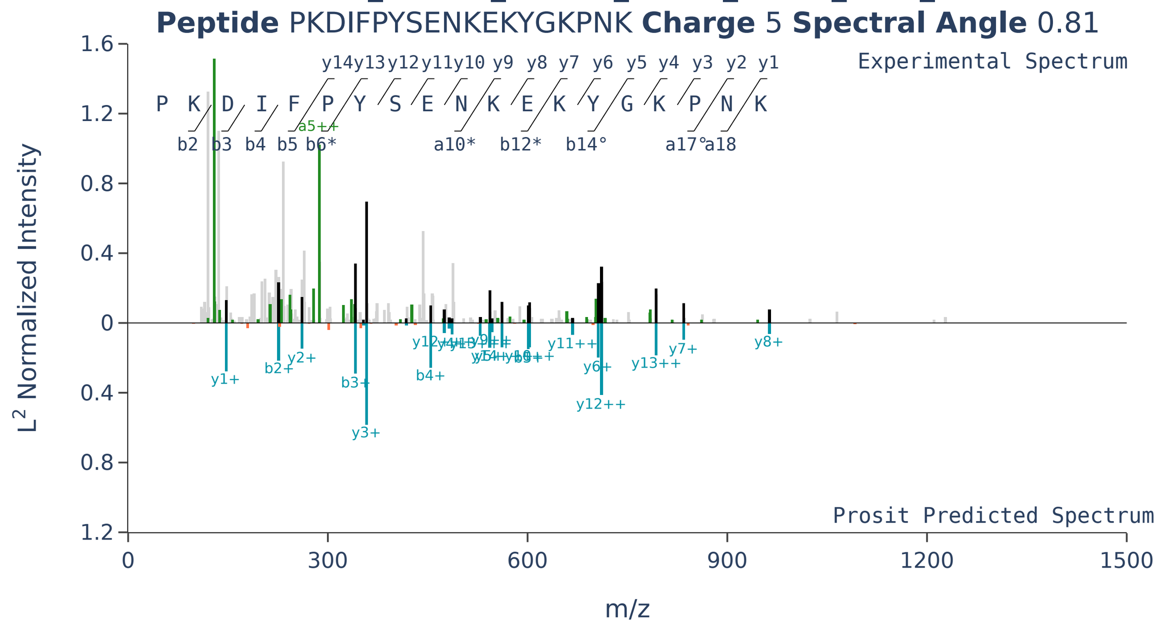


**Source** HRoetschke_271222_090123_HFGoe_LEDGF_b2R1_4h **Scan** 15439
**Peptide** HTEMITTLKKIRRF **Charge** 4 **Spectral Angle** 0.82

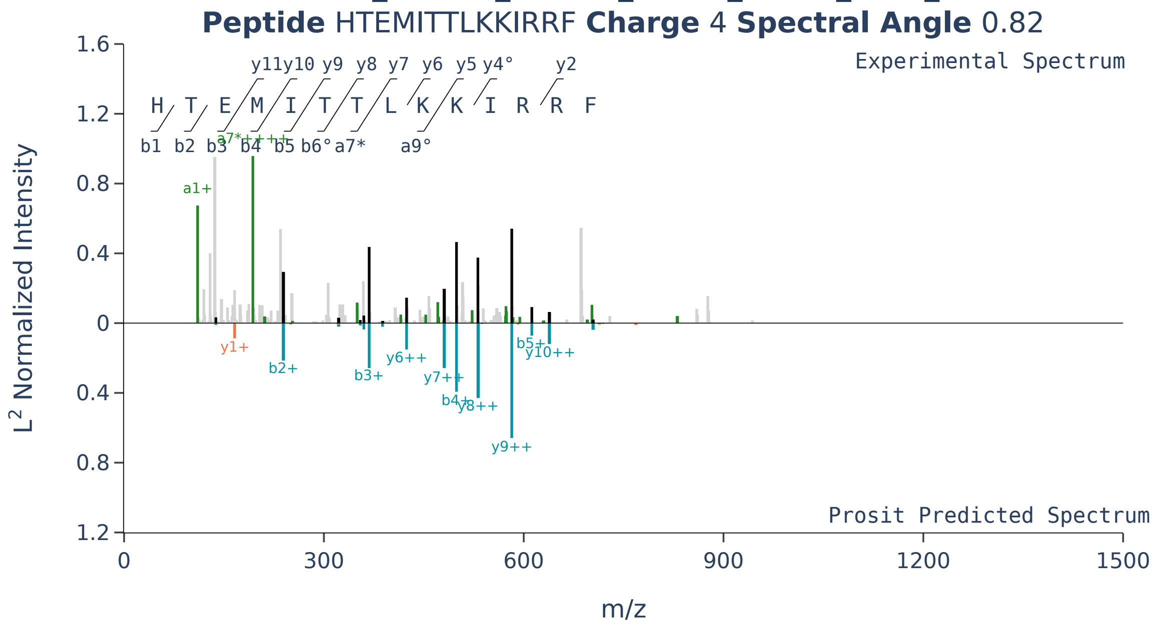


**Source** HRoetschke_271222_090123_HFGoe_LEDGF_b1R2_4h **Scan** 18155
**Peptide** EMITTLKKIRRFKVSQV **Charge** 3 **Spectral Angle** 0.86

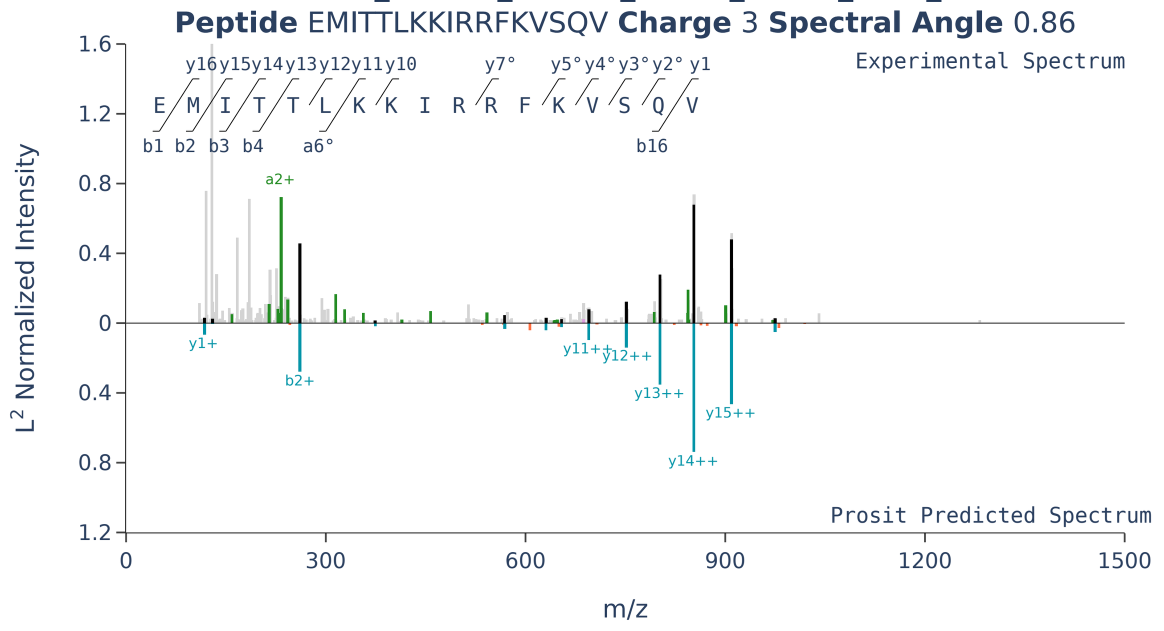


**Source** HRoetschke_271222_090123_HFGoe_LEDGF_b2R2_2h **Scan** 22337
**Peptide** KSTMLYNKFKNMF **Charge** 4 **Spectral Angle** 0.84

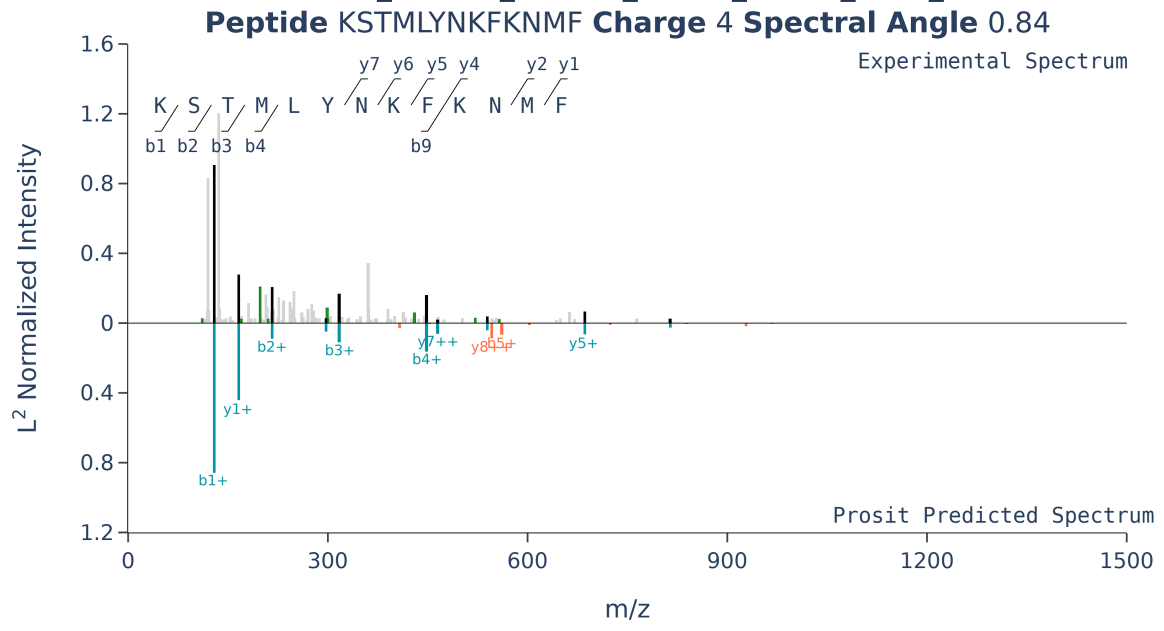


**Source** HRoetschke_271222_090123_HFGoe_LEDGF_b1R2_1h **Scan** 7565
**Peptide** LYNKFKN **Charge** 2 **Spectral Angle** 0.73

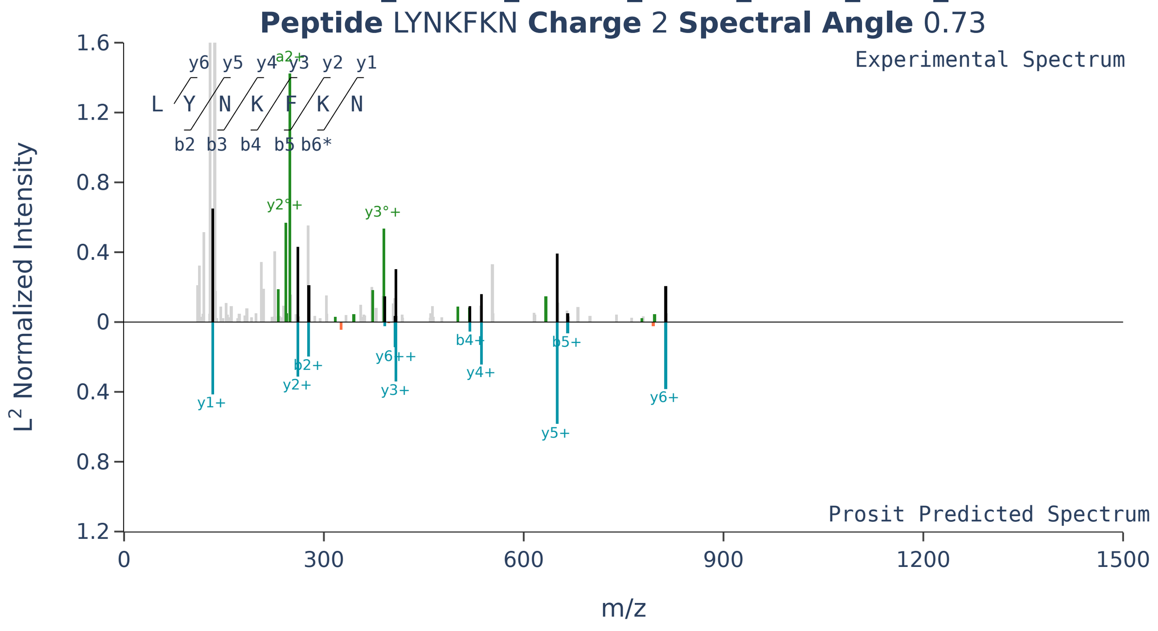


**Source** HRoetschke_271222_090123_HFGoe_LEDGF_b1R1_24h **Scan** 6099
**Peptide** LEKEQTGSKTLNGGSDAQDGNQPQH **Charge** 4 **Spectral Angle** 0.83

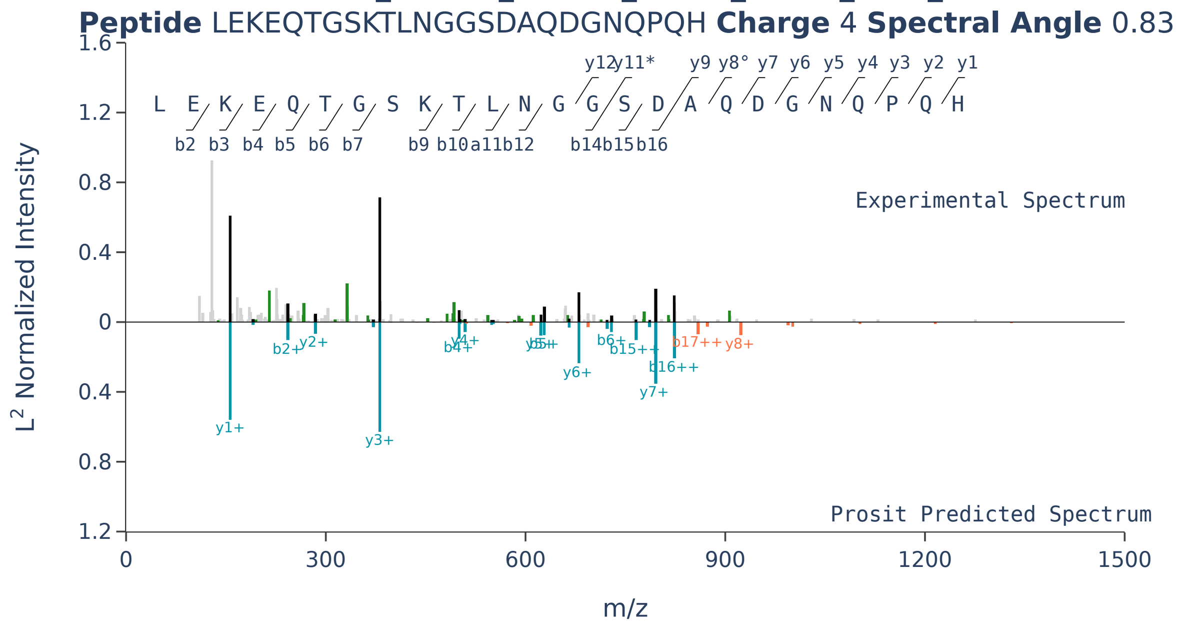


**Source** HRoetschke_271222_090123_HFGoe_LEDGF_b1R1_2h **Scan** 26796
**Peptide** KSTMLYNKFKNMFLVGEGD **Charge** 3 **Spectral Angle** 0.8

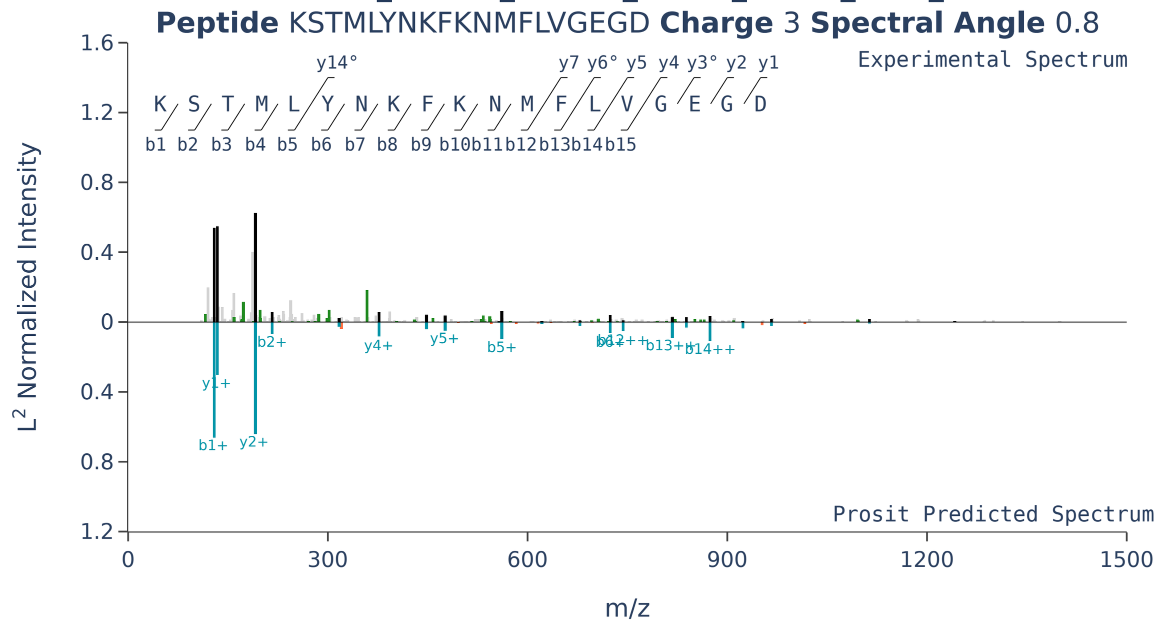


**Source** HRoetschke_271222_090123_HFGoe_LEDGF_b1R1_2h **Scan** 30457
**Peptide** STMLYNKFKNMFLVGE **Charge** 3 **Spectral Angle** 0.92

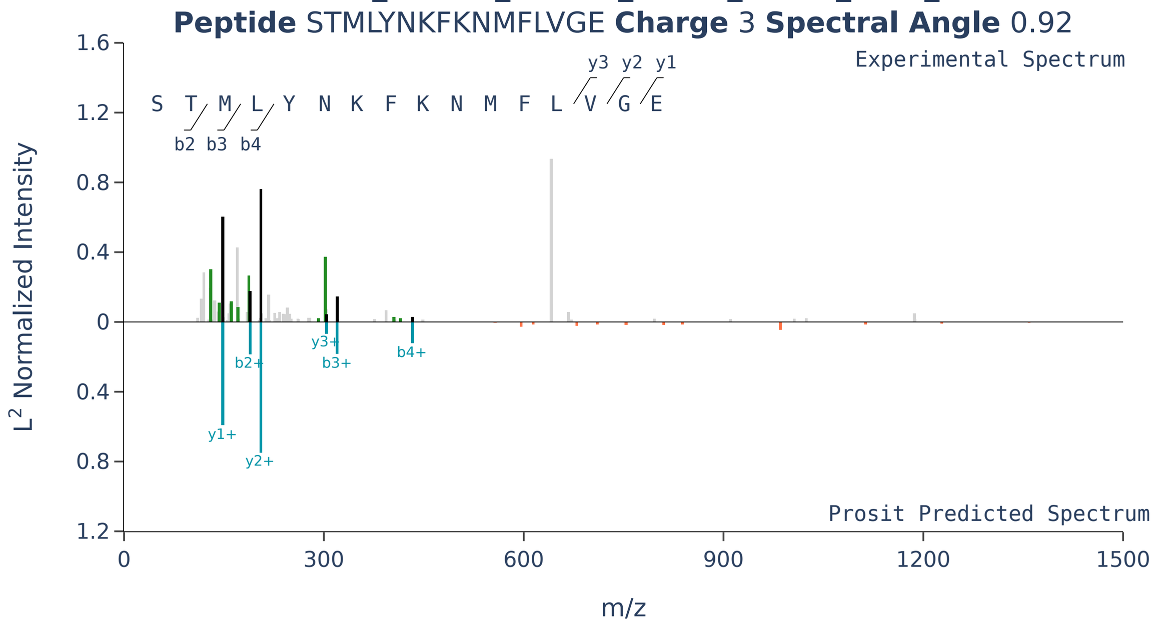


**Source** HRoetschke_271222_090123_HFGoe_LEDGF_b1R1_24h **Scan** 5296
**Peptide** SEEEGDDQEGEKKRKGGRNFQTA **Charge** 3 **Spectral Angle** 0.76

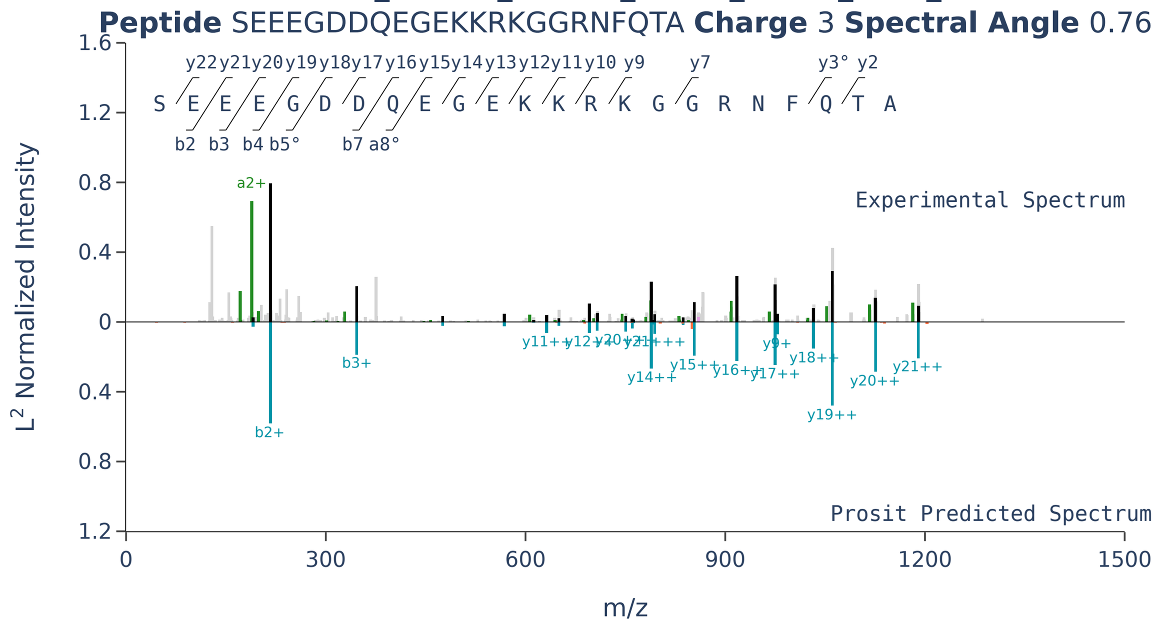


**Source** HRoetschke_271222_090123_HFGoe_LEDGF_b1R2_1h **Scan** 8964
**Peptide** SSDVEVEEKETSVSKE **Charge** 3 **Spectral Angle** 0.77

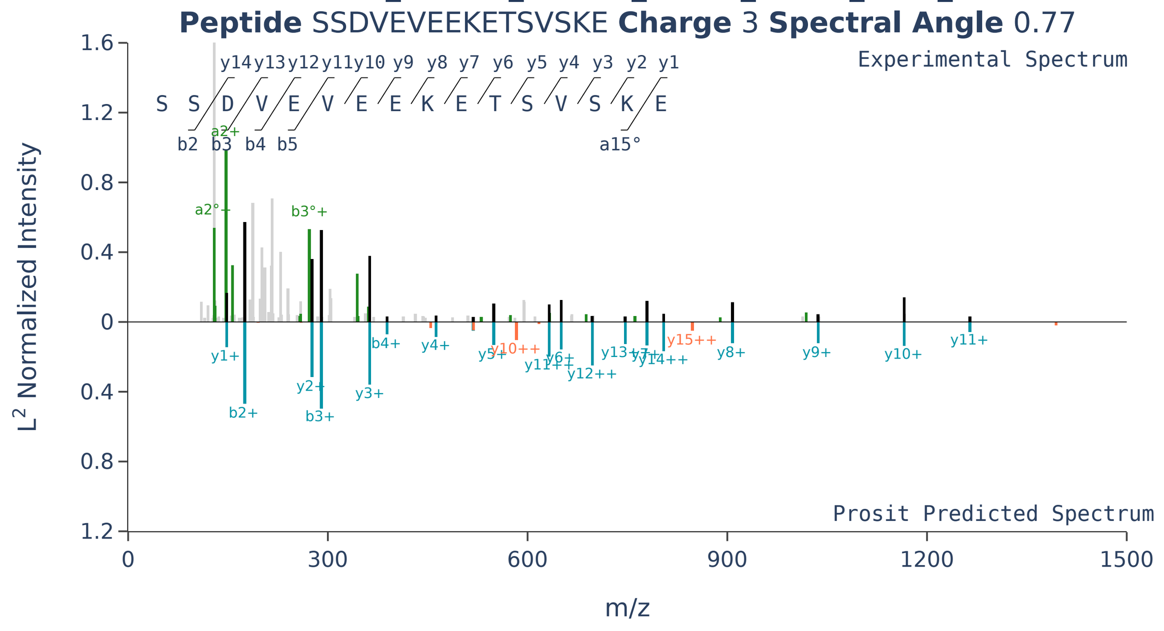

**Source** HRoetschke_271222_090123_HFGoe_LEDGF_b2R2_4h **Scan** 5883
**Peptide** TLNGGSDAQDGNQPQH **Charge** 2 **Spectral Angle** 0.72

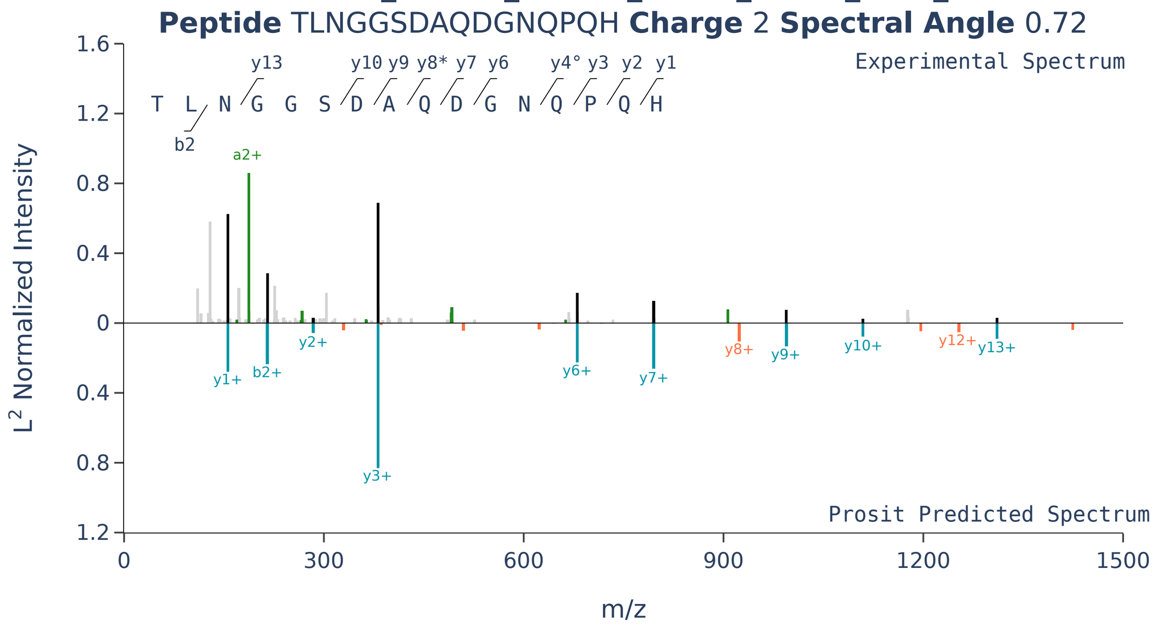


**Source** HRoetschke_271222_090123_HFGoe_LEDGF_b2R1_24h **Scan** 31890
**Peptide** AFLGPKDIFPY **Charge** 2 **Spectral Angle** 0.89

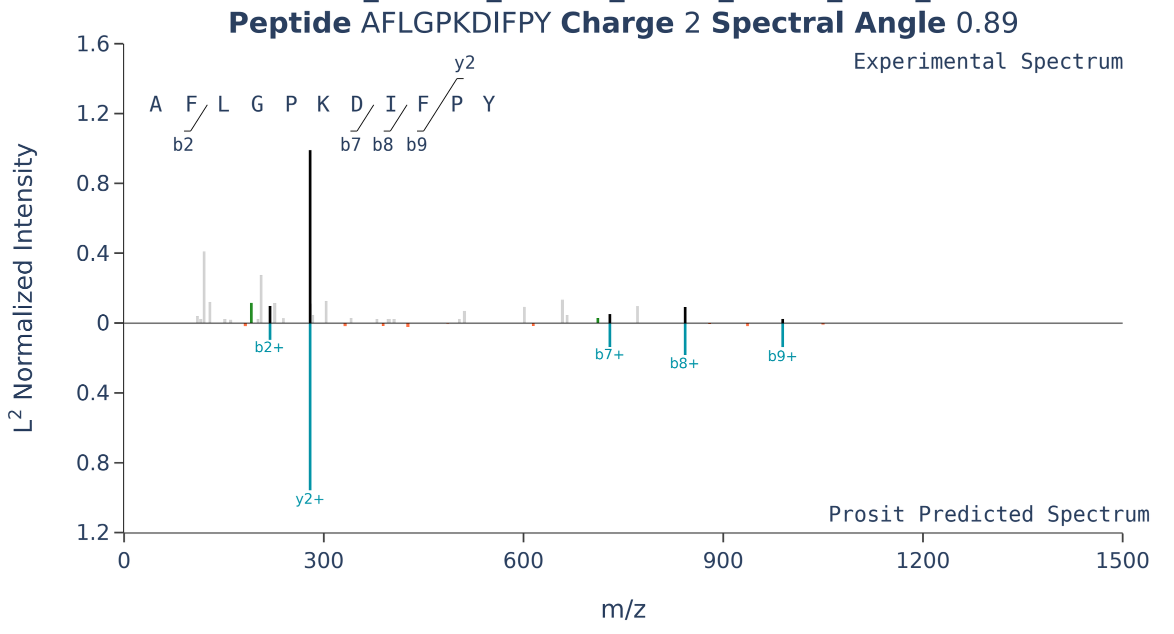


**Source** HRoetschke_271222_090123_HFGoe_LEDGF_b1R1_4h **Scan** 20453
**Peptide** KGFNEGLWEIDNNPKVKFSSQQAATKQSN **Charge** 4 **Spectral Angle** 0.86

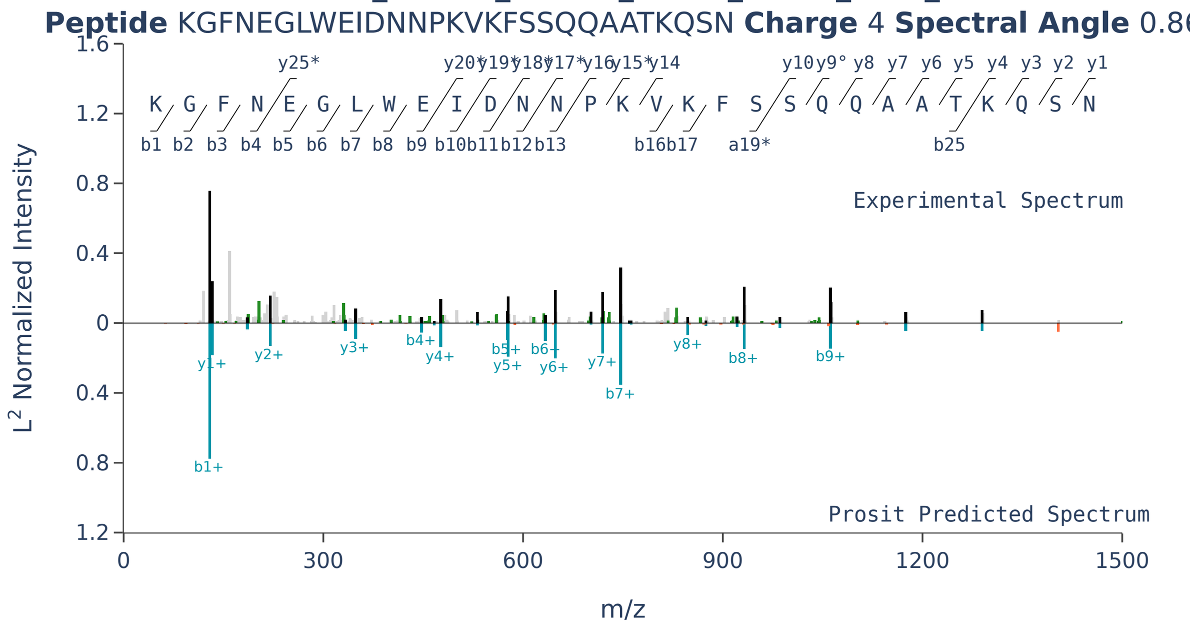


**Source** HRoetschke_271222_090123_HFGoe_LEDGF_b1R2_4h **Scan** 20306
**Peptide** KPGDLIFAKMKGYPHWPARVD **Charge** 5 **Spectral Angle** 0.87

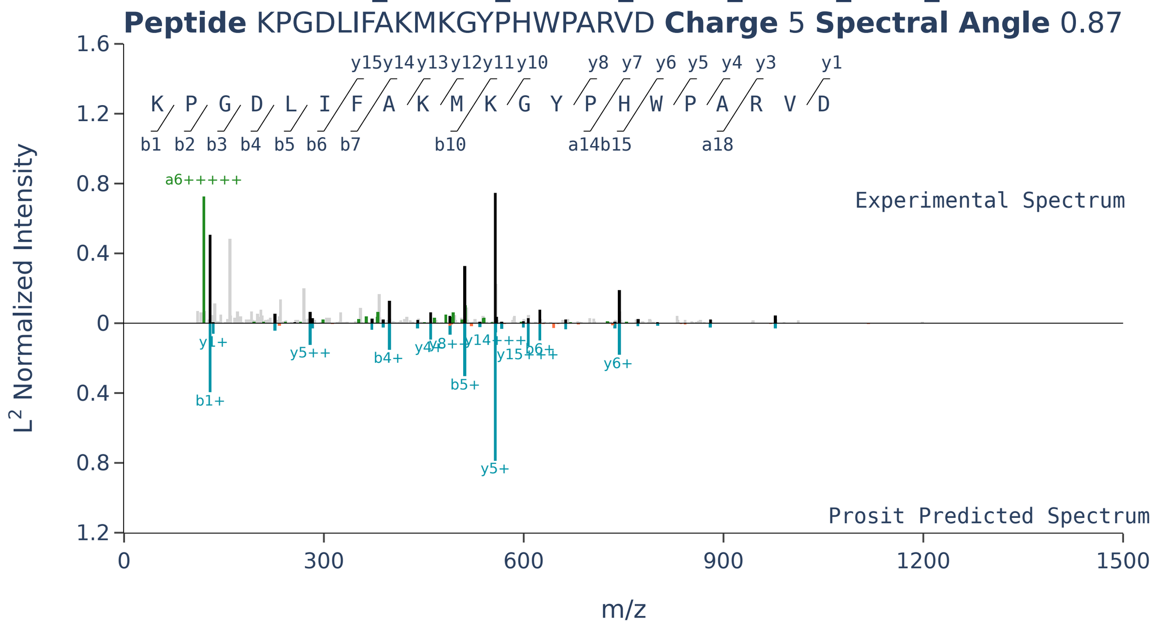


**Source** HRoetschke_271222_090123_HFGoe_LEDGF_b1R1_4h **Scan** 17236
**Peptide** AFLGPKDIFPYSENKEKYGKPNKRKGF **Charge** 5 **Spectral Angle** 0.8

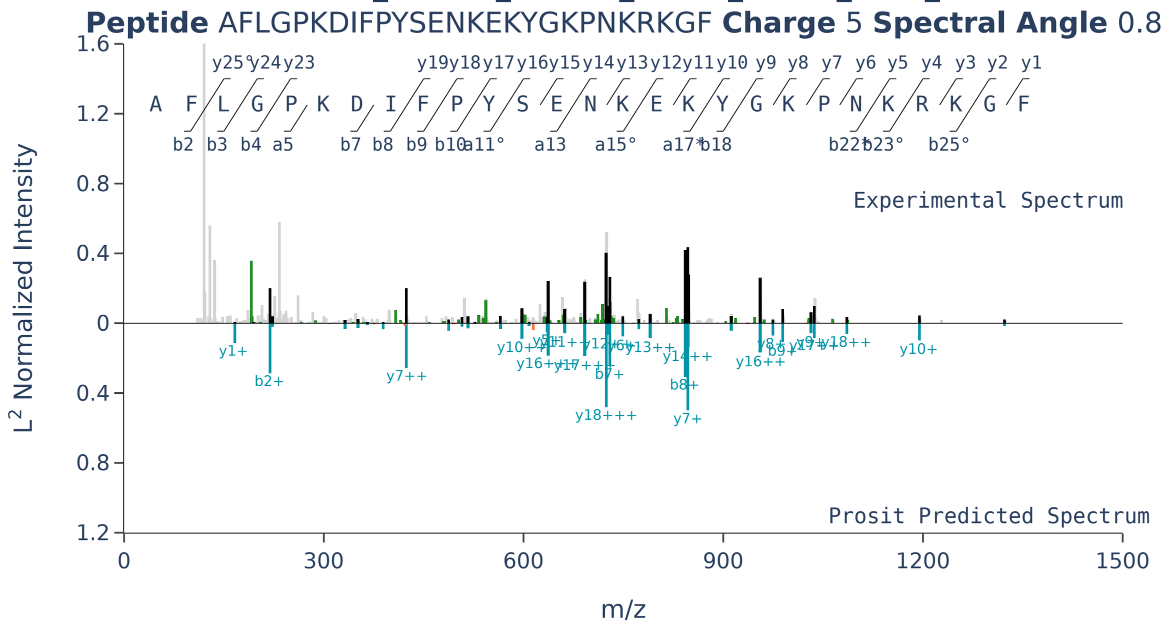


**Source** HRoetschke_271222_090123_HFGoe_LEDGF_b1R1_4h **Scan** 9589
**Peptide** EIDNNPKVKFSSQQAATKQSNASSD **Charge** 3 **Spectral Angle** 0.87

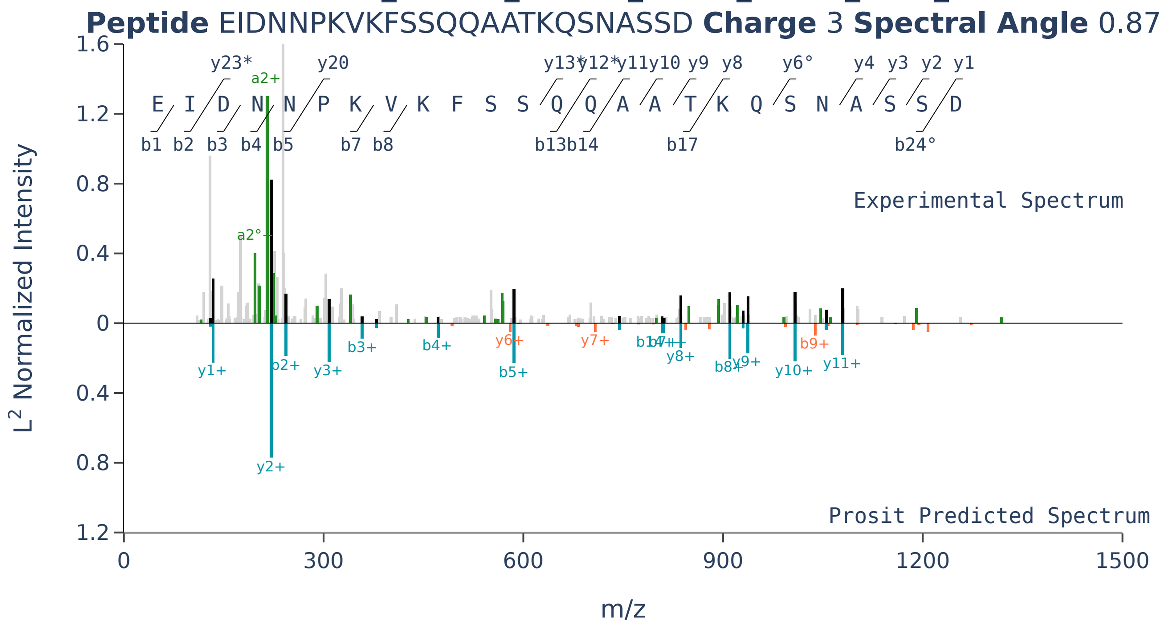


**Source** HRoetschke_271222_090123_HFGoe_LEDGF_b2R1_1h **Scan** 30840
**Peptide** DEVPDGAVKPPTNKLPIFFFGTHE **Charge** 3 **Spectral Angle** 0.91

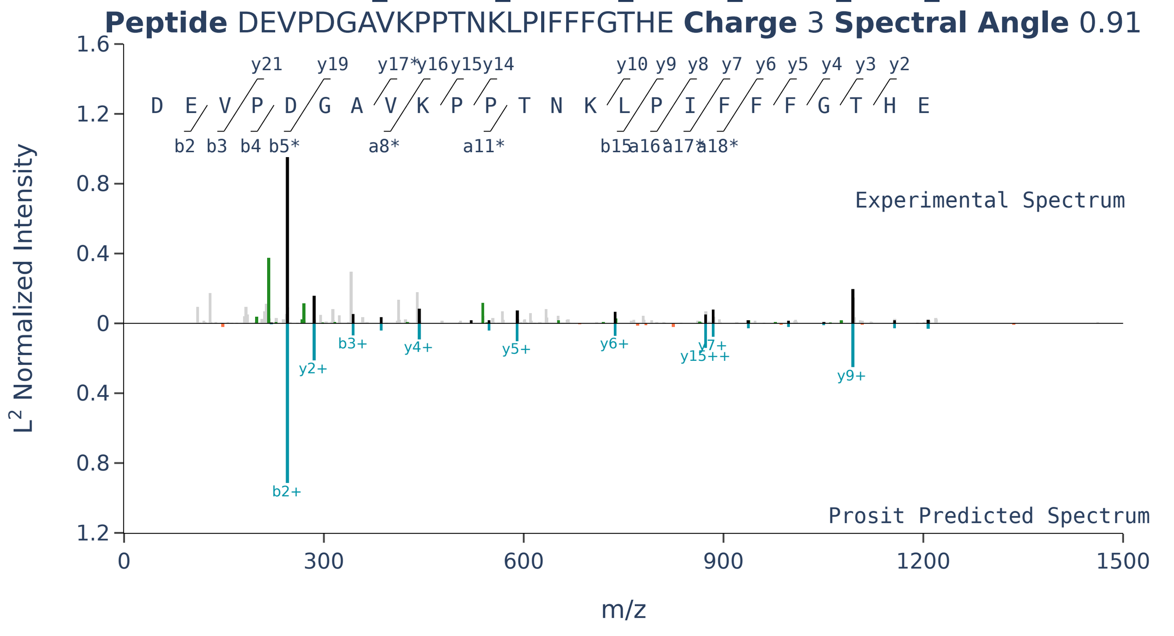


**Source** HRoetschke_271222_090123_HFGoe_LEDGF_b1R1_24h **Scan** 10970
**Peptide** KAEKQVETEEAGVVTTA **Charge** 2 **Spectral Angle** 0.76

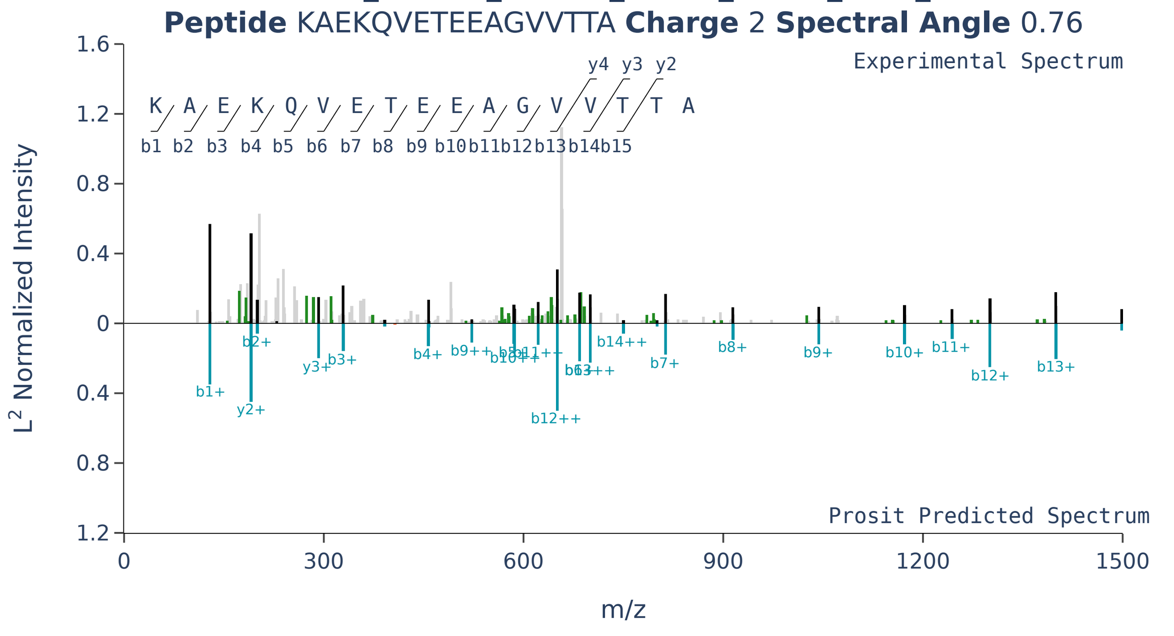


**Source** HRoetschke_271222_090123_HFGoe_LEDGF_b1R2_4h **Scan** 22349
**Peptide** QVIMEKSTMLYNKFKNM **Charge** 3 **Spectral Angle** 0.79

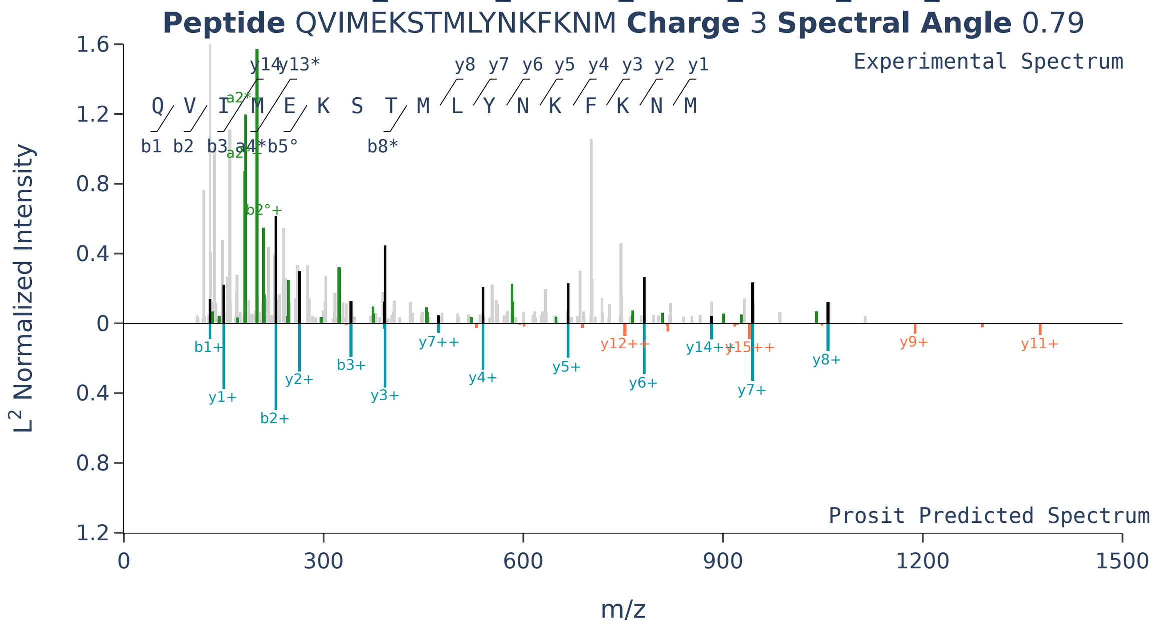


**Source** HRoetschke_271222_090123_HFGoe_LEDGF_b1R1_4h **Scan** 8350
**Peptide** EKKRETSMDSRLQRI **Charge** 4 **Spectral Angle** 0.83

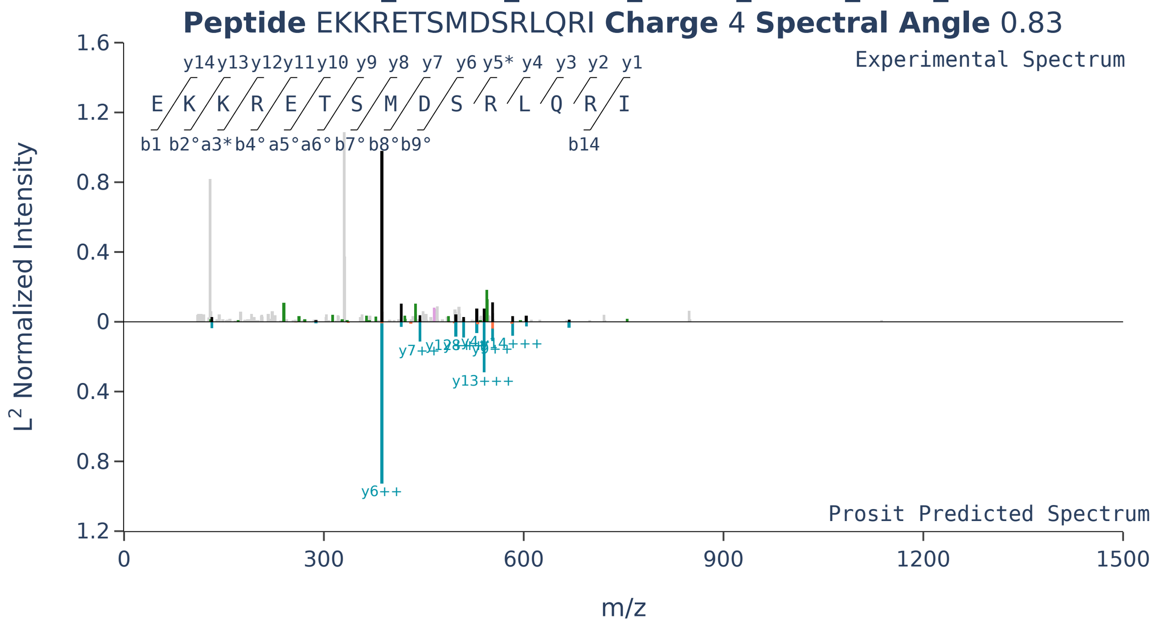


**Source** HRoetschke_271222_090123_HFGoe_LEDGF_b2R1_2h **Scan** 23828
**Peptide** VSQVIMEKSTMLY **Charge** 2 **Spectral Angle** 0.84

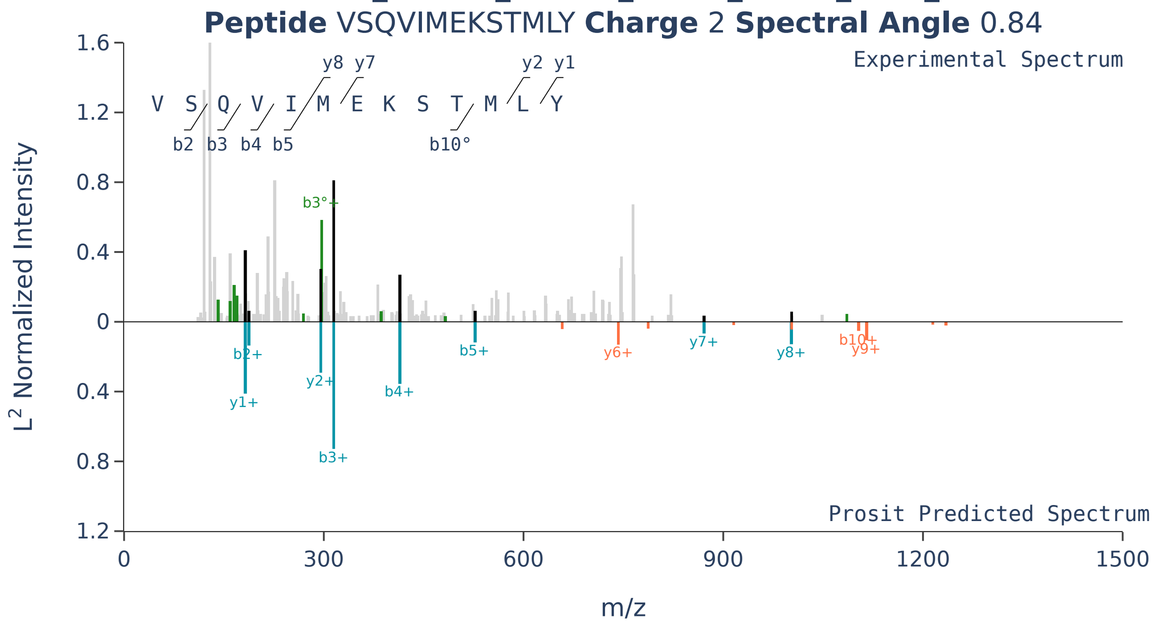


**Source** HRoetschke_271222_090123_HFGoe_LEDGF_b2R1_24h **Scan** 24245
**Peptide** TAFLGPKDIFPYSENKEKYG **Charge** 3 **Spectral Angle** 0.74

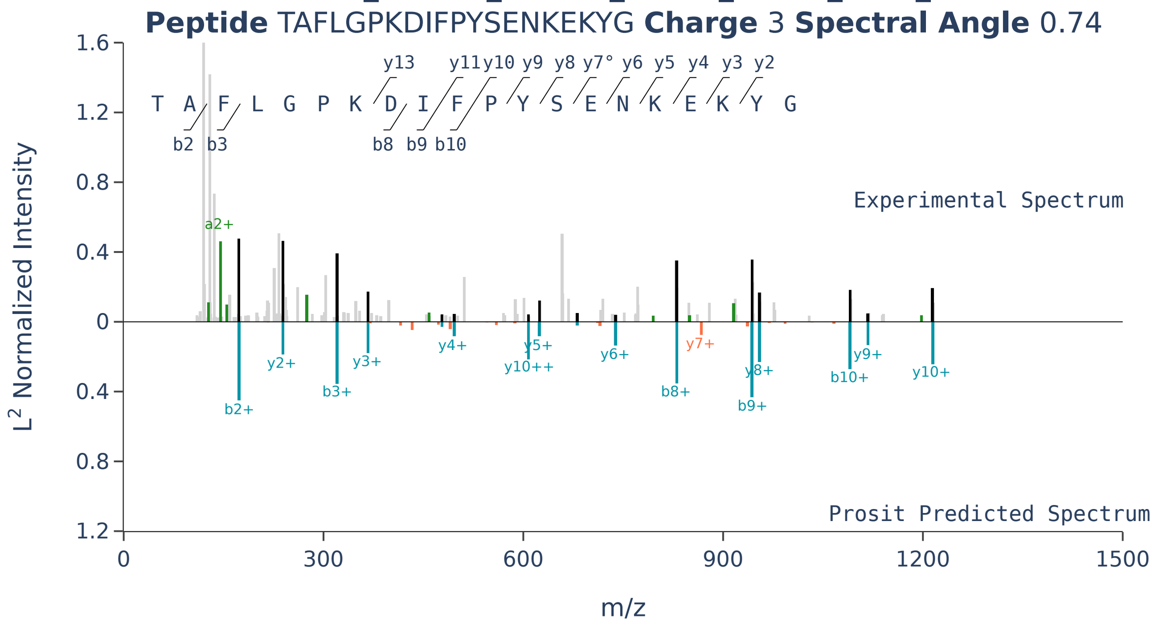


**Source** HRoetschke_271222_090123_HFGoe_LEDGF_b1R1_4h **Scan** 32973
**Peptide** DFKPGDLIF **Charge** 2 **Spectral Angle** 0.78

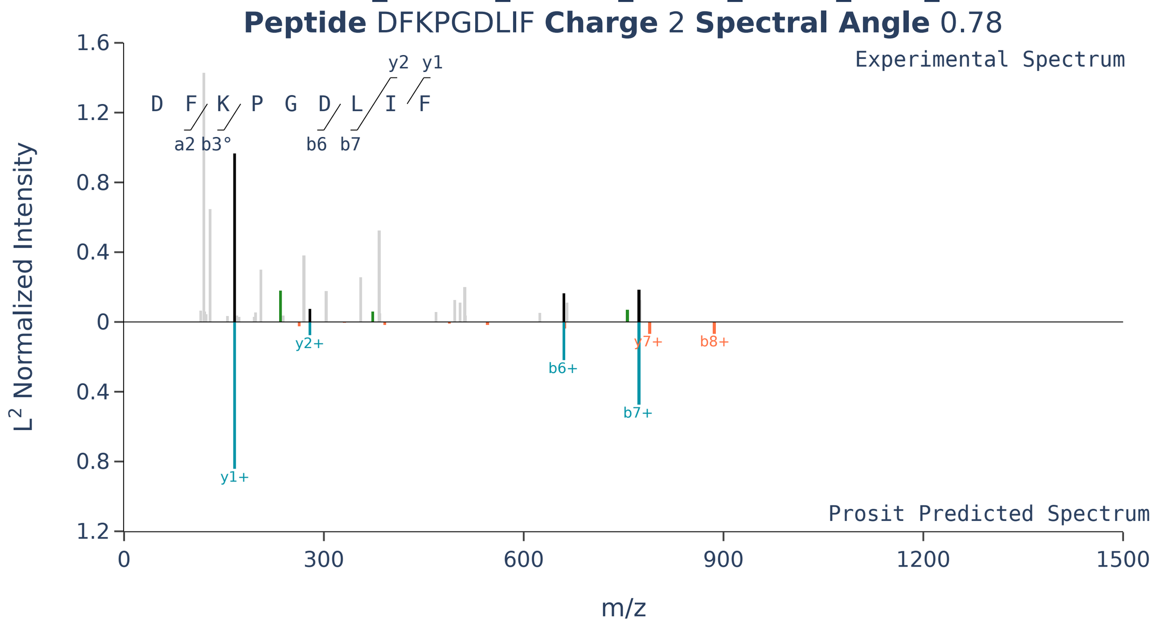


**Source** HRoetschke_271222_090123_HFGoe_LEDGF_b2R2_24h **Scan** 6968
**Peptide** FGTHETA **Charge** 2 **Spectral Angle** 0.75

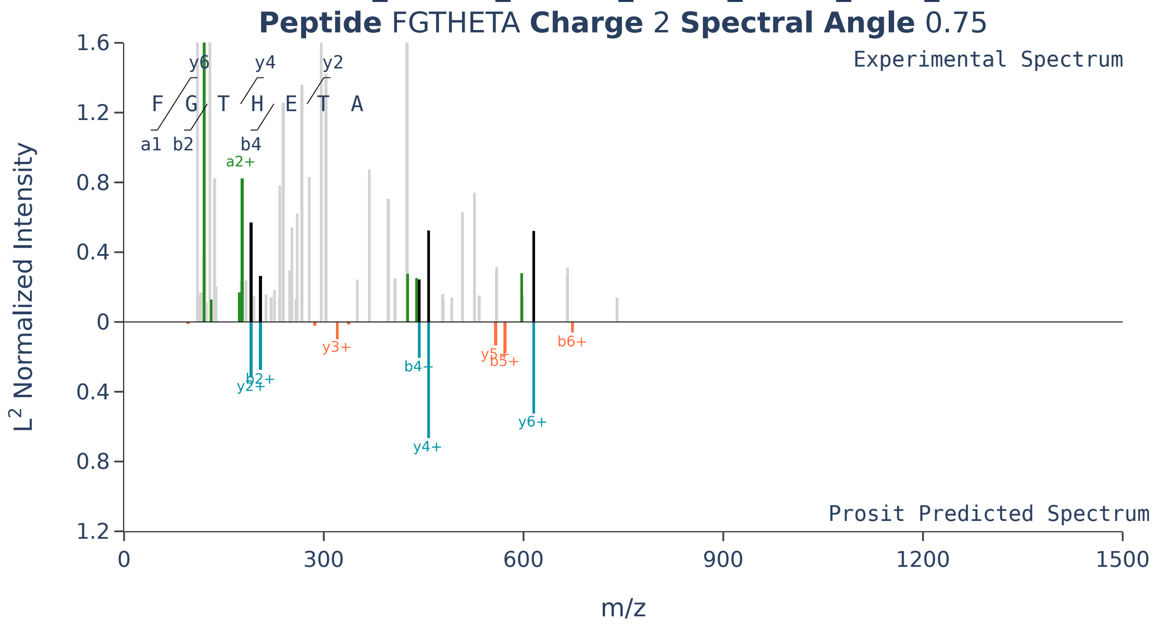


**Source** HRoetschke_271222_090123_HFGoe_LEDGF_b1R2_24h **Scan** 5853
**Peptide** EQTGSKTLNGGSDAQDGNQPQH **Charge** 3 **Spectral Angle** 0.77

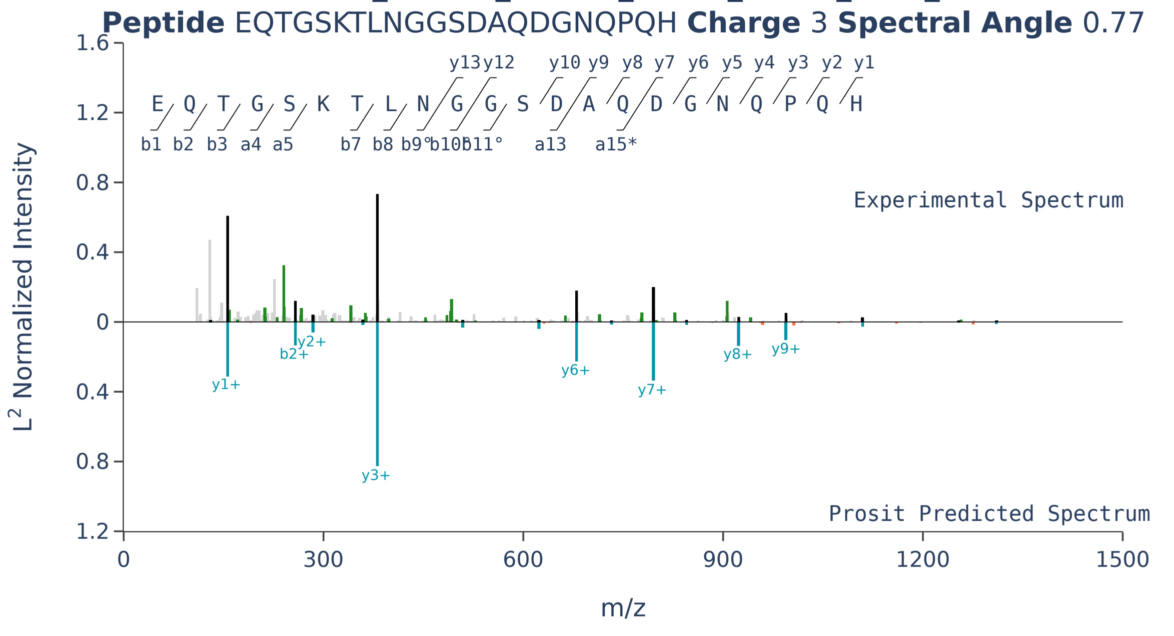

**Source** HRoetschke_271222_090123_HFGoe_LEDGF_b2R2_4h **Scan** 5528
**Peptide** TGSKTLNGGSDAQDGNQPQH **Charge** 3 **Spectral Angle** 0.73

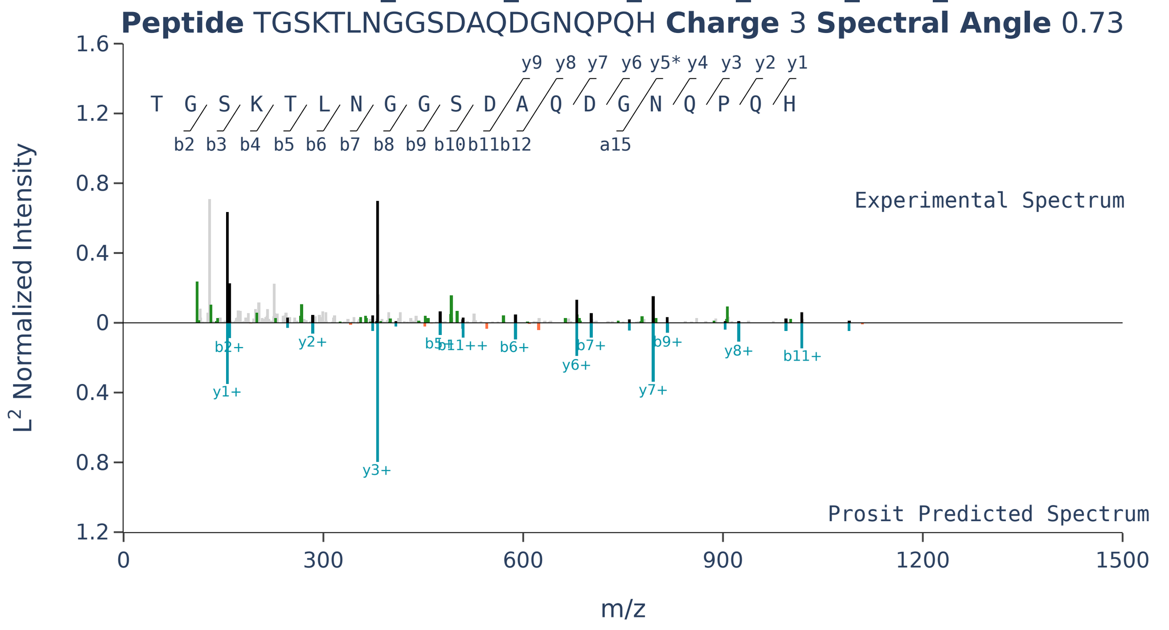


**Source** HRoetschke_271222_090123_HFGoe_LEDGF_b1R1_1h **Scan** 10410
**Peptide** TEVKIPKPRGRPKMV **Charge** 4 **Spectral Angle** 0.81

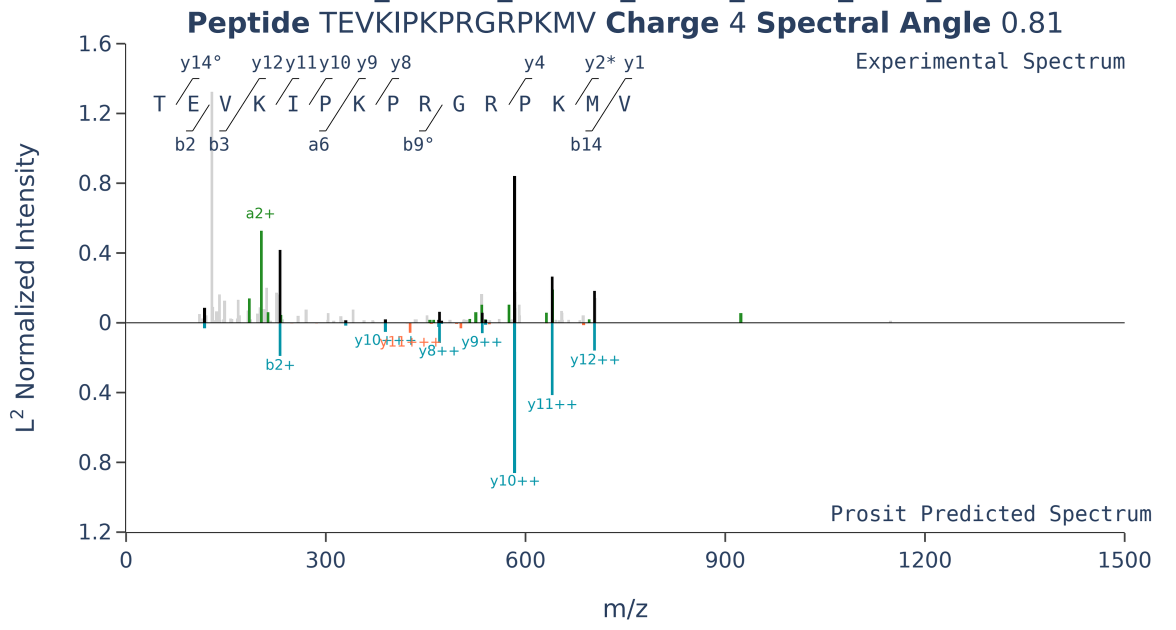


**Source** HRoetschke_271222_090123_HFGoe_LEDGF_b1R1_2h **Scan** 25638
**Peptide** FGTHETAFLGPKDIFPYSENKE **Charge** 4 **Spectral Angle** 0.75

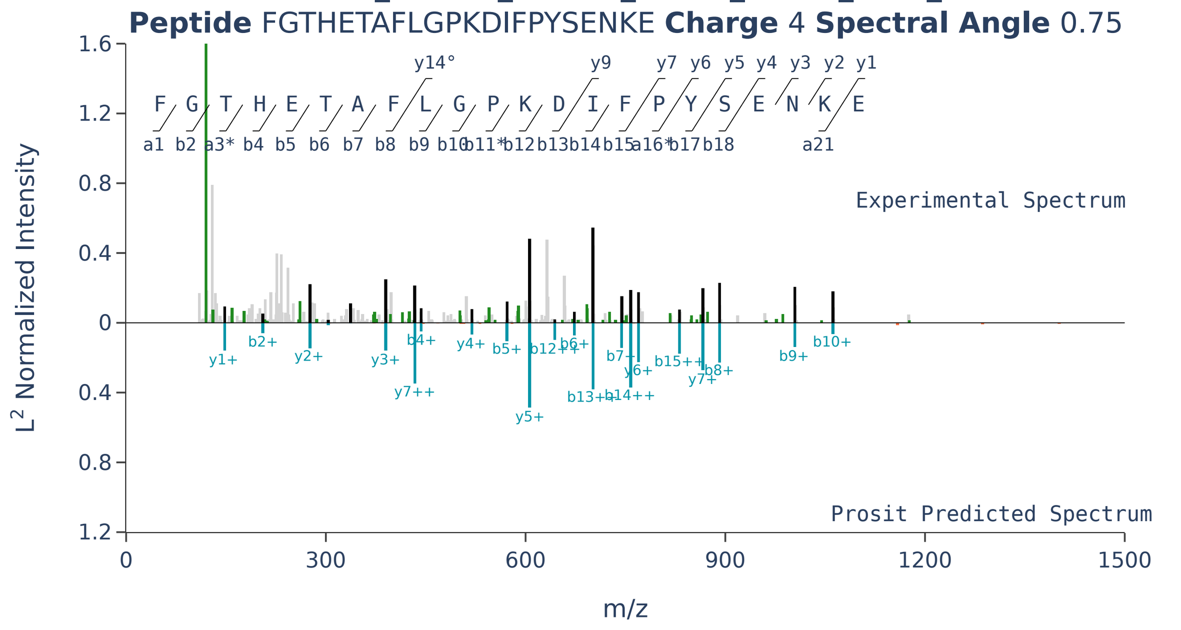


**Source** HRoetschke_271222_090123_HFGoe_LEDGF_b1R2_4h **Scan** 8406
**Peptide** SSQQAATKQSNASSDVEVE **Charge** 2 **Spectral Angle** 0.76

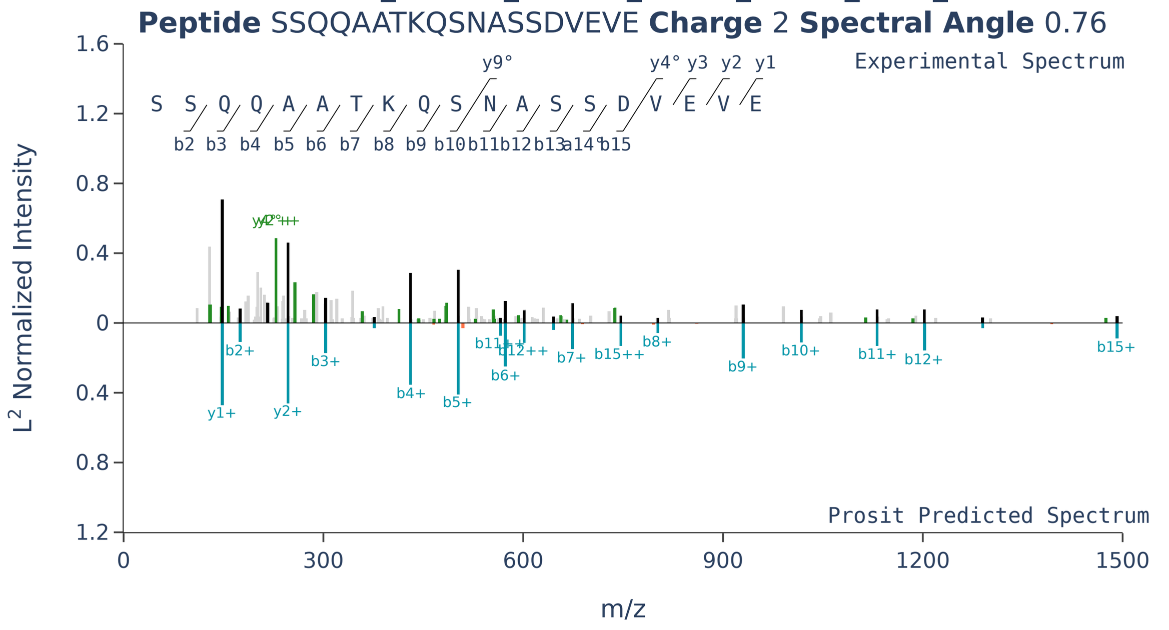


**Source** HRoetschke_271222_090123_HFGoe_LEDGF_b1R2_4h **Scan** 16830
**Peptide** FLGPKDIFPYSENKEKYGKPNKRKGFNE **Charge** 6 **Spectral Angle** 0.74

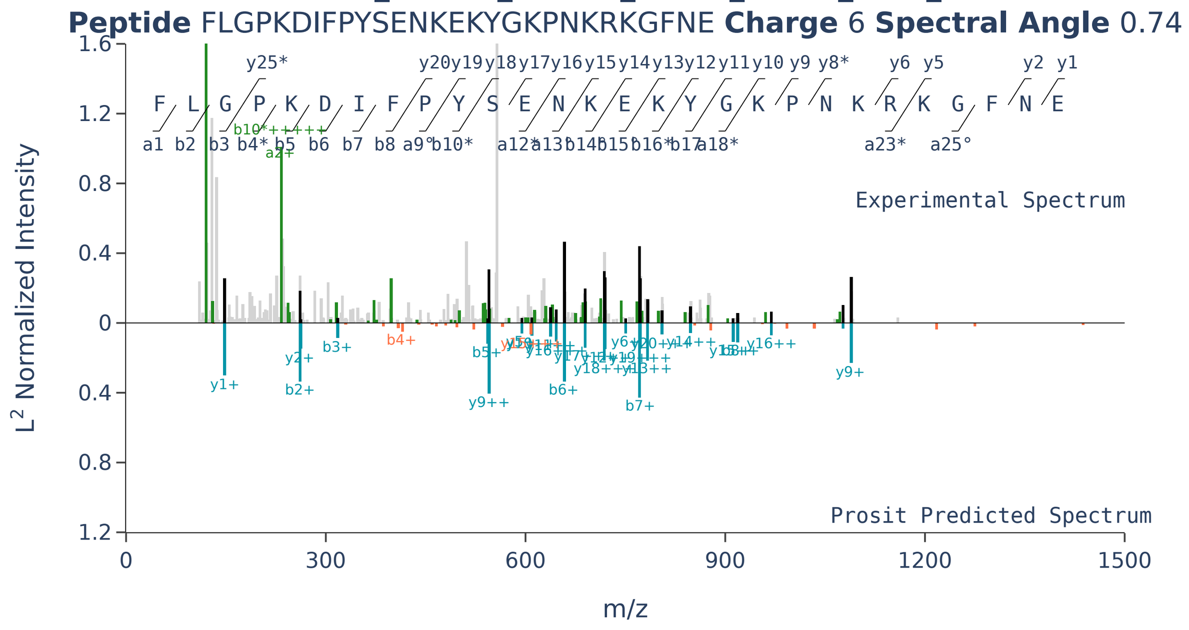


**Source** HRoetschke_271222_090123_HFGoe_LEDGF_b1R2_4h **Scan** 26786
**Peptide** GPGMTRDFKPGDLIF **Charge** 3 **Spectral Angle** 0.78

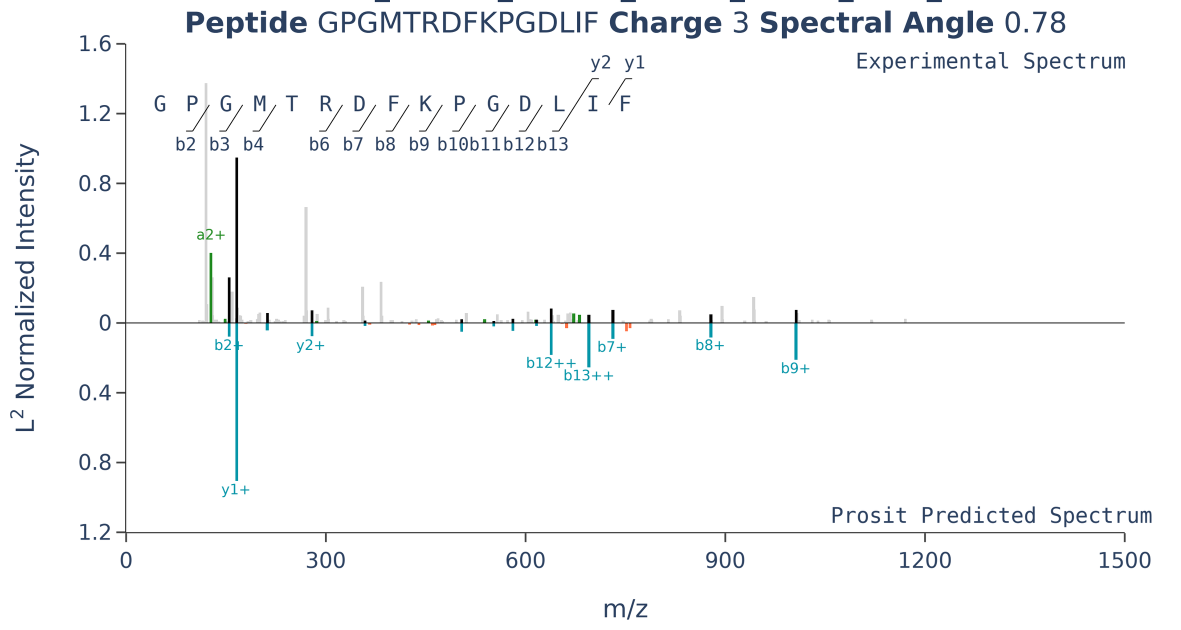


**Source** HRoetschke_271222_090123_HFGoe_LEDGF_b1R1_4h **Scan** 9021
**Peptide** DSKDNHEASTKKKPSSEERETEISL **Charge** 3 **Spectral Angle** 0.82

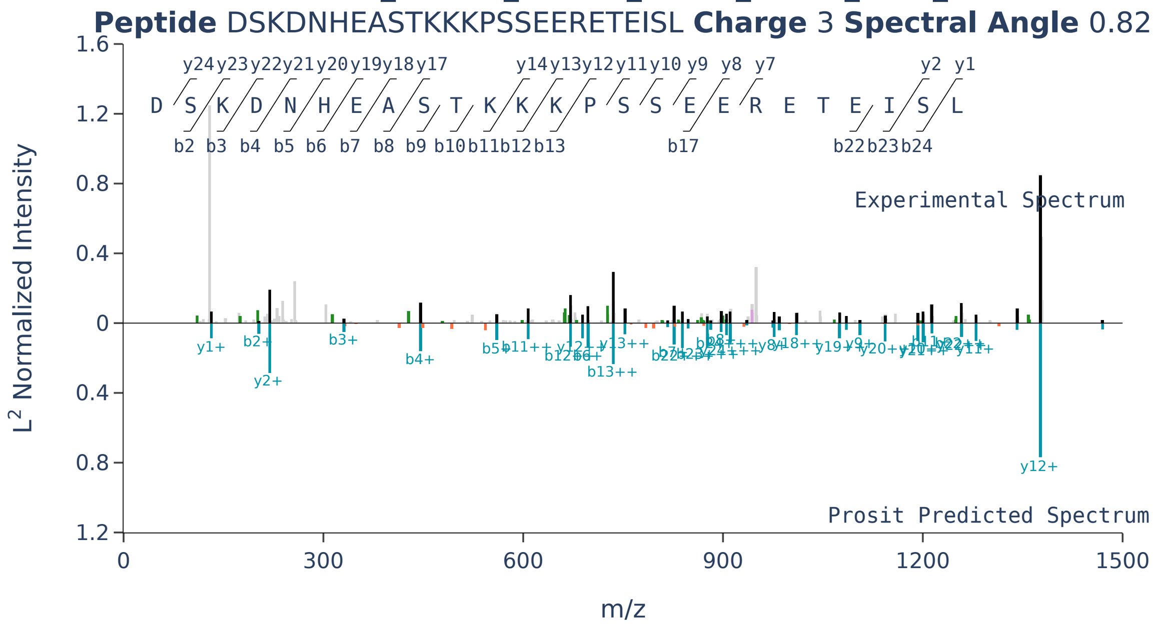


**Source** HRoetschke_271222_090123_HFGoe_LEDGF_b2R1_2h **Scan** 23031
**Peptide** EGLWEIDNNPKVKFSSQQA **Charge** 3 **Spectral Angle** 0.85

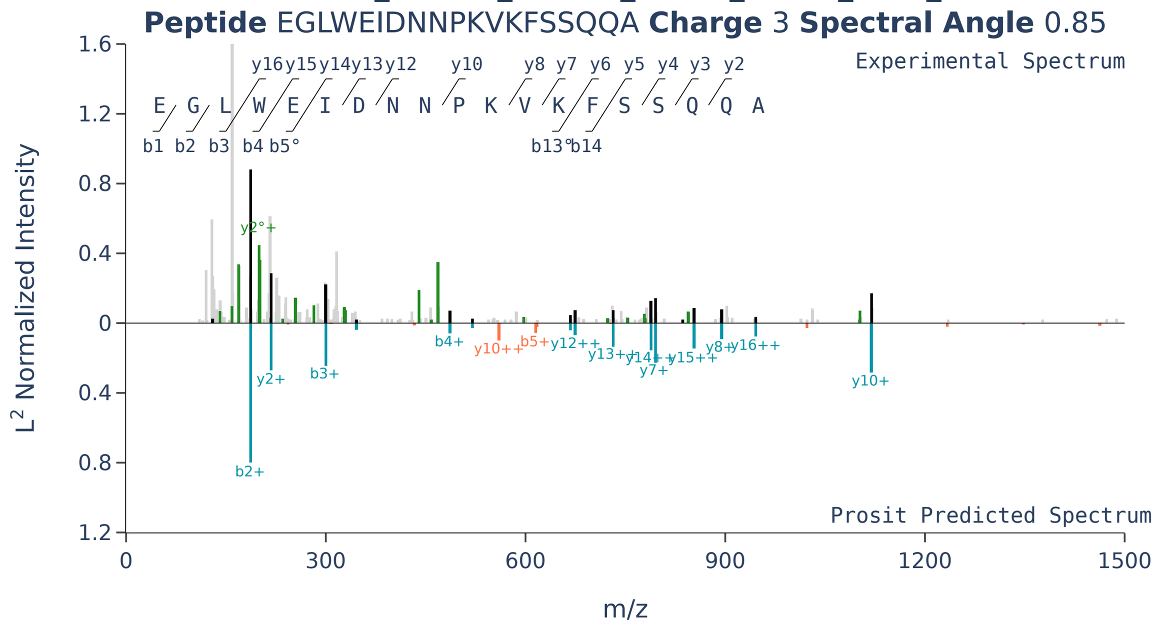


**Source** HRoetschke_271222_090123_HFGoe_LEDGF_b2R1_24h **Scan** 13520
**Peptide** DTDHEEKASNEDVTKAVDITTPKAAR **Charge** 5 **Spectral Angle** 0.86

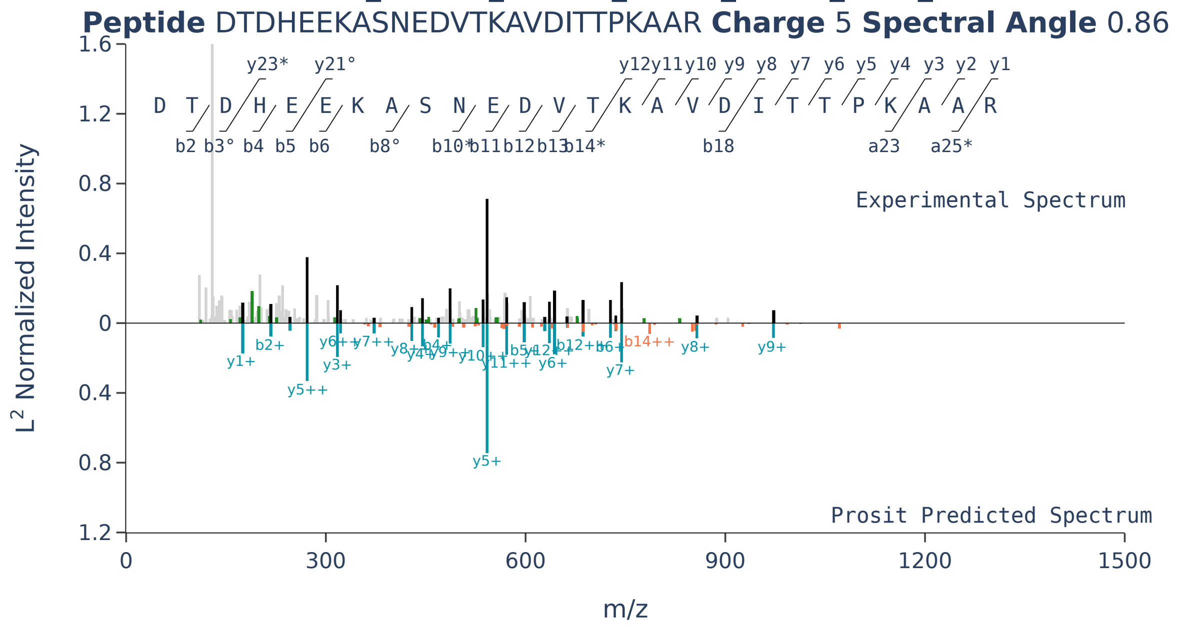


**Source** HRoetschke_271222_090123_HFGoe_LEDGF_b1R1_4h **Scan** 23333
**Peptide** KFKNMFLVG **Charge** 3 **Spectral Angle** 0.74

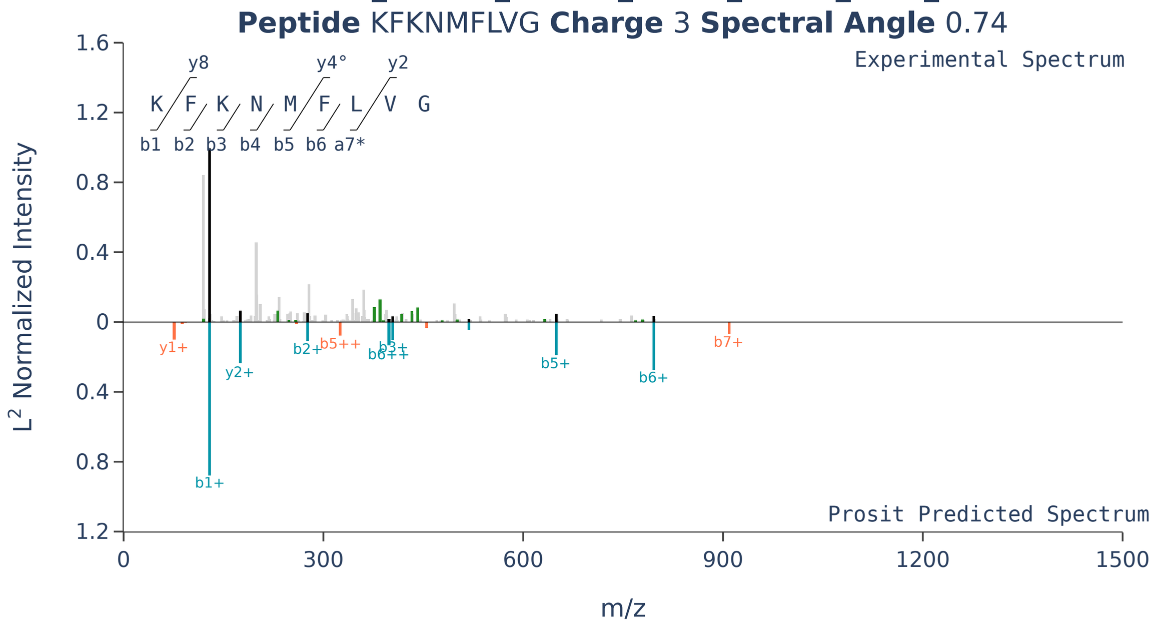


**Source** HRoetschke_271222_090123_HFGoe_LEDGF_b2R1_24h **Scan** 21442
**Peptide** SMDSRLQRIHAEIKNSLKID **Charge** 3 **Spectral Angle** 0.73

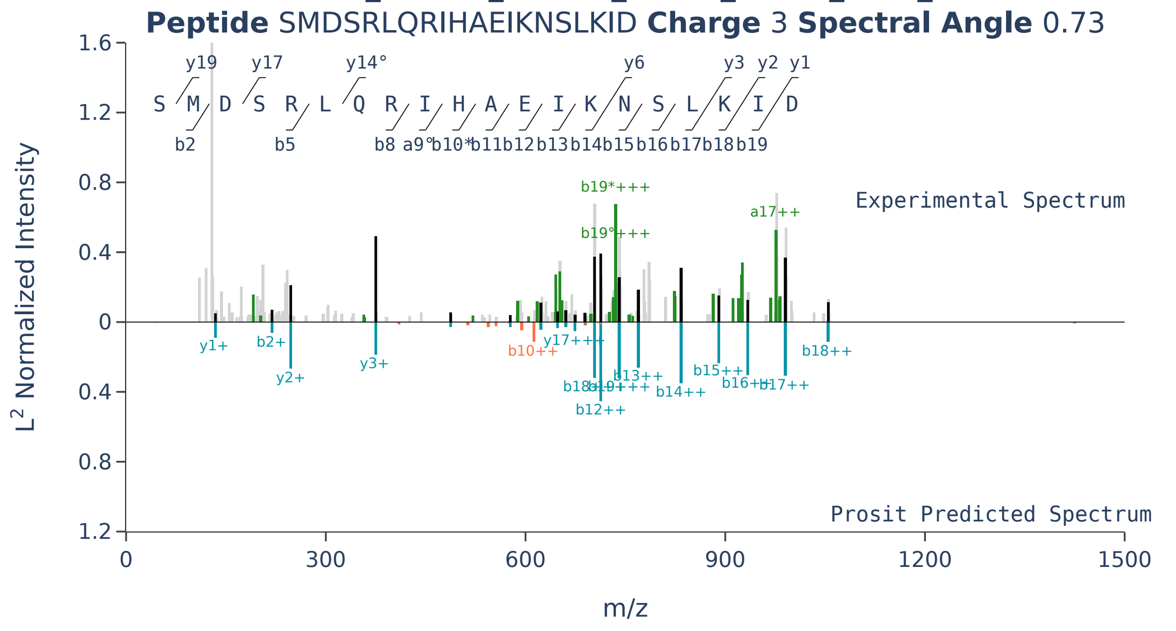


**Source** HRoetschke_271222_090123_HFGoe_LEDGF_b1R2_24h **Scan** 10325
**Peptide** RETSMDSRLQRIHA **Charge** 3 **Spectral Angle** 0.91

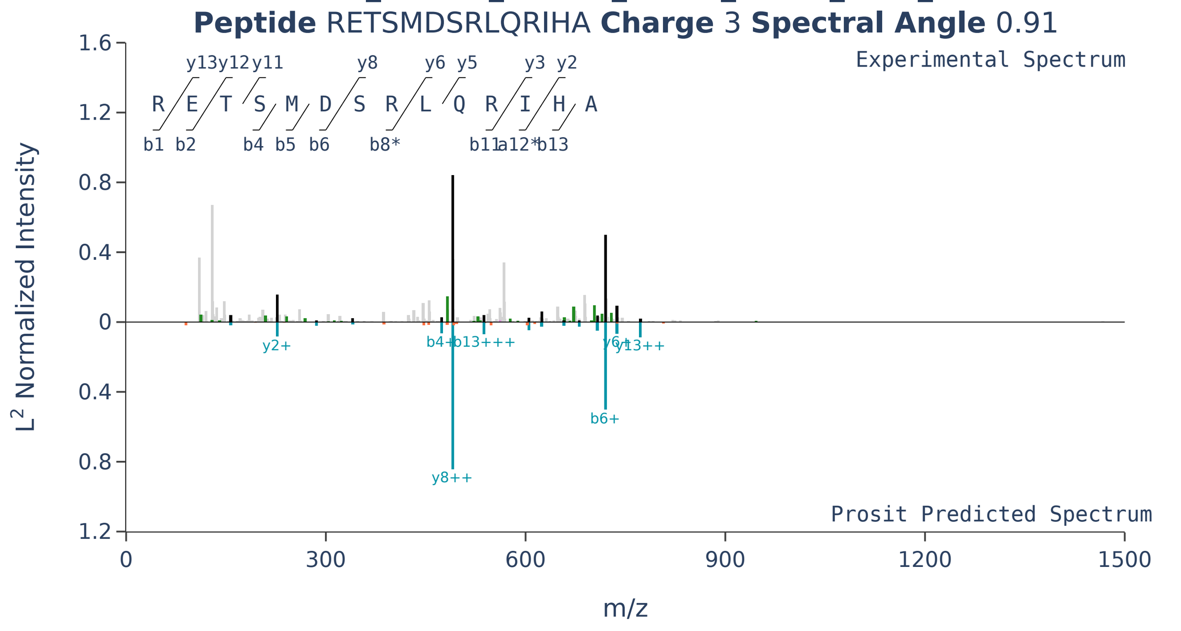


**Source** HRoetschke_271222_090123_HFGoe_LEDGF_b2R1_24h **Scan** 16171
**Peptide** AGVVTTATASVNLKVSPKRG **Charge** 4 **Spectral Angle** 0.71

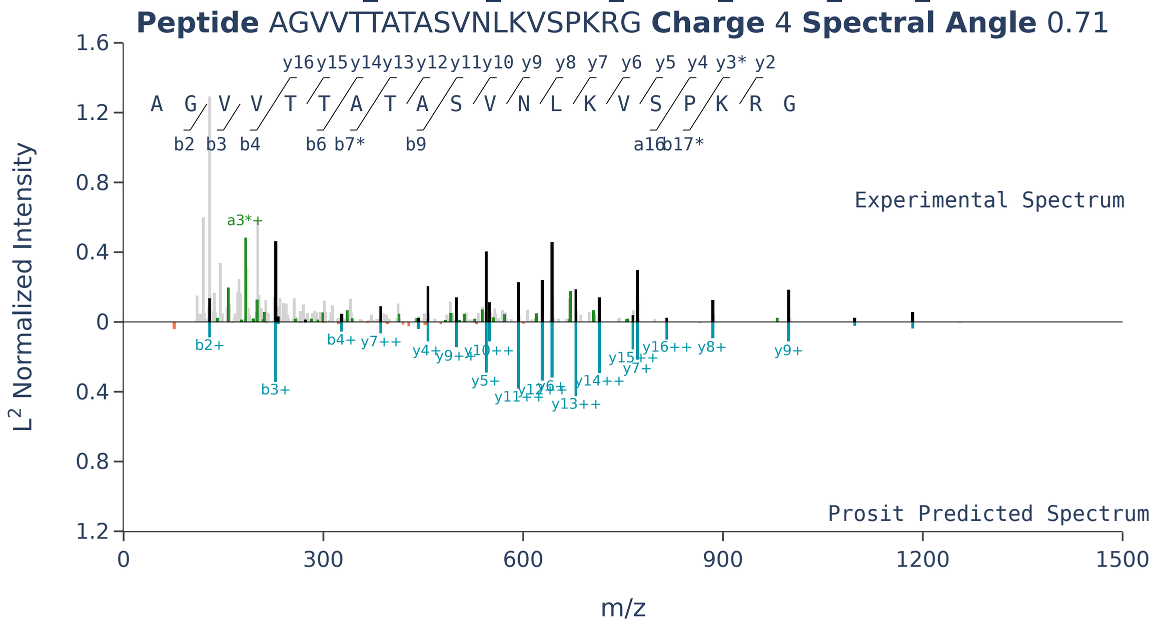


**Source** HRoetschke_271222_090123_HFGoe_LEDGF_b2R1_24h **Scan** 17613
**Peptide** EKQVETEEAGVVTTATASVNLKVSPKRGR **Charge** 4 **Spectral Angle** 0.9

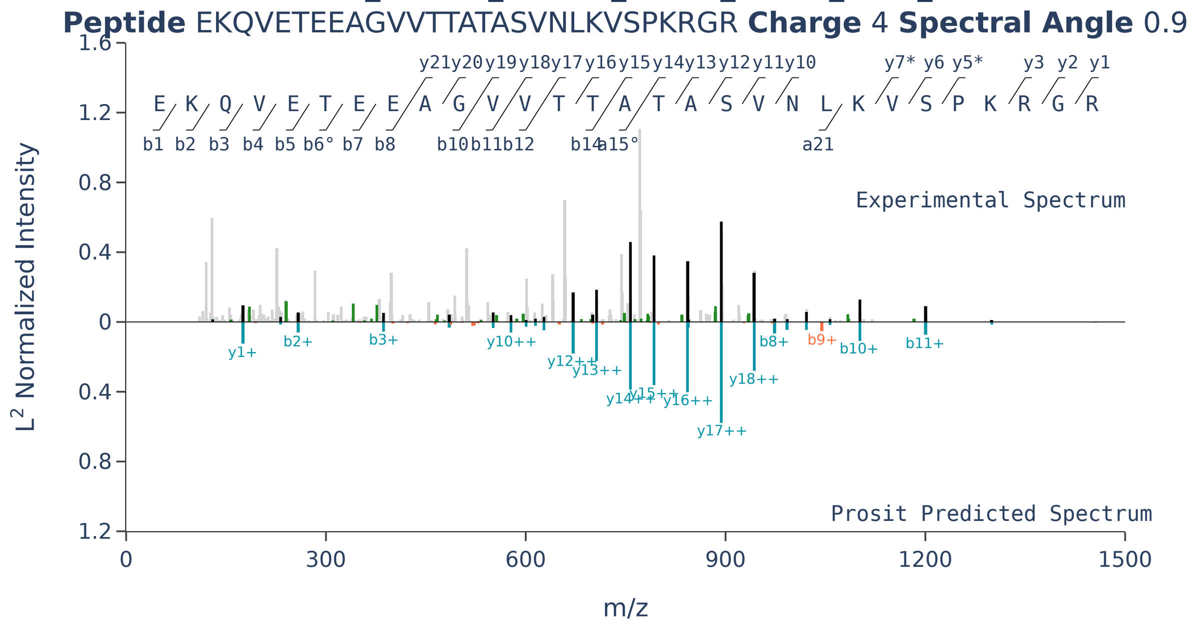


**Source** HRoetschke_271222_090123_HFGoe_LEDGF_b2R1_2h **Scan** 14695
**Peptide** IKNSLKI **Charge** 2 **Spectral Angle** 0.8

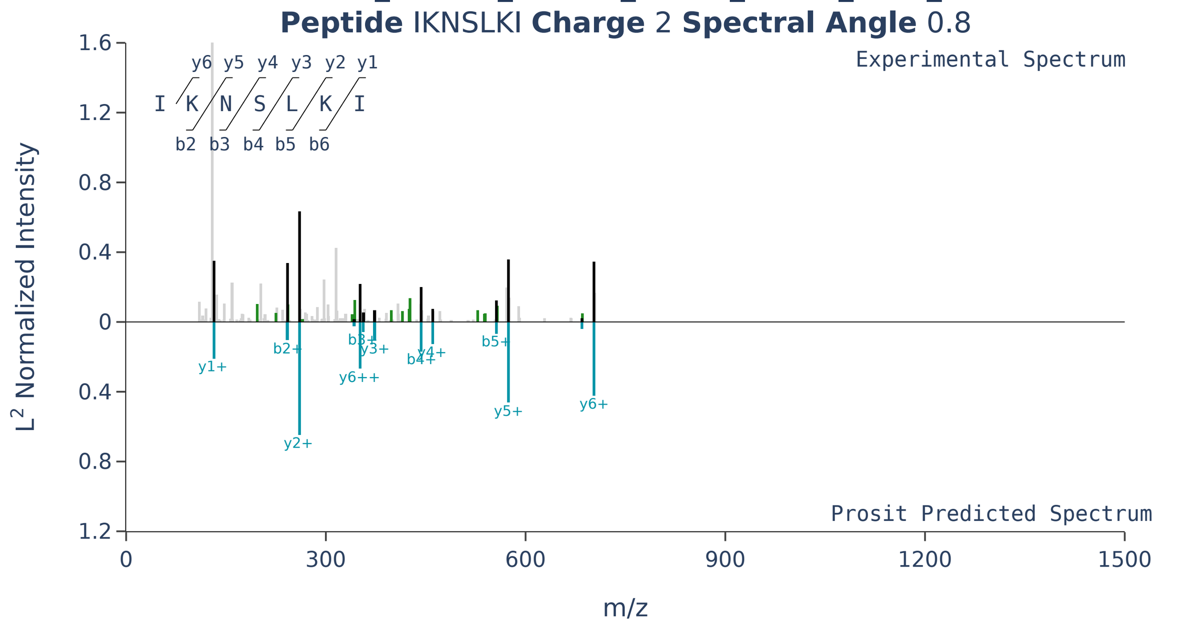

**Source** HRoetschke_271222_090123_HFGoe_LEDGF_b1R2_24h **Scan** 5900
**Peptide** KIPKPRGRPKM **Charge** 3 **Spectral Angle** 0.7

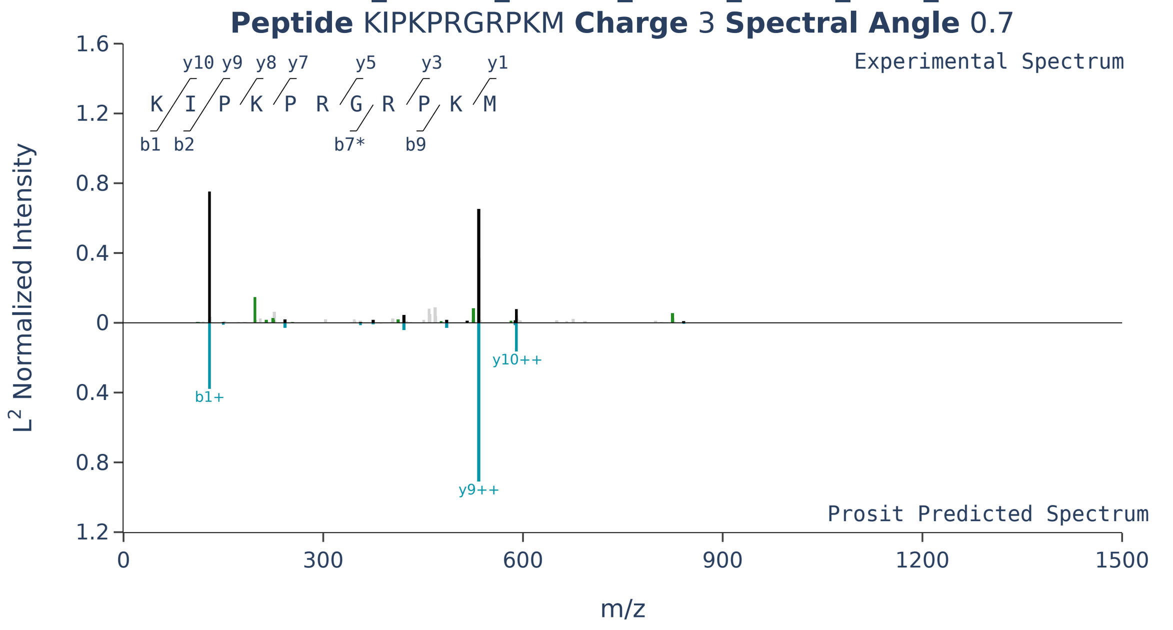


**Source** HRoetschke_271222_090123_HFGoe_LEDGF_b2R2_2h **Scan** 10417
**Peptide** MITTLKKIRRFK **Charge** 3 **Spectral Angle** 0.93

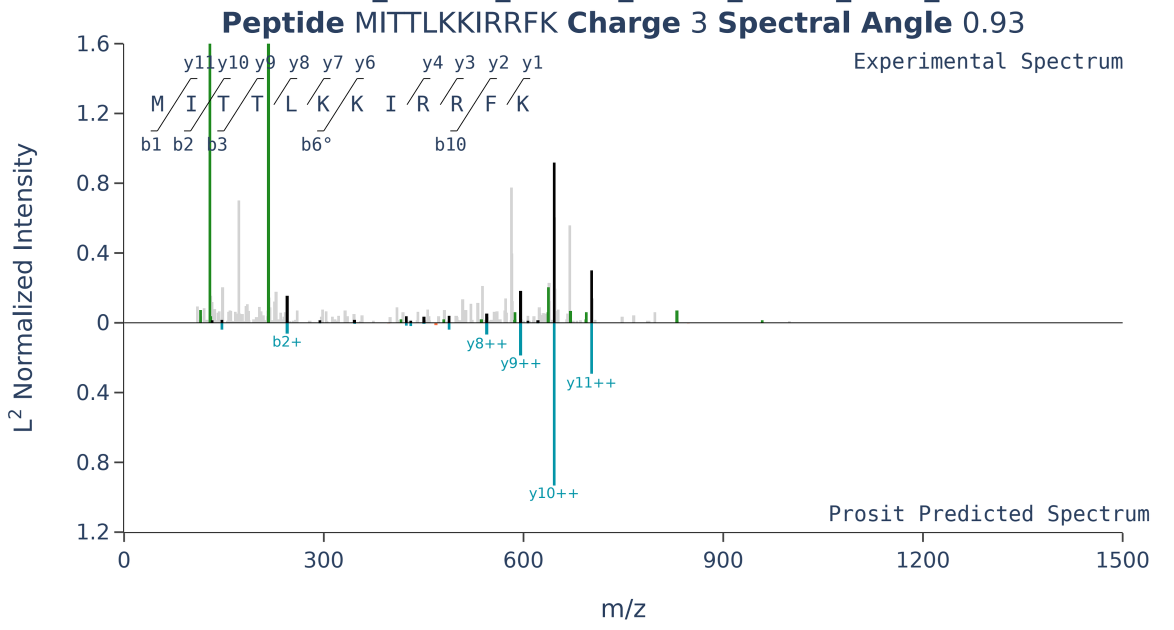


**Source** HRoetschke_271222_090123_HFGoe_LEDGF_b1R2_4h **Scan** 21623
**Peptide** VSQVIMEKSTMLYNKFK **Charge** 3 **Spectral Angle** 0.69

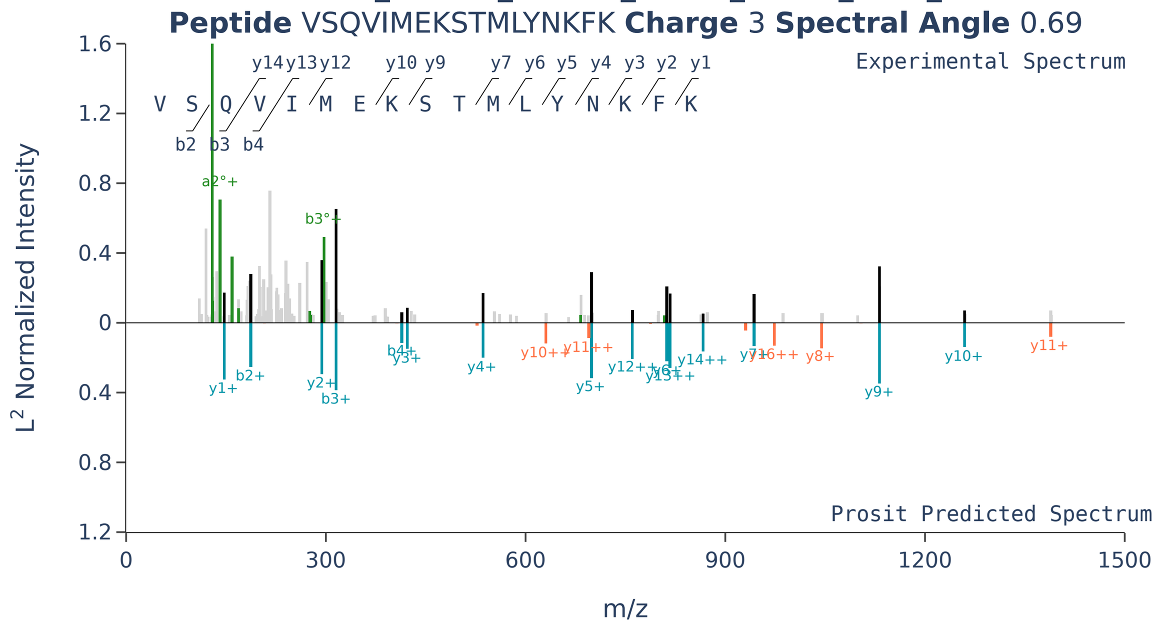


**Source** HRoetschke_271222_090123_HFGoe_LEDGF_b2R2_24h **Scan** 5156
**Peptide** SEEEGDDQEGEKKRKGGRNF **Charge** 3 **Spectral Angle** 0.77

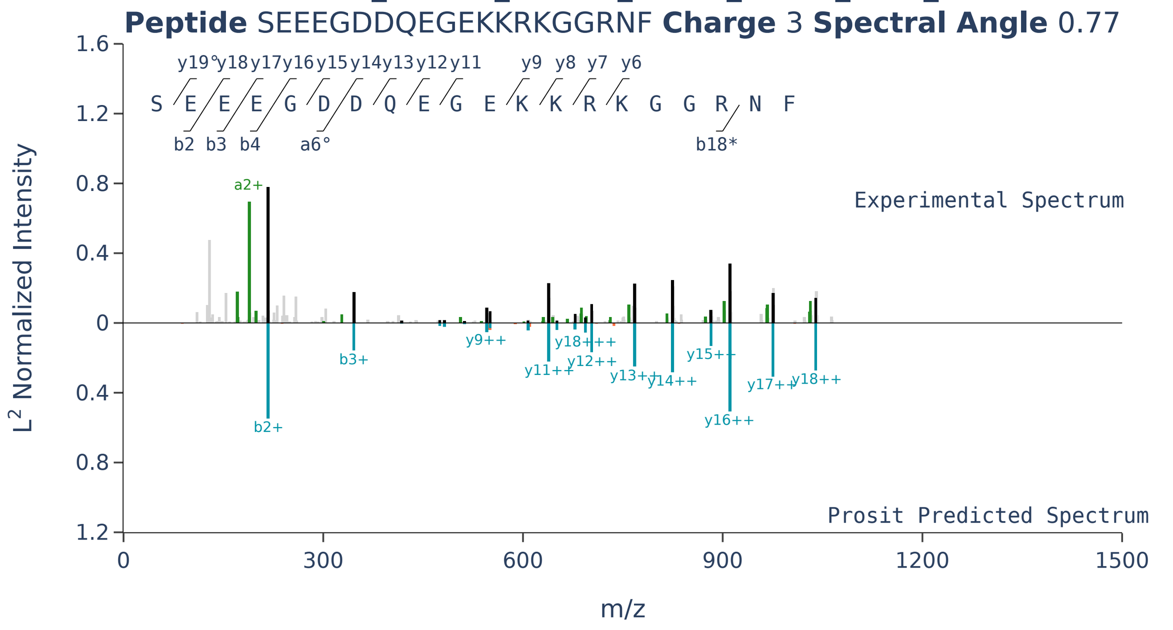


**Source** HRoetschke_271222_090123_HFGoe_LEDGF_b1R1_2h **Scan** 22613
**Peptide** ELASLQVTMQQAQKHTEMITTLKKIRR **Charge** 4 **Spectral Angle** 0.78

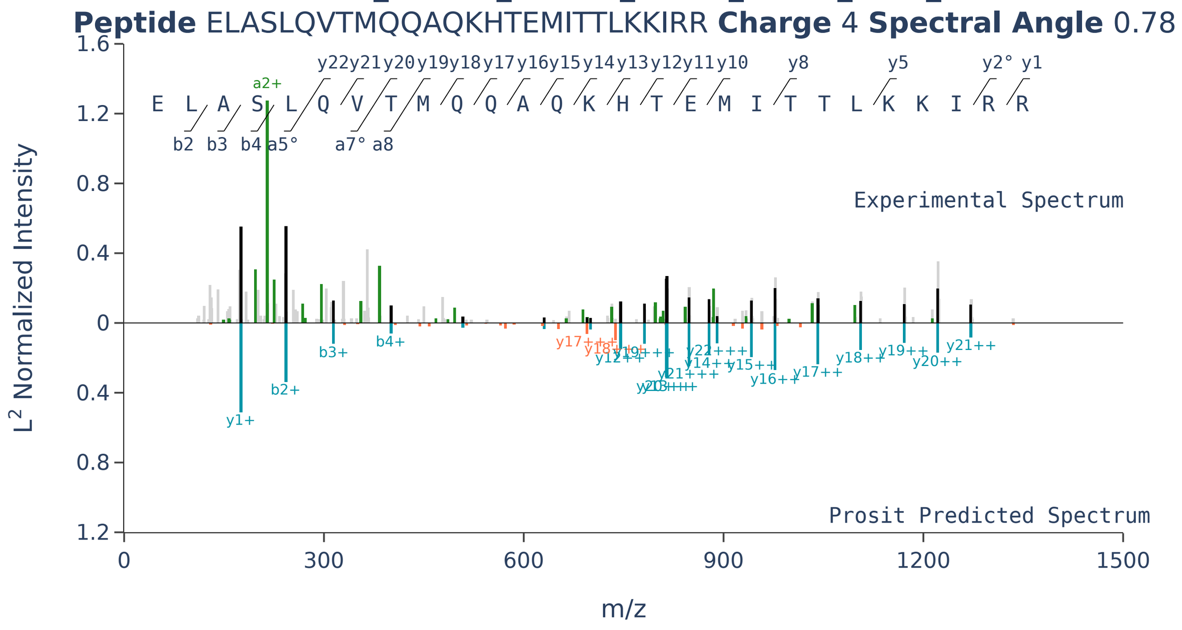


**Source** HRoetschke_271222_090123_HFGoe_LEDGF_b2R1_4h **Scan** 6691
**Peptide** NNPKVKFSSQQAATKQSNAS **Charge** 2 **Spectral Angle** 0.74

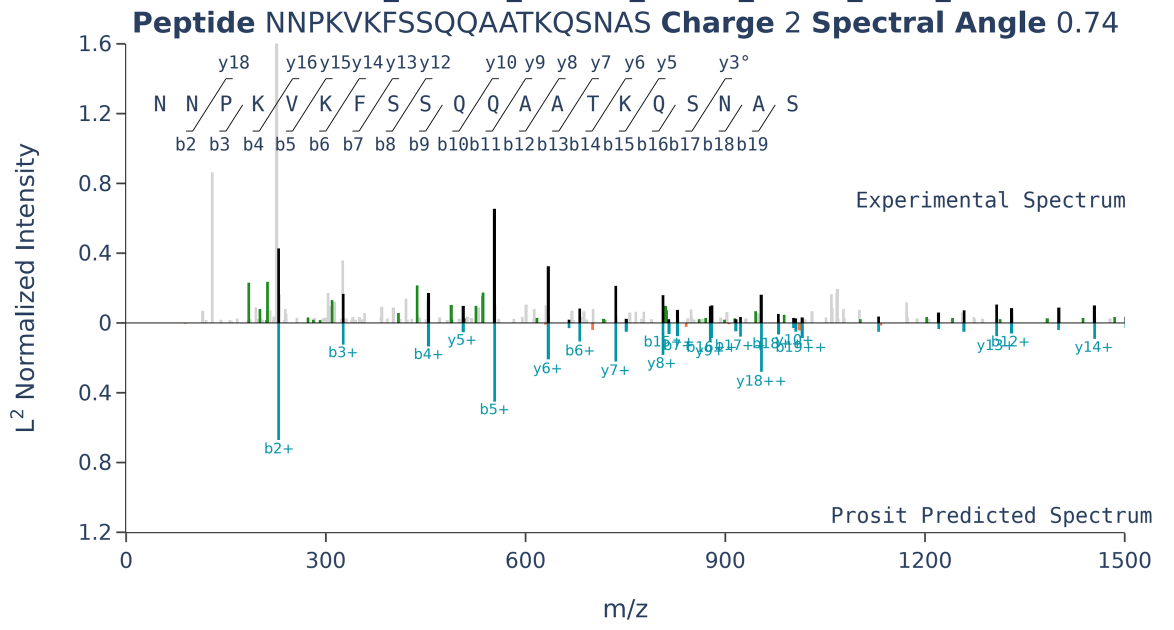


**Source** HRoetschke_271222_090123_HFGoe_LEDGF_b1R1_24h **Scan** 9930
**Peptide** KHTEMITTLK **Charge** 3 **Spectral Angle** 0.58

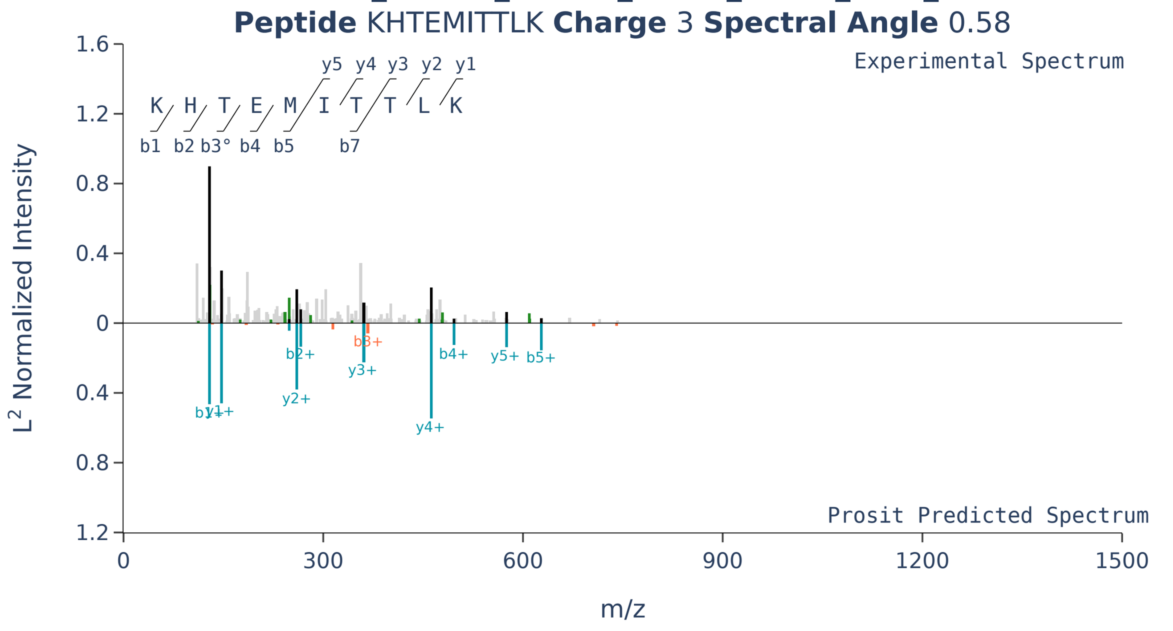


**Source** HRoetschke_271222_090123_HFGoe_LEDGF_b2R1_4h **Scan** 22616
**Peptide** VSQVIMEKSTML **Charge** 2 **Spectral Angle** 0.76

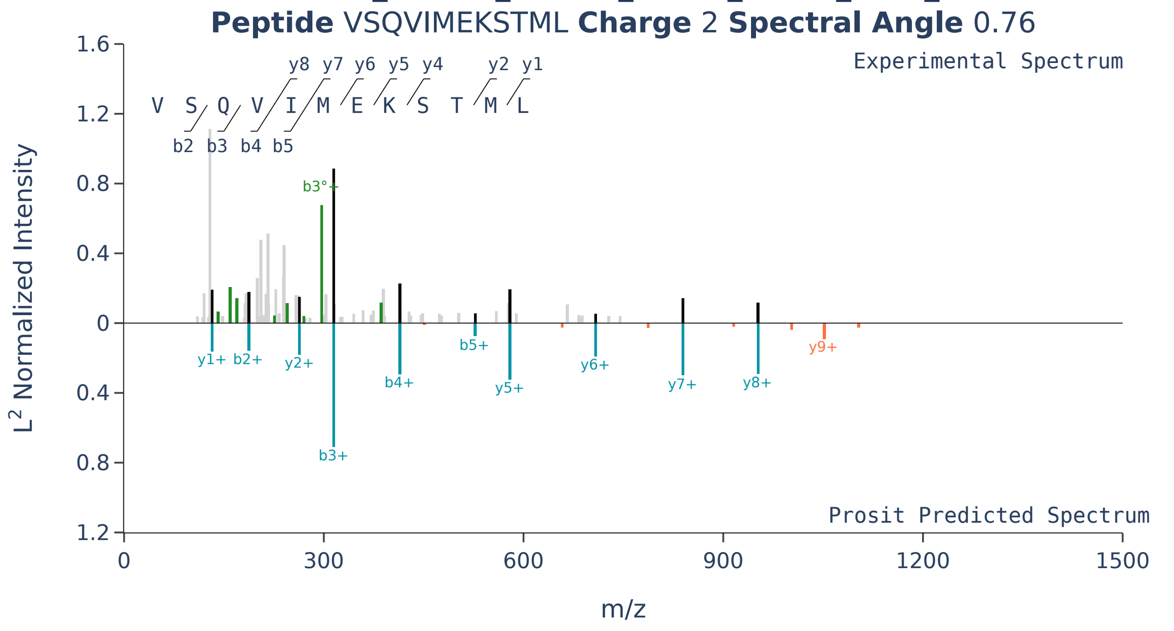


**Source** HRoetschke_271222_090123_HFGoe_LEDGF_b2R1_4h **Scan** 12009
**Peptide** SSDVEVEEKETSV **Charge** 2 **Spectral Angle** 0.82

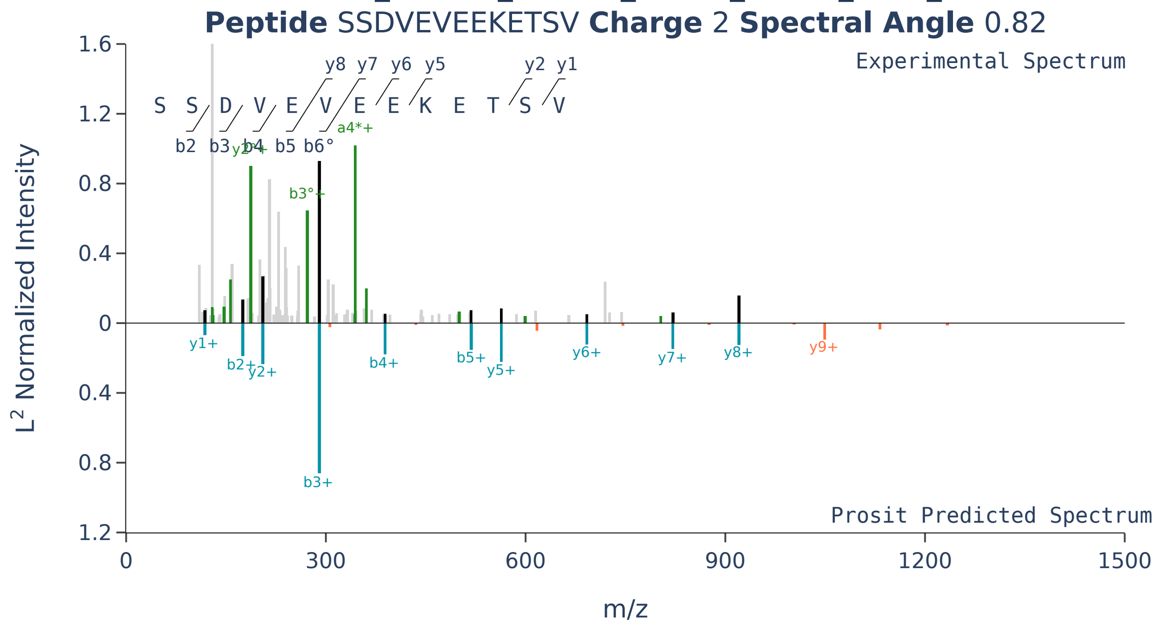


**Source** HRoetschke_271222_090123_HFGoe_LEDGF_b2R1_24h **Scan** 17666
**Peptide** LGPKDIFPYSENKEKYGK **Charge** 4 **Spectral Angle** 0.72

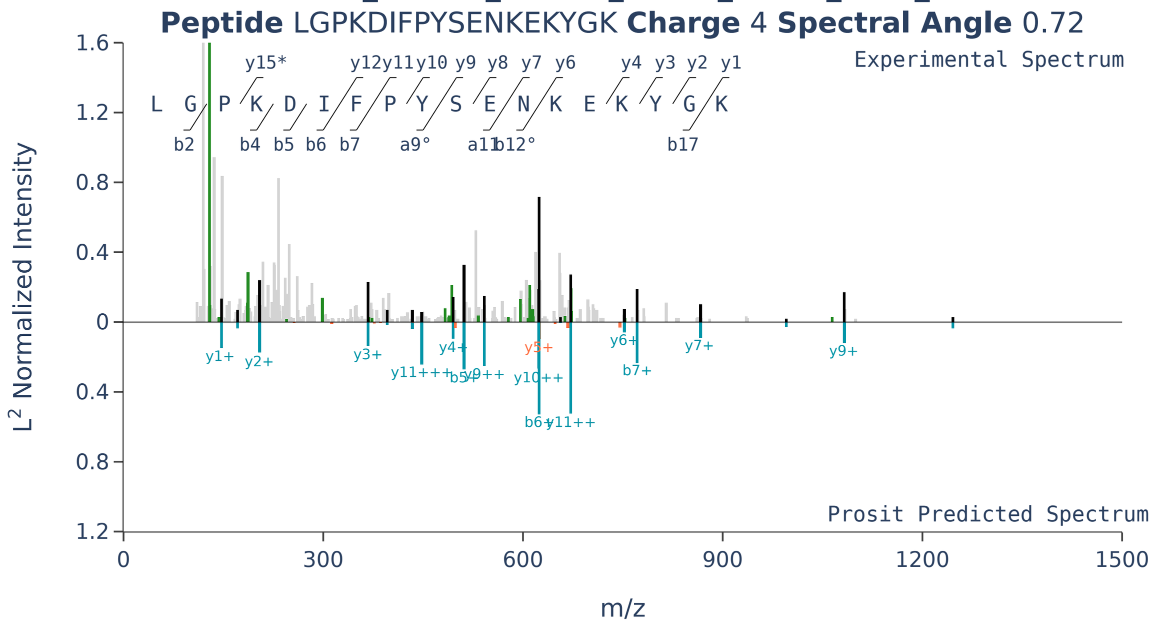


**Source** HRoetschke_271222_090123_HFGoe_LEDGF_b1R1_2h **Scan** 31282
**Peptide** DEVPDGAVKPPTNKLPIFFFGTHET **Charge** 3 **Spectral Angle** 0.91

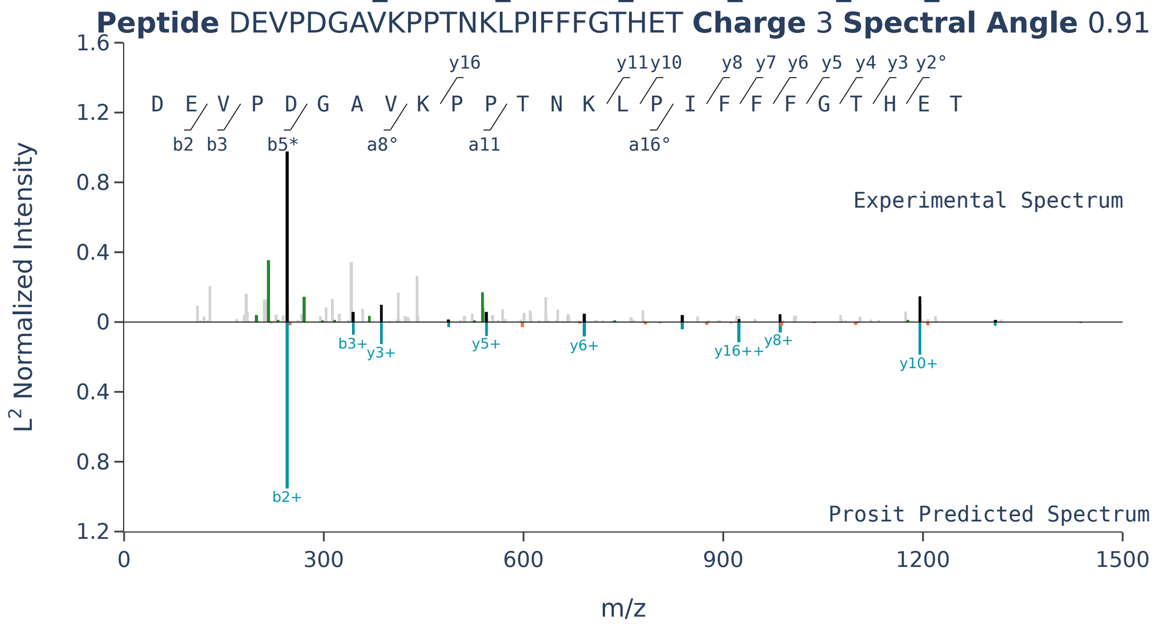


**Source** HRoetschke_271222_090123_HFGoe_LEDGF_b2R1_24h **Scan** 25694
**Peptide** KGFNEGLWEIDNNPKVKF **Charge** 3 **Spectral Angle** 0.75

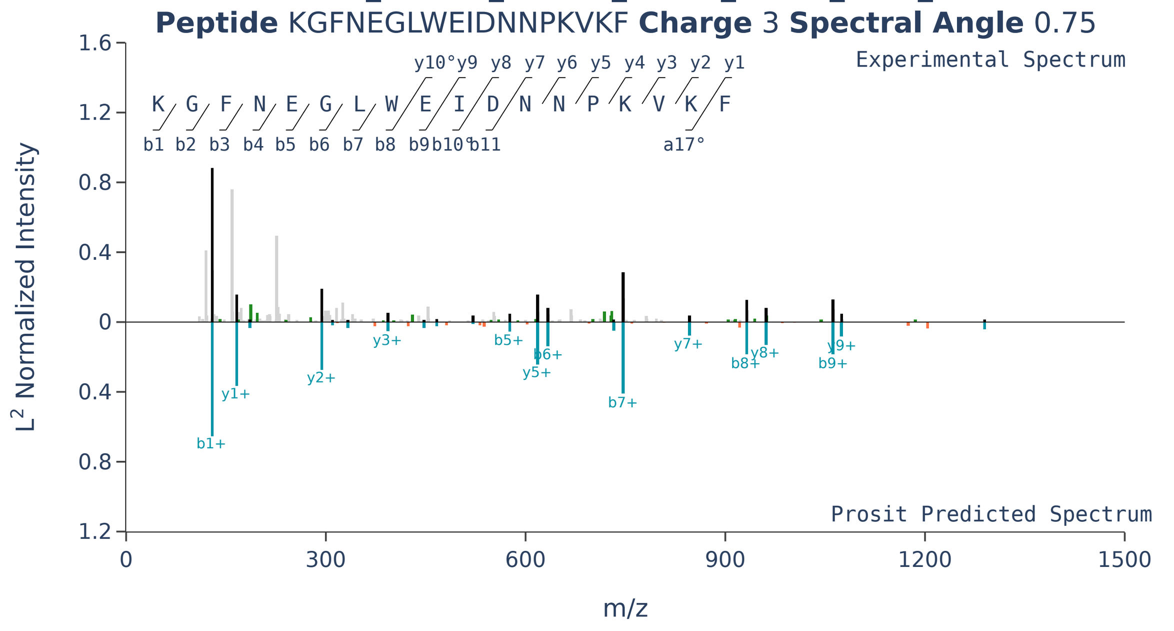


**Source** HRoetschke_271222_090123_HFGoe_LEDGF_b1R2_24h **Scan** 5403
**Peptide** SDIITEEDKSKKKGQEEKQPKKQ **Charge** 5 **Spectral Angle** 0.83

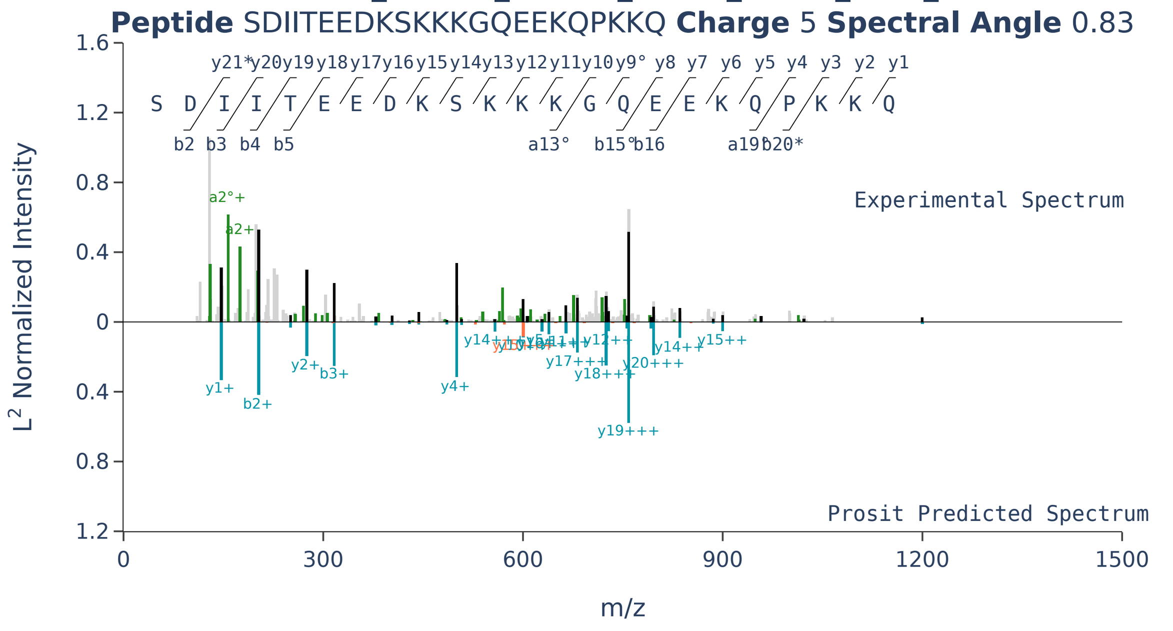


**Source** HRoetschke_271222_090123_HFGoe_LEDGF_b2R2_24h **Scan** 5633
**Peptide** KETSVSKEDTDHEEKASNEDVTKA **Charge** 3 **Spectral Angle** 0.92

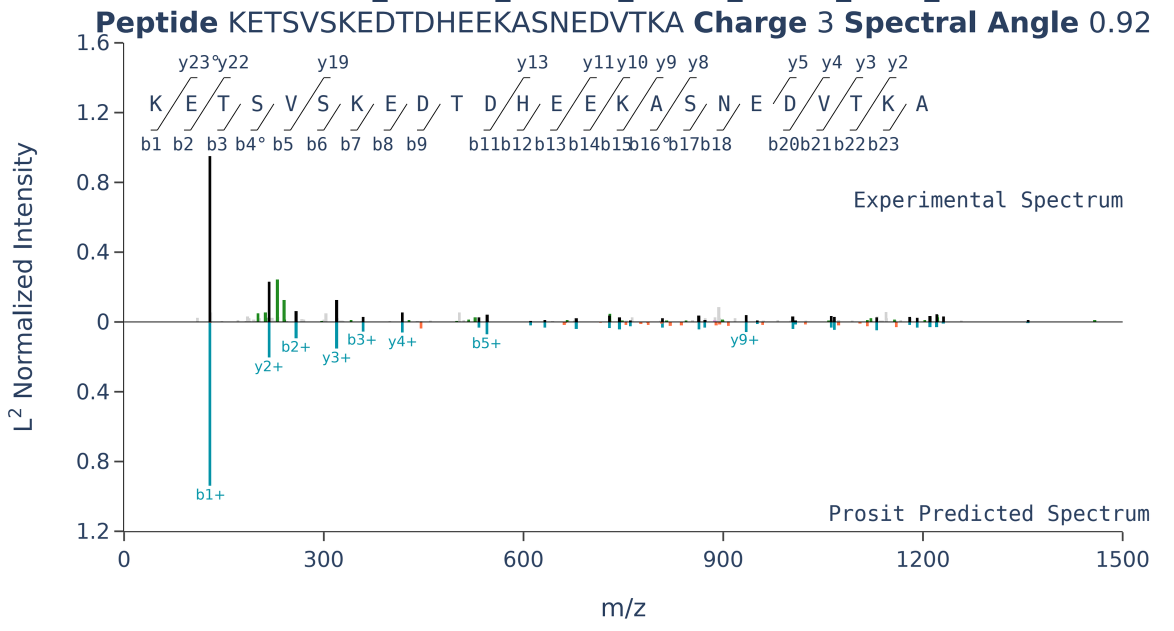


**Source** HRoetschke_271222_090123_HFGoe_LEDGF_b1R1_4h **Scan** 24789
**Peptide** SVITQVLNKSLAEQR **Charge** 3 **Spectral Angle** 0.69

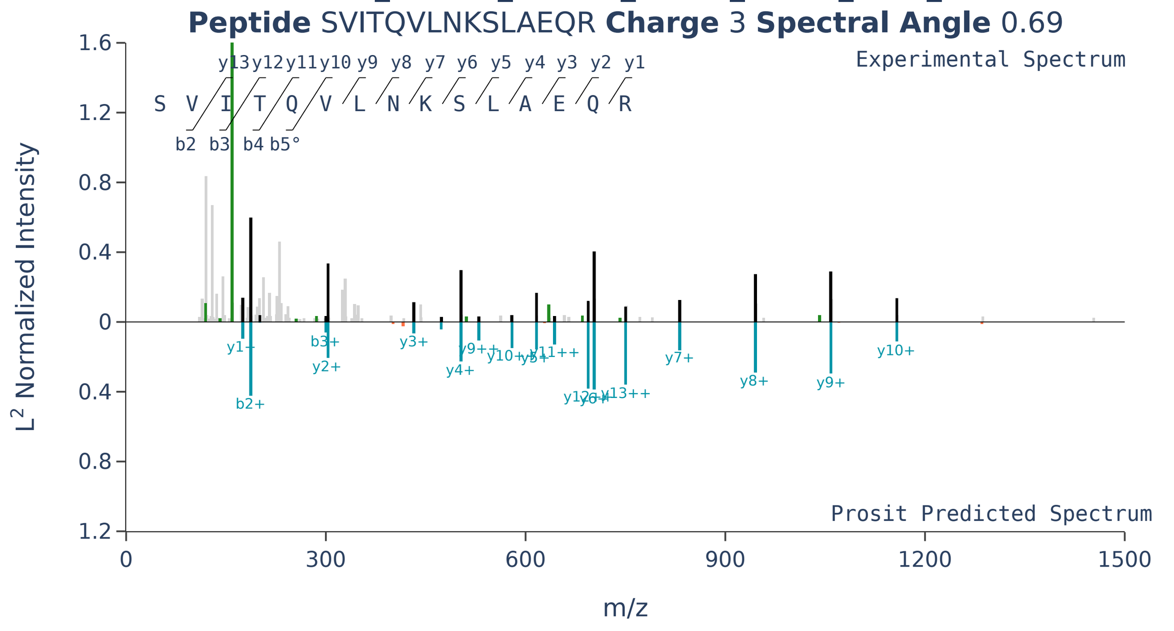

**Source** HRoetschke_271222_090123_HFGoe_LEDGF_b2R2_2h **Scan** 10908
**Peptide** IDNNPKVKFSSQQAATKQSNASSDV **Charge** 3 **Spectral Angle** 0.82

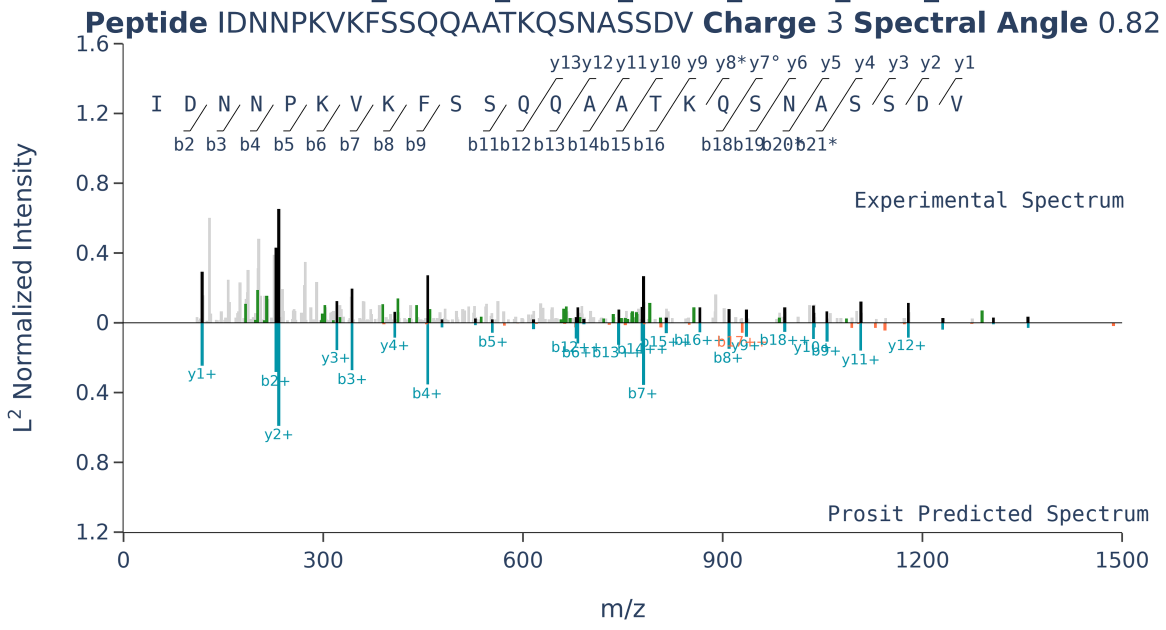


**Source** HRoetschke_271222_090123_HFGoe_LEDGF_b1R1_2h **Scan** 31964
**Peptide** VGEGDSVITQVLNKSL **Charge** 2 **Spectral Angle** 0.74

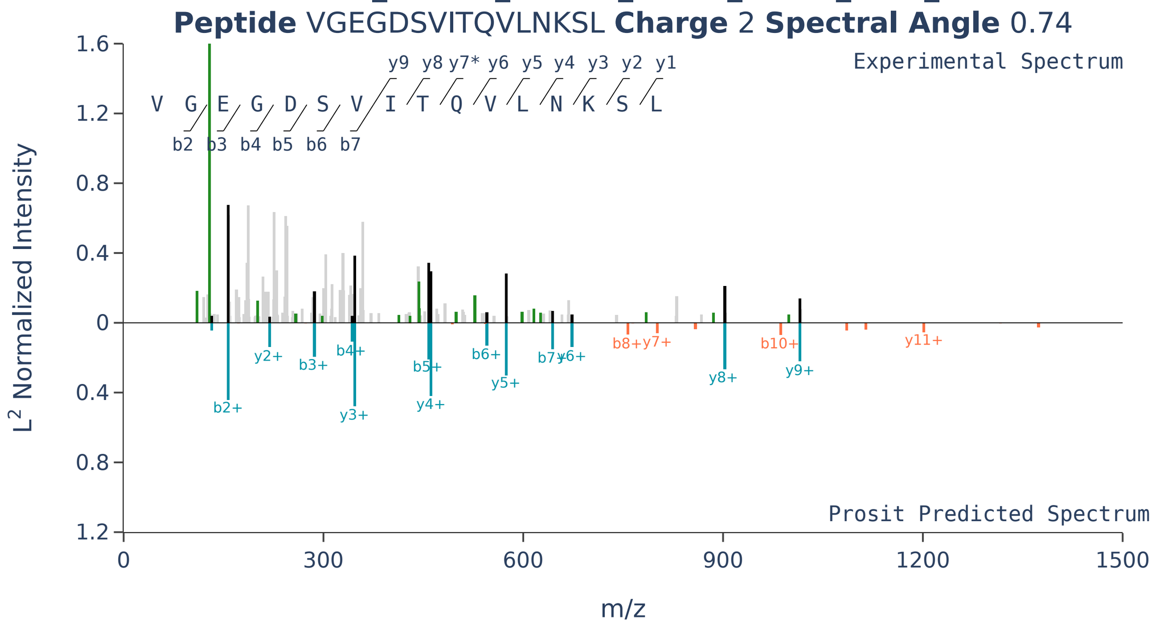


**Source** HRoetschke_271222_090123_HFGoe_LEDGF_b1R1_4h **Scan** 16989
**Peptide** FLGPKDIFPYSENKEKYGKPNKRKGF **Charge** 5 **Spectral Angle** 0.83

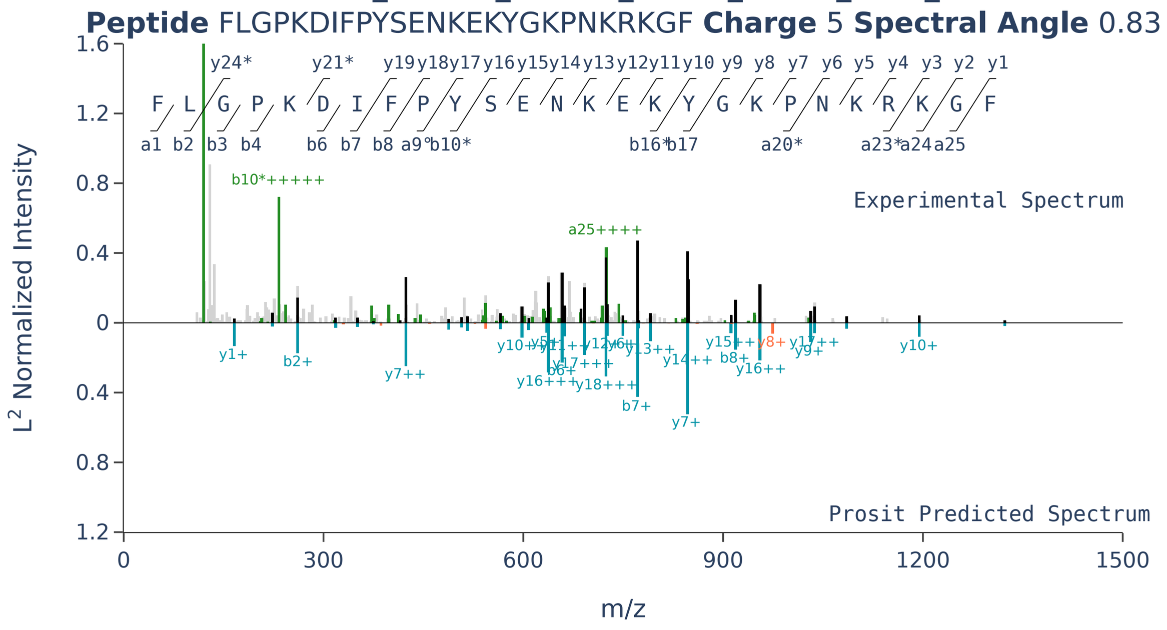


**Source** HRoetschke_271222_090123_HFGoe_LEDGF_b1R2_2h **Scan** 22475
**Peptide** NLDVNRCIEALD **Charge** 2 **Spectral Angle** 0.78

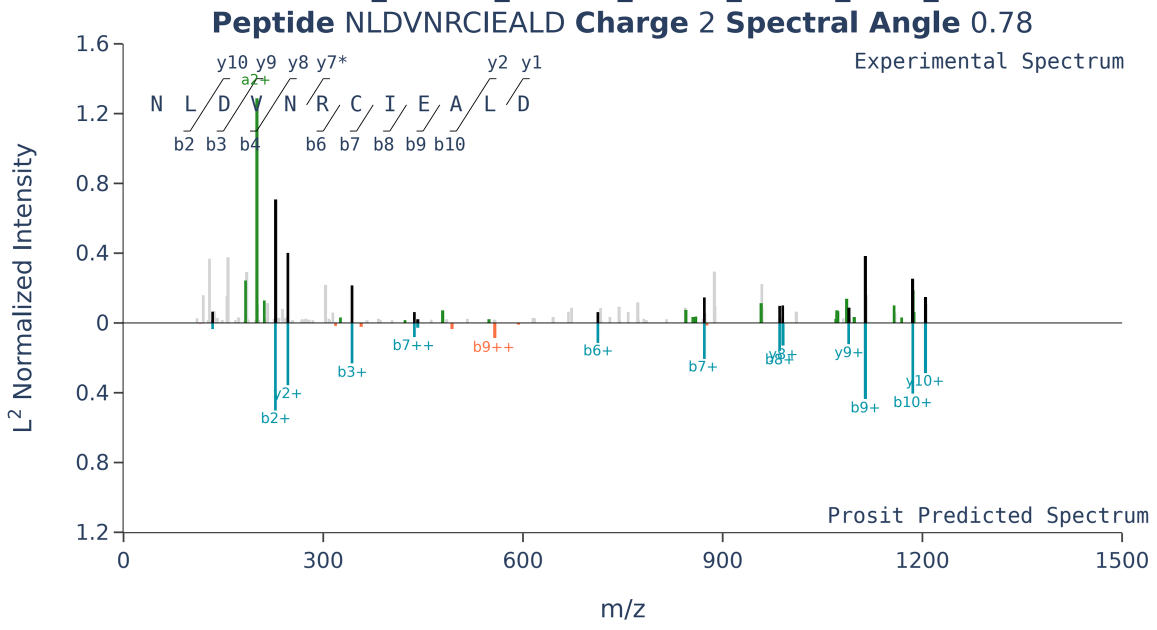


**Source** HRoetschke_271222_090123_HFGoe_LEDGF_b1R1_4h **Scan** 23902
**Peptide** AFLGPKDIFPYSENKEKYG **Charge** 4 **Spectral Angle** 0.71

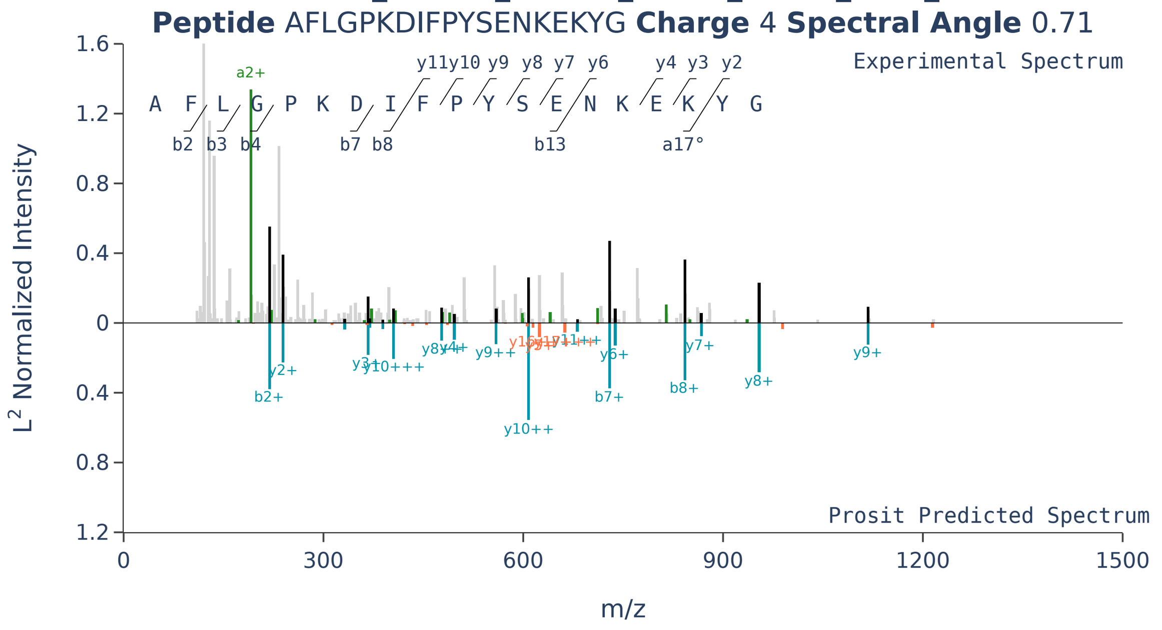


**Source** HRoetschke_271222_090123_HFGoe_LEDGF_b1R1_24h **Scan** 15838
**Peptide** WEIDNNPKVKFSSQQAATKQSNASSD **Charge** 3 **Spectral Angle** 0.88

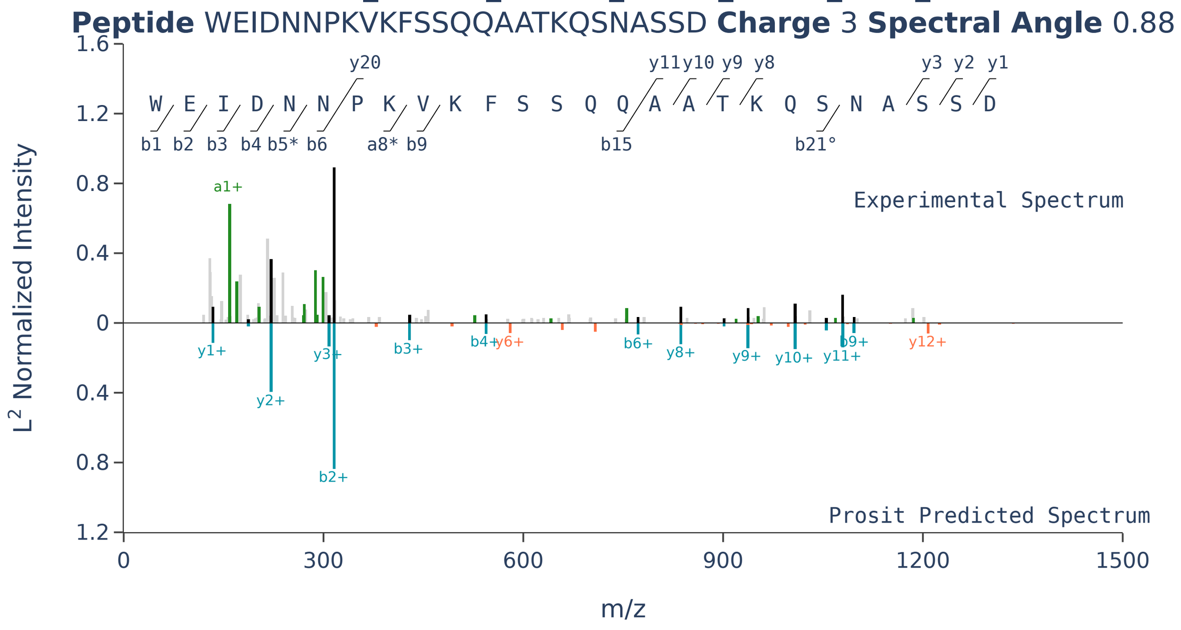


**Source** HRoetschke_271222_090123_HFGoe_LEDGF_b2R1_1h **Scan** 7529
**Peptide** RRFKVSQV **Charge** 2 **Spectral Angle** 0.8

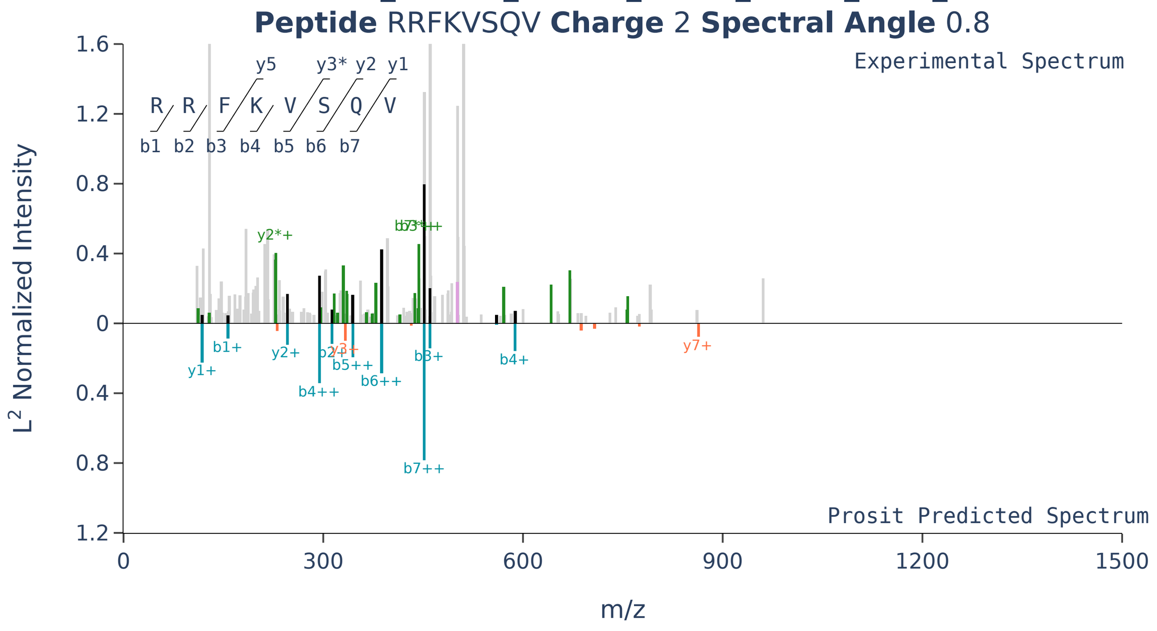


**Source** HRoetschke_271222_090123_HFGoe_LEDGF_b1R2_24h **Scan** 13819
**Peptide** EMITTLKKIRRFK **Charge** 3 **Spectral Angle** 0.91

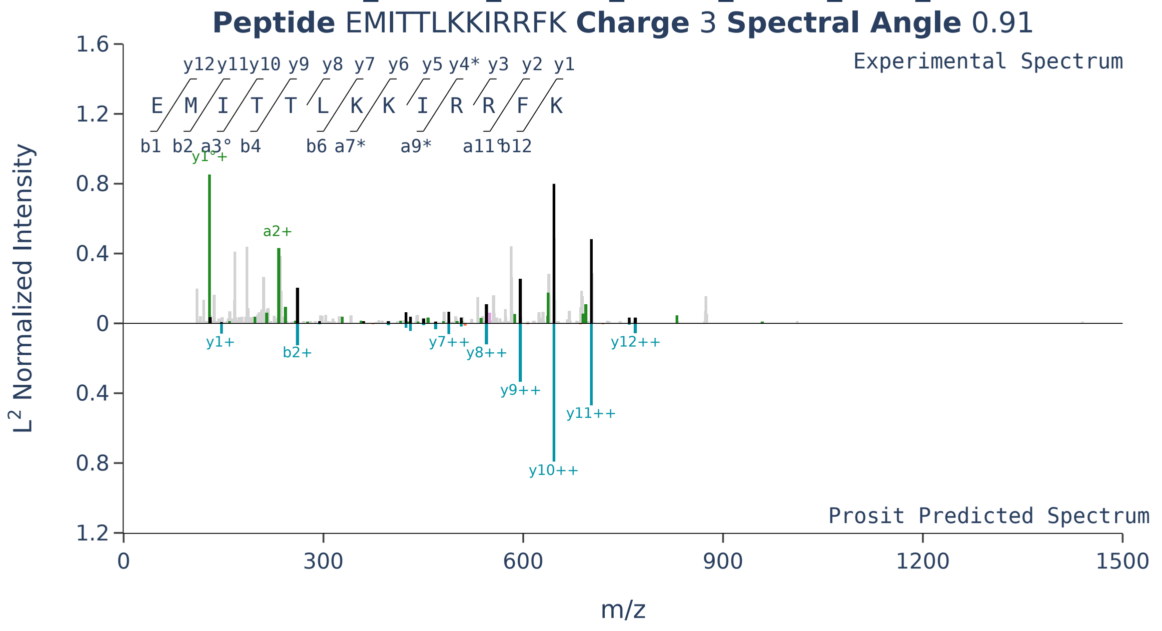


**Source** HRoetschke_271222_090123_HFGoe_LEDGF_b1R2_24h **Scan** 13232
**Peptide** MEKSTMLYNKFKN **Charge** 3 **Spectral Angle** 0.79

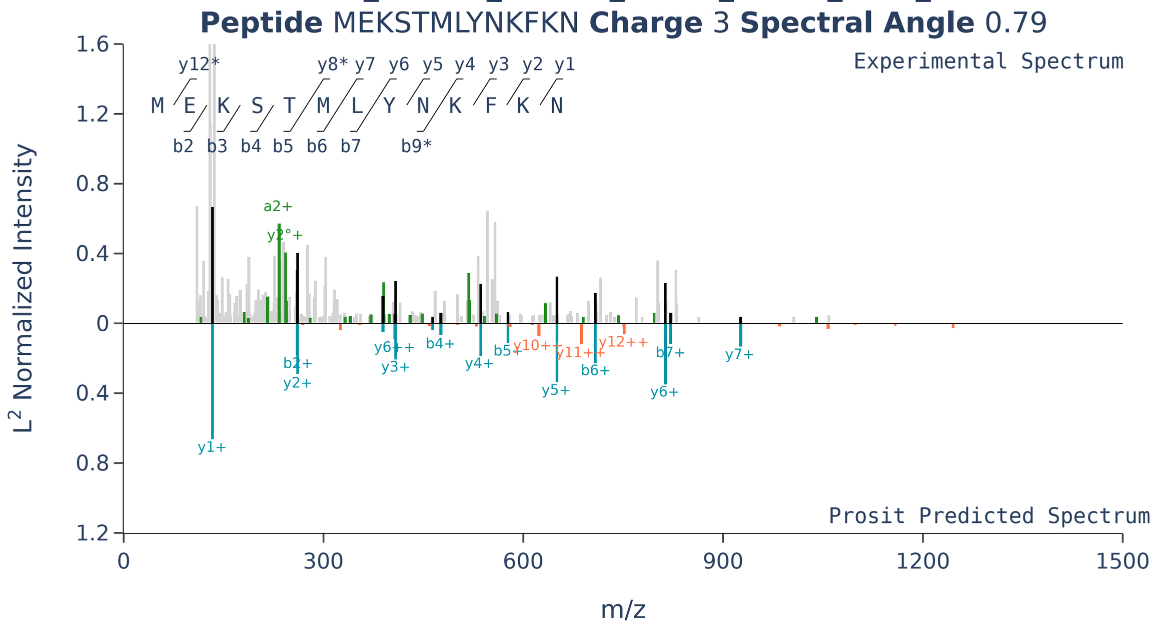


**Source** HRoetschke_271222_090123_HFGoe_LEDGF_b1R1_4h **Scan** 19109
**Peptide** SVITQVLNKSLAEQRQHEEANKTKD **Charge** 4 **Spectral Angle** 0.8

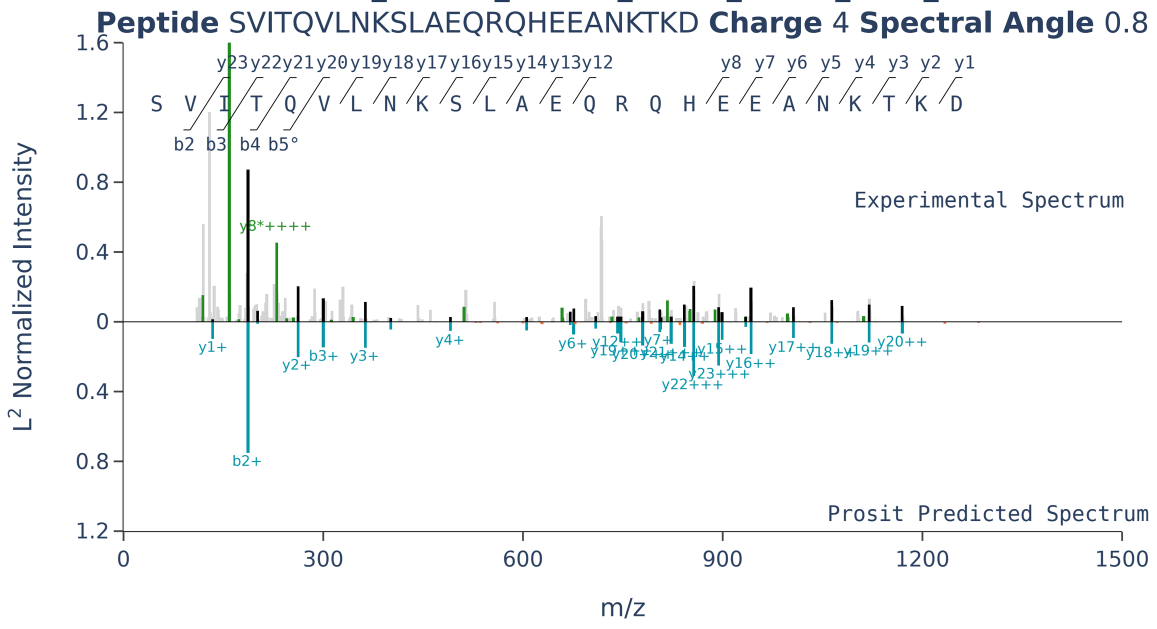


**Source** HRoetschke_271222_090123_HFGoe_LEDGF_b2R1_4h **Scan** 5480
**Peptide** DSKDNHEASTKKKPSSEERETEL **Charge** 4 **Spectral Angle** 0.77

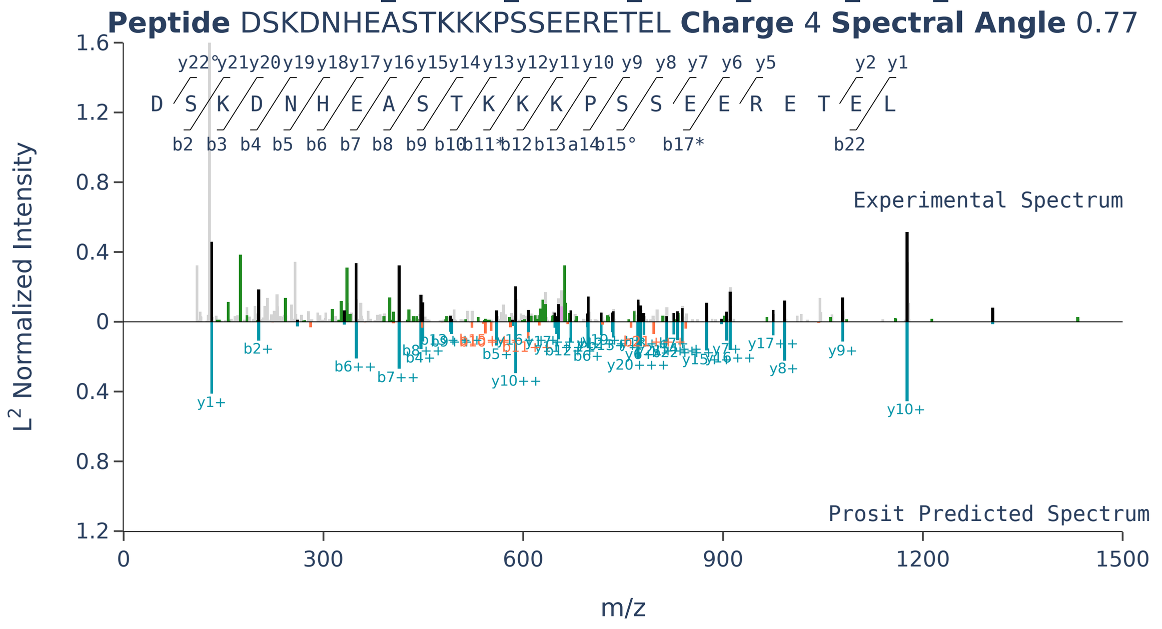


**Source** HRoetschke_271222_090123_HFGoe_LEDGF_b1R2_2h **Scan** 7848
**Peptide** DNNPKVKFSSQQAATKQS **Charge** 2 **Spectral Angle** 0.84

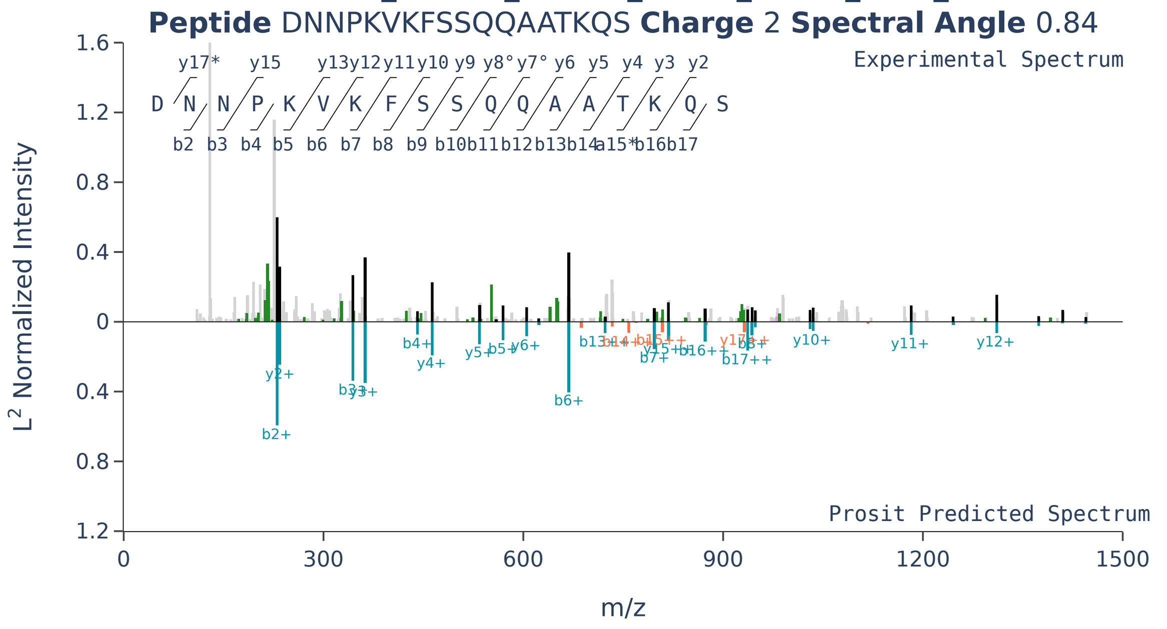


**Source** HRoetschke_271222_090123_HFGoe_LEDGF_b1R1_2h **Scan** 28222
**Peptide** GDSVITQVL **Charge** 2 **Spectral Angle** 0.85

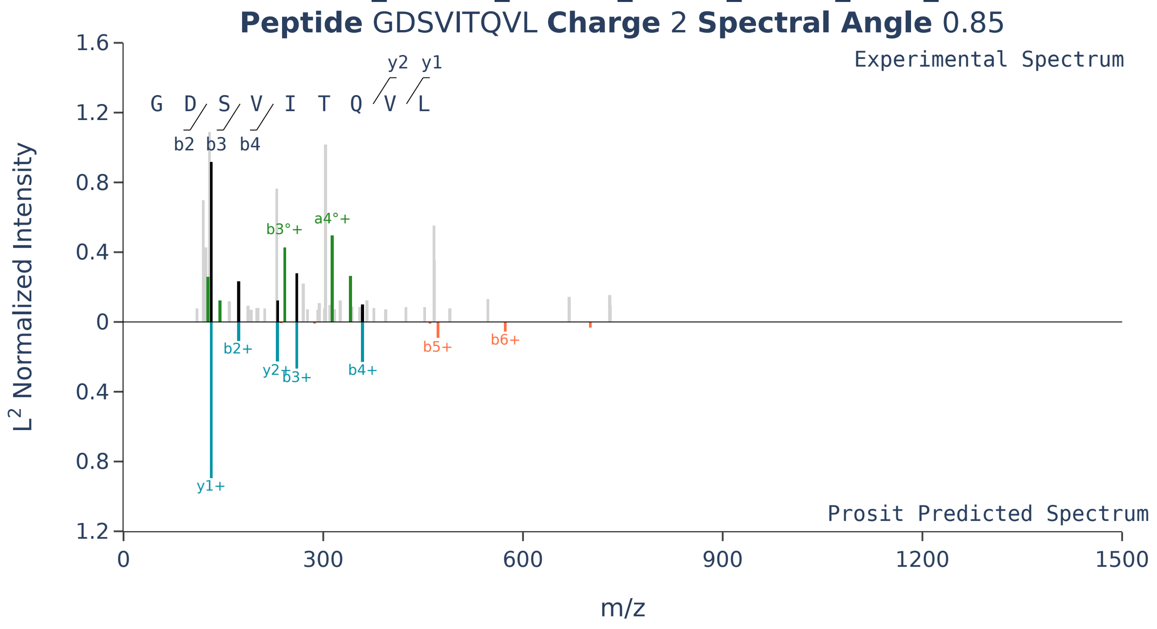


**Source** HRoetschke_271222_090123_HFGoe_LEDGF_b1R1_4h **Scan** 29674
**Peptide** PDGAVKPPTNKLPIFFFGTHET **Charge** 3 **Spectral Angle** 0.82

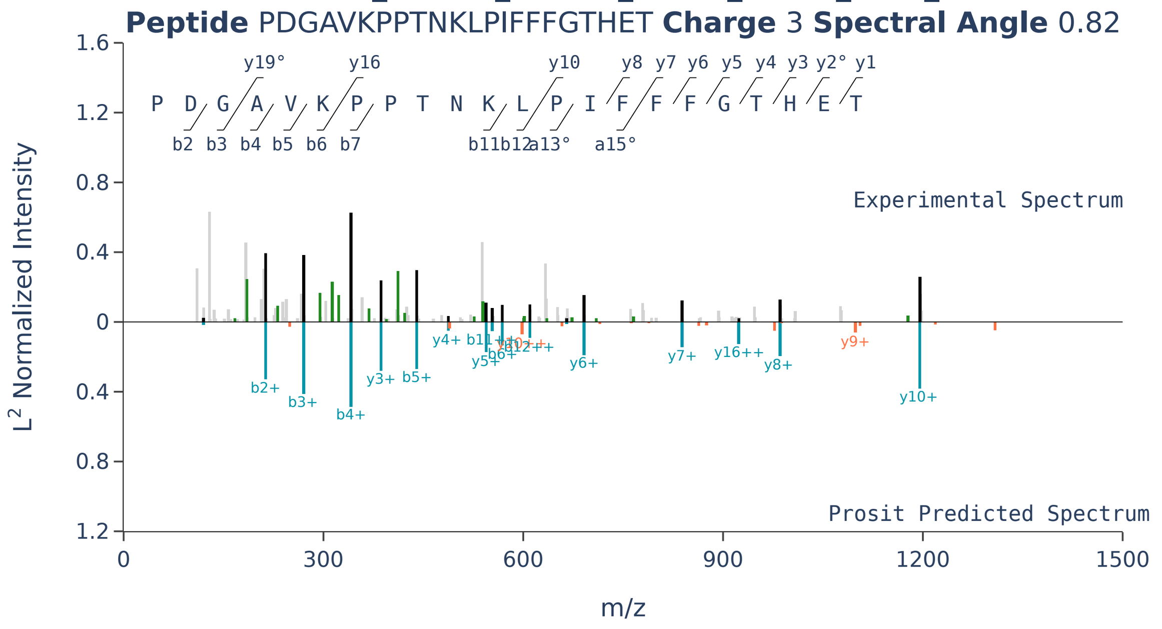


**Source** HRoetschke_271222_090123_HFGoe_LEDGF_b2R2_24h **Scan** 19947
**Peptide** QVIMEKSTMLYN **Charge** 2 **Spectral Angle** 0.86

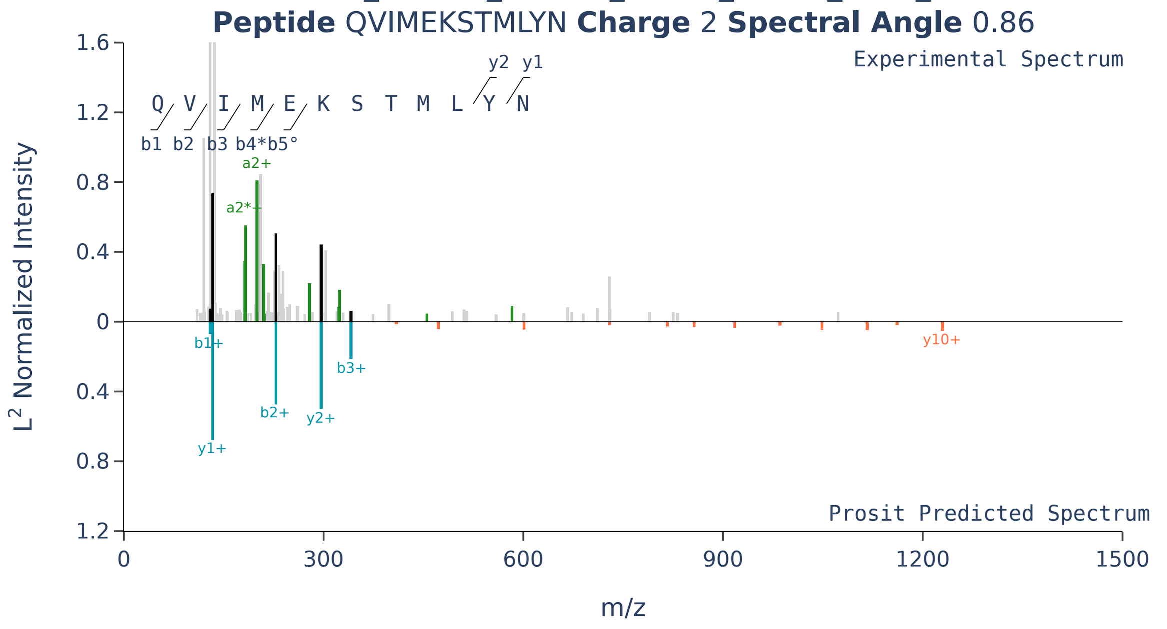

**Source** HRoetschke_271222_090123_HFGoe_LEDGF_b1R1_4h **Scan** 8235
**Peptide** DVTKAVDITTPKAARRGRK **Charge** 4 **Spectral Angle** 0.71

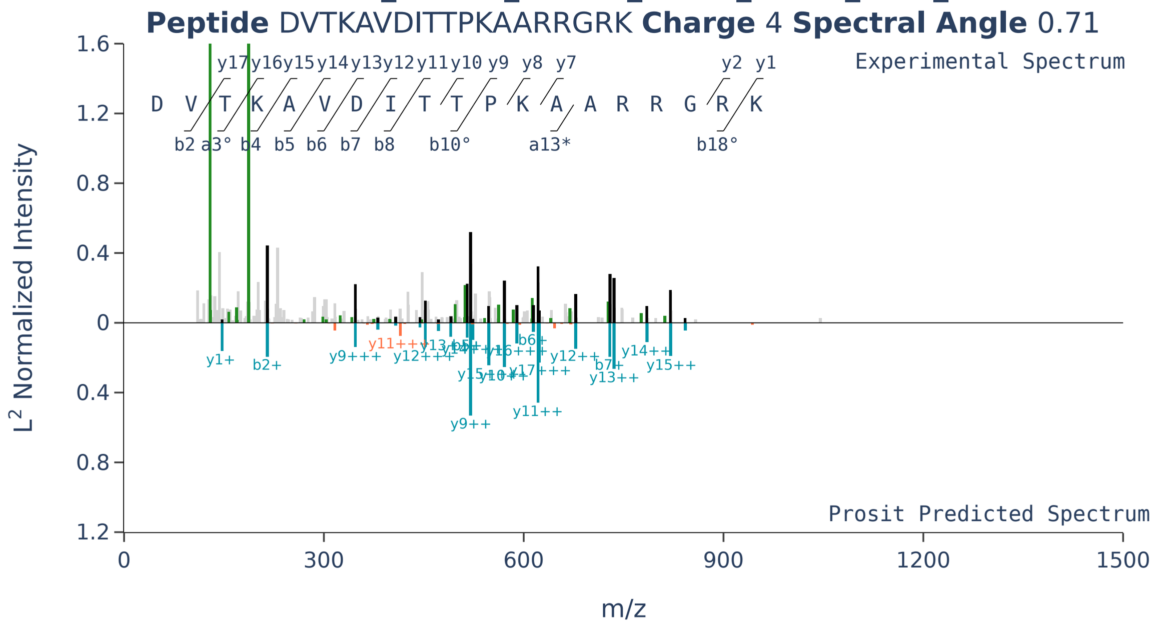


**Source** HRoetschke_271222_090123_HFGoe_LEDGF_b1R1_2h **Scan** 20547
**Peptide** ASLQVTMQQAQKHTEMITTLKKIR **Charge** 4 **Spectral Angle** 0.76

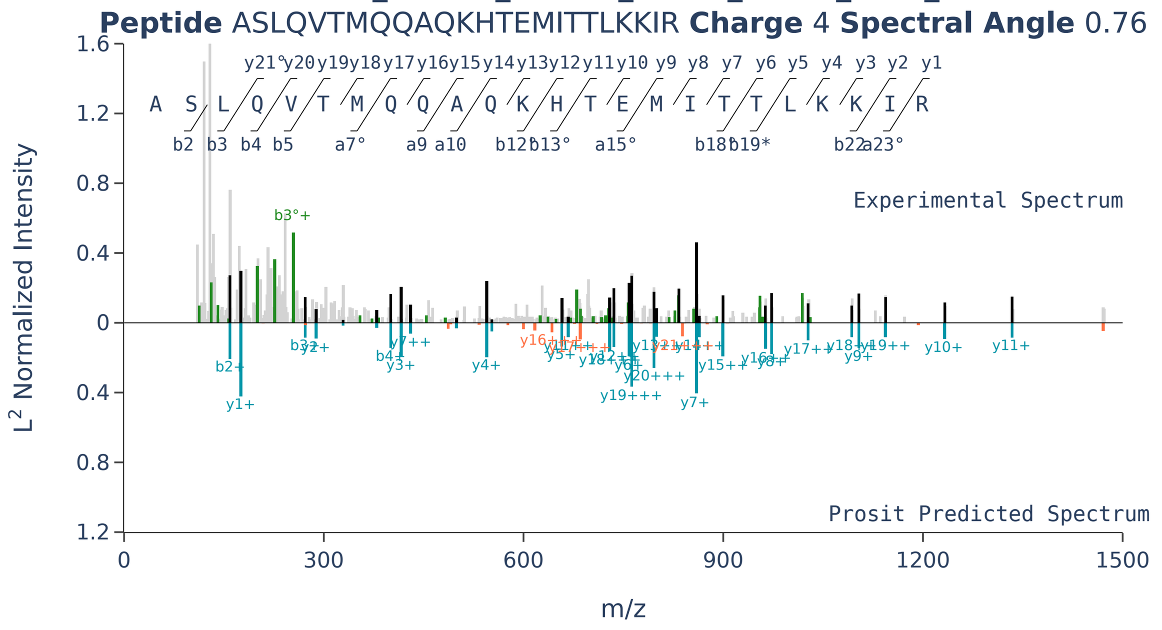


**Source** HRoetschke_271222_090123_HFGoe_LEDGF_b1R2_2h **Scan** 22865
**Peptide** KGFNEGLWEIDNNPKVKFSSQQAA **Charge** 4 **Spectral Angle** 0.89

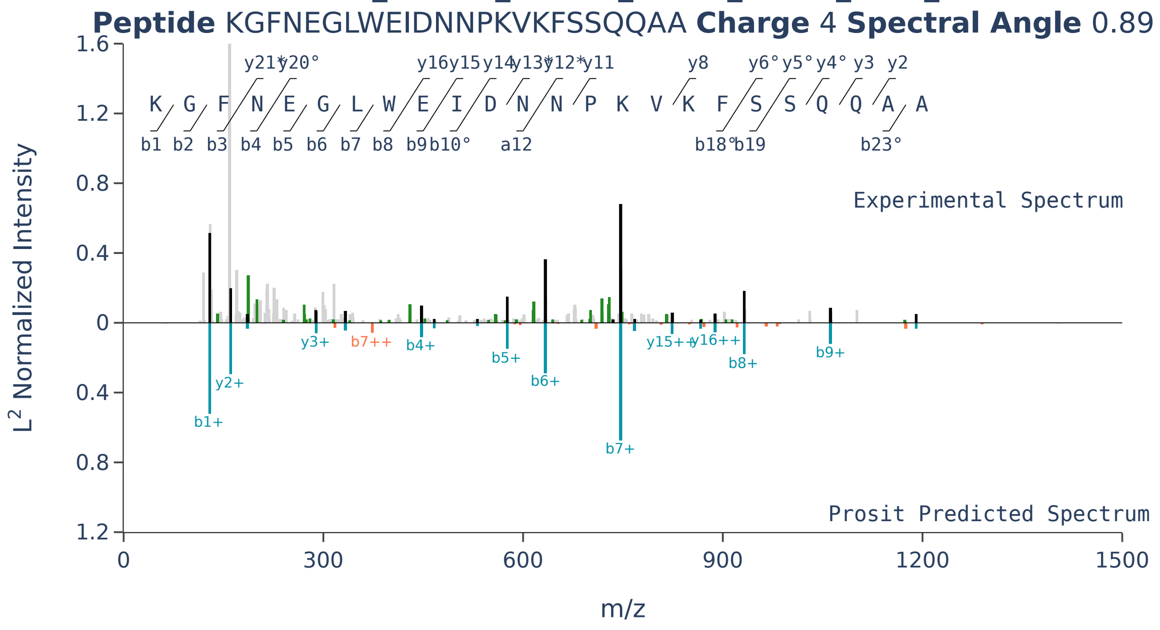


**Source** HRoetschke_271222_090123_HFGoe_LEDGF_b1R2_4h **Scan** 25813
**Peptide** ASLQVTMQQAQKHTEMITTL **Charge** 3 **Spectral Angle** 0.87

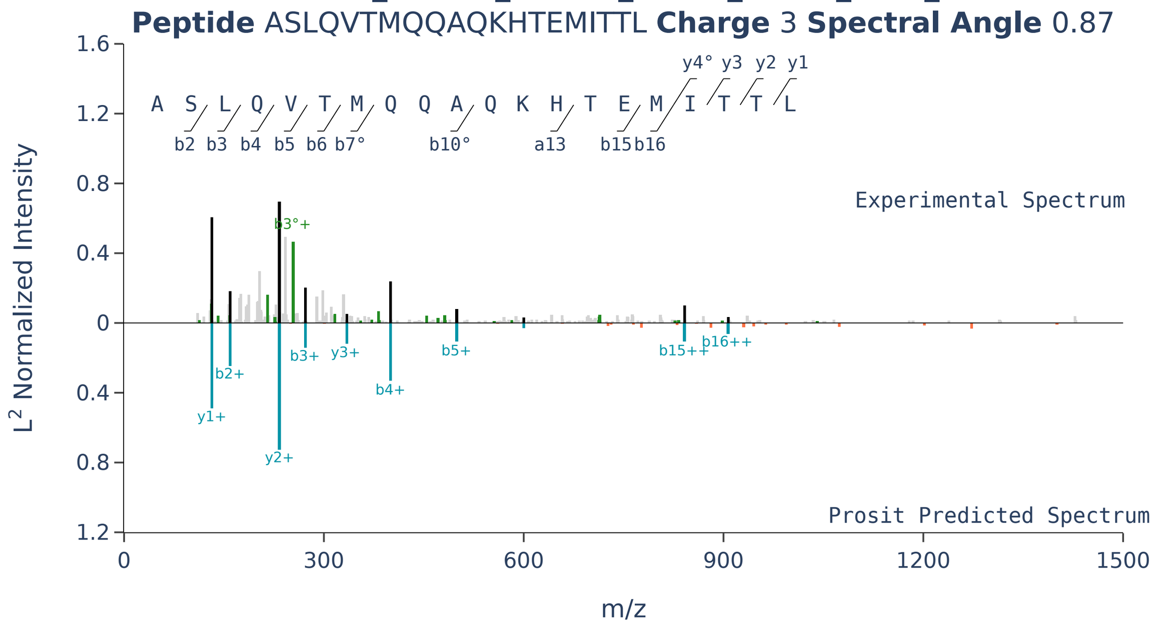


**Source** HRoetschke_271222_090123_HFGoe_LEDGF_b2R1_24h **Scan** 5508
**Peptide** VDITTPKAARRGRKRKAEKQVE **Charge** 4 **Spectral Angle** 0.77

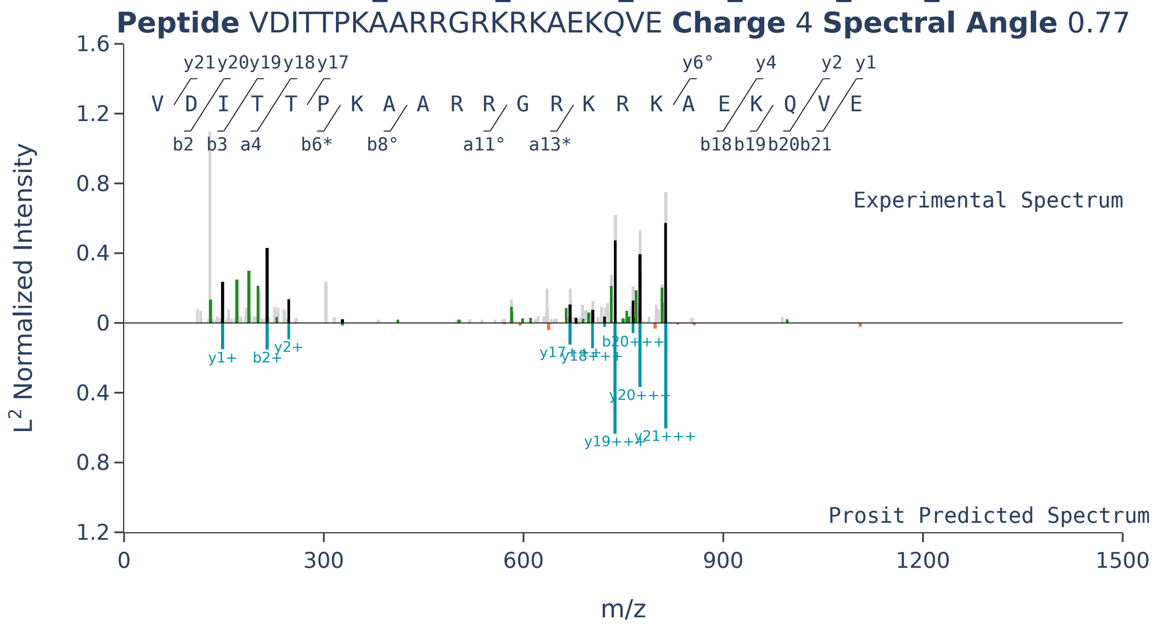


**Source** HRoetschke_271222_090123_HFGoe_LEDGF_b1R2_4h **Scan** 18457
**Peptide** WEIDNNPKVKFSSQQAAT **Charge** 2 **Spectral Angle** 0.89

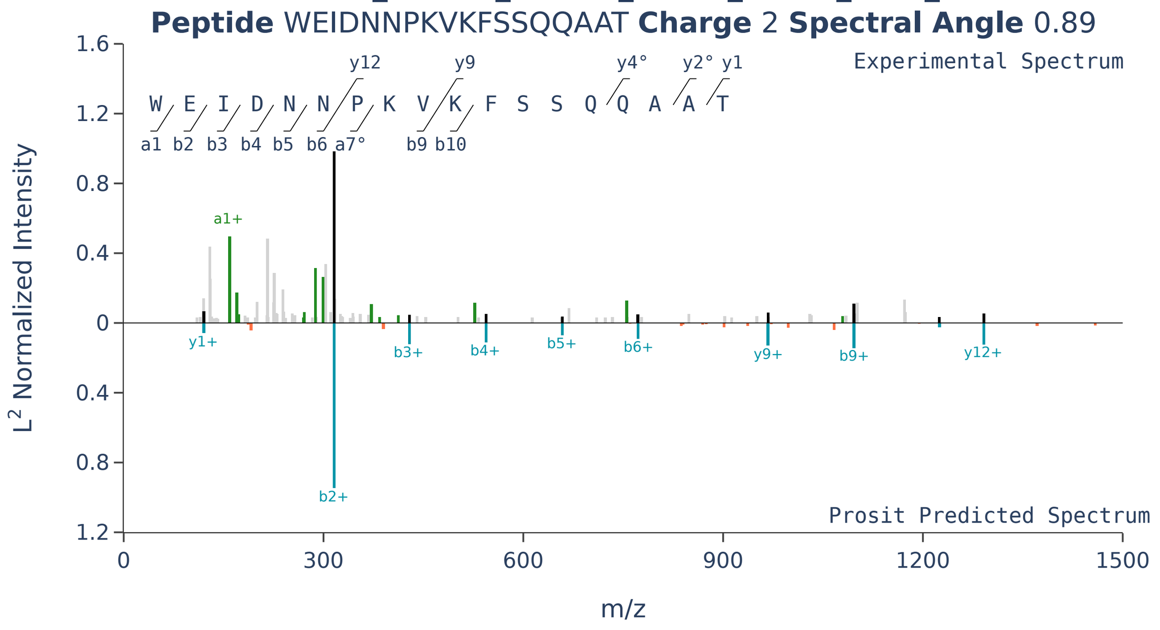


**Source** HRoetschke_271222_090123_HFGoe_LEDGF_b1R2_24h **Scan** 9842
**Peptide** AVKPPTNK **Charge** 2 **Spectral Angle** 0.95

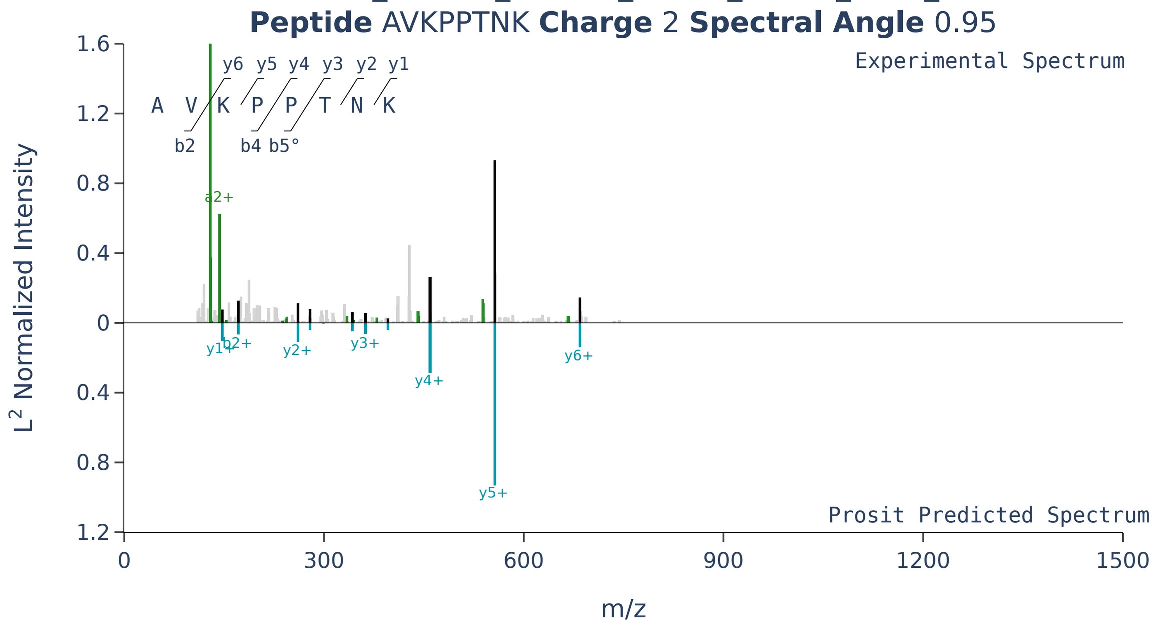


**Source** HRoetschke_271222_090123_HFGoe_LEDGF_b1R1_2h **Scan** 23971
**Peptide** GPGMTRDFKPGDLIFAKM **Charge** 2 **Spectral Angle** 0.68

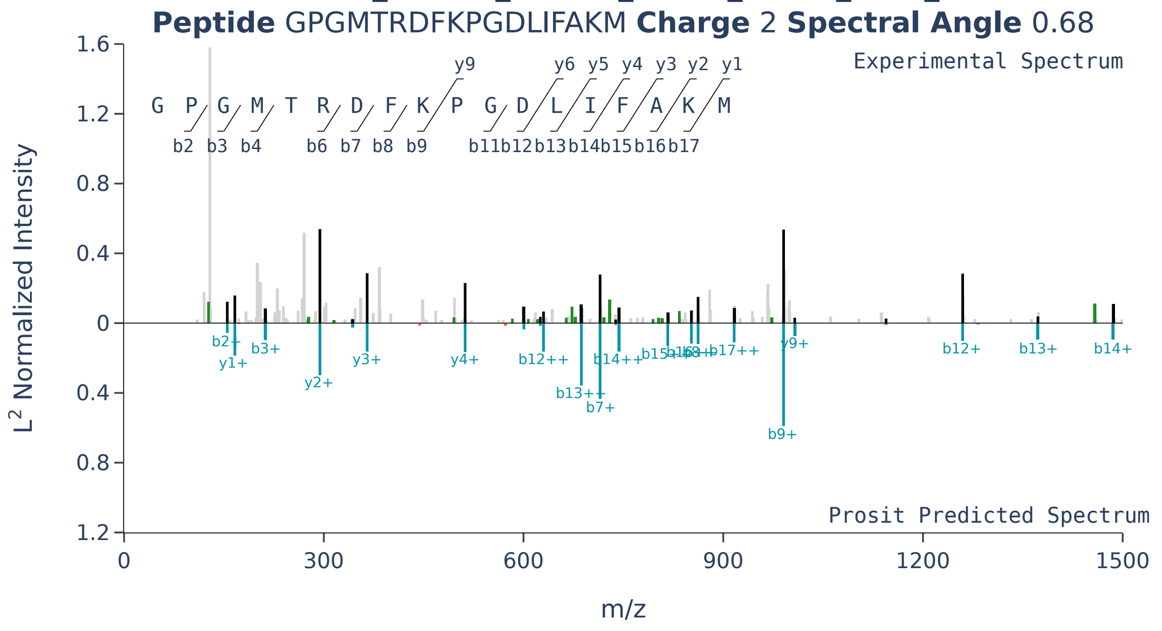


**Source** HRoetschke_271222_090123_HFGoe_LEDGF_b1R1_24h **Scan** 12516
**Peptide** NNPKVKF **Charge** 2 **Spectral Angle** 0.8

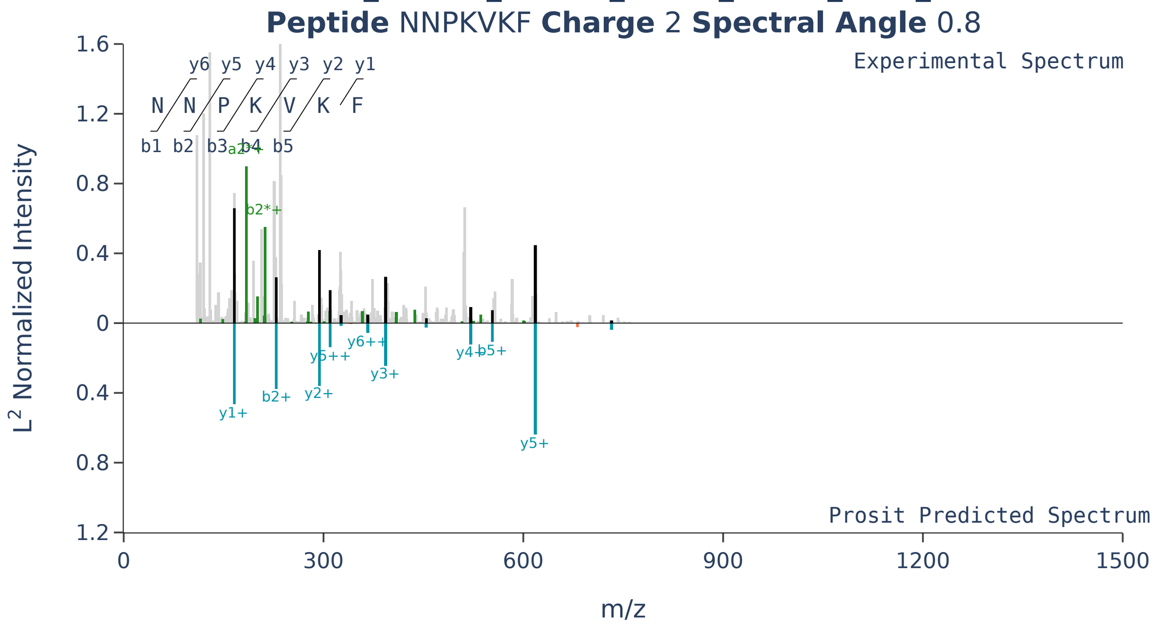


**Source** HRoetschke_271222_090123_HFGoe_LEDGF_b1R2_1h **Scan** 5059
**Peptide** EGKKPEVKKVEKKRETSMD **Charge** 3 **Spectral Angle** 0.92

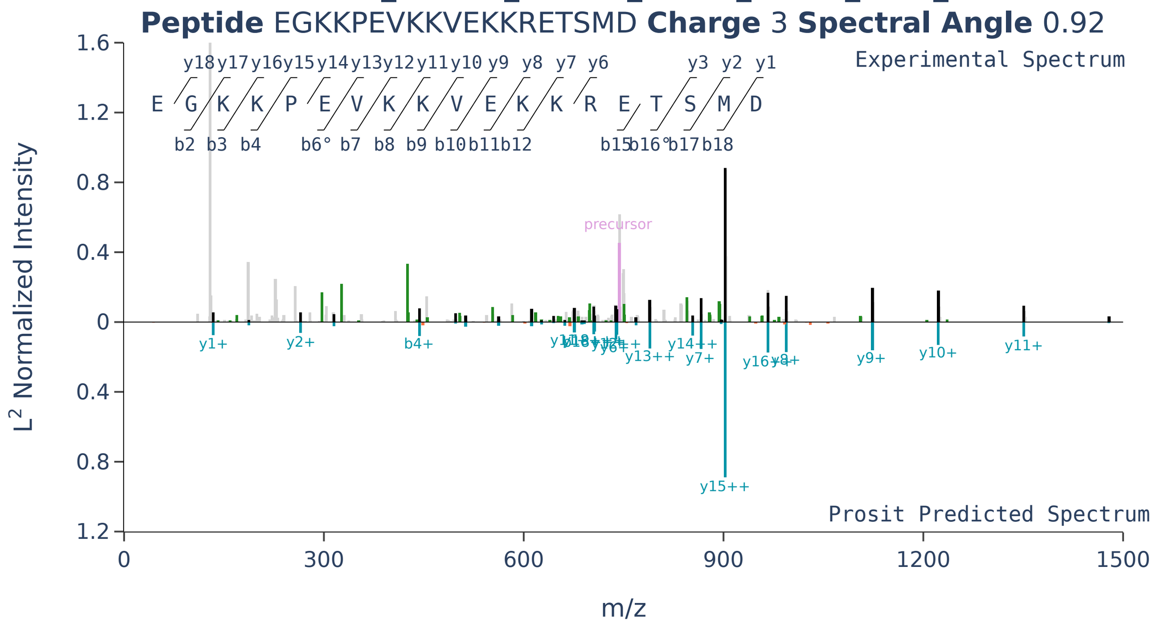


**Source** HRoetschke_271222_090123_HFGoe_LEDGF_b2R2_24h **Scan** 8197
**Peptide** QAATKQSNASSDVEVE **Charge** 2 **Spectral Angle** 0.74

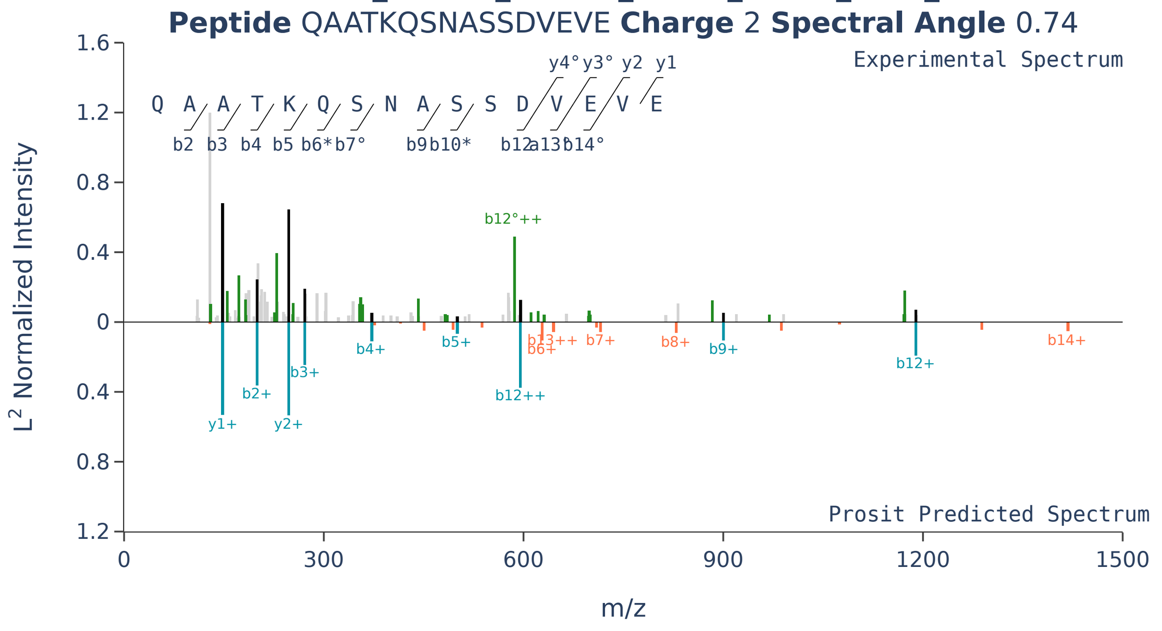


**Source** HRoetschke_271222_090123_HFGoe_LEDGF_b2R2_4h **Scan** 6225
**Peptide** FSSQQAATKQSNA **Charge** 2 **Spectral Angle** 0.78

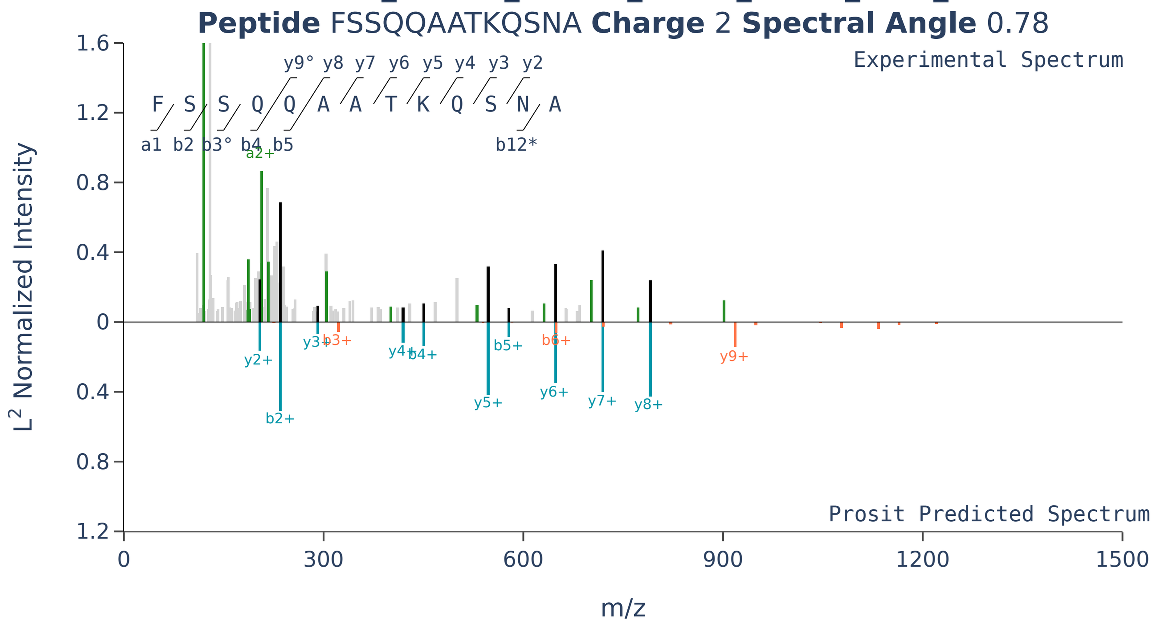


**Source** HRoetschke_271222_090123_HFGoe_LEDGF_b1R2_24h **Scan** 5163
**Peptide** TEQQNKDEGKKPEVKKVEKKRETSM **Charge** 5 **Spectral Angle** 0.71

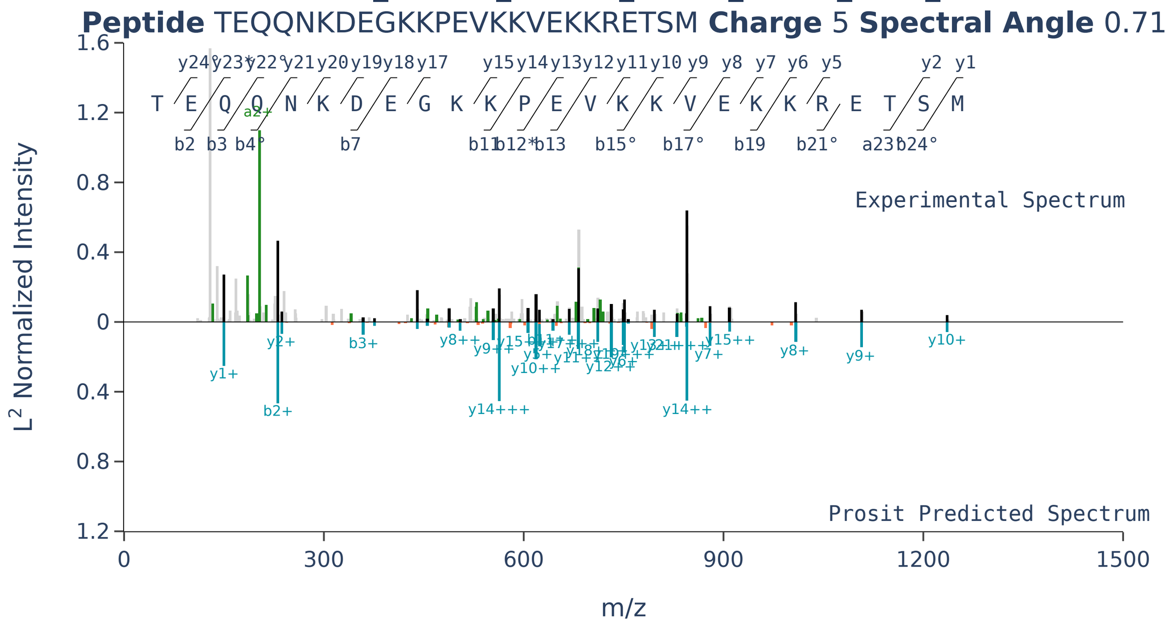


**Source** HRoetschke_271222_090123_HFGoe_LEDGF_b1R2_4h **Scan** 24979
**Peptide** EGLWEIDNNPKVKFS **Charge** 2 **Spectral Angle** 0.82

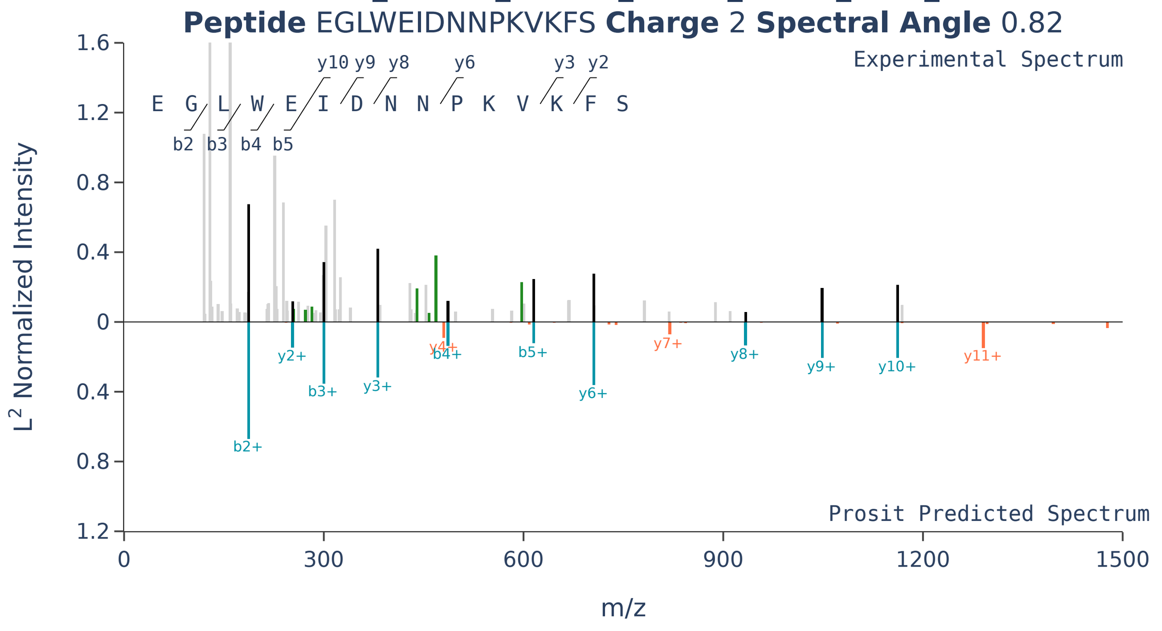


**Source** HRoetschke_271222_090123_HFGoe_LEDGF_b1R2_1h **Scan** 11326
**Peptide** SQQAATKQSNASSDVEVEEKETSV **Charge** 3 **Spectral Angle** 0.85

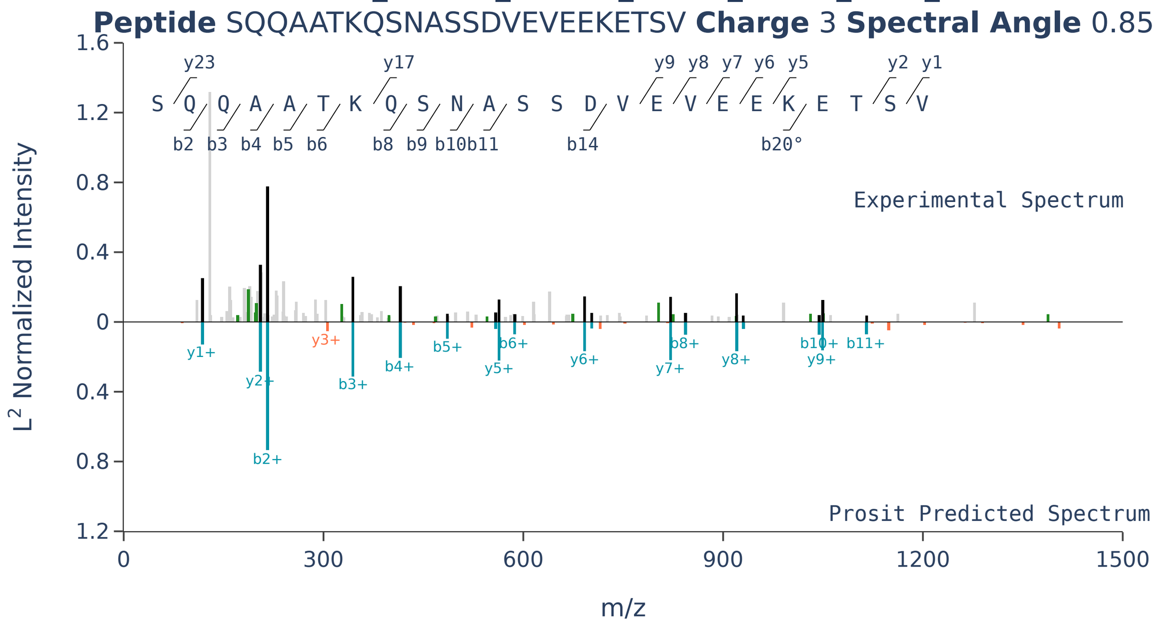

**Source** HRoetschke_271222_090123_HFGoe_LEDGF_b1R1_2h **Scan** 21412
**Peptide** GPGMTRDFKPGDLIFAKMK **Charge** 3 **Spectral Angle** 0.7

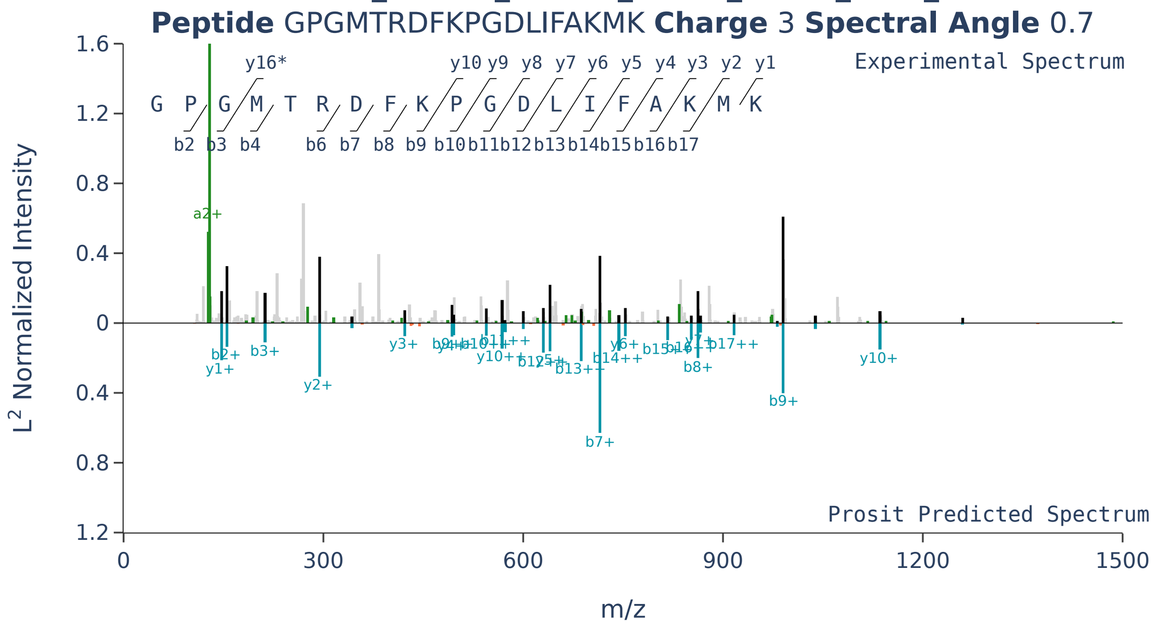


**Source** HRoetschke_271222_090123_HFGoe_LEDGF_b1R2_1h **Scan** 10534
**Peptide** ASTKKKPSSEERETEISLKDSTLDN **Charge** 5 **Spectral Angle** 0.82

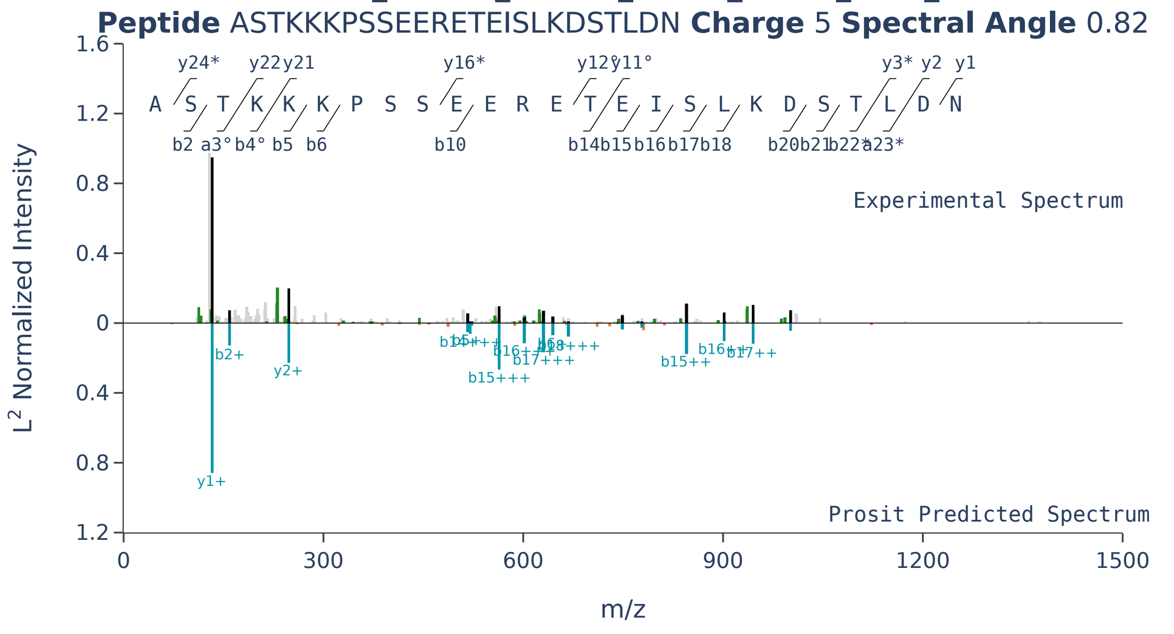


**Source** HRoetschke_271222_090123_HFGoe_LEDGF_b1R1_4h **Scan** 28623
**Peptide** GFNEGLWEIDNNPKVKF **Charge** 3 **Spectral Angle** 0.78

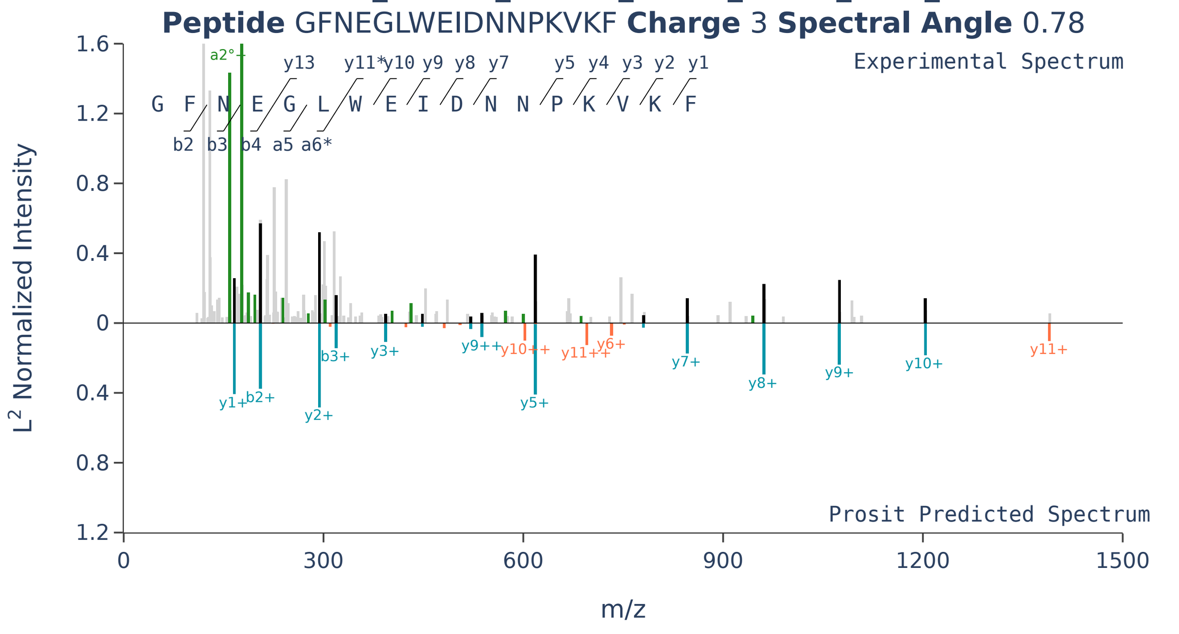


**Source** HRoetschke_271222_090123_HFGoe_LEDGF_b1R1_24h **Scan** 10505
**Peptide** GPKDIFPYSENKEKYGKPNKRKGFNE **Charge** 4 **Spectral Angle** 0.74

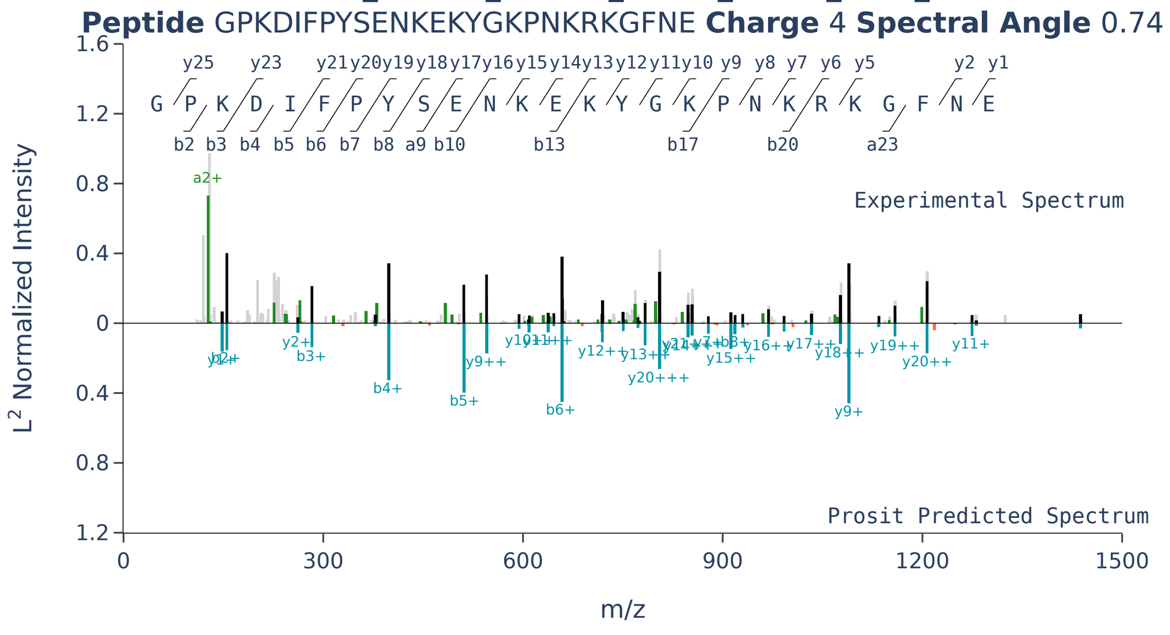


**Source** HRoetschke_271222_090123_HFGoe_LEDGF_b2R1_4h **Scan** 12267
**Peptide** KKIRRFKVSQVIME **Charge** 4 **Spectral Angle** 0.65

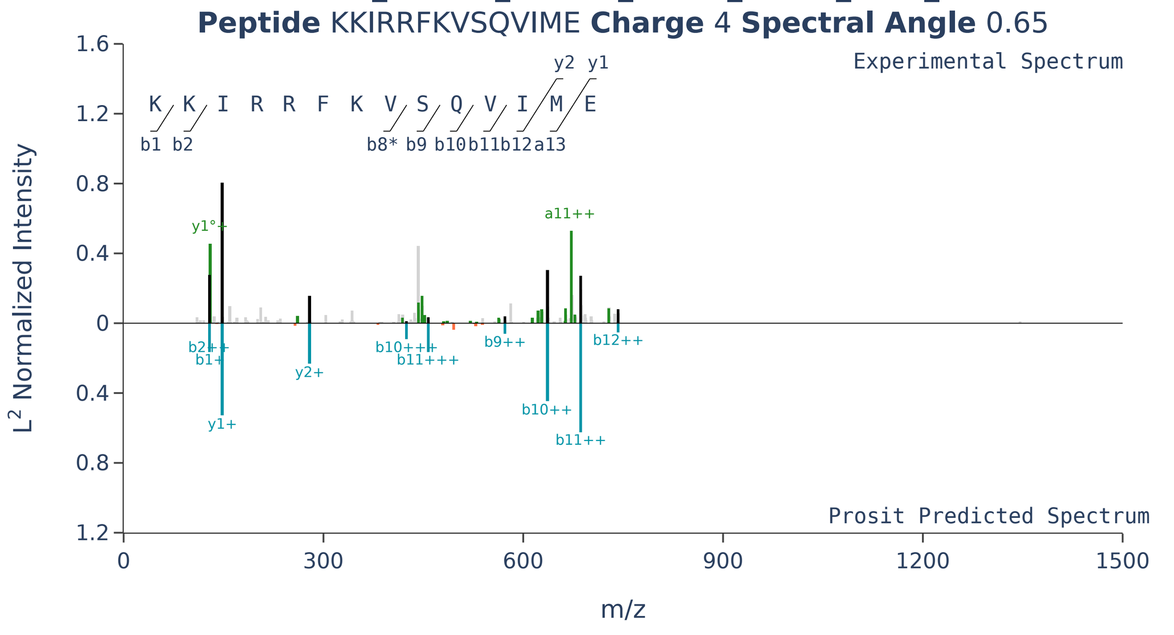


**Source** HRoetschke_271222_090123_HFGoe_LEDGF_b1R1_4h **Scan** 9020
**Peptide** SQQAATKQSNASSDVEVEEKETSVSKE **Charge** 3 **Spectral Angle** 0.87

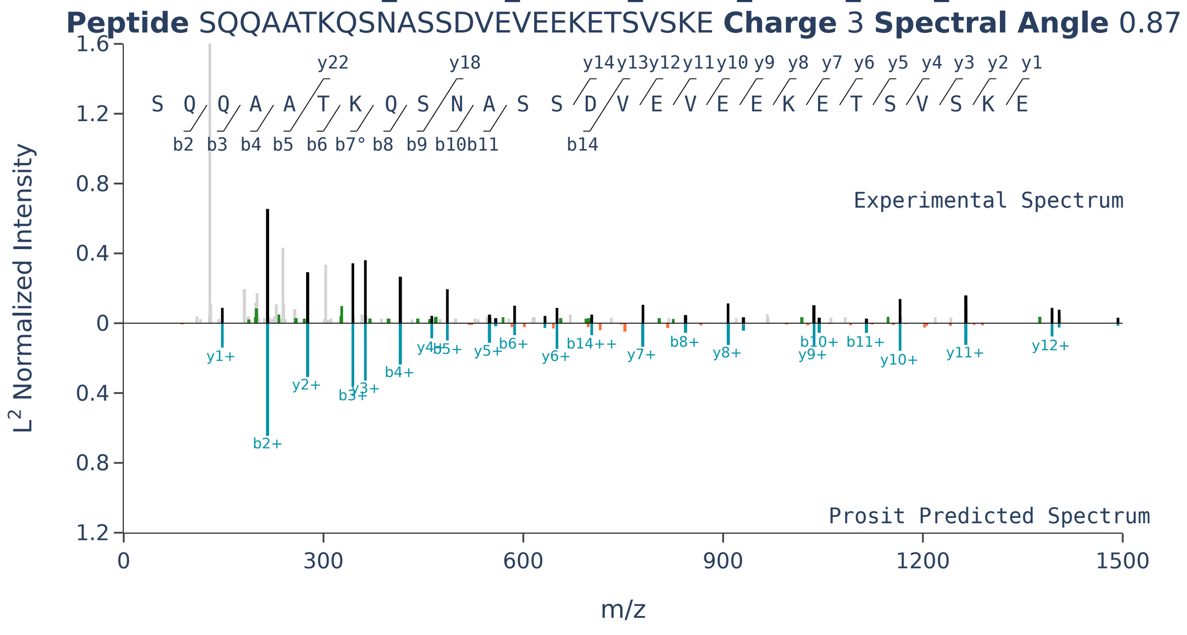


**Source** HRoetschke_271222_090123_HFGoe_LEDGF_b2R1_24h **Scan** 7014
**Peptide** KETSVSKEDTDHEEKASNEDVTKAVD **Charge** 3 **Spectral Angle** 0.86

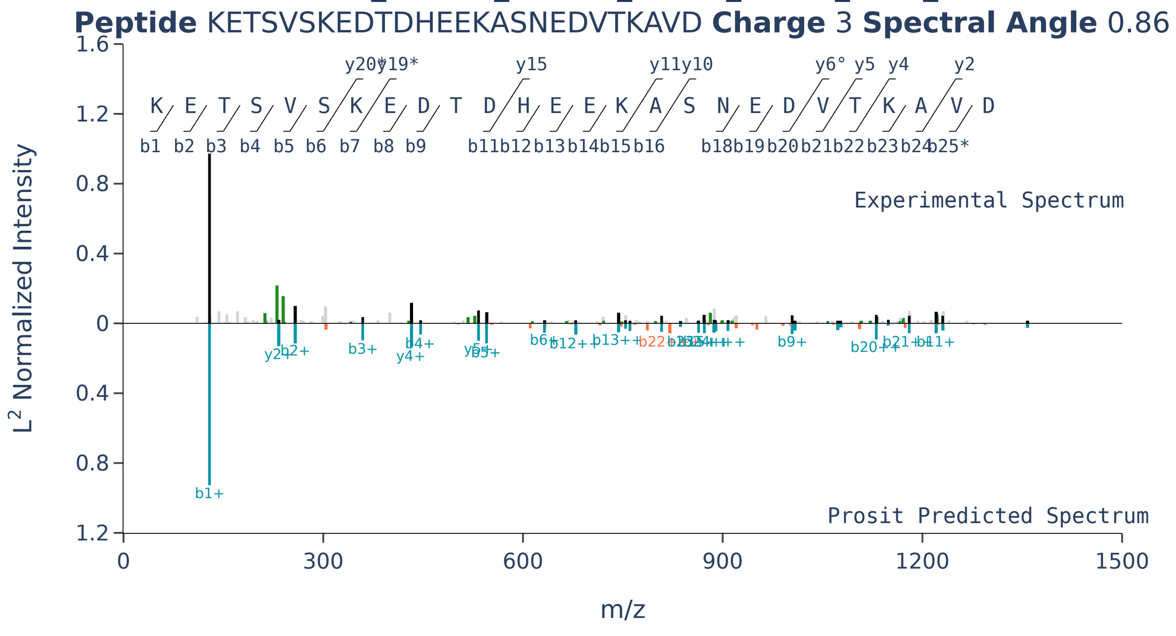


**Source** HRoetschke_271222_090123_HFGoe_LEDGF_b1R1_2h **Scan** 31400
**Peptide** DEVPDGAVKPPTNKLPIFFFGTHETA **Charge** 4 **Spectral Angle** 0.9

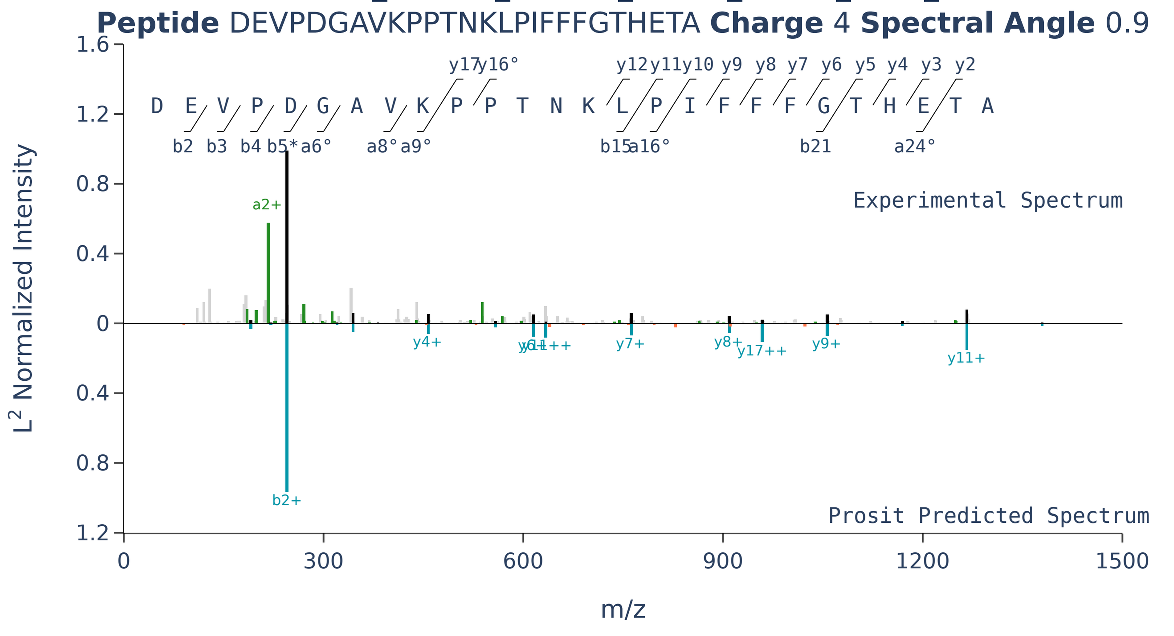


**Source** HRoetschke_271222_090123_HFGoe_LEDGF_b1R2_24h **Scan** 8184
**Peptide** EDVTKAVD **Charge** 1 **Spectral Angle** 0.86

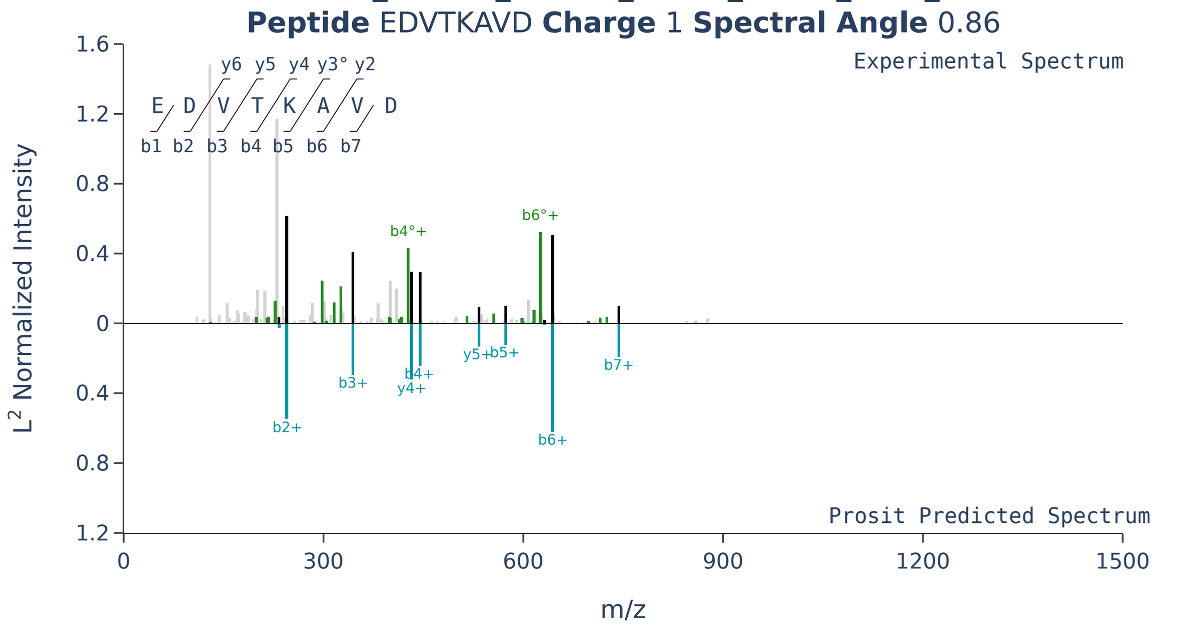


**Source** HRoetschke_271222_090123_HFGoe_LEDGF_b1R1_2h **Scan** 10138
**Peptide** KKIRRFKVSQVIMEKS **Charge** 4 **Spectral Angle** 0.78
[truncated: 725,307 more chars]
